# Supplementary material for: Systems-level proteomic models of cotton fiber development: a high-resolution data resource to analyze cell dynamics and trait engineering
Source: Plant Physiol. 2026 Jul 2;201(3):kiag318. doi: 10.1093/plphys/kiag318 (PMC13326644; doi:10.1093/plphys/kiag318)
Supplement: kiag318_Supplementary_Data [file Supplementary Data.pdf › Supplementary Data.pdf]

Supplementary Video S1. A movie of cotton flower and boll development (-5 to 53 DPA).

This video captures the phenotypic progression of cotton flowers and bolls from –5 to 53 days post anthesis (DPA). Anthesis occurs at 0 DPA, and boll opening is observed at 48 DPA. One sepal was removed from the flower to facilitate visualization. The proteomic sampling range (5–24 DPA) is highlighted within the developmental timeline.

Supplementary Figure S1. Enzyme coverage of metabolic pathways.

Cotton gene IDs were mapped onto metabolic pathways using Cellular Overview/Omics viewer at Plant Metabolic Network GraimondiiCyc v4.0.0 (https://pmn.plantcyc.org/overviewsWeb/celOv.shtml?orgid=GRAIMONDII).

1A. Pathway map of reproducible detected APO_T proteins (present in Figure 1C).

1B. Pathway map of reproducible detected P200 proteins (present in Figure 1C).
Because Plant Metabolic Network does not allow a search with a large number (>900) of gene IDs in a query, the two pathway mappings (1/2 and 2/2) were generated in the two separated searches.

1C. Pathway map of reproducible detected S200 proteins (present in Figure 1C).

1D. Pathway map of cytoplasmic proteins (P200 and S200) with informative profiles (present in Figure 2B).

1E. Pathway map of extracellular proteins (APO_T) with informative profiles (present in Figure 2B).

1A. Pathway map of reproducible detected APO_T proteins (present in Figure 1C).

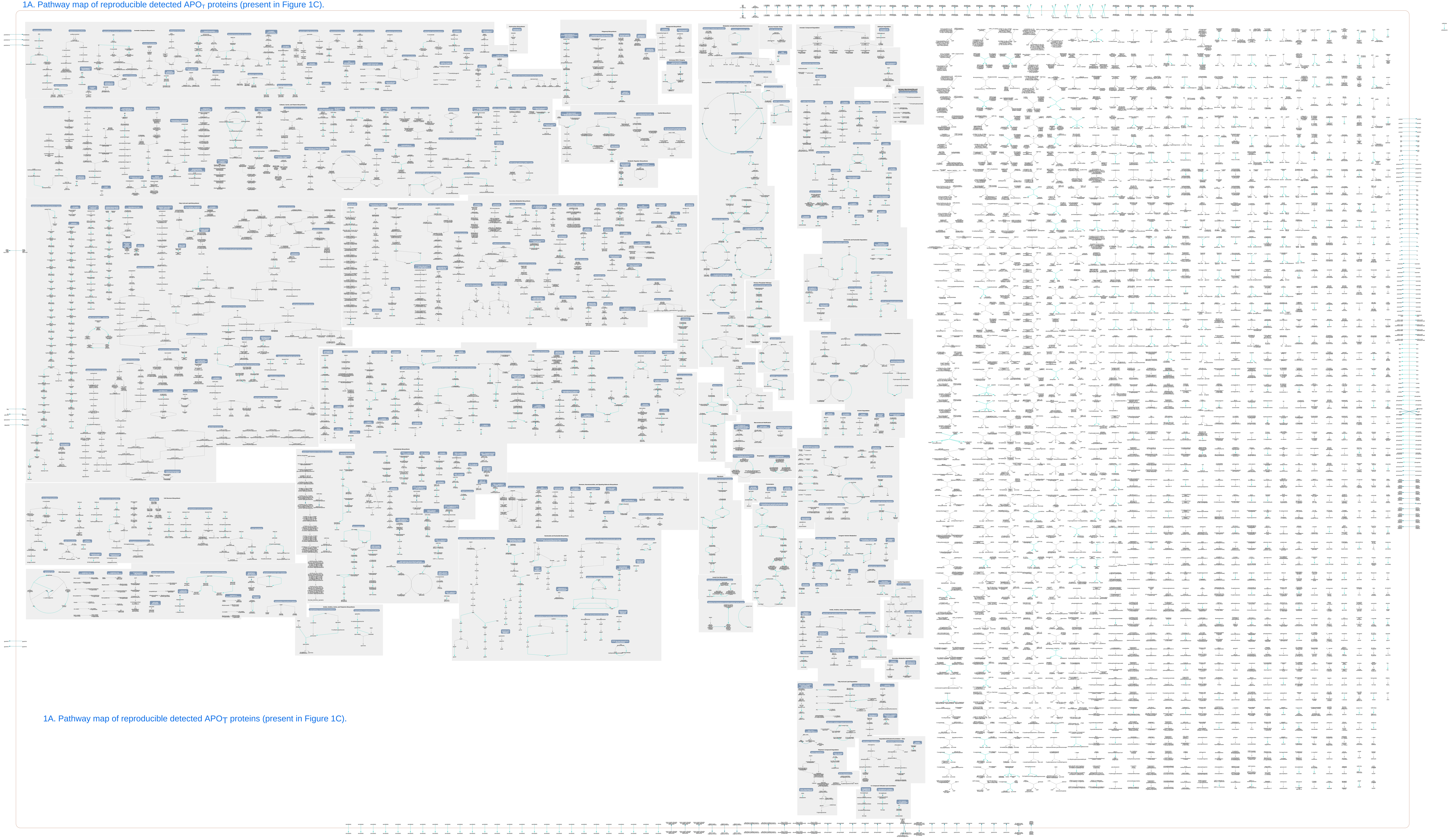
1A. Pathway map of reproducible detected APO_T proteins (present in Figure 1C).

1B. 1/2 Pathway map of reproducible detected P200 proteins (present in Figure 1C).

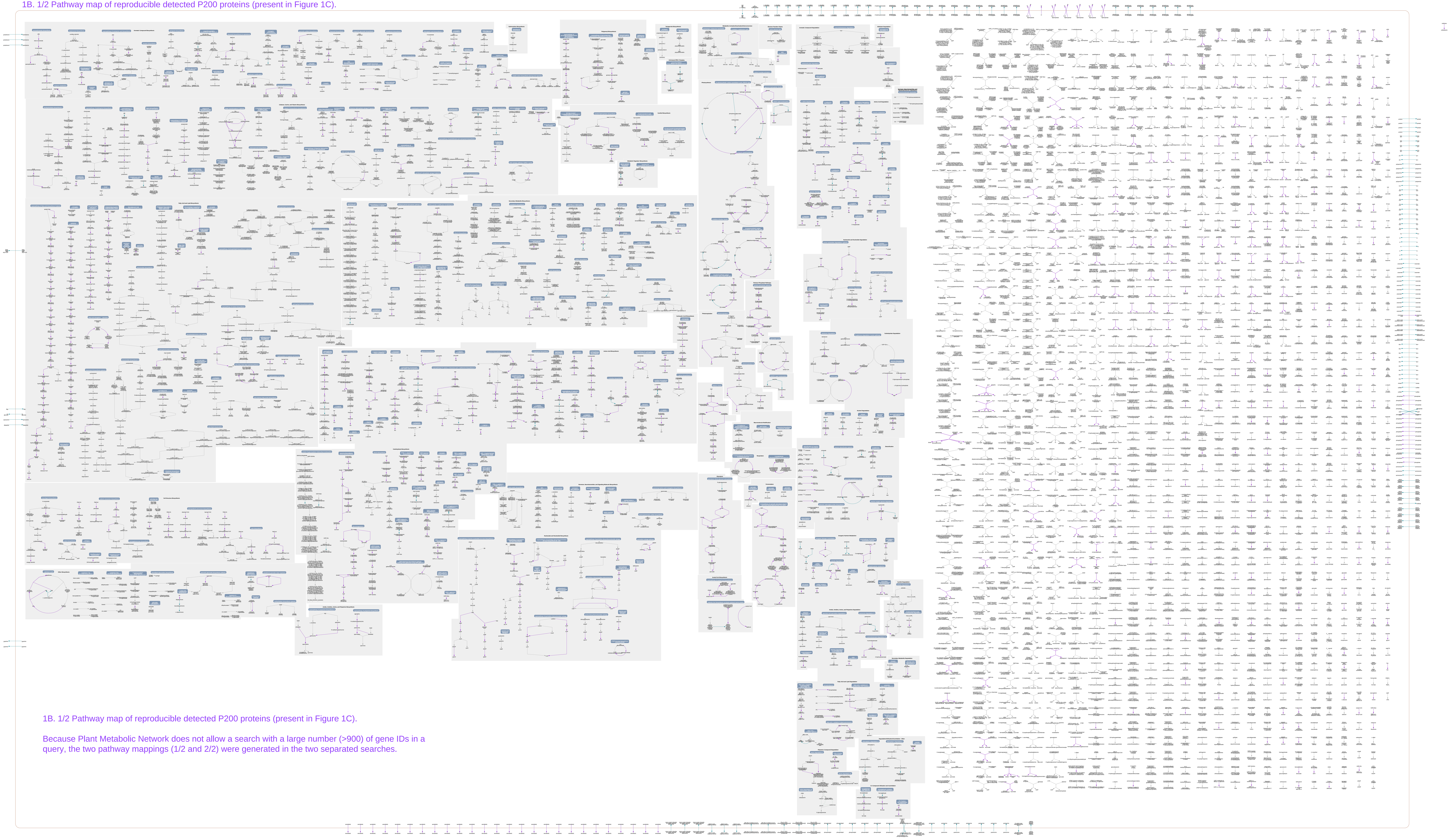

1B. 1/2 Pathway map of reproducible detected P200 proteins (present in Figure 1C).

Because Plant Metabolic Network does not allow a search with a large number (>900) of gene IDs in a query, the two pathway mappings (1/2 and 2/2) were generated in the two separated searches.

1B. 2/2 Pathway map of reproducible detected P200 proteins (present in Figure 1C).

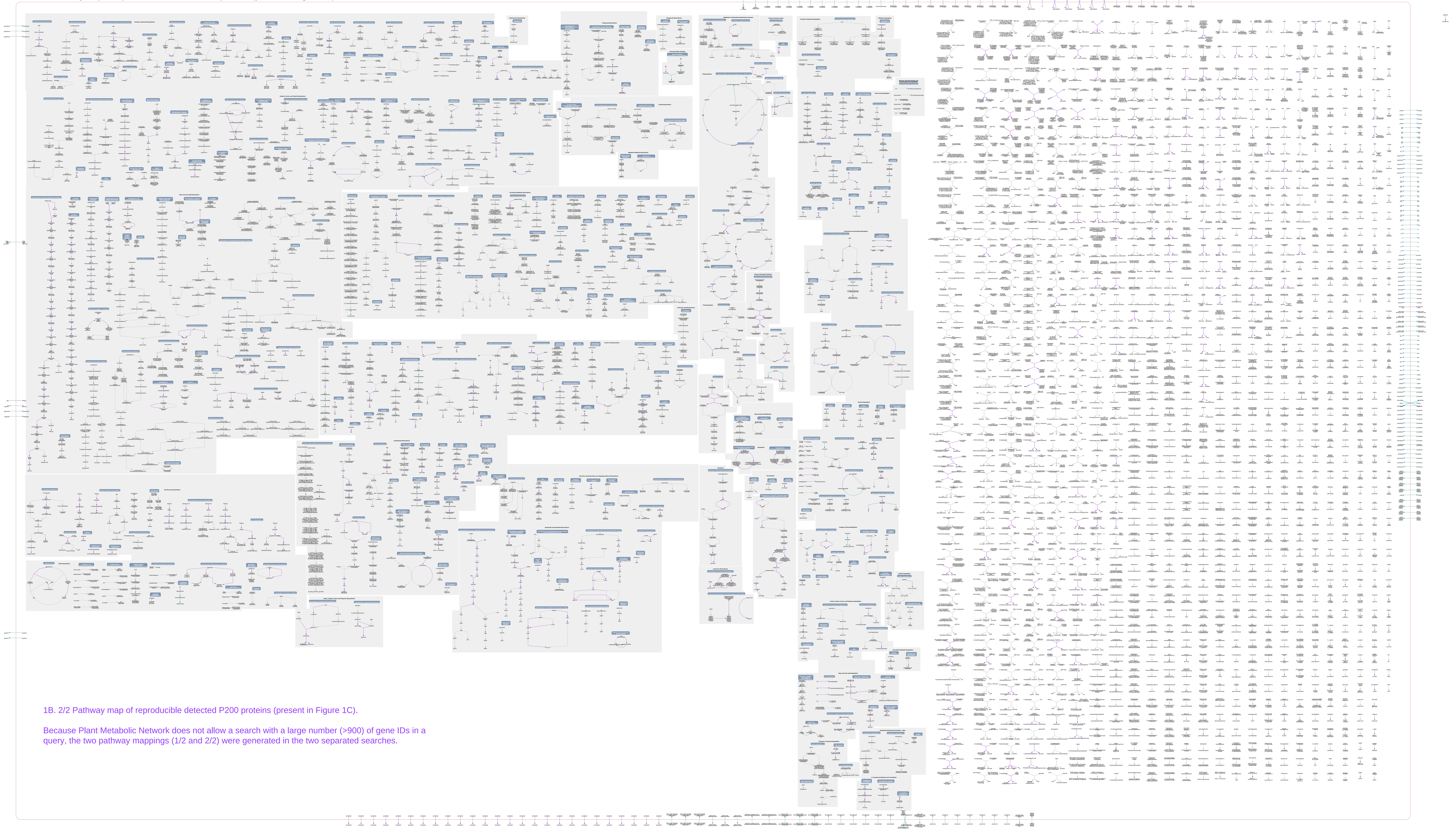

1B. 2/2 Pathway map of reproducible detected P200 proteins (present in Figure 1C).

Because Plant Metabolic Network does not allow a search with a large number (>900) of gene IDs in a query, the two pathway mappings (1/2 and 2/2) were generated in the two separated searches.

1C. Pathway map of reproducible detected S200 proteins (present in Figure 1C).

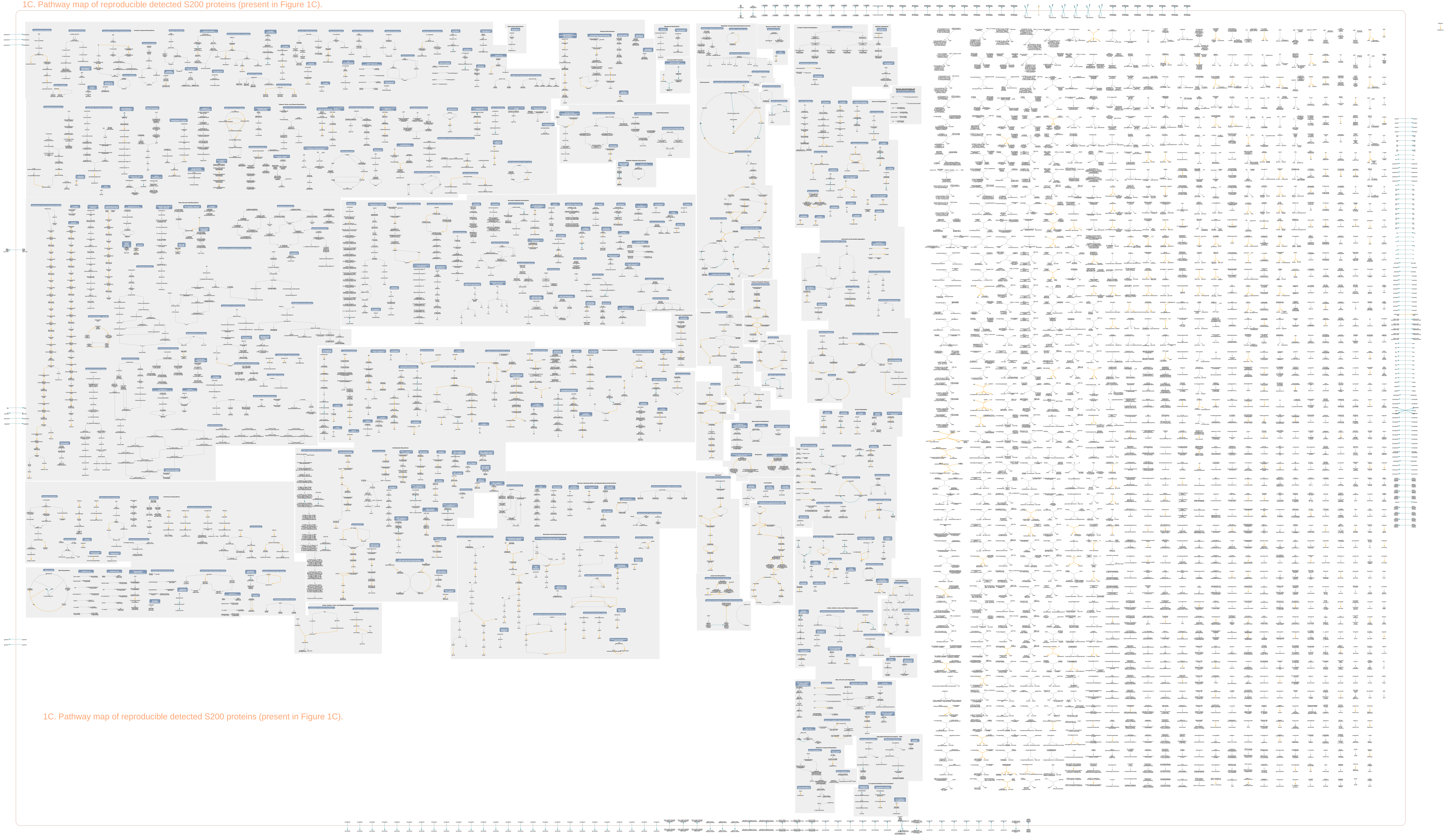

1C. Pathway map of reproducible detected S200 proteins (present in Figure 1C).

1D. Pathway map of cytoplasmic proteins (P200 and S200) with informative profiles (present in Figure 2B).

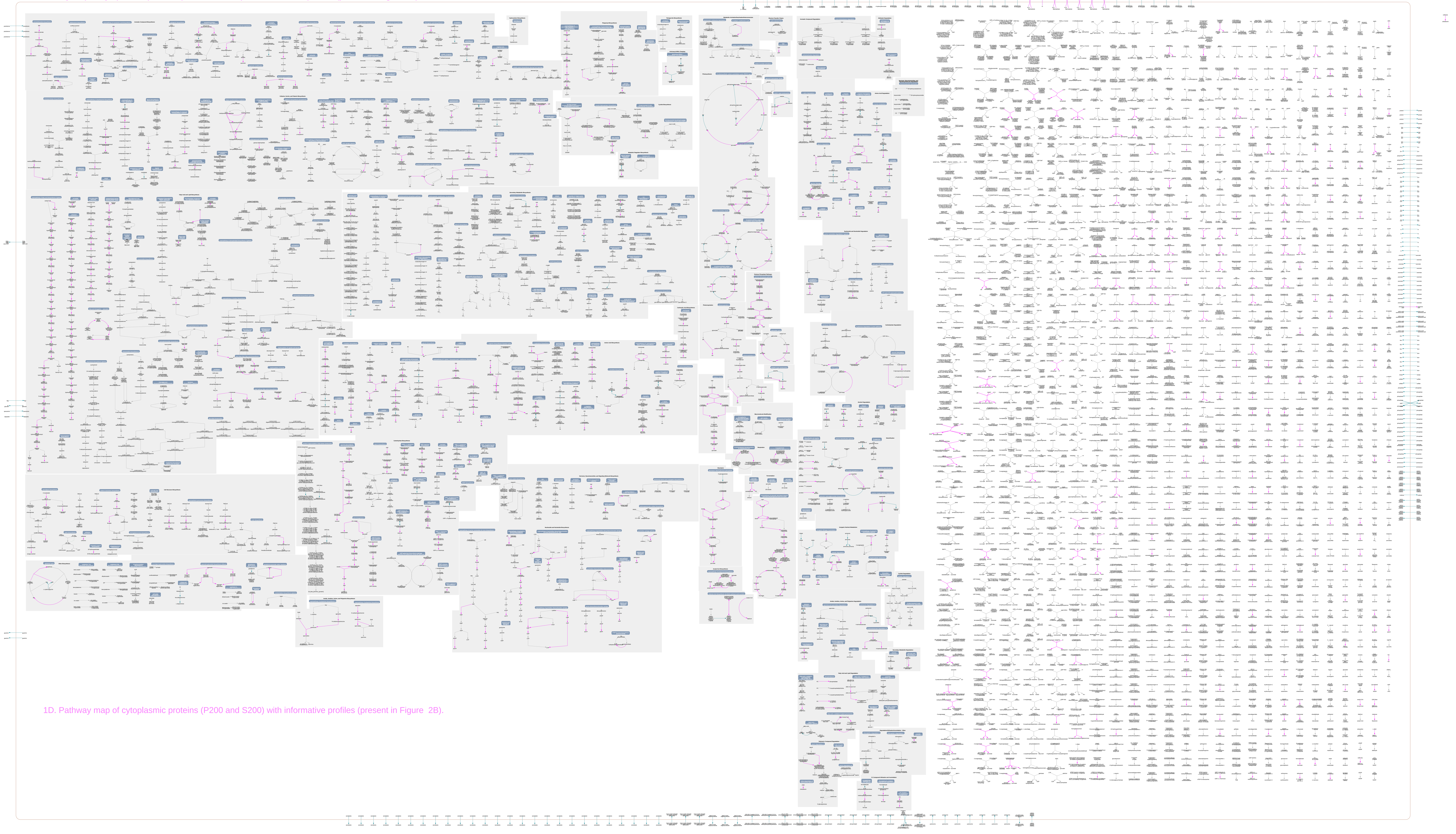

1D. Pathway map of cytoplasmic proteins (P200 and S200) with informative profiles (present in Figure 2B).

1E. Pathway map of extracellular proteins (APO$_T$) with informative profiles (present in Figure 2B).

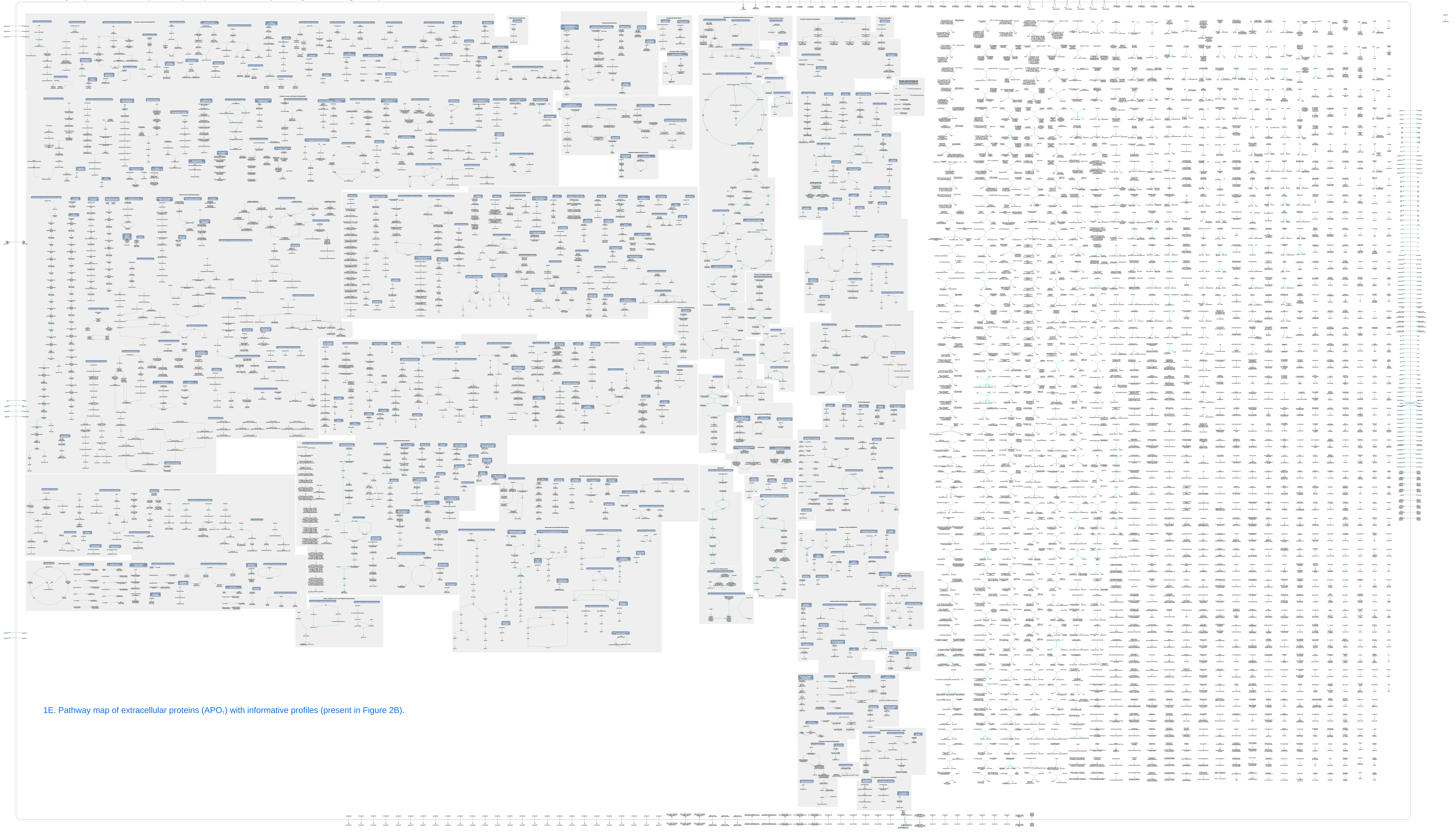

1E. Pathway map of extracellular proteins (APO$_T$) with informative profiles (present in Figure 2B).

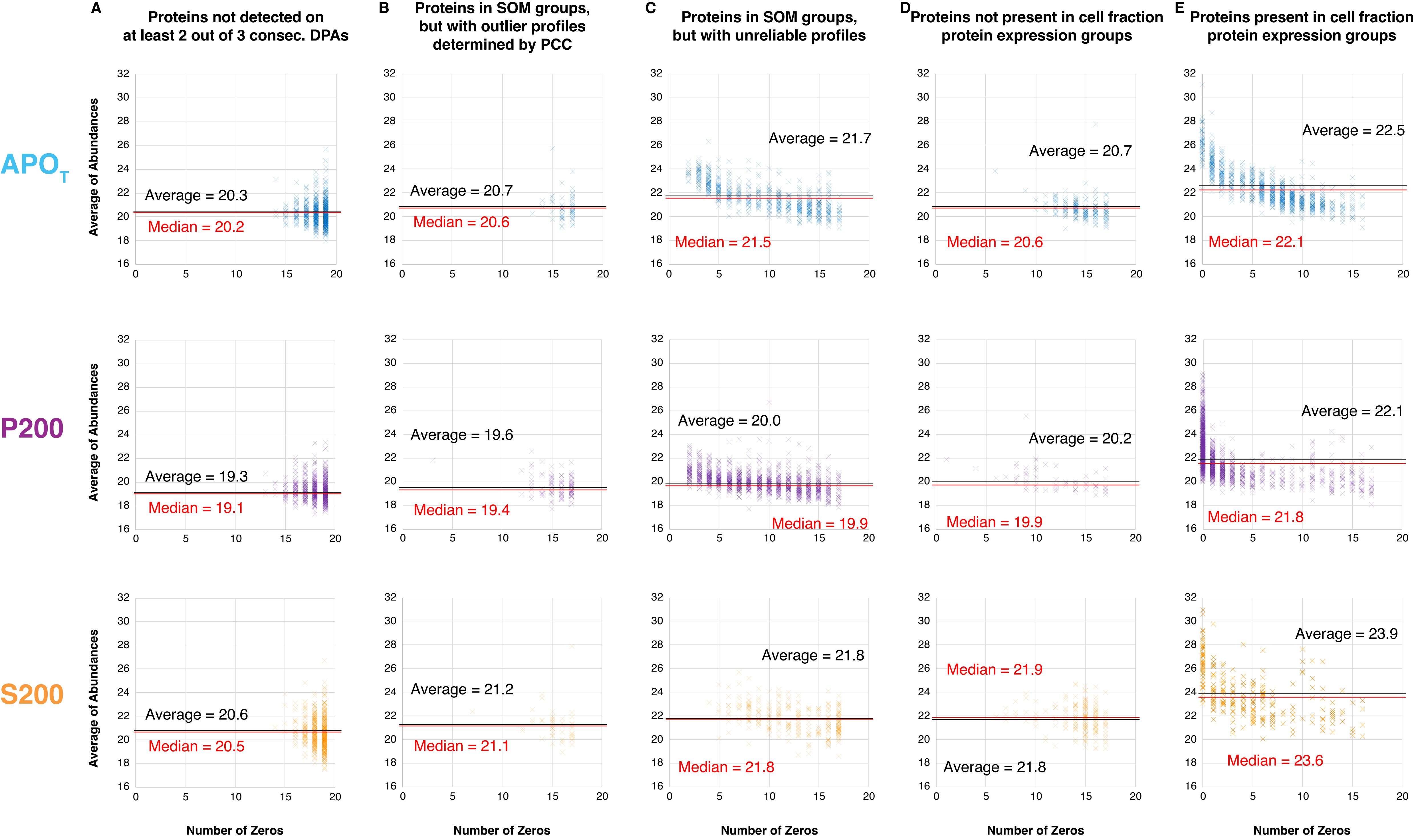

Supplementary Figure S2. Analysis of proteins removed at each data filtering step during the identification of reliable members.
A, Proteins that were not used in the SOM because they did not satisfy the criteria that 2 out 3 consecutive DPAs need to be adjacent. **B,** After SOM clustering, proteins that were identified as outliers by PCC of < 0.8 in each SOM group. **C,** Proteins that were defined as unreliable profiles. We scored a group as unreliable if the pattern reflected the overall pattern of protein coverage or if the pattern is dominated by patterns that are explained by the interpolation (e.g. 3 consecutive zeros defines the cluster). **D,** Proteins that were not present in the cell fraction protein expression groups. **E,** Proteins that are in the cell fraction protein expression groups.

Supplementary Figure S3. SOM grouping and profile filtering (APO_T).

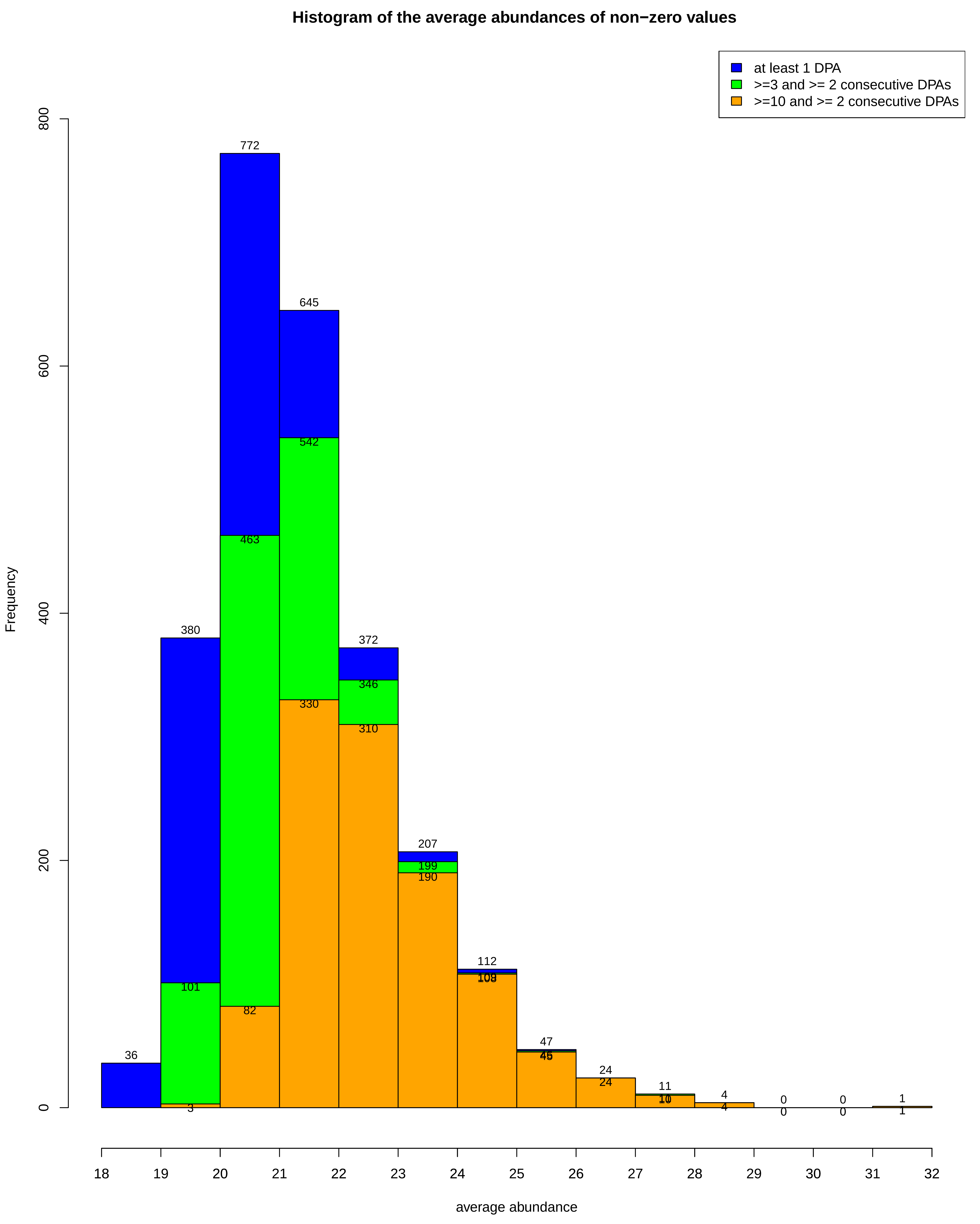

Histogram of the average abundances of non−zero values
at least 1 DPA
>=3 and >= 2 consecutive DPAs
>=10 and >= 2 consecutive DPAs
Frequency
average abundance
0
200
400
600
800
18
19
20
21
22
23
24
25
26
27
28
29
30
31
32
36
380
101
3
772
463
82
645
542
330
372
346
310
207
199
190
112
47
24
24
11
4
4
0
0
0
0
1
1

Codes

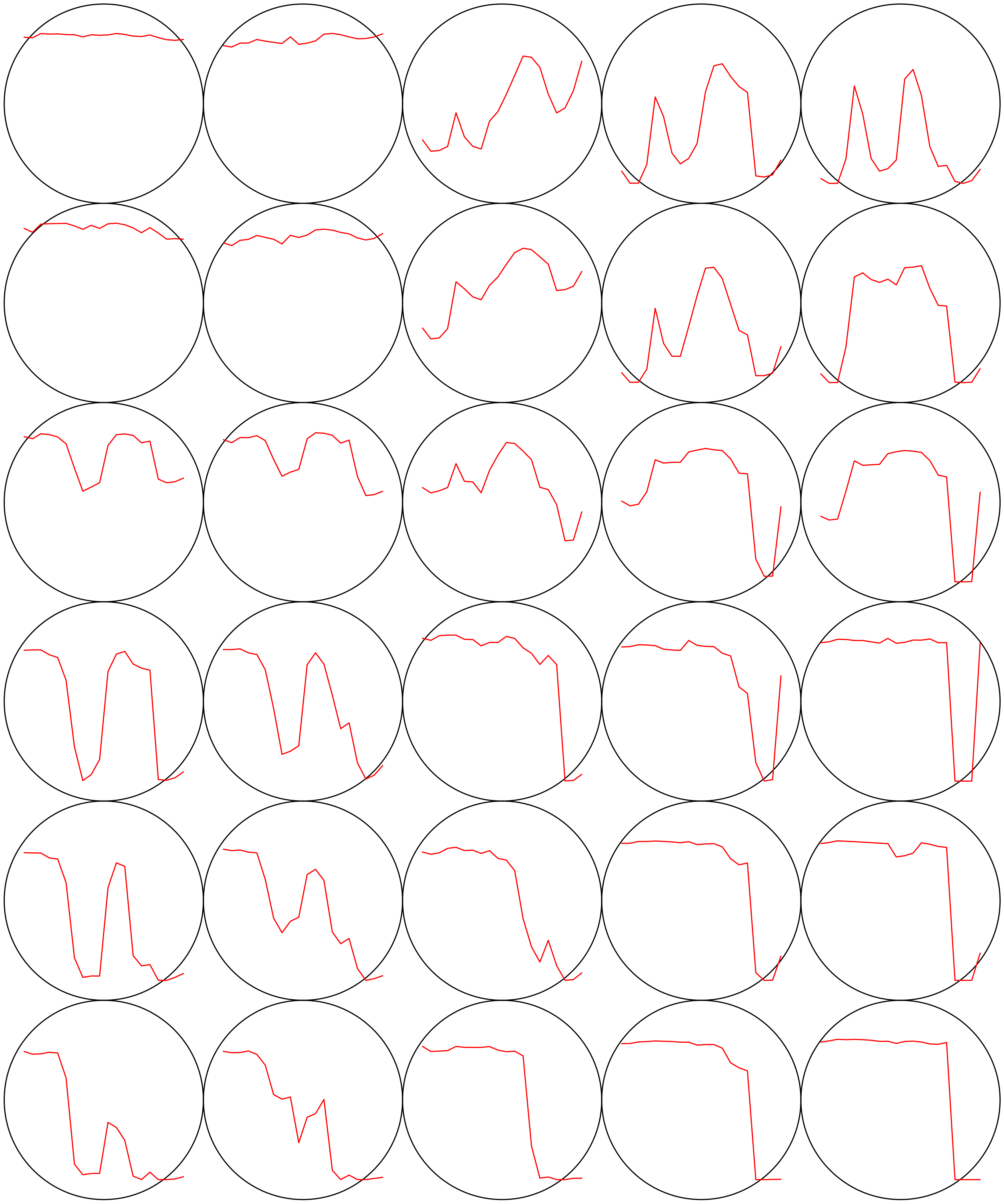

Count plot

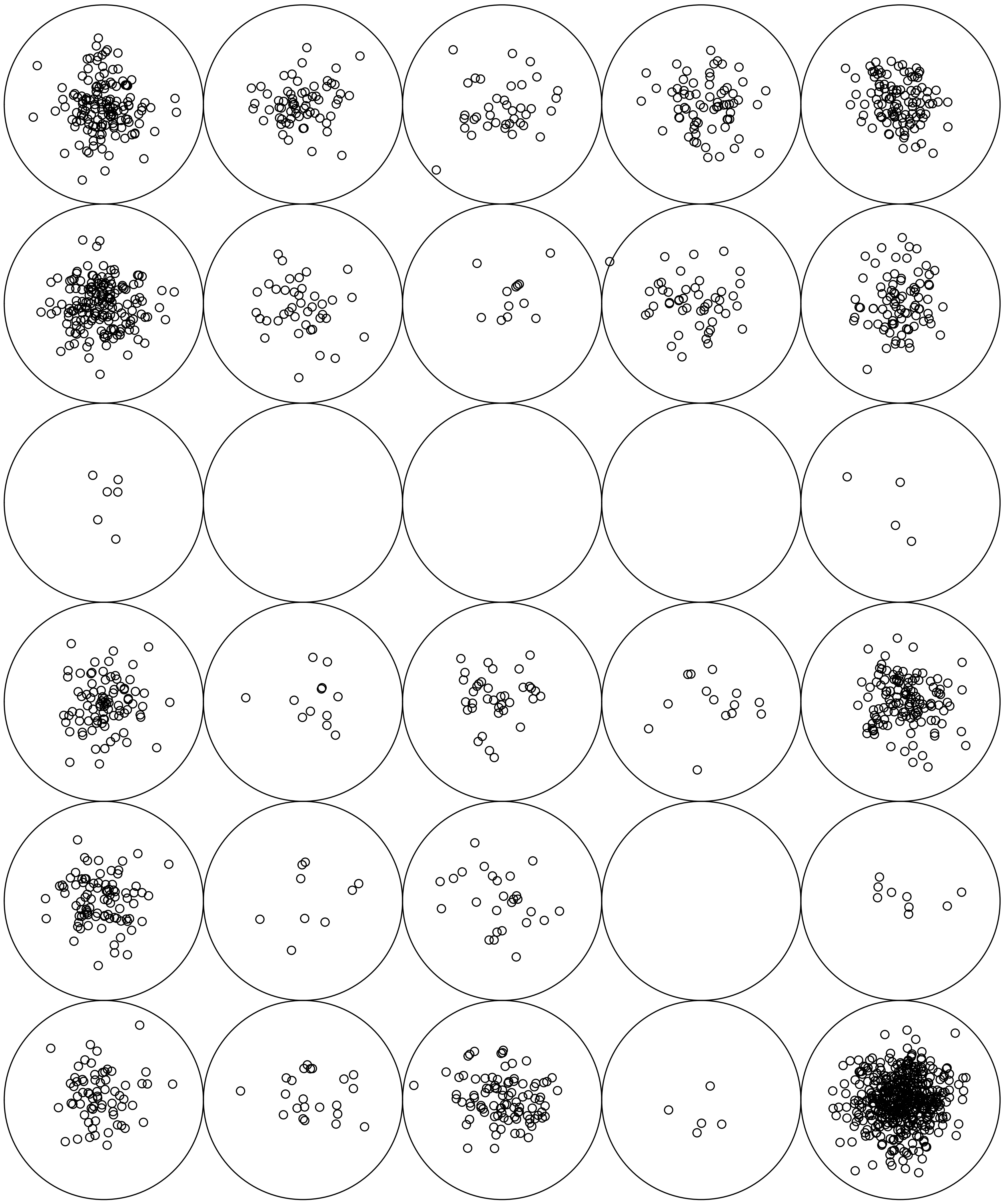

V1

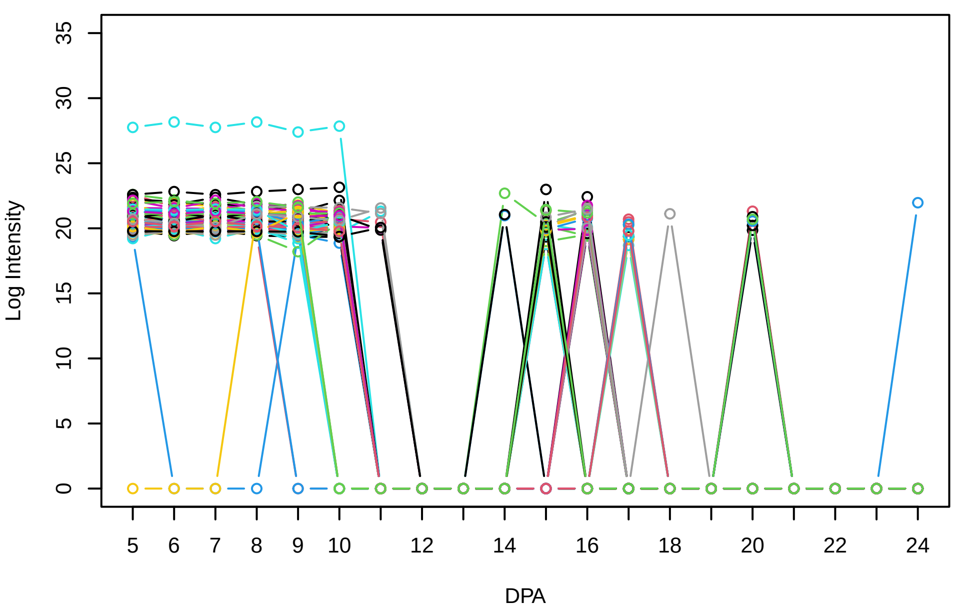

Histogram of the average abundances of non−zero values

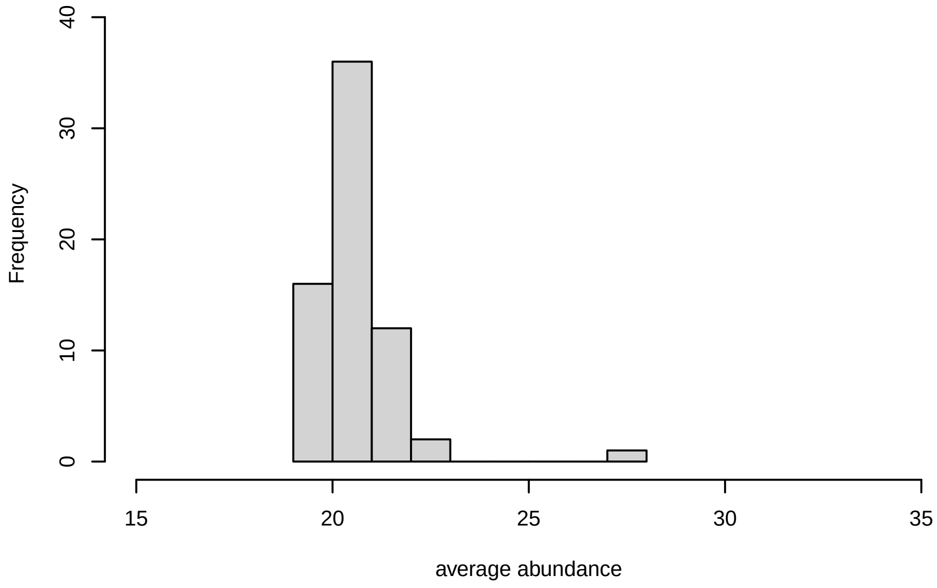

V1, after remove outlier and cutoff is 0.8

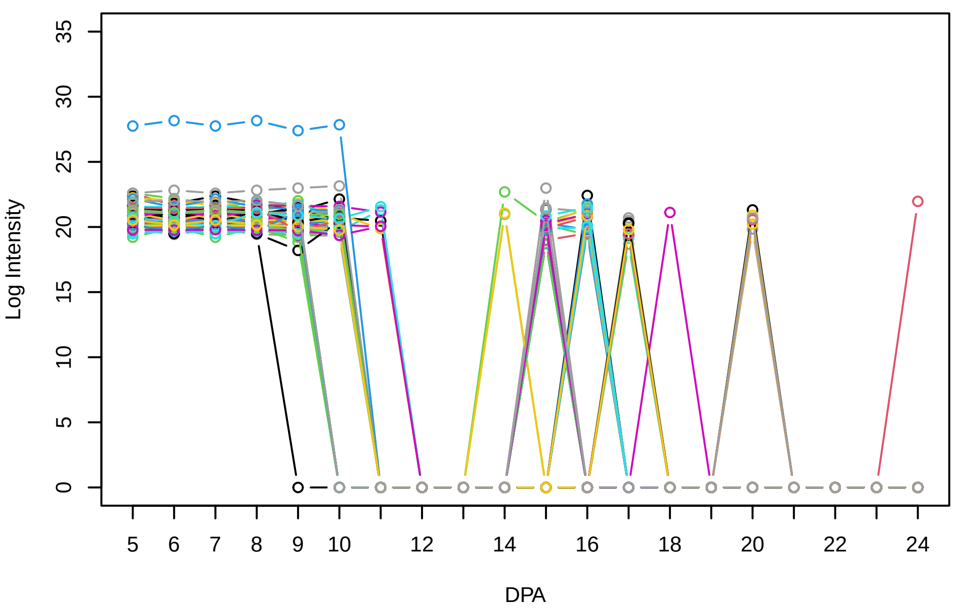

Histogram of the average abundances of non−outlier proteins

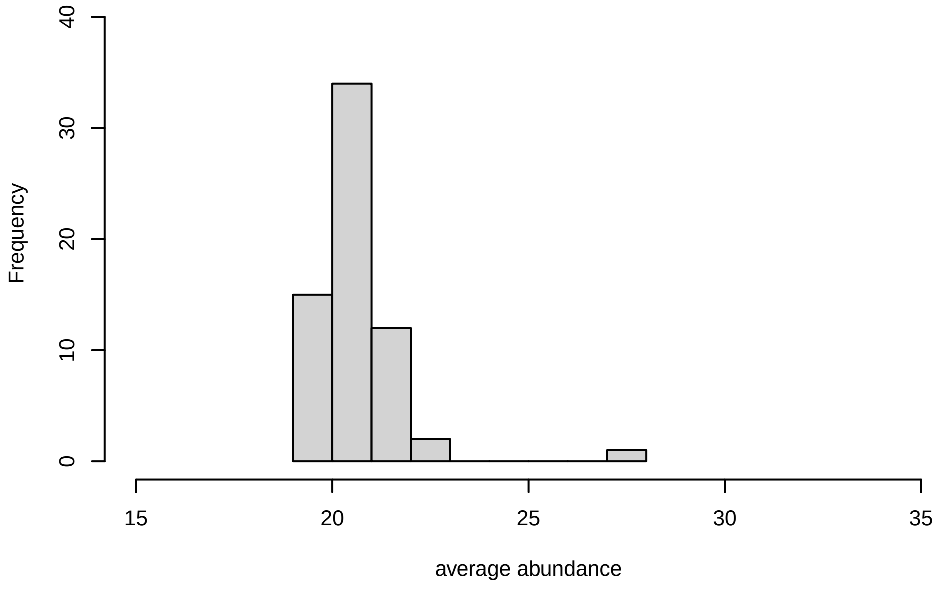

V1, after remove unreliable

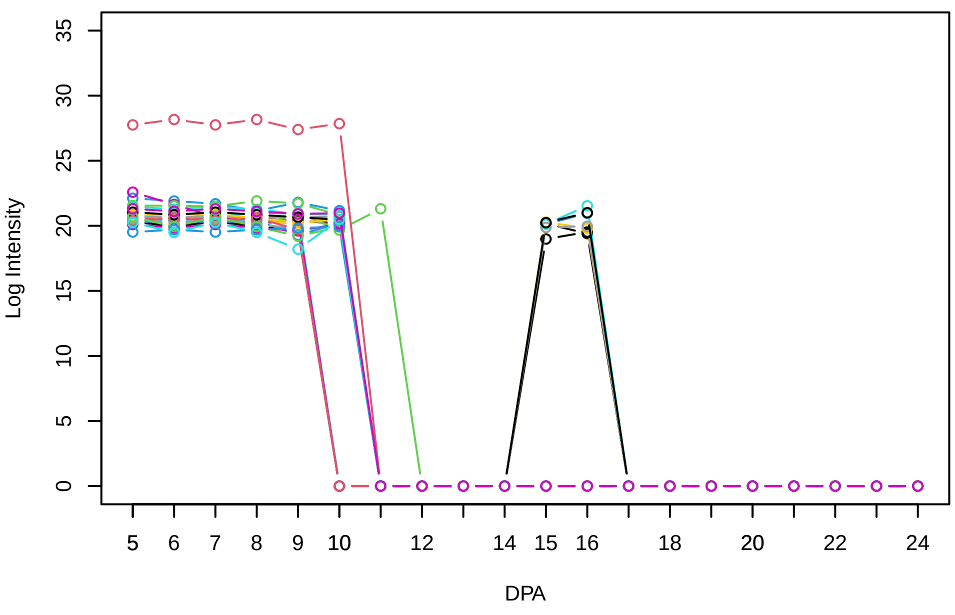

Histogram of the average abundances of reliable proteins

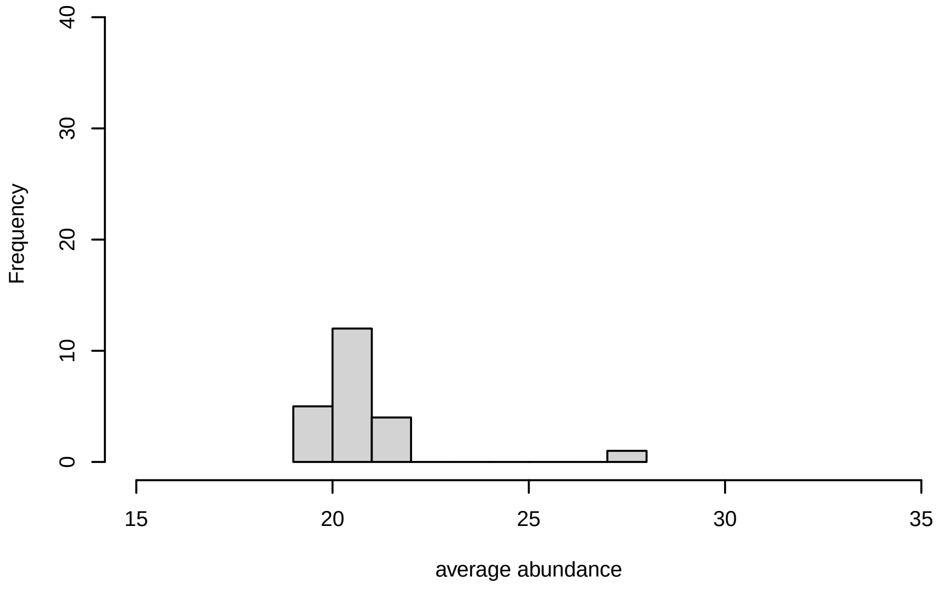

V1, after remove low abundance

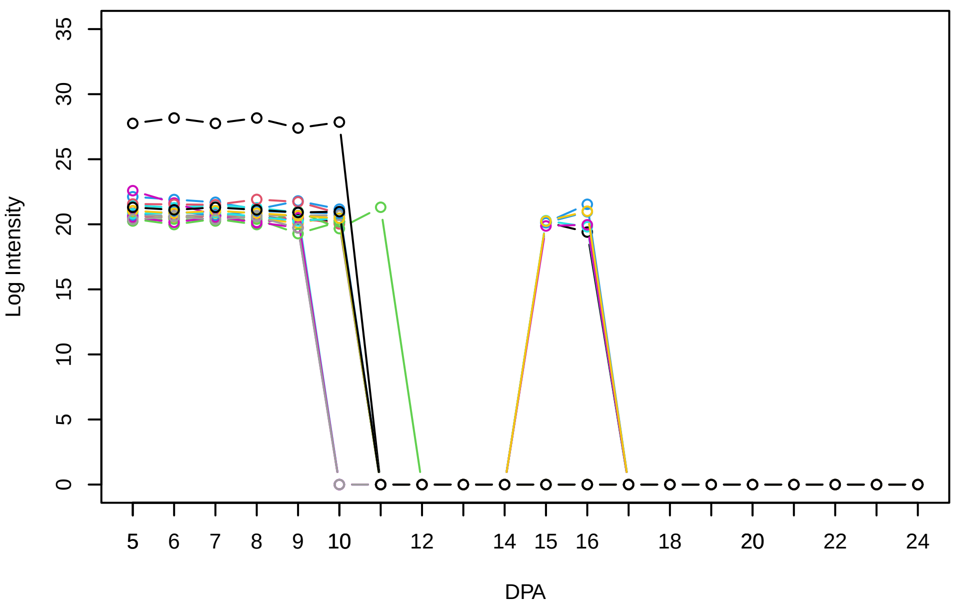

Histogram of the average abundances of high abundance proteins

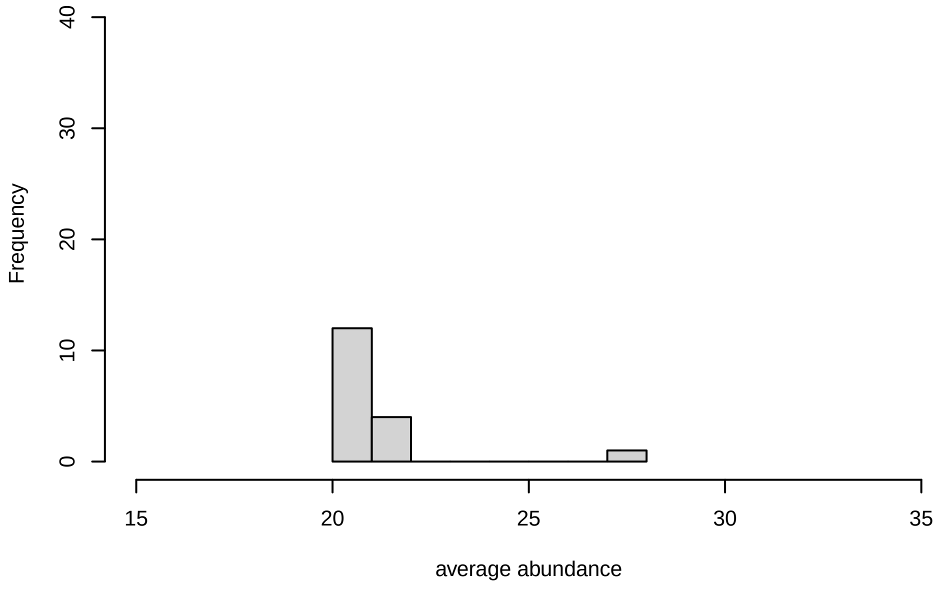

V2

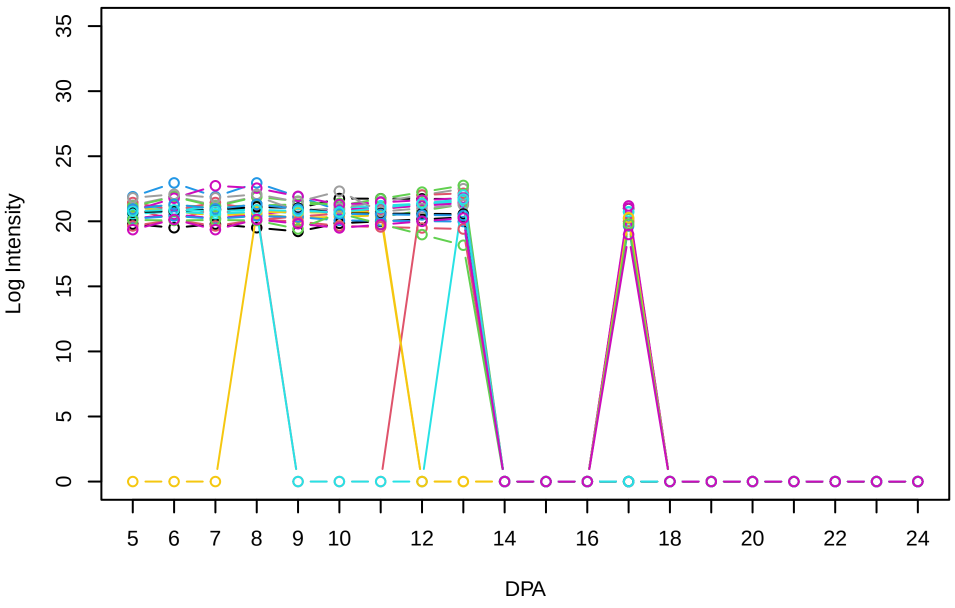

Histogram of the average abundances of non−zero values

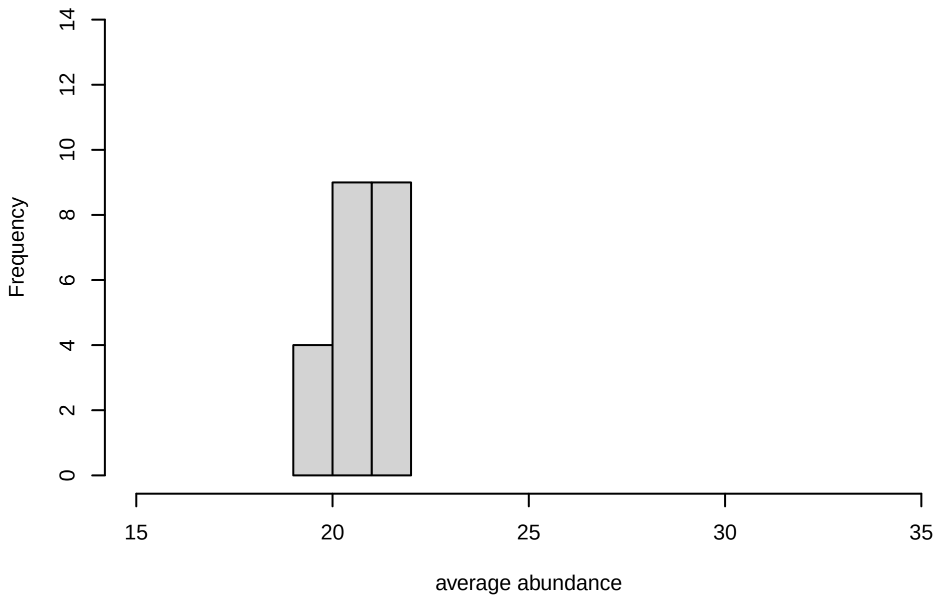

V2, after remove outlier and cutoff is 0.8

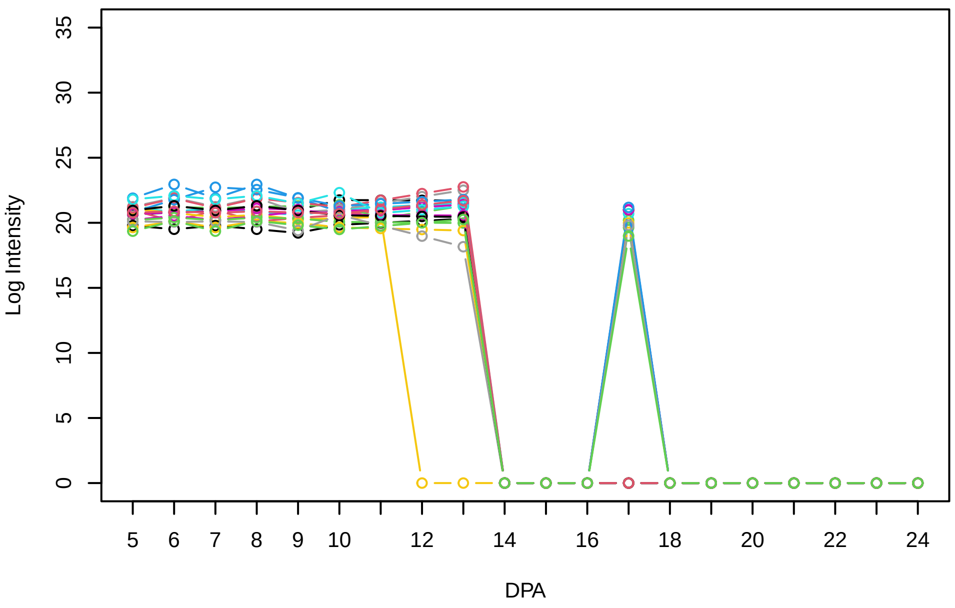

Histogram of the average abundances of non−outlier proteins

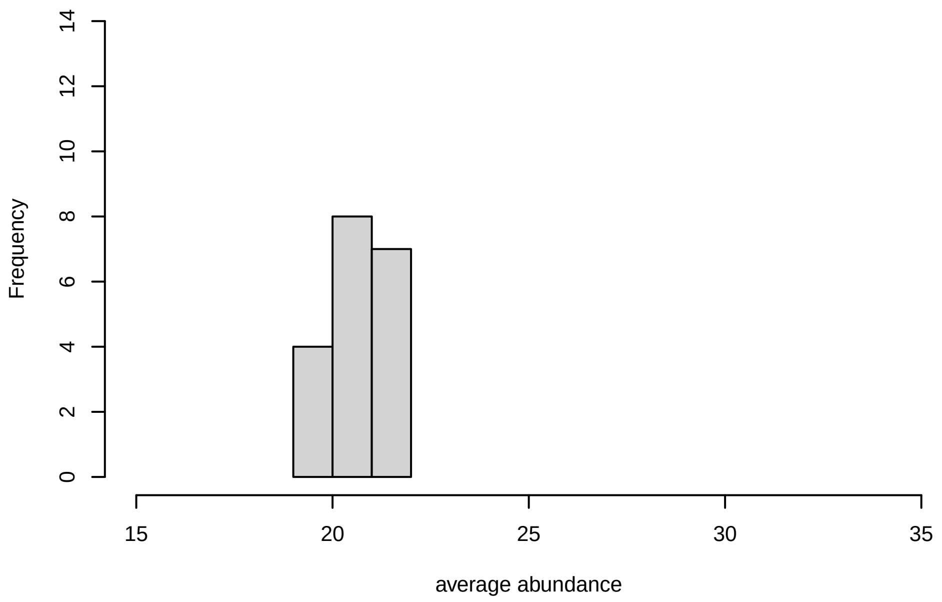

V2, after remove unreliable

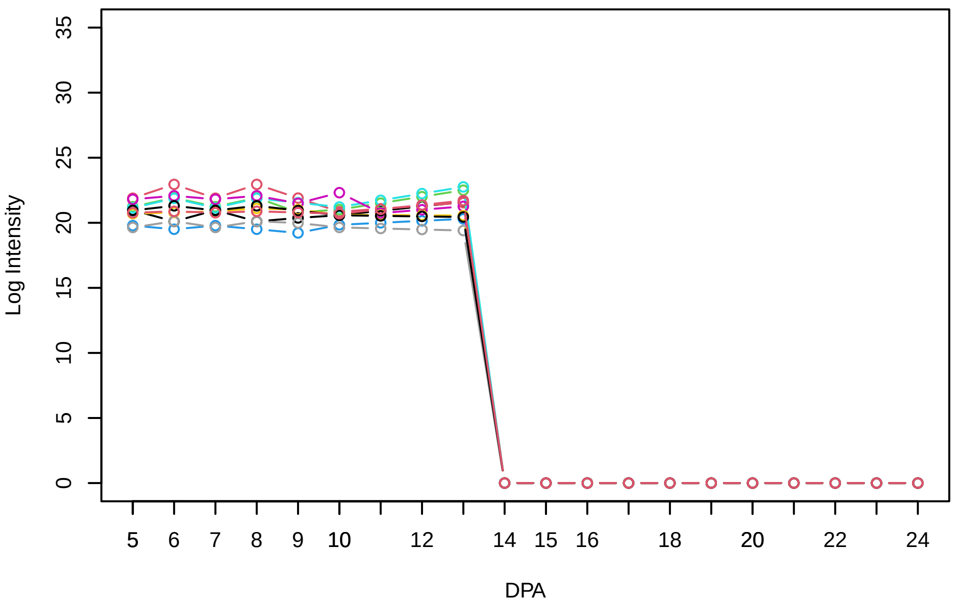

Histogram of the average abundances of reliable proteins

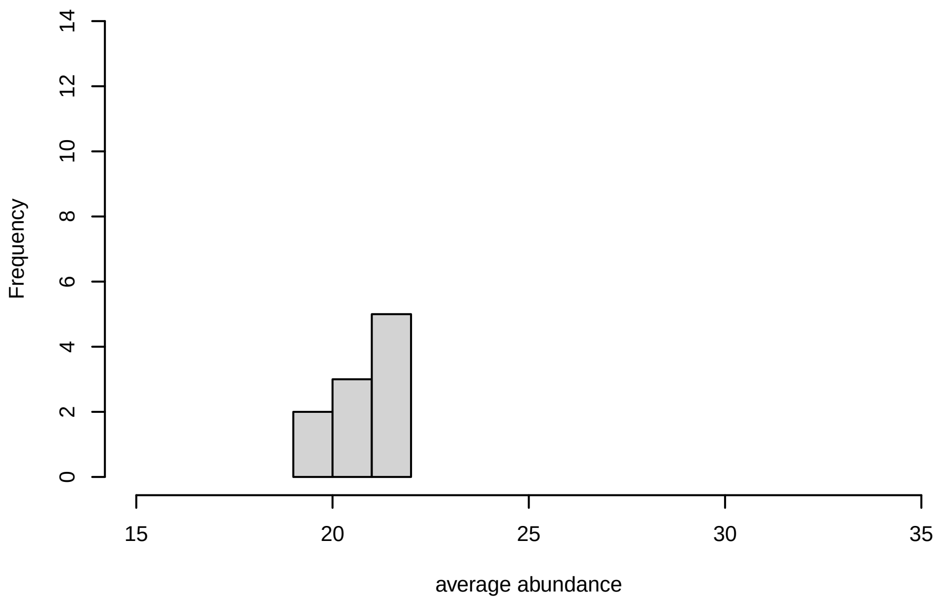

V2, after remove low abundance

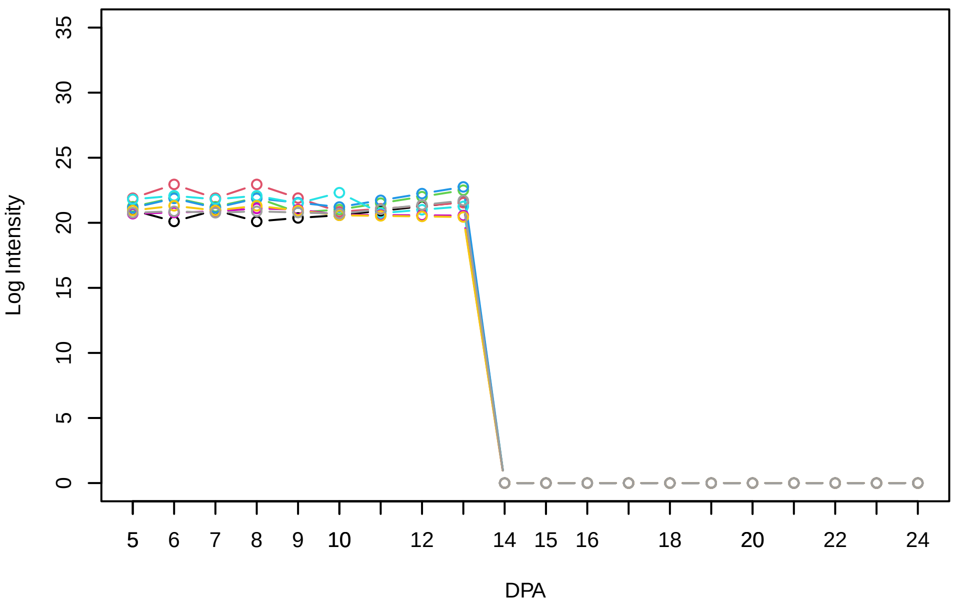

Histogram of the average abundances of high abundance proteins

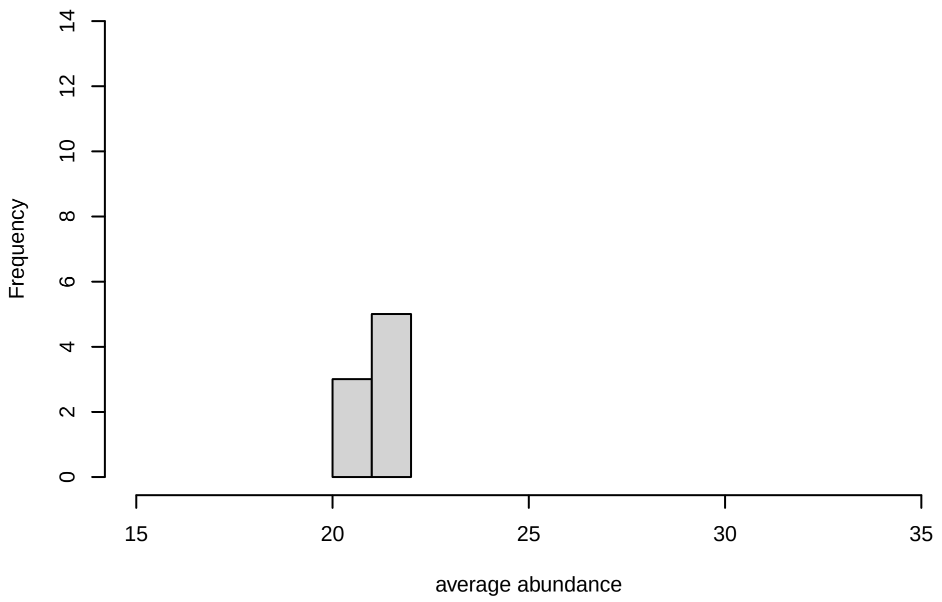

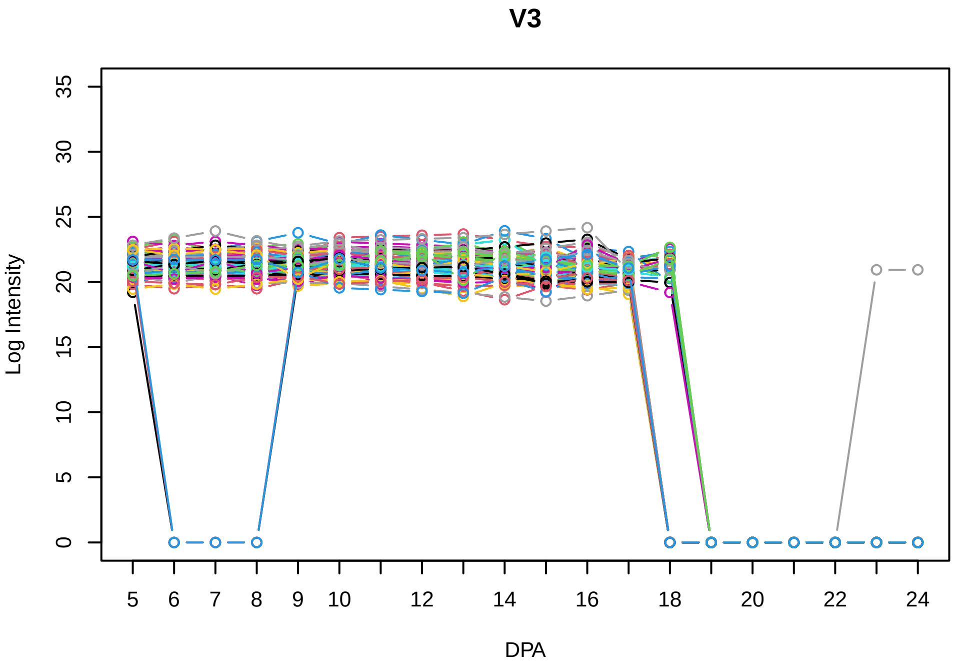
V3
Log Intensity
DPA

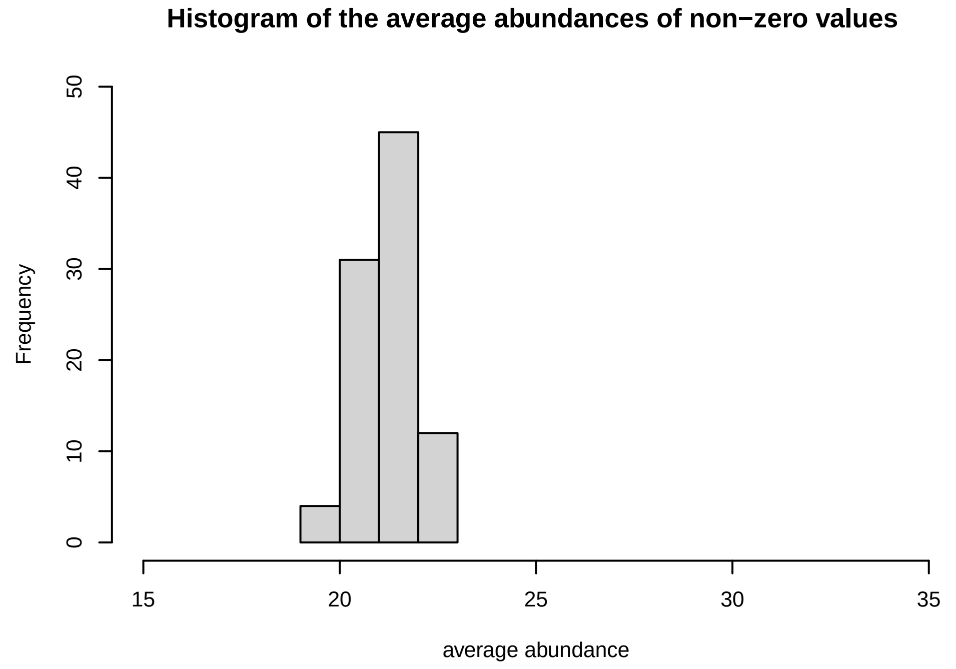
Histogram of the average abundances of non−zero values
Frequency
average abundance

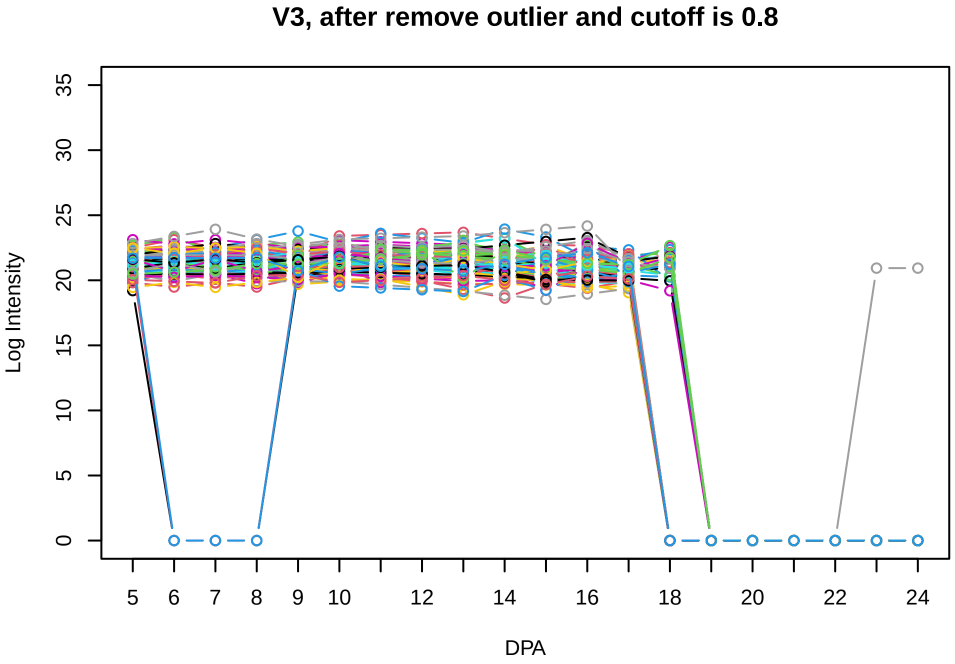
V3, after remove outlier and cutoff is 0.8
Log Intensity
DPA

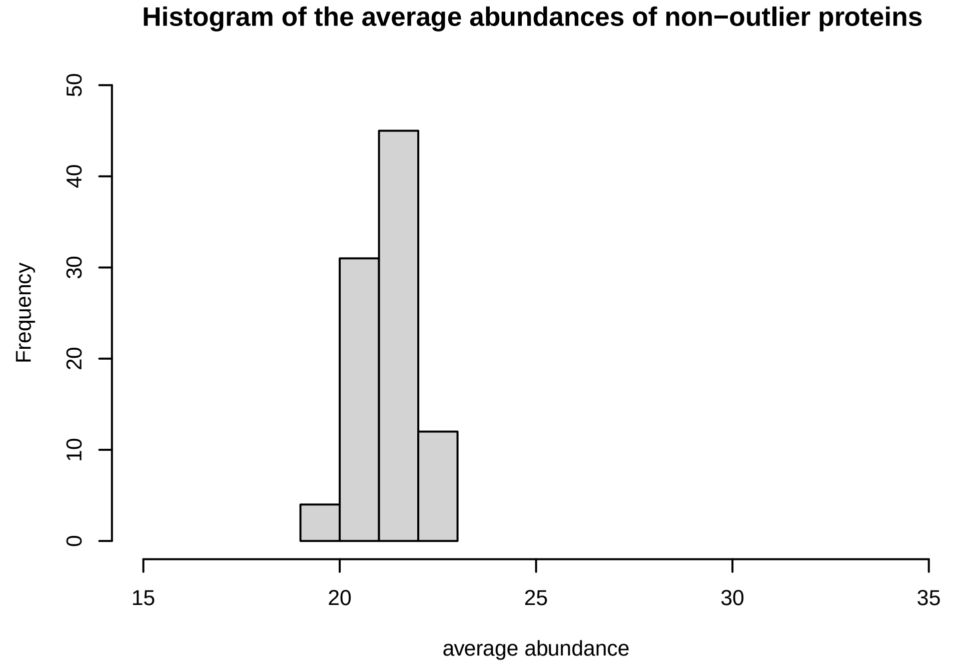
Histogram of the average abundances of non−outlier proteins
Frequency
average abundance

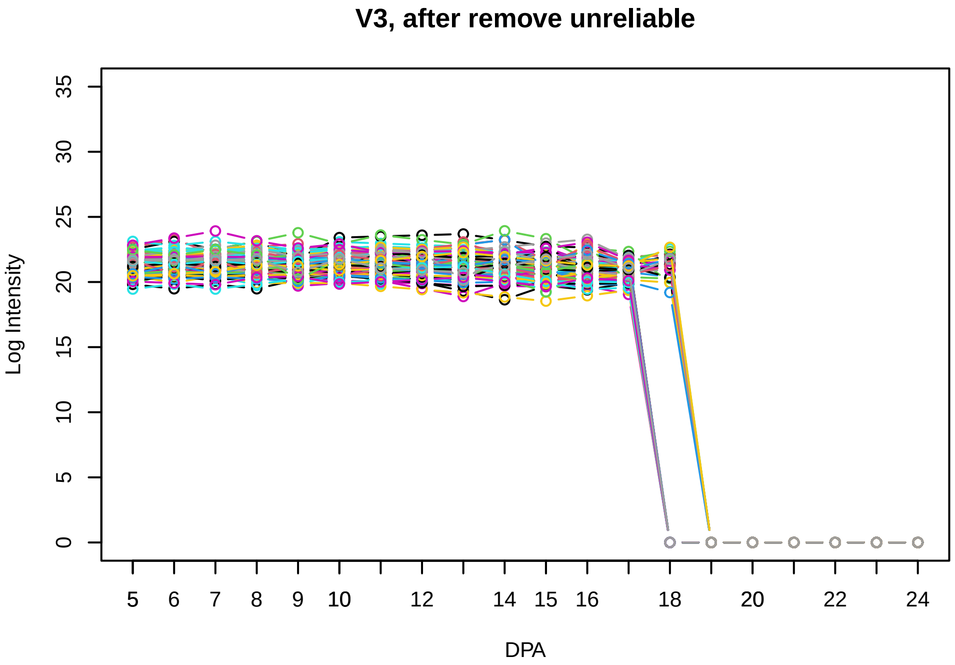
V3, after remove unreliable
Log Intensity
DPA

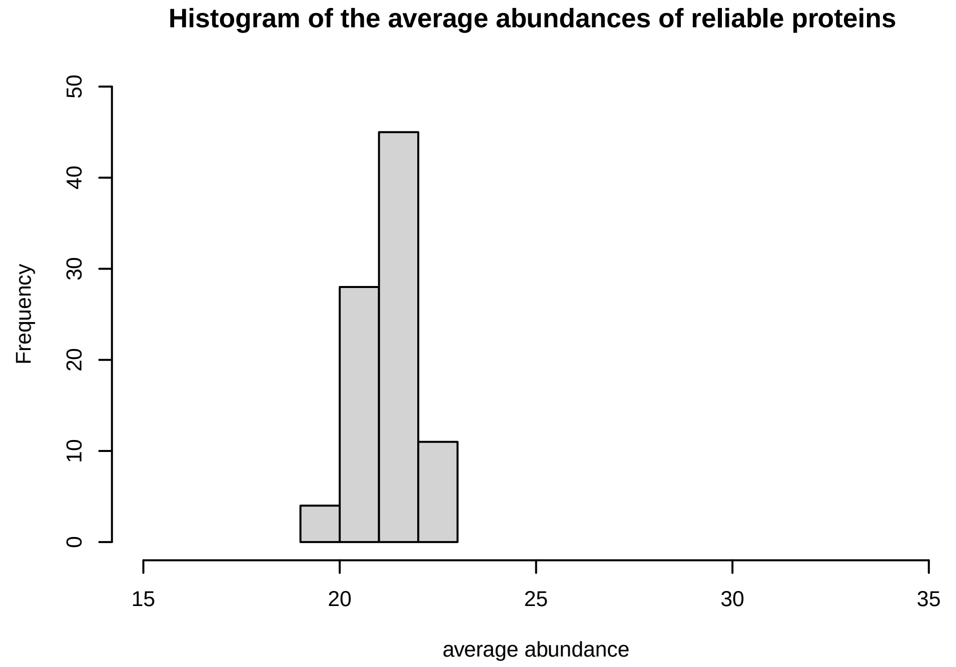
Histogram of the average abundances of reliable proteins
Frequency
average abundance

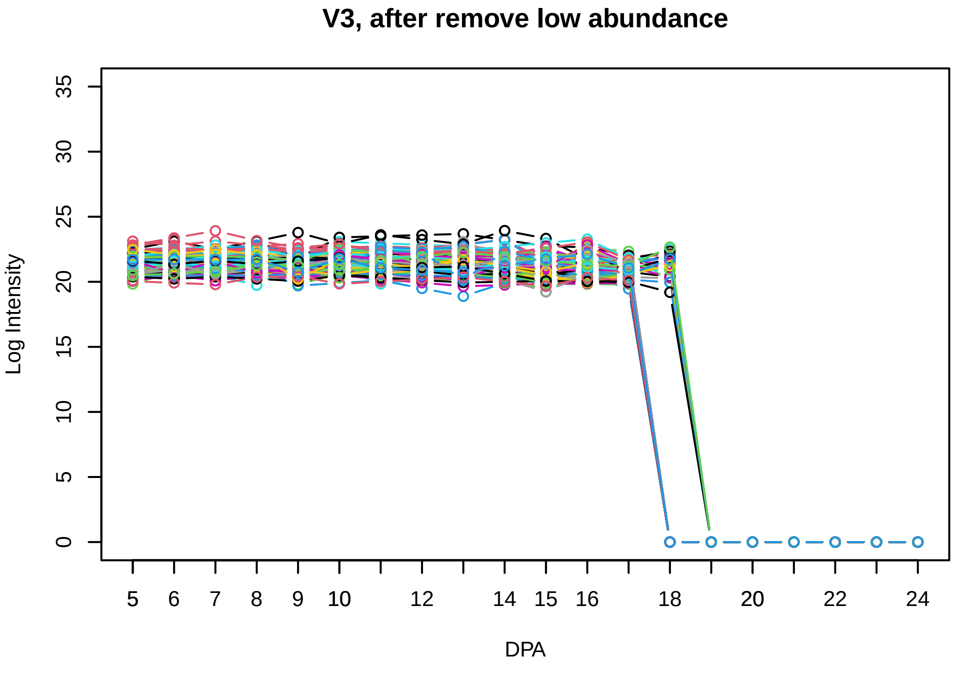
V3, after remove low abundance
Log Intensity
DPA

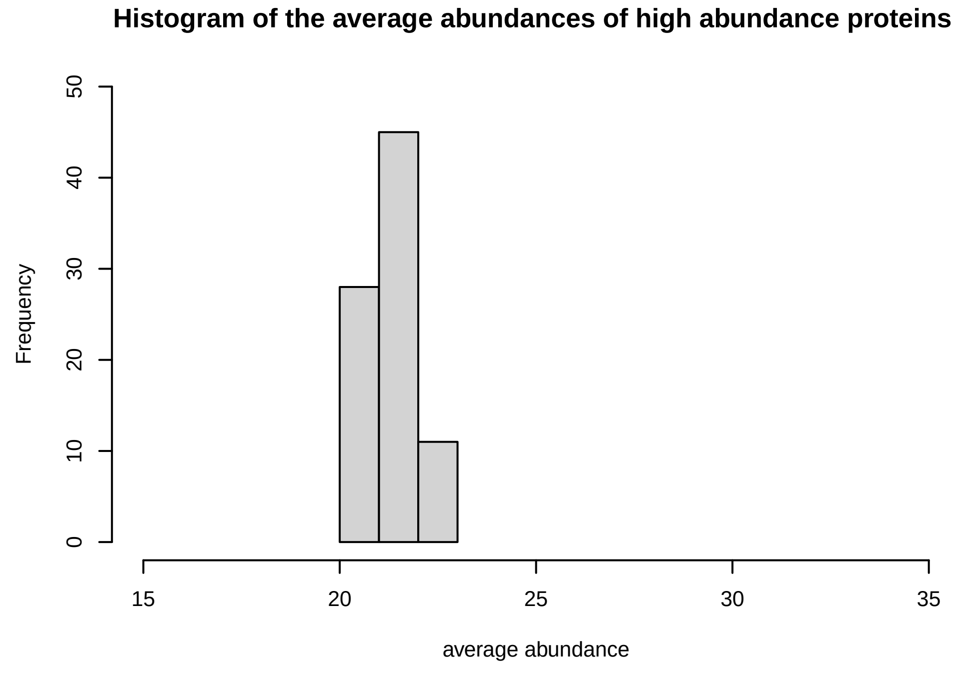
Histogram of the average abundances of high abundance proteins
Frequency
average abundance

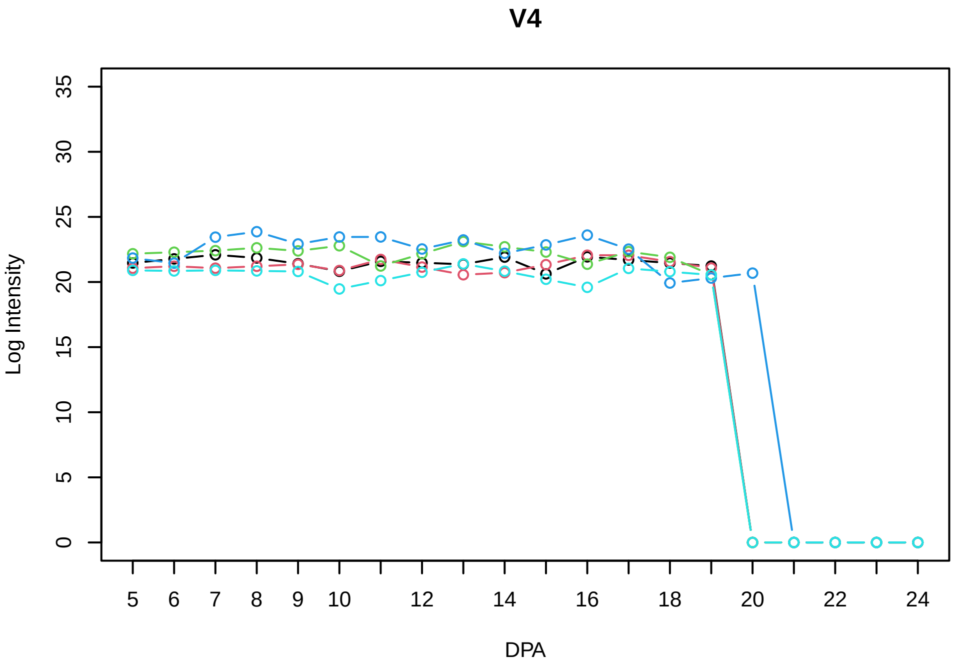
V4
Log Intensity
0 5 10 15 20 25 30 35
5 6 7 8 9 10 12 14 16 18 20 22 24
DPA

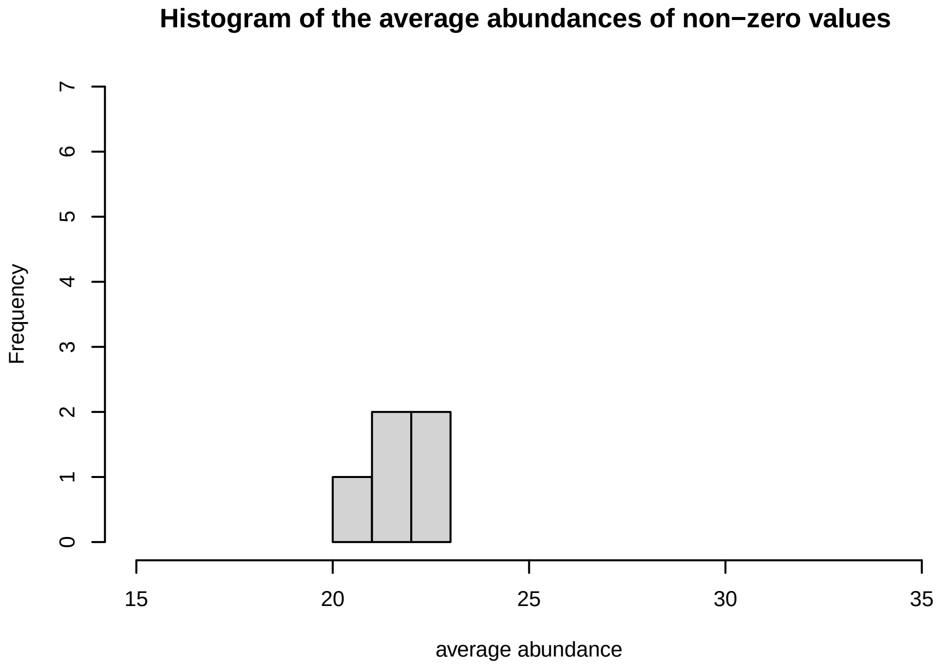
Histogram of the average abundances of non−zero values
Frequency
0 1 2 3 4 5 6 7
15 20 25 30 35
average abundance

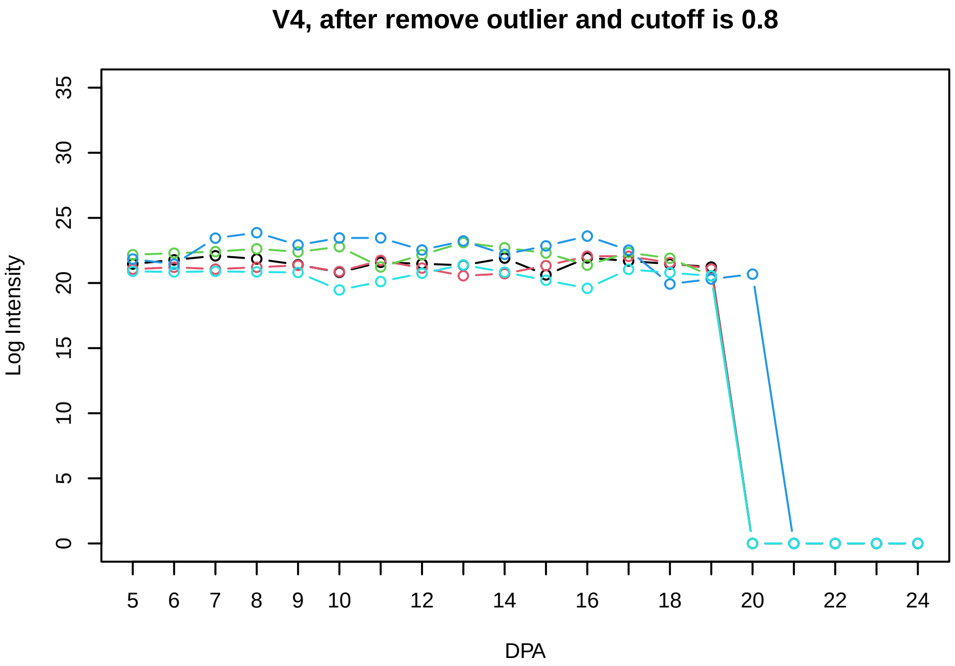
V4, after remove outlier and cutoff is 0.8
Log Intensity
0 5 10 15 20 25 30 35
5 6 7 8 9 10 12 14 16 18 20 22 24
DPA

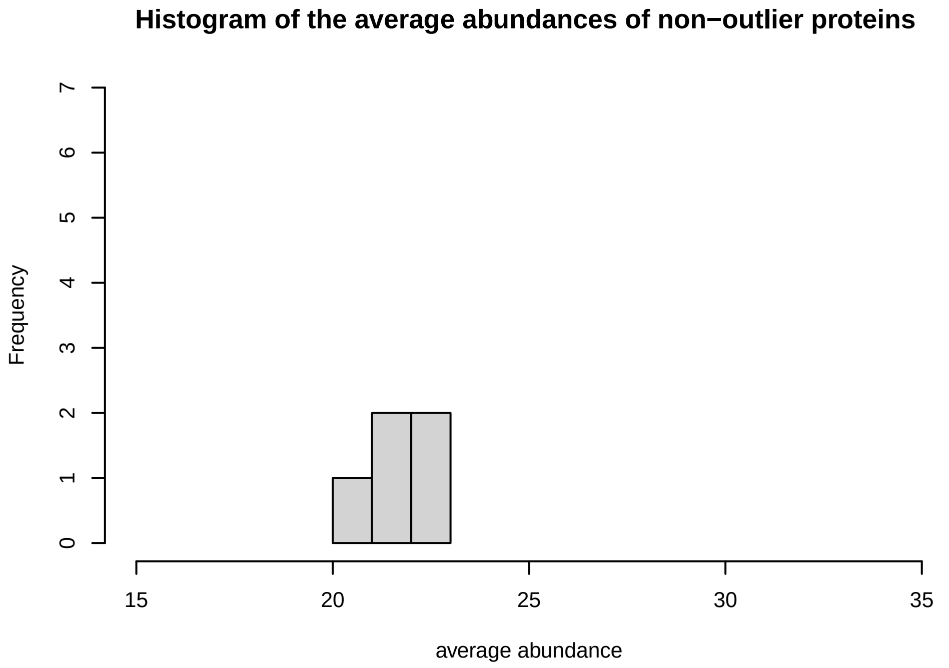
Histogram of the average abundances of non−outlier proteins
Frequency
0 1 2 3 4 5 6 7
15 20 25 30 35
average abundance

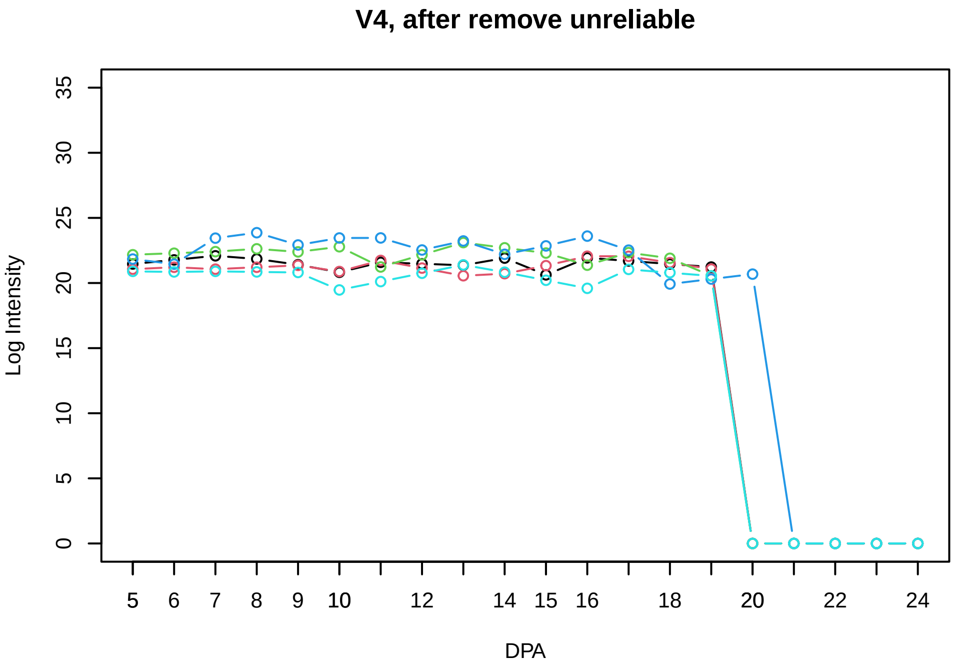
V4, after remove unreliable
Log Intensity
0 5 10 15 20 25 30 35
5 6 7 8 9 10 12 14 15 16 18 20 22 24
DPA

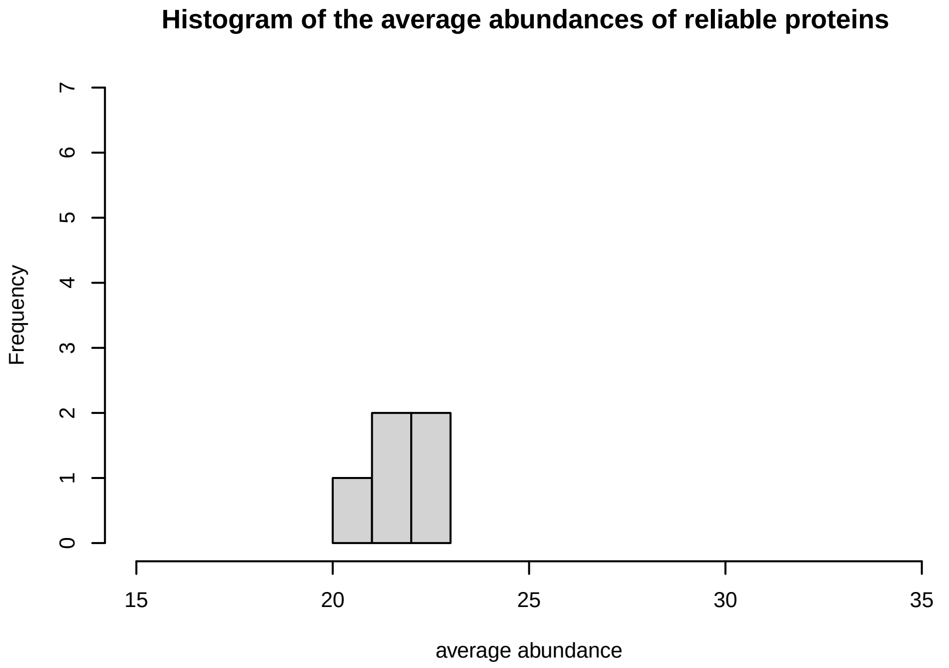
Histogram of the average abundances of reliable proteins
Frequency
0 1 2 3 4 5 6 7
15 20 25 30 35
average abundance

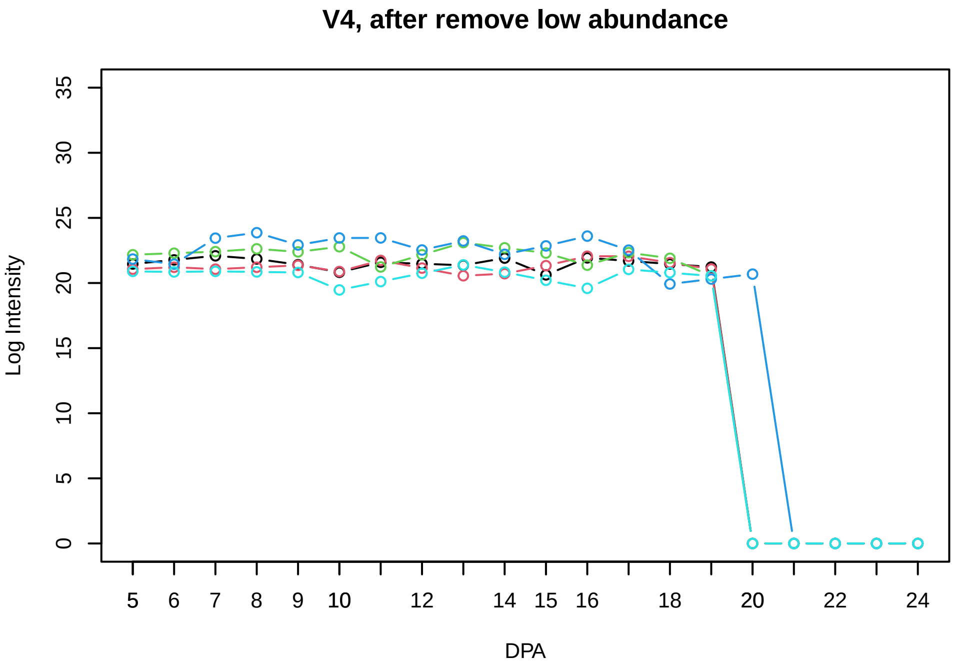
V4, after remove low abundance
Log Intensity
0 5 10 15 20 25 30 35
5 6 7 8 9 10 12 14 15 16 18 20 22 24
DPA

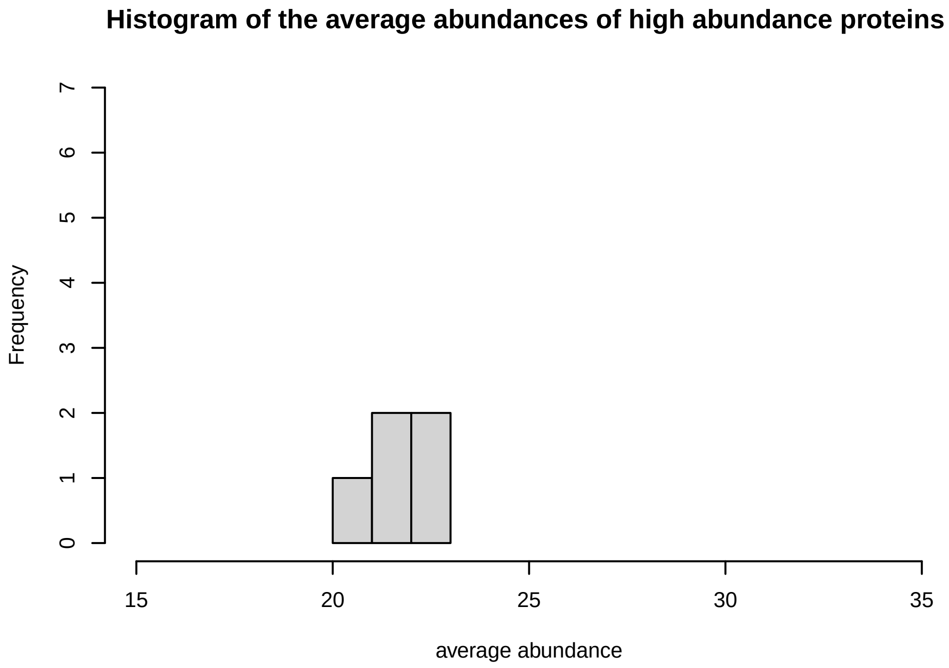
Histogram of the average abundances of high abundance proteins
Frequency
0 1 2 3 4 5 6 7
15 20 25 30 35
average abundance

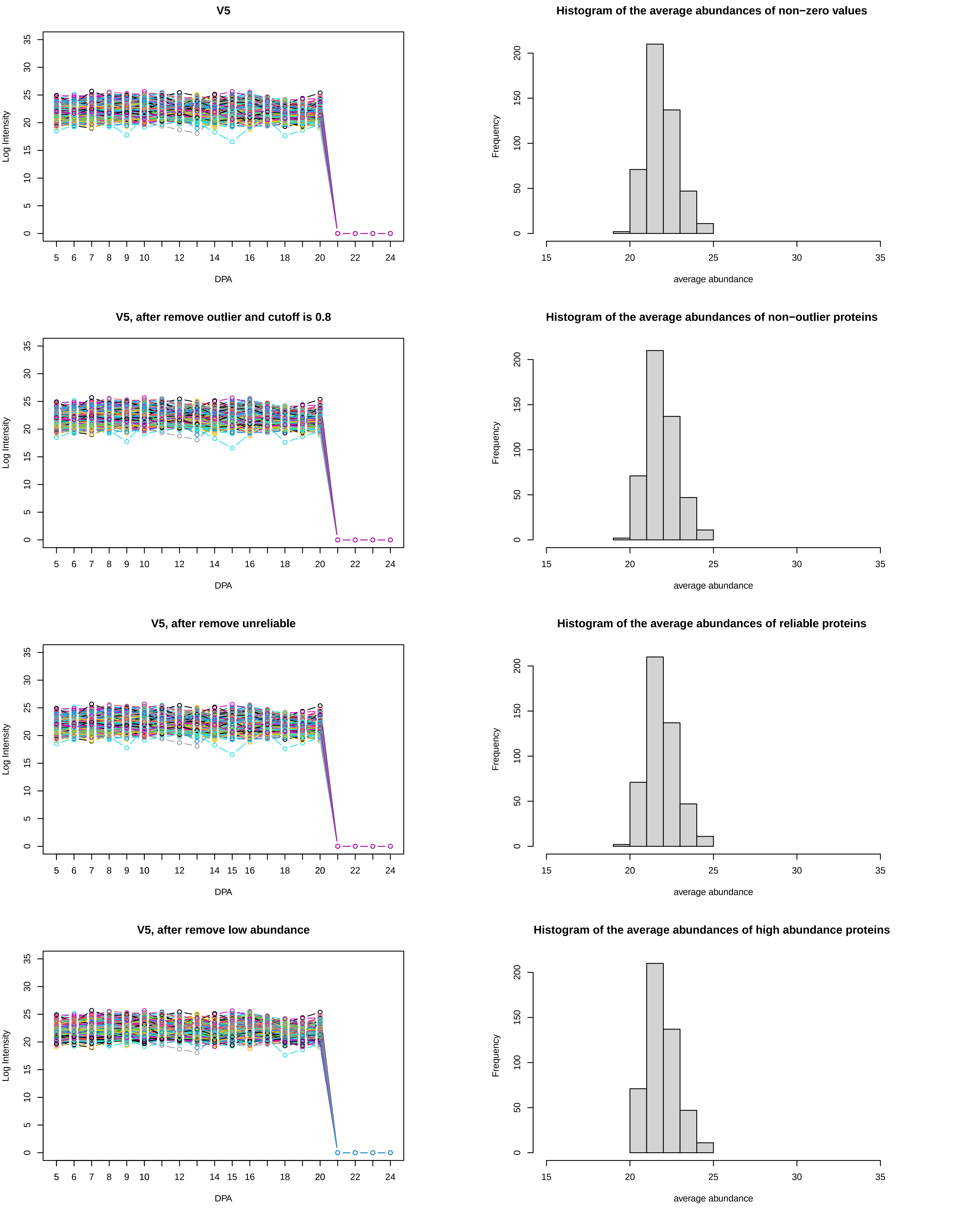
V5
Log Intensity
DPA
Histogram of the average abundances of non−zero values
Frequency
average abundance
V5, after remove outlier and cutoff is 0.8
Log Intensity
DPA
Histogram of the average abundances of non−outlier proteins
Frequency
average abundance
V5, after remove unreliable
Log Intensity
DPA
Histogram of the average abundances of reliable proteins
Frequency
average abundance
V5, after remove low abundance
Log Intensity
DPA
Histogram of the average abundances of high abundance proteins
Frequency
average abundance

V6

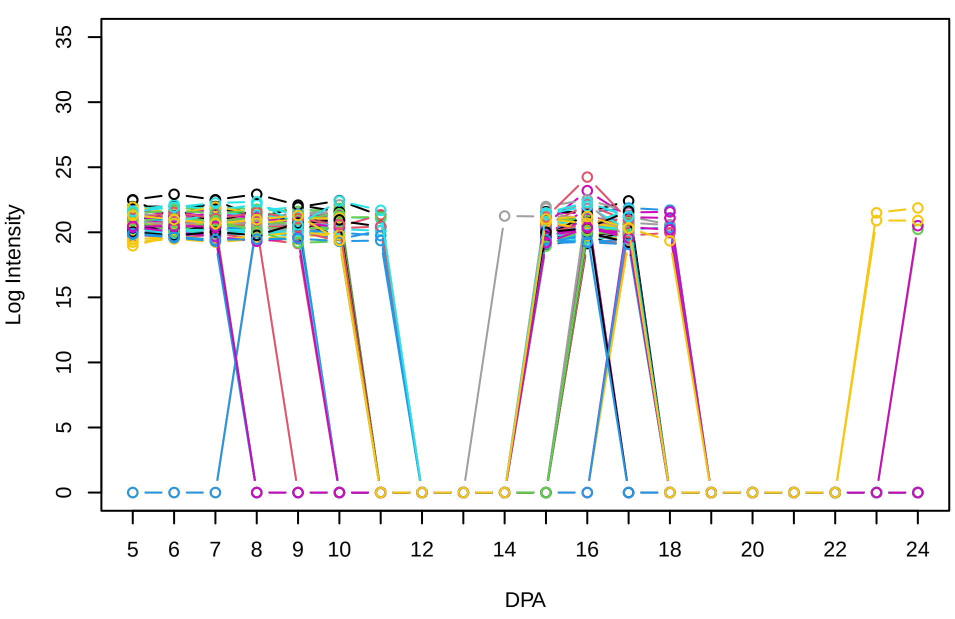

Histogram of the average abundances of non−zero values

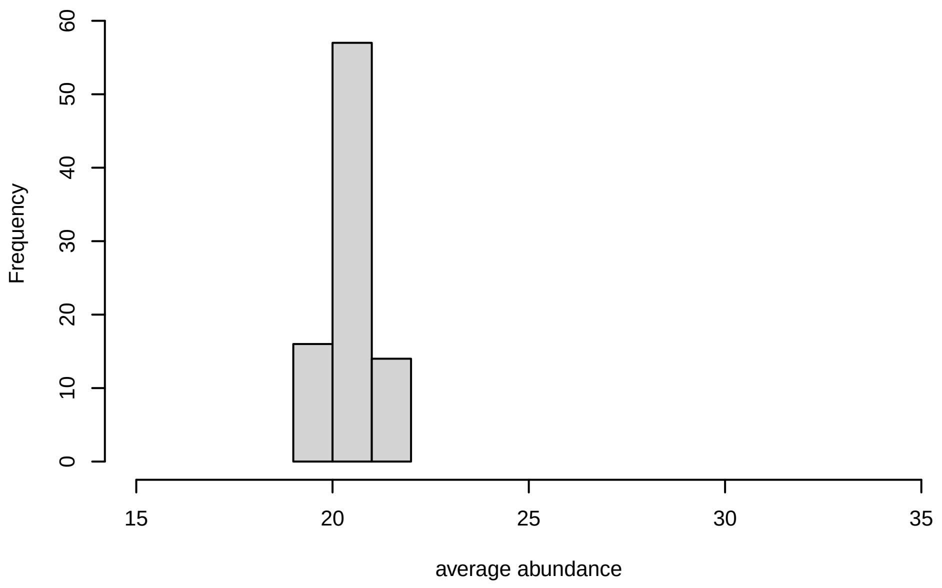

V6, after remove outlier and cutoff is 0.8

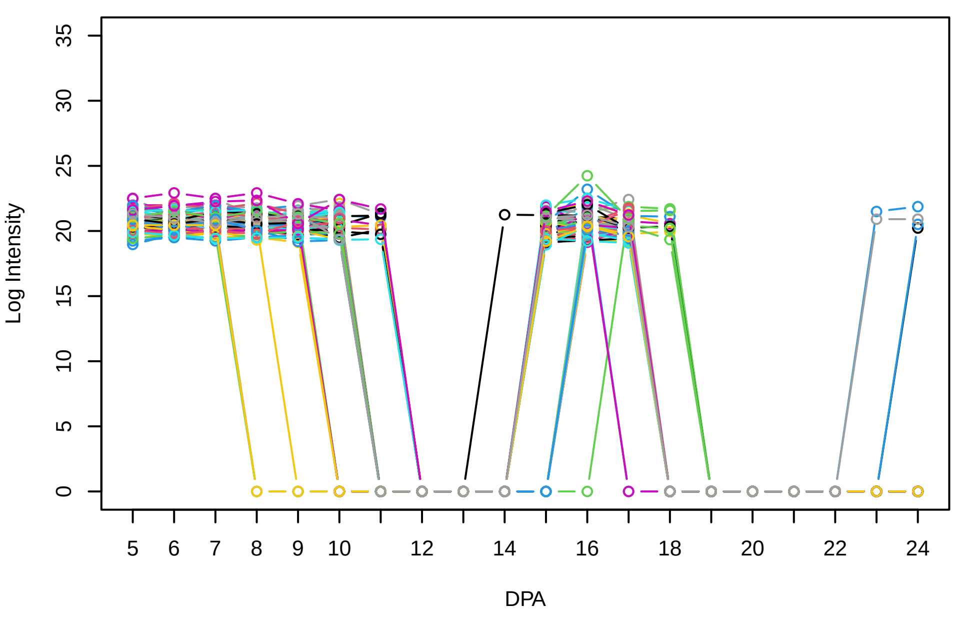

Histogram of the average abundances of non−outlier proteins

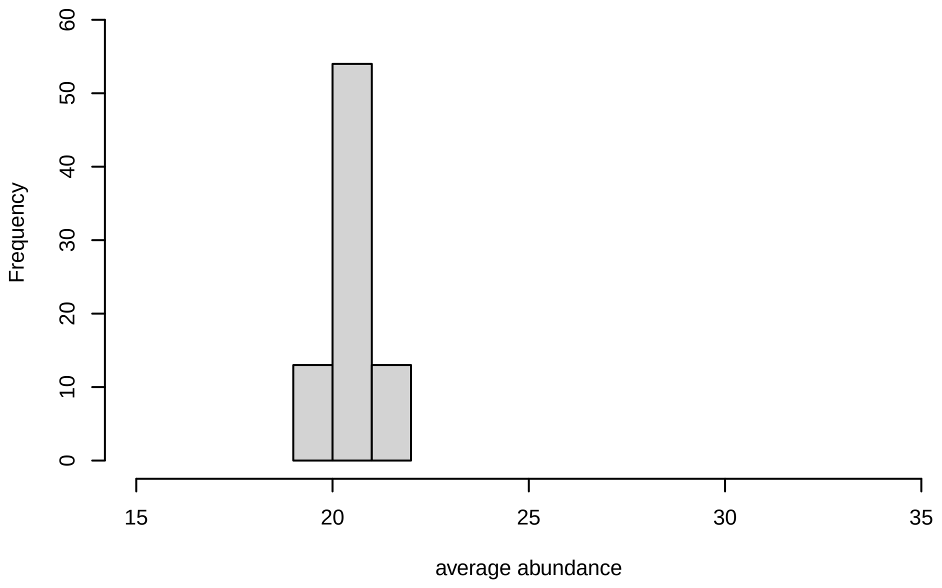

V6, after remove unreliable

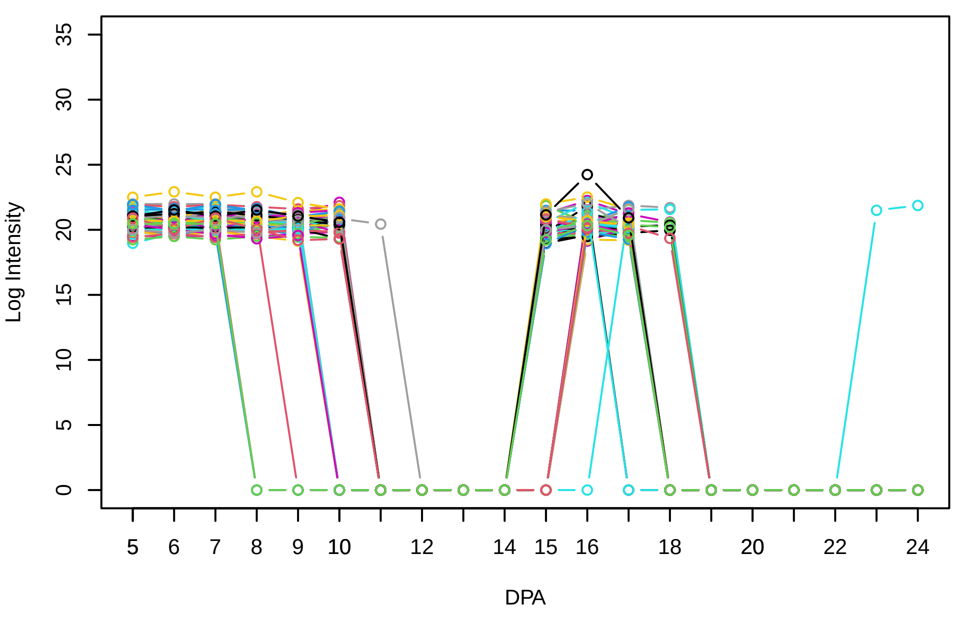

Histogram of the average abundances of reliable proteins

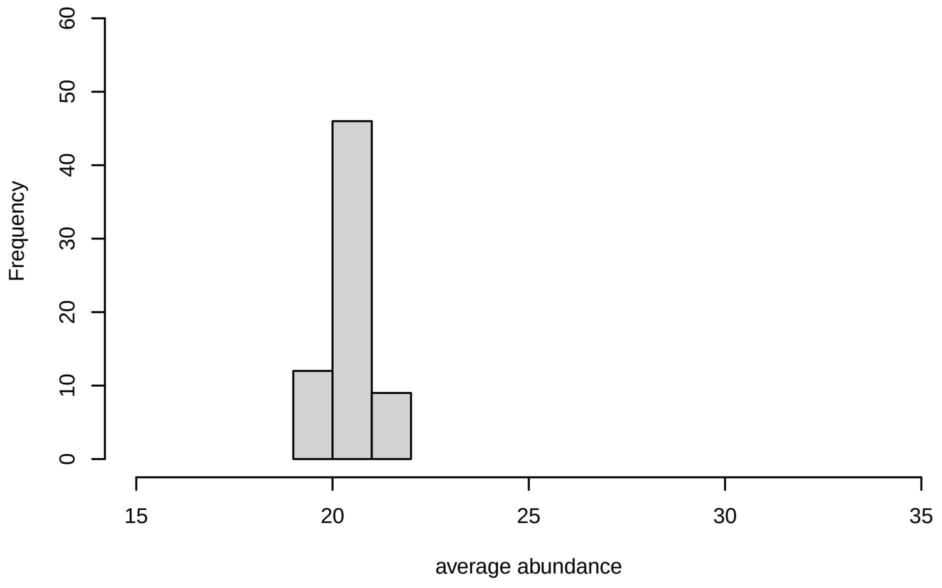

V6, after remove low abundance

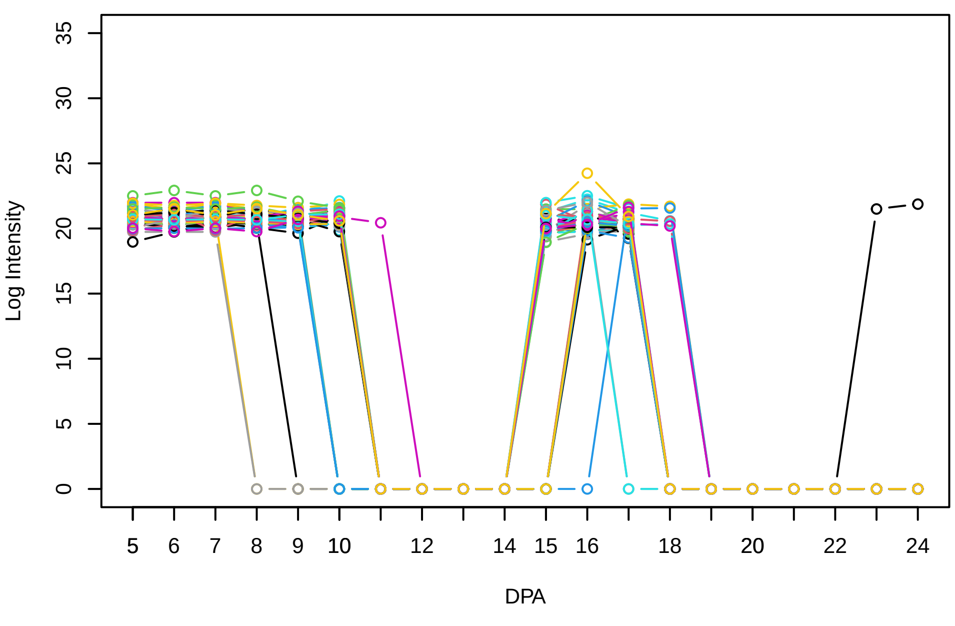

Histogram of the average abundances of high abundance proteins

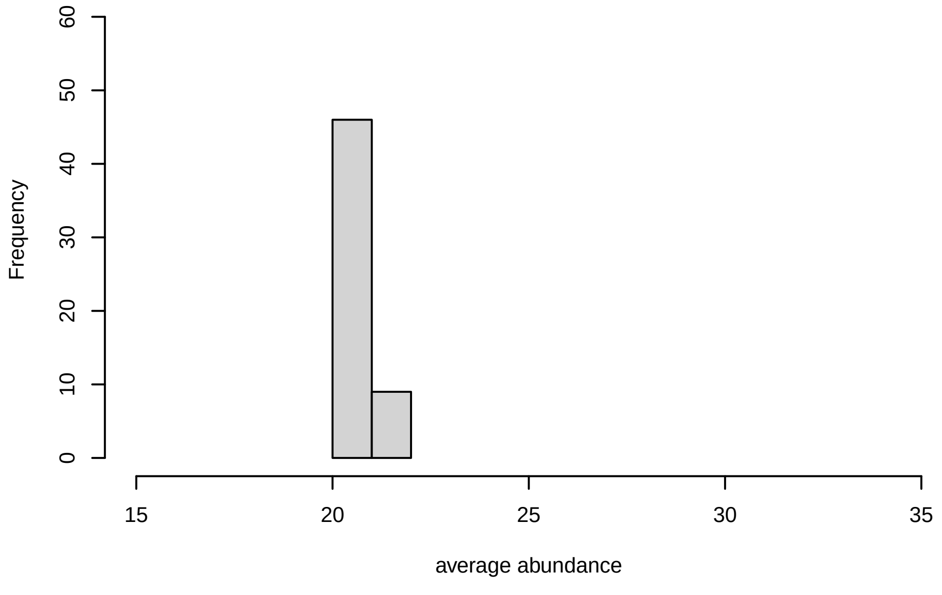

V7

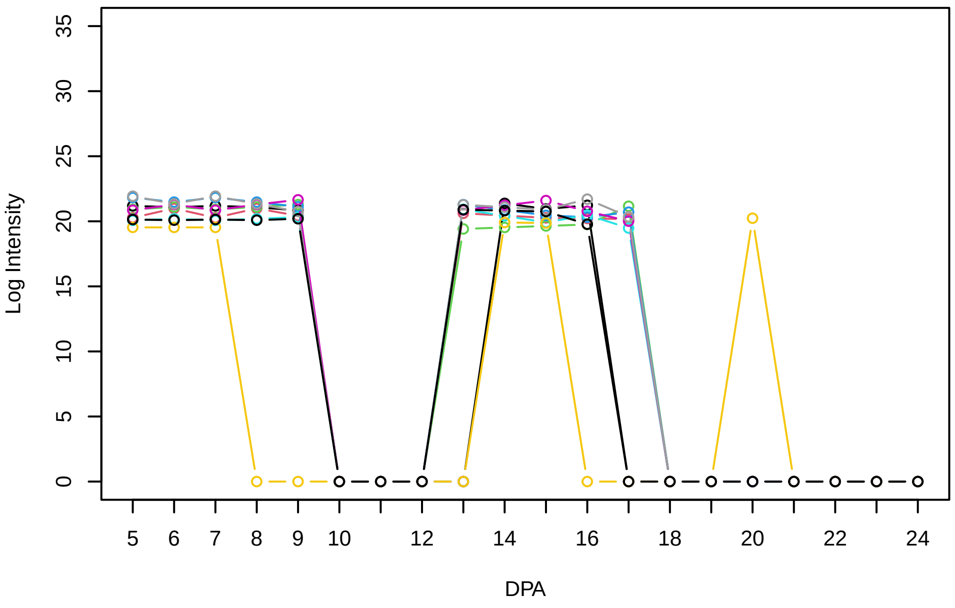

Histogram of the average abundances of non−zero values

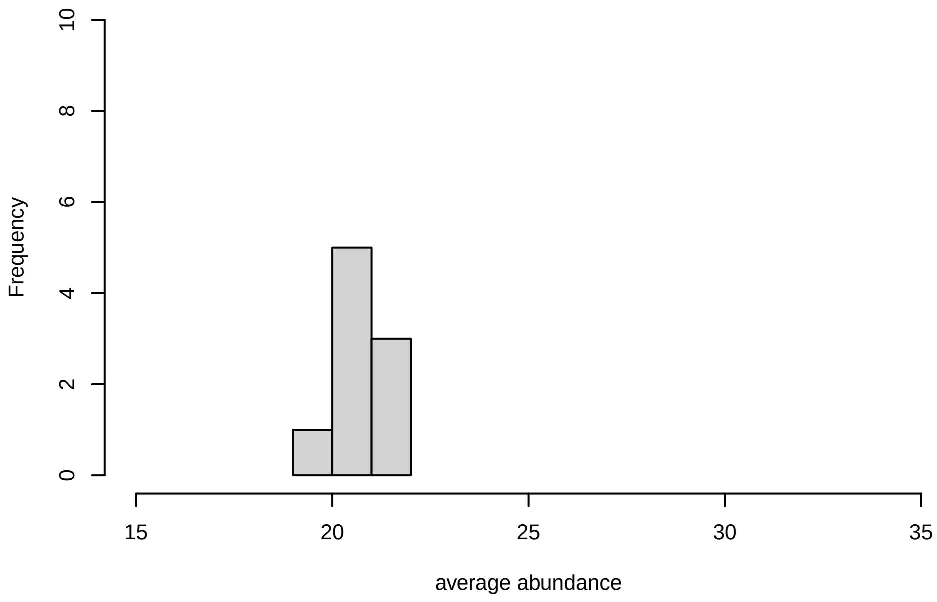

V7, after remove outlier and cutoff is 0.8

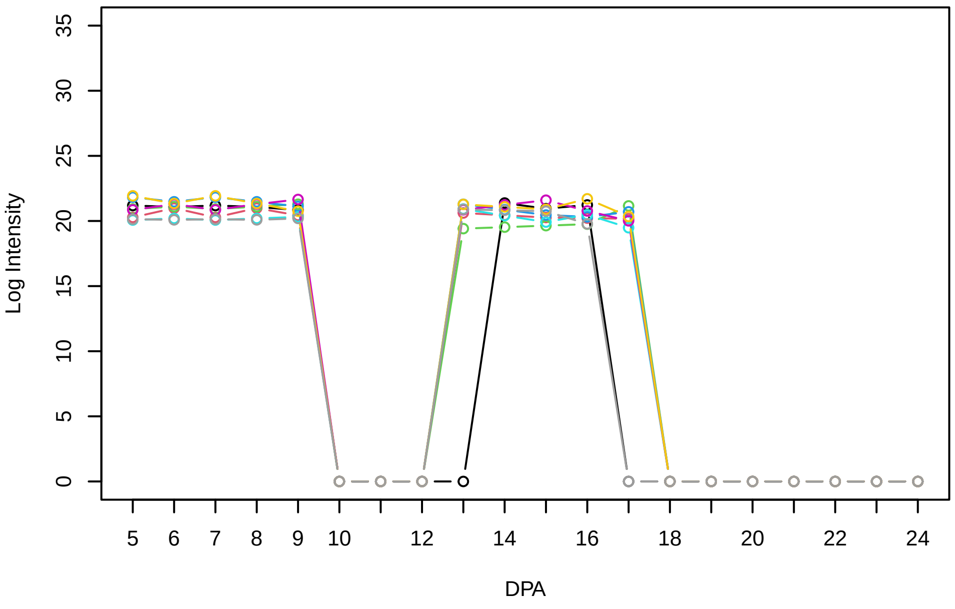

Histogram of the average abundances of non−outlier proteins

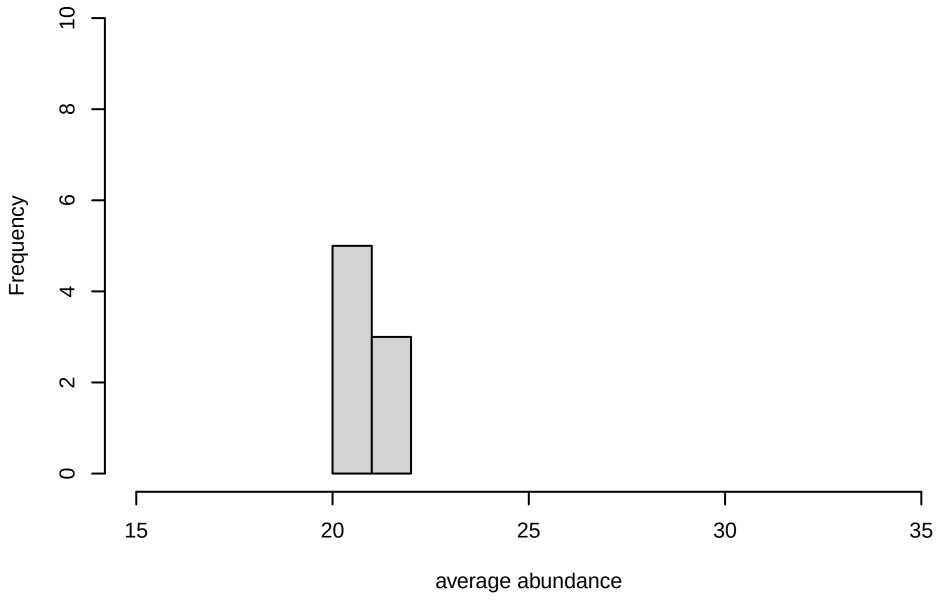

V7, after remove unreliable

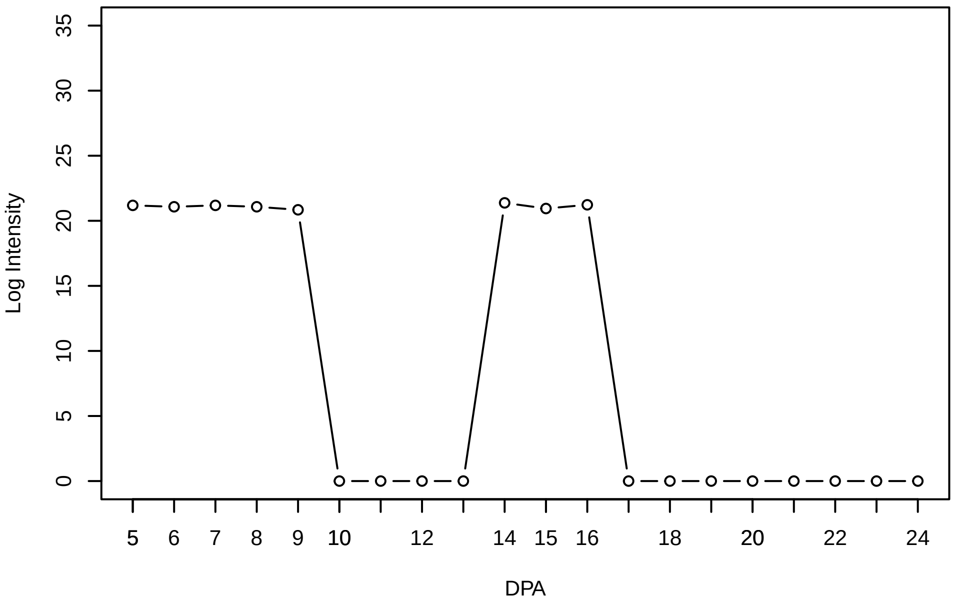

Histogram of the average abundances of reliable proteins

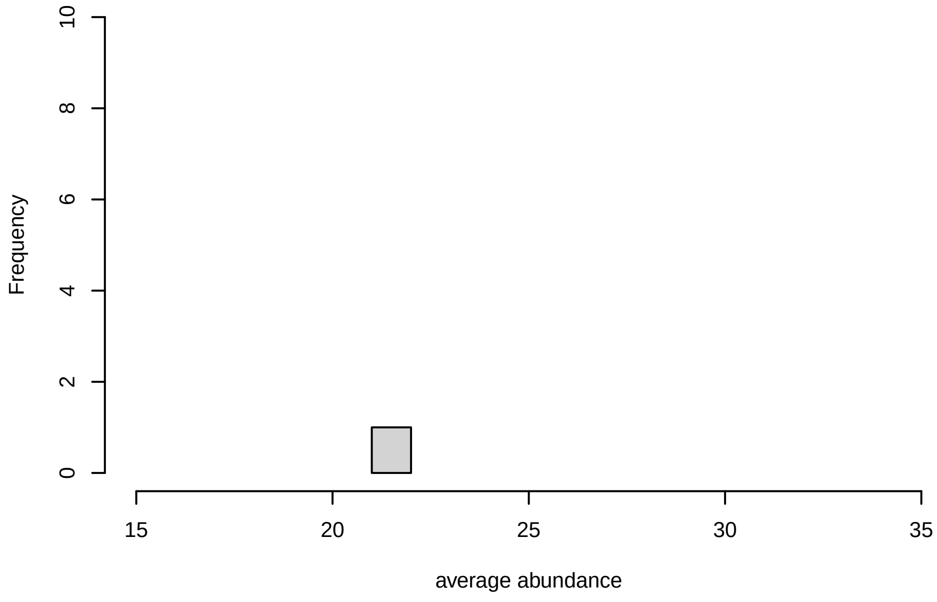

V7, after remove low abundance

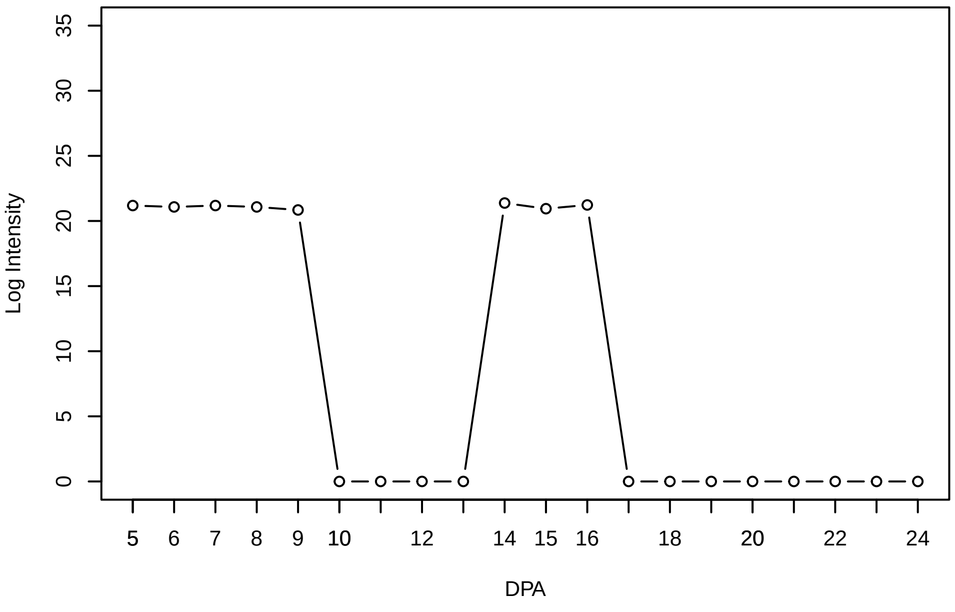

Histogram of the average abundances of high abundance proteins

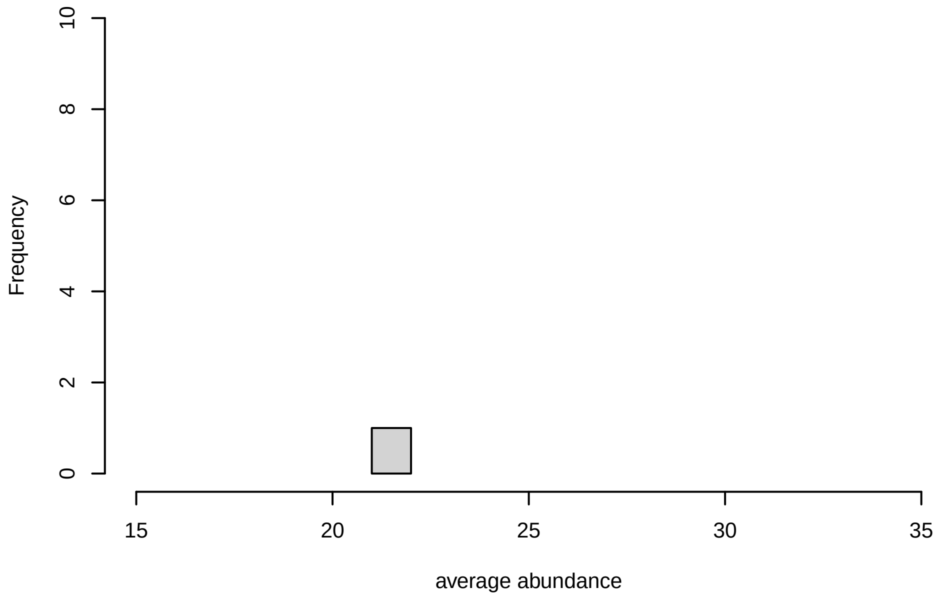

V8

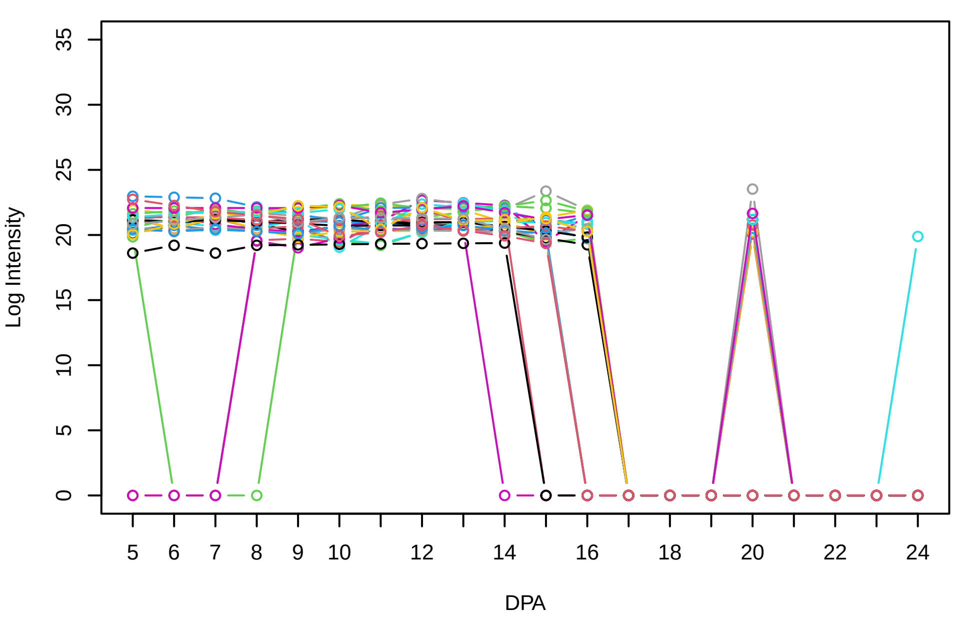

Histogram of the average abundances of non−zero values

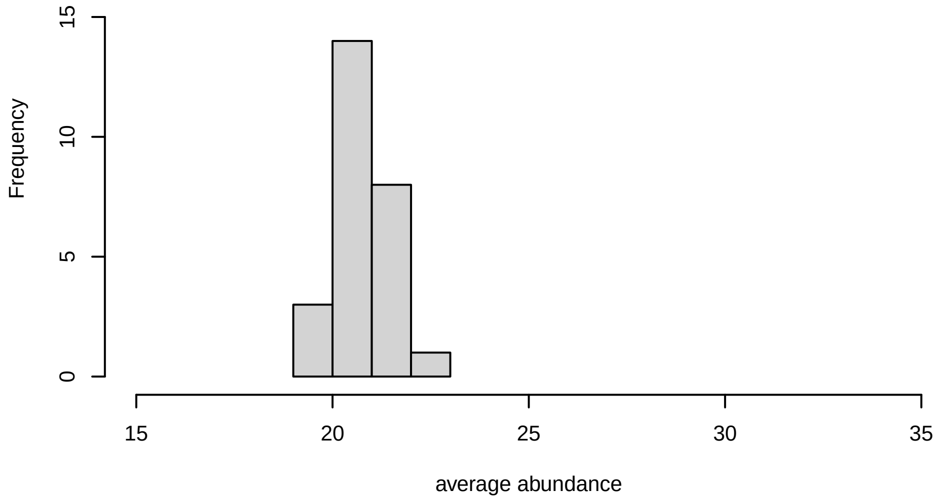

V8, after remove outlier and cutoff is 0.8

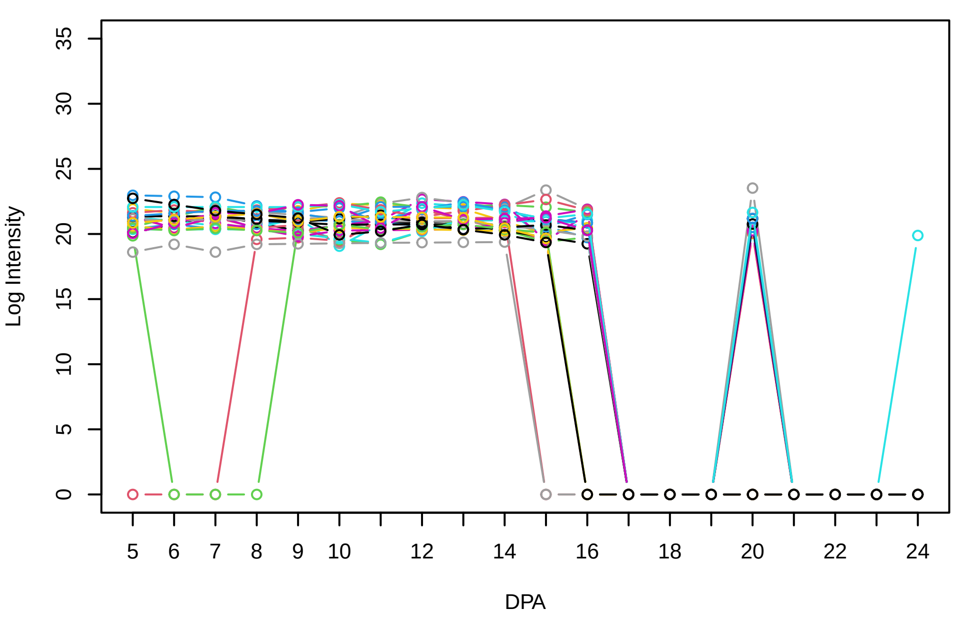

Histogram of the average abundances of non−outlier proteins

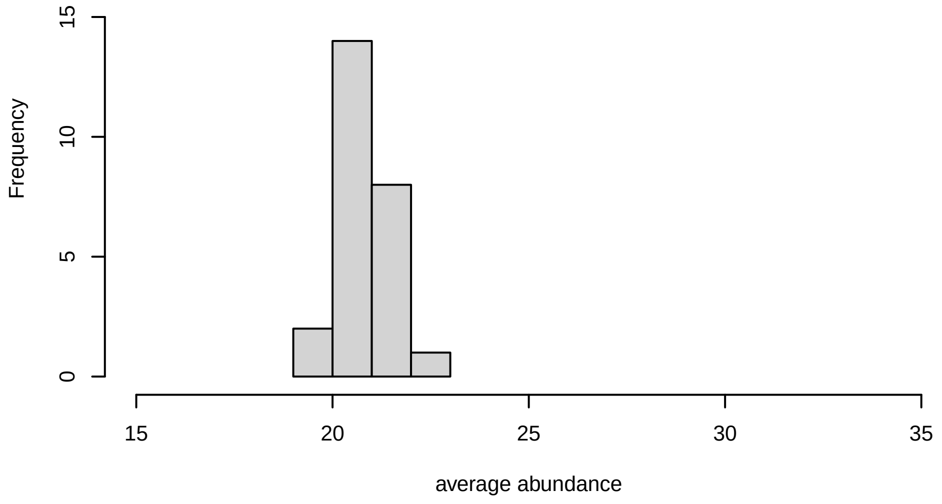

V8, after remove unreliable

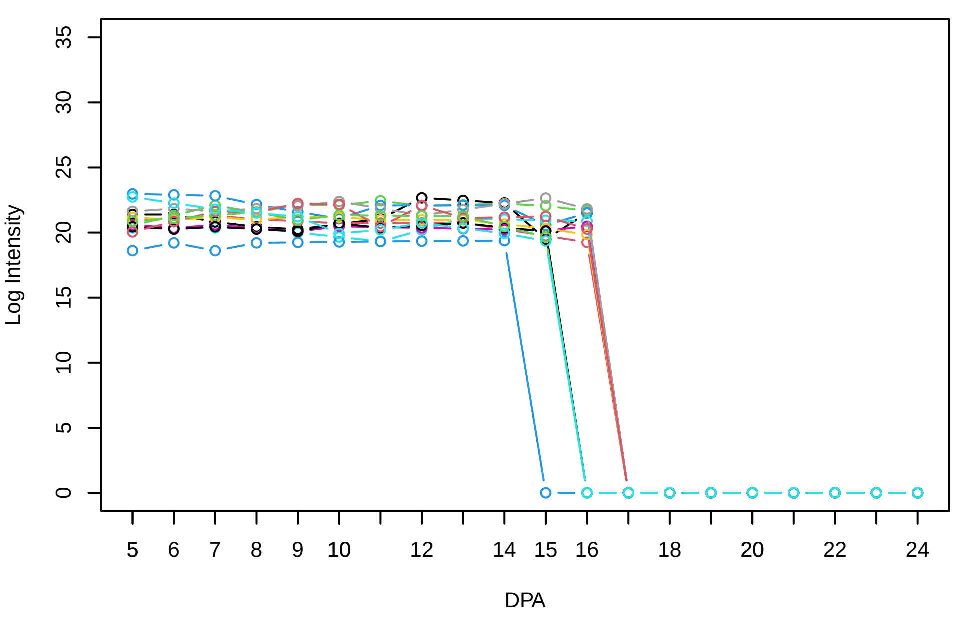

Histogram of the average abundances of reliable proteins

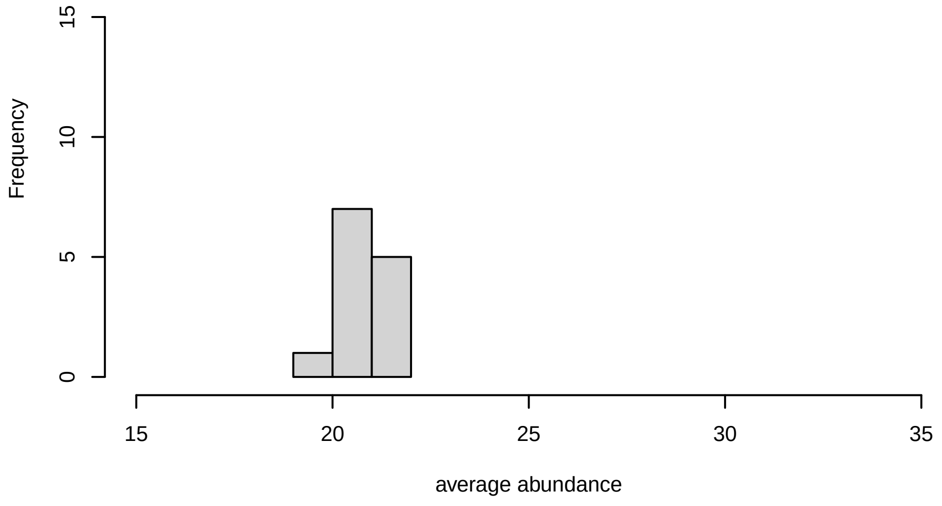

V8, after remove low abundance

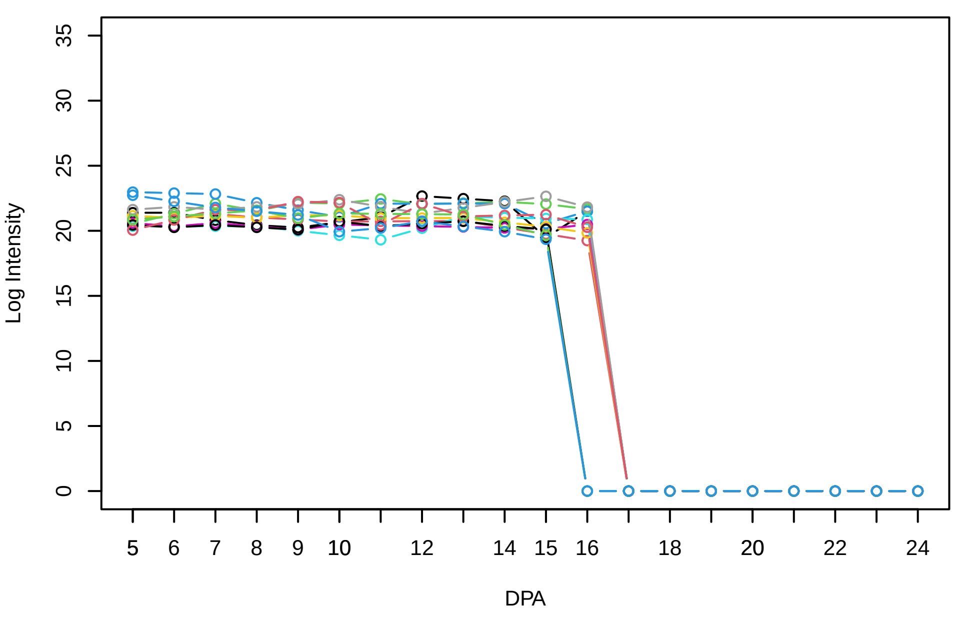

Histogram of the average abundances of high abundance proteins

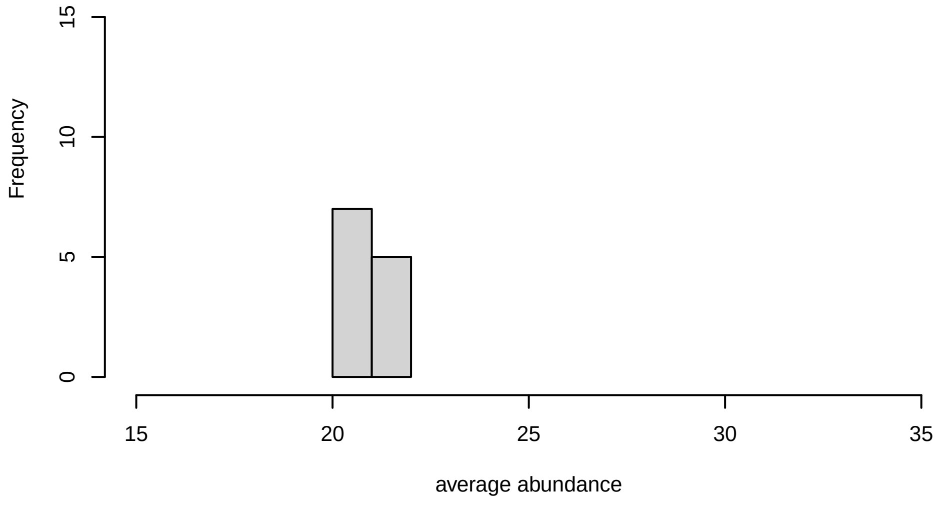

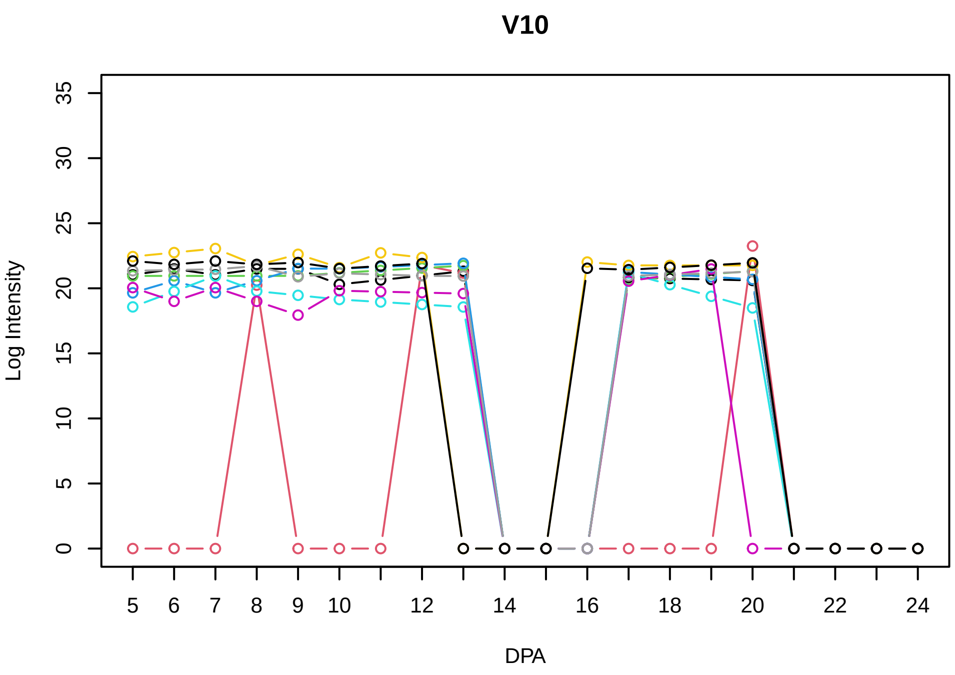
V10
Log Intensity
DPA
0
5
10
15
20
25
30
35
5
6
7
8
9
10
12
14
16
18
20
22
24

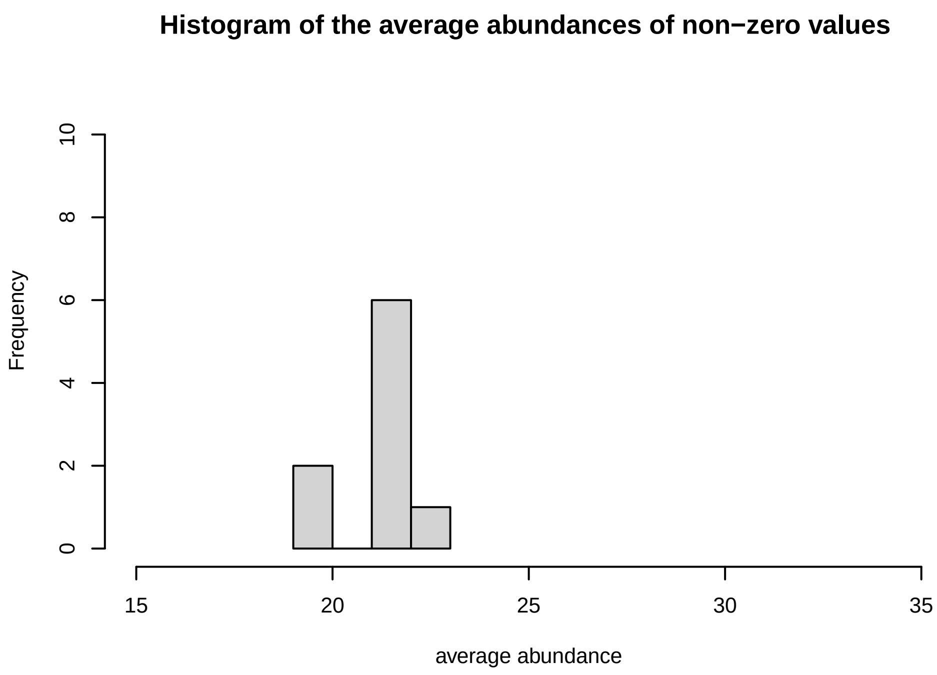
Histogram of the average abundances of non−zero values
Frequency
average abundance
0
2
4
6
8
10
15
20
25
30
35

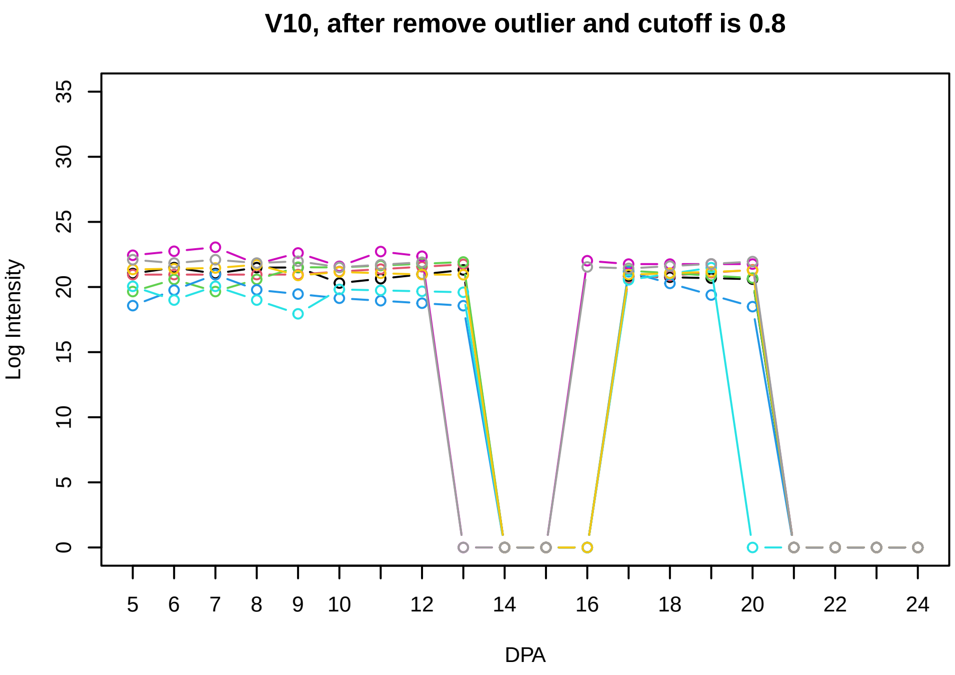
V10, after remove outlier and cutoff is 0.8
Log Intensity
DPA
0
5
10
15
20
25
30
35
5
6
7
8
9
10
12
14
16
18
20
22
24

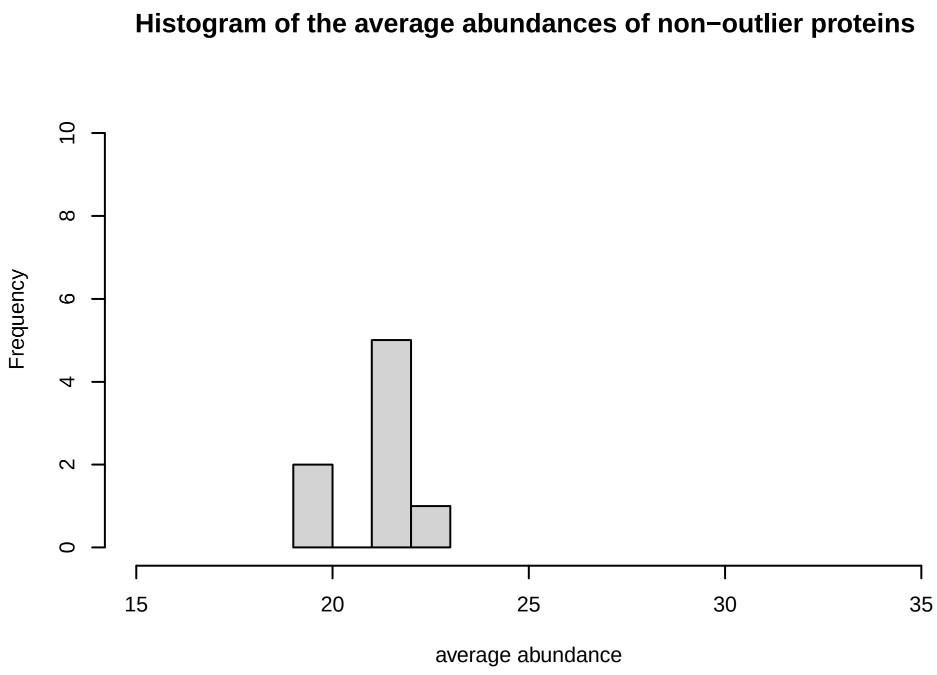
Histogram of the average abundances of non−outlier proteins
Frequency
average abundance
0
2
4
6
8
10
15
20
25
30
35

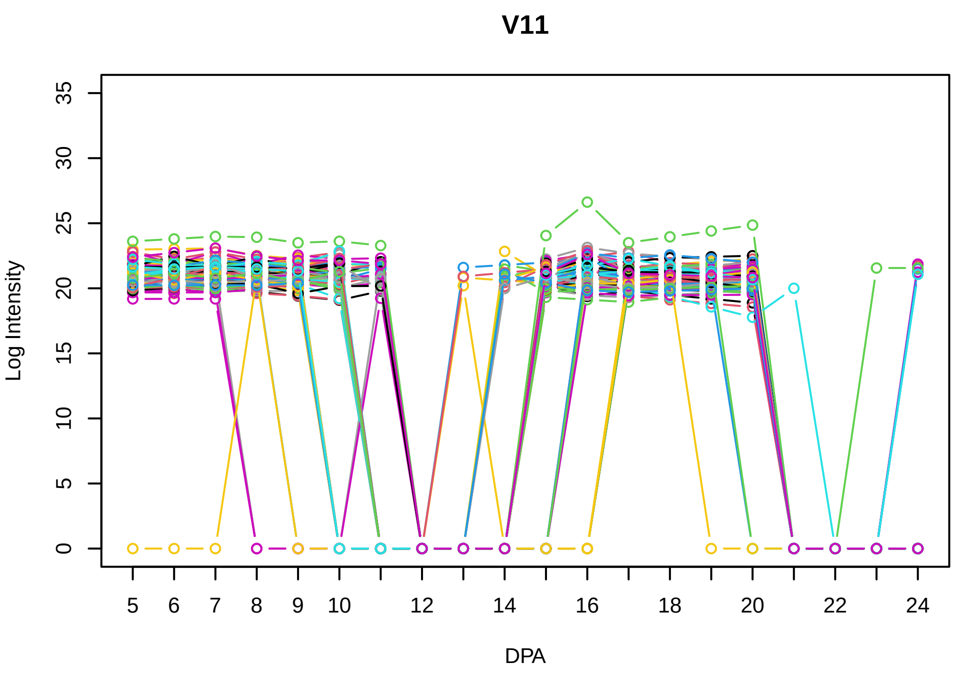
V11
Log Intensity
DPA

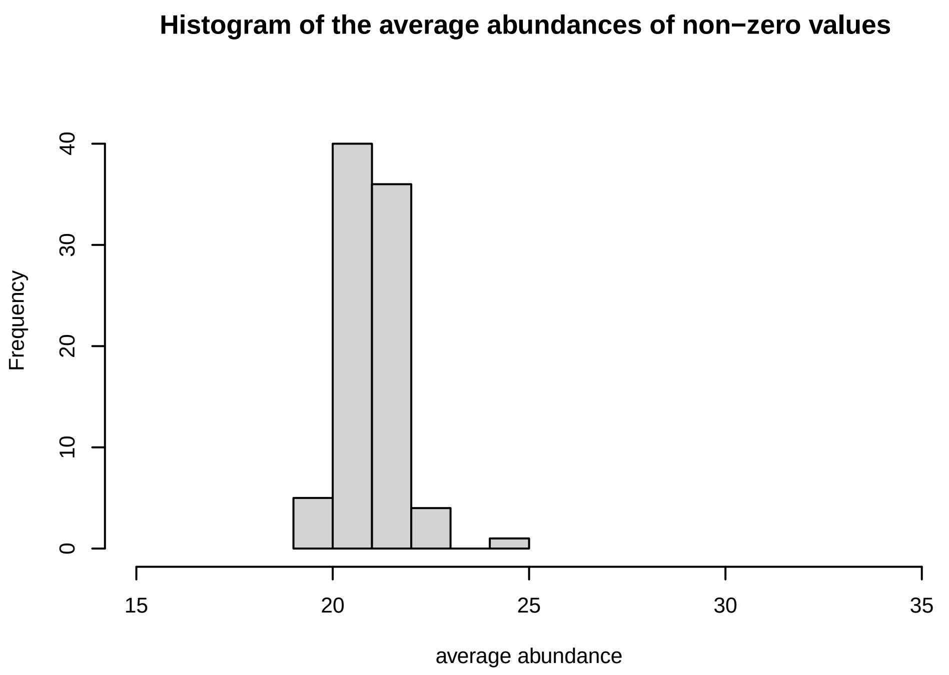
Histogram of the average abundances of non−zero values
Frequency
average abundance

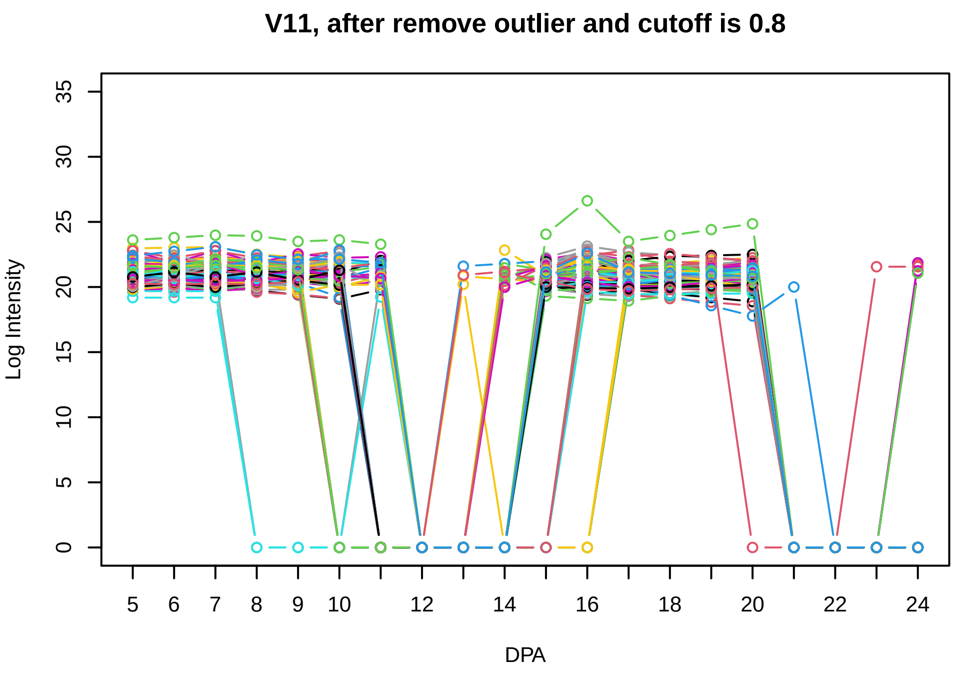
V11, after remove outlier and cutoff is 0.8
Log Intensity
DPA

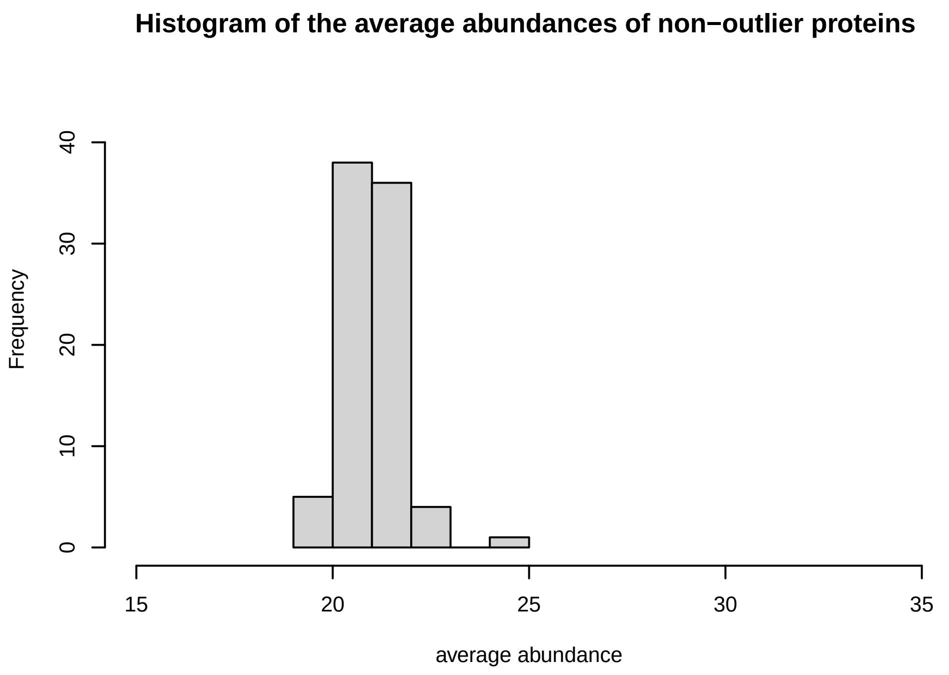
Histogram of the average abundances of non−outlier proteins
Frequency
average abundance

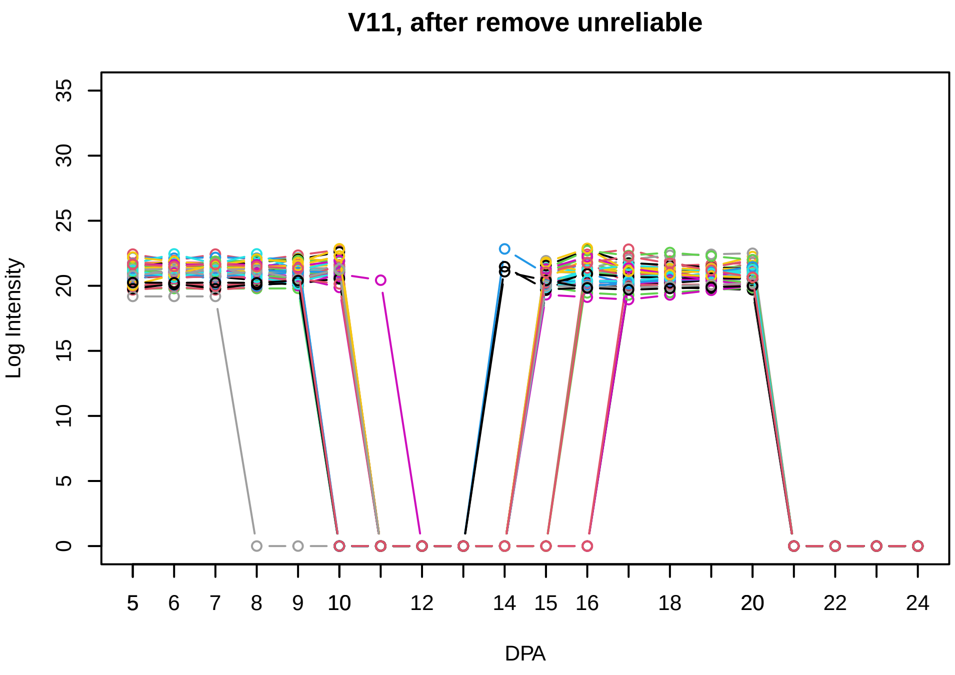
V11, after remove unreliable
Log Intensity
DPA

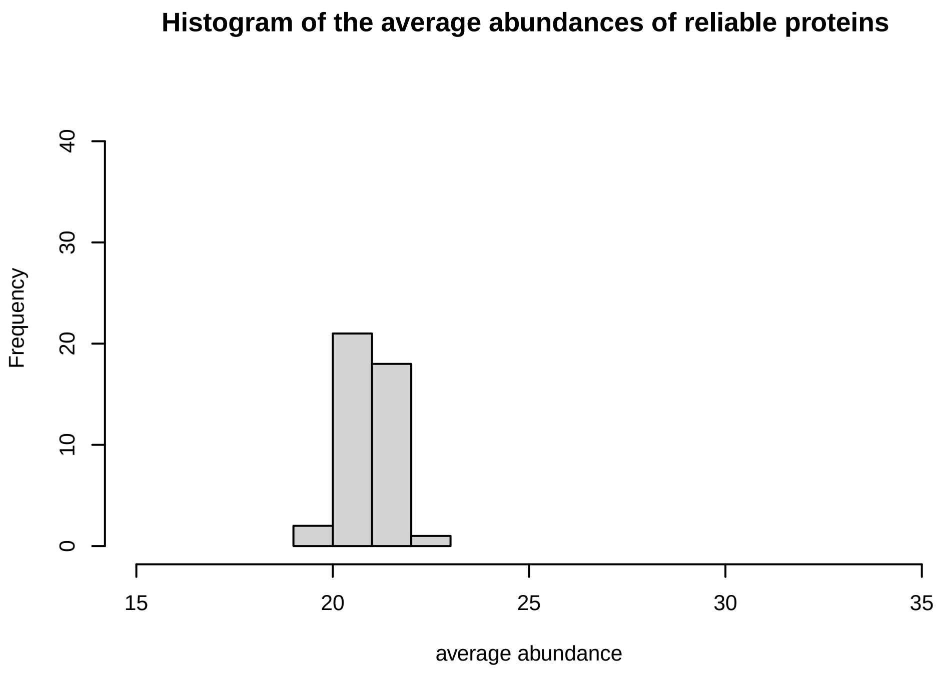
Histogram of the average abundances of reliable proteins
Frequency
average abundance

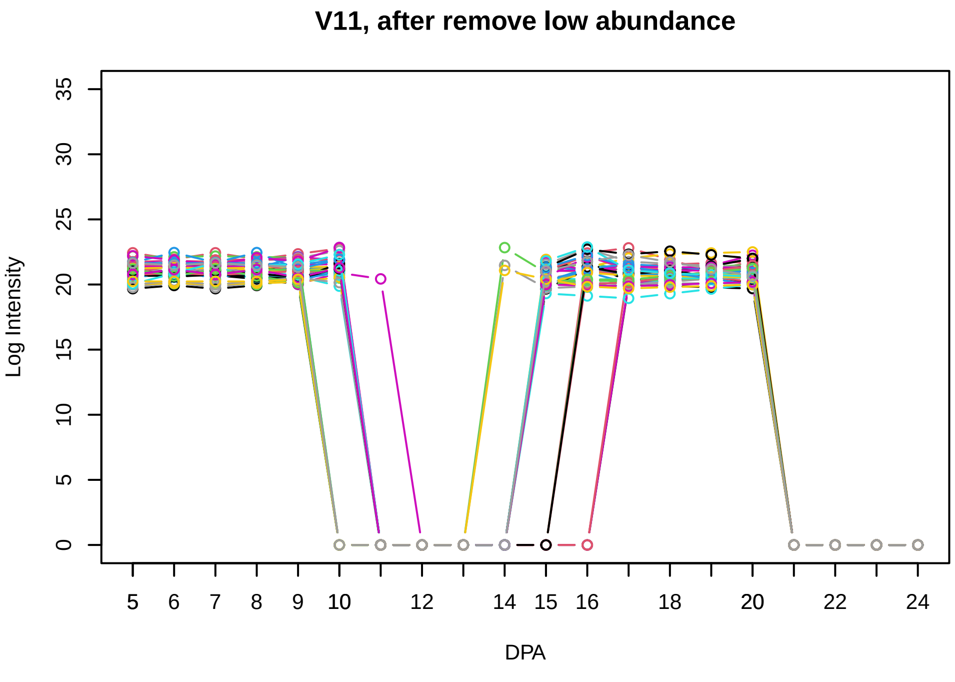
V11, after remove low abundance
Log Intensity
DPA

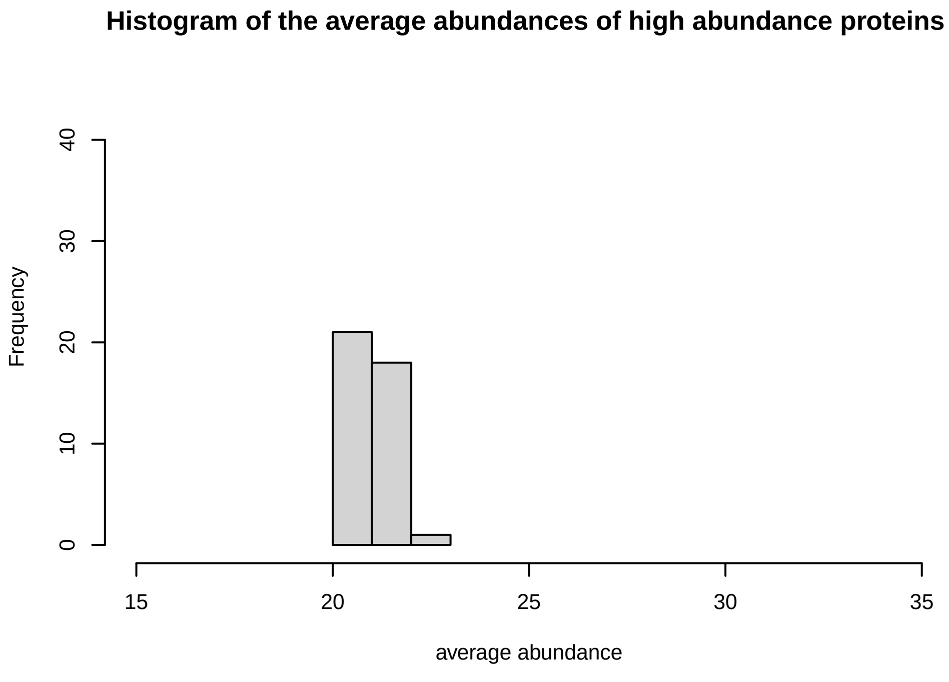
Histogram of the average abundances of high abundance proteins
Frequency
average abundance

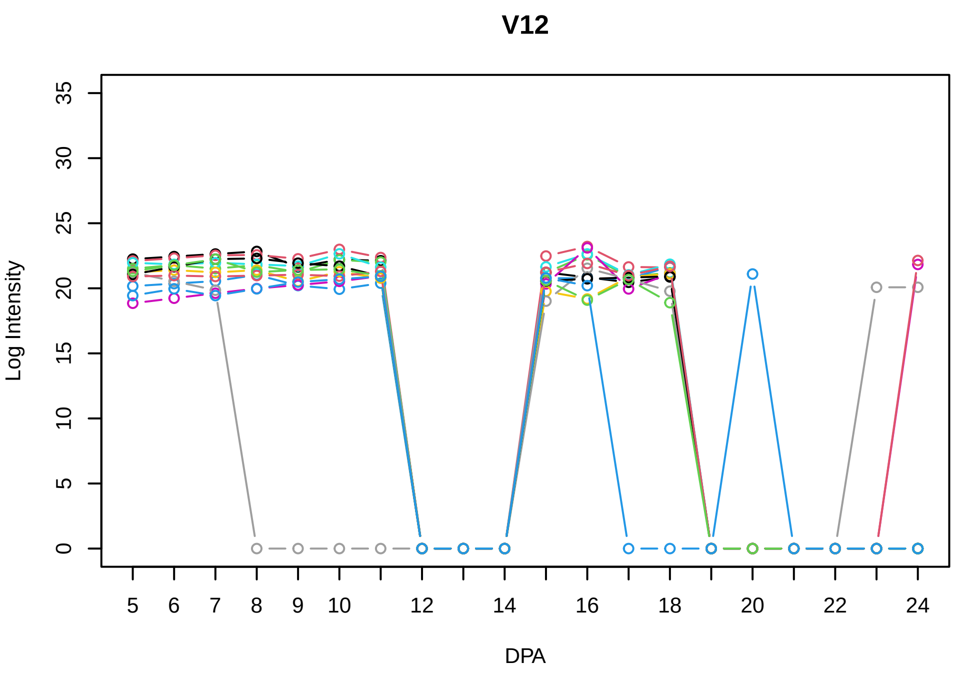
V12
Log Intensity
DPA
0
5
10
15
20
25
30
35
5 6 7 8 9 10 12 14 16 18 20 22 24

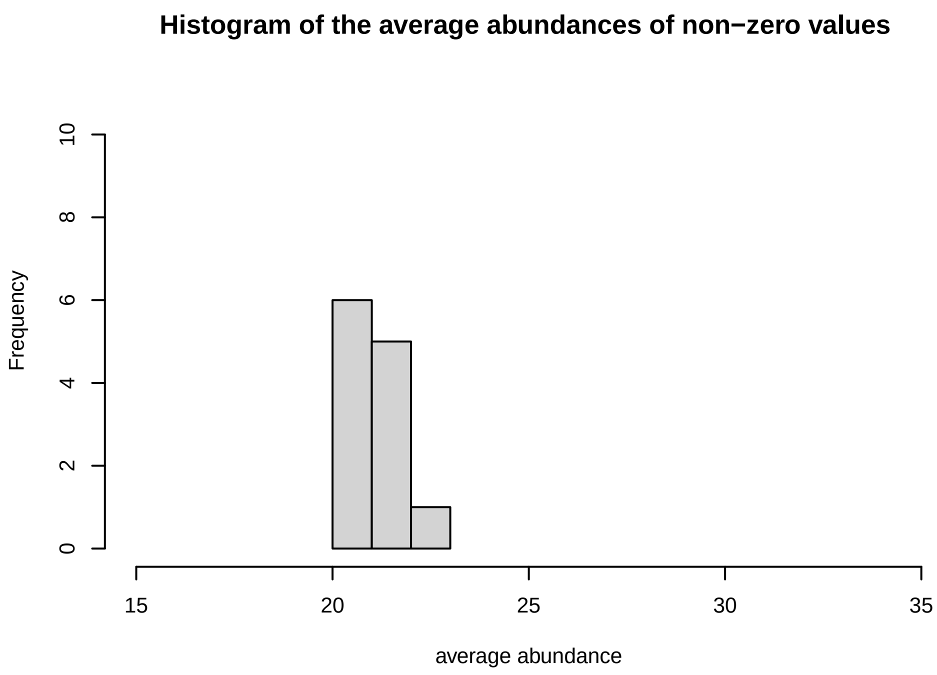
Histogram of the average abundances of non−zero values
Frequency
average abundance
0 2 4 6 8 10
15 20 25 30 35

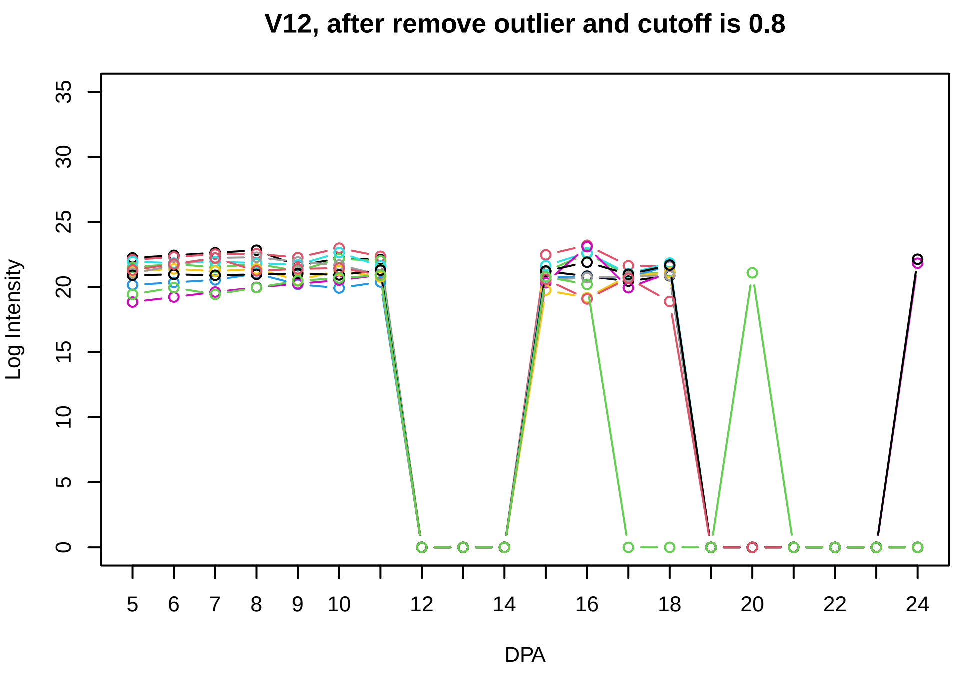
V12, after remove outlier and cutoff is 0.8
Log Intensity
DPA
0
5
10
15
20
25
30
35
5 6 7 8 9 10 12 14 16 18 20 22 24

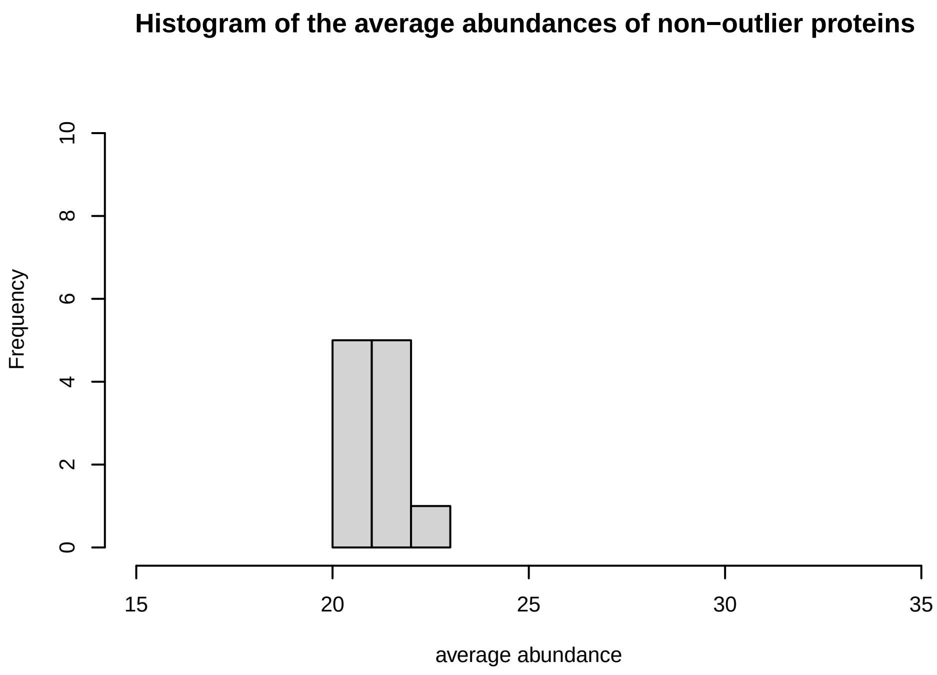
Histogram of the average abundances of non−outlier proteins
Frequency
average abundance
0 2 4 6 8 10
15 20 25 30 35

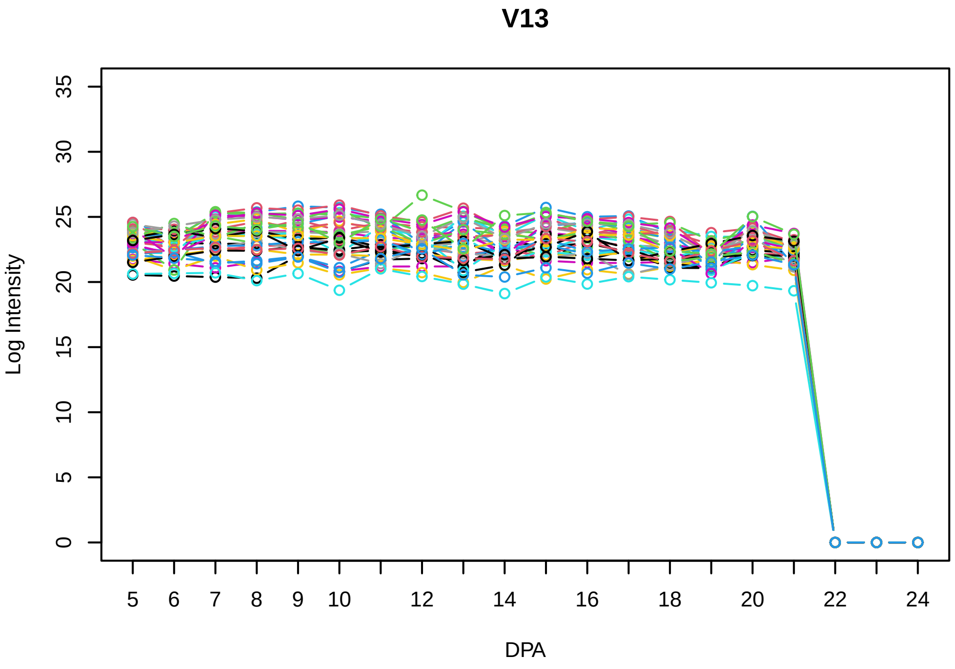
V13
Log Intensity
DPA

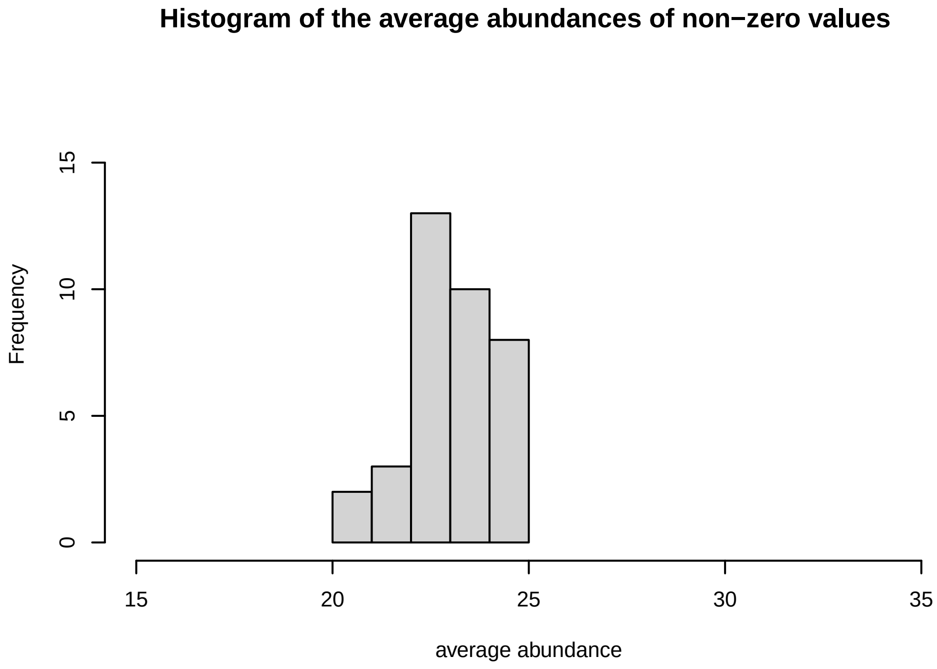
Histogram of the average abundances of non−zero values
Frequency
average abundance

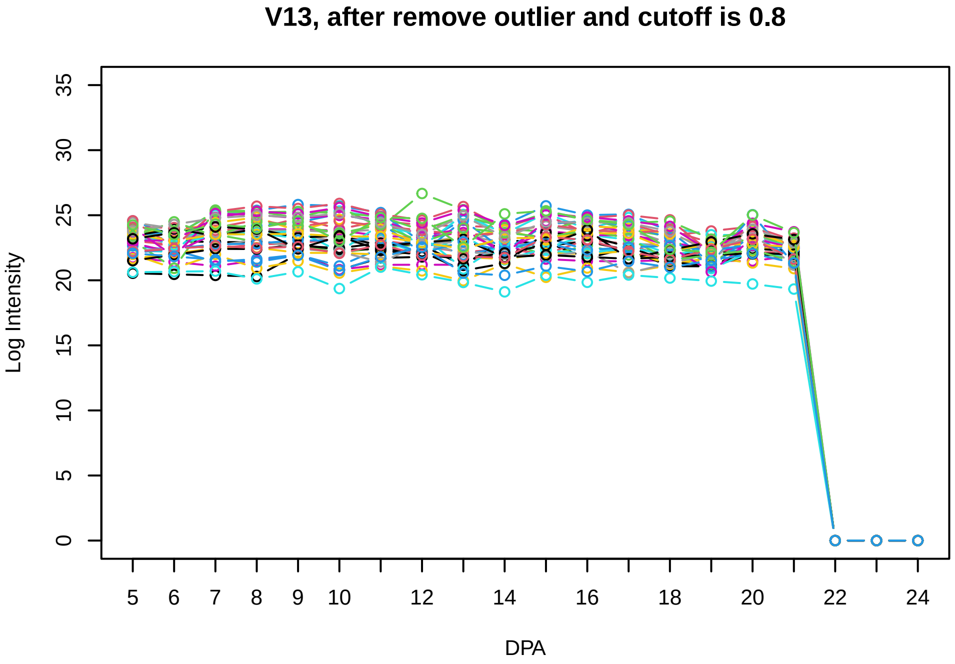
V13, after remove outlier and cutoff is 0.8
Log Intensity
DPA

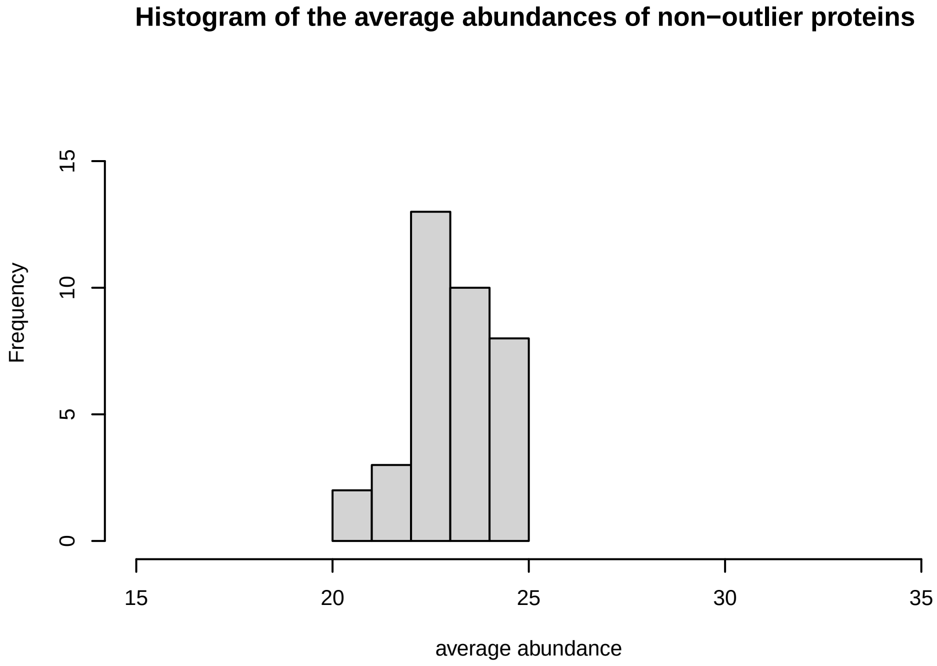
Histogram of the average abundances of non−outlier proteins
Frequency
average abundance

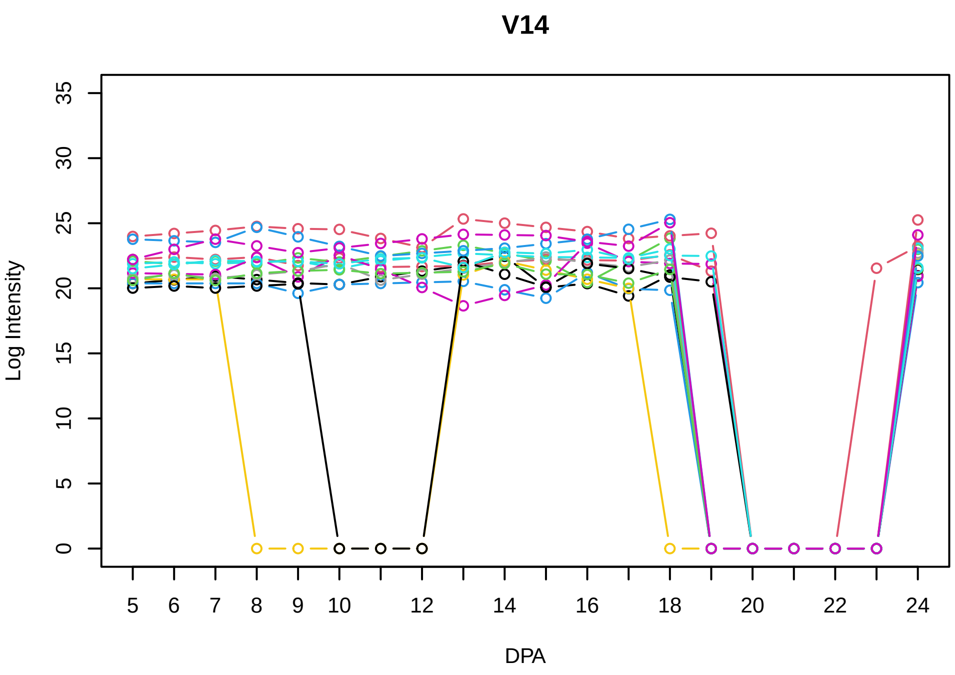
V14
Log Intensity
DPA
0
5
10
15
20
25
30
35
5 6 7 8 9 10 12 14 16 18 20 22 24

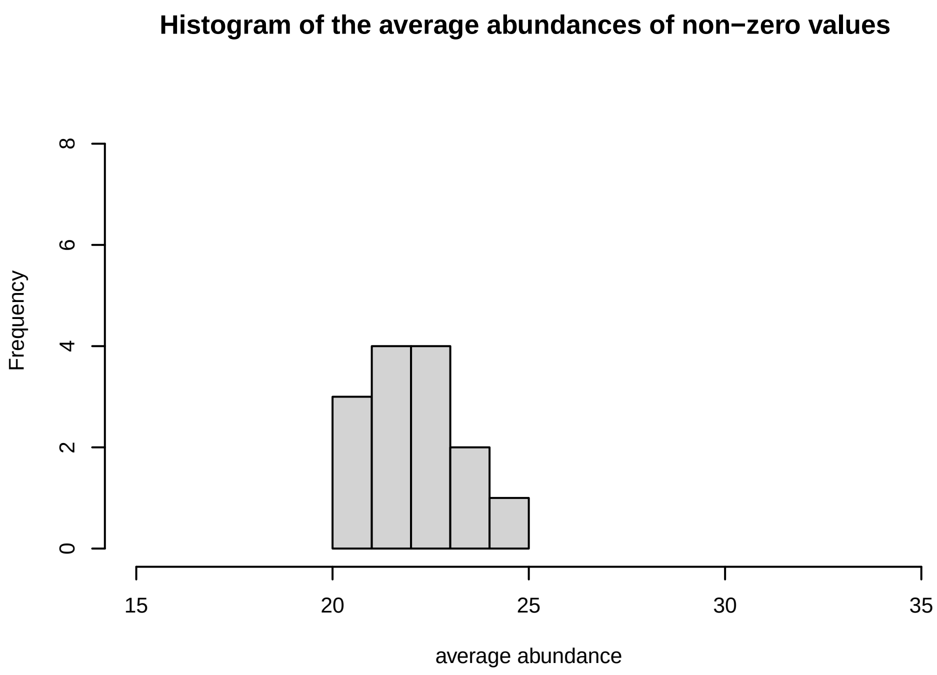
Histogram of the average abundances of non−zero values
Frequency
average abundance
0 2 4 6 8
15 20 25 30 35

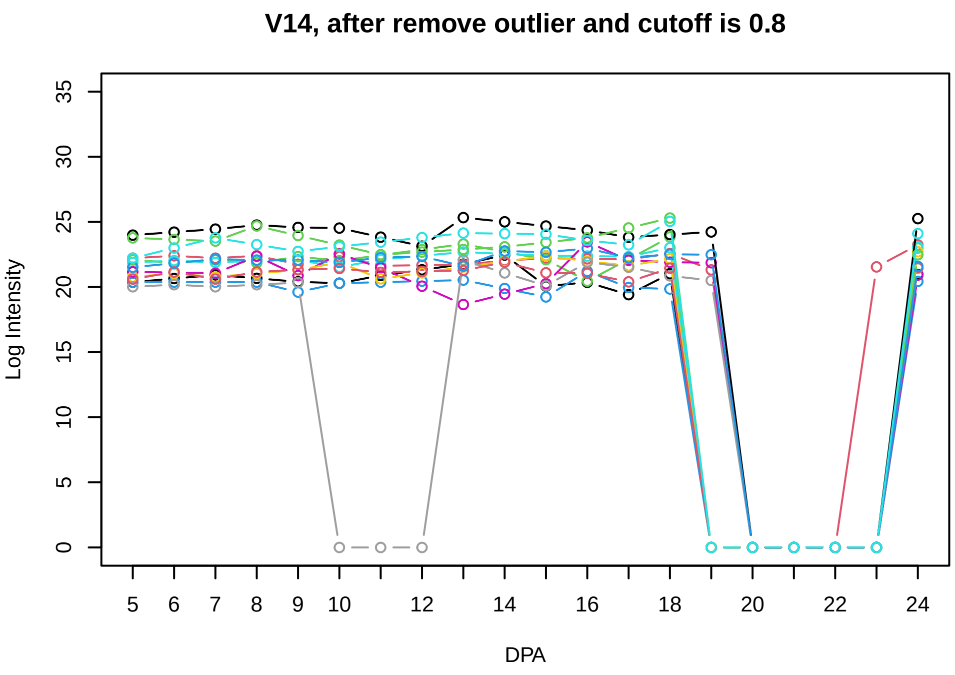
V14, after remove outlier and cutoff is 0.8
Log Intensity
DPA
0
5
10
15
20
25
30
35
5 6 7 8 9 10 12 14 16 18 20 22 24

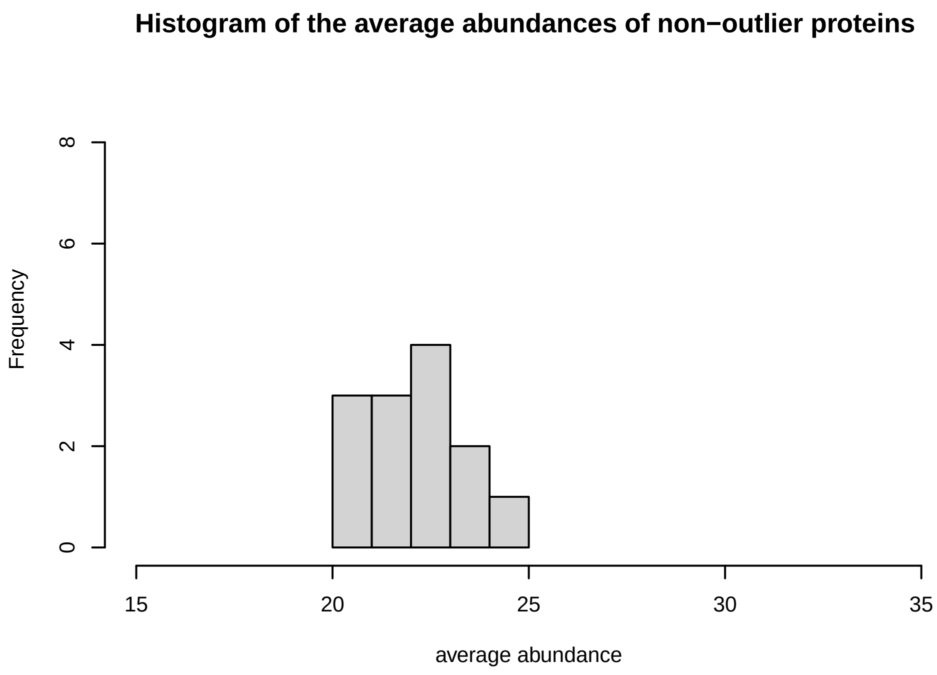
Histogram of the average abundances of non−outlier proteins
Frequency
average abundance
0 2 4 6 8
15 20 25 30 35

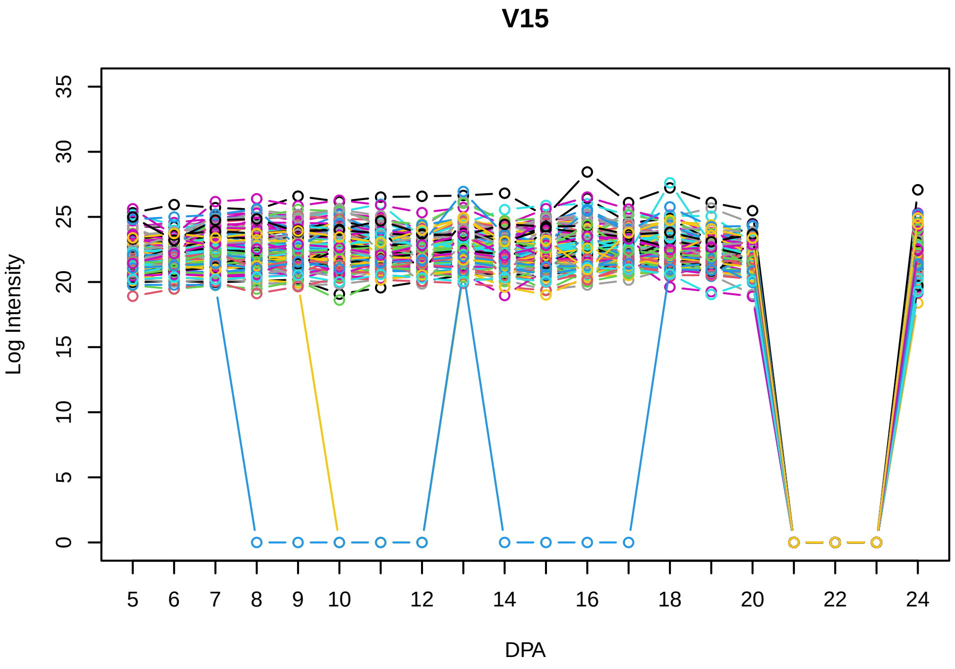
V15
Log Intensity
DPA
0
5
10
15
20
25
30
35
5
6
7
8
9
10
12
14
16
18
20
22
24

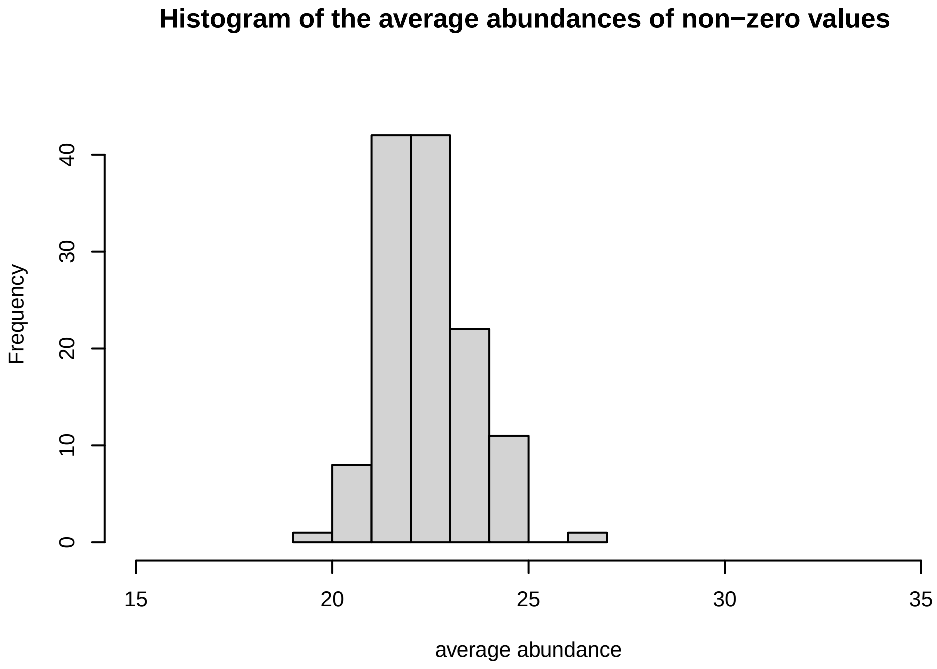
Histogram of the average abundances of non−zero values
Frequency
average abundance
0
10
20
30
40
15
20
25
30
35

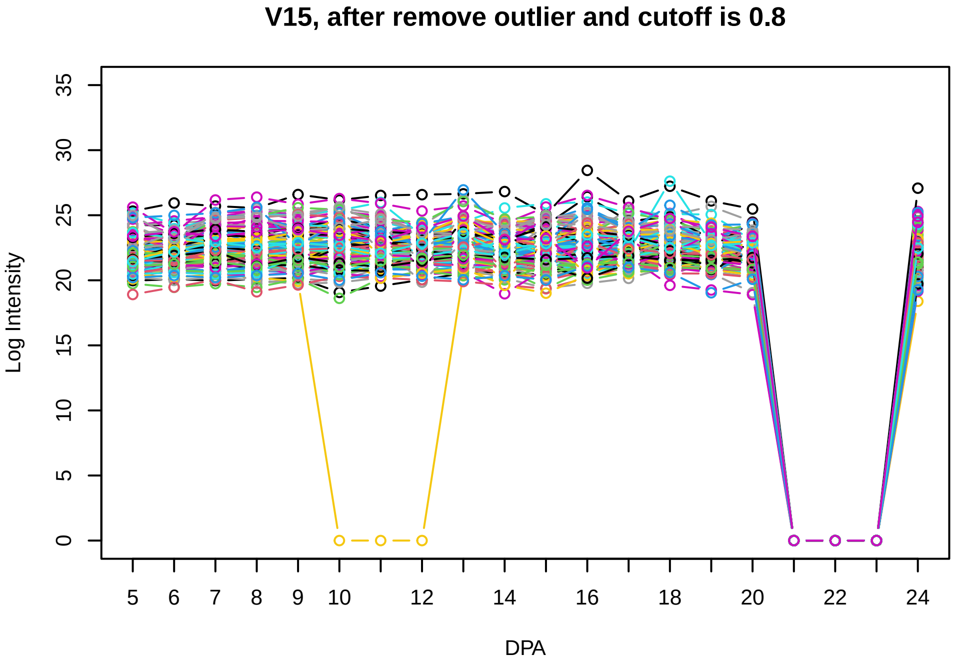
V15, after remove outlier and cutoff is 0.8
Log Intensity
DPA
0
5
10
15
20
25
30
35
5
6
7
8
9
10
12
14
16
18
20
22
24

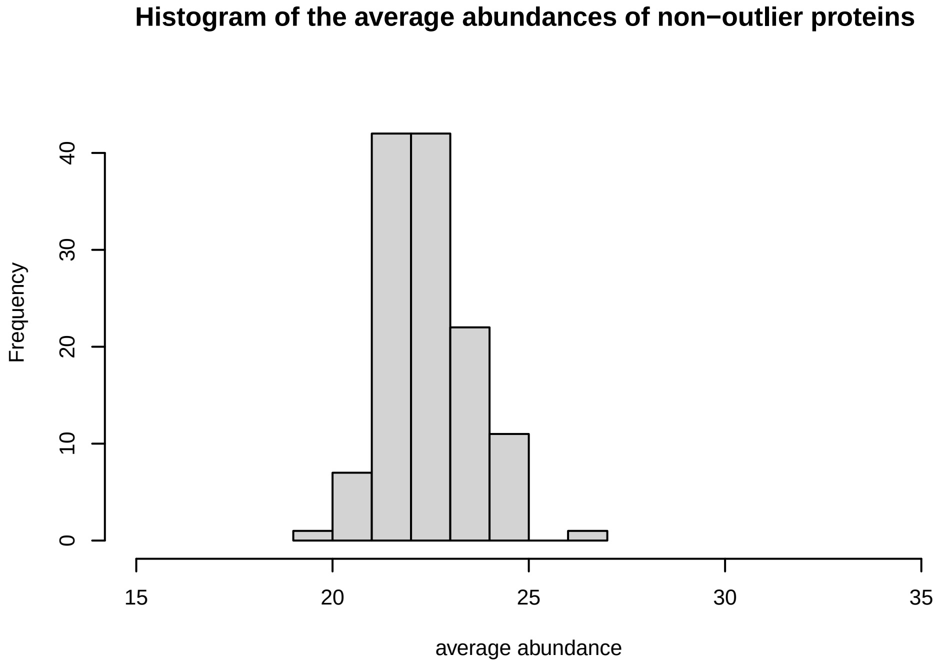
Histogram of the average abundances of non−outlier proteins
Frequency
average abundance
0
10
20
30
40
15
20
25
30
35

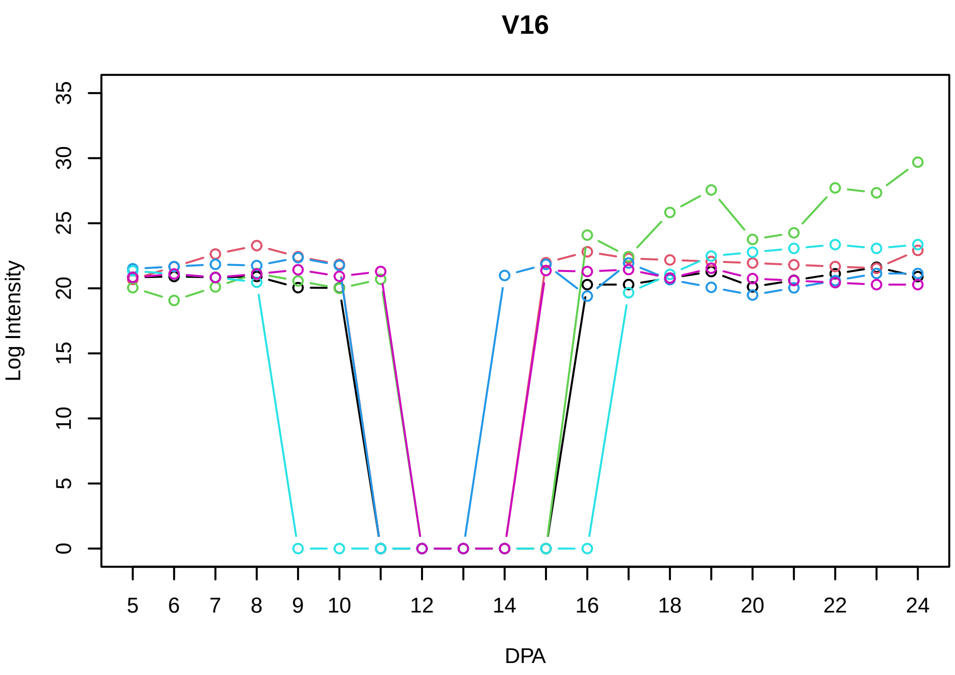
V16
Log Intensity
DPA
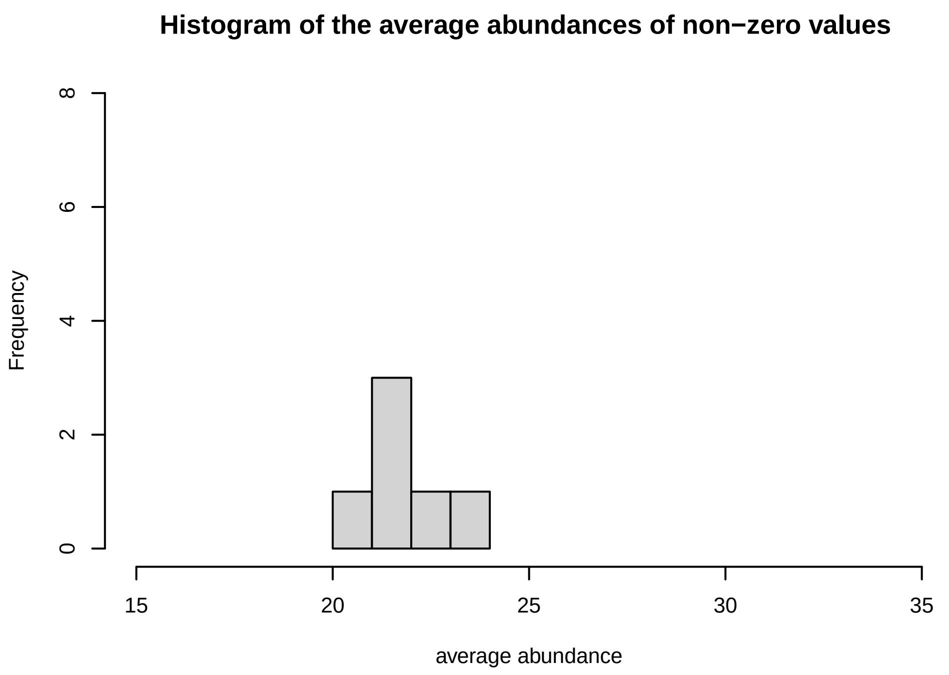
Histogram of the average abundances of non−zero values
Frequency
average abundance
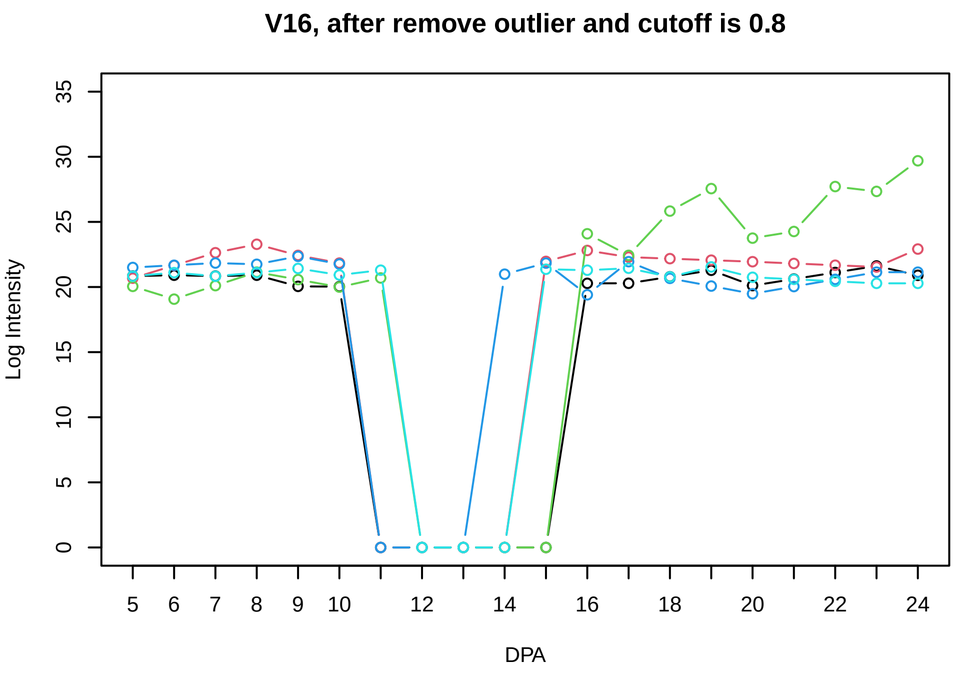
V16, after remove outlier and cutoff is 0.8
Log Intensity
DPA
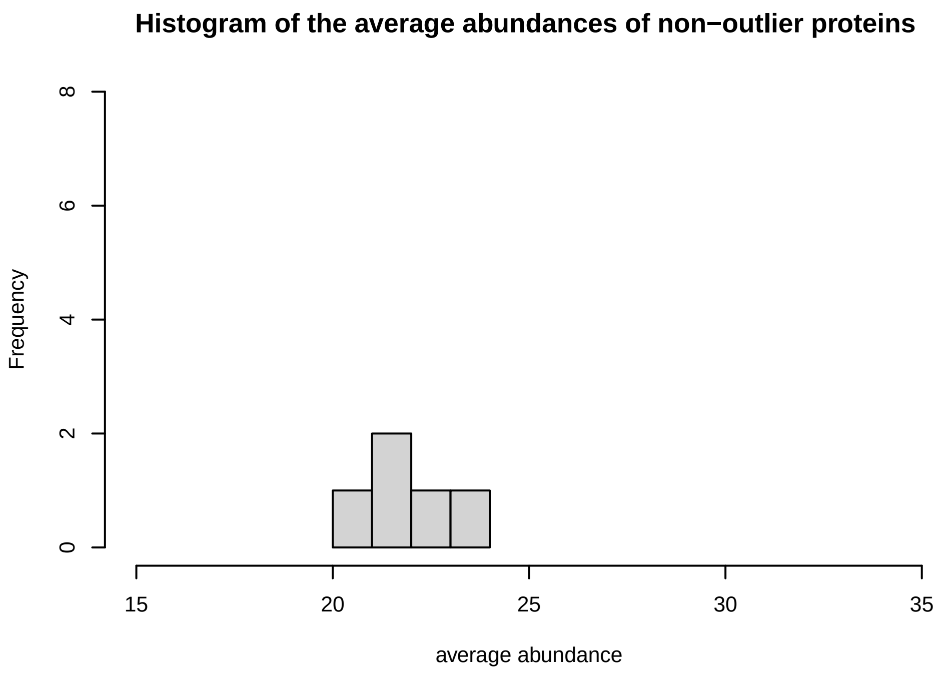
Histogram of the average abundances of non−outlier proteins
Frequency
average abundance
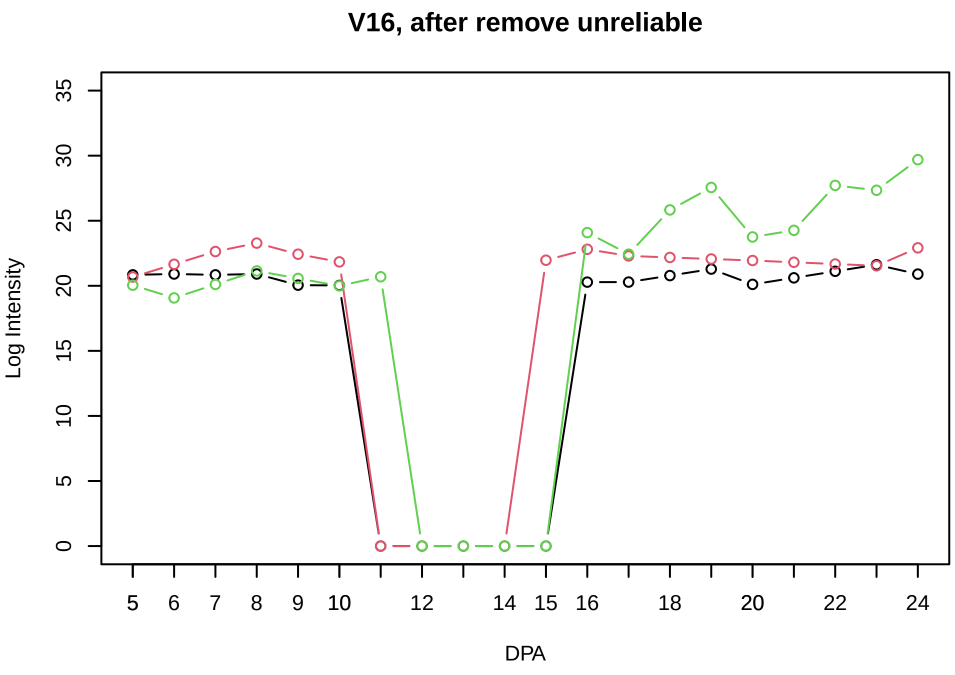
V16, after remove unreliable
Log Intensity
DPA
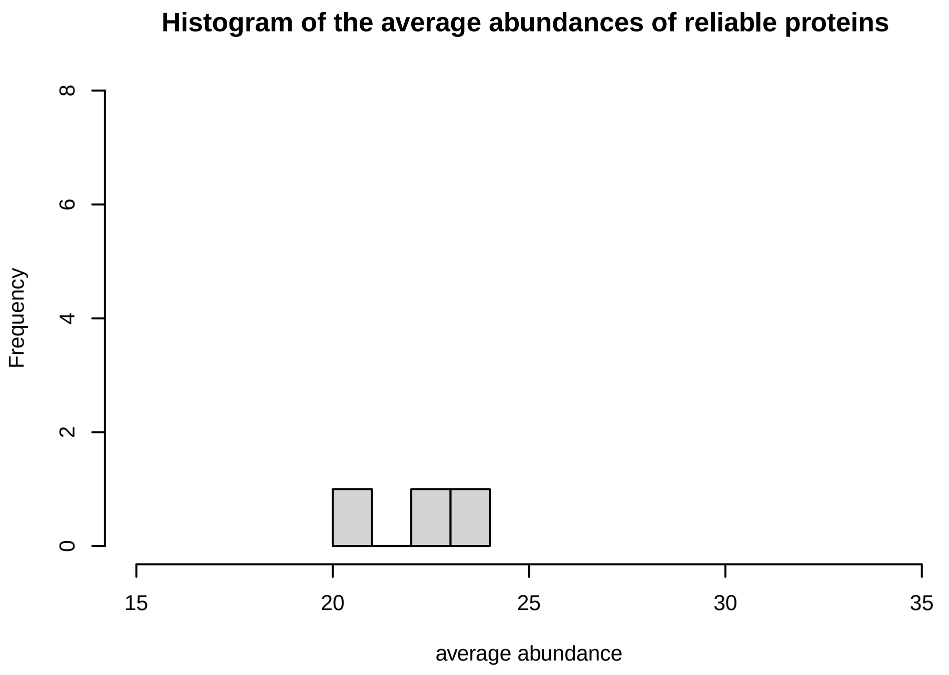
Histogram of the average abundances of reliable proteins
Frequency
average abundance
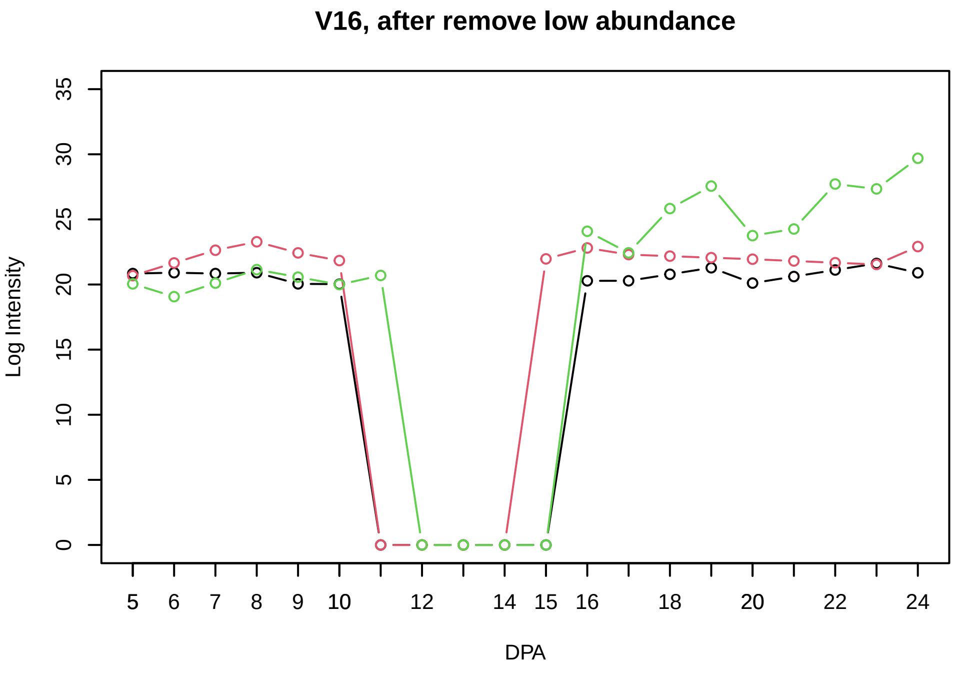
V16, after remove low abundance
Log Intensity
DPA
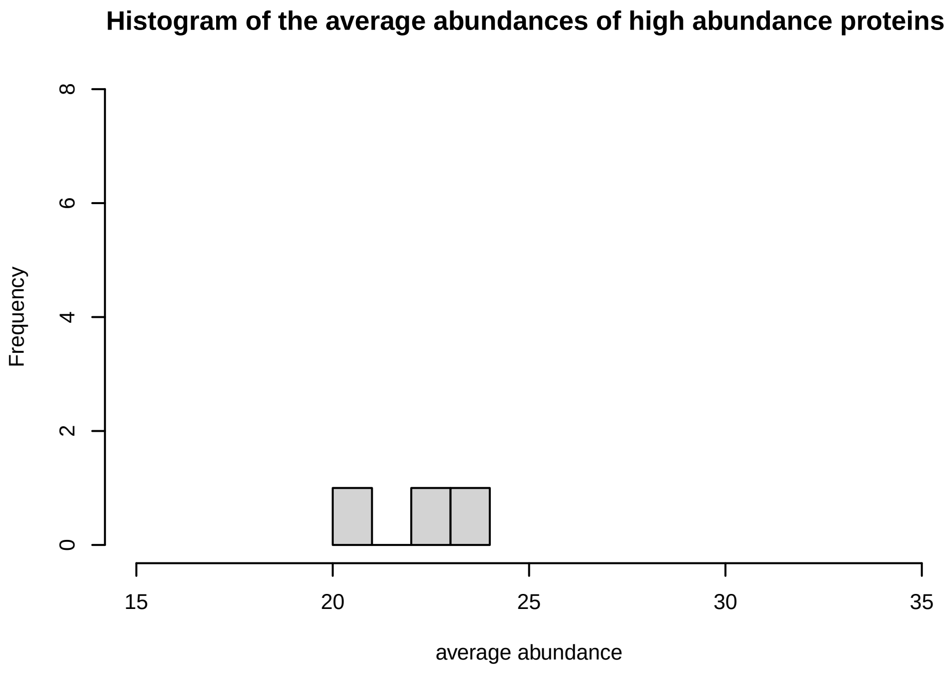
Histogram of the average abundances of high abundance proteins
Frequency
average abundance

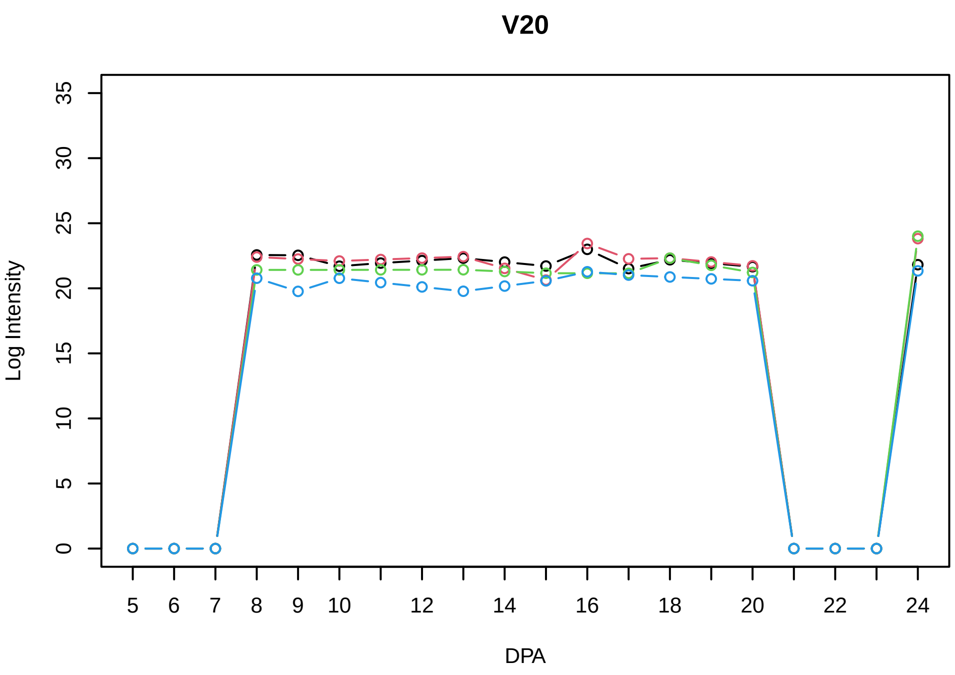
V20
Log Intensity
DPA
0
5
10
15
20
25
30
35
5
6
7
8
9
10
12
14
16
18
20
22
24

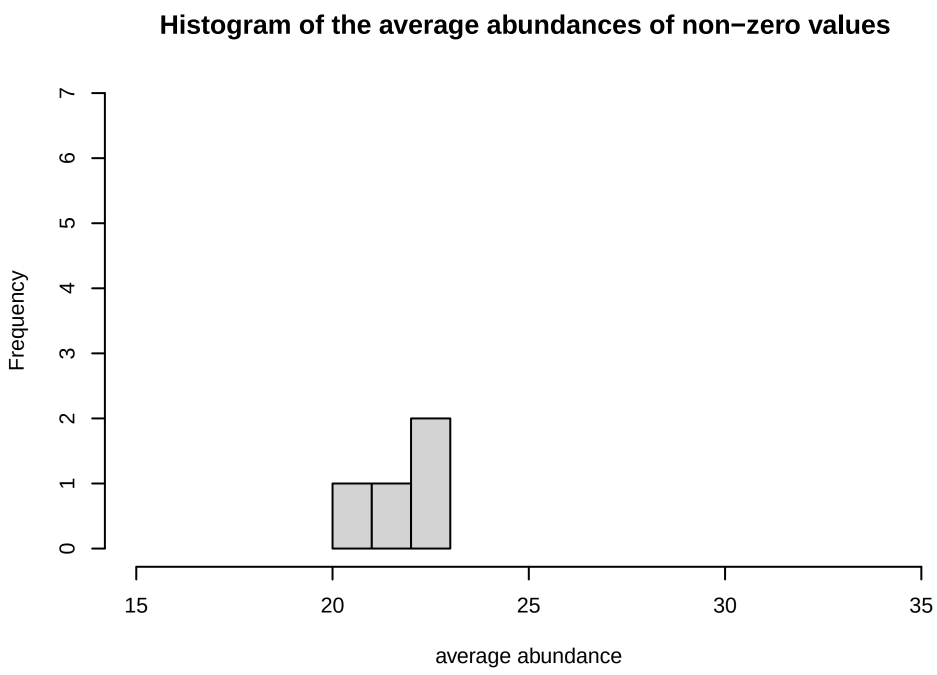
Histogram of the average abundances of non−zero values
Frequency
average abundance
0
1
2
3
4
5
6
7
15
20
25
30
35

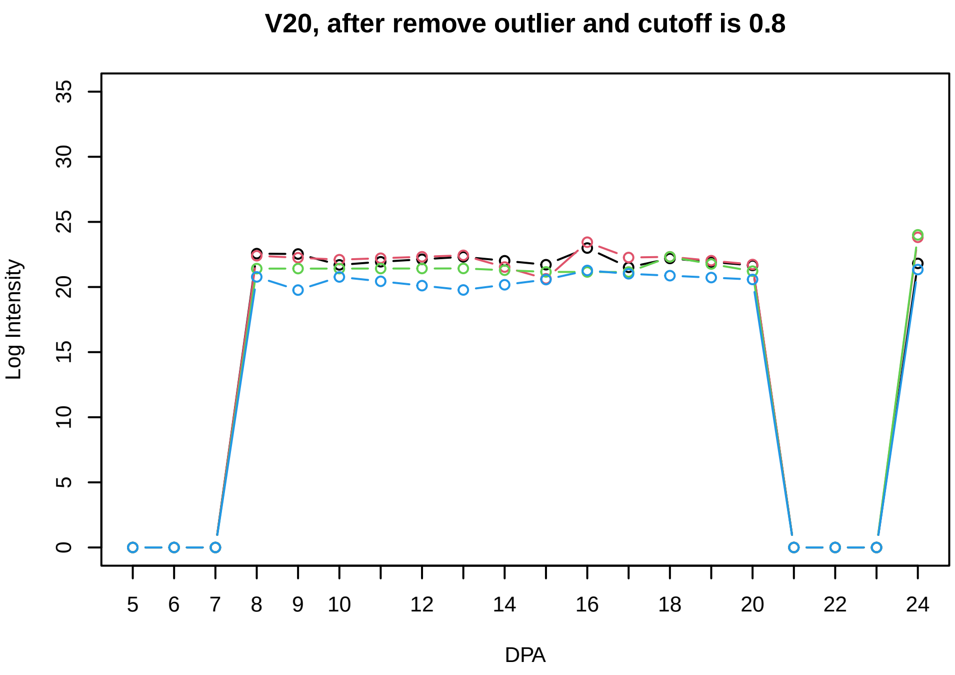
V20, after remove outlier and cutoff is 0.8
Log Intensity
DPA
0
5
10
15
20
25
30
35
5
6
7
8
9
10
12
14
16
18
20
22
24

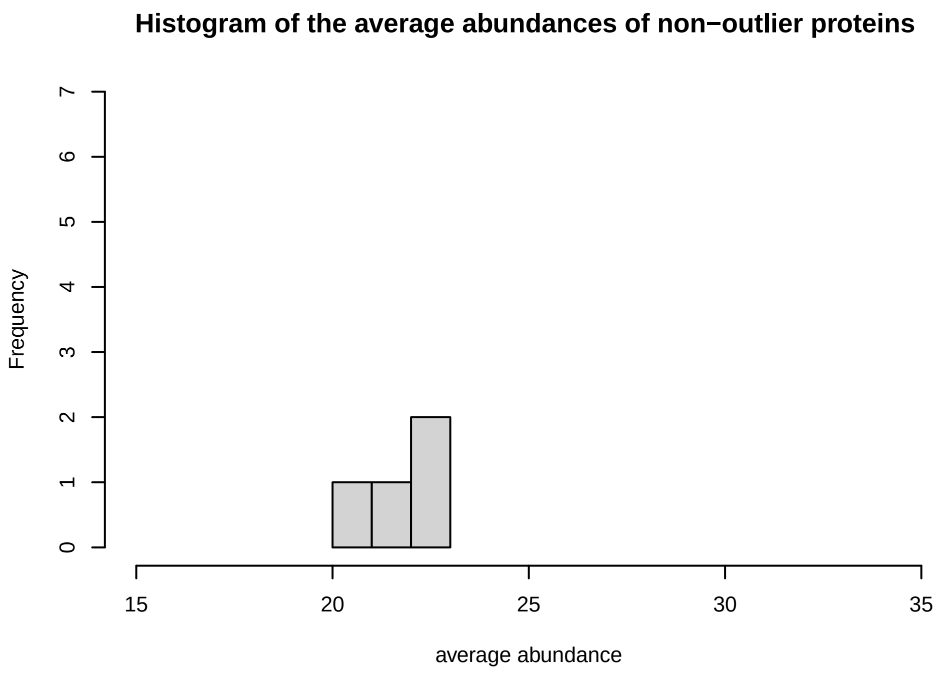
Histogram of the average abundances of non−outlier proteins
Frequency
average abundance
0
1
2
3
4
5
6
7
15
20
25
30
35

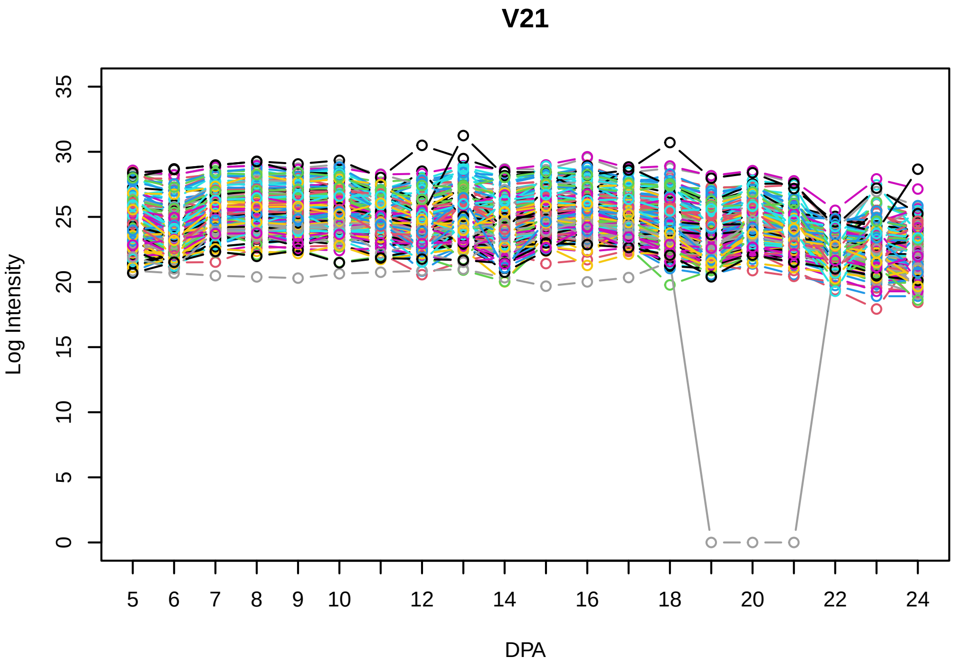
V21
Log Intensity
DPA

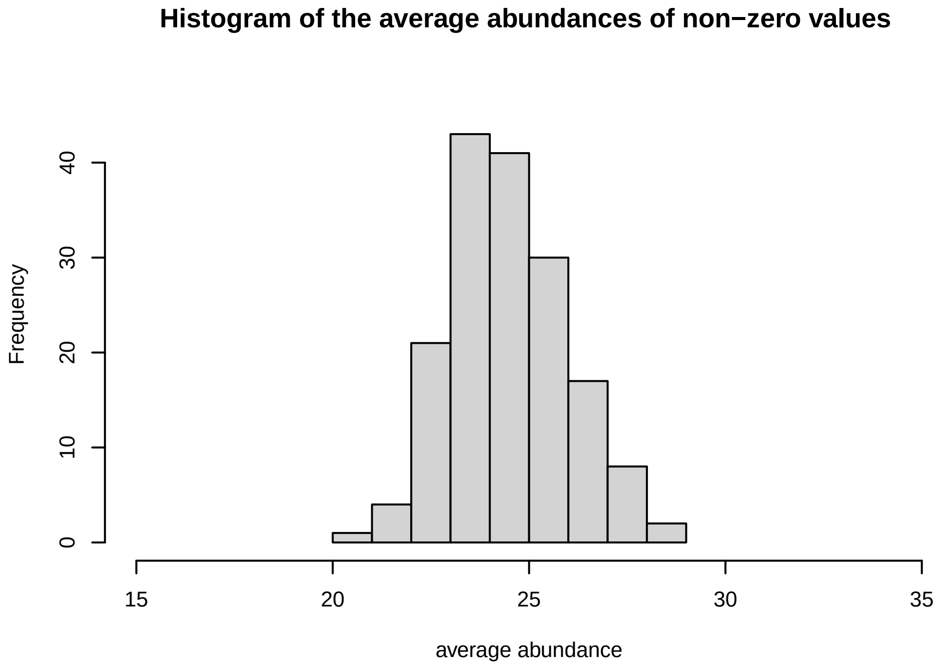
Histogram of the average abundances of non−zero values
Frequency
average abundance

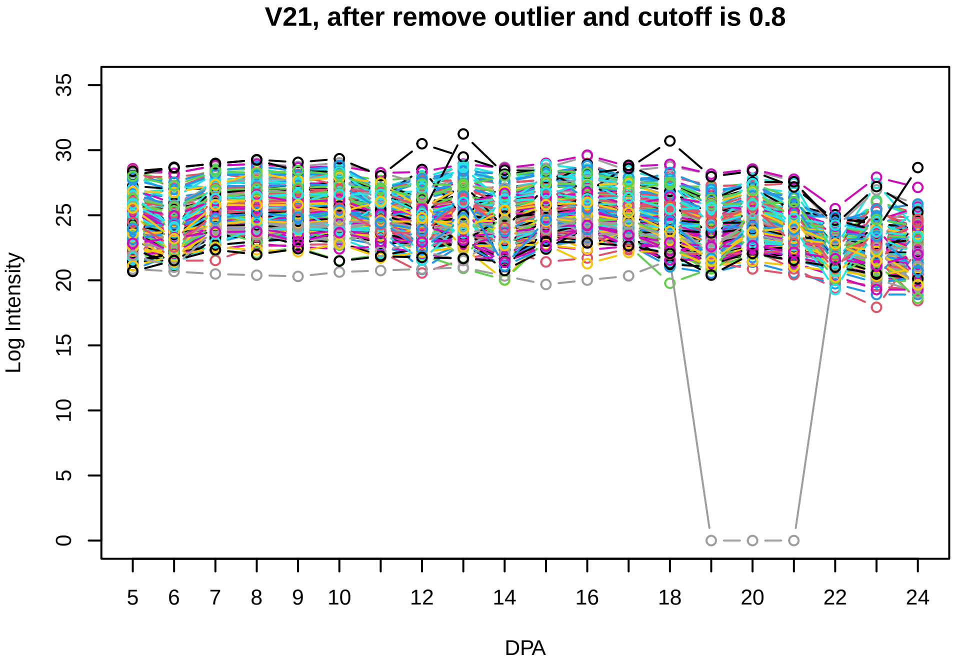
V21, after remove outlier and cutoff is 0.8
Log Intensity
DPA

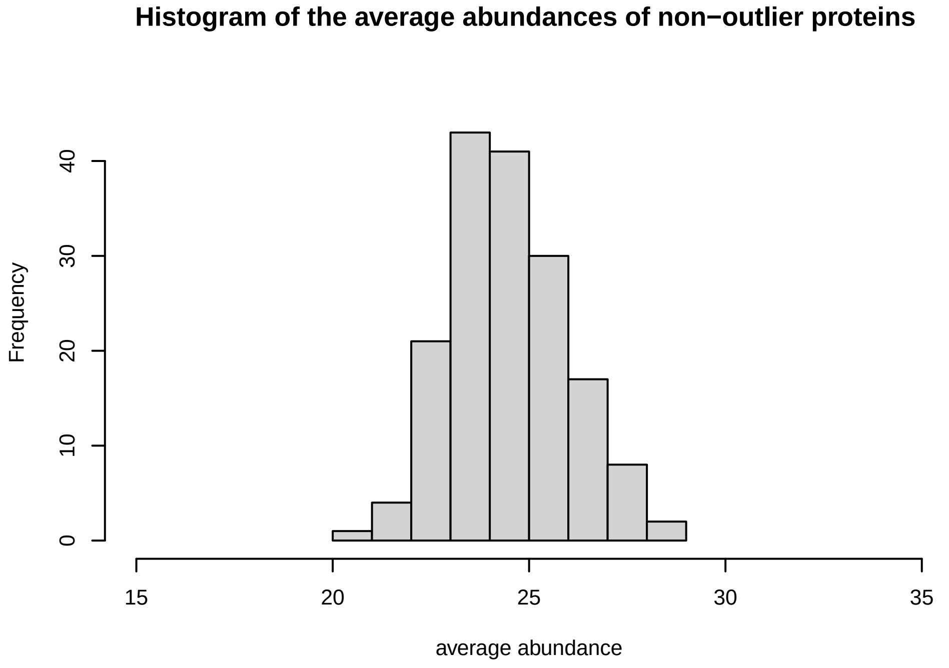
Histogram of the average abundances of non−outlier proteins
Frequency
average abundance

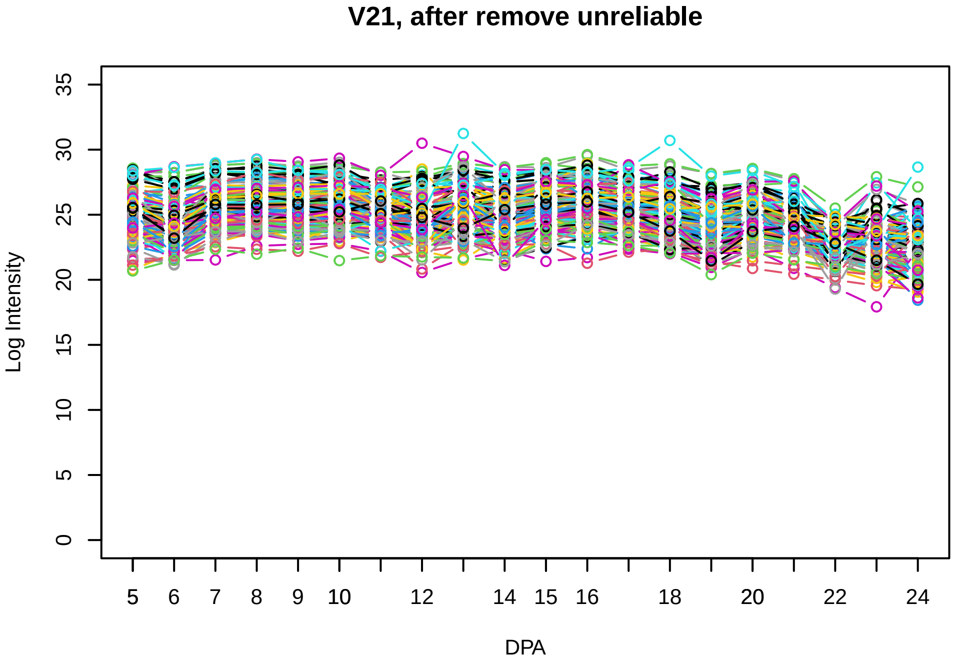
V21, after remove unreliable
Log Intensity
DPA

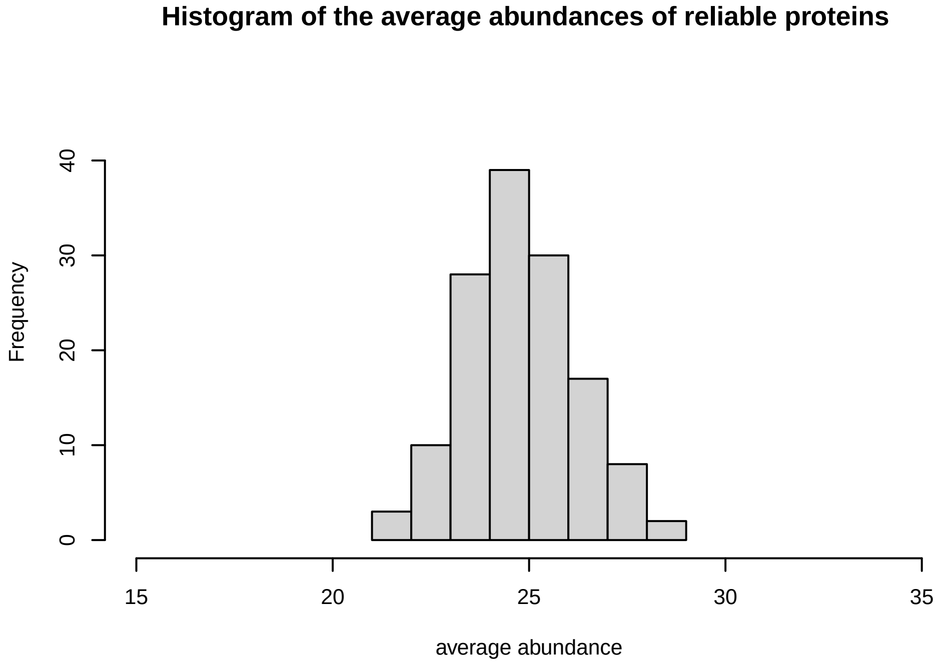
Histogram of the average abundances of reliable proteins
Frequency
average abundance

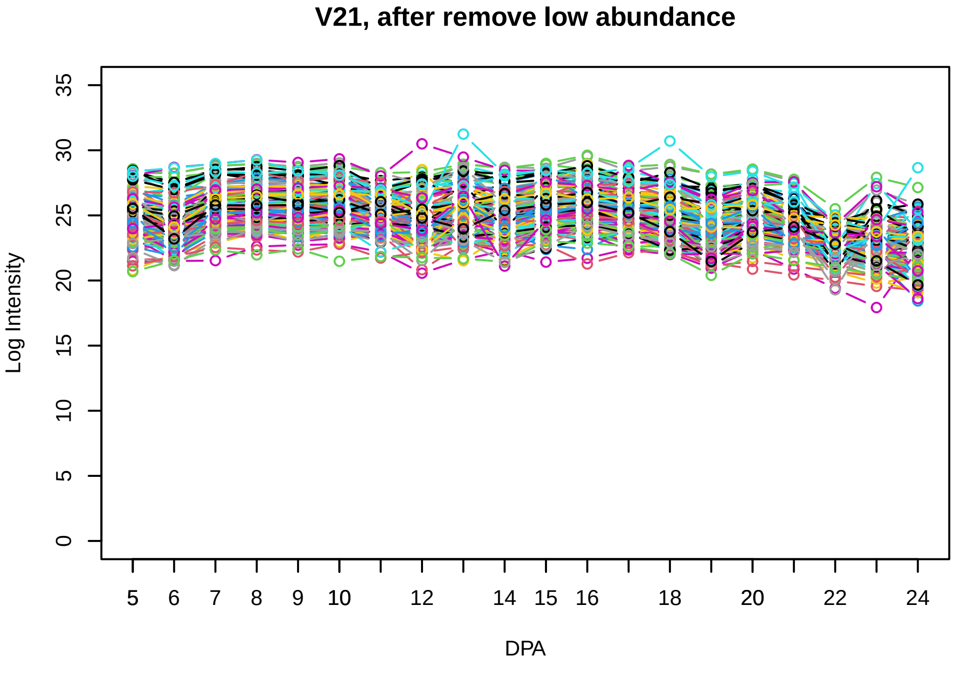
V21, after remove low abundance
Log Intensity
DPA

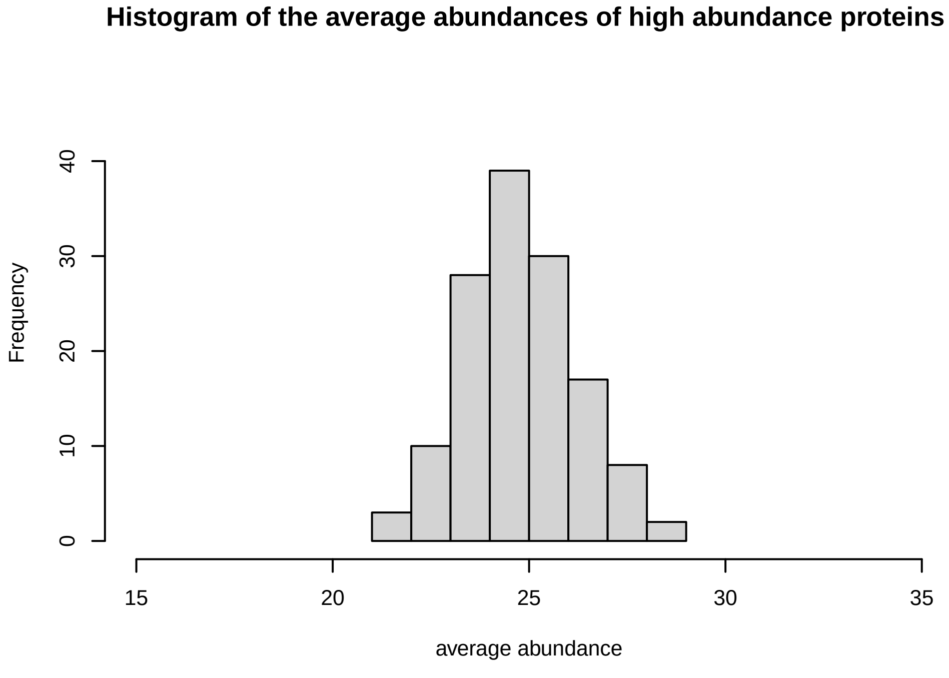
Histogram of the average abundances of high abundance proteins
Frequency
average abundance

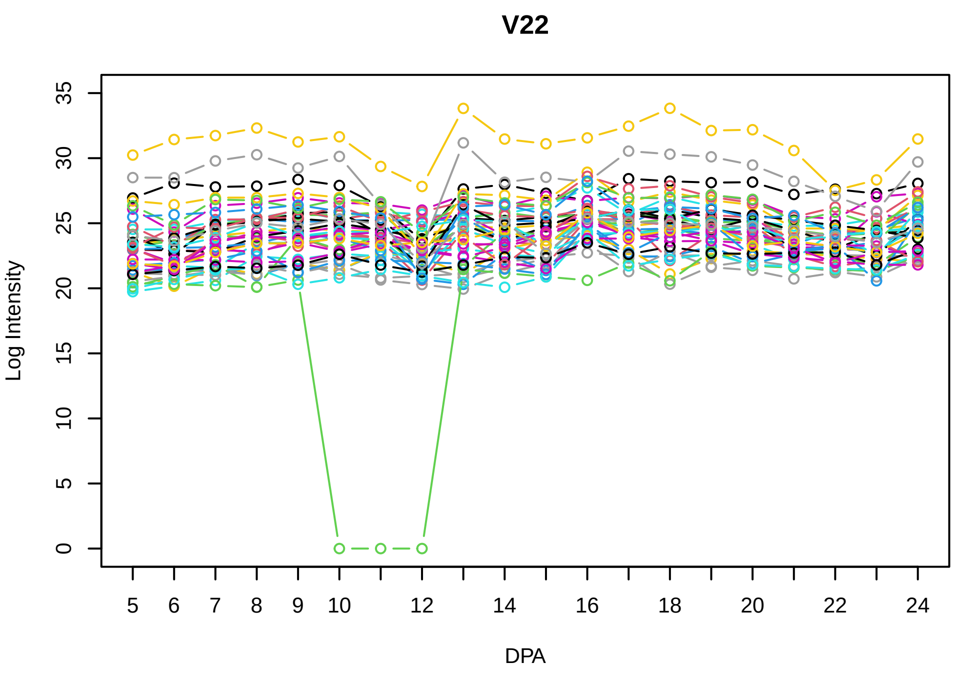
V22
Log Intensity
DPA
0 5 10 15 20 25 30 35
5 6 7 8 9 10 12 14 16 18 20 22 24

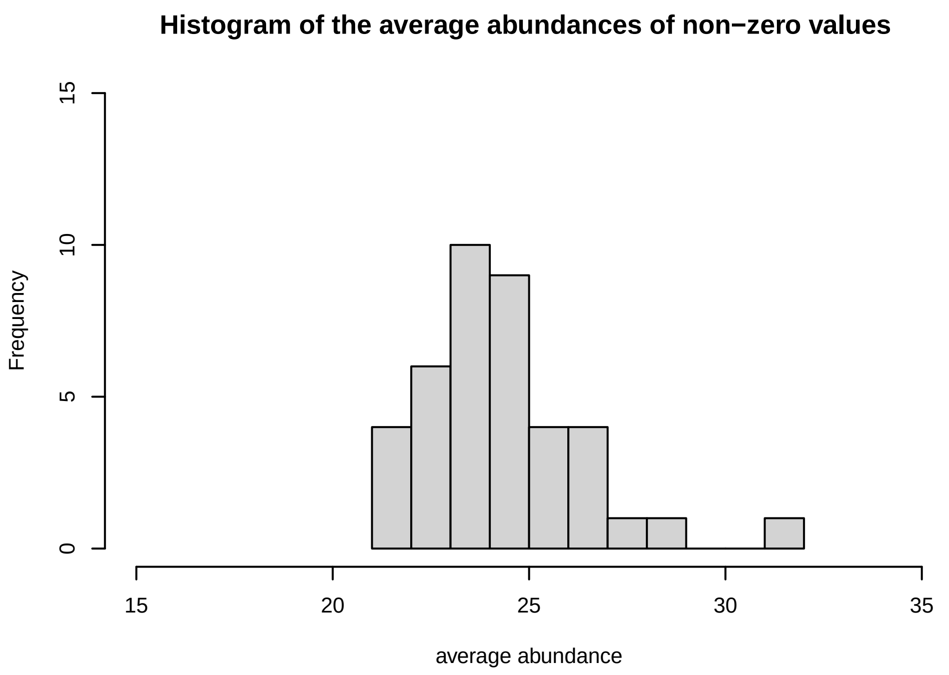
Histogram of the average abundances of non−zero values
Frequency
average abundance
0 5 10 15
15 20 25 30 35

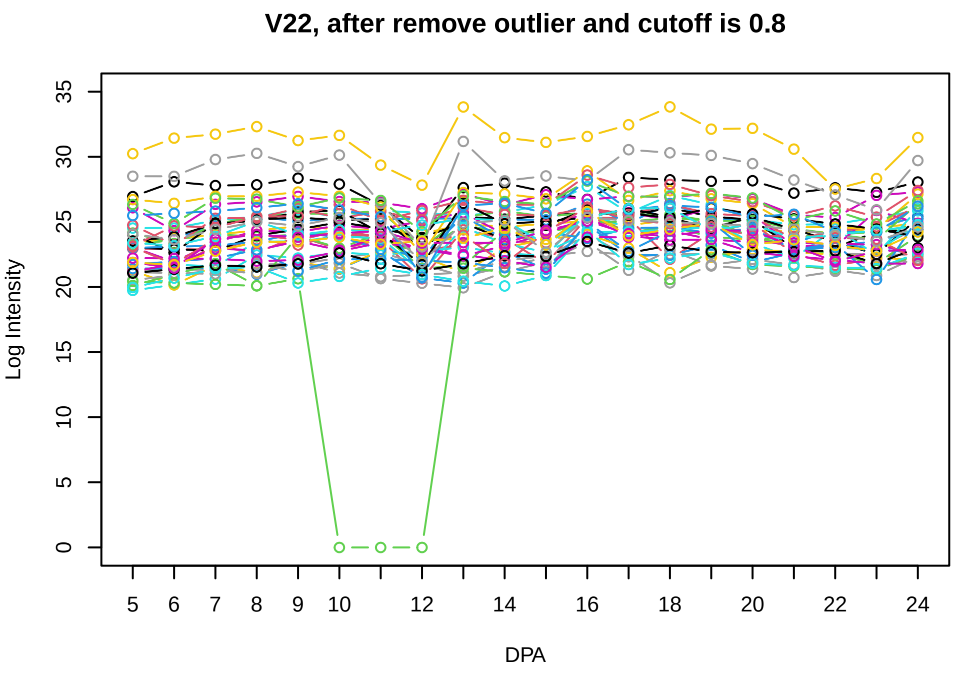
V22, after remove outlier and cutoff is 0.8
Log Intensity
DPA
0 5 10 15 20 25 30 35
5 6 7 8 9 10 12 14 16 18 20 22 24

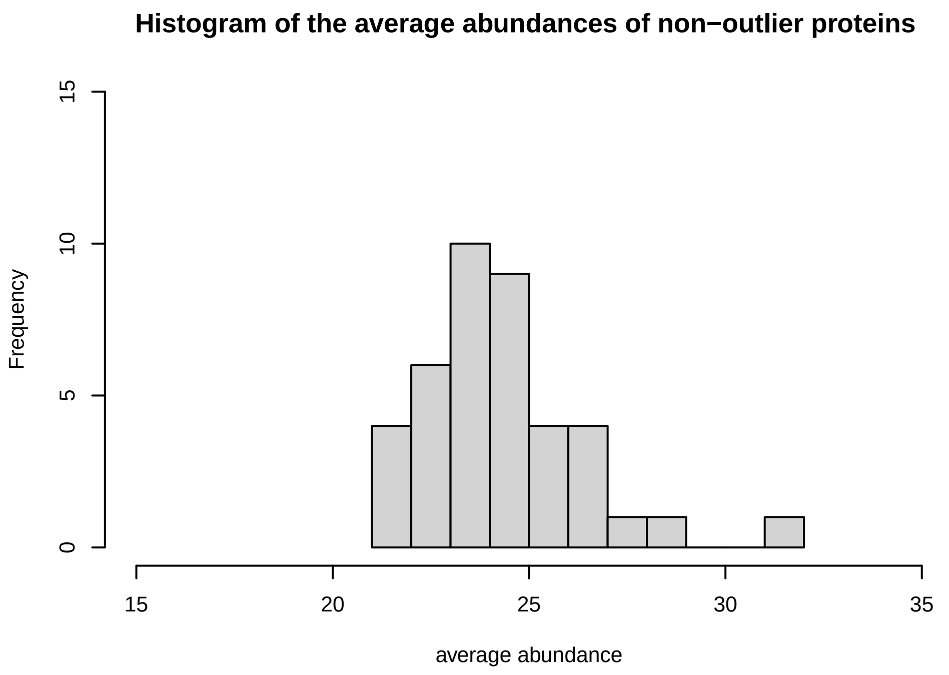
Histogram of the average abundances of non−outlier proteins
Frequency
average abundance
0 5 10 15
15 20 25 30 35

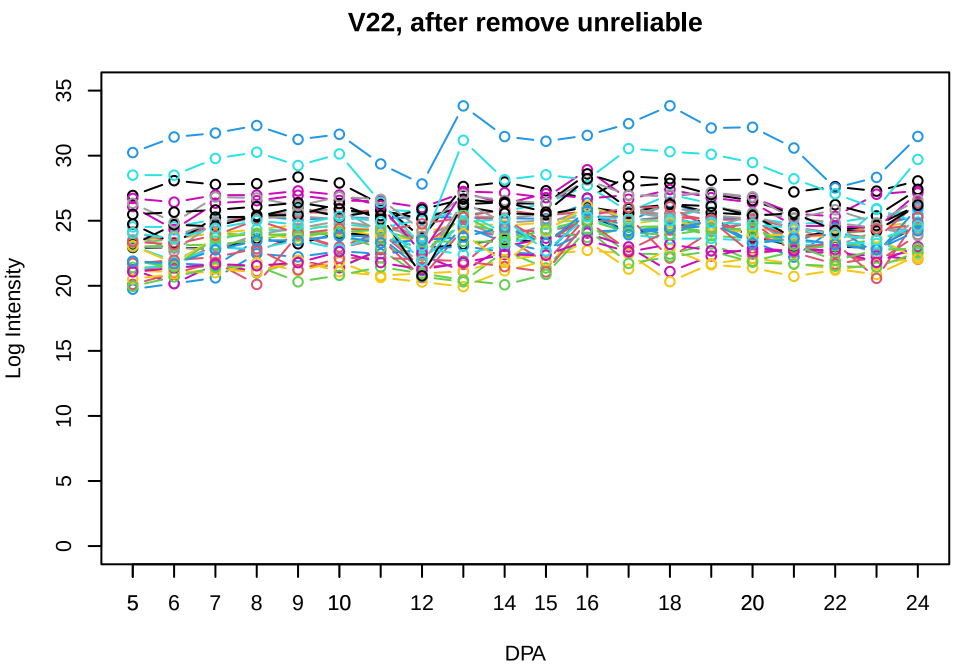
V22, after remove unreliable
Log Intensity
DPA
0 5 10 15 20 25 30 35
5 6 7 8 9 10 12 14 15 16 18 20 22 24

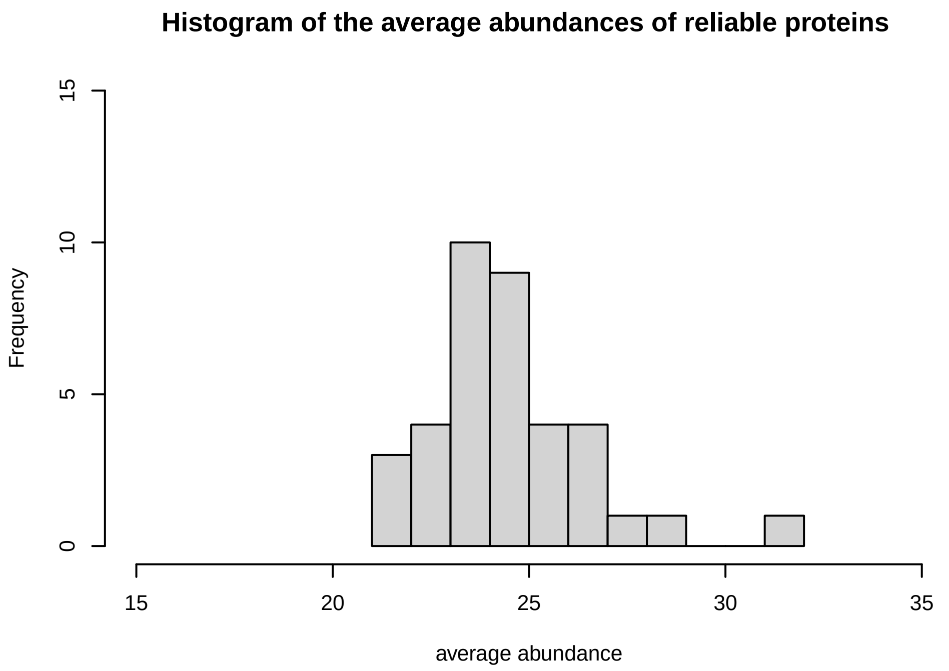
Histogram of the average abundances of reliable proteins
Frequency
average abundance
0 5 10 15
15 20 25 30 35

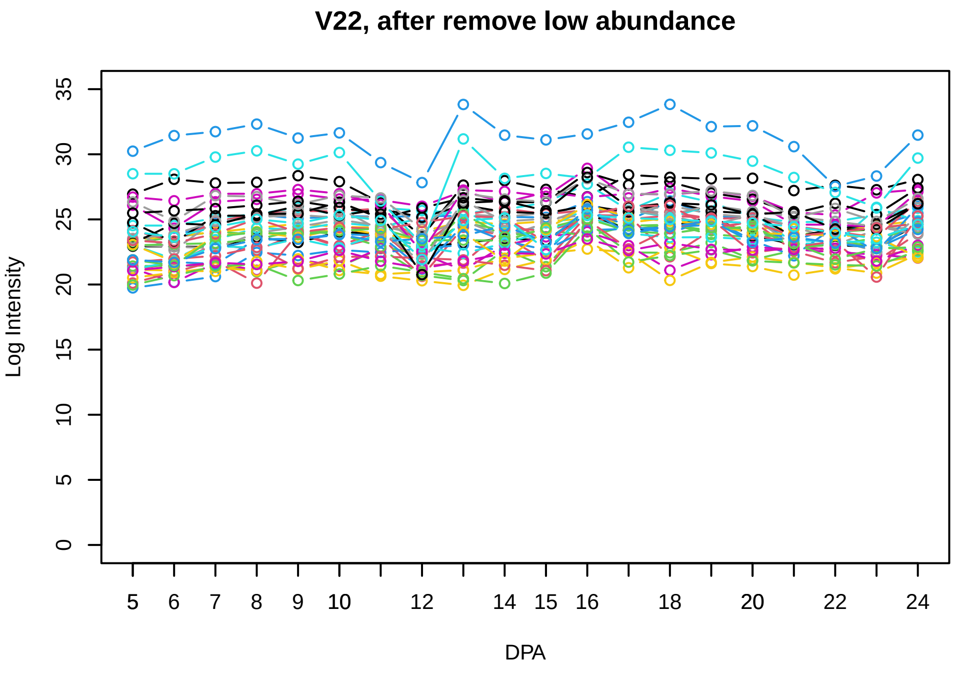
V22, after remove low abundance
Log Intensity
DPA
0 5 10 15 20 25 30 35
5 6 7 8 9 10 12 14 15 16 18 20 22 24

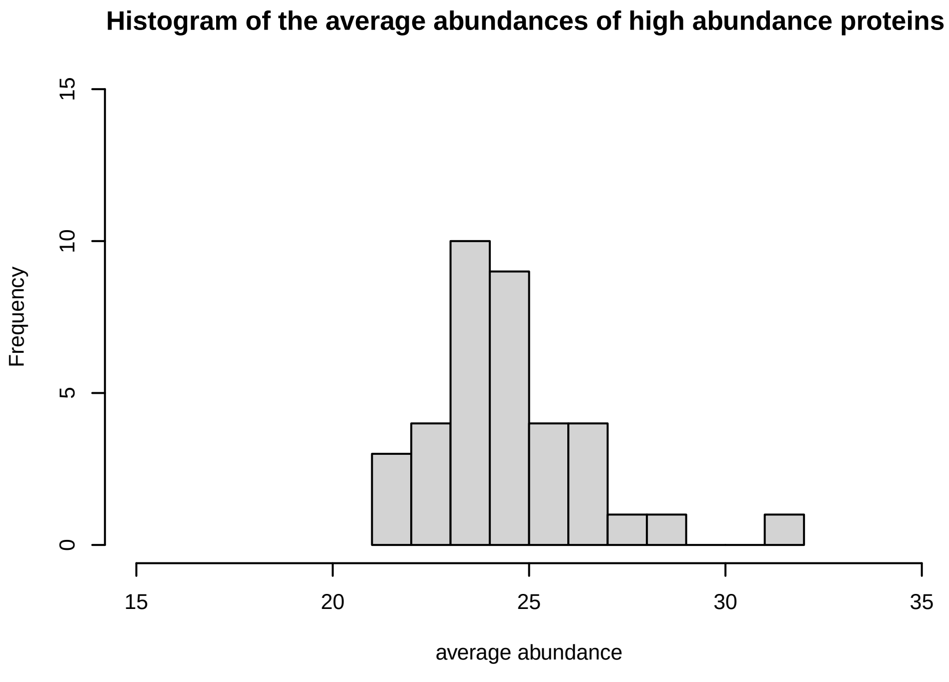
Histogram of the average abundances of high abundance proteins
Frequency
average abundance
0 5 10 15
15 20 25 30 35

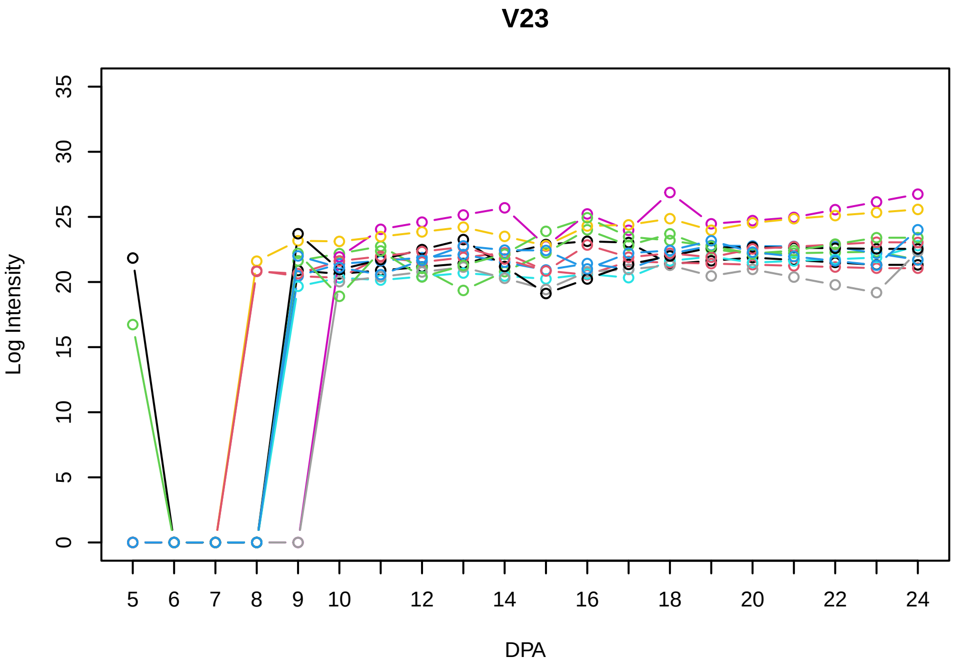
V23
Log Intensity
DPA
0 5 10 15 20 25 30 35
5 6 7 8 9 10 12 14 16 18 20 22 24

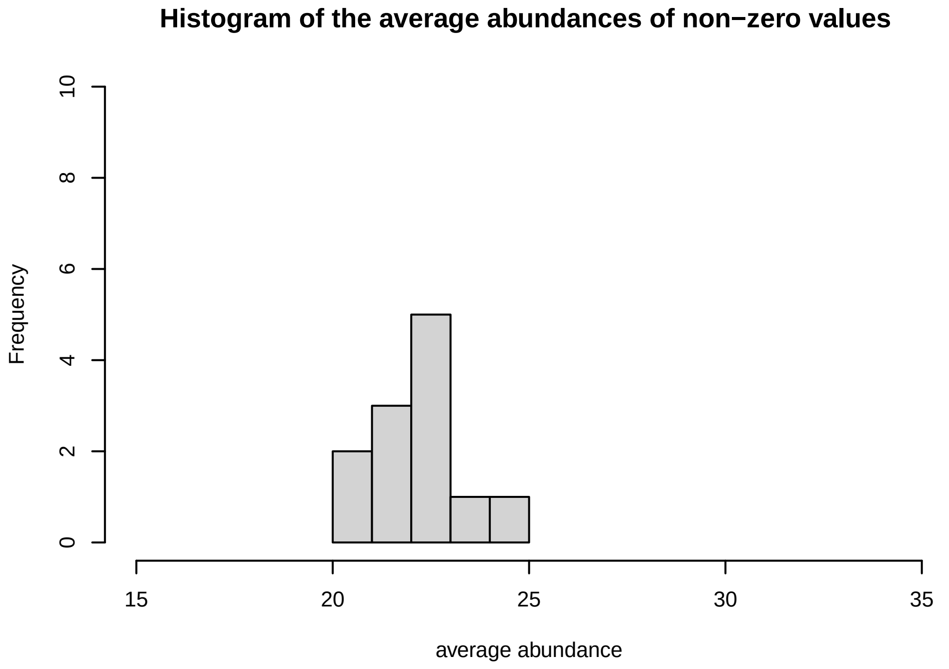
Histogram of the average abundances of non−zero values
Frequency
average abundance
0 2 4 6 8 10
15 20 25 30 35

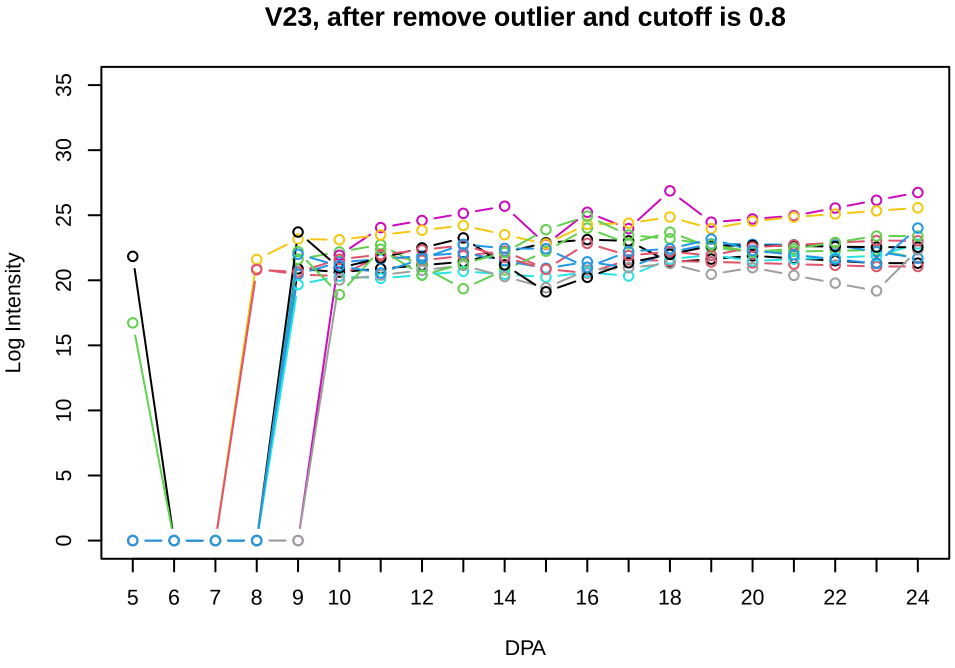
V23, after remove outlier and cutoff is 0.8
Log Intensity
DPA
0 5 10 15 20 25 30 35
5 6 7 8 9 10 12 14 16 18 20 22 24

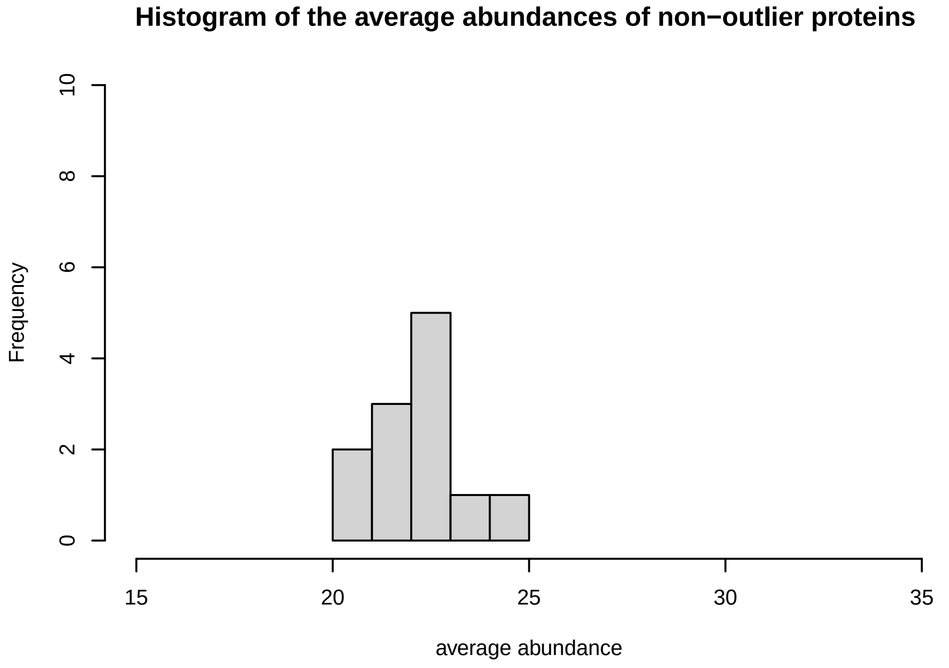
Histogram of the average abundances of non−outlier proteins
Frequency
average abundance
0 2 4 6 8 10
15 20 25 30 35

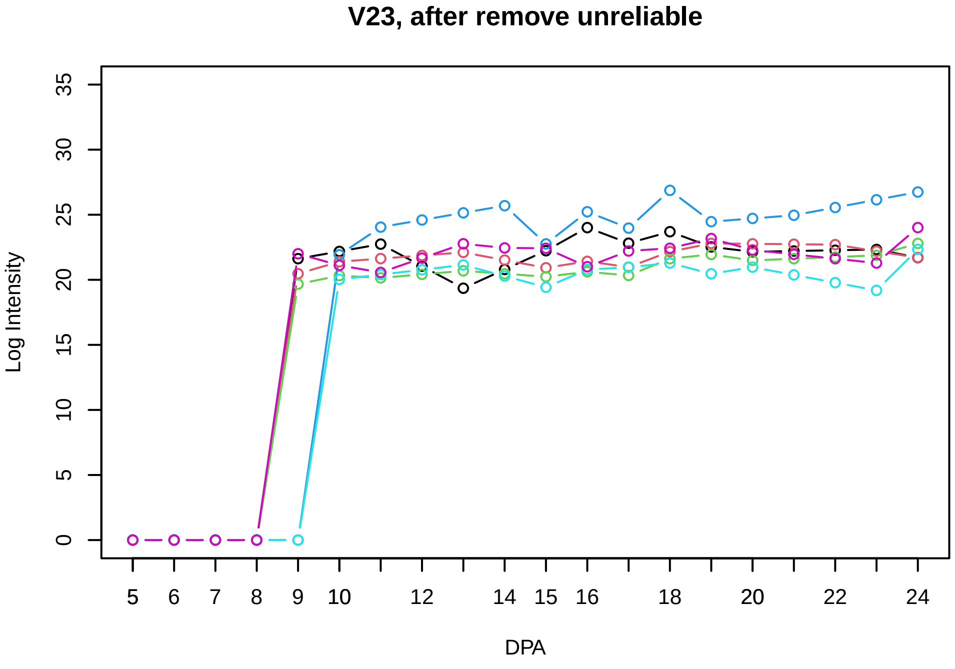
V23, after remove unreliable
Log Intensity
DPA
0 5 10 15 20 25 30 35
5 6 7 8 9 10 12 14 15 16 18 20 22 24

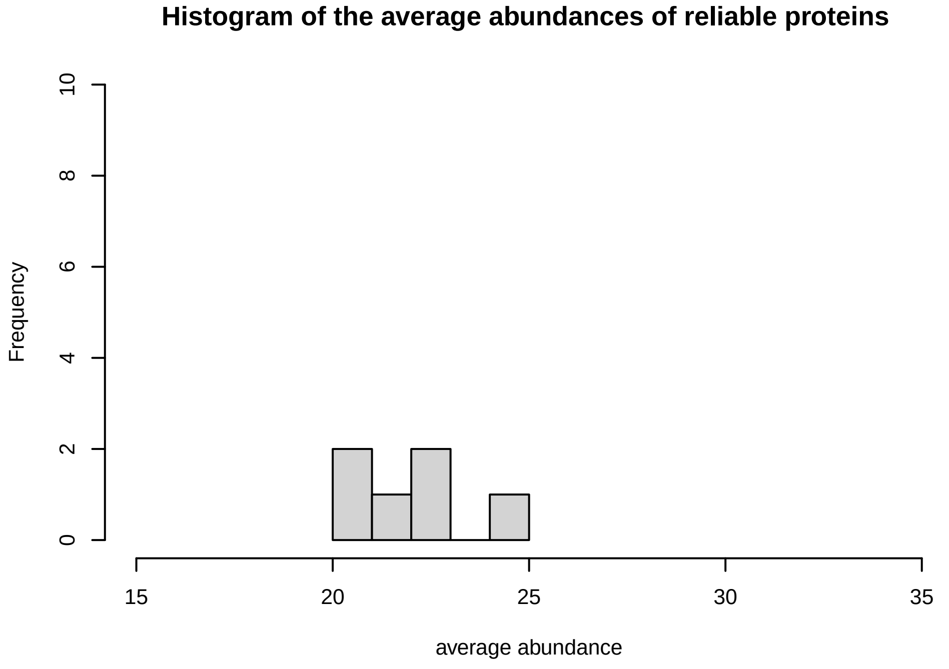
Histogram of the average abundances of reliable proteins
Frequency
average abundance
0 2 4 6 8 10
15 20 25 30 35

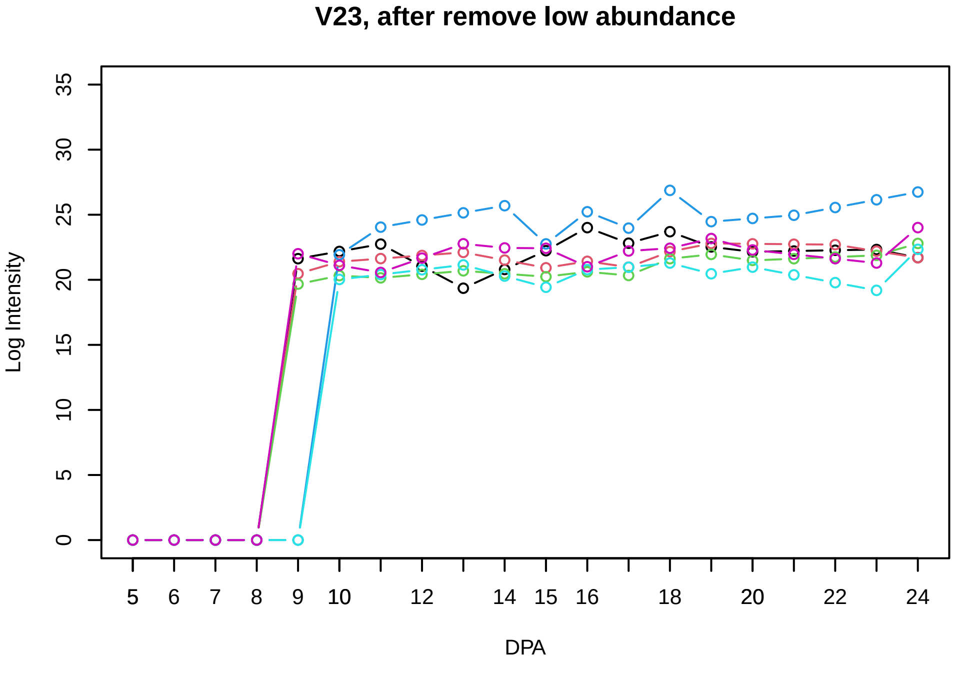
V23, after remove low abundance
Log Intensity
DPA
0 5 10 15 20 25 30 35
5 6 7 8 9 10 12 14 15 16 18 20 22 24

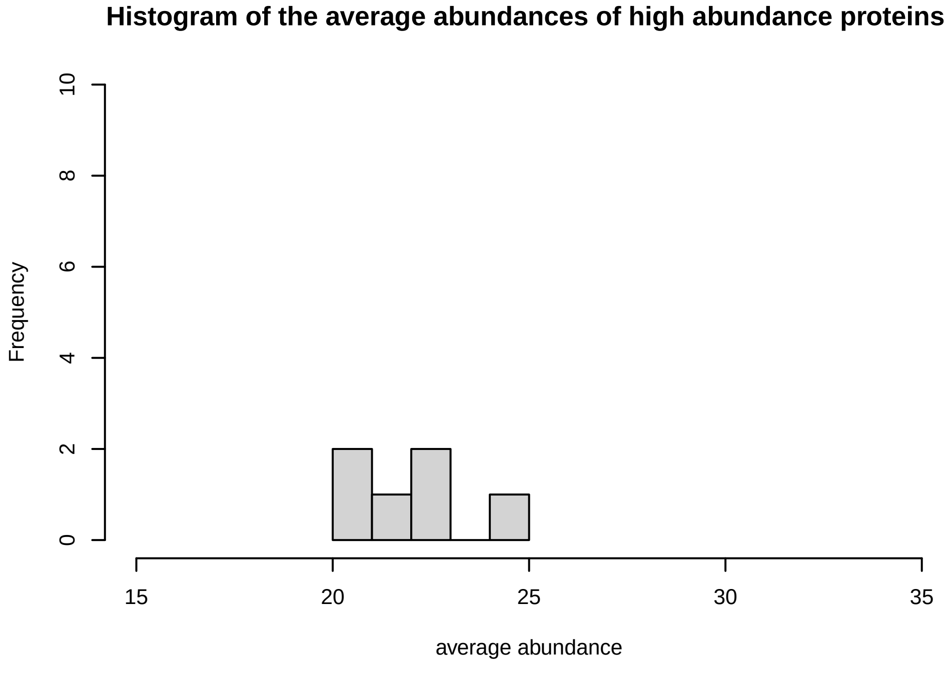
Histogram of the average abundances of high abundance proteins
Frequency
average abundance
0 2 4 6 8 10
15 20 25 30 35

V24

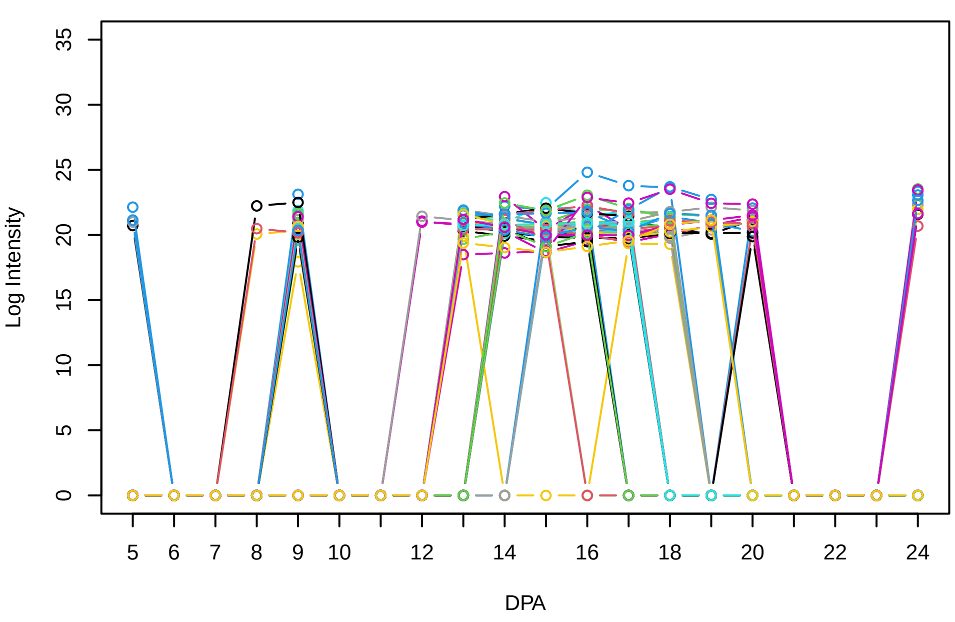

Histogram of the average abundances of non−zero values

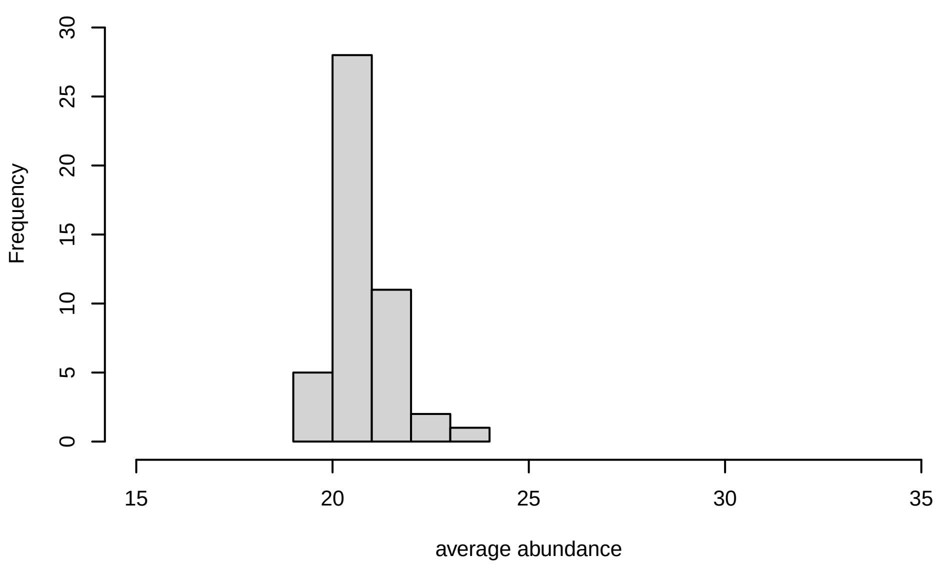

V24, after remove outlier and cutoff is 0.673441816473663

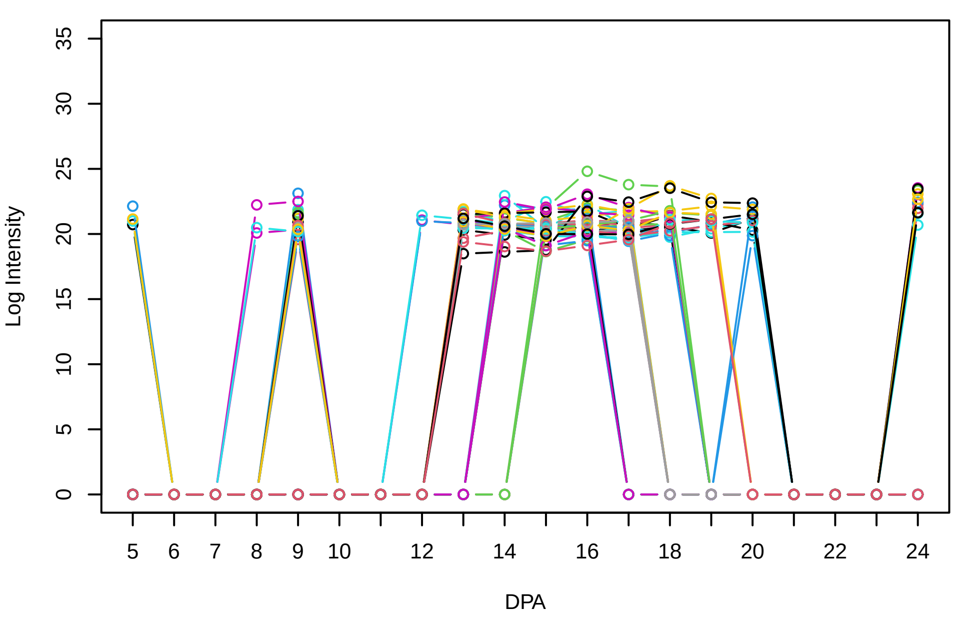

Histogram of the average abundances of non−outlier proteins

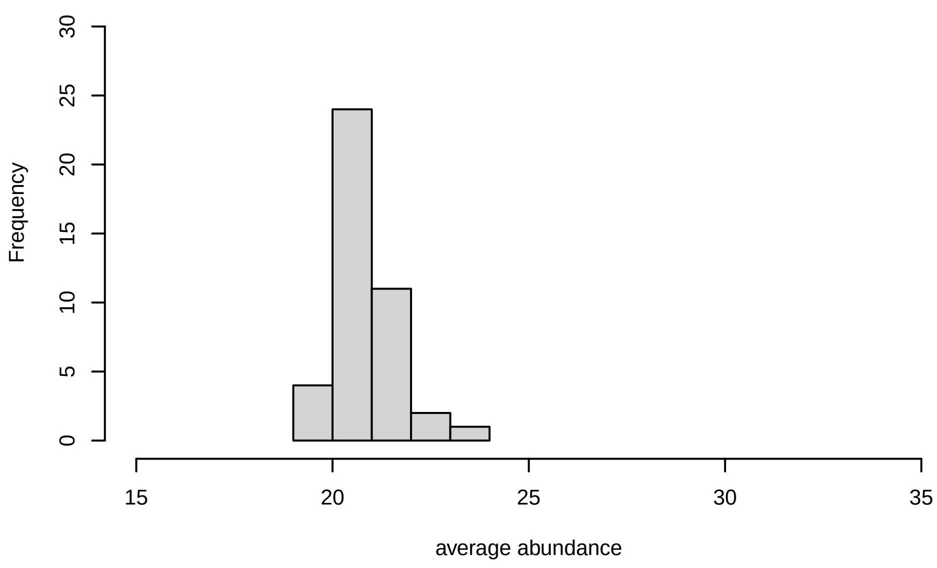

V24, after remove unreliable

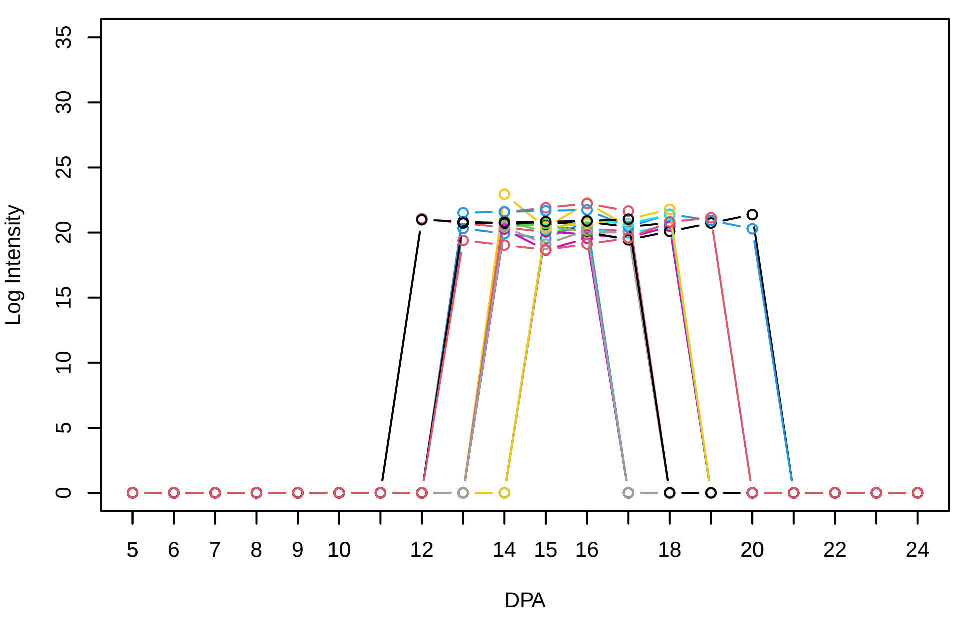

Histogram of the average abundances of reliable proteins

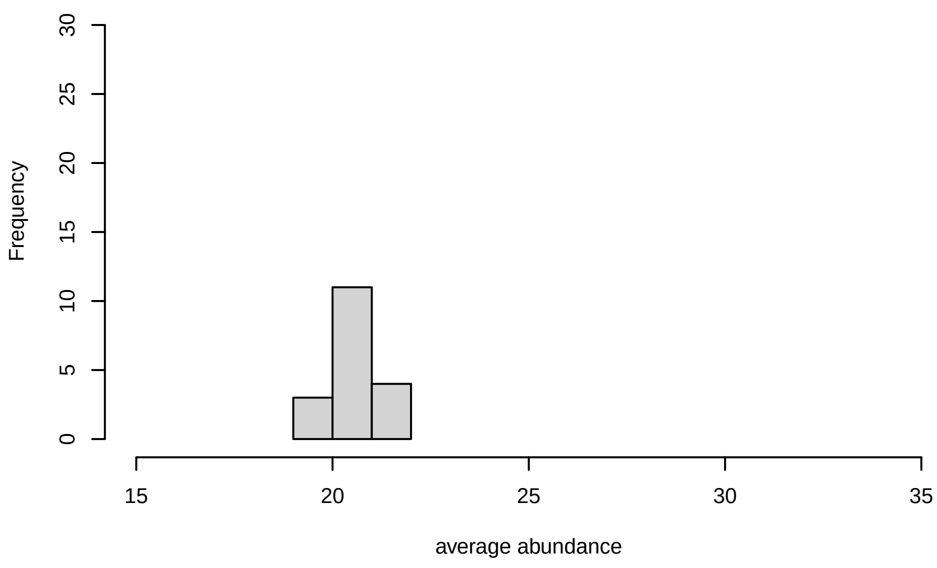

V24, after remove low abundance

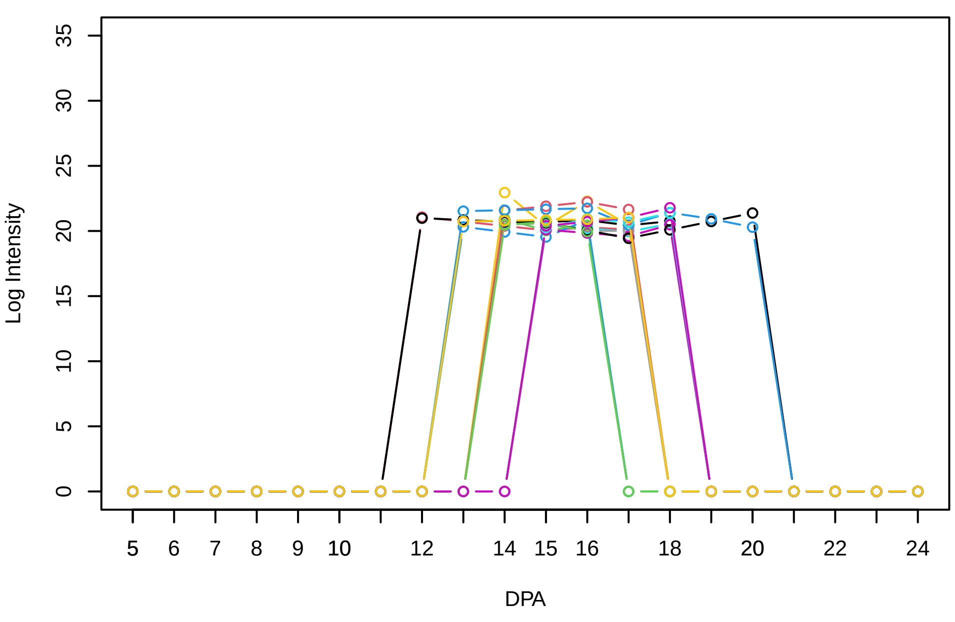

Histogram of the average abundances of high abundance proteins

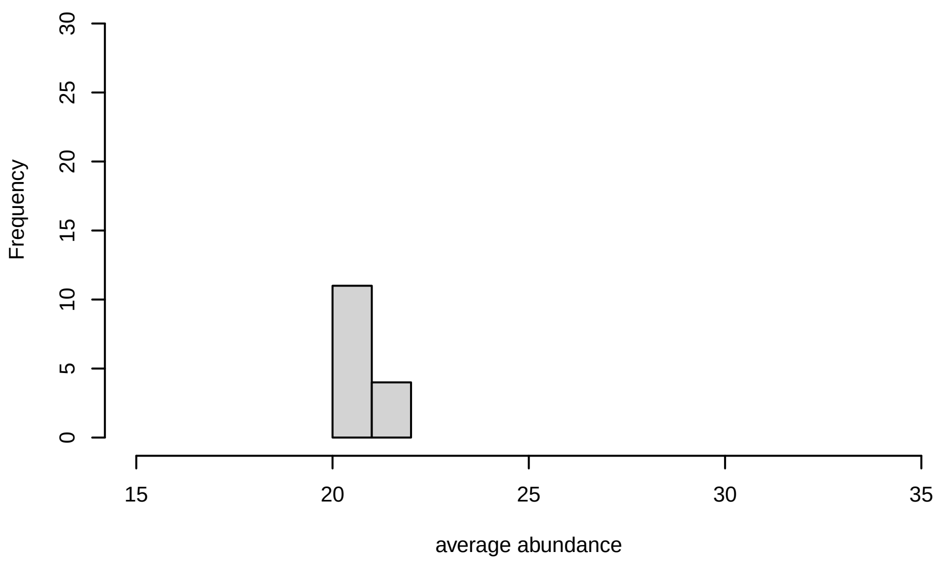

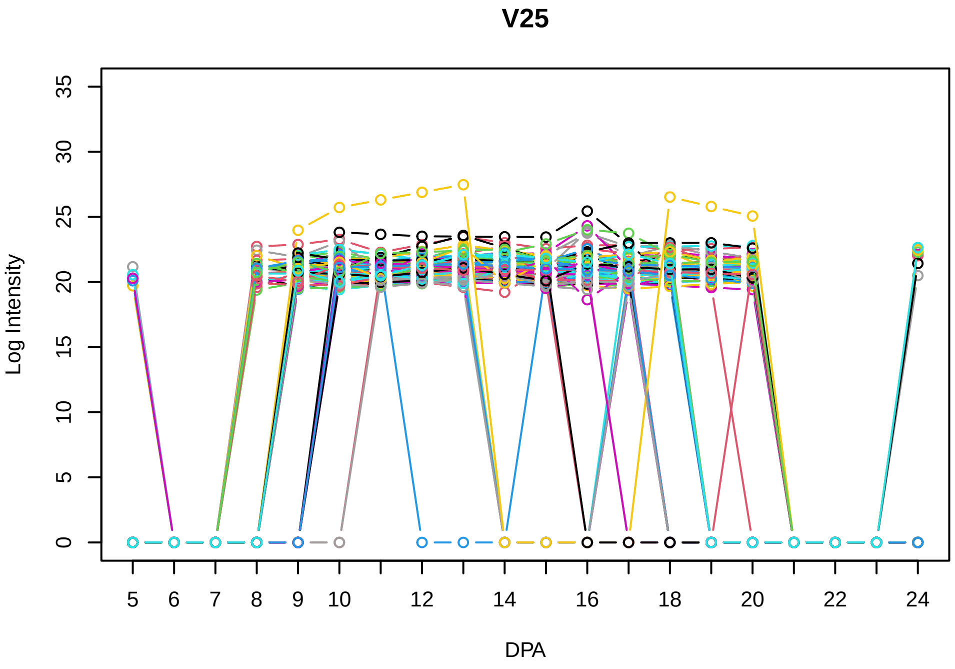
V25
Log Intensity
DPA

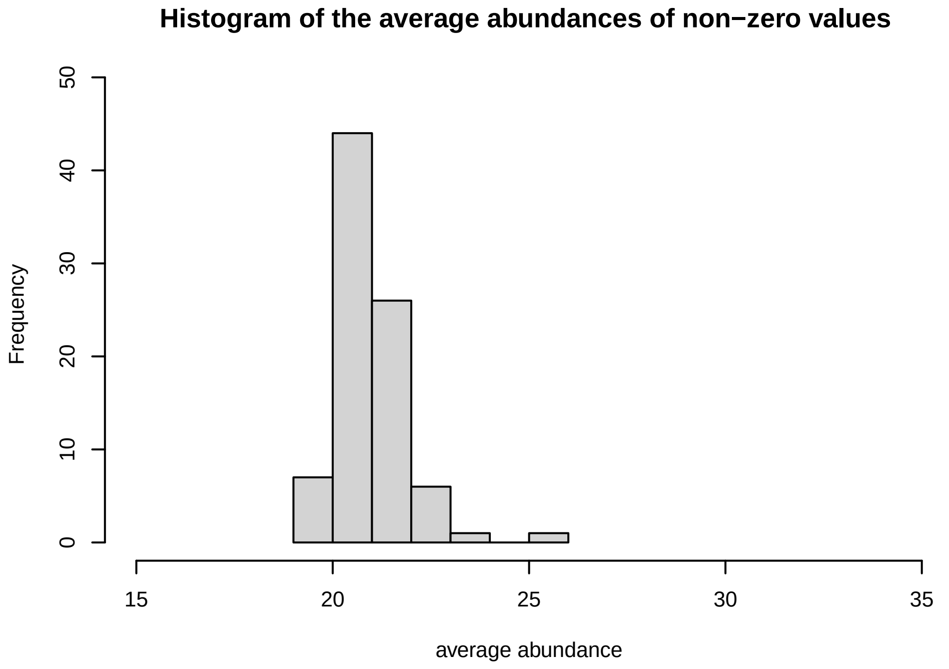
Histogram of the average abundances of non−zero values
Frequency
average abundance

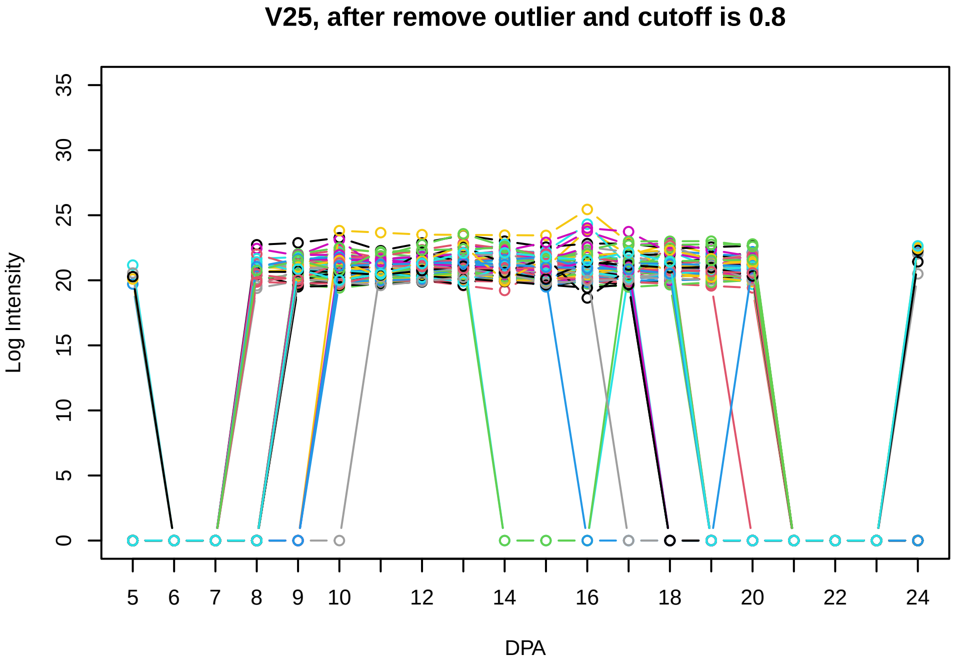
V25, after remove outlier and cutoff is 0.8
Log Intensity
DPA

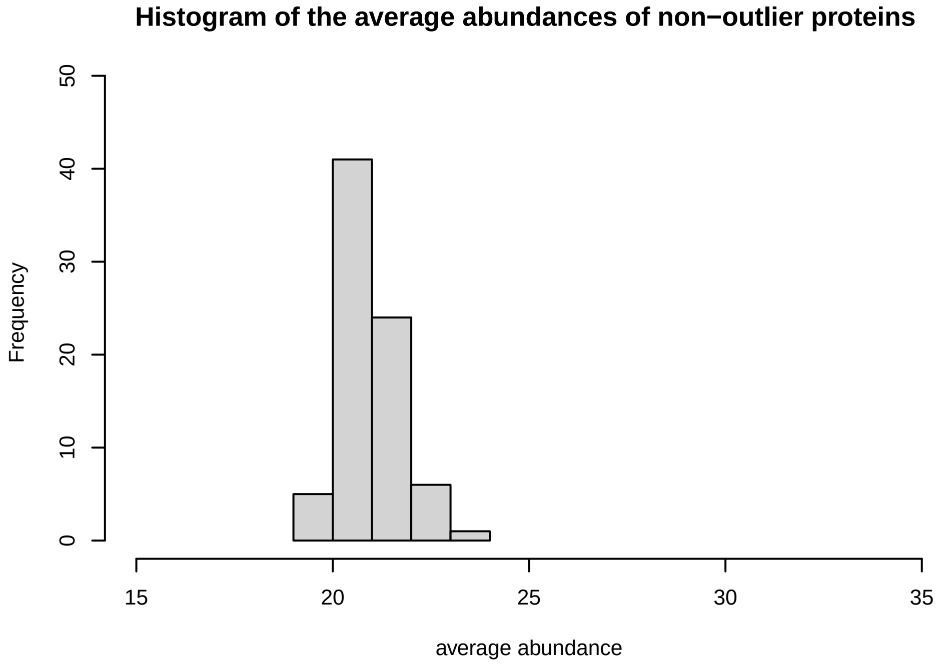
Histogram of the average abundances of non−outlier proteins
Frequency
average abundance

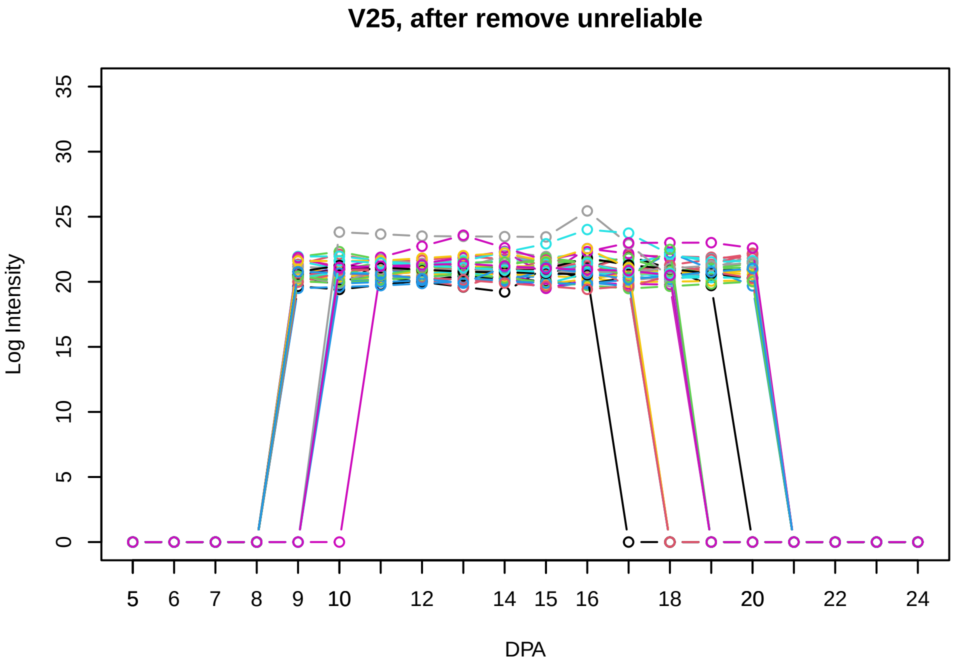
V25, after remove unreliable
Log Intensity
DPA

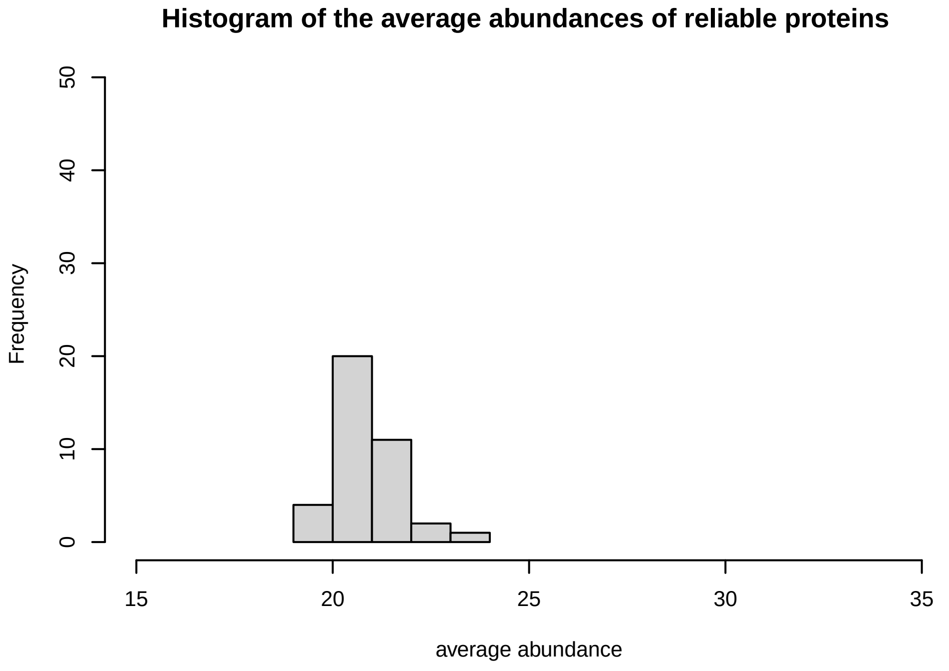
Histogram of the average abundances of reliable proteins
Frequency
average abundance

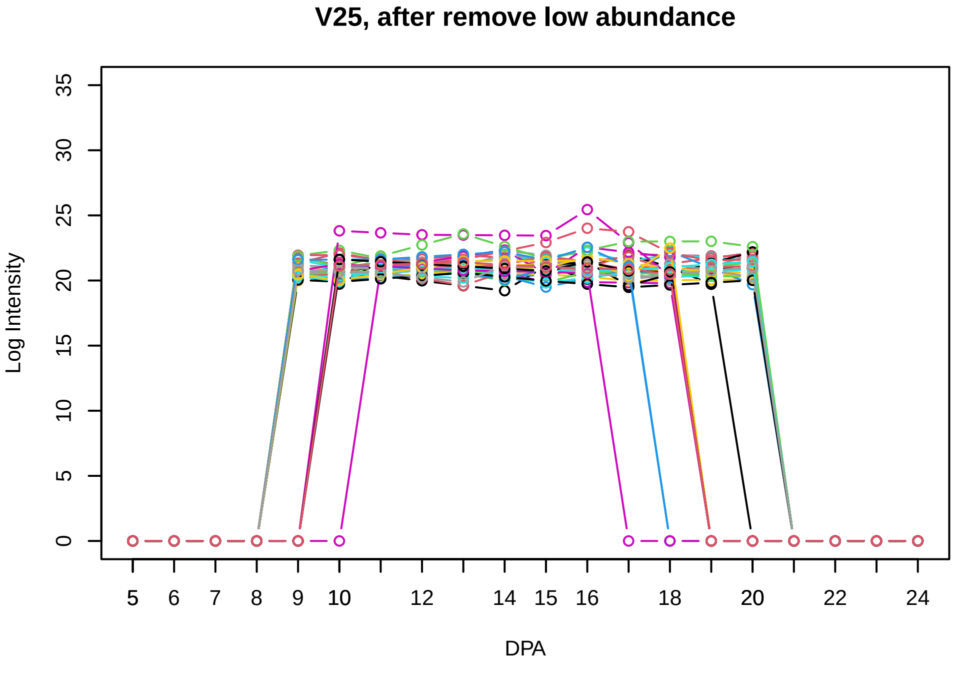
V25, after remove low abundance
Log Intensity
DPA

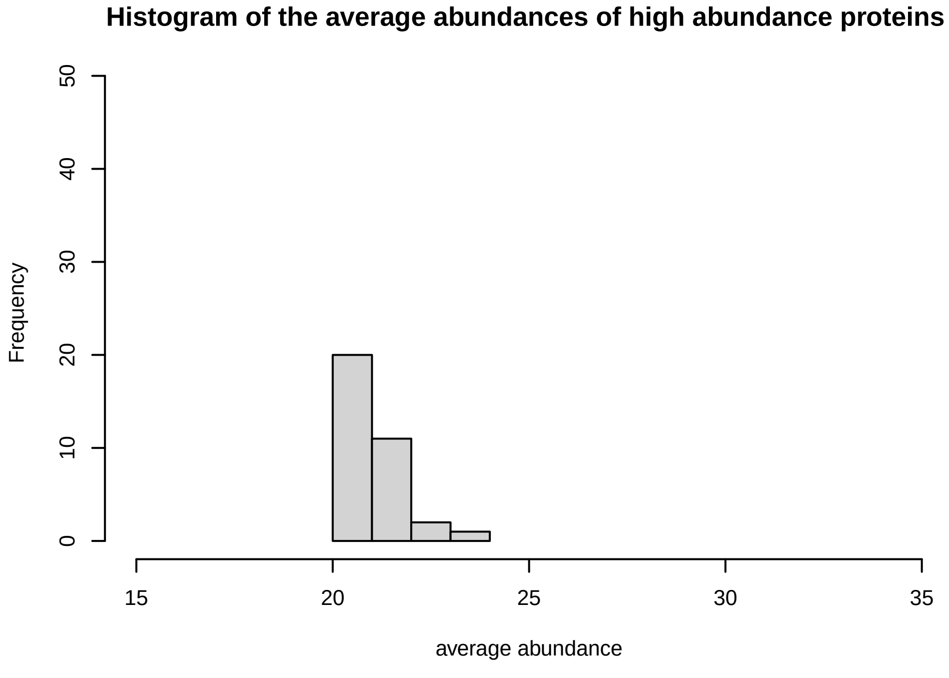
Histogram of the average abundances of high abundance proteins
Frequency
average abundance

V26

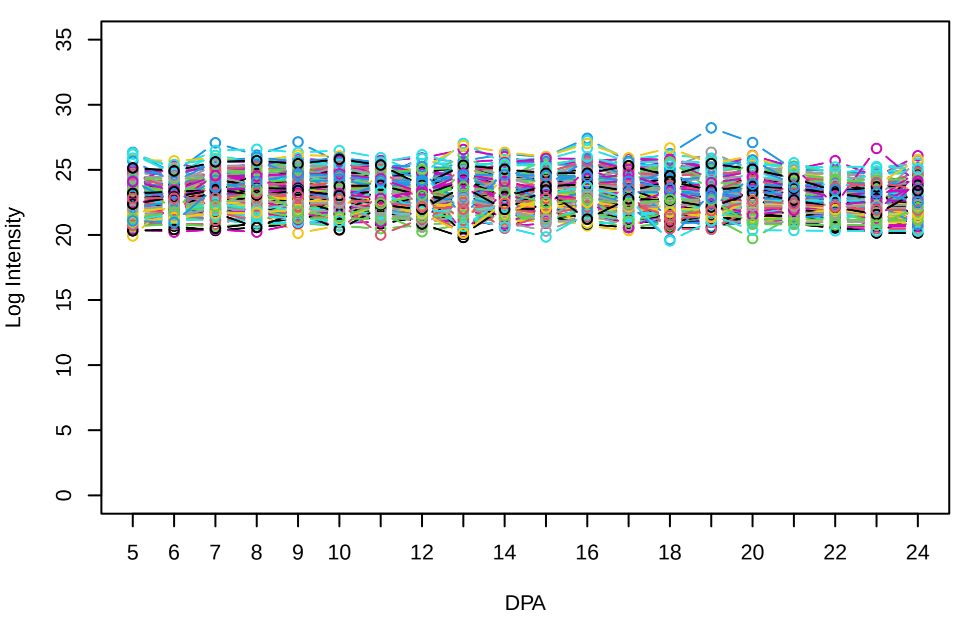

Histogram of the average abundances of non−zero values

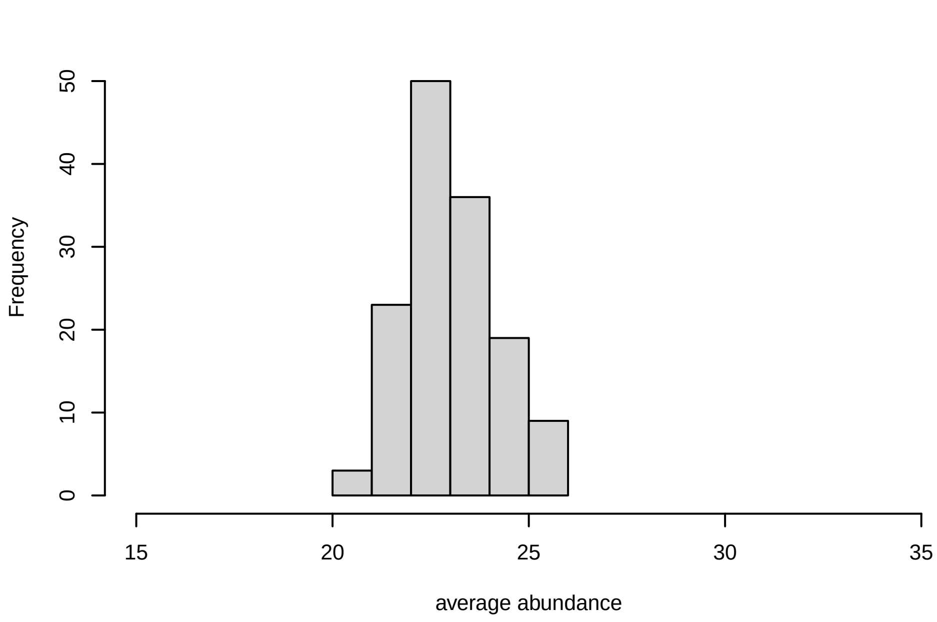

V26, after remove outlier and cutoff is 0.8

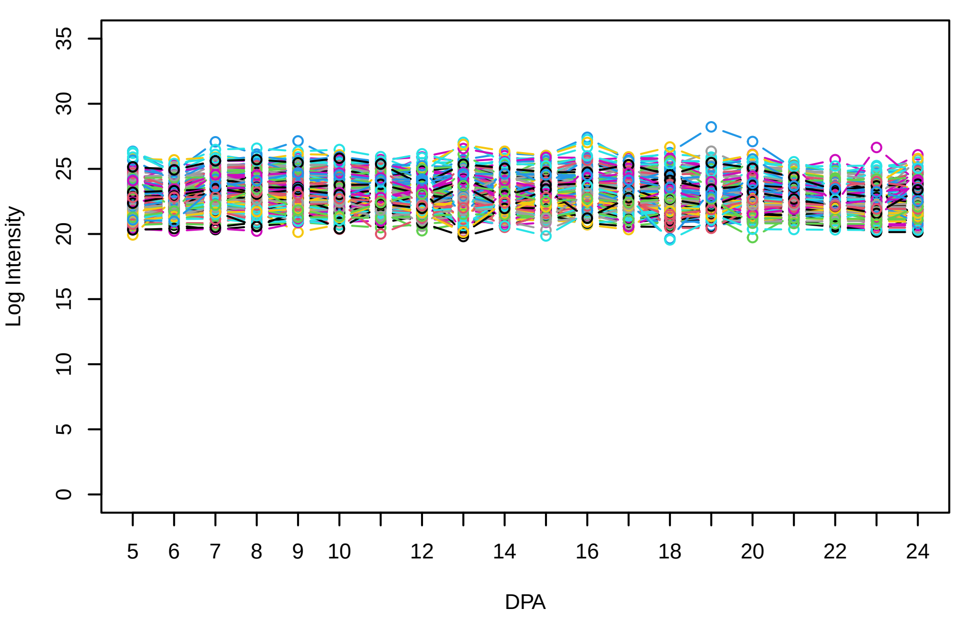

Histogram of the average abundances of non−outlier proteins

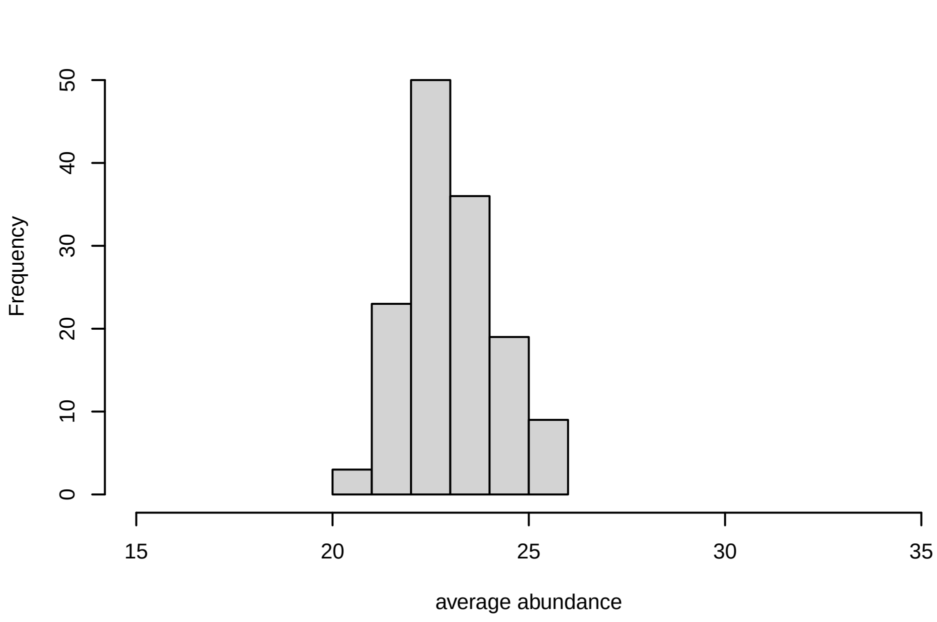

V26, after remove unreliable

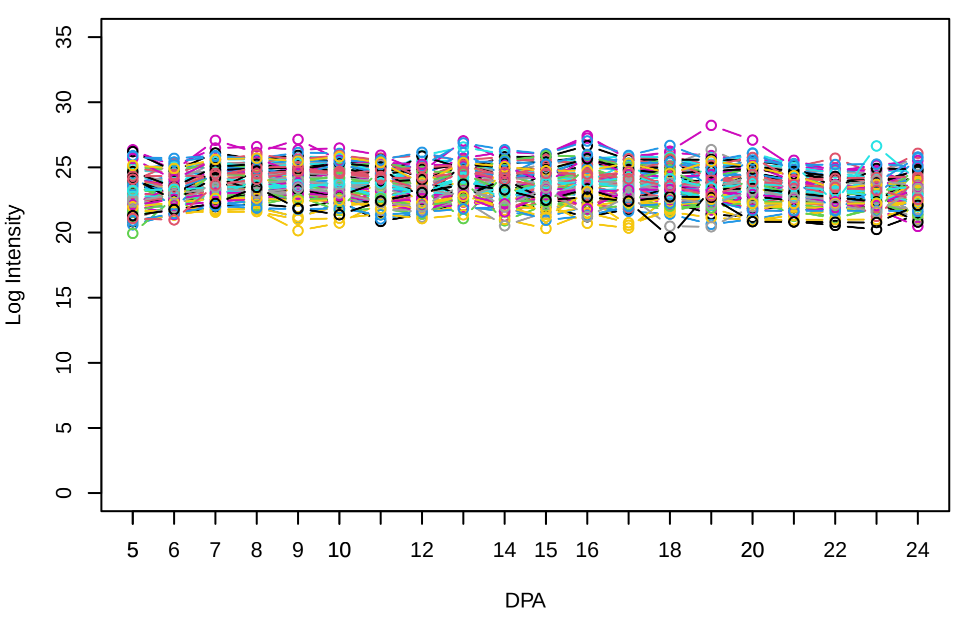

Histogram of the average abundances of reliable proteins

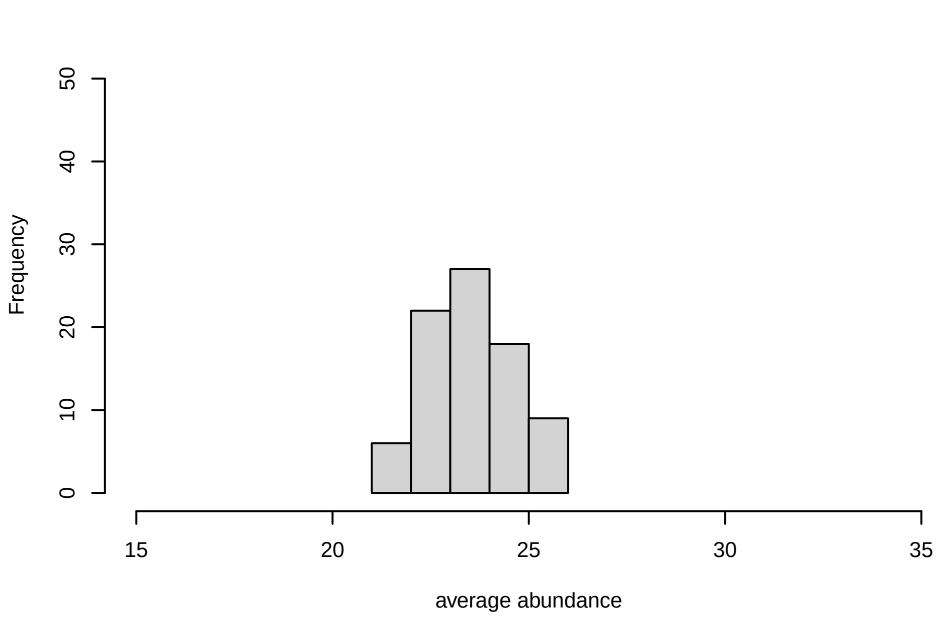

V26, after remove low abundance

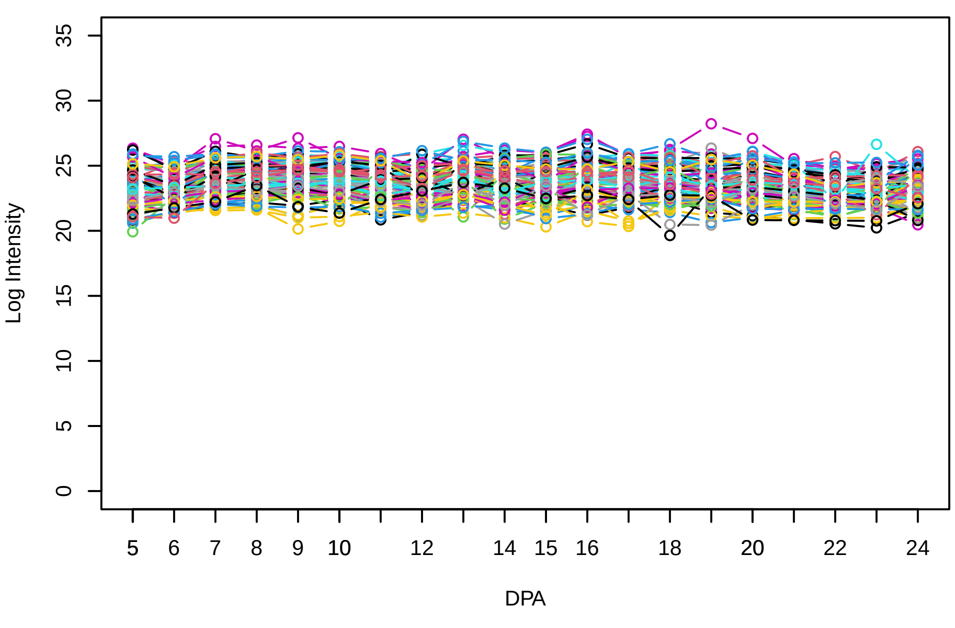

Histogram of the average abundances of high abundance proteins

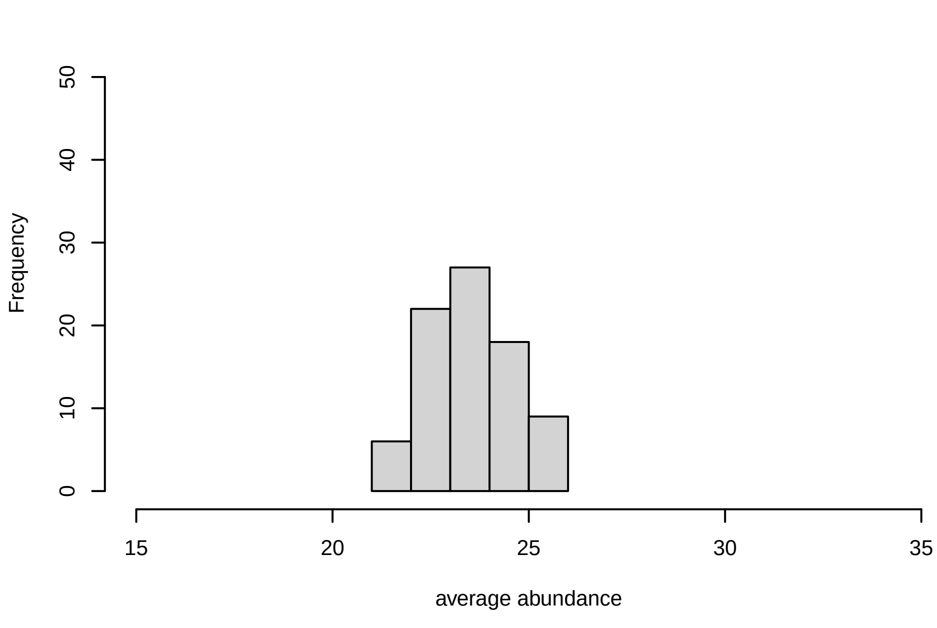

V27

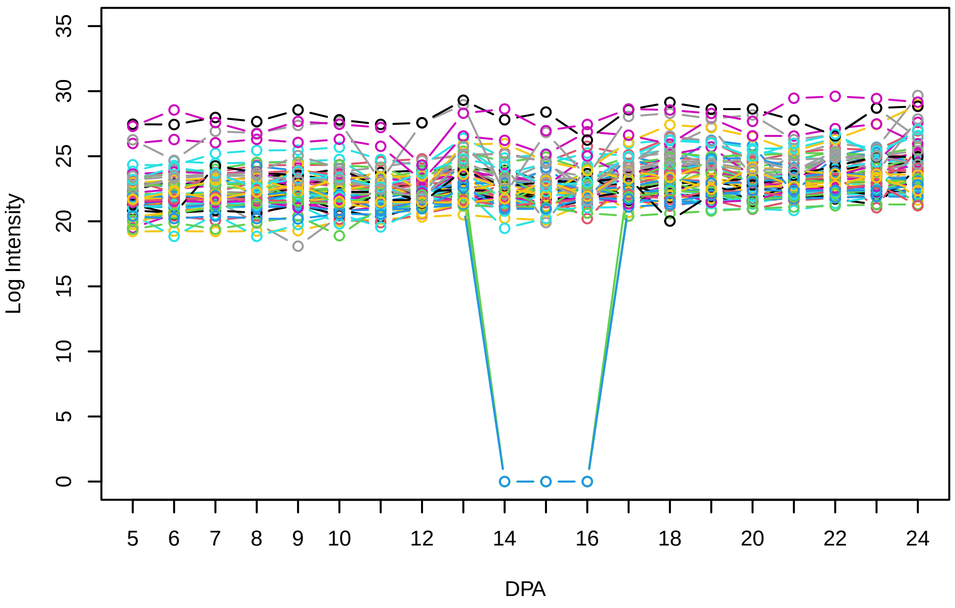

Histogram of the average abundances of non−zero values

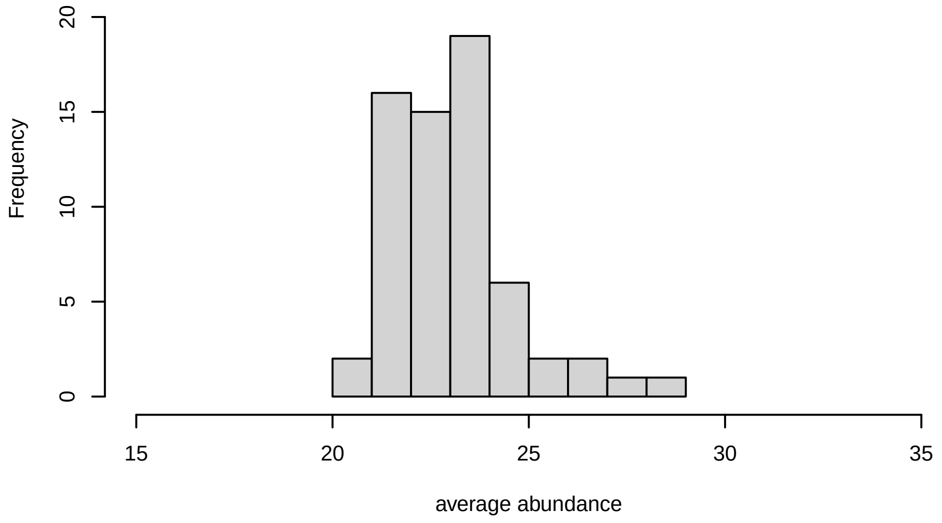

V27, after remove outlier and cutoff is 0.8

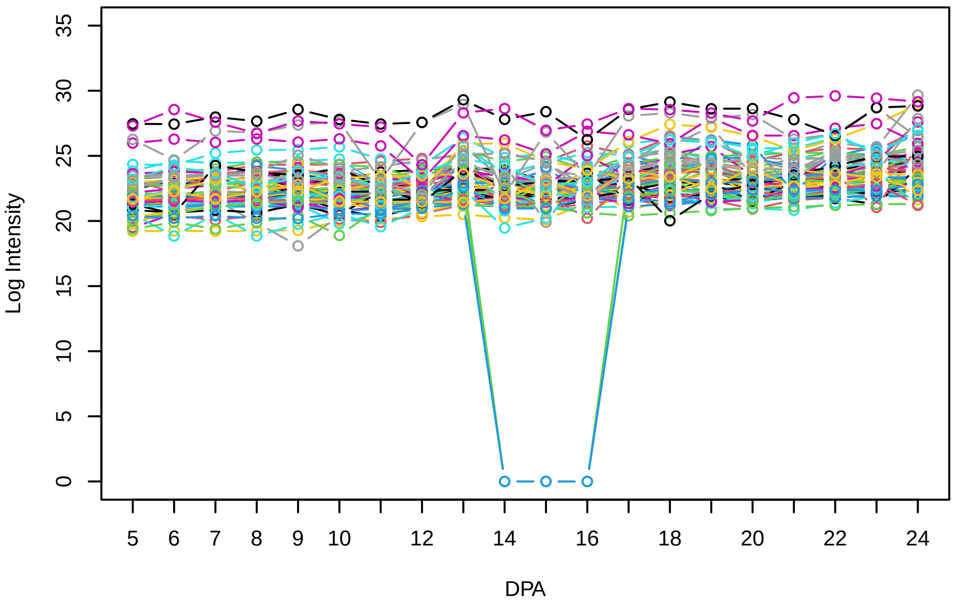

Histogram of the average abundances of non−outlier proteins

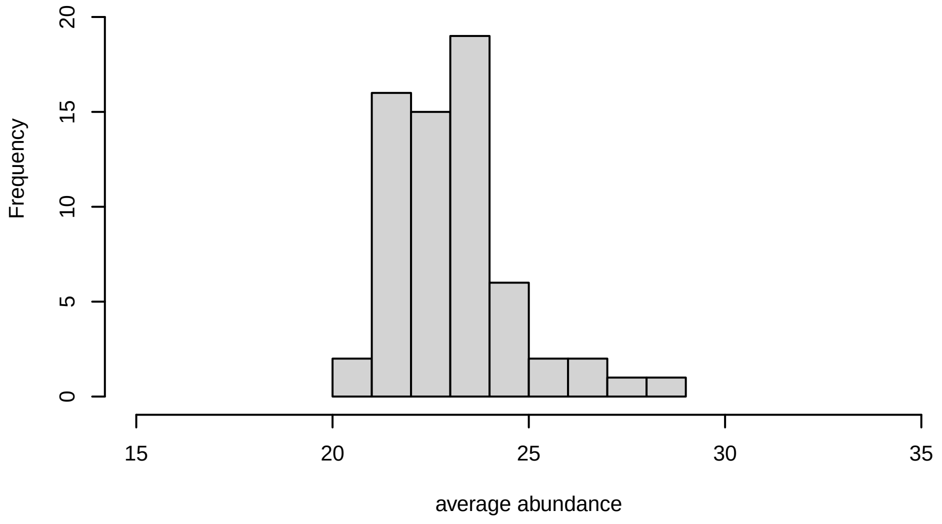

V27, after remove unreliable

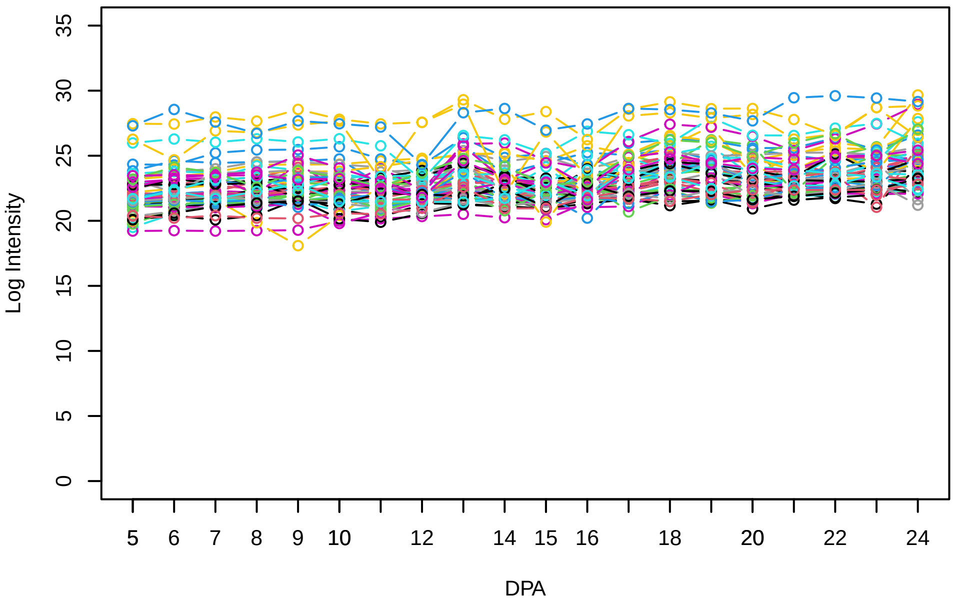

Histogram of the average abundances of reliable proteins

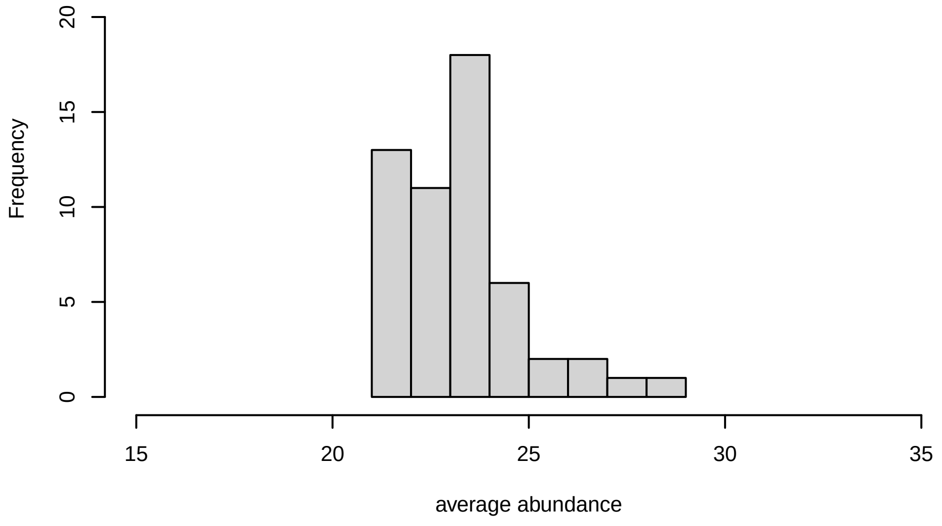

V27, after remove low abundance

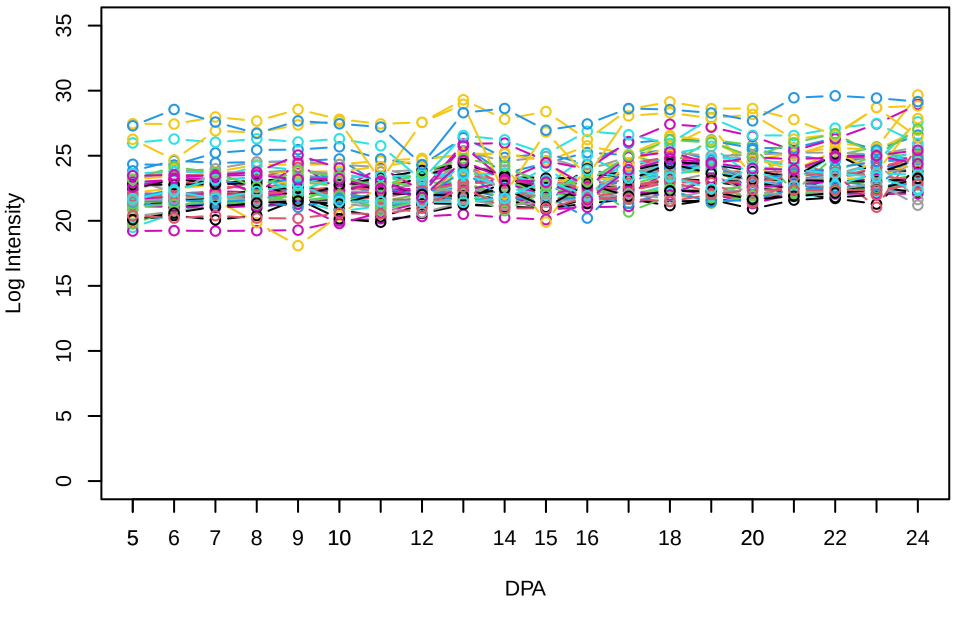

Histogram of the average abundances of high abundance proteins

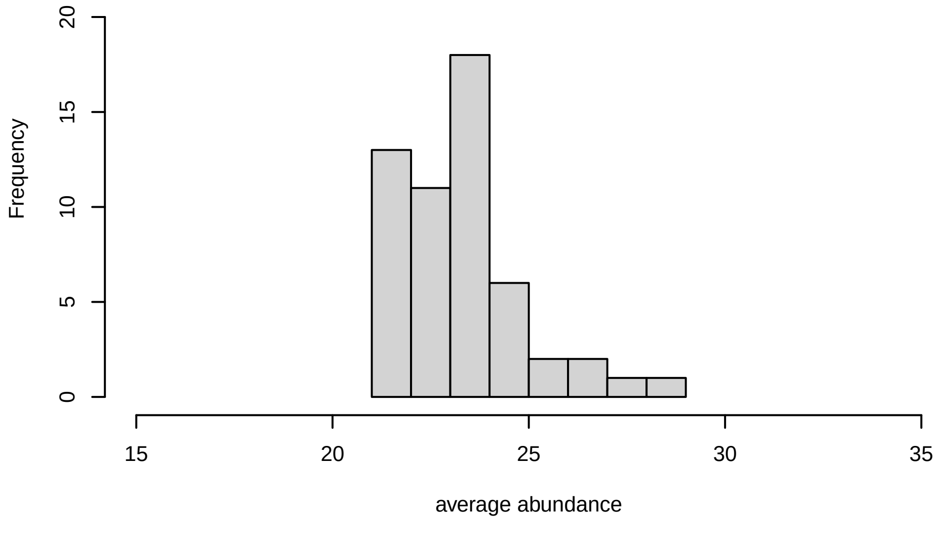

V28

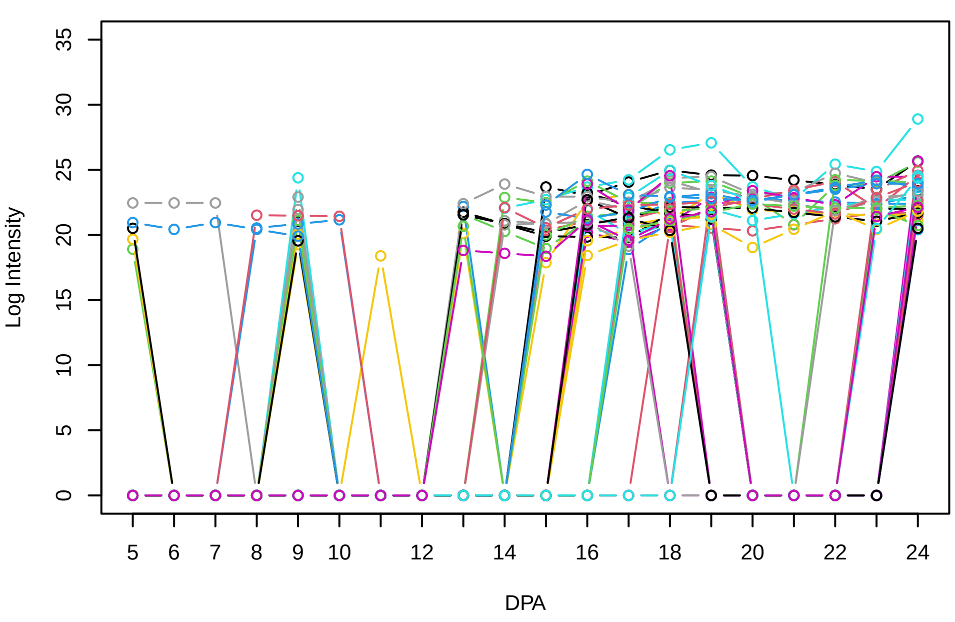

Histogram of the average abundances of non−zero values

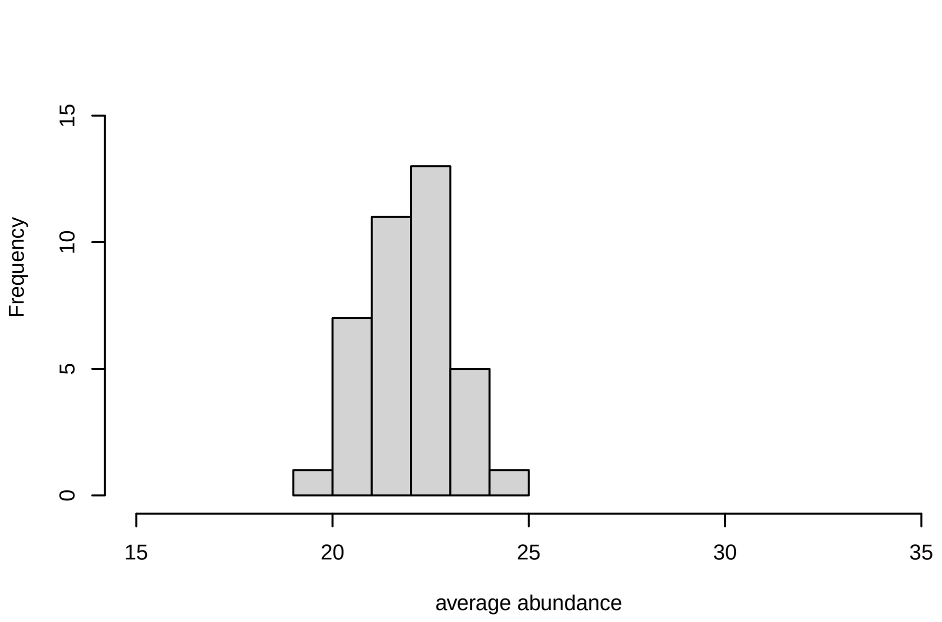

V28, after remove outlier and cutoff is 0.656944625404285

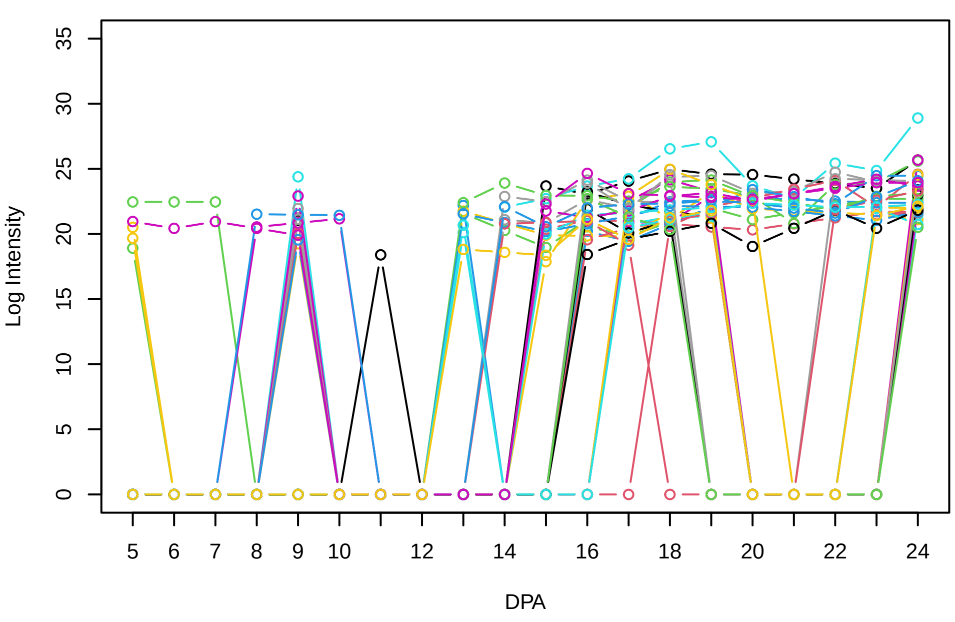

Histogram of the average abundances of non−outlier proteins

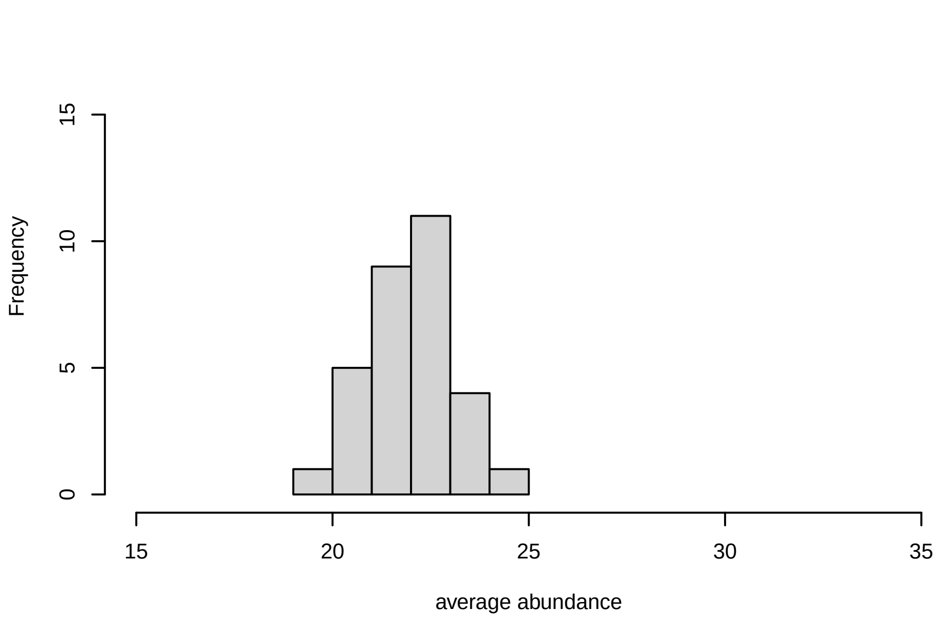

V28, after remove unreliable

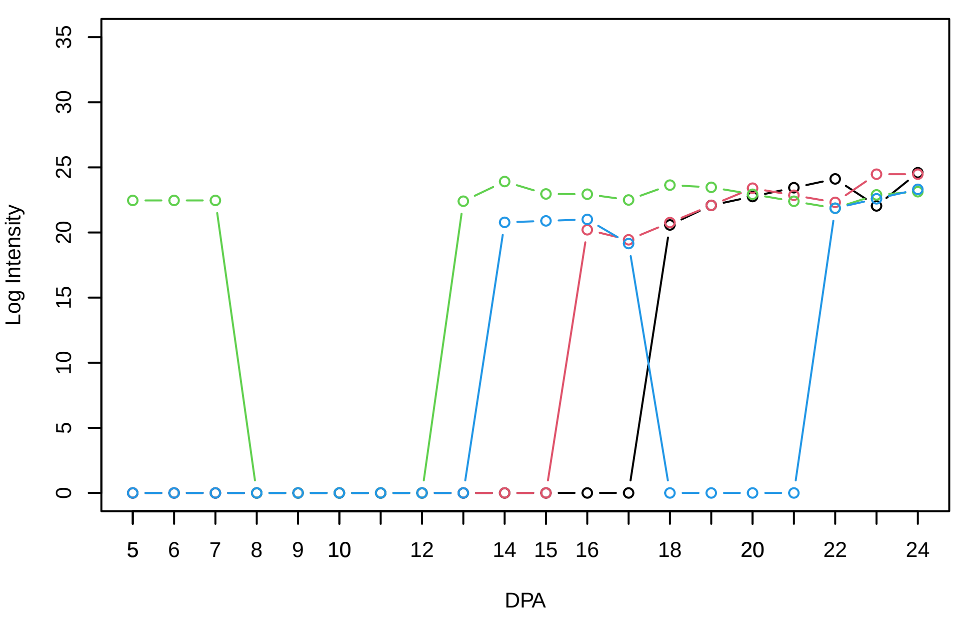

Histogram of the average abundances of reliable proteins

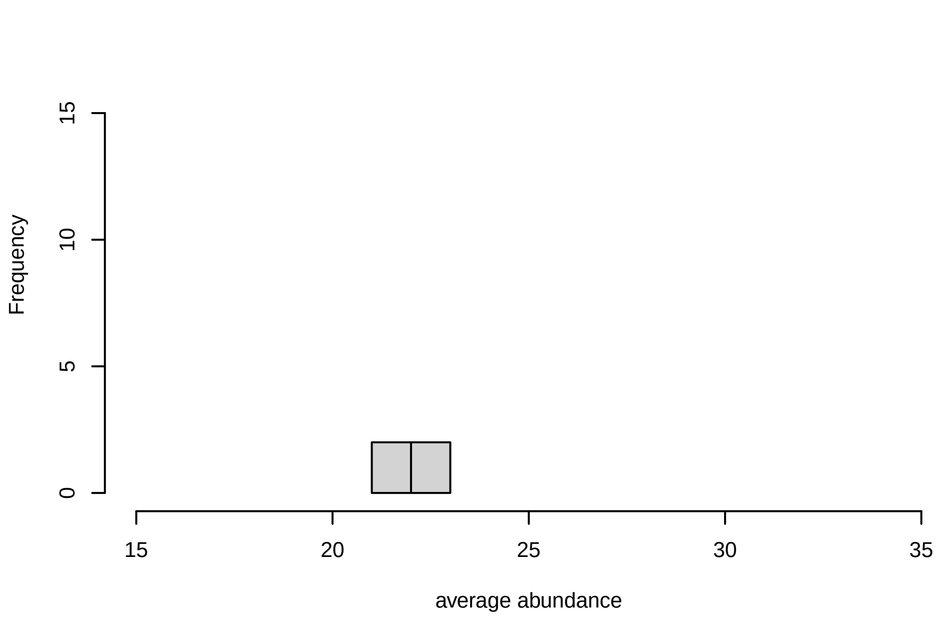

V28, after remove low abundance

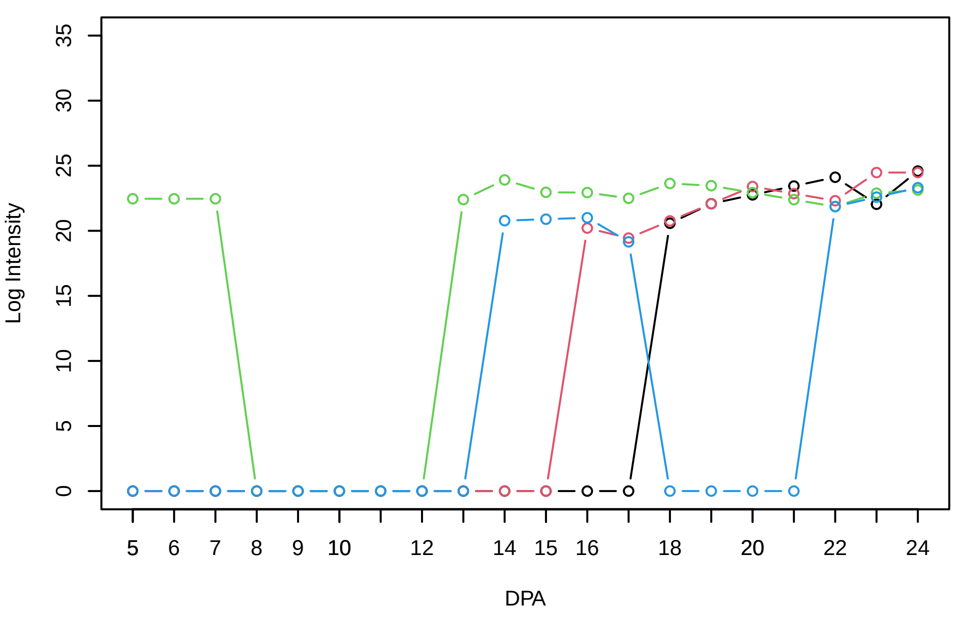

Histogram of the average abundances of high abundance proteins

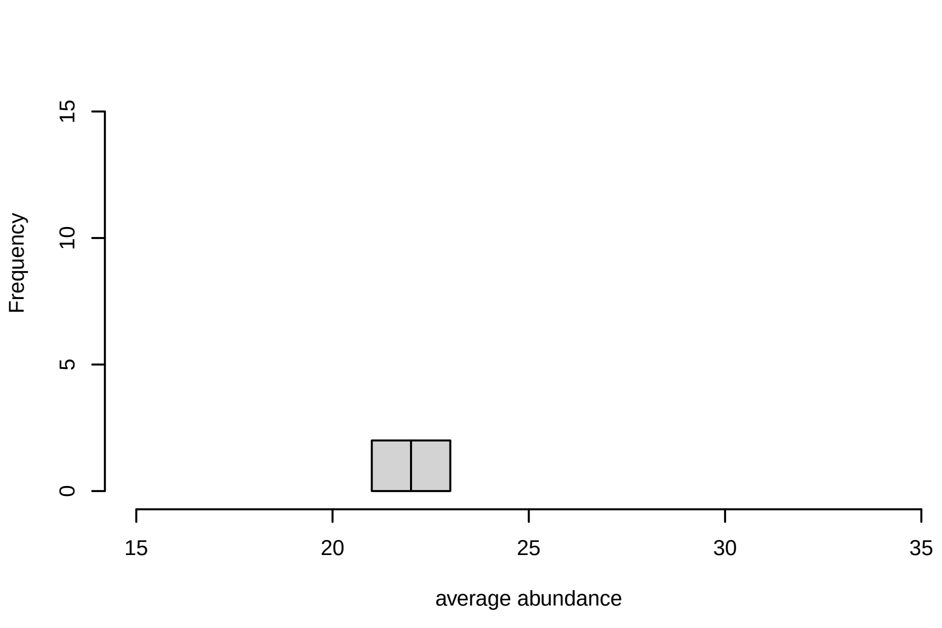

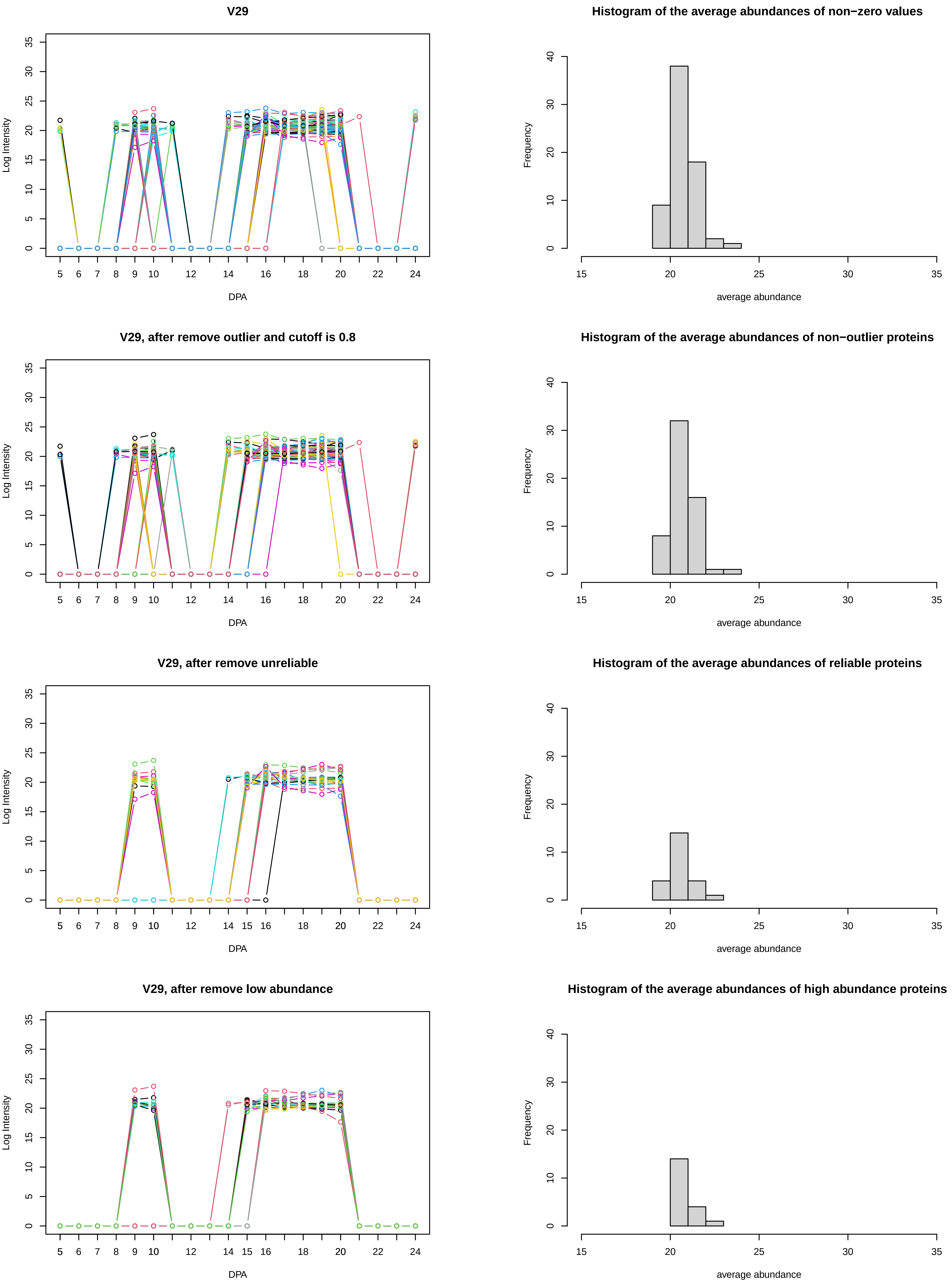
V29
Log Intensity
DPA
Histogram of the average abundances of non−zero values
Frequency
average abundance
V29, after remove outlier and cutoff is 0.8
Log Intensity
DPA
Histogram of the average abundances of non−outlier proteins
Frequency
average abundance
V29, after remove unreliable
Log Intensity
DPA
Histogram of the average abundances of reliable proteins
Frequency
average abundance
V29, after remove low abundance
Log Intensity
DPA
Histogram of the average abundances of high abundance proteins
Frequency
average abundance

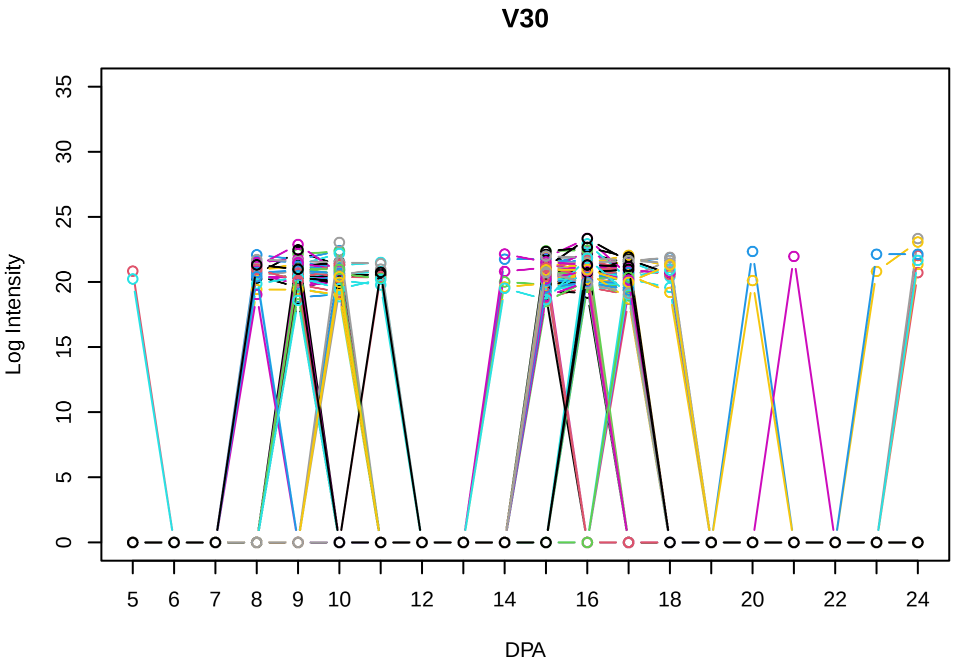
V30
Log Intensity
DPA

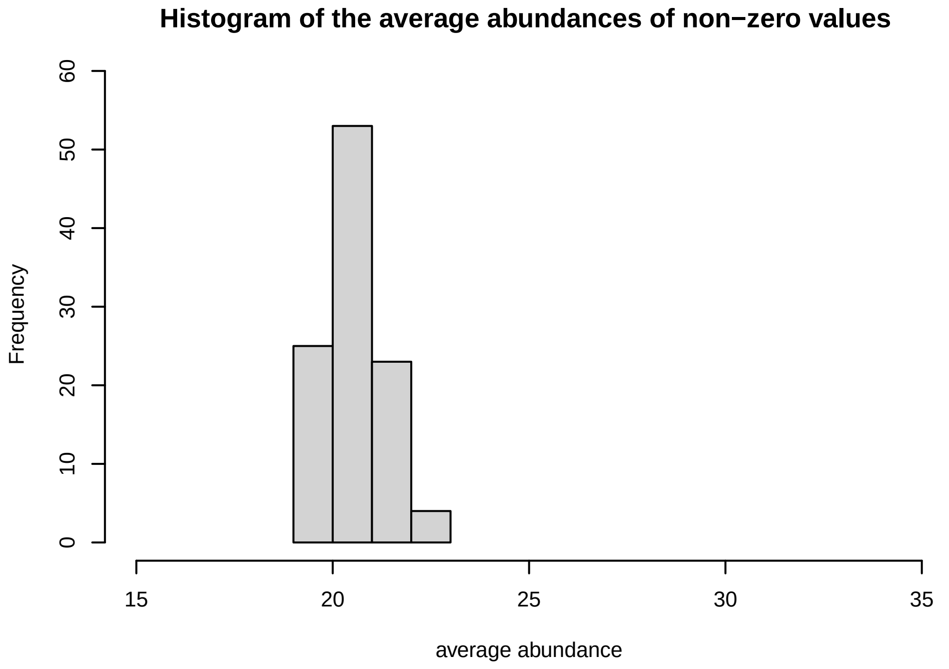
Histogram of the average abundances of non−zero values
Frequency
average abundance

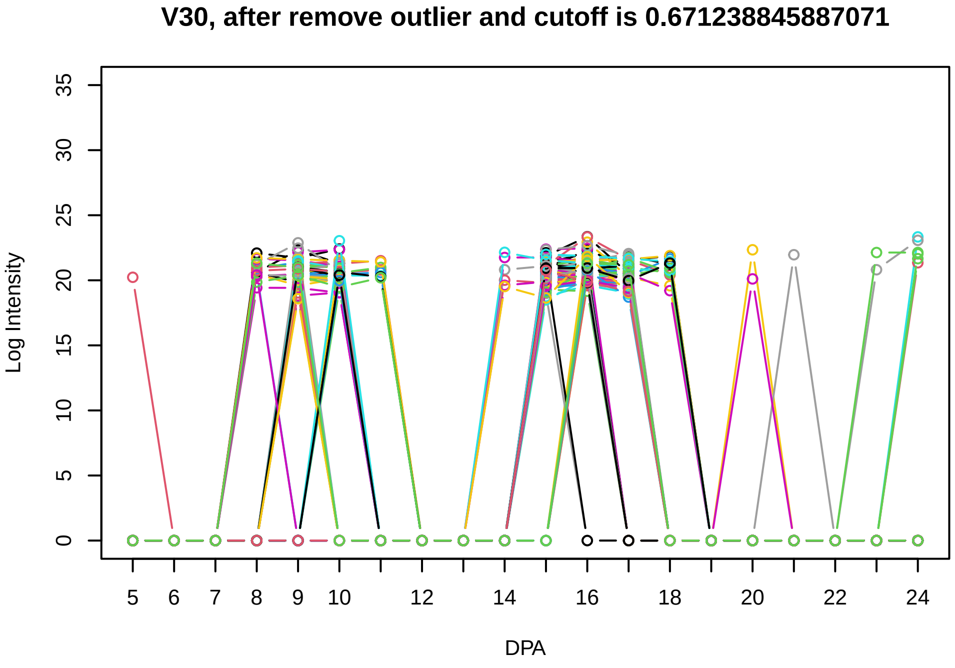
V30, after remove outlier and cutoff is 0.671238845887071
Log Intensity
DPA

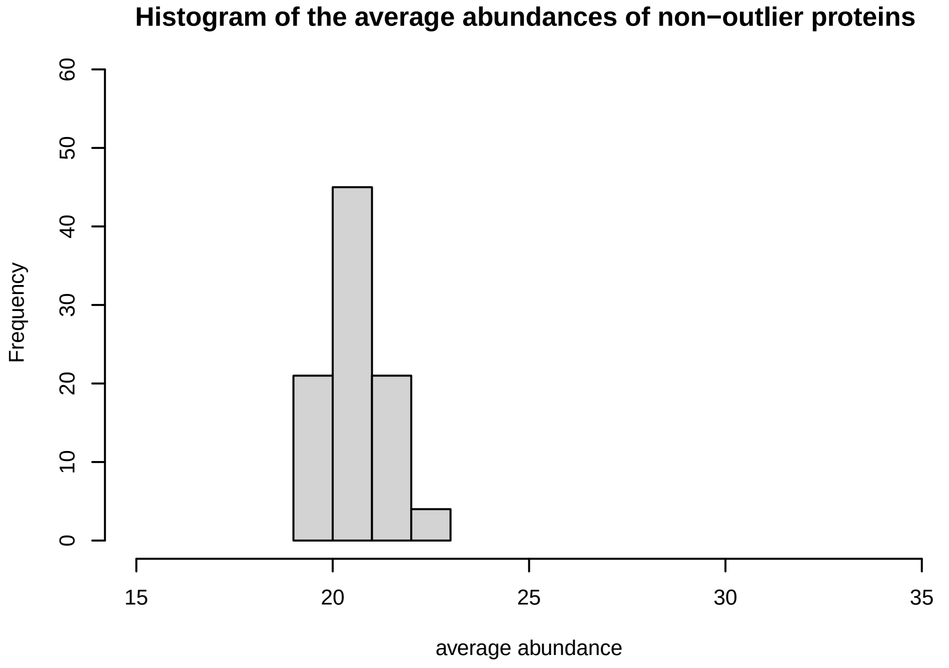
Histogram of the average abundances of non−outlier proteins
Frequency
average abundance

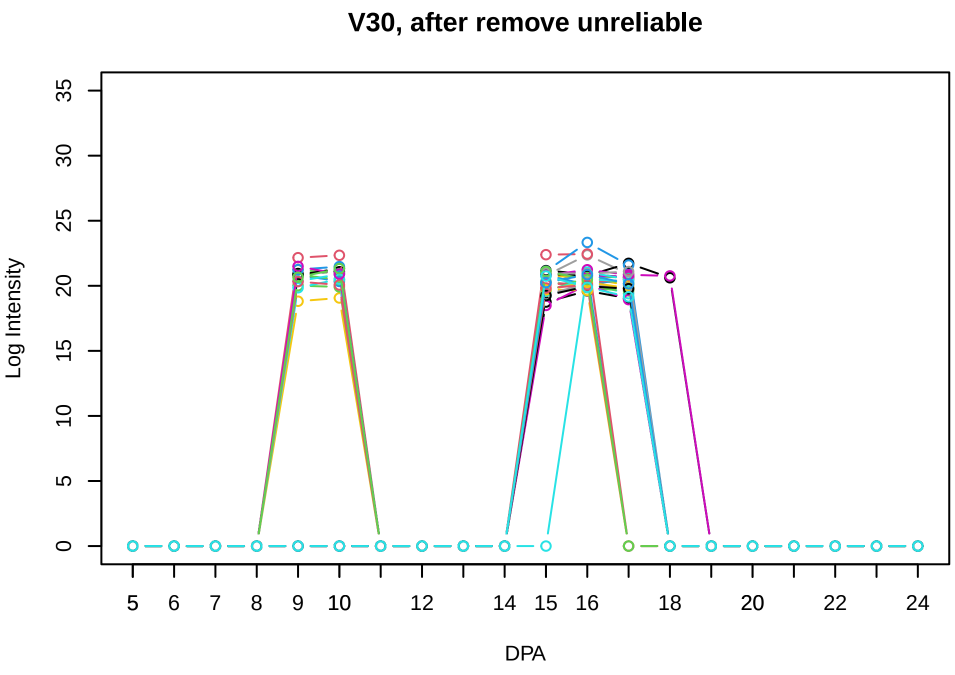
V30, after remove unreliable
Log Intensity
DPA

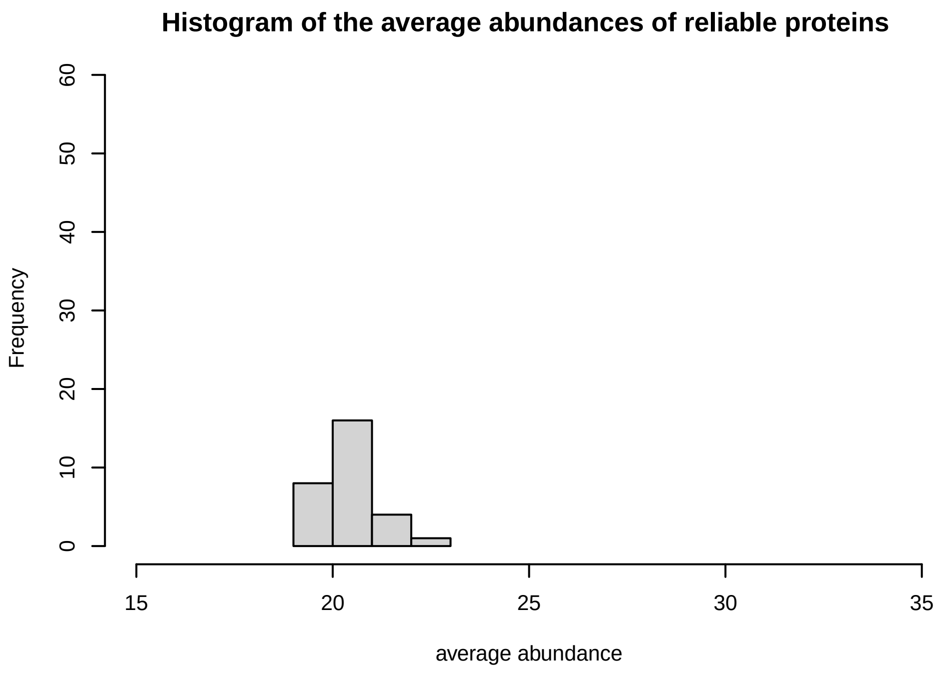
Histogram of the average abundances of reliable proteins
Frequency
average abundance

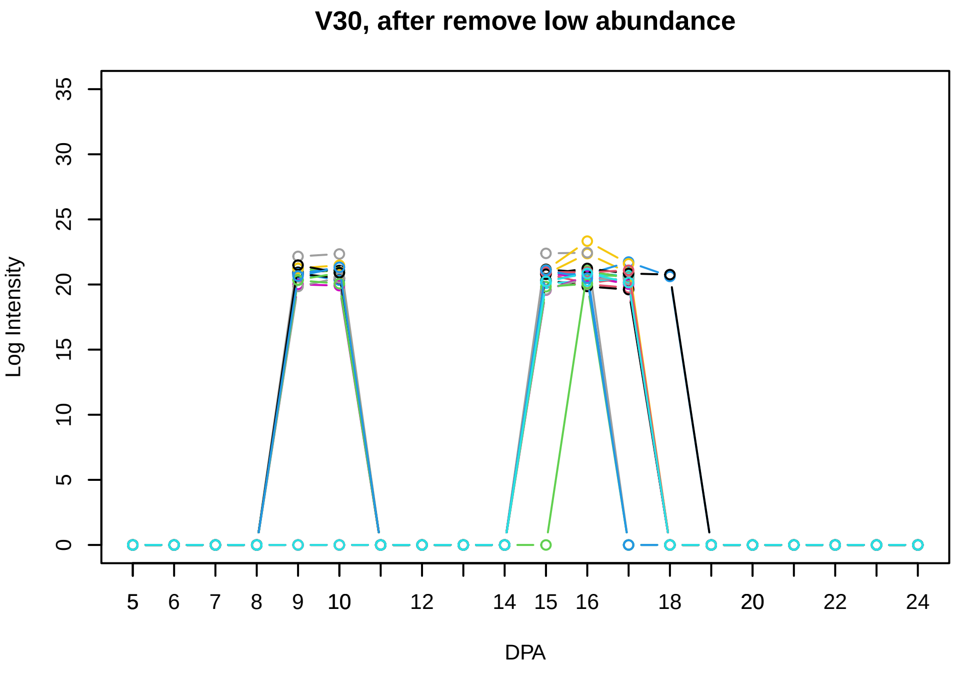
V30, after remove low abundance
Log Intensity
DPA

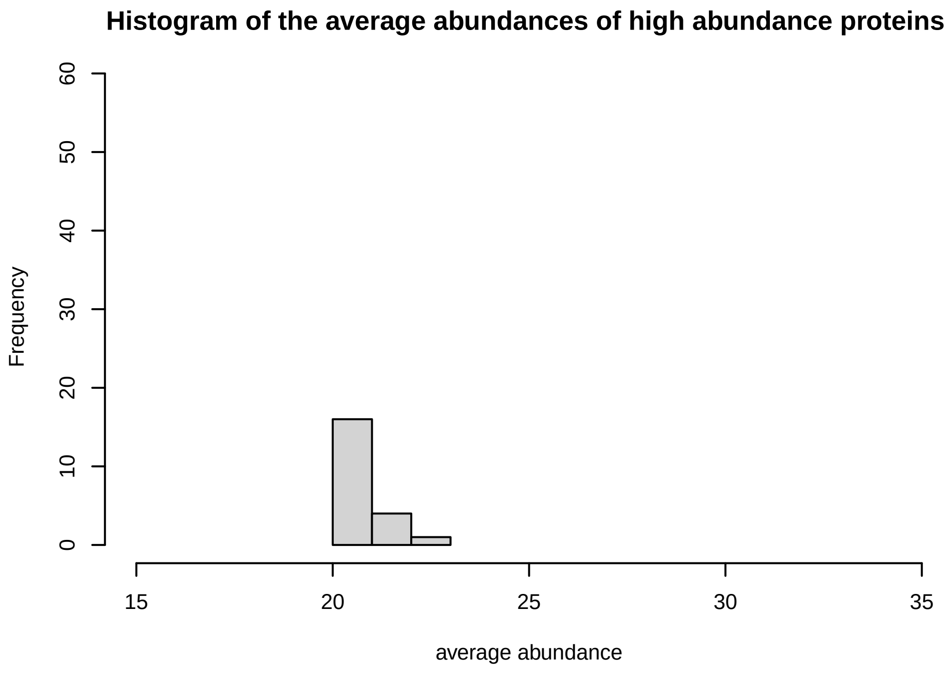
Histogram of the average abundances of high abundance proteins
Frequency
average abundance

Supplementary Figure S4. SOM grouping and profile filtering (P200).

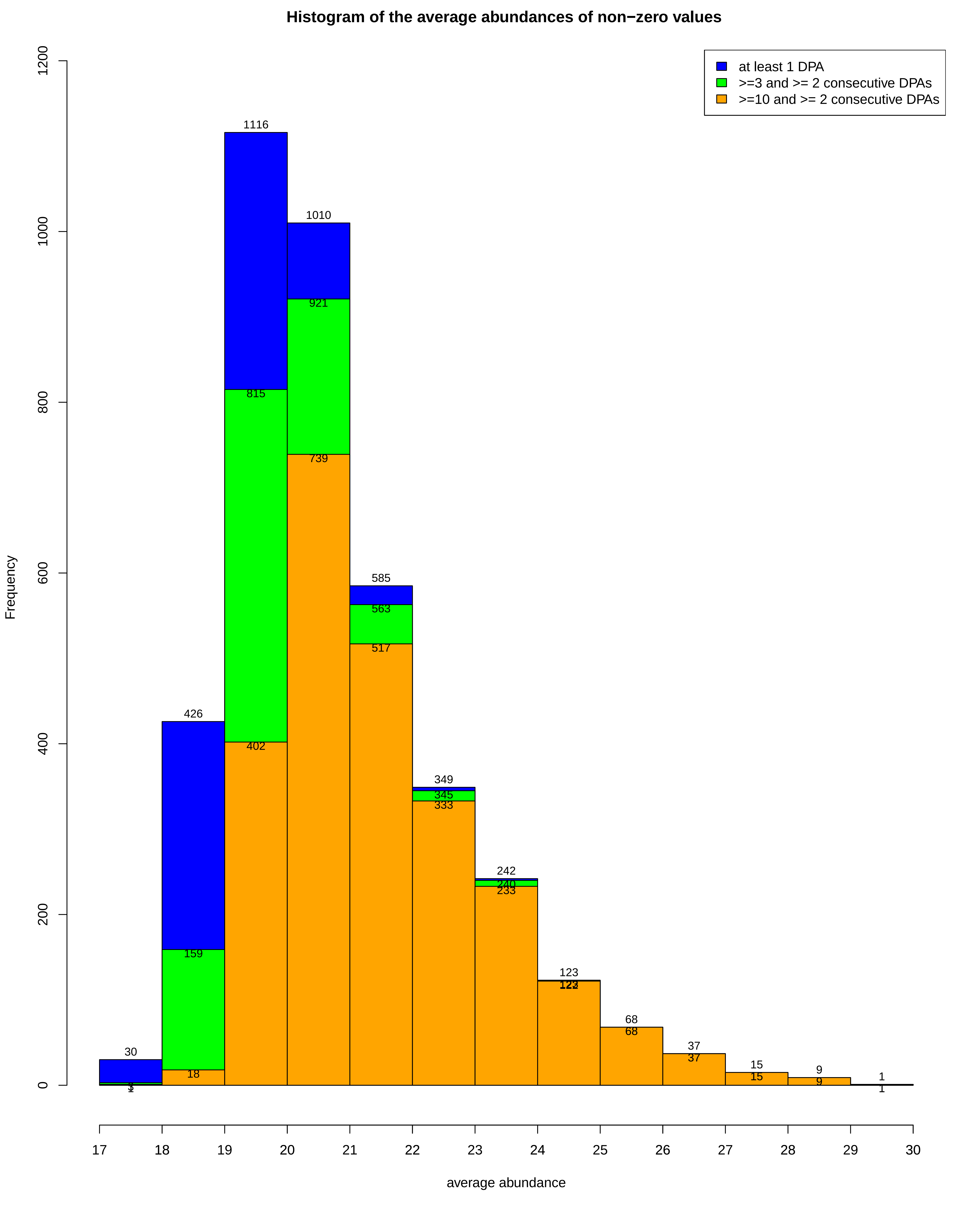

Histogram of the average abundances of non−zero values
at least 1 DPA
>=3 and >= 2 consecutive DPAs
>=10 and >= 2 consecutive DPAs
Frequency
0
200
400
600
800
1000
1200
average abundance
17
18
19
20
21
22
23
24
25
26
27
28
29
30
30
426
159
18
1116
815
402
1010
921
739
585
563
517
349
345
333
242
240
233
123
68
68
37
37
15
15
9
9
1
1

Codes

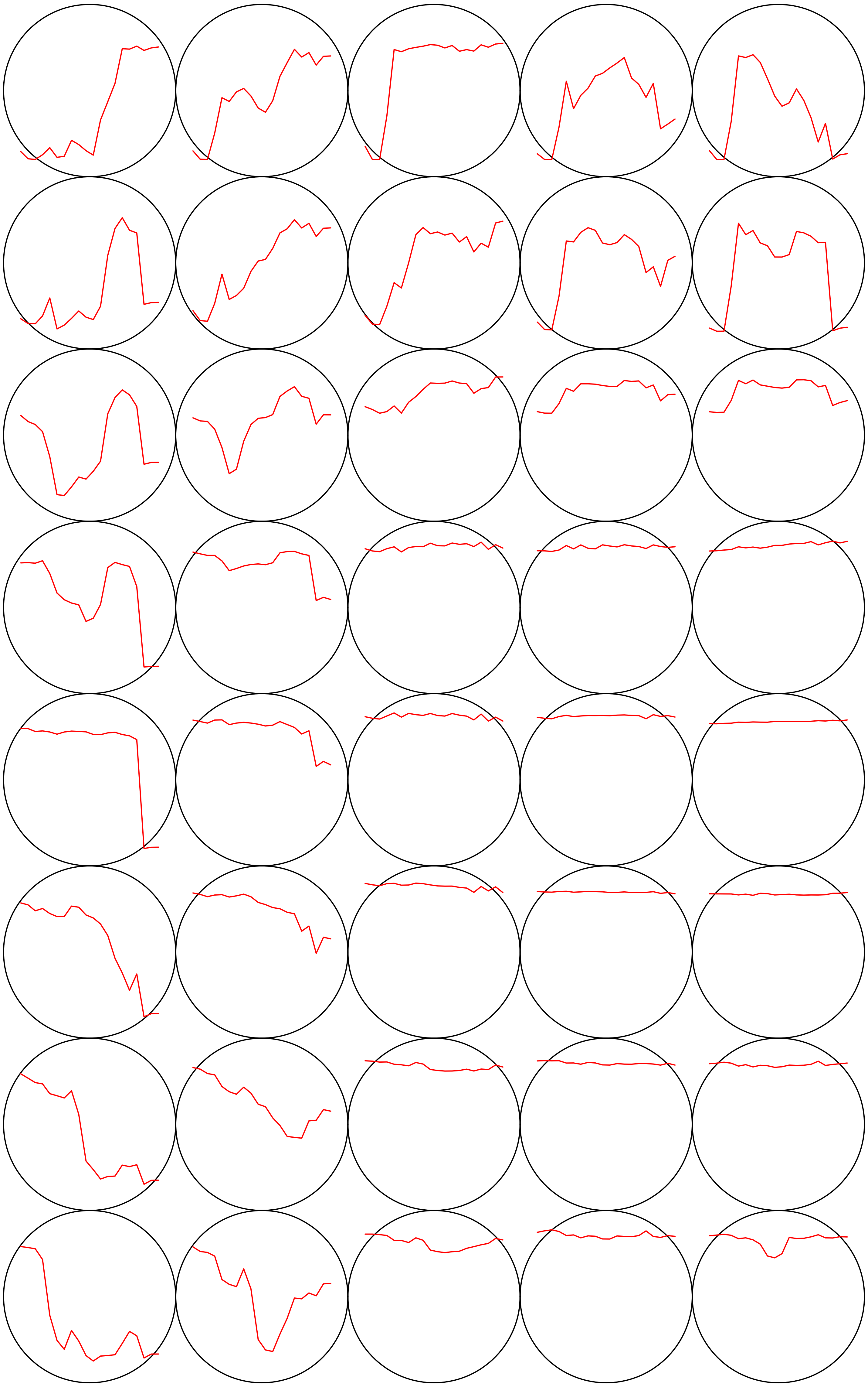

Count plot

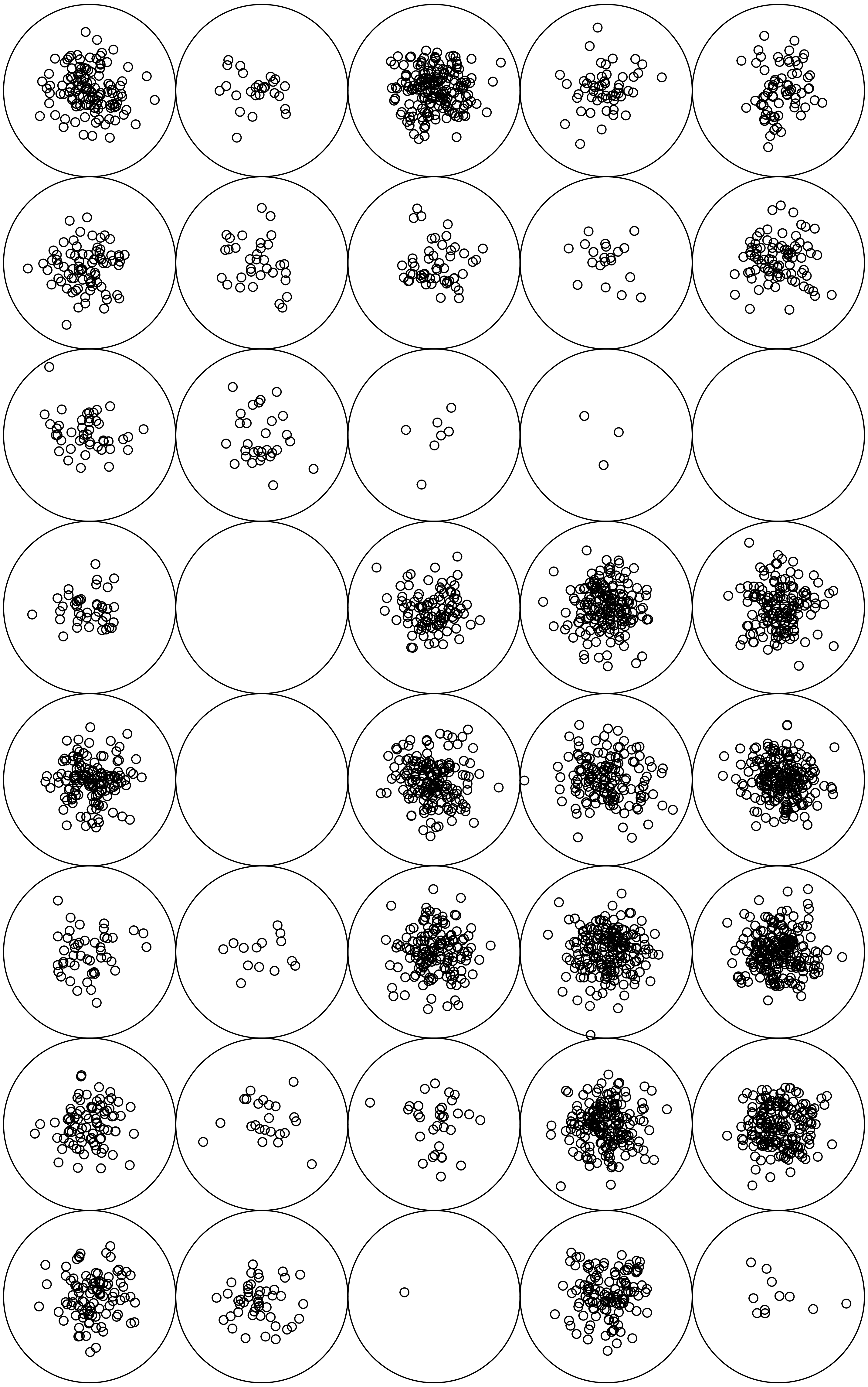

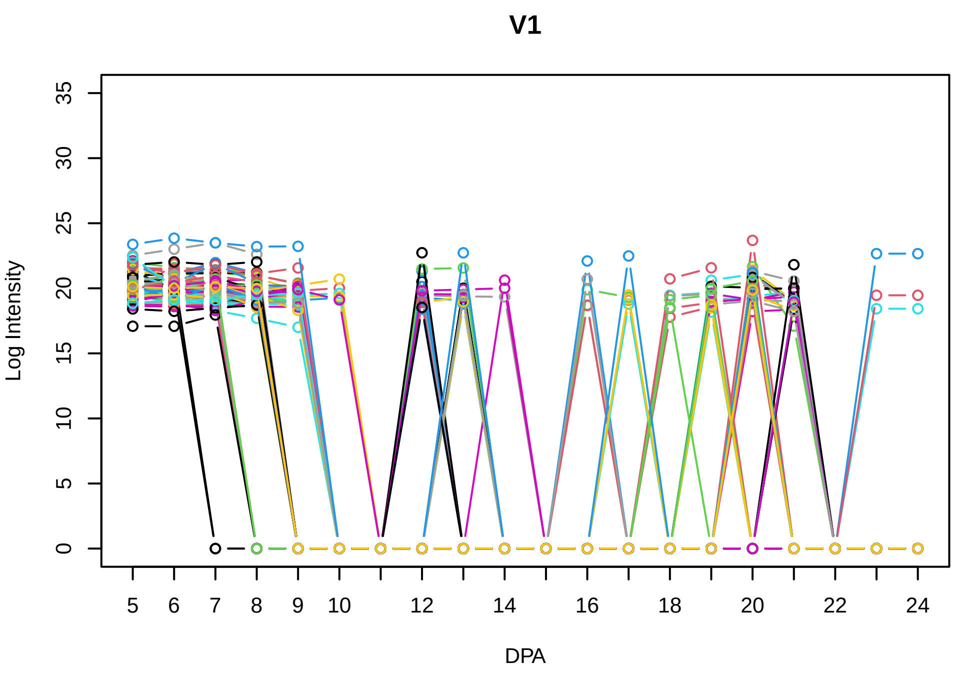
V1
Log Intensity
DPA

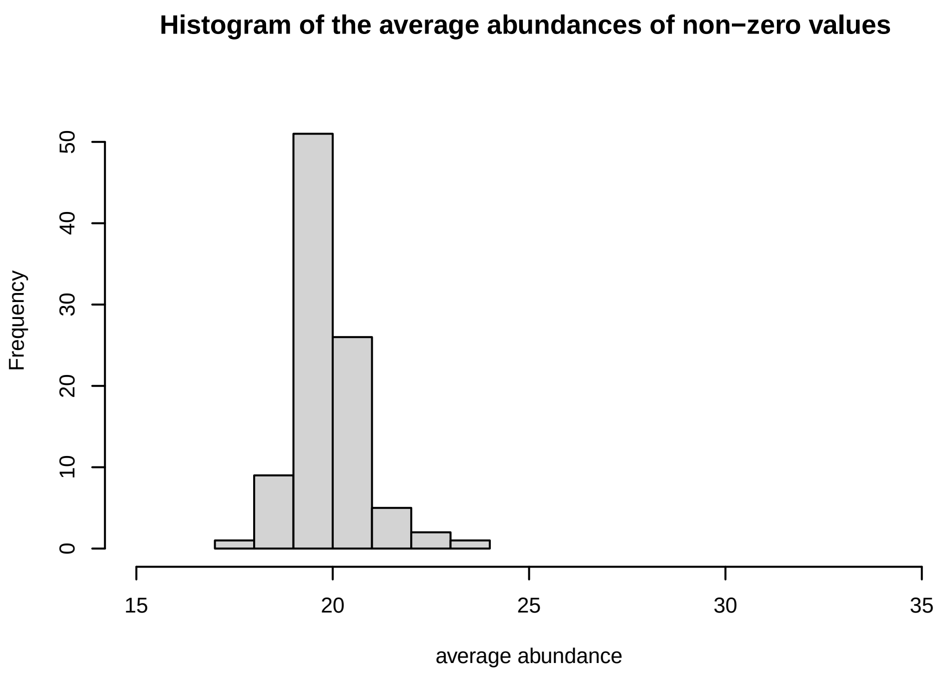
Histogram of the average abundances of non−zero values
Frequency
average abundance

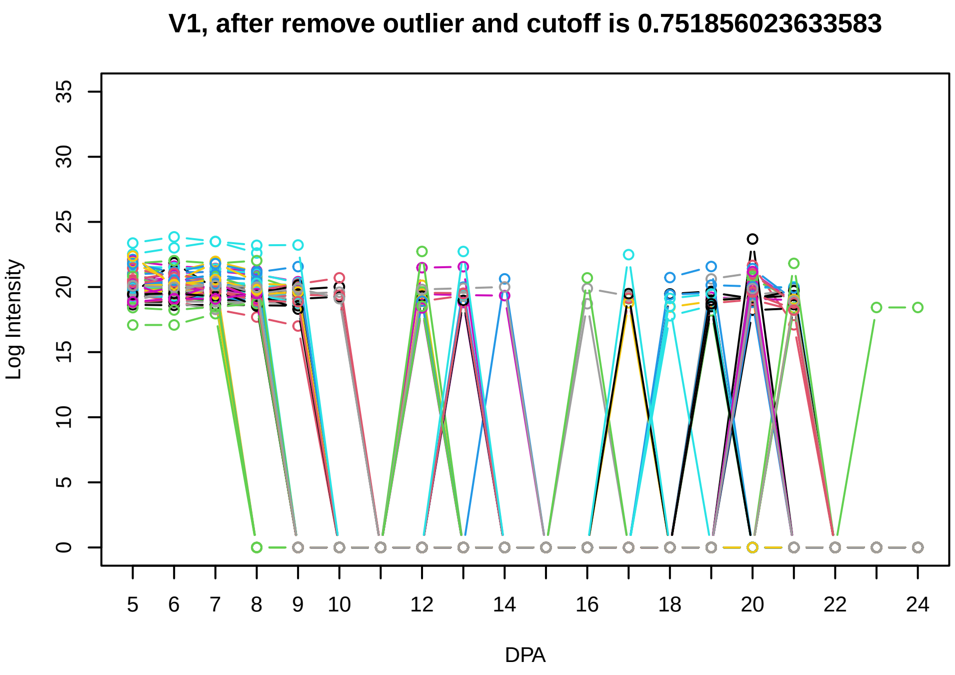
V1, after remove outlier and cutoff is 0.751856023633583
Log Intensity
DPA

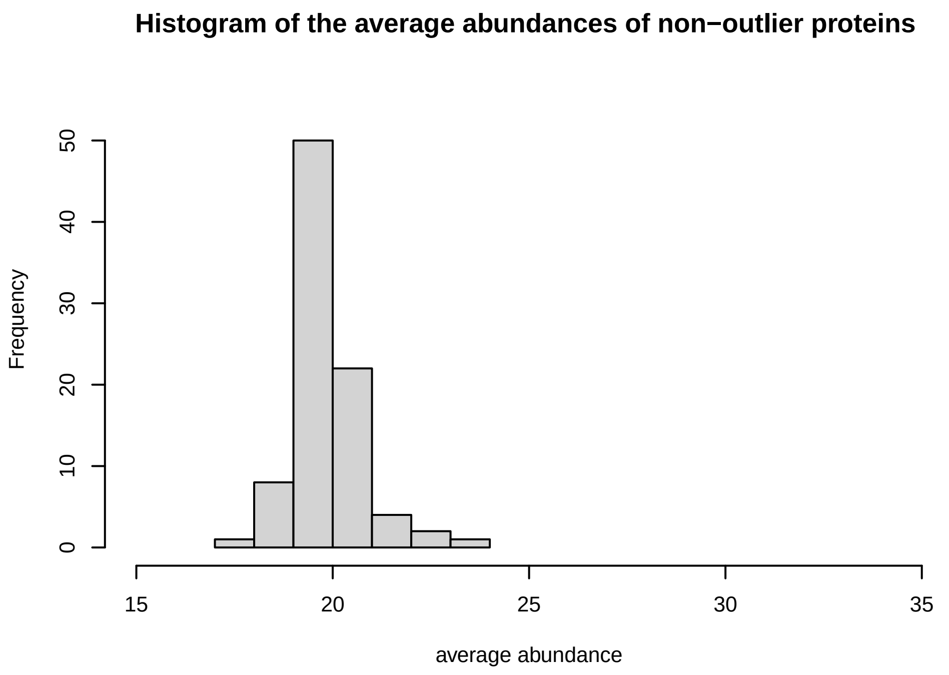
Histogram of the average abundances of non−outlier proteins
Frequency
average abundance

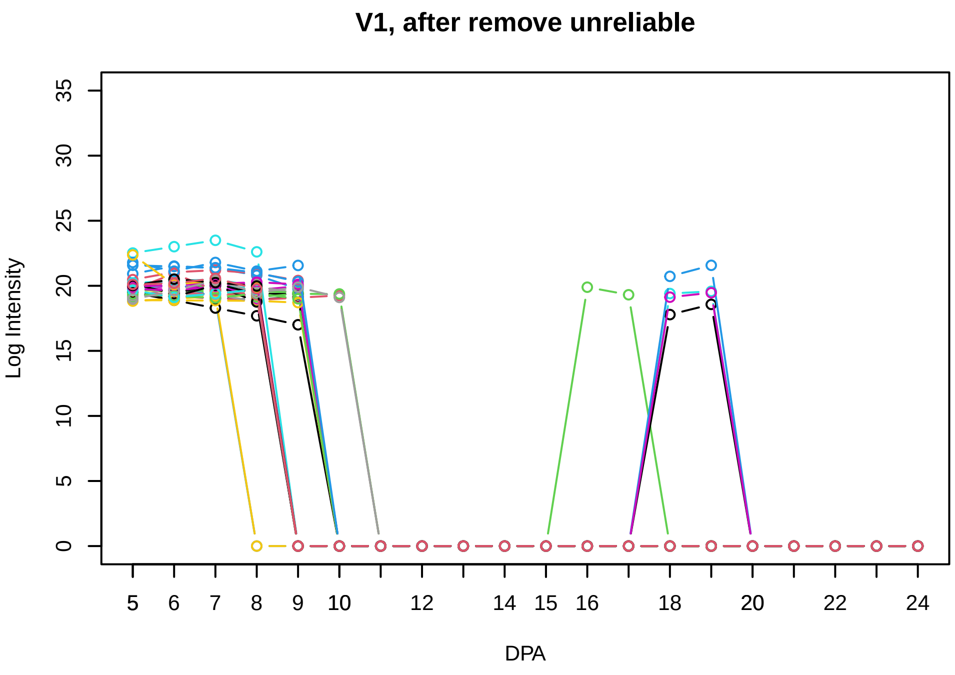
V1, after remove unreliable
Log Intensity
DPA

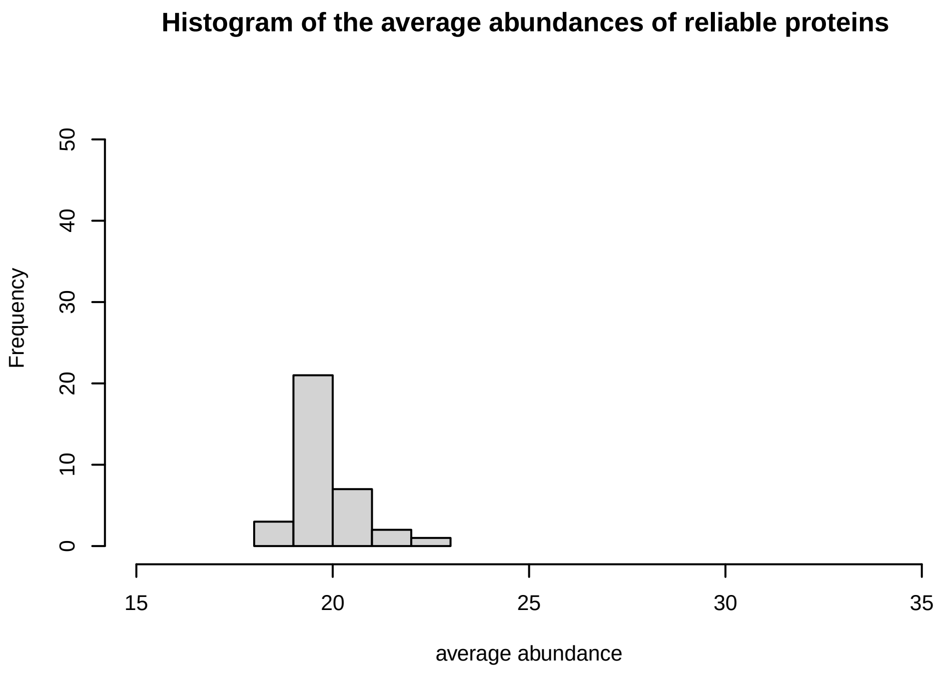
Histogram of the average abundances of reliable proteins
Frequency
average abundance

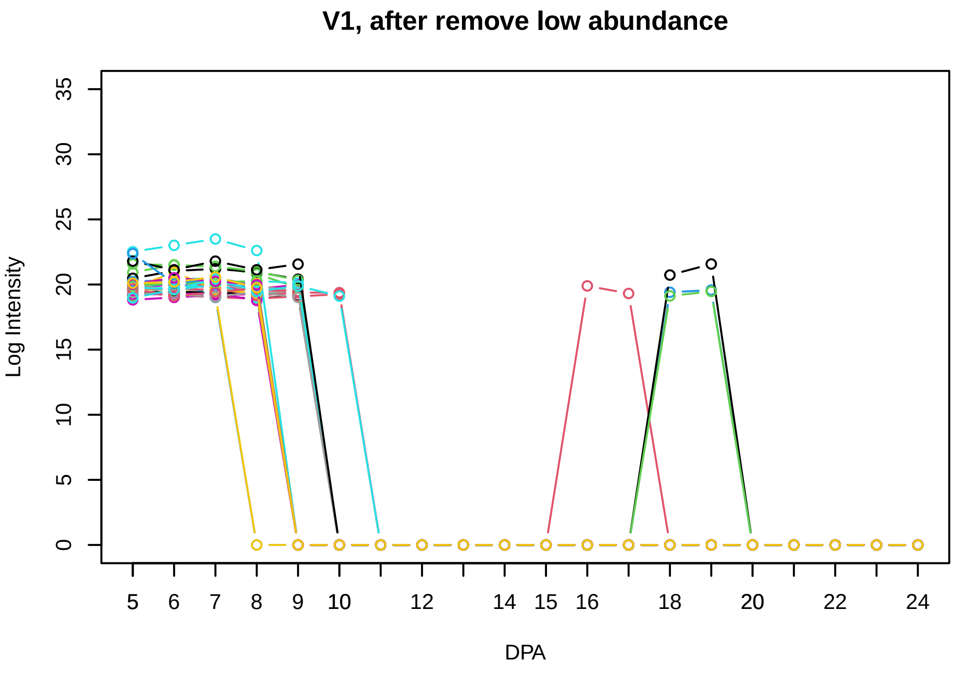
V1, after remove low abundance
Log Intensity
DPA

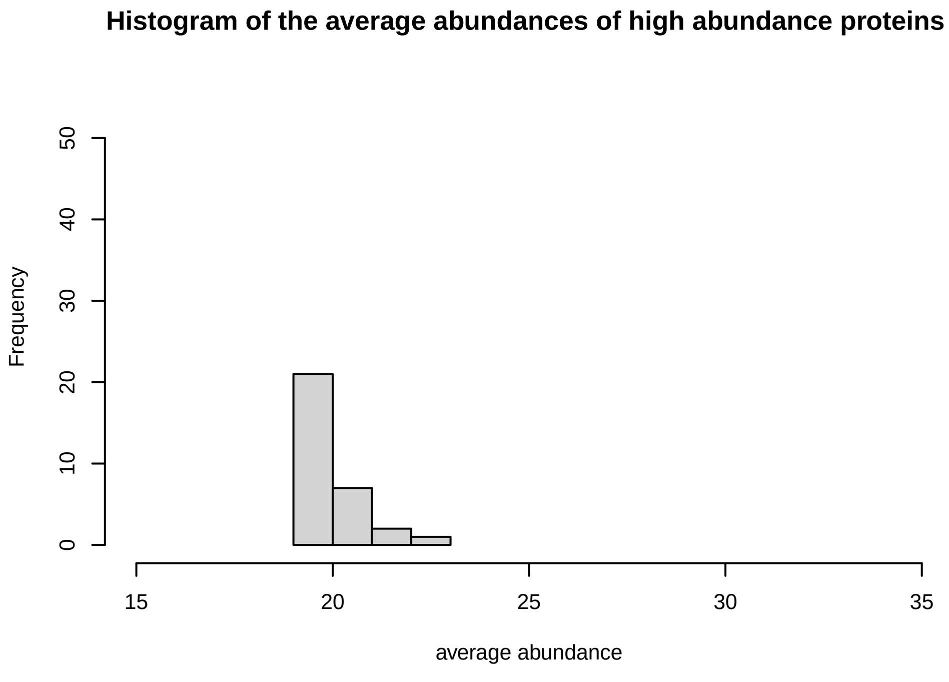
Histogram of the average abundances of high abundance proteins
Frequency
average abundance

V2

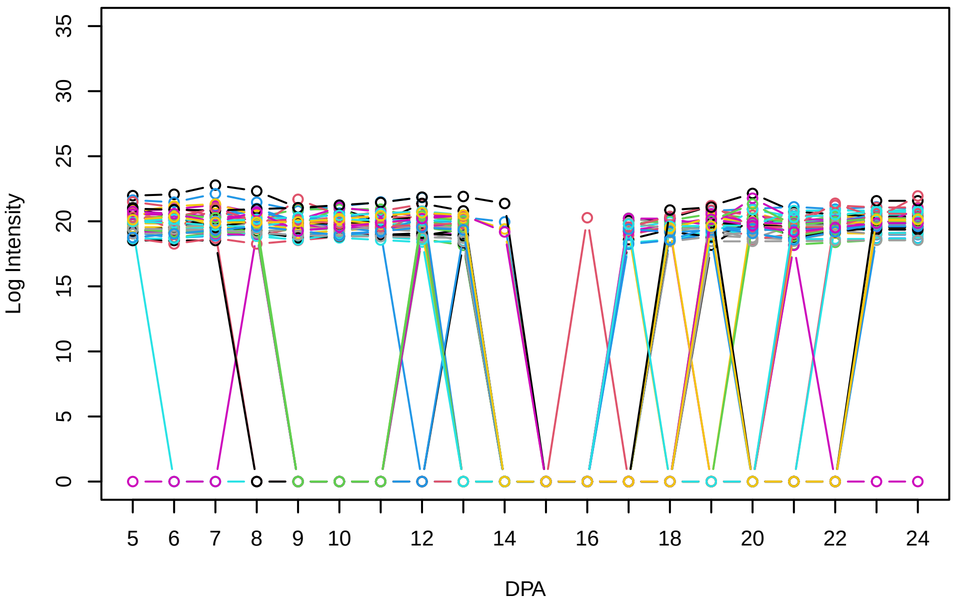

Histogram of the average abundances of non−zero values

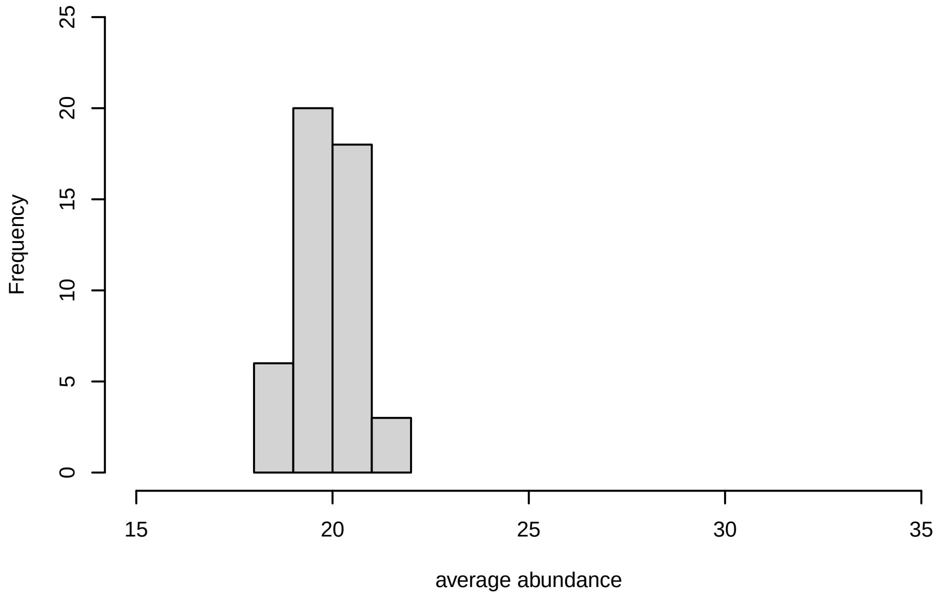

V2, after remove outlier and cutoff is 0.8

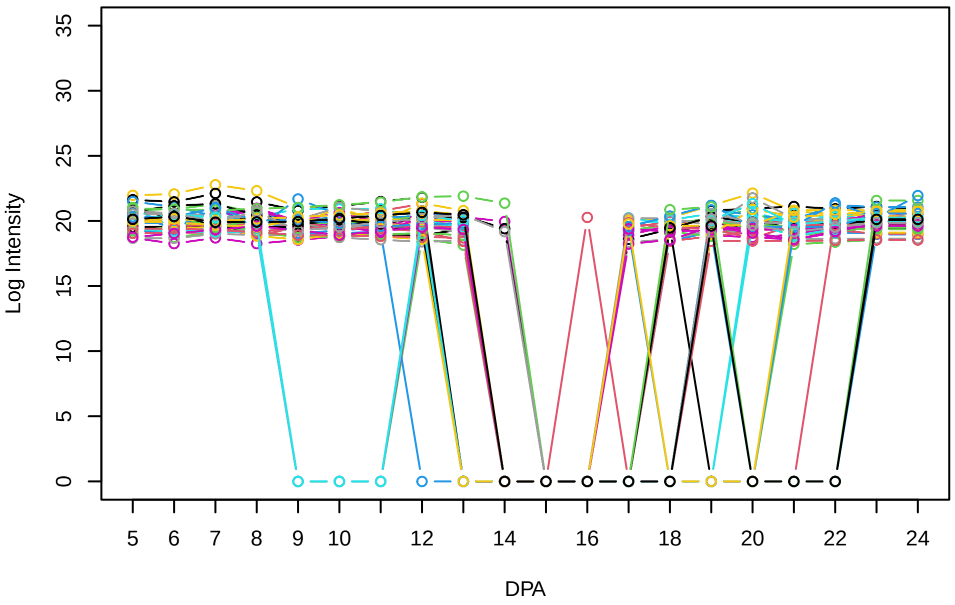

Histogram of the average abundances of non−outlier proteins

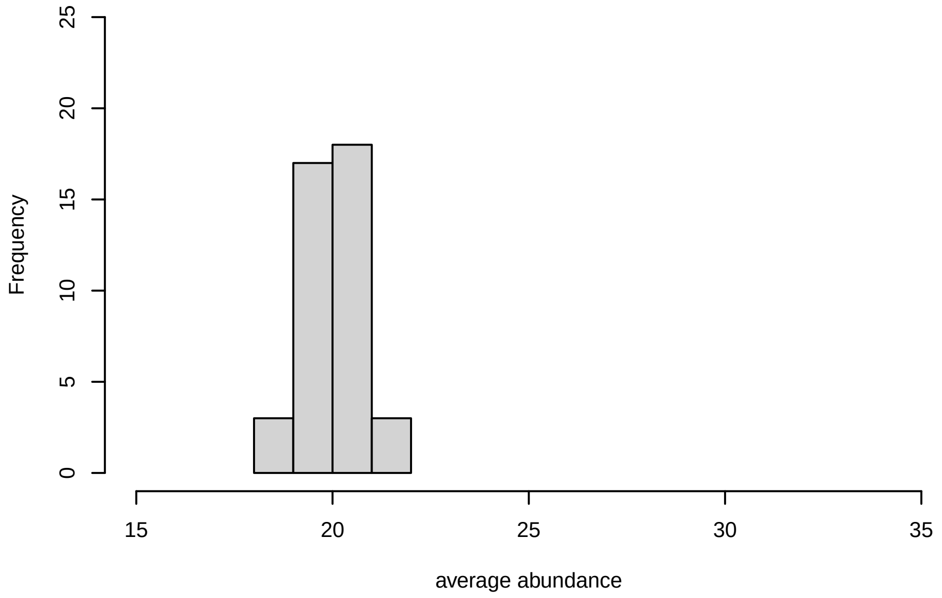

V2, after remove unreliable

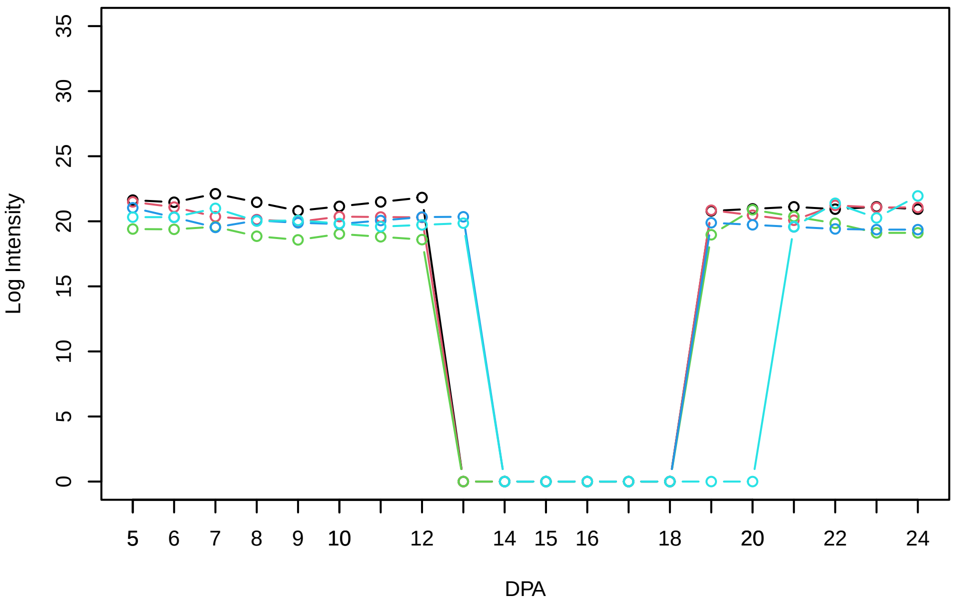

Histogram of the average abundances of reliable proteins

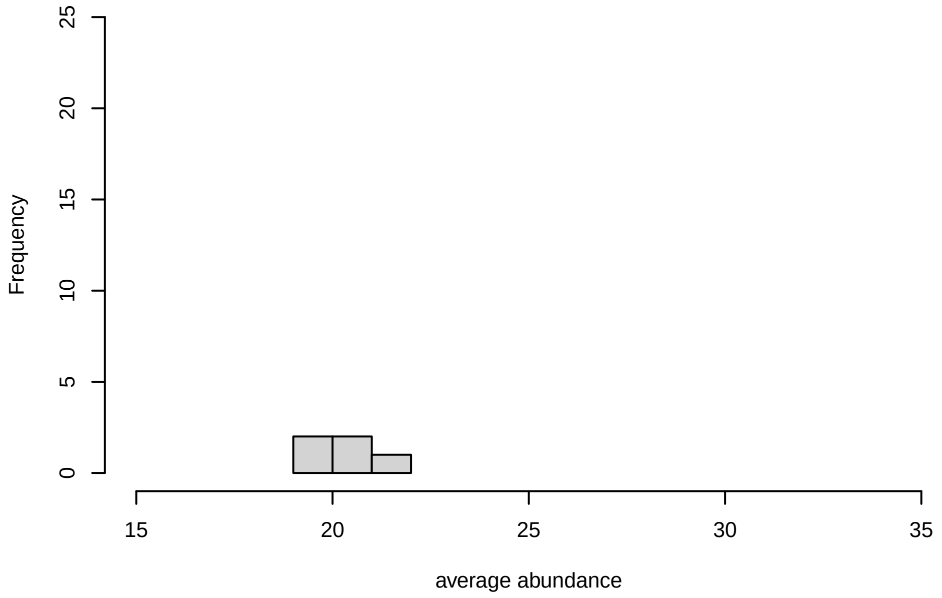

V2, after remove low abundance

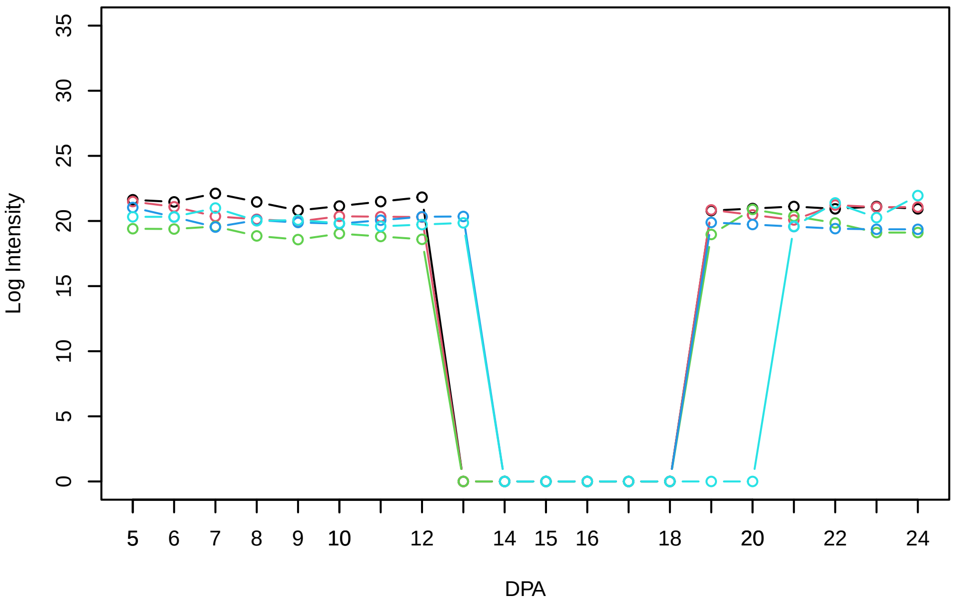

Histogram of the average abundances of high abundance proteins

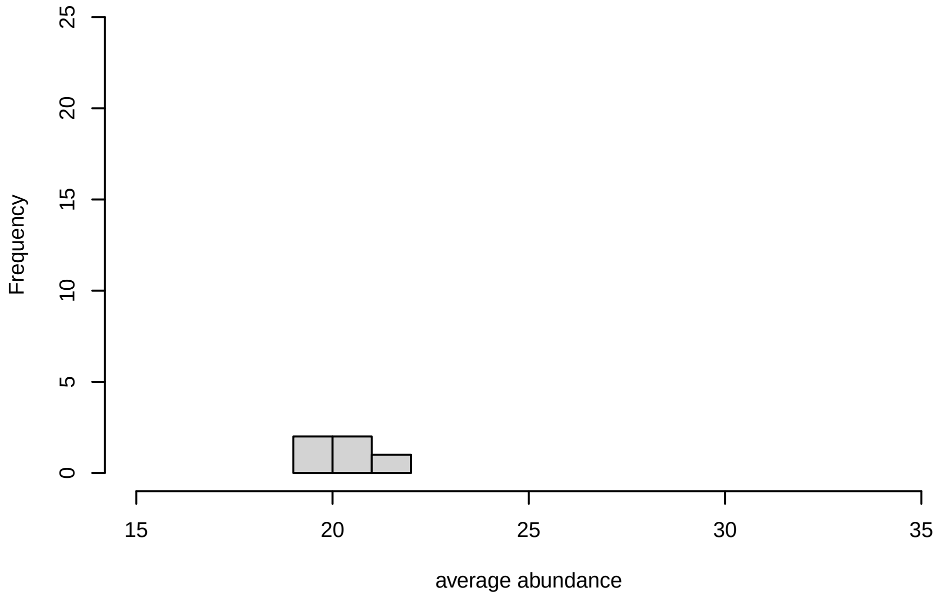

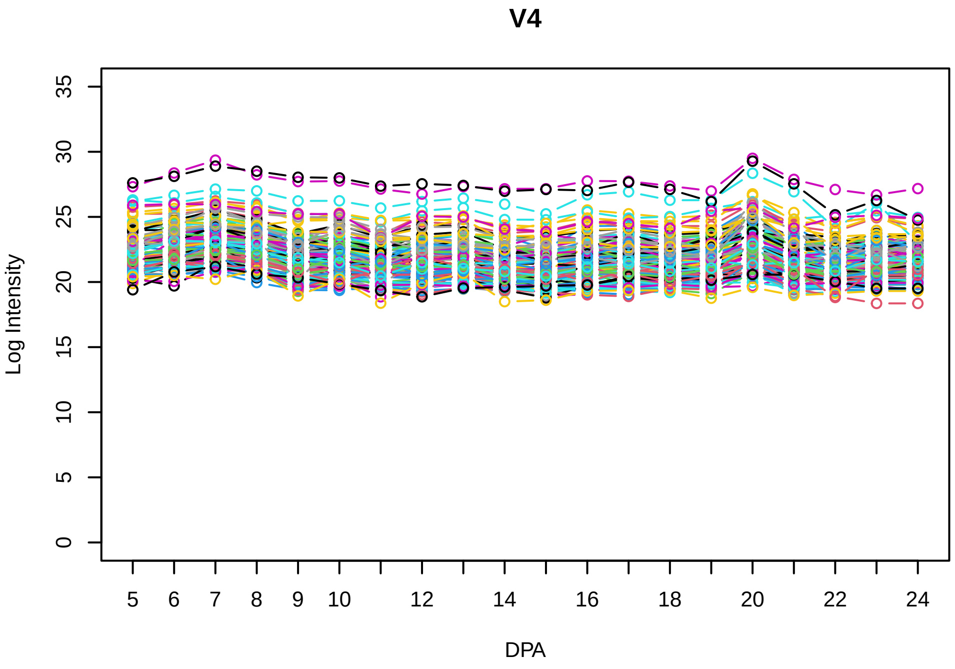
V4
Log Intensity
DPA

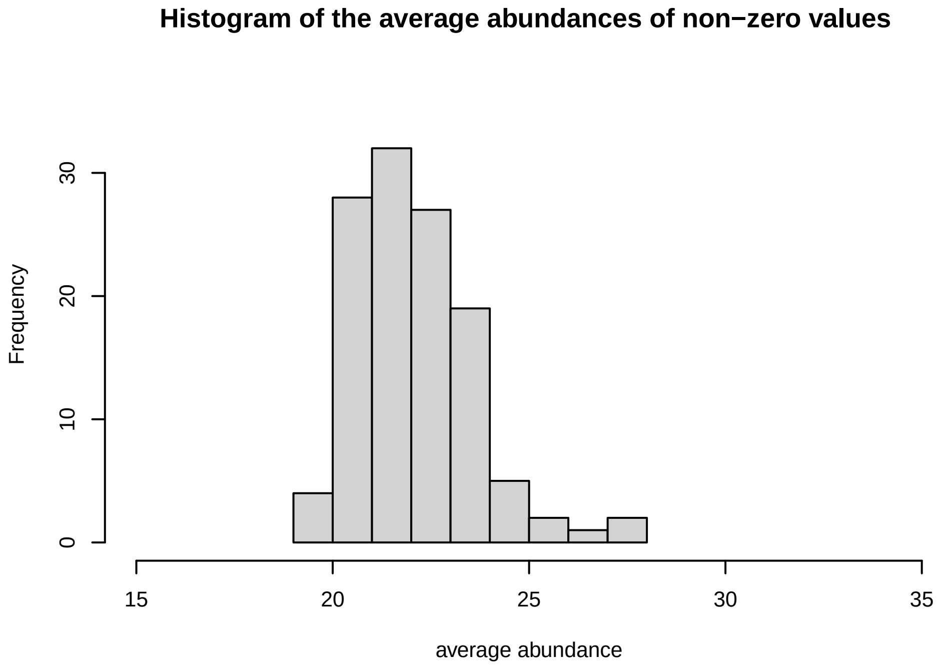
Histogram of the average abundances of non−zero values
Frequency
average abundance

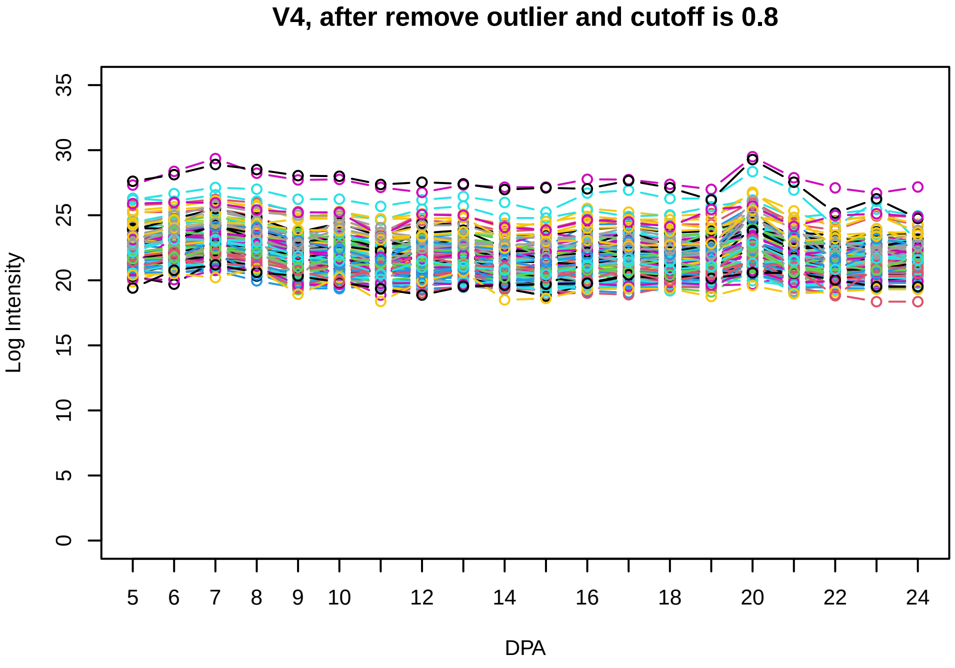
V4, after remove outlier and cutoff is 0.8
Log Intensity
DPA

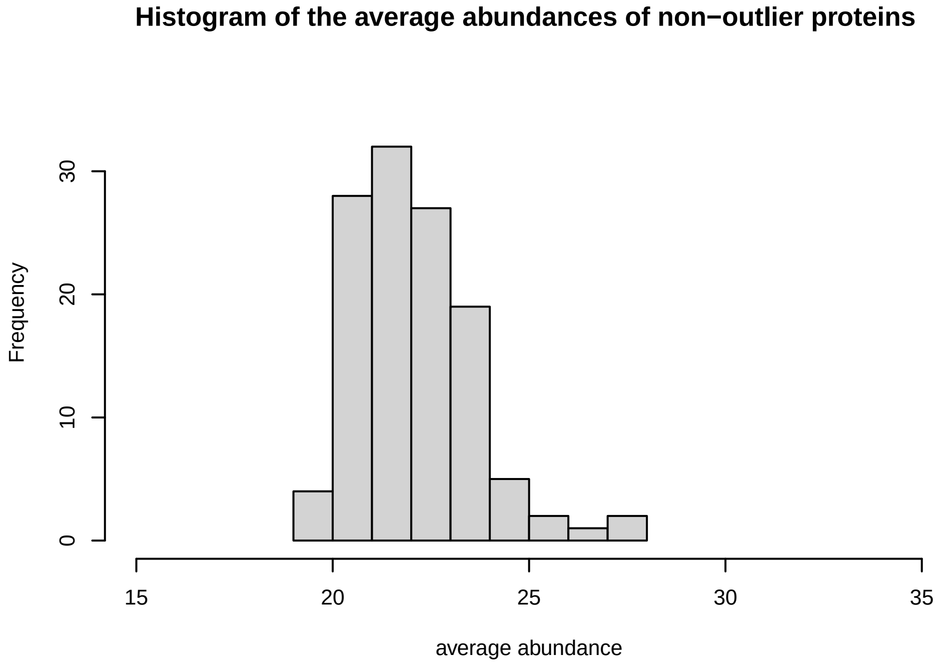
Histogram of the average abundances of non−outlier proteins
Frequency
average abundance

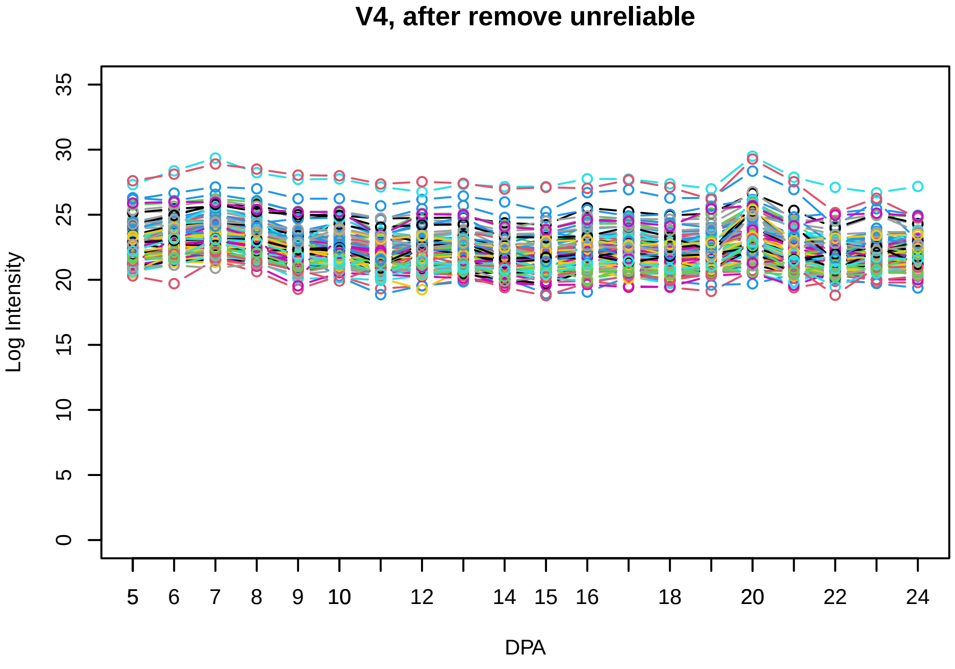
V4, after remove unreliable
Log Intensity
DPA

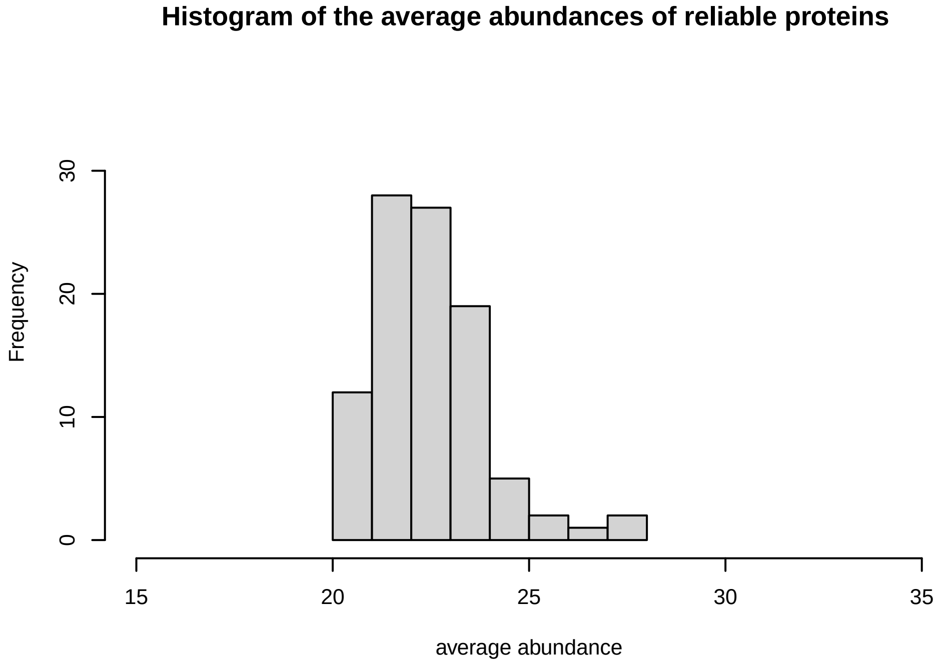
Histogram of the average abundances of reliable proteins
Frequency
average abundance

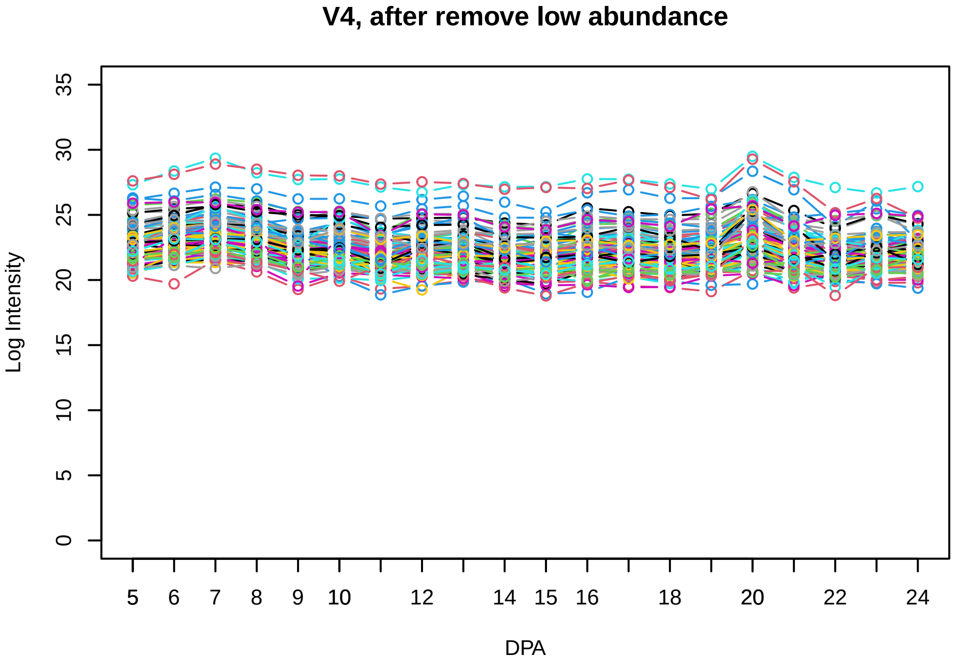
V4, after remove low abundance
Log Intensity
DPA

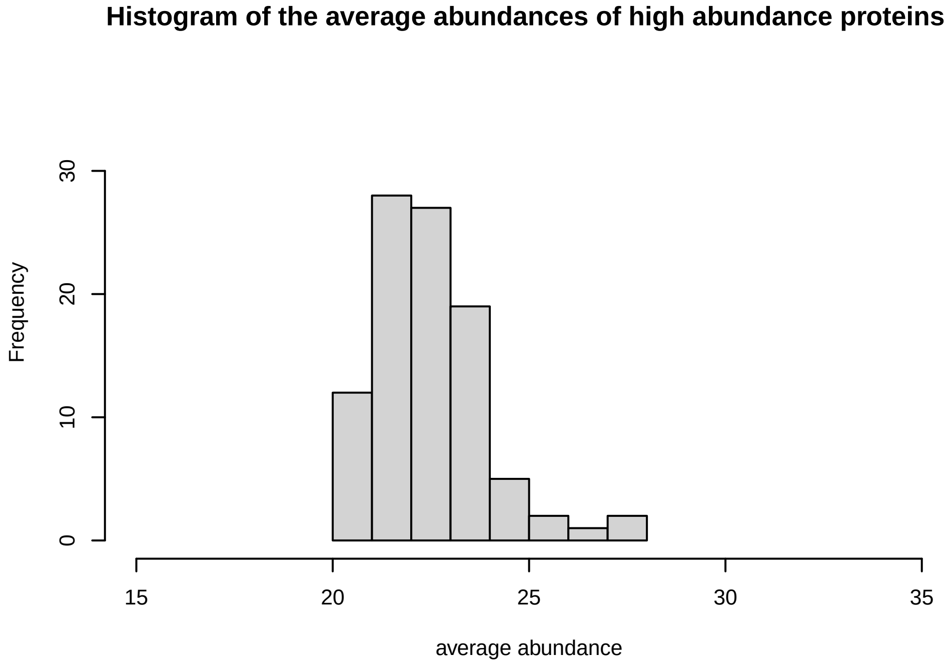
Histogram of the average abundances of high abundance proteins
Frequency
average abundance

V5

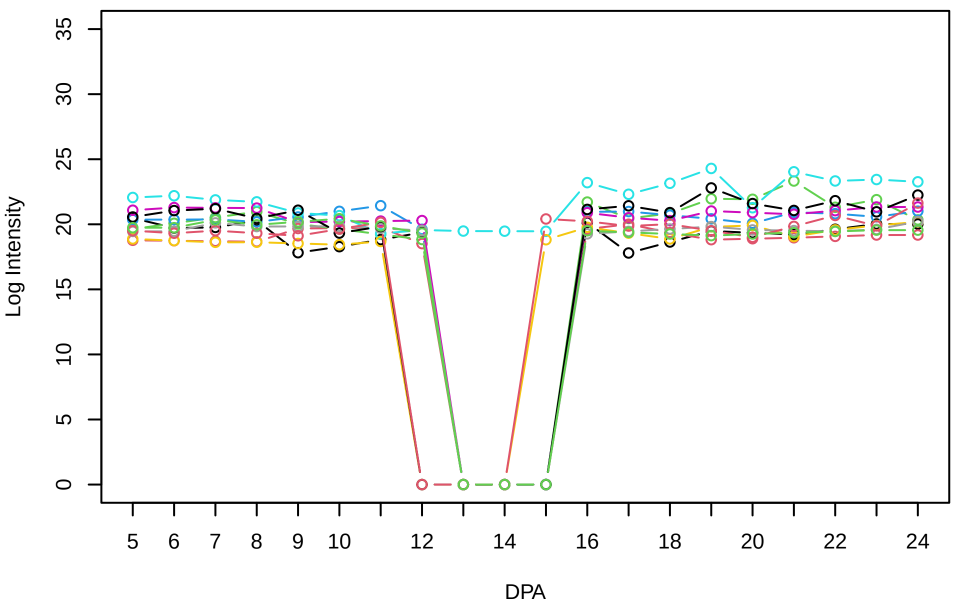

Histogram of the average abundances of non−zero values

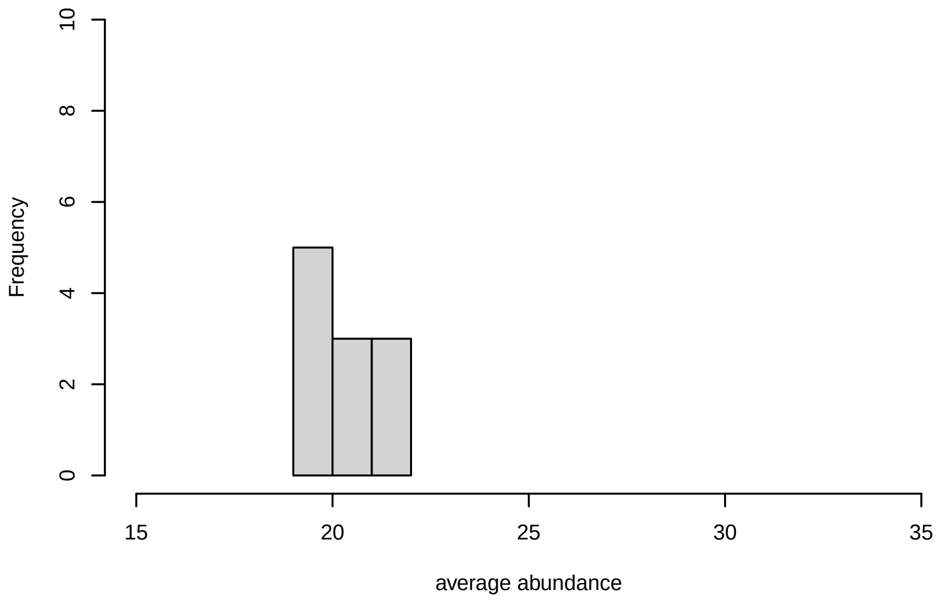

V5, after remove outlier and cutoff is 0.8

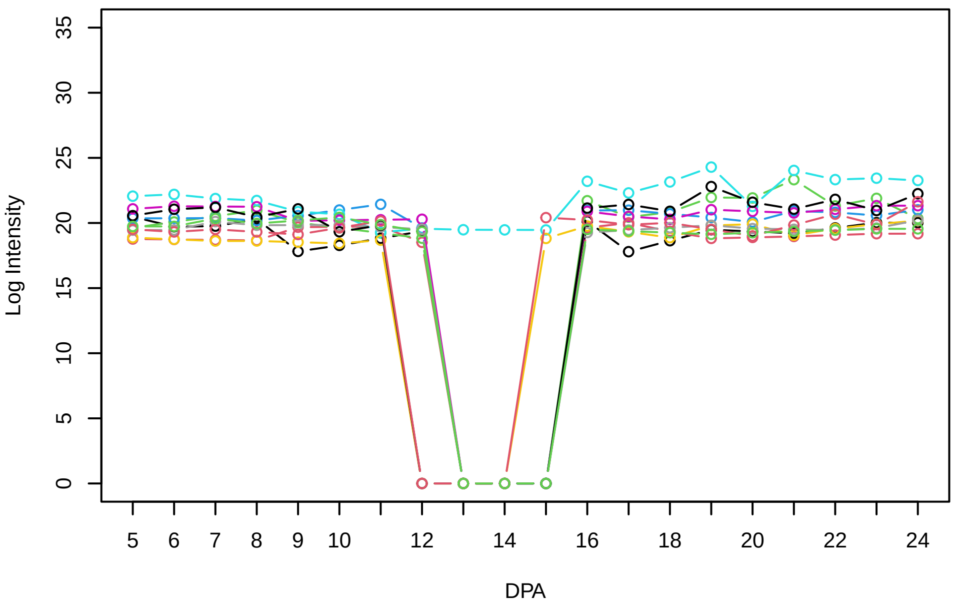

Histogram of the average abundances of non−outlier proteins

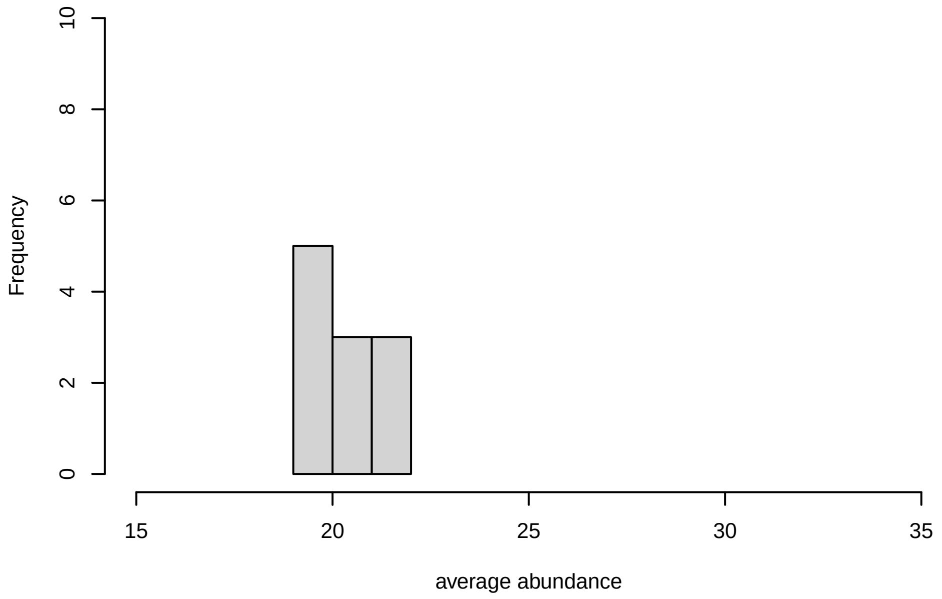

V5, after remove unreliable

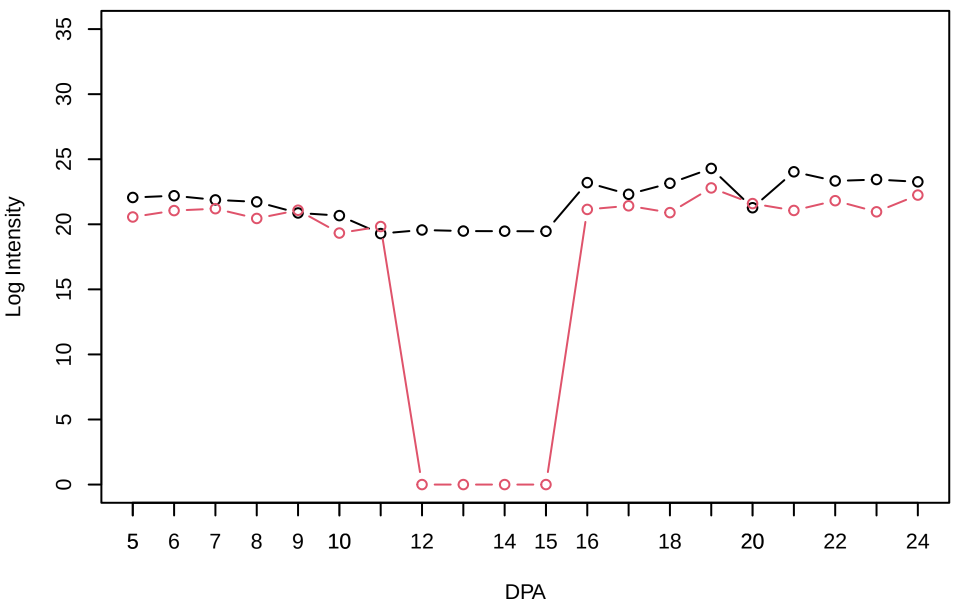

Histogram of the average abundances of reliable proteins

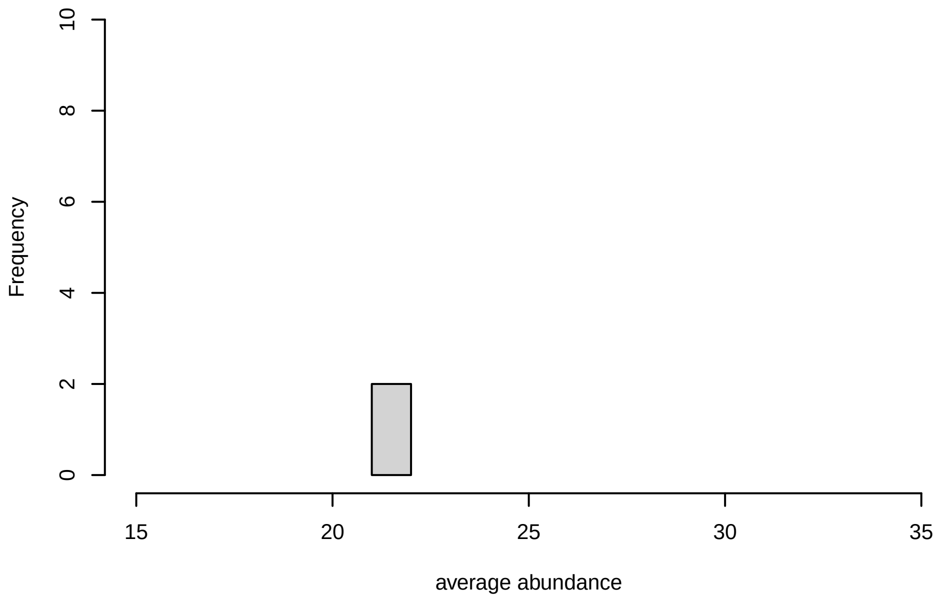

V5, after remove low abundance

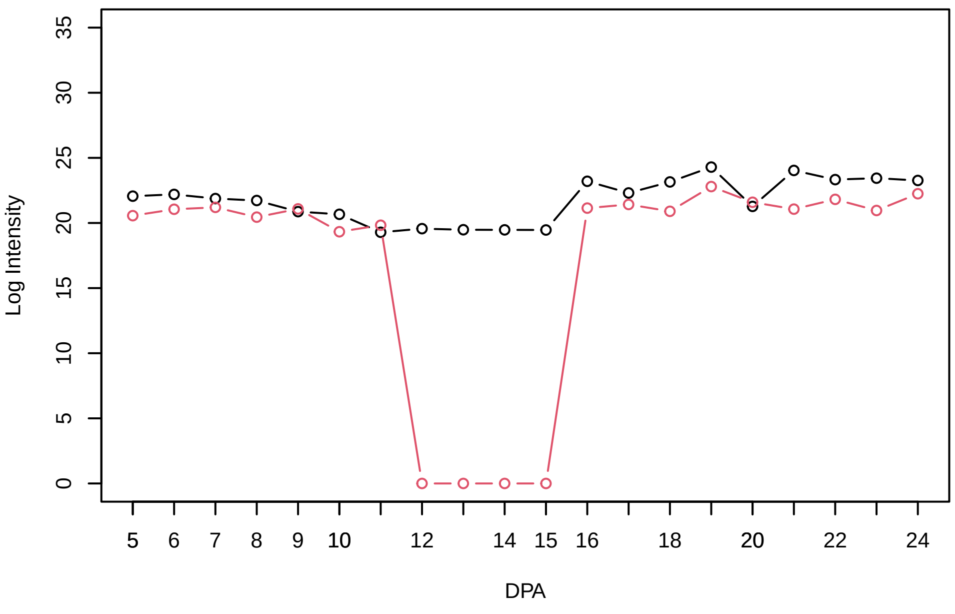

Histogram of the average abundances of high abundance proteins

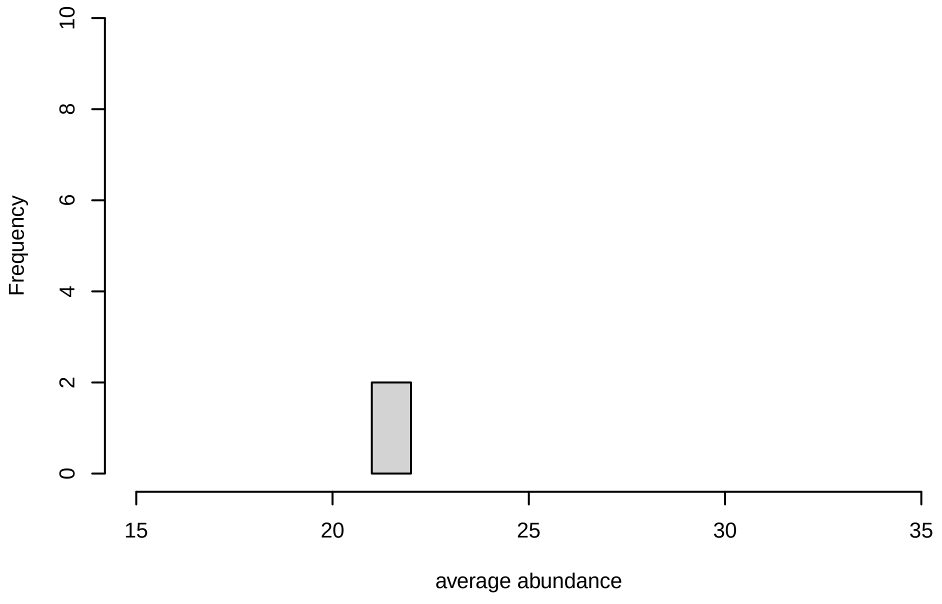

V6

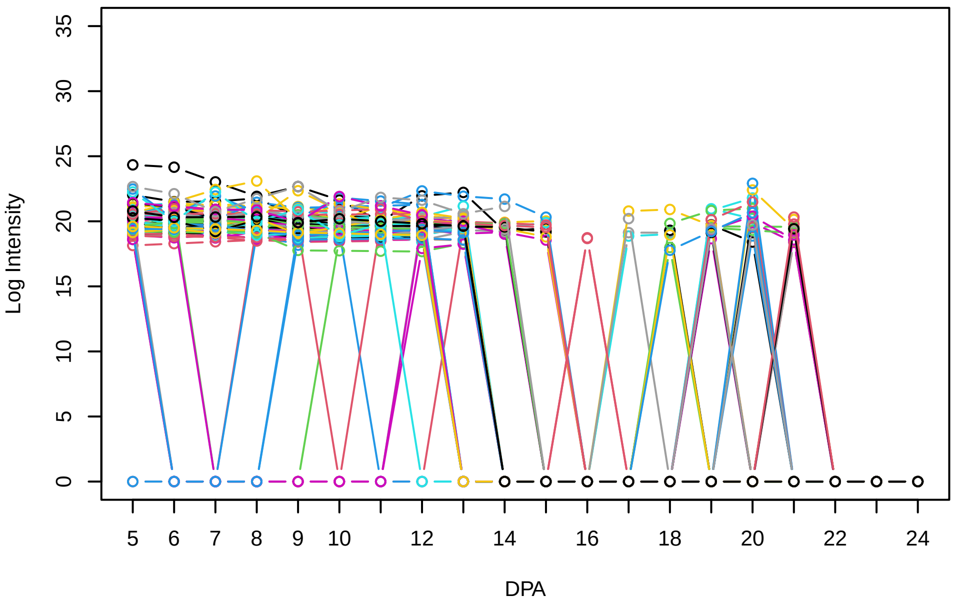

Histogram of the average abundances of non−zero values

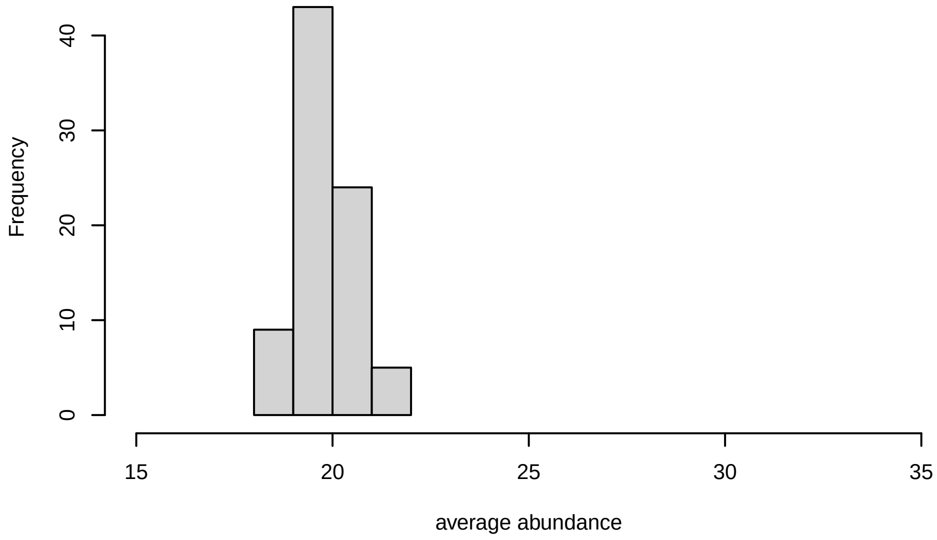

V6, after remove outlier and cutoff is 0.8

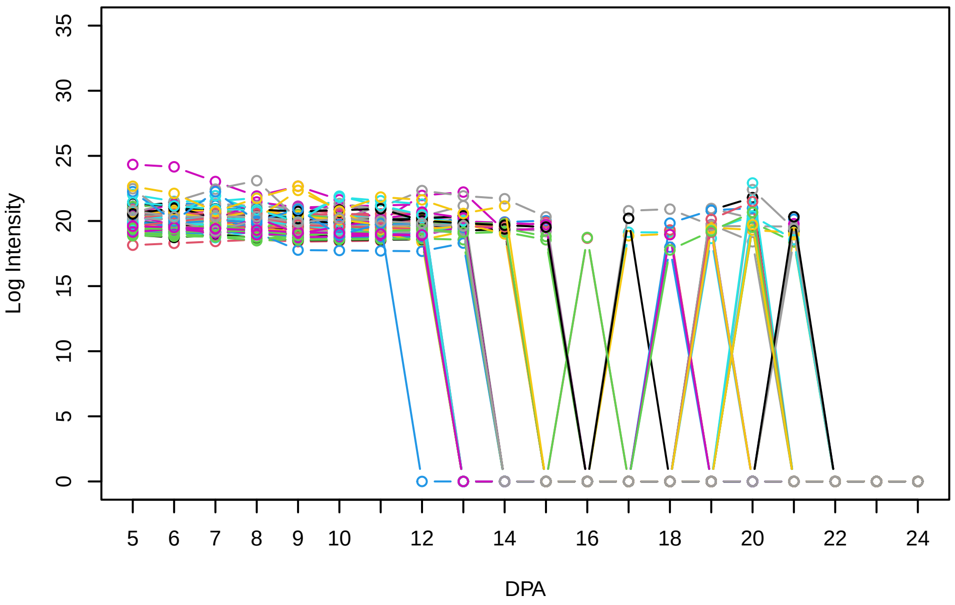

Histogram of the average abundances of non−outlier proteins

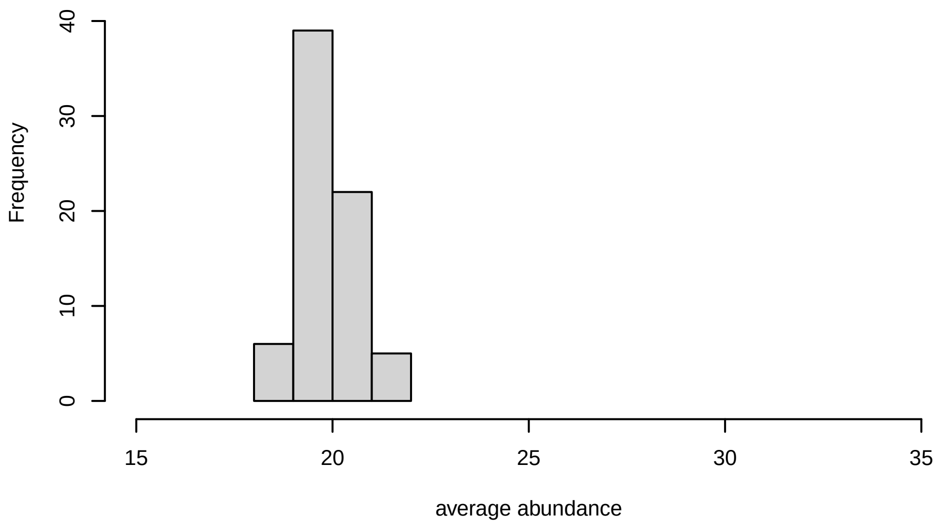

V6, after remove unreliable

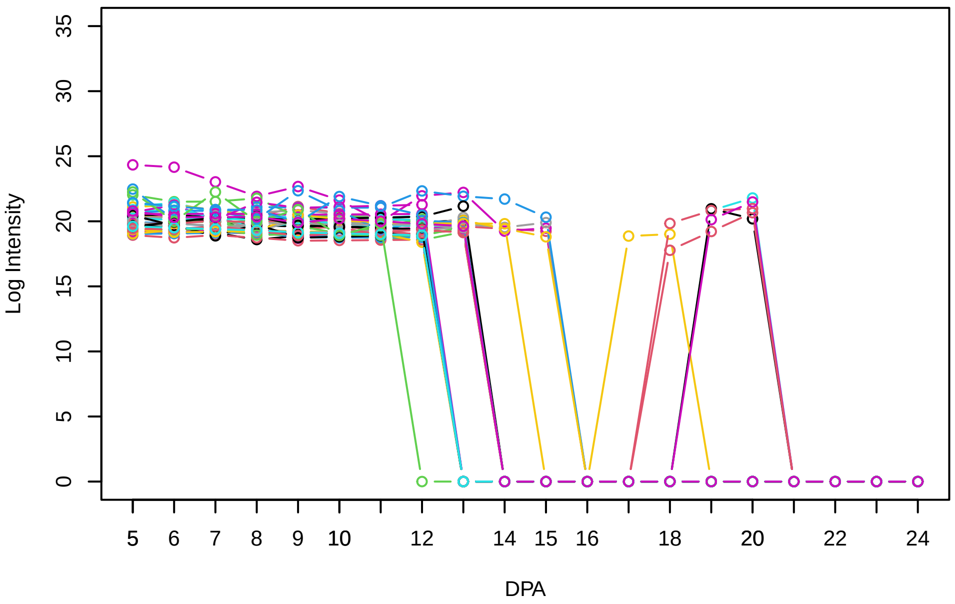

Histogram of the average abundances of reliable proteins

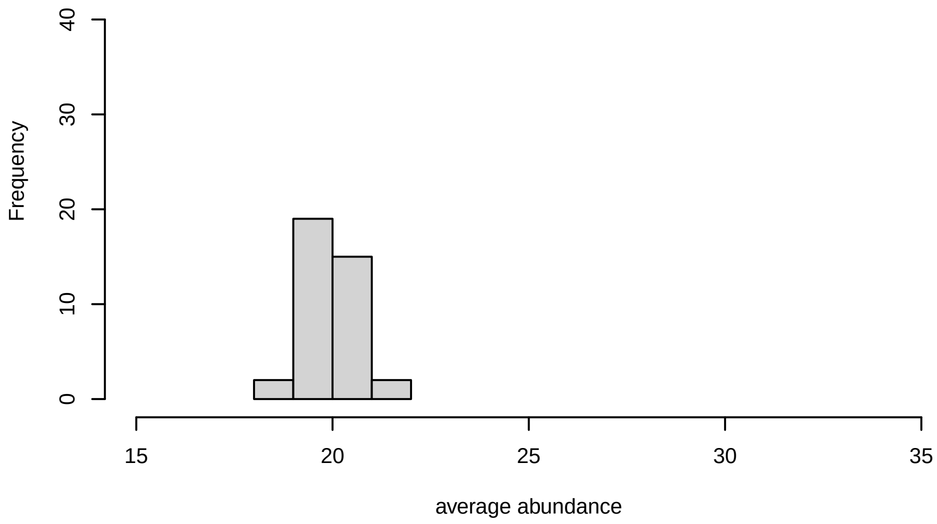

V6, after remove low abundance

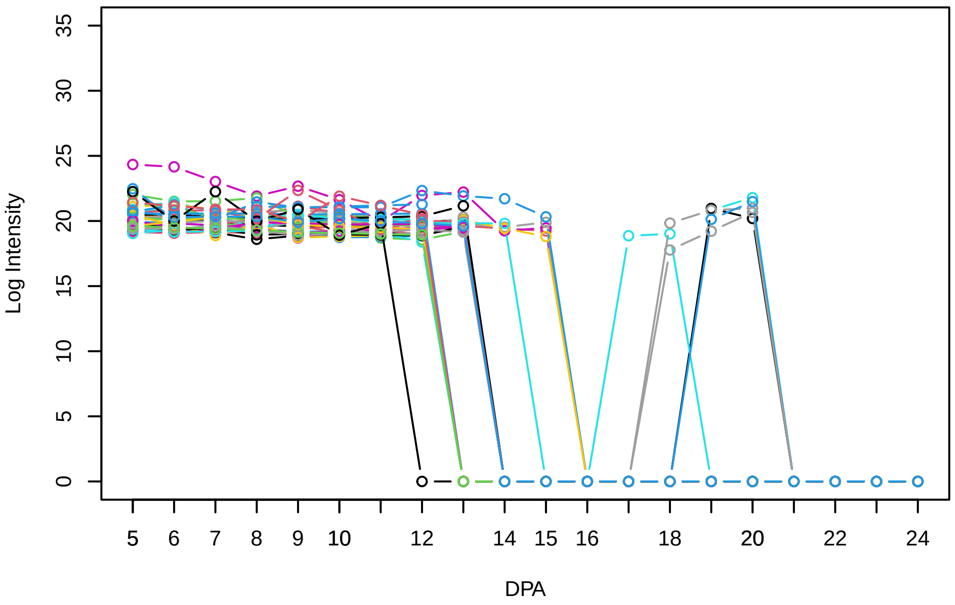

Histogram of the average abundances of high abundance proteins

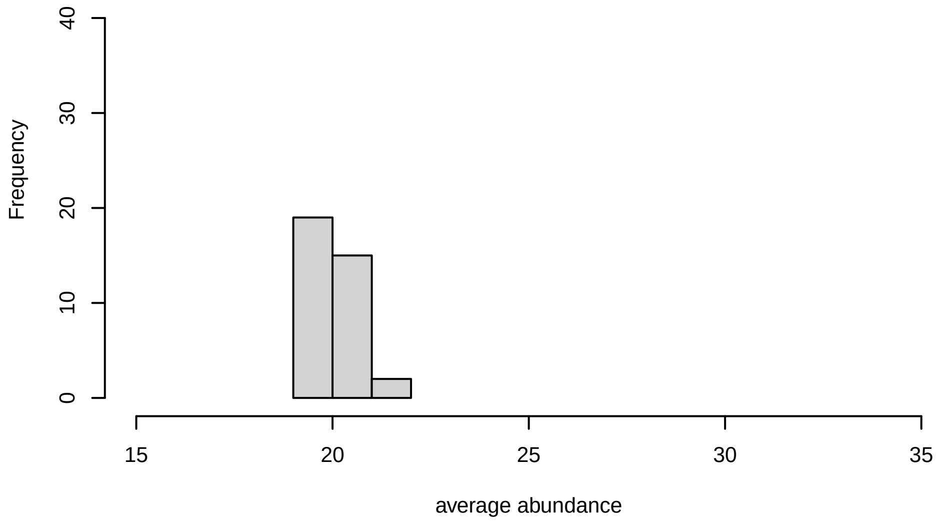

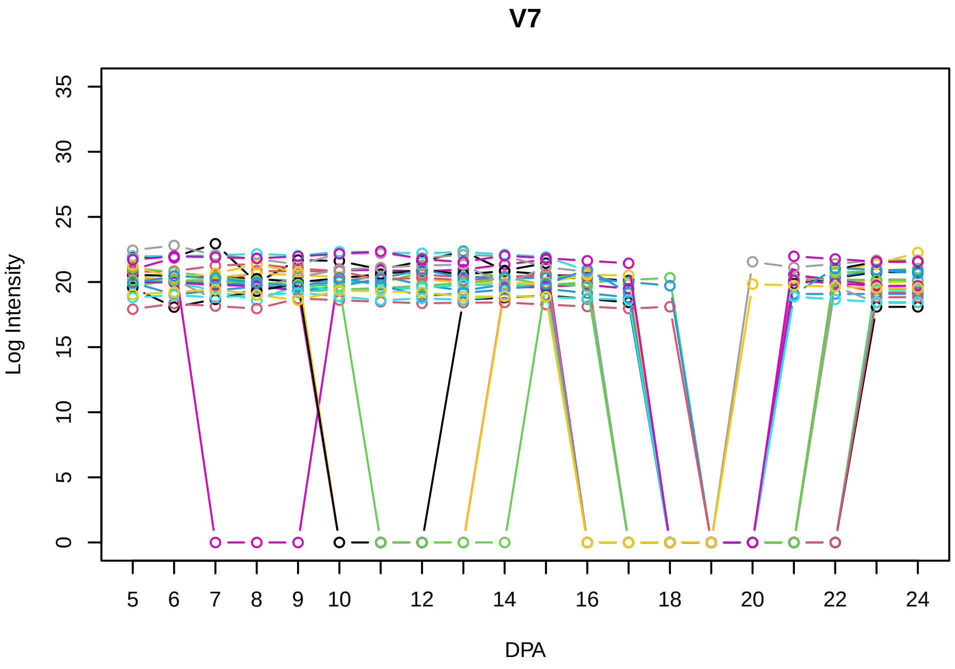
V7
Log Intensity
DPA
0 5 10 15 20 25 30 35
5 6 7 8 9 10 12 14 16 18 20 22 24

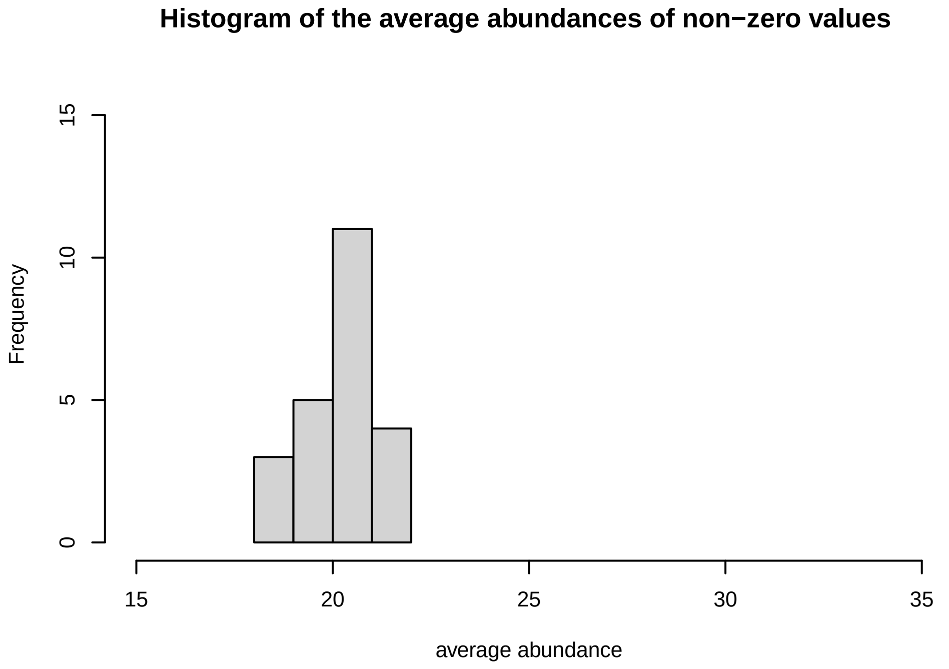
Histogram of the average abundances of non−zero values
Frequency
average abundance
0 5 10 15
15 20 25 30 35

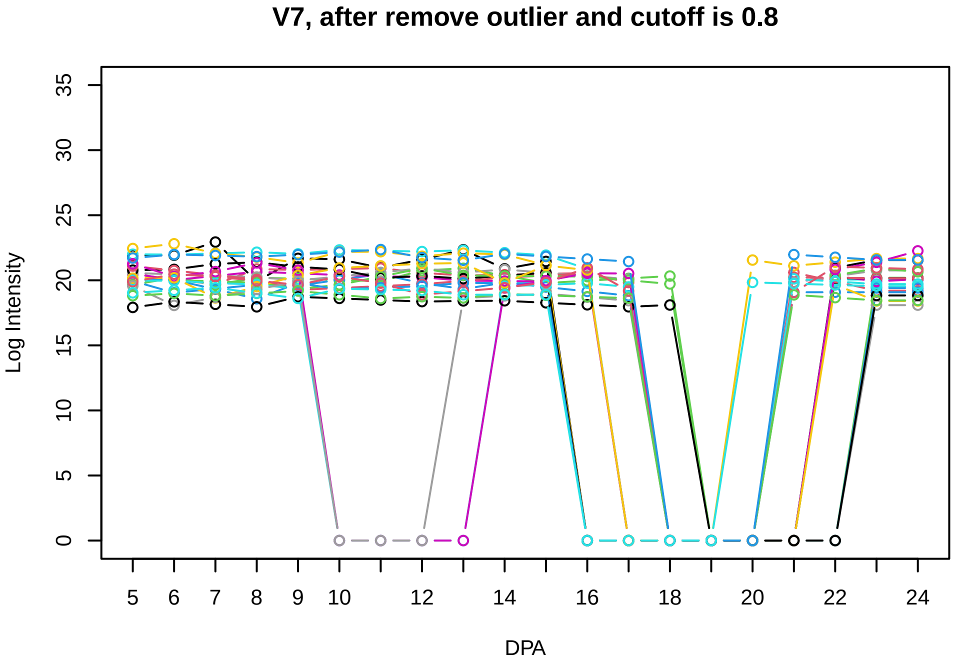
V7, after remove outlier and cutoff is 0.8
Log Intensity
DPA
0 5 10 15 20 25 30 35
5 6 7 8 9 10 12 14 16 18 20 22 24

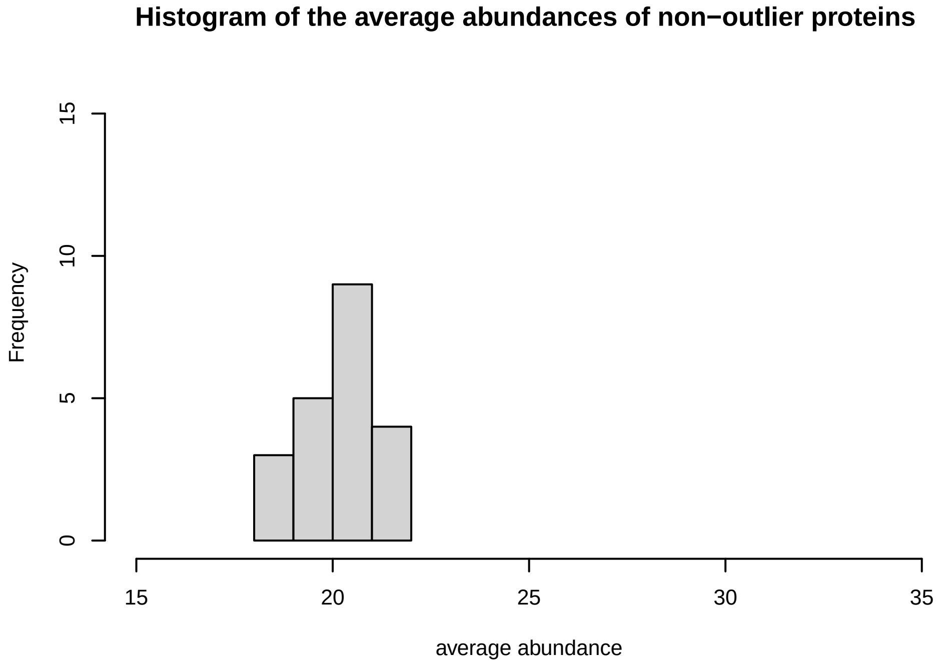
Histogram of the average abundances of non−outlier proteins
Frequency
average abundance
0 5 10 15
15 20 25 30 35

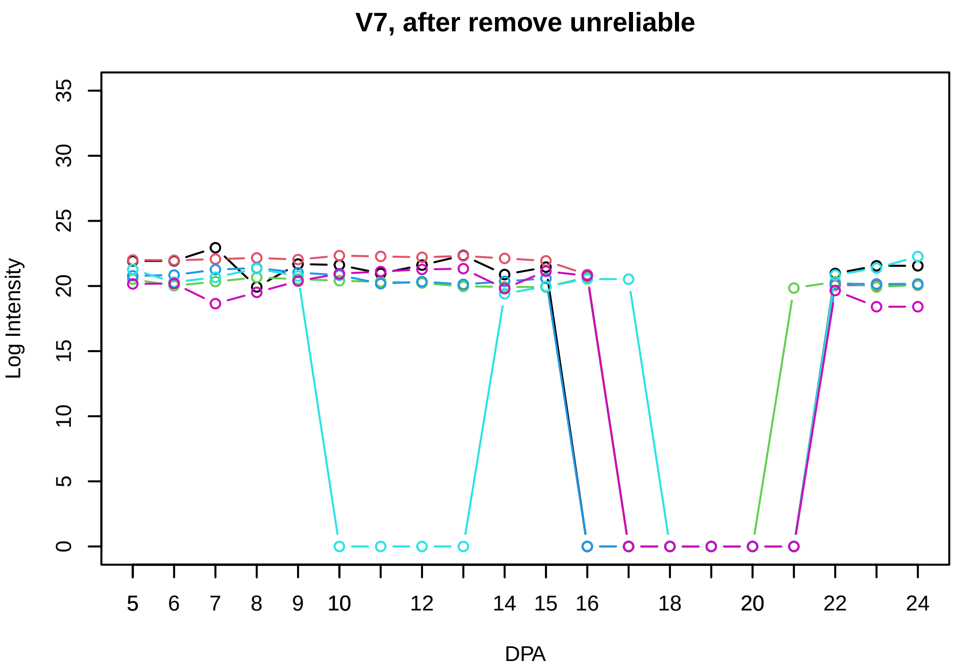
V7, after remove unreliable
Log Intensity
DPA
0 5 10 15 20 25 30 35
5 6 7 8 9 10 12 14 15 16 18 20 22 24

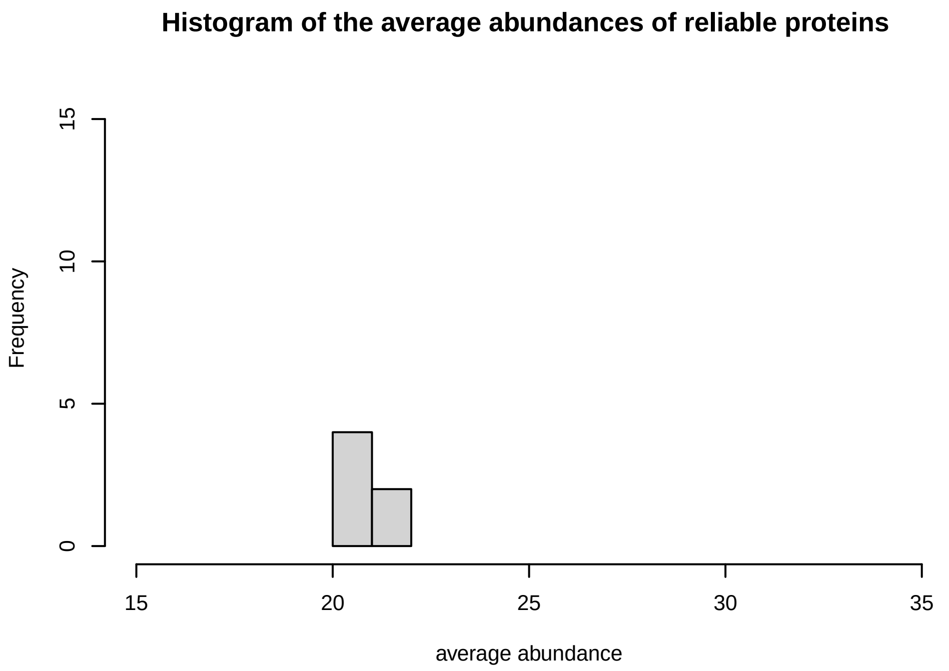
Histogram of the average abundances of reliable proteins
Frequency
average abundance
0 5 10 15
15 20 25 30 35

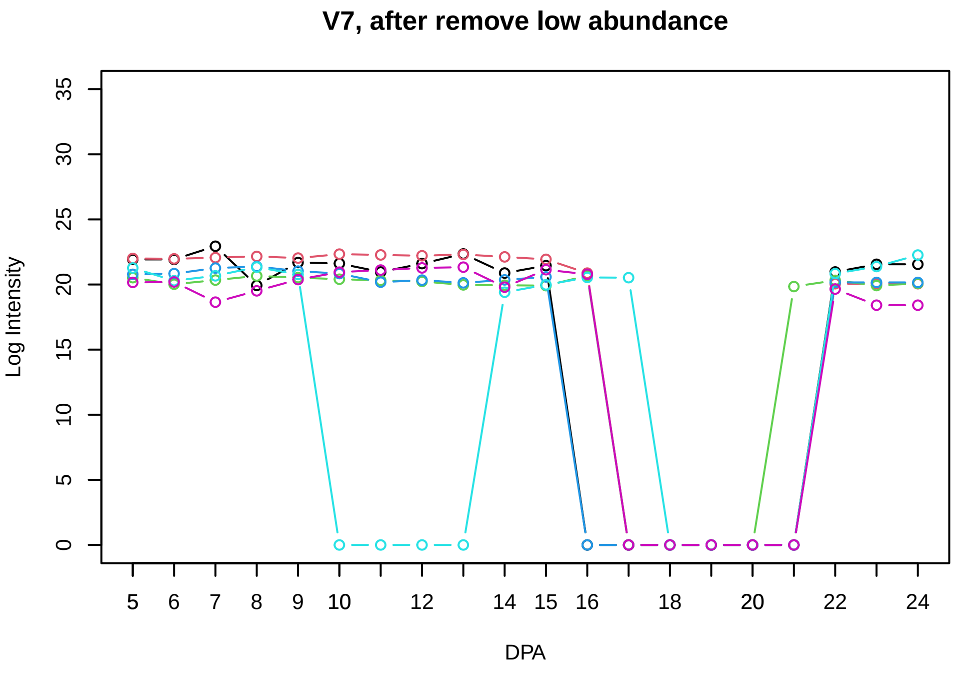
V7, after remove low abundance
Log Intensity
DPA
0 5 10 15 20 25 30 35
5 6 7 8 9 10 12 14 15 16 18 20 22 24

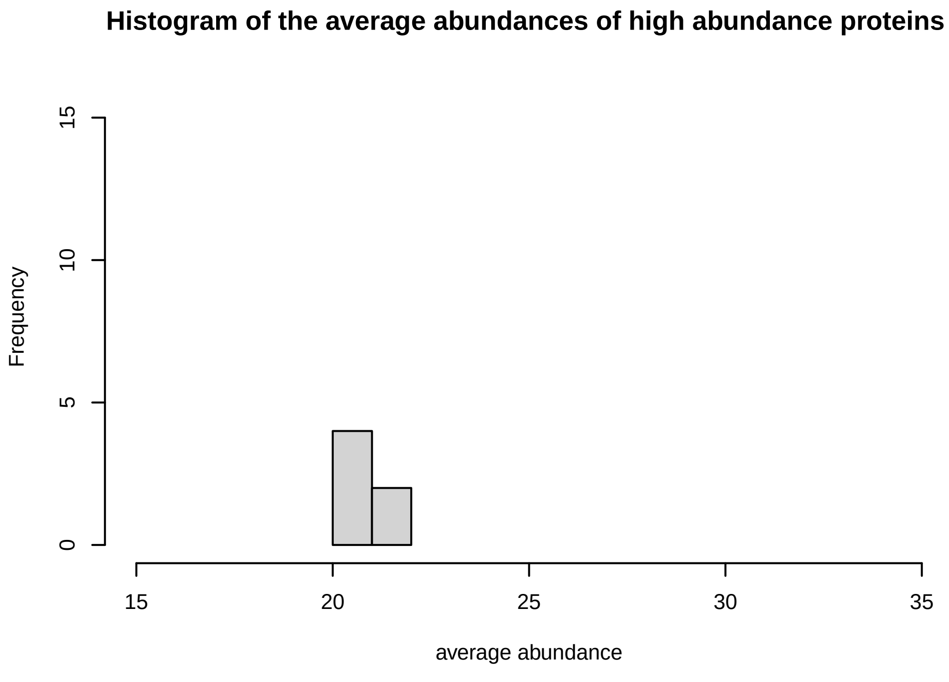
Histogram of the average abundances of high abundance proteins
Frequency
average abundance
0 5 10 15
15 20 25 30 35

V8

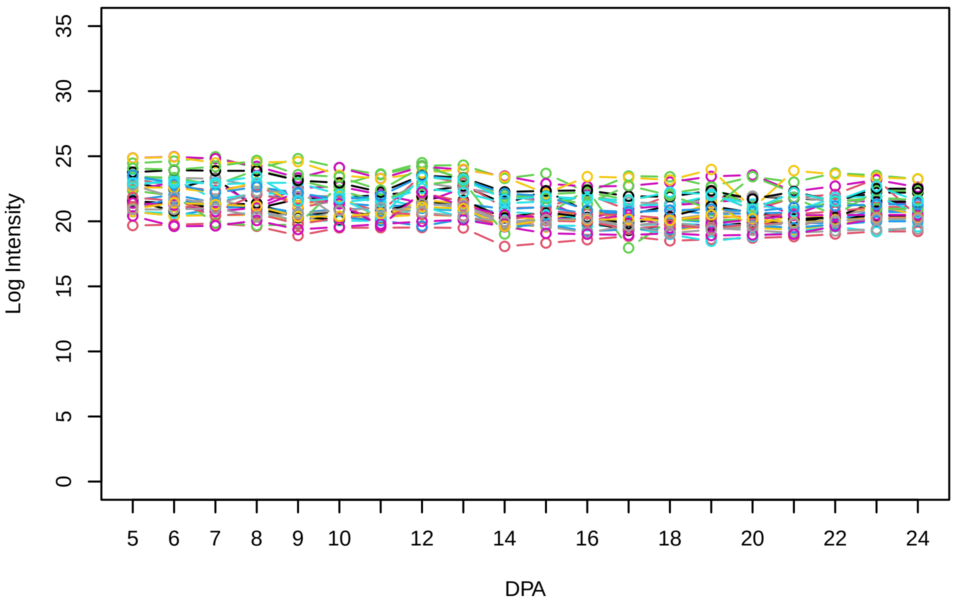

Histogram of the average abundances of non−zero values

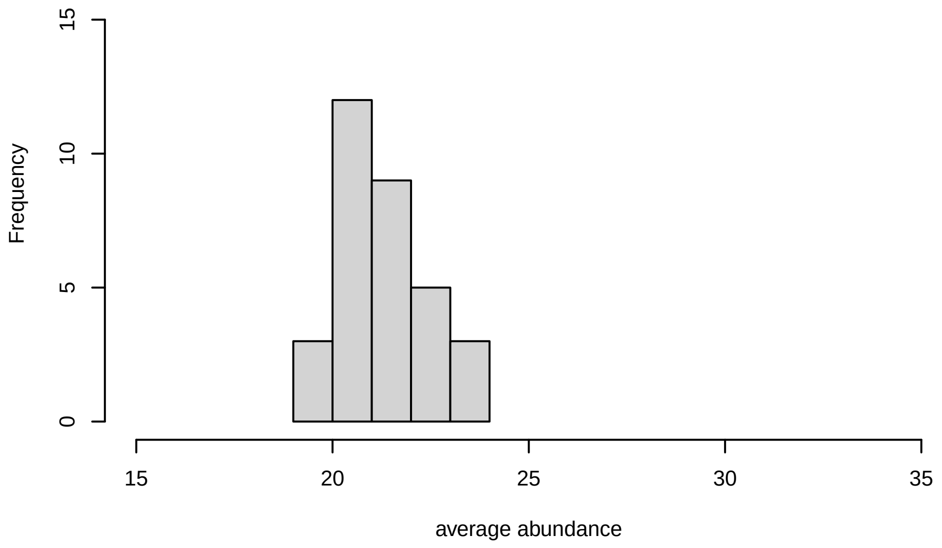

V8, after remove outlier and cutoff is 0.8

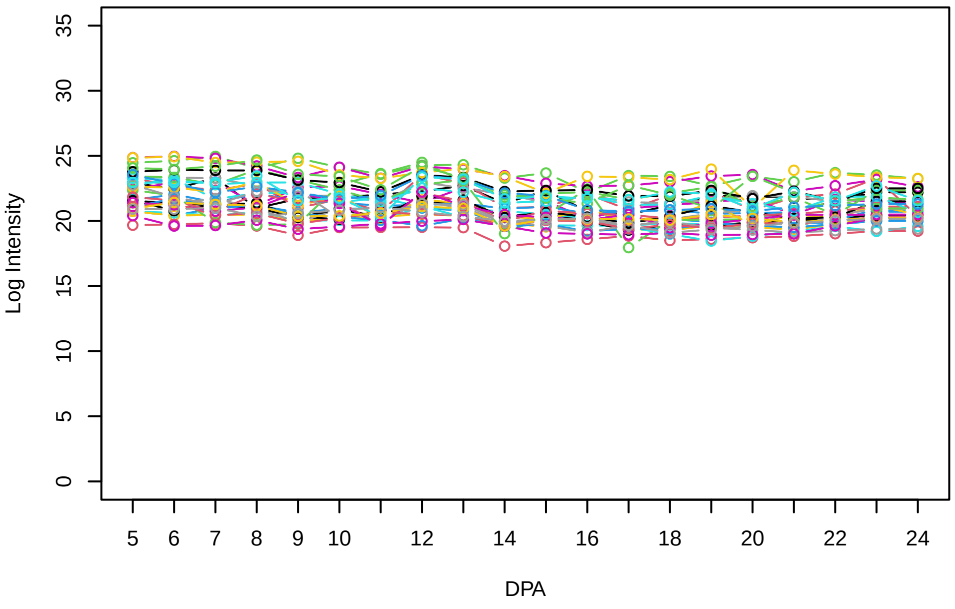

Histogram of the average abundances of non−outlier proteins

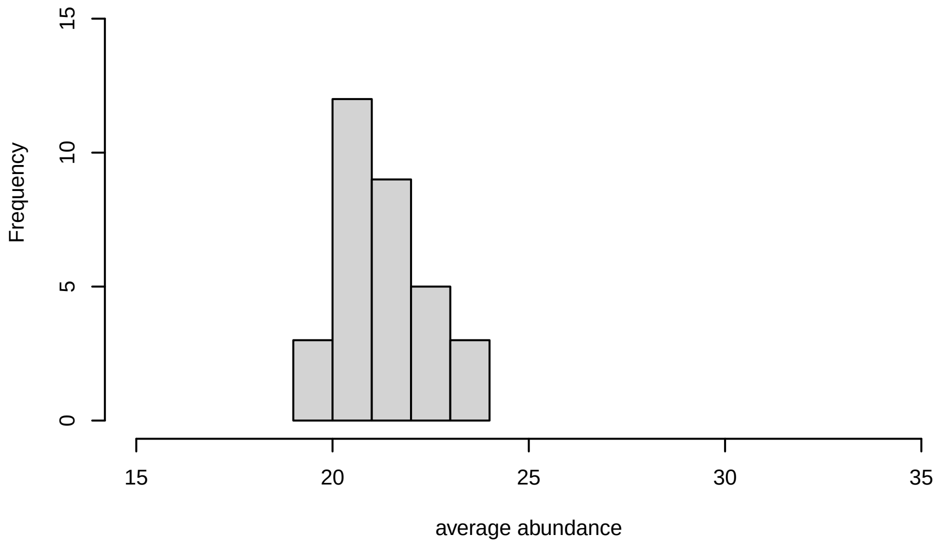

V8, after remove unreliable

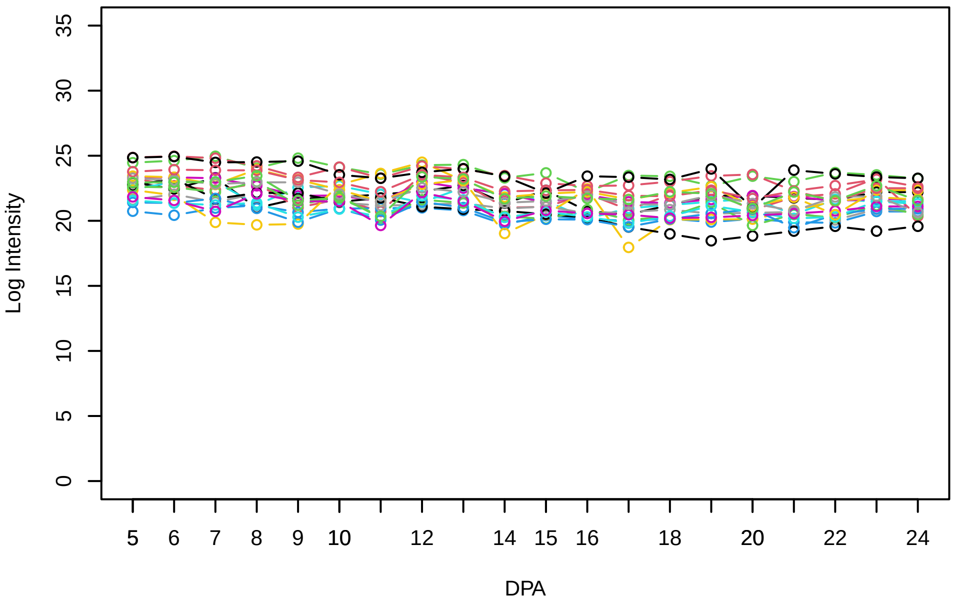

Histogram of the average abundances of reliable proteins

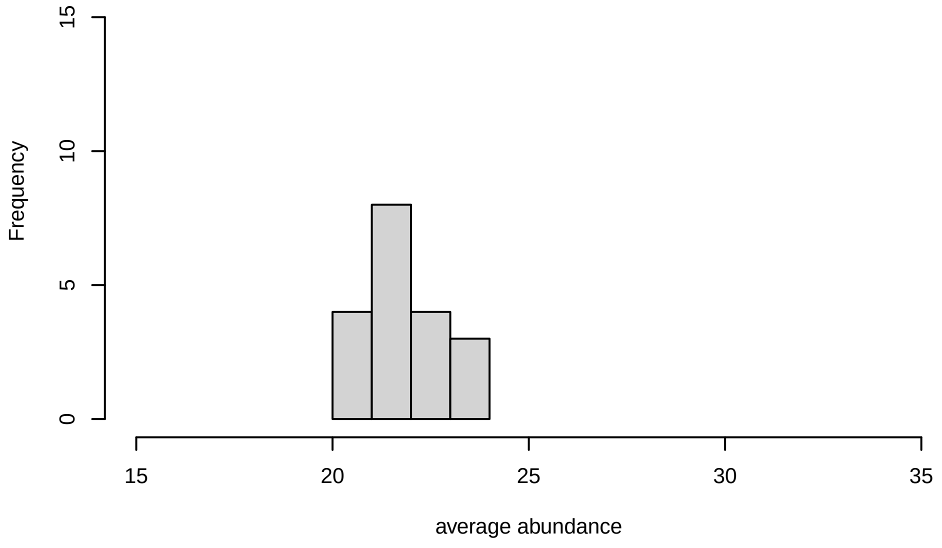

V8, after remove low abundance

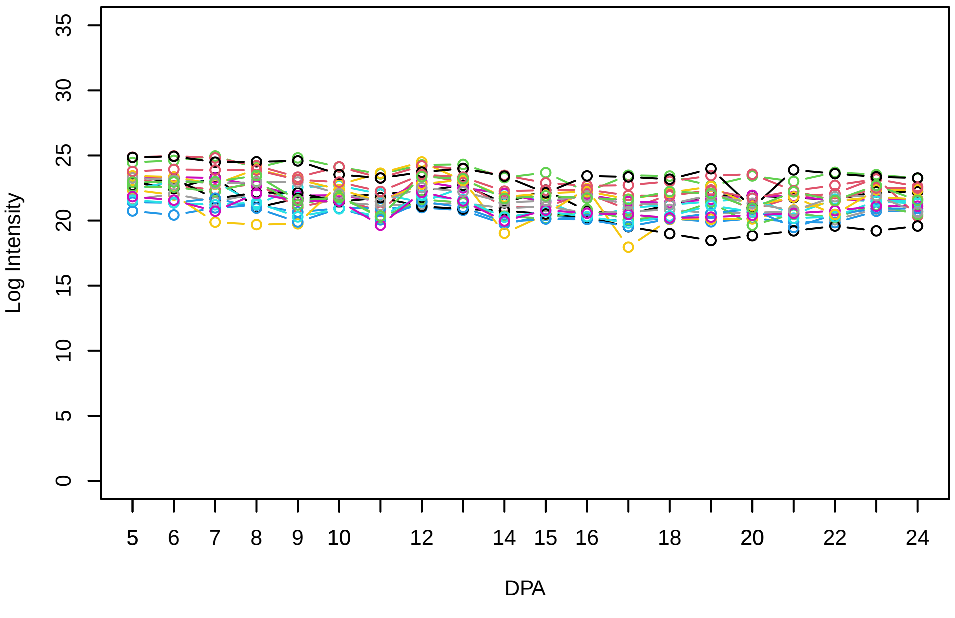

Histogram of the average abundances of high abundance proteins

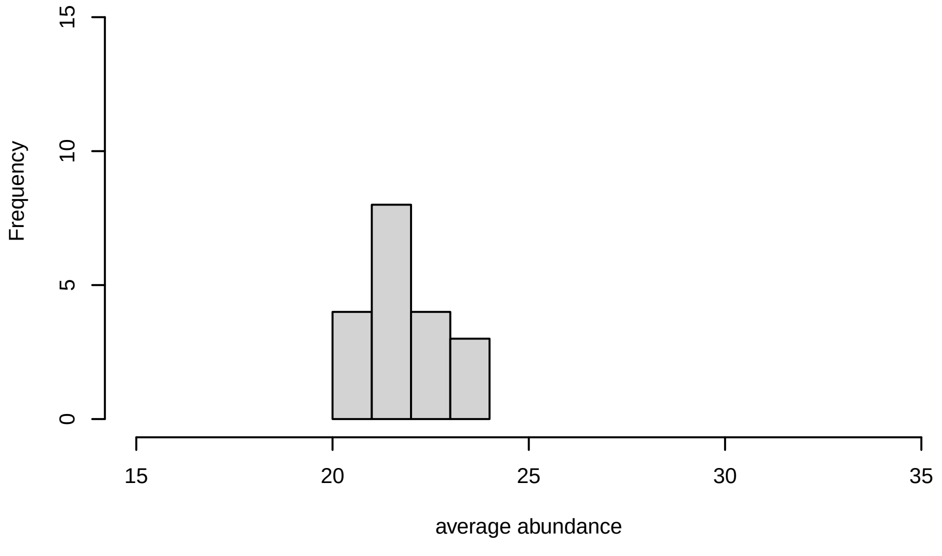

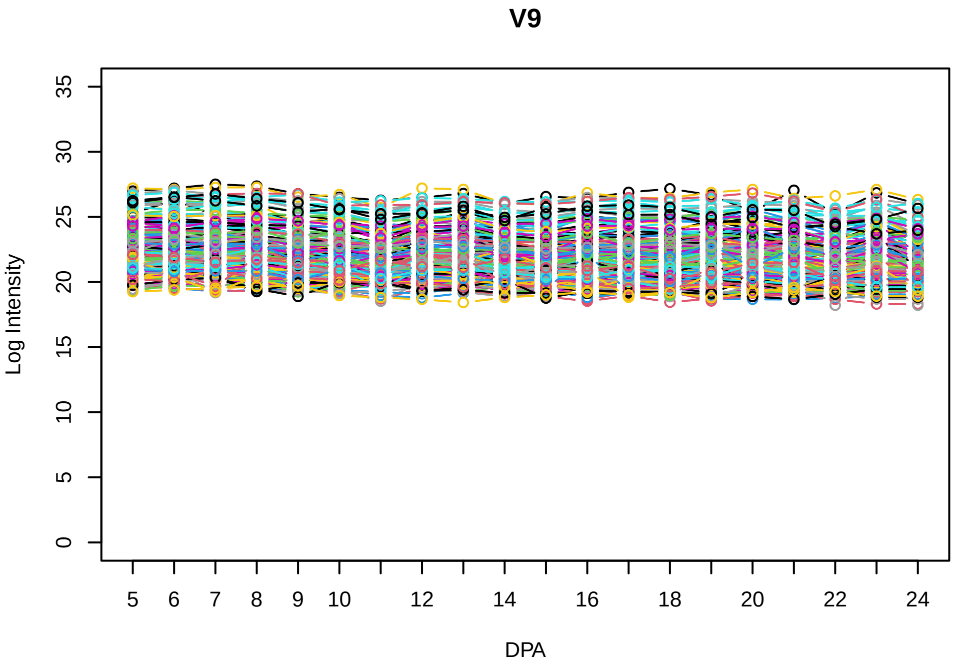
V9
Log Intensity
DPA
0 5 10 15 20 25 30 35
5 6 7 8 9 10 12 14 16 18 20 22 24

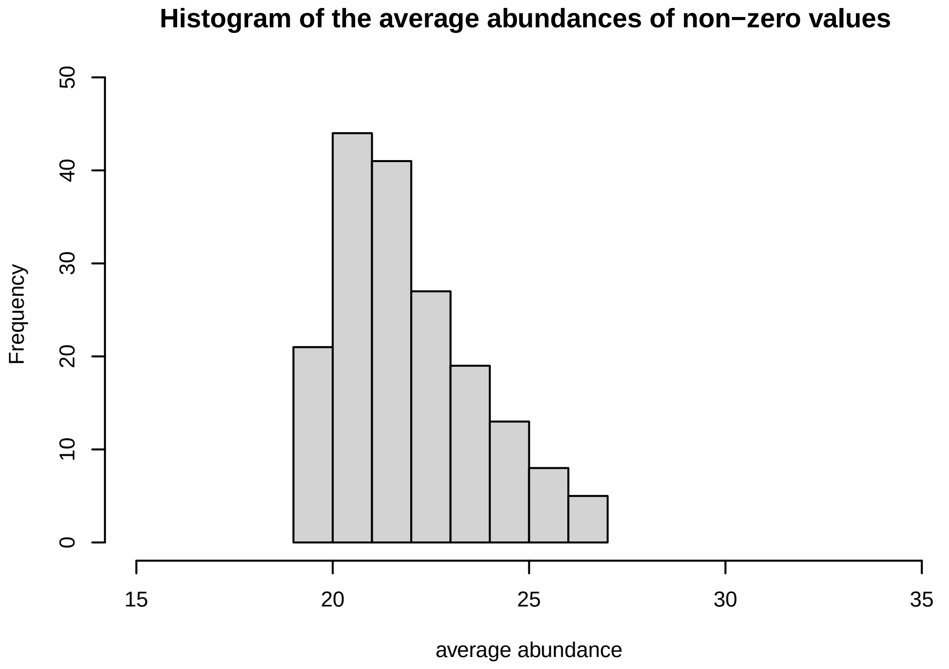
Histogram of the average abundances of non−zero values
Frequency
average abundance
0 10 20 30 40 50
15 20 25 30 35

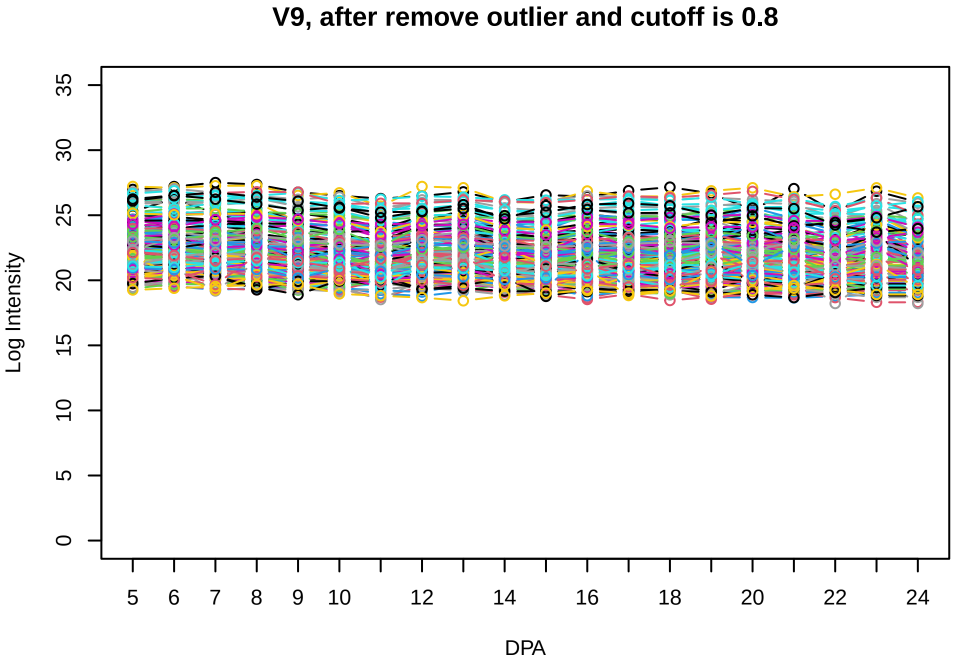
V9, after remove outlier and cutoff is 0.8
Log Intensity
DPA
0 5 10 15 20 25 30 35
5 6 7 8 9 10 12 14 16 18 20 22 24

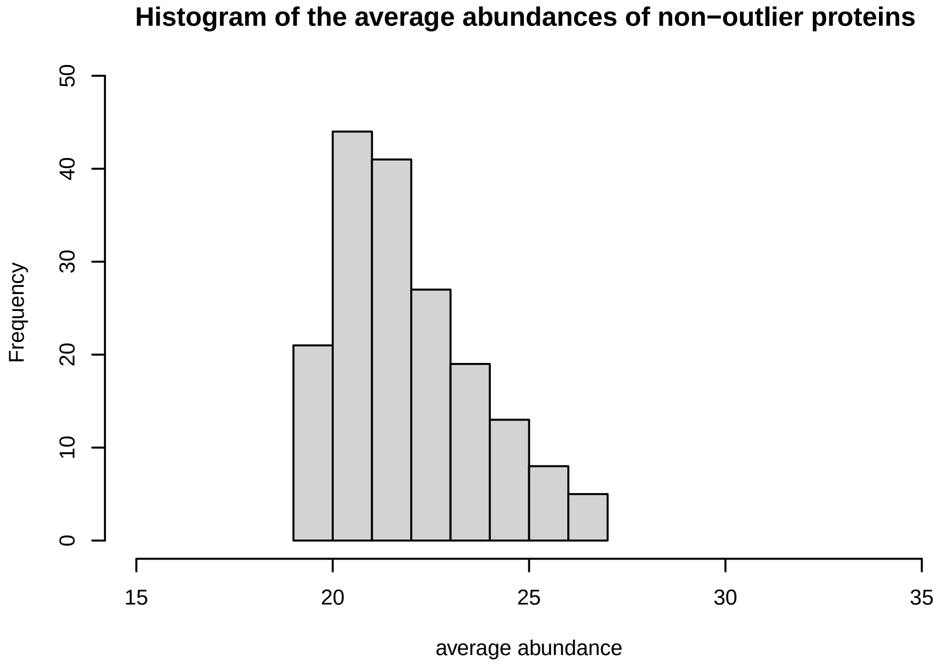
Histogram of the average abundances of non−outlier proteins
Frequency
average abundance
0 10 20 30 40 50
15 20 25 30 35

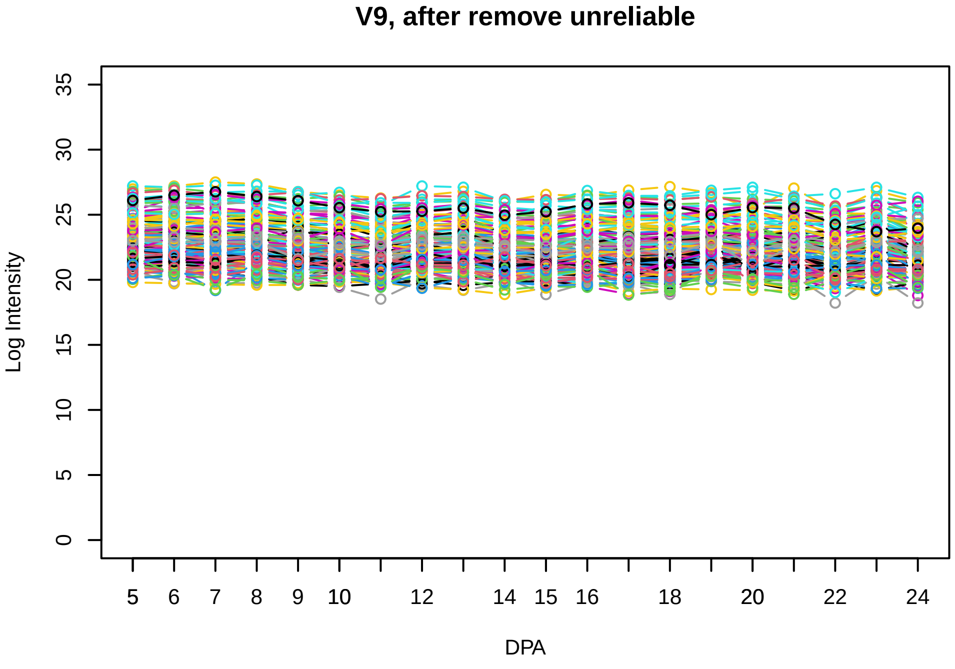
V9, after remove unreliable
Log Intensity
DPA
0 5 10 15 20 25 30 35
5 6 7 8 9 10 12 14 15 16 18 20 22 24

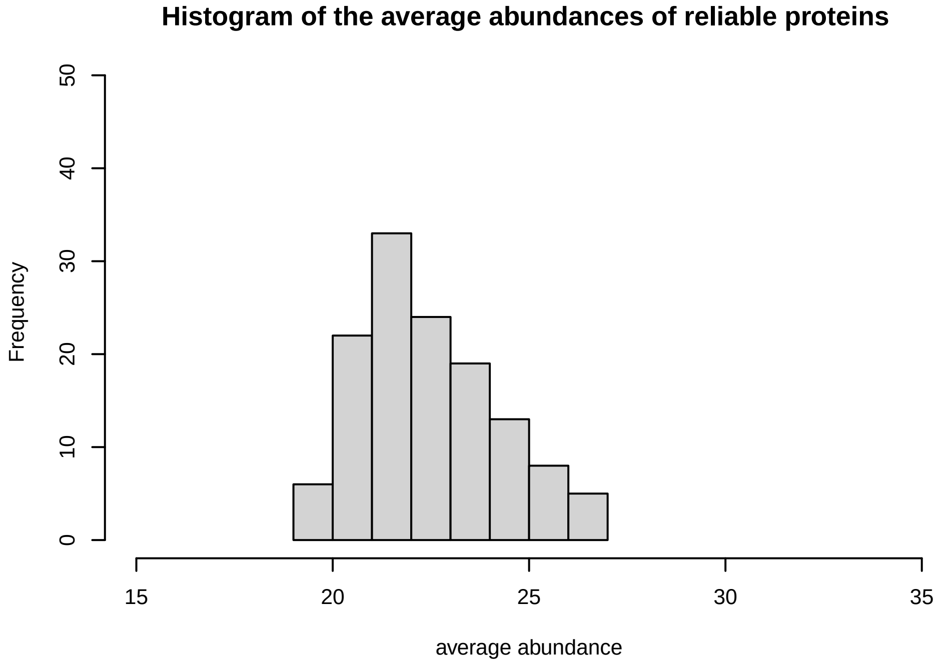
Histogram of the average abundances of reliable proteins
Frequency
average abundance
0 10 20 30 40 50
15 20 25 30 35

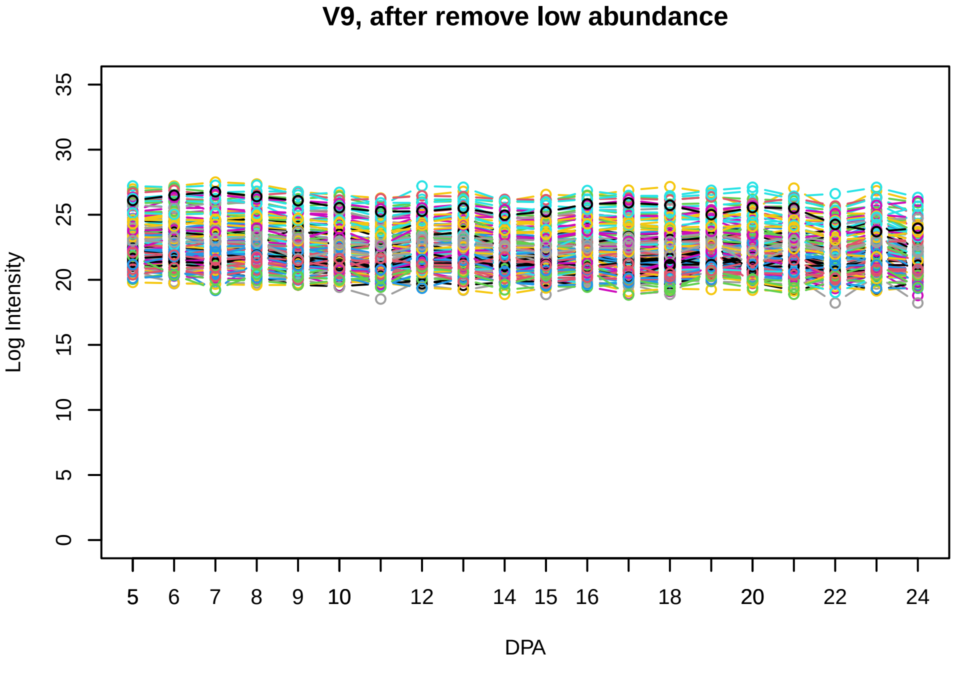
V9, after remove low abundance
Log Intensity
DPA
0 5 10 15 20 25 30 35
5 6 7 8 9 10 12 14 15 16 18 20 22 24

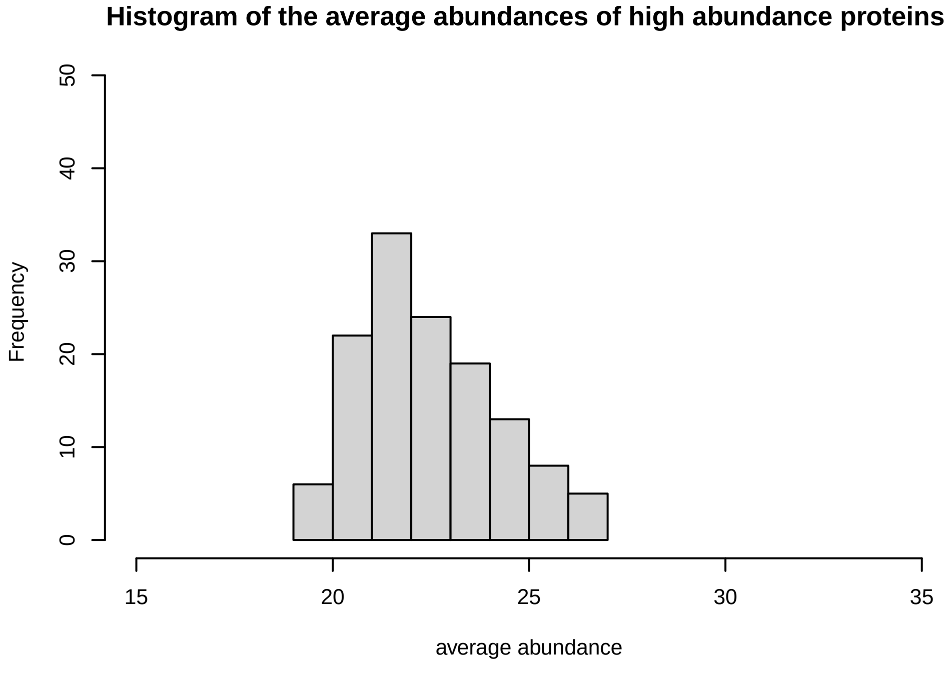
Histogram of the average abundances of high abundance proteins
Frequency
average abundance
0 10 20 30 40 50
15 20 25 30 35

V10

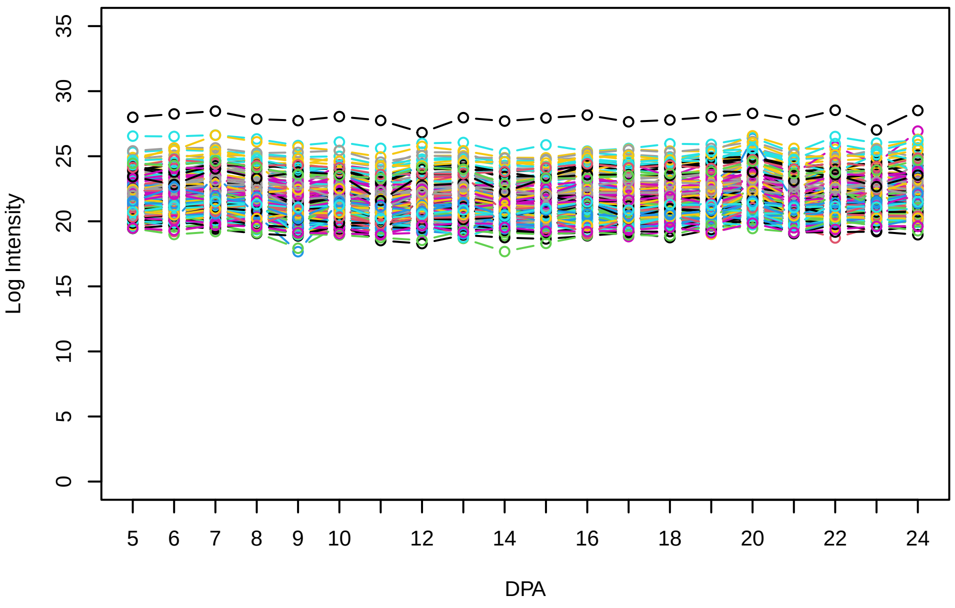

Histogram of the average abundances of non−zero values

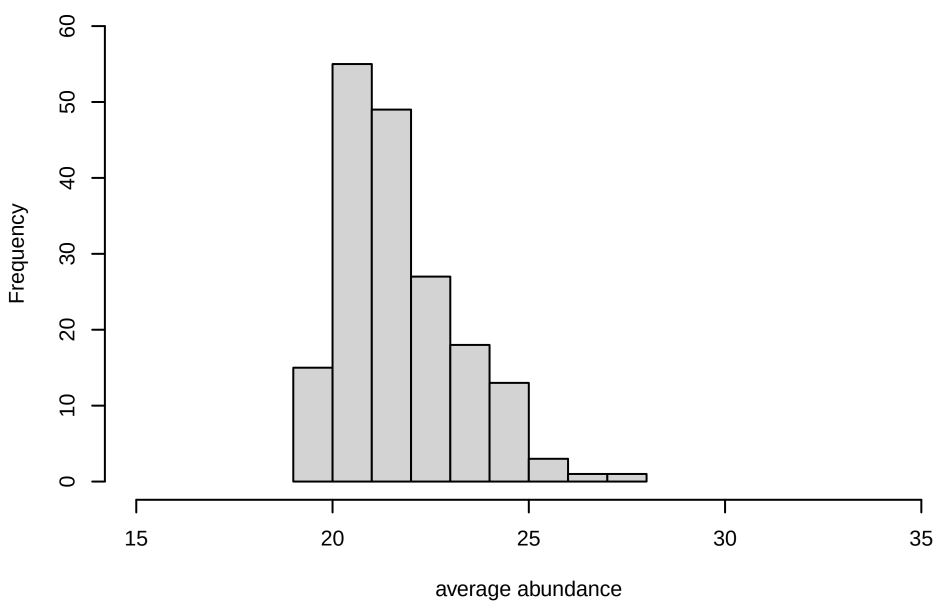

V10, after remove outlier and cutoff is 0.8

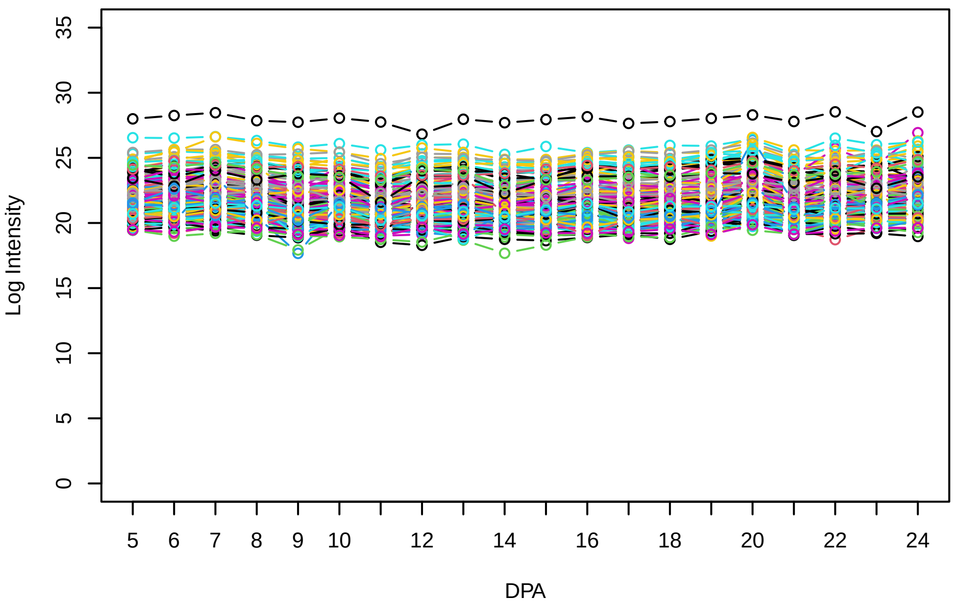

Histogram of the average abundances of non−outlier proteins

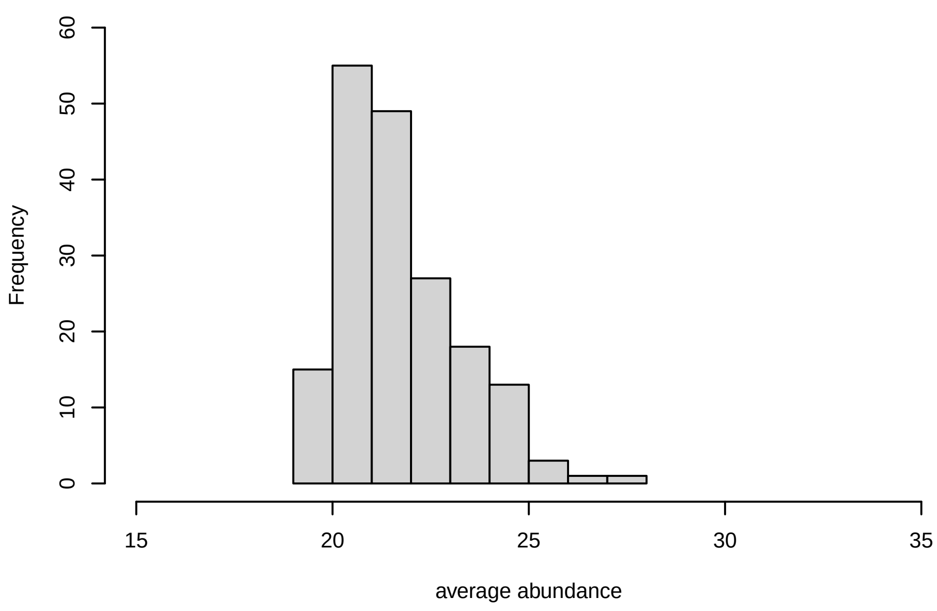

V10, after remove unreliable

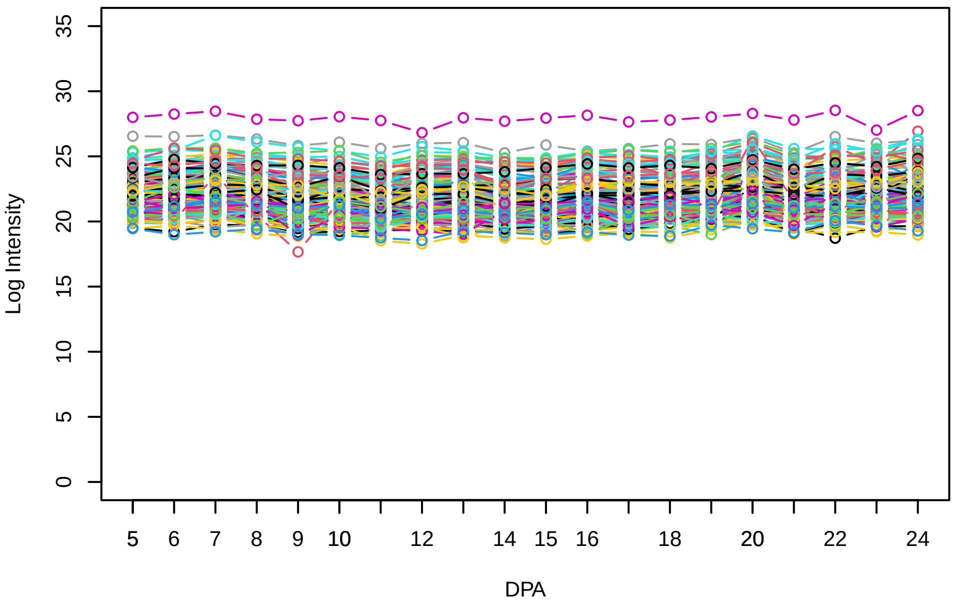

Histogram of the average abundances of reliable proteins

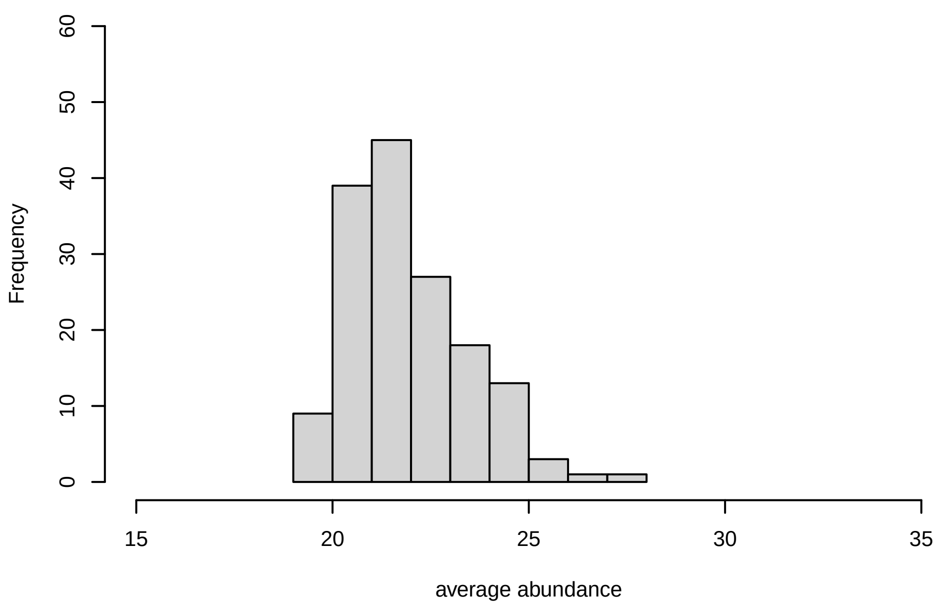

V10, after remove low abundance

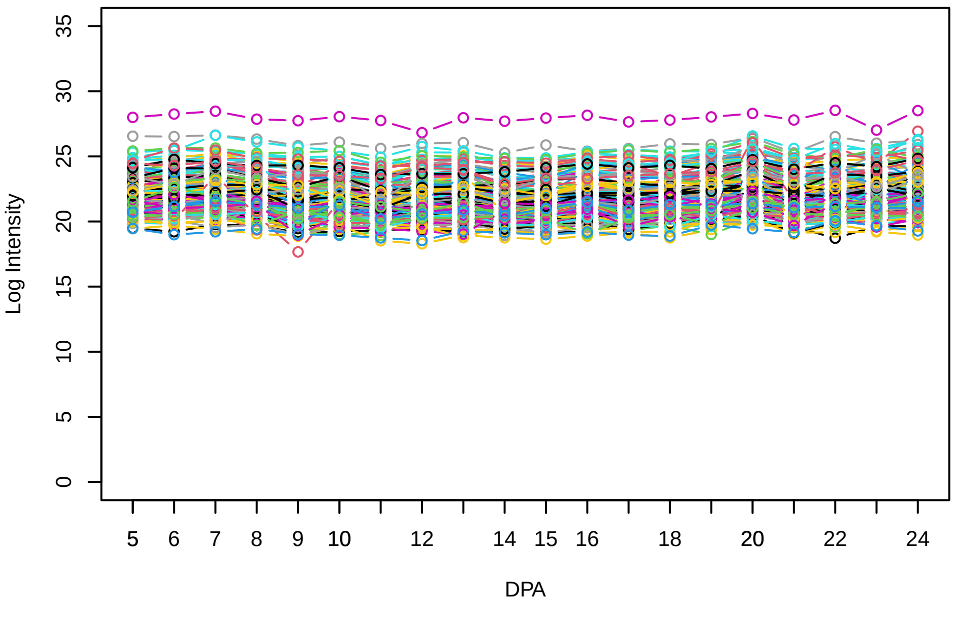

Histogram of the average abundances of high abundance proteins

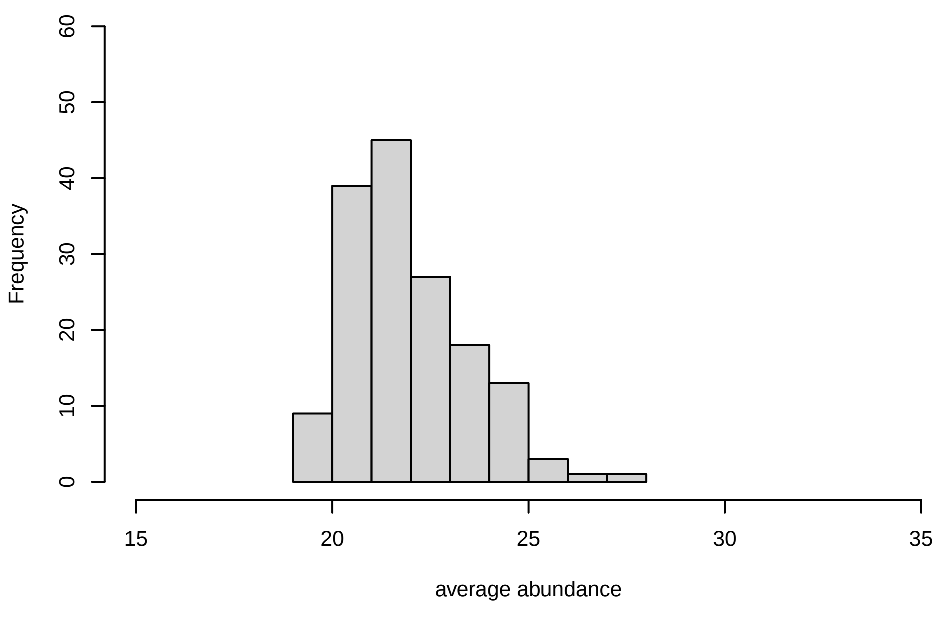

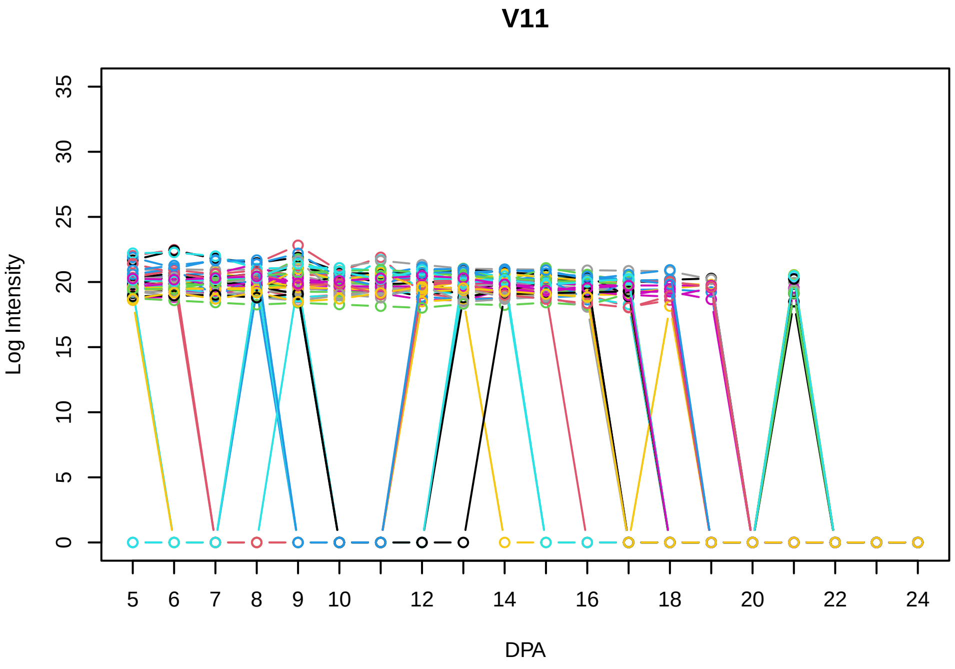
V11
Log Intensity
DPA

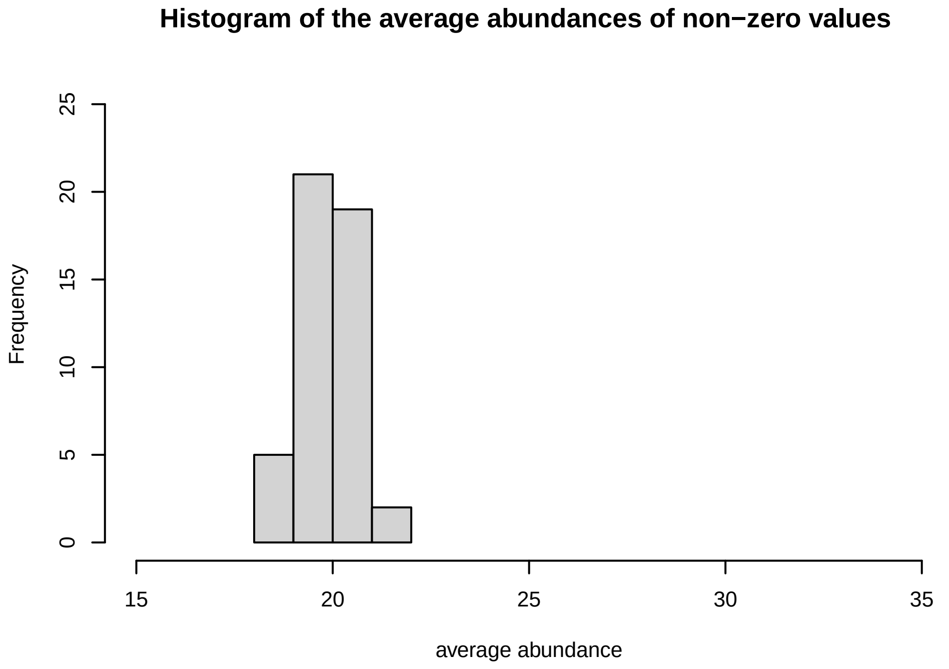
Histogram of the average abundances of non−zero values
Frequency
average abundance

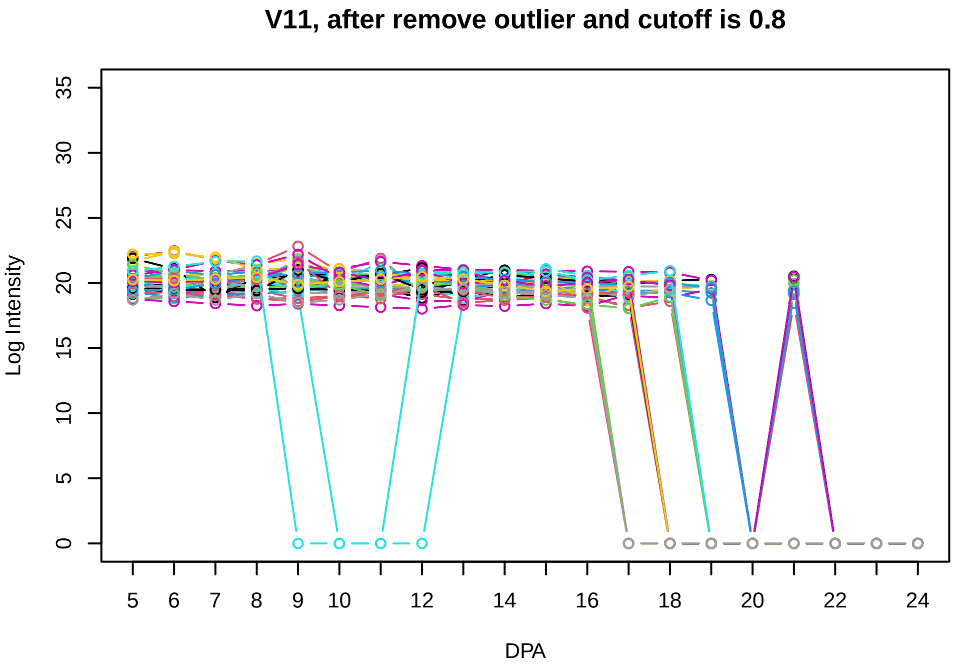
V11, after remove outlier and cutoff is 0.8
Log Intensity
DPA

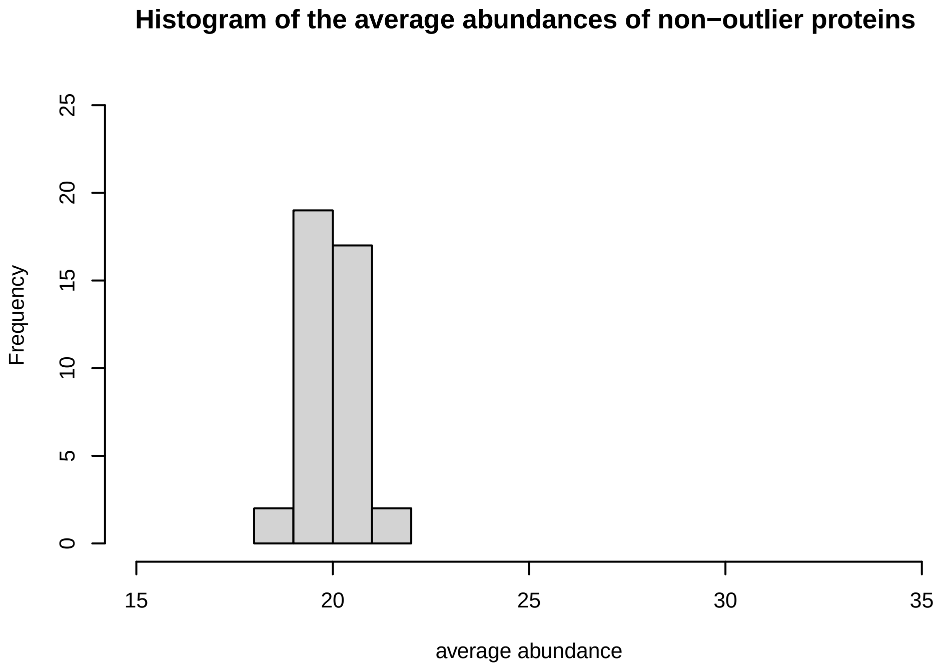
Histogram of the average abundances of non−outlier proteins
Frequency
average abundance

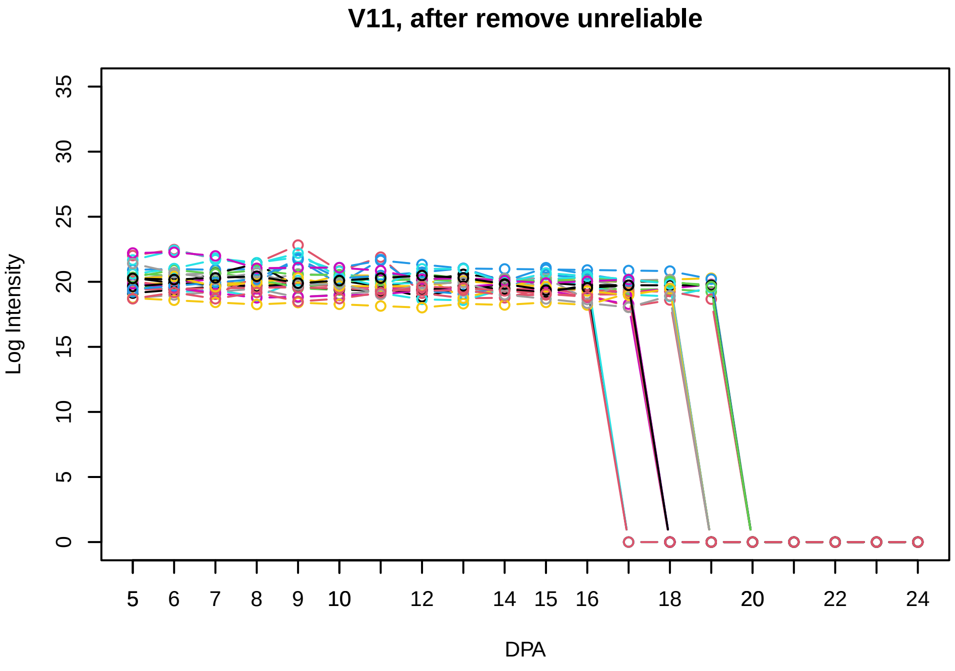
V11, after remove unreliable
Log Intensity
DPA

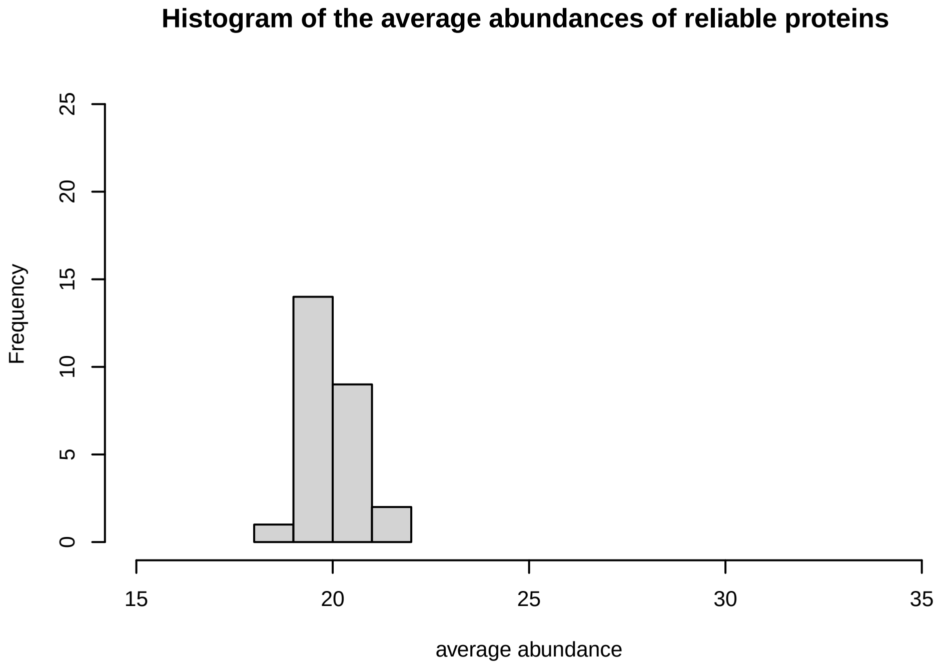
Histogram of the average abundances of reliable proteins
Frequency
average abundance

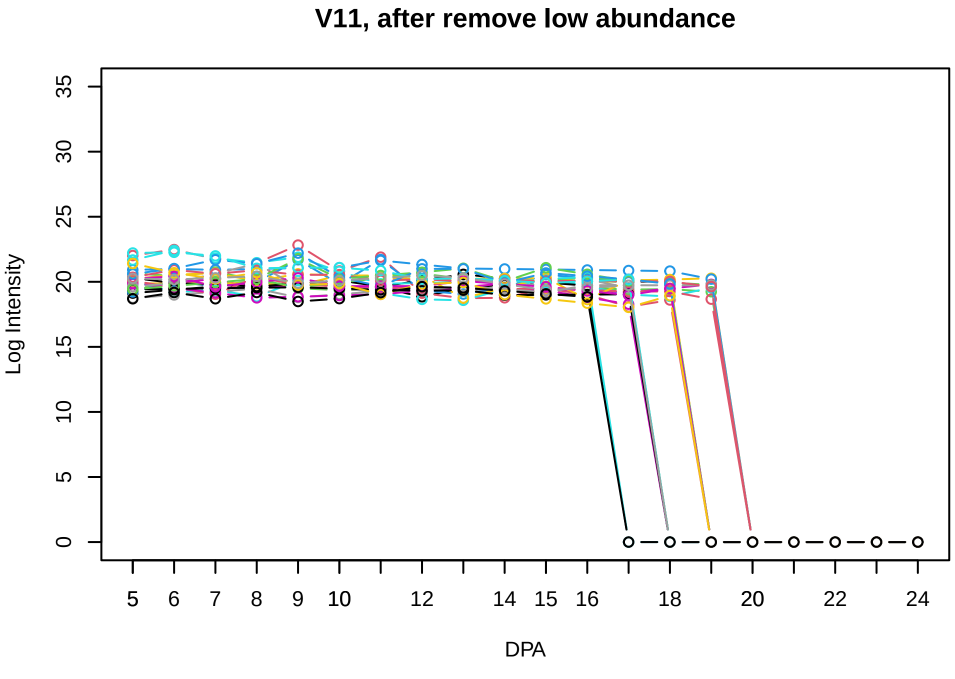
V11, after remove low abundance
Log Intensity
DPA

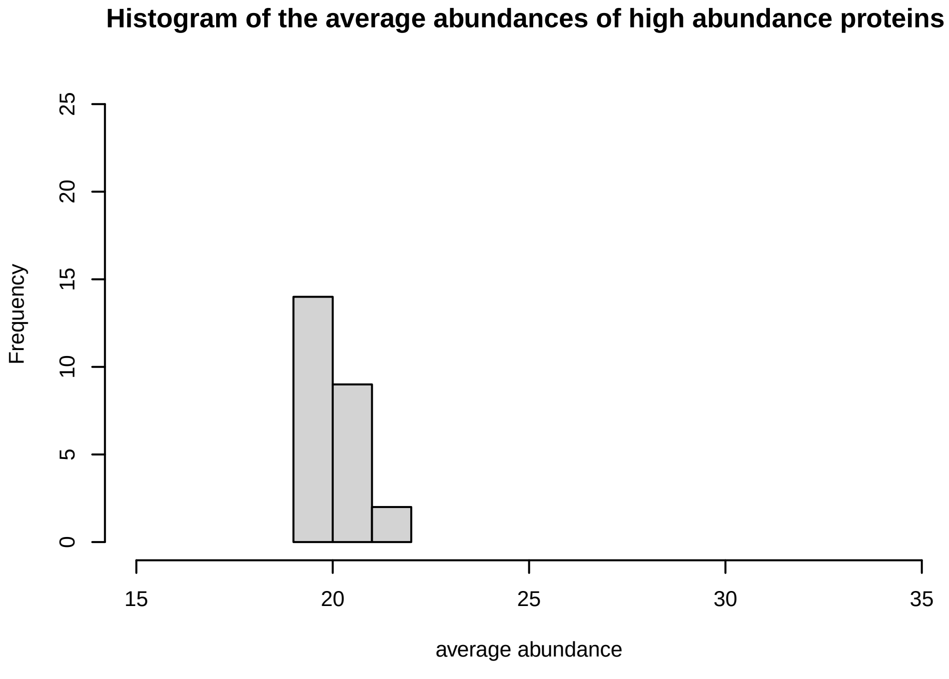
Histogram of the average abundances of high abundance proteins
Frequency
average abundance

V12

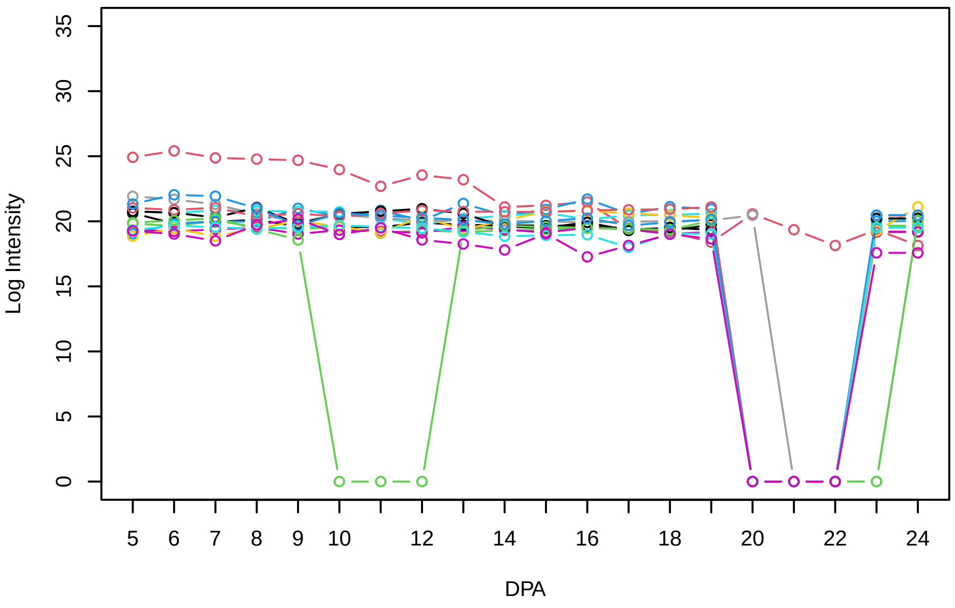

Histogram of the average abundances of non−zero values

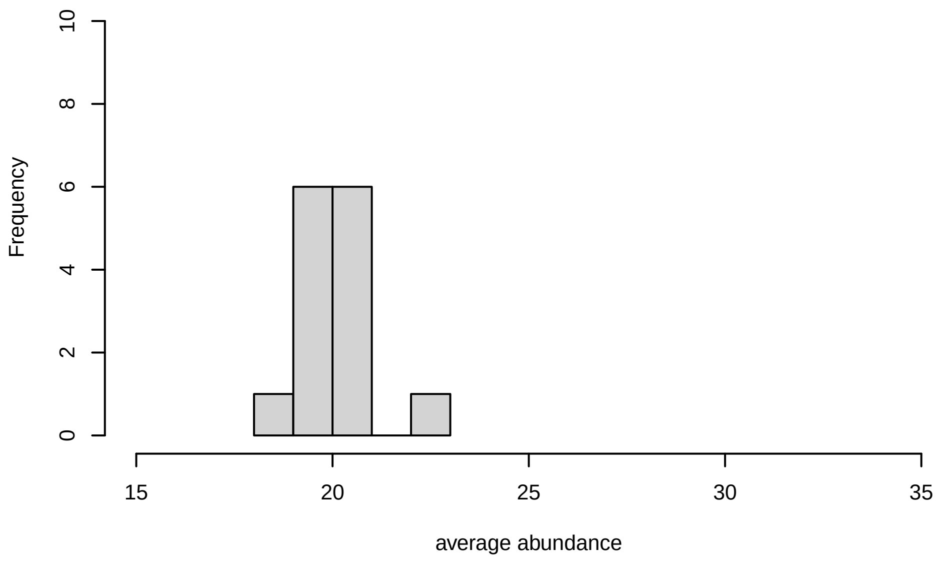

V12, after remove outlier and cutoff is 0.8

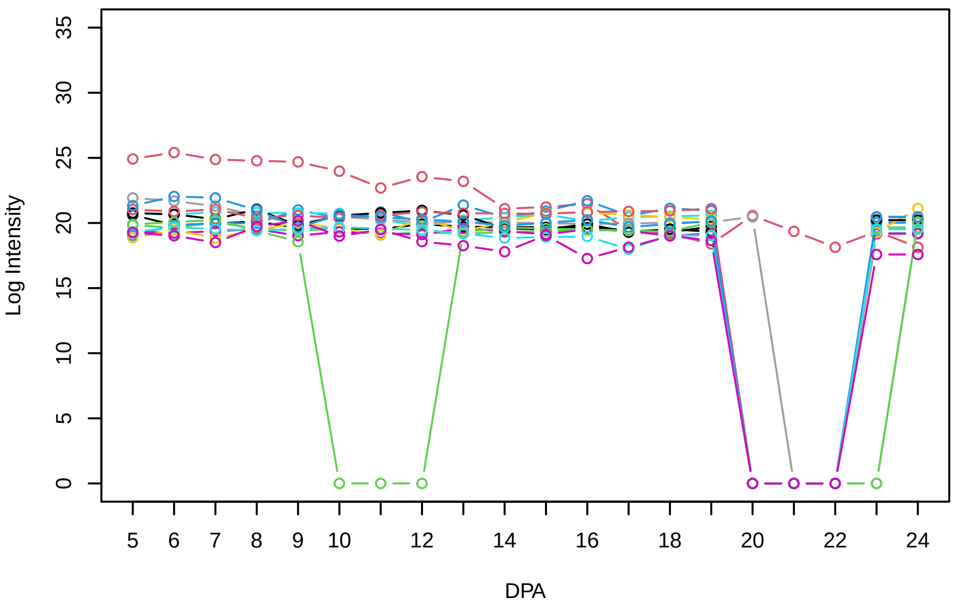

Histogram of the average abundances of non−outlier proteins

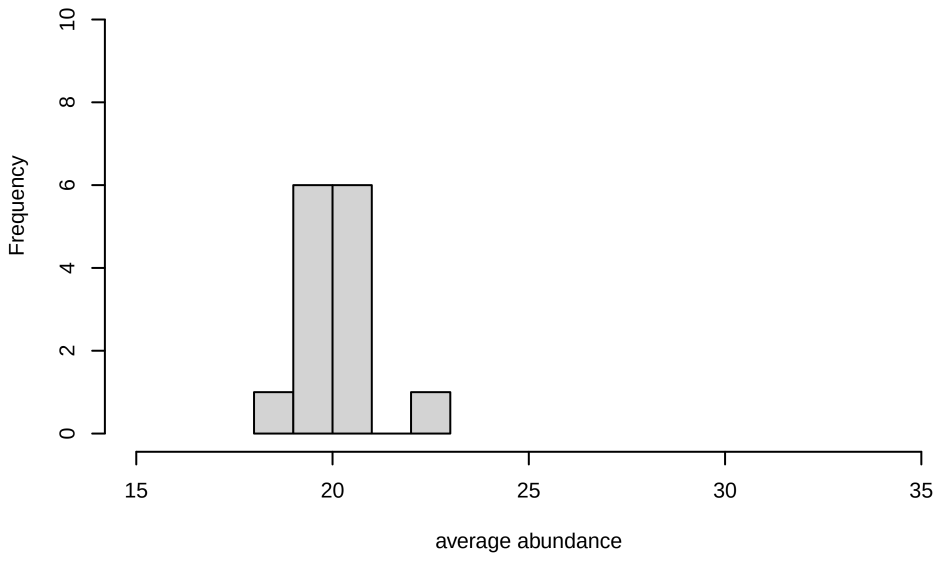

V12, after remove unreliable

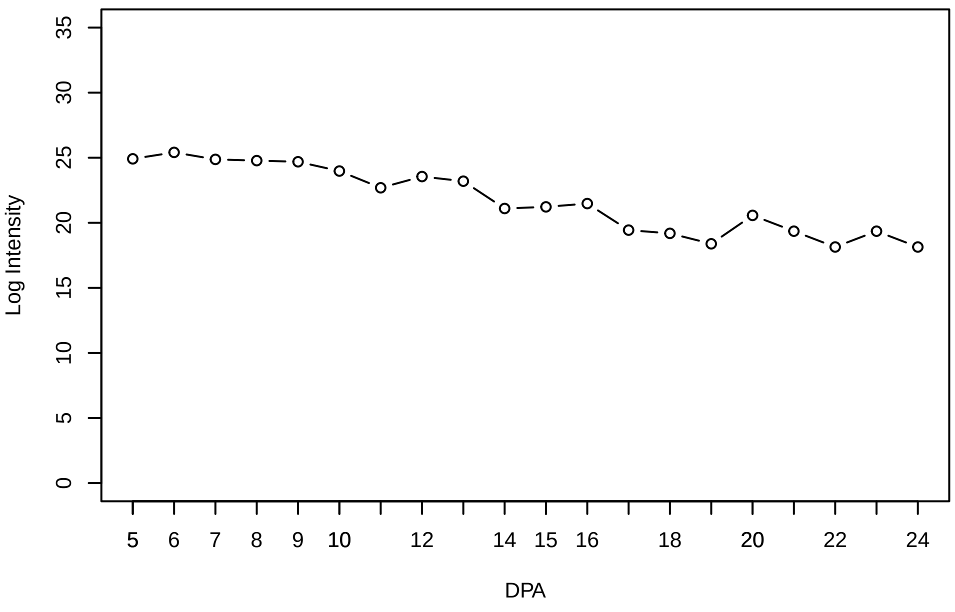

Histogram of the average abundances of reliable proteins

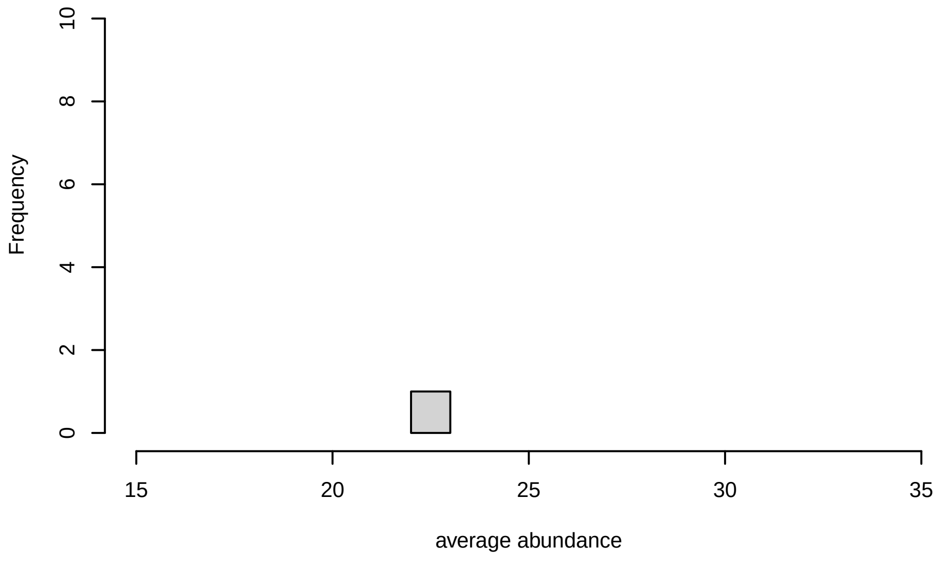

V12, after remove low abundance

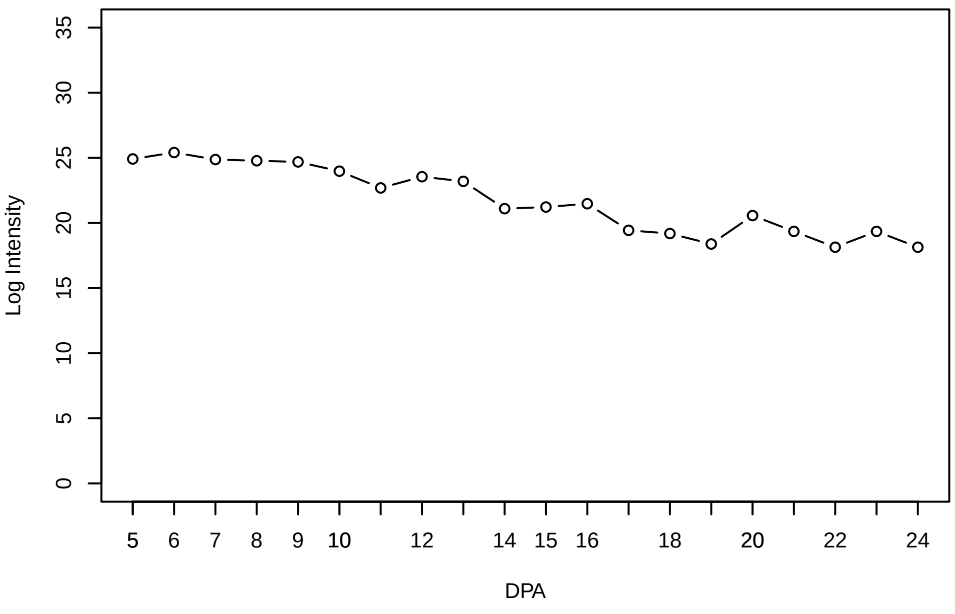

Histogram of the average abundances of high abundance proteins

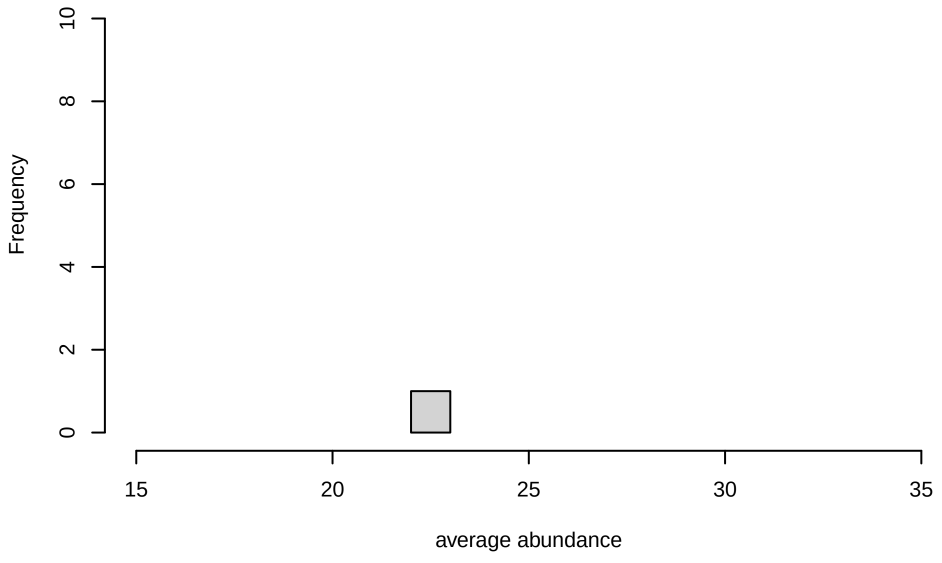

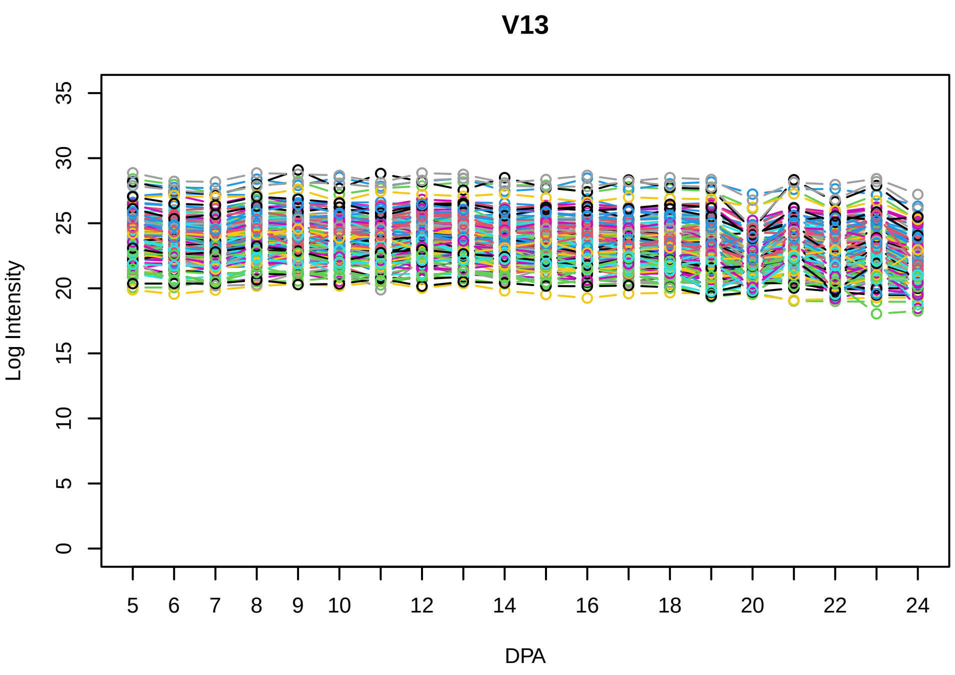
V13
Log Intensity
0 5 10 15 20 25 30 35
5 6 7 8 9 10 12 14 16 18 20 22 24
DPA

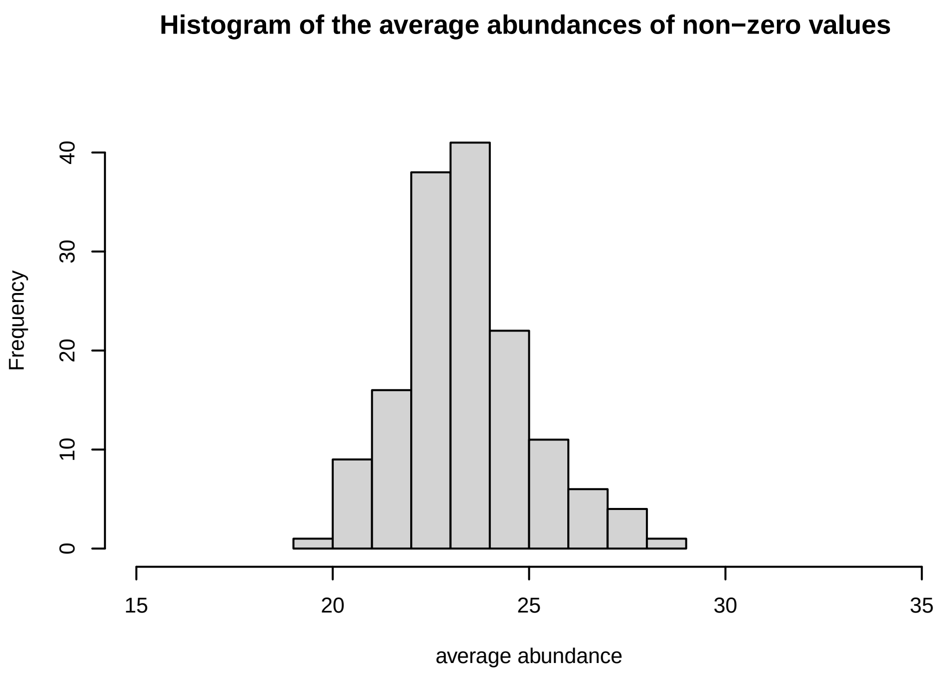
Histogram of the average abundances of non−zero values
Frequency
0 10 20 30 40
15 20 25 30 35
average abundance

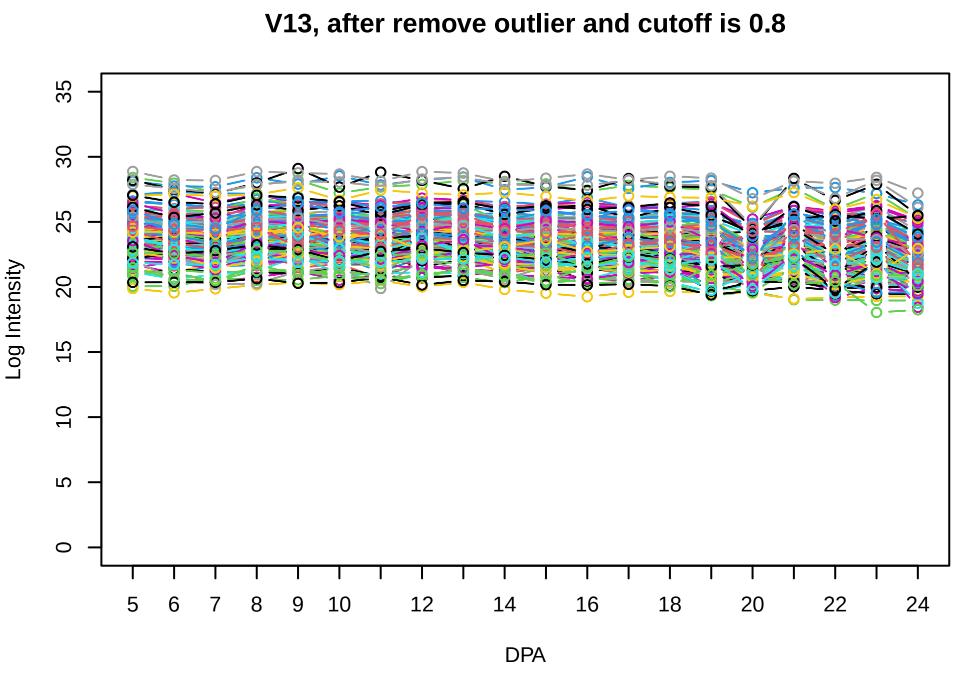
V13, after remove outlier and cutoff is 0.8
Log Intensity
0 5 10 15 20 25 30 35
5 6 7 8 9 10 12 14 16 18 20 22 24
DPA

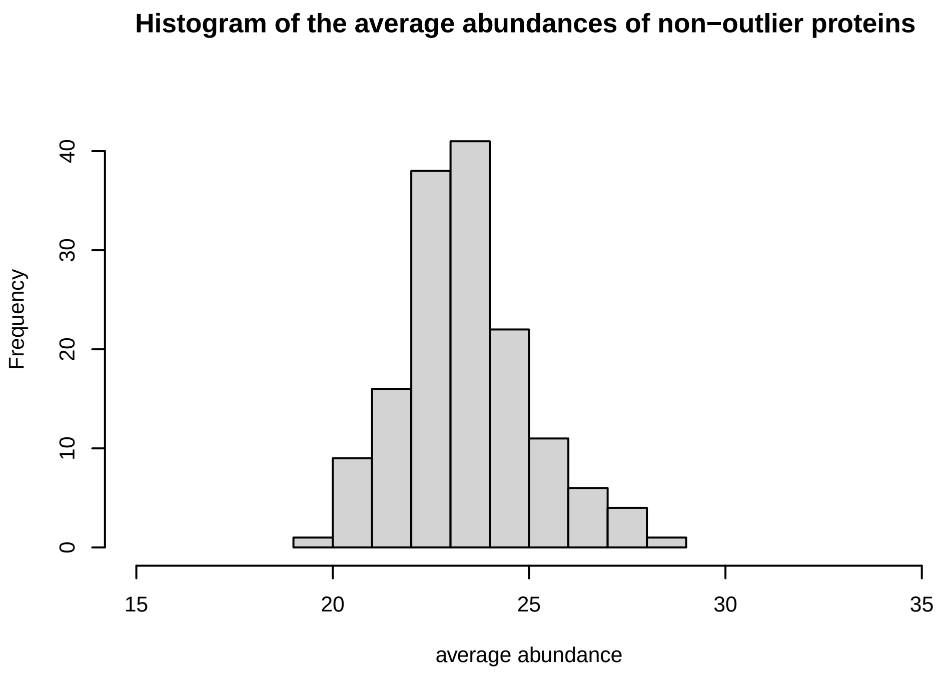
Histogram of the average abundances of non−outlier proteins
Frequency
0 10 20 30 40
15 20 25 30 35
average abundance

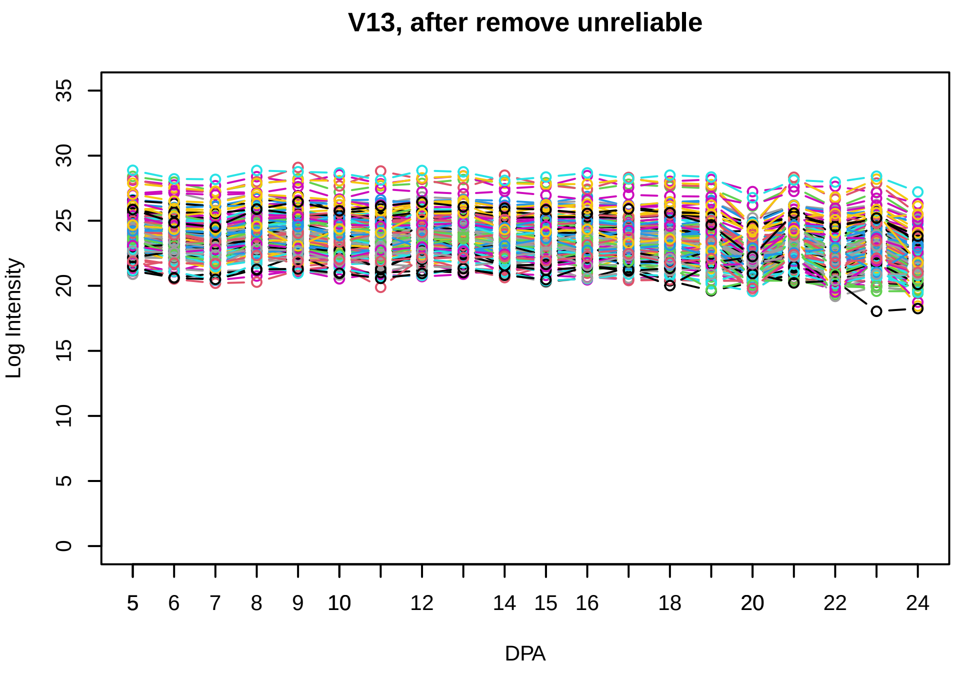
V13, after remove unreliable
Log Intensity
0 5 10 15 20 25 30 35
5 6 7 8 9 10 12 14 15 16 18 20 22 24
DPA

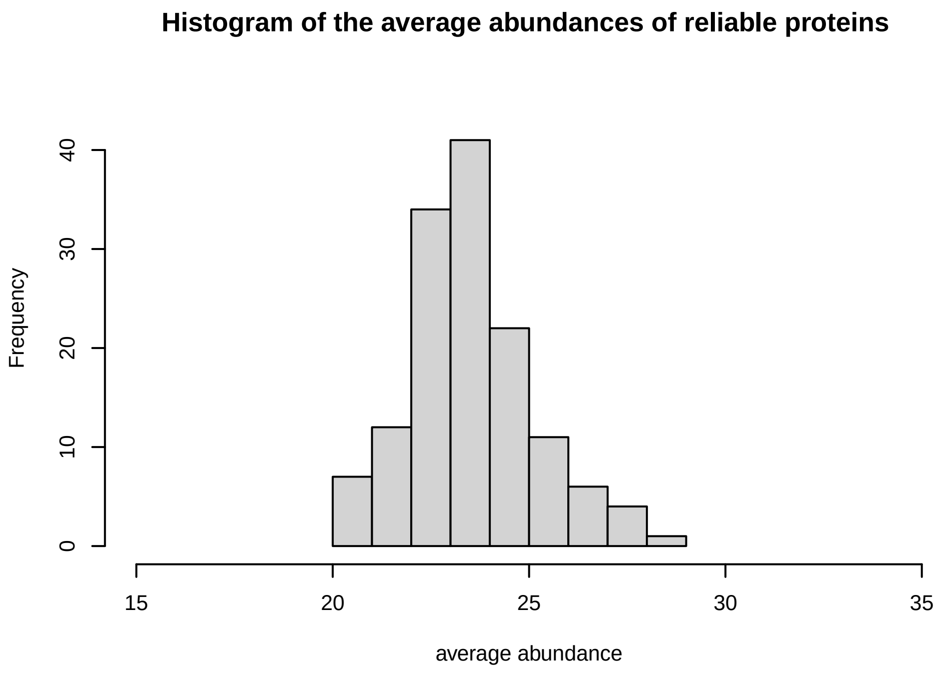
Histogram of the average abundances of reliable proteins
Frequency
0 10 20 30 40
15 20 25 30 35
average abundance

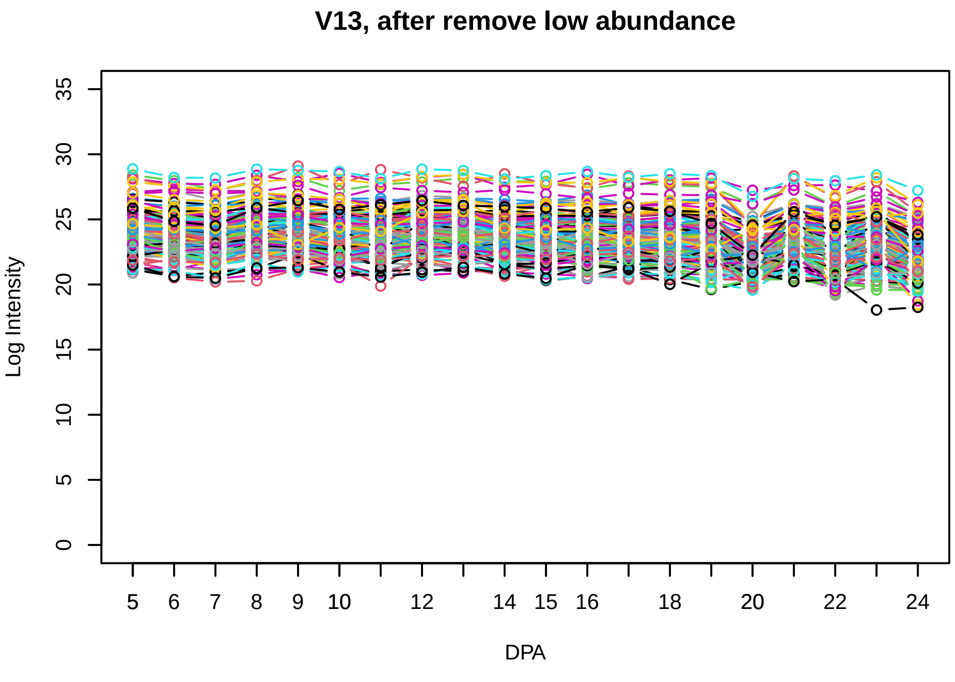
V13, after remove low abundance
Log Intensity
0 5 10 15 20 25 30 35
5 6 7 8 9 10 12 14 15 16 18 20 22 24
DPA

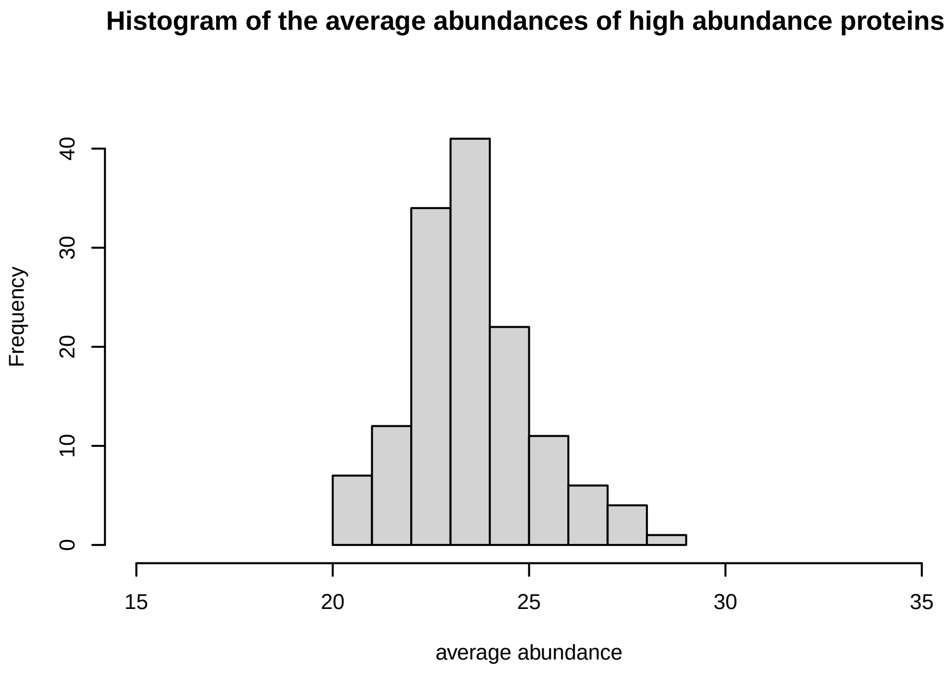
Histogram of the average abundances of high abundance proteins
Frequency
0 10 20 30 40
15 20 25 30 35
average abundance

V14

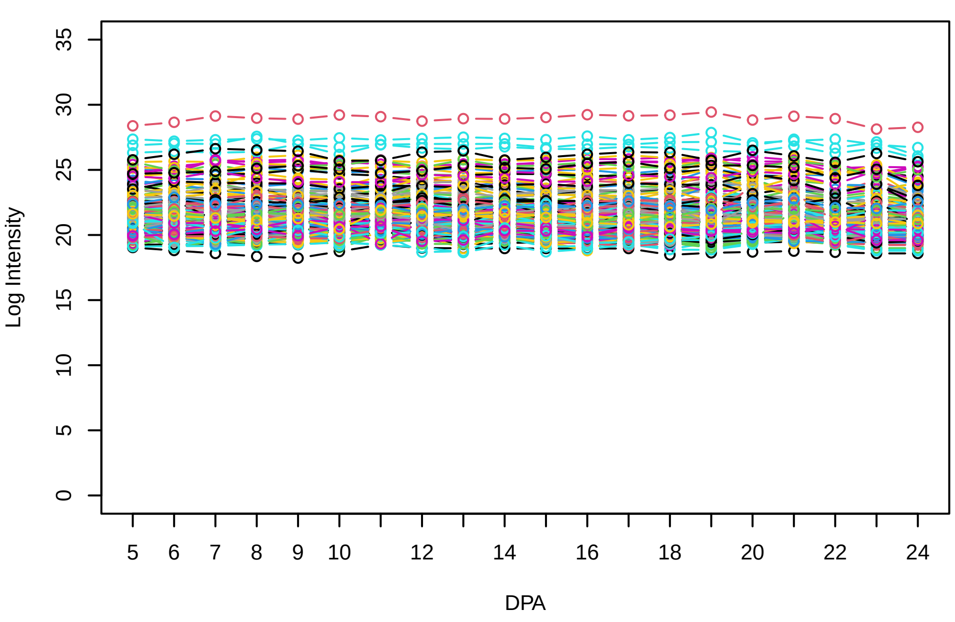

Histogram of the average abundances of non−zero values

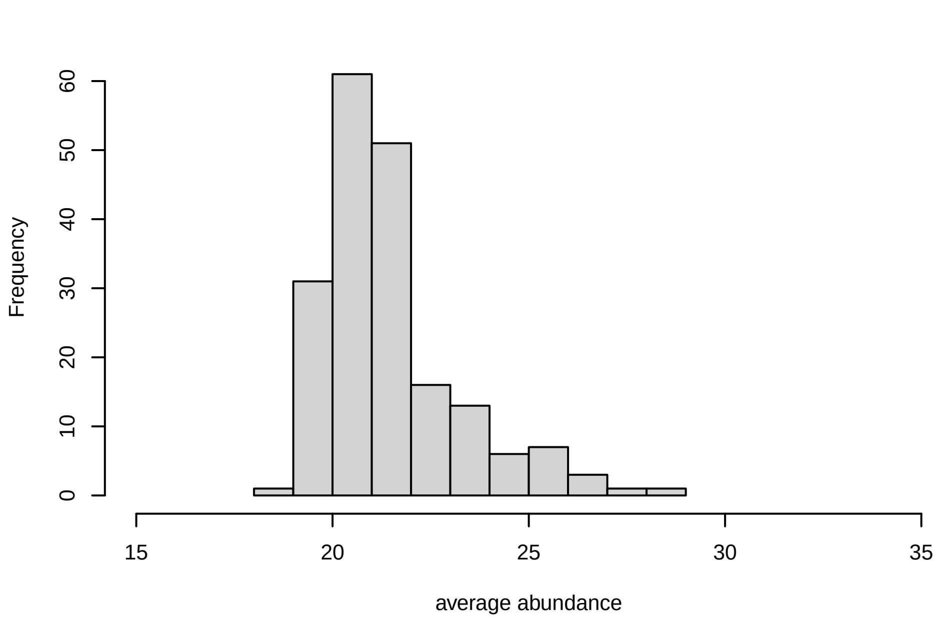

V14, after remove outlier and cutoff is 0.8

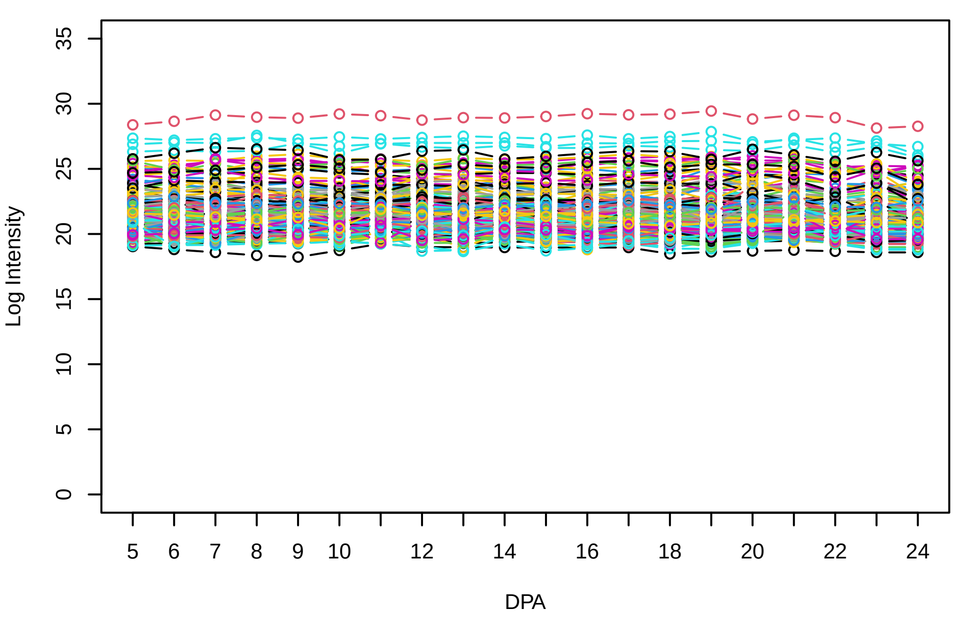

Histogram of the average abundances of non−outlier proteins

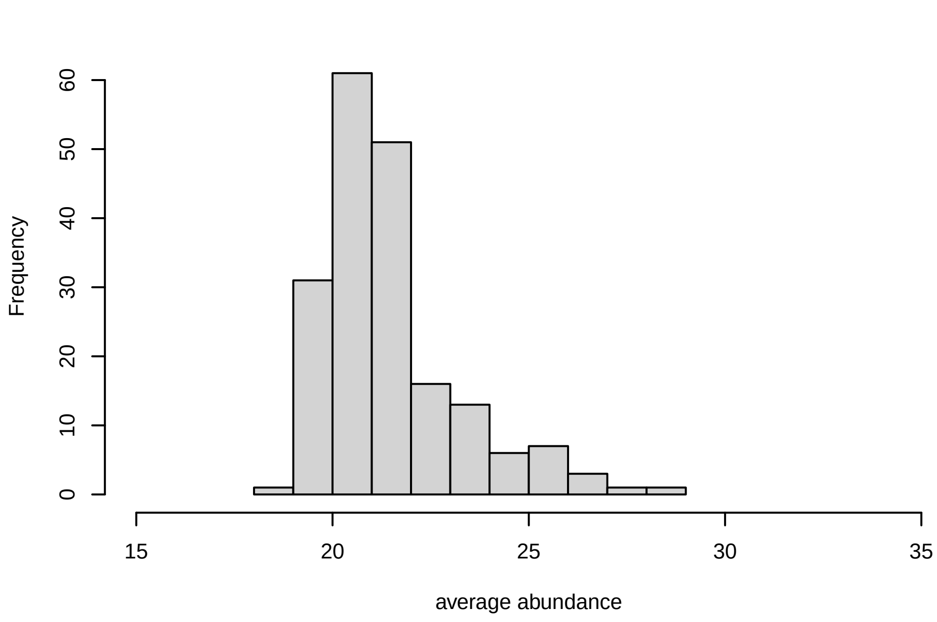

V14, after remove unreliable

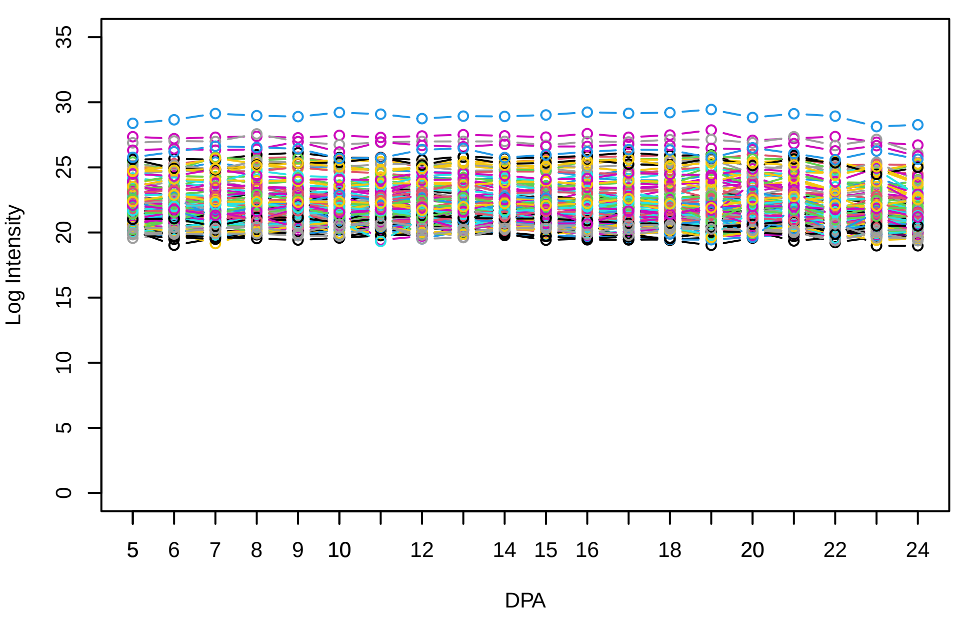

Histogram of the average abundances of reliable proteins

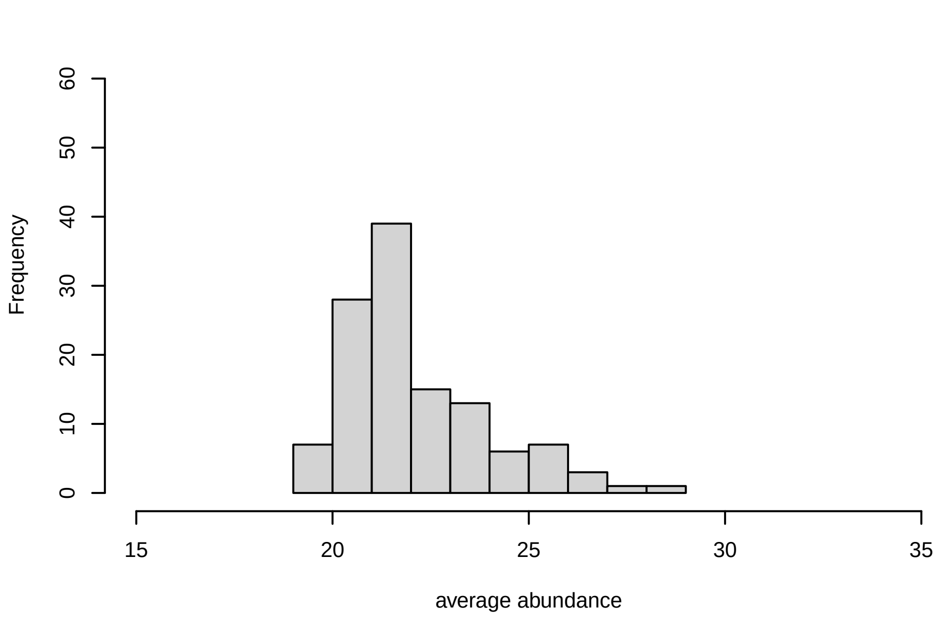

V14, after remove low abundance

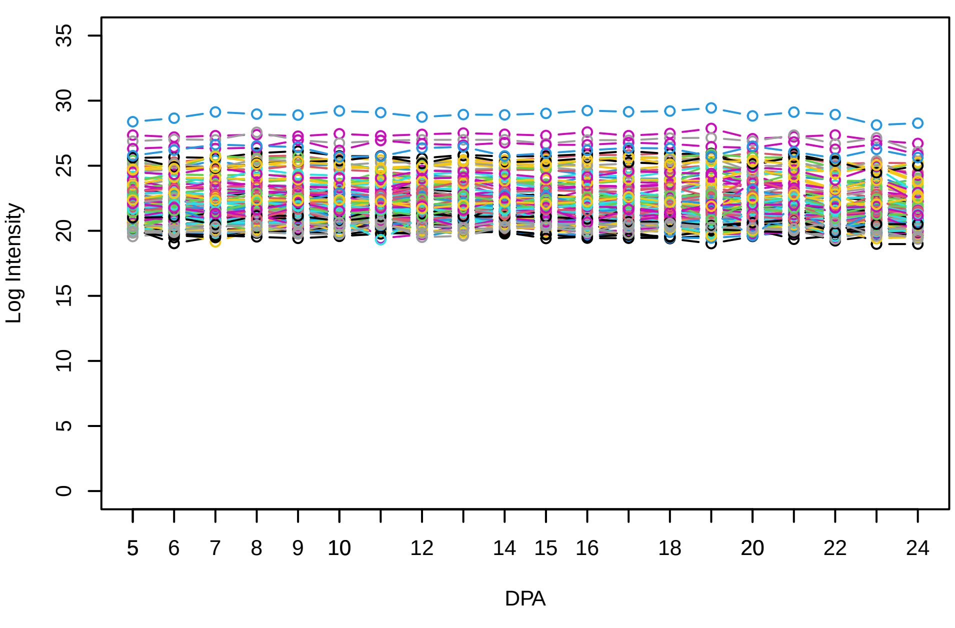

Histogram of the average abundances of high abundance proteins

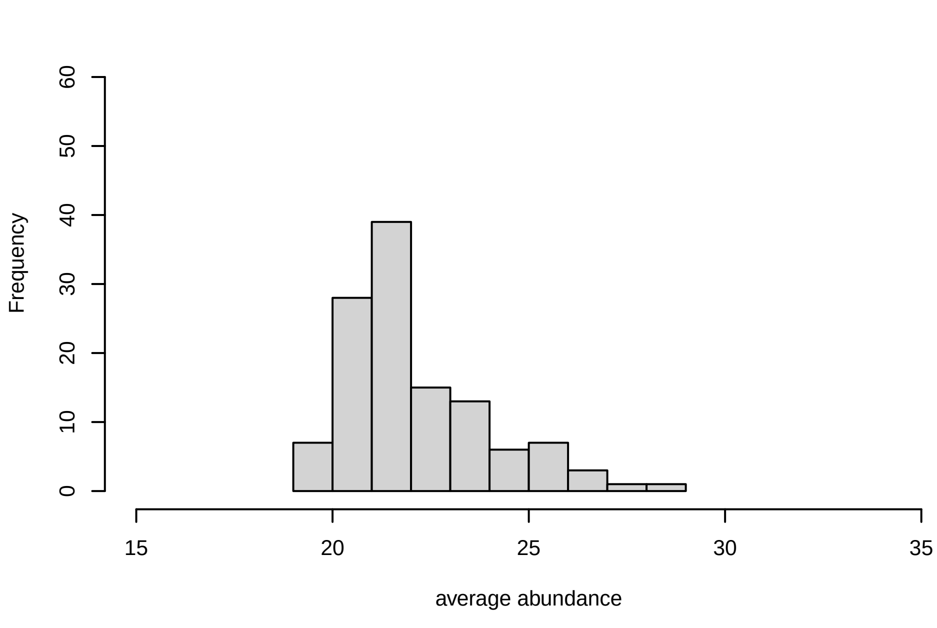

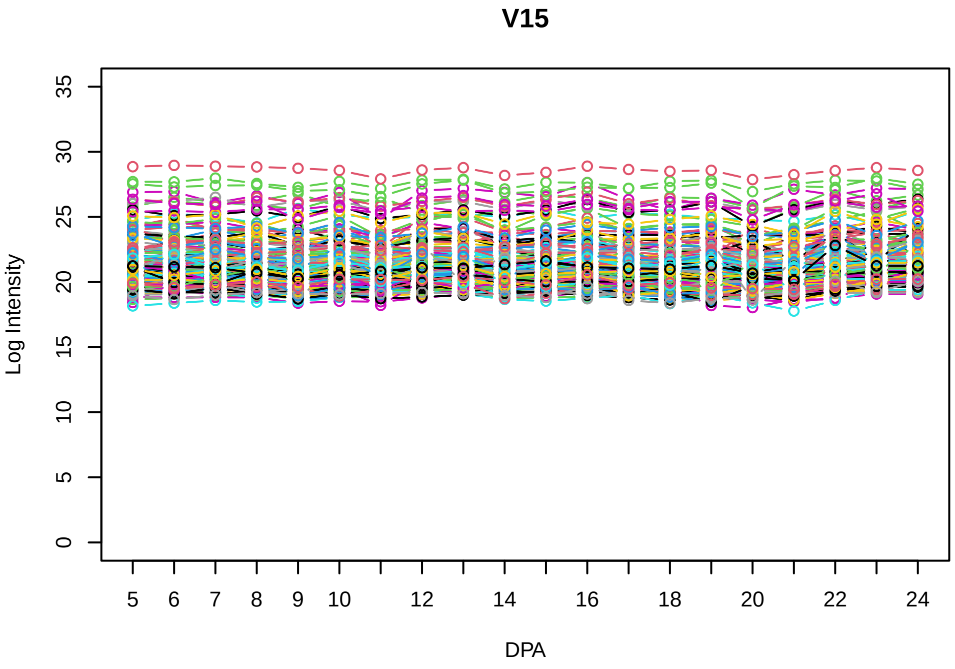
V15
Log Intensity
DPA

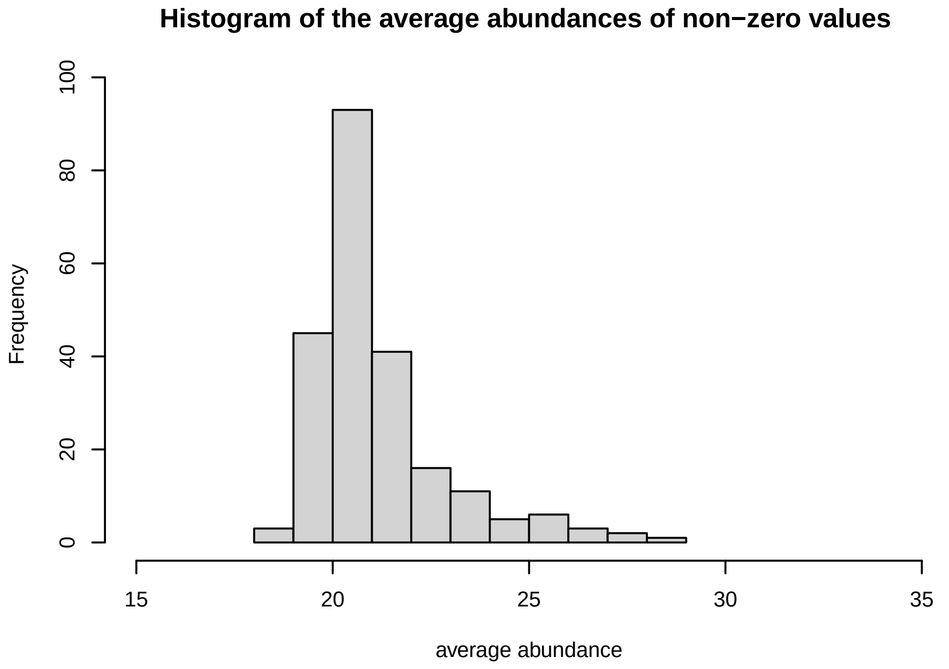
Histogram of the average abundances of non−zero values
Frequency
average abundance

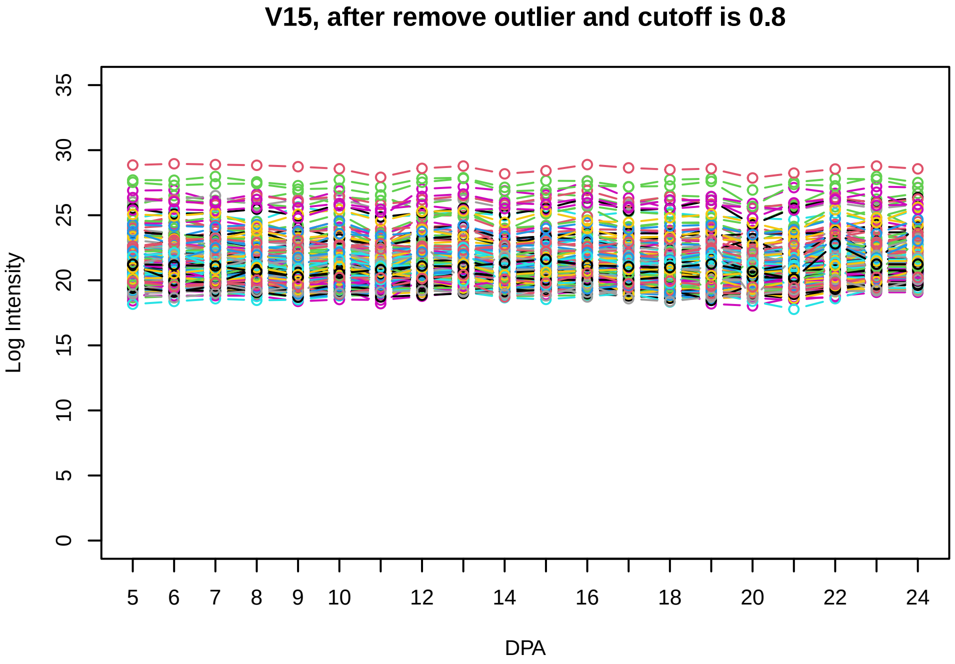
V15, after remove outlier and cutoff is 0.8
Log Intensity
DPA

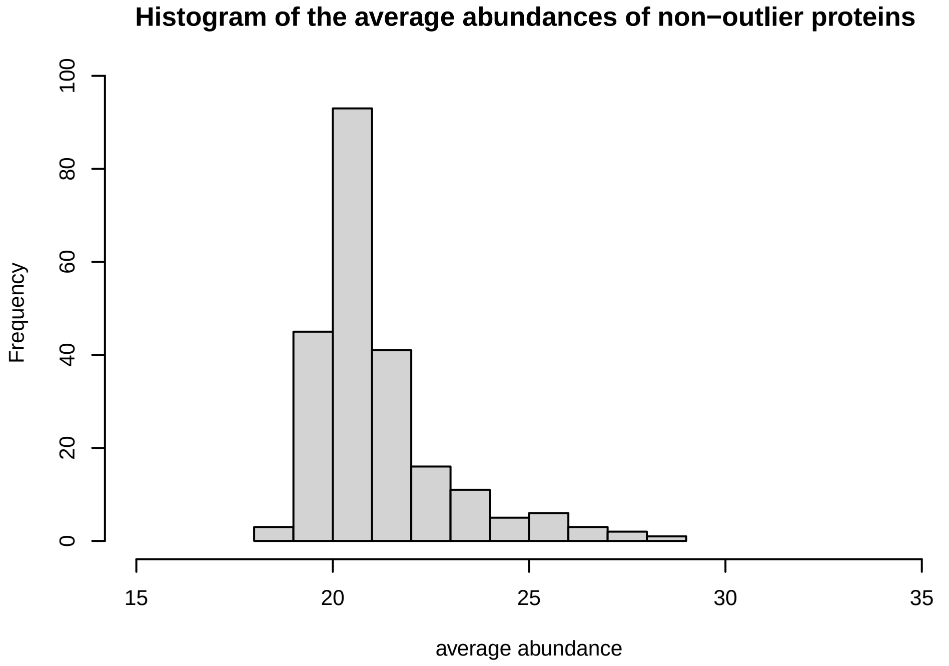
Histogram of the average abundances of non−outlier proteins
Frequency
average abundance

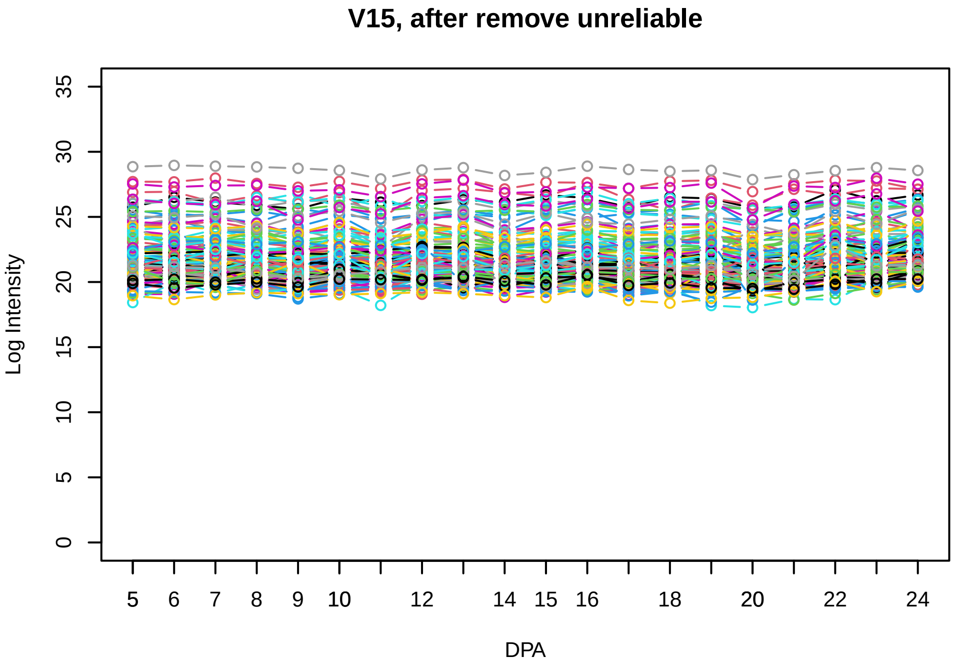
V15, after remove unreliable
Log Intensity
DPA

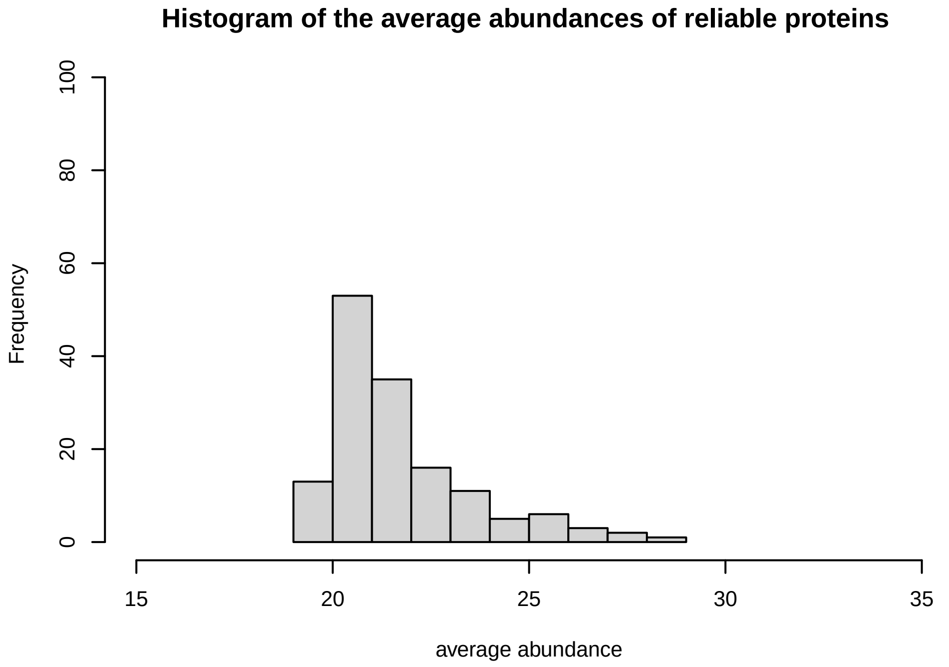
Histogram of the average abundances of reliable proteins
Frequency
average abundance

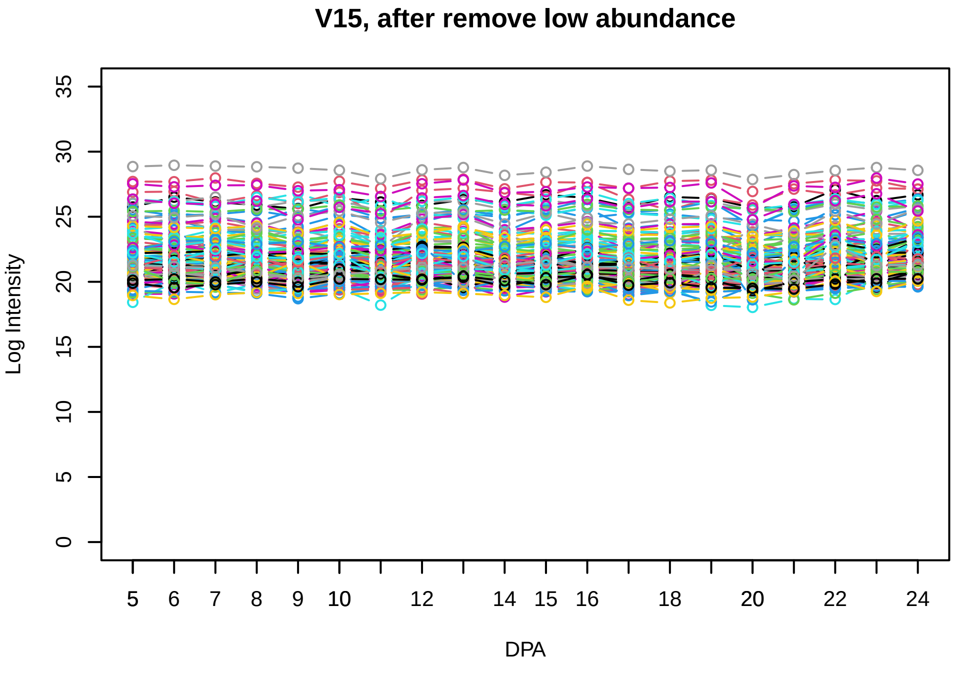
V15, after remove low abundance
Log Intensity
DPA

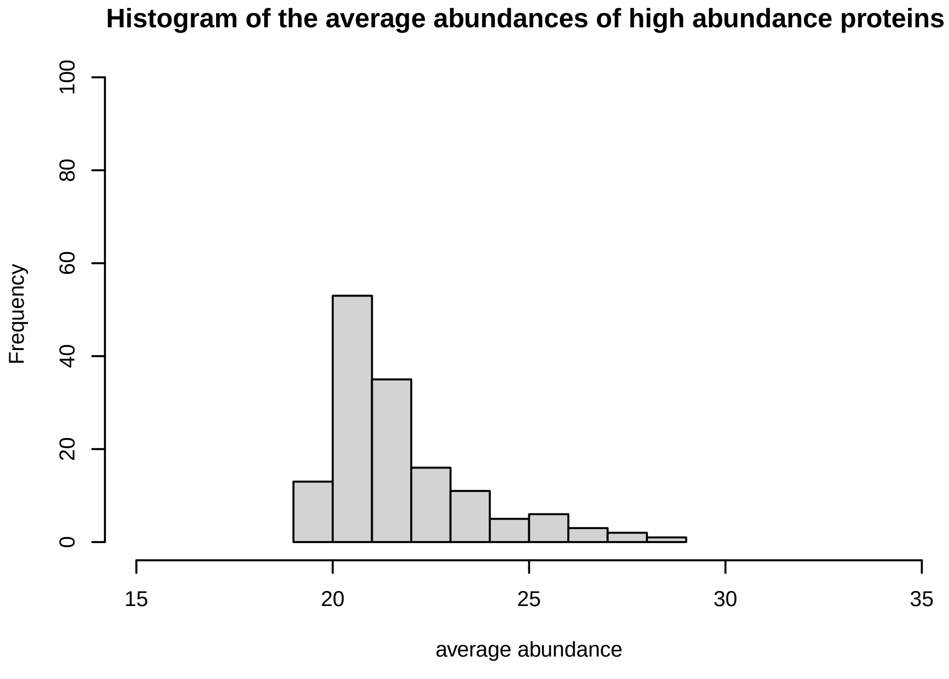
Histogram of the average abundances of high abundance proteins
Frequency
average abundance

V16

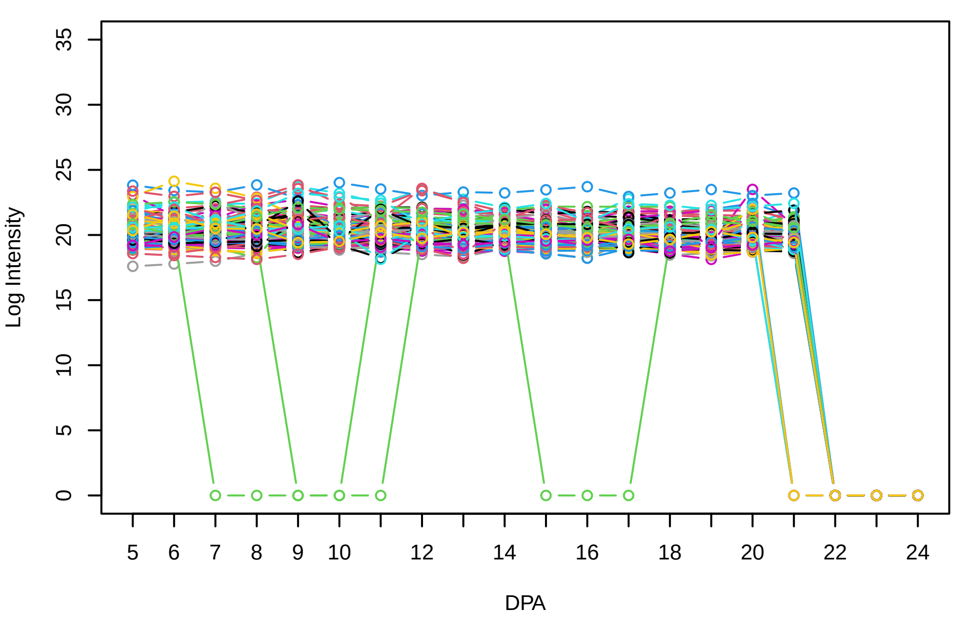

Histogram of the average abundances of non−zero values

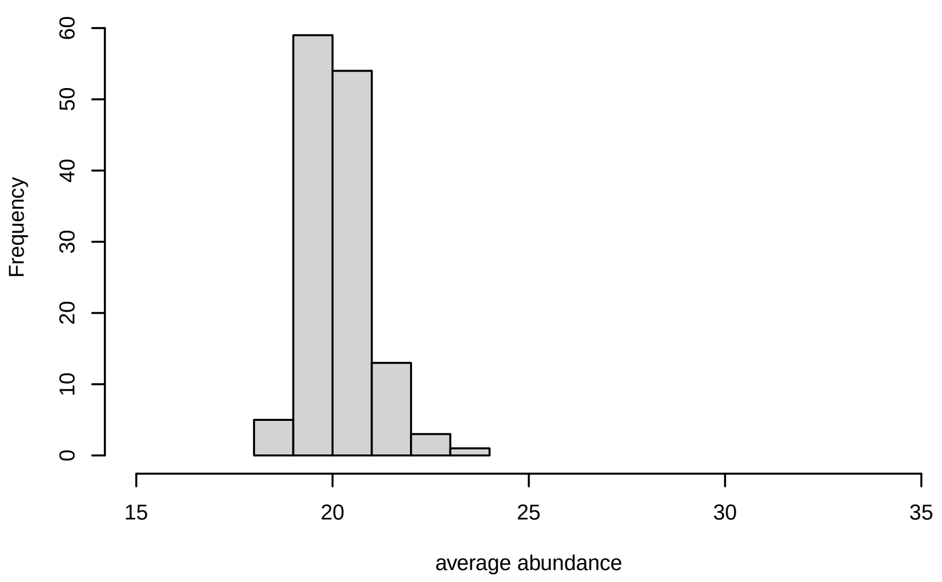

V16, after remove outlier and cutoff is 0.8

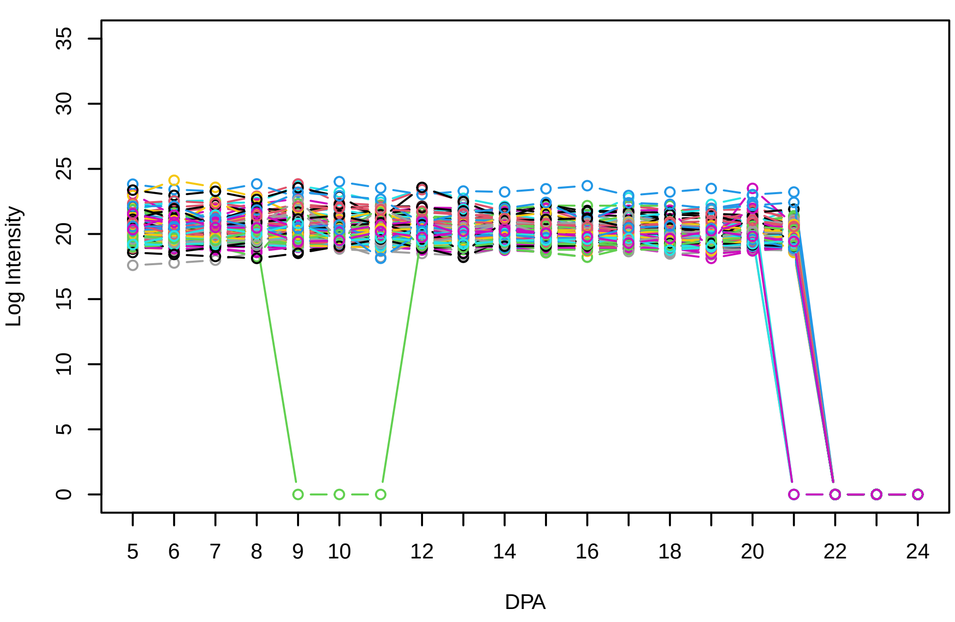

Histogram of the average abundances of non−outlier proteins

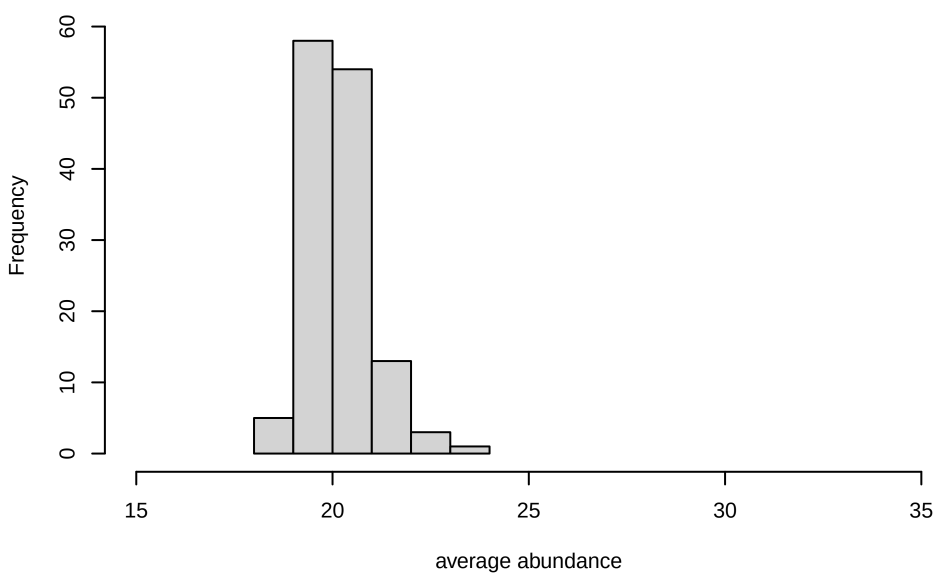

V16, after remove unreliable

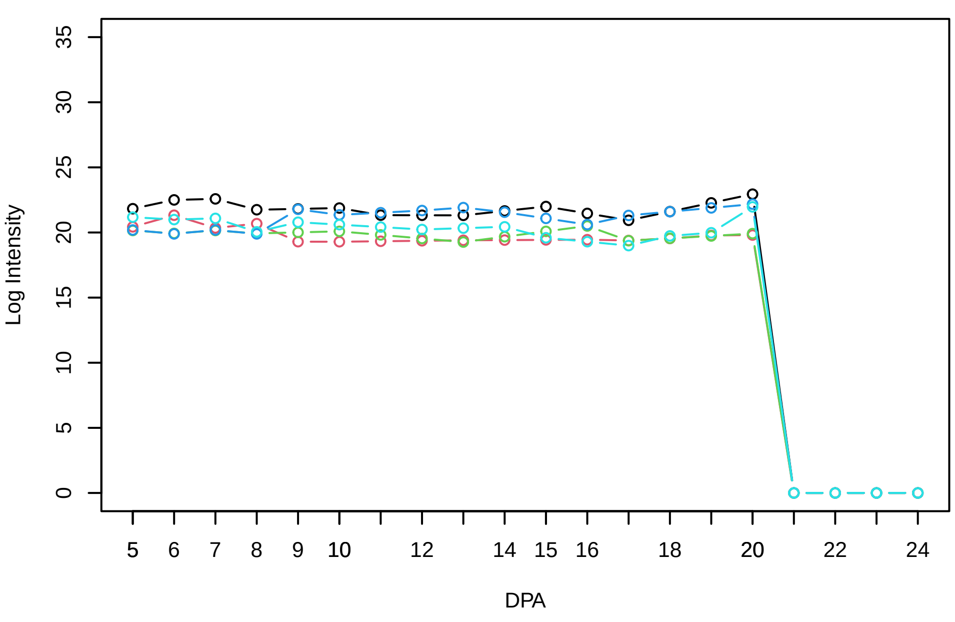

Histogram of the average abundances of reliable proteins

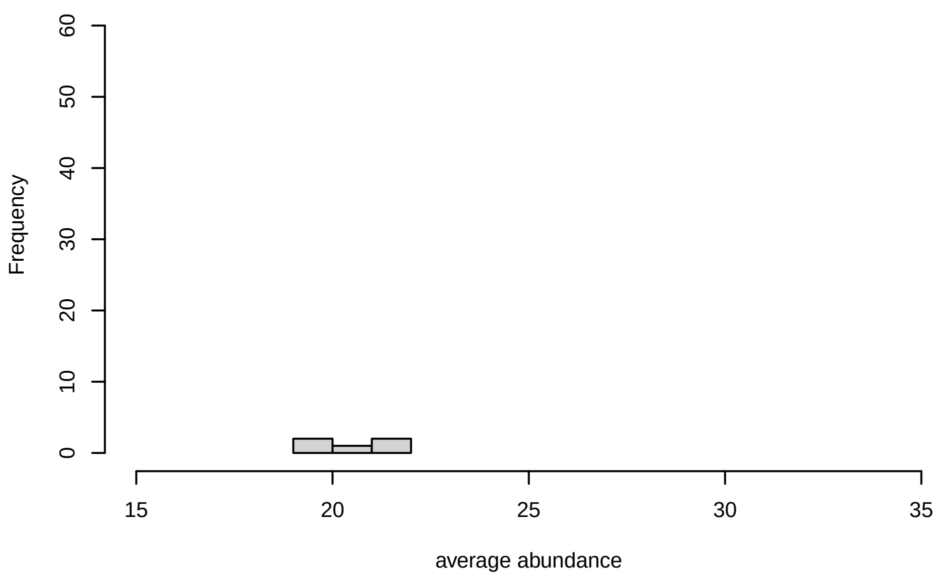

V16, after remove low abundance

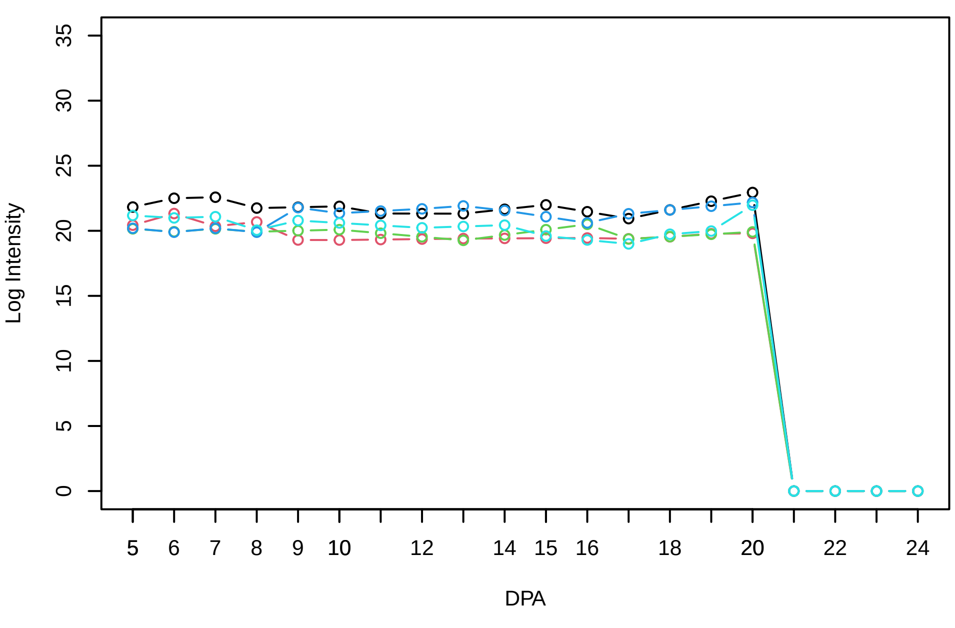

Histogram of the average abundances of high abundance proteins

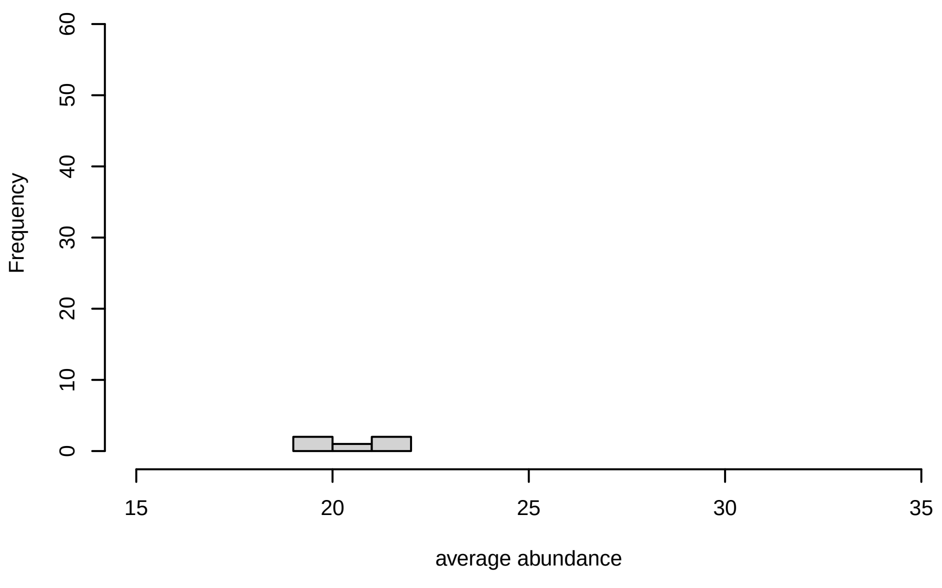

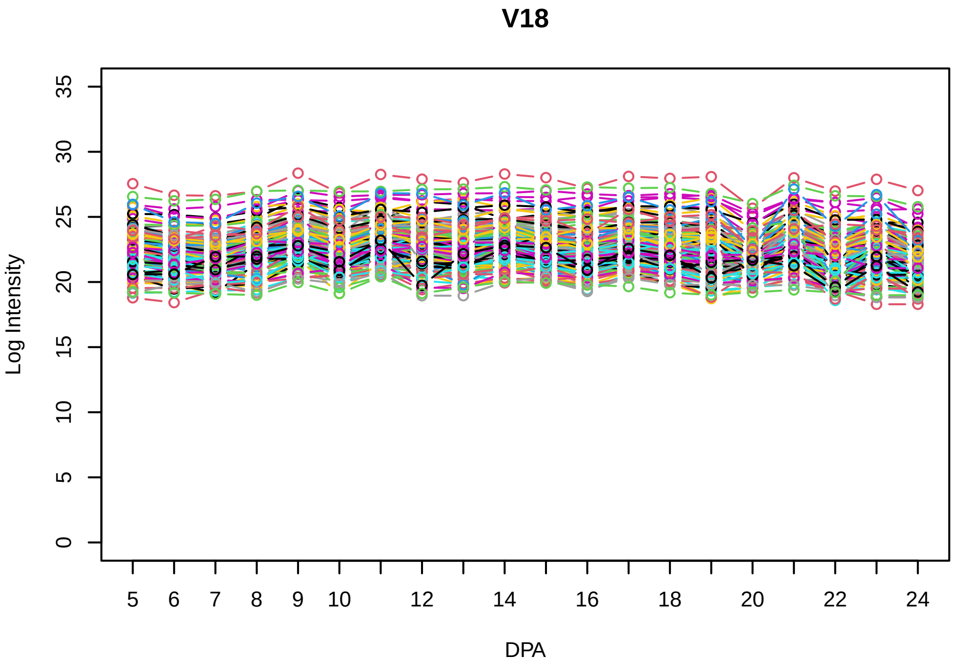
V18
Log Intensity
DPA
0
5
10
15
20
25
30
35
5
6
7
8
9
10
12
14
16
18
20
22
24
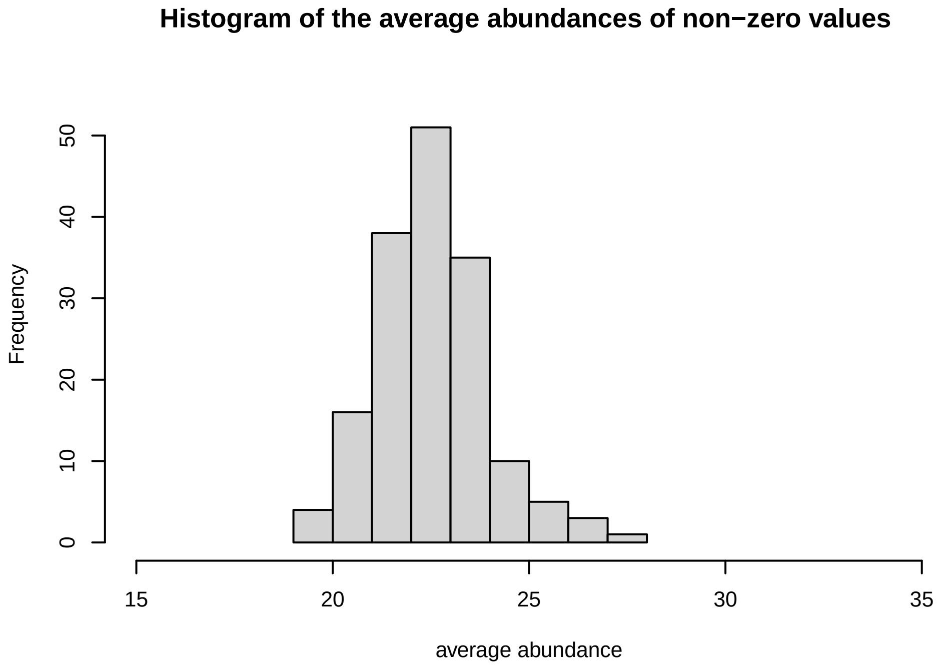
Histogram of the average abundances of non−zero values
Frequency
average abundance
0
10
20
30
40
50
15
20
25
30
35
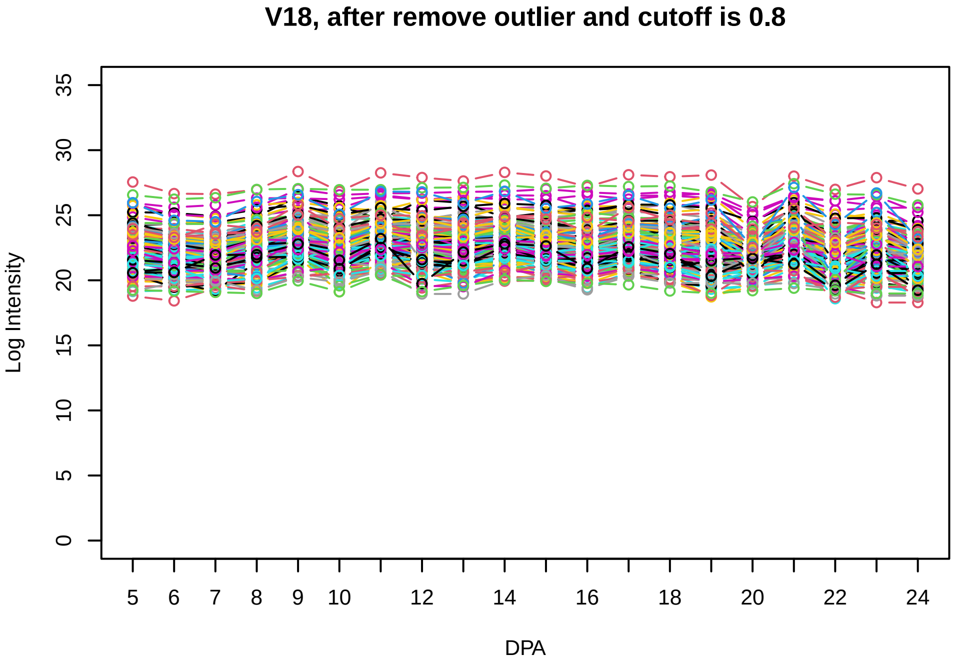
V18, after remove outlier and cutoff is 0.8
Log Intensity
DPA
0
5
10
15
20
25
30
35
5
6
7
8
9
10
12
14
16
18
20
22
24
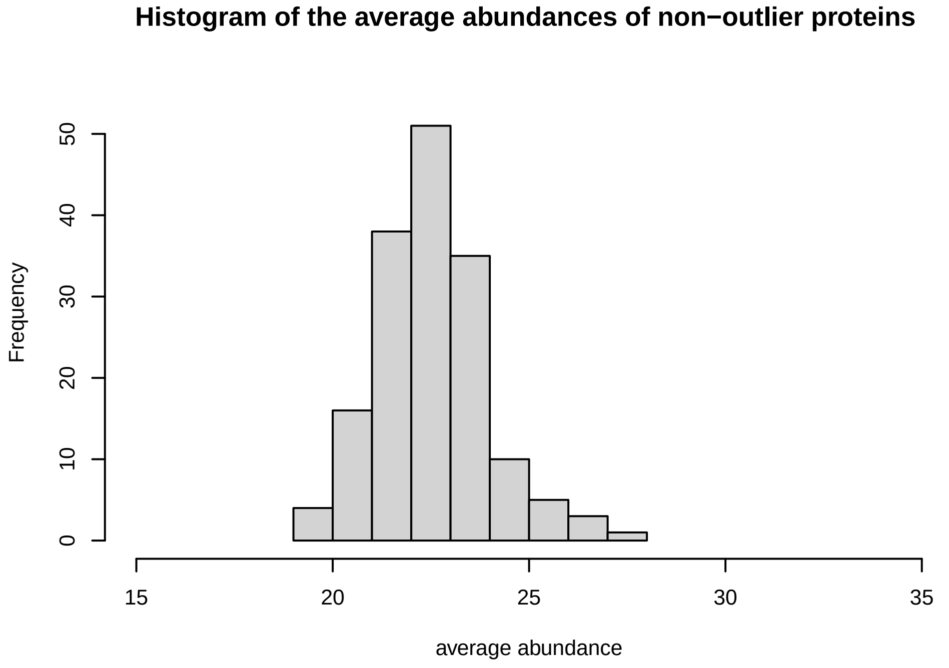
Histogram of the average abundances of non−outlier proteins
Frequency
average abundance
0
10
20
30
40
50
15
20
25
30
35
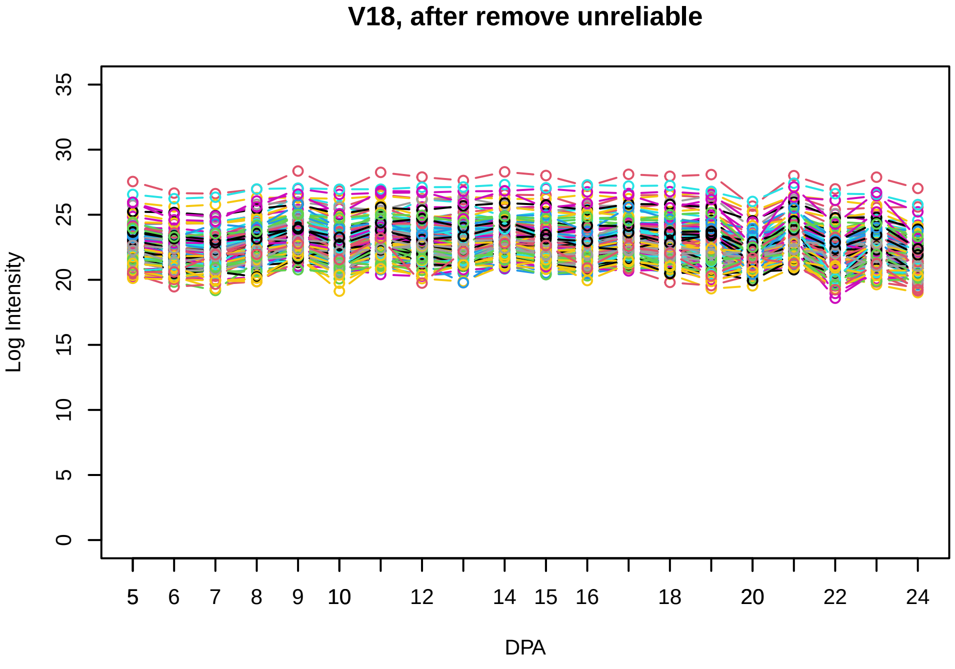
V18, after remove unreliable
Log Intensity
DPA
0
5
10
15
20
25
30
35
5
6
7
8
9
10
12
14
15
16
18
20
22
24
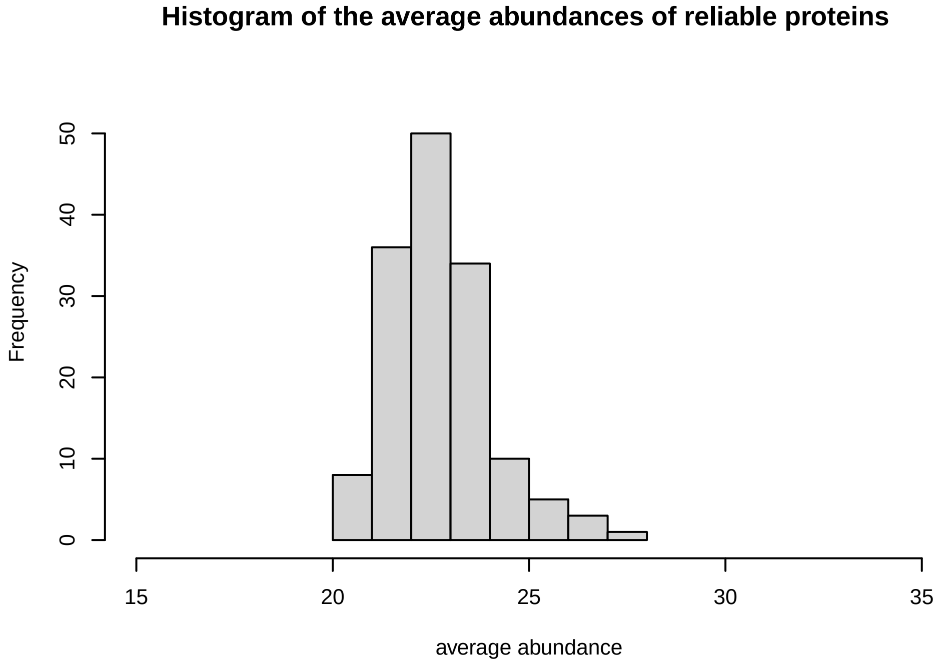
Histogram of the average abundances of reliable proteins
Frequency
average abundance
0
10
20
30
40
50
15
20
25
30
35
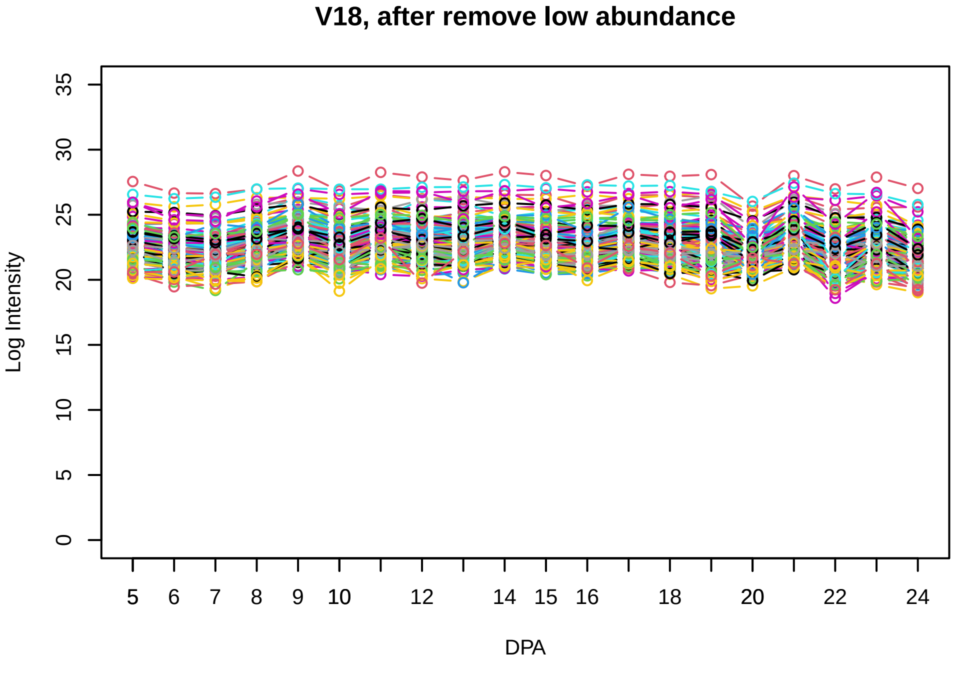
V18, after remove low abundance
Log Intensity
DPA
0
5
10
15
20
25
30
35
5
6
7
8
9
10
12
14
15
16
18
20
22
24
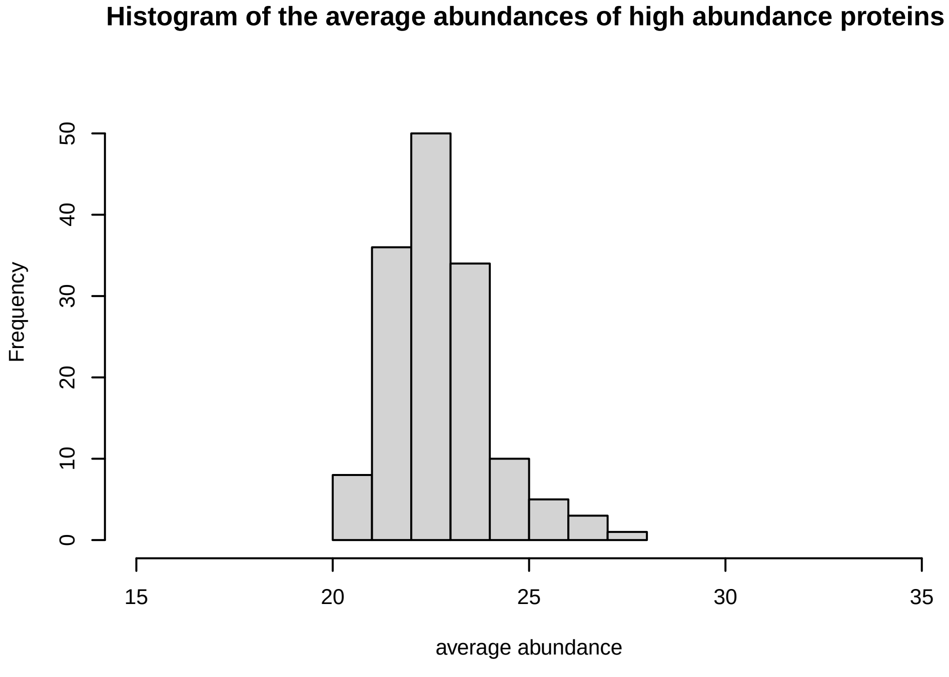
Histogram of the average abundances of high abundance proteins
Frequency
average abundance
0
10
20
30
40
50
15
20
25
30
35

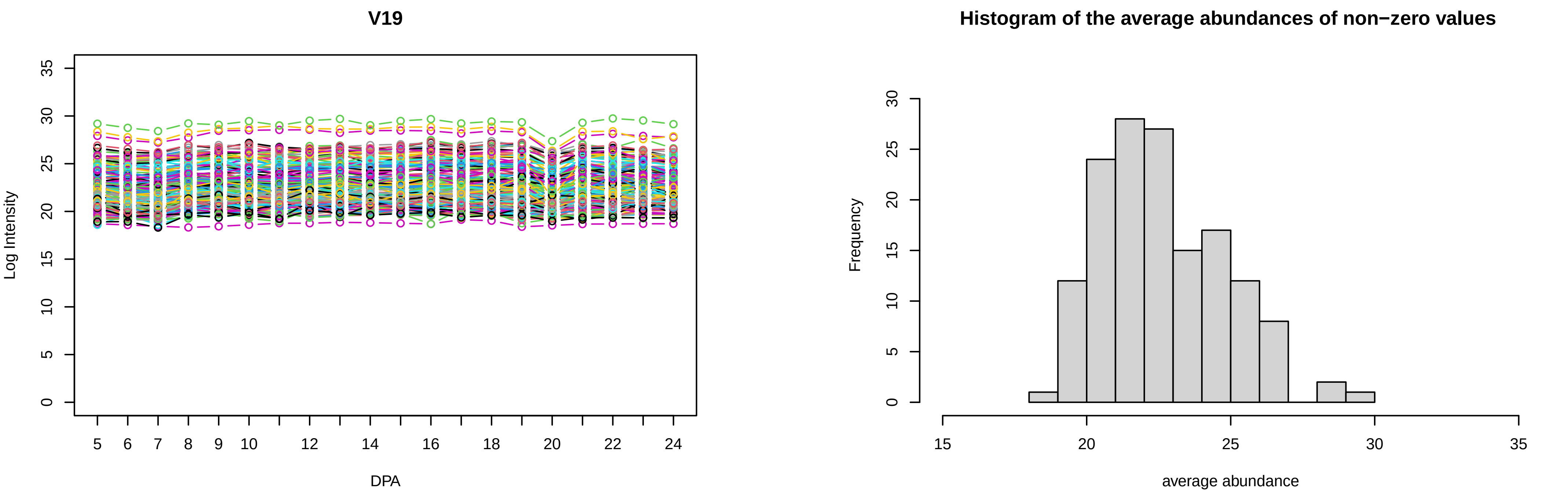
V19
Log Intensity
DPA
Histogram of the average abundances of non−zero values
Frequency
average abundance

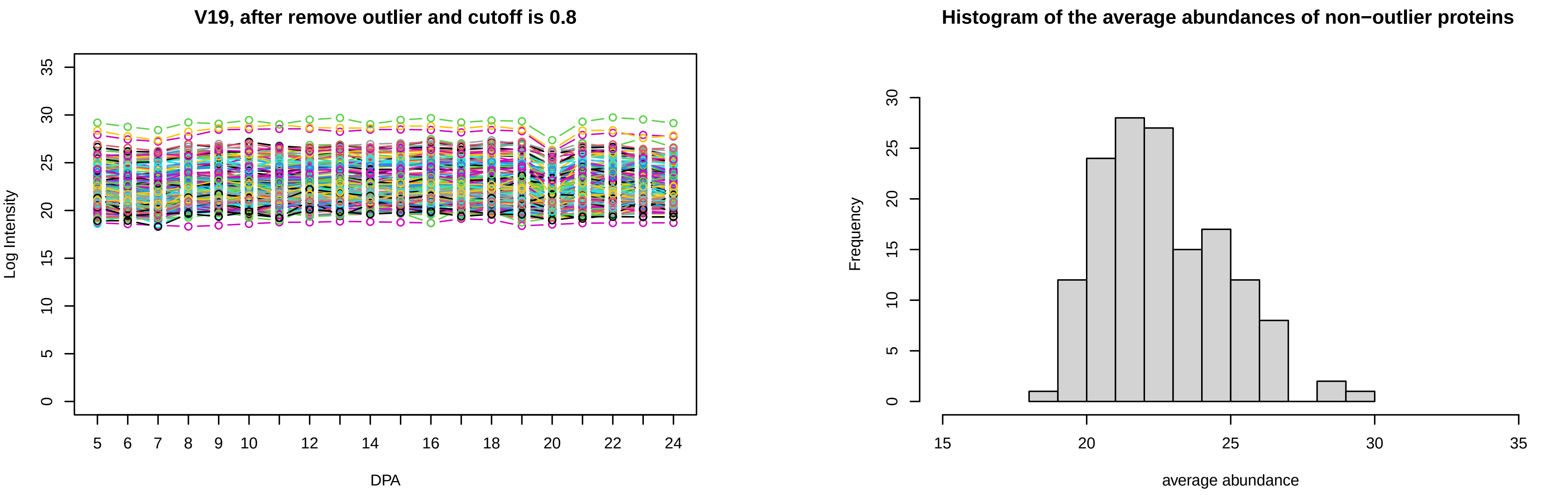
V19, after remove outlier and cutoff is 0.8
Log Intensity
DPA
Histogram of the average abundances of non−outlier proteins
Frequency
average abundance

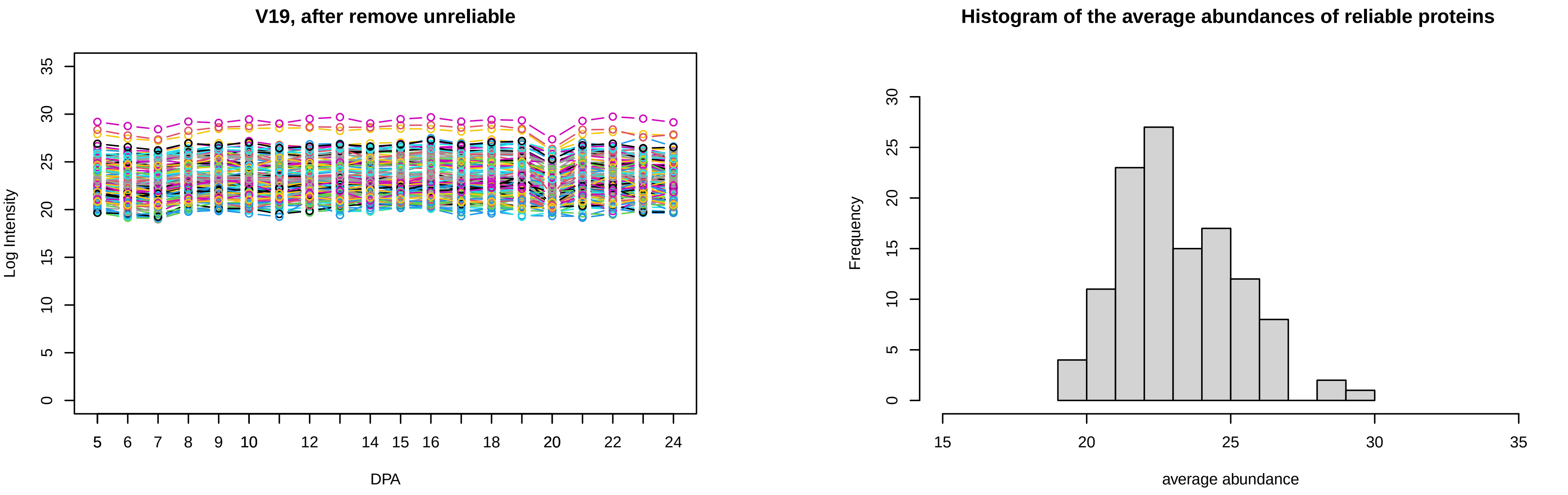
V19, after remove unreliable
Log Intensity
DPA
Histogram of the average abundances of reliable proteins
Frequency
average abundance

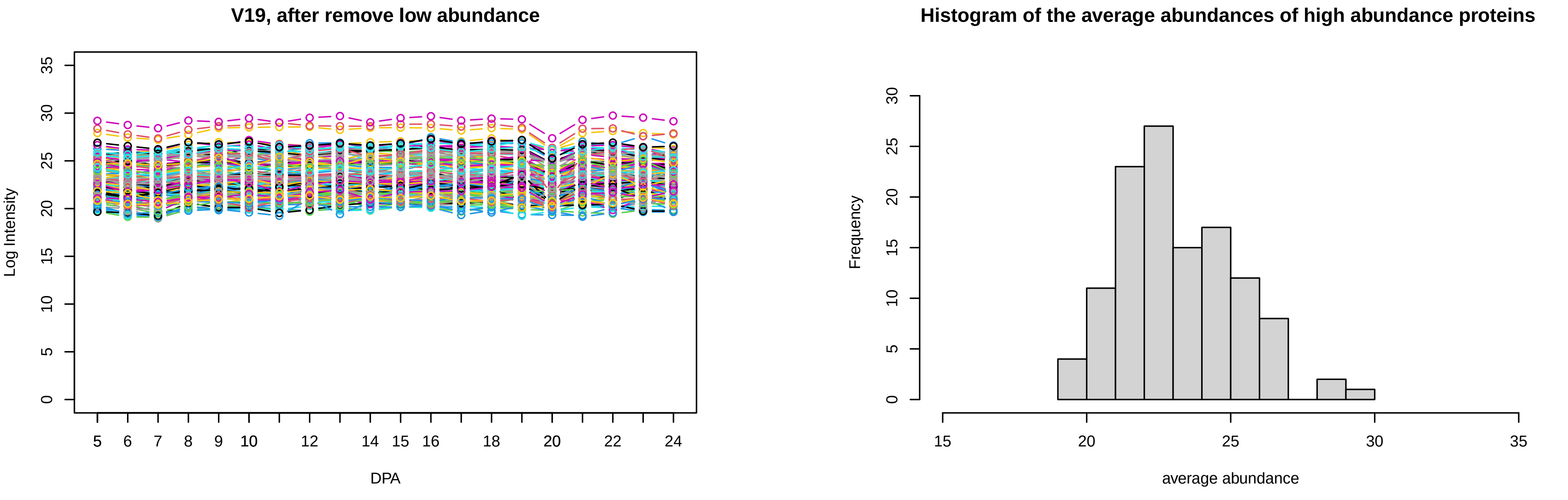
V19, after remove low abundance
Log Intensity
DPA
Histogram of the average abundances of high abundance proteins
Frequency
average abundance

V20

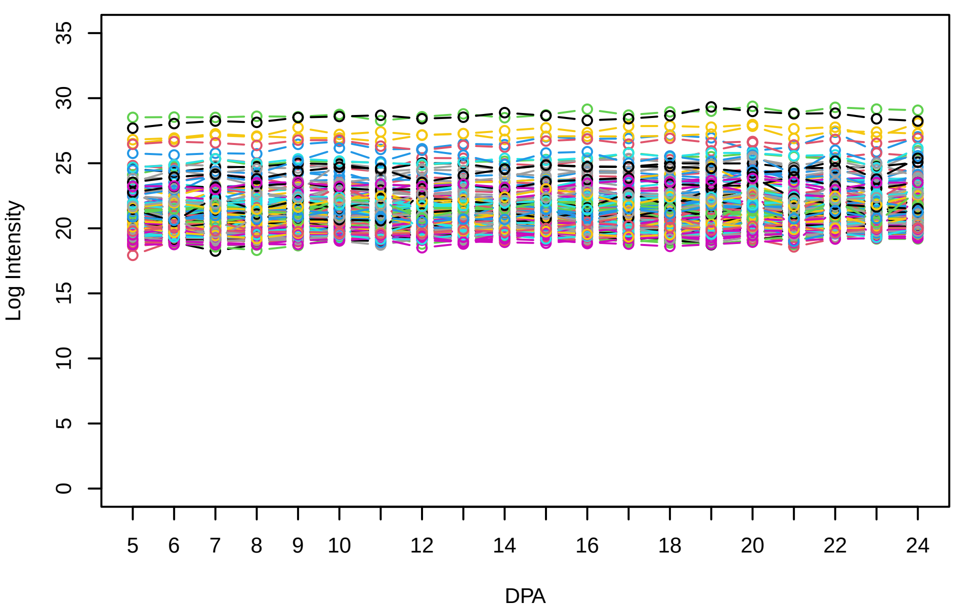

Histogram of the average abundances of non−zero values

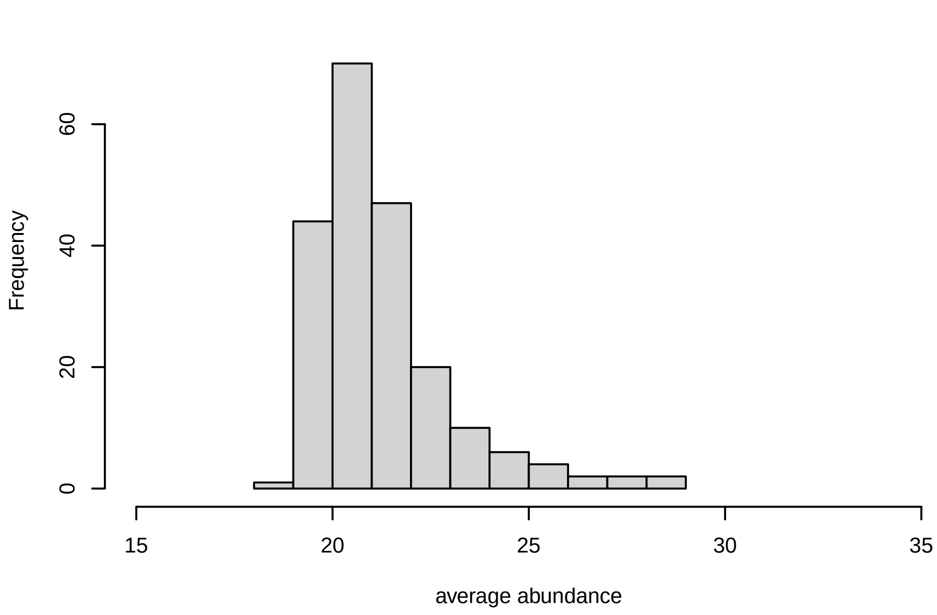

V20, after remove outlier and cutoff is 0.8

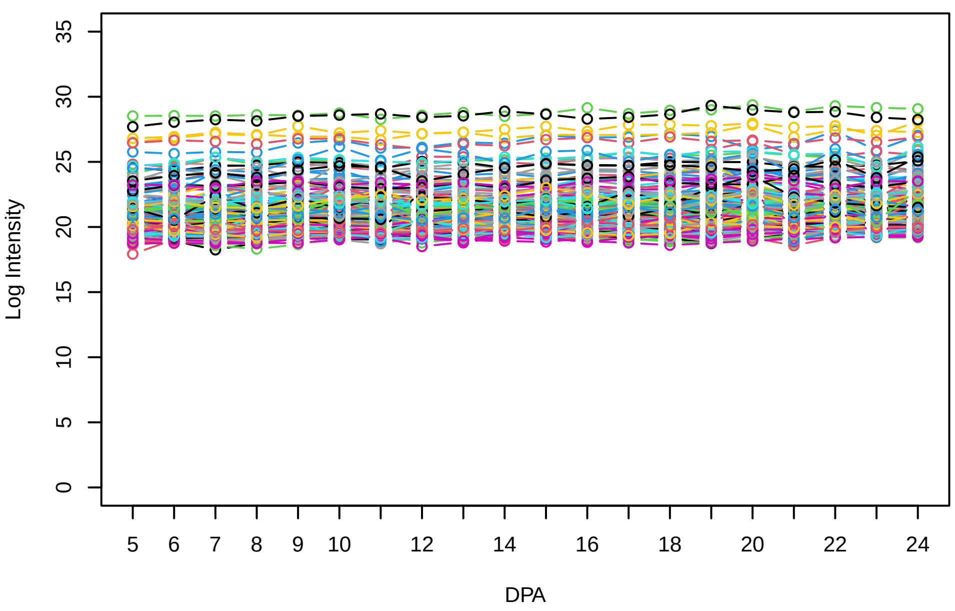

Histogram of the average abundances of non−outlier proteins

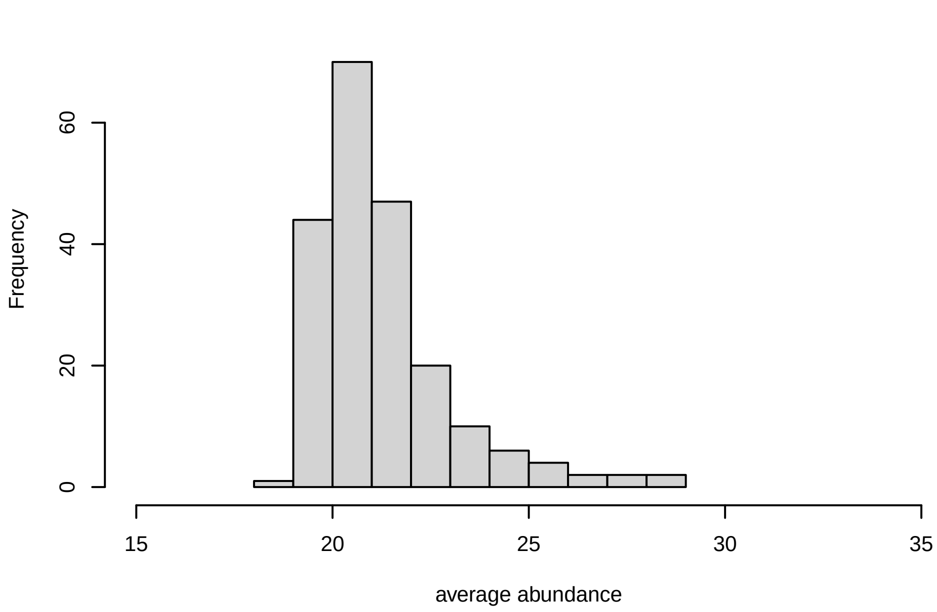

V20, after remove unreliable

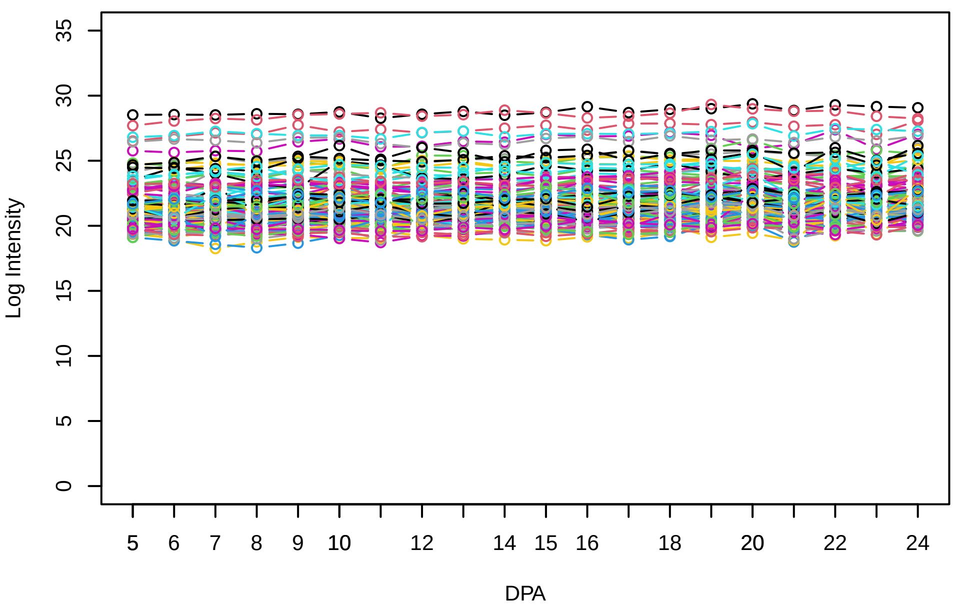

Histogram of the average abundances of reliable proteins

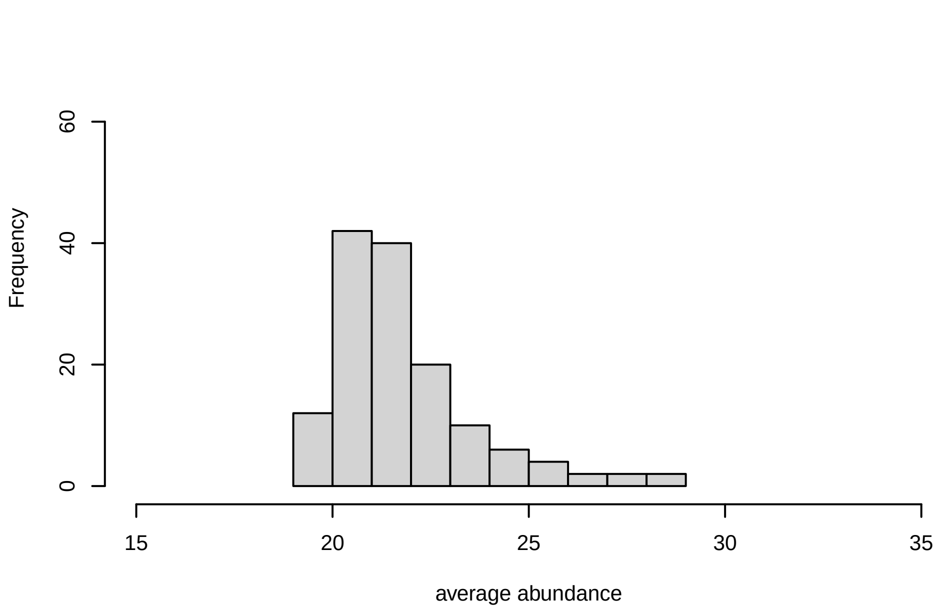

V20, after remove low abundance

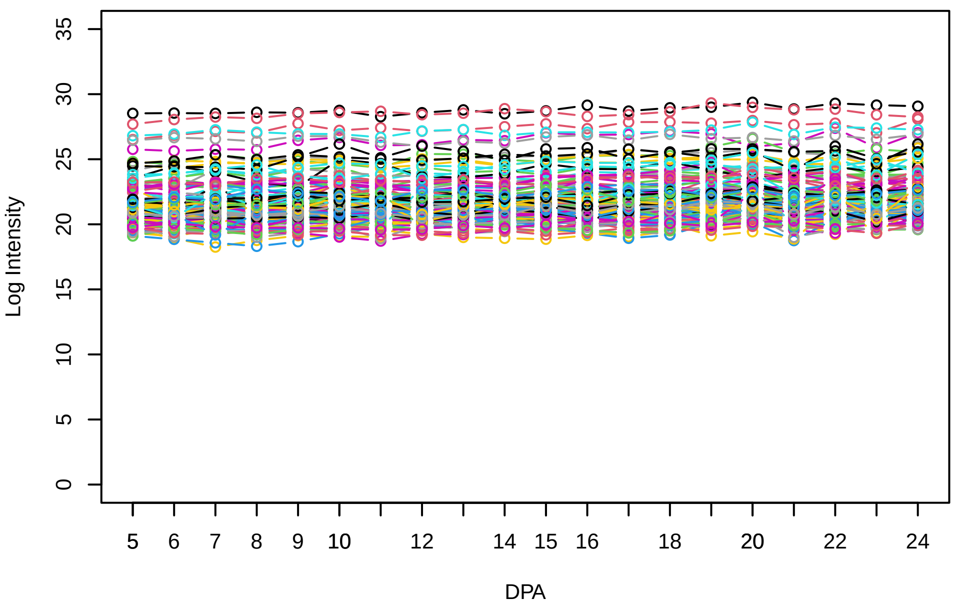

Histogram of the average abundances of high abundance proteins

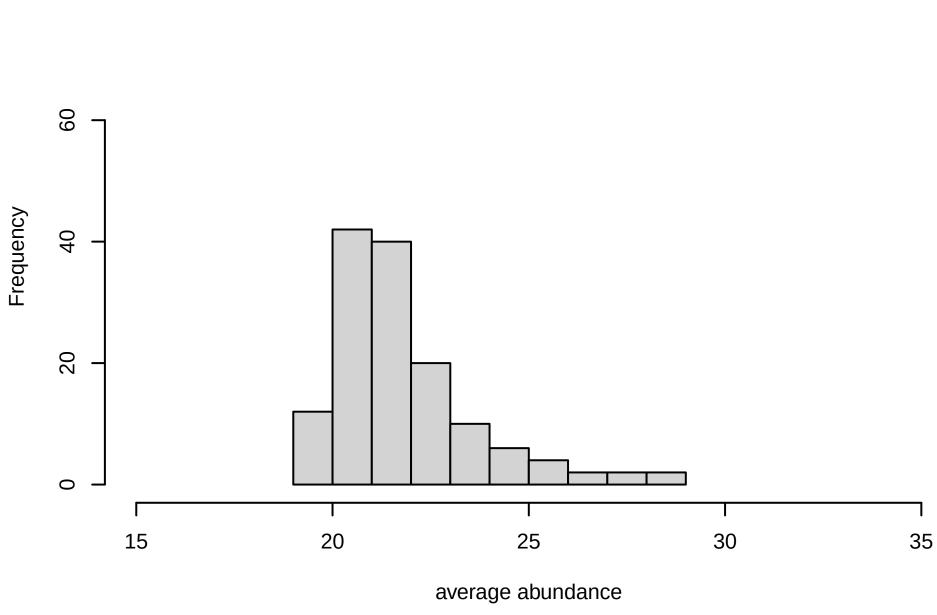

V21

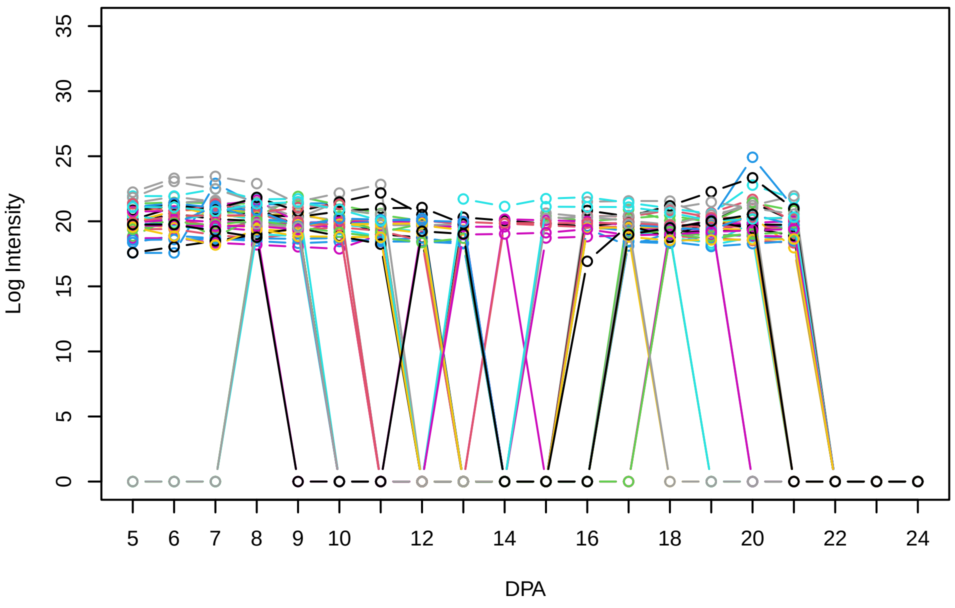

Histogram of the average abundances of non−zero values

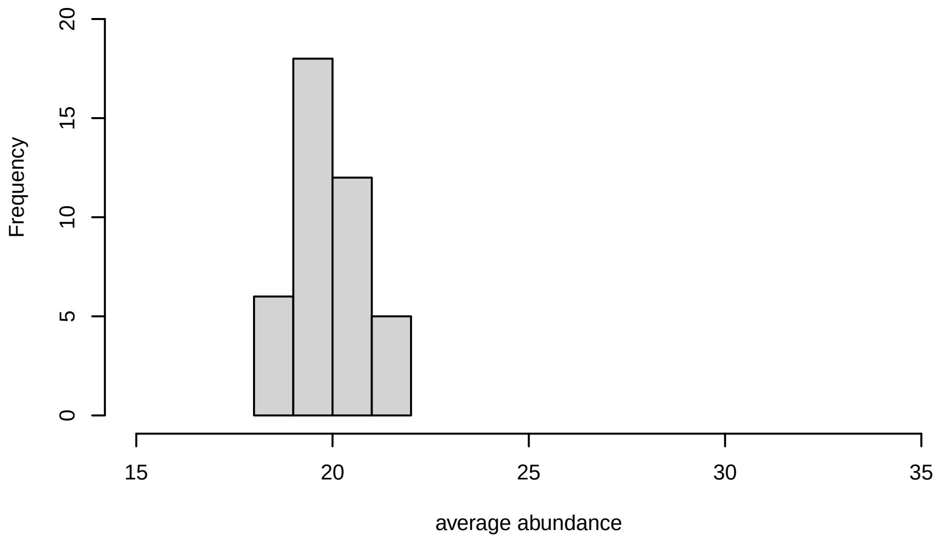

V21, after remove outlier and cutoff is 0.8

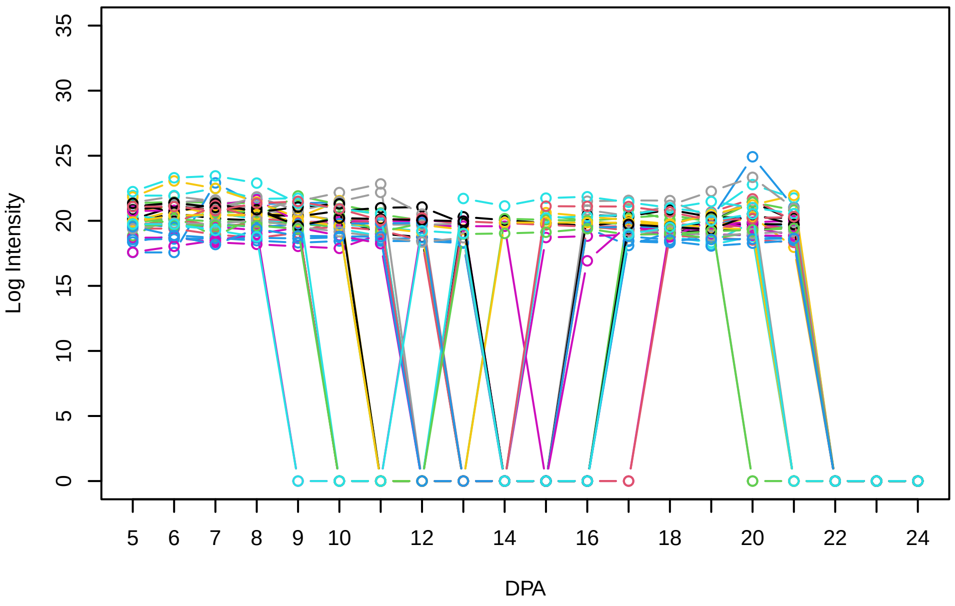

Histogram of the average abundances of non−outlier proteins

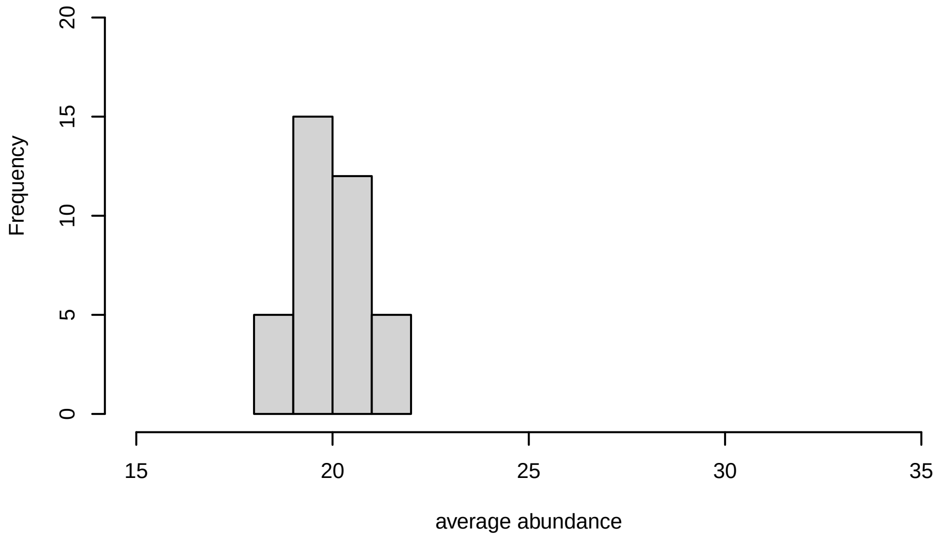

V21, after remove unreliable

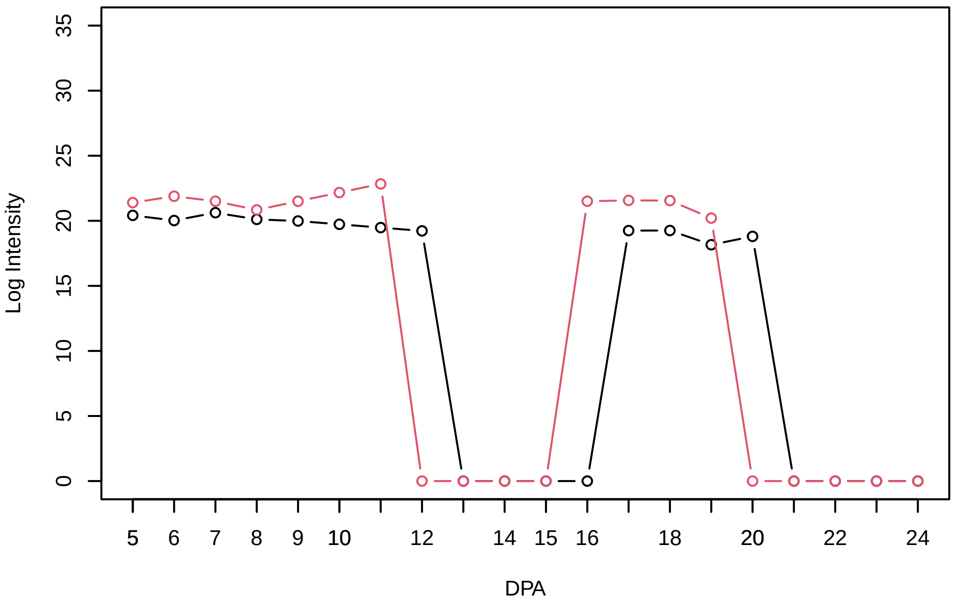

Histogram of the average abundances of reliable proteins

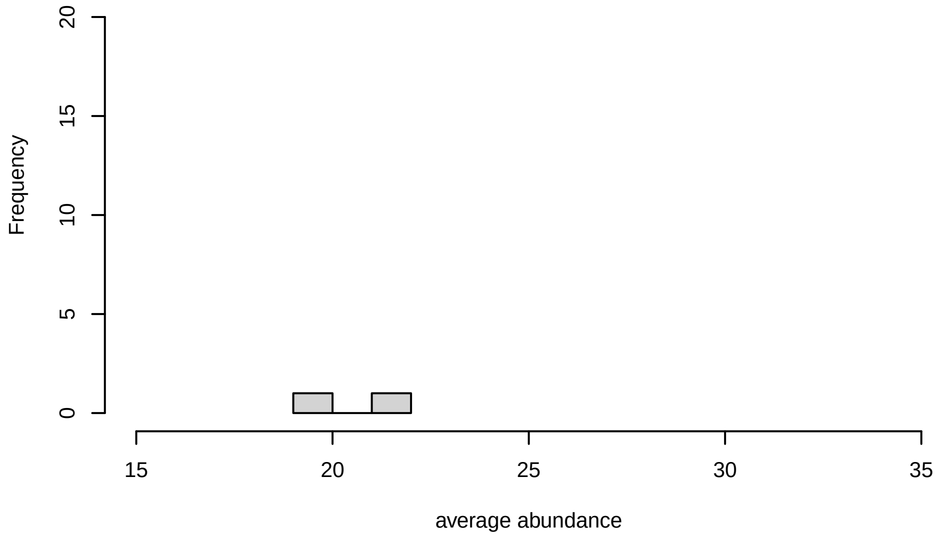

V21, after remove low abundance

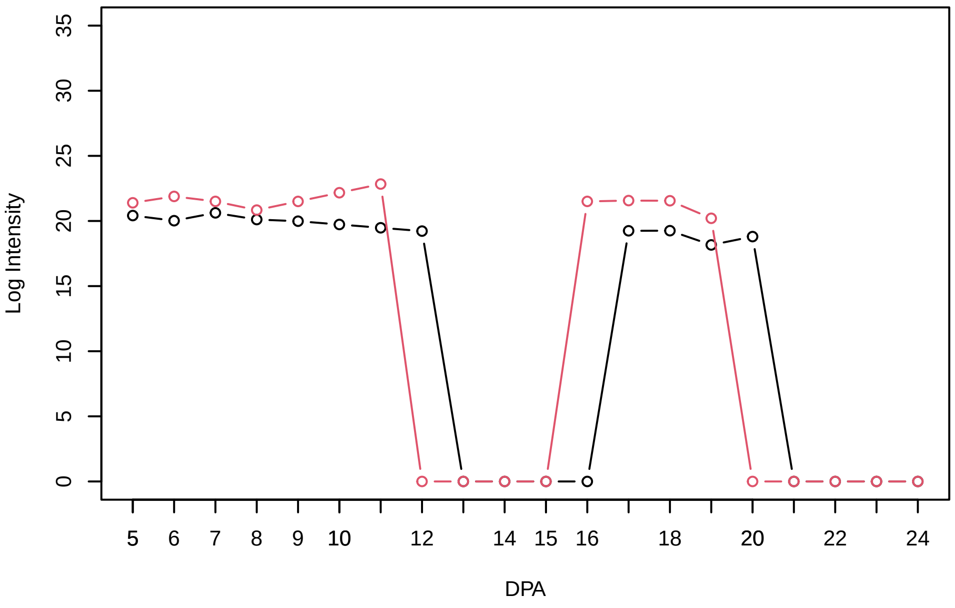

Histogram of the average abundances of high abundance proteins

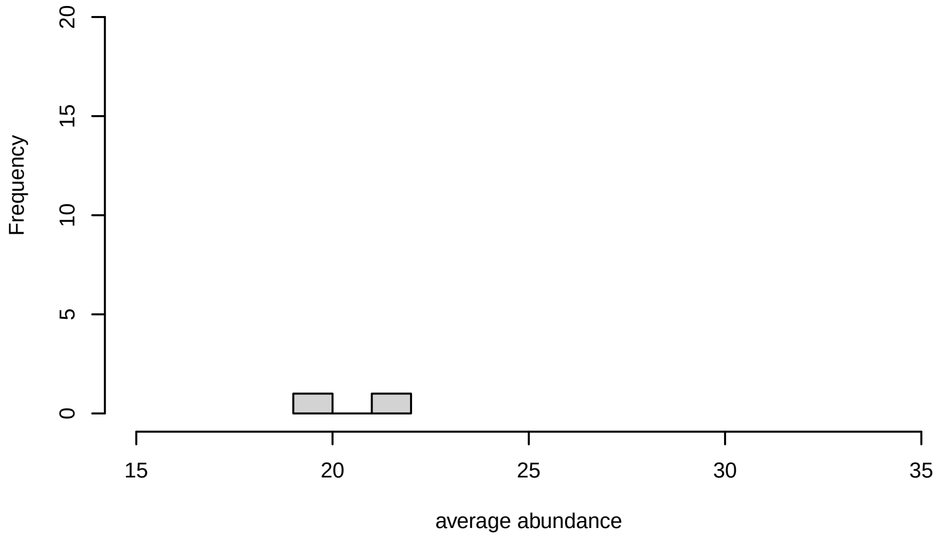

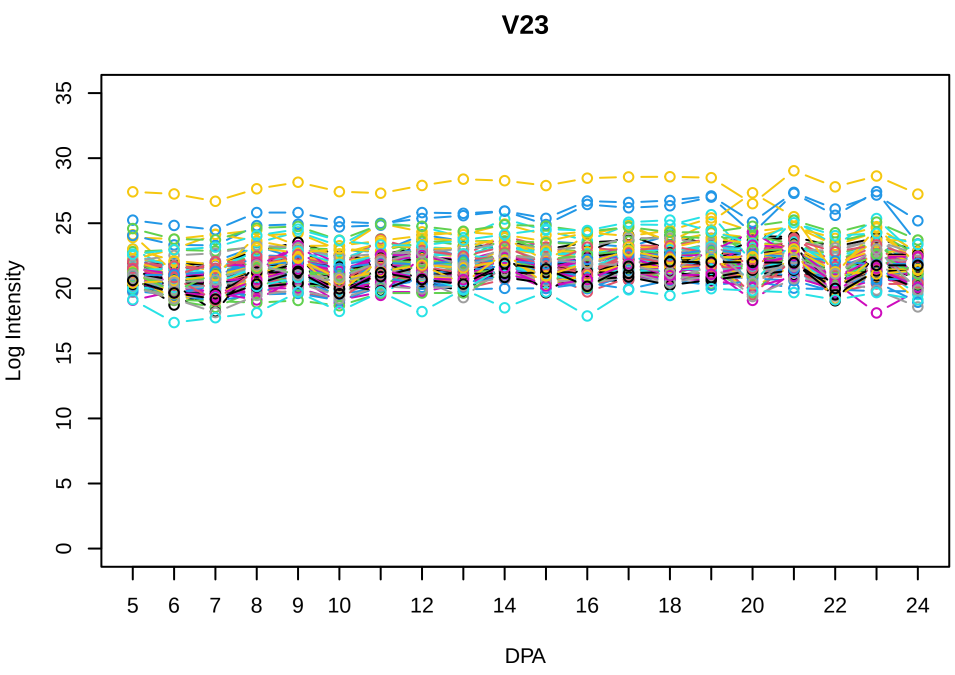
V23
Log Intensity
0 5 10 15 20 25 30 35
5 6 7 8 9 10 12 14 16 18 20 22 24
DPA

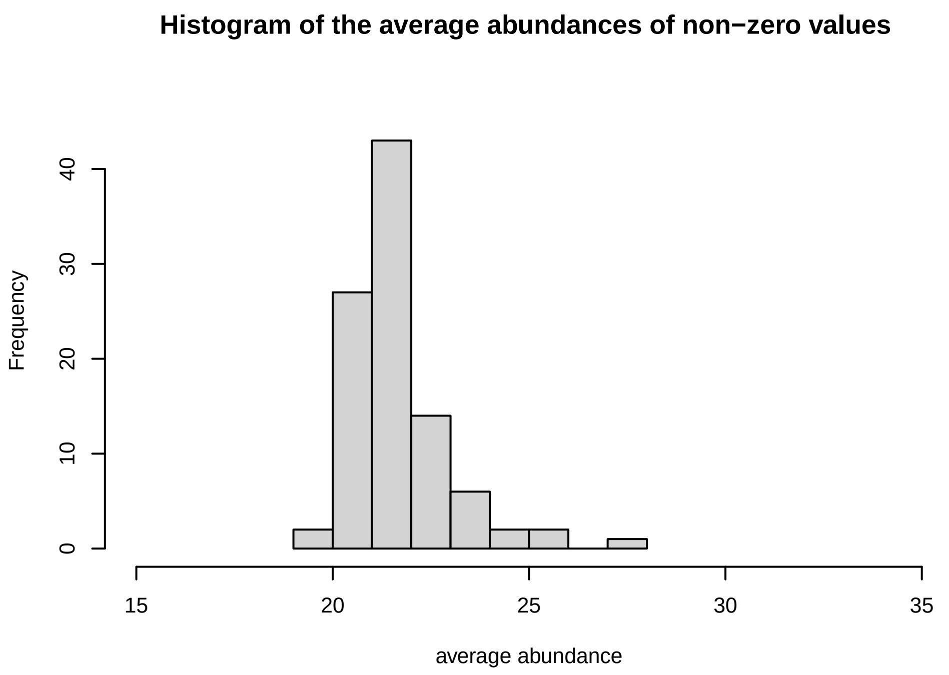
Histogram of the average abundances of non−zero values
Frequency
0 10 20 30 40
15 20 25 30 35
average abundance

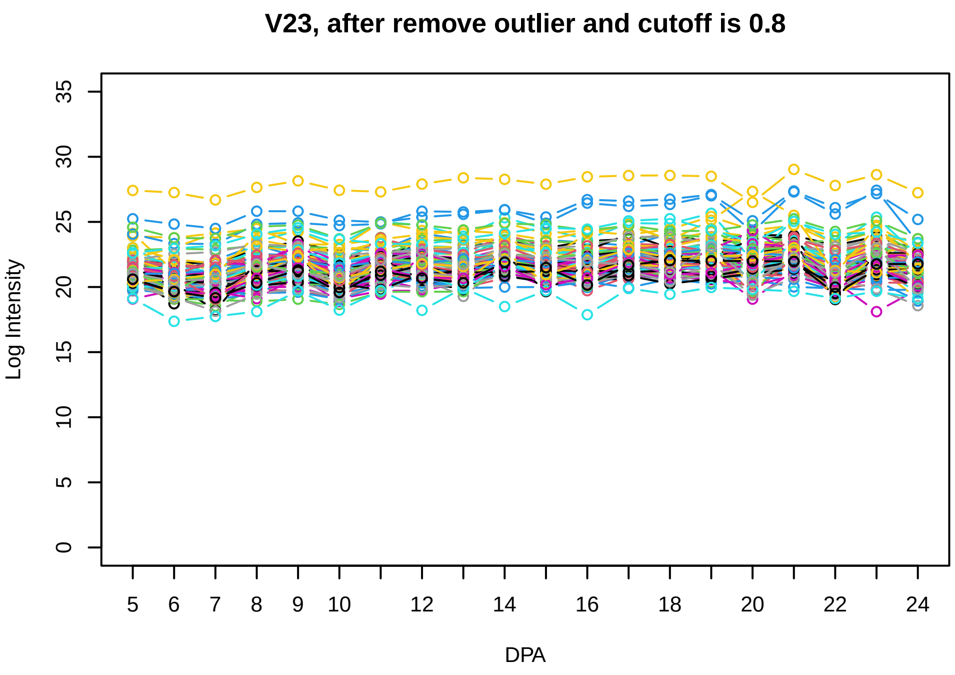
V23, after remove outlier and cutoff is 0.8
Log Intensity
0 5 10 15 20 25 30 35
5 6 7 8 9 10 12 14 16 18 20 22 24
DPA

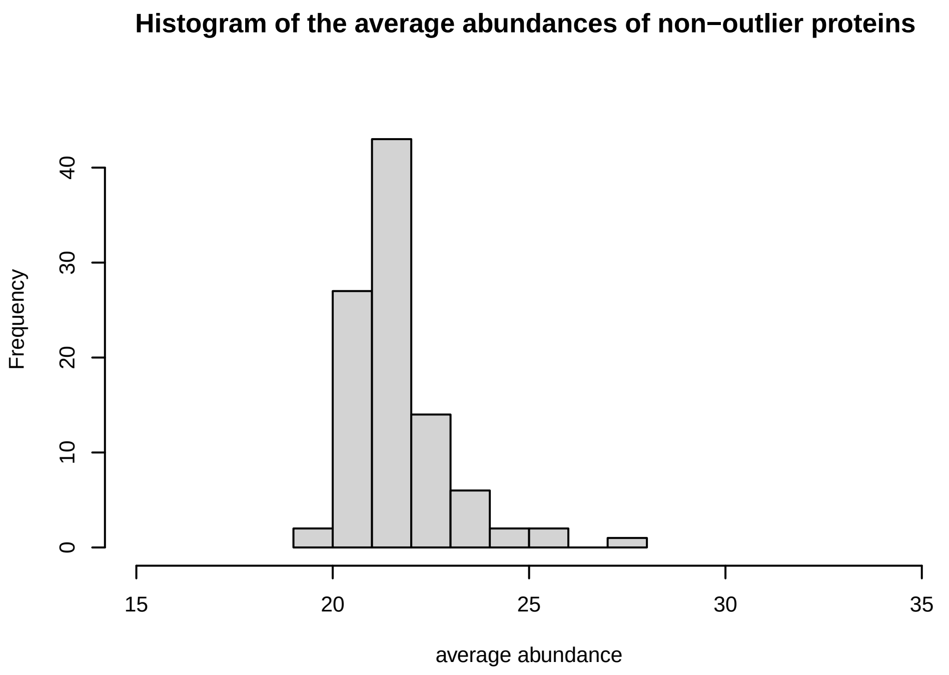
Histogram of the average abundances of non−outlier proteins
Frequency
0 10 20 30 40
15 20 25 30 35
average abundance

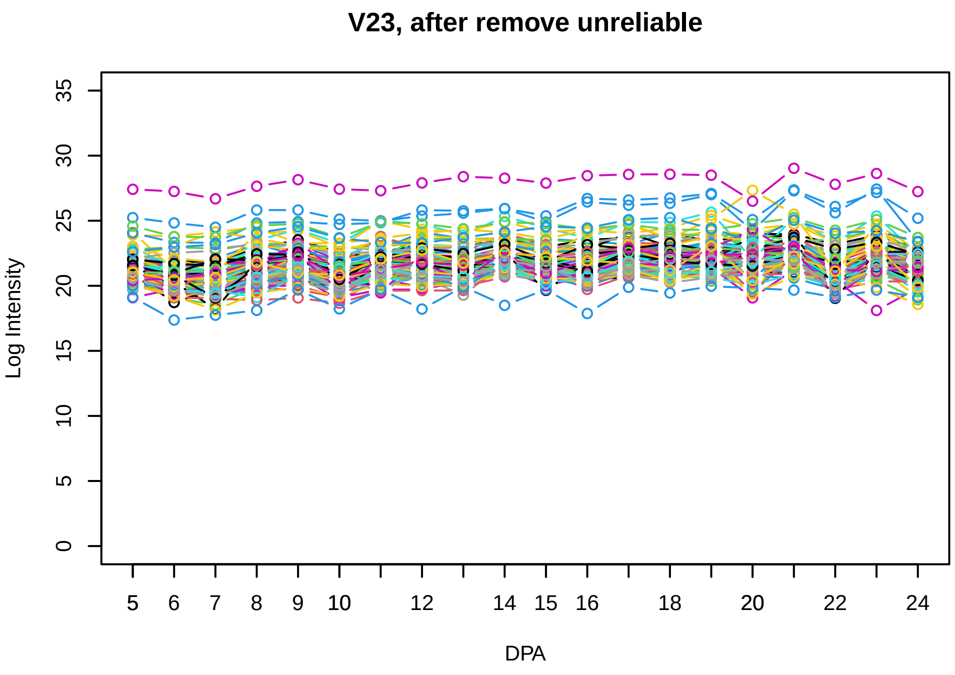
V23, after remove unreliable
Log Intensity
0 5 10 15 20 25 30 35
5 6 7 8 9 10 12 14 15 16 18 20 22 24
DPA

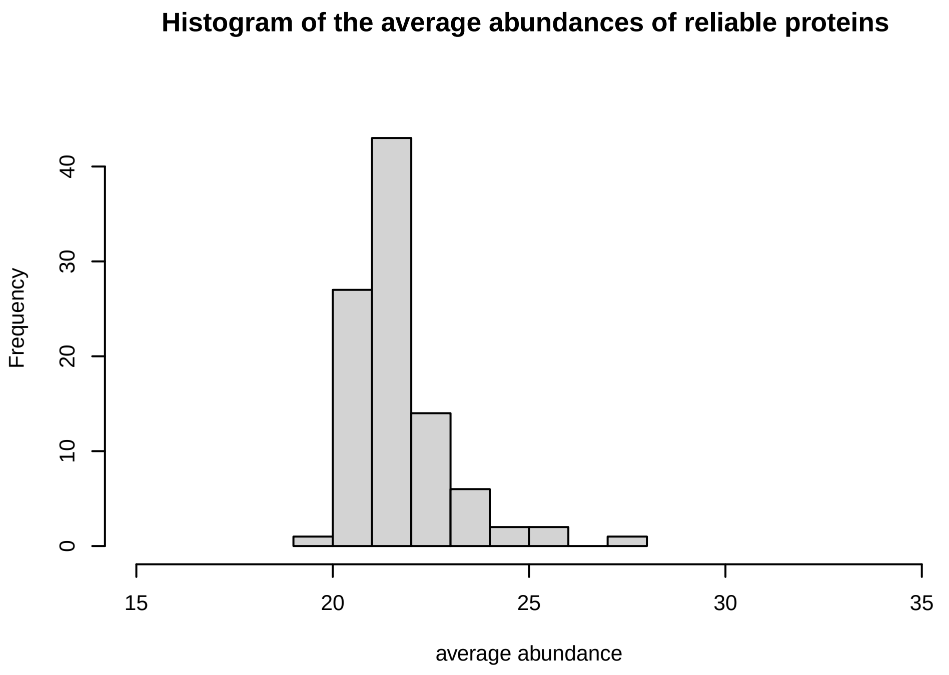
Histogram of the average abundances of reliable proteins
Frequency
0 10 20 30 40
15 20 25 30 35
average abundance

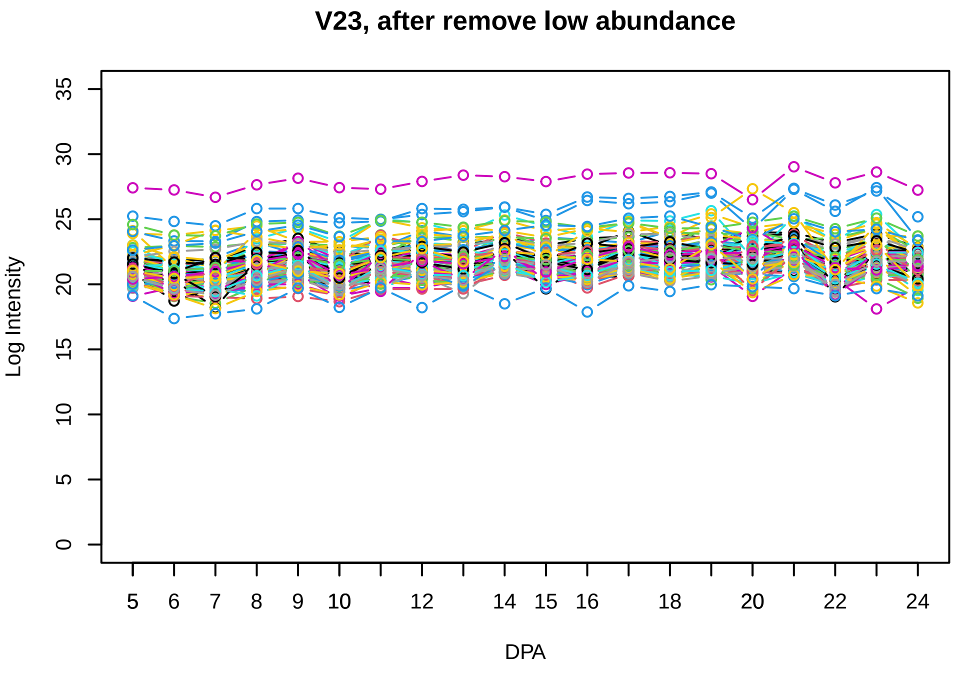
V23, after remove low abundance
Log Intensity
0 5 10 15 20 25 30 35
5 6 7 8 9 10 12 14 15 16 18 20 22 24
DPA

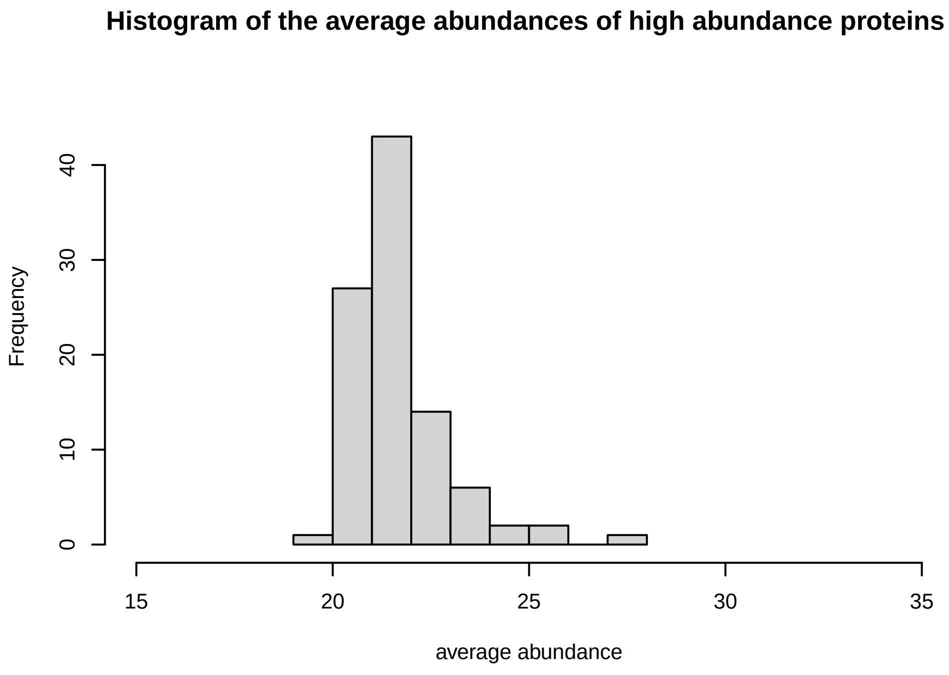
Histogram of the average abundances of high abundance proteins
Frequency
0 10 20 30 40
15 20 25 30 35
average abundance

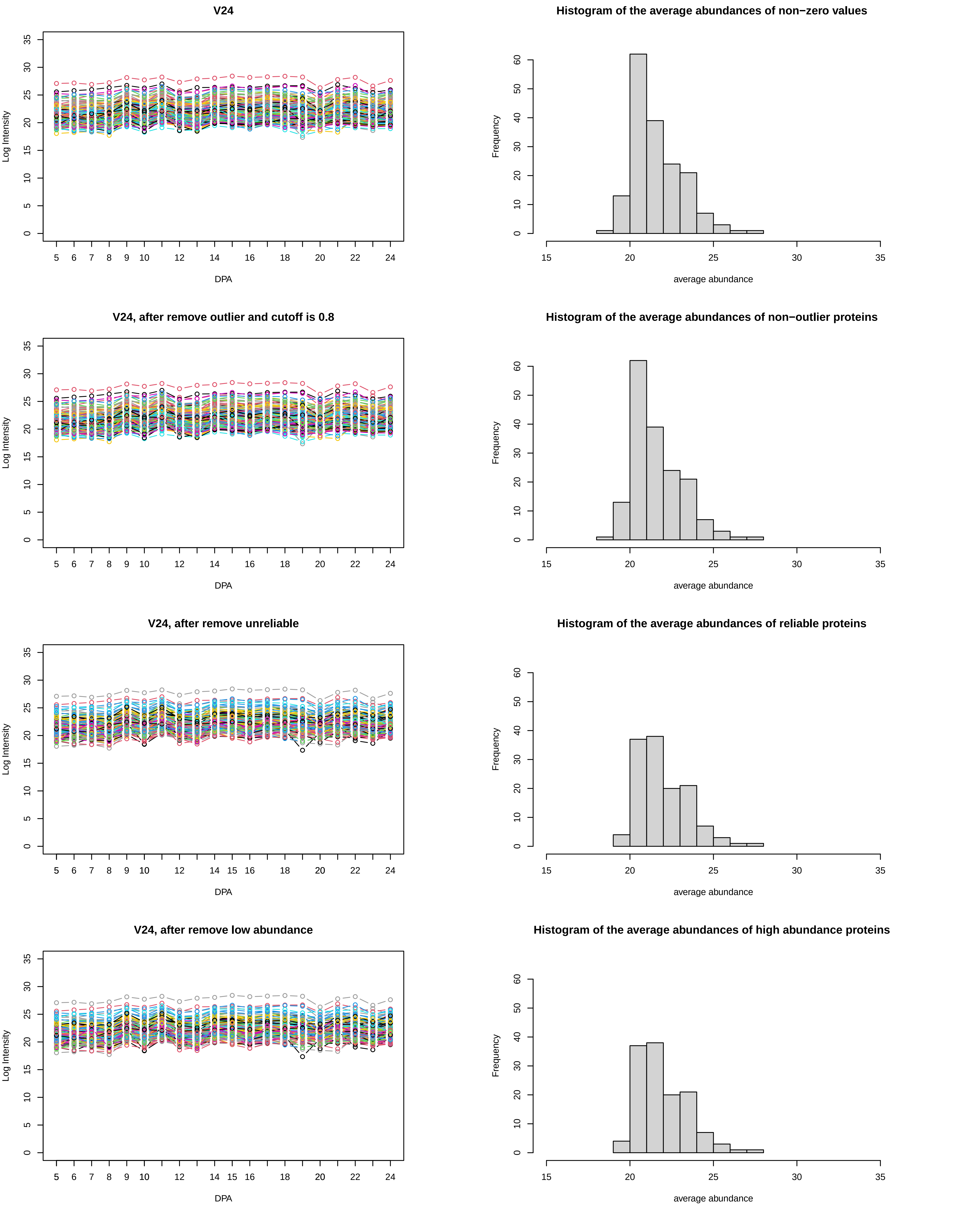
V24
Log Intensity
DPA
Histogram of the average abundances of non−zero values
Frequency
average abundance
V24, after remove outlier and cutoff is 0.8
Log Intensity
DPA
Histogram of the average abundances of non−outlier proteins
Frequency
average abundance
V24, after remove unreliable
Log Intensity
DPA
Histogram of the average abundances of reliable proteins
Frequency
average abundance
V24, after remove low abundance
Log Intensity
DPA
Histogram of the average abundances of high abundance proteins
Frequency
average abundance

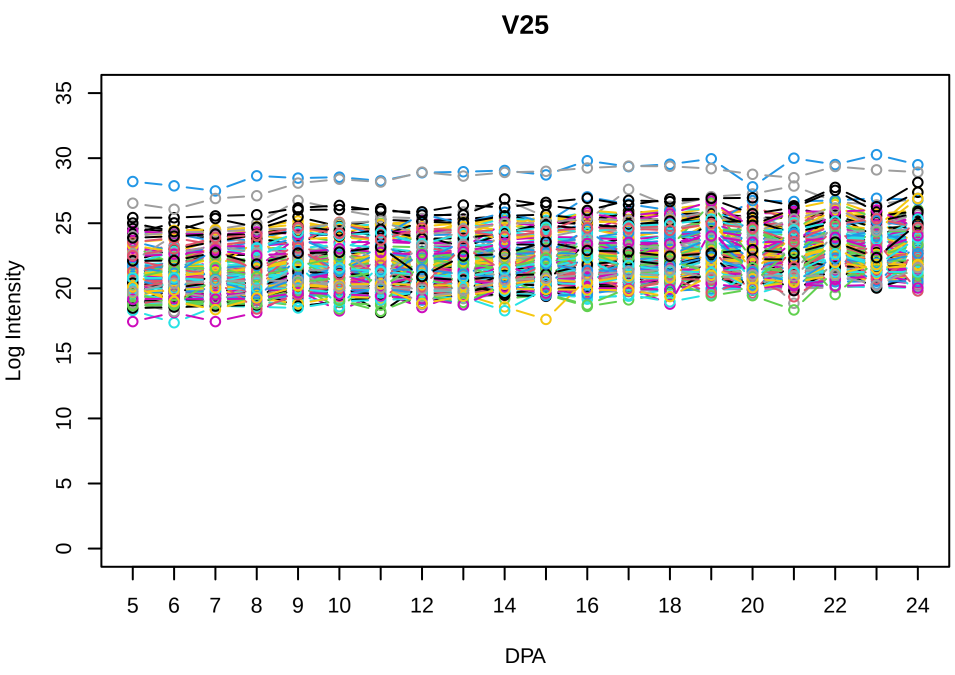
V25
Log Intensity
DPA

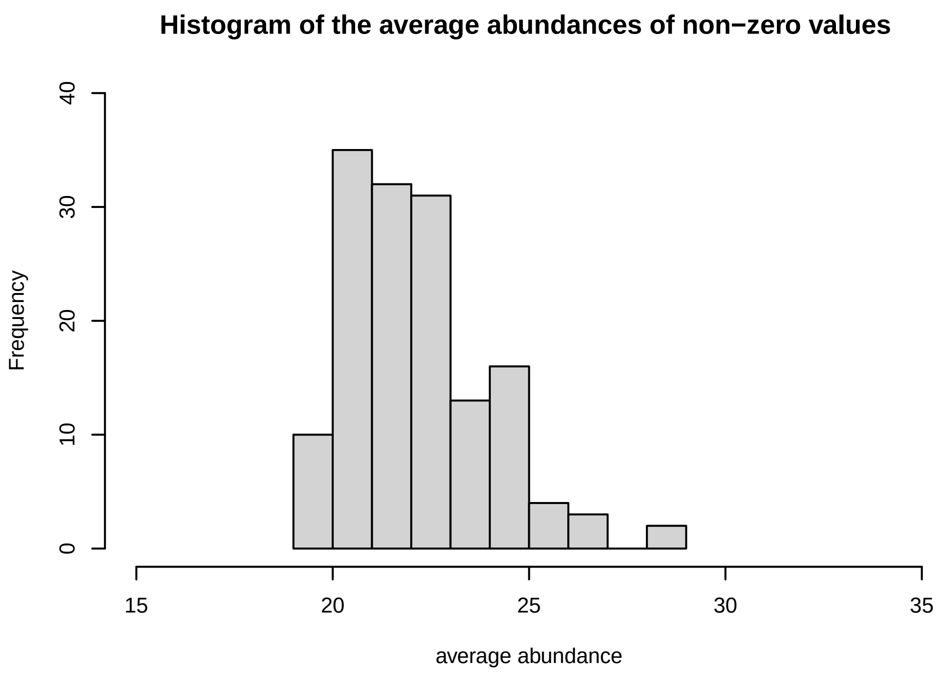
Histogram of the average abundances of non−zero values
Frequency
average abundance

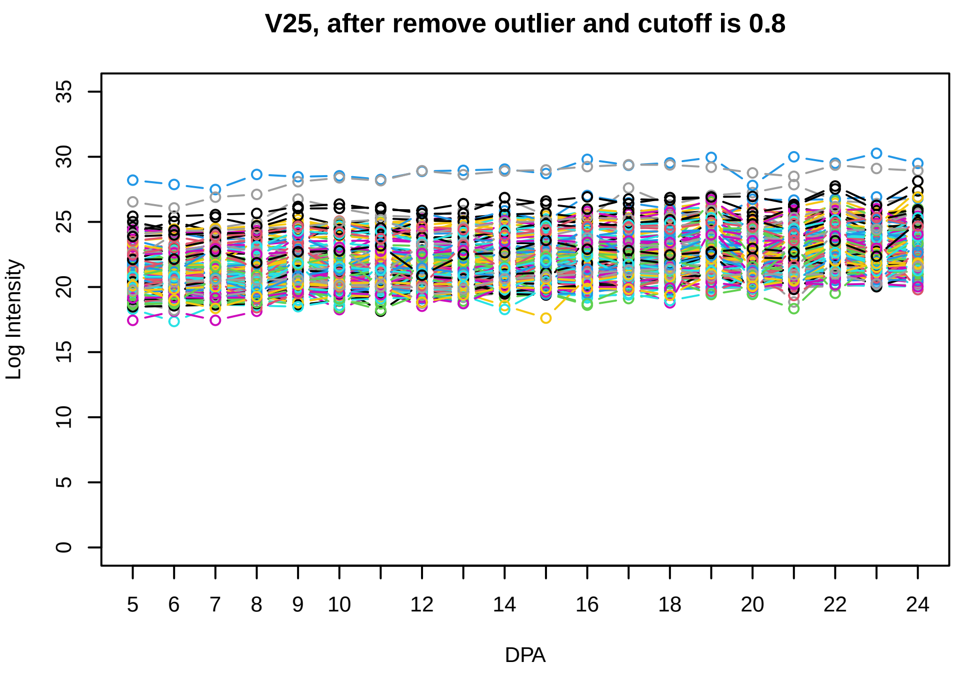
V25, after remove outlier and cutoff is 0.8
Log Intensity
DPA

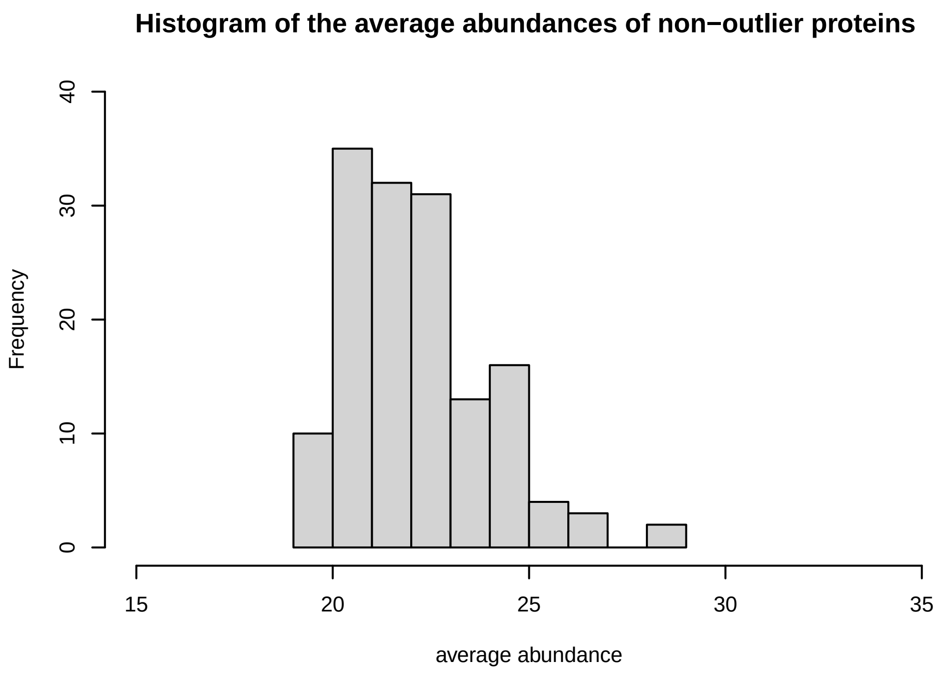
Histogram of the average abundances of non−outlier proteins
Frequency
average abundance

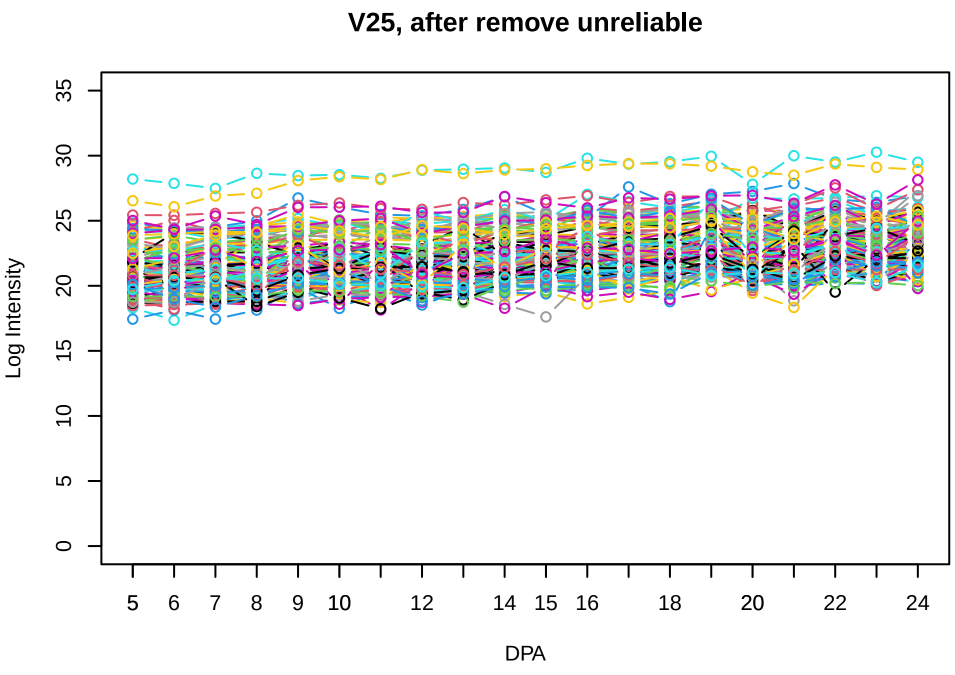
V25, after remove unreliable
Log Intensity
DPA

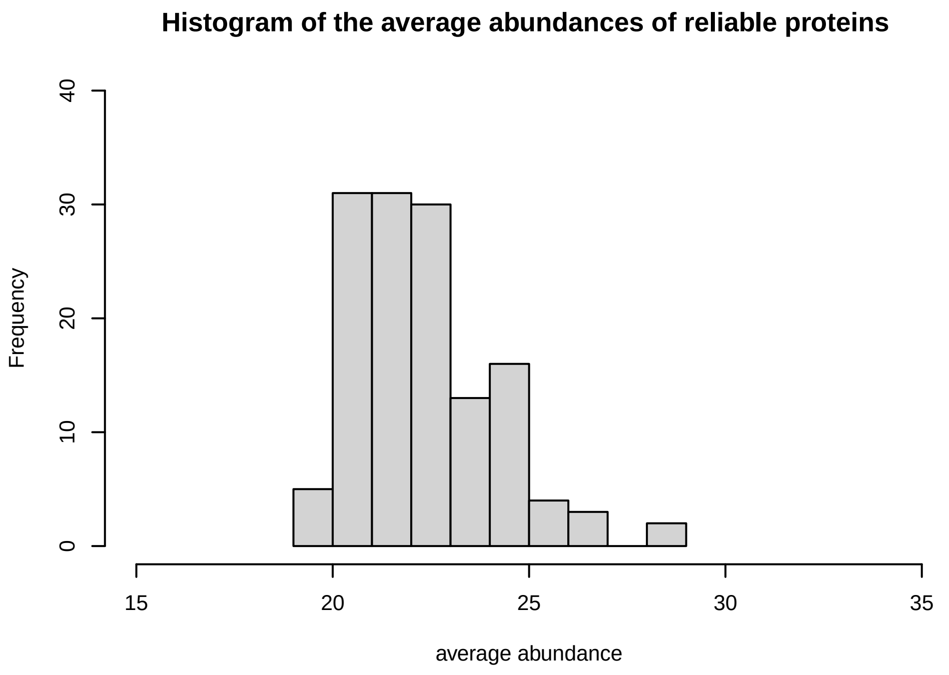
Histogram of the average abundances of reliable proteins
Frequency
average abundance

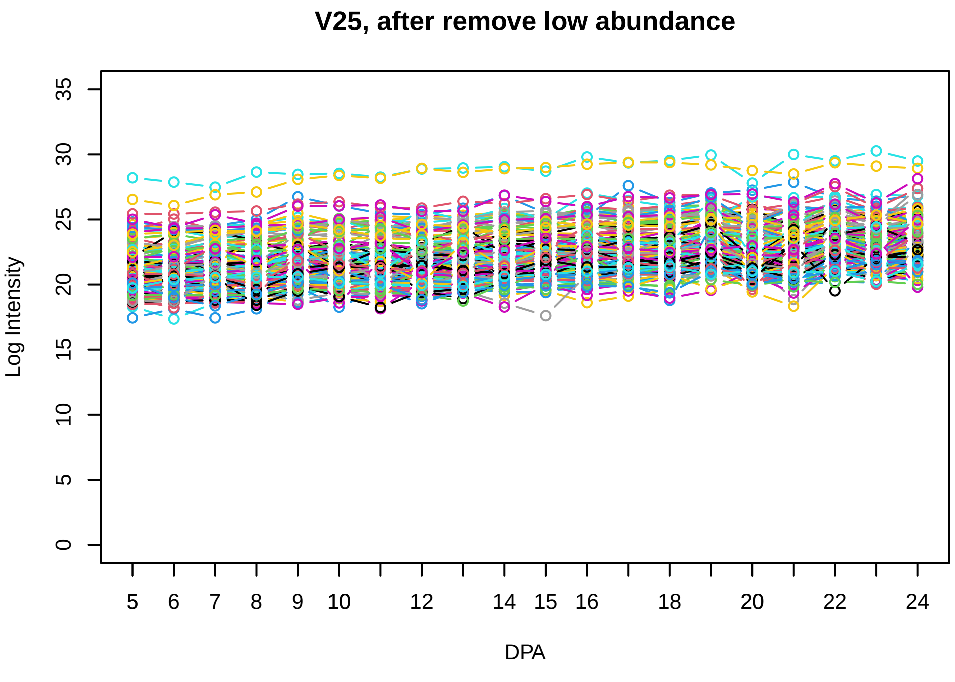
V25, after remove low abundance
Log Intensity
DPA

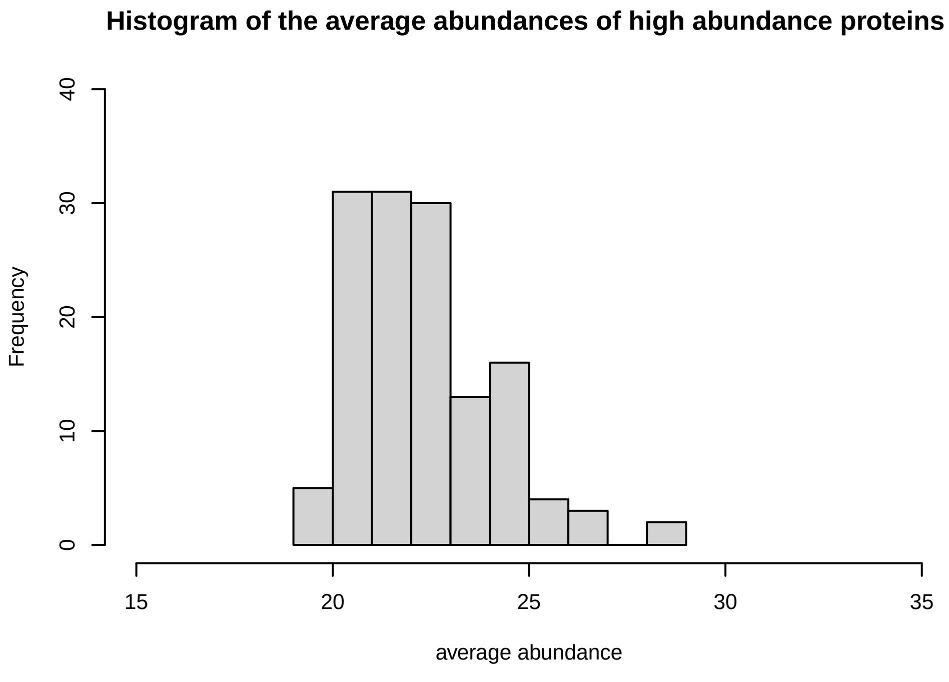
Histogram of the average abundances of high abundance proteins
Frequency
average abundance

V26

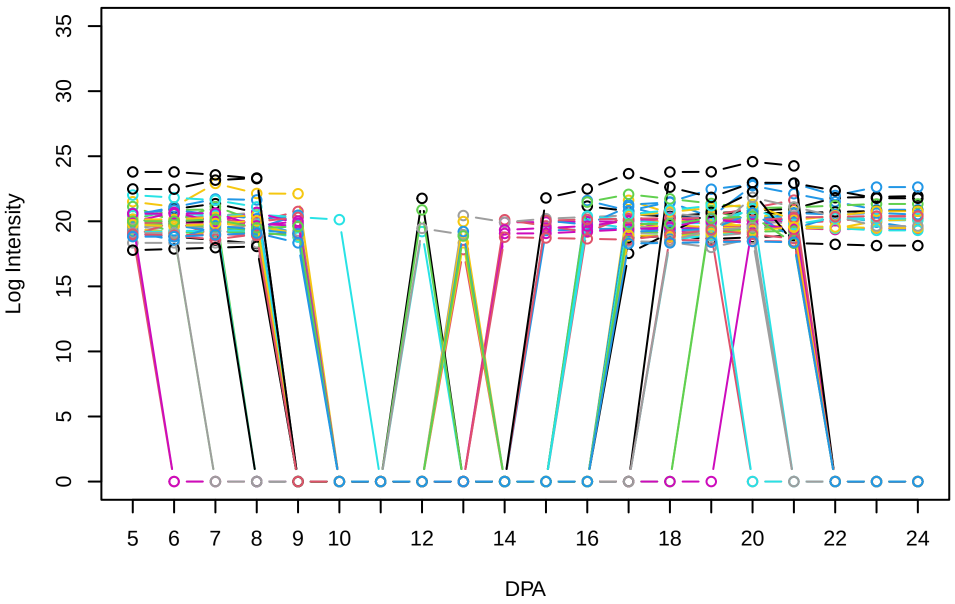

Histogram of the average abundances of non−zero values

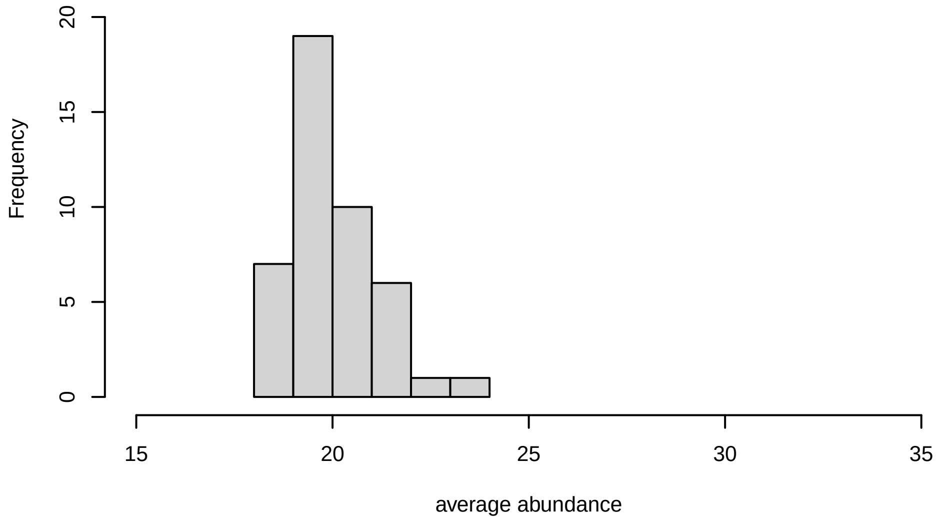

V26, after remove outlier and cutoff is 0.78944379457436

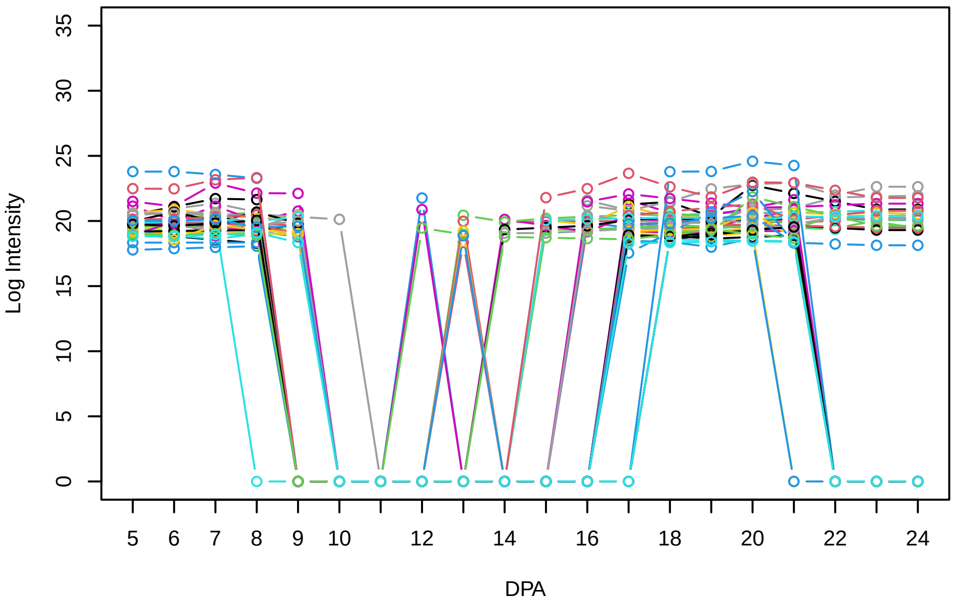

Histogram of the average abundances of non−outlier proteins

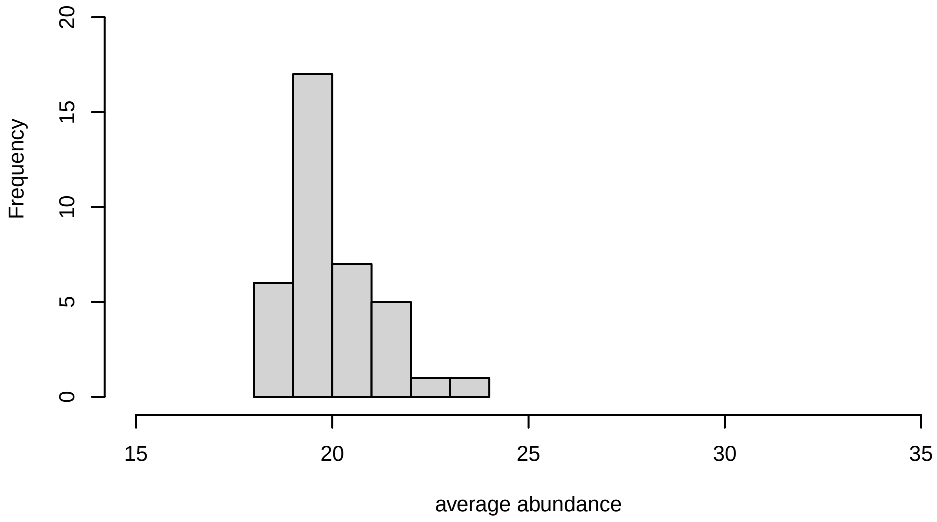

V26, after remove unreliable

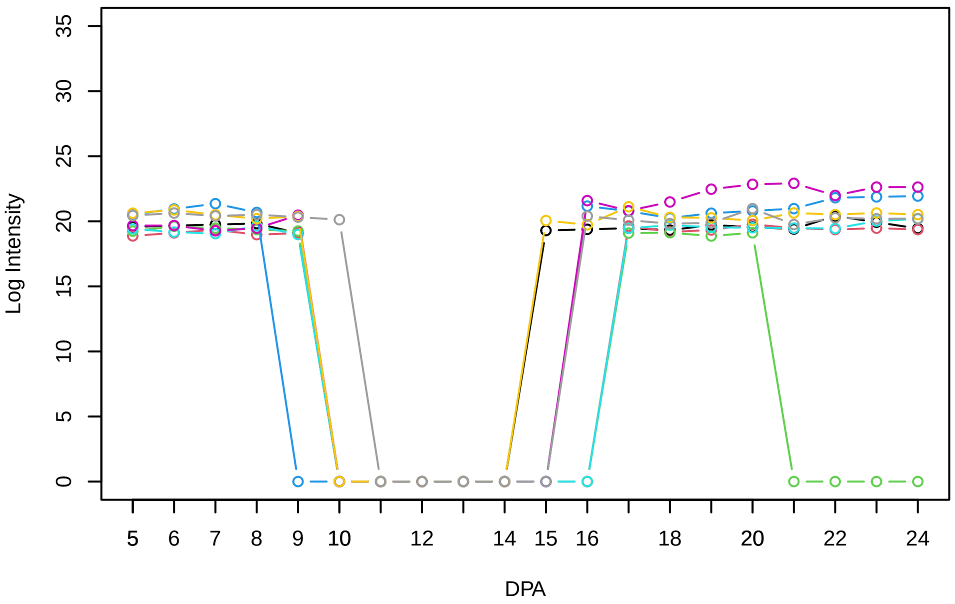

Histogram of the average abundances of reliable proteins

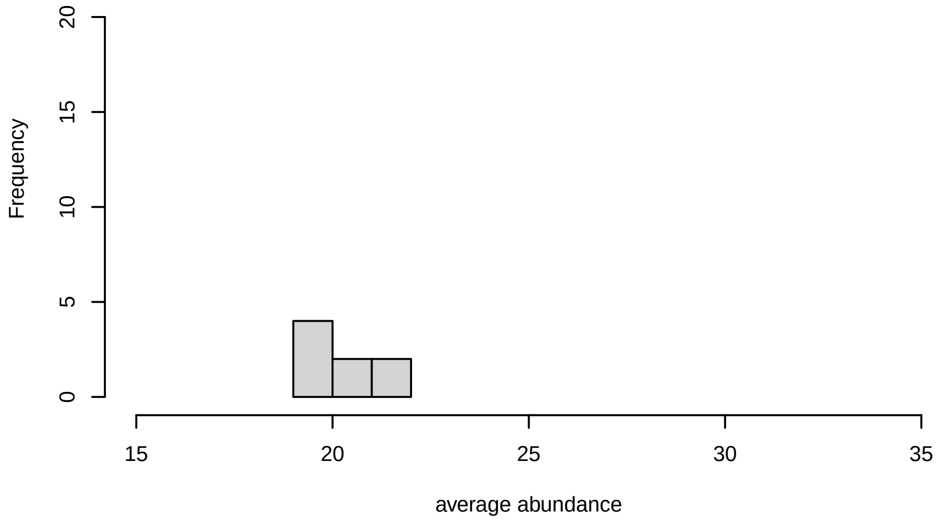

V26, after remove low abundance

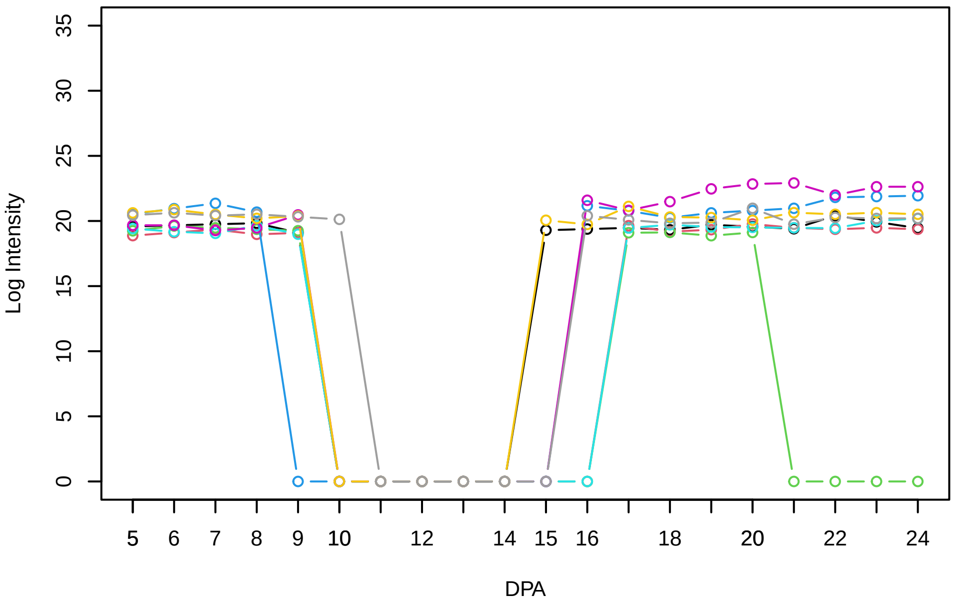

Histogram of the average abundances of high abundance proteins

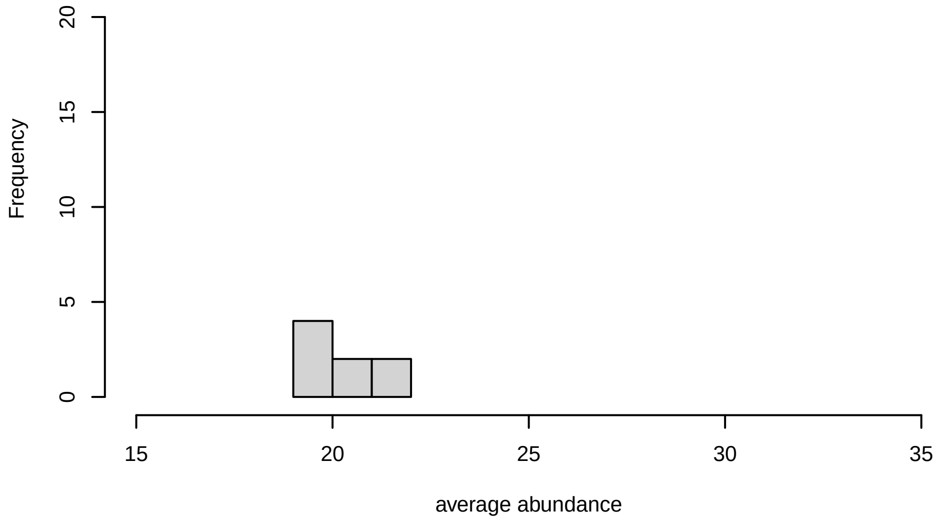

V27

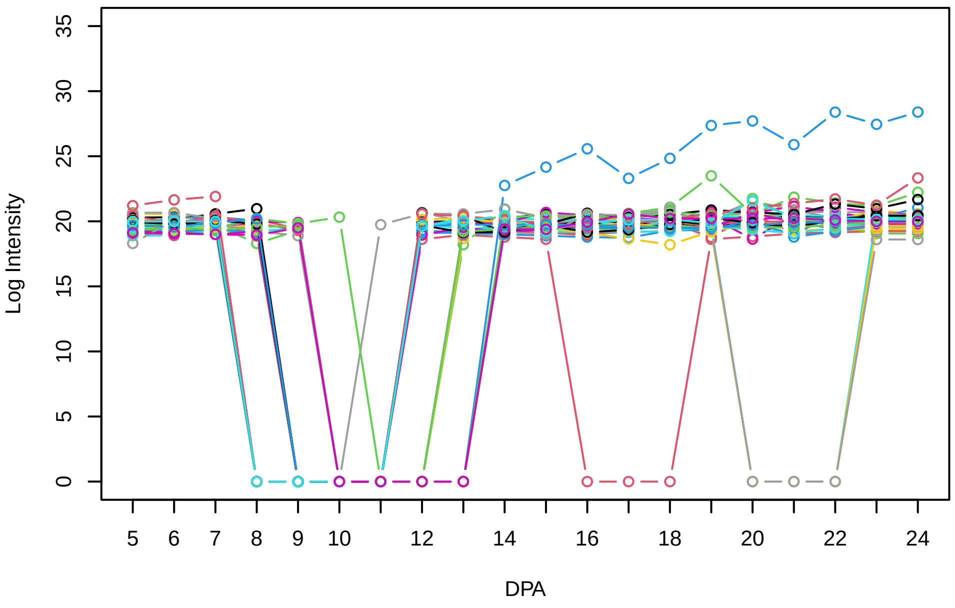

Histogram of the average abundances of non−zero values

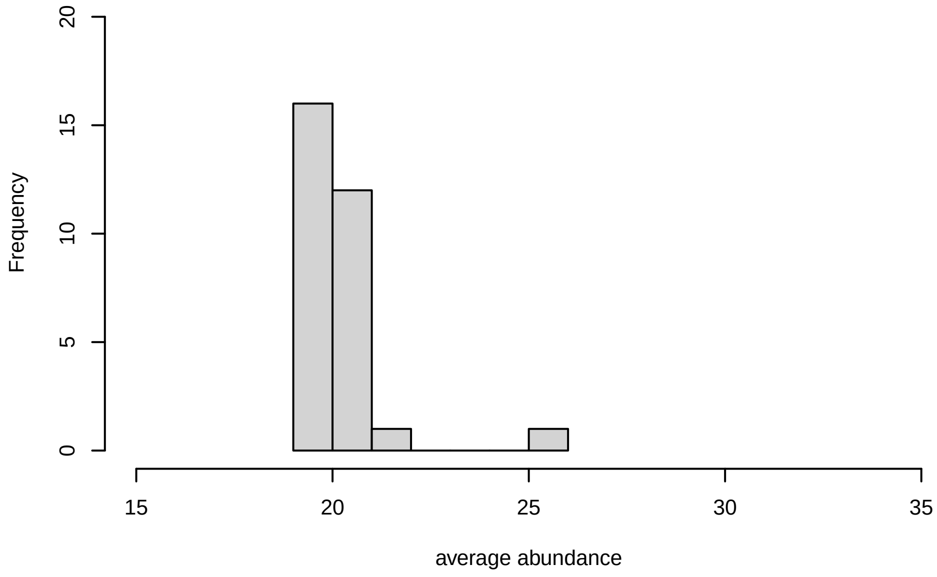

V27, after remove outlier and cutoff is 0.8

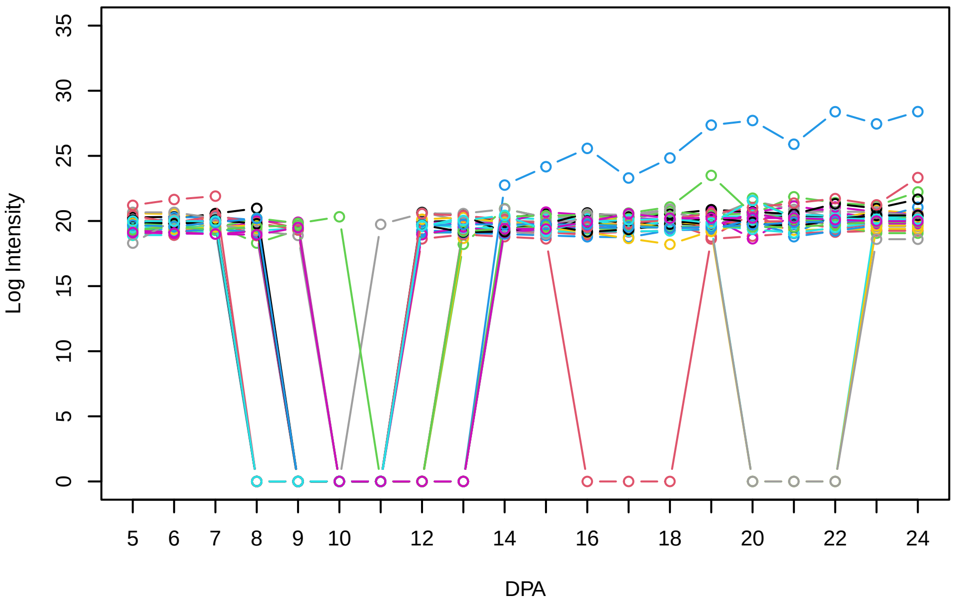

Histogram of the average abundances of non−outlier proteins

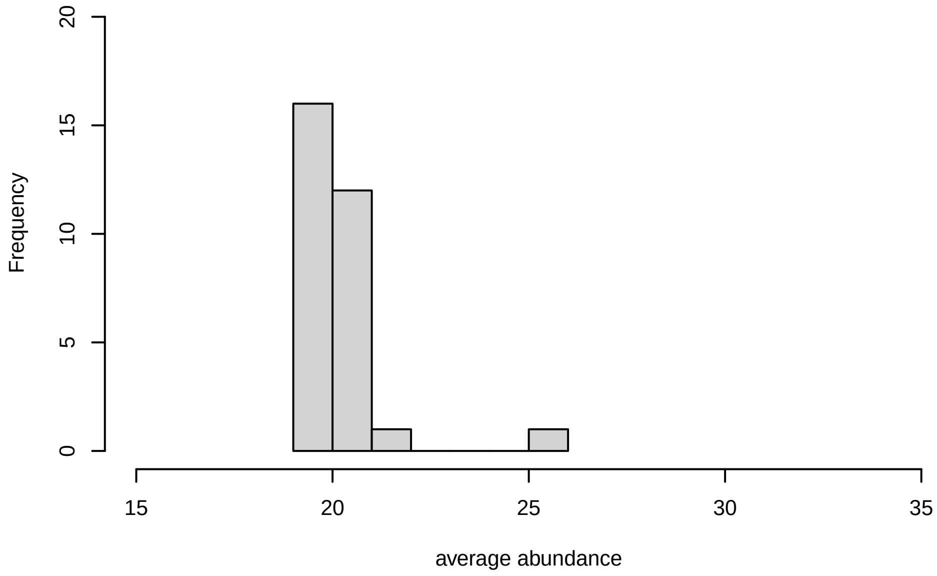

V27, after remove unreliable

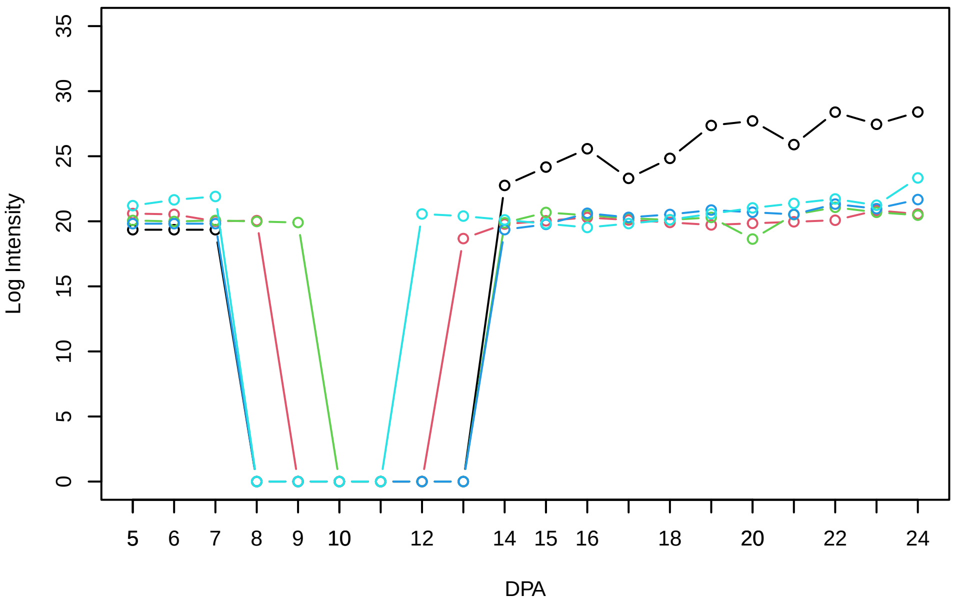

Histogram of the average abundances of reliable proteins

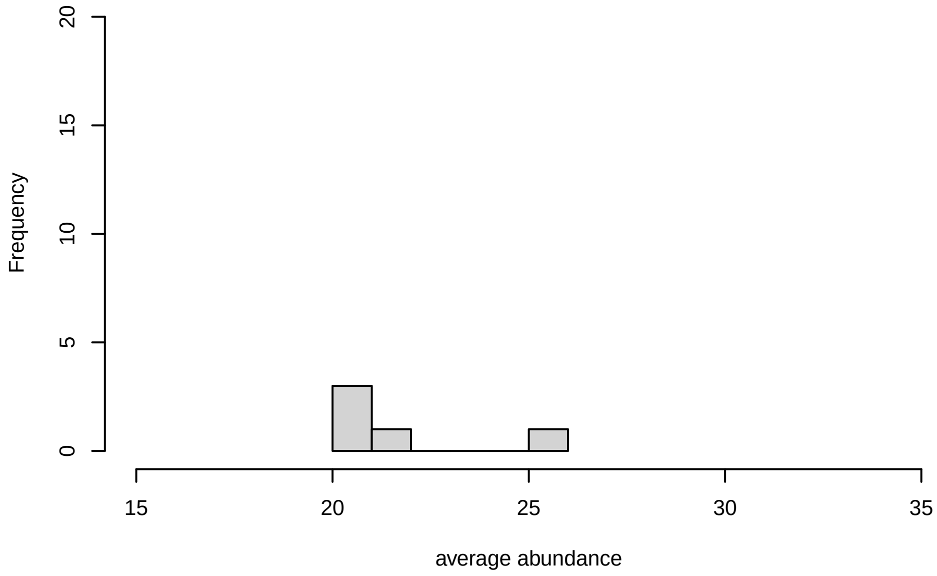

V27, after remove low abundance

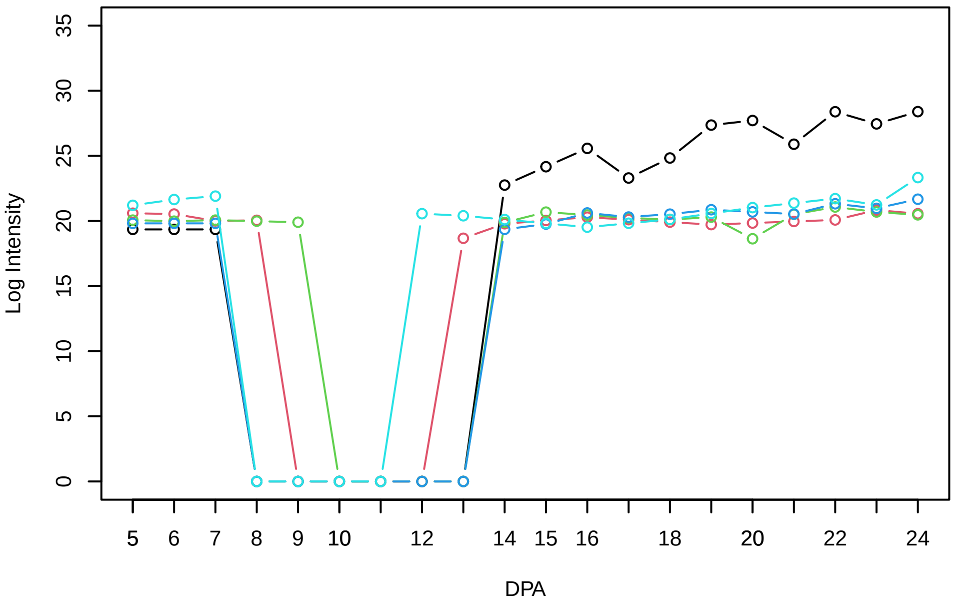

Histogram of the average abundances of high abundance proteins

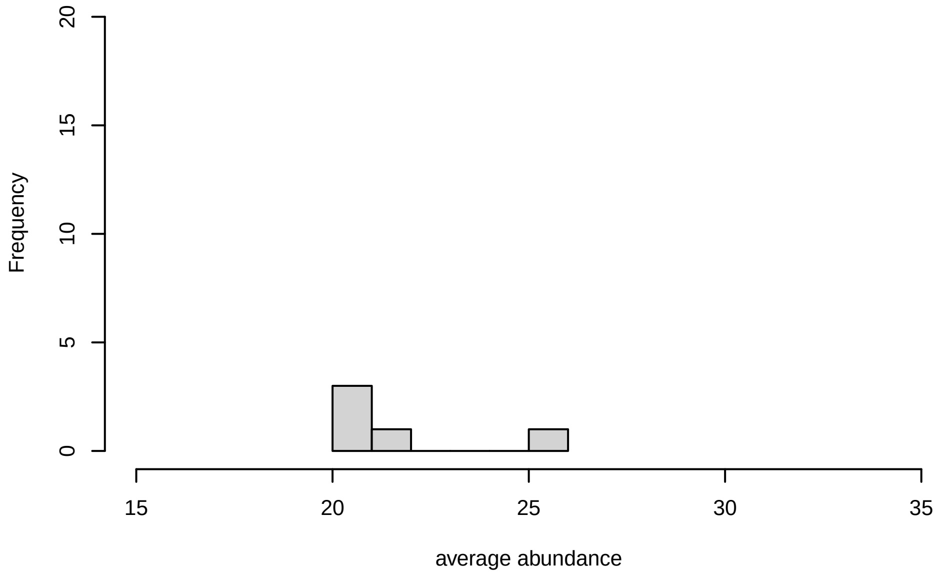

V28

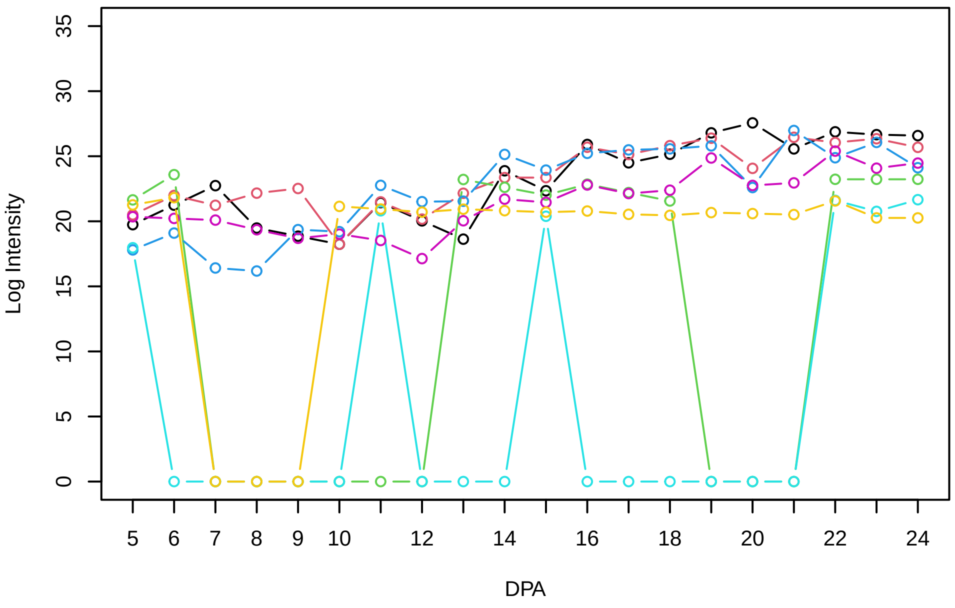

Histogram of the average abundances of non−zero values

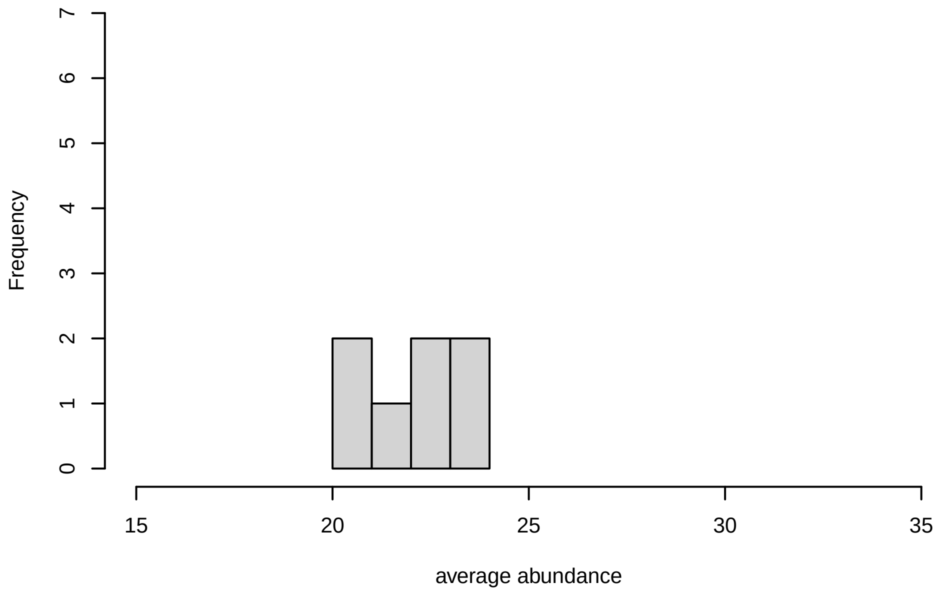

V28, after remove outlier and cutoff is 0.8

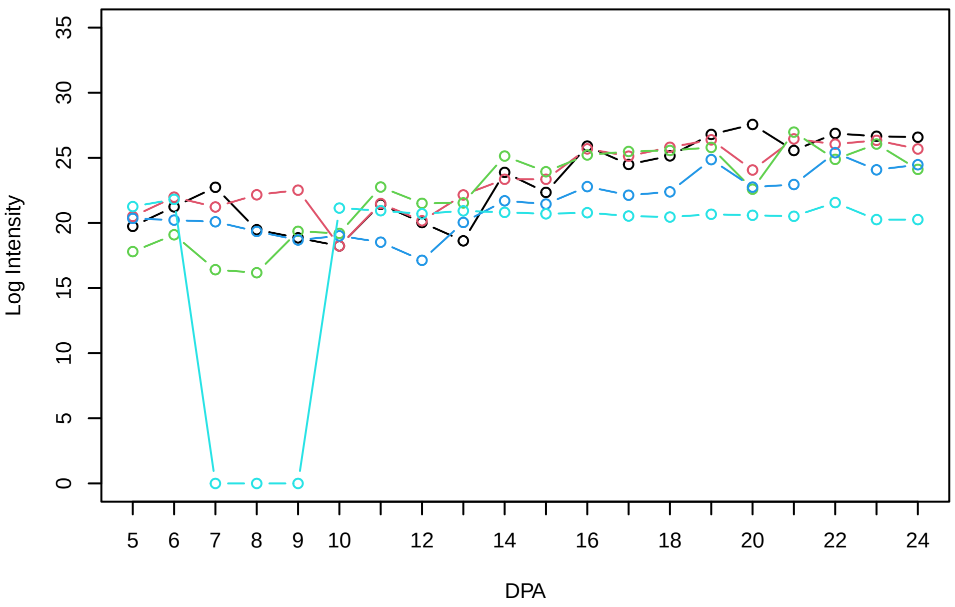

Histogram of the average abundances of non−outlier proteins

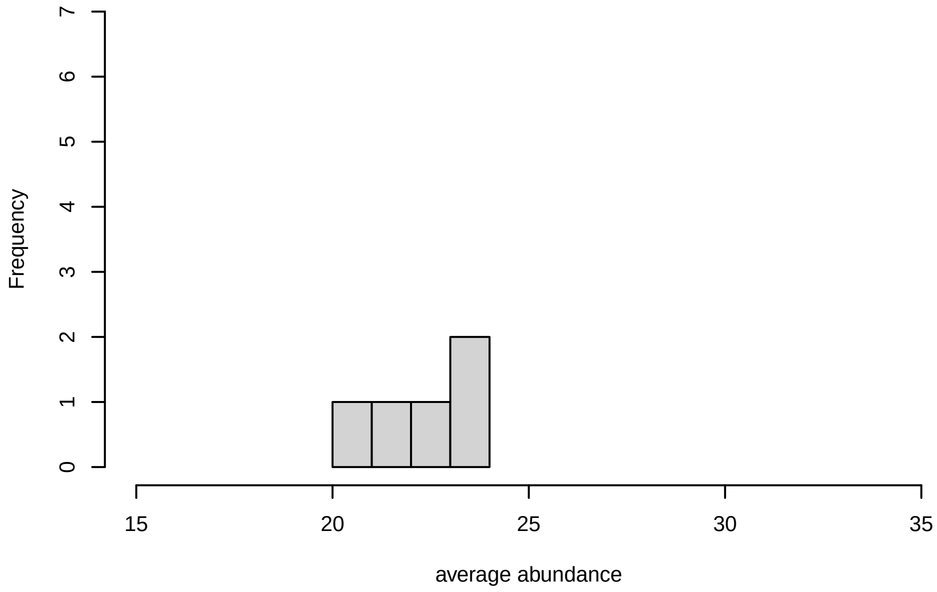

V28, after remove unreliable

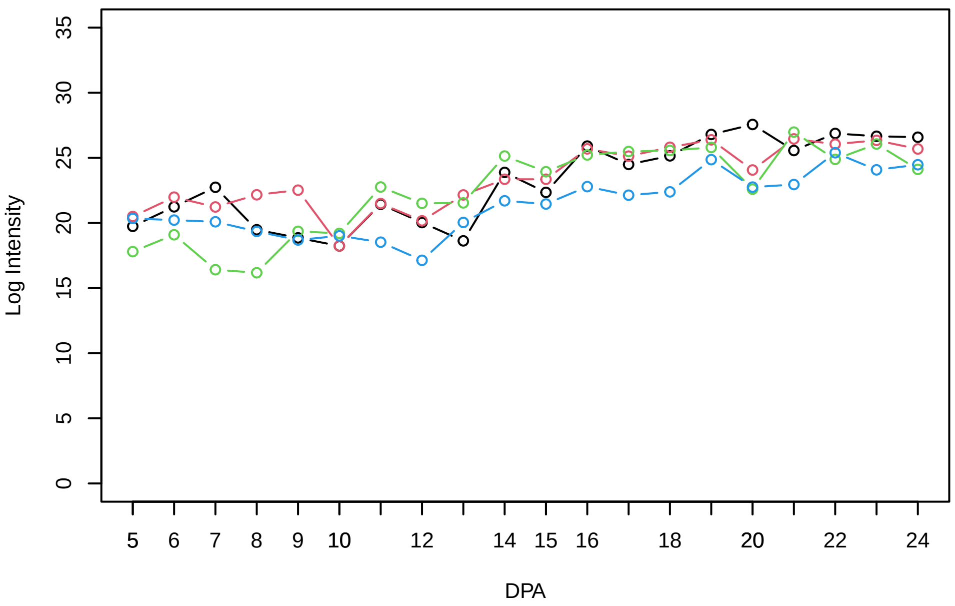

Histogram of the average abundances of reliable proteins

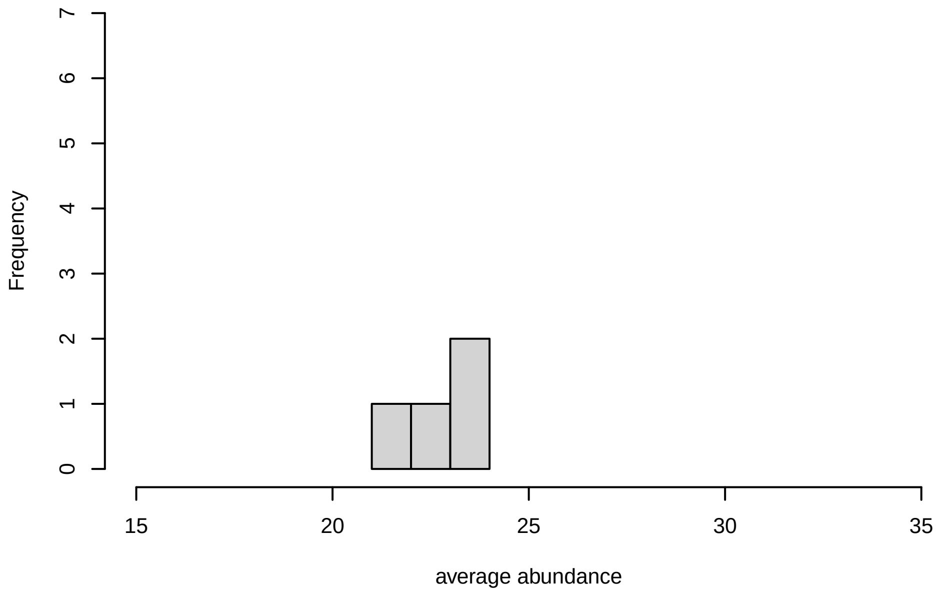

V28, after remove low abundance

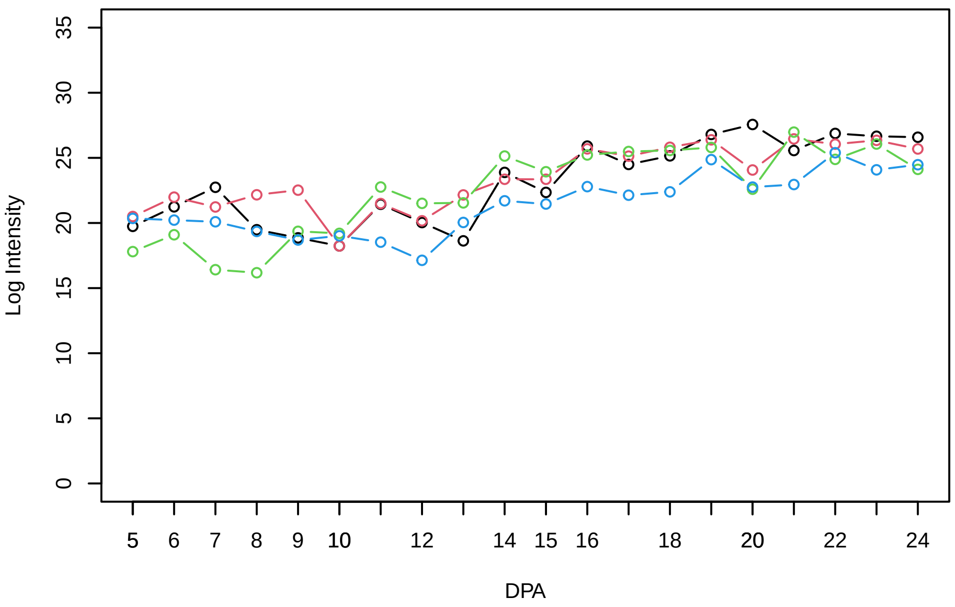

Histogram of the average abundances of high abundance proteins

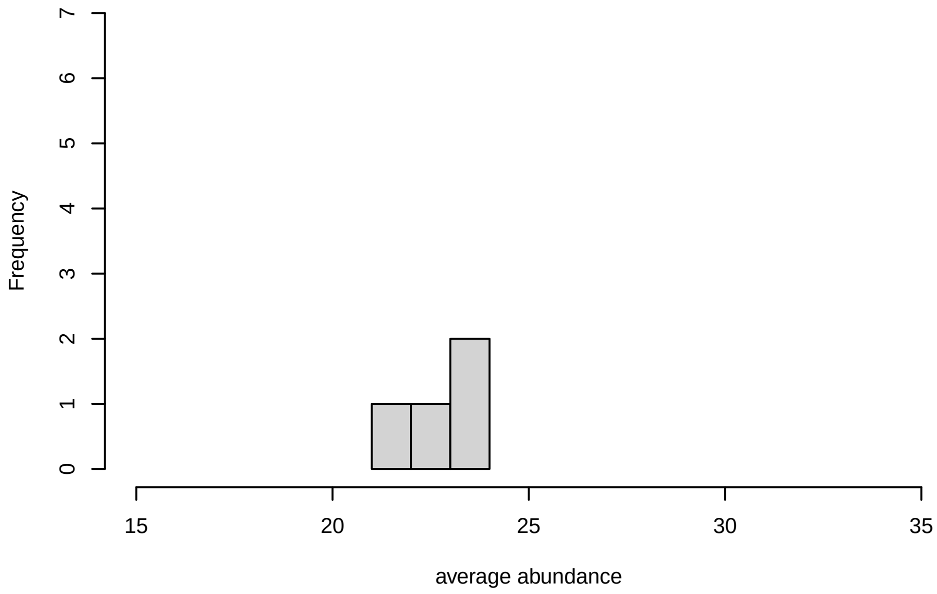

V29

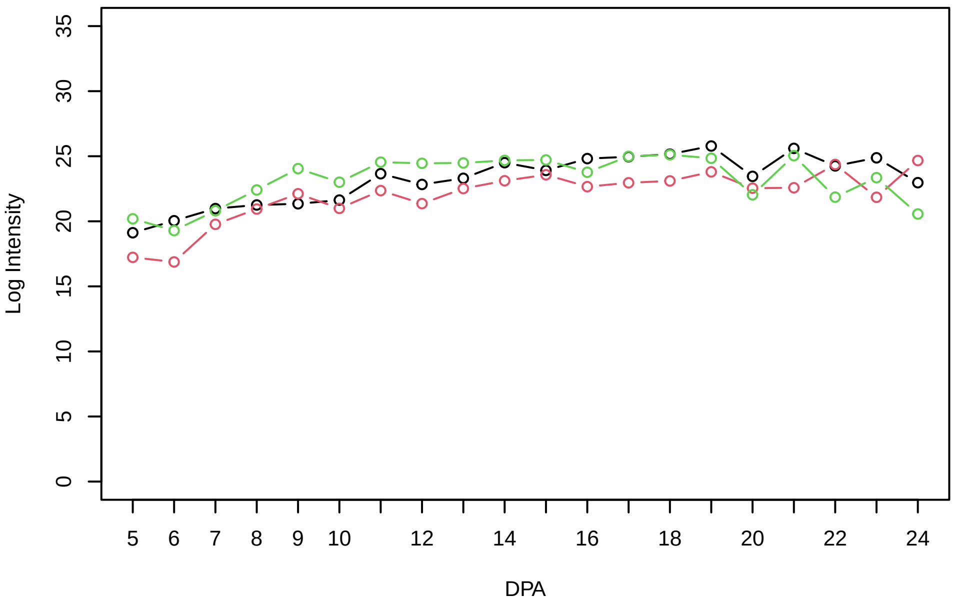

Histogram of the average abundances of non−zero values

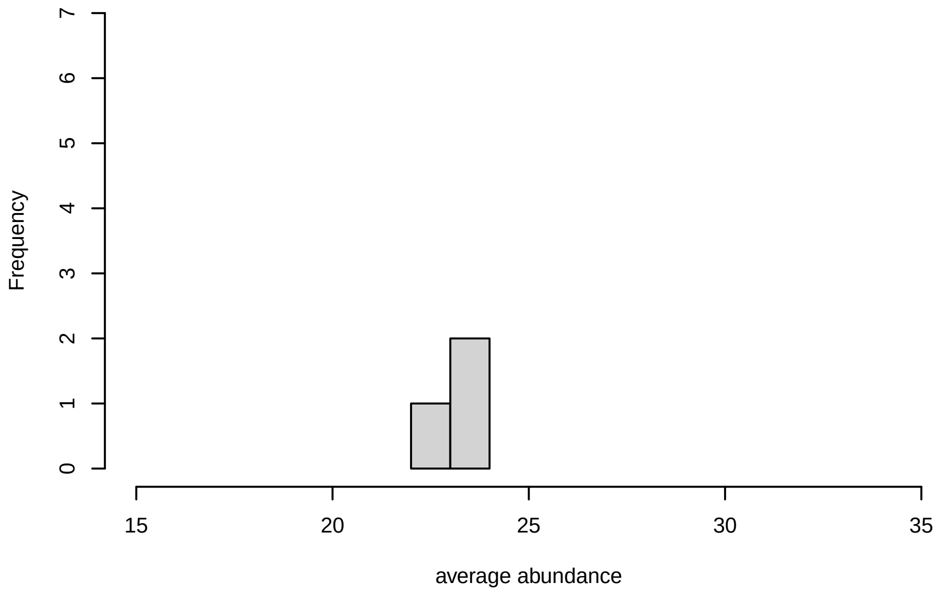

V29, after remove outlier and cutoff is 0.8

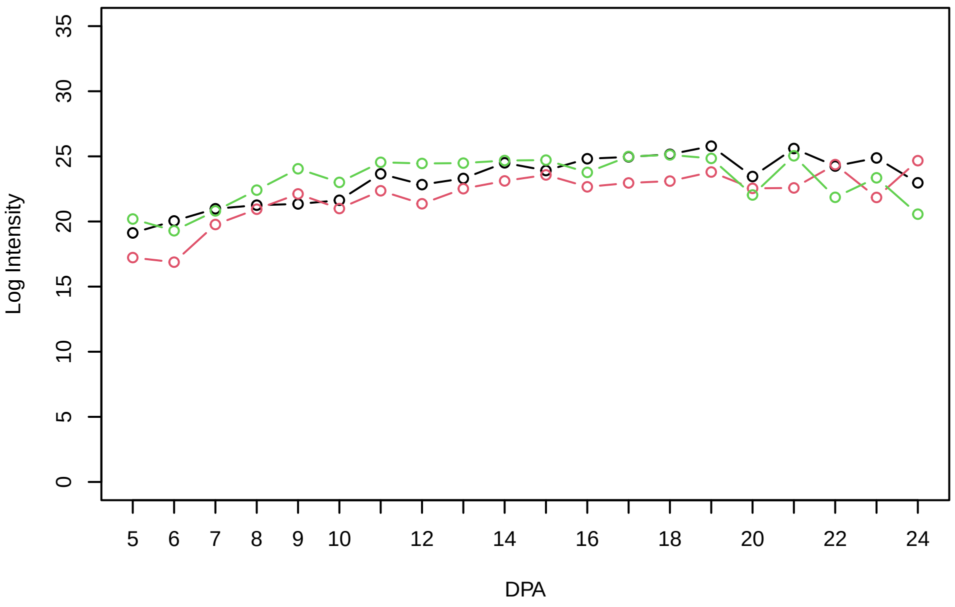

Histogram of the average abundances of non−outlier proteins

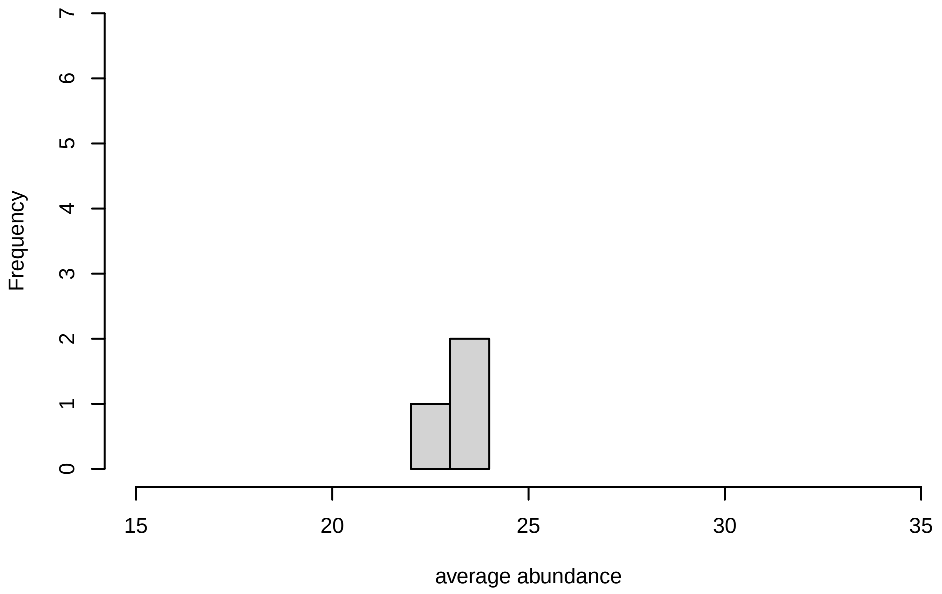

V29, after remove unreliable

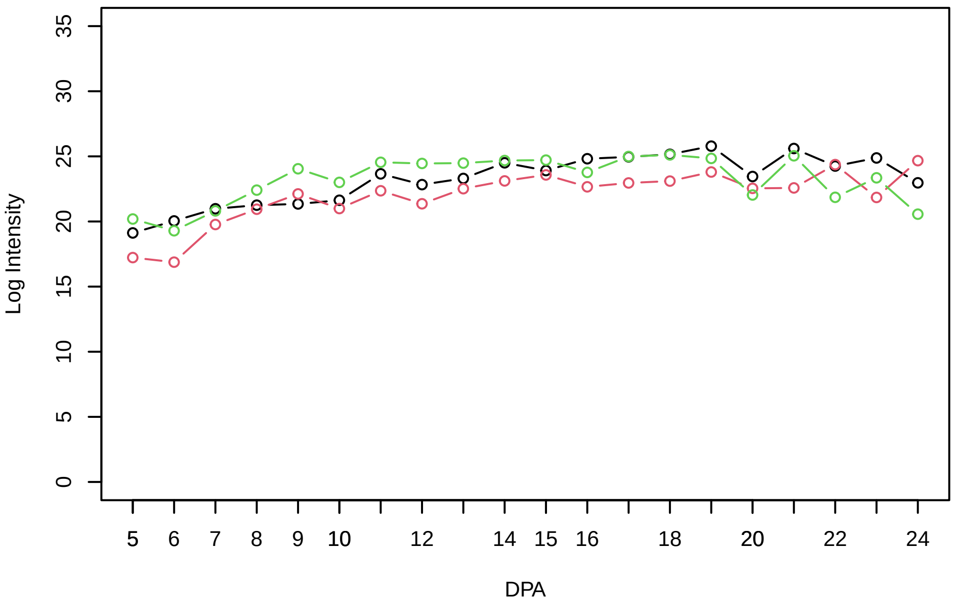

Histogram of the average abundances of reliable proteins

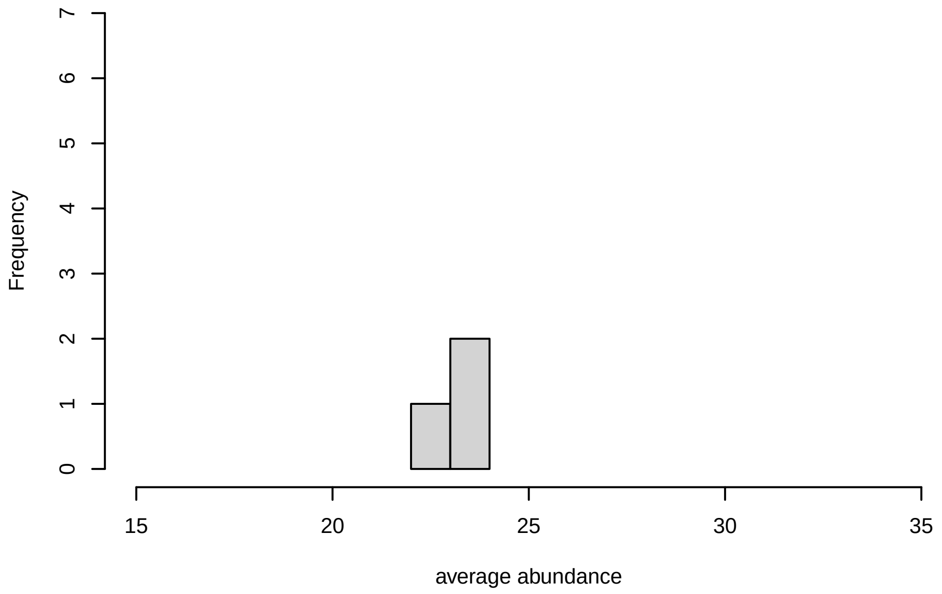

V29, after remove low abundance

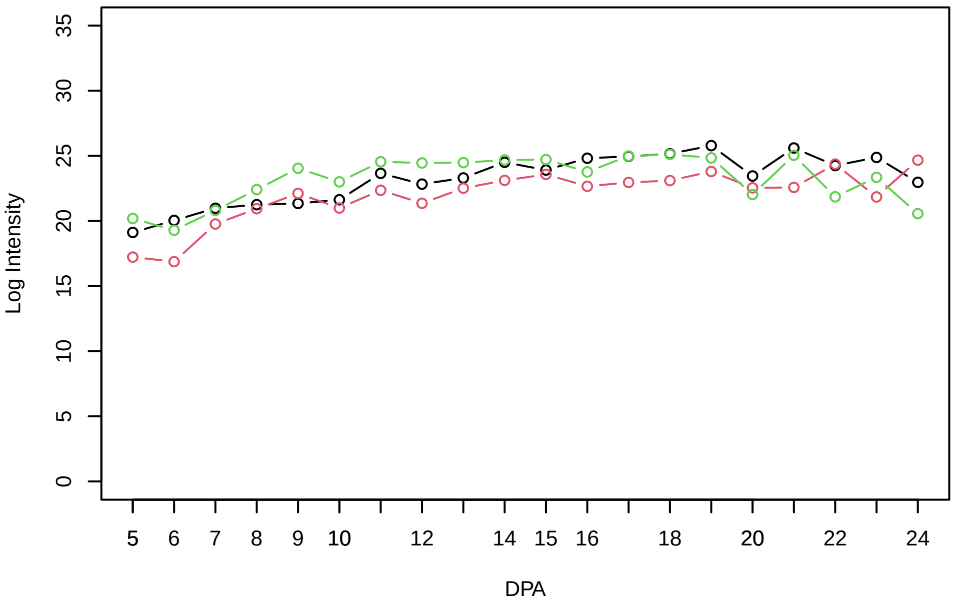

Histogram of the average abundances of high abundance proteins

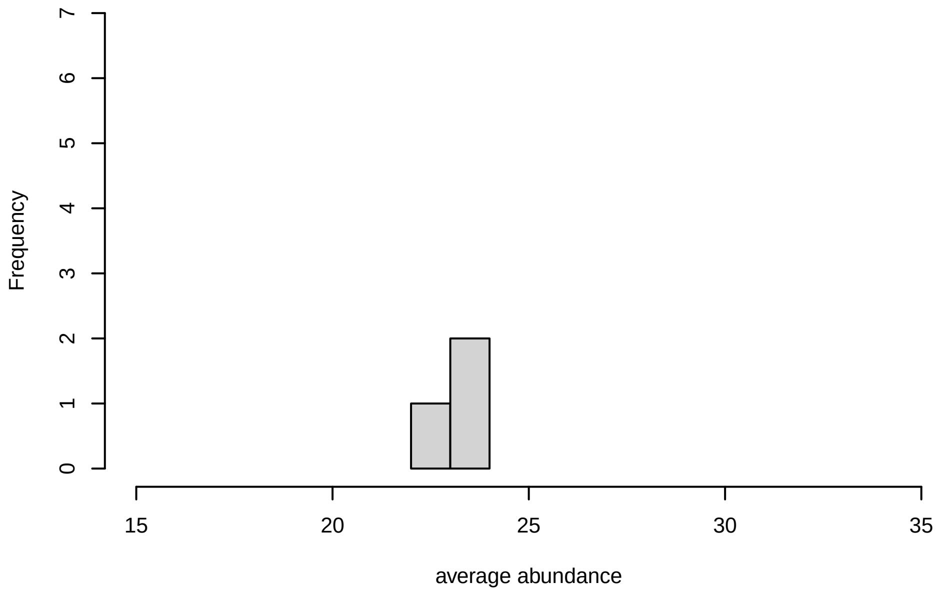

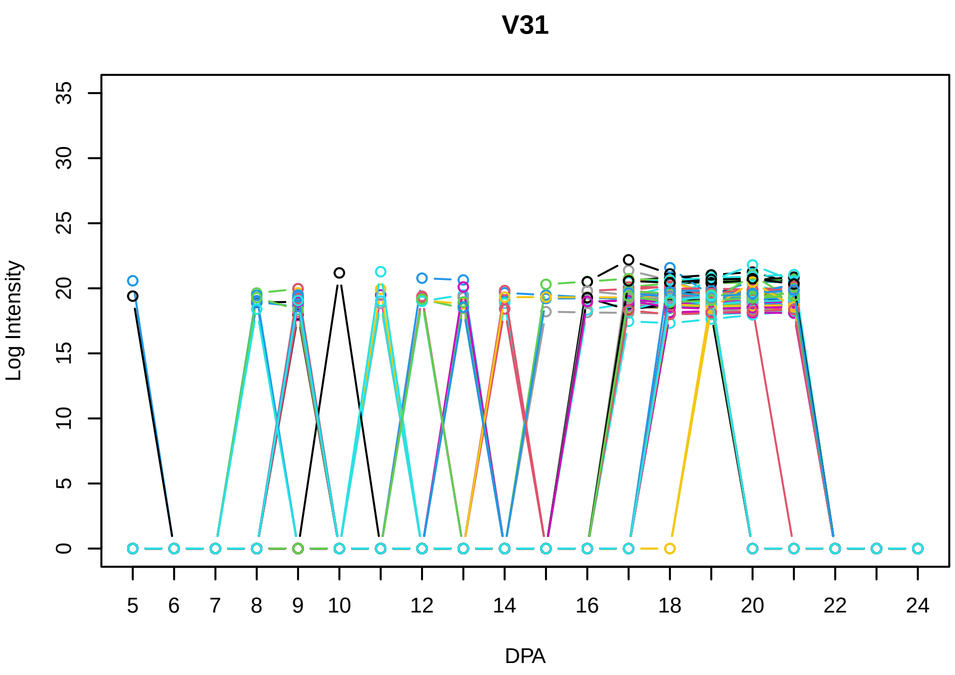
V31
Log Intensity
0
5
10
15
20
25
30
35
5
6
7
8
9
10
12
14
16
18
20
22
24
DPA

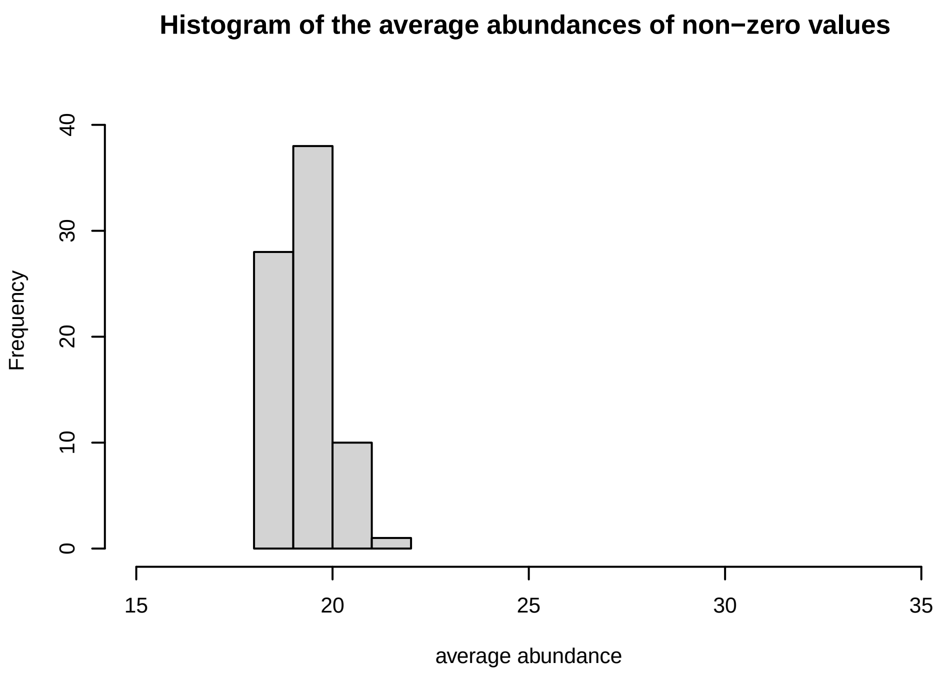
Histogram of the average abundances of non−zero values
Frequency
0
10
20
30
40
15
20
25
30
35
average abundance

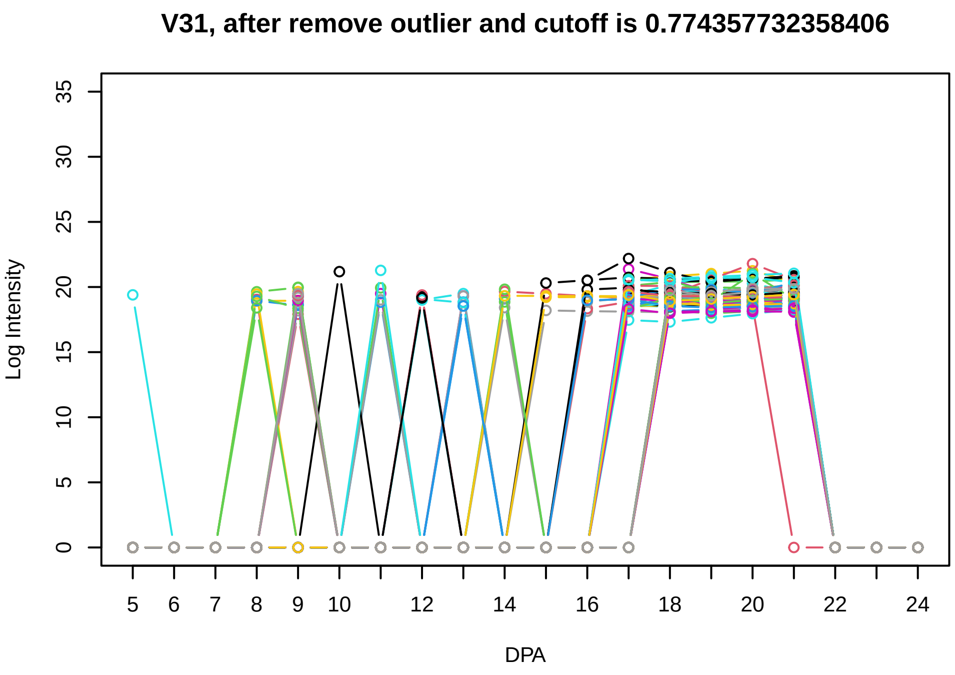
V31, after remove outlier and cutoff is 0.774357732358406
Log Intensity
0
5
10
15
20
25
30
35
5
6
7
8
9
10
12
14
16
18
20
22
24
DPA

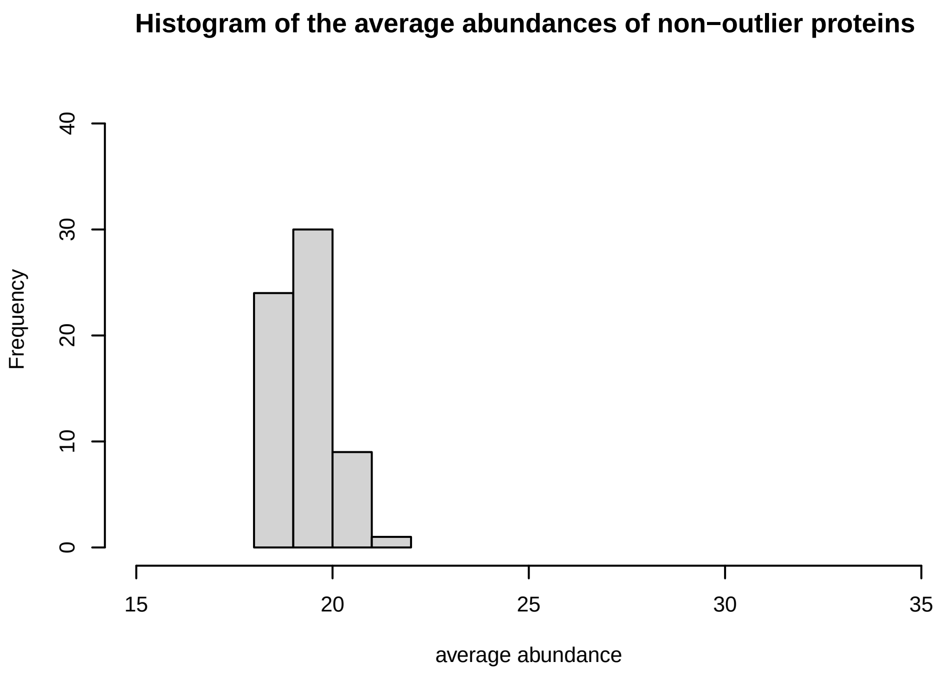
Histogram of the average abundances of non−outlier proteins
Frequency
0
10
20
30
40
15
20
25
30
35
average abundance

V32

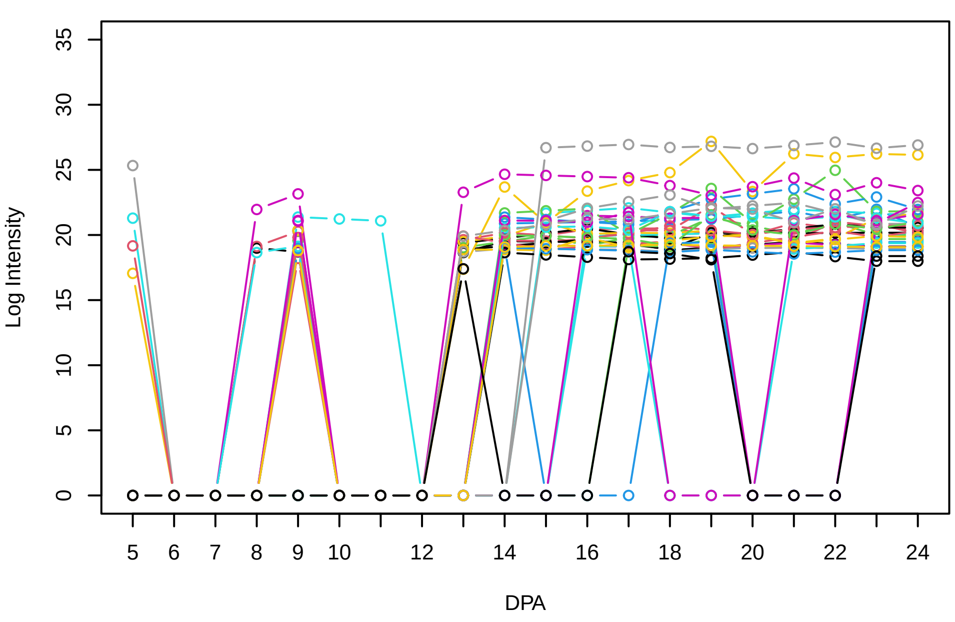

Histogram of the average abundances of non−zero values

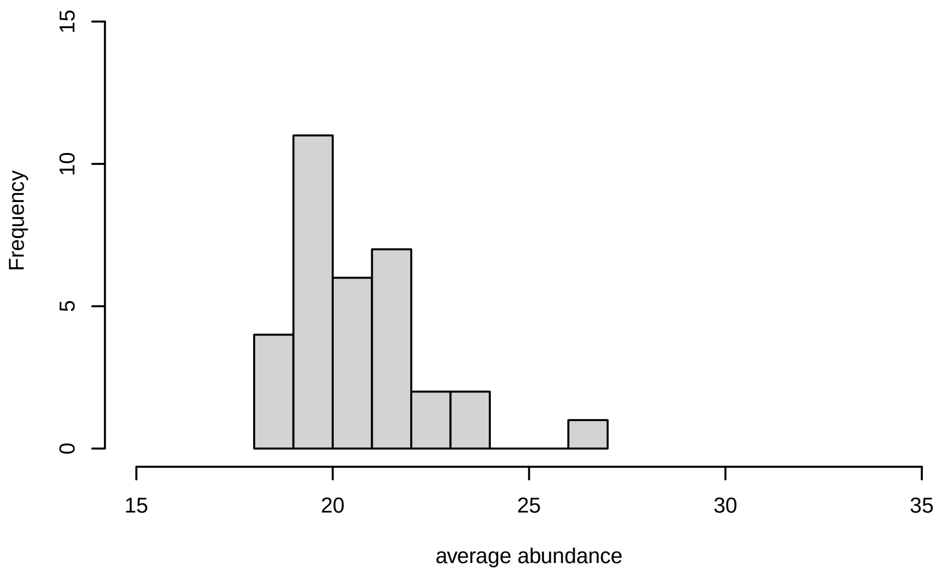

V32, after remove outlier and cutoff is 0.8

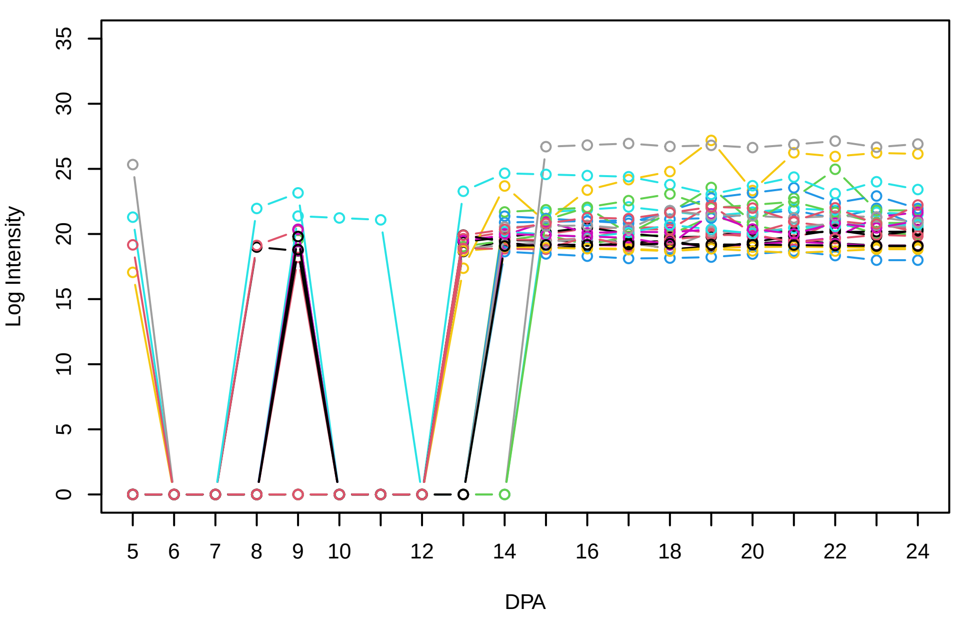

Histogram of the average abundances of non−outlier proteins

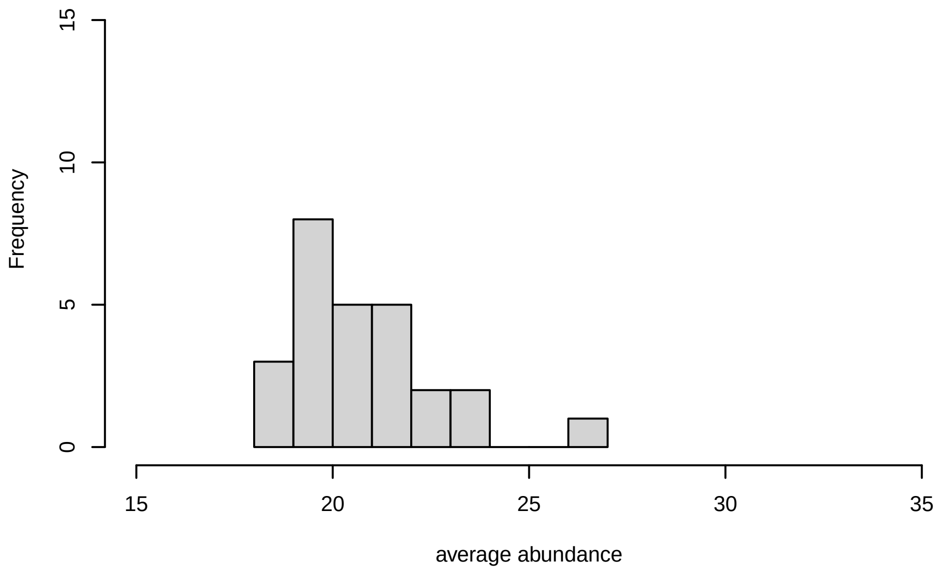

V32, after remove unreliable

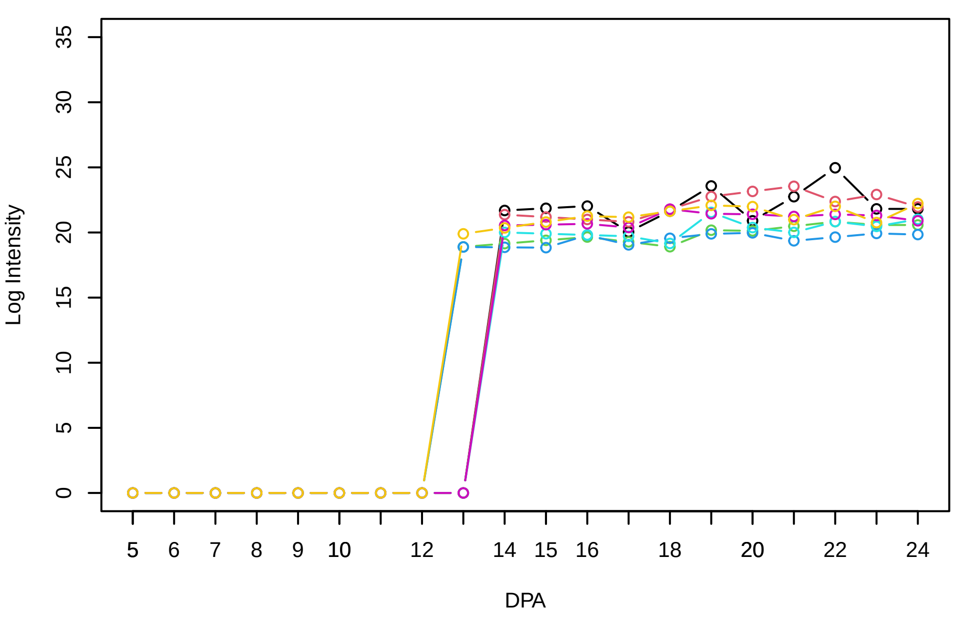

Histogram of the average abundances of reliable proteins

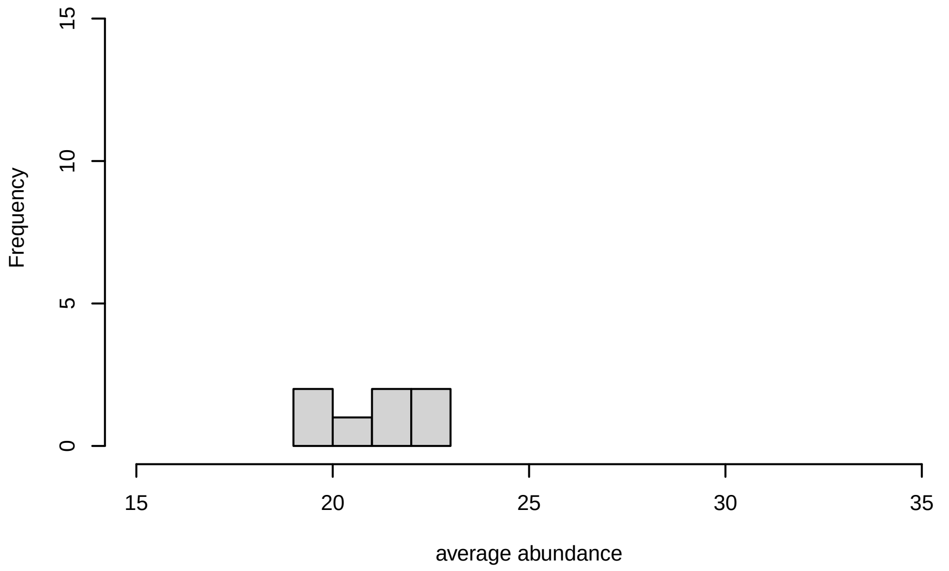

V32, after remove low abundance

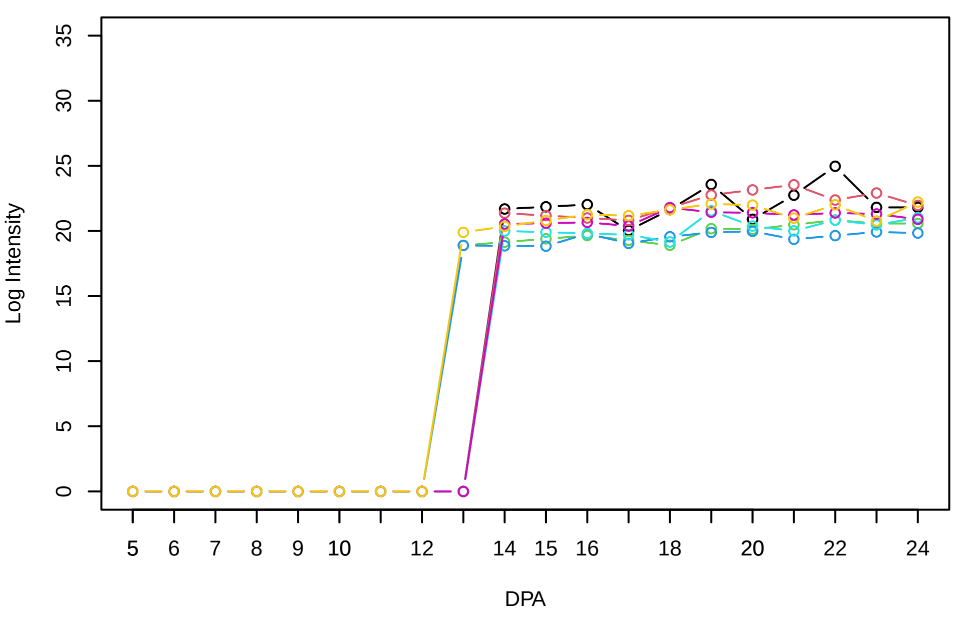

Histogram of the average abundances of high abundance proteins

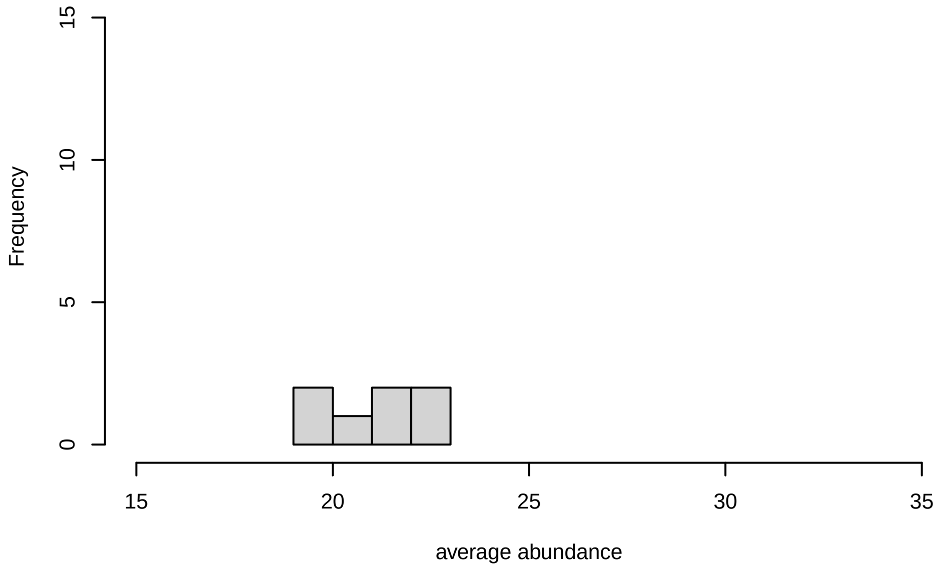

V33

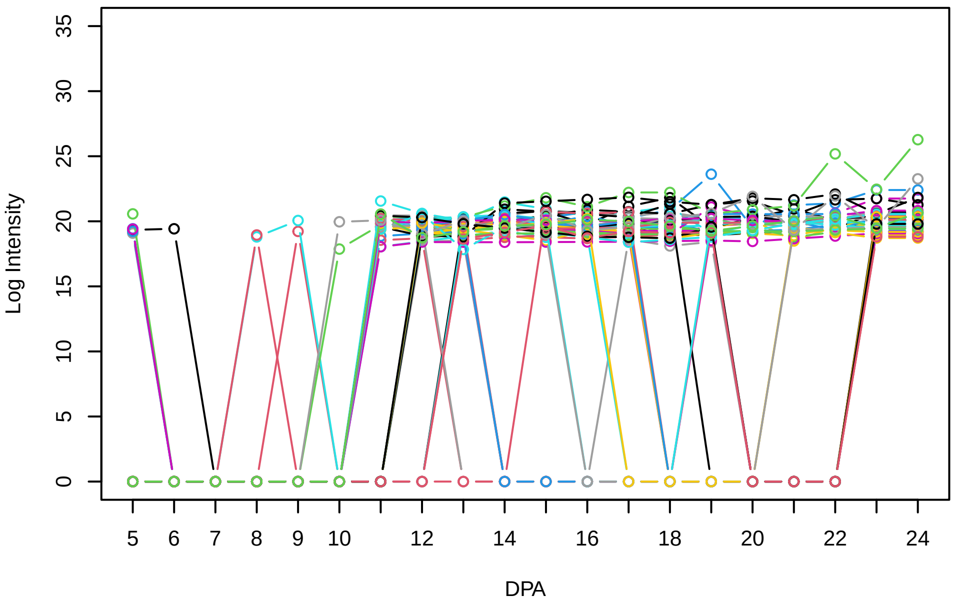

Histogram of the average abundances of non−zero values

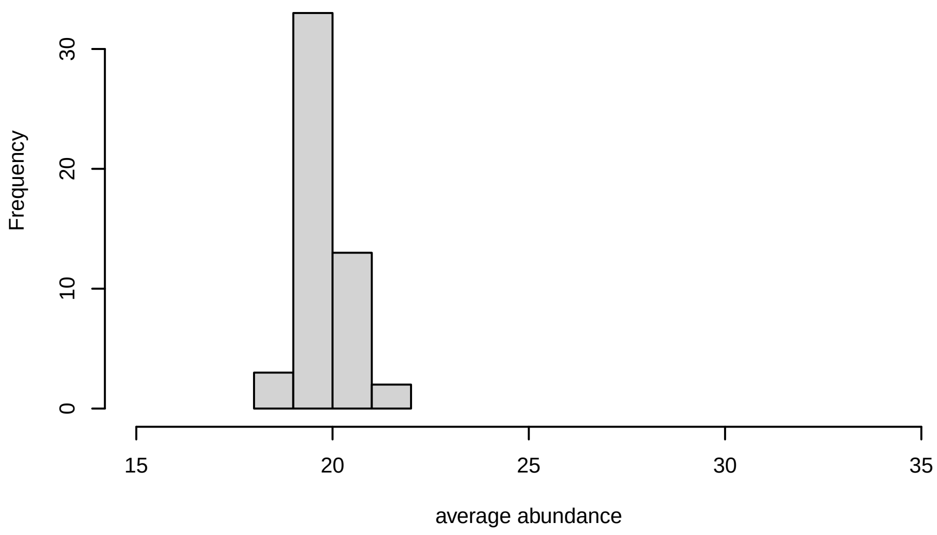

V33, after remove outlier and cutoff is 0.8

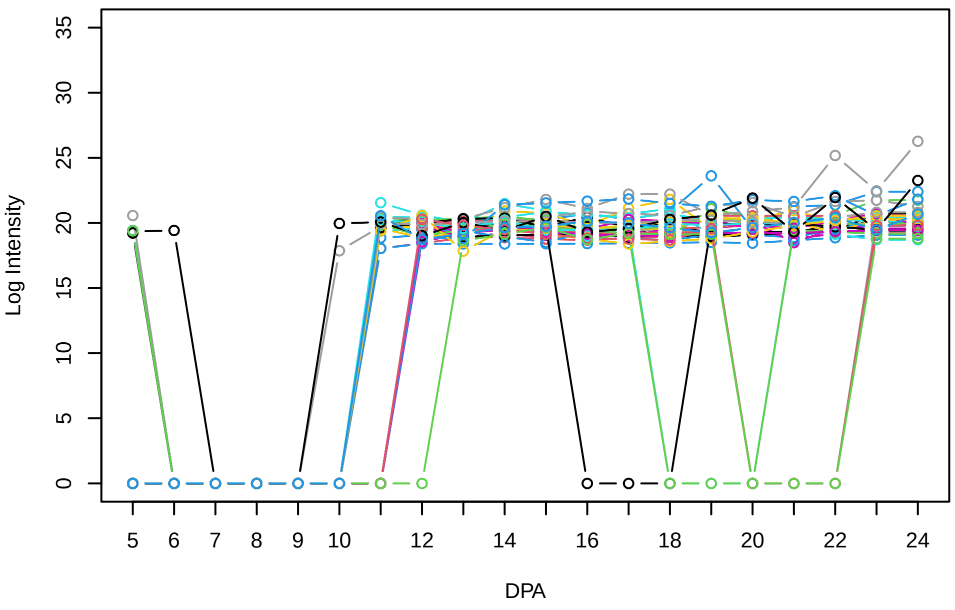

Histogram of the average abundances of non−outlier proteins

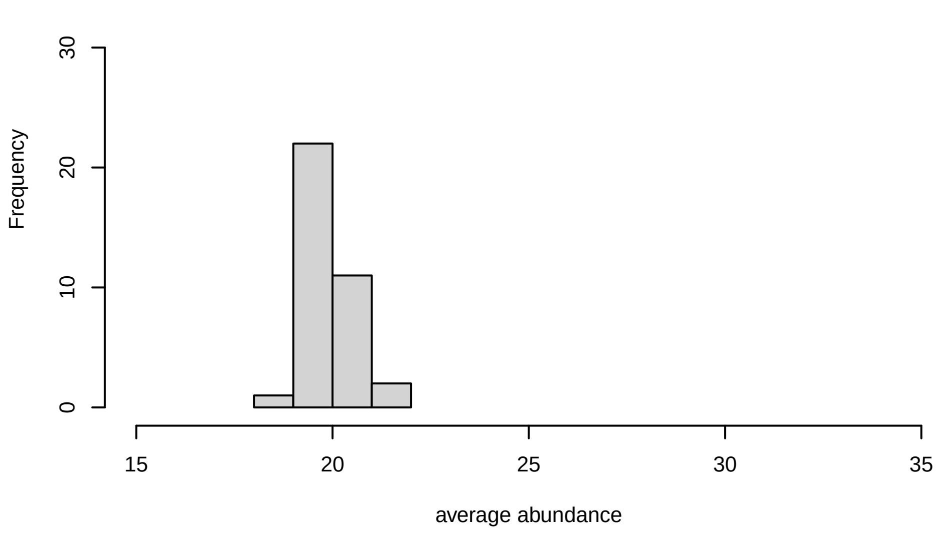

V33, after remove unreliable

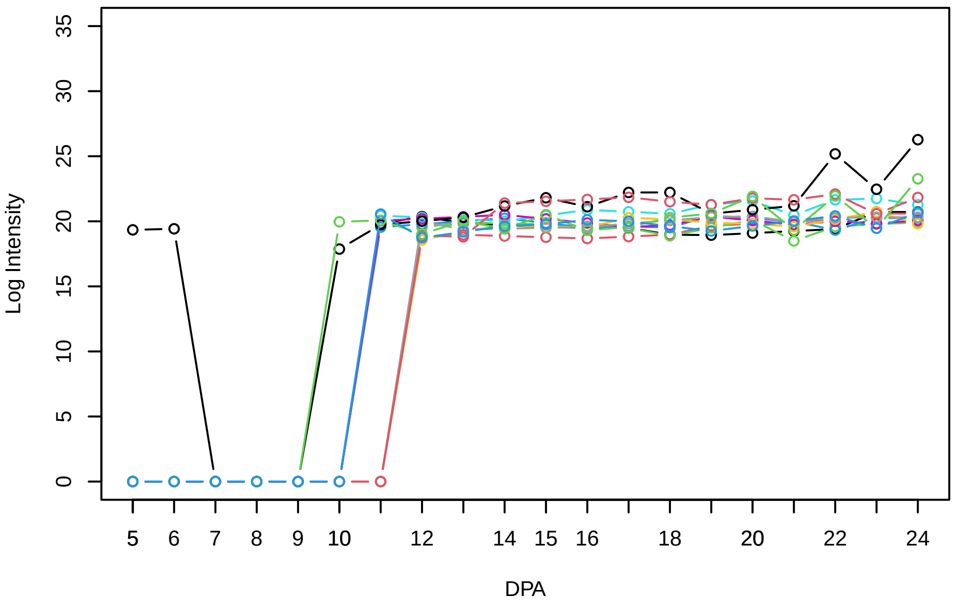

Histogram of the average abundances of reliable proteins

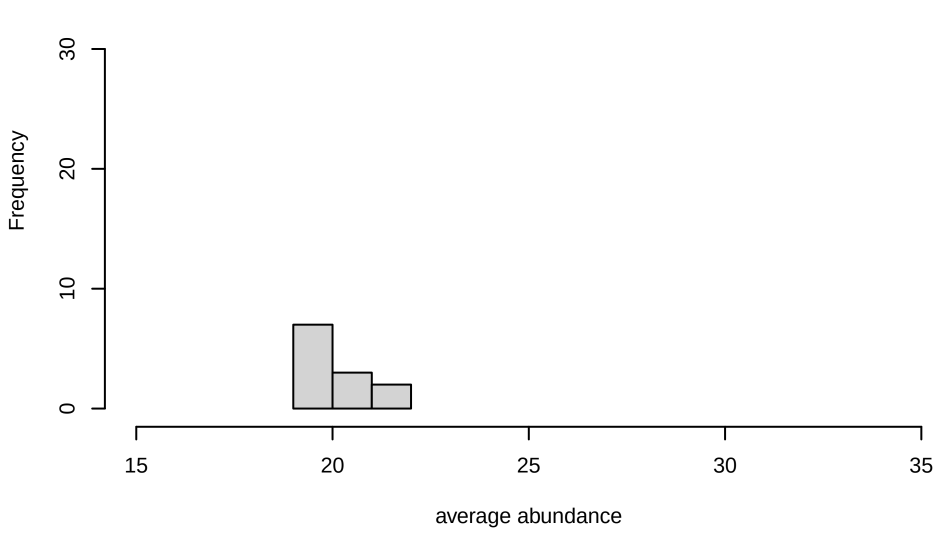

V33, after remove low abundance

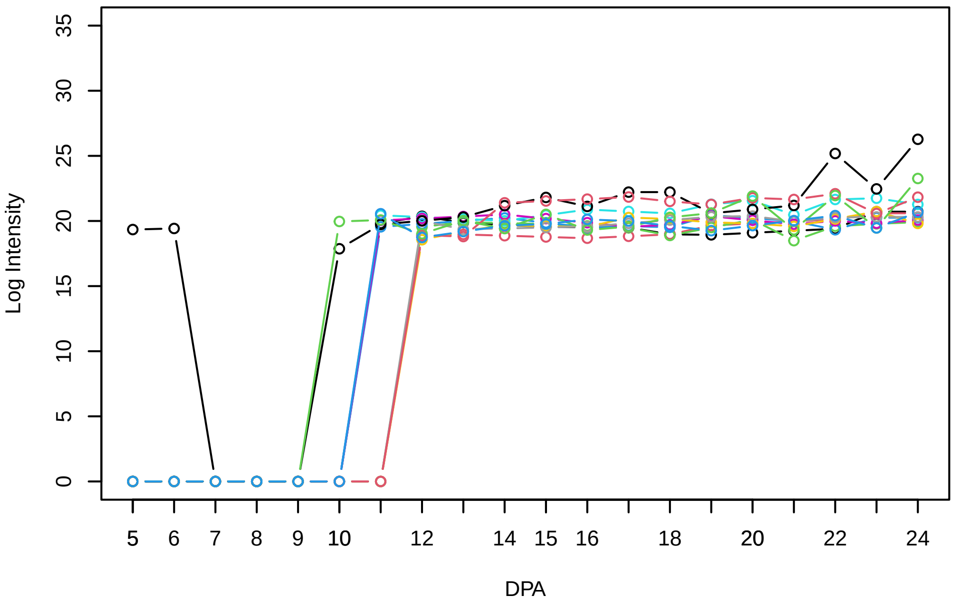

Histogram of the average abundances of high abundance proteins

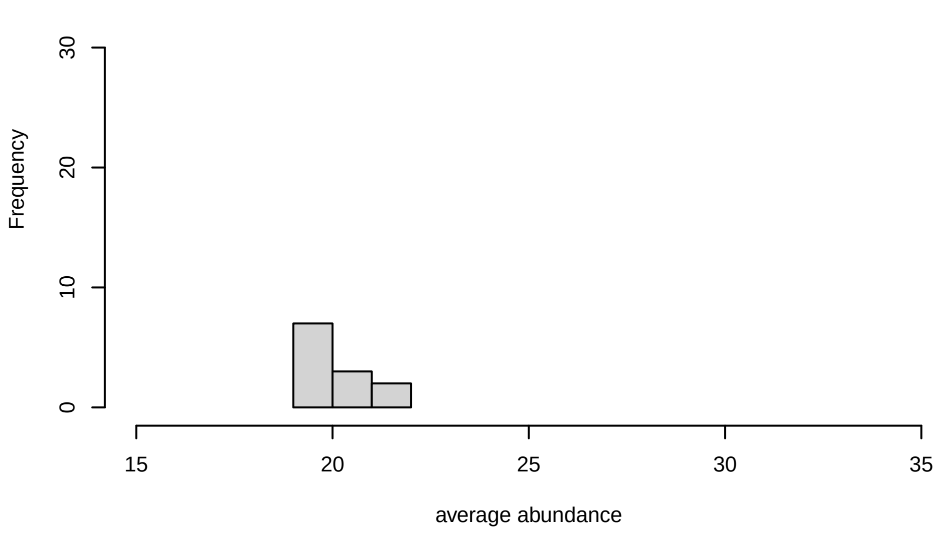

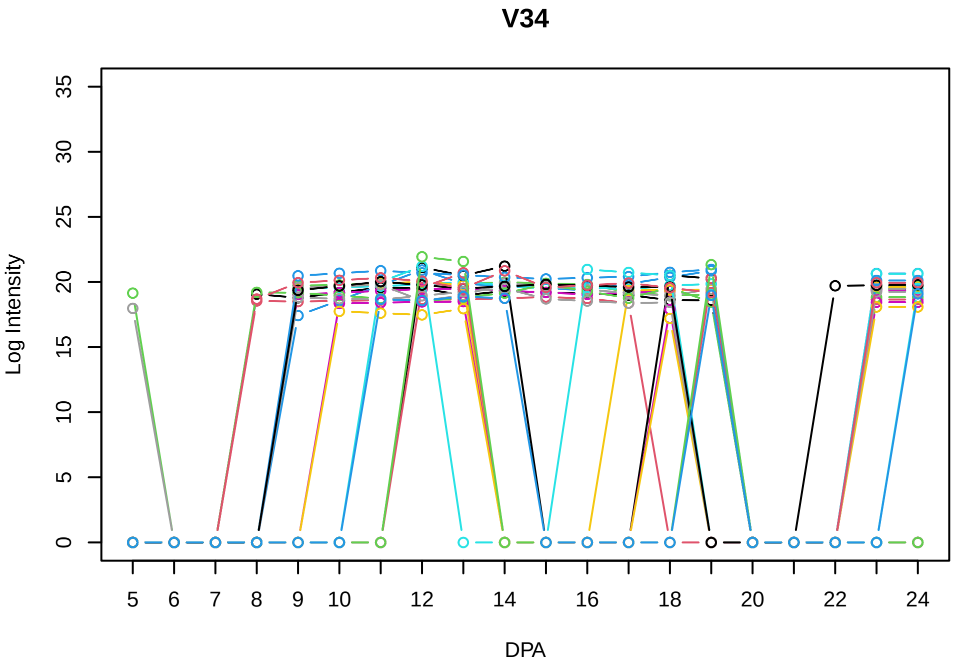
V34
Log Intensity
0
5
10
15
20
25
30
35
5
6
7
8
9
10
12
14
16
18
20
22
24
DPA

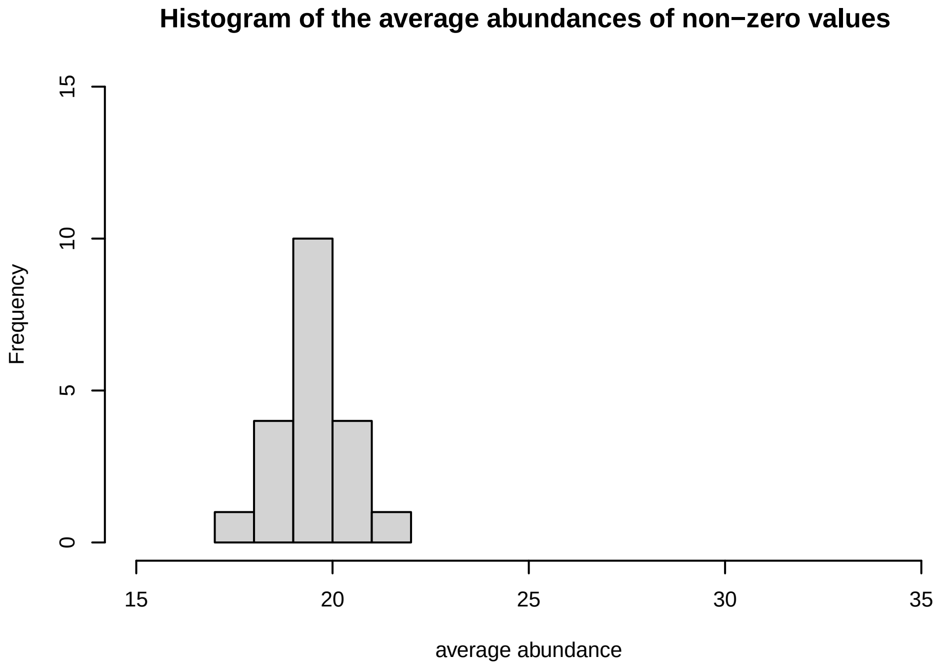
Histogram of the average abundances of non−zero values
Frequency
0
5
10
15
15
20
25
30
35
average abundance

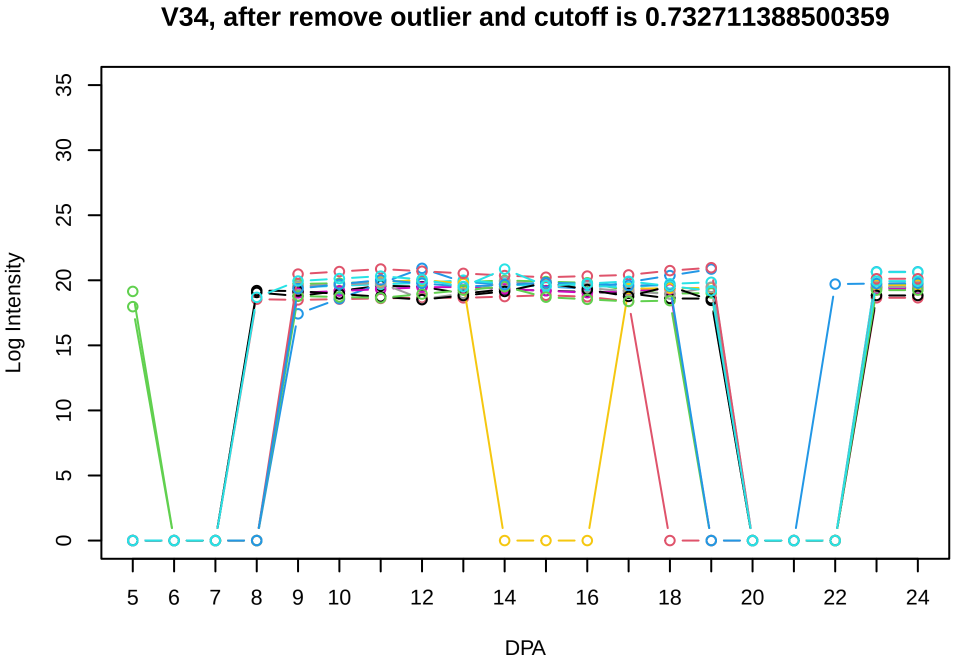
V34, after remove outlier and cutoff is 0.732711388500359
Log Intensity
0
5
10
15
20
25
30
35
5
6
7
8
9
10
12
14
16
18
20
22
24
DPA

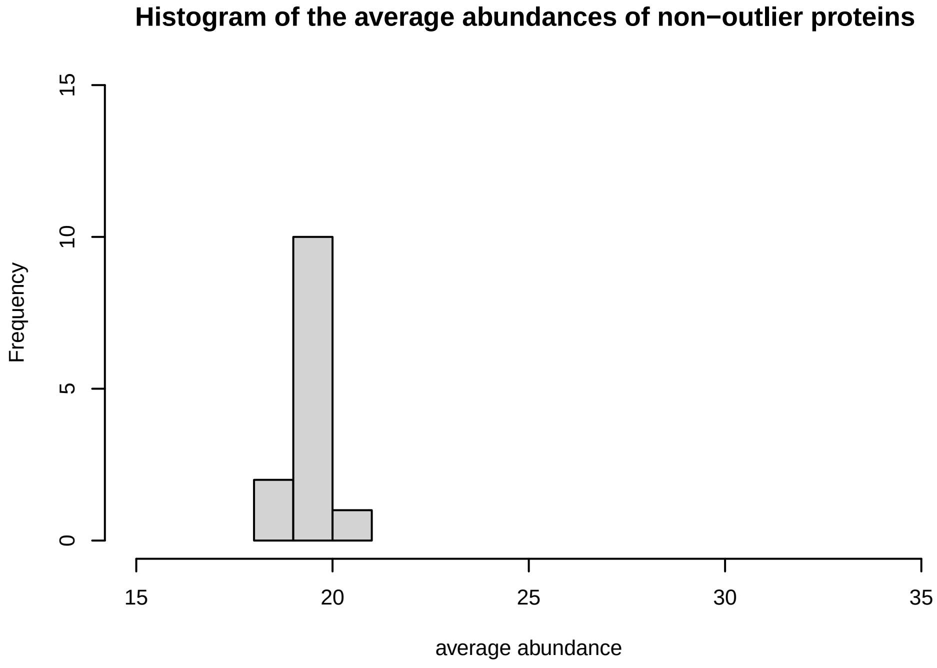
Histogram of the average abundances of non−outlier proteins
Frequency
0
5
10
15
15
20
25
30
35
average abundance

V35

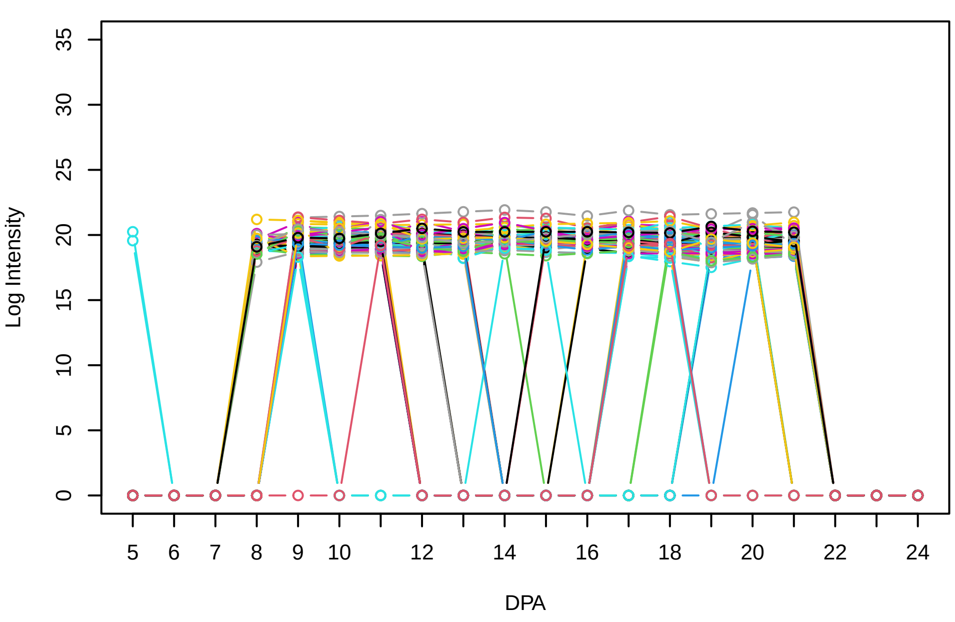

Histogram of the average abundances of non−zero values

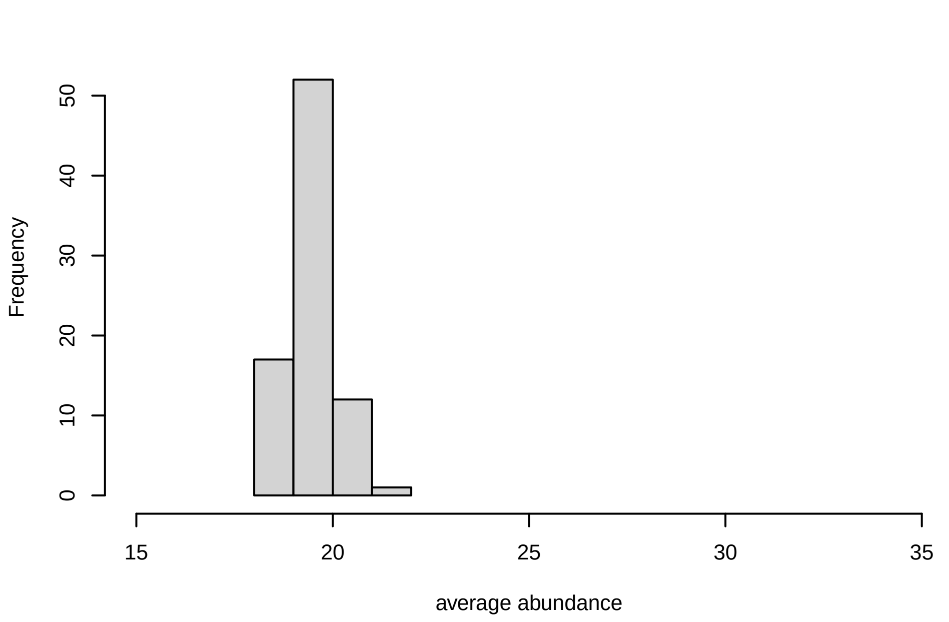

V35, after remove outlier and cutoff is 0.8

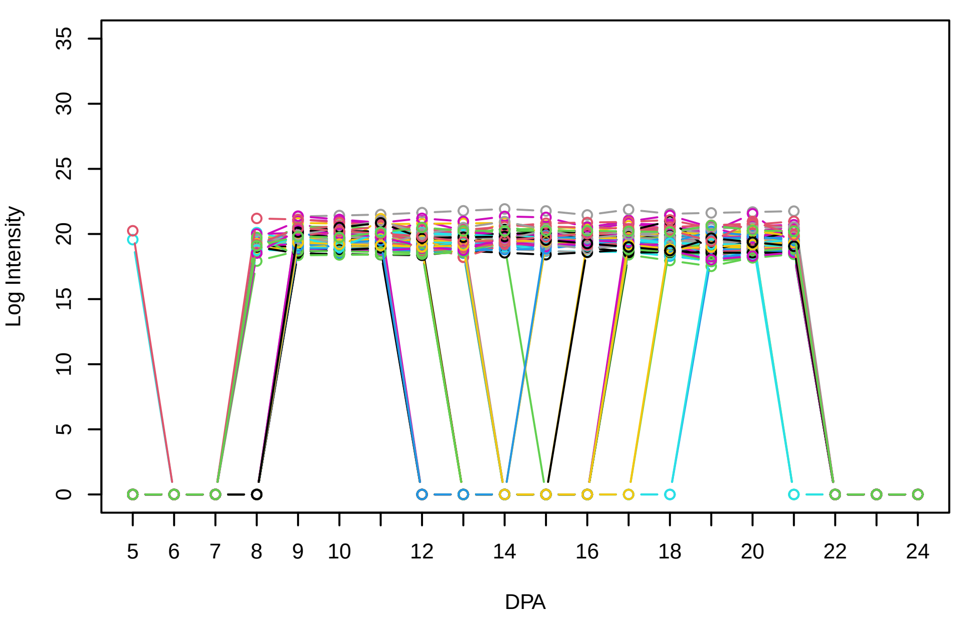

Histogram of the average abundances of non−outlier proteins

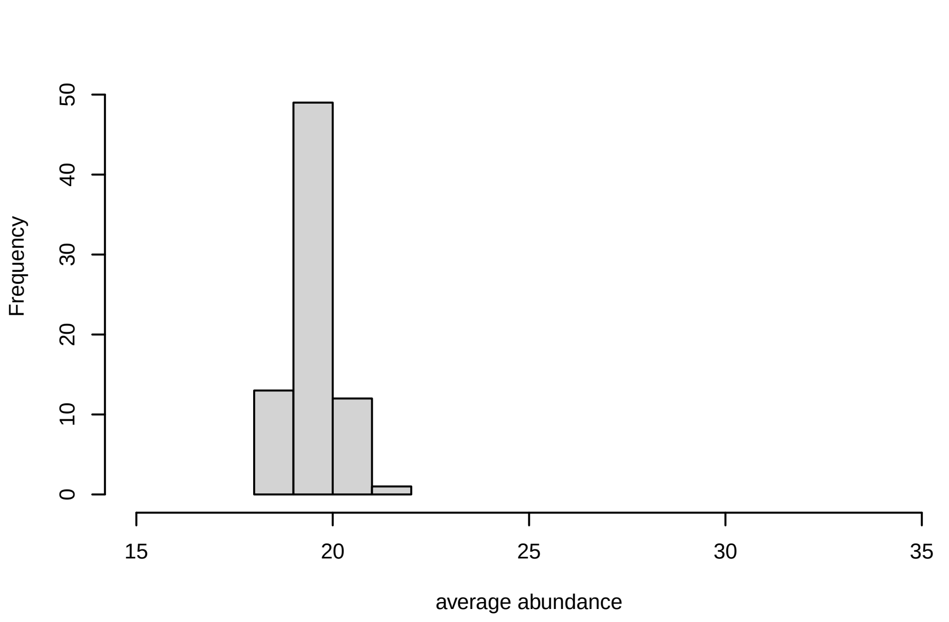

V35, after remove unreliable

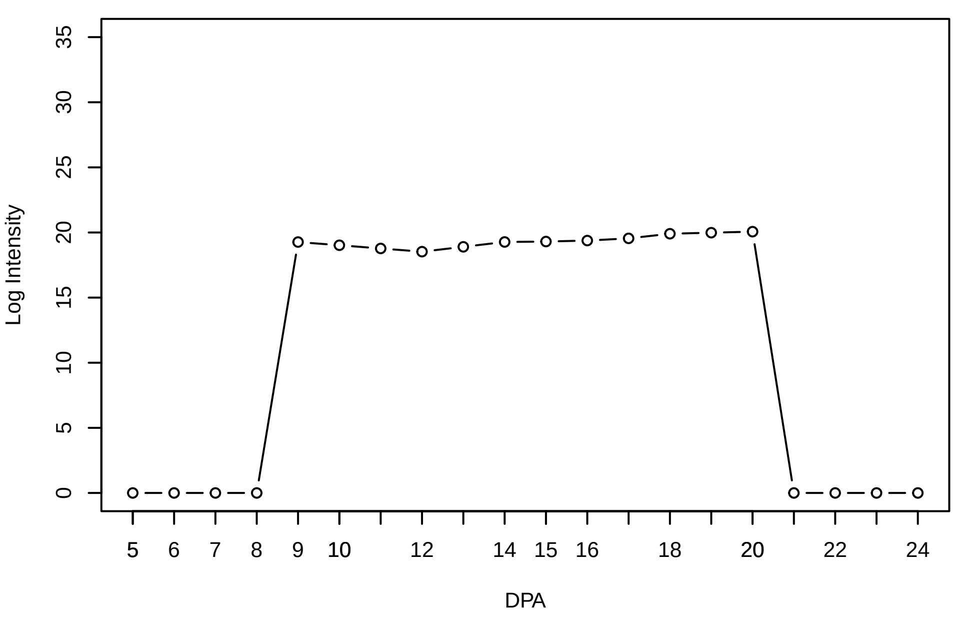

Histogram of the average abundances of reliable proteins

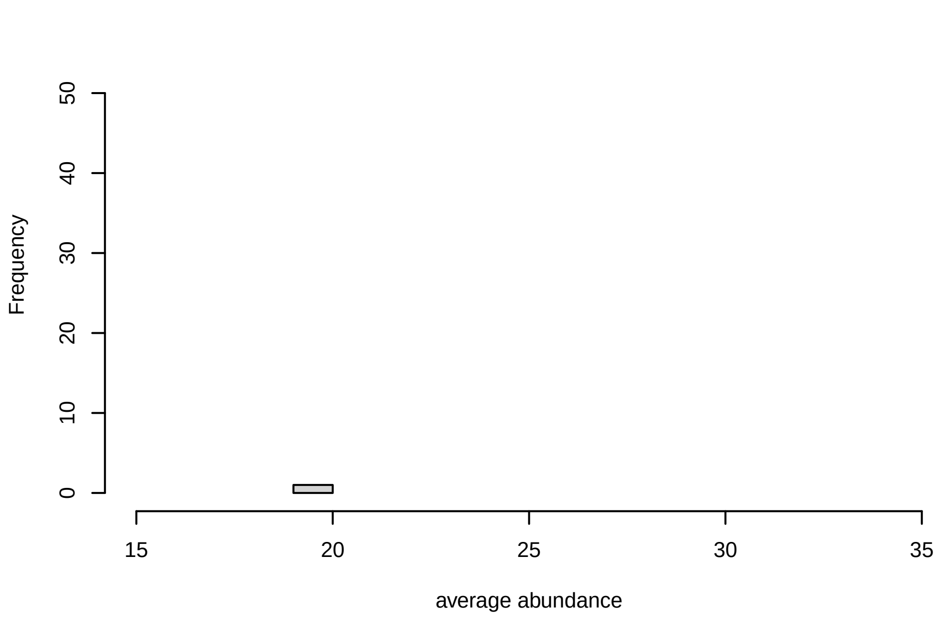

V35, after remove low abundance

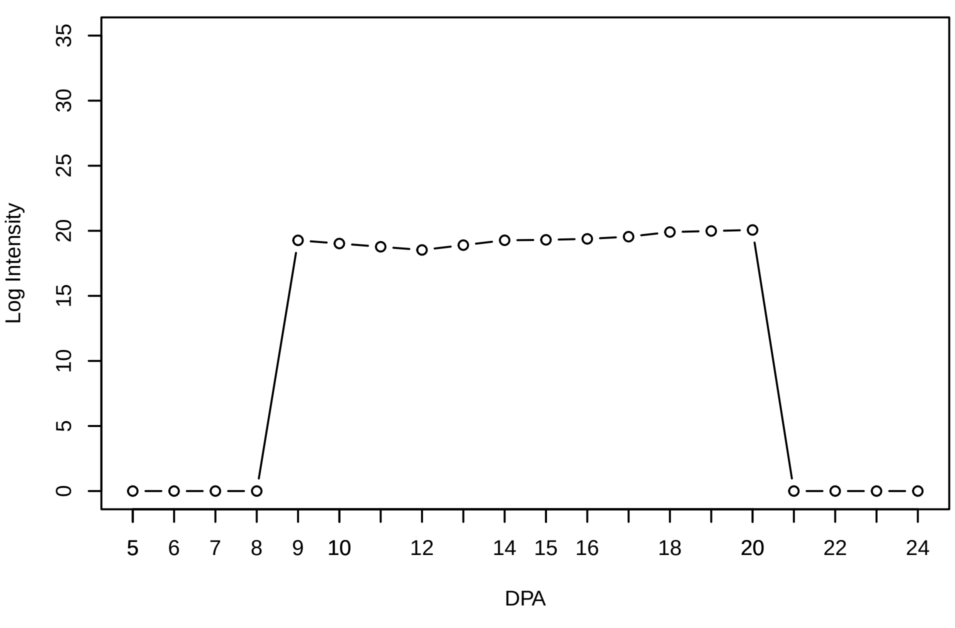

Histogram of the average abundances of high abundance proteins

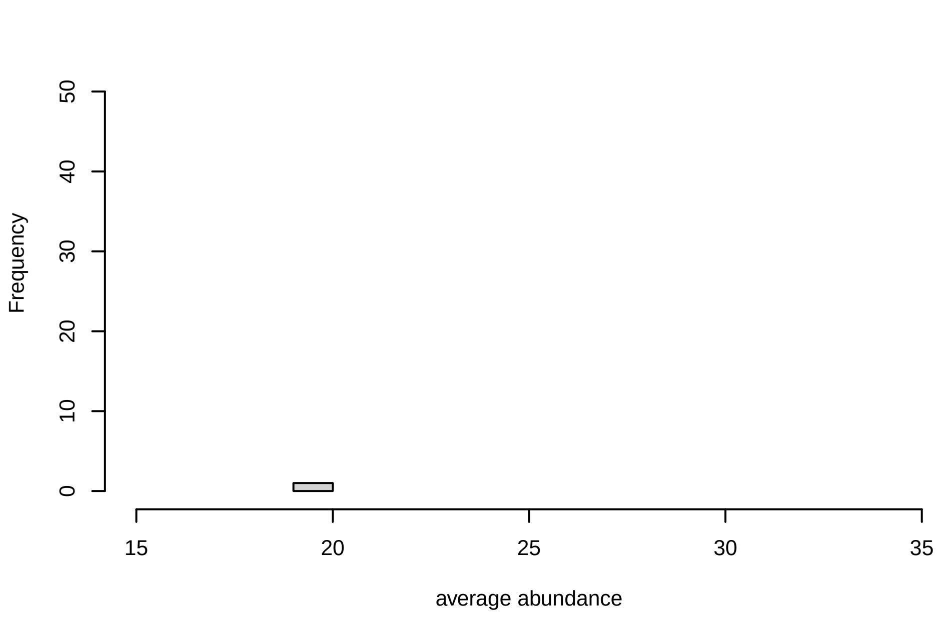

V36

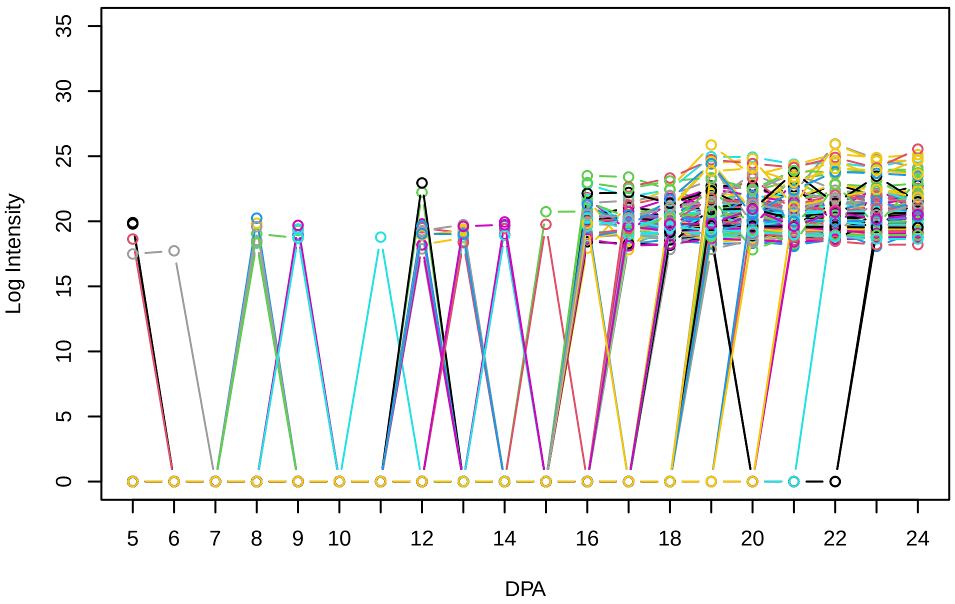

Histogram of the average abundances of non−zero values

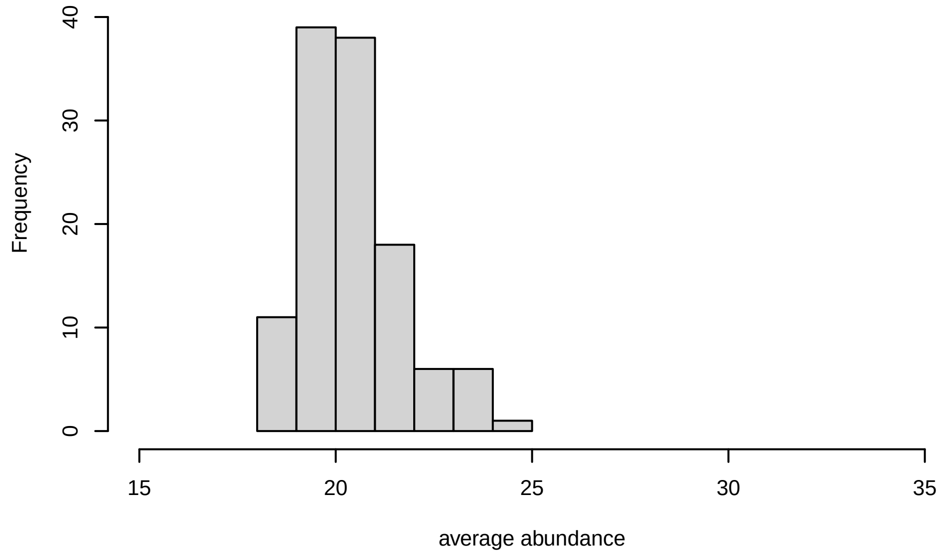

V36, after remove outlier and cutoff is 0.8

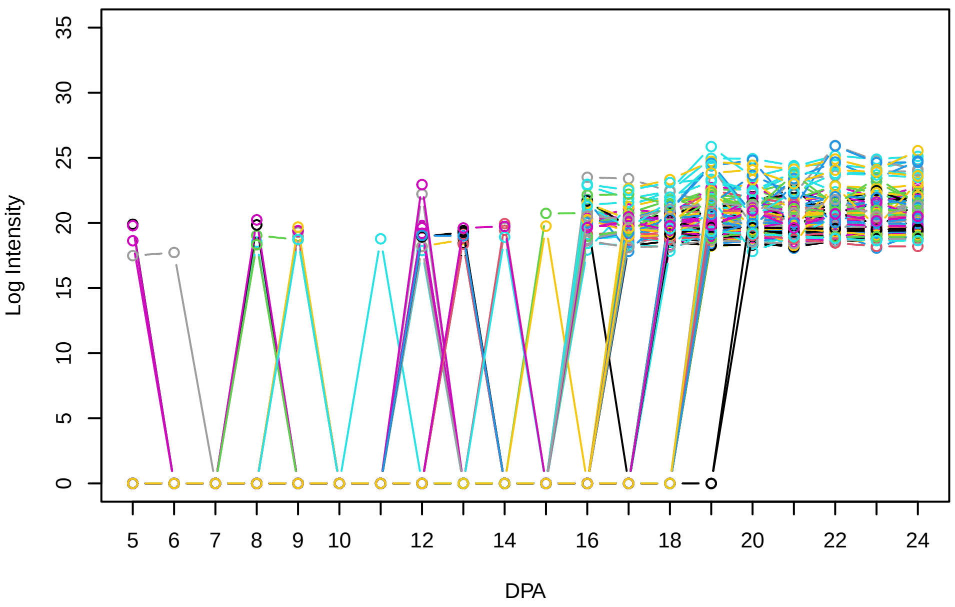

Histogram of the average abundances of non−outlier proteins

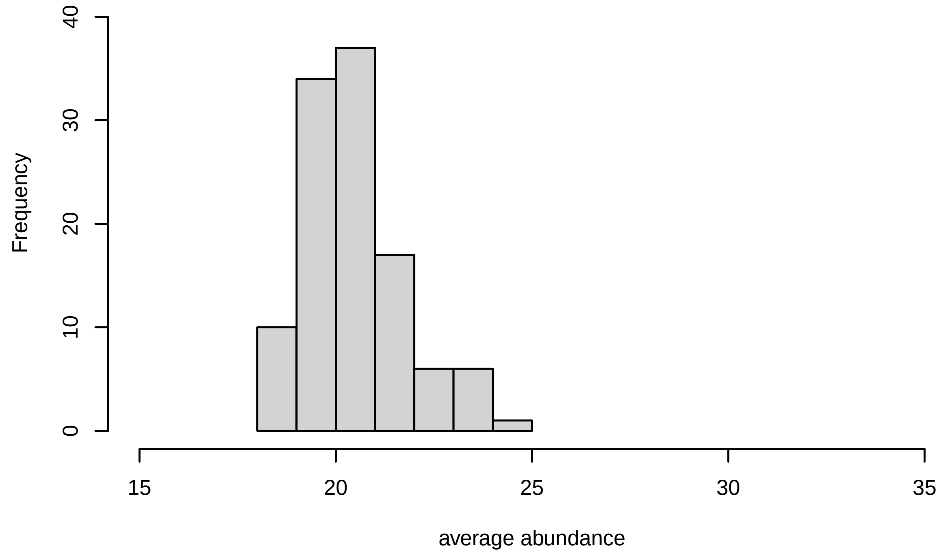

V36, after remove unreliable

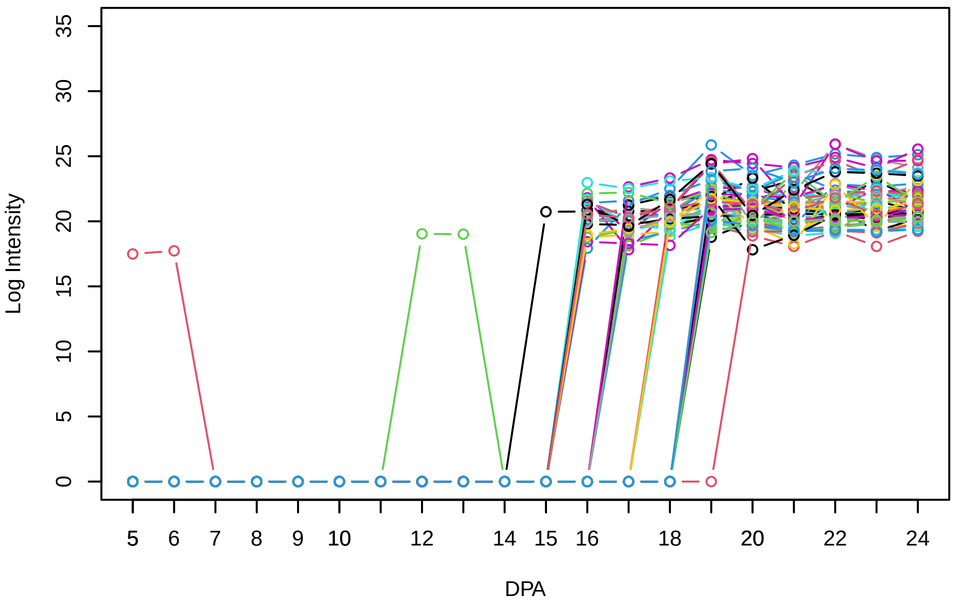

Histogram of the average abundances of reliable proteins

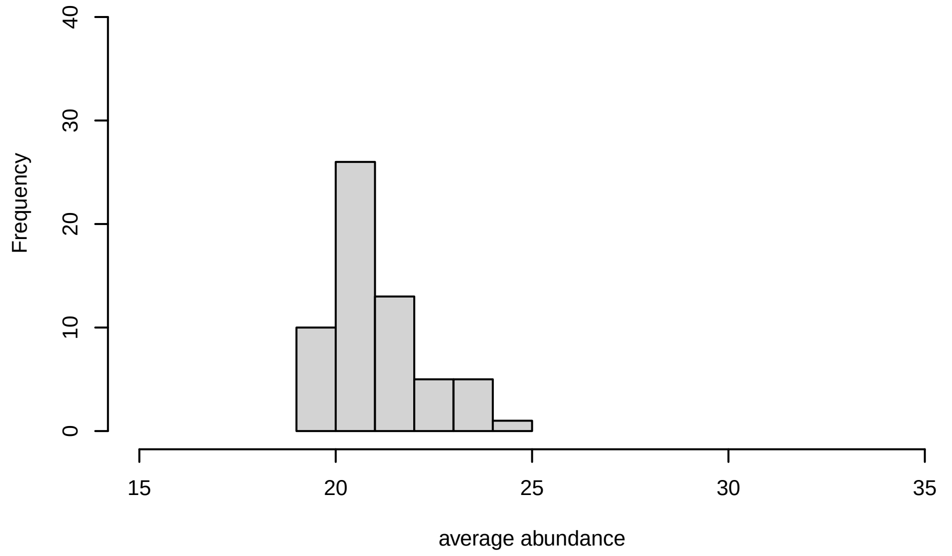

V36, after remove low abundance

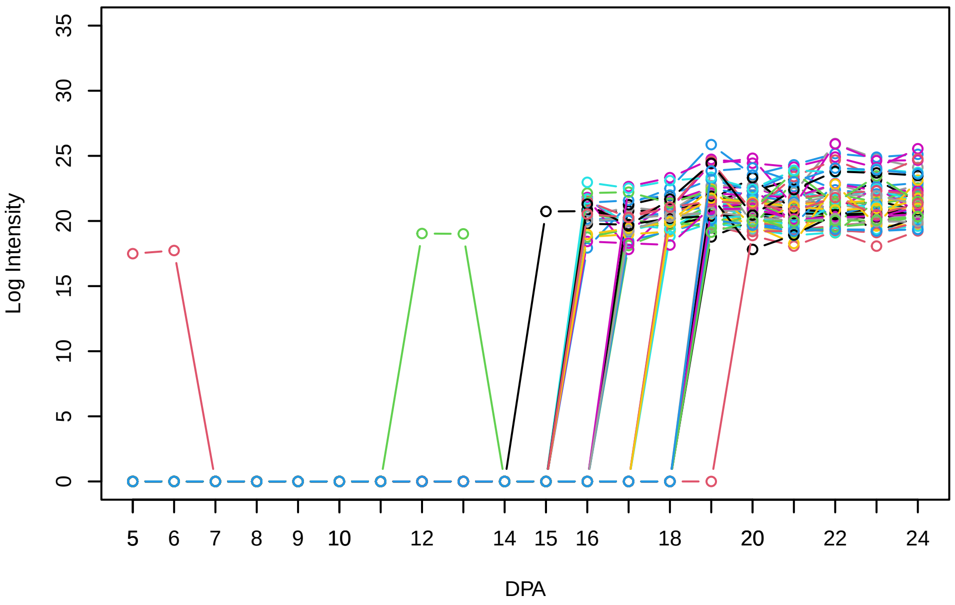

Histogram of the average abundances of high abundance proteins

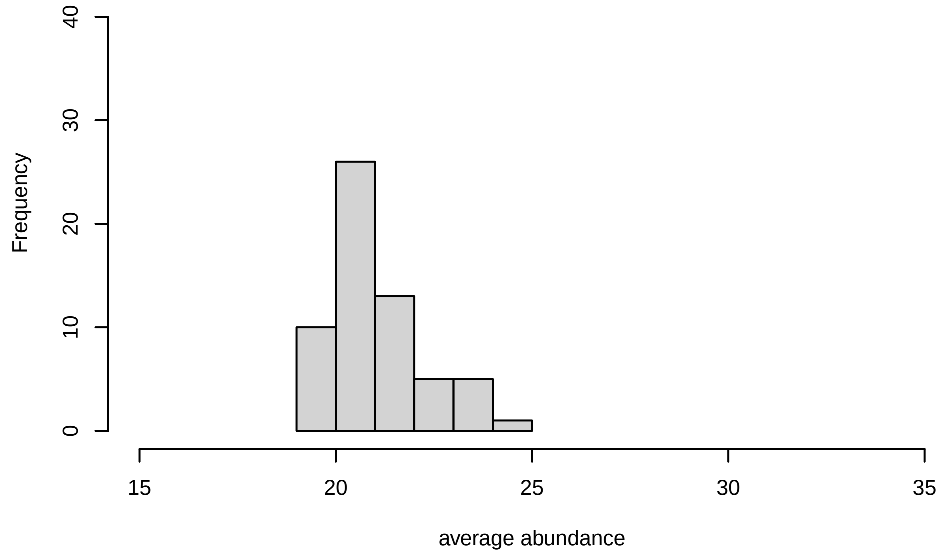

V37

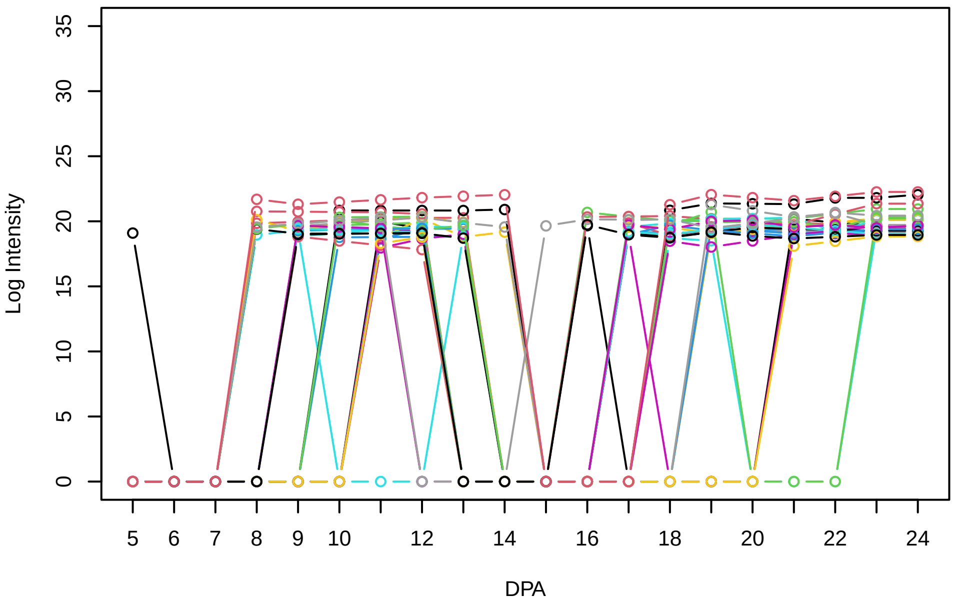

Histogram of the average abundances of non−zero values

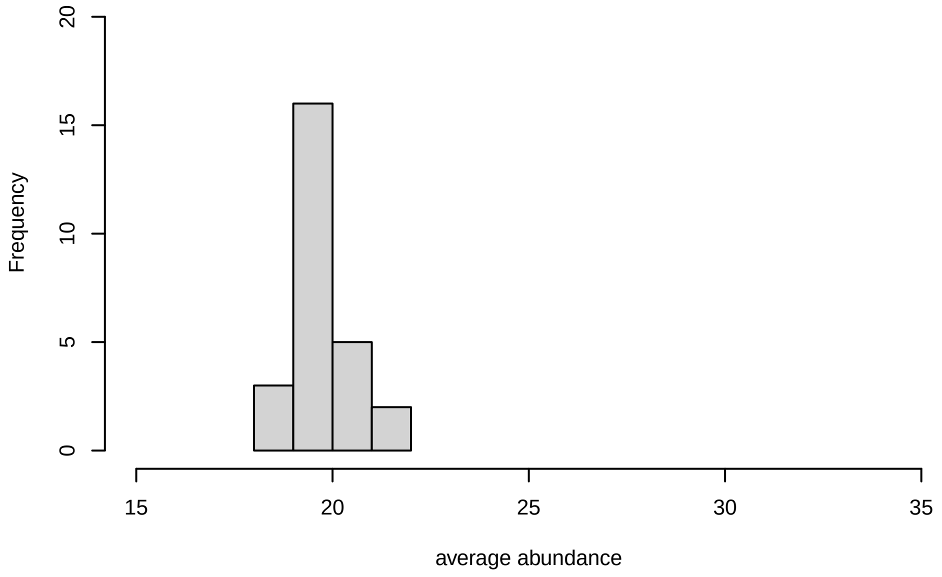

V37, after remove outlier and cutoff is 0.8

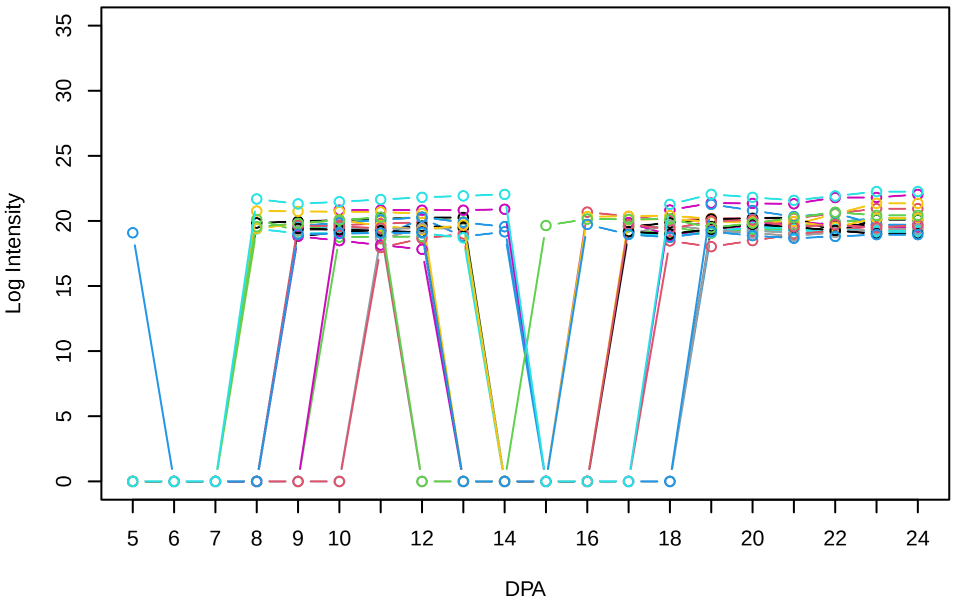

Histogram of the average abundances of non−outlier proteins

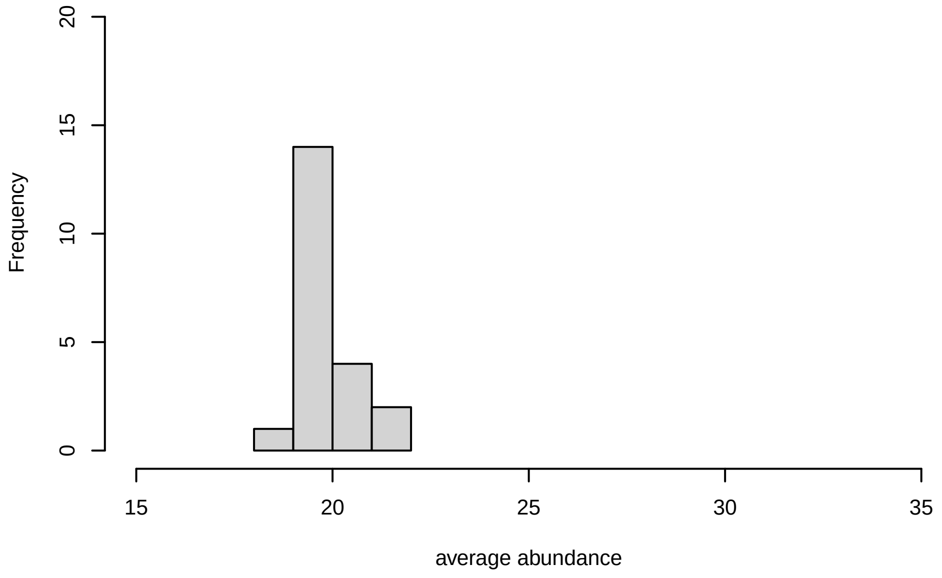

V37, after remove unreliable

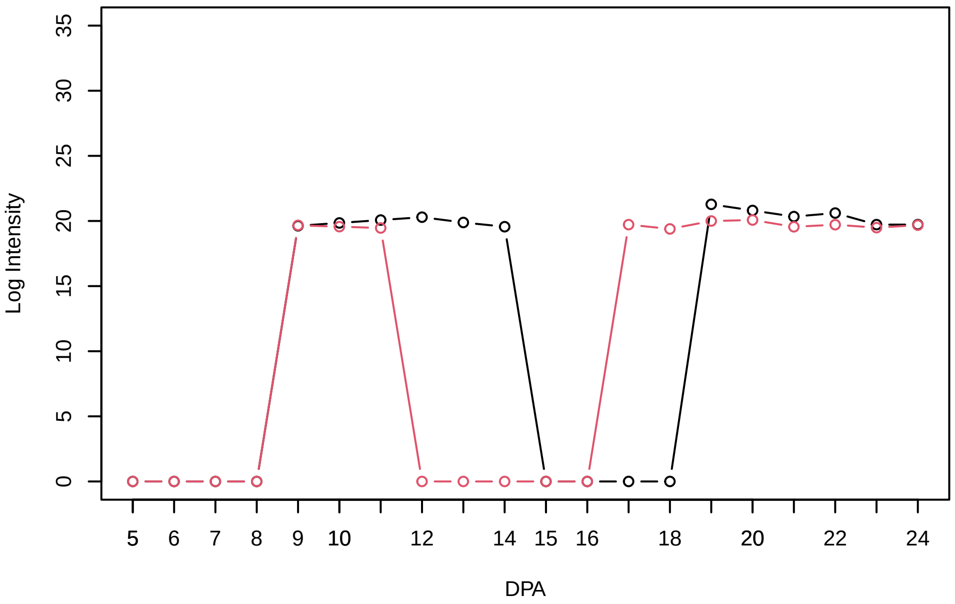

Histogram of the average abundances of reliable proteins

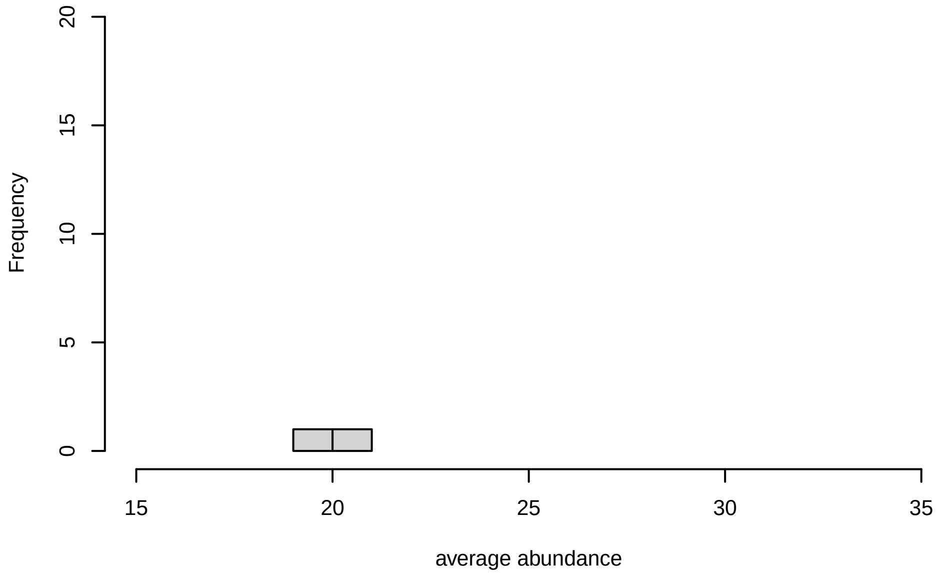

V37, after remove low abundance

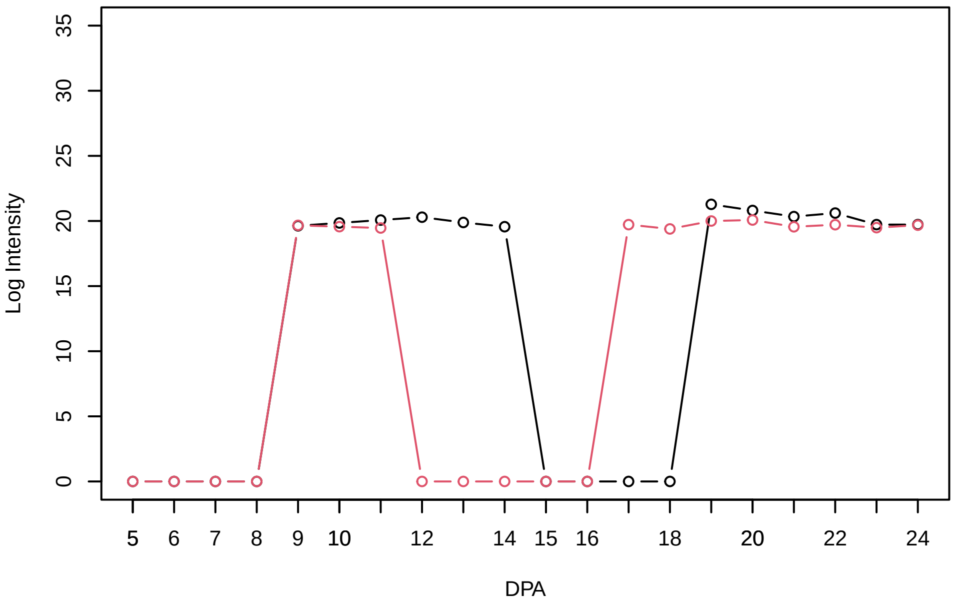

Histogram of the average abundances of high abundance proteins

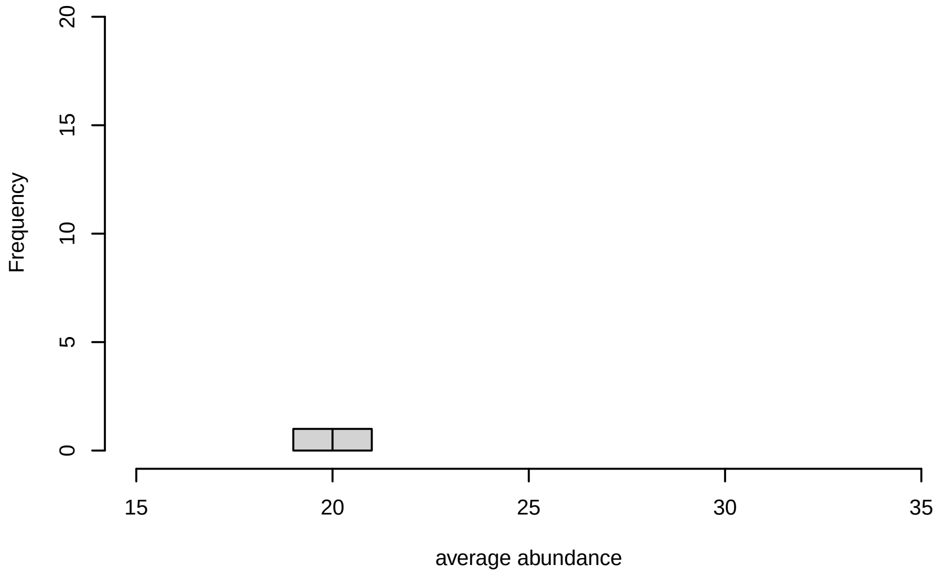

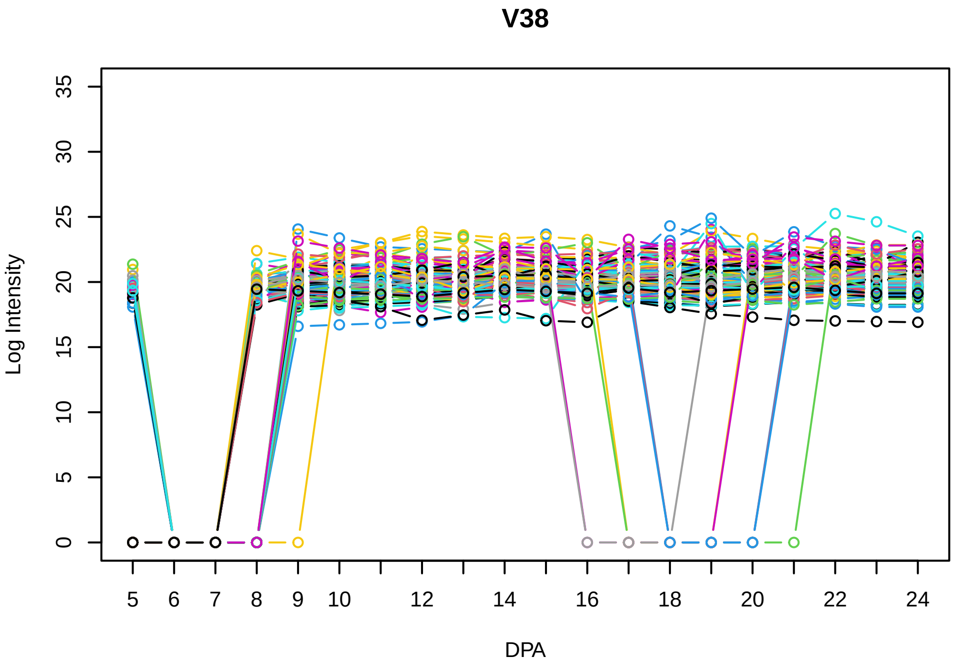
V38
Log Intensity
DPA

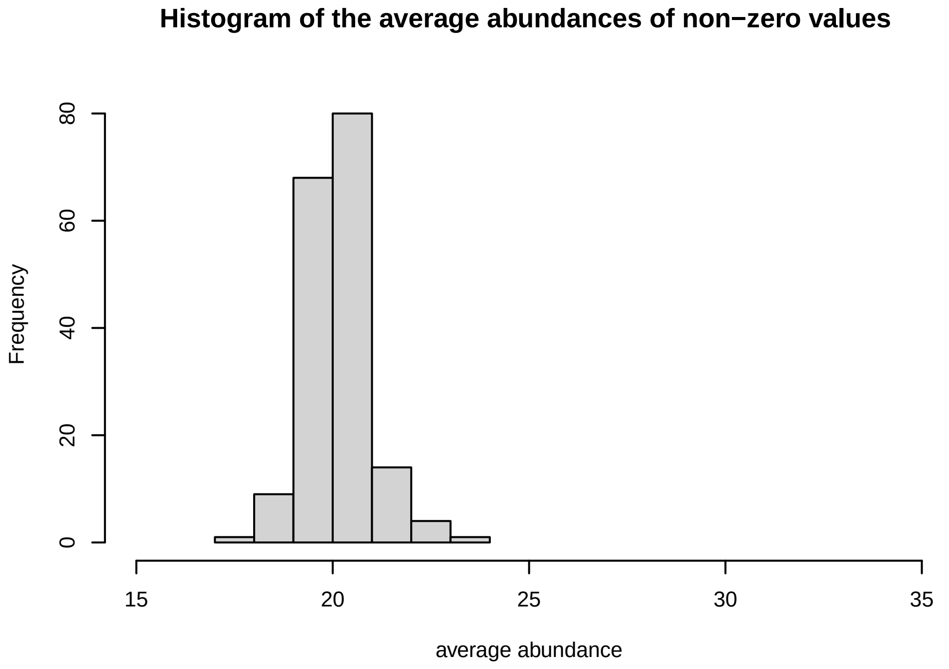
Histogram of the average abundances of non−zero values
Frequency
average abundance

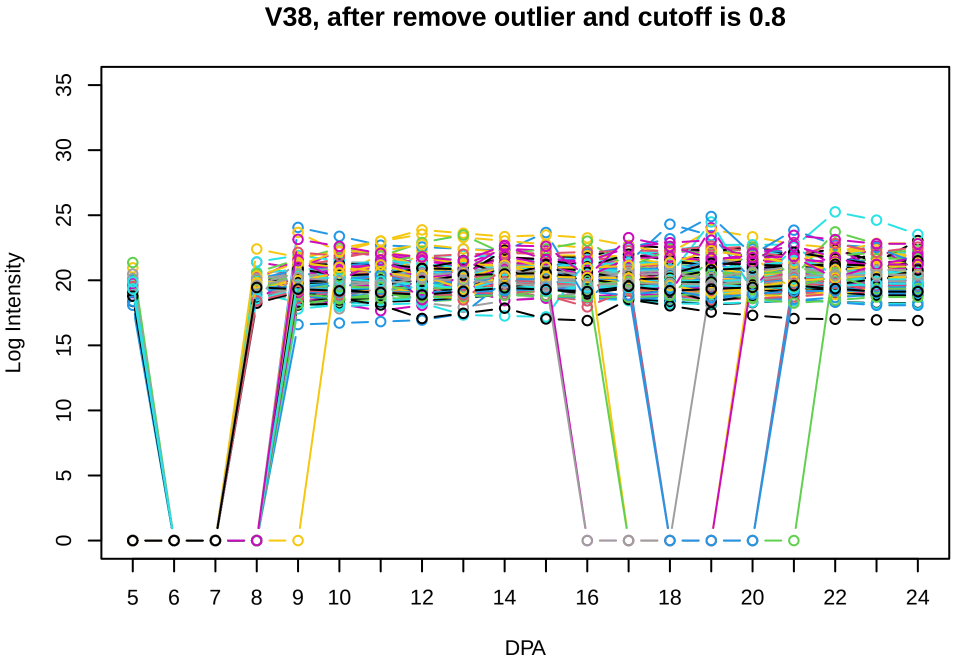
V38, after remove outlier and cutoff is 0.8
Log Intensity
DPA

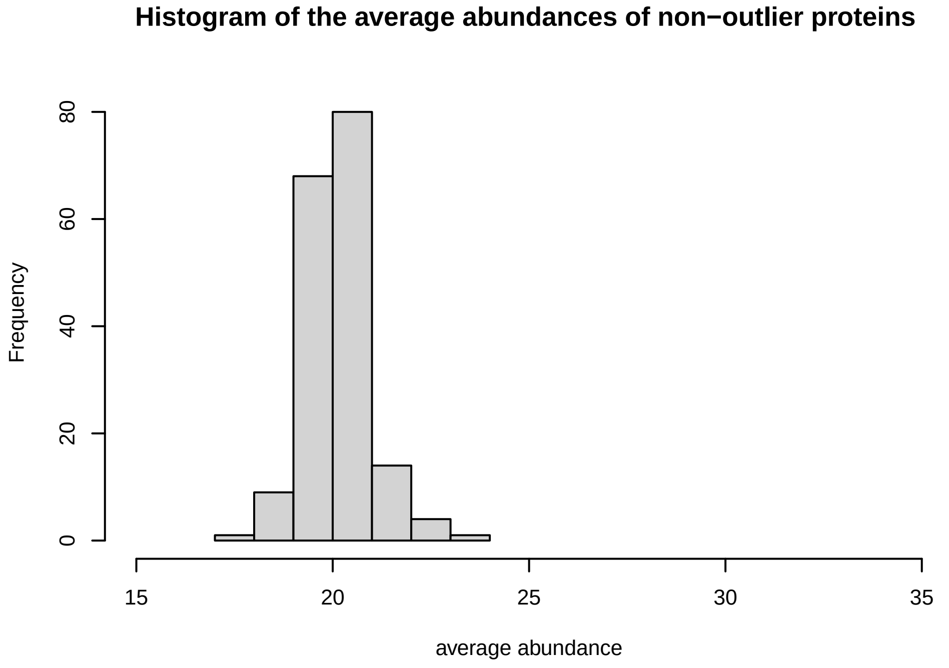
Histogram of the average abundances of non−outlier proteins
Frequency
average abundance

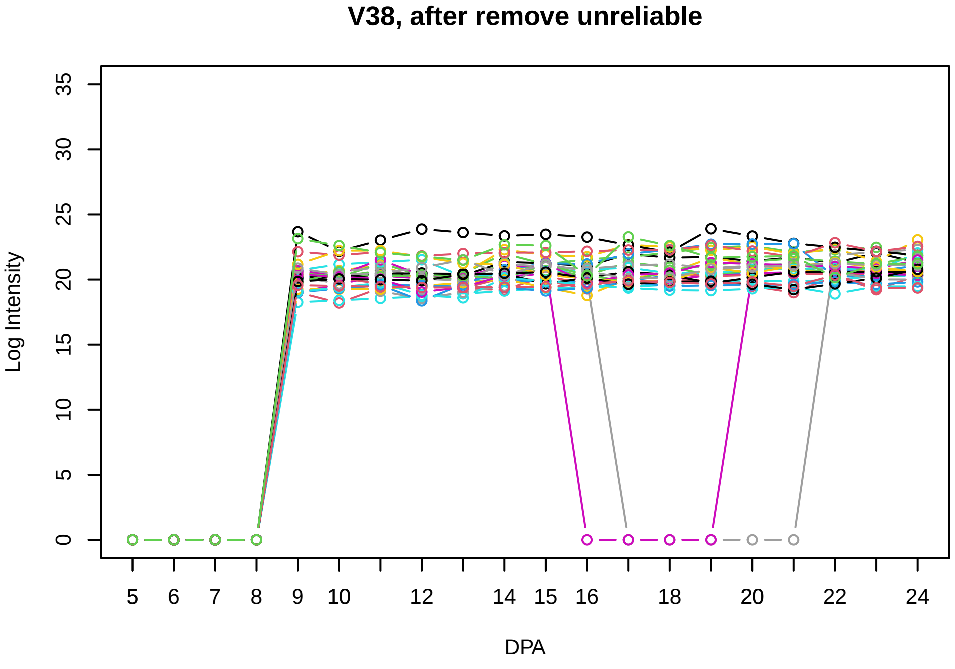
V38, after remove unreliable
Log Intensity
DPA

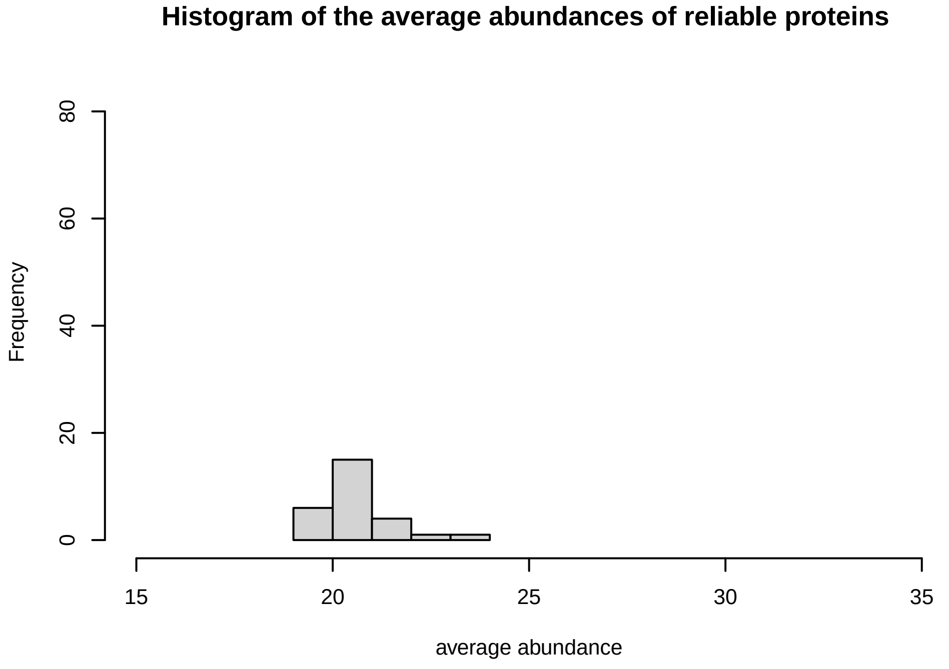
Histogram of the average abundances of reliable proteins
Frequency
average abundance

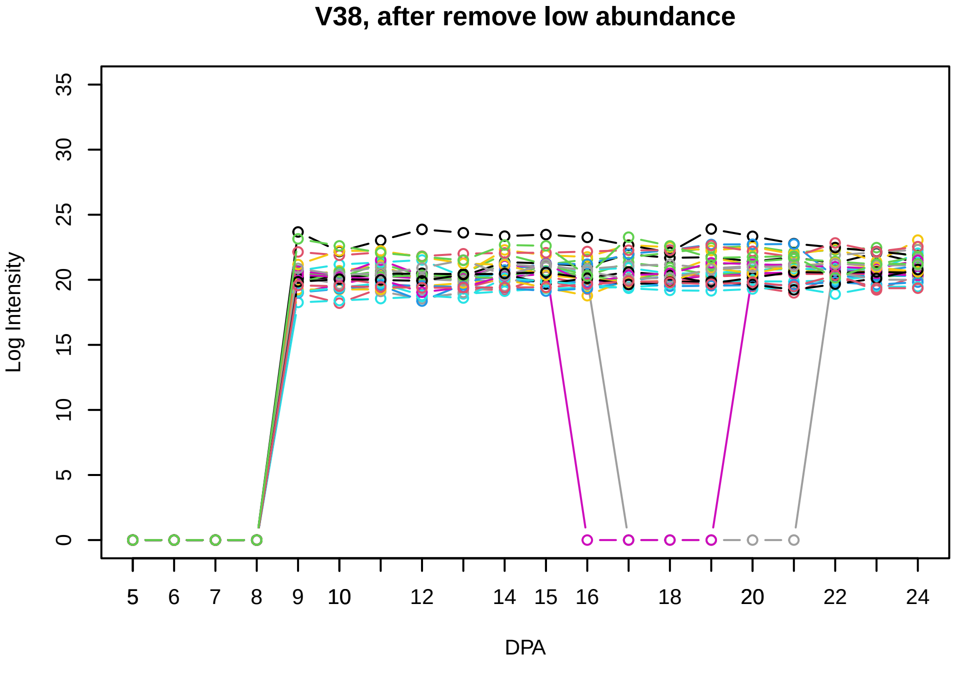
V38, after remove low abundance
Log Intensity
DPA

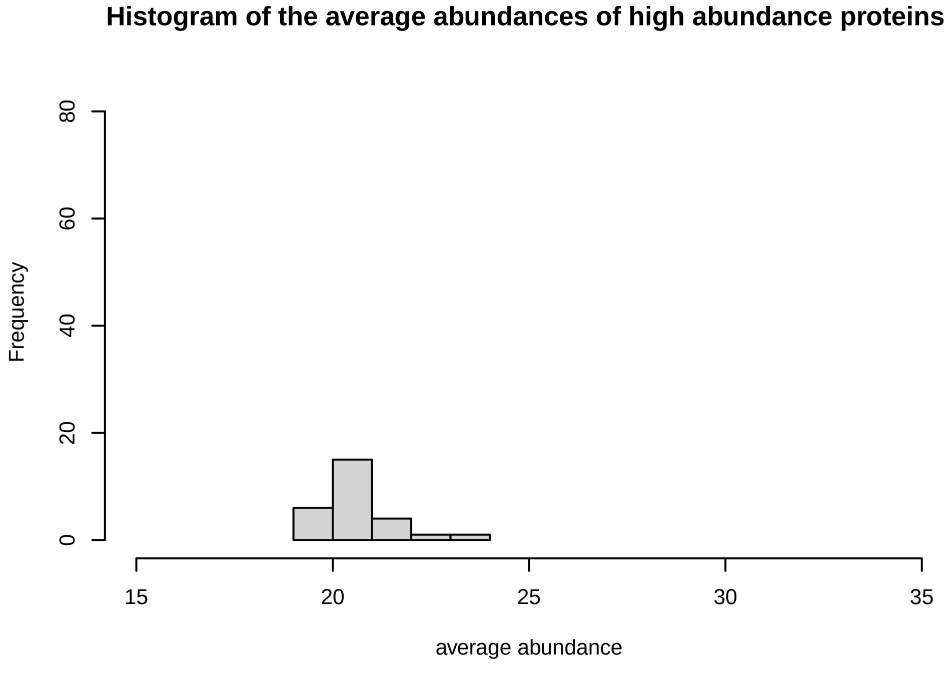
Histogram of the average abundances of high abundance proteins
Frequency
average abundance

V39

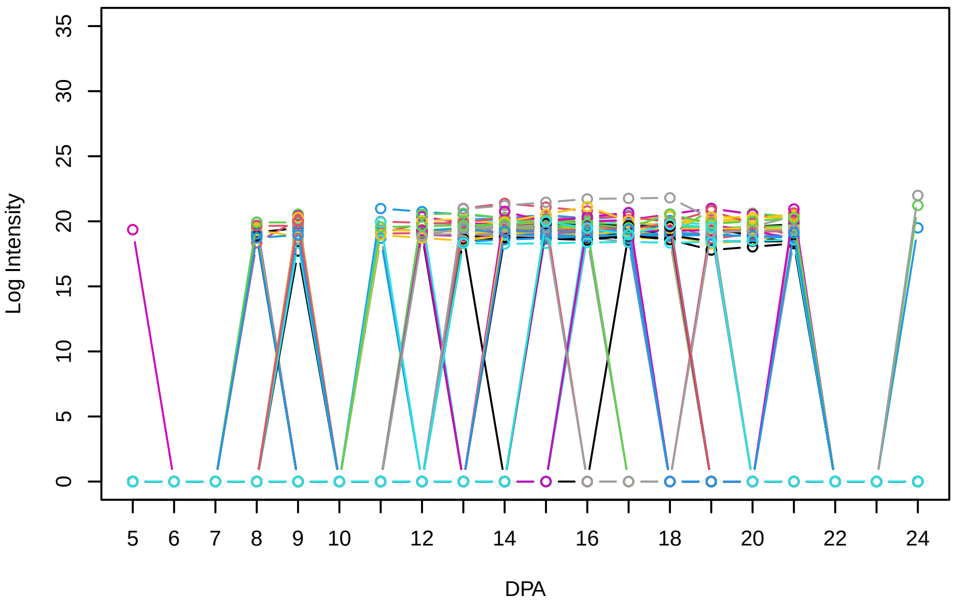

Histogram of the average abundances of non−zero values

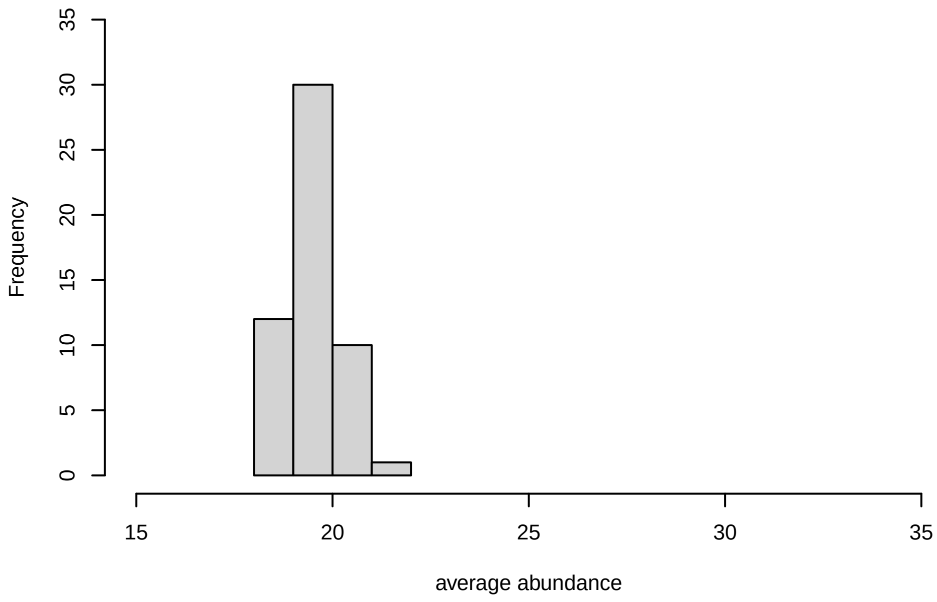

V39, after remove outlier and cutoff is 0.642679678568733

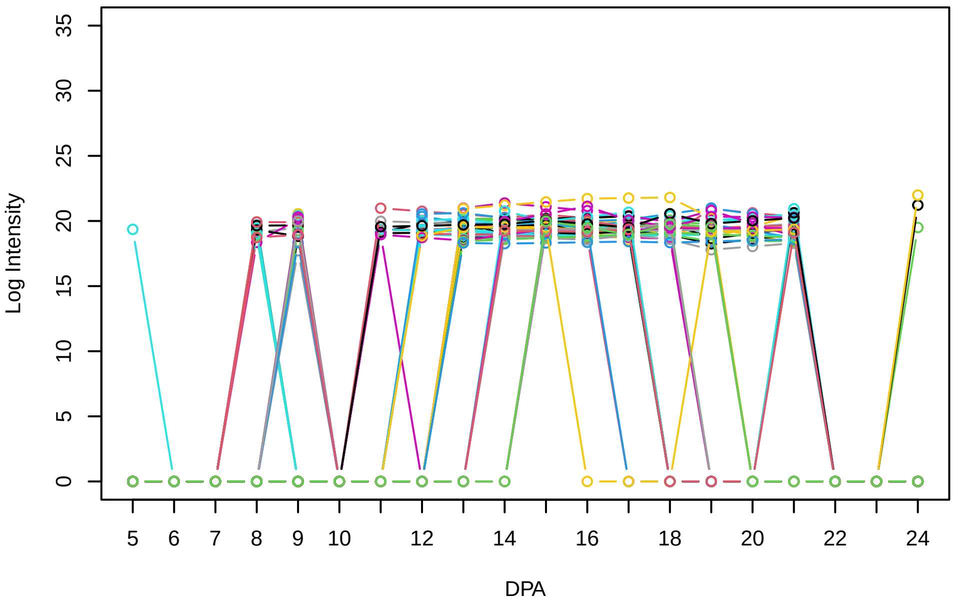

Histogram of the average abundances of non−outlier proteins

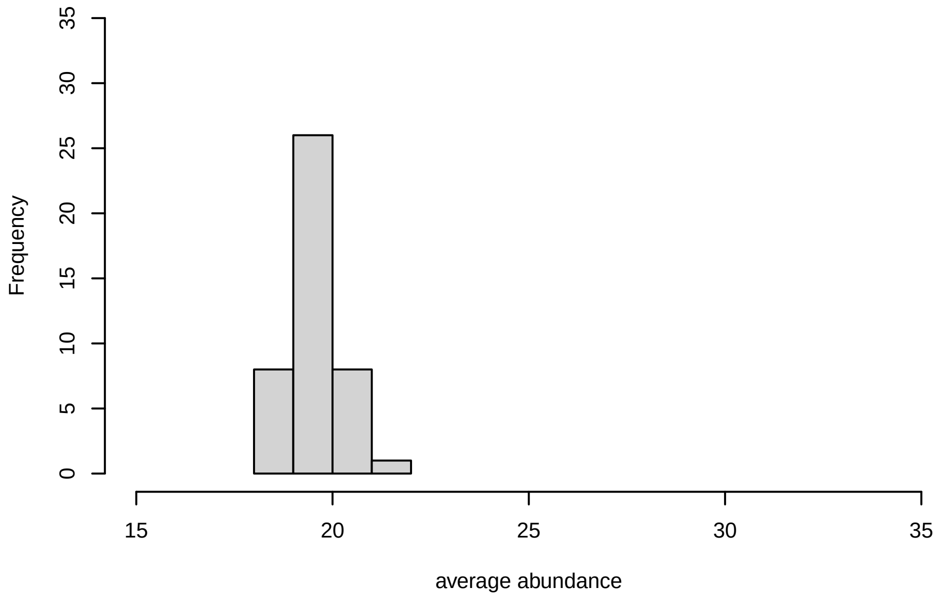

V39, after remove unreliable

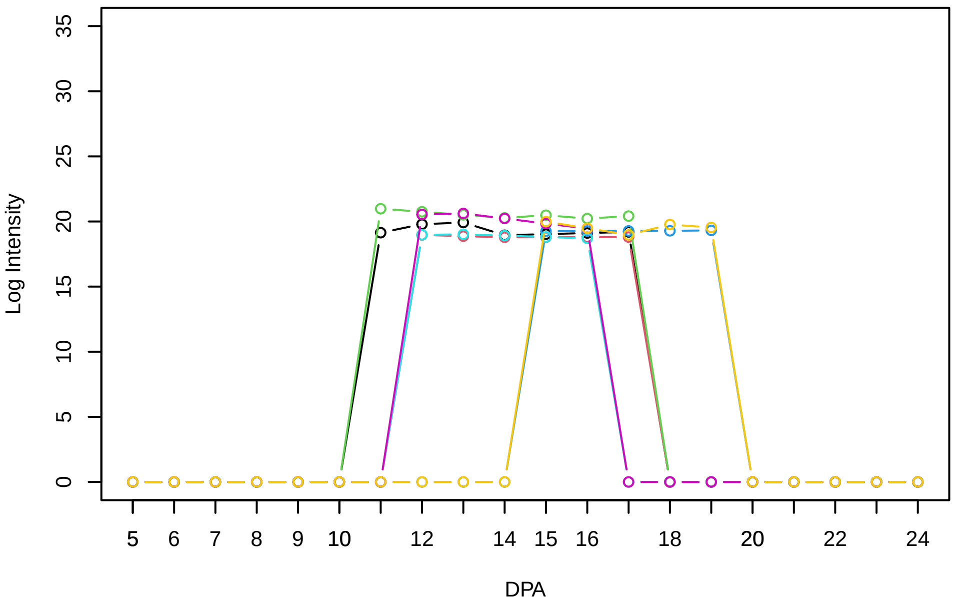

Histogram of the average abundances of reliable proteins

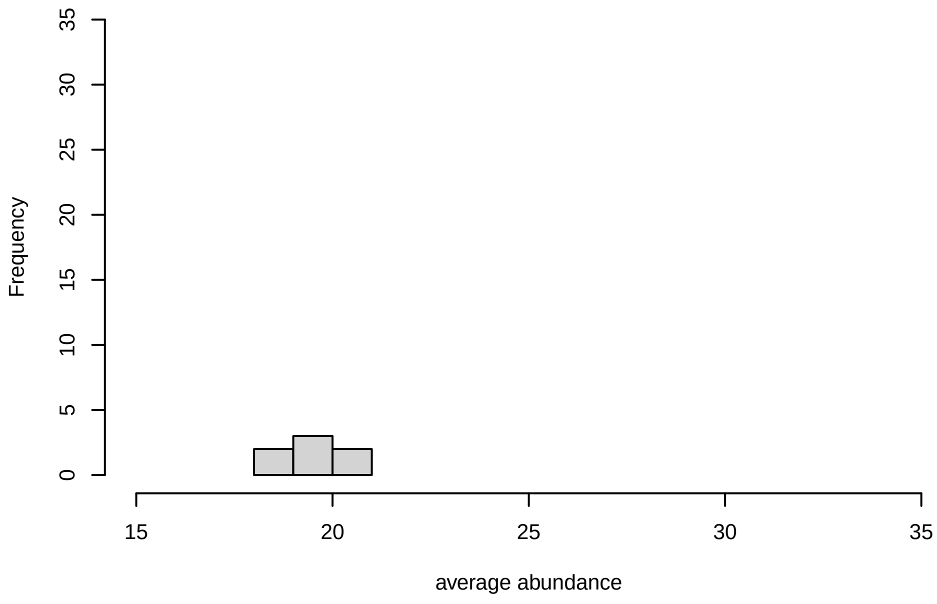

V39, after remove low abundance

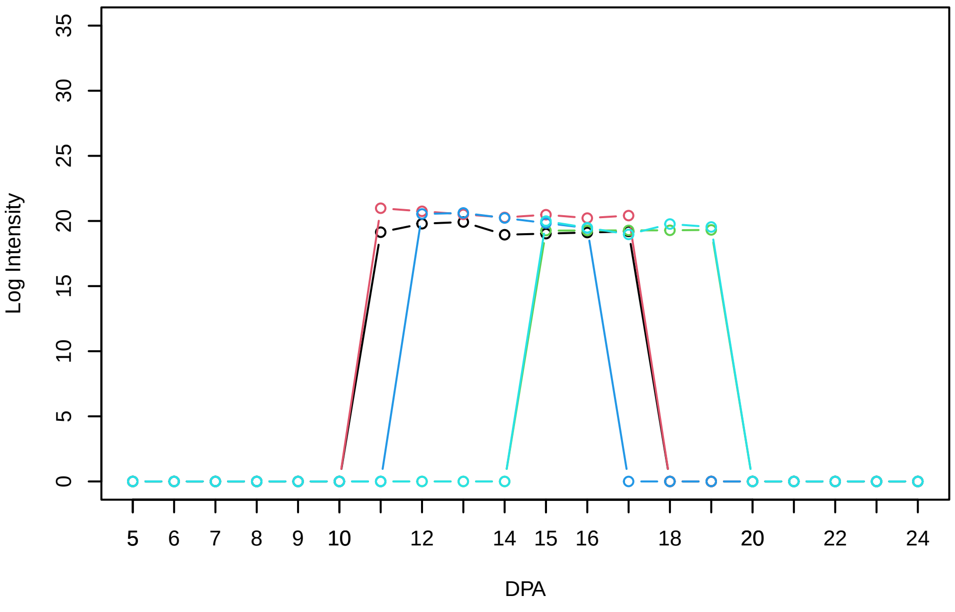

Histogram of the average abundances of high abundance proteins

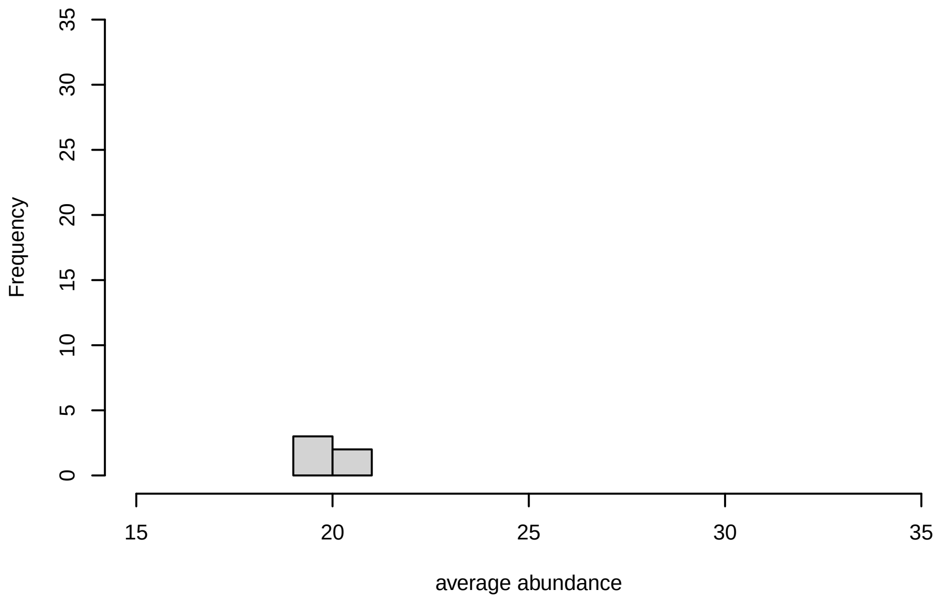

V40

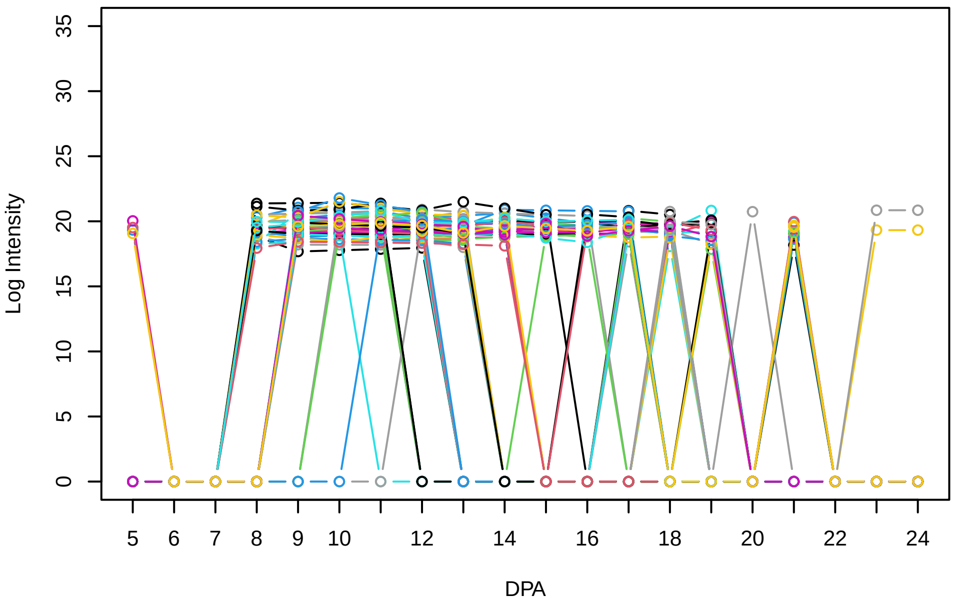

Histogram of the average abundances of non−zero values

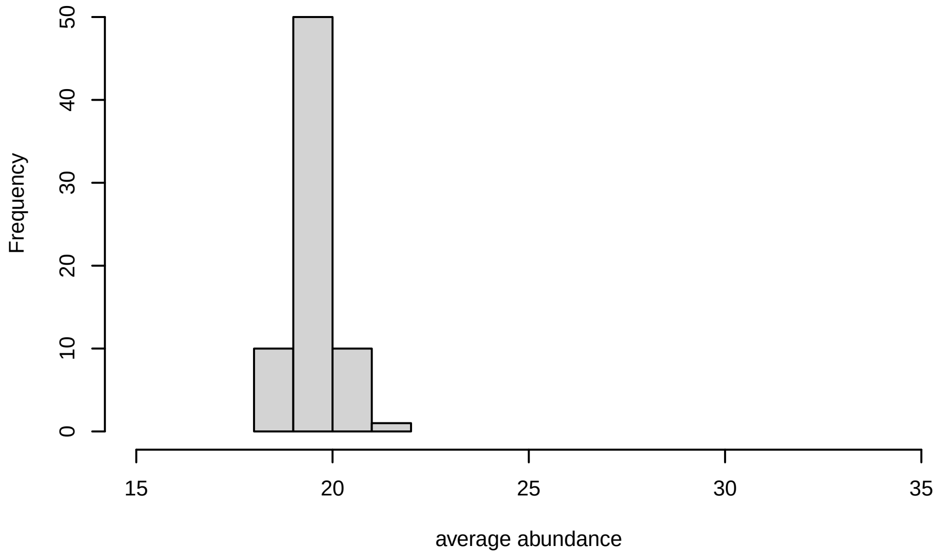

V40, after remove outlier and cutoff is 0.687304641725951

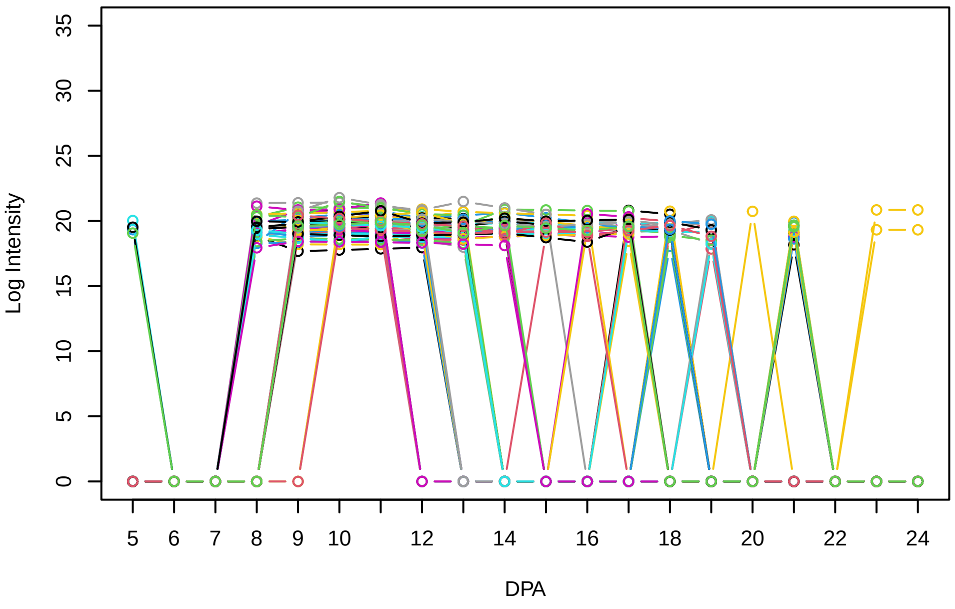

Histogram of the average abundances of non−outlier proteins

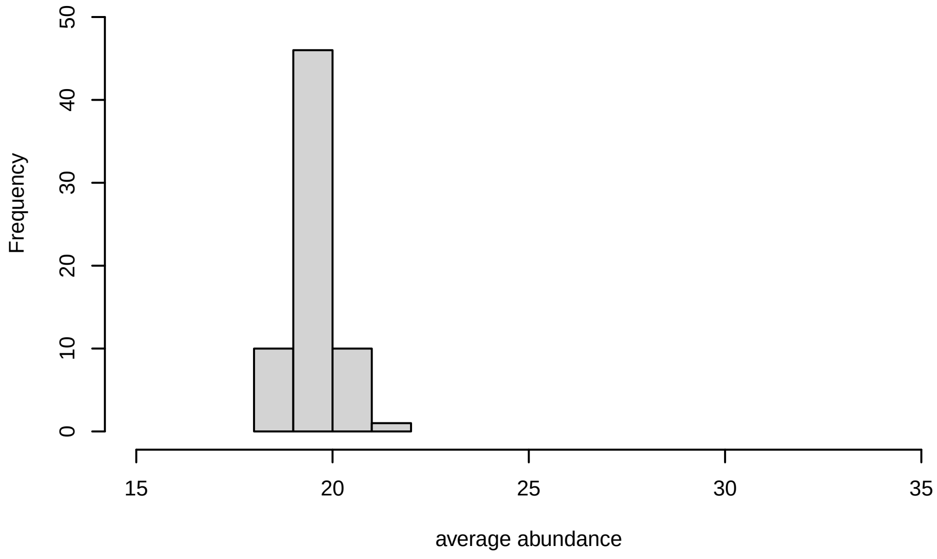

V40, after remove unreliable

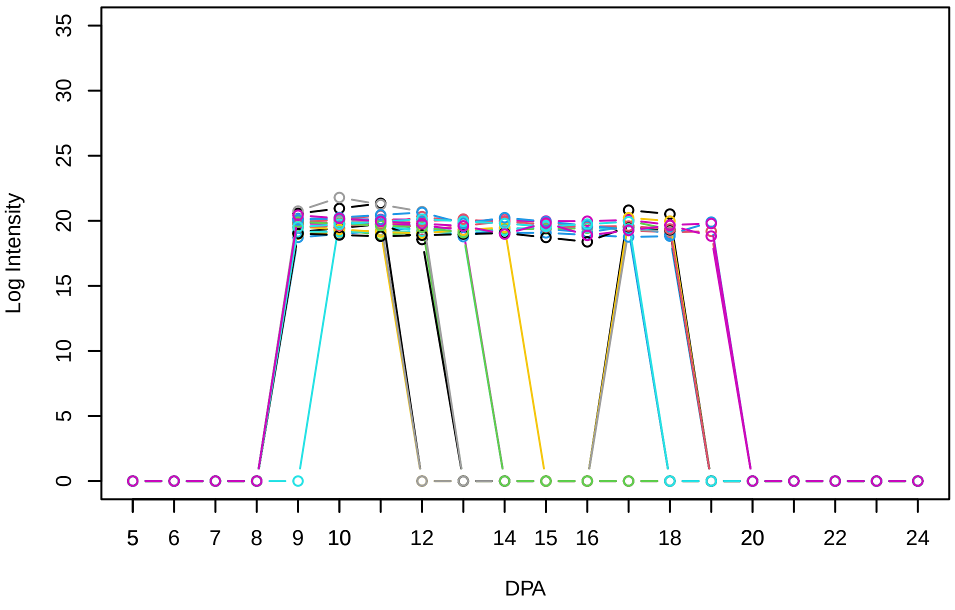

Histogram of the average abundances of reliable proteins

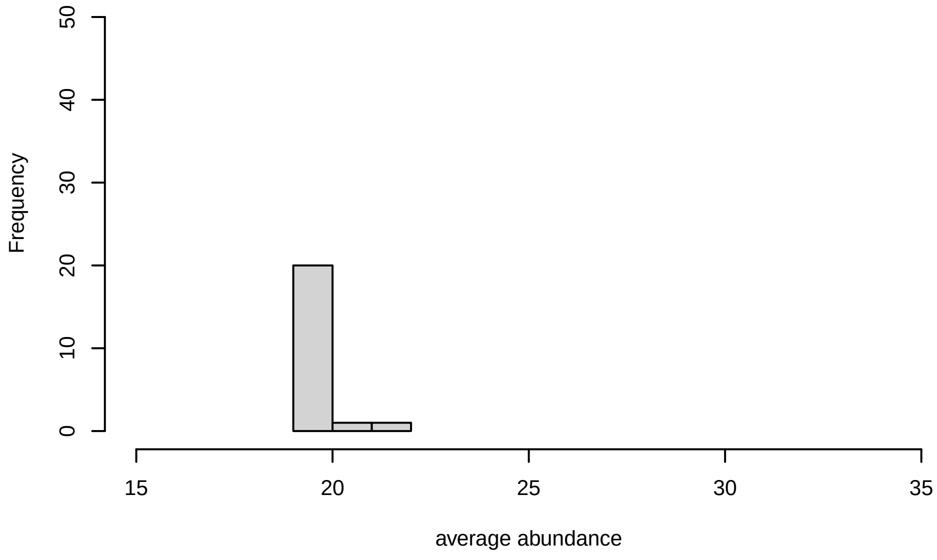

V40, after remove low abundance

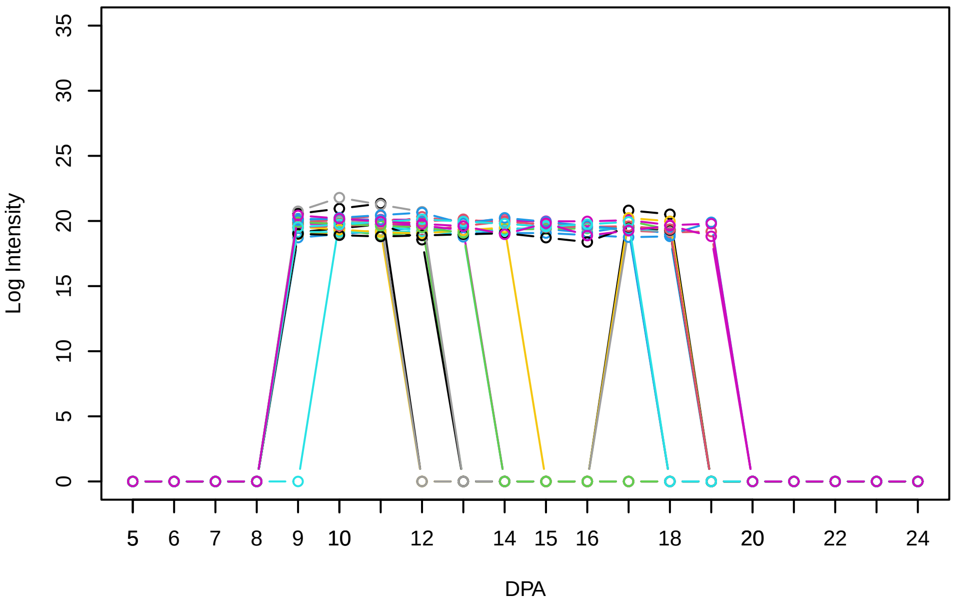

Histogram of the average abundances of high abundance proteins

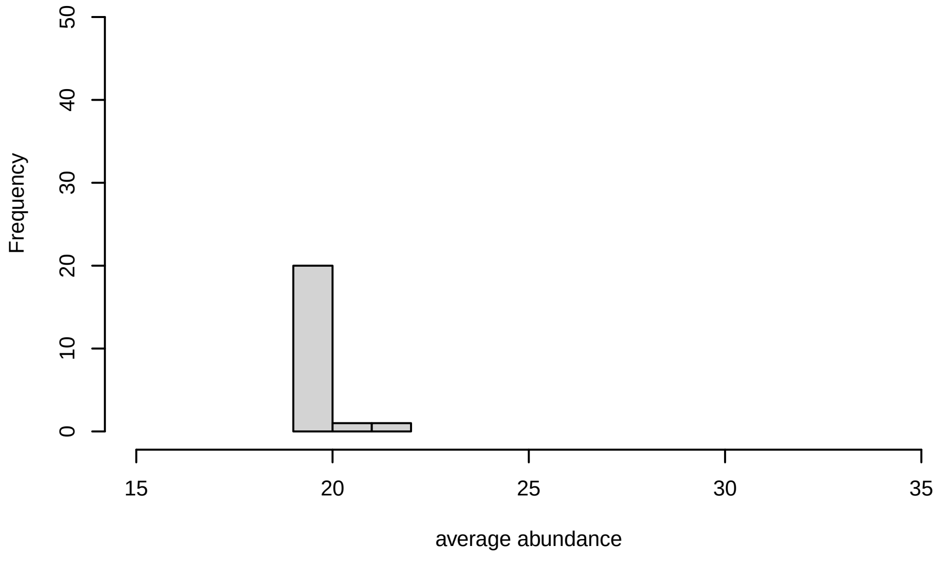

Supplementary Figure S5. SOM grouping and profile filtering (S200).

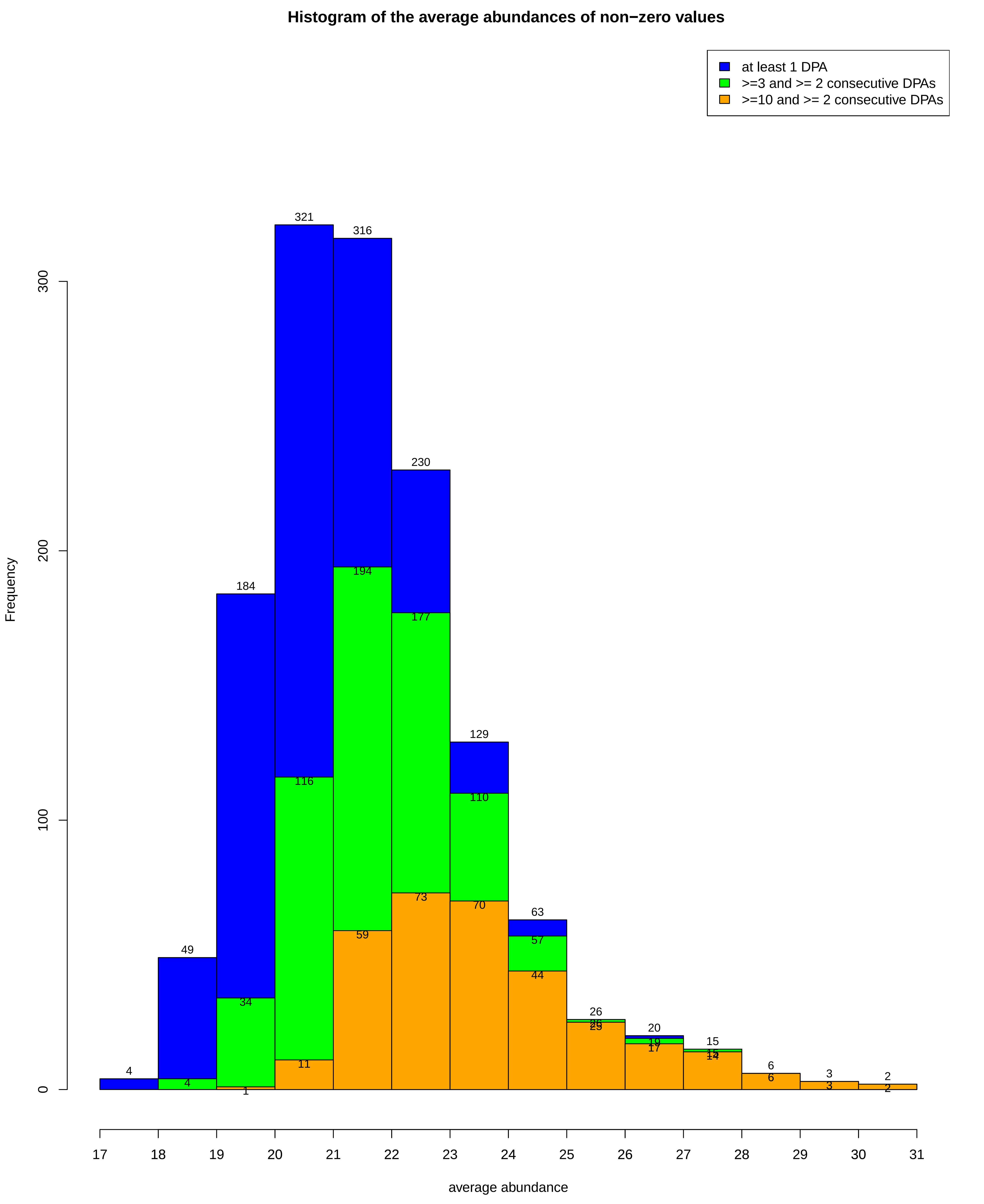

Histogram of the average abundances of non−zero values
at least 1 DPA
>=3 and >= 2 consecutive DPAs
>=10 and >= 2 consecutive DPAs
Frequency
0
100
200
300
4
49
4
184
34
1
321
116
11
316
194
59
230
177
73
129
110
70
63
57
44
26
25
20
19
17
15
14
6
6
3
3
2
2
17
18
19
20
21
22
23
24
25
26
27
28
29
30
31
average abundance

Codes

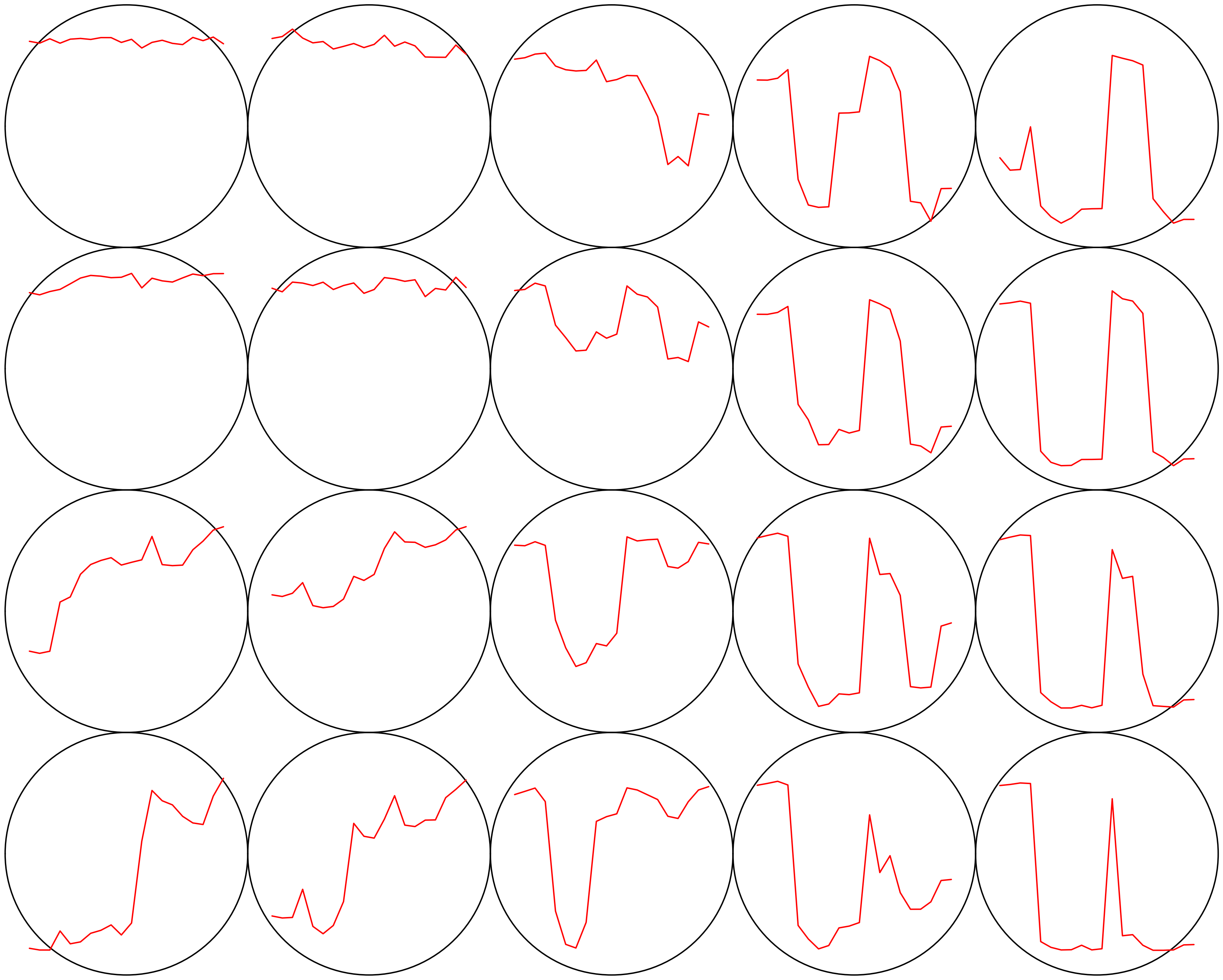

Count plot

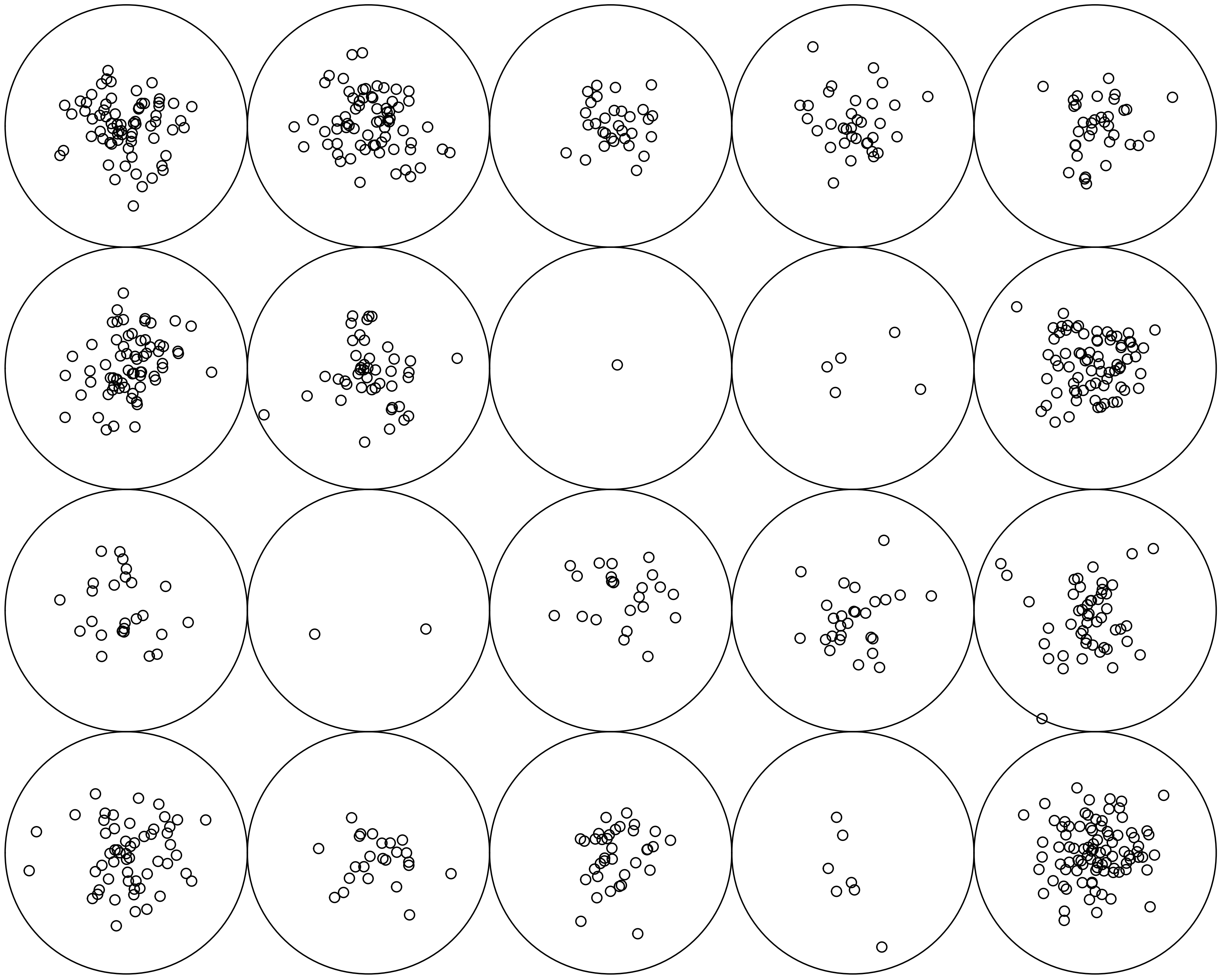

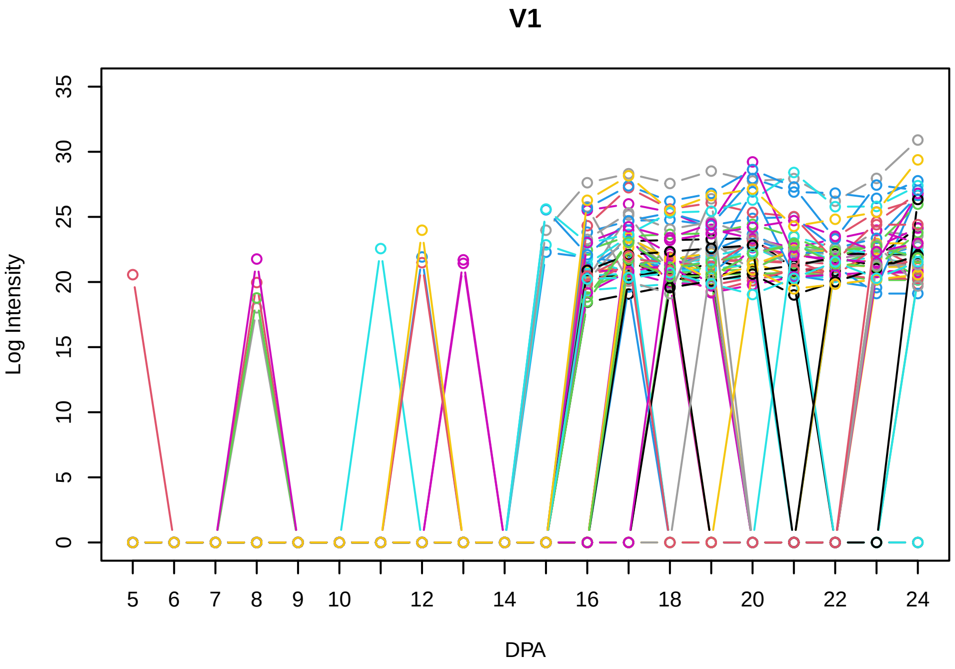
V1
Log Intensity
0
5
10
15
20
25
30
35
5 6 7 8 9 10 12 14 16 18 20 22 24
DPA

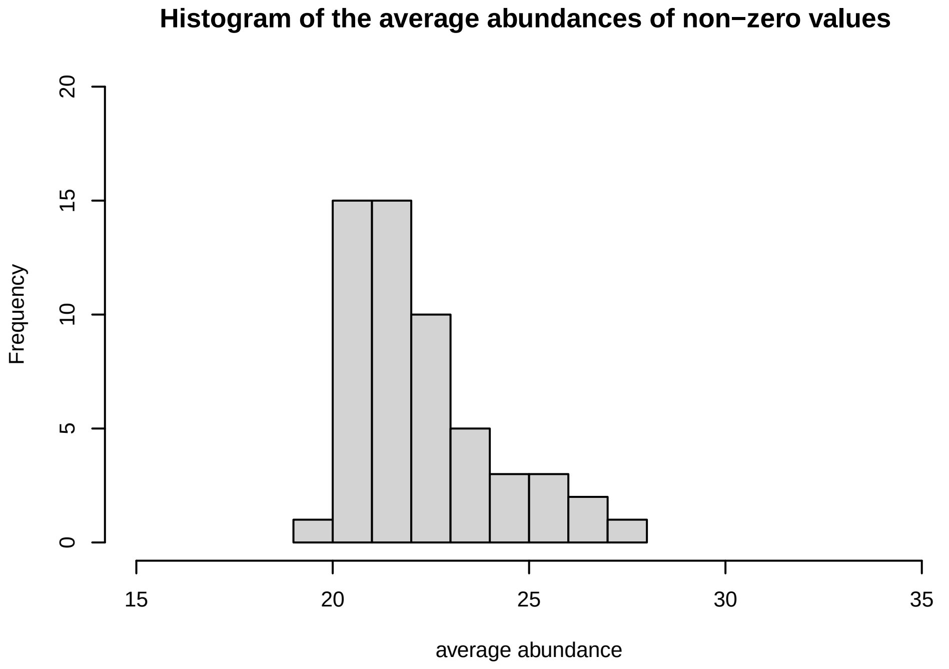
Histogram of the average abundances of non−zero values
Frequency
0
5
10
15
20
15 20 25 30 35
average abundance

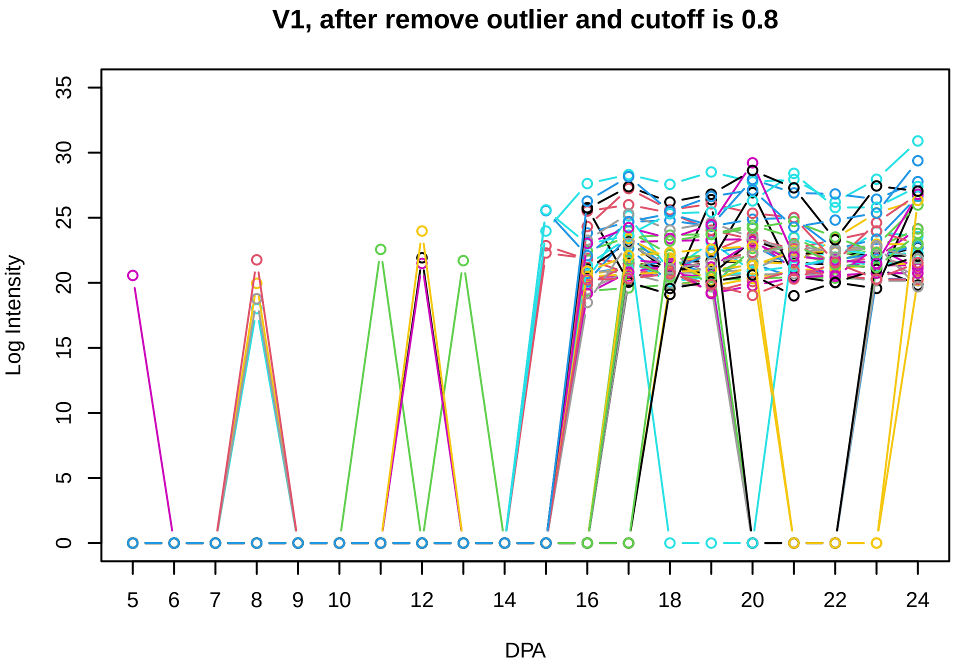
V1, after remove outlier and cutoff is 0.8
Log Intensity
0
5
10
15
20
25
30
35
5 6 7 8 9 10 12 14 16 18 20 22 24
DPA

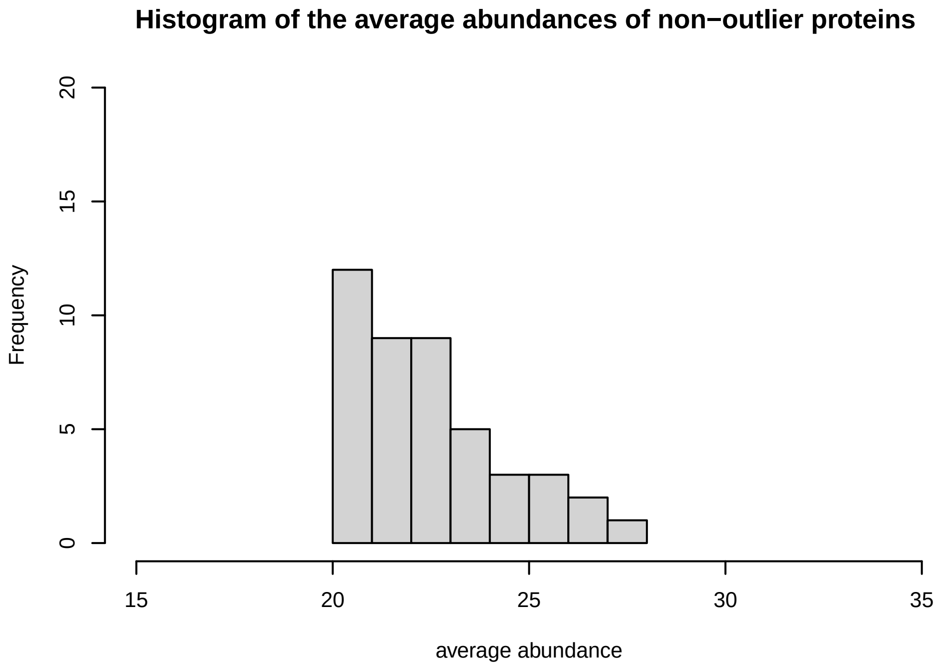
Histogram of the average abundances of non−outlier proteins
Frequency
0
5
10
15
20
15 20 25 30 35
average abundance

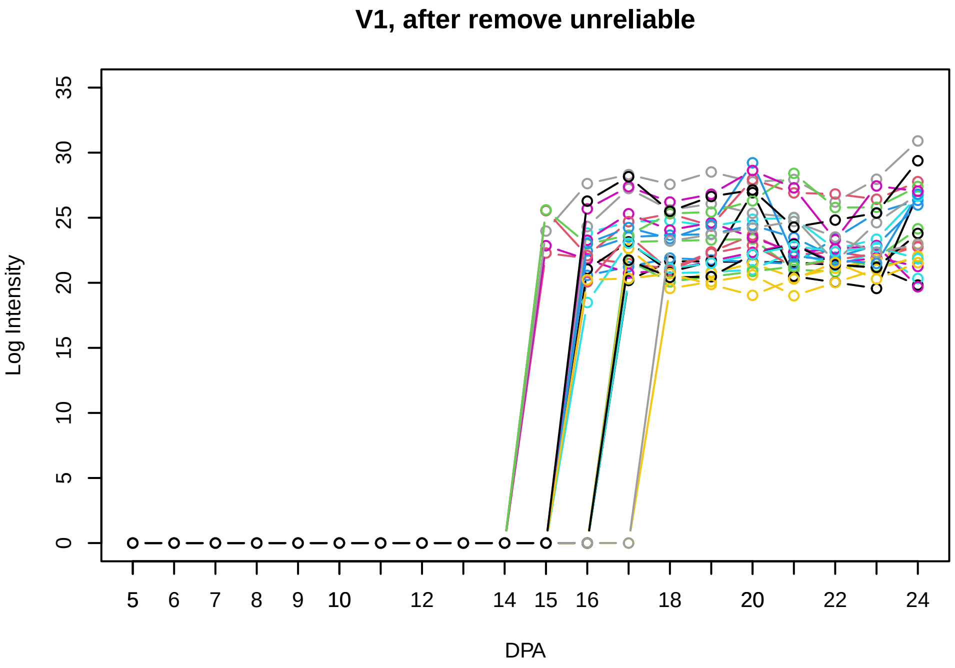
V1, after remove unreliable
Log Intensity
0
5
10
15
20
25
30
35
5 6 7 8 9 10 12 14 15 16 18 20 22 24
DPA

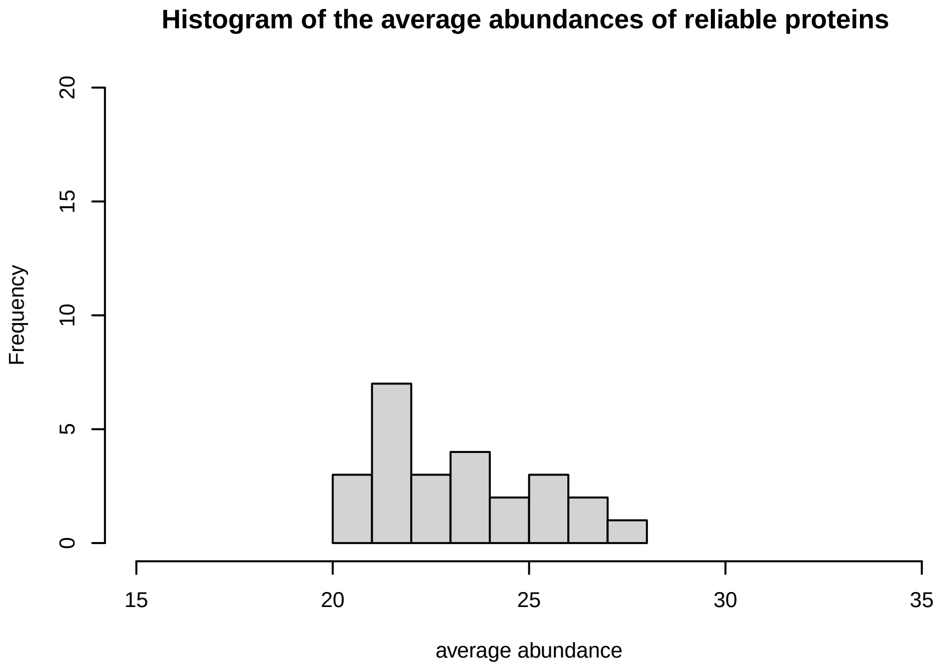
Histogram of the average abundances of reliable proteins
Frequency
0
5
10
15
20
15 20 25 30 35
average abundance

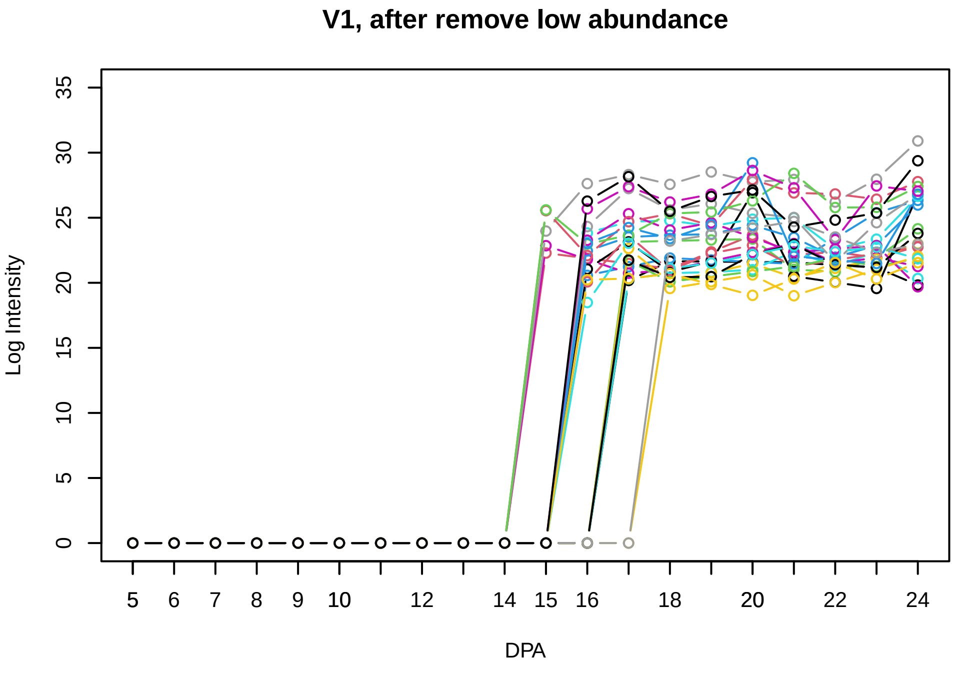
V1, after remove low abundance
Log Intensity
0
5
10
15
20
25
30
35
5 6 7 8 9 10 12 14 15 16 18 20 22 24
DPA

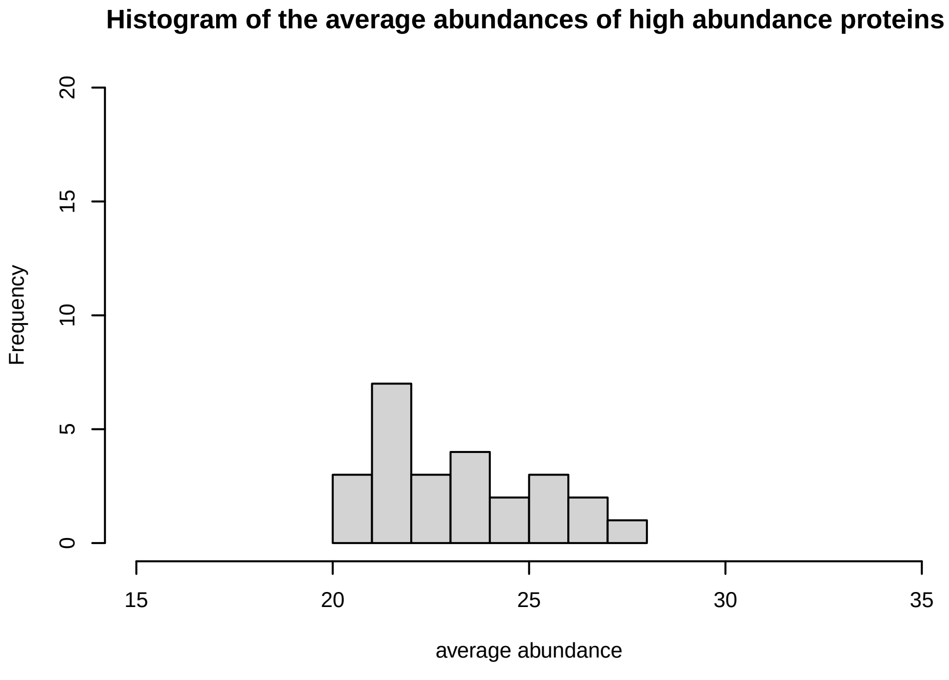
Histogram of the average abundances of high abundance proteins
Frequency
0
5
10
15
20
15 20 25 30 35
average abundance

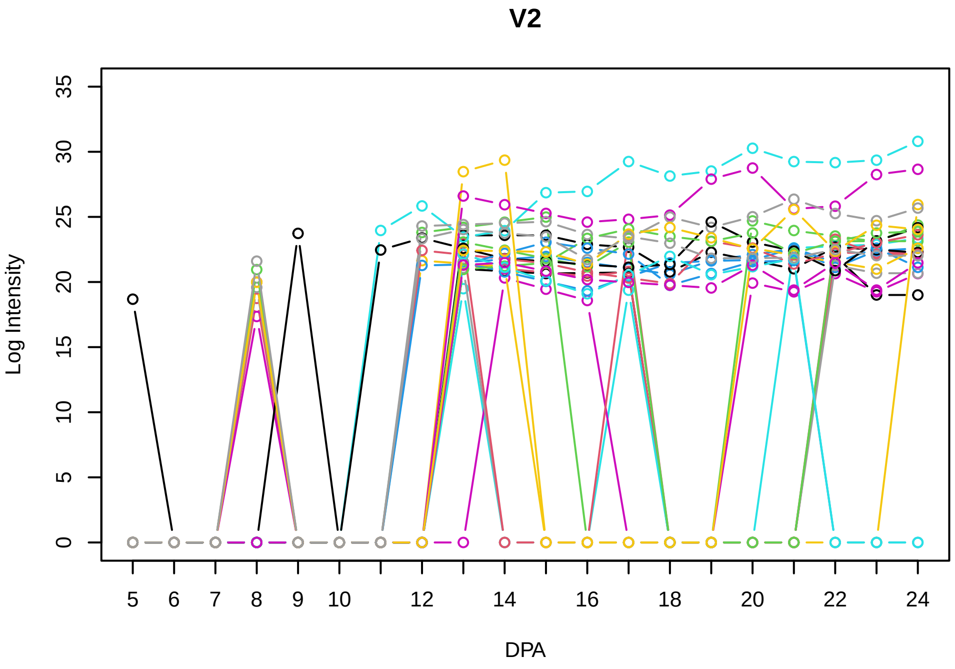
V2
Log Intensity
DPA

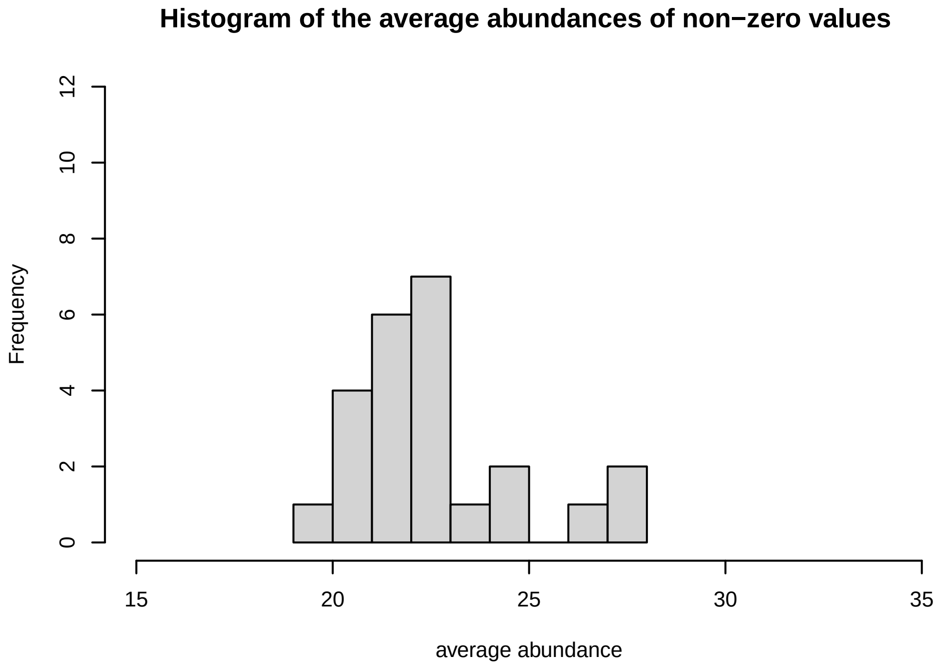
Histogram of the average abundances of non−zero values
Frequency
average abundance

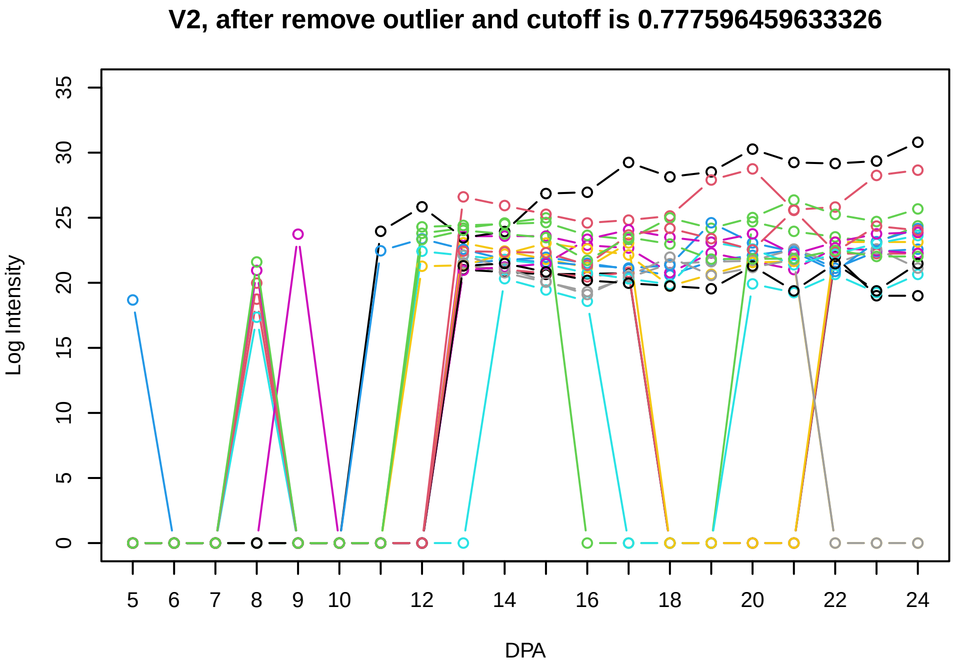
V2, after remove outlier and cutoff is 0.777596459633326
Log Intensity
DPA

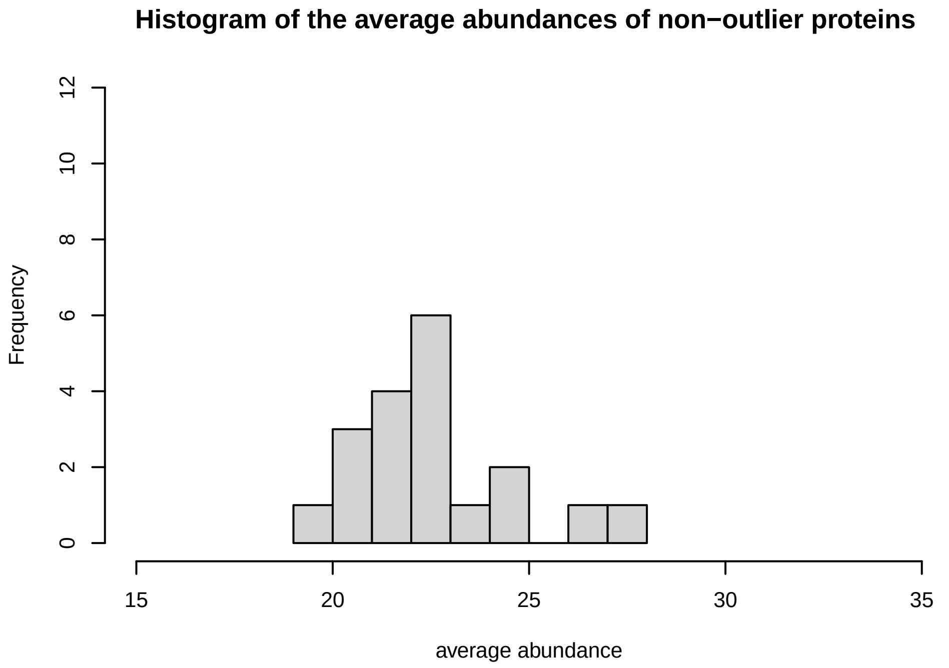
Histogram of the average abundances of non−outlier proteins
Frequency
average abundance

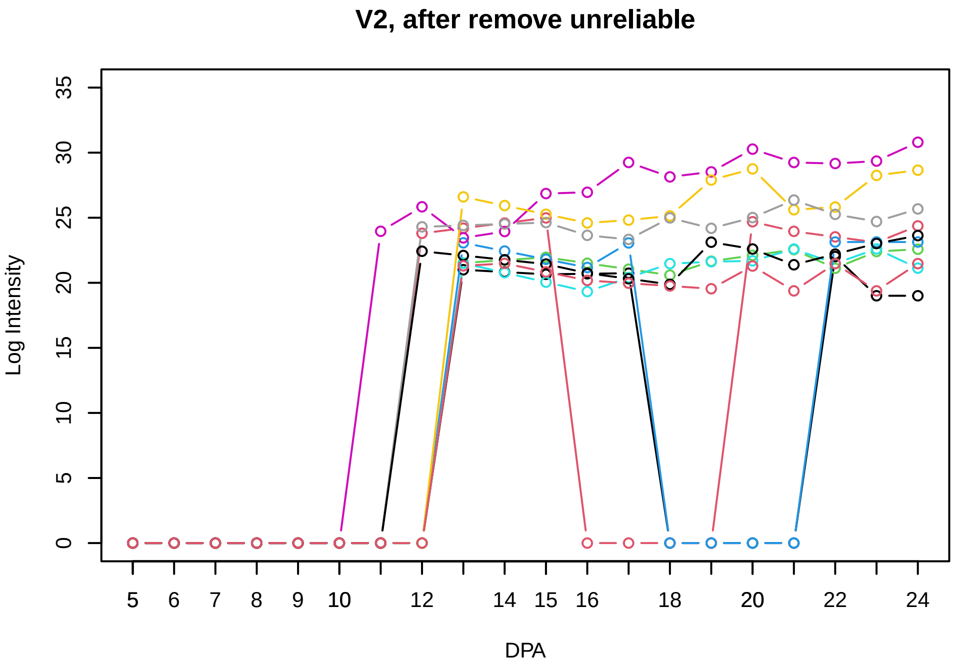
V2, after remove unreliable
Log Intensity
DPA

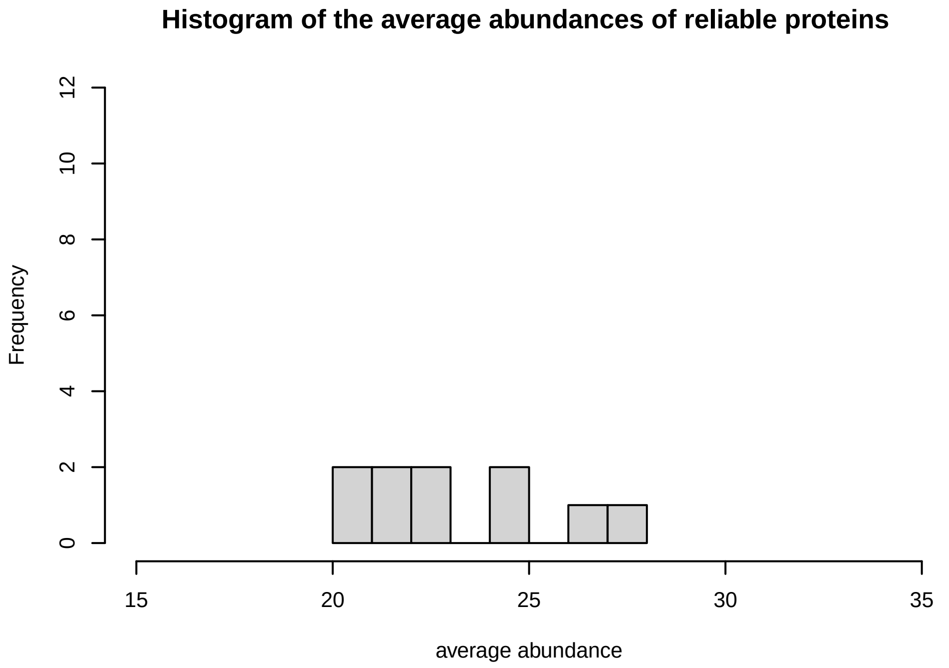
Histogram of the average abundances of reliable proteins
Frequency
average abundance

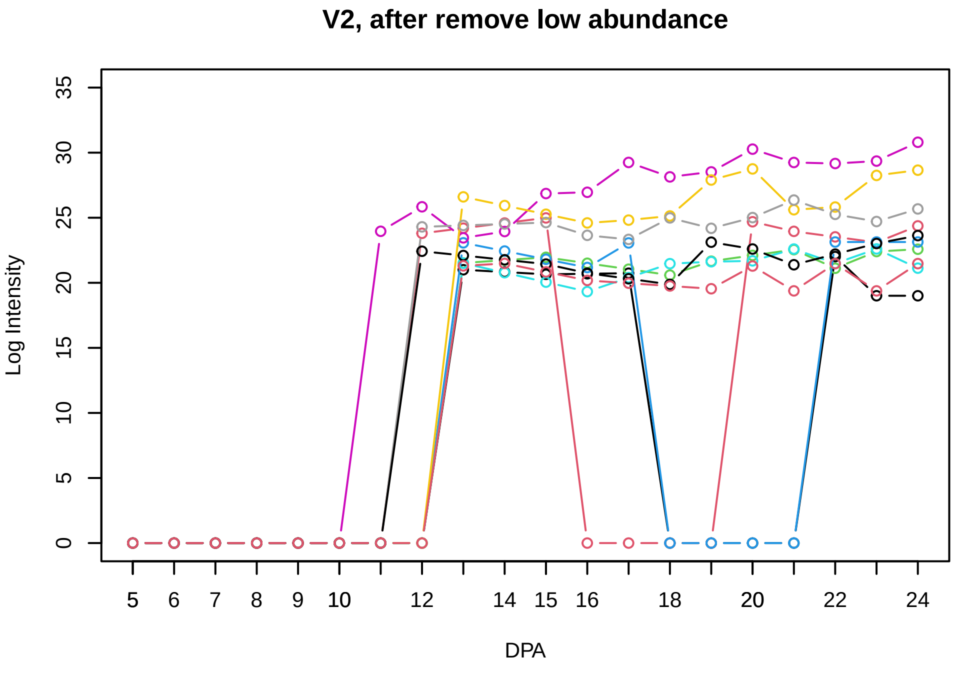
V2, after remove low abundance
Log Intensity
DPA

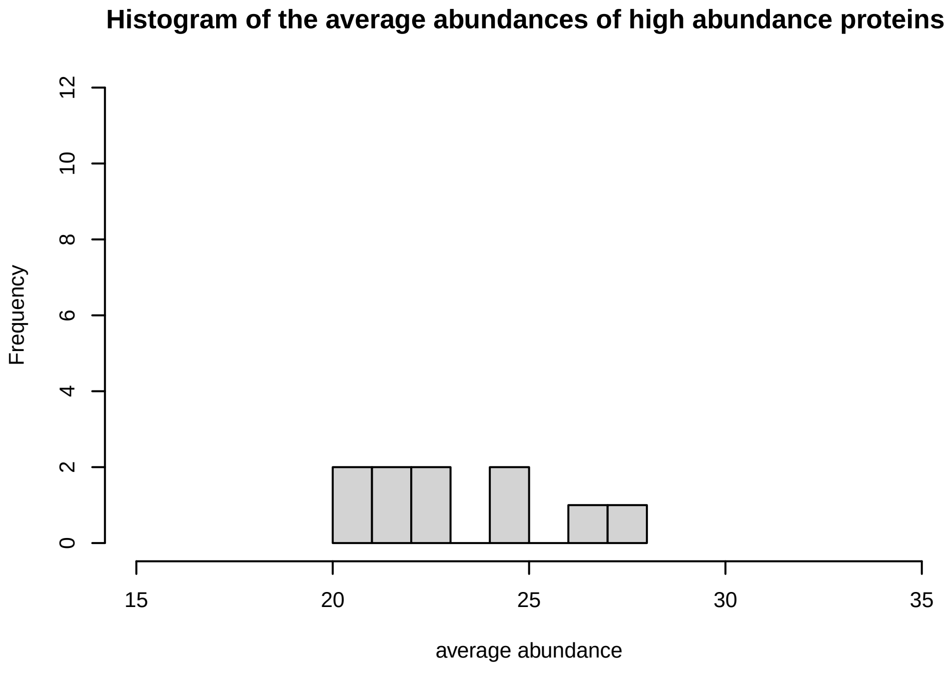
Histogram of the average abundances of high abundance proteins
Frequency
average abundance

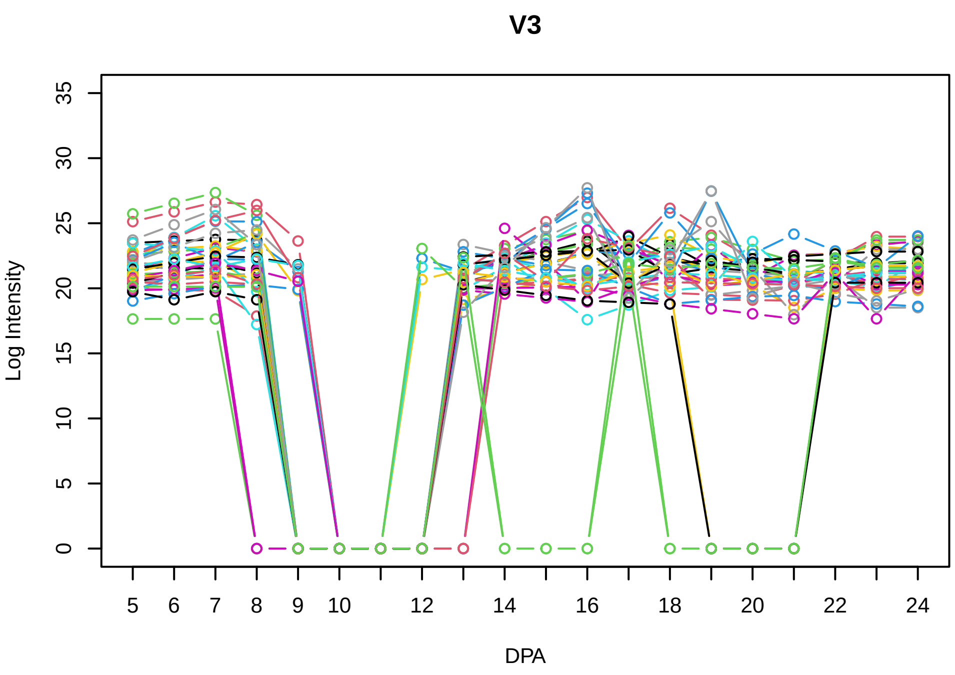
V3
Log Intensity
DPA

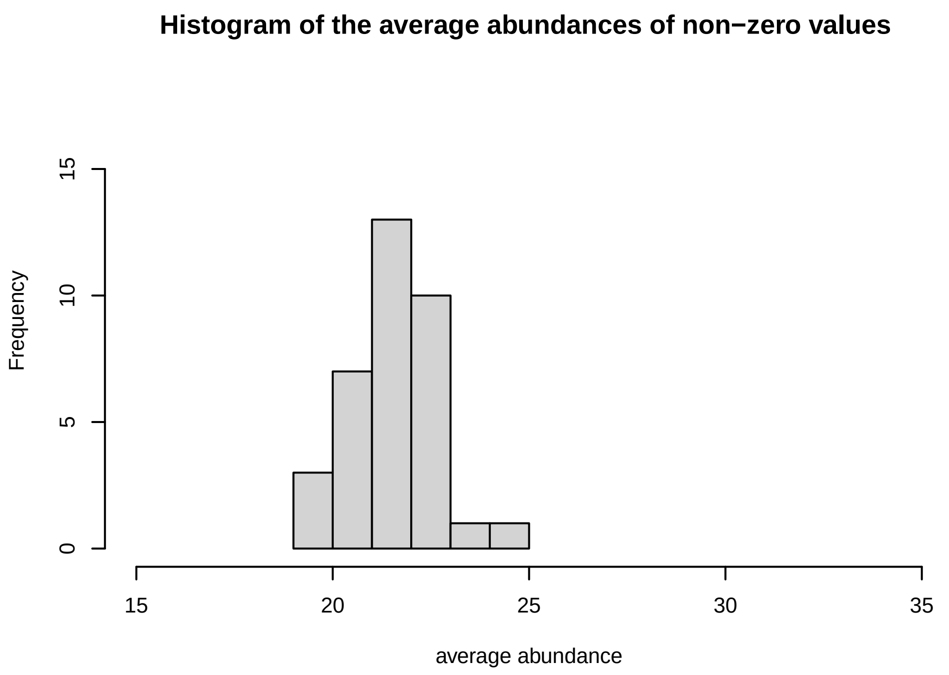
Histogram of the average abundances of non−zero values
Frequency
average abundance

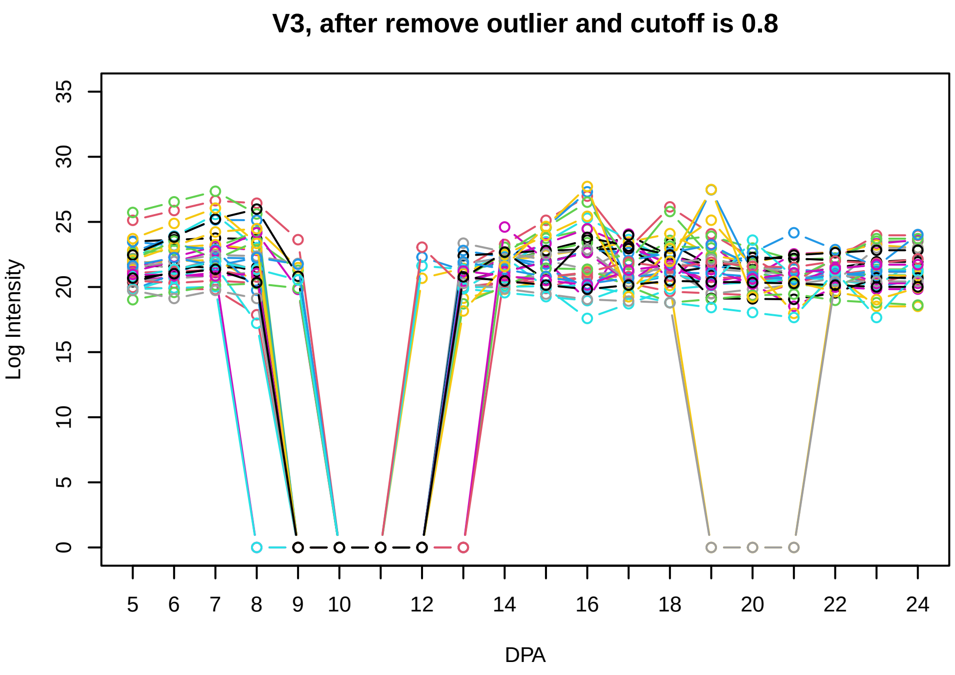
V3, after remove outlier and cutoff is 0.8
Log Intensity
DPA

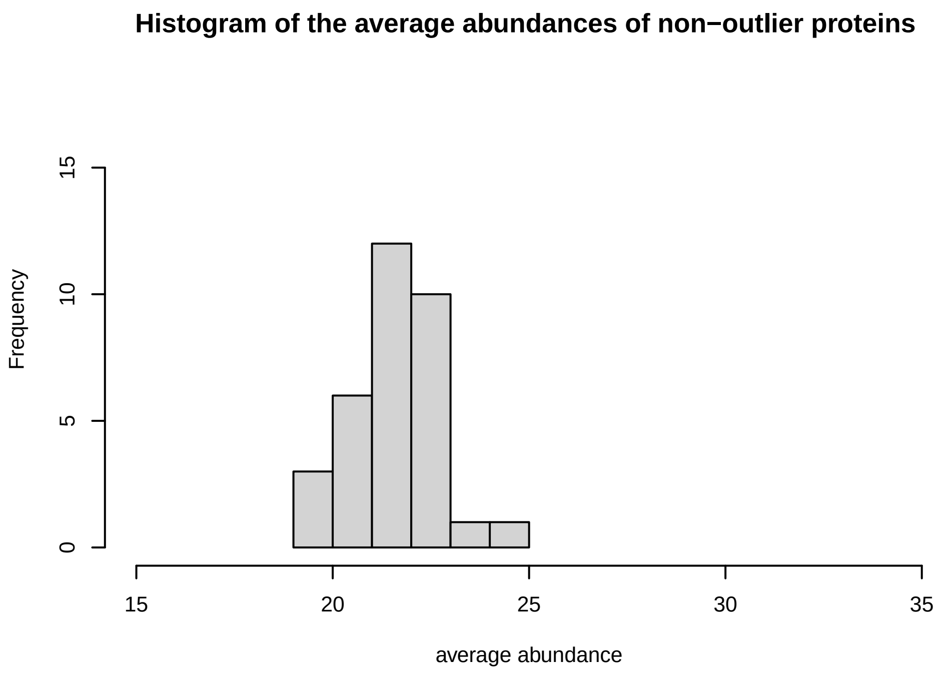
Histogram of the average abundances of non−outlier proteins
Frequency
average abundance

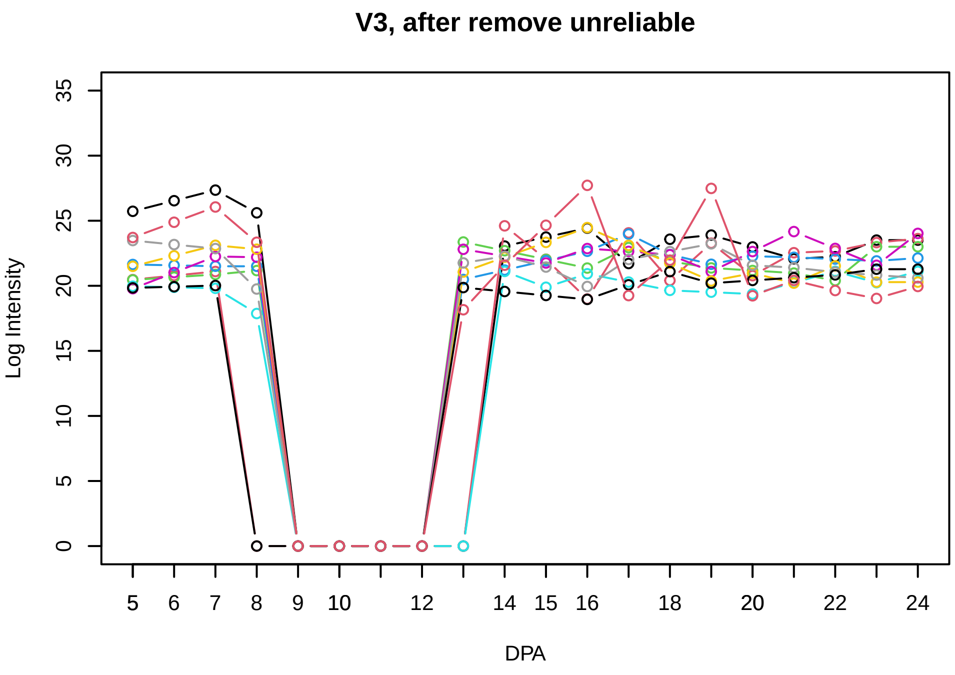
V3, after remove unreliable
Log Intensity
DPA

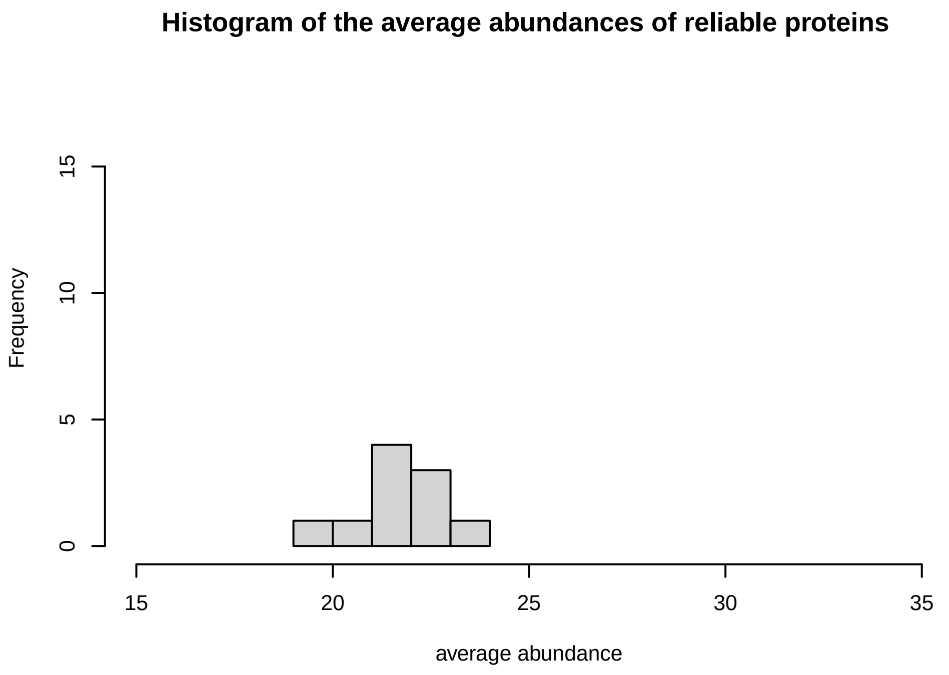
Histogram of the average abundances of reliable proteins
Frequency
average abundance

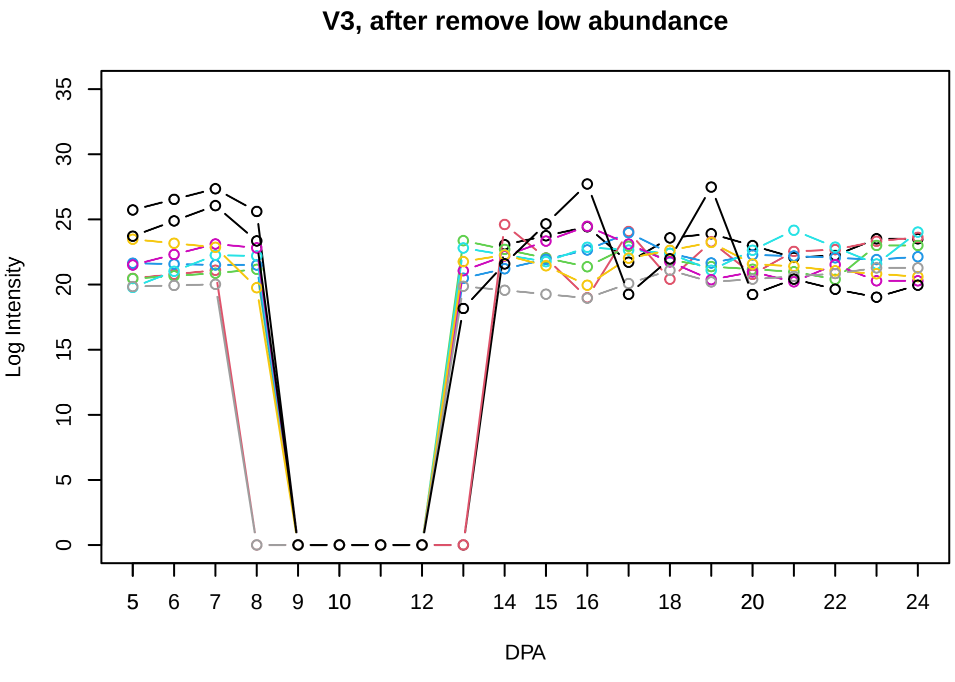
V3, after remove low abundance
Log Intensity
DPA

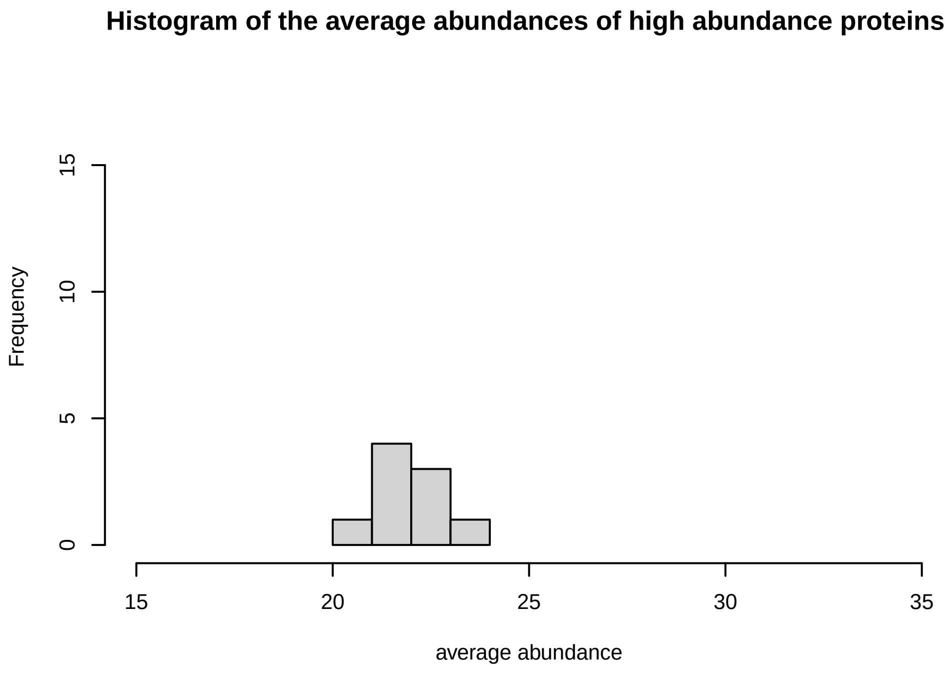
Histogram of the average abundances of high abundance proteins
Frequency
average abundance

V4

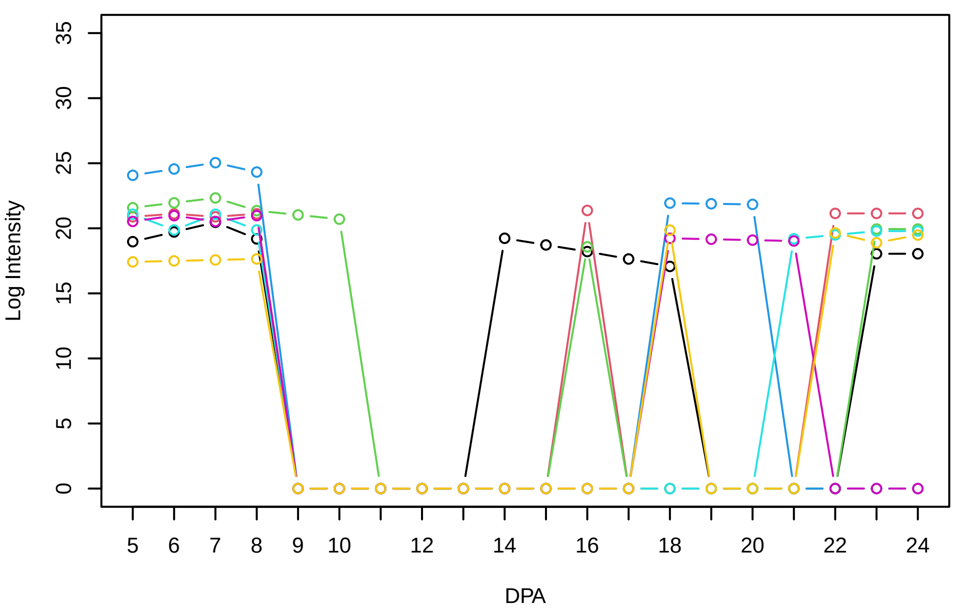

Histogram of the average abundances of non−zero values

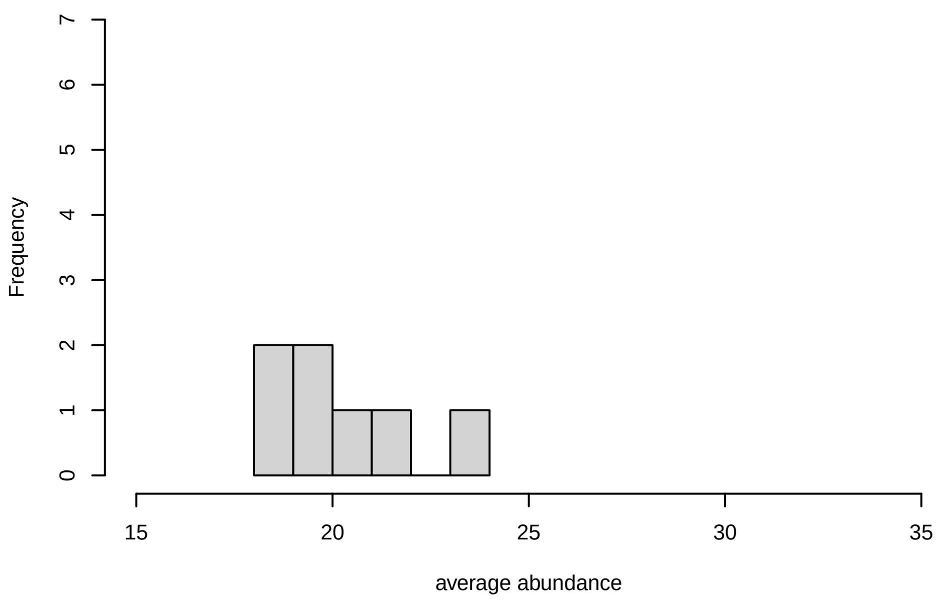

V4, after remove outlier and cutoff is 0.663396977903535

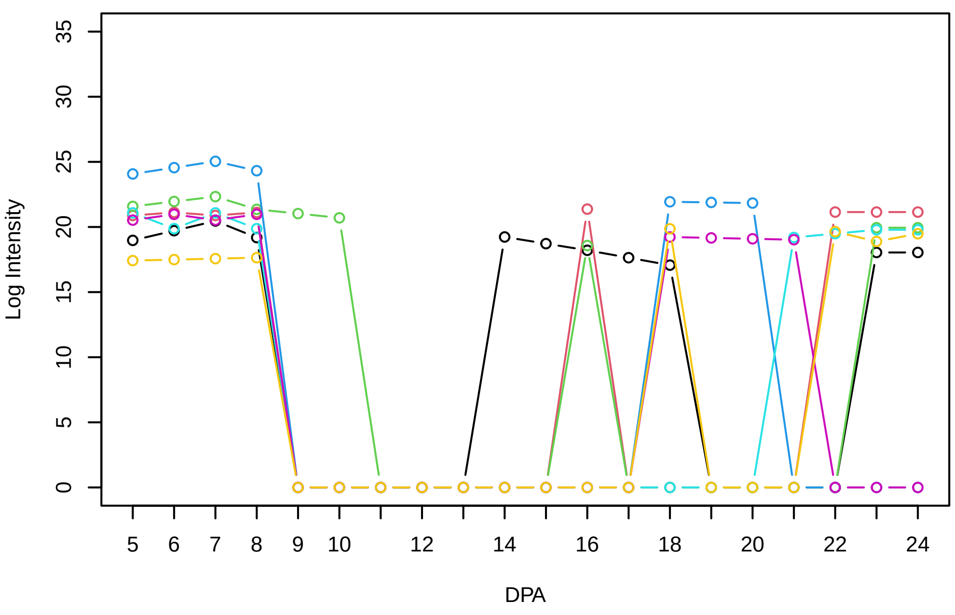

Histogram of the average abundances of non−outlier proteins

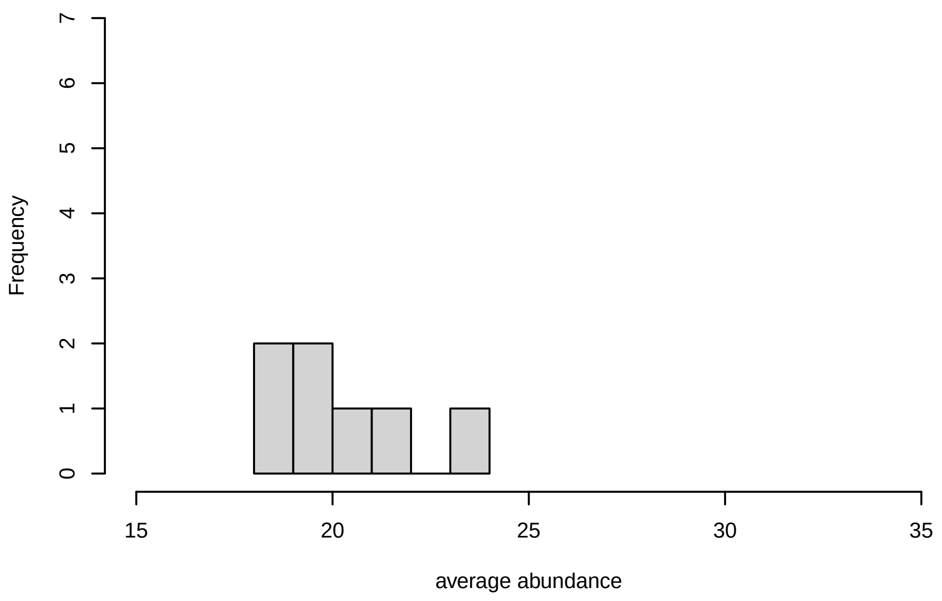

V4, after remove unreliable

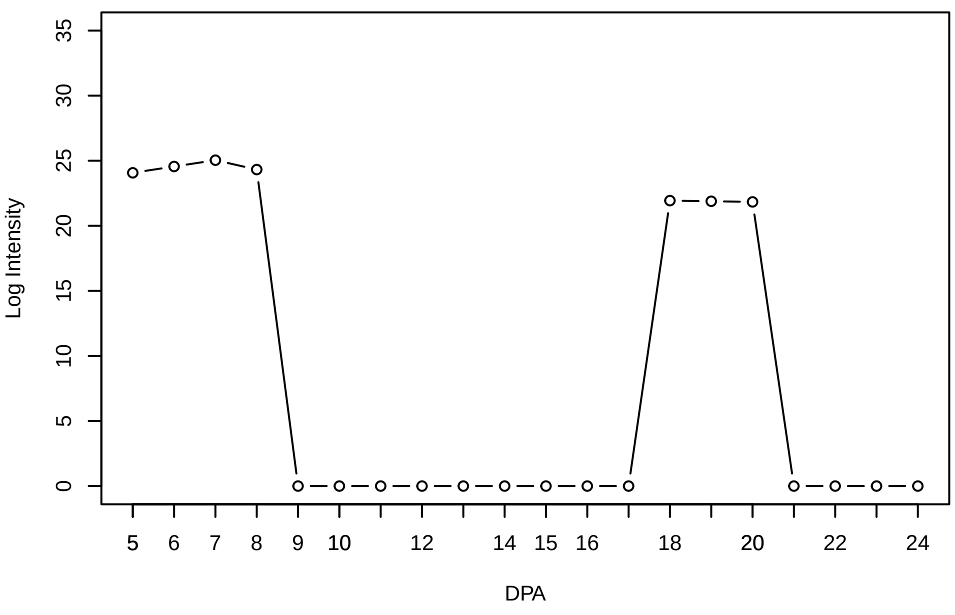

Histogram of the average abundances of reliable proteins

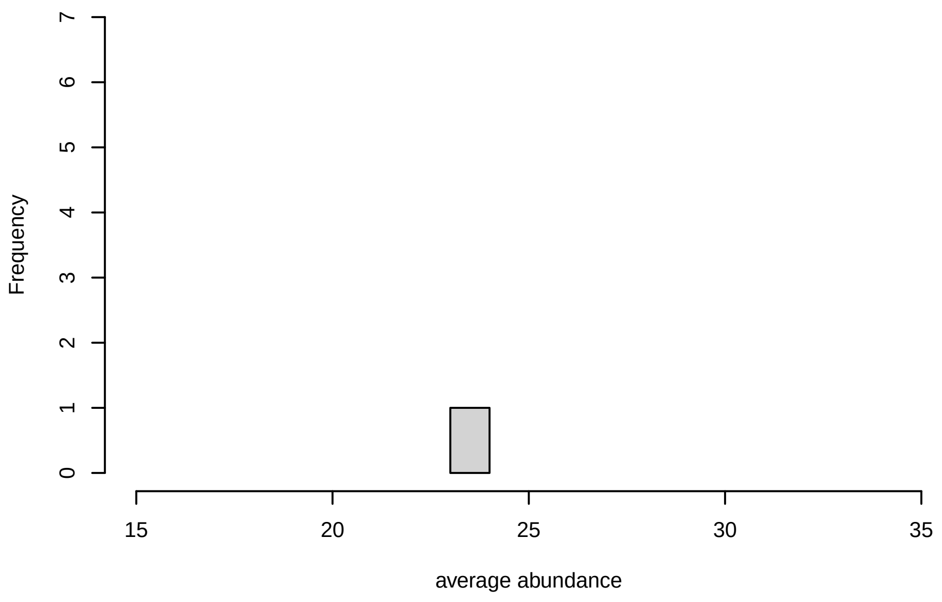

V4, after remove low abundance

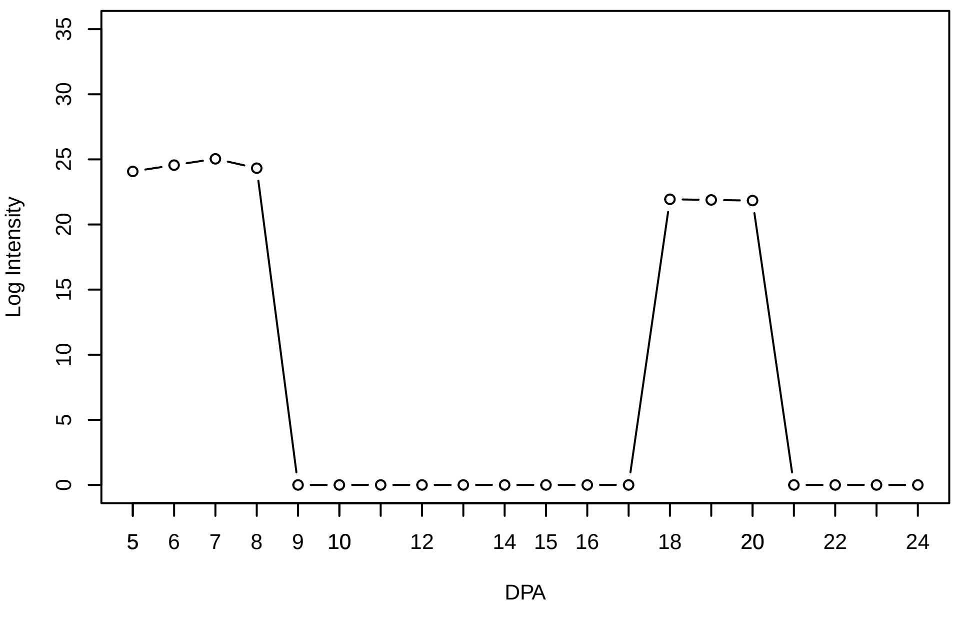

Histogram of the average abundances of high abundance proteins

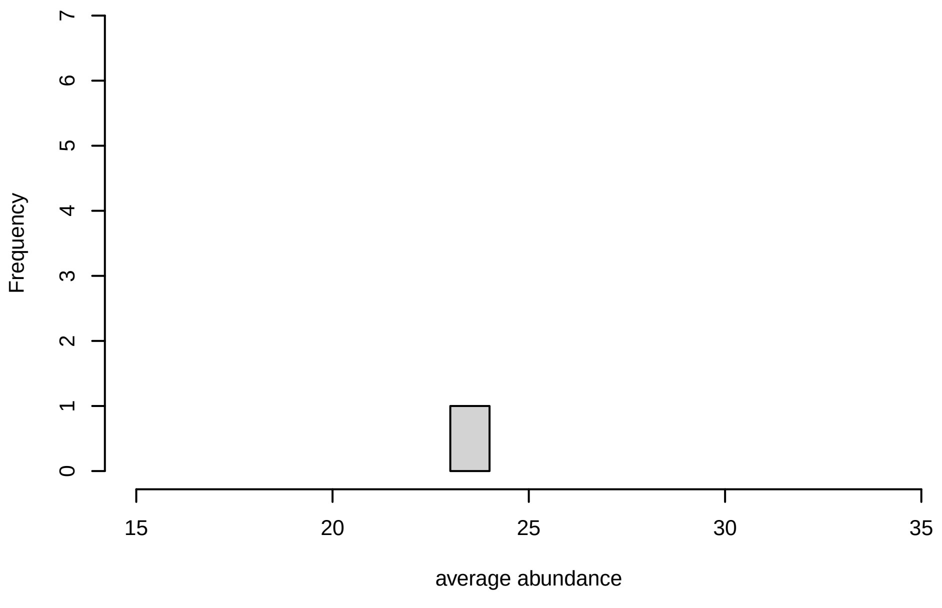

V5

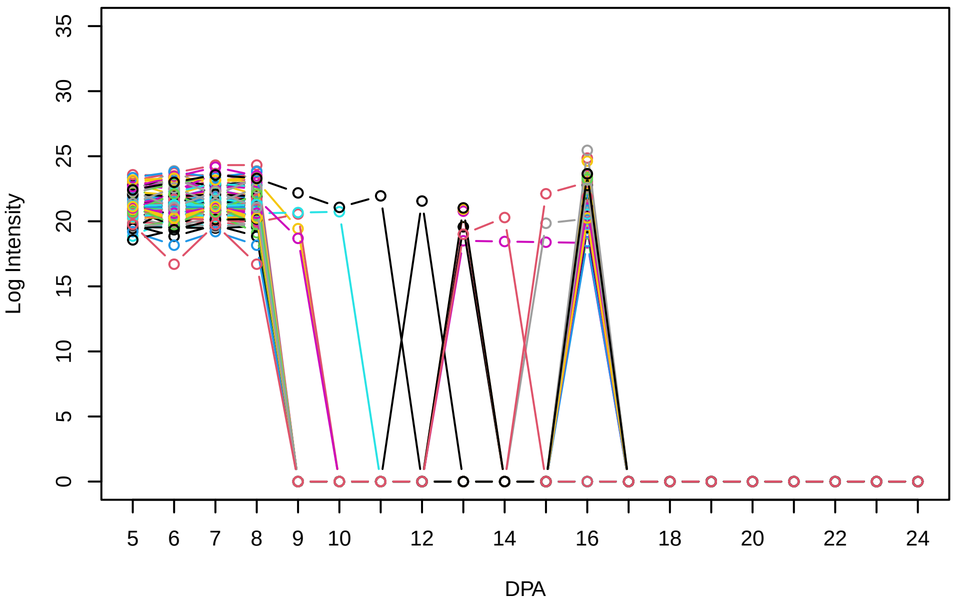

Histogram of the average abundances of non−zero values

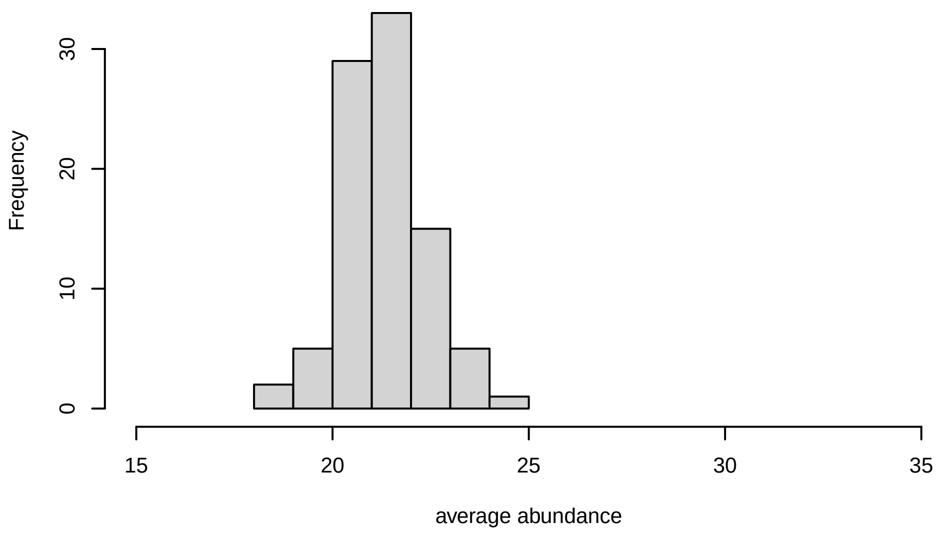

V5, after remove outlier and cutoff is 0.8

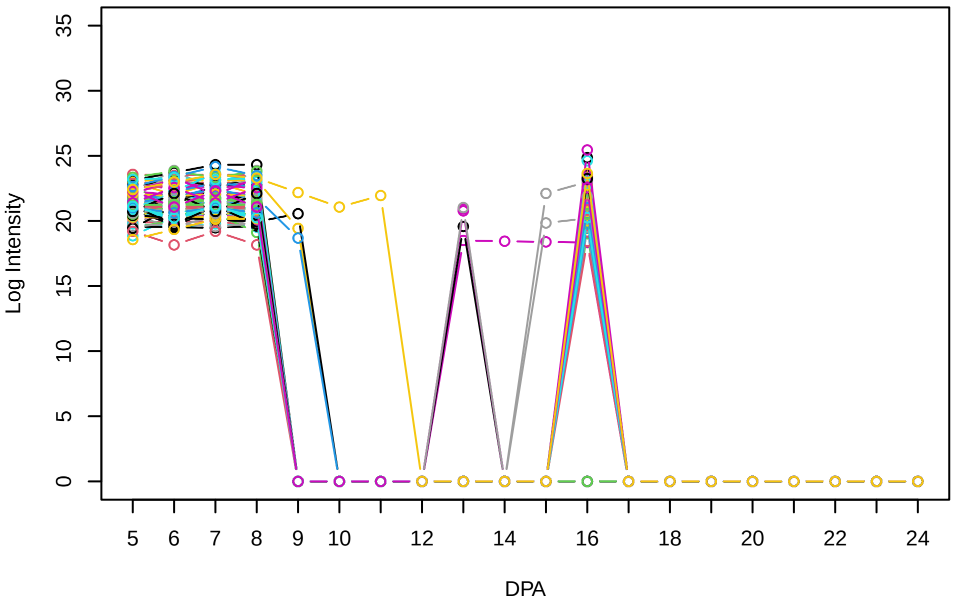

Histogram of the average abundances of non−outlier proteins

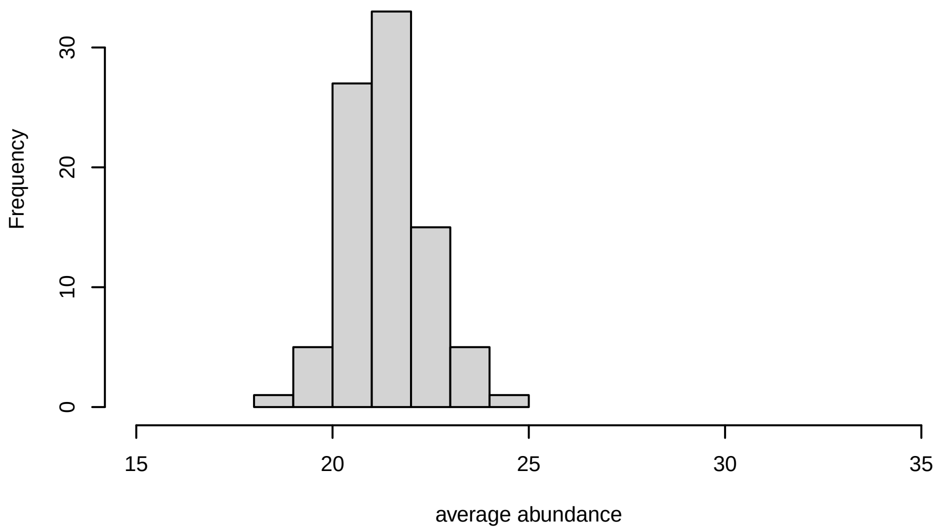

V5, after remove unreliable

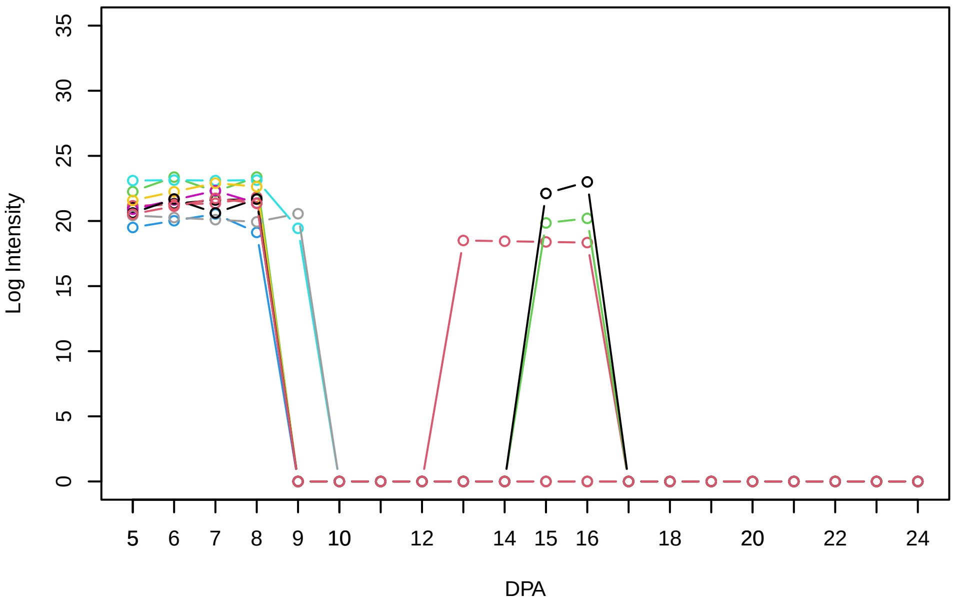

Histogram of the average abundances of reliable proteins

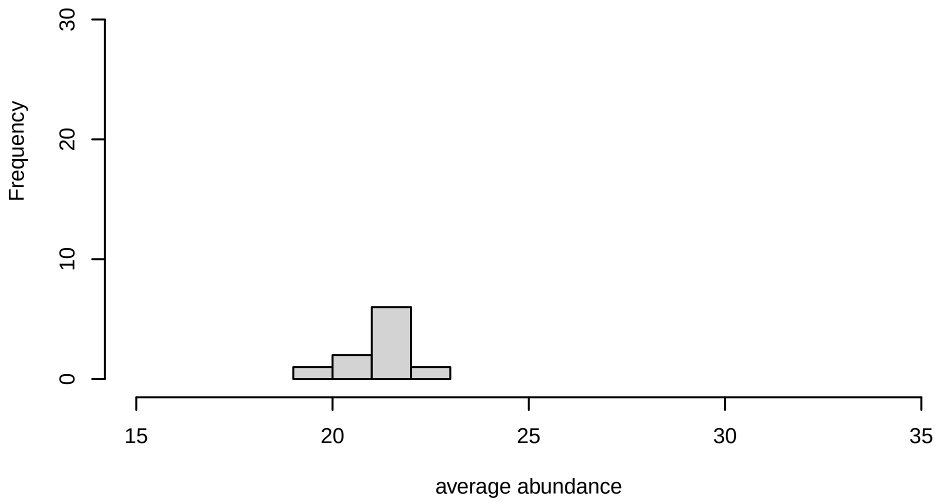

V5, after remove low abundance

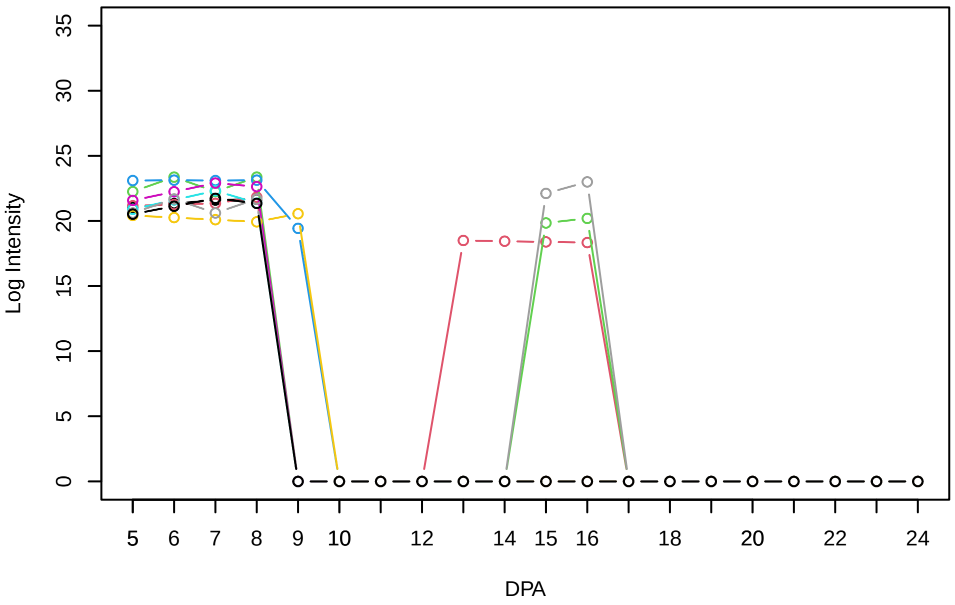

Histogram of the average abundances of high abundance proteins

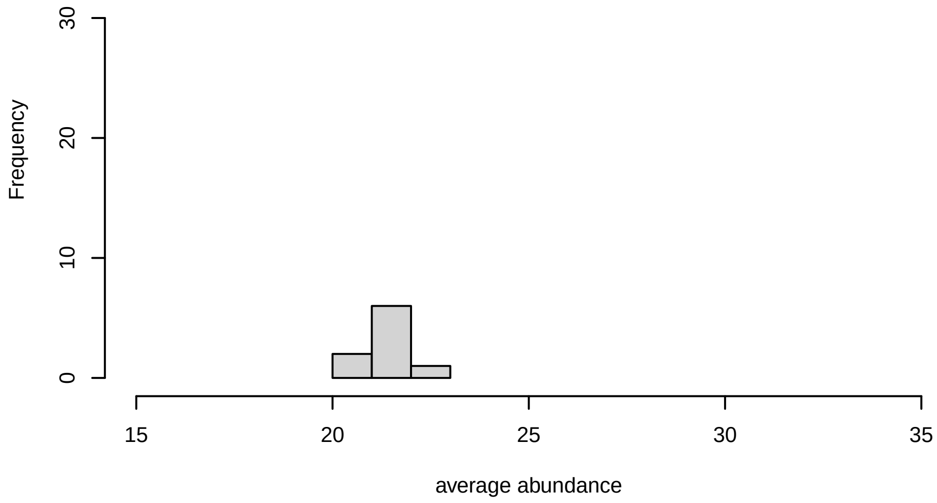

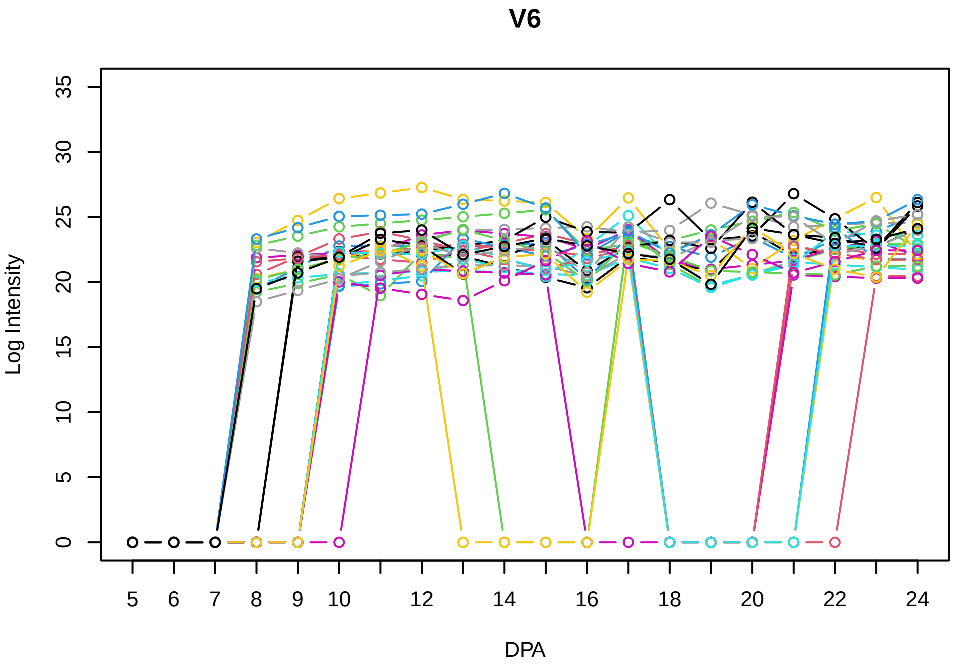
V6
Log Intensity
DPA
0 5 10 15 20 25 30 35
5 6 7 8 9 10 12 14 16 18 20 22 24

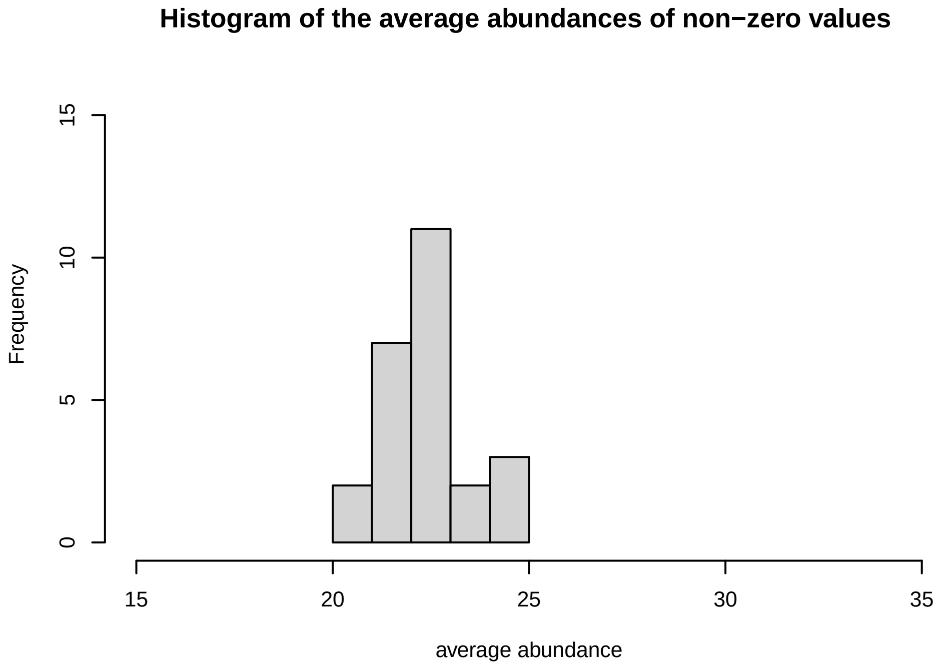
Histogram of the average abundances of non−zero values
Frequency
average abundance
0 5 10 15
15 20 25 30 35

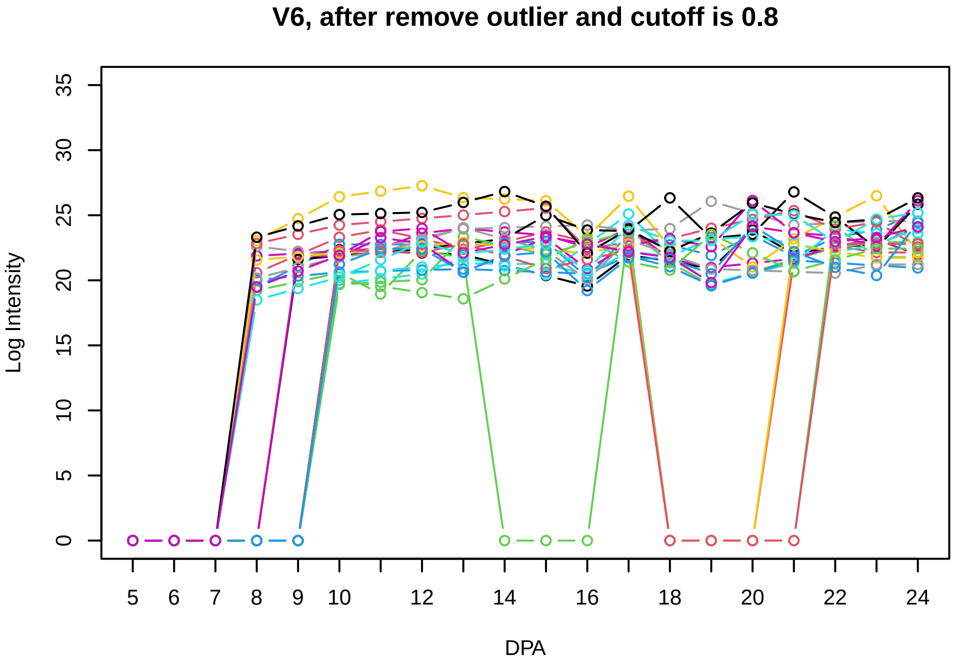
V6, after remove outlier and cutoff is 0.8
Log Intensity
DPA
0 5 10 15 20 25 30 35
5 6 7 8 9 10 12 14 16 18 20 22 24

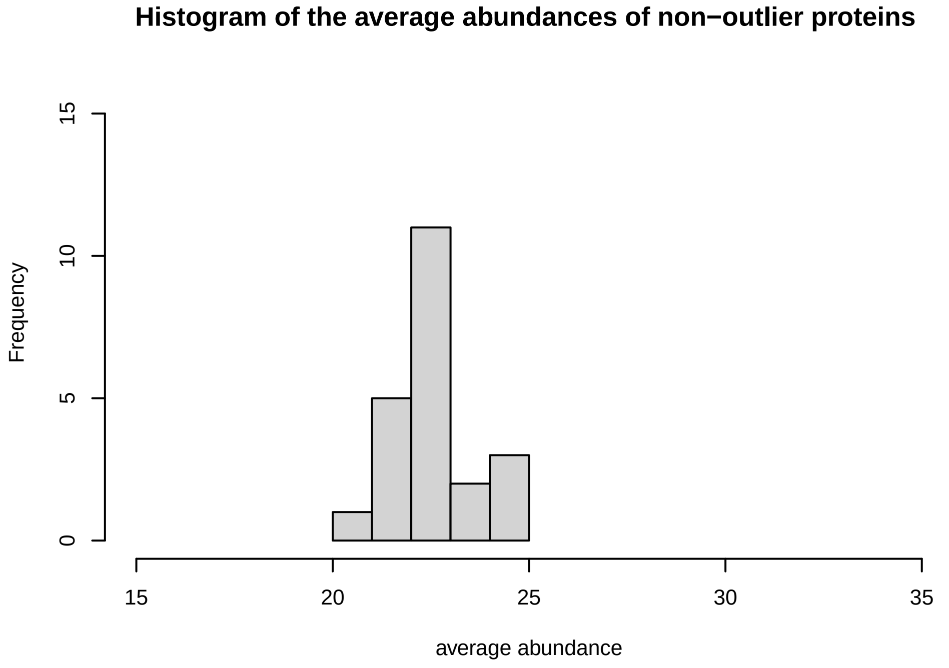
Histogram of the average abundances of non−outlier proteins
Frequency
average abundance
0 5 10 15
15 20 25 30 35

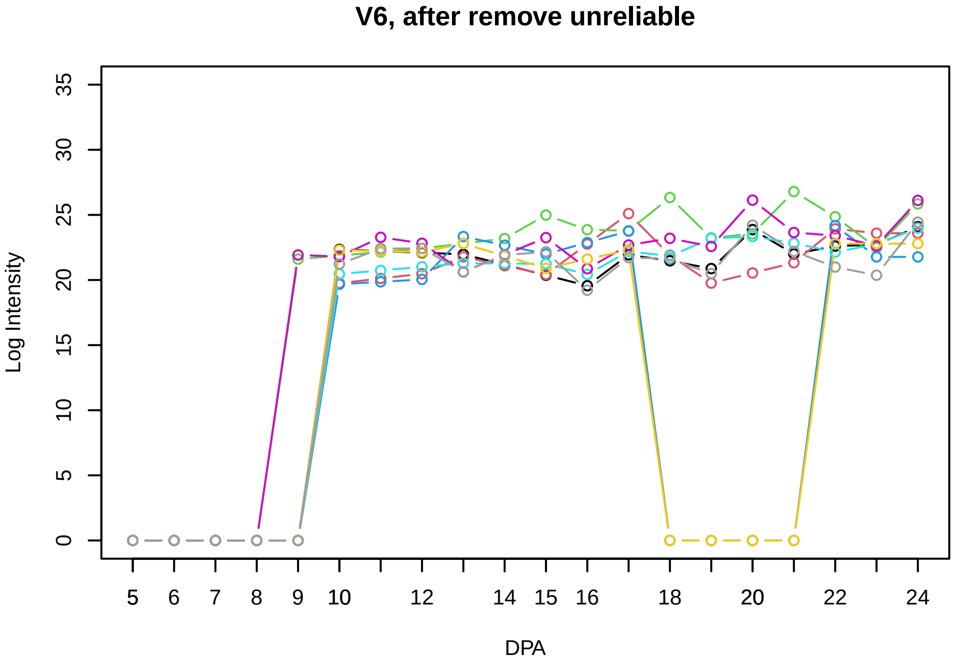
V6, after remove unreliable
Log Intensity
DPA
0 5 10 15 20 25 30 35
5 6 7 8 9 10 12 14 15 16 18 20 22 24

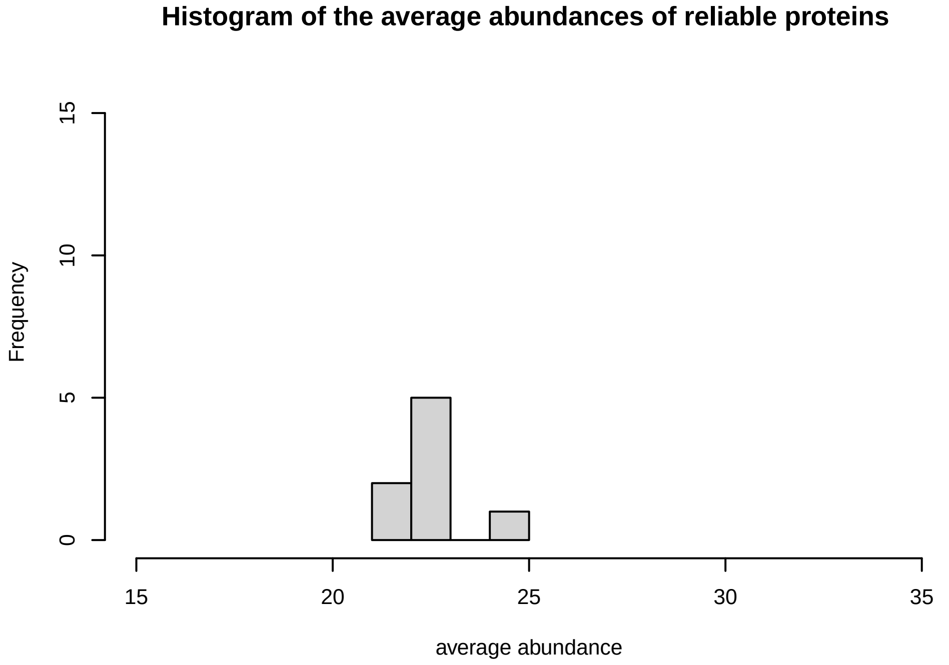
Histogram of the average abundances of reliable proteins
Frequency
average abundance
0 5 10 15
15 20 25 30 35

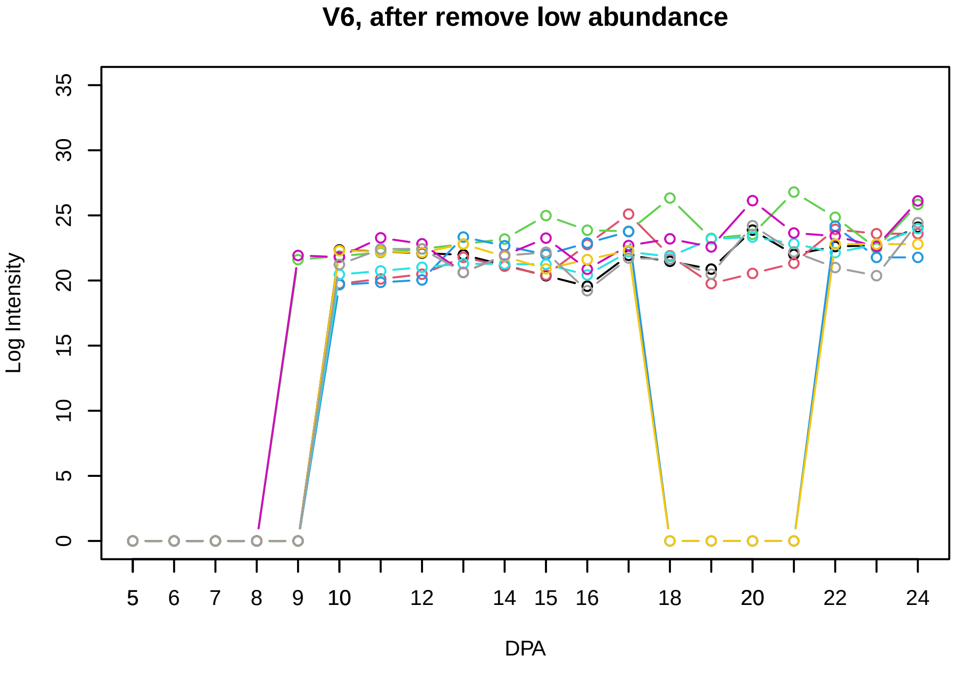
V6, after remove low abundance
Log Intensity
DPA
0 5 10 15 20 25 30 35
5 6 7 8 9 10 12 14 15 16 18 20 22 24

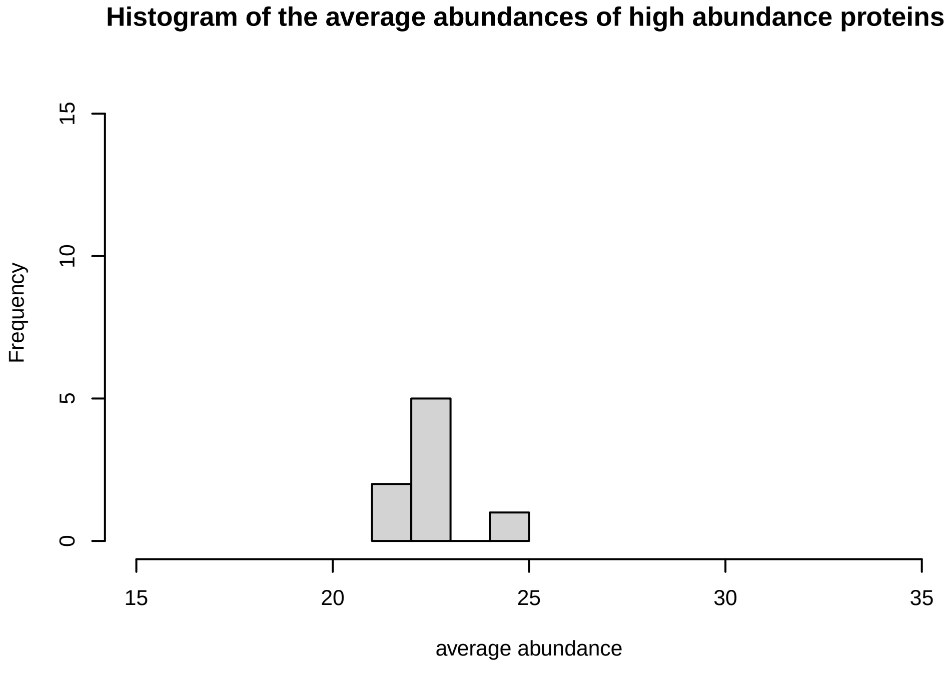
Histogram of the average abundances of high abundance proteins
Frequency
average abundance
0 5 10 15
15 20 25 30 35

V7

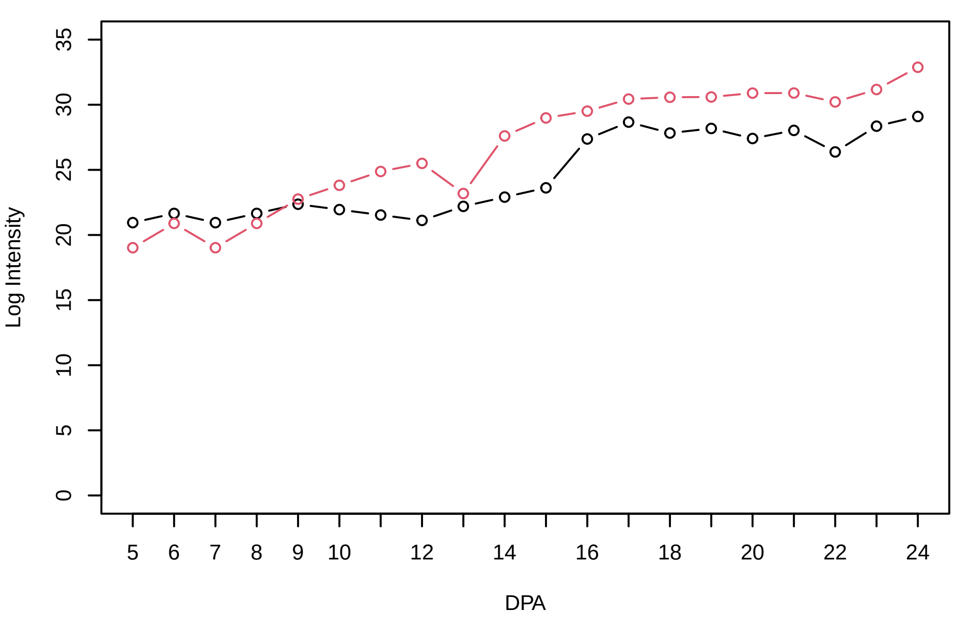

Histogram of the average abundances of non−zero values

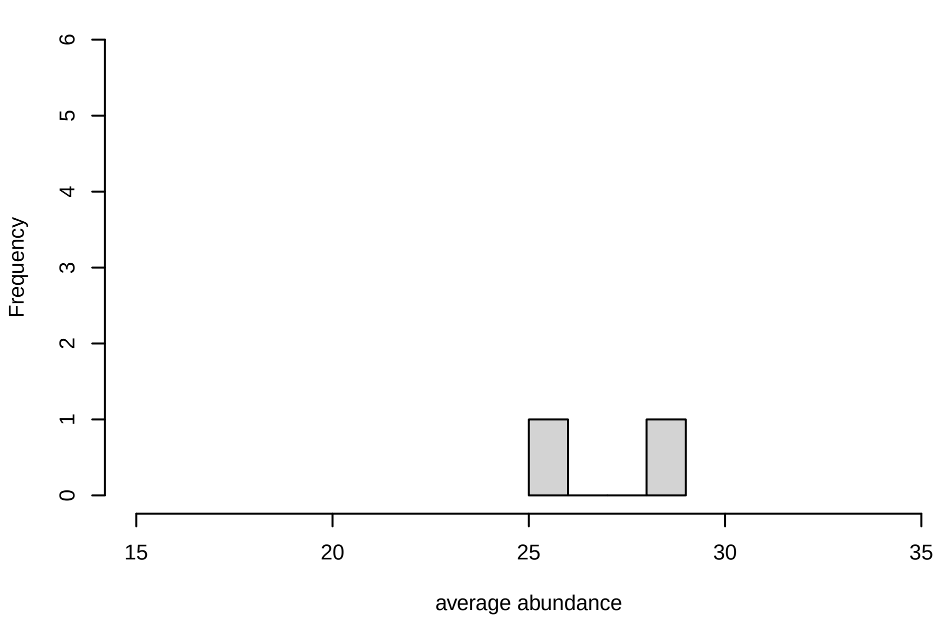

V7, after remove outlier and cutoff is 0.8

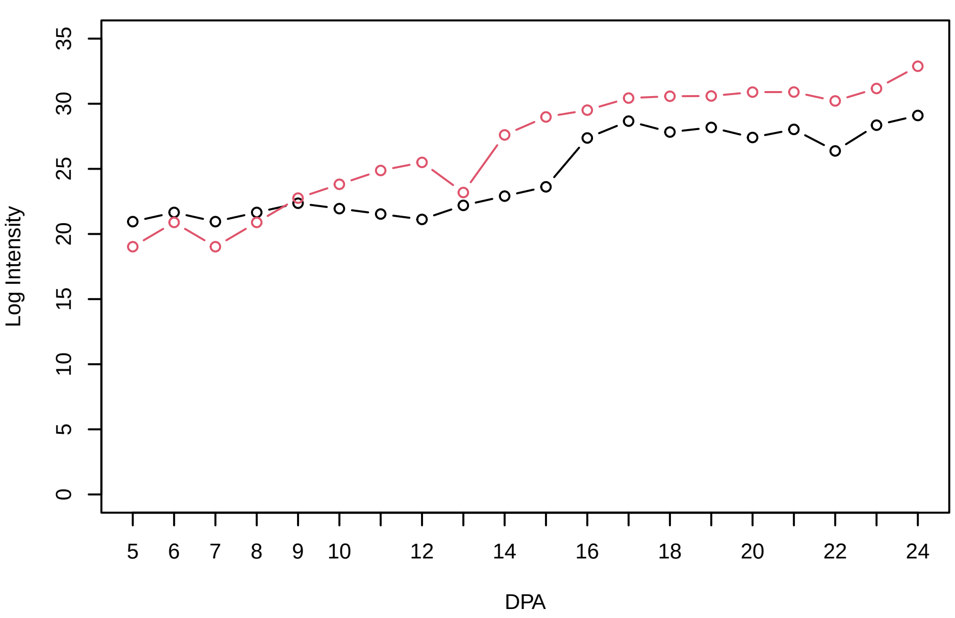

Histogram of the average abundances of non−outlier proteins

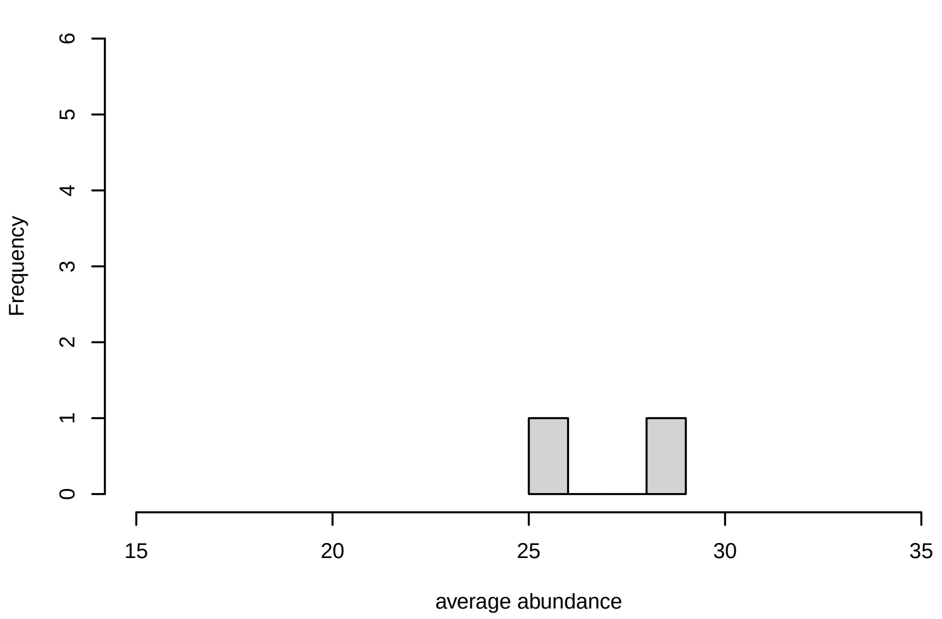

V7, after remove unreliable

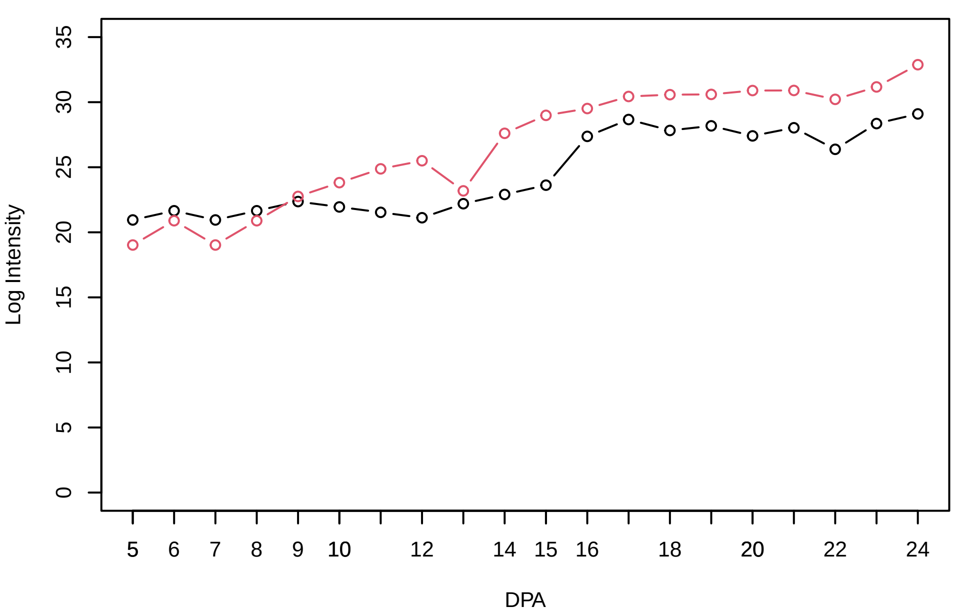

Histogram of the average abundances of reliable proteins

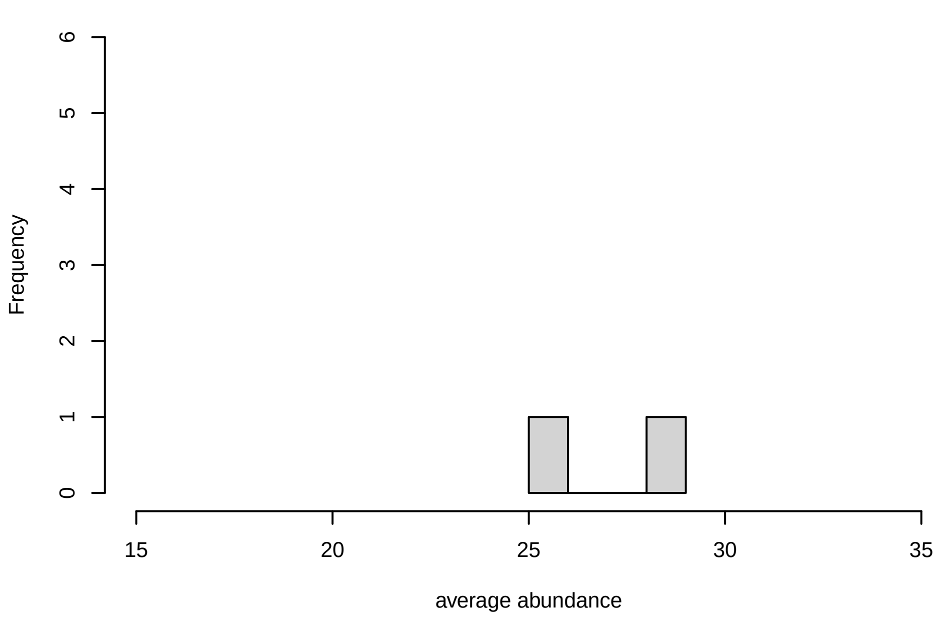

V7, after remove low abundance

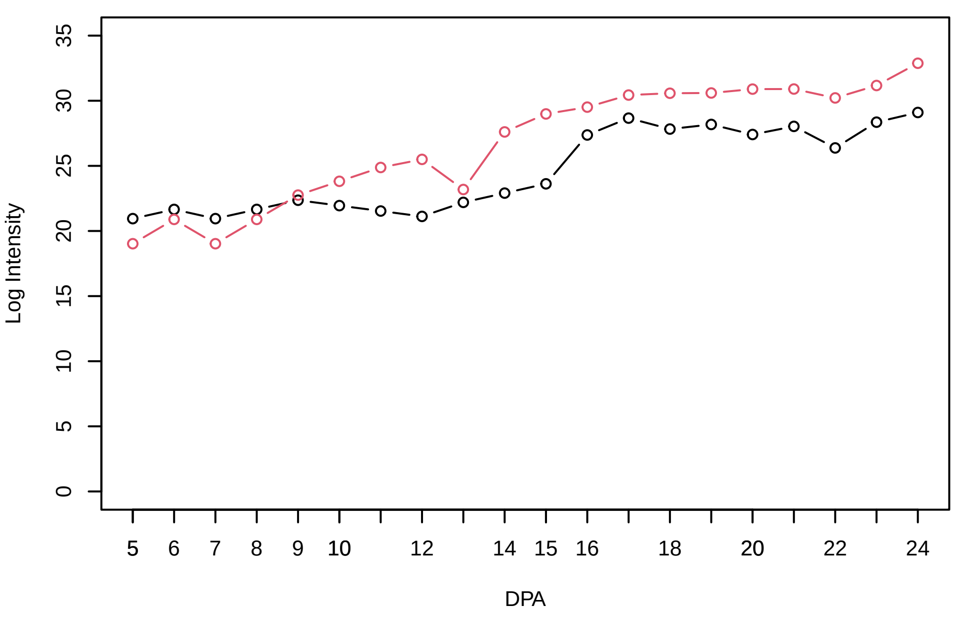

Histogram of the average abundances of high abundance proteins

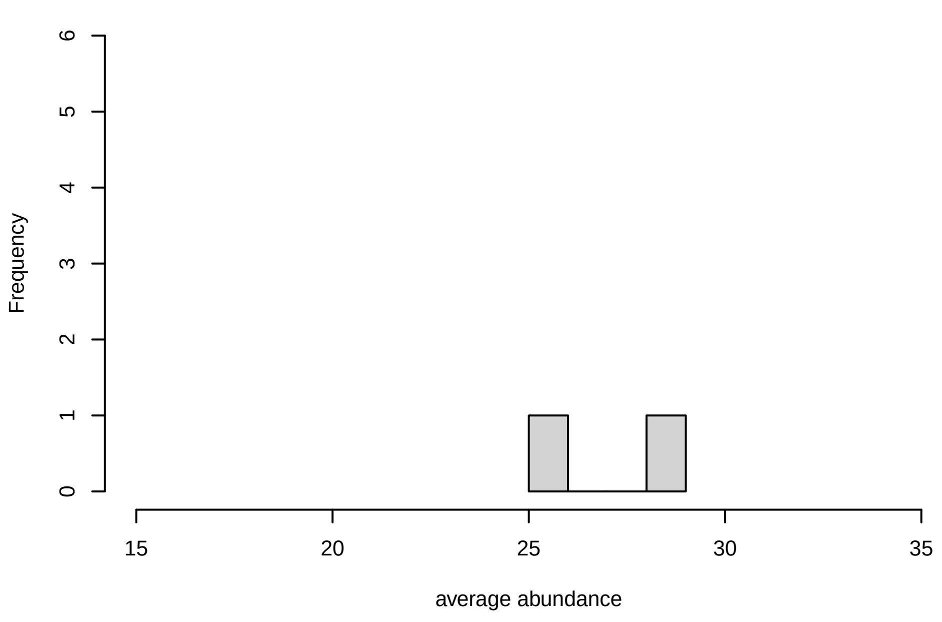

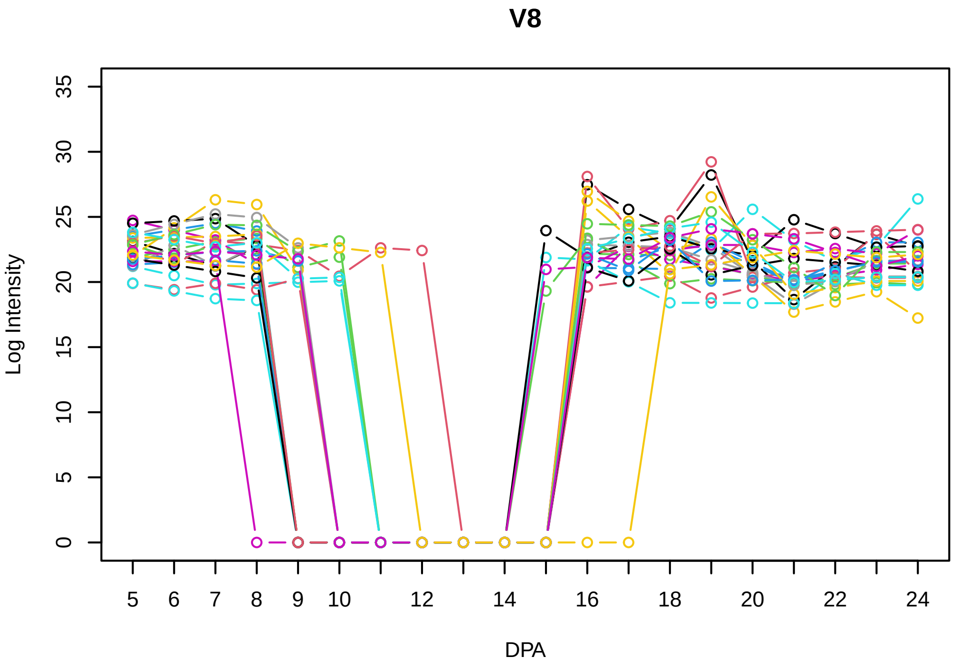
V8
Log Intensity
DPA

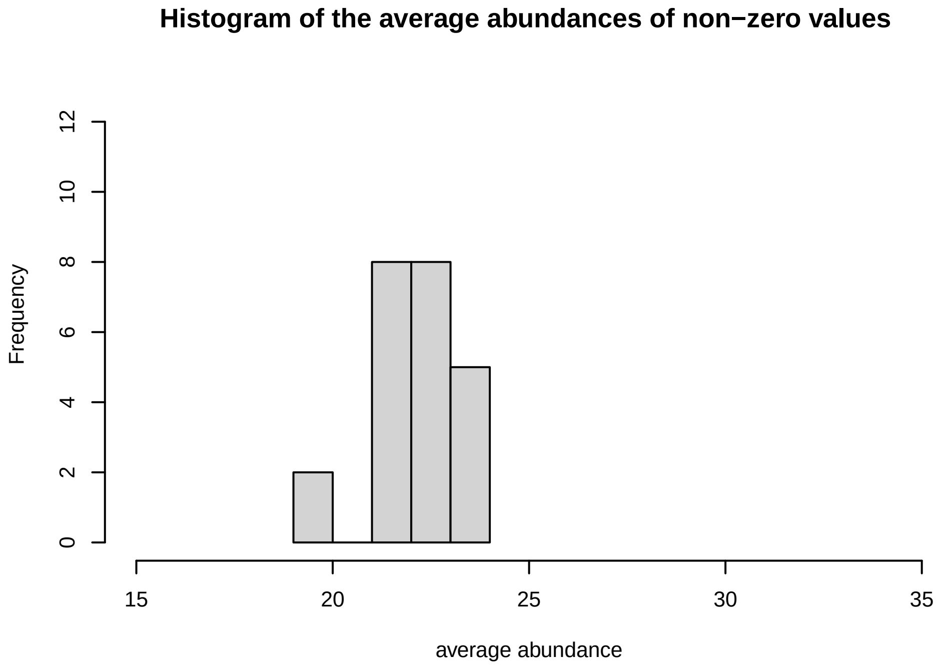
Histogram of the average abundances of non−zero values
Frequency
average abundance

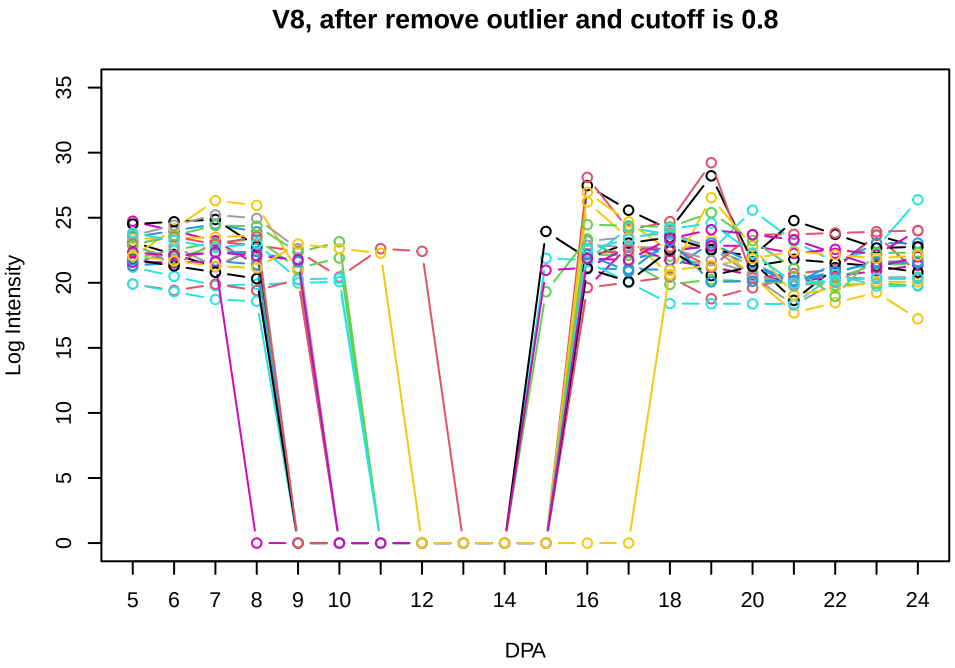
V8, after remove outlier and cutoff is 0.8
Log Intensity
DPA

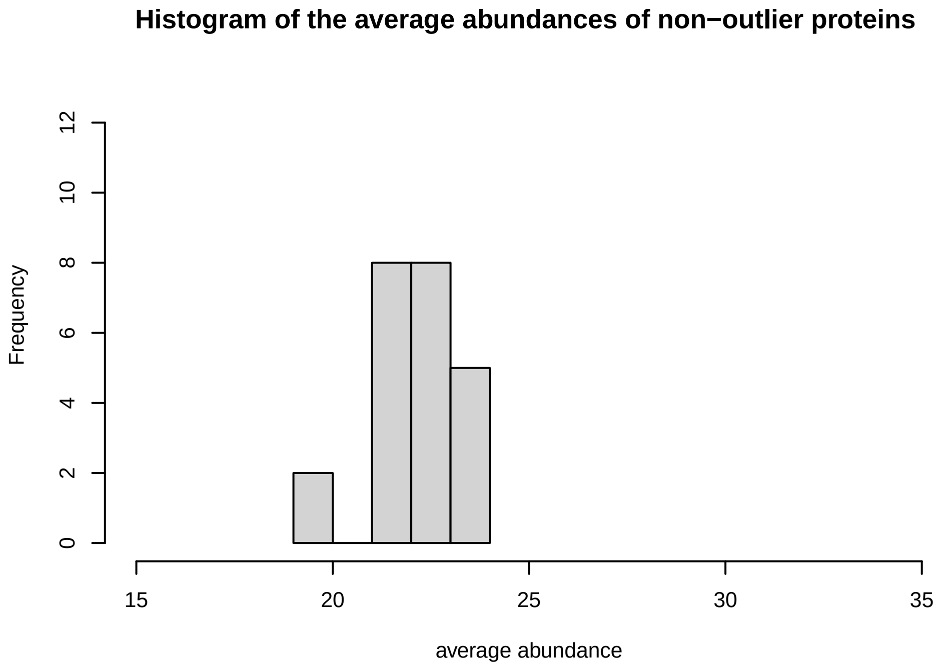
Histogram of the average abundances of non−outlier proteins
Frequency
average abundance

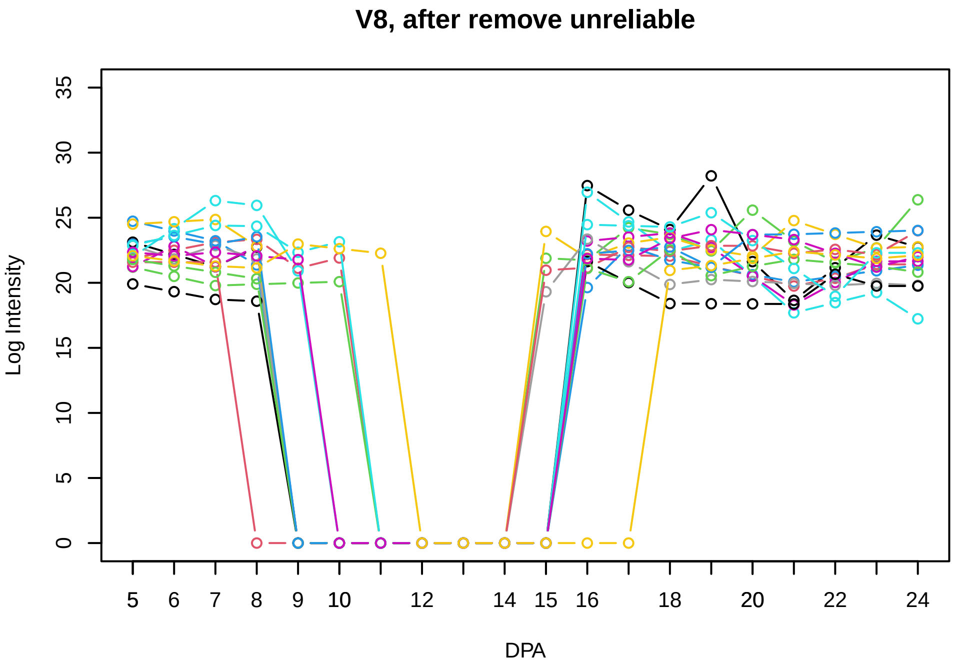
V8, after remove unreliable
Log Intensity
DPA

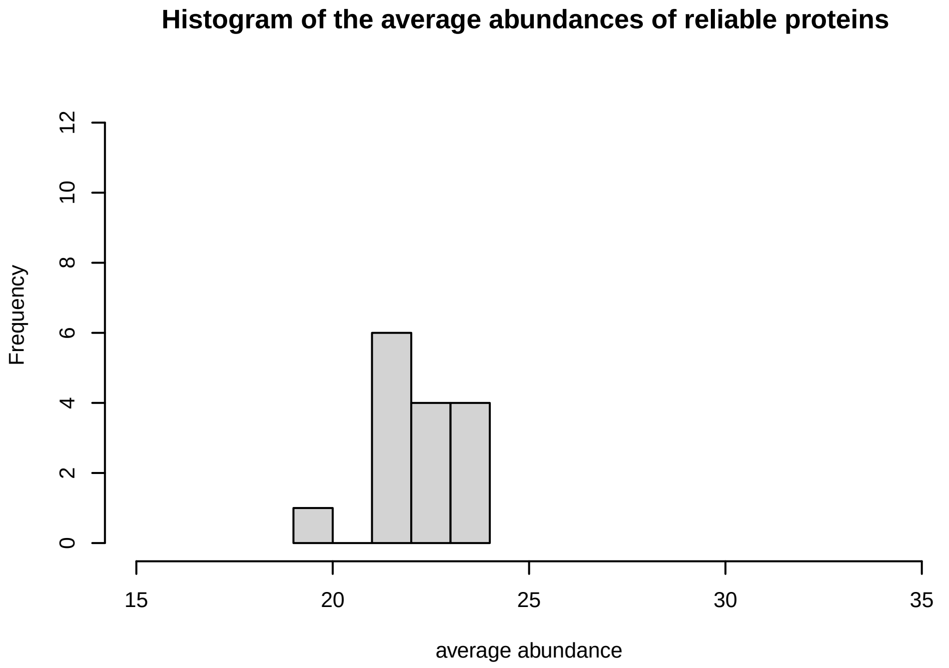
Histogram of the average abundances of reliable proteins
Frequency
average abundance

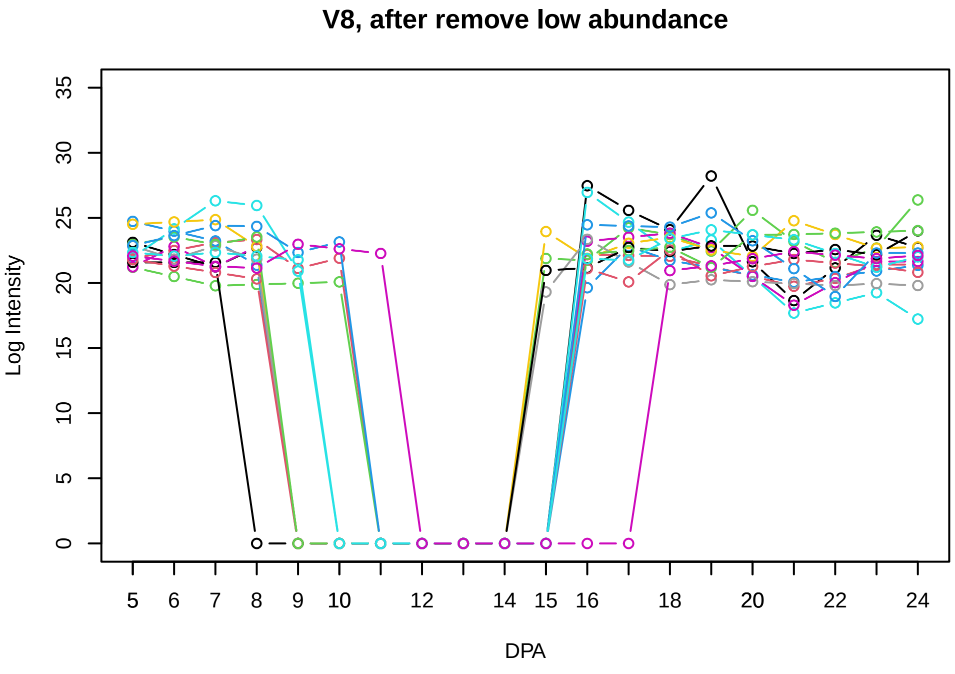
V8, after remove low abundance
Log Intensity
DPA

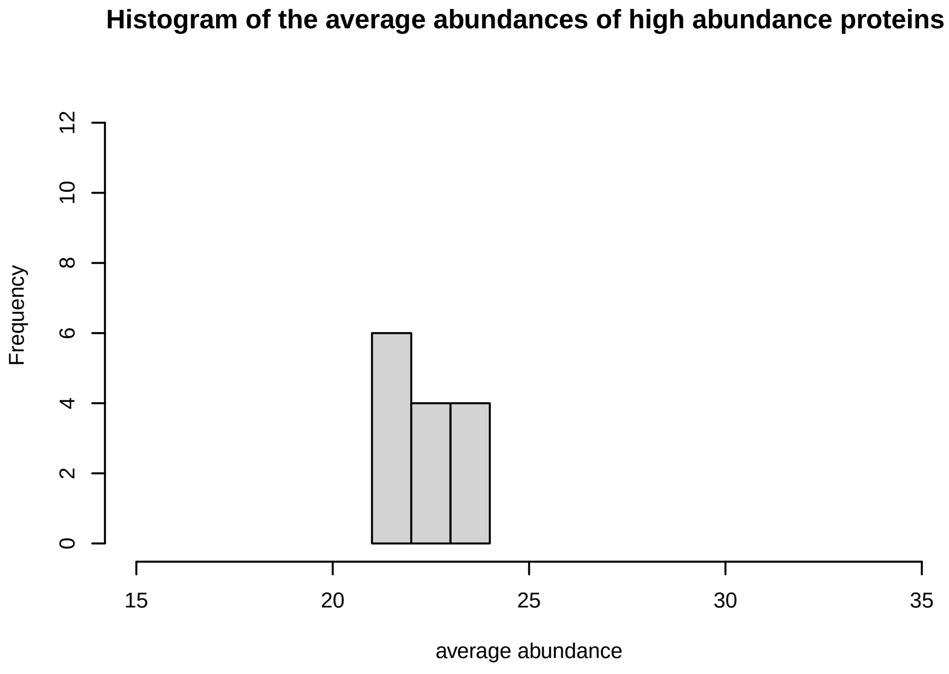
Histogram of the average abundances of high abundance proteins
Frequency
average abundance

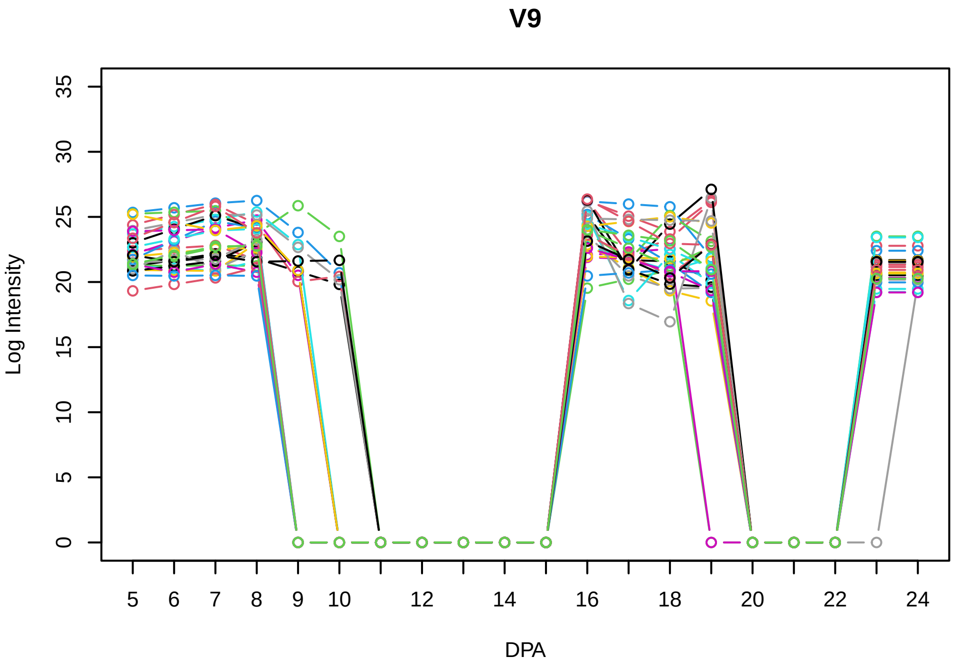
V9
Log Intensity
DPA
0
5
10
15
20
25
30
35
5
6
7
8
9
10
12
14
16
18
20
22
24

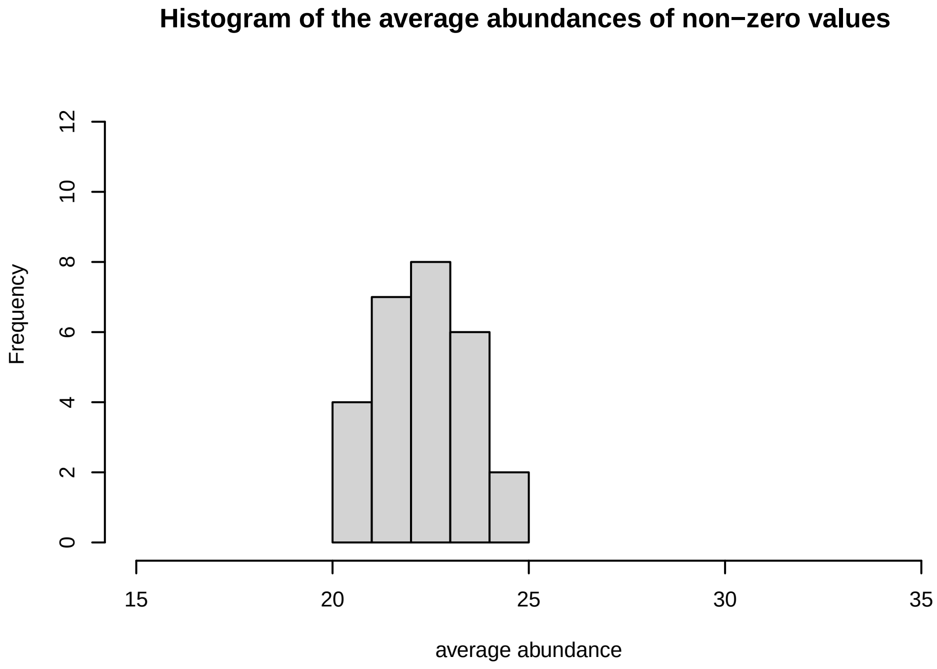
Histogram of the average abundances of non−zero values
Frequency
average abundance
0
2
4
6
8
10
12
15
20
25
30
35

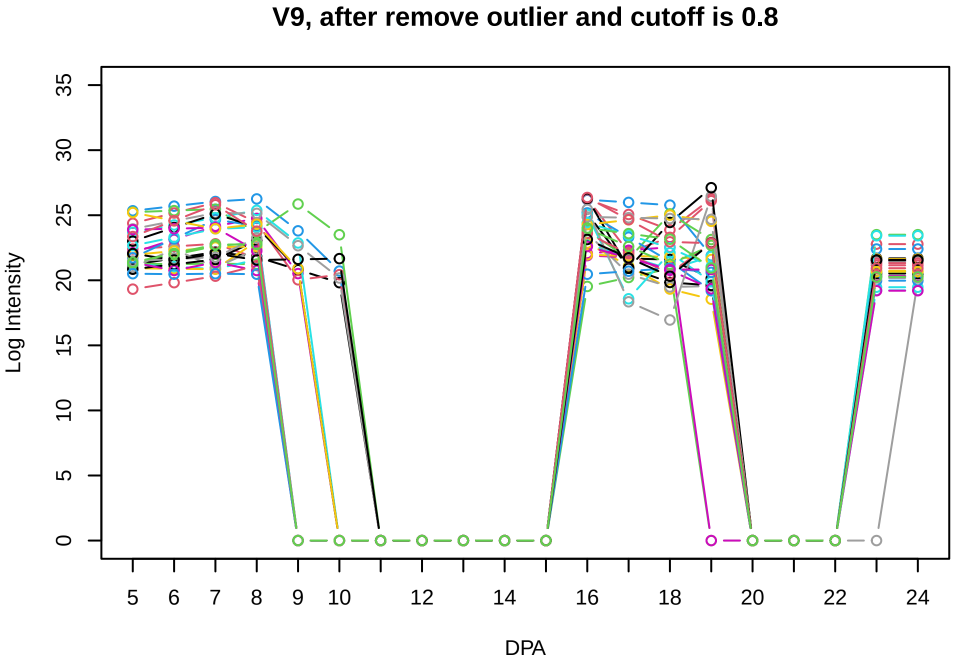
V9, after remove outlier and cutoff is 0.8
Log Intensity
DPA
0
5
10
15
20
25
30
35
5
6
7
8
9
10
12
14
16
18
20
22
24

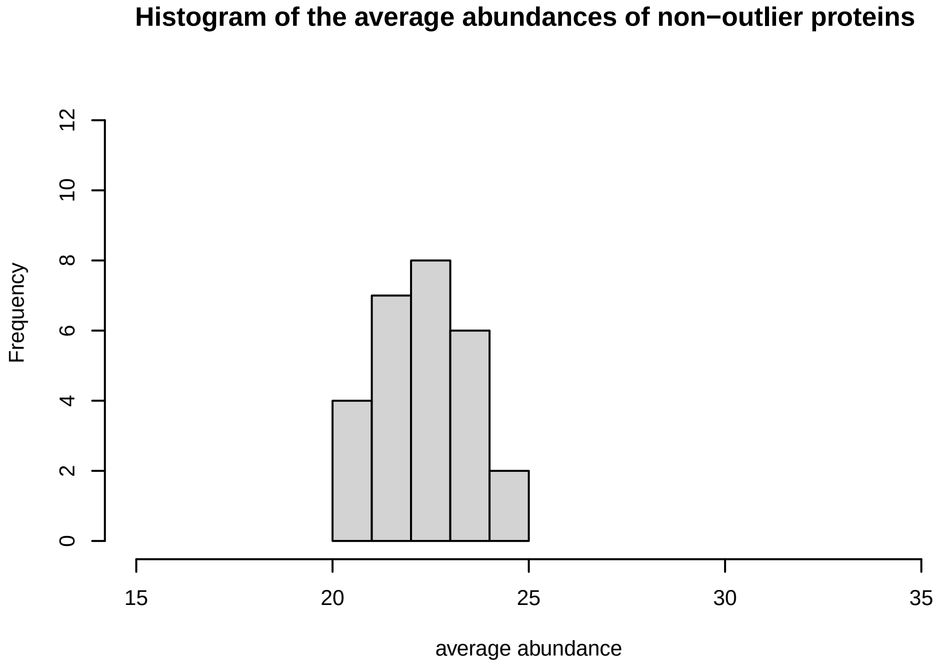
Histogram of the average abundances of non−outlier proteins
Frequency
average abundance
0
2
4
6
8
10
12
15
20
25
30
35

V10

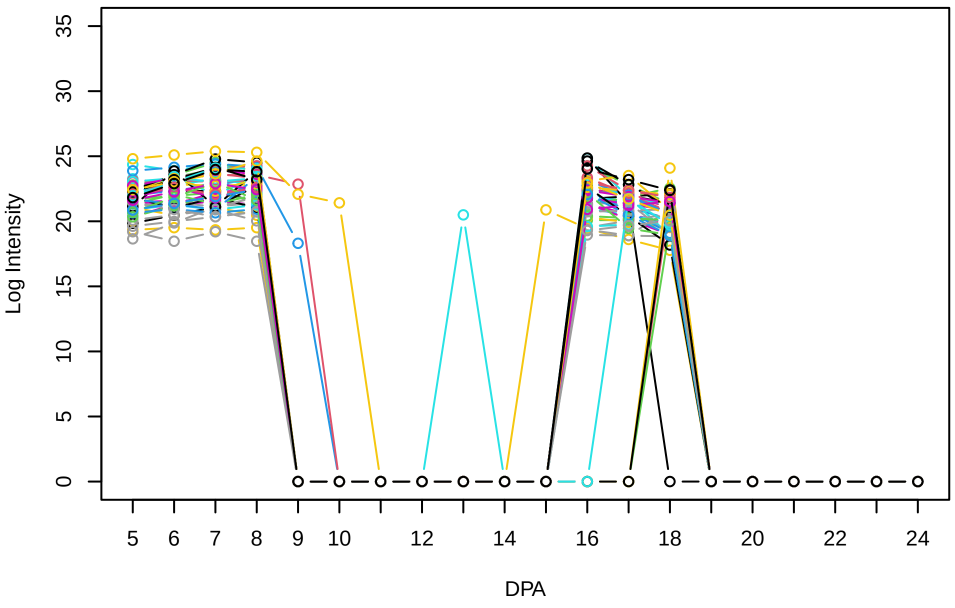

Histogram of the average abundances of non−zero values

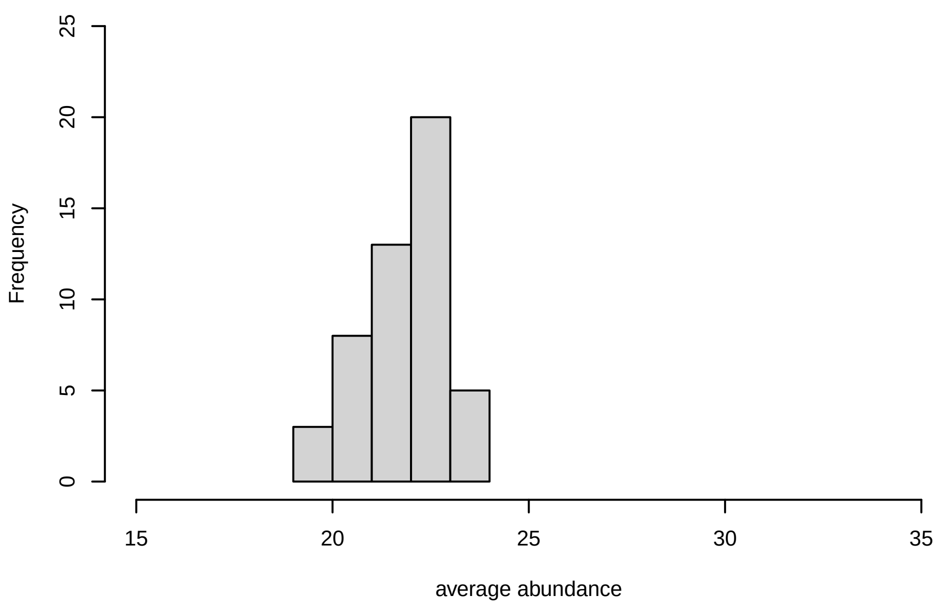

V10, after remove outlier and cutoff is 0.8

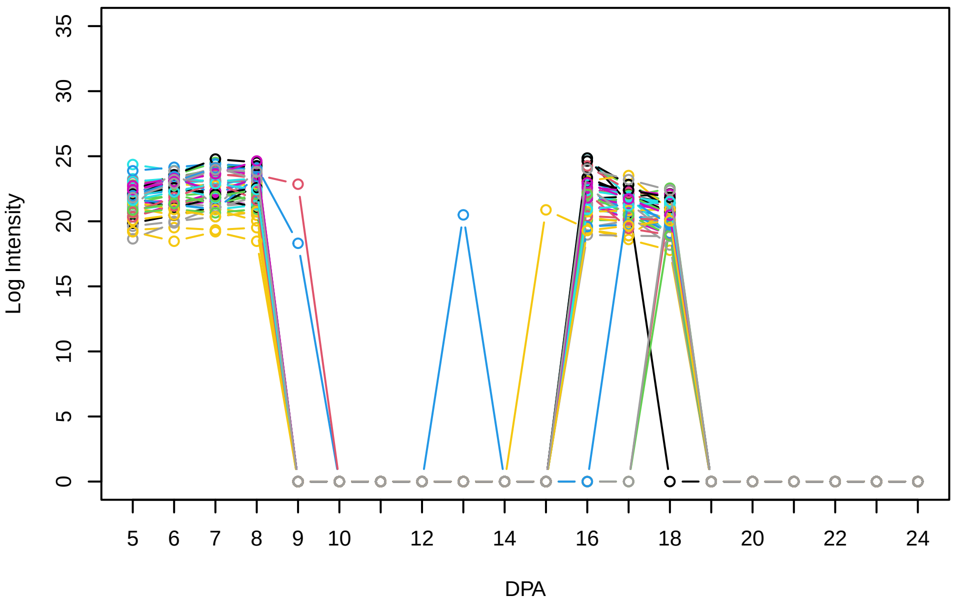

Histogram of the average abundances of non−outlier proteins

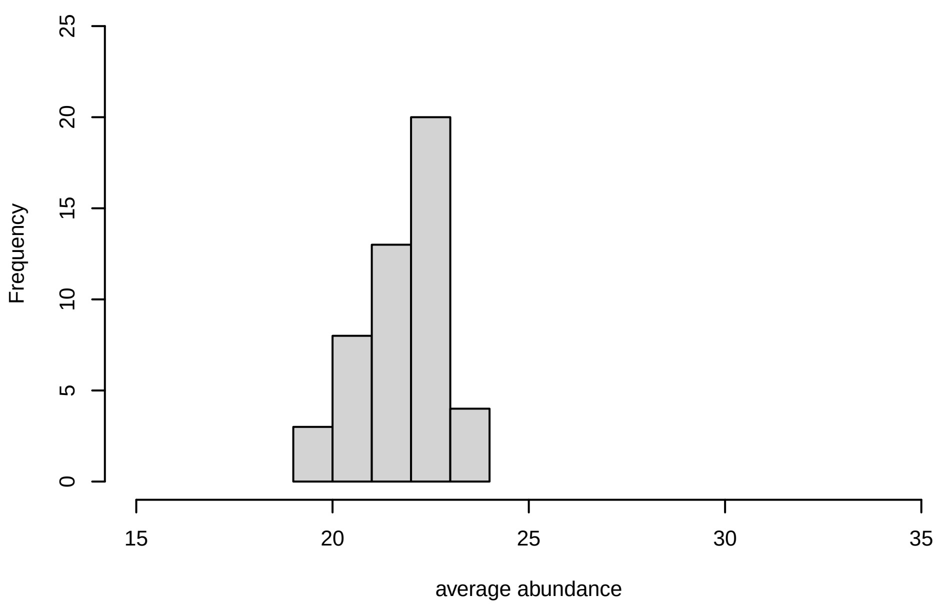

V10, after remove unreliable

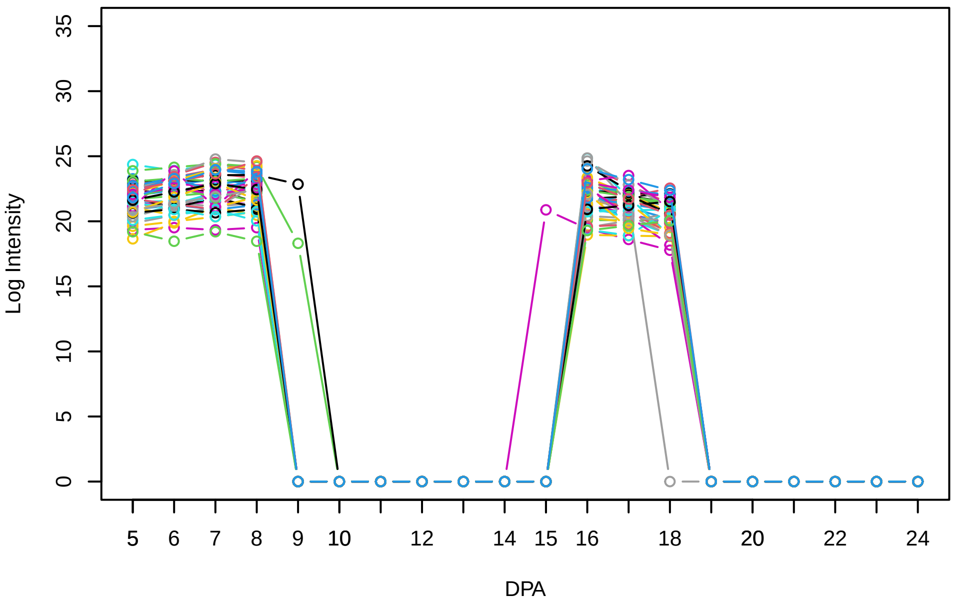

Histogram of the average abundances of reliable proteins

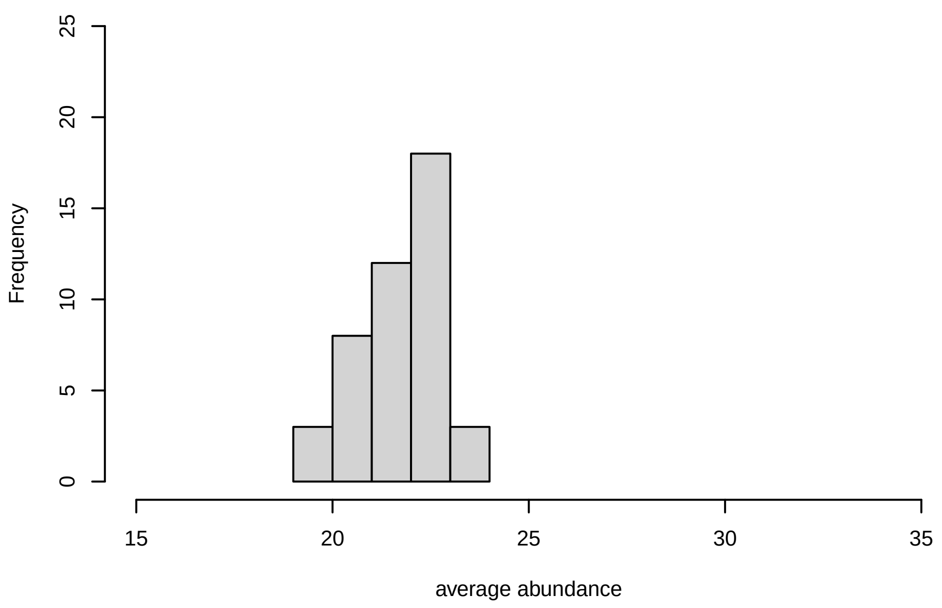

V10, after remove low abundance

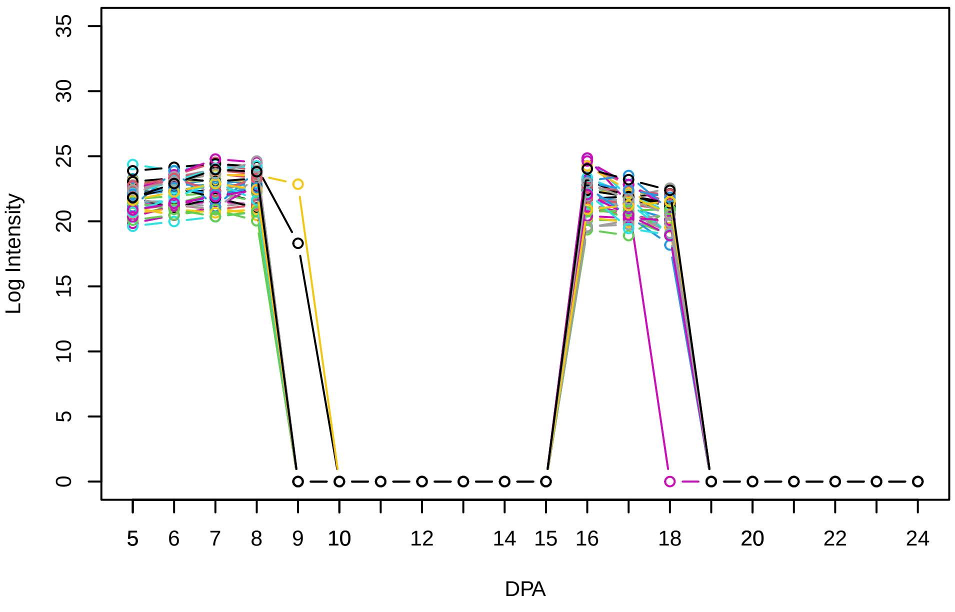

Histogram of the average abundances of high abundance proteins

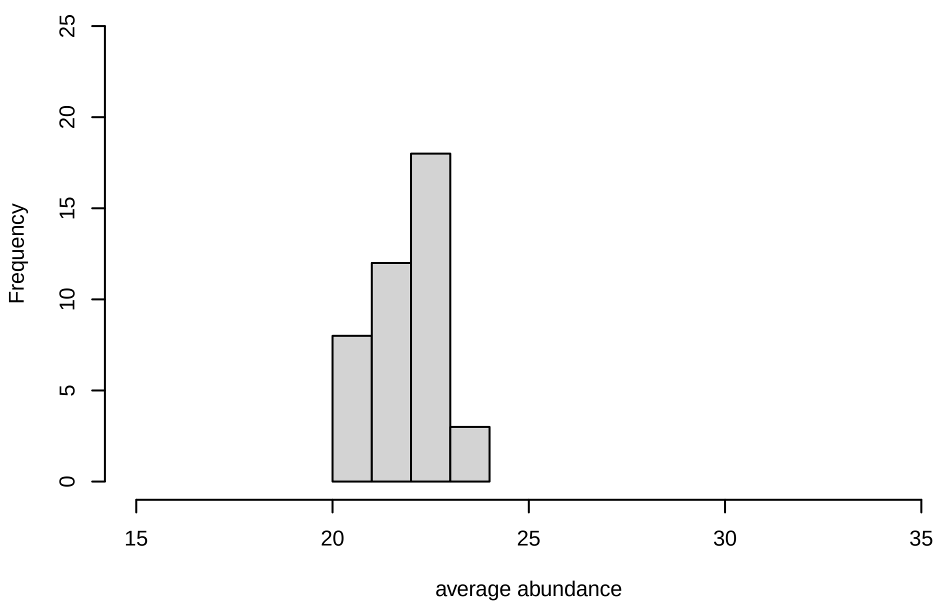

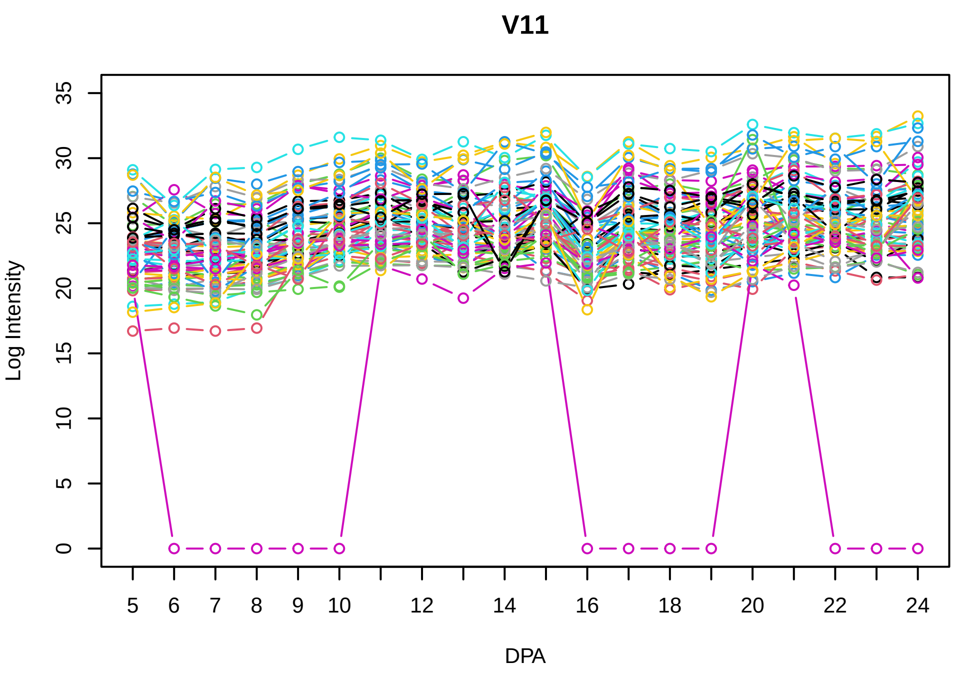
V11
Log Intensity
DPA

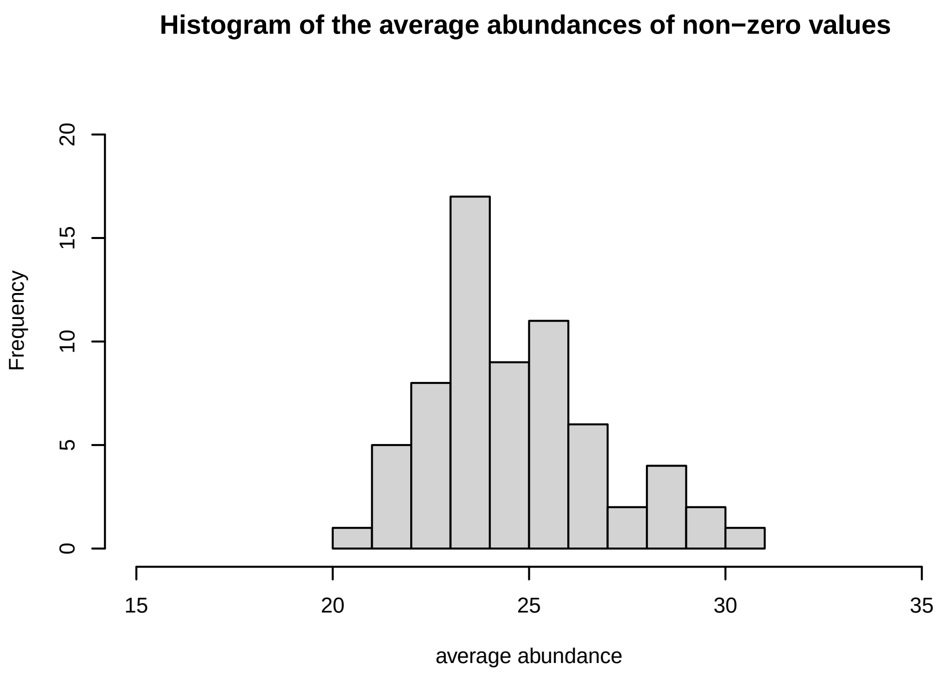
Histogram of the average abundances of non−zero values
Frequency
average abundance

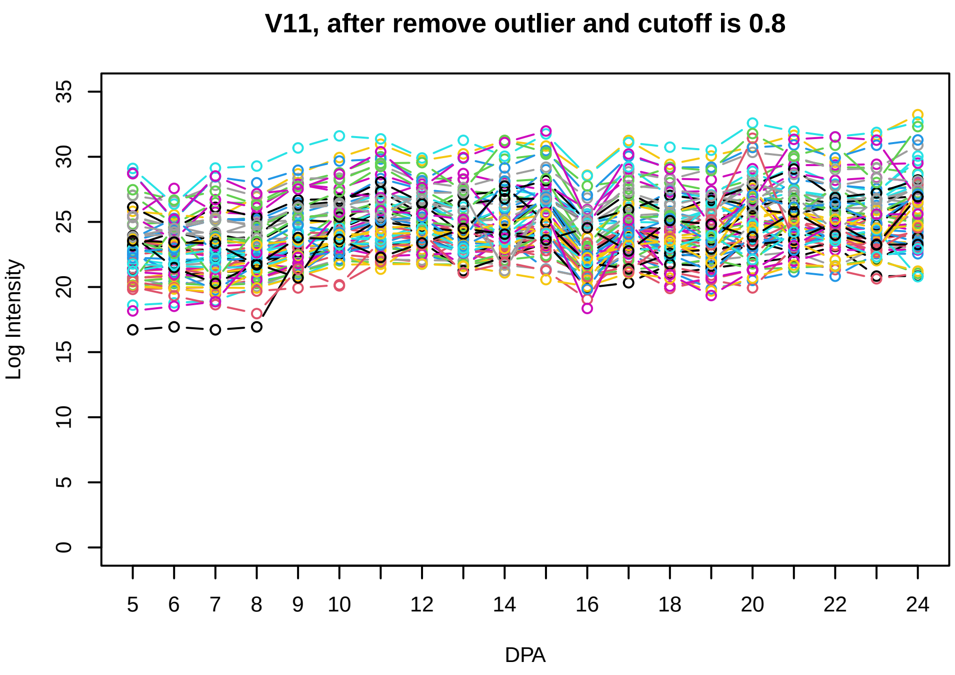
V11, after remove outlier and cutoff is 0.8
Log Intensity
DPA

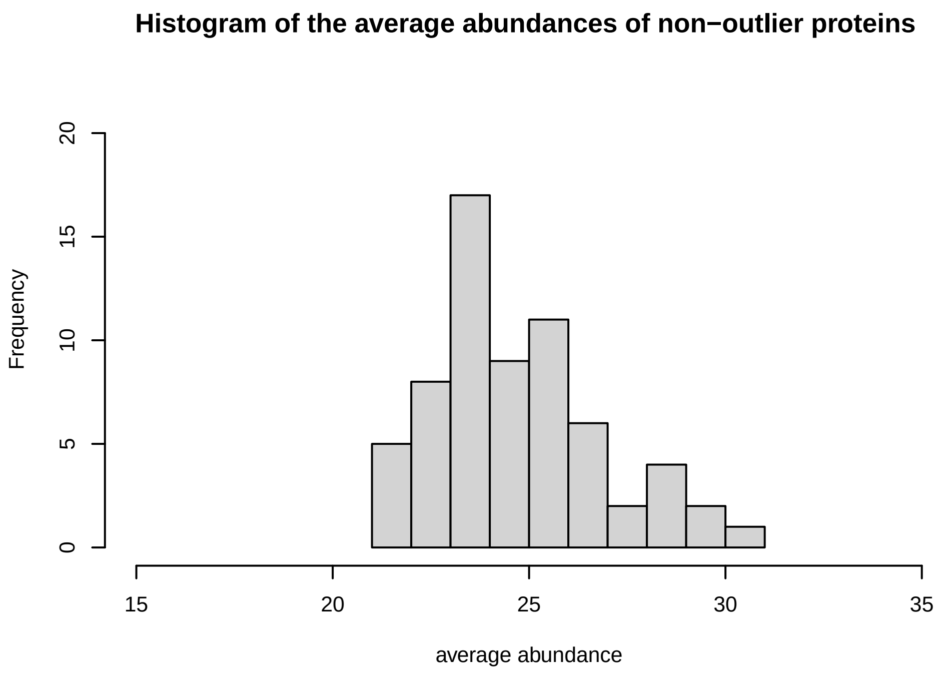
Histogram of the average abundances of non−outlier proteins
Frequency
average abundance

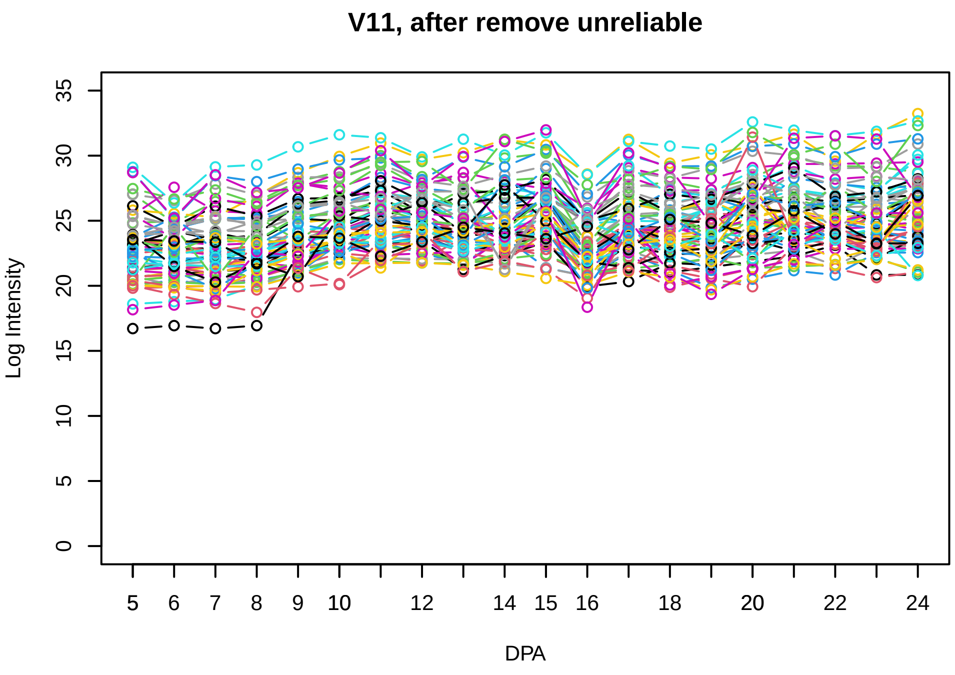
V11, after remove unreliable
Log Intensity
DPA

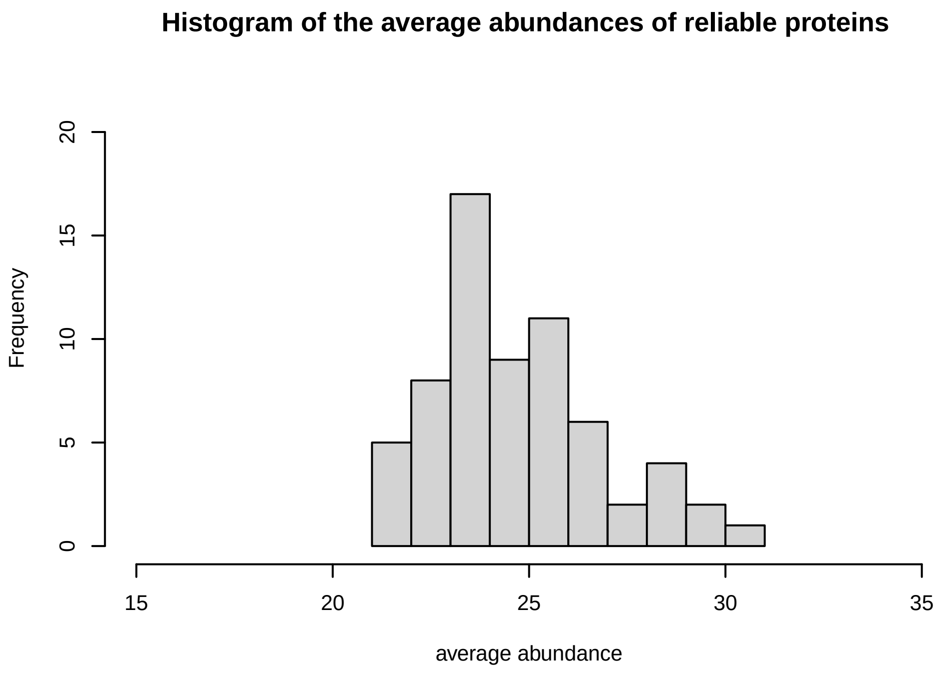
Histogram of the average abundances of reliable proteins
Frequency
average abundance

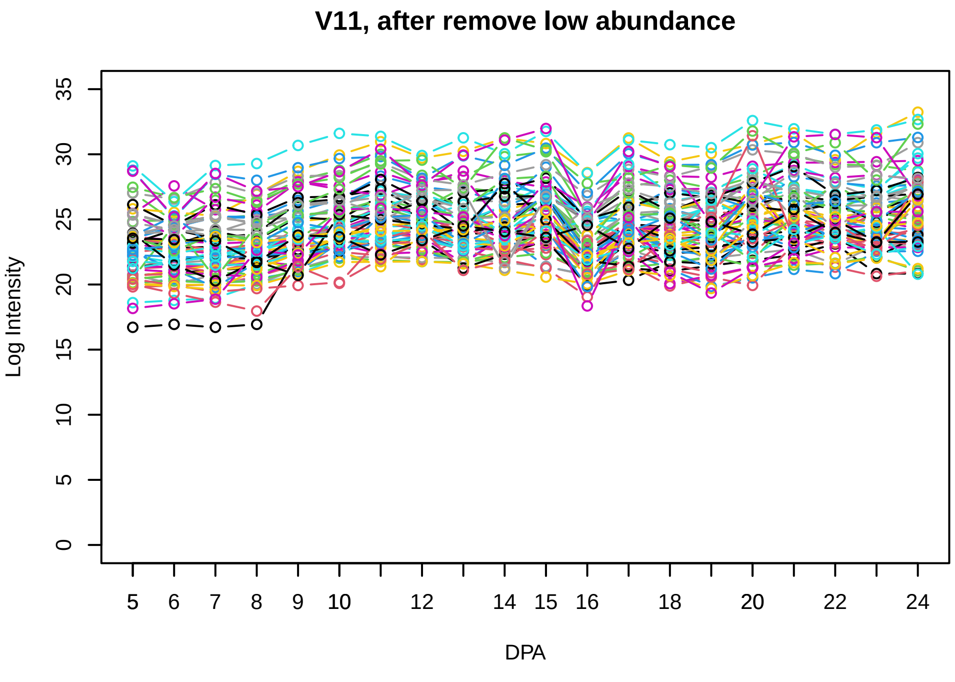
V11, after remove low abundance
Log Intensity
DPA

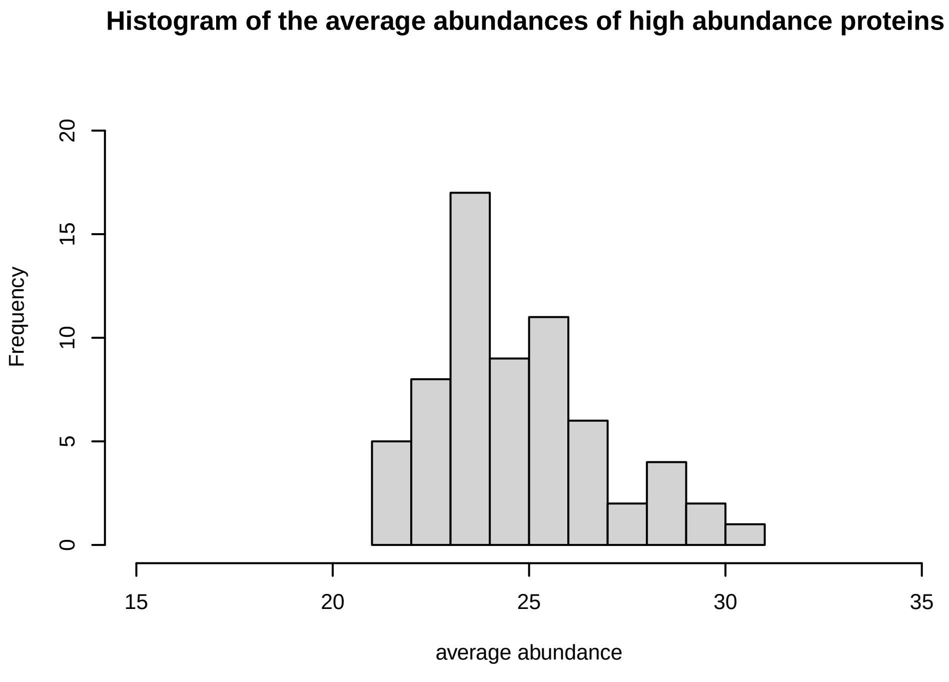
Histogram of the average abundances of high abundance proteins
Frequency
average abundance

V12

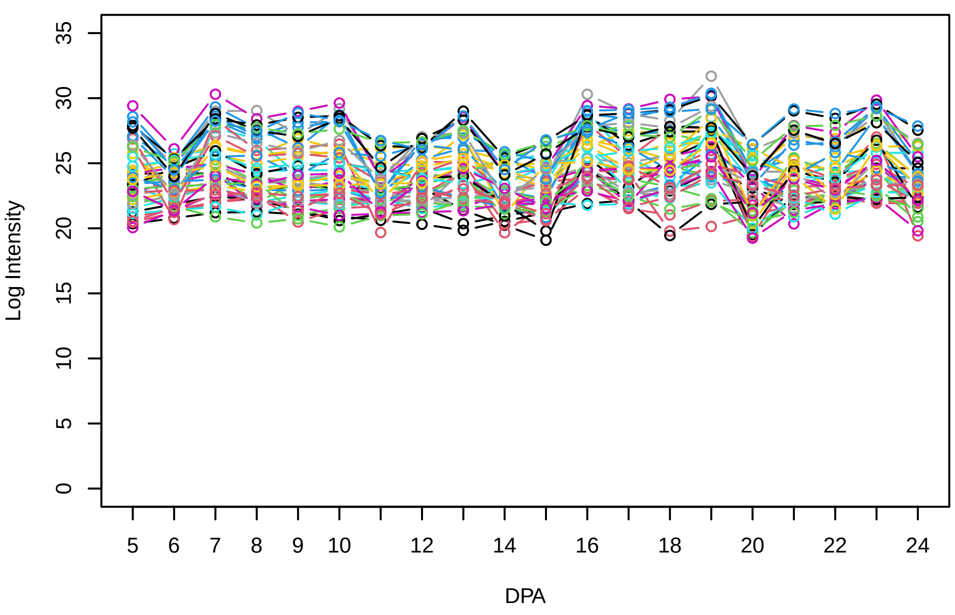

Histogram of the average abundances of non−zero values

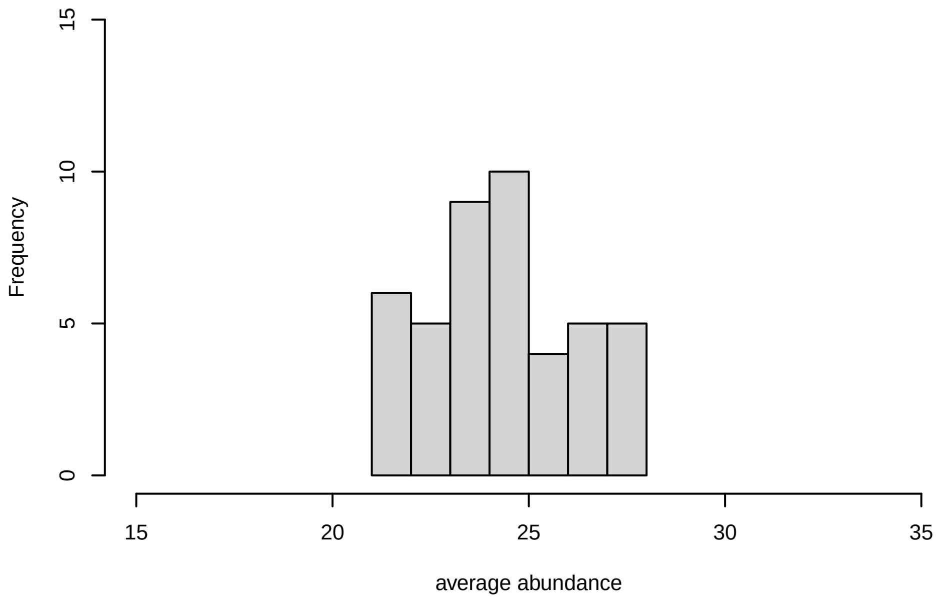

V12, after remove outlier and cutoff is 0.8

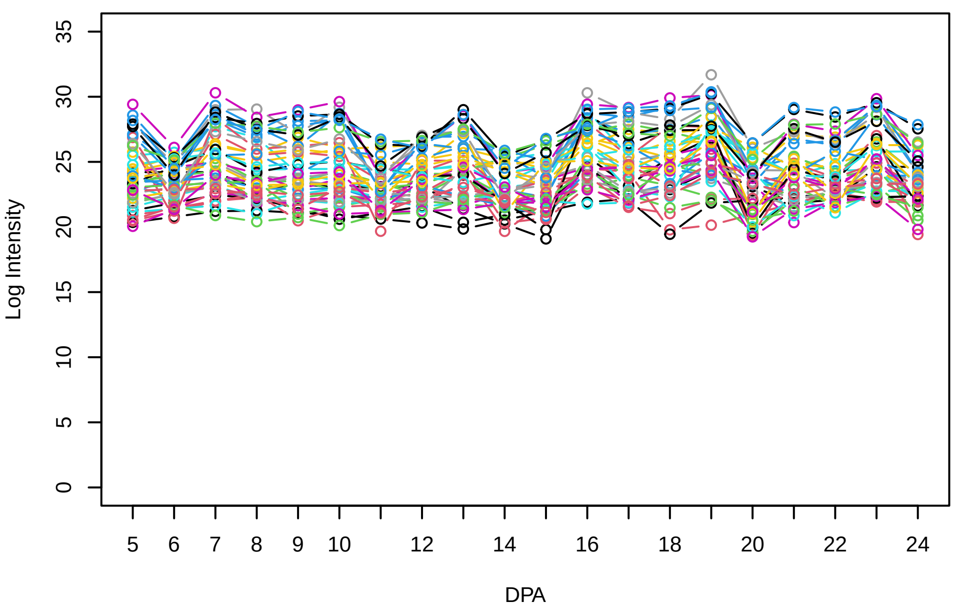

Histogram of the average abundances of non−outlier proteins

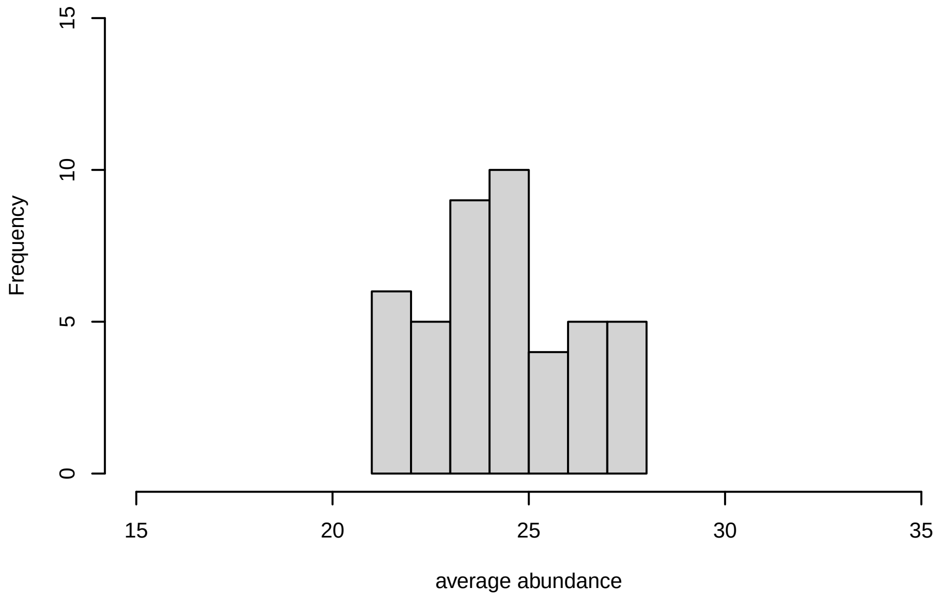

V12, after remove unreliable

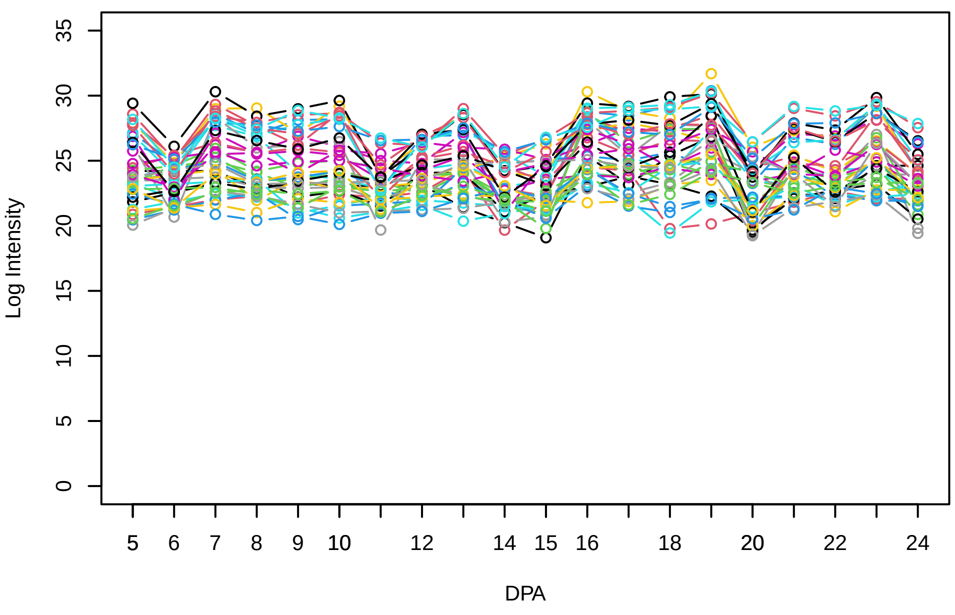

Histogram of the average abundances of reliable proteins

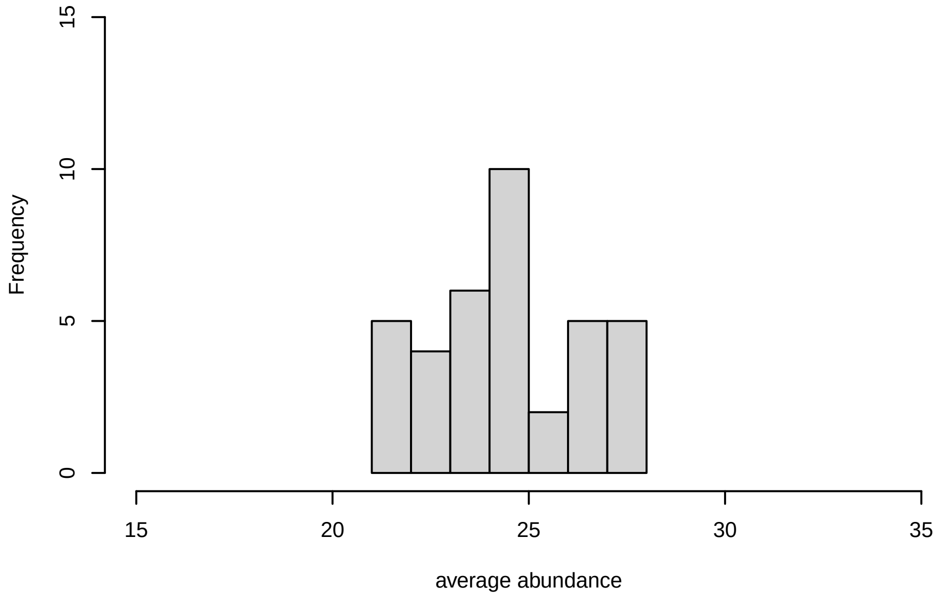

V12, after remove low abundance

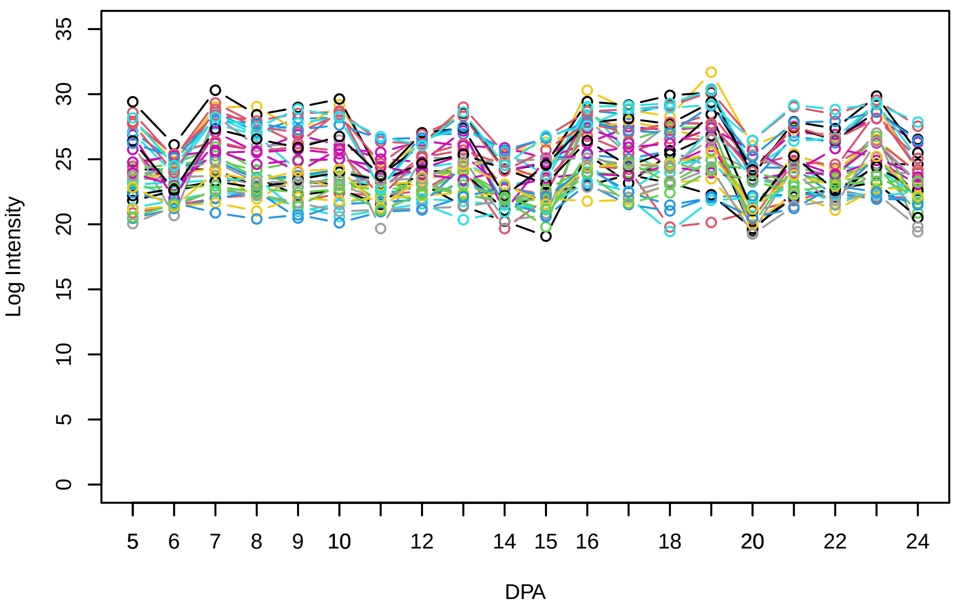

Histogram of the average abundances of high abundance proteins

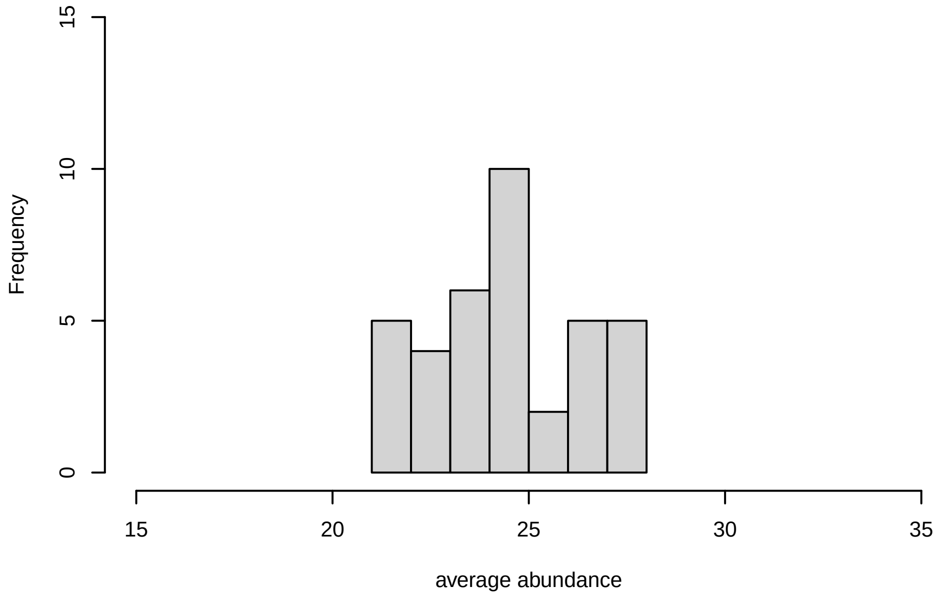

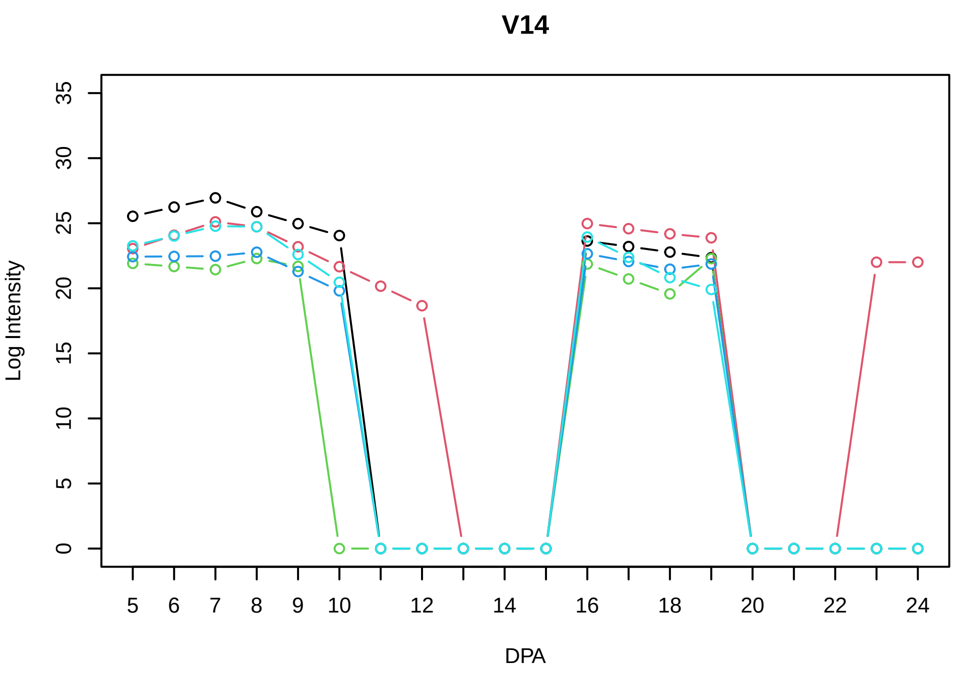
V14
Log Intensity
0 5 10 15 20 25 30 35
5 6 7 8 9 10 12 14 16 18 20 22 24
DPA

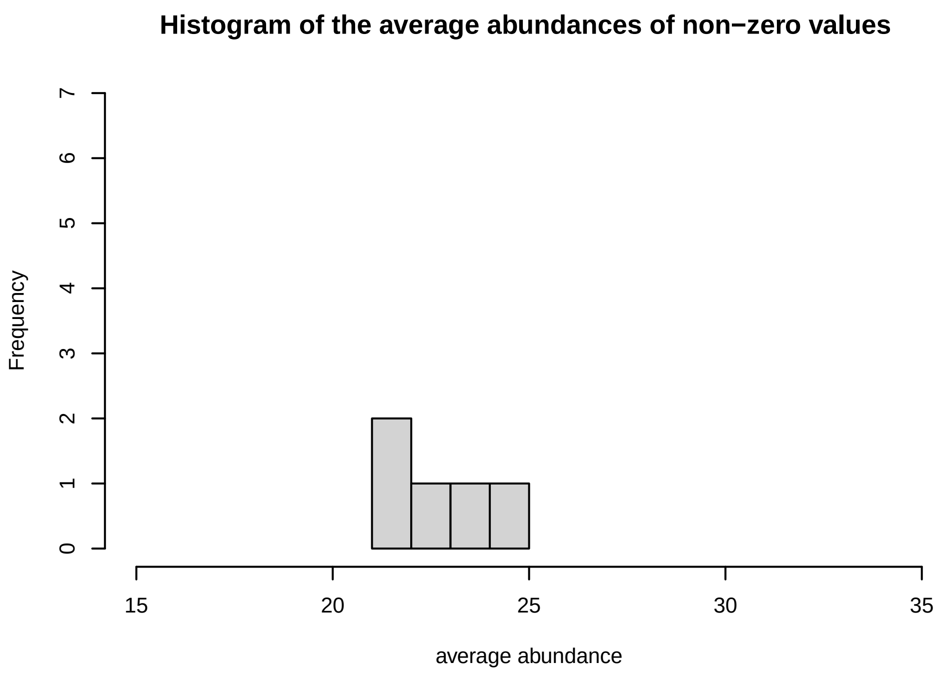
Histogram of the average abundances of non−zero values
Frequency
0 1 2 3 4 5 6 7
15 20 25 30 35
average abundance

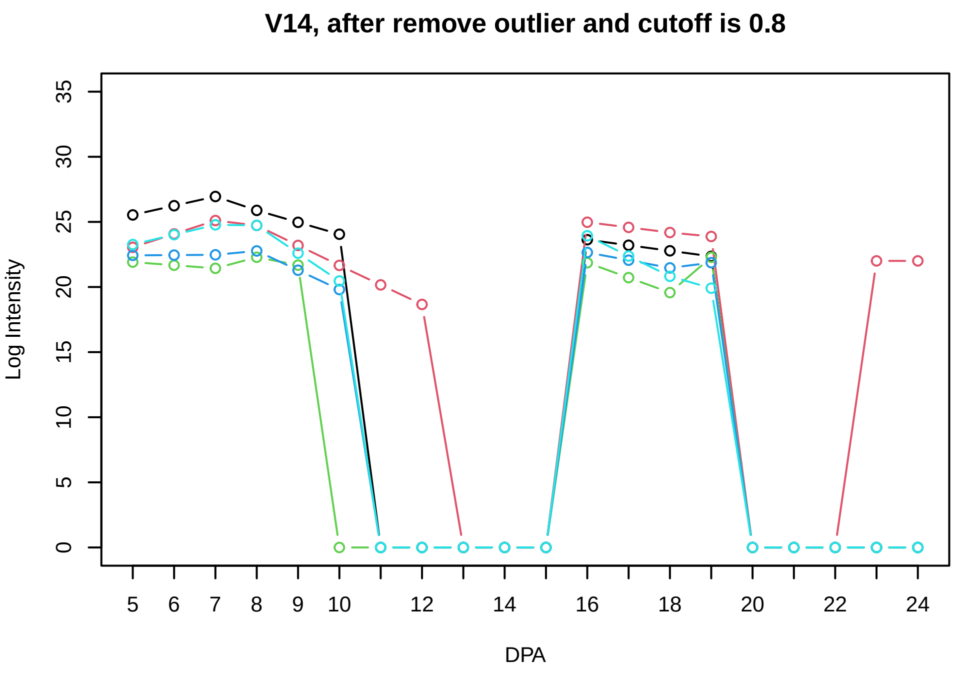
V14, after remove outlier and cutoff is 0.8
Log Intensity
0 5 10 15 20 25 30 35
5 6 7 8 9 10 12 14 16 18 20 22 24
DPA

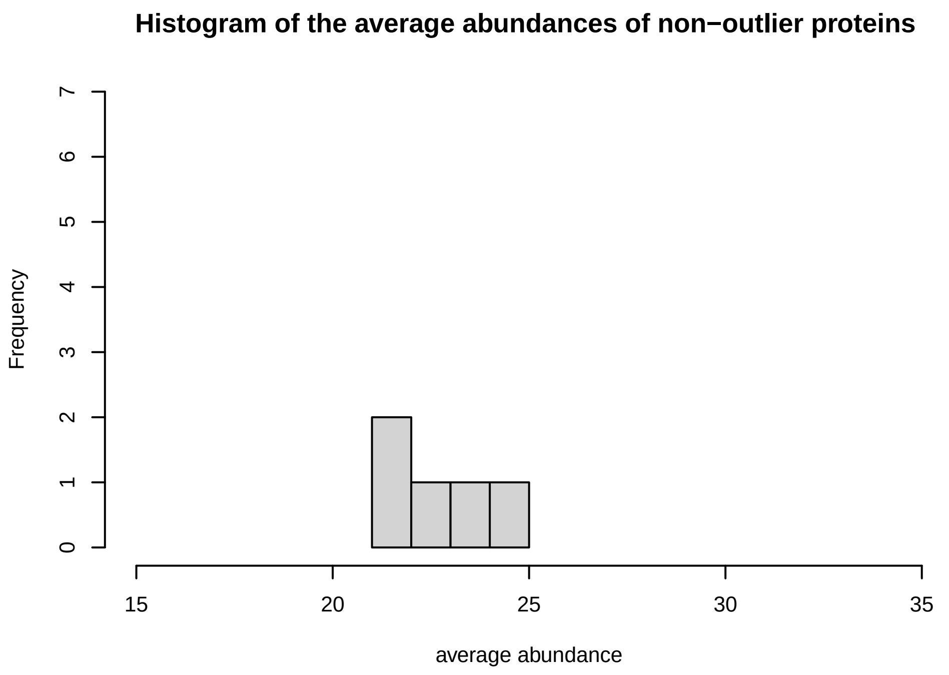
Histogram of the average abundances of non−outlier proteins
Frequency
0 1 2 3 4 5 6 7
15 20 25 30 35
average abundance

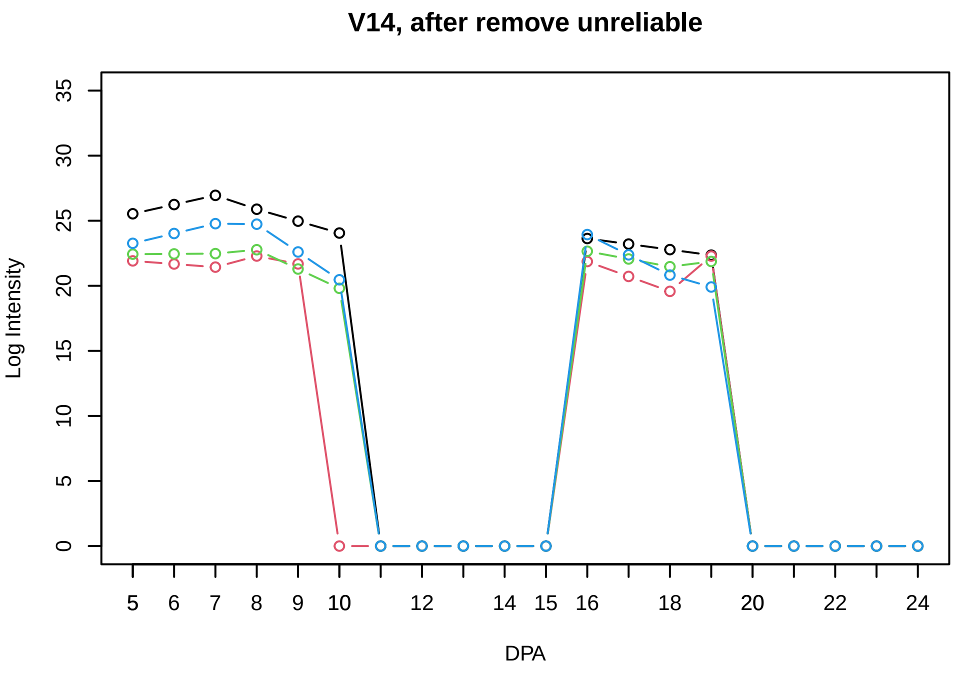
V14, after remove unreliable
Log Intensity
0 5 10 15 20 25 30 35
5 6 7 8 9 10 12 14 15 16 18 20 22 24
DPA

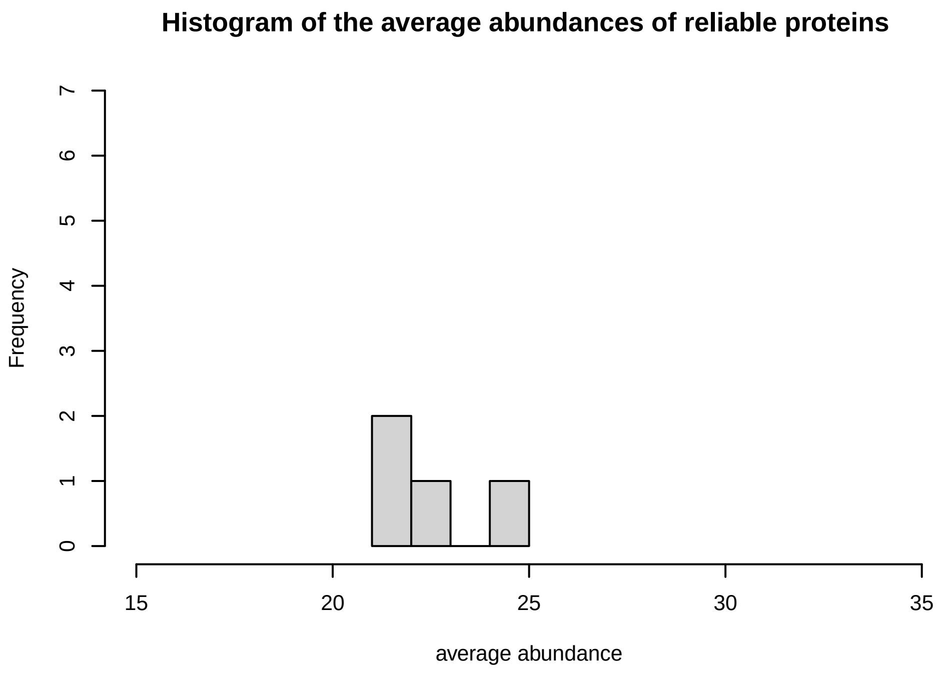
Histogram of the average abundances of reliable proteins
Frequency
0 1 2 3 4 5 6 7
15 20 25 30 35
average abundance

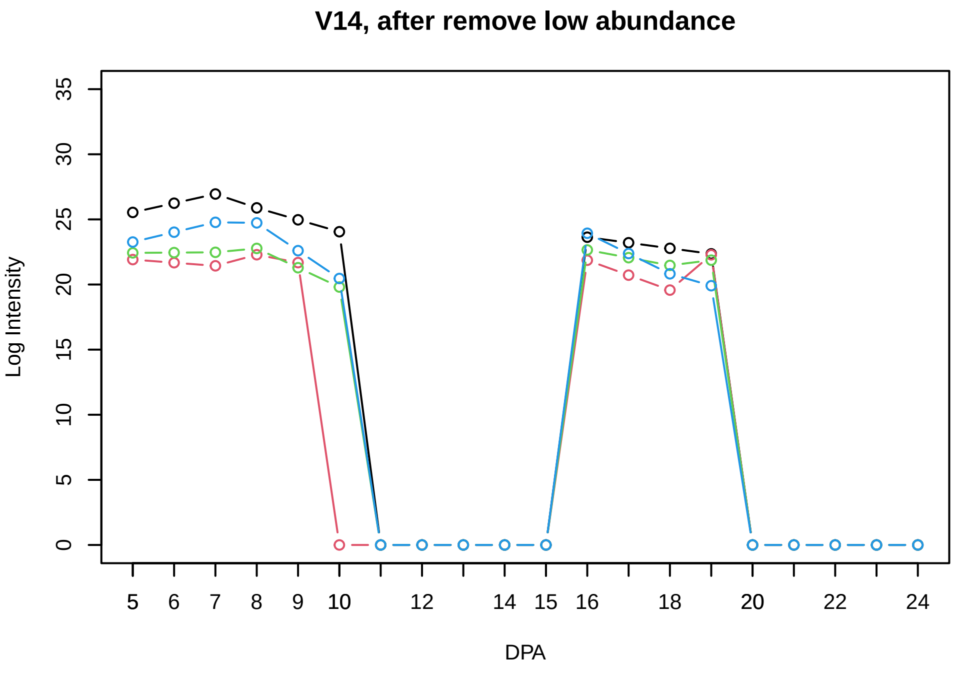
V14, after remove low abundance
Log Intensity
0 5 10 15 20 25 30 35
5 6 7 8 9 10 12 14 15 16 18 20 22 24
DPA

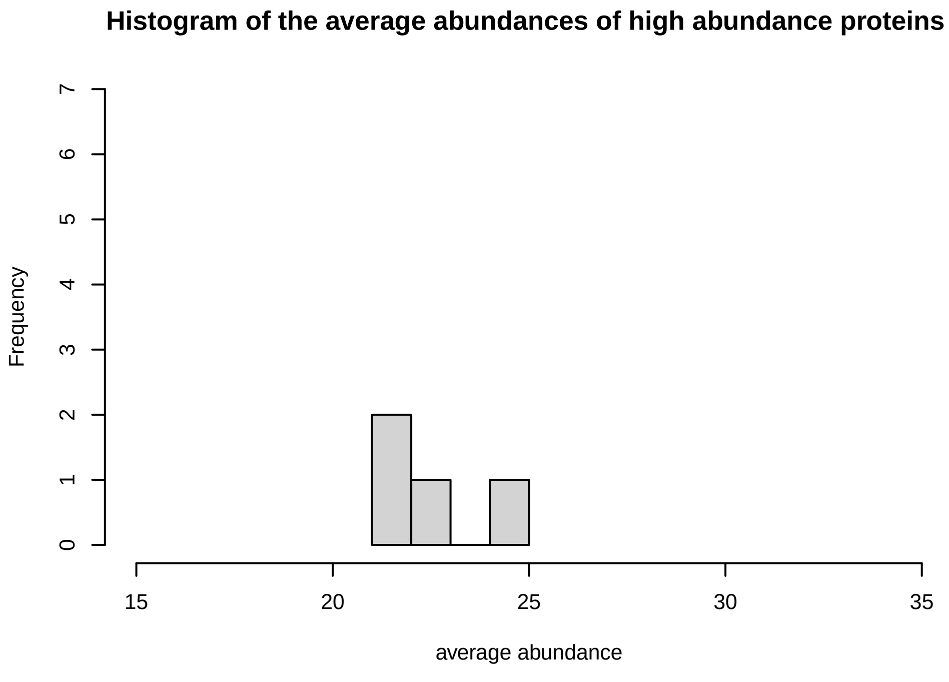
Histogram of the average abundances of high abundance proteins
Frequency
0 1 2 3 4 5 6 7
15 20 25 30 35
average abundance

V15

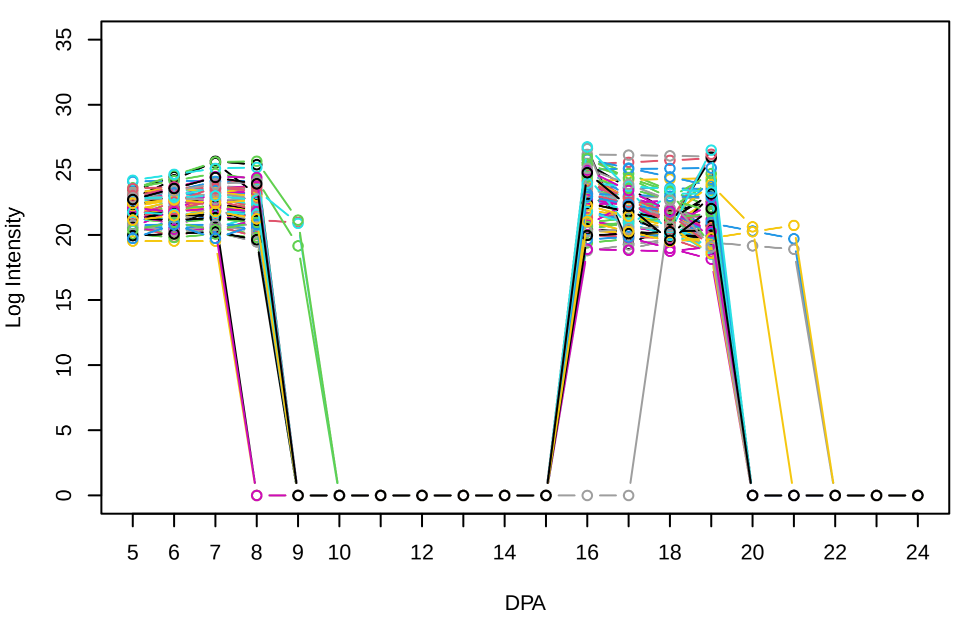

Histogram of the average abundances of non−zero values

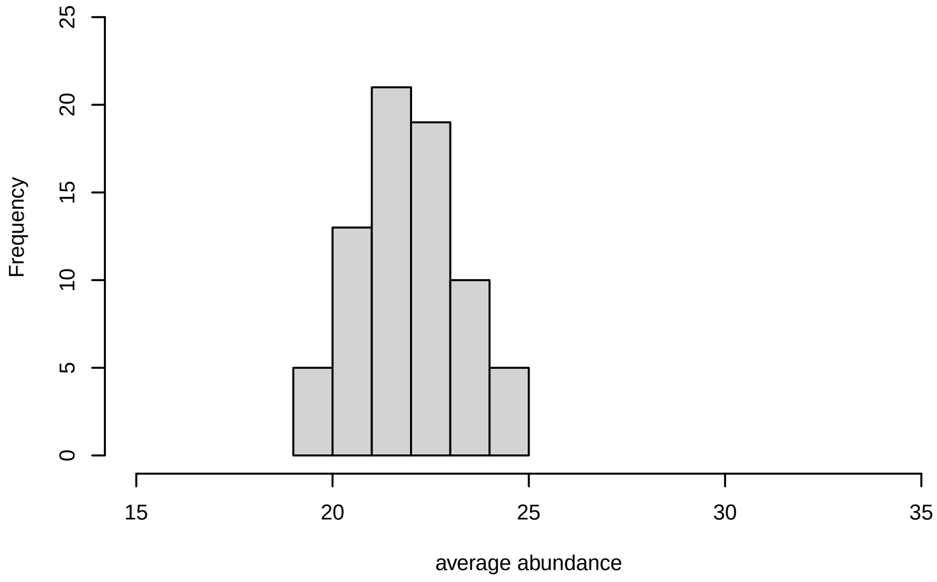

V15, after remove outlier and cutoff is 0.8

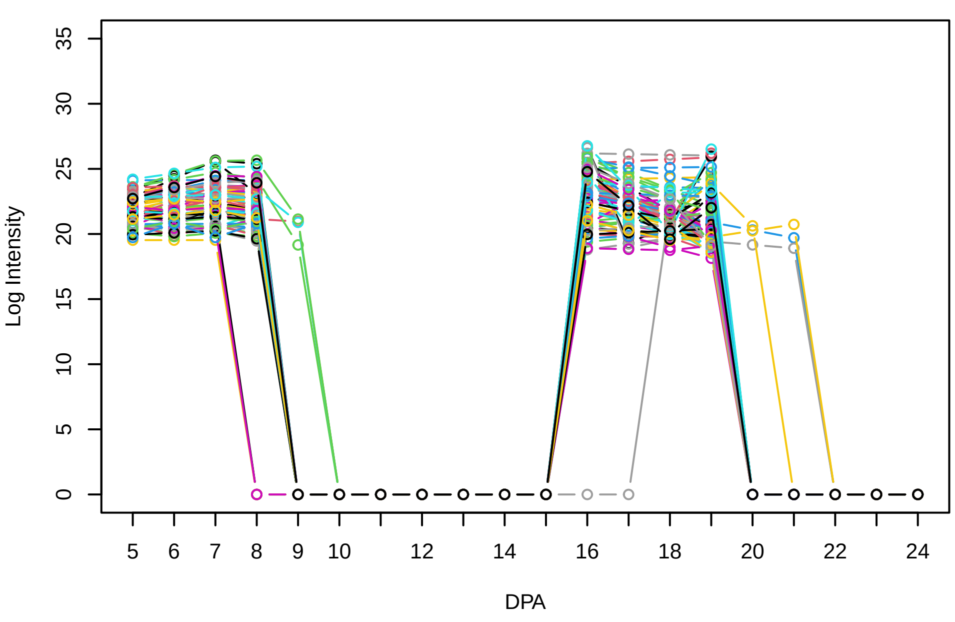

Histogram of the average abundances of non−outlier proteins

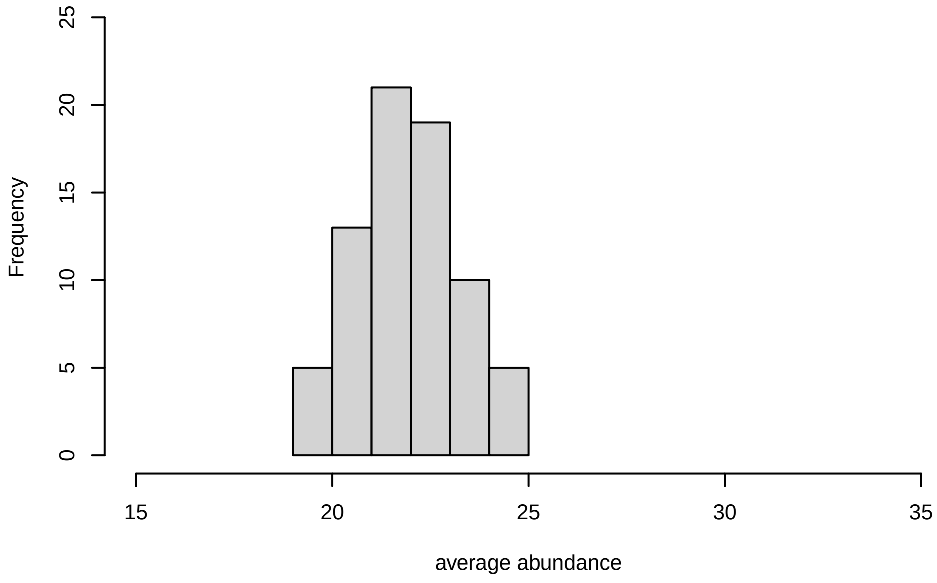

V15, after remove unreliable

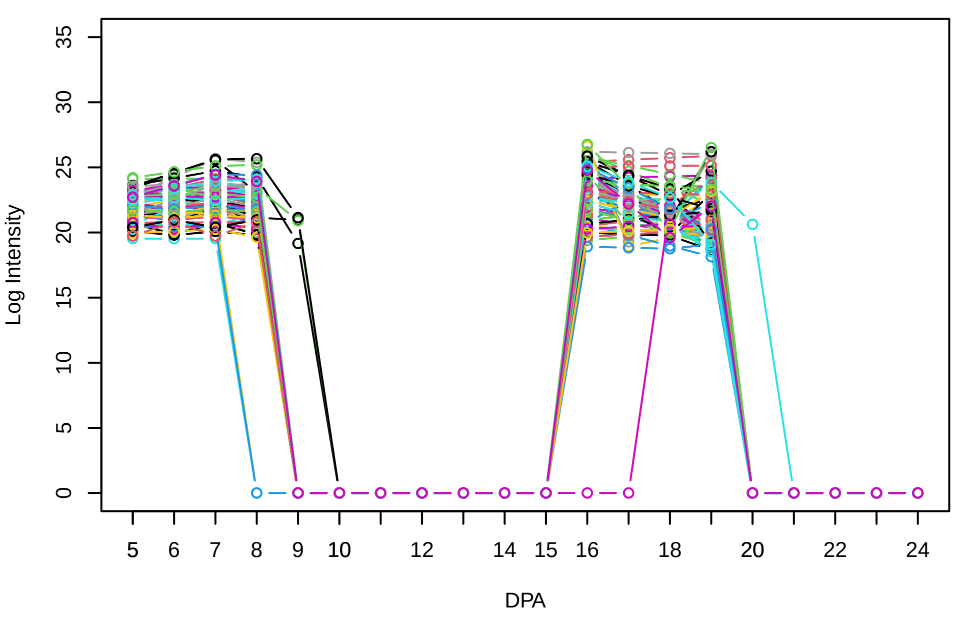

Histogram of the average abundances of reliable proteins

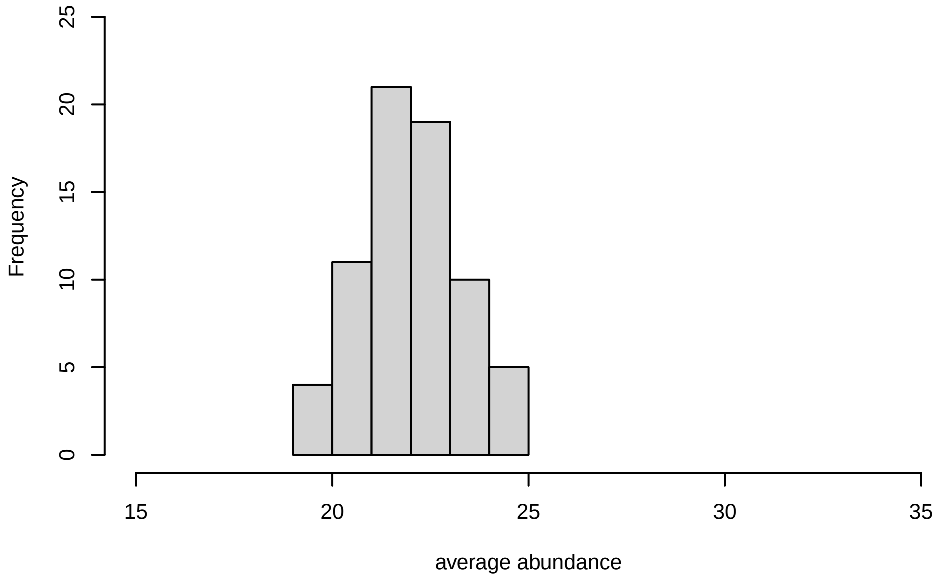

V15, after remove low abundance

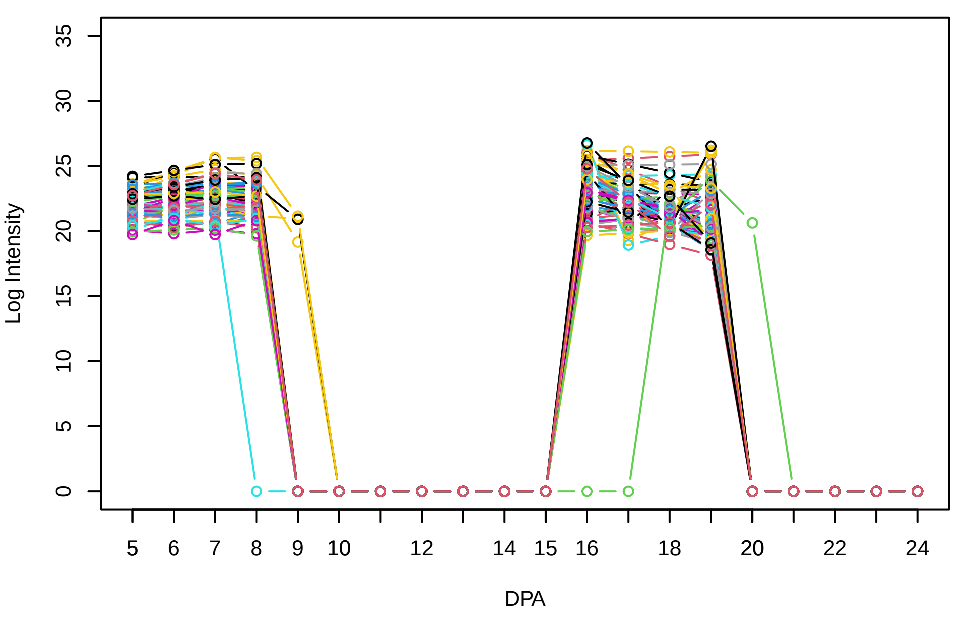

Histogram of the average abundances of high abundance proteins

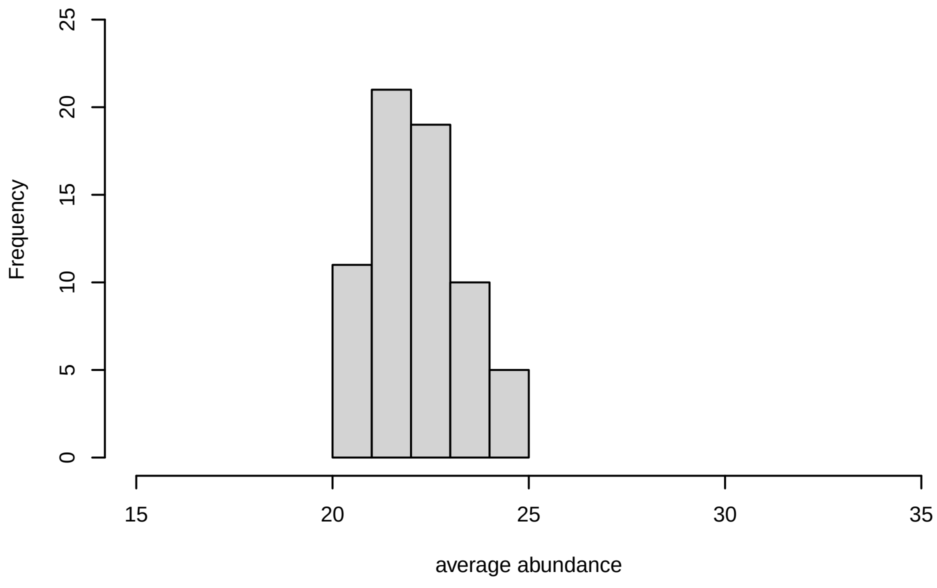

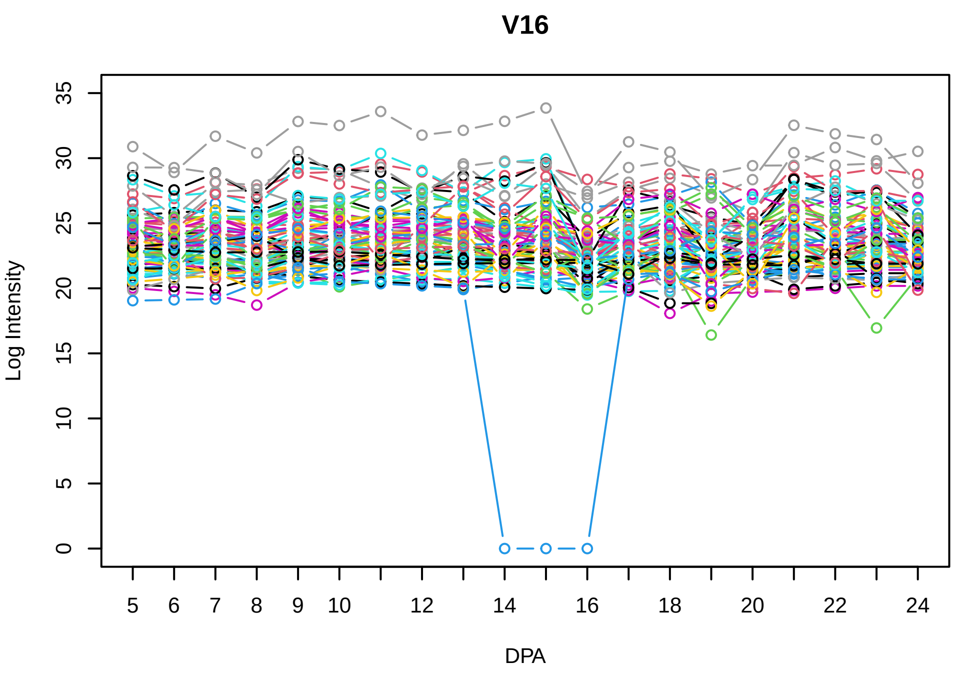
V16
Log Intensity
DPA

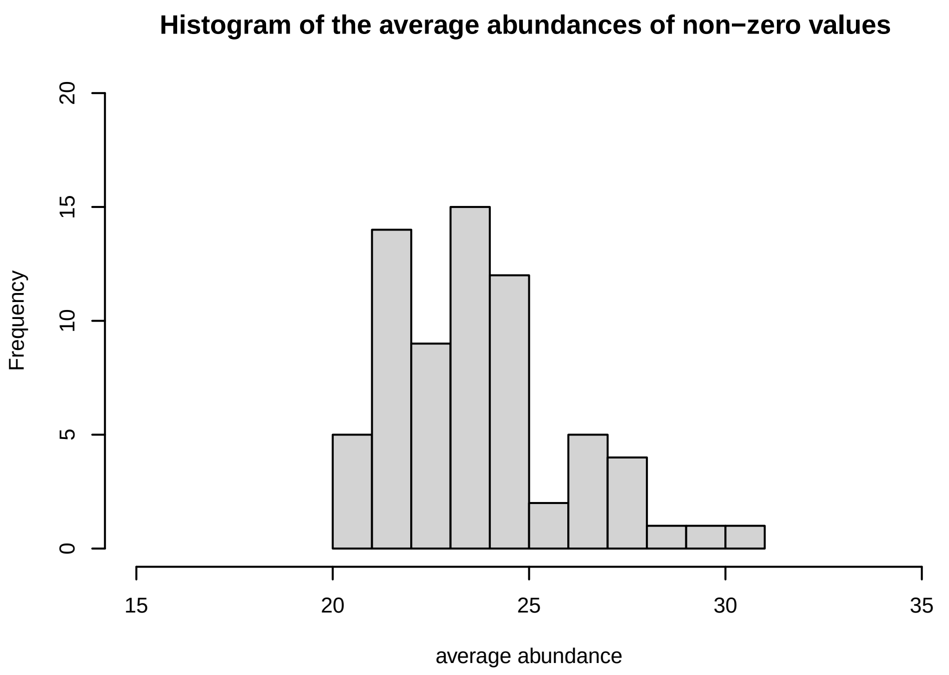
Histogram of the average abundances of non−zero values
Frequency
average abundance

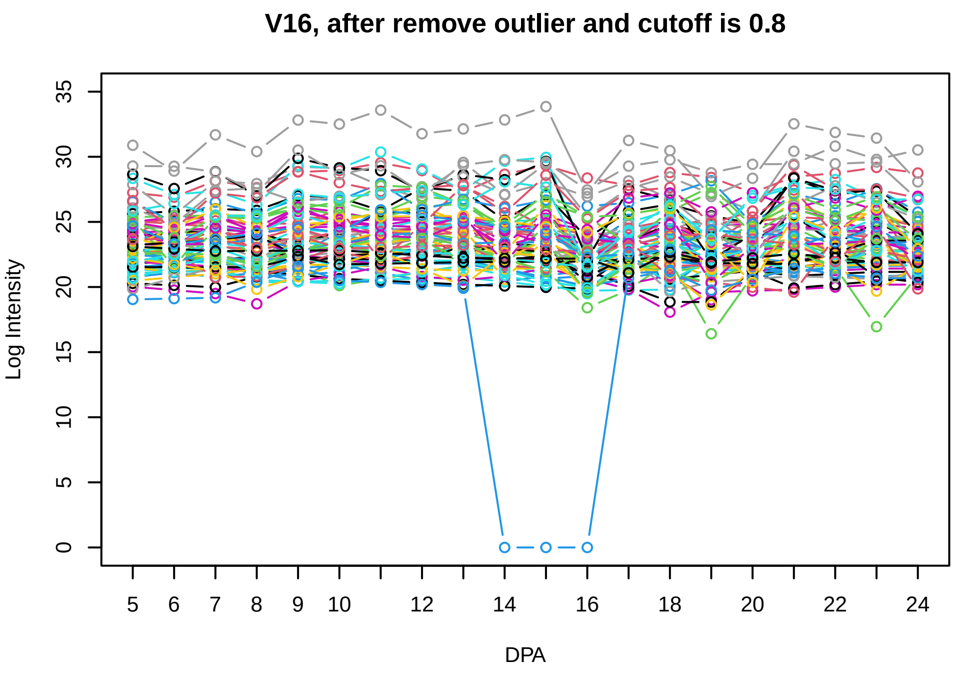
V16, after remove outlier and cutoff is 0.8
Log Intensity
DPA

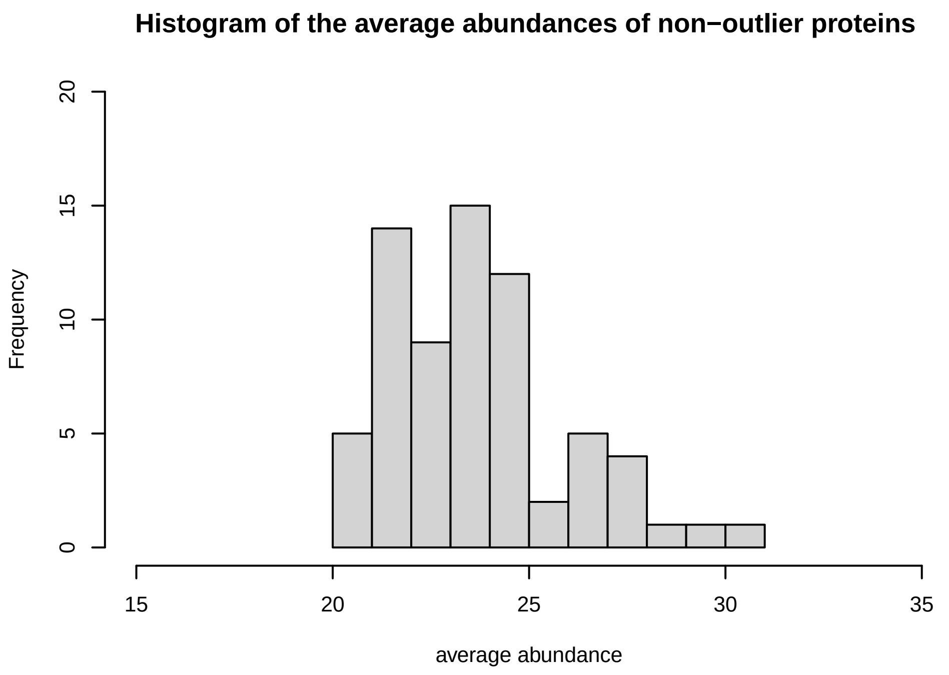
Histogram of the average abundances of non−outlier proteins
Frequency
average abundance

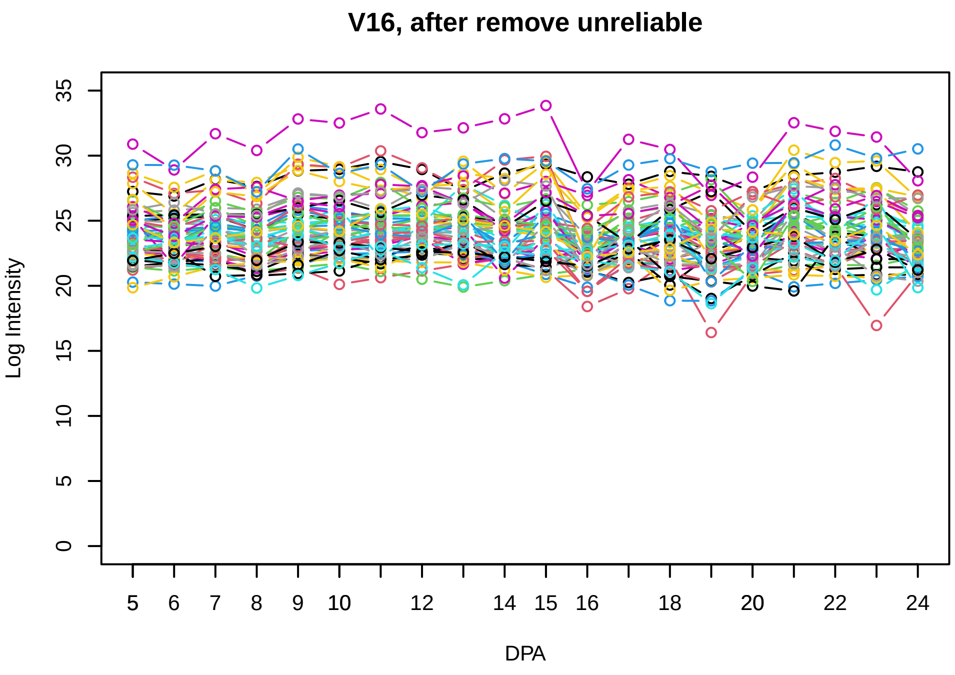
V16, after remove unreliable
Log Intensity
DPA

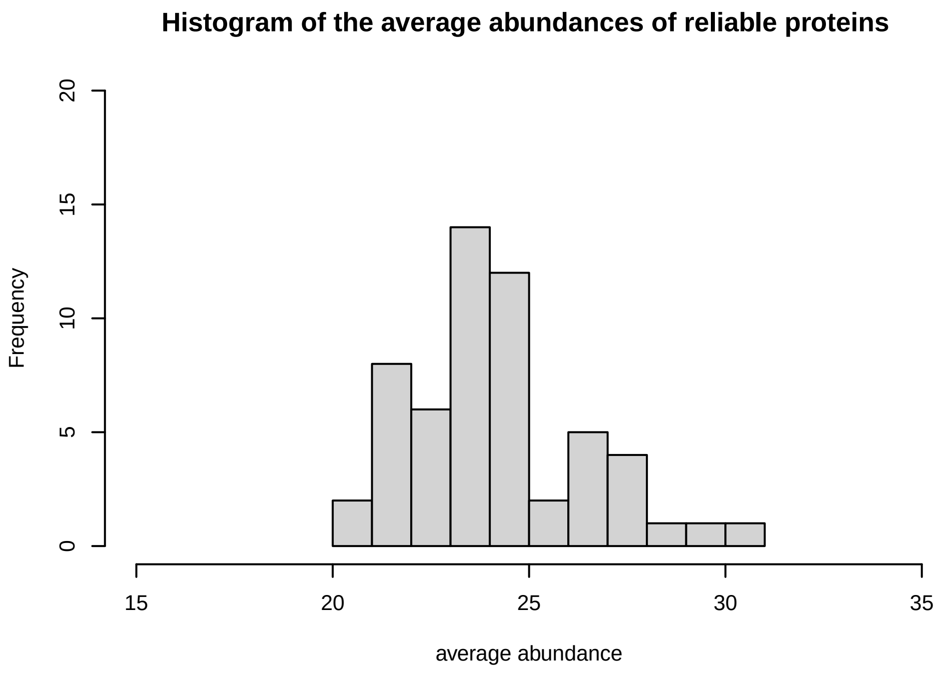
Histogram of the average abundances of reliable proteins
Frequency
average abundance

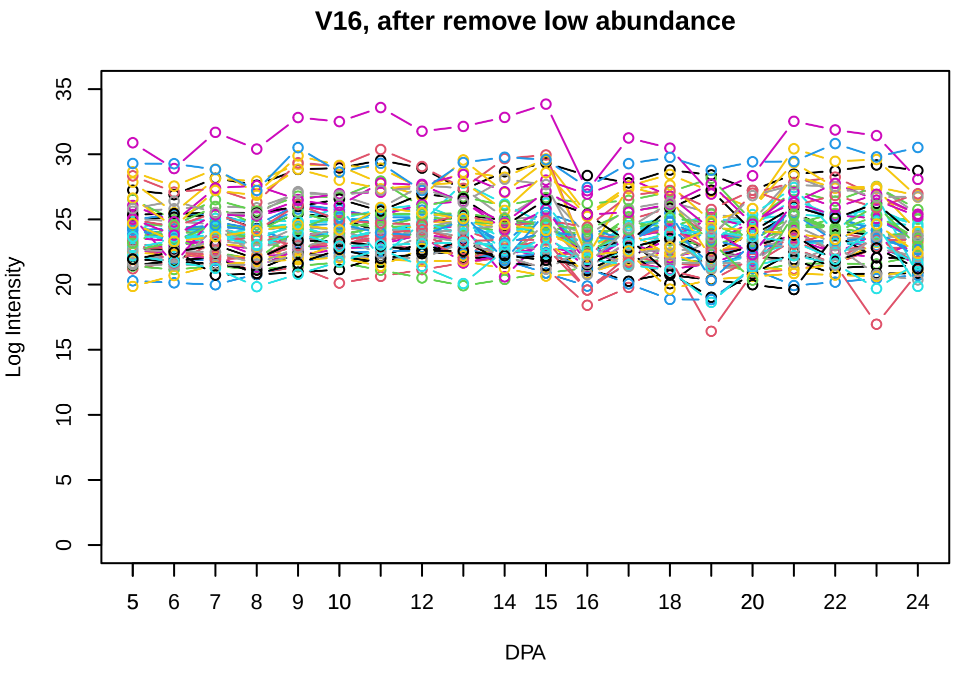
V16, after remove low abundance
Log Intensity
DPA

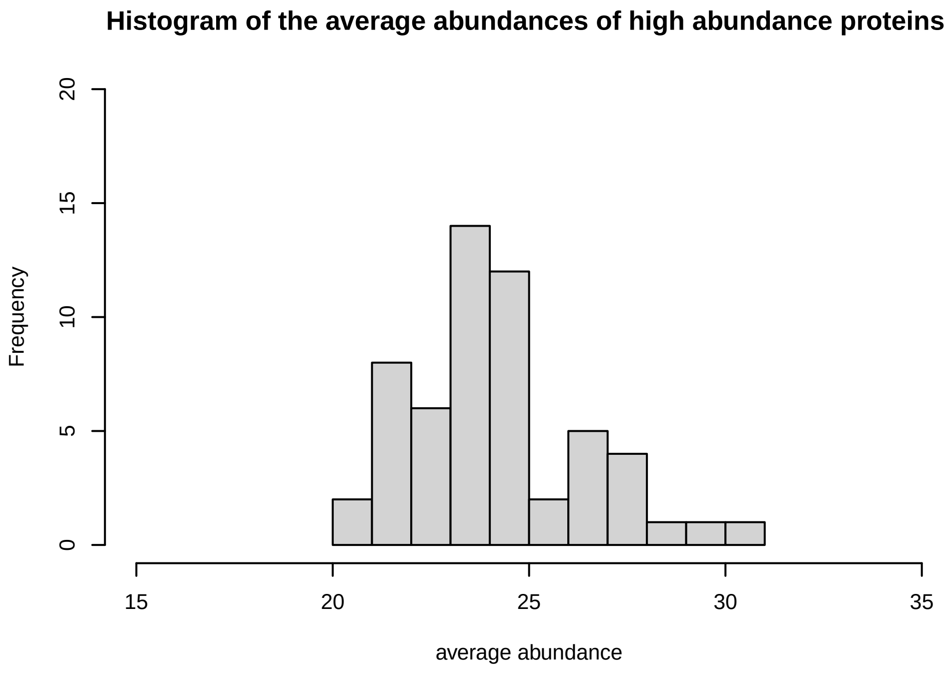
Histogram of the average abundances of high abundance proteins
Frequency
average abundance

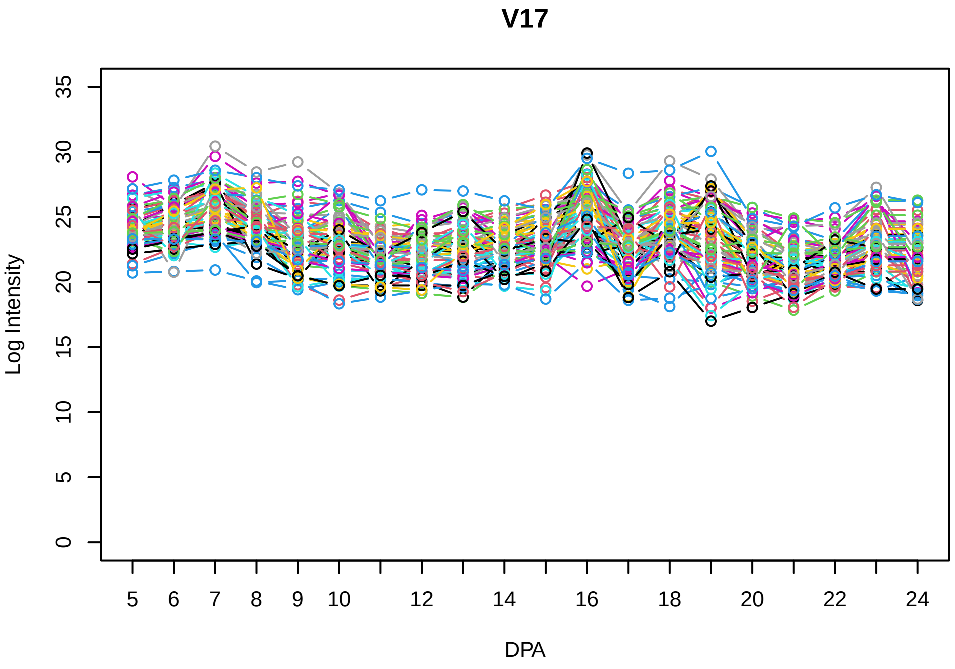
V17
Log Intensity
DPA

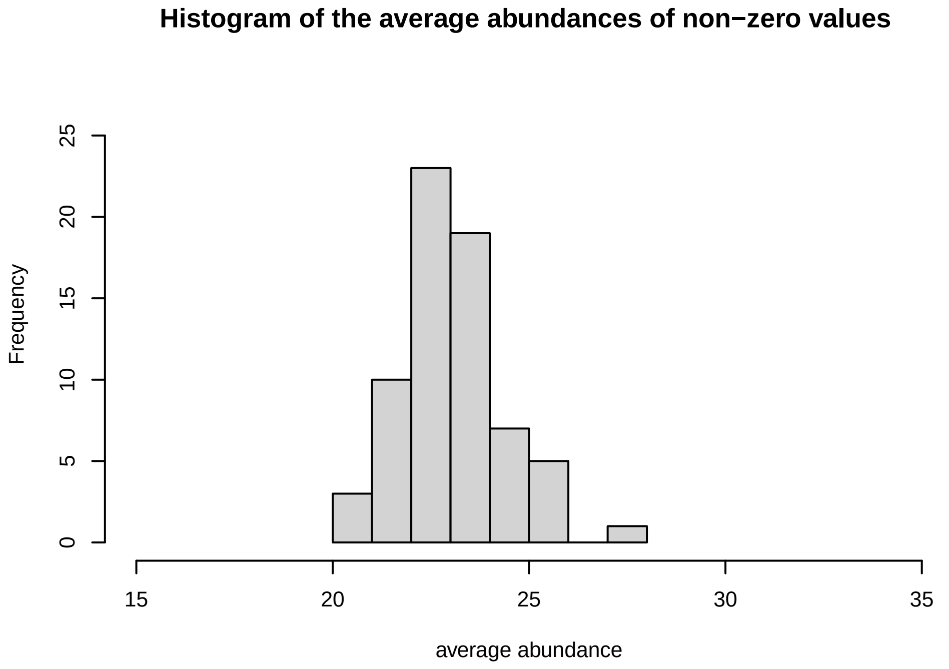
Histogram of the average abundances of non−zero values
Frequency
average abundance

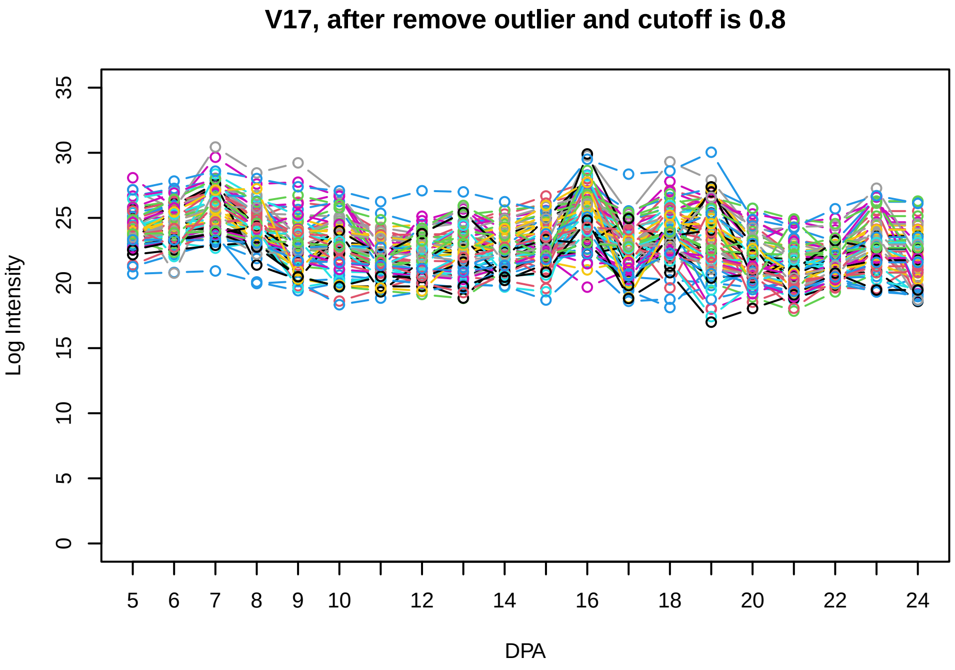
V17, after remove outlier and cutoff is 0.8
Log Intensity
DPA

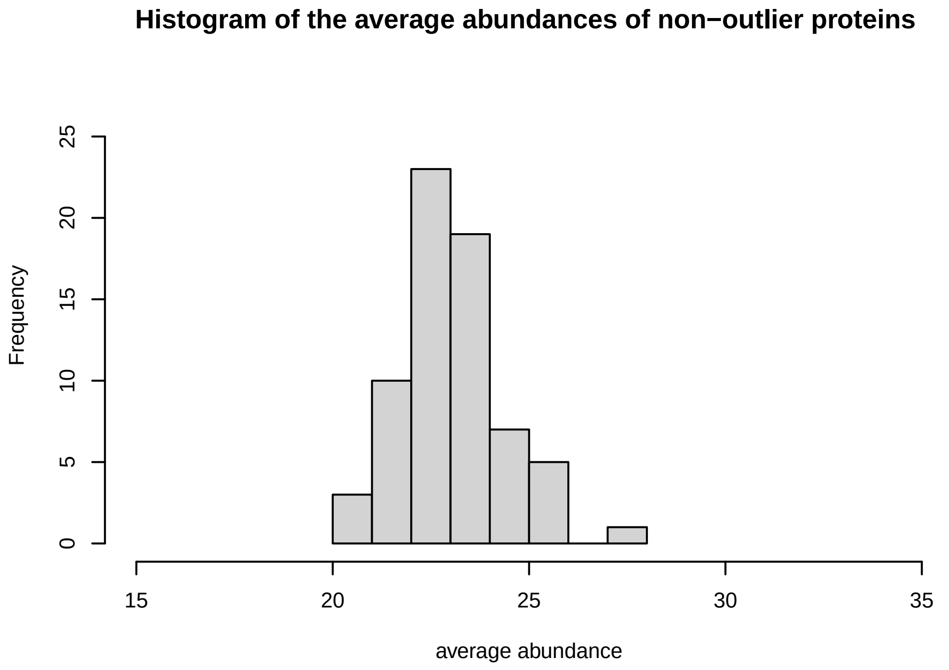
Histogram of the average abundances of non−outlier proteins
Frequency
average abundance

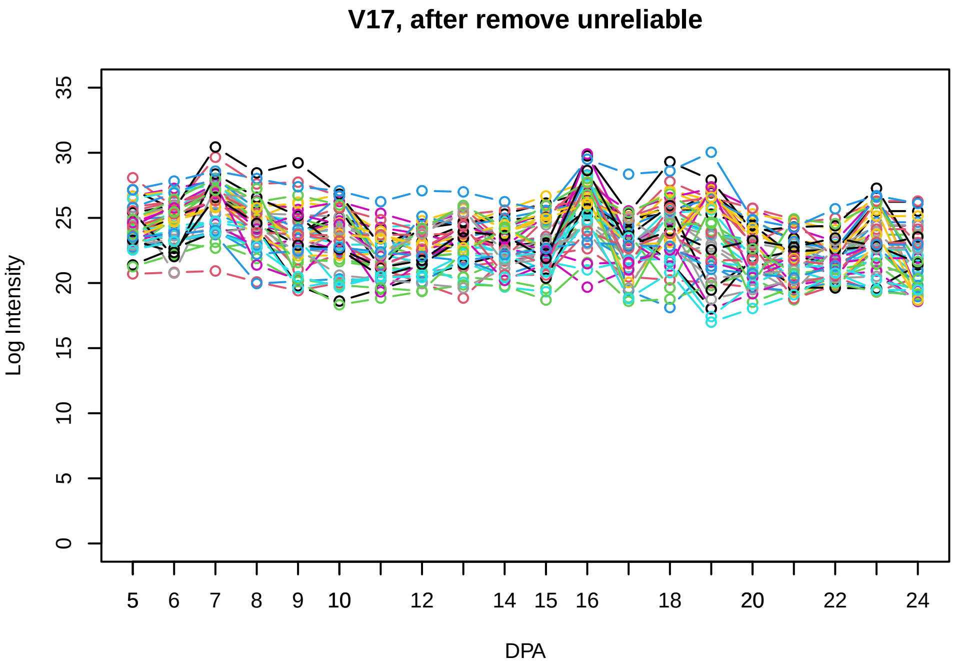
V17, after remove unreliable
Log Intensity
DPA

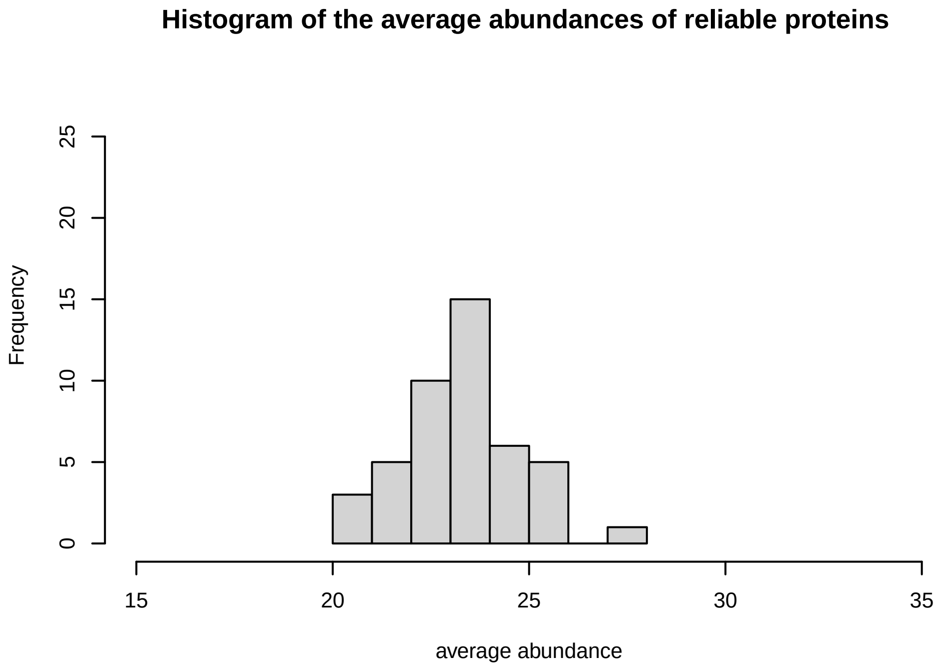
Histogram of the average abundances of reliable proteins
Frequency
average abundance

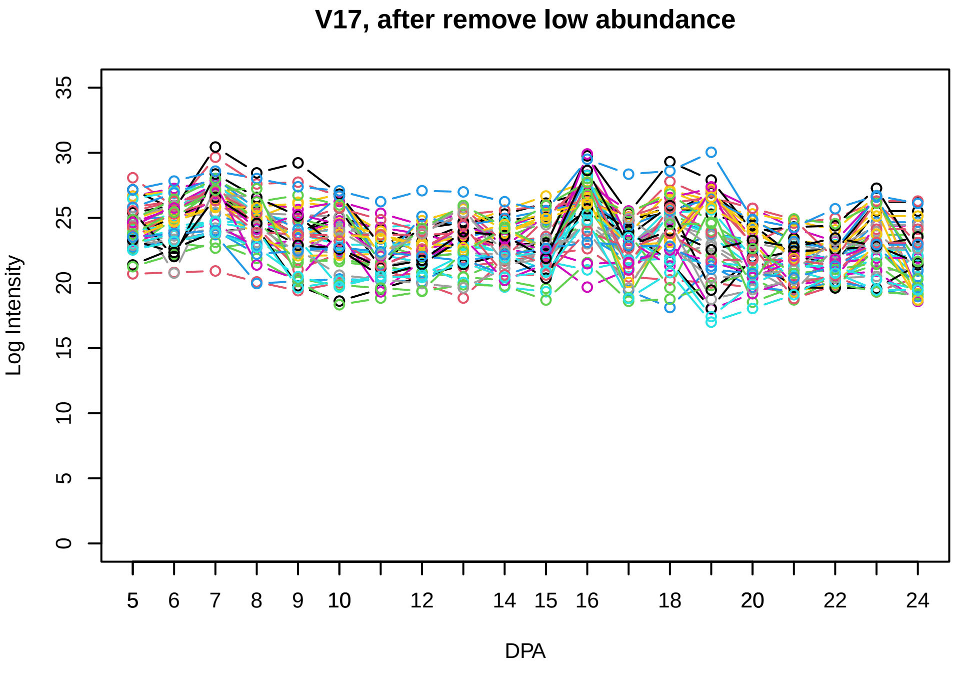
V17, after remove low abundance
Log Intensity
DPA

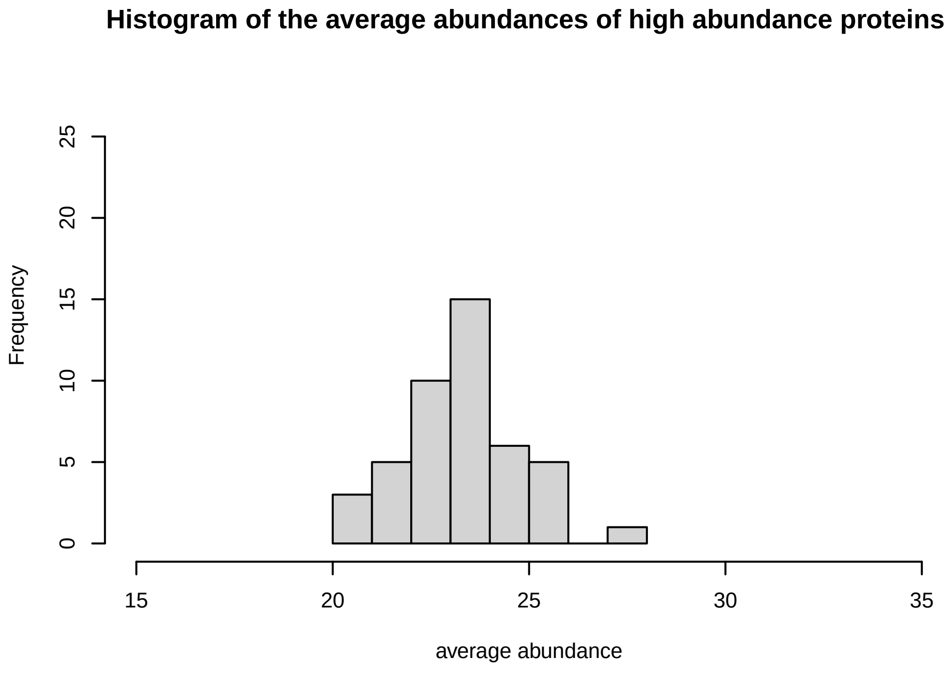
Histogram of the average abundances of high abundance proteins
Frequency
average abundance

V18

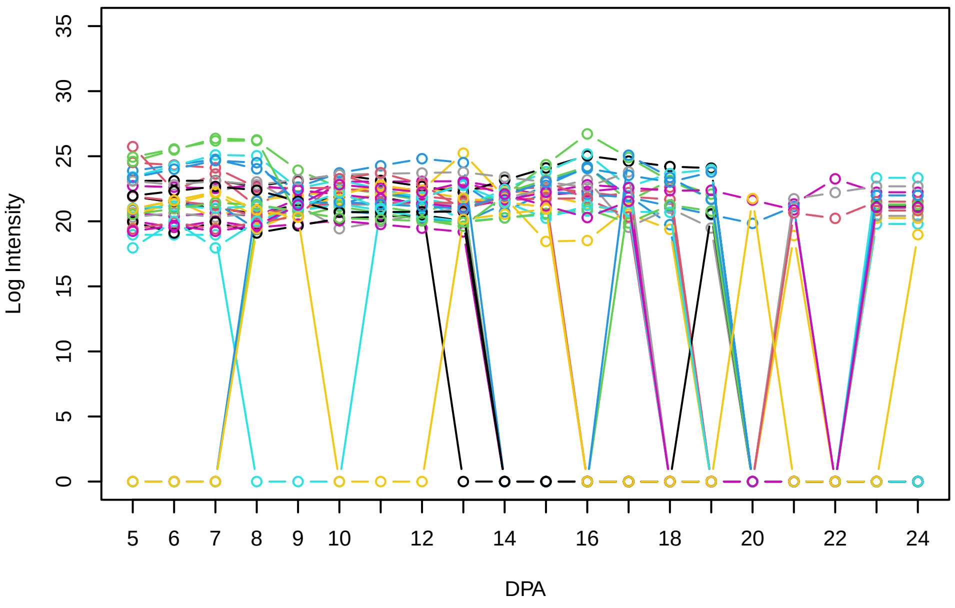

Histogram of the average abundances of non−zero values

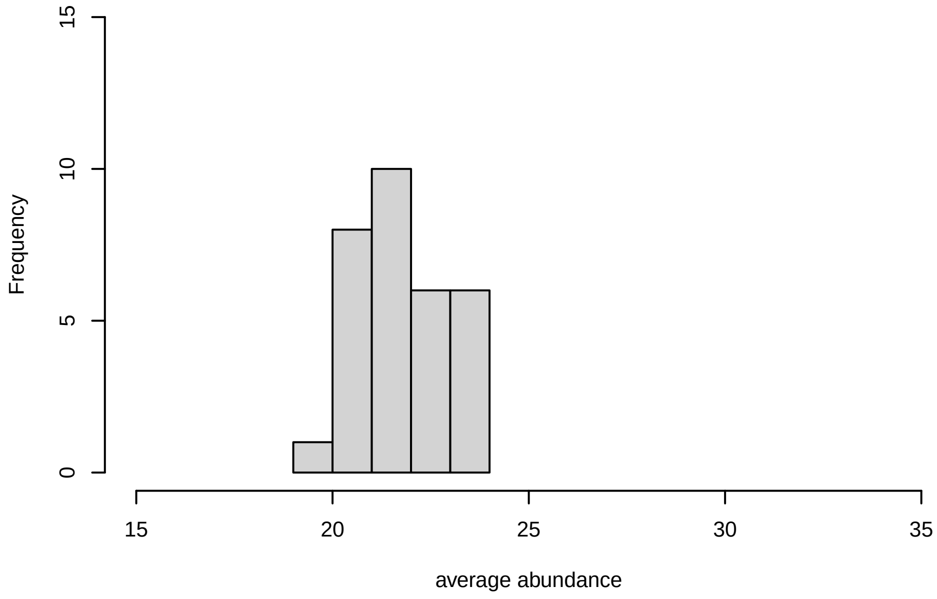

V18, after remove outlier and cutoff is 0.8

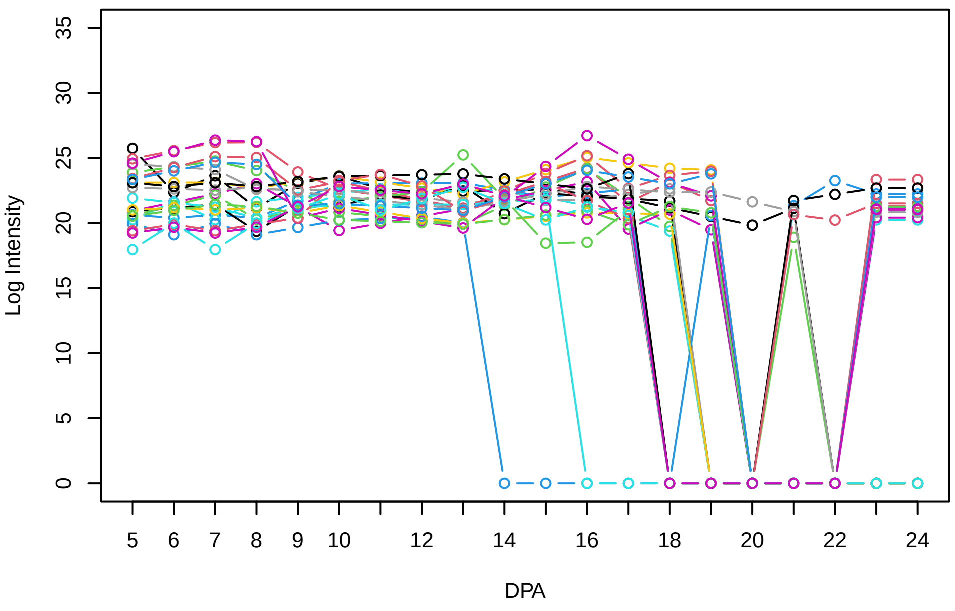

Histogram of the average abundances of non−outlier proteins

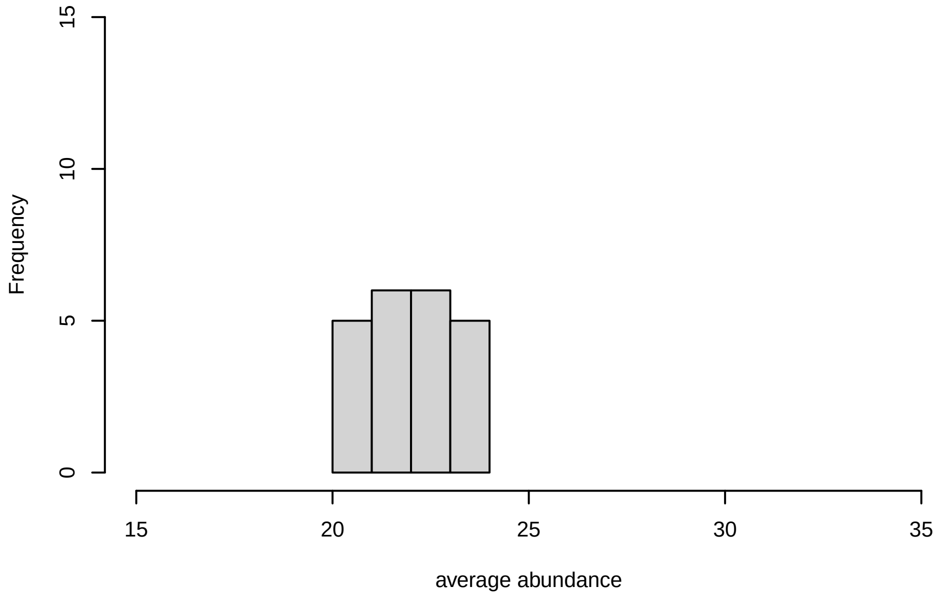

V18, after remove unreliable

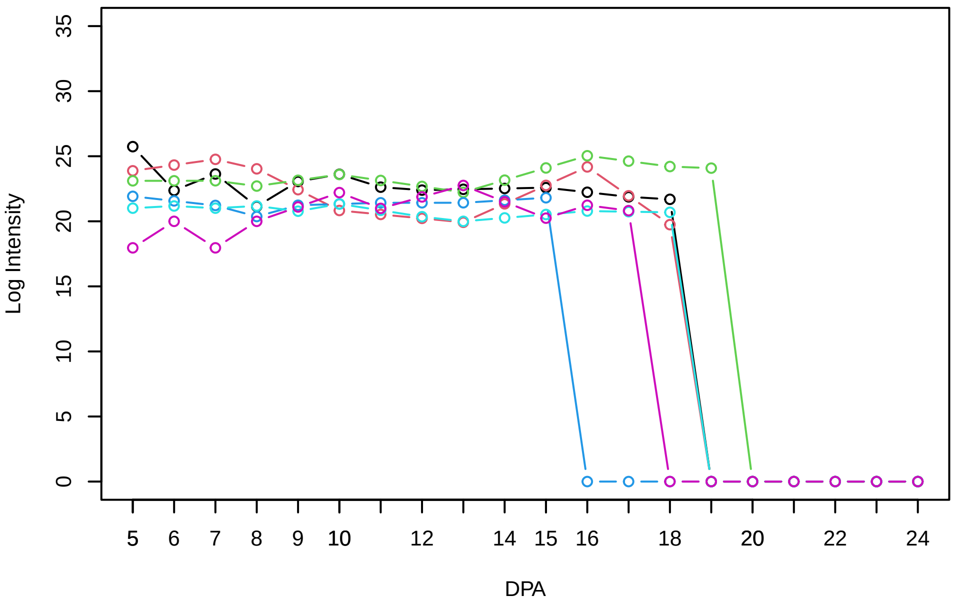

Histogram of the average abundances of reliable proteins

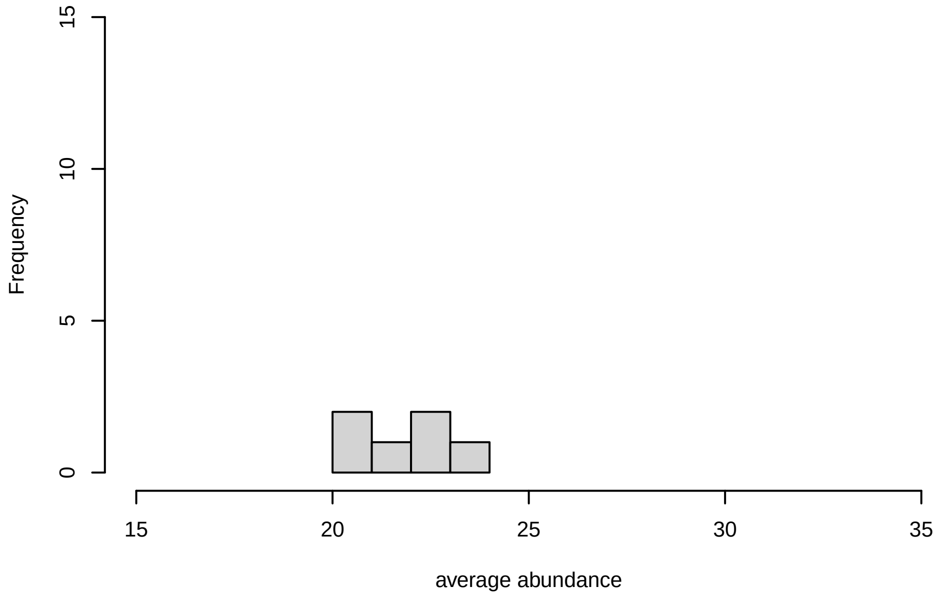

V18, after remove low abundance

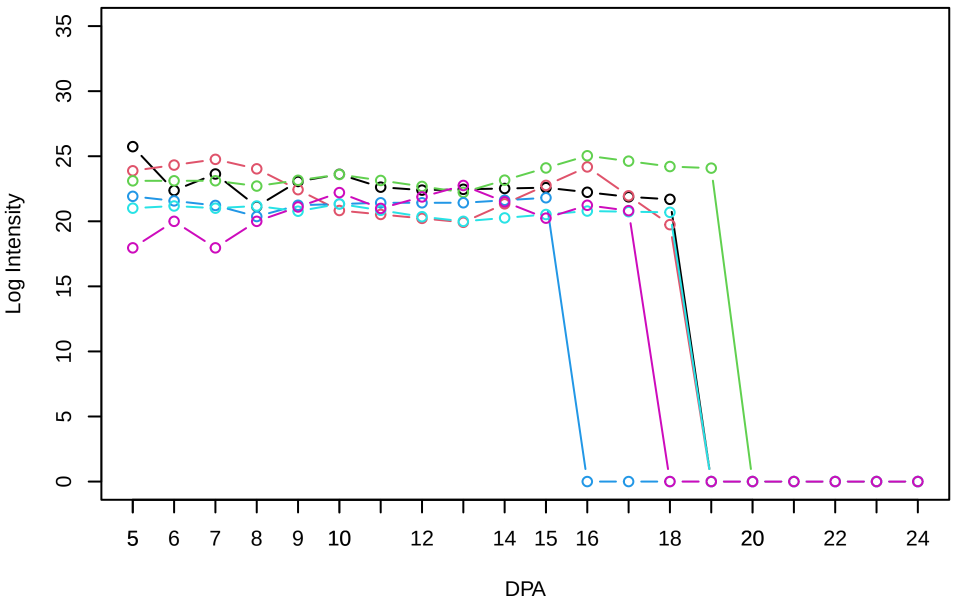

Histogram of the average abundances of high abundance proteins

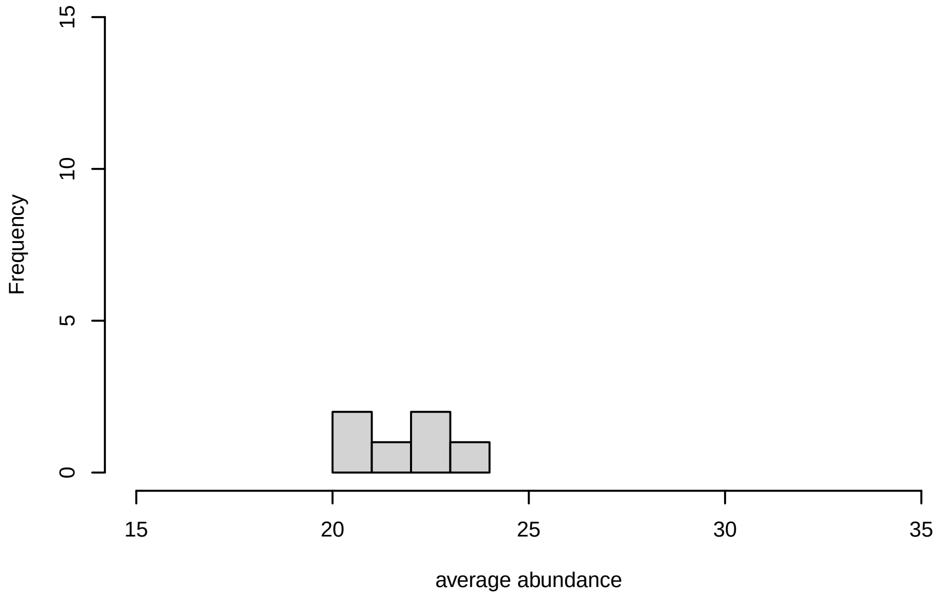

V19

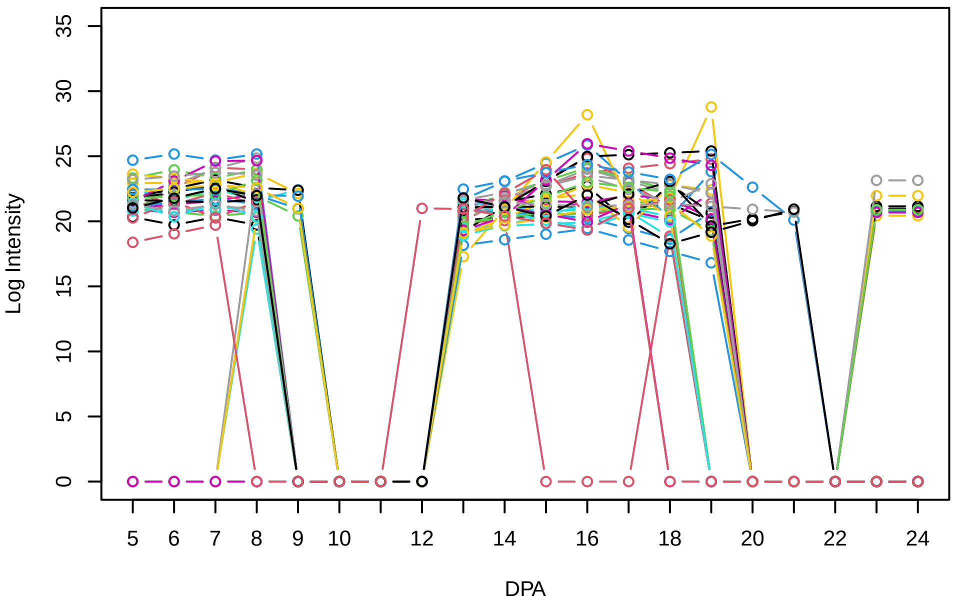

Histogram of the average abundances of non−zero values

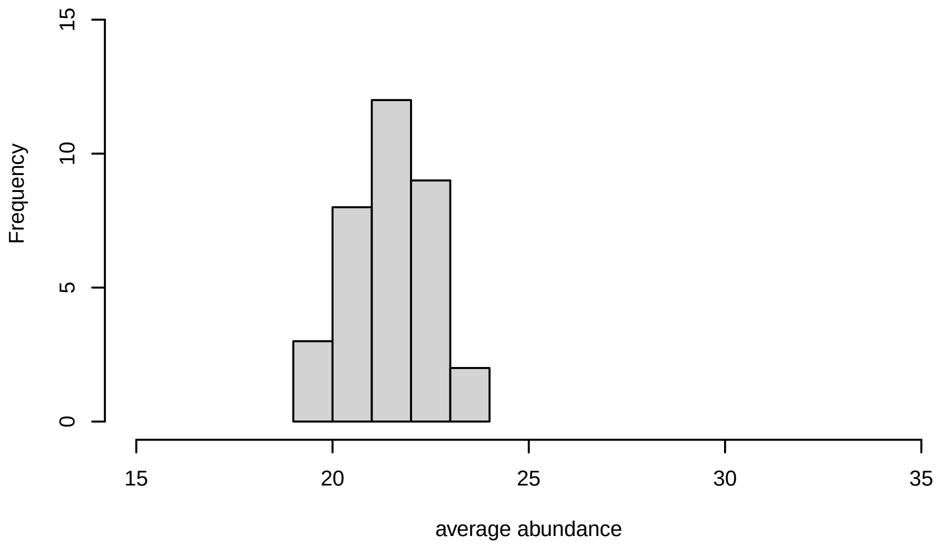

V19, after remove outlier and cutoff is 0.8

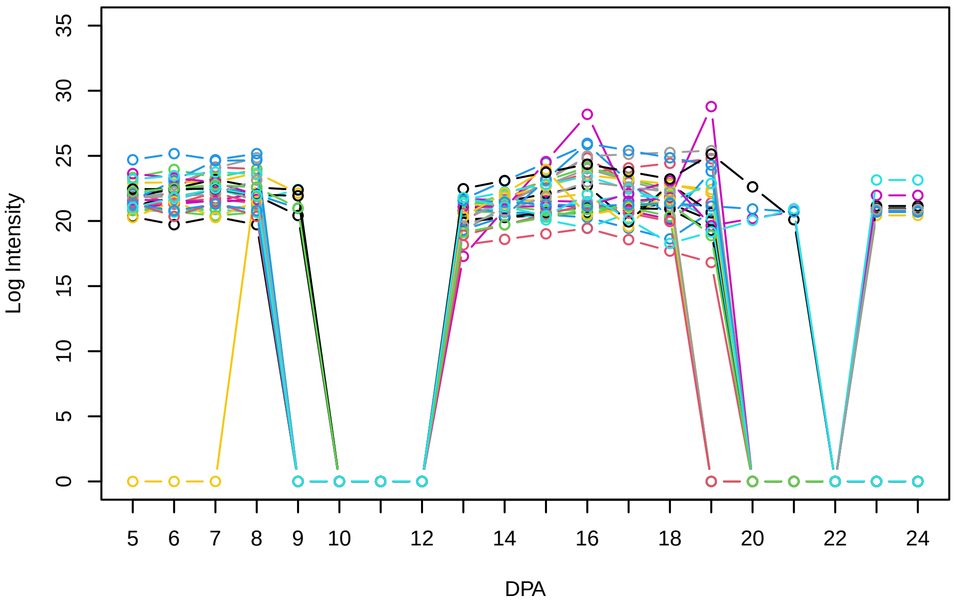

Histogram of the average abundances of non−outlier proteins

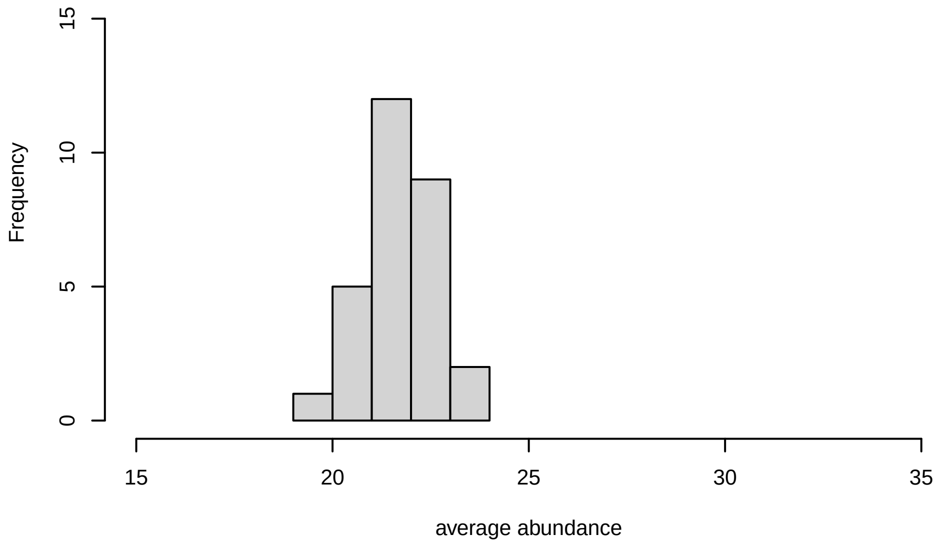

V19, after remove unreliable

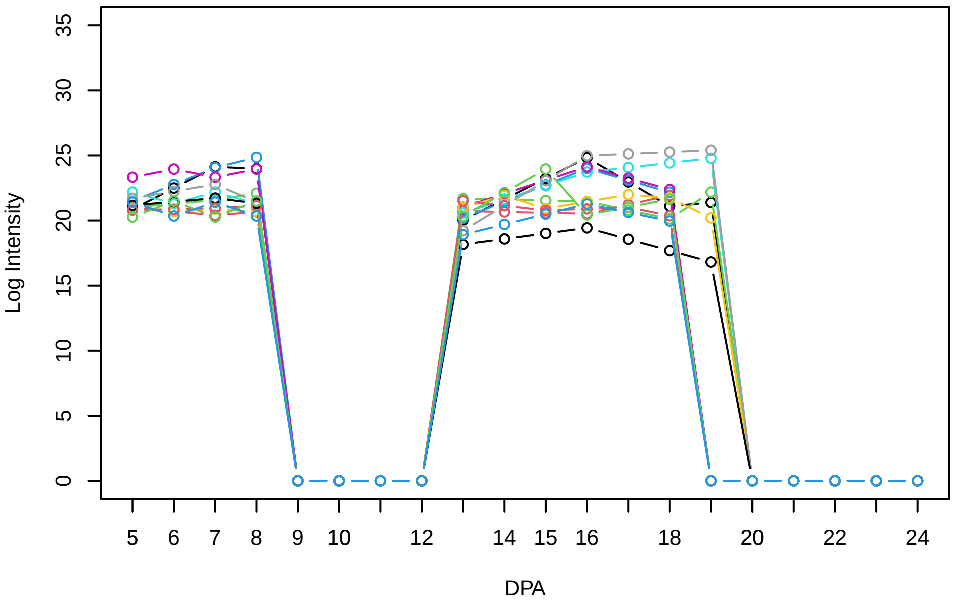

Histogram of the average abundances of reliable proteins

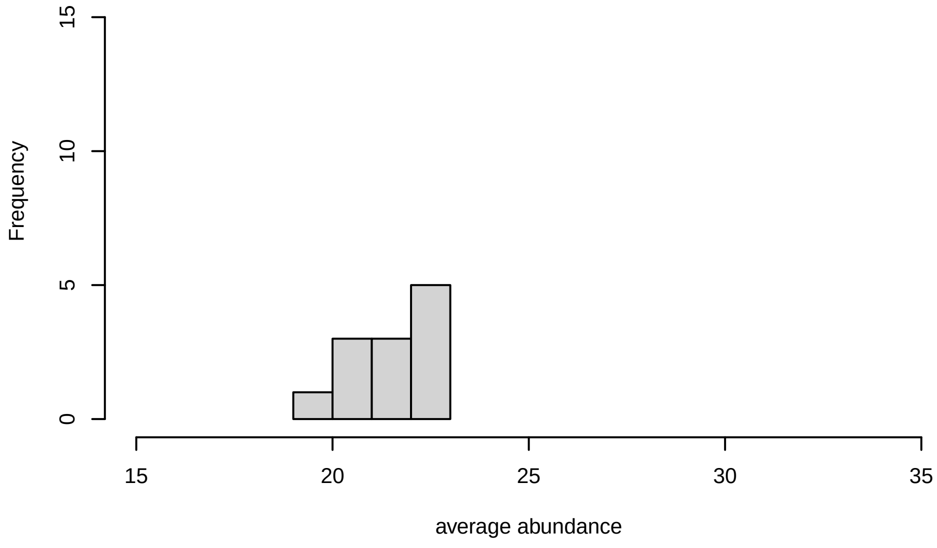

V19, after remove low abundance

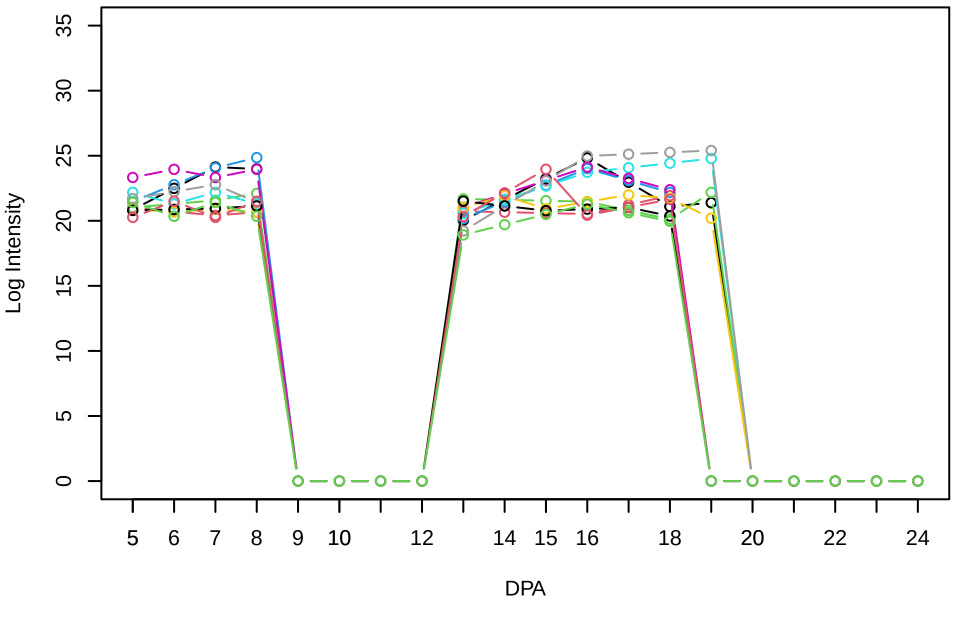

Histogram of the average abundances of high abundance proteins

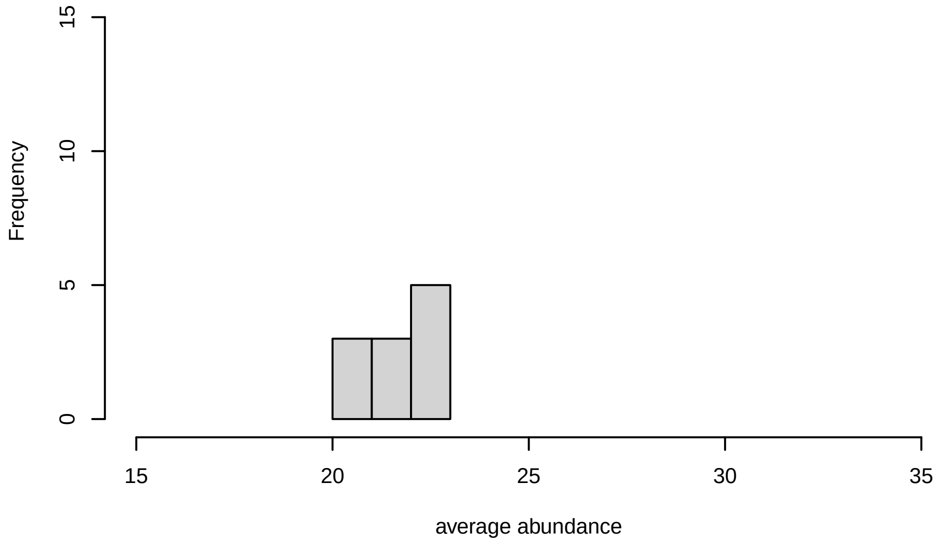

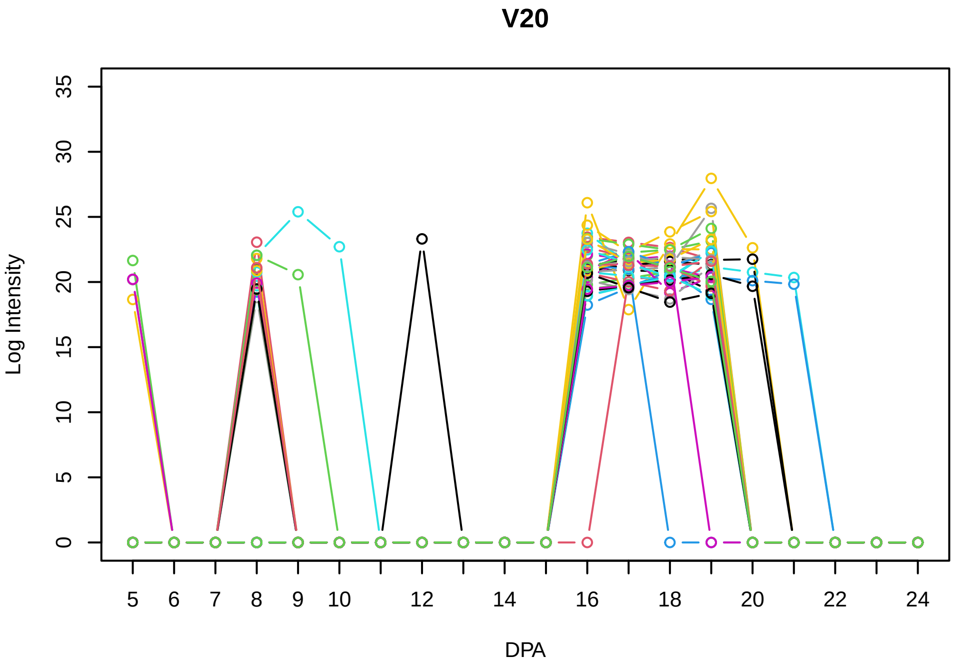
V20
Log Intensity
0 5 10 15 20 25 30 35
5 6 7 8 9 10 12 14 16 18 20 22 24
DPA

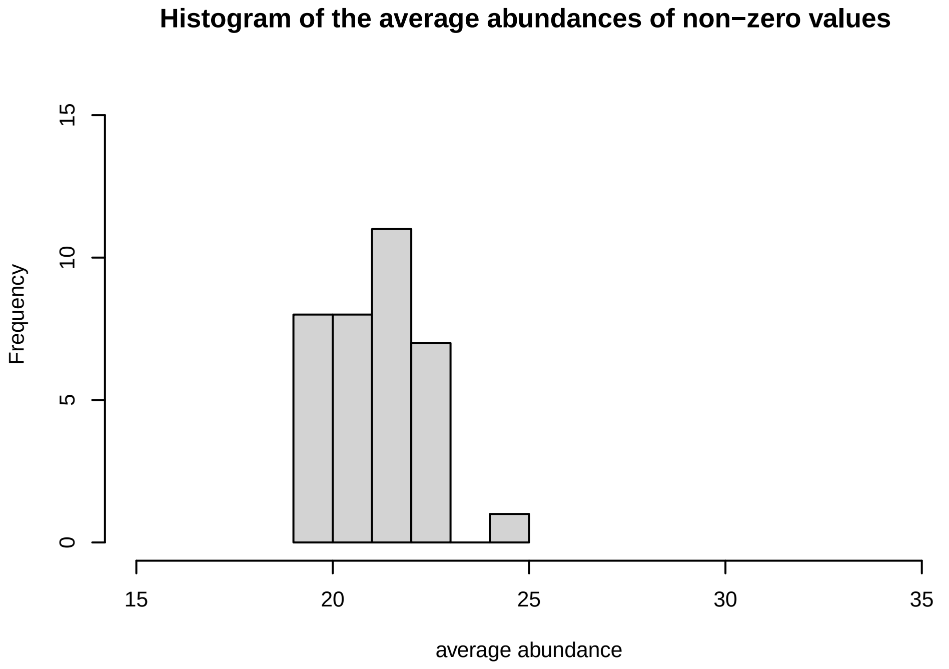
Histogram of the average abundances of non−zero values
Frequency
0 5 10 15
15 20 25 30 35
average abundance

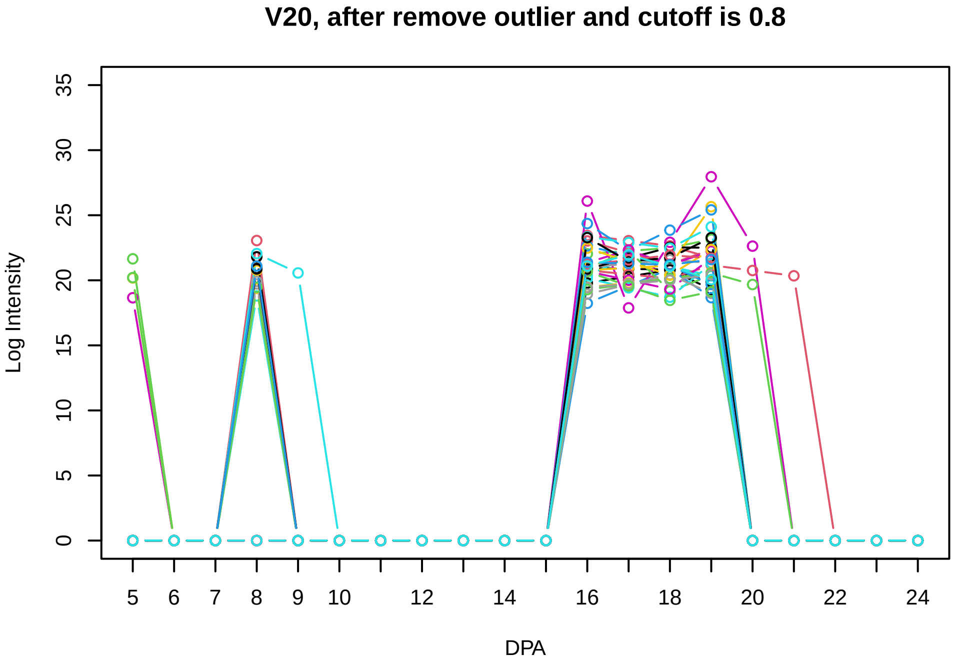
V20, after remove outlier and cutoff is 0.8
Log Intensity
0 5 10 15 20 25 30 35
5 6 7 8 9 10 12 14 16 18 20 22 24
DPA

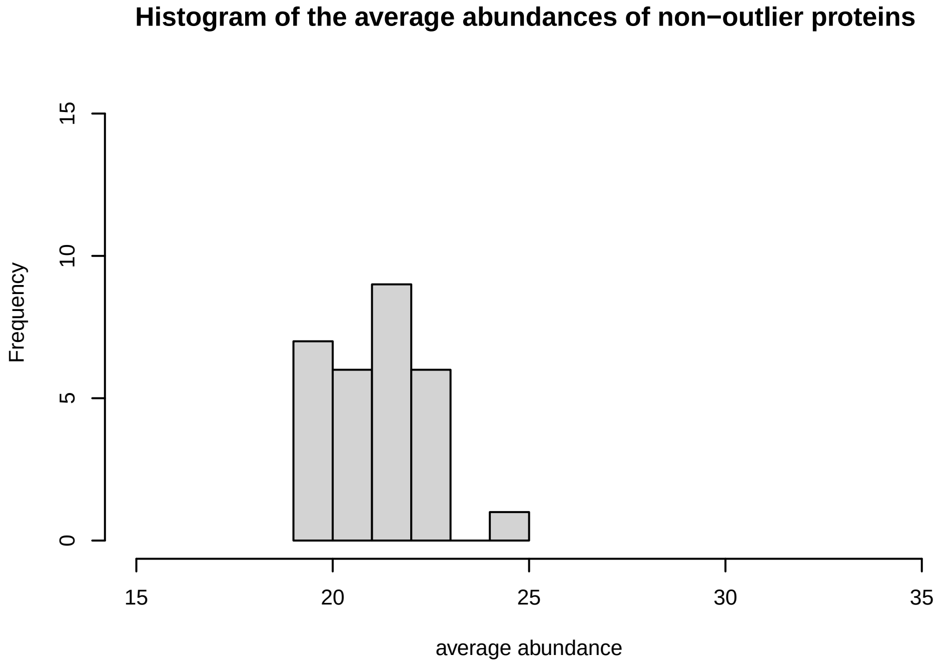
Histogram of the average abundances of non−outlier proteins
Frequency
0 5 10 15
15 20 25 30 35
average abundance

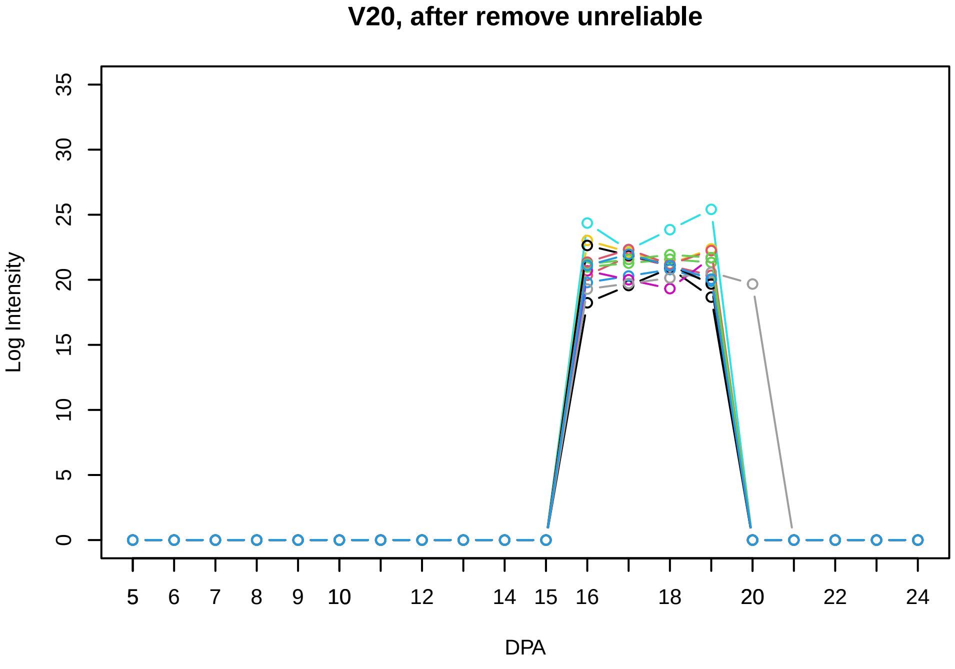
V20, after remove unreliable
Log Intensity
0 5 10 15 20 25 30 35
5 6 7 8 9 10 12 14 15 16 18 20 22 24
DPA

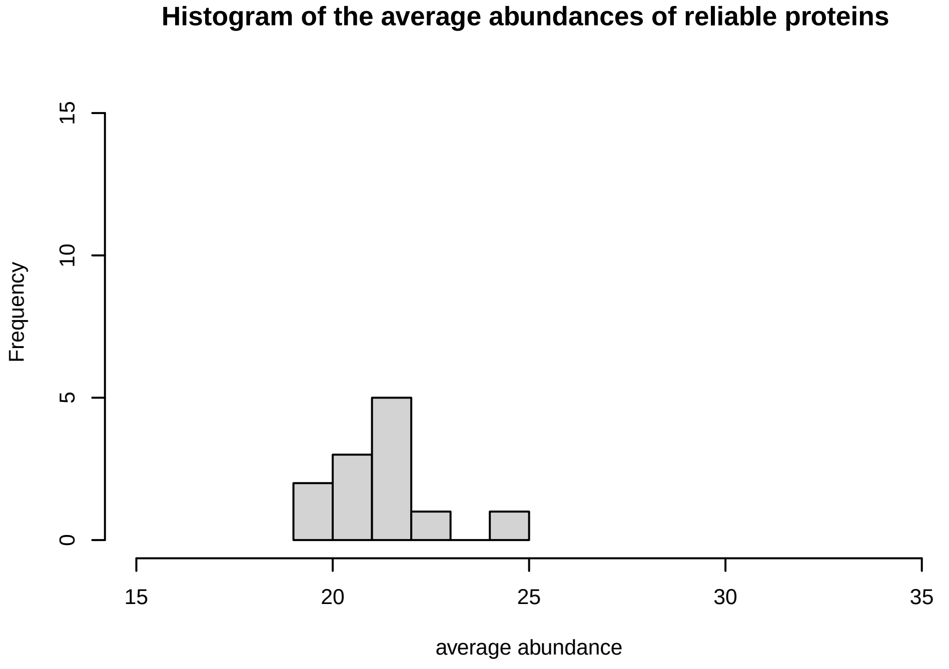
Histogram of the average abundances of reliable proteins
Frequency
0 5 10 15
15 20 25 30 35
average abundance

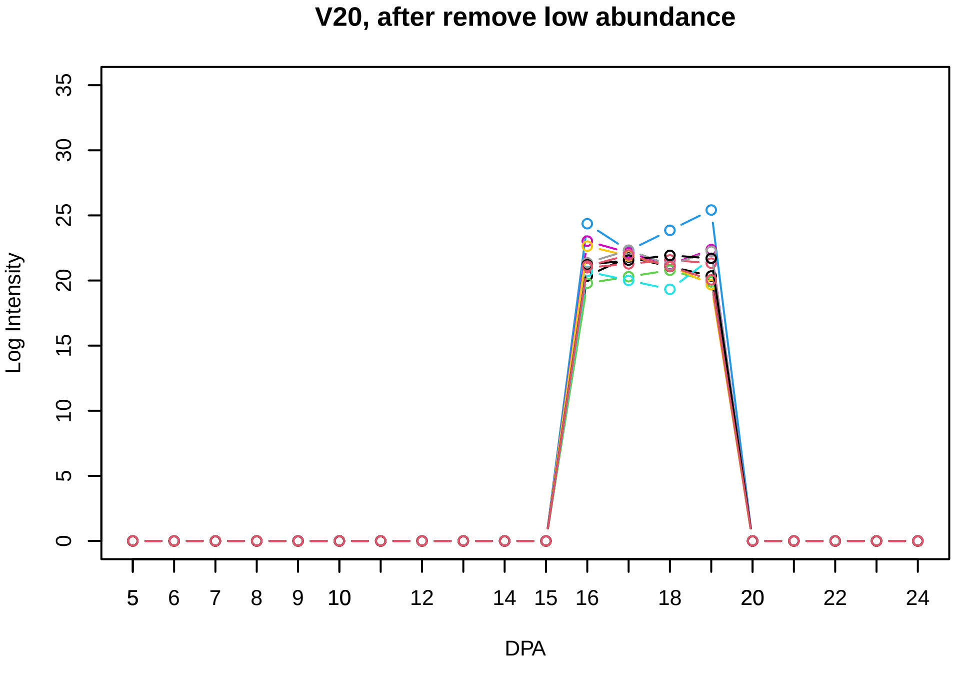
V20, after remove low abundance
Log Intensity
0 5 10 15 20 25 30 35
5 6 7 8 9 10 12 14 15 16 18 20 22 24
DPA

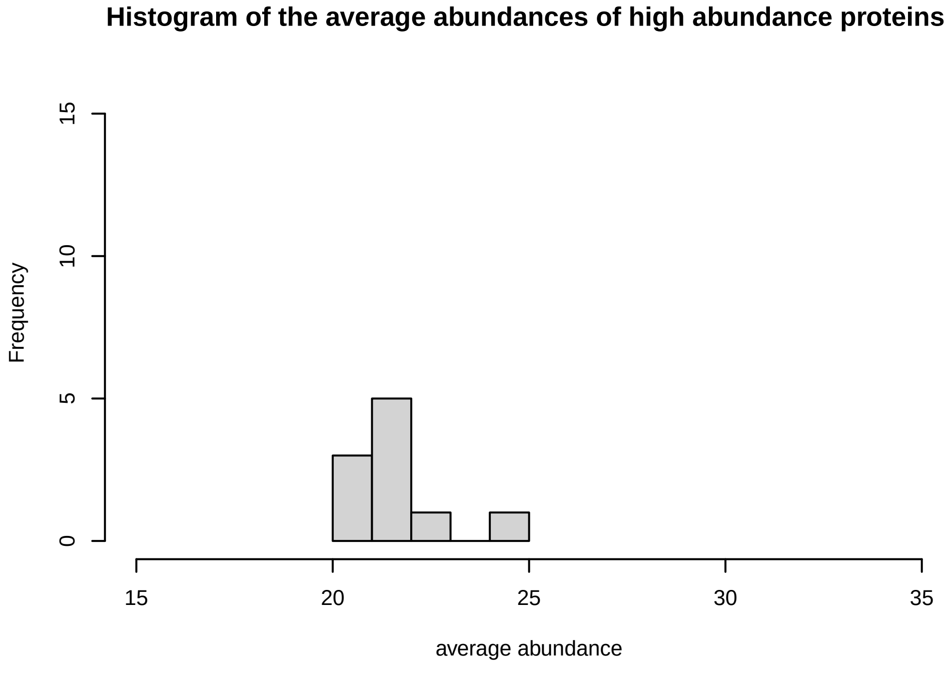
Histogram of the average abundances of high abundance proteins
Frequency
0 5 10 15
15 20 25 30 35
average abundance

Supplementary Figure S6. Merged SOM protein expression modules (9-cluster model).

Codes

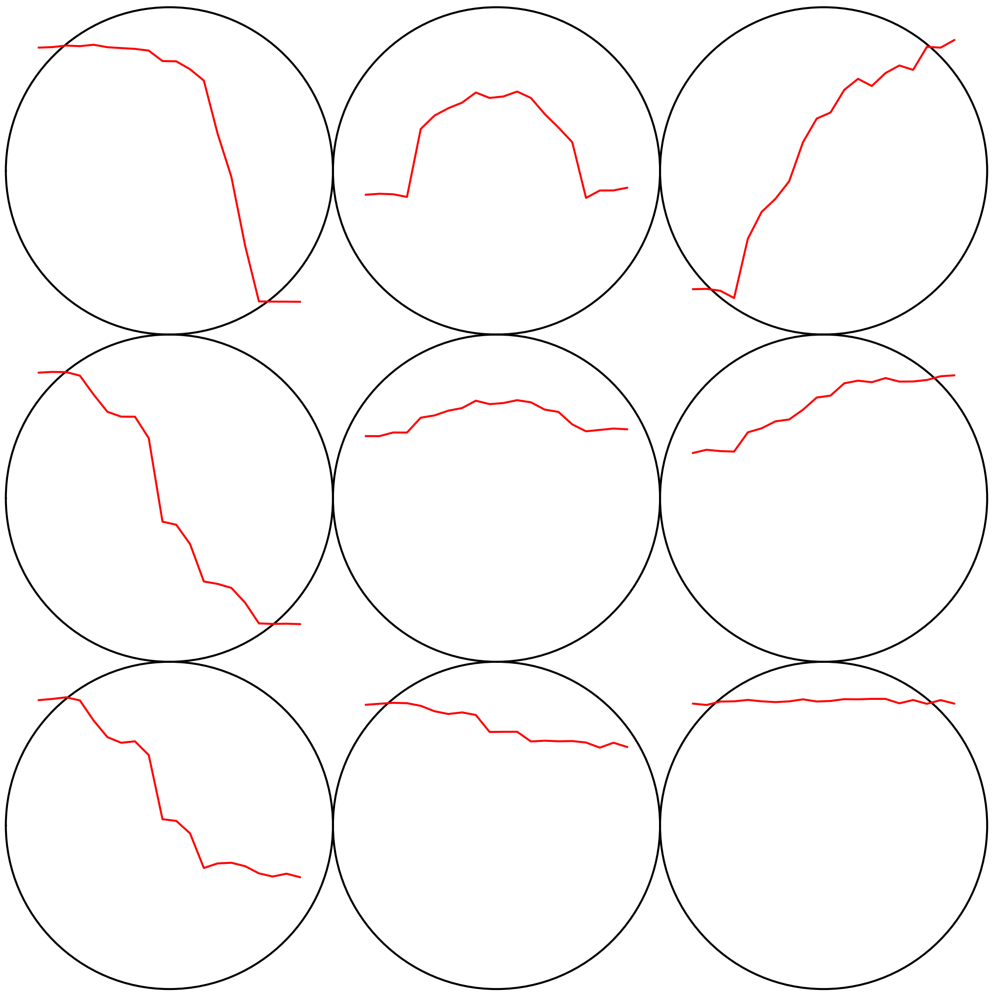

Count plot

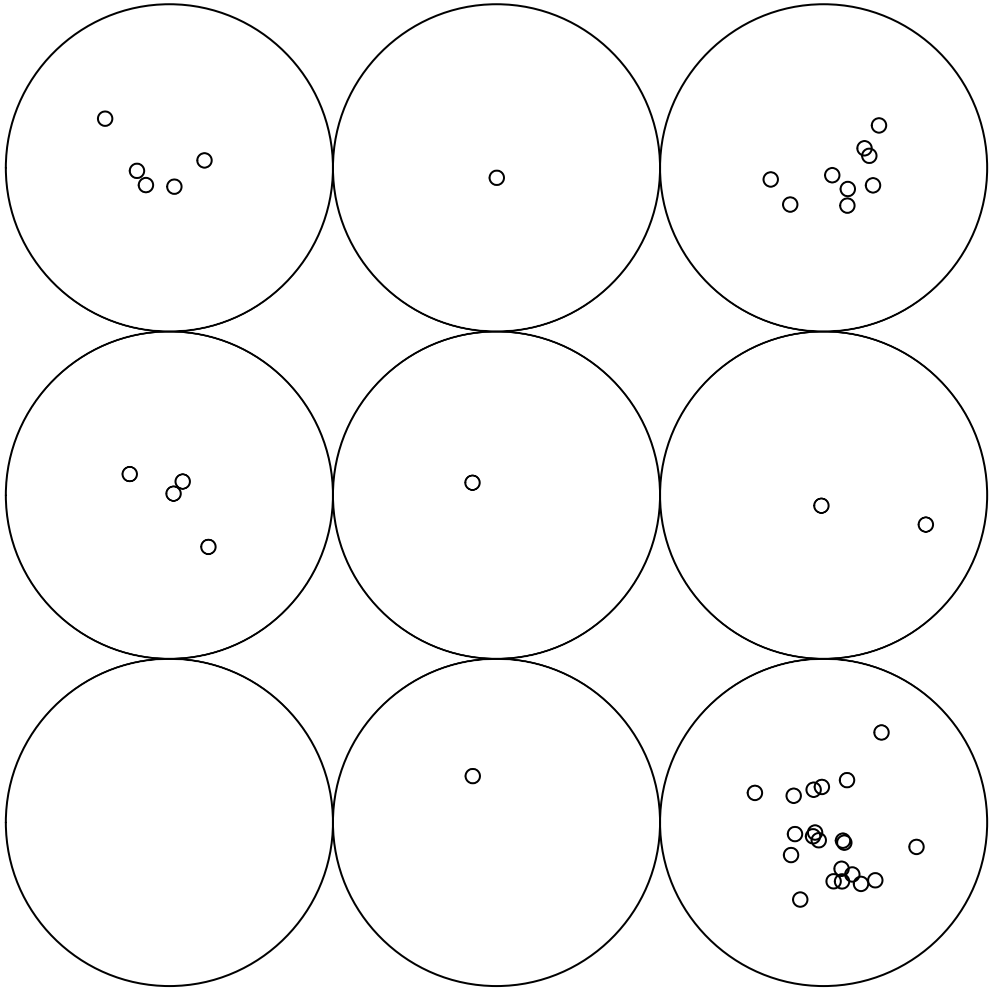

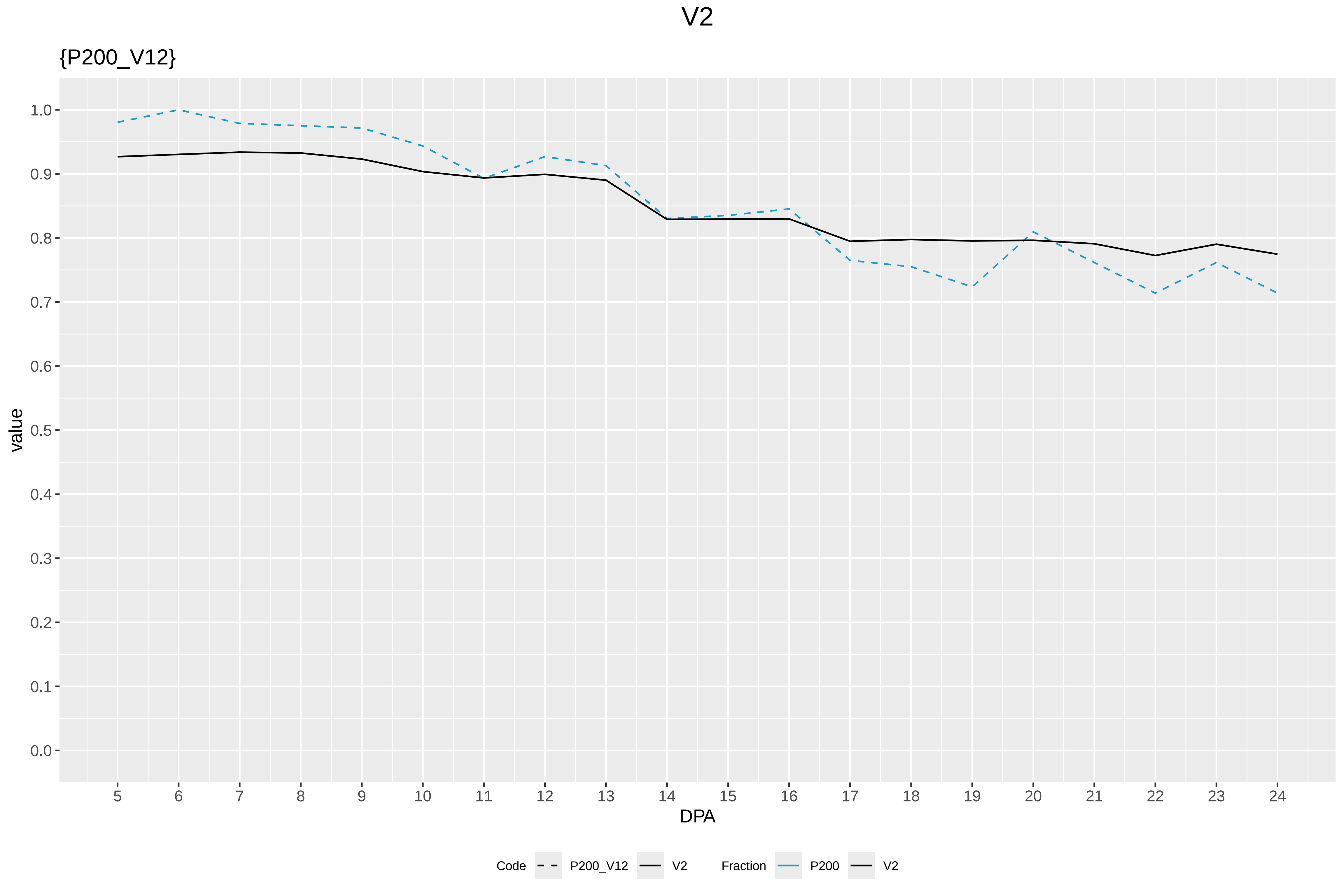
V2
{P200_V12}
value
1.0
0.9
0.8
0.7
0.6
0.5
0.4
0.3
0.2
0.1
0.0
5
6
7
8
9
10
11
12
13
14
15
16
17
18
19
20
21
22
23
24
DPA
Code
P200_V12
V2
Fraction
P200
V2

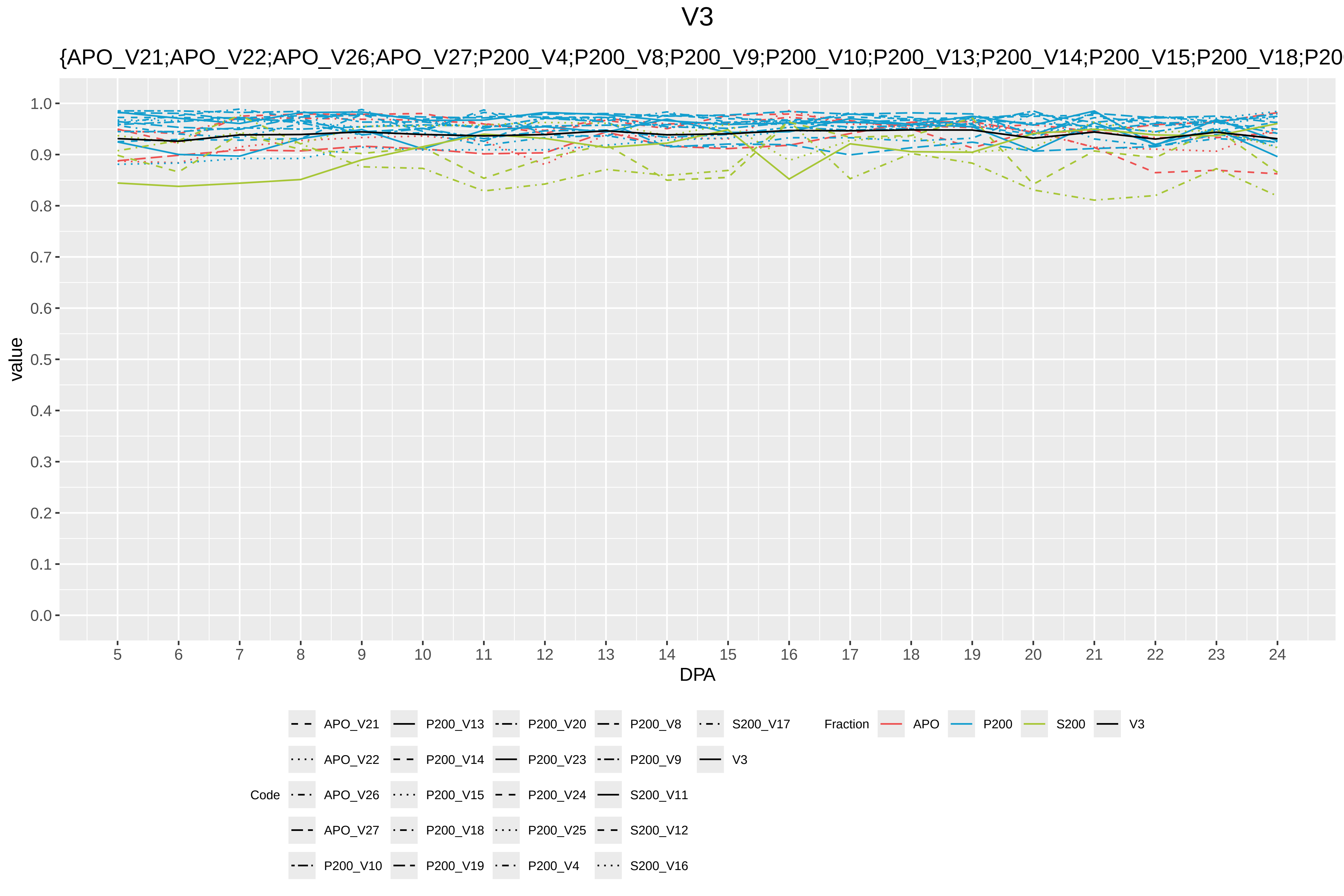

V3
{APO_V21;APO_V22;APO_V26;APO_V27;P200_V4;P200_V8;P200_V9;P200_V10;P200_V13;P200_V14;P200_V15;P200_V18;P20
value
1.0
0.9
0.8
0.7
0.6
0.5
0.4
0.3
0.2
0.1
0.0
5 6 7 8 9 10 11 12 13 14 15 16 17 18 19 20 21 22 23 24
DPA
Code
APO_V21
APO_V22
APO_V26
APO_V27
P200_V10
P200_V13
P200_V14
P200_V15
P200_V18
P200_V19
P200_V20
P200_V23
P200_V24
P200_V25
P200_V4
P200_V8
P200_V9
S200_V11
S200_V12
S200_V16
S200_V17
V3
Fraction
APO
P200
S200
V3

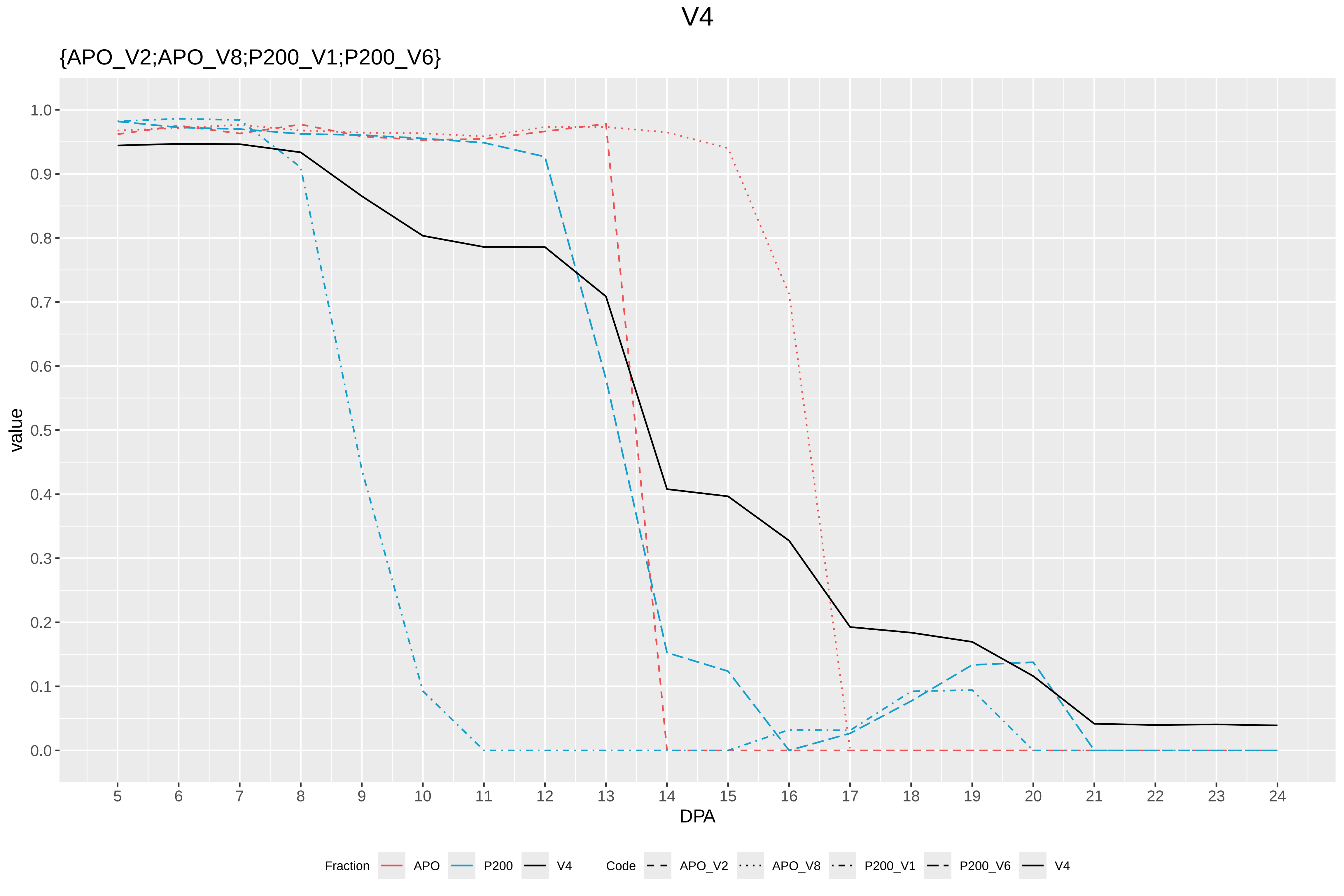
V4
{APO_V2;APO_V8;P200_V1;P200_V6}
value
1.0
0.9
0.8
0.7
0.6
0.5
0.4
0.3
0.2
0.1
0.0
5 6 7 8 9 10 11 12 13 14 15 16 17 18 19 20 21 22 23 24
DPA
Fraction
APO
P200
V4
Code
APO_V2
APO_V8
P200_V1
P200_V6
V4

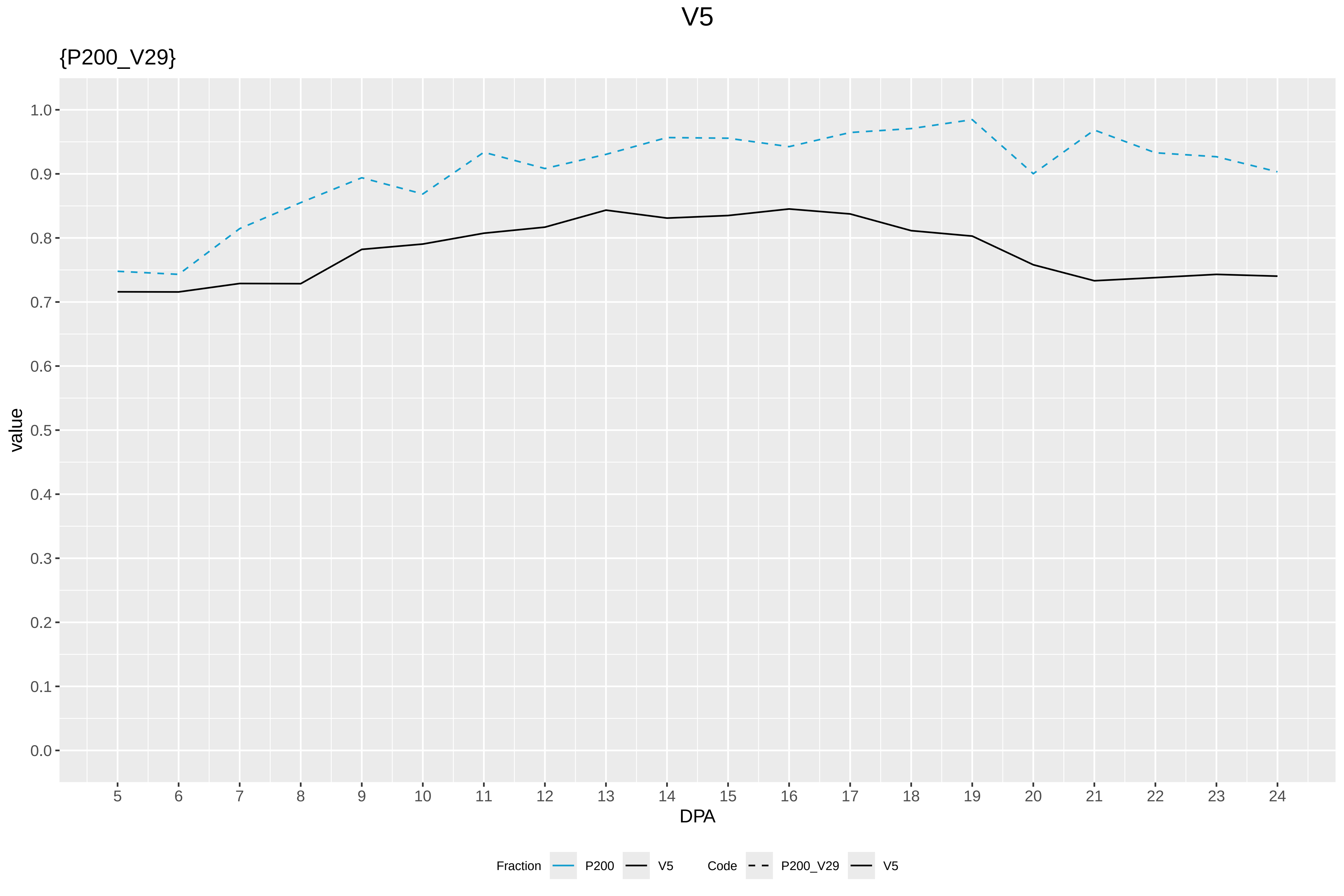

V5
{P200_V29}
value
1.0
0.9
0.8
0.7
0.6
0.5
0.4
0.3
0.2
0.1
0.0
5
6
7
8
9
10
11
12
13
14
15
16
17
18
19
20
21
22
23
24
DPA
Fraction
P200
V5
Code
P200_V29
V5

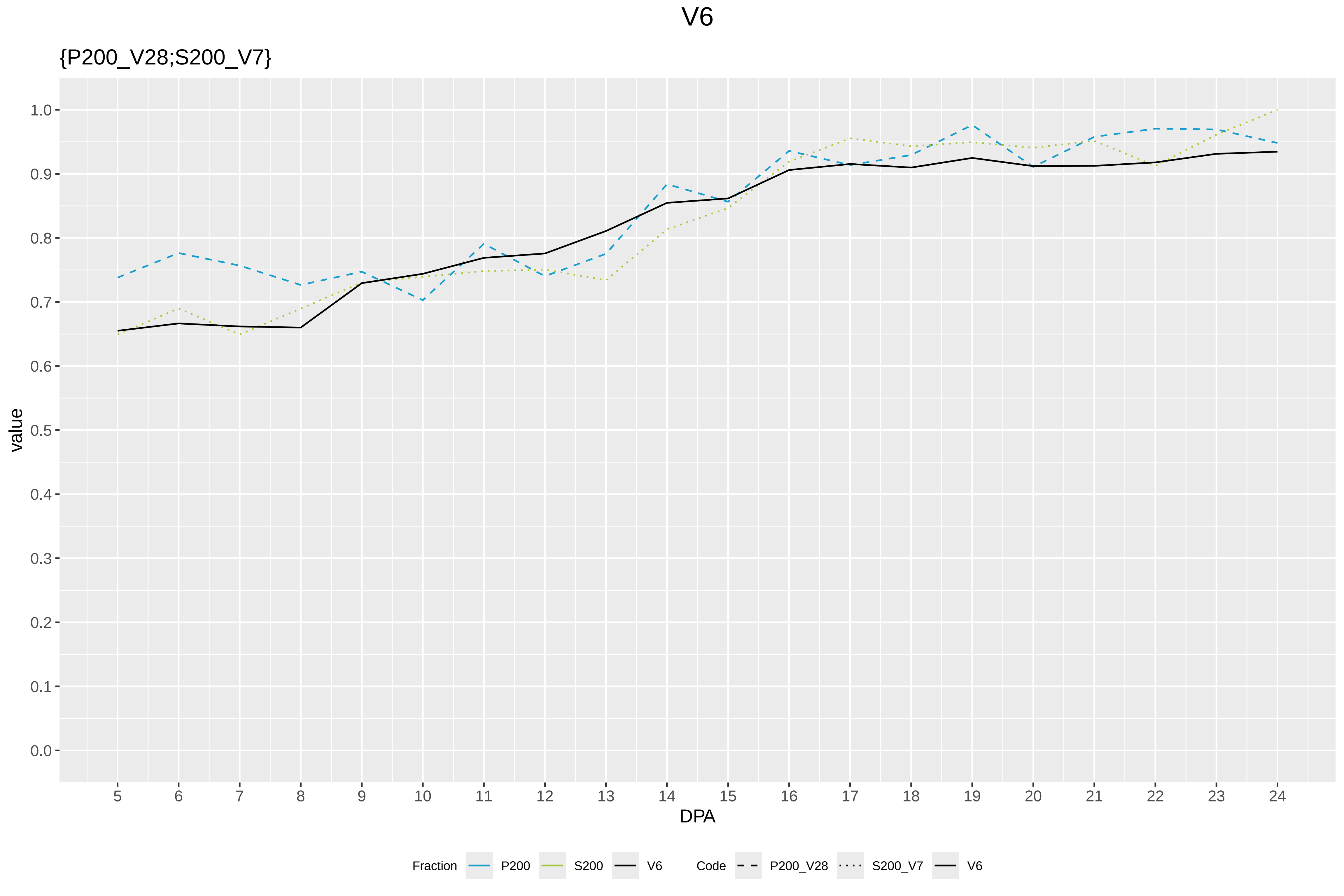
V6
{P200_V28;S200_V7}
value
1.0
0.9
0.8
0.7
0.6
0.5
0.4
0.3
0.2
0.1
0.0
5 6 7 8 9 10 11 12 13 14 15 16 17 18 19 20 21 22 23 24
DPA
Fraction P200 S200 V6
Code P200_V28 S200_V7 V6

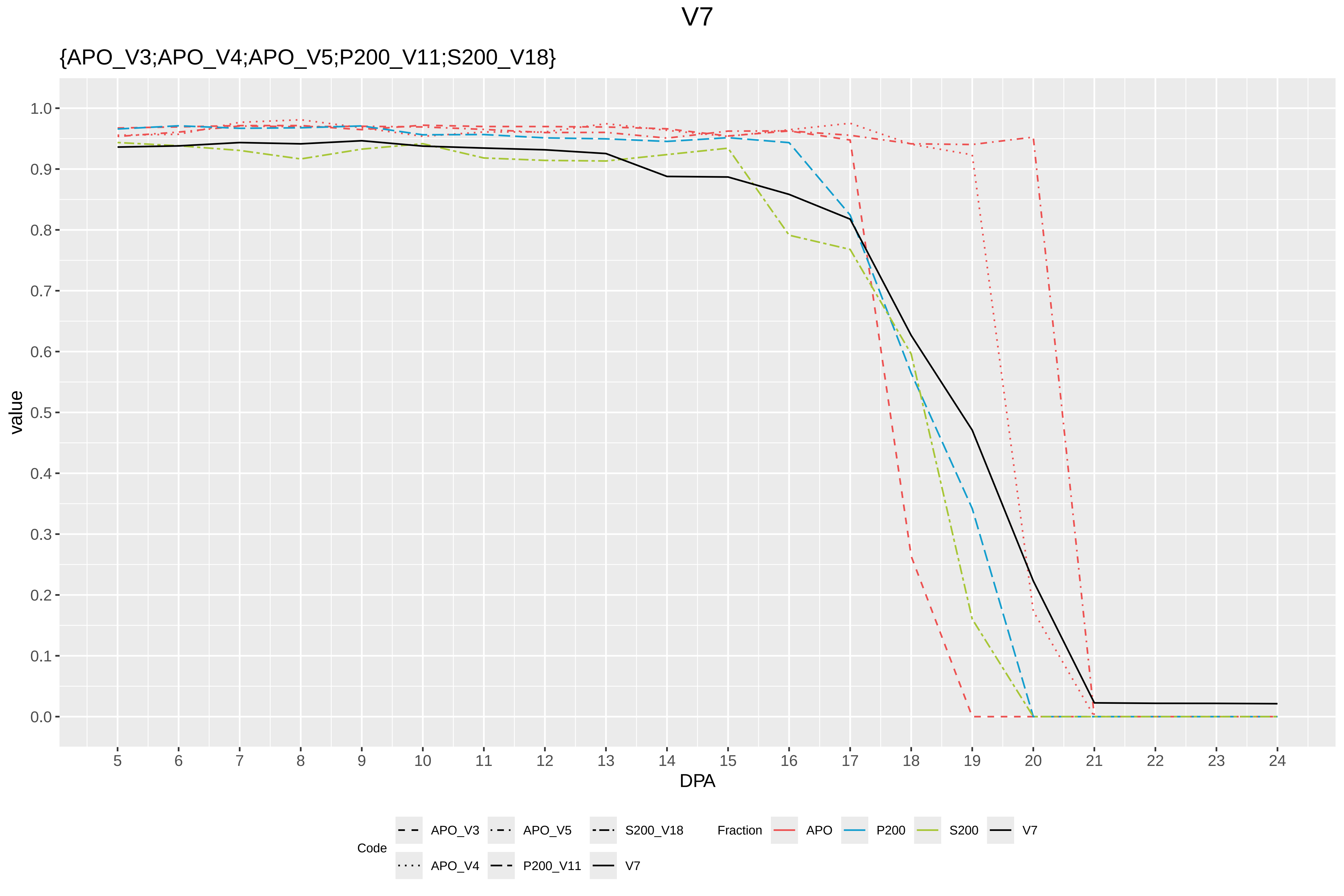

V7
{APO_V3;APO_V4;APO_V5;P200_V11;S200_V18}
value
1.0
0.9
0.8
0.7
0.6
0.5
0.4
0.3
0.2
0.1
0.0
5 6 7 8 9 10 11 12 13 14 15 16 17 18 19 20 21 22 23 24
DPA
Code
APO_V3
APO_V4
APO_V5
P200_V11
S200_V18
V7
Fraction
APO
P200
S200
V7

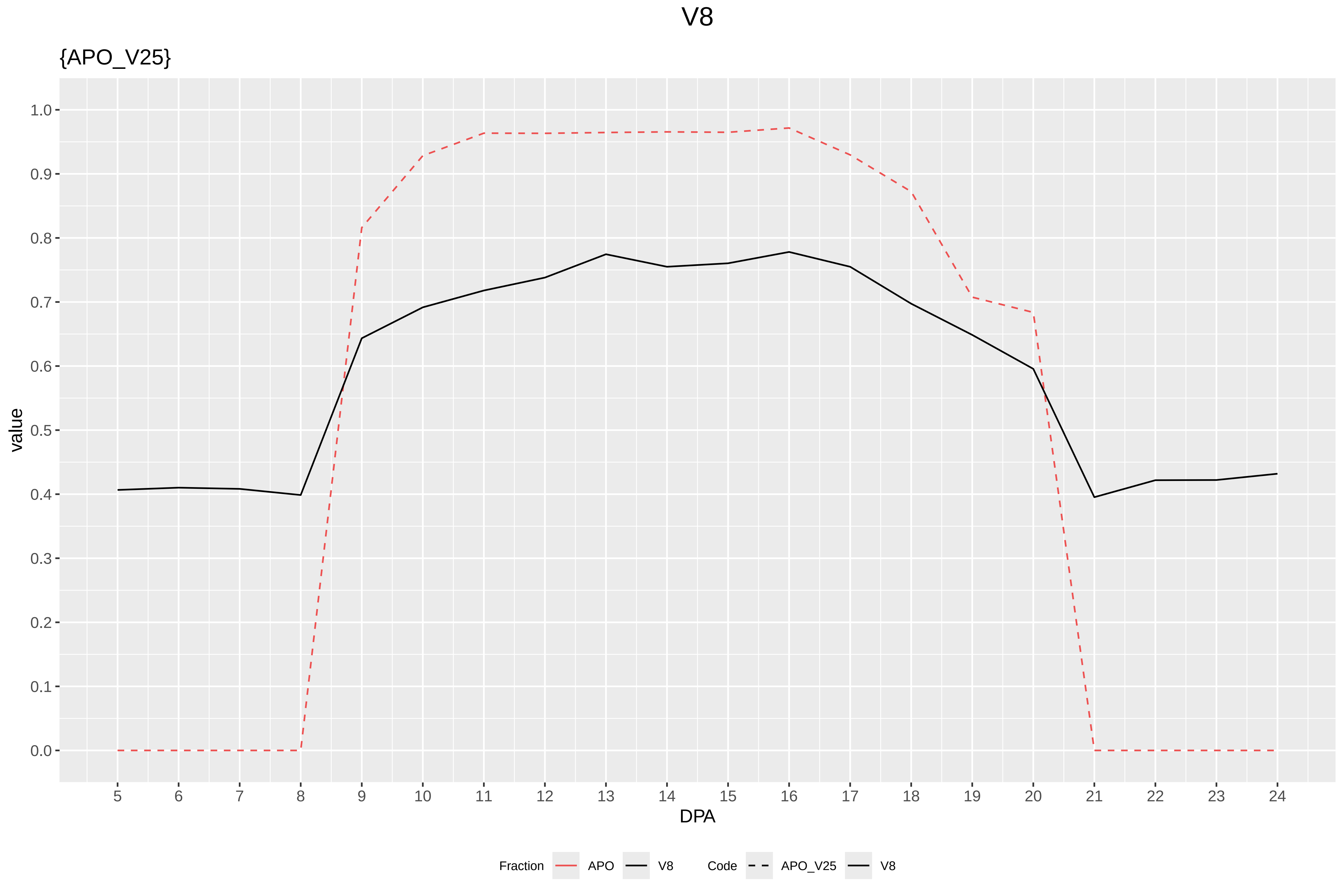

V8
{APO_V25}
value
1.0
0.9
0.8
0.7
0.6
0.5
0.4
0.3
0.2
0.1
0.0
5
6
7
8
9
10
11
12
13
14
15
16
17
18
19
20
21
22
23
24
DPA
Fraction
APO
V8
Code
APO_V25
V8

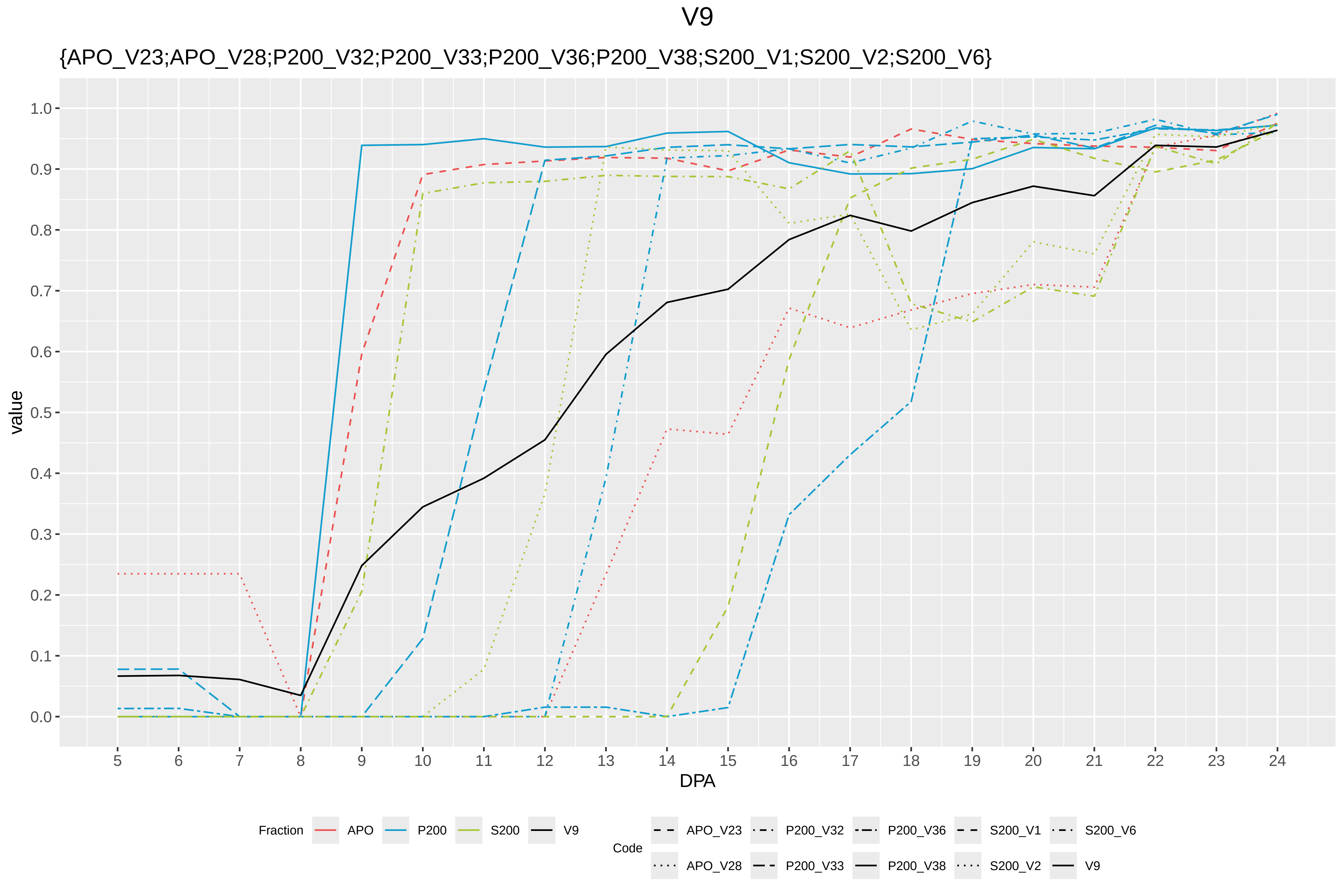
V9
{APO_V23;APO_V28;P200_V32;P200_V33;P200_V36;P200_V38;S200_V1;S200_V2;S200_V6}
value
1.0
0.9
0.8
0.7
0.6
0.5
0.4
0.3
0.2
0.1
0.0
5
6
7
8
9
10
11
12
13
14
15
16
17
18
19
20
21
22
23
24
DPA
Fraction
APO
P200
S200
V9
Code
APO_V23
APO_V28
P200_V32
P200_V33
P200_V36
P200_V38
S200_V1
S200_V2
S200_V6
V9

Supplementary Figure S7. Merged SOM protein expression modules (20-cluster model).

Codes

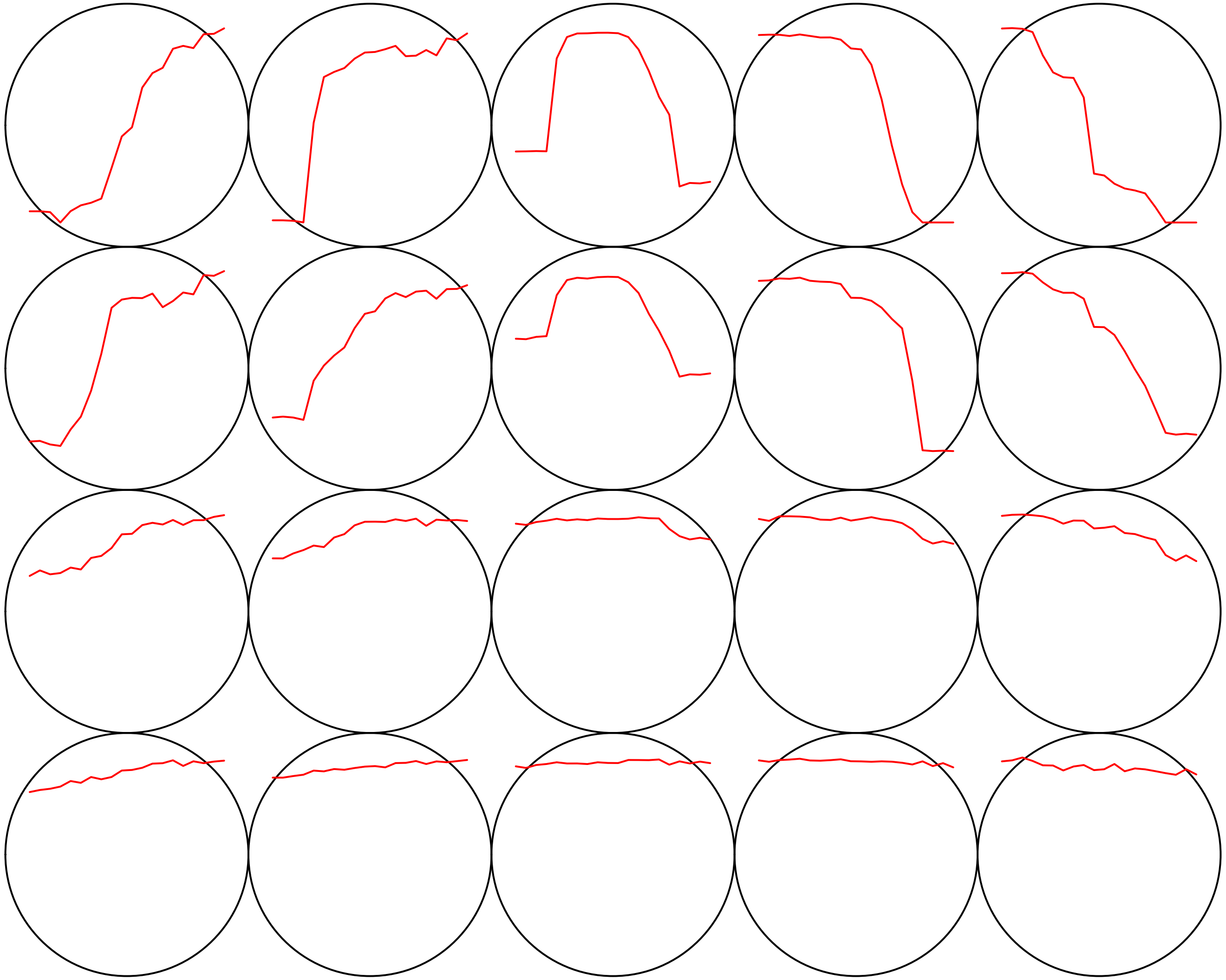

Count plot

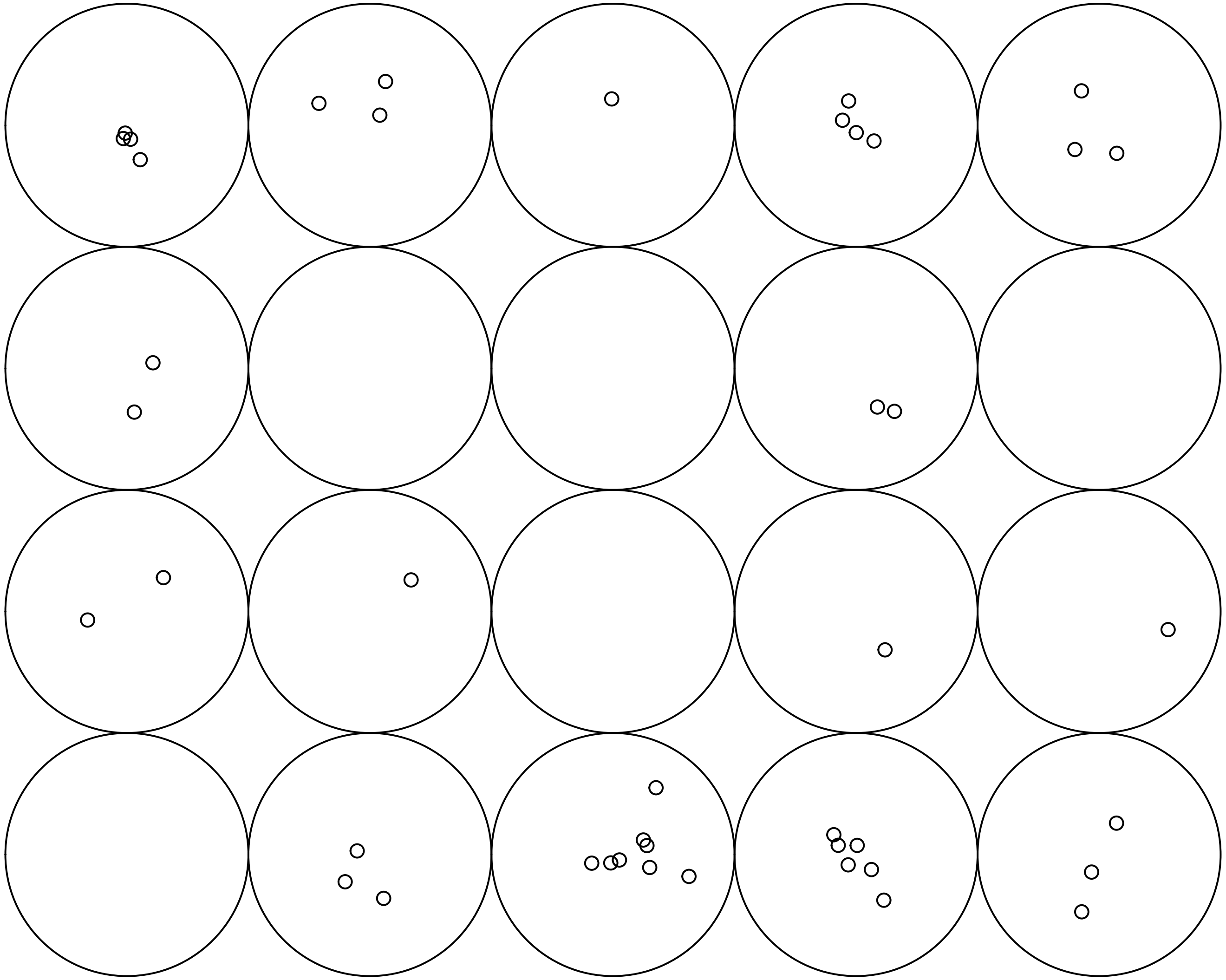

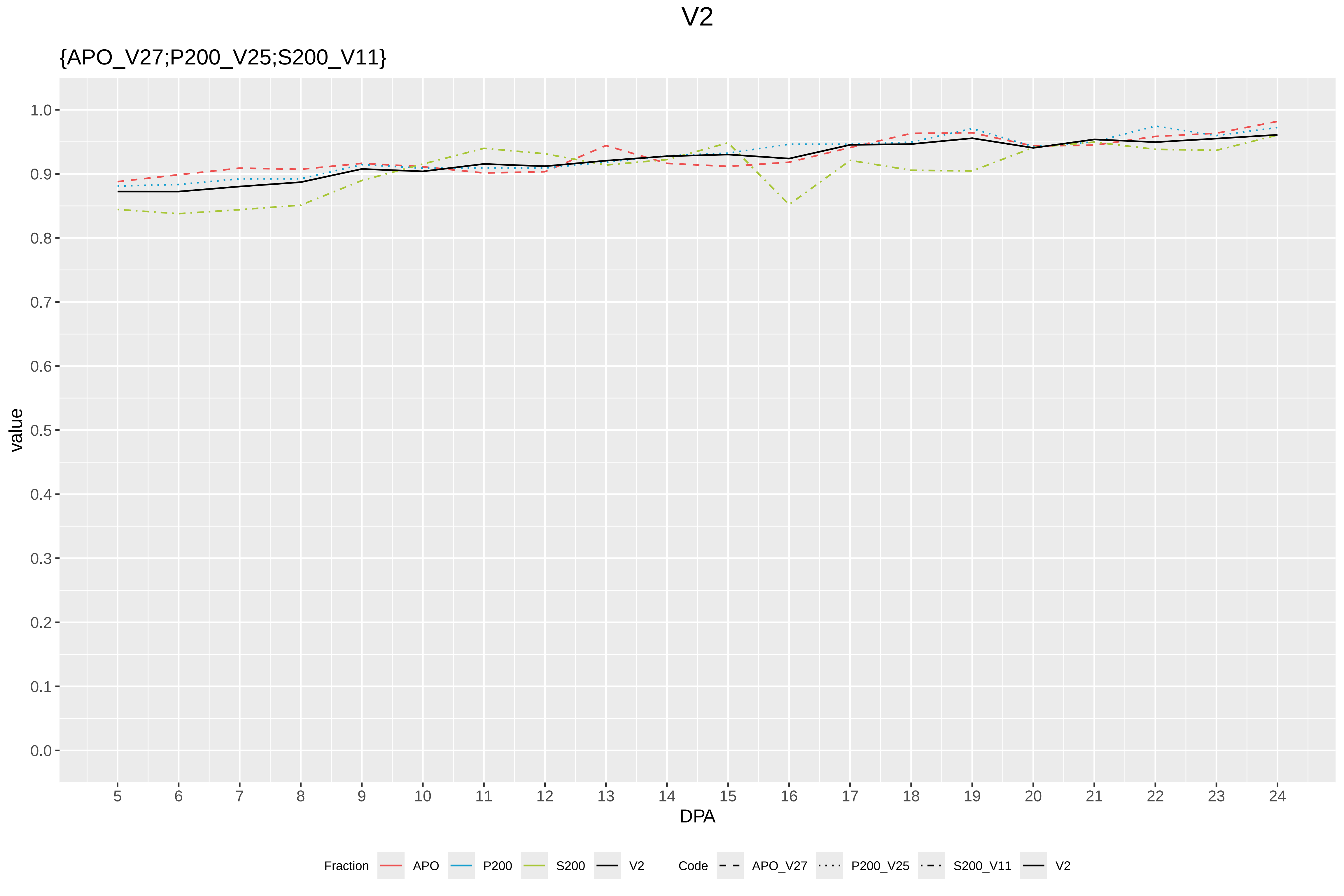

V2
{APO_V27;P200_V25;S200_V11}
1.0
0.9
0.8
0.7
0.6
0.5
0.4
0.3
0.2
0.1
0.0
value
5 6 7 8 9 10 11 12 13 14 15 16 17 18 19 20 21 22 23 24
DPA
Fraction APO P200 S200 V2
Code APO_V27 P200_V25 S200_V11 V2

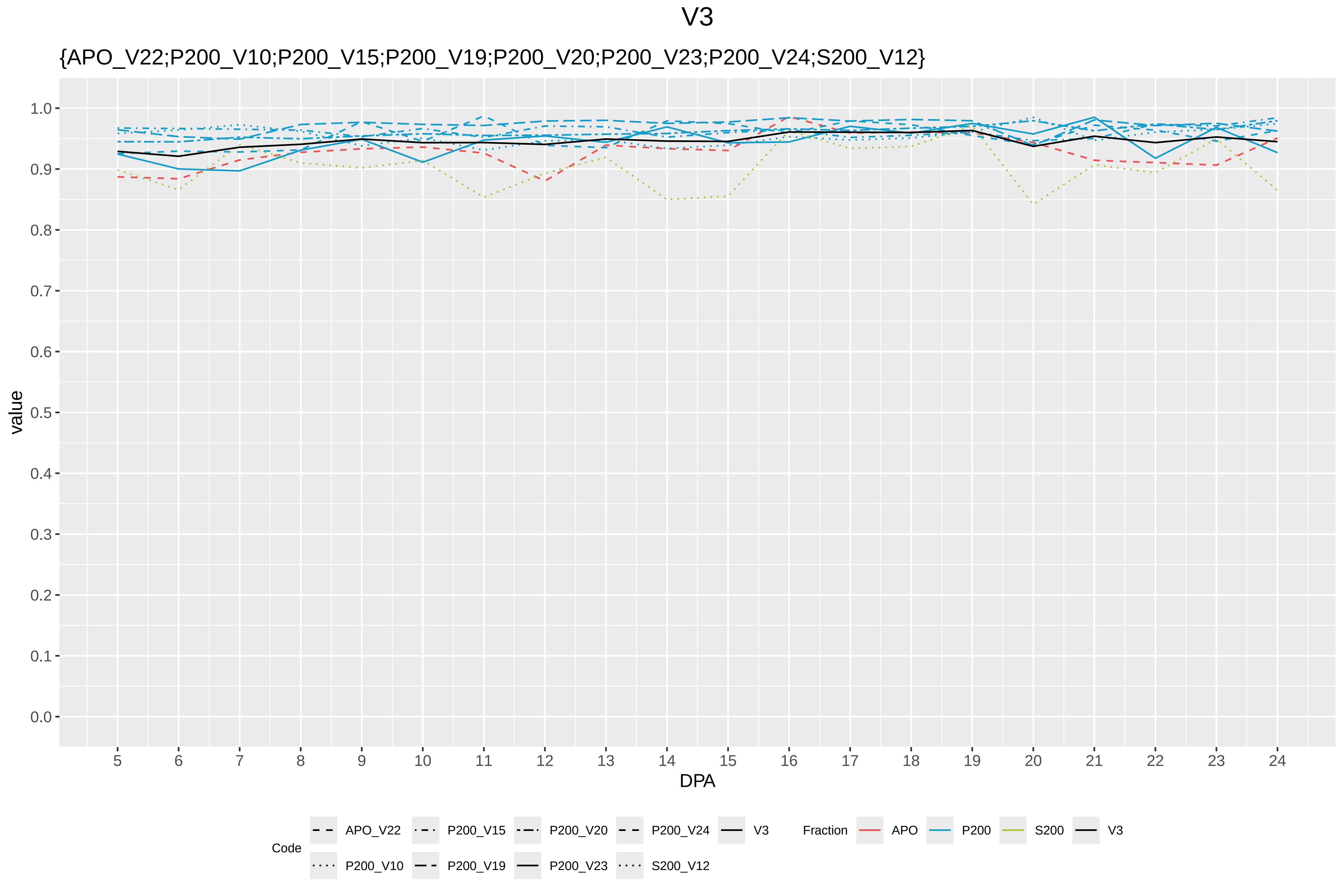
V3
{APO_V22;P200_V10;P200_V15;P200_V19;P200_V20;P200_V23;P200_V24;S200_V12}
value
1.0
0.9
0.8
0.7
0.6
0.5
0.4
0.3
0.2
0.1
0.0
5
6
7
8
9
10
11
12
13
14
15
16
17
18
19
20
21
22
23
24
DPA
Code
APO_V22
P200_V10
P200_V15
P200_V19
P200_V20
P200_V23
P200_V24
S200_V12
V3
Fraction
APO
P200
S200
V3

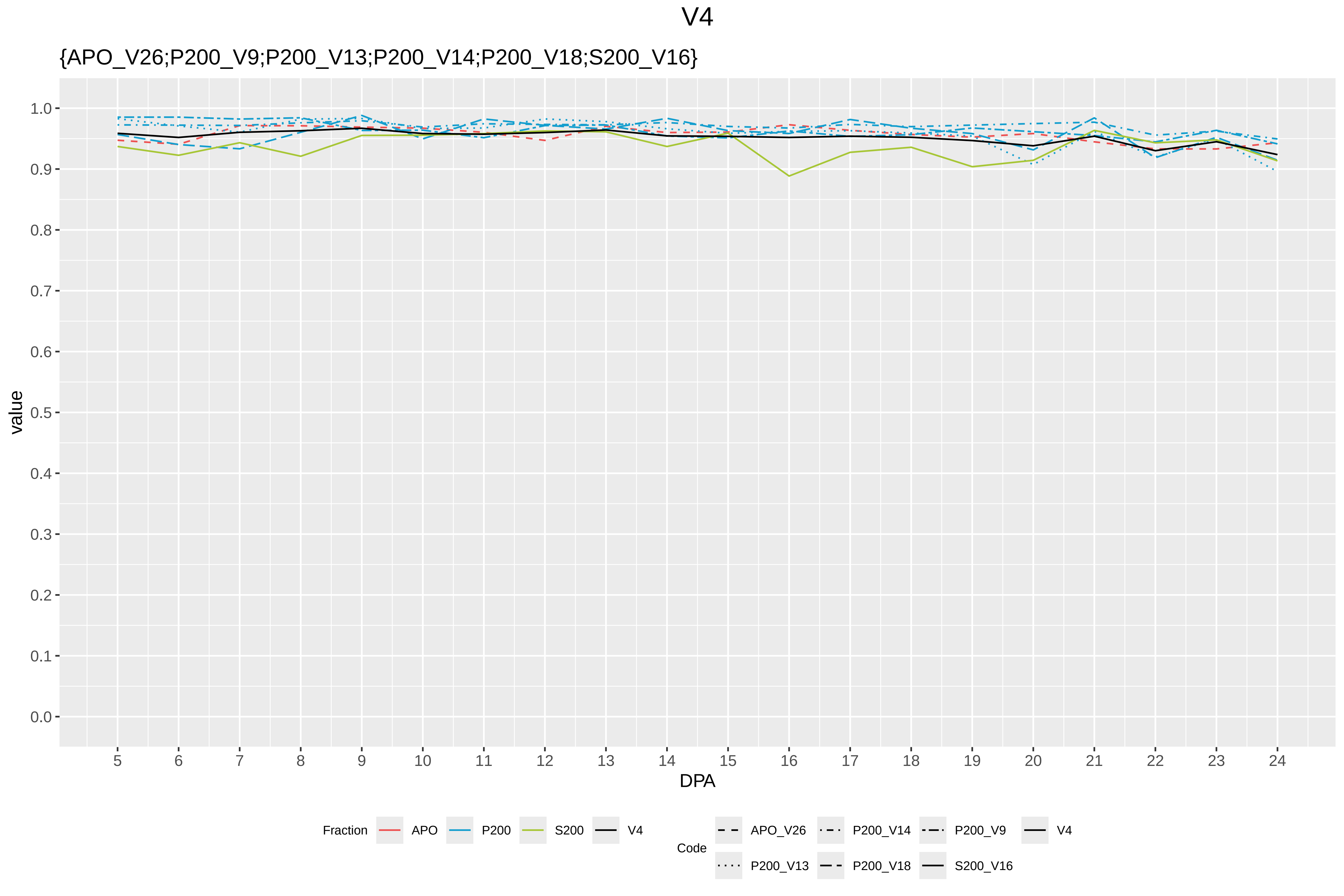

V4
{APO_V26;P200_V9;P200_V13;P200_V14;P200_V18;S200_V16}
value
1.0
0.9
0.8
0.7
0.6
0.5
0.4
0.3
0.2
0.1
0.0
5 6 7 8 9 10 11 12 13 14 15 16 17 18 19 20 21 22 23 24
DPA
Fraction
APO
P200
S200
V4
Code
APO_V26
P200_V14
P200_V9
V4
P200_V13
P200_V18
S200_V16

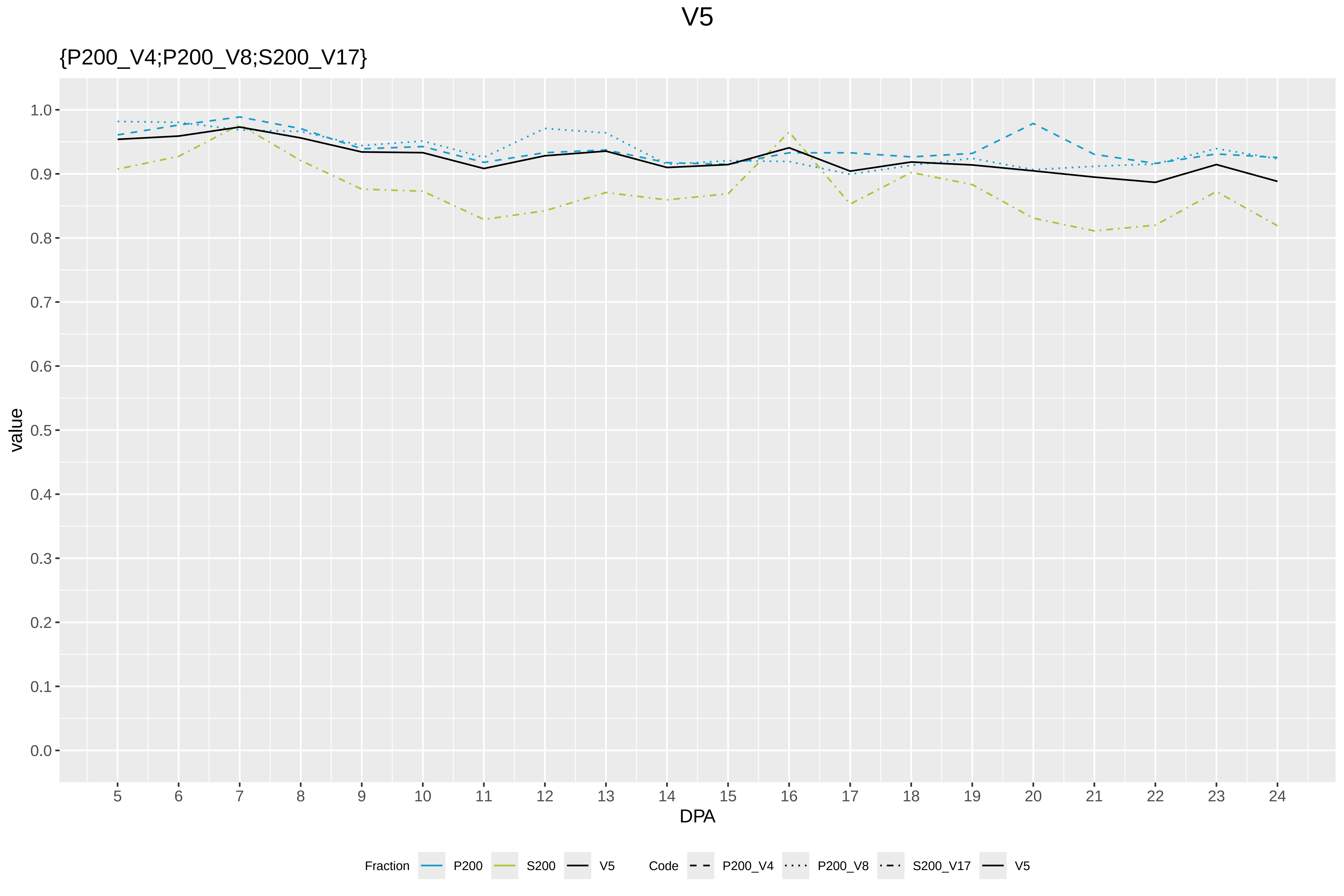
V5
{P200_V4;P200_V8;S200_V17}
value
DPA
1.0
0.9
0.8
0.7
0.6
0.5
0.4
0.3
0.2
0.1
0.0
5 6 7 8 9 10 11 12 13 14 15 16 17 18 19 20 21 22 23 24
Fraction P200 S200 V5
Code P200_V4 P200_V8 S200_V17 V5

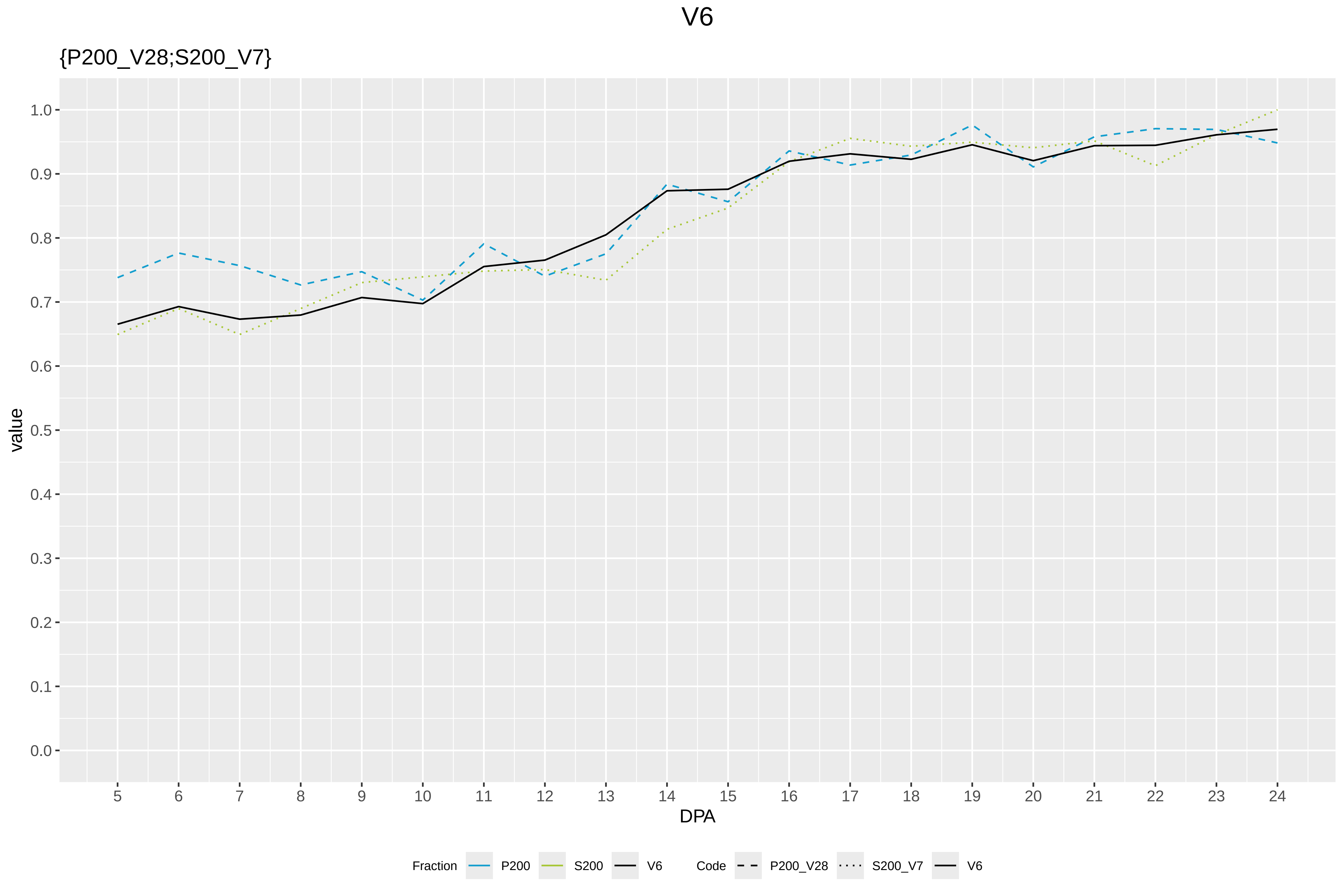
V6
{P200_V28;S200_V7}
value
1.0
0.9
0.8
0.7
0.6
0.5
0.4
0.3
0.2
0.1
0.0
5
6
7
8
9
10
11
12
13
14
15
16
17
18
19
20
21
22
23
24
DPA
Fraction
P200
S200
V6
Code
P200_V28
S200_V7
V6

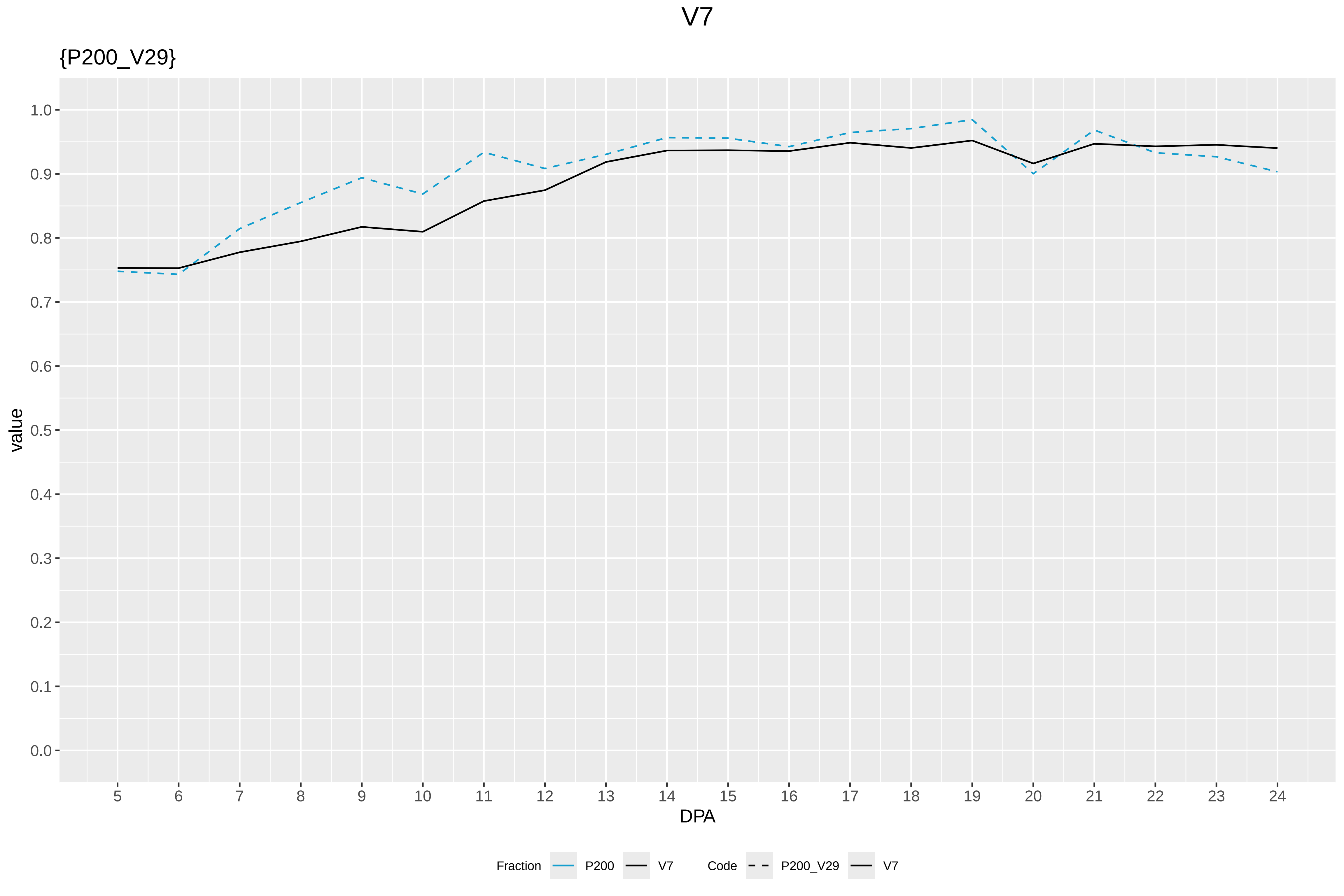
V7
{P200_V29}
value
1.0
0.9
0.8
0.7
0.6
0.5
0.4
0.3
0.2
0.1
0.0
5
6
7
8
9
10
11
12
13
14
15
16
17
18
19
20
21
22
23
24
DPA
Fraction
P200
V7
Code
P200_V29
V7

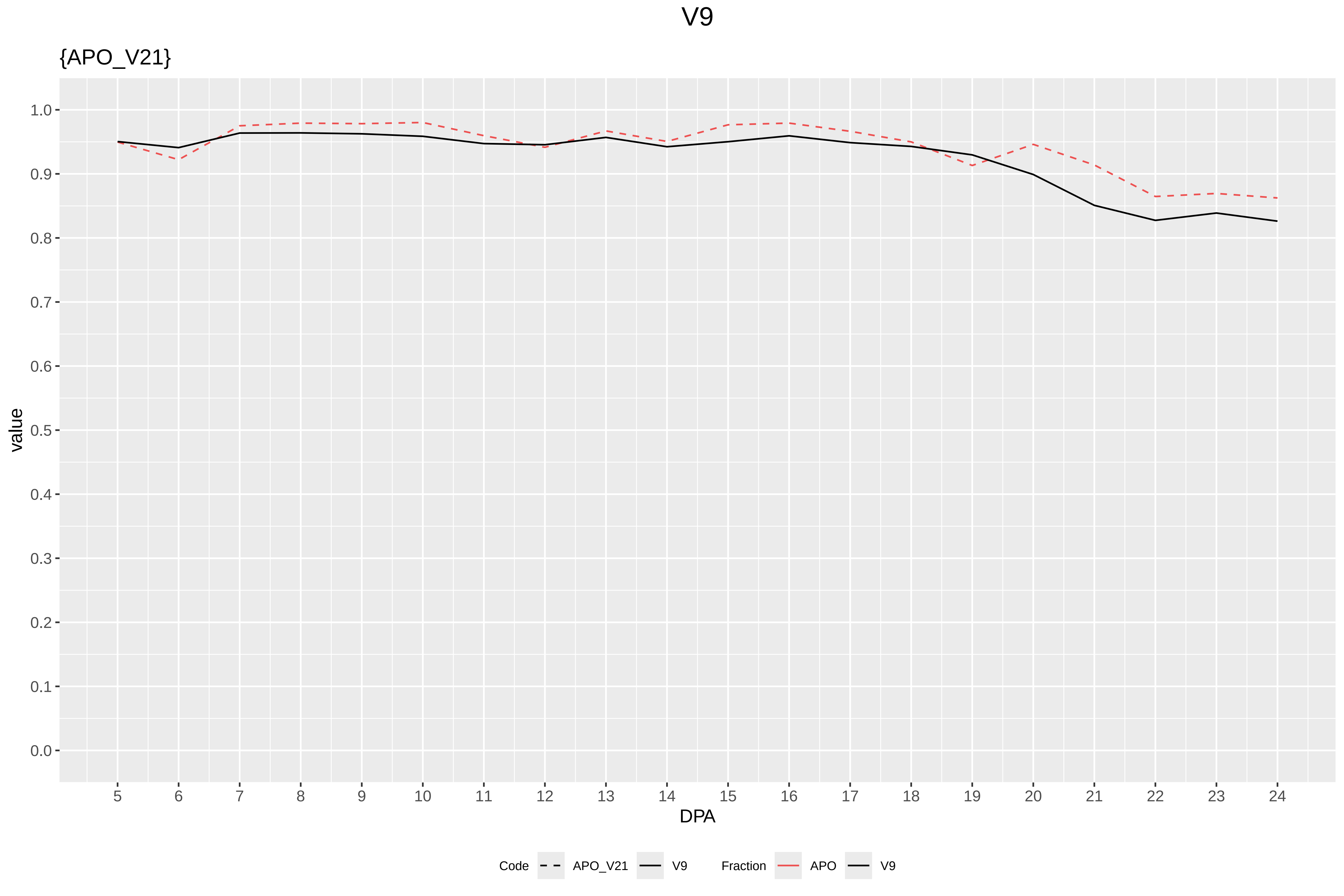

V9
{APO_V21}
value
1.0
0.9
0.8
0.7
0.6
0.5
0.4
0.3
0.2
0.1
0.0
5
6
7
8
9
10
11
12
13
14
15
16
17
18
19
20
21
22
23
24
DPA
Code
APO_V21
V9
Fraction
APO
V9

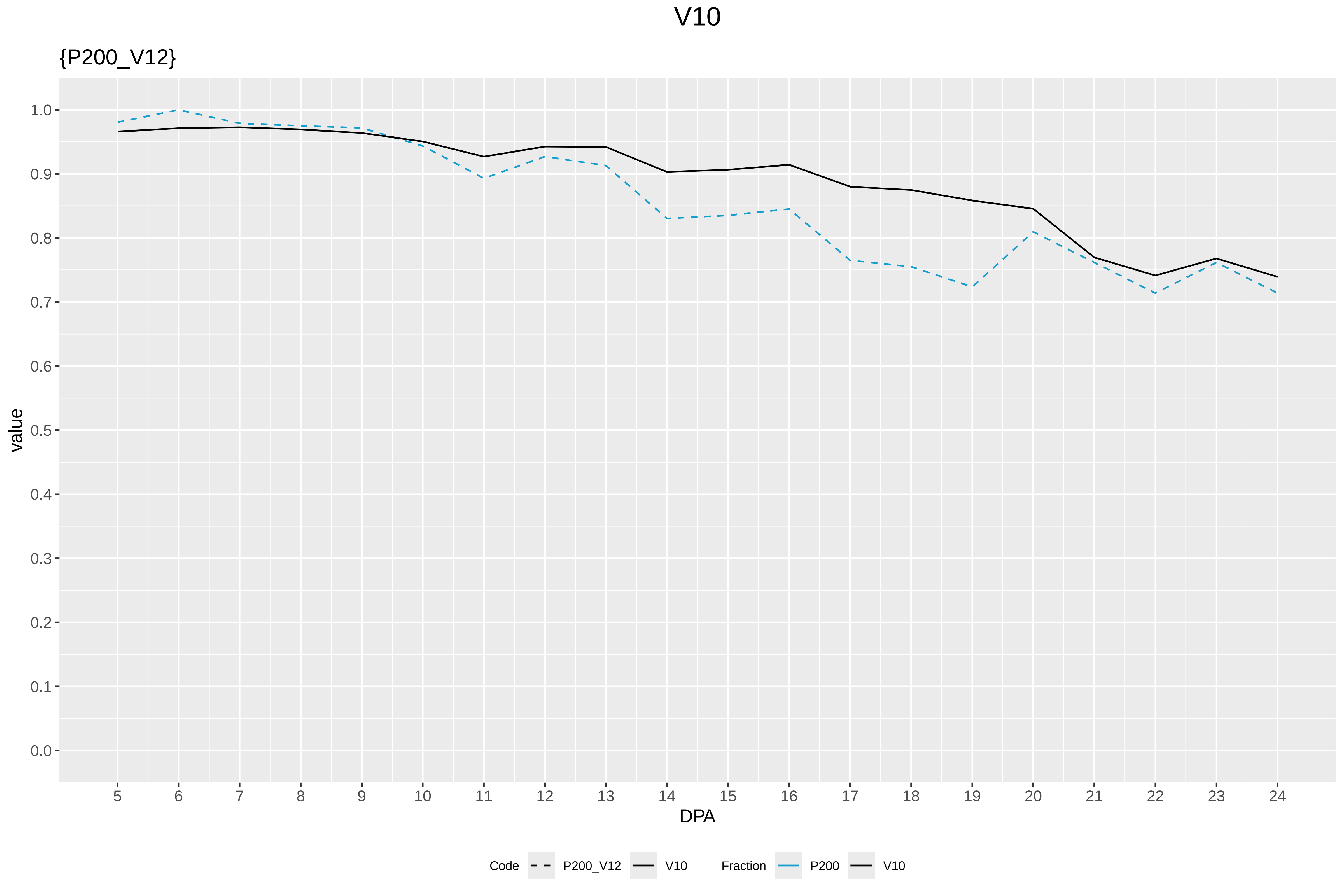

V10
{P200_V12}
value
1.0
0.9
0.8
0.7
0.6
0.5
0.4
0.3
0.2
0.1
0.0
5
6
7
8
9
10
11
12
13
14
15
16
17
18
19
20
21
22
23
24
DPA
Code
P200_V12
V10
Fraction
P200
V10

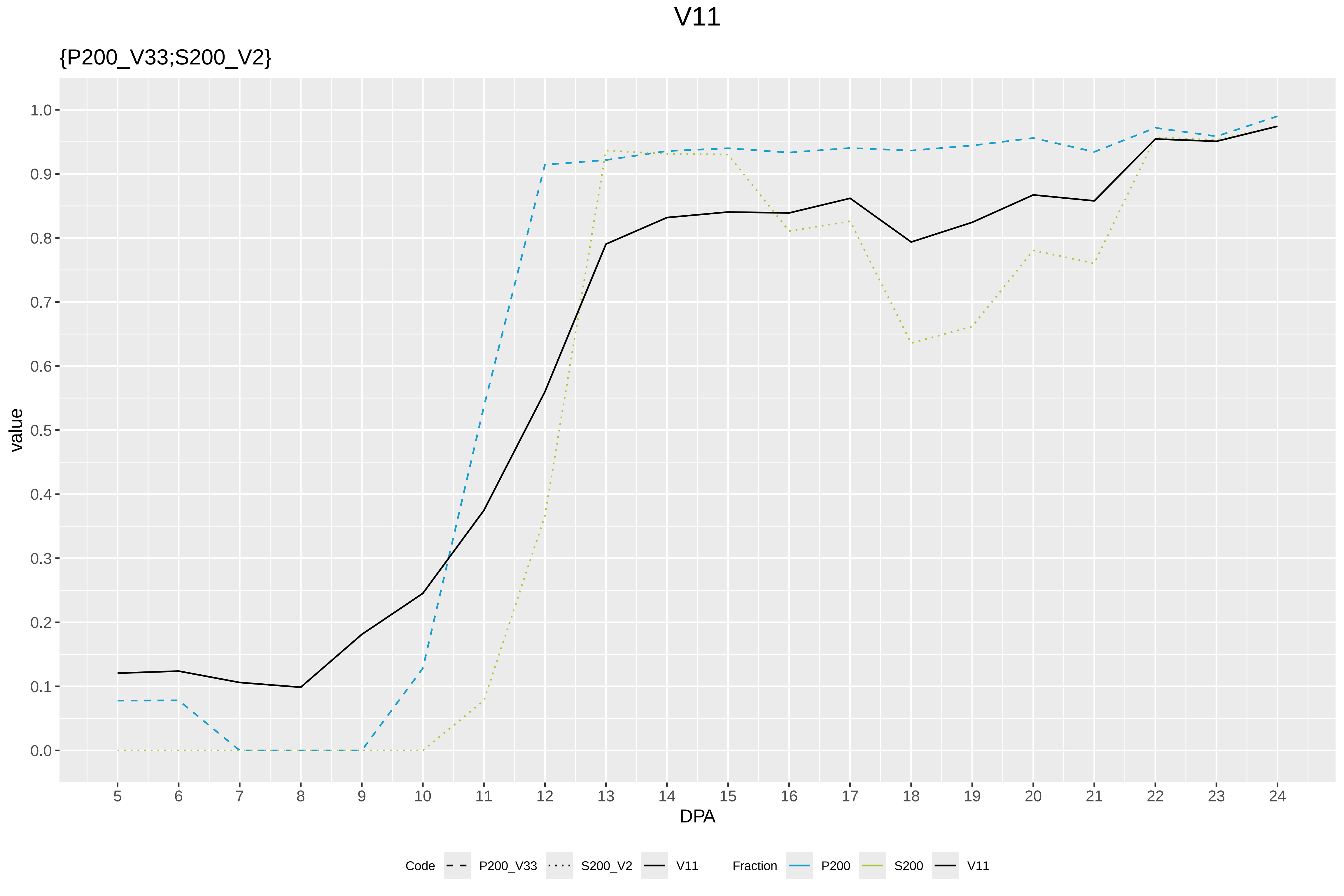

V11
{P200_V33;S200_V2}
value
1.0
0.9
0.8
0.7
0.6
0.5
0.4
0.3
0.2
0.1
0.0
5
6
7
8
9
10
11
12
13
14
15
16
17
18
19
20
21
22
23
24
DPA
Code
P200_V33
S200_V2
V11
Fraction
P200
S200
V11

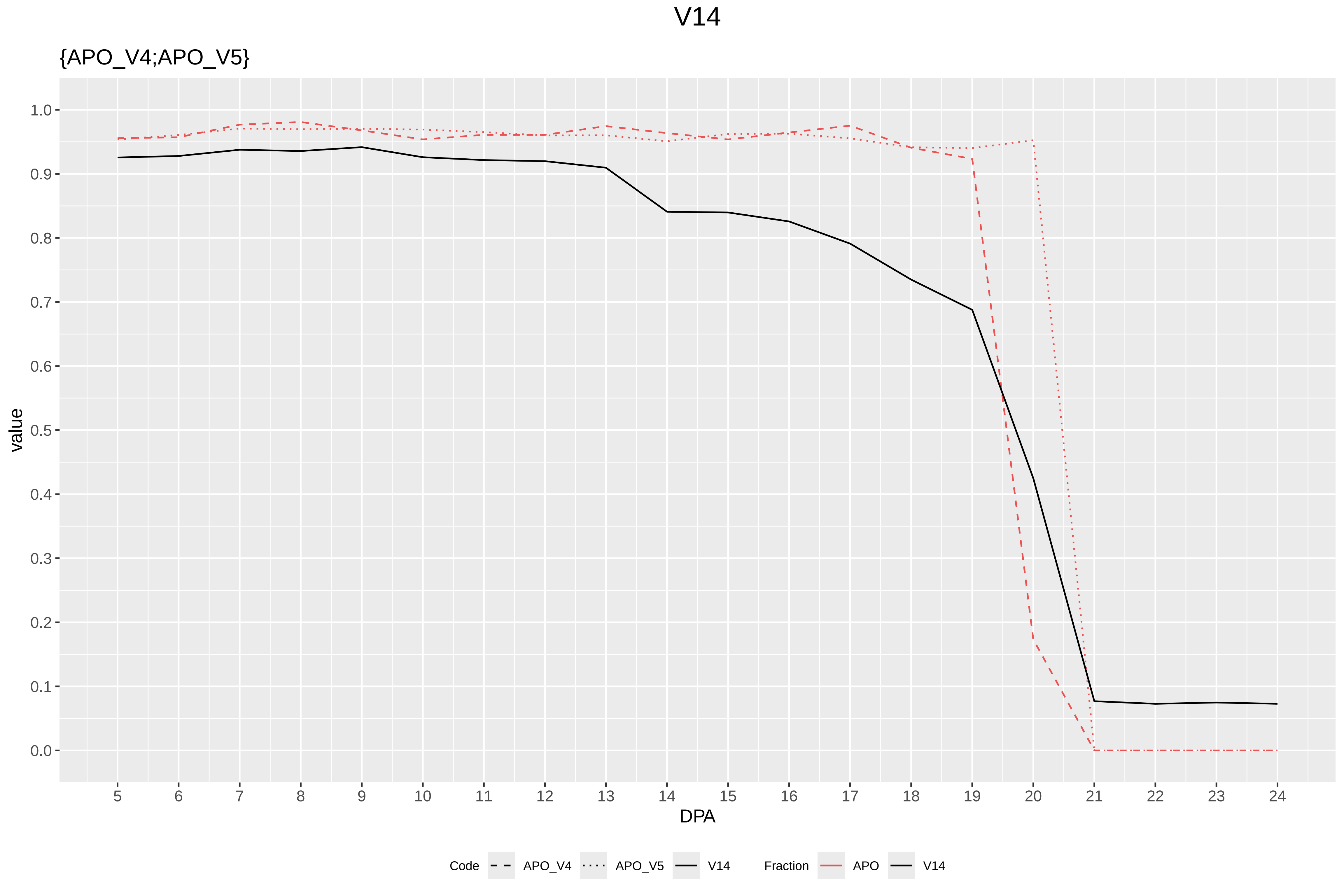

V14
{APO_V4;APO_V5}
value
DPA
1.0
0.9
0.8
0.7
0.6
0.5
0.4
0.3
0.2
0.1
0.0
5
6
7
8
9
10
11
12
13
14
15
16
17
18
19
20
21
22
23
24
Code
APO_V4
APO_V5
V14
Fraction
APO
V14

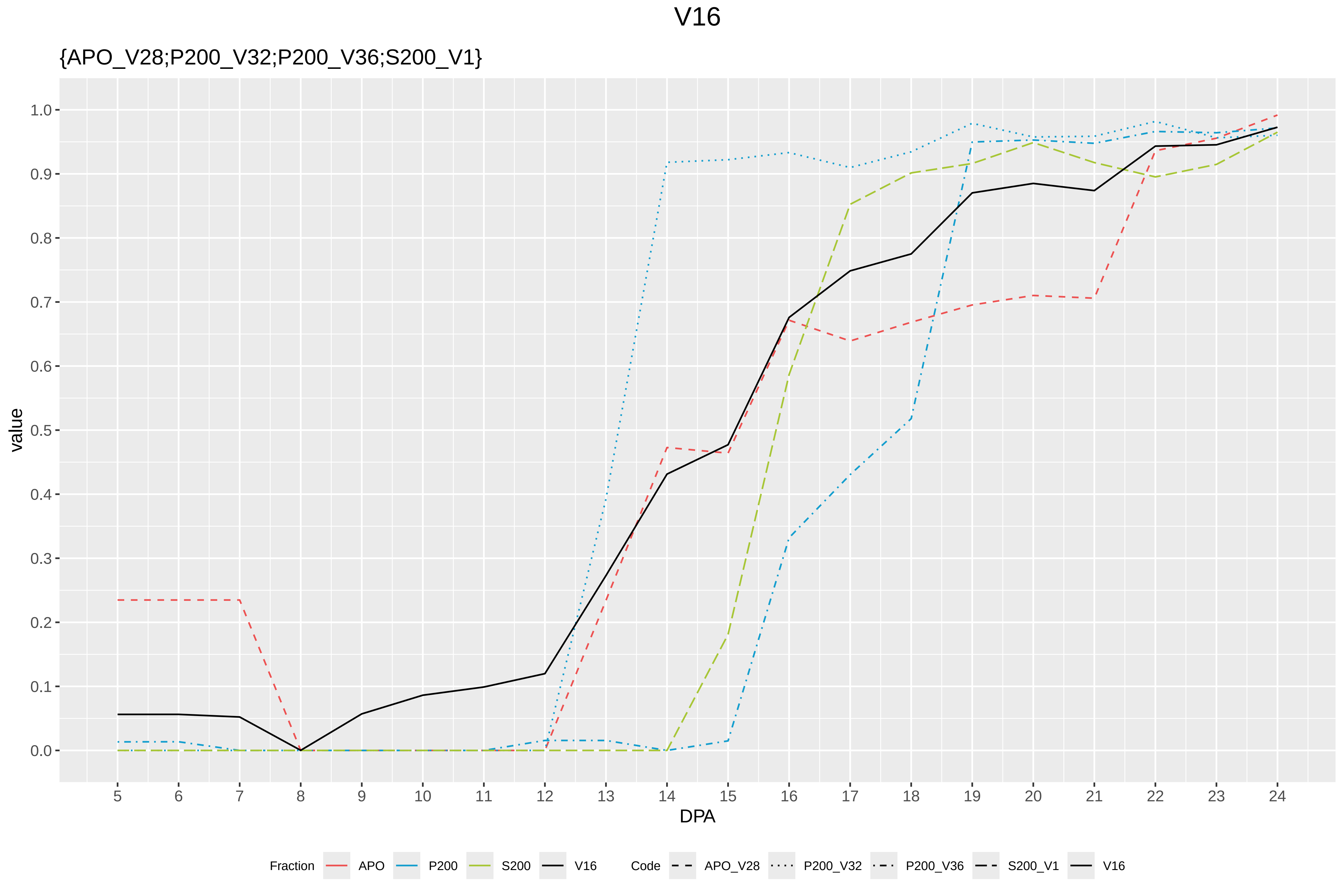

V16
{APO_V28;P200_V32;P200_V36;S200_V1}
value
DPA
1.0
0.9
0.8
0.7
0.6
0.5
0.4
0.3
0.2
0.1
0.0
5 6 7 8 9 10 11 12 13 14 15 16 17 18 19 20 21 22 23 24
Fraction APO P200 S200 V16
Code APO_V28 P200_V32 P200_V36 S200_V1 V16

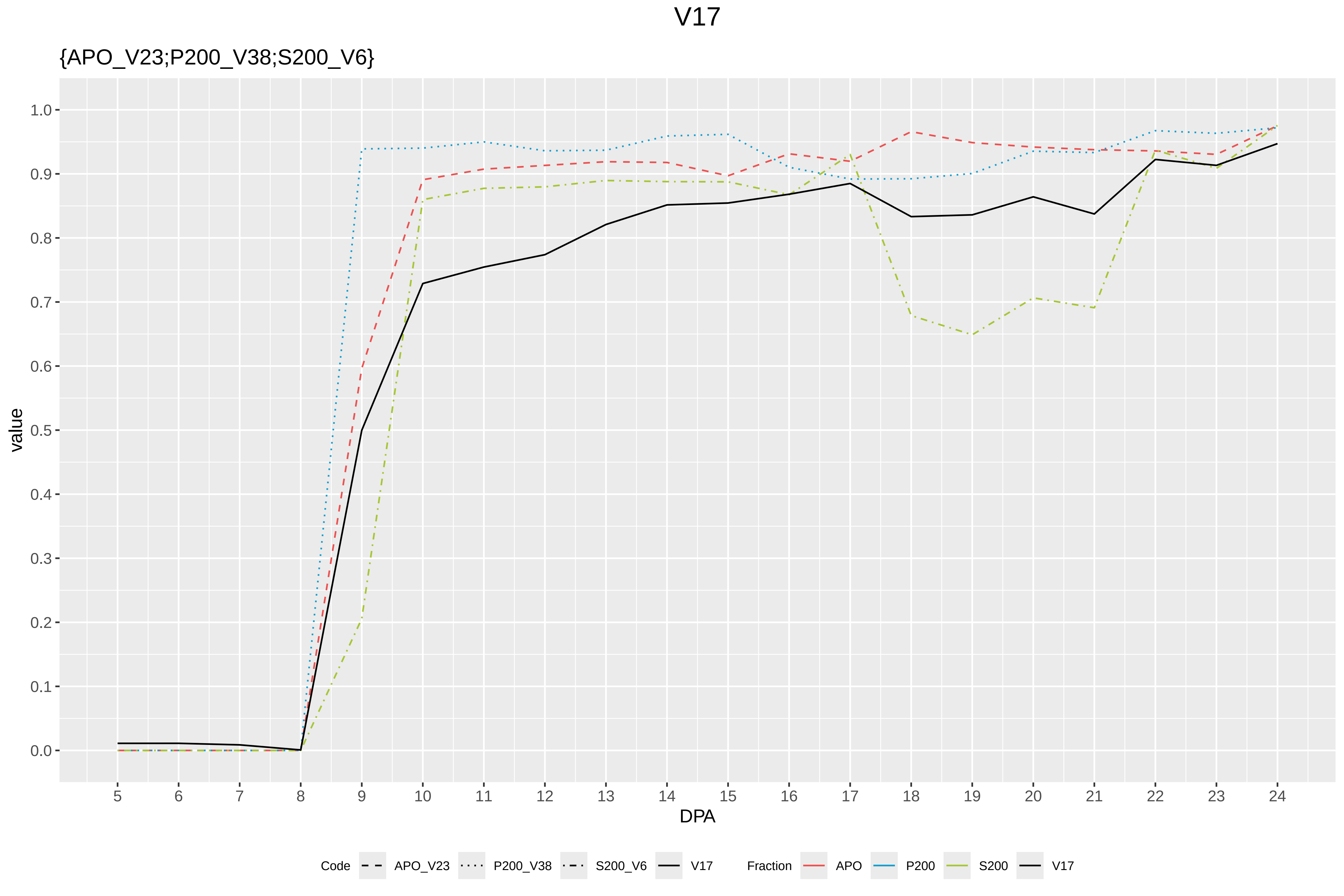

V17
{APO_V23;P200_V38;S200_V6}
value
1.0
0.9
0.8
0.7
0.6
0.5
0.4
0.3
0.2
0.1
0.0
5
6
7
8
9
10
11
12
13
14
15
16
17
18
19
20
21
22
23
24
DPA
Code
APO_V23
P200_V38
S200_V6
V17
Fraction
APO
P200
S200
V17

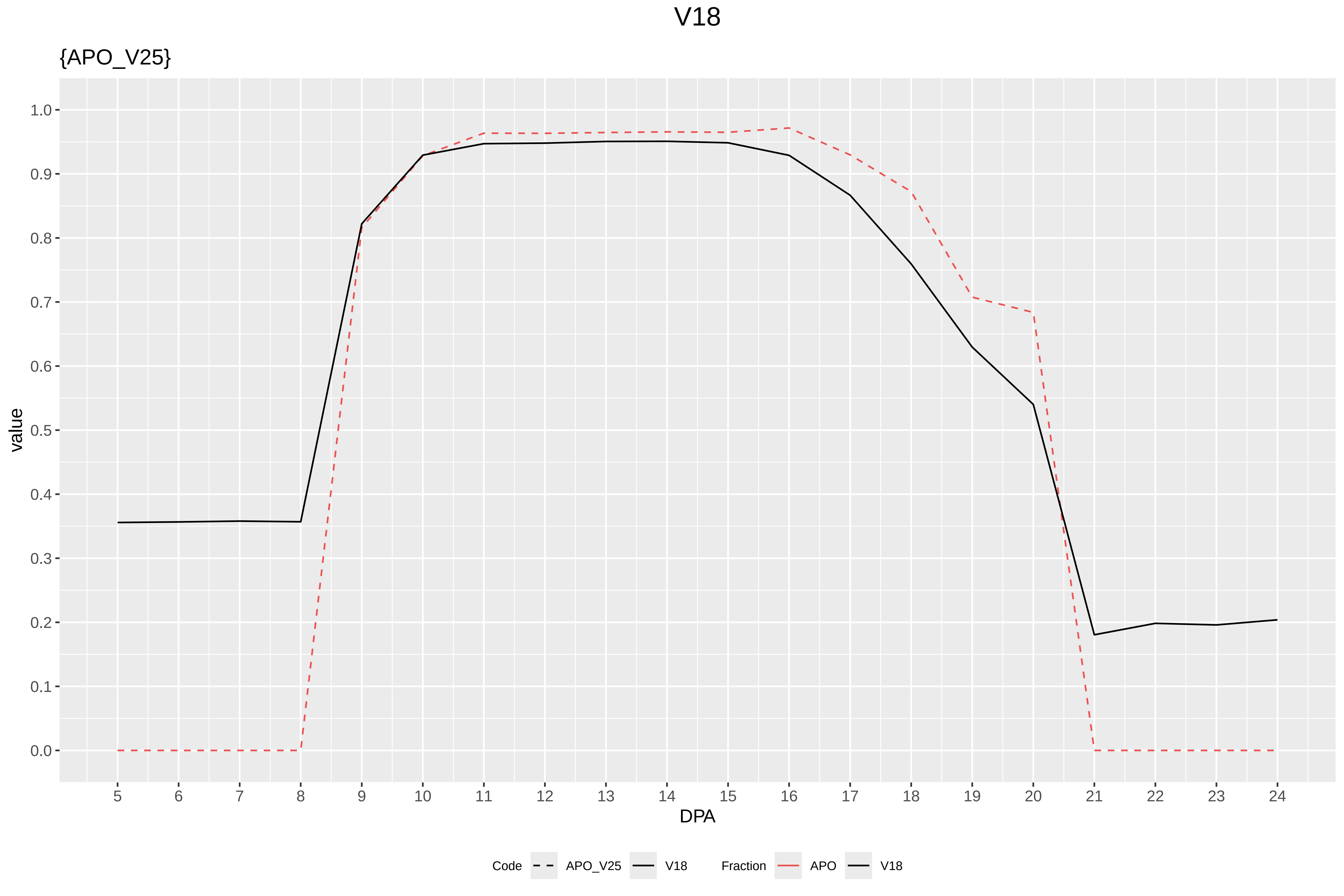
V18
{APO_V25}
value
1.0
0.9
0.8
0.7
0.6
0.5
0.4
0.3
0.2
0.1
0.0
5
6
7
8
9
10
11
12
13
14
15
16
17
18
19
20
21
22
23
24
DPA
Code
APO_V25
V18
Fraction
APO
V18

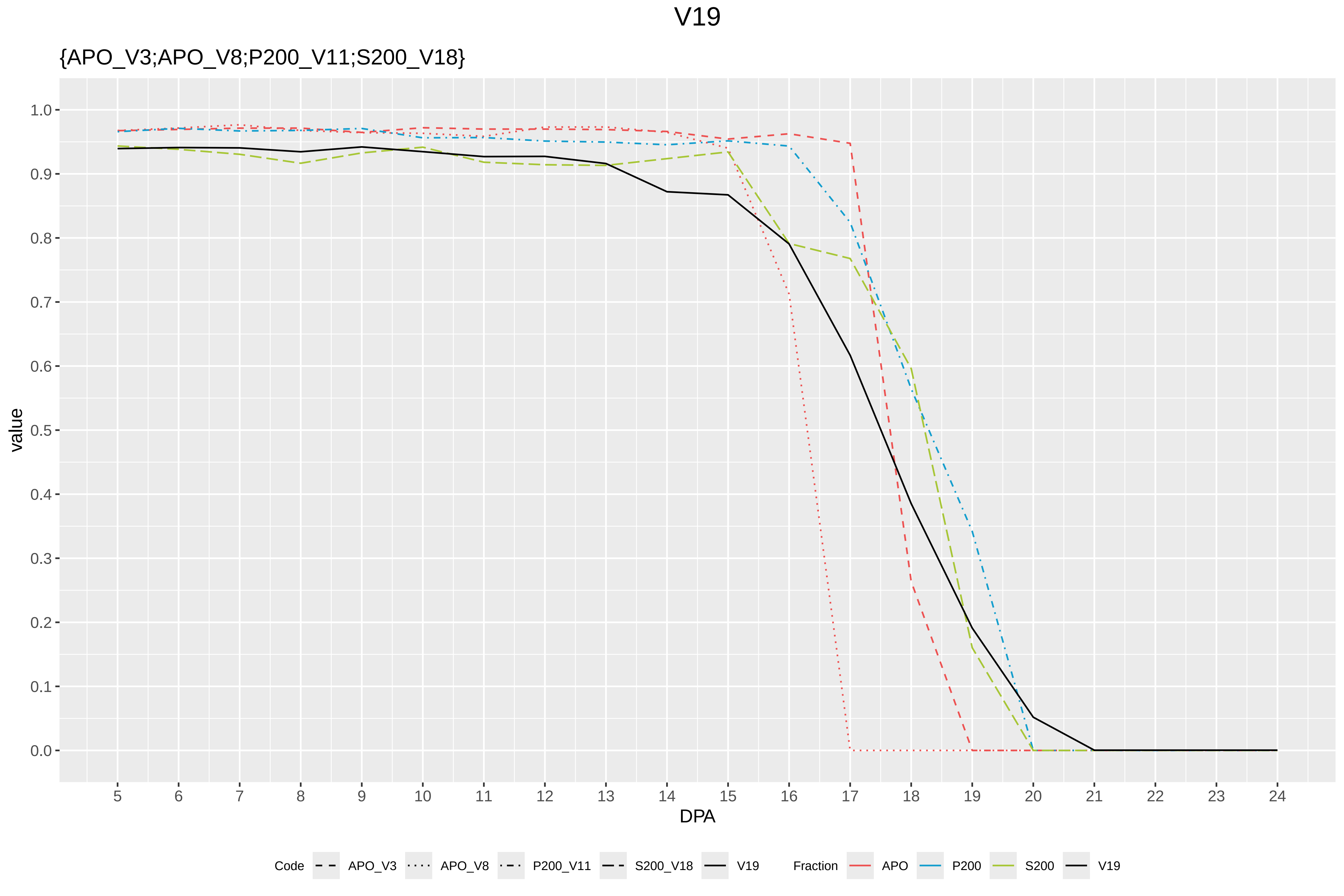

V19
{APO_V3;APO_V8;P200_V11;S200_V18}
value
1.0
0.9
0.8
0.7
0.6
0.5
0.4
0.3
0.2
0.1
0.0
5 6 7 8 9 10 11 12 13 14 15 16 17 18 19 20 21 22 23 24
DPA
Code
APO_V3
APO_V8
P200_V11
S200_V18
V19
Fraction
APO
P200
S200
V19

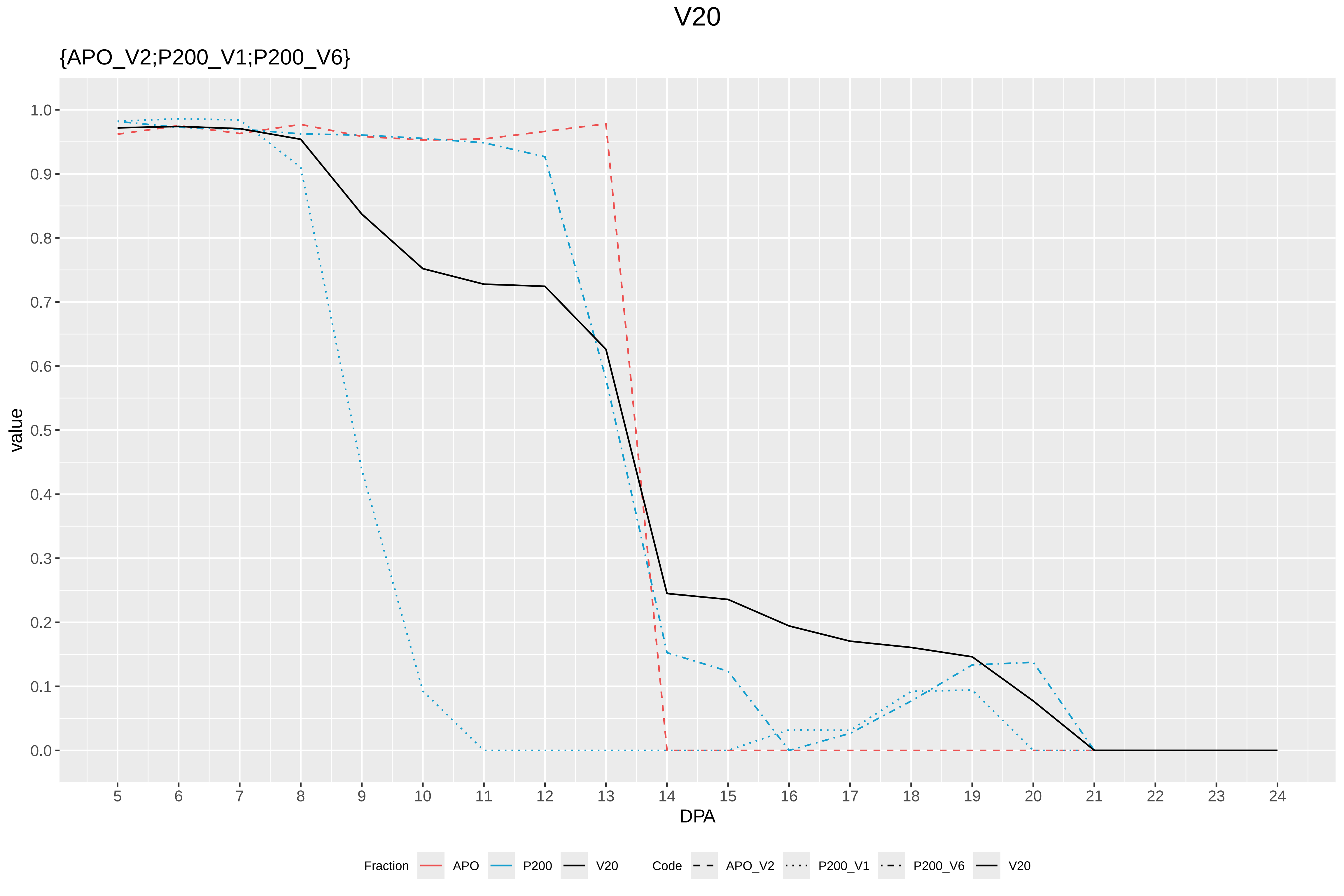
V20
{APO_V2;P200_V1;P200_V6}
value
1.0
0.9
0.8
0.7
0.6
0.5
0.4
0.3
0.2
0.1
0.0
5
6
7
8
9
10
11
12
13
14
15
16
17
18
19
20
21
22
23
24
DPA
Fraction
APO
P200
V20
Code
APO_V2
P200_V1
P200_V6
V20

Supplementary Figure S8. Merged SOM protein expression modules (30-cluster model).

Codes

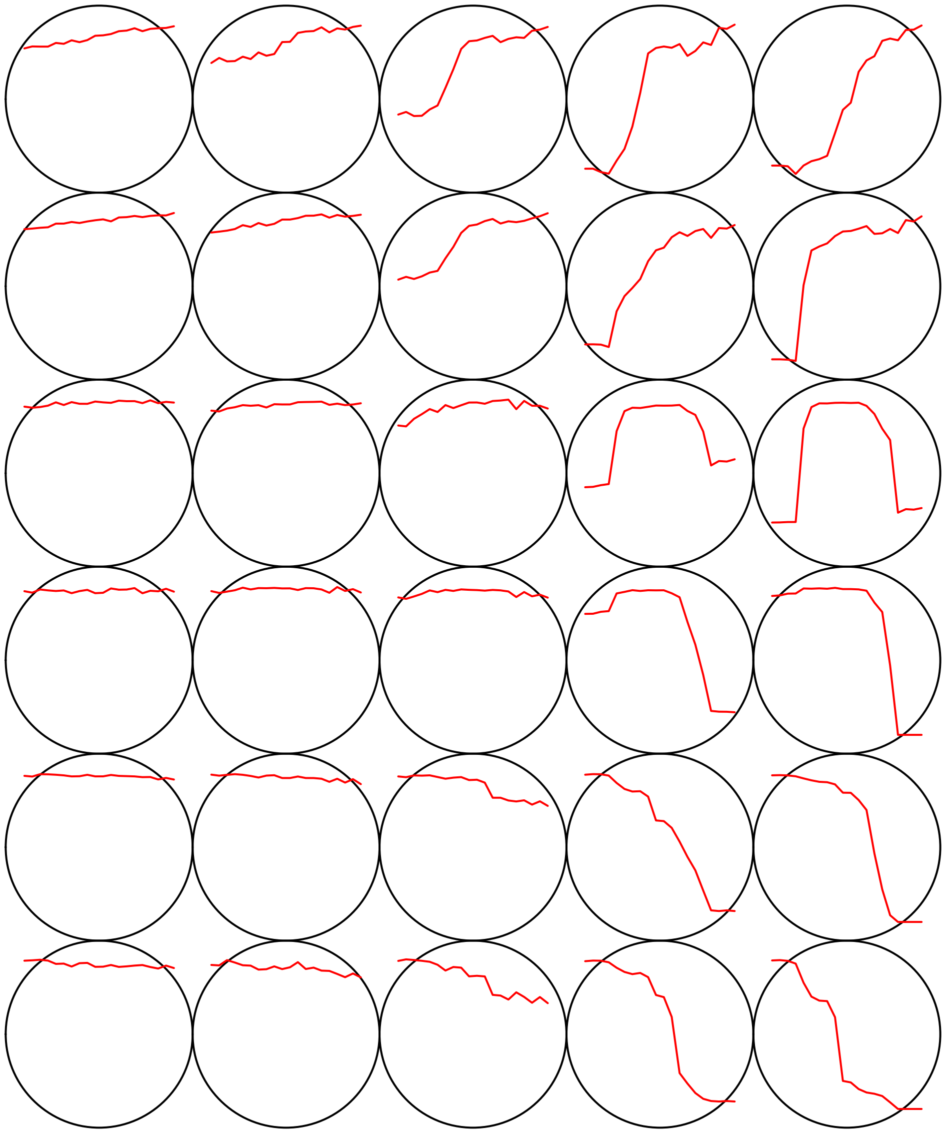

Count plot

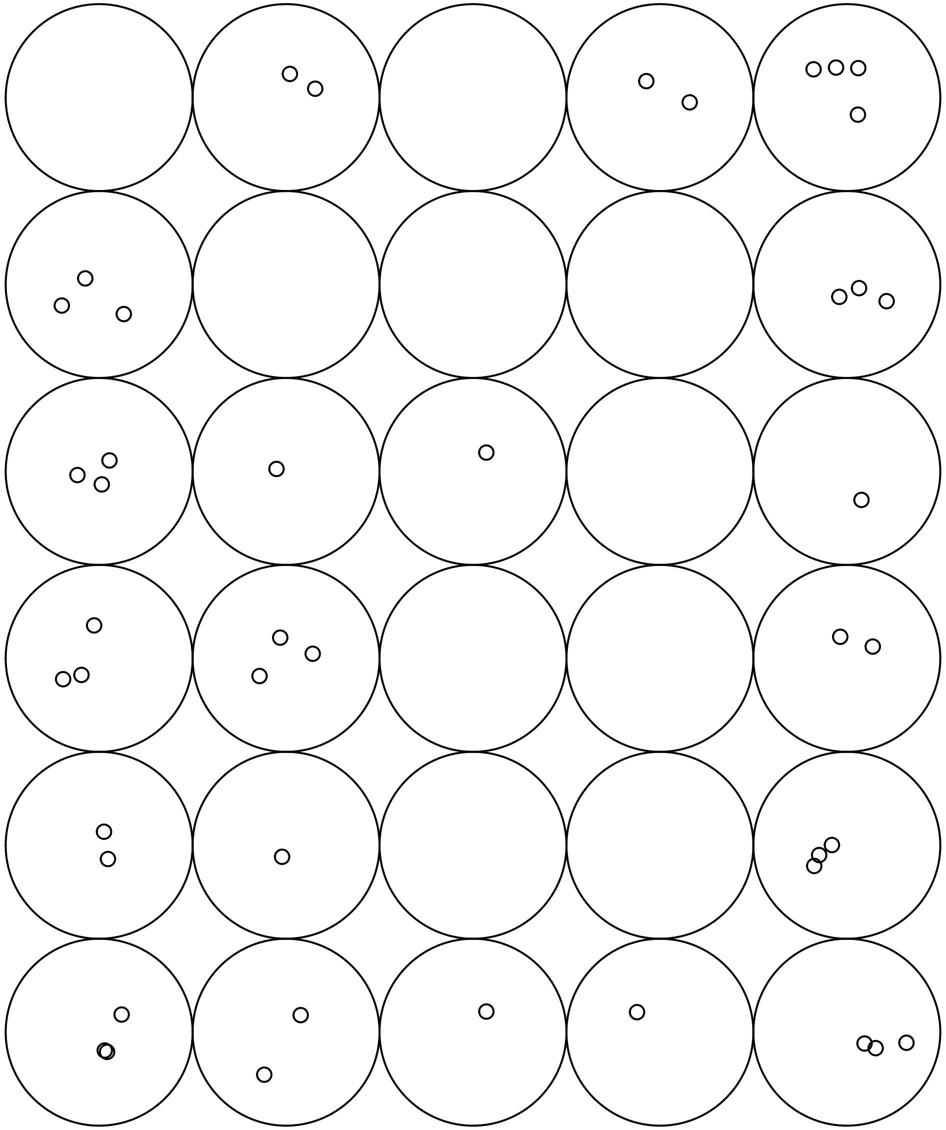

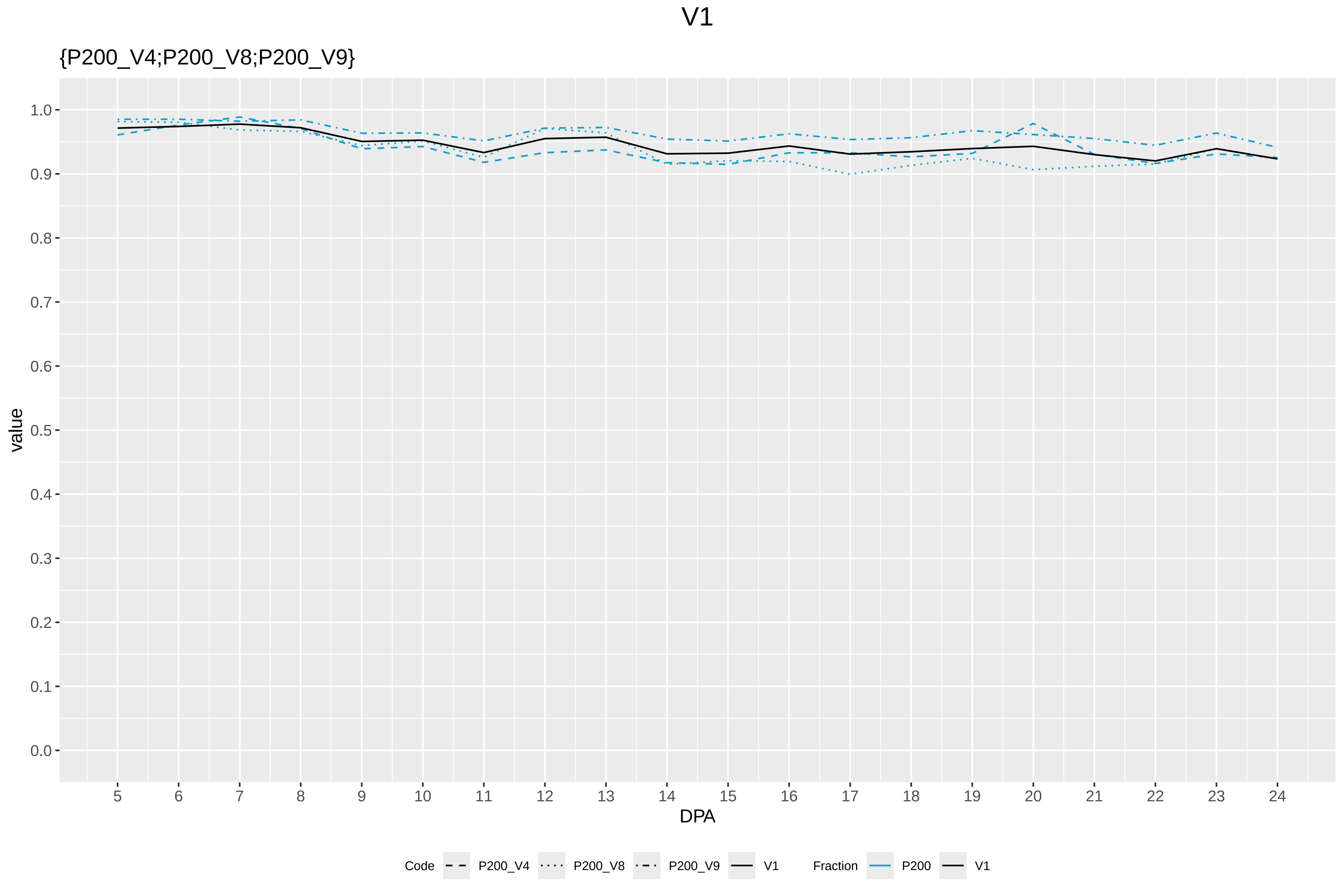
V1
{P200_V4;P200_V8;P200_V9}
value
0.0
0.1
0.2
0.3
0.4
0.5
0.6
0.7
0.8
0.9
1.0
5
6
7
8
9
10
11
12
13
14
15
16
17
18
19
20
21
22
23
24
DPA
Code
P200_V4
P200_V8
P200_V9
V1
Fraction
P200
V1

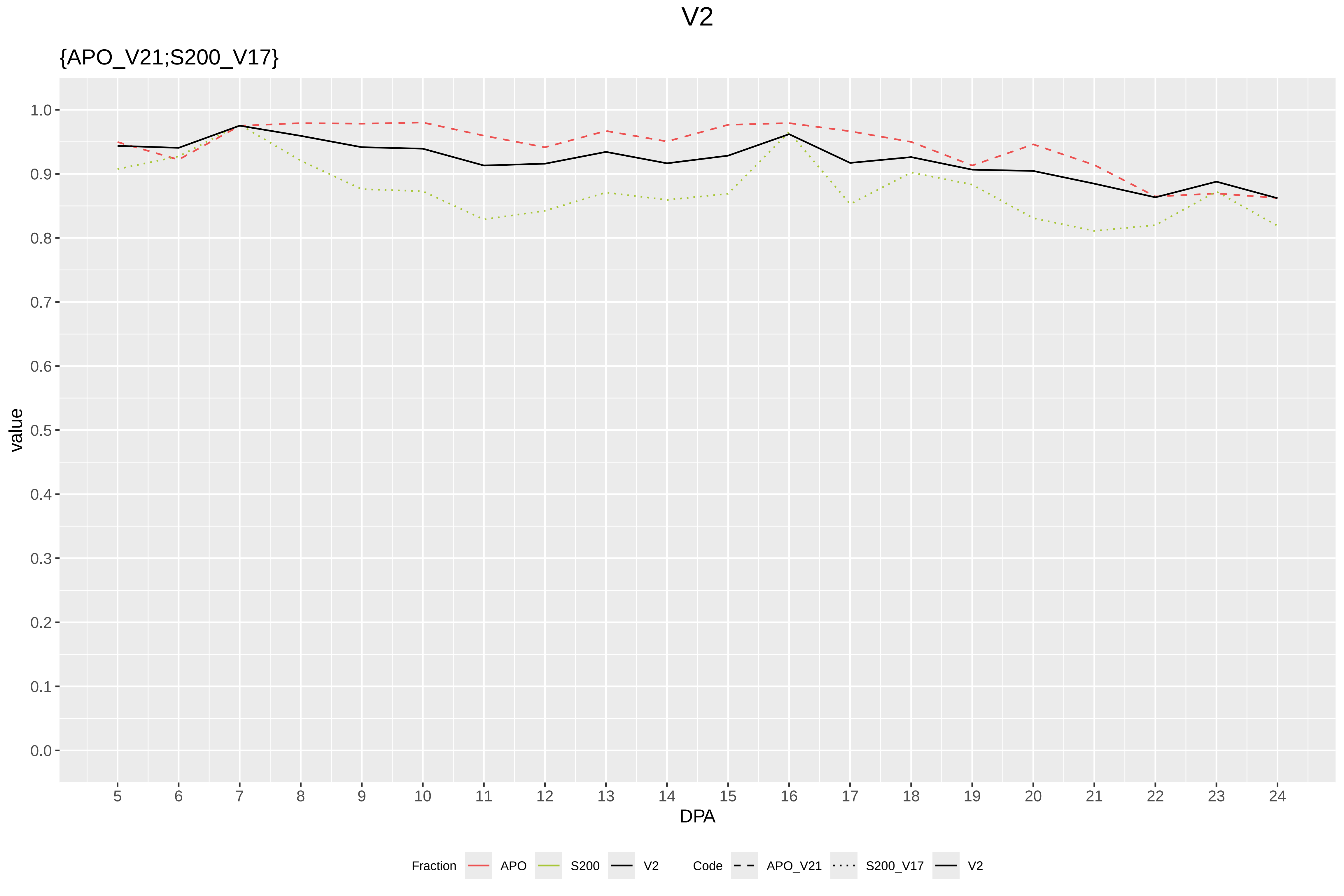
V2
{APO_V21;S200_V17}
value
1.0
0.9
0.8
0.7
0.6
0.5
0.4
0.3
0.2
0.1
0.0
5
6
7
8
9
10
11
12
13
14
15
16
17
18
19
20
21
22
23
24
DPA
Fraction
APO
S200
V2
Code
APO_V21
S200_V17
V2

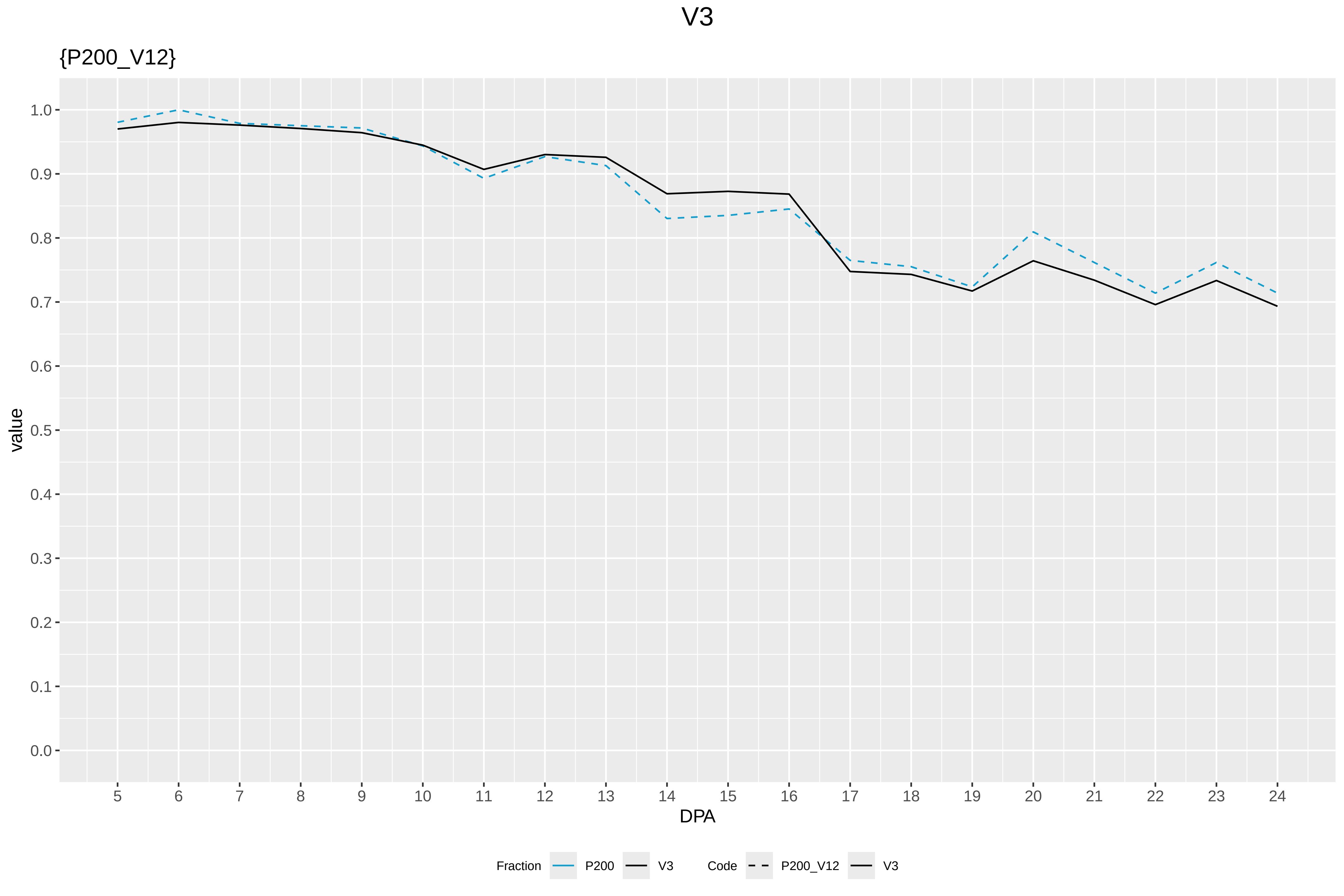

V3
{P200_V12}
value
1.0
0.9
0.8
0.7
0.6
0.5
0.4
0.3
0.2
0.1
0.0
5 6 7 8 9 10 11 12 13 14 15 16 17 18 19 20 21 22 23 24
DPA
Fraction
P200
V3
Code
P200_V12
V3

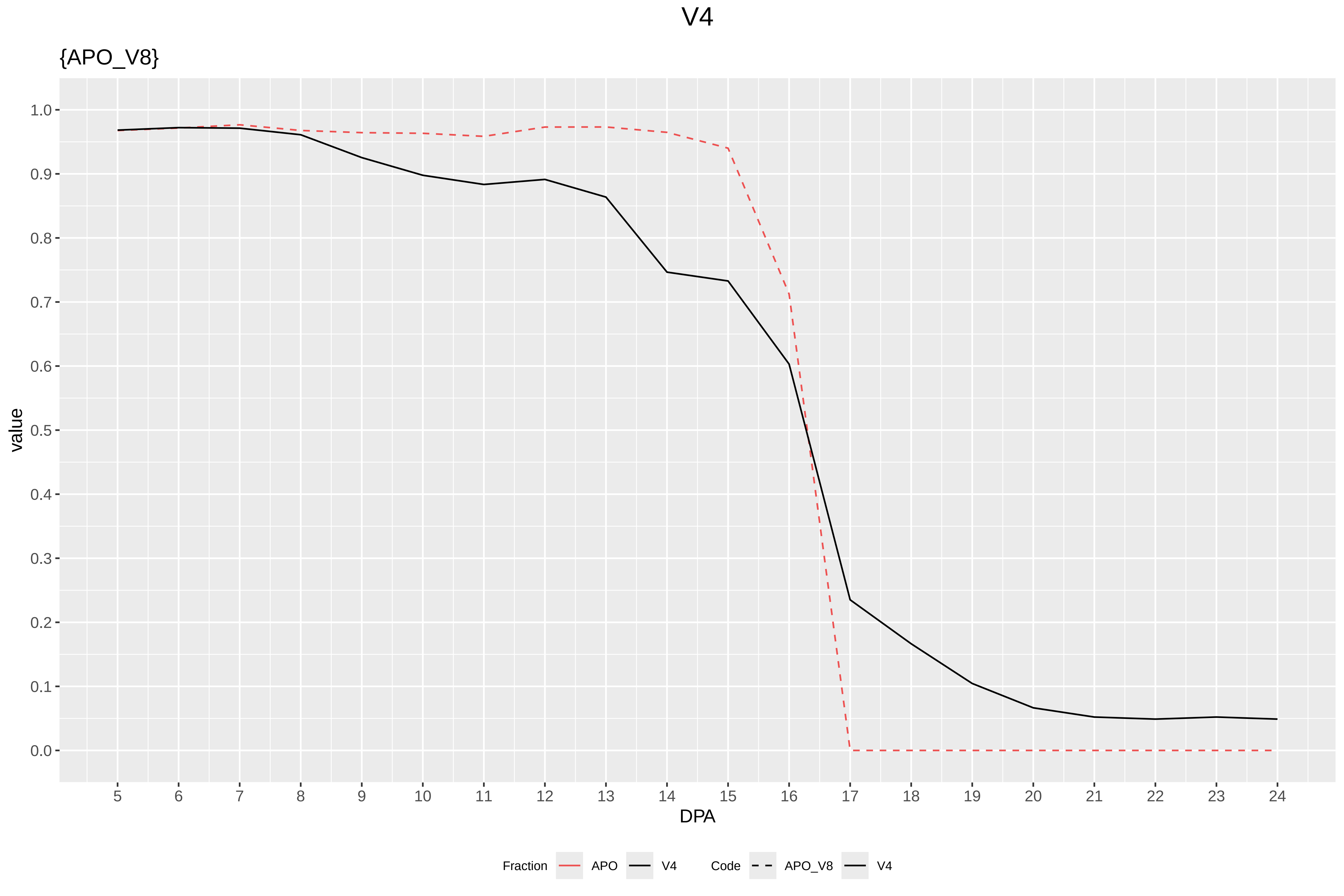

V4
{APO_V8}
value
1.0
0.9
0.8
0.7
0.6
0.5
0.4
0.3
0.2
0.1
0.0
5
6
7
8
9
10
11
12
13
14
15
16
17
18
19
20
21
22
23
24
DPA
Fraction
APO
V4
Code
APO_V8
V4

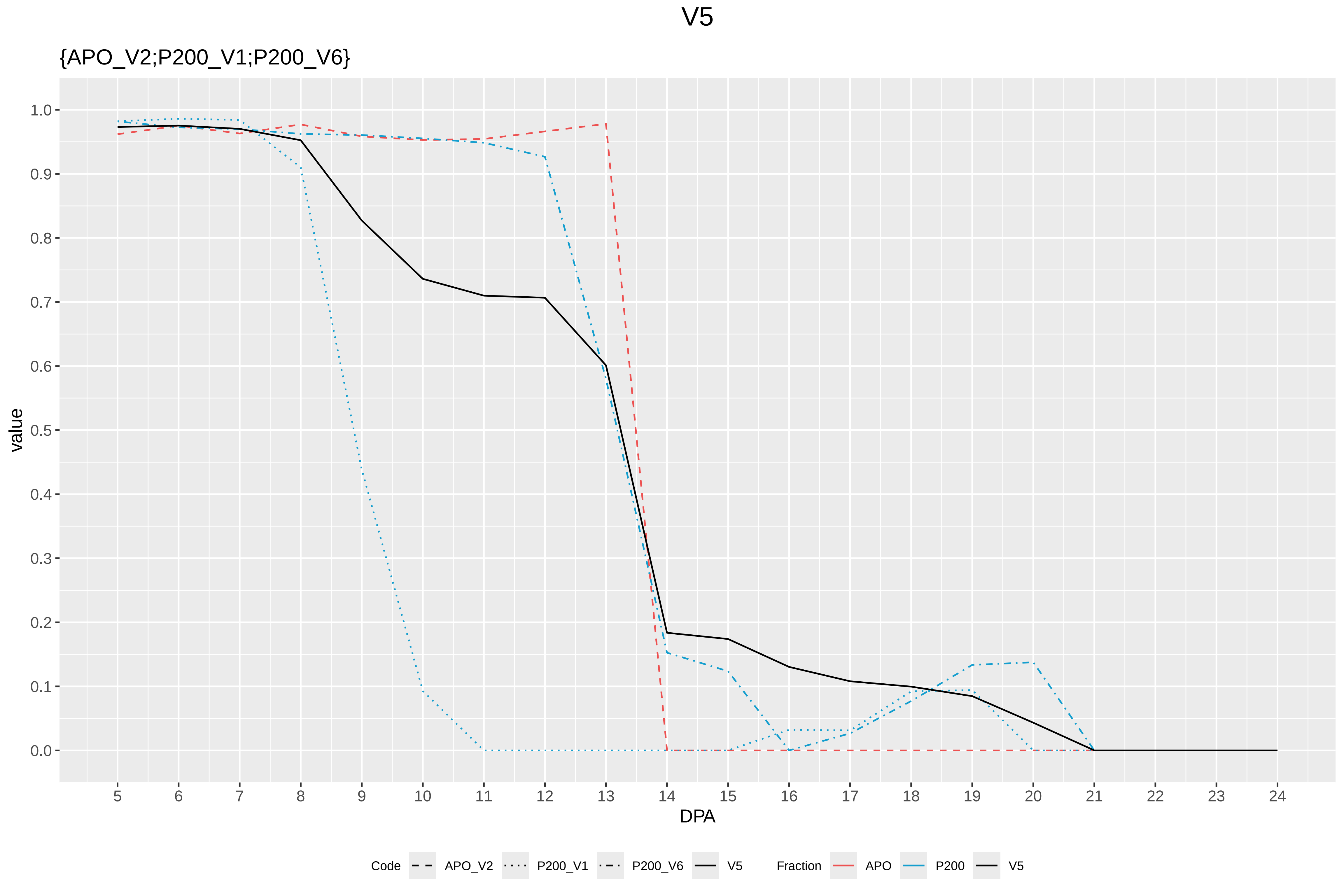
V5
{APO_V2;P200_V1;P200_V6}
value
1.0
0.9
0.8
0.7
0.6
0.5
0.4
0.3
0.2
0.1
0.0
5 6 7 8 9 10 11 12 13 14 15 16 17 18 19 20 21 22 23 24
DPA
Code
APO_V2
P200_V1
P200_V6
V5
Fraction
APO
P200
V5

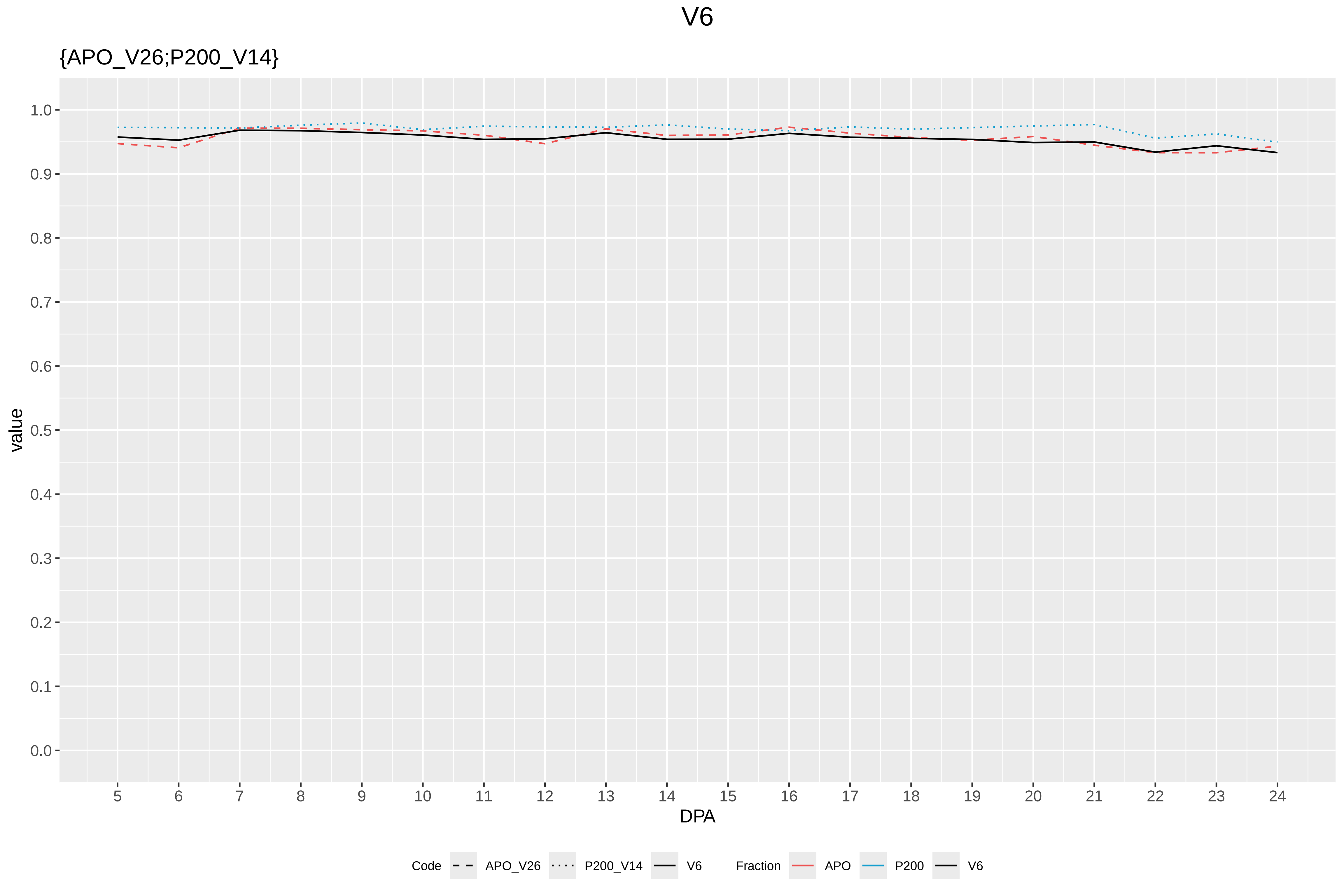
V6
{APO_V26;P200_V14}
value
1.0
0.9
0.8
0.7
0.6
0.5
0.4
0.3
0.2
0.1
0.0
5
6
7
8
9
10
11
12
13
14
15
16
17
18
19
20
21
22
23
24
DPA
Code
APO_V26
P200_V14
V6
Fraction
APO
P200
V6

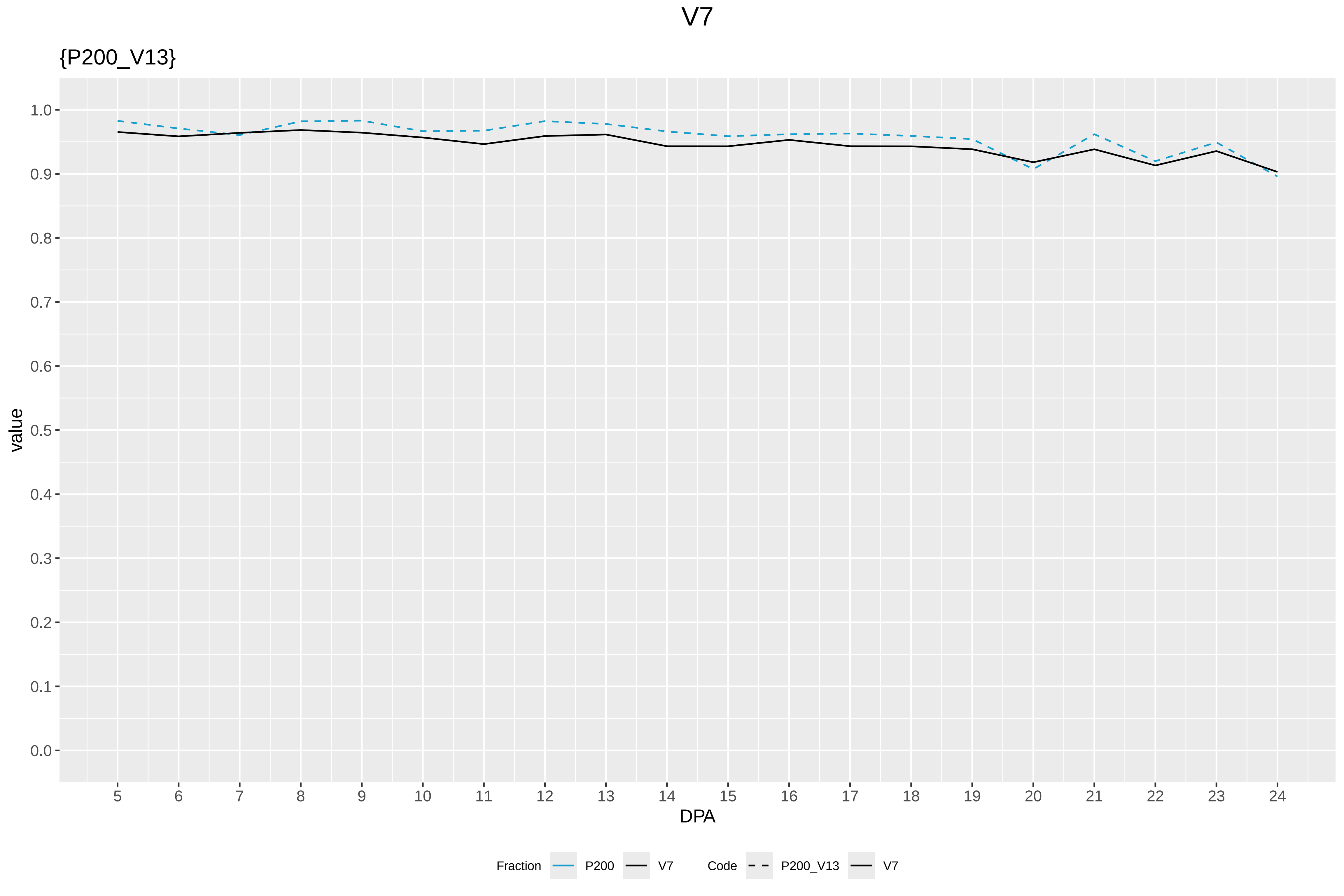

V7
{P200_V13}
value
1.0
0.9
0.8
0.7
0.6
0.5
0.4
0.3
0.2
0.1
0.0
5 6 7 8 9 10 11 12 13 14 15 16 17 18 19 20 21 22 23 24
DPA
Fraction
P200
V7
Code
P200_V13
V7

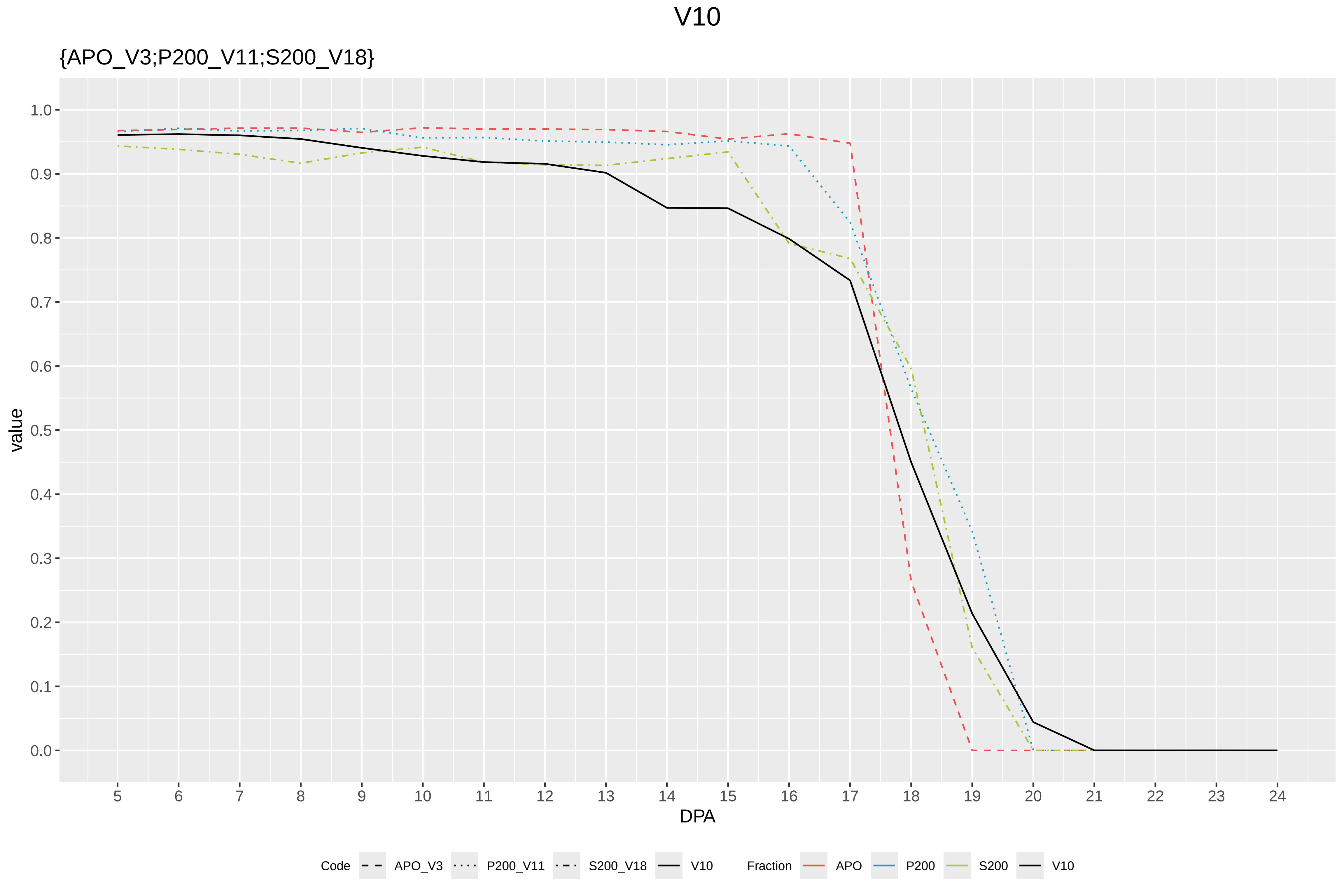
V10
{APO_V3;P200_V11;S200_V18}
value
DPA
1.0
0.9
0.8
0.7
0.6
0.5
0.4
0.3
0.2
0.1
0.0
5
6
7
8
9
10
11
12
13
14
15
16
17
18
19
20
21
22
23
24
Code
APO_V3
P200_V11
S200_V18
V10
Fraction
APO
P200
S200
V10

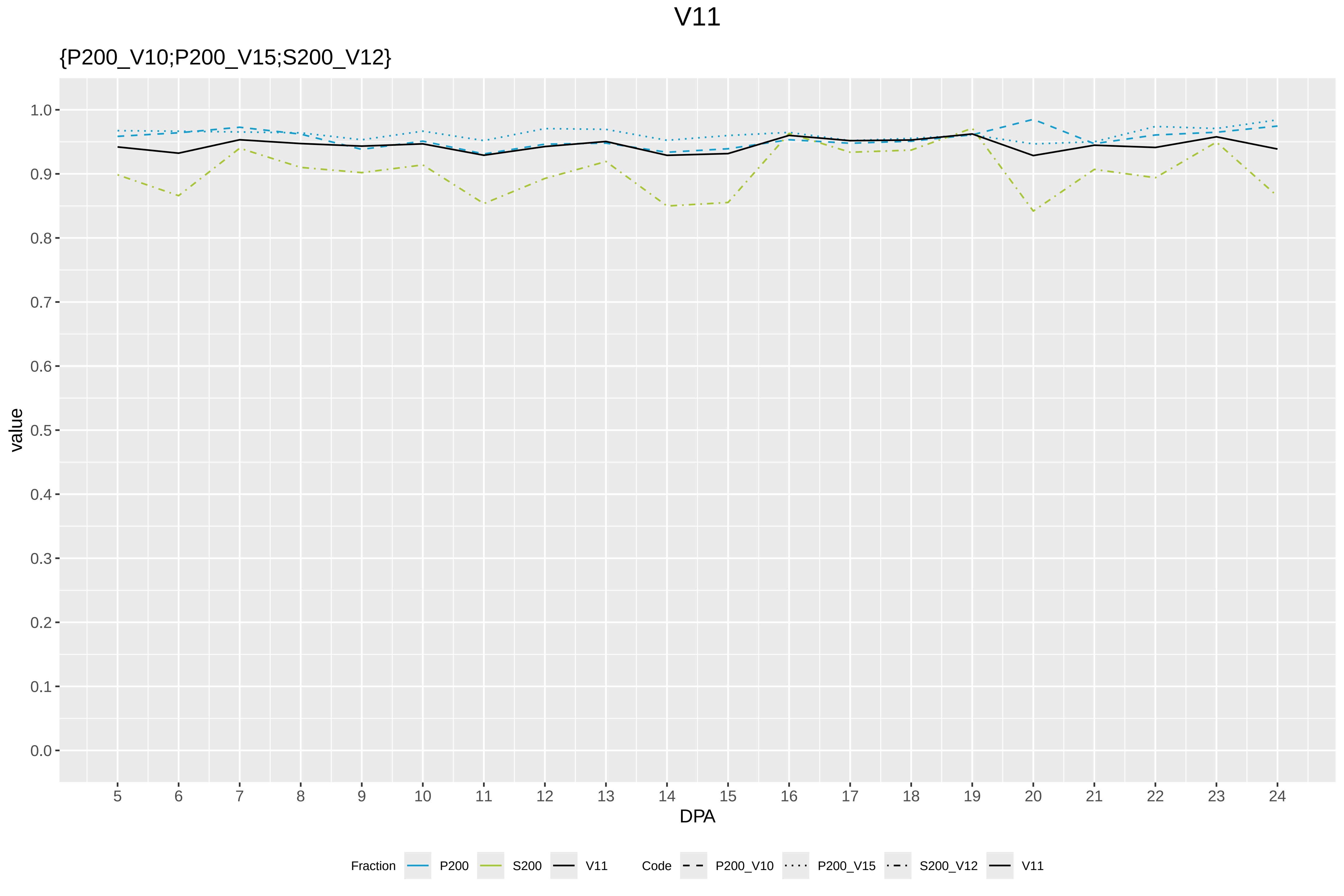
V11
{P200_V10;P200_V15;S200_V12}
value
DPA
0.0
0.1
0.2
0.3
0.4
0.5
0.6
0.7
0.8
0.9
1.0
5
6
7
8
9
10
11
12
13
14
15
16
17
18
19
20
21
22
23
24
Fraction
P200
S200
V11
Code
P200_V10
P200_V15
S200_V12
V11

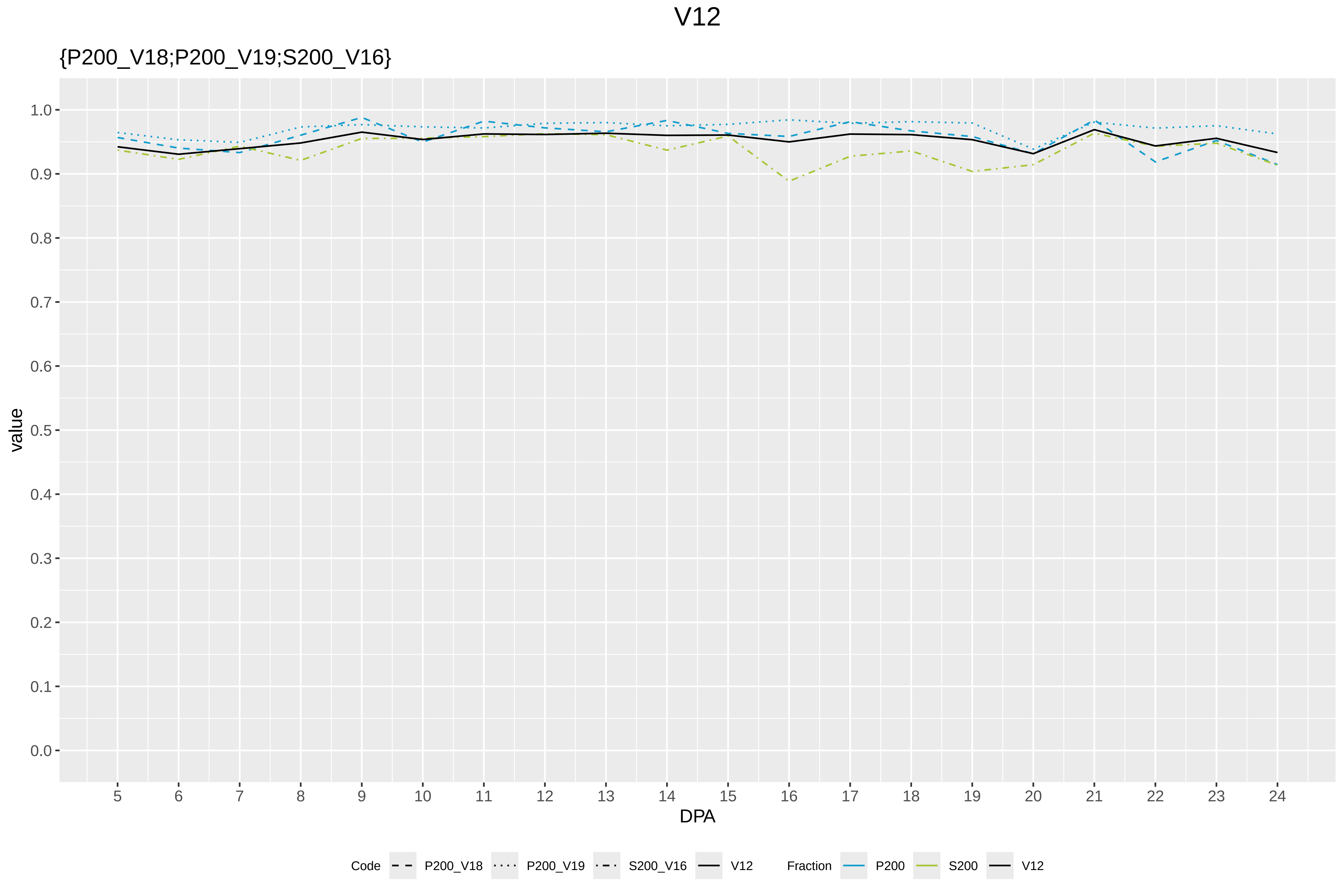

V12
{P200_V18;P200_V19;S200_V16}
value
1.0
0.9
0.8
0.7
0.6
0.5
0.4
0.3
0.2
0.1
0.0
5
6
7
8
9
10
11
12
13
14
15
16
17
18
19
20
21
22
23
24
DPA
Code
P200_V18
P200_V19
S200_V16
V12
Fraction
P200
S200
V12

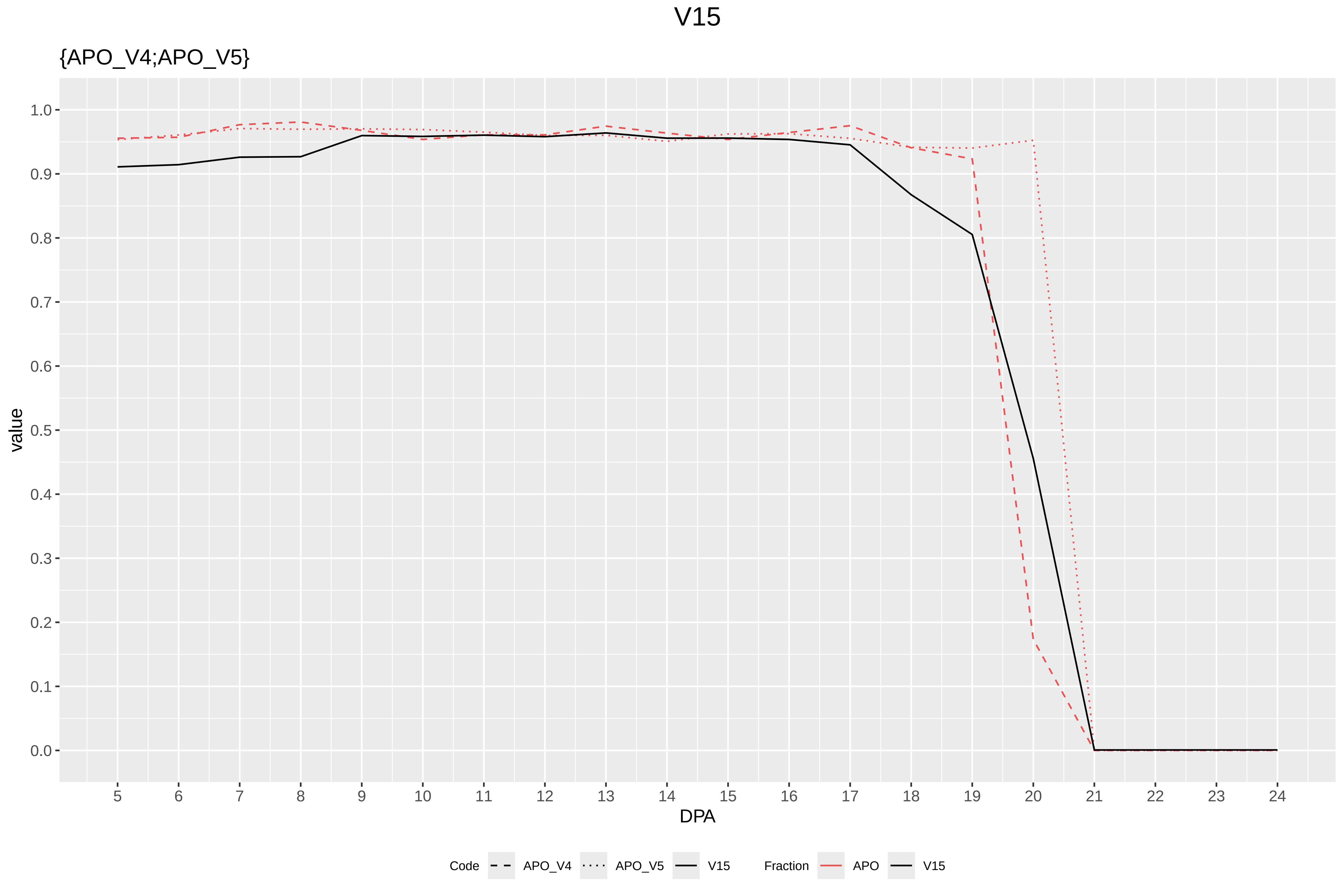

V15
{APO_V4;APO_V5}
value
1.0
0.9
0.8
0.7
0.6
0.5
0.4
0.3
0.2
0.1
0.0
5 6 7 8 9 10 11 12 13 14 15 16 17 18 19 20 21 22 23 24
DPA
Code
APO_V4
APO_V5
V15
Fraction
APO
V15

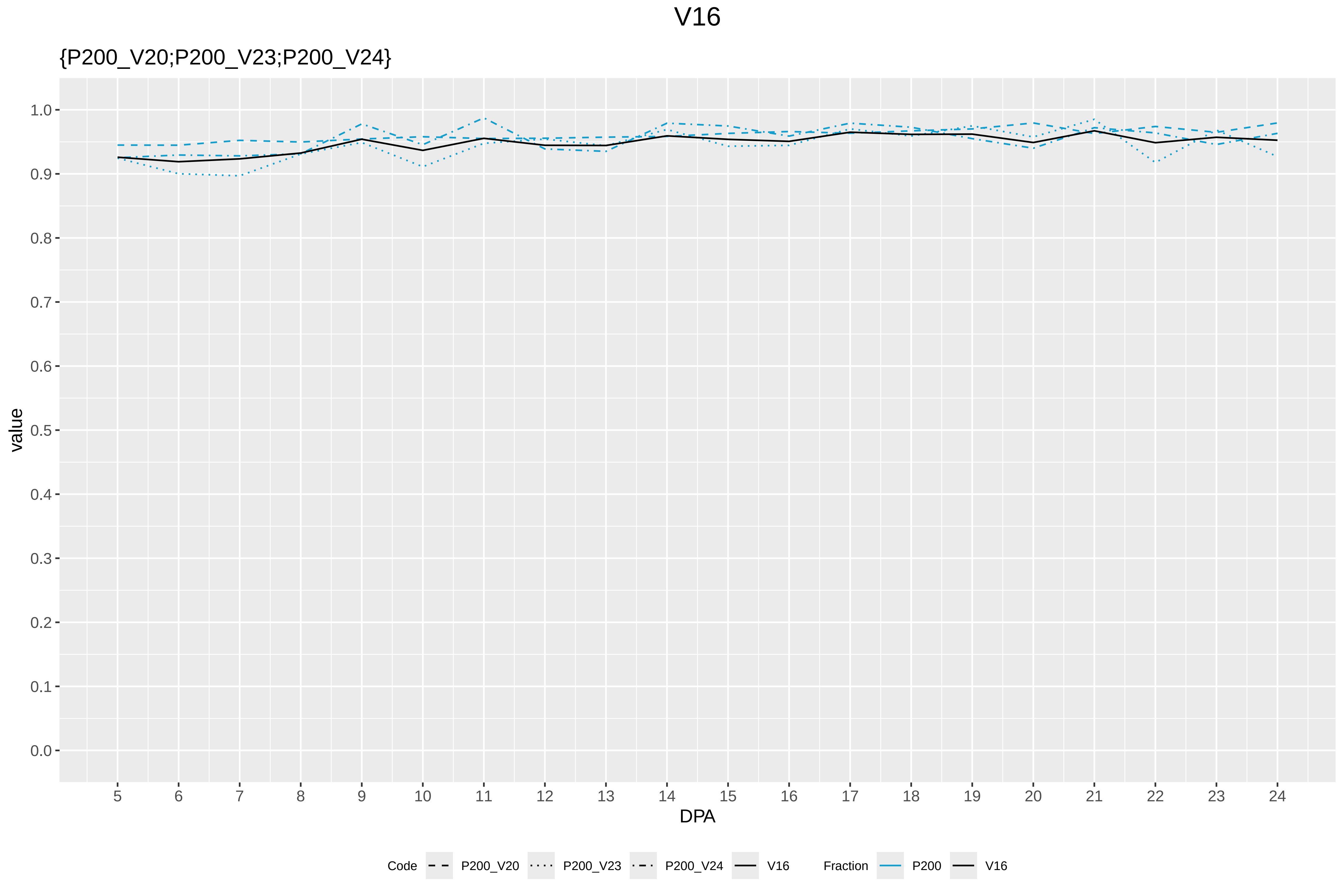
V16
{P200_V20;P200_V23;P200_V24}
value
1.0
0.9
0.8
0.7
0.6
0.5
0.4
0.3
0.2
0.1
0.0
5
6
7
8
9
10
11
12
13
14
15
16
17
18
19
20
21
22
23
24
DPA
Code
P200_V20
P200_V23
P200_V24
V16
Fraction
P200
V16

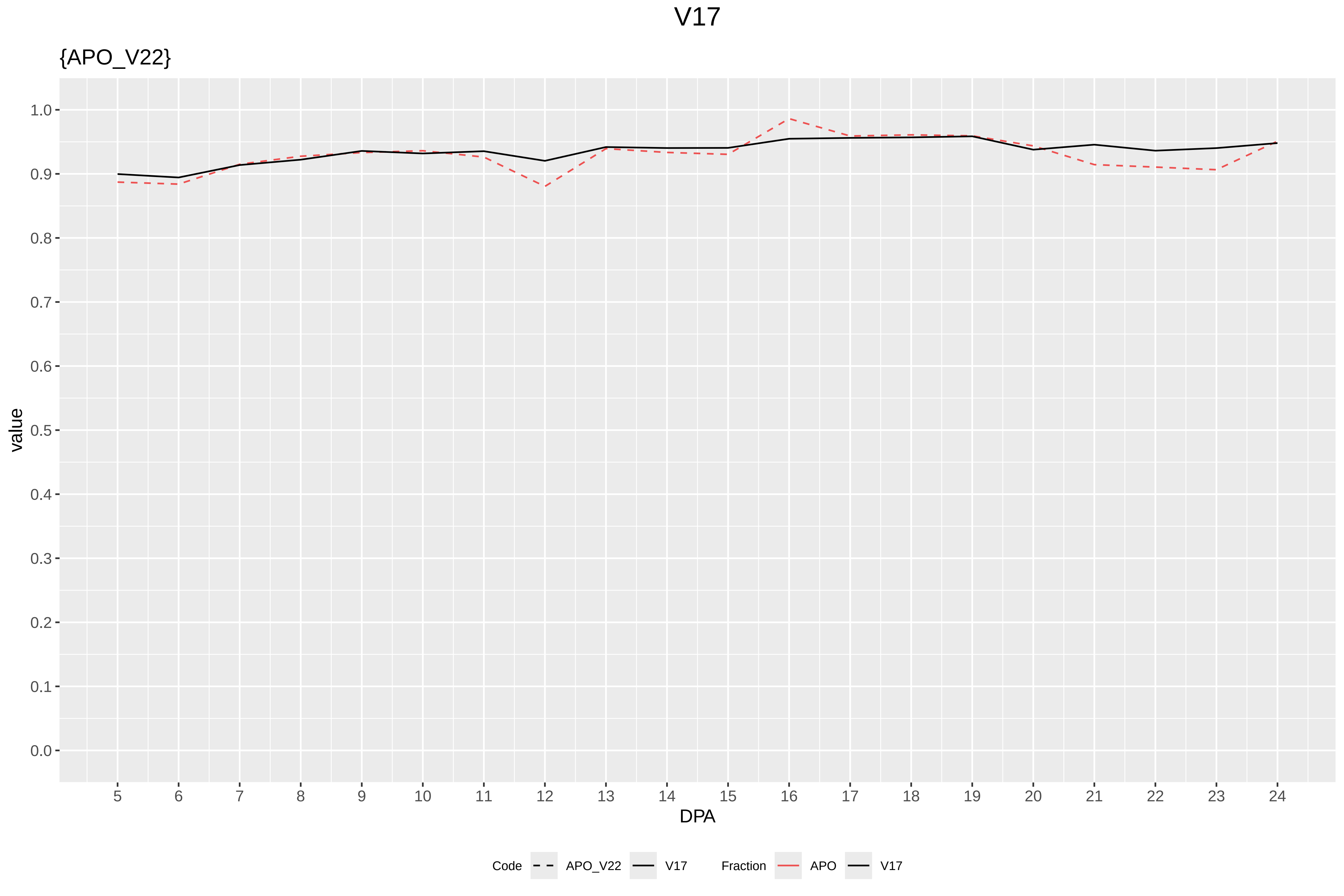
V17
{APO_V22}
value
1.0
0.9
0.8
0.7
0.6
0.5
0.4
0.3
0.2
0.1
0.0
5
6
7
8
9
10
11
12
13
14
15
16
17
18
19
20
21
22
23
24
DPA
Code
APO_V22
V17
Fraction
APO
V17

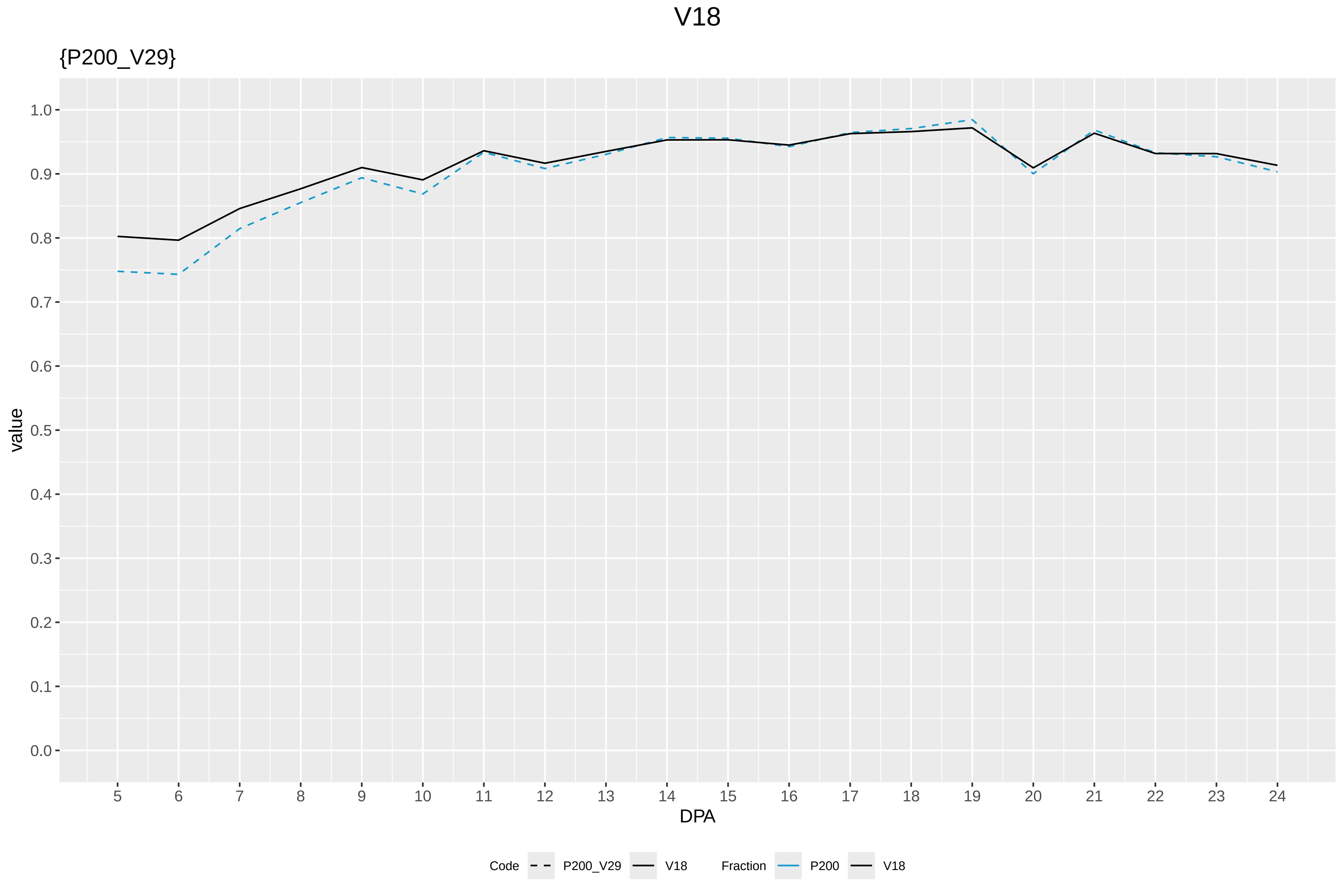

V18
{P200_V29}
value
1.0
0.9
0.8
0.7
0.6
0.5
0.4
0.3
0.2
0.1
0.0
5
6
7
8
9
10
11
12
13
14
15
16
17
18
19
20
21
22
23
24
DPA
Code
P200_V29
V18
Fraction
P200
V18

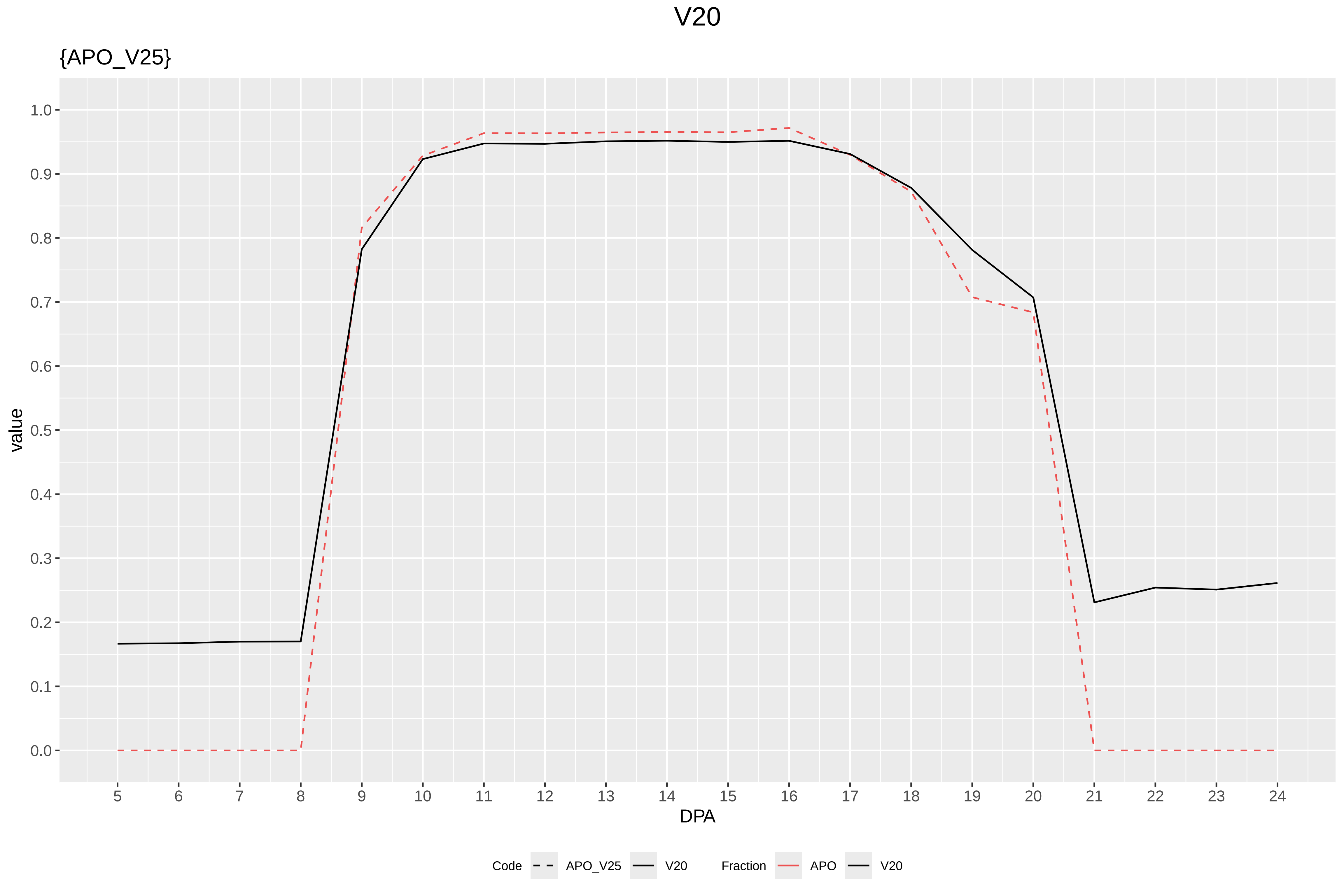

V20
{APO_V25}
value
1.0
0.9
0.8
0.7
0.6
0.5
0.4
0.3
0.2
0.1
0.0
5
6
7
8
9
10
11
12
13
14
15
16
17
18
19
20
21
22
23
24
DPA
Code
APO_V25
V20
Fraction
APO
V20

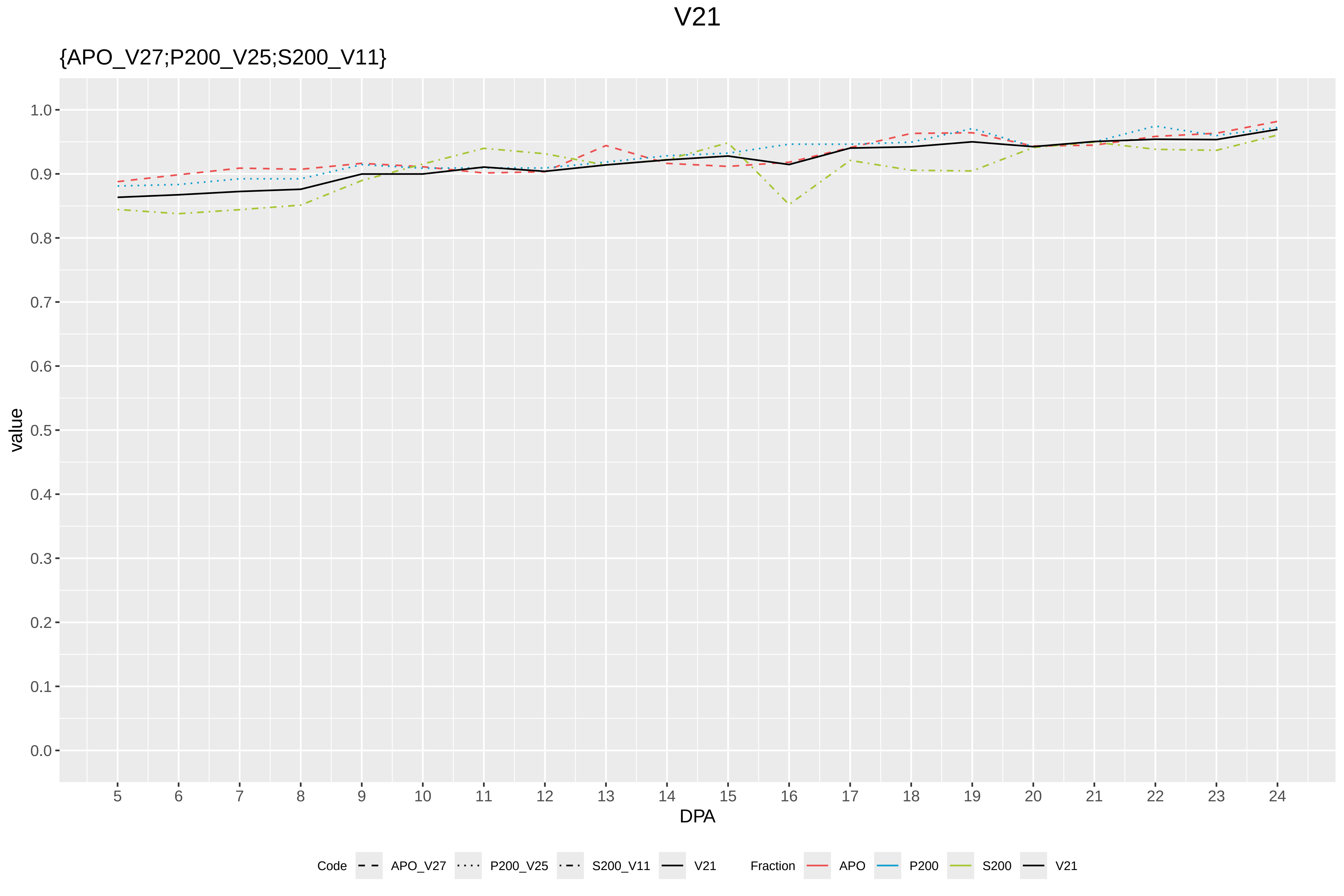

V21
{APO_V27;P200_V25;S200_V11}
value
DPA
1.0
0.9
0.8
0.7
0.6
0.5
0.4
0.3
0.2
0.1
0.0
5 6 7 8 9 10 11 12 13 14 15 16 17 18 19 20 21 22 23 24
Code
APO_V27
P200_V25
S200_V11
V21
Fraction
APO
P200
S200
V21

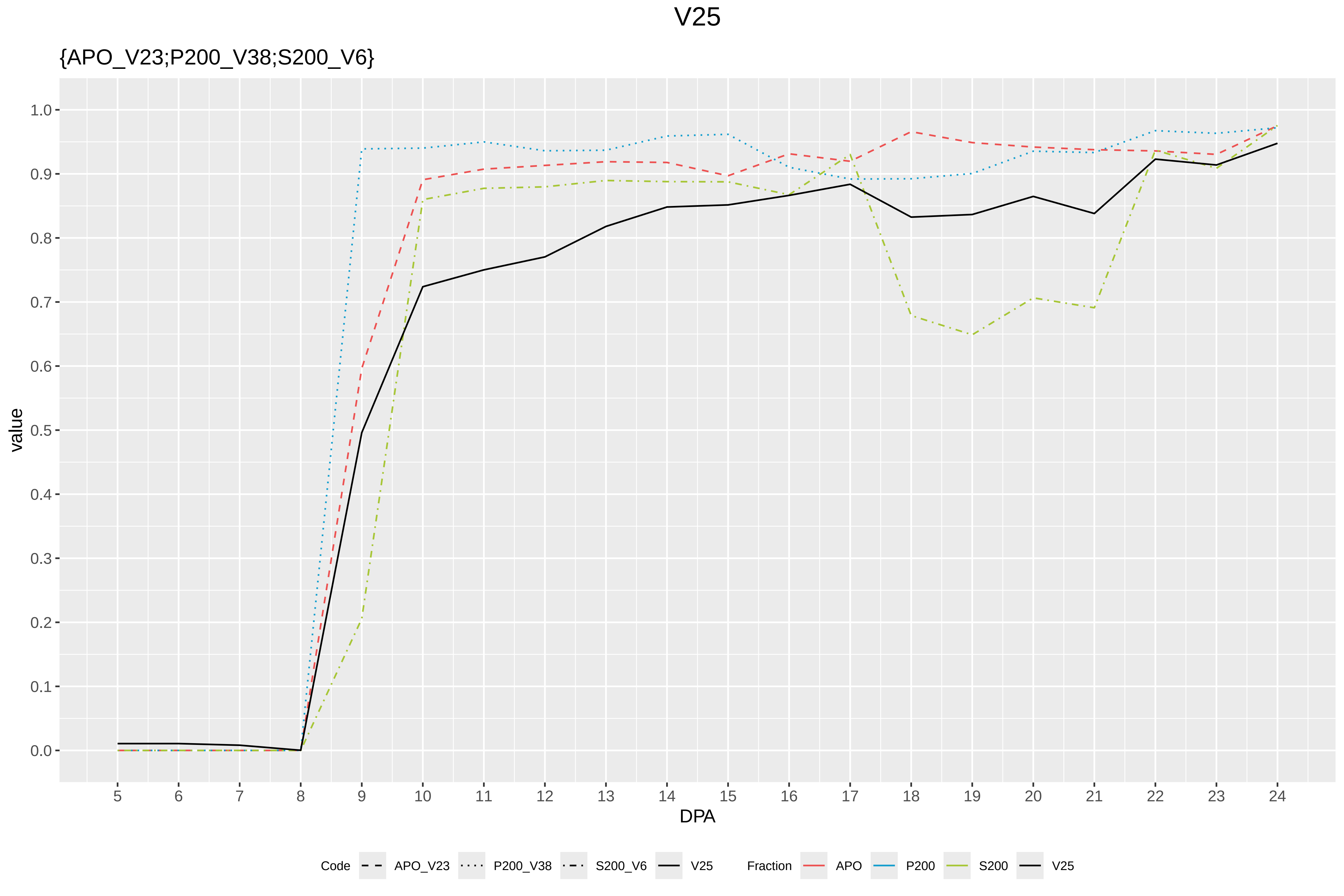

V25
{APO_V23;P200_V38;S200_V6}
value
1.0
0.9
0.8
0.7
0.6
0.5
0.4
0.3
0.2
0.1
0.0
5 6 7 8 9 10 11 12 13 14 15 16 17 18 19 20 21 22 23 24
DPA
Code
APO_V23
P200_V38
S200_V6
V25
Fraction
APO
P200
S200
V25

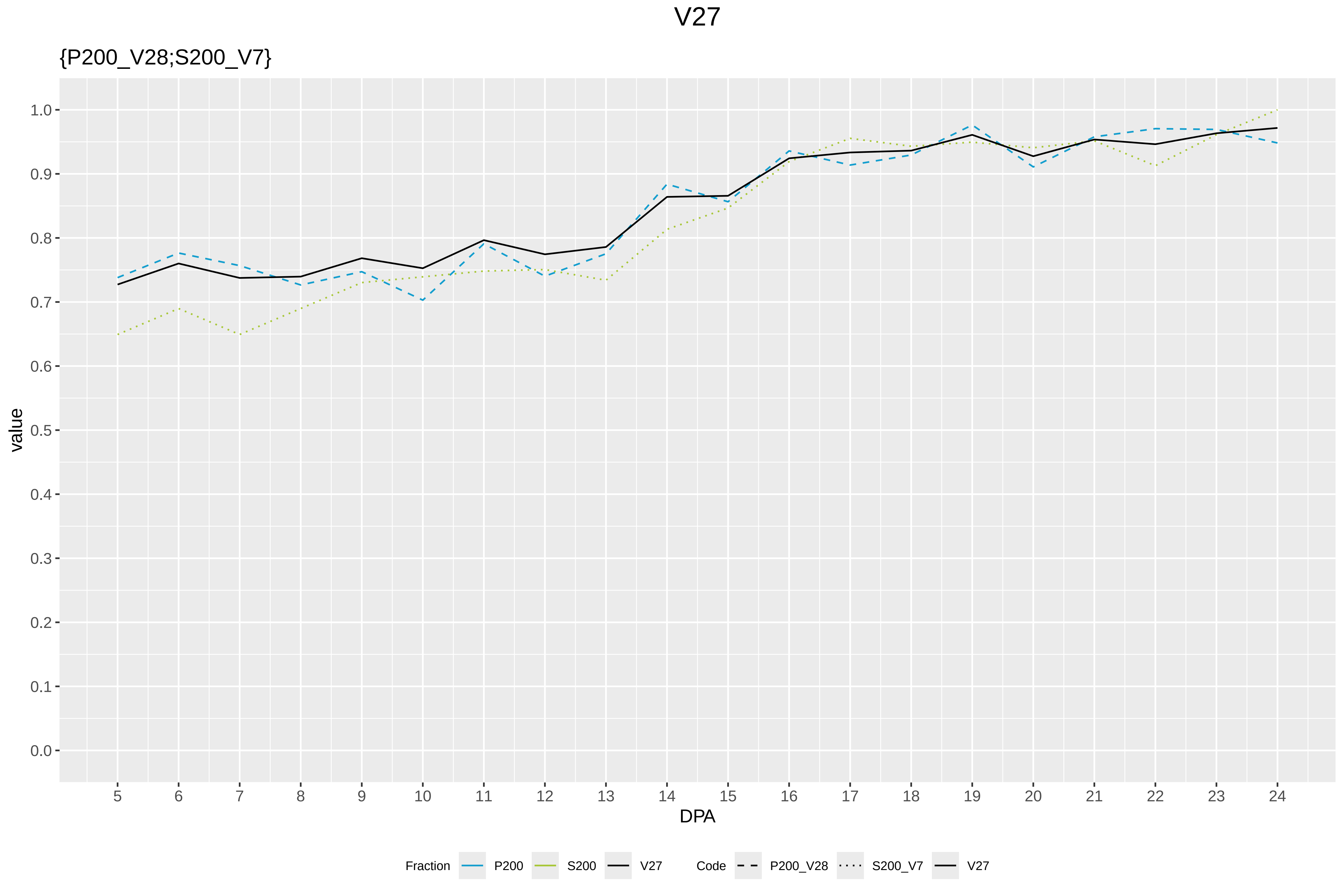
V27
{P200_V28;S200_V7}
value
DPA
1.0
0.9
0.8
0.7
0.6
0.5
0.4
0.3
0.2
0.1
0.0
5
6
7
8
9
10
11
12
13
14
15
16
17
18
19
20
21
22
23
24
Fraction
P200
S200
V27
Code
P200_V28
S200_V7
V27

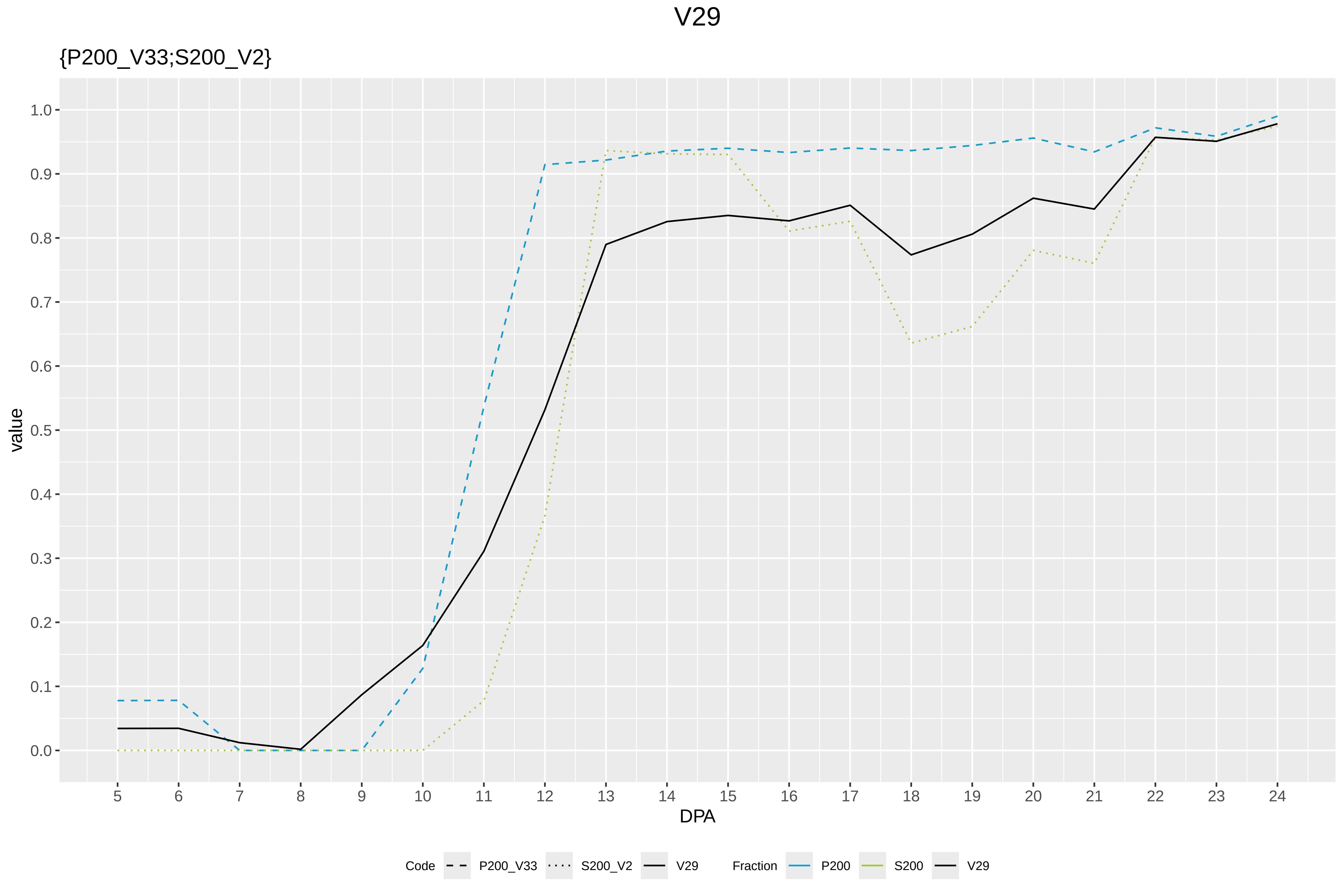
V29
{P200_V33;S200_V2}
value
DPA
1.0
0.9
0.8
0.7
0.6
0.5
0.4
0.3
0.2
0.1
0.0
5
6
7
8
9
10
11
12
13
14
15
16
17
18
19
20
21
22
23
24
Code
P200_V33
S200_V2
V29
Fraction
P200
S200
V29

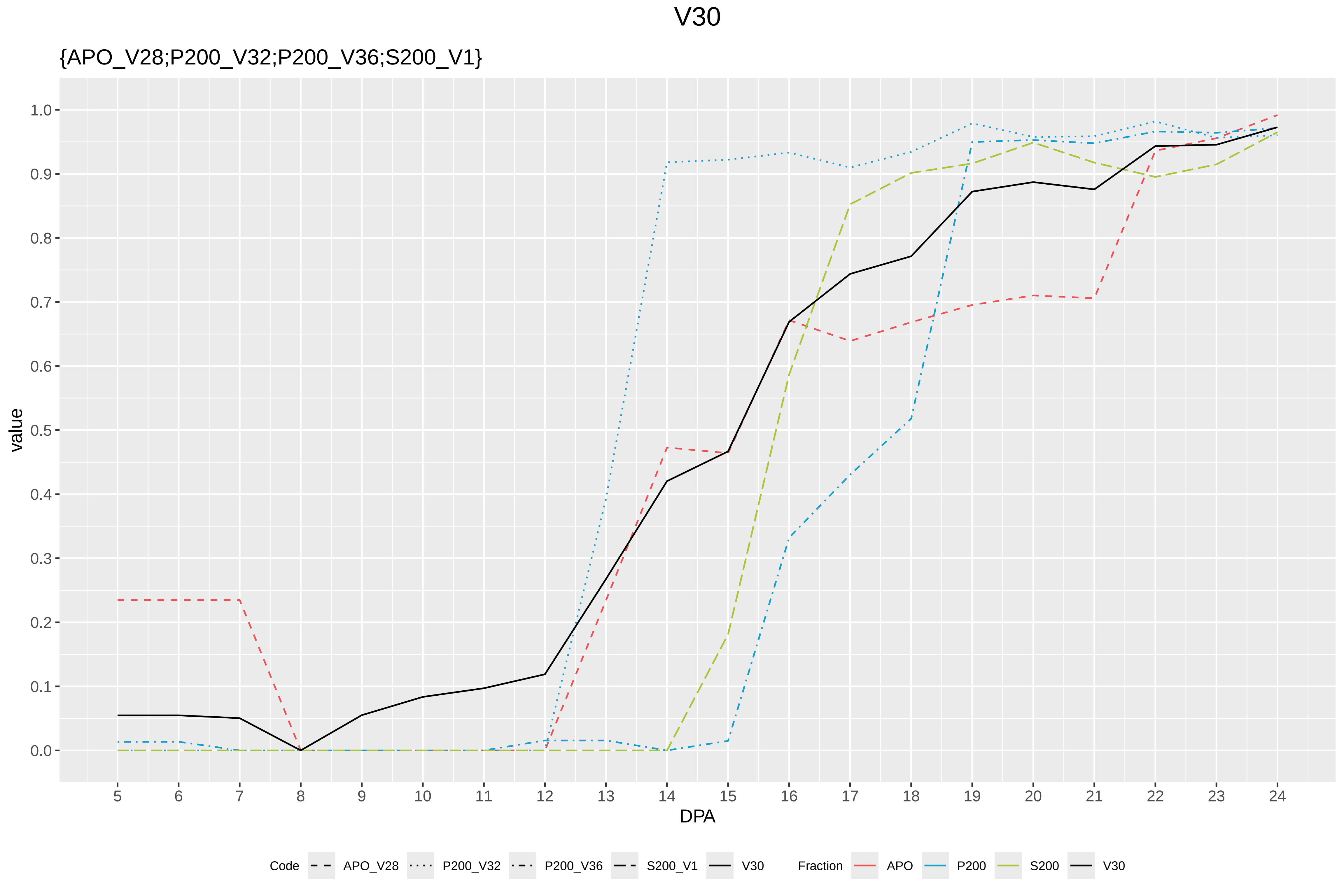

V30
{APO_V28;P200_V32;P200_V36;S200_V1}
value
1.0
0.9
0.8
0.7
0.6
0.5
0.4
0.3
0.2
0.1
0.0
5
6
7
8
9
10
11
12
13
14
15
16
17
18
19
20
21
22
23
24
DPA
Code
APO_V28
P200_V32
P200_V36
S200_V1
V30
Fraction
APO
P200
S200
V30

Supplementary Figure S9. Merged SOM protein expression modules (40-cluster model).

Codes

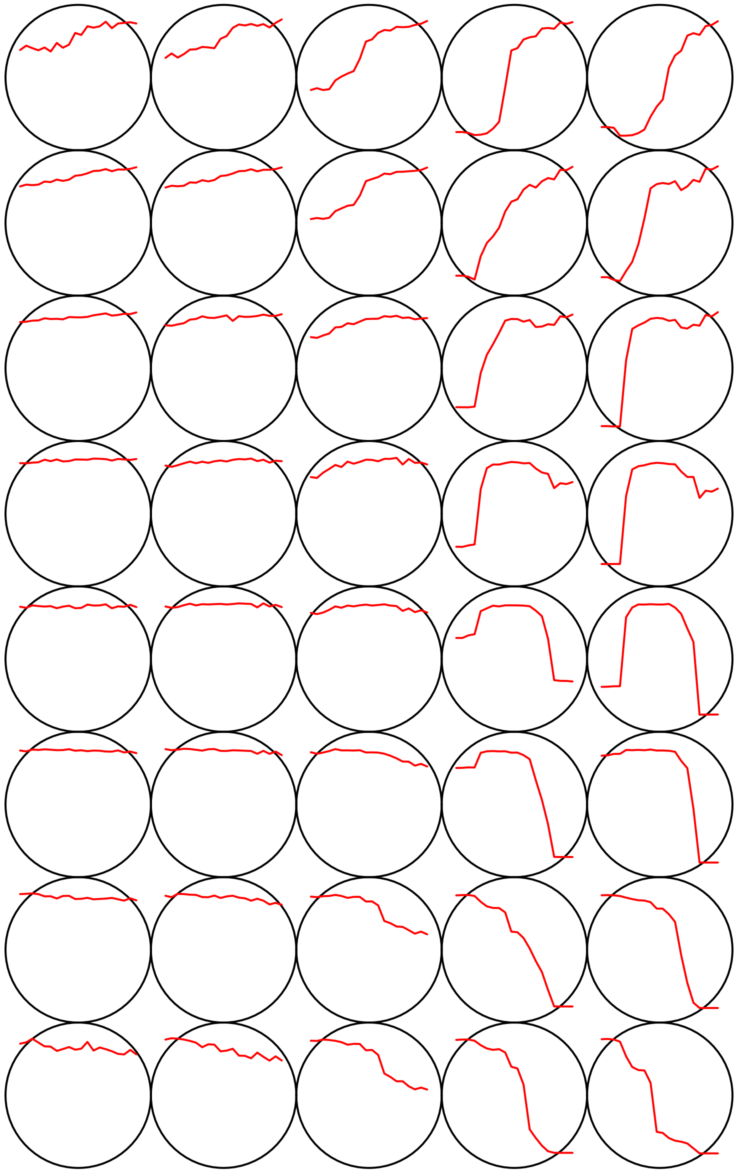

Count plot

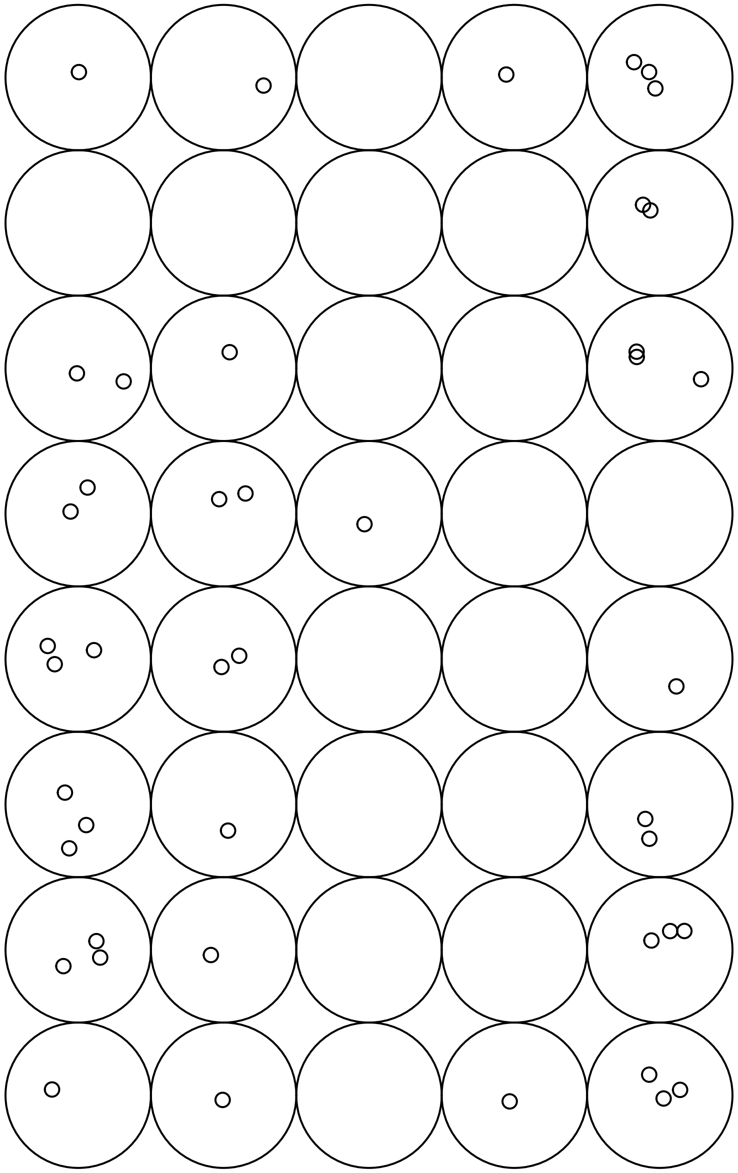

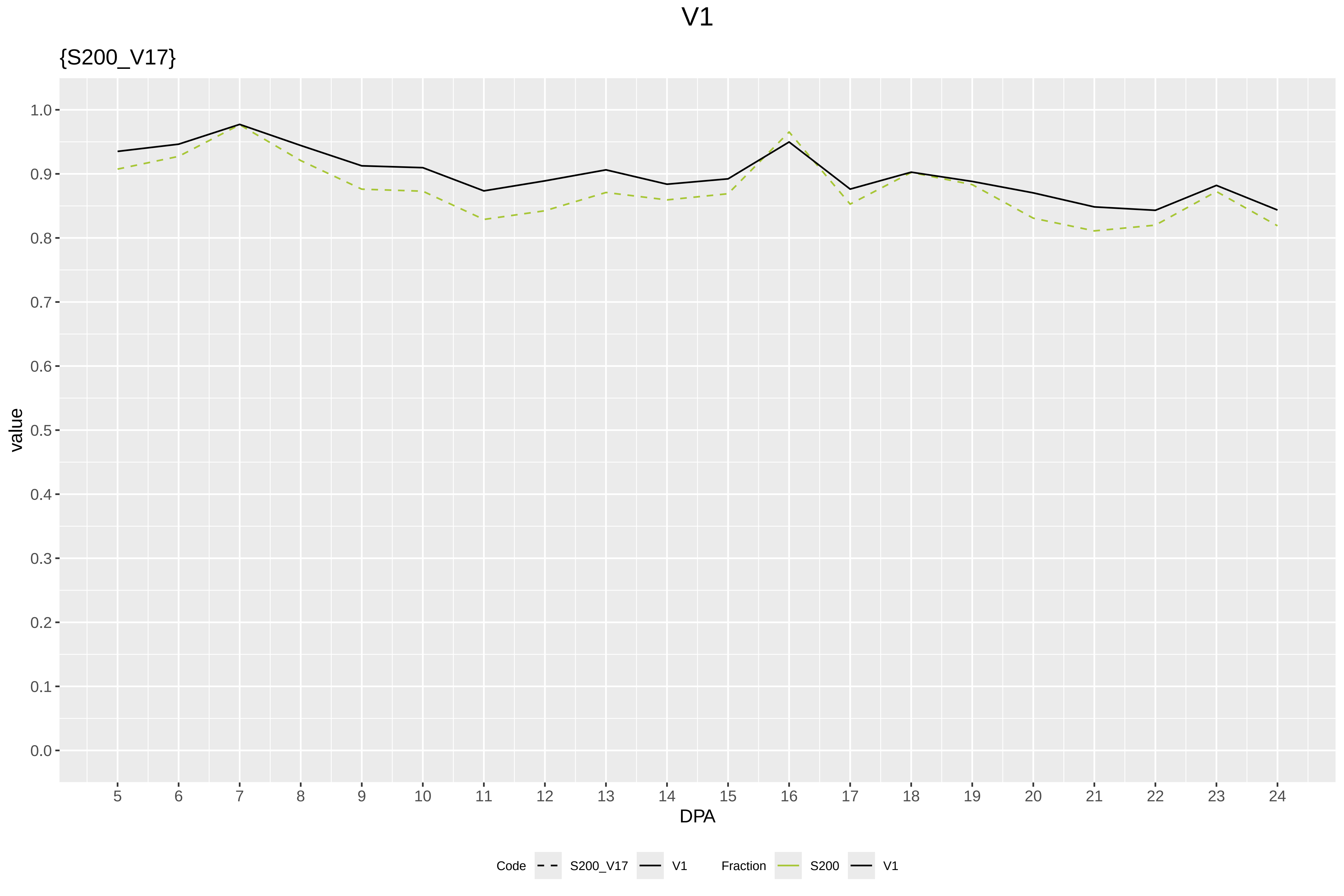

V1
{S200_V17}
value
1.0
0.9
0.8
0.7
0.6
0.5
0.4
0.3
0.2
0.1
0.0
5
6
7
8
9
10
11
12
13
14
15
16
17
18
19
20
21
22
23
24
DPA
Code
S200_V17
V1
Fraction
S200
V1

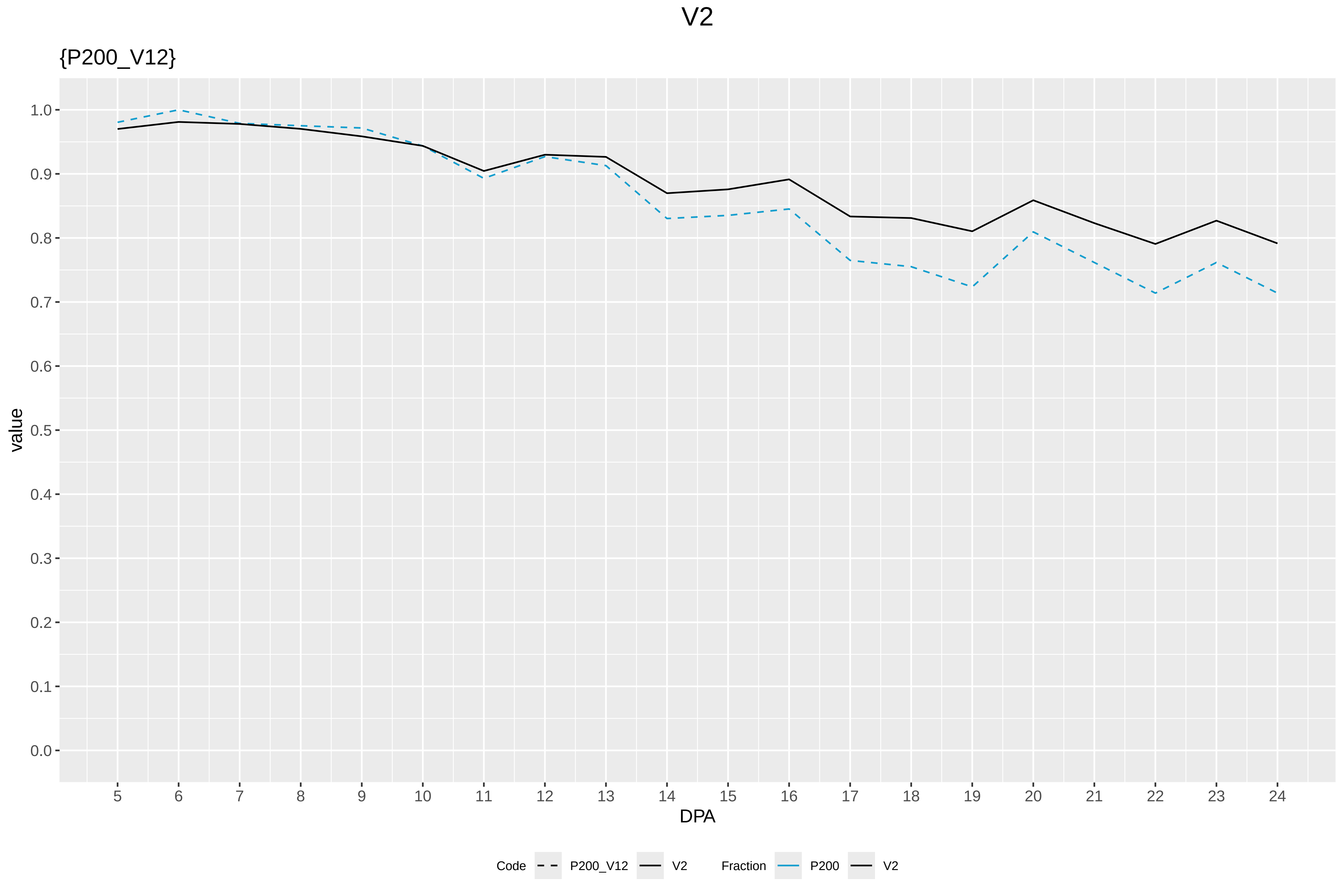

V2
{P200_V12}
value
1.0
0.9
0.8
0.7
0.6
0.5
0.4
0.3
0.2
0.1
0.0
5
6
7
8
9
10
11
12
13
14
15
16
17
18
19
20
21
22
23
24
DPA
Code
P200_V12
V2
Fraction
P200
V2

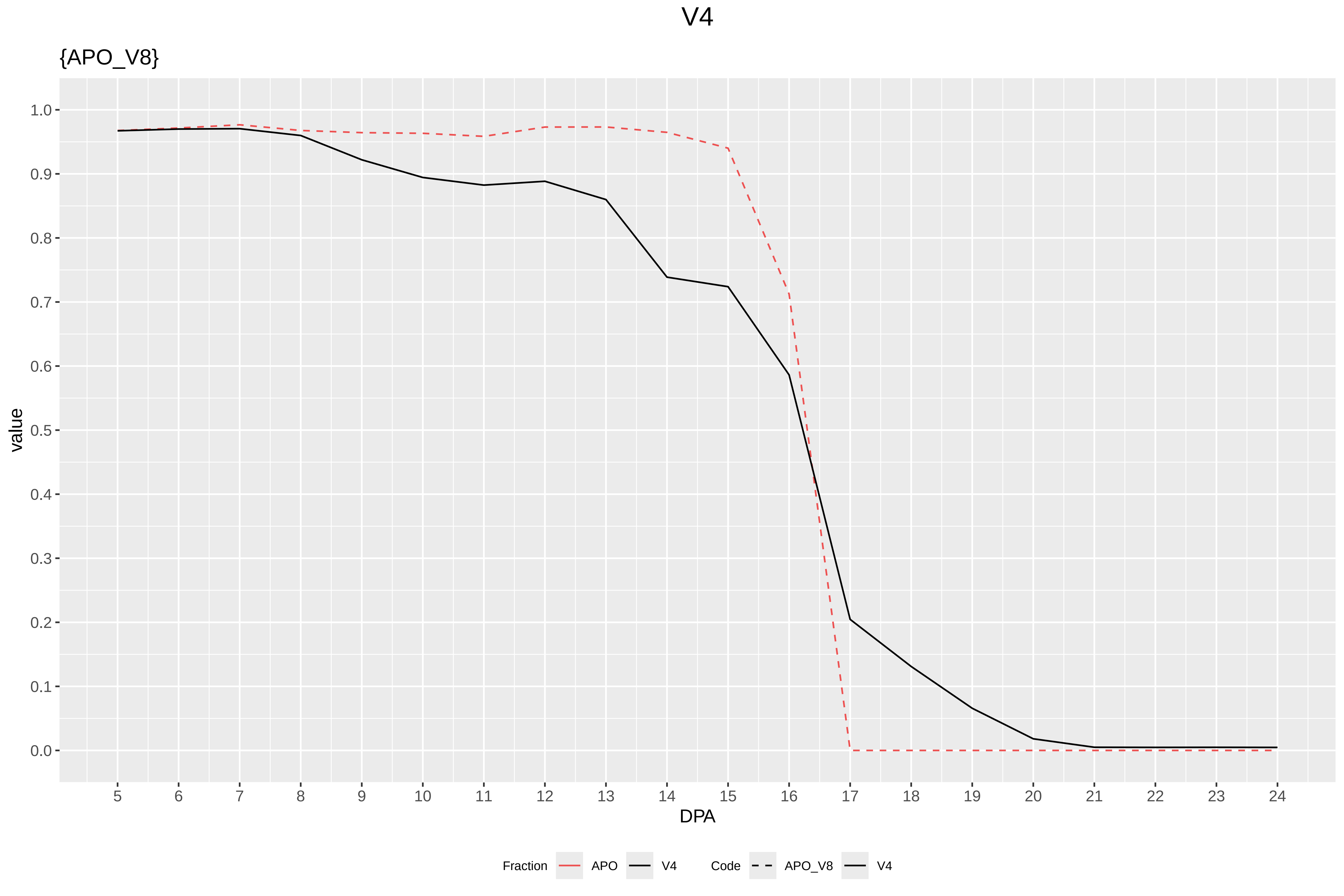

V4
{APO_V8}
value
DPA
1.0
0.9
0.8
0.7
0.6
0.5
0.4
0.3
0.2
0.1
0.0
5
6
7
8
9
10
11
12
13
14
15
16
17
18
19
20
21
22
23
24
Fraction
APO
V4
Code
APO_V8
V4

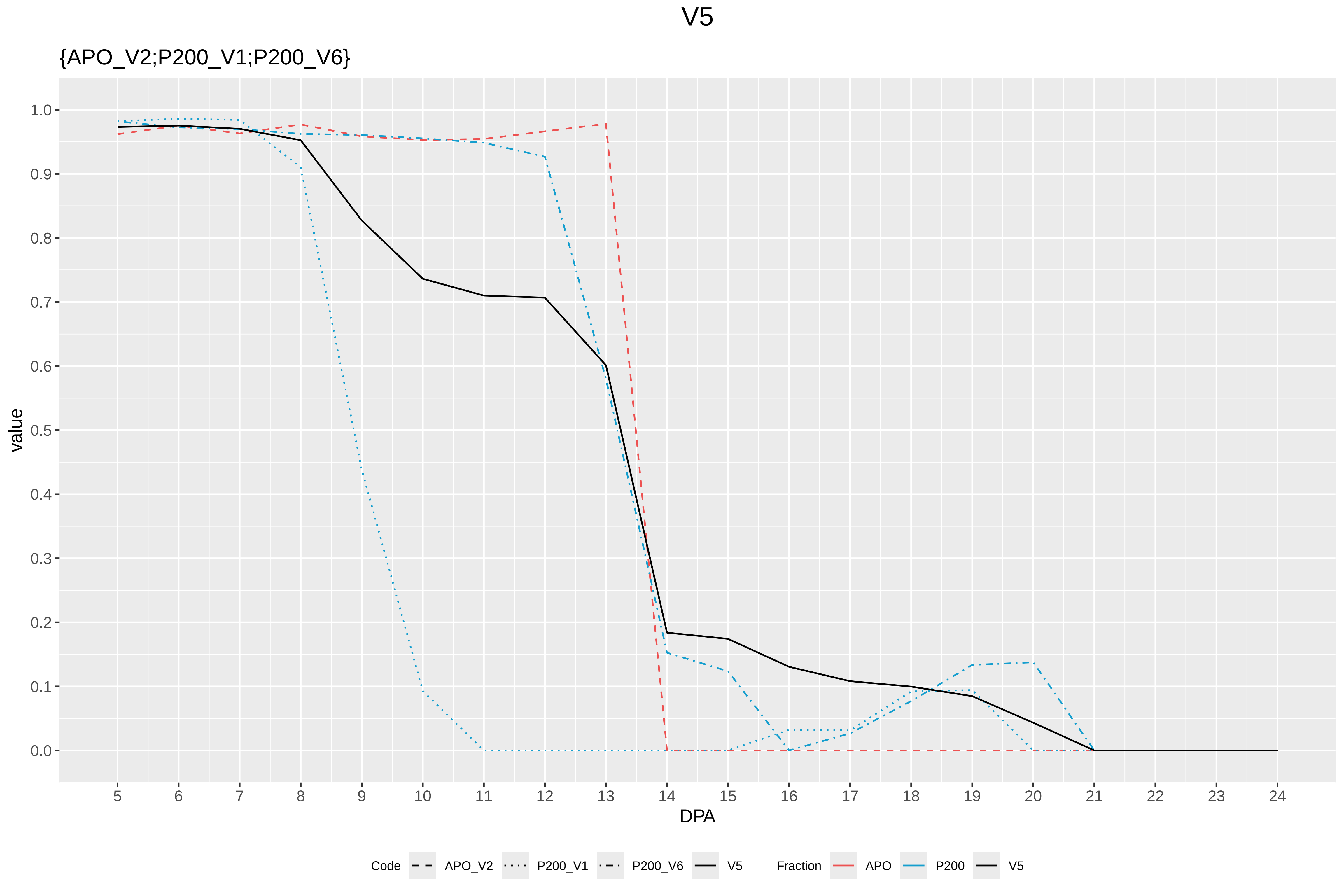

V5
{APO_V2;P200_V1;P200_V6}
value
1.0
0.9
0.8
0.7
0.6
0.5
0.4
0.3
0.2
0.1
0.0
5 6 7 8 9 10 11 12 13 14 15 16 17 18 19 20 21 22 23 24
DPA
Code
APO_V2
P200_V1
P200_V6
V5
Fraction
APO
P200
V5

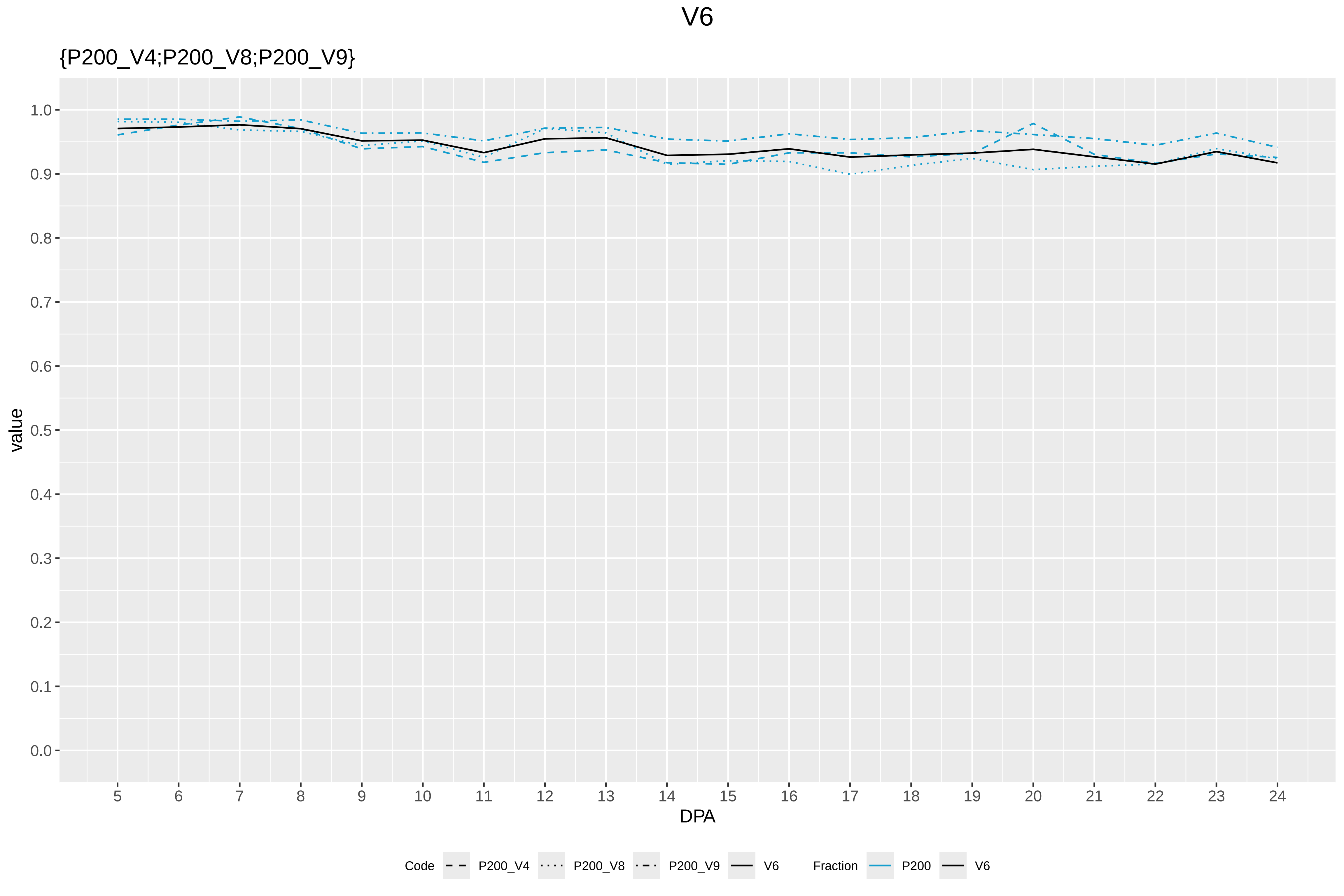

V6
{P200_V4;P200_V8;P200_V9}
value
1.0
0.9
0.8
0.7
0.6
0.5
0.4
0.3
0.2
0.1
0.0
5
6
7
8
9
10
11
12
13
14
15
16
17
18
19
20
21
22
23
24
DPA
Code
P200_V4
P200_V8
P200_V9
V6
Fraction
P200
V6

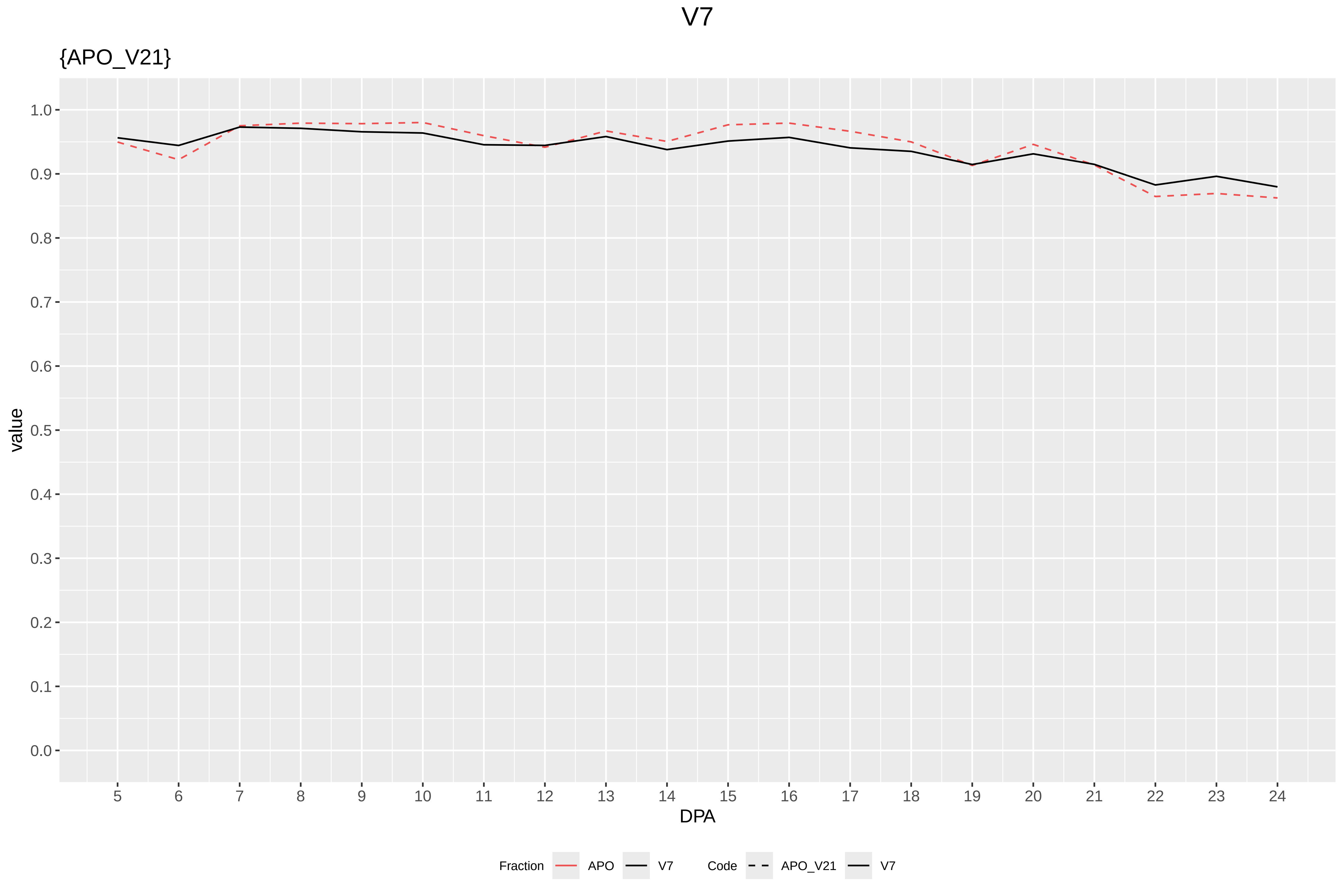
V7
{APO_V21}
value
1.0
0.9
0.8
0.7
0.6
0.5
0.4
0.3
0.2
0.1
0.0
5
6
7
8
9
10
11
12
13
14
15
16
17
18
19
20
21
22
23
24
DPA
Fraction
APO
V7
Code
APO_V21
V7

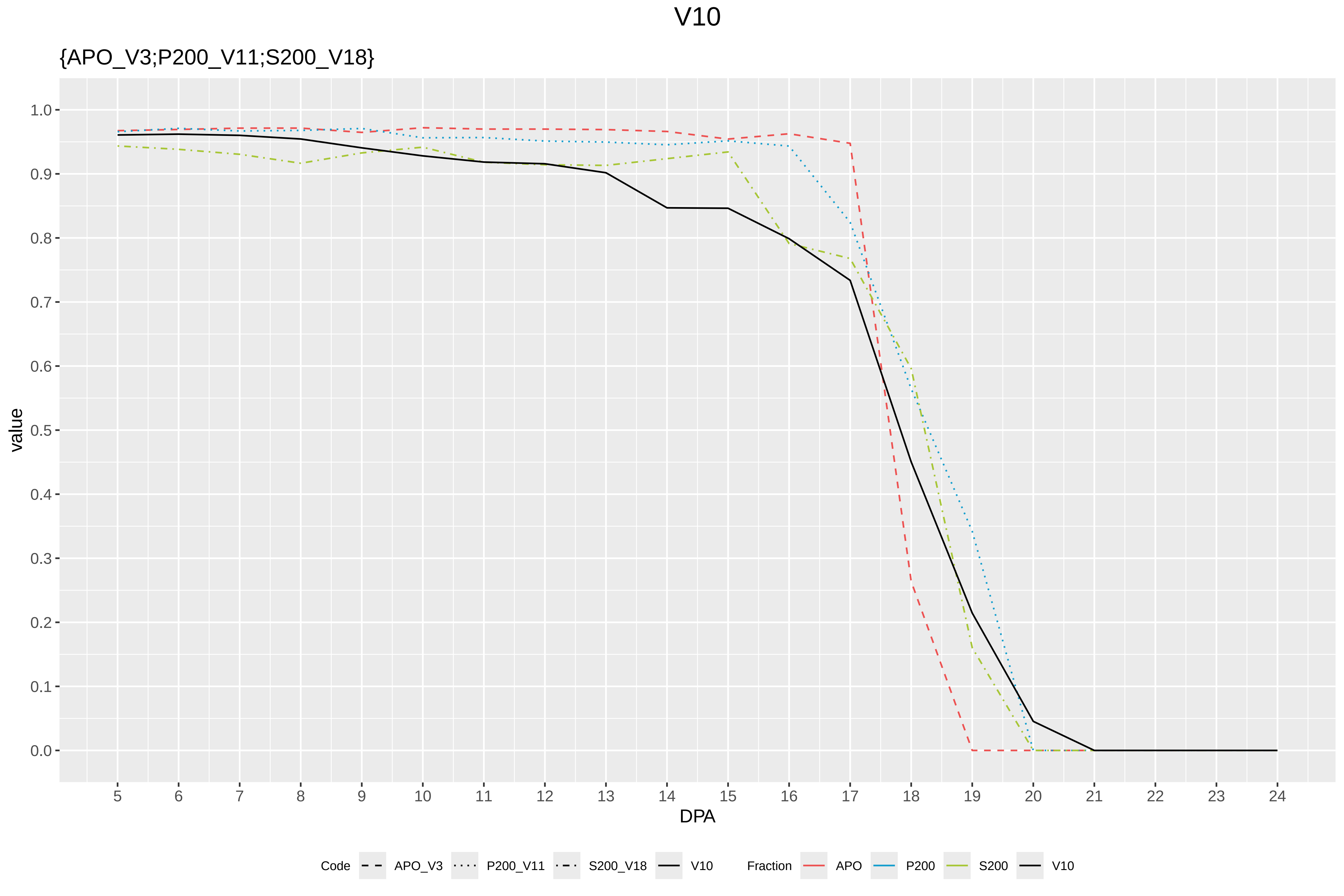

V10
{APO_V3;P200_V11;S200_V18}
value
1.0
0.9
0.8
0.7
0.6
0.5
0.4
0.3
0.2
0.1
0.0
5 6 7 8 9 10 11 12 13 14 15 16 17 18 19 20 21 22 23 24
DPA
Code APO_V3 P200_V11 S200_V18 V10
Fraction APO P200 S200 V10

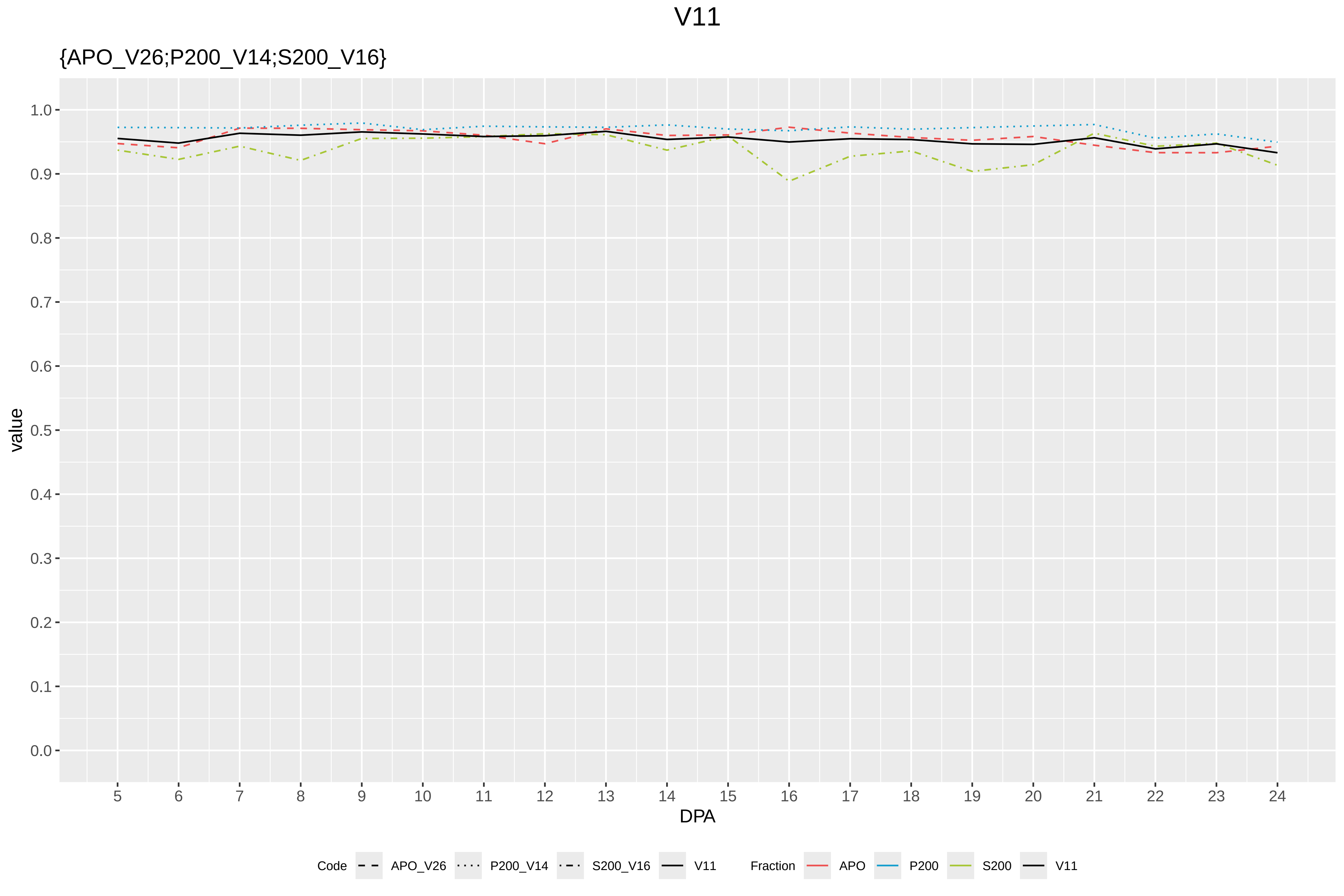
V11
{APO_V26;P200_V14;S200_V16}
value
1.0
0.9
0.8
0.7
0.6
0.5
0.4
0.3
0.2
0.1
0.0
5 6 7 8 9 10 11 12 13 14 15 16 17 18 19 20 21 22 23 24
DPA
Code
APO_V26
P200_V14
S200_V16
V11
Fraction
APO
P200
S200
V11

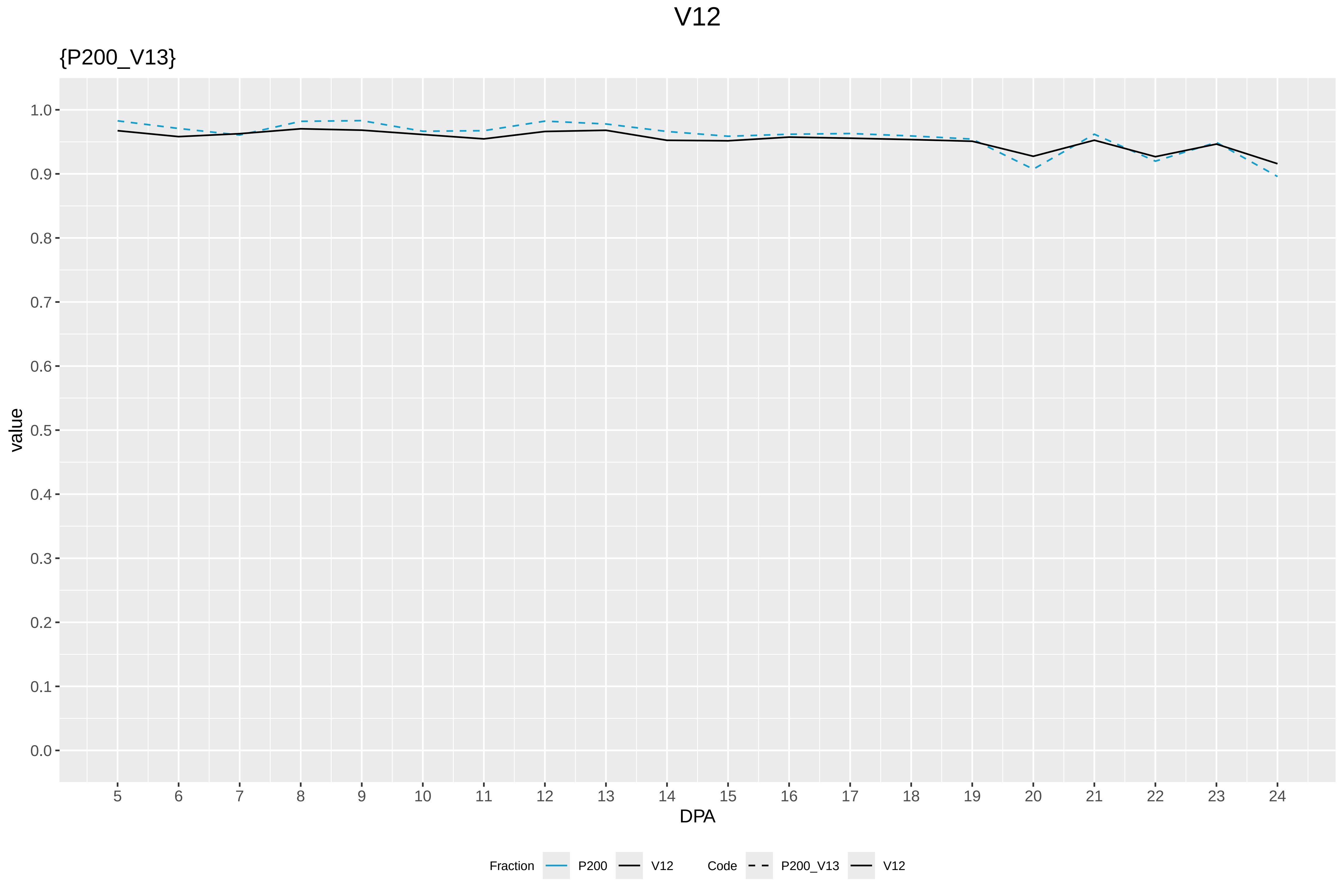

V12
{P200_V13}
value
1.0
0.9
0.8
0.7
0.6
0.5
0.4
0.3
0.2
0.1
0.0
5
6
7
8
9
10
11
12
13
14
15
16
17
18
19
20
21
22
23
24
DPA
Fraction
P200
V12
Code
P200_V13
V12

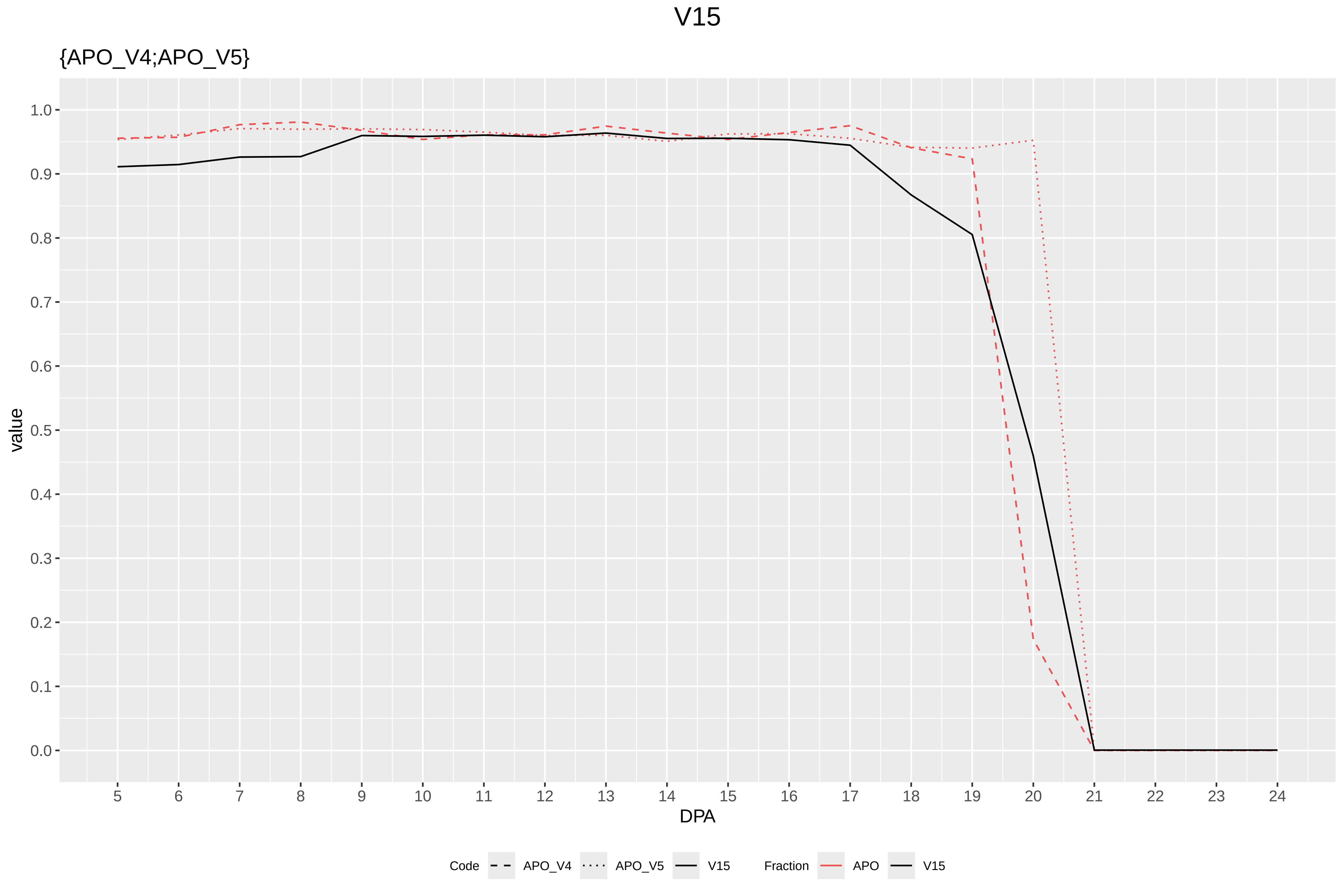
V15
{APO_V4;APO_V5}
value
1.0
0.9
0.8
0.7
0.6
0.5
0.4
0.3
0.2
0.1
0.0
5
6
7
8
9
10
11
12
13
14
15
16
17
18
19
20
21
22
23
24
DPA
Code
APO_V4
APO_V5
V15
Fraction
APO
V15

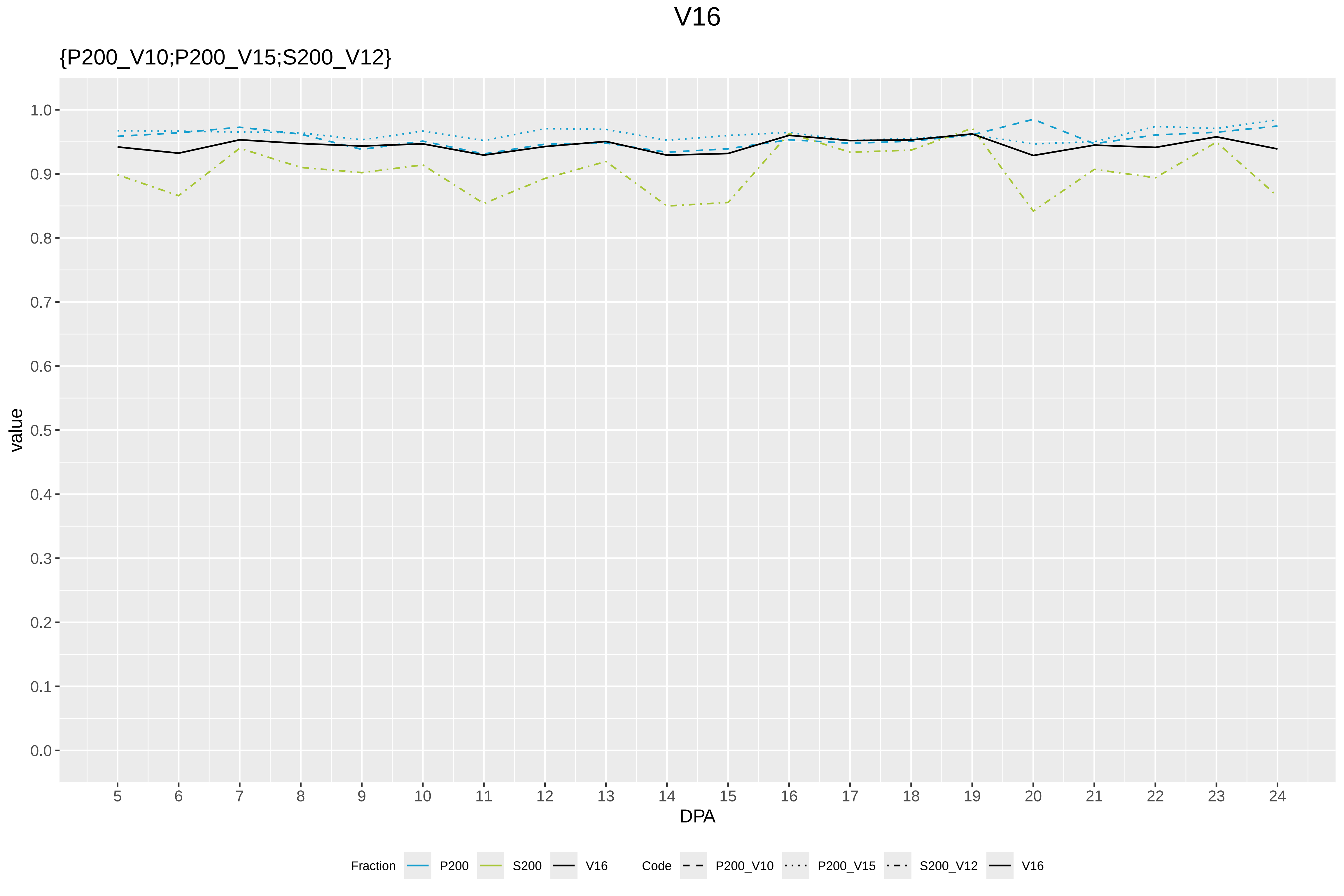
V16
{P200_V10;P200_V15;S200_V12}
value
1.0
0.9
0.8
0.7
0.6
0.5
0.4
0.3
0.2
0.1
0.0
5
6
7
8
9
10
11
12
13
14
15
16
17
18
19
20
21
22
23
24
DPA
Fraction
P200
S200
V16
Code
P200_V10
P200_V15
S200_V12
V16

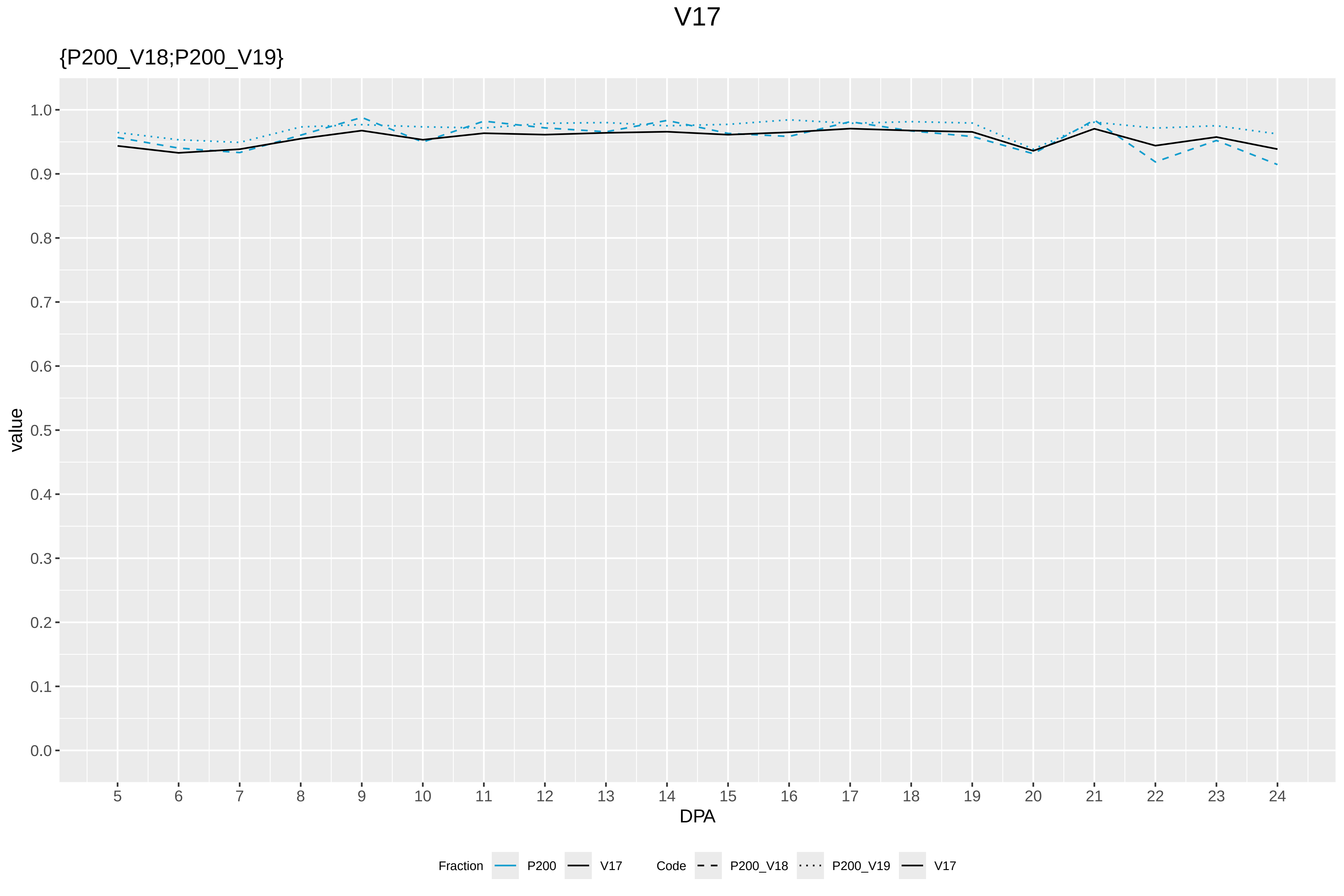
V17
{P200_V18;P200_V19}
1.0
0.9
0.8
0.7
0.6
0.5
0.4
0.3
0.2
0.1
0.0
value
5
6
7
8
9
10
11
12
13
14
15
16
17
18
19
20
21
22
23
24
DPA
Fraction
P200
V17
Code
P200_V18
P200_V19
V17

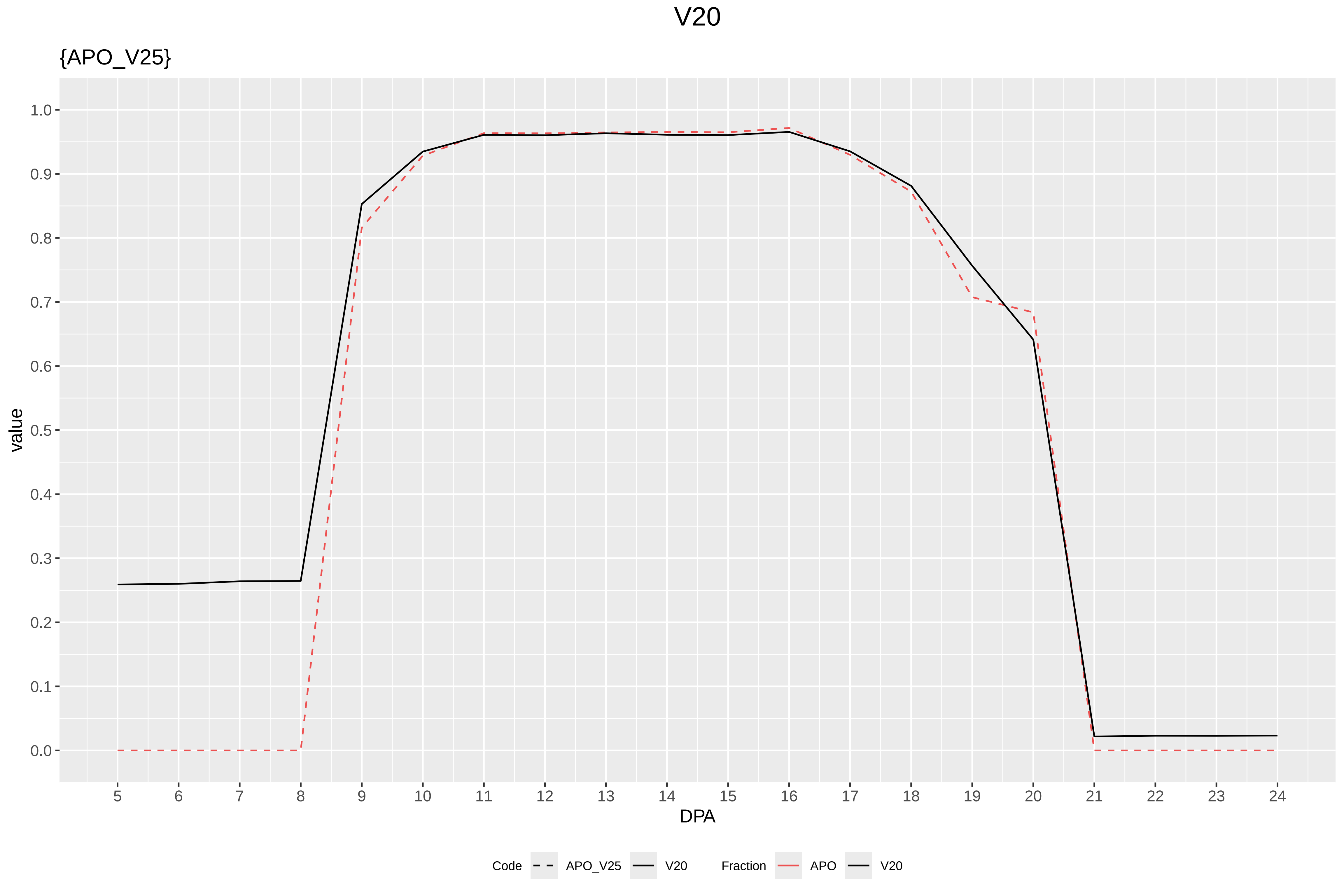
V20
{APO_V25}
value
1.0
0.9
0.8
0.7
0.6
0.5
0.4
0.3
0.2
0.1
0.0
5
6
7
8
9
10
11
12
13
14
15
16
17
18
19
20
21
22
23
24
DPA
Code
APO_V25
V20
Fraction
APO
V20

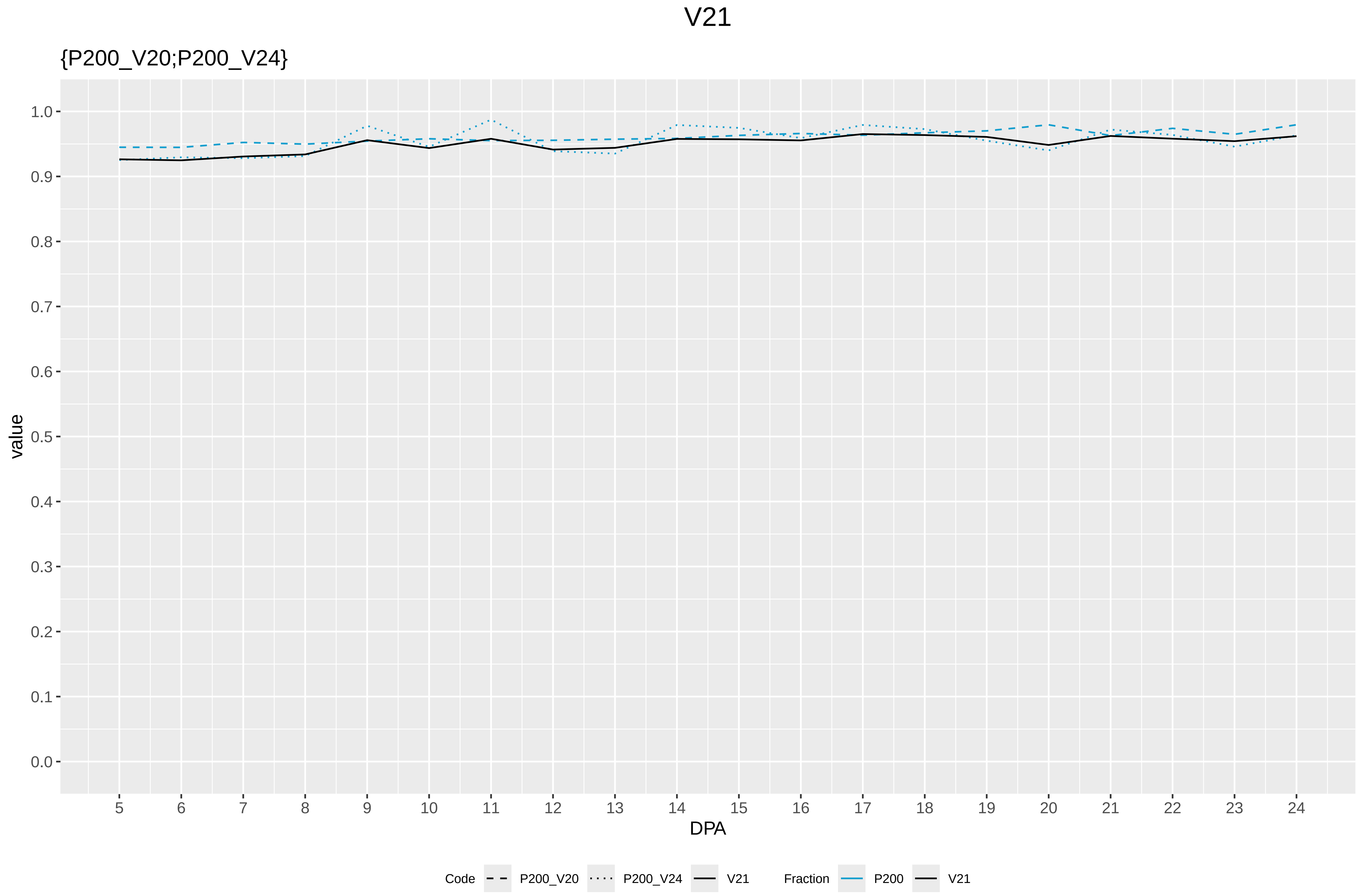
V21
{P200_V20;P200_V24}
value
DPA
1.0
0.9
0.8
0.7
0.6
0.5
0.4
0.3
0.2
0.1
0.0
5 6 7 8 9 10 11 12 13 14 15 16 17 18 19 20 21 22 23 24
Code
P200_V20
P200_V24
V21
Fraction
P200
V21

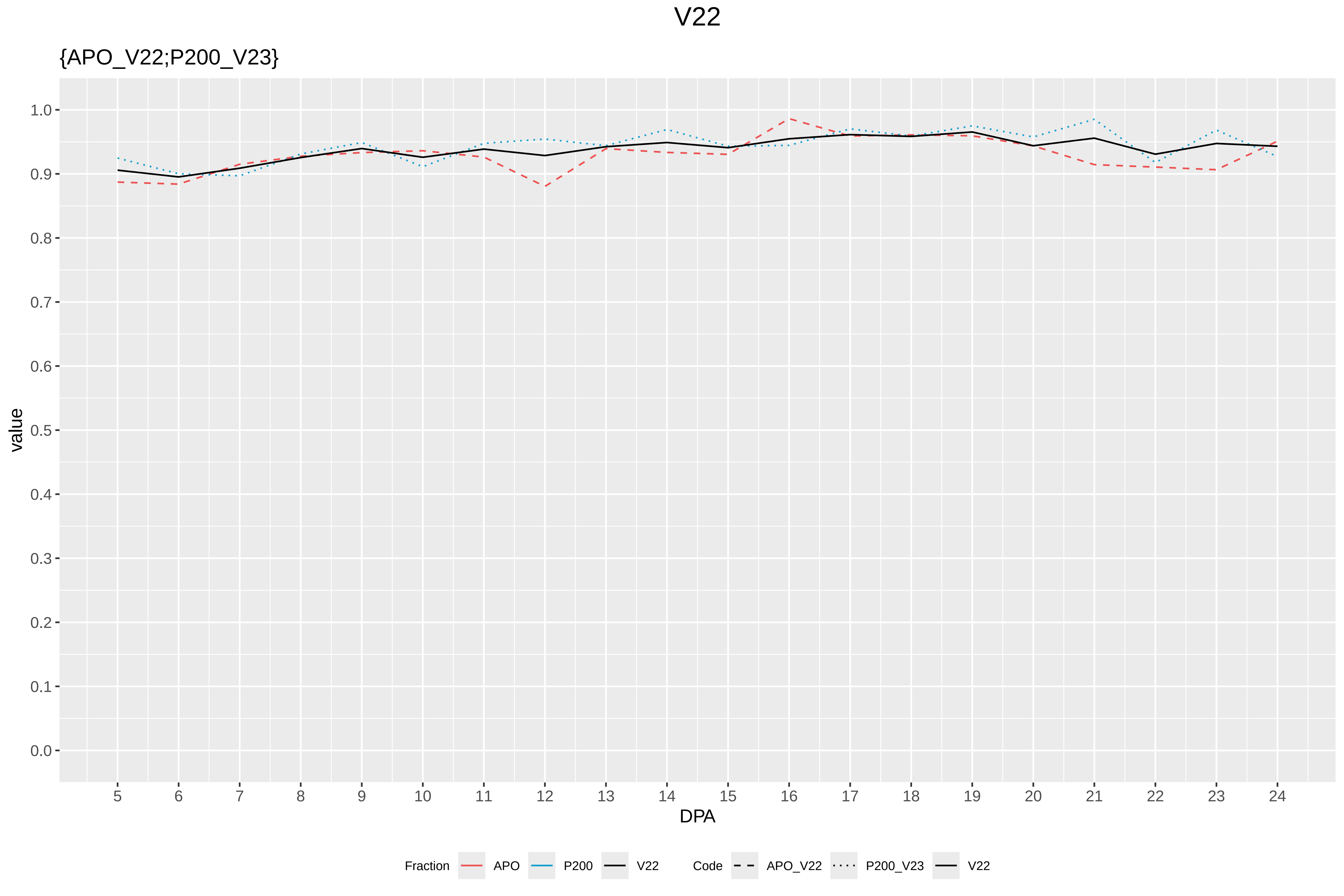

V22
{APO_V22;P200_V23}
value
1.0
0.9
0.8
0.7
0.6
0.5
0.4
0.3
0.2
0.1
0.0
5
6
7
8
9
10
11
12
13
14
15
16
17
18
19
20
21
22
23
24
DPA
Fraction
APO
P200
V22
Code
APO_V22
P200_V23
V22

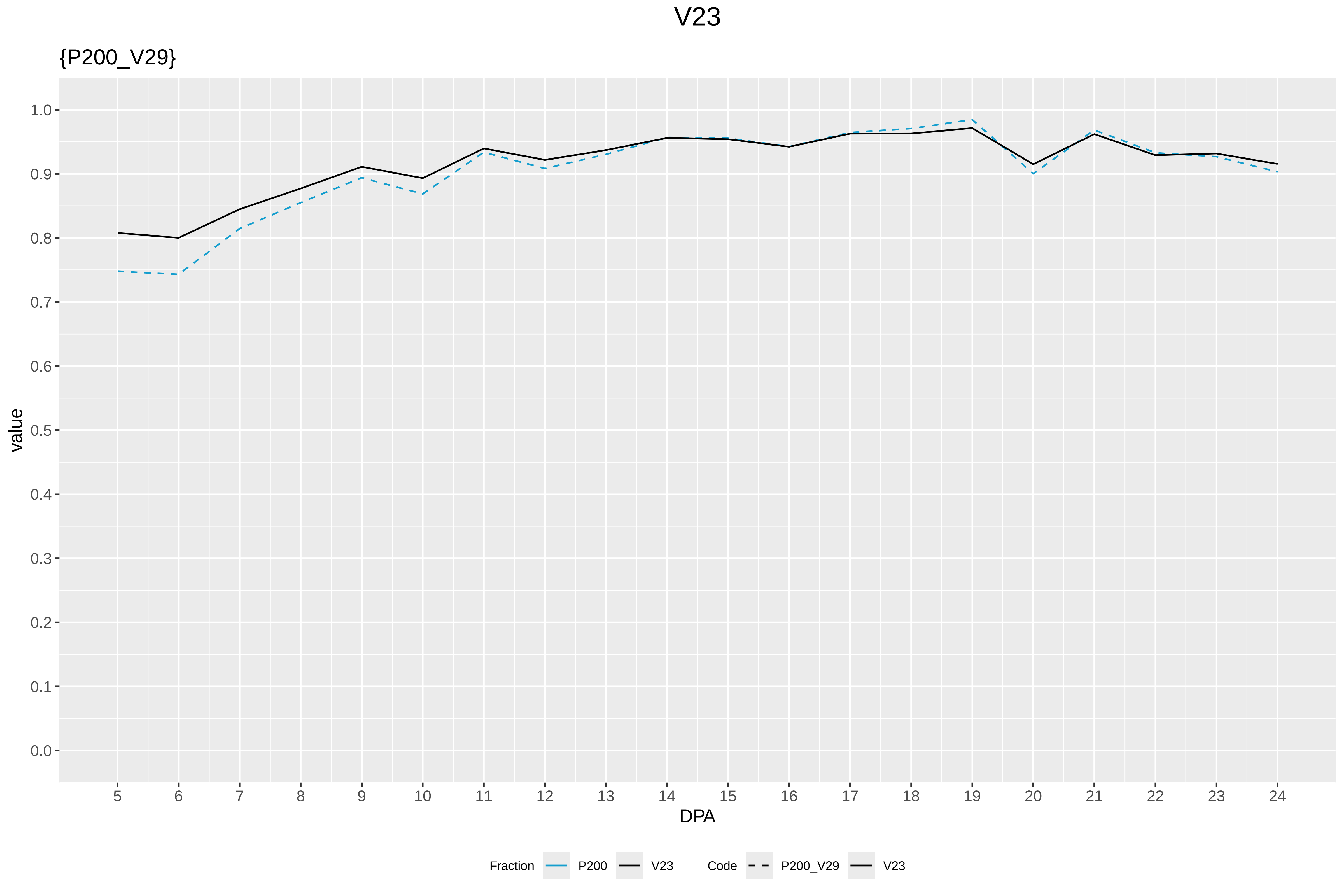
V23
{P200_V29}
value
1.0
0.9
0.8
0.7
0.6
0.5
0.4
0.3
0.2
0.1
0.0
5
6
7
8
9
10
11
12
13
14
15
16
17
18
19
20
21
22
23
24
DPA
Fraction
P200
V23
Code
P200_V29
V23

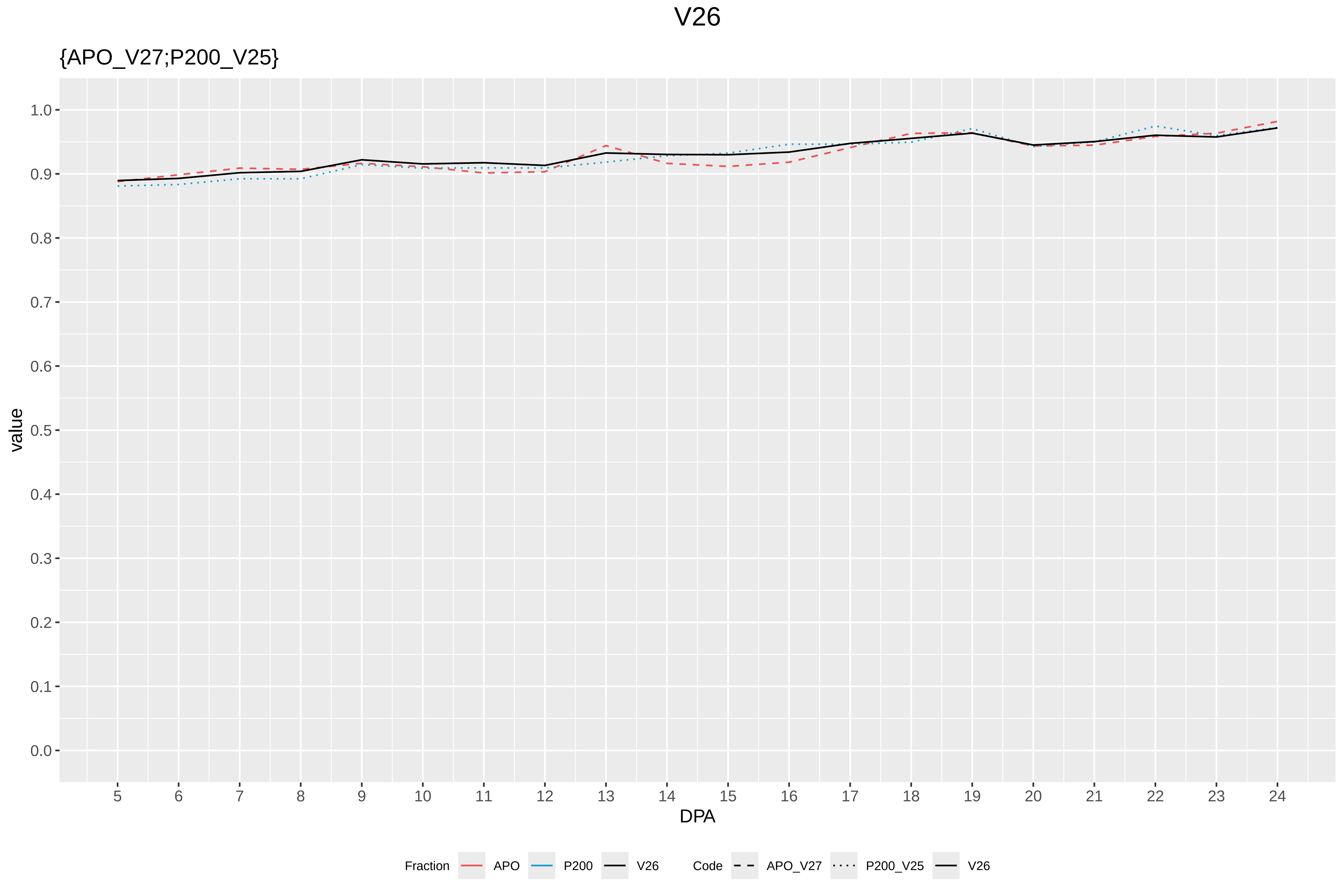

V26
{APO_V27;P200_V25}
value
1.0
0.9
0.8
0.7
0.6
0.5
0.4
0.3
0.2
0.1
0.0
5
6
7
8
9
10
11
12
13
14
15
16
17
18
19
20
21
22
23
24
DPA
Fraction
APO
P200
V26
Code
APO_V27
P200_V25
V26

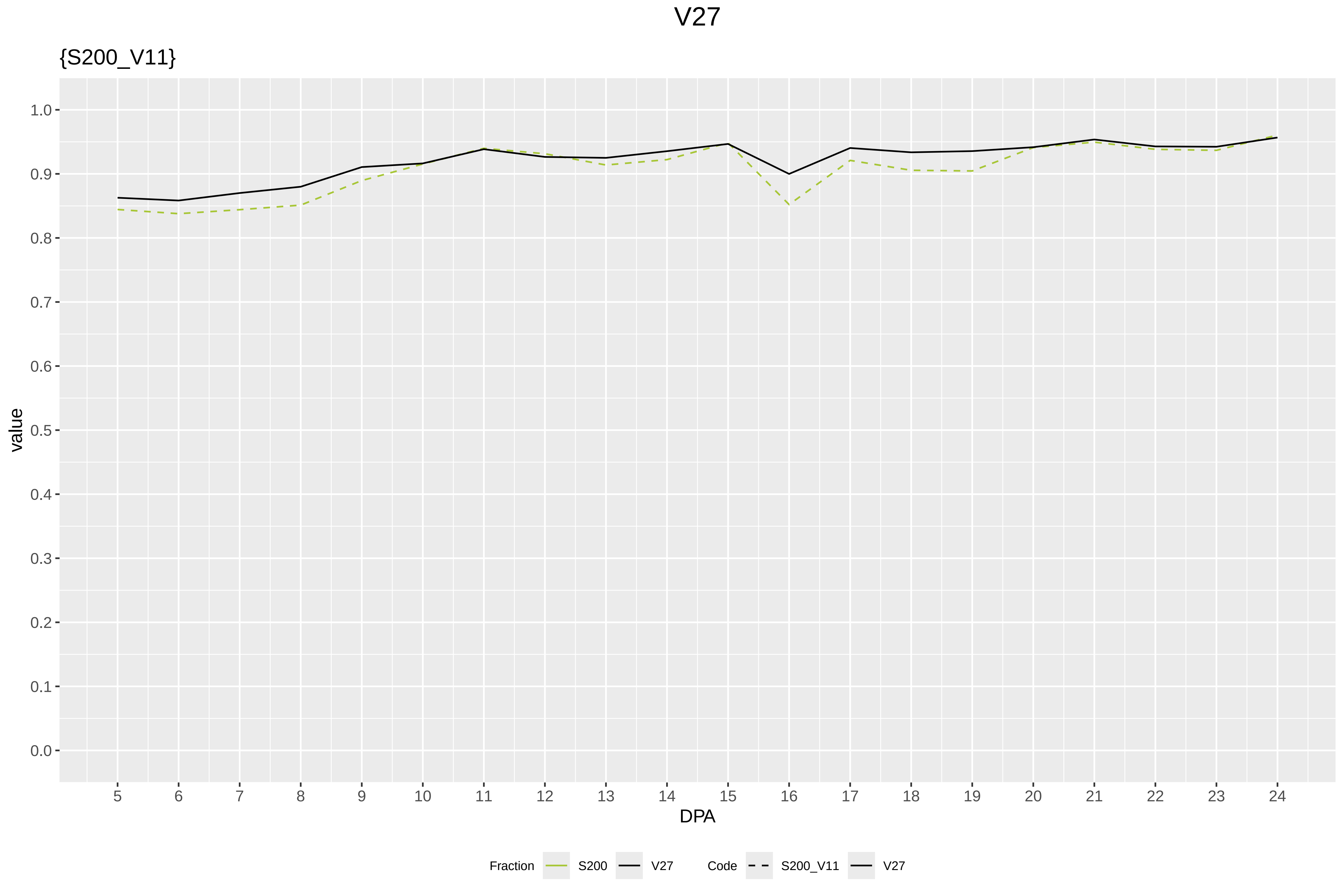
V27
{S200_V11}
1.0
0.9
0.8
0.7
0.6
0.5
0.4
0.3
0.2
0.1
0.0
value
5
6
7
8
9
10
11
12
13
14
15
16
17
18
19
20
21
22
23
24
DPA
Fraction
S200
V27
Code
S200_V11
V27

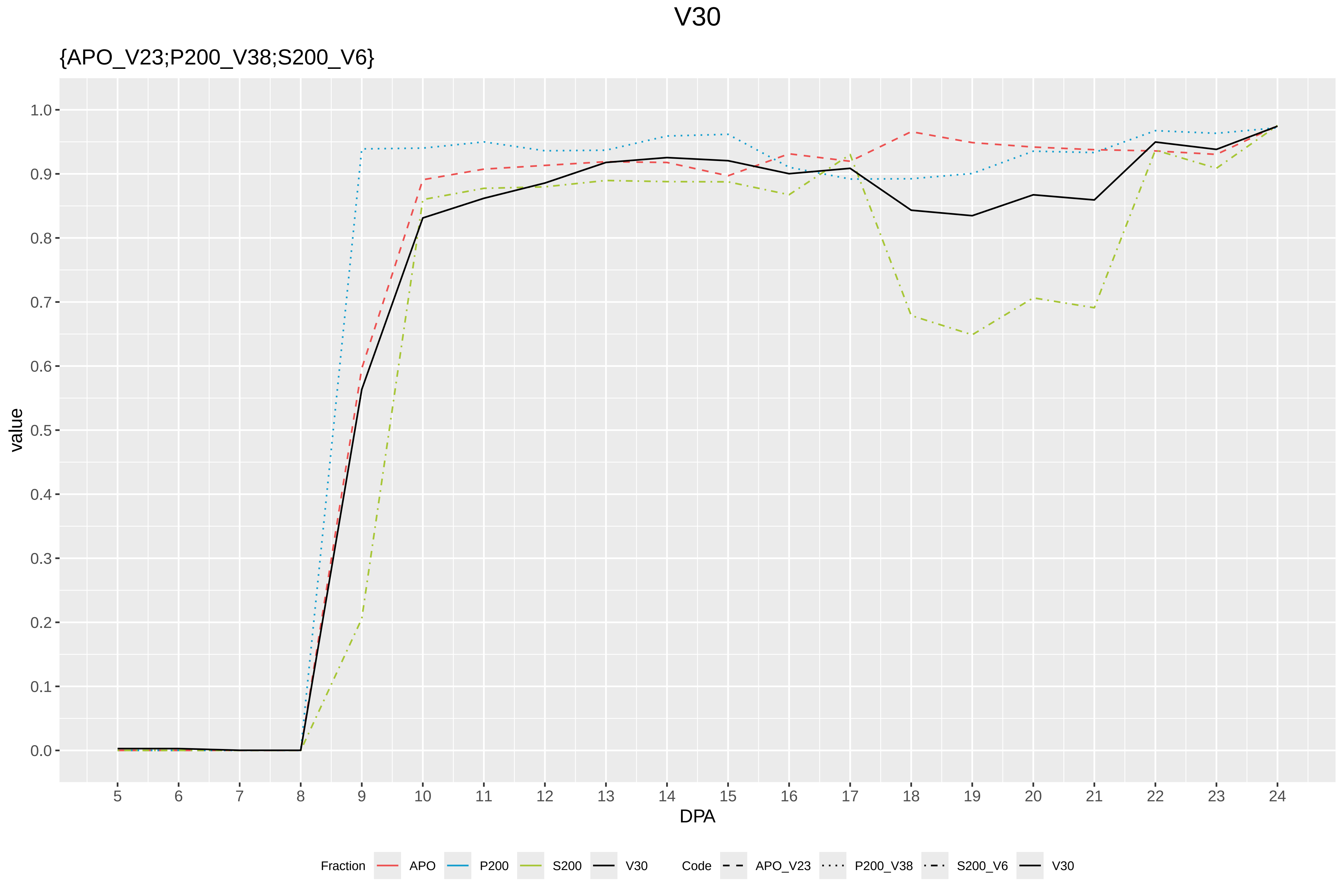
V30
{APO_V23;P200_V38;S200_V6}
value
1.0
0.9
0.8
0.7
0.6
0.5
0.4
0.3
0.2
0.1
0.0
5 6 7 8 9 10 11 12 13 14 15 16 17 18 19 20 21 22 23 24
DPA
Fraction APO P200 S200 V30
Code APO_V23 P200_V38 S200_V6 V30

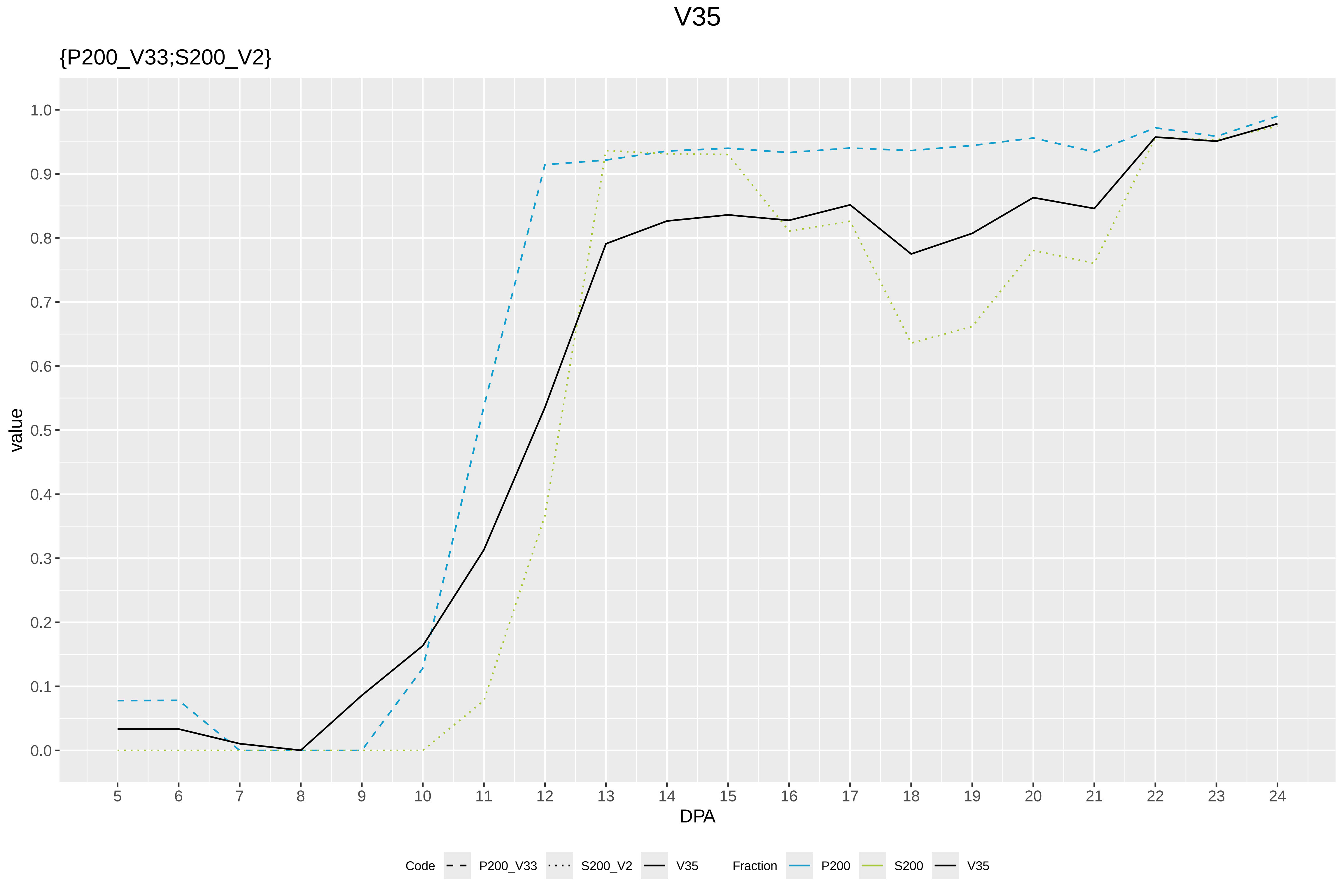

V35
{P200_V33;S200_V2}
value
1.0
0.9
0.8
0.7
0.6
0.5
0.4
0.3
0.2
0.1
0.0
5
6
7
8
9
10
11
12
13
14
15
16
17
18
19
20
21
22
23
24
DPA
Code
P200_V33
S200_V2
V35
Fraction
P200
S200
V35

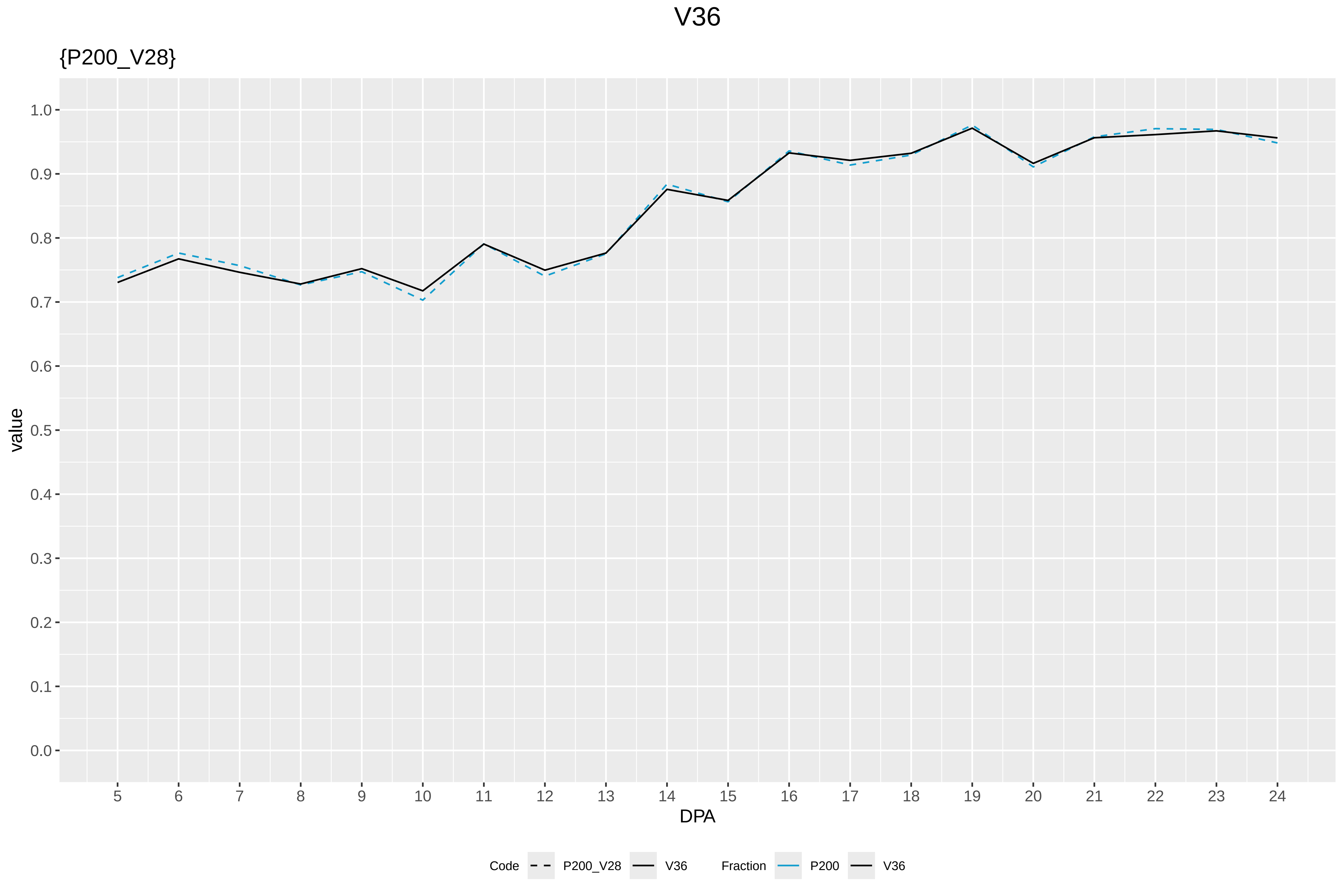

V36
{P200_V28}
value
DPA
1.0
0.9
0.8
0.7
0.6
0.5
0.4
0.3
0.2
0.1
0.0
5 6 7 8 9 10 11 12 13 14 15 16 17 18 19 20 21 22 23 24
Code
P200_V28
V36
Fraction
P200
V36

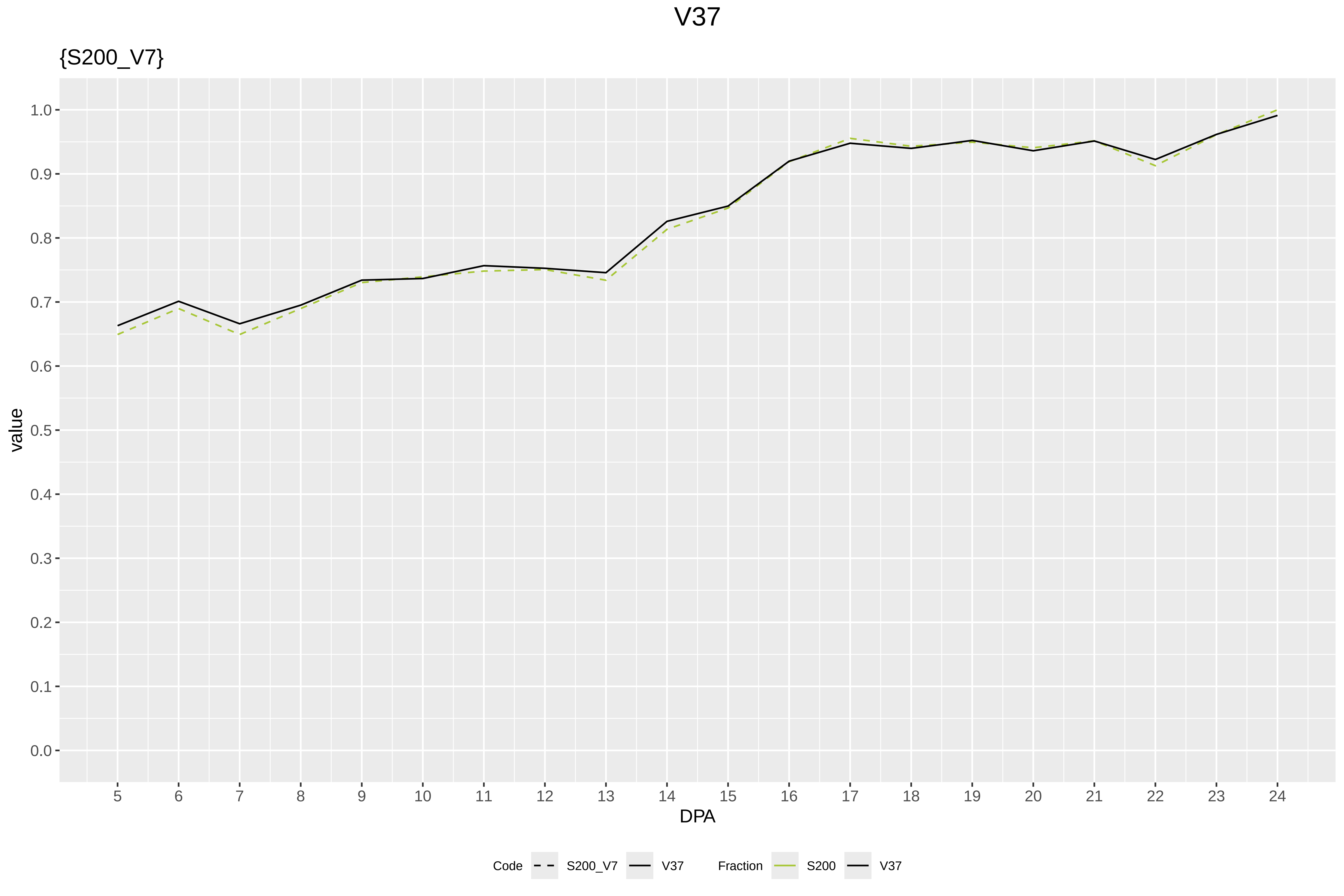
V37
{S200_V7}
value
1.0
0.9
0.8
0.7
0.6
0.5
0.4
0.3
0.2
0.1
0.0
5
6
7
8
9
10
11
12
13
14
15
16
17
18
19
20
21
22
23
24
DPA
Code
S200_V7
V37
Fraction
S200
V37

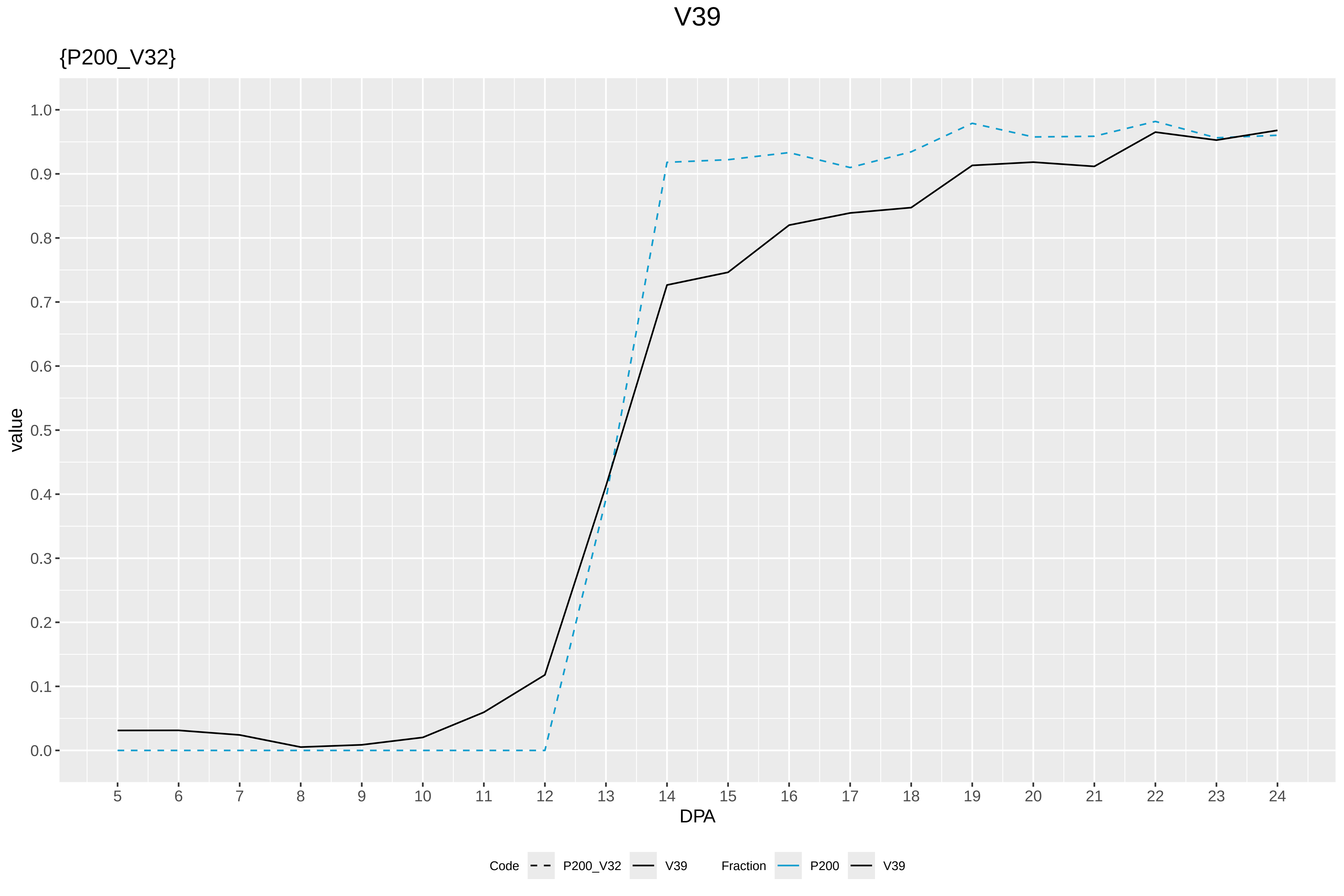

V39
{P200_V32}
value
1.0
0.9
0.8
0.7
0.6
0.5
0.4
0.3
0.2
0.1
0.0
5
6
7
8
9
10
11
12
13
14
15
16
17
18
19
20
21
22
23
24
DPA
Code
P200_V32
V39
Fraction
P200
V39

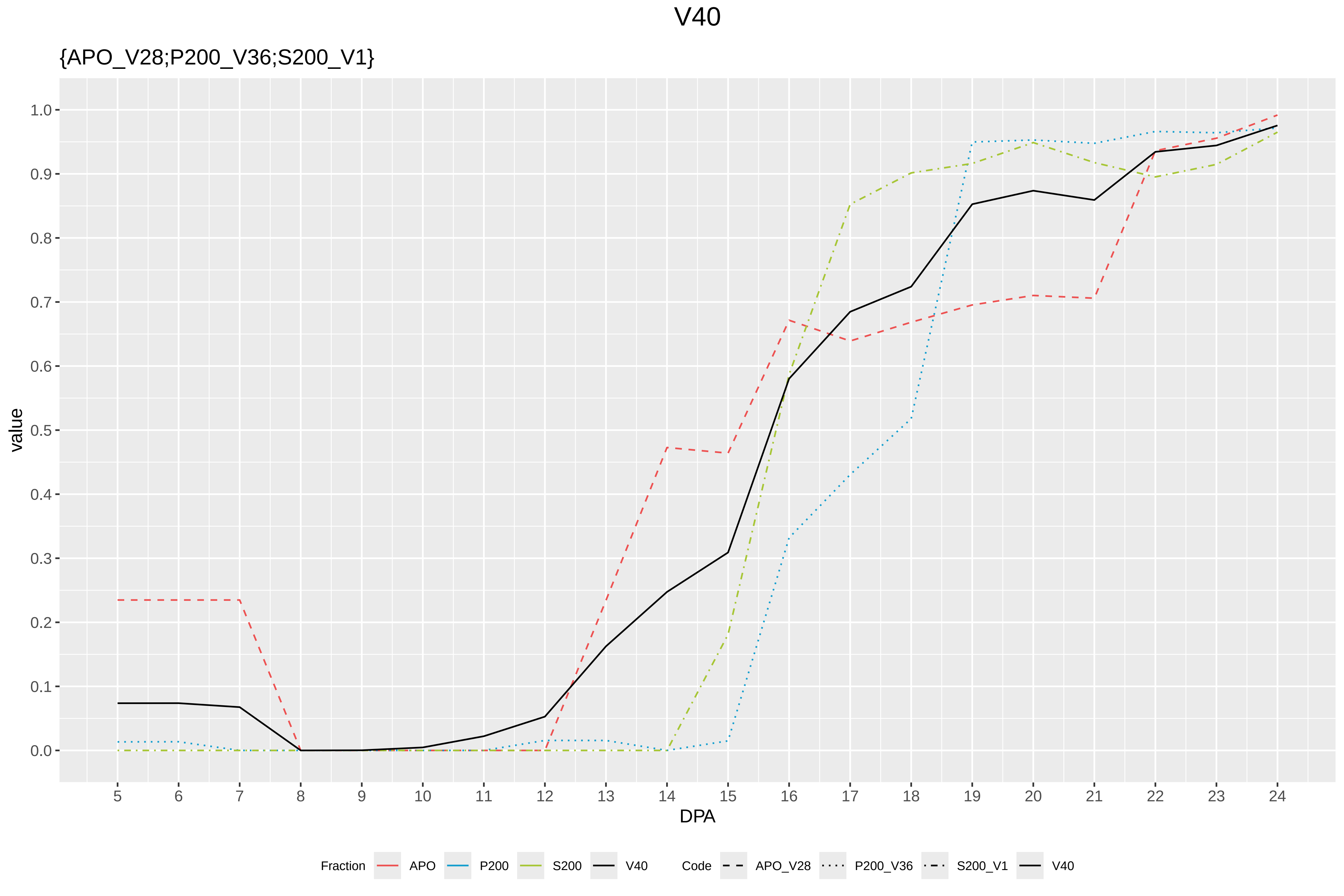

V40
{APO_V28;P200_V36;S200_V1}
1.0
0.9
0.8
0.7
0.6
0.5
0.4
0.3
0.2
0.1
0.0
value
5
6
7
8
9
10
11
12
13
14
15
16
17
18
19
20
21
22
23
24
DPA
Fraction
APO
P200
S200
V40
Code
APO_V28
P200_V36
S200_V1
V40

Supplementary Figure S10. Profiles of paired mRNAs and proteins (APO_T).

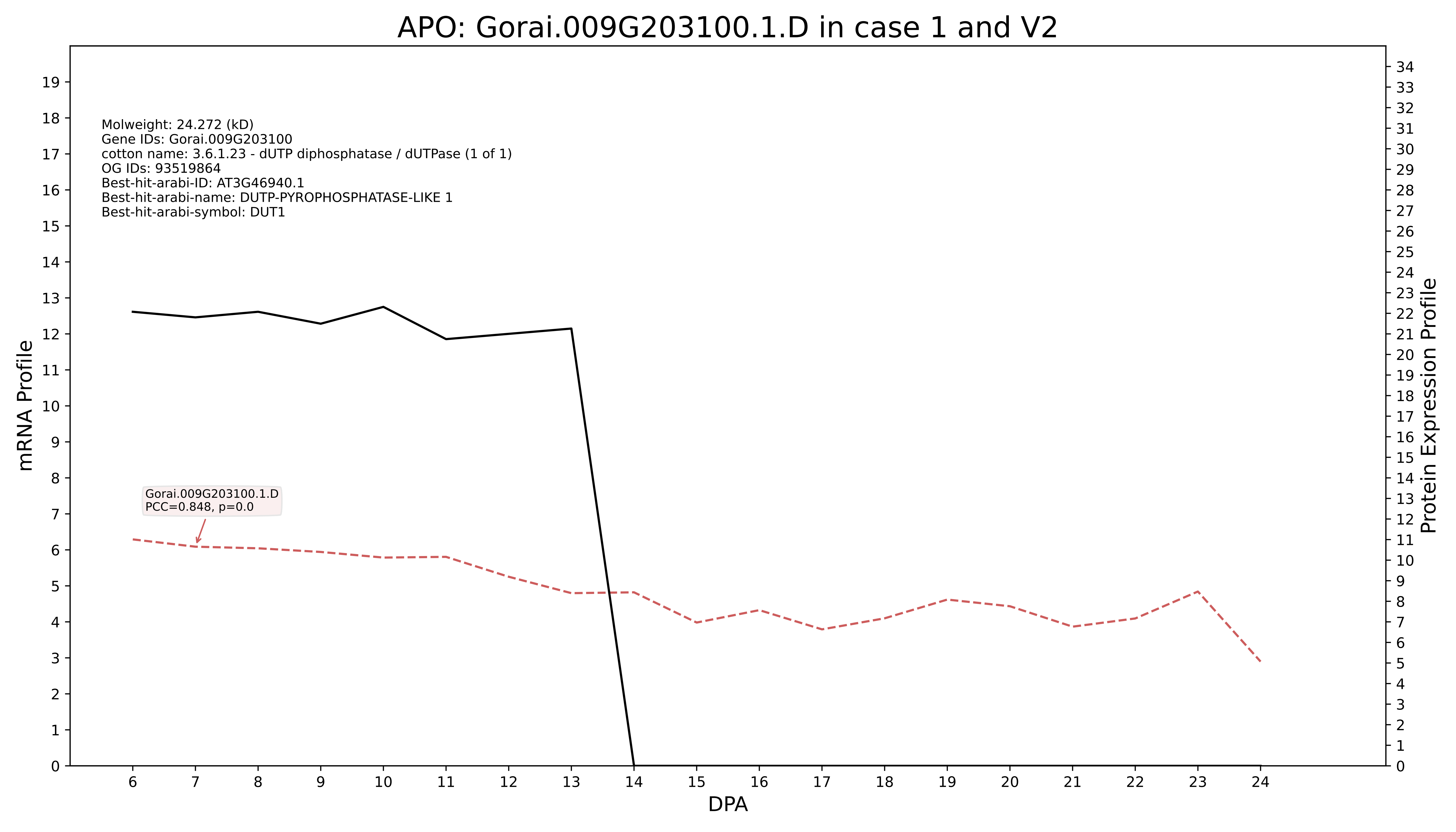
APO: Gorai.009G203100.1.D in case 1 and V2
Molweight: 24.272 (kD)
Gene IDs: Gorai.009G203100
cotton name: 3.6.1.23 - dUTP diphosphatase / dUTPase (1 of 1)
OG IDs: 93519864
Best-hit-arabi-ID: AT3G46940.1
Best-hit-arabi-name: DUTP-PYROPHOSPHATASE-LIKE 1
Best-hit-arabi-symbol: DUT1
Gorai.009G203100.1.D
PCC=0.848, p=0.0
mRNA Profile
Protein Expression Profile
DPA

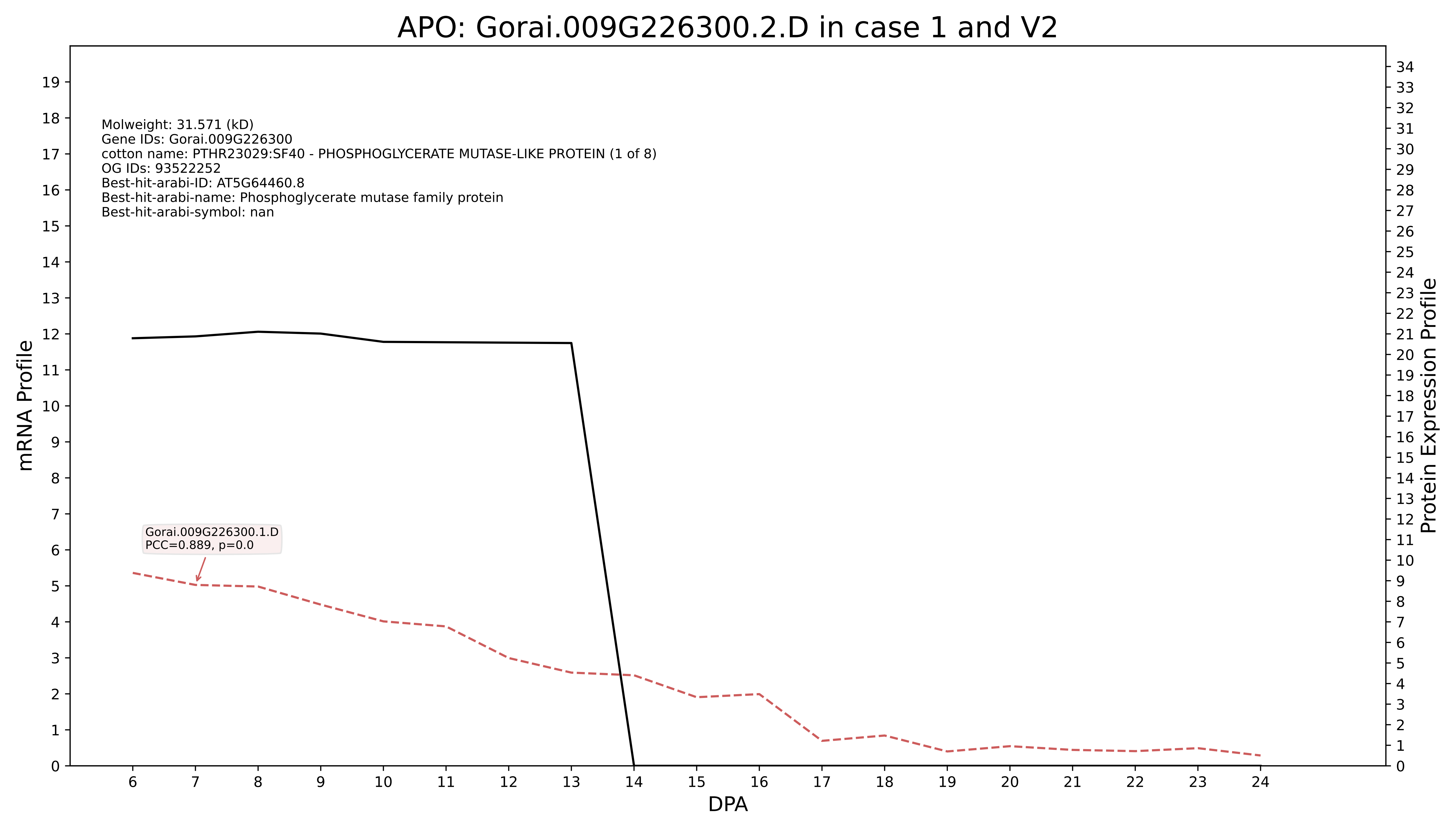

APO: Gorai.009G226300.2.D in case 1 and V2
Molweight: 31.571 (kD)
Gene IDs: Gorai.009G226300
cotton name: PTHR23029:SF40 - PHOSPHOGLYCERATE MUTASE-LIKE PROTEIN (1 of 8)
OG IDs: 93522252
Best-hit-arabi-ID: AT5G64460.8
Best-hit-arabi-name: Phosphoglycerate mutase family protein
Best-hit-arabi-symbol: nan
Gorai.009G226300.1.D
PCC=0.889, p=0.0
mRNA Profile
Protein Expression Profile
DPA

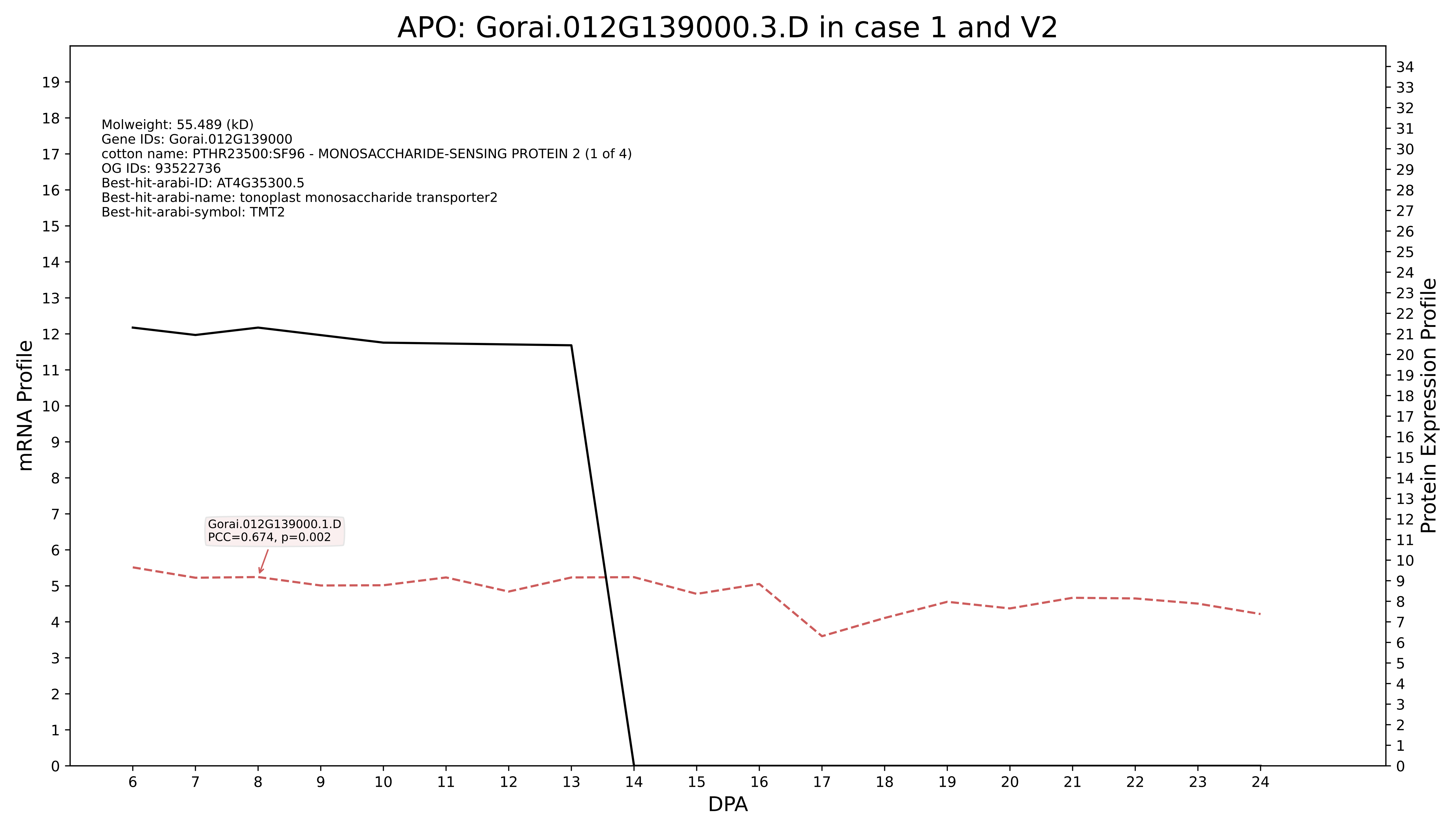

APO: Gorai.012G139000.3.D in case 1 and V2
Molweight: 55.489 (kD)
Gene IDs: Gorai.012G139000
cotton name: PTHR23500:SF96 - MONOSACCHARIDE-SENSING PROTEIN 2 (1 of 4)
OG IDs: 93522736
Best-hit-arabi-ID: AT4G35300.5
Best-hit-arabi-name: tonoplast monosaccharide transporter2
Best-hit-arabi-symbol: TMT2
Gorai.012G139000.1.D
PCC=0.674, p=0.002
mRNA Profile
Protein Expression Profile
DPA

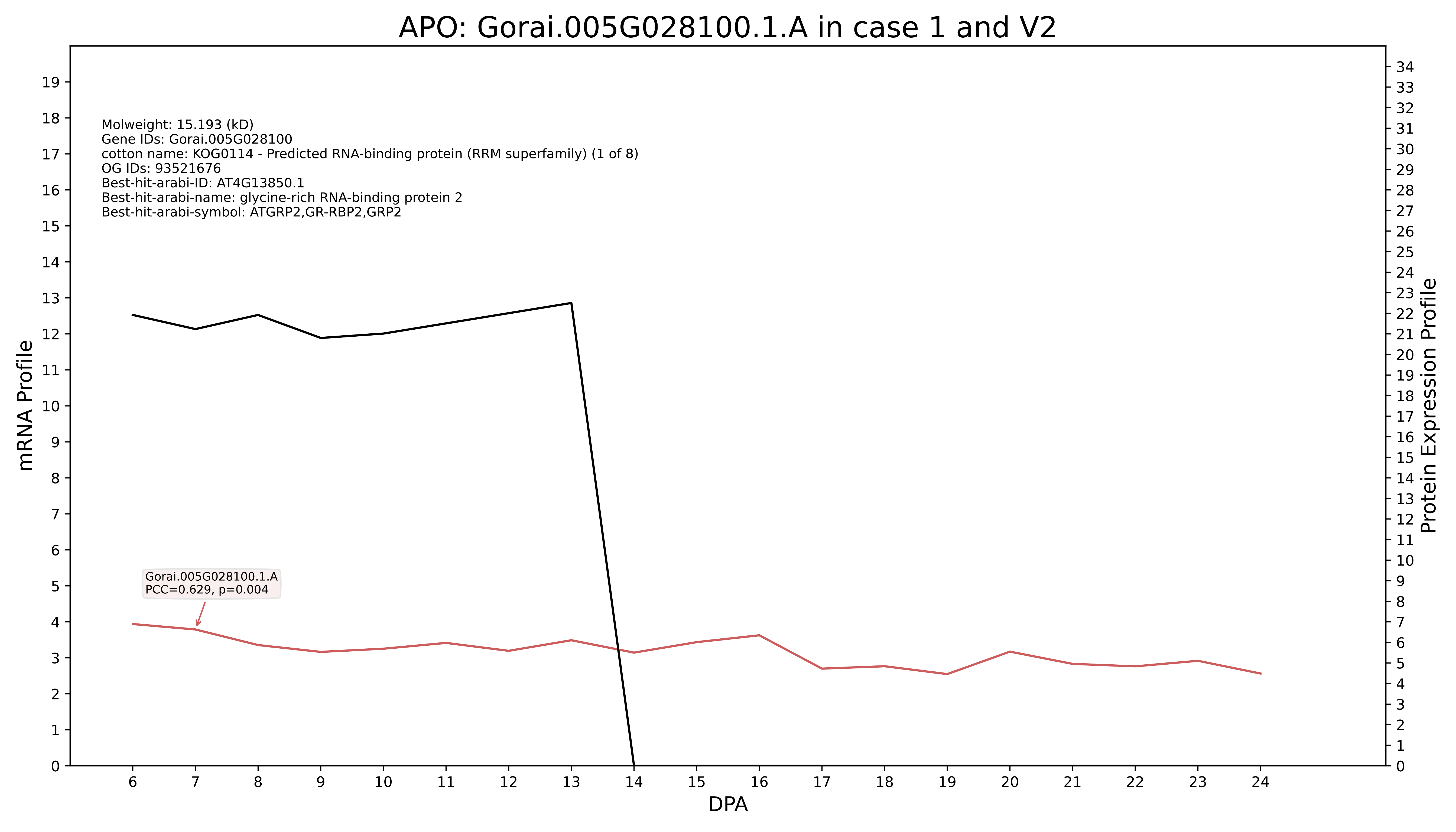
APO: Gorai.005G028100.1.A in case 1 and V2
Molweight: 15.193 (kD)
Gene IDs: Gorai.005G028100
cotton name: KOG0114 - Predicted RNA-binding protein (RRM superfamily) (1 of 8)
OG IDs: 93521676
Best-hit-arabi-ID: AT4G13850.1
Best-hit-arabi-name: glycine-rich RNA-binding protein 2
Best-hit-arabi-symbol: ATGRP2,GR-RBP2,GRP2
Gorai.005G028100.1.A
PCC=0.629, p=0.004
mRNA Profile
Protein Expression Profile
DPA

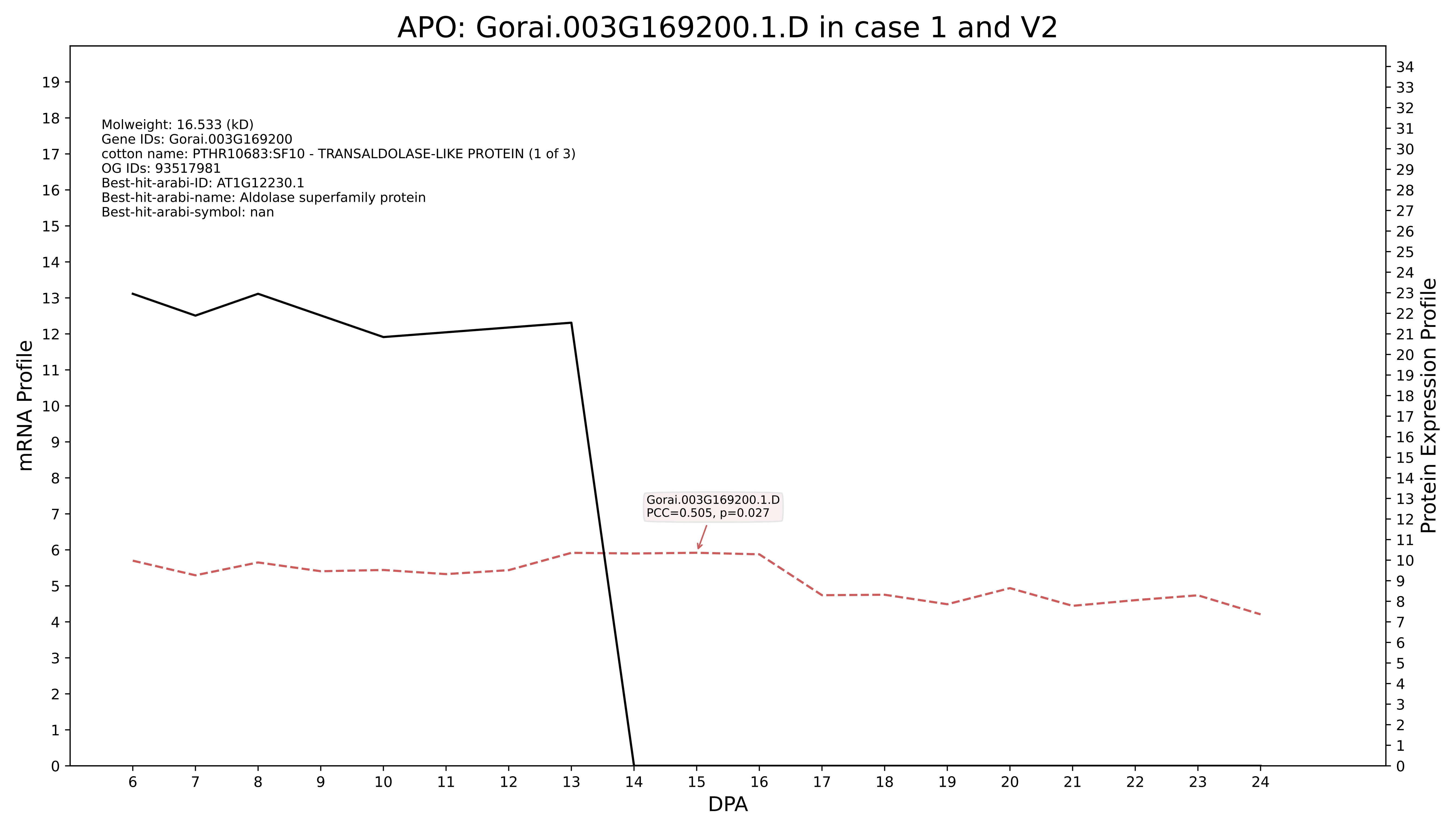
APO: Gorai.003G169200.1.D in case 1 and V2
Molweight: 16.533 (kD)
Gene IDs: Gorai.003G169200
cotton name: PTHR10683:SF10 - TRANSALDOLASE-LIKE PROTEIN (1 of 3)
OG IDs: 93517981
Best-hit-arabi-ID: AT1G12230.1
Best-hit-arabi-name: Aldolase superfamily protein
Best-hit-arabi-symbol: nan
Gorai.003G169200.1.D
PCC=0.505, p=0.027
mRNA Profile
Protein Expression Profile
DPA

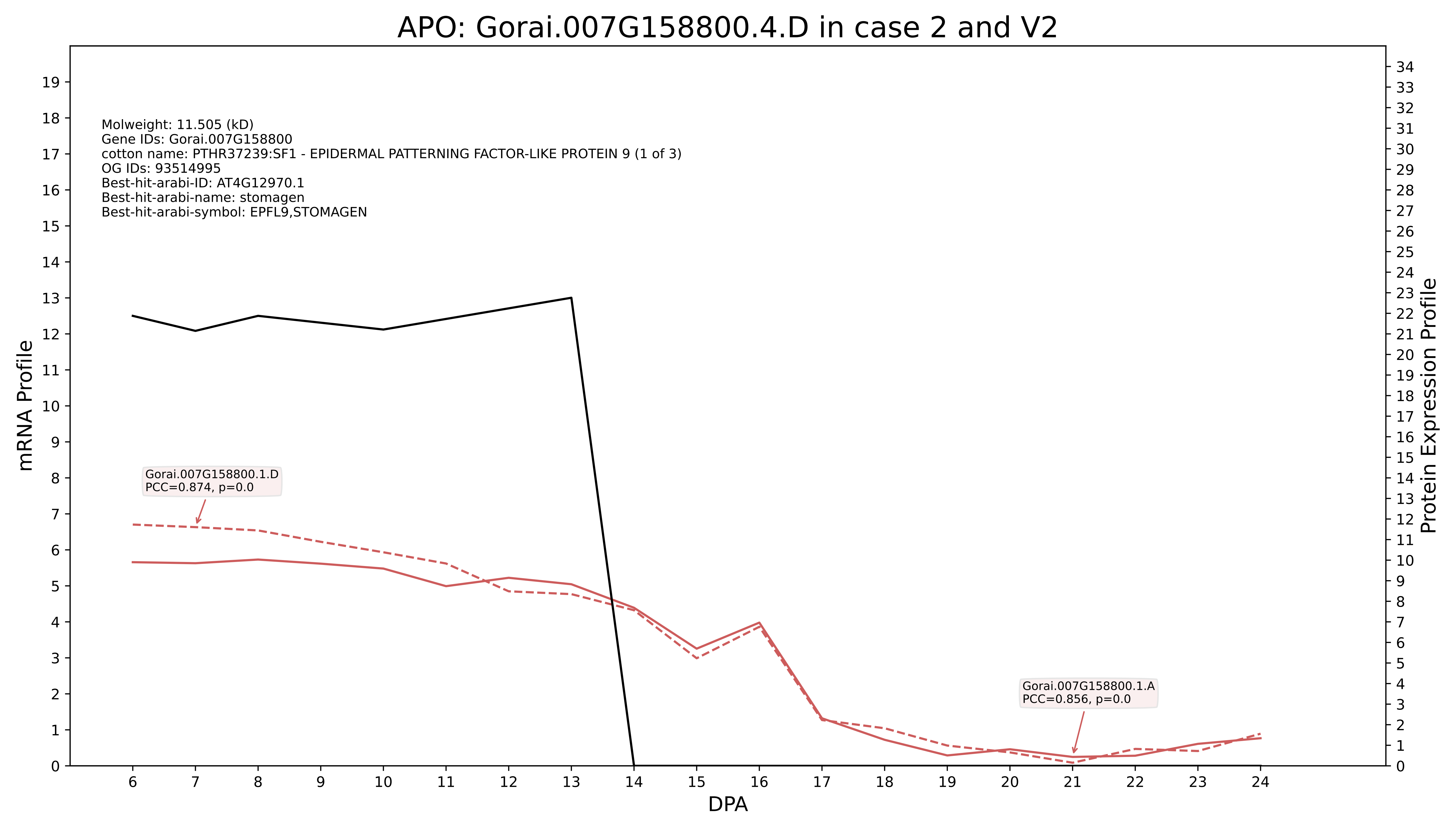

APO: Gorai.007G158800.4.D in case 2 and V2
Molweight: 11.505 (kD)
Gene IDs: Gorai.007G158800
cotton name: PTHR37239:SF1 - EPIDERMAL PATTERNING FACTOR-LIKE PROTEIN 9 (1 of 3)
OG IDs: 93514995
Best-hit-arabi-ID: AT4G12970.1
Best-hit-arabi-name: stomagen
Best-hit-arabi-symbol: EPFL9,STOMAGEN
Gorai.007G158800.1.D
PCC=0.874, p=0.0
Gorai.007G158800.1.A
PCC=0.856, p=0.0
mRNA Profile
Protein Expression Profile
DPA

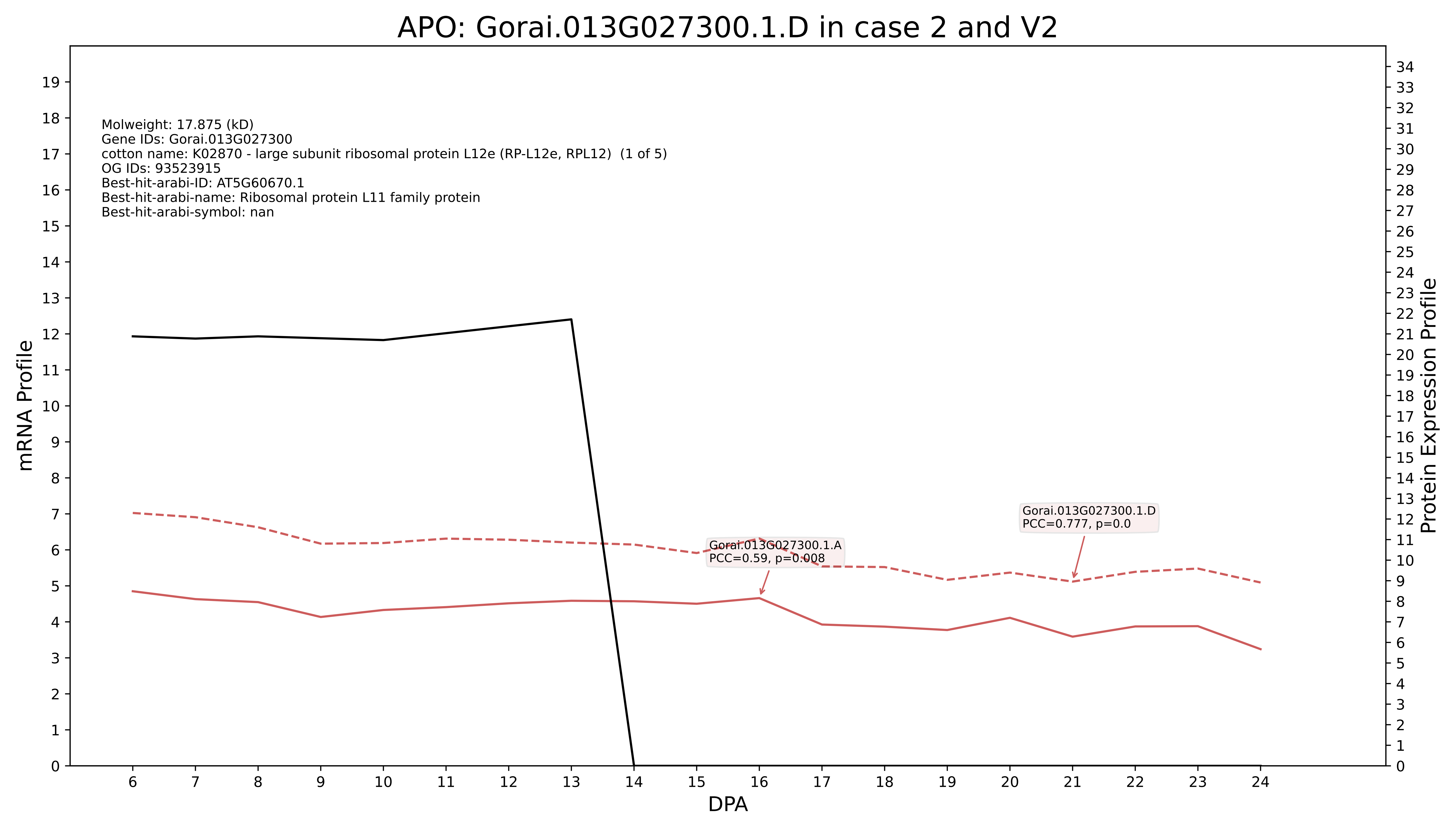

APO: Gorai.013G027300.1.D in case 2 and V2
Molweight: 17.875 (kD)
Gene IDs: Gorai.013G027300
cotton name: K02870 - large subunit ribosomal protein L12e (RP-L12e, RPL12) (1 of 5)
OG IDs: 93523915
Best-hit-arabi-ID: AT5G60670.1
Best-hit-arabi-name: Ribosomal protein L11 family protein
Best-hit-arabi-symbol: nan
Gorai.013G027300.1.A
PCC=0.59, p=0.008
Gorai.013G027300.1.D
PCC=0.777, p=0.0
mRNA Profile
Protein Expression Profile
DPA

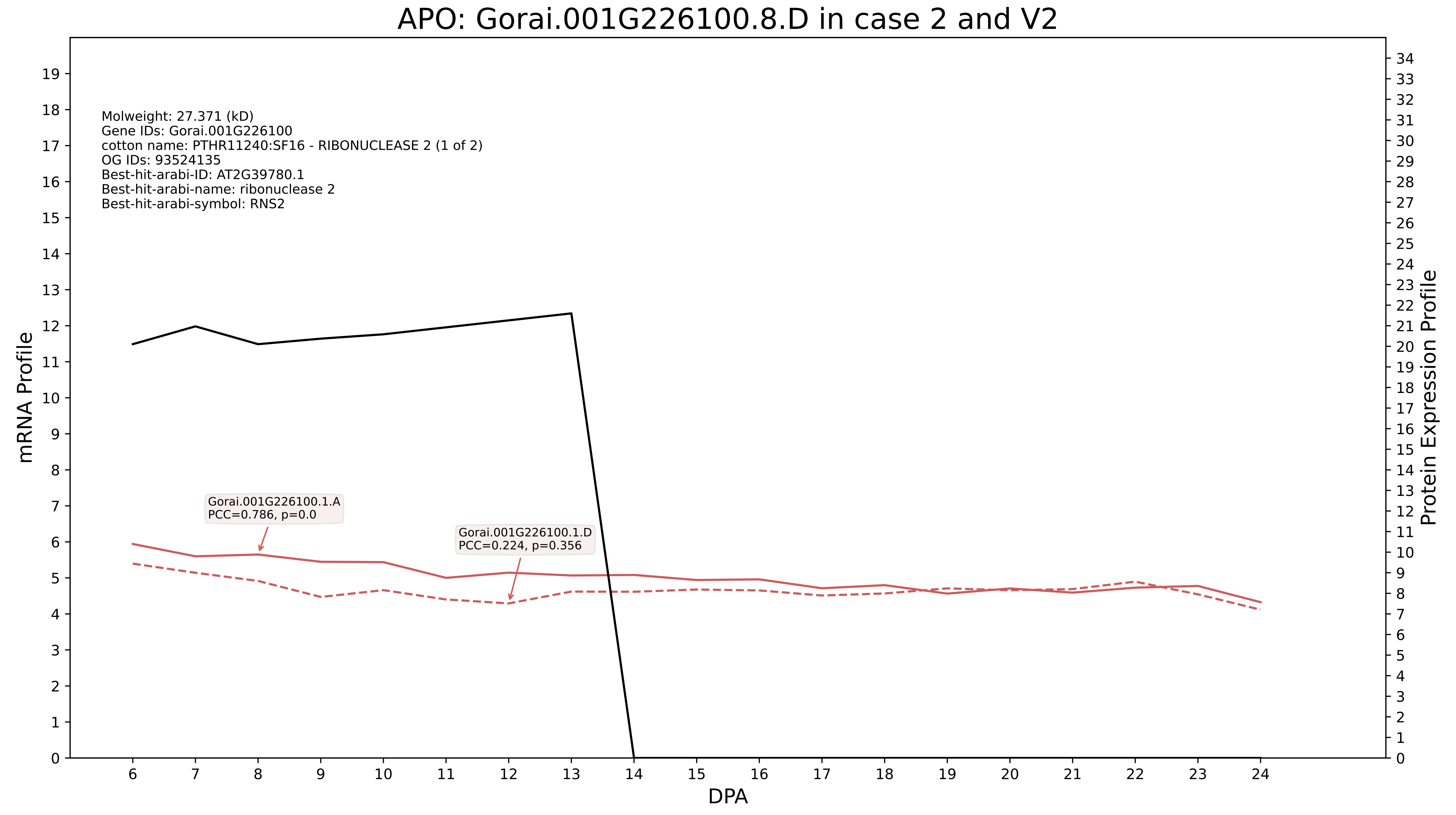
APO: Gorai.001G226100.8.D in case 2 and V2
Molweight: 27.371 (kD)
Gene IDs: Gorai.001G226100
cotton name: PTHR11240:SF16 - RIBONUCLEASE 2 (1 of 2)
OG IDs: 93524135
Best-hit-arabi-ID: AT2G39780.1
Best-hit-arabi-name: ribonuclease 2
Best-hit-arabi-symbol: RNS2
Gorai.001G226100.1.A
PCC=0.786, p=0.0
Gorai.001G226100.1.D
PCC=0.224, p=0.356
mRNA Profile
Protein Expression Profile
DPA
0
1
2
3
4
5
6
7
8
9
10
11
12
13
14
15
16
17
18
19
20
21
22
23
24
25
26
27
28
29
30
31
32
33
34

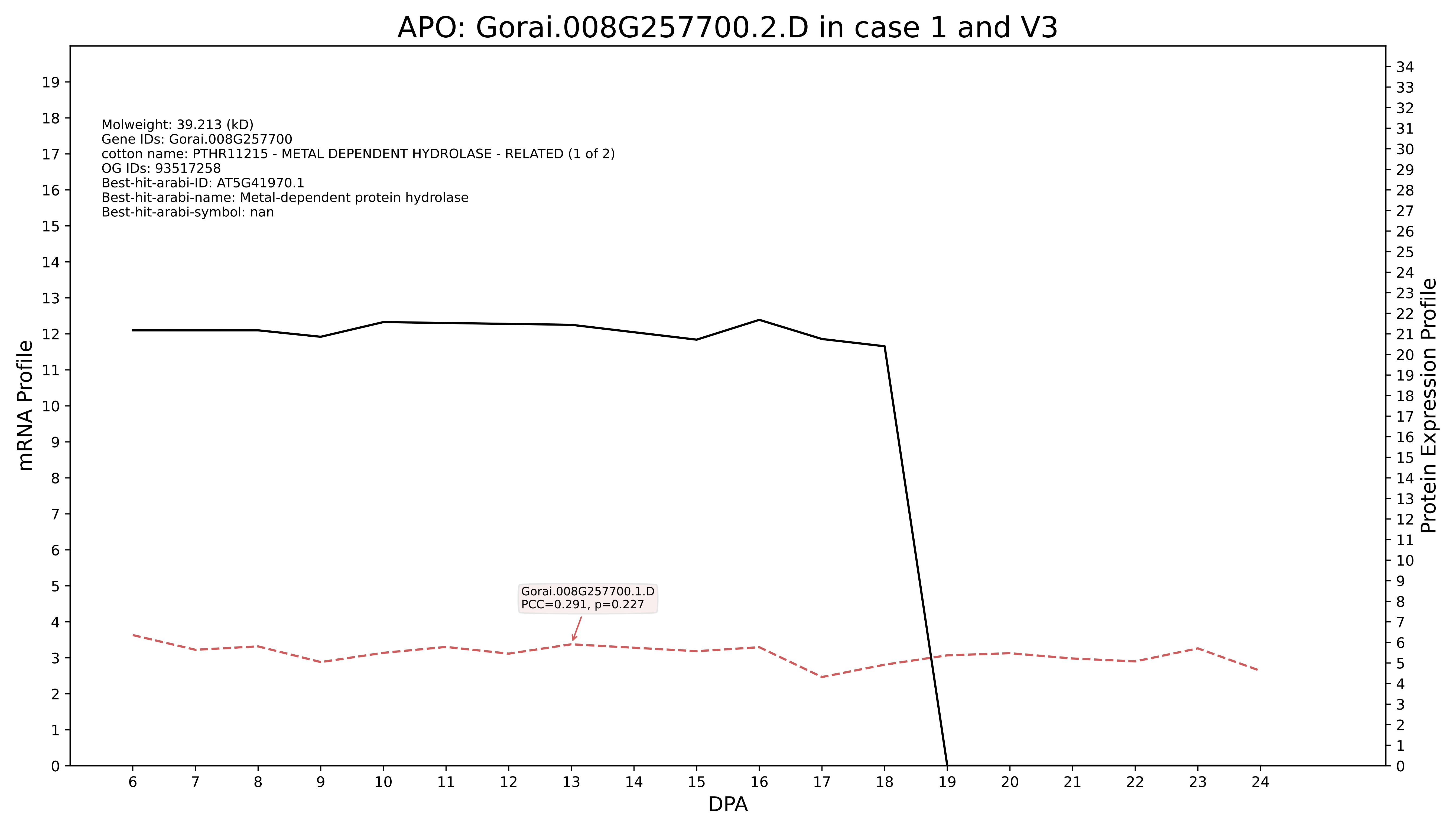

APO: Gorai.008G257700.2.D in case 1 and V3
Molweight: 39.213 (kD)
Gene IDs: Gorai.008G257700
cotton name: PTHR11215 - METAL DEPENDENT HYDROLASE - RELATED (1 of 2)
OG IDs: 93517258
Best-hit-arabi-ID: AT5G41970.1
Best-hit-arabi-name: Metal-dependent protein hydrolase
Best-hit-arabi-symbol: nan
Gorai.008G257700.1.D
PCC=0.291, p=0.227
mRNA Profile
Protein Expression Profile
DPA

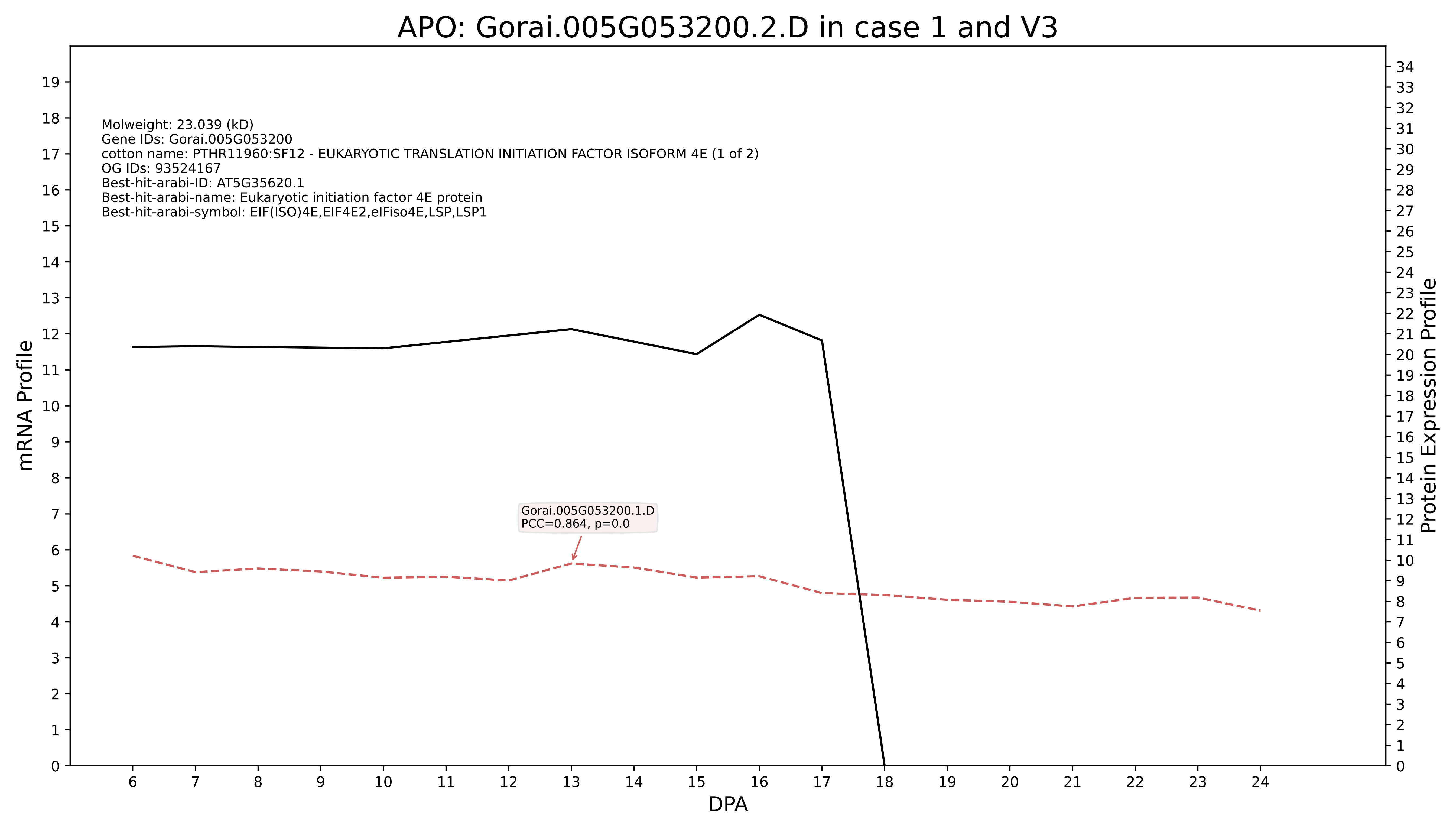

APO: Gorai.005G053200.2.D in case 1 and V3
Molweight: 23.039 (kD)
Gene IDs: Gorai.005G053200
cotton name: PTHR11960:SF12 - EUKARYOTIC TRANSLATION INITIATION FACTOR ISOFORM 4E (1 of 2)
OG IDs: 93524167
Best-hit-arabi-ID: AT5G35620.1
Best-hit-arabi-name: Eukaryotic initiation factor 4E protein
Best-hit-arabi-symbol: EIF(ISO)4E,EIF4E2,eIFiso4E,LSP,LSP1
Gorai.005G053200.1.D
PCC=0.864, p=0.0
mRNA Profile
Protein Expression Profile
DPA

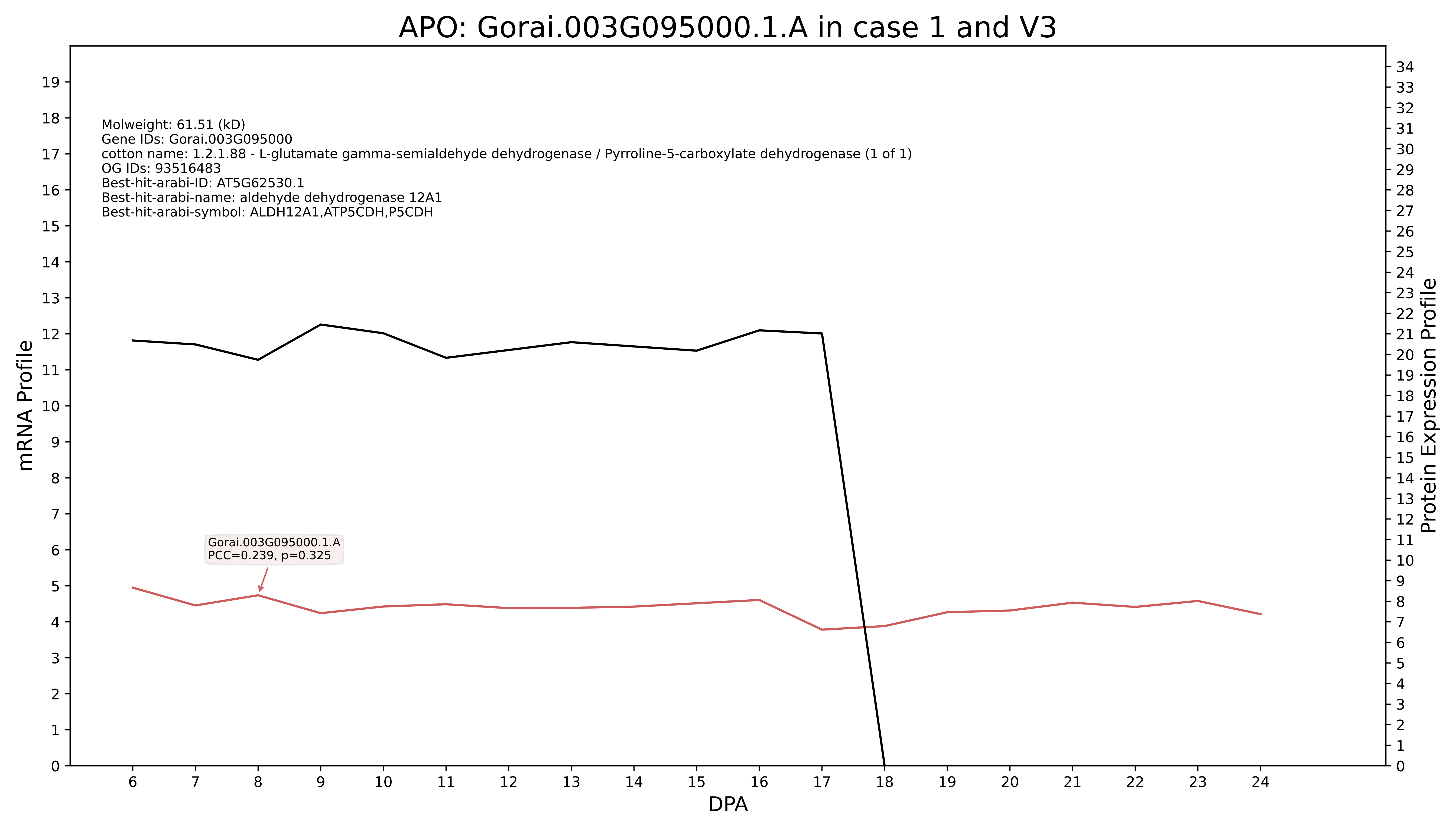
APO: Gorai.003G095000.1.A in case 1 and V3
Molweight: 61.51 (kD)
Gene IDs: Gorai.003G095000
cotton name: 1.2.1.88 - L-glutamate gamma-semialdehyde dehydrogenase / Pyrroline-5-carboxylate dehydrogenase (1 of 1)
OG IDs: 93516483
Best-hit-arabi-ID: AT5G62530.1
Best-hit-arabi-name: aldehyde dehydrogenase 12A1
Best-hit-arabi-symbol: ALDH12A1,ATP5CDH,P5CDH
Gorai.003G095000.1.A
PCC=0.239, p=0.325
mRNA Profile
Protein Expression Profile
DPA
0 1 2 3 4 5 6 7 8 9 10 11 12 13 14 15 16 17 18 19
0 1 2 3 4 5 6 7 8 9 10 11 12 13 14 15 16 17 18 19 20 21 22 23 24 25 26 27 28 29 30 31 32 33 34
6 7 8 9 10 11 12 13 14 15 16 17 18 19 20 21 22 23 24

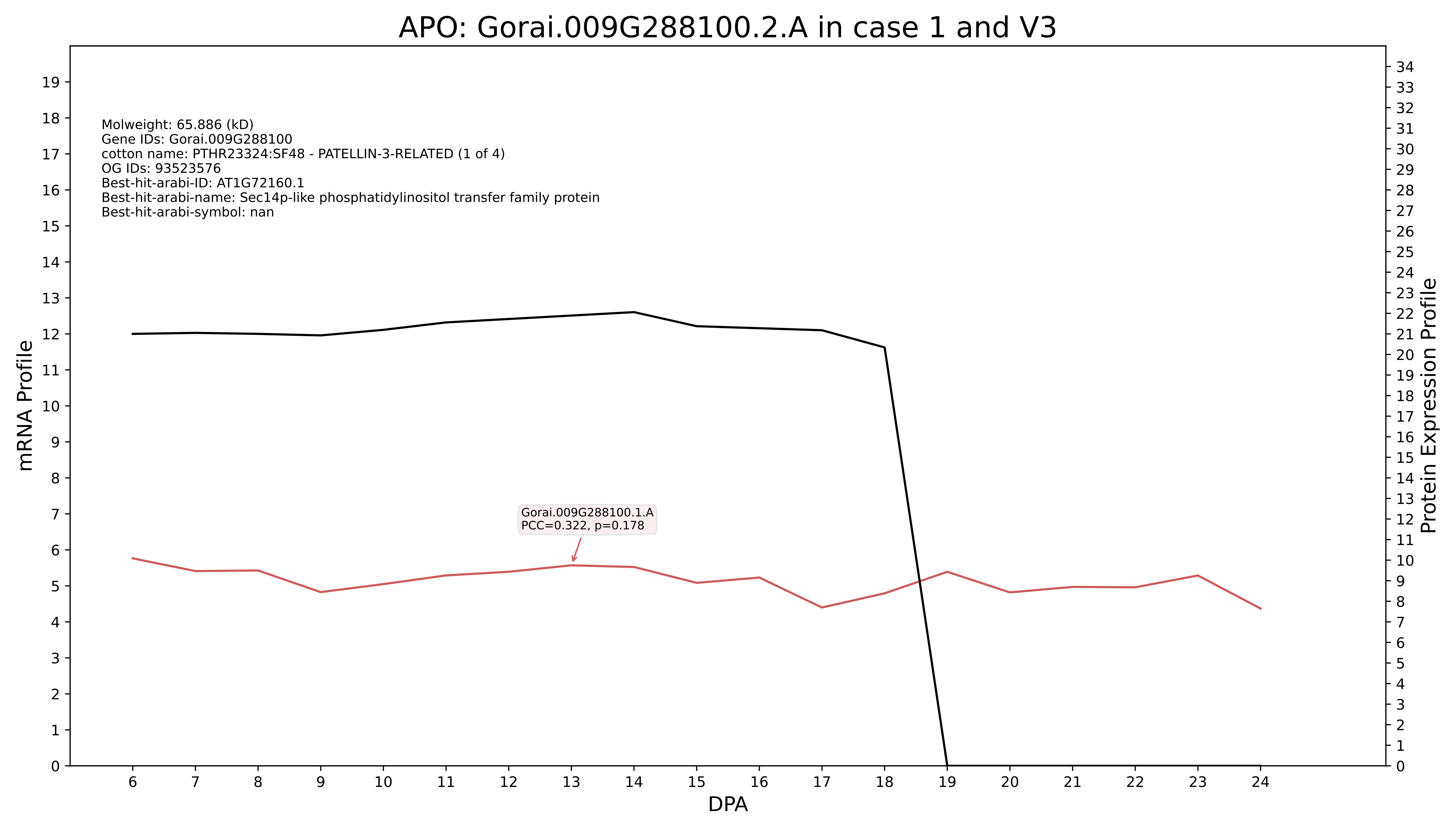
APO: Gorai.009G288100.2.A in case 1 and V3
Molweight: 65.886 (kD)
Gene IDs: Gorai.009G288100
cotton name: PTHR23324:SF48 - PATELLIN-3-RELATED (1 of 4)
OG IDs: 93523576
Best-hit-arabi-ID: AT1G72160.1
Best-hit-arabi-name: Sec14p-like phosphatidylinositol transfer family protein
Best-hit-arabi-symbol: nan
Gorai.009G288100.1.A
PCC=0.322, p=0.178
mRNA Profile
Protein Expression Profile
DPA

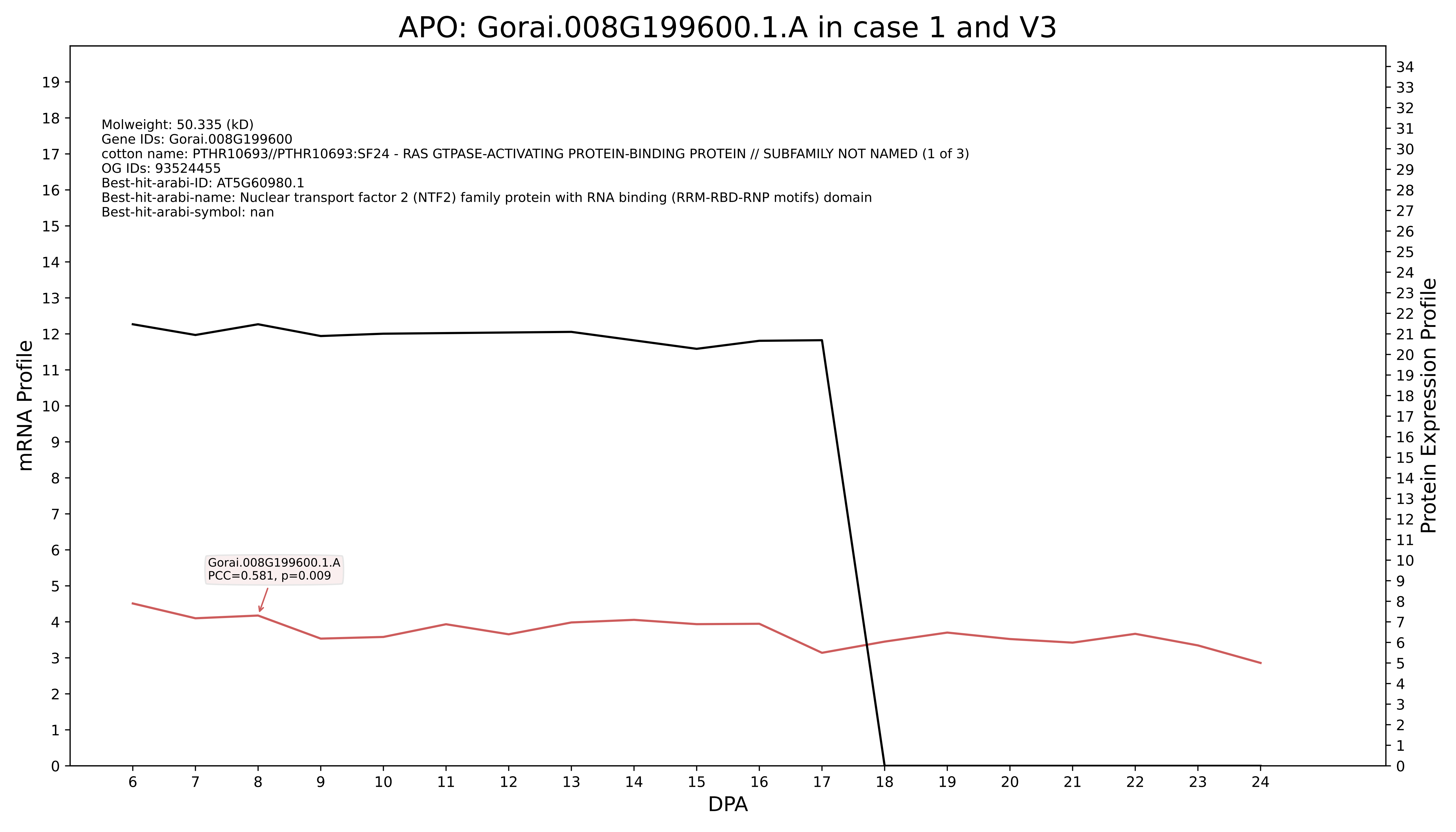
APO: Gorai.008G199600.1.A in case 1 and V3
Molweight: 50.335 (kD)
Gene IDs: Gorai.008G199600
cotton name: PTHR10693//PTHR10693:SF24 - RAS GTPASE-ACTIVATING PROTEIN-BINDING PROTEIN // SUBFAMILY NOT NAMED (1 of 3)
OG IDs: 93524455
Best-hit-arabi-ID: AT5G60980.1
Best-hit-arabi-name: Nuclear transport factor 2 (NTF2) family protein with RNA binding (RRM-RBD-RNP motifs) domain
Best-hit-arabi-symbol: nan
Gorai.008G199600.1.A
PCC=0.581, p=0.009
mRNA Profile
Protein Expression Profile
DPA

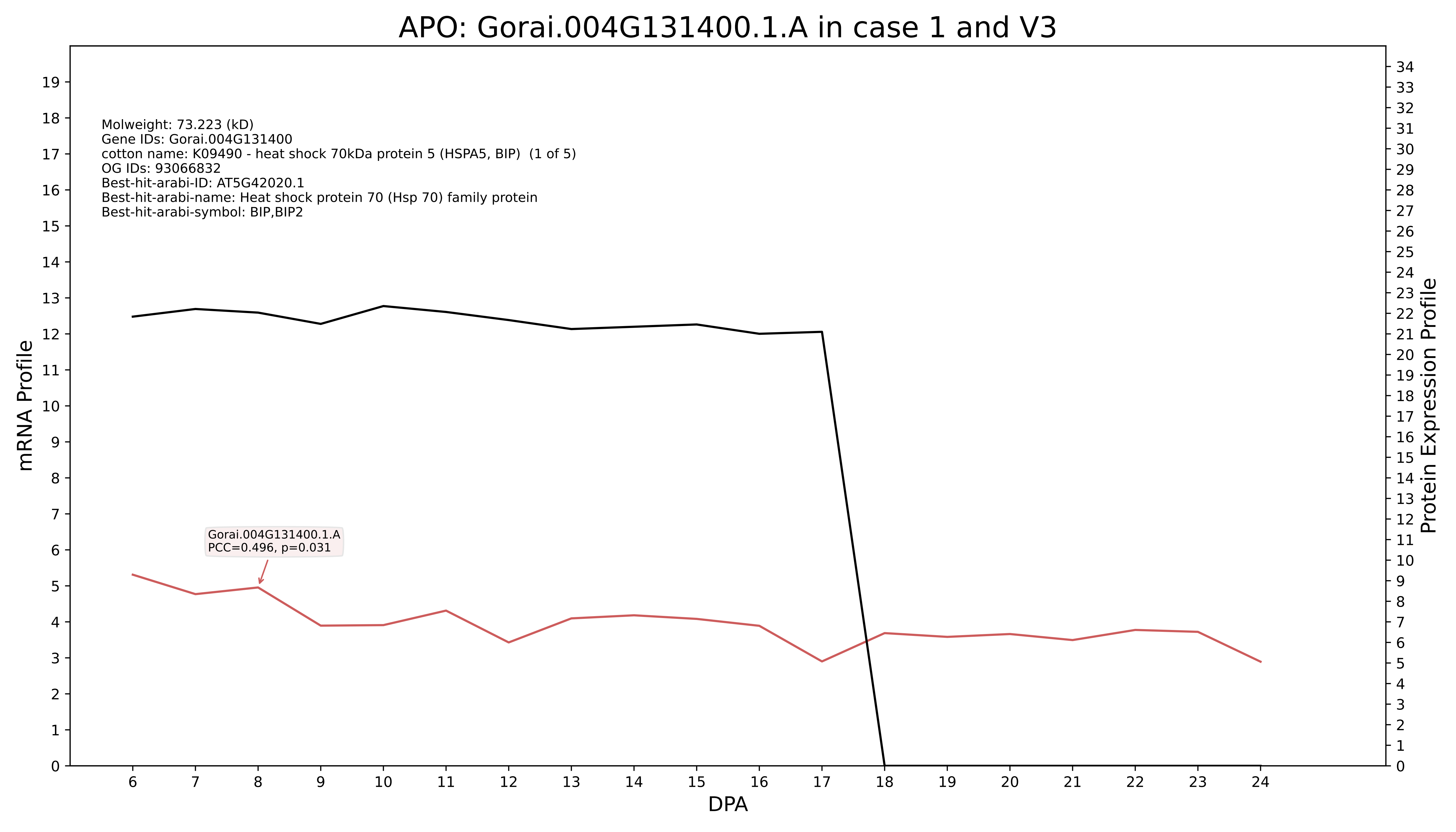

APO: Gorai.004G131400.1.A in case 1 and V3
Molweight: 73.223 (kD)
Gene IDs: Gorai.004G131400
cotton name: K09490 - heat shock 70kDa protein 5 (HSPA5, BIP) (1 of 5)
OG IDs: 93066832
Best-hit-arabi-ID: AT5G42020.1
Best-hit-arabi-name: Heat shock protein 70 (Hsp 70) family protein
Best-hit-arabi-symbol: BIP,BIP2
Gorai.004G131400.1.A
PCC=0.496, p=0.031
mRNA Profile
Protein Expression Profile
DPA

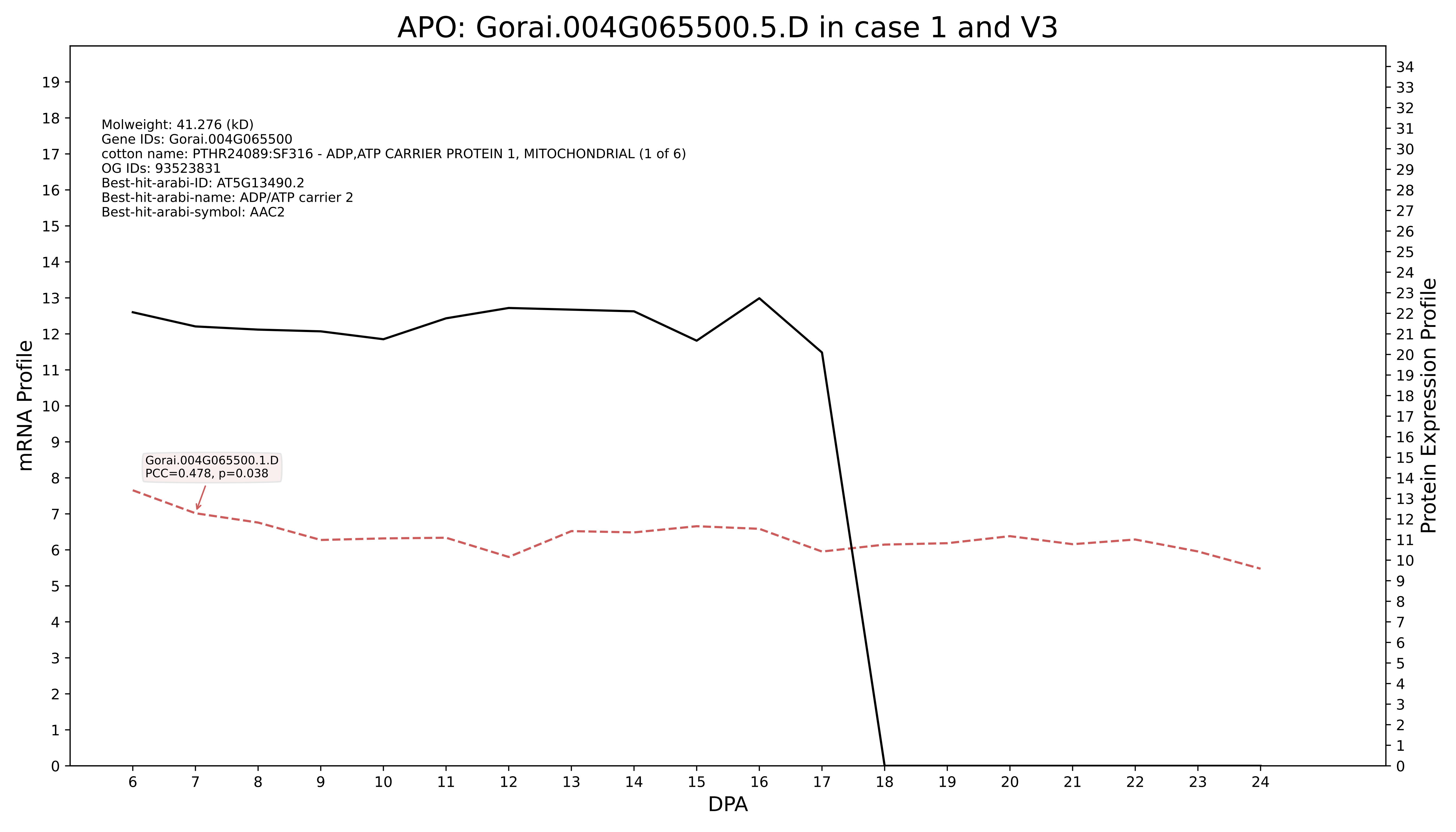
APO: Gorai.004G065500.5.D in case 1 and V3
Molweight: 41.276 (kD)
Gene IDs: Gorai.004G065500
cotton name: PTHR24089:SF316 - ADP,ATP CARRIER PROTEIN 1, MITOCHONDRIAL (1 of 6)
OG IDs: 93523831
Best-hit-arabi-ID: AT5G13490.2
Best-hit-arabi-name: ADP/ATP carrier 2
Best-hit-arabi-symbol: AAC2
Gorai.004G065500.1.D
PCC=0.478, p=0.038
mRNA Profile
Protein Expression Profile
DPA

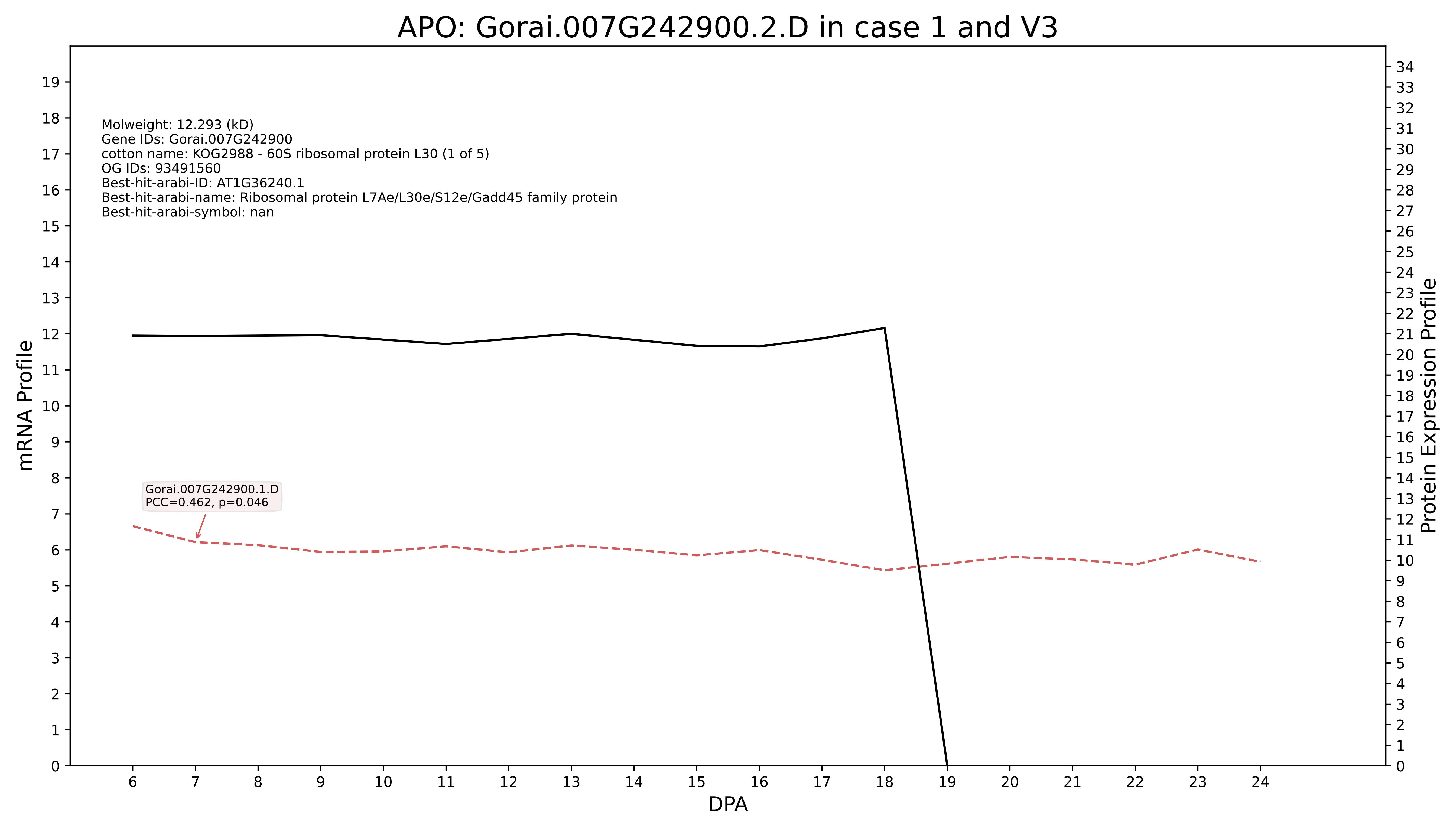
APO: Gorai.007G242900.2.D in case 1 and V3
Molweight: 12.293 (kD)
Gene IDs: Gorai.007G242900
cotton name: KOG2988 - 60S ribosomal protein L30 (1 of 5)
OG IDs: 93491560
Best-hit-arabi-ID: AT1G36240.1
Best-hit-arabi-name: Ribosomal protein L7Ae/L30e/S12e/Gadd45 family protein
Best-hit-arabi-symbol: nan
Gorai.007G242900.1.D
PCC=0.462, p=0.046
mRNA Profile
Protein Expression Profile
DPA

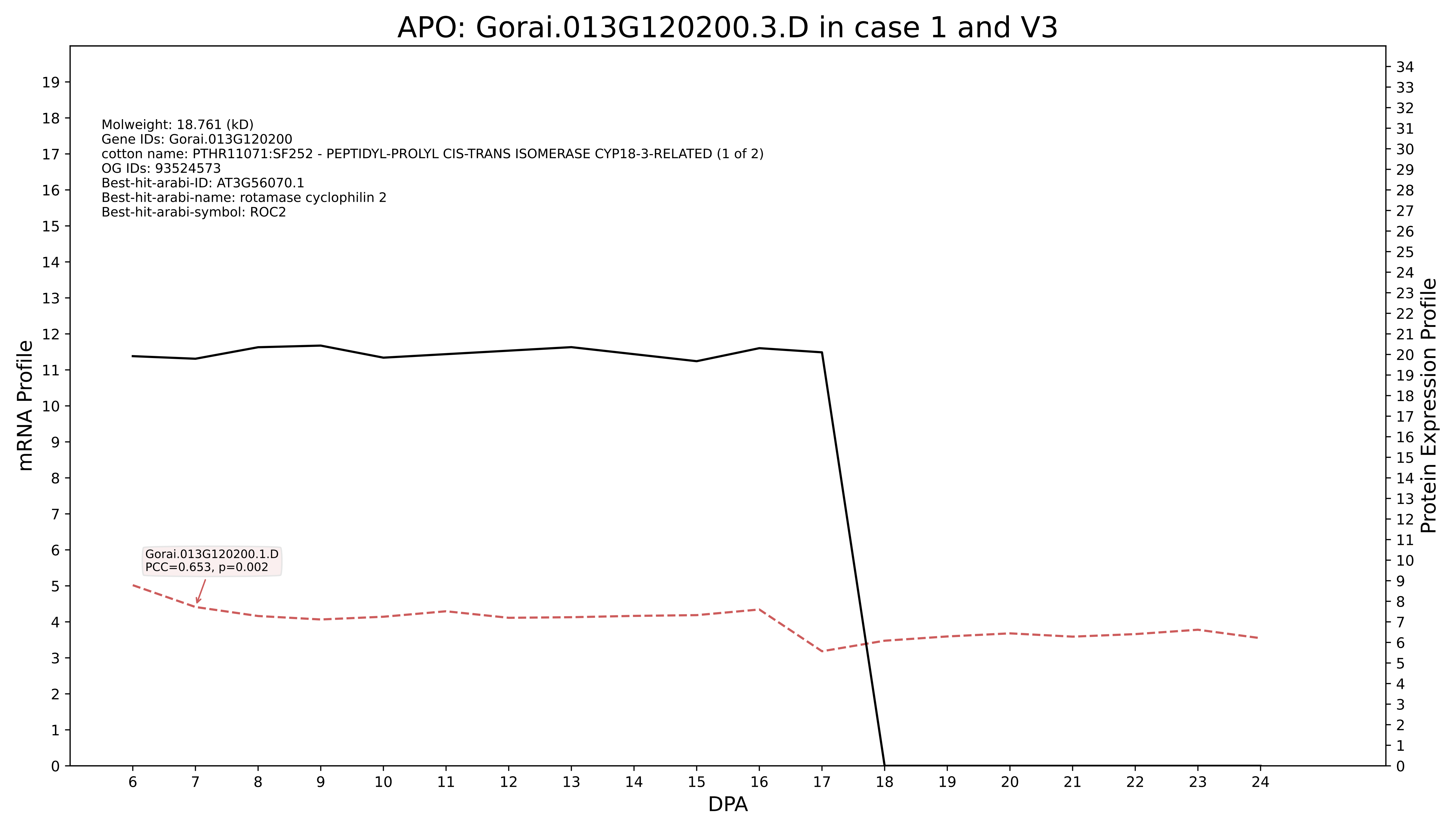

APO: Gorai.013G120200.3.D in case 1 and V3
Molweight: 18.761 (kD)
Gene IDs: Gorai.013G120200
cotton name: PTHR11071:SF252 - PEPTIDYL-PROLYL CIS-TRANS ISOMERASE CYP18-3-RELATED (1 of 2)
OG IDs: 93524573
Best-hit-arabi-ID: AT3G56070.1
Best-hit-arabi-name: rotamase cyclophilin 2
Best-hit-arabi-symbol: ROC2
Gorai.013G120200.1.D
PCC=0.653, p=0.002
mRNA Profile
Protein Expression Profile
DPA

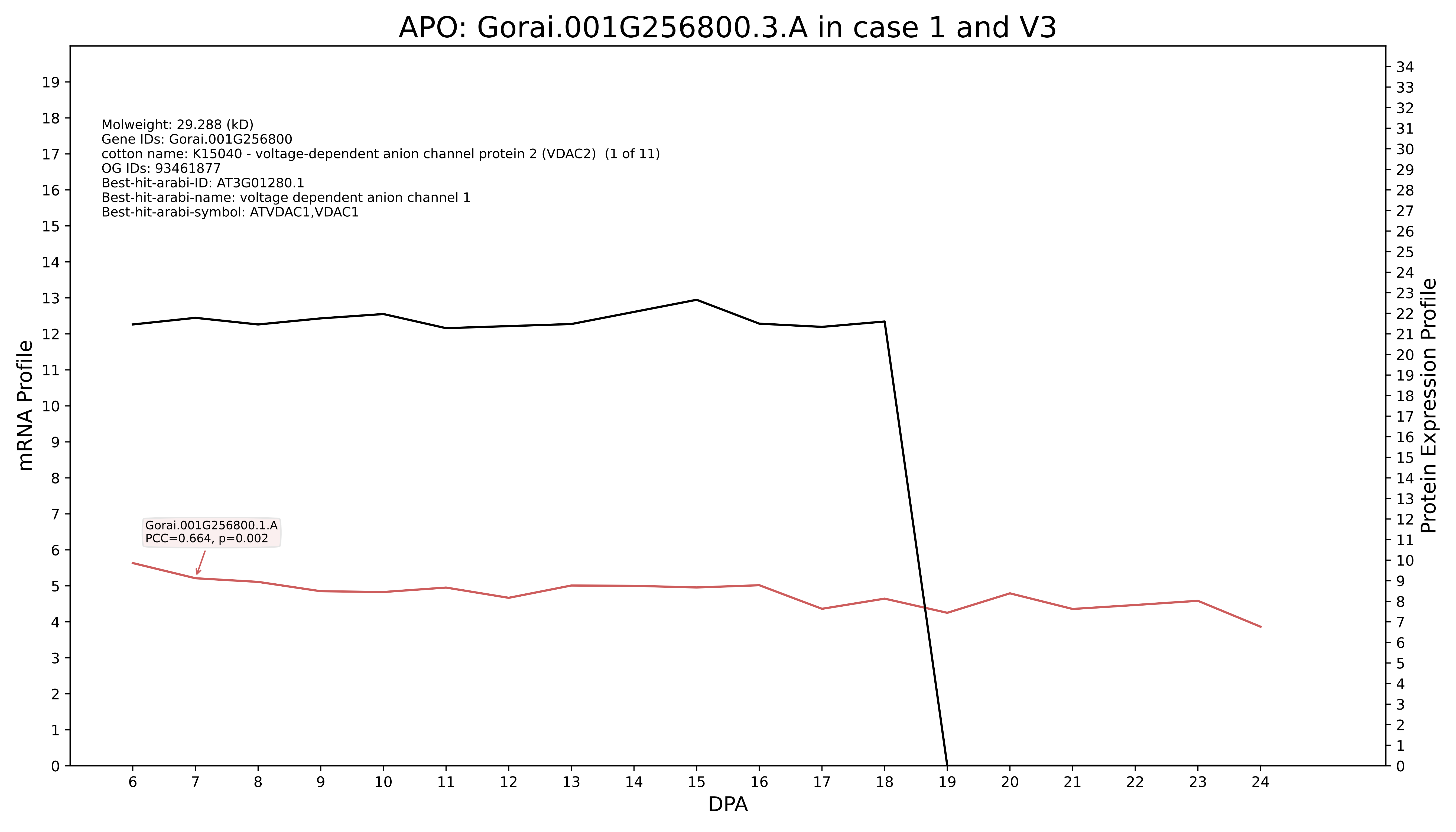
APO: Gorai.001G256800.3.A in case 1 and V3
Molweight: 29.288 (kD)
Gene IDs: Gorai.001G256800
cotton name: K15040 - voltage-dependent anion channel protein 2 (VDAC2) (1 of 11)
OG IDs: 93461877
Best-hit-arabi-ID: AT3G01280.1
Best-hit-arabi-name: voltage dependent anion channel 1
Best-hit-arabi-symbol: ATVDAC1,VDAC1
Gorai.001G256800.1.A
PCC=0.664, p=0.002
mRNA Profile
Protein Expression Profile
DPA

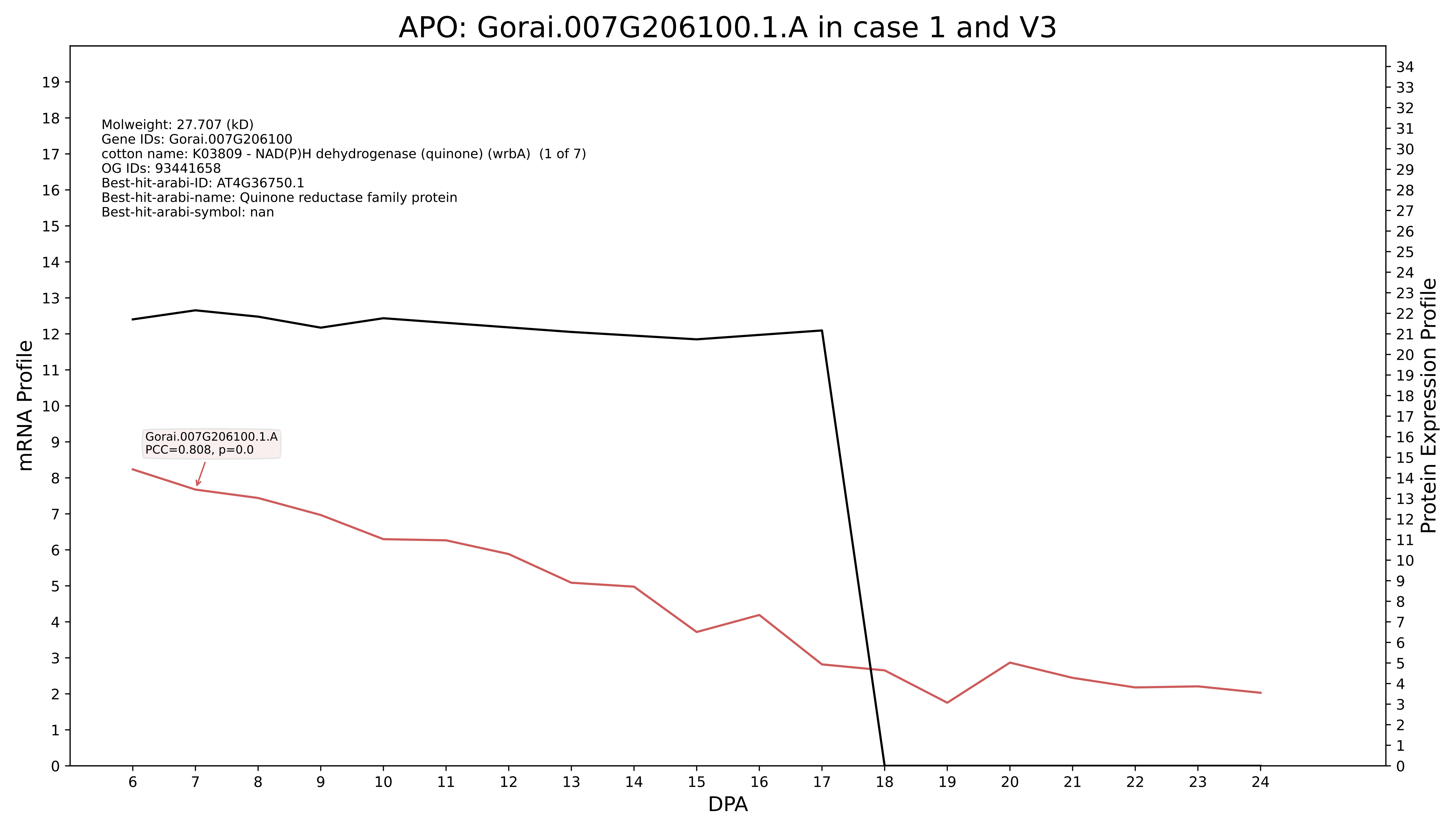
APO: Gorai.007G206100.1.A in case 1 and V3
Molweight: 27.707 (kD)
Gene IDs: Gorai.007G206100
cotton name: K03809 - NAD(P)H dehydrogenase (quinone) (wrbA) (1 of 7)
OG IDs: 93441658
Best-hit-arabi-ID: AT4G36750.1
Best-hit-arabi-name: Quinone reductase family protein
Best-hit-arabi-symbol: nan
Gorai.007G206100.1.A
PCC=0.808, p=0.0
mRNA Profile
Protein Expression Profile
DPA

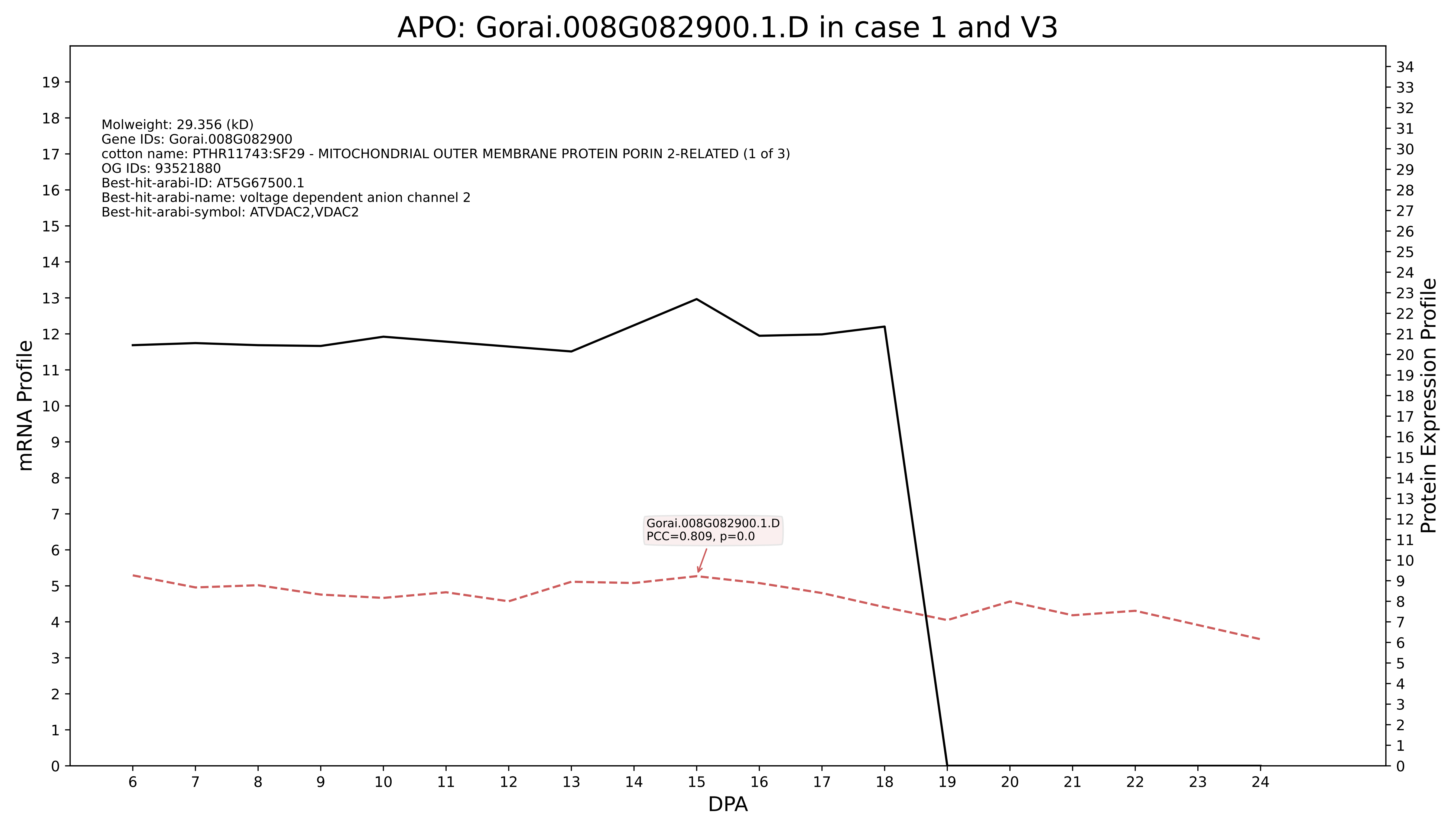
APO: Gorai.008G082900.1.D in case 1 and V3
Molweight: 29.356 (kD)
Gene IDs: Gorai.008G082900
cotton name: PTHR11743:SF29 - MITOCHONDRIAL OUTER MEMBRANE PROTEIN PORIN 2-RELATED (1 of 3)
OG IDs: 93521880
Best-hit-arabi-ID: AT5G67500.1
Best-hit-arabi-name: voltage dependent anion channel 2
Best-hit-arabi-symbol: ATVDAC2,VDAC2
Gorai.008G082900.1.D
PCC=0.809, p=0.0
mRNA Profile
Protein Expression Profile
DPA

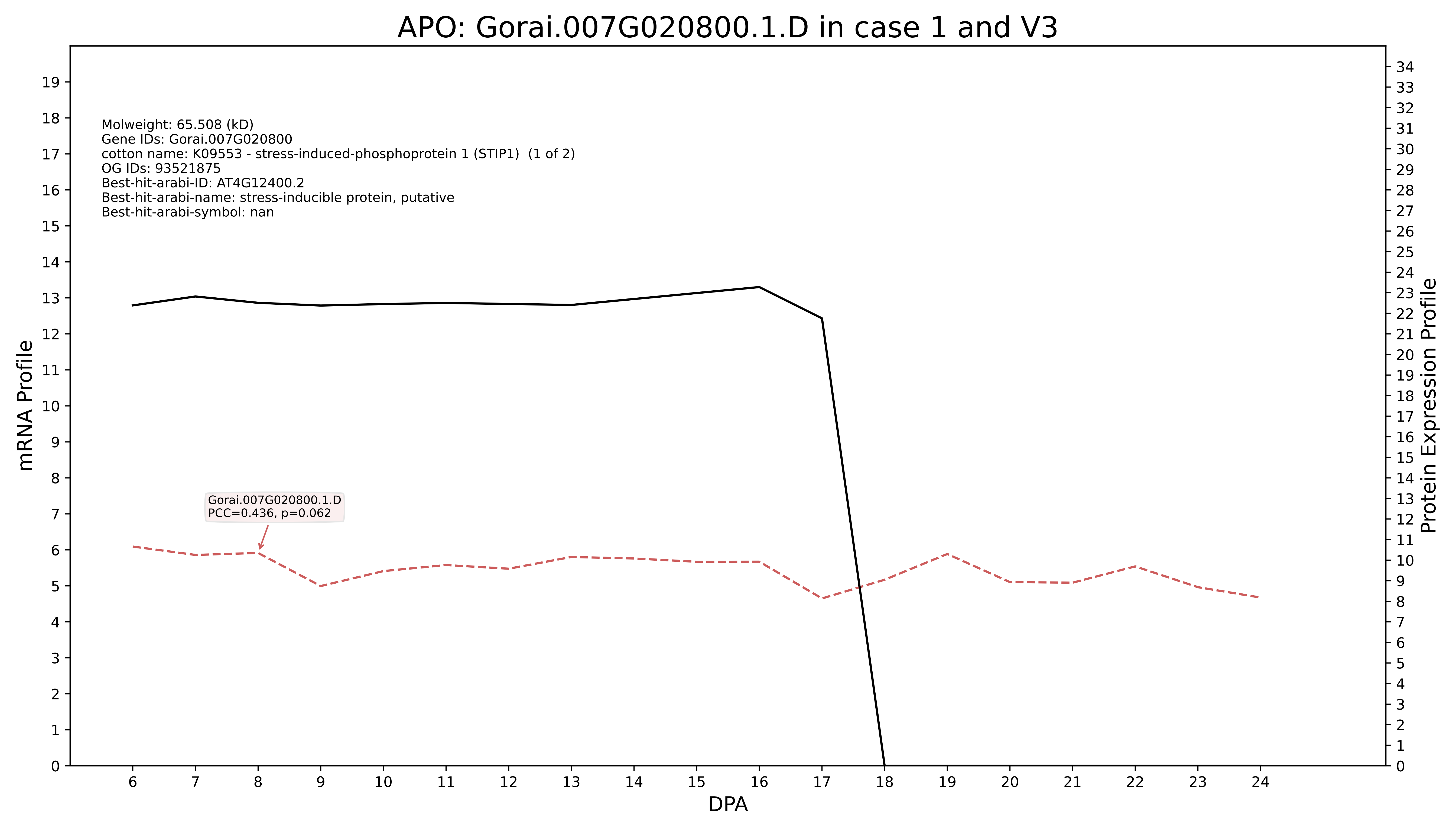

APO: Gorai.007G020800.1.D in case 1 and V3
Molweight: 65.508 (kD)
Gene IDs: Gorai.007G020800
cotton name: K09553 - stress-induced-phosphoprotein 1 (STIP1) (1 of 2)
OG IDs: 93521875
Best-hit-arabi-ID: AT4G12400.2
Best-hit-arabi-name: stress-inducible protein, putative
Best-hit-arabi-symbol: nan
Gorai.007G020800.1.D
PCC=0.436, p=0.062
mRNA Profile
Protein Expression Profile
DPA

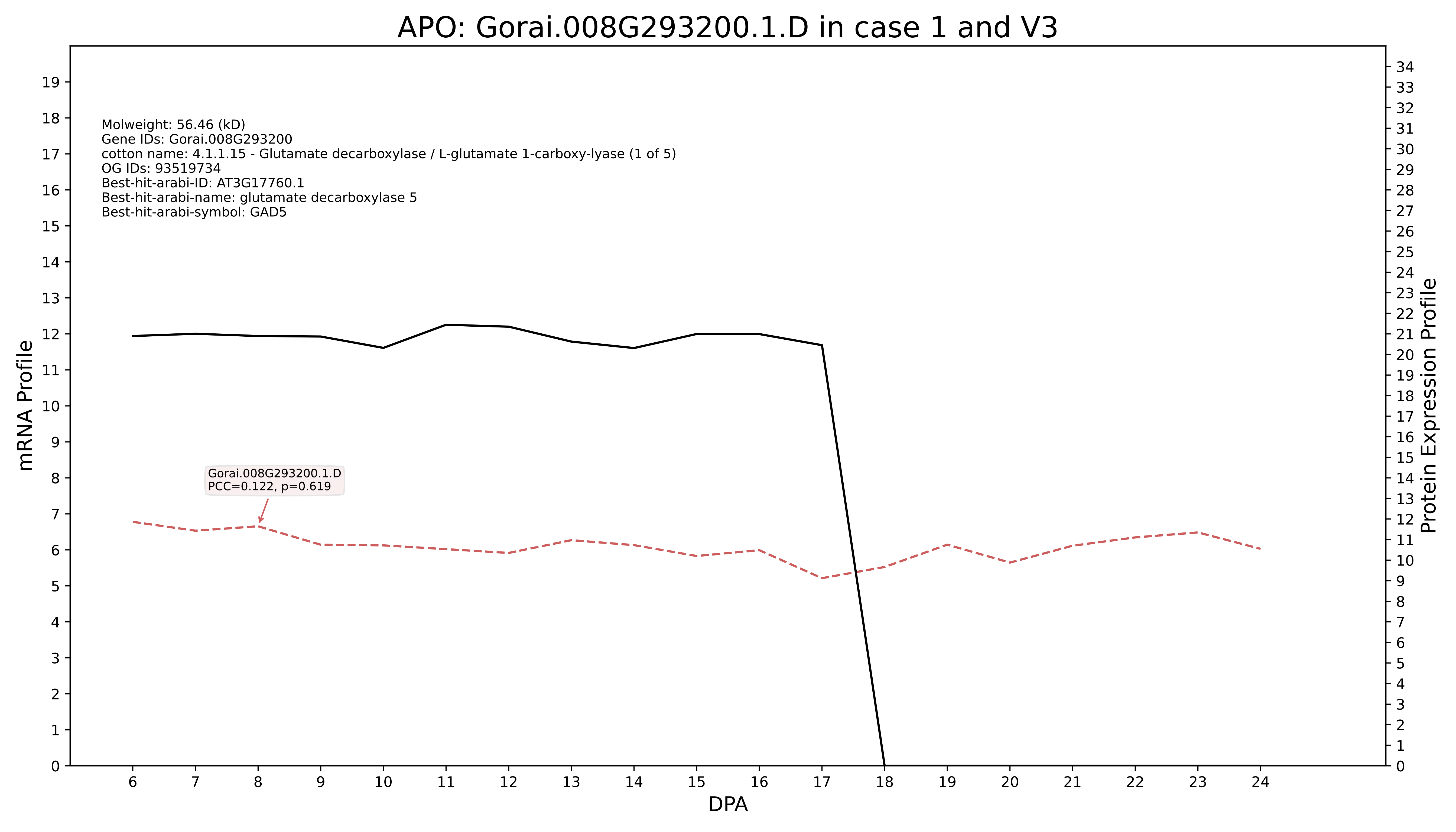
APO: Gorai.008G293200.1.D in case 1 and V3
Molweight: 56.46 (kD)
Gene IDs: Gorai.008G293200
cotton name: 4.1.1.15 - Glutamate decarboxylase / L-glutamate 1-carboxy-lyase (1 of 5)
OG IDs: 93519734
Best-hit-arabi-ID: AT3G17760.1
Best-hit-arabi-name: glutamate decarboxylase 5
Best-hit-arabi-symbol: GAD5
Gorai.008G293200.1.D
PCC=0.122, p=0.619
mRNA Profile
Protein Expression Profile
DPA

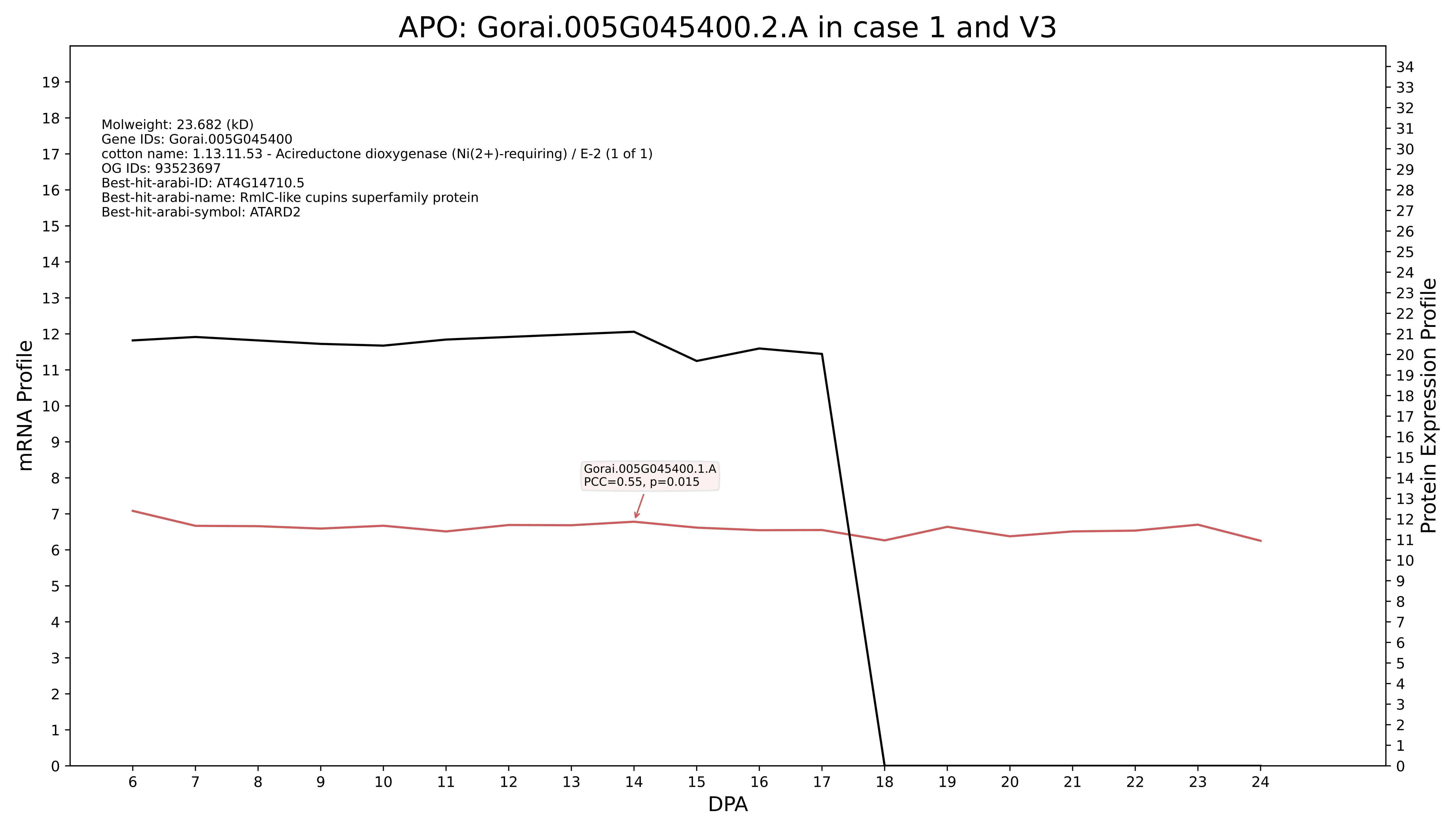
APO: Gorai.005G045400.2.A in case 1 and V3
Molweight: 23.682 (kD)
Gene IDs: Gorai.005G045400
cotton name: 1.13.11.53 - Acireductone dioxygenase (Ni(2+)-requiring) / E-2 (1 of 1)
OG IDs: 93523697
Best-hit-arabi-ID: AT4G14710.5
Best-hit-arabi-name: RmlC-like cupins superfamily protein
Best-hit-arabi-symbol: ATARD2
Gorai.005G045400.1.A
PCC=0.55, p=0.015
mRNA Profile
Protein Expression Profile
DPA

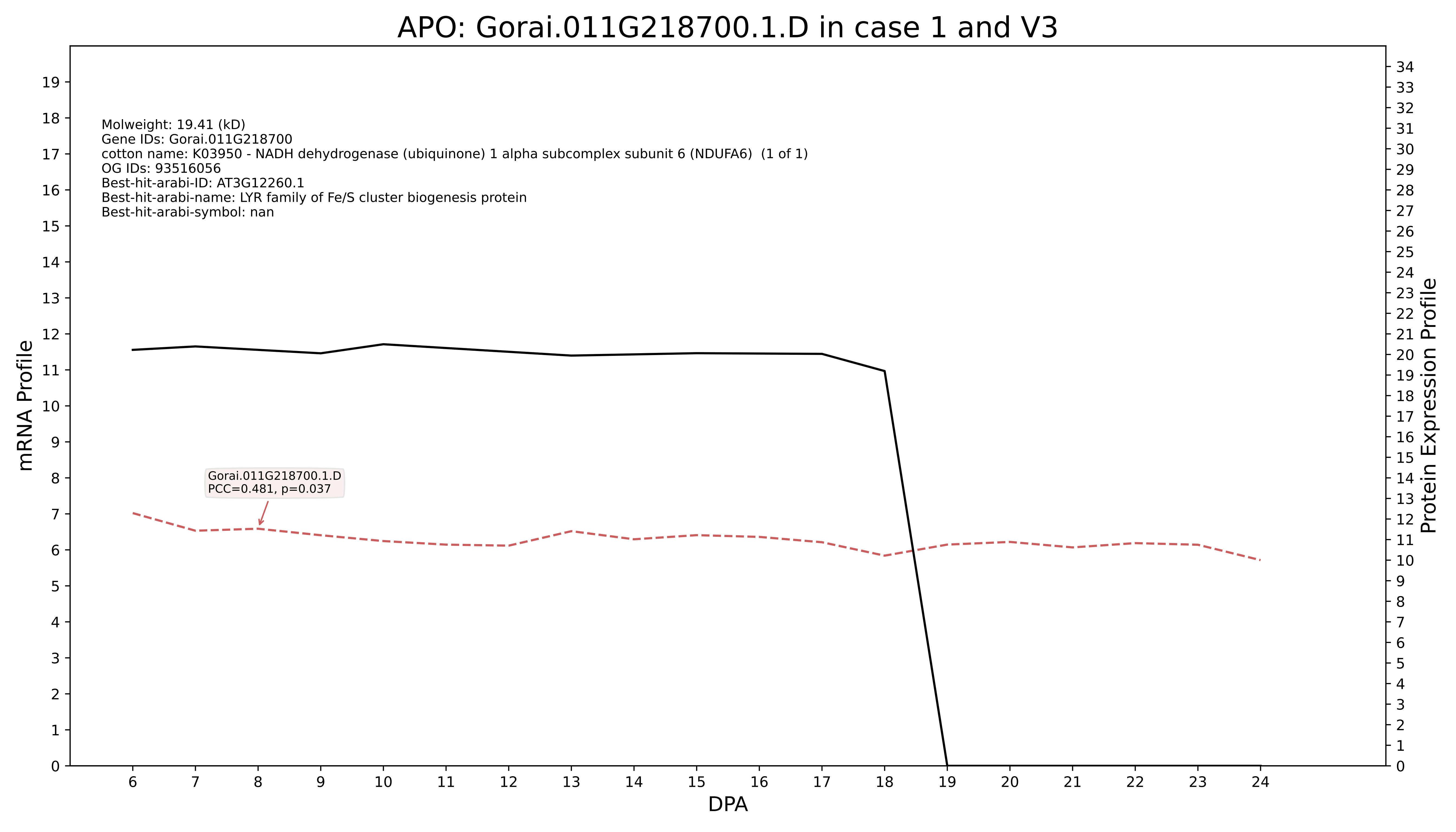
APO: Gorai.011G218700.1.D in case 1 and V3
Molweight: 19.41 (kD)
Gene IDs: Gorai.011G218700
cotton name: K03950 - NADH dehydrogenase (ubiquinone) 1 alpha subcomplex subunit 6 (NDUFA6) (1 of 1)
OG IDs: 93516056
Best-hit-arabi-ID: AT3G12260.1
Best-hit-arabi-name: LYR family of Fe/S cluster biogenesis protein
Best-hit-arabi-symbol: nan
Gorai.011G218700.1.D
PCC=0.481, p=0.037
mRNA Profile
Protein Expression Profile
DPA

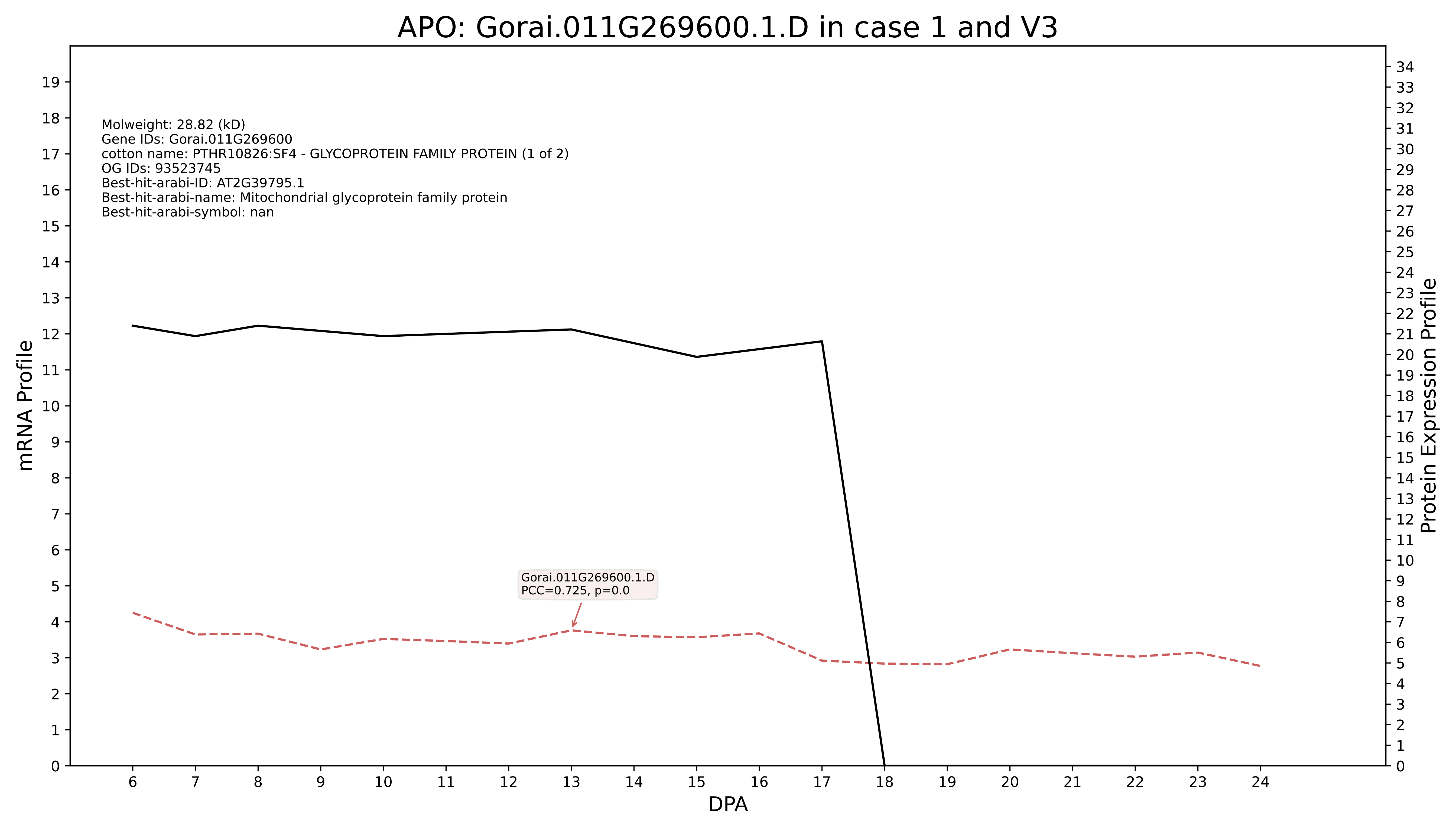

APO: Gorai.011G269600.1.D in case 1 and V3
Molweight: 28.82 (kD)
Gene IDs: Gorai.011G269600
cotton name: PTHR10826:SF4 - GLYCOPROTEIN FAMILY PROTEIN (1 of 2)
OG IDs: 93523745
Best-hit-arabi-ID: AT2G39795.1
Best-hit-arabi-name: Mitochondrial glycoprotein family protein
Best-hit-arabi-symbol: nan
Gorai.011G269600.1.D
PCC=0.725, p=0.0
mRNA Profile
Protein Expression Profile
DPA

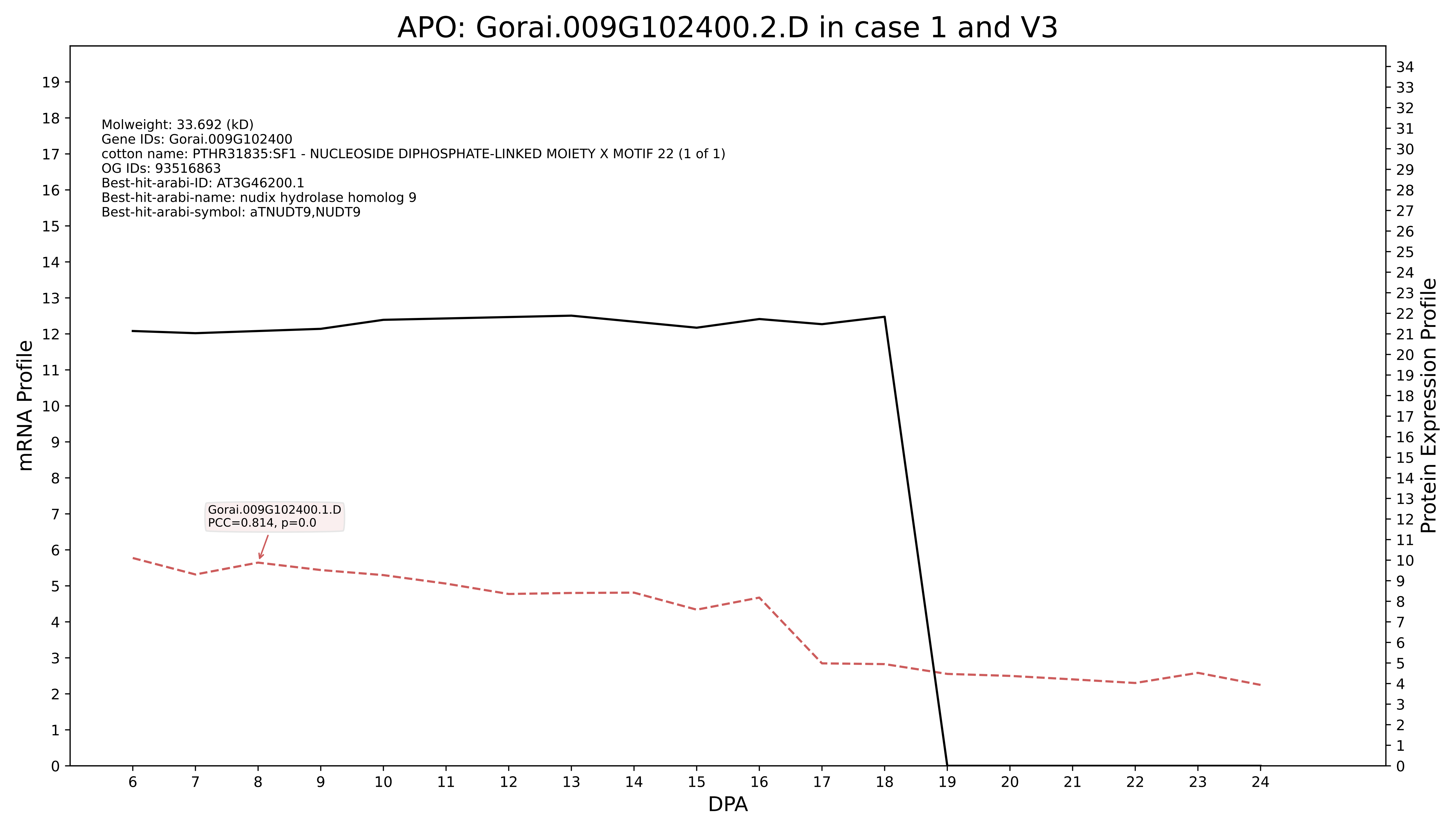

APO: Gorai.009G102400.2.D in case 1 and V3
Molweight: 33.692 (kD)
Gene IDs: Gorai.009G102400
cotton name: PTHR31835:SF1 - NUCLEOSIDE DIPHOSPHATE-LINKED MOIETY X MOTIF 22 (1 of 1)
OG IDs: 93516863
Best-hit-arabi-ID: AT3G46200.1
Best-hit-arabi-name: nudix hydrolase homolog 9
Best-hit-arabi-symbol: aTNUDT9,NUDT9
Gorai.009G102400.1.D
PCC=0.814, p=0.0
mRNA Profile
Protein Expression Profile
DPA

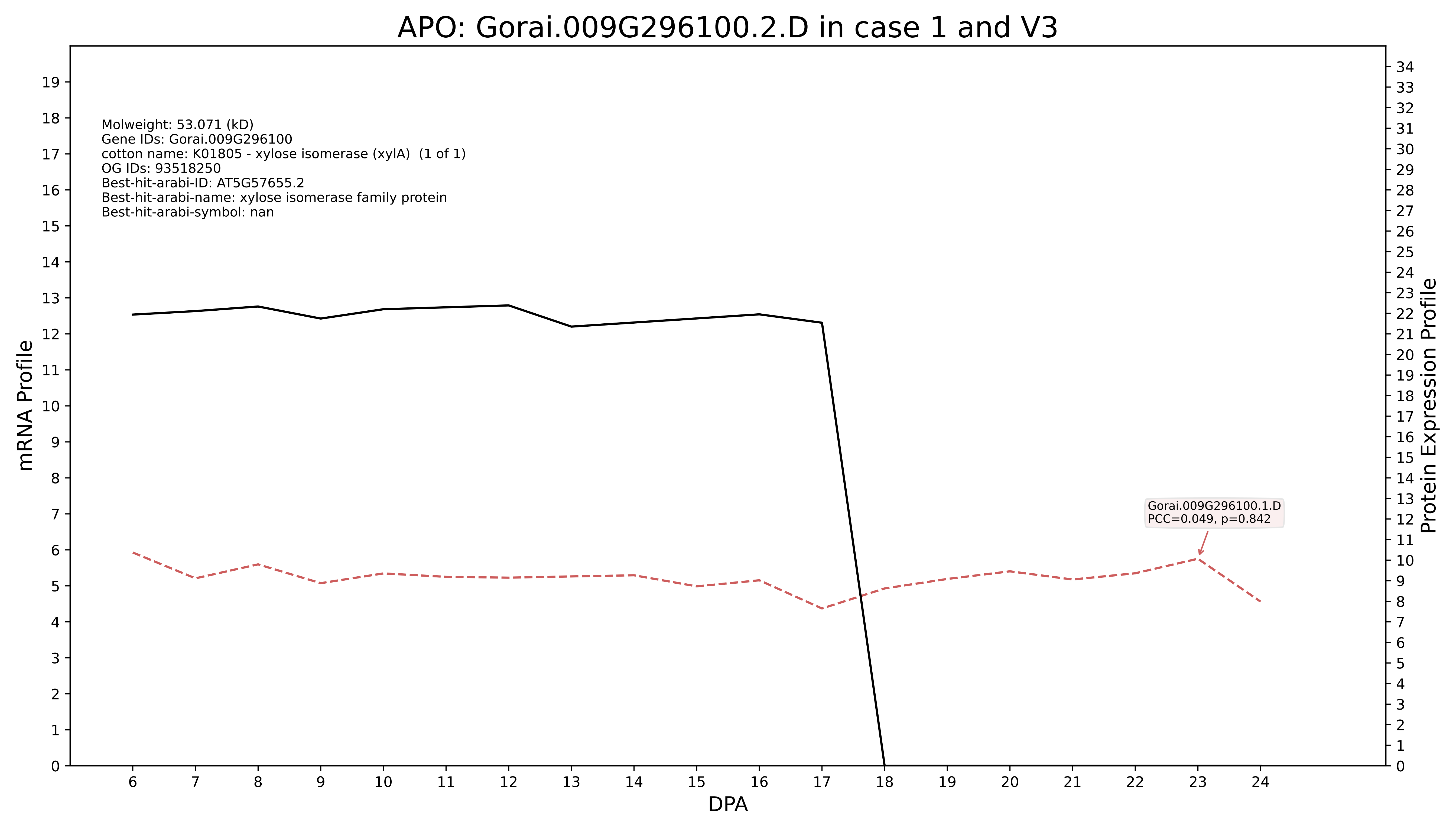
APO: Gorai.009G296100.2.D in case 1 and V3
Molweight: 53.071 (kD)
Gene IDs: Gorai.009G296100
cotton name: K01805 - xylose isomerase (xylA) (1 of 1)
OG IDs: 93518250
Best-hit-arabi-ID: AT5G57655.2
Best-hit-arabi-name: xylose isomerase family protein
Best-hit-arabi-symbol: nan
Gorai.009G296100.1.D
PCC=0.049, p=0.842
mRNA Profile
Protein Expression Profile
DPA

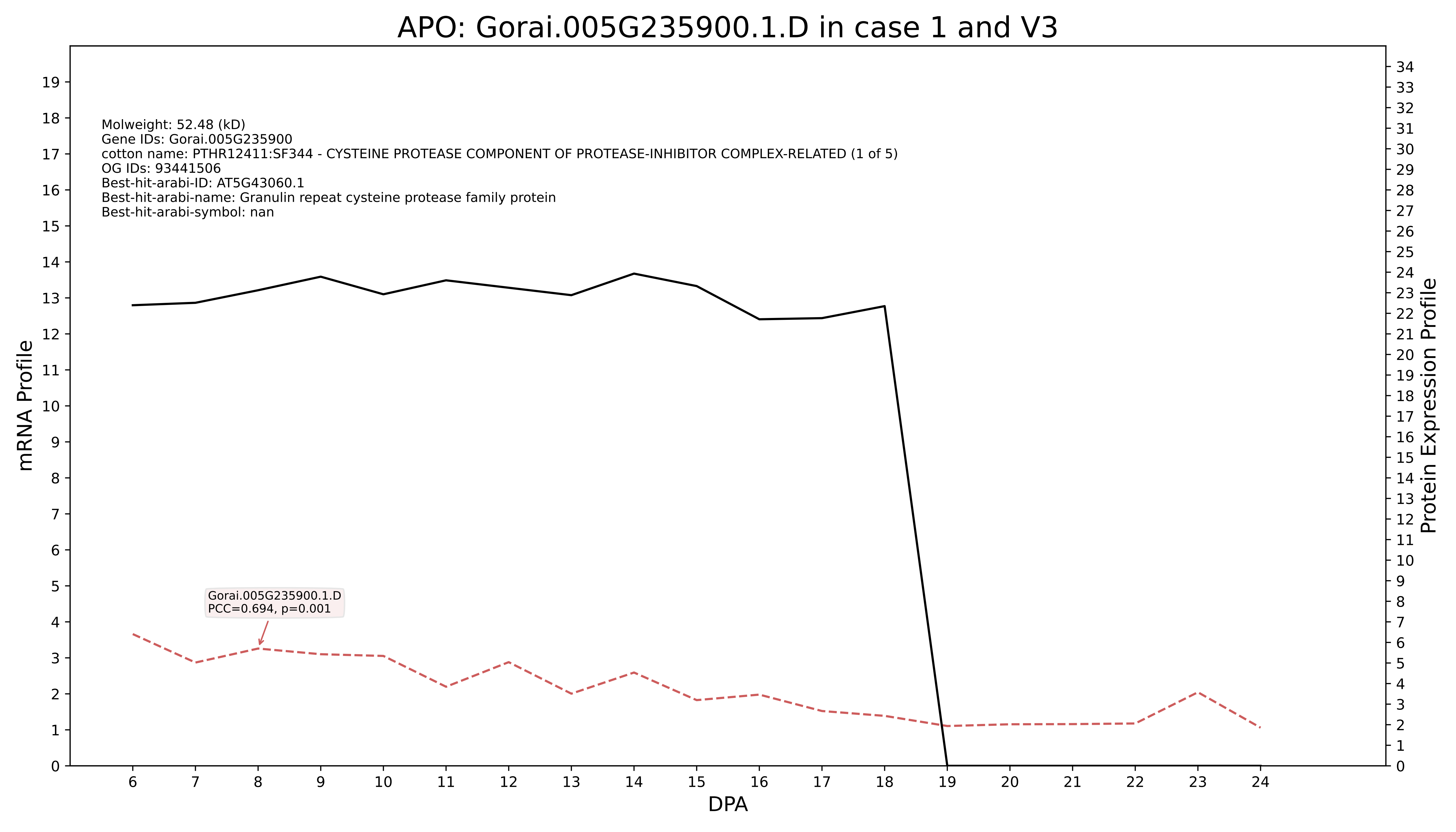

APO: Gorai.005G235900.1.D in case 1 and V3
Molweight: 52.48 (kD)
Gene IDs: Gorai.005G235900
cotton name: PTHR12411:SF344 - CYSTEINE PROTEASE COMPONENT OF PROTEASE-INHIBITOR COMPLEX-RELATED (1 of 5)
OG IDs: 93441506
Best-hit-arabi-ID: AT5G43060.1
Best-hit-arabi-name: Granulin repeat cysteine protease family protein
Best-hit-arabi-symbol: nan
Gorai.005G235900.1.D
PCC=0.694, p=0.001
mRNA Profile
Protein Expression Profile
DPA

APO: Gorai.013G077100.1.D in case 1 and V3

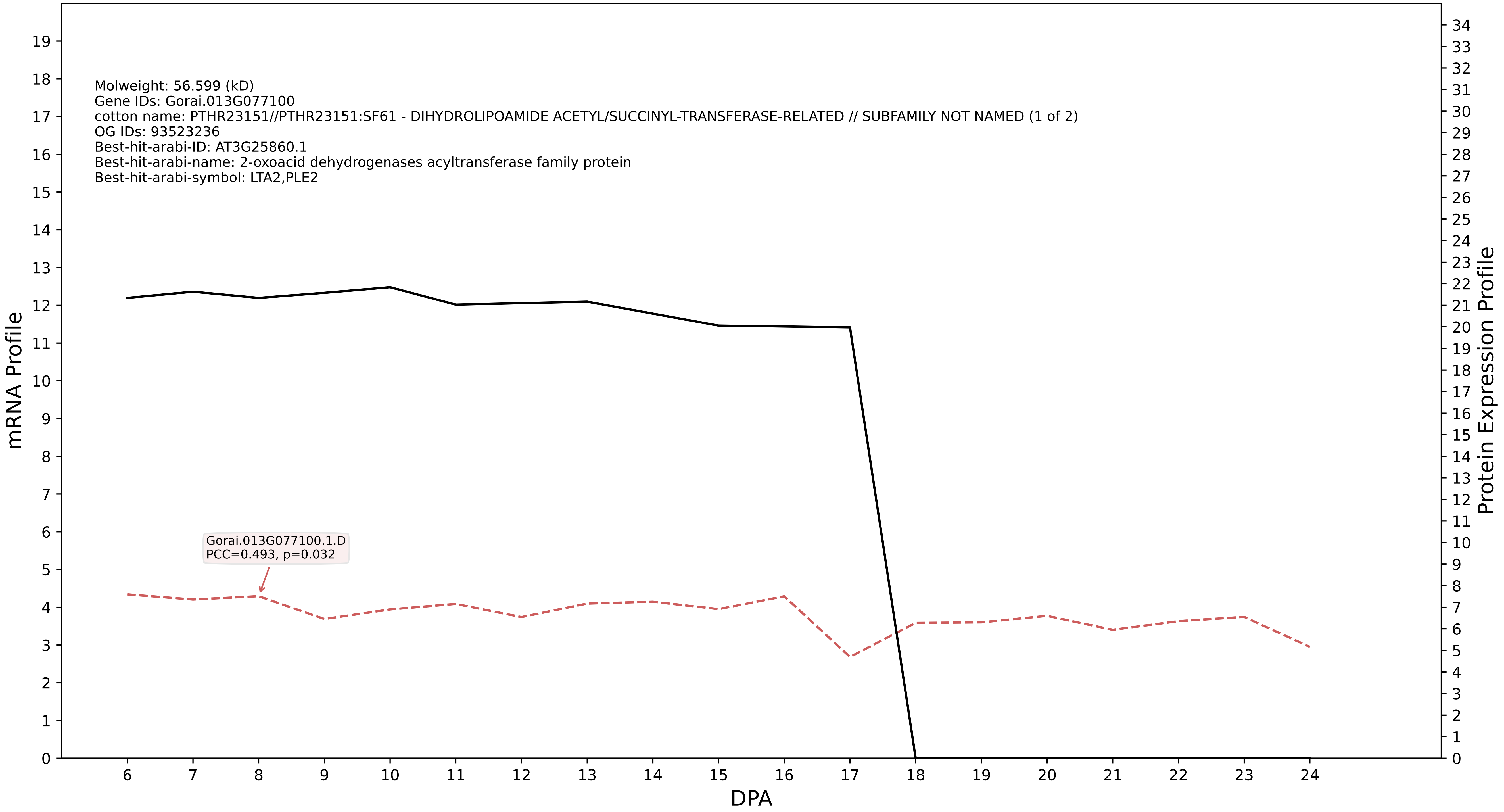

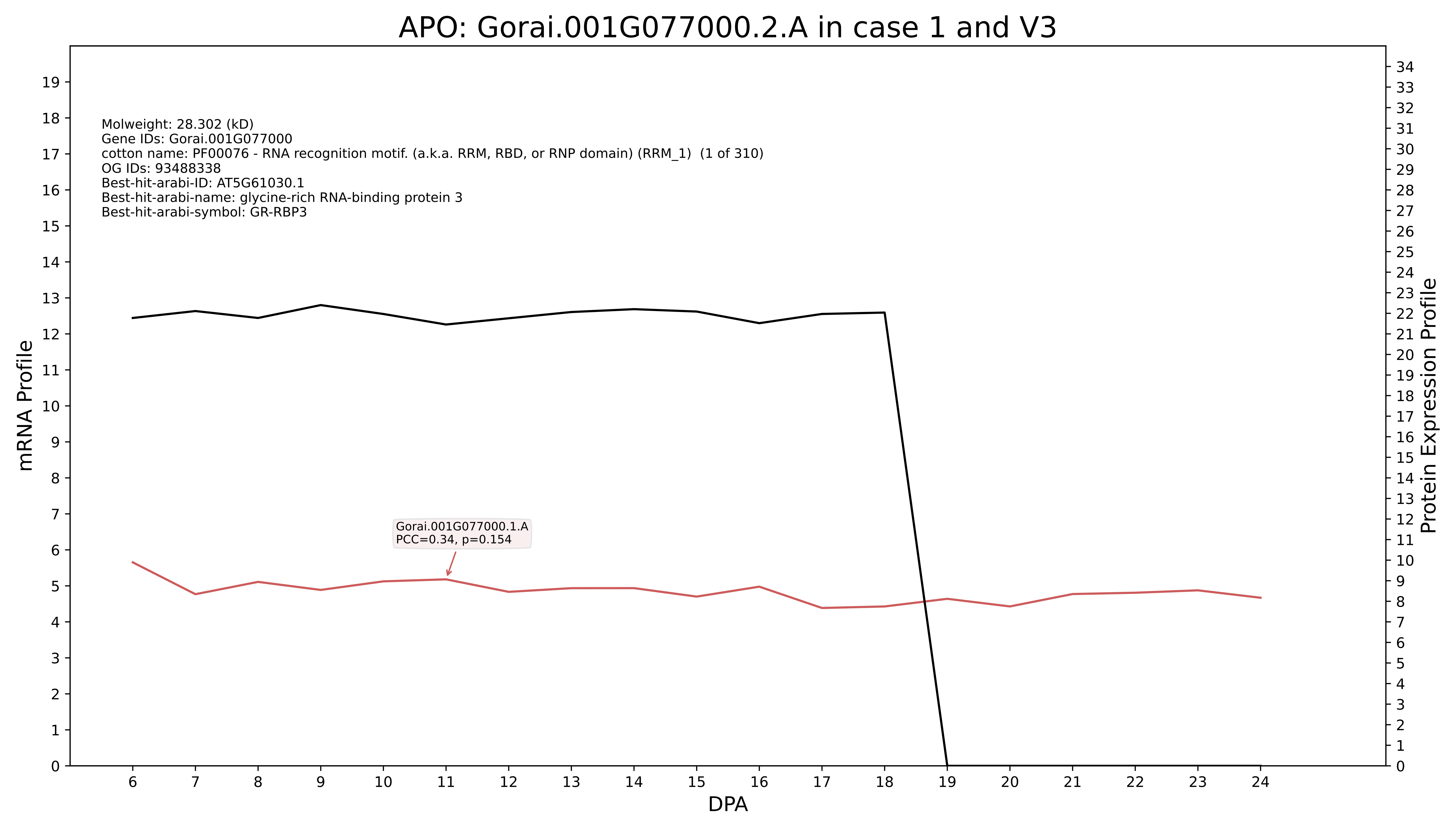

APO: Gorai.001G077000.2.A in case 1 and V3
Molweight: 28.302 (kD)
Gene IDs: Gorai.001G077000
cotton name: PF00076 - RNA recognition motif. (a.k.a. RRM, RBD, or RNP domain) (RRM_1) (1 of 310)
OG IDs: 93488338
Best-hit-arabi-ID: AT5G61030.1
Best-hit-arabi-name: glycine-rich RNA-binding protein 3
Best-hit-arabi-symbol: GR-RBP3
Gorai.001G077000.1.A
PCC=0.34, p=0.154
mRNA Profile
Protein Expression Profile
DPA

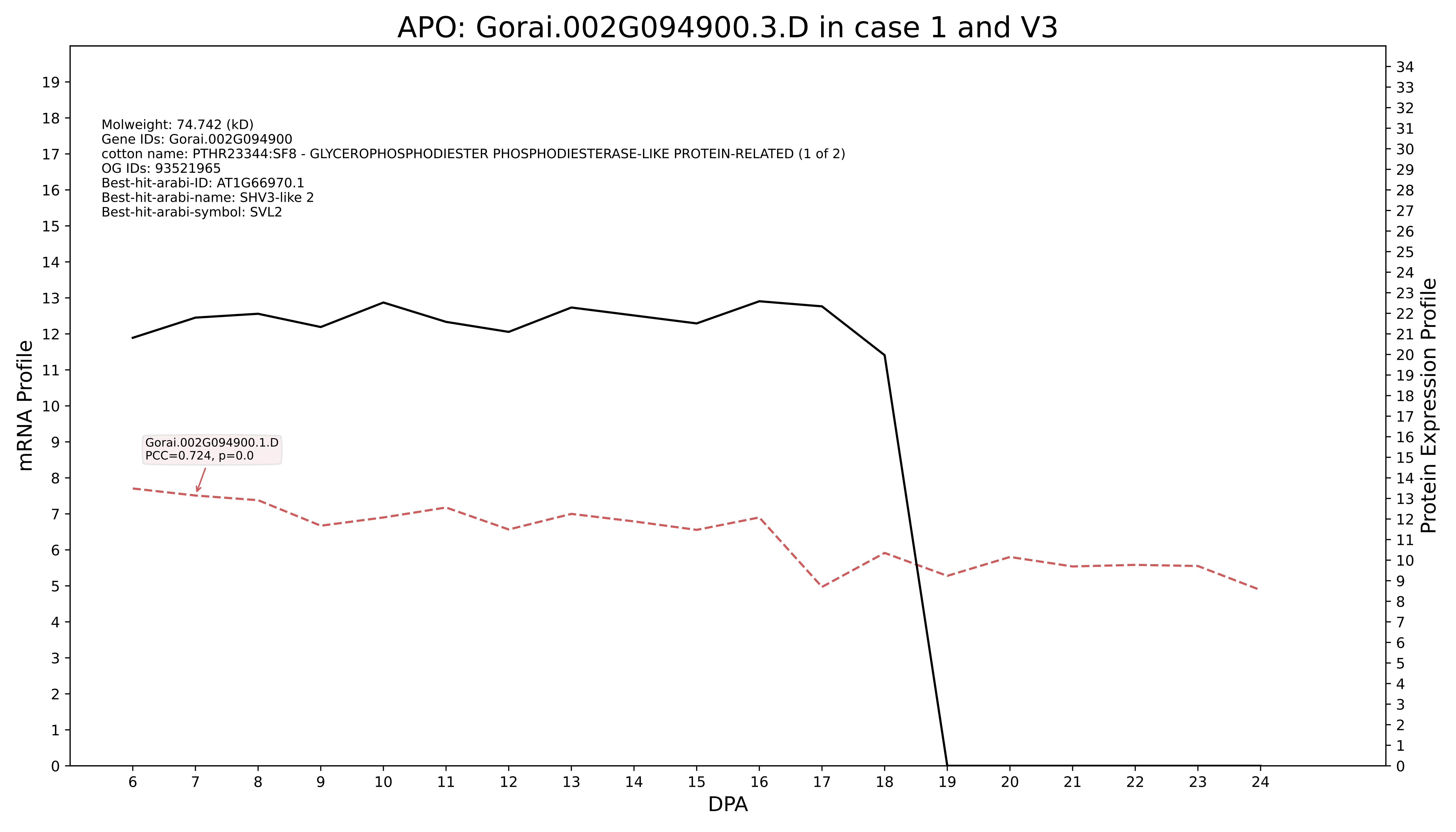
APO: Gorai.002G094900.3.D in case 1 and V3
Molweight: 74.742 (kD)
Gene IDs: Gorai.002G094900
cotton name: PTHR23344:SF8 - GLYCEROPHOSPHODIESTER PHOSPHODIESTERASE-LIKE PROTEIN-RELATED (1 of 2)
OG IDs: 93521965
Best-hit-arabi-ID: AT1G66970.1
Best-hit-arabi-name: SHV3-like 2
Best-hit-arabi-symbol: SVL2
Gorai.002G094900.1.D
PCC=0.724, p=0.0
mRNA Profile
Protein Expression Profile
DPA

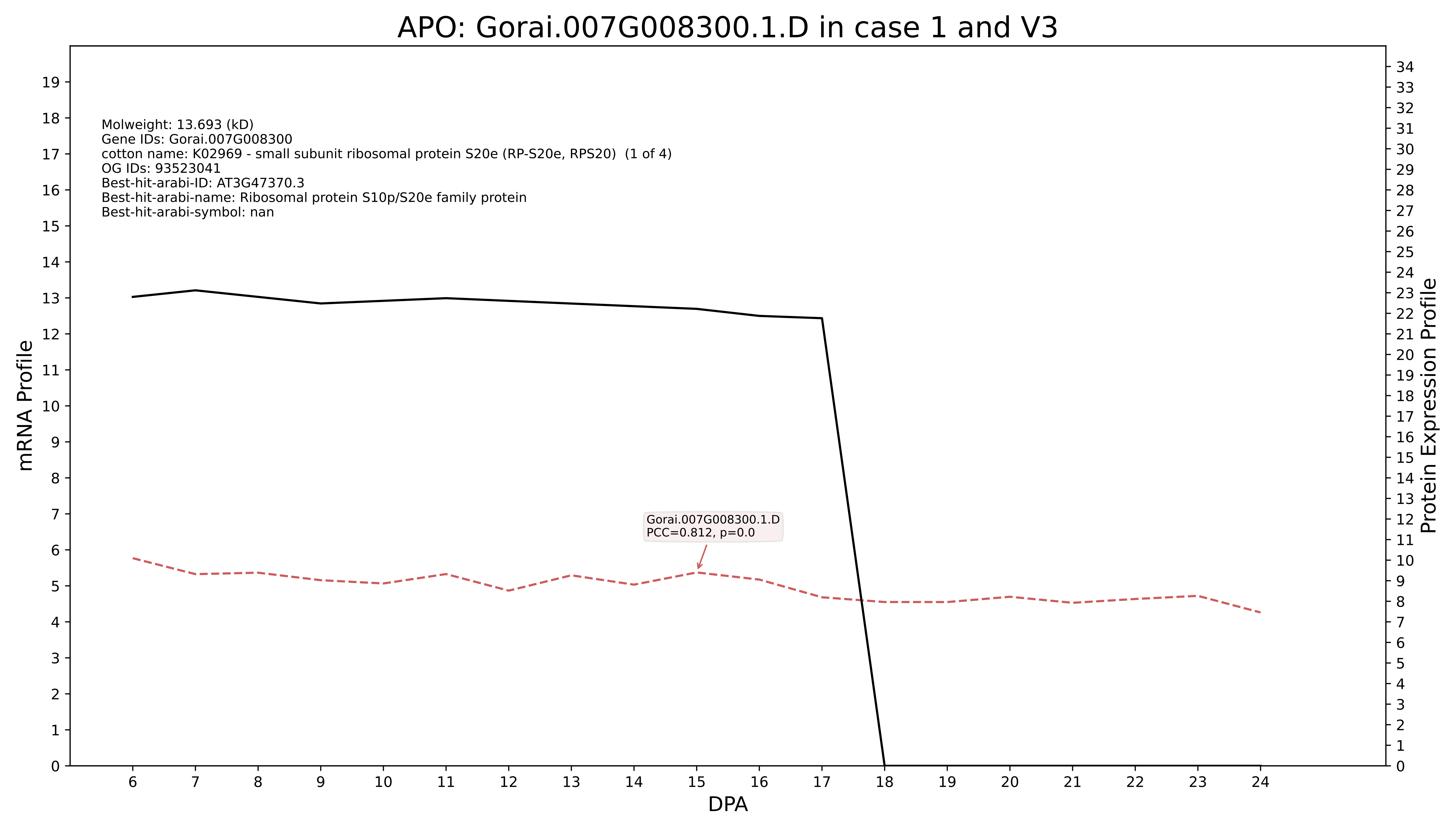
APO: Gorai.007G008300.1.D in case 1 and V3
Molweight: 13.693 (kD)
Gene IDs: Gorai.007G008300
cotton name: K02969 - small subunit ribosomal protein S20e (RP-S20e, RPS20) (1 of 4)
OG IDs: 93523041
Best-hit-arabi-ID: AT3G47370.3
Best-hit-arabi-name: Ribosomal protein S10p/S20e family protein
Best-hit-arabi-symbol: nan
Gorai.007G008300.1.D
PCC=0.812, p=0.0
mRNA Profile
Protein Expression Profile
DPA

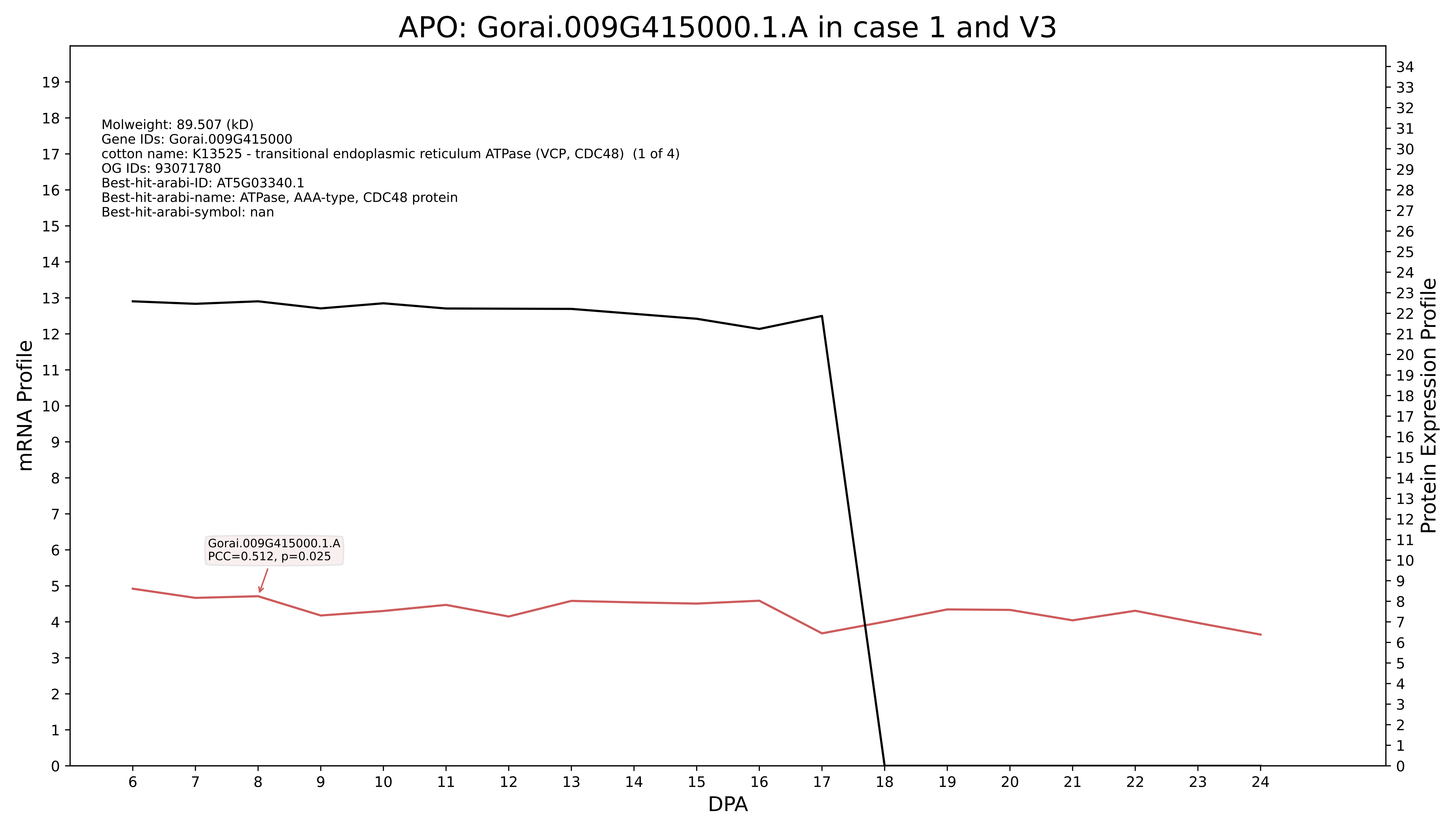
APO: Gorai.009G415000.1.A in case 1 and V3
Molweight: 89.507 (kD)
Gene IDs: Gorai.009G415000
cotton name: K13525 - transitional endoplasmic reticulum ATPase (VCP, CDC48) (1 of 4)
OG IDs: 93071780
Best-hit-arabi-ID: AT5G03340.1
Best-hit-arabi-name: ATPase, AAA-type, CDC48 protein
Best-hit-arabi-symbol: nan
Gorai.009G415000.1.A
PCC=0.512, p=0.025
mRNA Profile
Protein Expression Profile
DPA

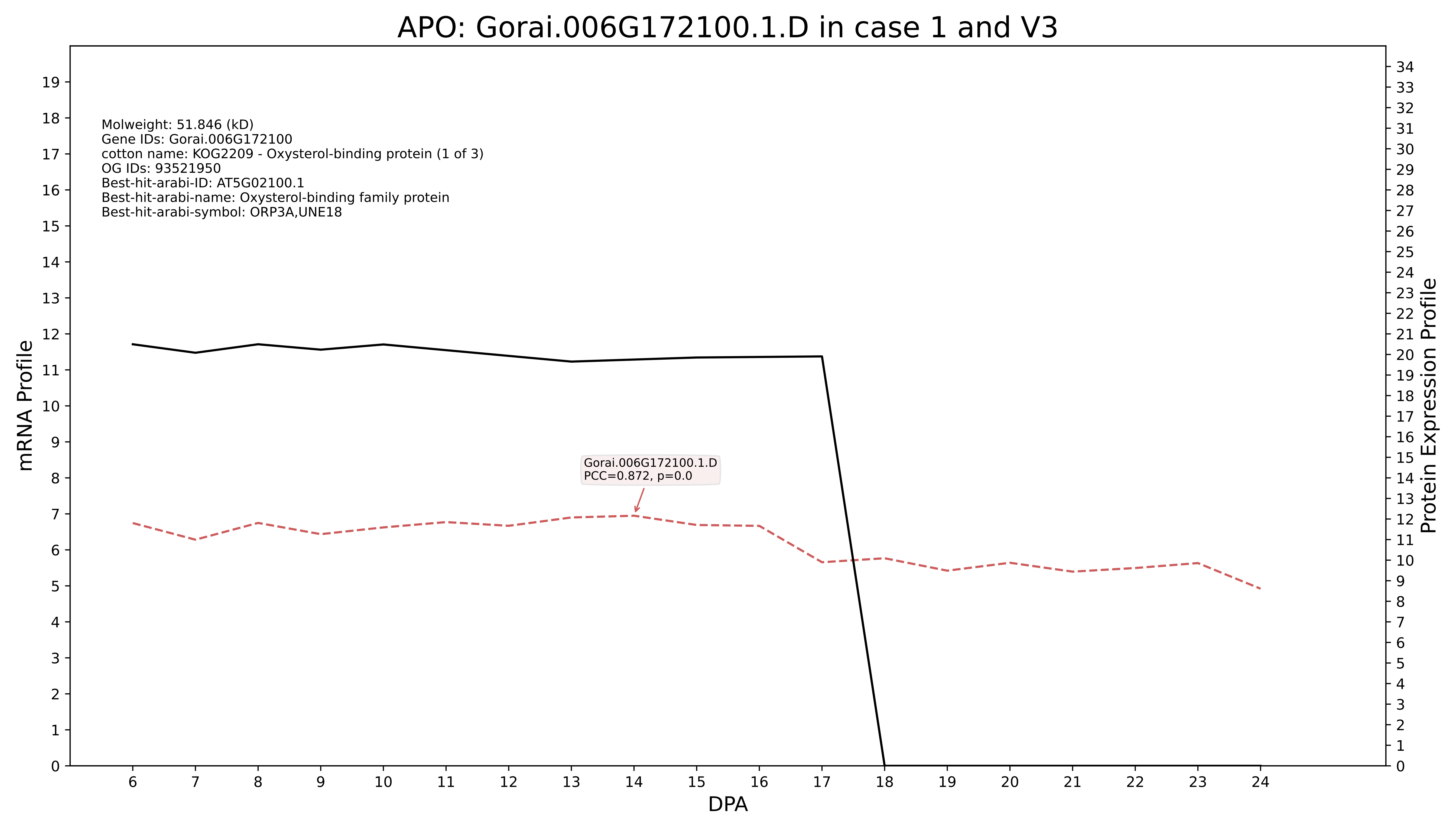

APO: Gorai.006G172100.1.D in case 1 and V3
Molweight: 51.846 (kD)
Gene IDs: Gorai.006G172100
cotton name: KOG2209 - Oxysterol-binding protein (1 of 3)
OG IDs: 93521950
Best-hit-arabi-ID: AT5G02100.1
Best-hit-arabi-name: Oxysterol-binding family protein
Best-hit-arabi-symbol: ORP3A,UNE18
Gorai.006G172100.1.D
PCC=0.872, p=0.0
mRNA Profile
Protein Expression Profile
DPA

APO: Gorai.002G060900.1.D in case 1 and V3

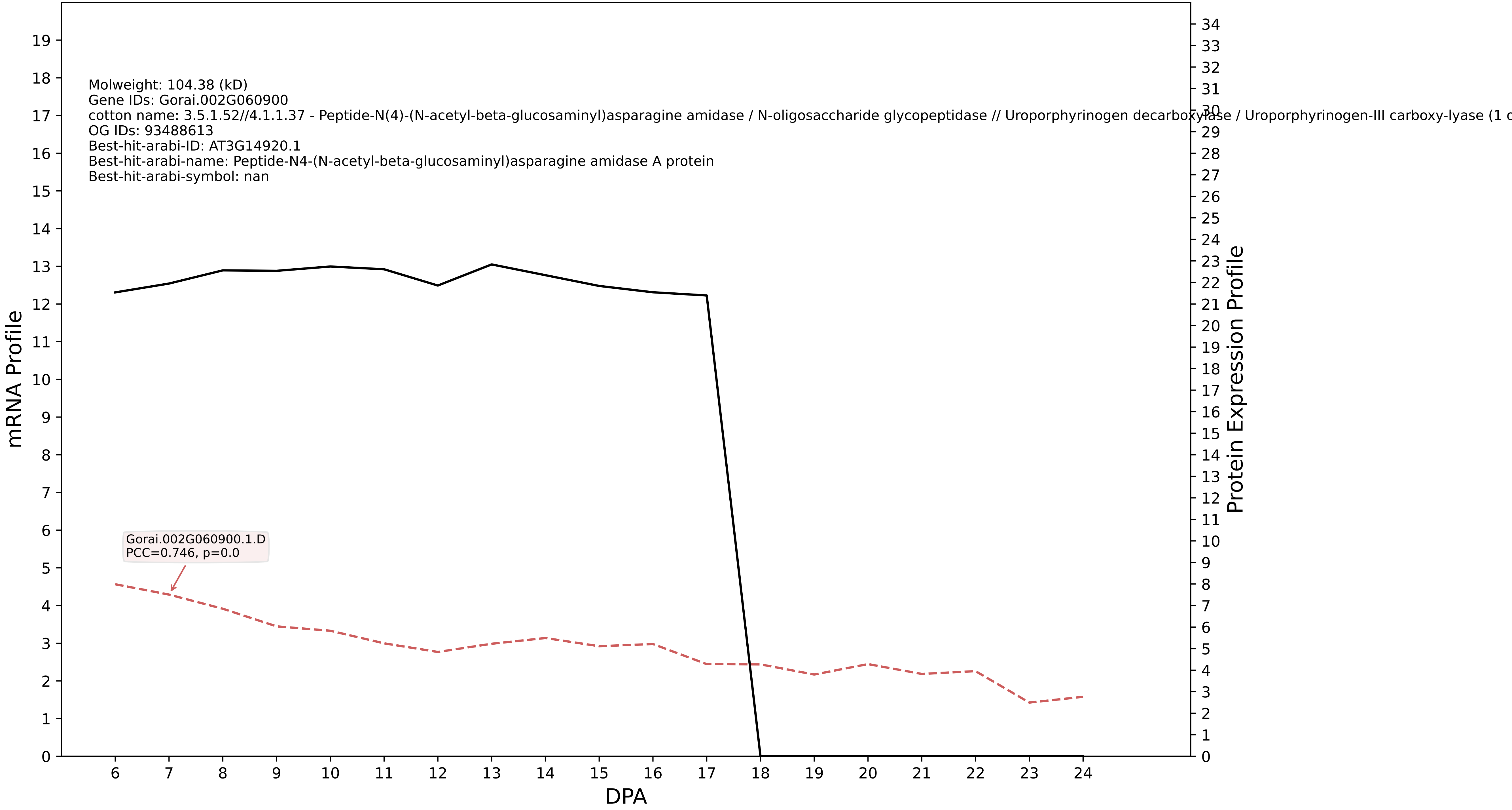

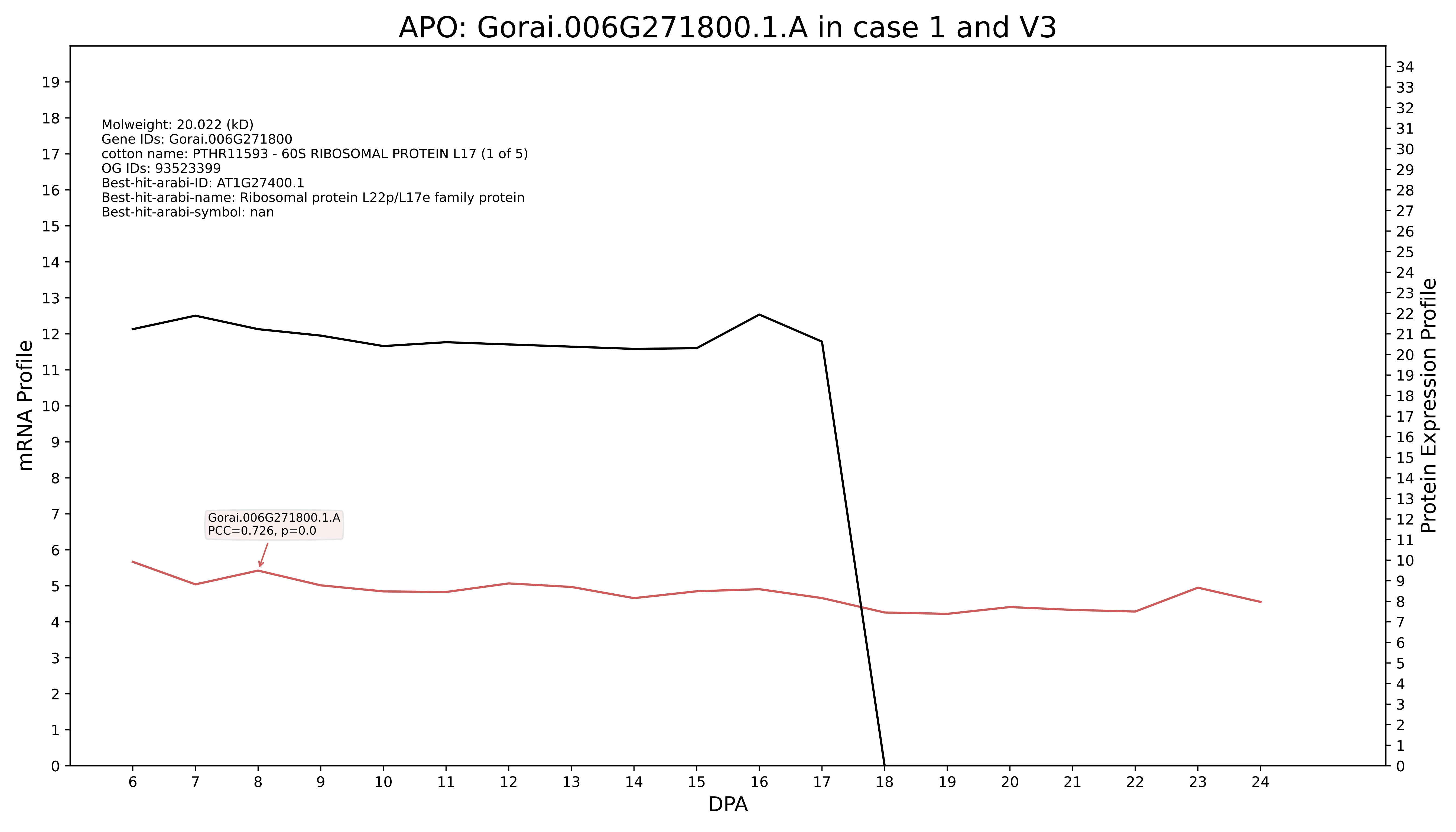
APO: Gorai.006G271800.1.A in case 1 and V3
Molweight: 20.022 (kD)
Gene IDs: Gorai.006G271800
cotton name: PTHR11593 - 60S RIBOSOMAL PROTEIN L17 (1 of 5)
OG IDs: 93523399
Best-hit-arabi-ID: AT1G27400.1
Best-hit-arabi-name: Ribosomal protein L22p/L17e family protein
Best-hit-arabi-symbol: nan
Gorai.006G271800.1.A
PCC=0.726, p=0.0
mRNA Profile
Protein Expression Profile
DPA
0
1
2
3
4
5
6
7
8
9
10
11
12
13
14
15
16
17
18
19
20
21
22
23
24
25
26
27
28
29
30
31
32
33
34

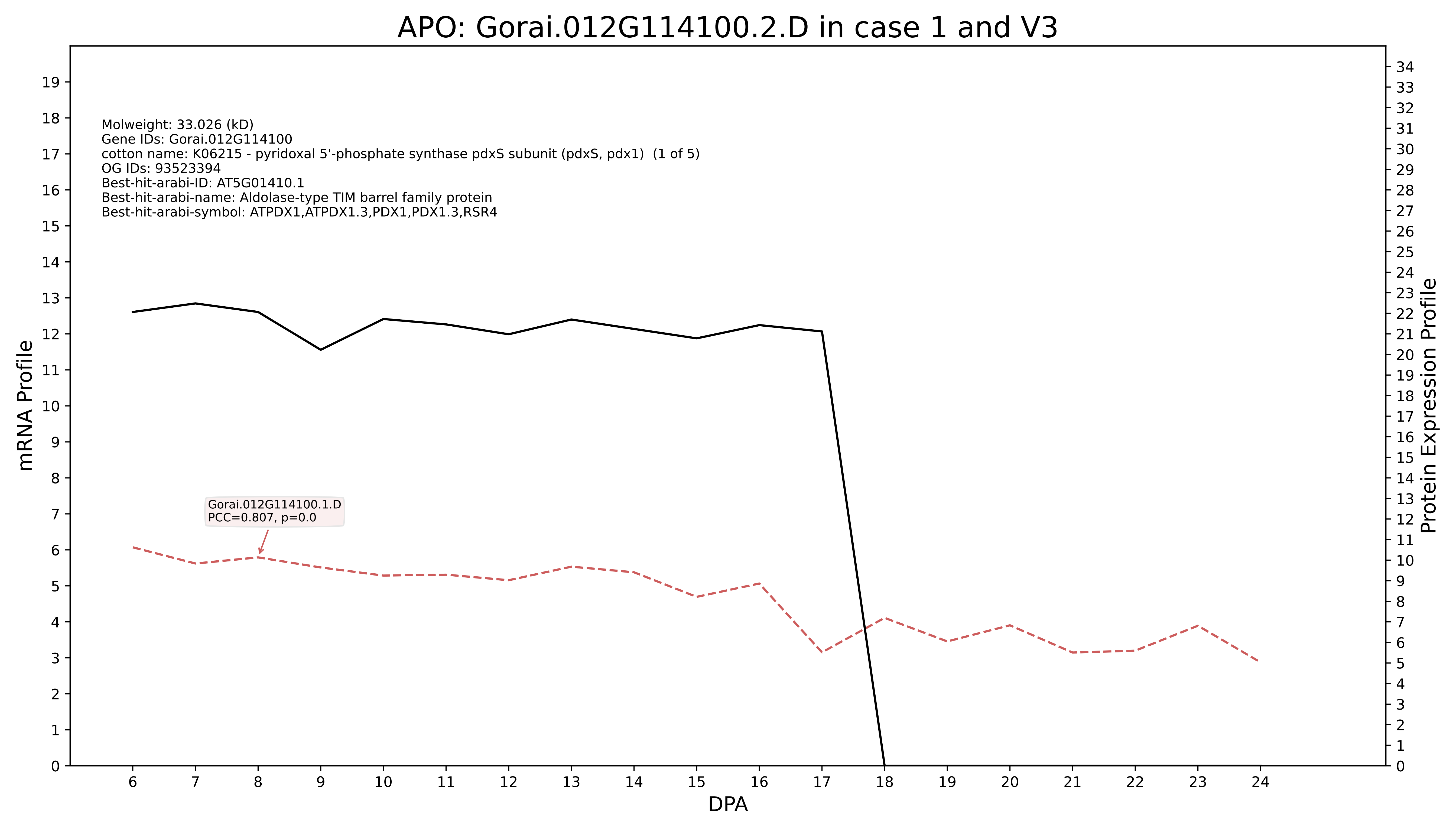

APO: Gorai.012G114100.2.D in case 1 and V3
Molweight: 33.026 (kD)
Gene IDs: Gorai.012G114100
cotton name: K06215 - pyridoxal 5'-phosphate synthase pdxS subunit (pdxS, pdx1) (1 of 5)
OG IDs: 93523394
Best-hit-arabi-ID: AT5G01410.1
Best-hit-arabi-name: Aldolase-type TIM barrel family protein
Best-hit-arabi-symbol: ATPDX1,ATPDX1.3,PDX1,PDX1.3,RSR4
Gorai.012G114100.1.D
PCC=0.807, p=0.0
mRNA Profile
Protein Expression Profile
DPA

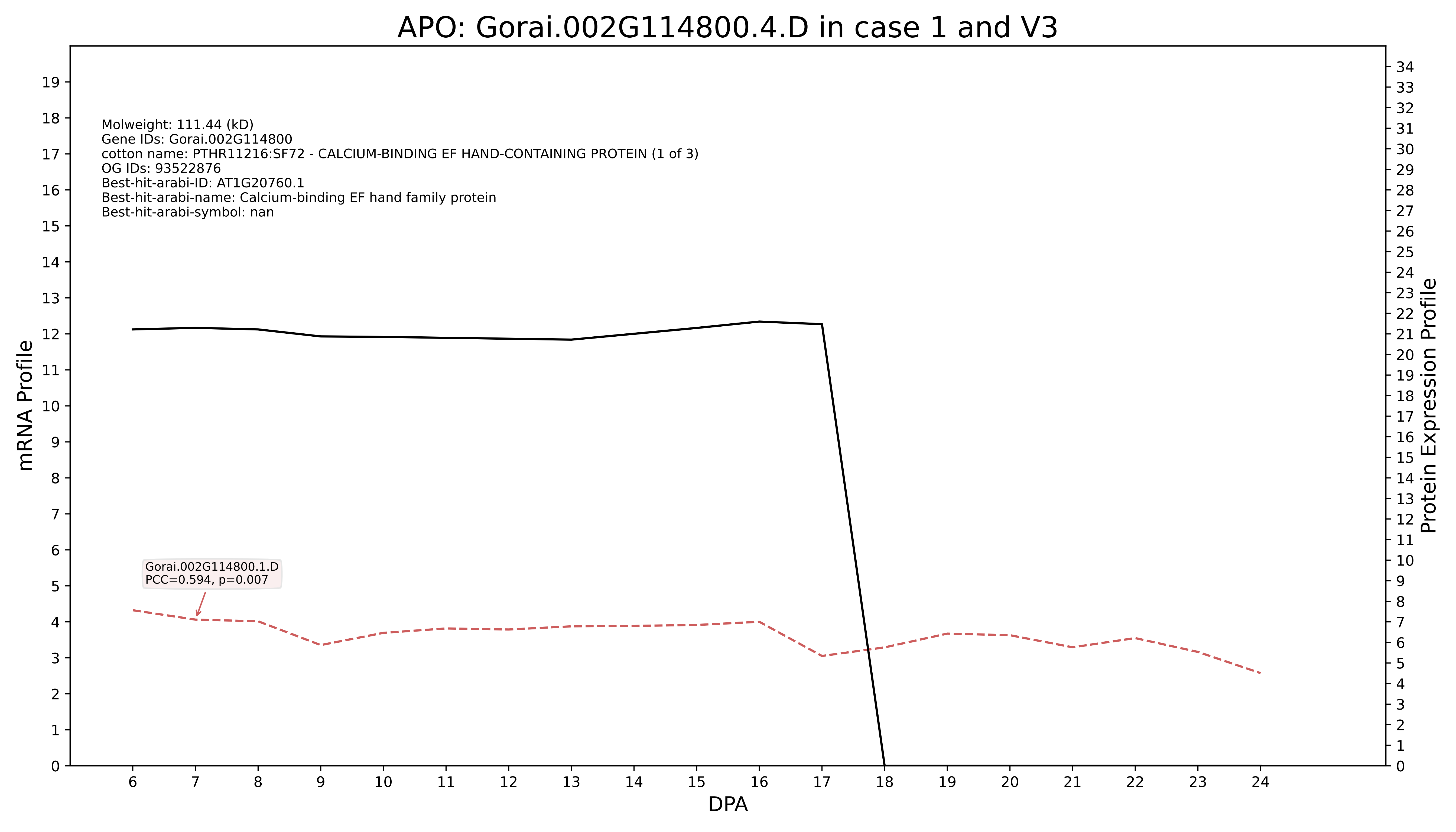

APO: Gorai.002G114800.4.D in case 1 and V3
Molweight: 111.44 (kD)
Gene IDs: Gorai.002G114800
cotton name: PTHR11216:SF72 - CALCIUM-BINDING EF HAND-CONTAINING PROTEIN (1 of 3)
OG IDs: 93522876
Best-hit-arabi-ID: AT1G20760.1
Best-hit-arabi-name: Calcium-binding EF hand family protein
Best-hit-arabi-symbol: nan
Gorai.002G114800.1.D
PCC=0.594, p=0.007
mRNA Profile
Protein Expression Profile
DPA

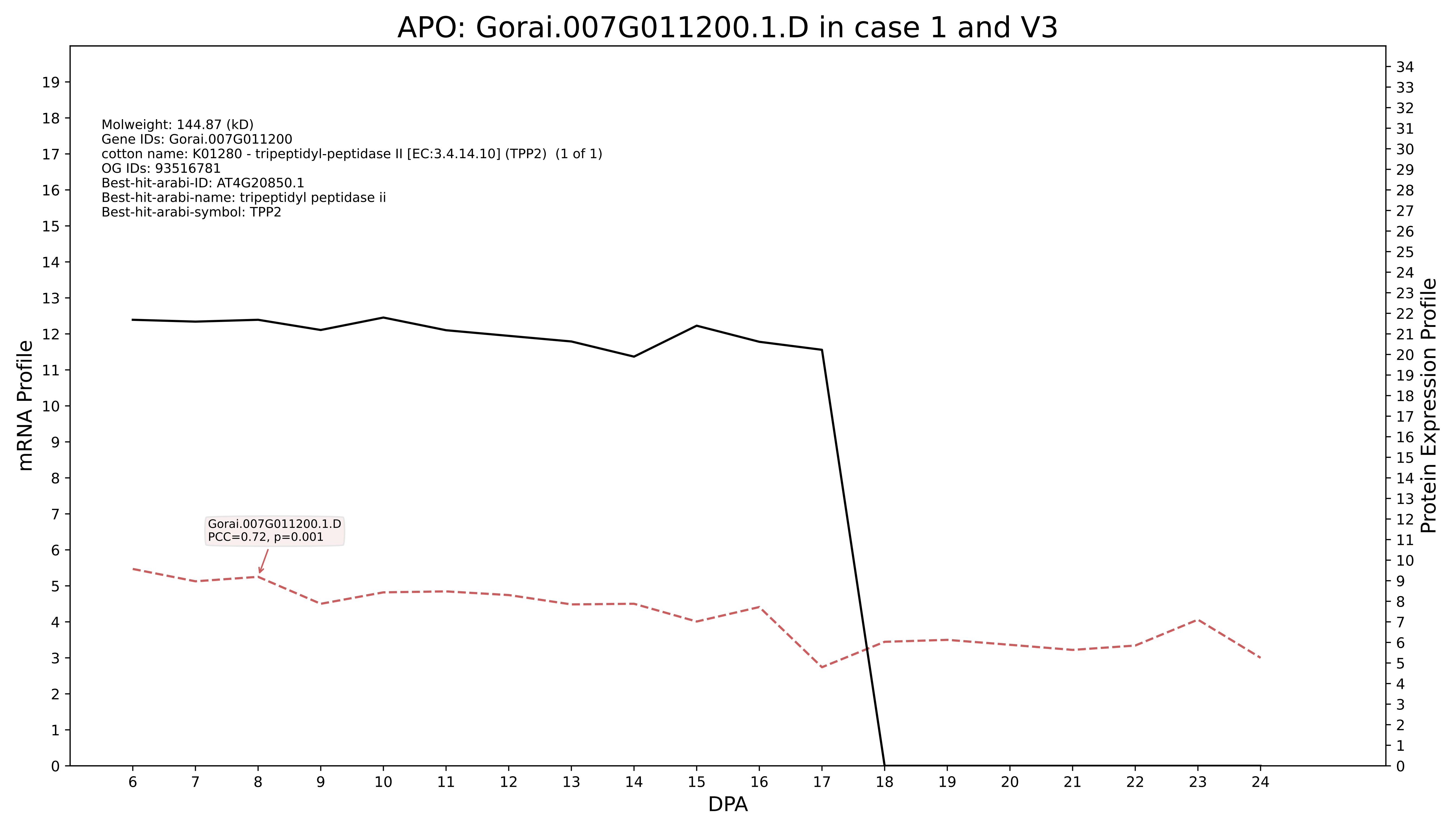
APO: Gorai.007G011200.1.D in case 1 and V3
Molweight: 144.87 (kD)
Gene IDs: Gorai.007G011200
cotton name: K01280 - tripeptidyl-peptidase II [EC:3.4.14.10] (TPP2) (1 of 1)
OG IDs: 93516781
Best-hit-arabi-ID: AT4G20850.1
Best-hit-arabi-name: tripeptidyl peptidase ii
Best-hit-arabi-symbol: TPP2
Gorai.007G011200.1.D
PCC=0.72, p=0.001
mRNA Profile
Protein Expression Profile
DPA

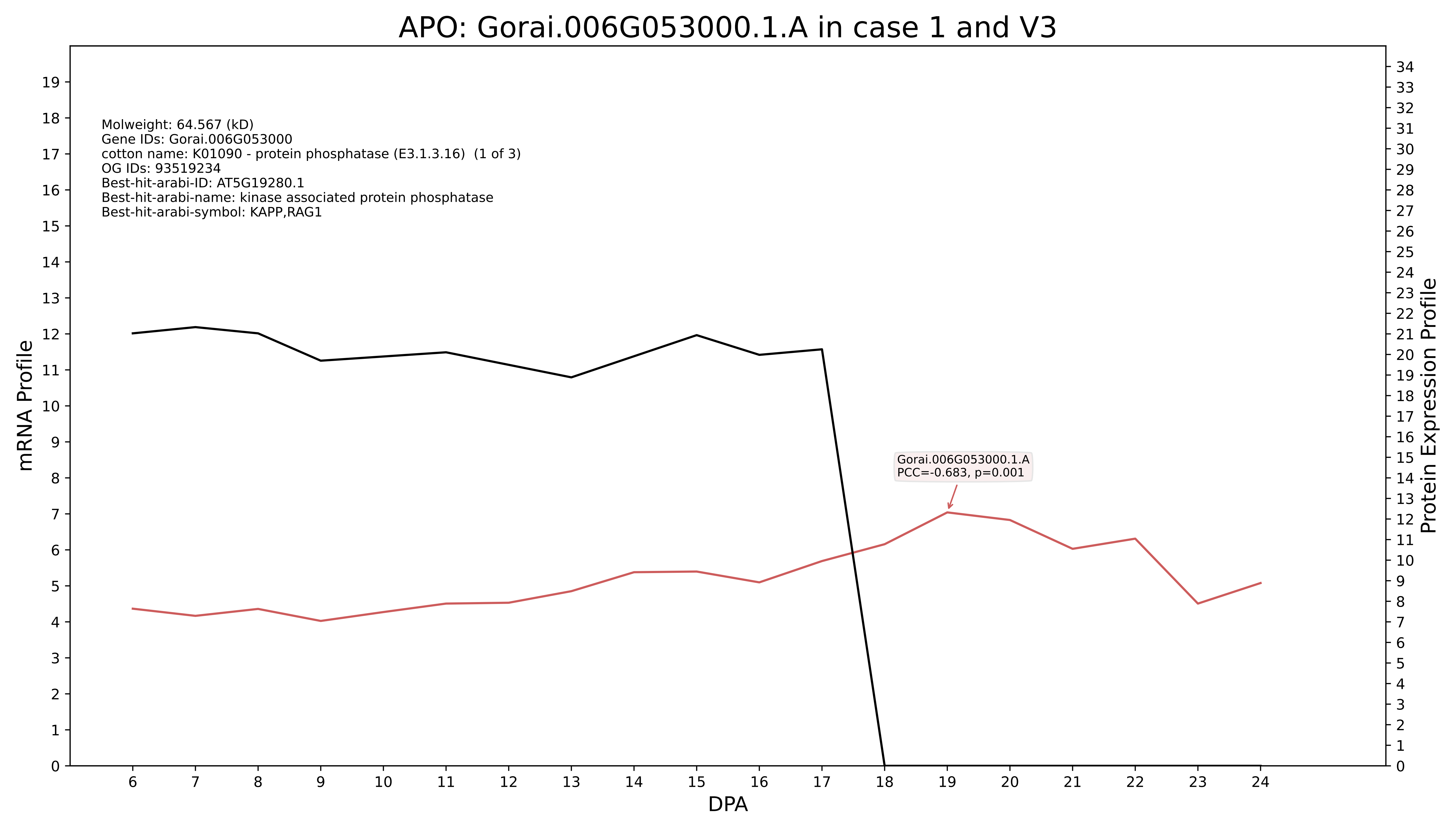
APO: Gorai.006G053000.1.A in case 1 and V3
Molweight: 64.567 (kD)
Gene IDs: Gorai.006G053000
cotton name: K01090 - protein phosphatase (E3.1.3.16) (1 of 3)
OG IDs: 93519234
Best-hit-arabi-ID: AT5G19280.1
Best-hit-arabi-name: kinase associated protein phosphatase
Best-hit-arabi-symbol: KAPP,RAG1
Gorai.006G053000.1.A
PCC=-0.683, p=0.001
mRNA Profile
Protein Expression Profile
DPA

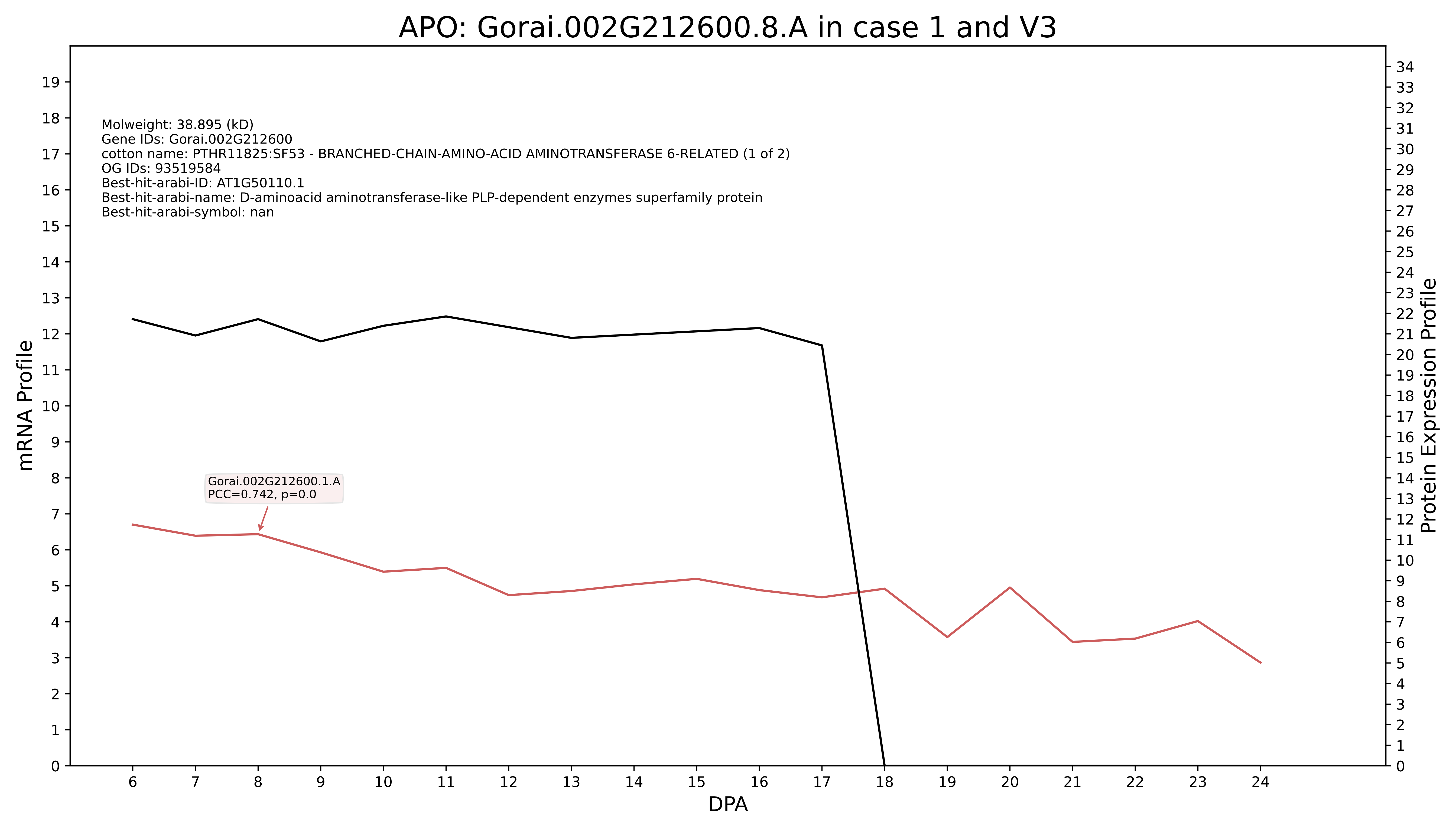
APO: Gorai.002G212600.8.A in case 1 and V3
Molweight: 38.895 (kD)
Gene IDs: Gorai.002G212600
cotton name: PTHR11825:SF53 - BRANCHED-CHAIN-AMINO-ACID AMINOTRANSFERASE 6-RELATED (1 of 2)
OG IDs: 93519584
Best-hit-arabi-ID: AT1G50110.1
Best-hit-arabi-name: D-aminoacid aminotransferase-like PLP-dependent enzymes superfamily protein
Best-hit-arabi-symbol: nan
Gorai.002G212600.1.A
PCC=0.742, p=0.0
mRNA Profile
Protein Expression Profile
DPA
0
1
2
3
4
5
6
7
8
9
10
11
12
13
14
15
16
17
18
19
0
1
2
3
4
5
6
7
8
9
10
11
12
13
14
15
16
17
18
19
20
21
22
23
24
25
26
27
28
29
30
31
32
33
34
6
7
8
9
10
11
12
13
14
15
16
17
18
19
20
21
22
23
24

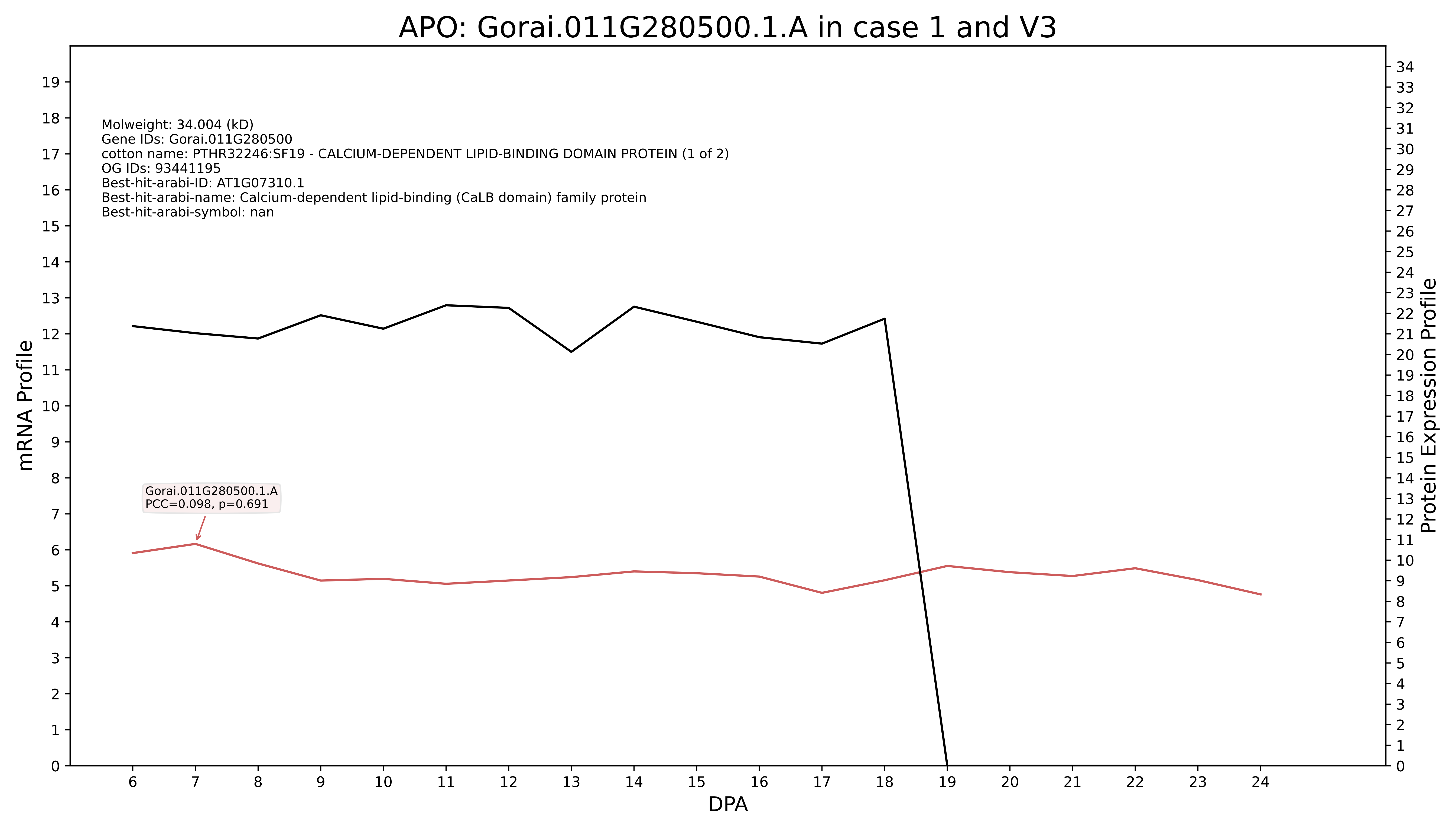
APO: Gorai.011G280500.1.A in case 1 and V3
Molweight: 34.004 (kD)
Gene IDs: Gorai.011G280500
cotton name: PTHR32246:SF19 - CALCIUM-DEPENDENT LIPID-BINDING DOMAIN PROTEIN (1 of 2)
OG IDs: 93441195
Best-hit-arabi-ID: AT1G07310.1
Best-hit-arabi-name: Calcium-dependent lipid-binding (CaLB domain) family protein
Best-hit-arabi-symbol: nan
Gorai.011G280500.1.A
PCC=0.098, p=0.691
mRNA Profile
Protein Expression Profile
DPA

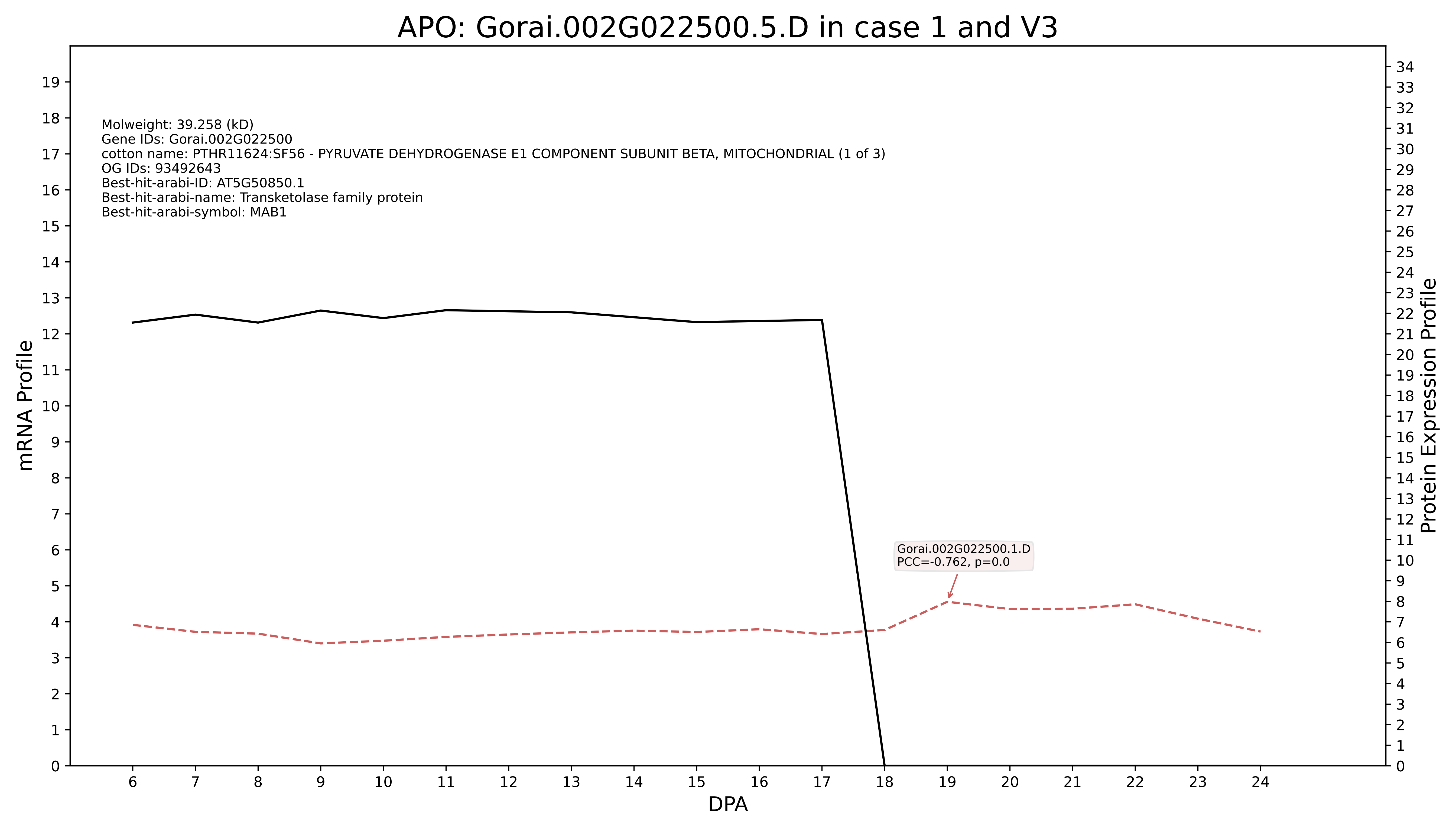

APO: Gorai.002G022500.5.D in case 1 and V3
Molweight: 39.258 (kD)
Gene IDs: Gorai.002G022500
cotton name: PTHR11624:SF56 - PYRUVATE DEHYDROGENASE E1 COMPONENT SUBUNIT BETA, MITOCHONDRIAL (1 of 3)
OG IDs: 93492643
Best-hit-arabi-ID: AT5G50850.1
Best-hit-arabi-name: Transketolase family protein
Best-hit-arabi-symbol: MAB1
Gorai.002G022500.1.D
PCC=-0.762, p=0.0
mRNA Profile
Protein Expression Profile
DPA

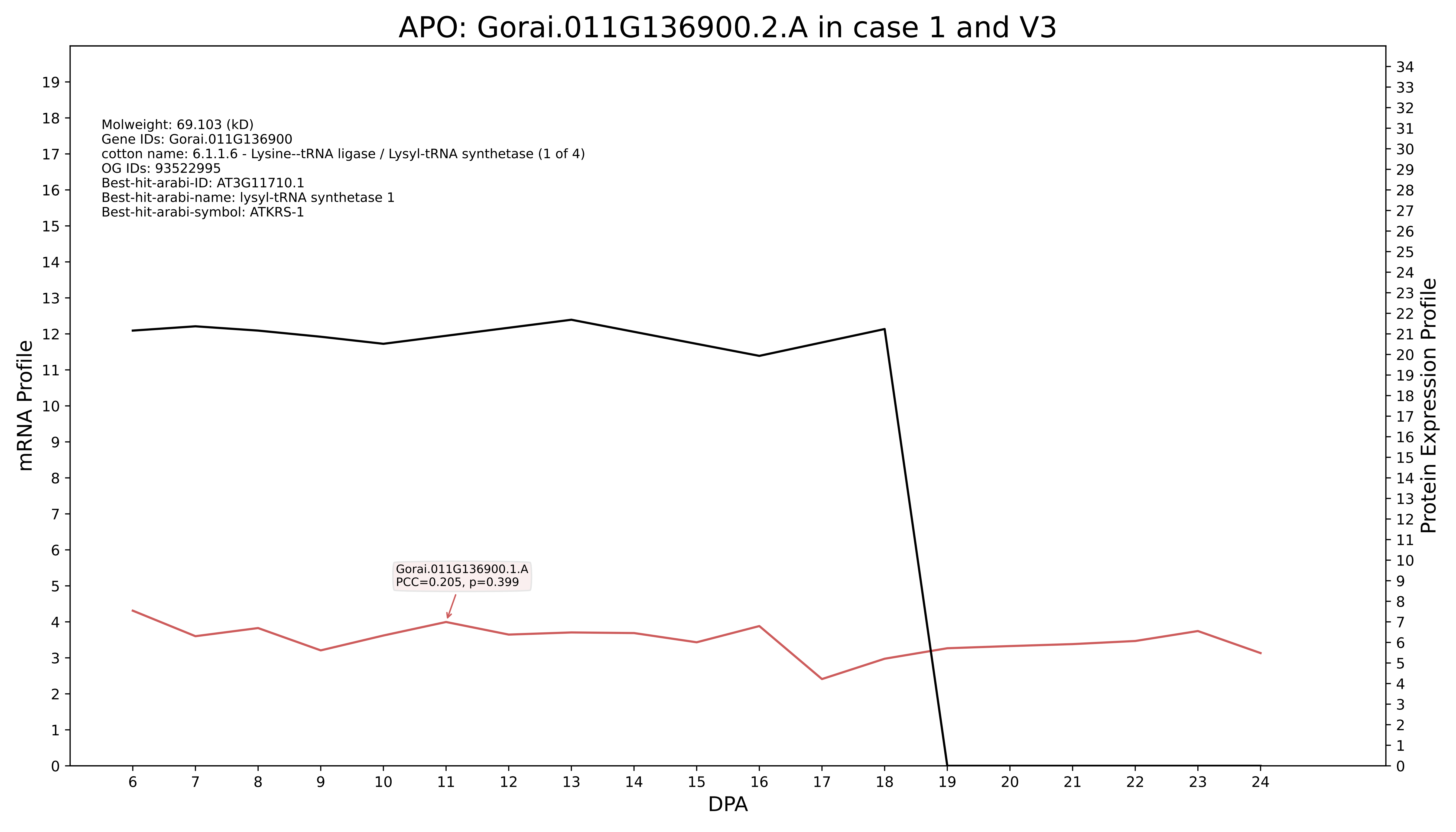
APO: Gorai.011G136900.2.A in case 1 and V3
Molweight: 69.103 (kD)
Gene IDs: Gorai.011G136900
cotton name: 6.1.1.6 - Lysine--tRNA ligase / Lysyl-tRNA synthetase (1 of 4)
OG IDs: 93522995
Best-hit-arabi-ID: AT3G11710.1
Best-hit-arabi-name: lysyl-tRNA synthetase 1
Best-hit-arabi-symbol: ATKRS-1
Gorai.011G136900.1.A
PCC=0.205, p=0.399
mRNA Profile
Protein Expression Profile
DPA

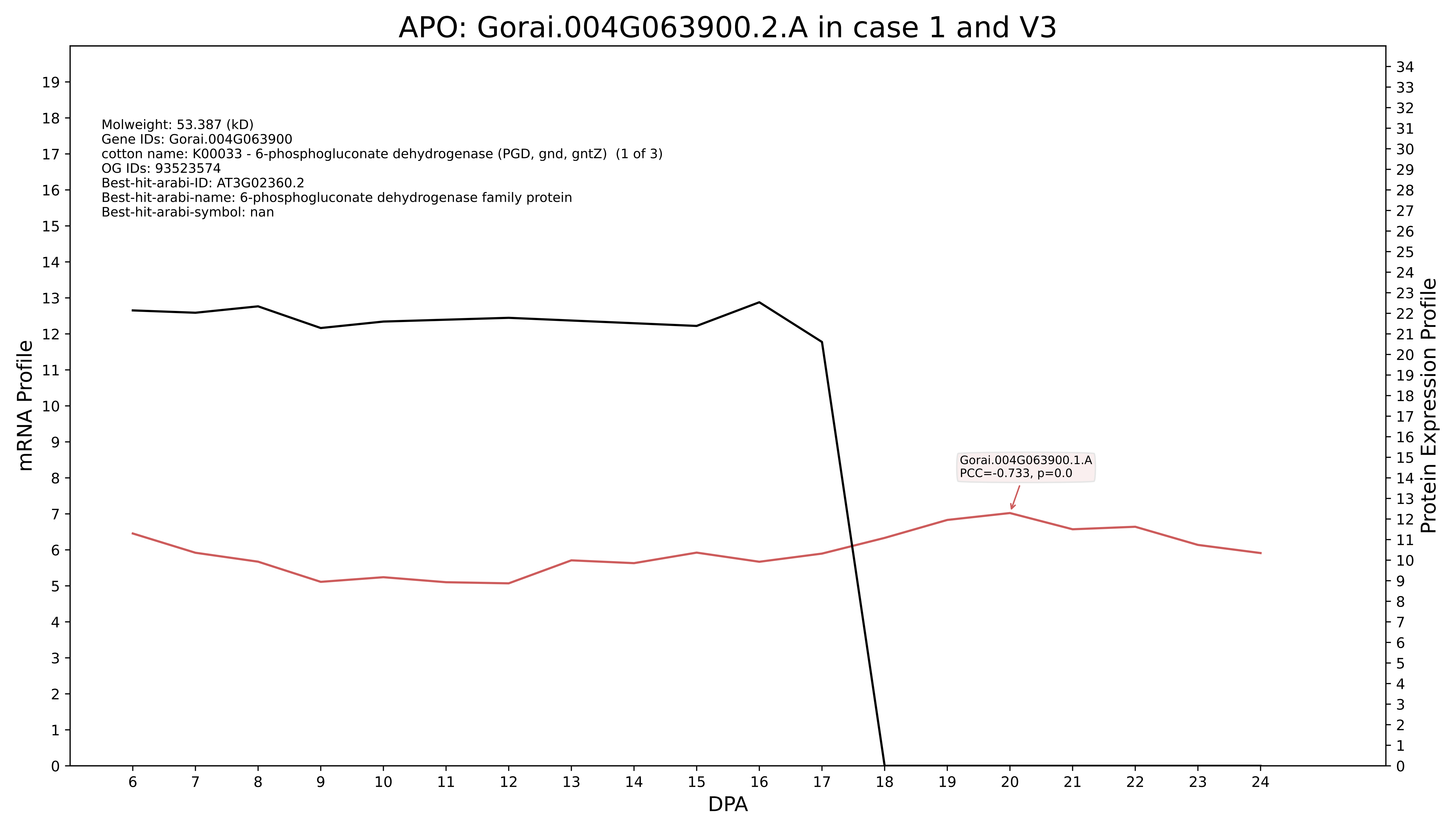

APO: Gorai.004G063900.2.A in case 1 and V3
Molweight: 53.387 (kD)
Gene IDs: Gorai.004G063900
cotton name: K00033 - 6-phosphogluconate dehydrogenase (PGD, gnd, gntZ) (1 of 3)
OG IDs: 93523574
Best-hit-arabi-ID: AT3G02360.2
Best-hit-arabi-name: 6-phosphogluconate dehydrogenase family protein
Best-hit-arabi-symbol: nan
Gorai.004G063900.1.A
PCC=-0.733, p=0.0
mRNA Profile
Protein Expression Profile
DPA

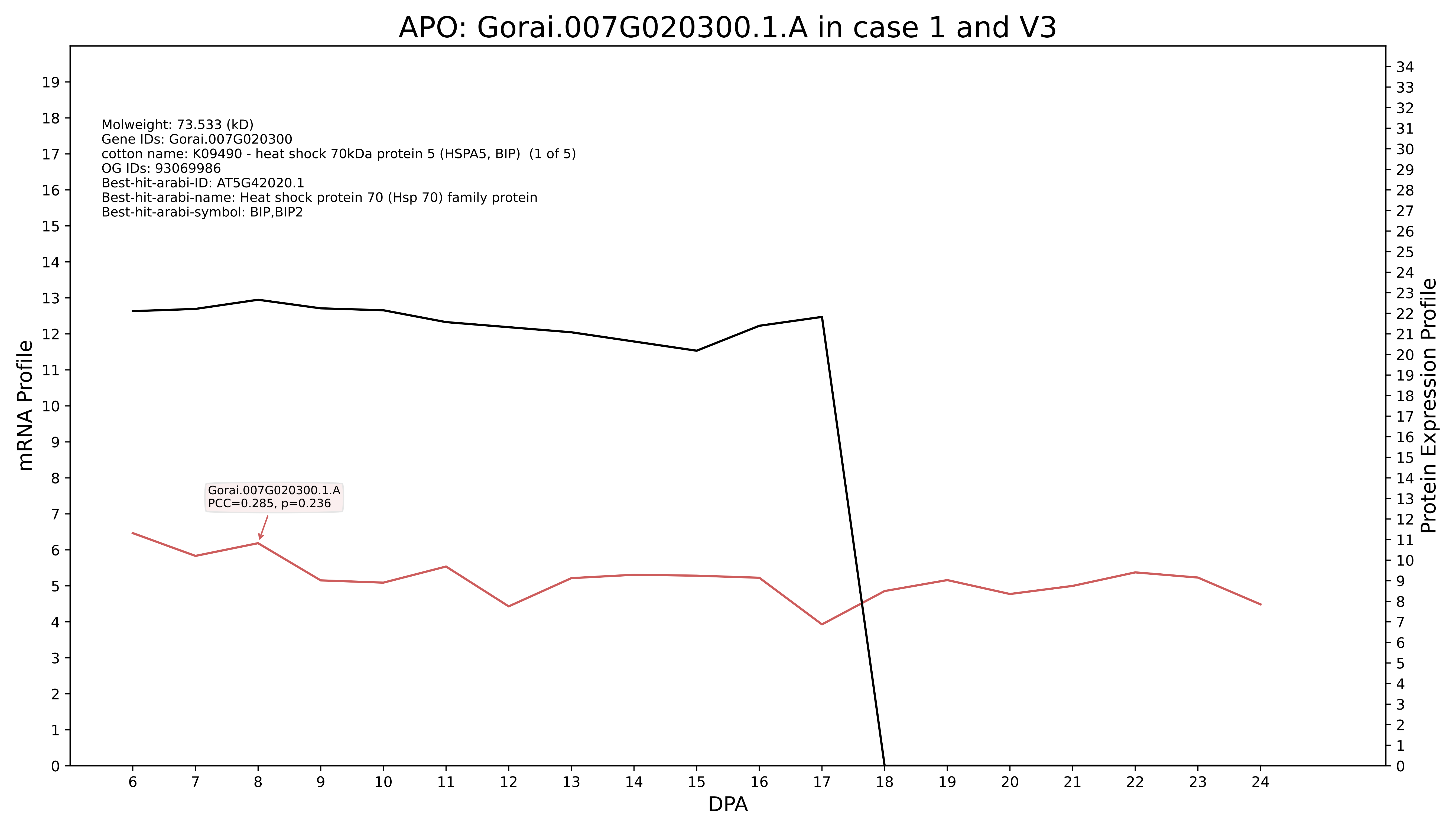
APO: Gorai.007G020300.1.A in case 1 and V3
Molweight: 73.533 (kD)
Gene IDs: Gorai.007G020300
cotton name: K09490 - heat shock 70kDa protein 5 (HSPA5, BIP) (1 of 5)
OG IDs: 93069986
Best-hit-arabi-ID: AT5G42020.1
Best-hit-arabi-name: Heat shock protein 70 (Hsp 70) family protein
Best-hit-arabi-symbol: BIP,BIP2
Gorai.007G020300.1.A
PCC=0.285, p=0.236
mRNA Profile
Protein Expression Profile
DPA

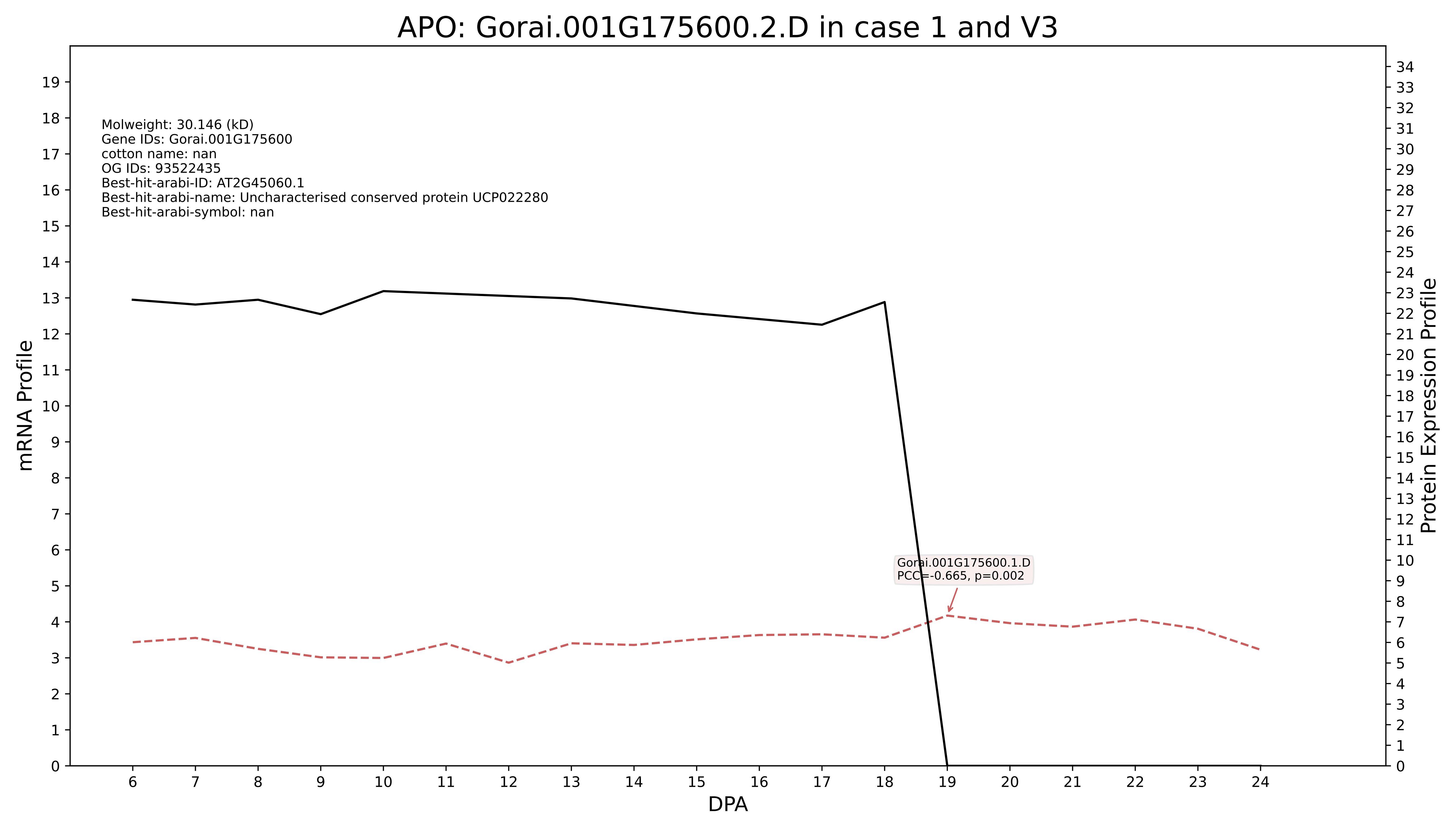
APO: Gorai.001G175600.2.D in case 1 and V3
Molweight: 30.146 (kD)
Gene IDs: Gorai.001G175600
cotton name: nan
OG IDs: 93522435
Best-hit-arabi-ID: AT2G45060.1
Best-hit-arabi-name: Uncharacterised conserved protein UCP022280
Best-hit-arabi-symbol: nan
Gorai.001G175600.1.D
PCC=-0.665, p=0.002
mRNA Profile
Protein Expression Profile
DPA

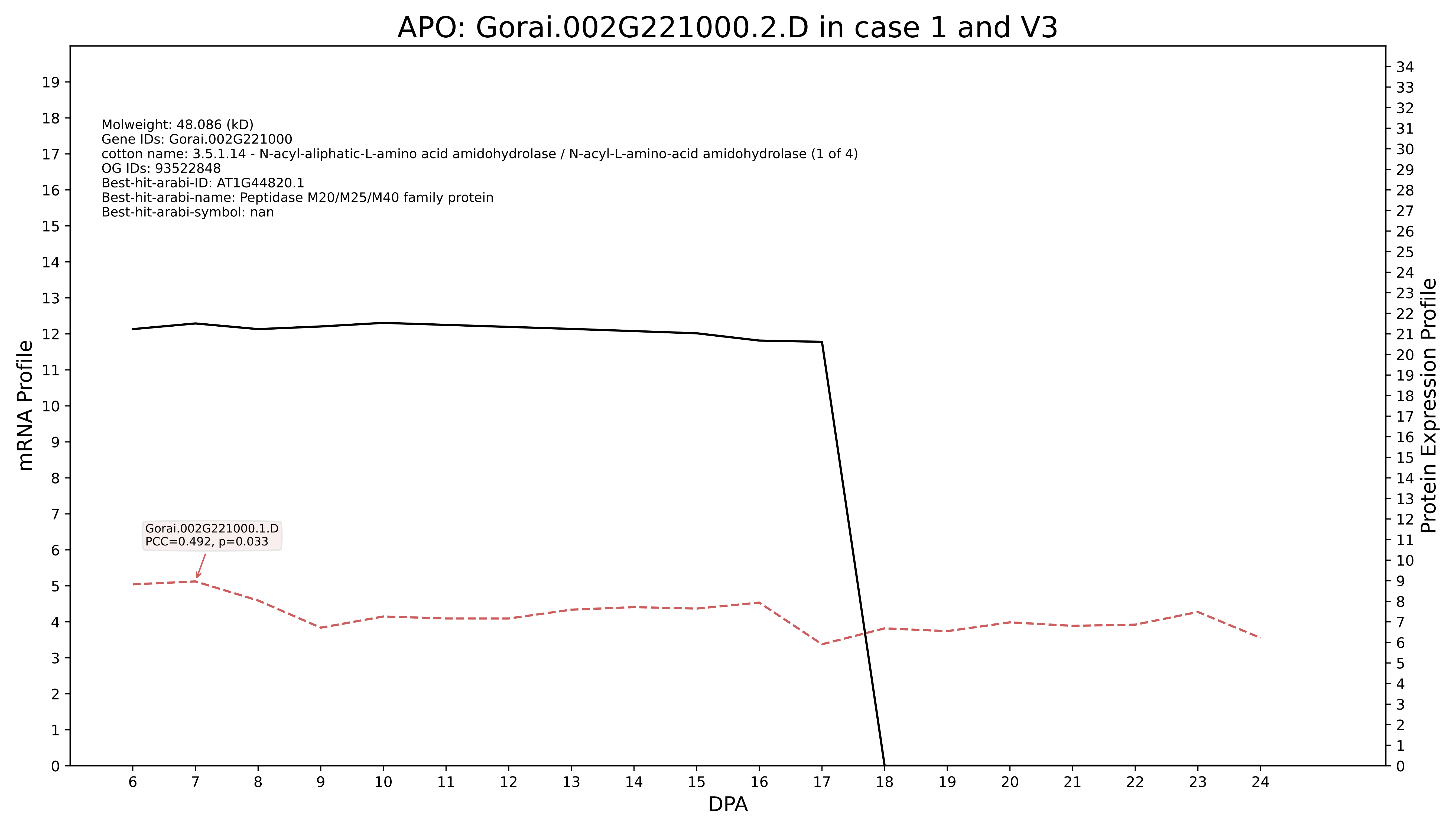

APO: Gorai.002G221000.2.D in case 1 and V3
Molweight: 48.086 (kD)
Gene IDs: Gorai.002G221000
cotton name: 3.5.1.14 - N-acyl-aliphatic-L-amino acid amidohydrolase / N-acyl-L-amino-acid amidohydrolase (1 of 4)
OG IDs: 93522848
Best-hit-arabi-ID: AT1G44820.1
Best-hit-arabi-name: Peptidase M20/M25/M40 family protein
Best-hit-arabi-symbol: nan
Gorai.002G221000.1.D
PCC=0.492, p=0.033
mRNA Profile
Protein Expression Profile
DPA

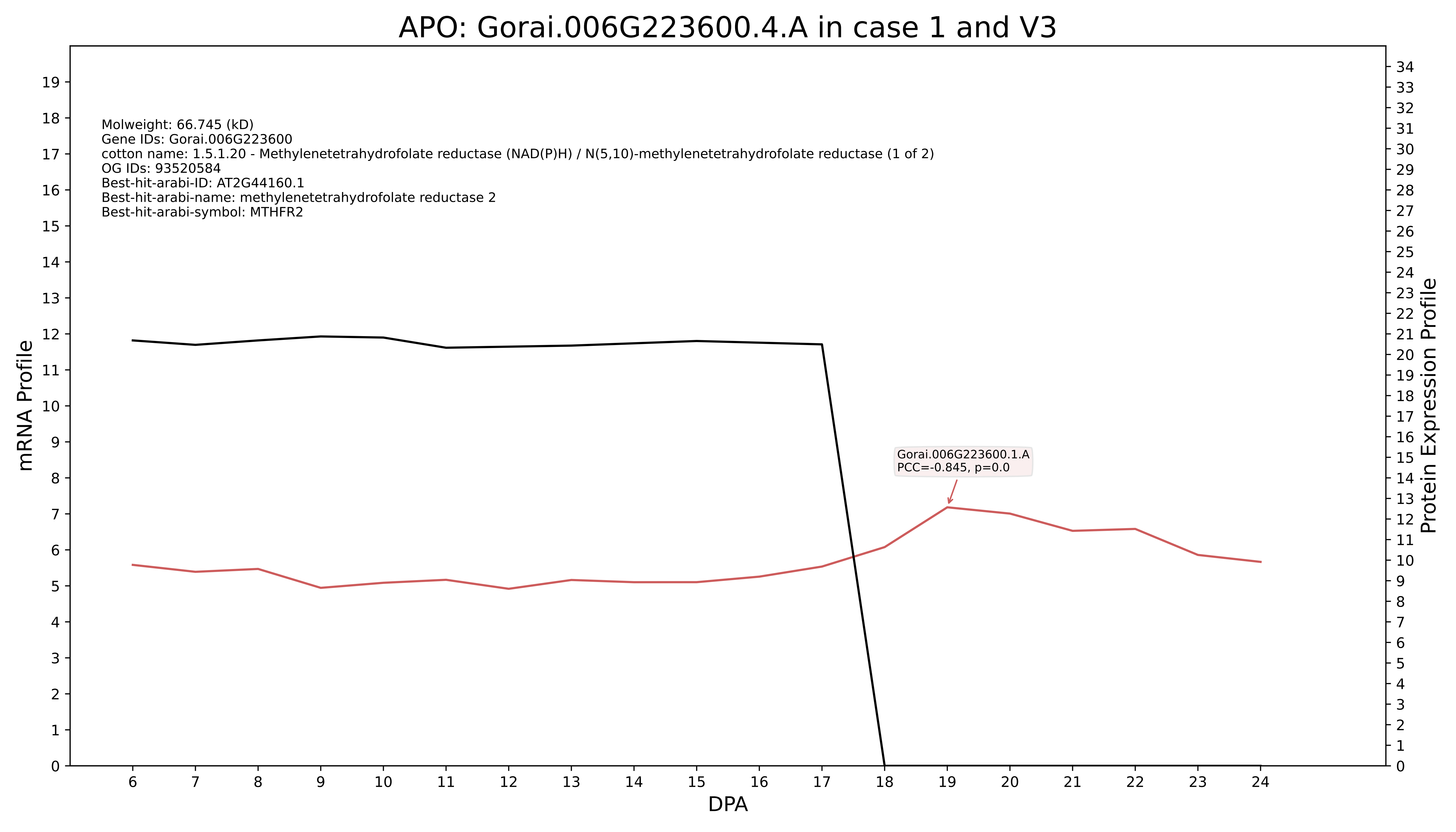
APO: Gorai.006G223600.4.A in case 1 and V3
Molweight: 66.745 (kD)
Gene IDs: Gorai.006G223600
cotton name: 1.5.1.20 - Methylenetetrahydrofolate reductase (NAD(P)H) / N(5,10)-methylenetetrahydrofolate reductase (1 of 2)
OG IDs: 93520584
Best-hit-arabi-ID: AT2G44160.1
Best-hit-arabi-name: methylenetetrahydrofolate reductase 2
Best-hit-arabi-symbol: MTHFR2
Gorai.006G223600.1.A
PCC=-0.845, p=0.0
mRNA Profile
Protein Expression Profile
DPA

APO: Gorai.006G147500.2.A in case 1 and V3

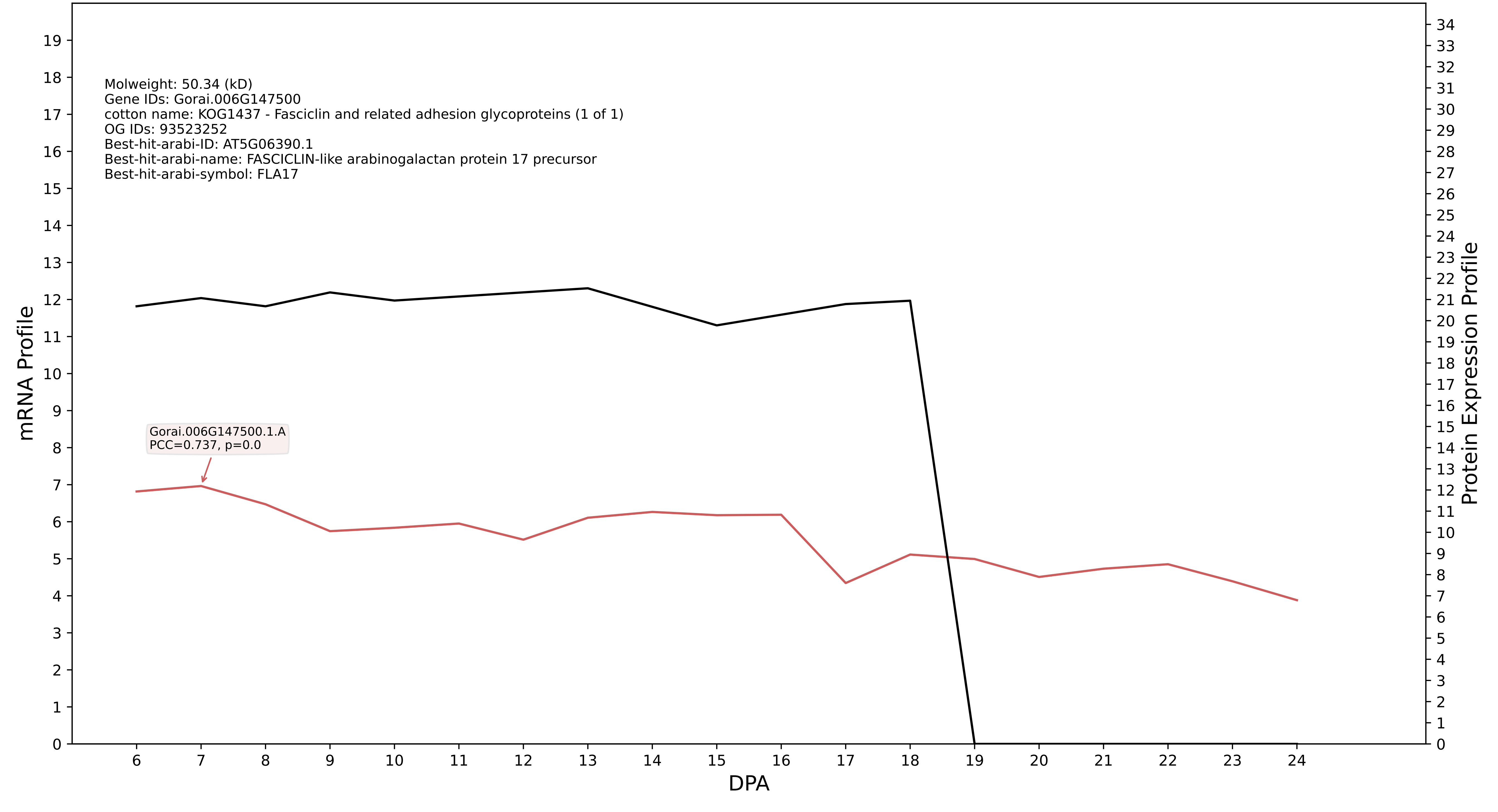

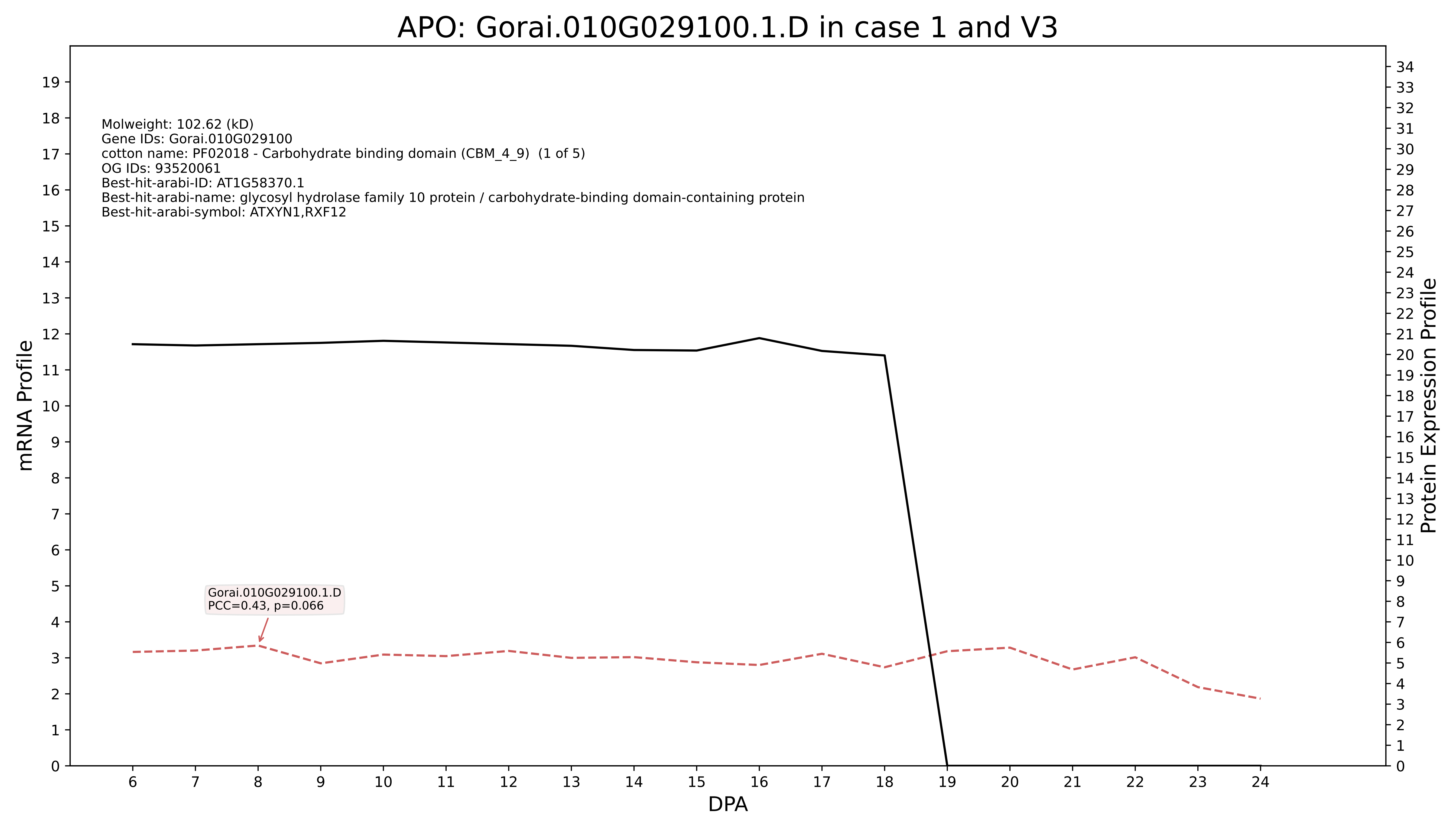

APO: Gorai.010G029100.1.D in case 1 and V3
Molweight: 102.62 (kD)
Gene IDs: Gorai.010G029100
cotton name: PF02018 - Carbohydrate binding domain (CBM_4_9) (1 of 5)
OG IDs: 93520061
Best-hit-arabi-ID: AT1G58370.1
Best-hit-arabi-name: glycosyl hydrolase family 10 protein / carbohydrate-binding domain-containing protein
Best-hit-arabi-symbol: ATXYN1,RXF12
Gorai.010G029100.1.D
PCC=0.43, p=0.066
mRNA Profile
Protein Expression Profile
DPA

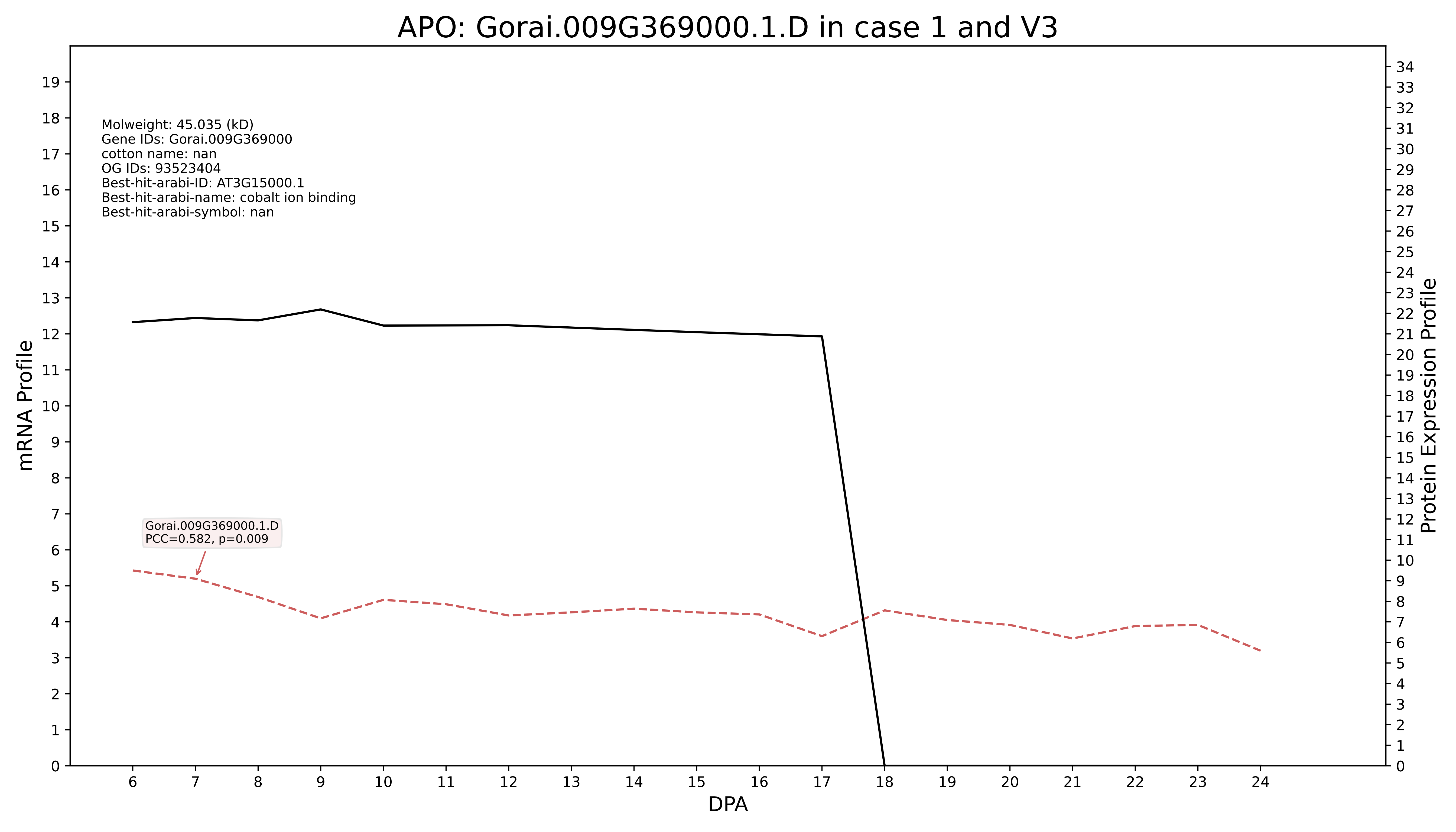
APO: Gorai.009G369000.1.D in case 1 and V3
Molweight: 45.035 (kD)
Gene IDs: Gorai.009G369000
cotton name: nan
OG IDs: 93523404
Best-hit-arabi-ID: AT3G15000.1
Best-hit-arabi-name: cobalt ion binding
Best-hit-arabi-symbol: nan
Gorai.009G369000.1.D
PCC=0.582, p=0.009
mRNA Profile
Protein Expression Profile
DPA

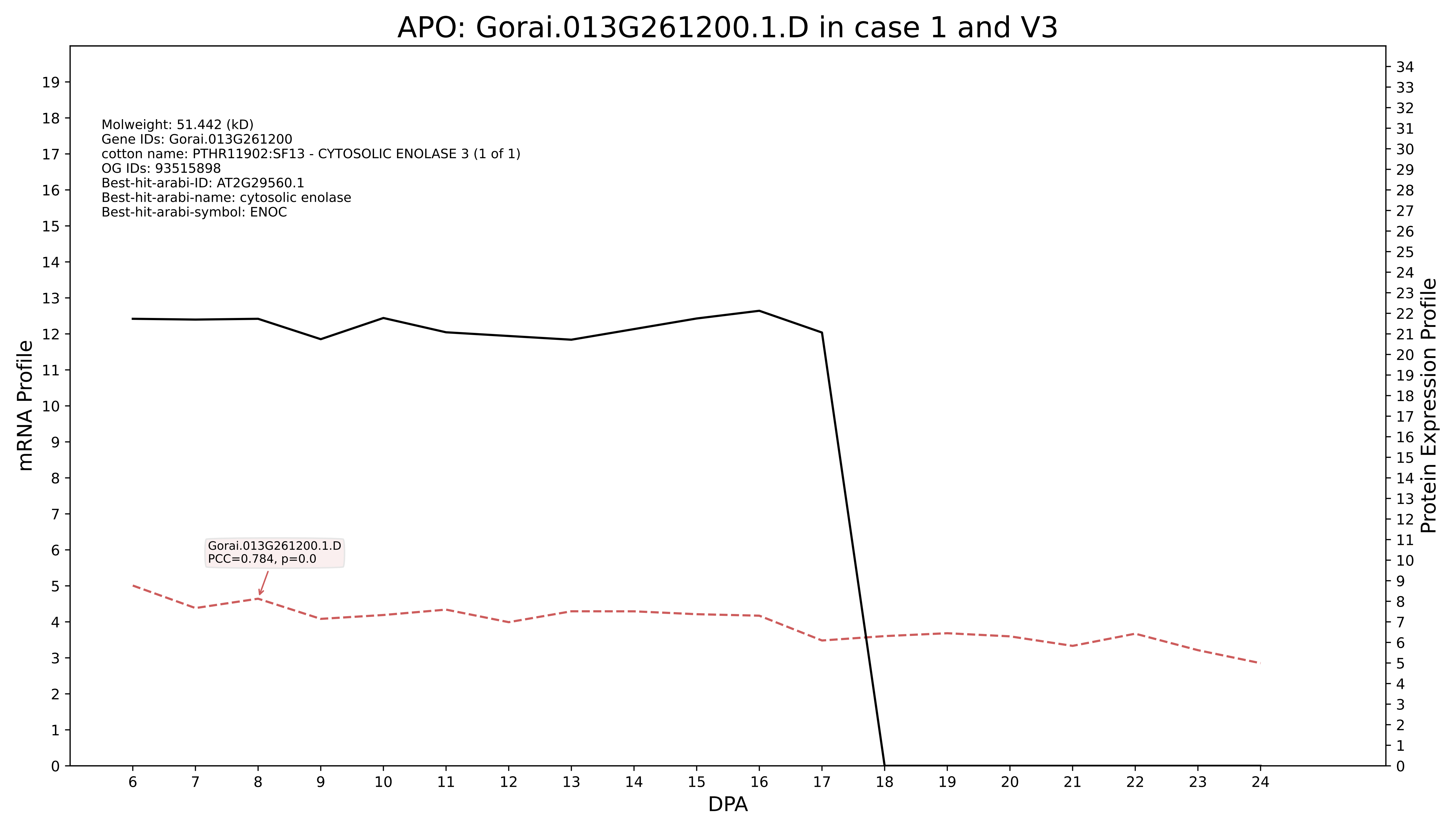
APO: Gorai.013G261200.1.D in case 1 and V3
Molweight: 51.442 (kD)
Gene IDs: Gorai.013G261200
cotton name: PTHR11902:SF13 - CYTOSOLIC ENOLASE 3 (1 of 1)
OG IDs: 93515898
Best-hit-arabi-ID: AT2G29560.1
Best-hit-arabi-name: cytosolic enolase
Best-hit-arabi-symbol: ENOC
Gorai.013G261200.1.D
PCC=0.784, p=0.0
mRNA Profile
Protein Expression Profile
DPA

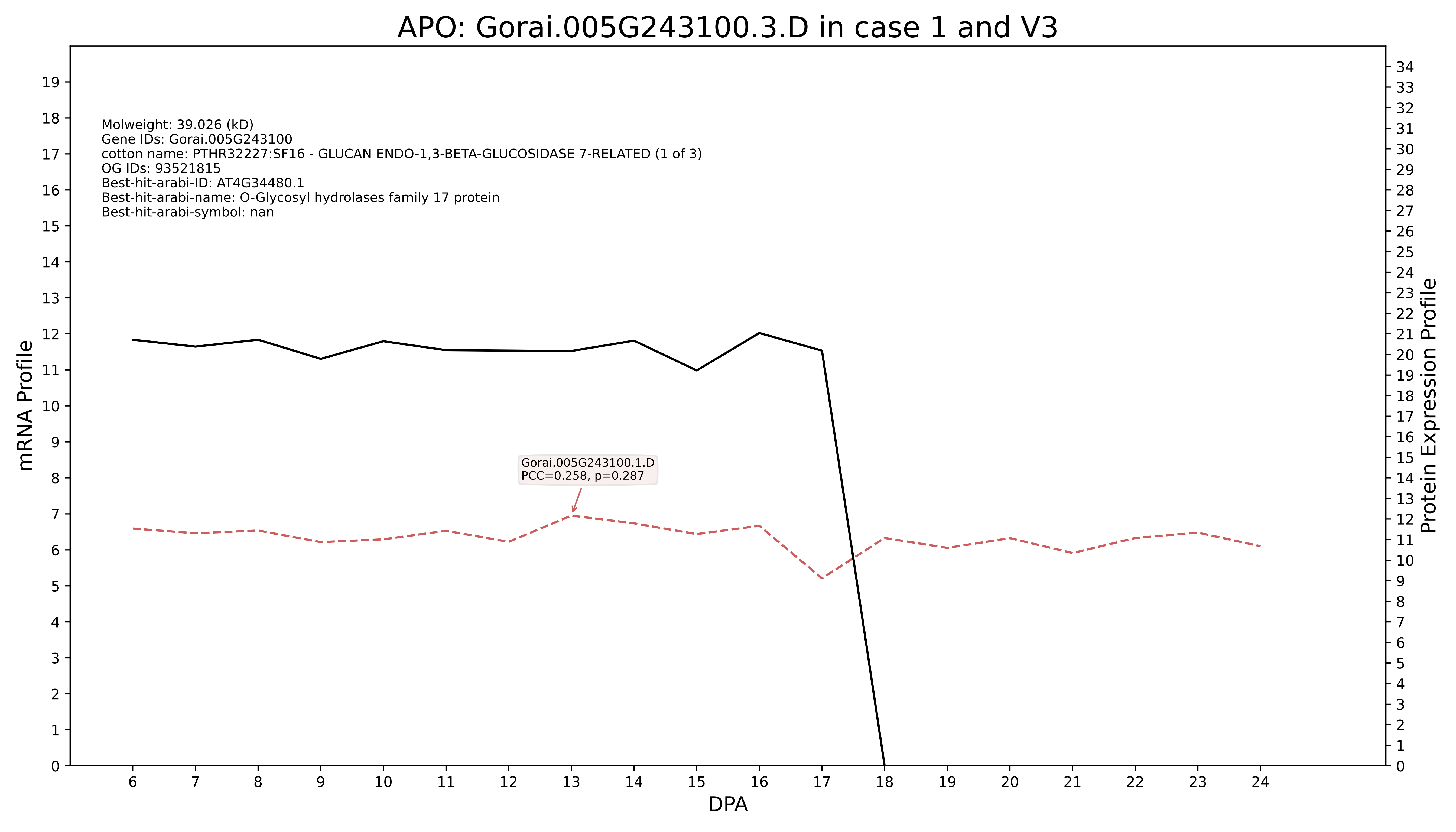

APO: Gorai.005G243100.3.D in case 1 and V3
Molweight: 39.026 (kD)
Gene IDs: Gorai.005G243100
cotton name: PTHR32227:SF16 - GLUCAN ENDO-1,3-BETA-GLUCOSIDASE 7-RELATED (1 of 3)
OG IDs: 93521815
Best-hit-arabi-ID: AT4G34480.1
Best-hit-arabi-name: O-Glycosyl hydrolases family 17 protein
Best-hit-arabi-symbol: nan
Gorai.005G243100.1.D
PCC=0.258, p=0.287
mRNA Profile
Protein Expression Profile
DPA

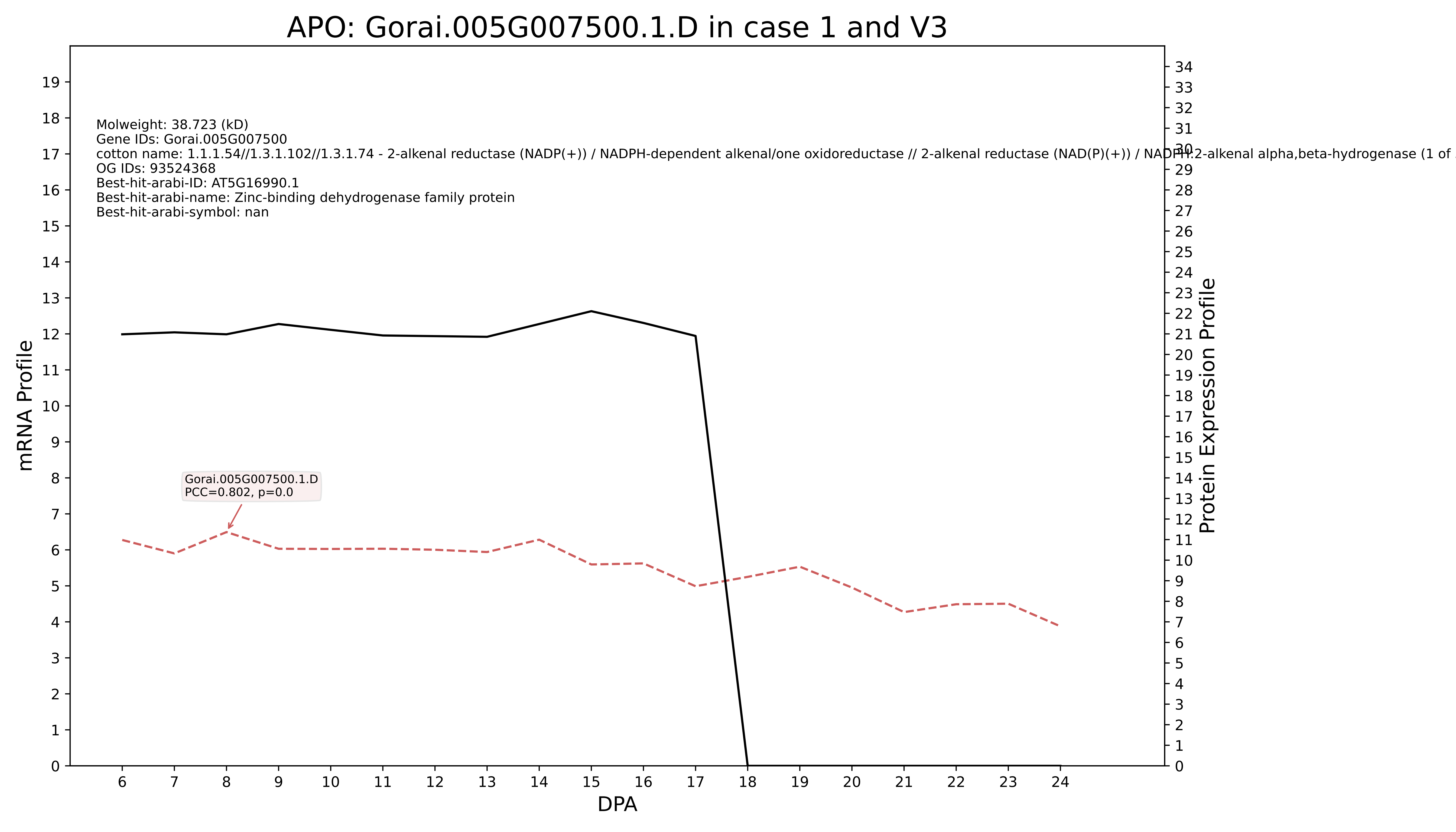

APO: Gorai.005G007500.1.D in case 1 and V3
Molweight: 38.723 (kD)
Gene IDs: Gorai.005G007500
cotton name: 1.1.1.54//1.3.1.102//1.3.1.74 - 2-alkenal reductase (NADP(+)) / NADPH-dependent alkenal/one oxidoreductase // 2-alkenal reductase (NAD(P)(+)) / NADPH:2-alkenal alpha,beta-hydrogenase (1 of
OG IDs: 93524368
Best-hit-arabi-ID: AT5G16990.1
Best-hit-arabi-name: Zinc-binding dehydrogenase family protein
Best-hit-arabi-symbol: nan
Gorai.005G007500.1.D
PCC=0.802, p=0.0
mRNA Profile
Protein Expression Profile
DPA

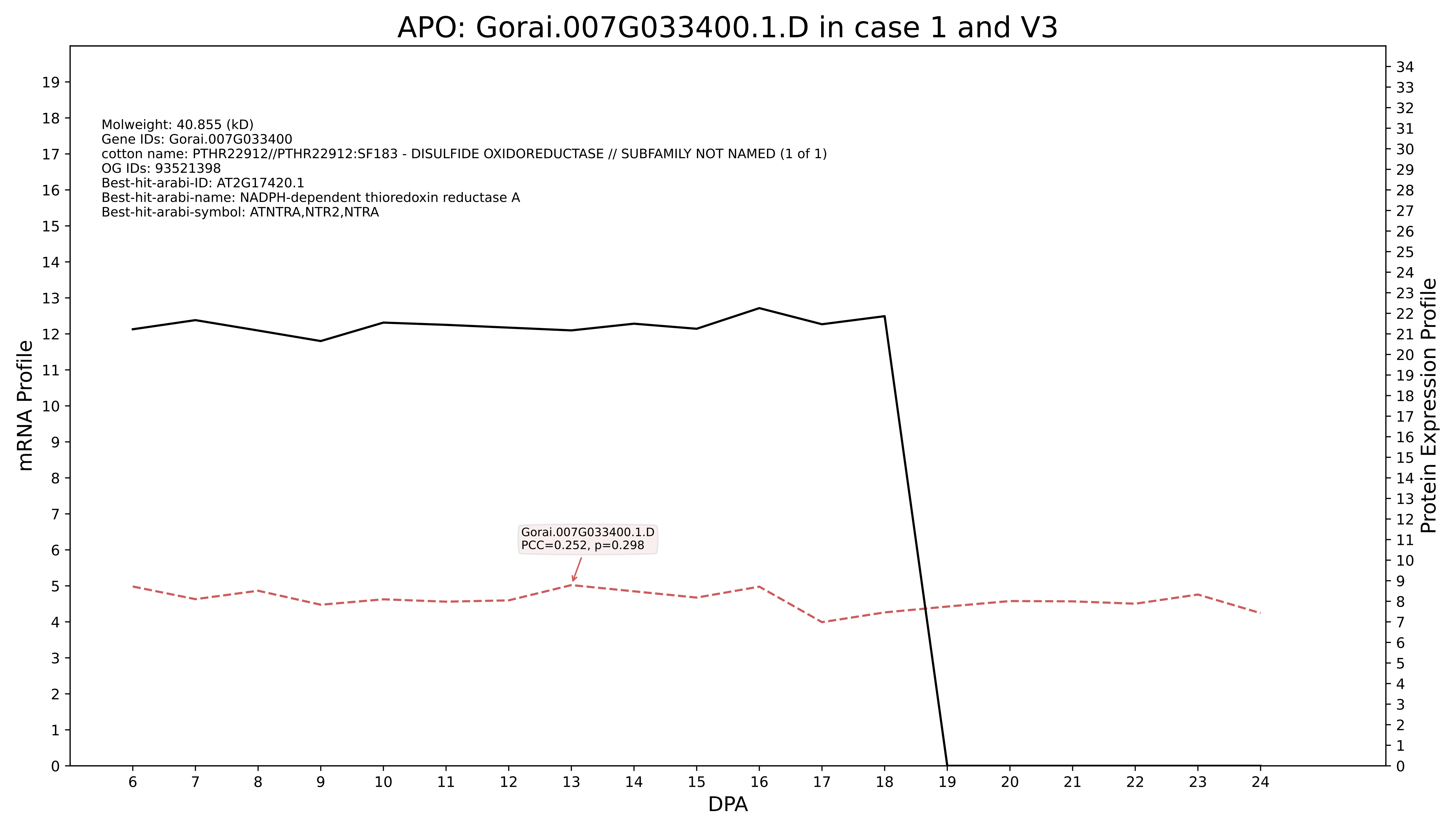
APO: Gorai.007G033400.1.D in case 1 and V3
Molweight: 40.855 (kD)
Gene IDs: Gorai.007G033400
cotton name: PTHR22912//PTHR22912:SF183 - DISULFIDE OXIDOREDUCTASE // SUBFAMILY NOT NAMED (1 of 1)
OG IDs: 93521398
Best-hit-arabi-ID: AT2G17420.1
Best-hit-arabi-name: NADPH-dependent thioredoxin reductase A
Best-hit-arabi-symbol: ATNTRA,NTR2,NTRA
Gorai.007G033400.1.D
PCC=0.252, p=0.298
mRNA Profile
Protein Expression Profile
DPA

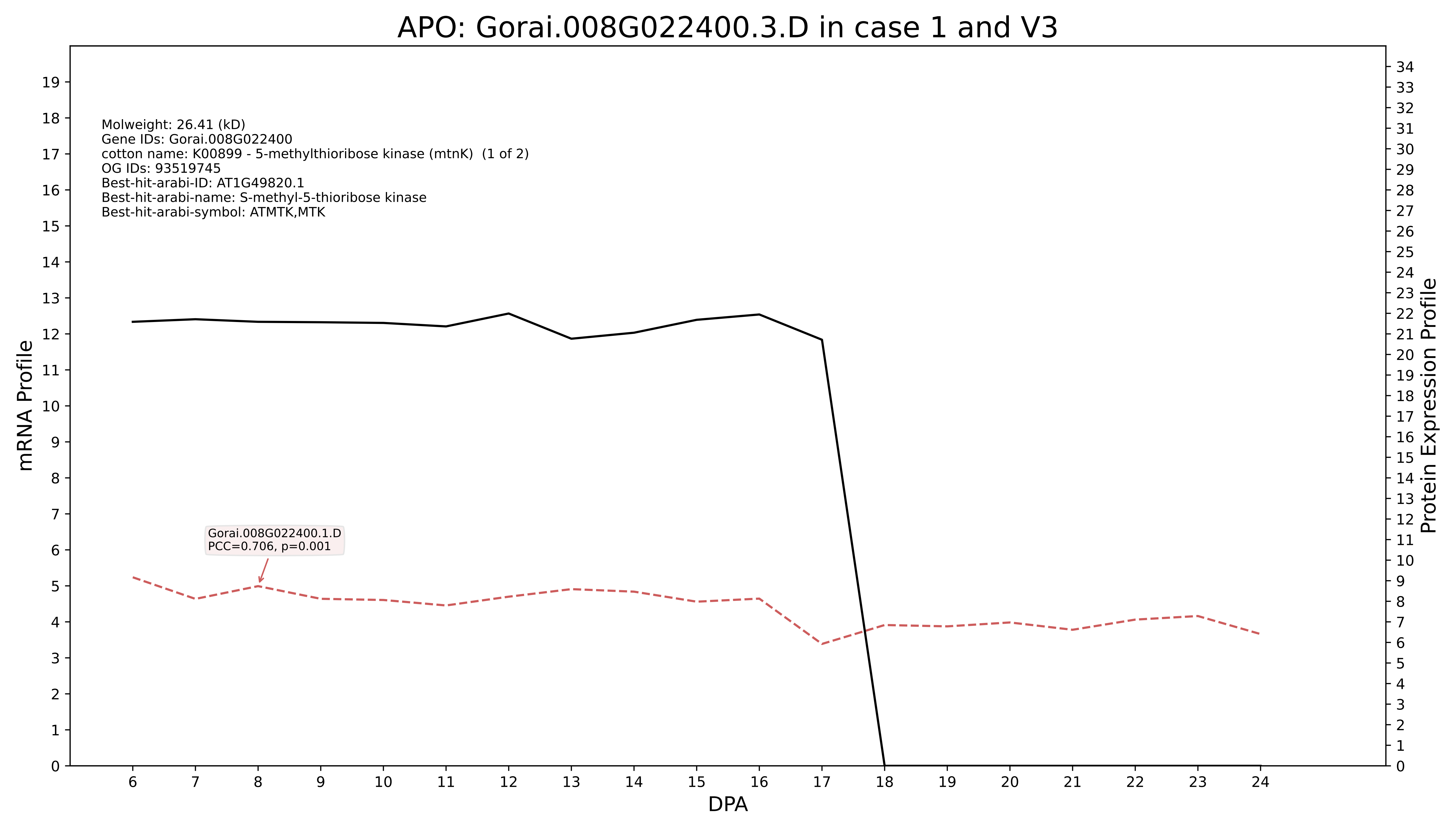
APO: Gorai.008G022400.3.D in case 1 and V3
Molweight: 26.41 (kD)
Gene IDs: Gorai.008G022400
cotton name: K00899 - 5-methylthioribose kinase (mtnK) (1 of 2)
OG IDs: 93519745
Best-hit-arabi-ID: AT1G49820.1
Best-hit-arabi-name: S-methyl-5-thioribose kinase
Best-hit-arabi-symbol: ATMTK,MTK
Gorai.008G022400.1.D
PCC=0.706, p=0.001
mRNA Profile
Protein Expression Profile
DPA

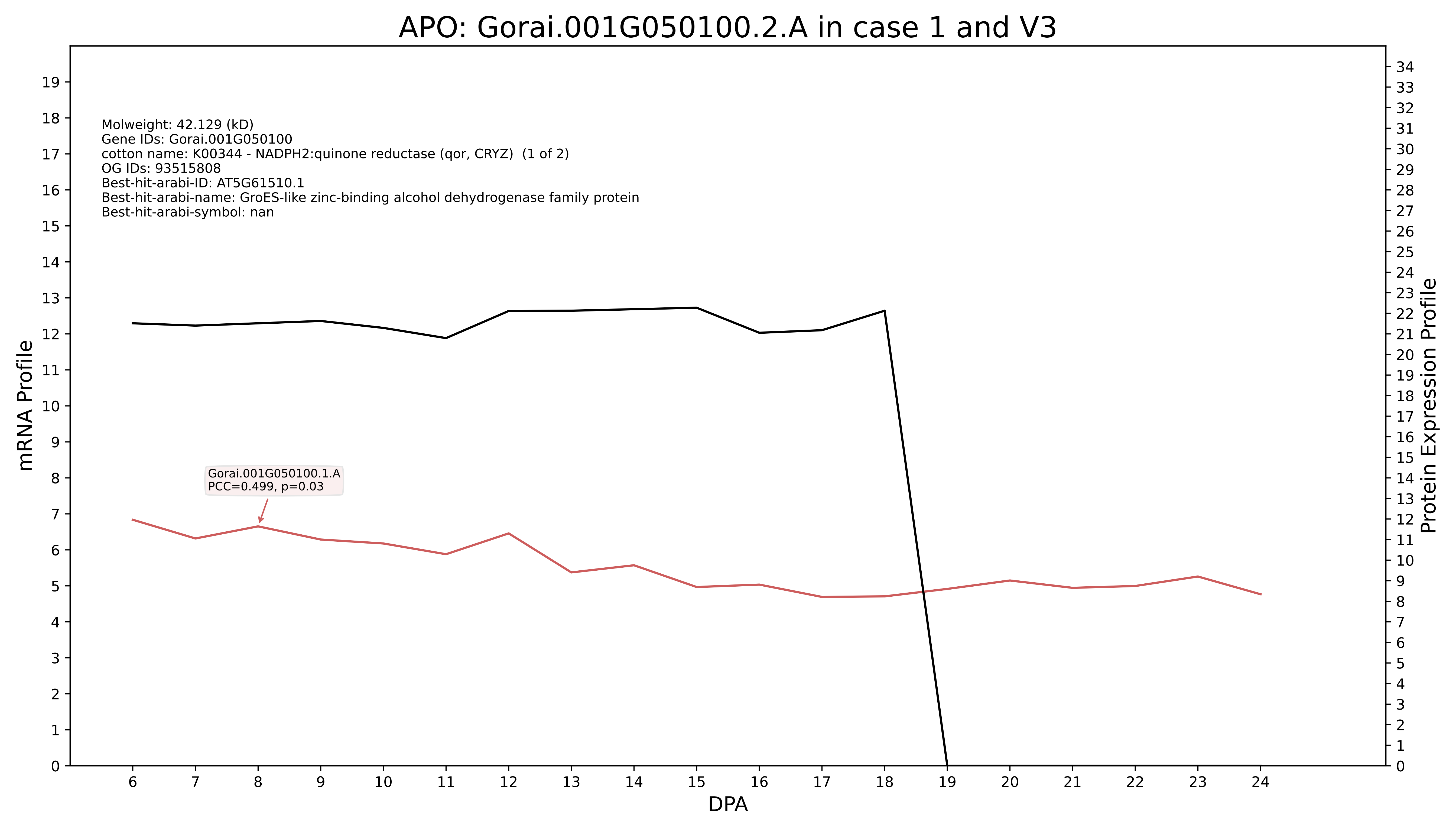
APO: Gorai.001G050100.2.A in case 1 and V3
Molweight: 42.129 (kD)
Gene IDs: Gorai.001G050100
cotton name: K00344 - NADPH2:quinone reductase (qor, CRYZ) (1 of 2)
OG IDs: 93515808
Best-hit-arabi-ID: AT5G61510.1
Best-hit-arabi-name: GroES-like zinc-binding alcohol dehydrogenase family protein
Best-hit-arabi-symbol: nan
Gorai.001G050100.1.A
PCC=0.499, p=0.03
mRNA Profile
Protein Expression Profile
DPA

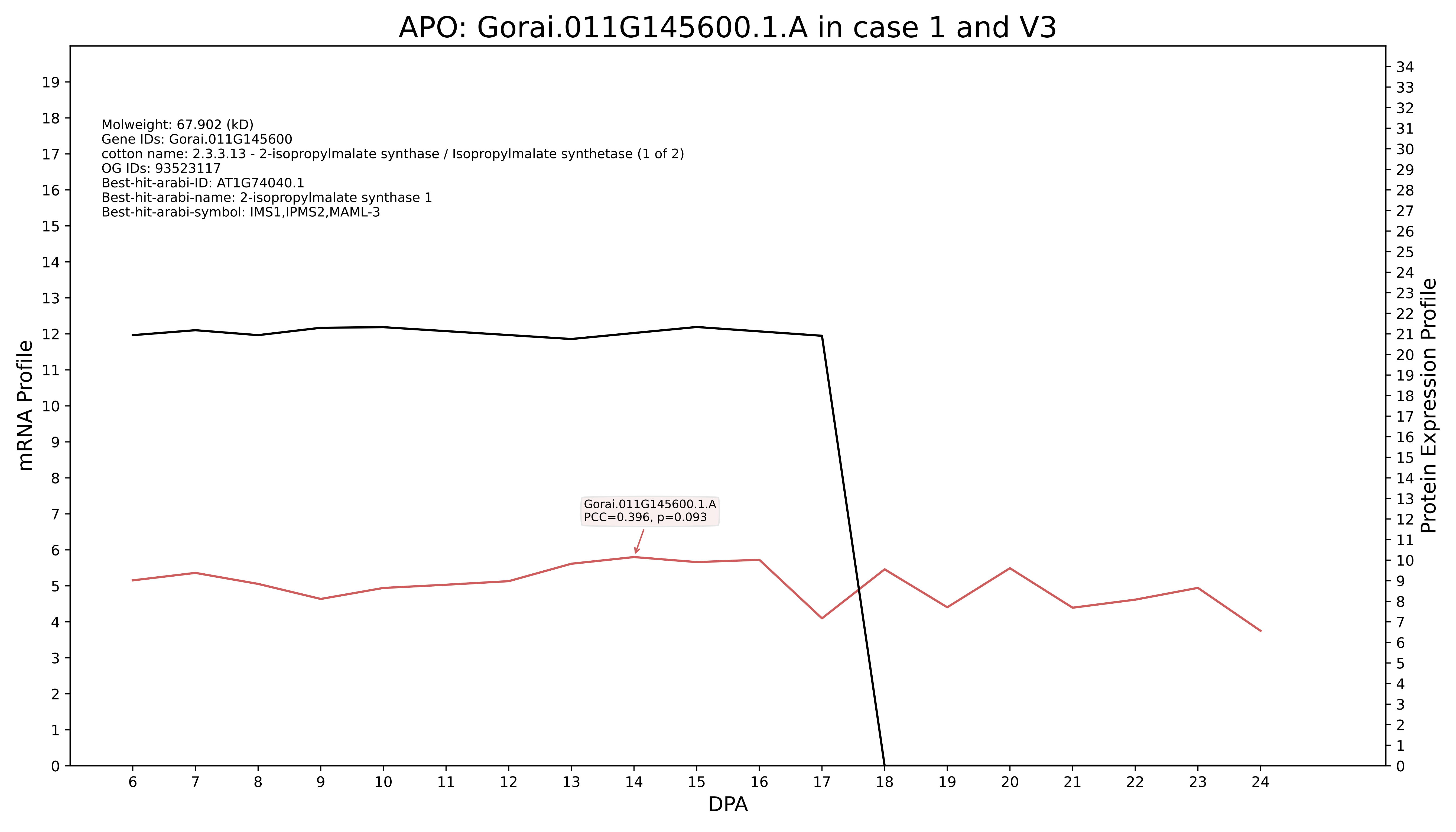

APO: Gorai.011G145600.1.A in case 1 and V3
Molweight: 67.902 (kD)
Gene IDs: Gorai.011G145600
cotton name: 2.3.3.13 - 2-isopropylmalate synthase / Isopropylmalate synthetase (1 of 2)
OG IDs: 93523117
Best-hit-arabi-ID: AT1G74040.1
Best-hit-arabi-name: 2-isopropylmalate synthase 1
Best-hit-arabi-symbol: IMS1,IPMS2,MAML-3
Gorai.011G145600.1.A
PCC=0.396, p=0.093
mRNA Profile
Protein Expression Profile
DPA

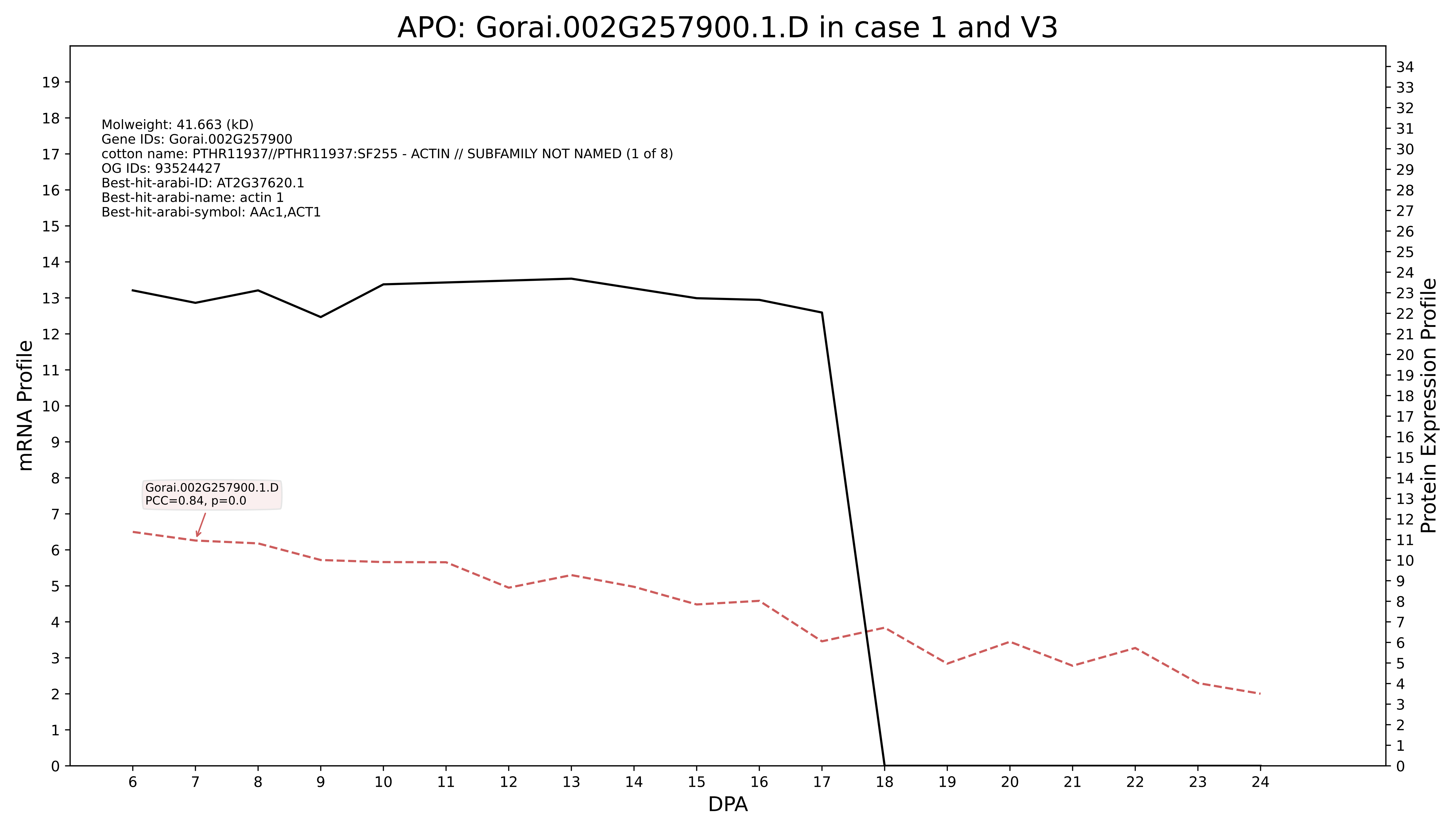

APO: Gorai.002G257900.1.D in case 1 and V3
Molweight: 41.663 (kD)
Gene IDs: Gorai.002G257900
cotton name: PTHR11937//PTHR11937:SF255 - ACTIN // SUBFAMILY NOT NAMED (1 of 8)
OG IDs: 93524427
Best-hit-arabi-ID: AT2G37620.1
Best-hit-arabi-name: actin 1
Best-hit-arabi-symbol: AAc1,ACT1
Gorai.002G257900.1.D
PCC=0.84, p=0.0
mRNA Profile
Protein Expression Profile
DPA

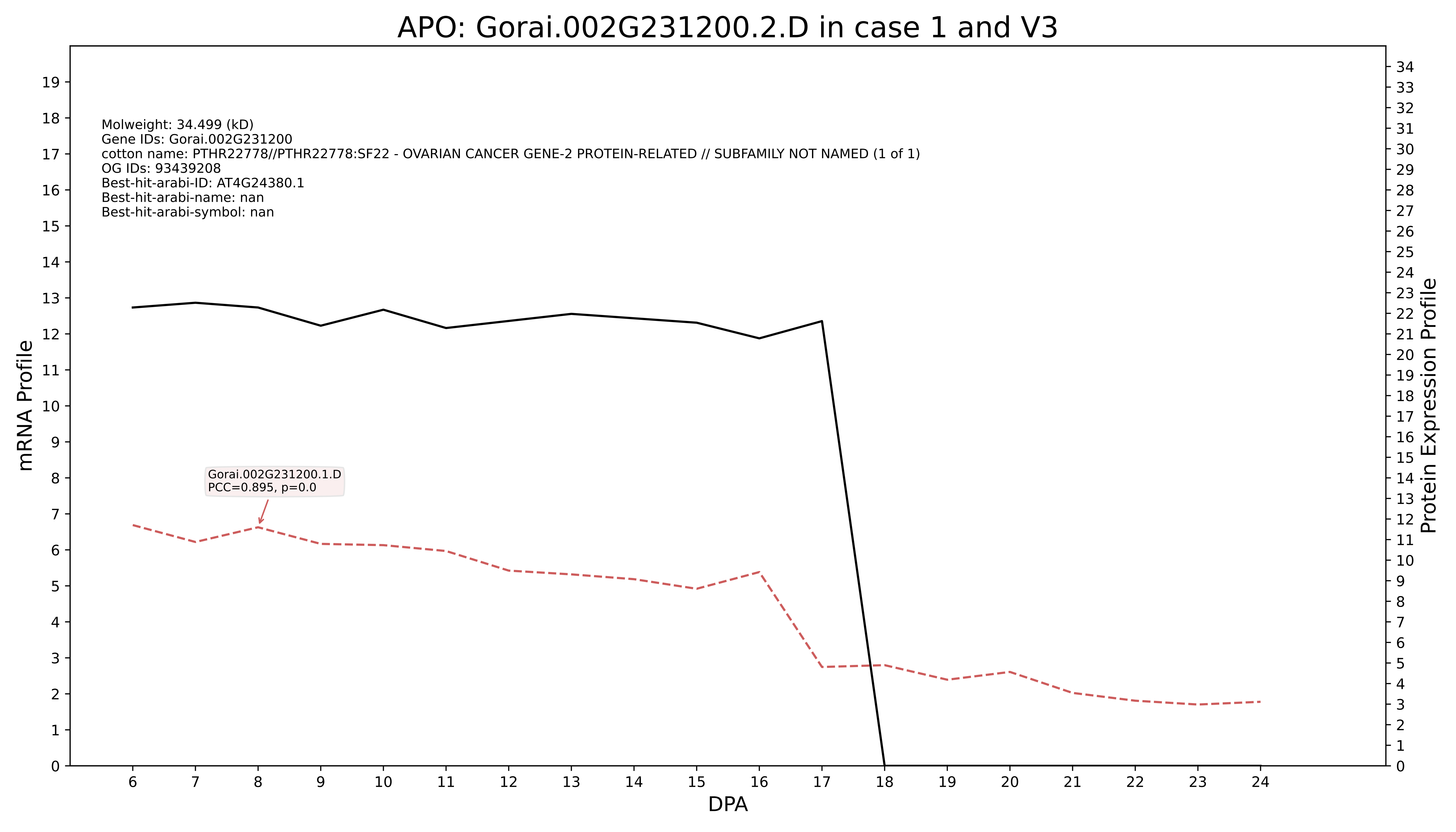

APO: Gorai.002G231200.2.D in case 1 and V3
Molweight: 34.499 (kD)
Gene IDs: Gorai.002G231200
cotton name: PTHR22778//PTHR22778:SF22 - OVARIAN CANCER GENE-2 PROTEIN-RELATED // SUBFAMILY NOT NAMED (1 of 1)
OG IDs: 93439208
Best-hit-arabi-ID: AT4G24380.1
Best-hit-arabi-name: nan
Best-hit-arabi-symbol: nan
Gorai.002G231200.1.D
PCC=0.895, p=0.0
mRNA Profile
Protein Expression Profile
DPA

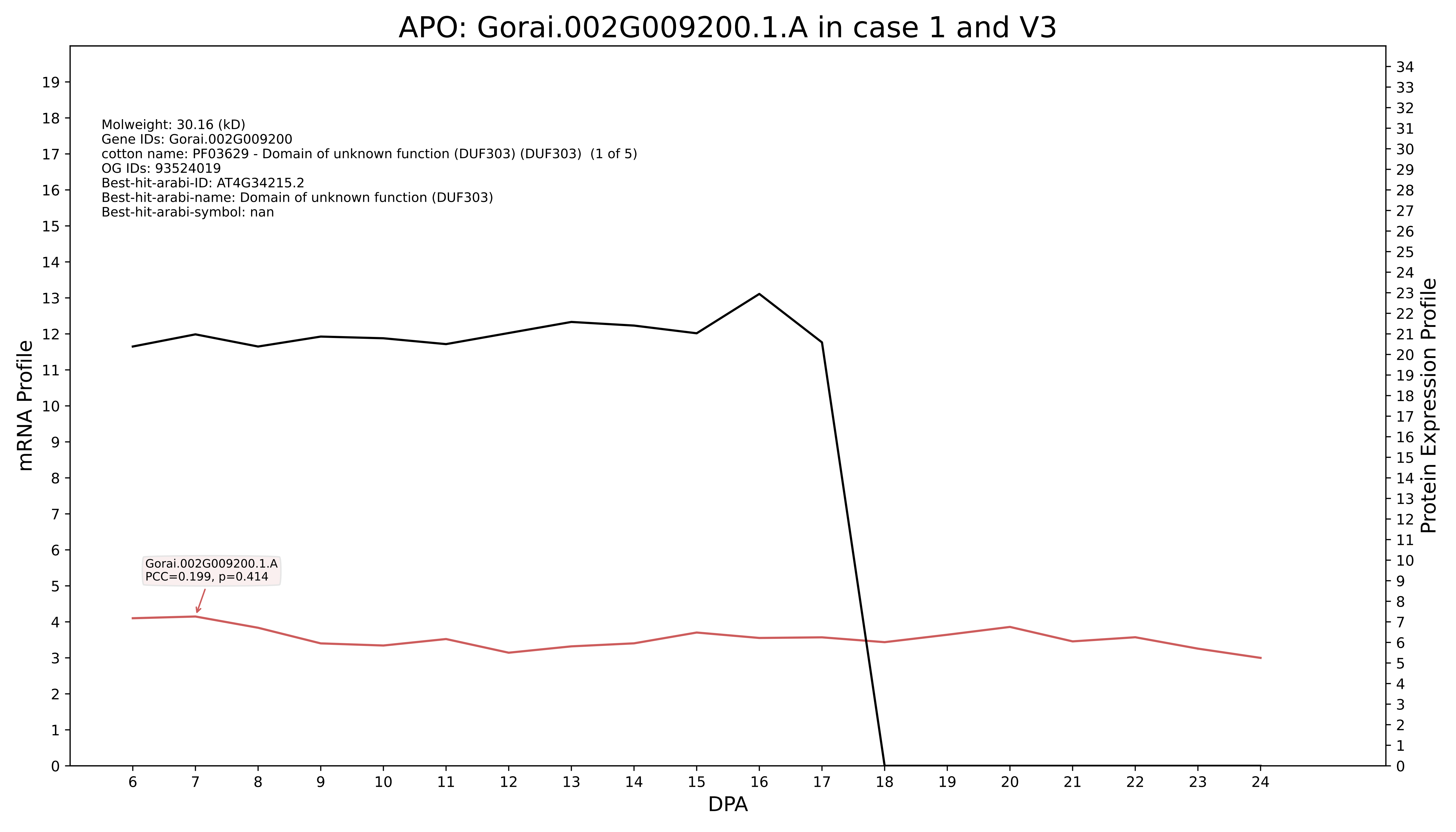
APO: Gorai.002G009200.1.A in case 1 and V3
Molweight: 30.16 (kD)
Gene IDs: Gorai.002G009200
cotton name: PF03629 - Domain of unknown function (DUF303) (DUF303) (1 of 5)
OG IDs: 93524019
Best-hit-arabi-ID: AT4G34215.2
Best-hit-arabi-name: Domain of unknown function (DUF303)
Best-hit-arabi-symbol: nan
Gorai.002G009200.1.A
PCC=0.199, p=0.414
mRNA Profile
Protein Expression Profile
DPA

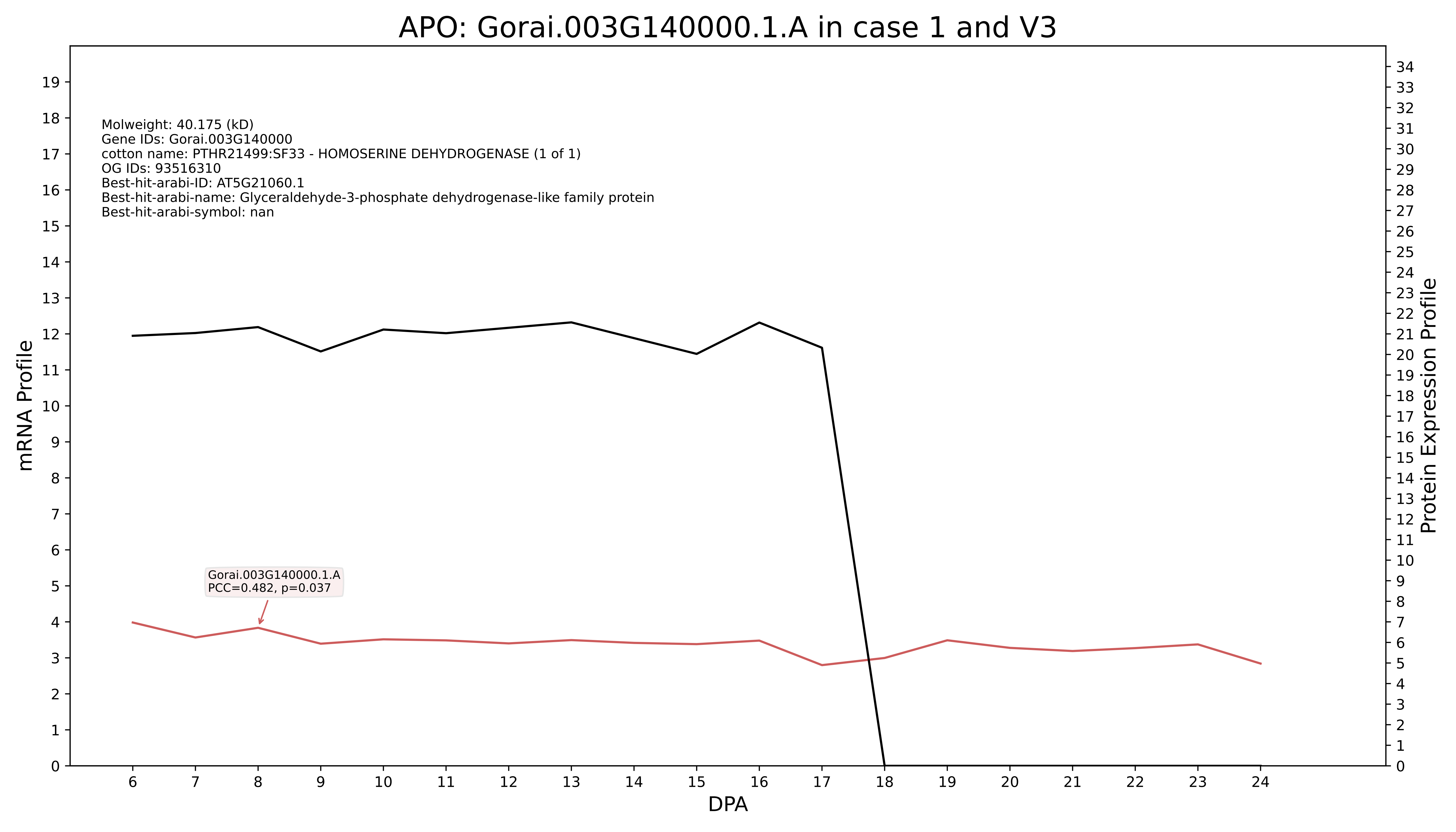

APO: Gorai.003G140000.1.A in case 1 and V3
Molweight: 40.175 (kD)
Gene IDs: Gorai.003G140000
cotton name: PTHR21499:SF33 - HOMOSERINE DEHYDROGENASE (1 of 1)
OG IDs: 93516310
Best-hit-arabi-ID: AT5G21060.1
Best-hit-arabi-name: Glyceraldehyde-3-phosphate dehydrogenase-like family protein
Best-hit-arabi-symbol: nan
Gorai.003G140000.1.A
PCC=0.482, p=0.037
mRNA Profile
Protein Expression Profile
DPA

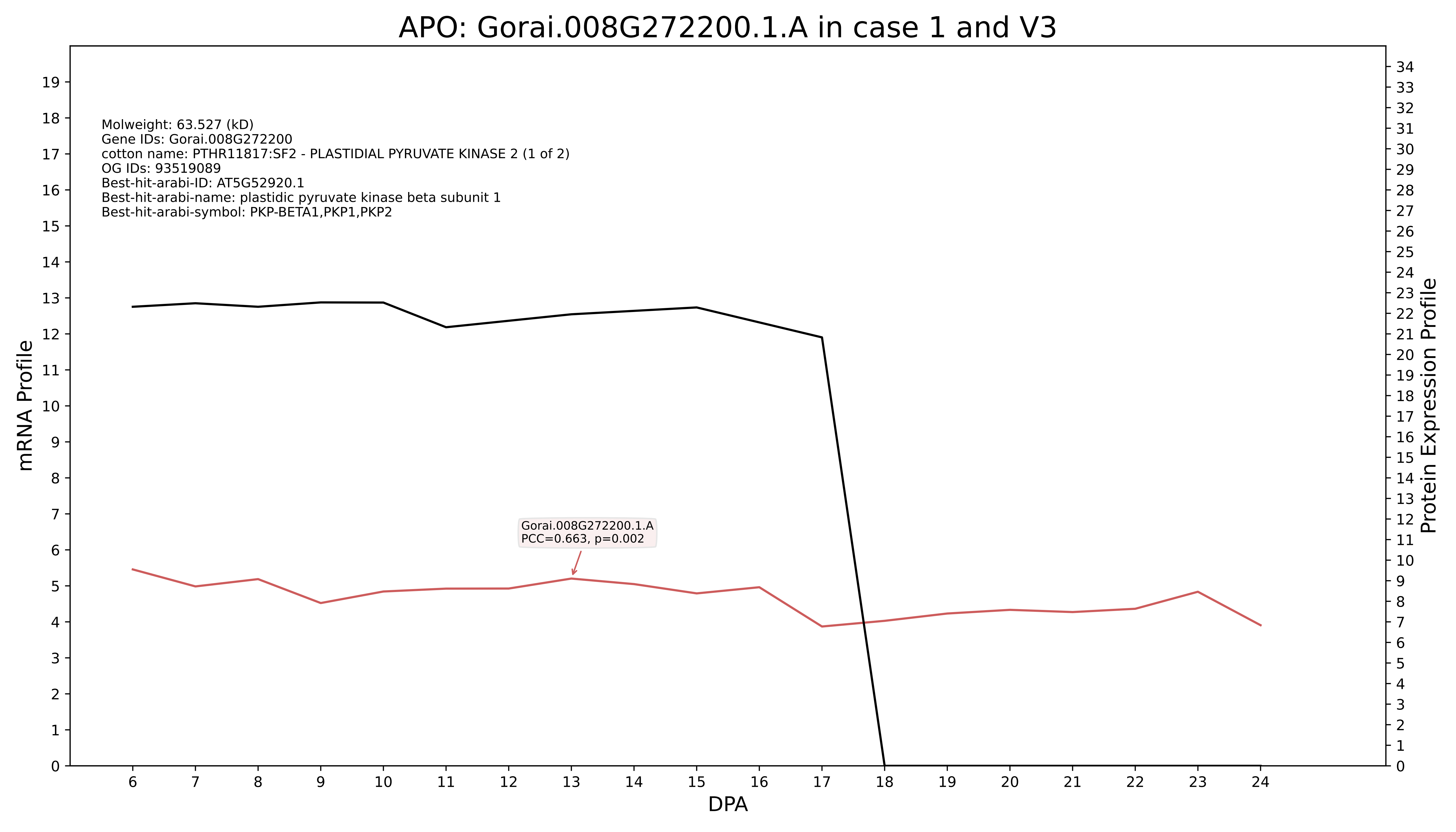
APO: Gorai.008G272200.1.A in case 1 and V3
Molweight: 63.527 (kD)
Gene IDs: Gorai.008G272200
cotton name: PTHR11817:SF2 - PLASTIDIAL PYRUVATE KINASE 2 (1 of 2)
OG IDs: 93519089
Best-hit-arabi-ID: AT5G52920.1
Best-hit-arabi-name: plastidic pyruvate kinase beta subunit 1
Best-hit-arabi-symbol: PKP-BETA1,PKP1,PKP2
Gorai.008G272200.1.A
PCC=0.663, p=0.002
mRNA Profile
Protein Expression Profile
DPA

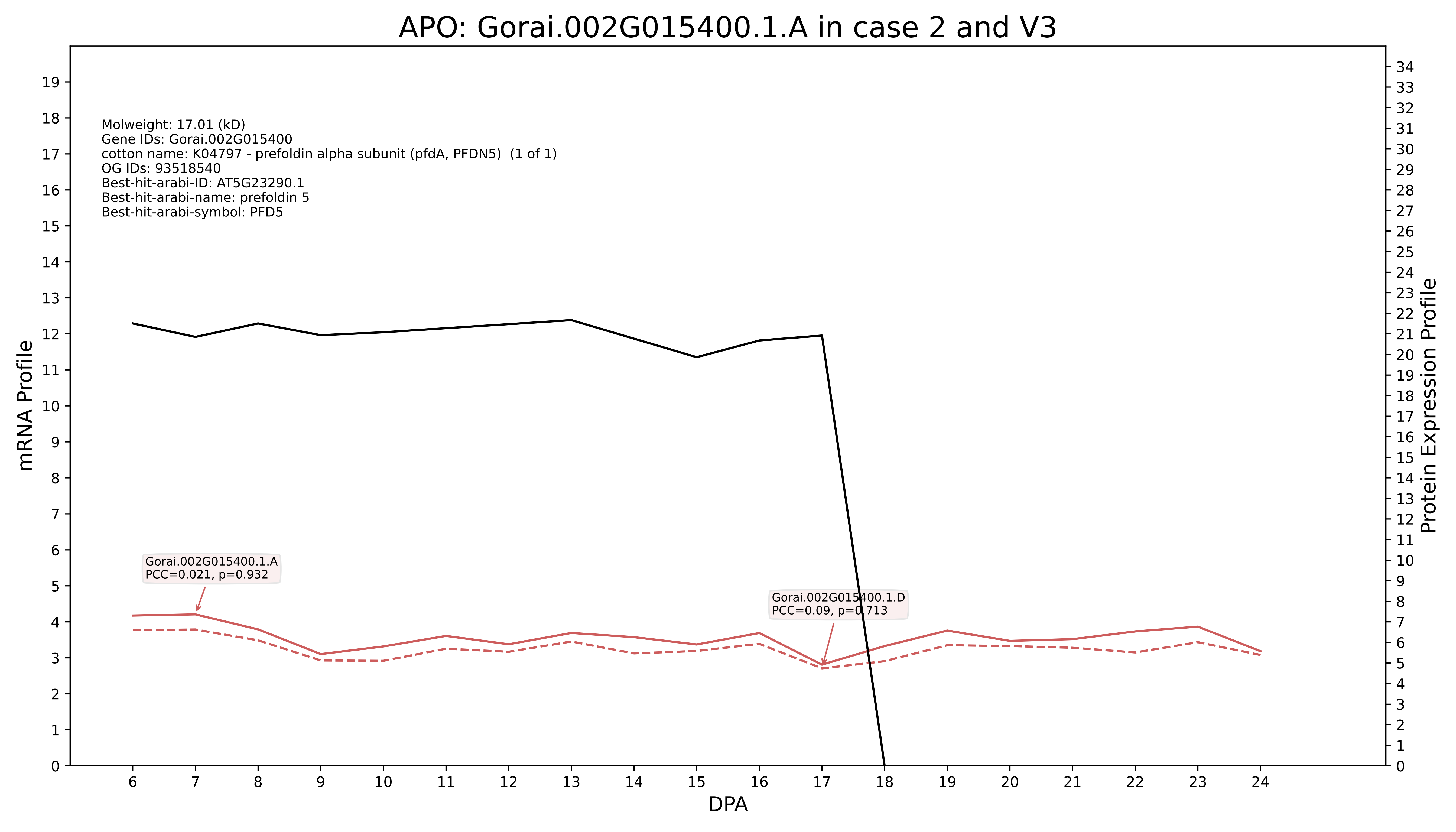
APO: Gorai.002G015400.1.A in case 2 and V3
Molweight: 17.01 (kD)
Gene IDs: Gorai.002G015400
cotton name: K04797 - prefoldin alpha subunit (pfdA, PFDN5) (1 of 1)
OG IDs: 93518540
Best-hit-arabi-ID: AT5G23290.1
Best-hit-arabi-name: prefoldin 5
Best-hit-arabi-symbol: PFD5
Gorai.002G015400.1.A
PCC=0.021, p=0.932
Gorai.002G015400.1.D
PCC=0.09, p=0.713
mRNA Profile
Protein Expression Profile
DPA

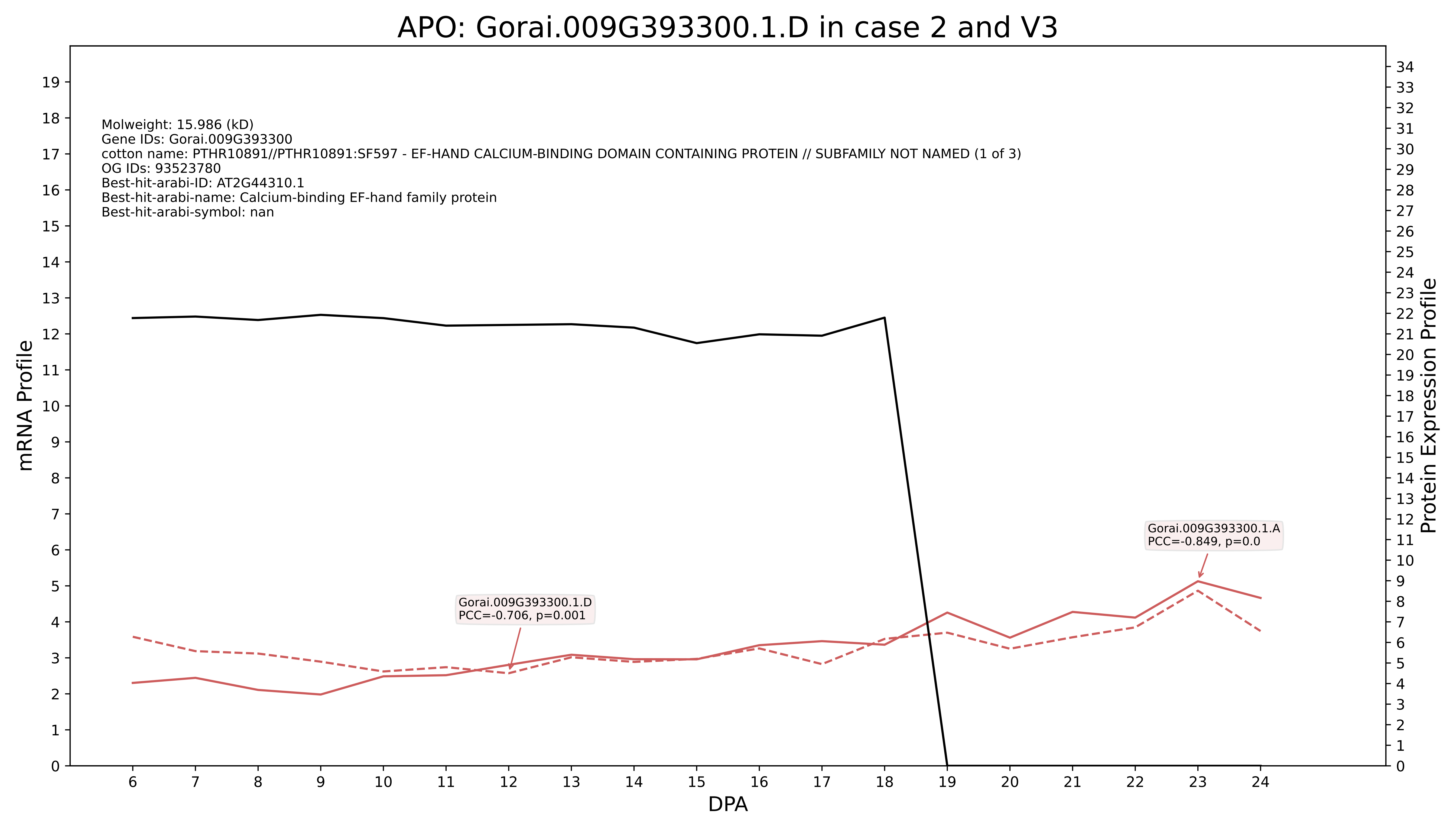

APO: Gorai.009G393300.1.D in case 2 and V3
Molweight: 15.986 (kD)
Gene IDs: Gorai.009G393300
cotton name: PTHR10891//PTHR10891:SF597 - EF-HAND CALCIUM-BINDING DOMAIN CONTAINING PROTEIN // SUBFAMILY NOT NAMED (1 of 3)
OG IDs: 93523780
Best-hit-arabi-ID: AT2G44310.1
Best-hit-arabi-name: Calcium-binding EF-hand family protein
Best-hit-arabi-symbol: nan
Gorai.009G393300.1.D
PCC=-0.706, p=0.001
Gorai.009G393300.1.A
PCC=-0.849, p=0.0
mRNA Profile
Protein Expression Profile
DPA

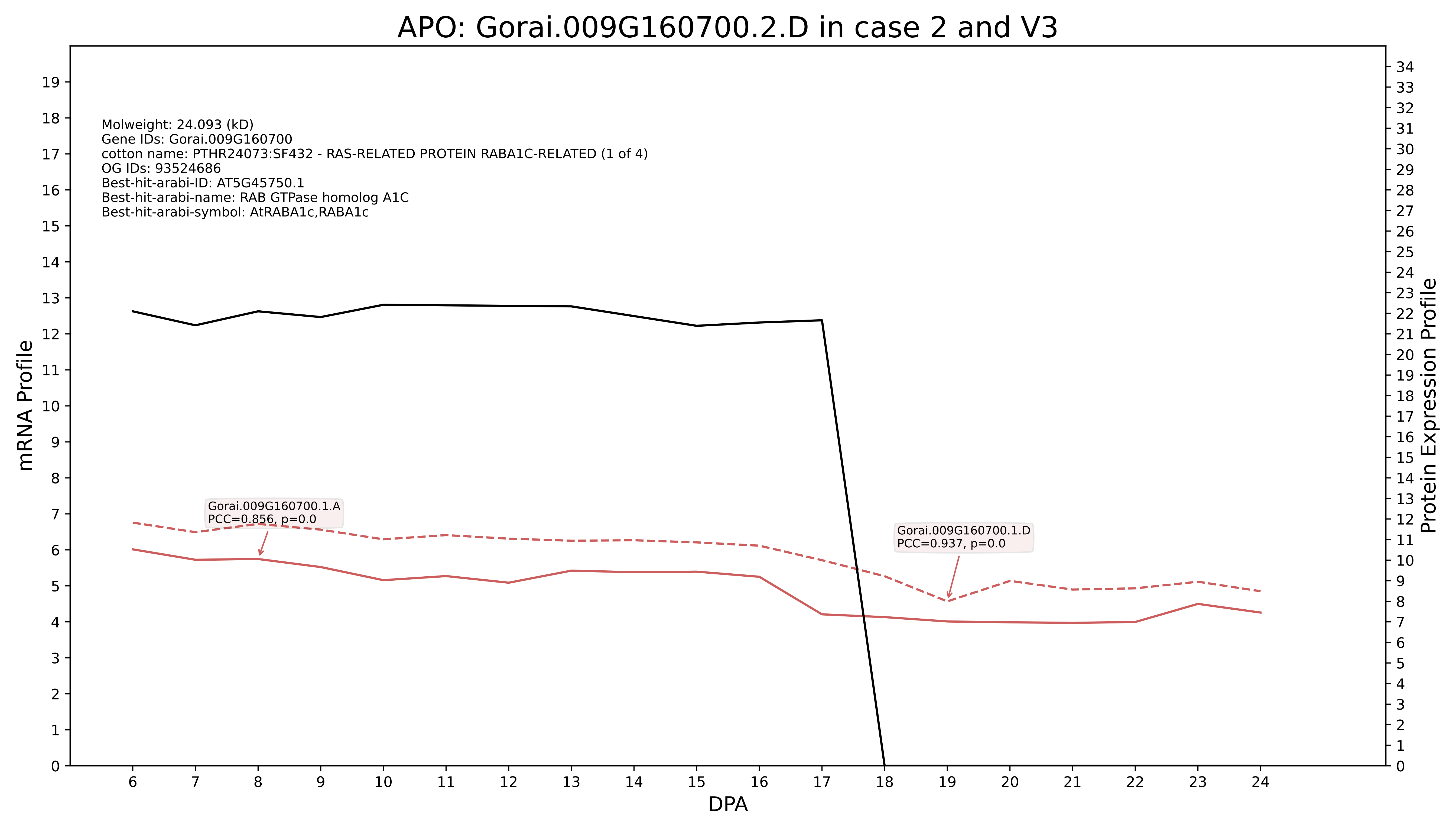

APO: Gorai.009G160700.2.D in case 2 and V3
Molweight: 24.093 (kD)
Gene IDs: Gorai.009G160700
cotton name: PTHR24073:SF432 - RAS-RELATED PROTEIN RABA1C-RELATED (1 of 4)
OG IDs: 93524686
Best-hit-arabi-ID: AT5G45750.1
Best-hit-arabi-name: RAB GTPase homolog A1C
Best-hit-arabi-symbol: AtRABA1c,RABA1c
Gorai.009G160700.1.A
PCC=0.856, p=0.0
Gorai.009G160700.1.D
PCC=0.937, p=0.0
mRNA Profile
Protein Expression Profile
DPA

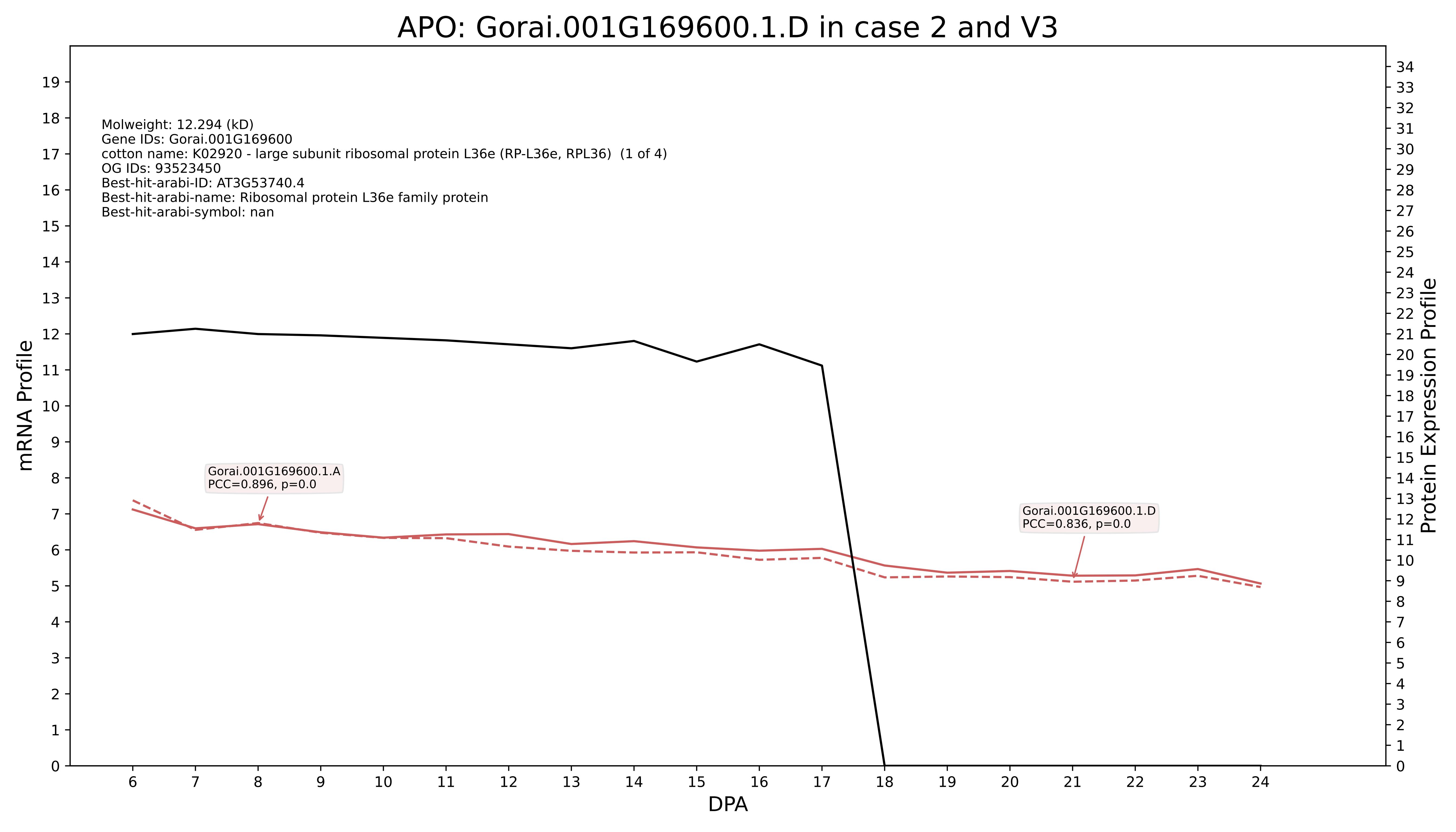

APO: Gorai.001G169600.1.D in case 2 and V3
Molweight: 12.294 (kD)
Gene IDs: Gorai.001G169600
cotton name: K02920 - large subunit ribosomal protein L36e (RP-L36e, RPL36) (1 of 4)
OG IDs: 93523450
Best-hit-arabi-ID: AT3G53740.4
Best-hit-arabi-name: Ribosomal protein L36e family protein
Best-hit-arabi-symbol: nan
Gorai.001G169600.1.A
PCC=0.896, p=0.0
Gorai.001G169600.1.D
PCC=0.836, p=0.0
mRNA Profile
Protein Expression Profile
DPA

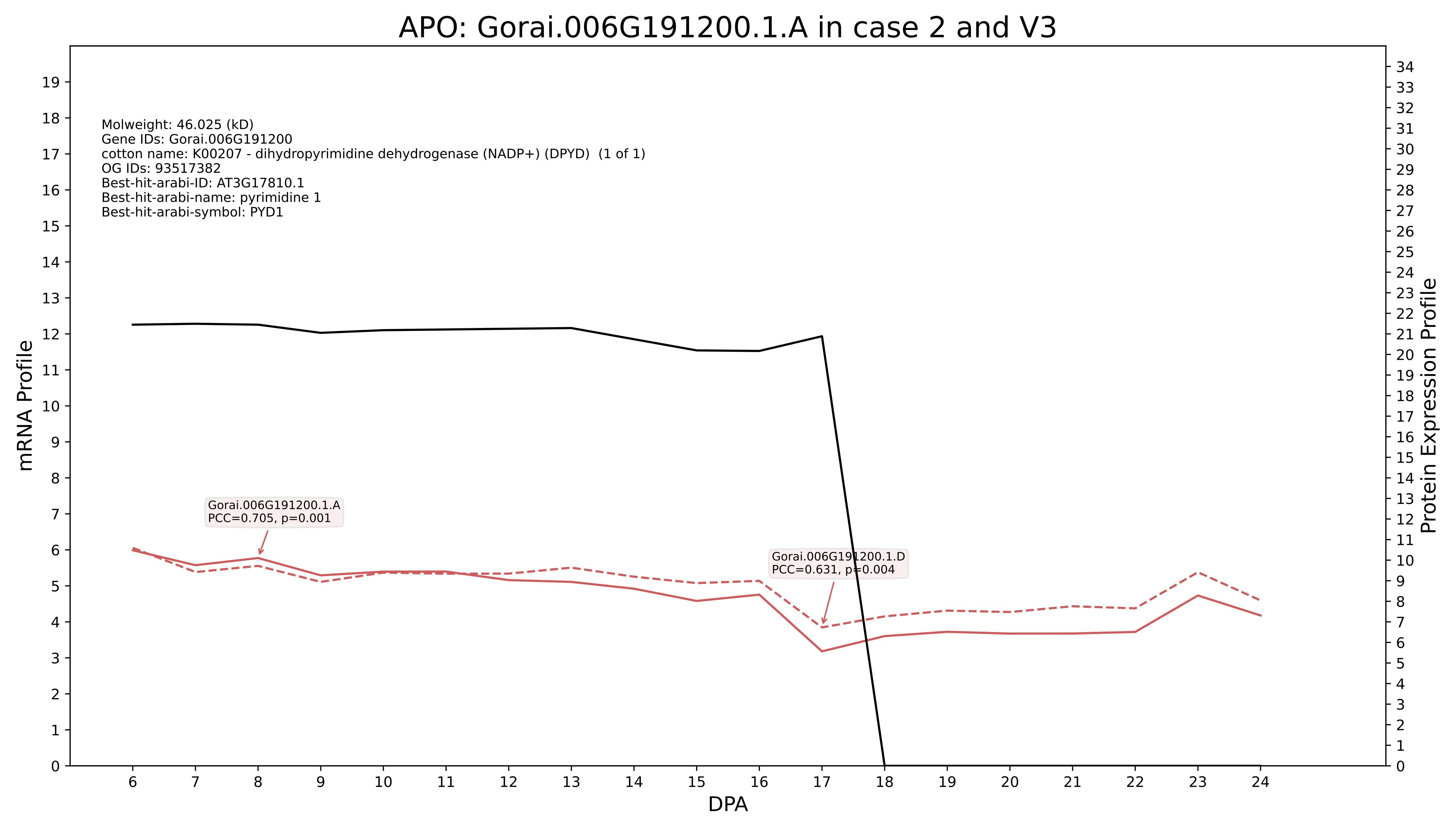
APO: Gorai.006G191200.1.A in case 2 and V3
Molweight: 46.025 (kD)
Gene IDs: Gorai.006G191200
cotton name: K00207 - dihydropyrimidine dehydrogenase (NADP+) (DPYD) (1 of 1)
OG IDs: 93517382
Best-hit-arabi-ID: AT3G17810.1
Best-hit-arabi-name: pyrimidine 1
Best-hit-arabi-symbol: PYD1
Gorai.006G191200.1.A
PCC=0.705, p=0.001
Gorai.006G191200.1.D
PCC=0.631, p=0.004
mRNA Profile
Protein Expression Profile
DPA

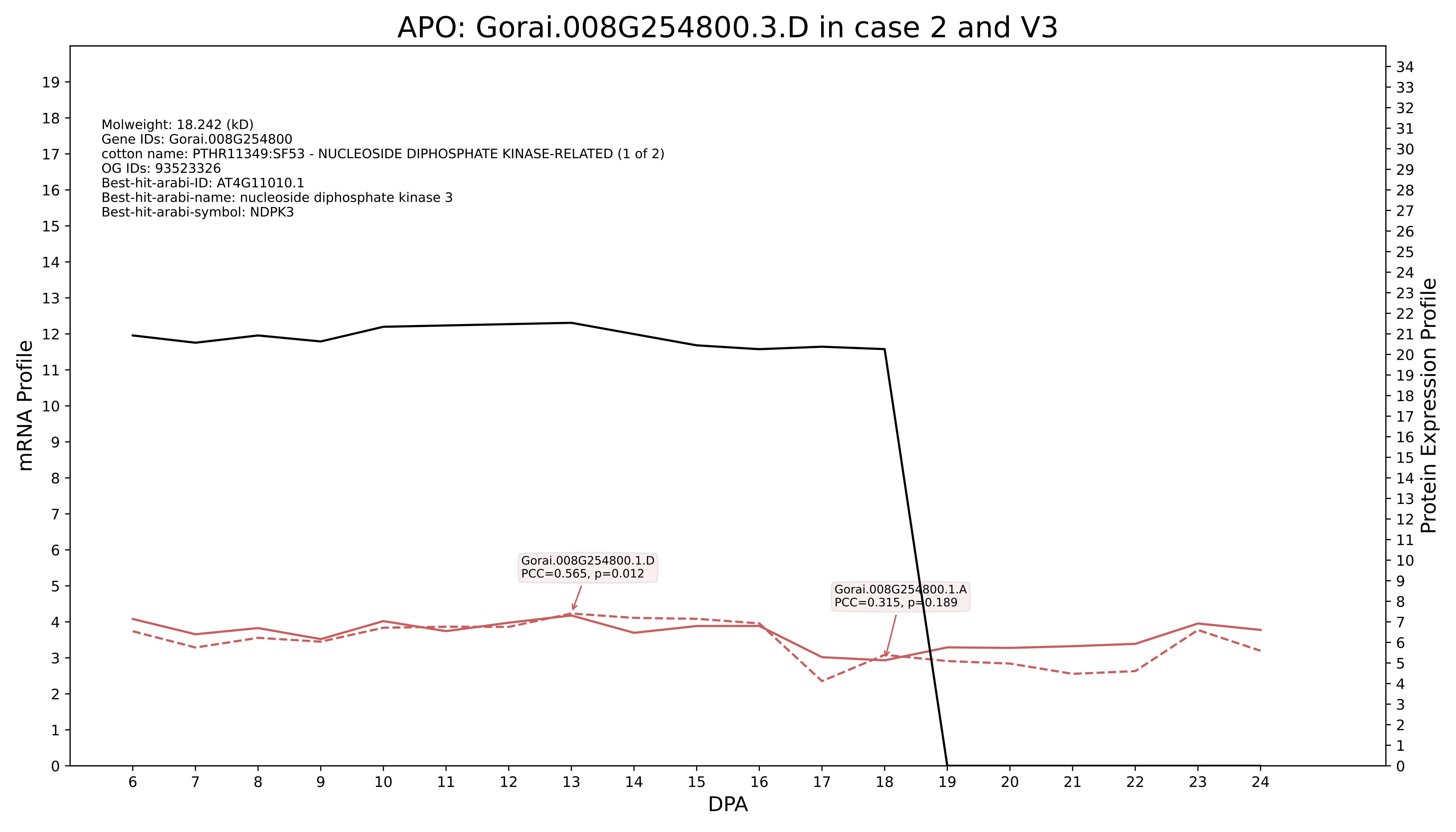
APO: Gorai.008G254800.3.D in case 2 and V3
Molweight: 18.242 (kD)
Gene IDs: Gorai.008G254800
cotton name: PTHR11349:SF53 - NUCLEOSIDE DIPHOSPHATE KINASE-RELATED (1 of 2)
OG IDs: 93523326
Best-hit-arabi-ID: AT4G11010.1
Best-hit-arabi-name: nucleoside diphosphate kinase 3
Best-hit-arabi-symbol: NDPK3
Gorai.008G254800.1.D
PCC=0.565, p=0.012
Gorai.008G254800.1.A
PCC=0.315, p=0.189
mRNA Profile
Protein Expression Profile
DPA

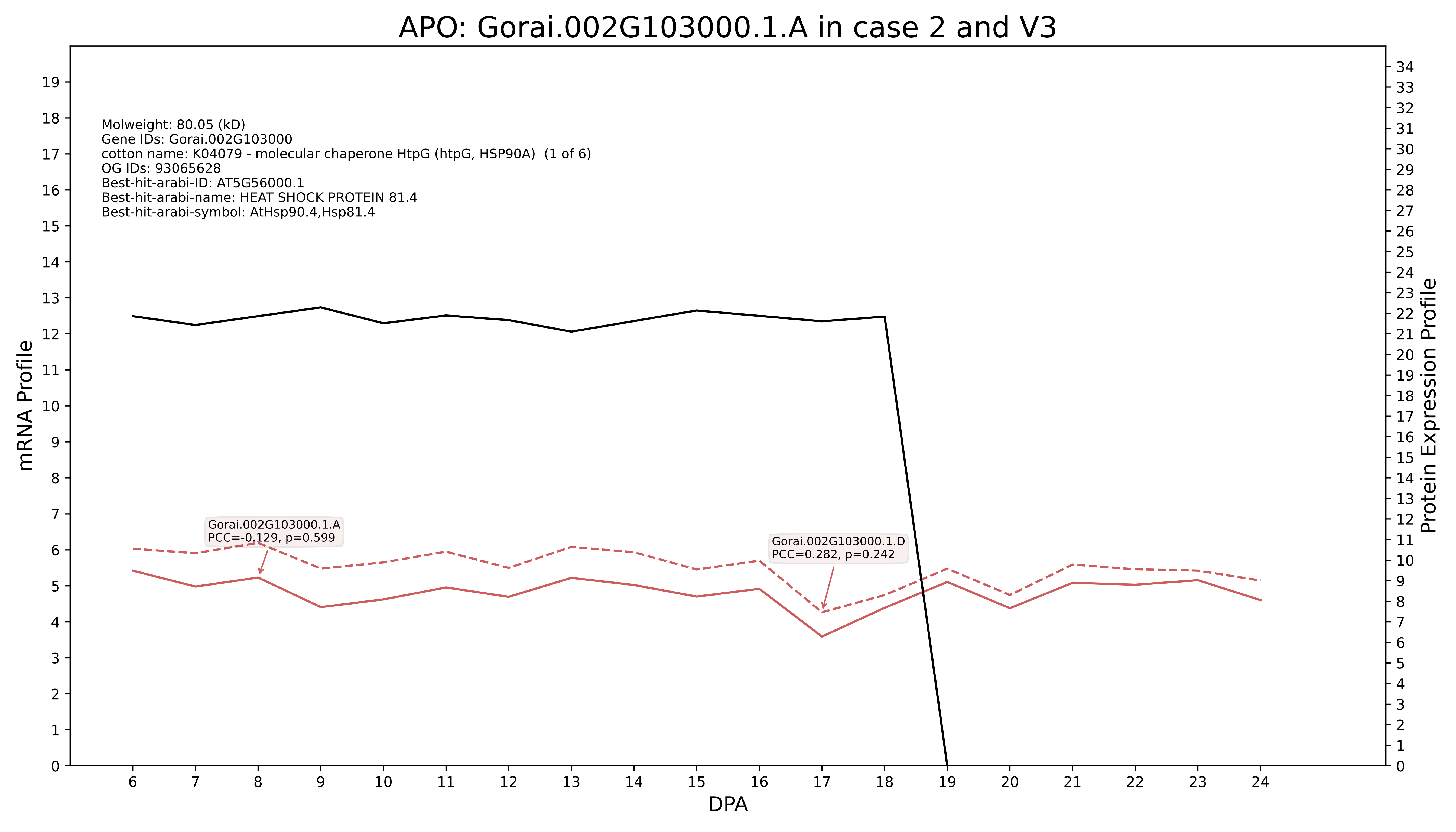
APO: Gorai.002G103000.1.A in case 2 and V3
Molweight: 80.05 (kD)
Gene IDs: Gorai.002G103000
cotton name: K04079 - molecular chaperone HtpG (htpG, HSP90A) (1 of 6)
OG IDs: 93065628
Best-hit-arabi-ID: AT5G56000.1
Best-hit-arabi-name: HEAT SHOCK PROTEIN 81.4
Best-hit-arabi-symbol: AtHsp90.4,Hsp81.4
Gorai.002G103000.1.A
PCC=-0.129, p=0.599
Gorai.002G103000.1.D
PCC=0.282, p=0.242
mRNA Profile
Protein Expression Profile
DPA
0
1
2
3
4
5
6
7
8
9
10
11
12
13
14
15
16
17
18
19
20
21
22
23
24
25
26
27
28
29
30
31
32
33
34

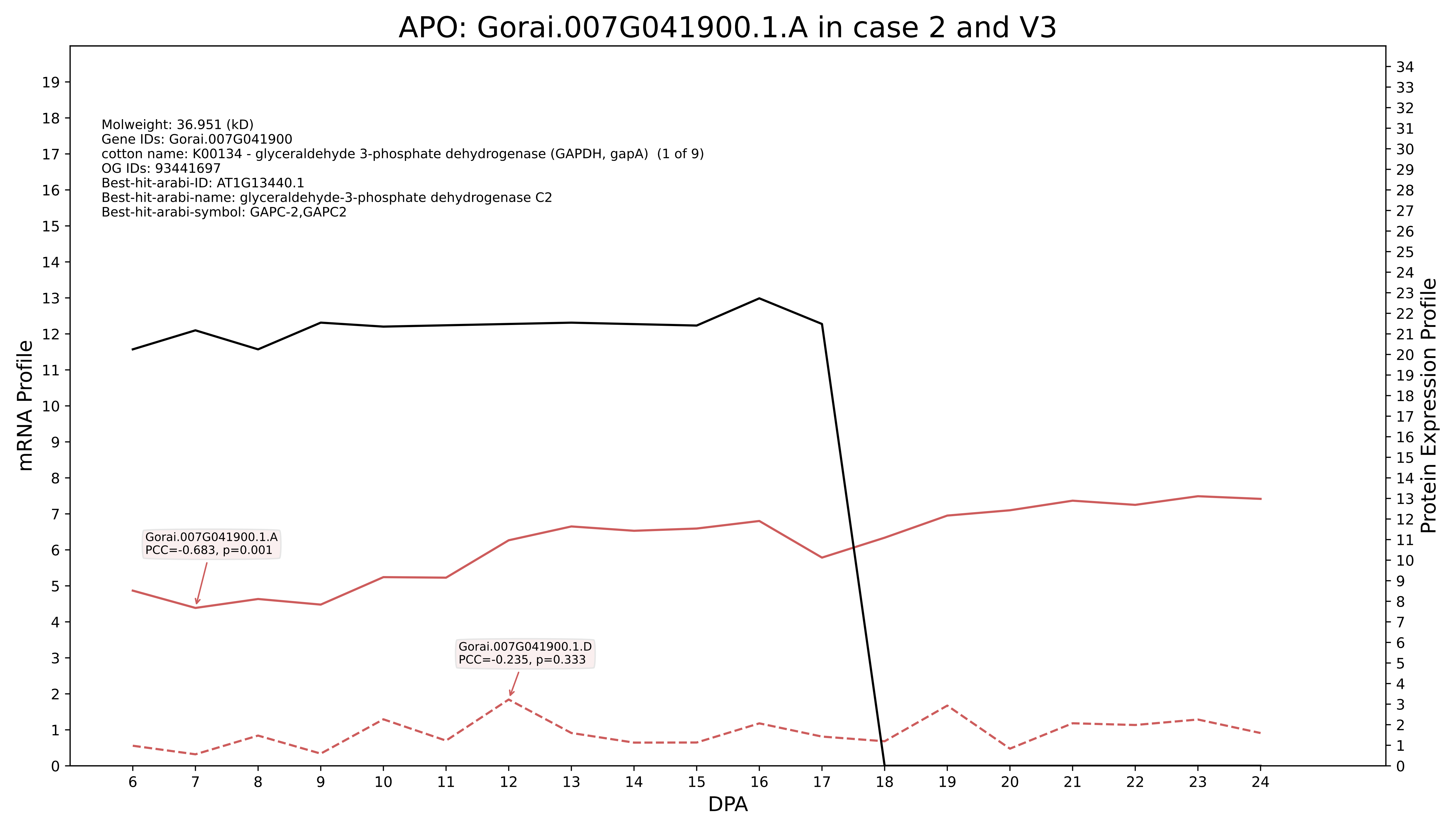
APO: Gorai.007G041900.1.A in case 2 and V3
Molweight: 36.951 (kD)
Gene IDs: Gorai.007G041900
cotton name: K00134 - glyceraldehyde 3-phosphate dehydrogenase (GAPDH, gapA) (1 of 9)
OG IDs: 93441697
Best-hit-arabi-ID: AT1G13440.1
Best-hit-arabi-name: glyceraldehyde-3-phosphate dehydrogenase C2
Best-hit-arabi-symbol: GAPC-2,GAPC2
Gorai.007G041900.1.A
PCC=-0.683, p=0.001
Gorai.007G041900.1.D
PCC=-0.235, p=0.333
mRNA Profile
Protein Expression Profile
DPA
0
1
2
3
4
5
6
7
8
9
10
11
12
13
14
15
16
17
18
19
20
21
22
23
24
25
26
27
28
29
30
31
32
33
34

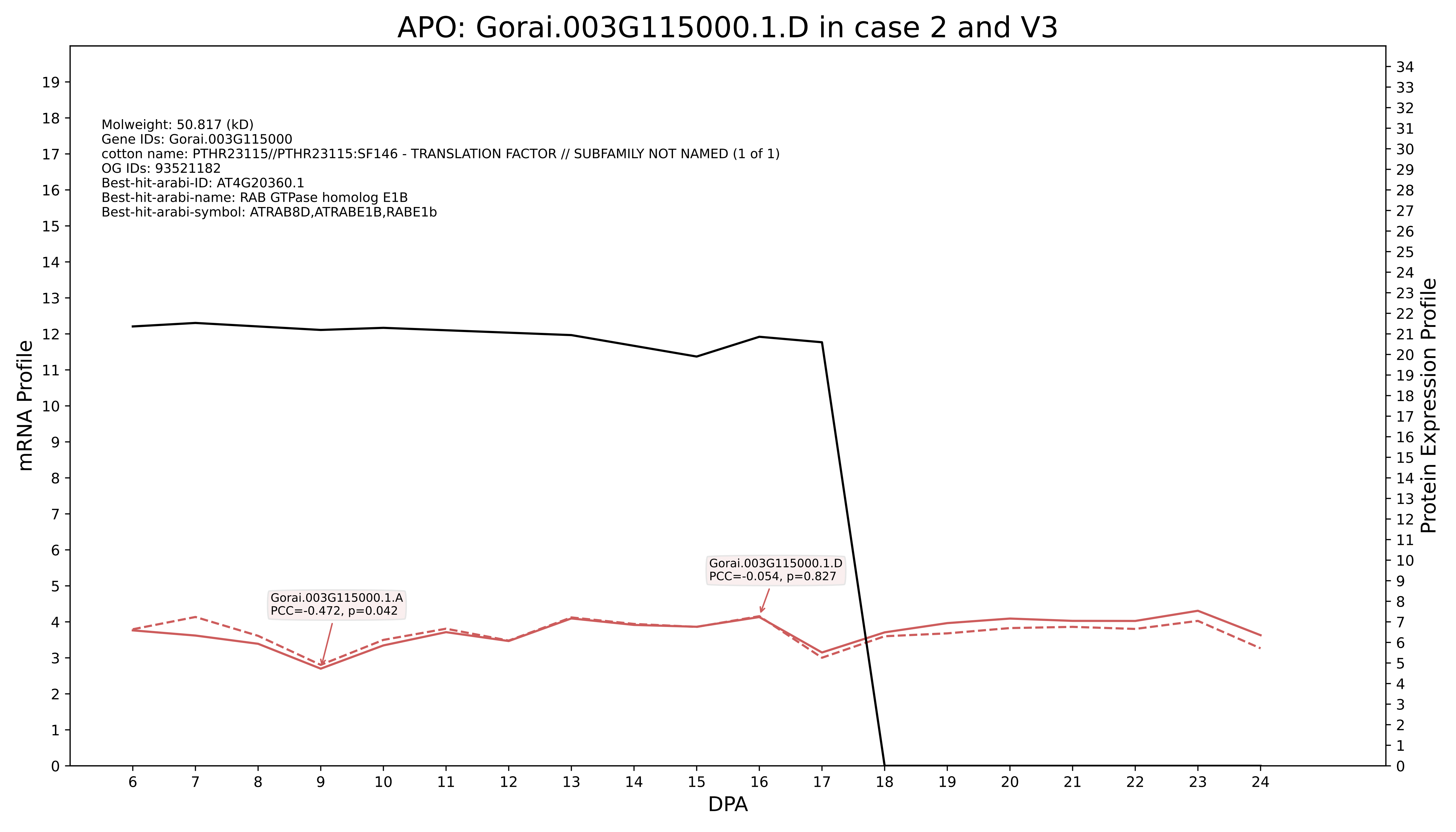
APO: Gorai.003G115000.1.D in case 2 and V3
Molweight: 50.817 (kD)
Gene IDs: Gorai.003G115000
cotton name: PTHR23115//PTHR23115:SF146 - TRANSLATION FACTOR // SUBFAMILY NOT NAMED (1 of 1)
OG IDs: 93521182
Best-hit-arabi-ID: AT4G20360.1
Best-hit-arabi-name: RAB GTPase homolog E1B
Best-hit-arabi-symbol: ATRAB8D,ATRABE1B,RABE1b
Gorai.003G115000.1.D
PCC=-0.054, p=0.827
Gorai.003G115000.1.A
PCC=-0.472, p=0.042
mRNA Profile
Protein Expression Profile
DPA

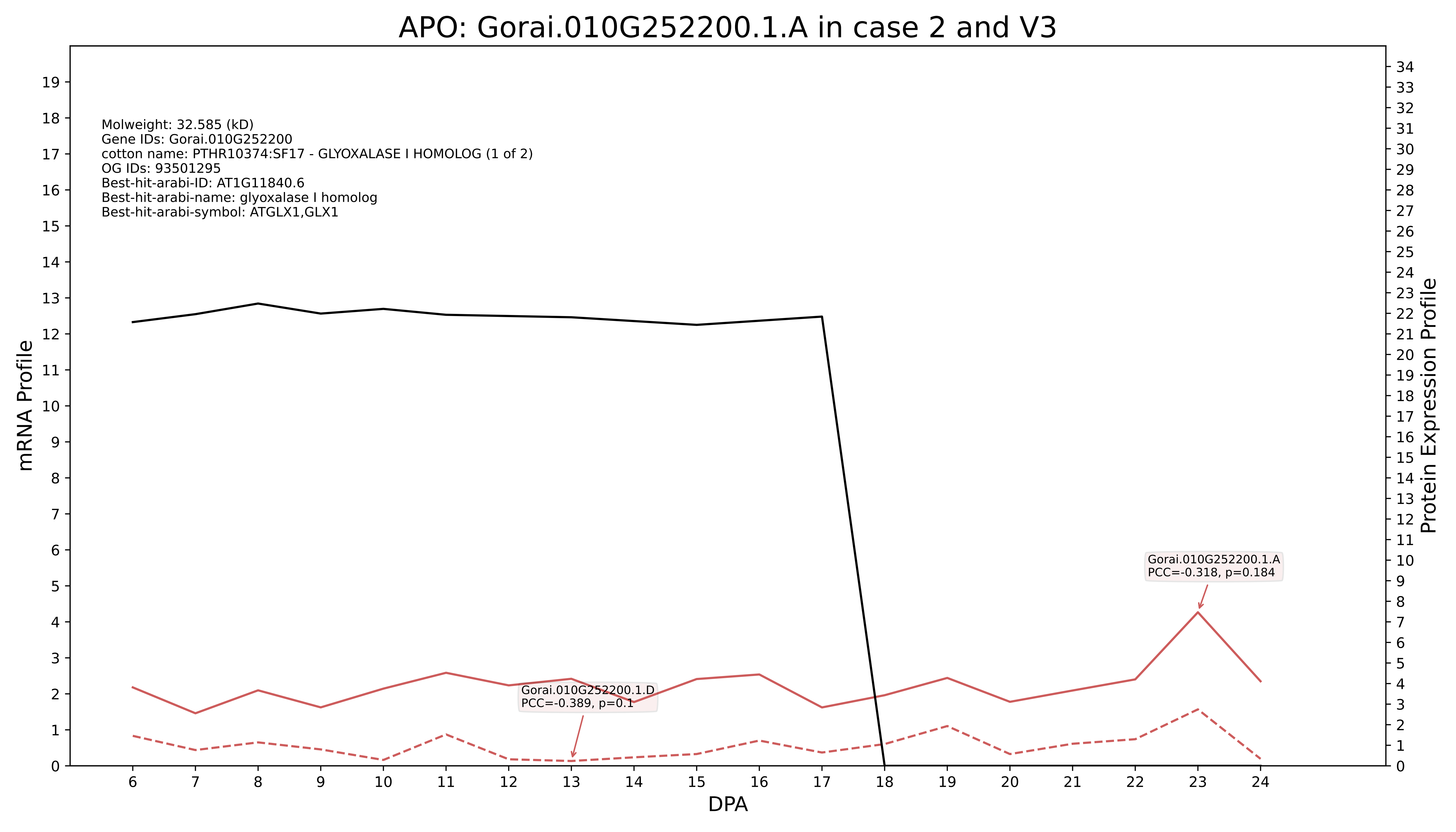

APO: Gorai.010G252200.1.A in case 2 and V3
Molweight: 32.585 (kD)
Gene IDs: Gorai.010G252200
cotton name: PTHR10374:SF17 - GLYOXALASE I HOMOLOG (1 of 2)
OG IDs: 93501295
Best-hit-arabi-ID: AT1G11840.6
Best-hit-arabi-name: glyoxalase I homolog
Best-hit-arabi-symbol: ATGLX1,GLX1
Gorai.010G252200.1.A
PCC=-0.318, p=0.184
Gorai.010G252200.1.D
PCC=-0.389, p=0.1
mRNA Profile
Protein Expression Profile
DPA

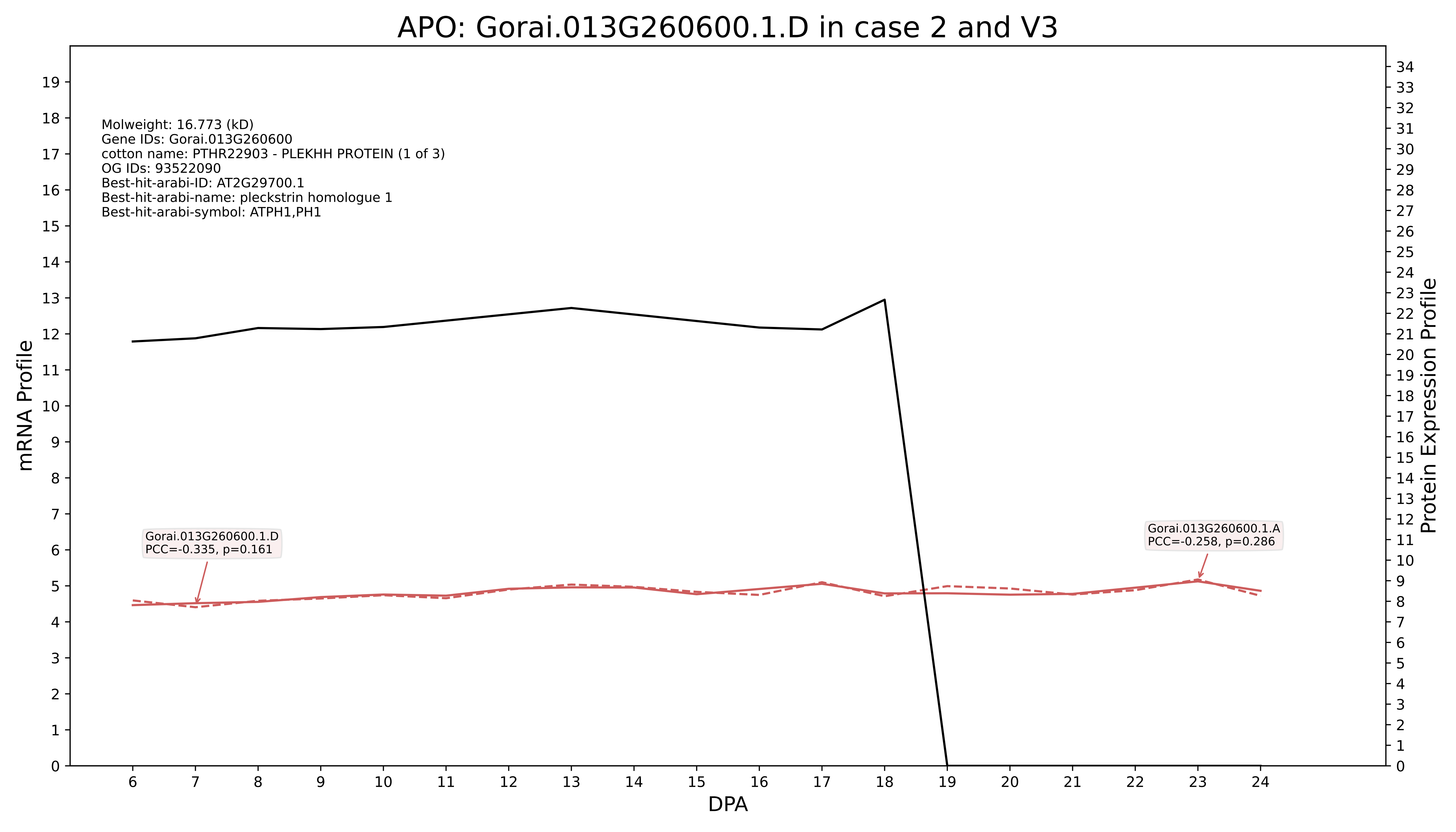
APO: Gorai.013G260600.1.D in case 2 and V3
Molweight: 16.773 (kD)
Gene IDs: Gorai.013G260600
cotton name: PTHR22903 - PLEKHH PROTEIN (1 of 3)
OG IDs: 93522090
Best-hit-arabi-ID: AT2G29700.1
Best-hit-arabi-name: pleckstrin homologue 1
Best-hit-arabi-symbol: ATPH1,PH1
Gorai.013G260600.1.D
PCC=-0.335, p=0.161
Gorai.013G260600.1.A
PCC=-0.258, p=0.286
mRNA Profile
Protein Expression Profile
DPA

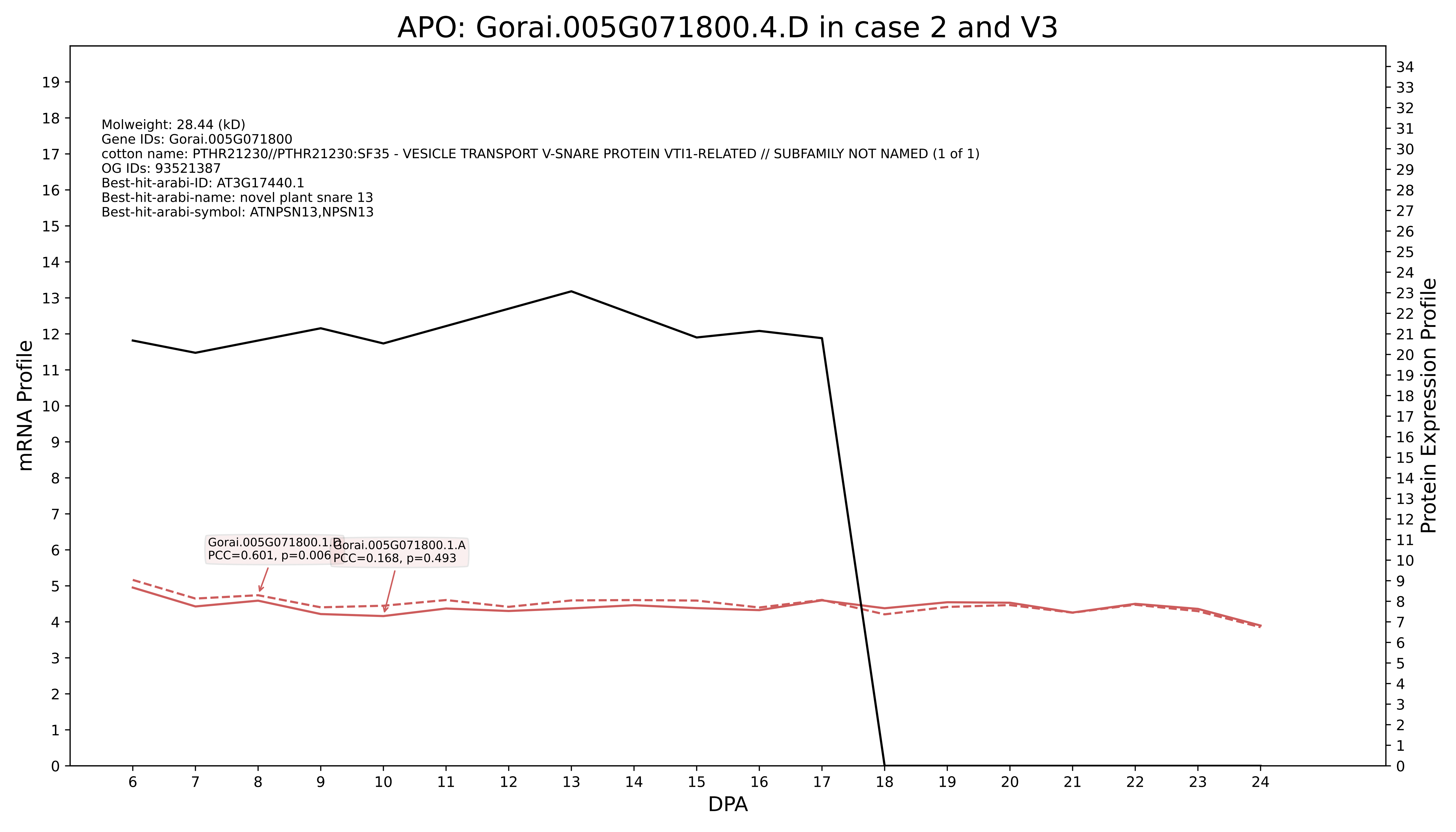
APO: Gorai.005G071800.4.D in case 2 and V3
Molweight: 28.44 (kD)
Gene IDs: Gorai.005G071800
cotton name: PTHR21230//PTHR21230:SF35 - VESICLE TRANSPORT V-SNARE PROTEIN VTI1-RELATED // SUBFAMILY NOT NAMED (1 of 1)
OG IDs: 93521387
Best-hit-arabi-ID: AT3G17440.1
Best-hit-arabi-name: novel plant snare 13
Best-hit-arabi-symbol: ATNPSN13,NPSN13
Gorai.005G071800.1.D
PCC=0.601, p=0.006
Gorai.005G071800.1.A
PCC=0.168, p=0.493
mRNA Profile
Protein Expression Profile
DPA

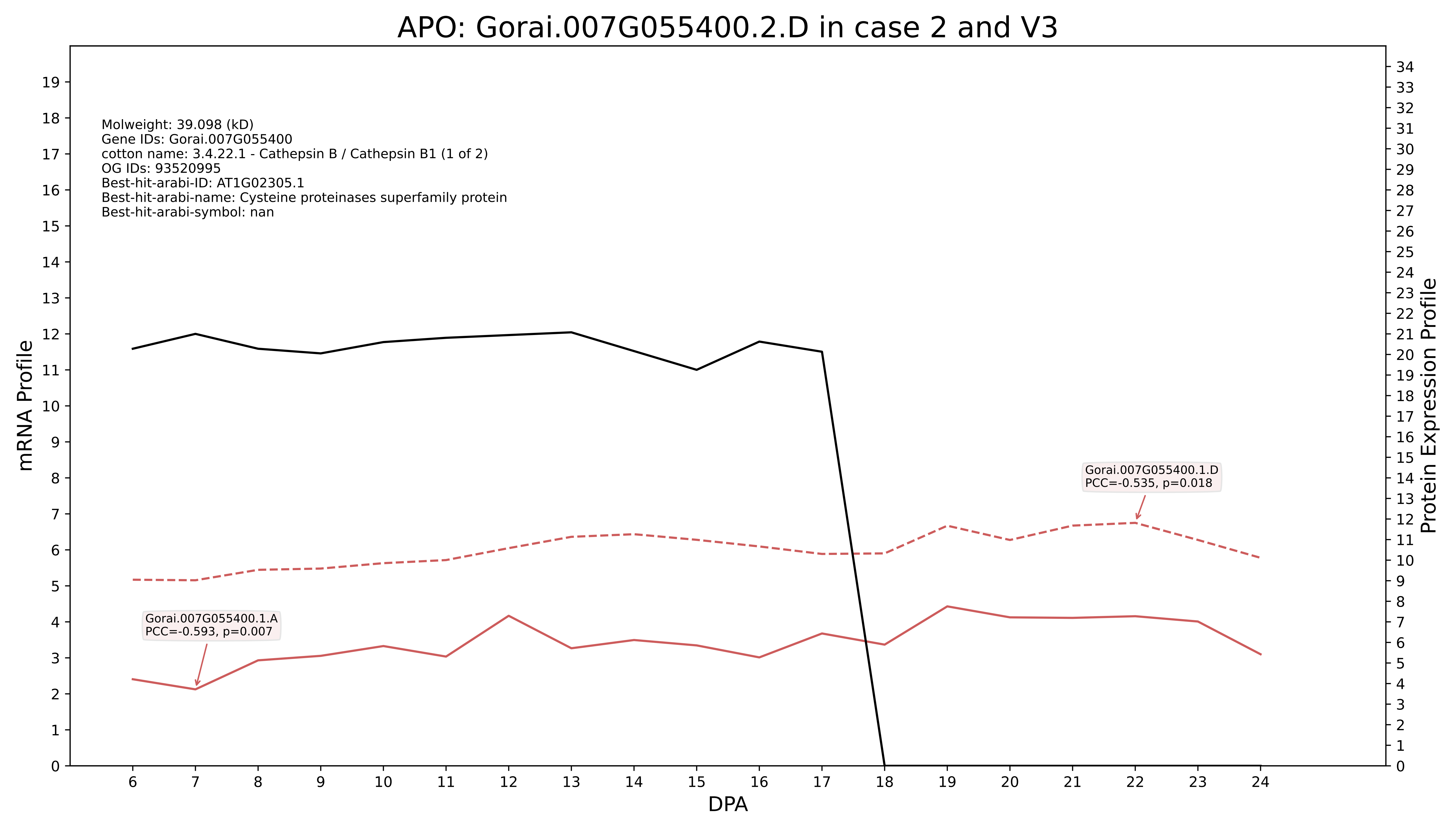
APO: Gorai.007G055400.2.D in case 2 and V3
Molweight: 39.098 (kD)
Gene IDs: Gorai.007G055400
cotton name: 3.4.22.1 - Cathepsin B / Cathepsin B1 (1 of 2)
OG IDs: 93520995
Best-hit-arabi-ID: AT1G02305.1
Best-hit-arabi-name: Cysteine proteinases superfamily protein
Best-hit-arabi-symbol: nan
Gorai.007G055400.1.D
PCC=-0.535, p=0.018
Gorai.007G055400.1.A
PCC=-0.593, p=0.007
mRNA Profile
Protein Expression Profile
DPA

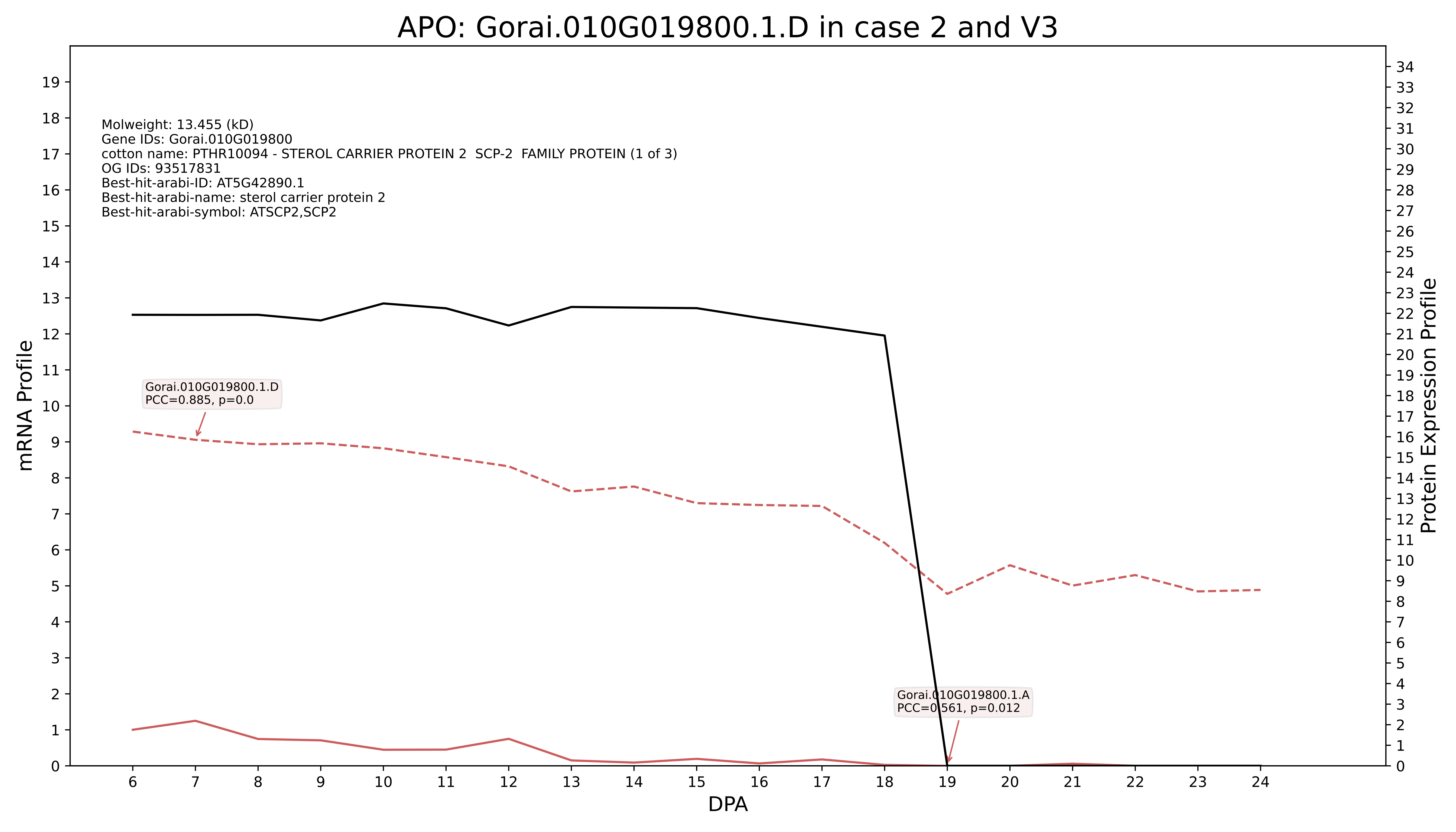

APO: Gorai.010G019800.1.D in case 2 and V3
Molweight: 13.455 (kD)
Gene IDs: Gorai.010G019800
cotton name: PTHR10094 - STEROL CARRIER PROTEIN 2 SCP-2 FAMILY PROTEIN (1 of 3)
OG IDs: 93517831
Best-hit-arabi-ID: AT5G42890.1
Best-hit-arabi-name: sterol carrier protein 2
Best-hit-arabi-symbol: ATSCP2,SCP2
Gorai.010G019800.1.D
PCC=0.885, p=0.0
Gorai.010G019800.1.A
PCC=0.561, p=0.012
mRNA Profile
Protein Expression Profile
DPA

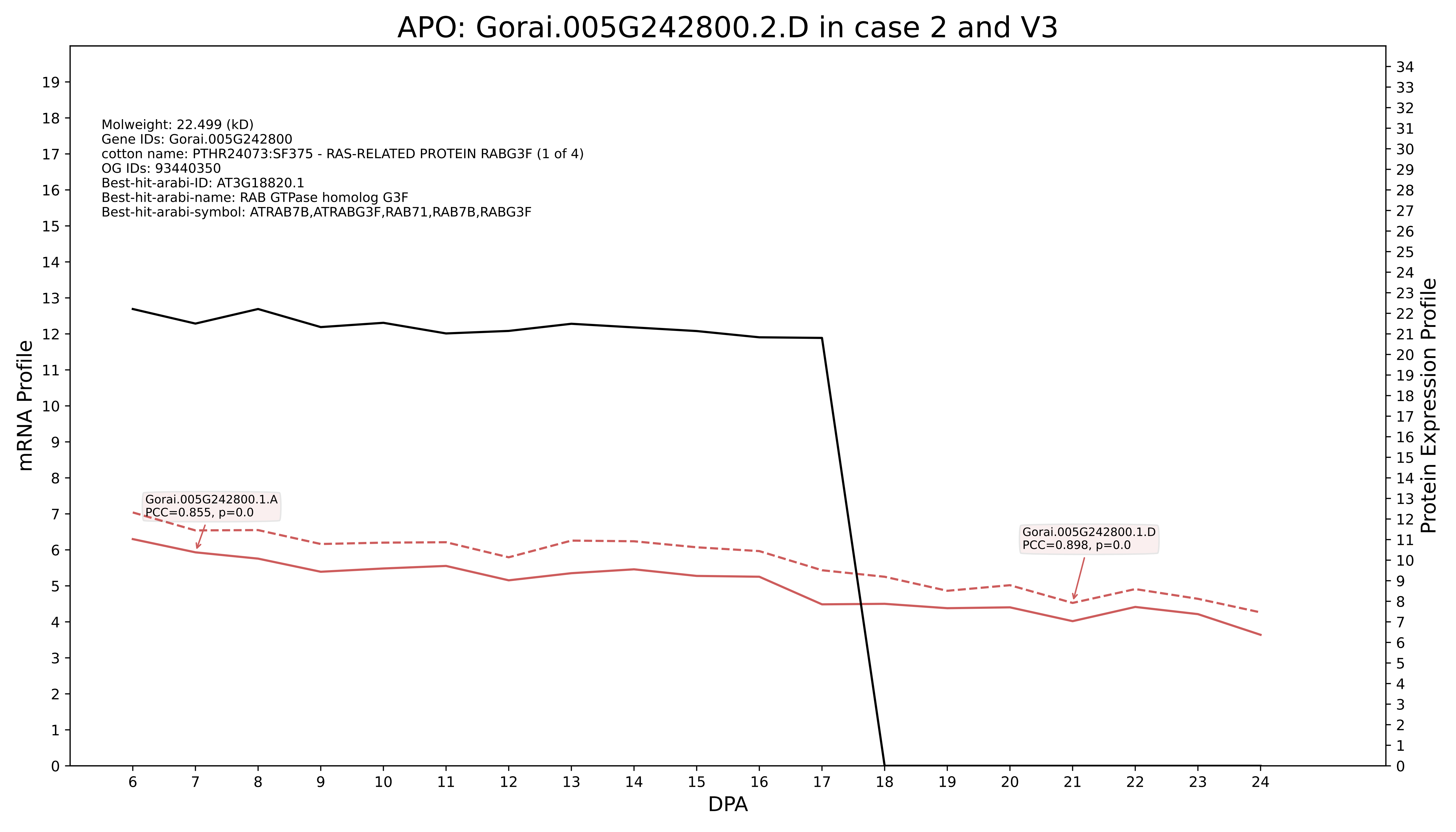

APO: Gorai.005G242800.2.D in case 2 and V3
Molweight: 22.499 (kD)
Gene IDs: Gorai.005G242800
cotton name: PTHR24073:SF375 - RAS-RELATED PROTEIN RABG3F (1 of 4)
OG IDs: 93440350
Best-hit-arabi-ID: AT3G18820.1
Best-hit-arabi-name: RAB GTPase homolog G3F
Best-hit-arabi-symbol: ATRAB7B,ATRABG3F,RAB71,RAB7B,RABG3F
Gorai.005G242800.1.A
PCC=0.855, p=0.0
Gorai.005G242800.1.D
PCC=0.898, p=0.0
mRNA Profile
Protein Expression Profile
DPA

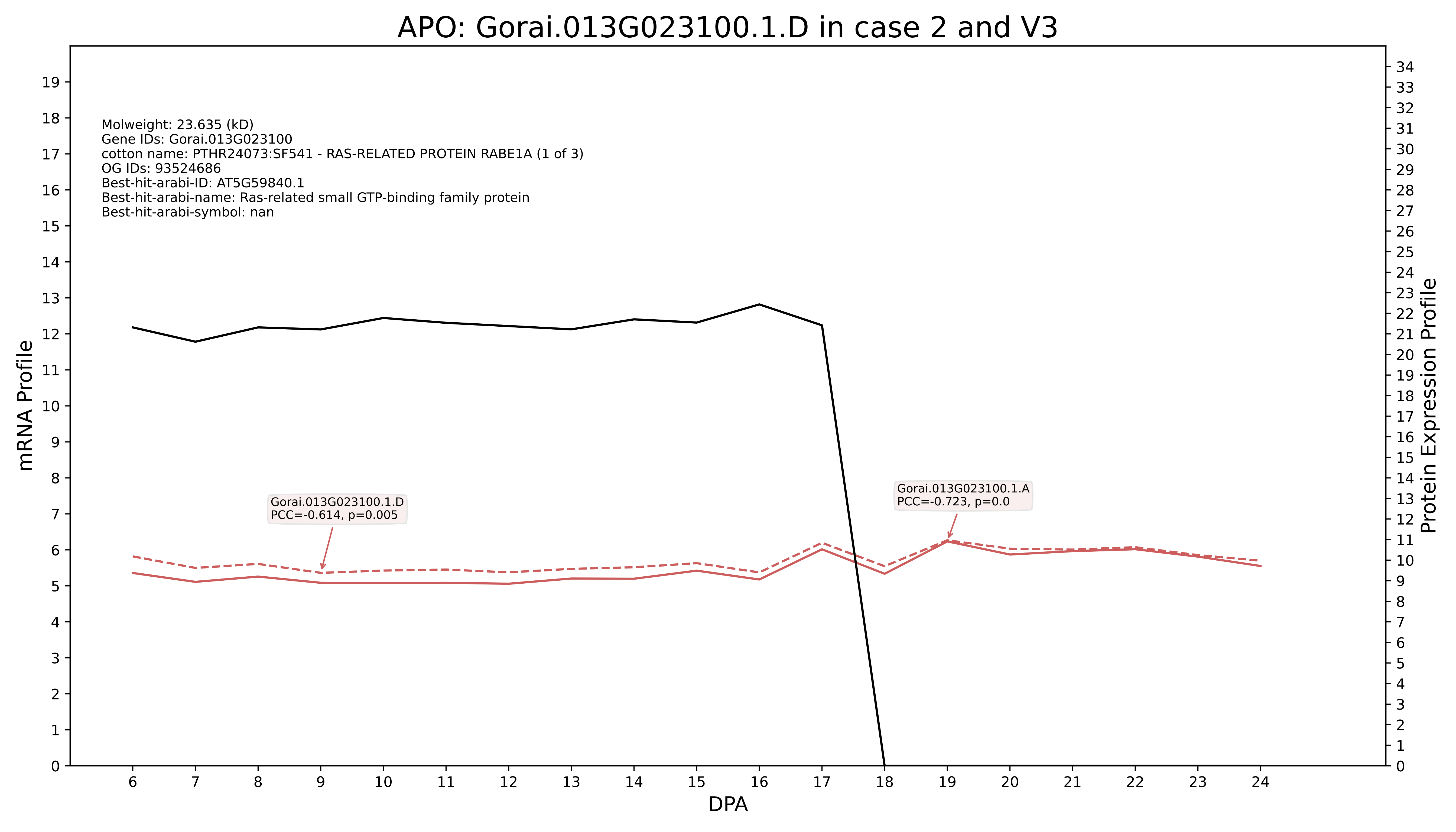

APO: Gorai.013G023100.1.D in case 2 and V3
Molweight: 23.635 (kD)
Gene IDs: Gorai.013G023100
cotton name: PTHR24073:SF541 - RAS-RELATED PROTEIN RABE1A (1 of 3)
OG IDs: 93524686
Best-hit-arabi-ID: AT5G59840.1
Best-hit-arabi-name: Ras-related small GTP-binding family protein
Best-hit-arabi-symbol: nan
Gorai.013G023100.1.D
PCC=-0.614, p=0.005
Gorai.013G023100.1.A
PCC=-0.723, p=0.0
mRNA Profile
Protein Expression Profile
DPA

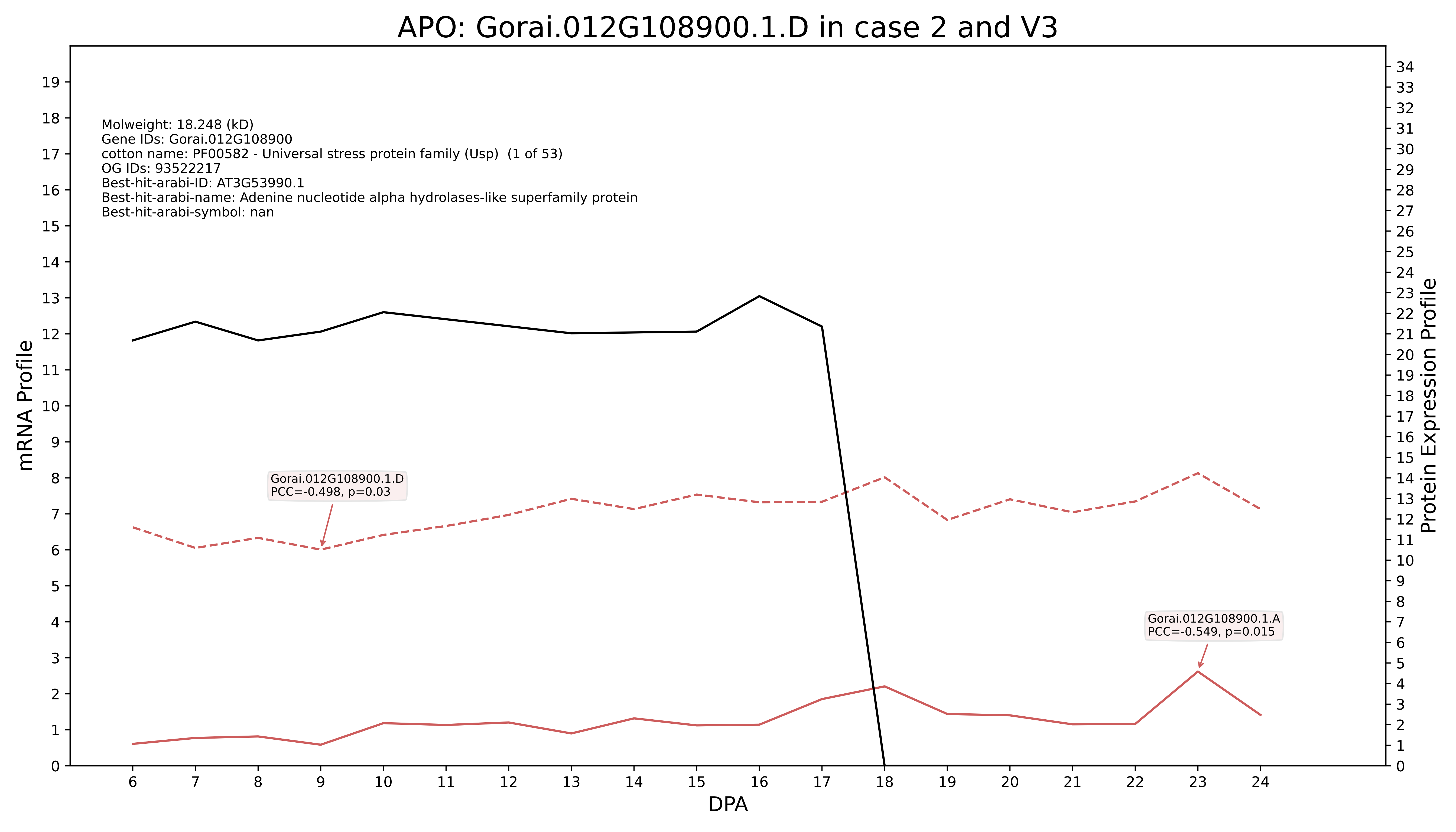
APO: Gorai.012G108900.1.D in case 2 and V3
Molweight: 18.248 (kD)
Gene IDs: Gorai.012G108900
cotton name: PF00582 - Universal stress protein family (Usp) (1 of 53)
OG IDs: 93522217
Best-hit-arabi-ID: AT3G53990.1
Best-hit-arabi-name: Adenine nucleotide alpha hydrolases-like superfamily protein
Best-hit-arabi-symbol: nan
Gorai.012G108900.1.D
PCC=-0.498, p=0.03
Gorai.012G108900.1.A
PCC=-0.549, p=0.015
mRNA Profile
Protein Expression Profile
DPA
0
1
2
3
4
5
6
7
8
9
10
11
12
13
14
15
16
17
18
19
20
21
22
23
24
25
26
27
28
29
30
31
32
33
34

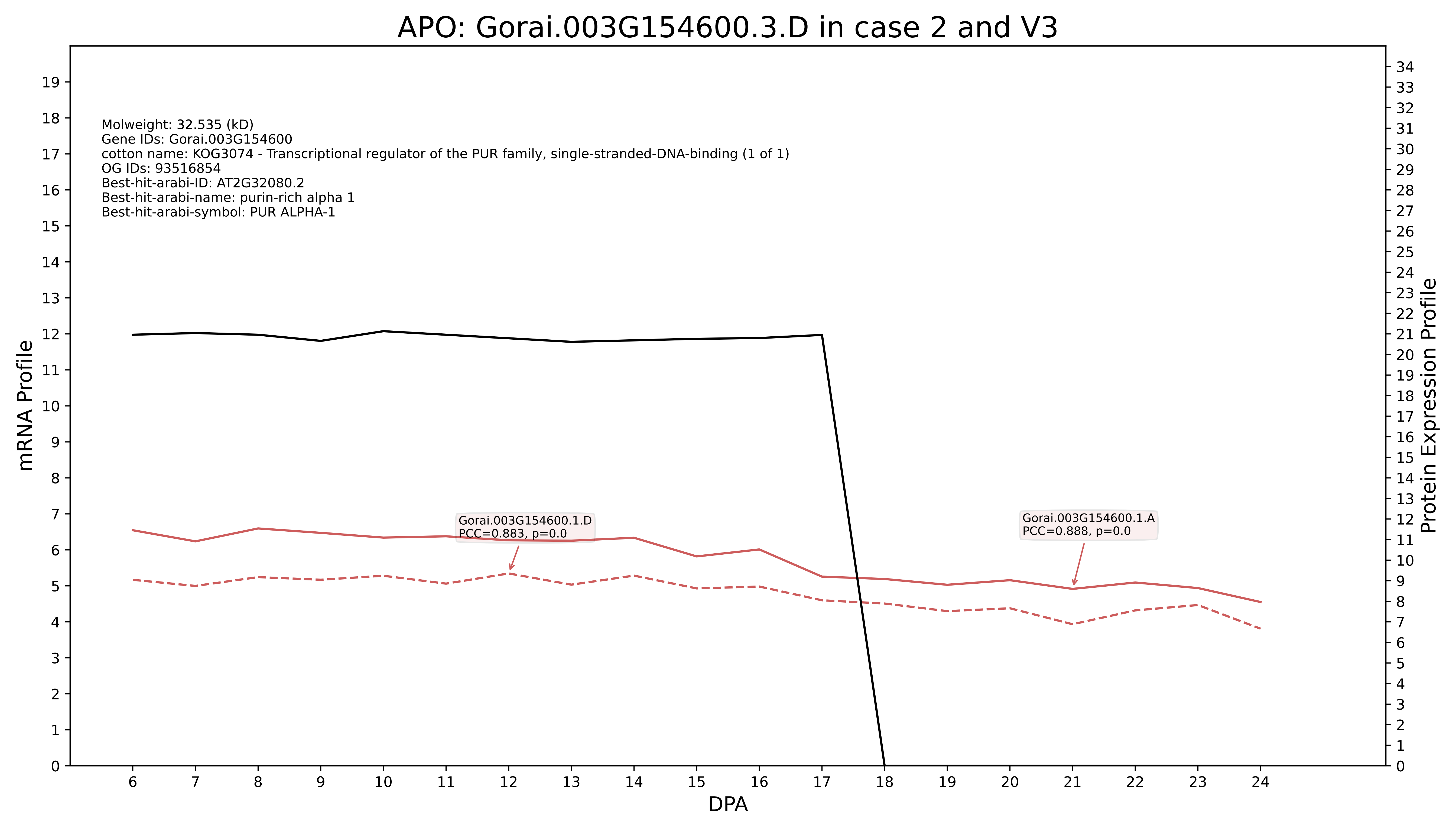
APO: Gorai.003G154600.3.D in case 2 and V3
Molweight: 32.535 (kD)
Gene IDs: Gorai.003G154600
cotton name: KOG3074 - Transcriptional regulator of the PUR family, single-stranded-DNA-binding (1 of 1)
OG IDs: 93516854
Best-hit-arabi-ID: AT2G32080.2
Best-hit-arabi-name: purin-rich alpha 1
Best-hit-arabi-symbol: PUR ALPHA-1
Gorai.003G154600.1.D
PCC=0.883, p=0.0
Gorai.003G154600.1.A
PCC=0.888, p=0.0
mRNA Profile
Protein Expression Profile
DPA

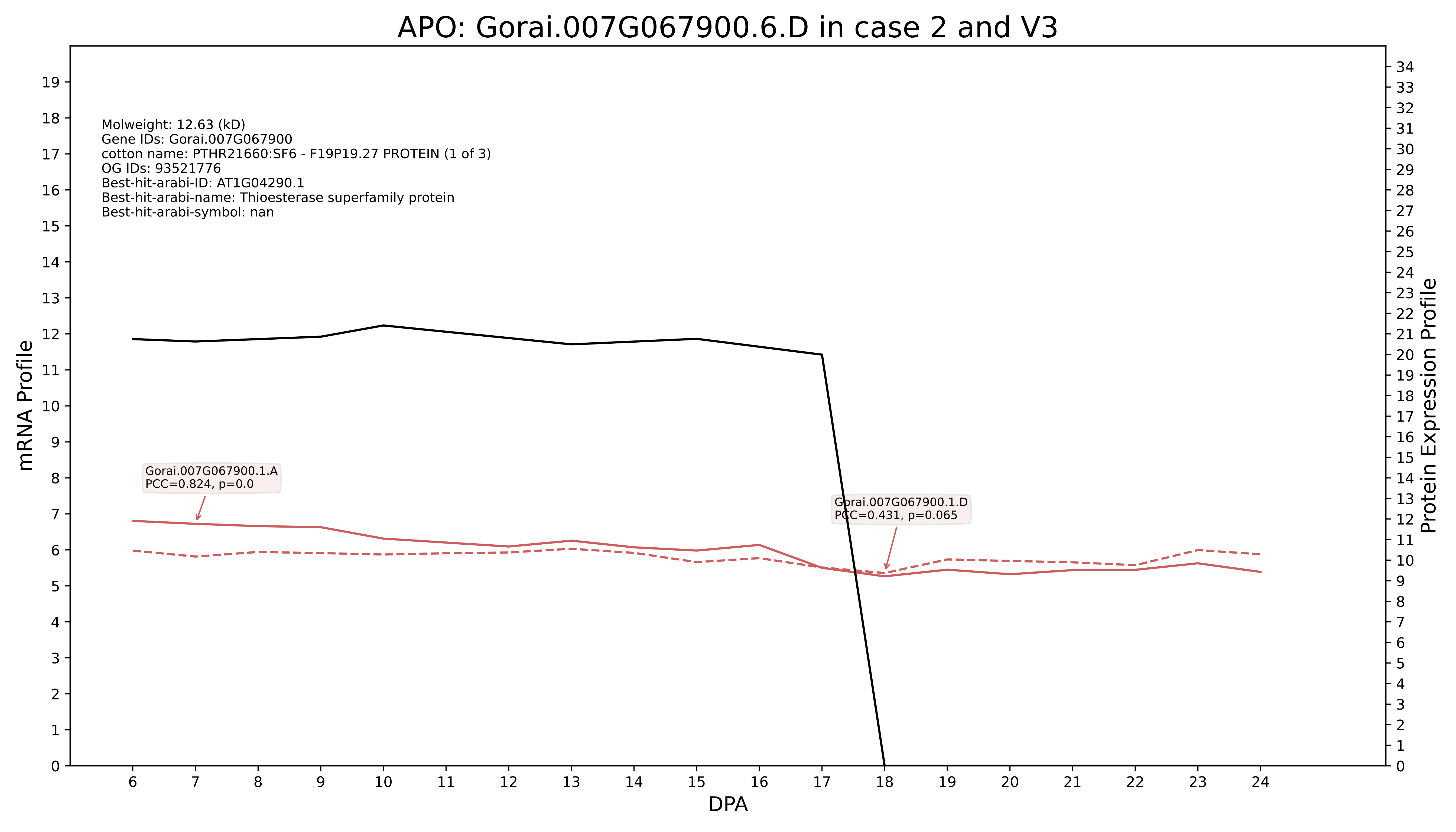
APO: Gorai.007G067900.6.D in case 2 and V3
Molweight: 12.63 (kD)
Gene IDs: Gorai.007G067900
cotton name: PTHR21660:SF6 - F19P19.27 PROTEIN (1 of 3)
OG IDs: 93521776
Best-hit-arabi-ID: AT1G04290.1
Best-hit-arabi-name: Thioesterase superfamily protein
Best-hit-arabi-symbol: nan
Gorai.007G067900.1.A
PCC=0.824, p=0.0
Gorai.007G067900.1.D
PCC=0.431, p=0.065
mRNA Profile
Protein Expression Profile
DPA

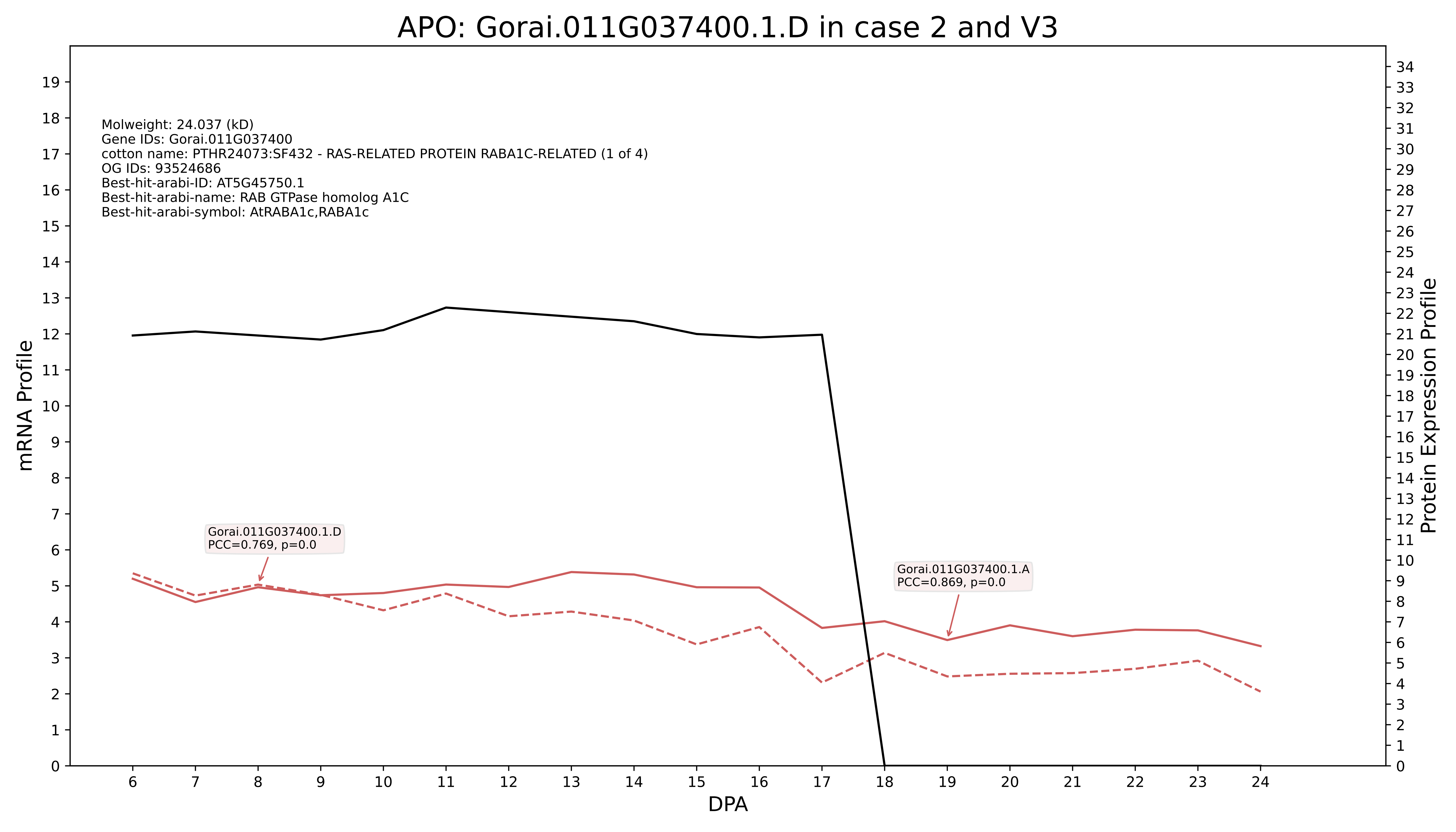
APO: Gorai.011G037400.1.D in case 2 and V3
Molweight: 24.037 (kD)
Gene IDs: Gorai.011G037400
cotton name: PTHR24073:SF432 - RAS-RELATED PROTEIN RABA1C-RELATED (1 of 4)
OG IDs: 93524686
Best-hit-arabi-ID: AT5G45750.1
Best-hit-arabi-name: RAB GTPase homolog A1C
Best-hit-arabi-symbol: AtRABA1c,RABA1c
Gorai.011G037400.1.D
PCC=0.769, p=0.0
Gorai.011G037400.1.A
PCC=0.869, p=0.0
mRNA Profile
Protein Expression Profile
DPA

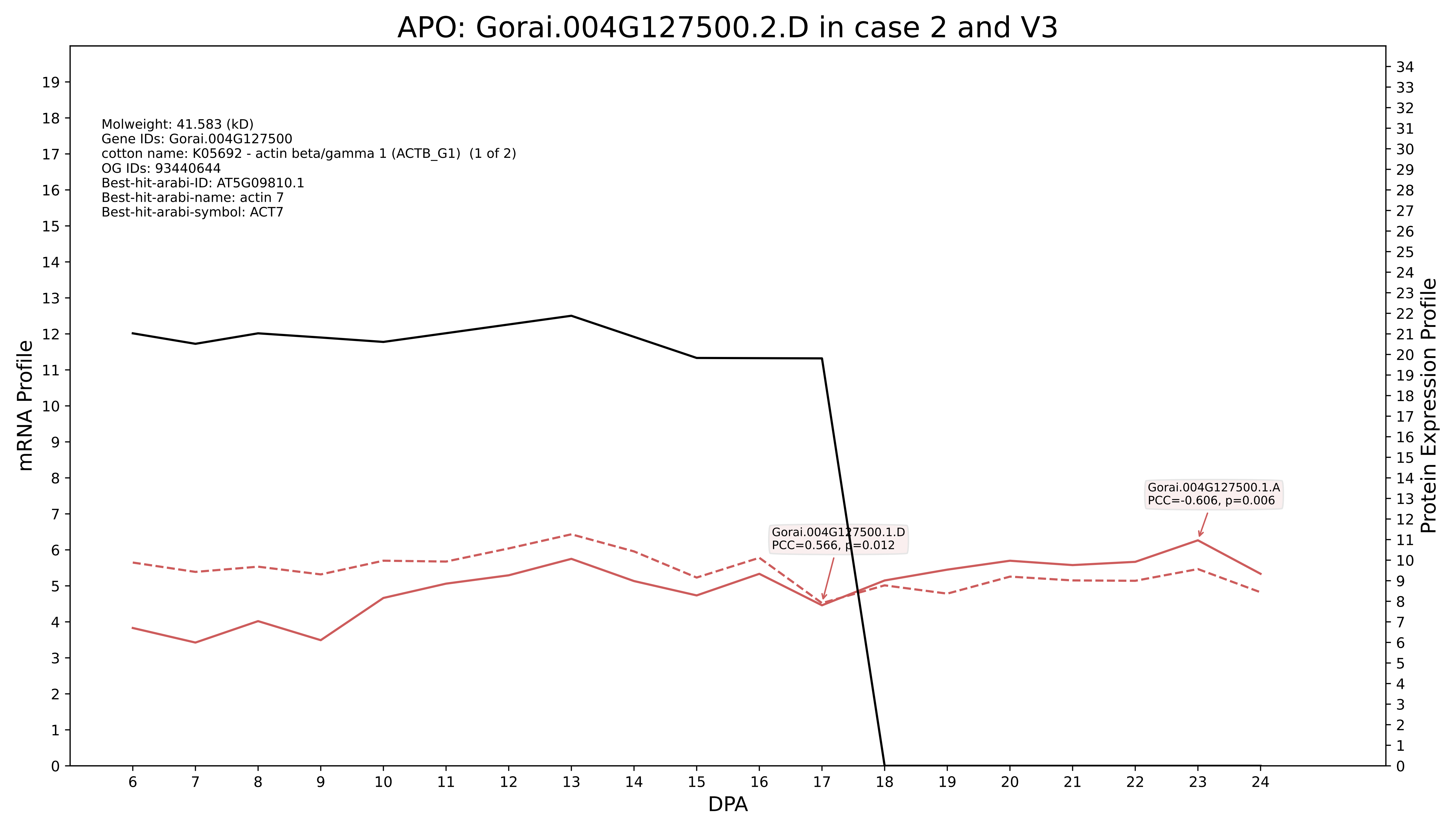
APO: Gorai.004G127500.2.D in case 2 and V3
Molweight: 41.583 (kD)
Gene IDs: Gorai.004G127500
cotton name: K05692 - actin beta/gamma 1 (ACTB_G1) (1 of 2)
OG IDs: 93440644
Best-hit-arabi-ID: AT5G09810.1
Best-hit-arabi-name: actin 7
Best-hit-arabi-symbol: ACT7
Gorai.004G127500.1.D
PCC=0.566, p=0.012
Gorai.004G127500.1.A
PCC=-0.606, p=0.006
mRNA Profile
Protein Expression Profile
DPA

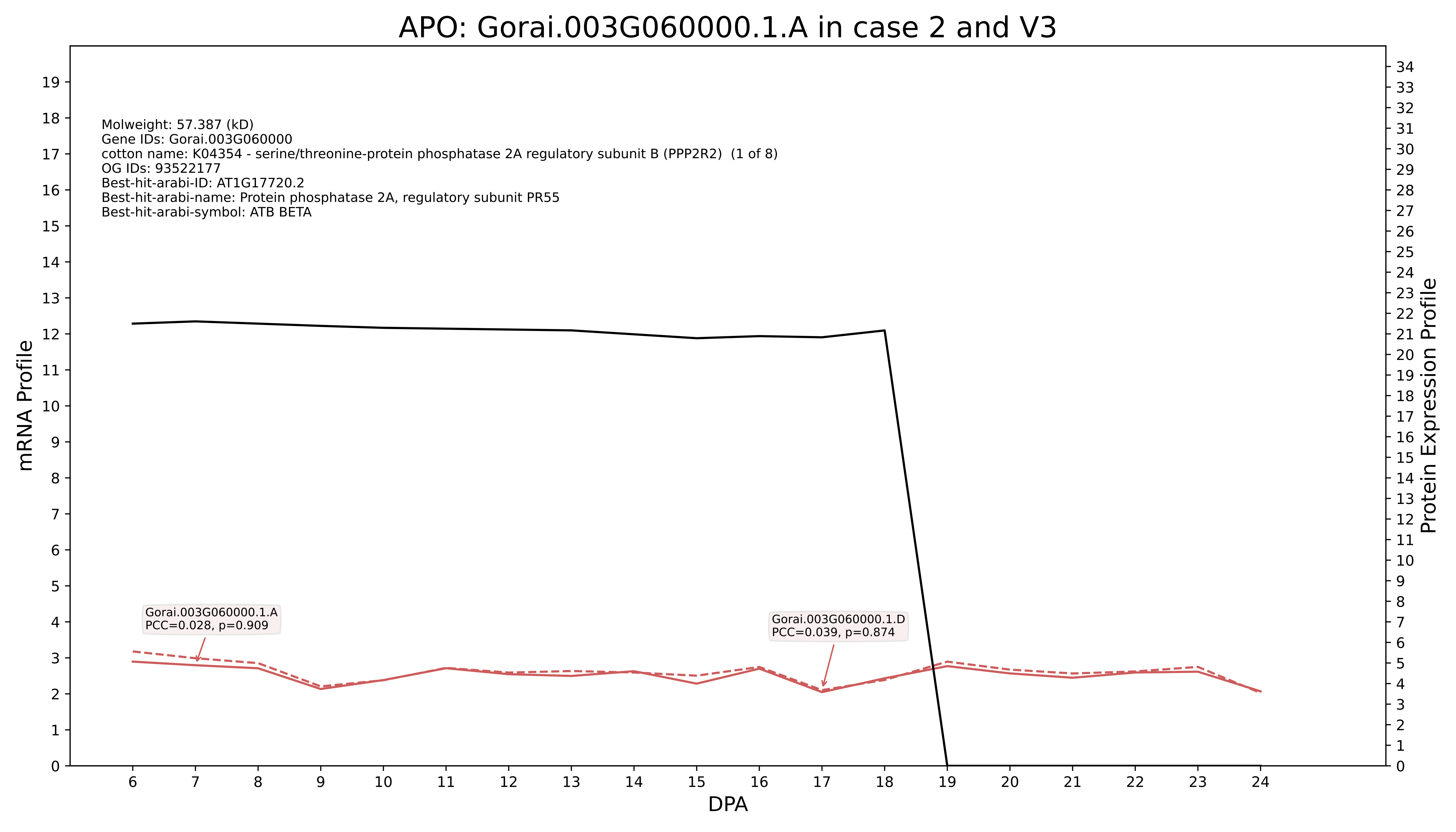

APO: Gorai.003G060000.1.A in case 2 and V3
Molweight: 57.387 (kD)
Gene IDs: Gorai.003G060000
cotton name: K04354 - serine/threonine-protein phosphatase 2A regulatory subunit B (PPP2R2) (1 of 8)
OG IDs: 93522177
Best-hit-arabi-ID: AT1G17720.2
Best-hit-arabi-name: Protein phosphatase 2A, regulatory subunit PR55
Best-hit-arabi-symbol: ATB BETA
Gorai.003G060000.1.A
PCC=0.028, p=0.909
Gorai.003G060000.1.D
PCC=0.039, p=0.874
mRNA Profile
Protein Expression Profile
DPA

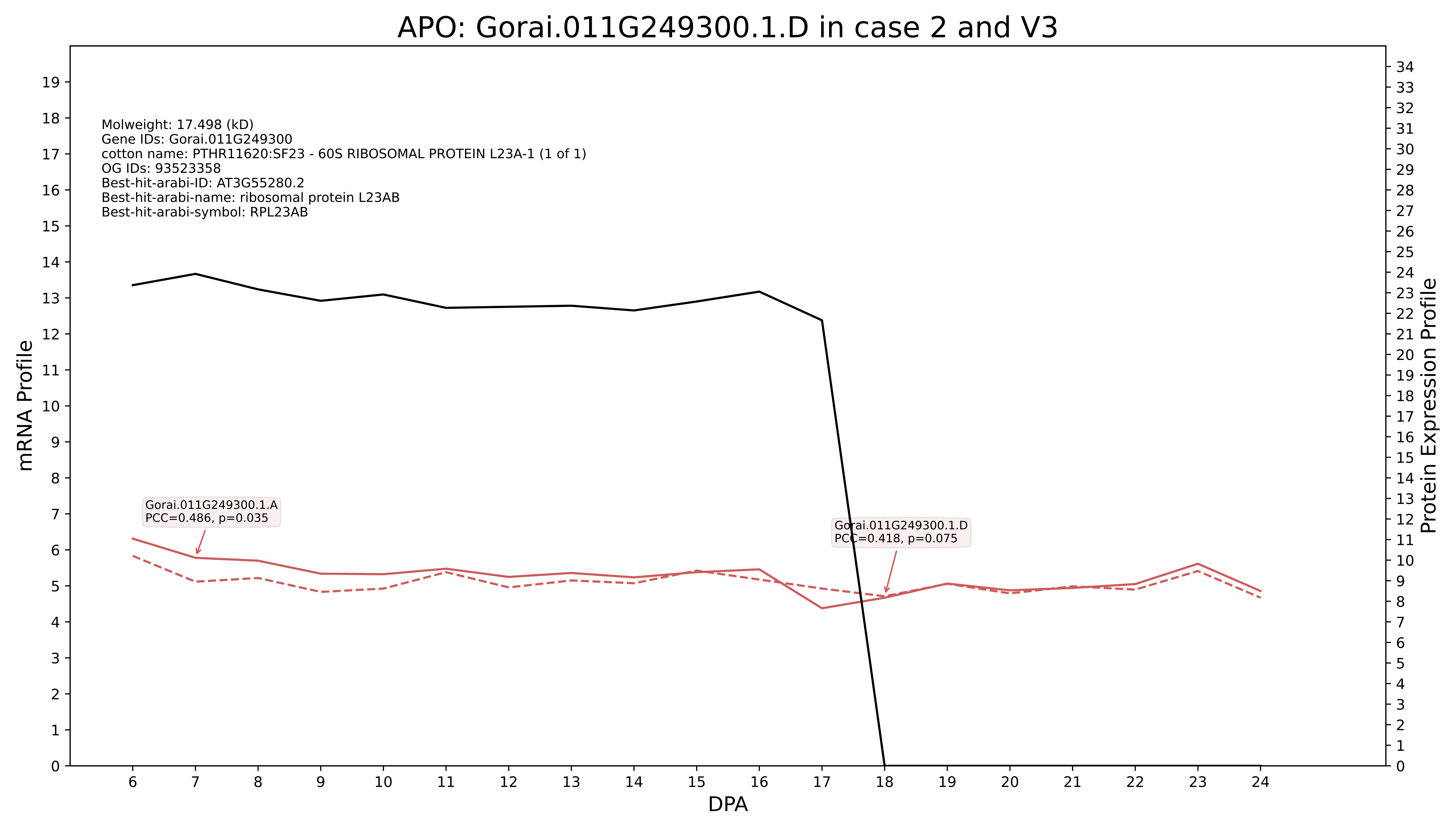

APO: Gorai.011G249300.1.D in case 2 and V3
Molweight: 17.498 (kD)
Gene IDs: Gorai.011G249300
cotton name: PTHR11620:SF23 - 60S RIBOSOMAL PROTEIN L23A-1 (1 of 1)
OG IDs: 93523358
Best-hit-arabi-ID: AT3G55280.2
Best-hit-arabi-name: ribosomal protein L23AB
Best-hit-arabi-symbol: RPL23AB
Gorai.011G249300.1.A
PCC=0.486, p=0.035
Gorai.011G249300.1.D
PCC=0.418, p=0.075
mRNA Profile
Protein Expression Profile
DPA

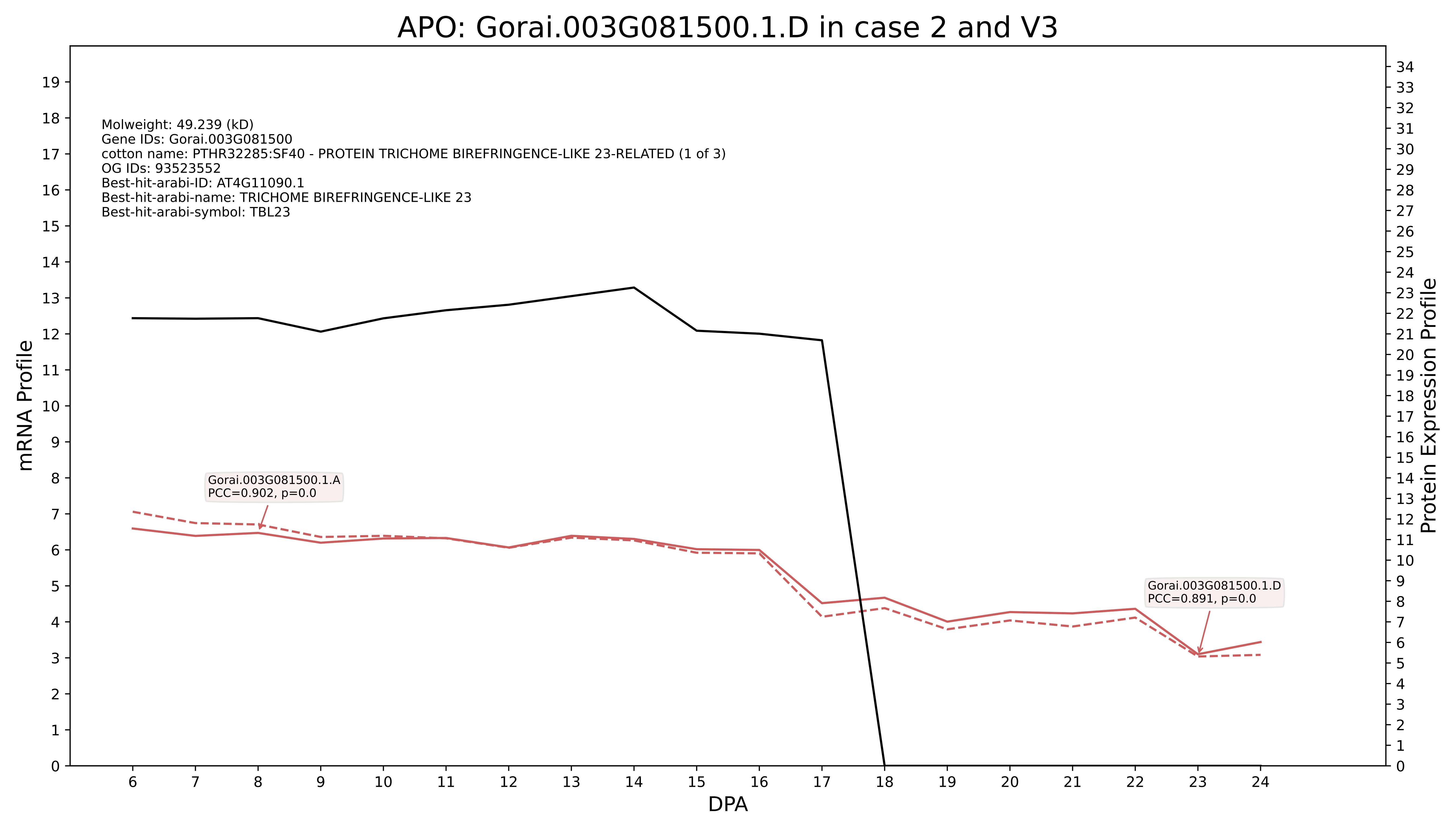
APO: Gorai.003G081500.1.D in case 2 and V3
Molweight: 49.239 (kD)
Gene IDs: Gorai.003G081500
cotton name: PTHR32285:SF40 - PROTEIN TRICHOME BIREFRINGENCE-LIKE 23-RELATED (1 of 3)
OG IDs: 93523552
Best-hit-arabi-ID: AT4G11090.1
Best-hit-arabi-name: TRICHOME BIREFRINGENCE-LIKE 23
Best-hit-arabi-symbol: TBL23
Gorai.003G081500.1.A
PCC=0.902, p=0.0
Gorai.003G081500.1.D
PCC=0.891, p=0.0
mRNA Profile
Protein Expression Profile
DPA

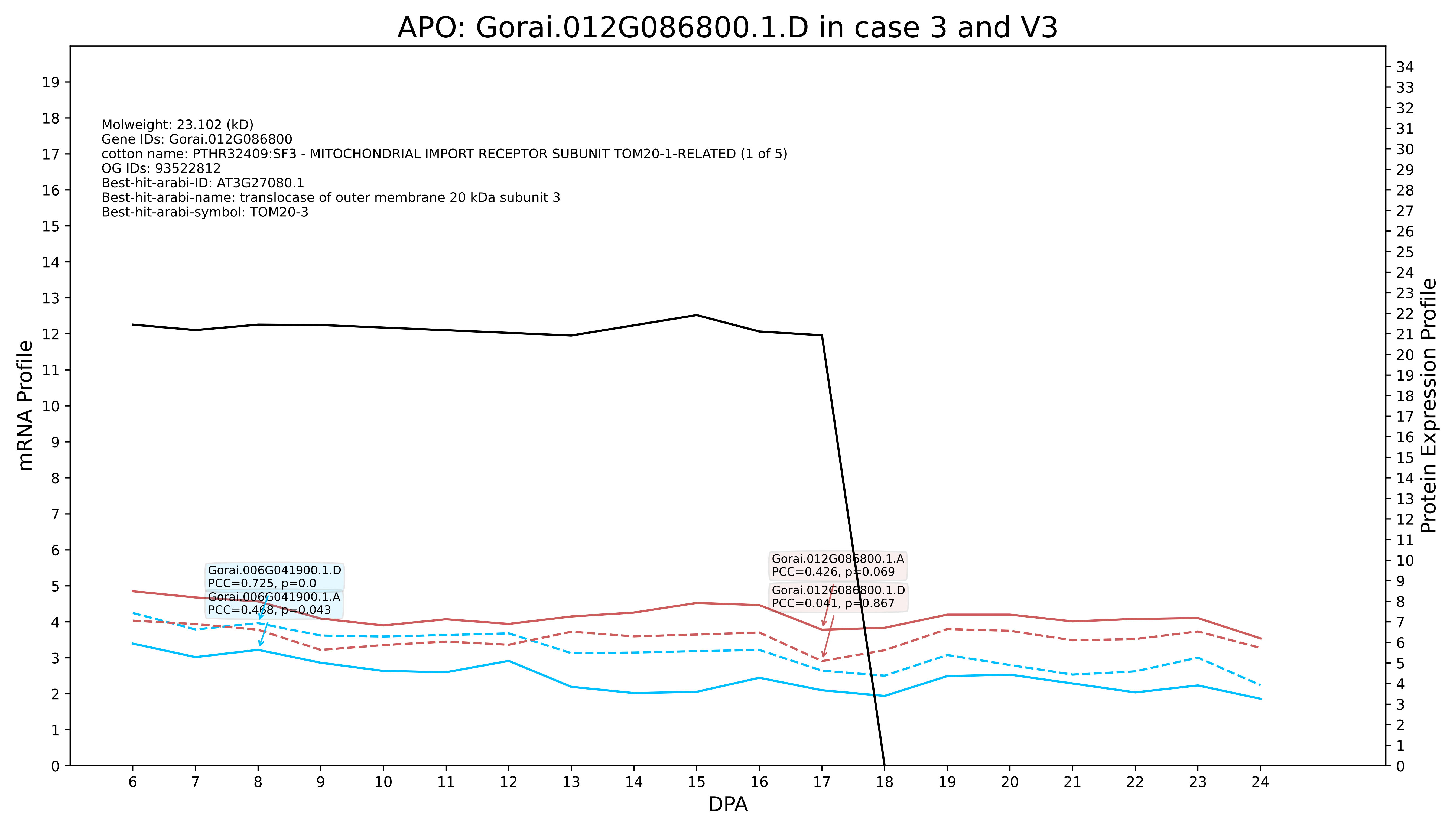

APO: Gorai.012G086800.1.D in case 3 and V3
Molweight: 23.102 (kD)
Gene IDs: Gorai.012G086800
cotton name: PTHR32409:SF3 - MITOCHONDRIAL IMPORT RECEPTOR SUBUNIT TOM20-1-RELATED (1 of 5)
OG IDs: 93522812
Best-hit-arabi-ID: AT3G27080.1
Best-hit-arabi-name: translocase of outer membrane 20 kDa subunit 3
Best-hit-arabi-symbol: TOM20-3
Gorai.006G041900.1.D
PCC=0.725, p=0.0
Gorai.006G041900.1.A
PCC=0.468, p=0.043
Gorai.012G086800.1.A
PCC=0.426, p=0.069
Gorai.012G086800.1.D
PCC=0.041, p=0.867
mRNA Profile
Protein Expression Profile
DPA

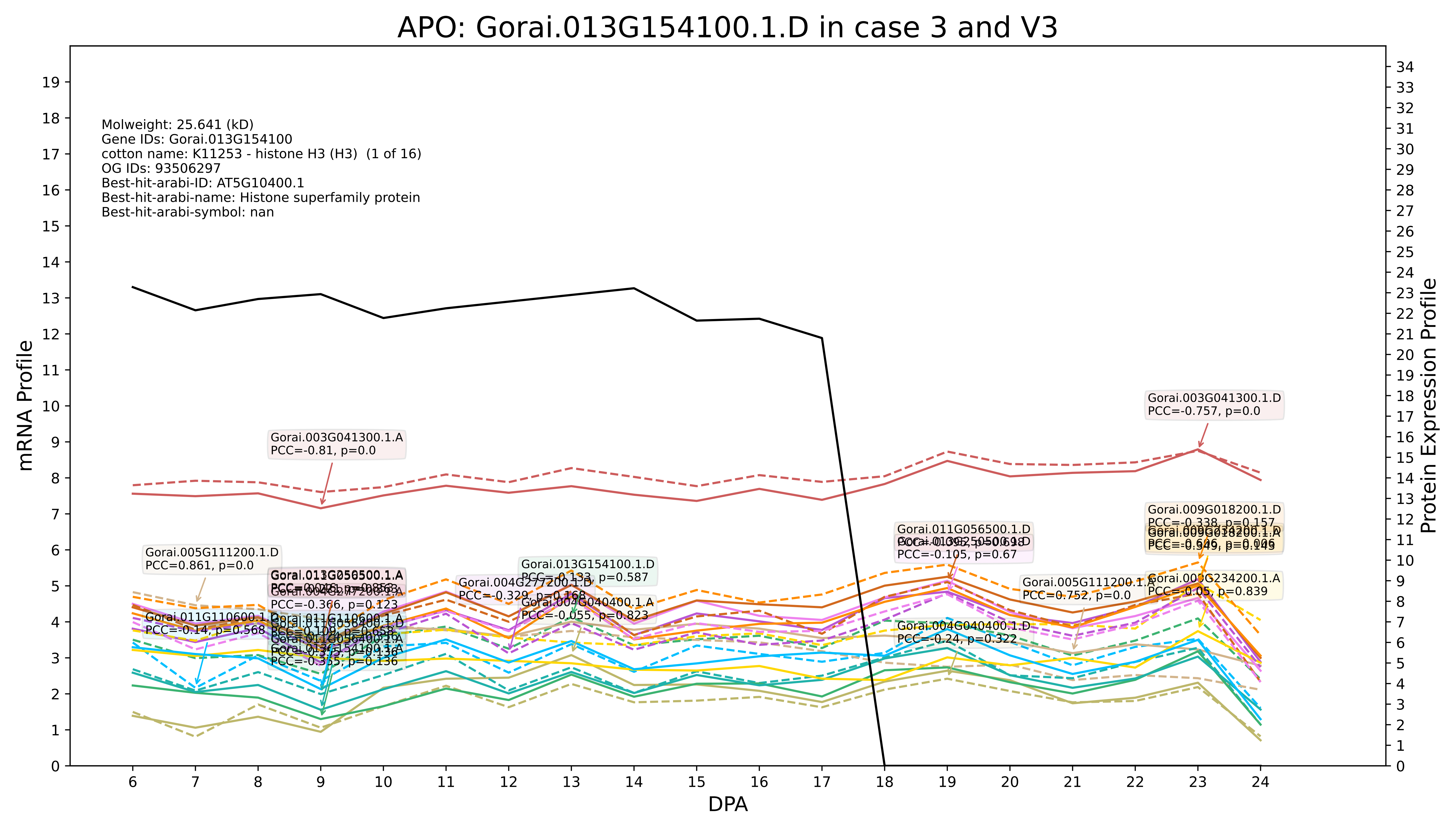
APO: Gorai.013G154100.1.D in case 3 and V3
Molweight: 25.641 (kD)
Gene IDs: Gorai.013G154100
cotton name: K11253 - histone H3 (H3) (1 of 16)
OG IDs: 93506297
Best-hit-arabi-ID: AT5G10400.1
Best-hit-arabi-name: Histone superfamily protein
Best-hit-arabi-symbol: nan
Gorai.003G041300.1.D
PCC=-0.757, p=0.0
Gorai.003G041300.1.A
PCC=-0.81, p=0.0
Gorai.005G111200.1.D
PCC=0.861, p=0.0
Gorai.013G154100.1.D
PCC=-0.133, p=0.587
Gorai.004G277200.1.D
PCC=-0.329, p=0.168
Gorai.004G040400.1.A
PCC=-0.055, p=0.823
Gorai.011G110600.1.D
PCC=-0.14, p=0.568
Gorai.011G056500.1.D
Gorai.005G111200.1.A
PCC=0.752, p=0.0
Gorai.004G040400.1.D
PCC=-0.24, p=0.322
Gorai.009G018200.1.D
PCC=-0.338, p=0.157
Gorai.009G234200.1.A
PCC=-0.05, p=0.839
mRNA Profile
Protein Expression Profile
DPA

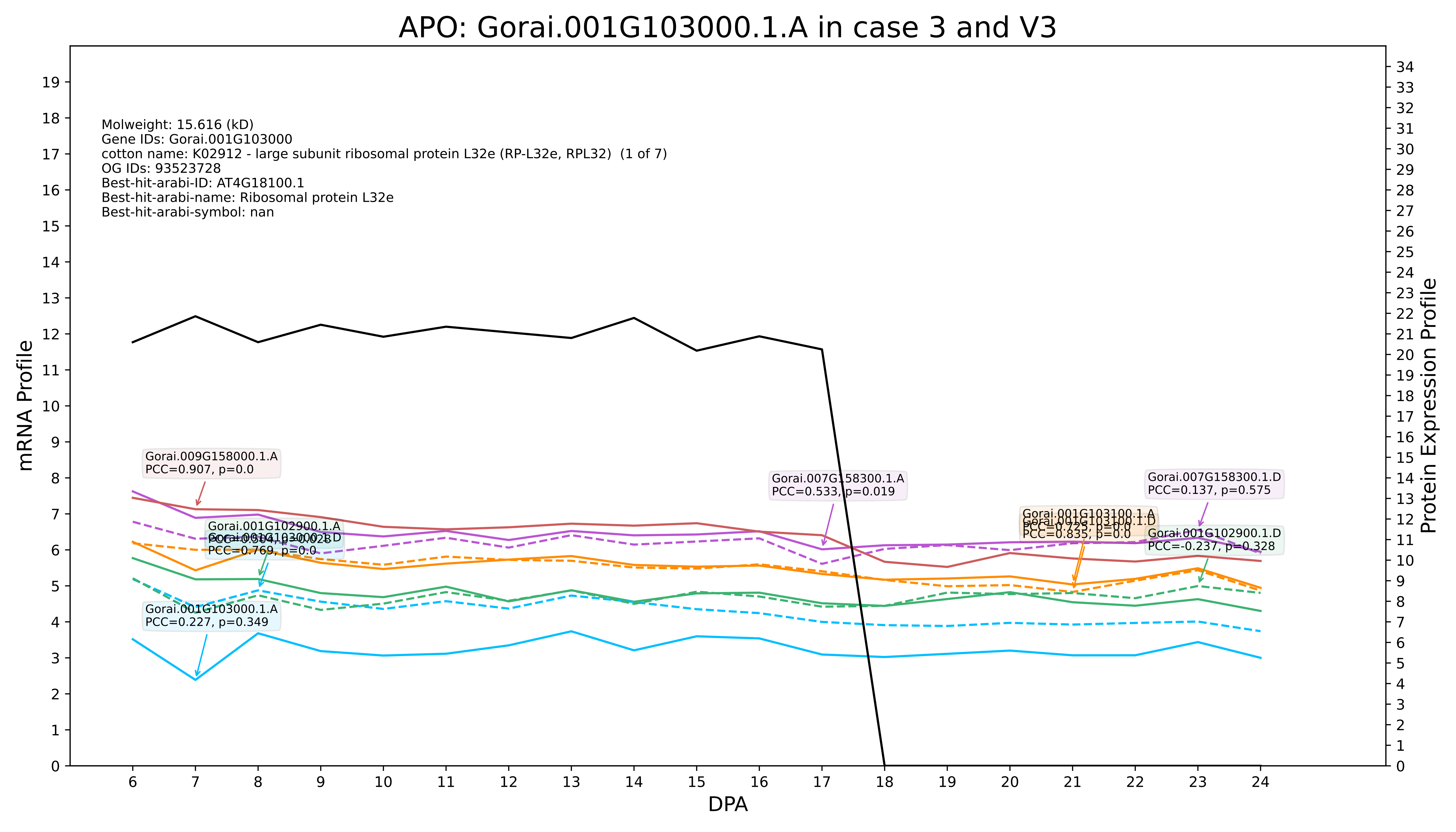
APO: Gorai.001G103000.1.A in case 3 and V3
Molweight: 15.616 (kD)
Gene IDs: Gorai.001G103000
cotton name: K02912 - large subunit ribosomal protein L32e (RP-L32e, RPL32) (1 of 7)
OG IDs: 93523728
Best-hit-arabi-ID: AT4G18100.1
Best-hit-arabi-name: Ribosomal protein L32e
Best-hit-arabi-symbol: nan
Gorai.009G158000.1.A
PCC=0.907, p=0.0
Gorai.001G102900.1.A
PCC=0.769, p=0.0
Gorai.001G103000.1.A
PCC=0.227, p=0.349
Gorai.007G158300.1.A
PCC=0.533, p=0.019
Gorai.007G158300.1.D
PCC=0.137, p=0.575
Gorai.001G103100.1.A
PCC=0.835, p=0.0
Gorai.001G102900.1.D
PCC=-0.237, p=0.328
mRNA Profile
Protein Expression Profile
DPA

APO: Gorai.007G300000.1.A in case 3 and V3

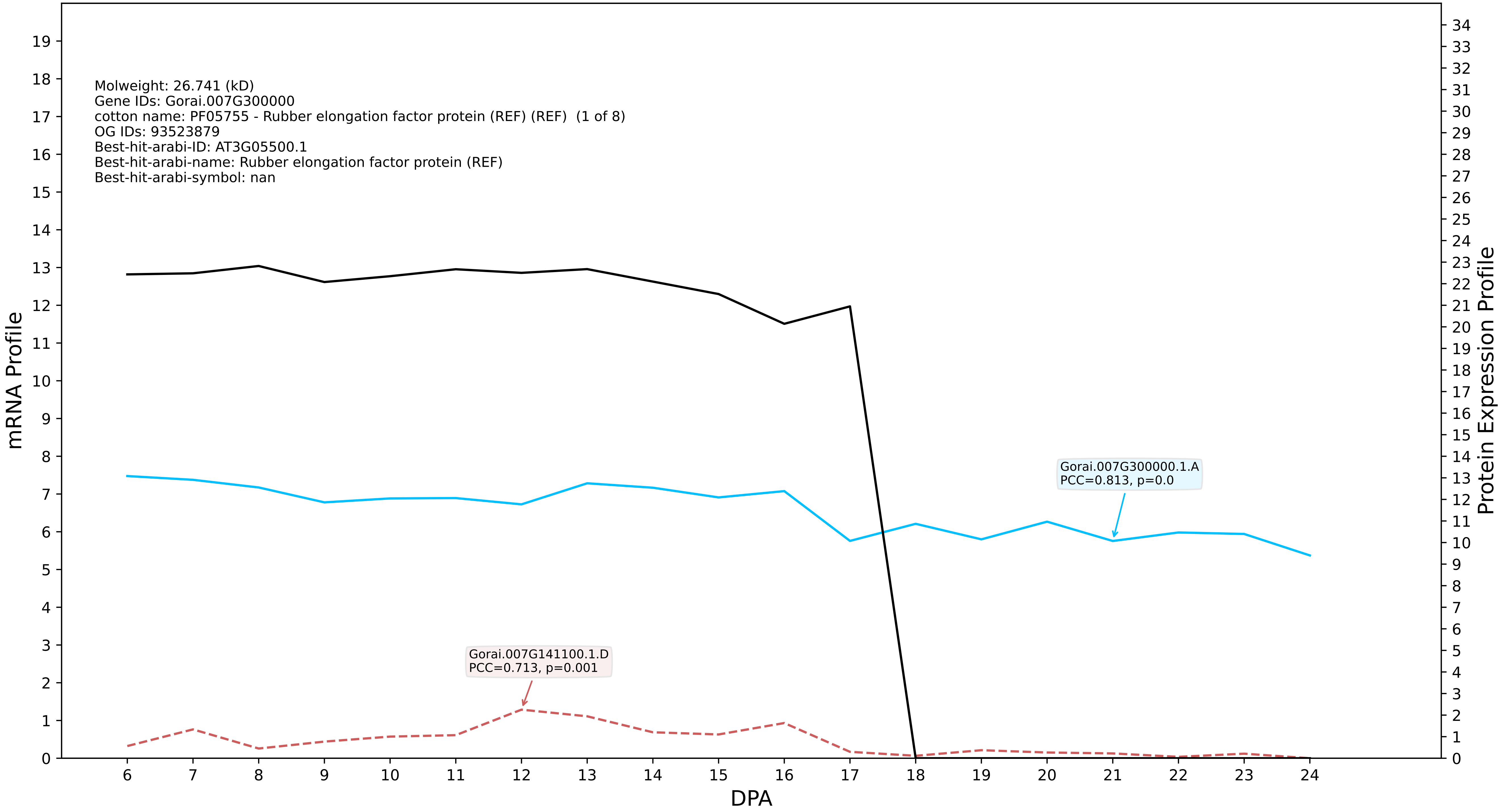

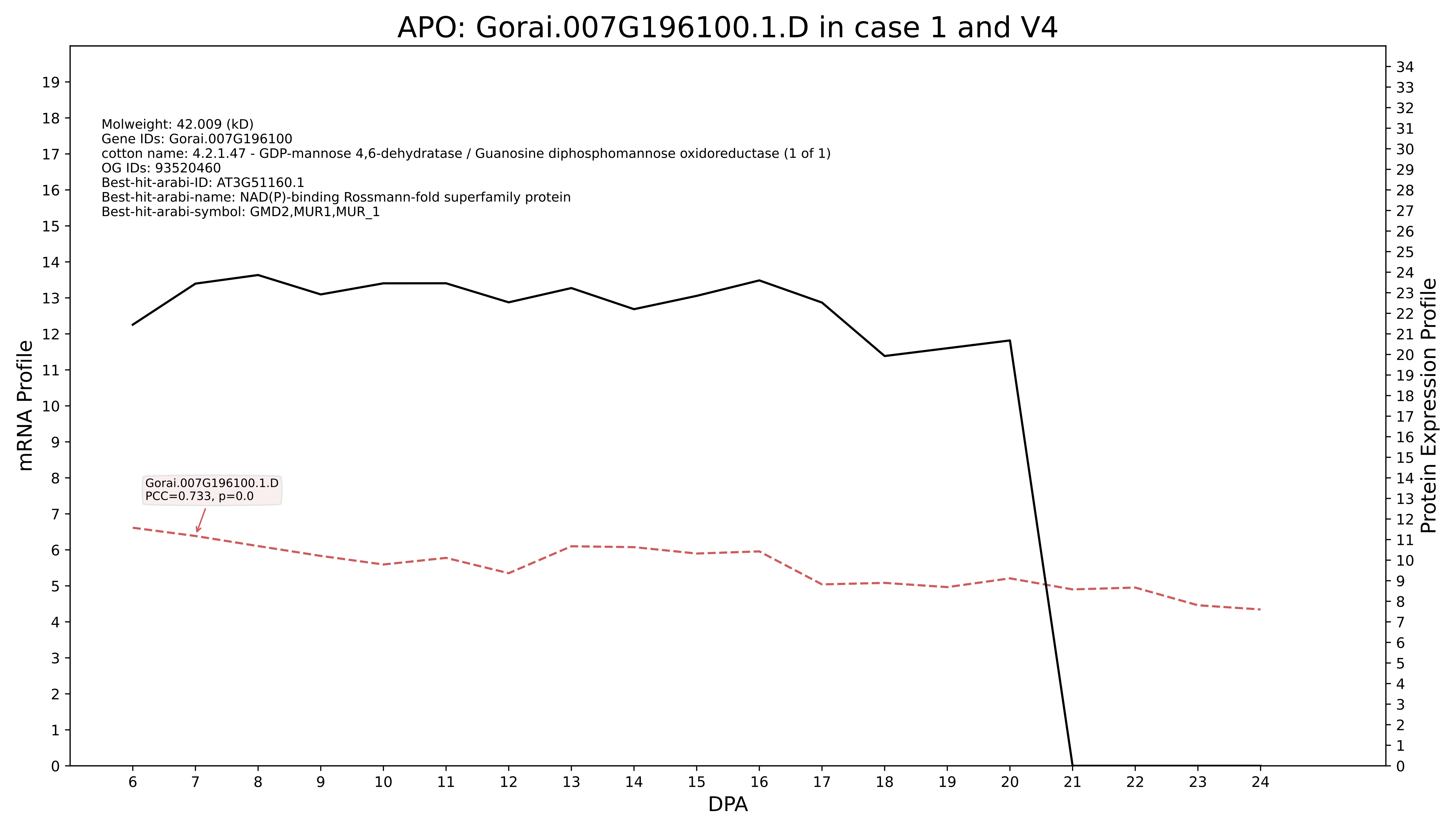
APO: Gorai.007G196100.1.D in case 1 and V4
Molweight: 42.009 (kD)
Gene IDs: Gorai.007G196100
cotton name: 4.2.1.47 - GDP-mannose 4,6-dehydratase / Guanosine diphosphomannose oxidoreductase (1 of 1)
OG IDs: 93520460
Best-hit-arabi-ID: AT3G51160.1
Best-hit-arabi-name: NAD(P)-binding Rossmann-fold superfamily protein
Best-hit-arabi-symbol: GMD2,MUR1,MUR_1
Gorai.007G196100.1.D
PCC=0.733, p=0.0
mRNA Profile
Protein Expression Profile
DPA
0
1
2
3
4
5
6
7
8
9
10
11
12
13
14
15
16
17
18
19
20
21
22
23
24
25
26
27
28
29
30
31
32
33
34

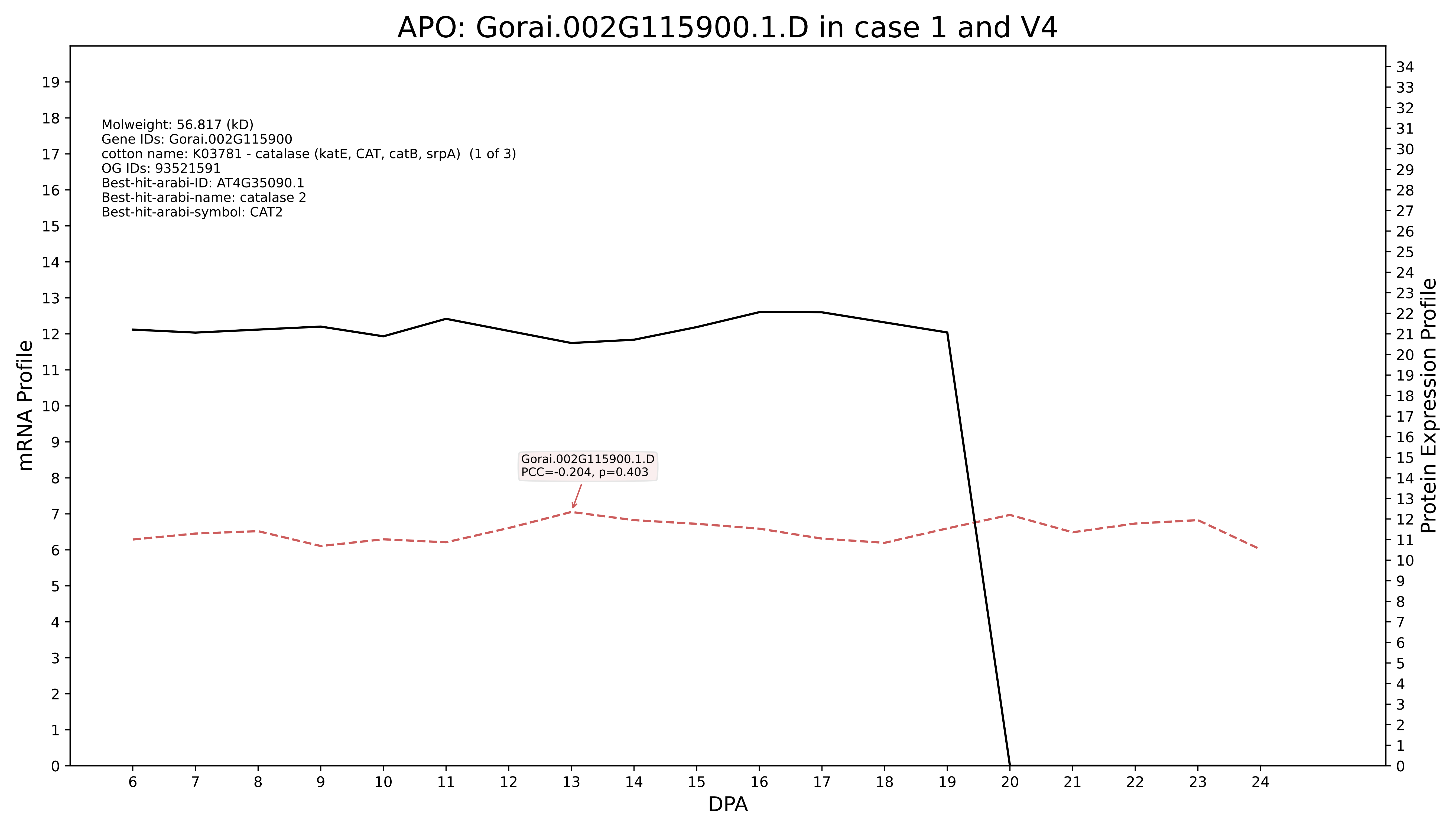
APO: Gorai.002G115900.1.D in case 1 and V4
Molweight: 56.817 (kD)
Gene IDs: Gorai.002G115900
cotton name: K03781 - catalase (katE, CAT, catB, srpA) (1 of 3)
OG IDs: 93521591
Best-hit-arabi-ID: AT4G35090.1
Best-hit-arabi-name: catalase 2
Best-hit-arabi-symbol: CAT2
Gorai.002G115900.1.D
PCC=-0.204, p=0.403
mRNA Profile
Protein Expression Profile
DPA

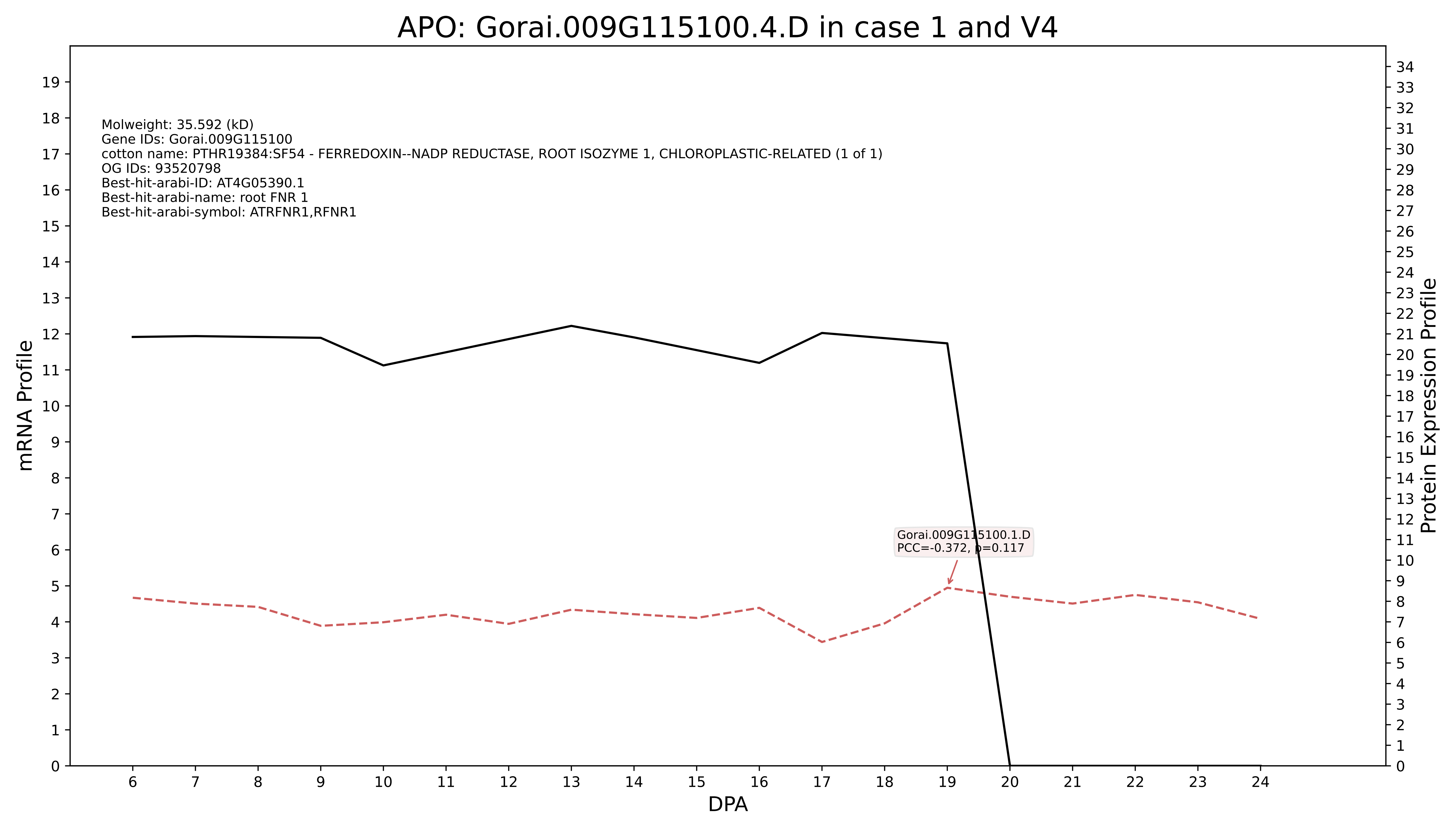
APO: Gorai.009G115100.4.D in case 1 and V4
Molweight: 35.592 (kD)
Gene IDs: Gorai.009G115100
cotton name: PTHR19384:SF54 - FERREDOXIN--NADP REDUCTASE, ROOT ISOZYME 1, CHLOROPLASTIC-RELATED (1 of 1)
OG IDs: 93520798
Best-hit-arabi-ID: AT4G05390.1
Best-hit-arabi-name: root FNR 1
Best-hit-arabi-symbol: ATRFNR1,RFNR1
Gorai.009G115100.1.D
PCC=-0.372, p=0.117
mRNA Profile
Protein Expression Profile
DPA

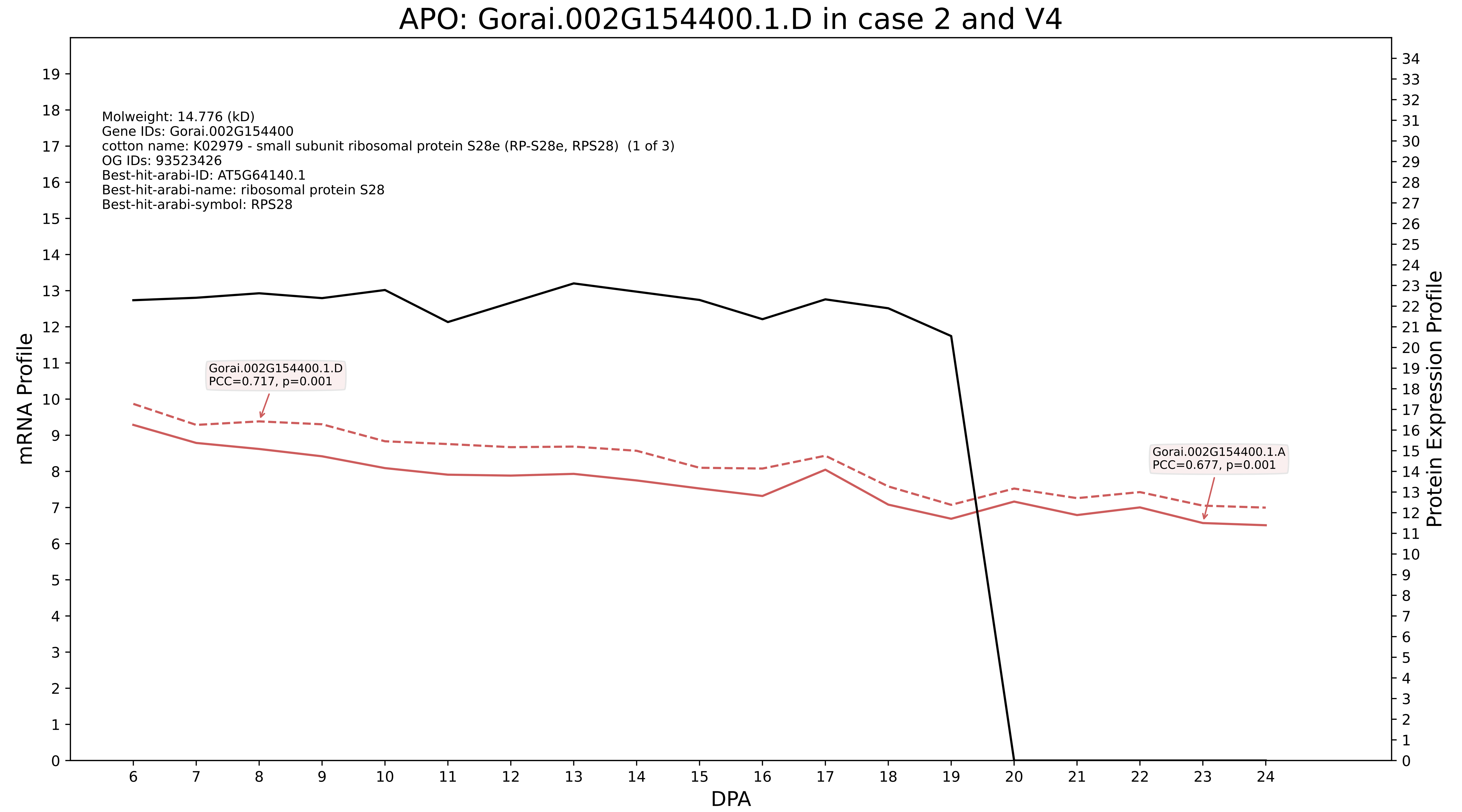
APO: Gorai.002G154400.1.D in case 2 and V4
Molweight: 14.776 (kD)
Gene IDs: Gorai.002G154400
cotton name: K02979 - small subunit ribosomal protein S28e (RP-S28e, RPS28) (1 of 3)
OG IDs: 93523426
Best-hit-arabi-ID: AT5G64140.1
Best-hit-arabi-name: ribosomal protein S28
Best-hit-arabi-symbol: RPS28
Gorai.002G154400.1.D
PCC=0.717, p=0.001
Gorai.002G154400.1.A
PCC=0.677, p=0.001
mRNA Profile
Protein Expression Profile
DPA

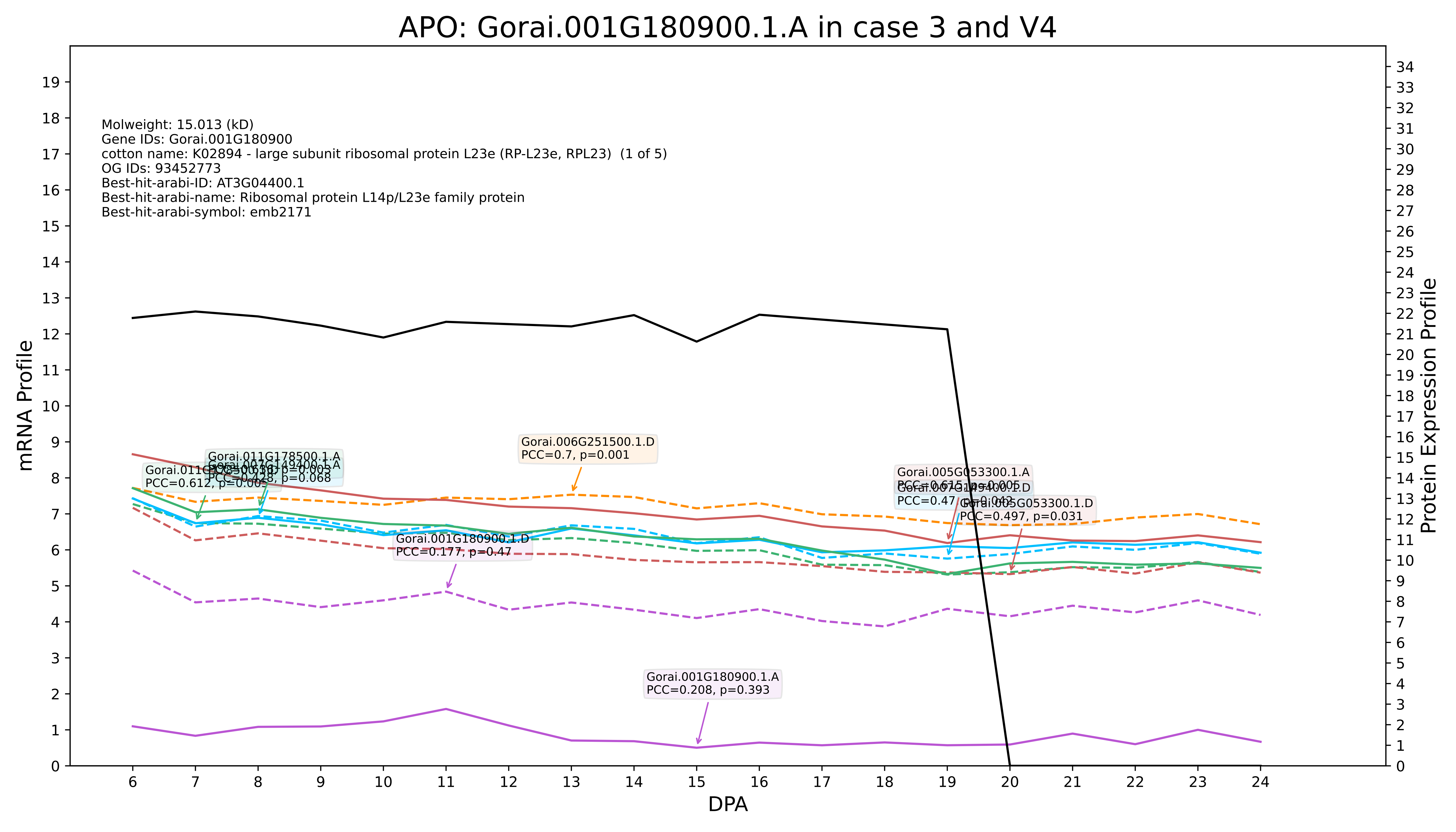
APO: Gorai.001G180900.1.A in case 3 and V4
Molweight: 15.013 (kD)
Gene IDs: Gorai.001G180900
cotton name: K02894 - large subunit ribosomal protein L23e (RP-L23e, RPL23) (1 of 5)
OG IDs: 93452773
Best-hit-arabi-ID: AT3G04400.1
Best-hit-arabi-name: Ribosomal protein L14p/L23e family protein
Best-hit-arabi-symbol: emb2171
Gorai.006G251500.1.D
PCC=0.7, p=0.001
Gorai.011G178500.1.A
Gorai.001G180900.1.D
PCC=0.177, p=0.47
Gorai.001G180900.1.A
PCC=0.208, p=0.393
Gorai.005G053300.1.A
Gorai.007G149400.1.D
PCC=0.47
Gorai.005G053300.1.D
PCC=0.497, p=0.031
mRNA Profile
Protein Expression Profile
DPA

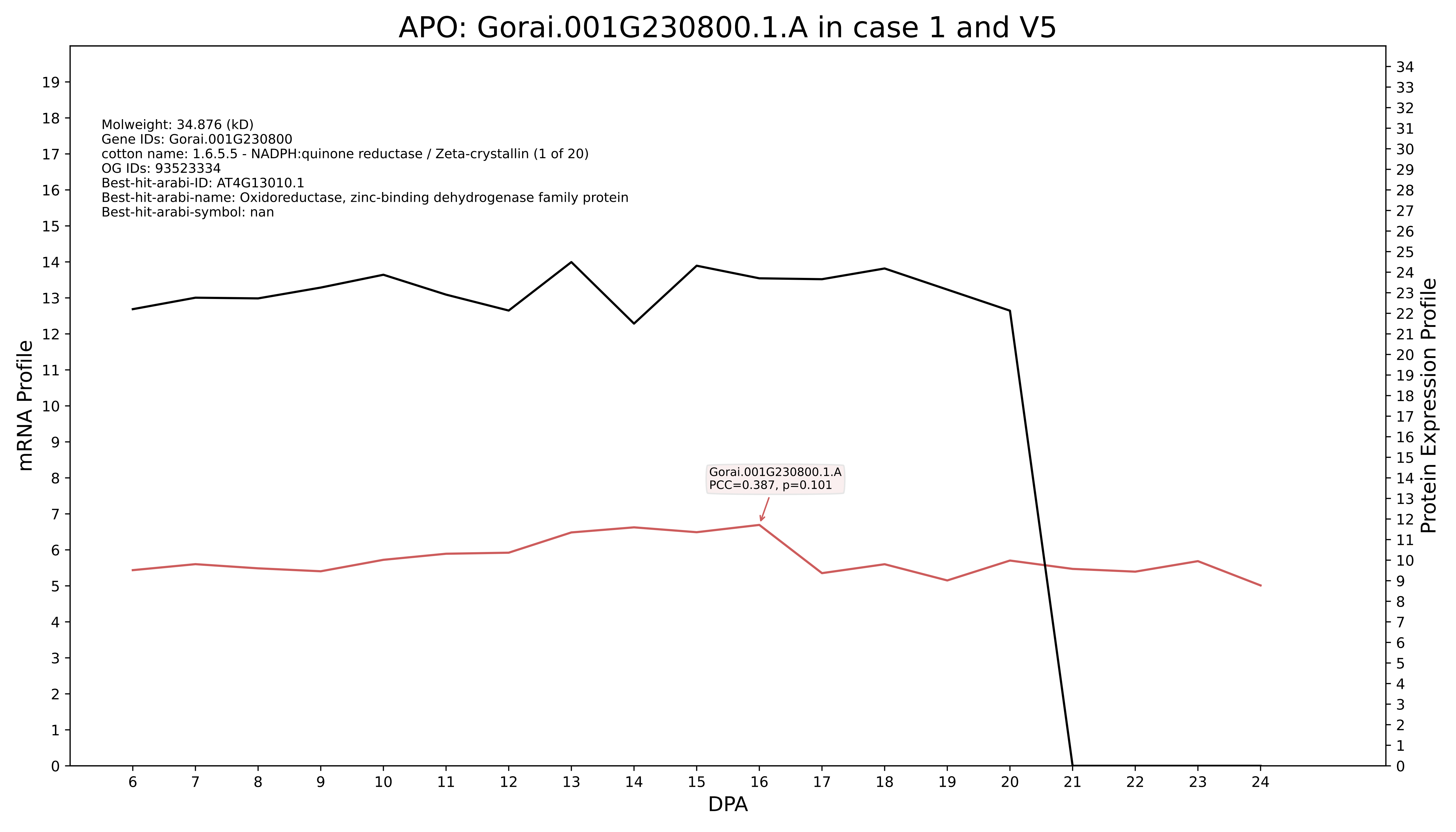
APO: Gorai.001G230800.1.A in case 1 and V5
Molweight: 34.876 (kD)
Gene IDs: Gorai.001G230800
cotton name: 1.6.5.5 - NADPH:quinone reductase / Zeta-crystallin (1 of 20)
OG IDs: 93523334
Best-hit-arabi-ID: AT4G13010.1
Best-hit-arabi-name: Oxidoreductase, zinc-binding dehydrogenase family protein
Best-hit-arabi-symbol: nan
Gorai.001G230800.1.A
PCC=0.387, p=0.101
mRNA Profile
Protein Expression Profile
DPA
0
1
2
3
4
5
6
7
8
9
10
11
12
13
14
15
16
17
18
19
20
21
22
23
24
25
26
27
28
29
30
31
32
33
34

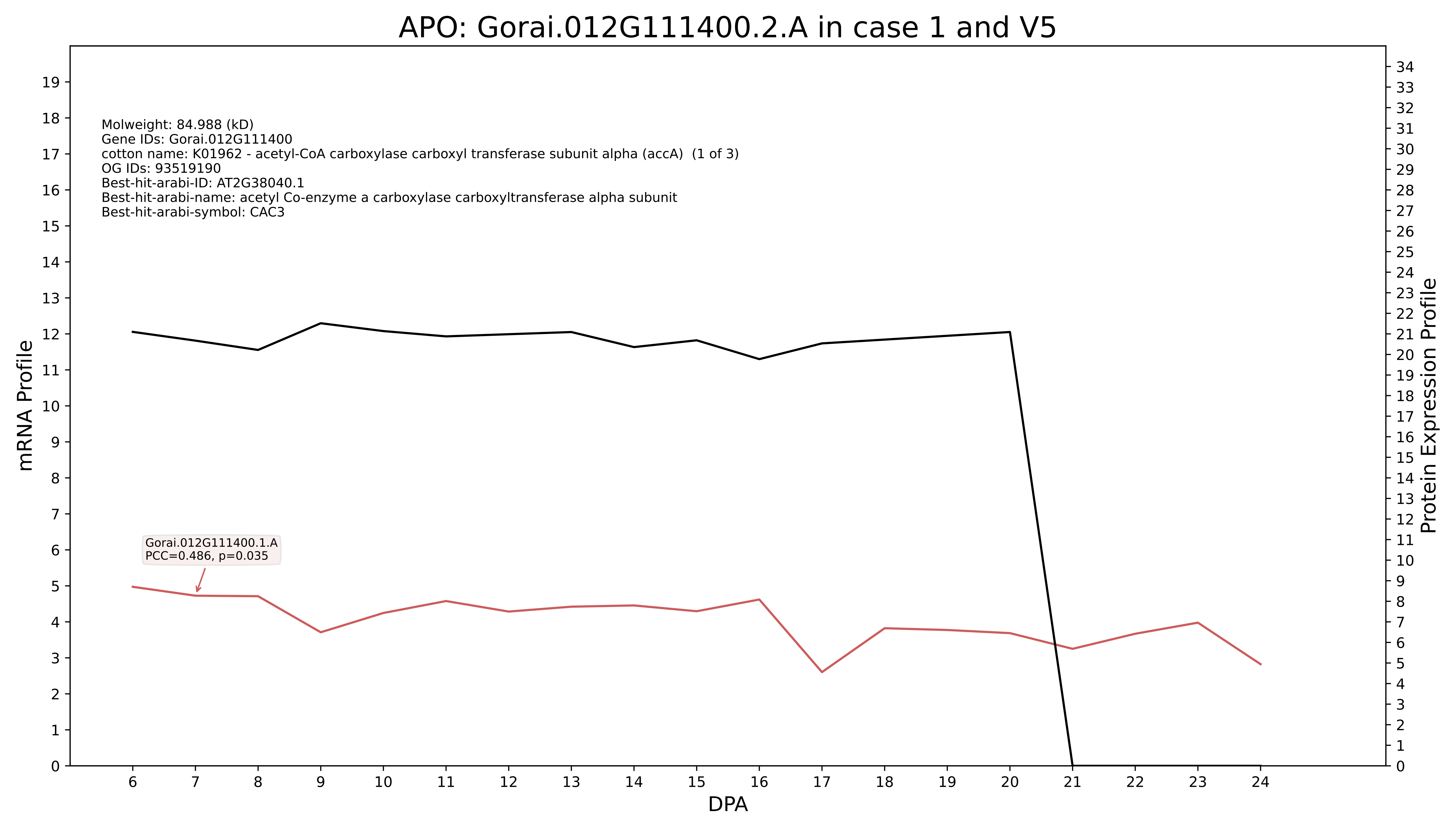
APO: Gorai.012G111400.2.A in case 1 and V5
Molweight: 84.988 (kD)
Gene IDs: Gorai.012G111400
cotton name: K01962 - acetyl-CoA carboxylase carboxyl transferase subunit alpha (accA) (1 of 3)
OG IDs: 93519190
Best-hit-arabi-ID: AT2G38040.1
Best-hit-arabi-name: acetyl Co-enzyme a carboxylase carboxyltransferase alpha subunit
Best-hit-arabi-symbol: CAC3
Gorai.012G111400.1.A
PCC=0.486, p=0.035
mRNA Profile
Protein Expression Profile
DPA

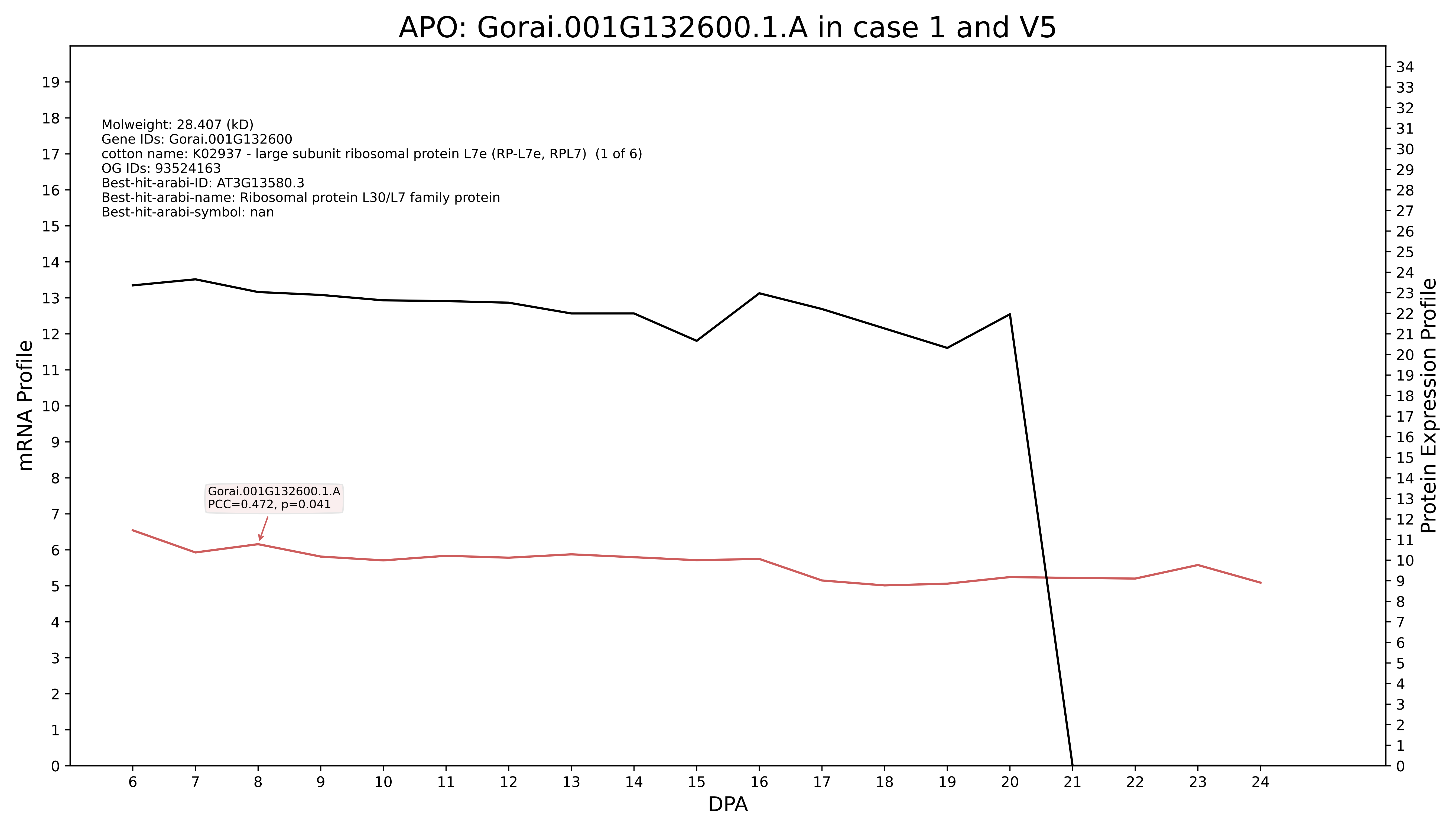
APO: Gorai.001G132600.1.A in case 1 and V5
Molweight: 28.407 (kD)
Gene IDs: Gorai.001G132600
cotton name: K02937 - large subunit ribosomal protein L7e (RP-L7e, RPL7) (1 of 6)
OG IDs: 93524163
Best-hit-arabi-ID: AT3G13580.3
Best-hit-arabi-name: Ribosomal protein L30/L7 family protein
Best-hit-arabi-symbol: nan
Gorai.001G132600.1.A
PCC=0.472, p=0.041
mRNA Profile
Protein Expression Profile
DPA

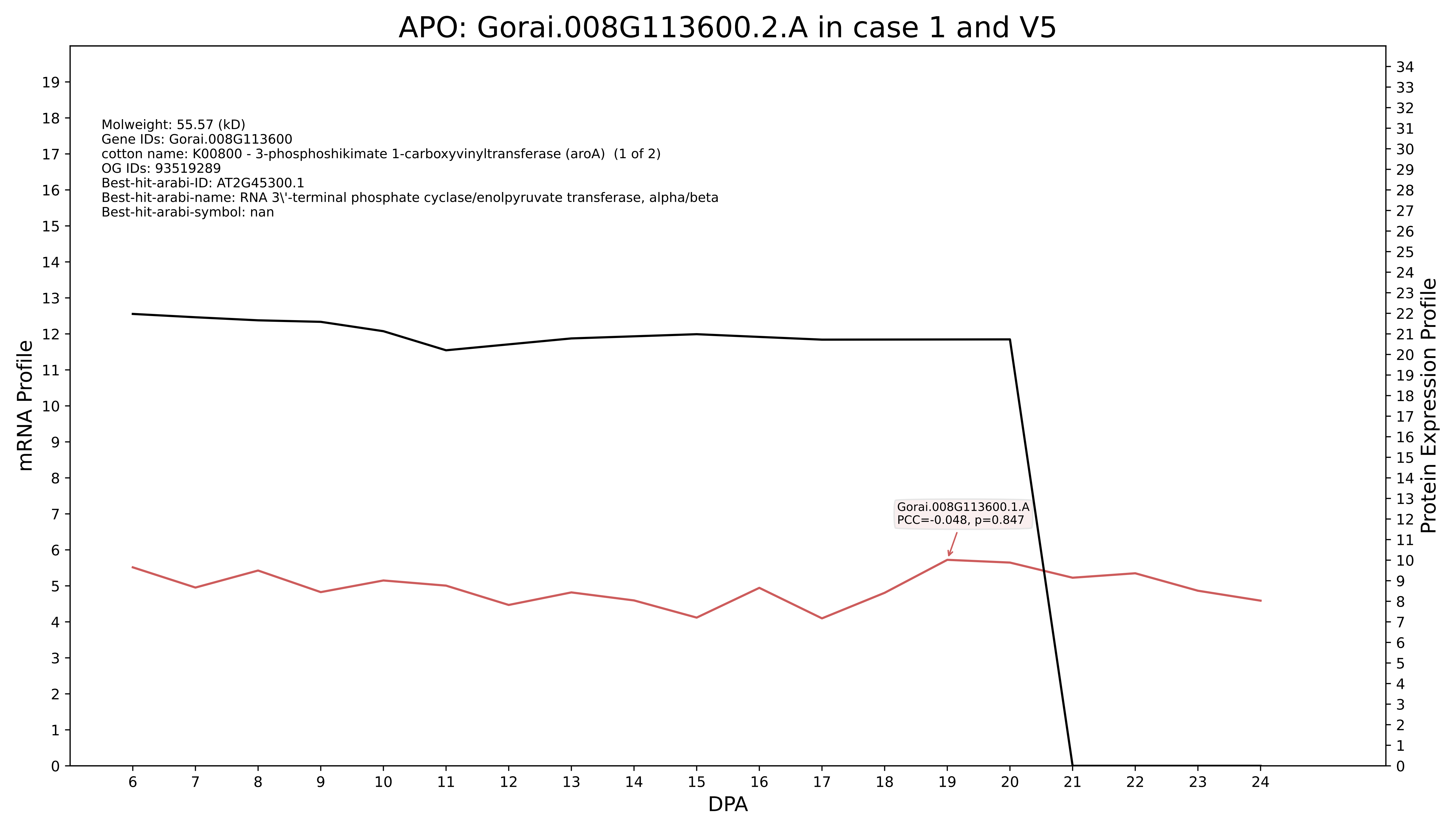

APO: Gorai.008G113600.2.A in case 1 and V5
Molweight: 55.57 (kD)
Gene IDs: Gorai.008G113600
cotton name: K00800 - 3-phosphoshikimate 1-carboxyvinyltransferase (aroA) (1 of 2)
OG IDs: 93519289
Best-hit-arabi-ID: AT2G45300.1
Best-hit-arabi-name: RNA 3\'-terminal phosphate cyclase/enolpyruvate transferase, alpha/beta
Best-hit-arabi-symbol: nan
Gorai.008G113600.1.A
PCC=-0.048, p=0.847
mRNA Profile
Protein Expression Profile
DPA

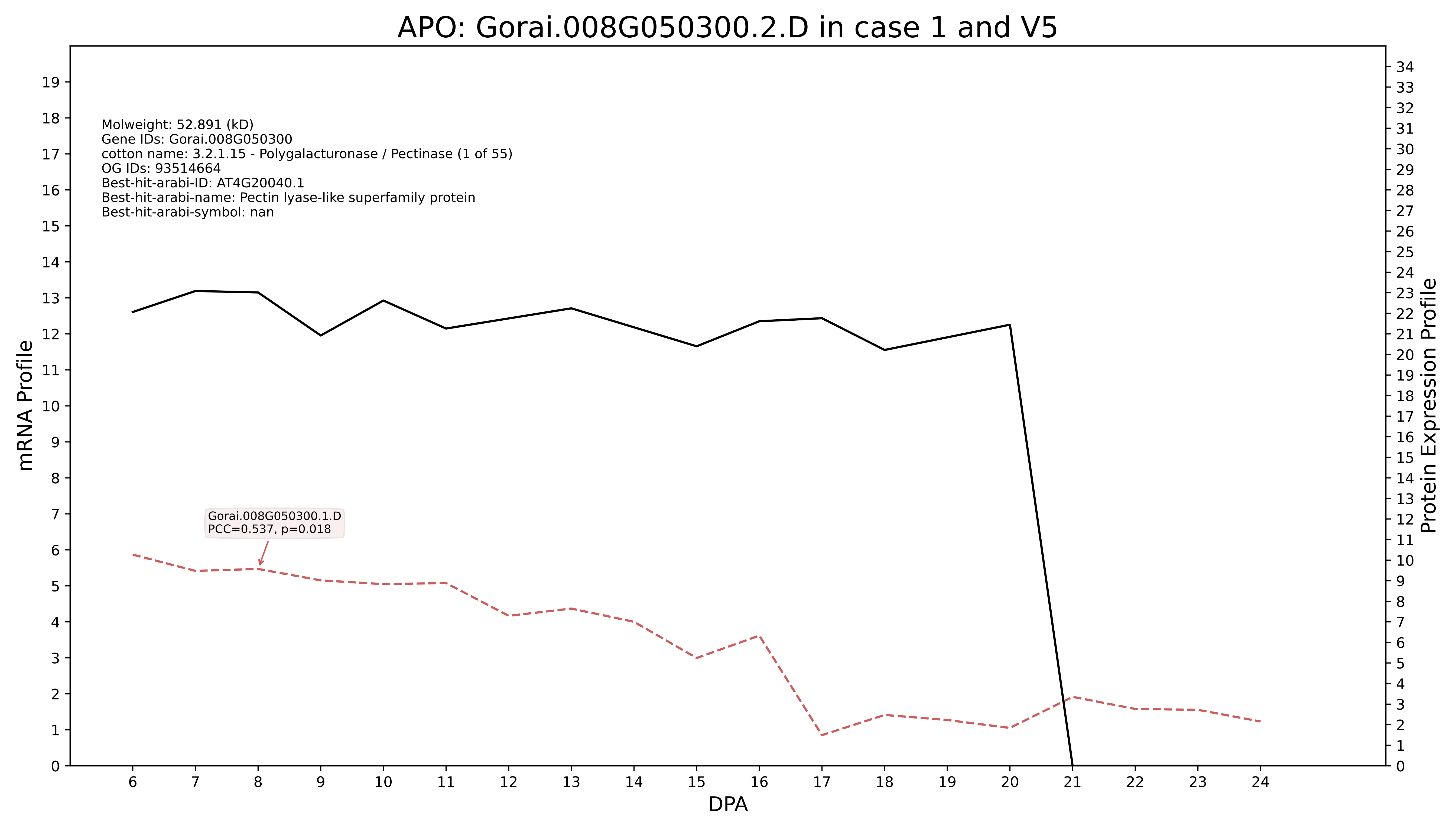
APO: Gorai.008G050300.2.D in case 1 and V5
Molweight: 52.891 (kD)
Gene IDs: Gorai.008G050300
cotton name: 3.2.1.15 - Polygalacturonase / Pectinase (1 of 55)
OG IDs: 93514664
Best-hit-arabi-ID: AT4G20040.1
Best-hit-arabi-name: Pectin lyase-like superfamily protein
Best-hit-arabi-symbol: nan
Gorai.008G050300.1.D
PCC=0.537, p=0.018
mRNA Profile
Protein Expression Profile
DPA

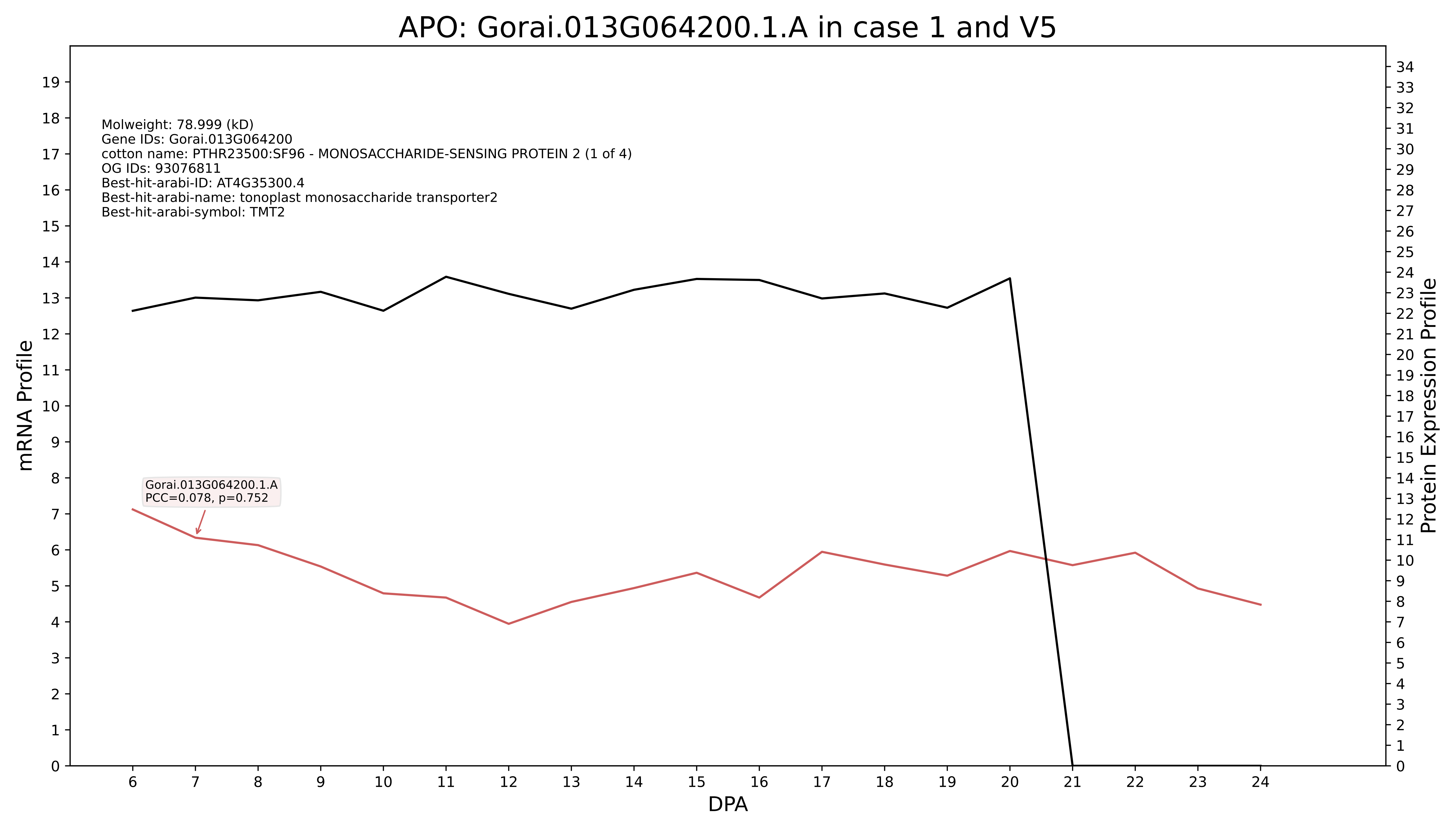
APO: Gorai.013G064200.1.A in case 1 and V5
Molweight: 78.999 (kD)
Gene IDs: Gorai.013G064200
cotton name: PTHR23500:SF96 - MONOSACCHARIDE-SENSING PROTEIN 2 (1 of 4)
OG IDs: 93076811
Best-hit-arabi-ID: AT4G35300.4
Best-hit-arabi-name: tonoplast monosaccharide transporter2
Best-hit-arabi-symbol: TMT2
Gorai.013G064200.1.A
PCC=0.078, p=0.752
mRNA Profile
Protein Expression Profile
DPA
0
1
2
3
4
5
6
7
8
9
10
11
12
13
14
15
16
17
18
19
20
21
22
23
24
25
26
27
28
29
30
31
32
33
34

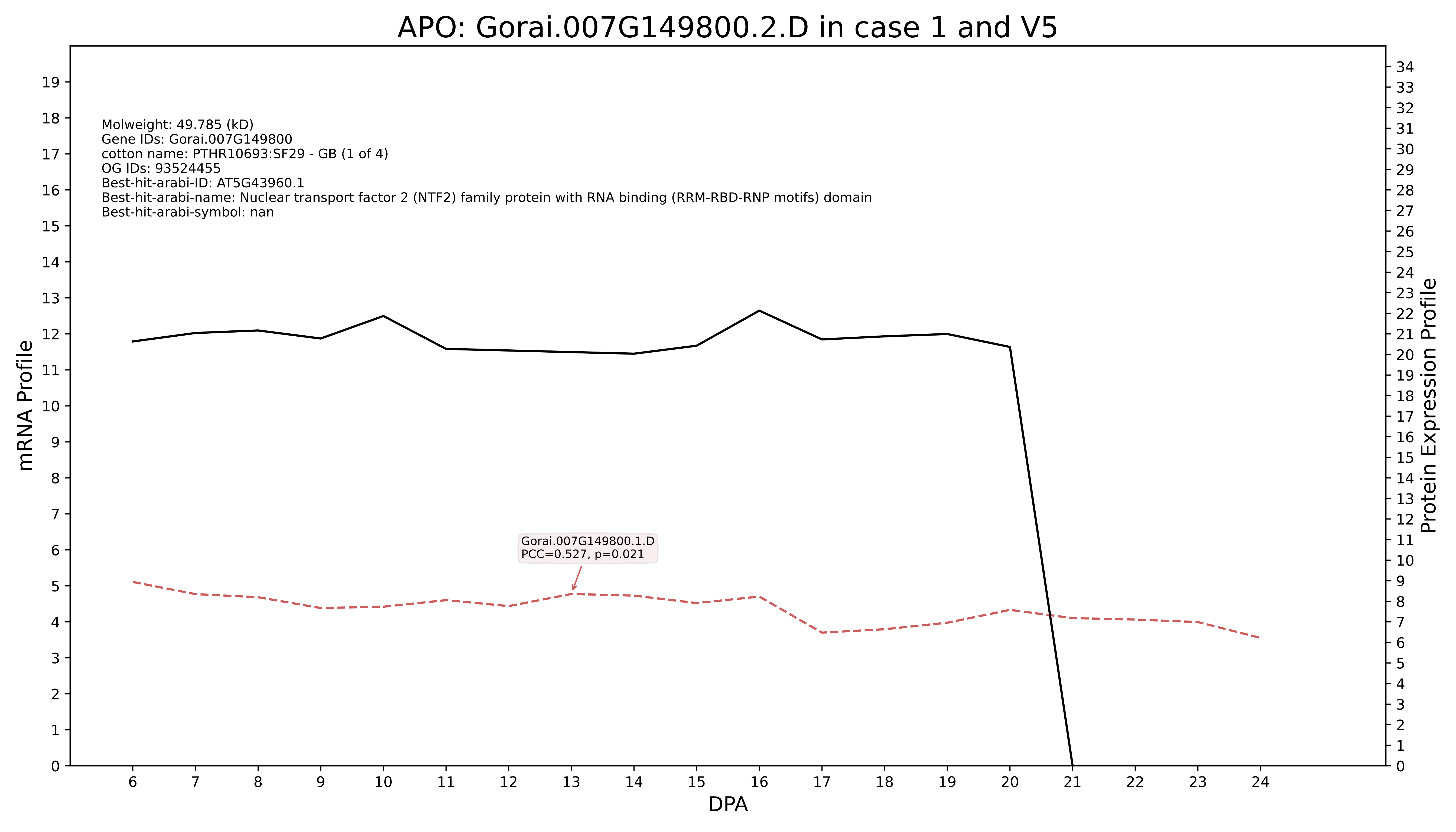

APO: Gorai.007G149800.2.D in case 1 and V5
Molweight: 49.785 (kD)
Gene IDs: Gorai.007G149800
cotton name: PTHR10693:SF29 - GB (1 of 4)
OG IDs: 93524455
Best-hit-arabi-ID: AT5G43960.1
Best-hit-arabi-name: Nuclear transport factor 2 (NTF2) family protein with RNA binding (RRM-RBD-RNP motifs) domain
Best-hit-arabi-symbol: nan
Gorai.007G149800.1.D
PCC=0.527, p=0.021
mRNA Profile
Protein Expression Profile
DPA

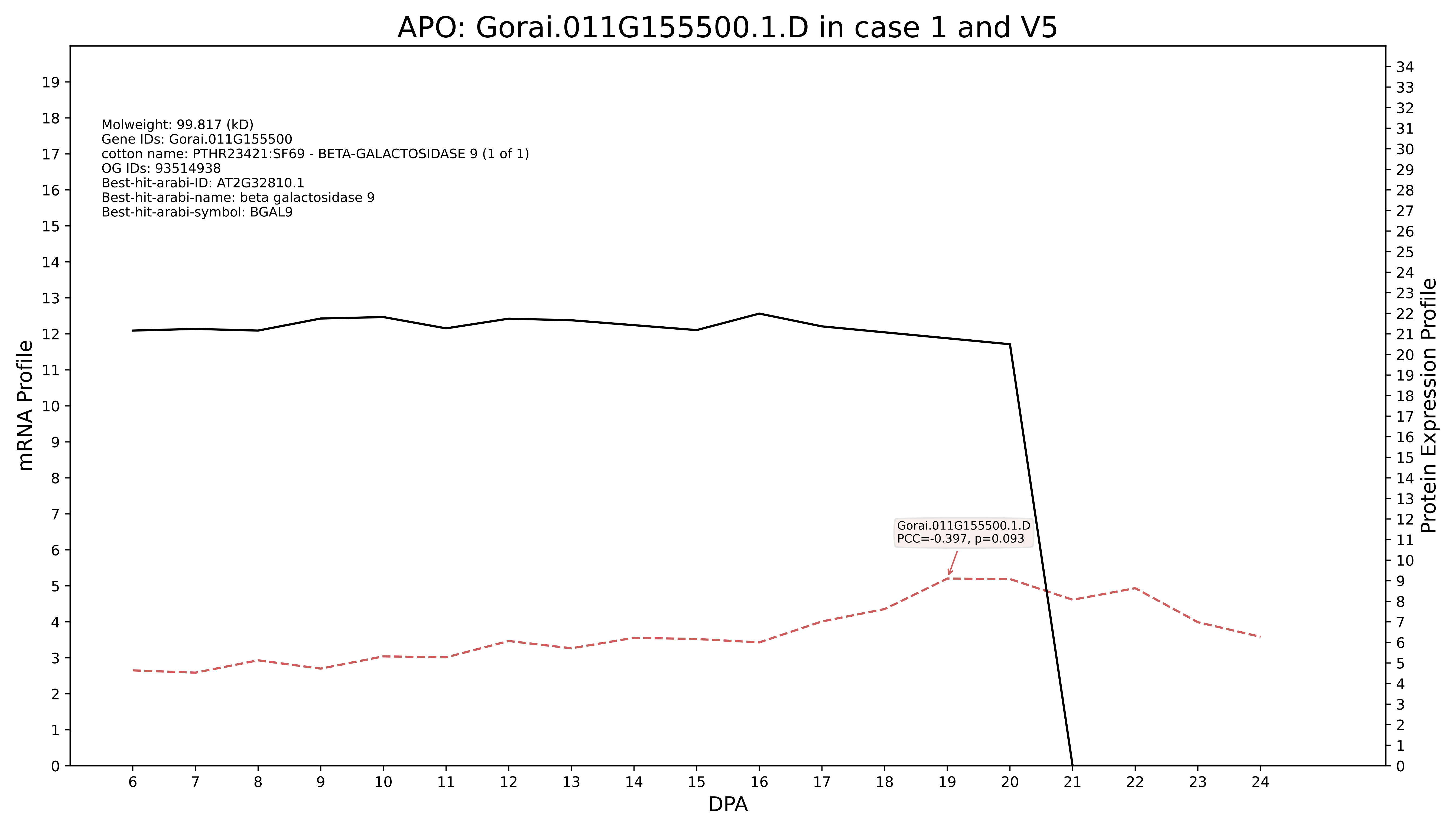
APO: Gorai.011G155500.1.D in case 1 and V5
Molweight: 99.817 (kD)
Gene IDs: Gorai.011G155500
cotton name: PTHR23421:SF69 - BETA-GALACTOSIDASE 9 (1 of 1)
OG IDs: 93514938
Best-hit-arabi-ID: AT2G32810.1
Best-hit-arabi-name: beta galactosidase 9
Best-hit-arabi-symbol: BGAL9
Gorai.011G155500.1.D
PCC=-0.397, p=0.093
mRNA Profile
Protein Expression Profile
DPA

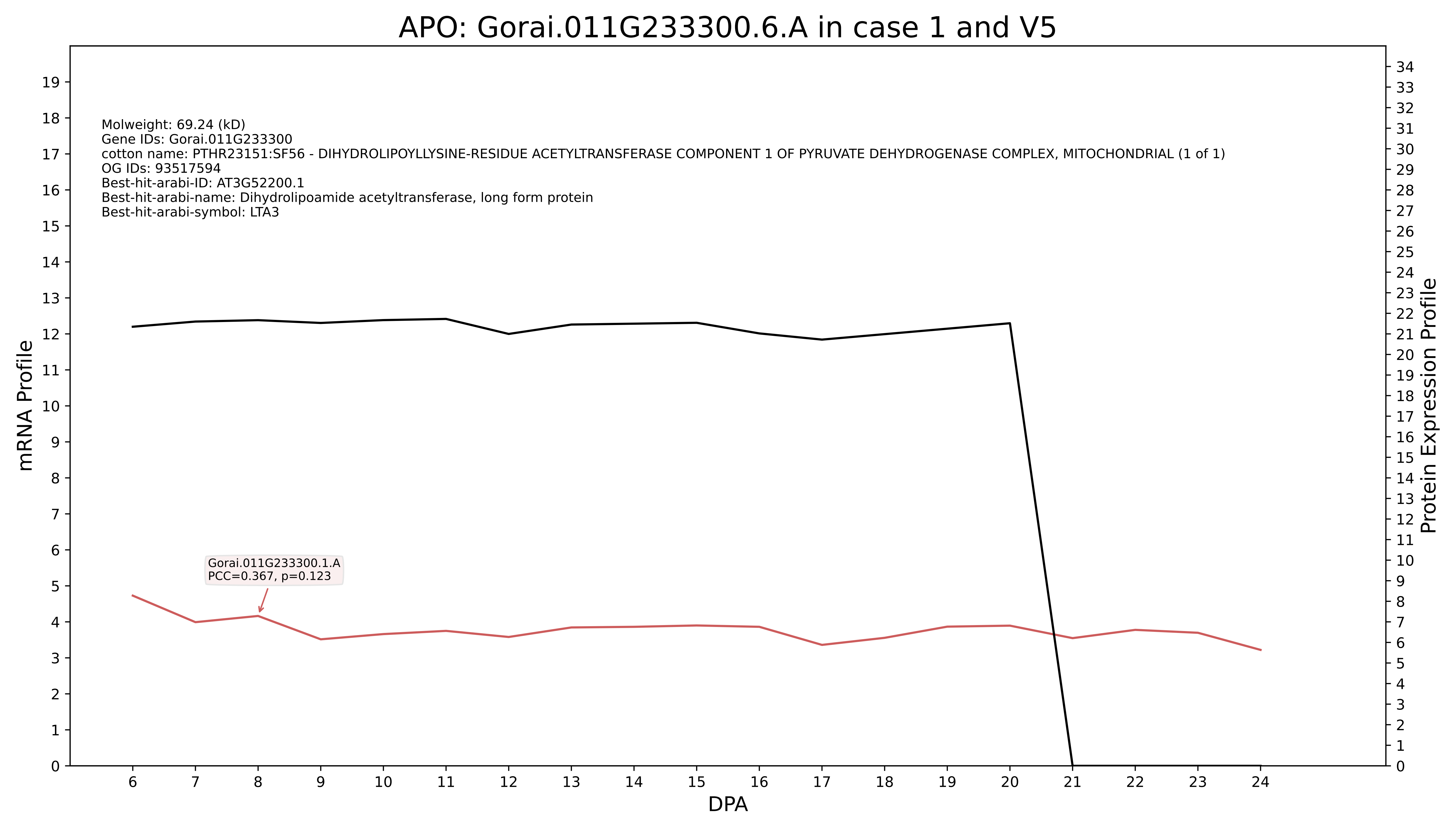

APO: Gorai.011G233300.6.A in case 1 and V5
Molweight: 69.24 (kD)
Gene IDs: Gorai.011G233300
cotton name: PTHR23151:SF56 - DIHYDROLIPOYLLYSINE-RESIDUE ACETYLTRANSFERASE COMPONENT 1 OF PYRUVATE DEHYDROGENASE COMPLEX, MITOCHONDRIAL (1 of 1)
OG IDs: 93517594
Best-hit-arabi-ID: AT3G52200.1
Best-hit-arabi-name: Dihydrolipoamide acetyltransferase, long form protein
Best-hit-arabi-symbol: LTA3
Gorai.011G233300.1.A
PCC=0.367, p=0.123
mRNA Profile
Protein Expression Profile
DPA

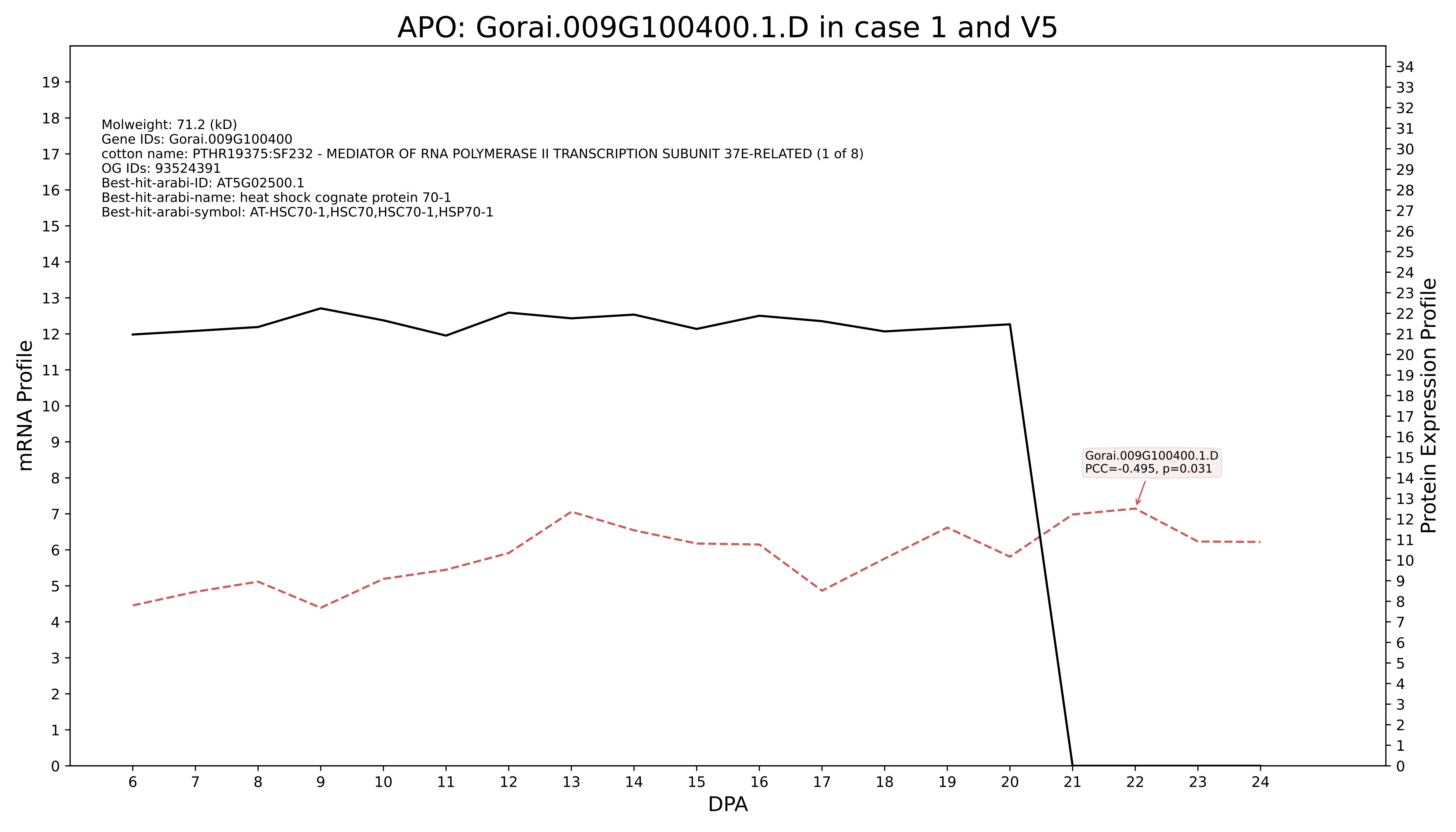
APO: Gorai.009G100400.1.D in case 1 and V5
Molweight: 71.2 (kD)
Gene IDs: Gorai.009G100400
cotton name: PTHR19375:SF232 - MEDIATOR OF RNA POLYMERASE II TRANSCRIPTION SUBUNIT 37E-RELATED (1 of 8)
OG IDs: 93524391
Best-hit-arabi-ID: AT5G02500.1
Best-hit-arabi-name: heat shock cognate protein 70-1
Best-hit-arabi-symbol: AT-HSC70-1,HSC70,HSC70-1,HSP70-1
Gorai.009G100400.1.D
PCC=-0.495, p=0.031
mRNA Profile
Protein Expression Profile
DPA

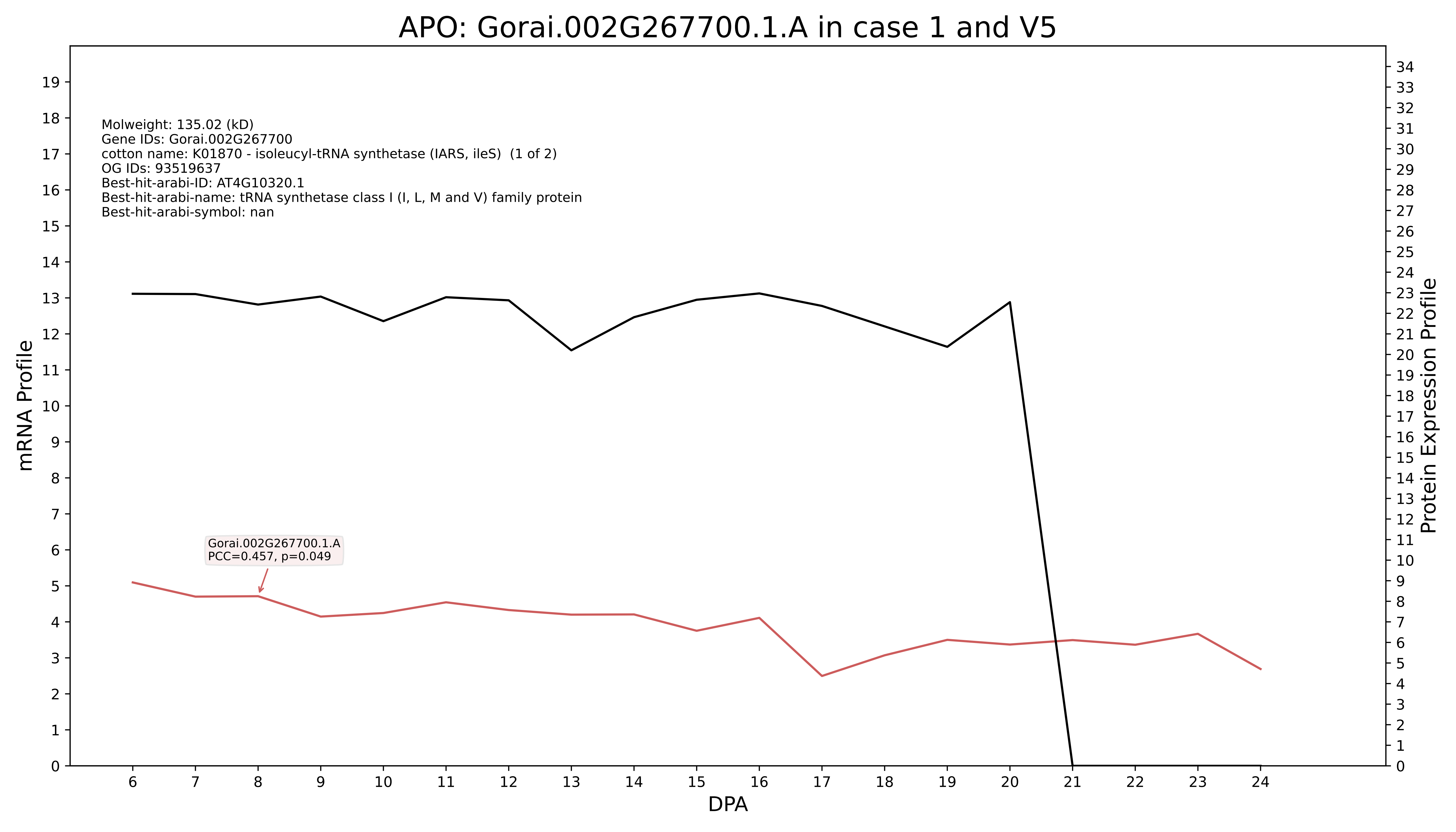
APO: Gorai.002G267700.1.A in case 1 and V5
Molweight: 135.02 (kD)
Gene IDs: Gorai.002G267700
cotton name: K01870 - isoleucyl-tRNA synthetase (IARS, ileS) (1 of 2)
OG IDs: 93519637
Best-hit-arabi-ID: AT4G10320.1
Best-hit-arabi-name: tRNA synthetase class I (I, L, M and V) family protein
Best-hit-arabi-symbol: nan
Gorai.002G267700.1.A
PCC=0.457, p=0.049
mRNA Profile
Protein Expression Profile
DPA

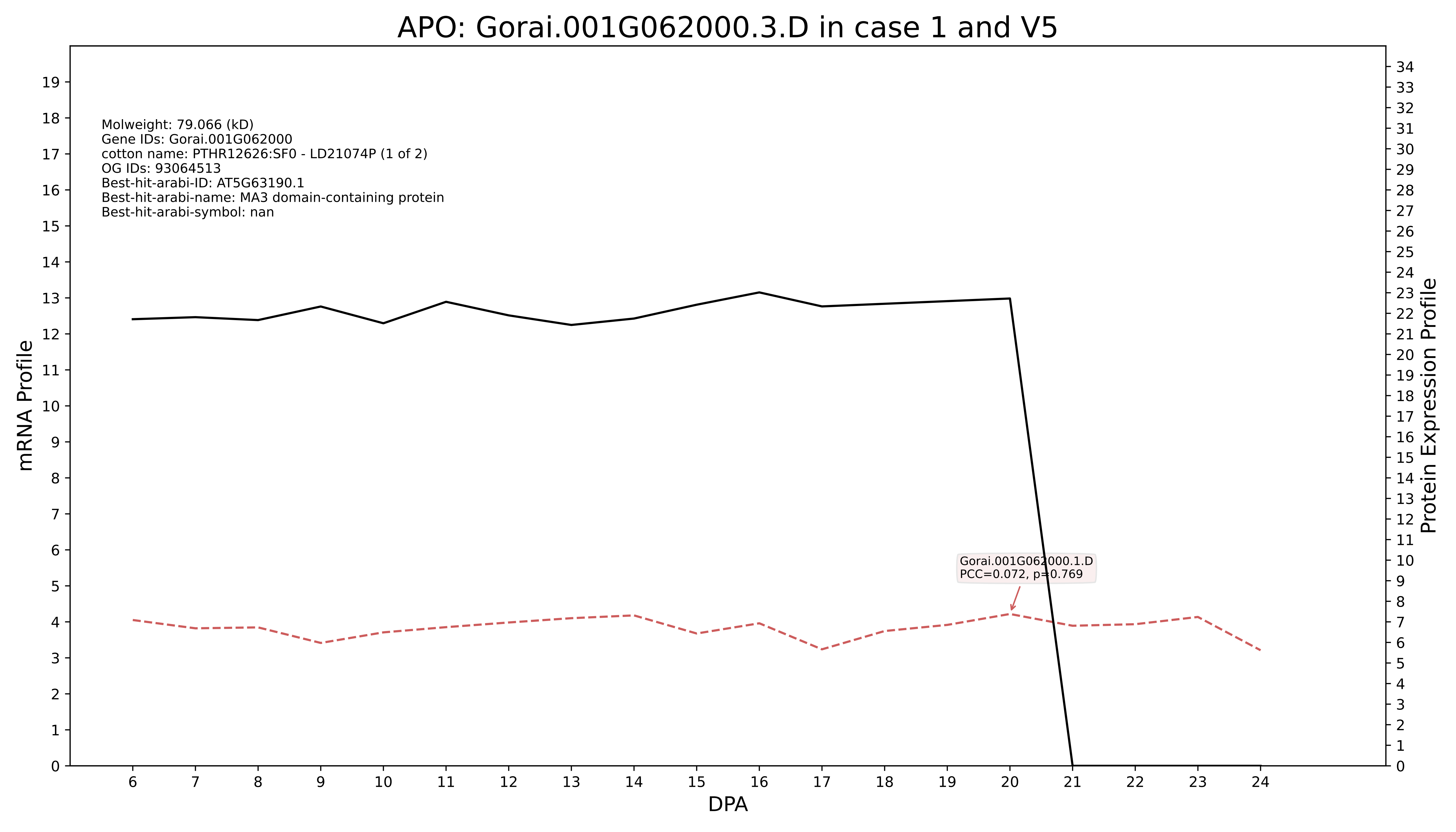

APO: Gorai.001G062000.3.D in case 1 and V5
Molweight: 79.066 (kD)
Gene IDs: Gorai.001G062000
cotton name: PTHR12626:SF0 - LD21074P (1 of 2)
OG IDs: 93064513
Best-hit-arabi-ID: AT5G63190.1
Best-hit-arabi-name: MA3 domain-containing protein
Best-hit-arabi-symbol: nan
Gorai.001G062000.1.D
PCC=0.072, p=0.769
mRNA Profile
Protein Expression Profile
DPA

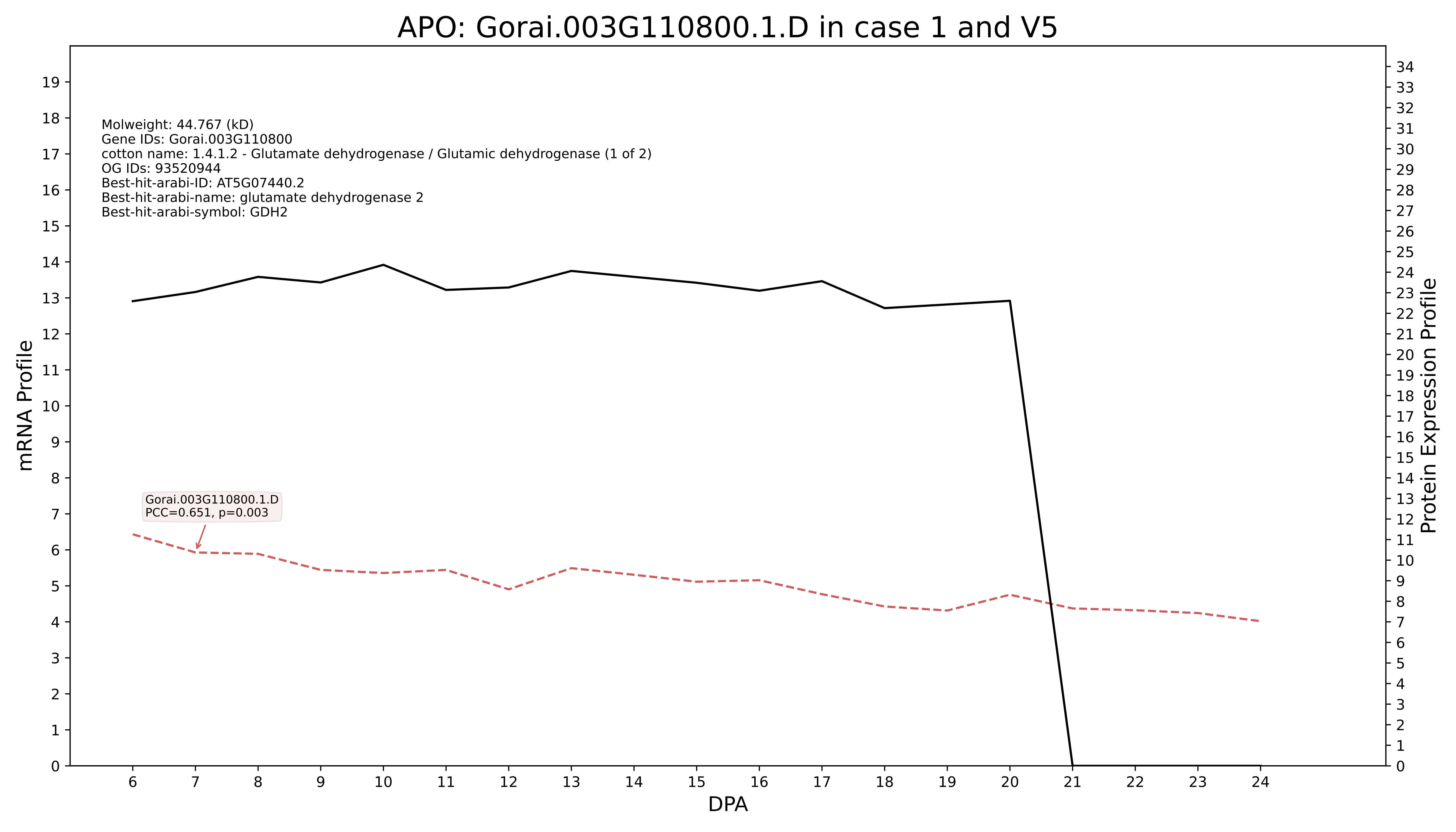
APO: Gorai.003G110800.1.D in case 1 and V5
Molweight: 44.767 (kD)
Gene IDs: Gorai.003G110800
cotton name: 1.4.1.2 - Glutamate dehydrogenase / Glutamic dehydrogenase (1 of 2)
OG IDs: 93520944
Best-hit-arabi-ID: AT5G07440.2
Best-hit-arabi-name: glutamate dehydrogenase 2
Best-hit-arabi-symbol: GDH2
Gorai.003G110800.1.D
PCC=0.651, p=0.003
mRNA Profile
Protein Expression Profile
DPA

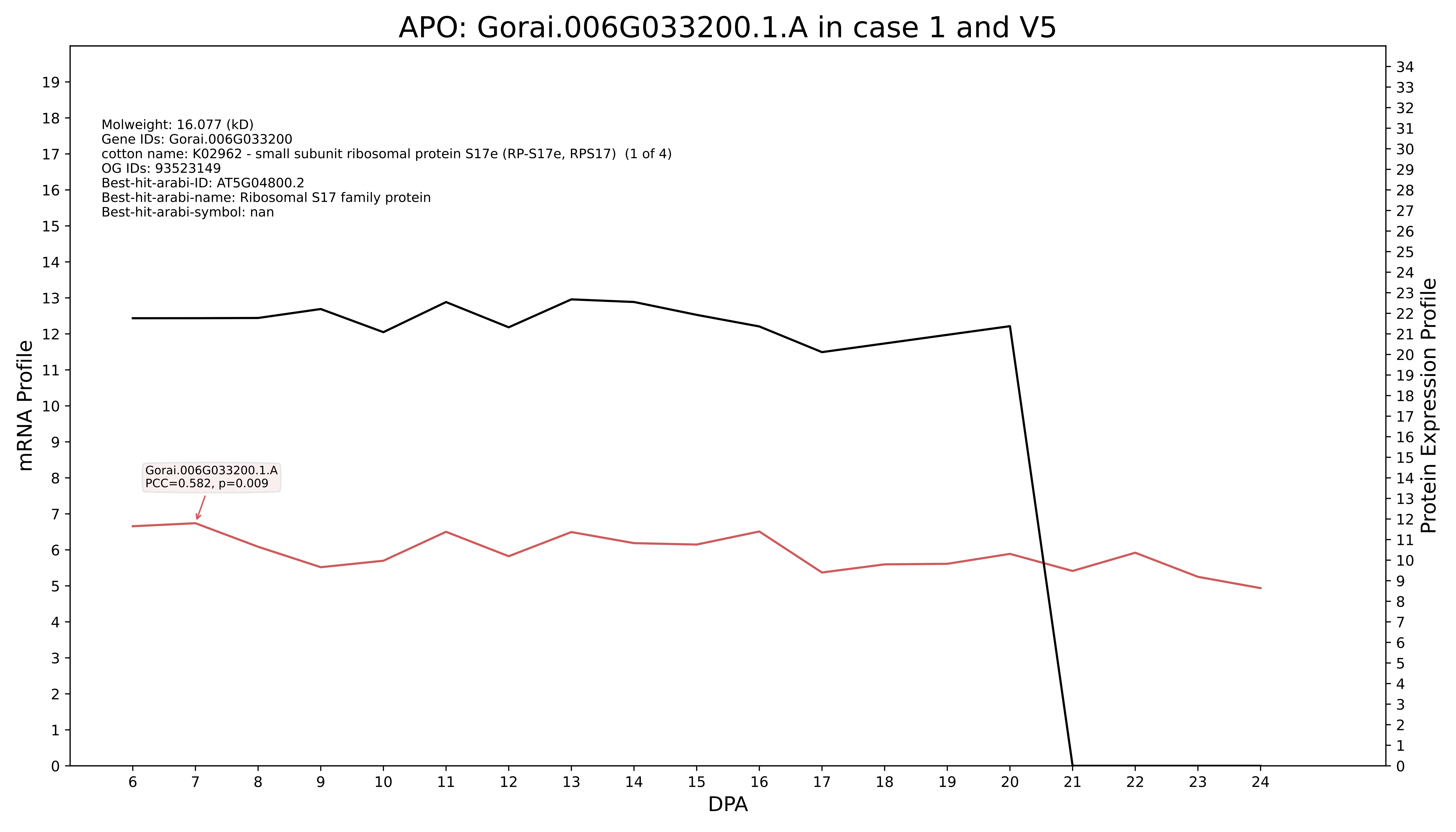

APO: Gorai.006G033200.1.A in case 1 and V5
Molweight: 16.077 (kD)
Gene IDs: Gorai.006G033200
cotton name: K02962 - small subunit ribosomal protein S17e (RP-S17e, RPS17) (1 of 4)
OG IDs: 93523149
Best-hit-arabi-ID: AT5G04800.2
Best-hit-arabi-name: Ribosomal S17 family protein
Best-hit-arabi-symbol: nan
Gorai.006G033200.1.A
PCC=0.582, p=0.009
mRNA Profile
Protein Expression Profile
DPA

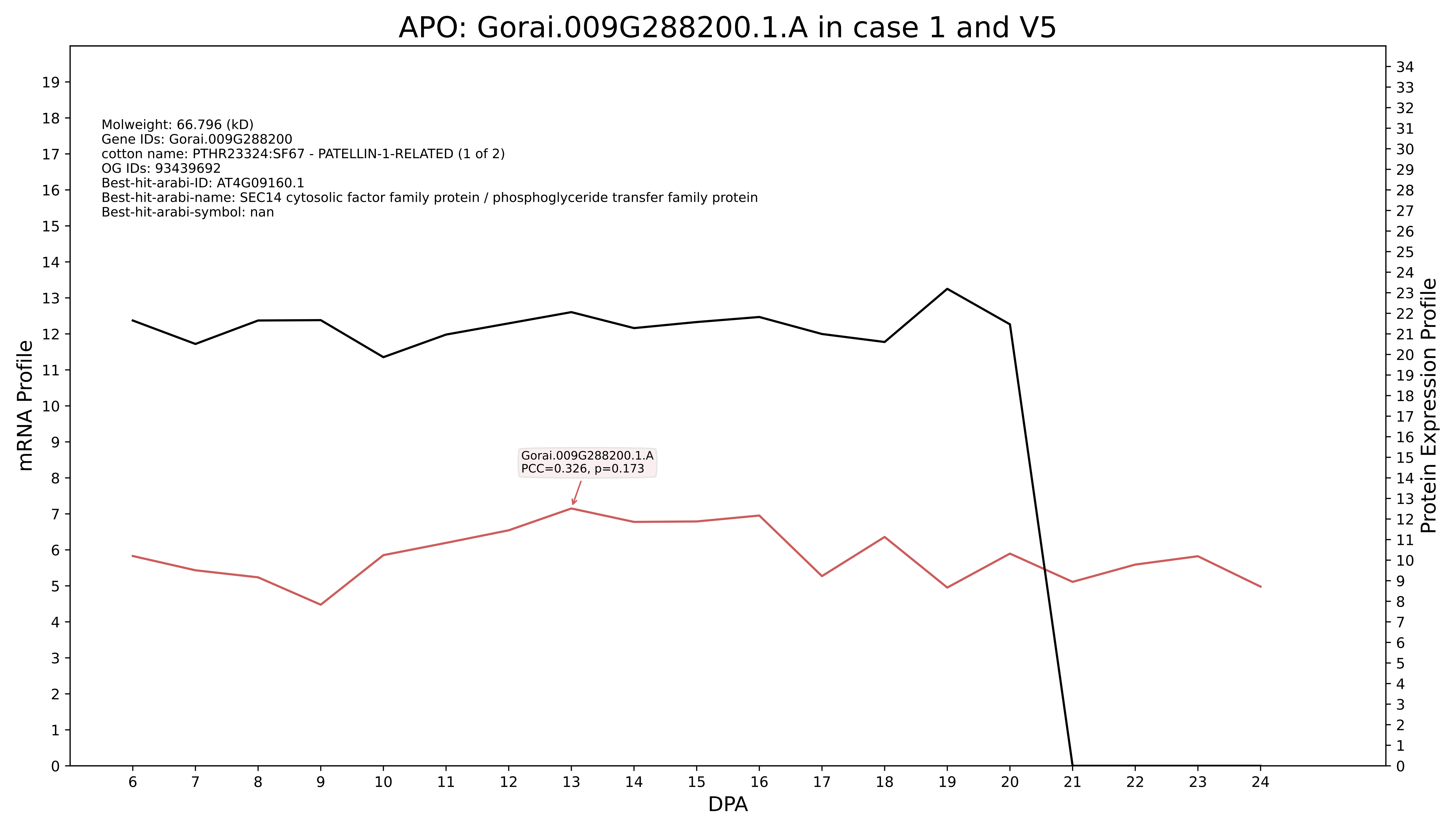
APO: Gorai.009G288200.1.A in case 1 and V5
Molweight: 66.796 (kD)
Gene IDs: Gorai.009G288200
cotton name: PTHR23324:SF67 - PATELLIN-1-RELATED (1 of 2)
OG IDs: 93439692
Best-hit-arabi-ID: AT4G09160.1
Best-hit-arabi-name: SEC14 cytosolic factor family protein / phosphoglyceride transfer family protein
Best-hit-arabi-symbol: nan
Gorai.009G288200.1.A
PCC=0.326, p=0.173
mRNA Profile
Protein Expression Profile
DPA

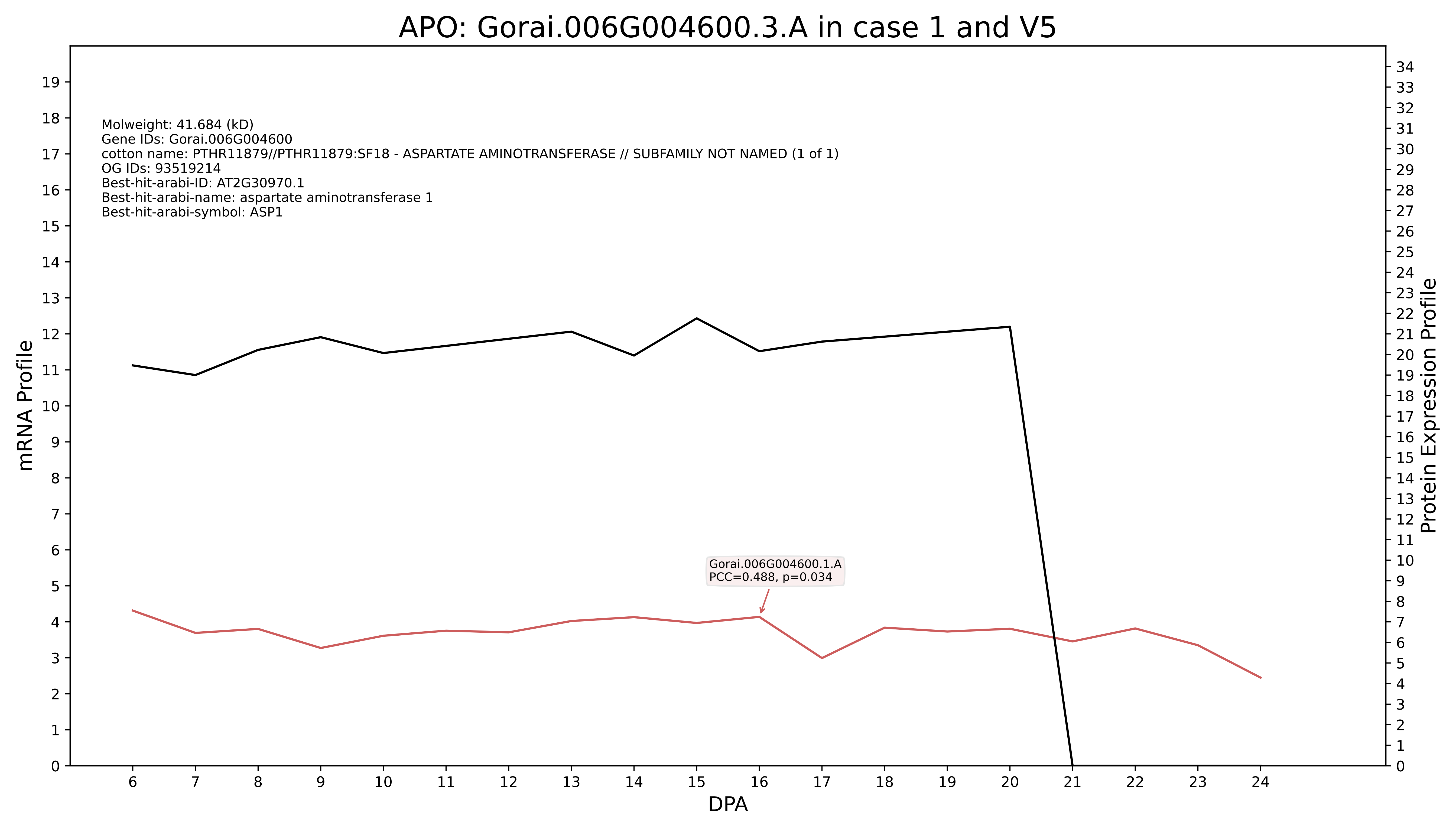
APO: Gorai.006G004600.3.A in case 1 and V5
Molweight: 41.684 (kD)
Gene IDs: Gorai.006G004600
cotton name: PTHR11879//PTHR11879:SF18 - ASPARTATE AMINOTRANSFERASE // SUBFAMILY NOT NAMED (1 of 1)
OG IDs: 93519214
Best-hit-arabi-ID: AT2G30970.1
Best-hit-arabi-name: aspartate aminotransferase 1
Best-hit-arabi-symbol: ASP1
Gorai.006G004600.1.A
PCC=0.488, p=0.034
mRNA Profile
Protein Expression Profile
DPA

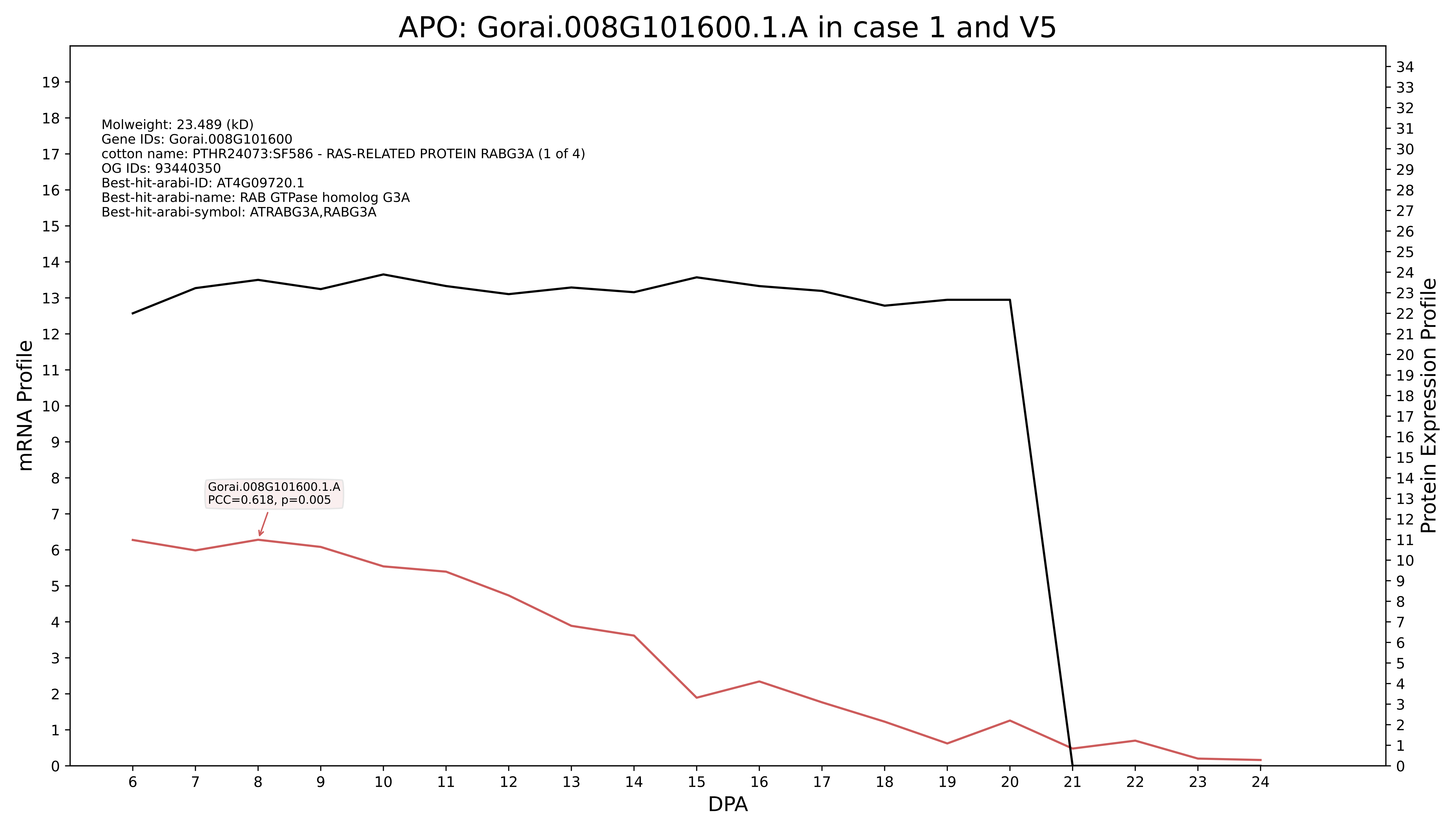
APO: Gorai.008G101600.1.A in case 1 and V5
Molweight: 23.489 (kD)
Gene IDs: Gorai.008G101600
cotton name: PTHR24073:SF586 - RAS-RELATED PROTEIN RABG3A (1 of 4)
OG IDs: 93440350
Best-hit-arabi-ID: AT4G09720.1
Best-hit-arabi-name: RAB GTPase homolog G3A
Best-hit-arabi-symbol: ATRABG3A,RABG3A
Gorai.008G101600.1.A
PCC=0.618, p=0.005
mRNA Profile
Protein Expression Profile
DPA

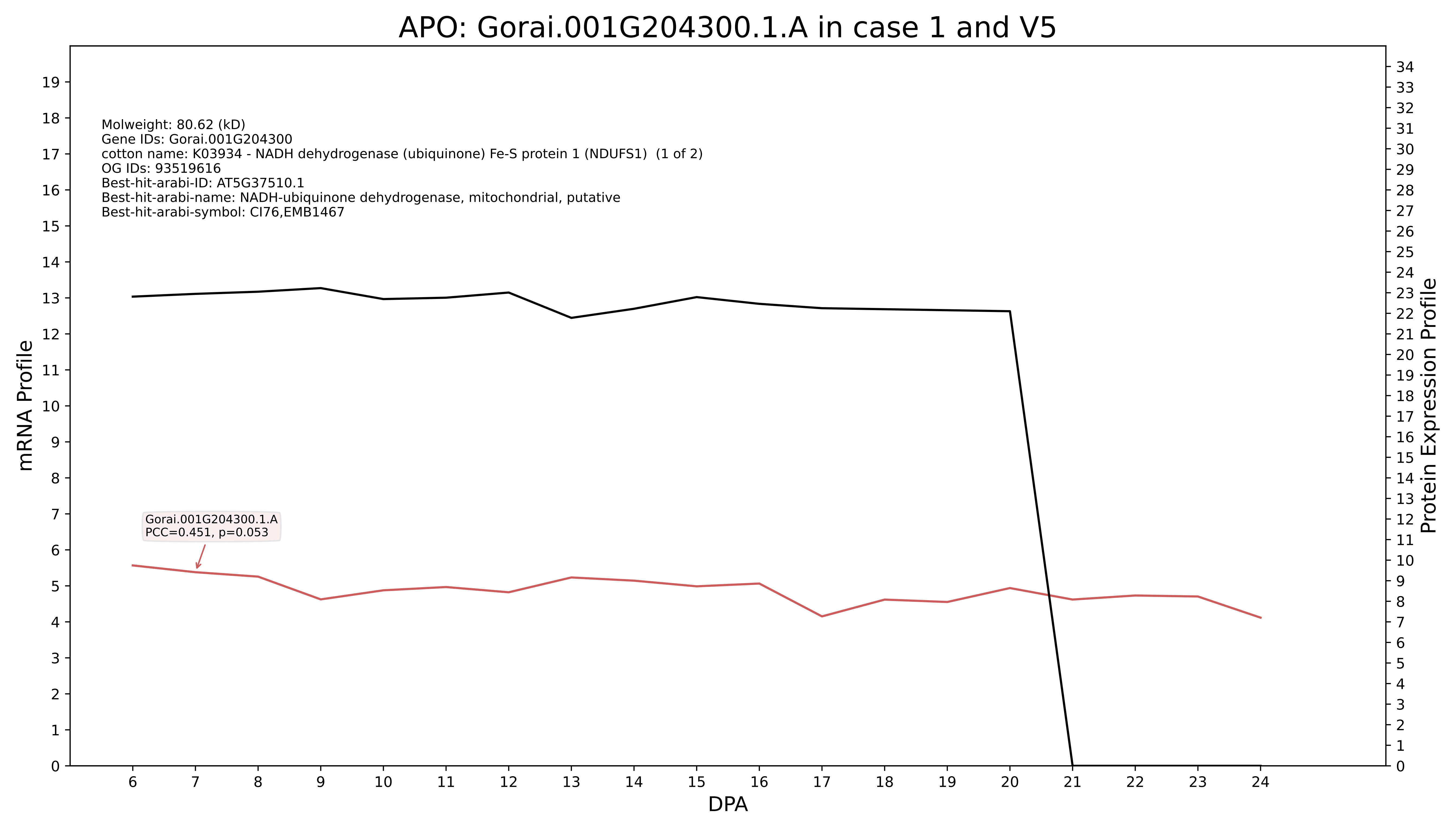
APO: Gorai.001G204300.1.A in case 1 and V5
Molweight: 80.62 (kD)
Gene IDs: Gorai.001G204300
cotton name: K03934 - NADH dehydrogenase (ubiquinone) Fe-S protein 1 (NDUFS1) (1 of 2)
OG IDs: 93519616
Best-hit-arabi-ID: AT5G37510.1
Best-hit-arabi-name: NADH-ubiquinone dehydrogenase, mitochondrial, putative
Best-hit-arabi-symbol: CI76,EMB1467
Gorai.001G204300.1.A
PCC=0.451, p=0.053
mRNA Profile
Protein Expression Profile
DPA

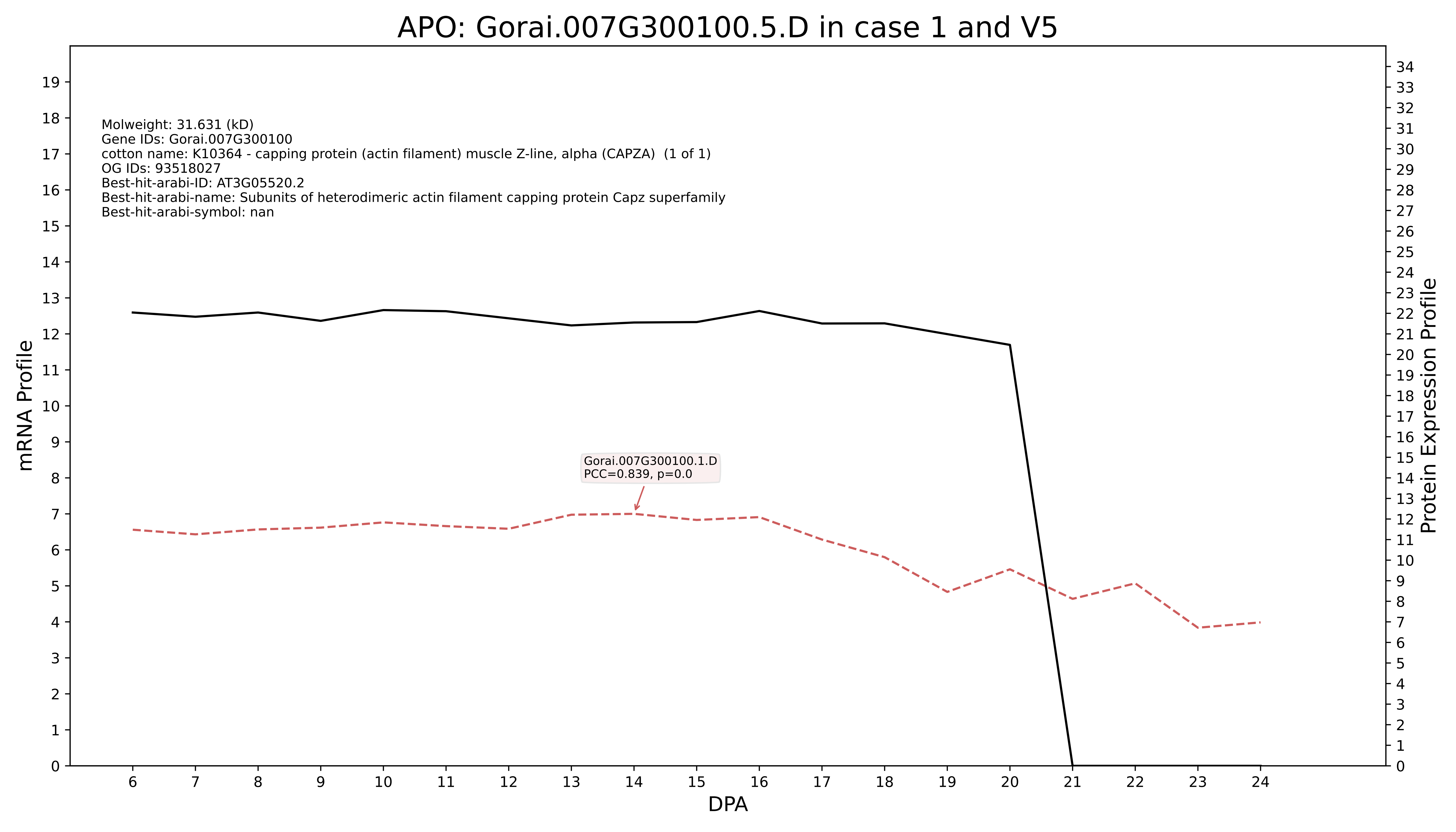
APO: Gorai.007G300100.5.D in case 1 and V5
Molweight: 31.631 (kD)
Gene IDs: Gorai.007G300100
cotton name: K10364 - capping protein (actin filament) muscle Z-line, alpha (CAPZA) (1 of 1)
OG IDs: 93518027
Best-hit-arabi-ID: AT3G05520.2
Best-hit-arabi-name: Subunits of heterodimeric actin filament capping protein Capz superfamily
Best-hit-arabi-symbol: nan
Gorai.007G300100.1.D
PCC=0.839, p=0.0
mRNA Profile
Protein Expression Profile
DPA
0
1
2
3
4
5
6
7
8
9
10
11
12
13
14
15
16
17
18
19
20
21
22
23
24
25
26
27
28
29
30
31
32
33
34

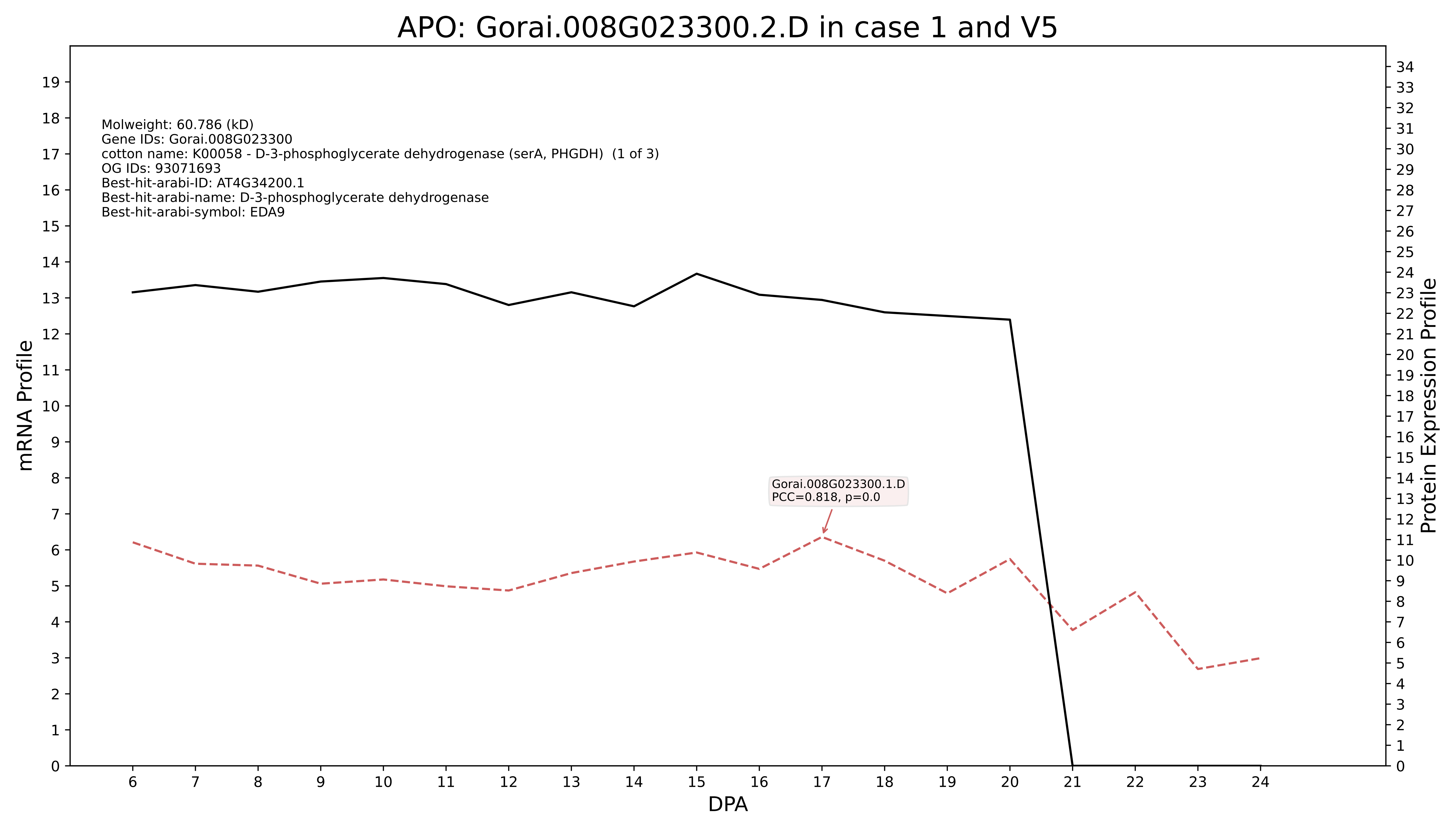
APO: Gorai.008G023300.2.D in case 1 and V5
Molweight: 60.786 (kD)
Gene IDs: Gorai.008G023300
cotton name: K00058 - D-3-phosphoglycerate dehydrogenase (serA, PHGDH) (1 of 3)
OG IDs: 93071693
Best-hit-arabi-ID: AT4G34200.1
Best-hit-arabi-name: D-3-phosphoglycerate dehydrogenase
Best-hit-arabi-symbol: EDA9
Gorai.008G023300.1.D
PCC=0.818, p=0.0
mRNA Profile
Protein Expression Profile
DPA

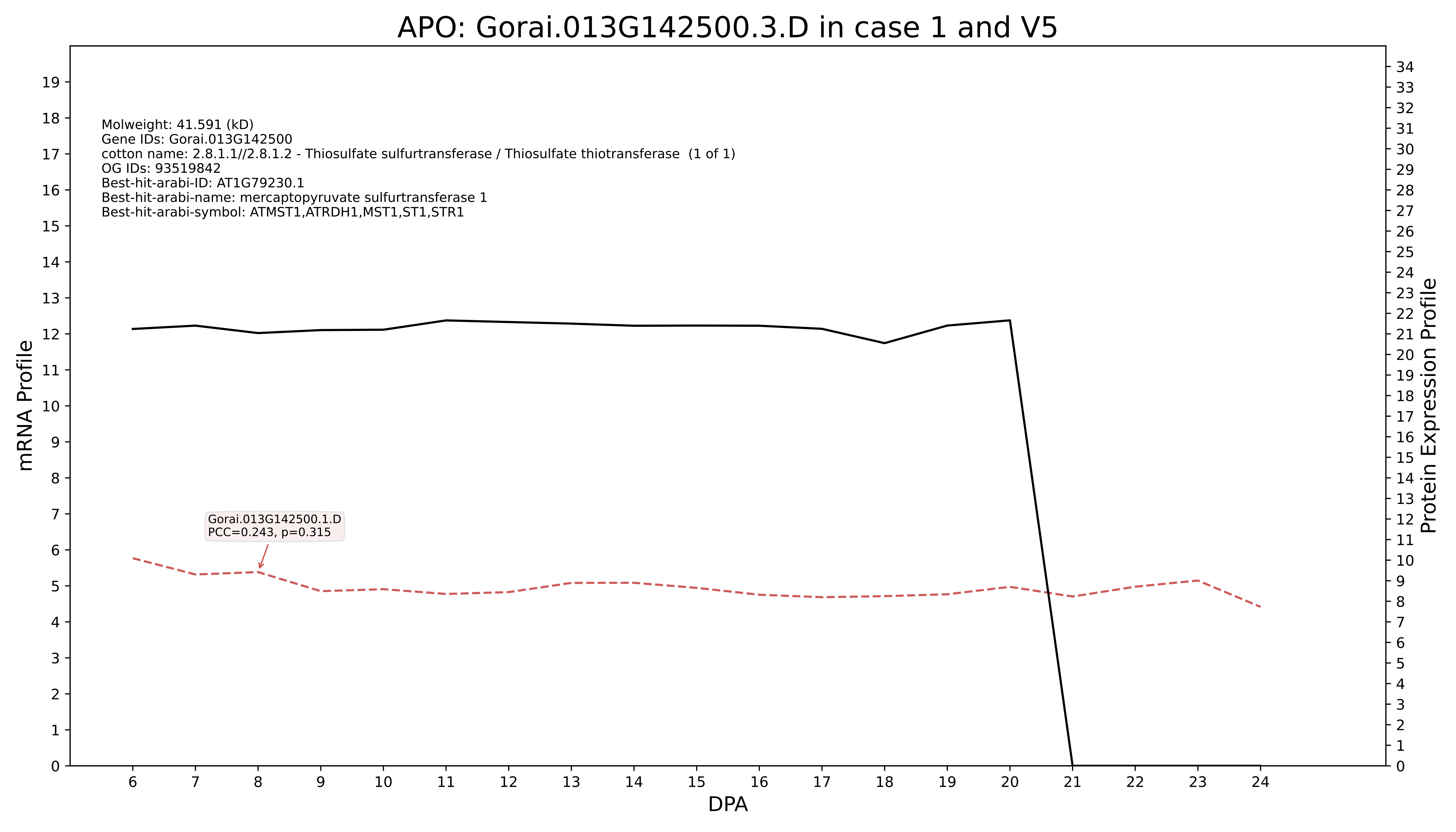

APO: Gorai.013G142500.3.D in case 1 and V5
Molweight: 41.591 (kD)
Gene IDs: Gorai.013G142500
cotton name: 2.8.1.1//2.8.1.2 - Thiosulfate sulfurtransferase / Thiosulfate thiotransferase (1 of 1)
OG IDs: 93519842
Best-hit-arabi-ID: AT1G79230.1
Best-hit-arabi-name: mercaptopyruvate sulfurtransferase 1
Best-hit-arabi-symbol: ATMST1,ATRDH1,MST1,ST1,STR1
Gorai.013G142500.1.D
PCC=0.243, p=0.315
mRNA Profile
Protein Expression Profile
DPA

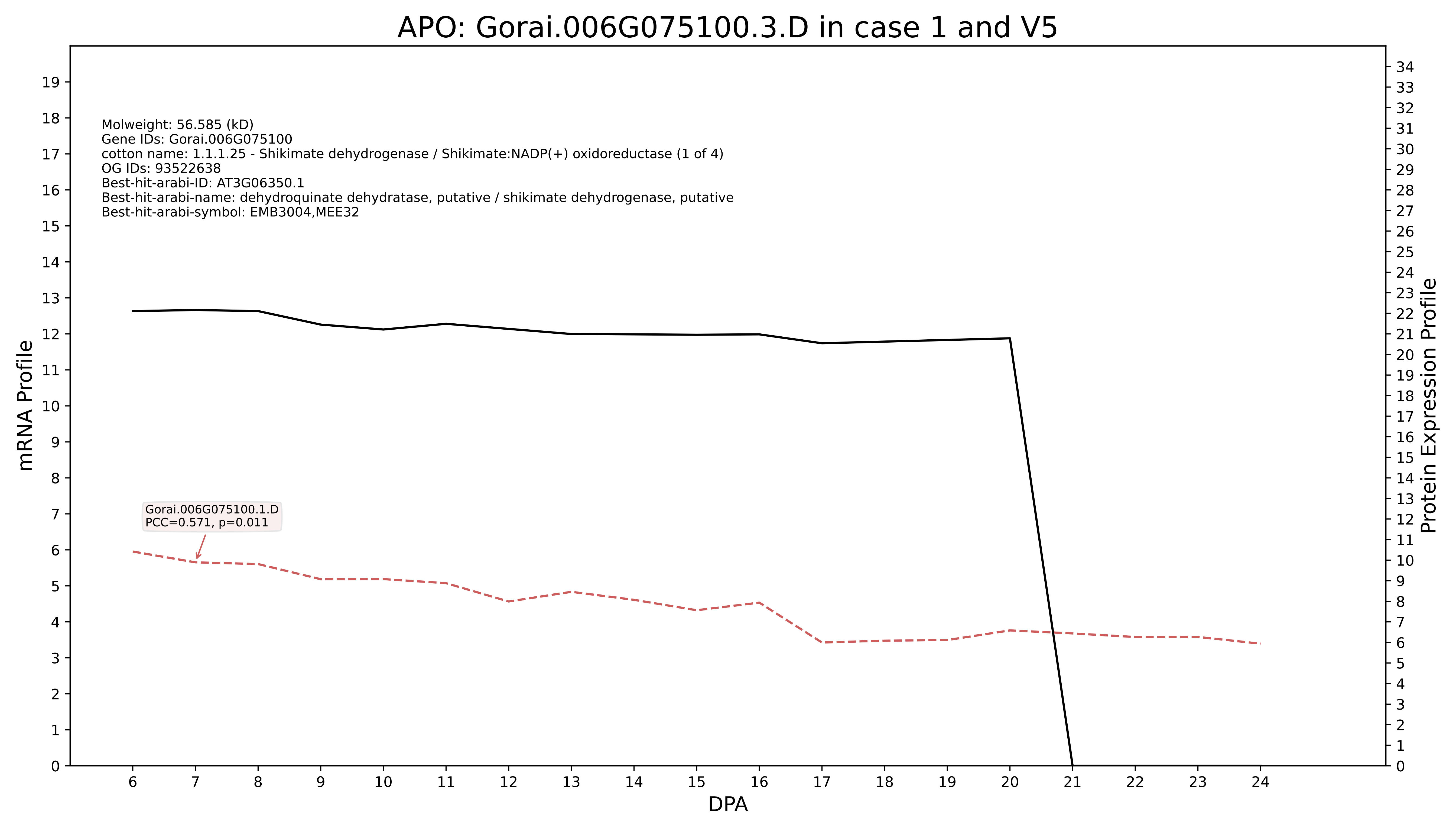

APO: Gorai.006G075100.3.D in case 1 and V5
Molweight: 56.585 (kD)
Gene IDs: Gorai.006G075100
cotton name: 1.1.1.25 - Shikimate dehydrogenase / Shikimate:NADP(+) oxidoreductase (1 of 4)
OG IDs: 93522638
Best-hit-arabi-ID: AT3G06350.1
Best-hit-arabi-name: dehydroquinate dehydratase, putative / shikimate dehydrogenase, putative
Best-hit-arabi-symbol: EMB3004,MEE32
Gorai.006G075100.1.D
PCC=0.571, p=0.011
mRNA Profile
Protein Expression Profile
DPA

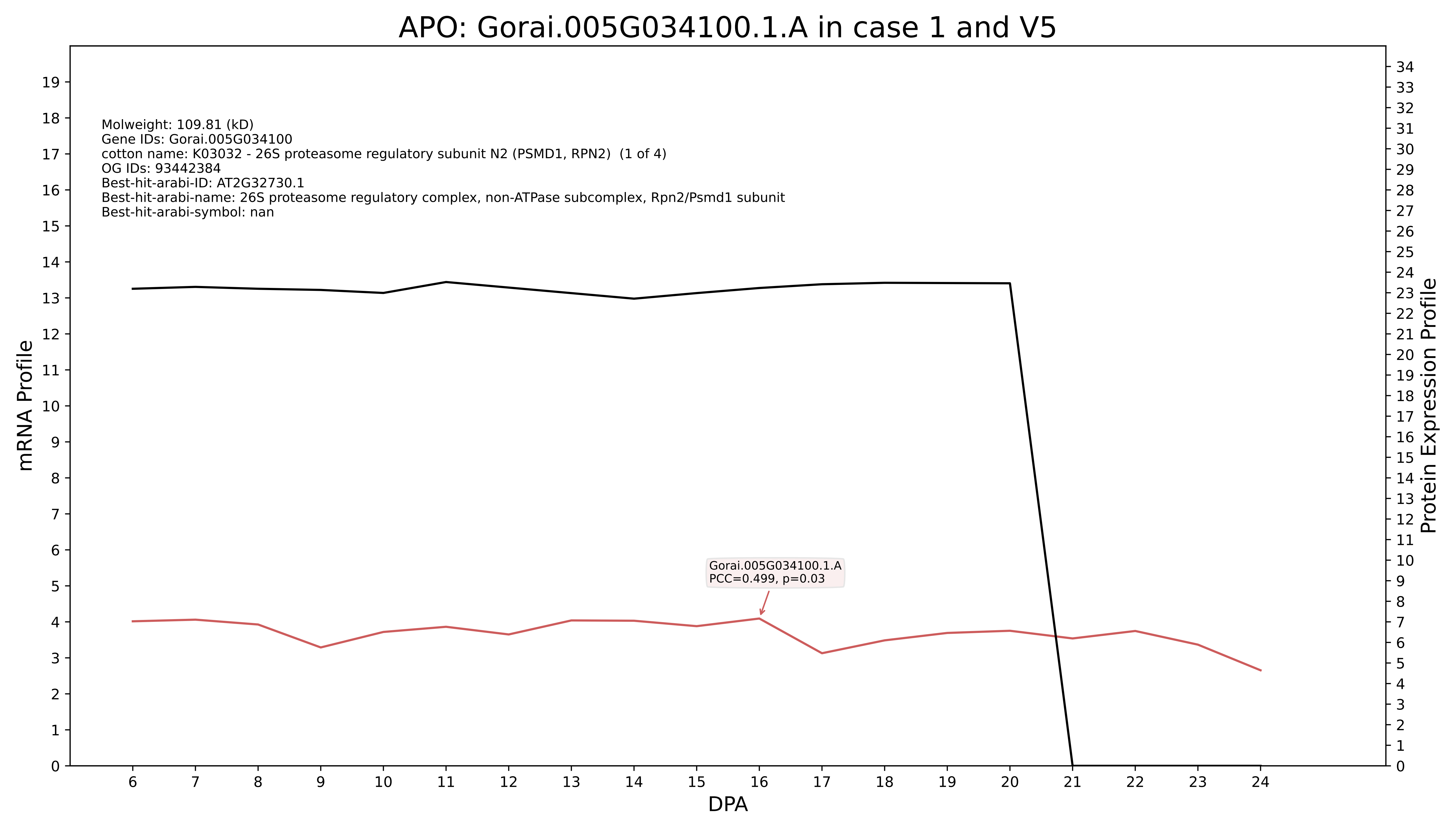
APO: Gorai.005G034100.1.A in case 1 and V5
Molweight: 109.81 (kD)
Gene IDs: Gorai.005G034100
cotton name: K03032 - 26S proteasome regulatory subunit N2 (PSMD1, RPN2) (1 of 4)
OG IDs: 93442384
Best-hit-arabi-ID: AT2G32730.1
Best-hit-arabi-name: 26S proteasome regulatory complex, non-ATPase subcomplex, Rpn2/Psmd1 subunit
Best-hit-arabi-symbol: nan
Gorai.005G034100.1.A
PCC=0.499, p=0.03
mRNA Profile
Protein Expression Profile
DPA

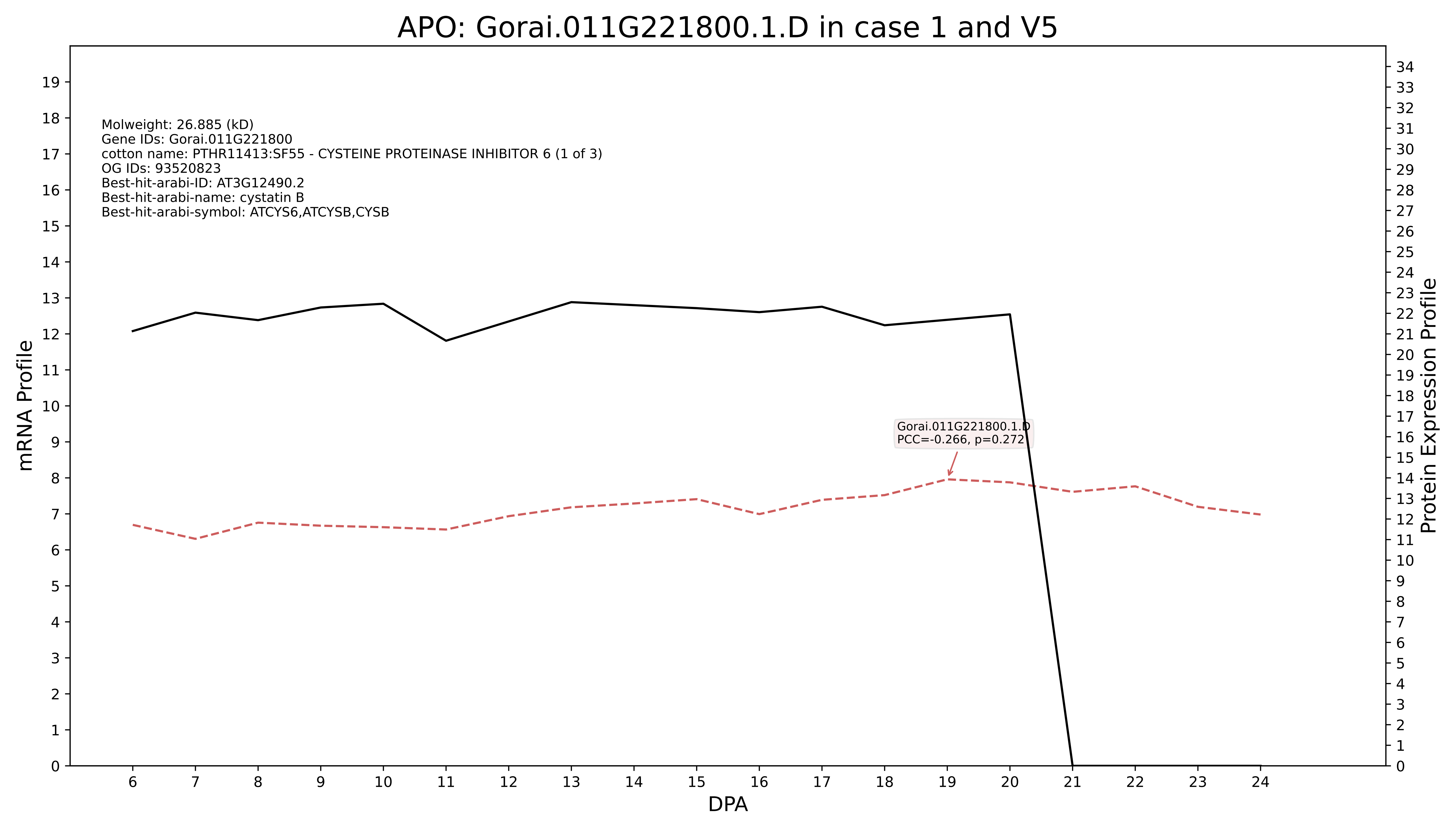
APO: Gorai.011G221800.1.D in case 1 and V5
Molweight: 26.885 (kD)
Gene IDs: Gorai.011G221800
cotton name: PTHR11413:SF55 - CYSTEINE PROTEINASE INHIBITOR 6 (1 of 3)
OG IDs: 93520823
Best-hit-arabi-ID: AT3G12490.2
Best-hit-arabi-name: cystatin B
Best-hit-arabi-symbol: ATCYS6,ATCYSB,CYSB
Gorai.011G221800.1.D
PCC=-0.266, p=0.272
mRNA Profile
Protein Expression Profile
DPA

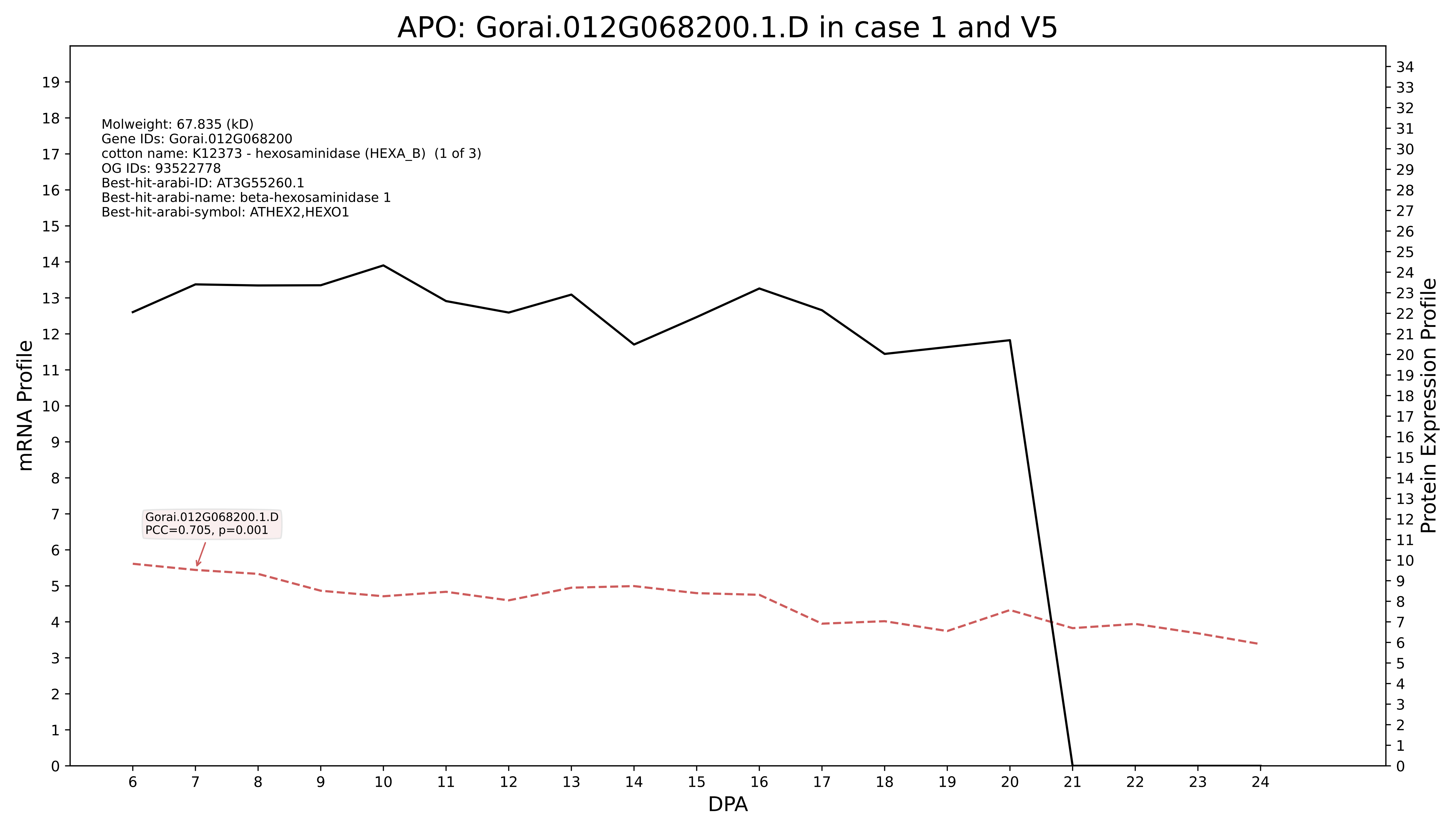
APO: Gorai.012G068200.1.D in case 1 and V5
Molweight: 67.835 (kD)
Gene IDs: Gorai.012G068200
cotton name: K12373 - hexosaminidase (HEXA_B) (1 of 3)
OG IDs: 93522778
Best-hit-arabi-ID: AT3G55260.1
Best-hit-arabi-name: beta-hexosaminidase 1
Best-hit-arabi-symbol: ATHEX2,HEXO1
Gorai.012G068200.1.D
PCC=0.705, p=0.001
mRNA Profile
Protein Expression Profile
DPA

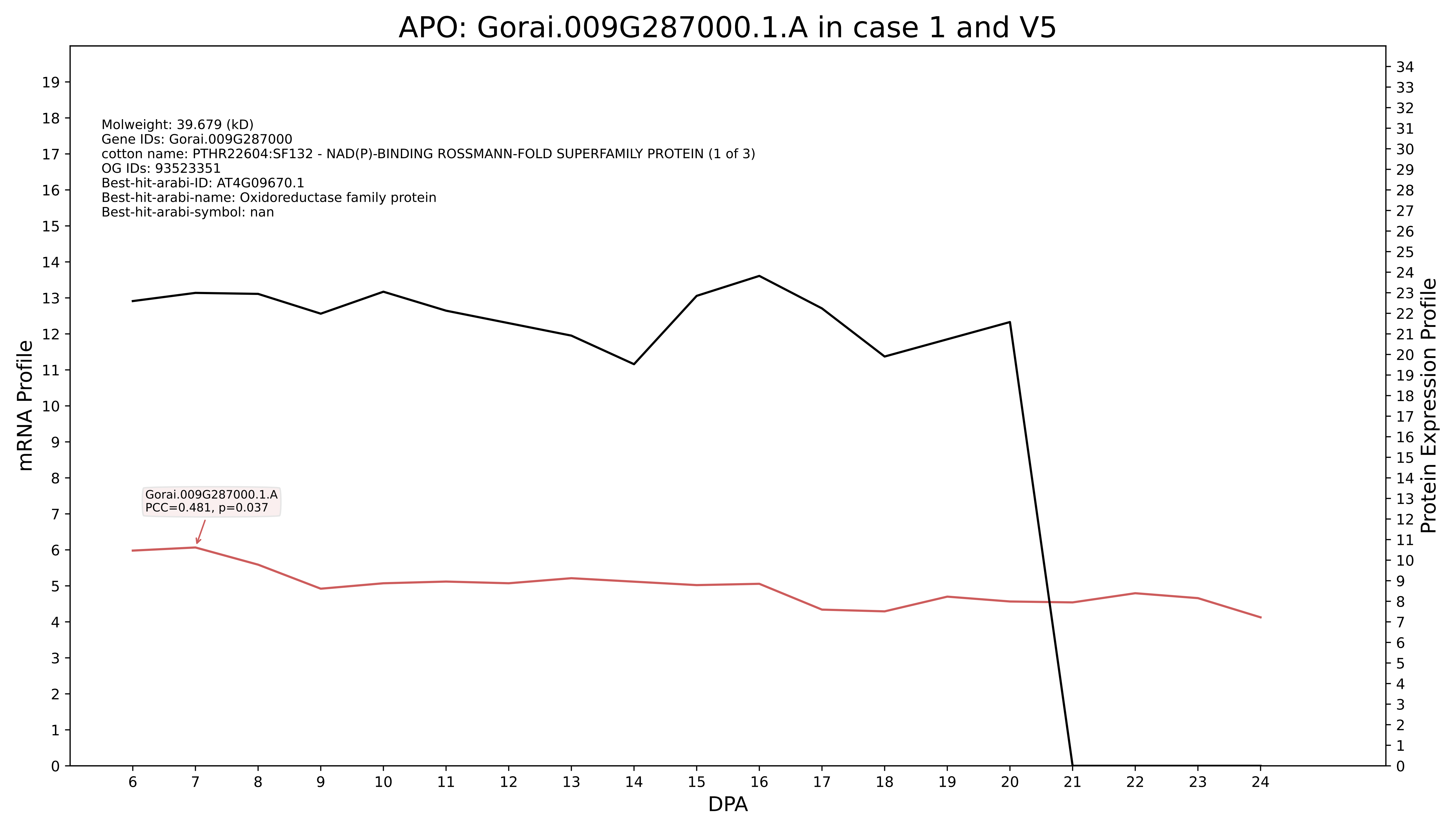
APO: Gorai.009G287000.1.A in case 1 and V5
Molweight: 39.679 (kD)
Gene IDs: Gorai.009G287000
cotton name: PTHR22604:SF132 - NAD(P)-BINDING ROSSMANN-FOLD SUPERFAMILY PROTEIN (1 of 3)
OG IDs: 93523351
Best-hit-arabi-ID: AT4G09670.1
Best-hit-arabi-name: Oxidoreductase family protein
Best-hit-arabi-symbol: nan
Gorai.009G287000.1.A
PCC=0.481, p=0.037
mRNA Profile
Protein Expression Profile
DPA

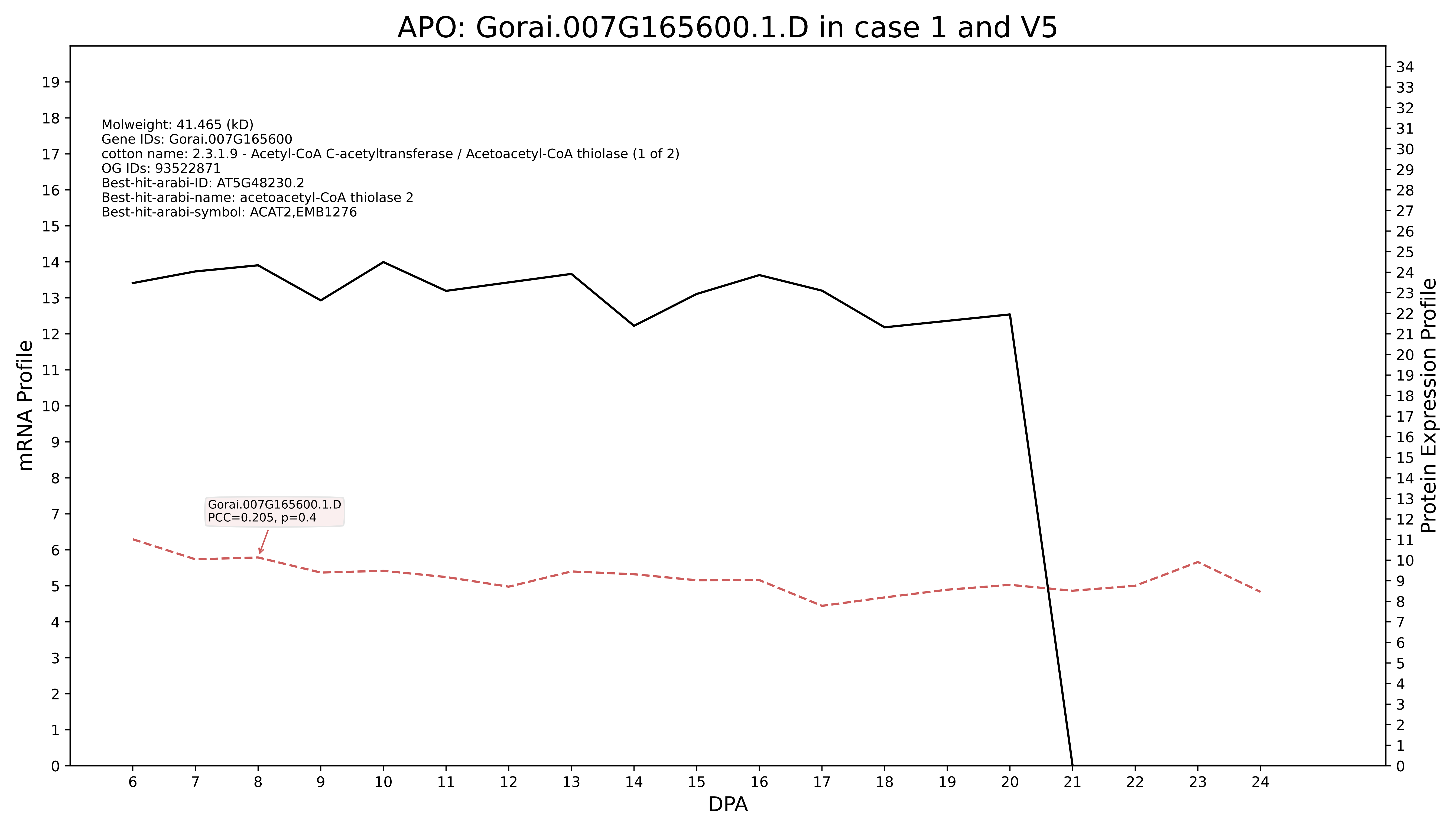
APO: Gorai.007G165600.1.D in case 1 and V5
Molweight: 41.465 (kD)
Gene IDs: Gorai.007G165600
cotton name: 2.3.1.9 - Acetyl-CoA C-acetyltransferase / Acetoacetyl-CoA thiolase (1 of 2)
OG IDs: 93522871
Best-hit-arabi-ID: AT5G48230.2
Best-hit-arabi-name: acetoacetyl-CoA thiolase 2
Best-hit-arabi-symbol: ACAT2,EMB1276
Gorai.007G165600.1.D
PCC=0.205, p=0.4
mRNA Profile
Protein Expression Profile
DPA
6
7
8
9
10
11
12
13
14
15
16
17
18
19
20
21
22
23
24

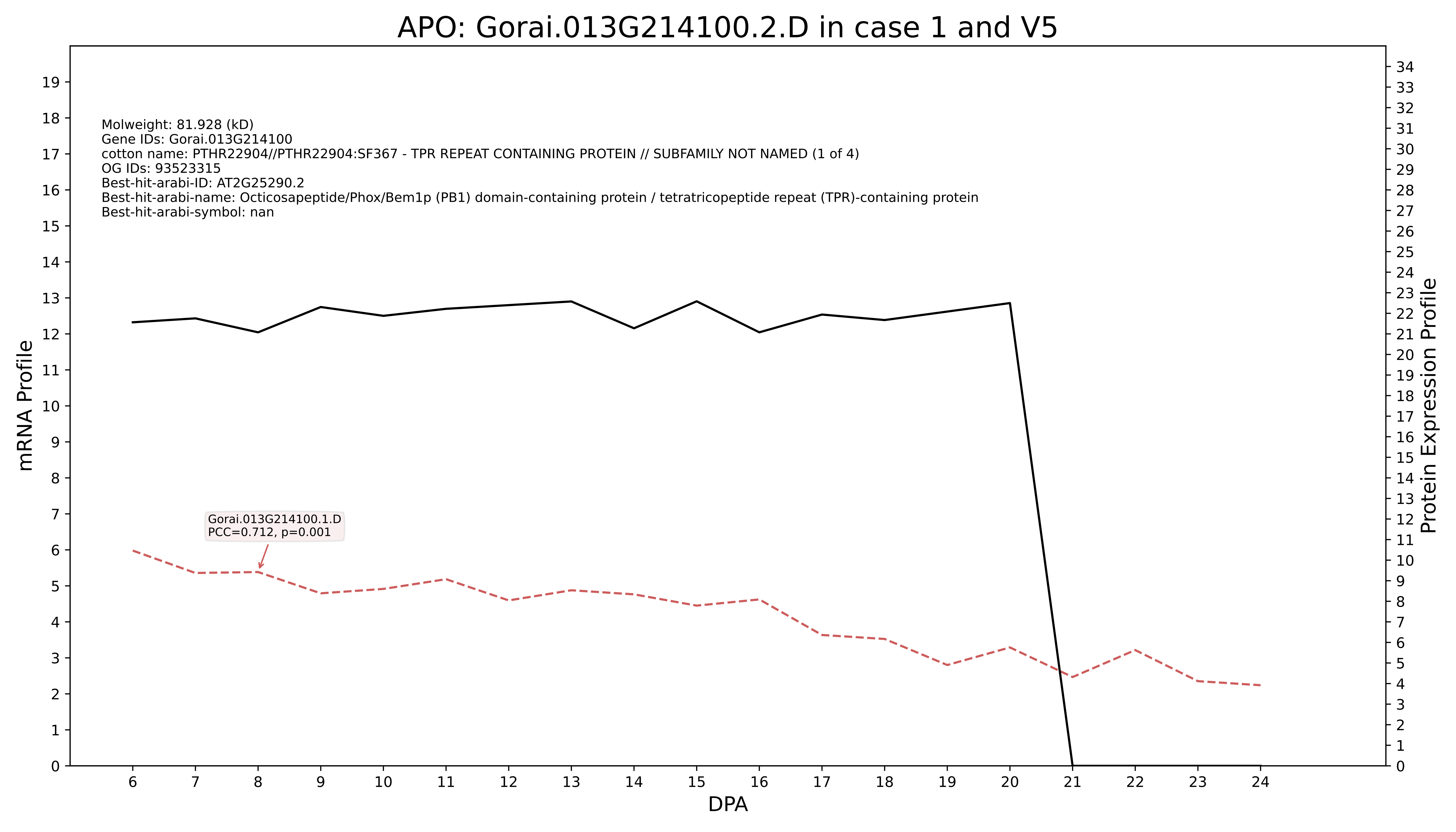
APO: Gorai.013G214100.2.D in case 1 and V5
Molweight: 81.928 (kD)
Gene IDs: Gorai.013G214100
cotton name: PTHR22904//PTHR22904:SF367 - TPR REPEAT CONTAINING PROTEIN // SUBFAMILY NOT NAMED (1 of 4)
OG IDs: 93523315
Best-hit-arabi-ID: AT2G25290.2
Best-hit-arabi-name: Octicosapeptide/Phox/Bem1p (PB1) domain-containing protein / tetratricopeptide repeat (TPR)-containing protein
Best-hit-arabi-symbol: nan
Gorai.013G214100.1.D
PCC=0.712, p=0.001
mRNA Profile
Protein Expression Profile
DPA

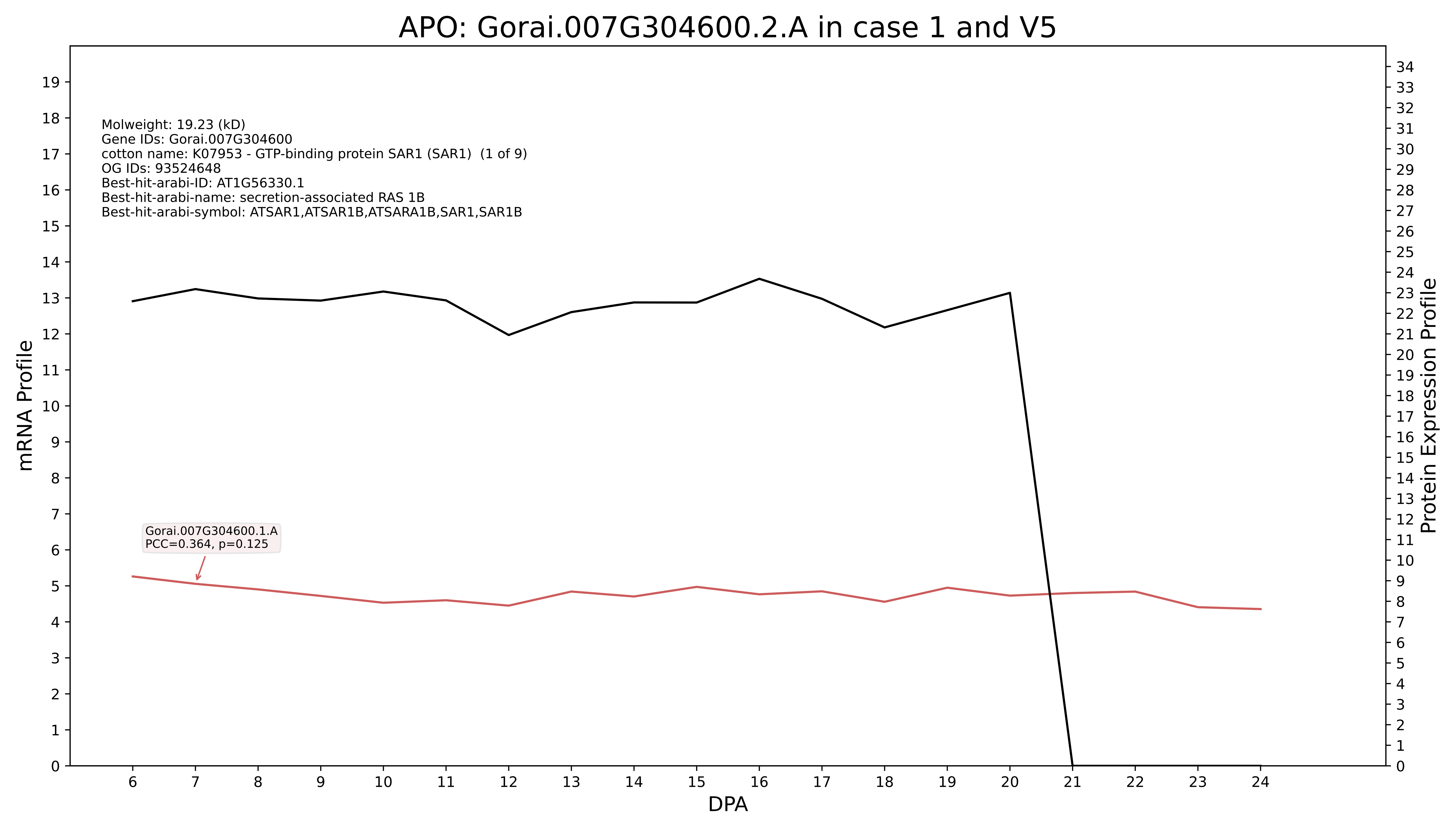
APO: Gorai.007G304600.2.A in case 1 and V5
Molweight: 19.23 (kD)
Gene IDs: Gorai.007G304600
cotton name: K07953 - GTP-binding protein SAR1 (SAR1) (1 of 9)
OG IDs: 93524648
Best-hit-arabi-ID: AT1G56330.1
Best-hit-arabi-name: secretion-associated RAS 1B
Best-hit-arabi-symbol: ATSAR1,ATSAR1B,ATSARA1B,SAR1,SAR1B
Gorai.007G304600.1.A
PCC=0.364, p=0.125
mRNA Profile
Protein Expression Profile
DPA

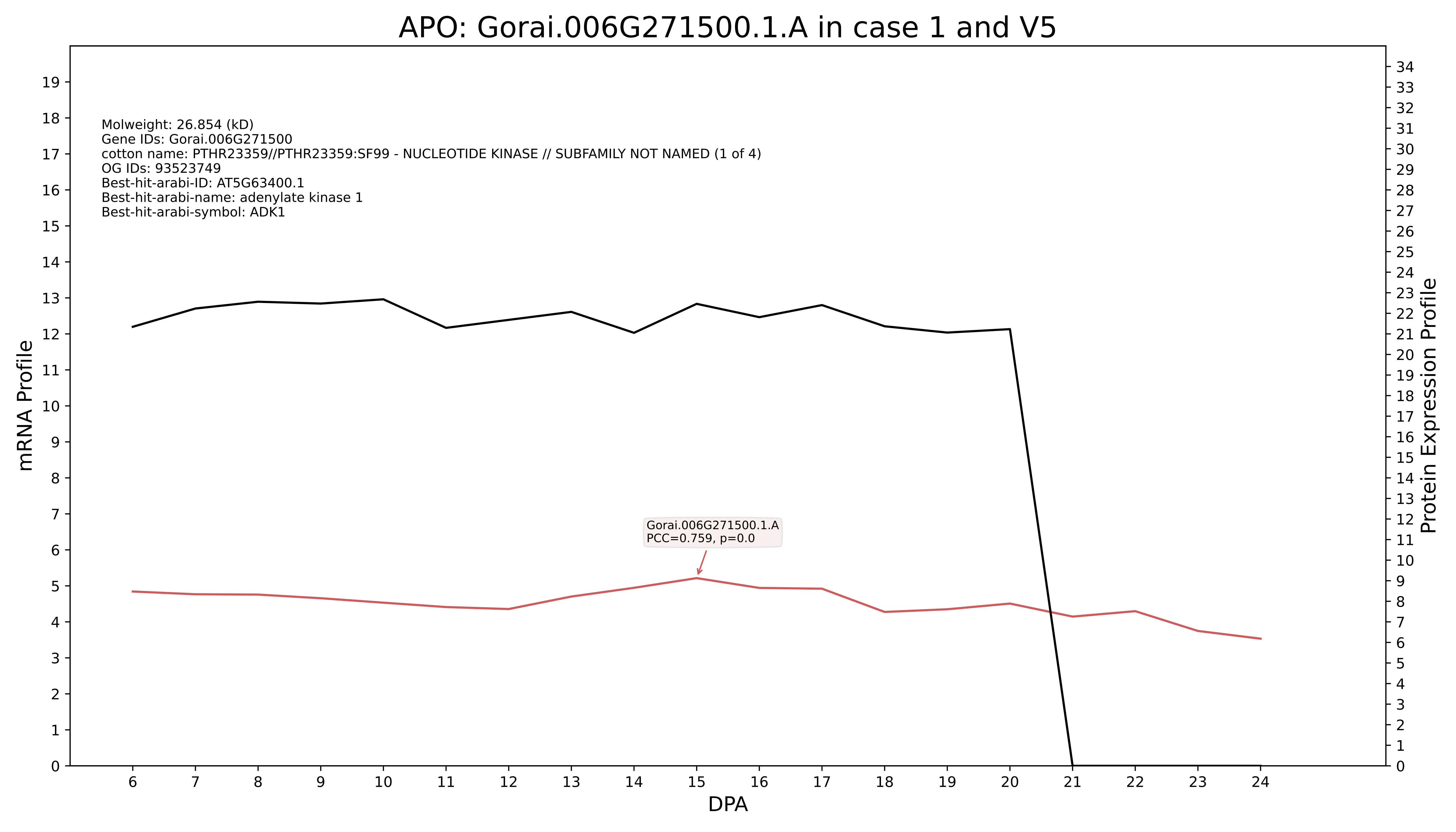

APO: Gorai.006G271500.1.A in case 1 and V5
Molweight: 26.854 (kD)
Gene IDs: Gorai.006G271500
cotton name: PTHR23359//PTHR23359:SF99 - NUCLEOTIDE KINASE // SUBFAMILY NOT NAMED (1 of 4)
OG IDs: 93523749
Best-hit-arabi-ID: AT5G63400.1
Best-hit-arabi-name: adenylate kinase 1
Best-hit-arabi-symbol: ADK1
Gorai.006G271500.1.A
PCC=0.759, p=0.0
mRNA Profile
Protein Expression Profile
DPA

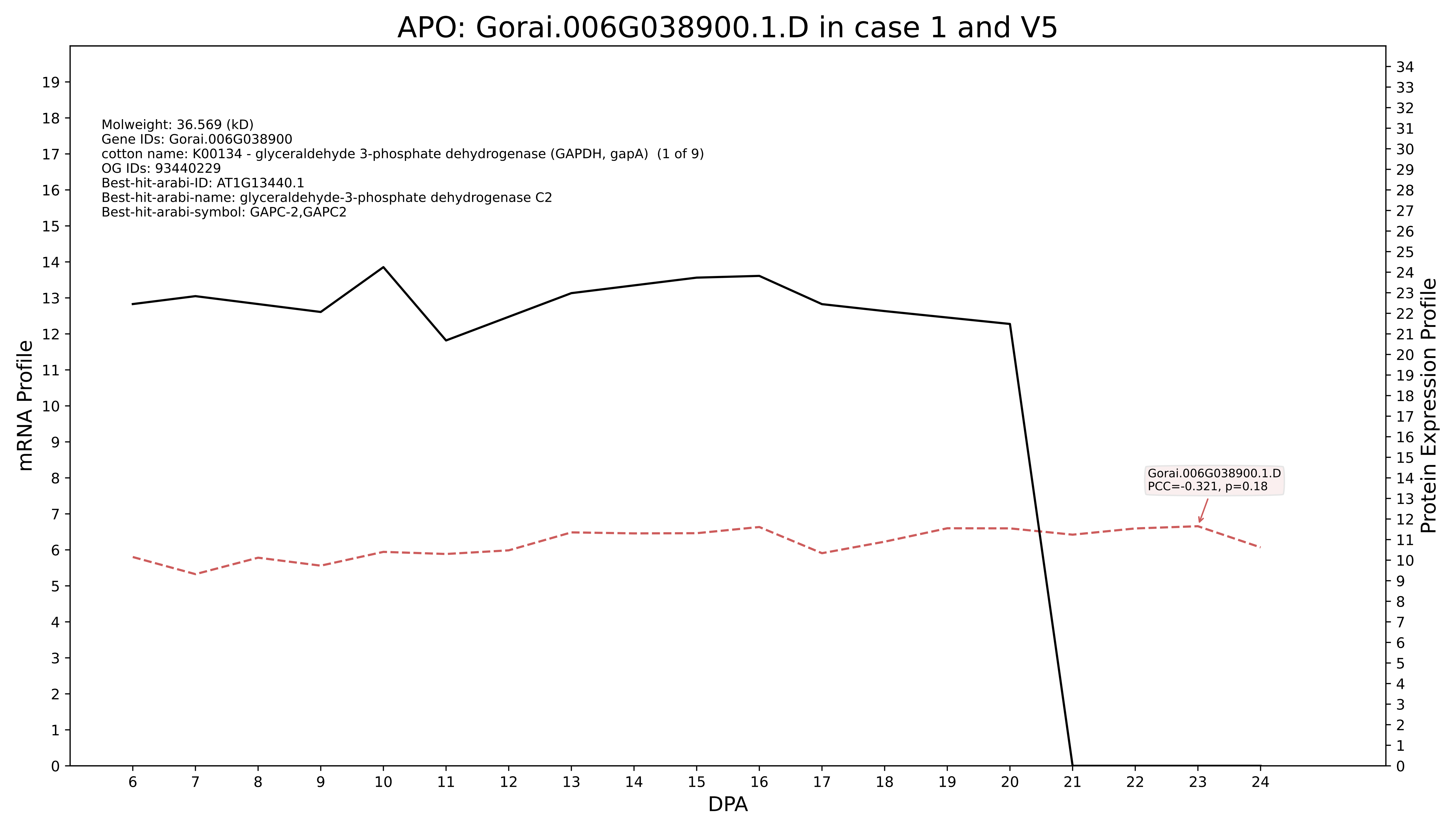

APO: Gorai.006G038900.1.D in case 1 and V5
Molweight: 36.569 (kD)
Gene IDs: Gorai.006G038900
cotton name: K00134 - glyceraldehyde 3-phosphate dehydrogenase (GAPDH, gapA) (1 of 9)
OG IDs: 93440229
Best-hit-arabi-ID: AT1G13440.1
Best-hit-arabi-name: glyceraldehyde-3-phosphate dehydrogenase C2
Best-hit-arabi-symbol: GAPC-2,GAPC2
Gorai.006G038900.1.D
PCC=-0.321, p=0.18
mRNA Profile
Protein Expression Profile
DPA

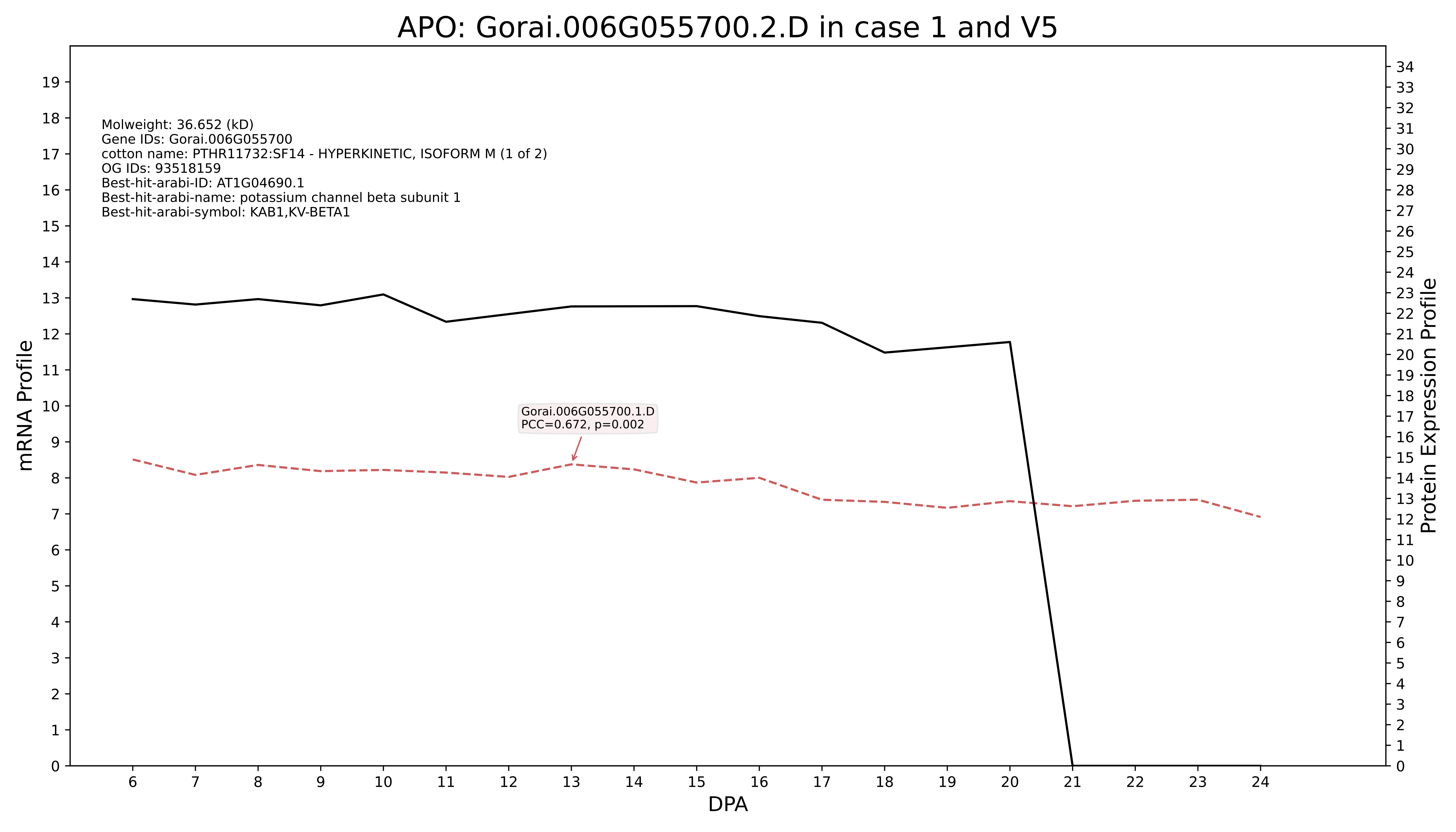

APO: Gorai.006G055700.2.D in case 1 and V5
Molweight: 36.652 (kD)
Gene IDs: Gorai.006G055700
cotton name: PTHR11732:SF14 - HYPERKINETIC, ISOFORM M (1 of 2)
OG IDs: 93518159
Best-hit-arabi-ID: AT1G04690.1
Best-hit-arabi-name: potassium channel beta subunit 1
Best-hit-arabi-symbol: KAB1,KV-BETA1
Gorai.006G055700.1.D
PCC=0.672, p=0.002
mRNA Profile
Protein Expression Profile
DPA

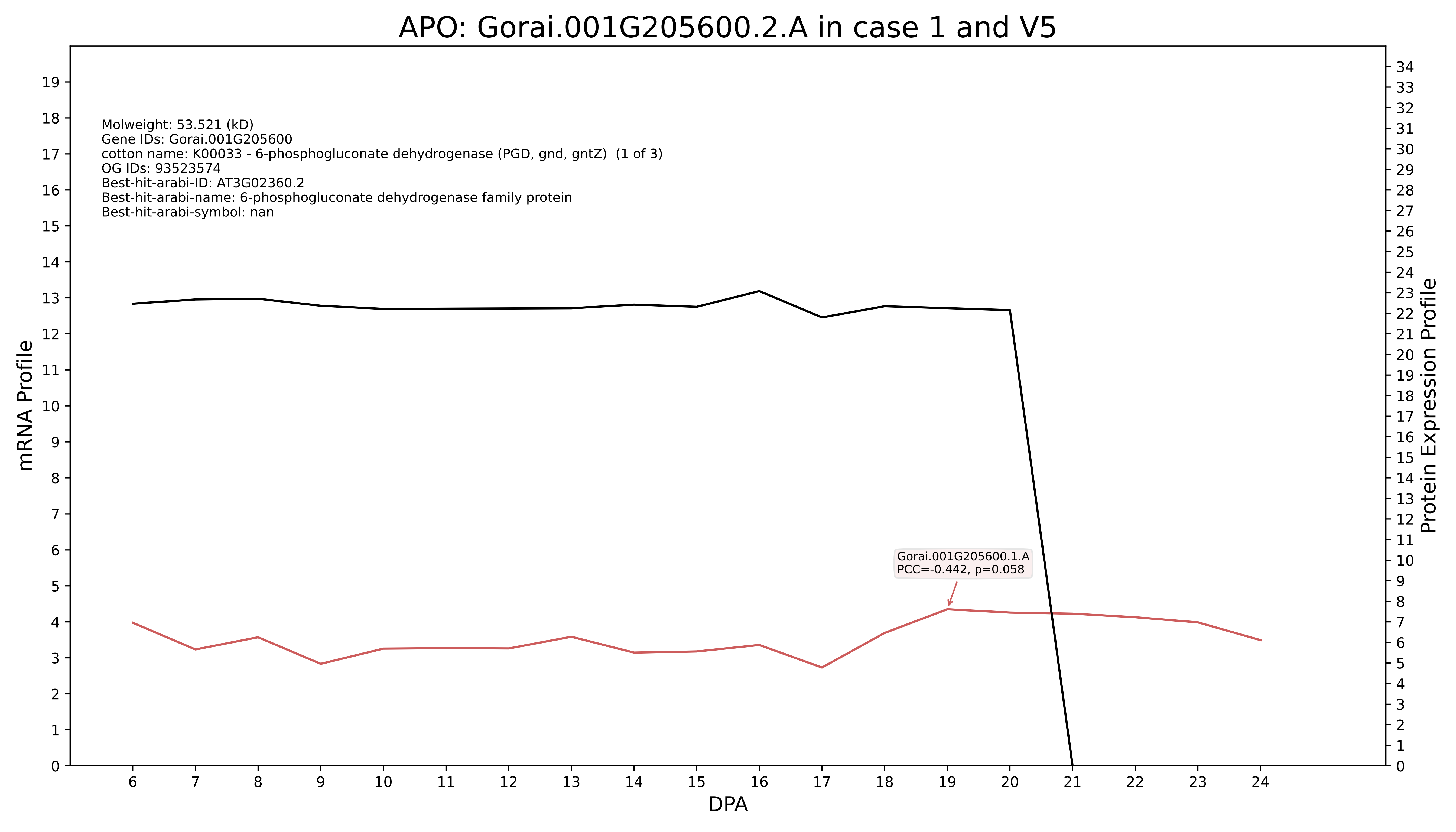
APO: Gorai.001G205600.2.A in case 1 and V5
Molweight: 53.521 (kD)
Gene IDs: Gorai.001G205600
cotton name: K00033 - 6-phosphogluconate dehydrogenase (PGD, gnd, gntZ) (1 of 3)
OG IDs: 93523574
Best-hit-arabi-ID: AT3G02360.2
Best-hit-arabi-name: 6-phosphogluconate dehydrogenase family protein
Best-hit-arabi-symbol: nan
Gorai.001G205600.1.A
PCC=-0.442, p=0.058
mRNA Profile
Protein Expression Profile
DPA

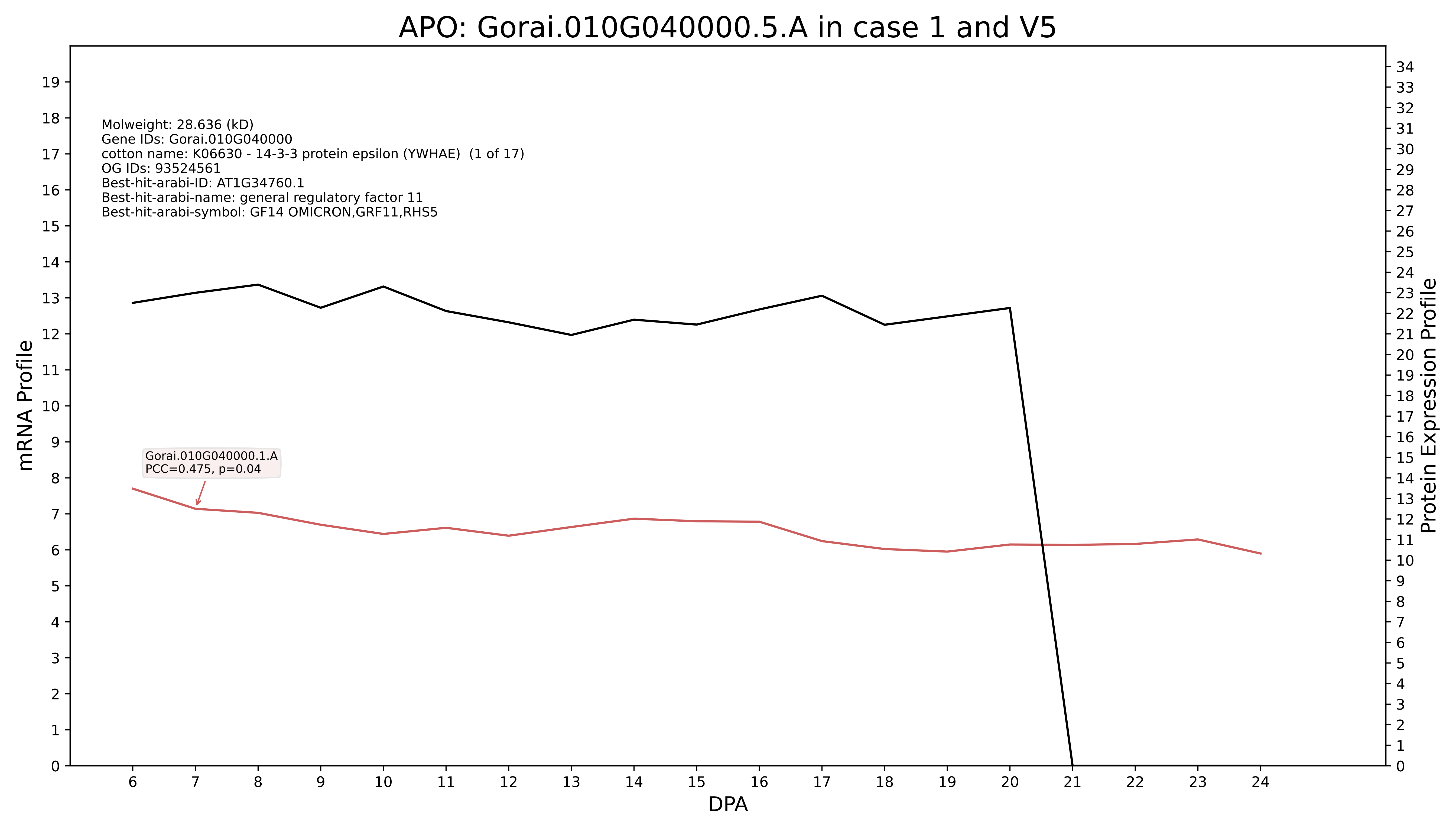

APO: Gorai.010G040000.5.A in case 1 and V5
Molweight: 28.636 (kD)
Gene IDs: Gorai.010G040000
cotton name: K06630 - 14-3-3 protein epsilon (YWHAE) (1 of 17)
OG IDs: 93524561
Best-hit-arabi-ID: AT1G34760.1
Best-hit-arabi-name: general regulatory factor 11
Best-hit-arabi-symbol: GF14 OMICRON,GRF11,RHS5
Gorai.010G040000.1.A
PCC=0.475, p=0.04
mRNA Profile
Protein Expression Profile
DPA

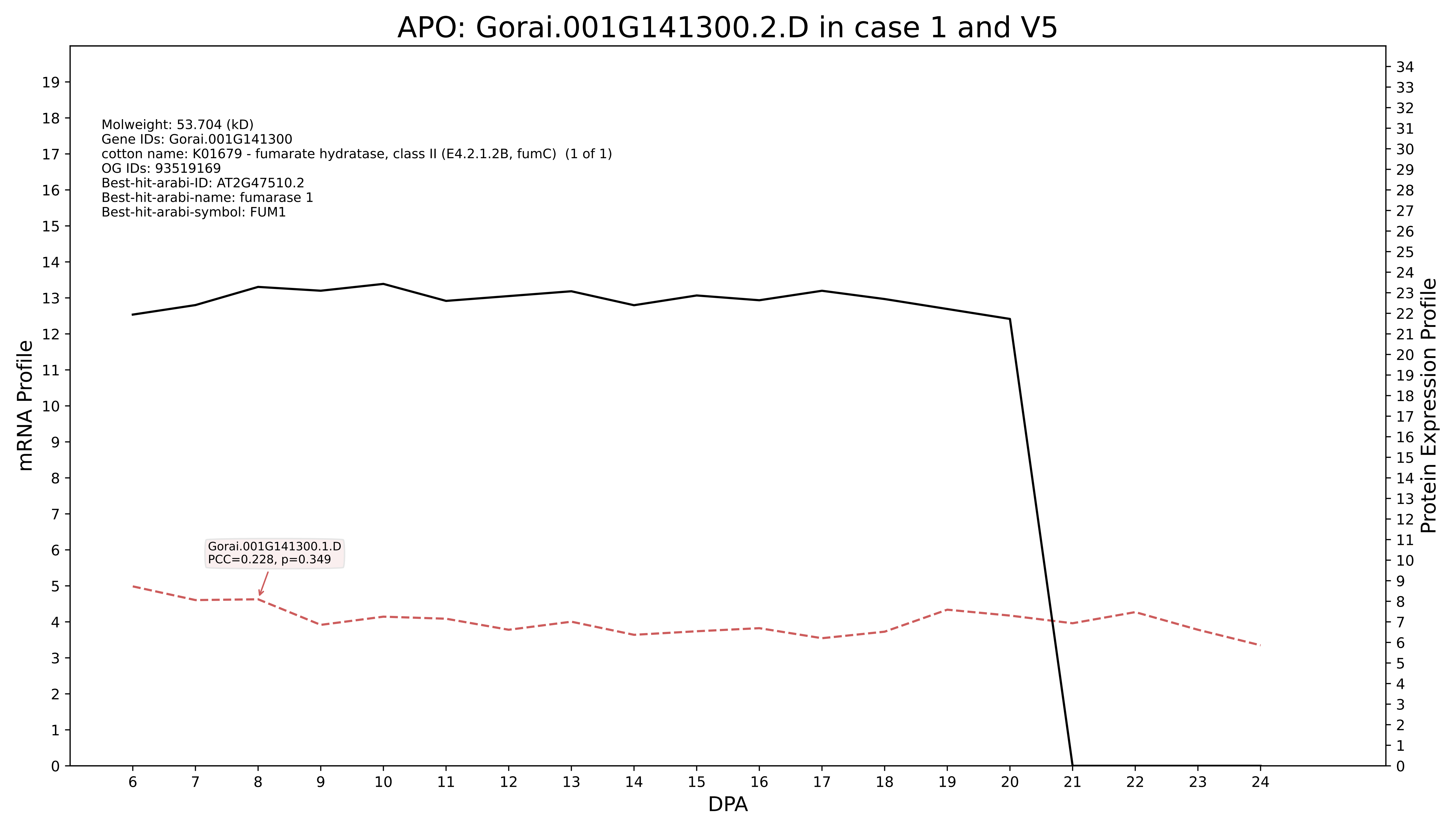
APO: Gorai.001G141300.2.D in case 1 and V5
Molweight: 53.704 (kD)
Gene IDs: Gorai.001G141300
cotton name: K01679 - fumarate hydratase, class II (E4.2.1.2B, fumC) (1 of 1)
OG IDs: 93519169
Best-hit-arabi-ID: AT2G47510.2
Best-hit-arabi-name: fumarase 1
Best-hit-arabi-symbol: FUM1
Gorai.001G141300.1.D
PCC=0.228, p=0.349
mRNA Profile
Protein Expression Profile
DPA

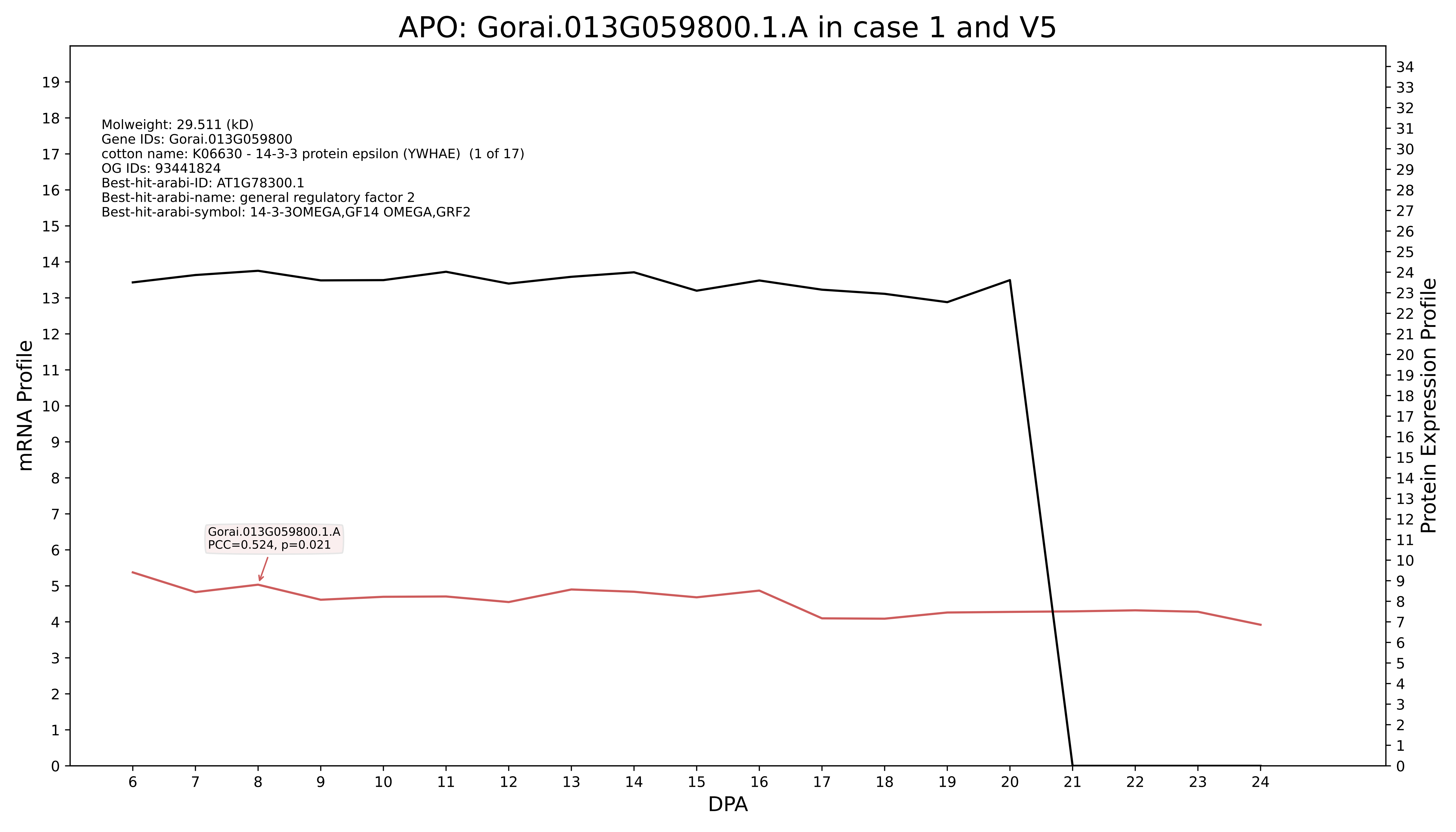

APO: Gorai.013G059800.1.A in case 1 and V5
Molweight: 29.511 (kD)
Gene IDs: Gorai.013G059800
cotton name: K06630 - 14-3-3 protein epsilon (YWHAE) (1 of 17)
OG IDs: 93441824
Best-hit-arabi-ID: AT1G78300.1
Best-hit-arabi-name: general regulatory factor 2
Best-hit-arabi-symbol: 14-3-3OMEGA,GF14 OMEGA,GRF2
Gorai.013G059800.1.A
PCC=0.524, p=0.021
mRNA Profile
Protein Expression Profile
DPA

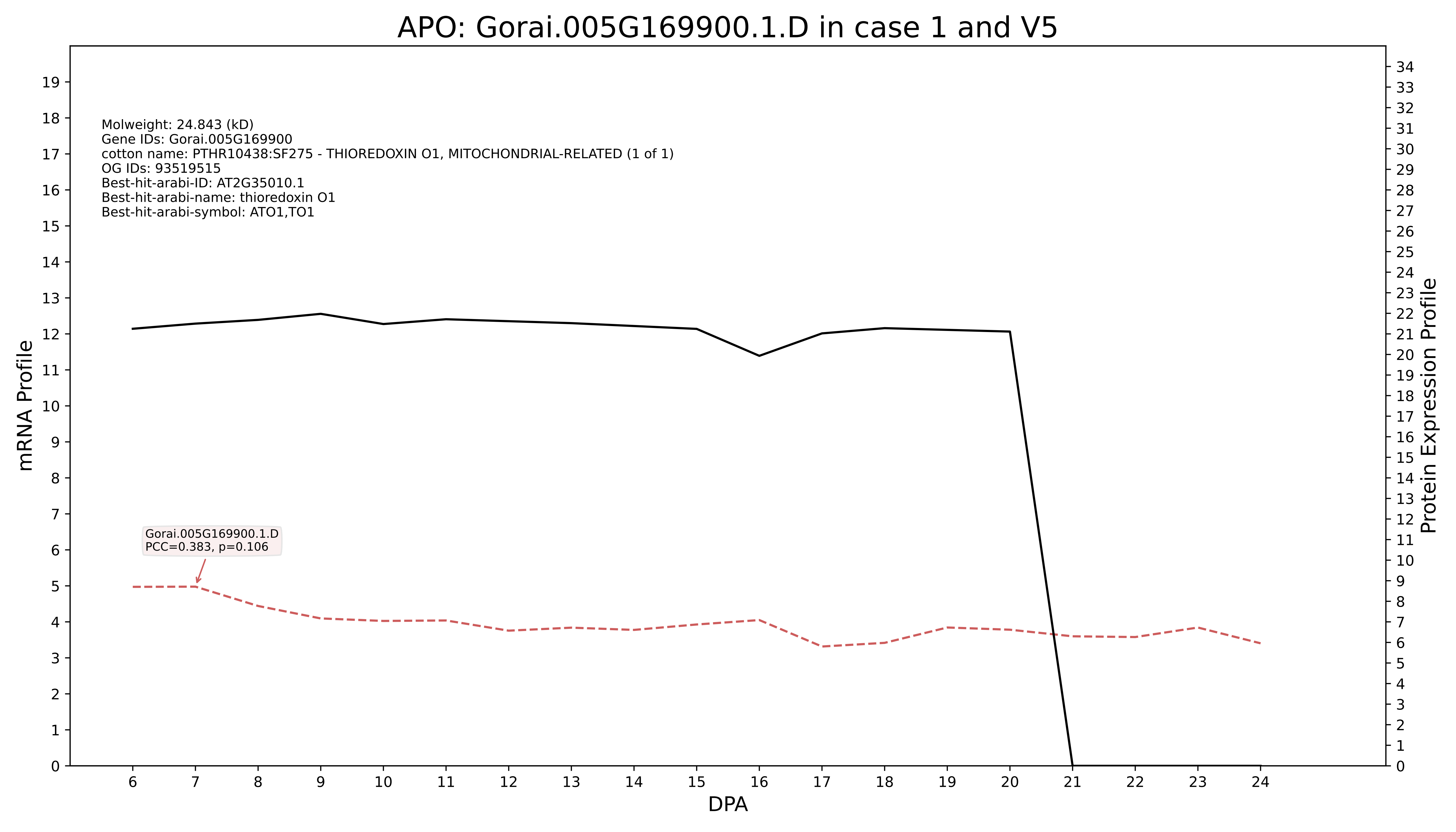
APO: Gorai.005G169900.1.D in case 1 and V5
Molweight: 24.843 (kD)
Gene IDs: Gorai.005G169900
cotton name: PTHR10438:SF275 - THIOREDOXIN O1, MITOCHONDRIAL-RELATED (1 of 1)
OG IDs: 93519515
Best-hit-arabi-ID: AT2G35010.1
Best-hit-arabi-name: thioredoxin O1
Best-hit-arabi-symbol: ATO1,TO1
Gorai.005G169900.1.D
PCC=0.383, p=0.106
mRNA Profile
Protein Expression Profile
DPA

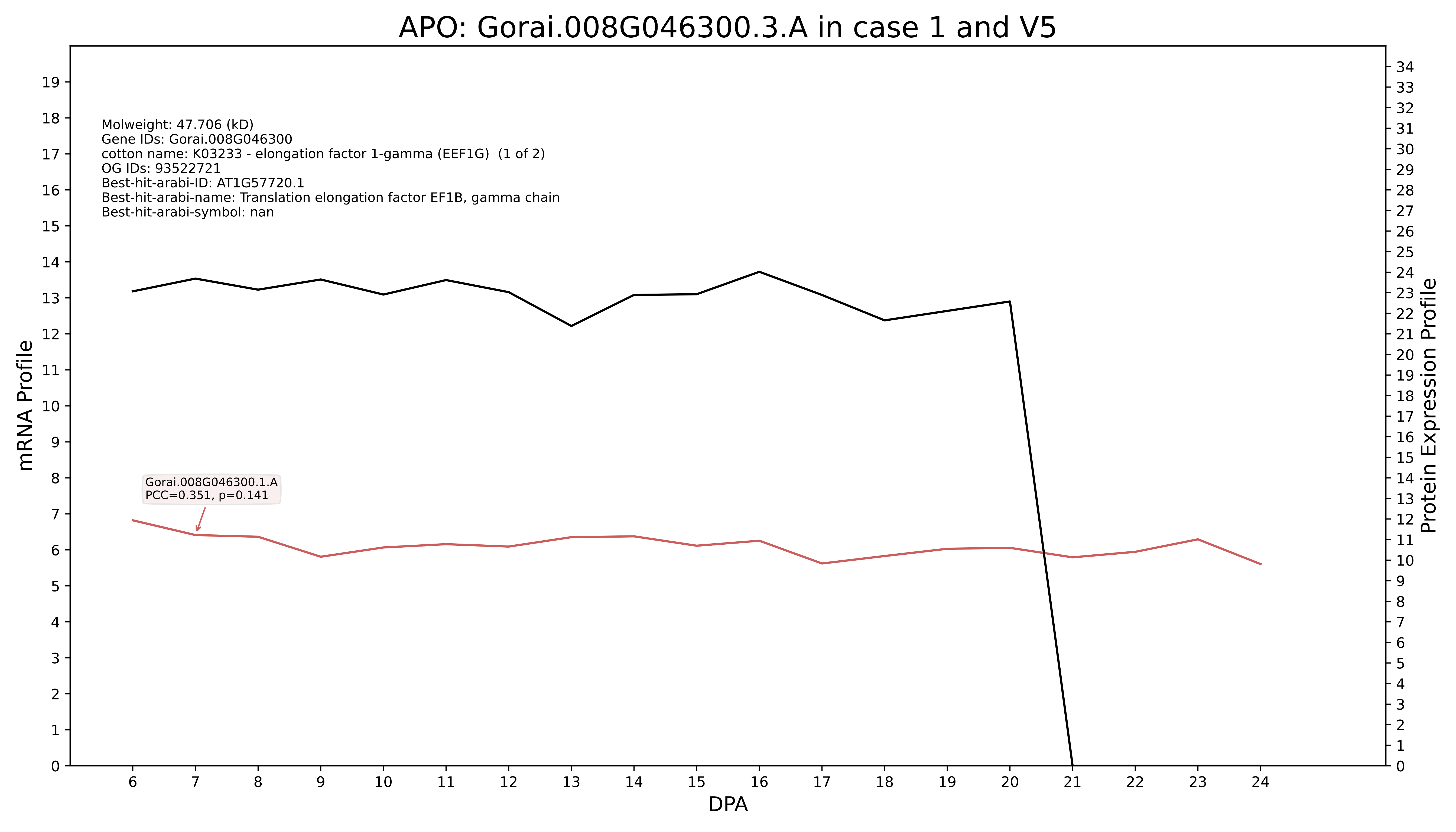
APO: Gorai.008G046300.3.A in case 1 and V5
Molweight: 47.706 (kD)
Gene IDs: Gorai.008G046300
cotton name: K03233 - elongation factor 1-gamma (EEF1G) (1 of 2)
OG IDs: 93522721
Best-hit-arabi-ID: AT1G57720.1
Best-hit-arabi-name: Translation elongation factor EF1B, gamma chain
Best-hit-arabi-symbol: nan
Gorai.008G046300.1.A
PCC=0.351, p=0.141
mRNA Profile
Protein Expression Profile
DPA
0
1
2
3
4
5
6
7
8
9
10
11
12
13
14
15
16
17
18
19
20
21
22
23
24
25
26
27
28
29
30
31
32
33
34

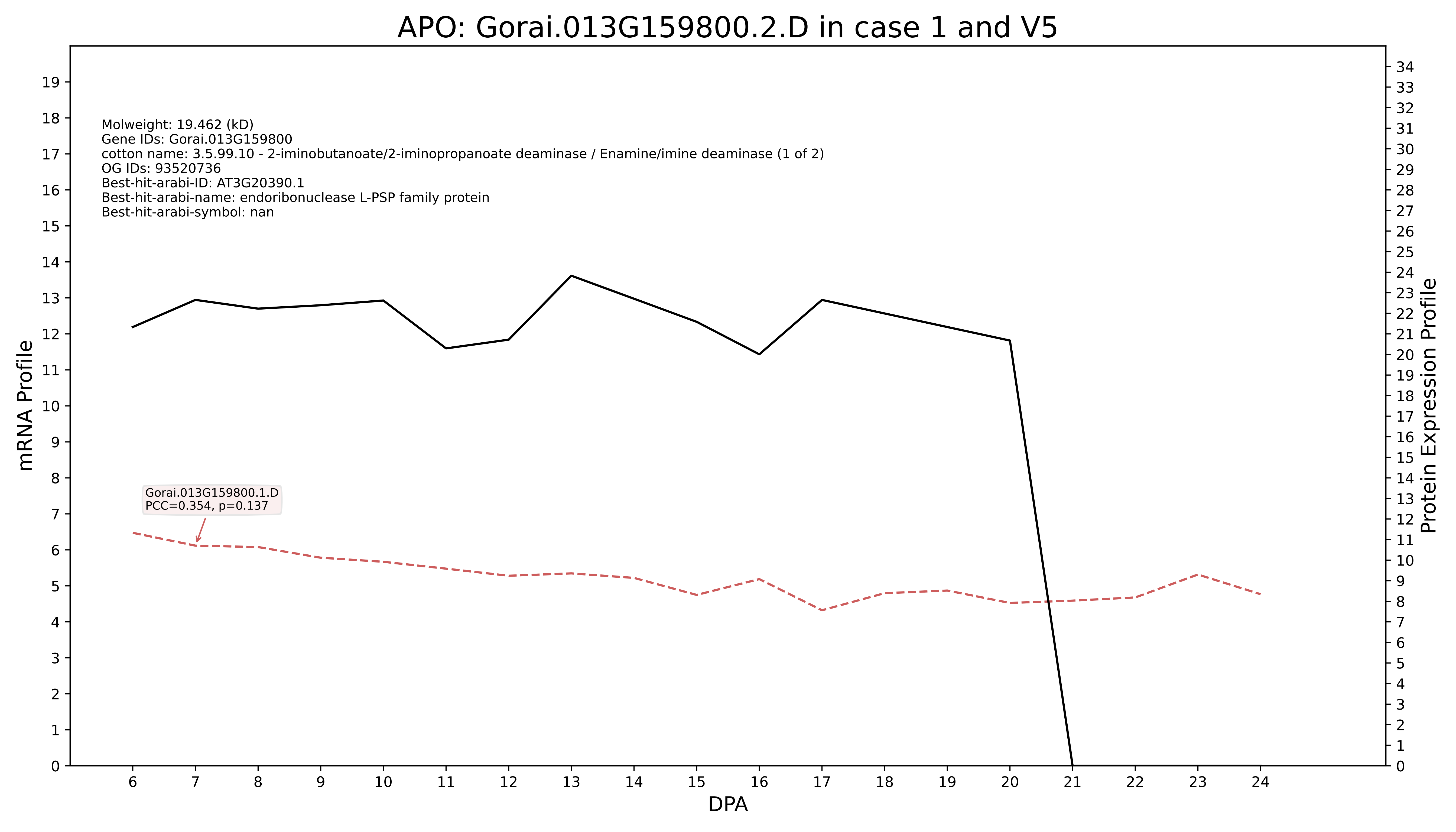
APO: Gorai.013G159800.2.D in case 1 and V5
Molweight: 19.462 (kD)
Gene IDs: Gorai.013G159800
cotton name: 3.5.99.10 - 2-iminobutanoate/2-iminopropanoate deaminase / Enamine/imine deaminase (1 of 2)
OG IDs: 93520736
Best-hit-arabi-ID: AT3G20390.1
Best-hit-arabi-name: endoribonuclease L-PSP family protein
Best-hit-arabi-symbol: nan
Gorai.013G159800.1.D
PCC=0.354, p=0.137
mRNA Profile
Protein Expression Profile
DPA

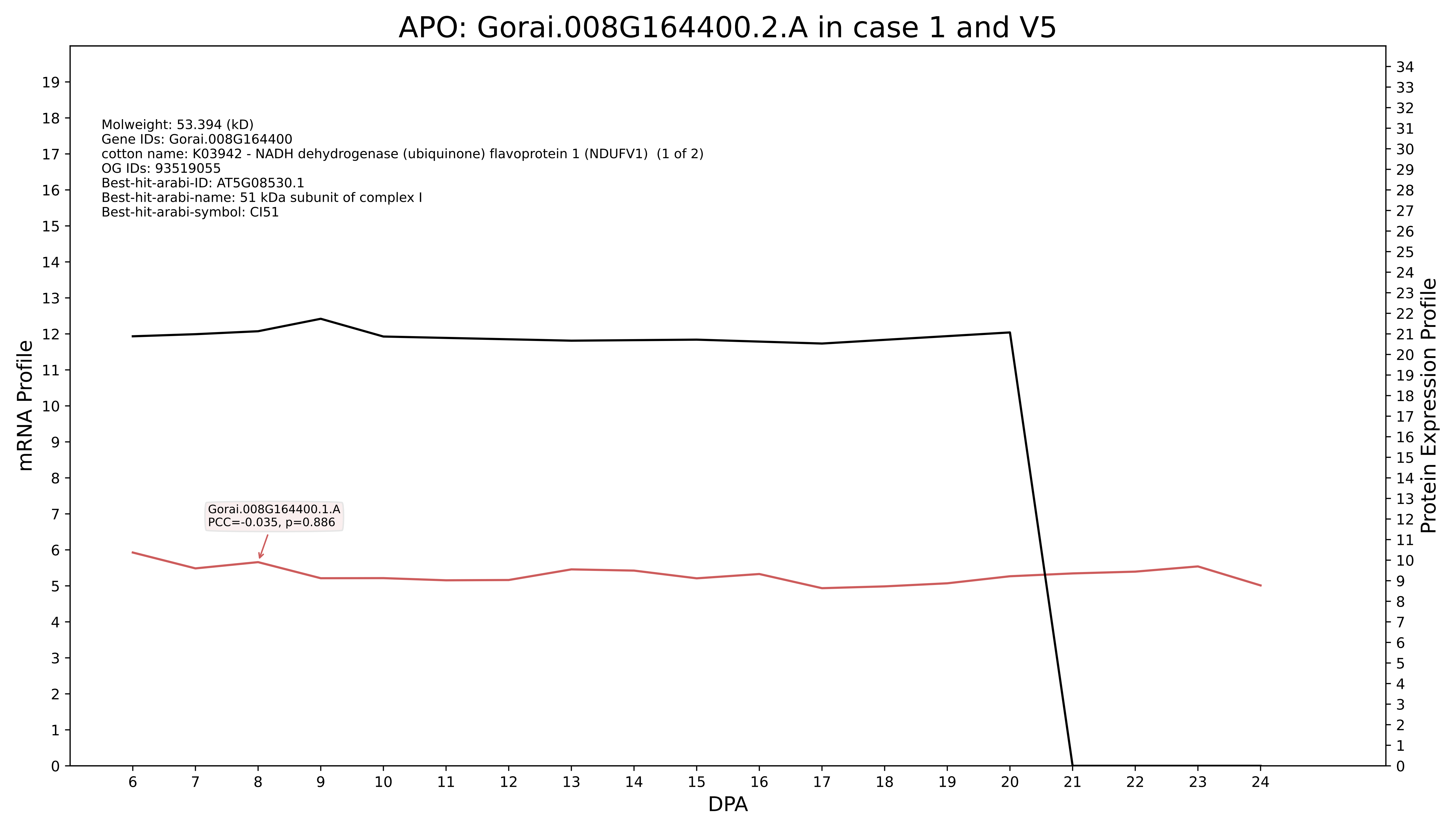

APO: Gorai.008G164400.2.A in case 1 and V5
Molweight: 53.394 (kD)
Gene IDs: Gorai.008G164400
cotton name: K03942 - NADH dehydrogenase (ubiquinone) flavoprotein 1 (NDUFV1) (1 of 2)
OG IDs: 93519055
Best-hit-arabi-ID: AT5G08530.1
Best-hit-arabi-name: 51 kDa subunit of complex I
Best-hit-arabi-symbol: CI51
Gorai.008G164400.1.A
PCC=-0.035, p=0.886
mRNA Profile
Protein Expression Profile
DPA

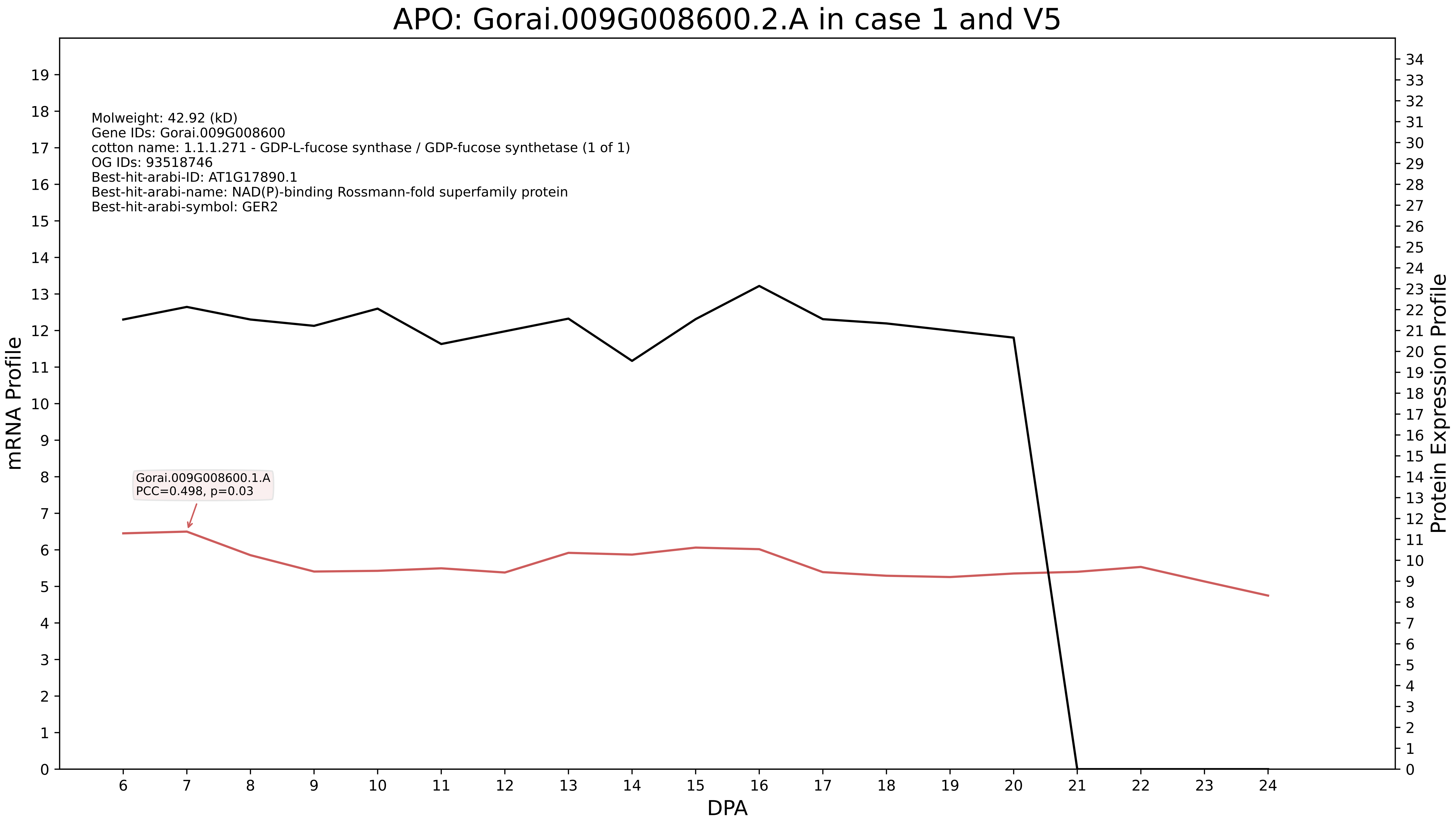
APO: Gorai.009G008600.2.A in case 1 and V5
Molweight: 42.92 (kD)
Gene IDs: Gorai.009G008600
cotton name: 1.1.1.271 - GDP-L-fucose synthase / GDP-fucose synthetase (1 of 1)
OG IDs: 93518746
Best-hit-arabi-ID: AT1G17890.1
Best-hit-arabi-name: NAD(P)-binding Rossmann-fold superfamily protein
Best-hit-arabi-symbol: GER2
Gorai.009G008600.1.A
PCC=0.498, p=0.03
mRNA Profile
Protein Expression Profile
DPA

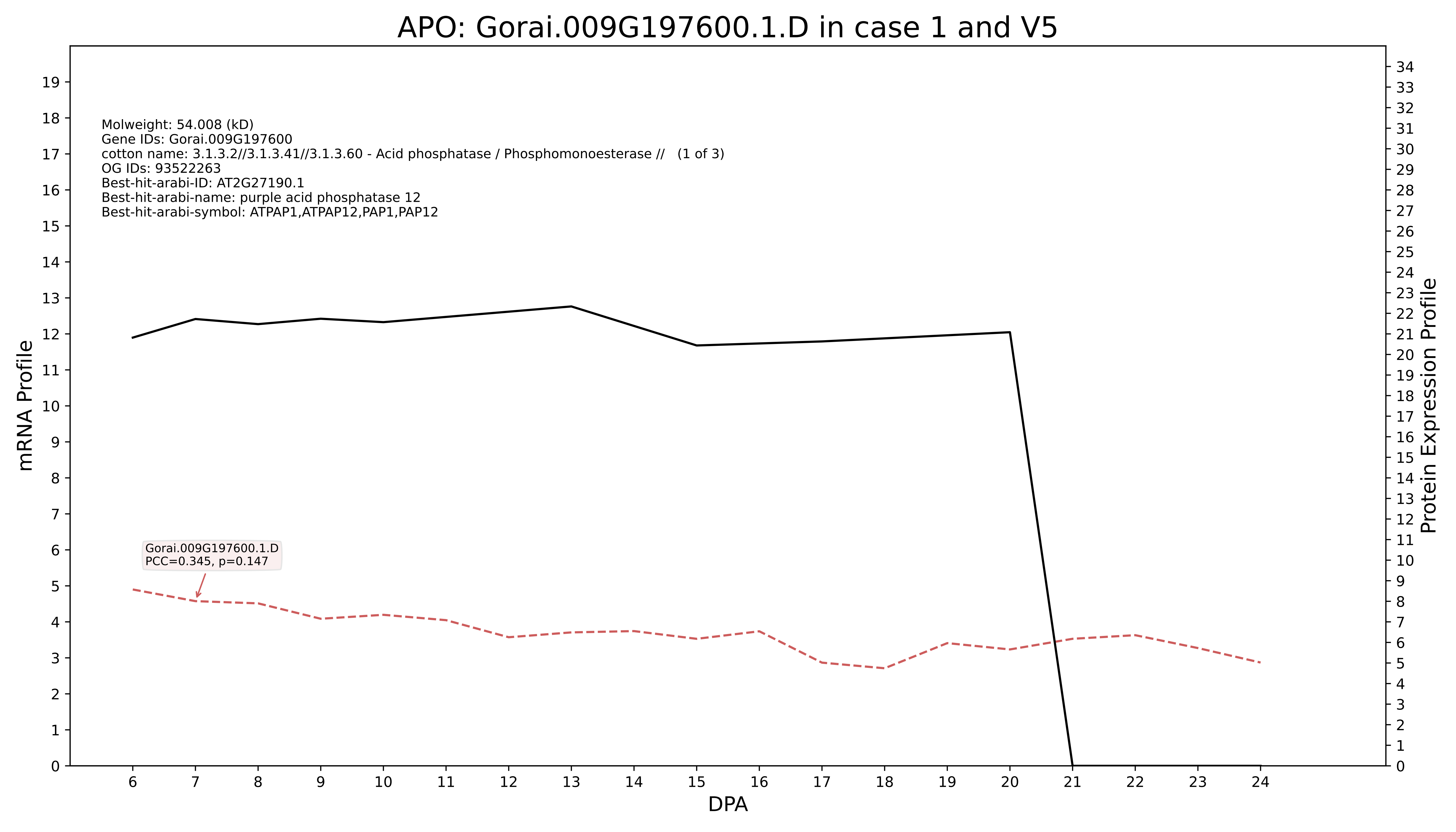

APO: Gorai.009G197600.1.D in case 1 and V5
Molweight: 54.008 (kD)
Gene IDs: Gorai.009G197600
cotton name: 3.1.3.2//3.1.3.41//3.1.3.60 - Acid phosphatase / Phosphomonoesterase // (1 of 3)
OG IDs: 93522263
Best-hit-arabi-ID: AT2G27190.1
Best-hit-arabi-name: purple acid phosphatase 12
Best-hit-arabi-symbol: ATPAP1,ATPAP12,PAP1,PAP12
Gorai.009G197600.1.D
PCC=0.345, p=0.147
mRNA Profile
Protein Expression Profile
DPA

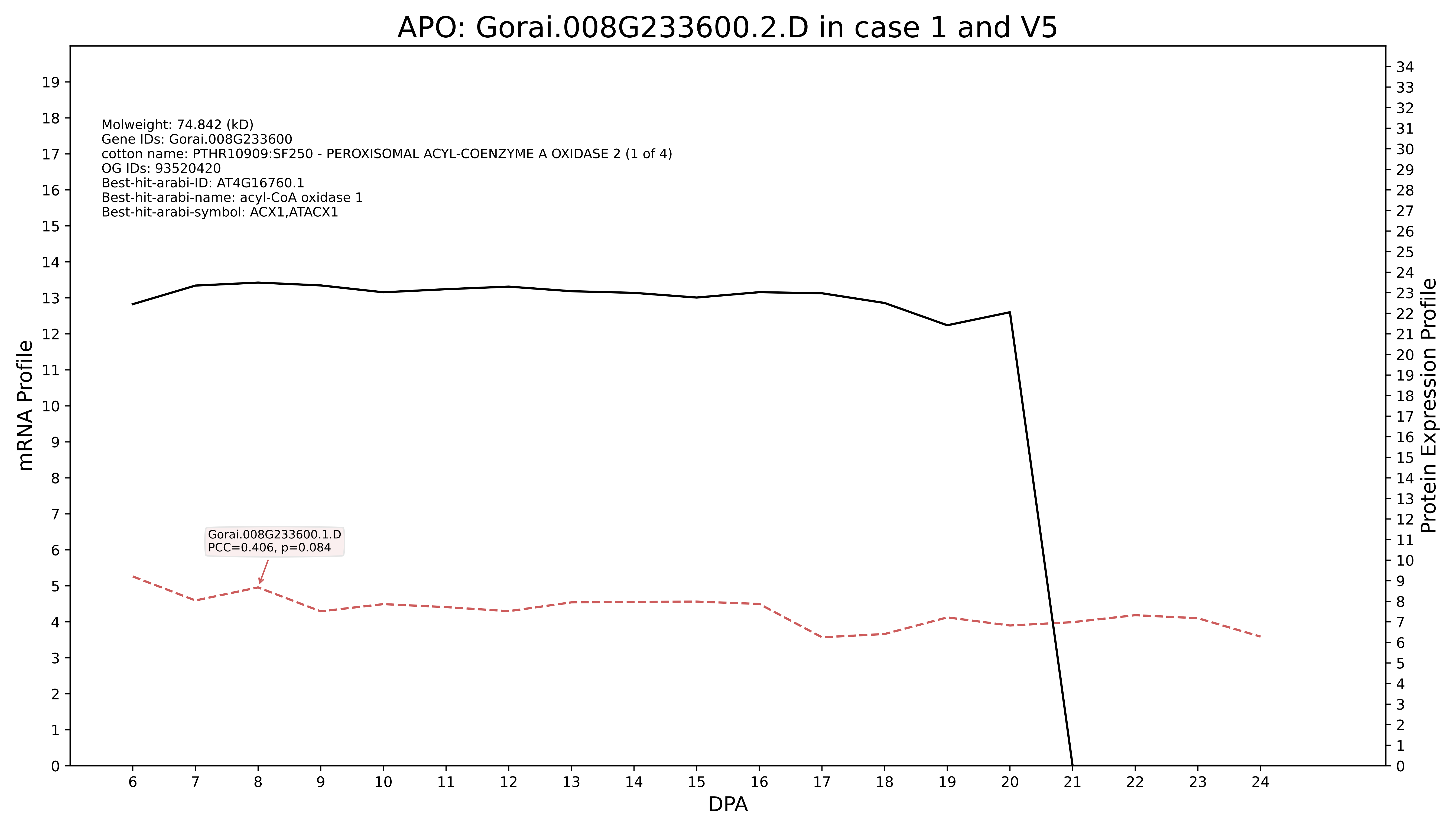
APO: Gorai.008G233600.2.D in case 1 and V5
Molweight: 74.842 (kD)
Gene IDs: Gorai.008G233600
cotton name: PTHR10909:SF250 - PEROXISOMAL ACYL-COENZYME A OXIDASE 2 (1 of 4)
OG IDs: 93520420
Best-hit-arabi-ID: AT4G16760.1
Best-hit-arabi-name: acyl-CoA oxidase 1
Best-hit-arabi-symbol: ACX1,ATACX1
Gorai.008G233600.1.D
PCC=0.406, p=0.084
mRNA Profile
Protein Expression Profile
DPA

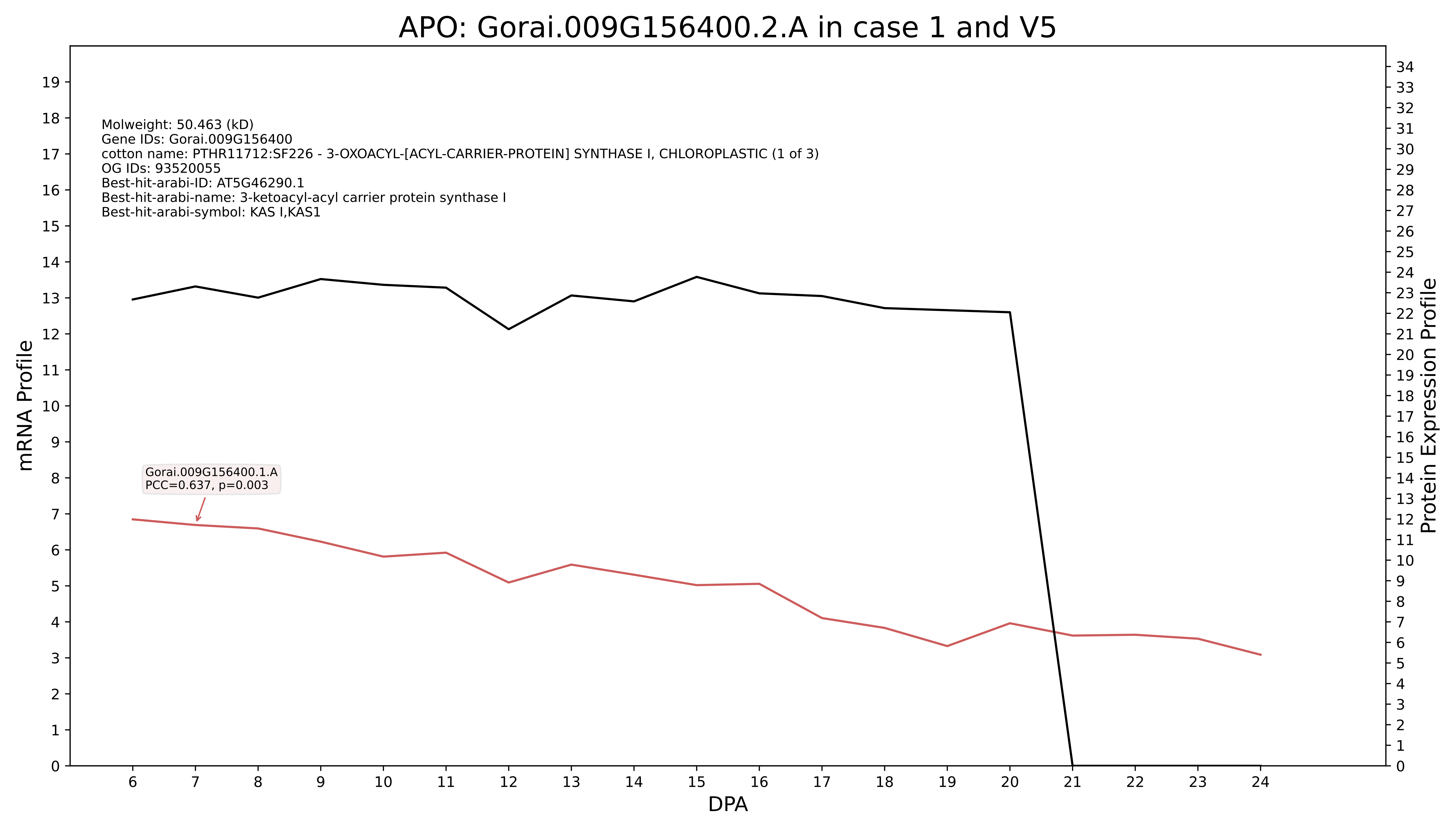

APO: Gorai.009G156400.2.A in case 1 and V5
Molweight: 50.463 (kD)
Gene IDs: Gorai.009G156400
cotton name: PTHR11712:SF226 - 3-OXOACYL-[ACYL-CARRIER-PROTEIN] SYNTHASE I, CHLOROPLASTIC (1 of 3)
OG IDs: 93520055
Best-hit-arabi-ID: AT5G46290.1
Best-hit-arabi-name: 3-ketoacyl-acyl carrier protein synthase I
Best-hit-arabi-symbol: KAS I,KAS1
Gorai.009G156400.1.A
PCC=0.637, p=0.003
mRNA Profile
Protein Expression Profile
DPA

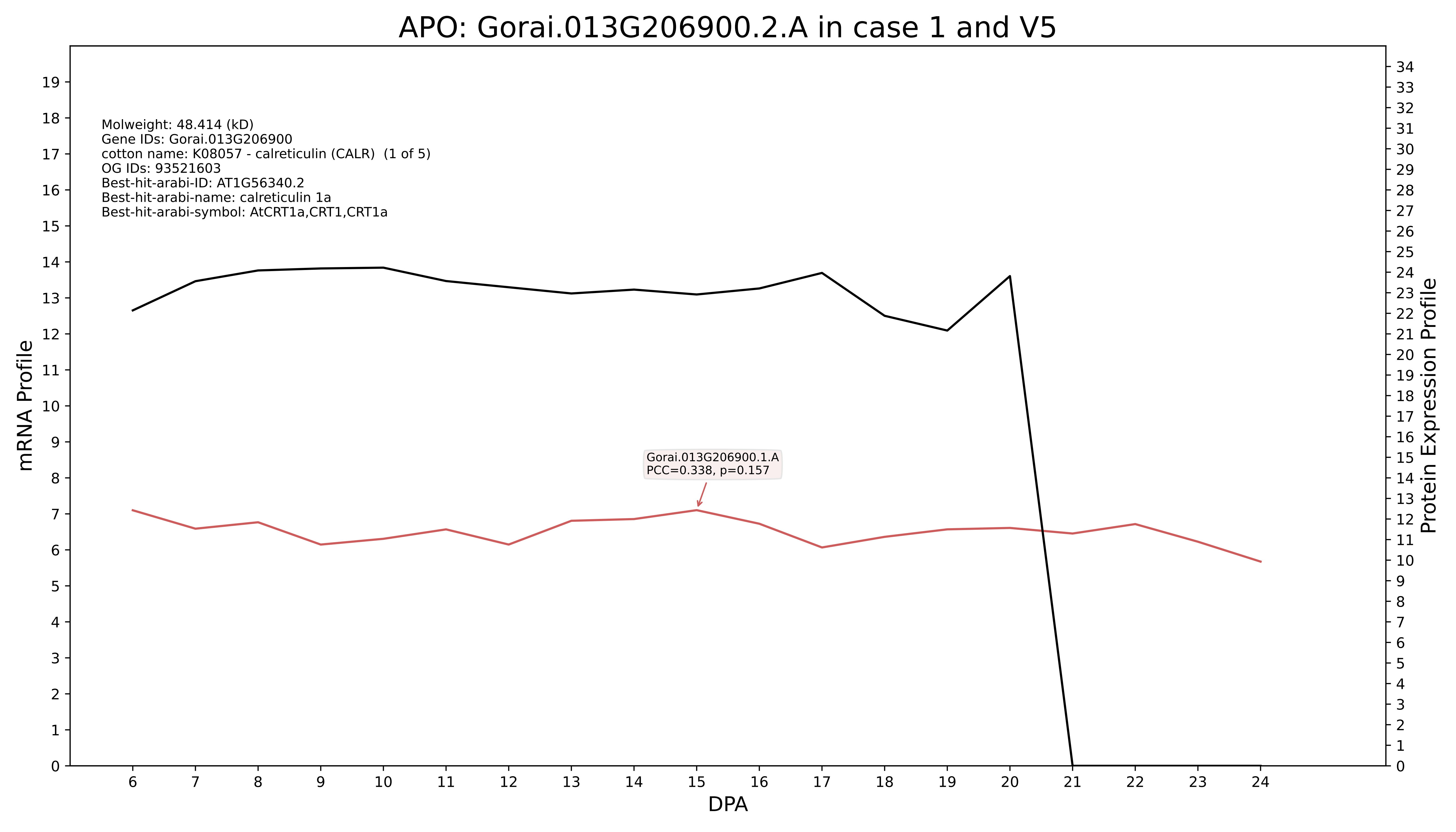
APO: Gorai.013G206900.2.A in case 1 and V5
Molweight: 48.414 (kD)
Gene IDs: Gorai.013G206900
cotton name: K08057 - calreticulin (CALR) (1 of 5)
OG IDs: 93521603
Best-hit-arabi-ID: AT1G56340.2
Best-hit-arabi-name: calreticulin 1a
Best-hit-arabi-symbol: AtCRT1a,CRT1,CRT1a
Gorai.013G206900.1.A
PCC=0.338, p=0.157
mRNA Profile
Protein Expression Profile
DPA

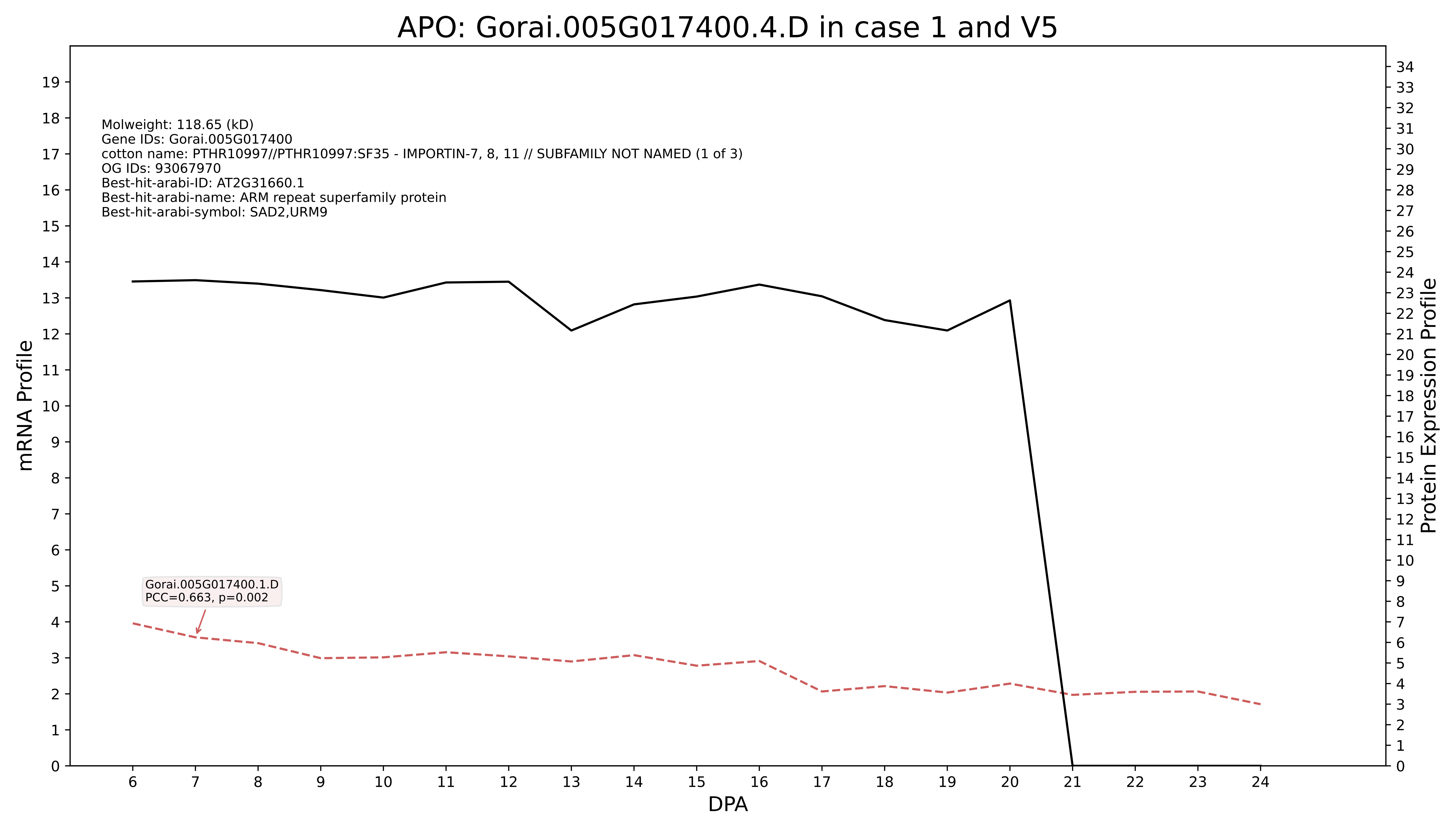

APO: Gorai.005G017400.4.D in case 1 and V5
Molweight: 118.65 (kD)
Gene IDs: Gorai.005G017400
cotton name: PTHR10997//PTHR10997:SF35 - IMPORTIN-7, 8, 11 // SUBFAMILY NOT NAMED (1 of 3)
OG IDs: 93067970
Best-hit-arabi-ID: AT2G31660.1
Best-hit-arabi-name: ARM repeat superfamily protein
Best-hit-arabi-symbol: SAD2,URM9
Gorai.005G017400.1.D
PCC=0.663, p=0.002
mRNA Profile
Protein Expression Profile
DPA

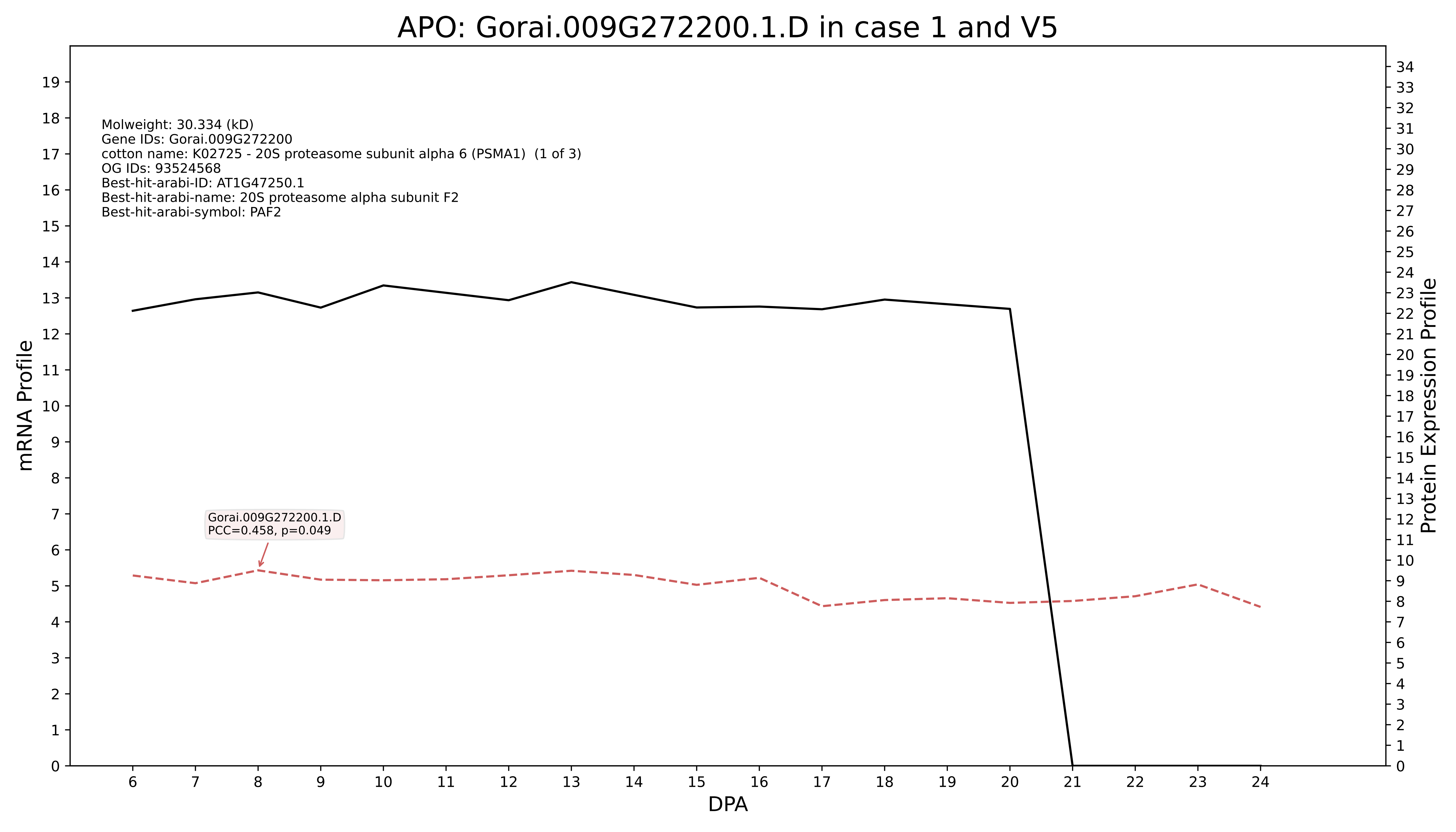

APO: Gorai.009G272200.1.D in case 1 and V5
Molweight: 30.334 (kD)
Gene IDs: Gorai.009G272200
cotton name: K02725 - 20S proteasome subunit alpha 6 (PSMA1) (1 of 3)
OG IDs: 93524568
Best-hit-arabi-ID: AT1G47250.1
Best-hit-arabi-name: 20S proteasome alpha subunit F2
Best-hit-arabi-symbol: PAF2
Gorai.009G272200.1.D
PCC=0.458, p=0.049
mRNA Profile
Protein Expression Profile
DPA

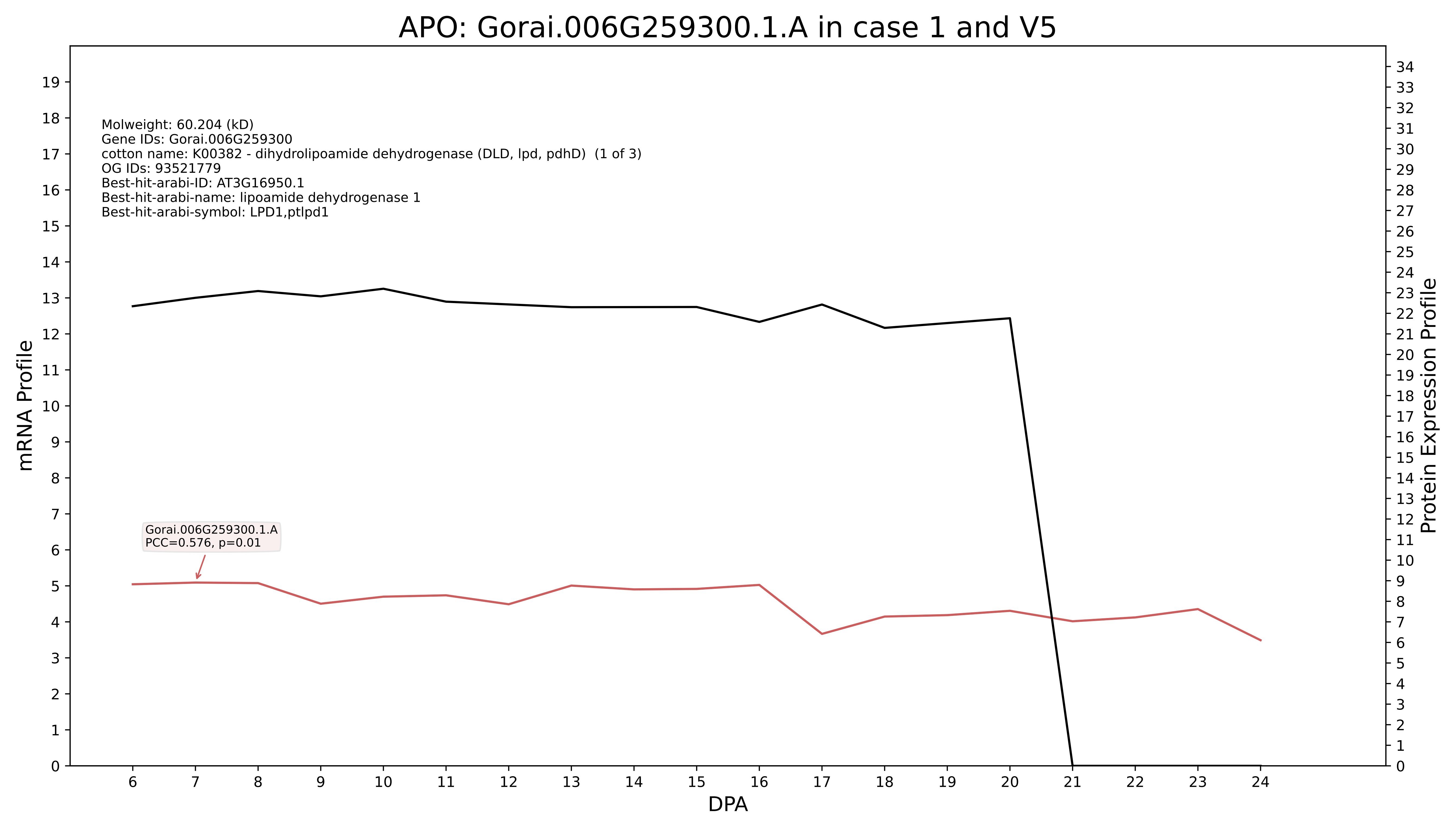
APO: Gorai.006G259300.1.A in case 1 and V5
Molweight: 60.204 (kD)
Gene IDs: Gorai.006G259300
cotton name: K00382 - dihydrolipoamide dehydrogenase (DLD, lpd, pdhD) (1 of 3)
OG IDs: 93521779
Best-hit-arabi-ID: AT3G16950.1
Best-hit-arabi-name: lipoamide dehydrogenase 1
Best-hit-arabi-symbol: LPD1,ptlpd1
Gorai.006G259300.1.A
PCC=0.576, p=0.01
mRNA Profile
Protein Expression Profile
DPA
0
1
2
3
4
5
6
7
8
9
10
11
12
13
14
15
16
17
18
19
20
21
22
23
24
25
26
27
28
29
30
31
32
33
34

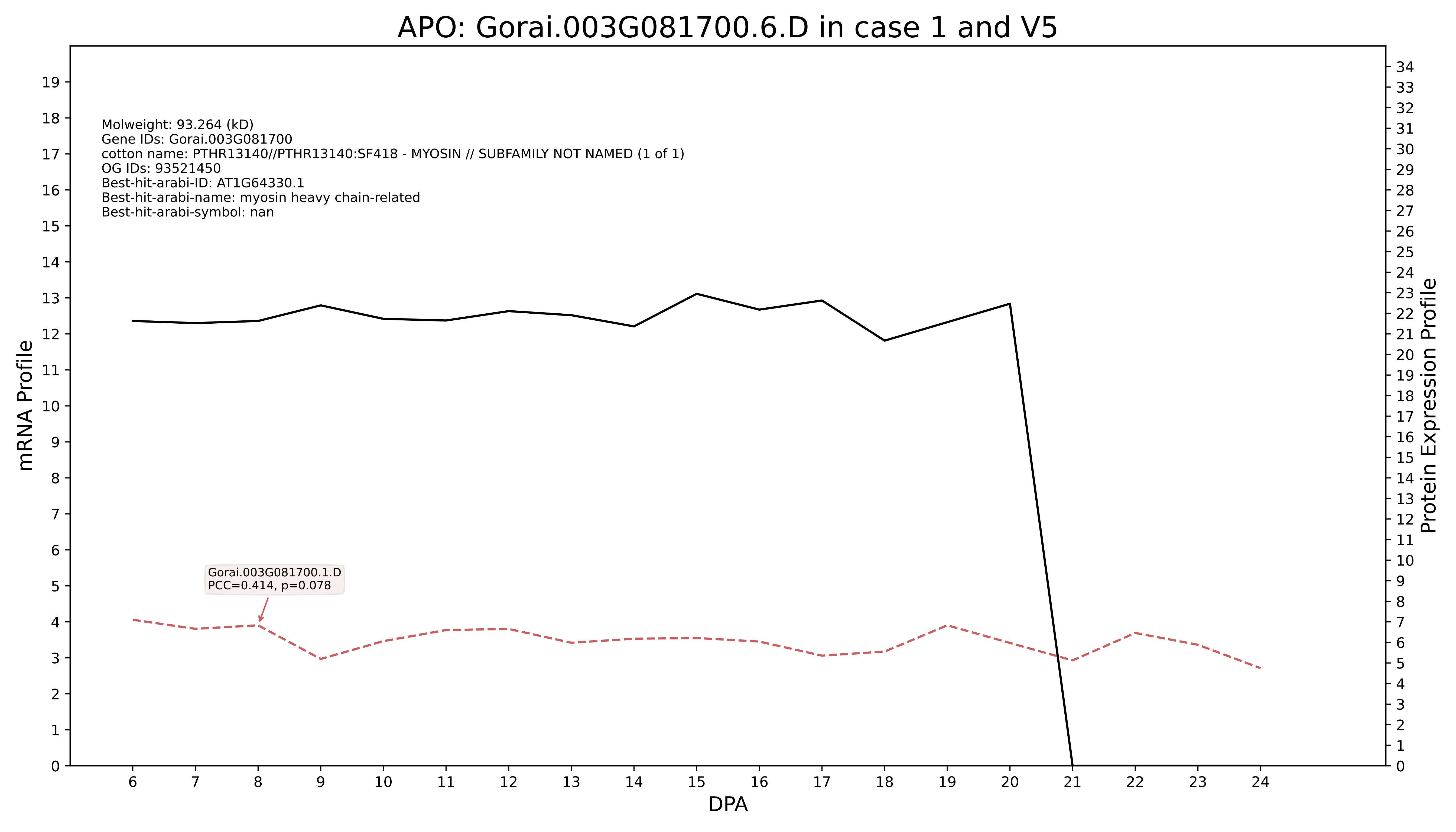

APO: Gorai.003G081700.6.D in case 1 and V5
Molweight: 93.264 (kD)
Gene IDs: Gorai.003G081700
cotton name: PTHR13140//PTHR13140:SF418 - MYOSIN // SUBFAMILY NOT NAMED (1 of 1)
OG IDs: 93521450
Best-hit-arabi-ID: AT1G64330.1
Best-hit-arabi-name: myosin heavy chain-related
Best-hit-arabi-symbol: nan
Gorai.003G081700.1.D
PCC=0.414, p=0.078
mRNA Profile
Protein Expression Profile
DPA

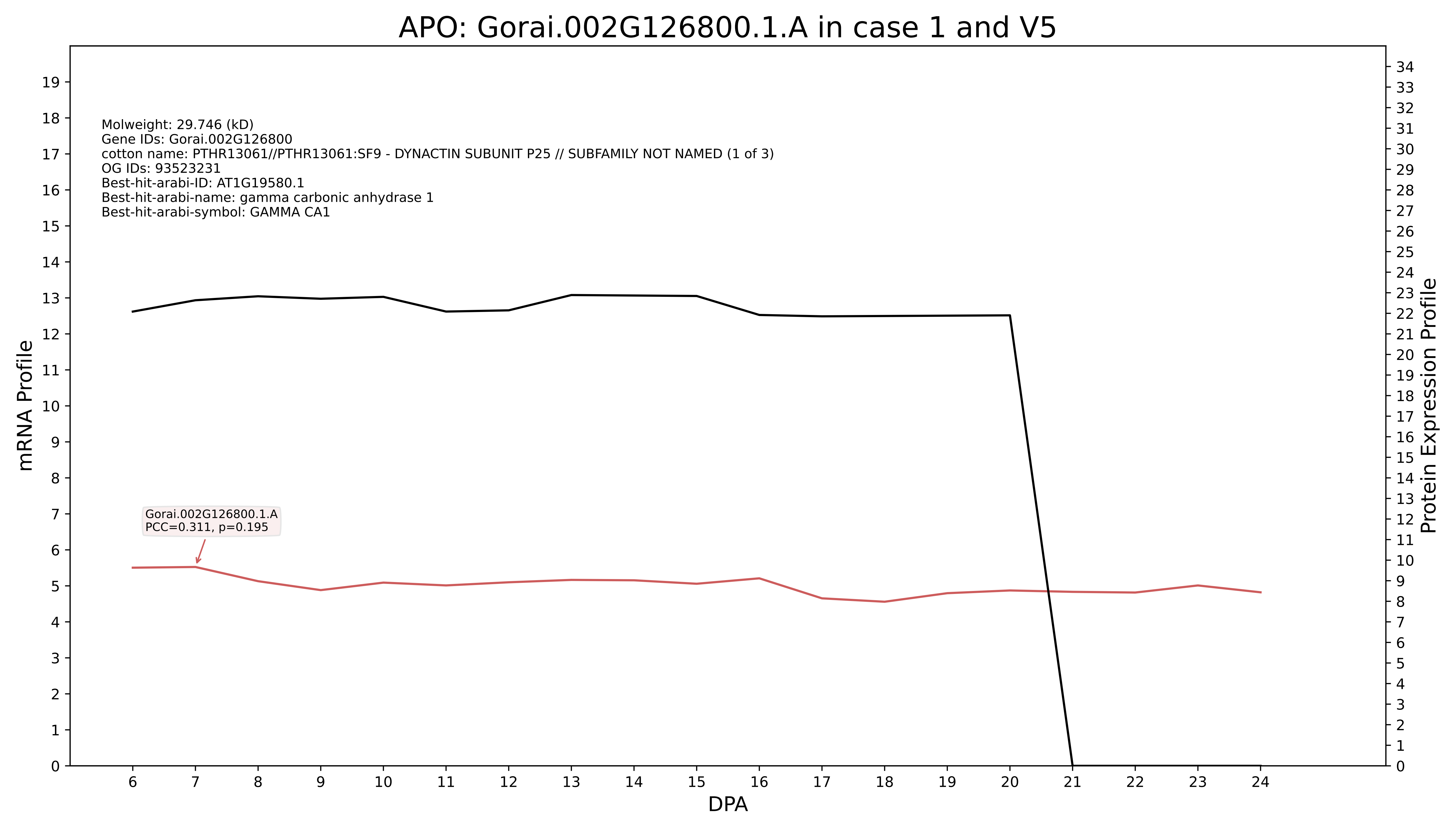
APO: Gorai.002G126800.1.A in case 1 and V5
Molweight: 29.746 (kD)
Gene IDs: Gorai.002G126800
cotton name: PTHR13061//PTHR13061:SF9 - DYNACTIN SUBUNIT P25 // SUBFAMILY NOT NAMED (1 of 3)
OG IDs: 93523231
Best-hit-arabi-ID: AT1G19580.1
Best-hit-arabi-name: gamma carbonic anhydrase 1
Best-hit-arabi-symbol: GAMMA CA1
Gorai.002G126800.1.A
PCC=0.311, p=0.195
mRNA Profile
Protein Expression Profile
DPA

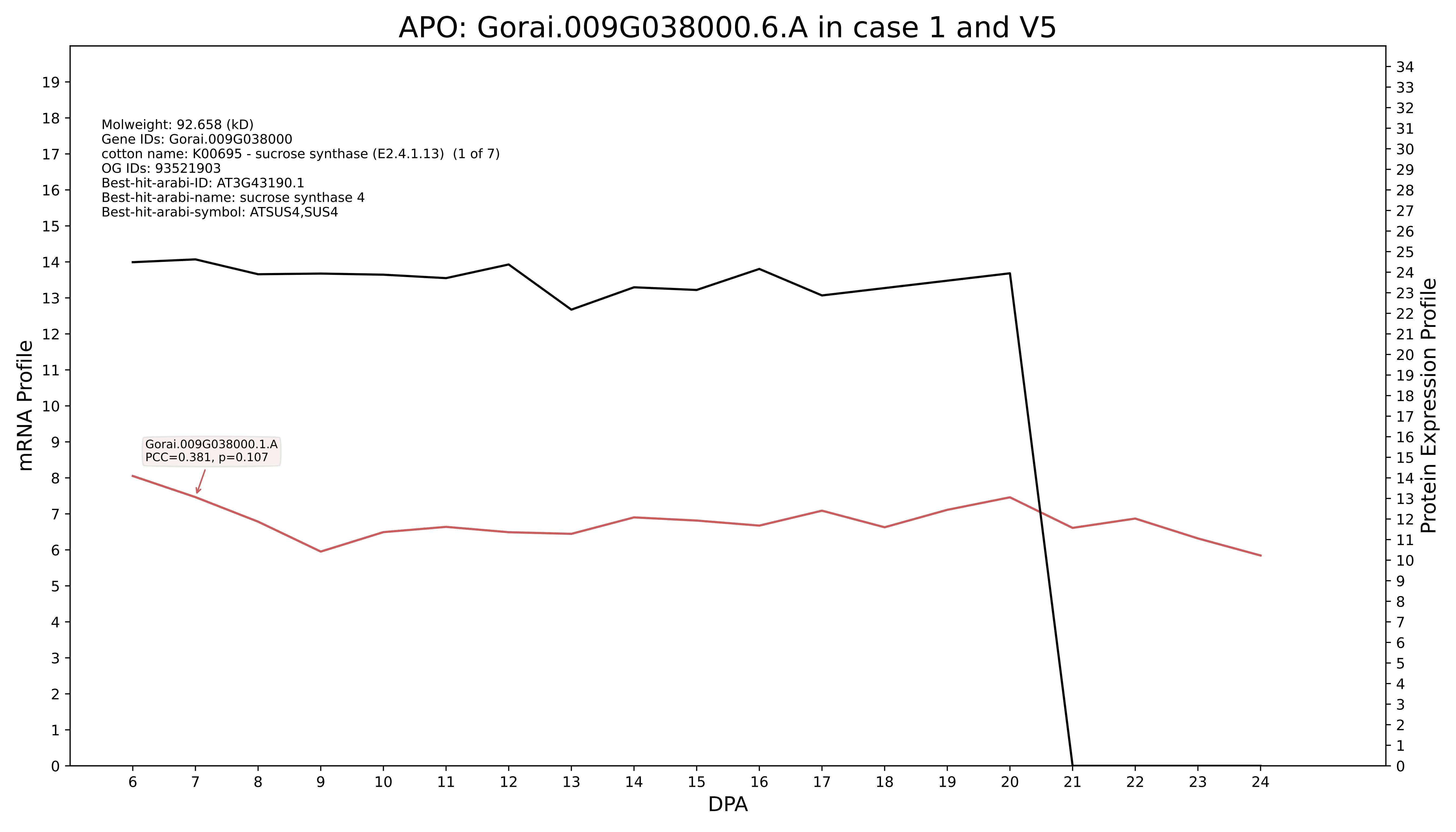
APO: Gorai.009G038000.6.A in case 1 and V5
Molweight: 92.658 (kD)
Gene IDs: Gorai.009G038000
cotton name: K00695 - sucrose synthase (E2.4.1.13) (1 of 7)
OG IDs: 93521903
Best-hit-arabi-ID: AT3G43190.1
Best-hit-arabi-name: sucrose synthase 4
Best-hit-arabi-symbol: ATSUS4,SUS4
Gorai.009G038000.1.A
PCC=0.381, p=0.107
mRNA Profile
Protein Expression Profile
DPA

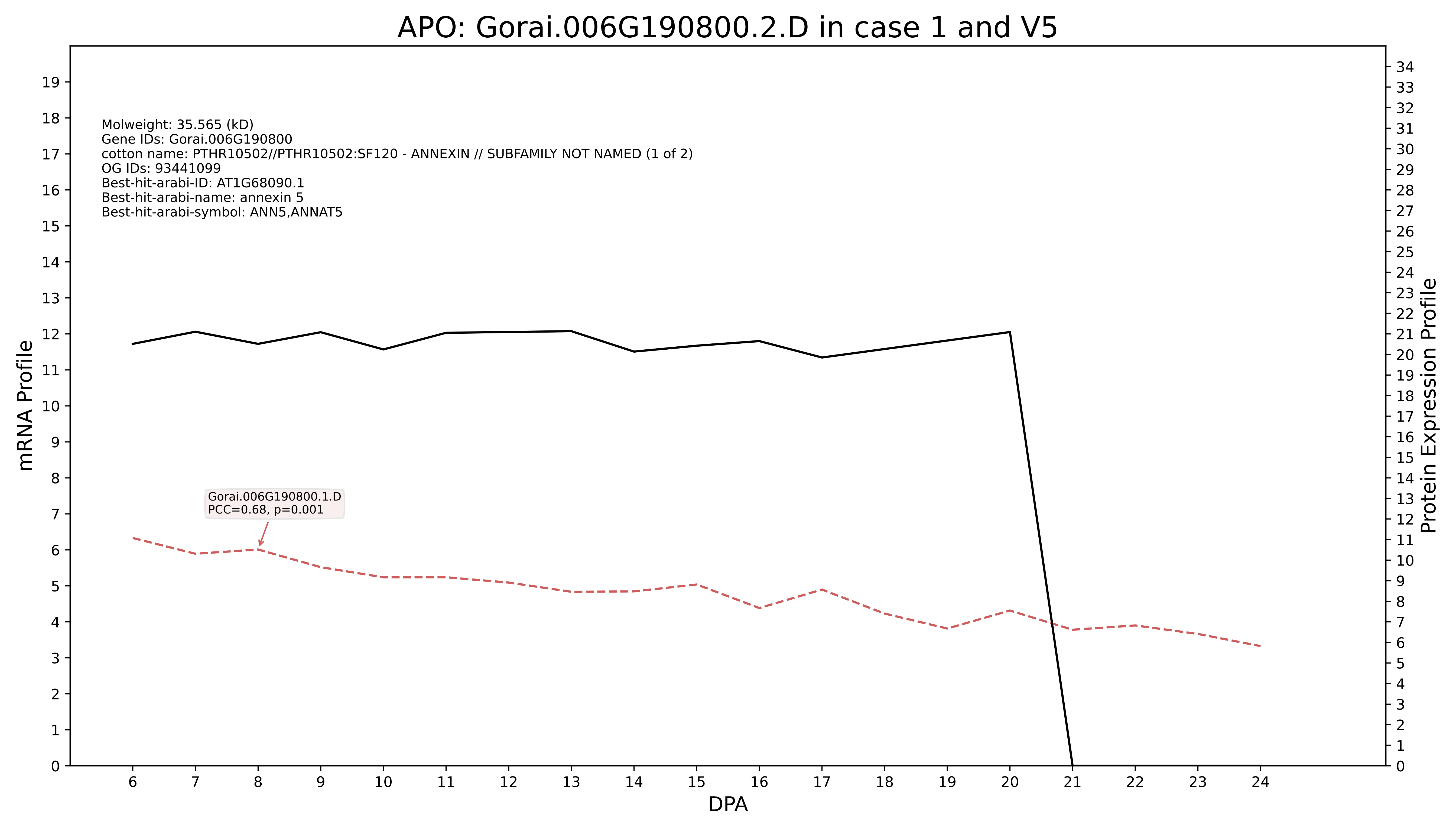

APO: Gorai.006G190800.2.D in case 1 and V5
Molweight: 35.565 (kD)
Gene IDs: Gorai.006G190800
cotton name: PTHR10502//PTHR10502:SF120 - ANNEXIN // SUBFAMILY NOT NAMED (1 of 2)
OG IDs: 93441099
Best-hit-arabi-ID: AT1G68090.1
Best-hit-arabi-name: annexin 5
Best-hit-arabi-symbol: ANN5,ANNAT5
Gorai.006G190800.1.D
PCC=0.68, p=0.001
mRNA Profile
Protein Expression Profile
DPA

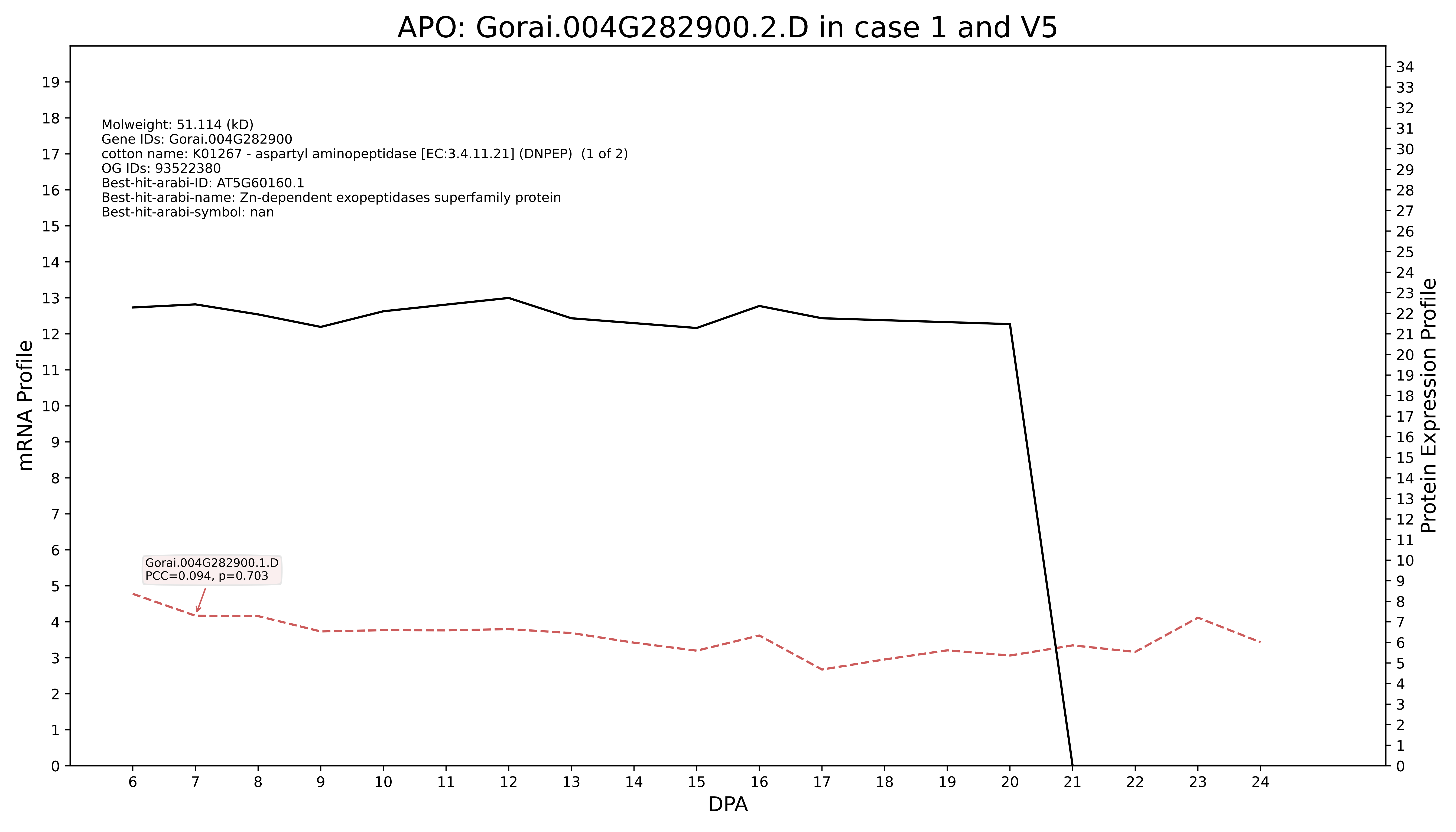
APO: Gorai.004G282900.2.D in case 1 and V5
Molweight: 51.114 (kD)
Gene IDs: Gorai.004G282900
cotton name: K01267 - aspartyl aminopeptidase [EC:3.4.11.21] (DNPEP) (1 of 2)
OG IDs: 93522380
Best-hit-arabi-ID: AT5G60160.1
Best-hit-arabi-name: Zn-dependent exopeptidases superfamily protein
Best-hit-arabi-symbol: nan
Gorai.004G282900.1.D
PCC=0.094, p=0.703
mRNA Profile
Protein Expression Profile
DPA
6
7
8
9
10
11
12
13
14
15
16
17
18
19
20
21
22
23
24

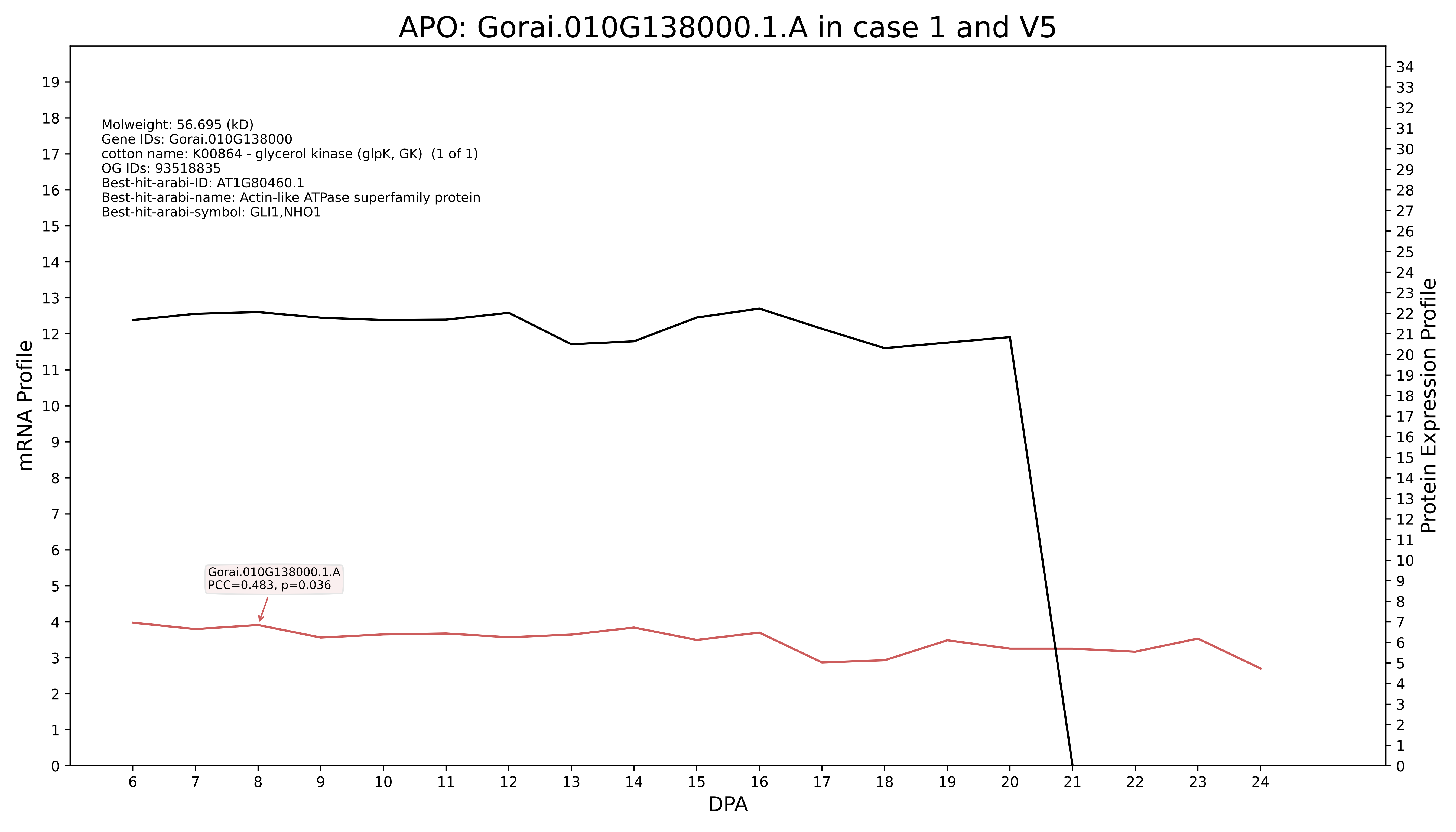

APO: Gorai.010G138000.1.A in case 1 and V5
Molweight: 56.695 (kD)
Gene IDs: Gorai.010G138000
cotton name: K00864 - glycerol kinase (glpK, GK) (1 of 1)
OG IDs: 93518835
Best-hit-arabi-ID: AT1G80460.1
Best-hit-arabi-name: Actin-like ATPase superfamily protein
Best-hit-arabi-symbol: GLI1,NHO1
Gorai.010G138000.1.A
PCC=0.483, p=0.036
mRNA Profile
Protein Expression Profile
DPA

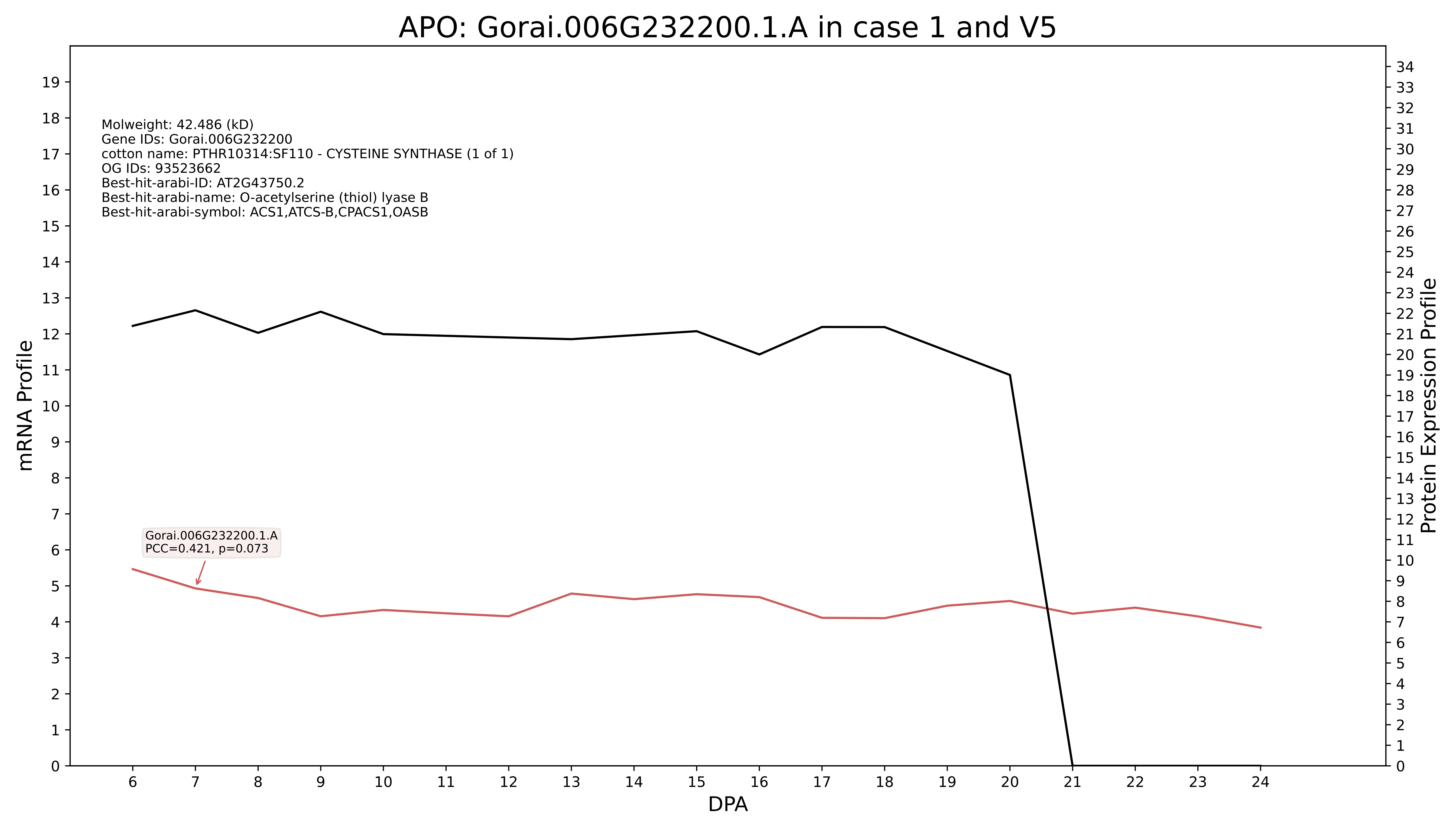
APO: Gorai.006G232200.1.A in case 1 and V5
Molweight: 42.486 (kD)
Gene IDs: Gorai.006G232200
cotton name: PTHR10314:SF110 - CYSTEINE SYNTHASE (1 of 1)
OG IDs: 93523662
Best-hit-arabi-ID: AT2G43750.2
Best-hit-arabi-name: O-acetylserine (thiol) lyase B
Best-hit-arabi-symbol: ACS1,ATCS-B,CPACS1,OASB
Gorai.006G232200.1.A
PCC=0.421, p=0.073
mRNA Profile
Protein Expression Profile
DPA

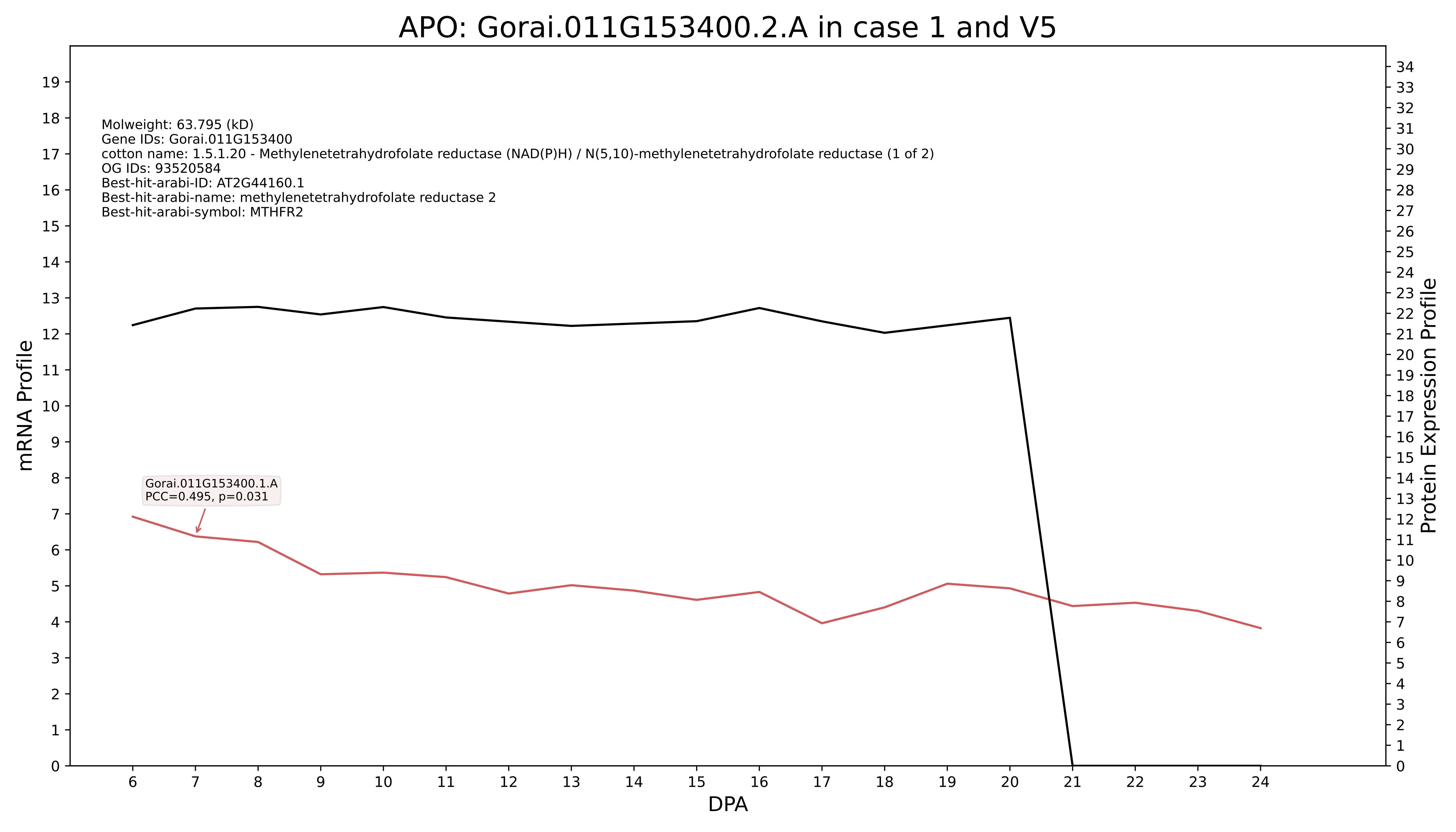
APO: Gorai.011G153400.2.A in case 1 and V5
Molweight: 63.795 (kD)
Gene IDs: Gorai.011G153400
cotton name: 1.5.1.20 - Methylenetetrahydrofolate reductase (NAD(P)H) / N(5,10)-methylenetetrahydrofolate reductase (1 of 2)
OG IDs: 93520584
Best-hit-arabi-ID: AT2G44160.1
Best-hit-arabi-name: methylenetetrahydrofolate reductase 2
Best-hit-arabi-symbol: MTHFR2
Gorai.011G153400.1.A
PCC=0.495, p=0.031
mRNA Profile
Protein Expression Profile
DPA

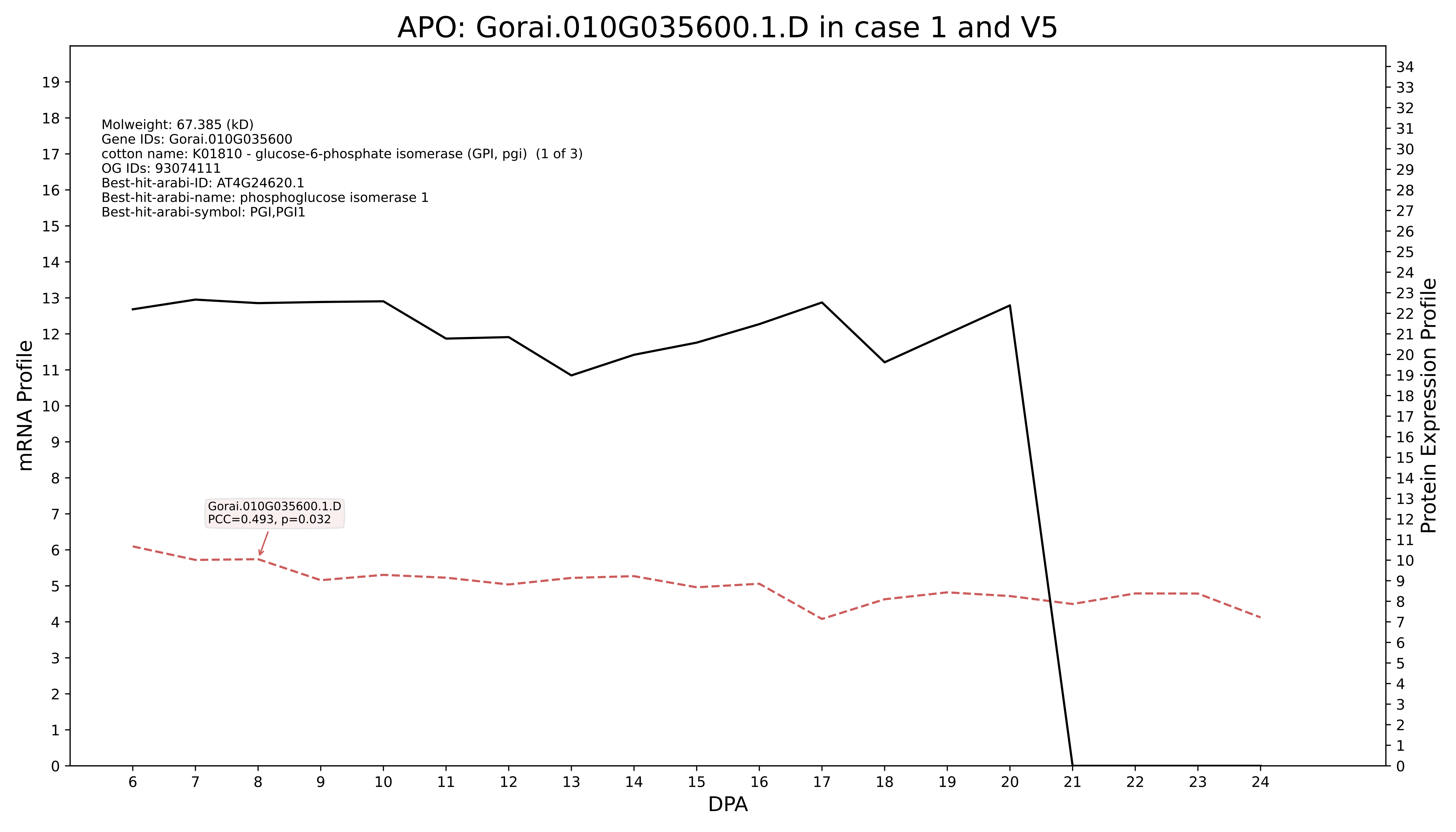
APO: Gorai.010G035600.1.D in case 1 and V5
Molweight: 67.385 (kD)
Gene IDs: Gorai.010G035600
cotton name: K01810 - glucose-6-phosphate isomerase (GPI, pgi) (1 of 3)
OG IDs: 93074111
Best-hit-arabi-ID: AT4G24620.1
Best-hit-arabi-name: phosphoglucose isomerase 1
Best-hit-arabi-symbol: PGI,PGI1
Gorai.010G035600.1.D
PCC=0.493, p=0.032
mRNA Profile
Protein Expression Profile
DPA

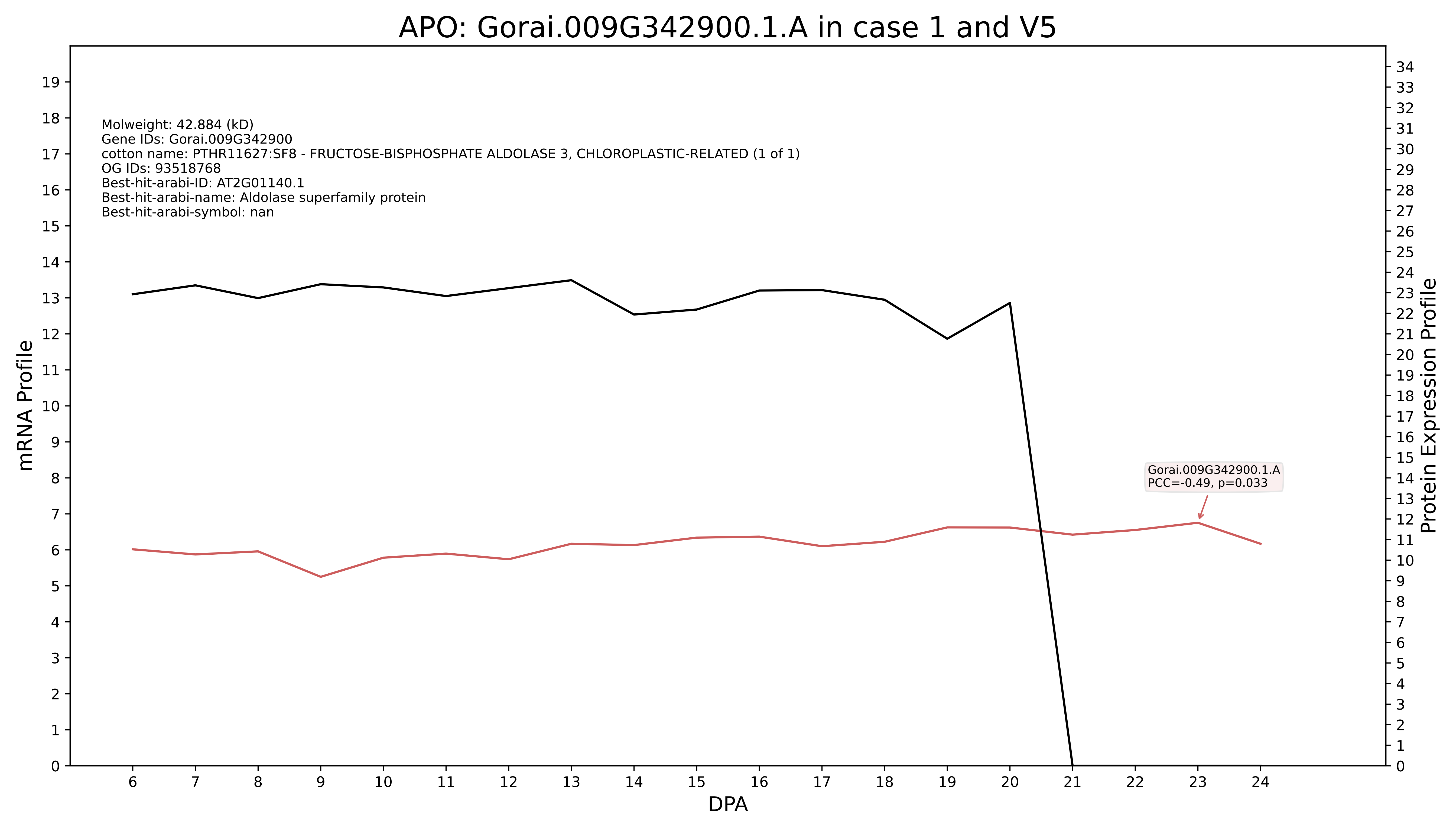

APO: Gorai.009G342900.1.A in case 1 and V5
Molweight: 42.884 (kD)
Gene IDs: Gorai.009G342900
cotton name: PTHR11627:SF8 - FRUCTOSE-BISPHOSPHATE ALDOLASE 3, CHLOROPLASTIC-RELATED (1 of 1)
OG IDs: 93518768
Best-hit-arabi-ID: AT2G01140.1
Best-hit-arabi-name: Aldolase superfamily protein
Best-hit-arabi-symbol: nan
Gorai.009G342900.1.A
PCC=-0.49, p=0.033
mRNA Profile
Protein Expression Profile
DPA

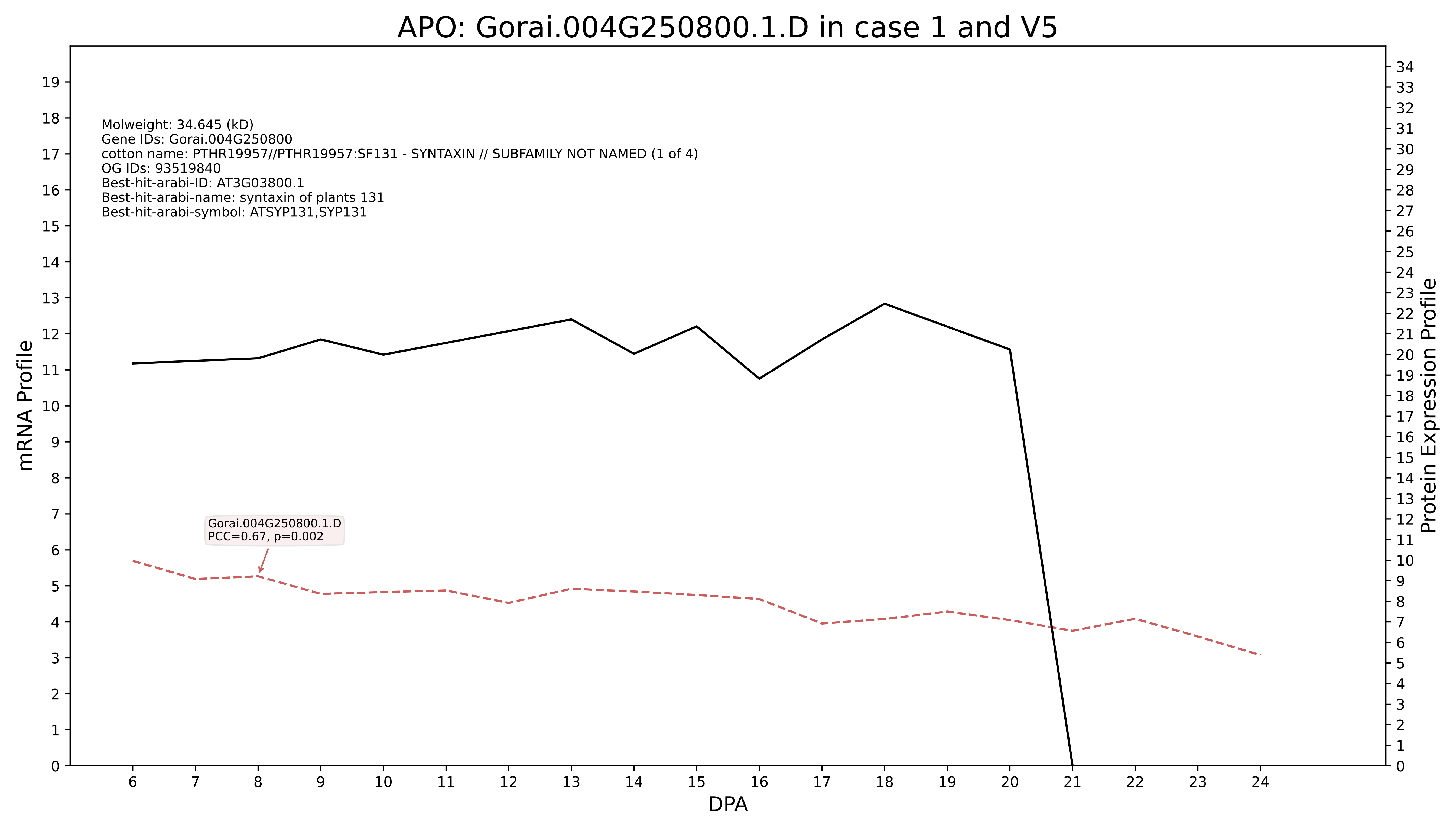
APO: Gorai.004G250800.1.D in case 1 and V5
Molweight: 34.645 (kD)
Gene IDs: Gorai.004G250800
cotton name: PTHR19957//PTHR19957:SF131 - SYNTAXIN // SUBFAMILY NOT NAMED (1 of 4)
OG IDs: 93519840
Best-hit-arabi-ID: AT3G03800.1
Best-hit-arabi-name: syntaxin of plants 131
Best-hit-arabi-symbol: ATSYP131,SYP131
Gorai.004G250800.1.D
PCC=0.67, p=0.002
mRNA Profile
Protein Expression Profile
DPA

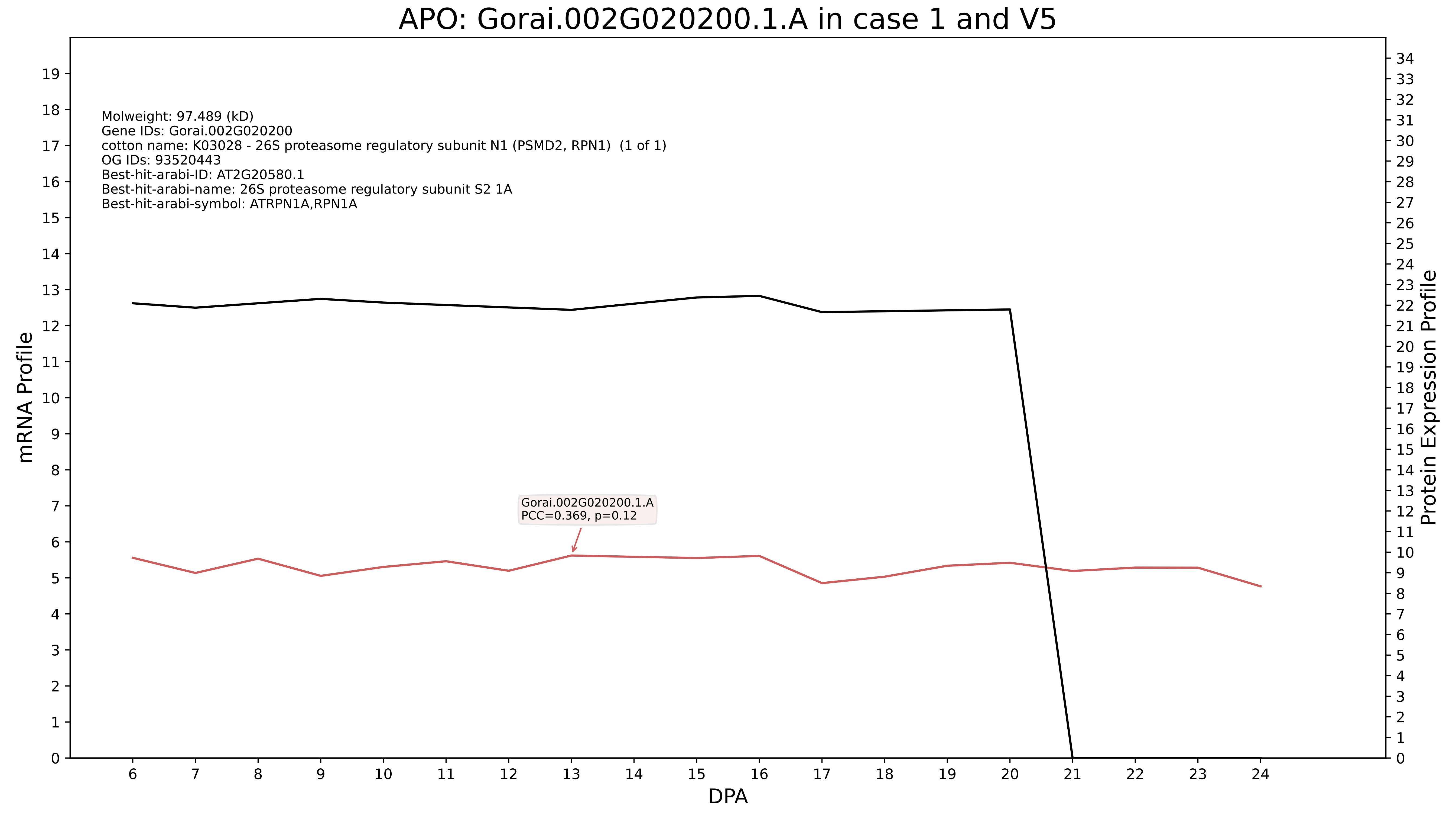
APO: Gorai.002G020200.1.A in case 1 and V5
Molweight: 97.489 (kD)
Gene IDs: Gorai.002G020200
cotton name: K03028 - 26S proteasome regulatory subunit N1 (PSMD2, RPN1) (1 of 1)
OG IDs: 93520443
Best-hit-arabi-ID: AT2G20580.1
Best-hit-arabi-name: 26S proteasome regulatory subunit S2 1A
Best-hit-arabi-symbol: ATRPN1A,RPN1A
Gorai.002G020200.1.A
PCC=0.369, p=0.12
mRNA Profile
Protein Expression Profile
DPA

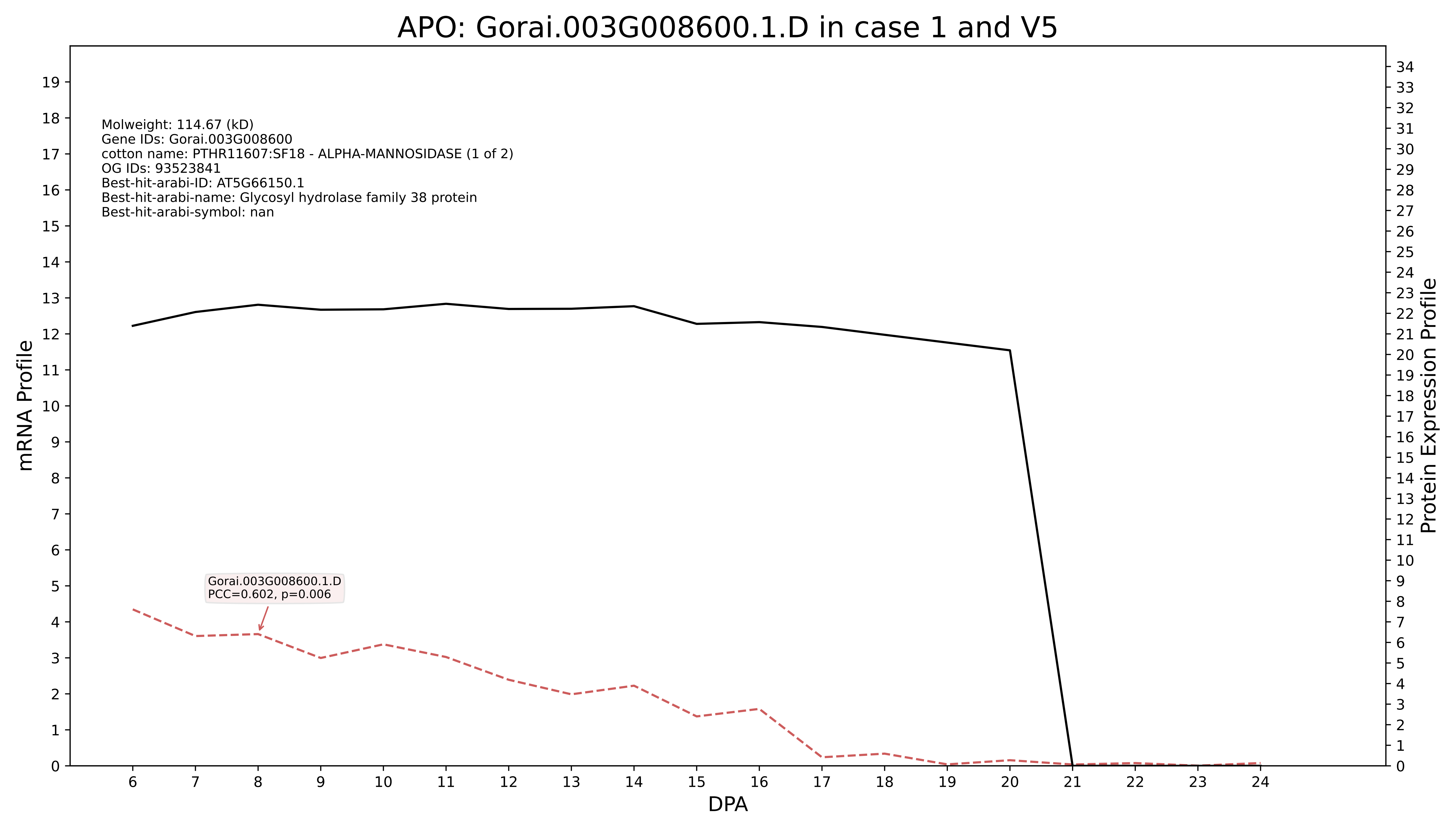
APO: Gorai.003G008600.1.D in case 1 and V5
Molweight: 114.67 (kD)
Gene IDs: Gorai.003G008600
cotton name: PTHR11607:SF18 - ALPHA-MANNOSIDASE (1 of 2)
OG IDs: 93523841
Best-hit-arabi-ID: AT5G66150.1
Best-hit-arabi-name: Glycosyl hydrolase family 38 protein
Best-hit-arabi-symbol: nan
Gorai.003G008600.1.D
PCC=0.602, p=0.006
mRNA Profile
Protein Expression Profile
DPA

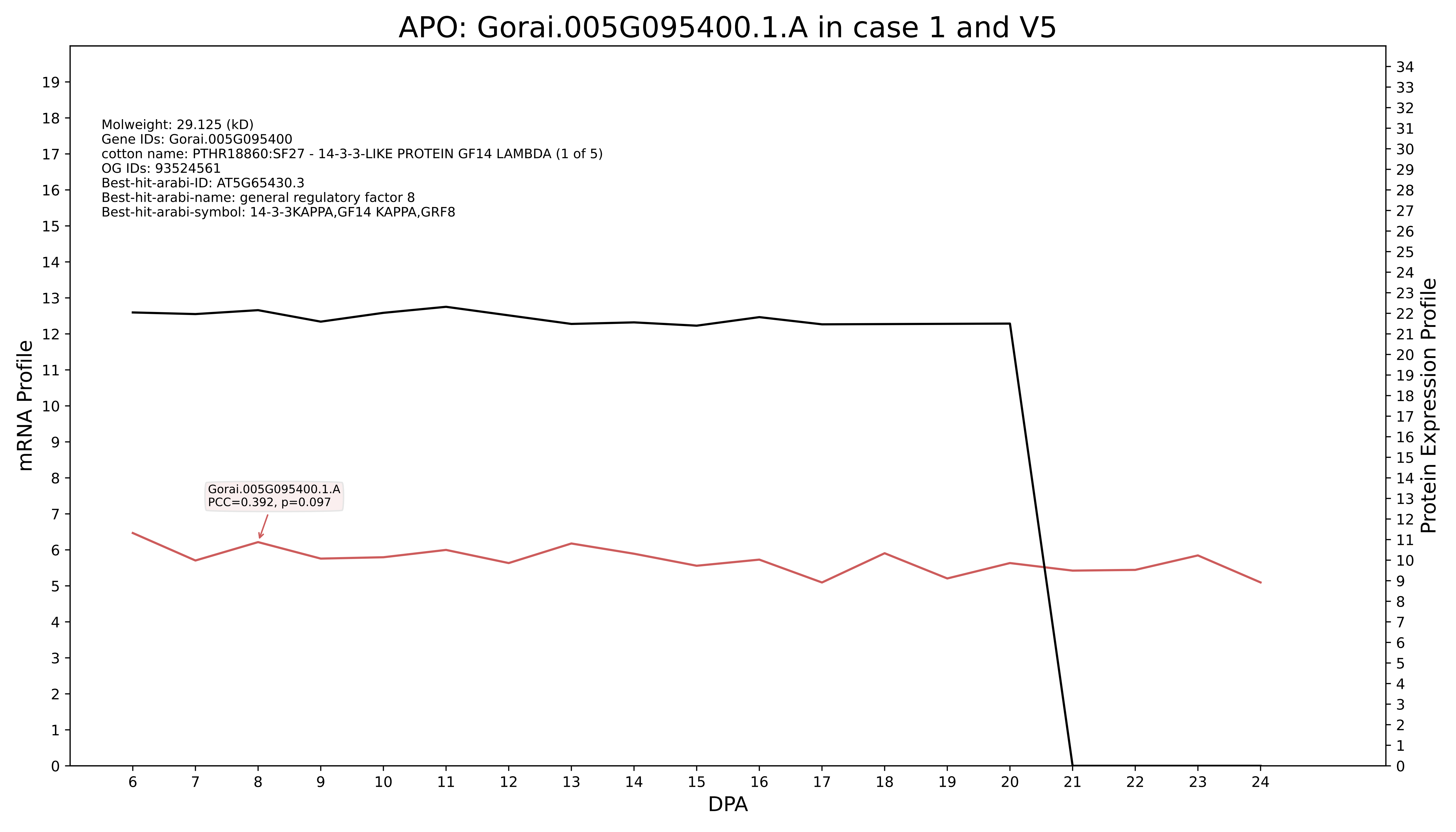
APO: Gorai.005G095400.1.A in case 1 and V5
Molweight: 29.125 (kD)
Gene IDs: Gorai.005G095400
cotton name: PTHR18860:SF27 - 14-3-3-LIKE PROTEIN GF14 LAMBDA (1 of 5)
OG IDs: 93524561
Best-hit-arabi-ID: AT5G65430.3
Best-hit-arabi-name: general regulatory factor 8
Best-hit-arabi-symbol: 14-3-3KAPPA,GF14 KAPPA,GRF8
Gorai.005G095400.1.A
PCC=0.392, p=0.097
mRNA Profile
Protein Expression Profile
DPA

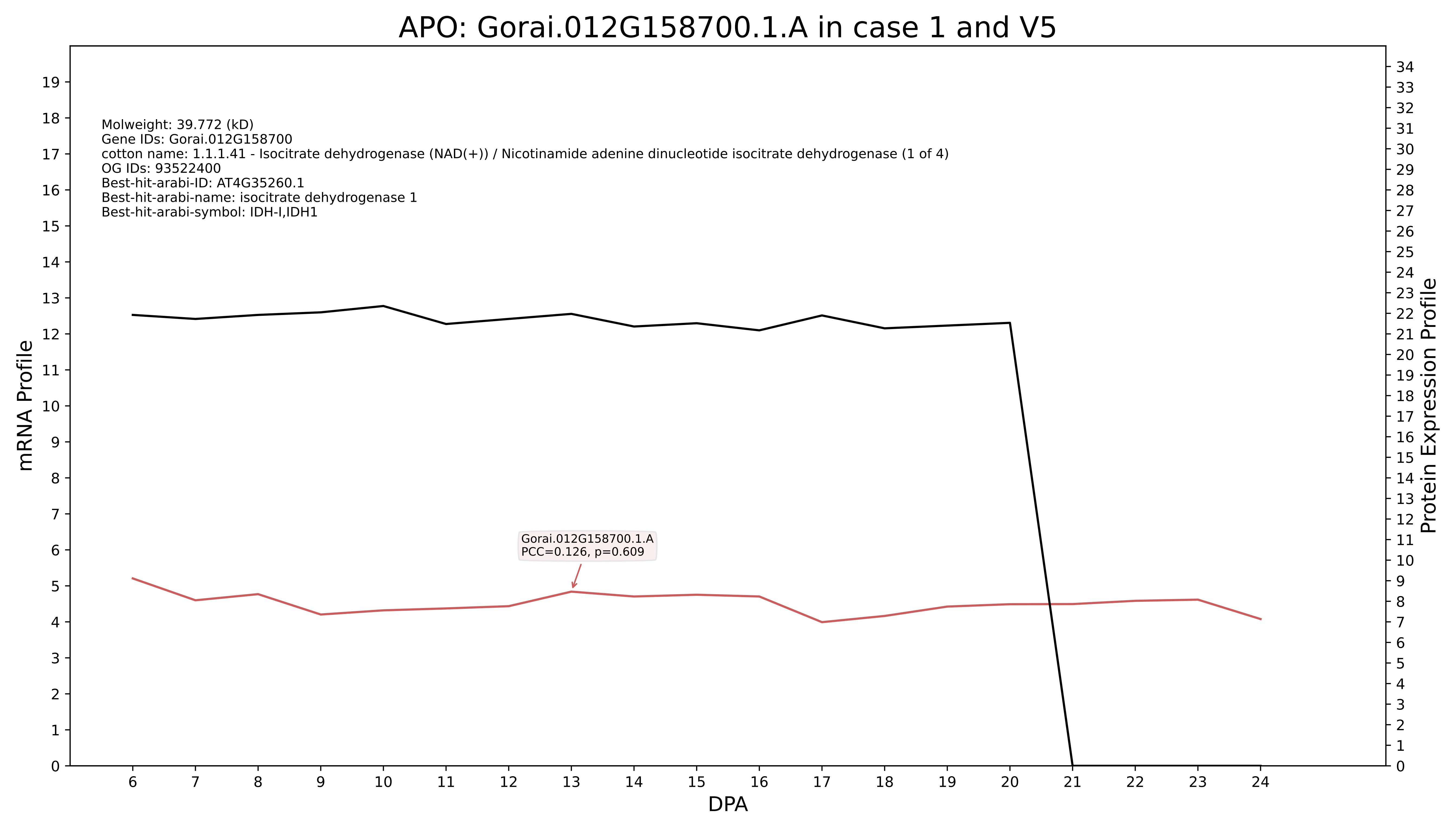

APO: Gorai.012G158700.1.A in case 1 and V5
Molweight: 39.772 (kD)
Gene IDs: Gorai.012G158700
cotton name: 1.1.1.41 - Isocitrate dehydrogenase (NAD(+)) / Nicotinamide adenine dinucleotide isocitrate dehydrogenase (1 of 4)
OG IDs: 93522400
Best-hit-arabi-ID: AT4G35260.1
Best-hit-arabi-name: isocitrate dehydrogenase 1
Best-hit-arabi-symbol: IDH-I,IDH1
Gorai.012G158700.1.A
PCC=0.126, p=0.609
mRNA Profile
Protein Expression Profile
DPA

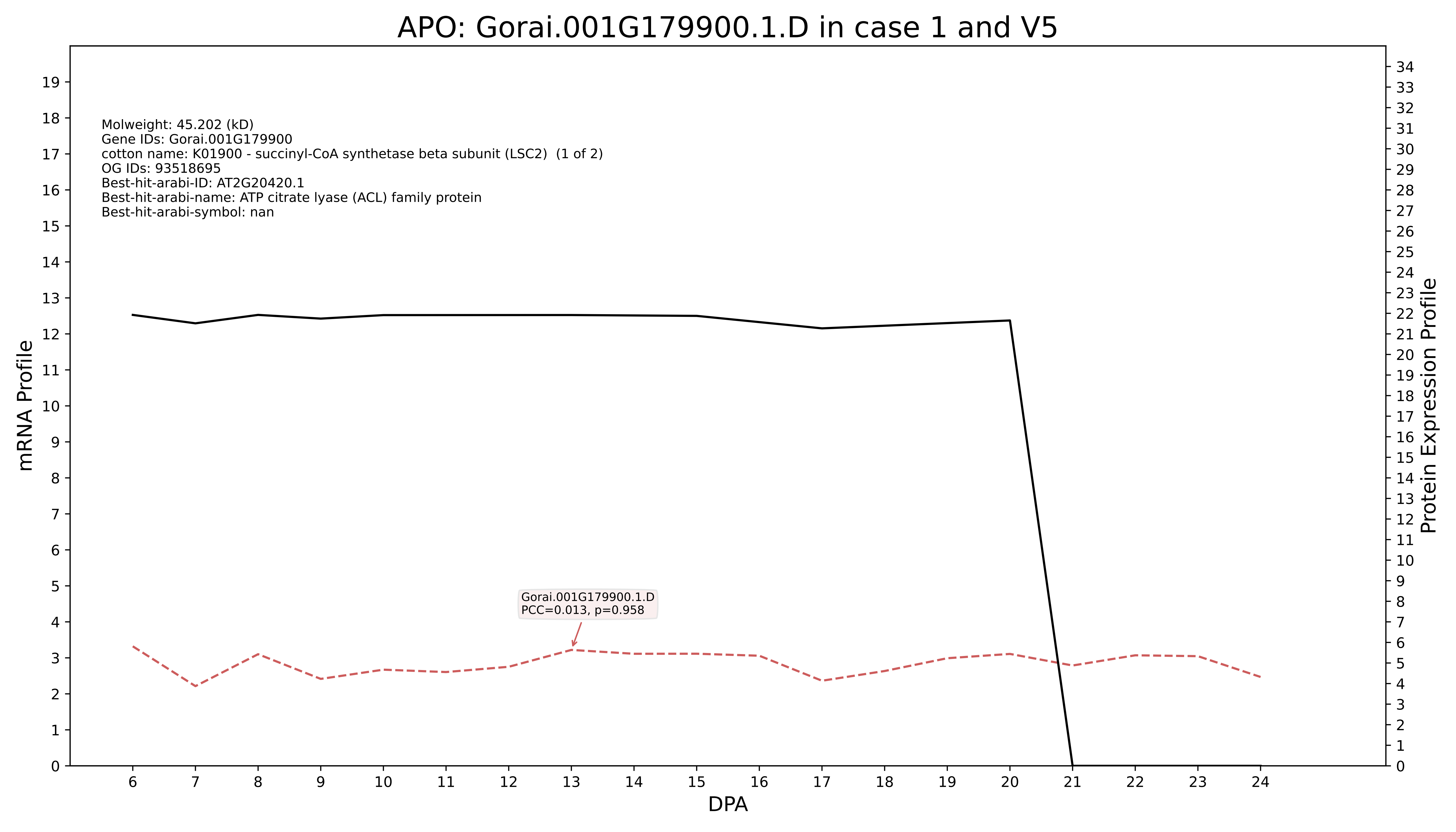

APO: Gorai.001G179900.1.D in case 1 and V5
Molweight: 45.202 (kD)
Gene IDs: Gorai.001G179900
cotton name: K01900 - succinyl-CoA synthetase beta subunit (LSC2) (1 of 2)
OG IDs: 93518695
Best-hit-arabi-ID: AT2G20420.1
Best-hit-arabi-name: ATP citrate lyase (ACL) family protein
Best-hit-arabi-symbol: nan
Gorai.001G179900.1.D
PCC=0.013, p=0.958
mRNA Profile
Protein Expression Profile
DPA

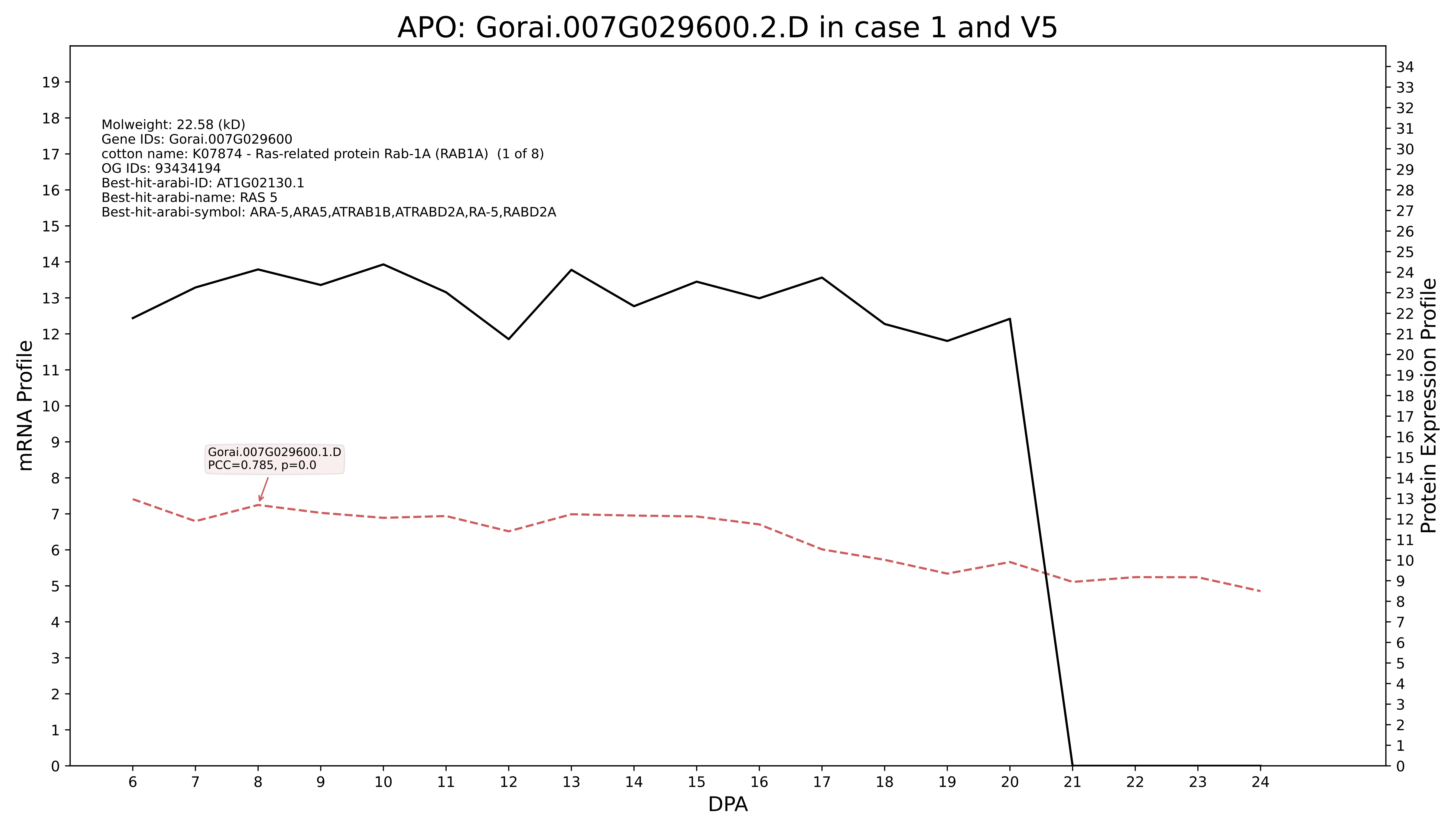

APO: Gorai.007G029600.2.D in case 1 and V5
Molweight: 22.58 (kD)
Gene IDs: Gorai.007G029600
cotton name: K07874 - Ras-related protein Rab-1A (RAB1A) (1 of 8)
OG IDs: 93434194
Best-hit-arabi-ID: AT1G02130.1
Best-hit-arabi-name: RAS 5
Best-hit-arabi-symbol: ARA-5,ARA5,ATRAB1B,ATRABD2A,RA-5,RABD2A
Gorai.007G029600.1.D
PCC=0.785, p=0.0
mRNA Profile
Protein Expression Profile
DPA

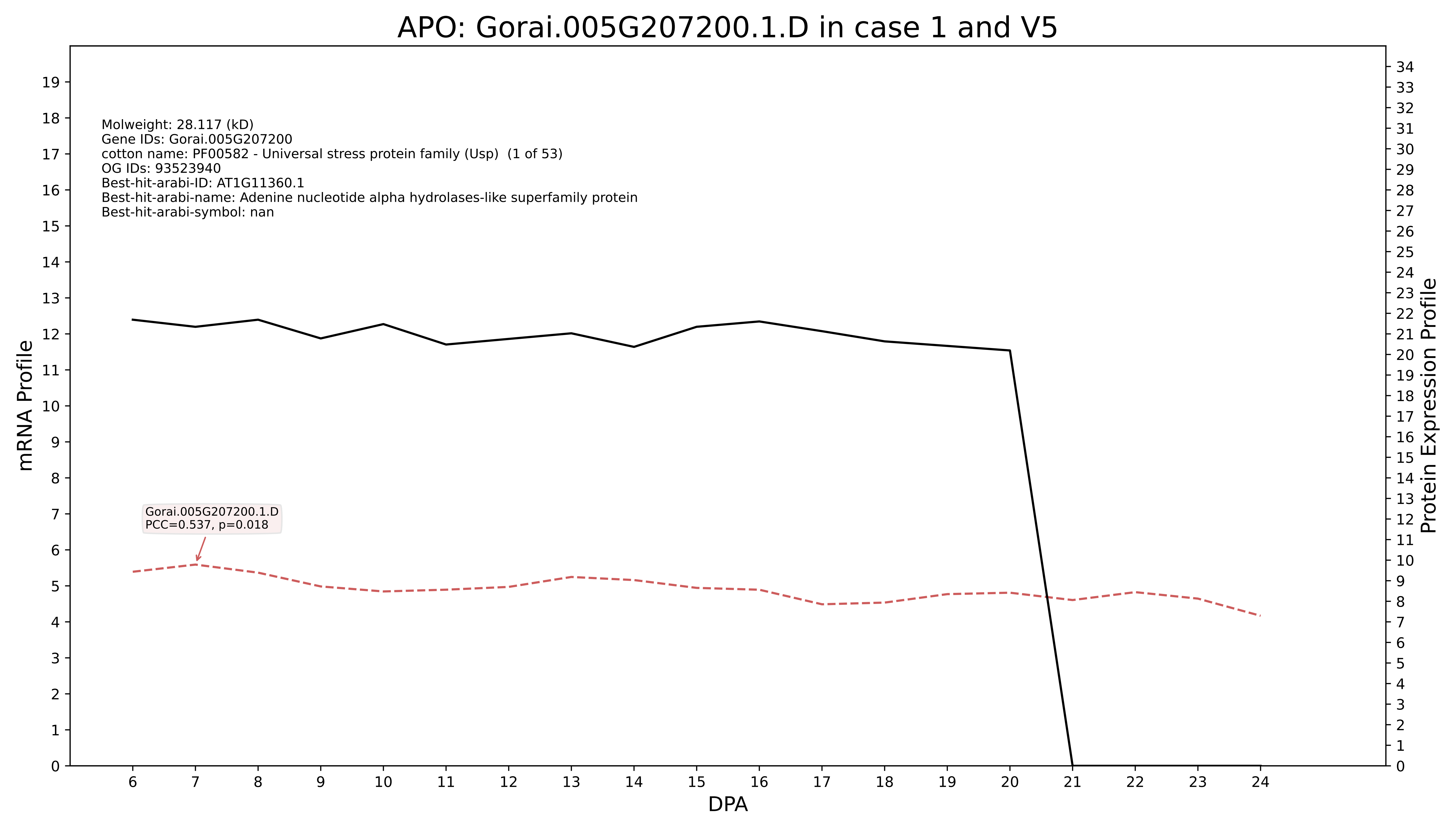

APO: Gorai.005G207200.1.D in case 1 and V5
Molweight: 28.117 (kD)
Gene IDs: Gorai.005G207200
cotton name: PF00582 - Universal stress protein family (Usp) (1 of 53)
OG IDs: 93523940
Best-hit-arabi-ID: AT1G11360.1
Best-hit-arabi-name: Adenine nucleotide alpha hydrolases-like superfamily protein
Best-hit-arabi-symbol: nan
Gorai.005G207200.1.D
PCC=0.537, p=0.018
mRNA Profile
Protein Expression Profile
DPA

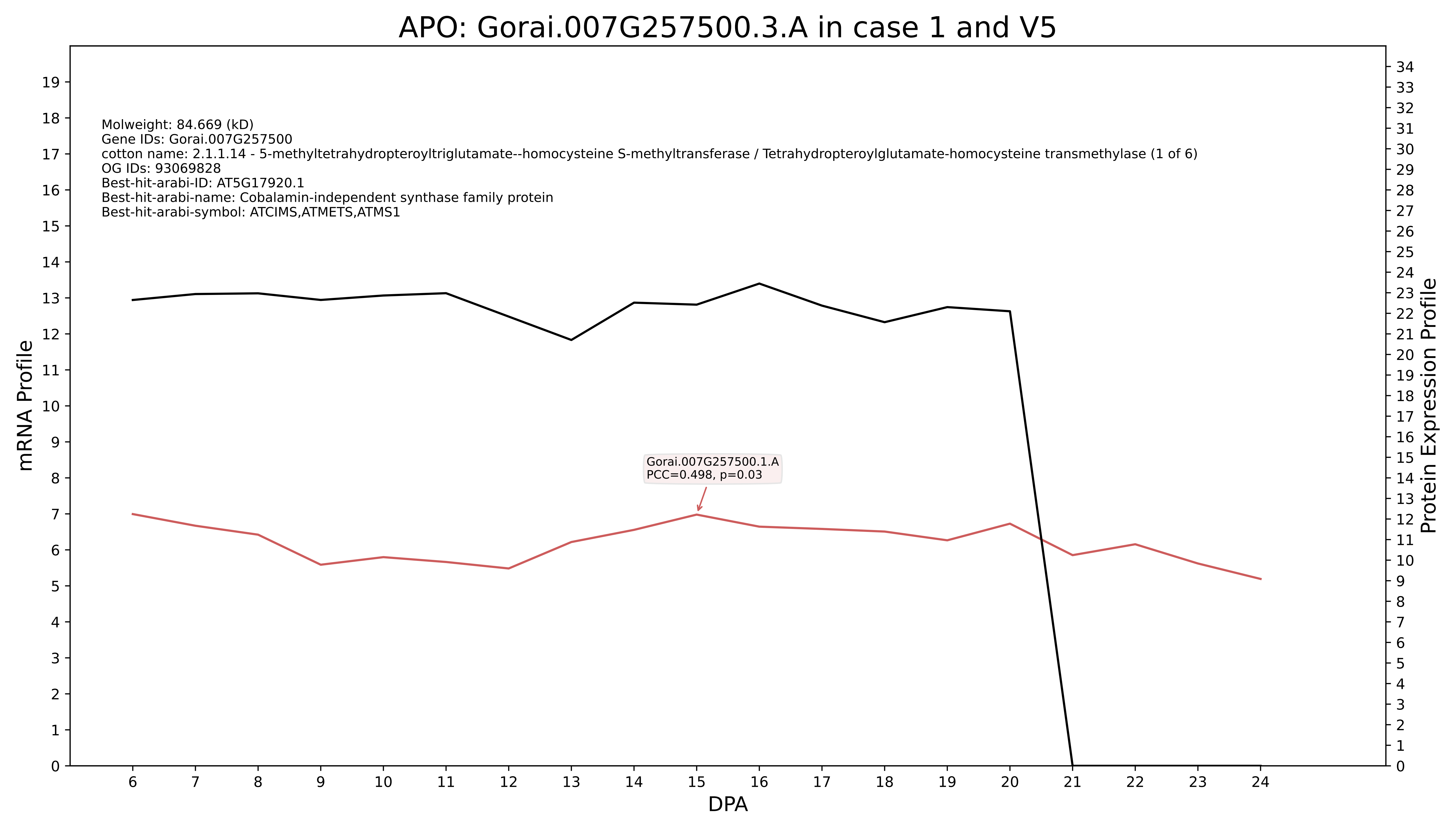
APO: Gorai.007G257500.3.A in case 1 and V5
Molweight: 84.669 (kD)
Gene IDs: Gorai.007G257500
cotton name: 2.1.1.14 - 5-methyltetrahydropteroyltriglutamate--homocysteine S-methyltransferase / Tetrahydropteroylglutamate-homocysteine transmethylase (1 of 6)
OG IDs: 93069828
Best-hit-arabi-ID: AT5G17920.1
Best-hit-arabi-name: Cobalamin-independent synthase family protein
Best-hit-arabi-symbol: ATCIMS,ATMETS,ATMS1
Gorai.007G257500.1.A
PCC=0.498, p=0.03
mRNA Profile
Protein Expression Profile
DPA

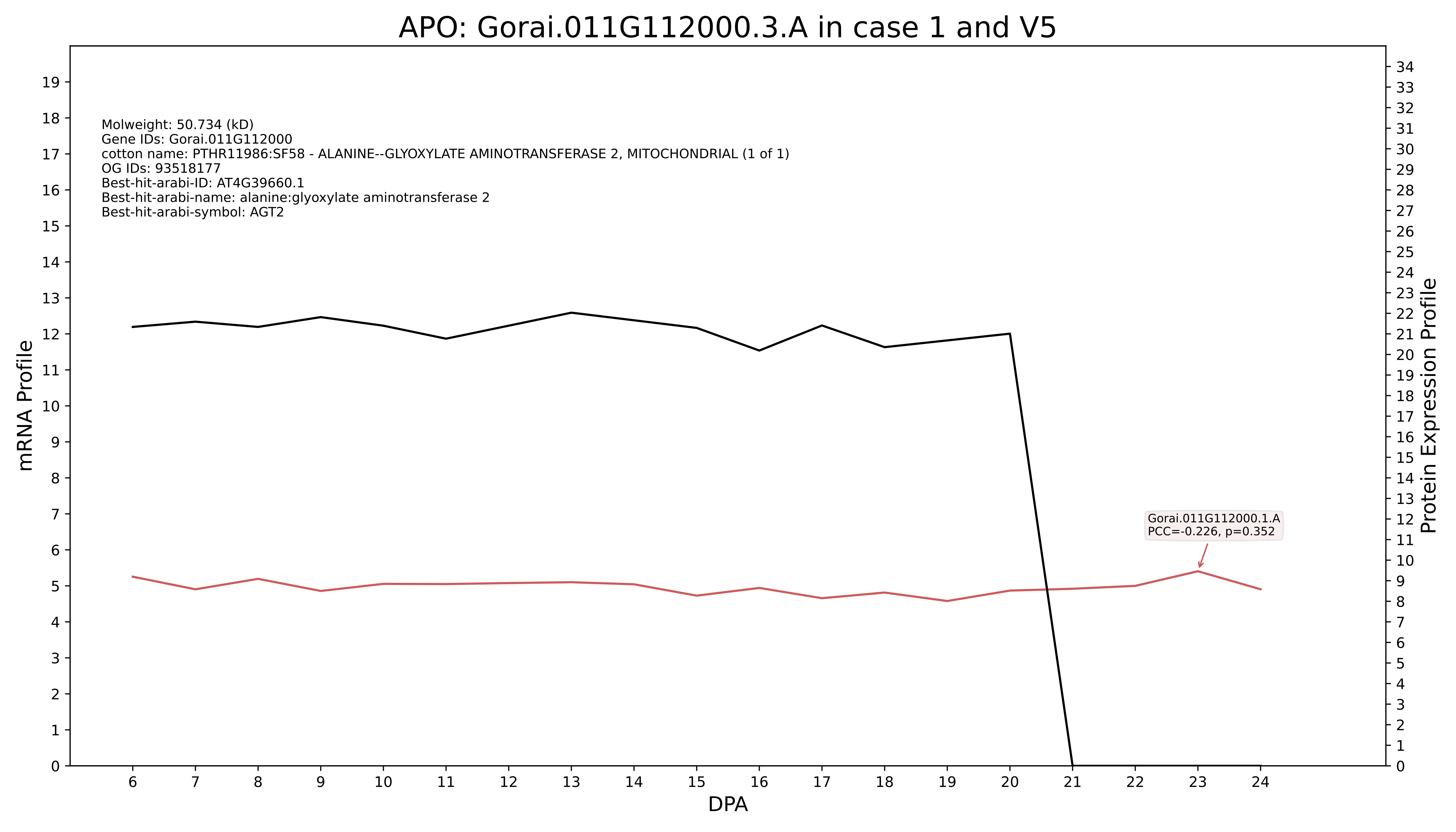
APO: Gorai.011G112000.3.A in case 1 and V5
Molweight: 50.734 (kD)
Gene IDs: Gorai.011G112000
cotton name: PTHR11986:SF58 - ALANINE--GLYOXYLATE AMINOTRANSFERASE 2, MITOCHONDRIAL (1 of 1)
OG IDs: 93518177
Best-hit-arabi-ID: AT4G39660.1
Best-hit-arabi-name: alanine:glyoxylate aminotransferase 2
Best-hit-arabi-symbol: AGT2
Gorai.011G112000.1.A
PCC=-0.226, p=0.352
mRNA Profile
Protein Expression Profile
DPA

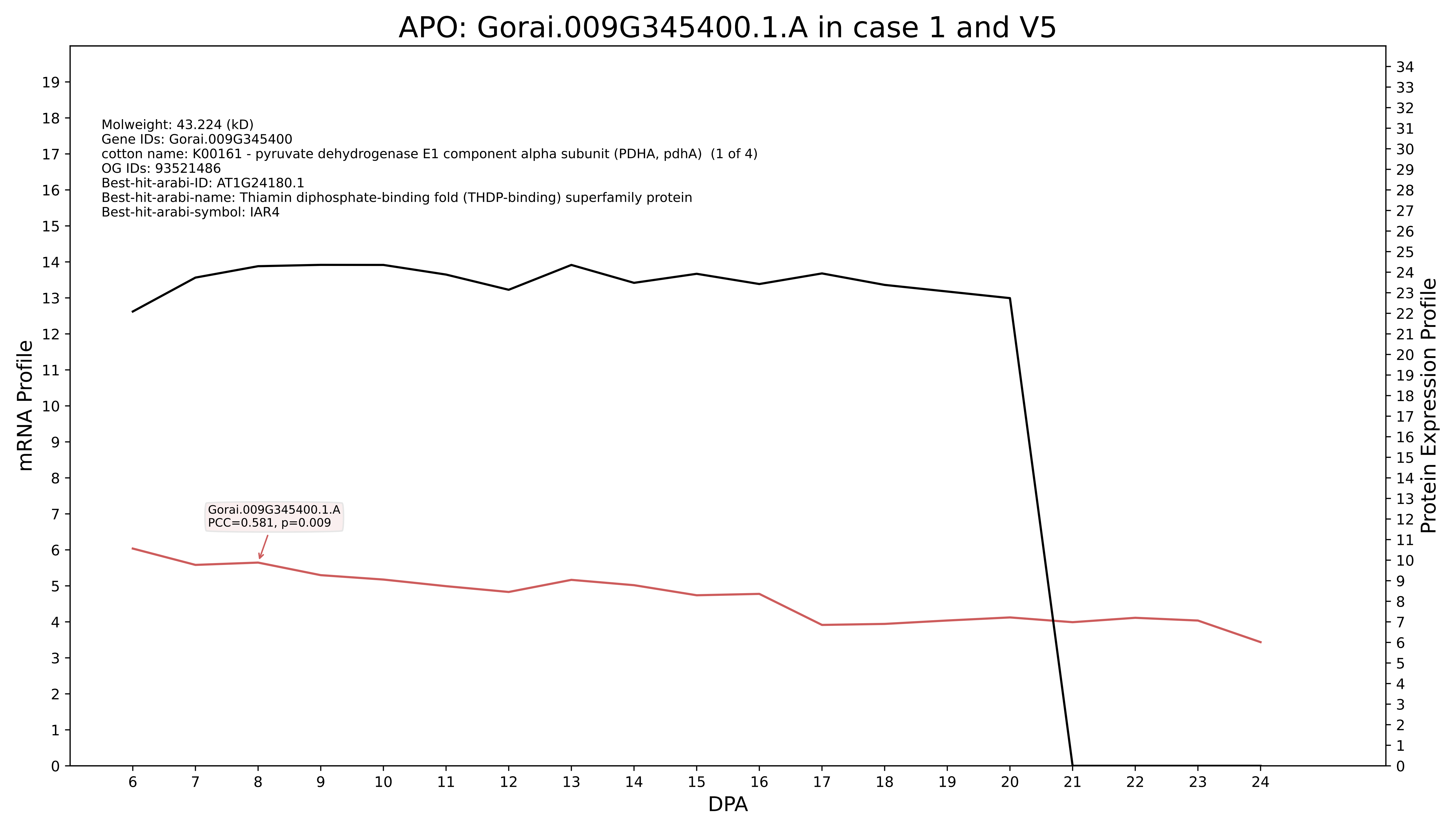
APO: Gorai.009G345400.1.A in case 1 and V5
Molweight: 43.224 (kD)
Gene IDs: Gorai.009G345400
cotton name: K00161 - pyruvate dehydrogenase E1 component alpha subunit (PDHA, pdhA) (1 of 4)
OG IDs: 93521486
Best-hit-arabi-ID: AT1G24180.1
Best-hit-arabi-name: Thiamin diphosphate-binding fold (THDP-binding) superfamily protein
Best-hit-arabi-symbol: IAR4
Gorai.009G345400.1.A
PCC=0.581, p=0.009
mRNA Profile
Protein Expression Profile
DPA

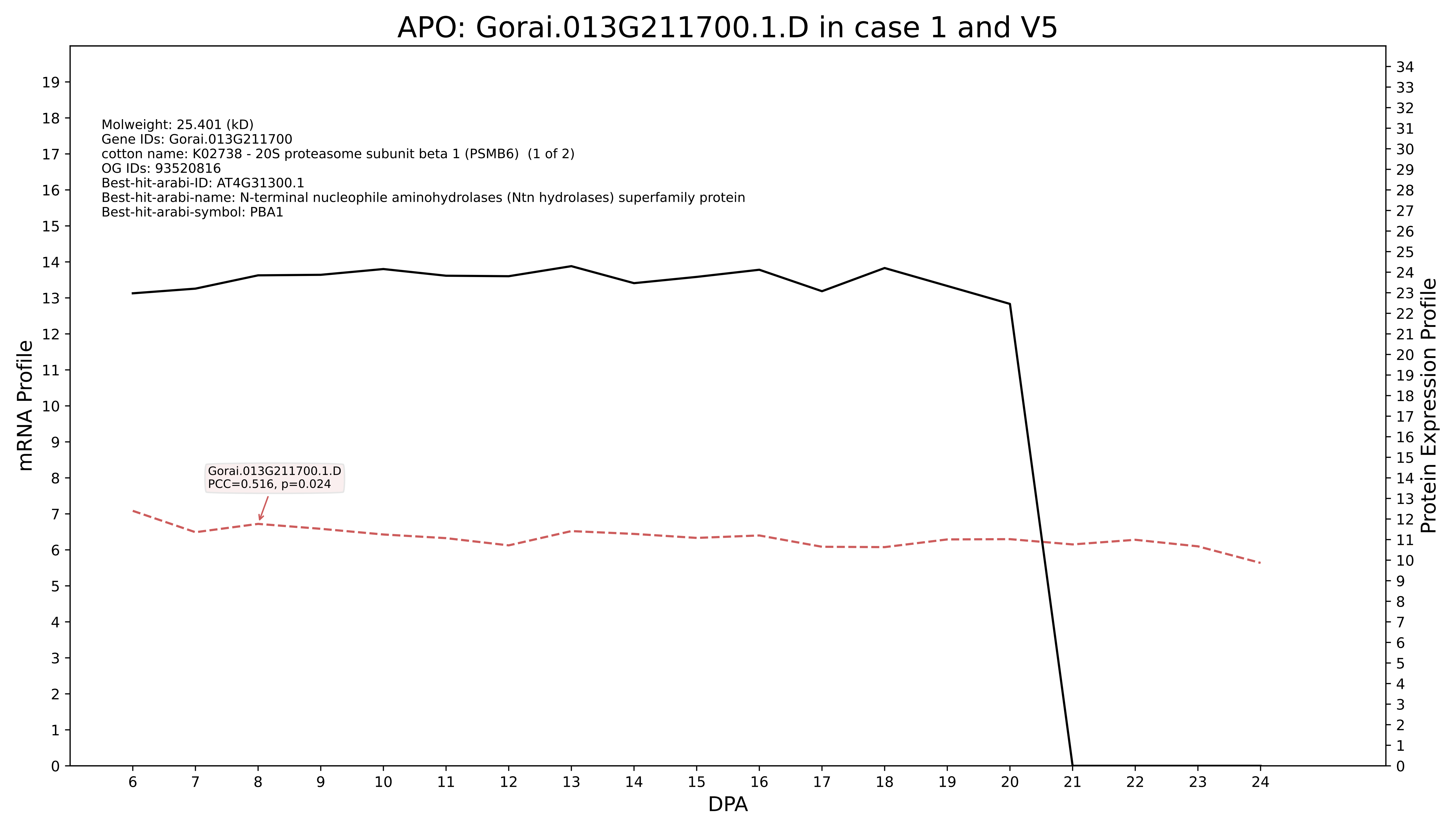
APO: Gorai.013G211700.1.D in case 1 and V5
Molweight: 25.401 (kD)
Gene IDs: Gorai.013G211700
cotton name: K02738 - 20S proteasome subunit beta 1 (PSMB6) (1 of 2)
OG IDs: 93520816
Best-hit-arabi-ID: AT4G31300.1
Best-hit-arabi-name: N-terminal nucleophile aminohydrolases (Ntn hydrolases) superfamily protein
Best-hit-arabi-symbol: PBA1
Gorai.013G211700.1.D
PCC=0.516, p=0.024
mRNA Profile
Protein Expression Profile
DPA

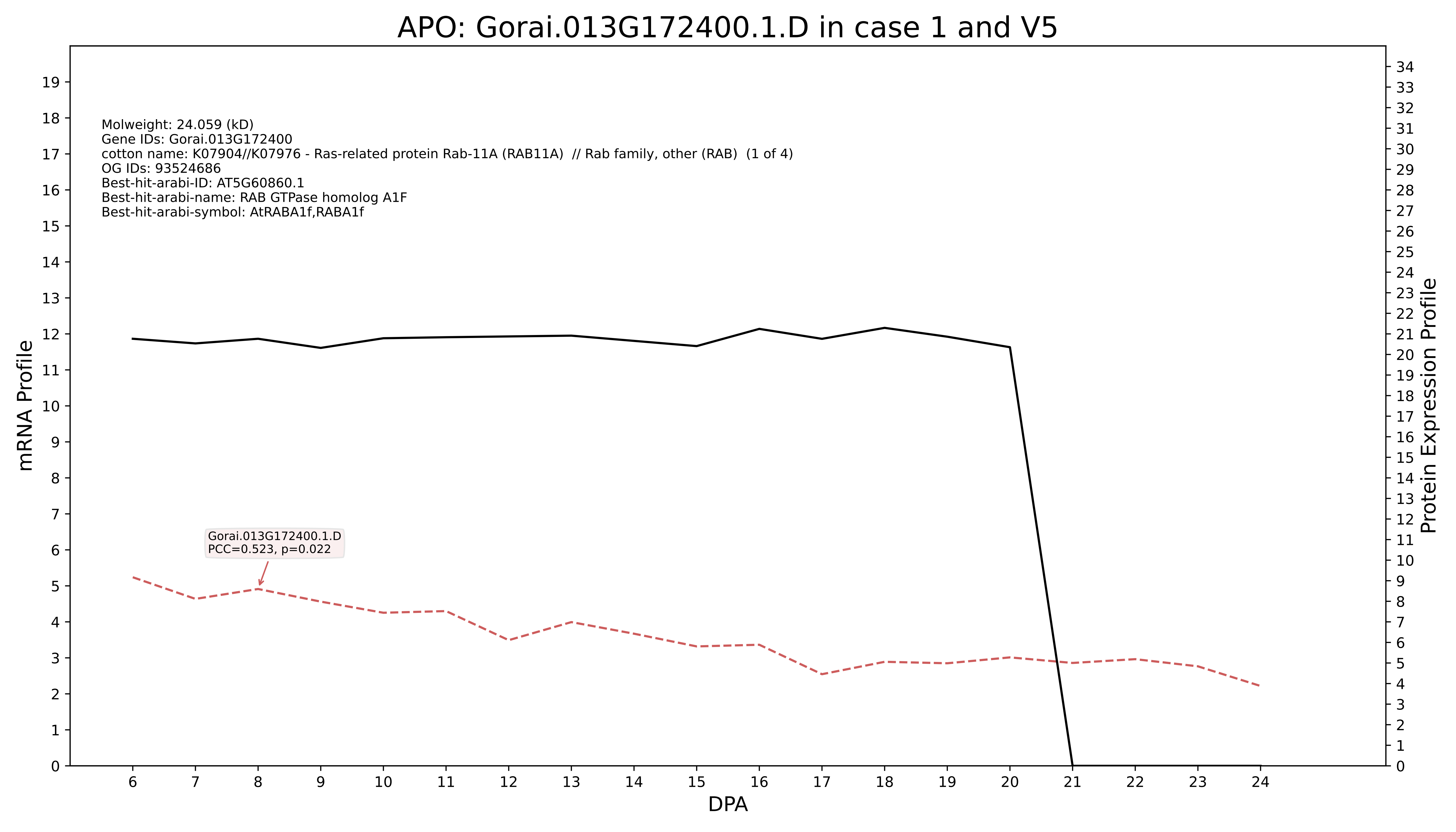
APO: Gorai.013G172400.1.D in case 1 and V5
Molweight: 24.059 (kD)
Gene IDs: Gorai.013G172400
cotton name: K07904//K07976 - Ras-related protein Rab-11A (RAB11A) // Rab family, other (RAB) (1 of 4)
OG IDs: 93524686
Best-hit-arabi-ID: AT5G60860.1
Best-hit-arabi-name: RAB GTPase homolog A1F
Best-hit-arabi-symbol: AtRABA1f,RABA1f
Gorai.013G172400.1.D
PCC=0.523, p=0.022
mRNA Profile
Protein Expression Profile
DPA

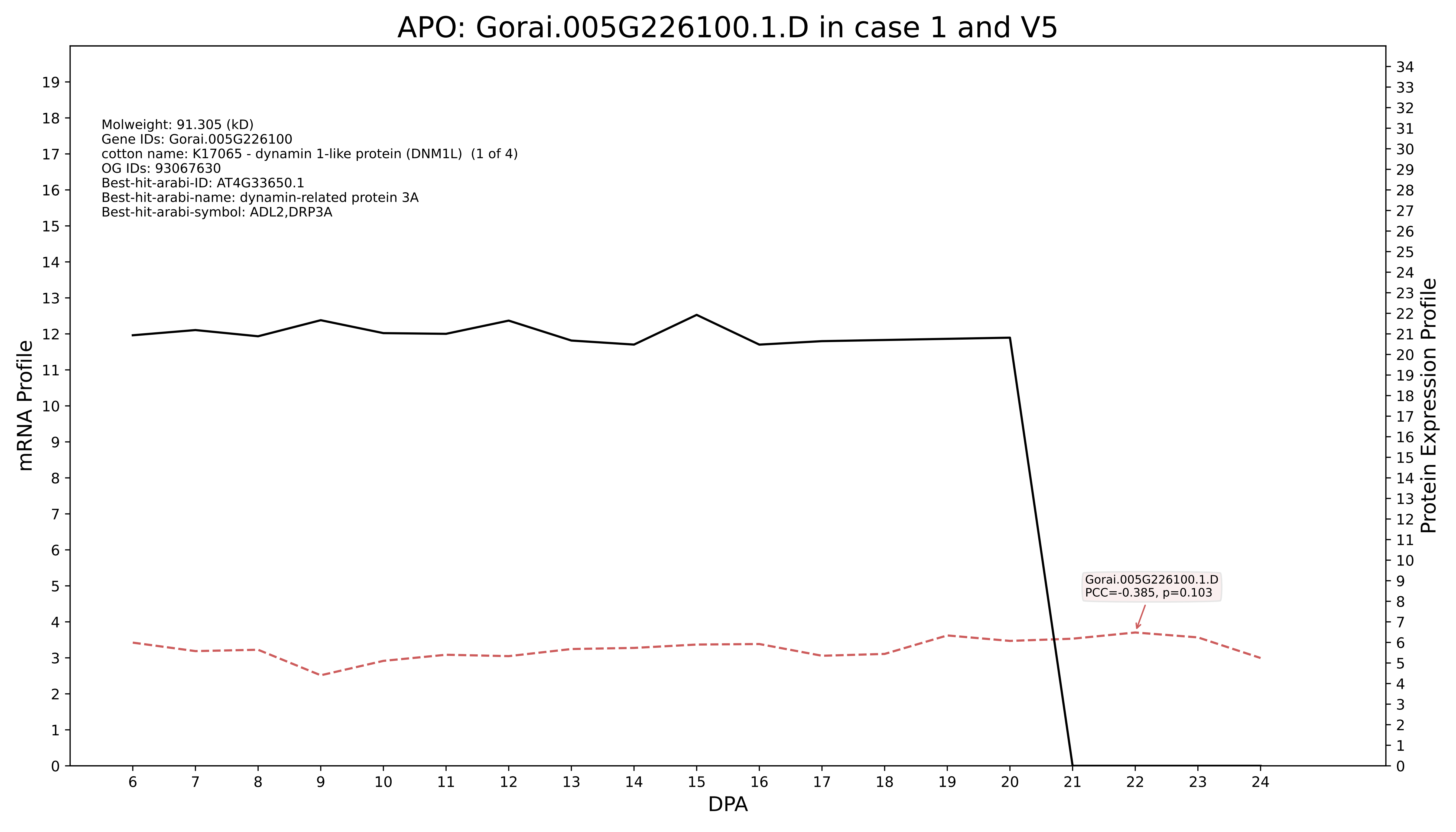

APO: Gorai.005G226100.1.D in case 1 and V5
Molweight: 91.305 (kD)
Gene IDs: Gorai.005G226100
cotton name: K17065 - dynamin 1-like protein (DNM1L) (1 of 4)
OG IDs: 93067630
Best-hit-arabi-ID: AT4G33650.1
Best-hit-arabi-name: dynamin-related protein 3A
Best-hit-arabi-symbol: ADL2,DRP3A
Gorai.005G226100.1.D
PCC=-0.385, p=0.103
mRNA Profile
Protein Expression Profile
DPA

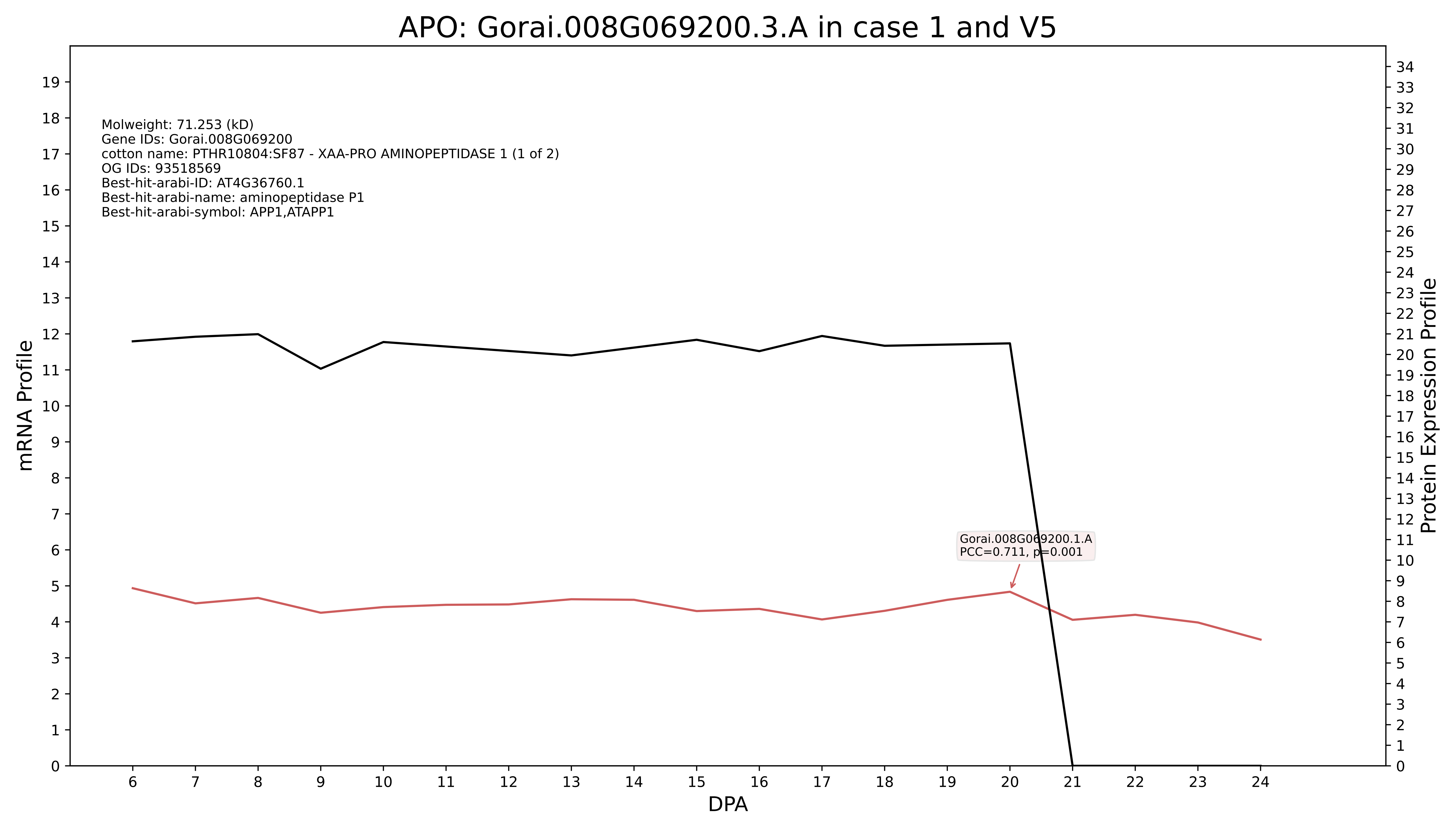

APO: Gorai.008G069200.3.A in case 1 and V5
Molweight: 71.253 (kD)
Gene IDs: Gorai.008G069200
cotton name: PTHR10804:SF87 - XAA-PRO AMINOPEPTIDASE 1 (1 of 2)
OG IDs: 93518569
Best-hit-arabi-ID: AT4G36760.1
Best-hit-arabi-name: aminopeptidase P1
Best-hit-arabi-symbol: APP1,ATAPP1
Gorai.008G069200.1.A
PCC=0.711, p=0.001
mRNA Profile
Protein Expression Profile
DPA

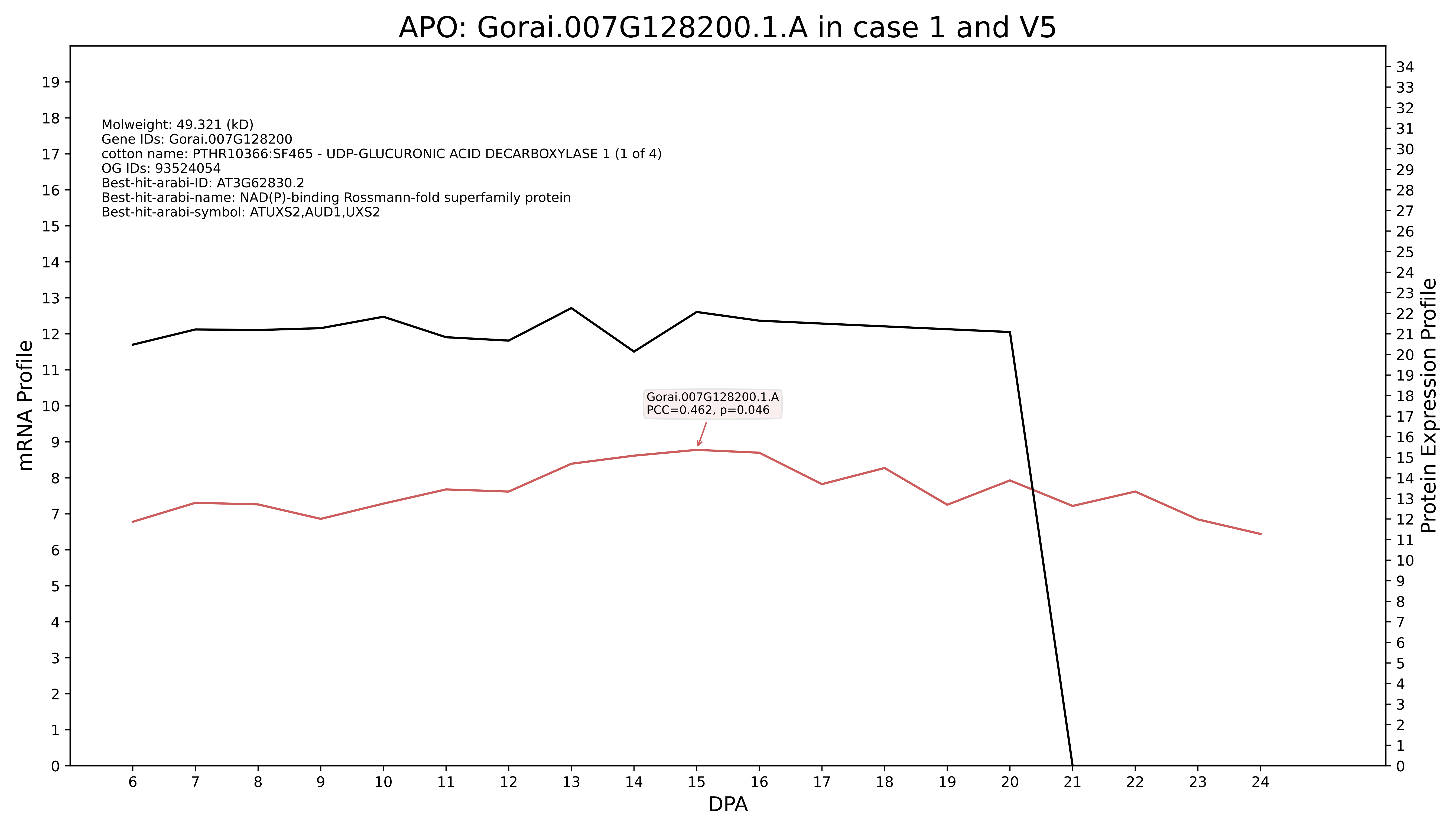
APO: Gorai.007G128200.1.A in case 1 and V5
Molweight: 49.321 (kD)
Gene IDs: Gorai.007G128200
cotton name: PTHR10366:SF465 - UDP-GLUCURONIC ACID DECARBOXYLASE 1 (1 of 4)
OG IDs: 93524054
Best-hit-arabi-ID: AT3G62830.2
Best-hit-arabi-name: NAD(P)-binding Rossmann-fold superfamily protein
Best-hit-arabi-symbol: ATUXS2,AUD1,UXS2
Gorai.007G128200.1.A
PCC=0.462, p=0.046
mRNA Profile
Protein Expression Profile
DPA

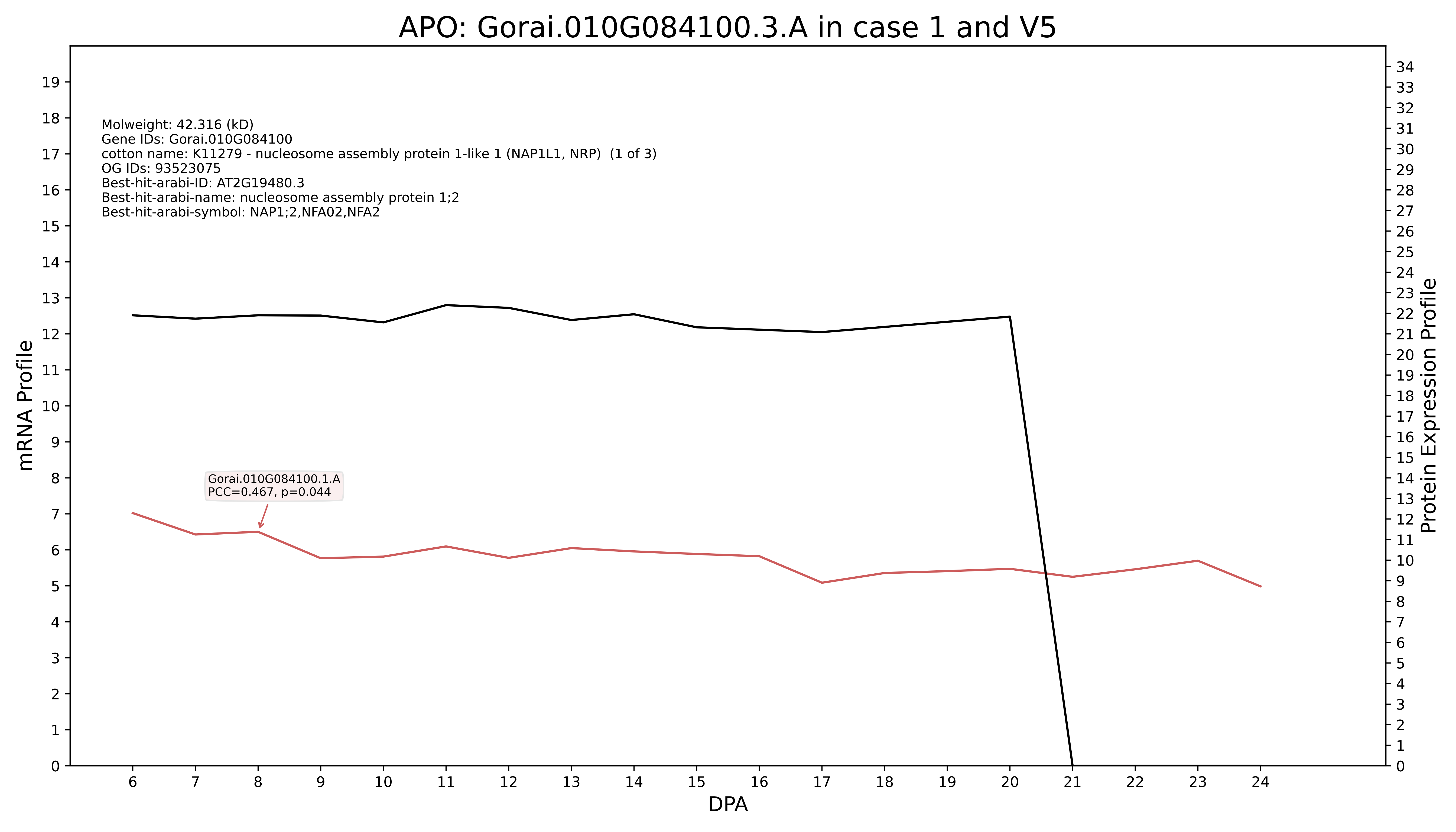
APO: Gorai.010G084100.3.A in case 1 and V5
Molweight: 42.316 (kD)
Gene IDs: Gorai.010G084100
cotton name: K11279 - nucleosome assembly protein 1-like 1 (NAP1L1, NRP) (1 of 3)
OG IDs: 93523075
Best-hit-arabi-ID: AT2G19480.3
Best-hit-arabi-name: nucleosome assembly protein 1;2
Best-hit-arabi-symbol: NAP1;2,NFA02,NFA2
Gorai.010G084100.1.A
PCC=0.467, p=0.044
mRNA Profile
Protein Expression Profile
DPA
0
1
2
3
4
5
6
7
8
9
10
11
12
13
14
15
16
17
18
19
20
21
22
23
24
25
26
27
28
29
30
31
32
33
34

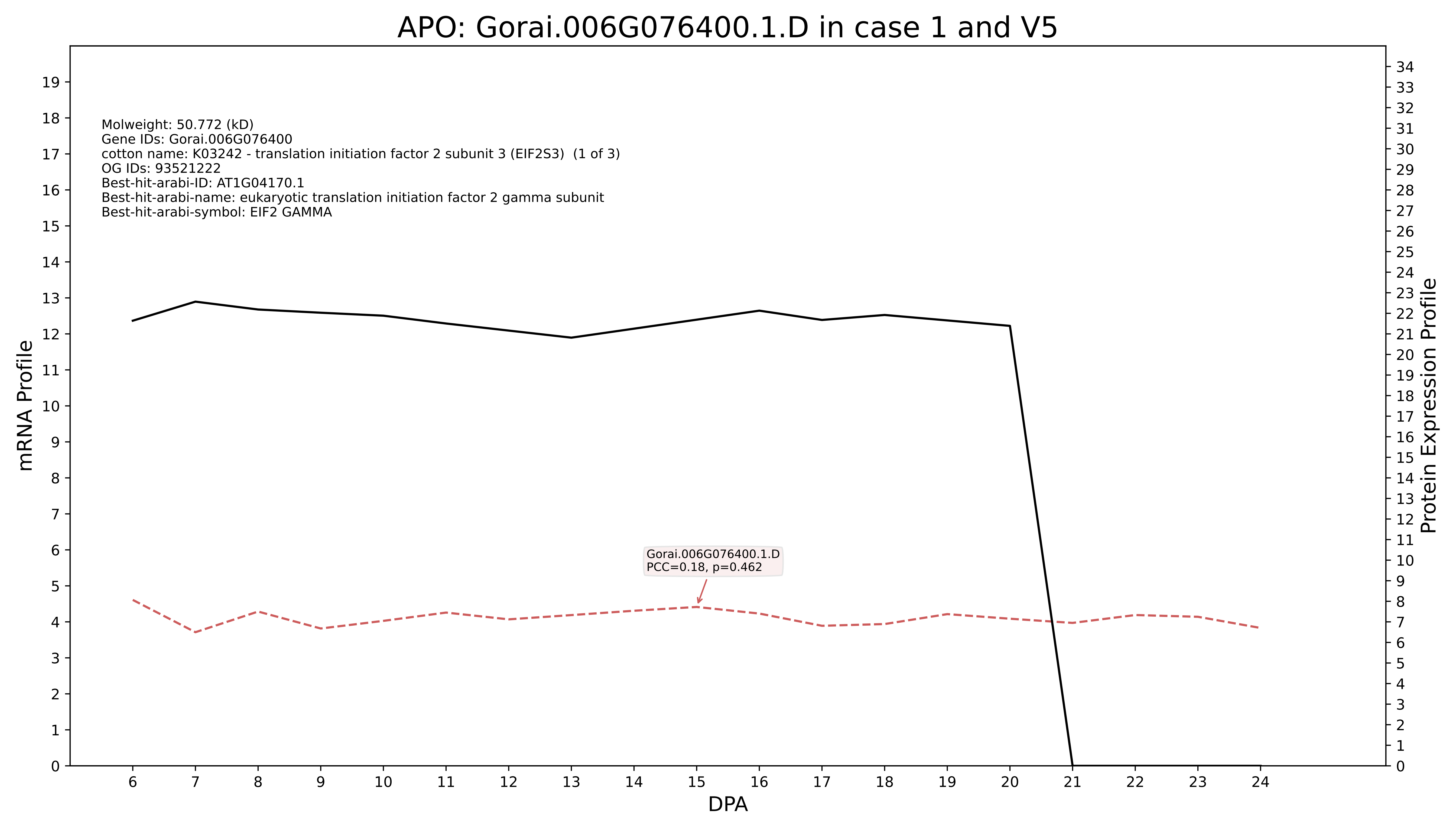

APO: Gorai.006G076400.1.D in case 1 and V5
Molweight: 50.772 (kD)
Gene IDs: Gorai.006G076400
cotton name: K03242 - translation initiation factor 2 subunit 3 (EIF2S3) (1 of 3)
OG IDs: 93521222
Best-hit-arabi-ID: AT1G04170.1
Best-hit-arabi-name: eukaryotic translation initiation factor 2 gamma subunit
Best-hit-arabi-symbol: EIF2 GAMMA
Gorai.006G076400.1.D
PCC=0.18, p=0.462
mRNA Profile
Protein Expression Profile
DPA

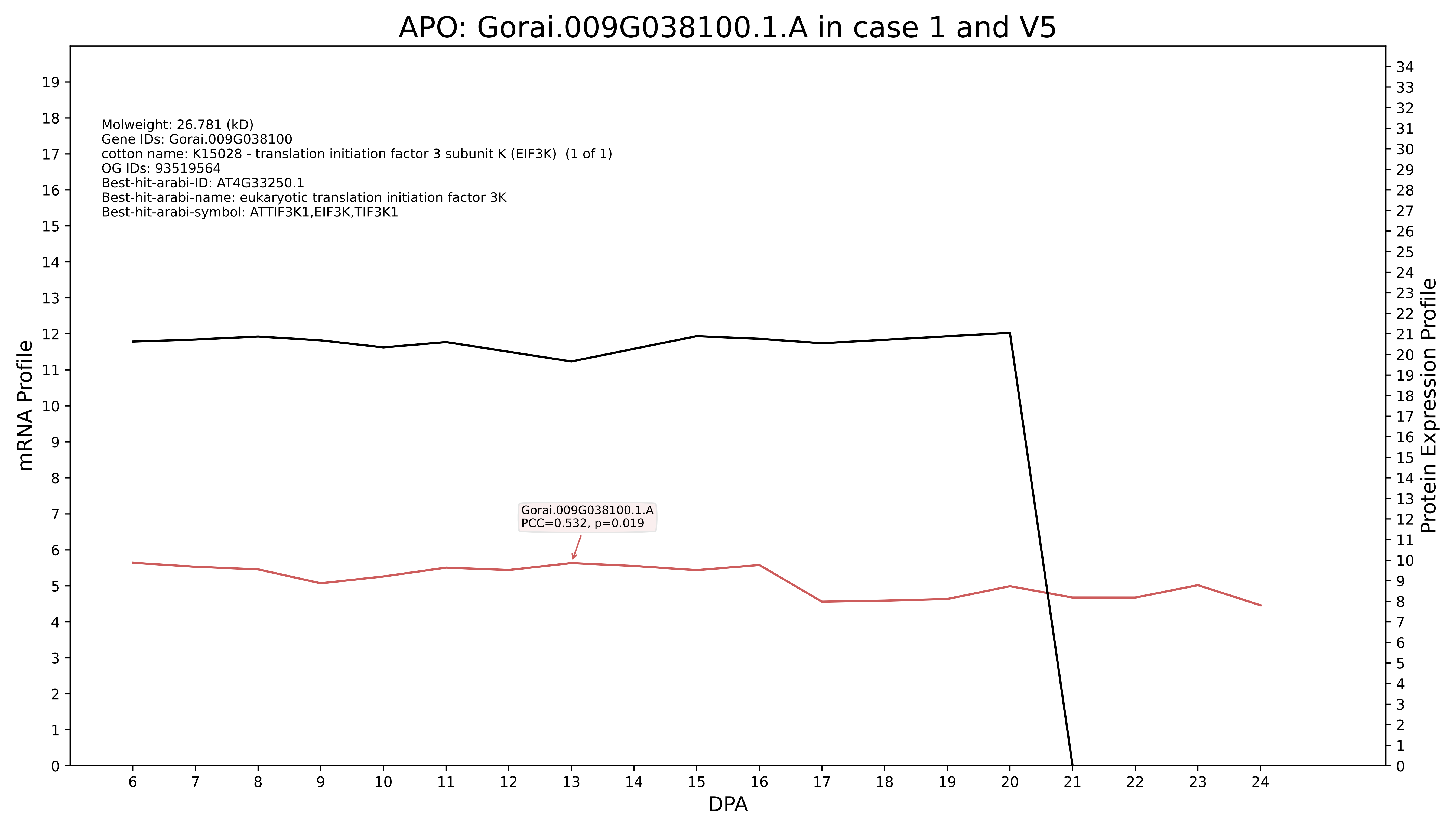

APO: Gorai.009G038100.1.A in case 1 and V5
Molweight: 26.781 (kD)
Gene IDs: Gorai.009G038100
cotton name: K15028 - translation initiation factor 3 subunit K (EIF3K) (1 of 1)
OG IDs: 93519564
Best-hit-arabi-ID: AT4G33250.1
Best-hit-arabi-name: eukaryotic translation initiation factor 3K
Best-hit-arabi-symbol: ATTIF3K1,EIF3K,TIF3K1
Gorai.009G038100.1.A
PCC=0.532, p=0.019
mRNA Profile
Protein Expression Profile
DPA

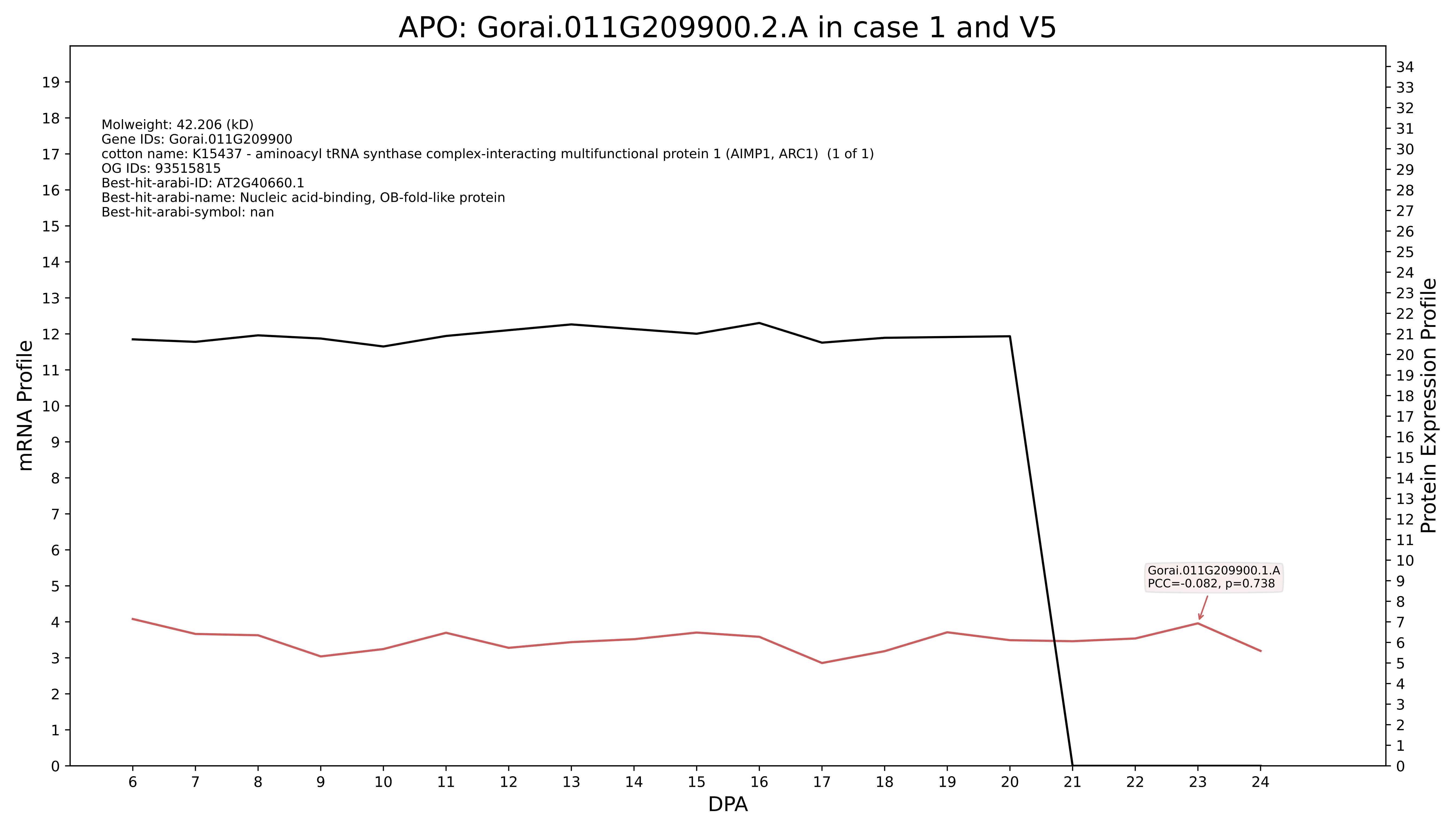
APO: Gorai.011G209900.2.A in case 1 and V5
Molweight: 42.206 (kD)
Gene IDs: Gorai.011G209900
cotton name: K15437 - aminoacyl tRNA synthase complex-interacting multifunctional protein 1 (AIMP1, ARC1) (1 of 1)
OG IDs: 93515815
Best-hit-arabi-ID: AT2G40660.1
Best-hit-arabi-name: Nucleic acid-binding, OB-fold-like protein
Best-hit-arabi-symbol: nan
Gorai.011G209900.1.A
PCC=-0.082, p=0.738
mRNA Profile
Protein Expression Profile
DPA

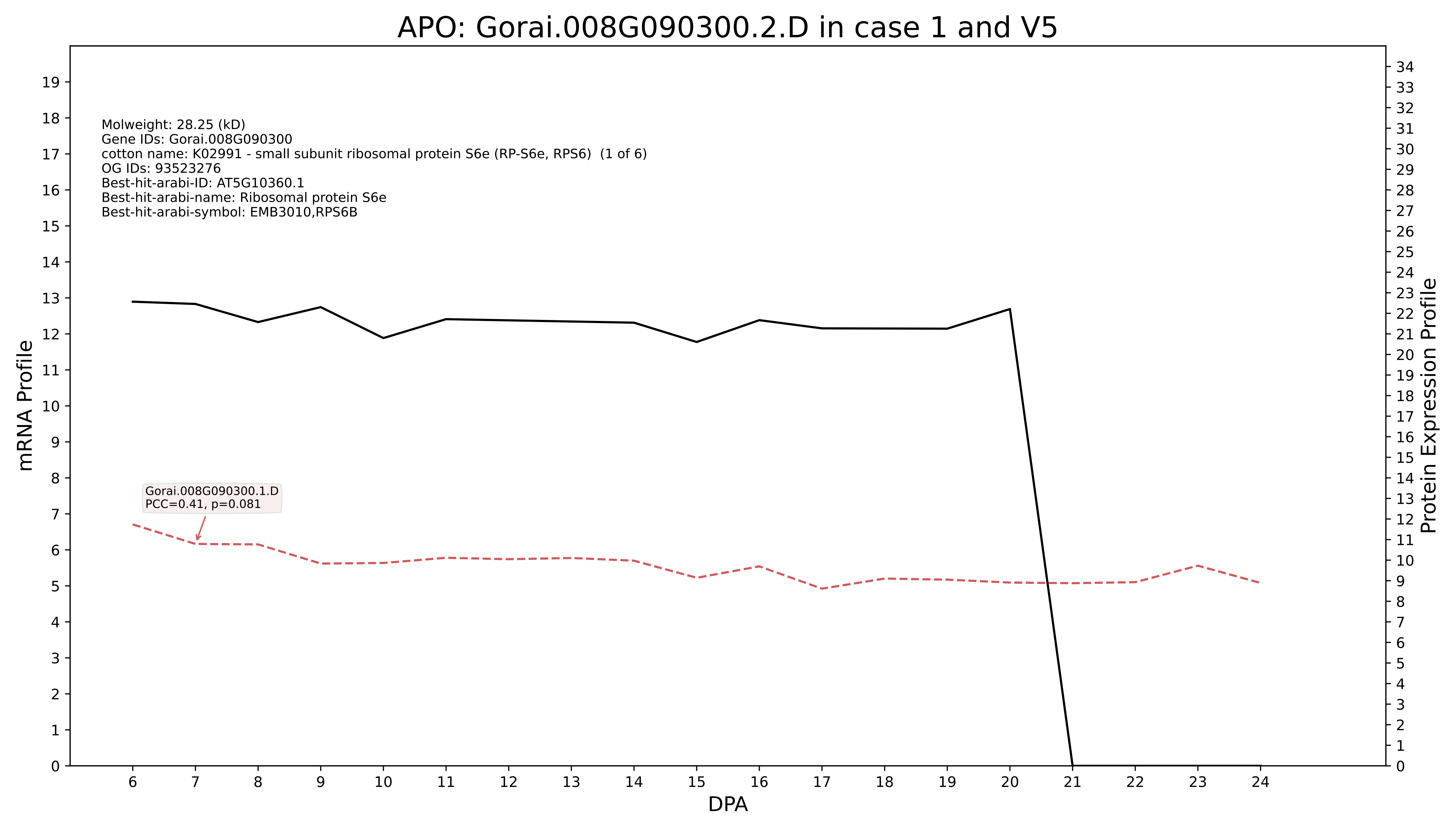

APO: Gorai.008G090300.2.D in case 1 and V5
Molweight: 28.25 (kD)
Gene IDs: Gorai.008G090300
cotton name: K02991 - small subunit ribosomal protein S6e (RP-S6e, RPS6) (1 of 6)
OG IDs: 93523276
Best-hit-arabi-ID: AT5G10360.1
Best-hit-arabi-name: Ribosomal protein S6e
Best-hit-arabi-symbol: EMB3010,RPS6B
Gorai.008G090300.1.D
PCC=0.41, p=0.081
mRNA Profile
Protein Expression Profile
DPA

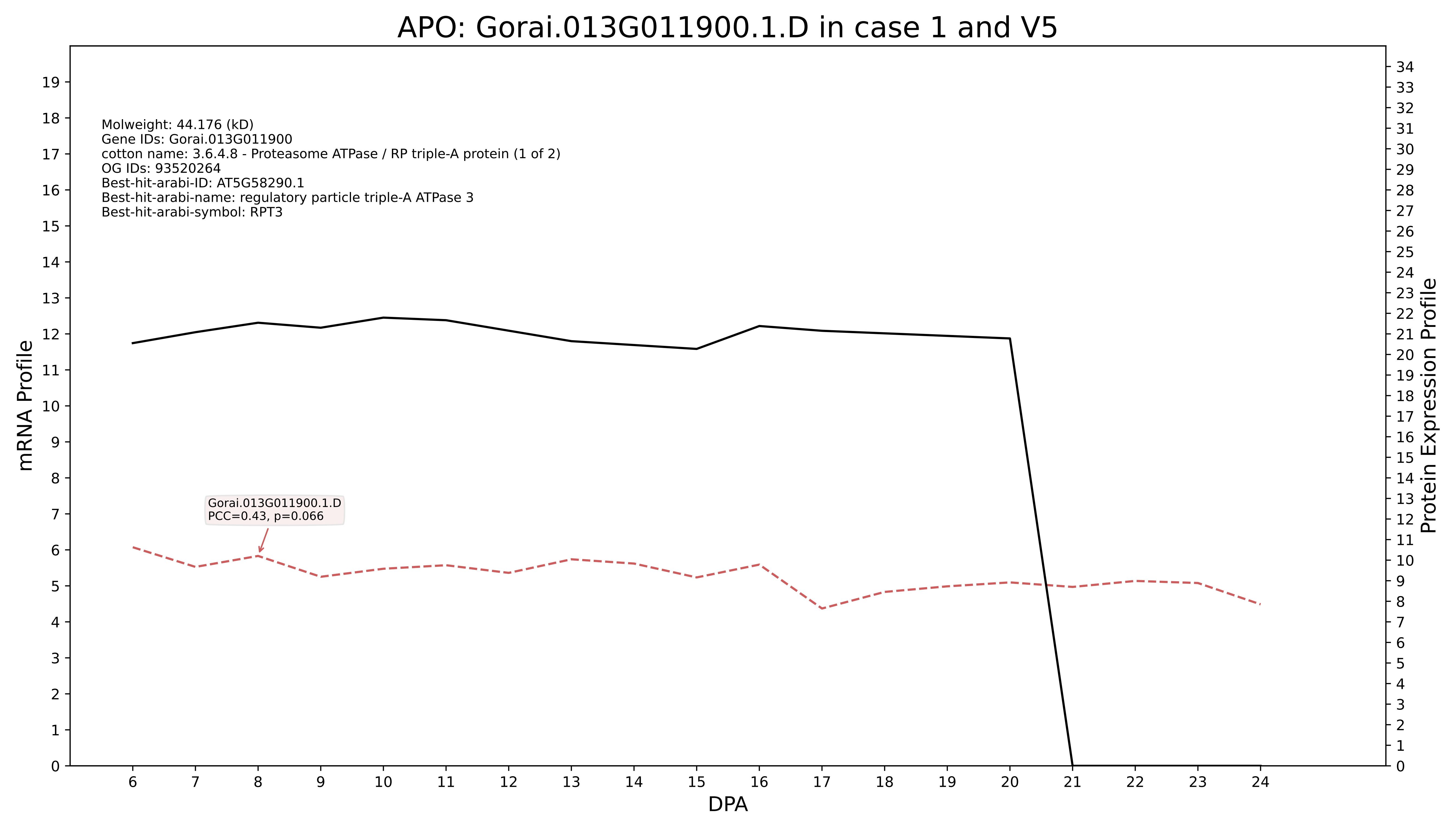
APO: Gorai.013G011900.1.D in case 1 and V5
Molweight: 44.176 (kD)
Gene IDs: Gorai.013G011900
cotton name: 3.6.4.8 - Proteasome ATPase / RP triple-A protein (1 of 2)
OG IDs: 93520264
Best-hit-arabi-ID: AT5G58290.1
Best-hit-arabi-name: regulatory particle triple-A ATPase 3
Best-hit-arabi-symbol: RPT3
Gorai.013G011900.1.D
PCC=0.43, p=0.066
mRNA Profile
Protein Expression Profile
DPA
0
1
2
3
4
5
6
7
8
9
10
11
12
13
14
15
16
17
18
19
20
21
22
23
24
25
26
27
28
29
30
31
32
33
34

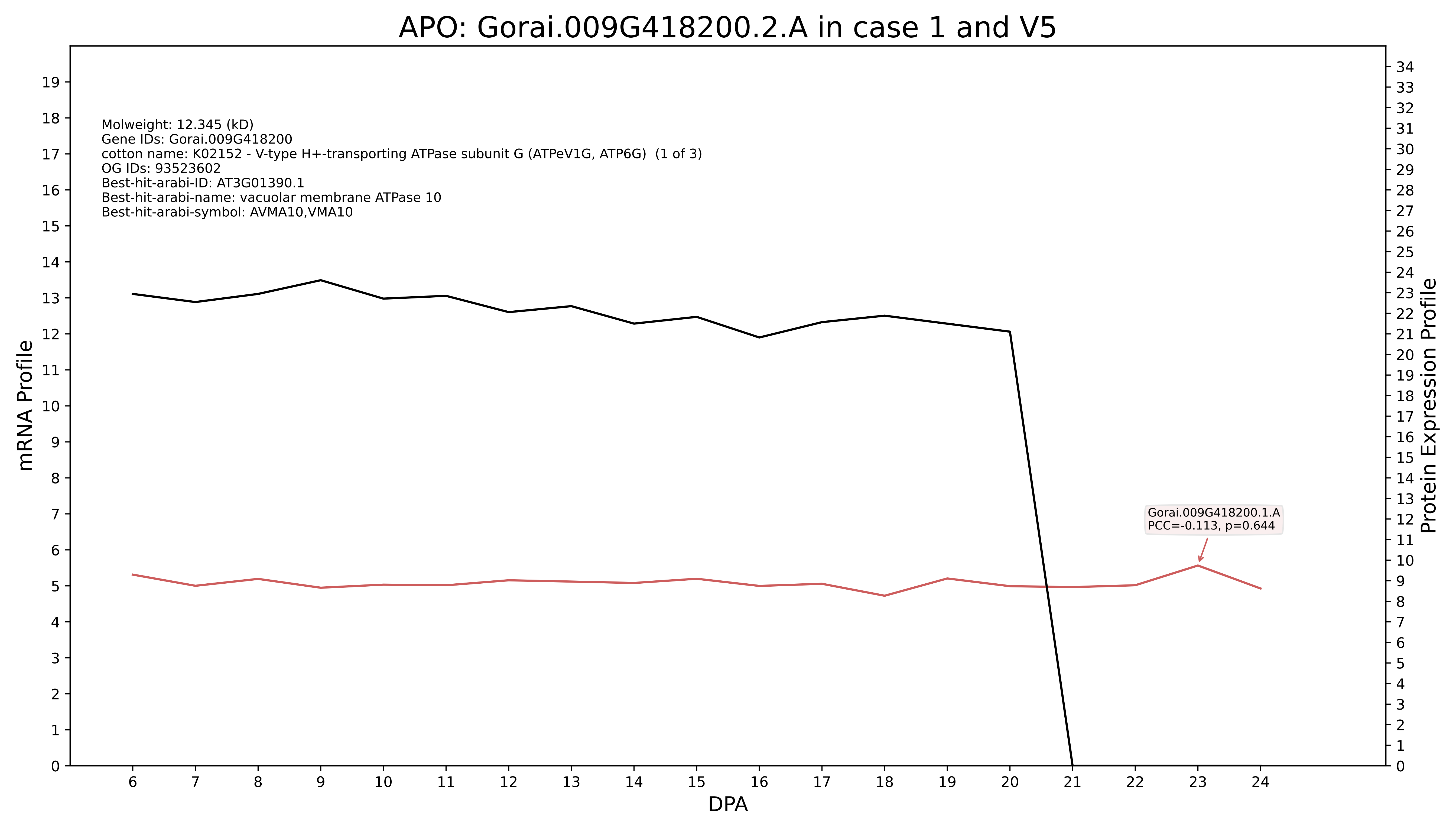
APO: Gorai.009G418200.2.A in case 1 and V5
Molweight: 12.345 (kD)
Gene IDs: Gorai.009G418200
cotton name: K02152 - V-type H+-transporting ATPase subunit G (ATPeV1G, ATP6G) (1 of 3)
OG IDs: 93523602
Best-hit-arabi-ID: AT3G01390.1
Best-hit-arabi-name: vacuolar membrane ATPase 10
Best-hit-arabi-symbol: AVMA10,VMA10
Gorai.009G418200.1.A
PCC=-0.113, p=0.644
mRNA Profile
Protein Expression Profile
DPA

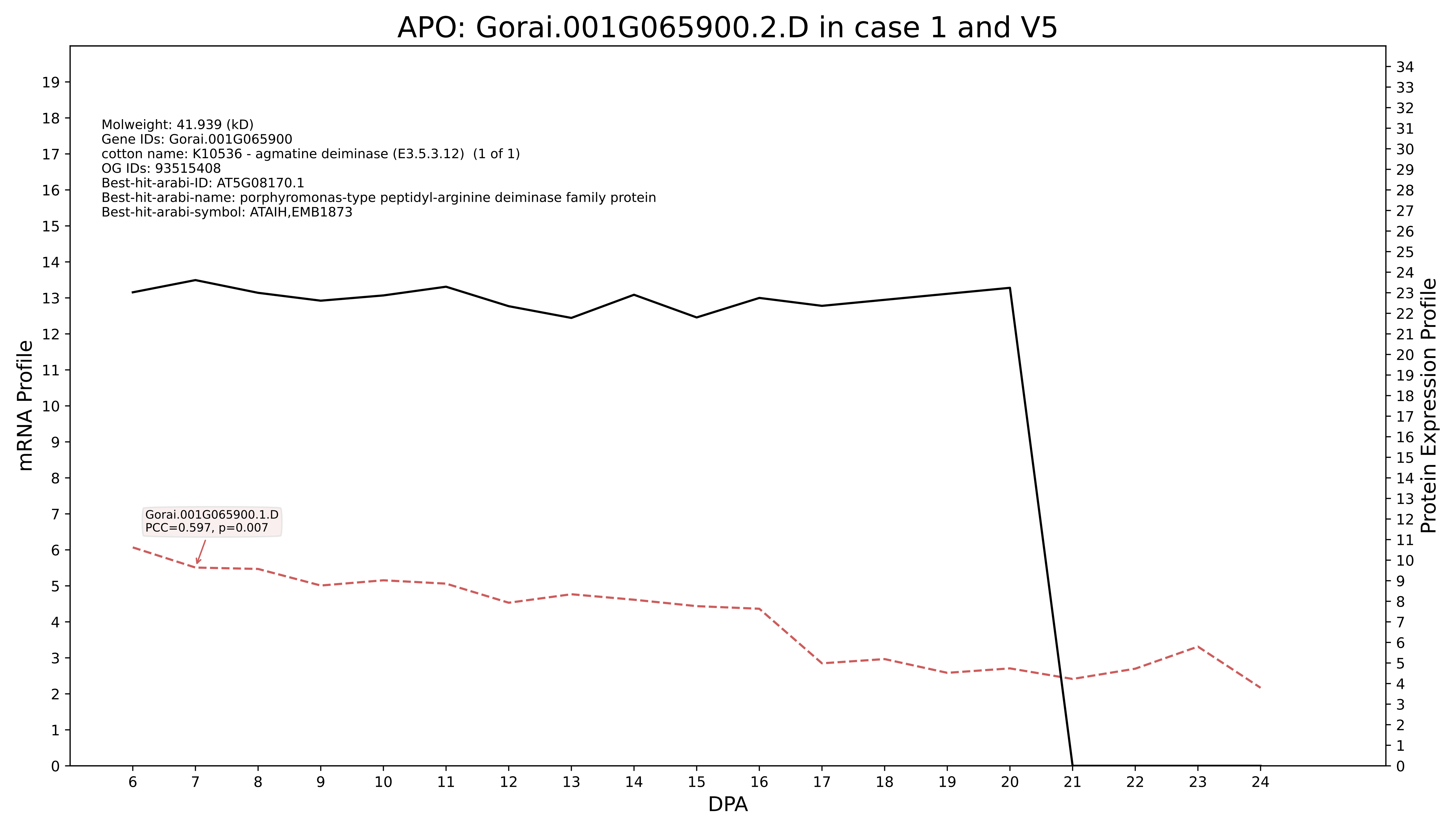

APO: Gorai.001G065900.2.D in case 1 and V5
Molweight: 41.939 (kD)
Gene IDs: Gorai.001G065900
cotton name: K10536 - agmatine deiminase (E3.5.3.12) (1 of 1)
OG IDs: 93515408
Best-hit-arabi-ID: AT5G08170.1
Best-hit-arabi-name: porphyromonas-type peptidyl-arginine deiminase family protein
Best-hit-arabi-symbol: ATAIH,EMB1873
Gorai.001G065900.1.D
PCC=0.597, p=0.007
mRNA Profile
Protein Expression Profile
DPA

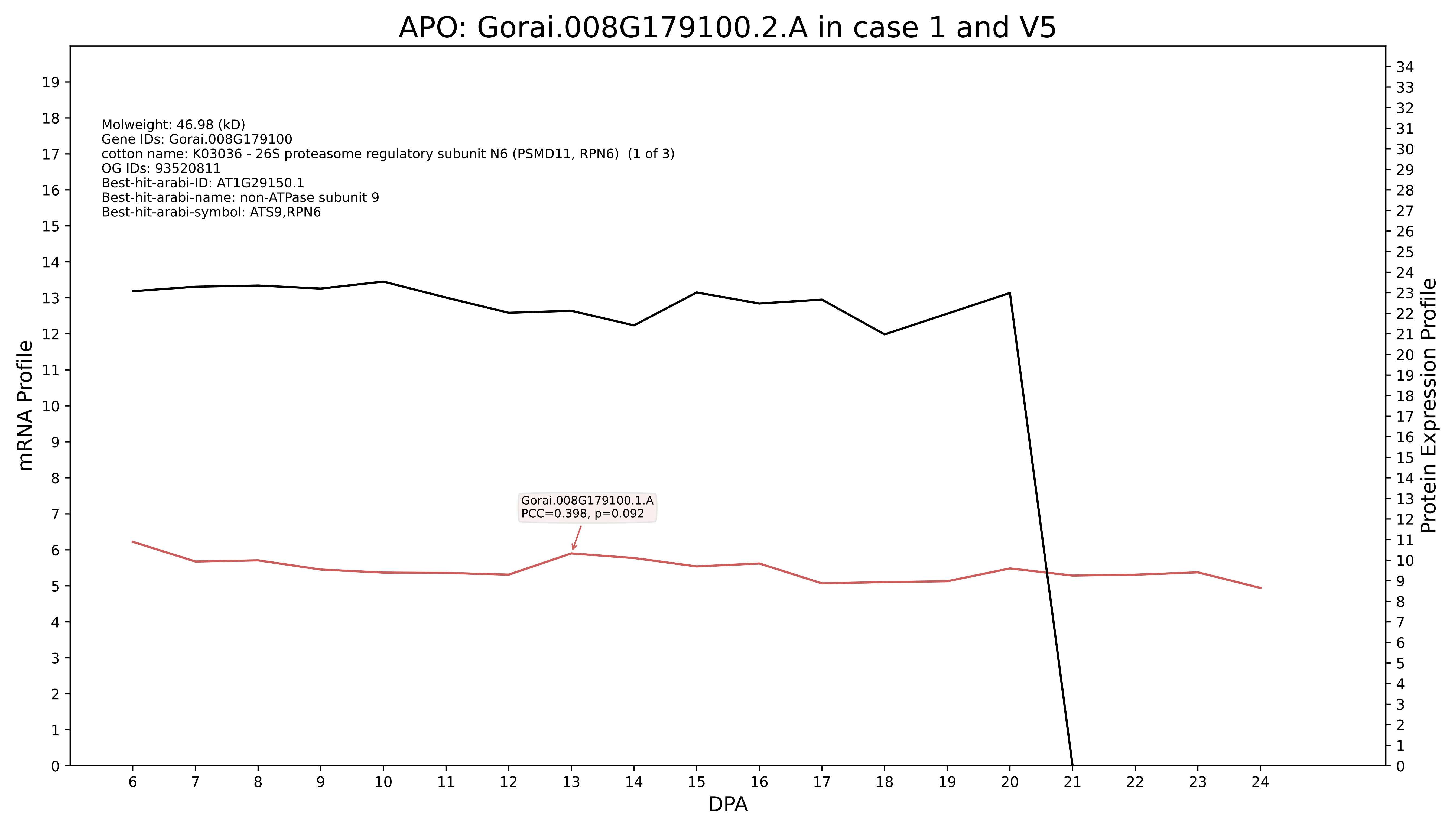
APO: Gorai.008G179100.2.A in case 1 and V5
Molweight: 46.98 (kD)
Gene IDs: Gorai.008G179100
cotton name: K03036 - 26S proteasome regulatory subunit N6 (PSMD11, RPN6) (1 of 3)
OG IDs: 93520811
Best-hit-arabi-ID: AT1G29150.1
Best-hit-arabi-name: non-ATPase subunit 9
Best-hit-arabi-symbol: ATS9,RPN6
Gorai.008G179100.1.A
PCC=0.398, p=0.092
mRNA Profile
Protein Expression Profile
DPA

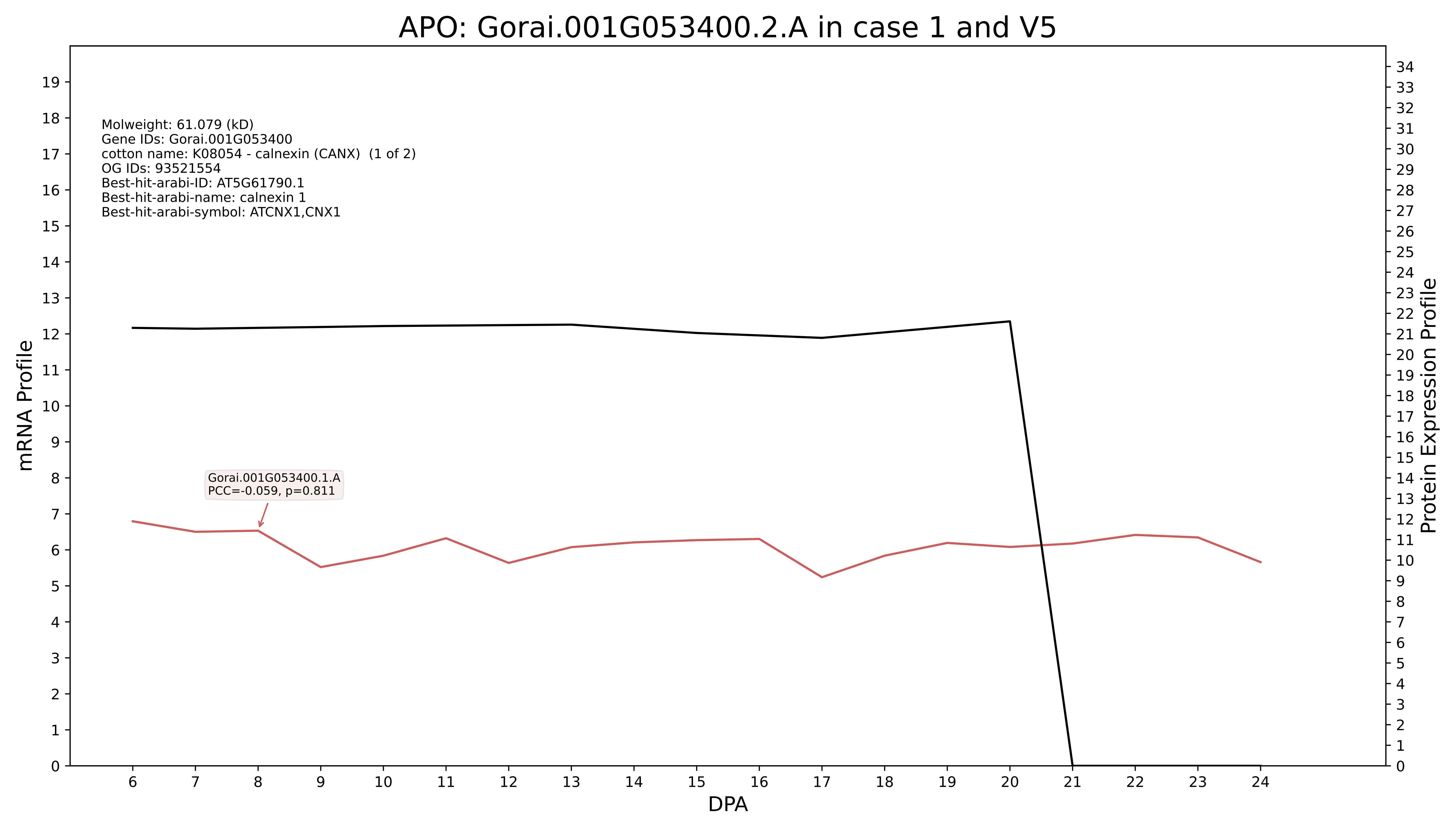
APO: Gorai.001G053400.2.A in case 1 and V5
Molweight: 61.079 (kD)
Gene IDs: Gorai.001G053400
cotton name: K08054 - calnexin (CANX) (1 of 2)
OG IDs: 93521554
Best-hit-arabi-ID: AT5G61790.1
Best-hit-arabi-name: calnexin 1
Best-hit-arabi-symbol: ATCNX1,CNX1
Gorai.001G053400.1.A
PCC=-0.059, p=0.811
mRNA Profile
Protein Expression Profile
DPA

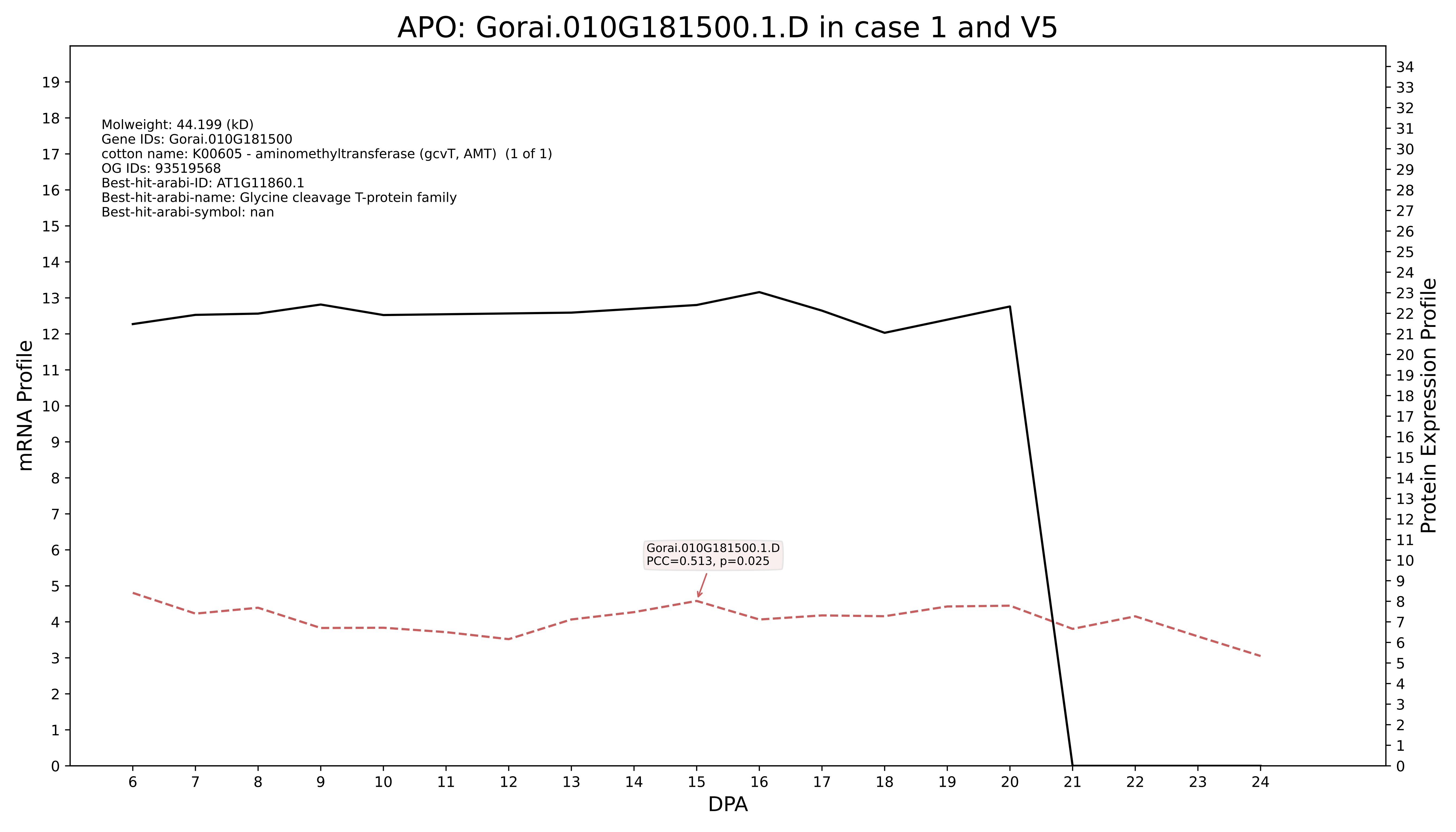
APO: Gorai.010G181500.1.D in case 1 and V5
Molweight: 44.199 (kD)
Gene IDs: Gorai.010G181500
cotton name: K00605 - aminomethyltransferase (gcvT, AMT) (1 of 1)
OG IDs: 93519568
Best-hit-arabi-ID: AT1G11860.1
Best-hit-arabi-name: Glycine cleavage T-protein family
Best-hit-arabi-symbol: nan
Gorai.010G181500.1.D
PCC=0.513, p=0.025
mRNA Profile
Protein Expression Profile
DPA

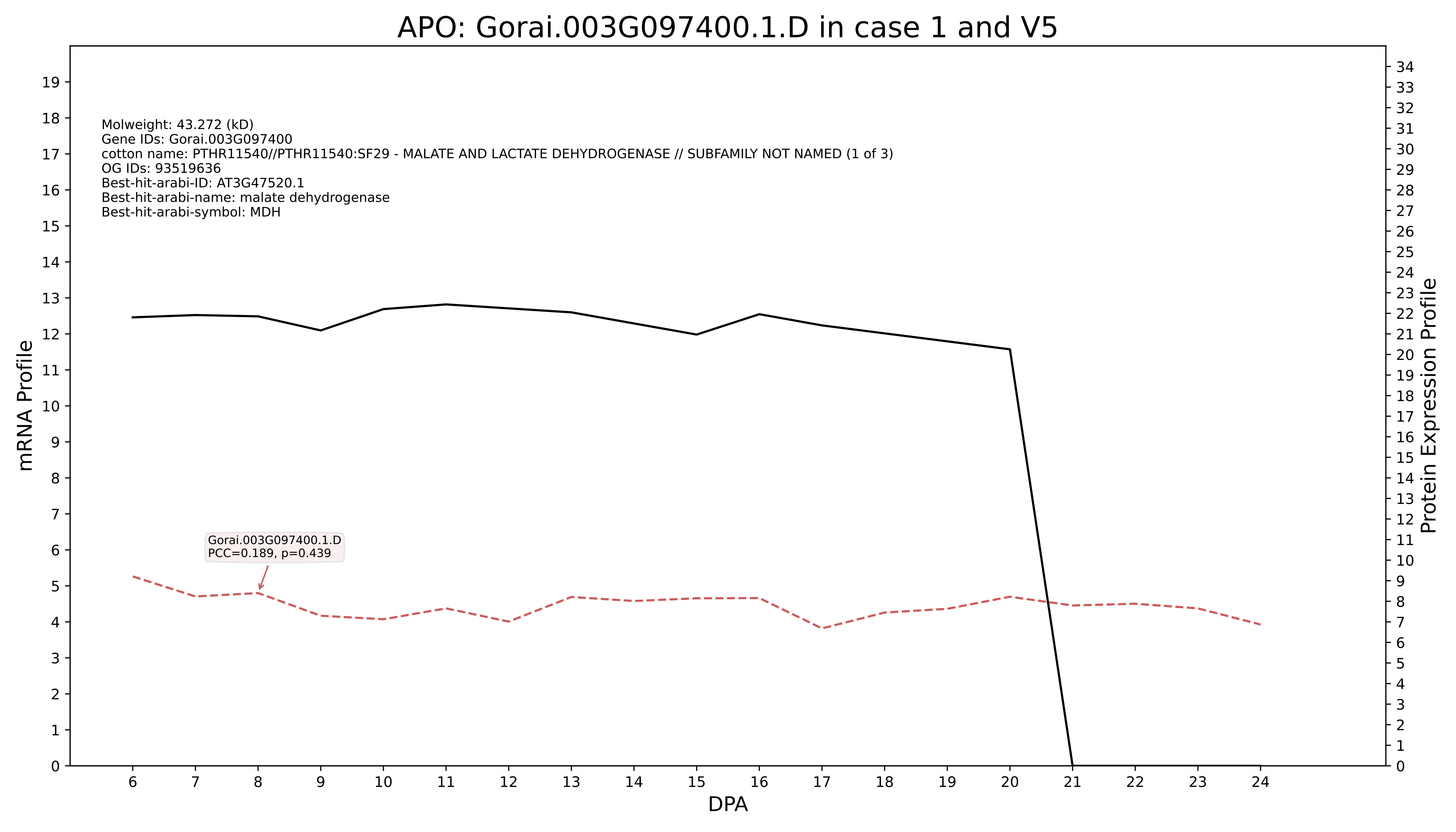

APO: Gorai.003G097400.1.D in case 1 and V5
Molweight: 43.272 (kD)
Gene IDs: Gorai.003G097400
cotton name: PTHR11540//PTHR11540:SF29 - MALATE AND LACTATE DEHYDROGENASE // SUBFAMILY NOT NAMED (1 of 3)
OG IDs: 93519636
Best-hit-arabi-ID: AT3G47520.1
Best-hit-arabi-name: malate dehydrogenase
Best-hit-arabi-symbol: MDH
Gorai.003G097400.1.D
PCC=0.189, p=0.439
mRNA Profile
Protein Expression Profile
DPA

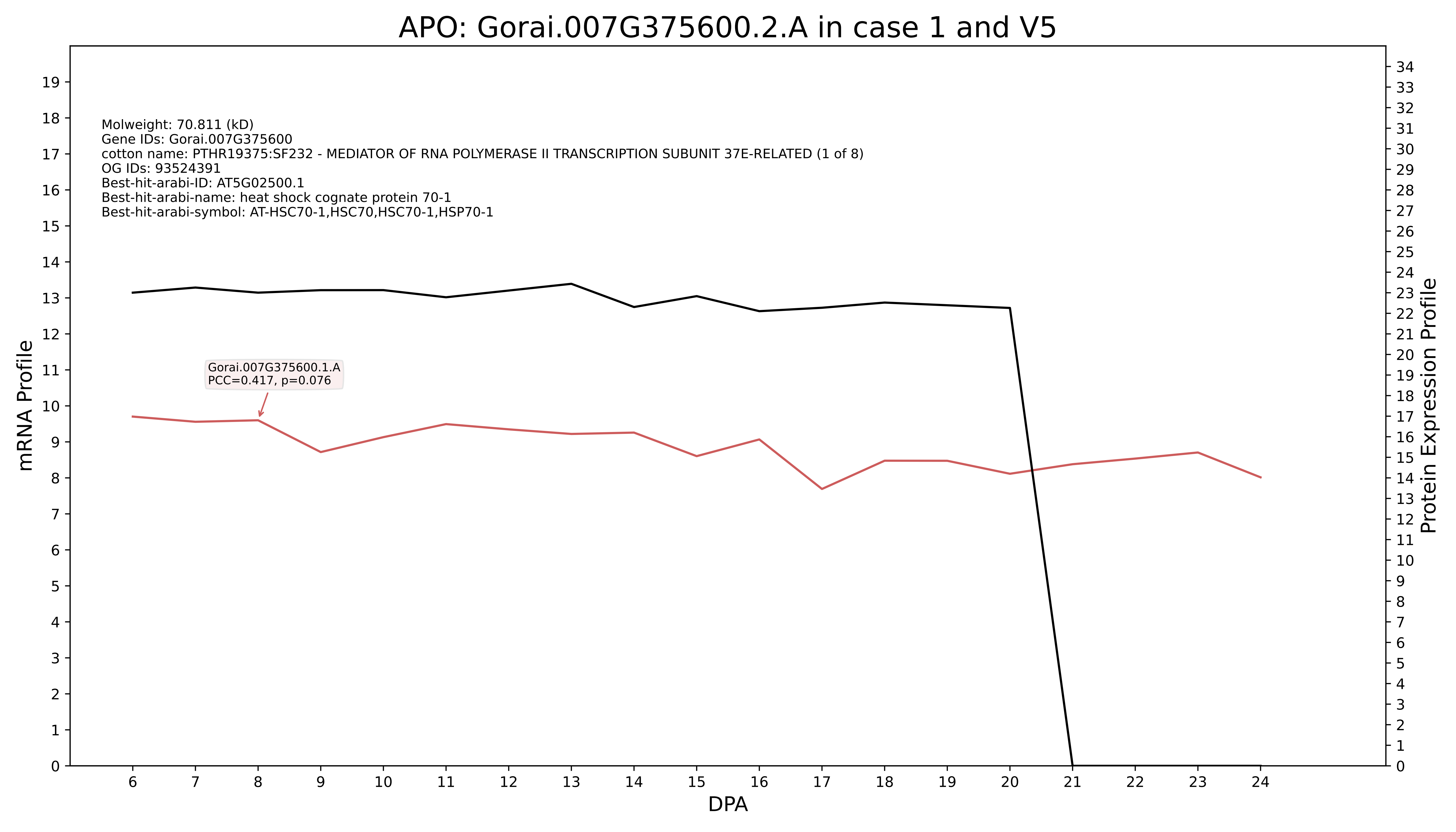
APO: Gorai.007G375600.2.A in case 1 and V5
Molweight: 70.811 (kD)
Gene IDs: Gorai.007G375600
cotton name: PTHR19375:SF232 - MEDIATOR OF RNA POLYMERASE II TRANSCRIPTION SUBUNIT 37E-RELATED (1 of 8)
OG IDs: 93524391
Best-hit-arabi-ID: AT5G02500.1
Best-hit-arabi-name: heat shock cognate protein 70-1
Best-hit-arabi-symbol: AT-HSC70-1,HSC70,HSC70-1,HSP70-1
Gorai.007G375600.1.A
PCC=0.417, p=0.076
mRNA Profile
Protein Expression Profile
DPA

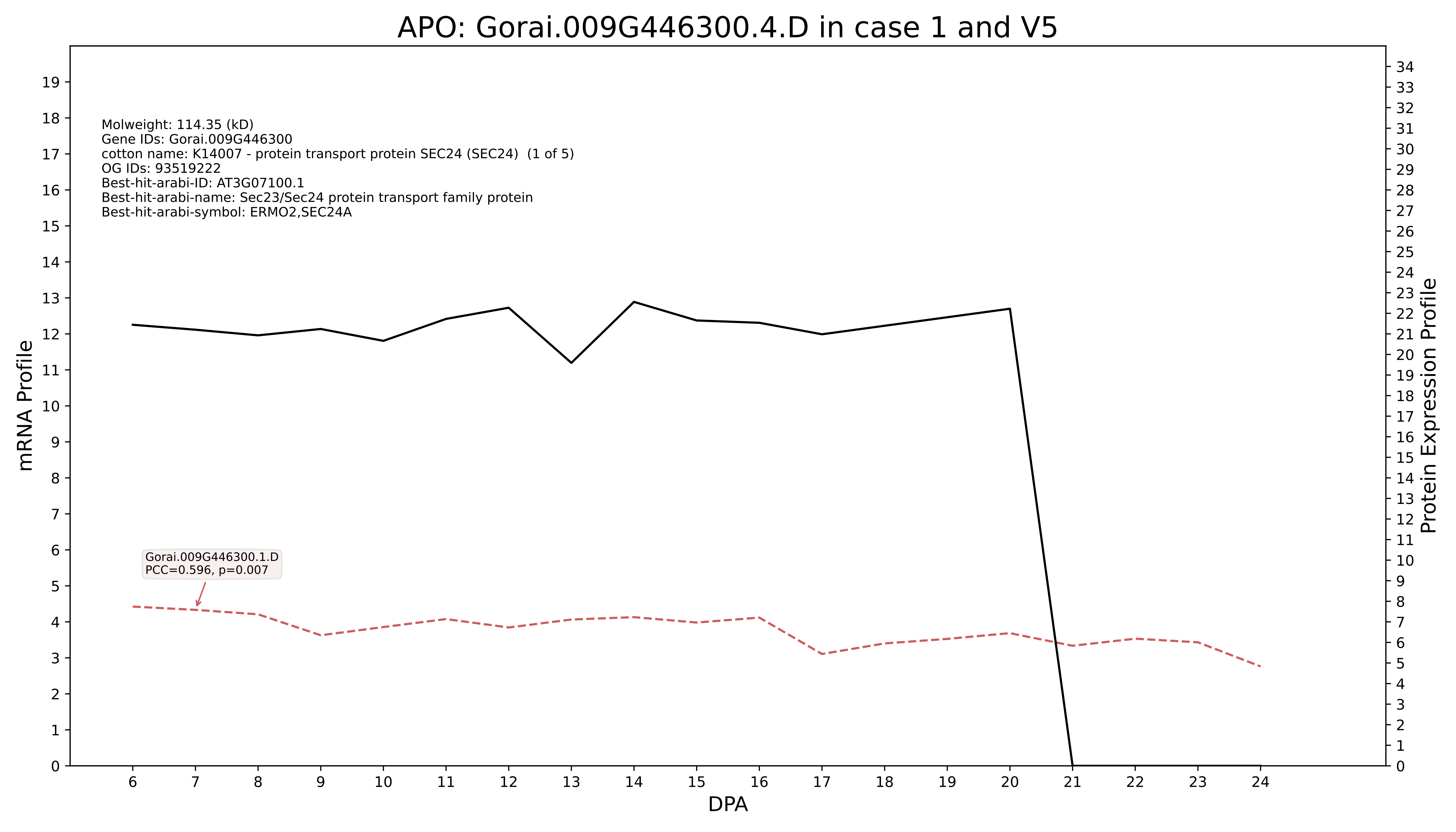

APO: Gorai.009G446300.4.D in case 1 and V5
Molweight: 114.35 (kD)
Gene IDs: Gorai.009G446300
cotton name: K14007 - protein transport protein SEC24 (SEC24) (1 of 5)
OG IDs: 93519222
Best-hit-arabi-ID: AT3G07100.1
Best-hit-arabi-name: Sec23/Sec24 protein transport family protein
Best-hit-arabi-symbol: ERMO2,SEC24A
Gorai.009G446300.1.D
PCC=0.596, p=0.007
mRNA Profile
Protein Expression Profile
DPA

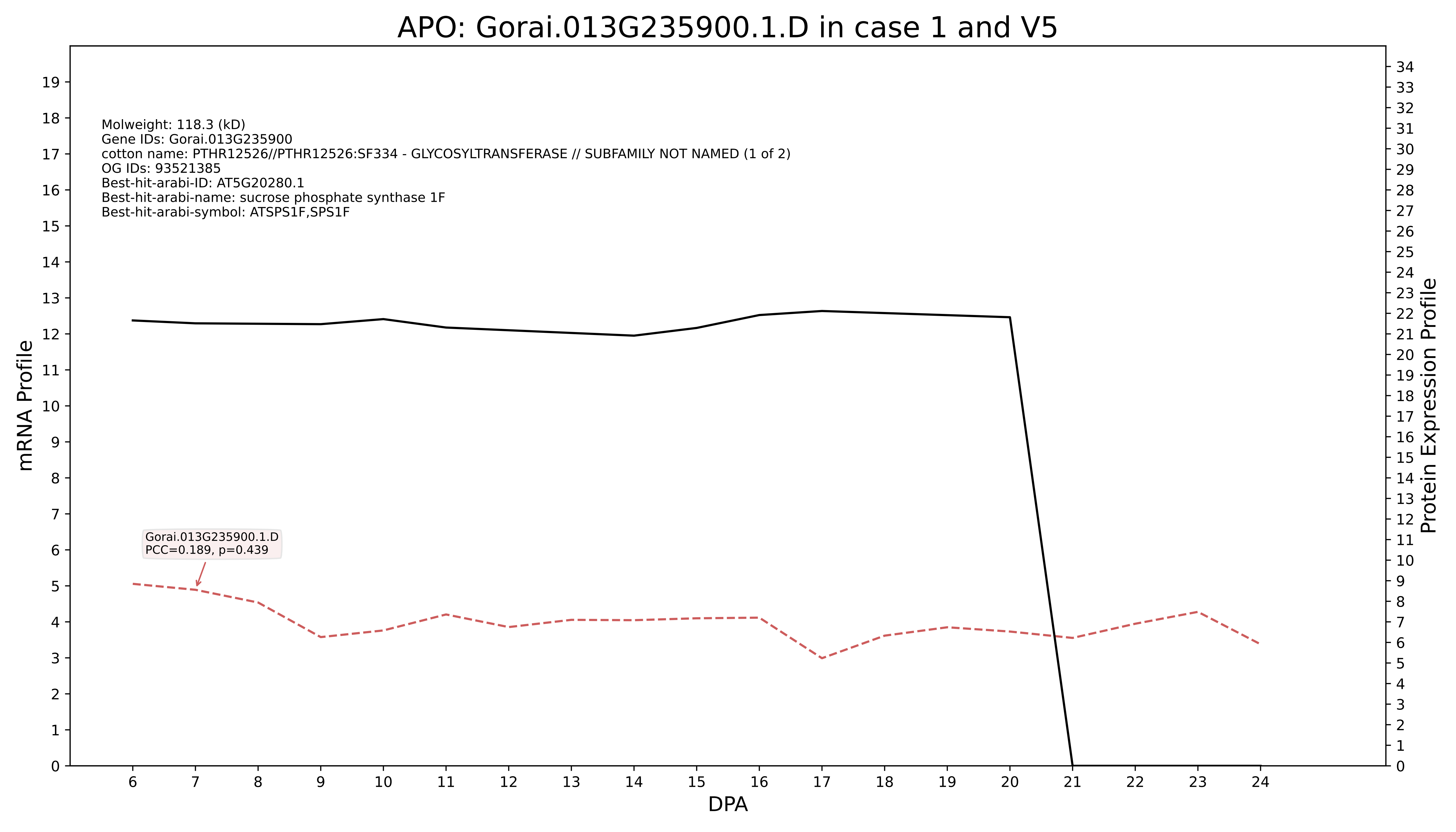

APO: Gorai.013G235900.1.D in case 1 and V5
Molweight: 118.3 (kD)
Gene IDs: Gorai.013G235900
cotton name: PTHR12526//PTHR12526:SF334 - GLYCOSYLTRANSFERASE // SUBFAMILY NOT NAMED (1 of 2)
OG IDs: 93521385
Best-hit-arabi-ID: AT5G20280.1
Best-hit-arabi-name: sucrose phosphate synthase 1F
Best-hit-arabi-symbol: ATSPS1F,SPS1F
Gorai.013G235900.1.D
PCC=0.189, p=0.439
mRNA Profile
Protein Expression Profile
DPA

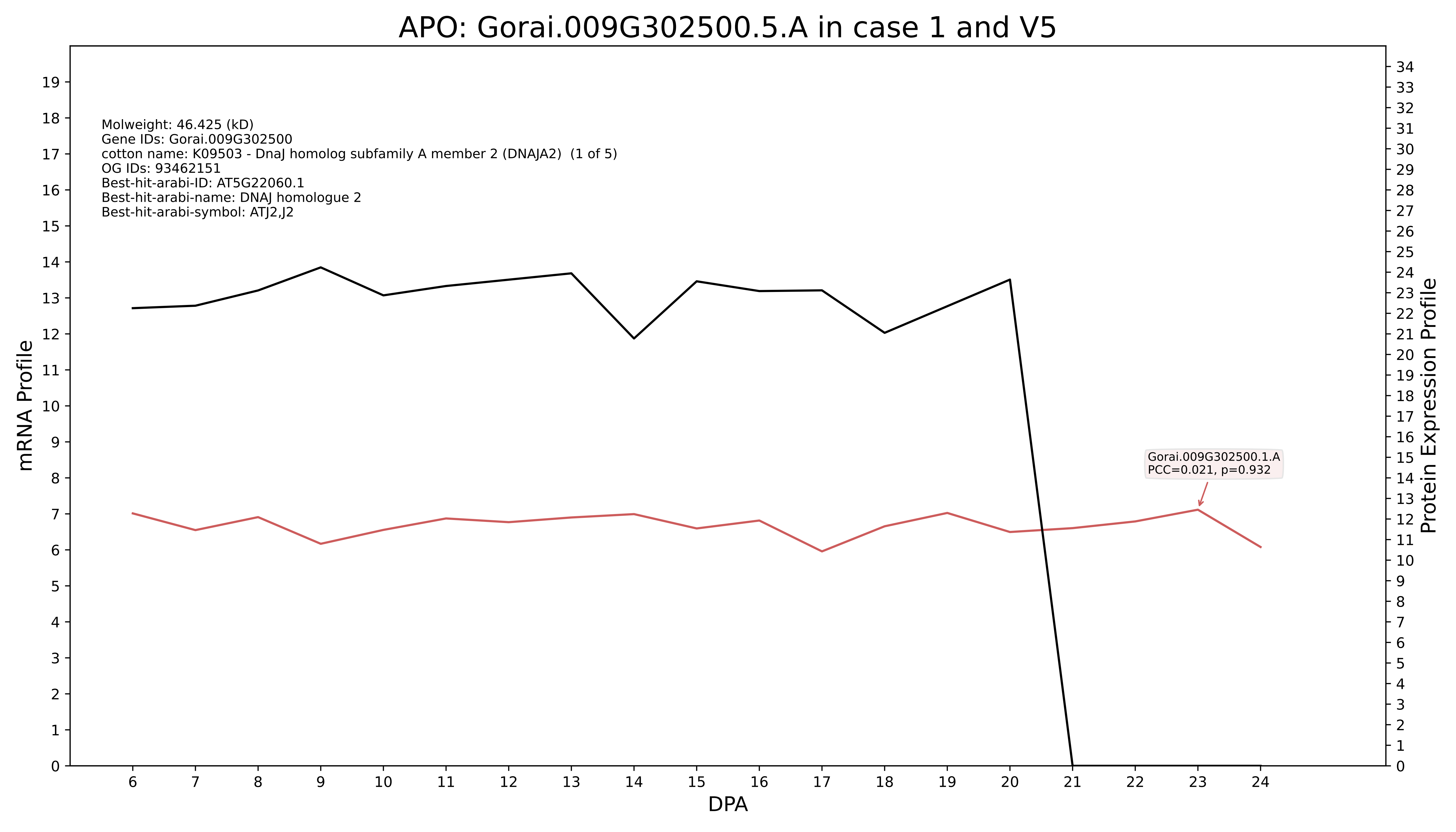
APO: Gorai.009G302500.5.A in case 1 and V5
Molweight: 46.425 (kD)
Gene IDs: Gorai.009G302500
cotton name: K09503 - DnaJ homolog subfamily A member 2 (DNAJA2) (1 of 5)
OG IDs: 93462151
Best-hit-arabi-ID: AT5G22060.1
Best-hit-arabi-name: DNAJ homologue 2
Best-hit-arabi-symbol: ATJ2,J2
Gorai.009G302500.1.A
PCC=0.021, p=0.932
mRNA Profile
Protein Expression Profile
DPA

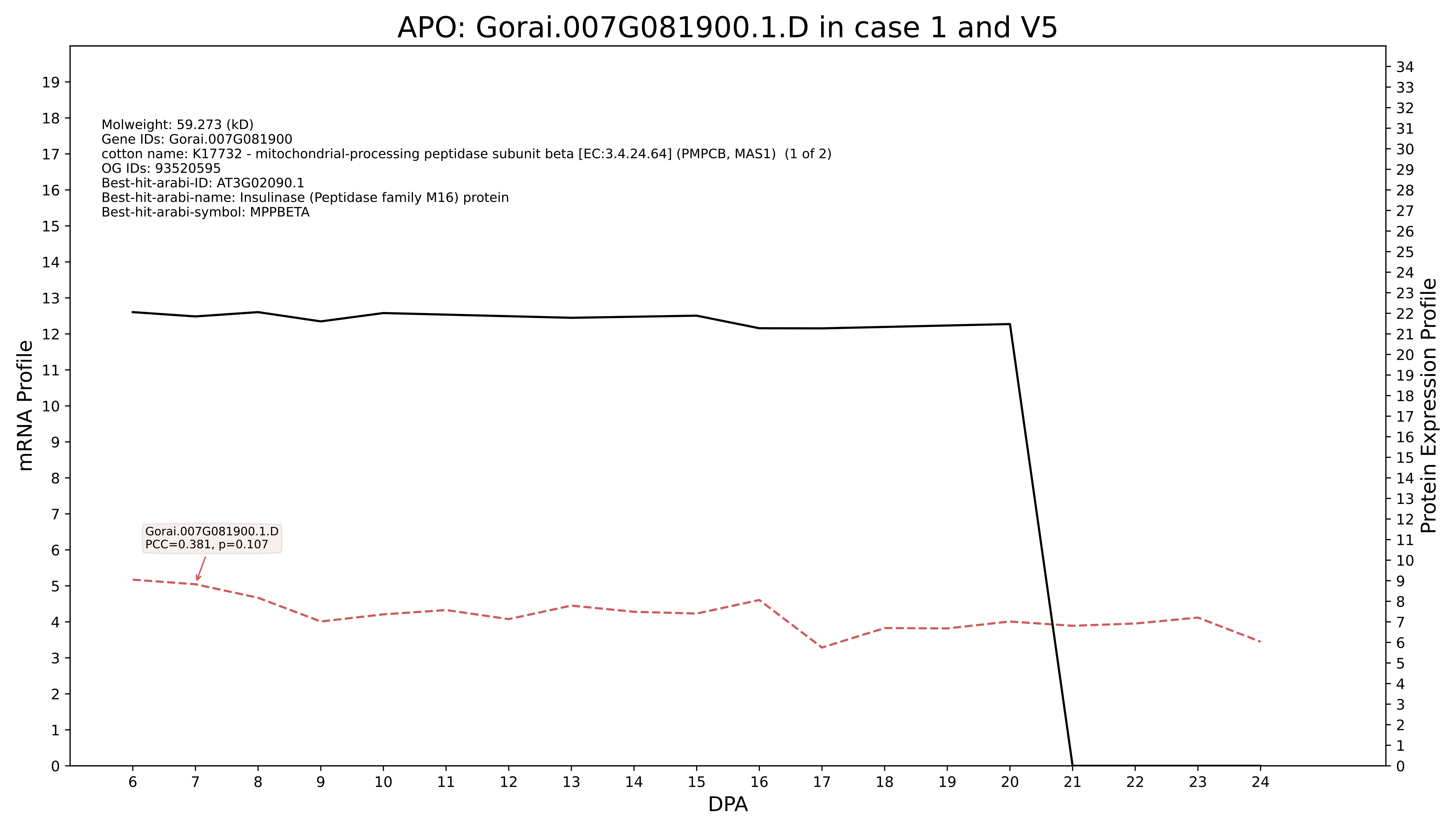

APO: Gorai.007G081900.1.D in case 1 and V5
Molweight: 59.273 (kD)
Gene IDs: Gorai.007G081900
cotton name: K17732 - mitochondrial-processing peptidase subunit beta [EC:3.4.24.64] (PMPCB, MAS1) (1 of 2)
OG IDs: 93520595
Best-hit-arabi-ID: AT3G02090.1
Best-hit-arabi-name: Insulinase (Peptidase family M16) protein
Best-hit-arabi-symbol: MPPBETA
Gorai.007G081900.1.D
PCC=0.381, p=0.107
mRNA Profile
Protein Expression Profile
DPA

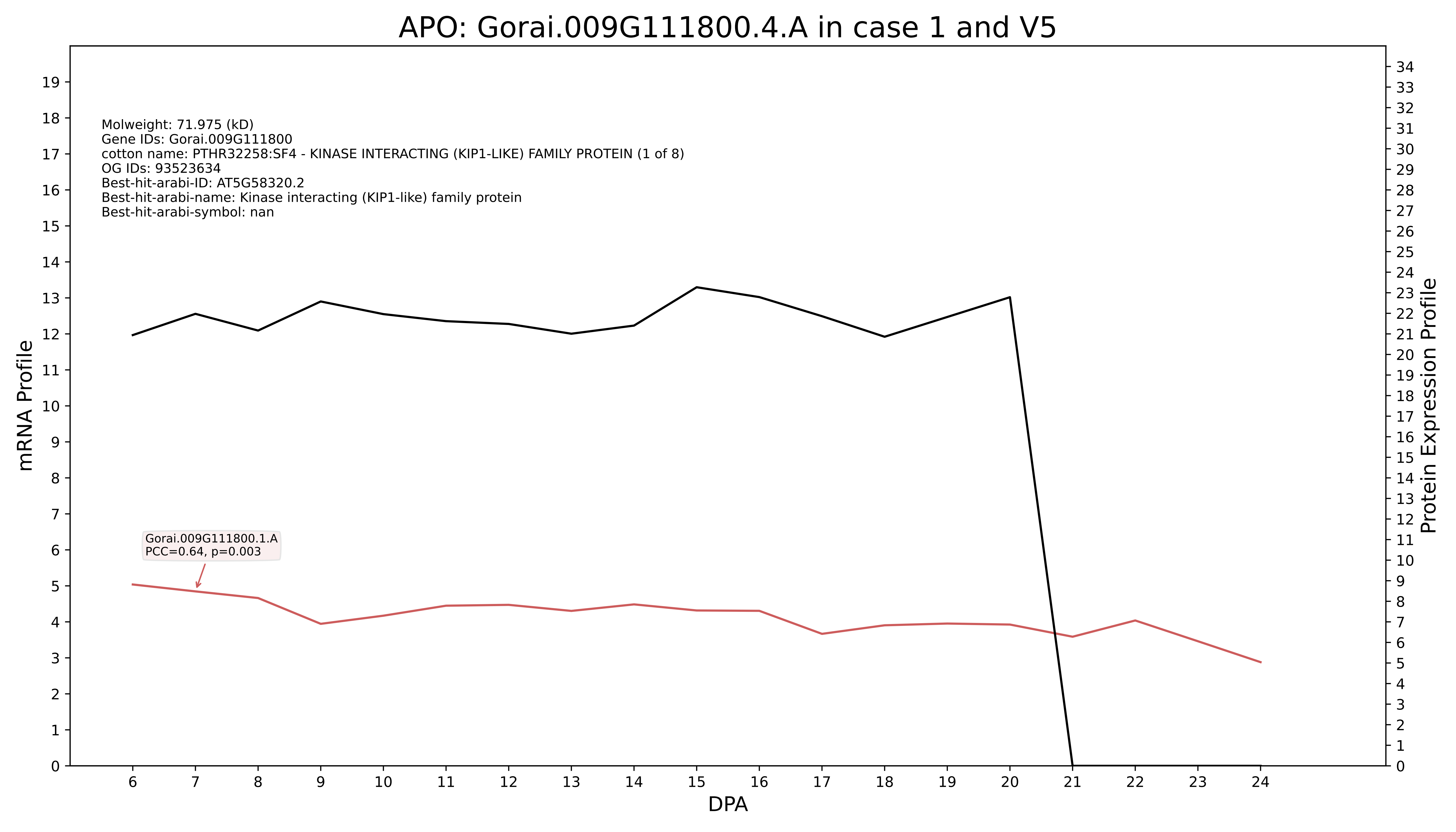
APO: Gorai.009G111800.4.A in case 1 and V5
Molweight: 71.975 (kD)
Gene IDs: Gorai.009G111800
cotton name: PTHR32258:SF4 - KINASE INTERACTING (KIP1-LIKE) FAMILY PROTEIN (1 of 8)
OG IDs: 93523634
Best-hit-arabi-ID: AT5G58320.2
Best-hit-arabi-name: Kinase interacting (KIP1-like) family protein
Best-hit-arabi-symbol: nan
Gorai.009G111800.1.A
PCC=0.64, p=0.003
mRNA Profile
Protein Expression Profile
DPA

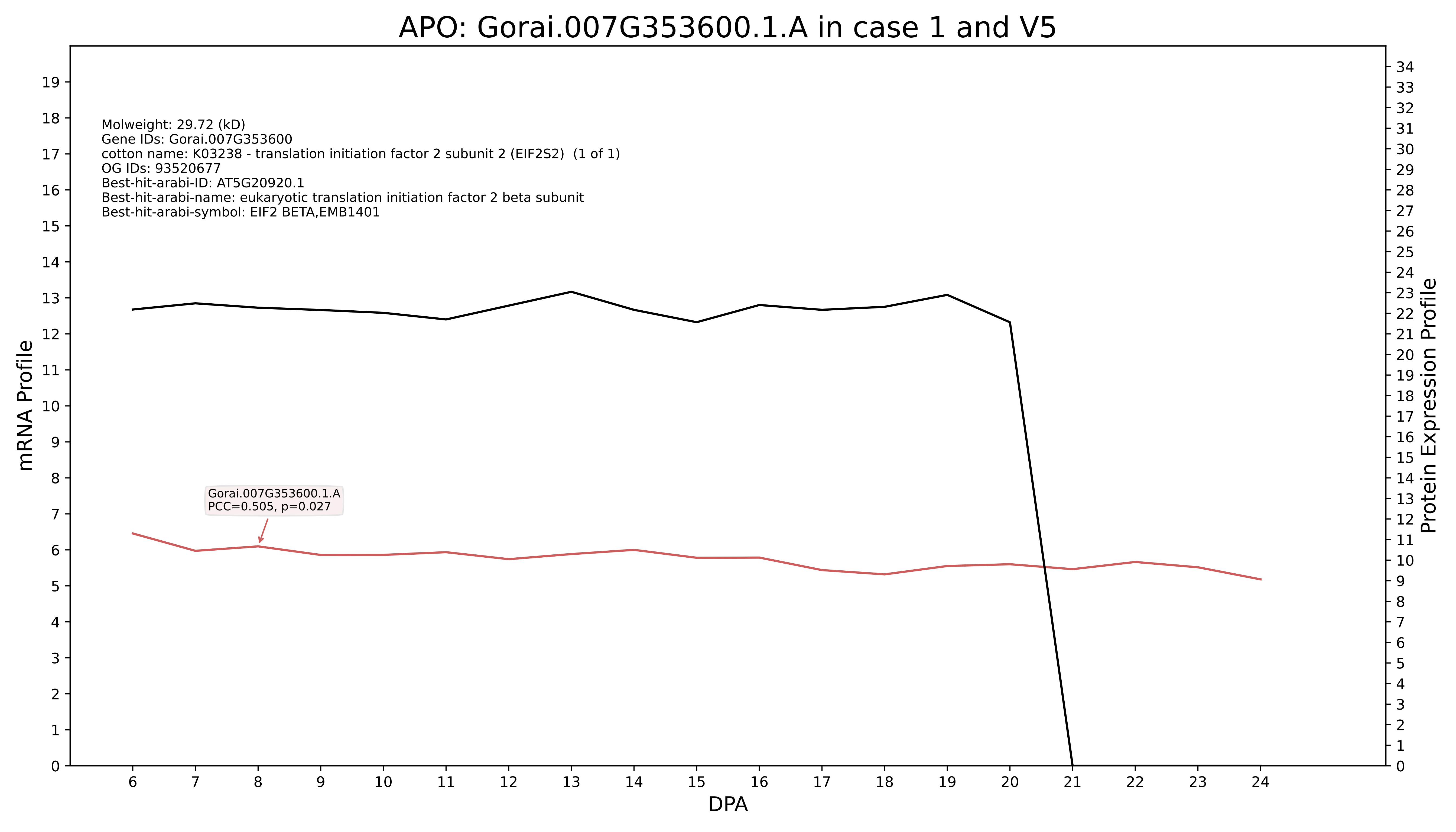
APO: Gorai.007G353600.1.A in case 1 and V5
Molweight: 29.72 (kD)
Gene IDs: Gorai.007G353600
cotton name: K03238 - translation initiation factor 2 subunit 2 (EIF2S2) (1 of 1)
OG IDs: 93520677
Best-hit-arabi-ID: AT5G20920.1
Best-hit-arabi-name: eukaryotic translation initiation factor 2 beta subunit
Best-hit-arabi-symbol: EIF2 BETA,EMB1401
Gorai.007G353600.1.A
PCC=0.505, p=0.027
mRNA Profile
Protein Expression Profile
DPA
0
1
2
3
4
5
6
7
8
9
10
11
12
13
14
15
16
17
18
19
20
21
22
23
24
25
26
27
28
29
30
31
32
33
34

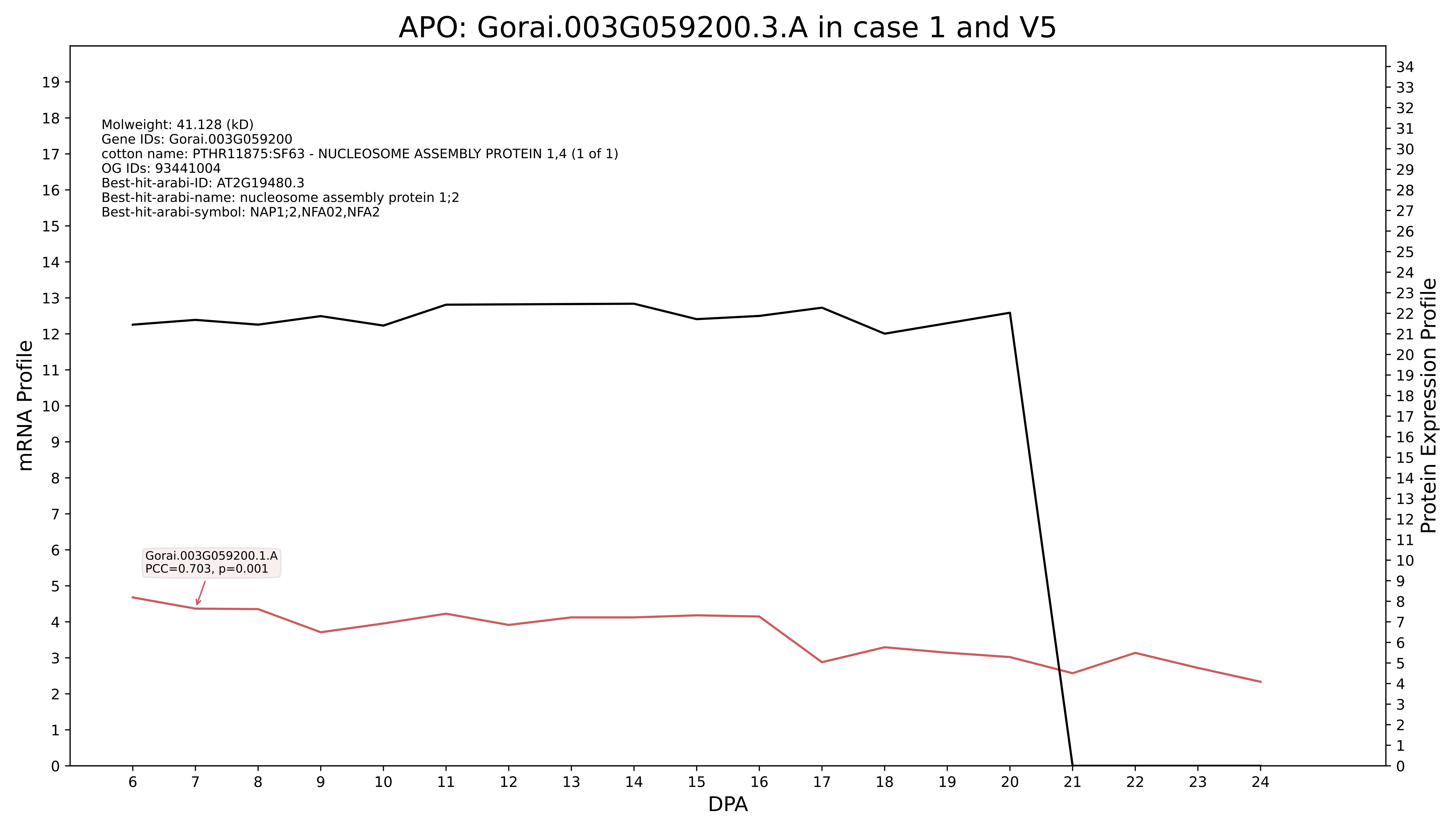

APO: Gorai.003G059200.3.A in case 1 and V5
Molweight: 41.128 (kD)
Gene IDs: Gorai.003G059200
cotton name: PTHR11875:SF63 - NUCLEOSOME ASSEMBLY PROTEIN 1,4 (1 of 1)
OG IDs: 93441004
Best-hit-arabi-ID: AT2G19480.3
Best-hit-arabi-name: nucleosome assembly protein 1;2
Best-hit-arabi-symbol: NAP1;2,NFA02,NFA2
Gorai.003G059200.1.A
PCC=0.703, p=0.001
mRNA Profile
Protein Expression Profile
DPA

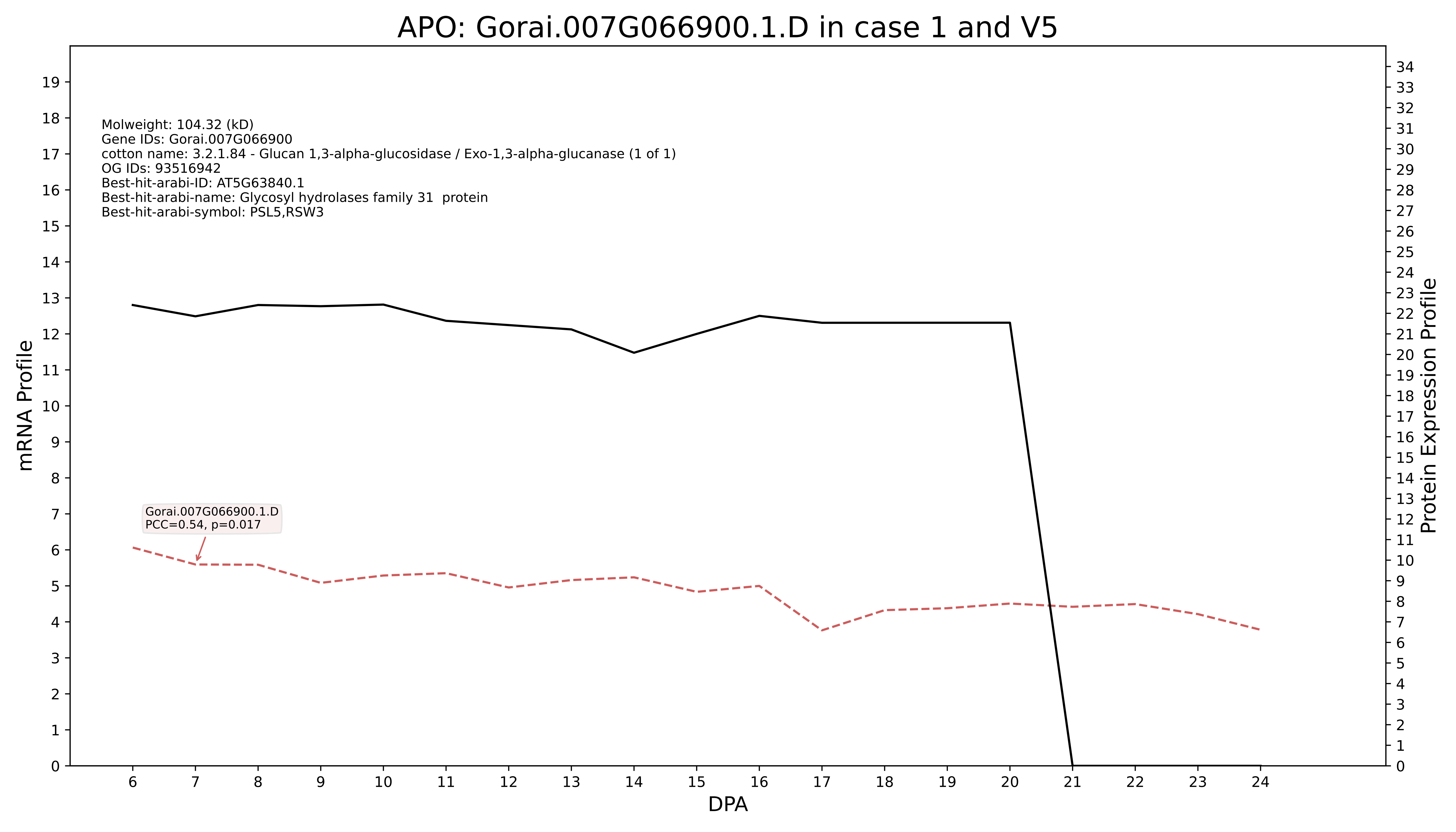

APO: Gorai.007G066900.1.D in case 1 and V5
Molweight: 104.32 (kD)
Gene IDs: Gorai.007G066900
cotton name: 3.2.1.84 - Glucan 1,3-alpha-glucosidase / Exo-1,3-alpha-glucanase (1 of 1)
OG IDs: 93516942
Best-hit-arabi-ID: AT5G63840.1
Best-hit-arabi-name: Glycosyl hydrolases family 31 protein
Best-hit-arabi-symbol: PSL5,RSW3
Gorai.007G066900.1.D
PCC=0.54, p=0.017
mRNA Profile
Protein Expression Profile
DPA

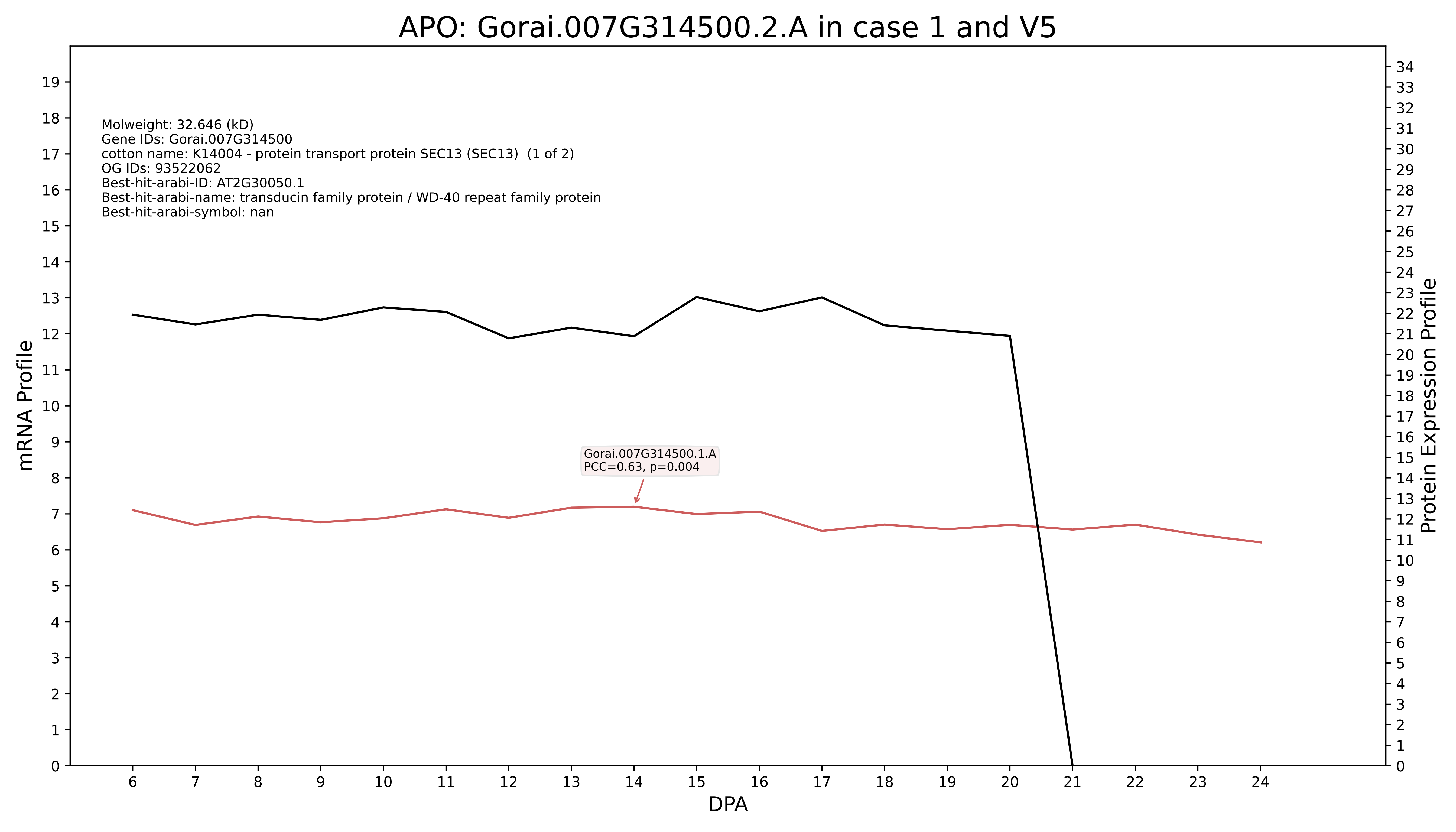

APO: Gorai.007G314500.2.A in case 1 and V5
Molweight: 32.646 (kD)
Gene IDs: Gorai.007G314500
cotton name: K14004 - protein transport protein SEC13 (SEC13) (1 of 2)
OG IDs: 93522062
Best-hit-arabi-ID: AT2G30050.1
Best-hit-arabi-name: transducin family protein / WD-40 repeat family protein
Best-hit-arabi-symbol: nan
Gorai.007G314500.1.A
PCC=0.63, p=0.004
mRNA Profile
Protein Expression Profile
DPA

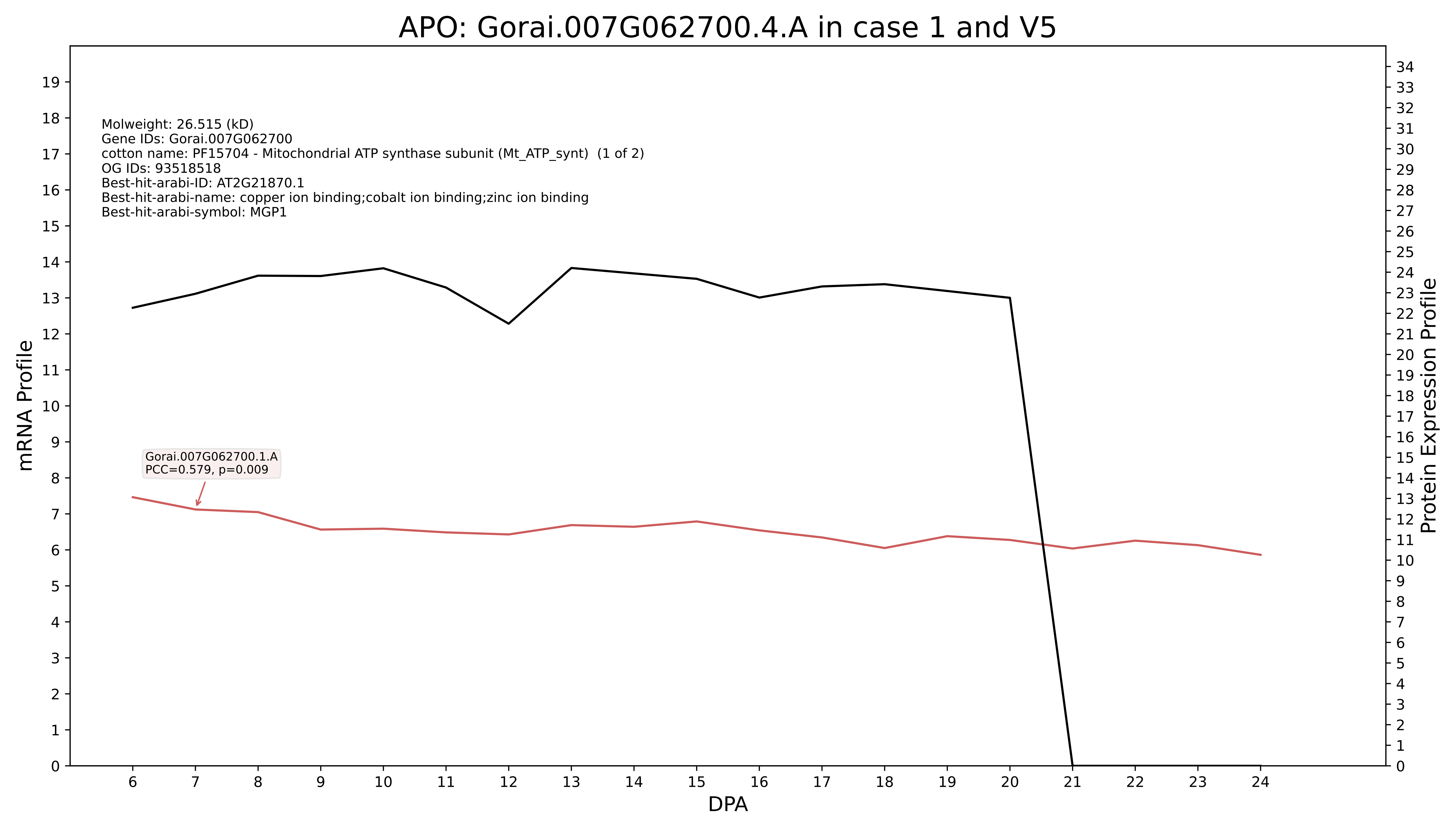
APO: Gorai.007G062700.4.A in case 1 and V5
Molweight: 26.515 (kD)
Gene IDs: Gorai.007G062700
cotton name: PF15704 - Mitochondrial ATP synthase subunit (Mt_ATP_synt) (1 of 2)
OG IDs: 93518518
Best-hit-arabi-ID: AT2G21870.1
Best-hit-arabi-name: copper ion binding;cobalt ion binding;zinc ion binding
Best-hit-arabi-symbol: MGP1
Gorai.007G062700.1.A
PCC=0.579, p=0.009
mRNA Profile
Protein Expression Profile
DPA

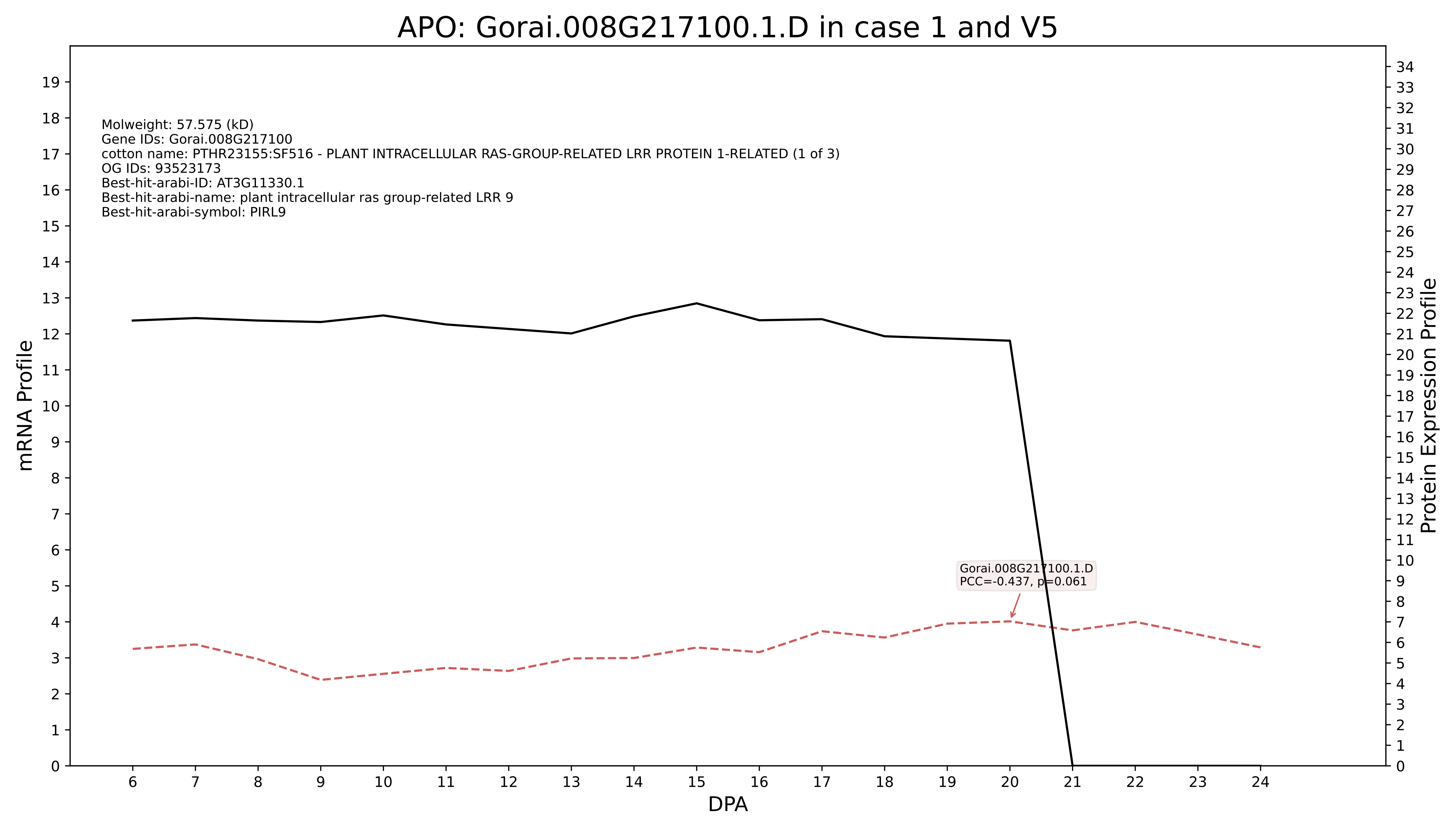
APO: Gorai.008G217100.1.D in case 1 and V5
Molweight: 57.575 (kD)
Gene IDs: Gorai.008G217100
cotton name: PTHR23155:SF516 - PLANT INTRACELLULAR RAS-GROUP-RELATED LRR PROTEIN 1-RELATED (1 of 3)
OG IDs: 93523173
Best-hit-arabi-ID: AT3G11330.1
Best-hit-arabi-name: plant intracellular ras group-related LRR 9
Best-hit-arabi-symbol: PIRL9
Gorai.008G217100.1.D
PCC=-0.437, p=0.061
mRNA Profile
Protein Expression Profile
DPA

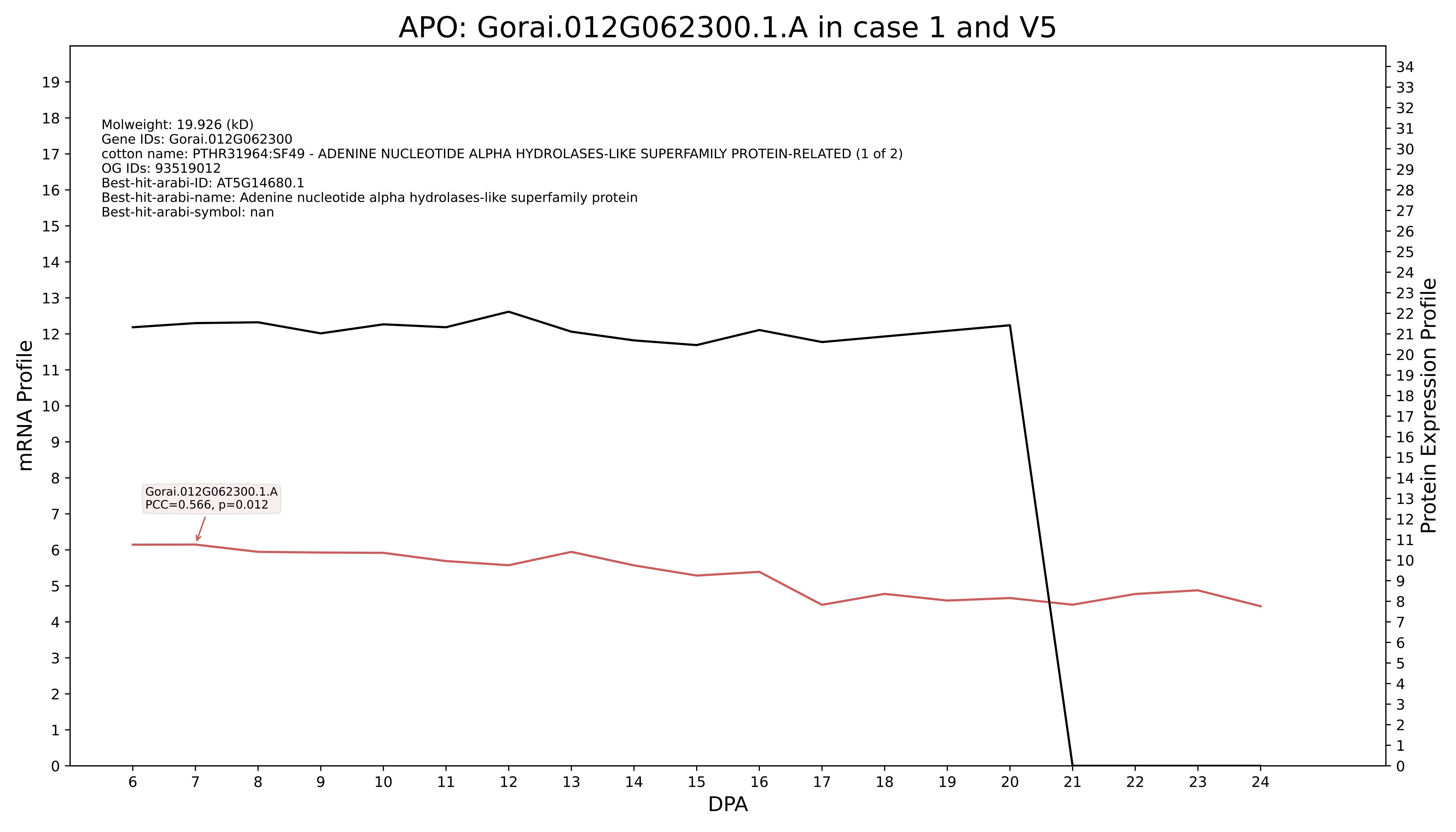
APO: Gorai.012G062300.1.A in case 1 and V5
Molweight: 19.926 (kD)
Gene IDs: Gorai.012G062300
cotton name: PTHR31964:SF49 - ADENINE NUCLEOTIDE ALPHA HYDROLASES-LIKE SUPERFAMILY PROTEIN-RELATED (1 of 2)
OG IDs: 93519012
Best-hit-arabi-ID: AT5G14680.1
Best-hit-arabi-name: Adenine nucleotide alpha hydrolases-like superfamily protein
Best-hit-arabi-symbol: nan
Gorai.012G062300.1.A
PCC=0.566, p=0.012
mRNA Profile
Protein Expression Profile
DPA

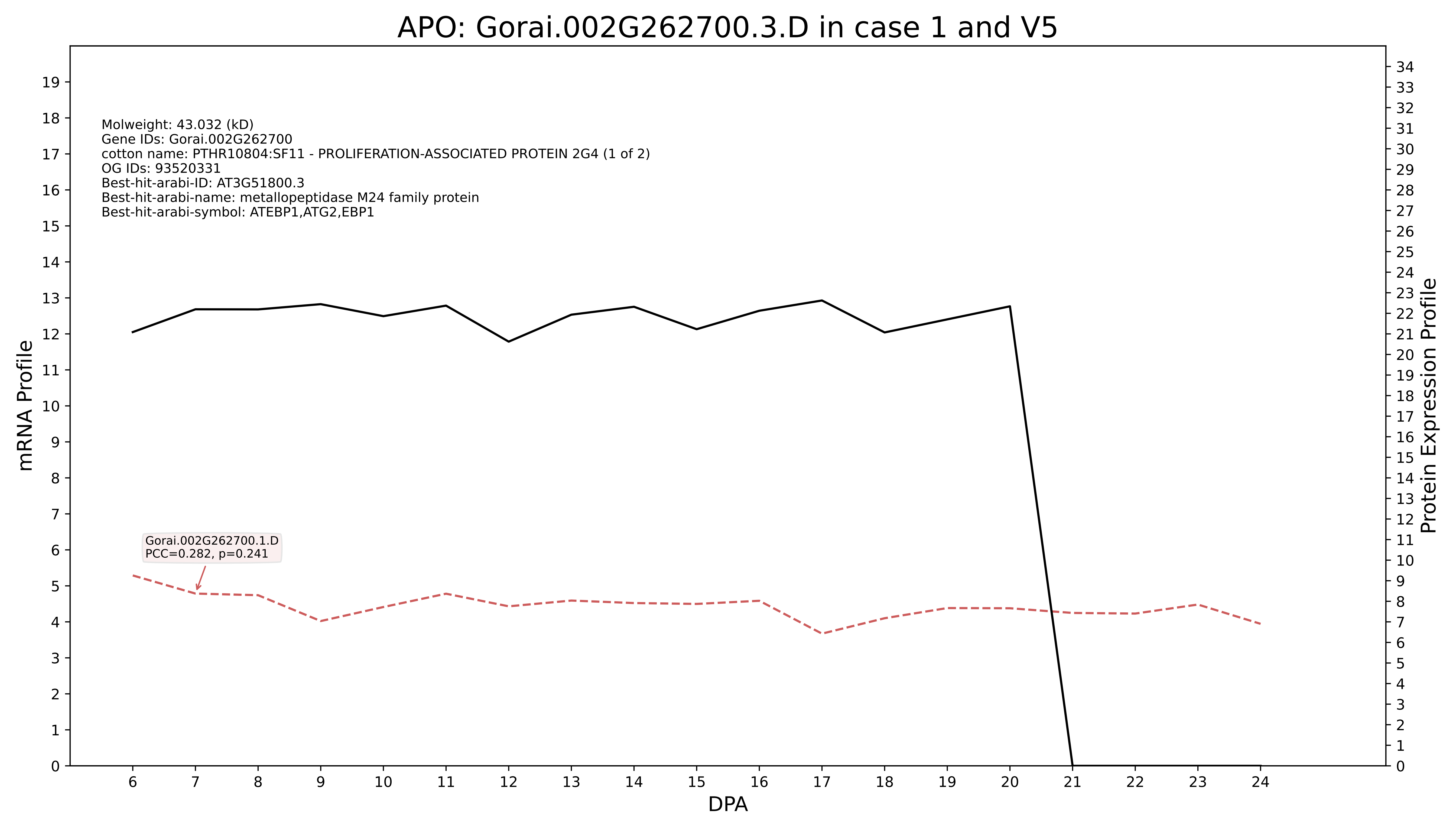

APO: Gorai.002G262700.3.D in case 1 and V5
Molweight: 43.032 (kD)
Gene IDs: Gorai.002G262700
cotton name: PTHR10804:SF11 - PROLIFERATION-ASSOCIATED PROTEIN 2G4 (1 of 2)
OG IDs: 93520331
Best-hit-arabi-ID: AT3G51800.3
Best-hit-arabi-name: metallopeptidase M24 family protein
Best-hit-arabi-symbol: ATEBP1,ATG2,EBP1
Gorai.002G262700.1.D
PCC=0.282, p=0.241
mRNA Profile
Protein Expression Profile
DPA

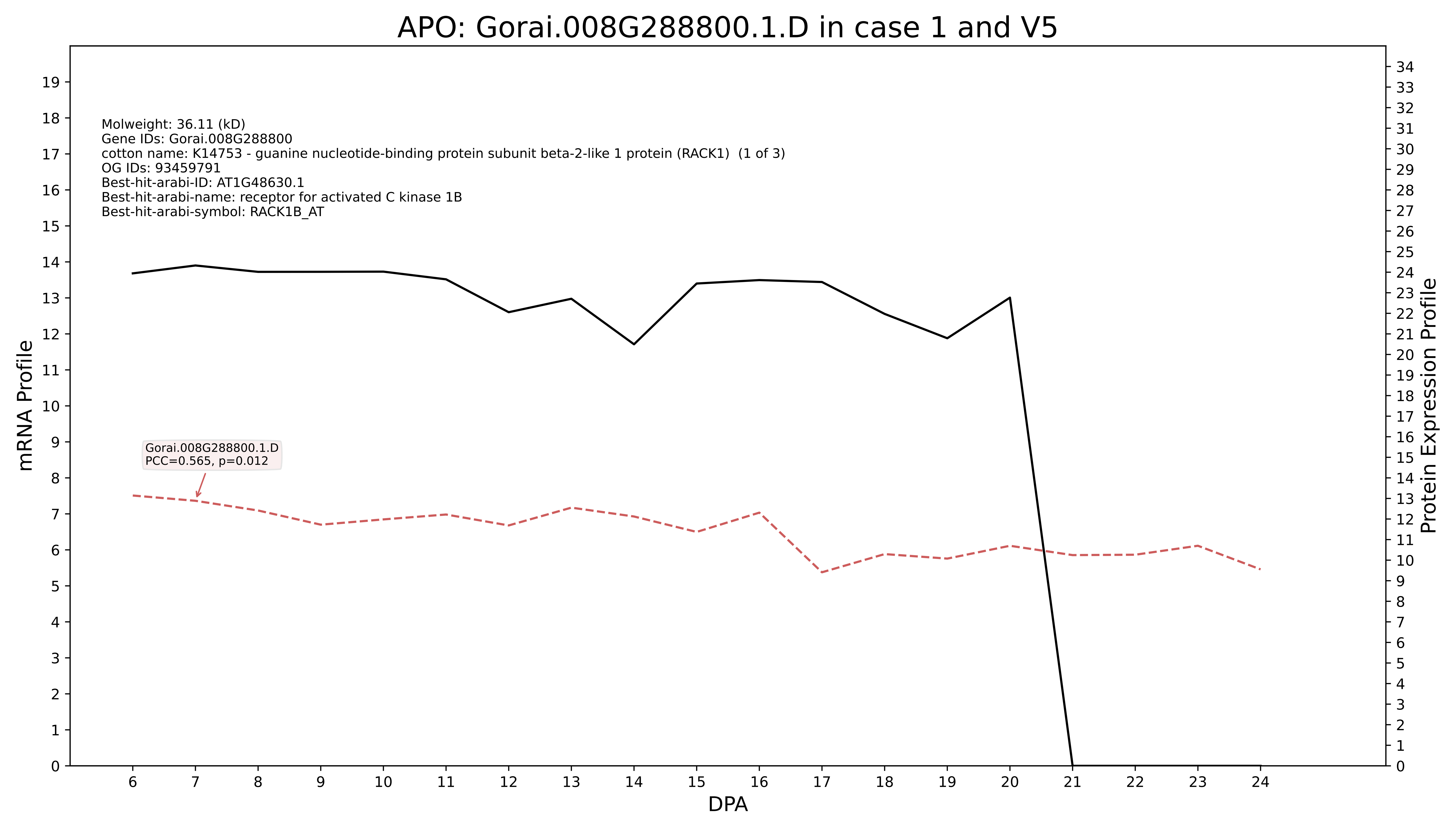
APO: Gorai.008G288800.1.D in case 1 and V5
Molweight: 36.11 (kD)
Gene IDs: Gorai.008G288800
cotton name: K14753 - guanine nucleotide-binding protein subunit beta-2-like 1 protein (RACK1) (1 of 3)
OG IDs: 93459791
Best-hit-arabi-ID: AT1G48630.1
Best-hit-arabi-name: receptor for activated C kinase 1B
Best-hit-arabi-symbol: RACK1B_AT
Gorai.008G288800.1.D
PCC=0.565, p=0.012
mRNA Profile
Protein Expression Profile
DPA

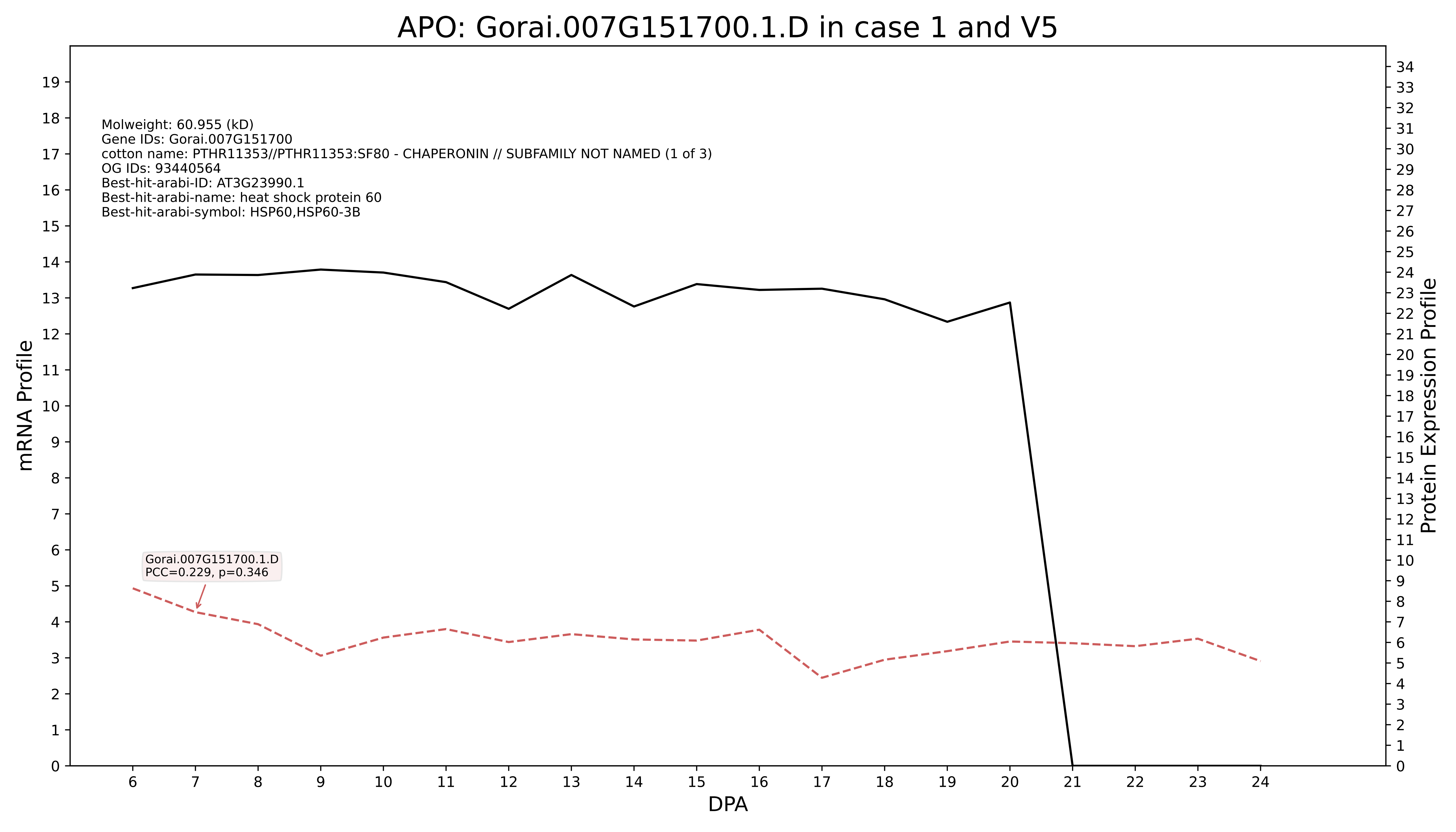
APO: Gorai.007G151700.1.D in case 1 and V5
Molweight: 60.955 (kD)
Gene IDs: Gorai.007G151700
cotton name: PTHR11353//PTHR11353:SF80 - CHAPERONIN // SUBFAMILY NOT NAMED (1 of 3)
OG IDs: 93440564
Best-hit-arabi-ID: AT3G23990.1
Best-hit-arabi-name: heat shock protein 60
Best-hit-arabi-symbol: HSP60,HSP60-3B
Gorai.007G151700.1.D
PCC=0.229, p=0.346
mRNA Profile
Protein Expression Profile
DPA
0
1
2
3
4
5
6
7
8
9
10
11
12
13
14
15
16
17
18
19
20
21
22
23
24
25
26
27
28
29
30
31
32
33
34

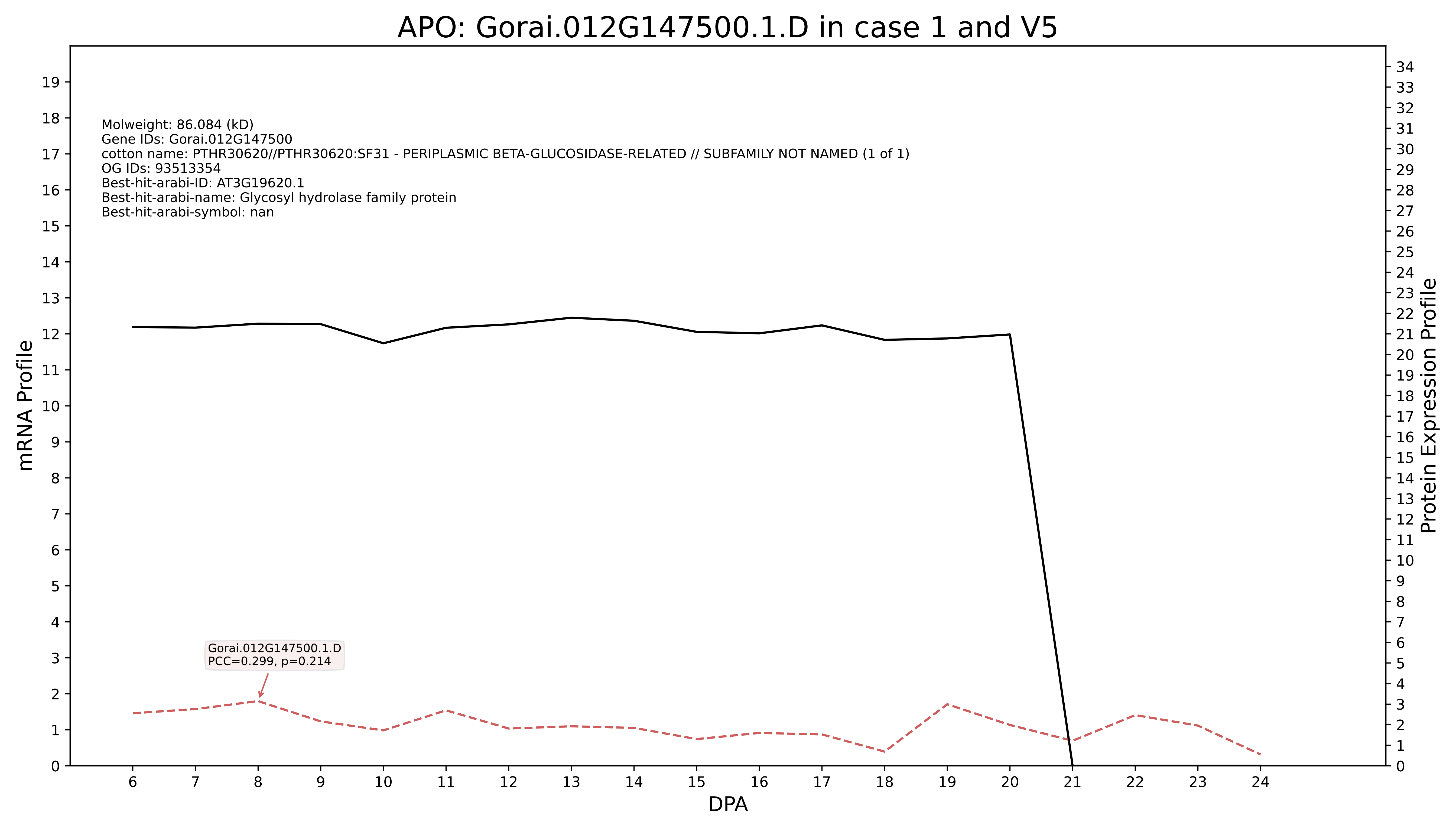

APO: Gorai.012G147500.1.D in case 1 and V5
Molweight: 86.084 (kD)
Gene IDs: Gorai.012G147500
cotton name: PTHR30620//PTHR30620:SF31 - PERIPLASMIC BETA-GLUCOSIDASE-RELATED // SUBFAMILY NOT NAMED (1 of 1)
OG IDs: 93513354
Best-hit-arabi-ID: AT3G19620.1
Best-hit-arabi-name: Glycosyl hydrolase family protein
Best-hit-arabi-symbol: nan
Gorai.012G147500.1.D
PCC=0.299, p=0.214
mRNA Profile
Protein Expression Profile
DPA

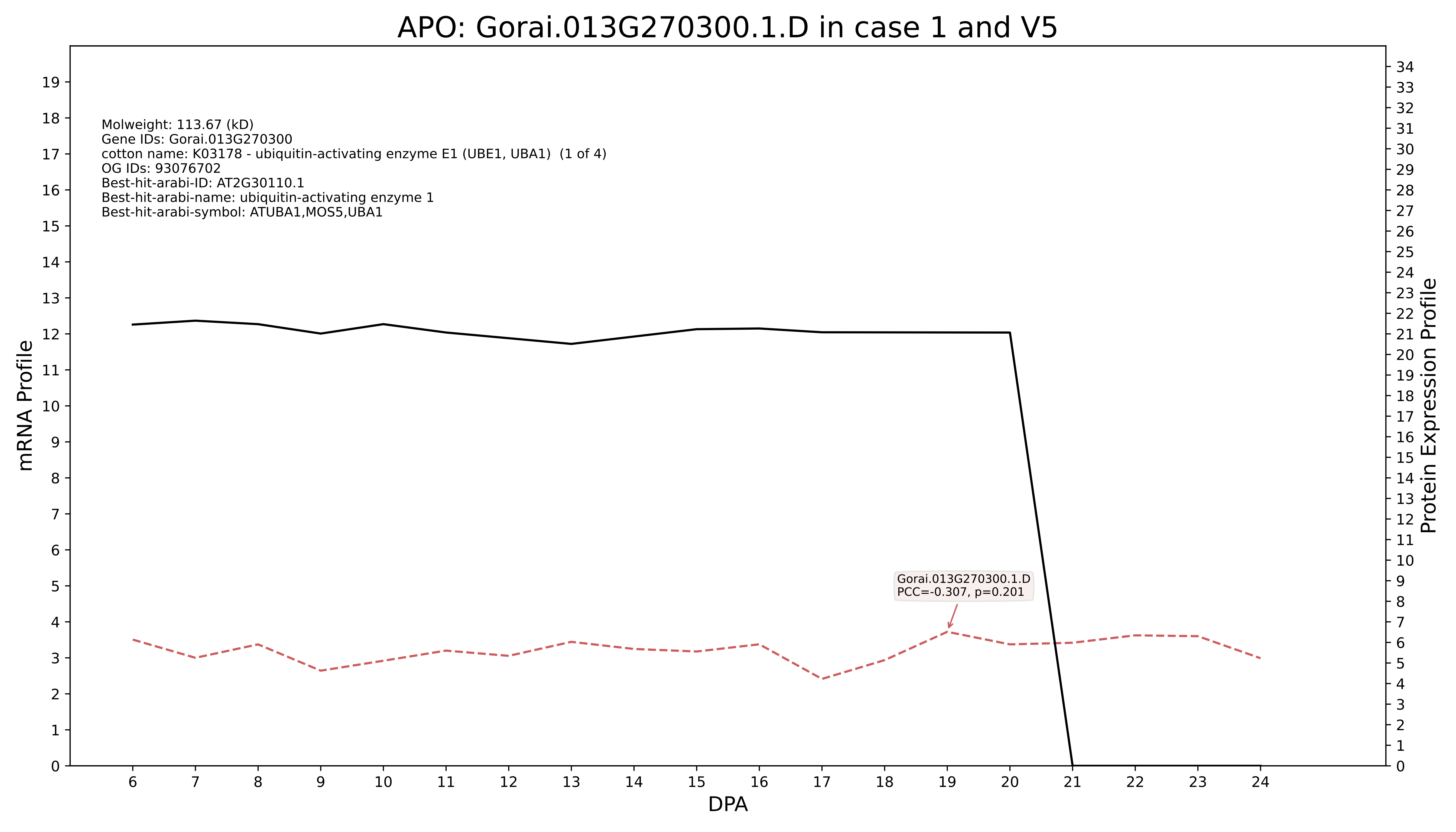
APO: Gorai.013G270300.1.D in case 1 and V5
Molweight: 113.67 (kD)
Gene IDs: Gorai.013G270300
cotton name: K03178 - ubiquitin-activating enzyme E1 (UBE1, UBA1) (1 of 4)
OG IDs: 93076702
Best-hit-arabi-ID: AT2G30110.1
Best-hit-arabi-name: ubiquitin-activating enzyme 1
Best-hit-arabi-symbol: ATUBA1,MOS5,UBA1
Gorai.013G270300.1.D
PCC=-0.307, p=0.201
mRNA Profile
Protein Expression Profile
DPA

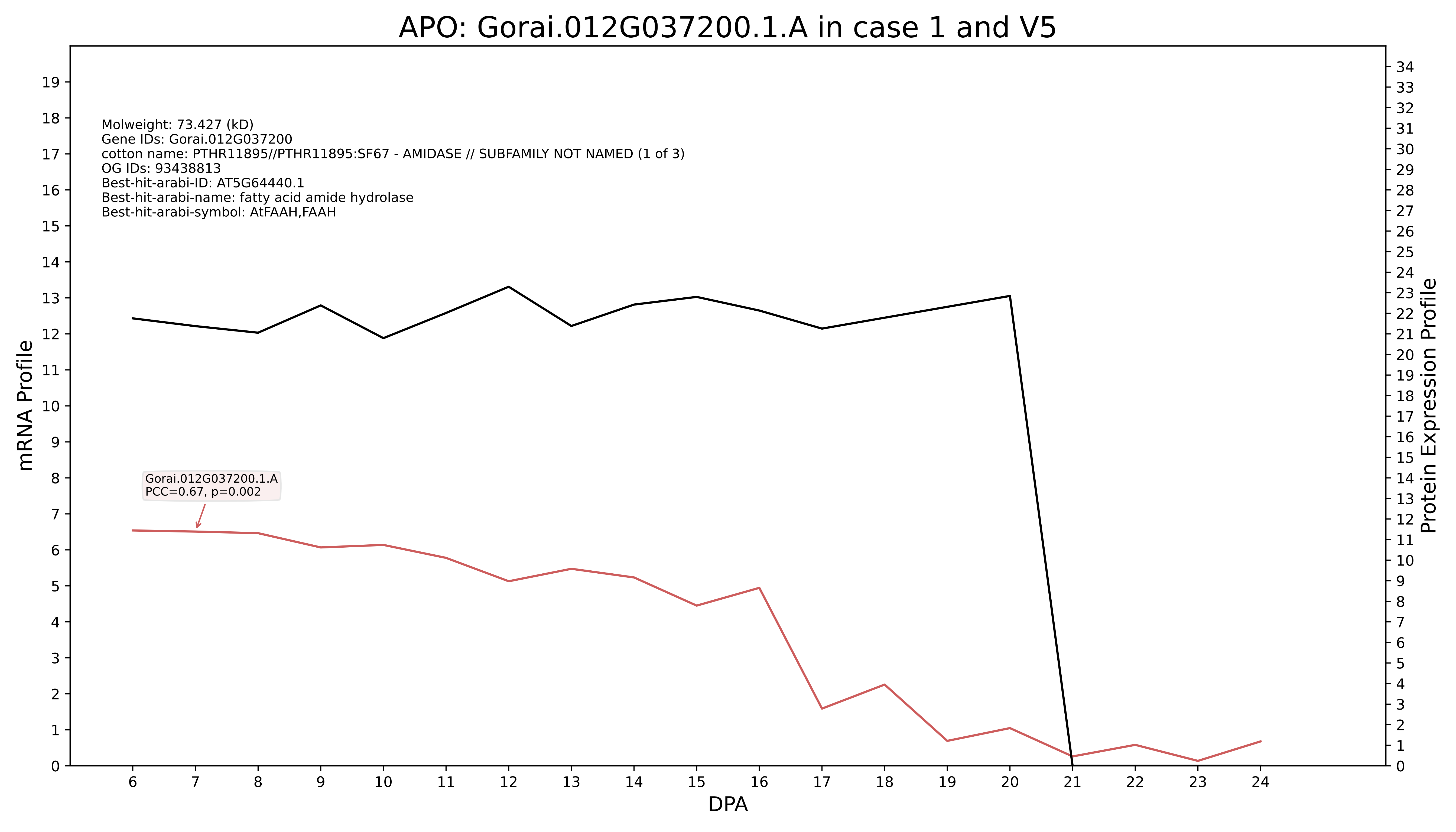
APO: Gorai.012G037200.1.A in case 1 and V5
Molweight: 73.427 (kD)
Gene IDs: Gorai.012G037200
cotton name: PTHR11895//PTHR11895:SF67 - AMIDASE // SUBFAMILY NOT NAMED (1 of 3)
OG IDs: 93438813
Best-hit-arabi-ID: AT5G64440.1
Best-hit-arabi-name: fatty acid amide hydrolase
Best-hit-arabi-symbol: AtFAAH,FAAH
Gorai.012G037200.1.A
PCC=0.67, p=0.002
mRNA Profile
Protein Expression Profile
DPA
0
1
2
3
4
5
6
7
8
9
10
11
12
13
14
15
16
17
18
19
20
21
22
23
24
25
26
27
28
29
30
31
32
33
34

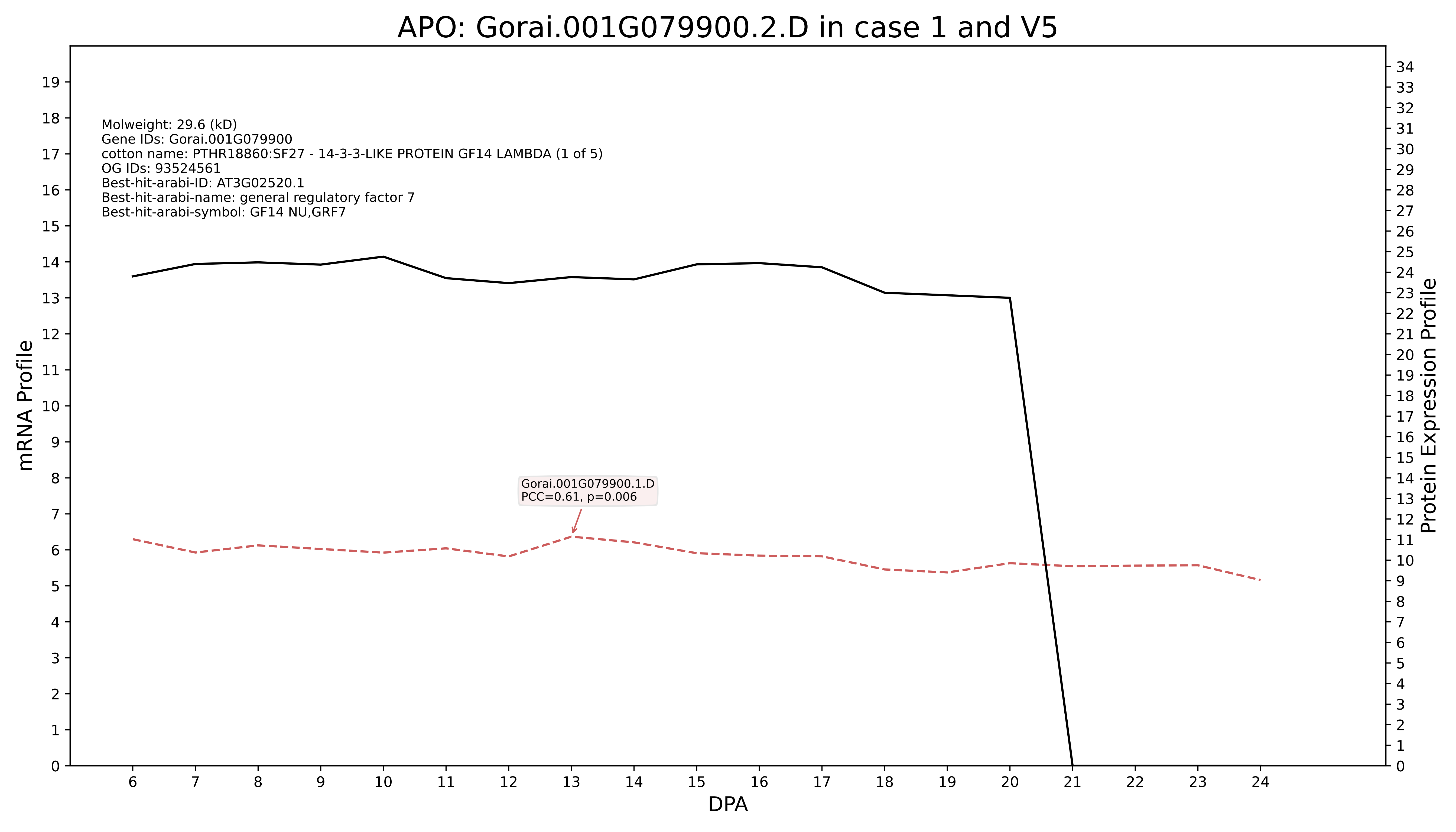
APO: Gorai.001G079900.2.D in case 1 and V5
Molweight: 29.6 (kD)
Gene IDs: Gorai.001G079900
cotton name: PTHR18860:SF27 - 14-3-3-LIKE PROTEIN GF14 LAMBDA (1 of 5)
OG IDs: 93524561
Best-hit-arabi-ID: AT3G02520.1
Best-hit-arabi-name: general regulatory factor 7
Best-hit-arabi-symbol: GF14 NU,GRF7
Gorai.001G079900.1.D
PCC=0.61, p=0.006
mRNA Profile
Protein Expression Profile
DPA

APO: Gorai.006G041700.5.D in case 1 and V5

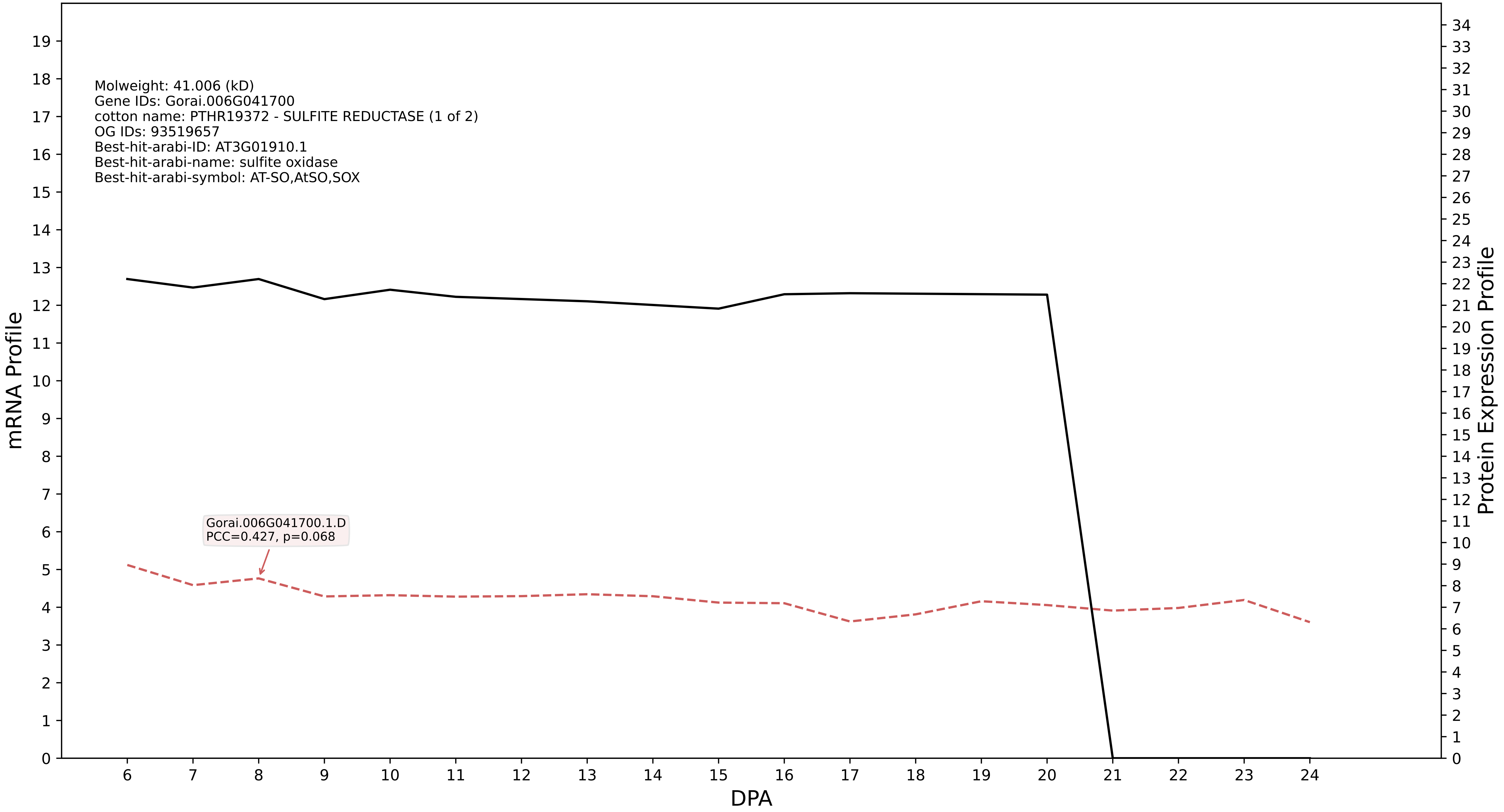

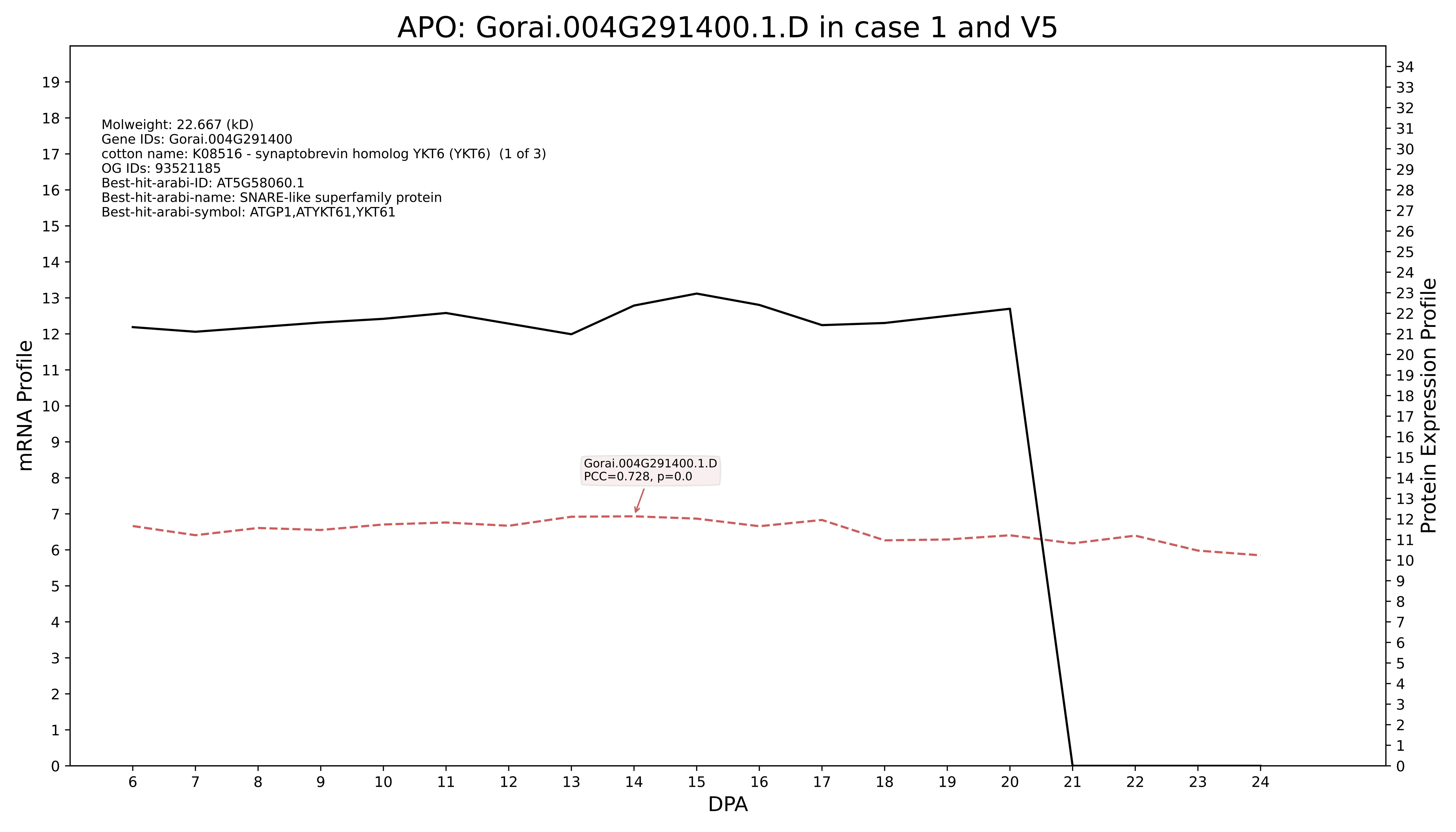

APO: Gorai.004G291400.1.D in case 1 and V5
Molweight: 22.667 (kD)
Gene IDs: Gorai.004G291400
cotton name: K08516 - synaptobrevin homolog YKT6 (YKT6) (1 of 3)
OG IDs: 93521185
Best-hit-arabi-ID: AT5G58060.1
Best-hit-arabi-name: SNARE-like superfamily protein
Best-hit-arabi-symbol: ATGP1,ATYKT61,YKT61
Gorai.004G291400.1.D
PCC=0.728, p=0.0
mRNA Profile
Protein Expression Profile
DPA

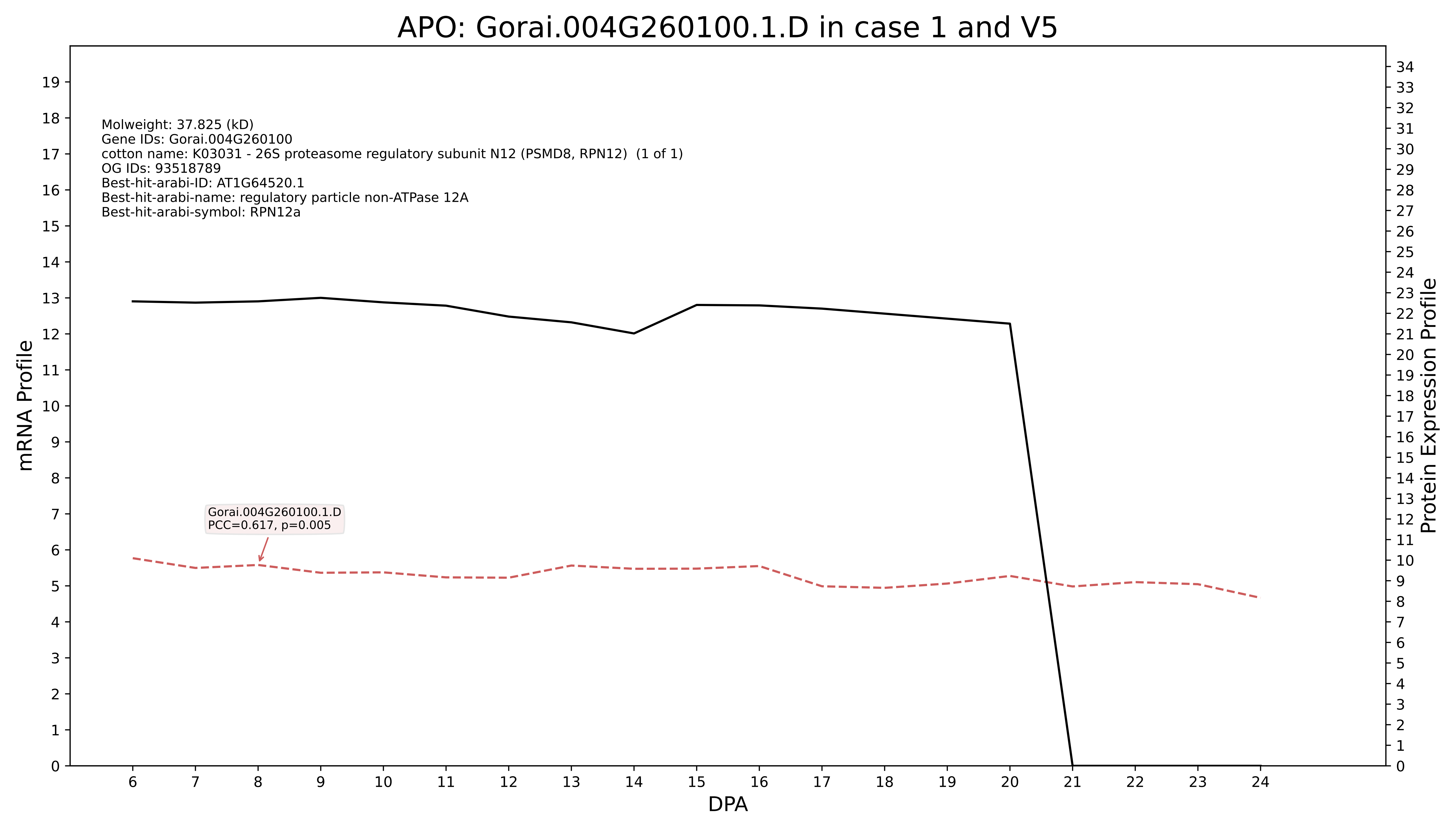

APO: Gorai.004G260100.1.D in case 1 and V5
Molweight: 37.825 (kD)
Gene IDs: Gorai.004G260100
cotton name: K03031 - 26S proteasome regulatory subunit N12 (PSMD8, RPN12) (1 of 1)
OG IDs: 93518789
Best-hit-arabi-ID: AT1G64520.1
Best-hit-arabi-name: regulatory particle non-ATPase 12A
Best-hit-arabi-symbol: RPN12a
Gorai.004G260100.1.D
PCC=0.617, p=0.005
mRNA Profile
Protein Expression Profile
DPA

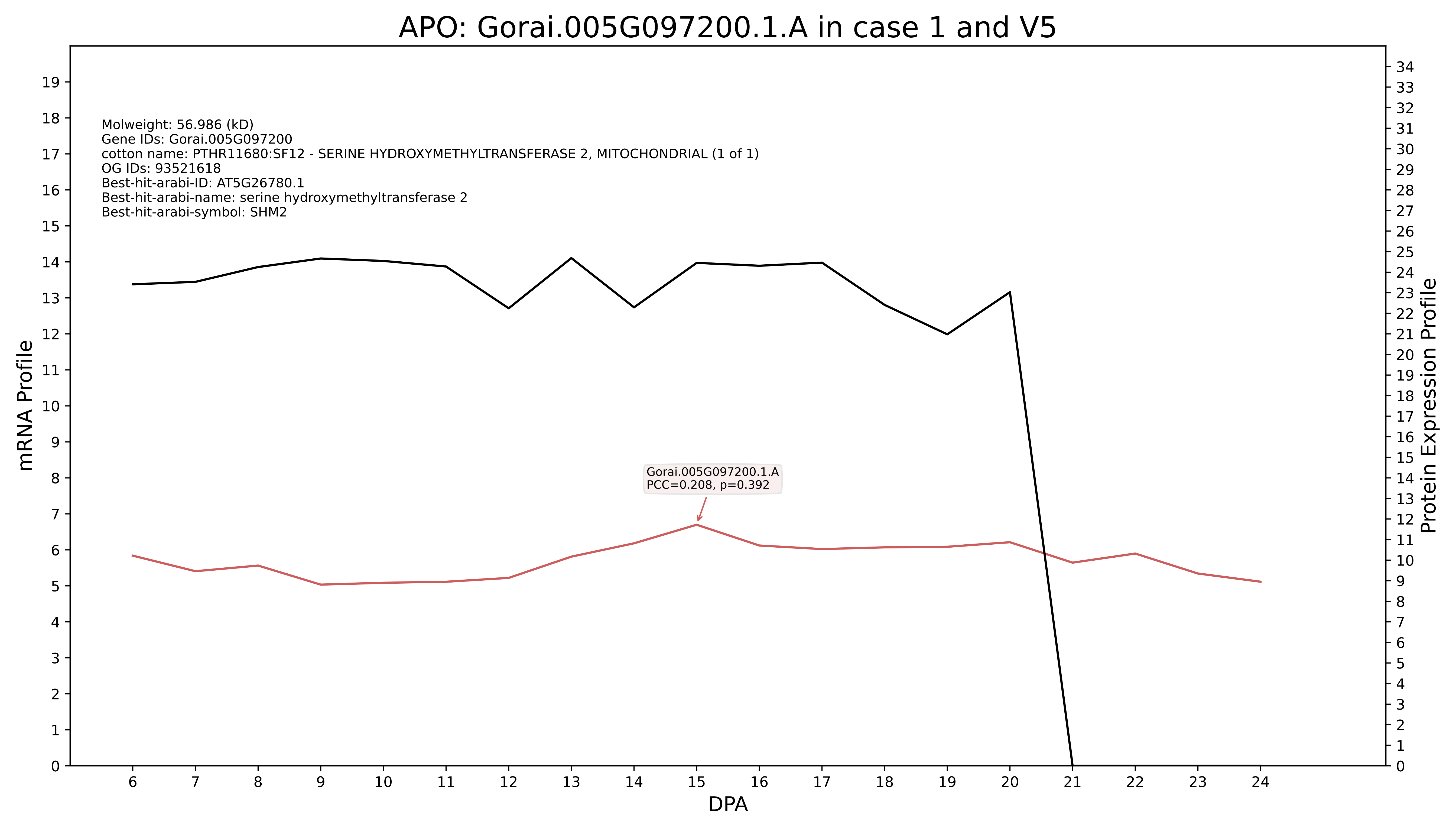
APO: Gorai.005G097200.1.A in case 1 and V5
Molweight: 56.986 (kD)
Gene IDs: Gorai.005G097200
cotton name: PTHR11680:SF12 - SERINE HYDROXYMETHYLTRANSFERASE 2, MITOCHONDRIAL (1 of 1)
OG IDs: 93521618
Best-hit-arabi-ID: AT5G26780.1
Best-hit-arabi-name: serine hydroxymethyltransferase 2
Best-hit-arabi-symbol: SHM2
Gorai.005G097200.1.A
PCC=0.208, p=0.392
mRNA Profile
Protein Expression Profile
DPA

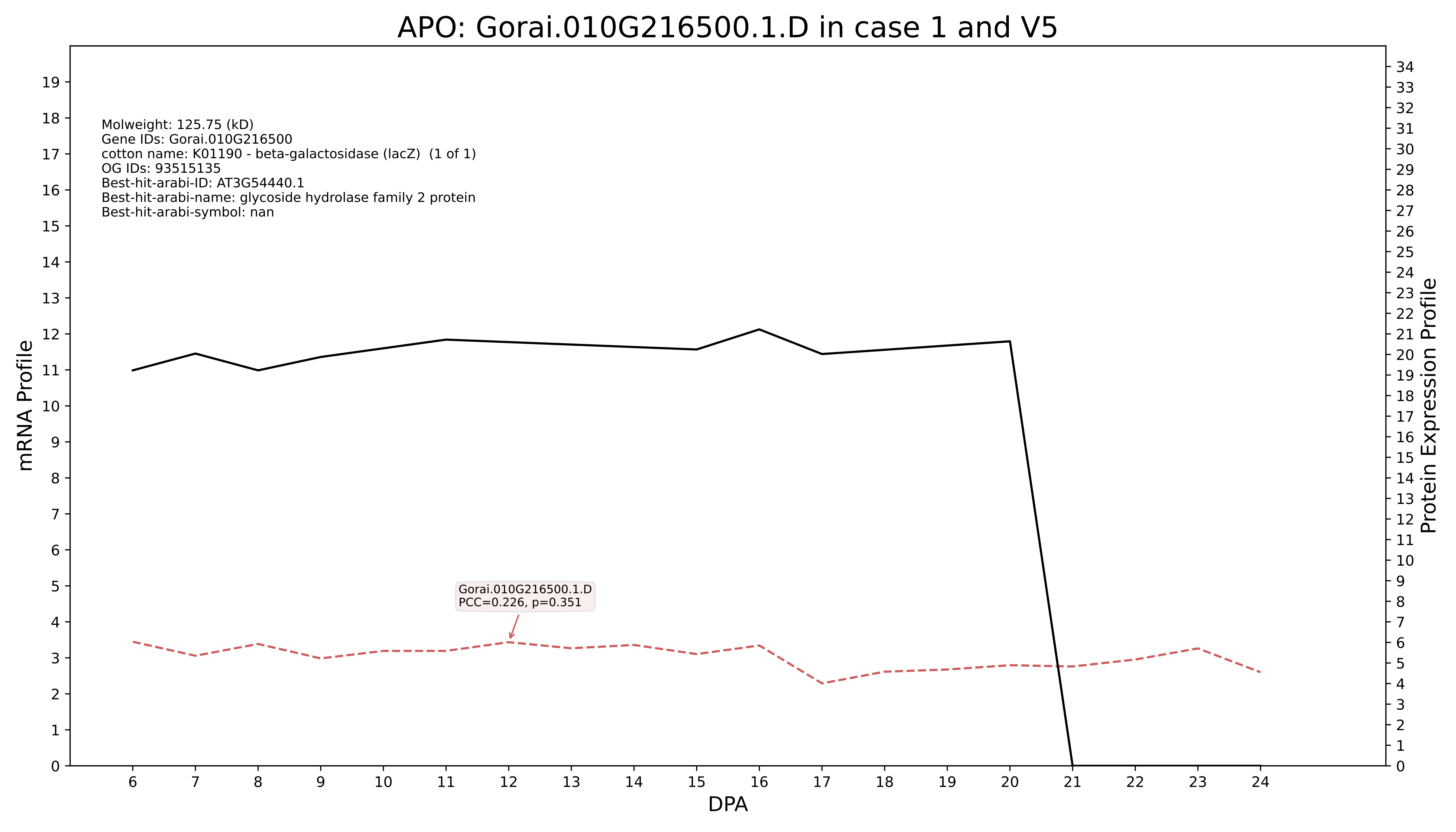

APO: Gorai.010G216500.1.D in case 1 and V5
Molweight: 125.75 (kD)
Gene IDs: Gorai.010G216500
cotton name: K01190 - beta-galactosidase (lacZ) (1 of 1)
OG IDs: 93515135
Best-hit-arabi-ID: AT3G54440.1
Best-hit-arabi-name: glycoside hydrolase family 2 protein
Best-hit-arabi-symbol: nan
Gorai.010G216500.1.D
PCC=0.226, p=0.351
mRNA Profile
Protein Expression Profile
DPA

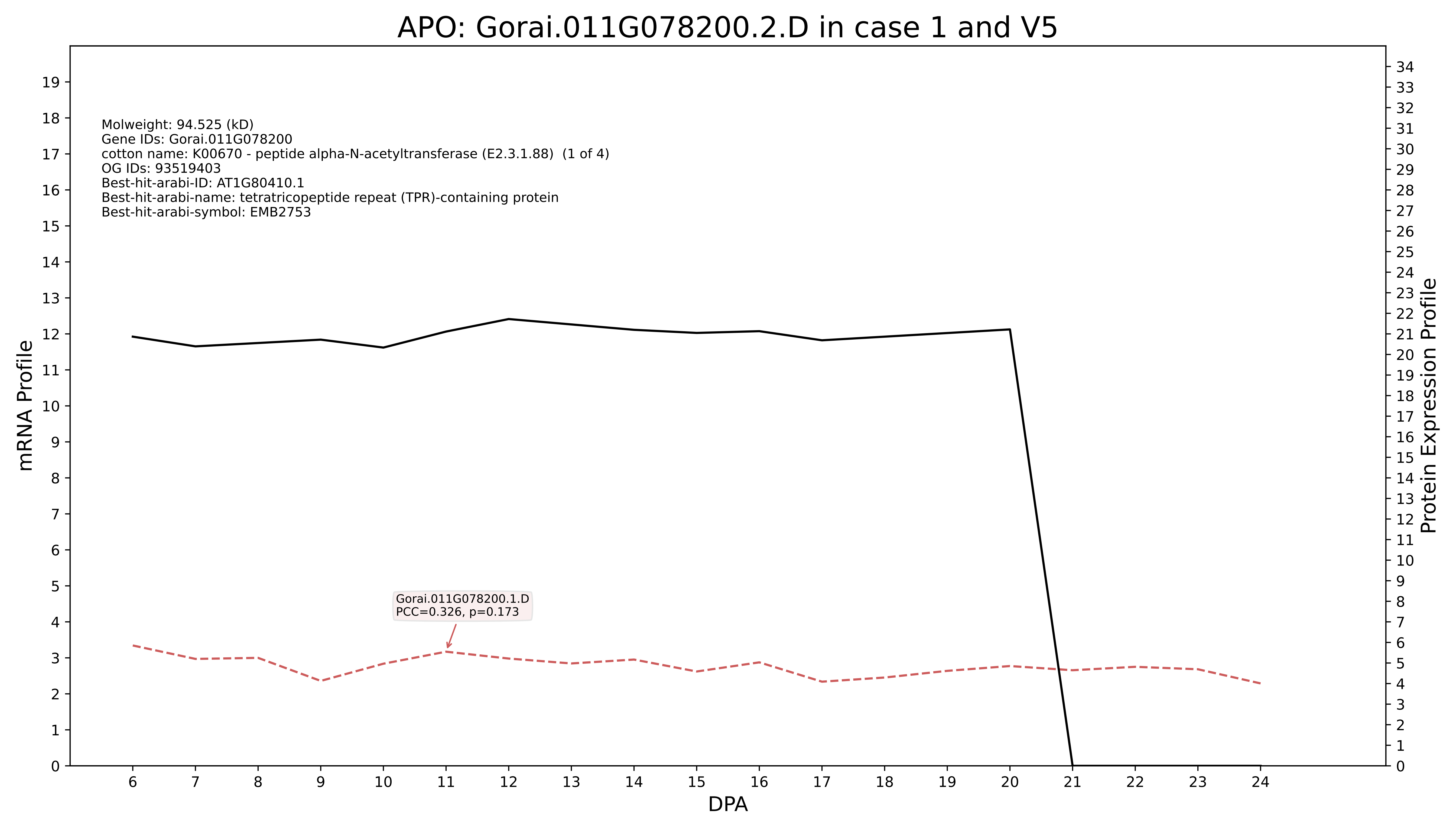

APO: Gorai.011G078200.2.D in case 1 and V5
Molweight: 94.525 (kD)
Gene IDs: Gorai.011G078200
cotton name: K00670 - peptide alpha-N-acetyltransferase (E2.3.1.88) (1 of 4)
OG IDs: 93519403
Best-hit-arabi-ID: AT1G80410.1
Best-hit-arabi-name: tetratricopeptide repeat (TPR)-containing protein
Best-hit-arabi-symbol: EMB2753
Gorai.011G078200.1.D
PCC=0.326, p=0.173
mRNA Profile
Protein Expression Profile
DPA

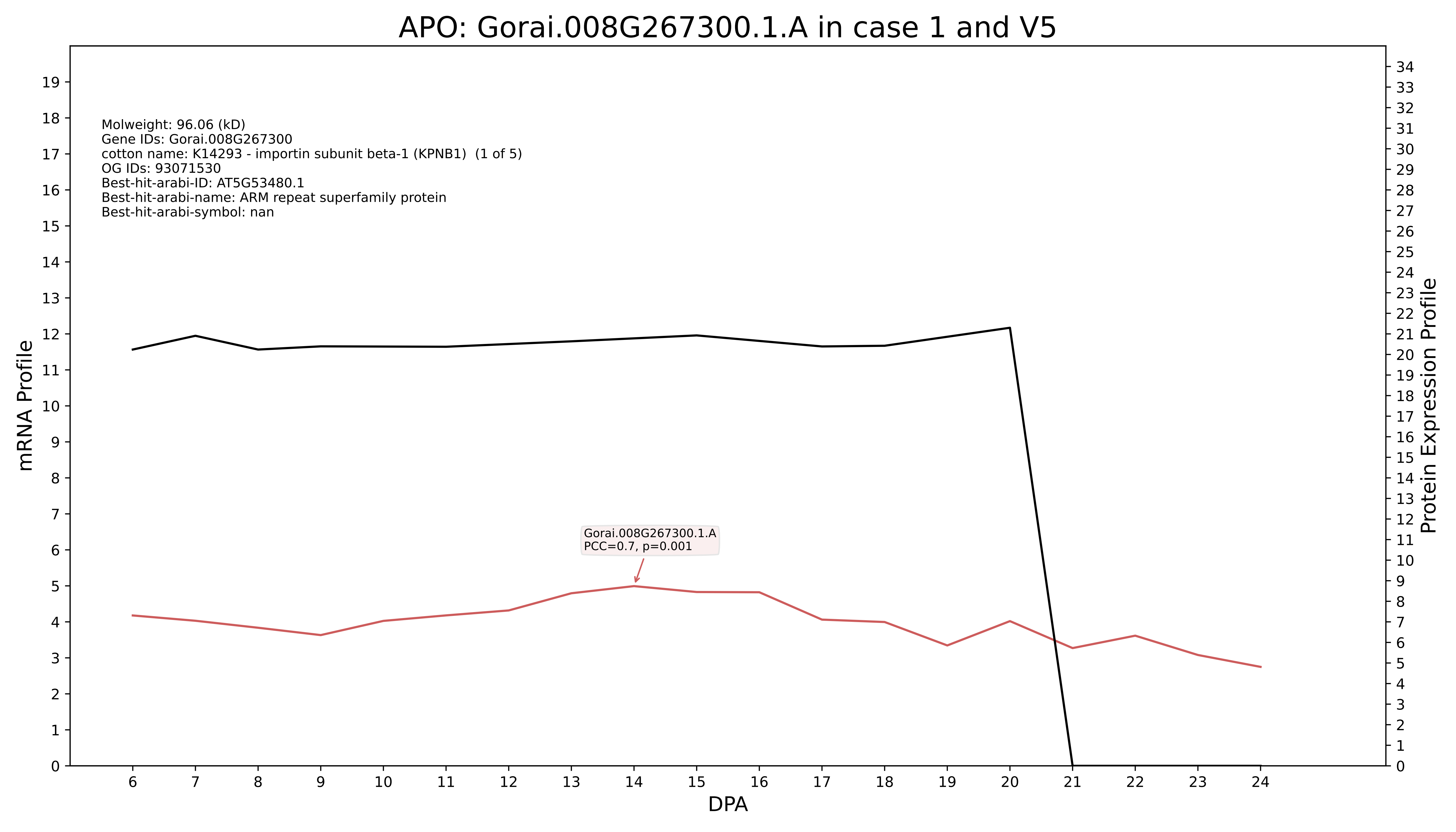

APO: Gorai.008G267300.1.A in case 1 and V5
Molweight: 96.06 (kD)
Gene IDs: Gorai.008G267300
cotton name: K14293 - importin subunit beta-1 (KPNB1) (1 of 5)
OG IDs: 93071530
Best-hit-arabi-ID: AT5G53480.1
Best-hit-arabi-name: ARM repeat superfamily protein
Best-hit-arabi-symbol: nan
Gorai.008G267300.1.A
PCC=0.7, p=0.001
mRNA Profile
Protein Expression Profile
DPA

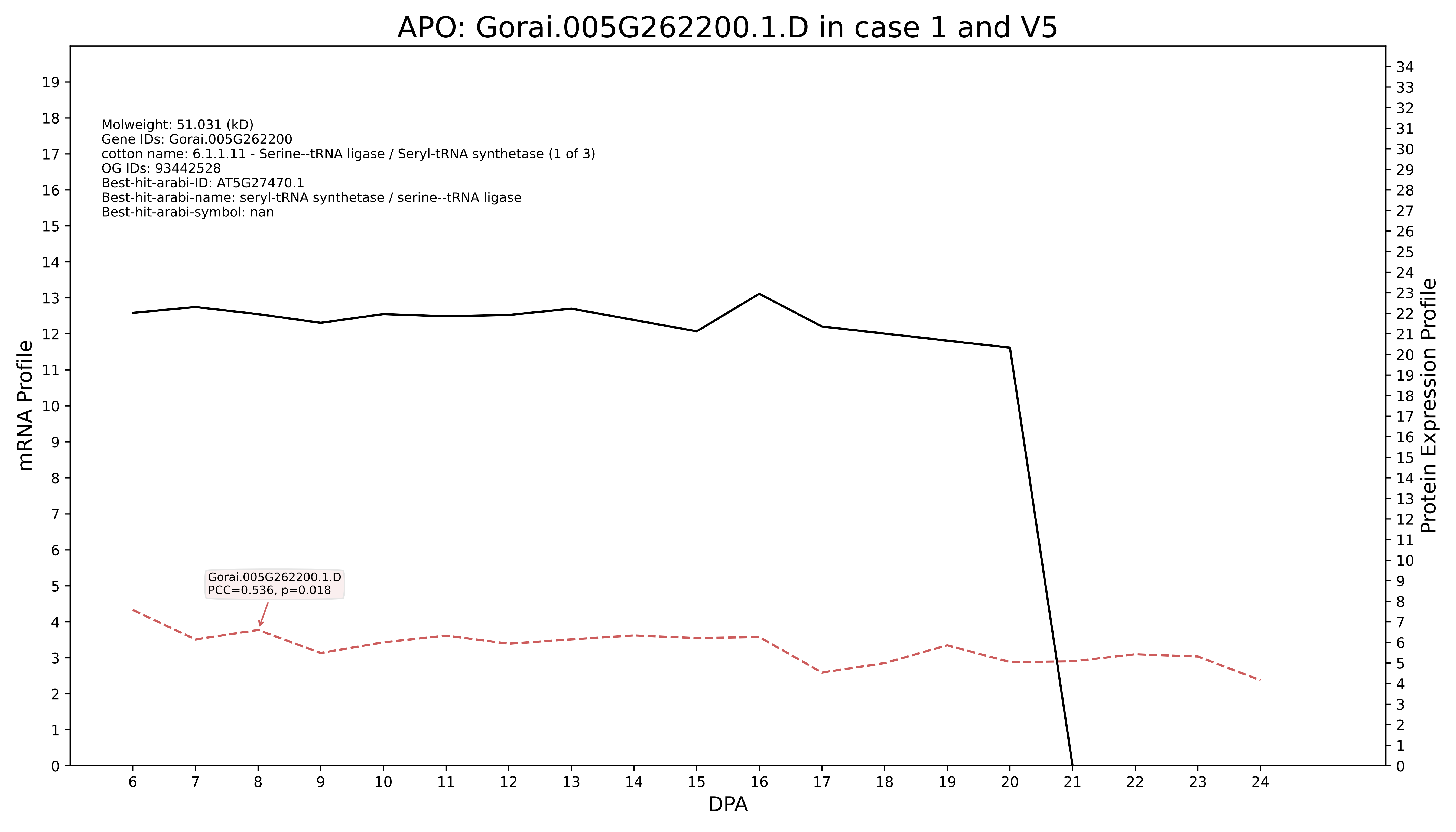
APO: Gorai.005G262200.1.D in case 1 and V5
Molweight: 51.031 (kD)
Gene IDs: Gorai.005G262200
cotton name: 6.1.1.11 - Serine--tRNA ligase / Seryl-tRNA synthetase (1 of 3)
OG IDs: 93442528
Best-hit-arabi-ID: AT5G27470.1
Best-hit-arabi-name: seryl-tRNA synthetase / serine--tRNA ligase
Best-hit-arabi-symbol: nan
Gorai.005G262200.1.D
PCC=0.536, p=0.018
mRNA Profile
Protein Expression Profile
DPA

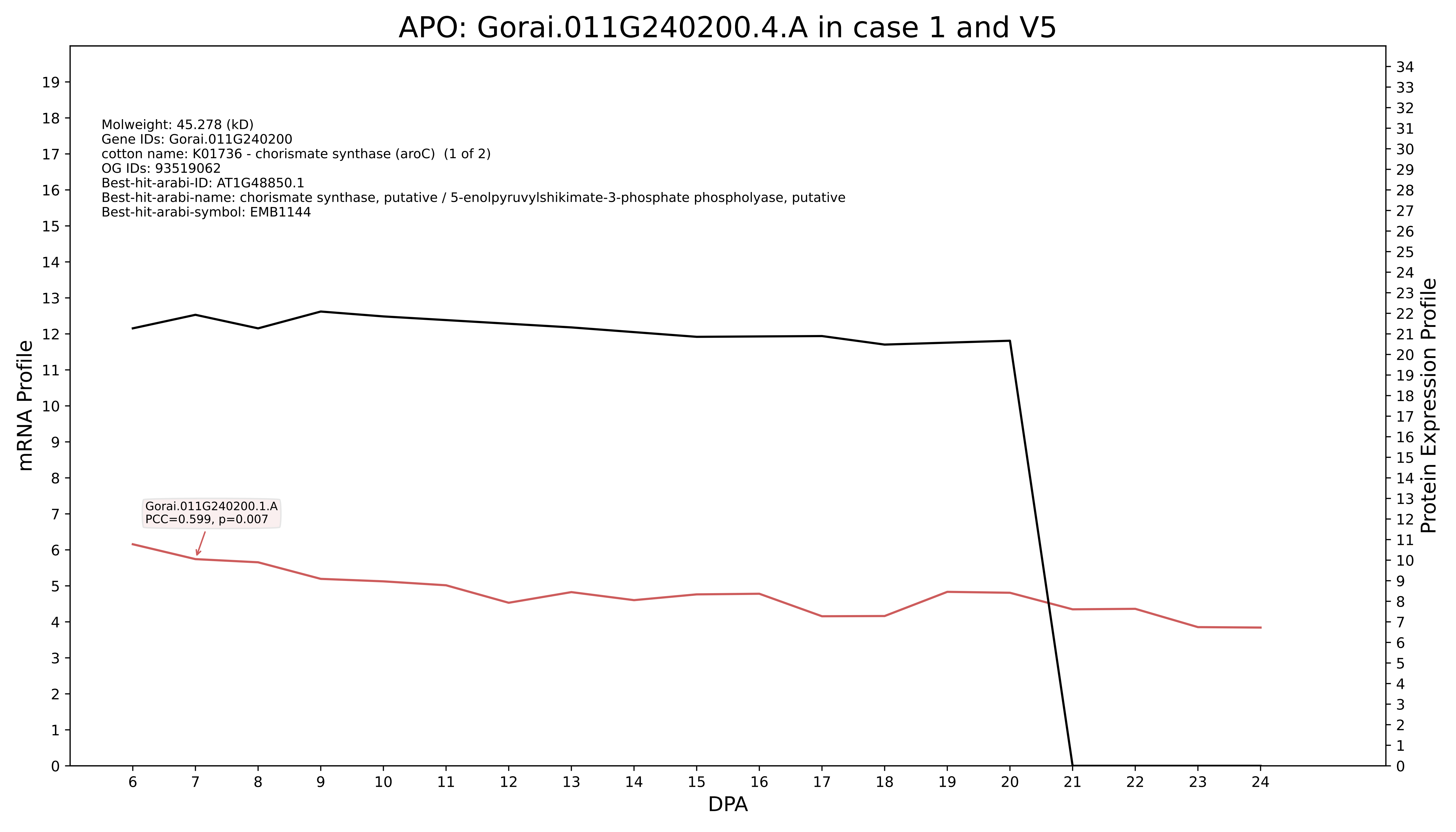
APO: Gorai.011G240200.4.A in case 1 and V5
Molweight: 45.278 (kD)
Gene IDs: Gorai.011G240200
cotton name: K01736 - chorismate synthase (aroC) (1 of 2)
OG IDs: 93519062
Best-hit-arabi-ID: AT1G48850.1
Best-hit-arabi-name: chorismate synthase, putative / 5-enolpyruvylshikimate-3-phosphate phospholyase, putative
Best-hit-arabi-symbol: EMB1144
Gorai.011G240200.1.A
PCC=0.599, p=0.007
mRNA Profile
Protein Expression Profile
DPA

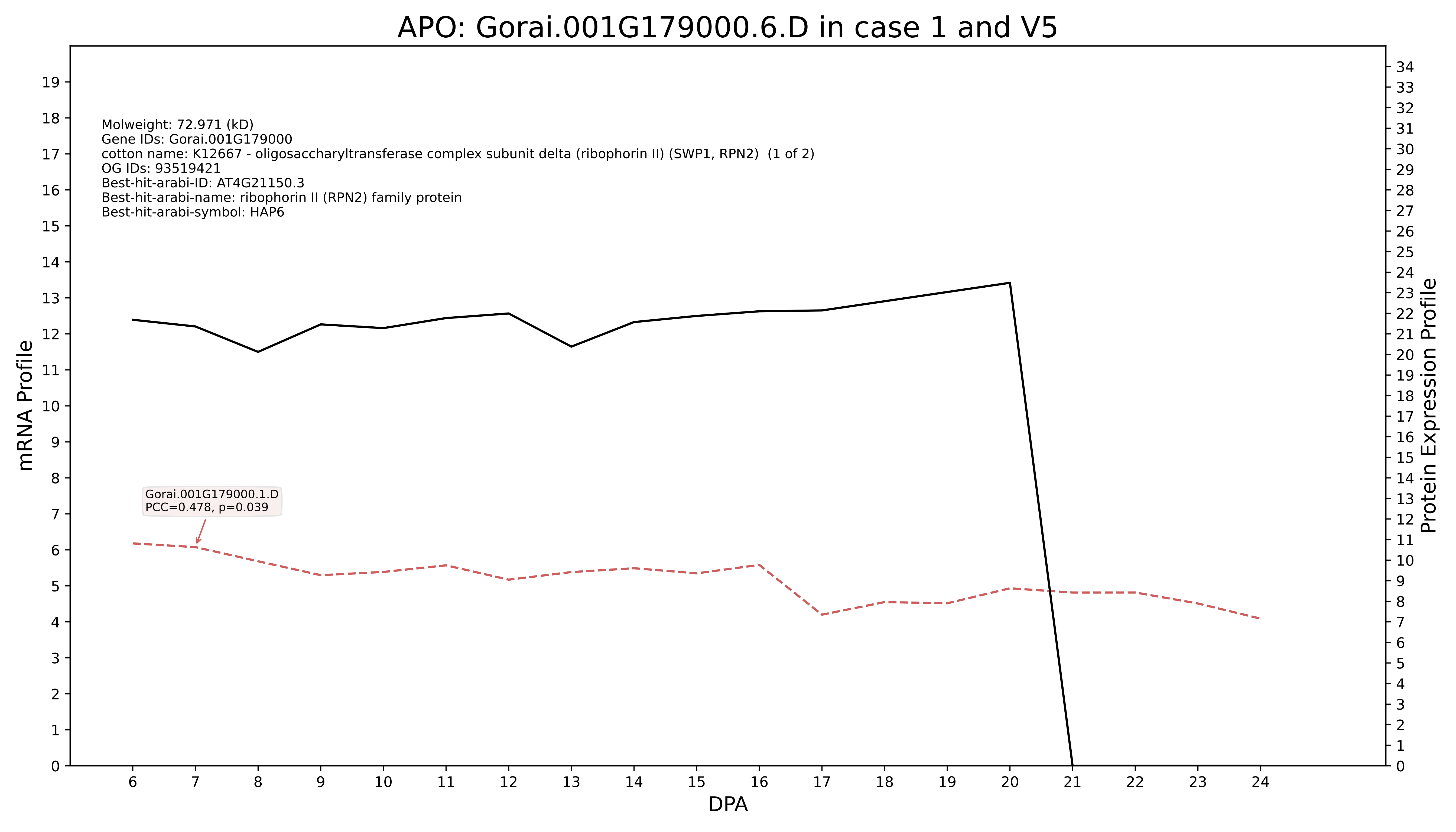

APO: Gorai.001G179000.6.D in case 1 and V5
Molweight: 72.971 (kD)
Gene IDs: Gorai.001G179000
cotton name: K12667 - oligosaccharyltransferase complex subunit delta (ribophorin II) (SWP1, RPN2) (1 of 2)
OG IDs: 93519421
Best-hit-arabi-ID: AT4G21150.3
Best-hit-arabi-name: ribophorin II (RPN2) family protein
Best-hit-arabi-symbol: HAP6
Gorai.001G179000.1.D
PCC=0.478, p=0.039
mRNA Profile
Protein Expression Profile
DPA

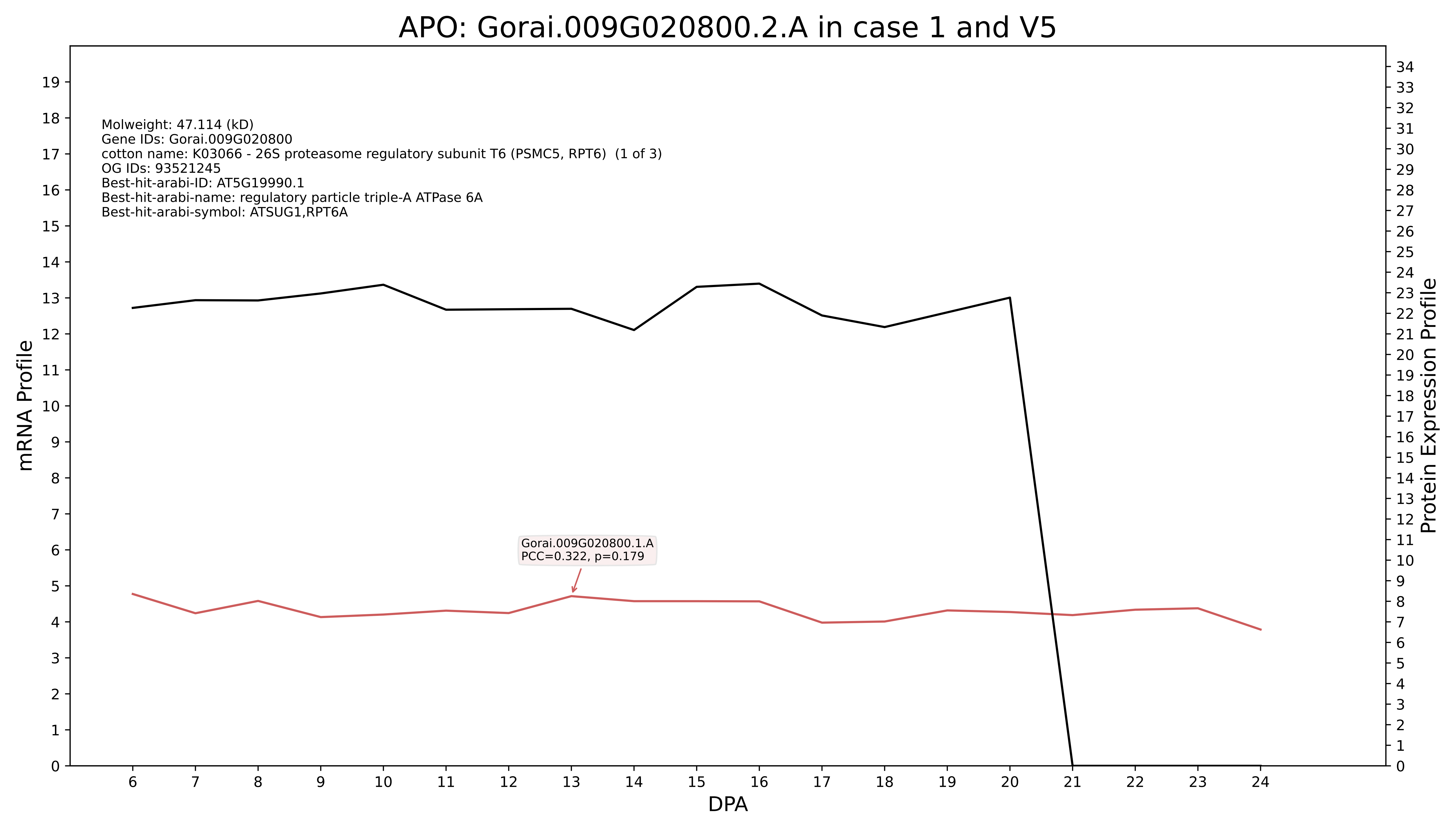
APO: Gorai.009G020800.2.A in case 1 and V5
Molweight: 47.114 (kD)
Gene IDs: Gorai.009G020800
cotton name: K03066 - 26S proteasome regulatory subunit T6 (PSMC5, RPT6) (1 of 3)
OG IDs: 93521245
Best-hit-arabi-ID: AT5G19990.1
Best-hit-arabi-name: regulatory particle triple-A ATPase 6A
Best-hit-arabi-symbol: ATSUG1,RPT6A
Gorai.009G020800.1.A
PCC=0.322, p=0.179
mRNA Profile
Protein Expression Profile
DPA
0
1
2
3
4
5
6
7
8
9
10
11
12
13
14
15
16
17
18
19
20
21
22
23
24
25
26
27
28
29
30
31
32
33
34

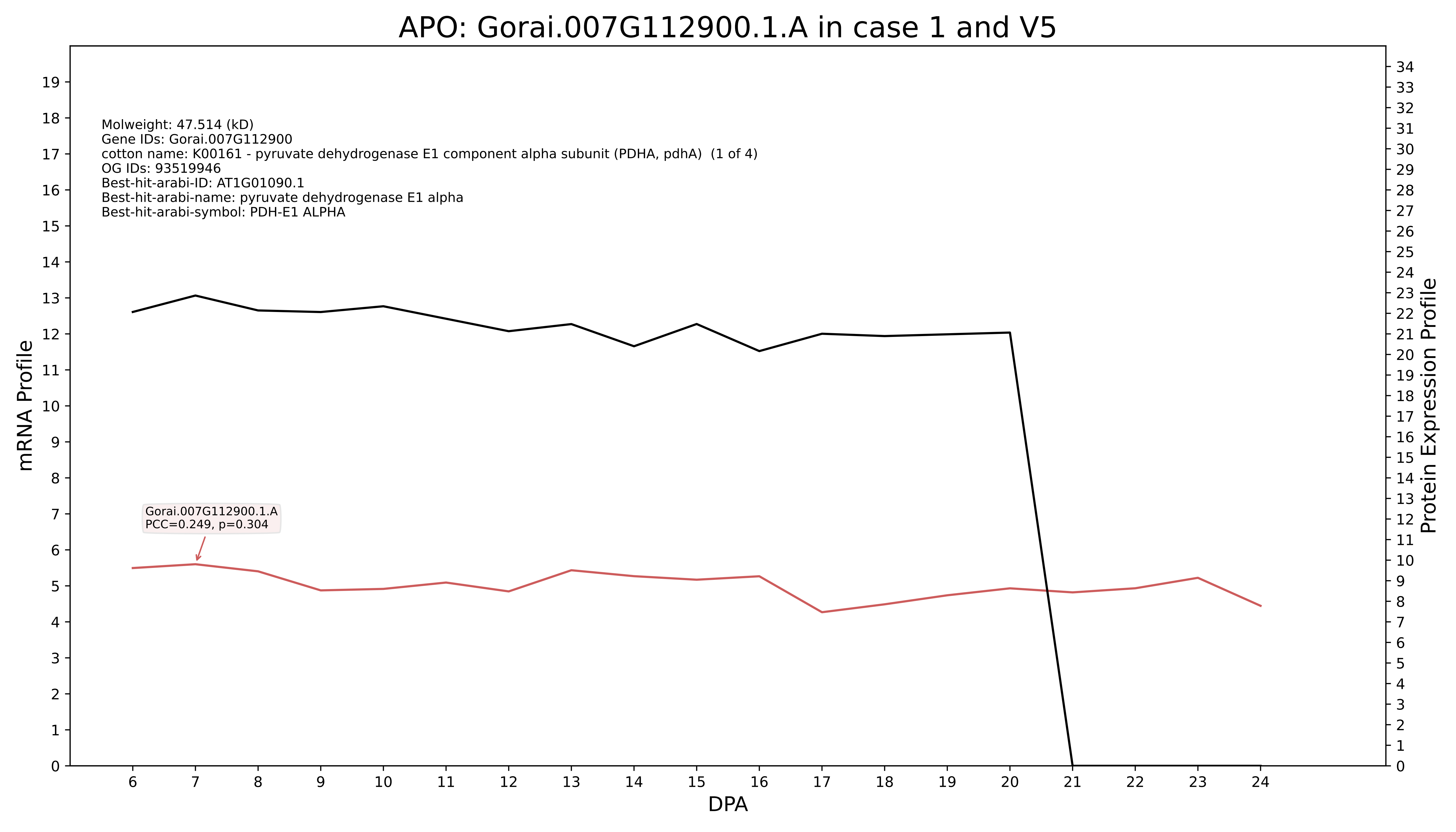

APO: Gorai.007G112900.1.A in case 1 and V5
Molweight: 47.514 (kD)
Gene IDs: Gorai.007G112900
cotton name: K00161 - pyruvate dehydrogenase E1 component alpha subunit (PDHA, pdhA) (1 of 4)
OG IDs: 93519946
Best-hit-arabi-ID: AT1G01090.1
Best-hit-arabi-name: pyruvate dehydrogenase E1 alpha
Best-hit-arabi-symbol: PDH-E1 ALPHA
Gorai.007G112900.1.A
PCC=0.249, p=0.304
mRNA Profile
Protein Expression Profile
DPA

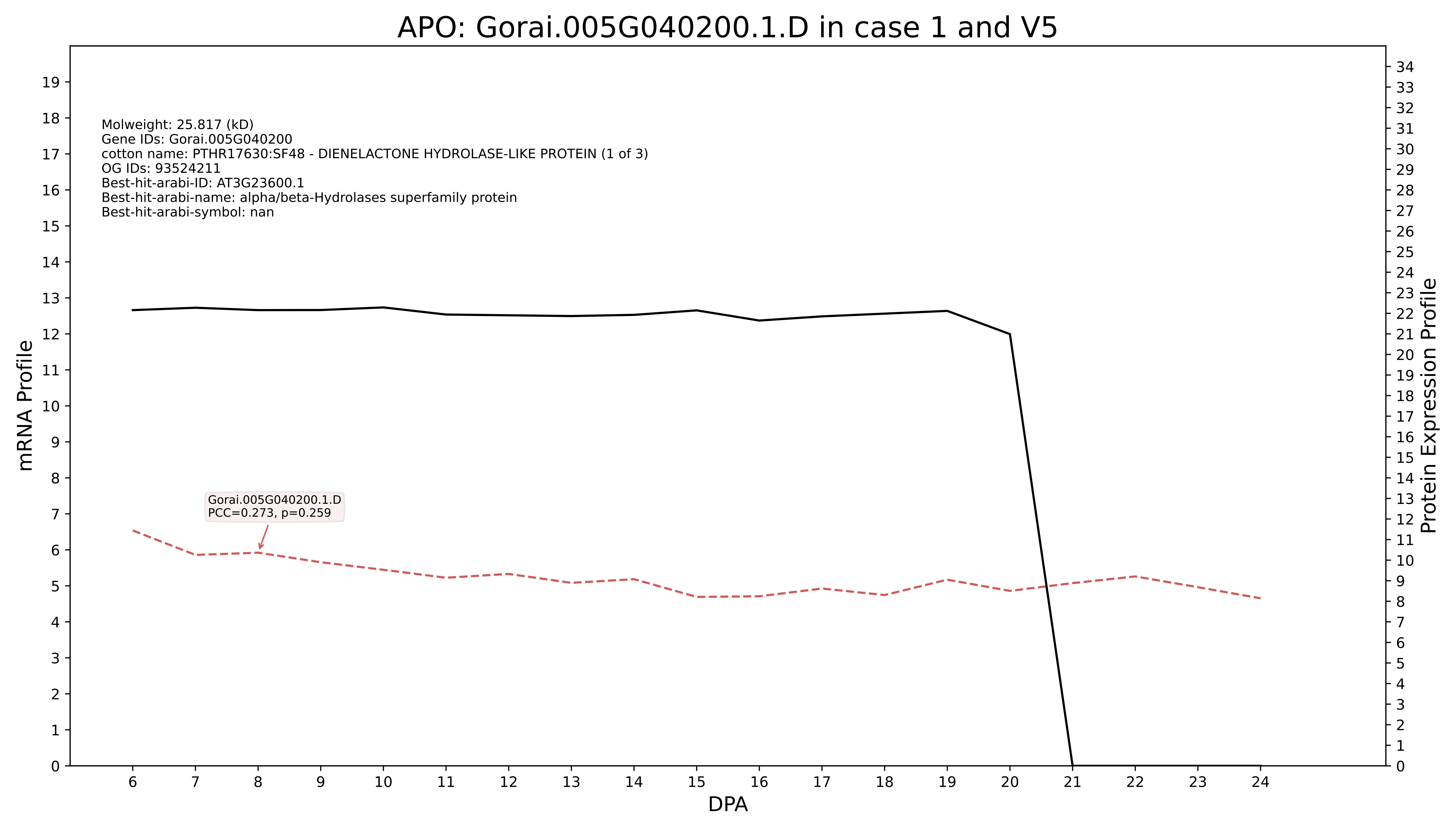

APO: Gorai.005G040200.1.D in case 1 and V5
Molweight: 25.817 (kD)
Gene IDs: Gorai.005G040200
cotton name: PTHR17630:SF48 - DIENELACTONE HYDROLASE-LIKE PROTEIN (1 of 3)
OG IDs: 93524211
Best-hit-arabi-ID: AT3G23600.1
Best-hit-arabi-name: alpha/beta-Hydrolases superfamily protein
Best-hit-arabi-symbol: nan
Gorai.005G040200.1.D
PCC=0.273, p=0.259
mRNA Profile
Protein Expression Profile
DPA

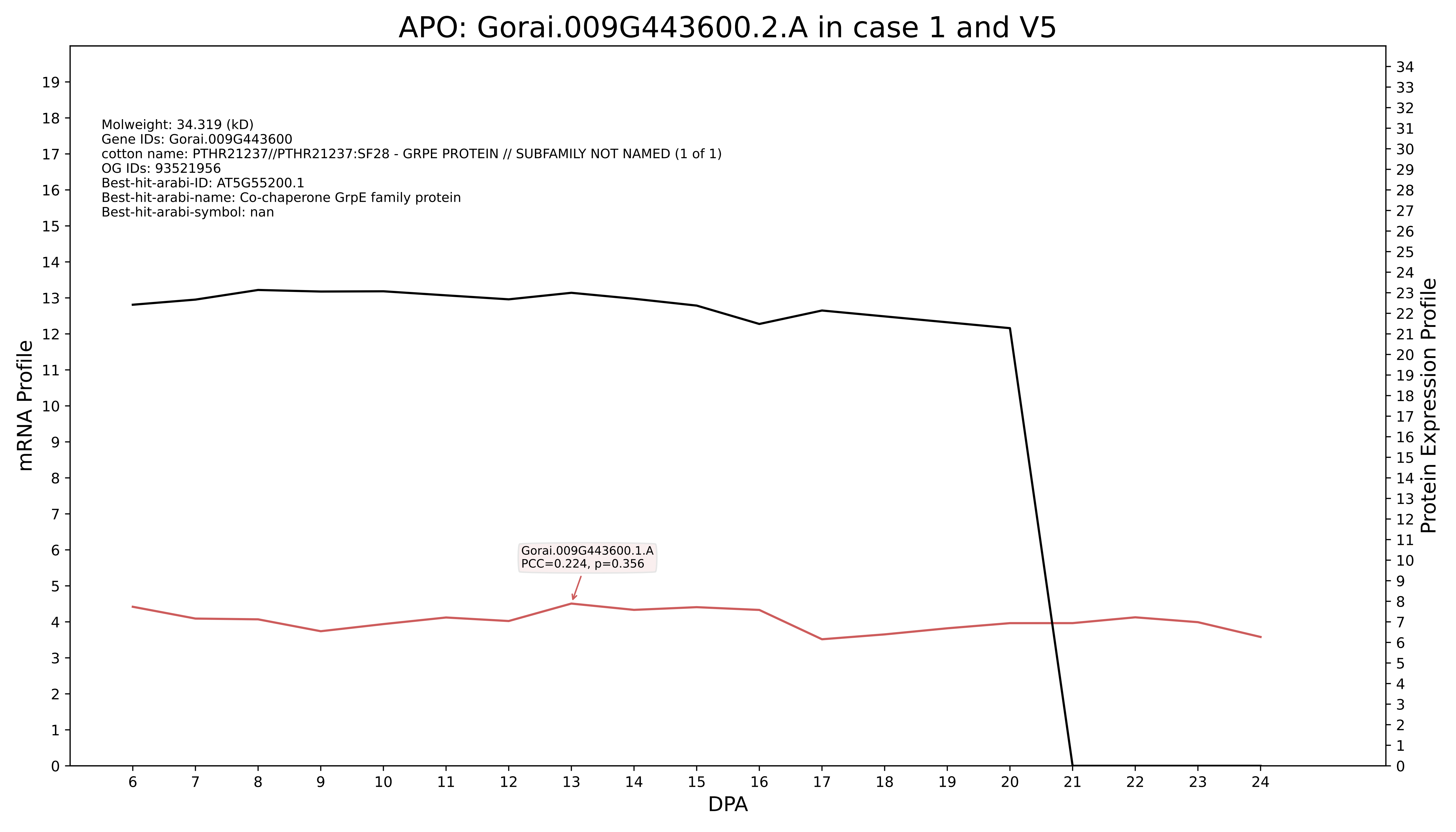

APO: Gorai.009G443600.2.A in case 1 and V5
Molweight: 34.319 (kD)
Gene IDs: Gorai.009G443600
cotton name: PTHR21237//PTHR21237:SF28 - GRPE PROTEIN // SUBFAMILY NOT NAMED (1 of 1)
OG IDs: 93521956
Best-hit-arabi-ID: AT5G55200.1
Best-hit-arabi-name: Co-chaperone GrpE family protein
Best-hit-arabi-symbol: nan
Gorai.009G443600.1.A
PCC=0.224, p=0.356
mRNA Profile
Protein Expression Profile
DPA

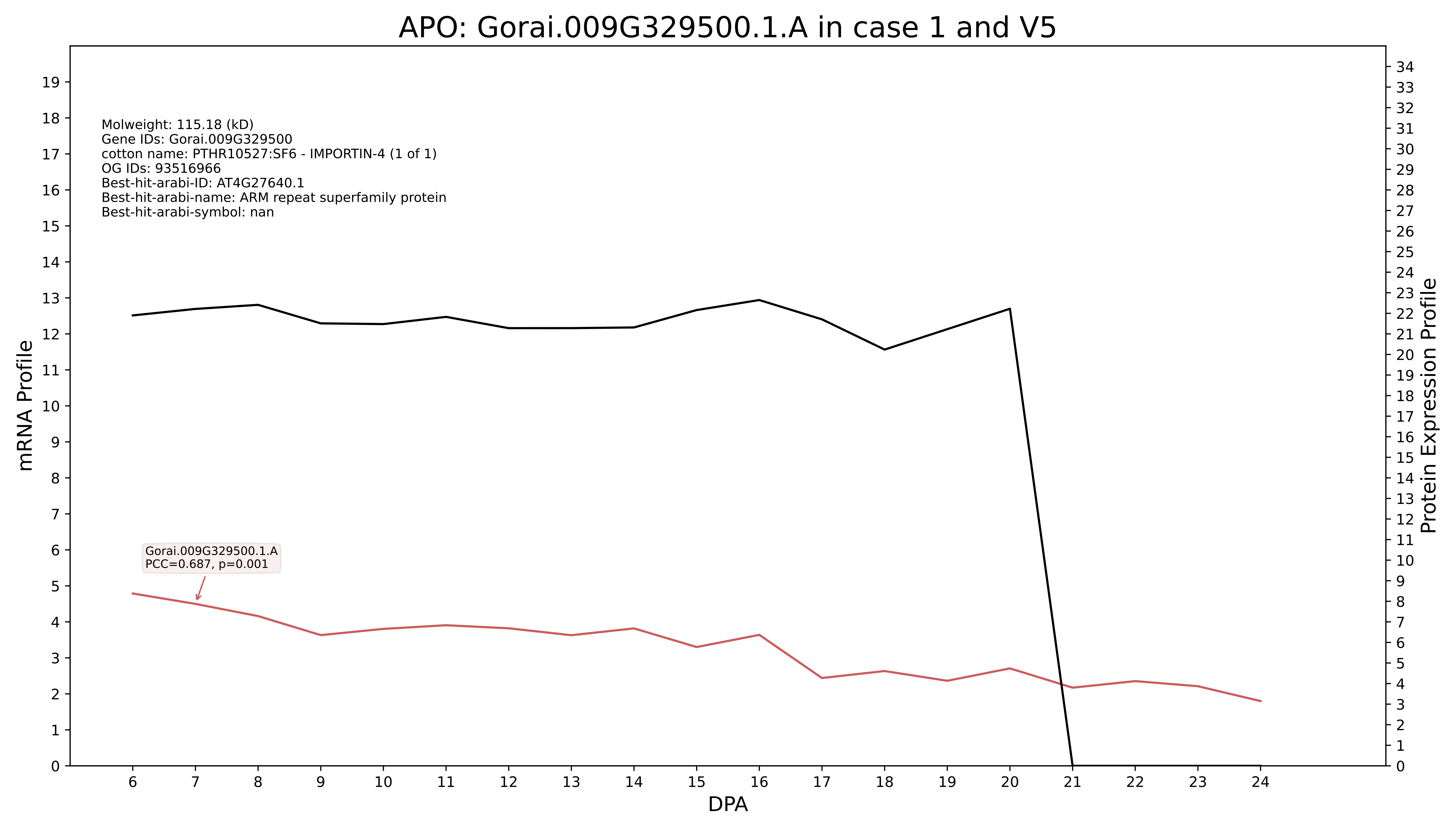

APO: Gorai.009G329500.1.A in case 1 and V5
Molweight: 115.18 (kD)
Gene IDs: Gorai.009G329500
cotton name: PTHR10527:SF6 - IMPORTIN-4 (1 of 1)
OG IDs: 93516966
Best-hit-arabi-ID: AT4G27640.1
Best-hit-arabi-name: ARM repeat superfamily protein
Best-hit-arabi-symbol: nan
Gorai.009G329500.1.A
PCC=0.687, p=0.001
mRNA Profile
Protein Expression Profile
DPA

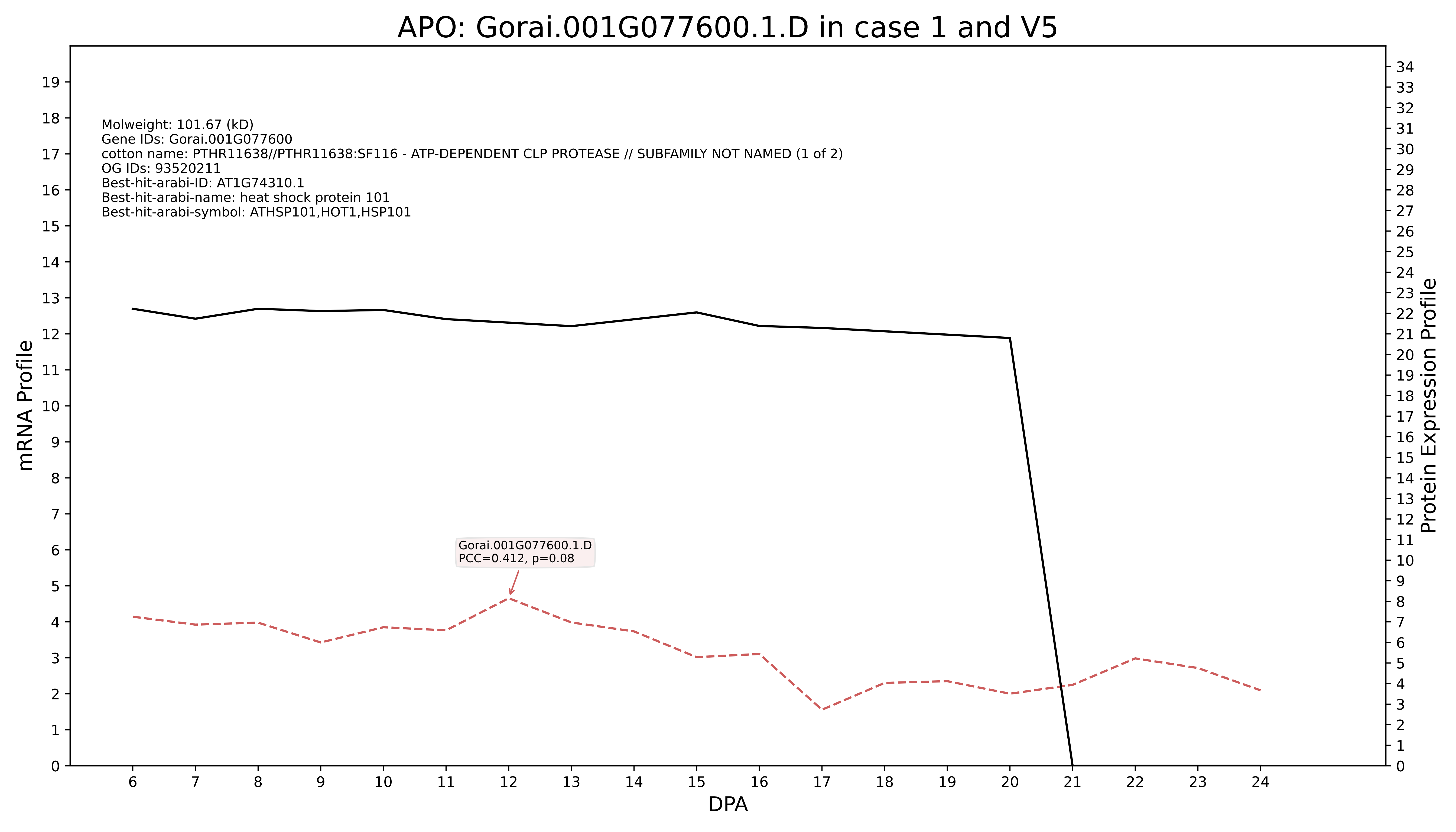
APO: Gorai.001G077600.1.D in case 1 and V5
Molweight: 101.67 (kD)
Gene IDs: Gorai.001G077600
cotton name: PTHR11638//PTHR11638:SF116 - ATP-DEPENDENT CLP PROTEASE // SUBFAMILY NOT NAMED (1 of 2)
OG IDs: 93520211
Best-hit-arabi-ID: AT1G74310.1
Best-hit-arabi-name: heat shock protein 101
Best-hit-arabi-symbol: ATHSP101,HOT1,HSP101
Gorai.001G077600.1.D
PCC=0.412, p=0.08
mRNA Profile
Protein Expression Profile
DPA

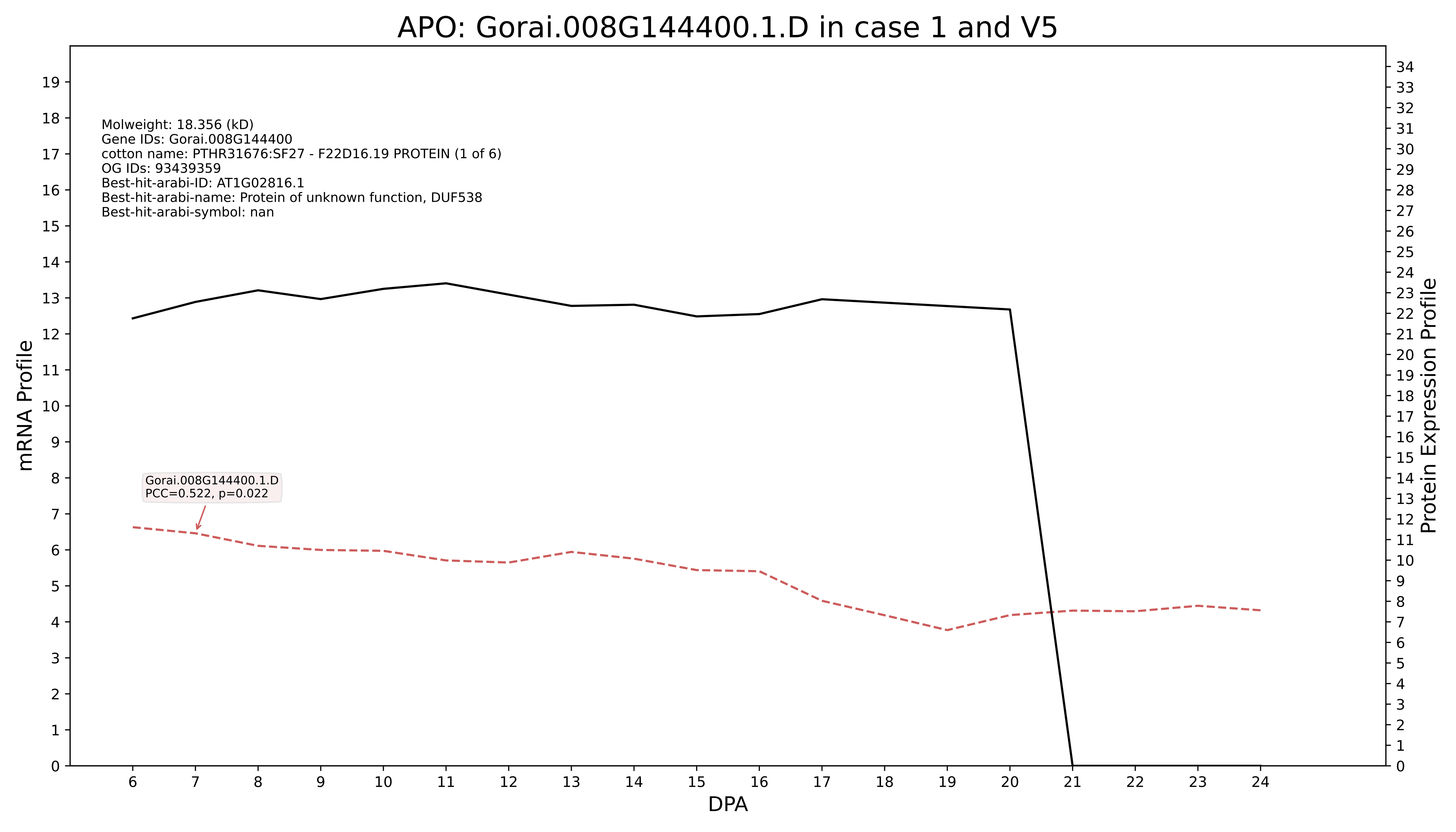
APO: Gorai.008G144400.1.D in case 1 and V5
Molweight: 18.356 (kD)
Gene IDs: Gorai.008G144400
cotton name: PTHR31676:SF27 - F22D16.19 PROTEIN (1 of 6)
OG IDs: 93439359
Best-hit-arabi-ID: AT1G02816.1
Best-hit-arabi-name: Protein of unknown function, DUF538
Best-hit-arabi-symbol: nan
Gorai.008G144400.1.D
PCC=0.522, p=0.022
mRNA Profile
Protein Expression Profile
DPA

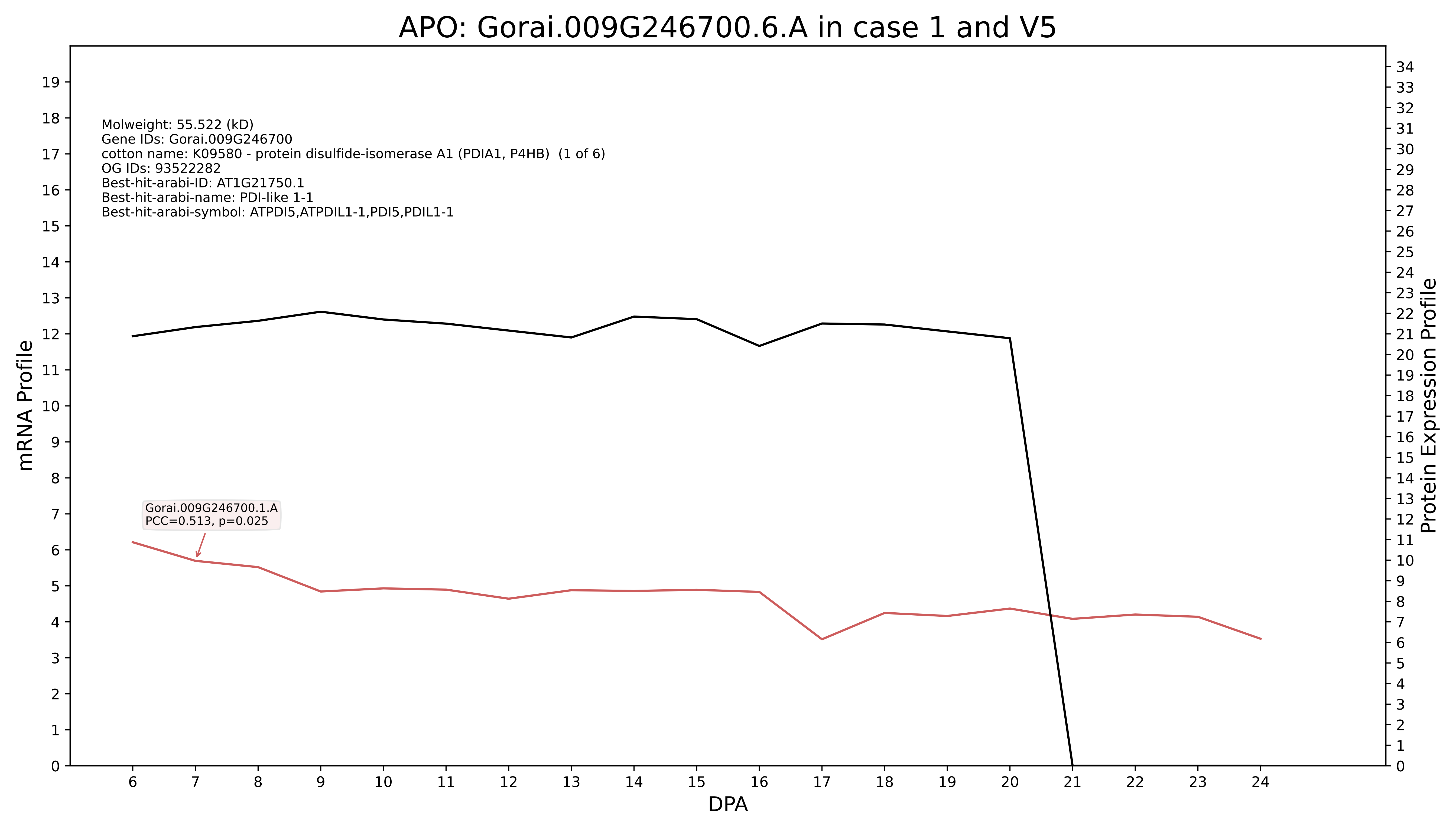
APO: Gorai.009G246700.6.A in case 1 and V5
Molweight: 55.522 (kD)
Gene IDs: Gorai.009G246700
cotton name: K09580 - protein disulfide-isomerase A1 (PDIA1, P4HB) (1 of 6)
OG IDs: 93522282
Best-hit-arabi-ID: AT1G21750.1
Best-hit-arabi-name: PDI-like 1-1
Best-hit-arabi-symbol: ATPDI5,ATPDIL1-1,PDI5,PDIL1-1
Gorai.009G246700.1.A
PCC=0.513, p=0.025
mRNA Profile
Protein Expression Profile
DPA
0
1
2
3
4
5
6
7
8
9
10
11
12
13
14
15
16
17
18
19
20
21
22
23
24
25
26
27
28
29
30
31
32
33
34

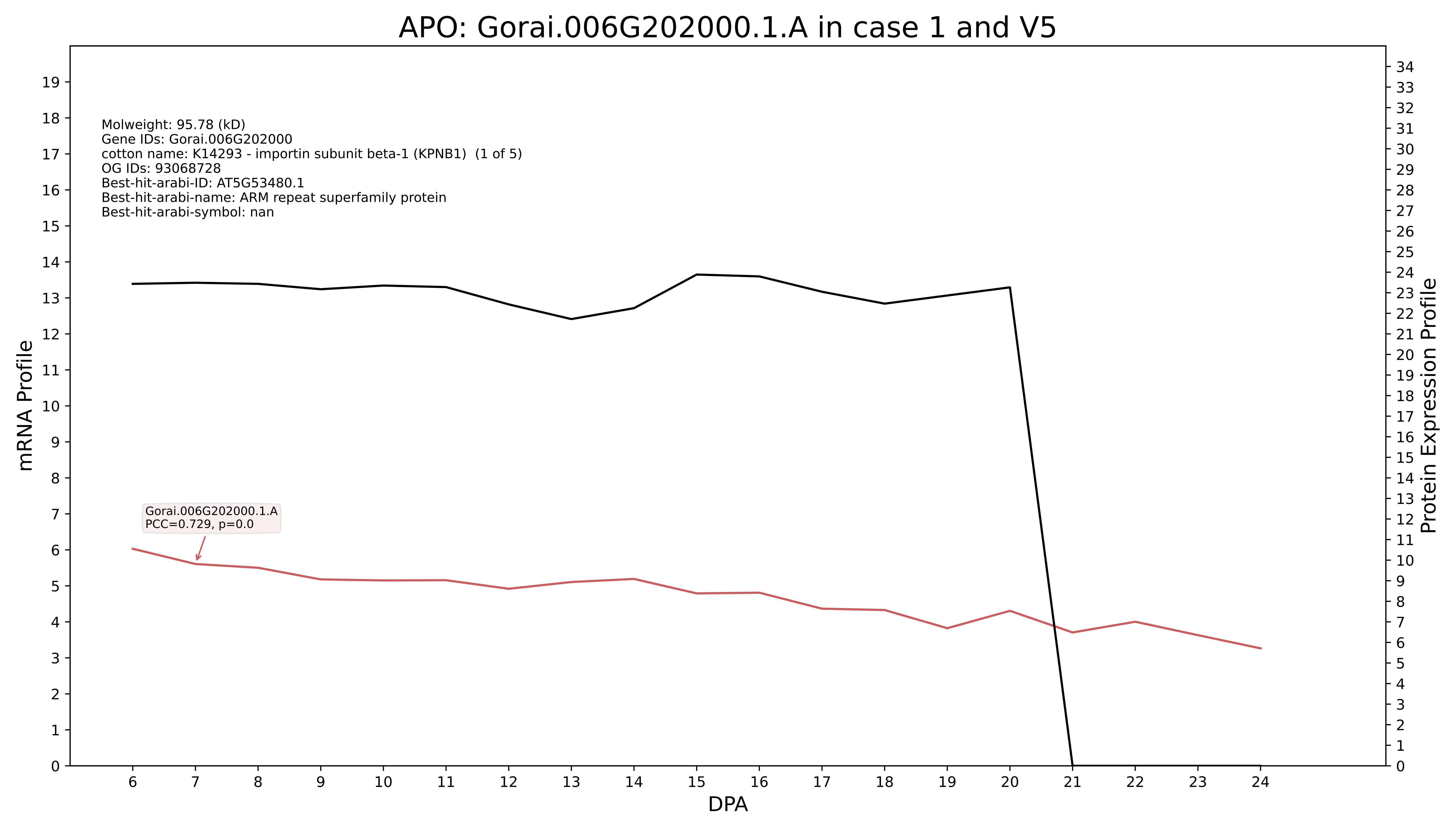

APO: Gorai.006G202000.1.A in case 1 and V5
Molweight: 95.78 (kD)
Gene IDs: Gorai.006G202000
cotton name: K14293 - importin subunit beta-1 (KPNB1) (1 of 5)
OG IDs: 93068728
Best-hit-arabi-ID: AT5G53480.1
Best-hit-arabi-name: ARM repeat superfamily protein
Best-hit-arabi-symbol: nan
Gorai.006G202000.1.A
PCC=0.729, p=0.0
mRNA Profile
Protein Expression Profile
DPA

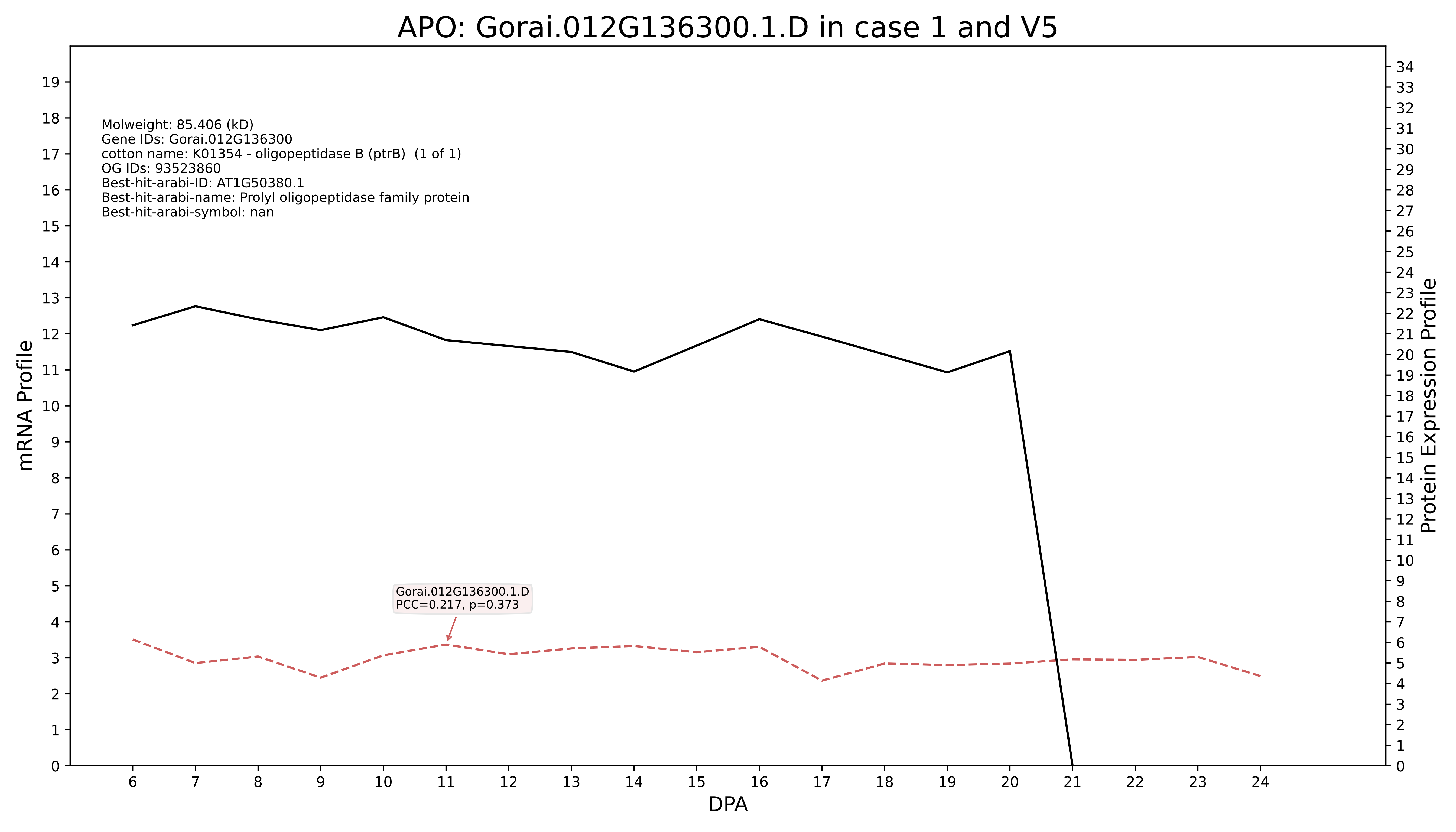

APO: Gorai.012G136300.1.D in case 1 and V5
Molweight: 85.406 (kD)
Gene IDs: Gorai.012G136300
cotton name: K01354 - oligopeptidase B (ptrB) (1 of 1)
OG IDs: 93523860
Best-hit-arabi-ID: AT1G50380.1
Best-hit-arabi-name: Prolyl oligopeptidase family protein
Best-hit-arabi-symbol: nan
Gorai.012G136300.1.D
PCC=0.217, p=0.373
mRNA Profile
Protein Expression Profile
DPA

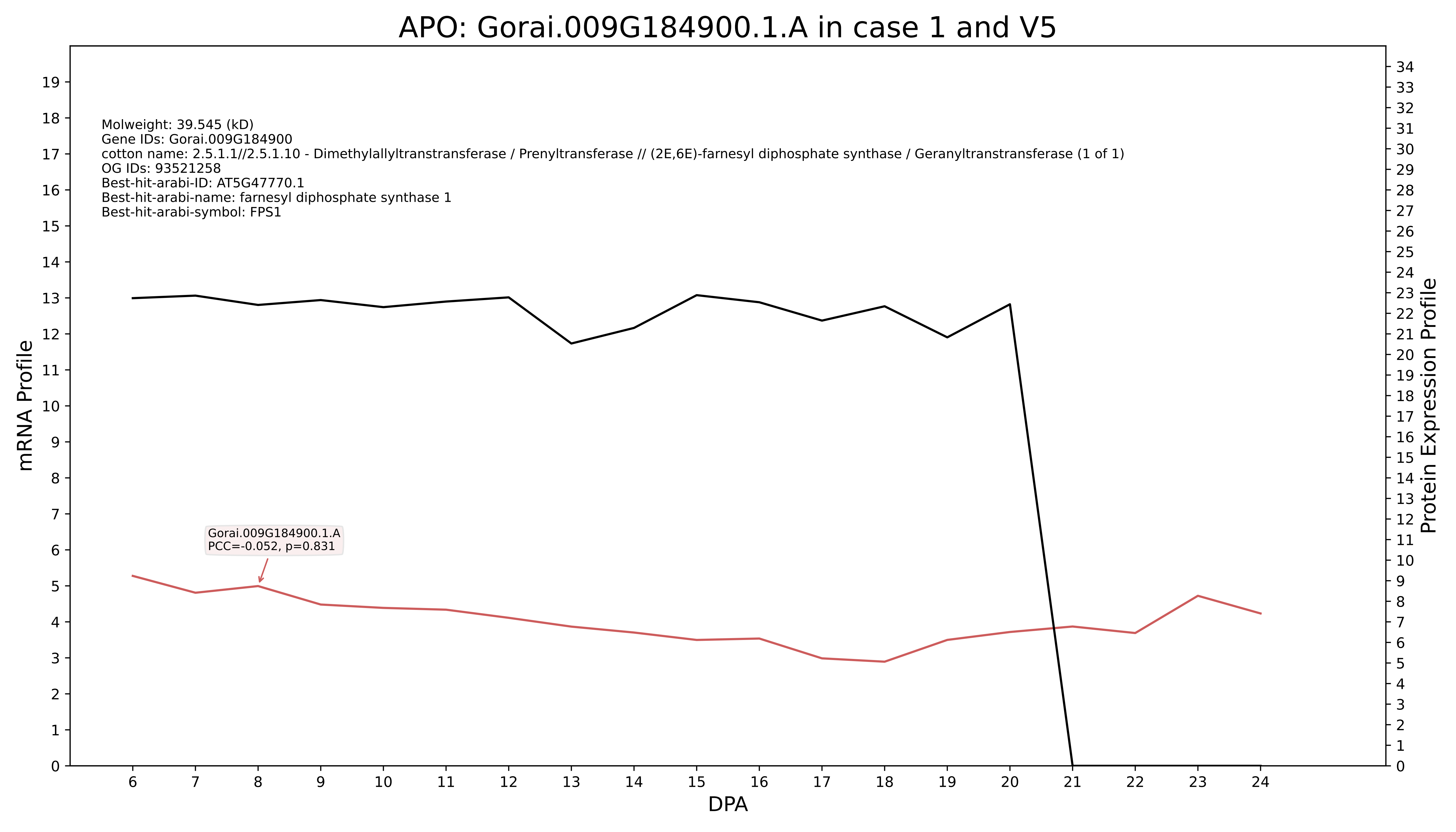
APO: Gorai.009G184900.1.A in case 1 and V5
Molweight: 39.545 (kD)
Gene IDs: Gorai.009G184900
cotton name: 2.5.1.1//2.5.1.10 - Dimethylallyltranstransferase / Prenyltransferase // (2E,6E)-farnesyl diphosphate synthase / Geranyltranstransferase (1 of 1)
OG IDs: 93521258
Best-hit-arabi-ID: AT5G47770.1
Best-hit-arabi-name: farnesyl diphosphate synthase 1
Best-hit-arabi-symbol: FPS1
Gorai.009G184900.1.A
PCC=-0.052, p=0.831
mRNA Profile
Protein Expression Profile
DPA

APO: Gorai.011G207000.1.A in case 1 and V5

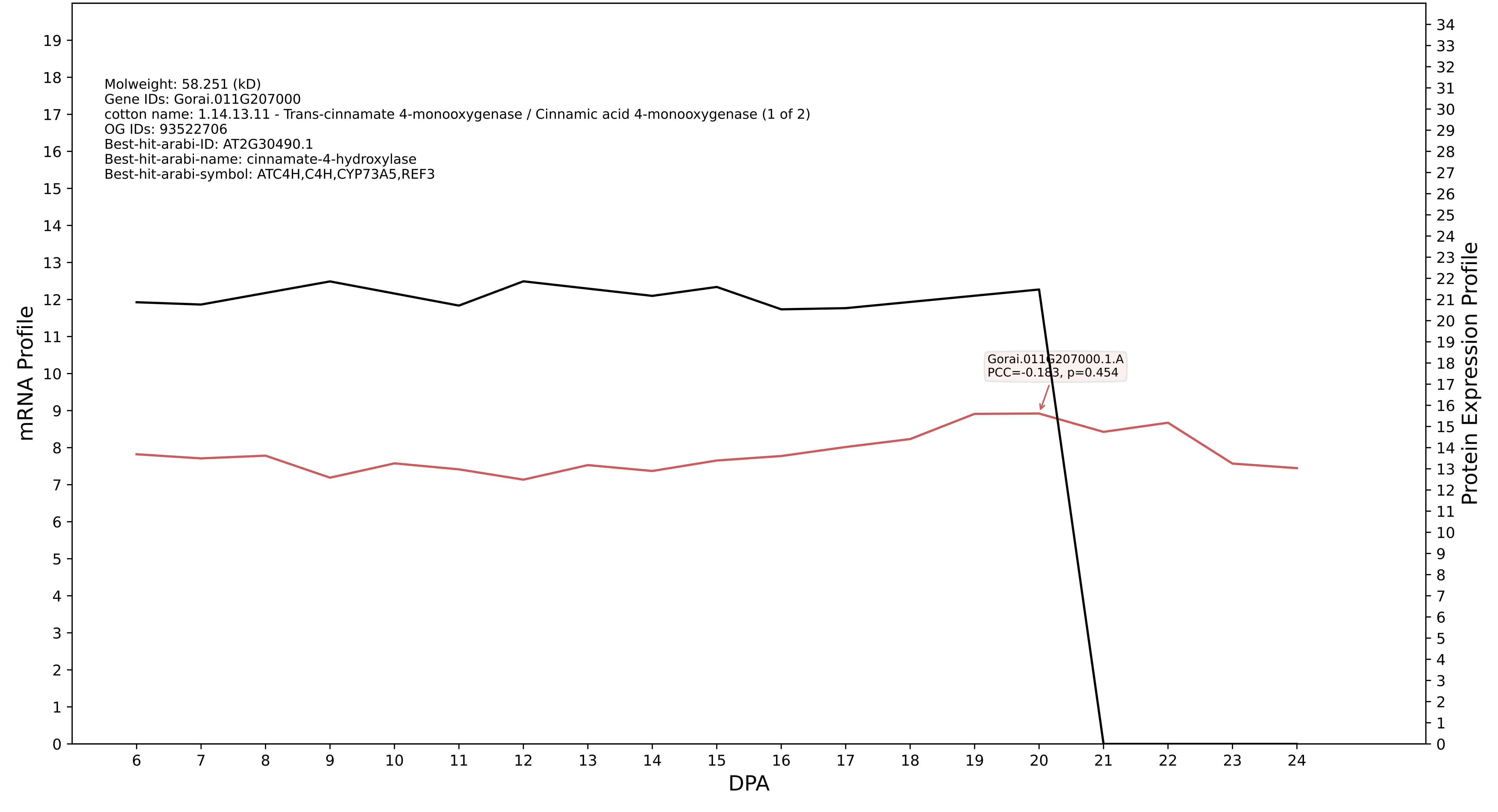

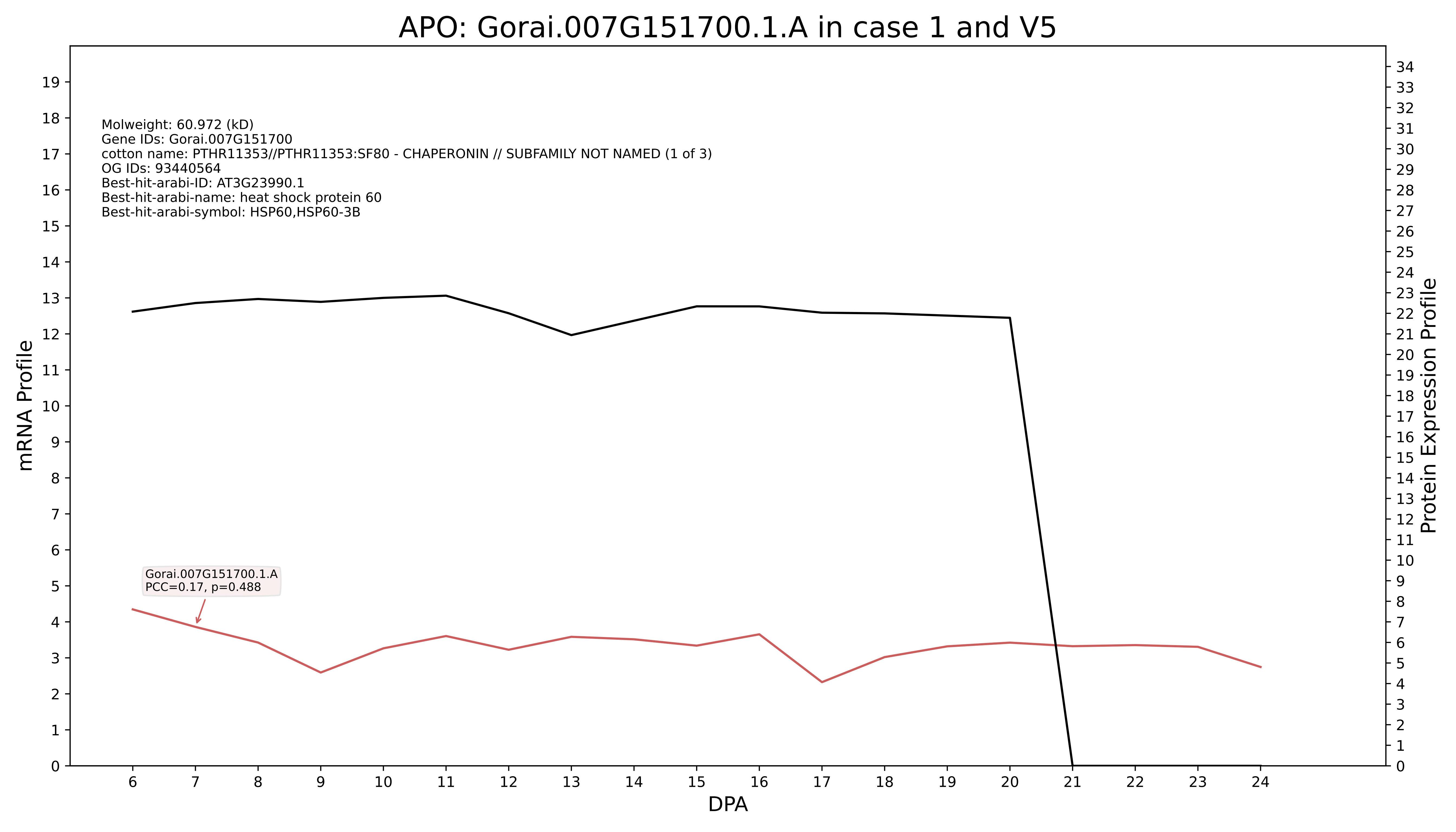
APO: Gorai.007G151700.1.A in case 1 and V5
Molweight: 60.972 (kD)
Gene IDs: Gorai.007G151700
cotton name: PTHR11353//PTHR11353:SF80 - CHAPERONIN // SUBFAMILY NOT NAMED (1 of 3)
OG IDs: 93440564
Best-hit-arabi-ID: AT3G23990.1
Best-hit-arabi-name: heat shock protein 60
Best-hit-arabi-symbol: HSP60,HSP60-3B
Gorai.007G151700.1.A
PCC=0.17, p=0.488
mRNA Profile
Protein Expression Profile
DPA
0
1
2
3
4
5
6
7
8
9
10
11
12
13
14
15
16
17
18
19
20
21
22
23
24
25
26
27
28
29
30
31
32
33
34

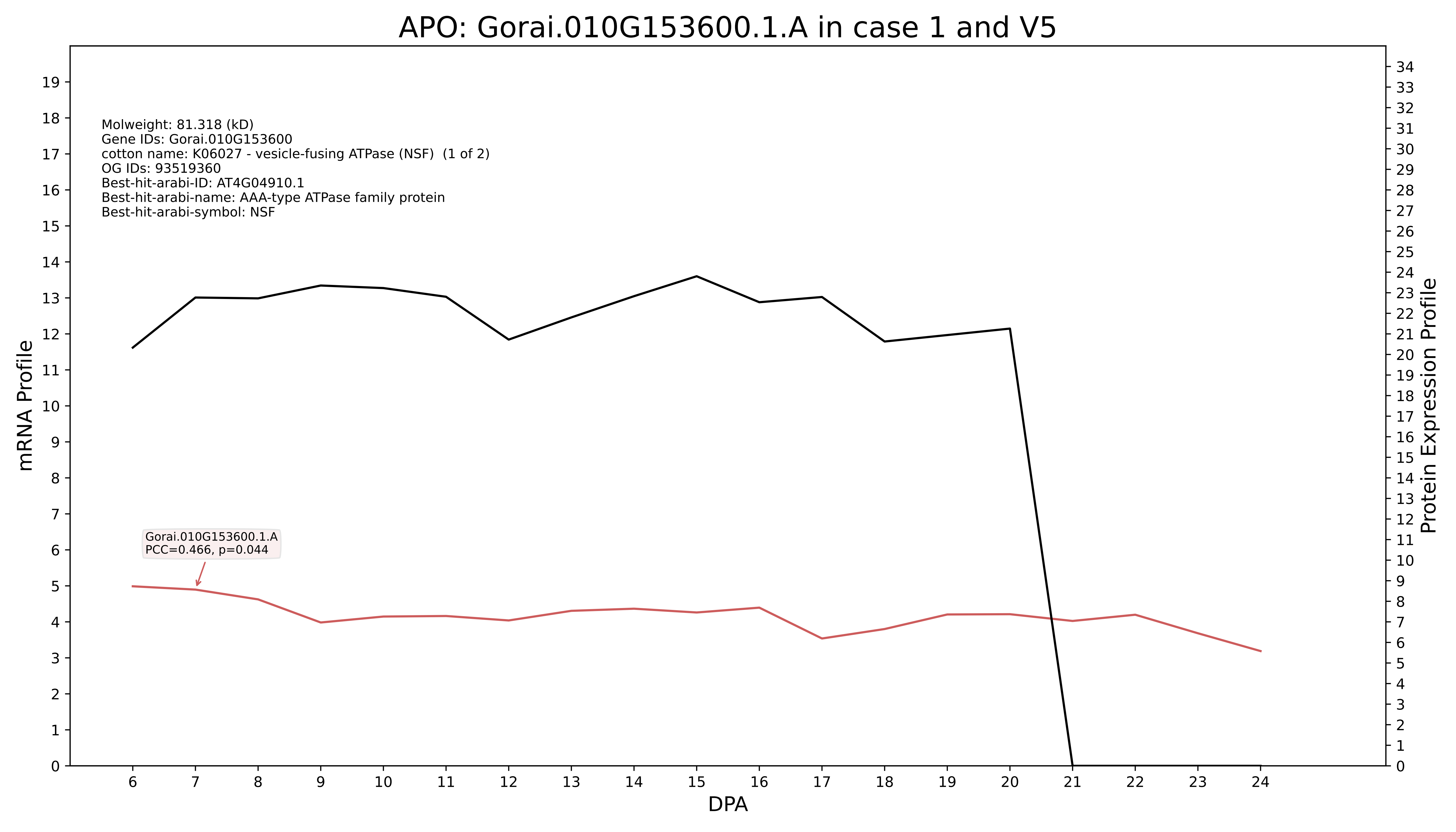
APO: Gorai.010G153600.1.A in case 1 and V5
Molweight: 81.318 (kD)
Gene IDs: Gorai.010G153600
cotton name: K06027 - vesicle-fusing ATPase (NSF) (1 of 2)
OG IDs: 93519360
Best-hit-arabi-ID: AT4G04910.1
Best-hit-arabi-name: AAA-type ATPase family protein
Best-hit-arabi-symbol: NSF
Gorai.010G153600.1.A
PCC=0.466, p=0.044
mRNA Profile
Protein Expression Profile
DPA
0
1
2
3
4
5
6
7
8
9
10
11
12
13
14
15
16
17
18
19
20
21
22
23
24
25
26
27
28
29
30
31
32
33
34

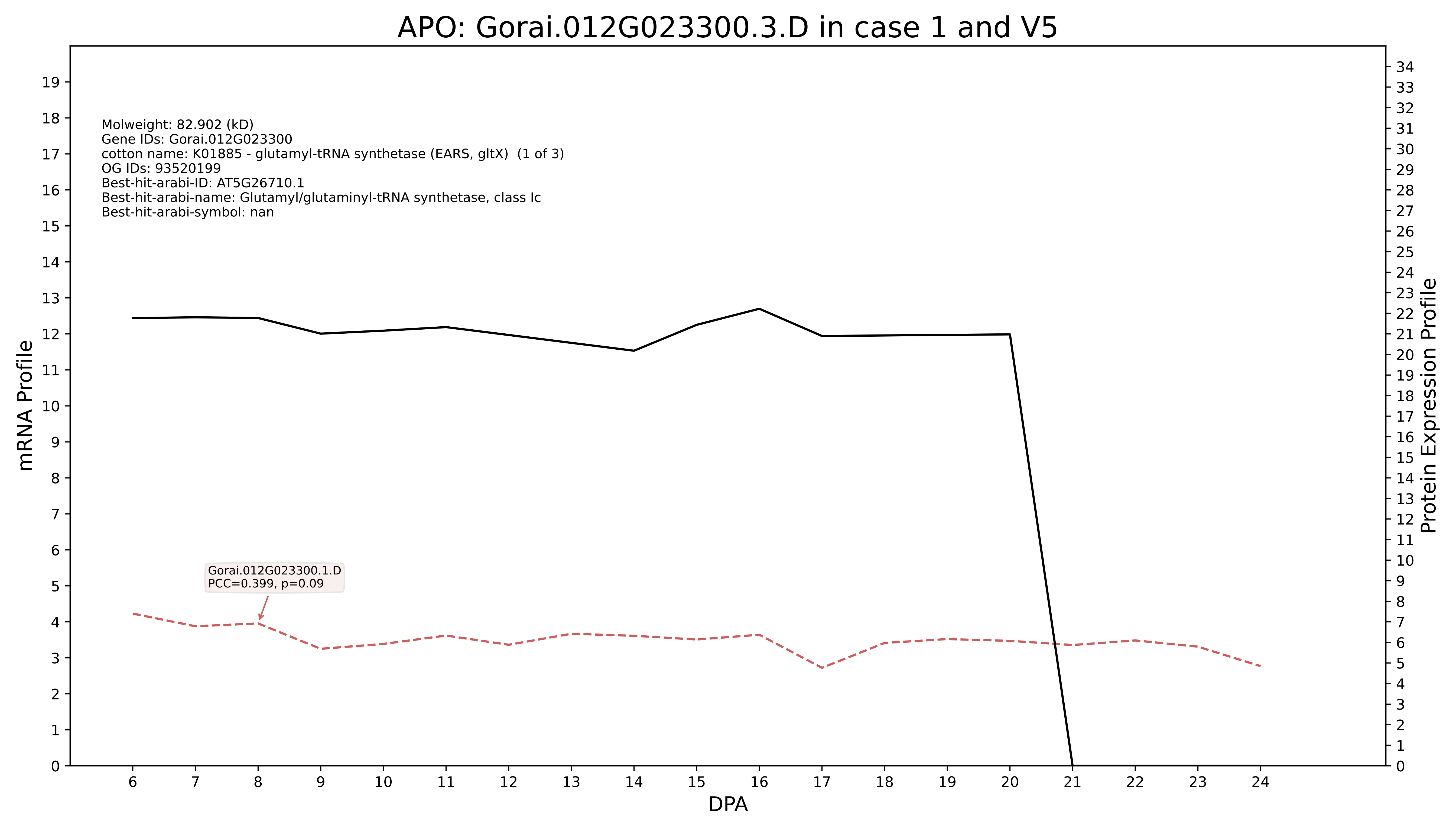
APO: Gorai.012G023300.3.D in case 1 and V5
Molweight: 82.902 (kD)
Gene IDs: Gorai.012G023300
cotton name: K01885 - glutamyl-tRNA synthetase (EARS, gltX) (1 of 3)
OG IDs: 93520199
Best-hit-arabi-ID: AT5G26710.1
Best-hit-arabi-name: Glutamyl/glutaminyl-tRNA synthetase, class Ic
Best-hit-arabi-symbol: nan
Gorai.012G023300.1.D
PCC=0.399, p=0.09
mRNA Profile
Protein Expression Profile
DPA

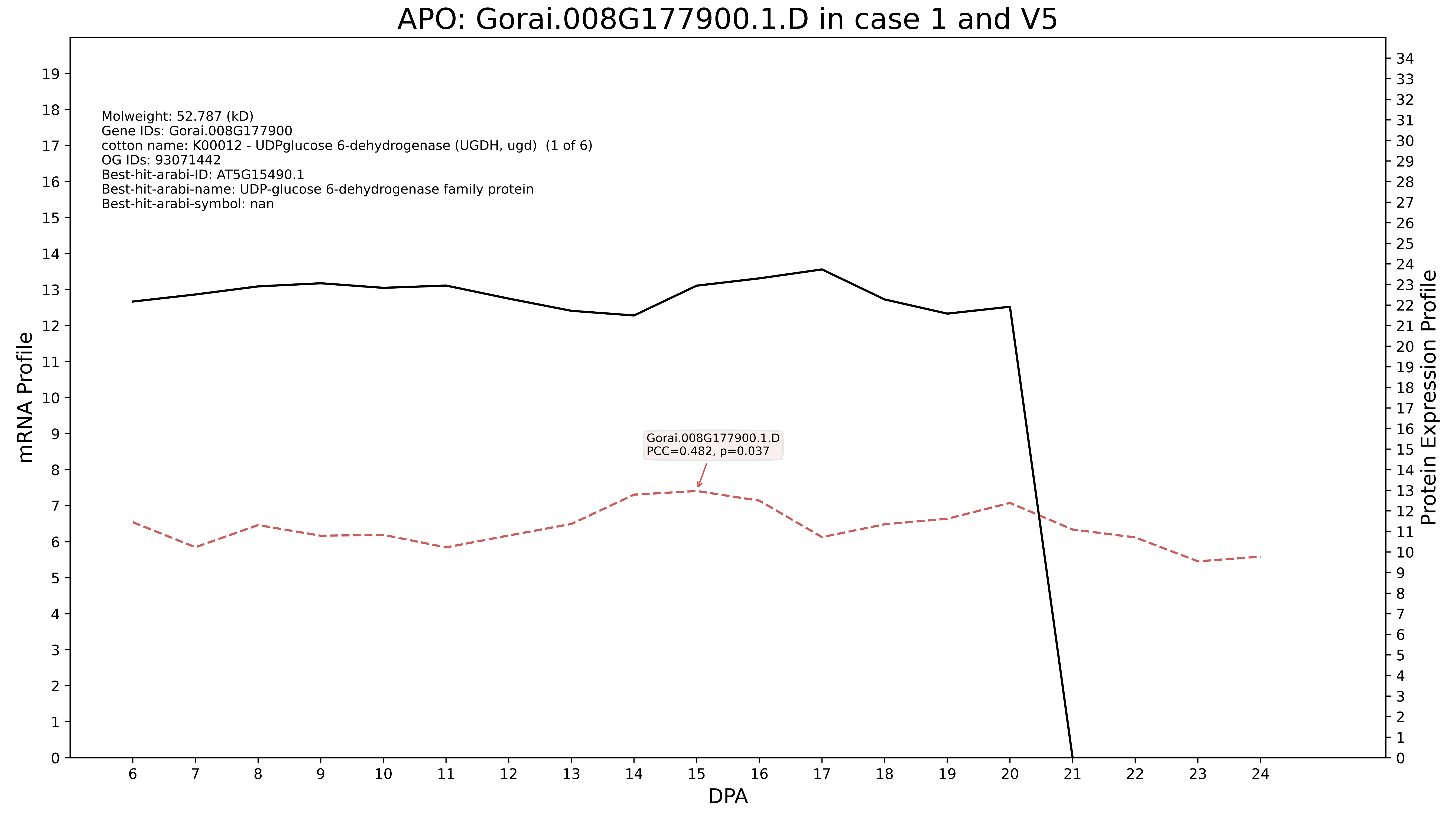
APO: Gorai.008G177900.1.D in case 1 and V5
Molweight: 52.787 (kD)
Gene IDs: Gorai.008G177900
cotton name: K00012 - UDPglucose 6-dehydrogenase (UGDH, ugd) (1 of 6)
OG IDs: 93071442
Best-hit-arabi-ID: AT5G15490.1
Best-hit-arabi-name: UDP-glucose 6-dehydrogenase family protein
Best-hit-arabi-symbol: nan
Gorai.008G177900.1.D
PCC=0.482, p=0.037
mRNA Profile
Protein Expression Profile
DPA

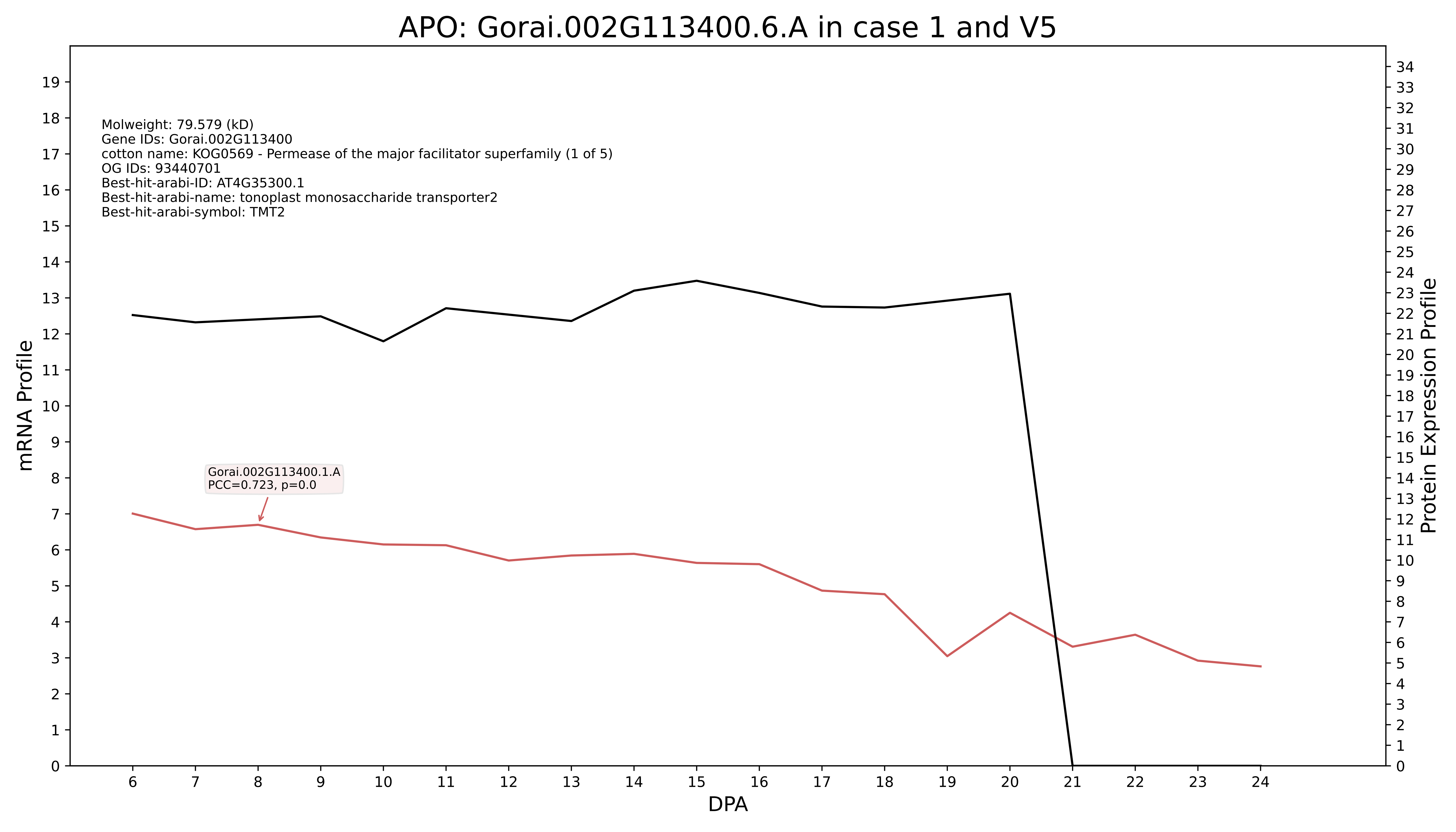

APO: Gorai.002G113400.6.A in case 1 and V5
Molweight: 79.579 (kD)
Gene IDs: Gorai.002G113400
cotton name: KOG0569 - Permease of the major facilitator superfamily (1 of 5)
OG IDs: 93440701
Best-hit-arabi-ID: AT4G35300.1
Best-hit-arabi-name: tonoplast monosaccharide transporter2
Best-hit-arabi-symbol: TMT2
Gorai.002G113400.1.A
PCC=0.723, p=0.0
mRNA Profile
Protein Expression Profile
DPA

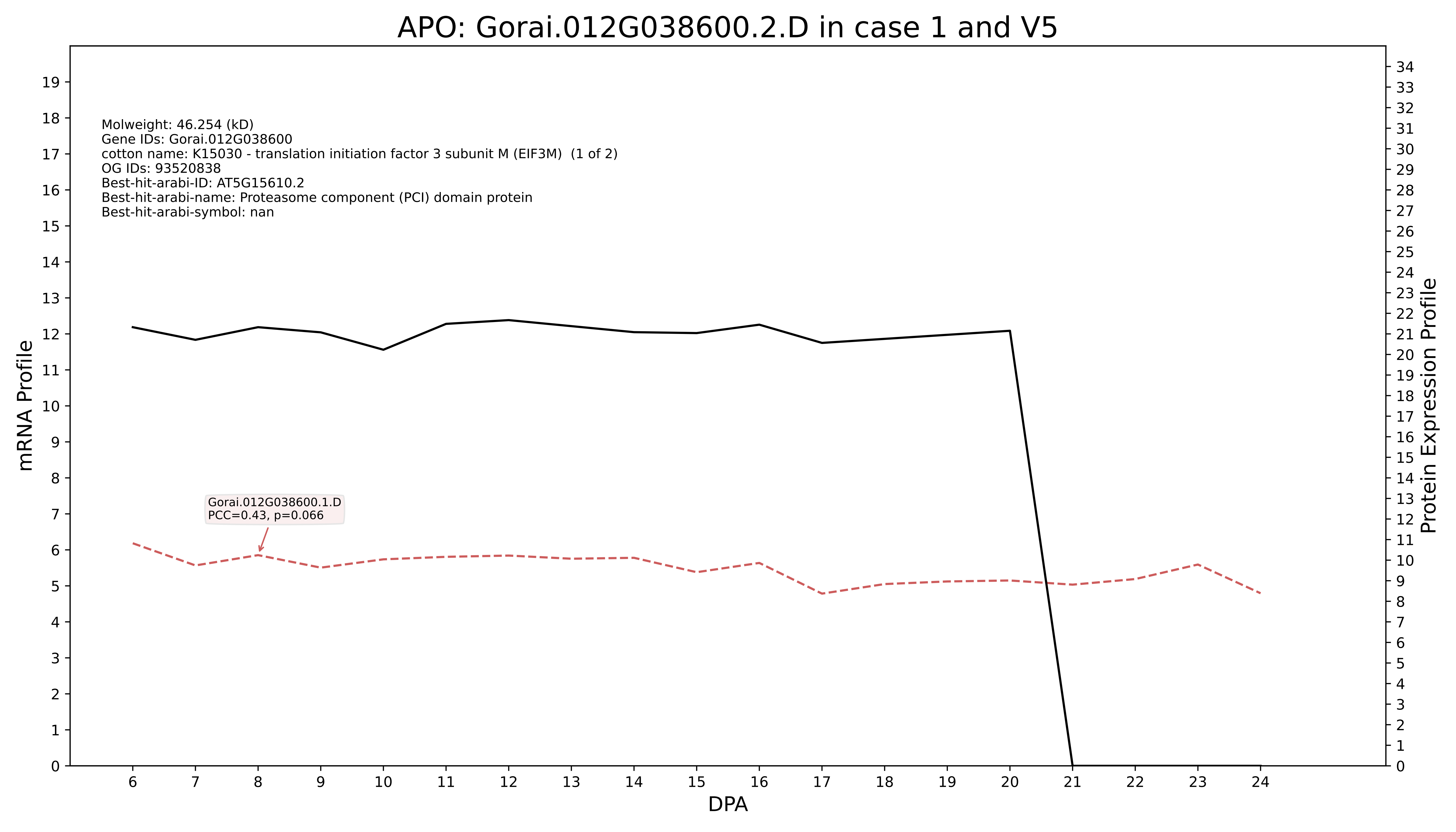
APO: Gorai.012G038600.2.D in case 1 and V5
Molweight: 46.254 (kD)
Gene IDs: Gorai.012G038600
cotton name: K15030 - translation initiation factor 3 subunit M (EIF3M) (1 of 2)
OG IDs: 93520838
Best-hit-arabi-ID: AT5G15610.2
Best-hit-arabi-name: Proteasome component (PCI) domain protein
Best-hit-arabi-symbol: nan
Gorai.012G038600.1.D
PCC=0.43, p=0.066
mRNA Profile
Protein Expression Profile
DPA

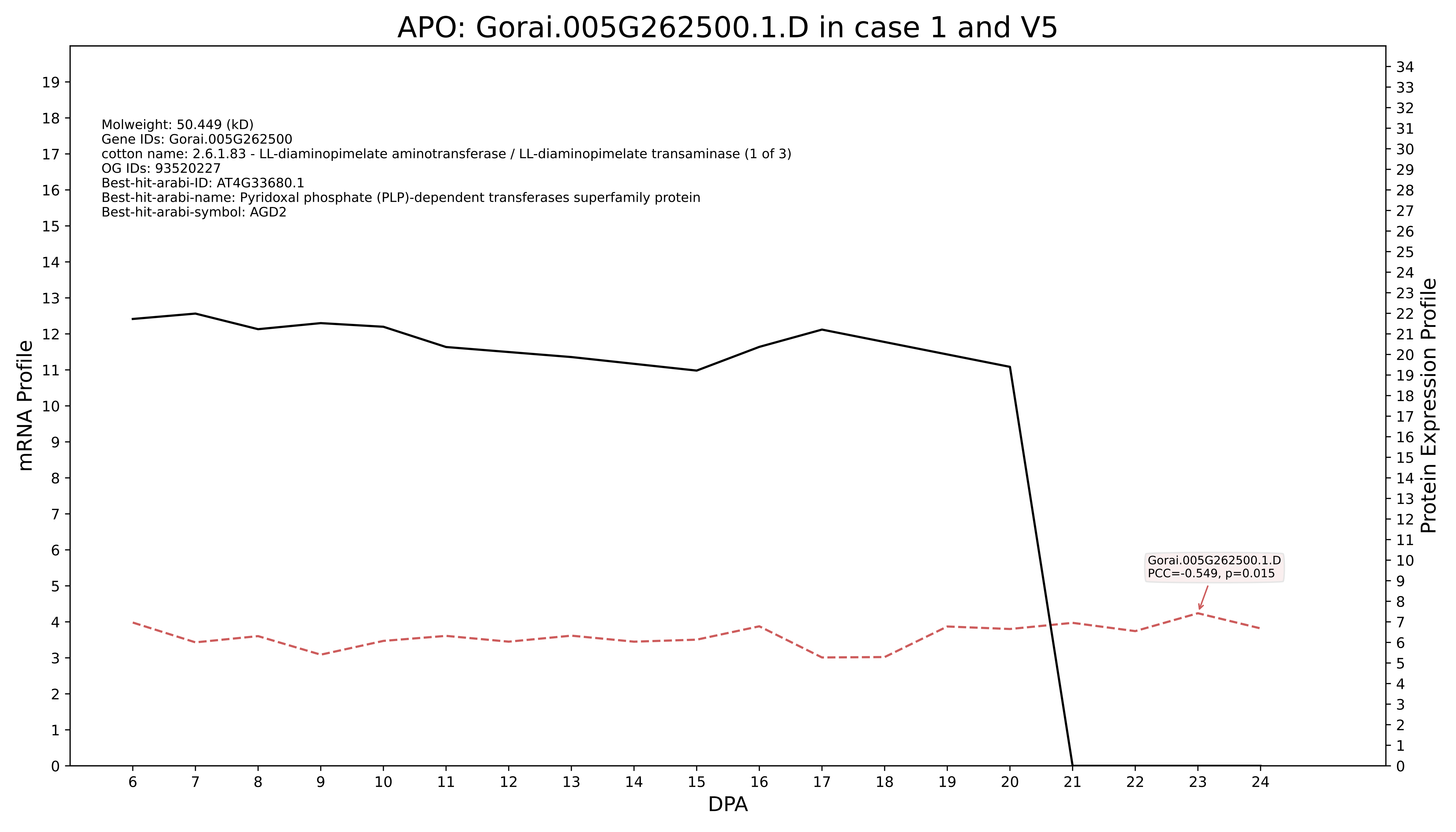

APO: Gorai.005G262500.1.D in case 1 and V5
Molweight: 50.449 (kD)
Gene IDs: Gorai.005G262500
cotton name: 2.6.1.83 - LL-diaminopimelate aminotransferase / LL-diaminopimelate transaminase (1 of 3)
OG IDs: 93520227
Best-hit-arabi-ID: AT4G33680.1
Best-hit-arabi-name: Pyridoxal phosphate (PLP)-dependent transferases superfamily protein
Best-hit-arabi-symbol: AGD2
Gorai.005G262500.1.D
PCC=-0.549, p=0.015
mRNA Profile
Protein Expression Profile
DPA

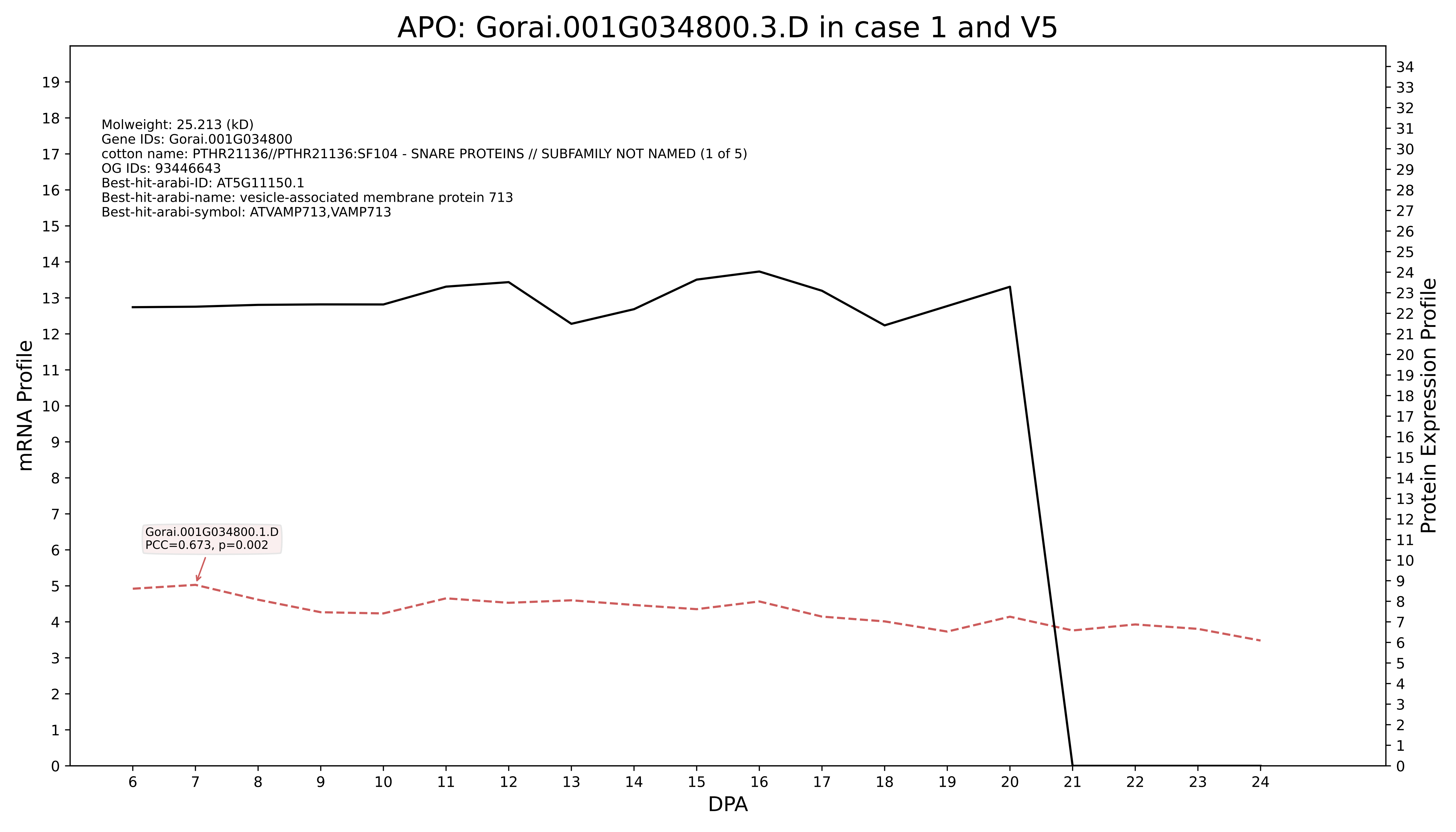
APO: Gorai.001G034800.3.D in case 1 and V5
Molweight: 25.213 (kD)
Gene IDs: Gorai.001G034800
cotton name: PTHR21136//PTHR21136:SF104 - SNARE PROTEINS // SUBFAMILY NOT NAMED (1 of 5)
OG IDs: 93446643
Best-hit-arabi-ID: AT5G11150.1
Best-hit-arabi-name: vesicle-associated membrane protein 713
Best-hit-arabi-symbol: ATVAMP713,VAMP713
Gorai.001G034800.1.D
PCC=0.673, p=0.002
mRNA Profile
Protein Expression Profile
DPA

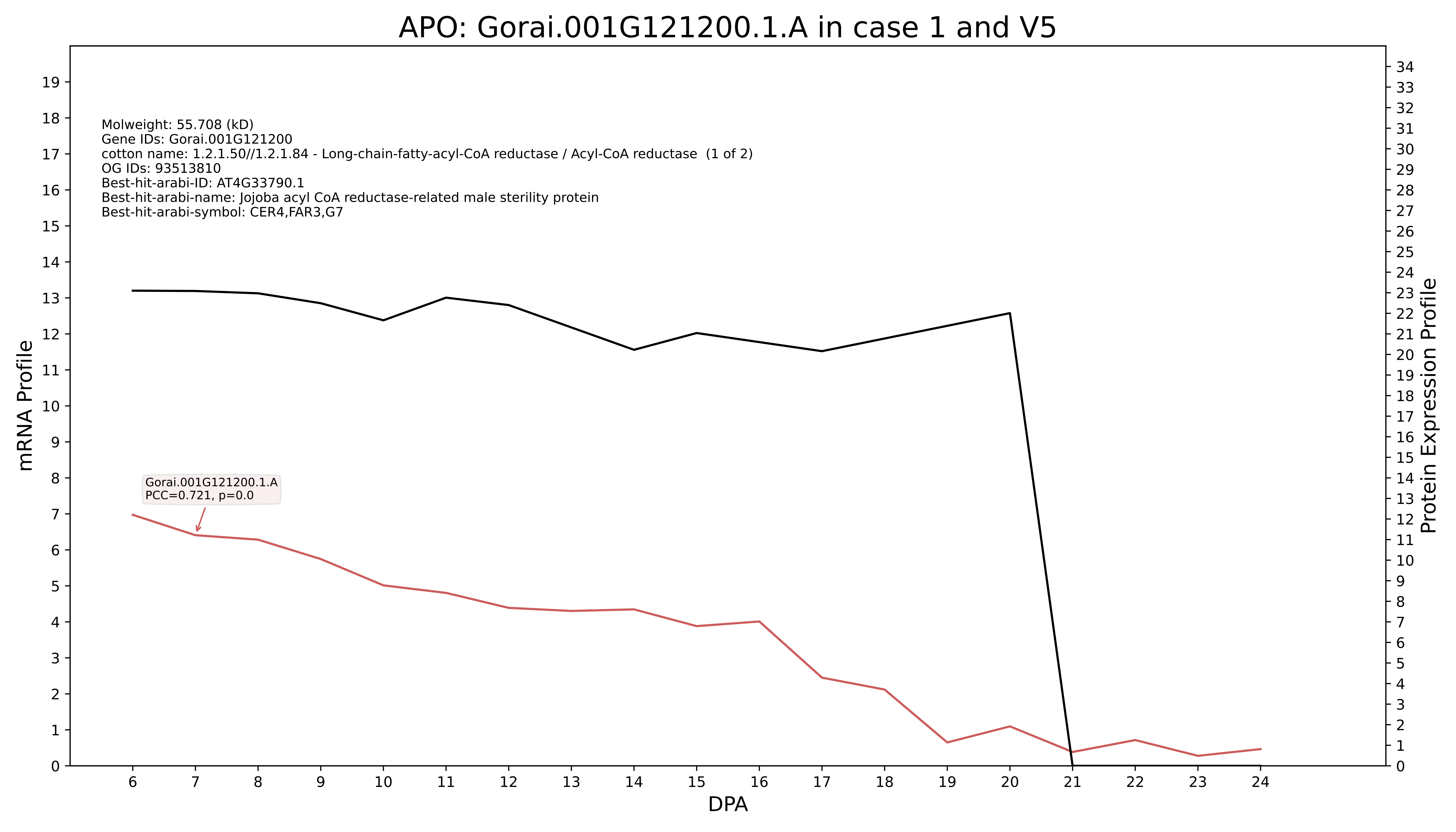
APO: Gorai.001G121200.1.A in case 1 and V5
Molweight: 55.708 (kD)
Gene IDs: Gorai.001G121200
cotton name: 1.2.1.50//1.2.1.84 - Long-chain-fatty-acyl-CoA reductase / Acyl-CoA reductase (1 of 2)
OG IDs: 93513810
Best-hit-arabi-ID: AT4G33790.1
Best-hit-arabi-name: Jojoba acyl CoA reductase-related male sterility protein
Best-hit-arabi-symbol: CER4,FAR3,G7
Gorai.001G121200.1.A
PCC=0.721, p=0.0
mRNA Profile
Protein Expression Profile
DPA

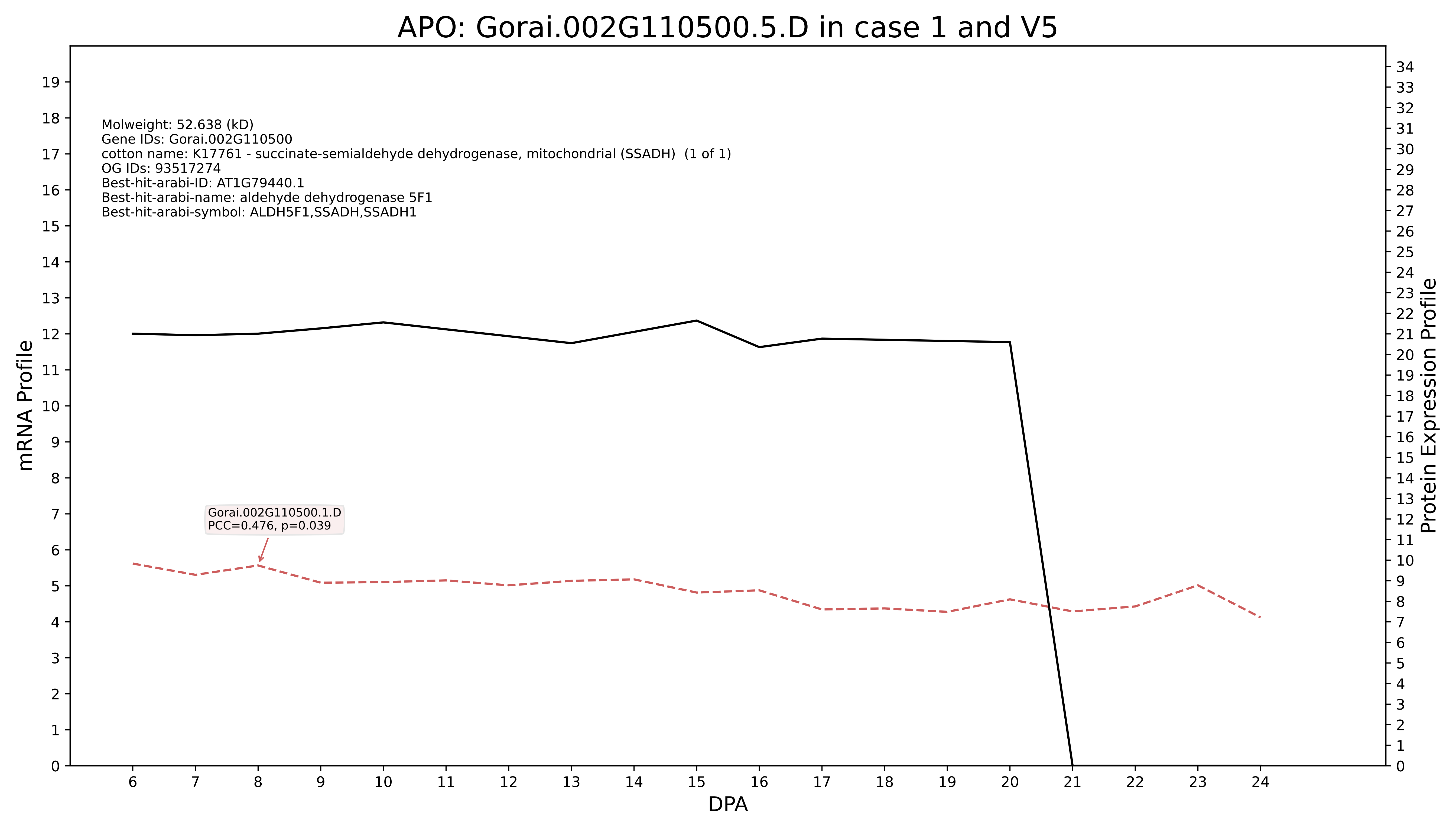
APO: Gorai.002G110500.5.D in case 1 and V5
Molweight: 52.638 (kD)
Gene IDs: Gorai.002G110500
cotton name: K17761 - succinate-semialdehyde dehydrogenase, mitochondrial (SSADH) (1 of 1)
OG IDs: 93517274
Best-hit-arabi-ID: AT1G79440.1
Best-hit-arabi-name: aldehyde dehydrogenase 5F1
Best-hit-arabi-symbol: ALDH5F1,SSADH,SSADH1
Gorai.002G110500.1.D
PCC=0.476, p=0.039
mRNA Profile
Protein Expression Profile
DPA

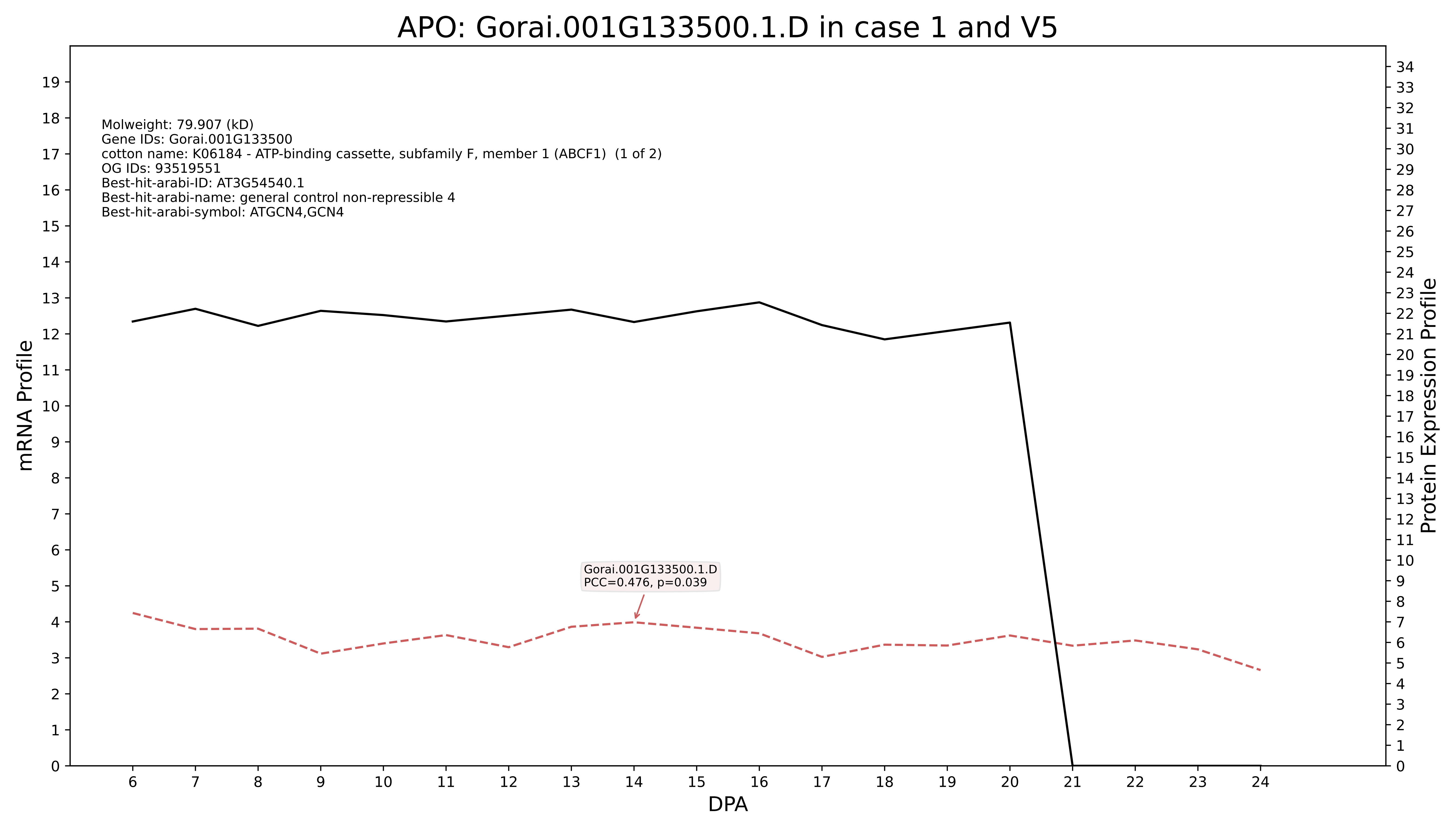

APO: Gorai.001G133500.1.D in case 1 and V5
Molweight: 79.907 (kD)
Gene IDs: Gorai.001G133500
cotton name: K06184 - ATP-binding cassette, subfamily F, member 1 (ABCF1) (1 of 2)
OG IDs: 93519551
Best-hit-arabi-ID: AT3G54540.1
Best-hit-arabi-name: general control non-repressible 4
Best-hit-arabi-symbol: ATGCN4,GCN4
Gorai.001G133500.1.D
PCC=0.476, p=0.039
mRNA Profile
Protein Expression Profile
DPA

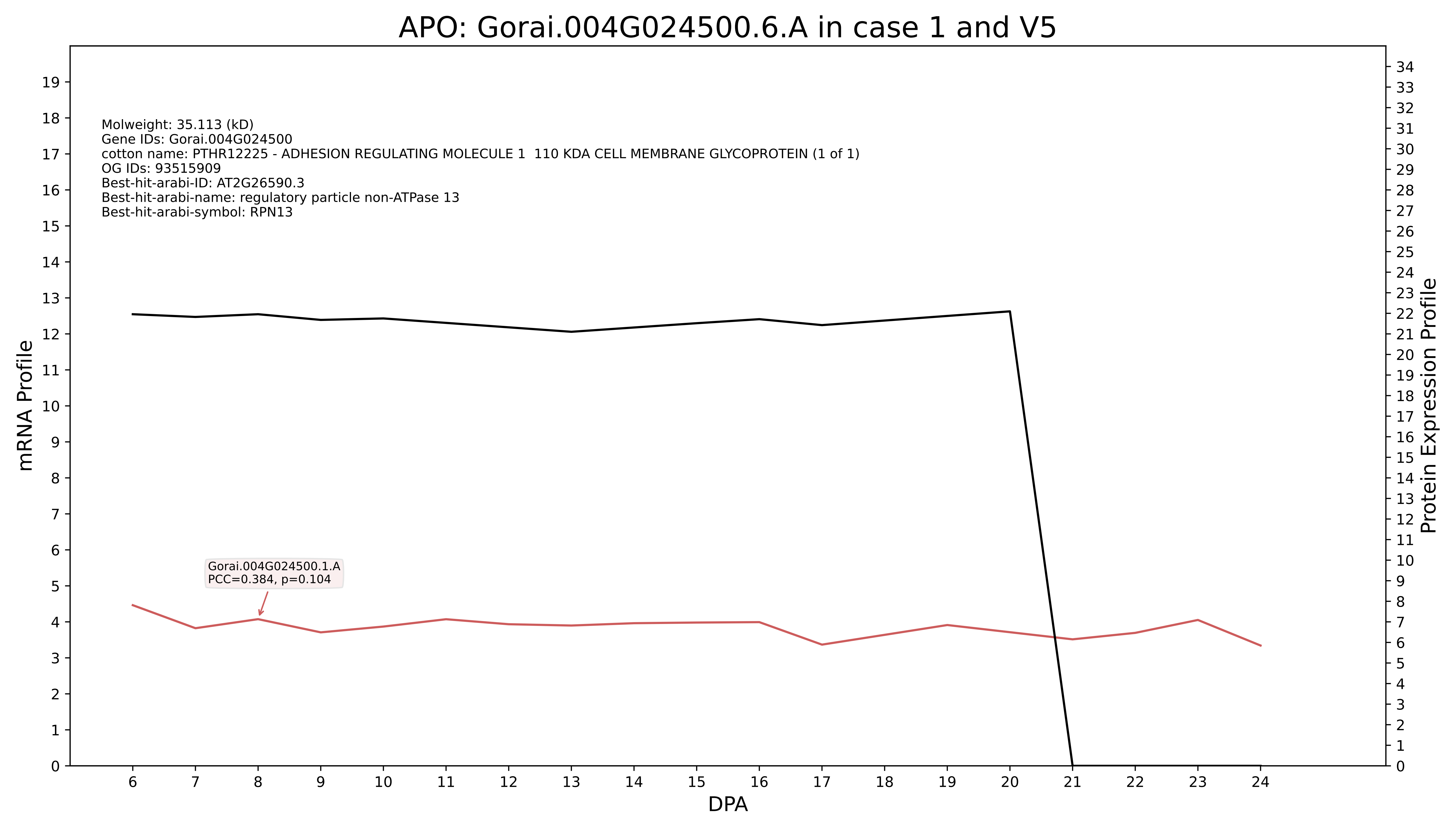
APO: Gorai.004G024500.6.A in case 1 and V5
Molweight: 35.113 (kD)
Gene IDs: Gorai.004G024500
cotton name: PTHR12225 - ADHESION REGULATING MOLECULE 1 110 KDA CELL MEMBRANE GLYCOPROTEIN (1 of 1)
OG IDs: 93515909
Best-hit-arabi-ID: AT2G26590.3
Best-hit-arabi-name: regulatory particle non-ATPase 13
Best-hit-arabi-symbol: RPN13
Gorai.004G024500.1.A
PCC=0.384, p=0.104
mRNA Profile
Protein Expression Profile
DPA

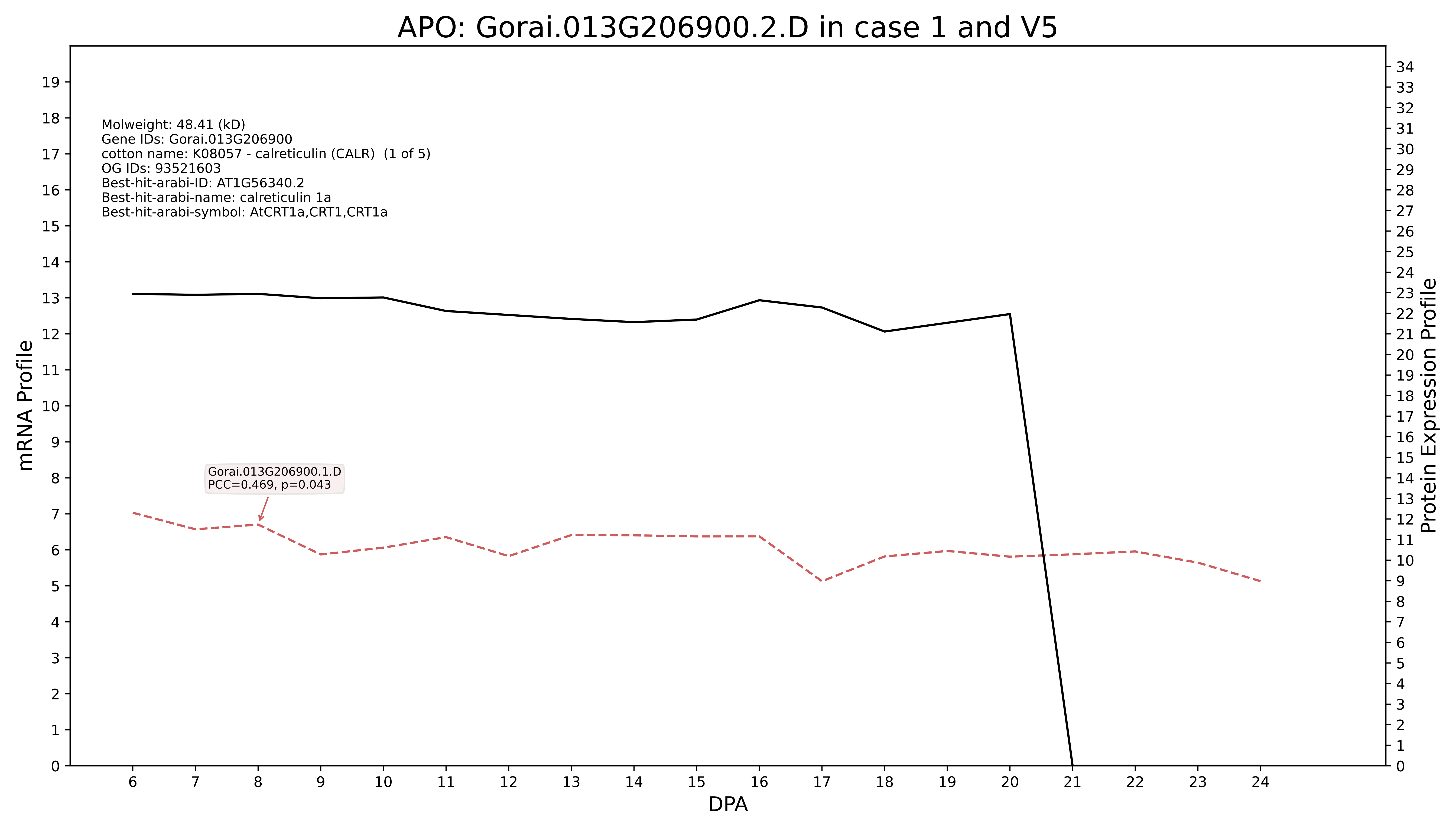

APO: Gorai.013G206900.2.D in case 1 and V5
Molweight: 48.41 (kD)
Gene IDs: Gorai.013G206900
cotton name: K08057 - calreticulin (CALR) (1 of 5)
OG IDs: 93521603
Best-hit-arabi-ID: AT1G56340.2
Best-hit-arabi-name: calreticulin 1a
Best-hit-arabi-symbol: AtCRT1a,CRT1,CRT1a
Gorai.013G206900.1.D
PCC=0.469, p=0.043
mRNA Profile
Protein Expression Profile
DPA

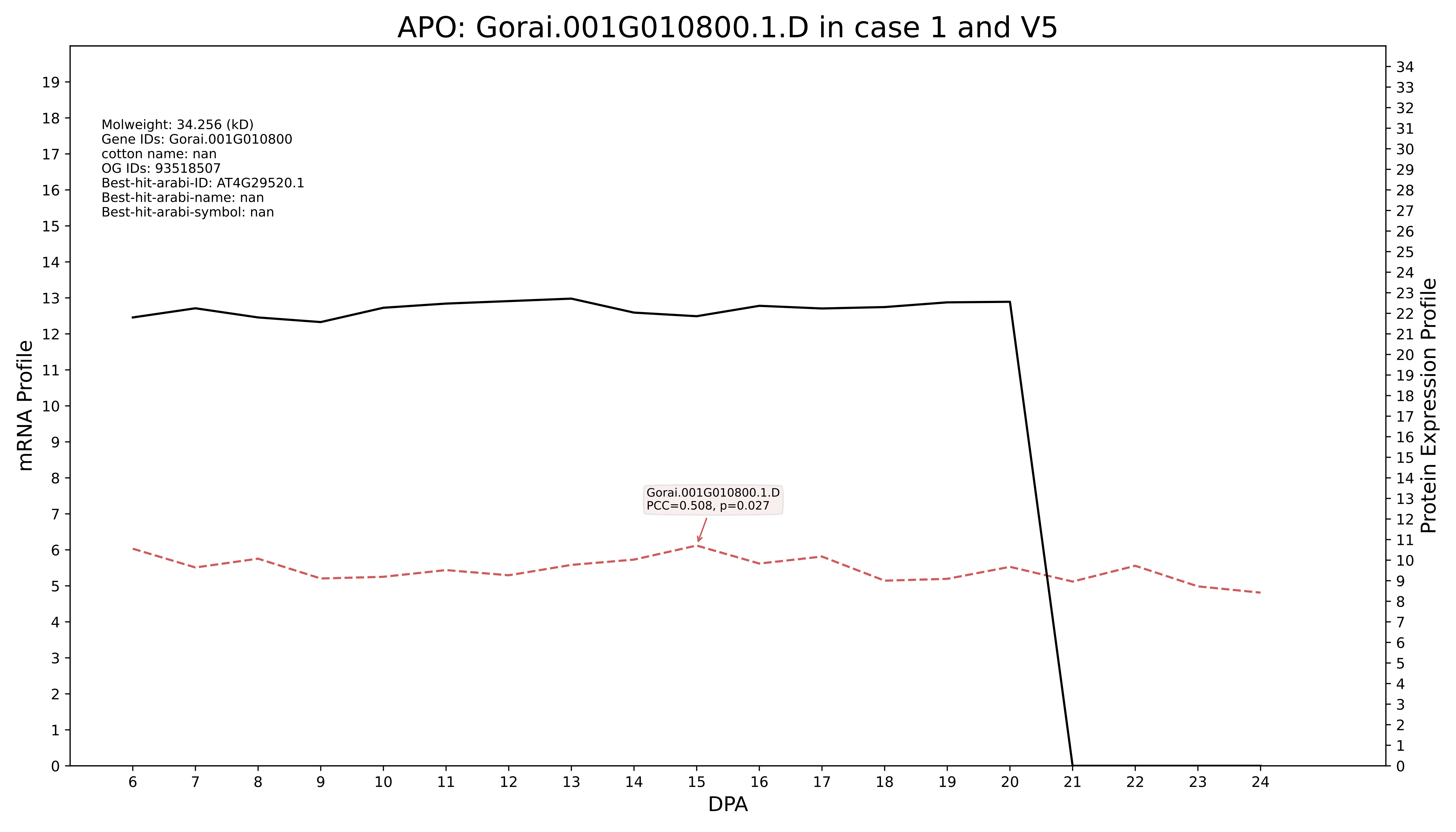

APO: Gorai.001G010800.1.D in case 1 and V5
Molweight: 34.256 (kD)
Gene IDs: Gorai.001G010800
cotton name: nan
OG IDs: 93518507
Best-hit-arabi-ID: AT4G29520.1
Best-hit-arabi-name: nan
Best-hit-arabi-symbol: nan
Gorai.001G010800.1.D
PCC=0.508, p=0.027
mRNA Profile
Protein Expression Profile
DPA

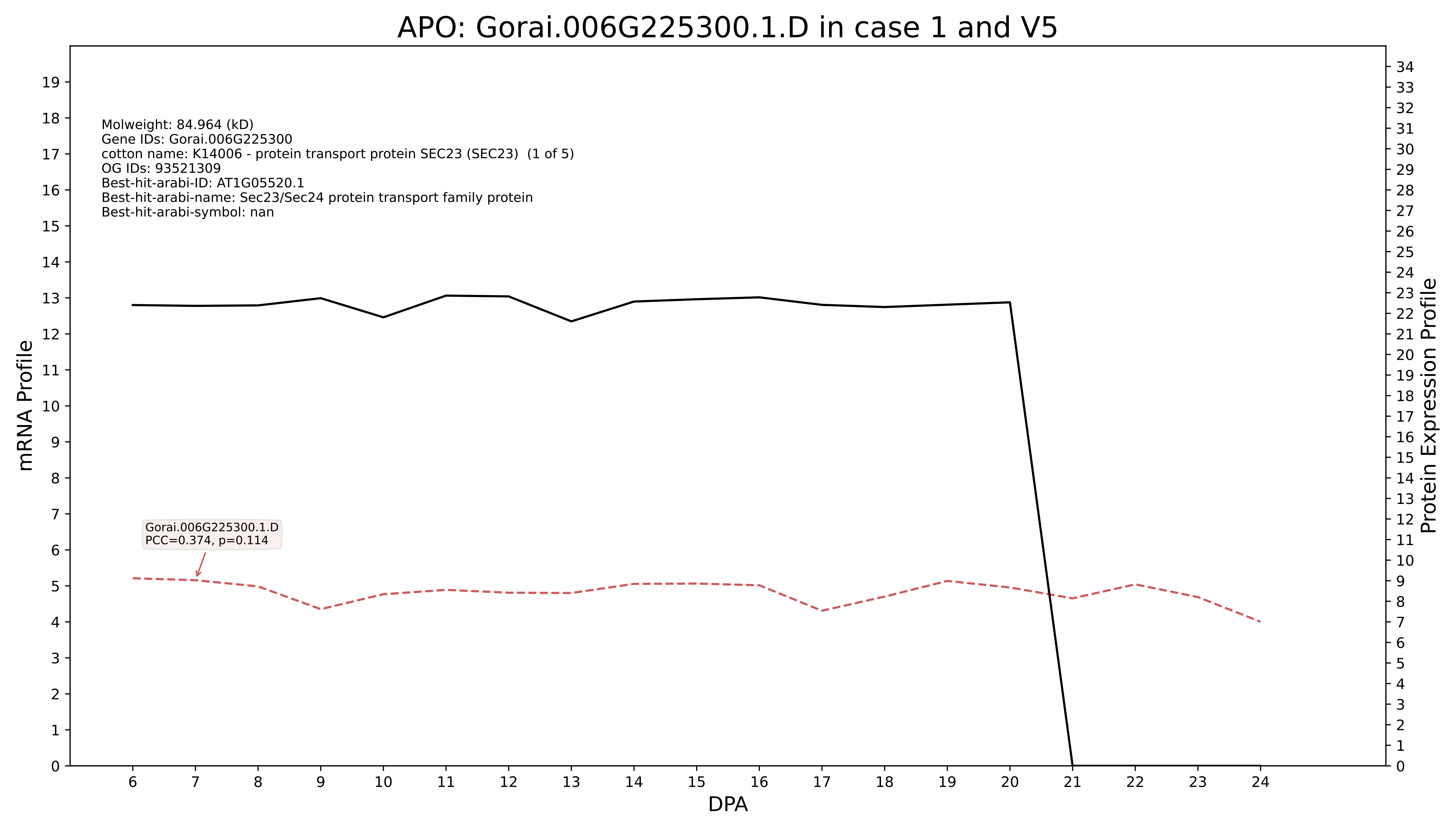

APO: Gorai.006G225300.1.D in case 1 and V5
Molweight: 84.964 (kD)
Gene IDs: Gorai.006G225300
cotton name: K14006 - protein transport protein SEC23 (SEC23) (1 of 5)
OG IDs: 93521309
Best-hit-arabi-ID: AT1G05520.1
Best-hit-arabi-name: Sec23/Sec24 protein transport family protein
Best-hit-arabi-symbol: nan
Gorai.006G225300.1.D
PCC=0.374, p=0.114
mRNA Profile
Protein Expression Profile
DPA

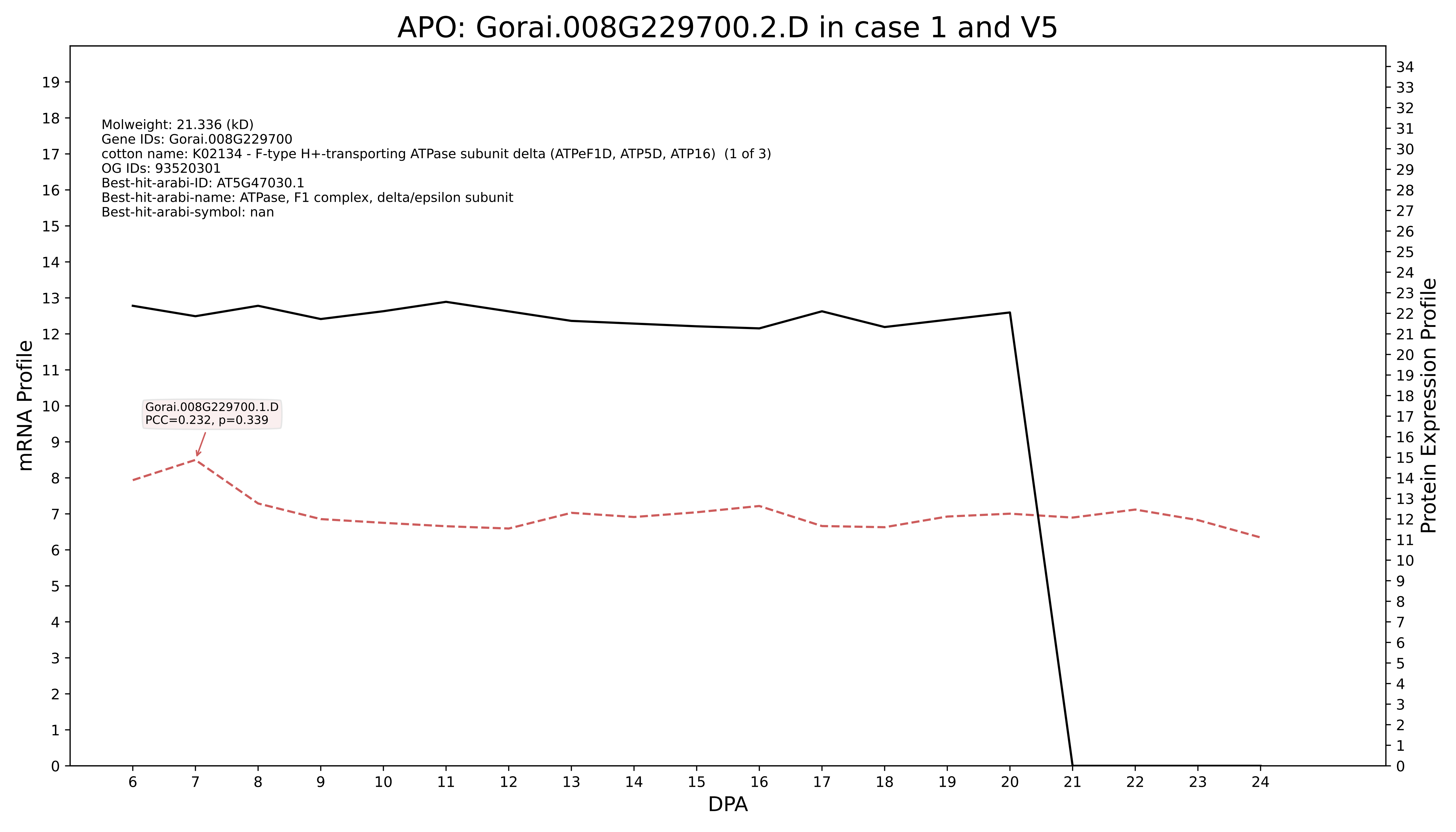
APO: Gorai.008G229700.2.D in case 1 and V5
Molweight: 21.336 (kD)
Gene IDs: Gorai.008G229700
cotton name: K02134 - F-type H+-transporting ATPase subunit delta (ATPeF1D, ATP5D, ATP16) (1 of 3)
OG IDs: 93520301
Best-hit-arabi-ID: AT5G47030.1
Best-hit-arabi-name: ATPase, F1 complex, delta/epsilon subunit
Best-hit-arabi-symbol: nan
Gorai.008G229700.1.D
PCC=0.232, p=0.339
mRNA Profile
Protein Expression Profile
DPA

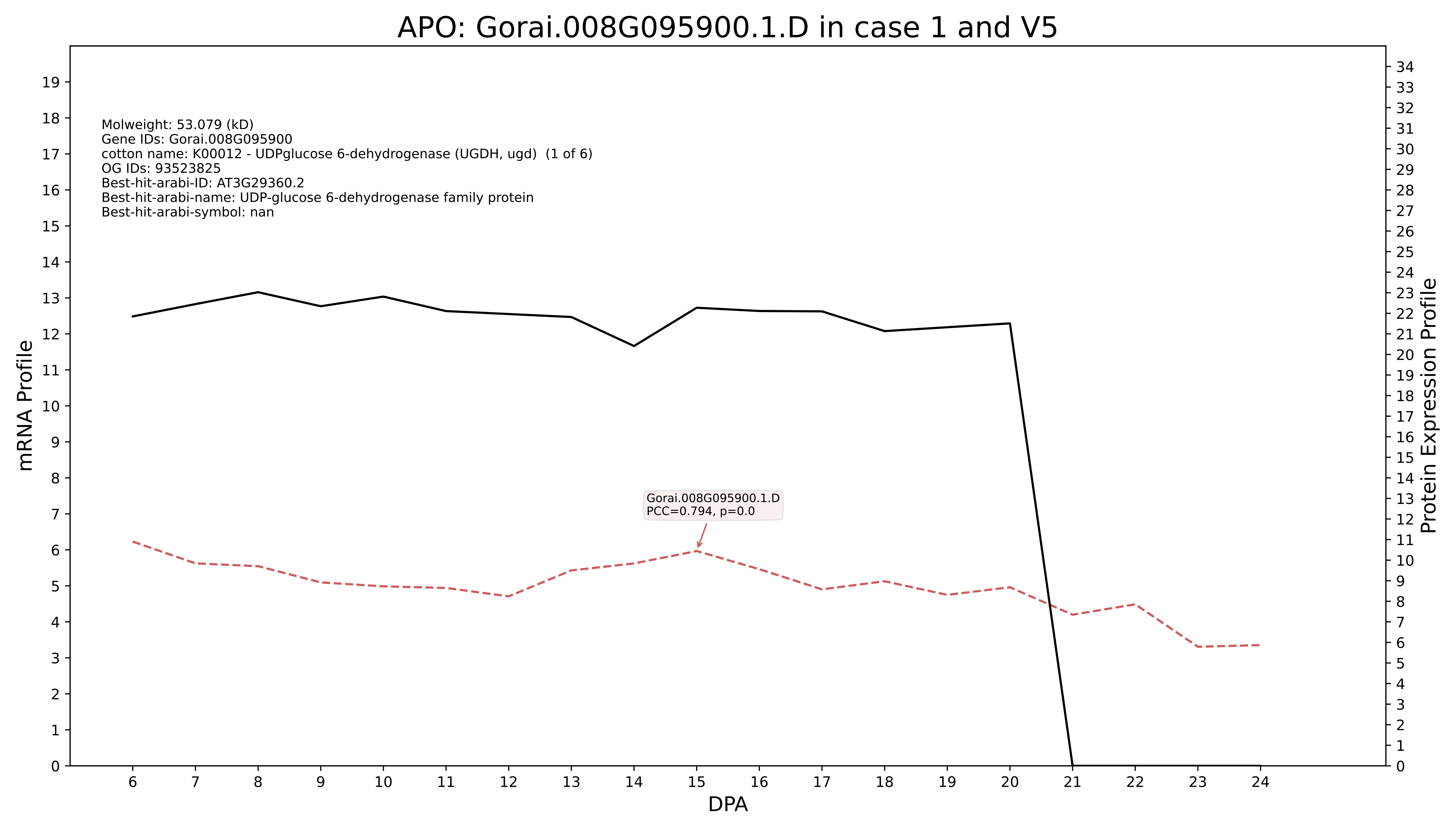

APO: Gorai.008G095900.1.D in case 1 and V5
Molweight: 53.079 (kD)
Gene IDs: Gorai.008G095900
cotton name: K00012 - UDPglucose 6-dehydrogenase (UGDH, ugd) (1 of 6)
OG IDs: 93523825
Best-hit-arabi-ID: AT3G29360.2
Best-hit-arabi-name: UDP-glucose 6-dehydrogenase family protein
Best-hit-arabi-symbol: nan
Gorai.008G095900.1.D
PCC=0.794, p=0.0
mRNA Profile
Protein Expression Profile
DPA

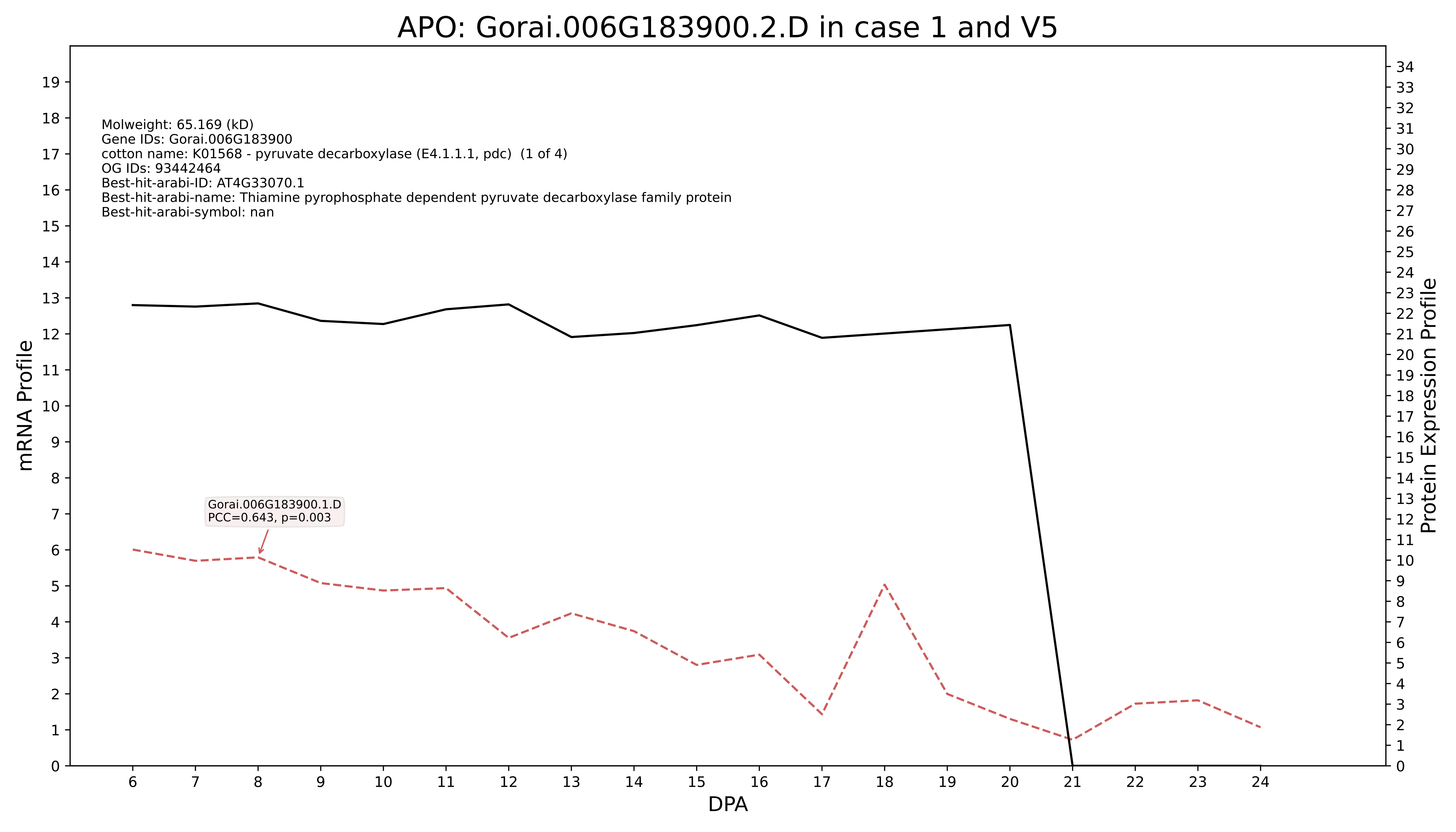
APO: Gorai.006G183900.2.D in case 1 and V5
Molweight: 65.169 (kD)
Gene IDs: Gorai.006G183900
cotton name: K01568 - pyruvate decarboxylase (E4.1.1.1, pdc) (1 of 4)
OG IDs: 93442464
Best-hit-arabi-ID: AT4G33070.1
Best-hit-arabi-name: Thiamine pyrophosphate dependent pyruvate decarboxylase family protein
Best-hit-arabi-symbol: nan
Gorai.006G183900.1.D
PCC=0.643, p=0.003
mRNA Profile
Protein Expression Profile
DPA

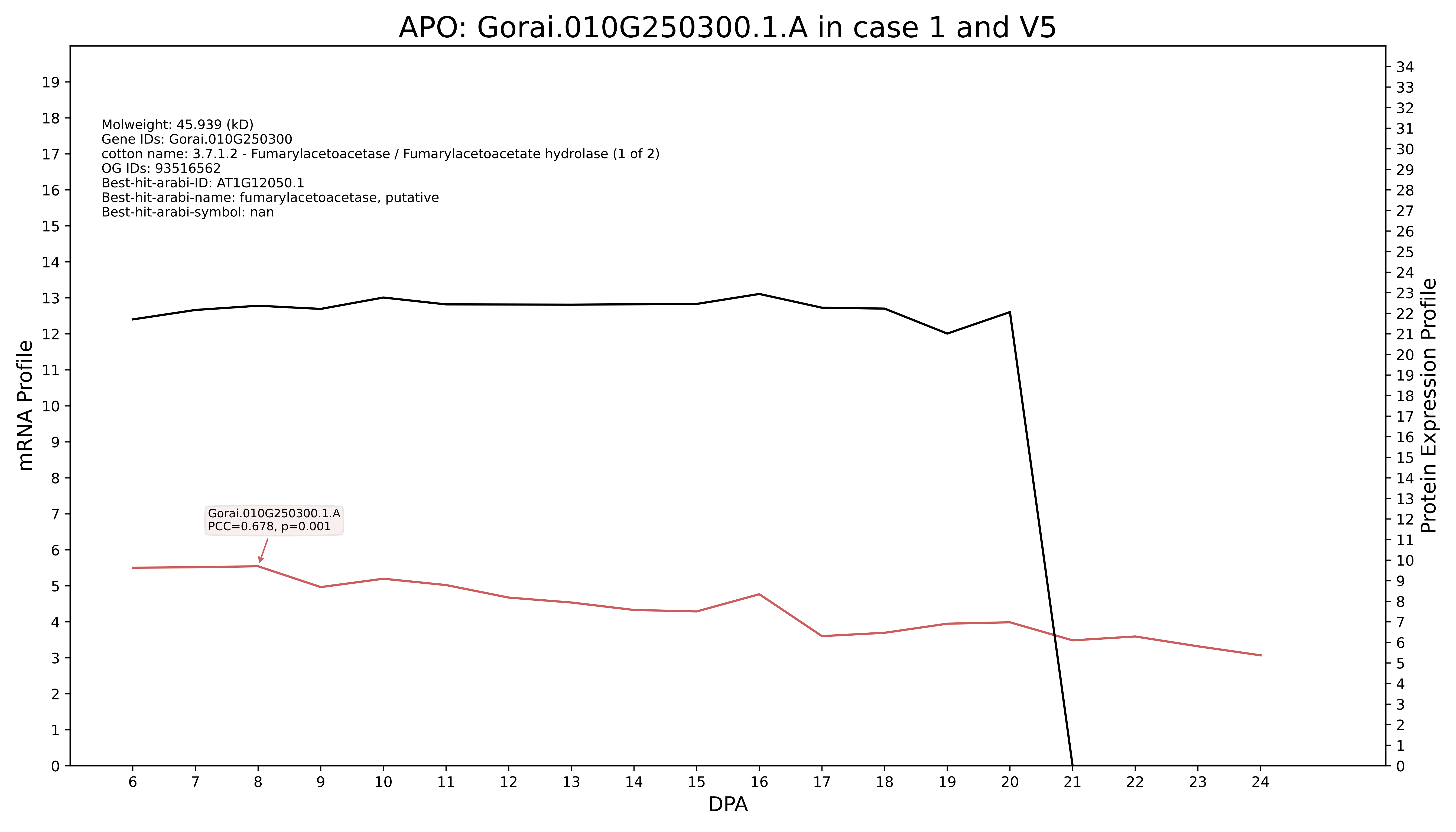
APO: Gorai.010G250300.1.A in case 1 and V5
Molweight: 45.939 (kD)
Gene IDs: Gorai.010G250300
cotton name: 3.7.1.2 - Fumarylacetoacetase / Fumarylacetoacetate hydrolase (1 of 2)
OG IDs: 93516562
Best-hit-arabi-ID: AT1G12050.1
Best-hit-arabi-name: fumarylacetoacetase, putative
Best-hit-arabi-symbol: nan
Gorai.010G250300.1.A
PCC=0.678, p=0.001
mRNA Profile
Protein Expression Profile
DPA

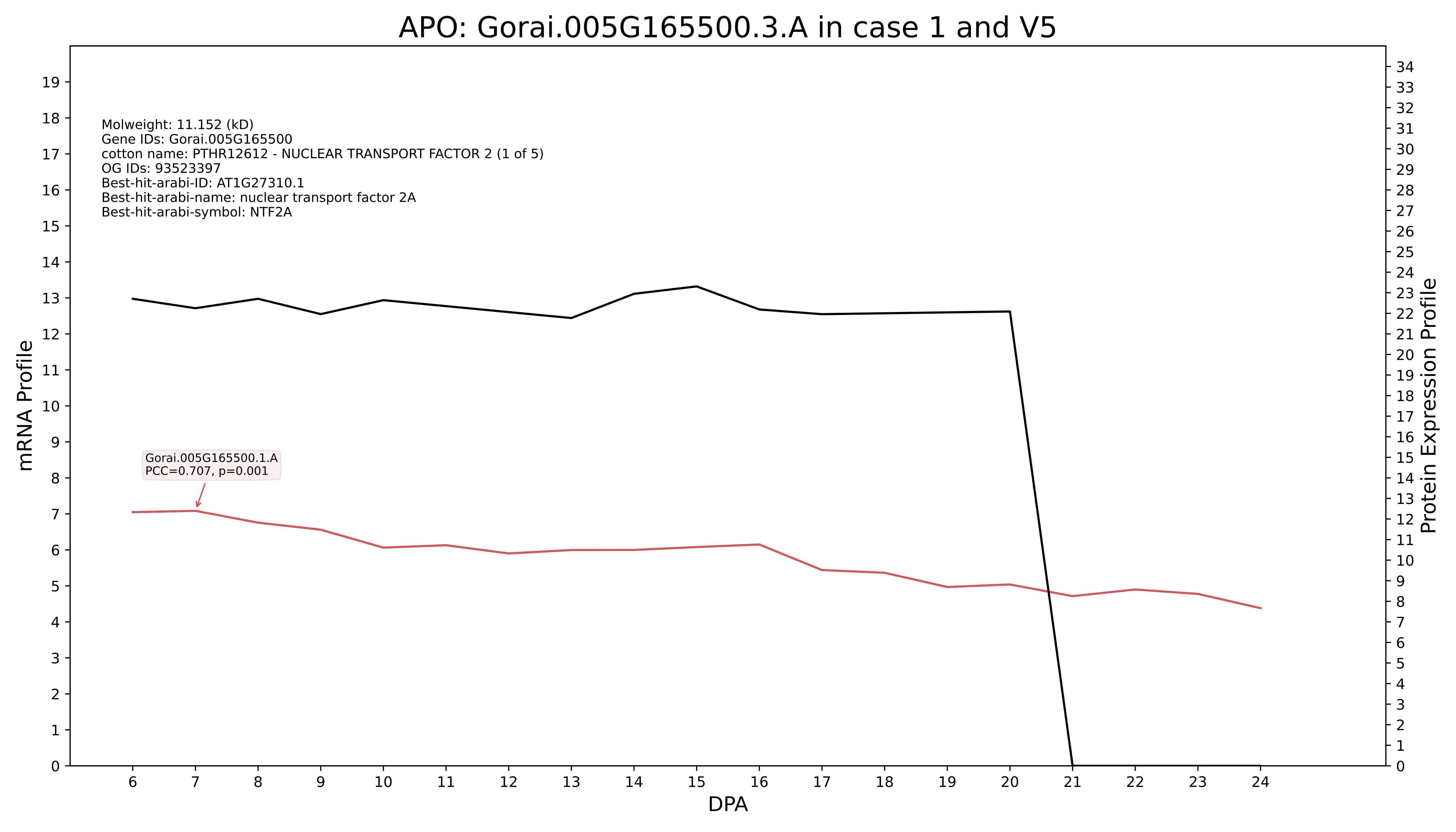

APO: Gorai.005G165500.3.A in case 1 and V5
Molweight: 11.152 (kD)
Gene IDs: Gorai.005G165500
cotton name: PTHR12612 - NUCLEAR TRANSPORT FACTOR 2 (1 of 5)
OG IDs: 93523397
Best-hit-arabi-ID: AT1G27310.1
Best-hit-arabi-name: nuclear transport factor 2A
Best-hit-arabi-symbol: NTF2A
Gorai.005G165500.1.A
PCC=0.707, p=0.001
mRNA Profile
Protein Expression Profile
DPA

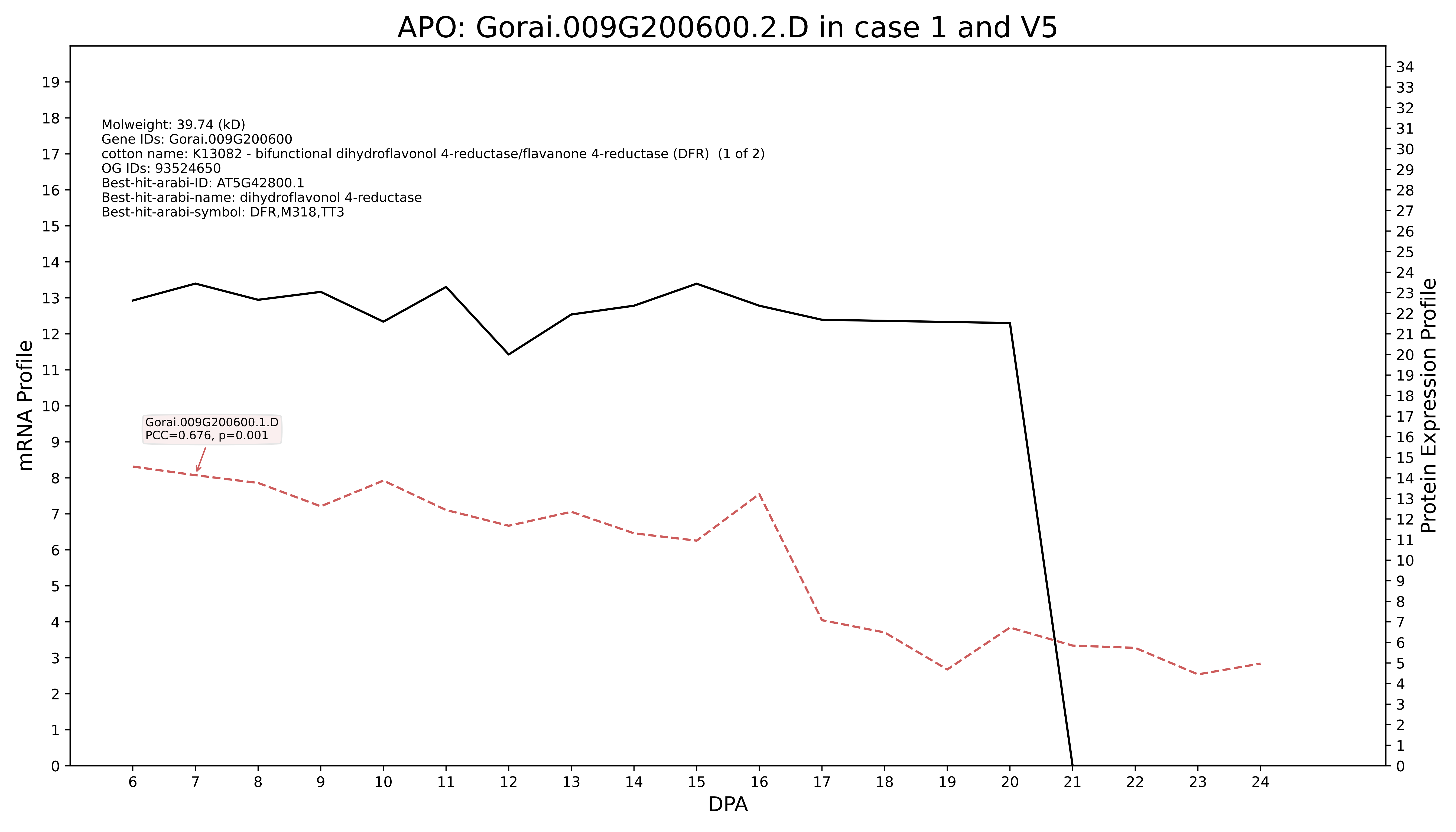

APO: Gorai.009G200600.2.D in case 1 and V5
Molweight: 39.74 (kD)
Gene IDs: Gorai.009G200600
cotton name: K13082 - bifunctional dihydroflavonol 4-reductase/flavanone 4-reductase (DFR) (1 of 2)
OG IDs: 93524650
Best-hit-arabi-ID: AT5G42800.1
Best-hit-arabi-name: dihydroflavonol 4-reductase
Best-hit-arabi-symbol: DFR,M318,TT3
Gorai.009G200600.1.D
PCC=0.676, p=0.001
mRNA Profile
Protein Expression Profile
DPA

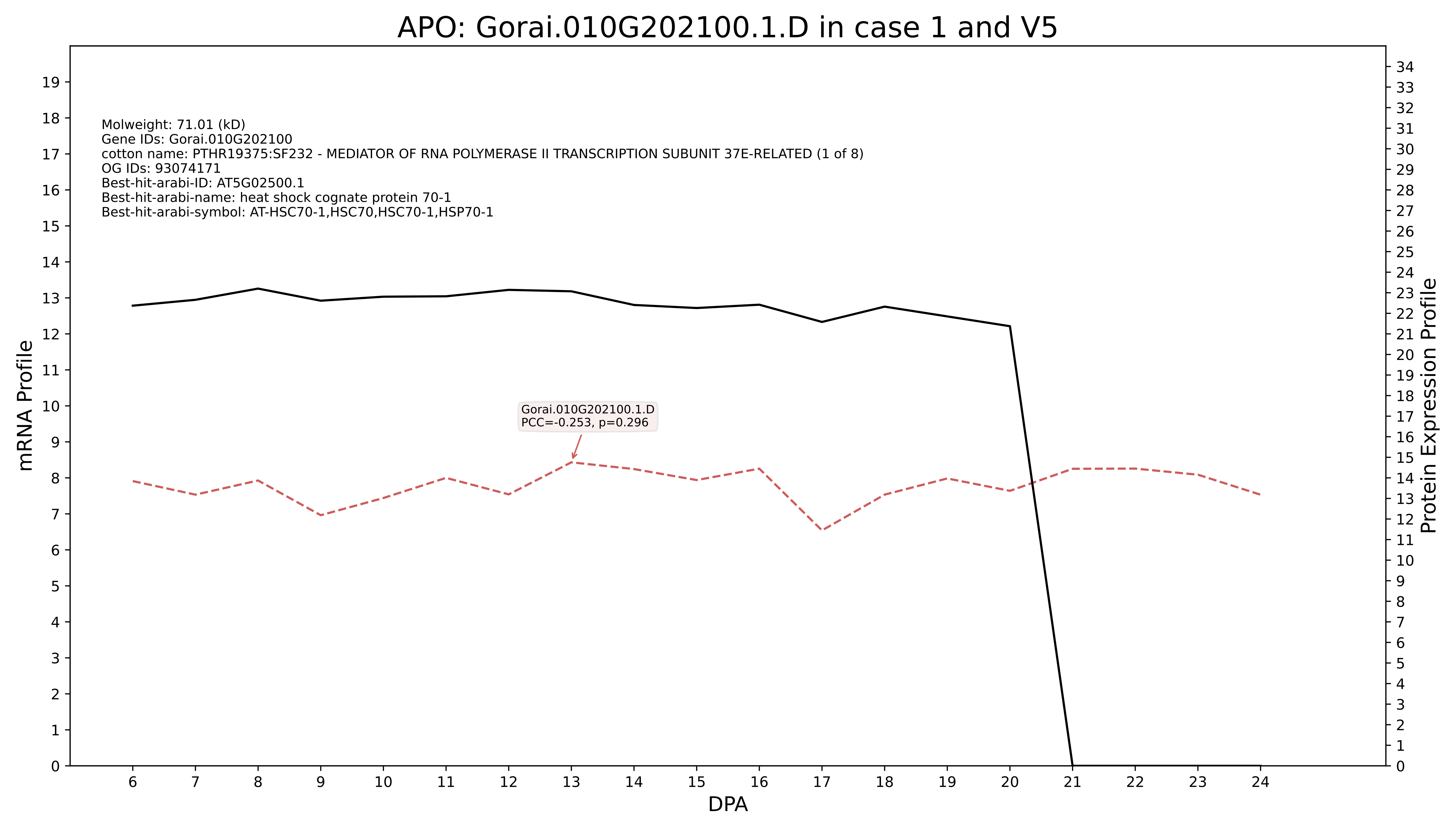
APO: Gorai.010G202100.1.D in case 1 and V5
Molweight: 71.01 (kD)
Gene IDs: Gorai.010G202100
cotton name: PTHR19375:SF232 - MEDIATOR OF RNA POLYMERASE II TRANSCRIPTION SUBUNIT 37E-RELATED (1 of 8)
OG IDs: 93074171
Best-hit-arabi-ID: AT5G02500.1
Best-hit-arabi-name: heat shock cognate protein 70-1
Best-hit-arabi-symbol: AT-HSC70-1,HSC70,HSC70-1,HSP70-1
Gorai.010G202100.1.D
PCC=-0.253, p=0.296
mRNA Profile
Protein Expression Profile
DPA

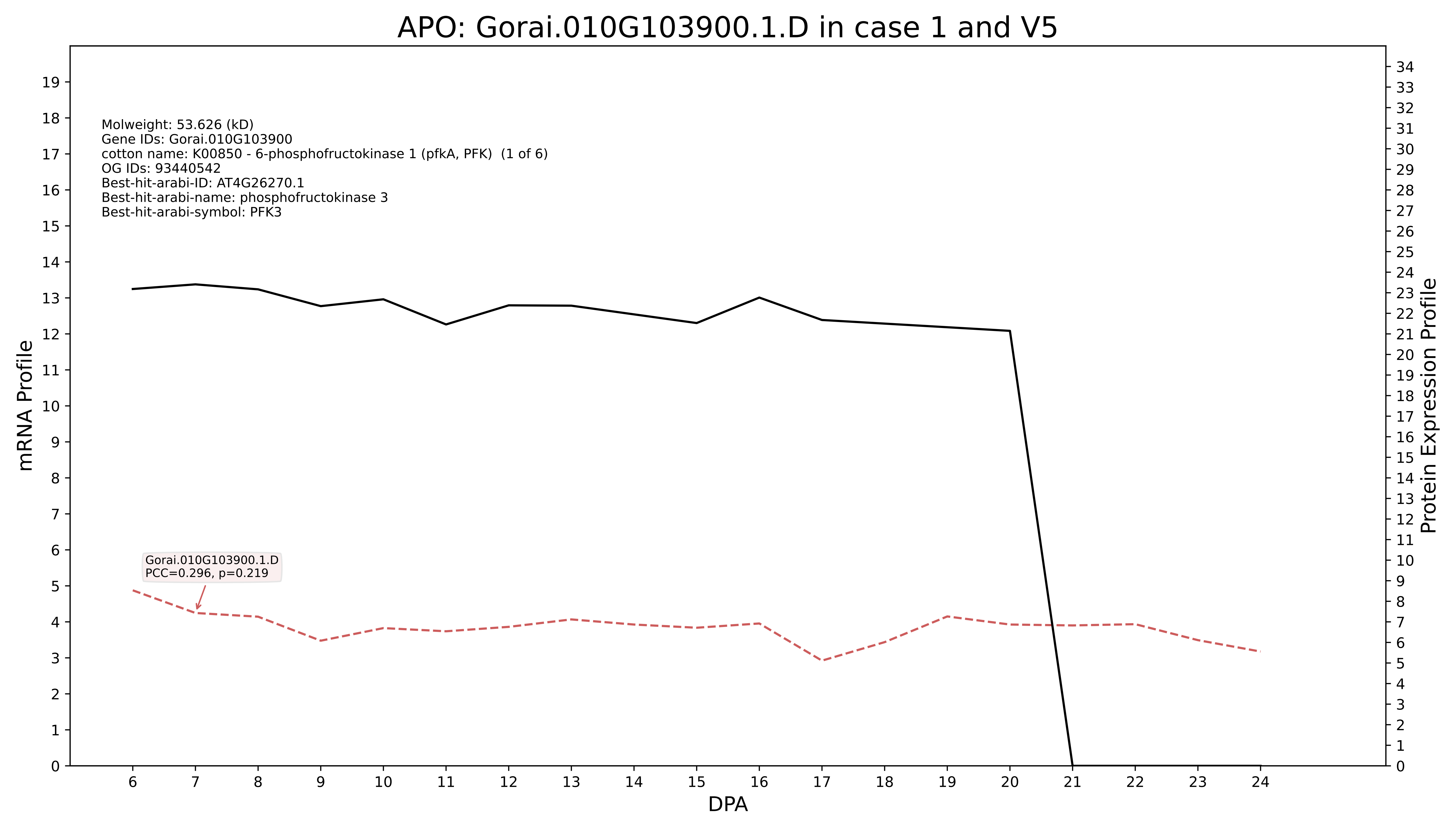
APO: Gorai.010G103900.1.D in case 1 and V5
Molweight: 53.626 (kD)
Gene IDs: Gorai.010G103900
cotton name: K00850 - 6-phosphofructokinase 1 (pfkA, PFK) (1 of 6)
OG IDs: 93440542
Best-hit-arabi-ID: AT4G26270.1
Best-hit-arabi-name: phosphofructokinase 3
Best-hit-arabi-symbol: PFK3
Gorai.010G103900.1.D
PCC=0.296, p=0.219
mRNA Profile
Protein Expression Profile
DPA

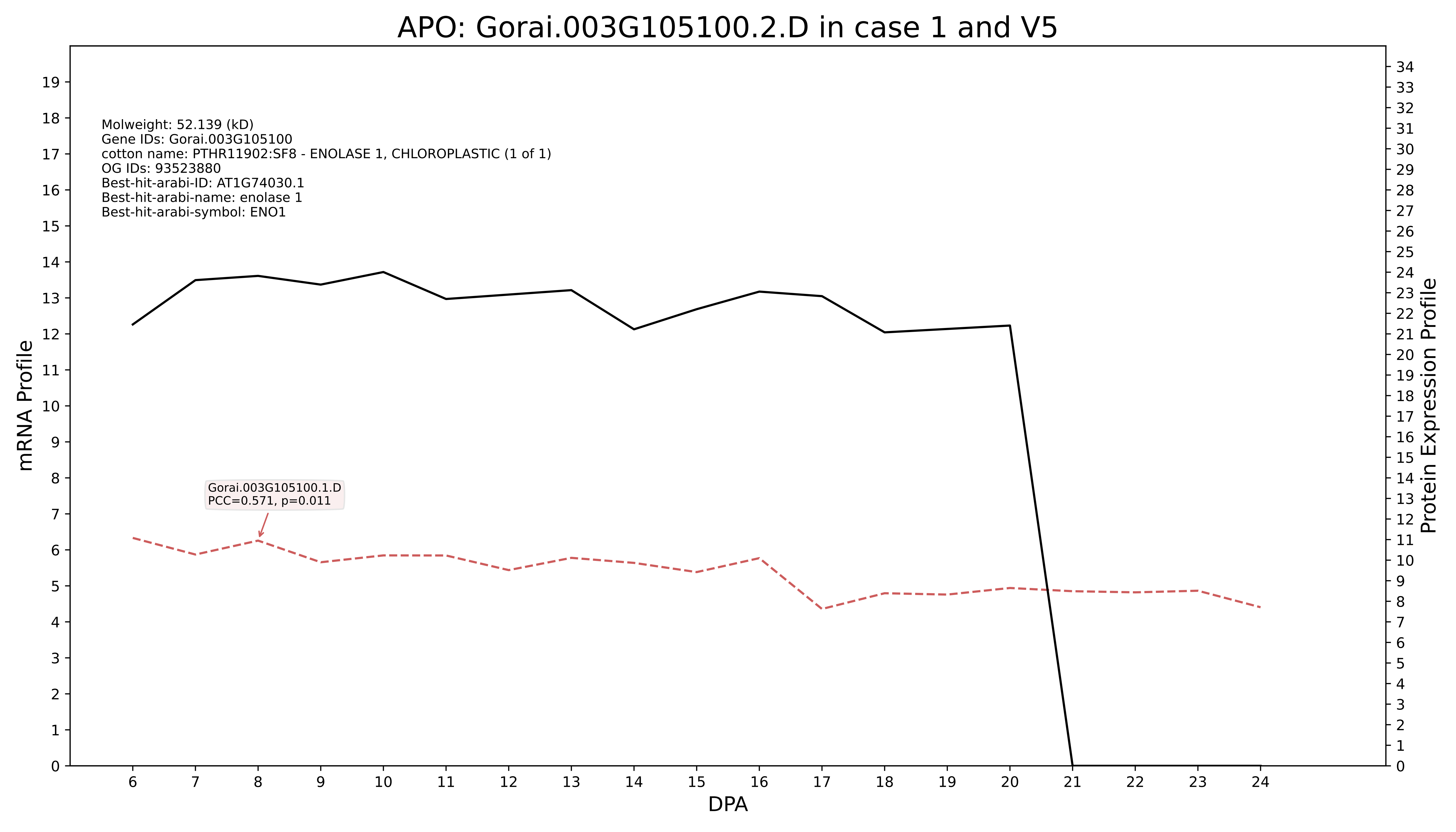
APO: Gorai.003G105100.2.D in case 1 and V5
Molweight: 52.139 (kD)
Gene IDs: Gorai.003G105100
cotton name: PTHR11902:SF8 - ENOLASE 1, CHLOROPLASTIC (1 of 1)
OG IDs: 93523880
Best-hit-arabi-ID: AT1G74030.1
Best-hit-arabi-name: enolase 1
Best-hit-arabi-symbol: ENO1
Gorai.003G105100.1.D
PCC=0.571, p=0.011
mRNA Profile
Protein Expression Profile
DPA

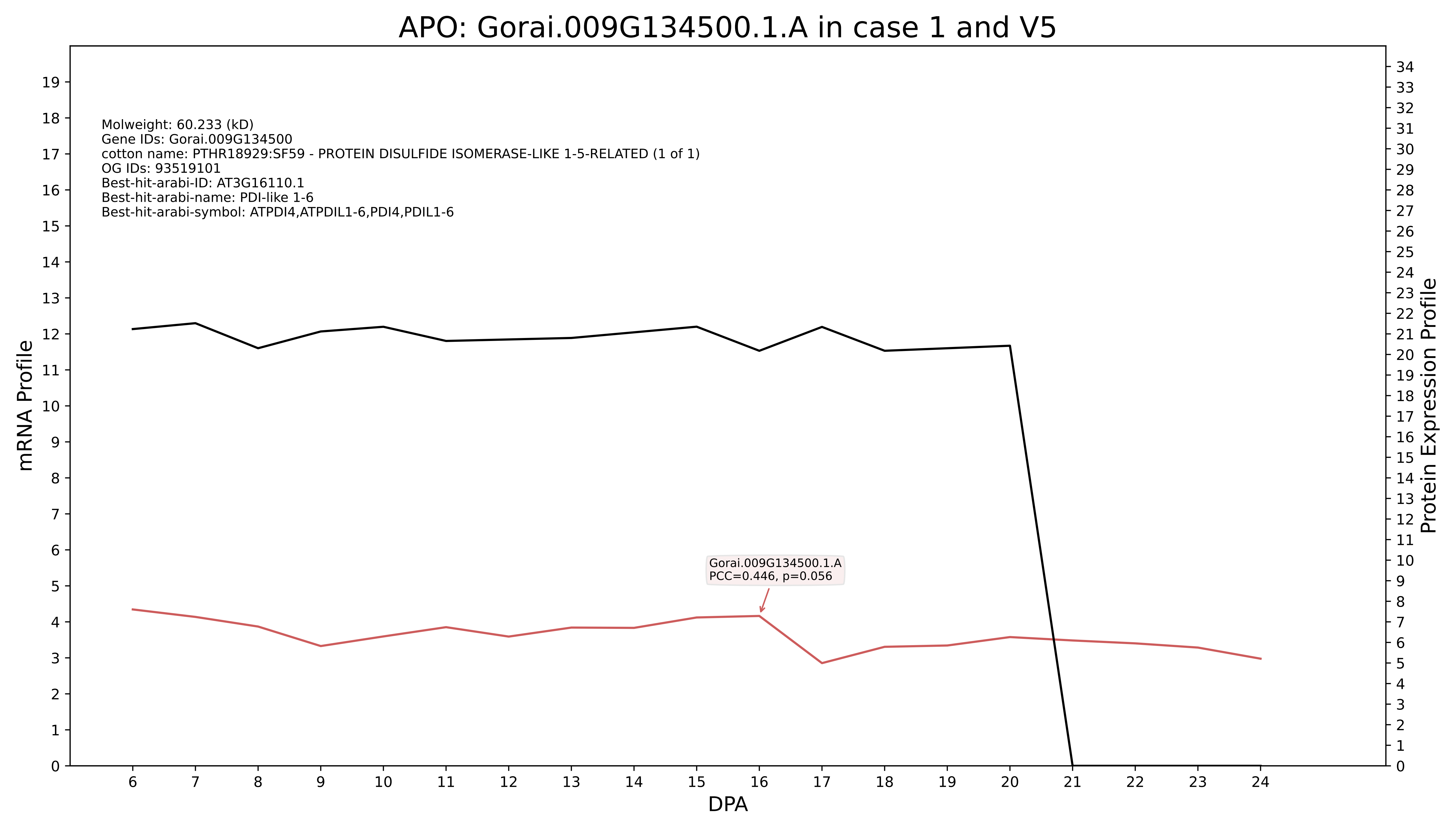
APO: Gorai.009G134500.1.A in case 1 and V5
Molweight: 60.233 (kD)
Gene IDs: Gorai.009G134500
cotton name: PTHR18929:SF59 - PROTEIN DISULFIDE ISOMERASE-LIKE 1-5-RELATED (1 of 1)
OG IDs: 93519101
Best-hit-arabi-ID: AT3G16110.1
Best-hit-arabi-name: PDI-like 1-6
Best-hit-arabi-symbol: ATPDI4,ATPDIL1-6,PDI4,PDIL1-6
Gorai.009G134500.1.A
PCC=0.446, p=0.056
mRNA Profile
Protein Expression Profile
DPA

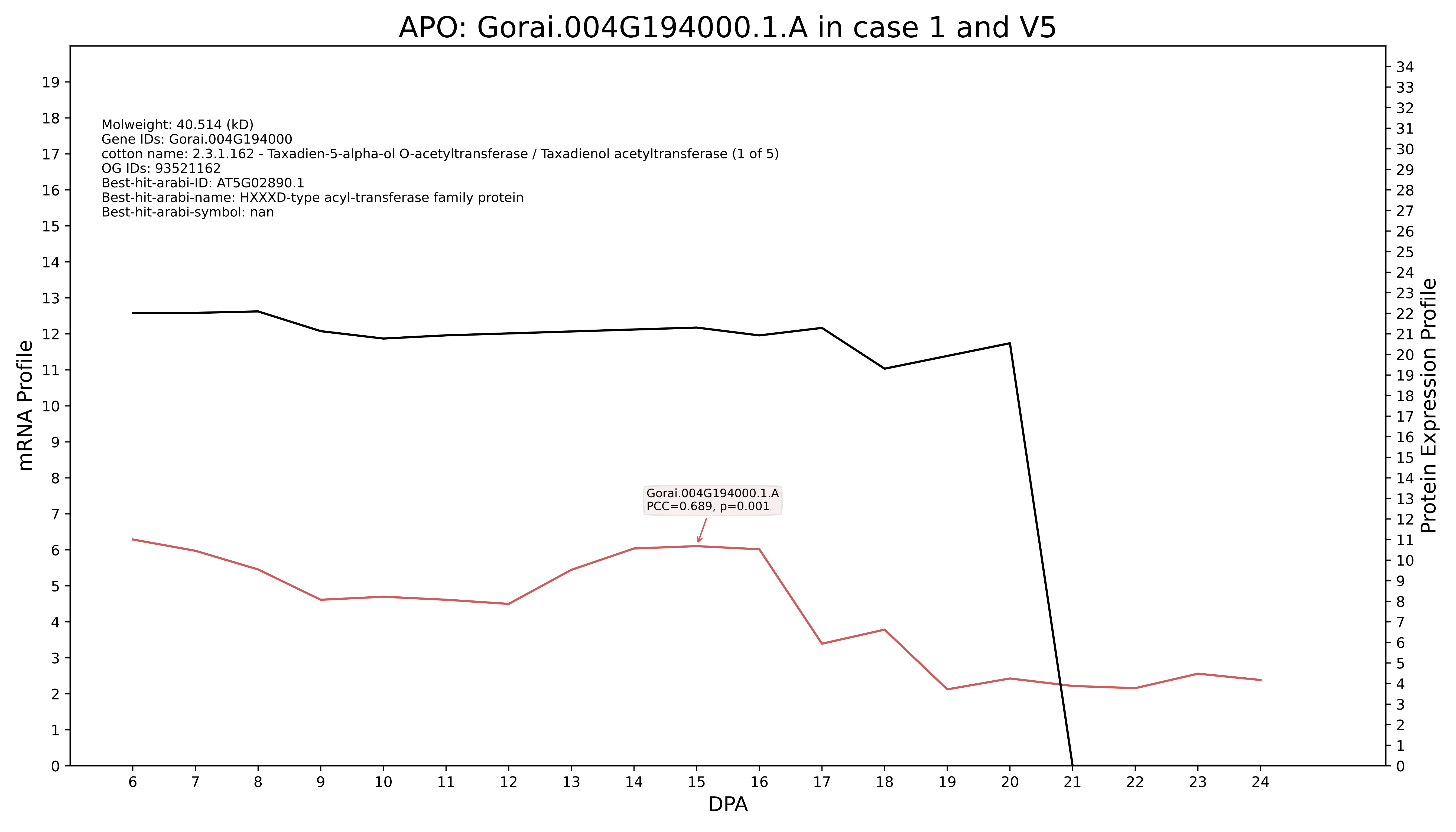
APO: Gorai.004G194000.1.A in case 1 and V5
Molweight: 40.514 (kD)
Gene IDs: Gorai.004G194000
cotton name: 2.3.1.162 - Taxadien-5-alpha-ol O-acetyltransferase / Taxadienol acetyltransferase (1 of 5)
OG IDs: 93521162
Best-hit-arabi-ID: AT5G02890.1
Best-hit-arabi-name: HXXXD-type acyl-transferase family protein
Best-hit-arabi-symbol: nan
Gorai.004G194000.1.A
PCC=0.689, p=0.001
mRNA Profile
Protein Expression Profile
DPA

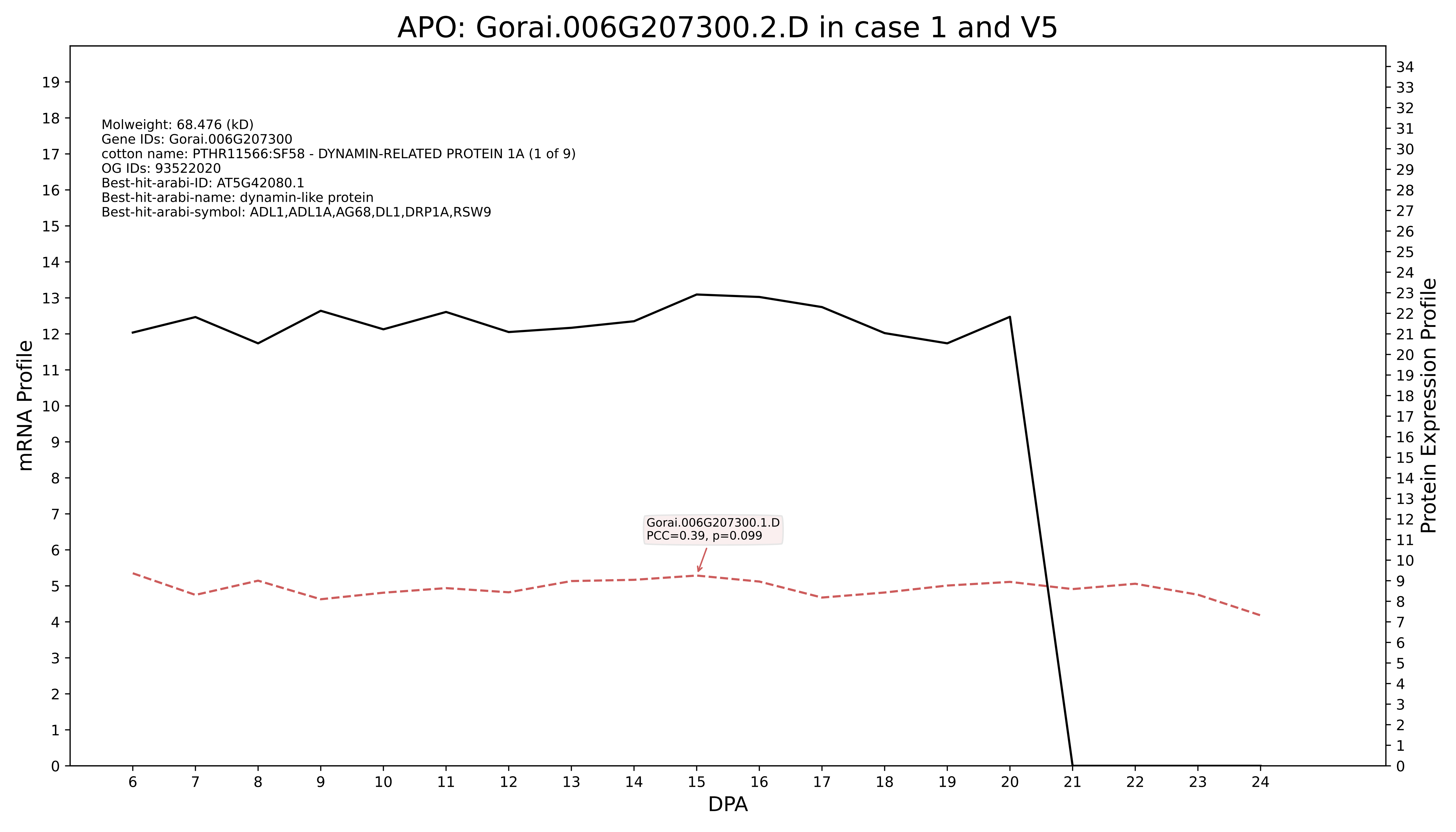
APO: Gorai.006G207300.2.D in case 1 and V5
Molweight: 68.476 (kD)
Gene IDs: Gorai.006G207300
cotton name: PTHR11566:SF58 - DYNAMIN-RELATED PROTEIN 1A (1 of 9)
OG IDs: 93522020
Best-hit-arabi-ID: AT5G42080.1
Best-hit-arabi-name: dynamin-like protein
Best-hit-arabi-symbol: ADL1,ADL1A,AG68,DL1,DRP1A,RSW9
Gorai.006G207300.1.D
PCC=0.39, p=0.099
mRNA Profile
Protein Expression Profile
DPA

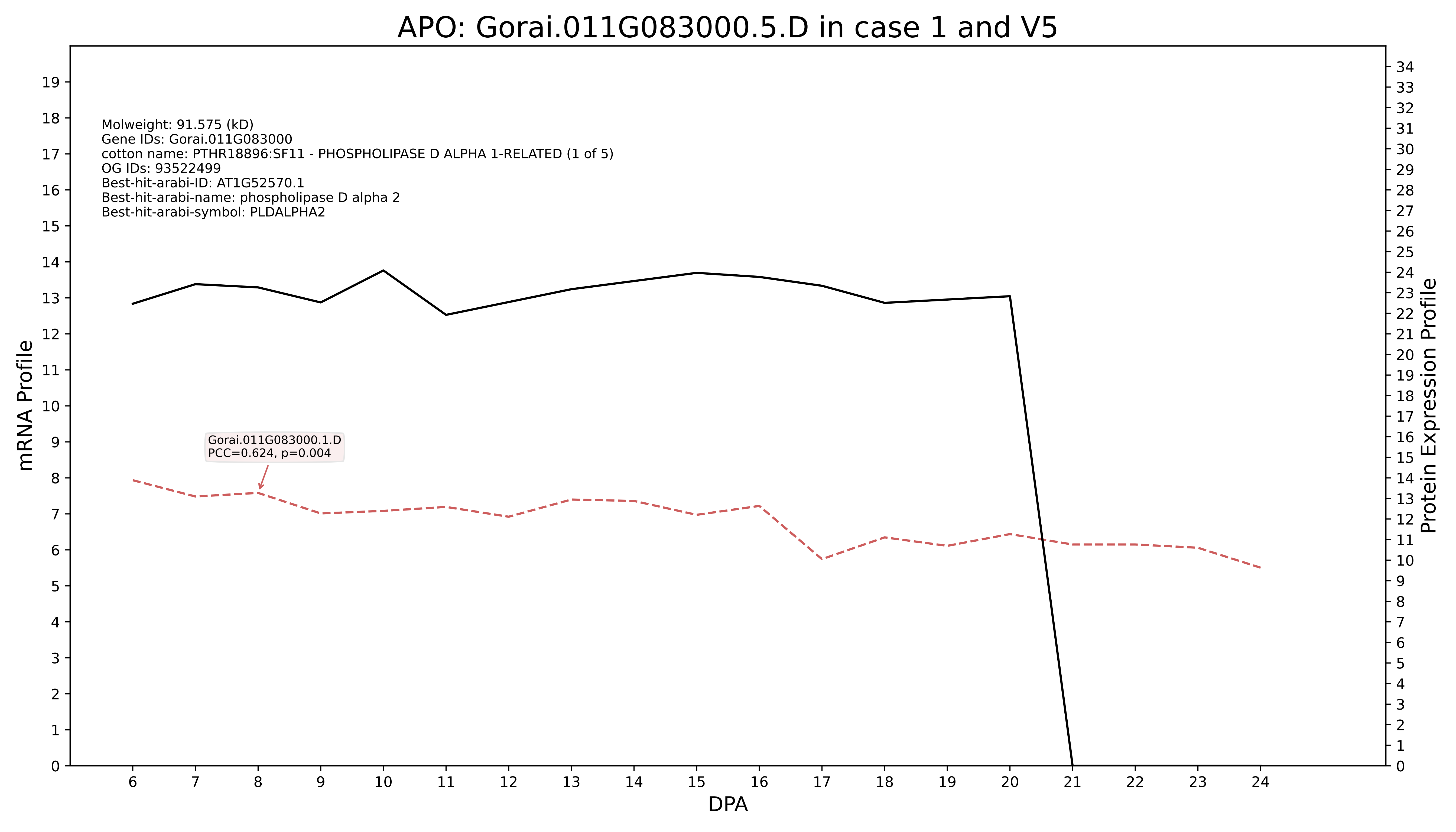
APO: Gorai.011G083000.5.D in case 1 and V5
Molweight: 91.575 (kD)
Gene IDs: Gorai.011G083000
cotton name: PTHR18896:SF11 - PHOSPHOLIPASE D ALPHA 1-RELATED (1 of 5)
OG IDs: 93522499
Best-hit-arabi-ID: AT1G52570.1
Best-hit-arabi-name: phospholipase D alpha 2
Best-hit-arabi-symbol: PLDALPHA2
Gorai.011G083000.1.D
PCC=0.624, p=0.004
mRNA Profile
Protein Expression Profile
DPA
0
1
2
3
4
5
6
7
8
9
10
11
12
13
14
15
16
17
18
19
20
21
22
23
24
25
26
27
28
29
30
31
32
33
34

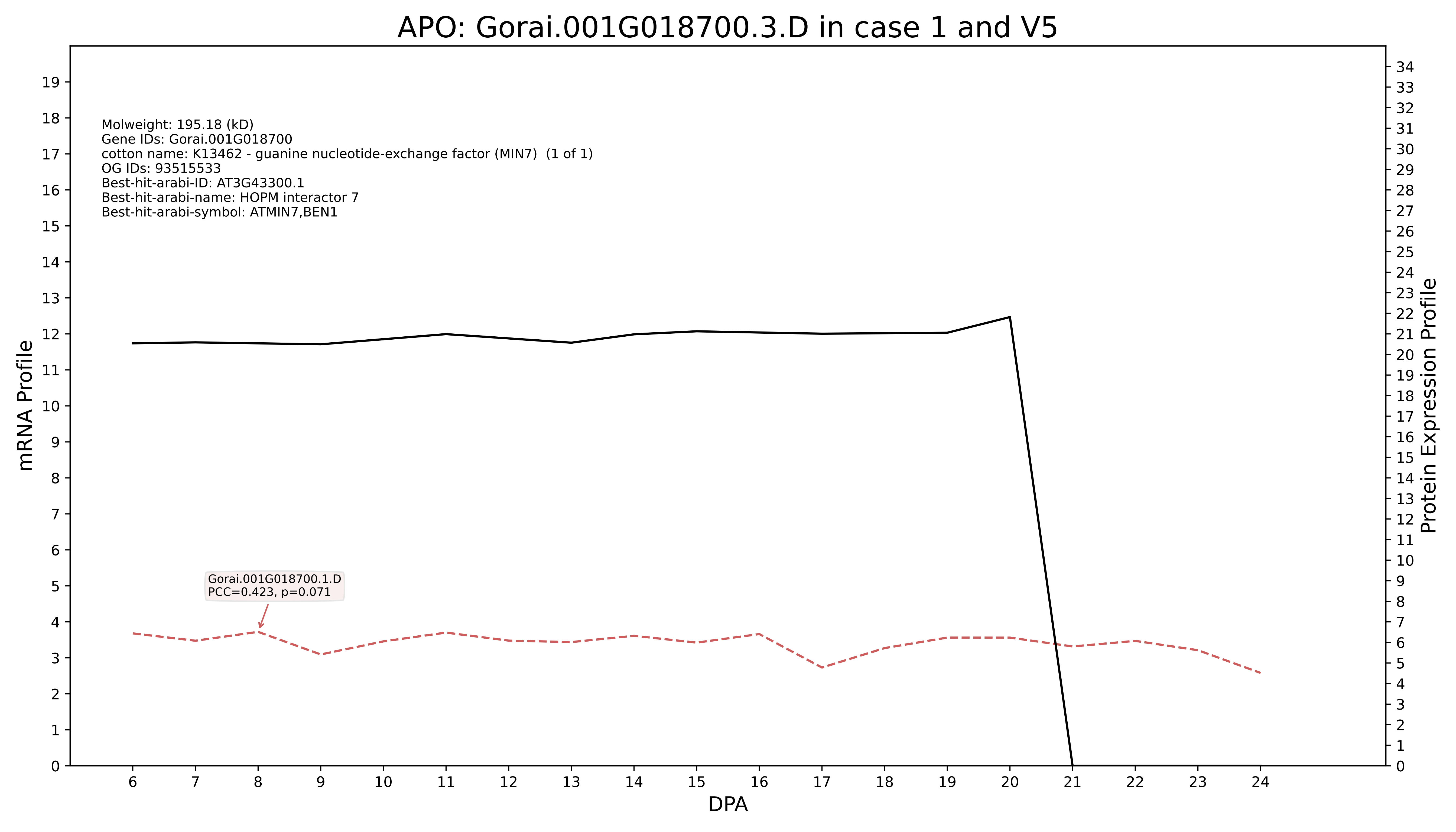
APO: Gorai.001G018700.3.D in case 1 and V5
Molweight: 195.18 (kD)
Gene IDs: Gorai.001G018700
cotton name: K13462 - guanine nucleotide-exchange factor (MIN7) (1 of 1)
OG IDs: 93515533
Best-hit-arabi-ID: AT3G43300.1
Best-hit-arabi-name: HOPM interactor 7
Best-hit-arabi-symbol: ATMIN7,BEN1
Gorai.001G018700.1.D
PCC=0.423, p=0.071
mRNA Profile
Protein Expression Profile
DPA

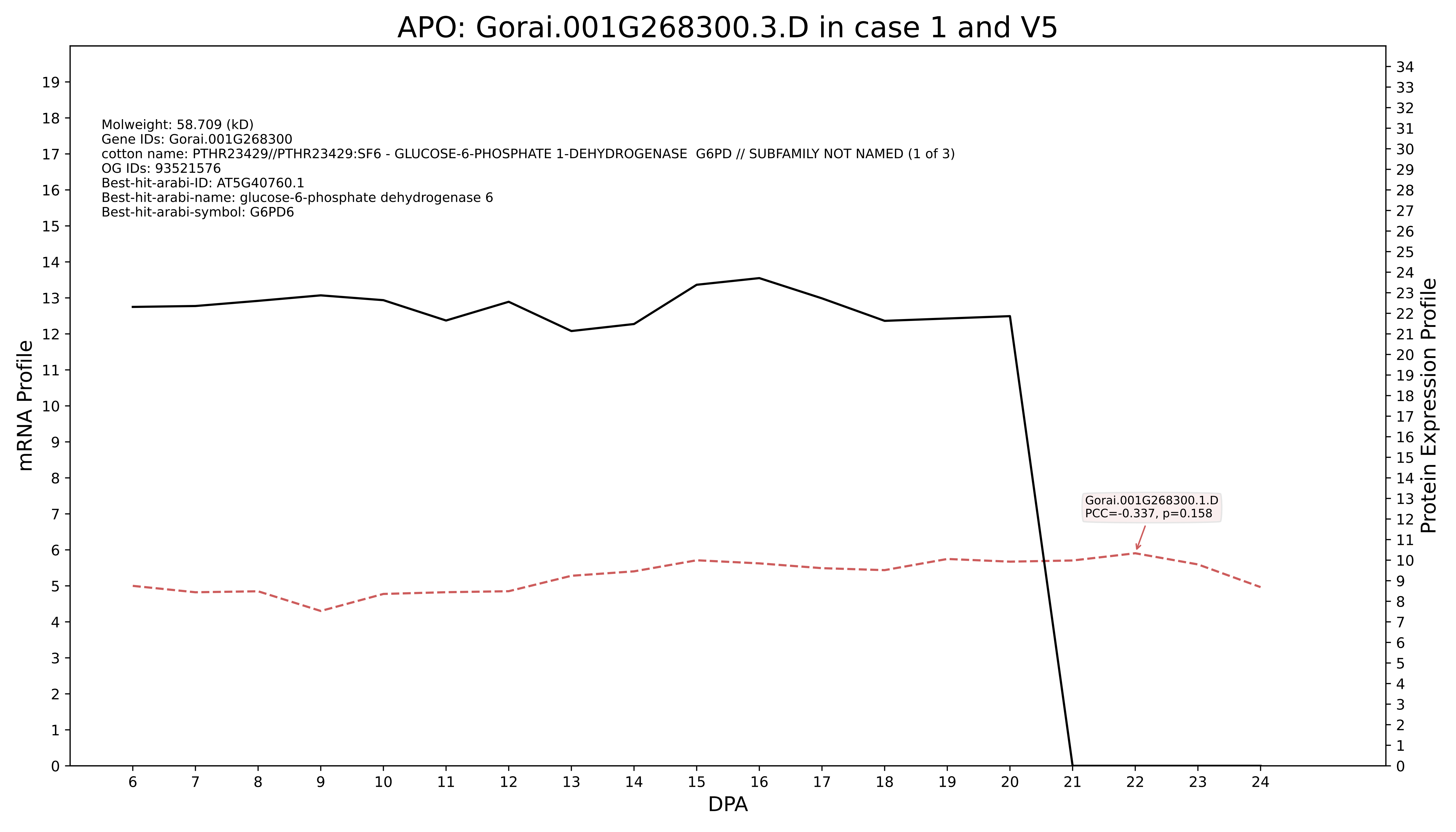

APO: Gorai.001G268300.3.D in case 1 and V5
Molweight: 58.709 (kD)
Gene IDs: Gorai.001G268300
cotton name: PTHR23429//PTHR23429:SF6 - GLUCOSE-6-PHOSPHATE 1-DEHYDROGENASE G6PD // SUBFAMILY NOT NAMED (1 of 3)
OG IDs: 93521576
Best-hit-arabi-ID: AT5G40760.1
Best-hit-arabi-name: glucose-6-phosphate dehydrogenase 6
Best-hit-arabi-symbol: G6PD6
Gorai.001G268300.1.D
PCC=-0.337, p=0.158
mRNA Profile
Protein Expression Profile
DPA

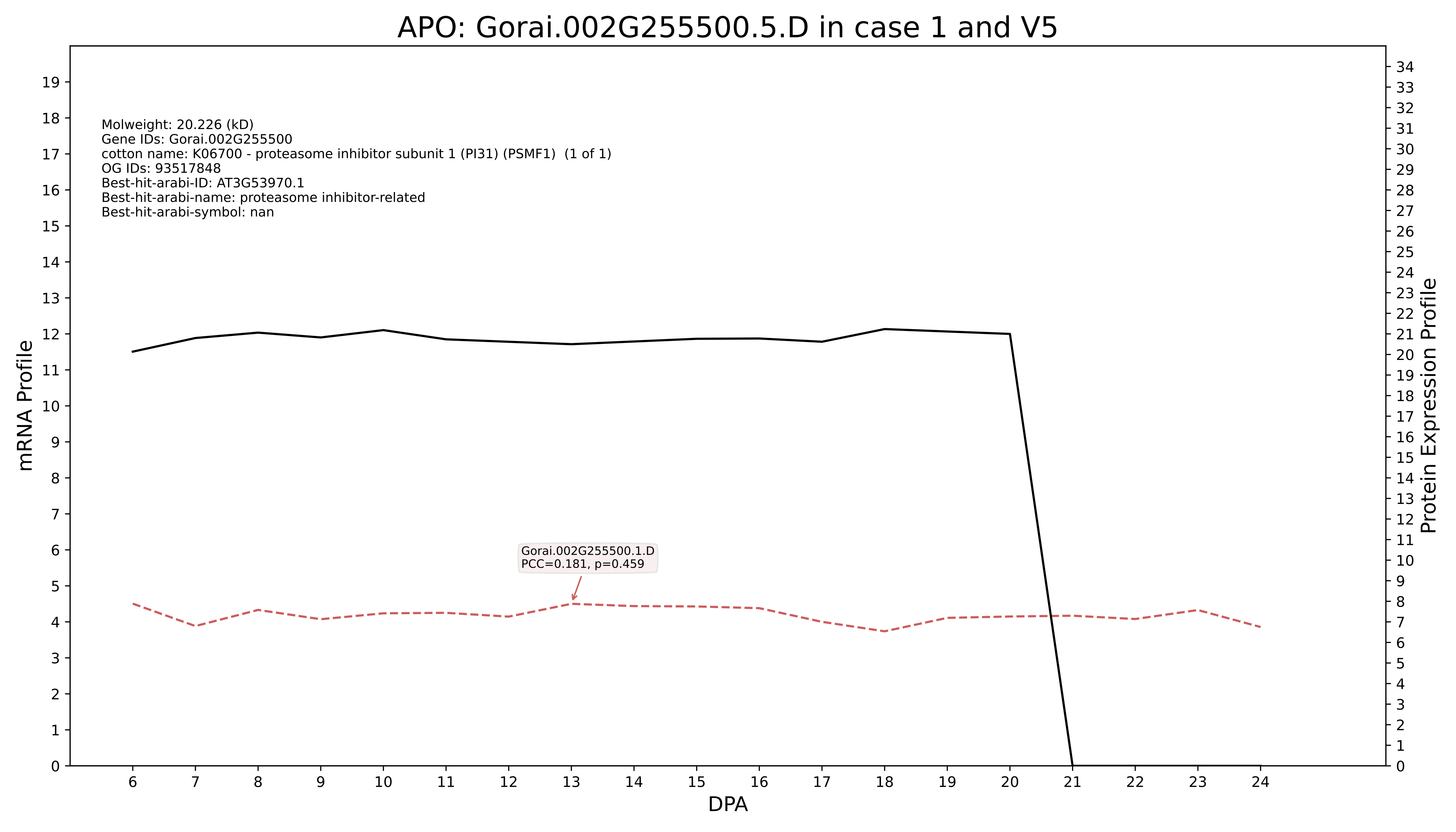
APO: Gorai.002G255500.5.D in case 1 and V5
Molweight: 20.226 (kD)
Gene IDs: Gorai.002G255500
cotton name: K06700 - proteasome inhibitor subunit 1 (PI31) (PSMF1) (1 of 1)
OG IDs: 93517848
Best-hit-arabi-ID: AT3G53970.1
Best-hit-arabi-name: proteasome inhibitor-related
Best-hit-arabi-symbol: nan
Gorai.002G255500.1.D
PCC=0.181, p=0.459
mRNA Profile
Protein Expression Profile
DPA

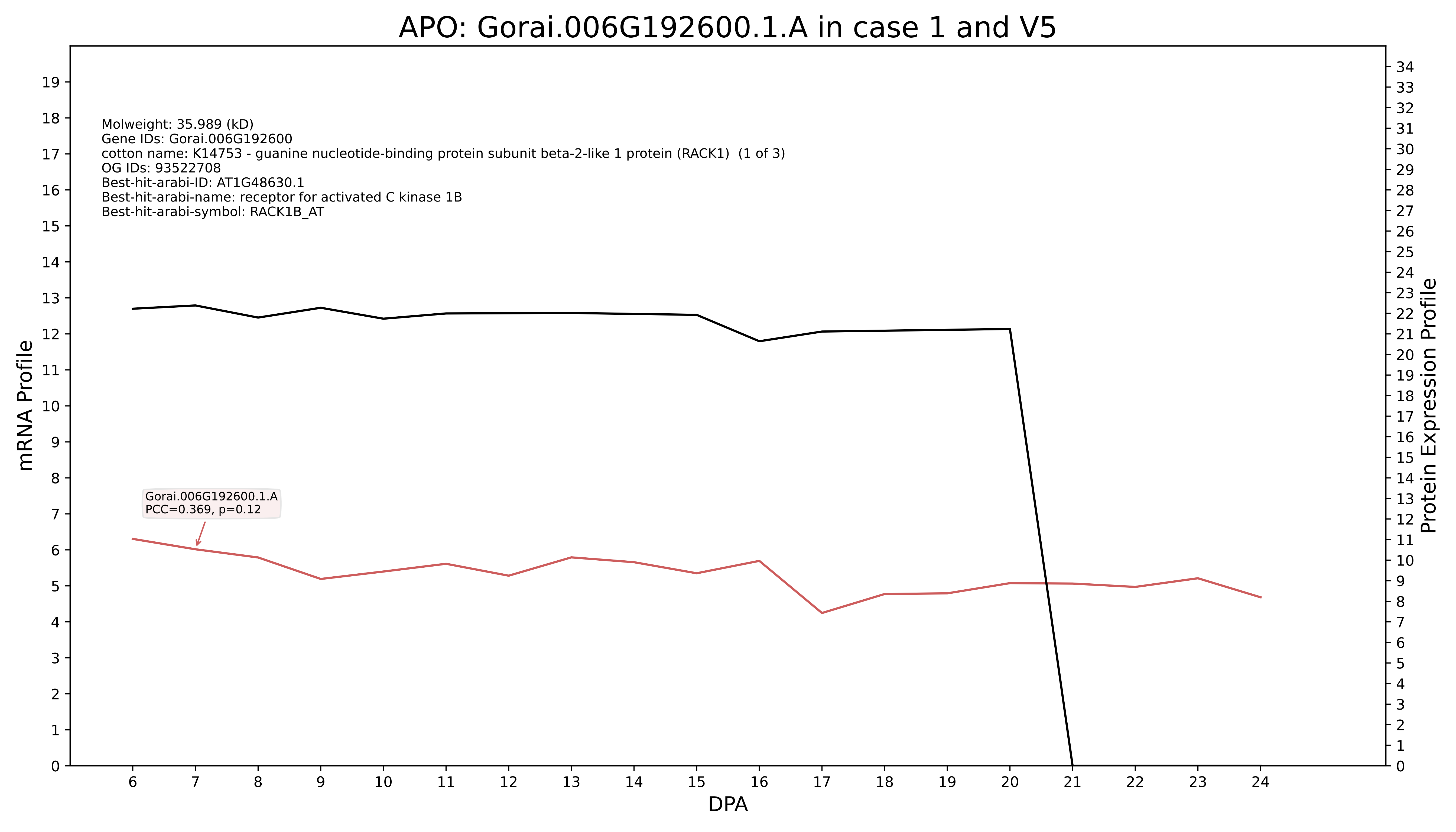
APO: Gorai.006G192600.1.A in case 1 and V5
Molweight: 35.989 (kD)
Gene IDs: Gorai.006G192600
cotton name: K14753 - guanine nucleotide-binding protein subunit beta-2-like 1 protein (RACK1) (1 of 3)
OG IDs: 93522708
Best-hit-arabi-ID: AT1G48630.1
Best-hit-arabi-name: receptor for activated C kinase 1B
Best-hit-arabi-symbol: RACK1B_AT
Gorai.006G192600.1.A
PCC=0.369, p=0.12
mRNA Profile
Protein Expression Profile
DPA
0
1
2
3
4
5
6
7
8
9
10
11
12
13
14
15
16
17
18
19
20
21
22
23
24
25
26
27
28
29
30
31
32
33
34

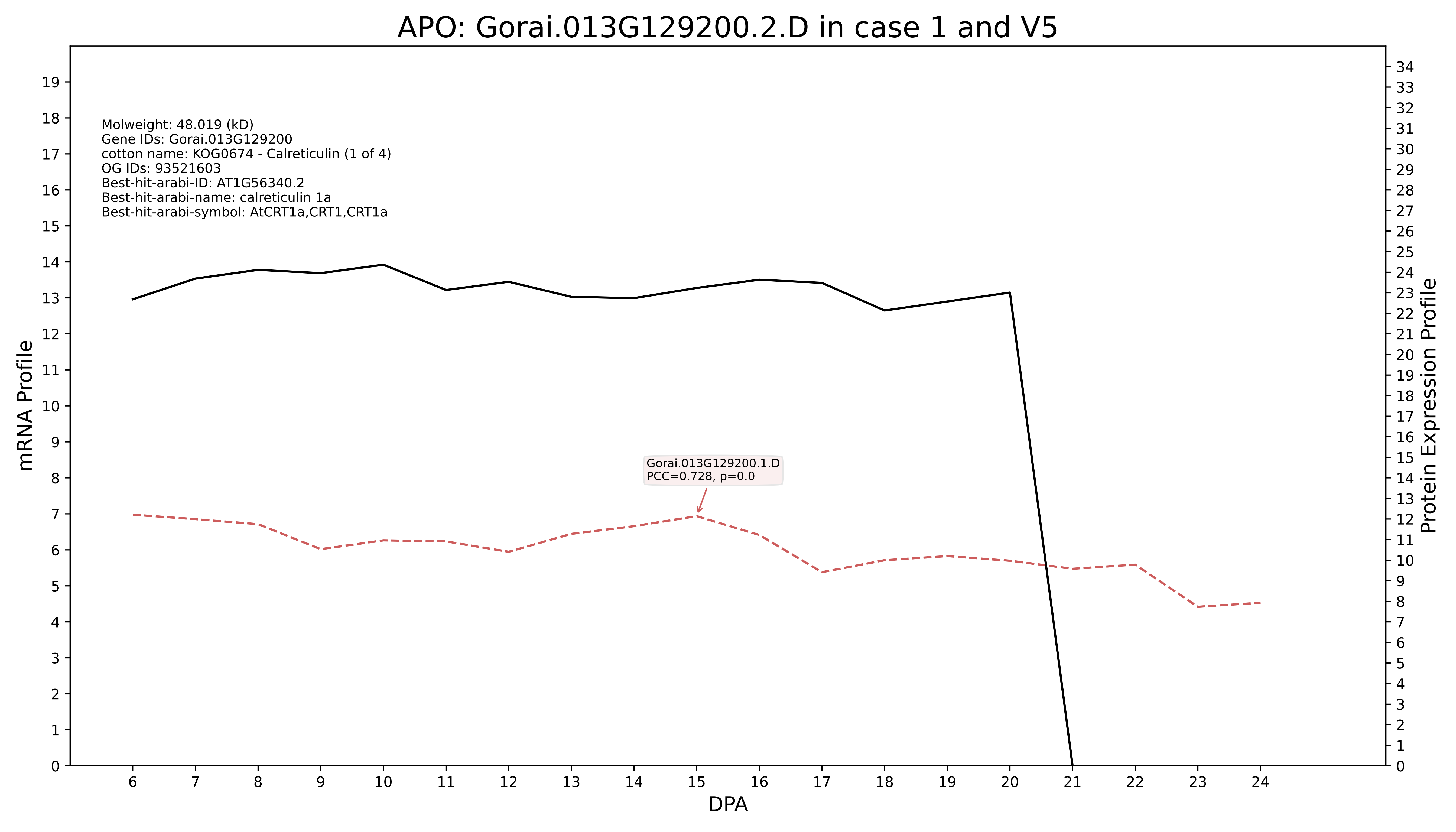
APO: Gorai.013G129200.2.D in case 1 and V5
Molweight: 48.019 (kD)
Gene IDs: Gorai.013G129200
cotton name: KOG0674 - Calreticulin (1 of 4)
OG IDs: 93521603
Best-hit-arabi-ID: AT1G56340.2
Best-hit-arabi-name: calreticulin 1a
Best-hit-arabi-symbol: AtCRT1a,CRT1,CRT1a
Gorai.013G129200.1.D
PCC=0.728, p=0.0
mRNA Profile
Protein Expression Profile
DPA
0
1
2
3
4
5
6
7
8
9
10
11
12
13
14
15
16
17
18
19
20
21
22
23
24
25
26
27
28
29
30
31
32
33
34

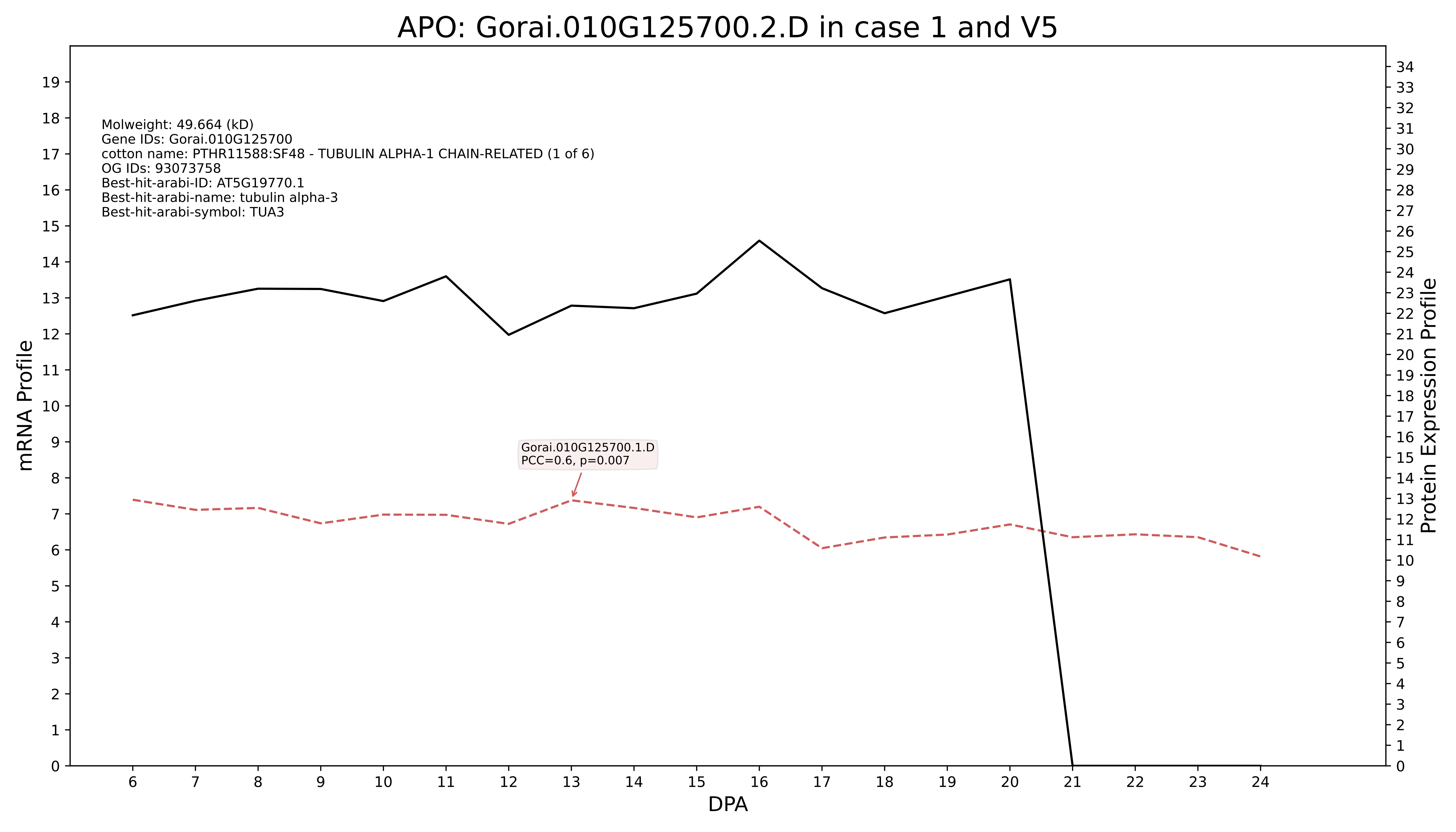

APO: Gorai.010G125700.2.D in case 1 and V5
Molweight: 49.664 (kD)
Gene IDs: Gorai.010G125700
cotton name: PTHR11588:SF48 - TUBULIN ALPHA-1 CHAIN-RELATED (1 of 6)
OG IDs: 93073758
Best-hit-arabi-ID: AT5G19770.1
Best-hit-arabi-name: tubulin alpha-3
Best-hit-arabi-symbol: TUA3
Gorai.010G125700.1.D
PCC=0.6, p=0.007
mRNA Profile
Protein Expression Profile
DPA

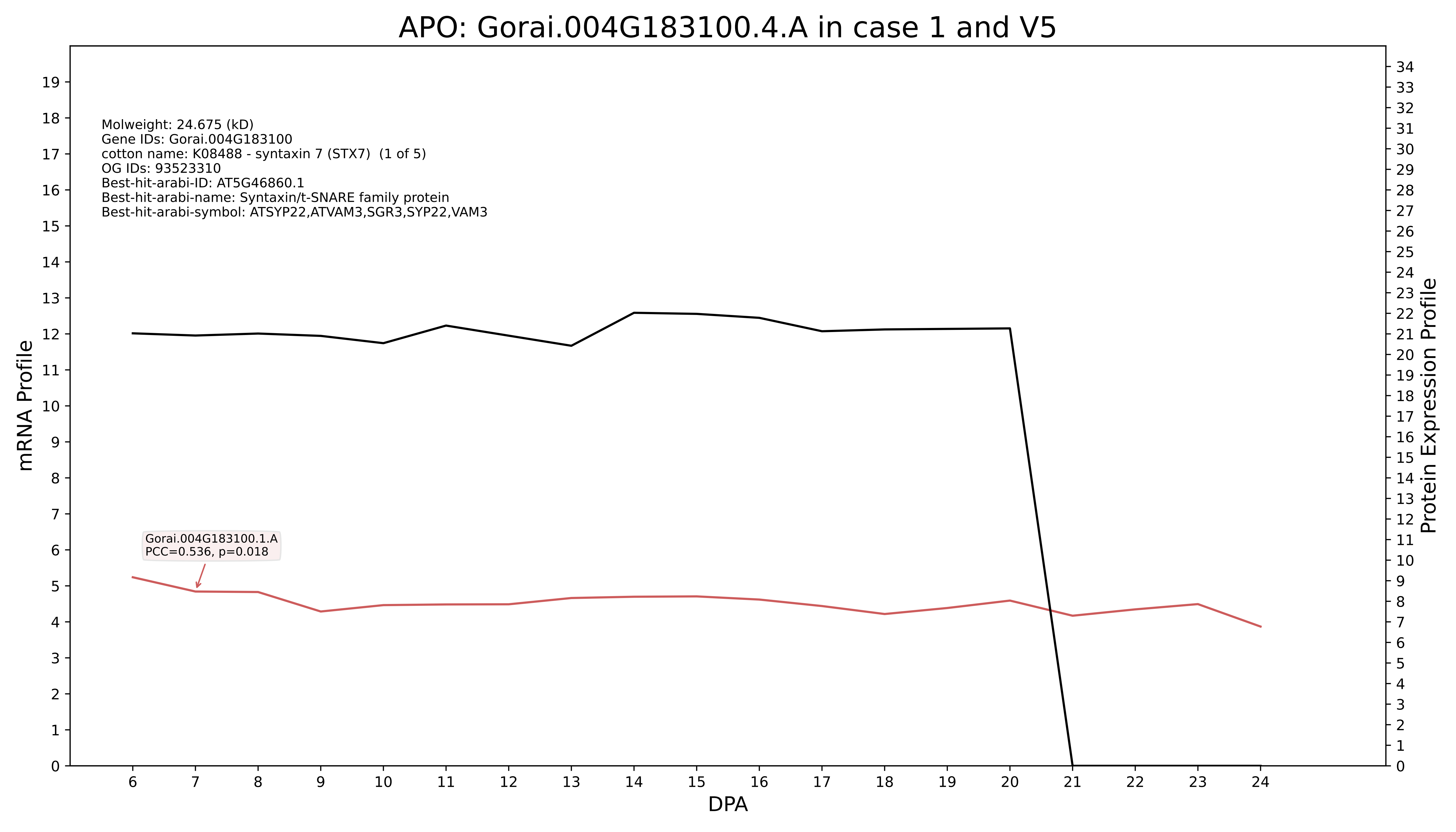
APO: Gorai.004G183100.4.A in case 1 and V5
Molweight: 24.675 (kD)
Gene IDs: Gorai.004G183100
cotton name: K08488 - syntaxin 7 (STX7) (1 of 5)
OG IDs: 93523310
Best-hit-arabi-ID: AT5G46860.1
Best-hit-arabi-name: Syntaxin/t-SNARE family protein
Best-hit-arabi-symbol: ATSYP22,ATVAM3,SGR3,SYP22,VAM3
Gorai.004G183100.1.A
PCC=0.536, p=0.018
mRNA Profile
Protein Expression Profile
DPA

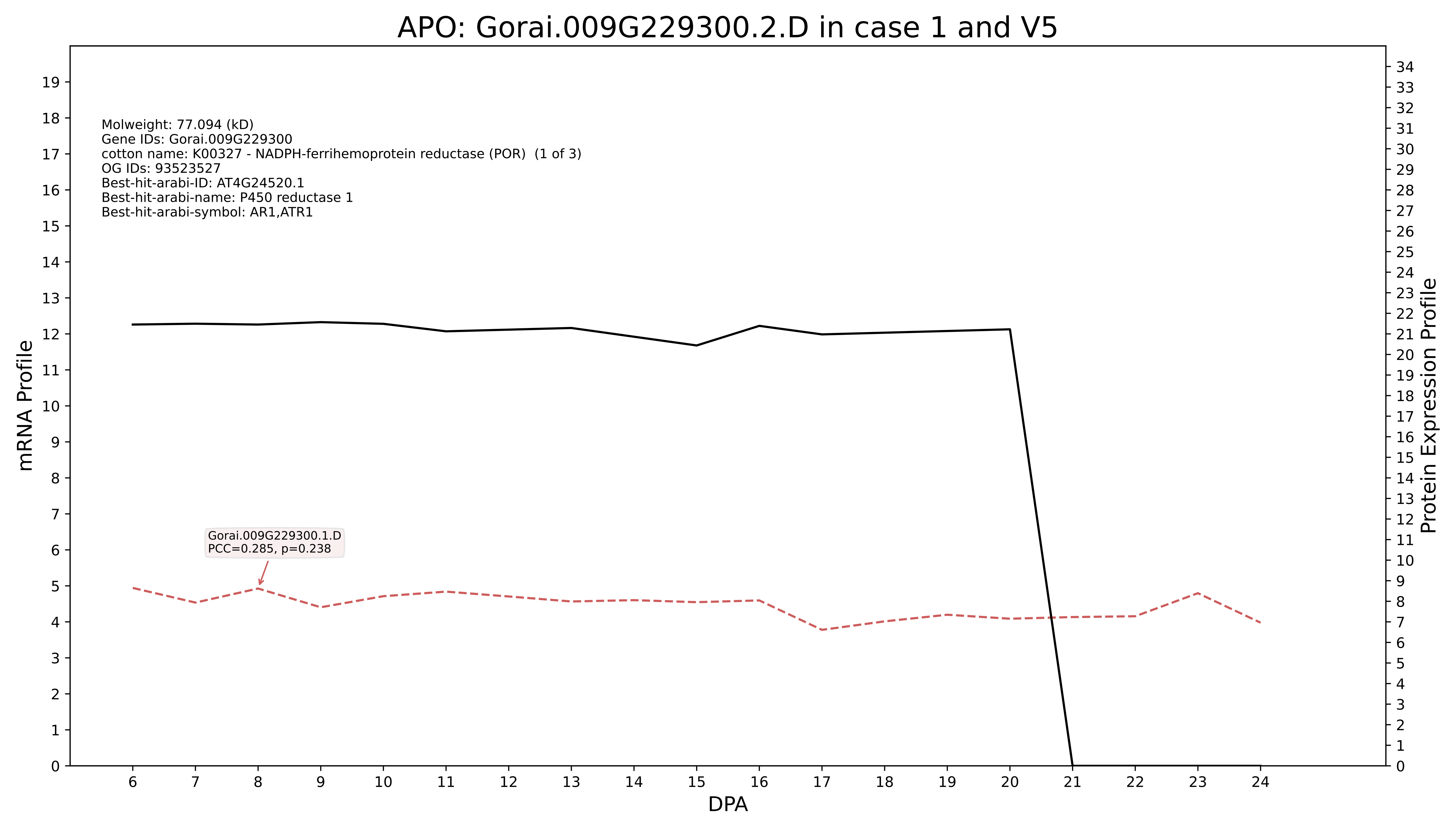
APO: Gorai.009G229300.2.D in case 1 and V5
Molweight: 77.094 (kD)
Gene IDs: Gorai.009G229300
cotton name: K00327 - NADPH-ferrihemoprotein reductase (POR) (1 of 3)
OG IDs: 93523527
Best-hit-arabi-ID: AT4G24520.1
Best-hit-arabi-name: P450 reductase 1
Best-hit-arabi-symbol: AR1,ATR1
Gorai.009G229300.1.D
PCC=0.285, p=0.238
mRNA Profile
Protein Expression Profile
DPA

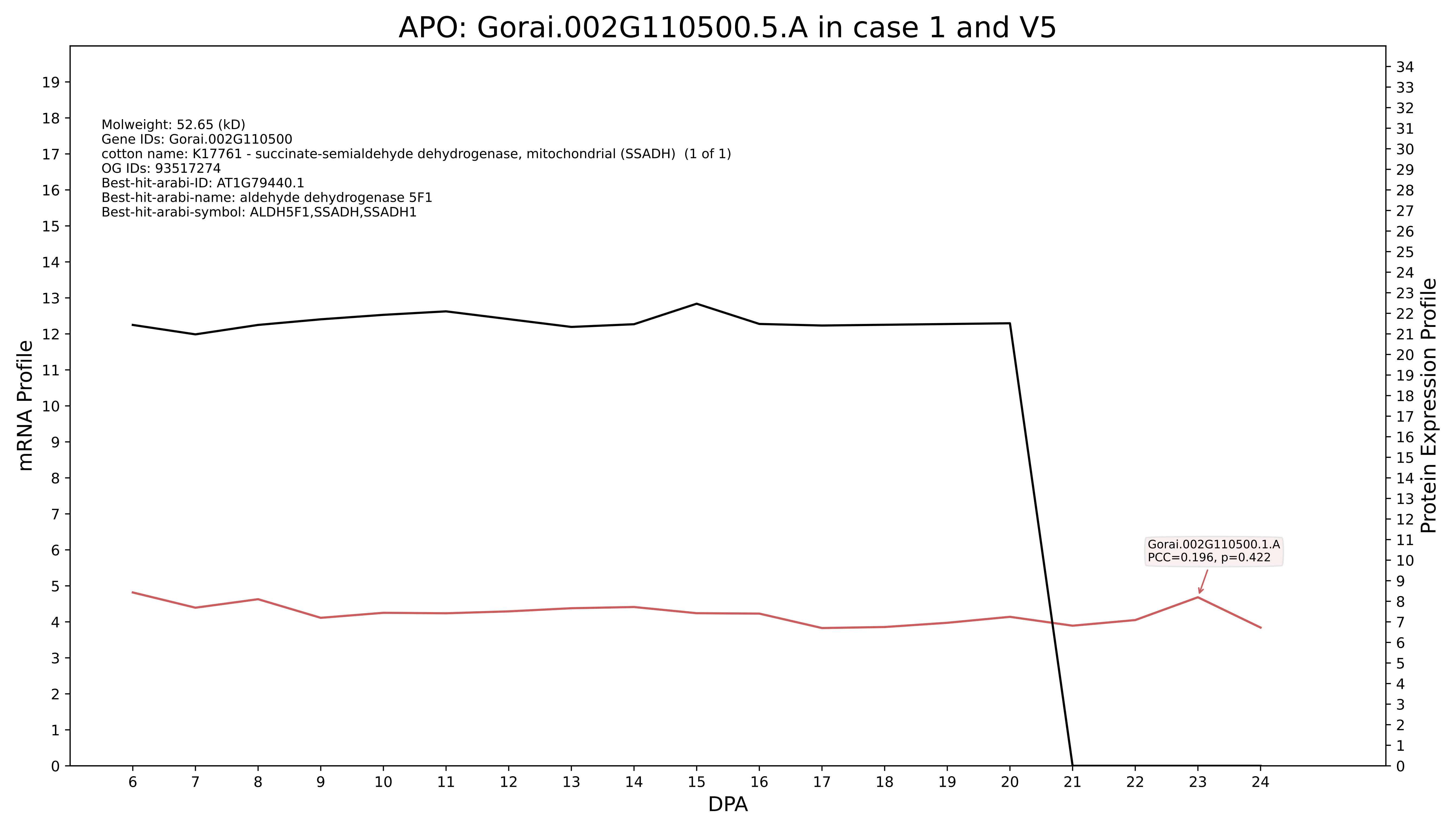

APO: Gorai.002G110500.5.A in case 1 and V5
Molweight: 52.65 (kD)
Gene IDs: Gorai.002G110500
cotton name: K17761 - succinate-semialdehyde dehydrogenase, mitochondrial (SSADH) (1 of 1)
OG IDs: 93517274
Best-hit-arabi-ID: AT1G79440.1
Best-hit-arabi-name: aldehyde dehydrogenase 5F1
Best-hit-arabi-symbol: ALDH5F1,SSADH,SSADH1
Gorai.002G110500.1.A
PCC=0.196, p=0.422
mRNA Profile
Protein Expression Profile
DPA

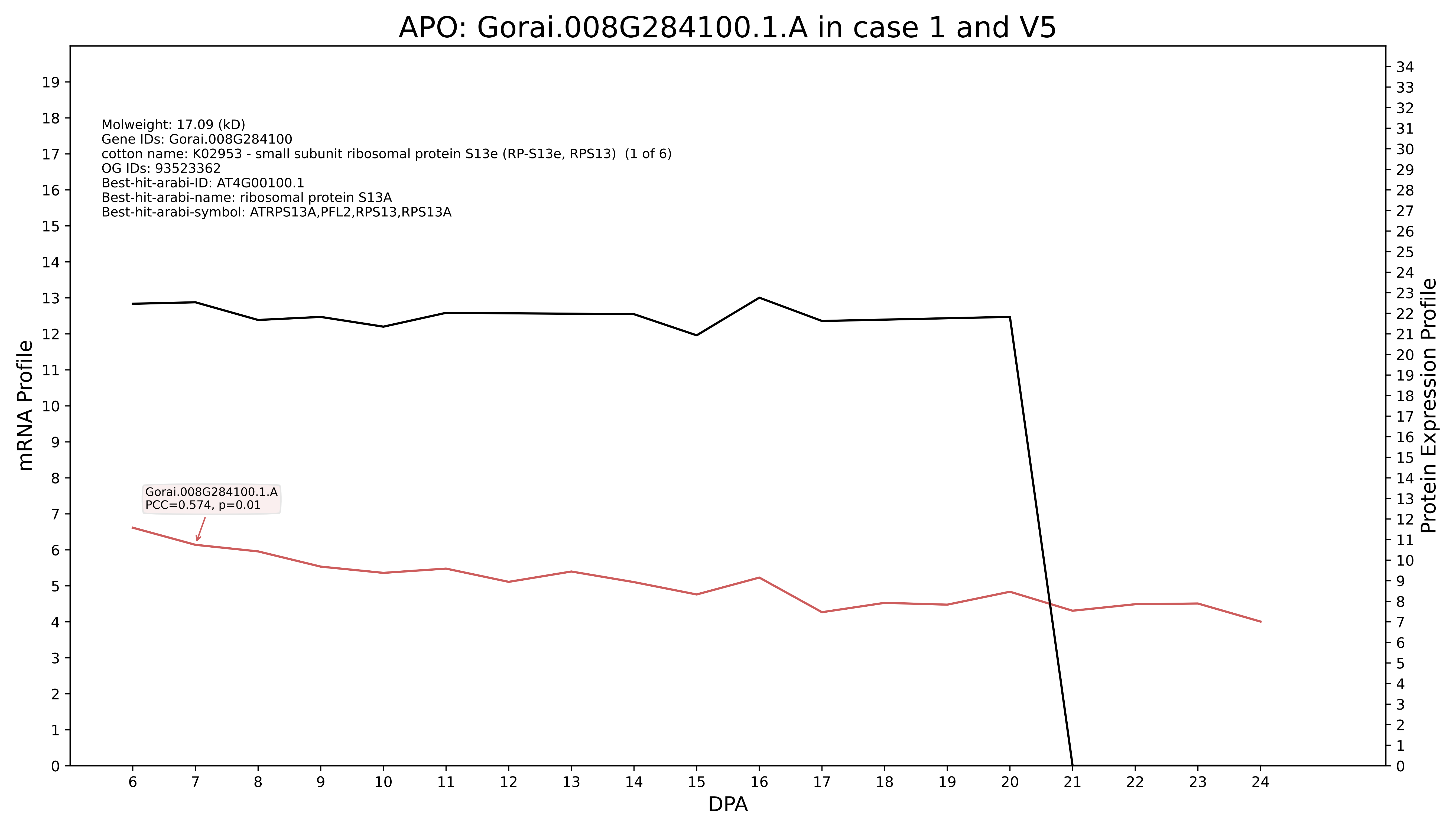
APO: Gorai.008G284100.1.A in case 1 and V5
Molweight: 17.09 (kD)
Gene IDs: Gorai.008G284100
cotton name: K02953 - small subunit ribosomal protein S13e (RP-S13e, RPS13) (1 of 6)
OG IDs: 93523362
Best-hit-arabi-ID: AT4G00100.1
Best-hit-arabi-name: ribosomal protein S13A
Best-hit-arabi-symbol: ATRPS13A,PFL2,RPS13,RPS13A
Gorai.008G284100.1.A
PCC=0.574, p=0.01
mRNA Profile
Protein Expression Profile
DPA

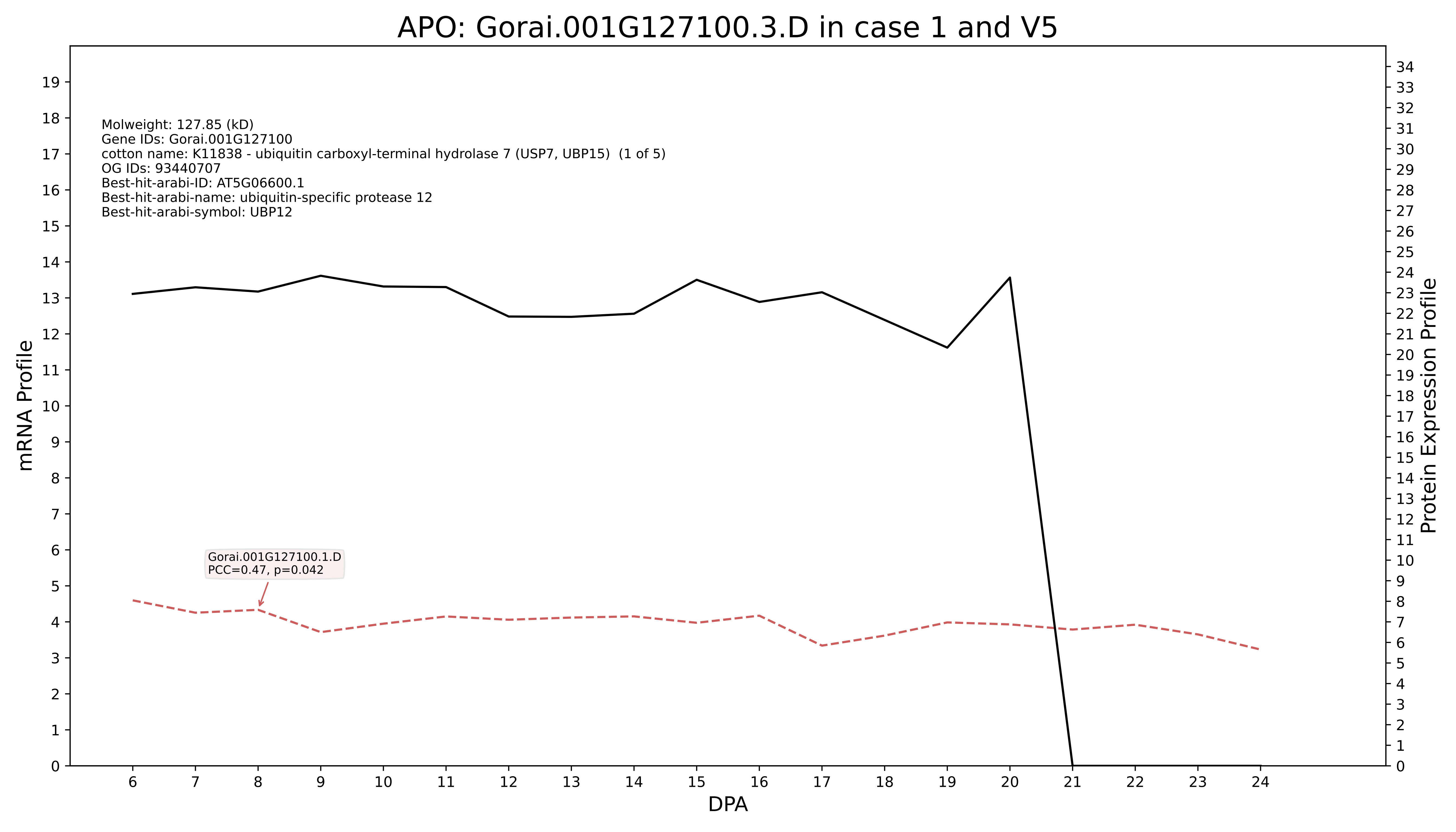
APO: Gorai.001G127100.3.D in case 1 and V5
Molweight: 127.85 (kD)
Gene IDs: Gorai.001G127100
cotton name: K11838 - ubiquitin carboxyl-terminal hydrolase 7 (USP7, UBP15) (1 of 5)
OG IDs: 93440707
Best-hit-arabi-ID: AT5G06600.1
Best-hit-arabi-name: ubiquitin-specific protease 12
Best-hit-arabi-symbol: UBP12
Gorai.001G127100.1.D
PCC=0.47, p=0.042
mRNA Profile
Protein Expression Profile
DPA

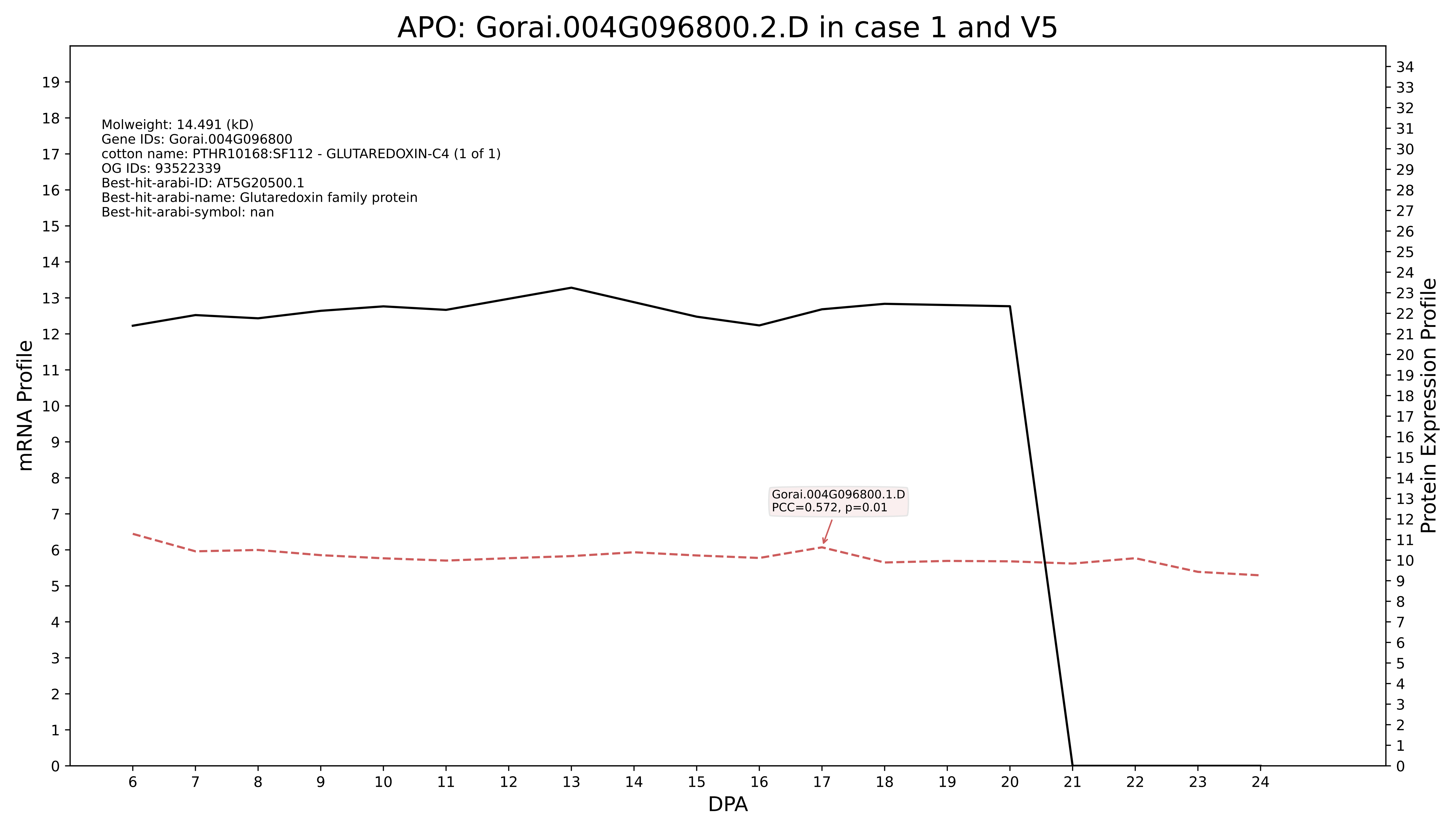
APO: Gorai.004G096800.2.D in case 1 and V5
Molweight: 14.491 (kD)
Gene IDs: Gorai.004G096800
cotton name: PTHR10168:SF112 - GLUTAREDOXIN-C4 (1 of 1)
OG IDs: 93522339
Best-hit-arabi-ID: AT5G20500.1
Best-hit-arabi-name: Glutaredoxin family protein
Best-hit-arabi-symbol: nan
Gorai.004G096800.1.D
PCC=0.572, p=0.01
mRNA Profile
Protein Expression Profile
DPA

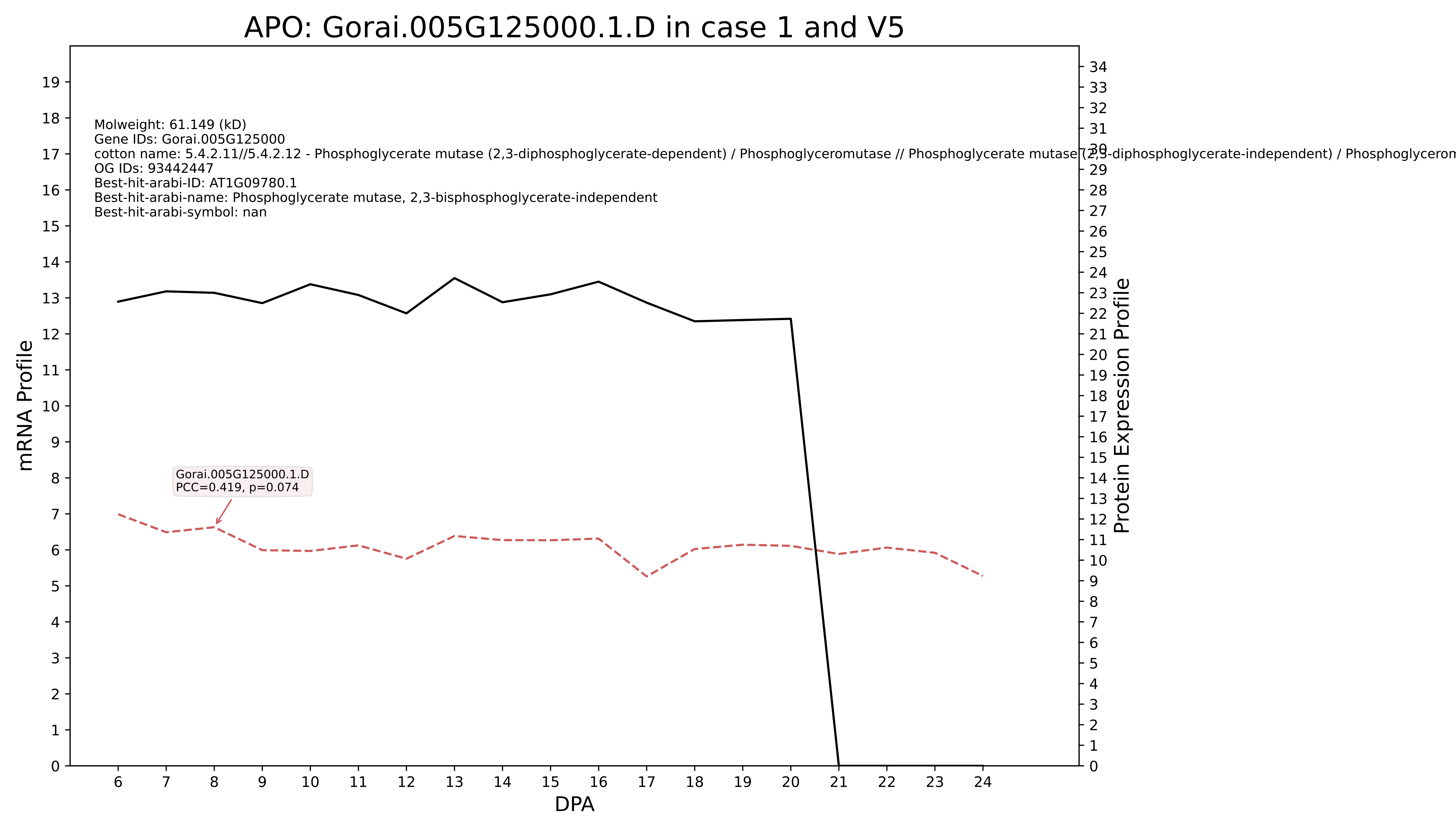
APO: Gorai.005G125000.1.D in case 1 and V5
Molweight: 61.149 (kD)
Gene IDs: Gorai.005G125000
cotton name: 5.4.2.11//5.4.2.12 - Phosphoglycerate mutase (2,3-diphosphoglycerate-dependent) / Phosphoglyceromutase // Phosphoglycerate mutase (2,3-diphosphoglycerate-independent) / Phosphoglyceron
OG IDs: 93442447
Best-hit-arabi-ID: AT1G09780.1
Best-hit-arabi-name: Phosphoglycerate mutase, 2,3-bisphosphoglycerate-independent
Best-hit-arabi-symbol: nan
Gorai.005G125000.1.D
PCC=0.419, p=0.074
mRNA Profile
Protein Expression Profile
DPA

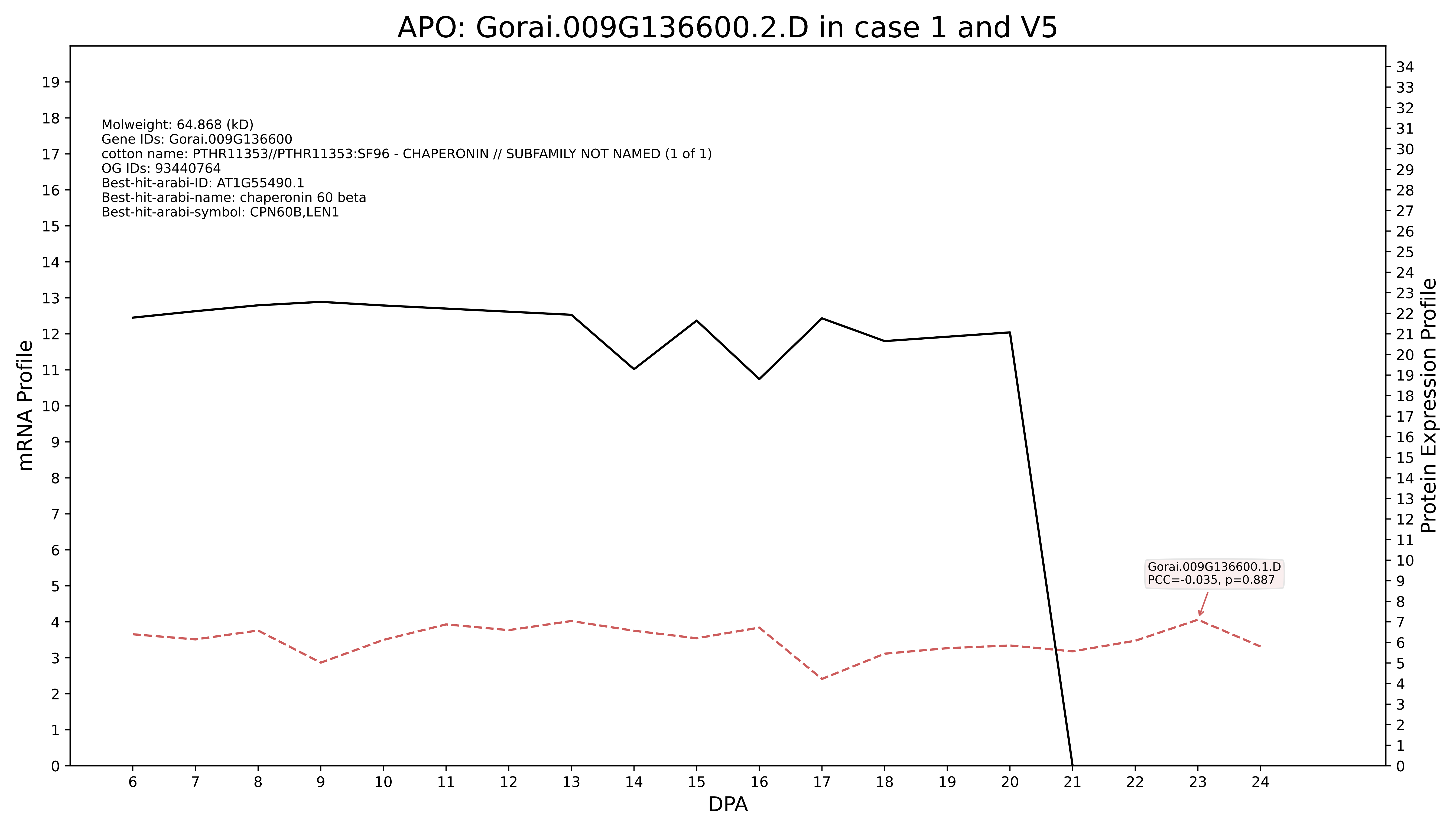

APO: Gorai.009G136600.2.D in case 1 and V5
Molweight: 64.868 (kD)
Gene IDs: Gorai.009G136600
cotton name: PTHR11353//PTHR11353:SF96 - CHAPERONIN // SUBFAMILY NOT NAMED (1 of 1)
OG IDs: 93440764
Best-hit-arabi-ID: AT1G55490.1
Best-hit-arabi-name: chaperonin 60 beta
Best-hit-arabi-symbol: CPN60B,LEN1
Gorai.009G136600.1.D
PCC=-0.035, p=0.887
mRNA Profile
Protein Expression Profile
DPA

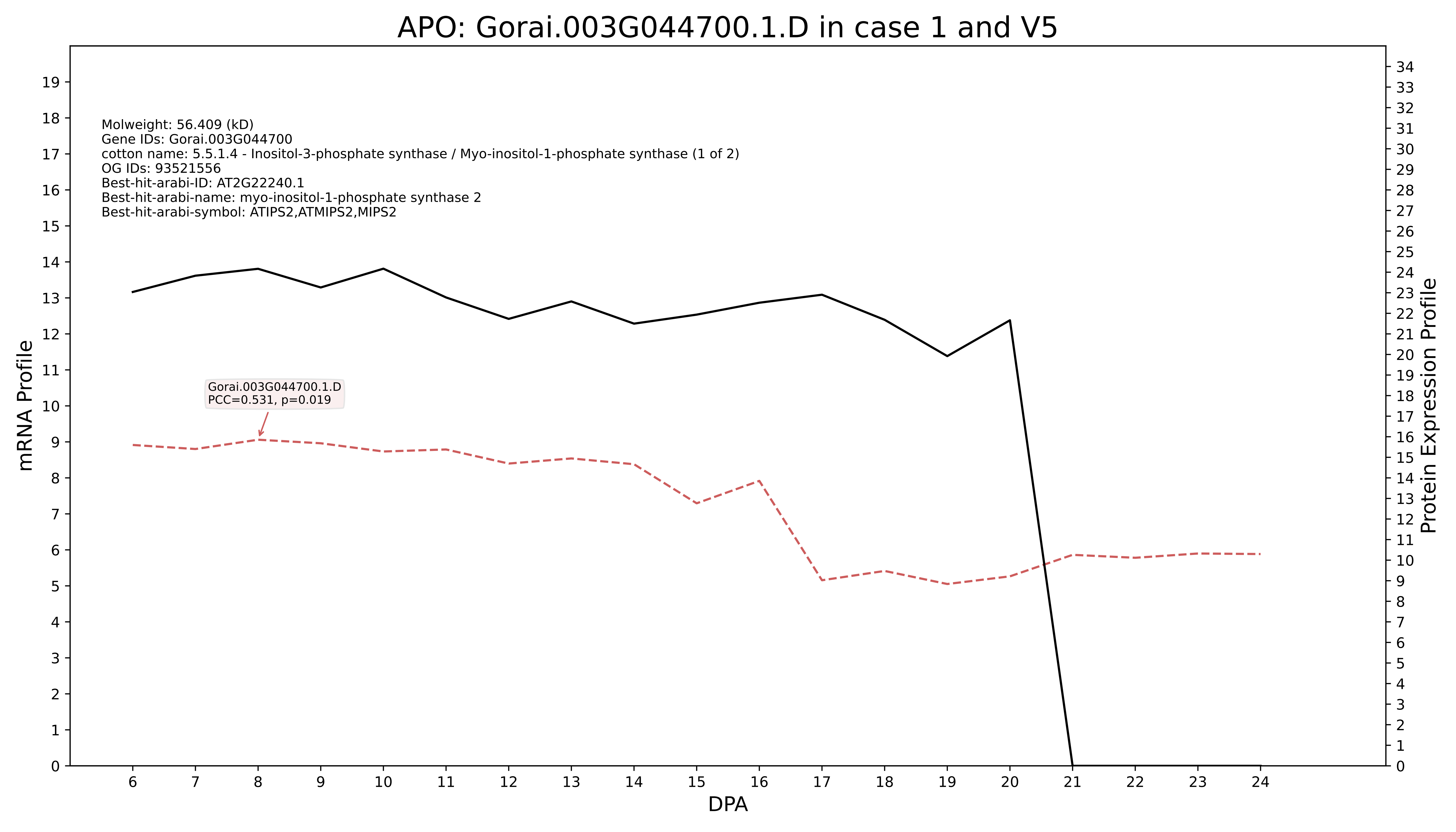
APO: Gorai.003G044700.1.D in case 1 and V5
Molweight: 56.409 (kD)
Gene IDs: Gorai.003G044700
cotton name: 5.5.1.4 - Inositol-3-phosphate synthase / Myo-inositol-1-phosphate synthase (1 of 2)
OG IDs: 93521556
Best-hit-arabi-ID: AT2G22240.1
Best-hit-arabi-name: myo-inositol-1-phosphate synthase 2
Best-hit-arabi-symbol: ATIPS2,ATMIPS2,MIPS2
Gorai.003G044700.1.D
PCC=0.531, p=0.019
mRNA Profile
Protein Expression Profile
DPA

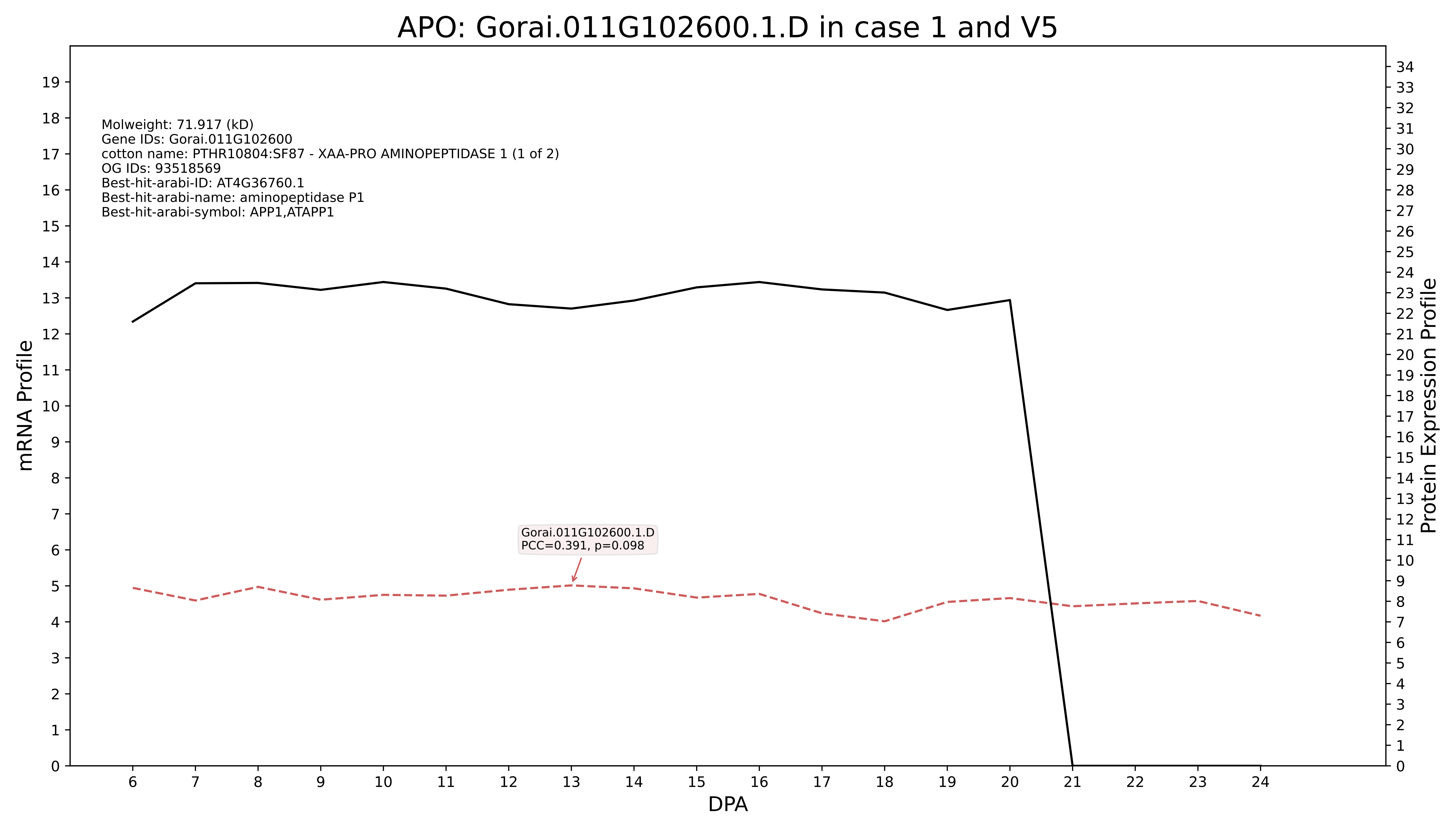
APO: Gorai.011G102600.1.D in case 1 and V5
Molweight: 71.917 (kD)
Gene IDs: Gorai.011G102600
cotton name: PTHR10804:SF87 - XAA-PRO AMINOPEPTIDASE 1 (1 of 2)
OG IDs: 93518569
Best-hit-arabi-ID: AT4G36760.1
Best-hit-arabi-name: aminopeptidase P1
Best-hit-arabi-symbol: APP1,ATAPP1
Gorai.011G102600.1.D
PCC=0.391, p=0.098
mRNA Profile
Protein Expression Profile
DPA

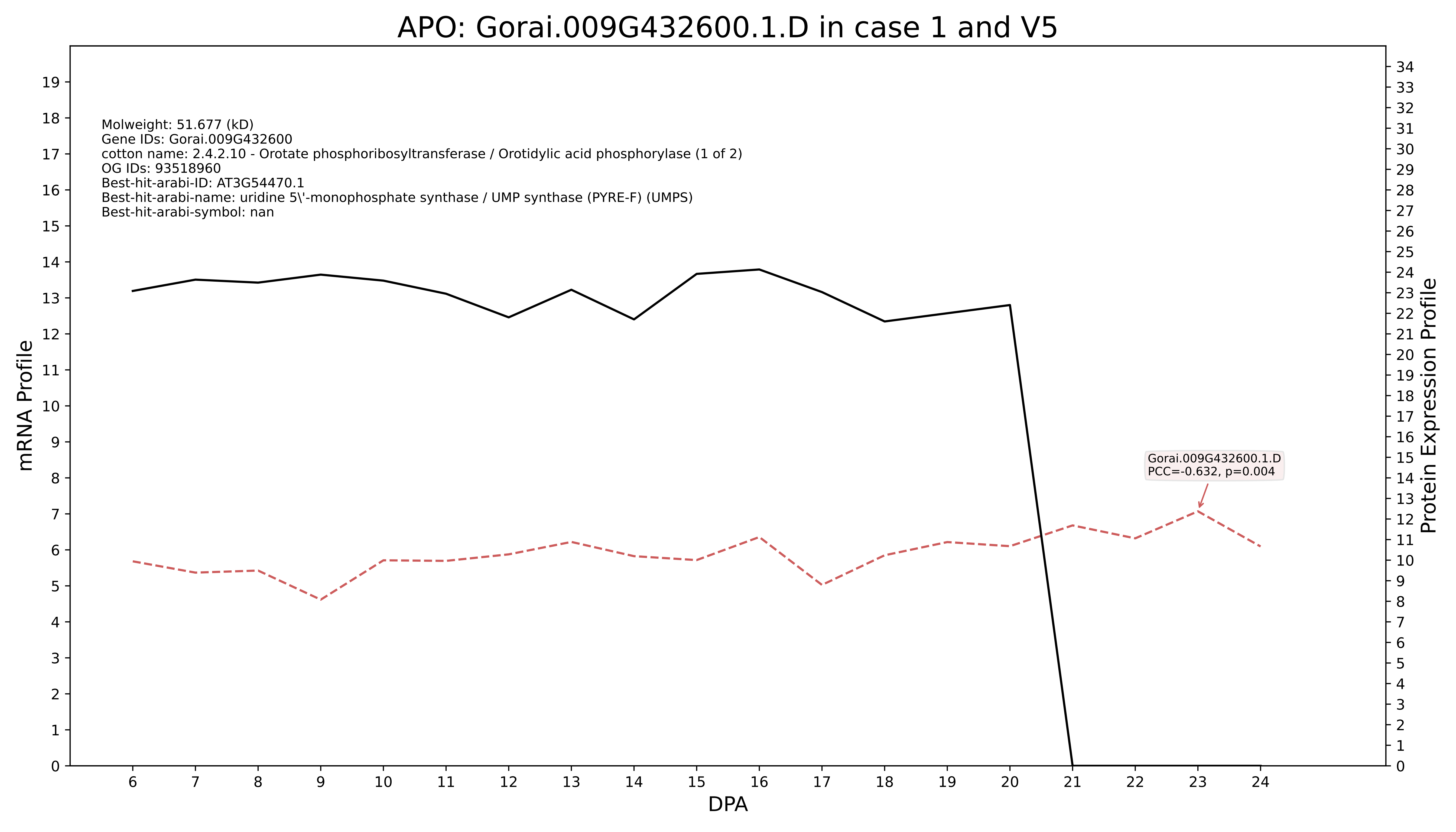
APO: Gorai.009G432600.1.D in case 1 and V5
Molweight: 51.677 (kD)
Gene IDs: Gorai.009G432600
cotton name: 2.4.2.10 - Orotate phosphoribosyltransferase / Orotidylic acid phosphorylase (1 of 2)
OG IDs: 93518960
Best-hit-arabi-ID: AT3G54470.1
Best-hit-arabi-name: uridine 5\'-monophosphate synthase / UMP synthase (PYRE-F) (UMPS)
Best-hit-arabi-symbol: nan
Gorai.009G432600.1.D
PCC=-0.632, p=0.004
mRNA Profile
Protein Expression Profile
DPA
0
1
2
3
4
5
6
7
8
9
10
11
12
13
14
15
16
17
18
19
20
21
22
23
24
25
26
27
28
29
30
31
32
33
34

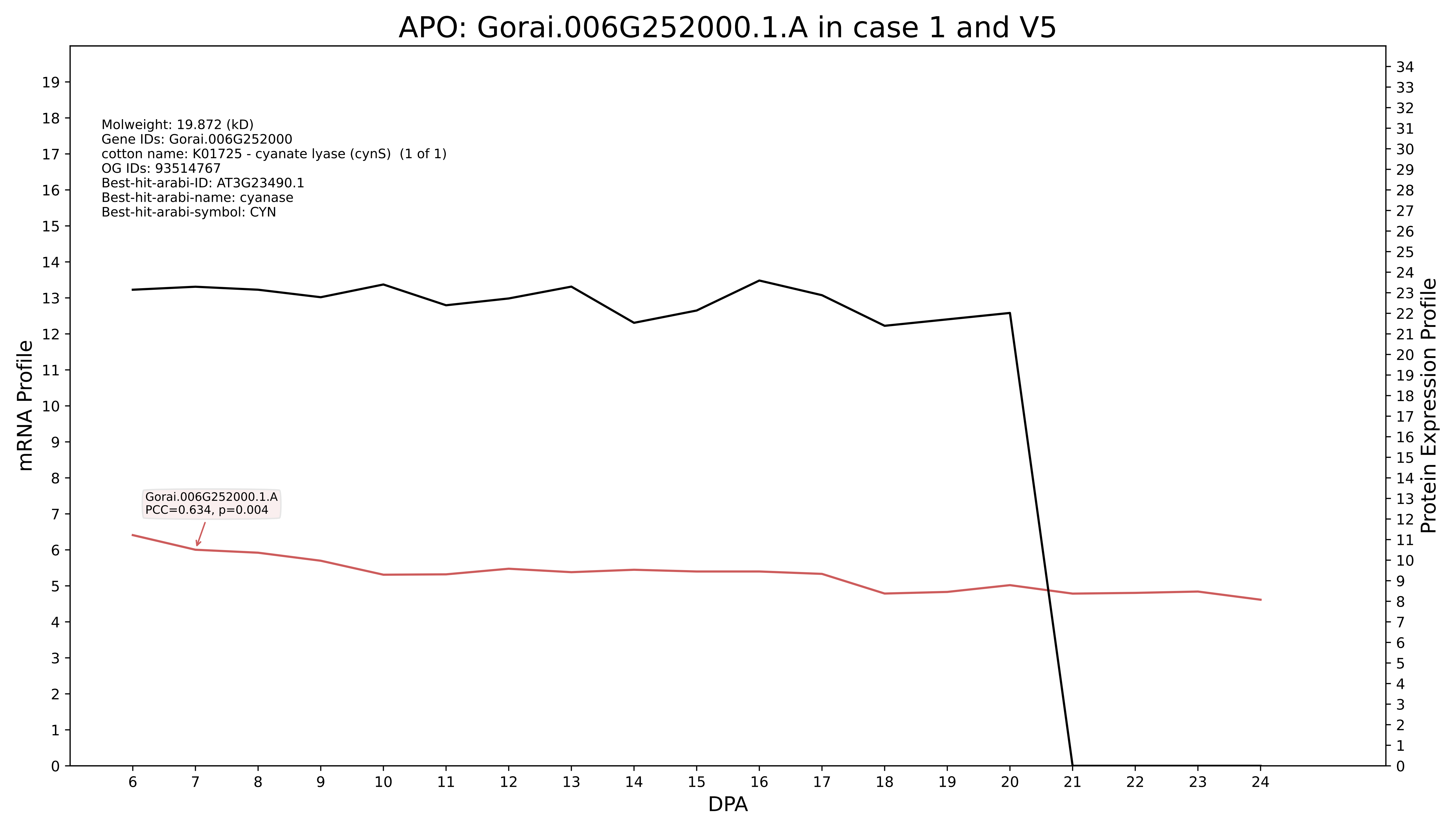
APO: Gorai.006G252000.1.A in case 1 and V5
Molweight: 19.872 (kD)
Gene IDs: Gorai.006G252000
cotton name: K01725 - cyanate lyase (cynS) (1 of 1)
OG IDs: 93514767
Best-hit-arabi-ID: AT3G23490.1
Best-hit-arabi-name: cyanase
Best-hit-arabi-symbol: CYN
Gorai.006G252000.1.A
PCC=0.634, p=0.004
mRNA Profile
Protein Expression Profile
DPA

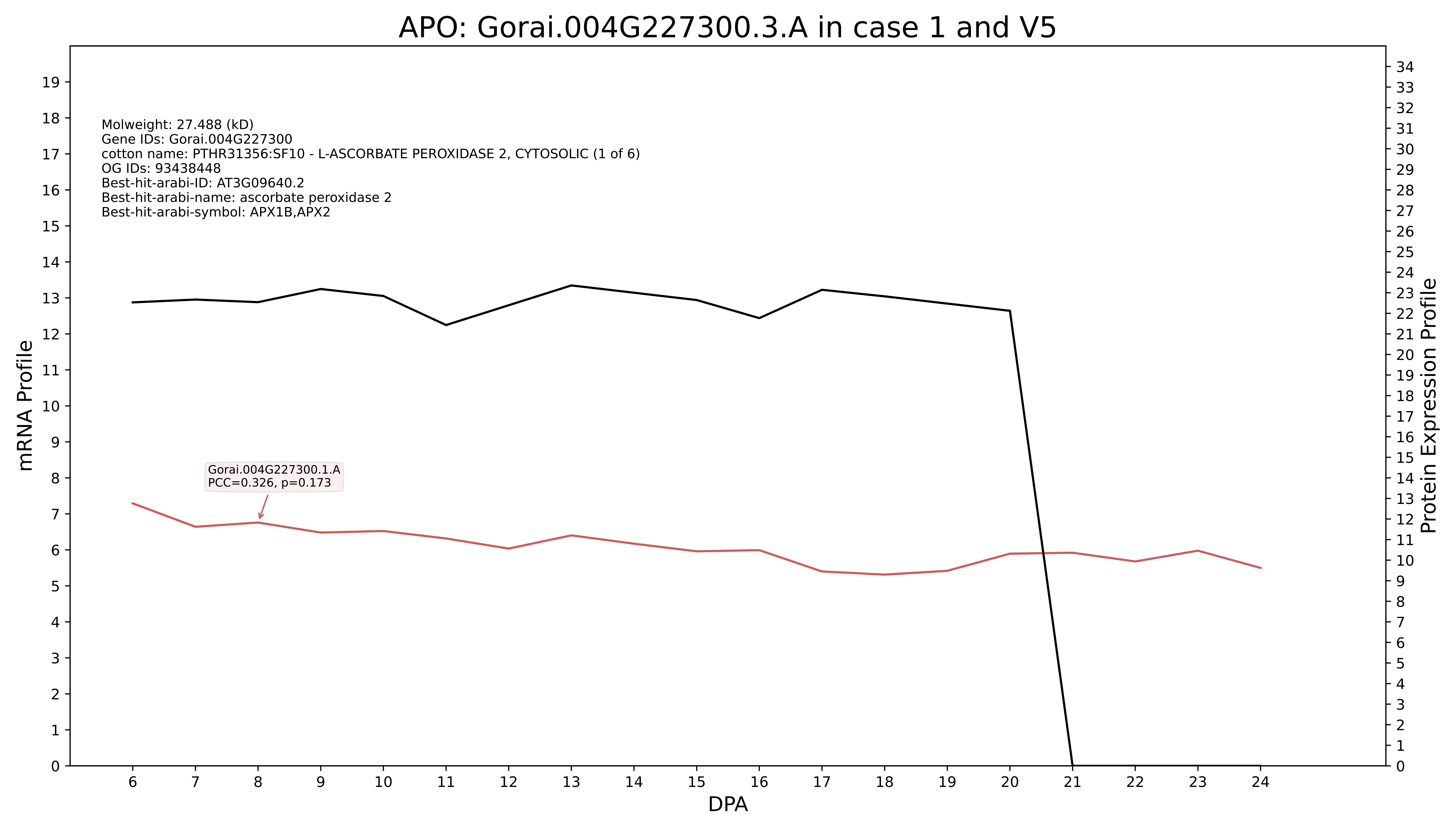

APO: Gorai.004G227300.3.A in case 1 and V5
Molweight: 27.488 (kD)
Gene IDs: Gorai.004G227300
cotton name: PTHR31356:SF10 - L-ASCORBATE PEROXIDASE 2, CYTOSOLIC (1 of 6)
OG IDs: 93438448
Best-hit-arabi-ID: AT3G09640.2
Best-hit-arabi-name: ascorbate peroxidase 2
Best-hit-arabi-symbol: APX1B,APX2
Gorai.004G227300.1.A
PCC=0.326, p=0.173
mRNA Profile
Protein Expression Profile
DPA

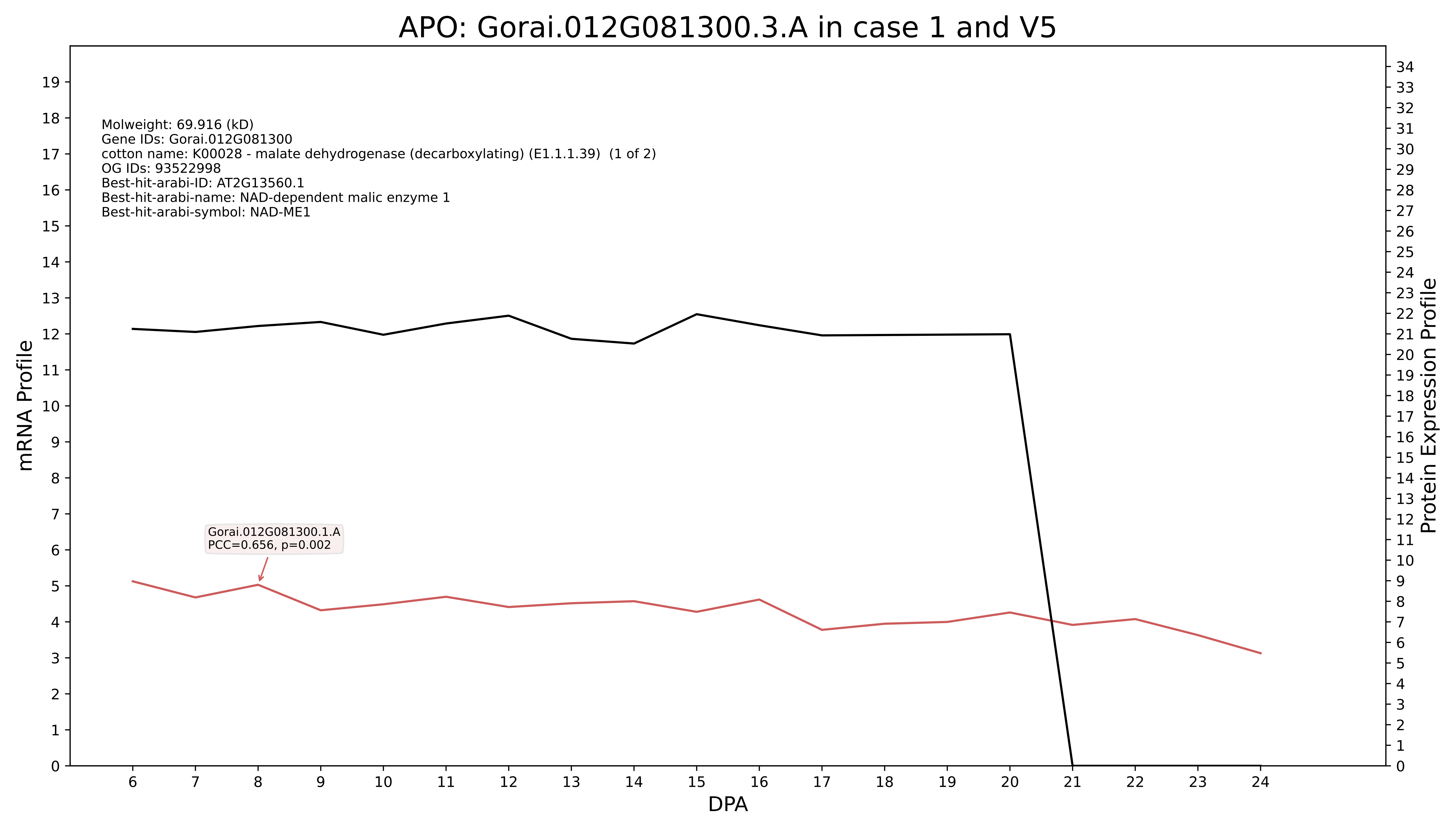
APO: Gorai.012G081300.3.A in case 1 and V5
Molweight: 69.916 (kD)
Gene IDs: Gorai.012G081300
cotton name: K00028 - malate dehydrogenase (decarboxylating) (E1.1.1.39) (1 of 2)
OG IDs: 93522998
Best-hit-arabi-ID: AT2G13560.1
Best-hit-arabi-name: NAD-dependent malic enzyme 1
Best-hit-arabi-symbol: NAD-ME1
Gorai.012G081300.1.A
PCC=0.656, p=0.002
mRNA Profile
Protein Expression Profile
DPA
0
1
2
3
4
5
6
7
8
9
10
11
12
13
14
15
16
17
18
19
20
21
22
23
24
25
26
27
28
29
30
31
32
33
34

APO: Gorai.011G269100.1.A in case 1 and V5

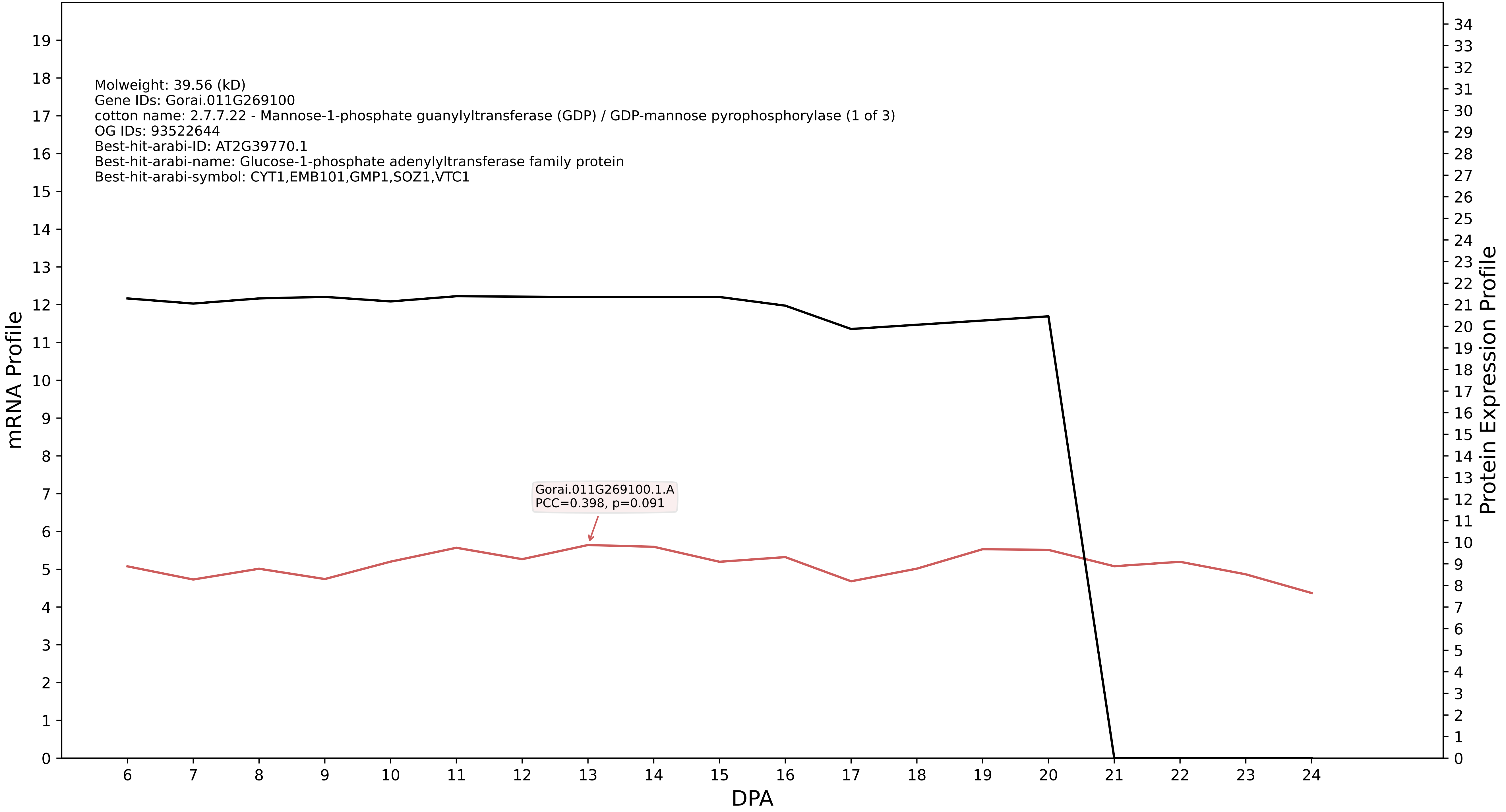

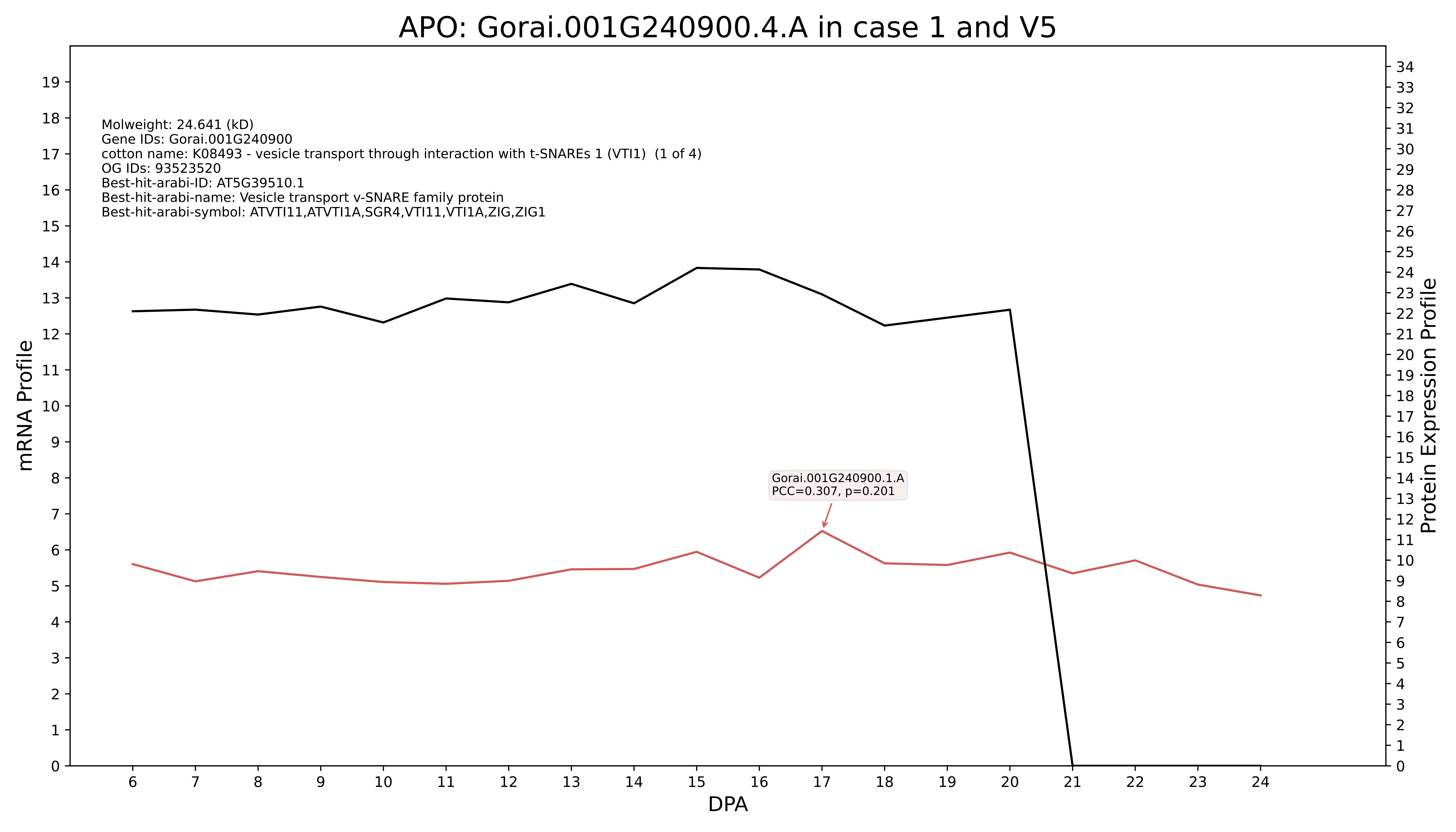

APO: Gorai.001G240900.4.A in case 1 and V5
Molweight: 24.641 (kD)
Gene IDs: Gorai.001G240900
cotton name: K08493 - vesicle transport through interaction with t-SNAREs 1 (VTI1) (1 of 4)
OG IDs: 93523520
Best-hit-arabi-ID: AT5G39510.1
Best-hit-arabi-name: Vesicle transport v-SNARE family protein
Best-hit-arabi-symbol: ATVTI11,ATVTI1A,SGR4,VTI11,VTI1A,ZIG,ZIG1
Gorai.001G240900.1.A
PCC=0.307, p=0.201
mRNA Profile
Protein Expression Profile
DPA

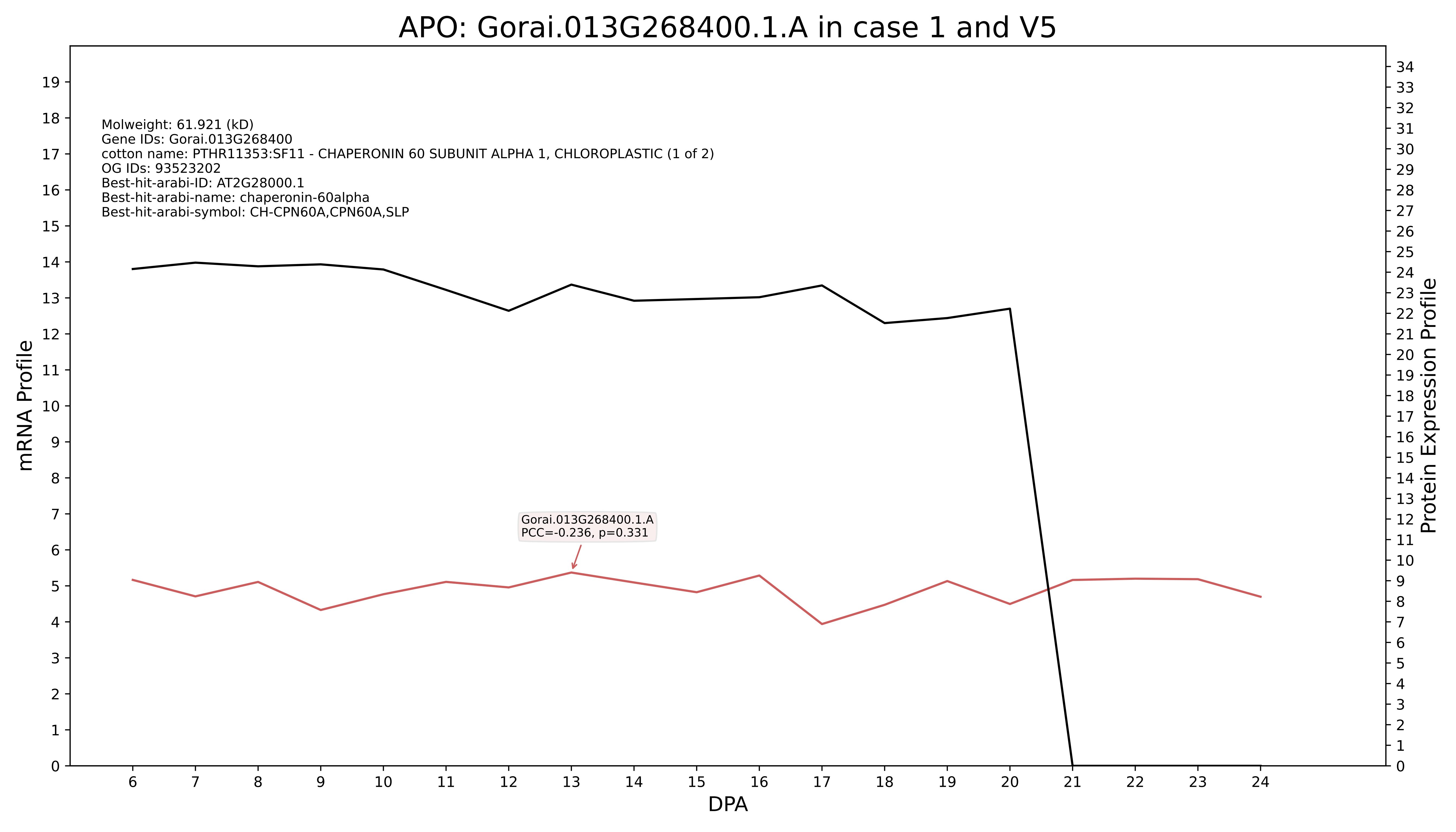

APO: Gorai.013G268400.1.A in case 1 and V5
Molweight: 61.921 (kD)
Gene IDs: Gorai.013G268400
cotton name: PTHR11353:SF11 - CHAPERONIN 60 SUBUNIT ALPHA 1, CHLOROPLASTIC (1 of 2)
OG IDs: 93523202
Best-hit-arabi-ID: AT2G28000.1
Best-hit-arabi-name: chaperonin-60alpha
Best-hit-arabi-symbol: CH-CPN60A,CPN60A,SLP
Gorai.013G268400.1.A
PCC=-0.236, p=0.331
mRNA Profile
Protein Expression Profile
DPA

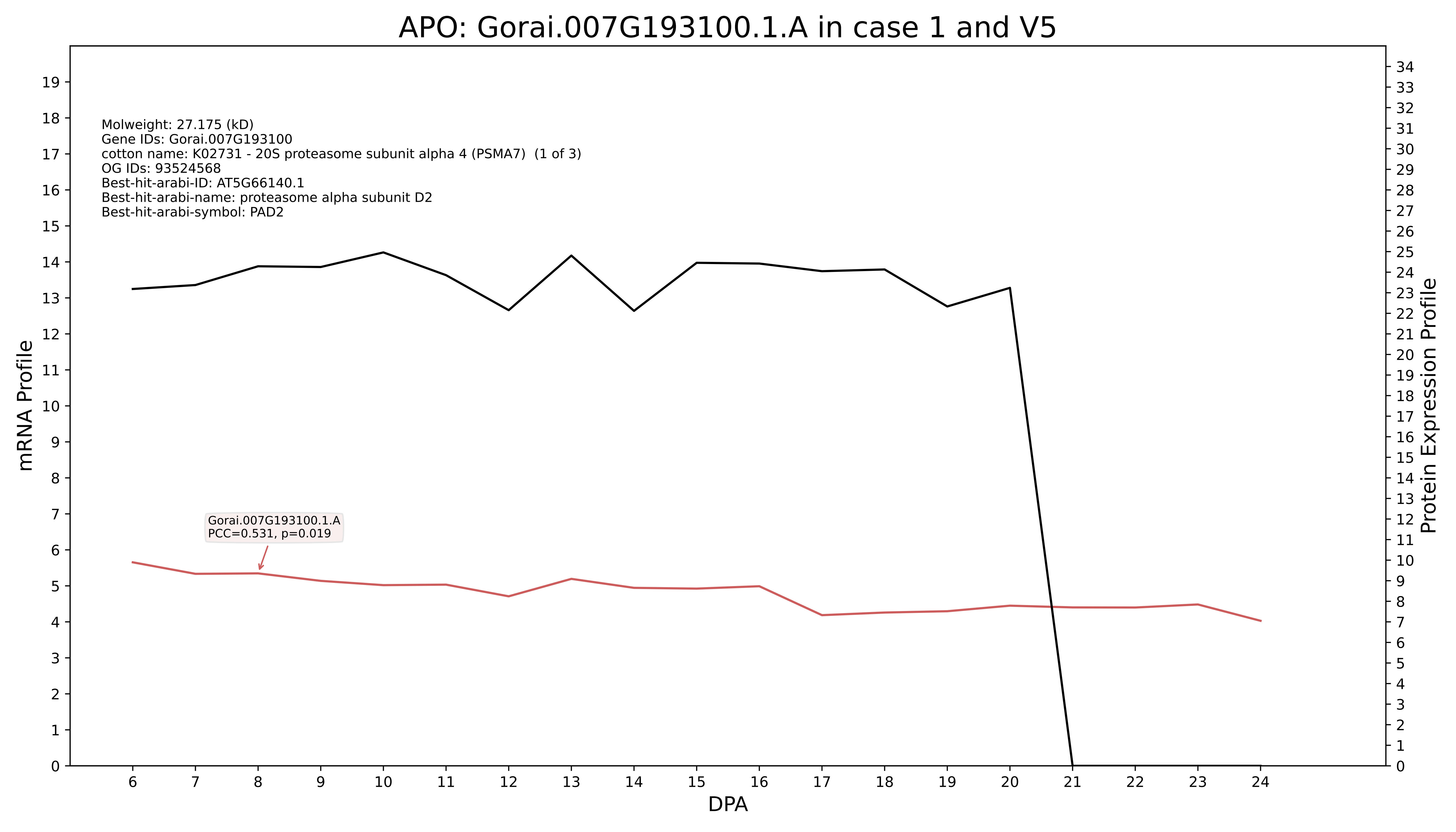

APO: Gorai.007G193100.1.A in case 1 and V5
Molweight: 27.175 (kD)
Gene IDs: Gorai.007G193100
cotton name: K02731 - 20S proteasome subunit alpha 4 (PSMA7) (1 of 3)
OG IDs: 93524568
Best-hit-arabi-ID: AT5G66140.1
Best-hit-arabi-name: proteasome alpha subunit D2
Best-hit-arabi-symbol: PAD2
Gorai.007G193100.1.A
PCC=0.531, p=0.019
mRNA Profile
Protein Expression Profile
DPA

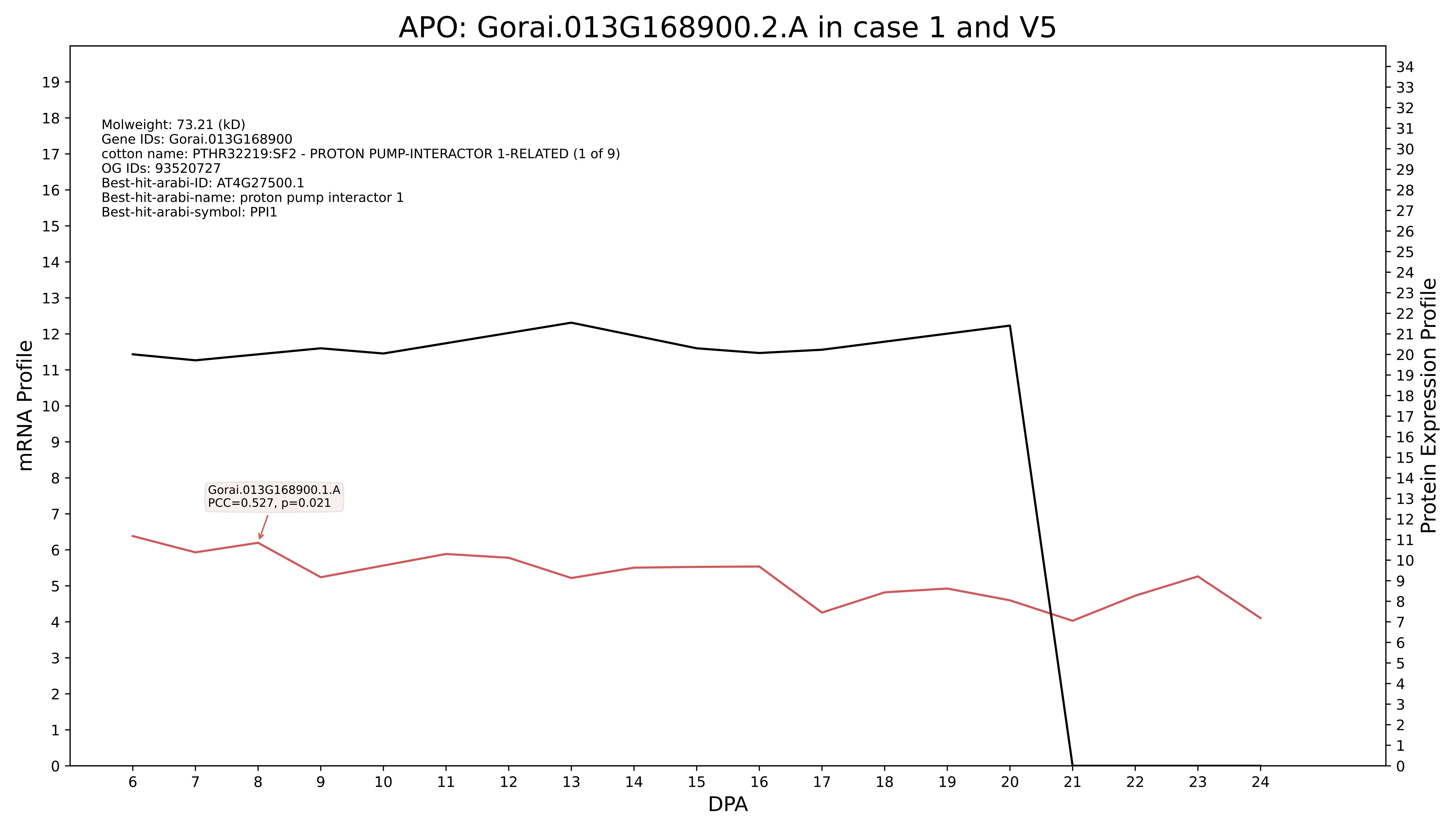

APO: Gorai.013G168900.2.A in case 1 and V5
Molweight: 73.21 (kD)
Gene IDs: Gorai.013G168900
cotton name: PTHR32219:SF2 - PROTON PUMP-INTERACTOR 1-RELATED (1 of 9)
OG IDs: 93520727
Best-hit-arabi-ID: AT4G27500.1
Best-hit-arabi-name: proton pump interactor 1
Best-hit-arabi-symbol: PPI1
Gorai.013G168900.1.A
PCC=0.527, p=0.021
mRNA Profile
Protein Expression Profile
DPA

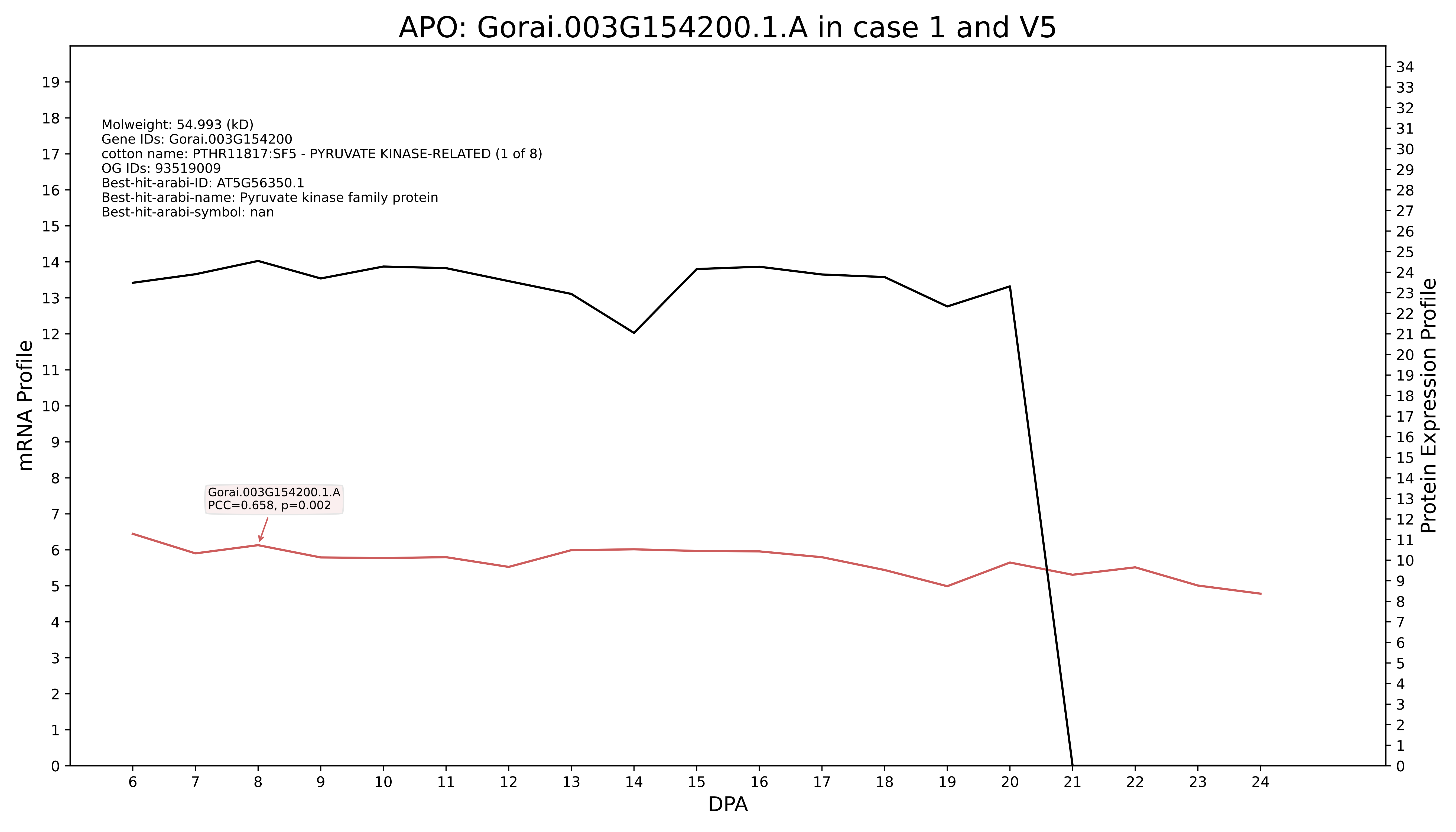
APO: Gorai.003G154200.1.A in case 1 and V5
Molweight: 54.993 (kD)
Gene IDs: Gorai.003G154200
cotton name: PTHR11817:SF5 - PYRUVATE KINASE-RELATED (1 of 8)
OG IDs: 93519009
Best-hit-arabi-ID: AT5G56350.1
Best-hit-arabi-name: Pyruvate kinase family protein
Best-hit-arabi-symbol: nan
Gorai.003G154200.1.A
PCC=0.658, p=0.002
mRNA Profile
Protein Expression Profile
DPA

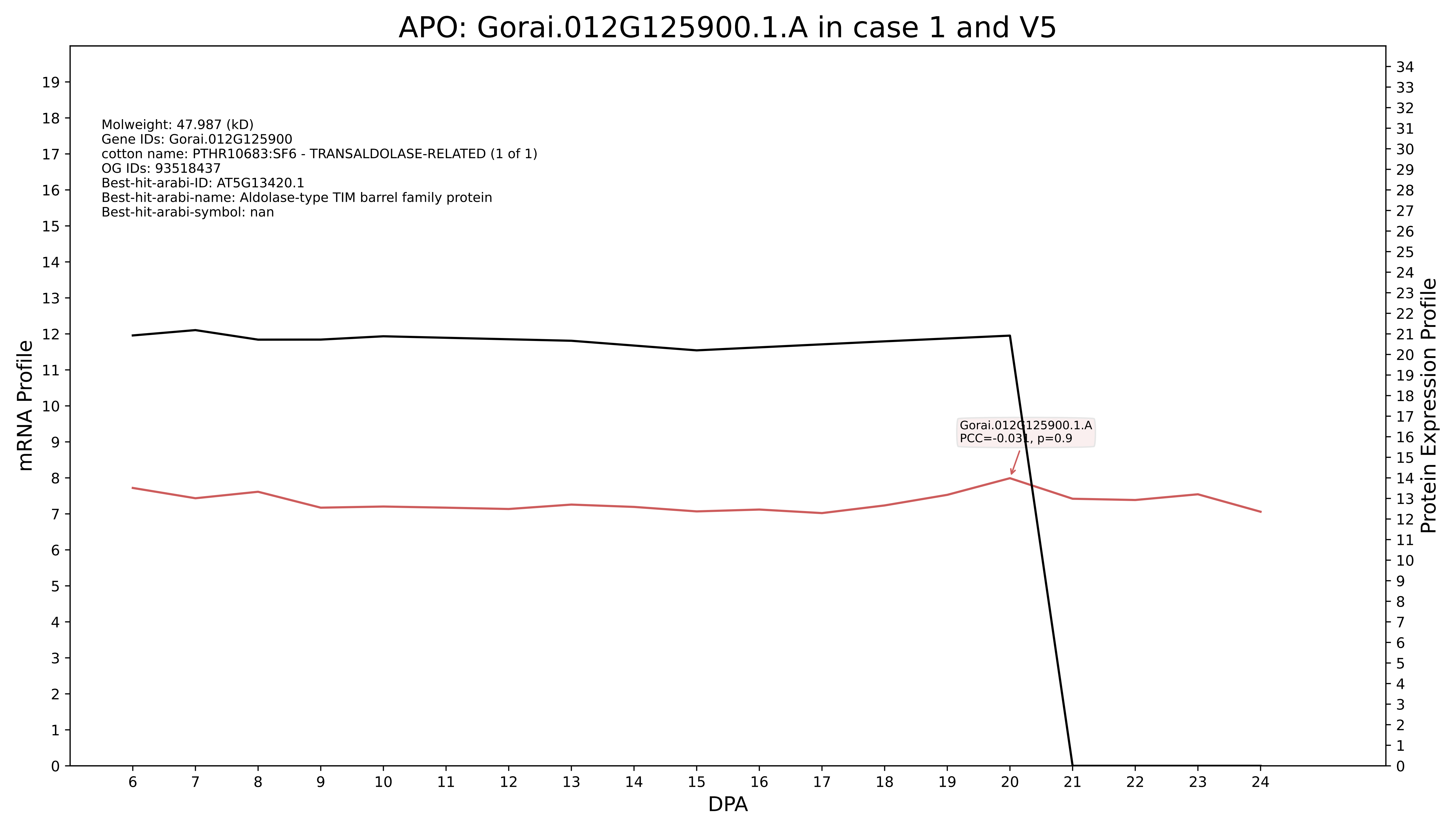

APO: Gorai.012G125900.1.A in case 1 and V5
Molweight: 47.987 (kD)
Gene IDs: Gorai.012G125900
cotton name: PTHR10683:SF6 - TRANSALDOLASE-RELATED (1 of 1)
OG IDs: 93518437
Best-hit-arabi-ID: AT5G13420.1
Best-hit-arabi-name: Aldolase-type TIM barrel family protein
Best-hit-arabi-symbol: nan
Gorai.012G125900.1.A
PCC=-0.031, p=0.9
mRNA Profile
Protein Expression Profile
DPA

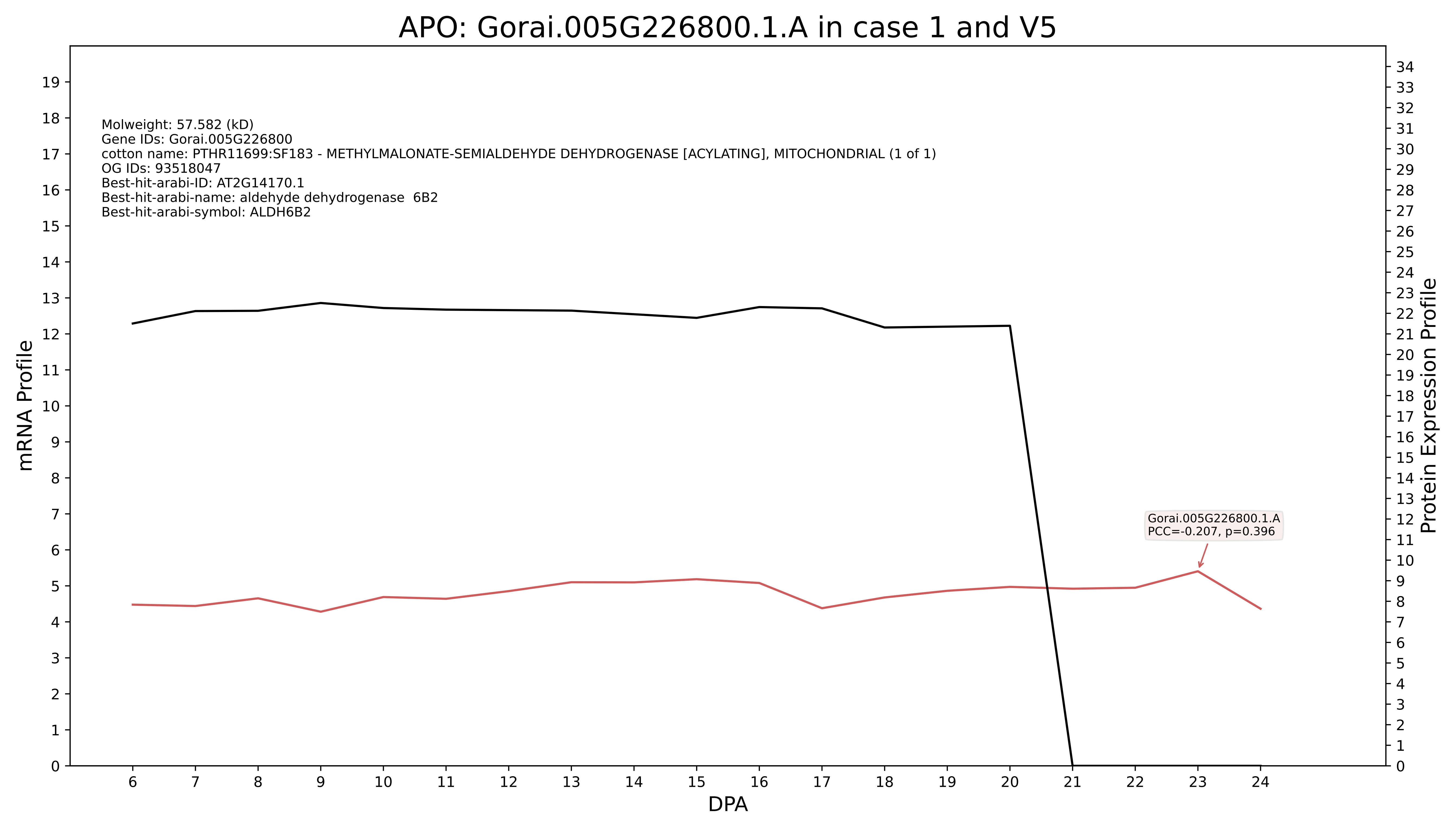
APO: Gorai.005G226800.1.A in case 1 and V5
Molweight: 57.582 (kD)
Gene IDs: Gorai.005G226800
cotton name: PTHR11699:SF183 - METHYLMALONATE-SEMIALDEHYDE DEHYDROGENASE [ACYLATING], MITOCHONDRIAL (1 of 1)
OG IDs: 93518047
Best-hit-arabi-ID: AT2G14170.1
Best-hit-arabi-name: aldehyde dehydrogenase 6B2
Best-hit-arabi-symbol: ALDH6B2
Gorai.005G226800.1.A
PCC=-0.207, p=0.396
mRNA Profile
Protein Expression Profile
DPA
0
1
2
3
4
5
6
7
8
9
10
11
12
13
14
15
16
17
18
19
20
21
22
23
24
25
26
27
28
29
30
31
32
33
34

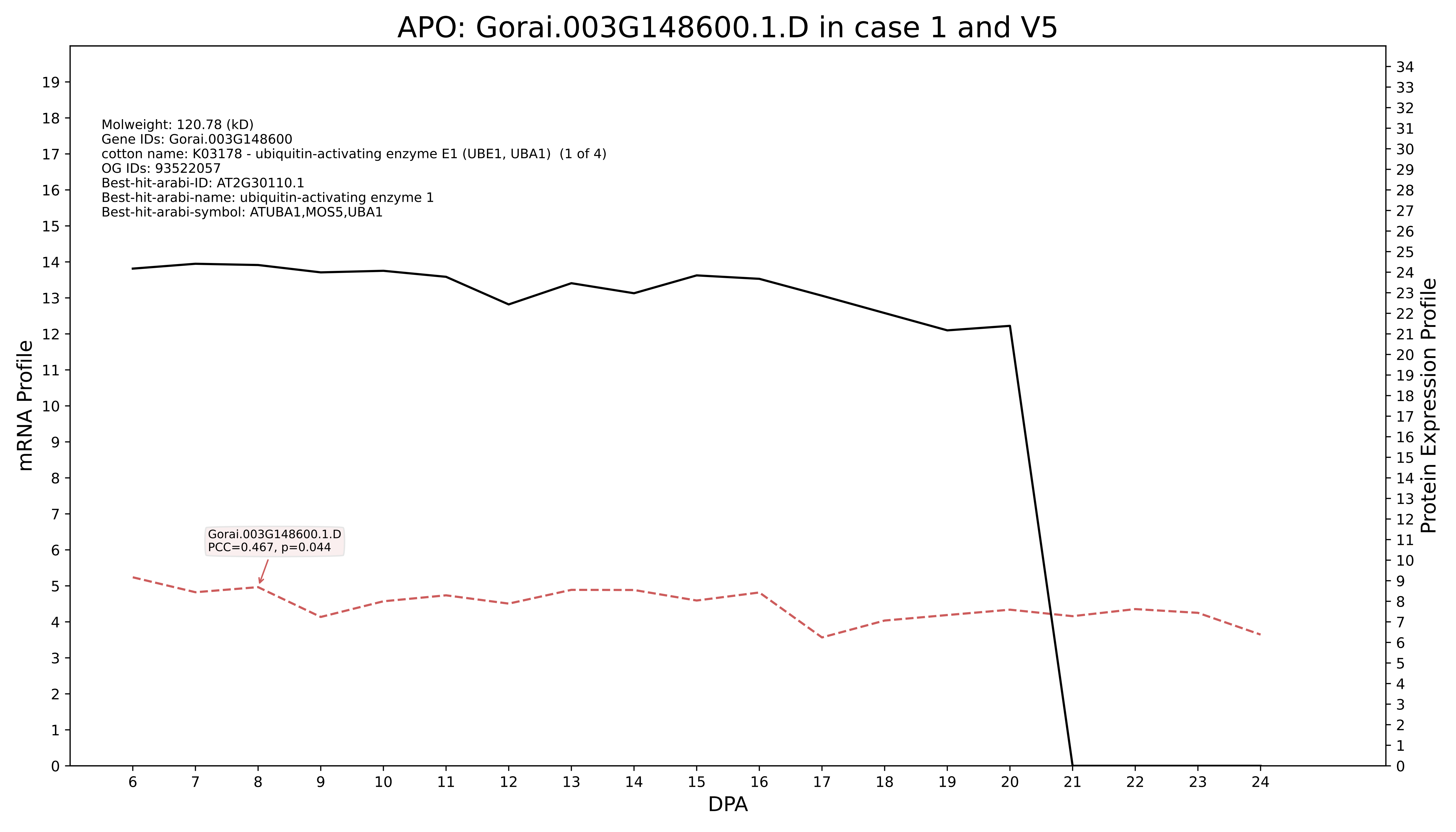
APO: Gorai.003G148600.1.D in case 1 and V5
Molweight: 120.78 (kD)
Gene IDs: Gorai.003G148600
cotton name: K03178 - ubiquitin-activating enzyme E1 (UBE1, UBA1) (1 of 4)
OG IDs: 93522057
Best-hit-arabi-ID: AT2G30110.1
Best-hit-arabi-name: ubiquitin-activating enzyme 1
Best-hit-arabi-symbol: ATUBA1,MOS5,UBA1
Gorai.003G148600.1.D
PCC=0.467, p=0.044
mRNA Profile
Protein Expression Profile
DPA

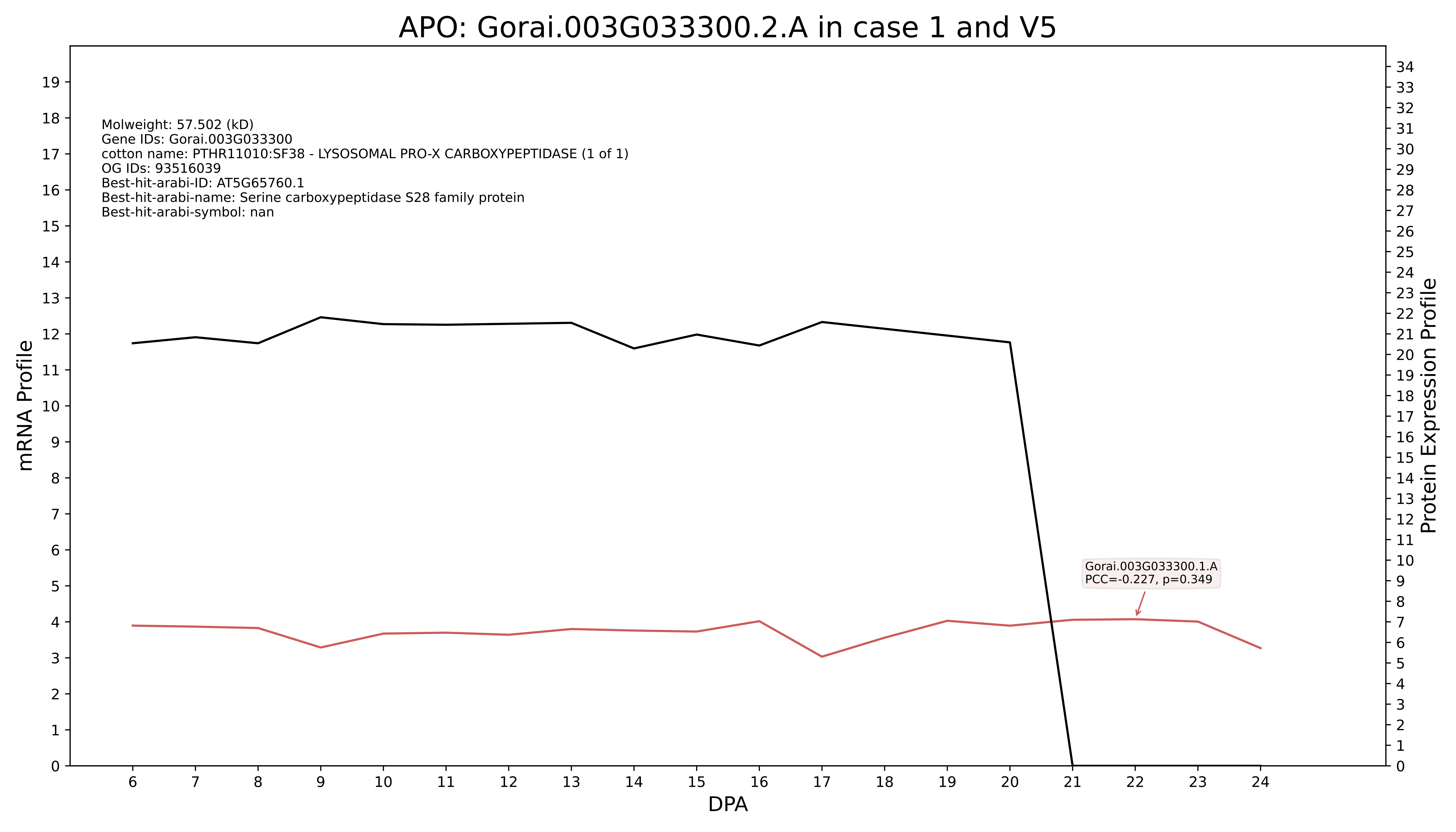

APO: Gorai.003G033300.2.A in case 1 and V5
Molweight: 57.502 (kD)
Gene IDs: Gorai.003G033300
cotton name: PTHR11010:SF38 - LYSOSOMAL PRO-X CARBOXYPEPTIDASE (1 of 1)
OG IDs: 93516039
Best-hit-arabi-ID: AT5G65760.1
Best-hit-arabi-name: Serine carboxypeptidase S28 family protein
Best-hit-arabi-symbol: nan
Gorai.003G033300.1.A
PCC=-0.227, p=0.349
mRNA Profile
Protein Expression Profile
DPA

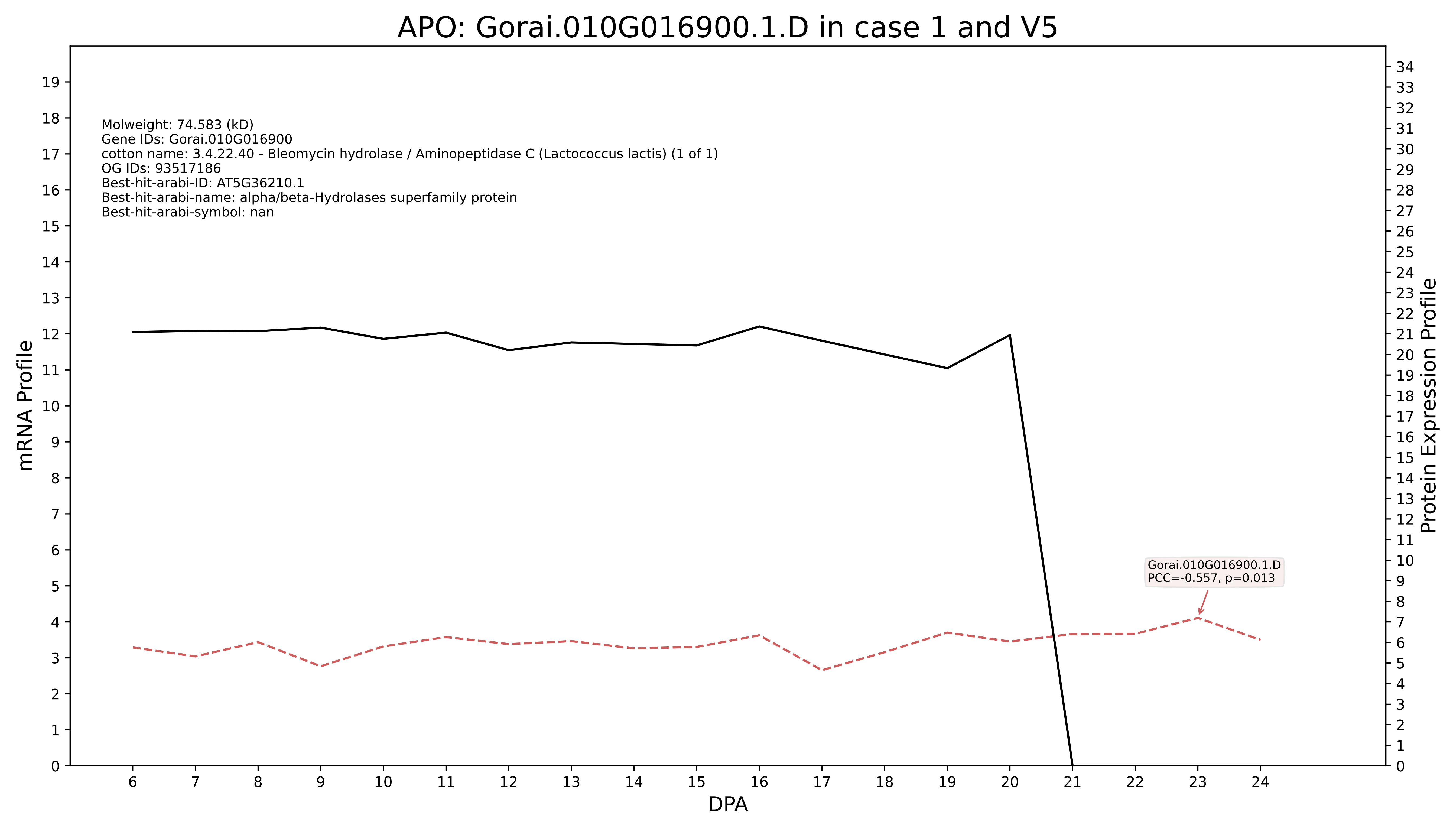

APO: Gorai.010G016900.1.D in case 1 and V5
Molweight: 74.583 (kD)
Gene IDs: Gorai.010G016900
cotton name: 3.4.22.40 - Bleomycin hydrolase / Aminopeptidase C (Lactococcus lactis) (1 of 1)
OG IDs: 93517186
Best-hit-arabi-ID: AT5G36210.1
Best-hit-arabi-name: alpha/beta-Hydrolases superfamily protein
Best-hit-arabi-symbol: nan
Gorai.010G016900.1.D
PCC=-0.557, p=0.013
mRNA Profile
Protein Expression Profile
DPA

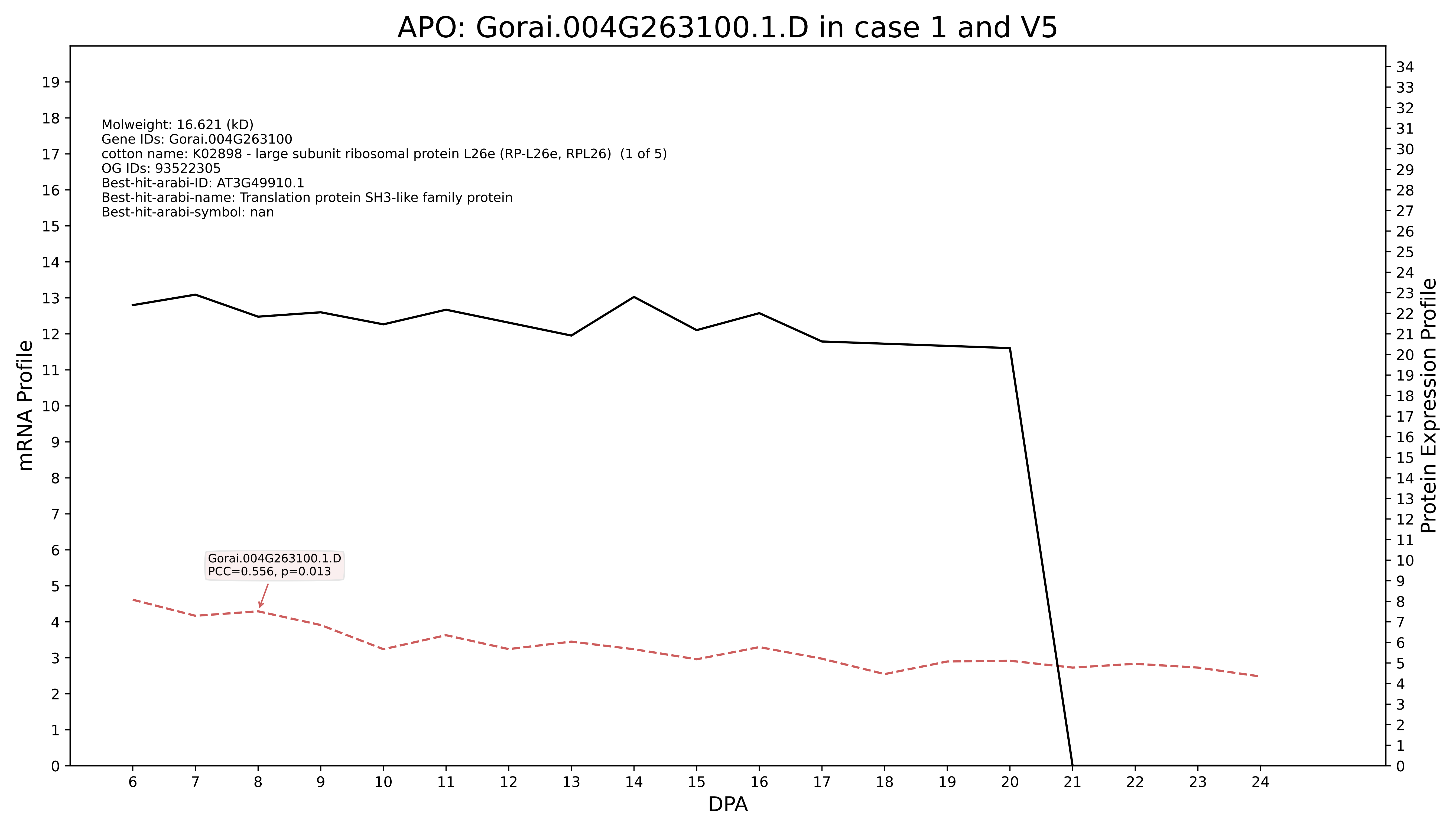
APO: Gorai.004G263100.1.D in case 1 and V5
Molweight: 16.621 (kD)
Gene IDs: Gorai.004G263100
cotton name: K02898 - large subunit ribosomal protein L26e (RP-L26e, RPL26) (1 of 5)
OG IDs: 93522305
Best-hit-arabi-ID: AT3G49910.1
Best-hit-arabi-name: Translation protein SH3-like family protein
Best-hit-arabi-symbol: nan
Gorai.004G263100.1.D
PCC=0.556, p=0.013
mRNA Profile
Protein Expression Profile
DPA

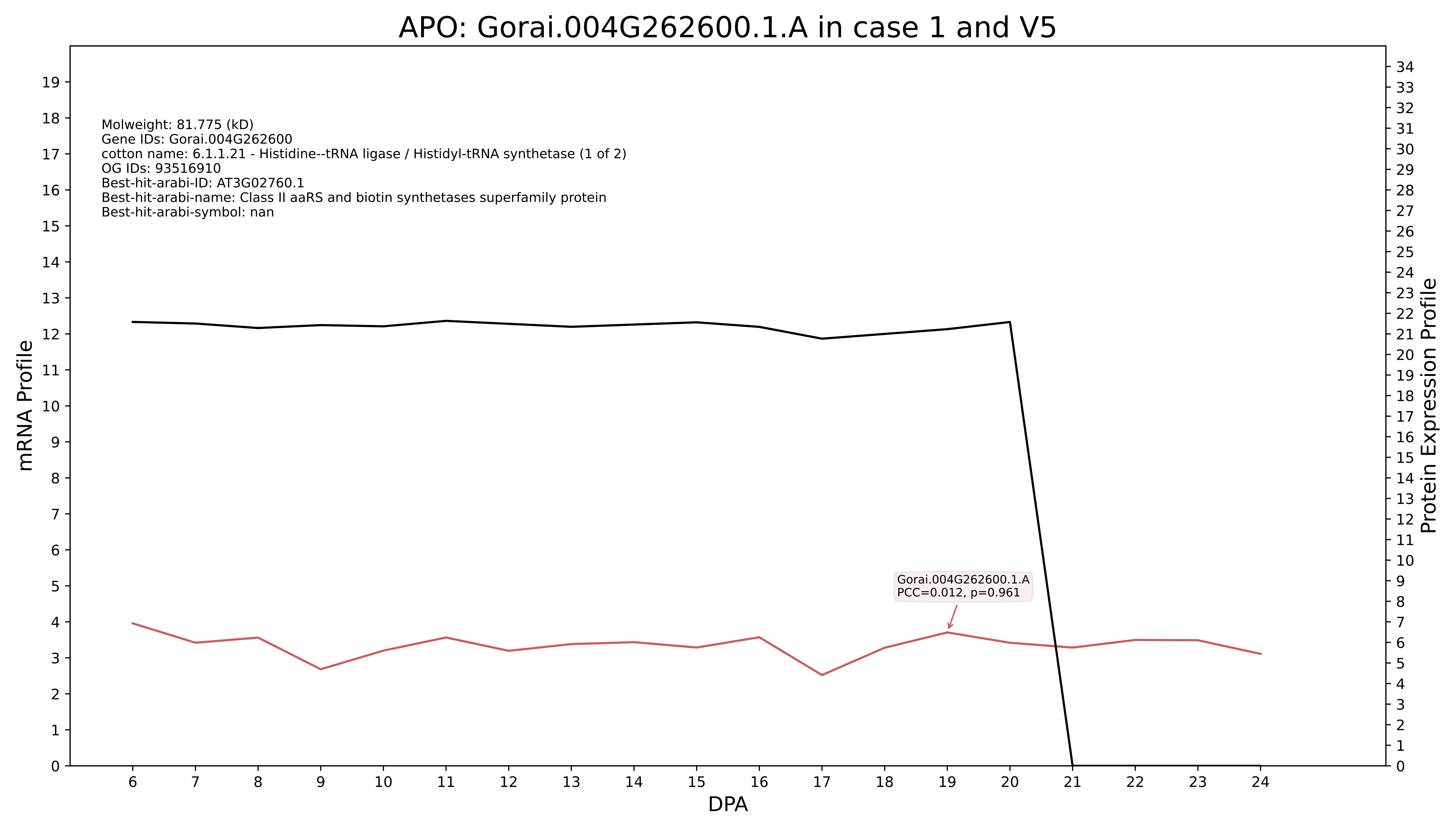
APO: Gorai.004G262600.1.A in case 1 and V5
Molweight: 81.775 (kD)
Gene IDs: Gorai.004G262600
cotton name: 6.1.1.21 - Histidine--tRNA ligase / Histidyl-tRNA synthetase (1 of 2)
OG IDs: 93516910
Best-hit-arabi-ID: AT3G02760.1
Best-hit-arabi-name: Class II aaRS and biotin synthetases superfamily protein
Best-hit-arabi-symbol: nan
Gorai.004G262600.1.A
PCC=0.012, p=0.961
mRNA Profile
Protein Expression Profile
DPA

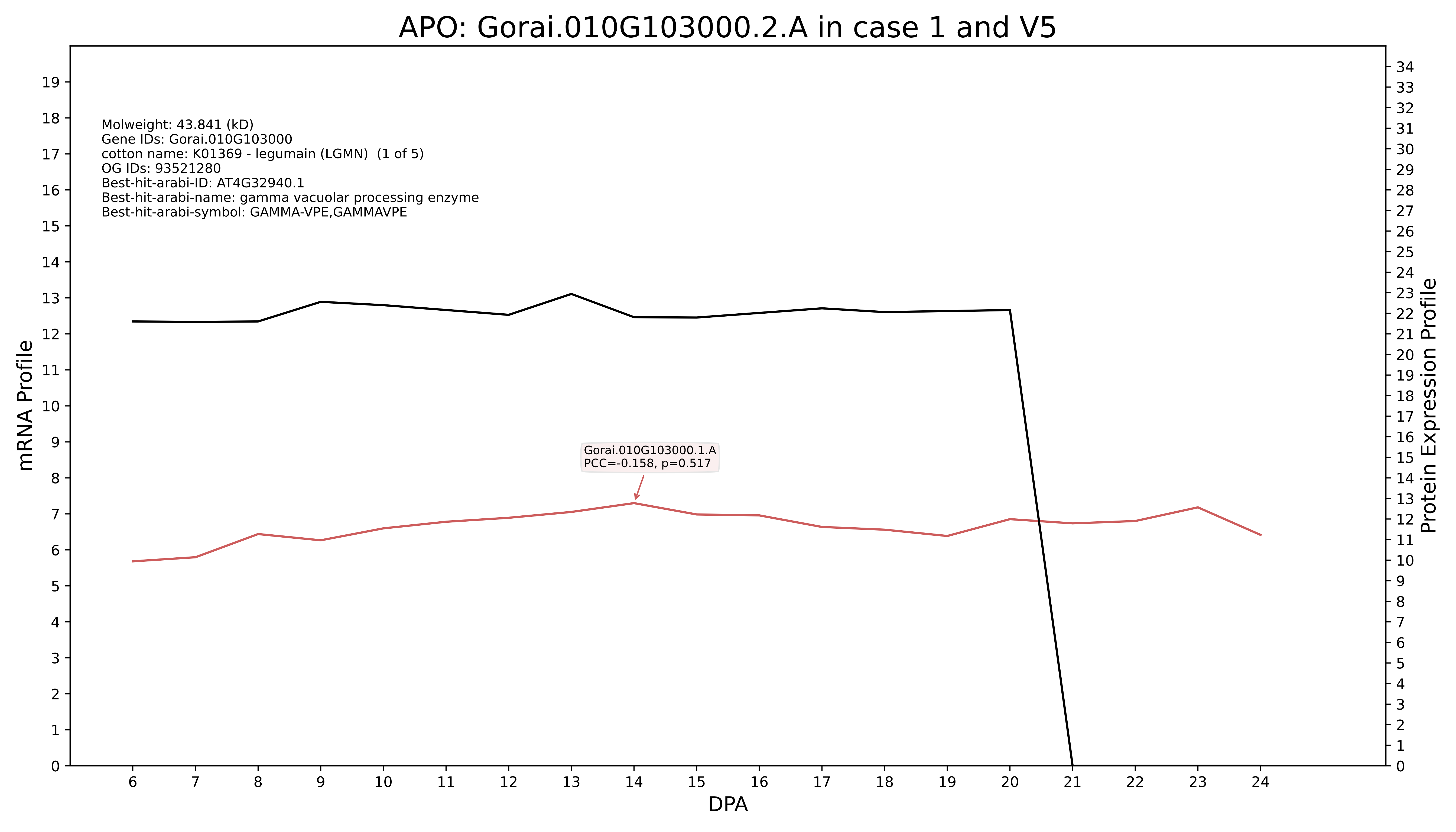
APO: Gorai.010G103000.2.A in case 1 and V5
Molweight: 43.841 (kD)
Gene IDs: Gorai.010G103000
cotton name: K01369 - legumain (LGMN) (1 of 5)
OG IDs: 93521280
Best-hit-arabi-ID: AT4G32940.1
Best-hit-arabi-name: gamma vacuolar processing enzyme
Best-hit-arabi-symbol: GAMMA-VPE,GAMMAVPE
Gorai.010G103000.1.A
PCC=-0.158, p=0.517
mRNA Profile
Protein Expression Profile
DPA

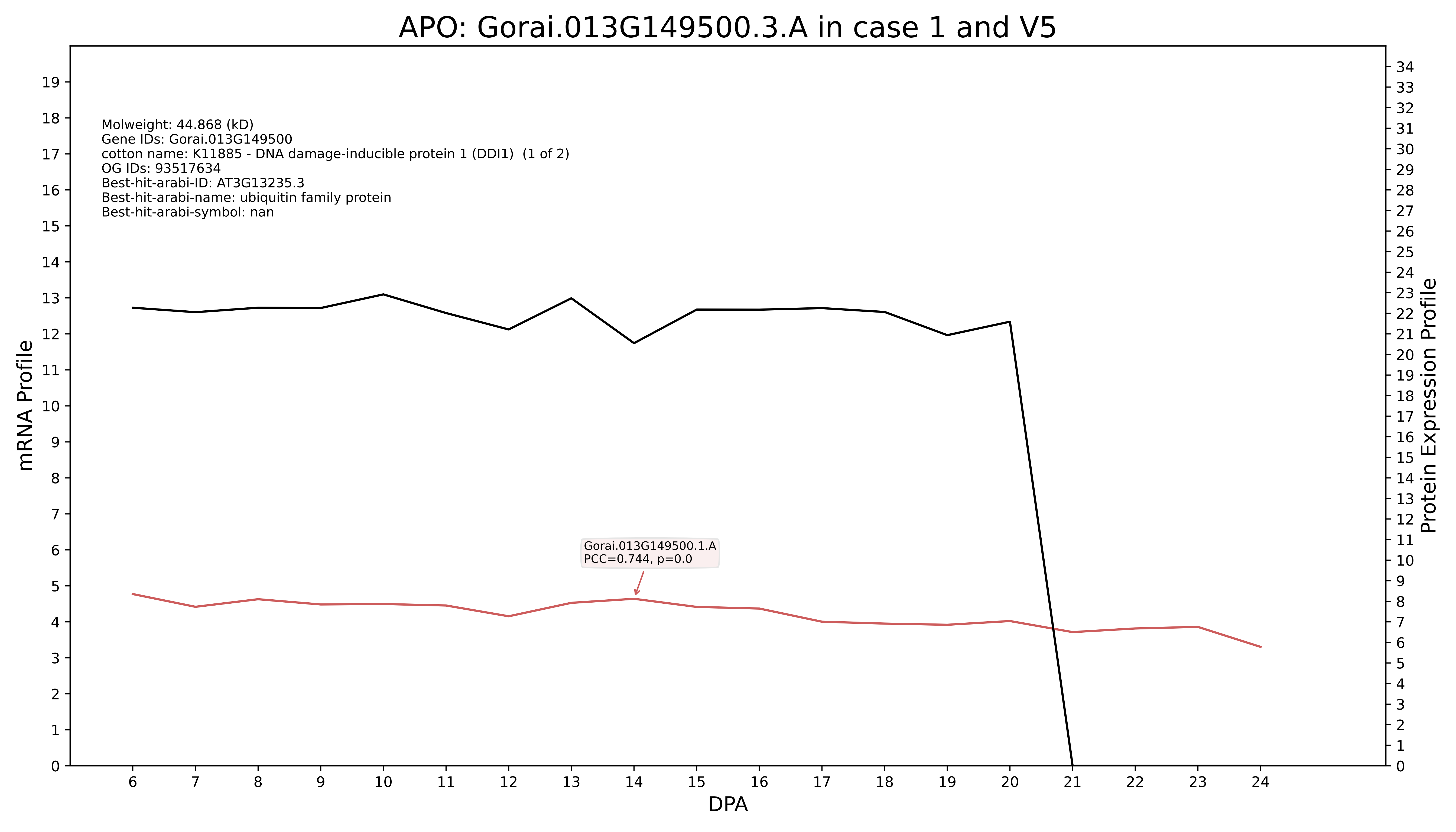
APO: Gorai.013G149500.3.A in case 1 and V5
Molweight: 44.868 (kD)
Gene IDs: Gorai.013G149500
cotton name: K11885 - DNA damage-inducible protein 1 (DDI1) (1 of 2)
OG IDs: 93517634
Best-hit-arabi-ID: AT3G13235.3
Best-hit-arabi-name: ubiquitin family protein
Best-hit-arabi-symbol: nan
Gorai.013G149500.1.A
PCC=0.744, p=0.0
mRNA Profile
Protein Expression Profile
DPA

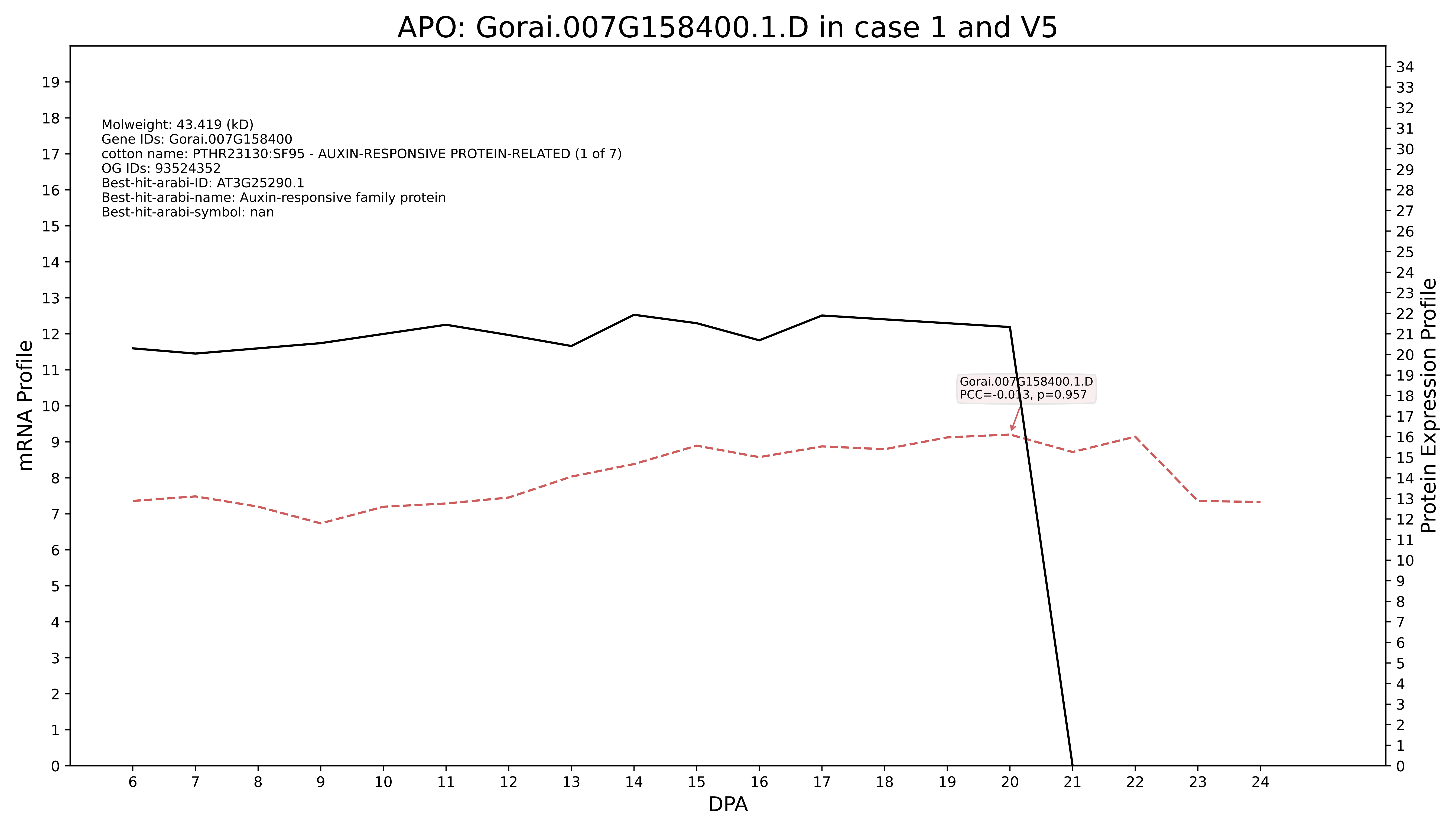

APO: Gorai.007G158400.1.D in case 1 and V5
Molweight: 43.419 (kD)
Gene IDs: Gorai.007G158400
cotton name: PTHR23130:SF95 - AUXIN-RESPONSIVE PROTEIN-RELATED (1 of 7)
OG IDs: 93524352
Best-hit-arabi-ID: AT3G25290.1
Best-hit-arabi-name: Auxin-responsive family protein
Best-hit-arabi-symbol: nan
Gorai.007G158400.1.D
PCC=-0.013, p=0.957
mRNA Profile
Protein Expression Profile
DPA

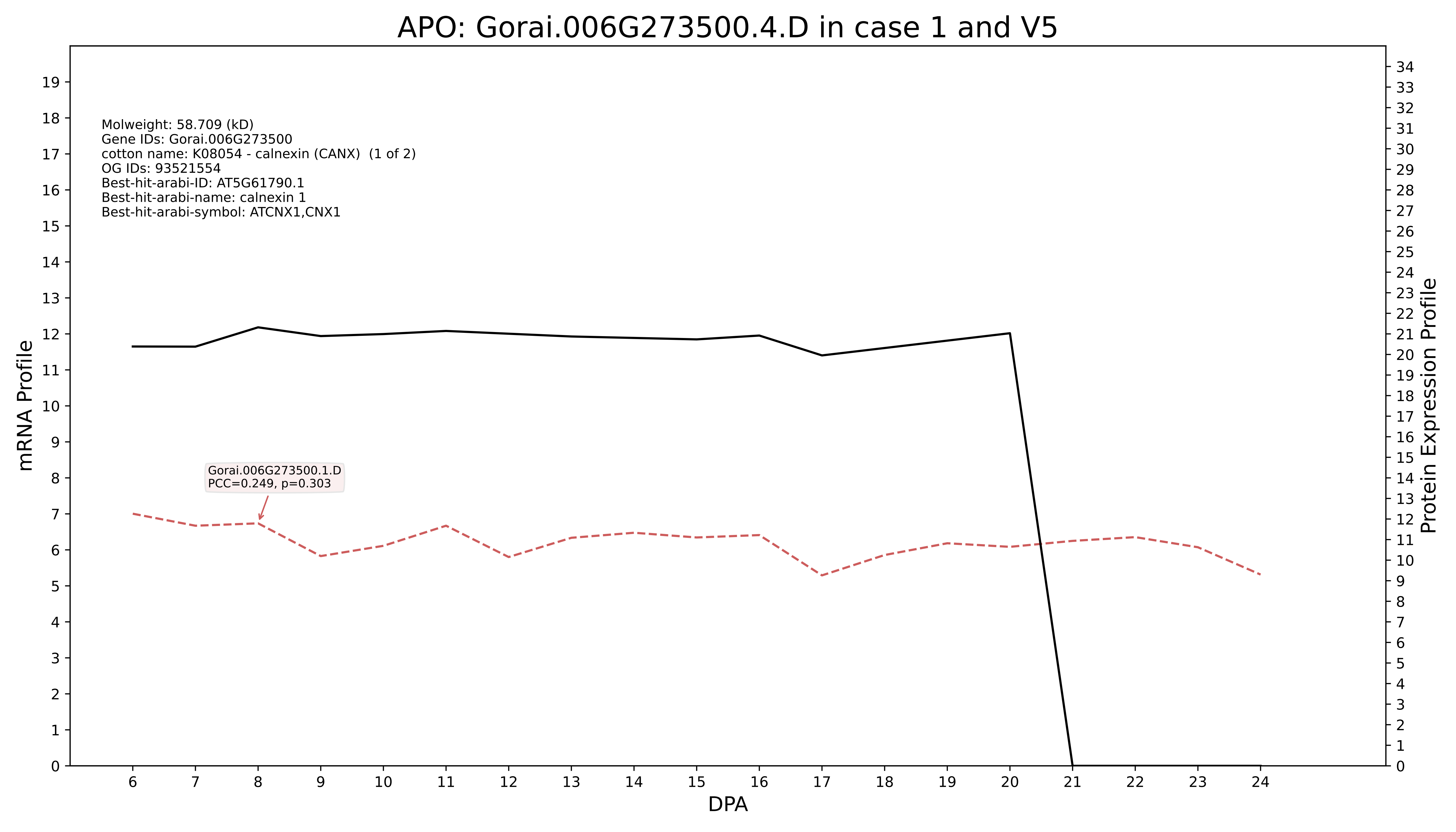
APO: Gorai.006G273500.4.D in case 1 and V5
Molweight: 58.709 (kD)
Gene IDs: Gorai.006G273500
cotton name: K08054 - calnexin (CANX) (1 of 2)
OG IDs: 93521554
Best-hit-arabi-ID: AT5G61790.1
Best-hit-arabi-name: calnexin 1
Best-hit-arabi-symbol: ATCNX1,CNX1
Gorai.006G273500.1.D
PCC=0.249, p=0.303
mRNA Profile
Protein Expression Profile
DPA

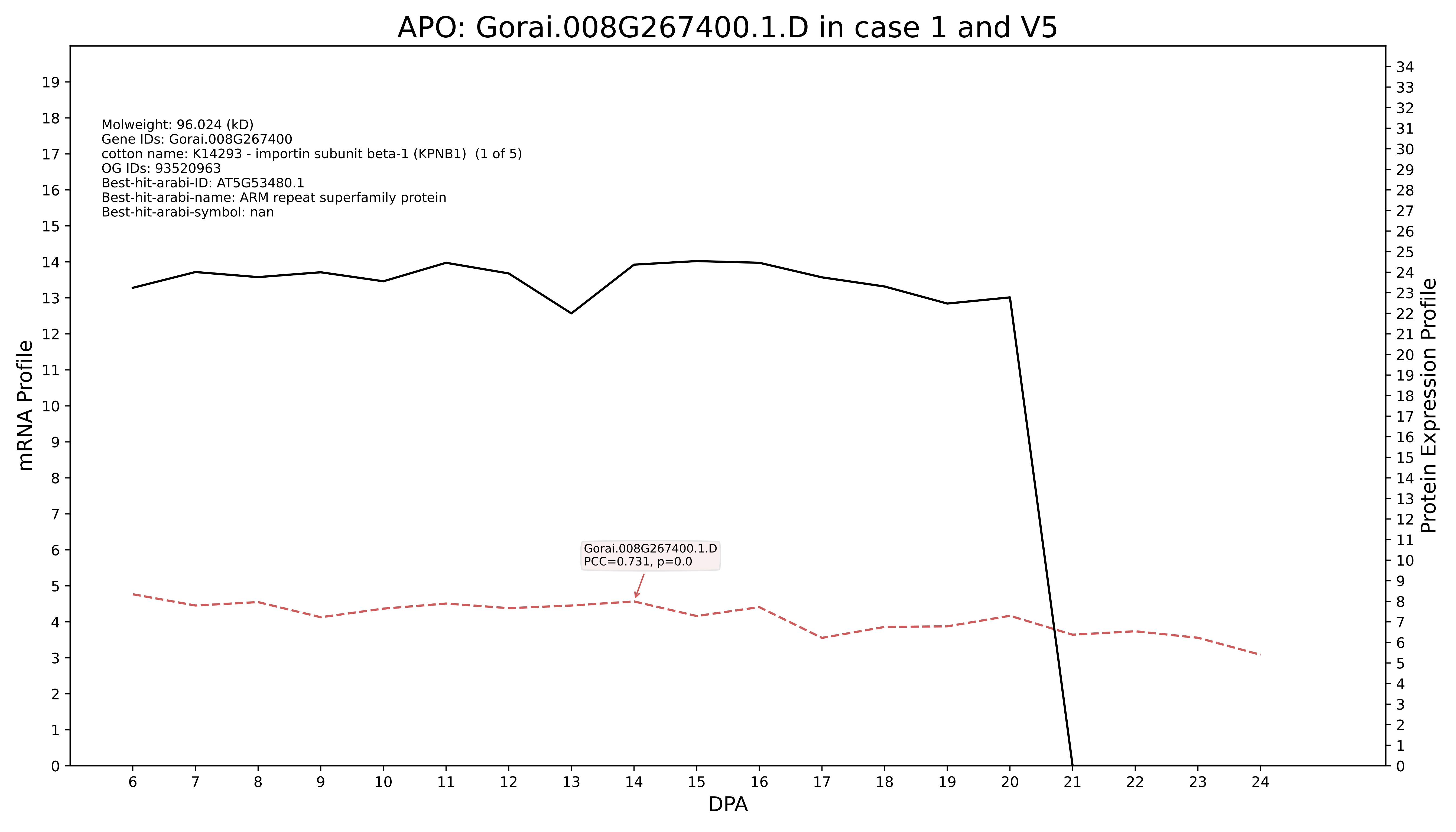

APO: Gorai.008G267400.1.D in case 1 and V5
Molweight: 96.024 (kD)
Gene IDs: Gorai.008G267400
cotton name: K14293 - importin subunit beta-1 (KPNB1) (1 of 5)
OG IDs: 93520963
Best-hit-arabi-ID: AT5G53480.1
Best-hit-arabi-name: ARM repeat superfamily protein
Best-hit-arabi-symbol: nan
Gorai.008G267400.1.D
PCC=0.731, p=0.0
mRNA Profile
Protein Expression Profile
DPA

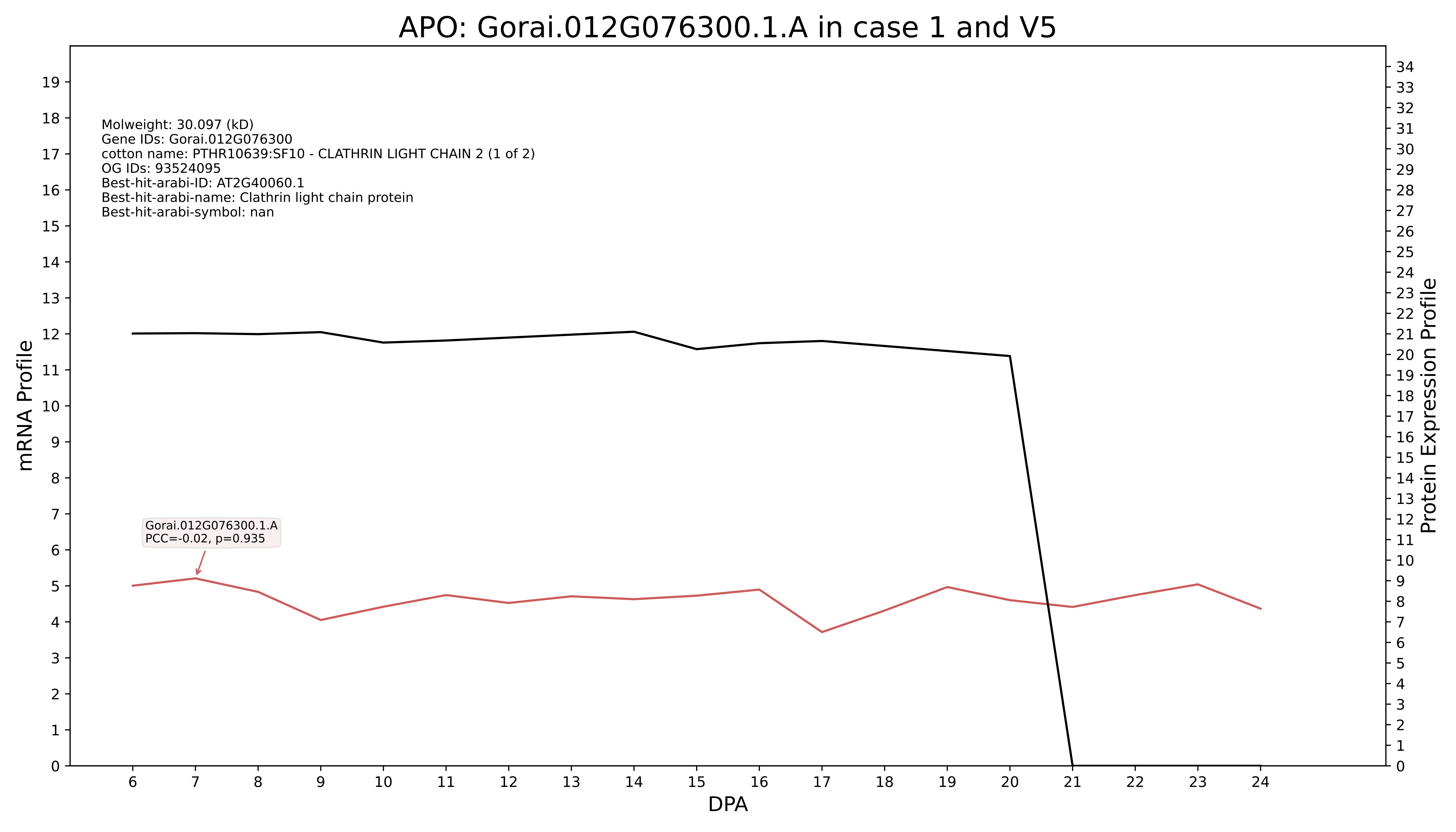
APO: Gorai.012G076300.1.A in case 1 and V5
Molweight: 30.097 (kD)
Gene IDs: Gorai.012G076300
cotton name: PTHR10639:SF10 - CLATHRIN LIGHT CHAIN 2 (1 of 2)
OG IDs: 93524095
Best-hit-arabi-ID: AT2G40060.1
Best-hit-arabi-name: Clathrin light chain protein
Best-hit-arabi-symbol: nan
Gorai.012G076300.1.A
PCC=-0.02, p=0.935
mRNA Profile
Protein Expression Profile
DPA

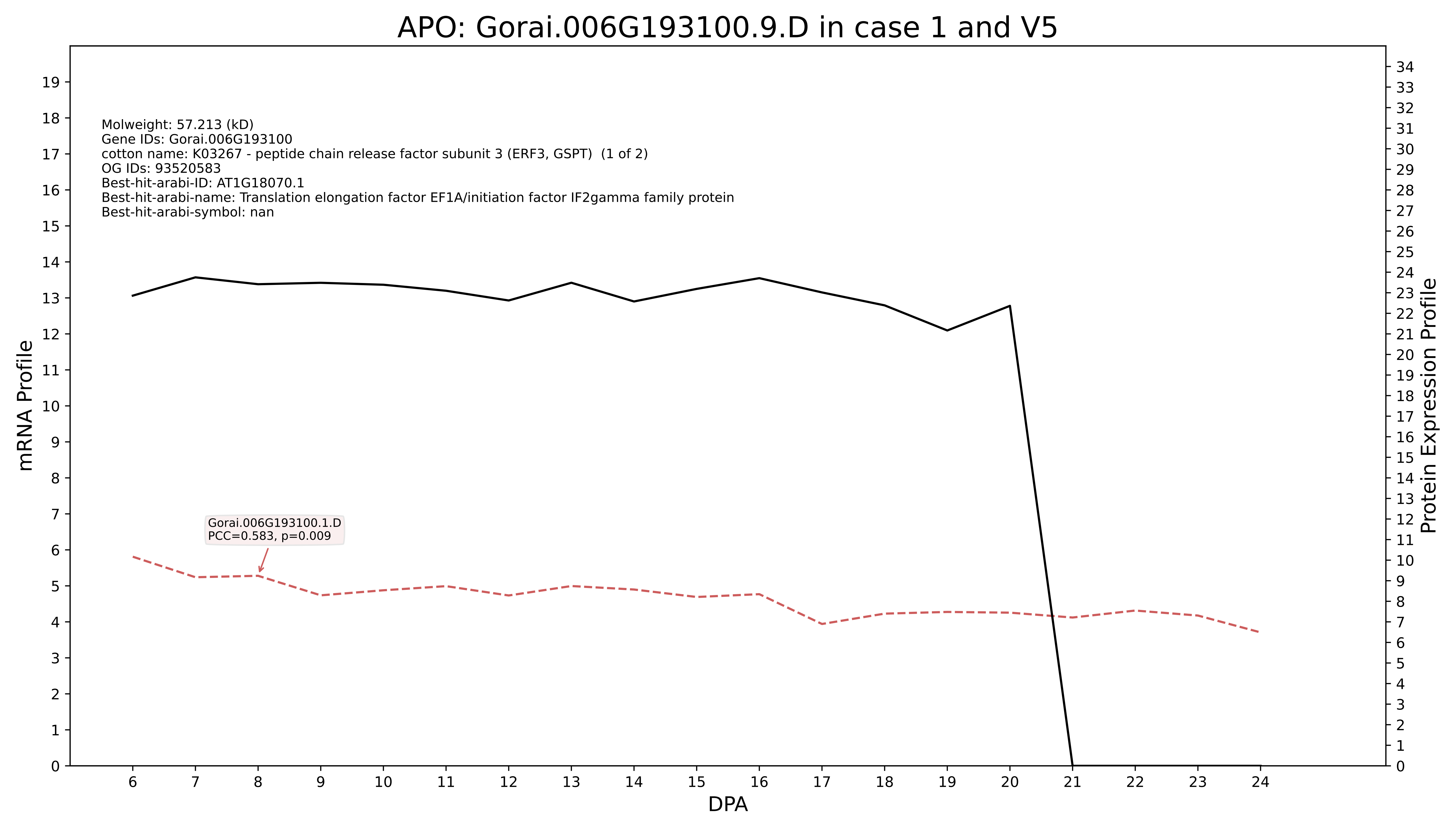
APO: Gorai.006G193100.9.D in case 1 and V5
Molweight: 57.213 (kD)
Gene IDs: Gorai.006G193100
cotton name: K03267 - peptide chain release factor subunit 3 (ERF3, GSPT) (1 of 2)
OG IDs: 93520583
Best-hit-arabi-ID: AT1G18070.1
Best-hit-arabi-name: Translation elongation factor EF1A/initiation factor IF2gamma family protein
Best-hit-arabi-symbol: nan
Gorai.006G193100.1.D
PCC=0.583, p=0.009
mRNA Profile
Protein Expression Profile
DPA
0
1
2
3
4
5
6
7
8
9
10
11
12
13
14
15
16
17
18
19
6
7
8
9
10
11
12
13
14
15
16
17
18
19
20
21
22
23
24
0
1
2
3
4
5
6
7
8
9
10
11
12
13
14
15
16
17
18
19
20
21
22
23
24
25
26
27
28
29
30
31
32
33
34

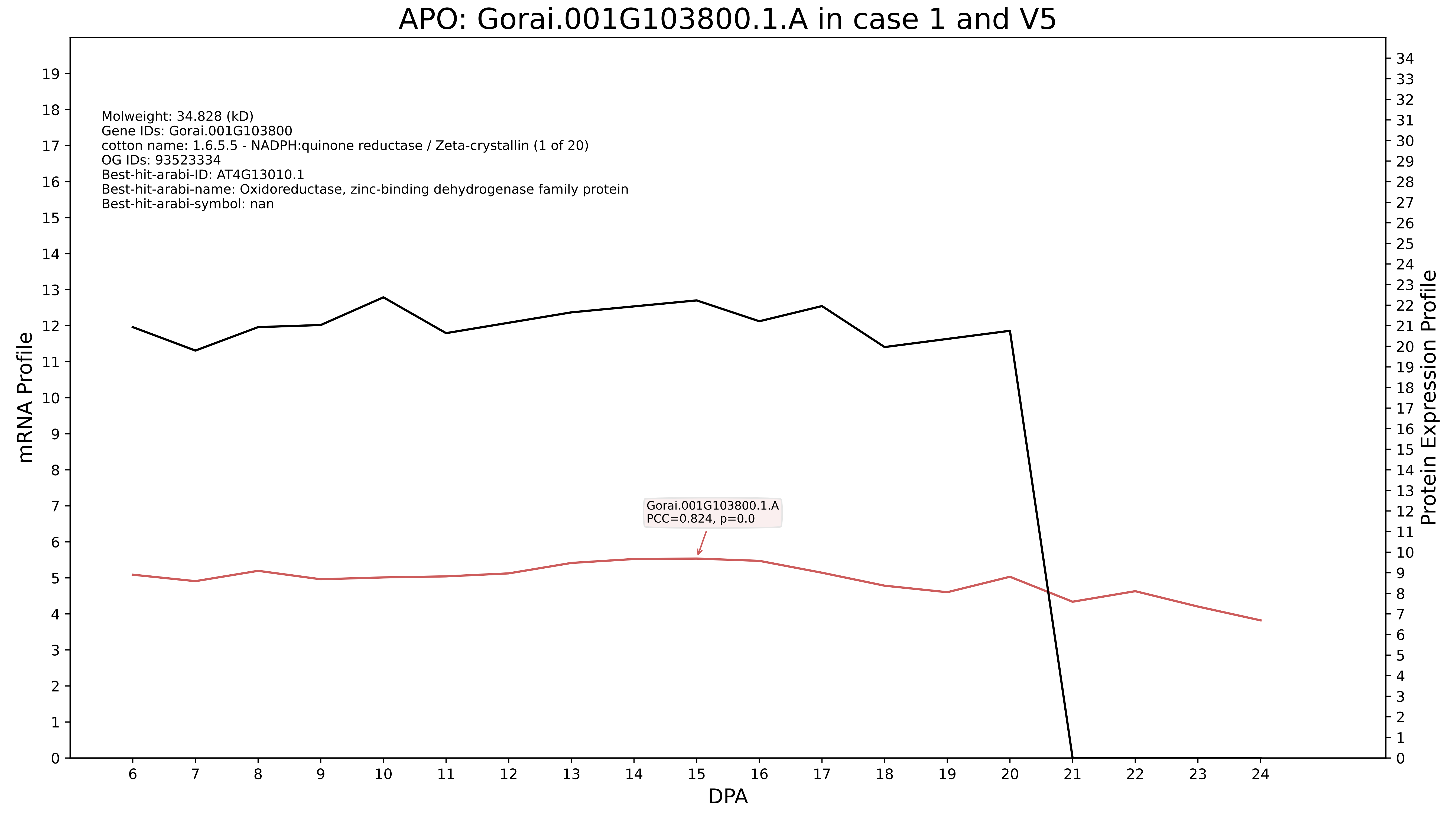
APO: Gorai.001G103800.1.A in case 1 and V5
Molweight: 34.828 (kD)
Gene IDs: Gorai.001G103800
cotton name: 1.6.5.5 - NADPH:quinone reductase / Zeta-crystallin (1 of 20)
OG IDs: 93523334
Best-hit-arabi-ID: AT4G13010.1
Best-hit-arabi-name: Oxidoreductase, zinc-binding dehydrogenase family protein
Best-hit-arabi-symbol: nan
Gorai.001G103800.1.A
PCC=0.824, p=0.0
mRNA Profile
Protein Expression Profile
DPA

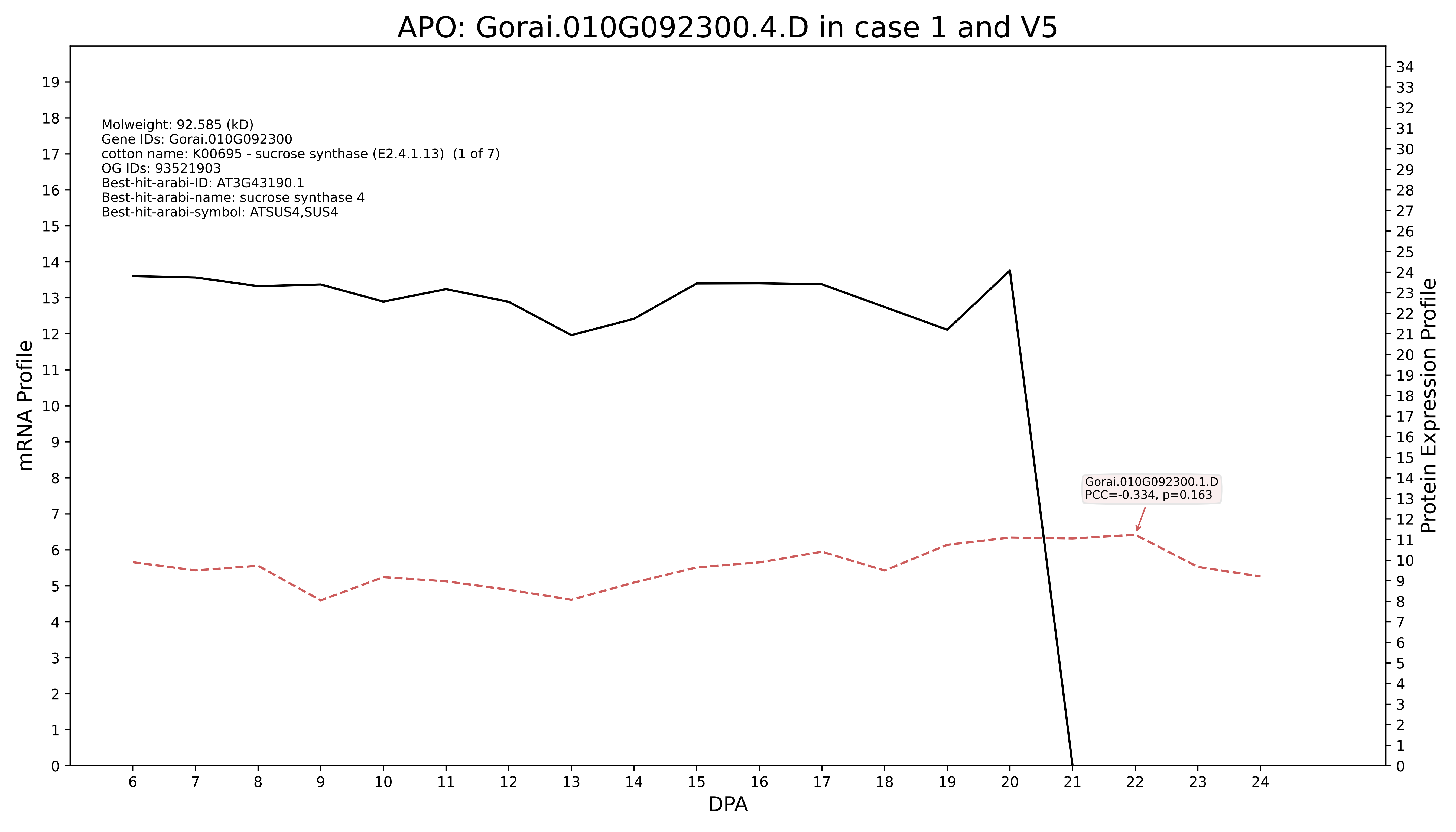

APO: Gorai.010G092300.4.D in case 1 and V5
Molweight: 92.585 (kD)
Gene IDs: Gorai.010G092300
cotton name: K00695 - sucrose synthase (E2.4.1.13) (1 of 7)
OG IDs: 93521903
Best-hit-arabi-ID: AT3G43190.1
Best-hit-arabi-name: sucrose synthase 4
Best-hit-arabi-symbol: ATSUS4,SUS4
Gorai.010G092300.1.D
PCC=-0.334, p=0.163
mRNA Profile
Protein Expression Profile
DPA

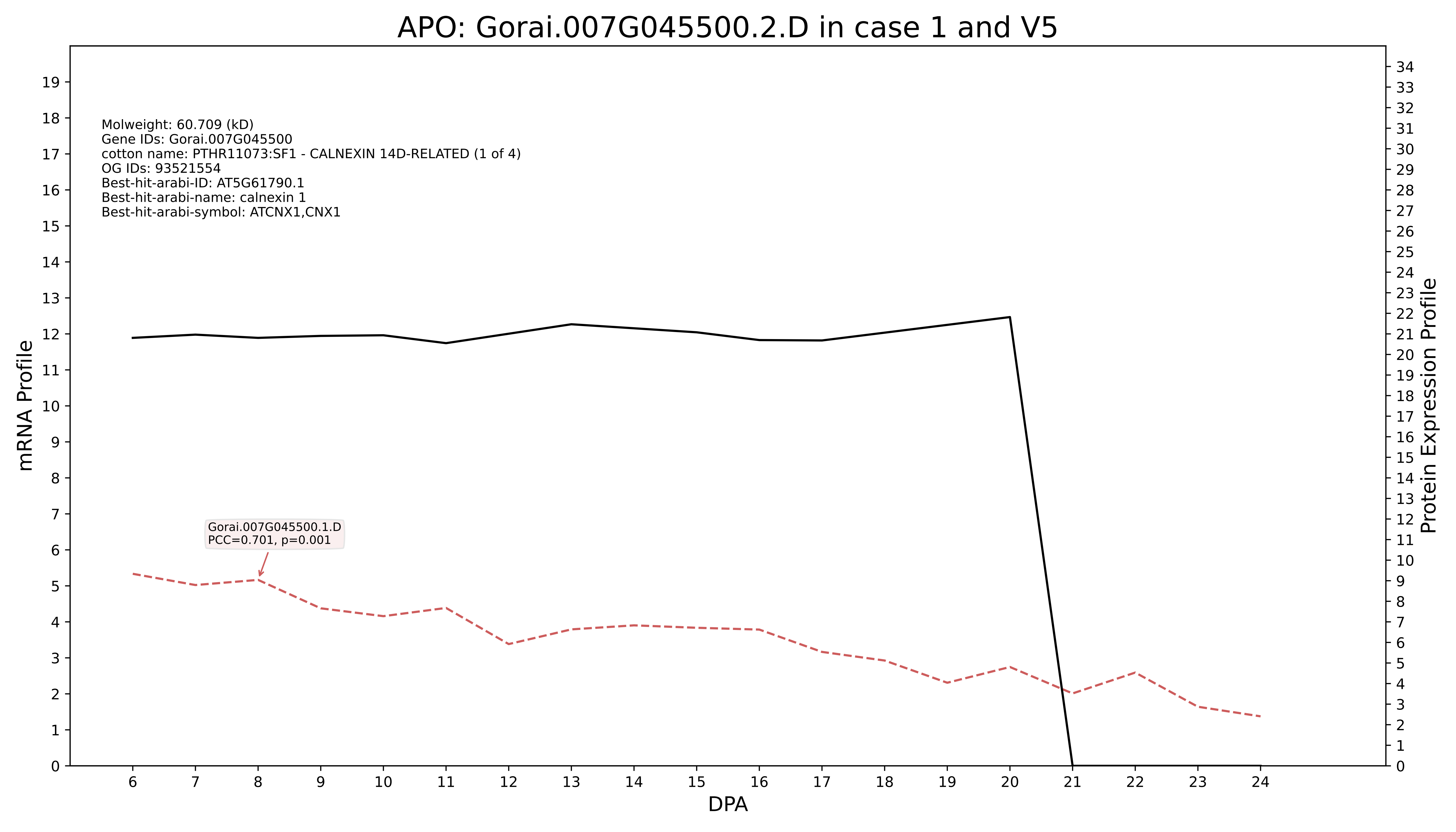
APO: Gorai.007G045500.2.D in case 1 and V5
Molweight: 60.709 (kD)
Gene IDs: Gorai.007G045500
cotton name: PTHR11073:SF1 - CALNEXIN 14D-RELATED (1 of 4)
OG IDs: 93521554
Best-hit-arabi-ID: AT5G61790.1
Best-hit-arabi-name: calnexin 1
Best-hit-arabi-symbol: ATCNX1,CNX1
Gorai.007G045500.1.D
PCC=0.701, p=0.001
mRNA Profile
Protein Expression Profile
DPA

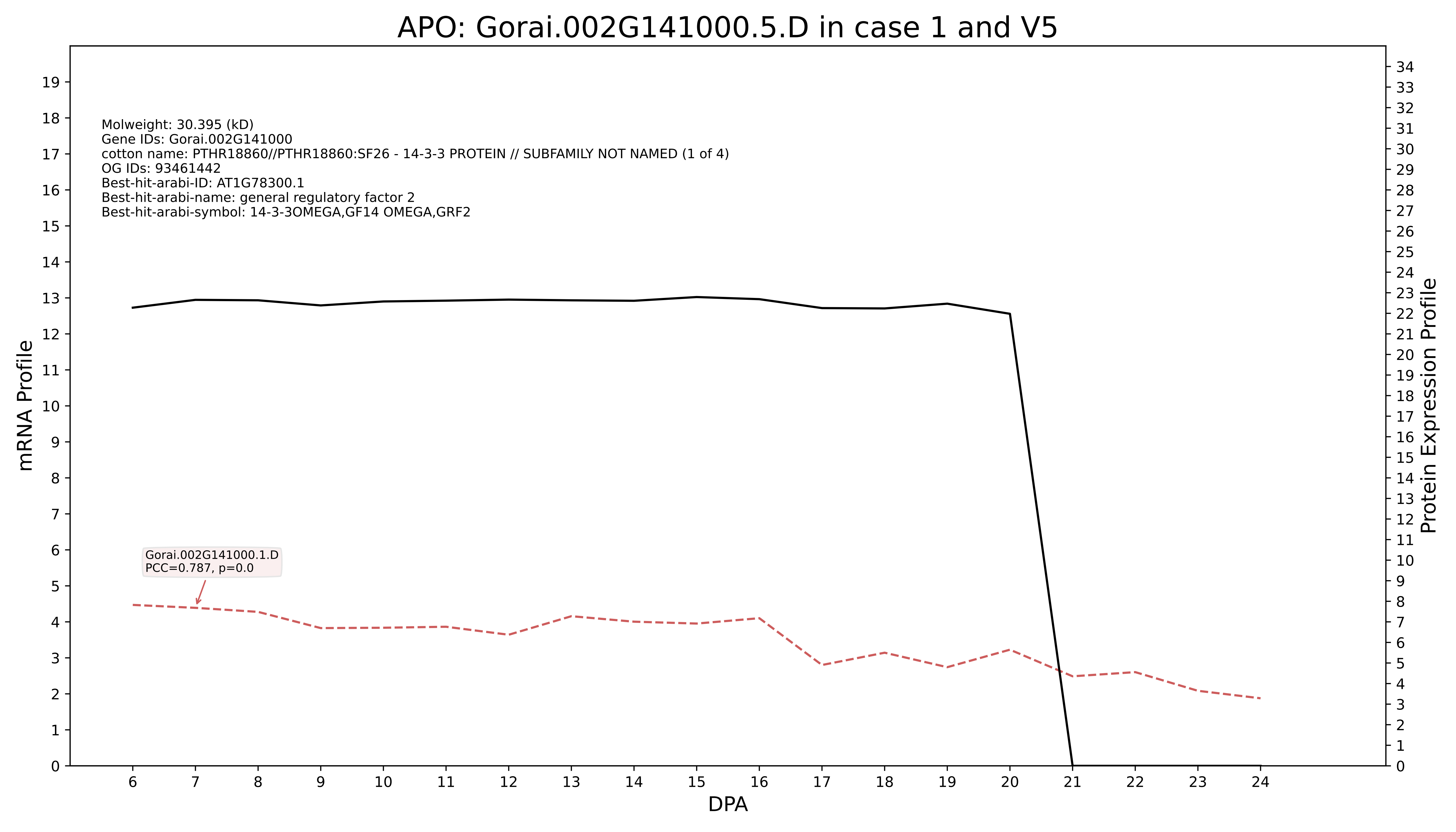

APO: Gorai.002G141000.5.D in case 1 and V5
Molweight: 30.395 (kD)
Gene IDs: Gorai.002G141000
cotton name: PTHR18860//PTHR18860:SF26 - 14-3-3 PROTEIN // SUBFAMILY NOT NAMED (1 of 4)
OG IDs: 93461442
Best-hit-arabi-ID: AT1G78300.1
Best-hit-arabi-name: general regulatory factor 2
Best-hit-arabi-symbol: 14-3-3OMEGA,GF14 OMEGA,GRF2
Gorai.002G141000.1.D
PCC=0.787, p=0.0
mRNA Profile
Protein Expression Profile
DPA

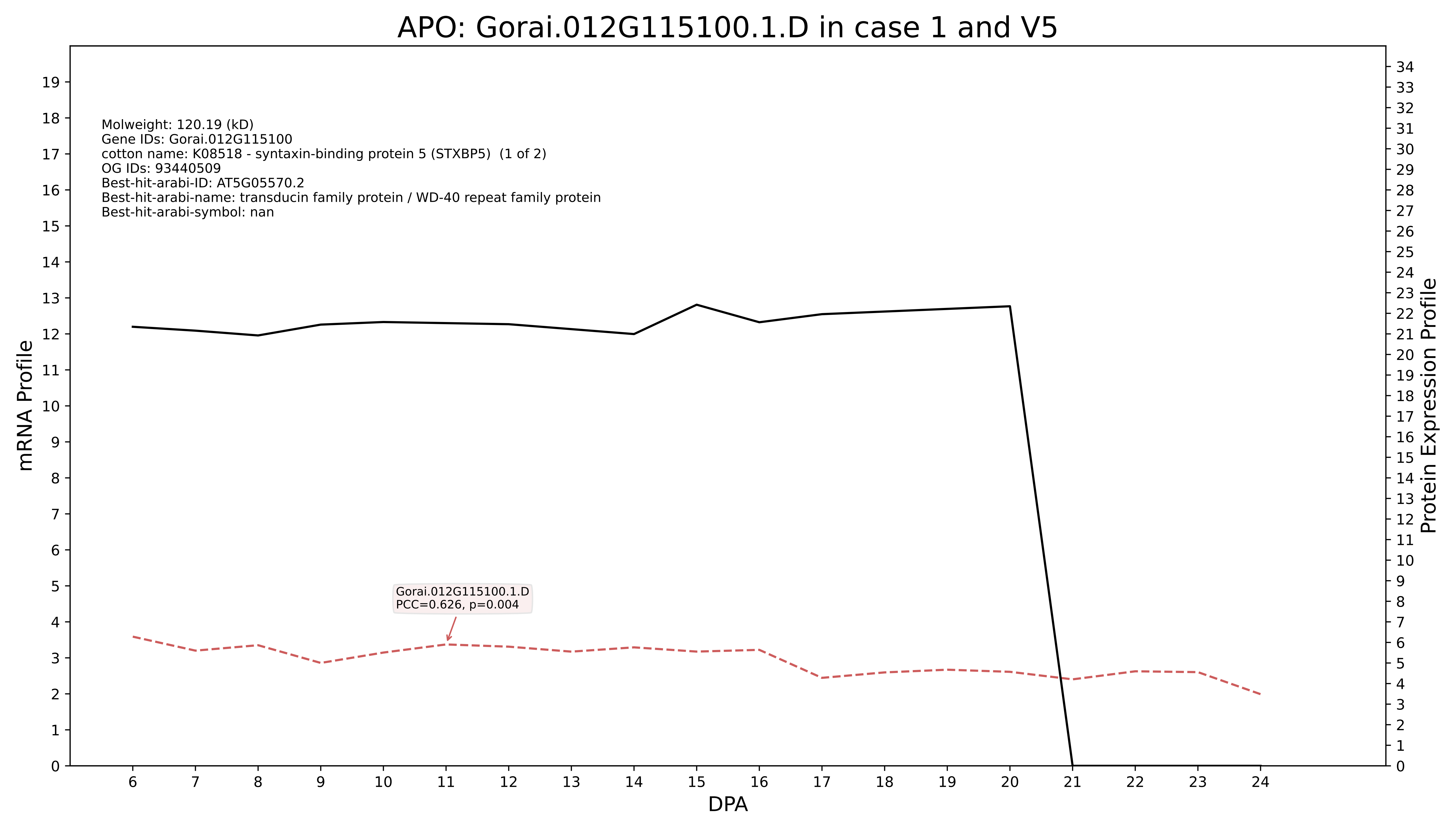
APO: Gorai.012G115100.1.D in case 1 and V5
Molweight: 120.19 (kD)
Gene IDs: Gorai.012G115100
cotton name: K08518 - syntaxin-binding protein 5 (STXBP5) (1 of 2)
OG IDs: 93440509
Best-hit-arabi-ID: AT5G05570.2
Best-hit-arabi-name: transducin family protein / WD-40 repeat family protein
Best-hit-arabi-symbol: nan
Gorai.012G115100.1.D
PCC=0.626, p=0.004
mRNA Profile
Protein Expression Profile
DPA

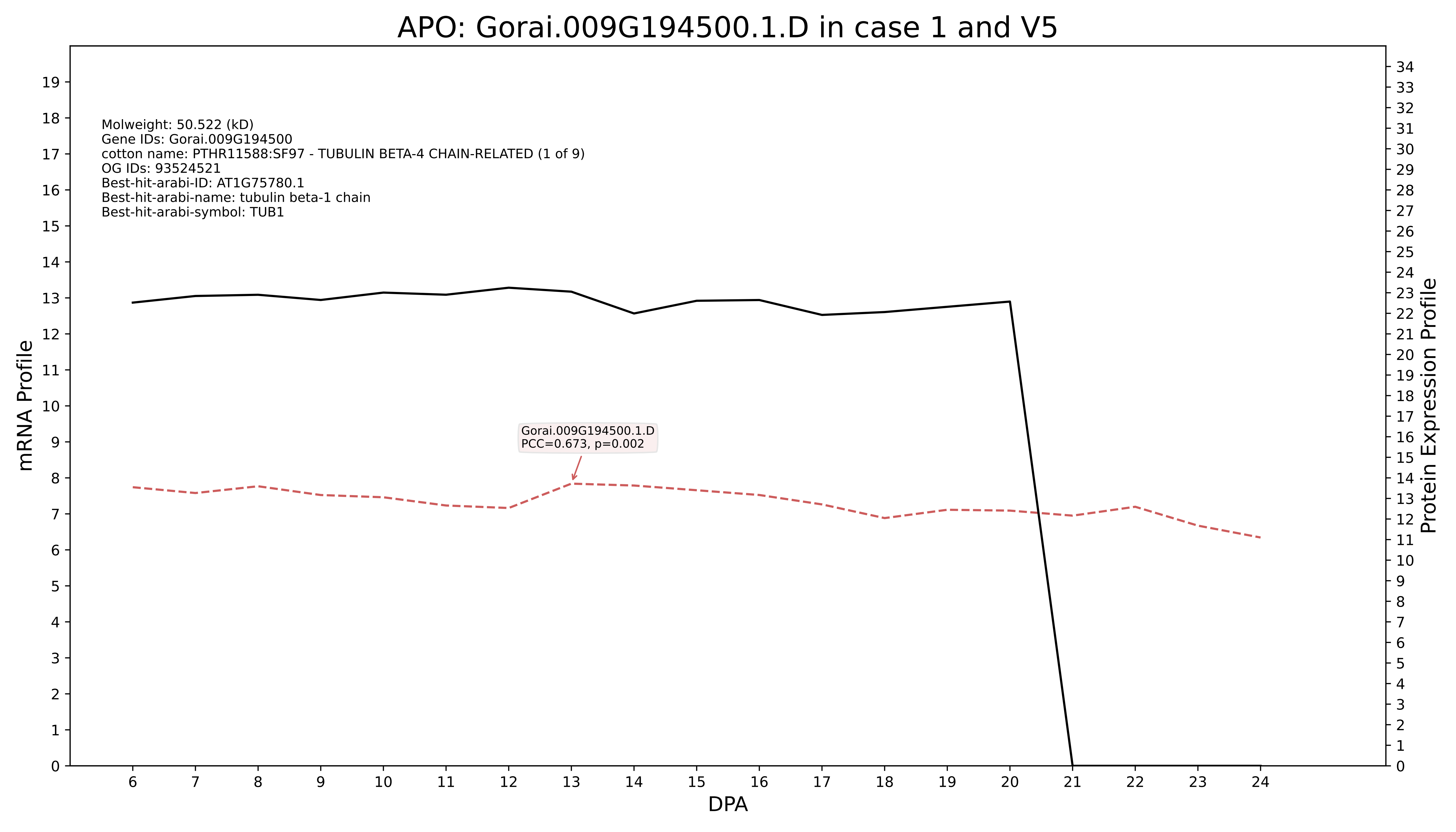
APO: Gorai.009G194500.1.D in case 1 and V5
Molweight: 50.522 (kD)
Gene IDs: Gorai.009G194500
cotton name: PTHR11588:SF97 - TUBULIN BETA-4 CHAIN-RELATED (1 of 9)
OG IDs: 93524521
Best-hit-arabi-ID: AT1G75780.1
Best-hit-arabi-name: tubulin beta-1 chain
Best-hit-arabi-symbol: TUB1
Gorai.009G194500.1.D
PCC=0.673, p=0.002
mRNA Profile
Protein Expression Profile
DPA

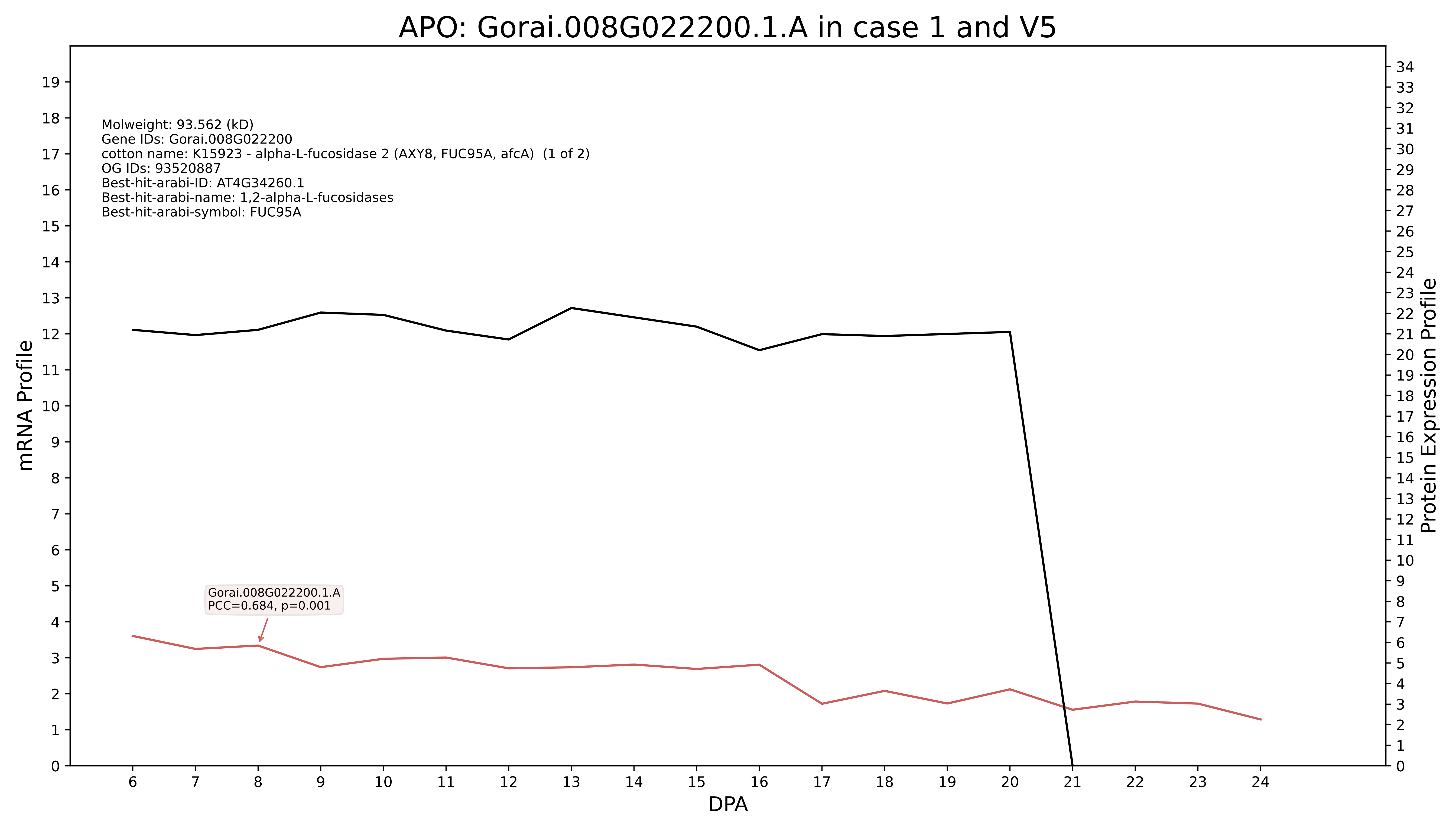

APO: Gorai.008G022200.1.A in case 1 and V5
Molweight: 93.562 (kD)
Gene IDs: Gorai.008G022200
cotton name: K15923 - alpha-L-fucosidase 2 (AXY8, FUC95A, afcA) (1 of 2)
OG IDs: 93520887
Best-hit-arabi-ID: AT4G34260.1
Best-hit-arabi-name: 1,2-alpha-L-fucosidases
Best-hit-arabi-symbol: FUC95A
Gorai.008G022200.1.A
PCC=0.684, p=0.001
mRNA Profile
Protein Expression Profile
DPA

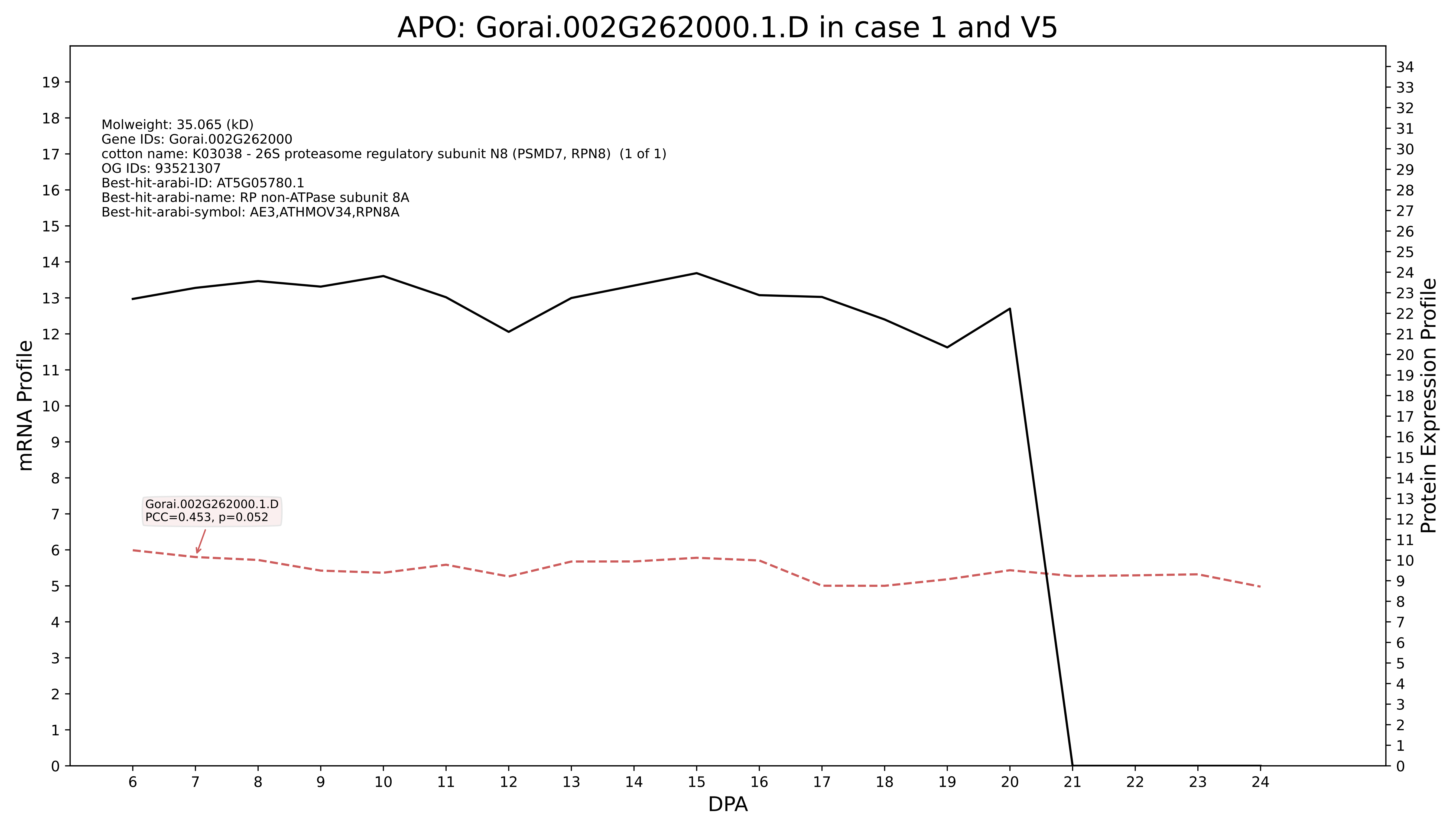

APO: Gorai.002G262000.1.D in case 1 and V5
Molweight: 35.065 (kD)
Gene IDs: Gorai.002G262000
cotton name: K03038 - 26S proteasome regulatory subunit N8 (PSMD7, RPN8) (1 of 1)
OG IDs: 93521307
Best-hit-arabi-ID: AT5G05780.1
Best-hit-arabi-name: RP non-ATPase subunit 8A
Best-hit-arabi-symbol: AE3,ATHMOV34,RPN8A
Gorai.002G262000.1.D
PCC=0.453, p=0.052
mRNA Profile
Protein Expression Profile
DPA

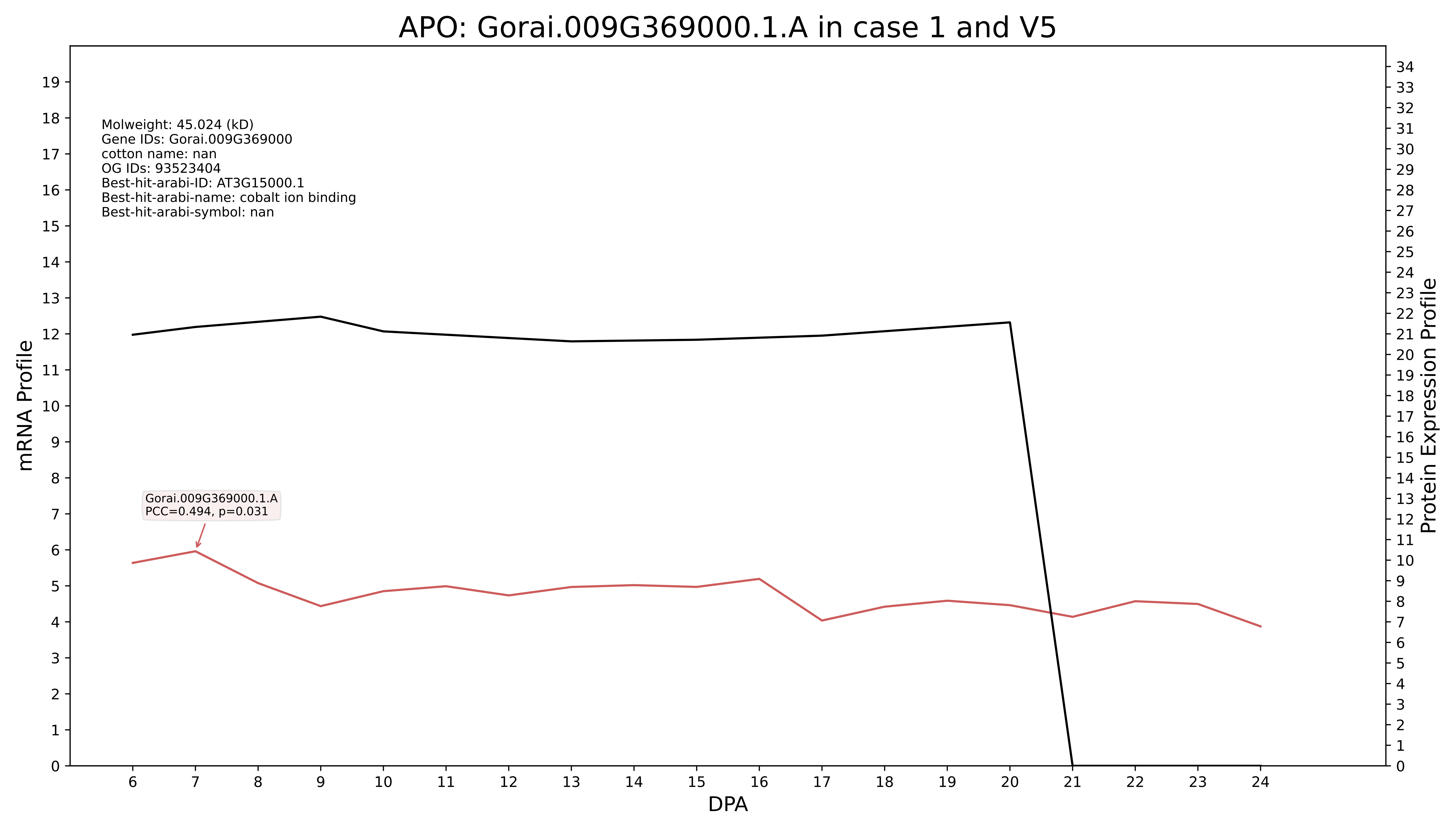
APO: Gorai.009G369000.1.A in case 1 and V5
Molweight: 45.024 (kD)
Gene IDs: Gorai.009G369000
cotton name: nan
OG IDs: 93523404
Best-hit-arabi-ID: AT3G15000.1
Best-hit-arabi-name: cobalt ion binding
Best-hit-arabi-symbol: nan
Gorai.009G369000.1.A
PCC=0.494, p=0.031
mRNA Profile
Protein Expression Profile
DPA
0
1
2
3
4
5
6
7
8
9
10
11
12
13
14
15
16
17
18
19
20
21
22
23
24
25
26
27
28
29
30
31
32
33
34

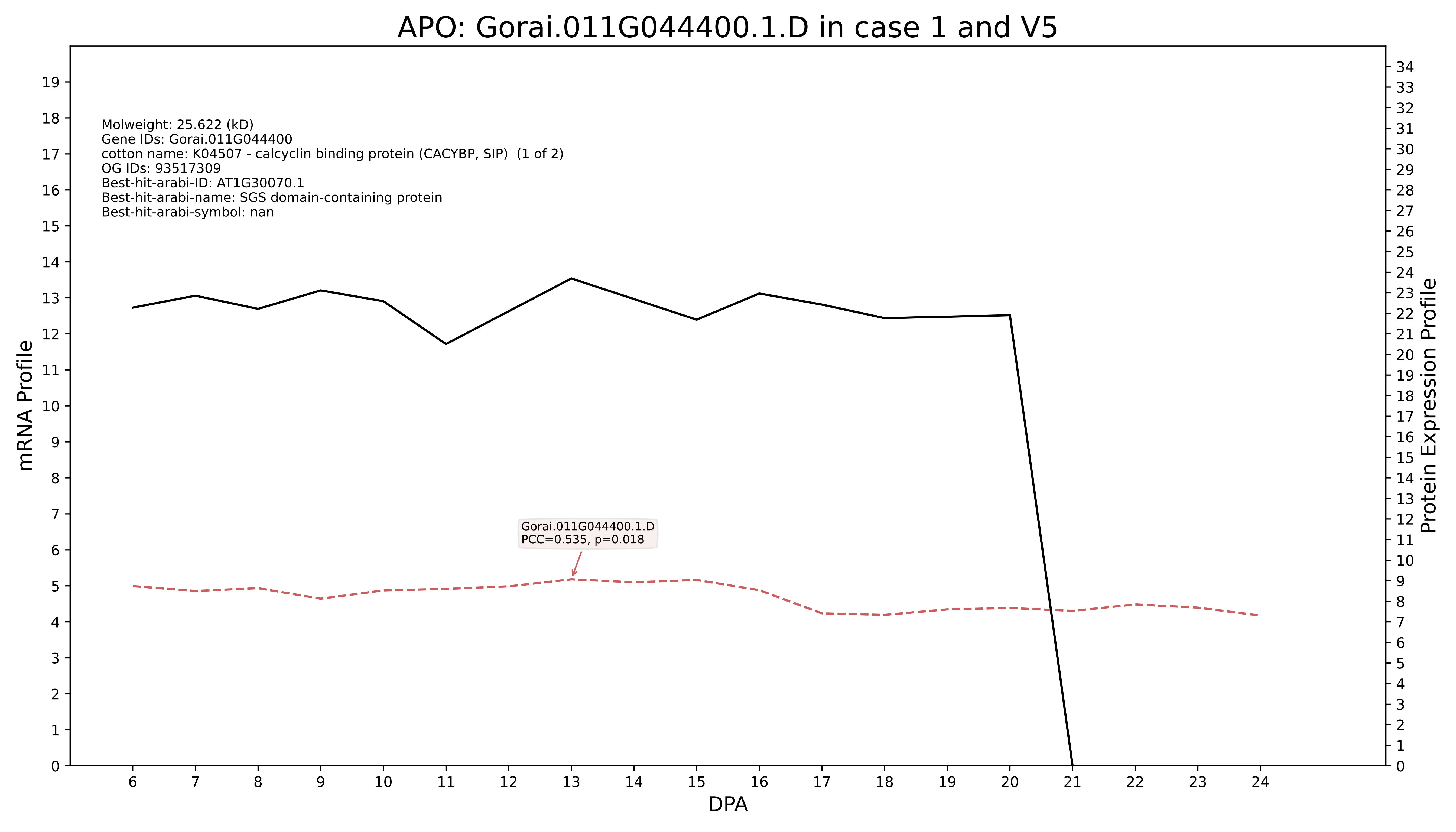
APO: Gorai.011G044400.1.D in case 1 and V5
Molweight: 25.622 (kD)
Gene IDs: Gorai.011G044400
cotton name: K04507 - calcyclin binding protein (CACYBP, SIP) (1 of 2)
OG IDs: 93517309
Best-hit-arabi-ID: AT1G30070.1
Best-hit-arabi-name: SGS domain-containing protein
Best-hit-arabi-symbol: nan
Gorai.011G044400.1.D
PCC=0.535, p=0.018
mRNA Profile
Protein Expression Profile
DPA

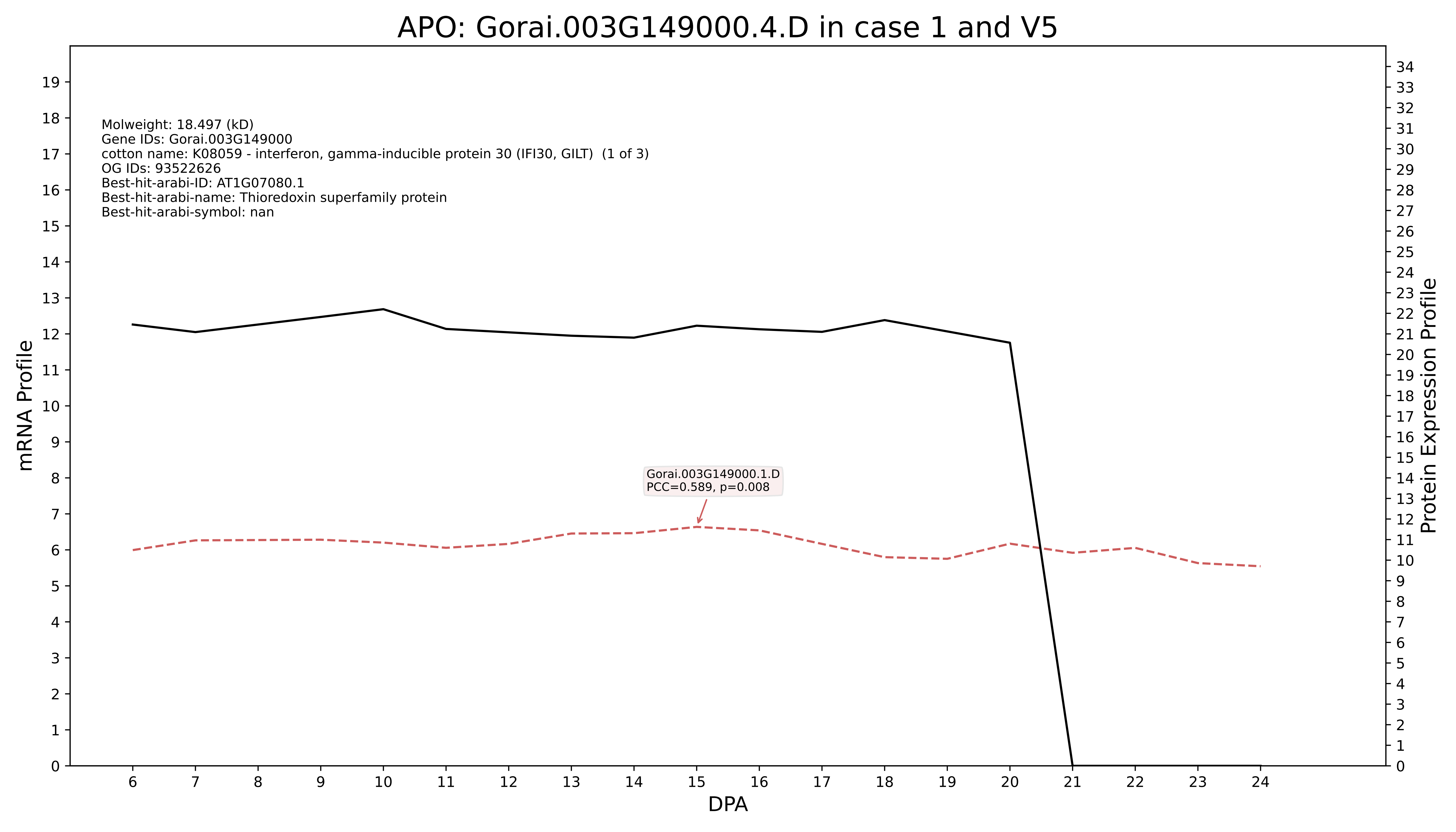

APO: Gorai.003G149000.4.D in case 1 and V5
Molweight: 18.497 (kD)
Gene IDs: Gorai.003G149000
cotton name: K08059 - interferon, gamma-inducible protein 30 (IFI30, GILT) (1 of 3)
OG IDs: 93522626
Best-hit-arabi-ID: AT1G07080.1
Best-hit-arabi-name: Thioredoxin superfamily protein
Best-hit-arabi-symbol: nan
Gorai.003G149000.1.D
PCC=0.589, p=0.008
mRNA Profile
Protein Expression Profile
DPA

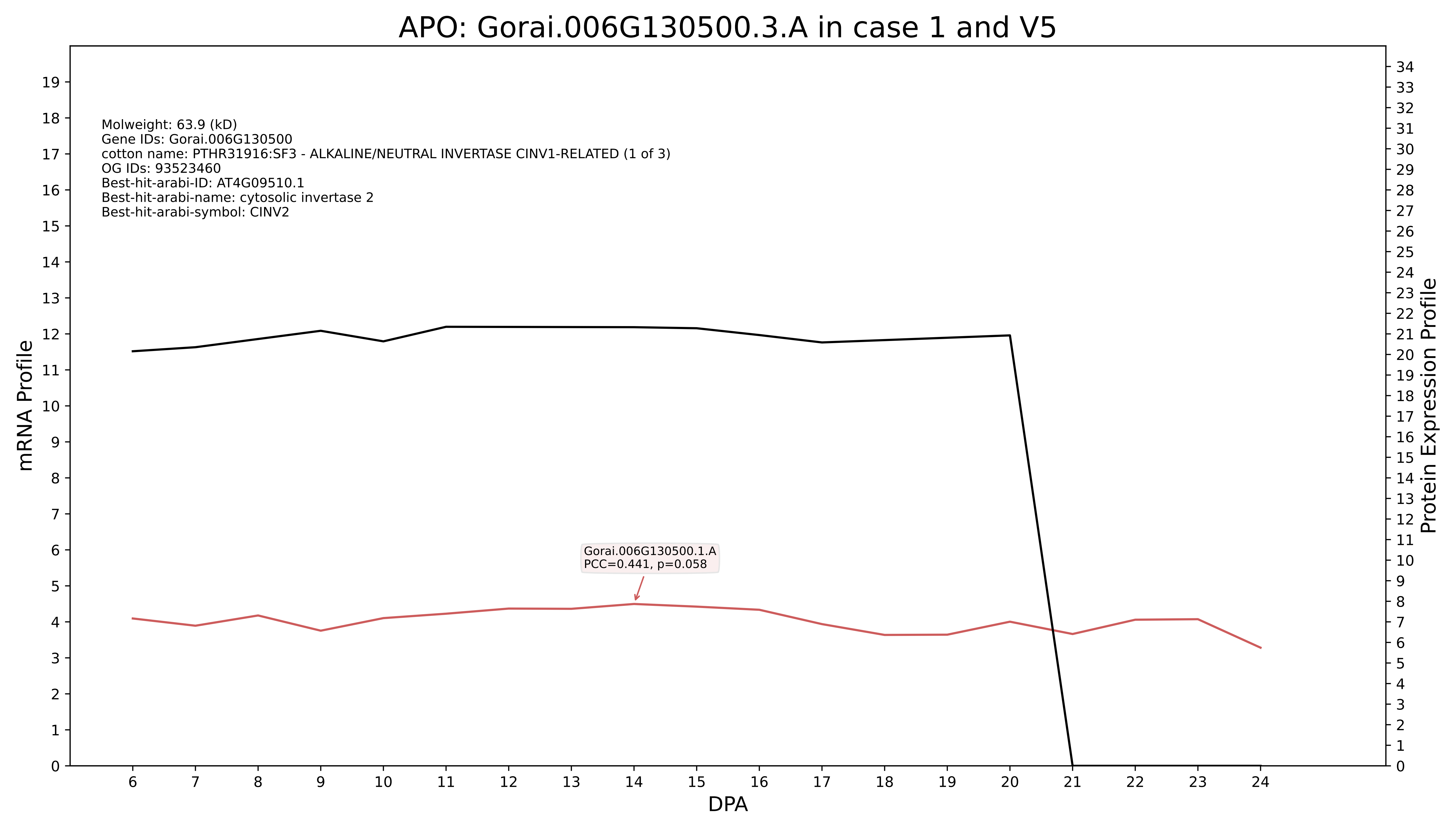

APO: Gorai.006G130500.3.A in case 1 and V5
Molweight: 63.9 (kD)
Gene IDs: Gorai.006G130500
cotton name: PTHR31916:SF3 - ALKALINE/NEUTRAL INVERTASE CINV1-RELATED (1 of 3)
OG IDs: 93523460
Best-hit-arabi-ID: AT4G09510.1
Best-hit-arabi-name: cytosolic invertase 2
Best-hit-arabi-symbol: CINV2
Gorai.006G130500.1.A
PCC=0.441, p=0.058
mRNA Profile
Protein Expression Profile
DPA

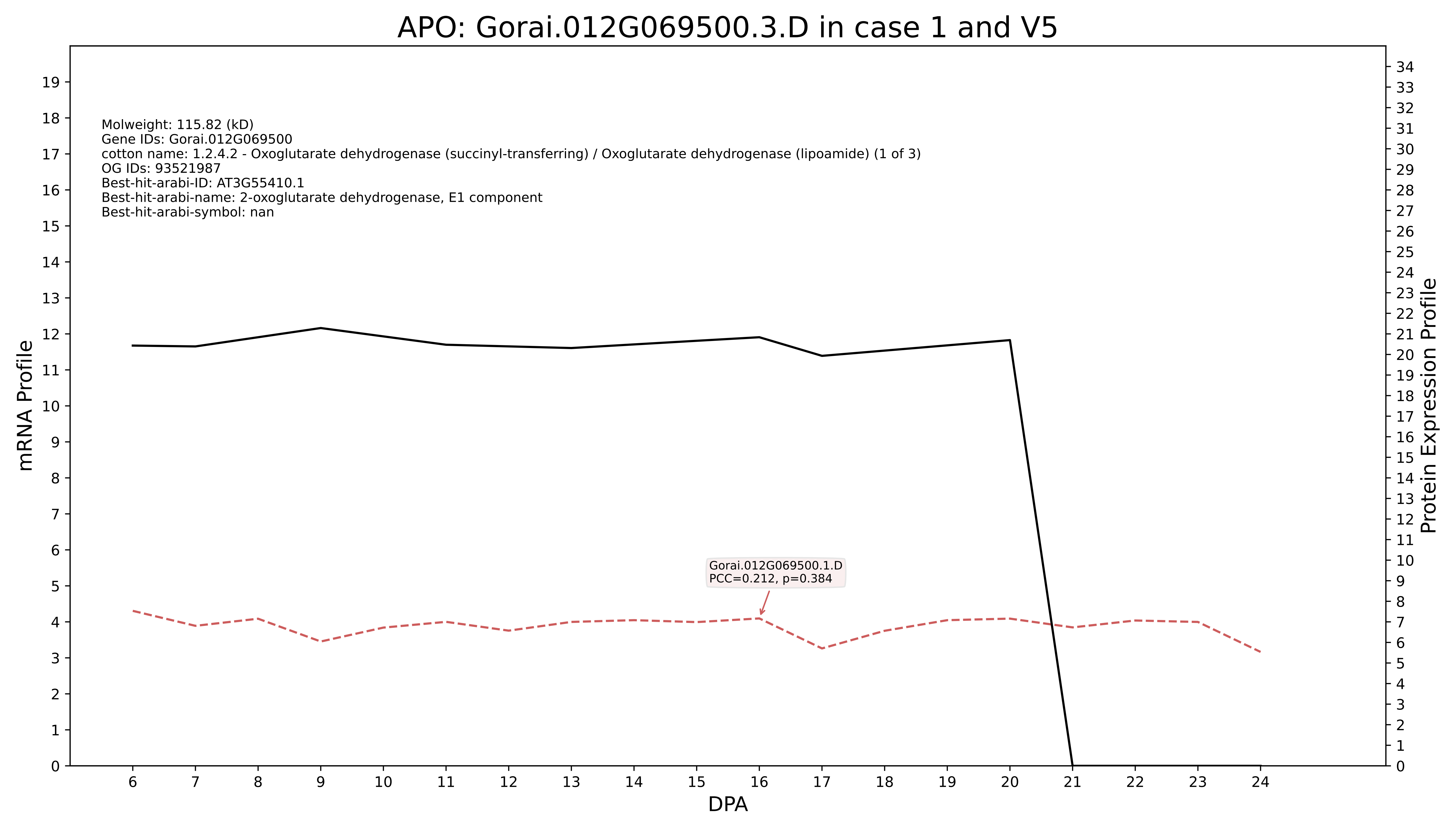
APO: Gorai.012G069500.3.D in case 1 and V5
Molweight: 115.82 (kD)
Gene IDs: Gorai.012G069500
cotton name: 1.2.4.2 - Oxoglutarate dehydrogenase (succinyl-transferring) / Oxoglutarate dehydrogenase (lipoamide) (1 of 3)
OG IDs: 93521987
Best-hit-arabi-ID: AT3G55410.1
Best-hit-arabi-name: 2-oxoglutarate dehydrogenase, E1 component
Best-hit-arabi-symbol: nan
Gorai.012G069500.1.D
PCC=0.212, p=0.384
mRNA Profile
Protein Expression Profile
DPA

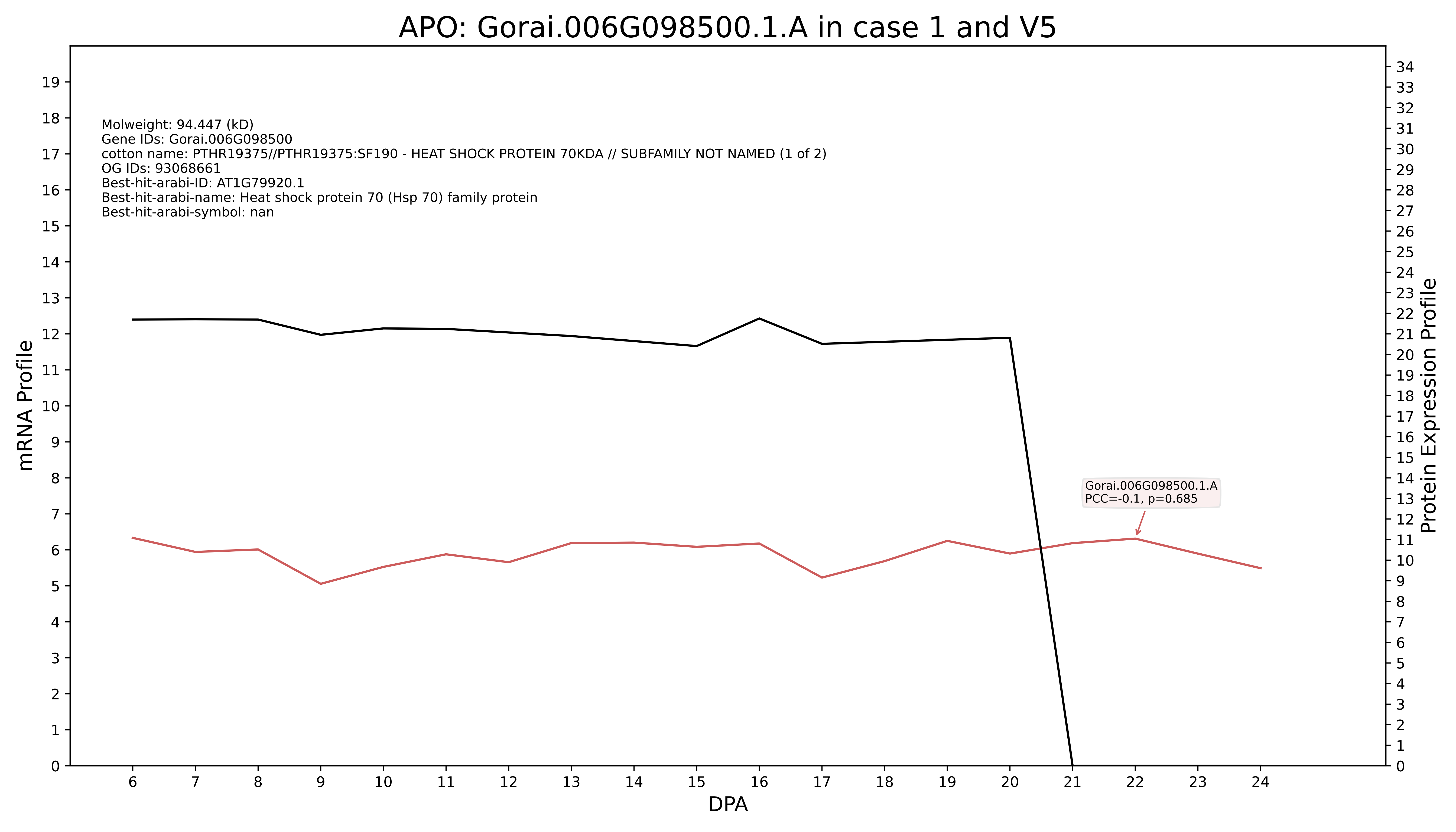
APO: Gorai.006G098500.1.A in case 1 and V5
Molweight: 94.447 (kD)
Gene IDs: Gorai.006G098500
cotton name: PTHR19375//PTHR19375:SF190 - HEAT SHOCK PROTEIN 70KDA // SUBFAMILY NOT NAMED (1 of 2)
OG IDs: 93068661
Best-hit-arabi-ID: AT1G79920.1
Best-hit-arabi-name: Heat shock protein 70 (Hsp 70) family protein
Best-hit-arabi-symbol: nan
Gorai.006G098500.1.A
PCC=-0.1, p=0.685
mRNA Profile
Protein Expression Profile
DPA

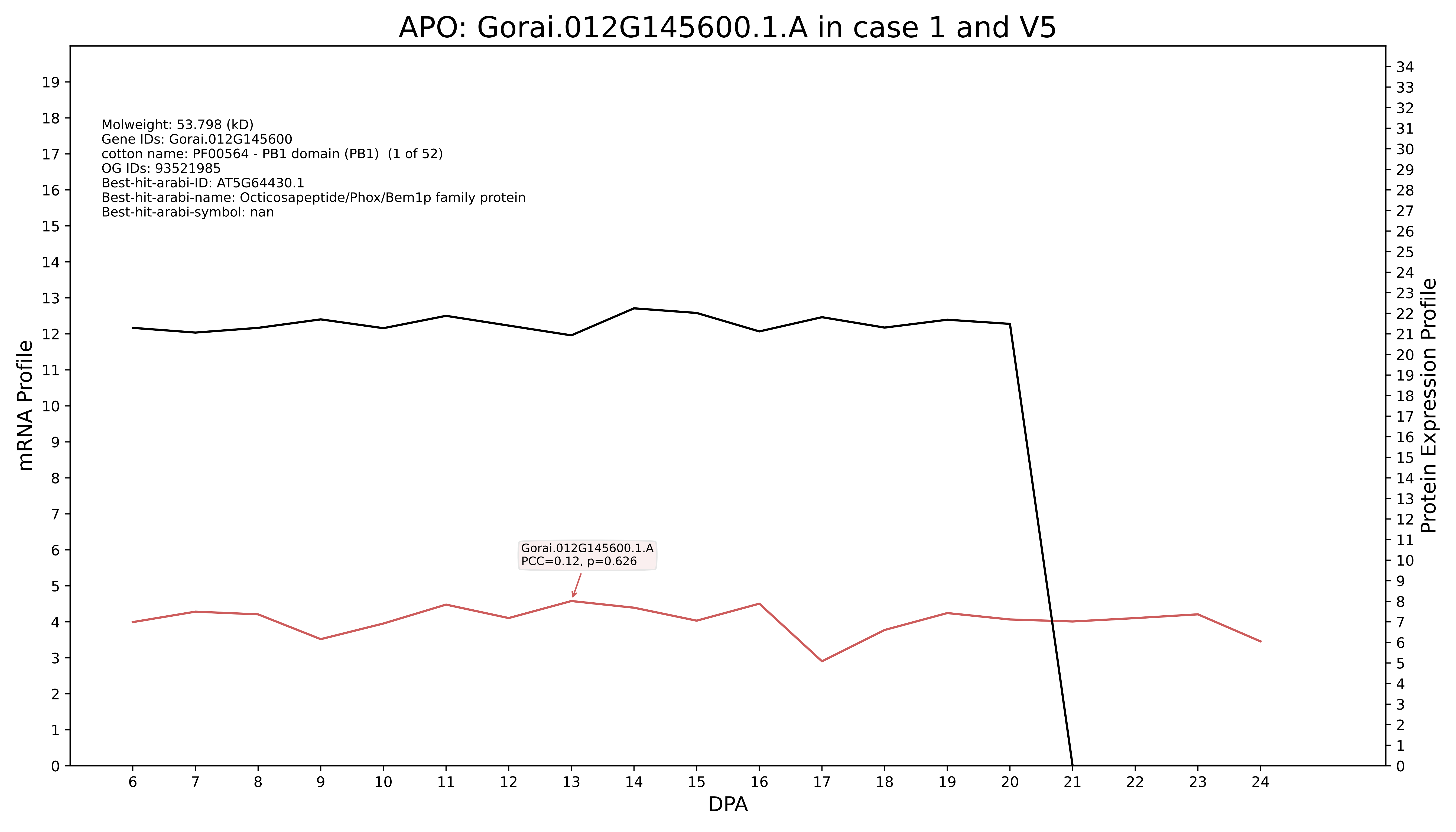

APO: Gorai.012G145600.1.A in case 1 and V5
Molweight: 53.798 (kD)
Gene IDs: Gorai.012G145600
cotton name: PF00564 - PB1 domain (PB1) (1 of 52)
OG IDs: 93521985
Best-hit-arabi-ID: AT5G64430.1
Best-hit-arabi-name: Octicosapeptide/Phox/Bem1p family protein
Best-hit-arabi-symbol: nan
Gorai.012G145600.1.A
PCC=0.12, p=0.626
mRNA Profile
Protein Expression Profile
DPA

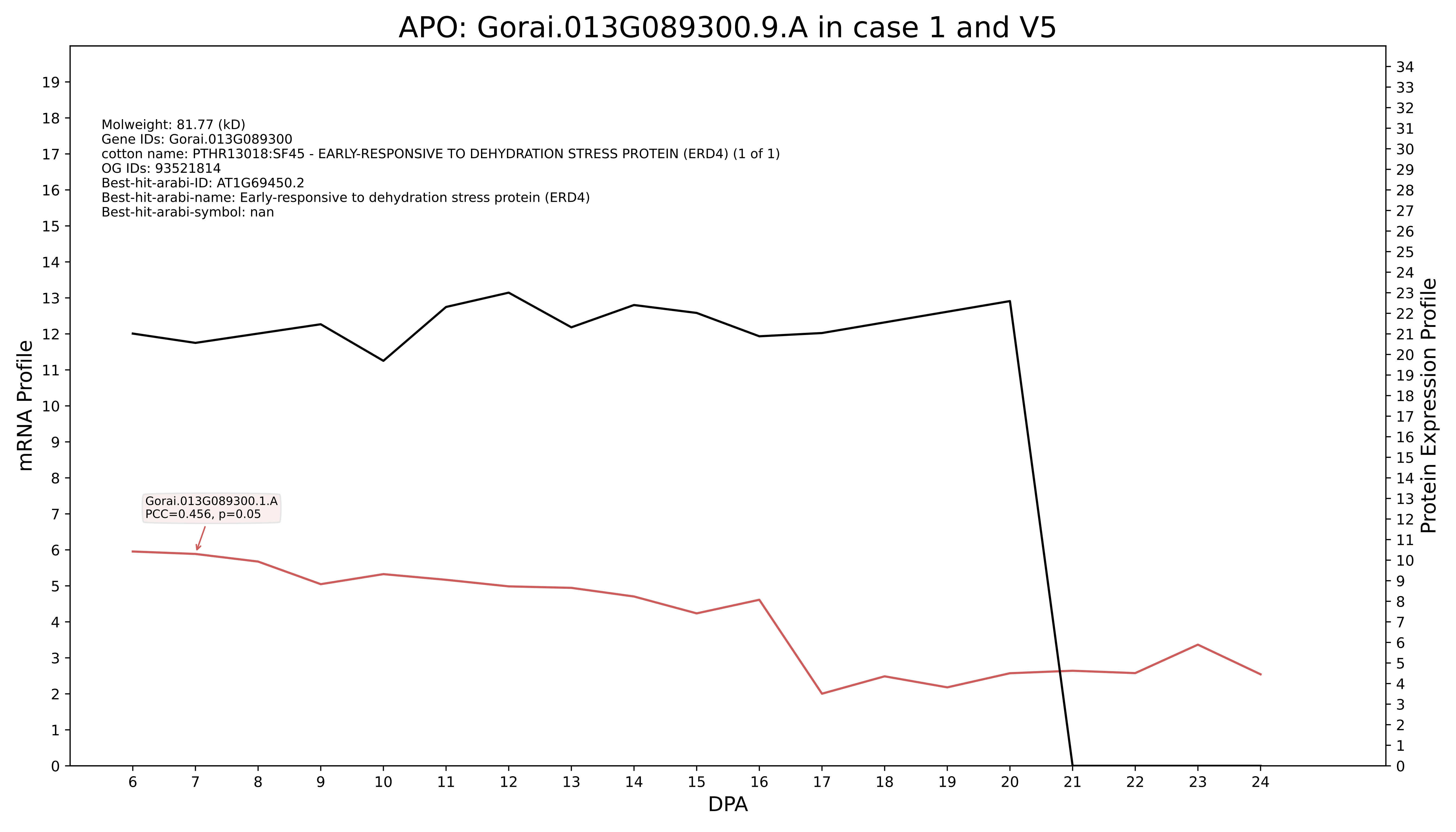
APO: Gorai.013G089300.9.A in case 1 and V5
Molweight: 81.77 (kD)
Gene IDs: Gorai.013G089300
cotton name: PTHR13018:SF45 - EARLY-RESPONSIVE TO DEHYDRATION STRESS PROTEIN (ERD4) (1 of 1)
OG IDs: 93521814
Best-hit-arabi-ID: AT1G69450.2
Best-hit-arabi-name: Early-responsive to dehydration stress protein (ERD4)
Best-hit-arabi-symbol: nan
Gorai.013G089300.1.A
PCC=0.456, p=0.05
mRNA Profile
Protein Expression Profile
DPA

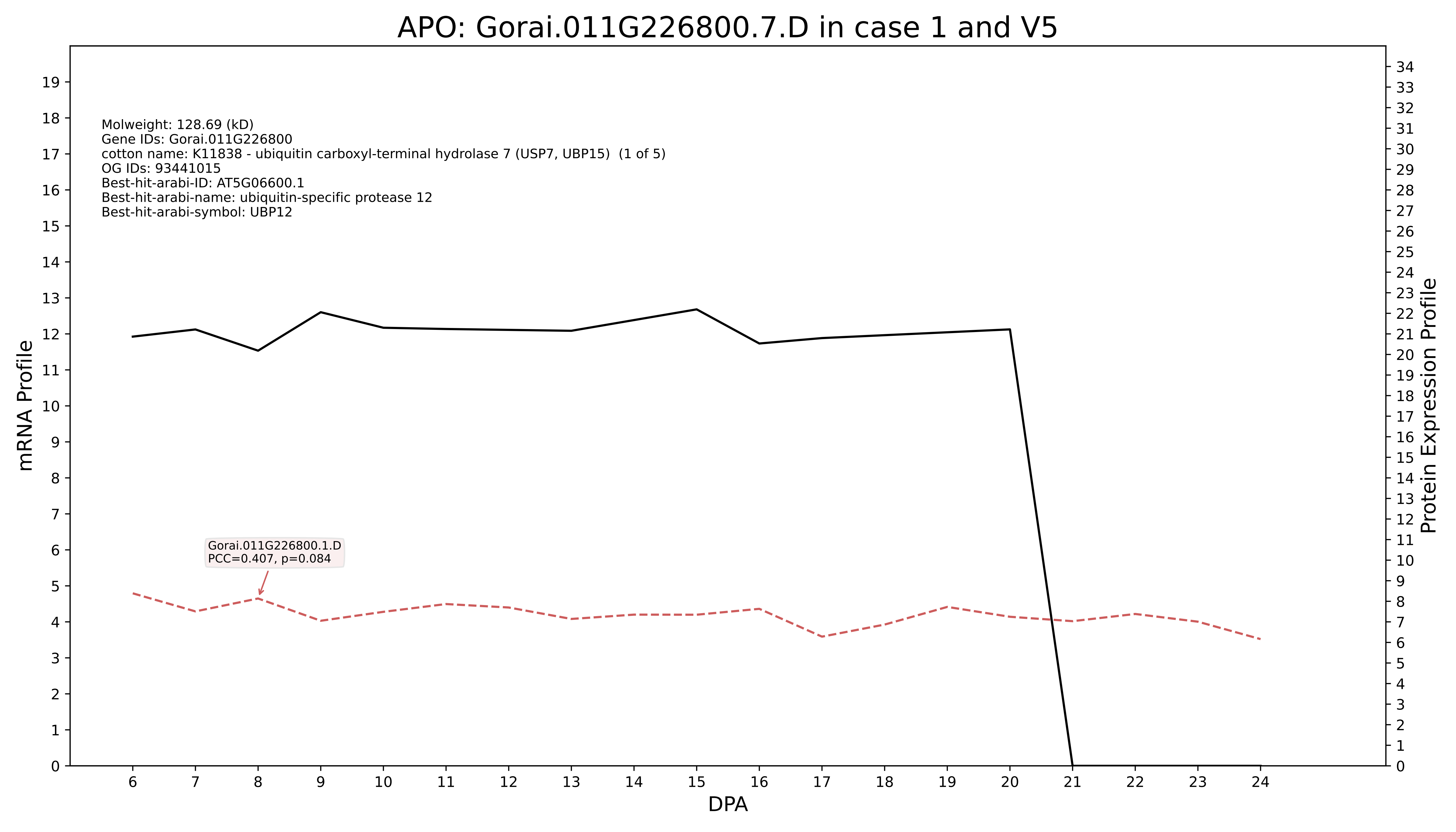
APO: Gorai.011G226800.7.D in case 1 and V5
Molweight: 128.69 (kD)
Gene IDs: Gorai.011G226800
cotton name: K11838 - ubiquitin carboxyl-terminal hydrolase 7 (USP7, UBP15) (1 of 5)
OG IDs: 93441015
Best-hit-arabi-ID: AT5G06600.1
Best-hit-arabi-name: ubiquitin-specific protease 12
Best-hit-arabi-symbol: UBP12
Gorai.011G226800.1.D
PCC=0.407, p=0.084
mRNA Profile
Protein Expression Profile
DPA

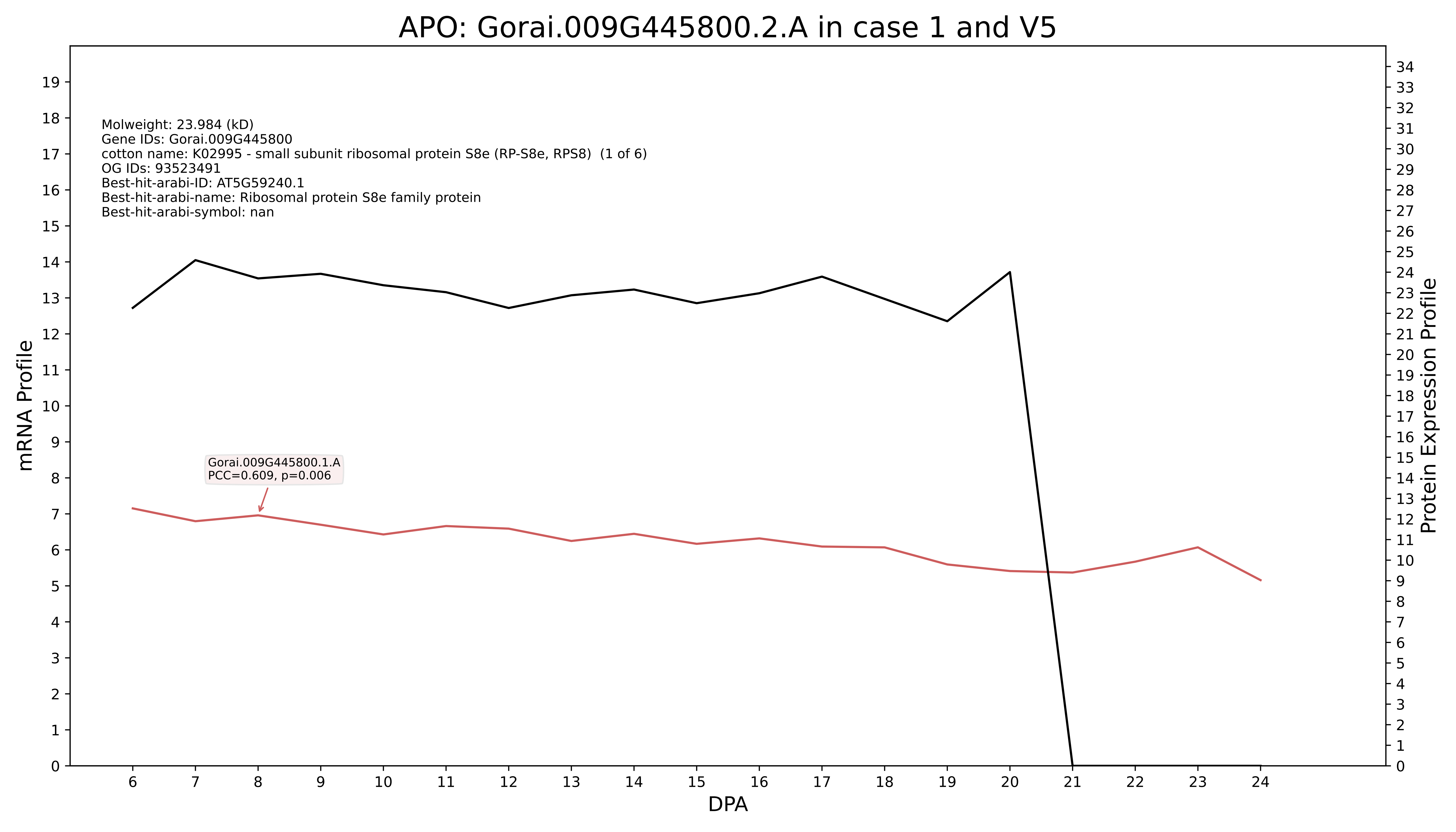

APO: Gorai.009G445800.2.A in case 1 and V5
Molweight: 23.984 (kD)
Gene IDs: Gorai.009G445800
cotton name: K02995 - small subunit ribosomal protein S8e (RP-S8e, RPS8) (1 of 6)
OG IDs: 93523491
Best-hit-arabi-ID: AT5G59240.1
Best-hit-arabi-name: Ribosomal protein S8e family protein
Best-hit-arabi-symbol: nan
Gorai.009G445800.1.A
PCC=0.609, p=0.006
mRNA Profile
Protein Expression Profile
DPA

APO: Gorai.007G014400.1.A in case 1 and V5

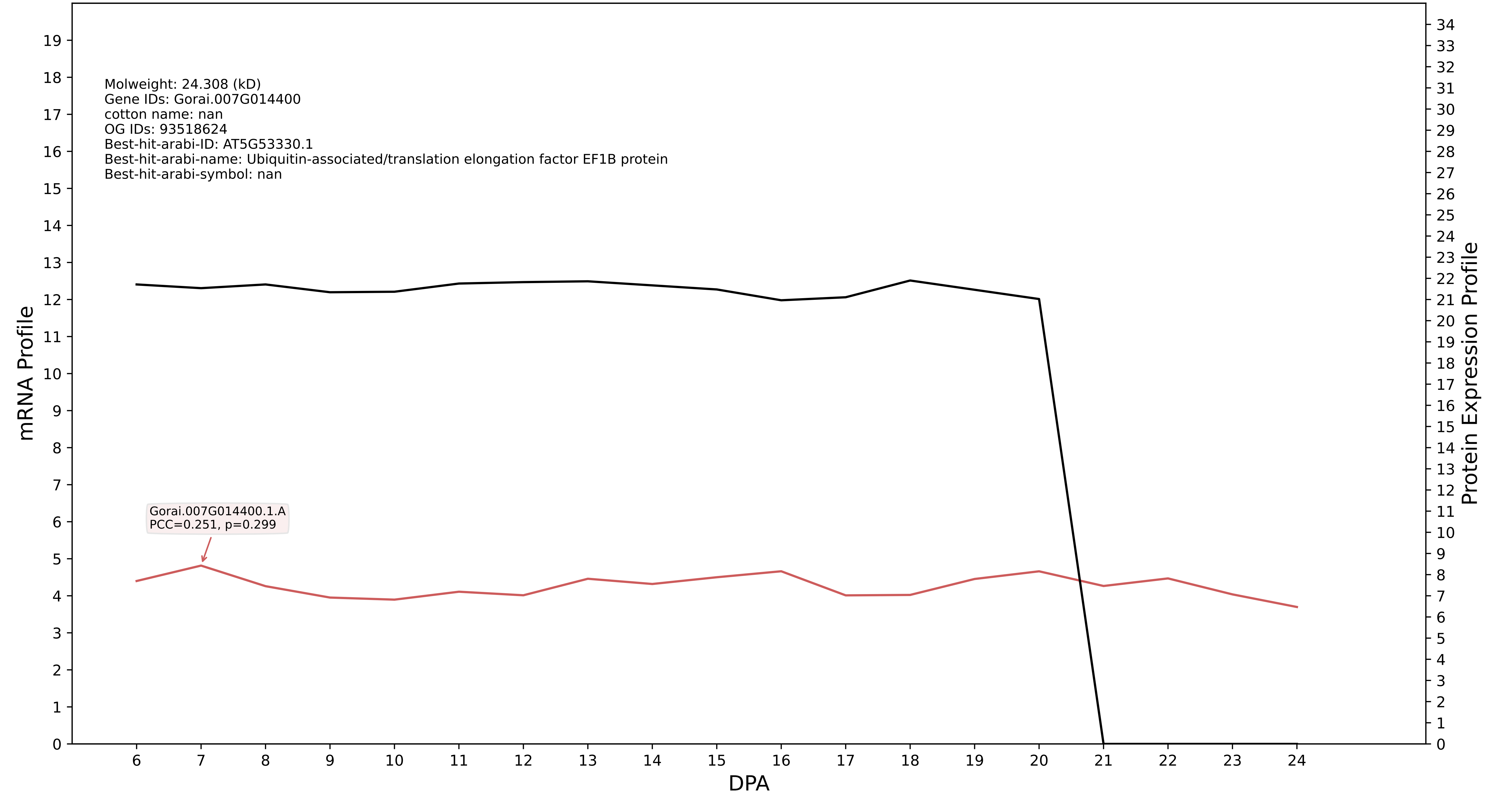

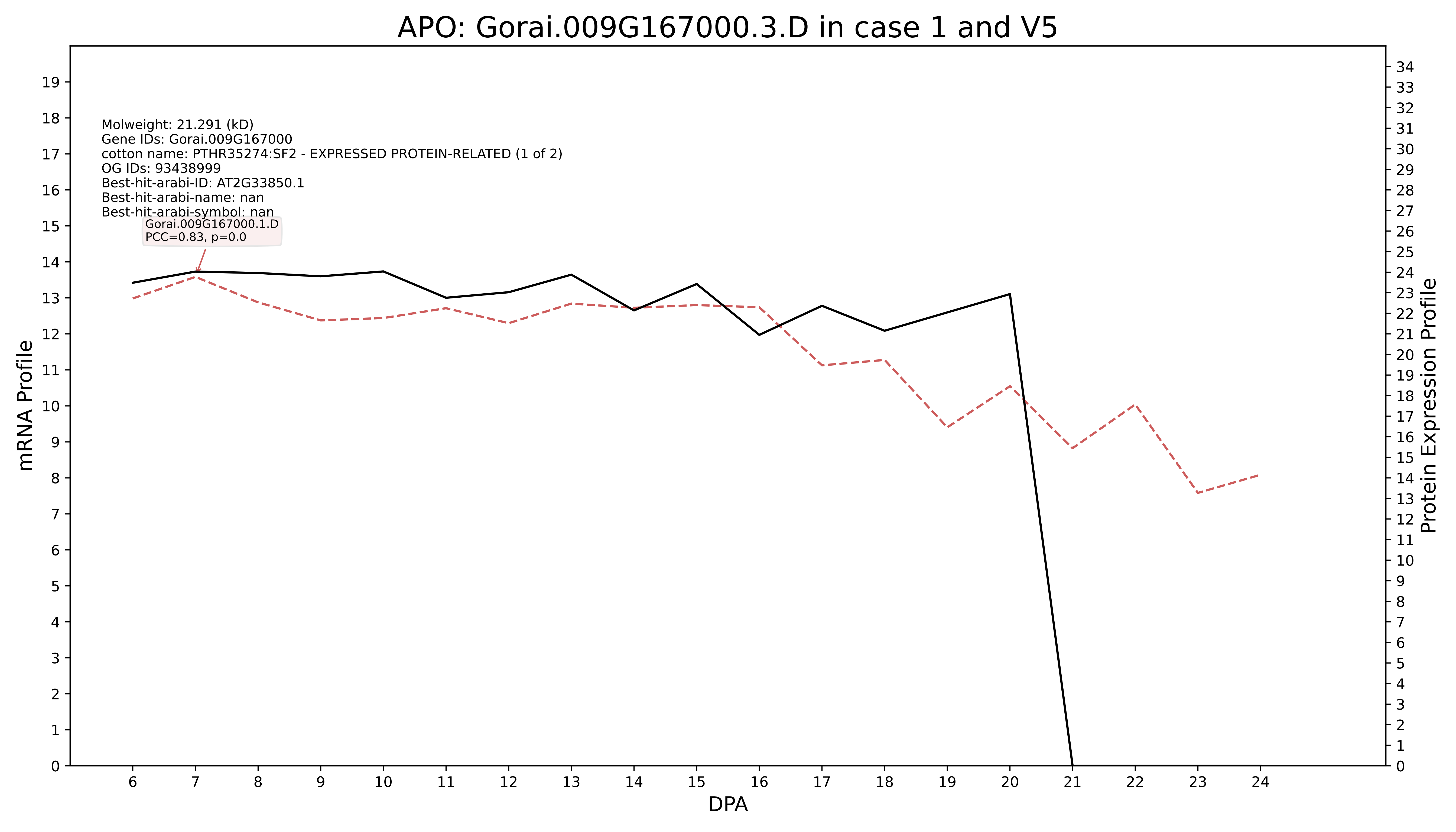
APO: Gorai.009G167000.3.D in case 1 and V5
Molweight: 21.291 (kD)
Gene IDs: Gorai.009G167000
cotton name: PTHR35274:SF2 - EXPRESSED PROTEIN-RELATED (1 of 2)
OG IDs: 93438999
Best-hit-arabi-ID: AT2G33850.1
Best-hit-arabi-name: nan
Best-hit-arabi-symbol: nan
Gorai.009G167000.1.D
PCC=0.83, p=0.0
mRNA Profile
Protein Expression Profile
DPA

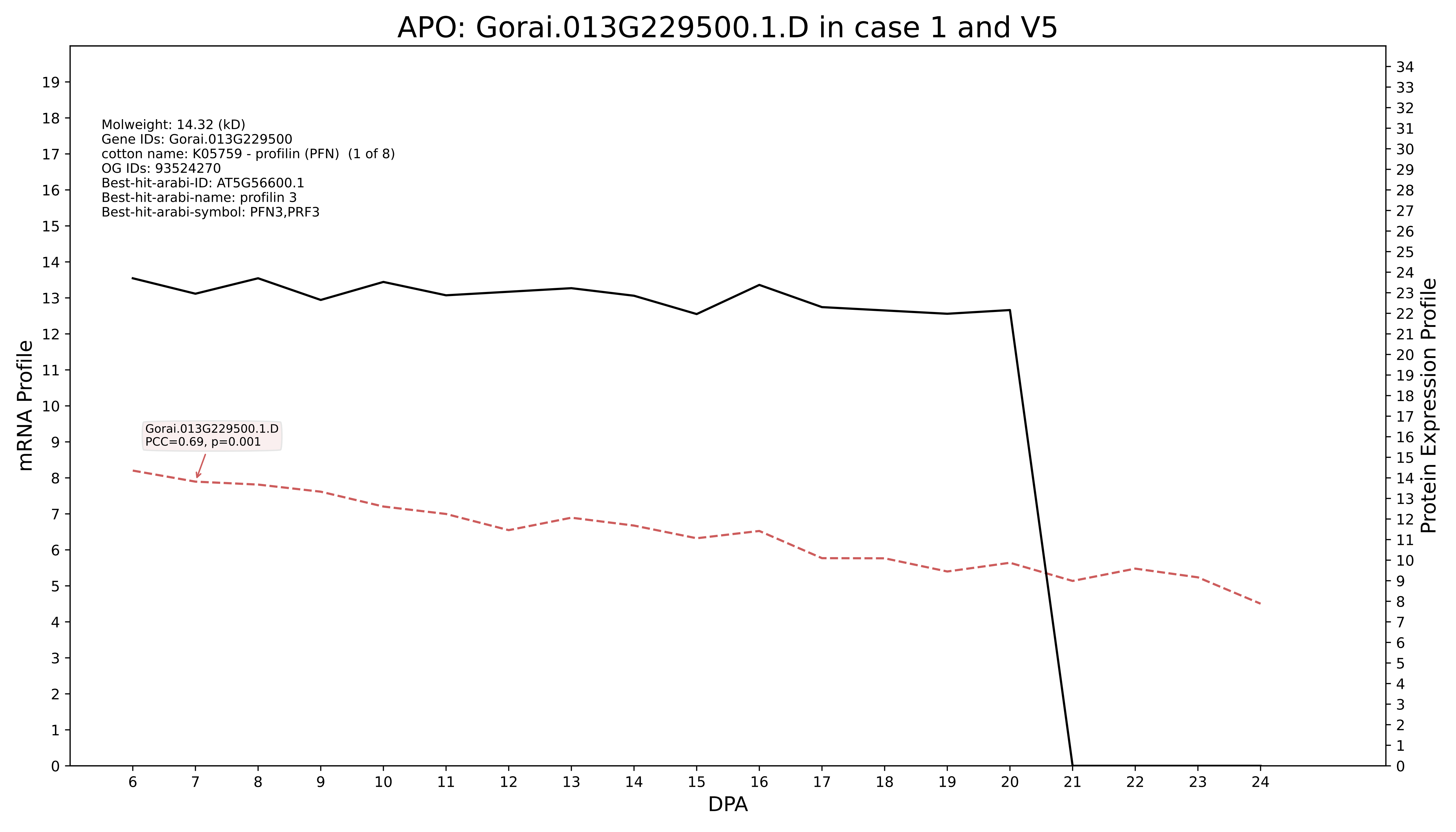
APO: Gorai.013G229500.1.D in case 1 and V5
Molweight: 14.32 (kD)
Gene IDs: Gorai.013G229500
cotton name: K05759 - profilin (PFN) (1 of 8)
OG IDs: 93524270
Best-hit-arabi-ID: AT5G56600.1
Best-hit-arabi-name: profilin 3
Best-hit-arabi-symbol: PFN3,PRF3
Gorai.013G229500.1.D
PCC=0.69, p=0.001
mRNA Profile
Protein Expression Profile
DPA

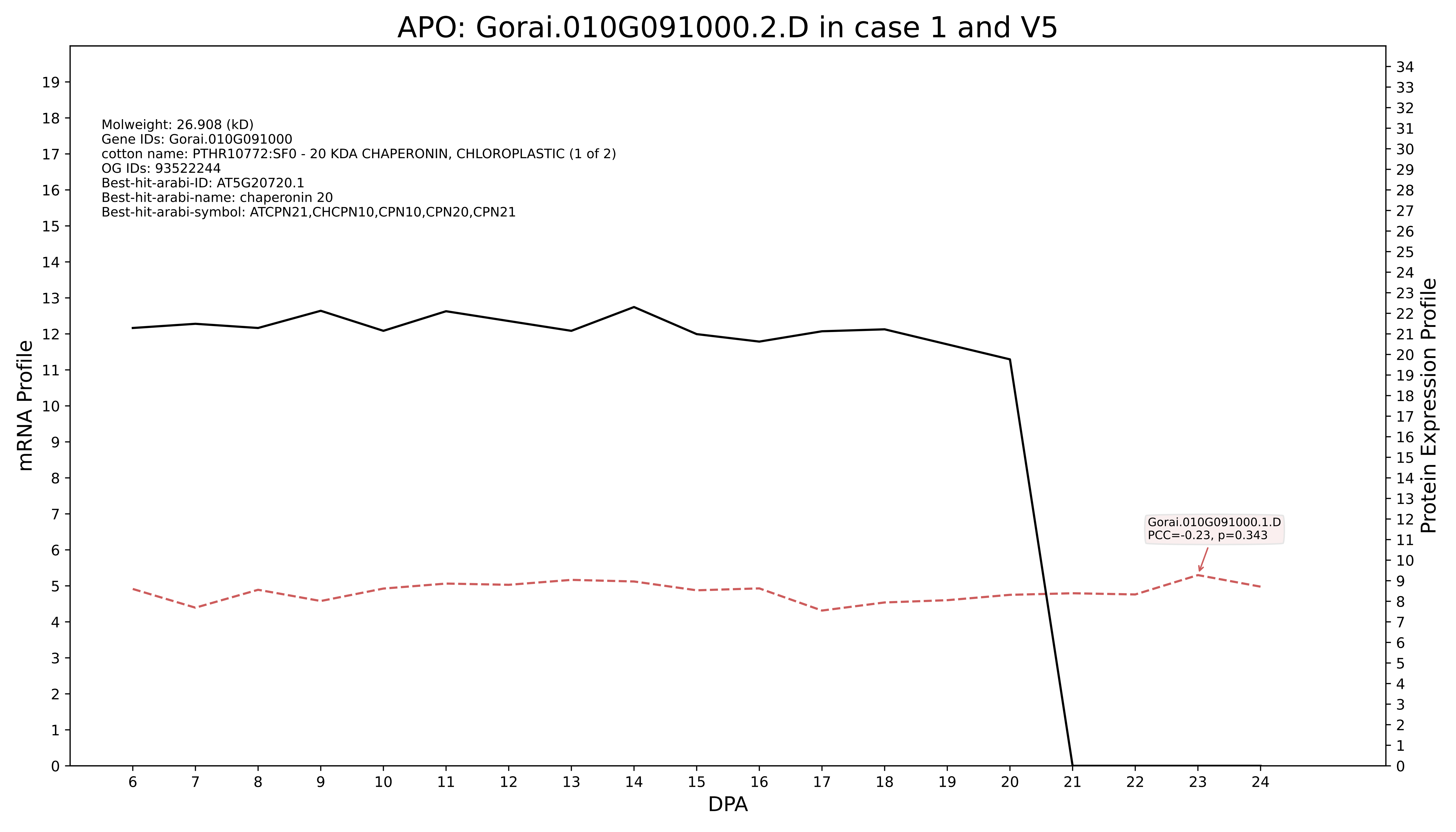
APO: Gorai.010G091000.2.D in case 1 and V5
Molweight: 26.908 (kD)
Gene IDs: Gorai.010G091000
cotton name: PTHR10772:SF0 - 20 KDA CHAPERONIN, CHLOROPLASTIC (1 of 2)
OG IDs: 93522244
Best-hit-arabi-ID: AT5G20720.1
Best-hit-arabi-name: chaperonin 20
Best-hit-arabi-symbol: ATCPN21,CHCPN10,CPN10,CPN20,CPN21
Gorai.010G091000.1.D
PCC=-0.23, p=0.343
mRNA Profile
Protein Expression Profile
DPA

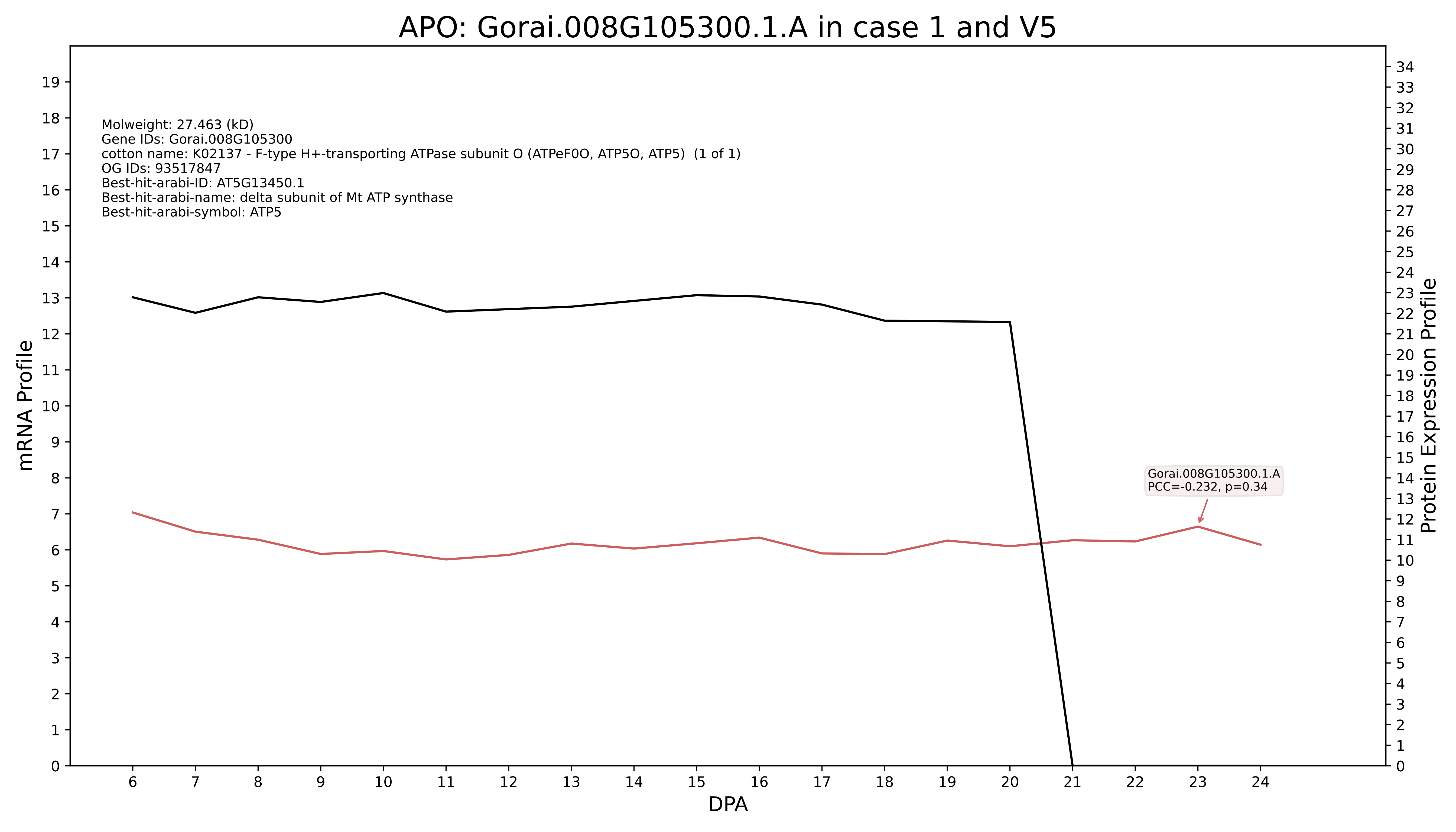
APO: Gorai.008G105300.1.A in case 1 and V5
Molweight: 27.463 (kD)
Gene IDs: Gorai.008G105300
cotton name: K02137 - F-type H+-transporting ATPase subunit O (ATPeF0O, ATP5O, ATP5) (1 of 1)
OG IDs: 93517847
Best-hit-arabi-ID: AT5G13450.1
Best-hit-arabi-name: delta subunit of Mt ATP synthase
Best-hit-arabi-symbol: ATP5
Gorai.008G105300.1.A
PCC=-0.232, p=0.34
mRNA Profile
Protein Expression Profile
DPA

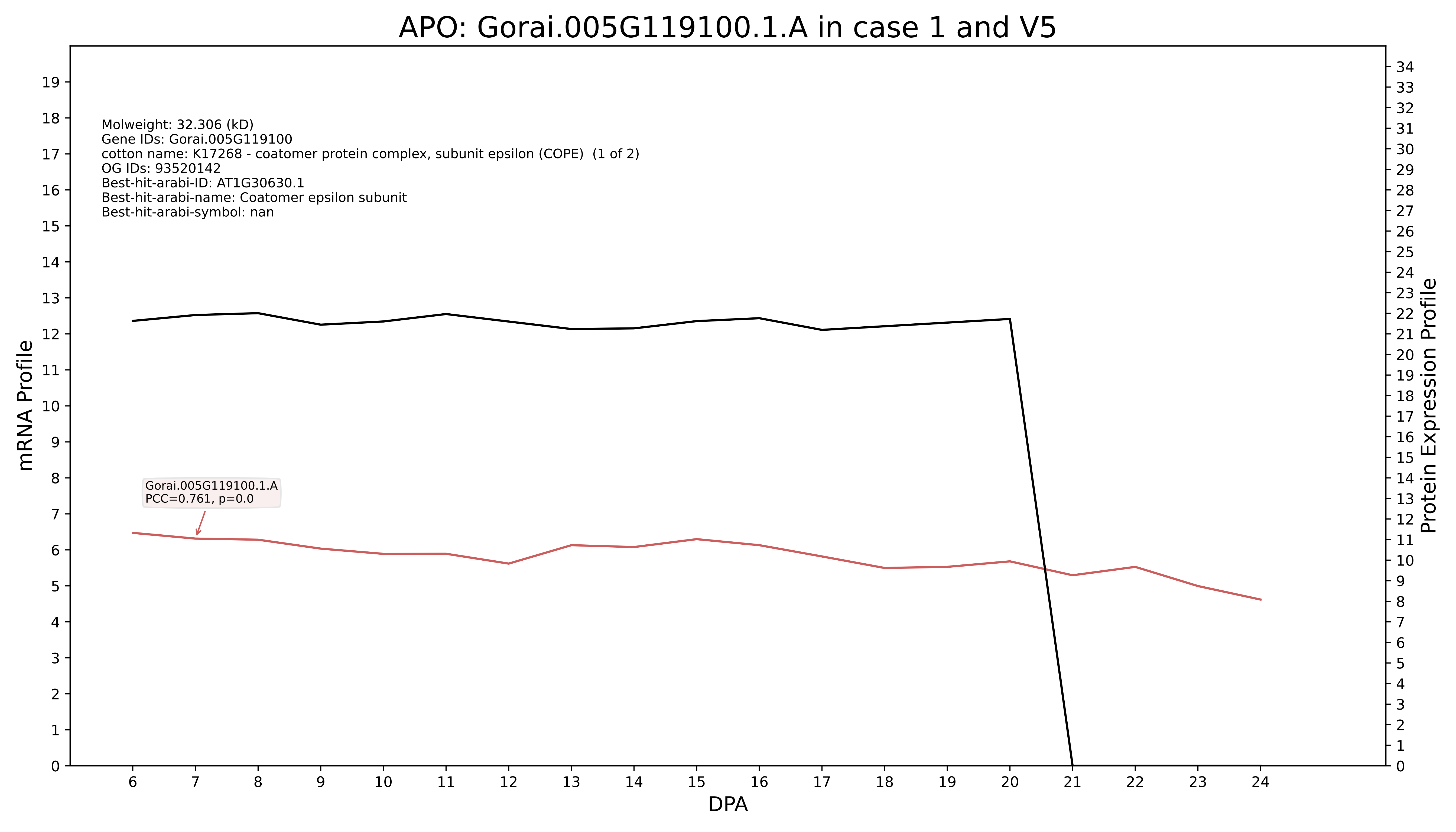

APO: Gorai.005G119100.1.A in case 1 and V5
Molweight: 32.306 (kD)
Gene IDs: Gorai.005G119100
cotton name: K17268 - coatomer protein complex, subunit epsilon (COPE) (1 of 2)
OG IDs: 93520142
Best-hit-arabi-ID: AT1G30630.1
Best-hit-arabi-name: Coatomer epsilon subunit
Best-hit-arabi-symbol: nan
Gorai.005G119100.1.A
PCC=0.761, p=0.0
mRNA Profile
Protein Expression Profile
DPA

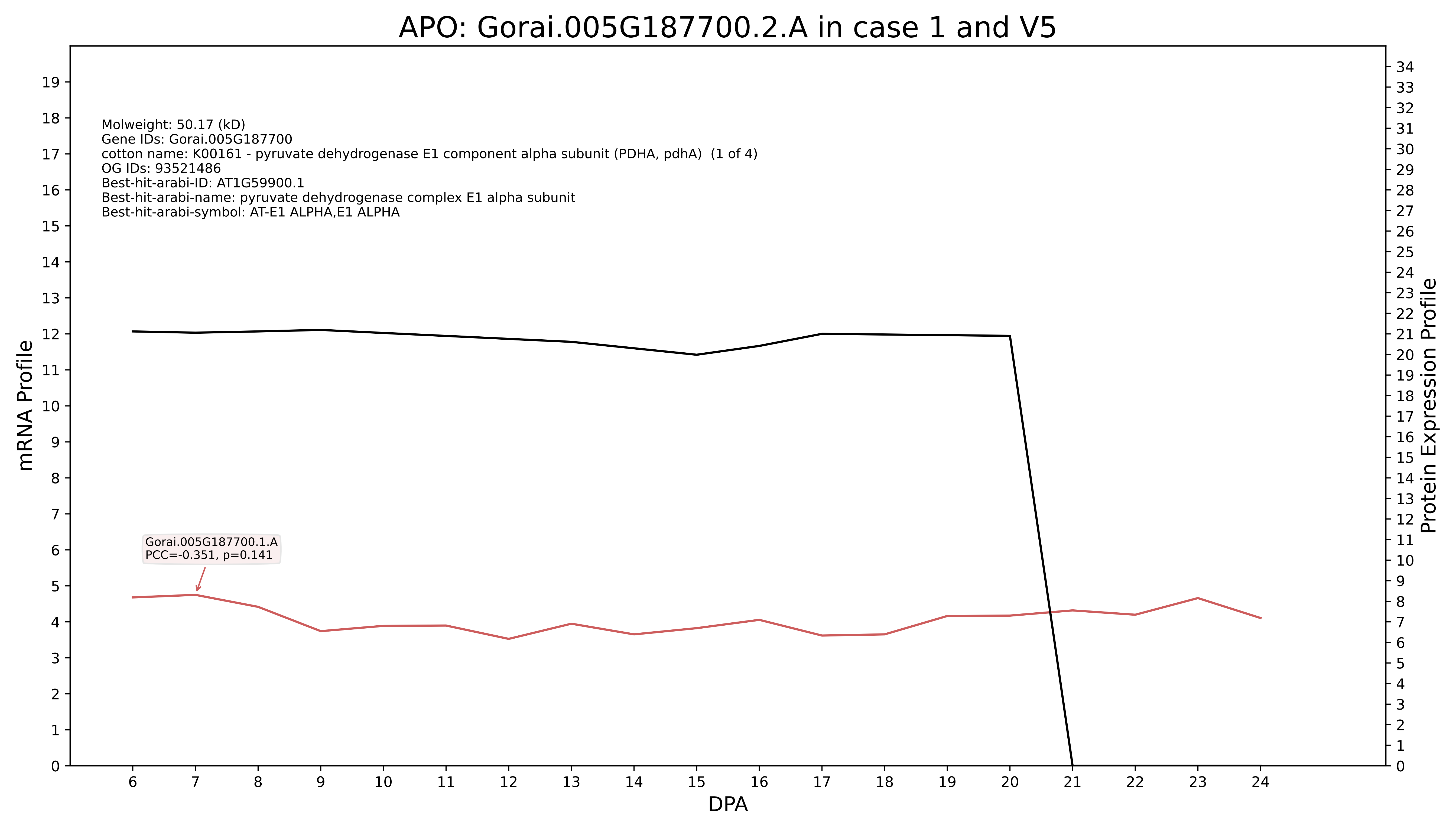

APO: Gorai.005G187700.2.A in case 1 and V5
Molweight: 50.17 (kD)
Gene IDs: Gorai.005G187700
cotton name: K00161 - pyruvate dehydrogenase E1 component alpha subunit (PDHA, pdhA) (1 of 4)
OG IDs: 93521486
Best-hit-arabi-ID: AT1G59900.1
Best-hit-arabi-name: pyruvate dehydrogenase complex E1 alpha subunit
Best-hit-arabi-symbol: AT-E1 ALPHA,E1 ALPHA
Gorai.005G187700.1.A
PCC=-0.351, p=0.141
mRNA Profile
Protein Expression Profile
DPA

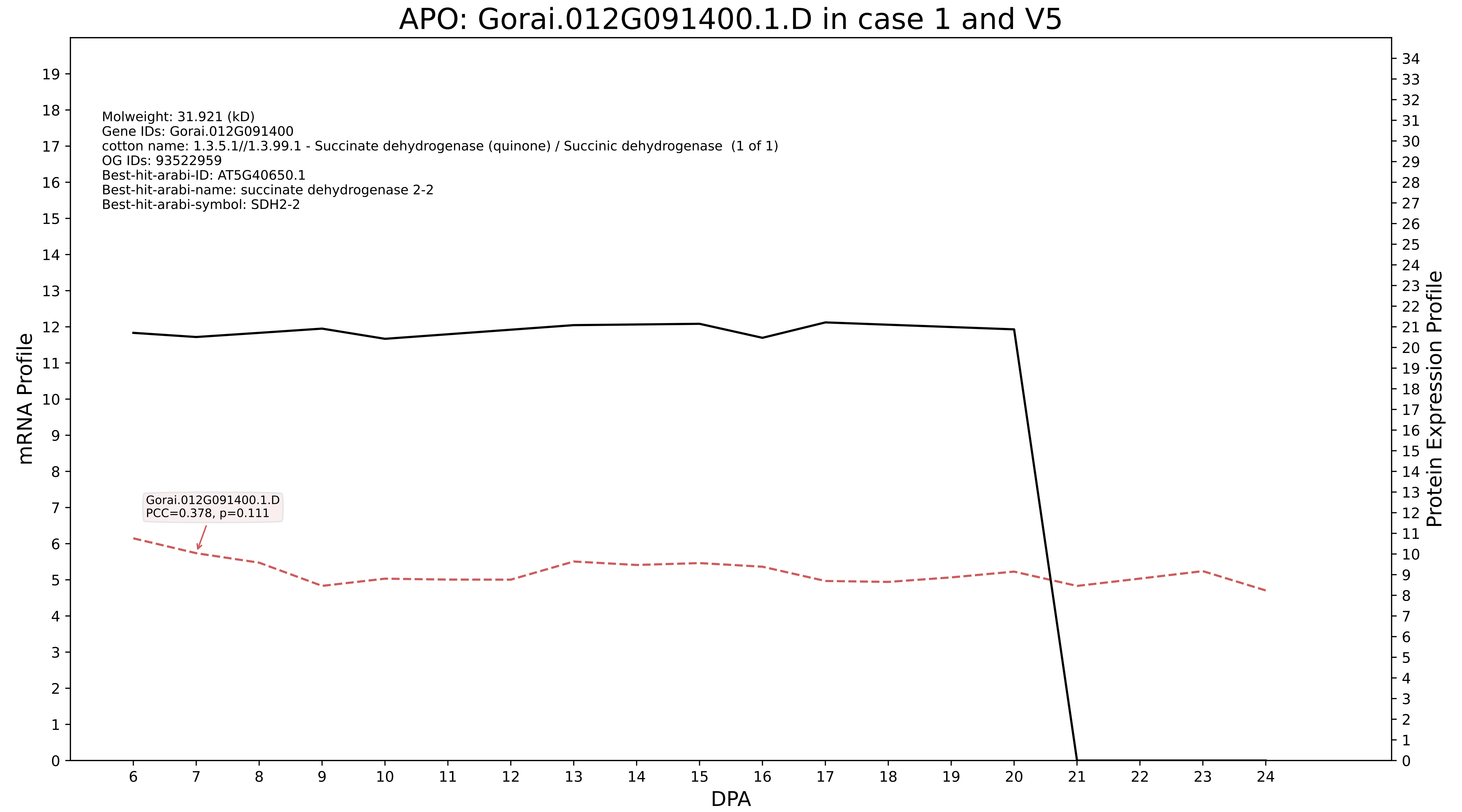
APO: Gorai.012G091400.1.D in case 1 and V5
Molweight: 31.921 (kD)
Gene IDs: Gorai.012G091400
cotton name: 1.3.5.1//1.3.99.1 - Succinate dehydrogenase (quinone) / Succinic dehydrogenase (1 of 1)
OG IDs: 93522959
Best-hit-arabi-ID: AT5G40650.1
Best-hit-arabi-name: succinate dehydrogenase 2-2
Best-hit-arabi-symbol: SDH2-2
Gorai.012G091400.1.D
PCC=0.378, p=0.111
mRNA Profile
Protein Expression Profile
DPA

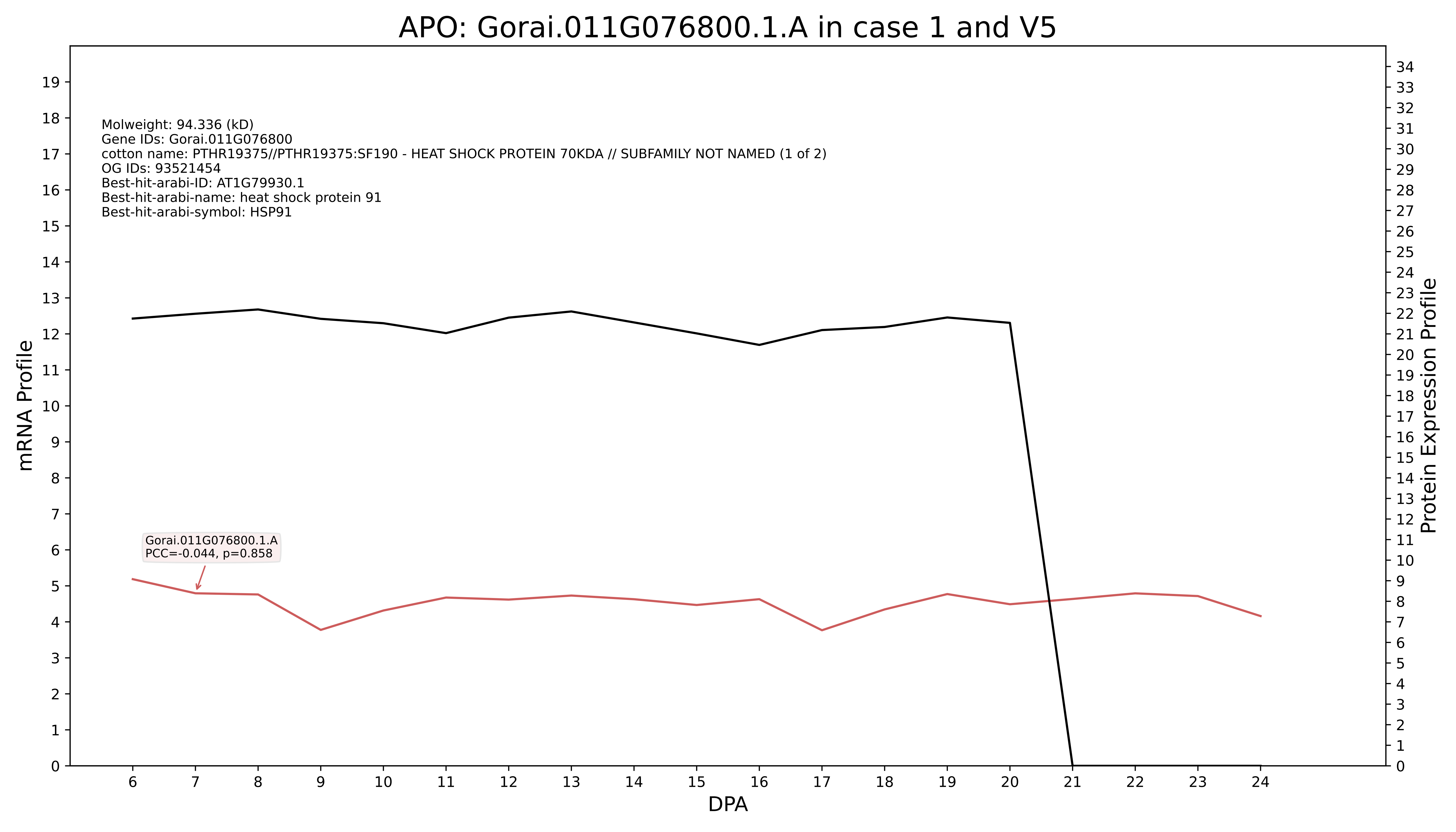

APO: Gorai.011G076800.1.A in case 1 and V5
Molweight: 94.336 (kD)
Gene IDs: Gorai.011G076800
cotton name: PTHR19375//PTHR19375:SF190 - HEAT SHOCK PROTEIN 70KDA // SUBFAMILY NOT NAMED (1 of 2)
OG IDs: 93521454
Best-hit-arabi-ID: AT1G79930.1
Best-hit-arabi-name: heat shock protein 91
Best-hit-arabi-symbol: HSP91
Gorai.011G076800.1.A
PCC=-0.044, p=0.858
mRNA Profile
Protein Expression Profile
DPA

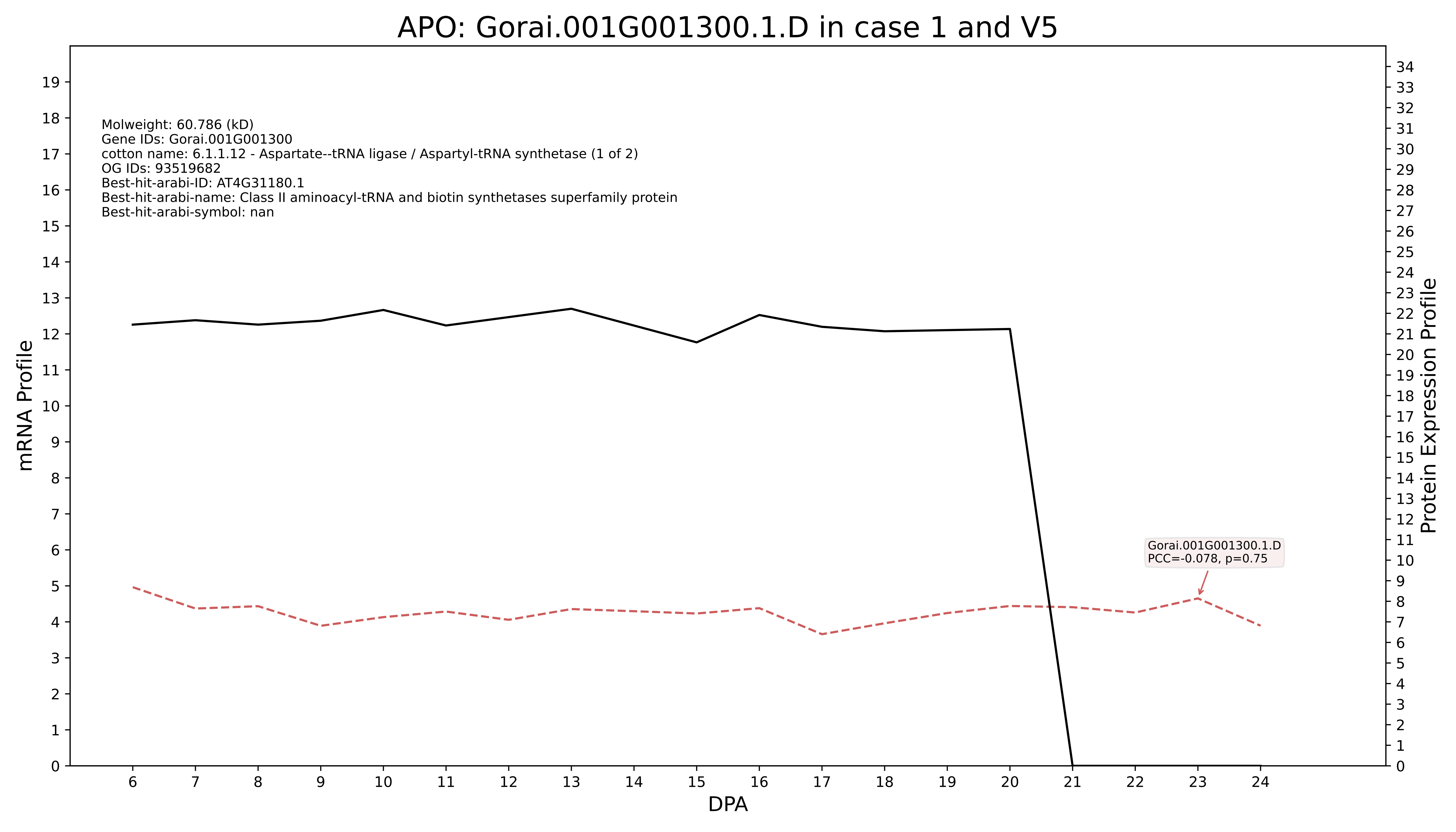
APO: Gorai.001G001300.1.D in case 1 and V5
Molweight: 60.786 (kD)
Gene IDs: Gorai.001G001300
cotton name: 6.1.1.12 - Aspartate--tRNA ligase / Aspartyl-tRNA synthetase (1 of 2)
OG IDs: 93519682
Best-hit-arabi-ID: AT4G31180.1
Best-hit-arabi-name: Class II aminoacyl-tRNA and biotin synthetases superfamily protein
Best-hit-arabi-symbol: nan
Gorai.001G001300.1.D
PCC=-0.078, p=0.75
mRNA Profile
Protein Expression Profile
DPA

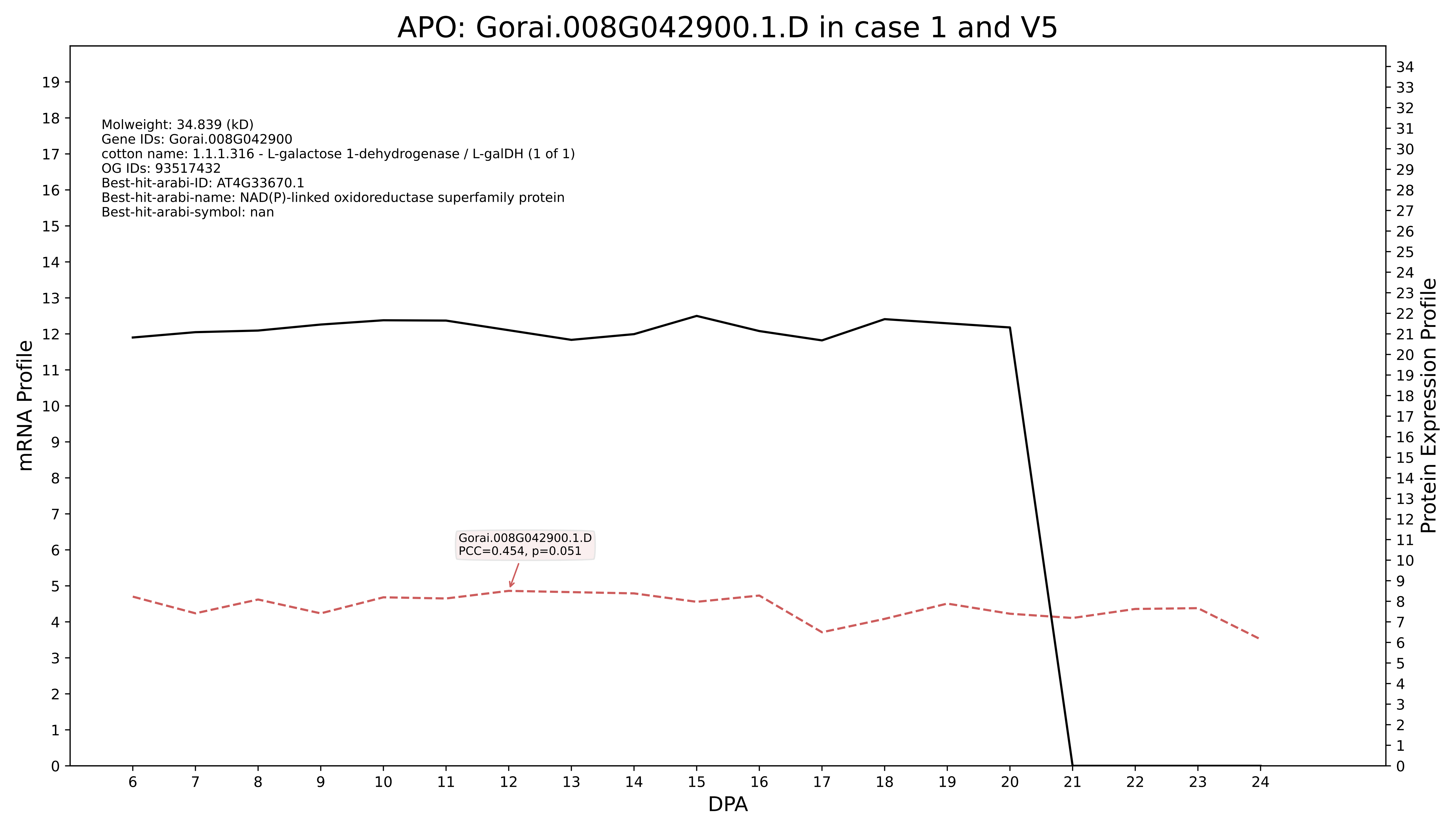
APO: Gorai.008G042900.1.D in case 1 and V5
Molweight: 34.839 (kD)
Gene IDs: Gorai.008G042900
cotton name: 1.1.1.316 - L-galactose 1-dehydrogenase / L-galDH (1 of 1)
OG IDs: 93517432
Best-hit-arabi-ID: AT4G33670.1
Best-hit-arabi-name: NAD(P)-linked oxidoreductase superfamily protein
Best-hit-arabi-symbol: nan
Gorai.008G042900.1.D
PCC=0.454, p=0.051
mRNA Profile
Protein Expression Profile
DPA
0
1
2
3
4
5
6
7
8
9
10
11
12
13
14
15
16
17
18
19
20
21
22
23
24
25
26
27
28
29
30
31
32
33
34

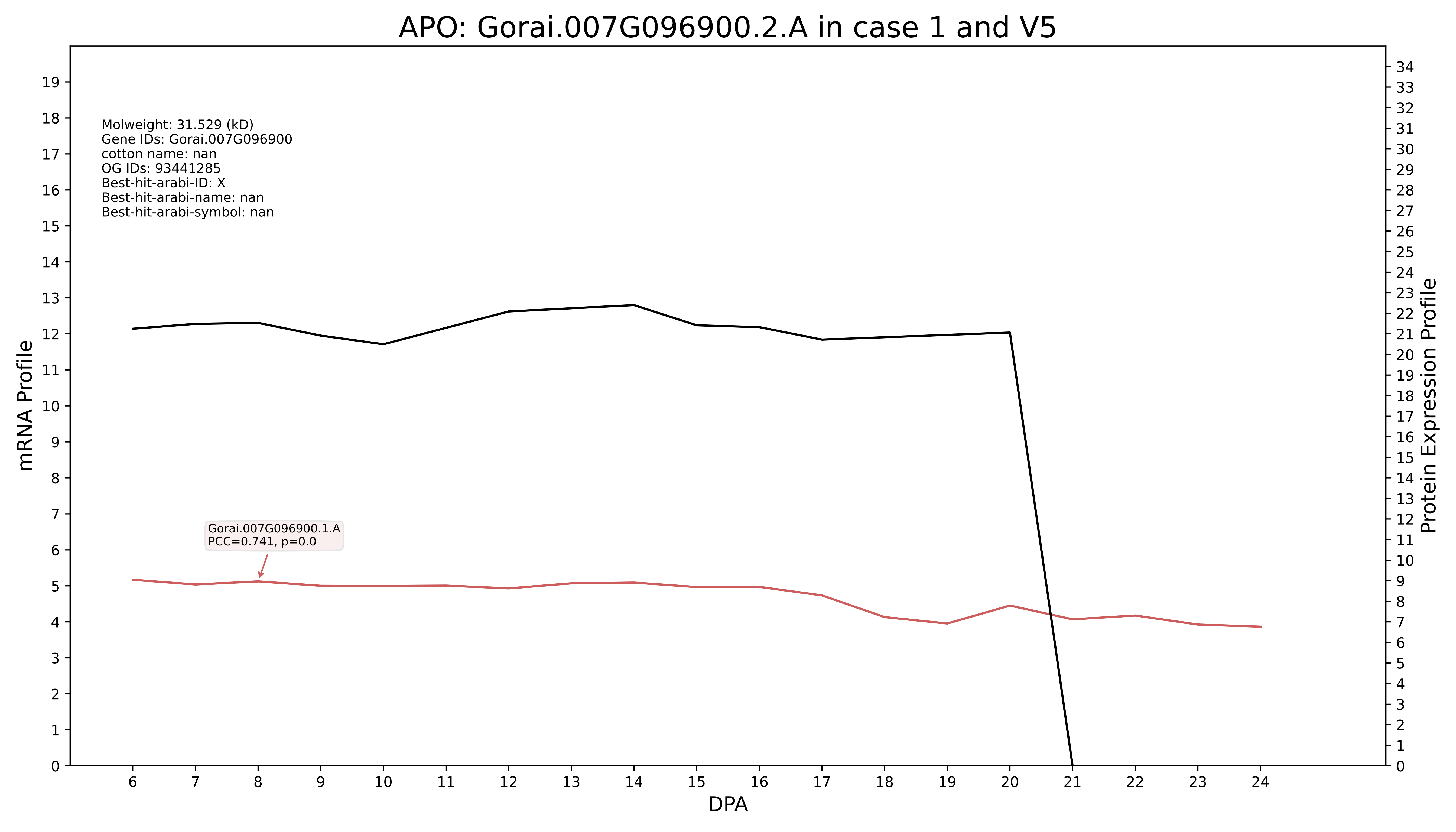
APO: Gorai.007G096900.2.A in case 1 and V5
Molweight: 31.529 (kD)
Gene IDs: Gorai.007G096900
cotton name: nan
OG IDs: 93441285
Best-hit-arabi-ID: X
Best-hit-arabi-name: nan
Best-hit-arabi-symbol: nan
Gorai.007G096900.1.A
PCC=0.741, p=0.0
mRNA Profile
Protein Expression Profile
DPA

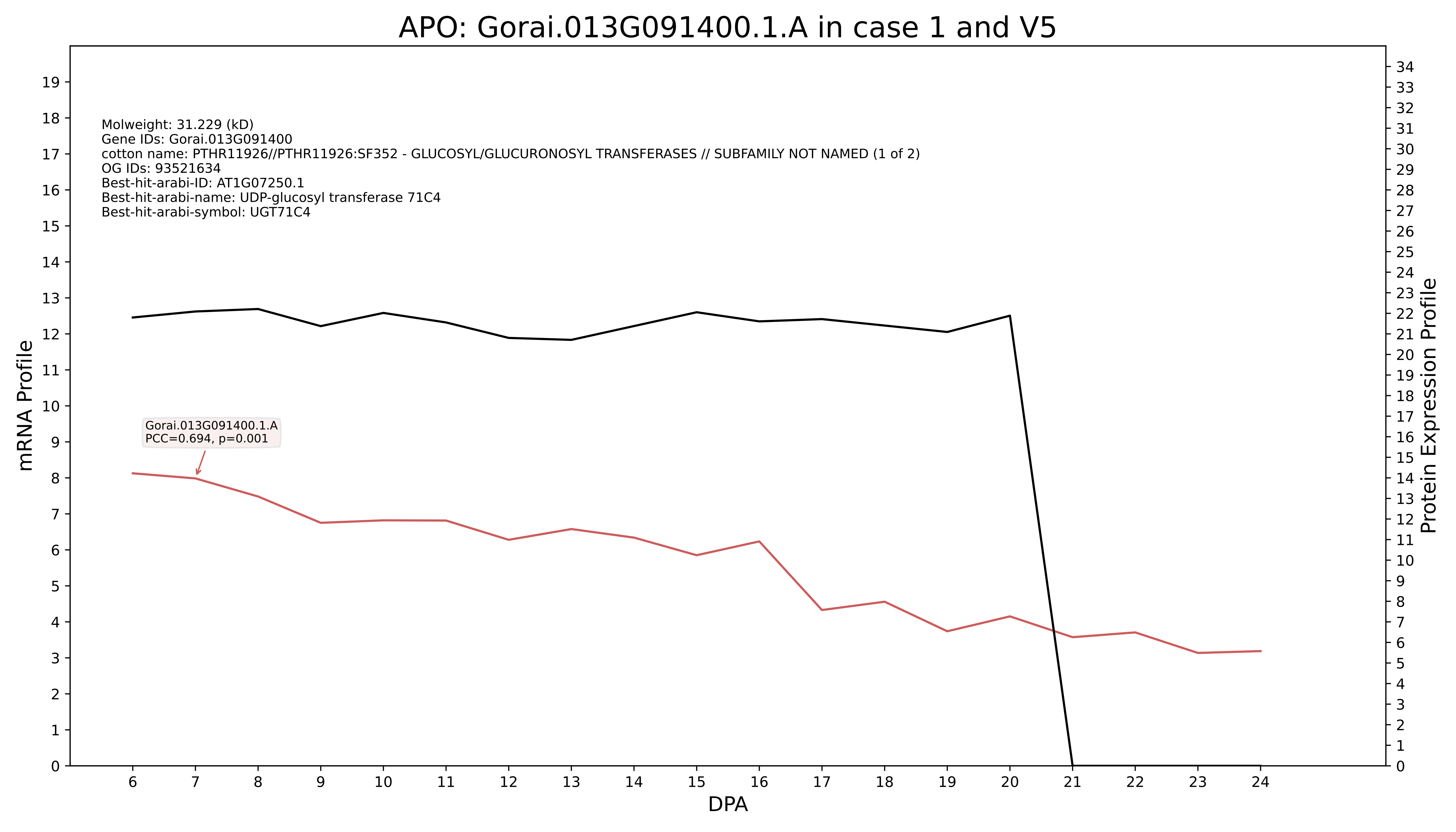

APO: Gorai.013G091400.1.A in case 1 and V5
Molweight: 31.229 (kD)
Gene IDs: Gorai.013G091400
cotton name: PTHR11926//PTHR11926:SF352 - GLUCOSYL/GLUCURONOSYL TRANSFERASES // SUBFAMILY NOT NAMED (1 of 2)
OG IDs: 93521634
Best-hit-arabi-ID: AT1G07250.1
Best-hit-arabi-name: UDP-glucosyl transferase 71C4
Best-hit-arabi-symbol: UGT71C4
Gorai.013G091400.1.A
PCC=0.694, p=0.001
mRNA Profile
Protein Expression Profile
DPA

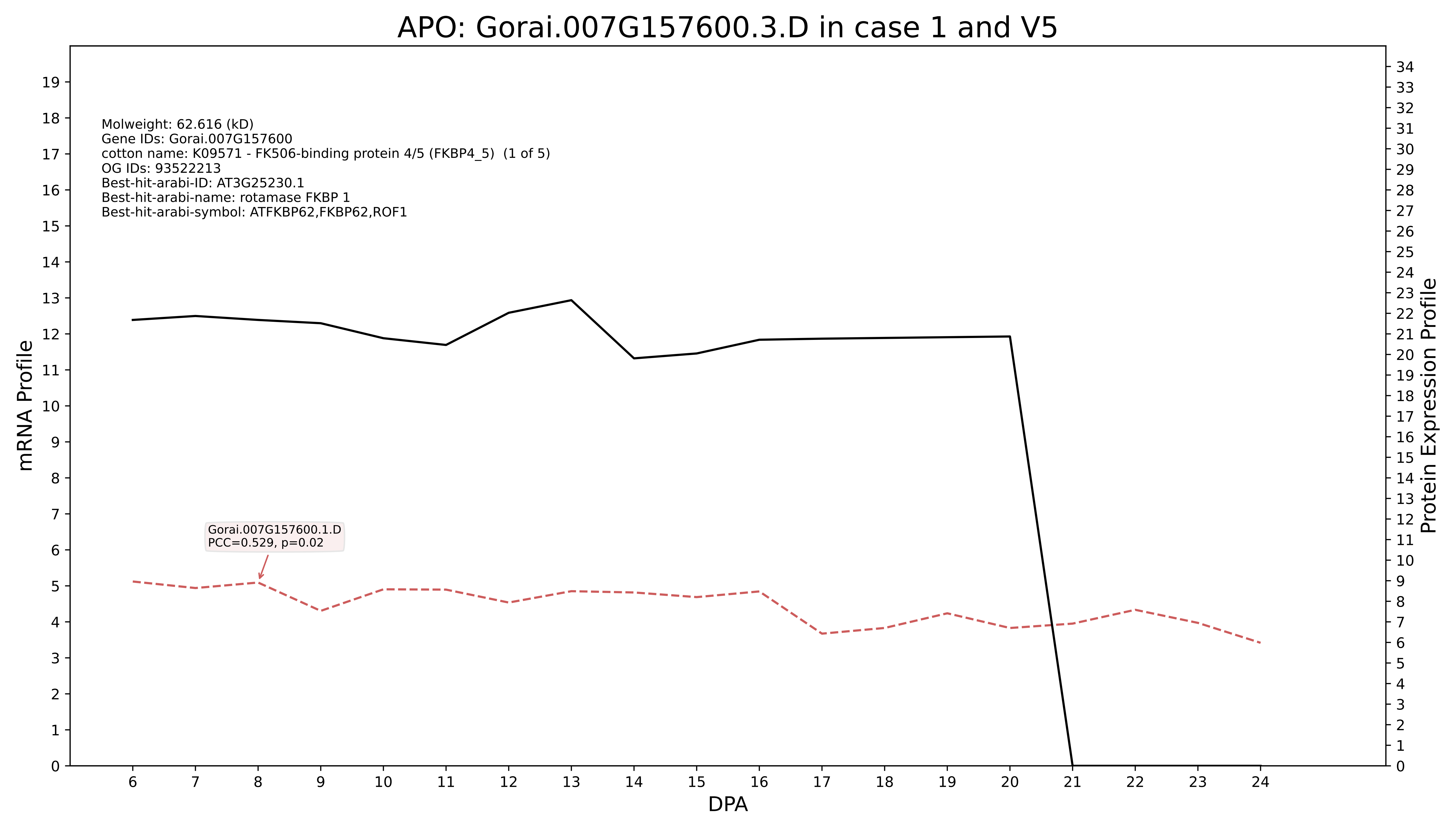
APO: Gorai.007G157600.3.D in case 1 and V5
Molweight: 62.616 (kD)
Gene IDs: Gorai.007G157600
cotton name: K09571 - FK506-binding protein 4/5 (FKBP4_5) (1 of 5)
OG IDs: 93522213
Best-hit-arabi-ID: AT3G25230.1
Best-hit-arabi-name: rotamase FKBP 1
Best-hit-arabi-symbol: ATFKBP62,FKBP62,ROF1
Gorai.007G157600.1.D
PCC=0.529, p=0.02
mRNA Profile
Protein Expression Profile
DPA

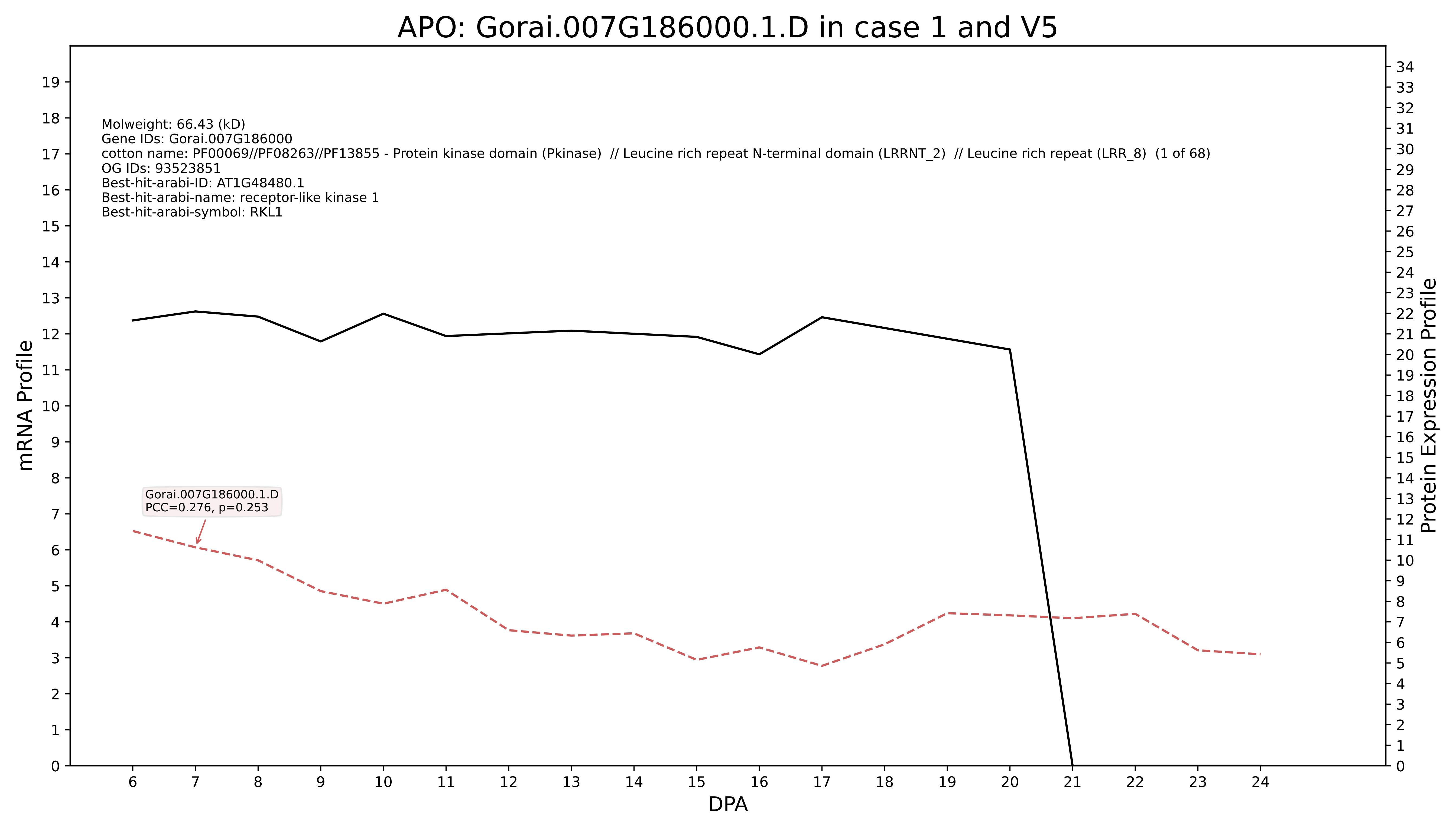
APO: Gorai.007G186000.1.D in case 1 and V5
Molweight: 66.43 (kD)
Gene IDs: Gorai.007G186000
cotton name: PF00069//PF08263//PF13855 - Protein kinase domain (Pkinase) // Leucine rich repeat N-terminal domain (LRRNT_2) // Leucine rich repeat (LRR_8) (1 of 68)
OG IDs: 93523851
Best-hit-arabi-ID: AT1G48480.1
Best-hit-arabi-name: receptor-like kinase 1
Best-hit-arabi-symbol: RKL1
Gorai.007G186000.1.D
PCC=0.276, p=0.253
mRNA Profile
Protein Expression Profile
DPA

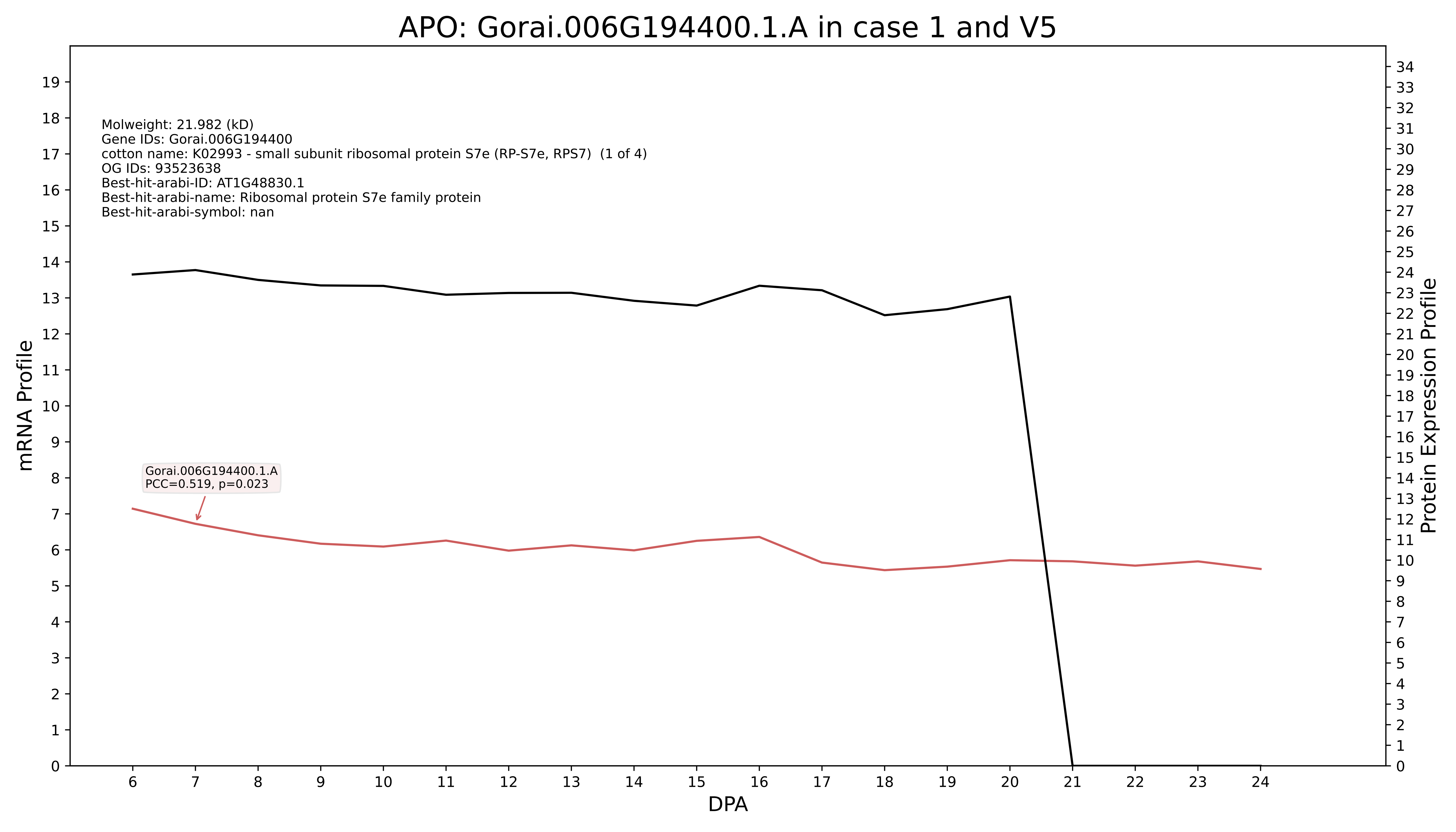

APO: Gorai.006G194400.1.A in case 1 and V5
Molweight: 21.982 (kD)
Gene IDs: Gorai.006G194400
cotton name: K02993 - small subunit ribosomal protein S7e (RP-S7e, RPS7) (1 of 4)
OG IDs: 93523638
Best-hit-arabi-ID: AT1G48830.1
Best-hit-arabi-name: Ribosomal protein S7e family protein
Best-hit-arabi-symbol: nan
Gorai.006G194400.1.A
PCC=0.519, p=0.023
mRNA Profile
Protein Expression Profile
DPA

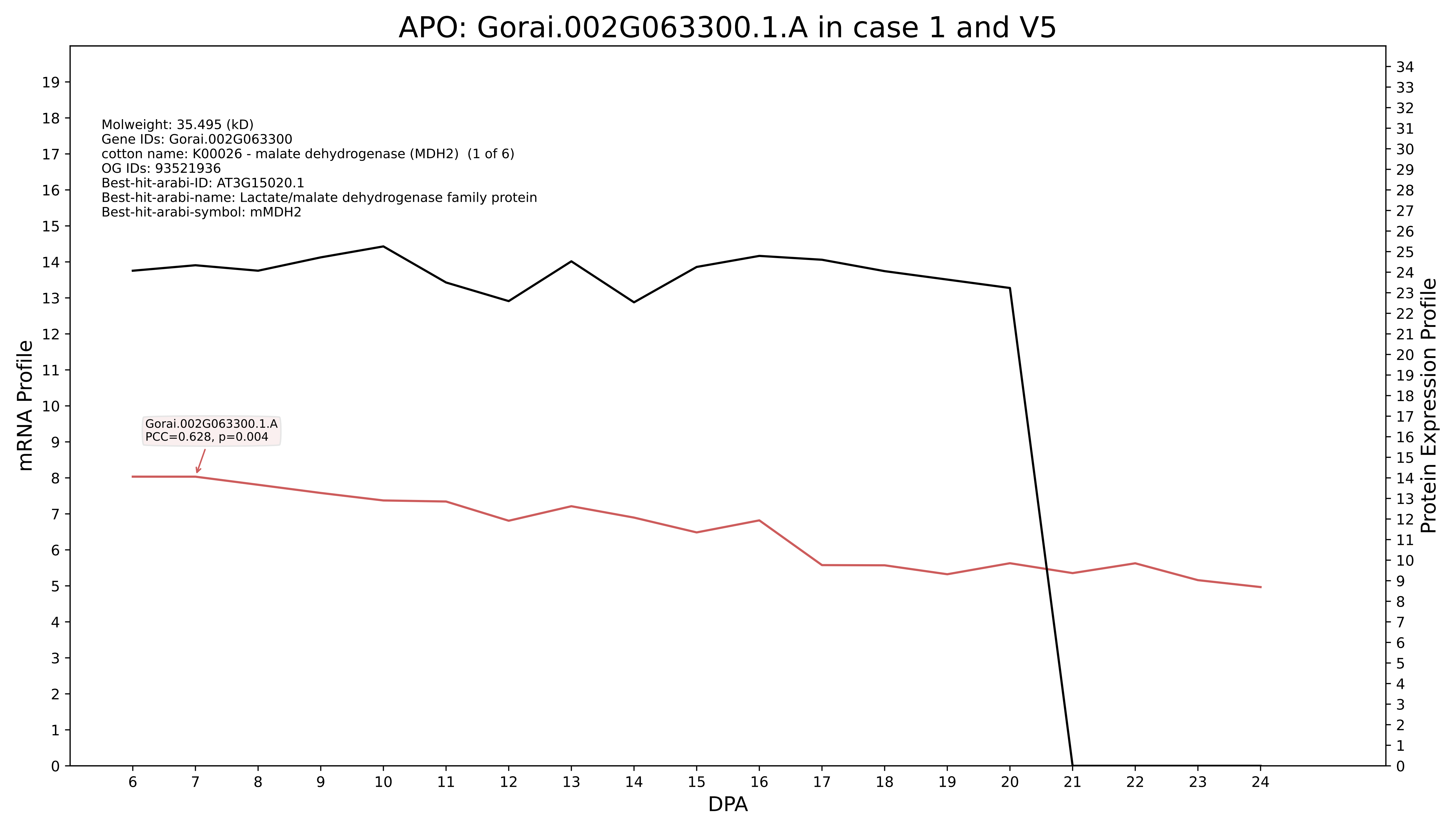

APO: Gorai.002G063300.1.A in case 1 and V5
Molweight: 35.495 (kD)
Gene IDs: Gorai.002G063300
cotton name: K00026 - malate dehydrogenase (MDH2) (1 of 6)
OG IDs: 93521936
Best-hit-arabi-ID: AT3G15020.1
Best-hit-arabi-name: Lactate/malate dehydrogenase family protein
Best-hit-arabi-symbol: mMDH2
Gorai.002G063300.1.A
PCC=0.628, p=0.004
mRNA Profile
Protein Expression Profile
DPA

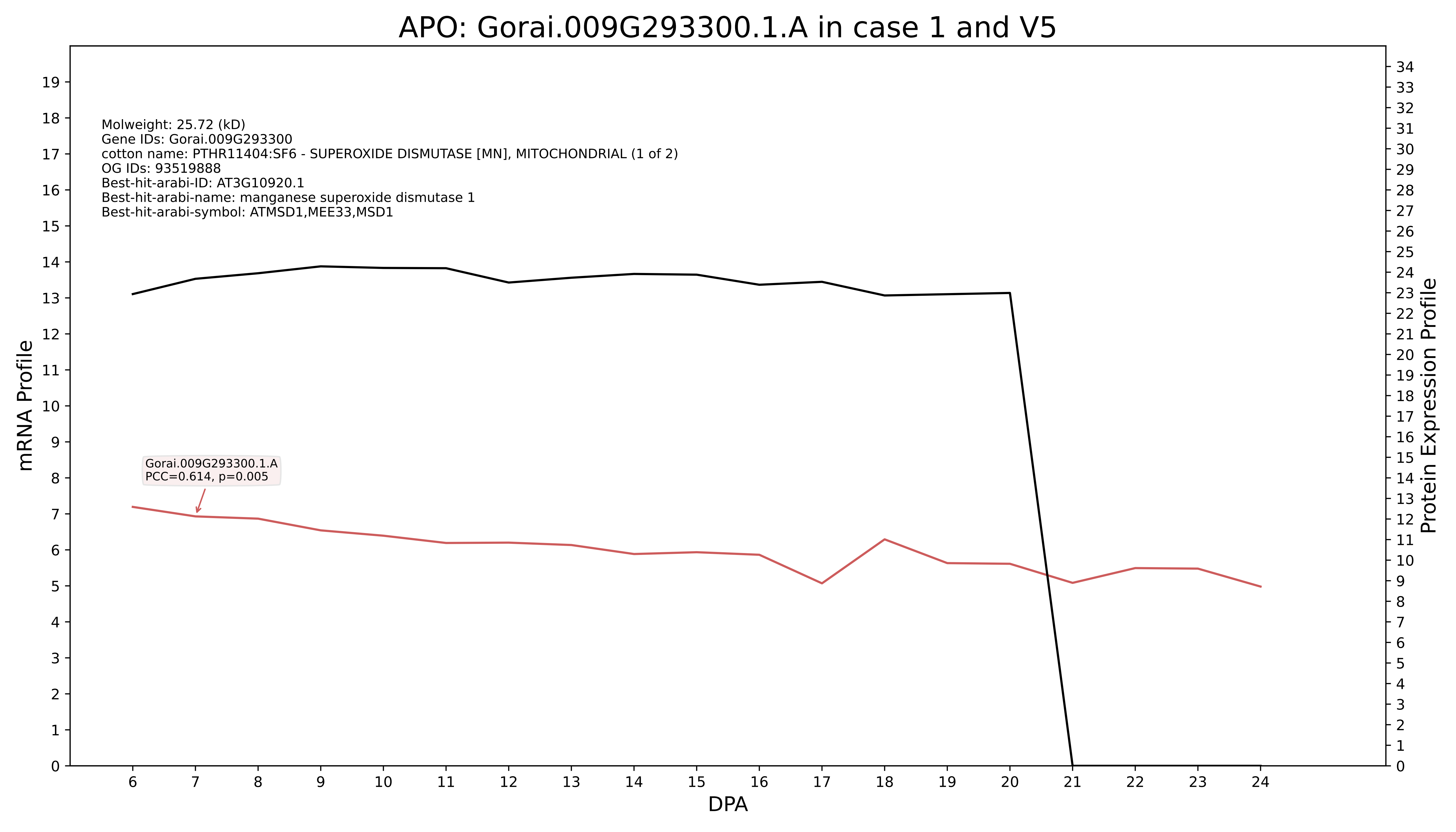
APO: Gorai.009G293300.1.A in case 1 and V5
Molweight: 25.72 (kD)
Gene IDs: Gorai.009G293300
cotton name: PTHR11404:SF6 - SUPEROXIDE DISMUTASE [MN], MITOCHONDRIAL (1 of 2)
OG IDs: 93519888
Best-hit-arabi-ID: AT3G10920.1
Best-hit-arabi-name: manganese superoxide dismutase 1
Best-hit-arabi-symbol: ATMSD1,MEE33,MSD1
Gorai.009G293300.1.A
PCC=0.614, p=0.005
mRNA Profile
Protein Expression Profile
DPA

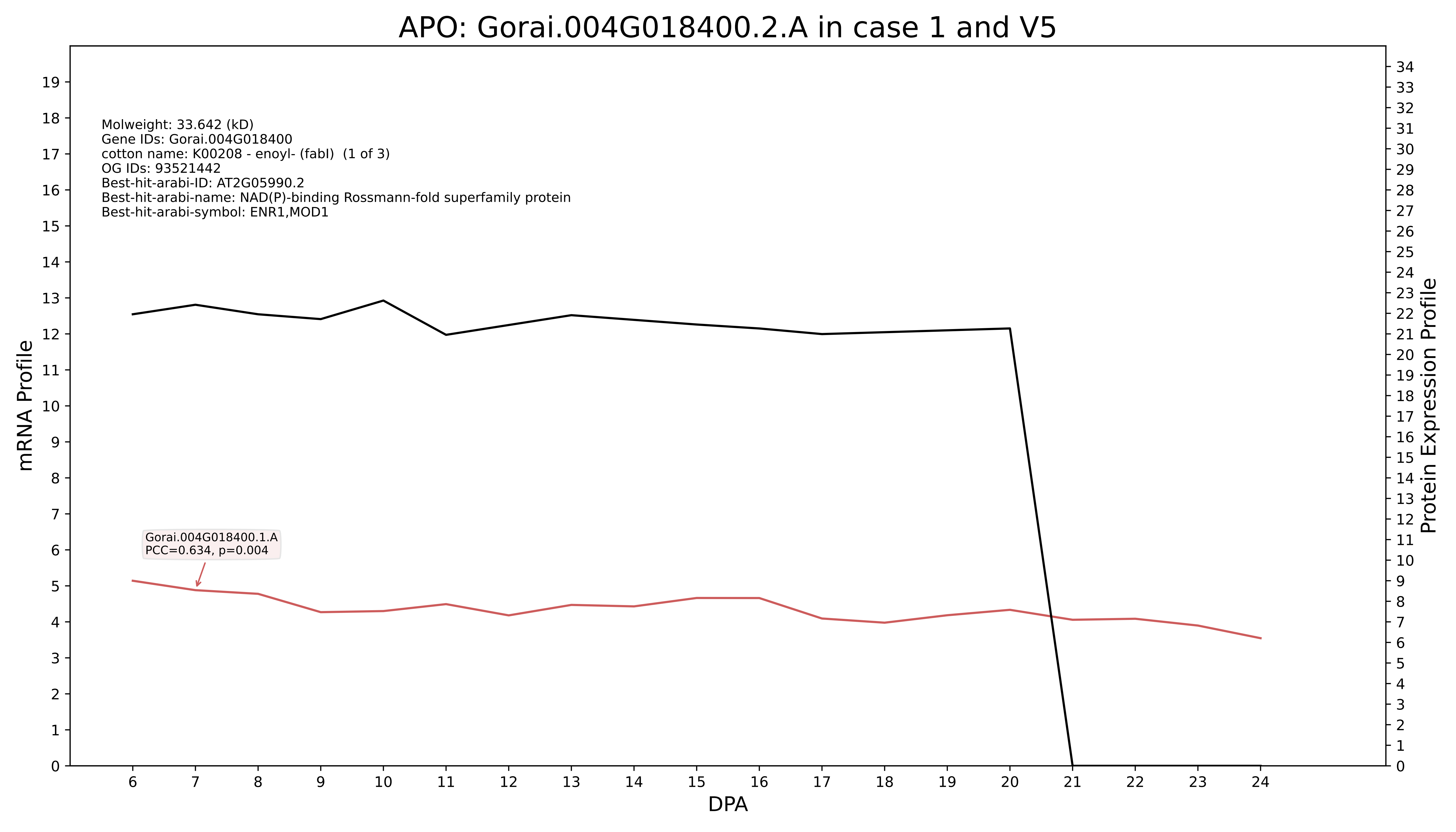

APO: Gorai.004G018400.2.A in case 1 and V5
Molweight: 33.642 (kD)
Gene IDs: Gorai.004G018400
cotton name: K00208 - enoyl- (fabI) (1 of 3)
OG IDs: 93521442
Best-hit-arabi-ID: AT2G05990.2
Best-hit-arabi-name: NAD(P)-binding Rossmann-fold superfamily protein
Best-hit-arabi-symbol: ENR1,MOD1
Gorai.004G018400.1.A
PCC=0.634, p=0.004
mRNA Profile
Protein Expression Profile
DPA
6 7 8 9 10 11 12 13 14 15 16 17 18 19 20 21 22 23 24
0 1 2 3 4 5 6 7 8 9 10 11 12 13 14 15 16 17 18 19
0 1 2 3 4 5 6 7 8 9 10 11 12 13 14 15 16 17 18 19 20 21 22 23 24 25 26 27 28 29 30 31 32 33 34

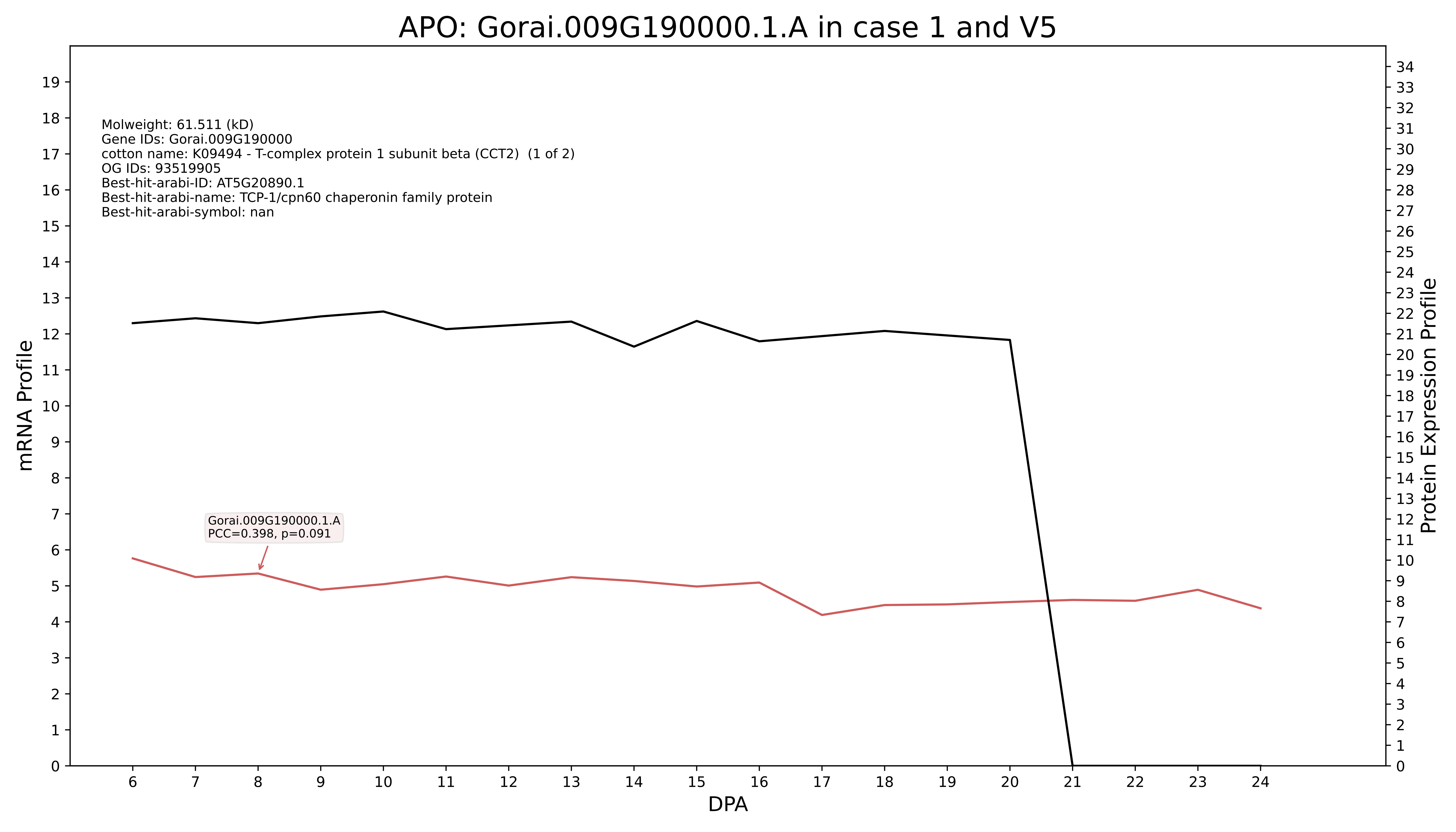

APO: Gorai.009G190000.1.A in case 1 and V5
Molweight: 61.511 (kD)
Gene IDs: Gorai.009G190000
cotton name: K09494 - T-complex protein 1 subunit beta (CCT2) (1 of 2)
OG IDs: 93519905
Best-hit-arabi-ID: AT5G20890.1
Best-hit-arabi-name: TCP-1/cpn60 chaperonin family protein
Best-hit-arabi-symbol: nan
Gorai.009G190000.1.A
PCC=0.398, p=0.091
mRNA Profile
Protein Expression Profile
DPA

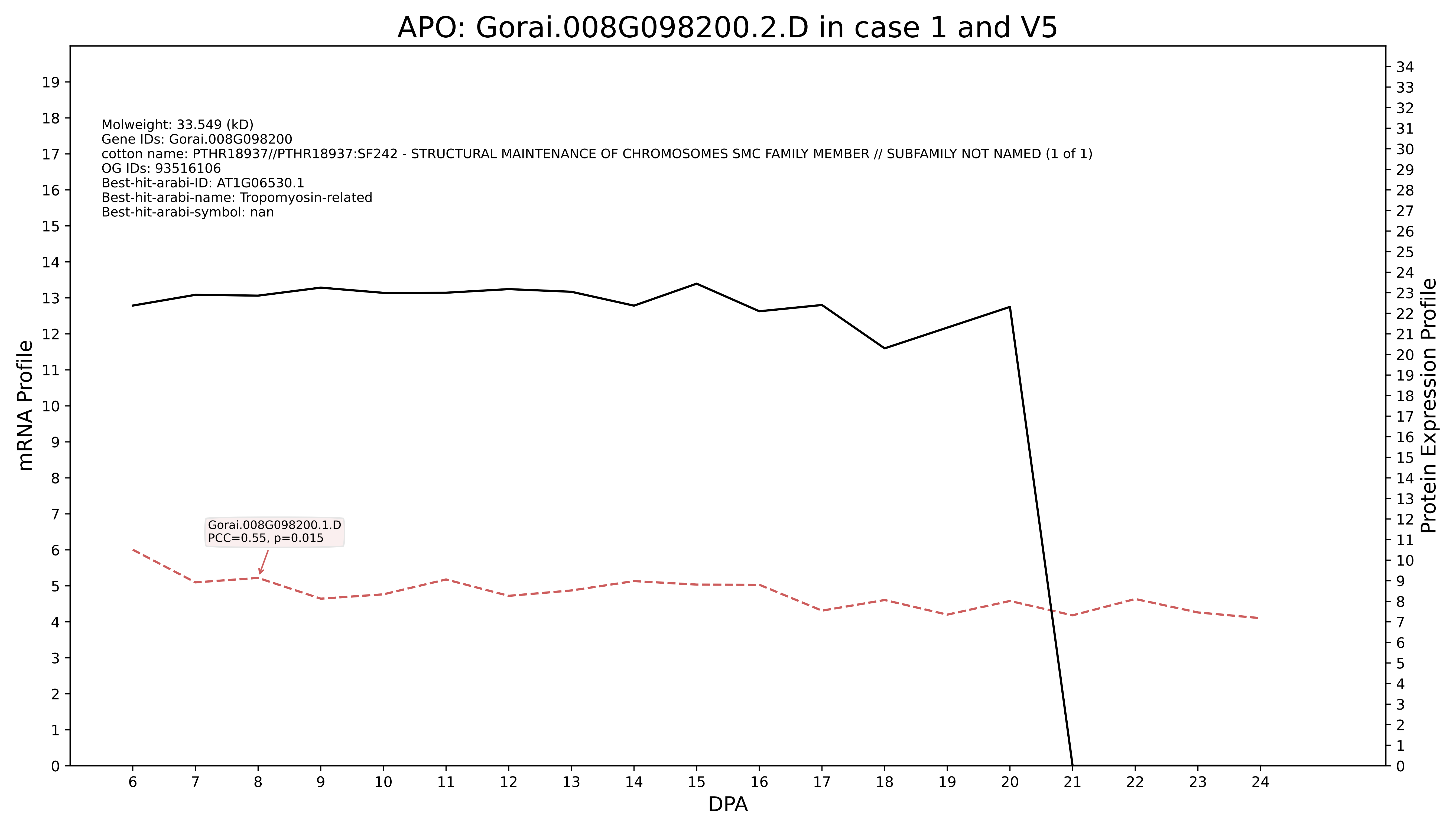
APO: Gorai.008G098200.2.D in case 1 and V5
Molweight: 33.549 (kD)
Gene IDs: Gorai.008G098200
cotton name: PTHR18937//PTHR18937:SF242 - STRUCTURAL MAINTENANCE OF CHROMOSOMES SMC FAMILY MEMBER // SUBFAMILY NOT NAMED (1 of 1)
OG IDs: 93516106
Best-hit-arabi-ID: AT1G06530.1
Best-hit-arabi-name: Tropomyosin-related
Best-hit-arabi-symbol: nan
Gorai.008G098200.1.D
PCC=0.55, p=0.015
mRNA Profile
Protein Expression Profile
DPA

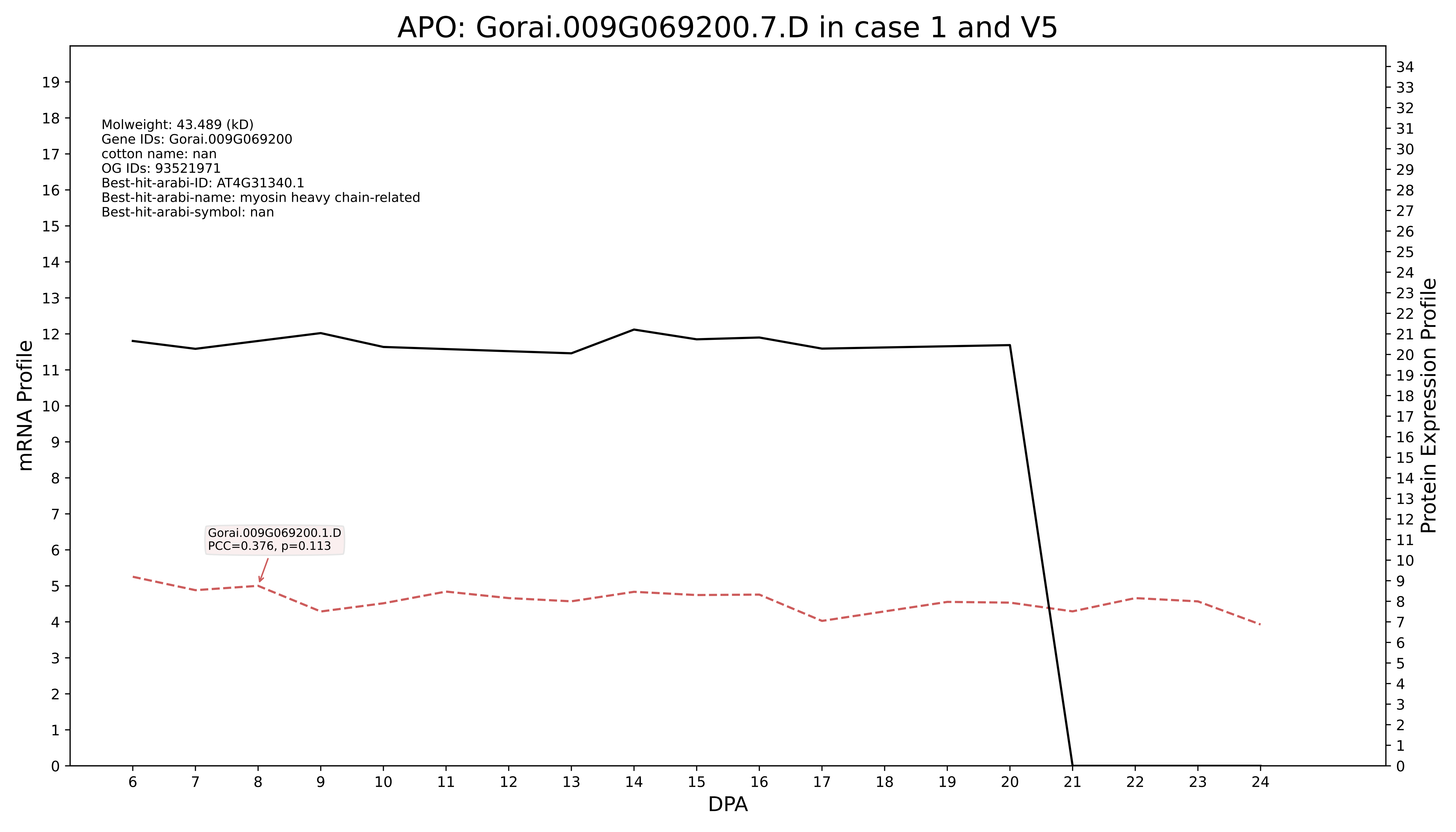

APO: Gorai.009G069200.7.D in case 1 and V5
Molweight: 43.489 (kD)
Gene IDs: Gorai.009G069200
cotton name: nan
OG IDs: 93521971
Best-hit-arabi-ID: AT4G31340.1
Best-hit-arabi-name: myosin heavy chain-related
Best-hit-arabi-symbol: nan
Gorai.009G069200.1.D
PCC=0.376, p=0.113
mRNA Profile
Protein Expression Profile
DPA

APO: Gorai.002G128400.6.D in case 1 and V5

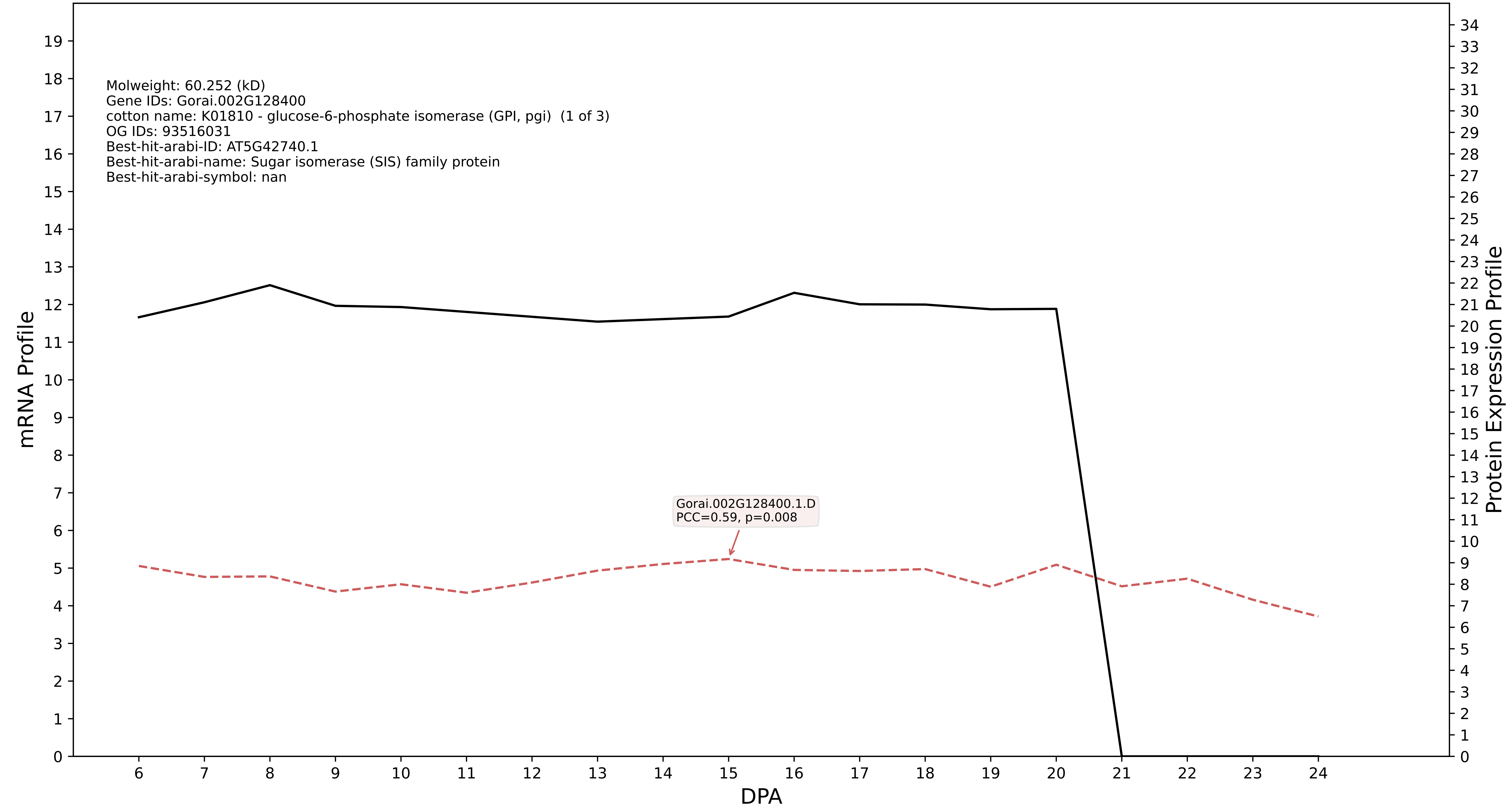

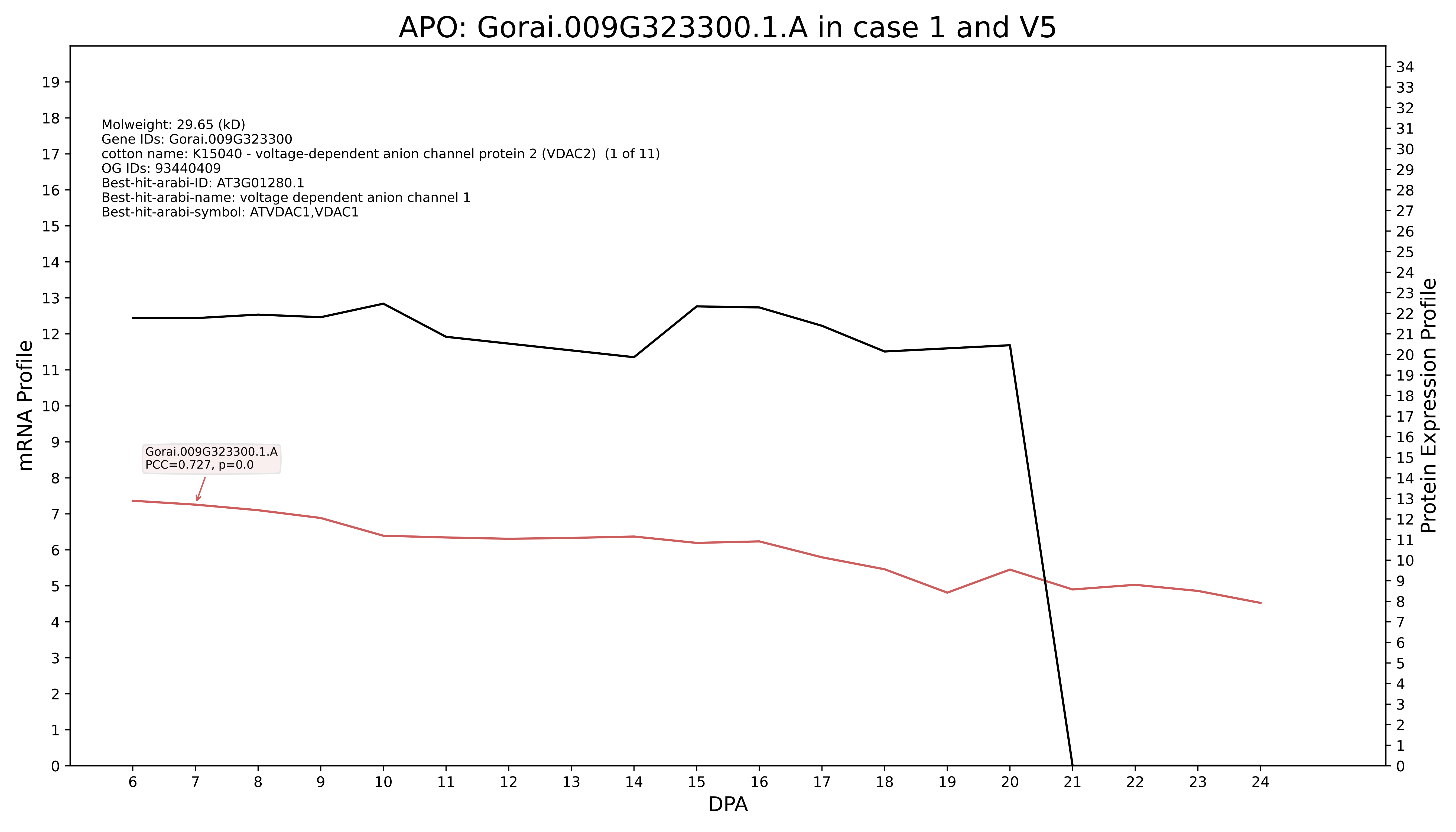
APO: Gorai.009G323300.1.A in case 1 and V5
Molweight: 29.65 (kD)
Gene IDs: Gorai.009G323300
cotton name: K15040 - voltage-dependent anion channel protein 2 (VDAC2) (1 of 11)
OG IDs: 93440409
Best-hit-arabi-ID: AT3G01280.1
Best-hit-arabi-name: voltage dependent anion channel 1
Best-hit-arabi-symbol: ATVDAC1,VDAC1
Gorai.009G323300.1.A
PCC=0.727, p=0.0
mRNA Profile
Protein Expression Profile
DPA

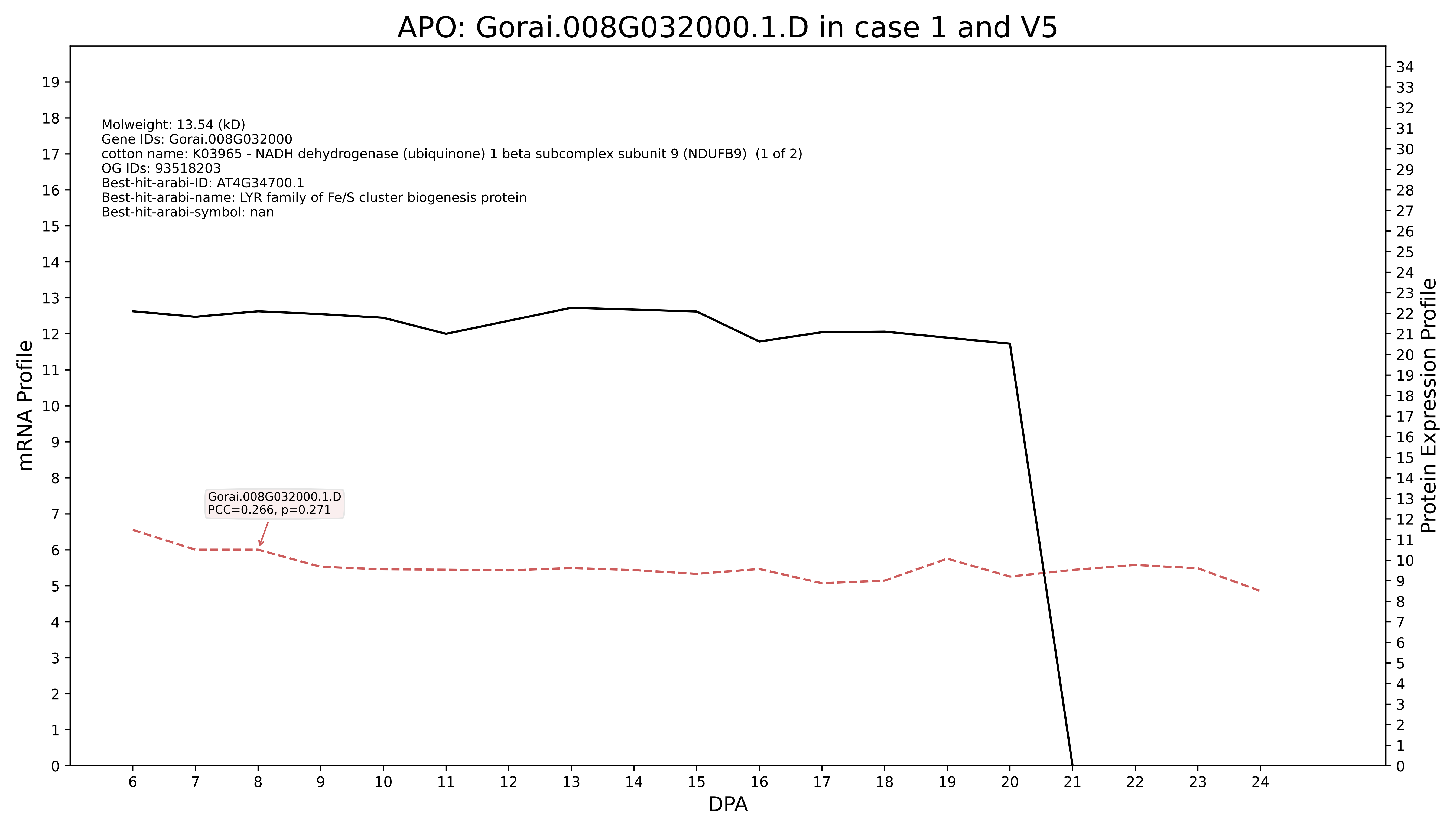
APO: Gorai.008G032000.1.D in case 1 and V5
Molweight: 13.54 (kD)
Gene IDs: Gorai.008G032000
cotton name: K03965 - NADH dehydrogenase (ubiquinone) 1 beta subcomplex subunit 9 (NDUFB9) (1 of 2)
OG IDs: 93518203
Best-hit-arabi-ID: AT4G34700.1
Best-hit-arabi-name: LYR family of Fe/S cluster biogenesis protein
Best-hit-arabi-symbol: nan
Gorai.008G032000.1.D
PCC=0.266, p=0.271
mRNA Profile
Protein Expression Profile
DPA

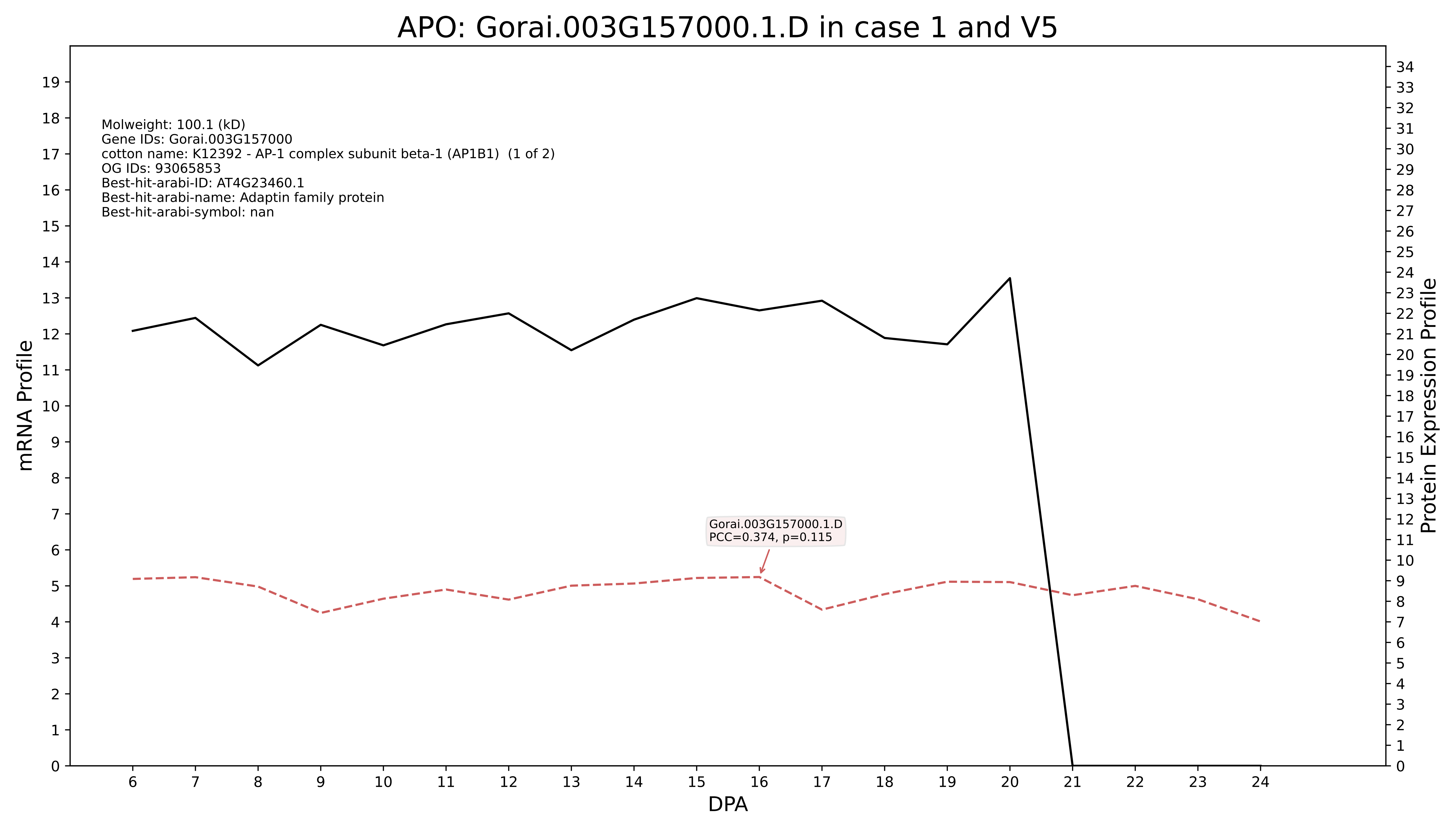
APO: Gorai.003G157000.1.D in case 1 and V5
Molweight: 100.1 (kD)
Gene IDs: Gorai.003G157000
cotton name: K12392 - AP-1 complex subunit beta-1 (AP1B1) (1 of 2)
OG IDs: 93065853
Best-hit-arabi-ID: AT4G23460.1
Best-hit-arabi-name: Adaptin family protein
Best-hit-arabi-symbol: nan
Gorai.003G157000.1.D
PCC=0.374, p=0.115
mRNA Profile
Protein Expression Profile
DPA

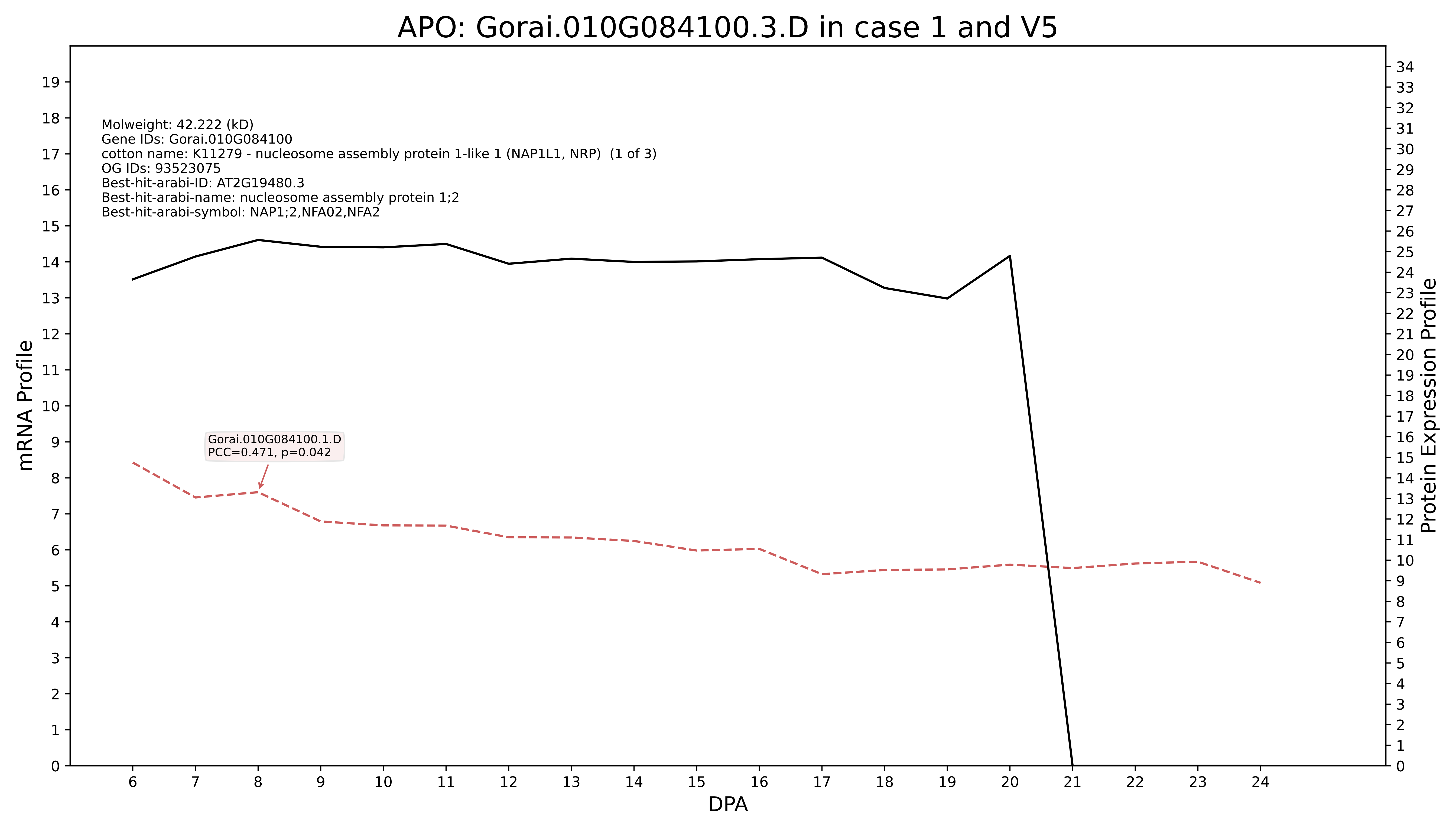

APO: Gorai.010G084100.3.D in case 1 and V5
Molweight: 42.222 (kD)
Gene IDs: Gorai.010G084100
cotton name: K11279 - nucleosome assembly protein 1-like 1 (NAP1L1, NRP) (1 of 3)
OG IDs: 93523075
Best-hit-arabi-ID: AT2G19480.3
Best-hit-arabi-name: nucleosome assembly protein 1;2
Best-hit-arabi-symbol: NAP1;2,NFA02,NFA2
Gorai.010G084100.1.D
PCC=0.471, p=0.042
mRNA Profile
Protein Expression Profile
DPA

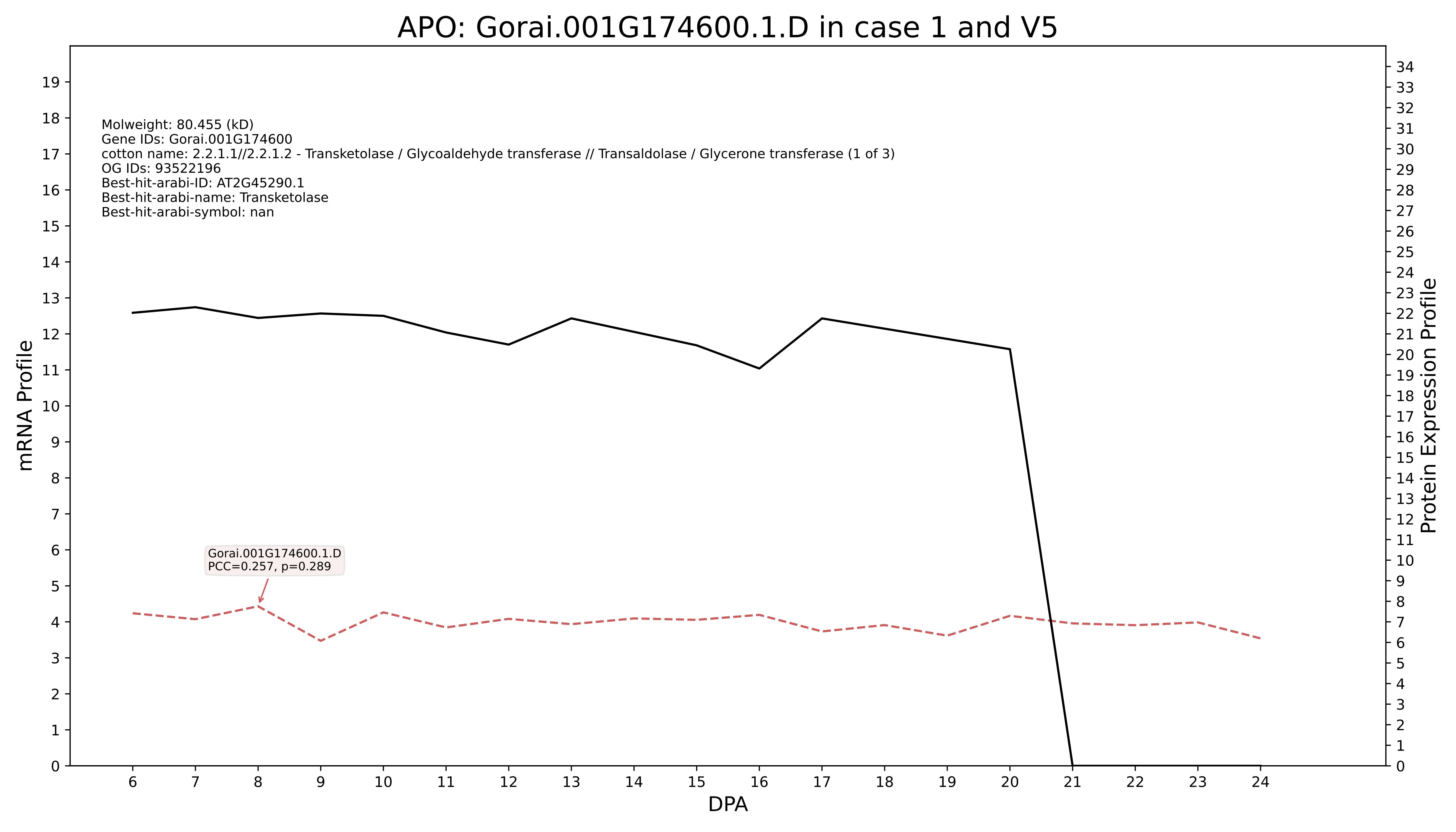
APO: Gorai.001G174600.1.D in case 1 and V5
Molweight: 80.455 (kD)
Gene IDs: Gorai.001G174600
cotton name: 2.2.1.1//2.2.1.2 - Transketolase / Glycoaldehyde transferase // Transaldolase / Glycerone transferase (1 of 3)
OG IDs: 93522196
Best-hit-arabi-ID: AT2G45290.1
Best-hit-arabi-name: Transketolase
Best-hit-arabi-symbol: nan
Gorai.001G174600.1.D
PCC=0.257, p=0.289
mRNA Profile
Protein Expression Profile
DPA

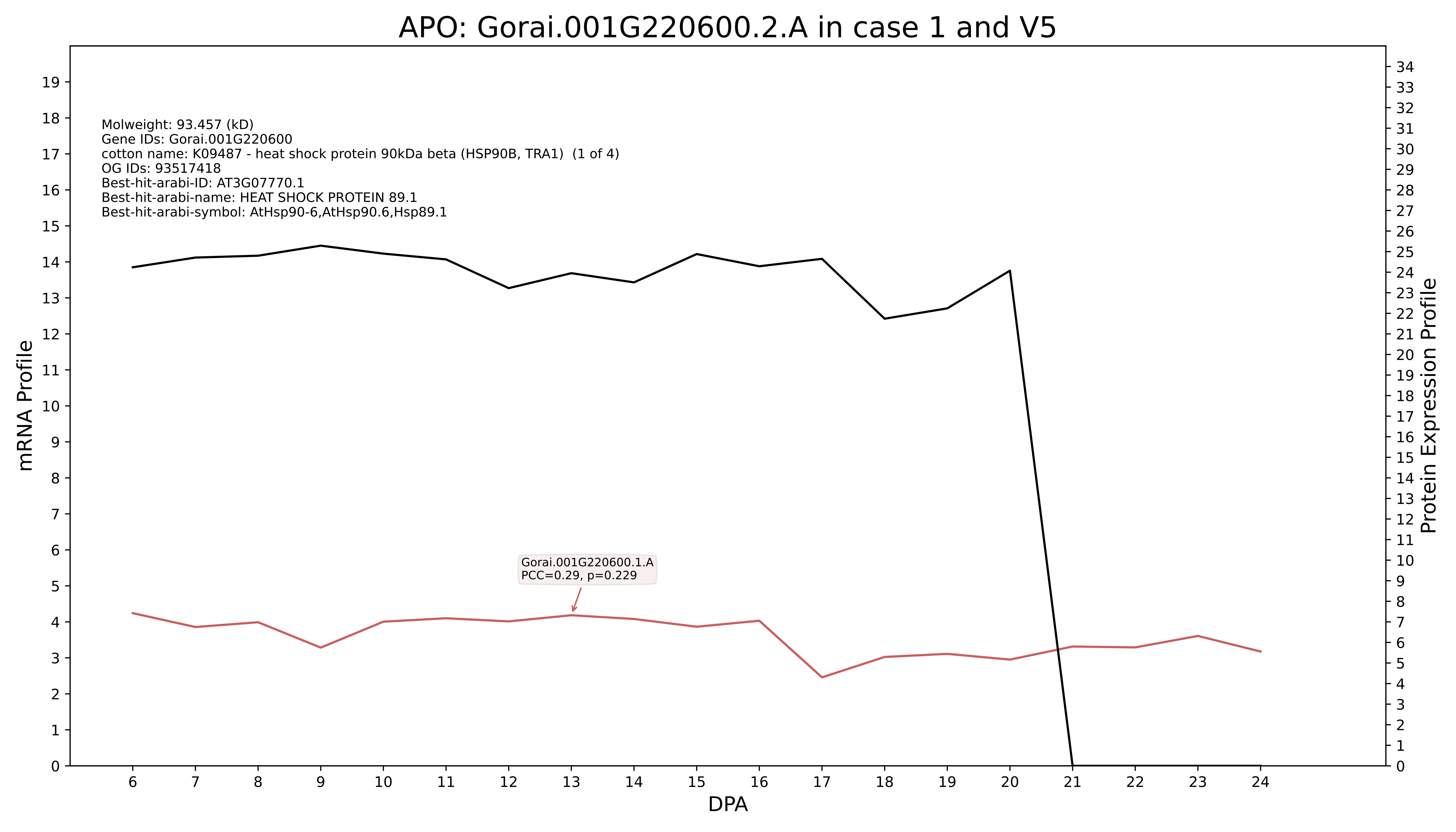
APO: Gorai.001G220600.2.A in case 1 and V5
Molweight: 93.457 (kD)
Gene IDs: Gorai.001G220600
cotton name: K09487 - heat shock protein 90kDa beta (HSP90B, TRA1) (1 of 4)
OG IDs: 93517418
Best-hit-arabi-ID: AT3G07770.1
Best-hit-arabi-name: HEAT SHOCK PROTEIN 89.1
Best-hit-arabi-symbol: AtHsp90-6,AtHsp90.6,Hsp89.1
Gorai.001G220600.1.A
PCC=0.29, p=0.229
mRNA Profile
Protein Expression Profile
DPA
0
1
2
3
4
5
6
7
8
9
10
11
12
13
14
15
16
17
18
19
0
1
2
3
4
5
6
7
8
9
10
11
12
13
14
15
16
17
18
19
20
21
22
23
24
25
26
27
28
29
30
31
32
33
34
6
7
8
9
10
11
12
13
14
15
16
17
18
19
20
21
22
23
24

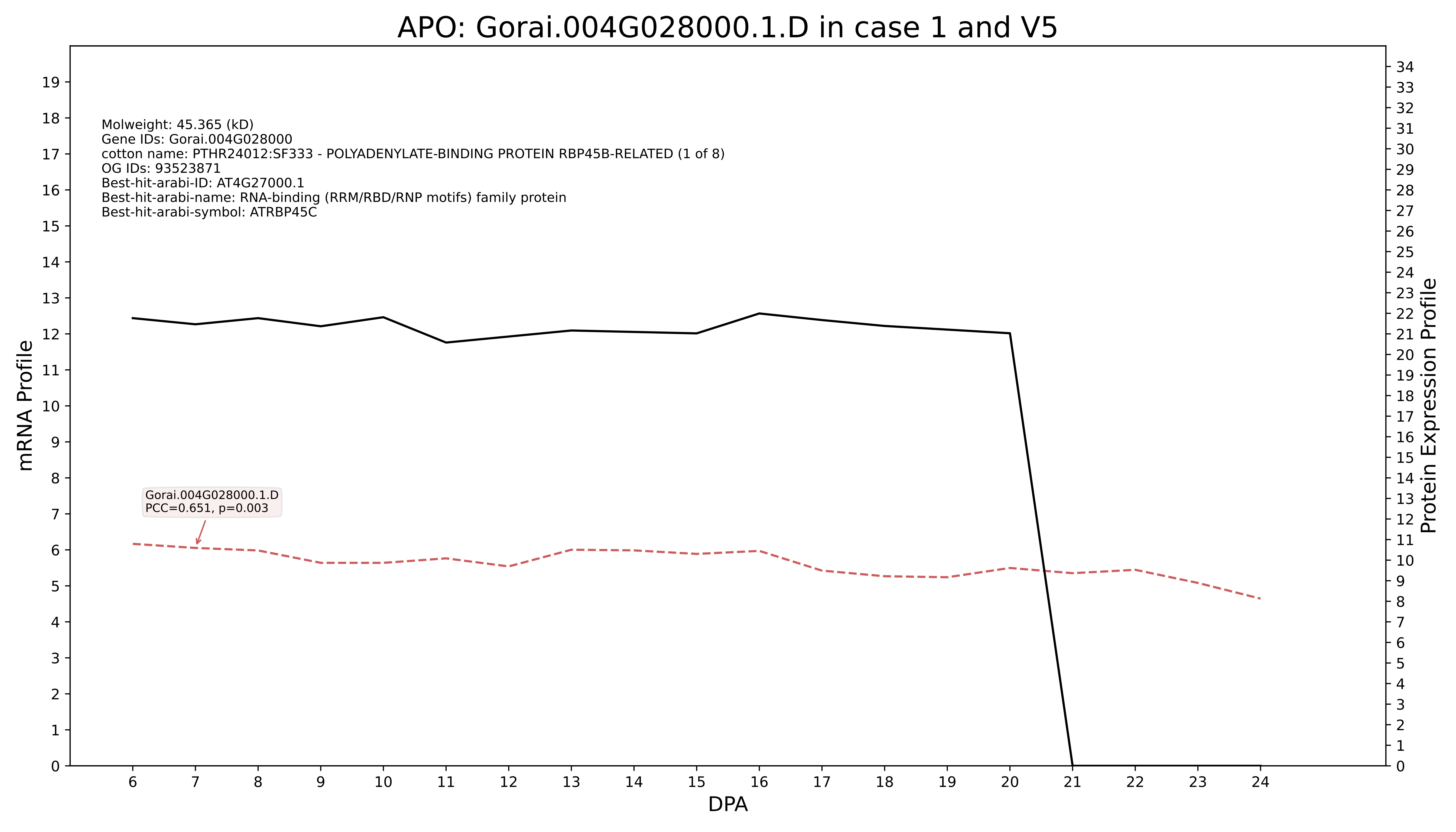
APO: Gorai.004G028000.1.D in case 1 and V5
Molweight: 45.365 (kD)
Gene IDs: Gorai.004G028000
cotton name: PTHR24012:SF333 - POLYADENYLATE-BINDING PROTEIN RBP45B-RELATED (1 of 8)
OG IDs: 93523871
Best-hit-arabi-ID: AT4G27000.1
Best-hit-arabi-name: RNA-binding (RRM/RBD/RNP motifs) family protein
Best-hit-arabi-symbol: ATRBP45C
Gorai.004G028000.1.D
PCC=0.651, p=0.003
mRNA Profile
Protein Expression Profile
DPA

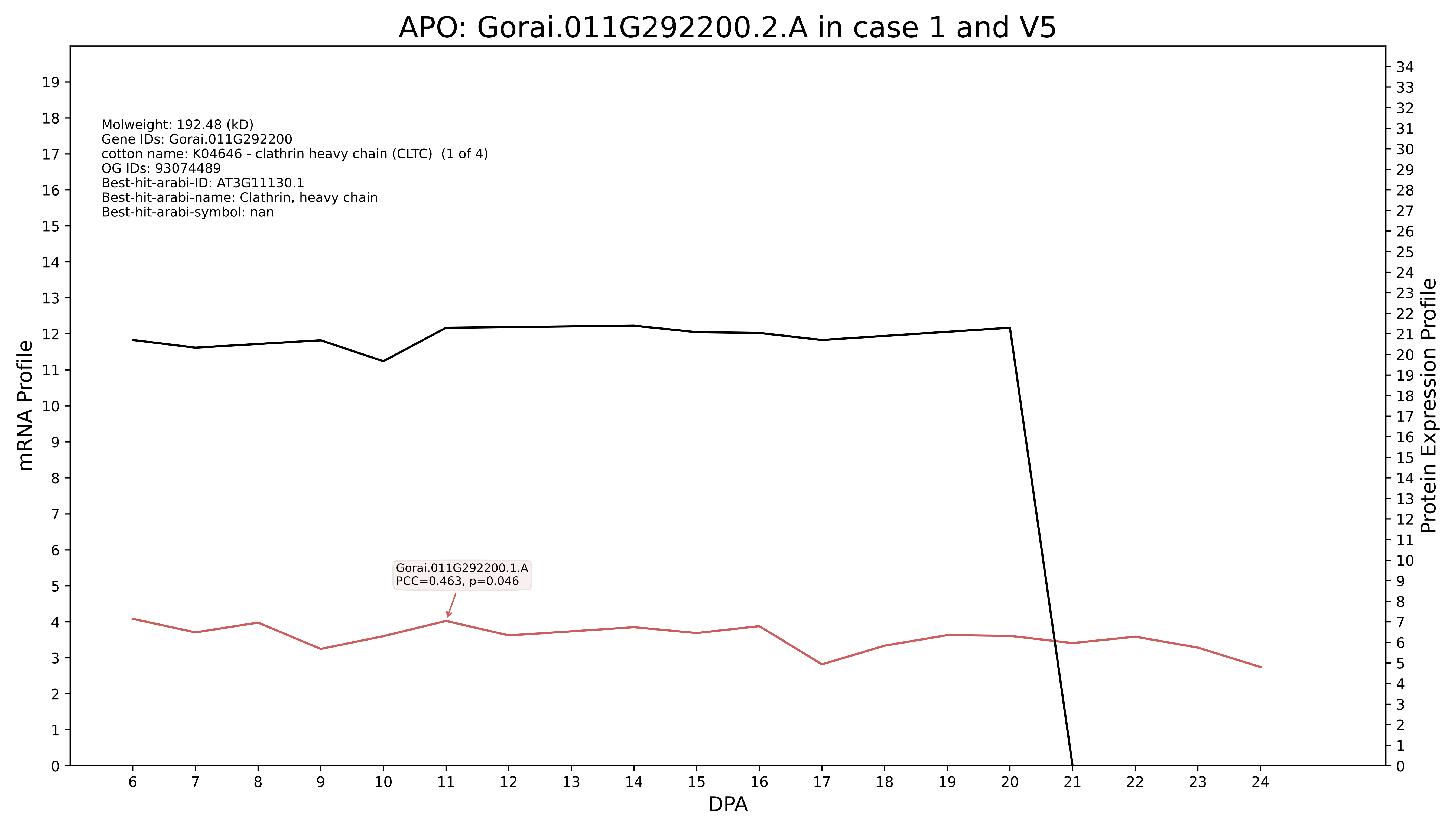
APO: Gorai.011G292200.2.A in case 1 and V5
Molweight: 192.48 (kD)
Gene IDs: Gorai.011G292200
cotton name: K04646 - clathrin heavy chain (CLTC) (1 of 4)
OG IDs: 93074489
Best-hit-arabi-ID: AT3G11130.1
Best-hit-arabi-name: Clathrin, heavy chain
Best-hit-arabi-symbol: nan
Gorai.011G292200.1.A
PCC=0.463, p=0.046
mRNA Profile
Protein Expression Profile
DPA

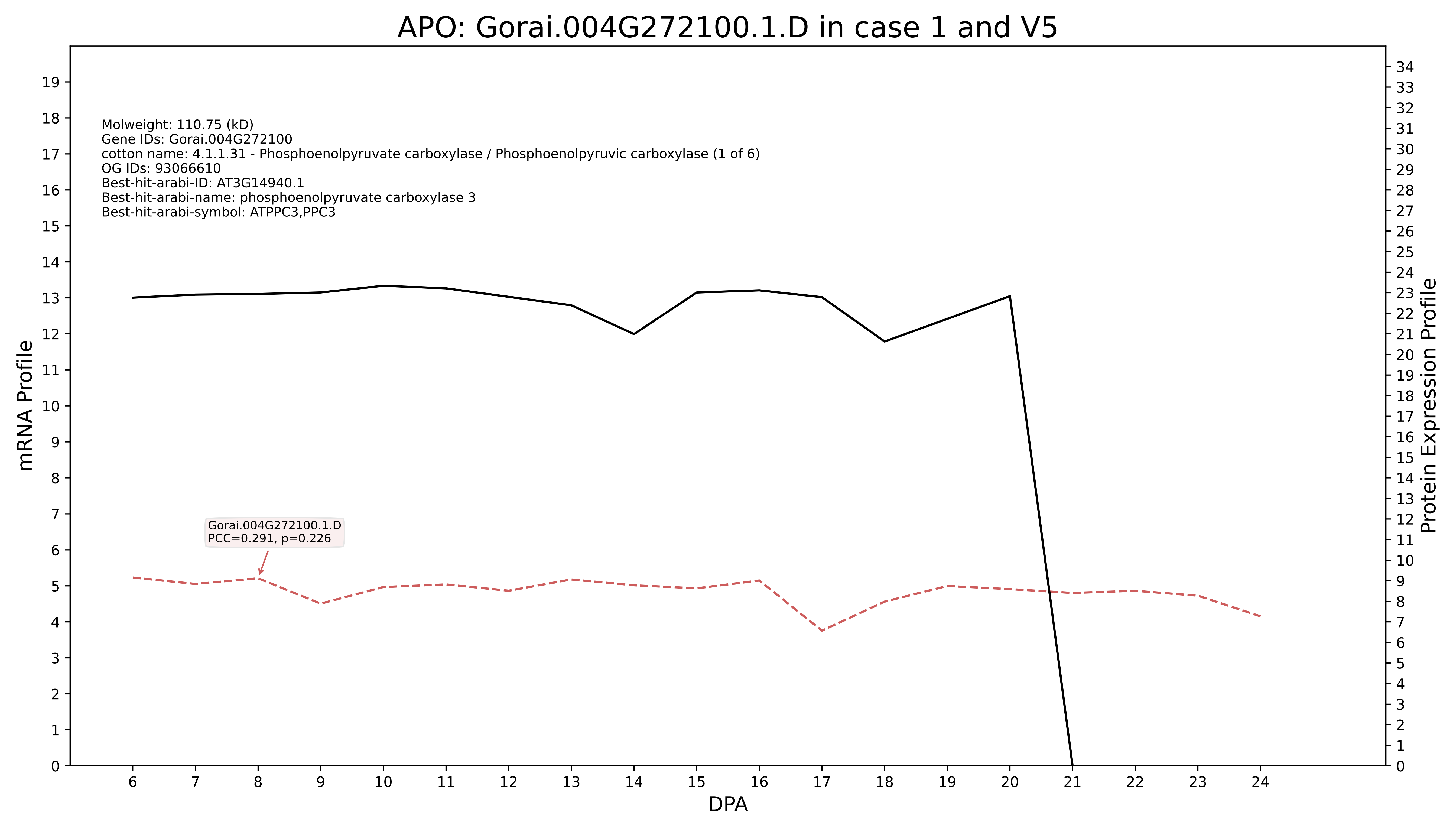
APO: Gorai.004G272100.1.D in case 1 and V5
Molweight: 110.75 (kD)
Gene IDs: Gorai.004G272100
cotton name: 4.1.1.31 - Phosphoenolpyruvate carboxylase / Phosphoenolpyruvic carboxylase (1 of 6)
OG IDs: 93066610
Best-hit-arabi-ID: AT3G14940.1
Best-hit-arabi-name: phosphoenolpyruvate carboxylase 3
Best-hit-arabi-symbol: ATPPC3,PPC3
Gorai.004G272100.1.D
PCC=0.291, p=0.226
mRNA Profile
Protein Expression Profile
DPA

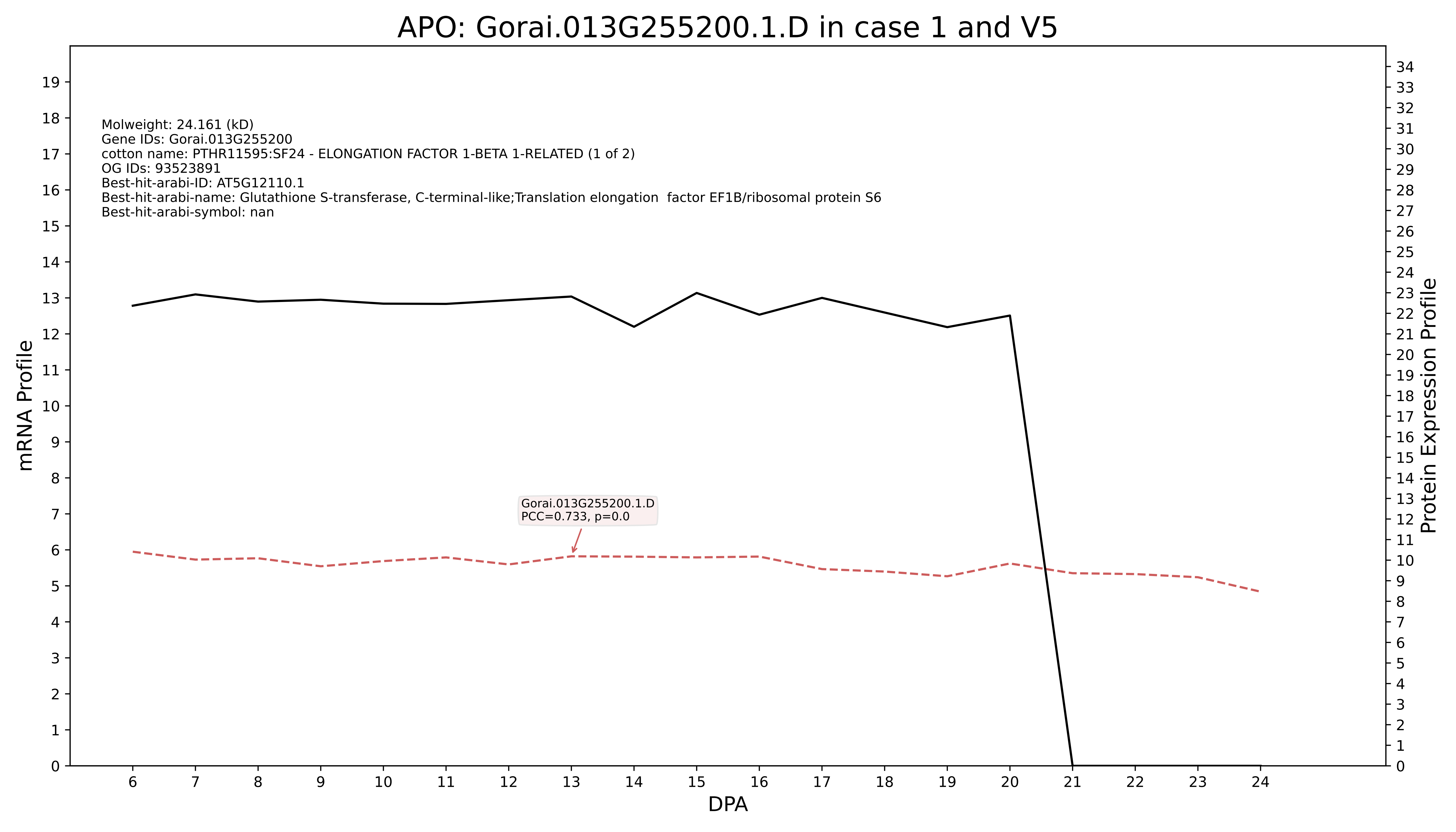

APO: Gorai.013G255200.1.D in case 1 and V5
Molweight: 24.161 (kD)
Gene IDs: Gorai.013G255200
cotton name: PTHR11595:SF24 - ELONGATION FACTOR 1-BETA 1-RELATED (1 of 2)
OG IDs: 93523891
Best-hit-arabi-ID: AT5G12110.1
Best-hit-arabi-name: Glutathione S-transferase, C-terminal-like;Translation elongation factor EF1B/ribosomal protein S6
Best-hit-arabi-symbol: nan
Gorai.013G255200.1.D
PCC=0.733, p=0.0
mRNA Profile
Protein Expression Profile
DPA

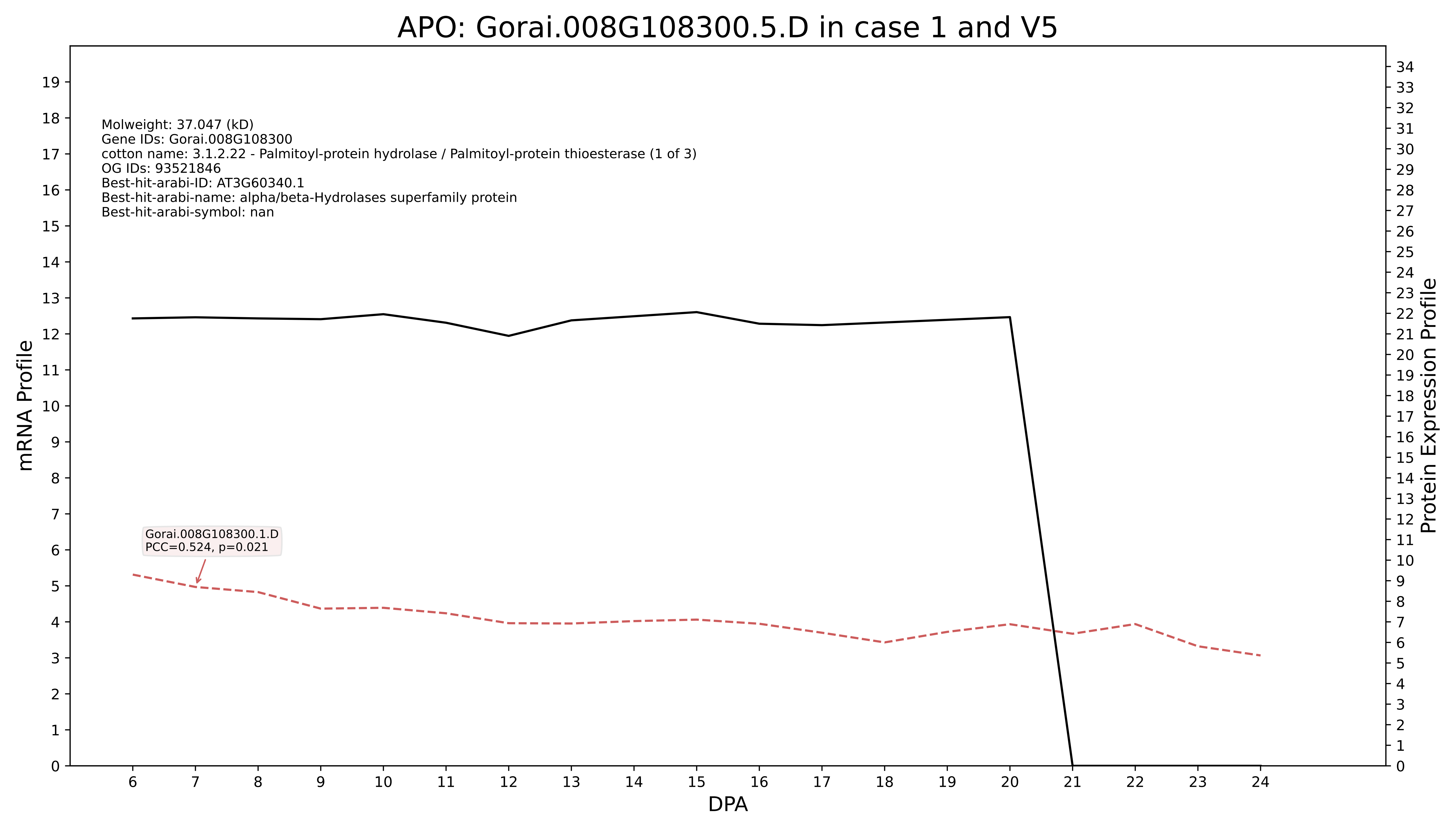
APO: Gorai.008G108300.5.D in case 1 and V5
Molweight: 37.047 (kD)
Gene IDs: Gorai.008G108300
cotton name: 3.1.2.22 - Palmitoyl-protein hydrolase / Palmitoyl-protein thioesterase (1 of 3)
OG IDs: 93521846
Best-hit-arabi-ID: AT3G60340.1
Best-hit-arabi-name: alpha/beta-Hydrolases superfamily protein
Best-hit-arabi-symbol: nan
Gorai.008G108300.1.D
PCC=0.524, p=0.021
mRNA Profile
Protein Expression Profile
DPA

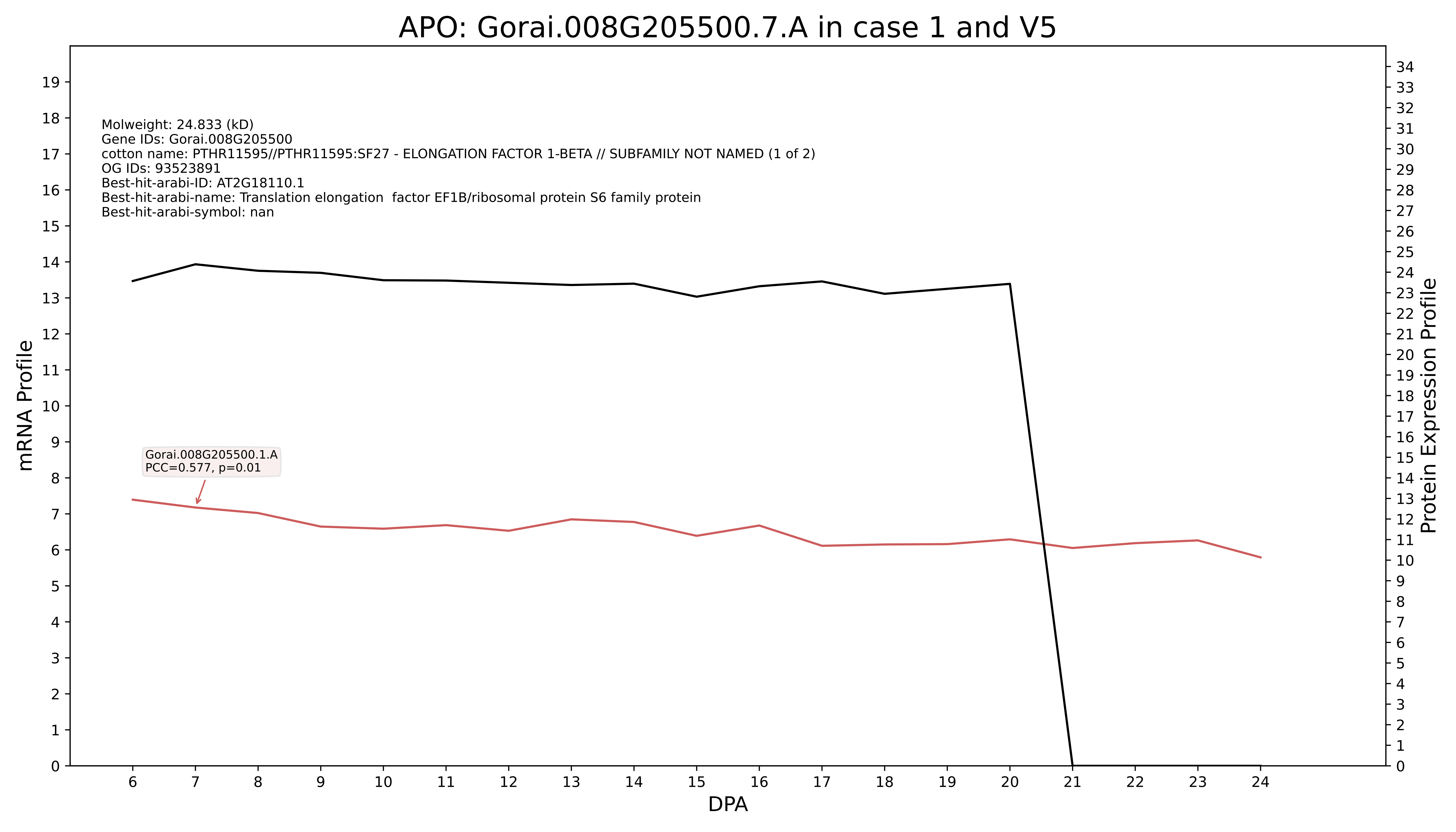

APO: Gorai.008G205500.7.A in case 1 and V5
Molweight: 24.833 (kD)
Gene IDs: Gorai.008G205500
cotton name: PTHR11595//PTHR11595:SF27 - ELONGATION FACTOR 1-BETA // SUBFAMILY NOT NAMED (1 of 2)
OG IDs: 93523891
Best-hit-arabi-ID: AT2G18110.1
Best-hit-arabi-name: Translation elongation factor EF1B/ribosomal protein S6 family protein
Best-hit-arabi-symbol: nan
Gorai.008G205500.1.A
PCC=0.577, p=0.01
mRNA Profile
Protein Expression Profile
DPA

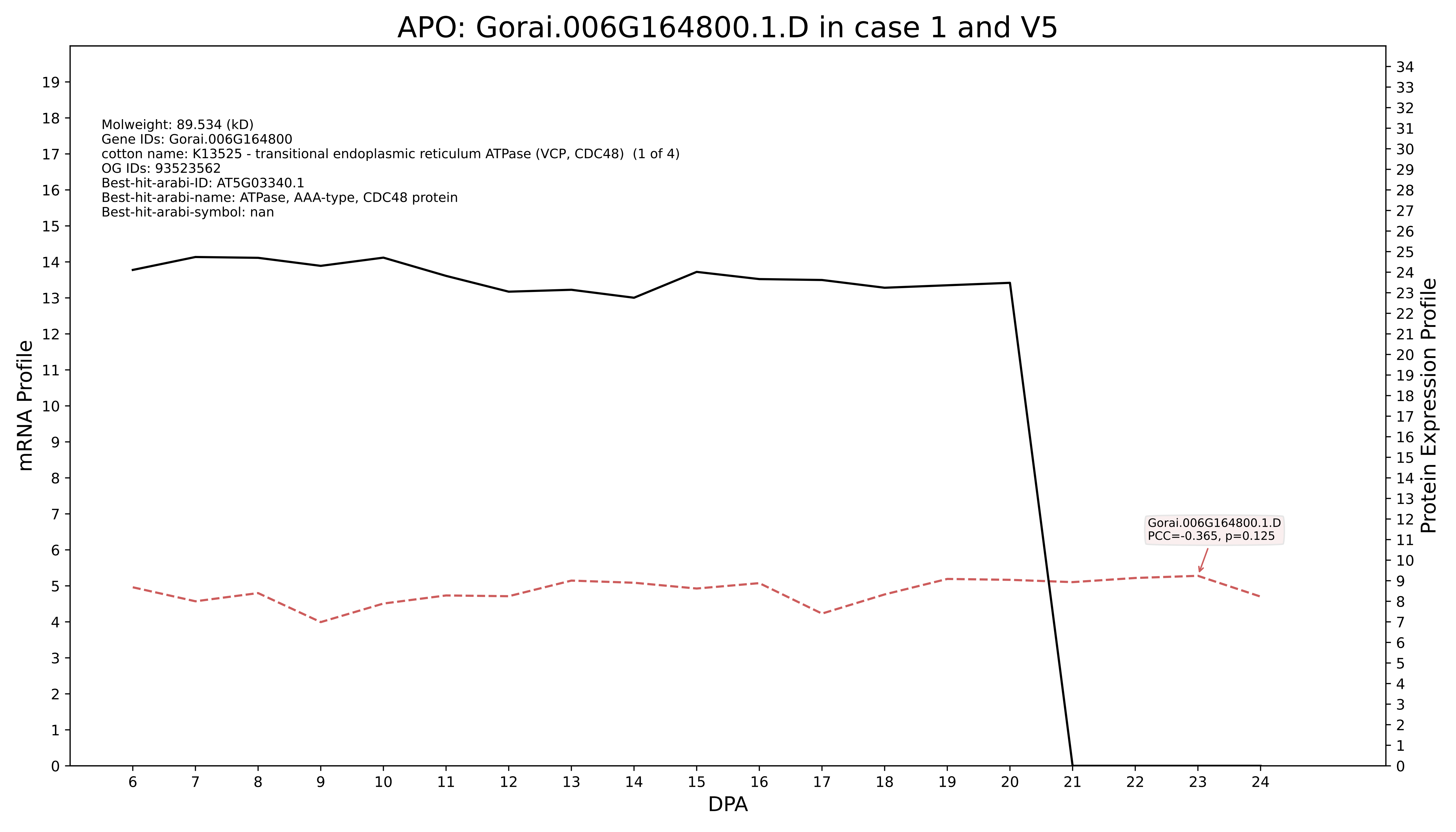

APO: Gorai.006G164800.1.D in case 1 and V5
Molweight: 89.534 (kD)
Gene IDs: Gorai.006G164800
cotton name: K13525 - transitional endoplasmic reticulum ATPase (VCP, CDC48) (1 of 4)
OG IDs: 93523562
Best-hit-arabi-ID: AT5G03340.1
Best-hit-arabi-name: ATPase, AAA-type, CDC48 protein
Best-hit-arabi-symbol: nan
Gorai.006G164800.1.D
PCC=-0.365, p=0.125
mRNA Profile
Protein Expression Profile
DPA

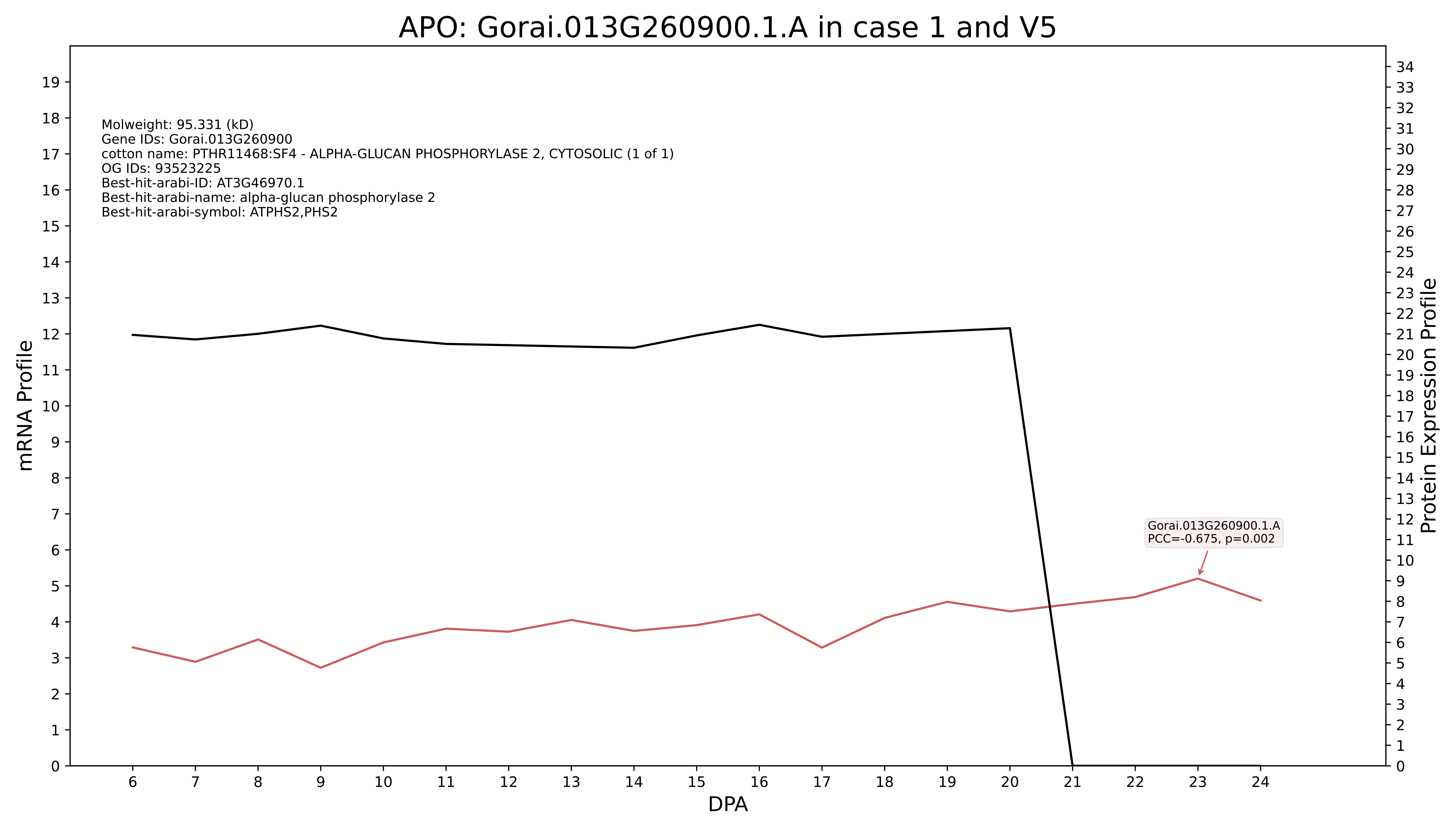

APO: Gorai.013G260900.1.A in case 1 and V5
Molweight: 95.331 (kD)
Gene IDs: Gorai.013G260900
cotton name: PTHR11468:SF4 - ALPHA-GLUCAN PHOSPHORYLASE 2, CYTOSOLIC (1 of 1)
OG IDs: 93523225
Best-hit-arabi-ID: AT3G46970.1
Best-hit-arabi-name: alpha-glucan phosphorylase 2
Best-hit-arabi-symbol: ATPHS2,PHS2
Gorai.013G260900.1.A
PCC=-0.675, p=0.002
mRNA Profile
Protein Expression Profile
DPA

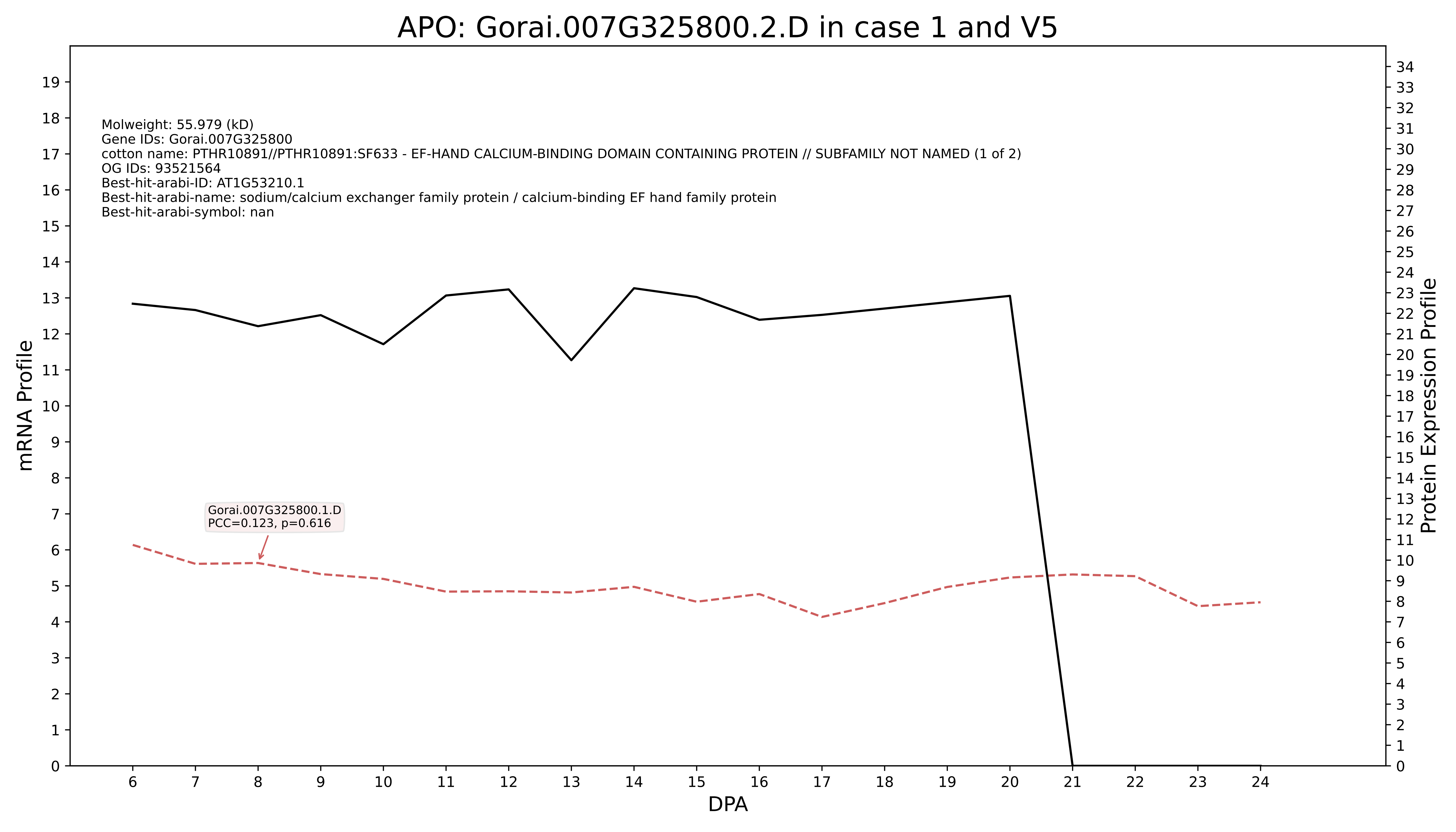
APO: Gorai.007G325800.2.D in case 1 and V5
Molweight: 55.979 (kD)
Gene IDs: Gorai.007G325800
cotton name: PTHR10891//PTHR10891:SF633 - EF-HAND CALCIUM-BINDING DOMAIN CONTAINING PROTEIN // SUBFAMILY NOT NAMED (1 of 2)
OG IDs: 93521564
Best-hit-arabi-ID: AT1G53210.1
Best-hit-arabi-name: sodium/calcium exchanger family protein / calcium-binding EF hand family protein
Best-hit-arabi-symbol: nan
Gorai.007G325800.1.D
PCC=0.123, p=0.616
mRNA Profile
Protein Expression Profile
DPA

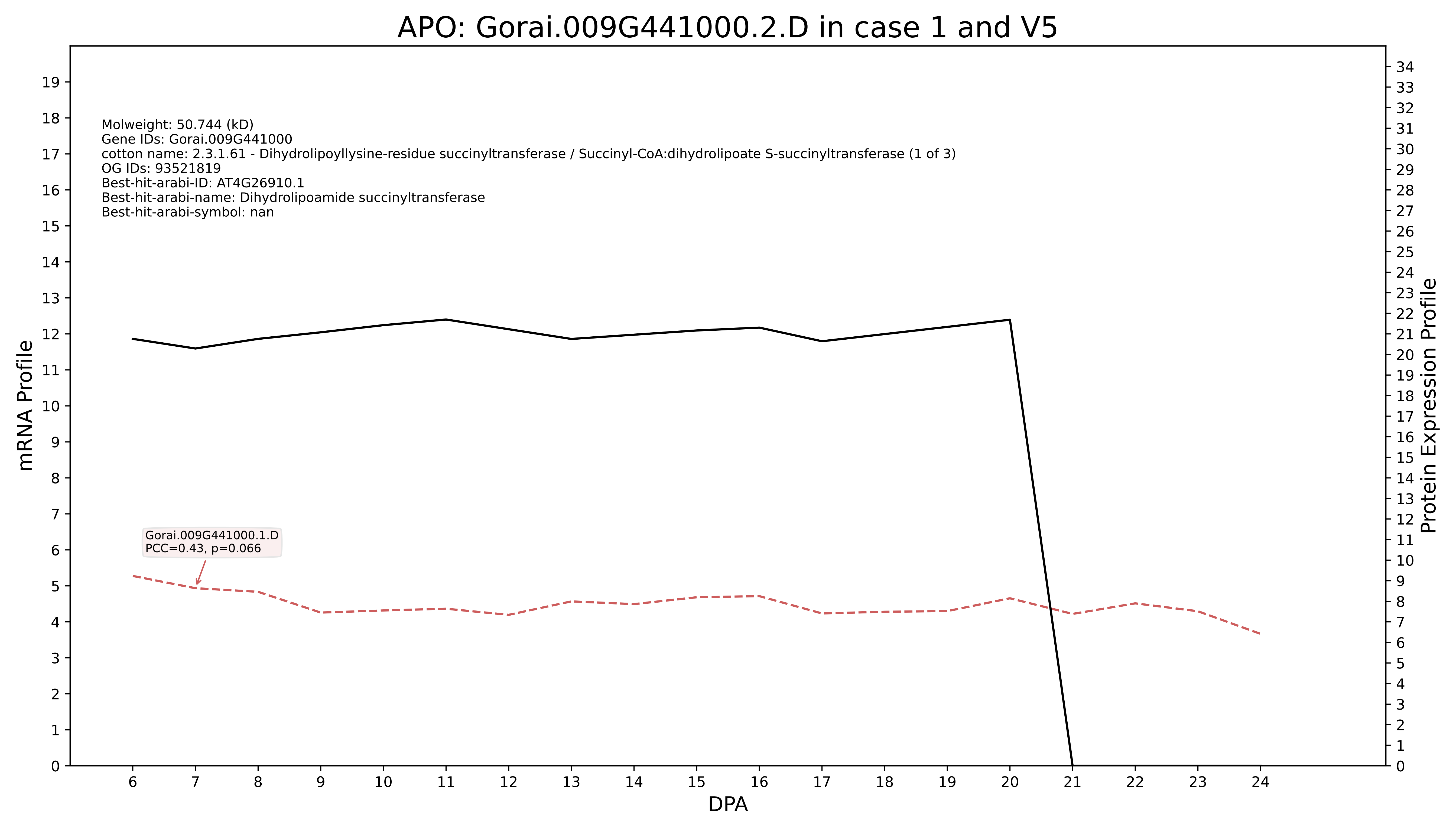

APO: Gorai.009G441000.2.D in case 1 and V5
Molweight: 50.744 (kD)
Gene IDs: Gorai.009G441000
cotton name: 2.3.1.61 - Dihydrolipoyllysine-residue succinyltransferase / Succinyl-CoA:dihydrolipoate S-succinyltransferase (1 of 3)
OG IDs: 93521819
Best-hit-arabi-ID: AT4G26910.1
Best-hit-arabi-name: Dihydrolipoamide succinyltransferase
Best-hit-arabi-symbol: nan
Gorai.009G441000.1.D
PCC=0.43, p=0.066
mRNA Profile
Protein Expression Profile
DPA

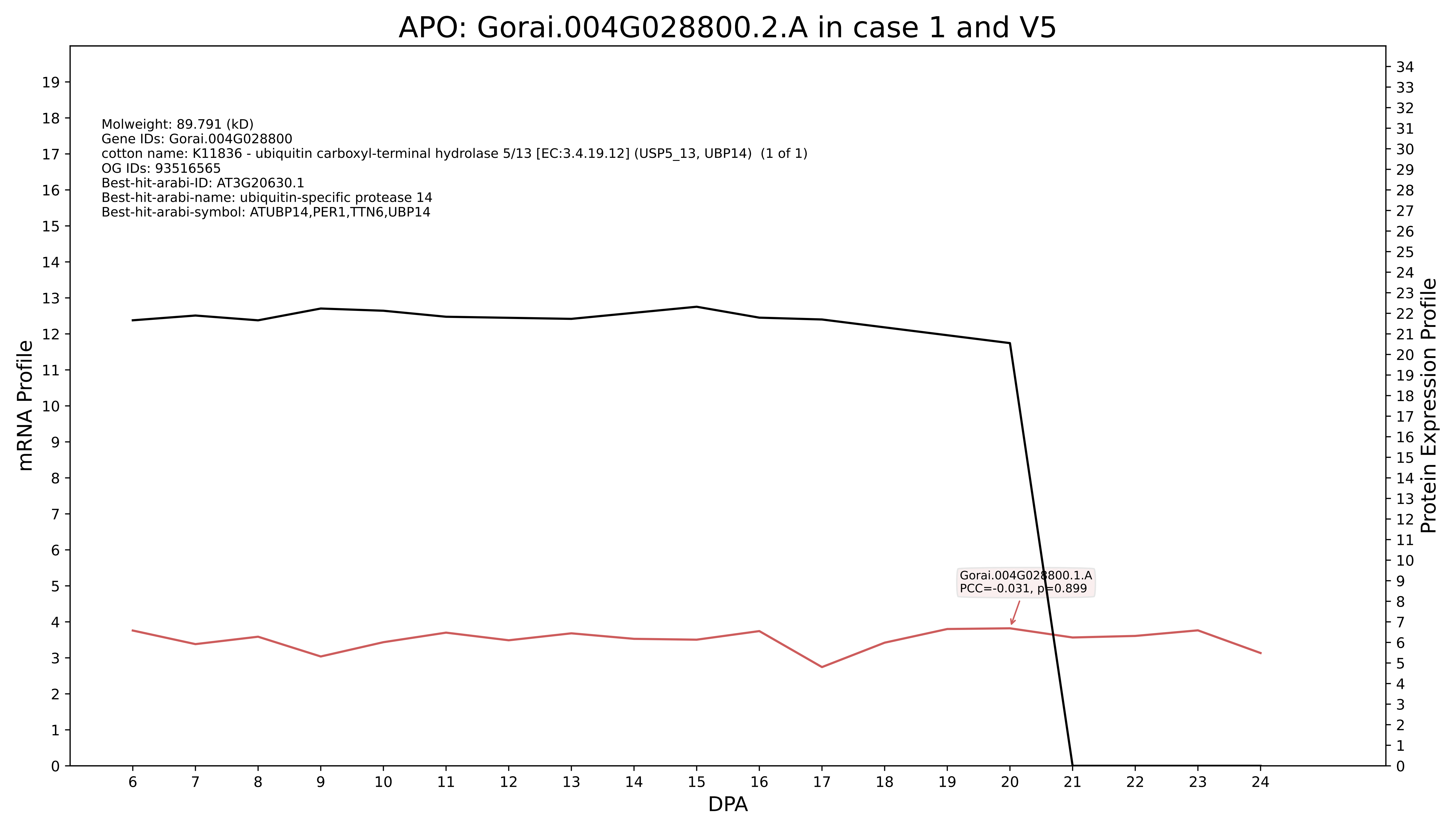
APO: Gorai.004G028800.2.A in case 1 and V5
Molweight: 89.791 (kD)
Gene IDs: Gorai.004G028800
cotton name: K11836 - ubiquitin carboxyl-terminal hydrolase 5/13 [EC:3.4.19.12] (USP5_13, UBP14) (1 of 1)
OG IDs: 93516565
Best-hit-arabi-ID: AT3G20630.1
Best-hit-arabi-name: ubiquitin-specific protease 14
Best-hit-arabi-symbol: ATUBP14,PER1,TTN6,UBP14
Gorai.004G028800.1.A
PCC=-0.031, p=0.899
mRNA Profile
Protein Expression Profile
DPA

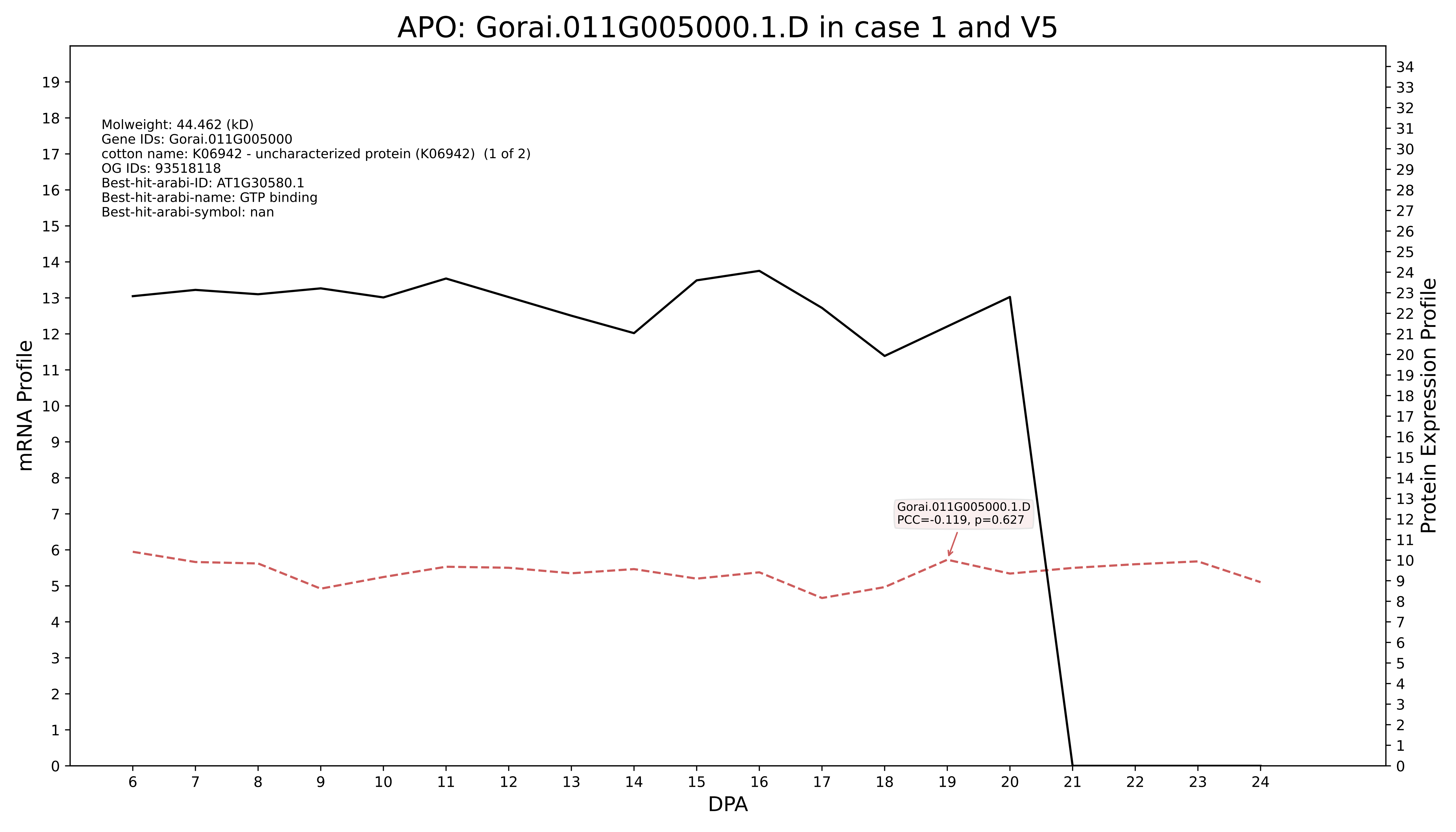
APO: Gorai.011G005000.1.D in case 1 and V5
Molweight: 44.462 (kD)
Gene IDs: Gorai.011G005000
cotton name: K06942 - uncharacterized protein (K06942) (1 of 2)
OG IDs: 93518118
Best-hit-arabi-ID: AT1G30580.1
Best-hit-arabi-name: GTP binding
Best-hit-arabi-symbol: nan
Gorai.011G005000.1.D
PCC=-0.119, p=0.627
mRNA Profile
Protein Expression Profile
DPA

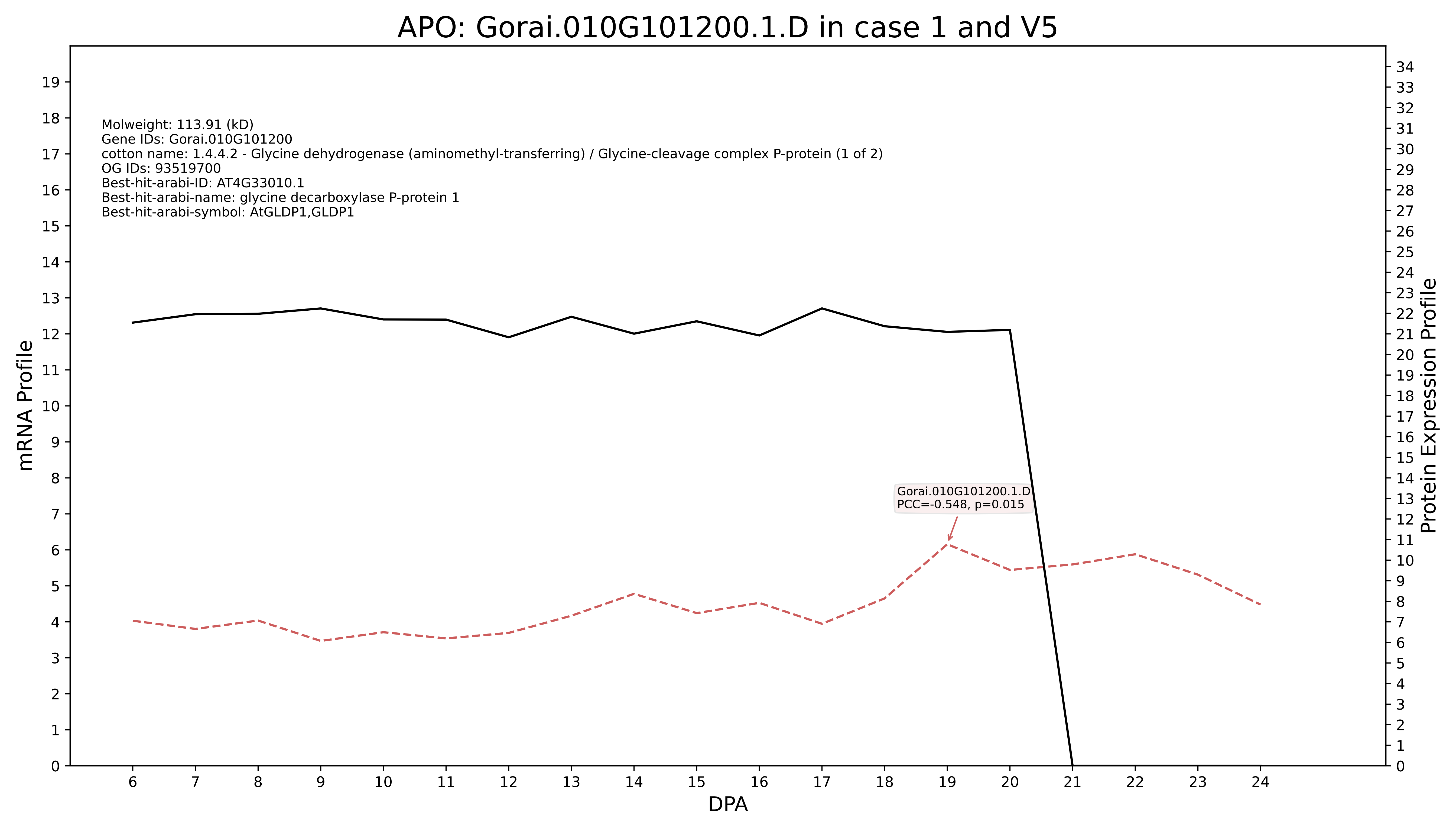
APO: Gorai.010G101200.1.D in case 1 and V5
Molweight: 113.91 (kD)
Gene IDs: Gorai.010G101200
cotton name: 1.4.4.2 - Glycine dehydrogenase (aminomethyl-transferring) / Glycine-cleavage complex P-protein (1 of 2)
OG IDs: 93519700
Best-hit-arabi-ID: AT4G33010.1
Best-hit-arabi-name: glycine decarboxylase P-protein 1
Best-hit-arabi-symbol: AtGLDP1,GLDP1
Gorai.010G101200.1.D
PCC=-0.548, p=0.015
mRNA Profile
Protein Expression Profile
DPA

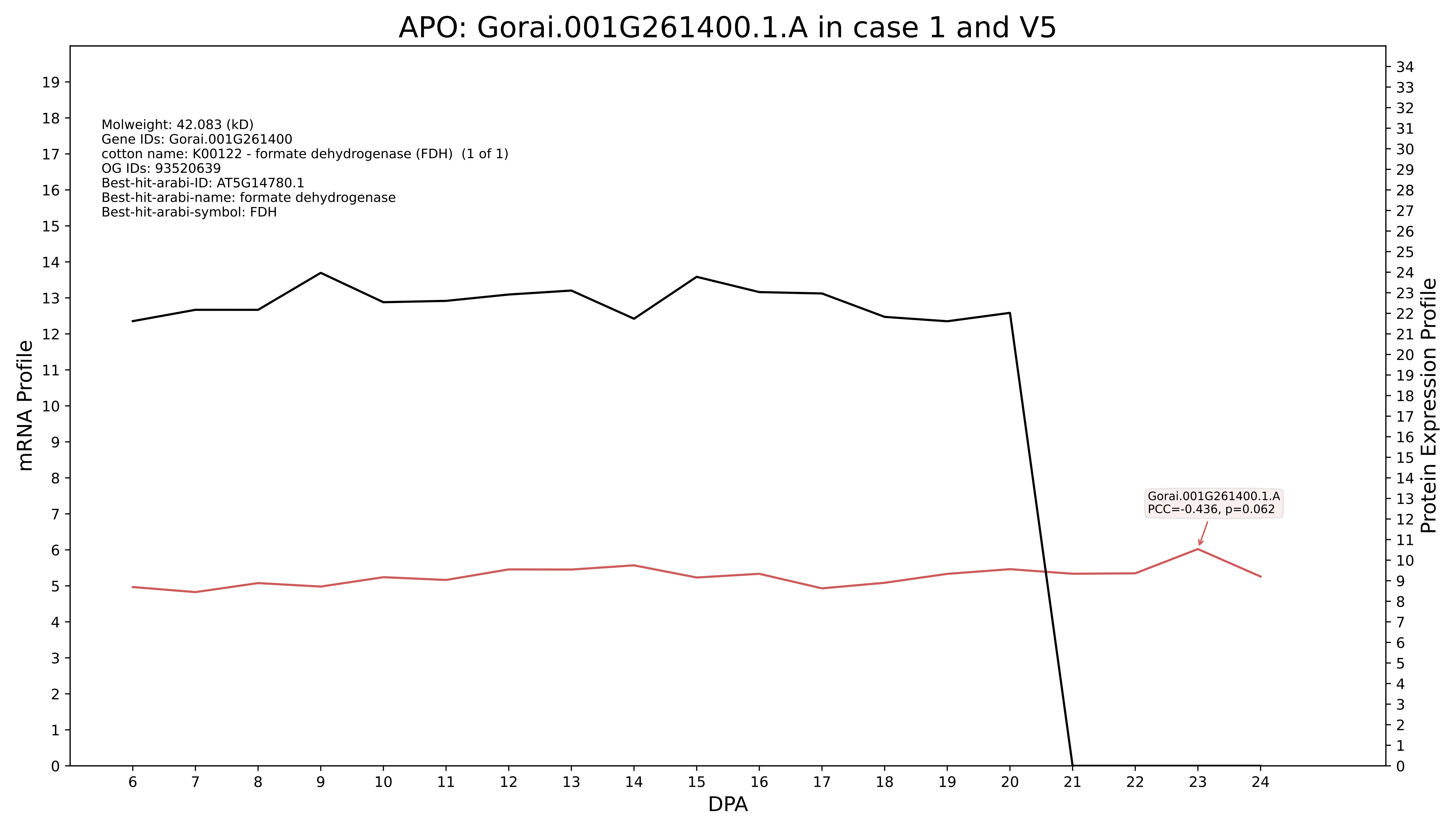
APO: Gorai.001G261400.1.A in case 1 and V5
Molweight: 42.083 (kD)
Gene IDs: Gorai.001G261400
cotton name: K00122 - formate dehydrogenase (FDH) (1 of 1)
OG IDs: 93520639
Best-hit-arabi-ID: AT5G14780.1
Best-hit-arabi-name: formate dehydrogenase
Best-hit-arabi-symbol: FDH
Gorai.001G261400.1.A
PCC=-0.436, p=0.062
mRNA Profile
Protein Expression Profile
DPA

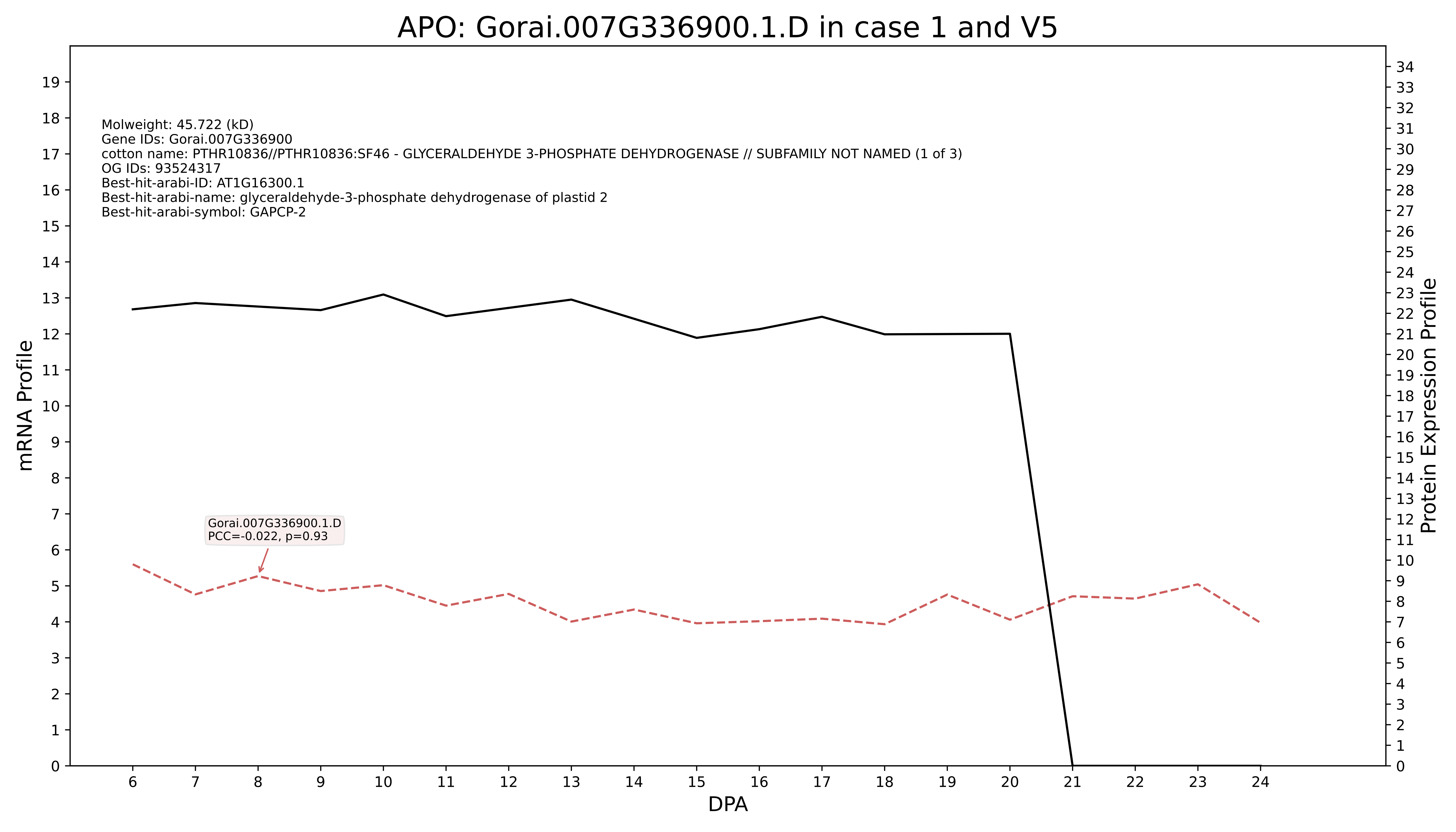
APO: Gorai.007G336900.1.D in case 1 and V5
Molweight: 45.722 (kD)
Gene IDs: Gorai.007G336900
cotton name: PTHR10836//PTHR10836:SF46 - GLYCERALDEHYDE 3-PHOSPHATE DEHYDROGENASE // SUBFAMILY NOT NAMED (1 of 3)
OG IDs: 93524317
Best-hit-arabi-ID: AT1G16300.1
Best-hit-arabi-name: glyceraldehyde-3-phosphate dehydrogenase of plastid 2
Best-hit-arabi-symbol: GAPCP-2
Gorai.007G336900.1.D
PCC=-0.022, p=0.93
mRNA Profile
Protein Expression Profile
DPA

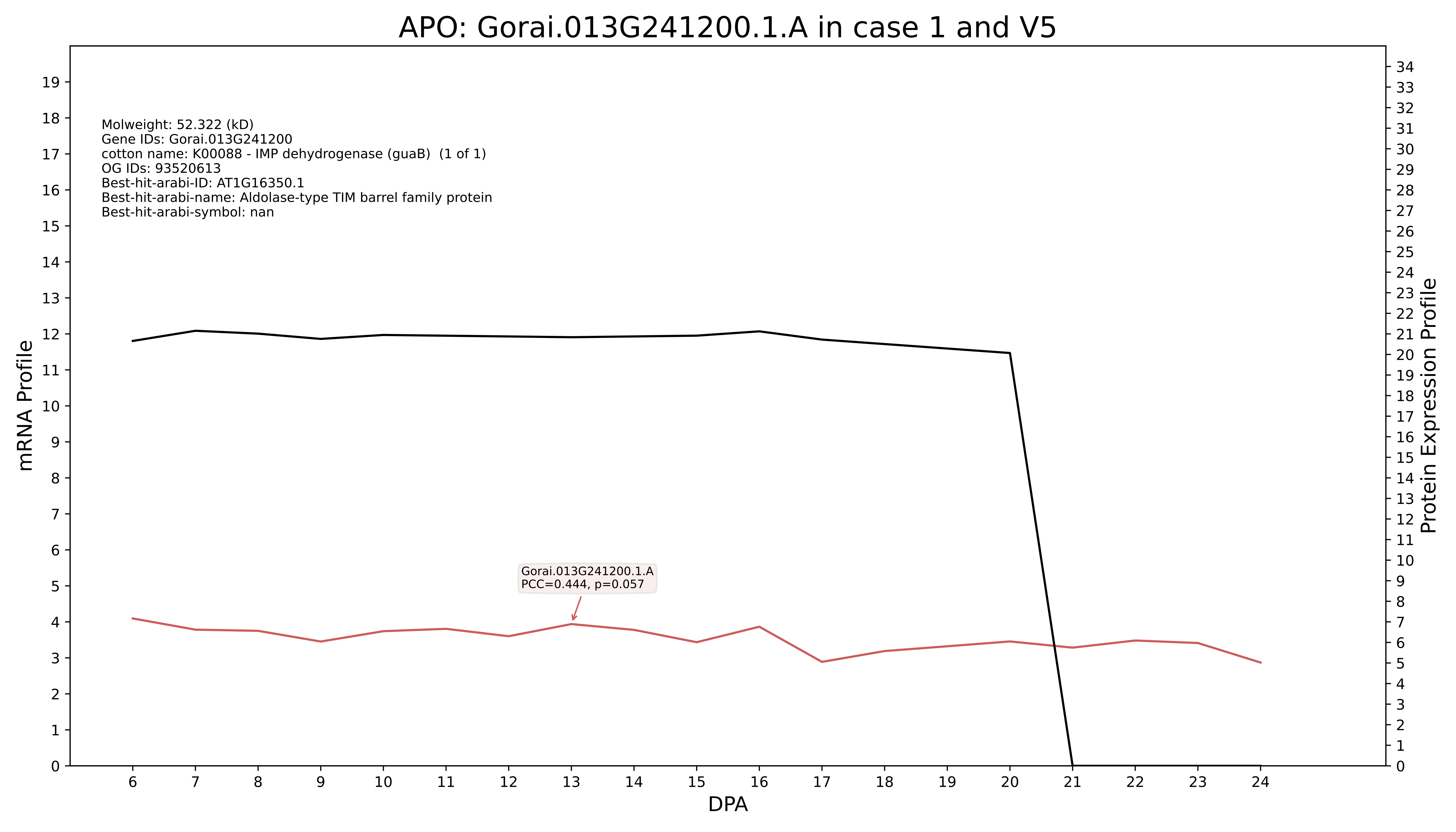
APO: Gorai.013G241200.1.A in case 1 and V5
Molweight: 52.322 (kD)
Gene IDs: Gorai.013G241200
cotton name: K00088 - IMP dehydrogenase (guaB) (1 of 1)
OG IDs: 93520613
Best-hit-arabi-ID: AT1G16350.1
Best-hit-arabi-name: Aldolase-type TIM barrel family protein
Best-hit-arabi-symbol: nan
Gorai.013G241200.1.A
PCC=0.444, p=0.057
mRNA Profile
Protein Expression Profile
DPA

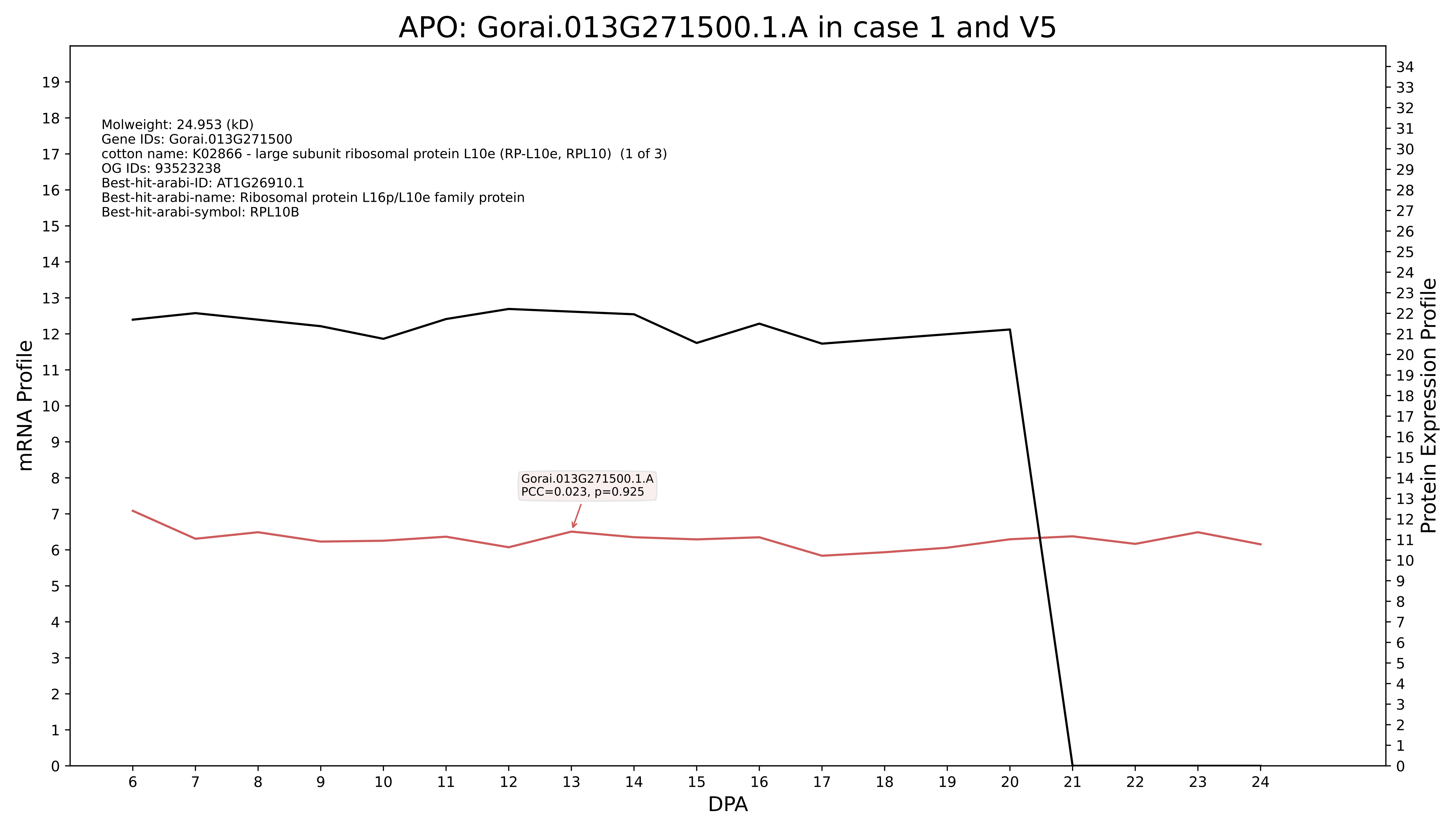
APO: Gorai.013G271500.1.A in case 1 and V5
Molweight: 24.953 (kD)
Gene IDs: Gorai.013G271500
cotton name: K02866 - large subunit ribosomal protein L10e (RP-L10e, RPL10) (1 of 3)
OG IDs: 93523238
Best-hit-arabi-ID: AT1G26910.1
Best-hit-arabi-name: Ribosomal protein L16p/L10e family protein
Best-hit-arabi-symbol: RPL10B
Gorai.013G271500.1.A
PCC=0.023, p=0.925
mRNA Profile
Protein Expression Profile
DPA

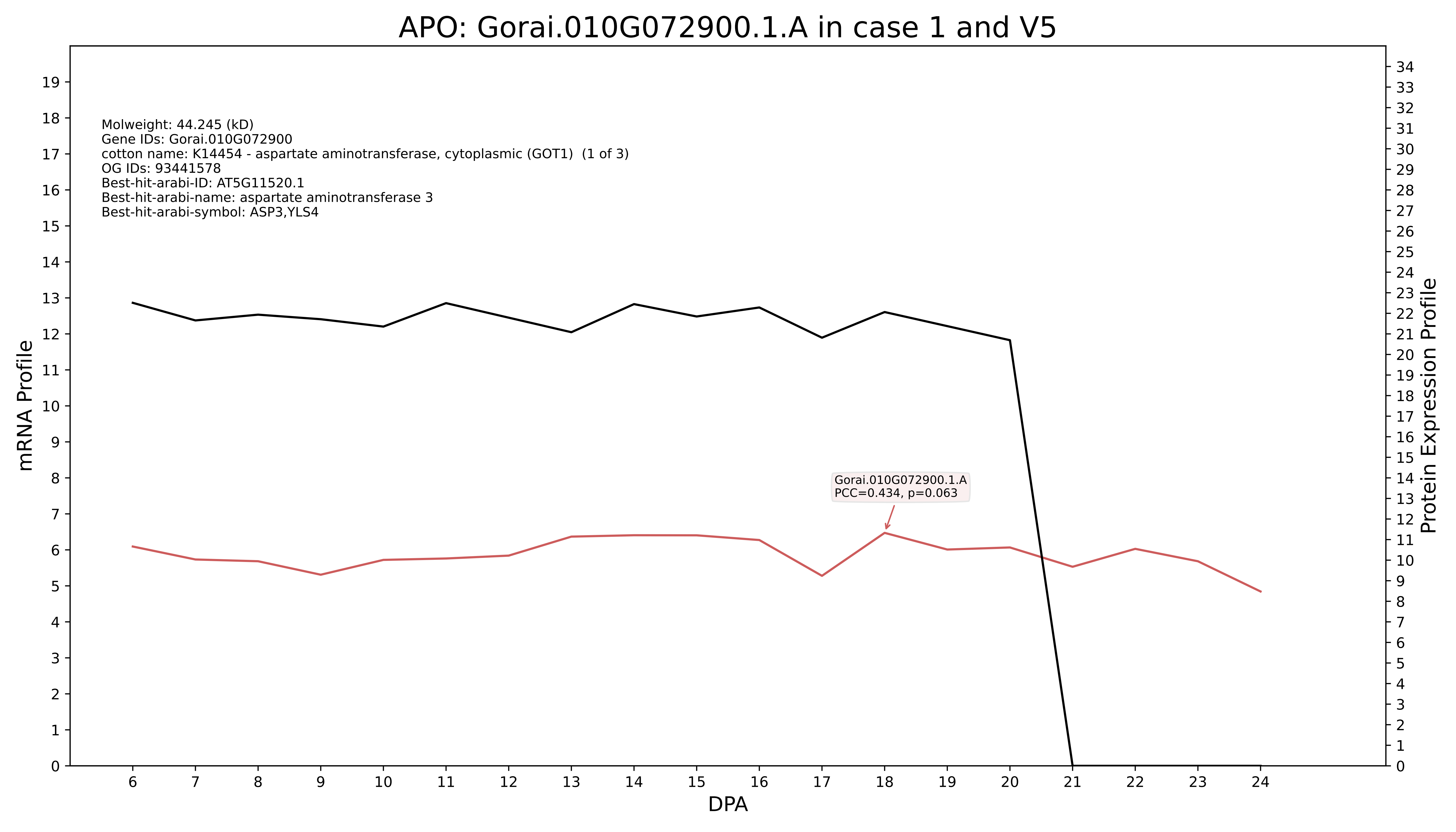
APO: Gorai.010G072900.1.A in case 1 and V5
Molweight: 44.245 (kD)
Gene IDs: Gorai.010G072900
cotton name: K14454 - aspartate aminotransferase, cytoplasmic (GOT1) (1 of 3)
OG IDs: 93441578
Best-hit-arabi-ID: AT5G11520.1
Best-hit-arabi-name: aspartate aminotransferase 3
Best-hit-arabi-symbol: ASP3,YLS4
Gorai.010G072900.1.A
PCC=0.434, p=0.063
mRNA Profile
Protein Expression Profile
DPA

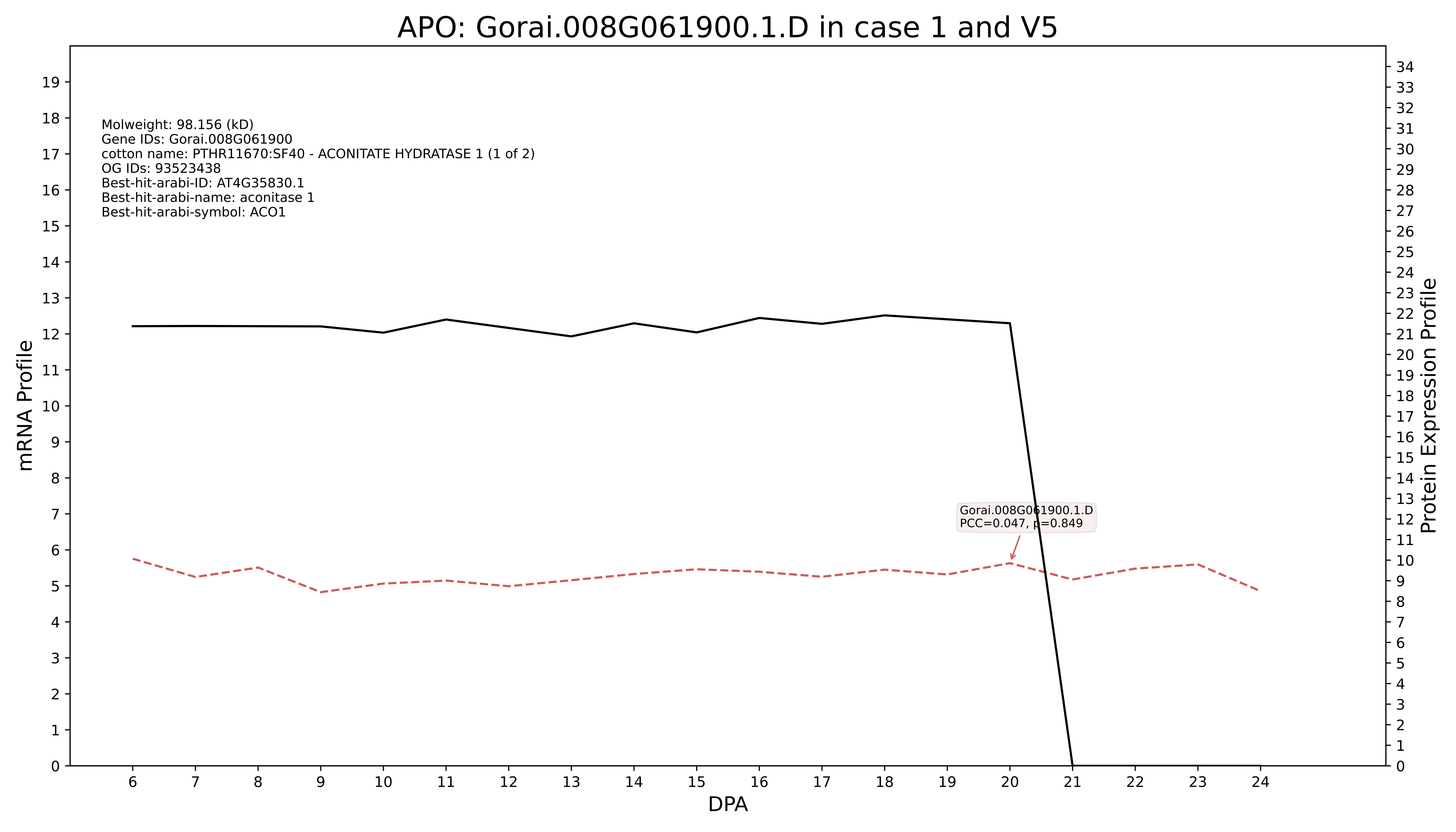
APO: Gorai.008G061900.1.D in case 1 and V5
Molweight: 98.156 (kD)
Gene IDs: Gorai.008G061900
cotton name: PTHR11670:SF40 - ACONITATE HYDRATASE 1 (1 of 2)
OG IDs: 93523438
Best-hit-arabi-ID: AT4G35830.1
Best-hit-arabi-name: aconitase 1
Best-hit-arabi-symbol: ACO1
Gorai.008G061900.1.D
PCC=0.047, p=0.849
mRNA Profile
Protein Expression Profile
DPA
0 1 2 3 4 5 6 7 8 9 10 11 12 13 14 15 16 17 18 19
0 1 2 3 4 5 6 7 8 9 10 11 12 13 14 15 16 17 18 19 20 21 22 23 24 25 26 27 28 29 30 31 32 33 34
6 7 8 9 10 11 12 13 14 15 16 17 18 19 20 21 22 23 24

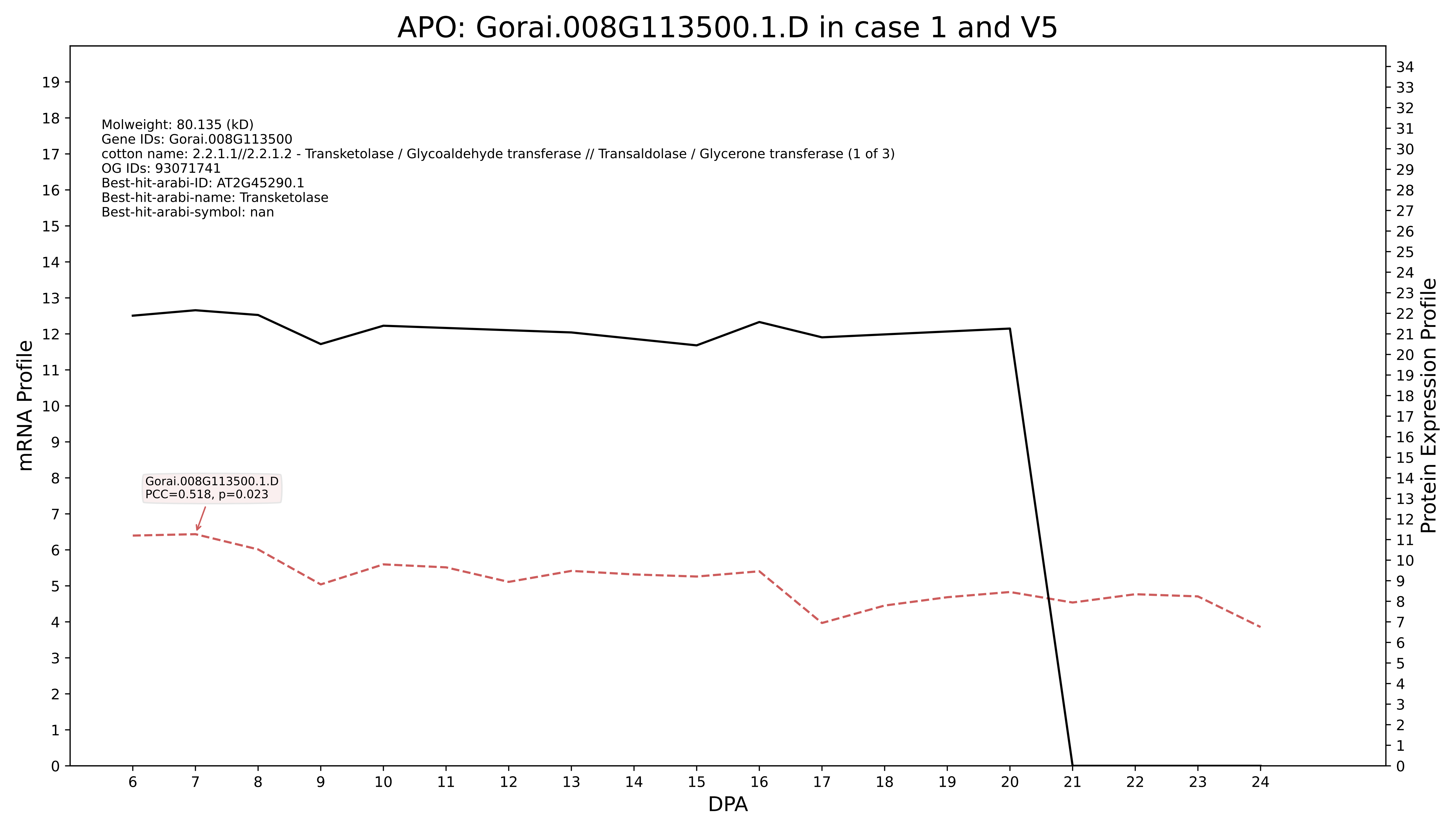
APO: Gorai.008G113500.1.D in case 1 and V5
Molweight: 80.135 (kD)
Gene IDs: Gorai.008G113500
cotton name: 2.2.1.1//2.2.1.2 - Transketolase / Glycoaldehyde transferase // Transaldolase / Glycerone transferase (1 of 3)
OG IDs: 93071741
Best-hit-arabi-ID: AT2G45290.1
Best-hit-arabi-name: Transketolase
Best-hit-arabi-symbol: nan
Gorai.008G113500.1.D
PCC=0.518, p=0.023
mRNA Profile
Protein Expression Profile
DPA

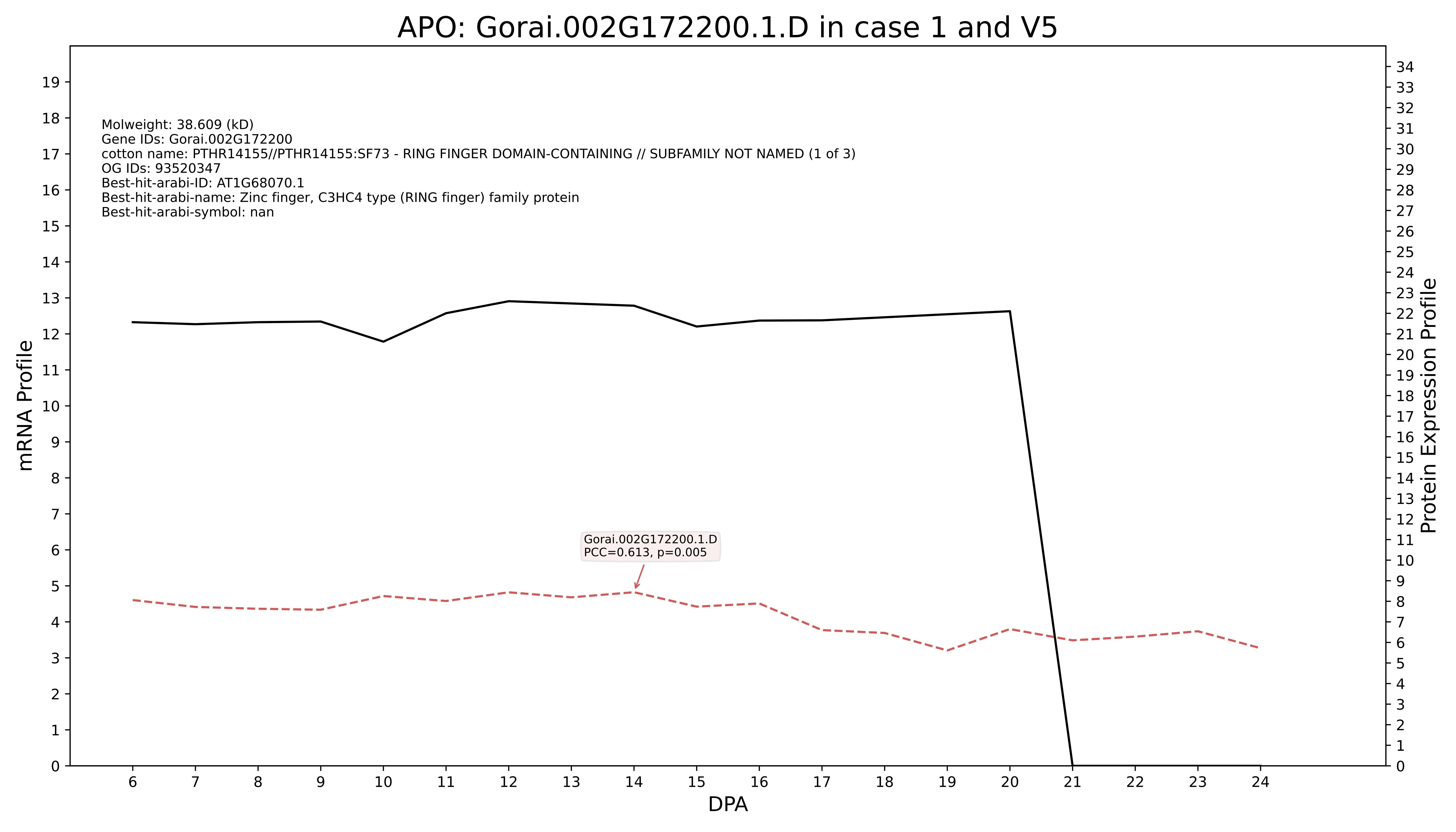
APO: Gorai.002G172200.1.D in case 1 and V5
Molweight: 38.609 (kD)
Gene IDs: Gorai.002G172200
cotton name: PTHR14155//PTHR14155:SF73 - RING FINGER DOMAIN-CONTAINING // SUBFAMILY NOT NAMED (1 of 3)
OG IDs: 93520347
Best-hit-arabi-ID: AT1G68070.1
Best-hit-arabi-name: Zinc finger, C3HC4 type (RING finger) family protein
Best-hit-arabi-symbol: nan
Gorai.002G172200.1.D
PCC=0.613, p=0.005
mRNA Profile
Protein Expression Profile
DPA

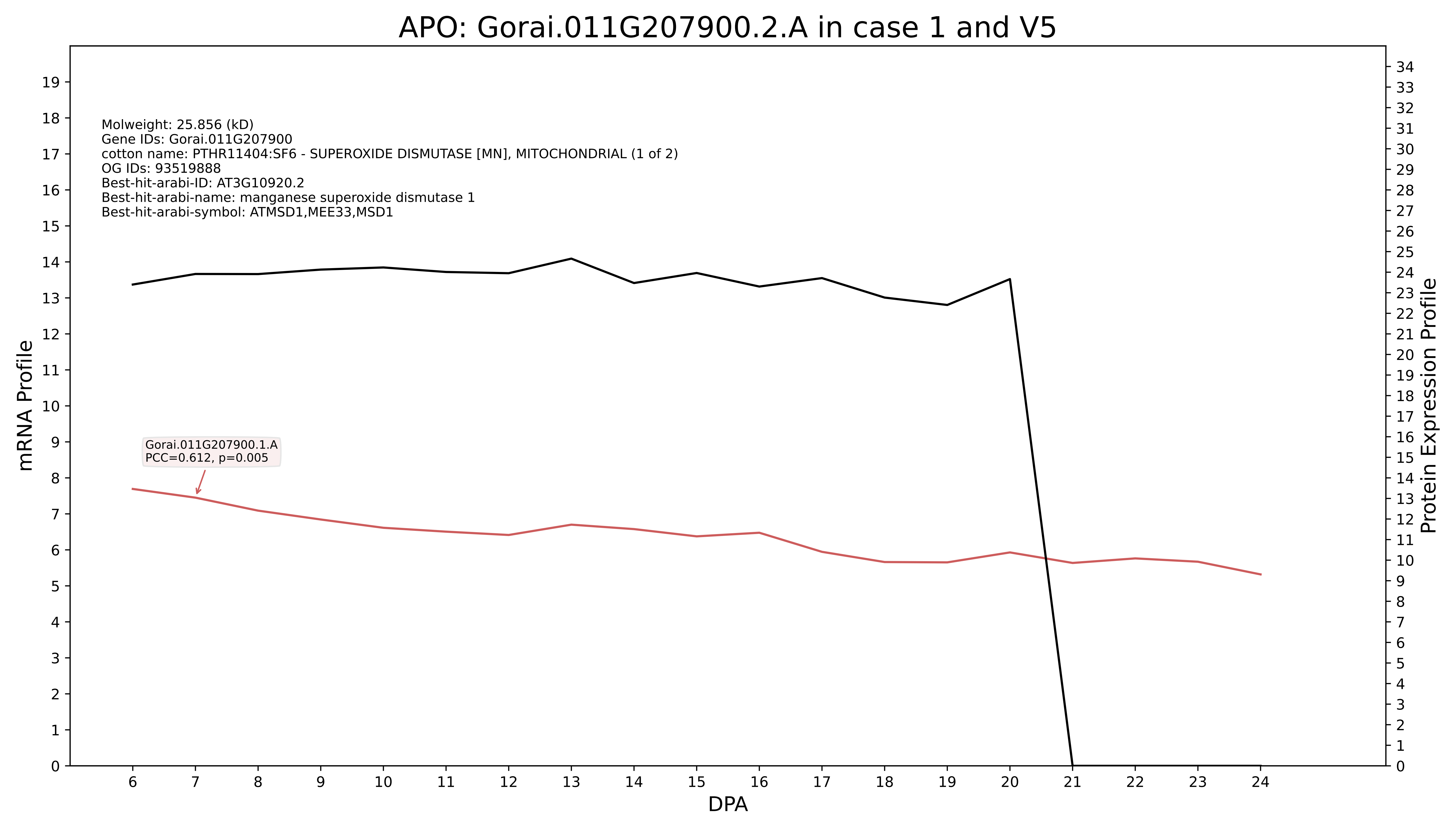
APO: Gorai.011G207900.2.A in case 1 and V5
Molweight: 25.856 (kD)
Gene IDs: Gorai.011G207900
cotton name: PTHR11404:SF6 - SUPEROXIDE DISMUTASE [MN], MITOCHONDRIAL (1 of 2)
OG IDs: 93519888
Best-hit-arabi-ID: AT3G10920.2
Best-hit-arabi-name: manganese superoxide dismutase 1
Best-hit-arabi-symbol: ATMSD1,MEE33,MSD1
Gorai.011G207900.1.A
PCC=0.612, p=0.005
mRNA Profile
Protein Expression Profile
DPA
0
1
2
3
4
5
6
7
8
9
10
11
12
13
14
15
16
17
18
19
20
21
22
23
24
25
26
27
28
29
30
31
32
33
34

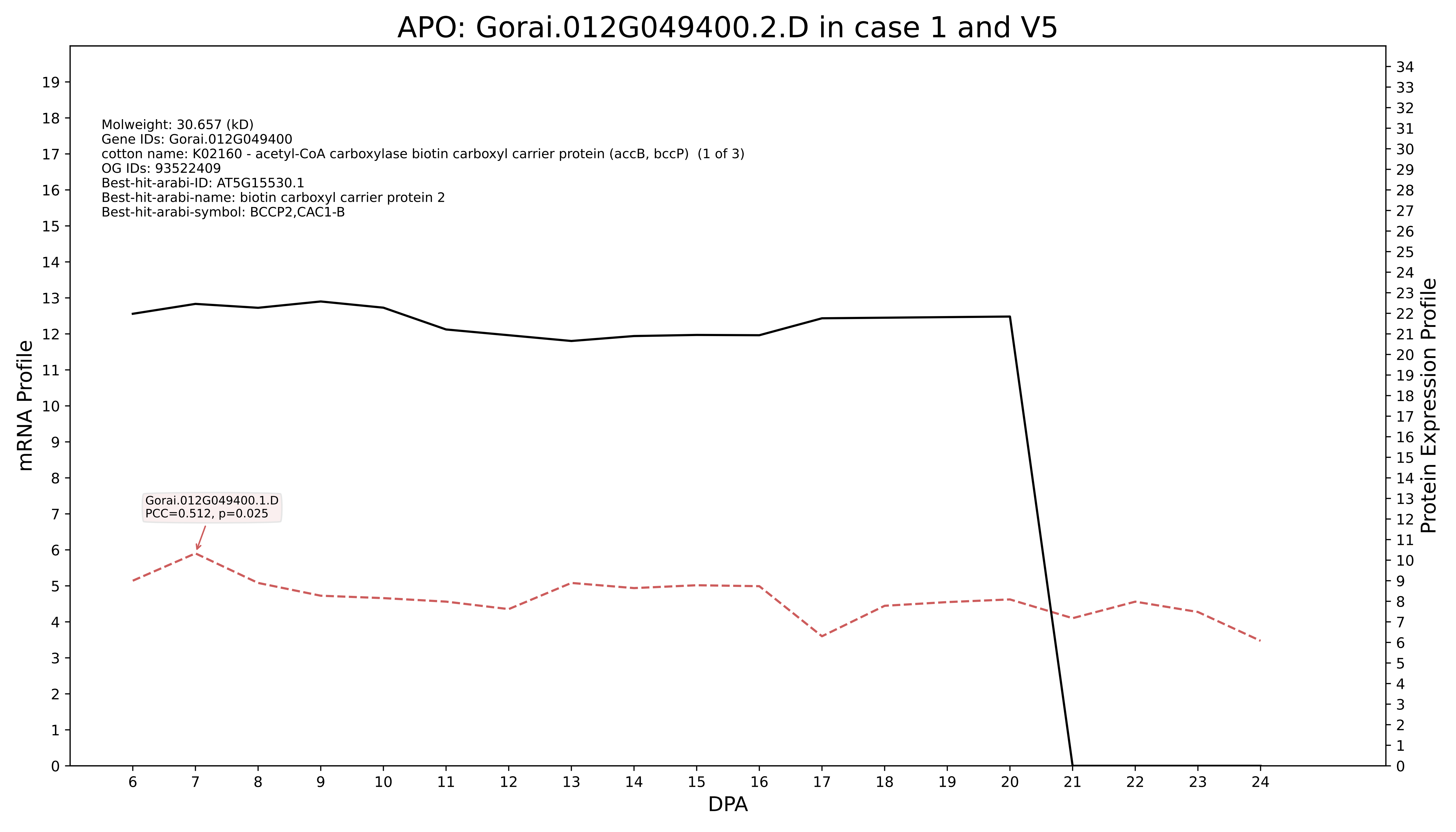
APO: Gorai.012G049400.2.D in case 1 and V5
Molweight: 30.657 (kD)
Gene IDs: Gorai.012G049400
cotton name: K02160 - acetyl-CoA carboxylase biotin carboxyl carrier protein (accB, bccP) (1 of 3)
OG IDs: 93522409
Best-hit-arabi-ID: AT5G15530.1
Best-hit-arabi-name: biotin carboxyl carrier protein 2
Best-hit-arabi-symbol: BCCP2,CAC1-B
Gorai.012G049400.1.D
PCC=0.512, p=0.025
mRNA Profile
Protein Expression Profile
DPA
0
1
2
3
4
5
6
7
8
9
10
11
12
13
14
15
16
17
18
19
20
21
22
23
24
25
26
27
28
29
30
31
32
33
34

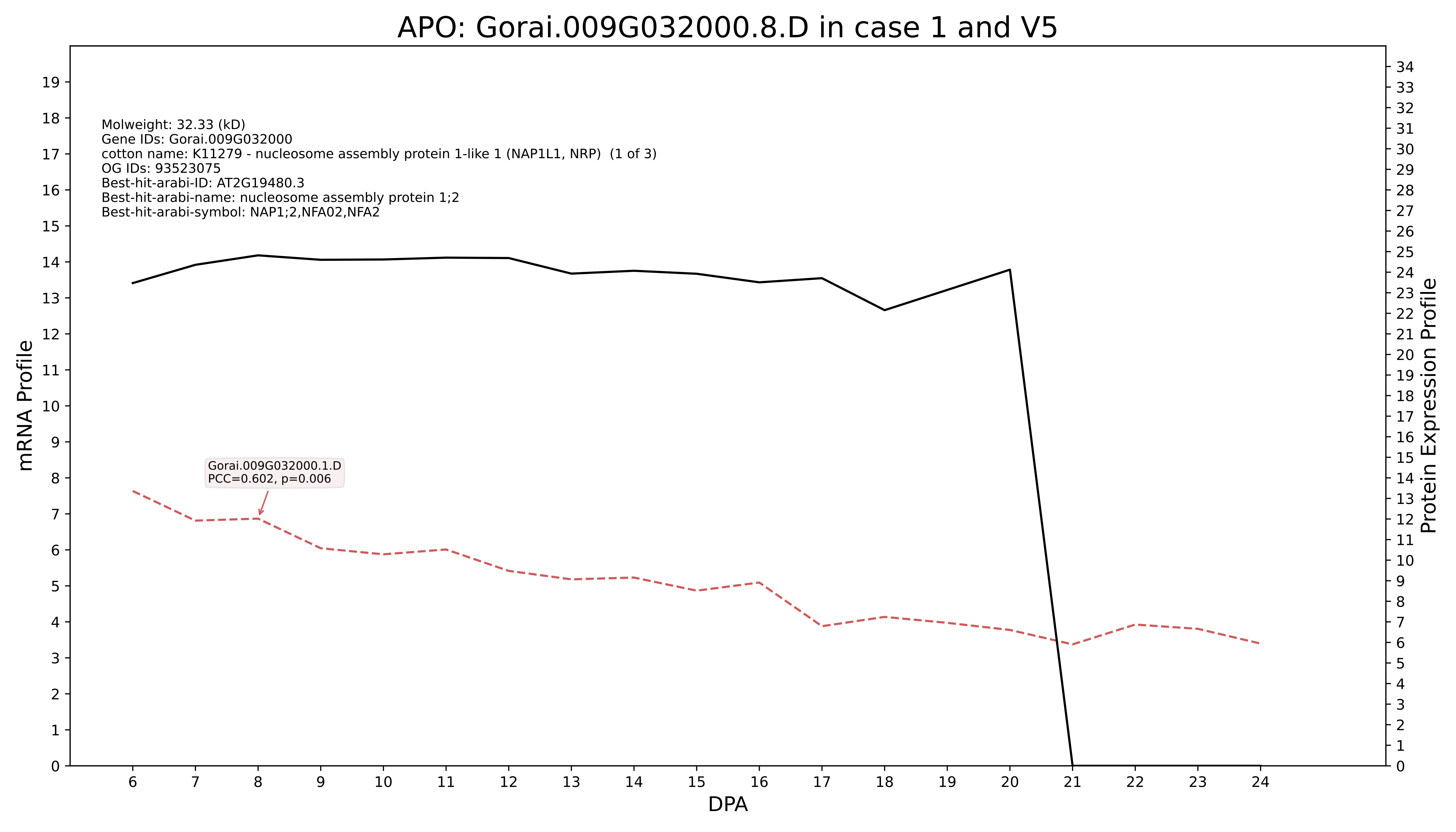
APO: Gorai.009G032000.8.D in case 1 and V5
Molweight: 32.33 (kD)
Gene IDs: Gorai.009G032000
cotton name: K11279 - nucleosome assembly protein 1-like 1 (NAP1L1, NRP) (1 of 3)
OG IDs: 93523075
Best-hit-arabi-ID: AT2G19480.3
Best-hit-arabi-name: nucleosome assembly protein 1;2
Best-hit-arabi-symbol: NAP1;2,NFA02,NFA2
Gorai.009G032000.1.D
PCC=0.602, p=0.006
mRNA Profile
Protein Expression Profile
DPA

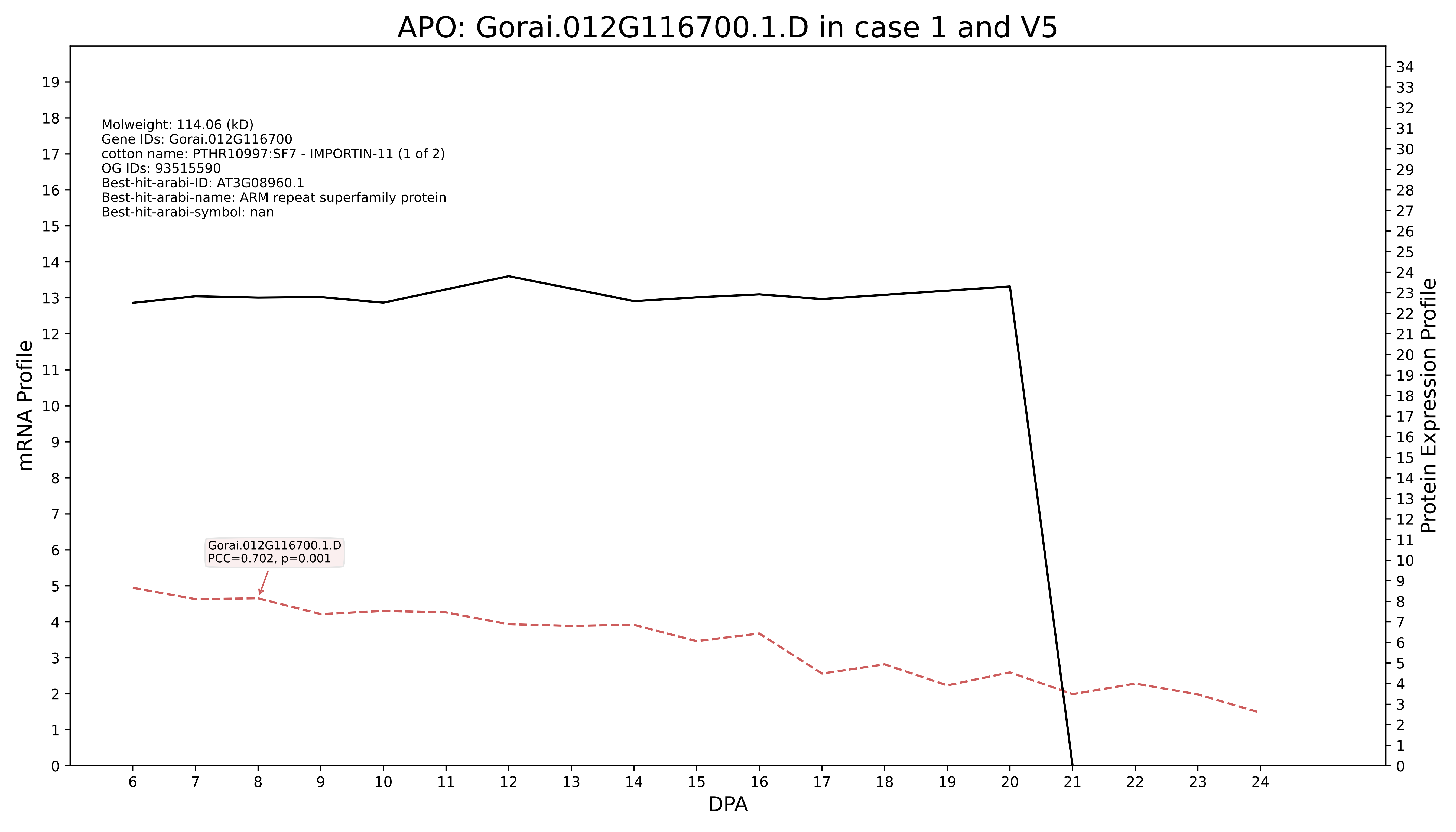

APO: Gorai.012G116700.1.D in case 1 and V5
Molweight: 114.06 (kD)
Gene IDs: Gorai.012G116700
cotton name: PTHR10997:SF7 - IMPORTIN-11 (1 of 2)
OG IDs: 93515590
Best-hit-arabi-ID: AT3G08960.1
Best-hit-arabi-name: ARM repeat superfamily protein
Best-hit-arabi-symbol: nan
Gorai.012G116700.1.D
PCC=0.702, p=0.001
mRNA Profile
Protein Expression Profile
DPA

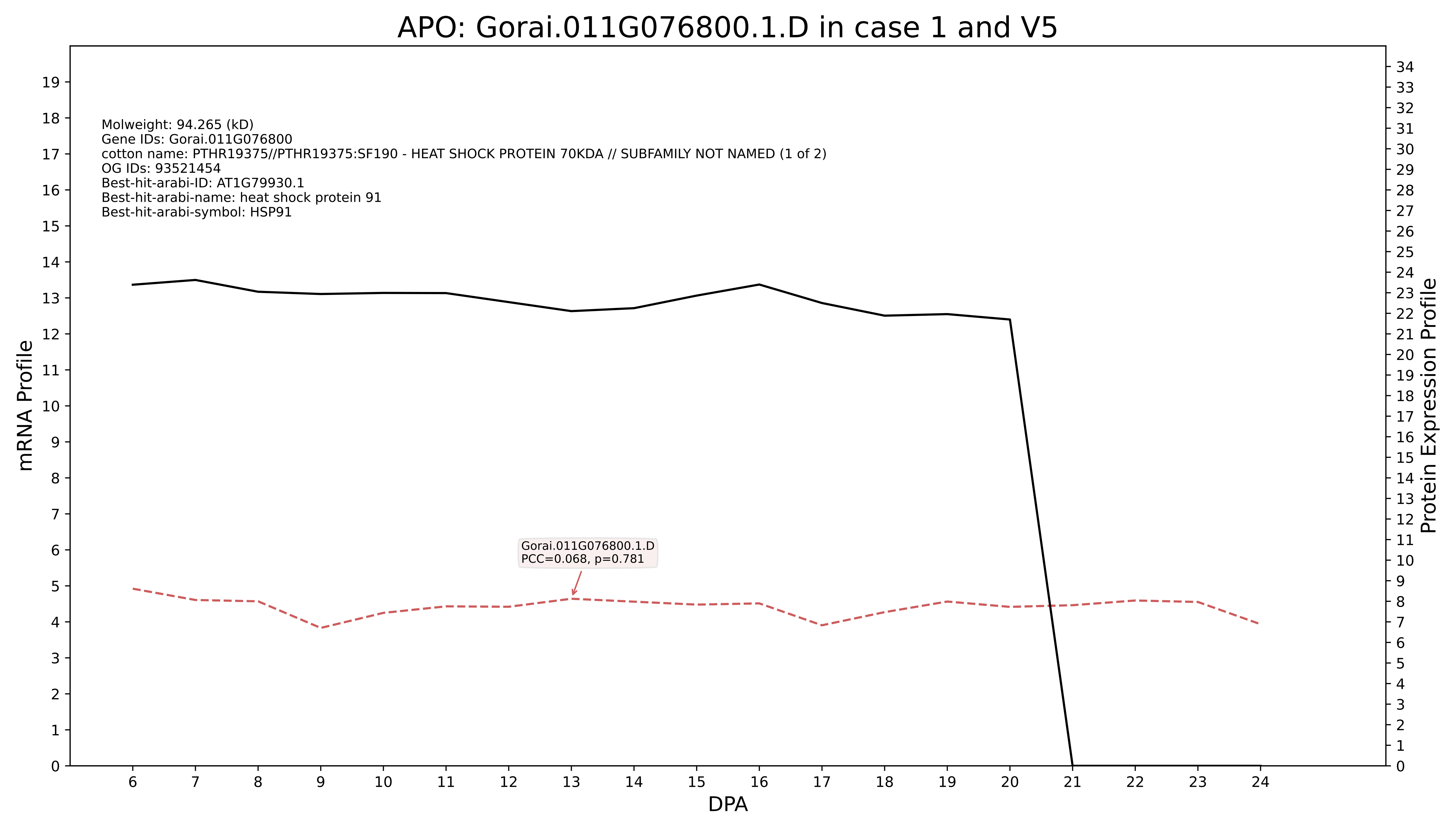
APO: Gorai.011G076800.1.D in case 1 and V5
Molweight: 94.265 (kD)
Gene IDs: Gorai.011G076800
cotton name: PTHR19375//PTHR19375:SF190 - HEAT SHOCK PROTEIN 70KDA // SUBFAMILY NOT NAMED (1 of 2)
OG IDs: 93521454
Best-hit-arabi-ID: AT1G79930.1
Best-hit-arabi-name: heat shock protein 91
Best-hit-arabi-symbol: HSP91
Gorai.011G076800.1.D
PCC=0.068, p=0.781
mRNA Profile
Protein Expression Profile
DPA

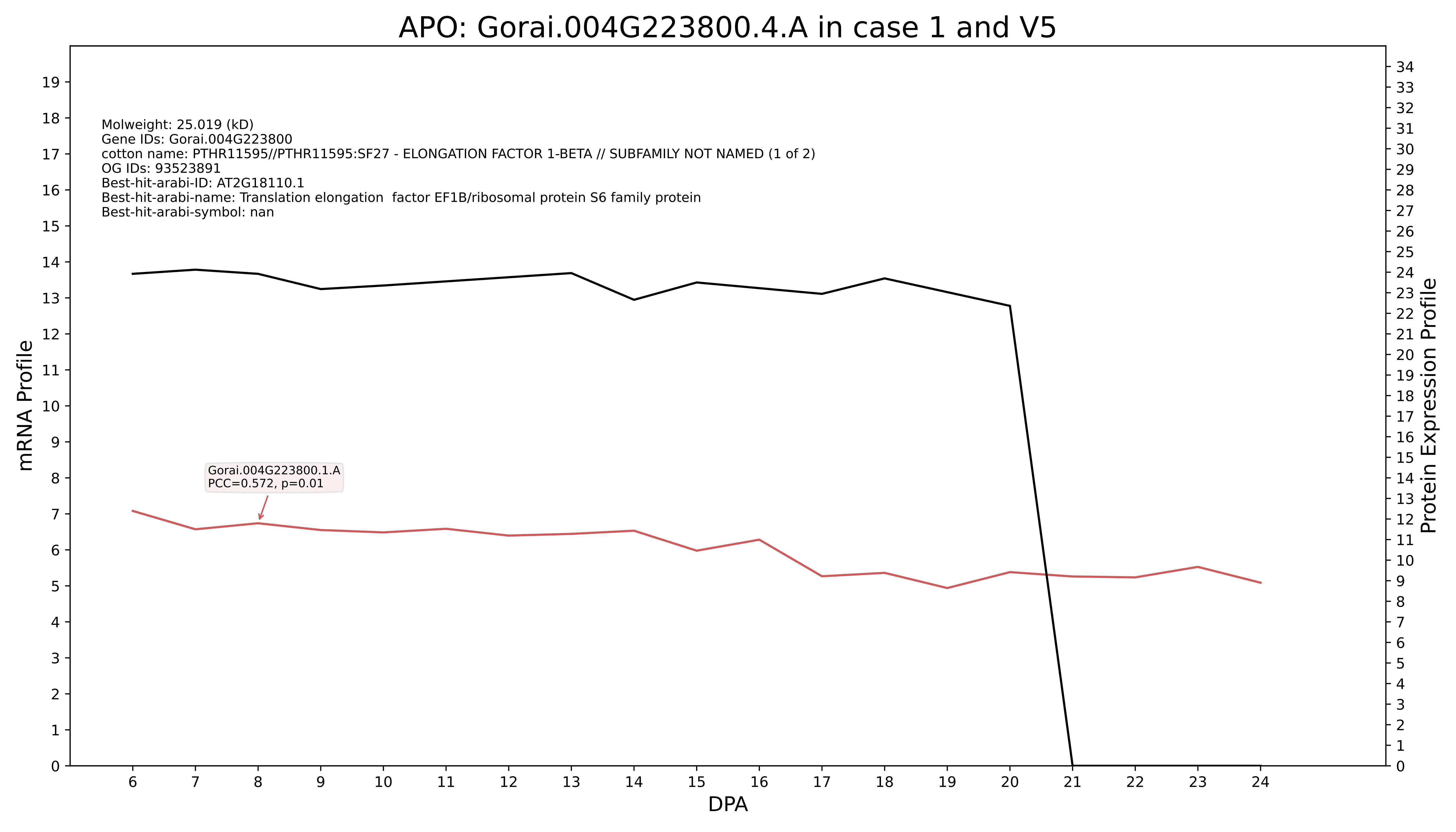

APO: Gorai.004G223800.4.A in case 1 and V5
Molweight: 25.019 (kD)
Gene IDs: Gorai.004G223800
cotton name: PTHR11595//PTHR11595:SF27 - ELONGATION FACTOR 1-BETA // SUBFAMILY NOT NAMED (1 of 2)
OG IDs: 93523891
Best-hit-arabi-ID: AT2G18110.1
Best-hit-arabi-name: Translation elongation factor EF1B/ribosomal protein S6 family protein
Best-hit-arabi-symbol: nan
Gorai.004G223800.1.A
PCC=0.572, p=0.01
mRNA Profile
Protein Expression Profile
DPA

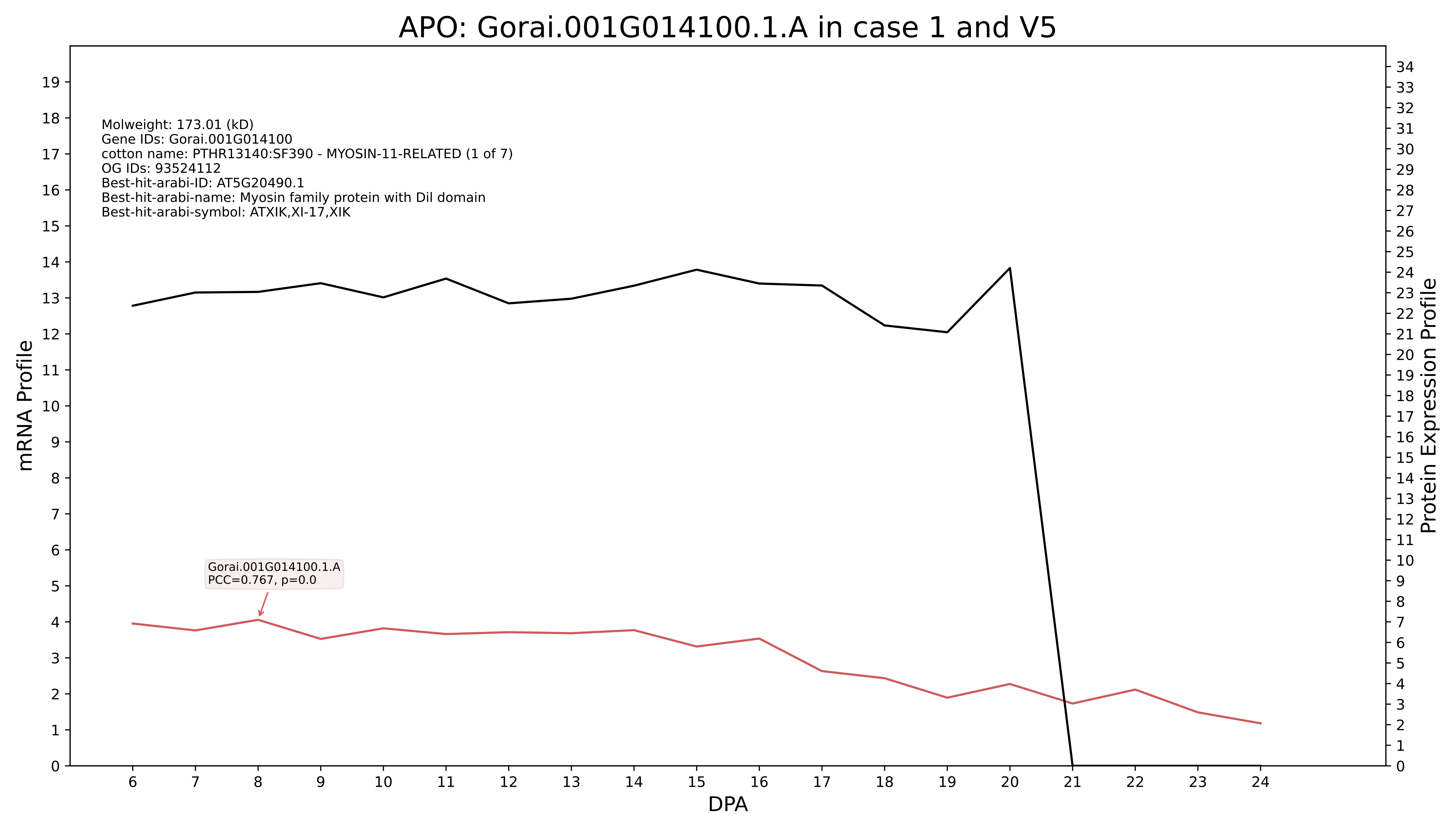

APO: Gorai.001G014100.1.A in case 1 and V5
Molweight: 173.01 (kD)
Gene IDs: Gorai.001G014100
cotton name: PTHR13140:SF390 - MYOSIN-11-RELATED (1 of 7)
OG IDs: 93524112
Best-hit-arabi-ID: AT5G20490.1
Best-hit-arabi-name: Myosin family protein with Dil domain
Best-hit-arabi-symbol: ATXIK,XI-17,XIK
Gorai.001G014100.1.A
PCC=0.767, p=0.0
mRNA Profile
Protein Expression Profile
DPA

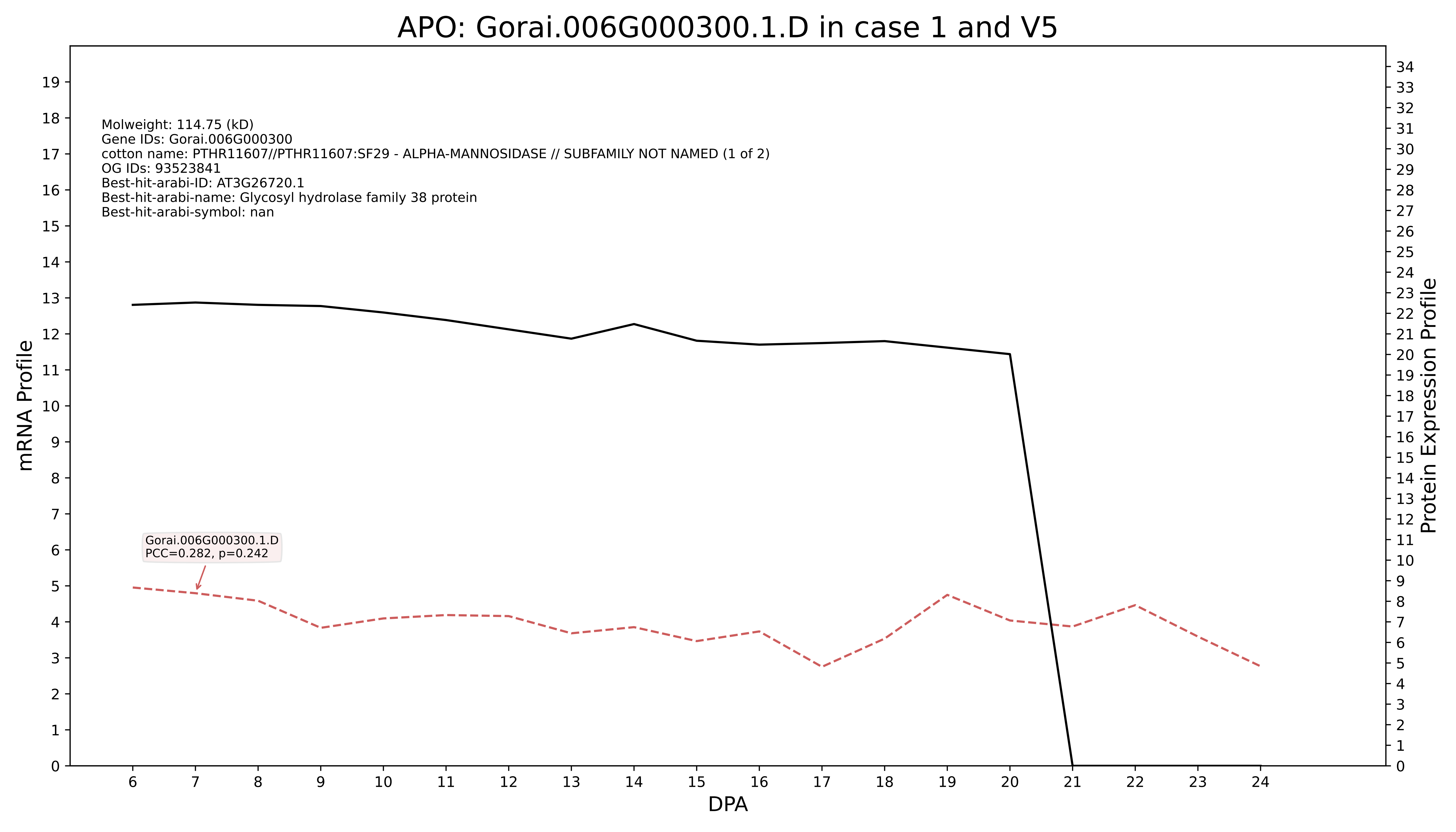
APO: Gorai.006G000300.1.D in case 1 and V5
Molweight: 114.75 (kD)
Gene IDs: Gorai.006G000300
cotton name: PTHR11607//PTHR11607:SF29 - ALPHA-MANNOSIDASE // SUBFAMILY NOT NAMED (1 of 2)
OG IDs: 93523841
Best-hit-arabi-ID: AT3G26720.1
Best-hit-arabi-name: Glycosyl hydrolase family 38 protein
Best-hit-arabi-symbol: nan
Gorai.006G000300.1.D
PCC=0.282, p=0.242
mRNA Profile
Protein Expression Profile
DPA

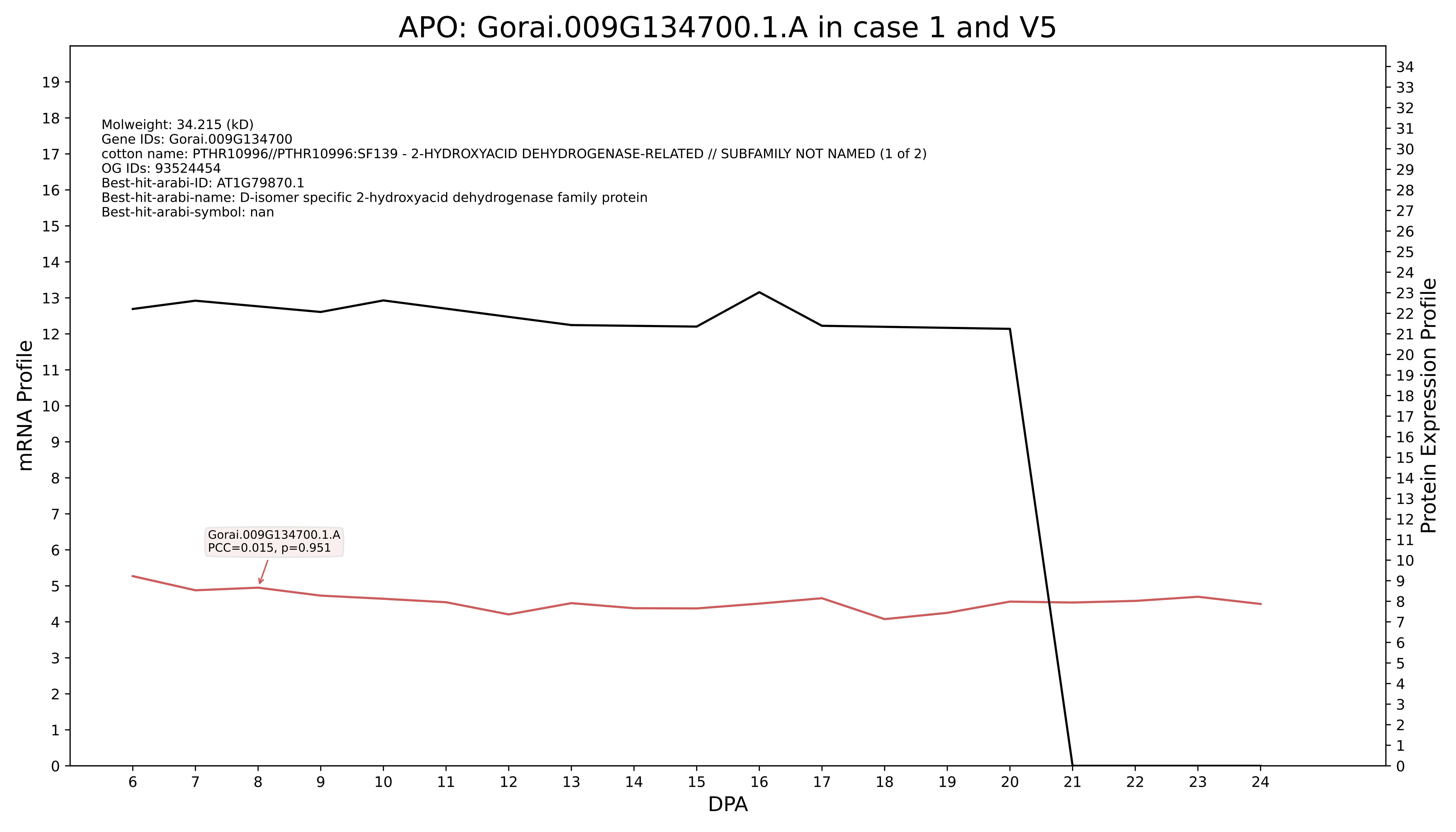
APO: Gorai.009G134700.1.A in case 1 and V5
Molweight: 34.215 (kD)
Gene IDs: Gorai.009G134700
cotton name: PTHR10996//PTHR10996:SF139 - 2-HYDROXYACID DEHYDROGENASE-RELATED // SUBFAMILY NOT NAMED (1 of 2)
OG IDs: 93524454
Best-hit-arabi-ID: AT1G79870.1
Best-hit-arabi-name: D-isomer specific 2-hydroxyacid dehydrogenase family protein
Best-hit-arabi-symbol: nan
Gorai.009G134700.1.A
PCC=0.015, p=0.951
mRNA Profile
Protein Expression Profile
DPA

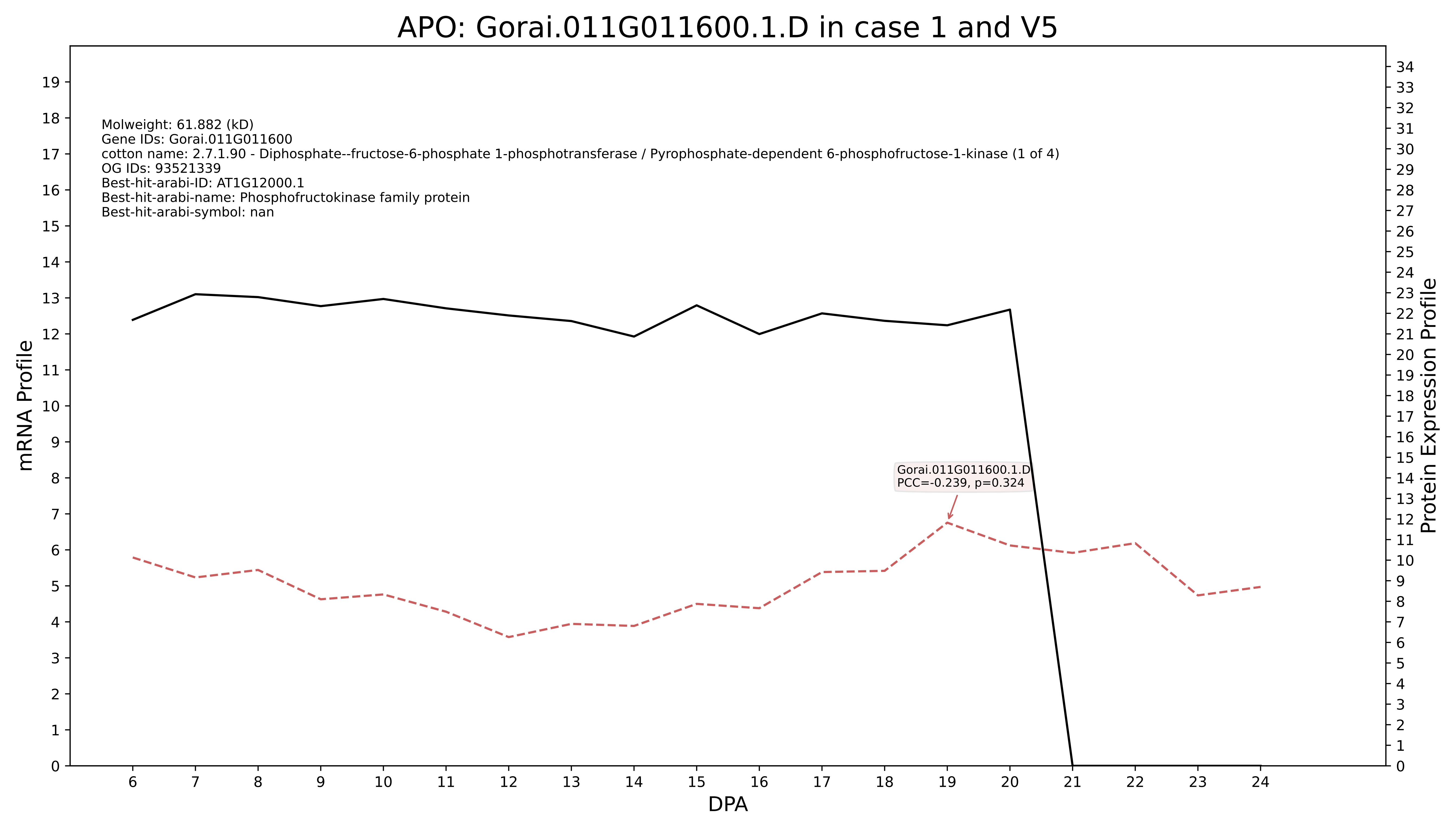
APO: Gorai.011G011600.1.D in case 1 and V5
Molweight: 61.882 (kD)
Gene IDs: Gorai.011G011600
cotton name: 2.7.1.90 - Diphosphate--fructose-6-phosphate 1-phosphotransferase / Pyrophosphate-dependent 6-phosphofructose-1-kinase (1 of 4)
OG IDs: 93521339
Best-hit-arabi-ID: AT1G12000.1
Best-hit-arabi-name: Phosphofructokinase family protein
Best-hit-arabi-symbol: nan
Gorai.011G011600.1.D
PCC=-0.239, p=0.324
mRNA Profile
Protein Expression Profile
DPA

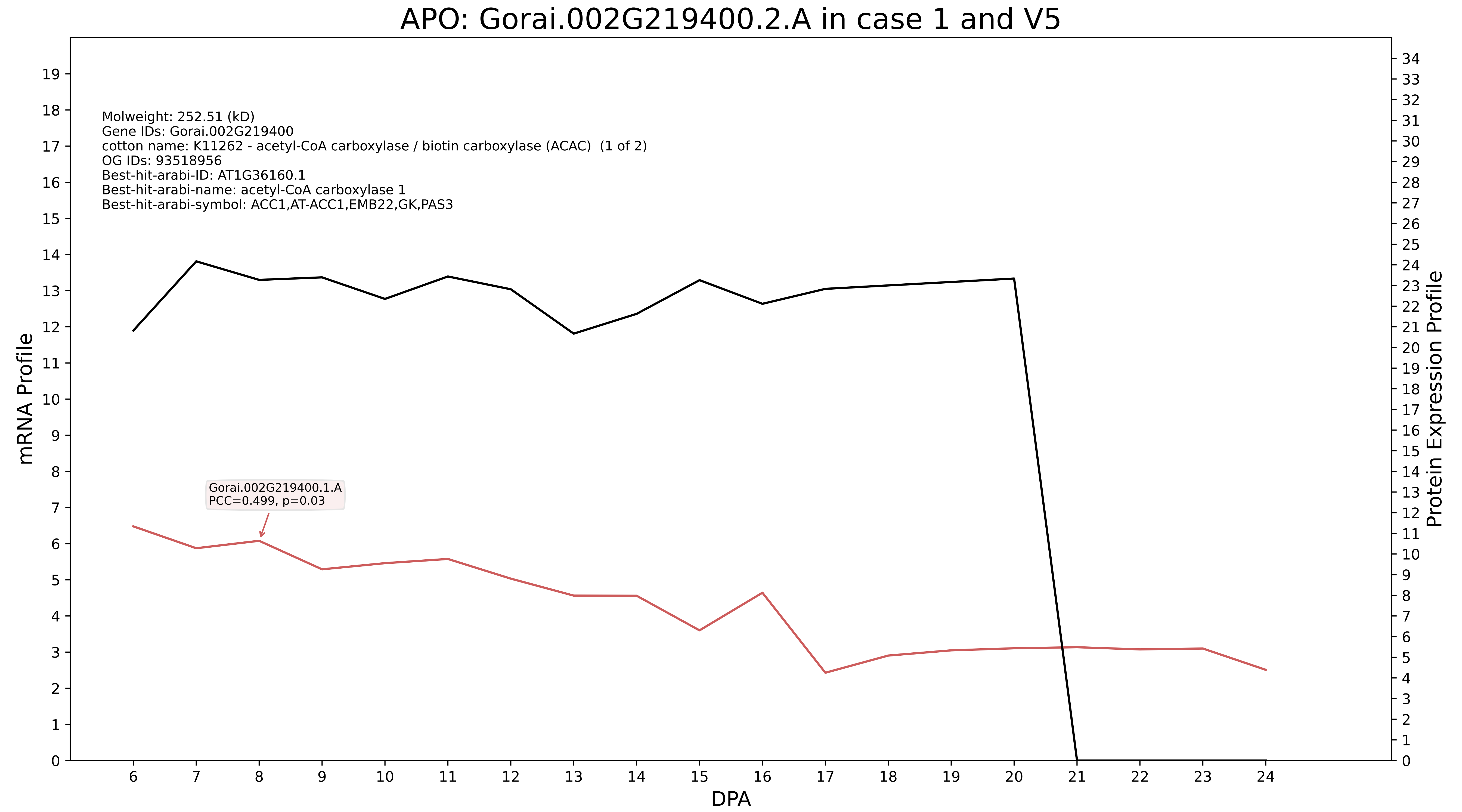
APO: Gorai.002G219400.2.A in case 1 and V5
Molweight: 252.51 (kD)
Gene IDs: Gorai.002G219400
cotton name: K11262 - acetyl-CoA carboxylase / biotin carboxylase (ACAC) (1 of 2)
OG IDs: 93518956
Best-hit-arabi-ID: AT1G36160.1
Best-hit-arabi-name: acetyl-CoA carboxylase 1
Best-hit-arabi-symbol: ACC1,AT-ACC1,EMB22,GK,PAS3
Gorai.002G219400.1.A
PCC=0.499, p=0.03
mRNA Profile
Protein Expression Profile
DPA
0
1
2
3
4
5
6
7
8
9
10
11
12
13
14
15
16
17
18
19
20
21
22
23
24
25
26
27
28
29
30
31
32
33
34

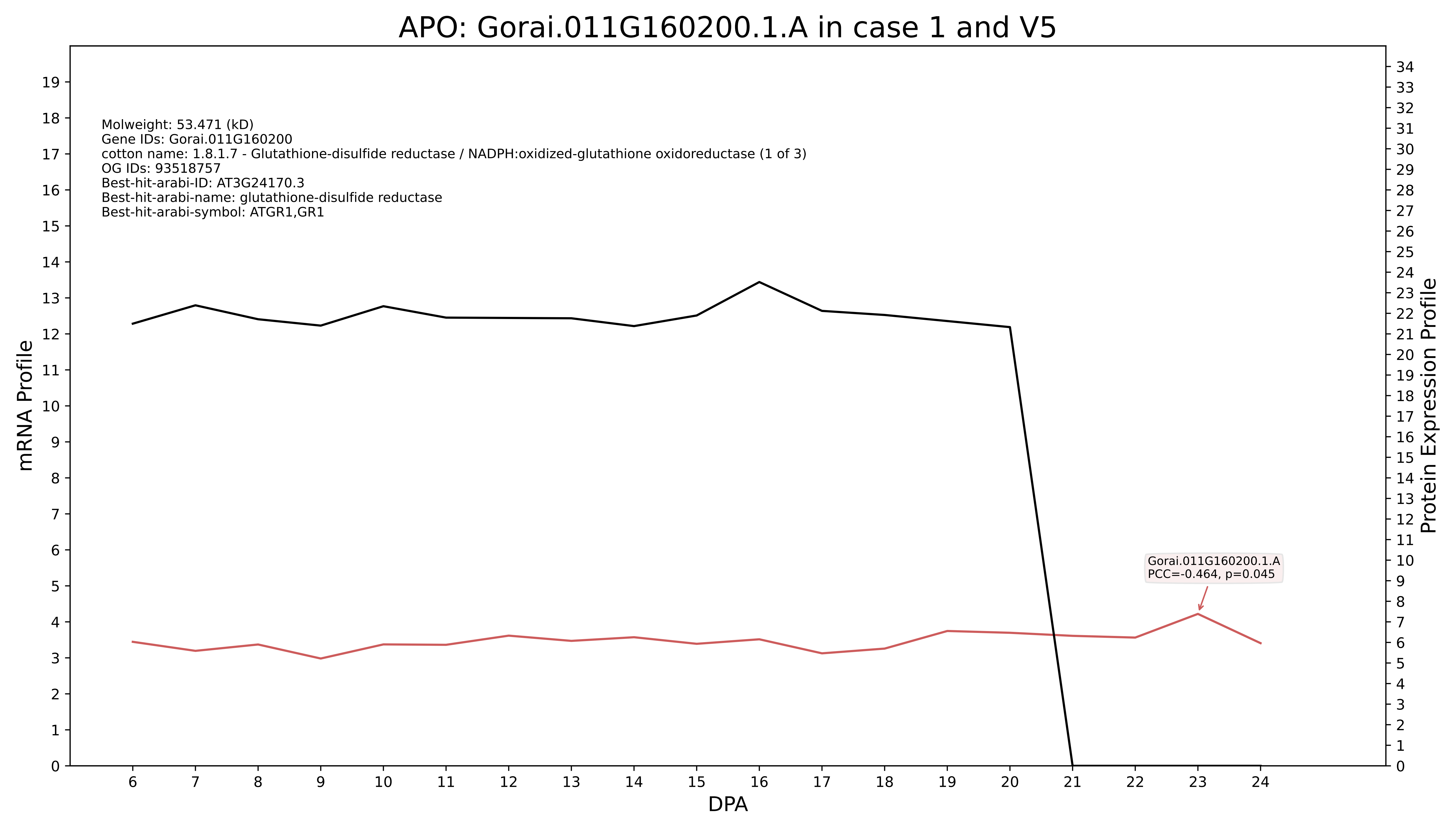
APO: Gorai.011G160200.1.A in case 1 and V5
Molweight: 53.471 (kD)
Gene IDs: Gorai.011G160200
cotton name: 1.8.1.7 - Glutathione-disulfide reductase / NADPH:oxidized-glutathione oxidoreductase (1 of 3)
OG IDs: 93518757
Best-hit-arabi-ID: AT3G24170.3
Best-hit-arabi-name: glutathione-disulfide reductase
Best-hit-arabi-symbol: ATGR1,GR1
Gorai.011G160200.1.A
PCC=-0.464, p=0.045
mRNA Profile
Protein Expression Profile
DPA
0
1
2
3
4
5
6
7
8
9
10
11
12
13
14
15
16
17
18
19
20
21
22
23
24
25
26
27
28
29
30
31
32
33
34

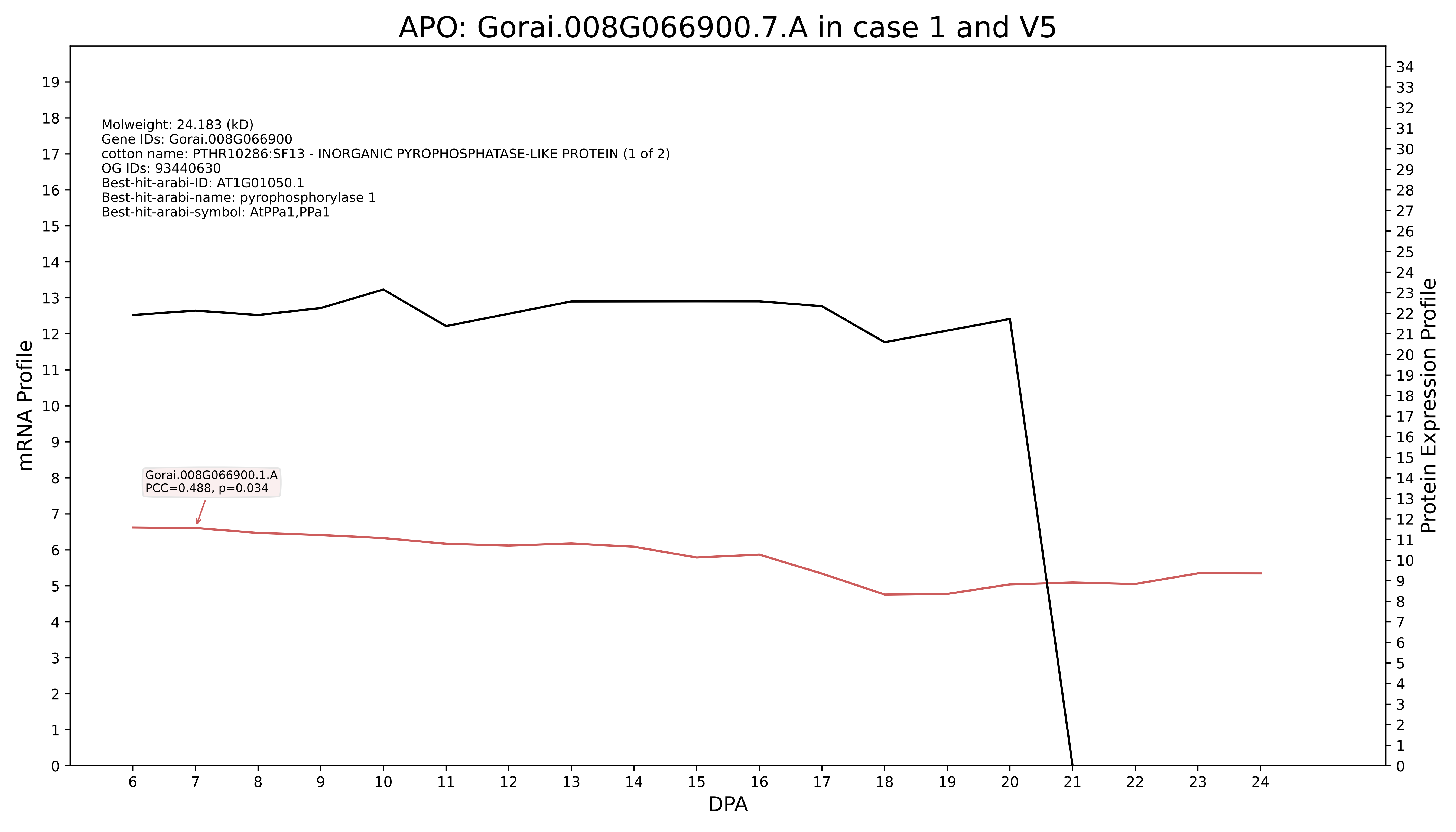

APO: Gorai.008G066900.7.A in case 1 and V5
Molweight: 24.183 (kD)
Gene IDs: Gorai.008G066900
cotton name: PTHR10286:SF13 - INORGANIC PYROPHOSPHATASE-LIKE PROTEIN (1 of 2)
OG IDs: 93440630
Best-hit-arabi-ID: AT1G01050.1
Best-hit-arabi-name: pyrophosphorylase 1
Best-hit-arabi-symbol: AtPPa1,PPa1
Gorai.008G066900.1.A
PCC=0.488, p=0.034
mRNA Profile
Protein Expression Profile
DPA

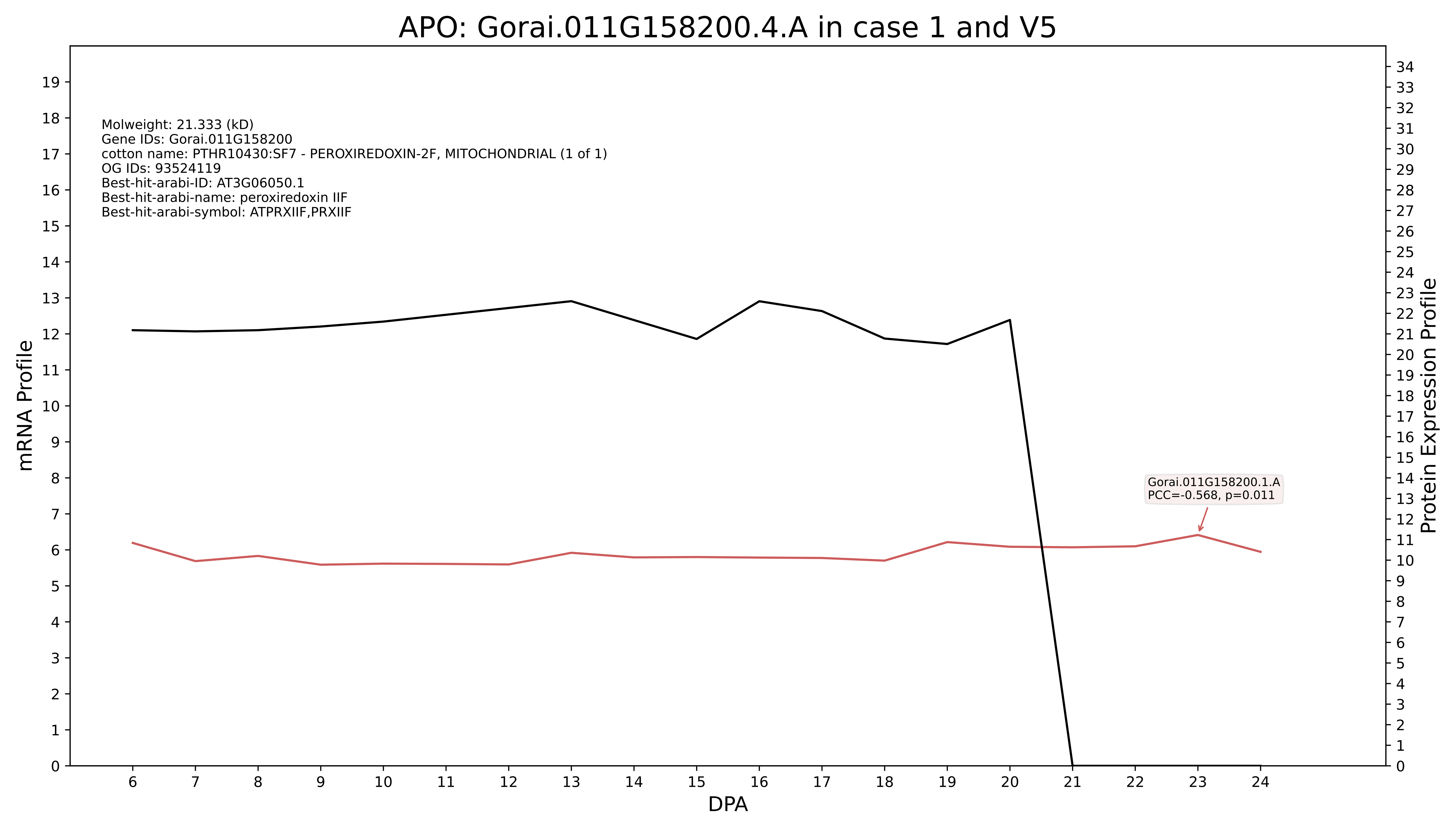
APO: Gorai.011G158200.4.A in case 1 and V5
Molweight: 21.333 (kD)
Gene IDs: Gorai.011G158200
cotton name: PTHR10430:SF7 - PEROXIREDOXIN-2F, MITOCHONDRIAL (1 of 1)
OG IDs: 93524119
Best-hit-arabi-ID: AT3G06050.1
Best-hit-arabi-name: peroxiredoxin IIF
Best-hit-arabi-symbol: ATPRXIIF,PRXIIF
Gorai.011G158200.1.A
PCC=-0.568, p=0.011
mRNA Profile
Protein Expression Profile
DPA
0
1
2
3
4
5
6
7
8
9
10
11
12
13
14
15
16
17
18
19
20
21
22
23
24
25
26
27
28
29
30
31
32
33
34

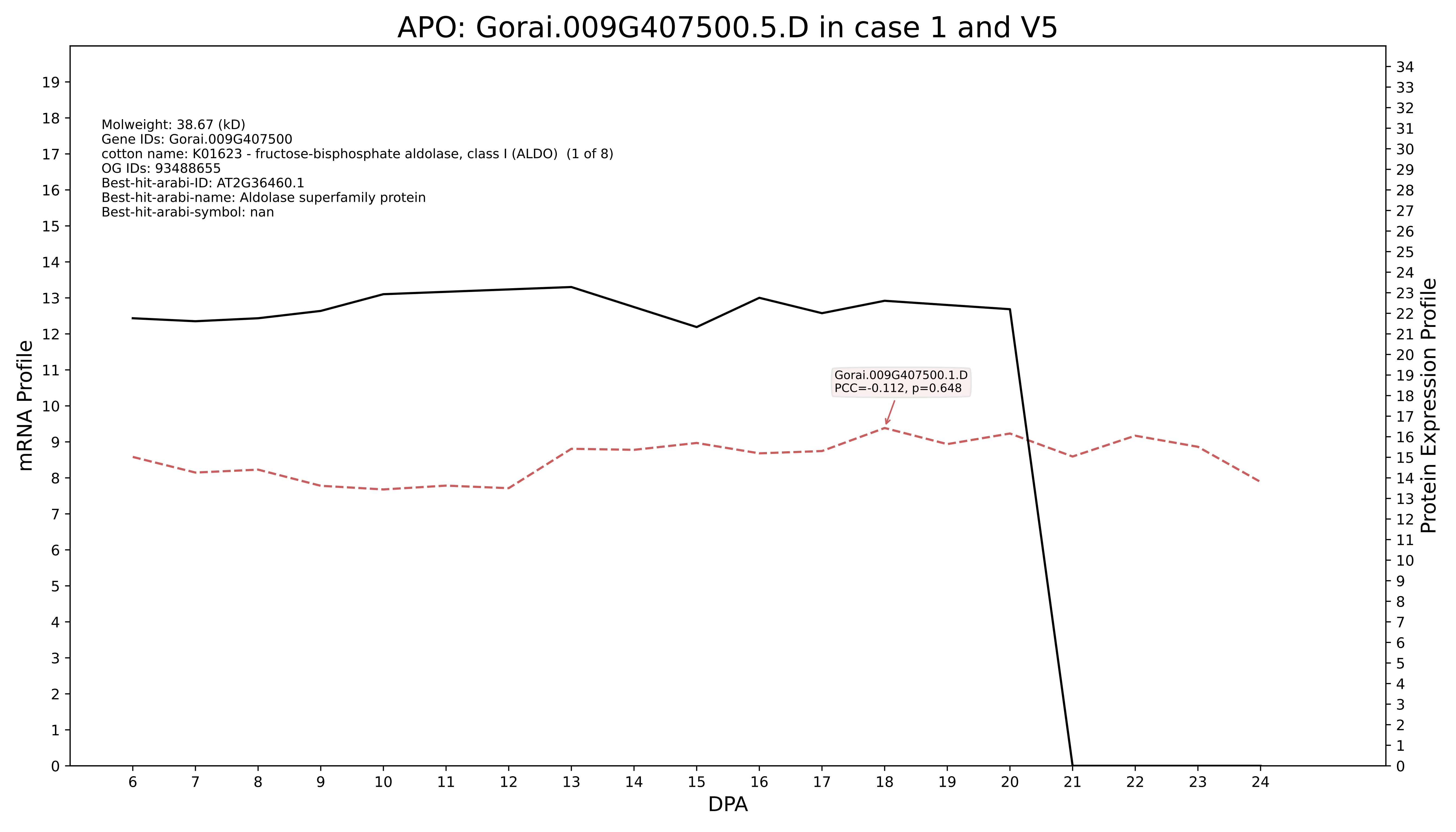

APO: Gorai.009G407500.5.D in case 1 and V5
Molweight: 38.67 (kD)
Gene IDs: Gorai.009G407500
cotton name: K01623 - fructose-bisphosphate aldolase, class I (ALDO) (1 of 8)
OG IDs: 93488655
Best-hit-arabi-ID: AT2G36460.1
Best-hit-arabi-name: Aldolase superfamily protein
Best-hit-arabi-symbol: nan
Gorai.009G407500.1.D
PCC=-0.112, p=0.648
mRNA Profile
Protein Expression Profile
DPA

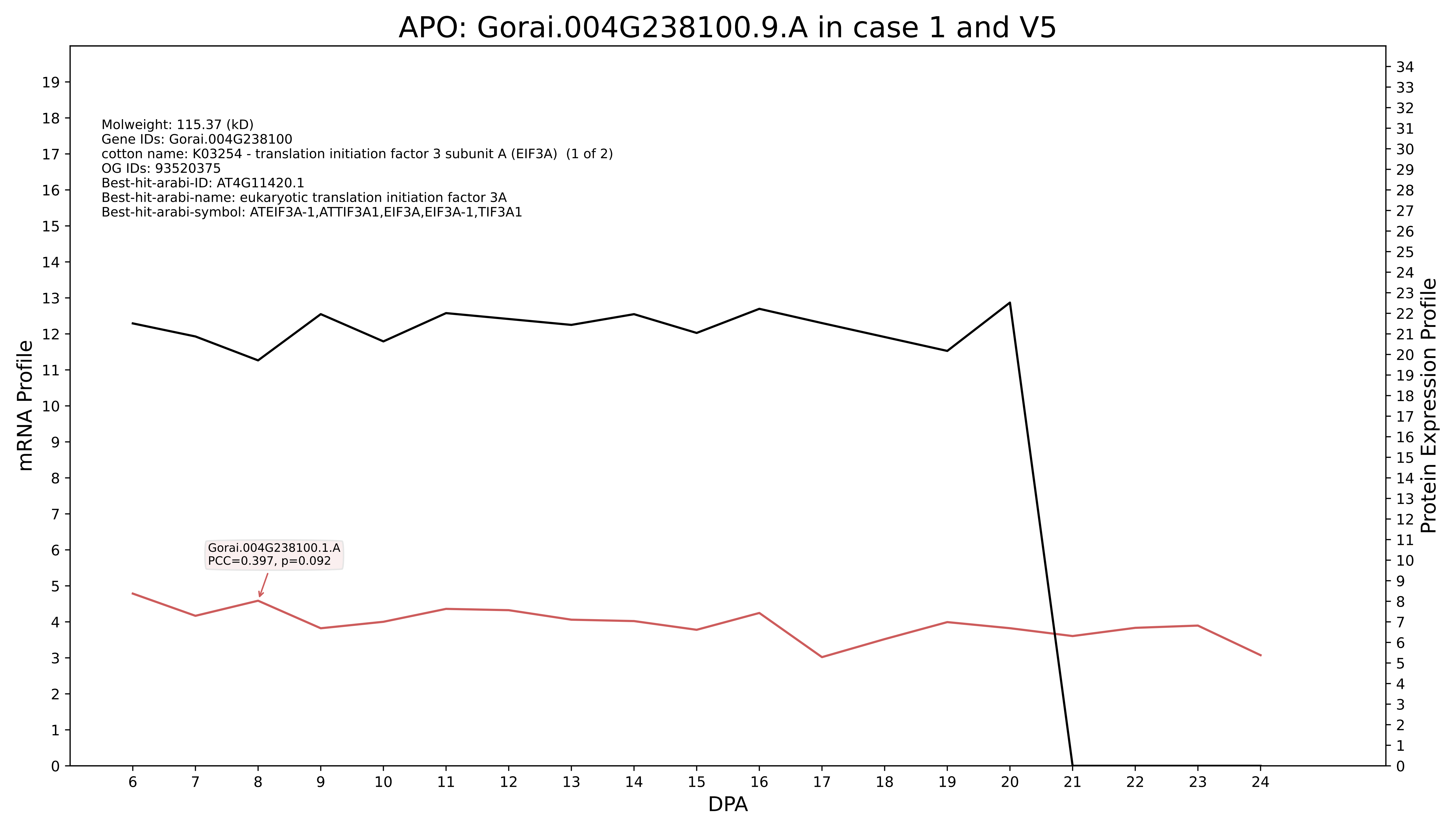

APO: Gorai.004G238100.9.A in case 1 and V5
Molweight: 115.37 (kD)
Gene IDs: Gorai.004G238100
cotton name: K03254 - translation initiation factor 3 subunit A (EIF3A) (1 of 2)
OG IDs: 93520375
Best-hit-arabi-ID: AT4G11420.1
Best-hit-arabi-name: eukaryotic translation initiation factor 3A
Best-hit-arabi-symbol: ATEIF3A-1,ATTIF3A1,EIF3A,EIF3A-1,TIF3A1
Gorai.004G238100.1.A
PCC=0.397, p=0.092
mRNA Profile
Protein Expression Profile
DPA

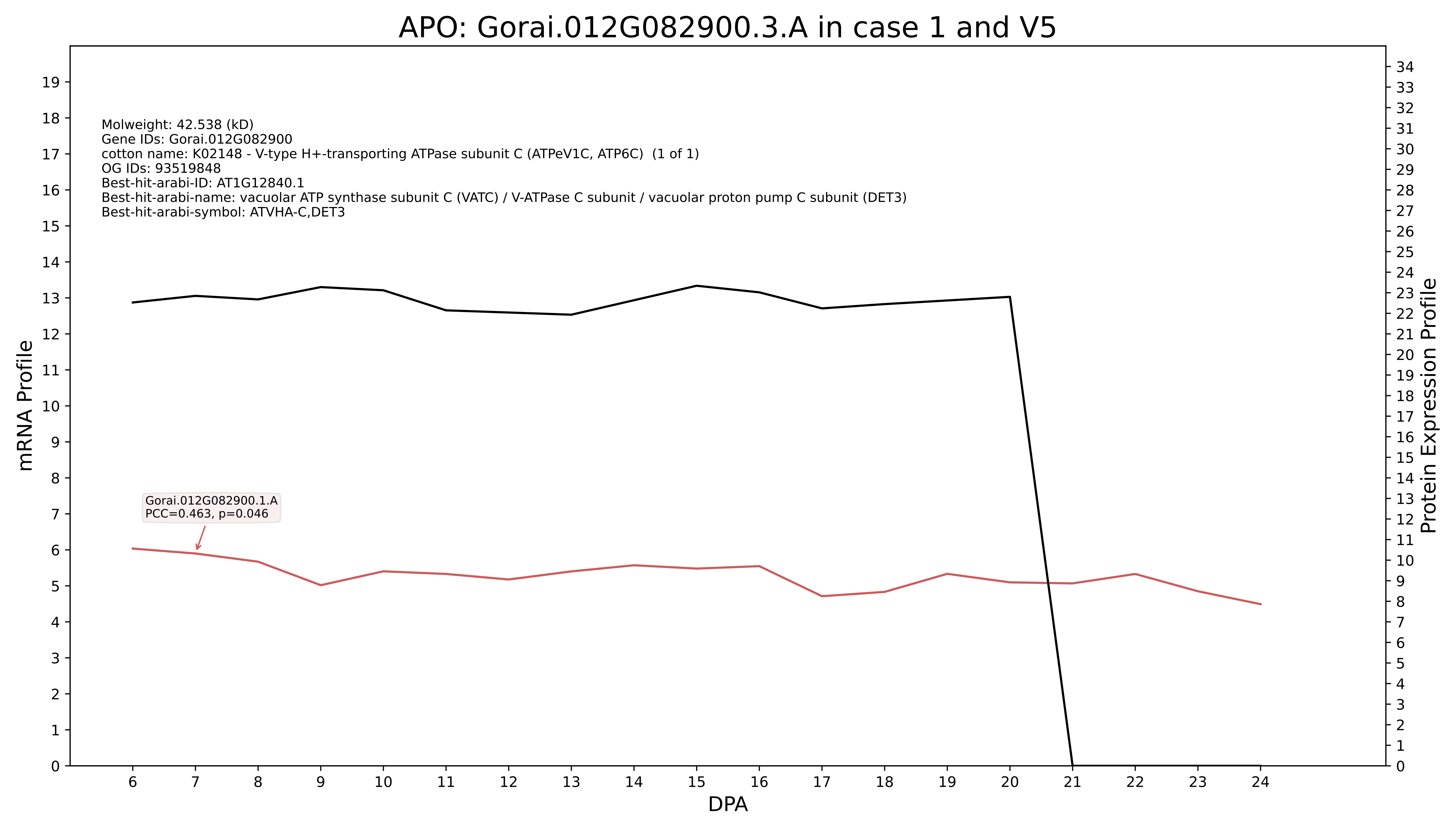
APO: Gorai.012G082900.3.A in case 1 and V5
Molweight: 42.538 (kD)
Gene IDs: Gorai.012G082900
cotton name: K02148 - V-type H+-transporting ATPase subunit C (ATPeV1C, ATP6C) (1 of 1)
OG IDs: 93519848
Best-hit-arabi-ID: AT1G12840.1
Best-hit-arabi-name: vacuolar ATP synthase subunit C (VATC) / V-ATPase C subunit / vacuolar proton pump C subunit (DET3)
Best-hit-arabi-symbol: ATVHA-C,DET3
Gorai.012G082900.1.A
PCC=0.463, p=0.046
mRNA Profile
Protein Expression Profile
DPA
0
1
2
3
4
5
6
7
8
9
10
11
12
13
14
15
16
17
18
19
20
21
22
23
24
25
26
27
28
29
30
31
32
33
34

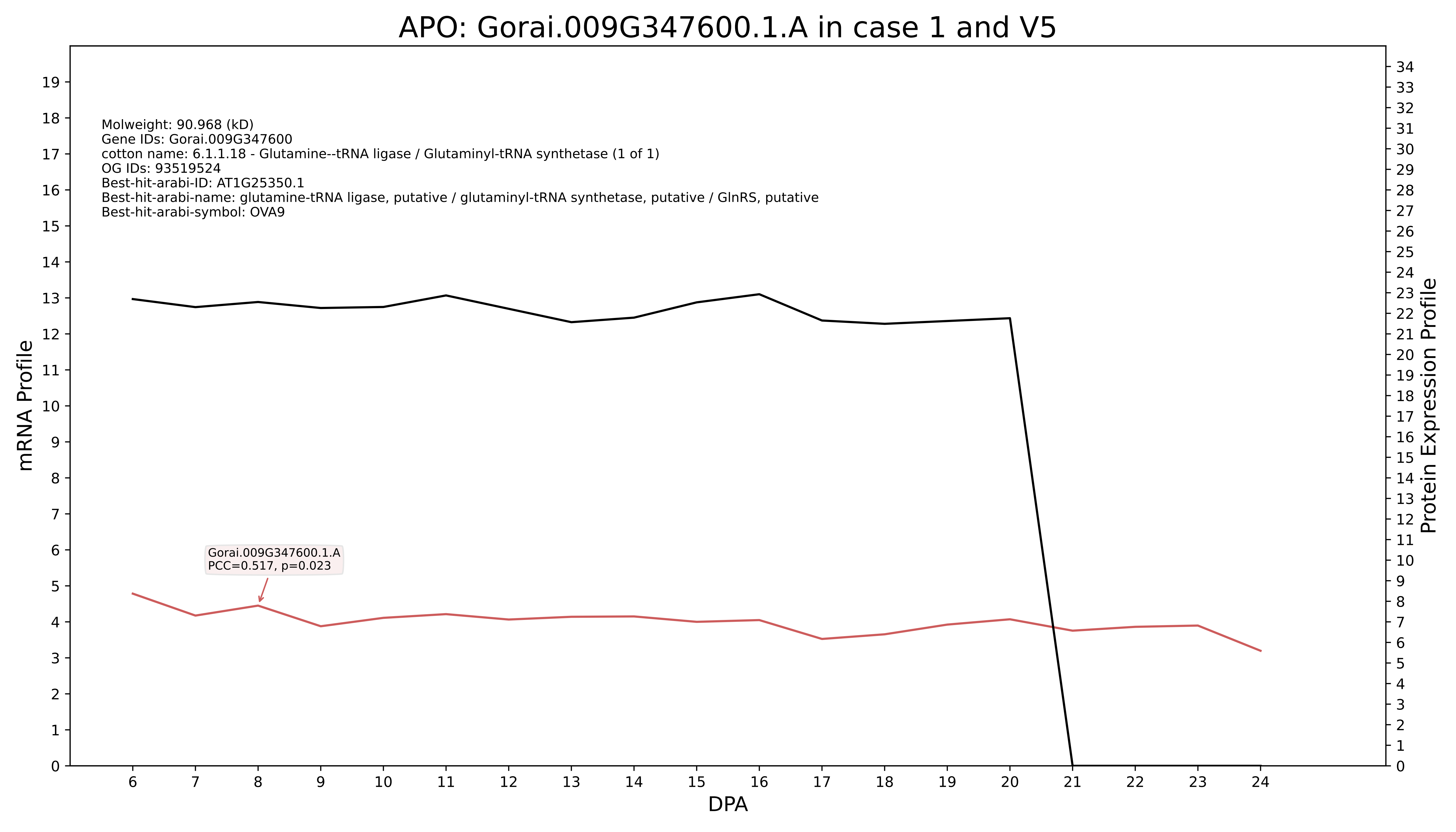
APO: Gorai.009G347600.1.A in case 1 and V5
Molweight: 90.968 (kD)
Gene IDs: Gorai.009G347600
cotton name: 6.1.1.18 - Glutamine--tRNA ligase / Glutaminyl-tRNA synthetase (1 of 1)
OG IDs: 93519524
Best-hit-arabi-ID: AT1G25350.1
Best-hit-arabi-name: glutamine-tRNA ligase, putative / glutaminyl-tRNA synthetase, putative / GlnRS, putative
Best-hit-arabi-symbol: OVA9
Gorai.009G347600.1.A
PCC=0.517, p=0.023
mRNA Profile
Protein Expression Profile
DPA

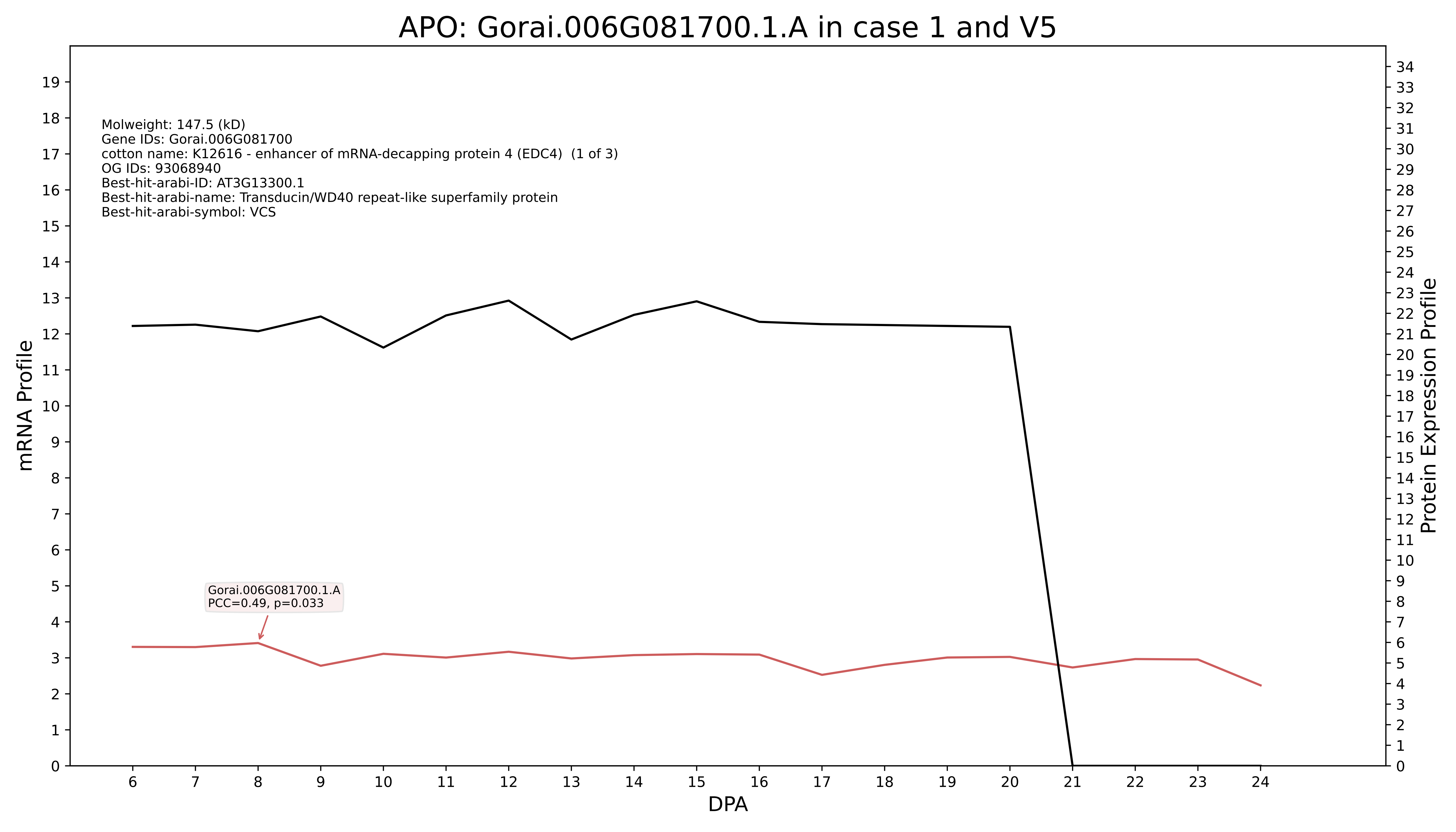
APO: Gorai.006G081700.1.A in case 1 and V5
Molweight: 147.5 (kD)
Gene IDs: Gorai.006G081700
cotton name: K12616 - enhancer of mRNA-decapping protein 4 (EDC4) (1 of 3)
OG IDs: 93068940
Best-hit-arabi-ID: AT3G13300.1
Best-hit-arabi-name: Transducin/WD40 repeat-like superfamily protein
Best-hit-arabi-symbol: VCS
Gorai.006G081700.1.A
PCC=0.49, p=0.033
mRNA Profile
Protein Expression Profile
DPA

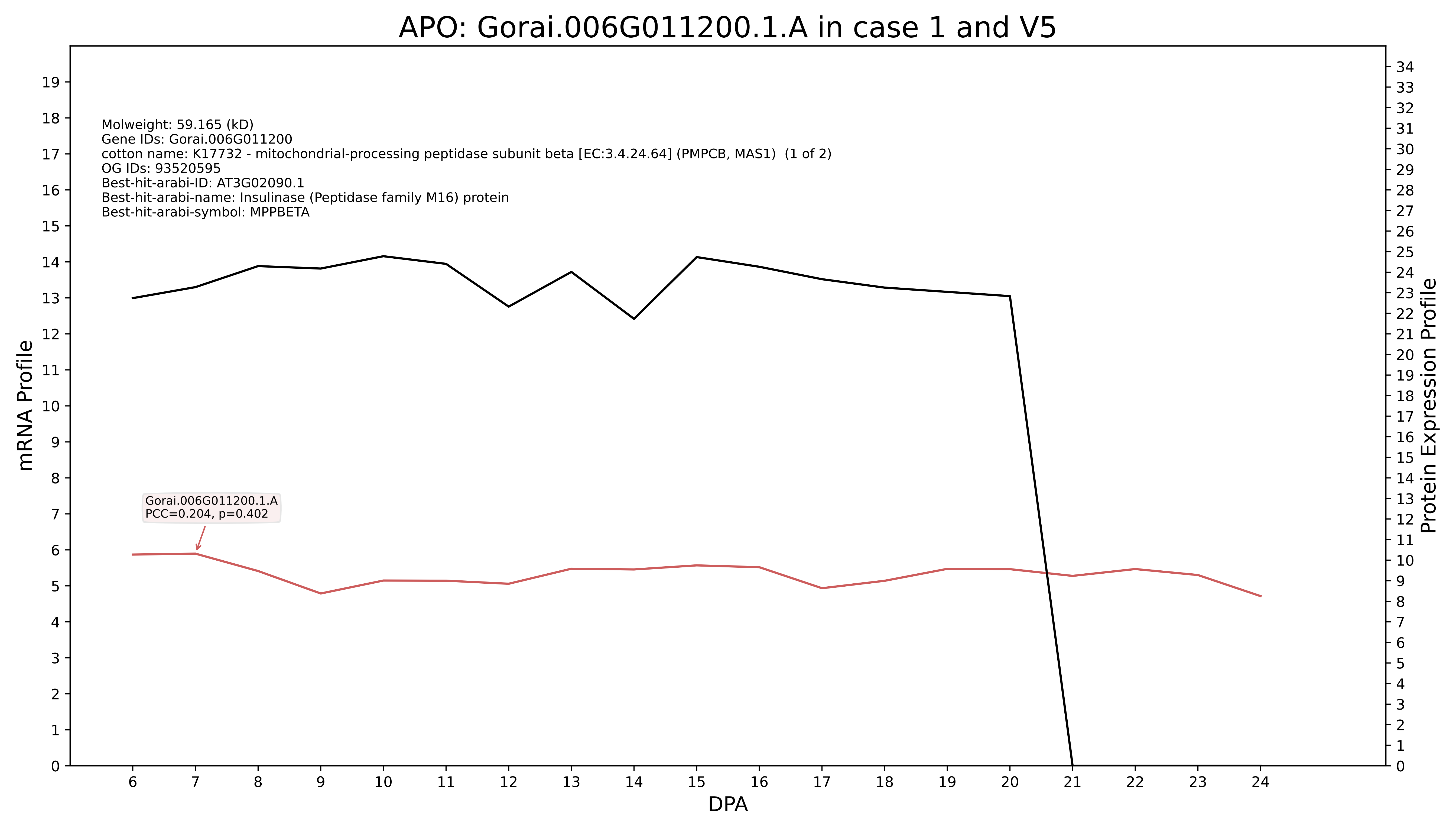
APO: Gorai.006G011200.1.A in case 1 and V5
Molweight: 59.165 (kD)
Gene IDs: Gorai.006G011200
cotton name: K17732 - mitochondrial-processing peptidase subunit beta [EC:3.4.24.64] (PMPCB, MAS1) (1 of 2)
OG IDs: 93520595
Best-hit-arabi-ID: AT3G02090.1
Best-hit-arabi-name: Insulinase (Peptidase family M16) protein
Best-hit-arabi-symbol: MPPBETA
Gorai.006G011200.1.A
PCC=0.204, p=0.402
mRNA Profile
Protein Expression Profile
DPA

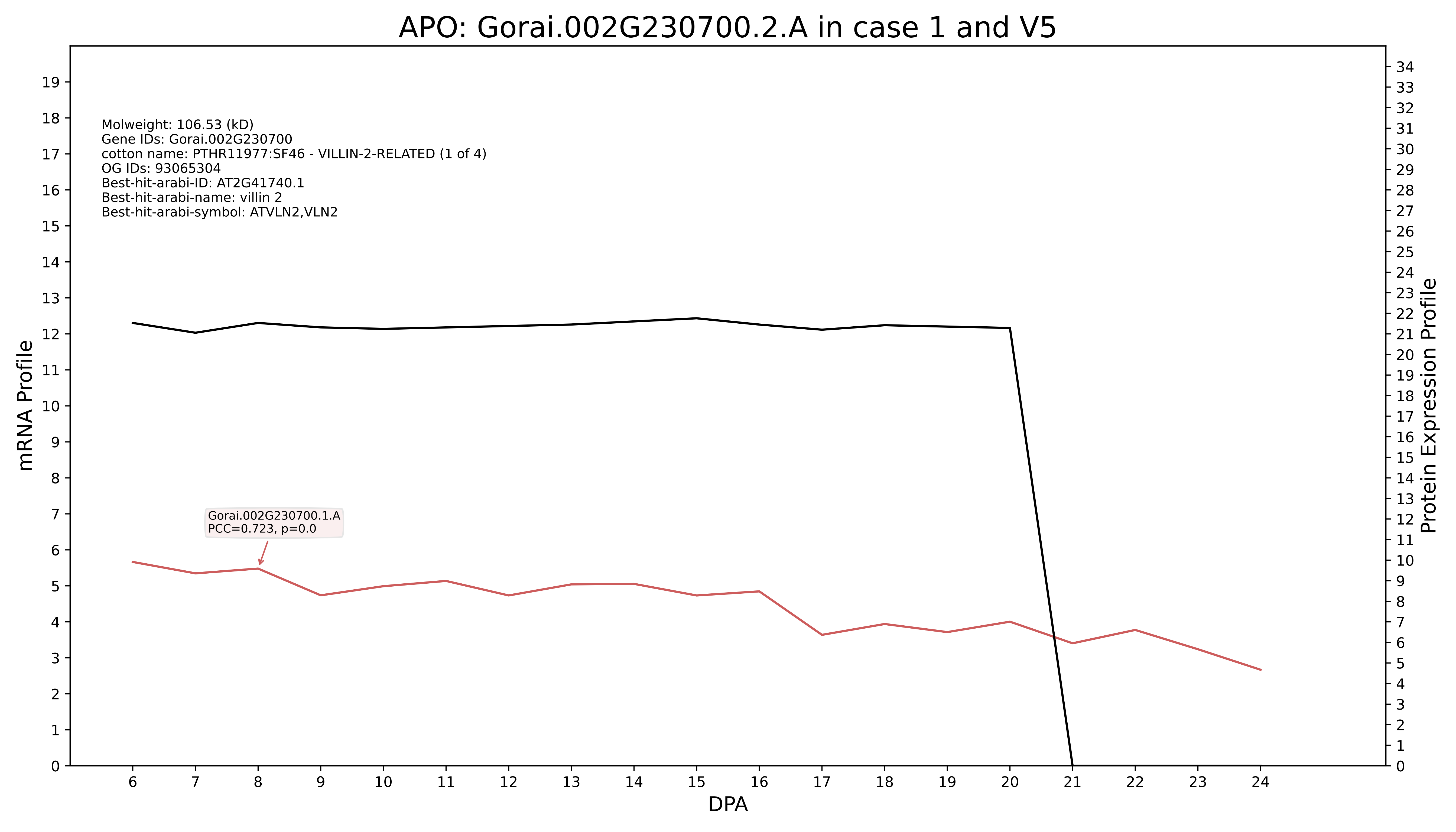

APO: Gorai.002G230700.2.A in case 1 and V5
Molweight: 106.53 (kD)
Gene IDs: Gorai.002G230700
cotton name: PTHR11977:SF46 - VILLIN-2-RELATED (1 of 4)
OG IDs: 93065304
Best-hit-arabi-ID: AT2G41740.1
Best-hit-arabi-name: villin 2
Best-hit-arabi-symbol: ATVLN2,VLN2
Gorai.002G230700.1.A
PCC=0.723, p=0.0
mRNA Profile
Protein Expression Profile
DPA

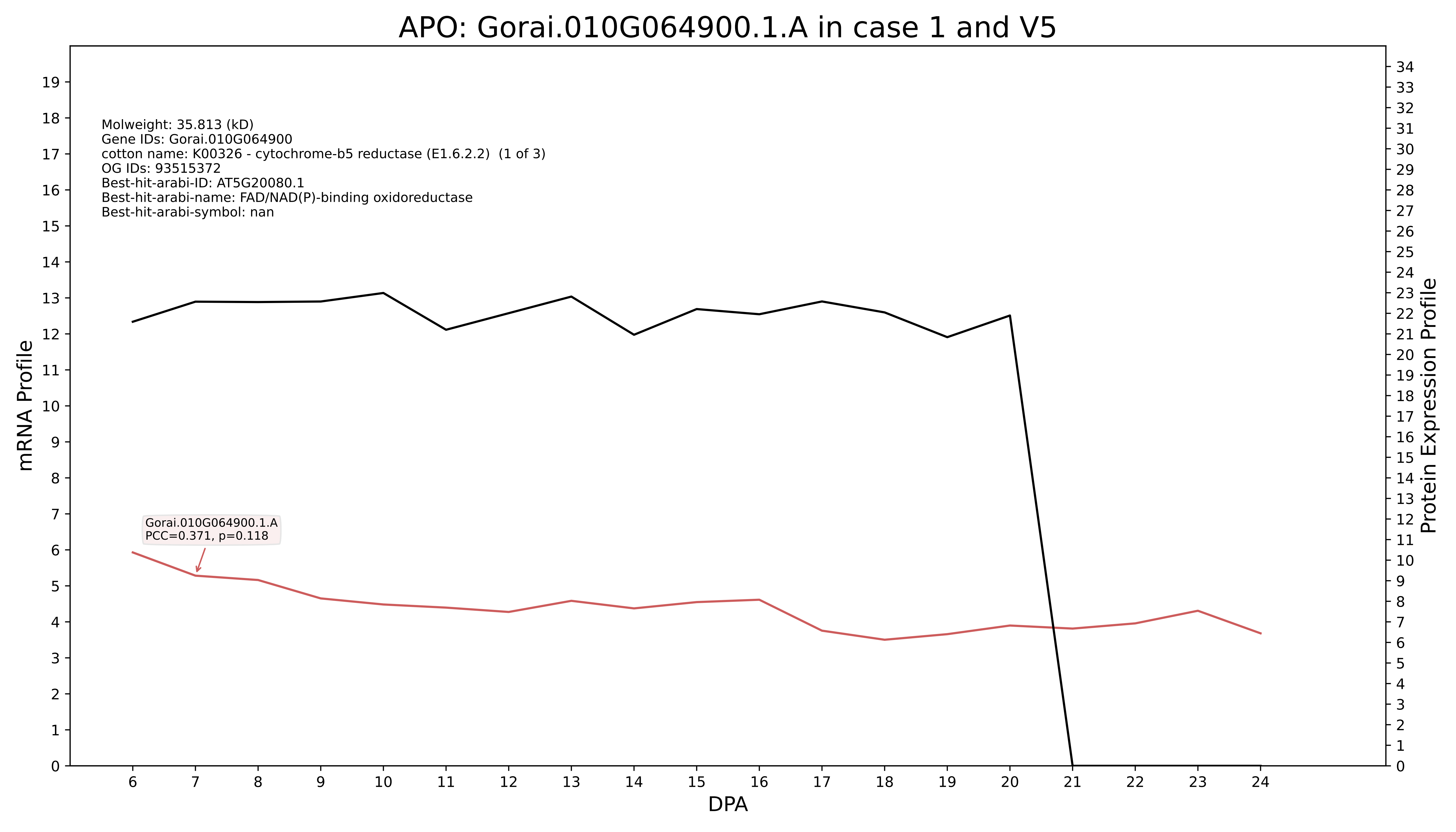

APO: Gorai.010G064900.1.A in case 1 and V5
Molweight: 35.813 (kD)
Gene IDs: Gorai.010G064900
cotton name: K00326 - cytochrome-b5 reductase (E1.6.2.2) (1 of 3)
OG IDs: 93515372
Best-hit-arabi-ID: AT5G20080.1
Best-hit-arabi-name: FAD/NAD(P)-binding oxidoreductase
Best-hit-arabi-symbol: nan
Gorai.010G064900.1.A
PCC=0.371, p=0.118
mRNA Profile
Protein Expression Profile
DPA

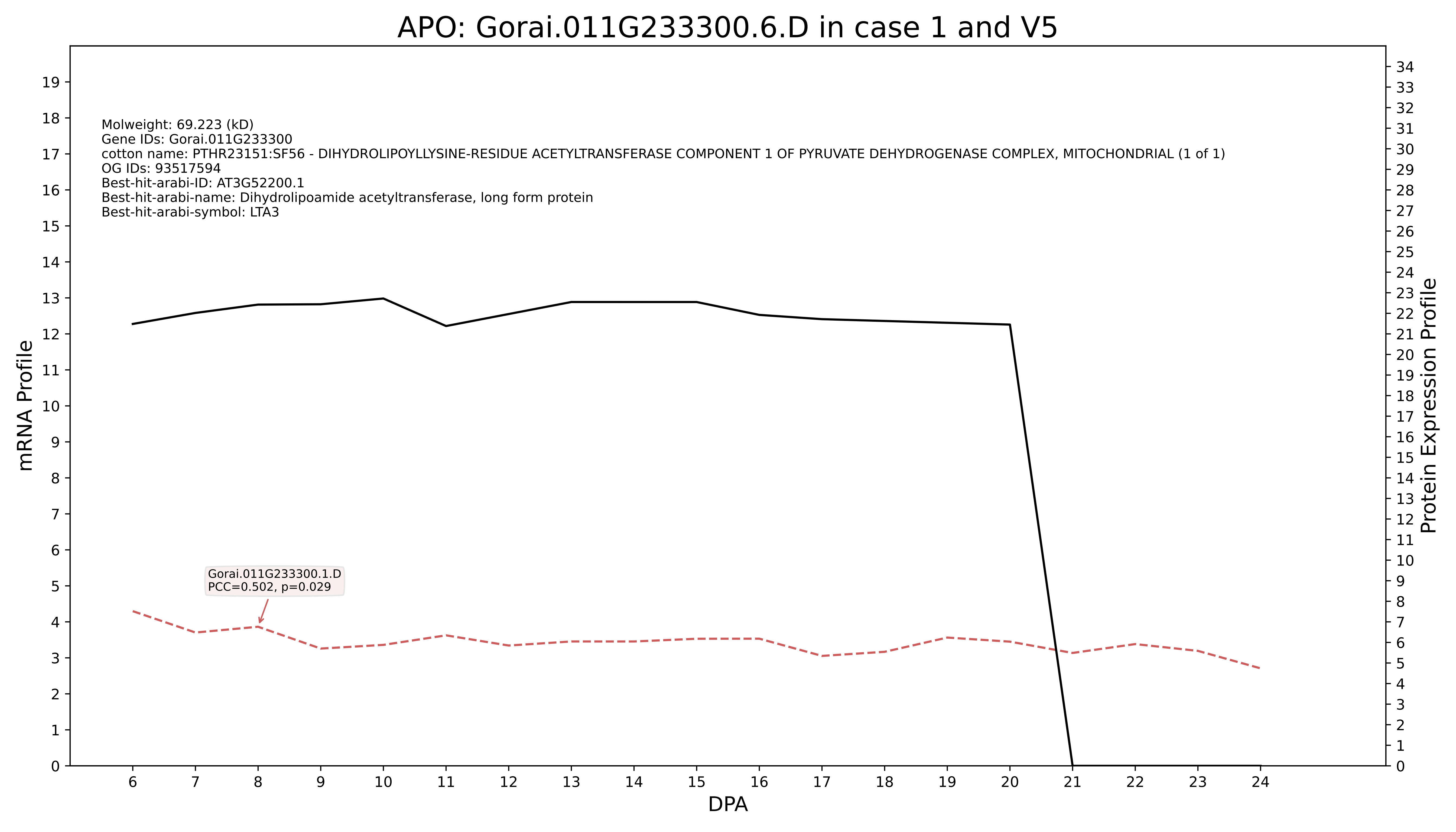

APO: Gorai.011G233300.6.D in case 1 and V5
Molweight: 69.223 (kD)
Gene IDs: Gorai.011G233300
cotton name: PTHR23151:SF56 - DIHYDROLIPOYLLYSINE-RESIDUE ACETYLTRANSFERASE COMPONENT 1 OF PYRUVATE DEHYDROGENASE COMPLEX, MITOCHONDRIAL (1 of 1)
OG IDs: 93517594
Best-hit-arabi-ID: AT3G52200.1
Best-hit-arabi-name: Dihydrolipoamide acetyltransferase, long form protein
Best-hit-arabi-symbol: LTA3
Gorai.011G233300.1.D
PCC=0.502, p=0.029
mRNA Profile
Protein Expression Profile
DPA

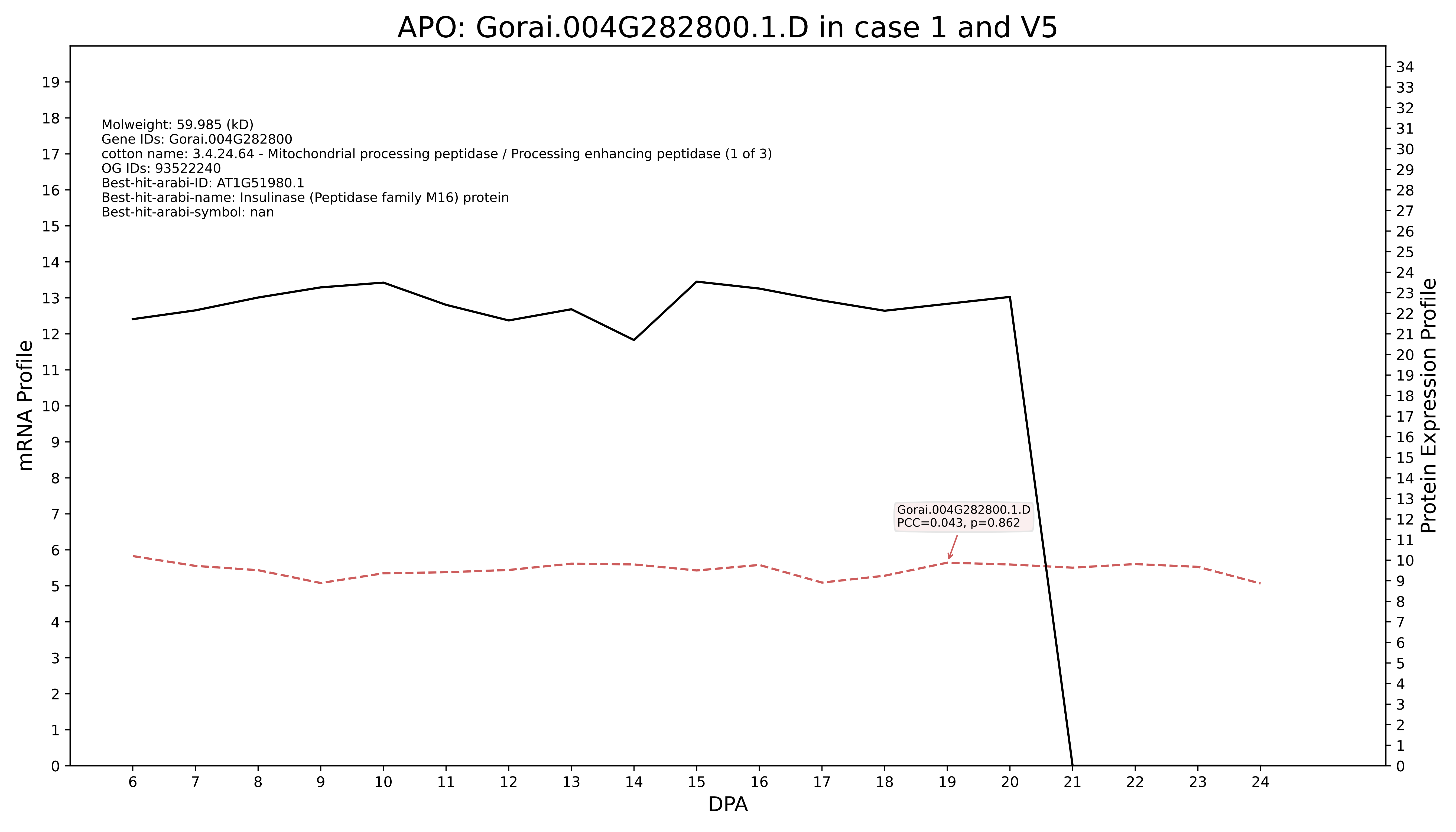

APO: Gorai.004G282800.1.D in case 1 and V5
Molweight: 59.985 (kD)
Gene IDs: Gorai.004G282800
cotton name: 3.4.24.64 - Mitochondrial processing peptidase / Processing enhancing peptidase (1 of 3)
OG IDs: 93522240
Best-hit-arabi-ID: AT1G51980.1
Best-hit-arabi-name: Insulinase (Peptidase family M16) protein
Best-hit-arabi-symbol: nan
Gorai.004G282800.1.D
PCC=0.043, p=0.862
mRNA Profile
Protein Expression Profile
DPA

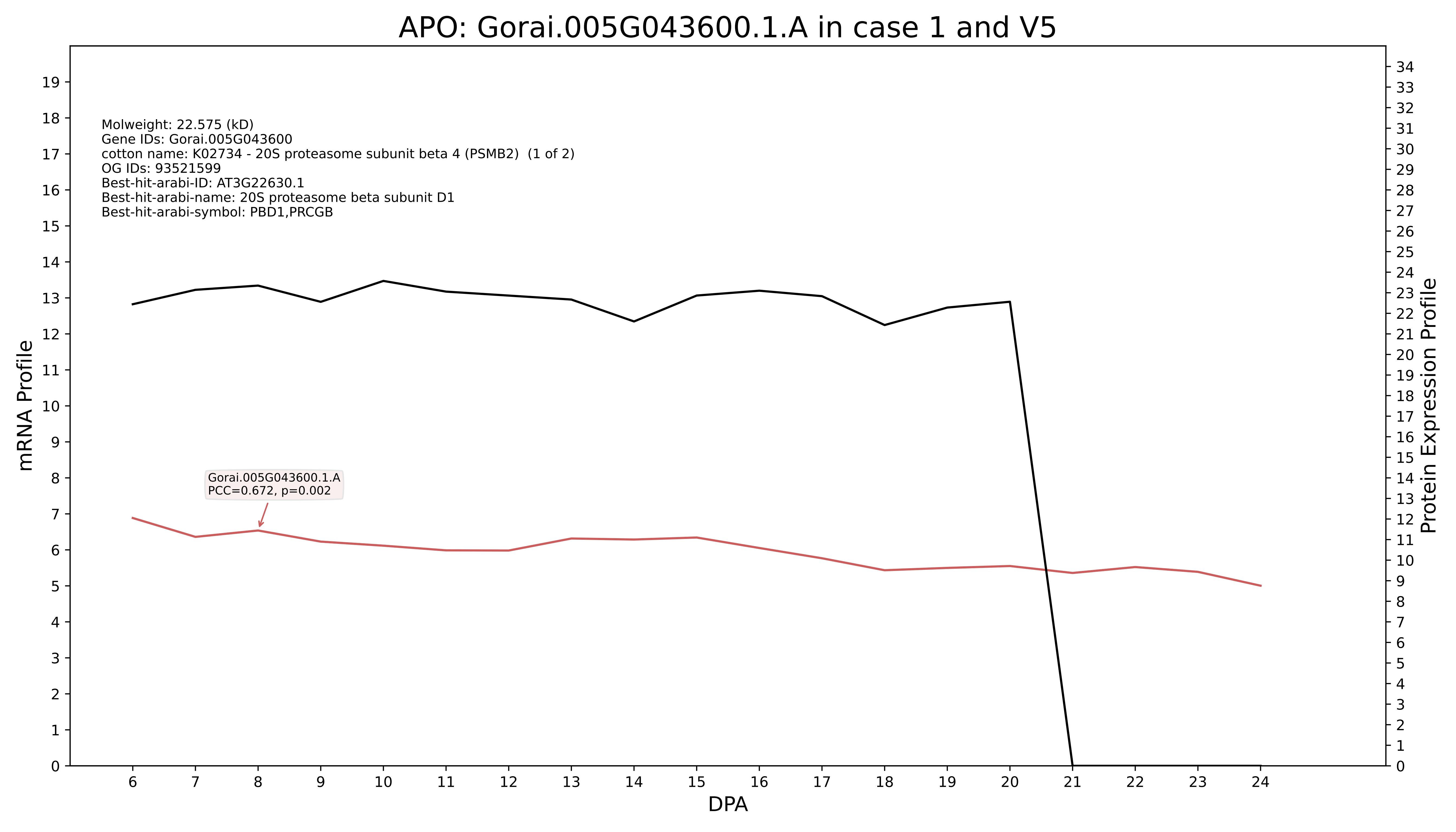

APO: Gorai.005G043600.1.A in case 1 and V5
Molweight: 22.575 (kD)
Gene IDs: Gorai.005G043600
cotton name: K02734 - 20S proteasome subunit beta 4 (PSMB2) (1 of 2)
OG IDs: 93521599
Best-hit-arabi-ID: AT3G22630.1
Best-hit-arabi-name: 20S proteasome beta subunit D1
Best-hit-arabi-symbol: PBD1,PRCGB
Gorai.005G043600.1.A
PCC=0.672, p=0.002
mRNA Profile
Protein Expression Profile
DPA

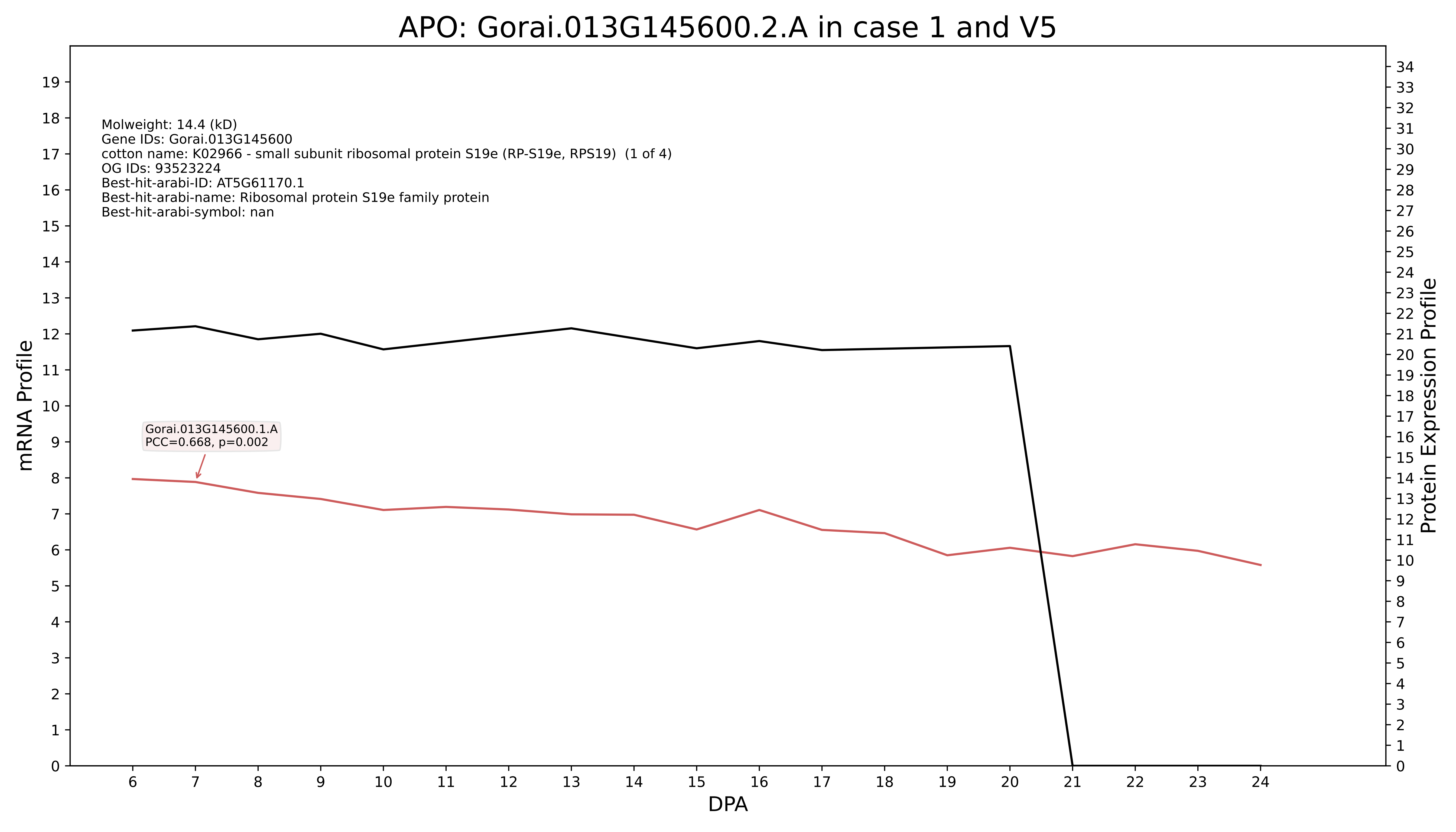

APO: Gorai.013G145600.2.A in case 1 and V5
Molweight: 14.4 (kD)
Gene IDs: Gorai.013G145600
cotton name: K02966 - small subunit ribosomal protein S19e (RP-S19e, RPS19) (1 of 4)
OG IDs: 93523224
Best-hit-arabi-ID: AT5G61170.1
Best-hit-arabi-name: Ribosomal protein S19e family protein
Best-hit-arabi-symbol: nan
Gorai.013G145600.1.A
PCC=0.668, p=0.002
mRNA Profile
Protein Expression Profile
DPA

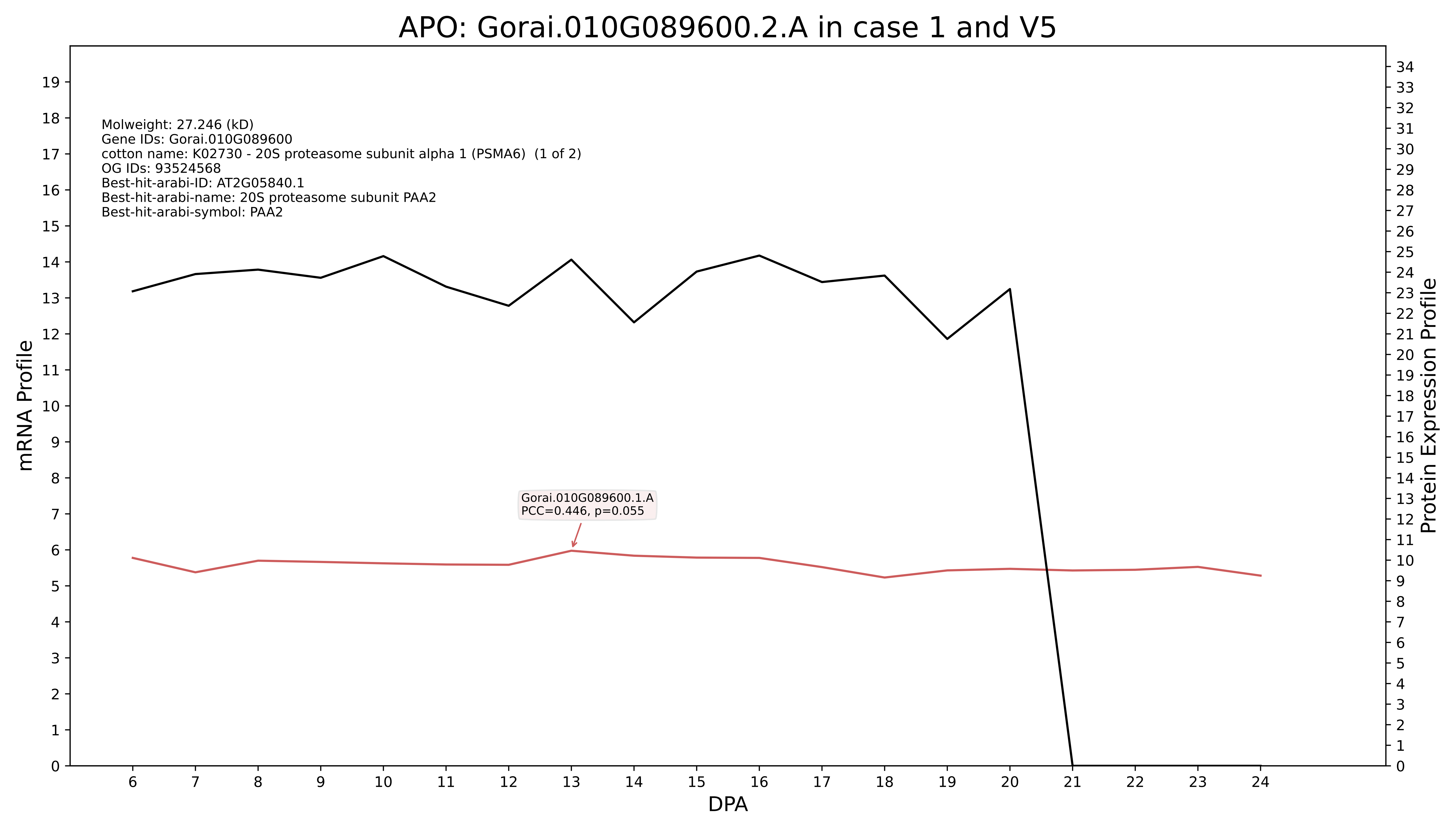

APO: Gorai.010G089600.2.A in case 1 and V5
Molweight: 27.246 (kD)
Gene IDs: Gorai.010G089600
cotton name: K02730 - 20S proteasome subunit alpha 1 (PSMA6) (1 of 2)
OG IDs: 93524568
Best-hit-arabi-ID: AT2G05840.1
Best-hit-arabi-name: 20S proteasome subunit PAA2
Best-hit-arabi-symbol: PAA2
Gorai.010G089600.1.A
PCC=0.446, p=0.055
mRNA Profile
Protein Expression Profile
DPA

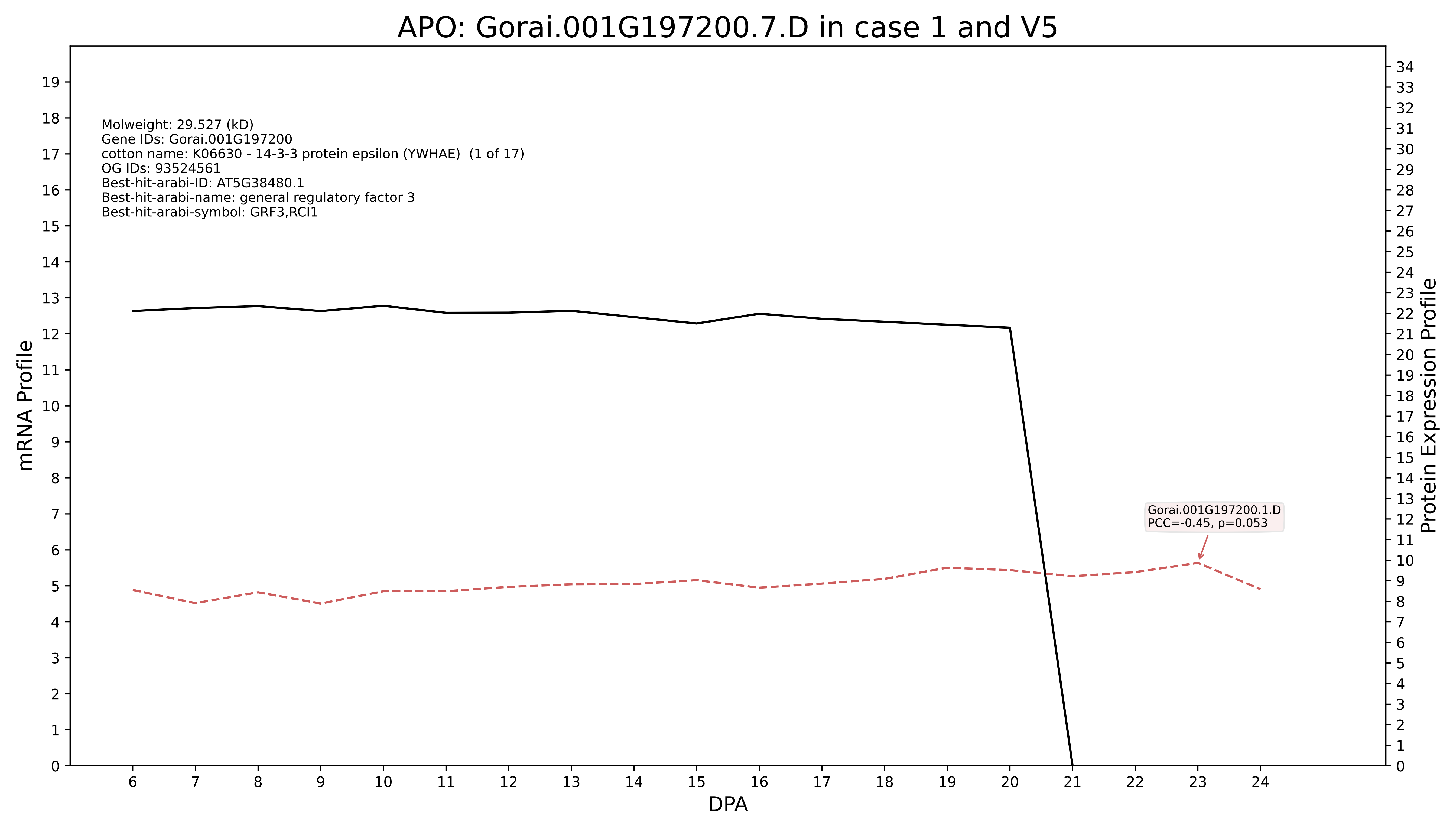
APO: Gorai.001G197200.7.D in case 1 and V5
Molweight: 29.527 (kD)
Gene IDs: Gorai.001G197200
cotton name: K06630 - 14-3-3 protein epsilon (YWHAE) (1 of 17)
OG IDs: 93524561
Best-hit-arabi-ID: AT5G38480.1
Best-hit-arabi-name: general regulatory factor 3
Best-hit-arabi-symbol: GRF3,RCI1
Gorai.001G197200.1.D
PCC=-0.45, p=0.053
mRNA Profile
Protein Expression Profile
DPA

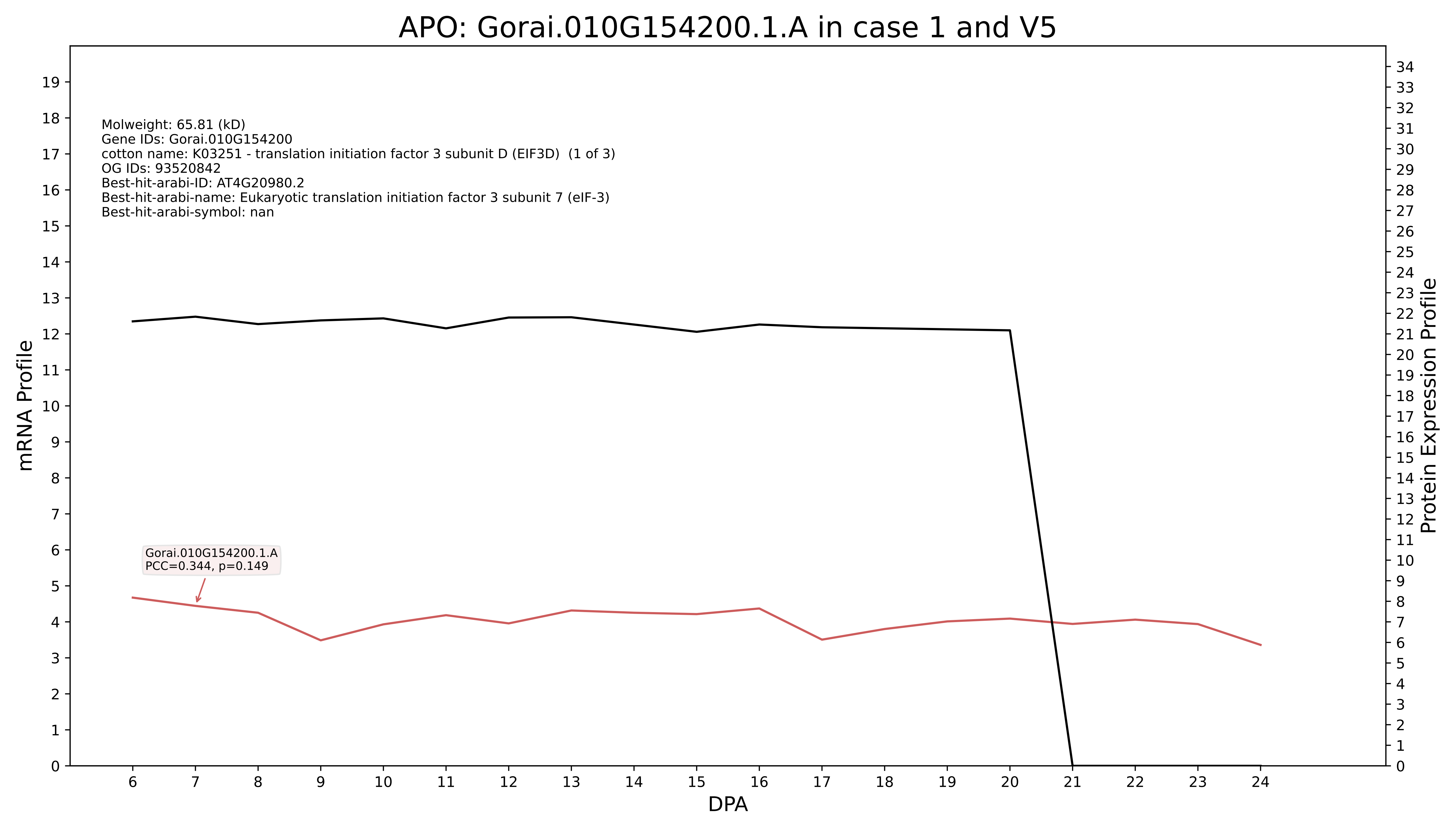

APO: Gorai.010G154200.1.A in case 1 and V5
Molweight: 65.81 (kD)
Gene IDs: Gorai.010G154200
cotton name: K03251 - translation initiation factor 3 subunit D (EIF3D) (1 of 3)
OG IDs: 93520842
Best-hit-arabi-ID: AT4G20980.2
Best-hit-arabi-name: Eukaryotic translation initiation factor 3 subunit 7 (eIF-3)
Best-hit-arabi-symbol: nan
Gorai.010G154200.1.A
PCC=0.344, p=0.149
mRNA Profile
Protein Expression Profile
DPA

APO: Gorai.008G046400.1.A in case 1 and V5

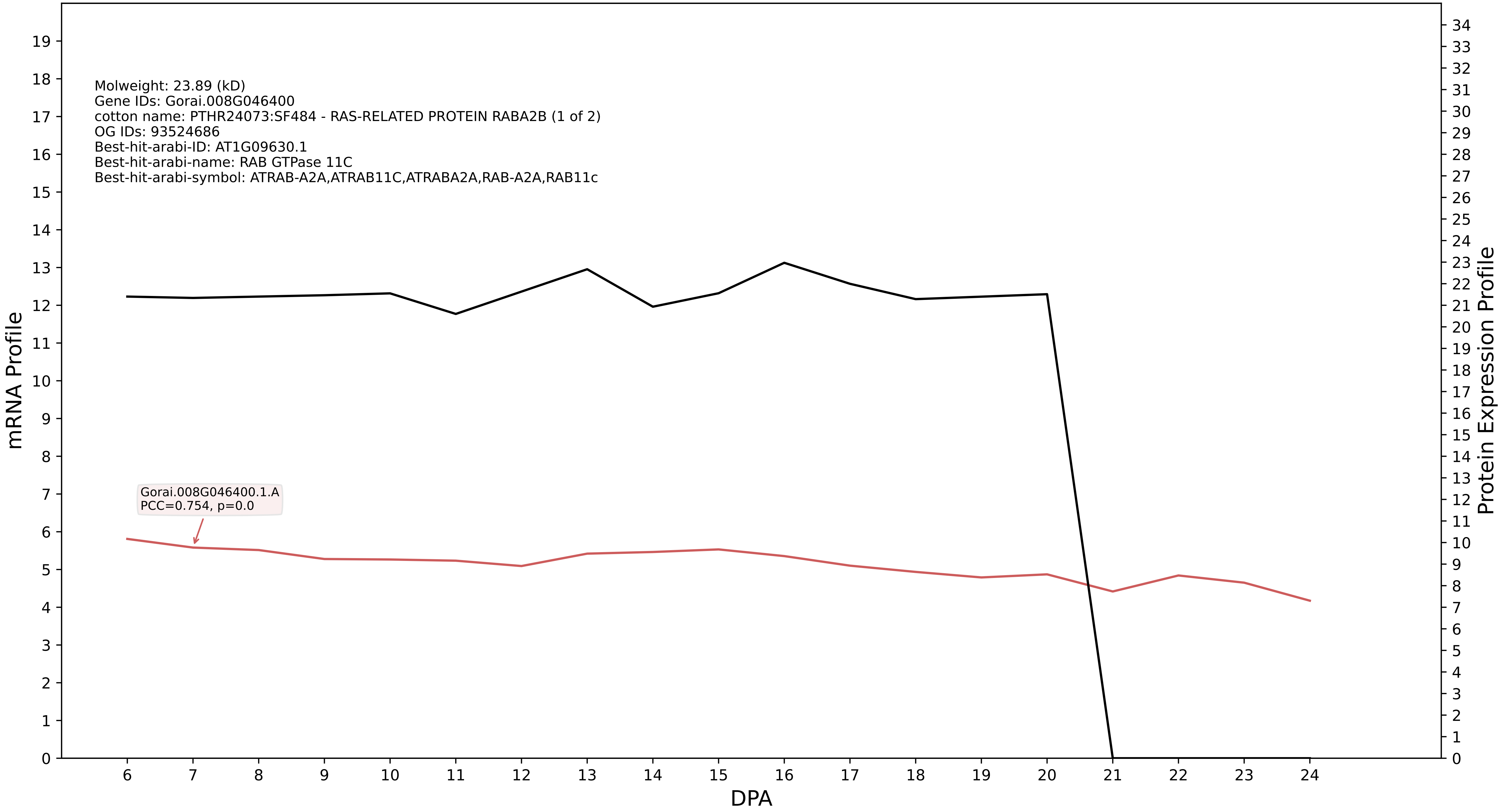

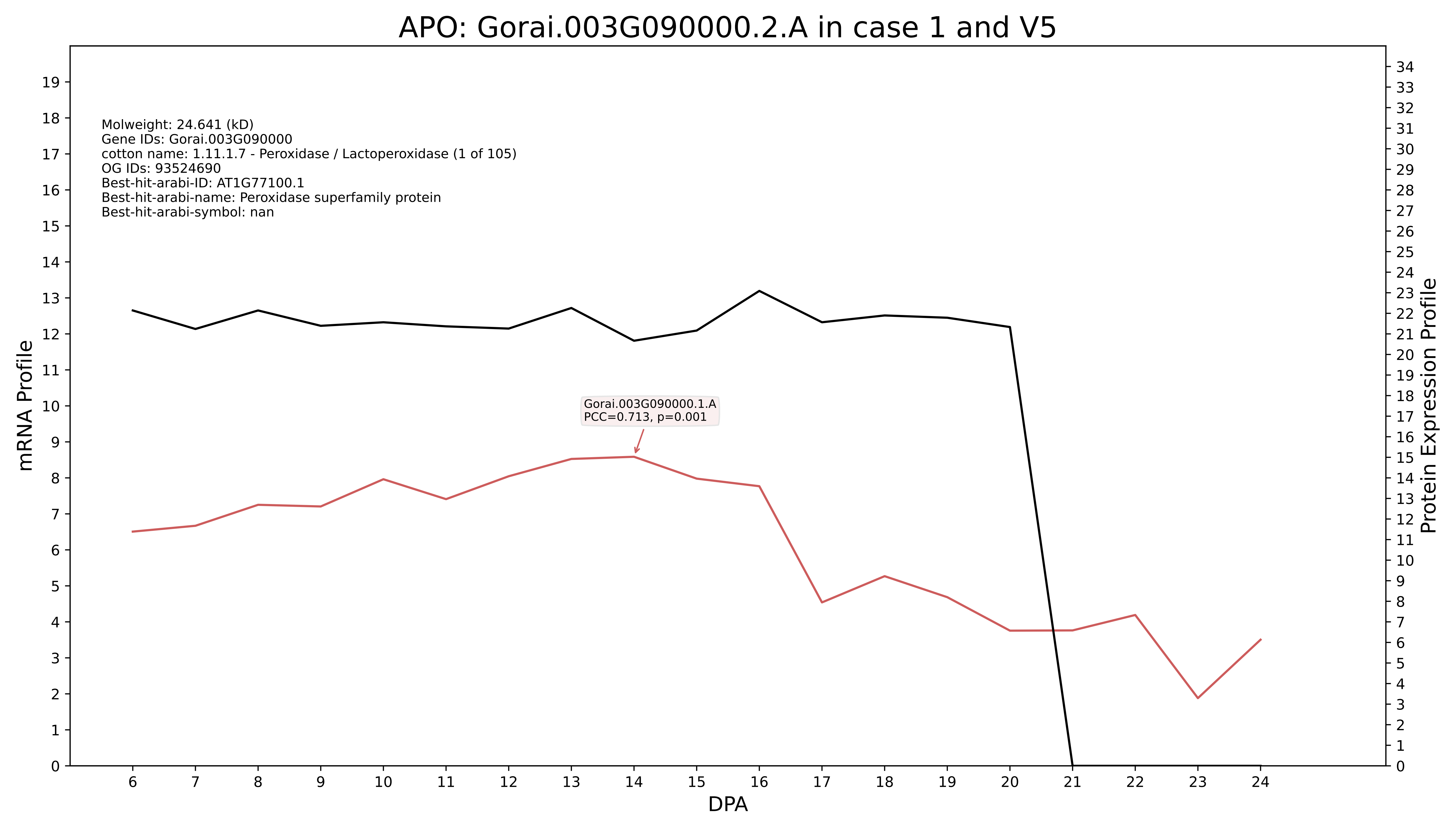

APO: Gorai.003G090000.2.A in case 1 and V5
Molweight: 24.641 (kD)
Gene IDs: Gorai.003G090000
cotton name: 1.11.1.7 - Peroxidase / Lactoperoxidase (1 of 105)
OG IDs: 93524690
Best-hit-arabi-ID: AT1G77100.1
Best-hit-arabi-name: Peroxidase superfamily protein
Best-hit-arabi-symbol: nan
Gorai.003G090000.1.A
PCC=0.713, p=0.001
mRNA Profile
Protein Expression Profile
DPA

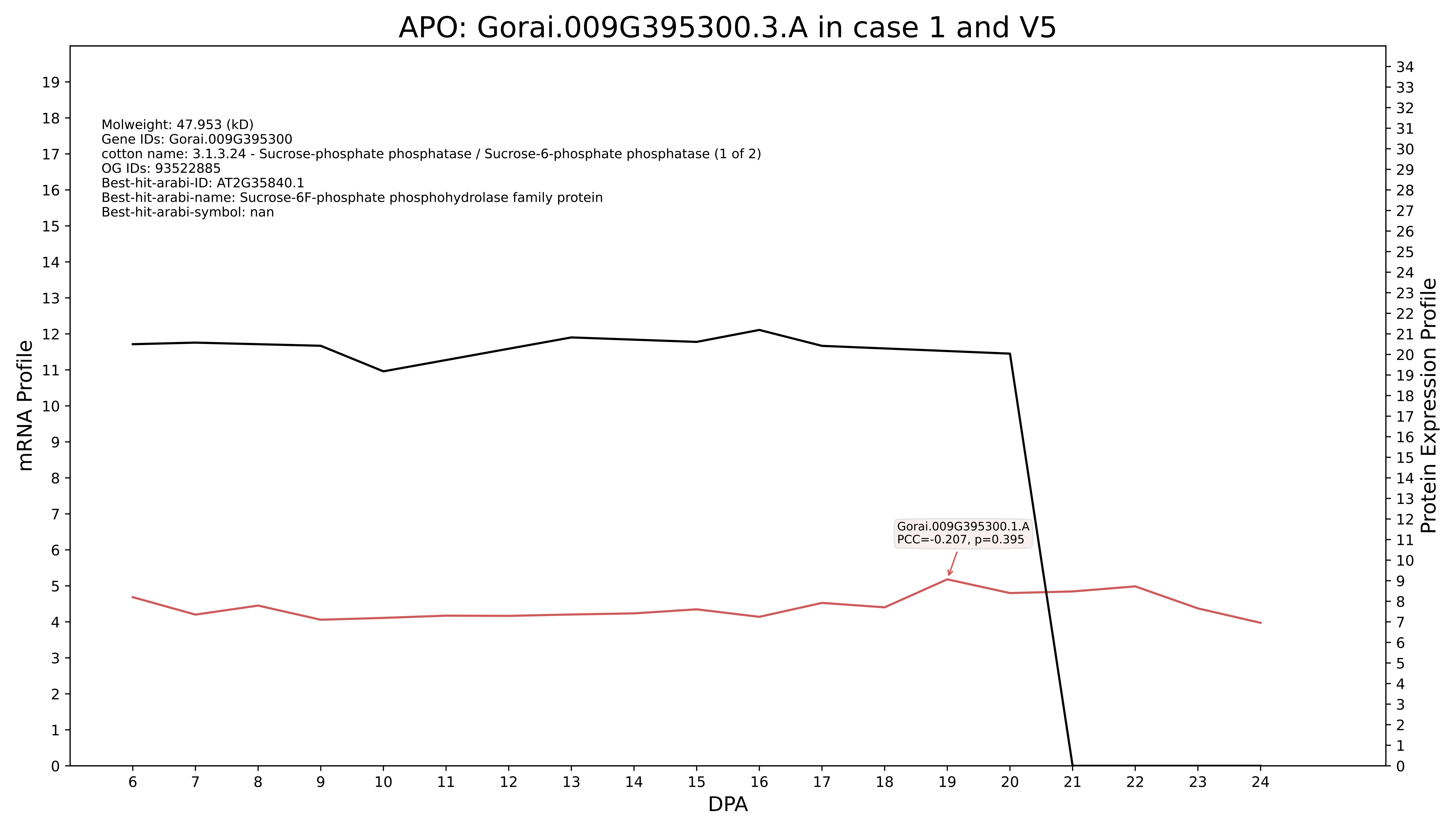
APO: Gorai.009G395300.3.A in case 1 and V5
Molweight: 47.953 (kD)
Gene IDs: Gorai.009G395300
cotton name: 3.1.3.24 - Sucrose-phosphate phosphatase / Sucrose-6-phosphate phosphatase (1 of 2)
OG IDs: 93522885
Best-hit-arabi-ID: AT2G35840.1
Best-hit-arabi-name: Sucrose-6F-phosphate phosphohydrolase family protein
Best-hit-arabi-symbol: nan
Gorai.009G395300.1.A
PCC=-0.207, p=0.395
mRNA Profile
Protein Expression Profile
DPA

APO: Gorai.005G012900.1.D in case 1 and V5

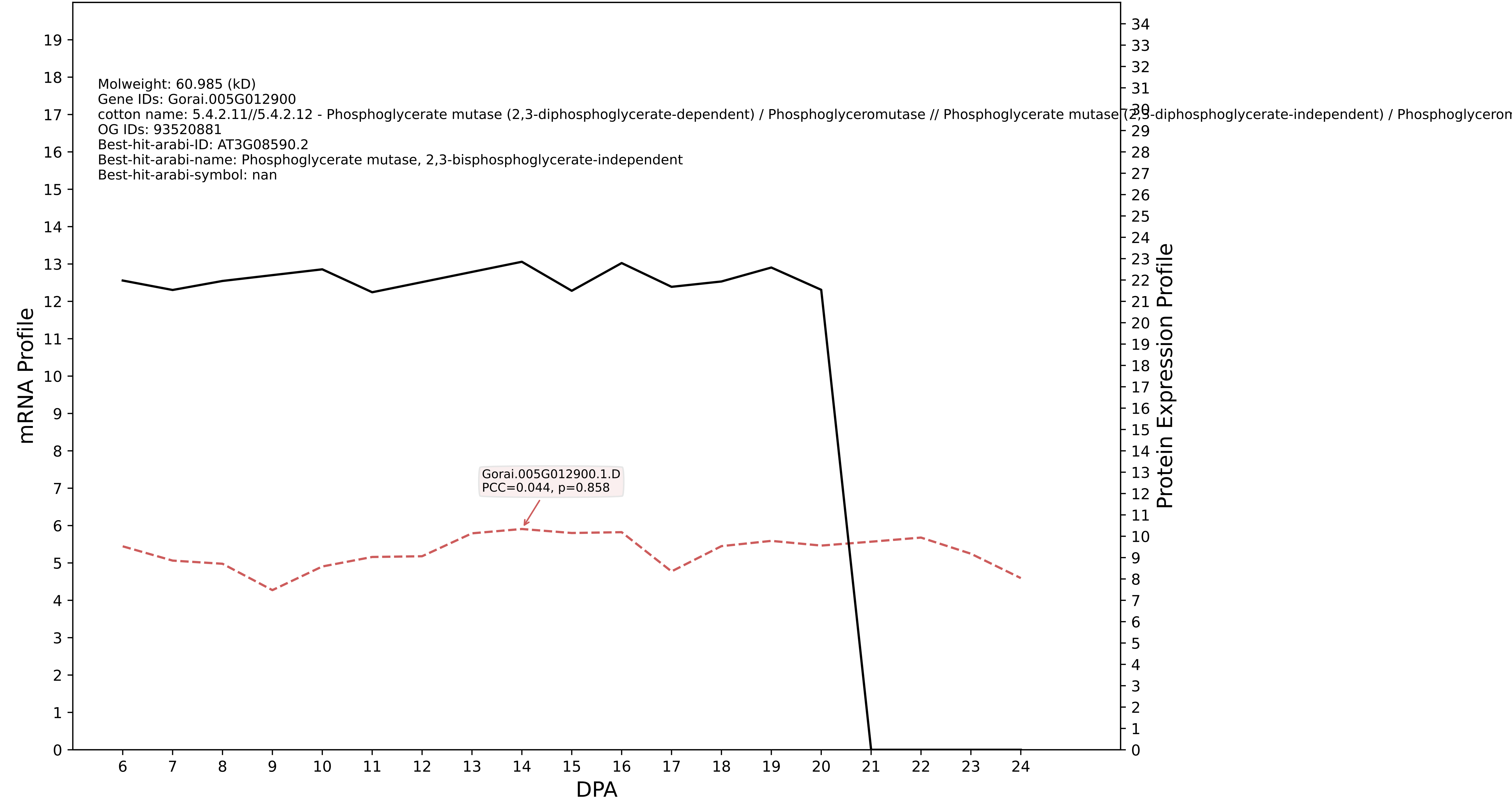

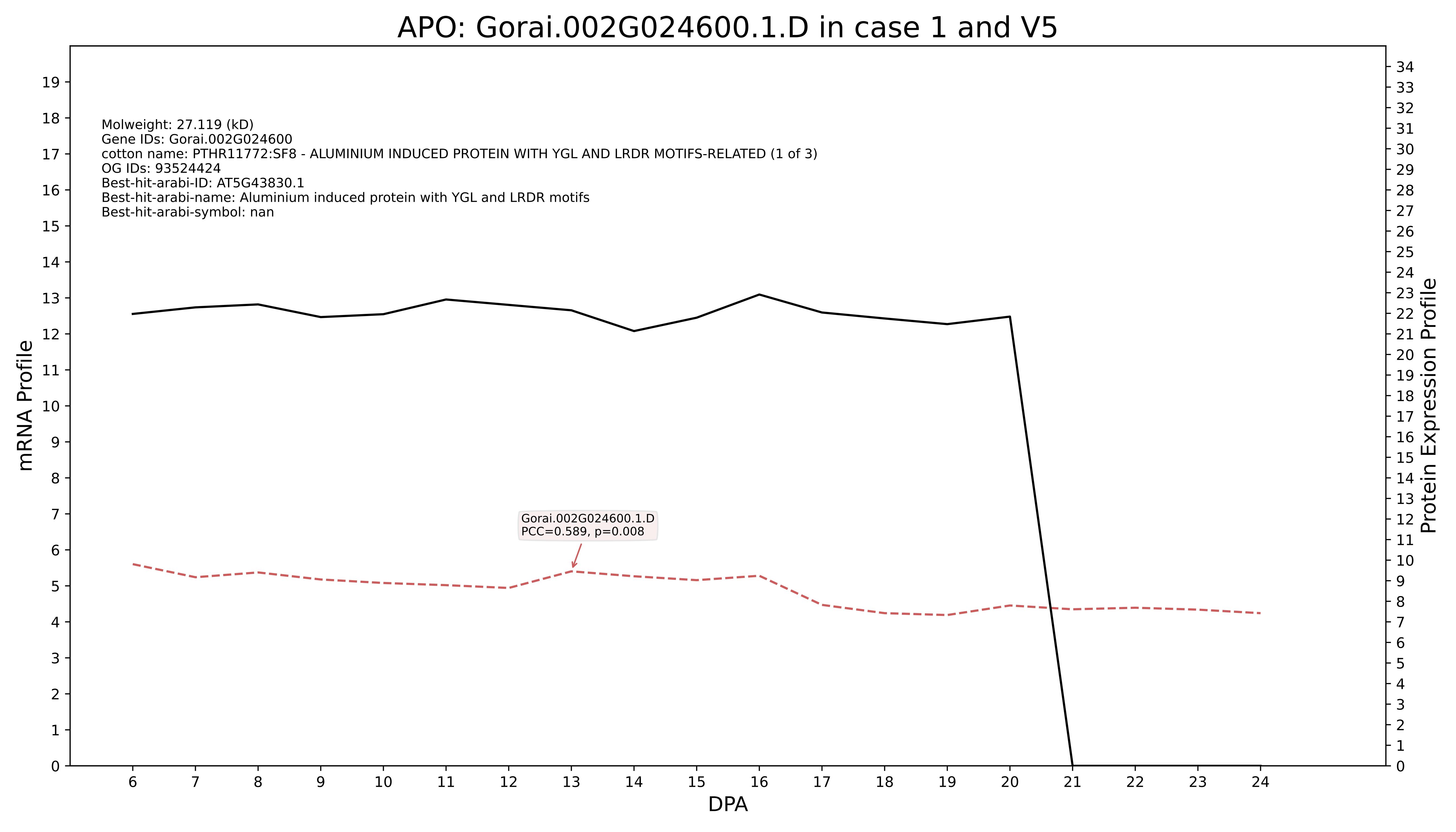

APO: Gorai.002G024600.1.D in case 1 and V5
Molweight: 27.119 (kD)
Gene IDs: Gorai.002G024600
cotton name: PTHR11772:SF8 - ALUMINIUM INDUCED PROTEIN WITH YGL AND LRDR MOTIFS-RELATED (1 of 3)
OG IDs: 93524424
Best-hit-arabi-ID: AT5G43830.1
Best-hit-arabi-name: Aluminium induced protein with YGL and LRDR motifs
Best-hit-arabi-symbol: nan
Gorai.002G024600.1.D
PCC=0.589, p=0.008
mRNA Profile
Protein Expression Profile
DPA

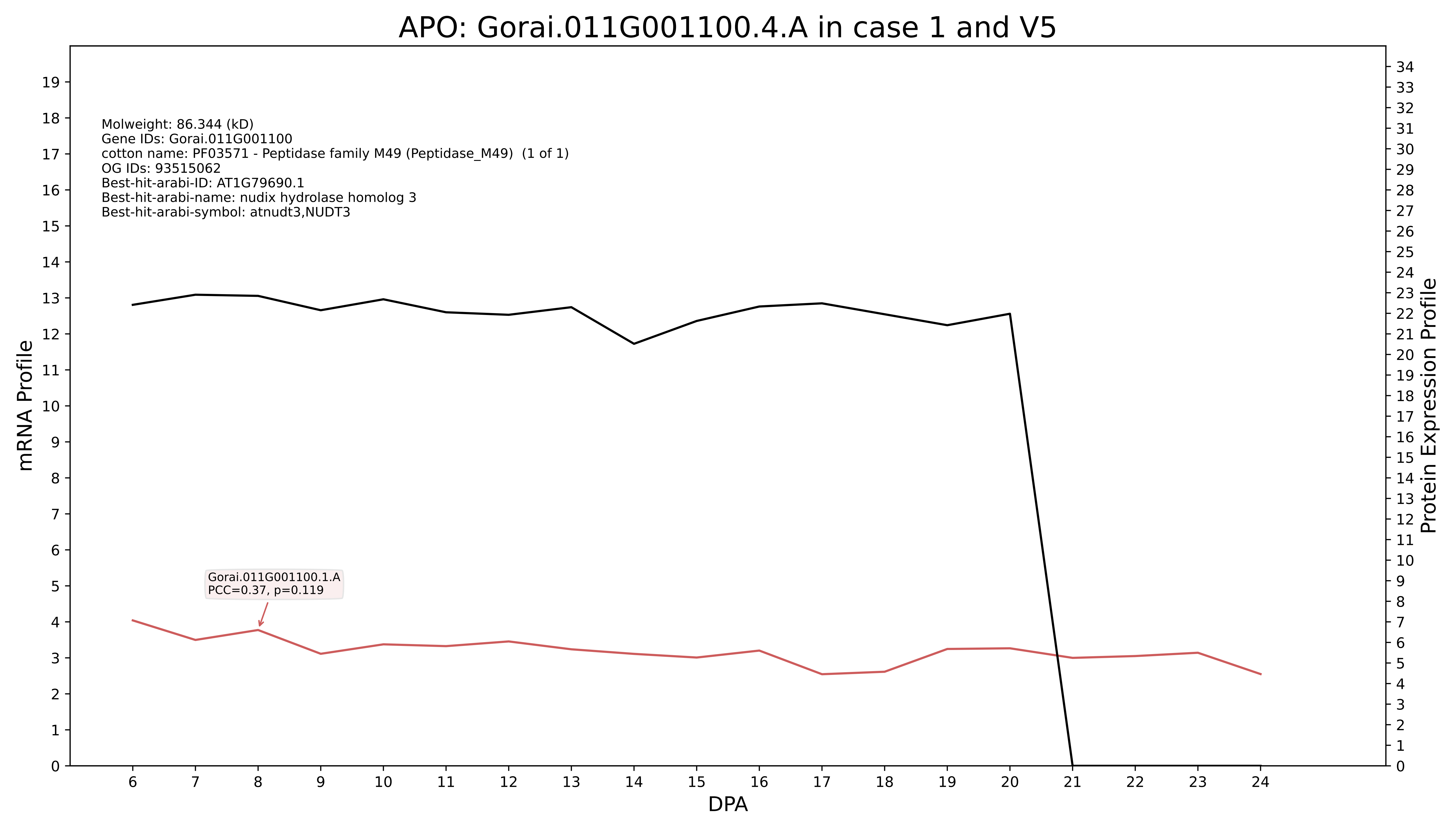
APO: Gorai.011G001100.4.A in case 1 and V5
Molweight: 86.344 (kD)
Gene IDs: Gorai.011G001100
cotton name: PF03571 - Peptidase family M49 (Peptidase_M49) (1 of 1)
OG IDs: 93515062
Best-hit-arabi-ID: AT1G79690.1
Best-hit-arabi-name: nudix hydrolase homolog 3
Best-hit-arabi-symbol: atnudt3,NUDT3
Gorai.011G001100.1.A
PCC=0.37, p=0.119
mRNA Profile
Protein Expression Profile
DPA

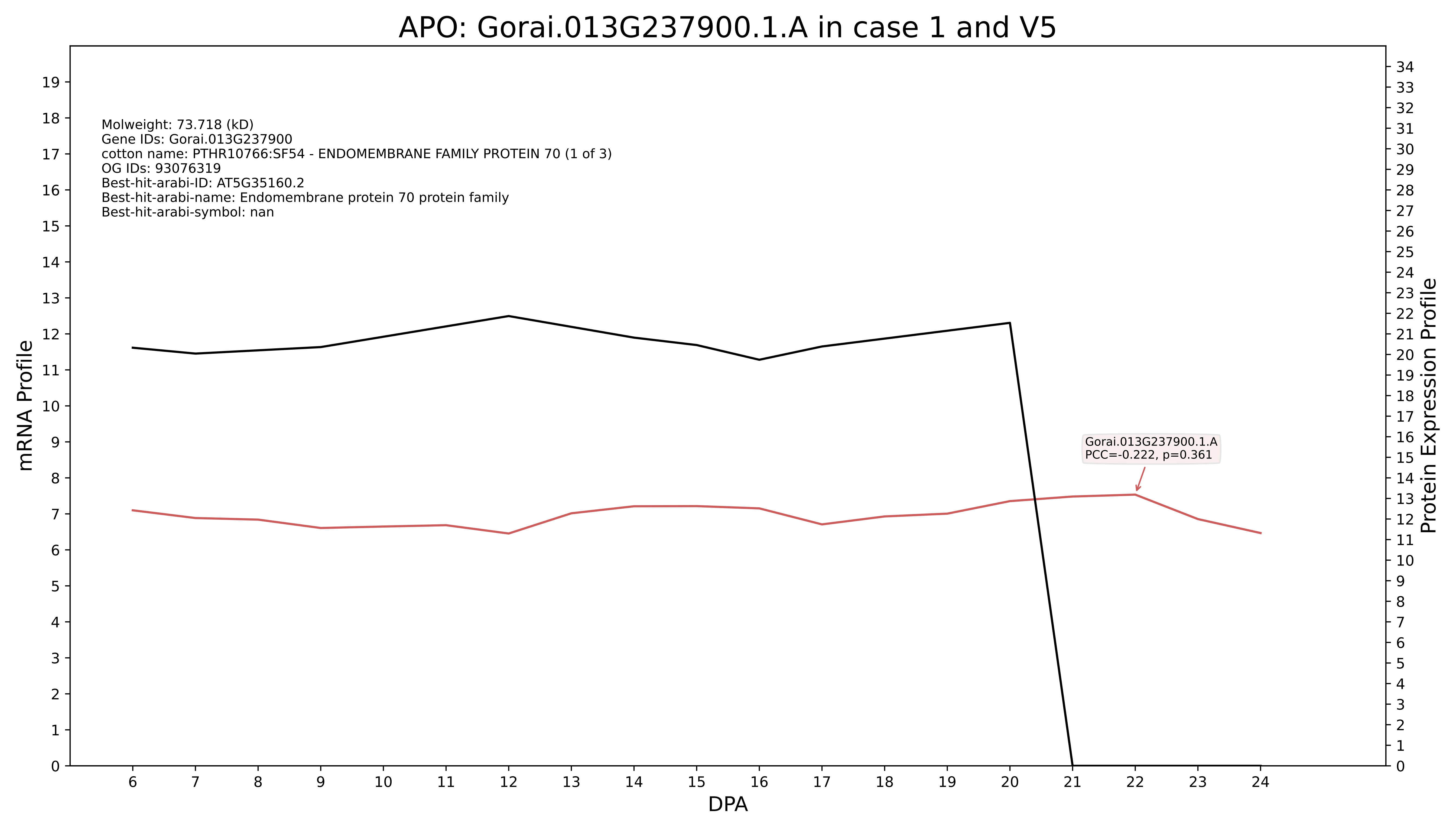

APO: Gorai.013G237900.1.A in case 1 and V5
Molweight: 73.718 (kD)
Gene IDs: Gorai.013G237900
cotton name: PTHR10766:SF54 - ENDOMEMBRANE FAMILY PROTEIN 70 (1 of 3)
OG IDs: 93076319
Best-hit-arabi-ID: AT5G35160.2
Best-hit-arabi-name: Endomembrane protein 70 protein family
Best-hit-arabi-symbol: nan
Gorai.013G237900.1.A
PCC=-0.222, p=0.361
mRNA Profile
Protein Expression Profile
DPA

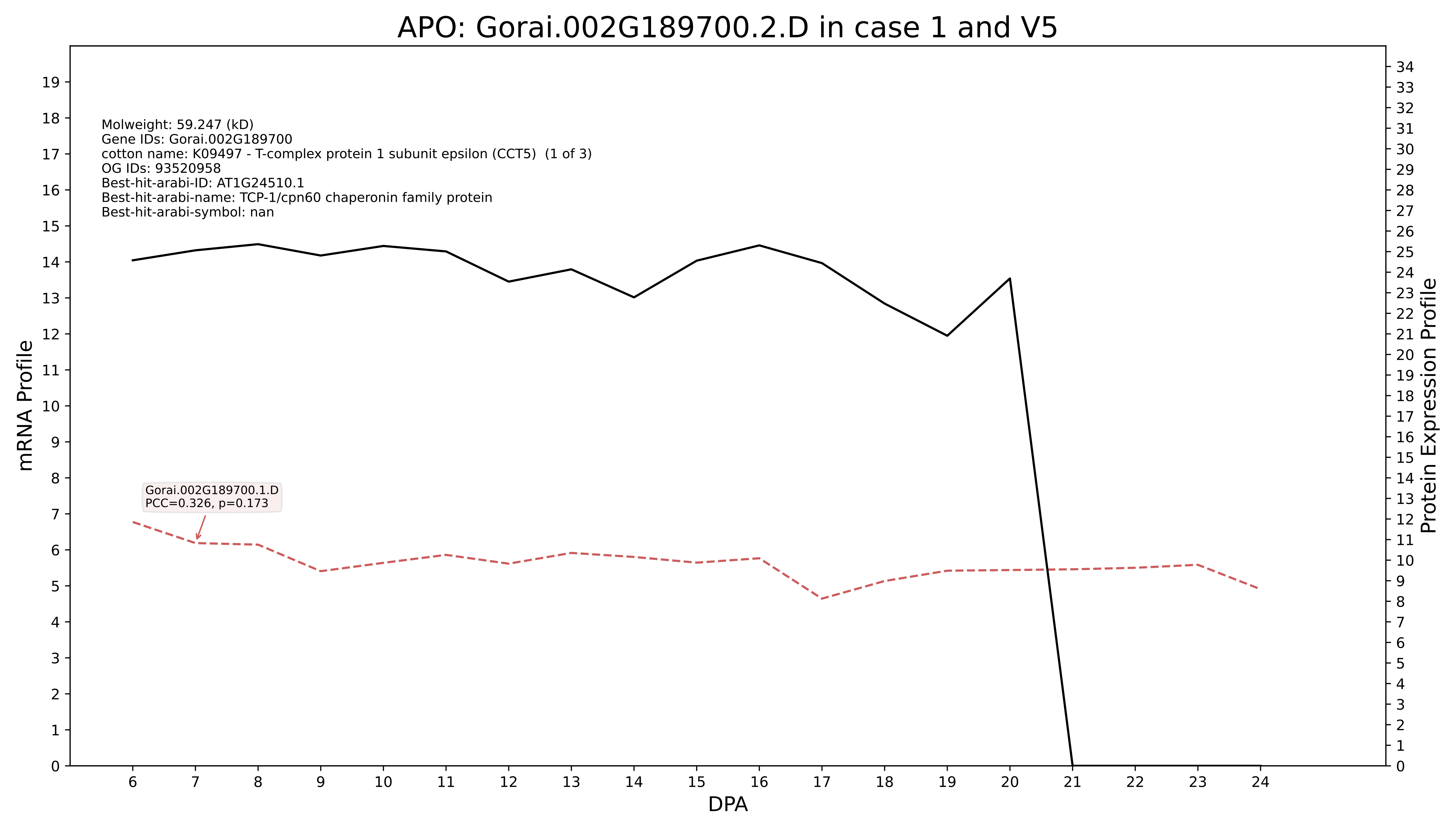
APO: Gorai.002G189700.2.D in case 1 and V5
Molweight: 59.247 (kD)
Gene IDs: Gorai.002G189700
cotton name: K09497 - T-complex protein 1 subunit epsilon (CCT5) (1 of 3)
OG IDs: 93520958
Best-hit-arabi-ID: AT1G24510.1
Best-hit-arabi-name: TCP-1/cpn60 chaperonin family protein
Best-hit-arabi-symbol: nan
Gorai.002G189700.1.D
PCC=0.326, p=0.173
mRNA Profile
Protein Expression Profile
DPA

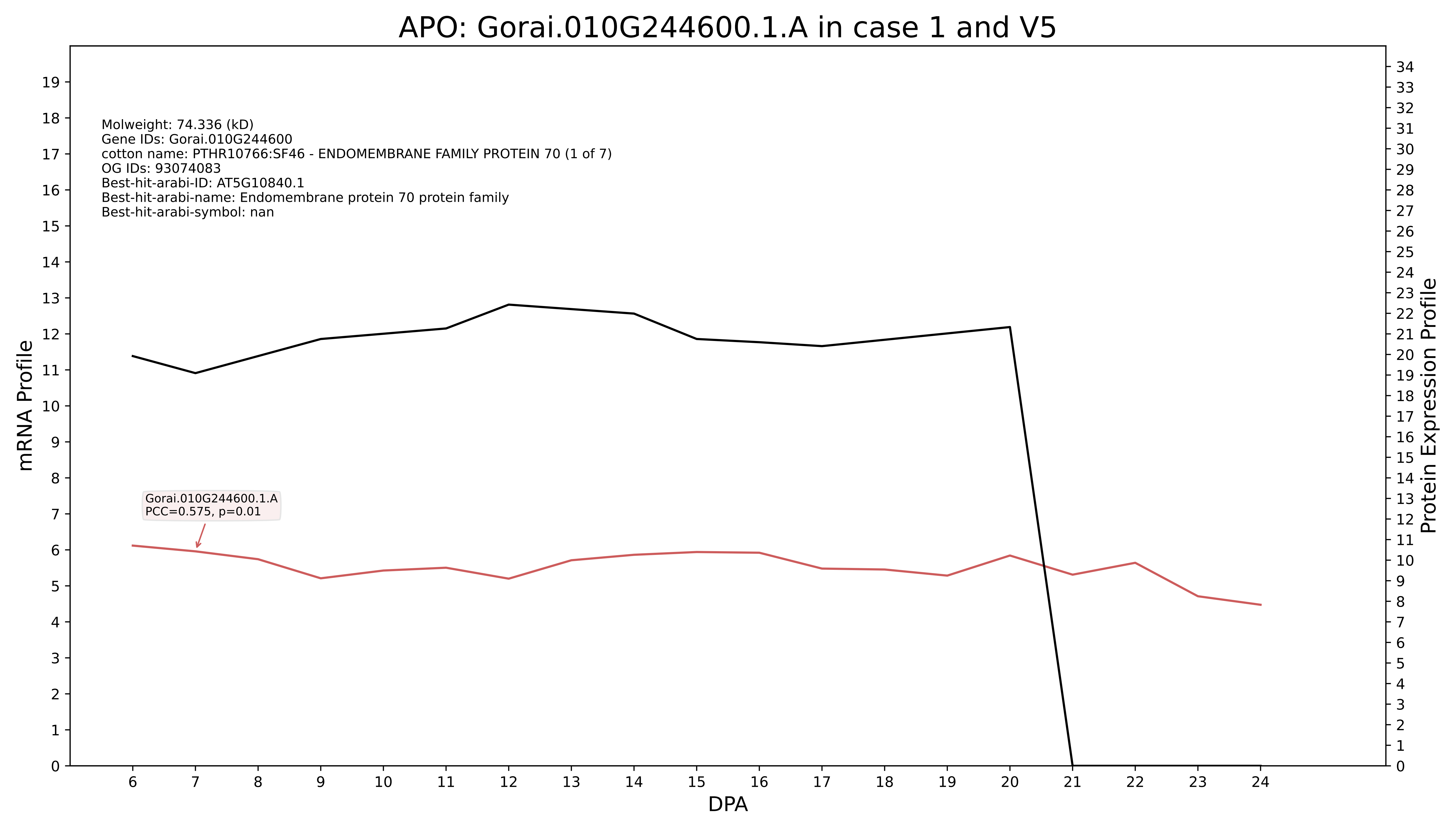

APO: Gorai.010G244600.1.A in case 1 and V5
Molweight: 74.336 (kD)
Gene IDs: Gorai.010G244600
cotton name: PTHR10766:SF46 - ENDOMEMBRANE FAMILY PROTEIN 70 (1 of 7)
OG IDs: 93074083
Best-hit-arabi-ID: AT5G10840.1
Best-hit-arabi-name: Endomembrane protein 70 protein family
Best-hit-arabi-symbol: nan
Gorai.010G244600.1.A
PCC=0.575, p=0.01
mRNA Profile
Protein Expression Profile
DPA

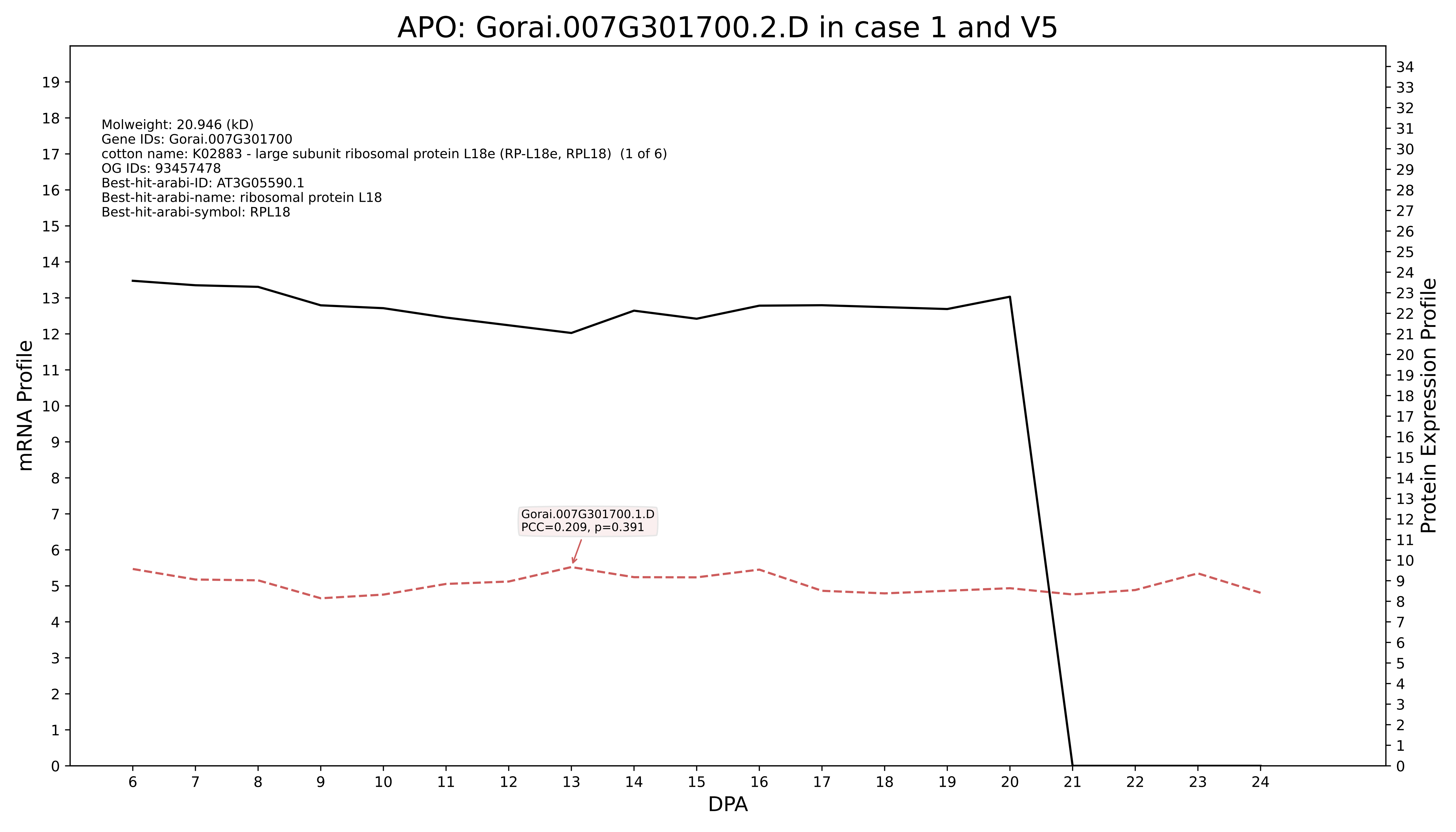

APO: Gorai.007G301700.2.D in case 1 and V5
Molweight: 20.946 (kD)
Gene IDs: Gorai.007G301700
cotton name: K02883 - large subunit ribosomal protein L18e (RP-L18e, RPL18) (1 of 6)
OG IDs: 93457478
Best-hit-arabi-ID: AT3G05590.1
Best-hit-arabi-name: ribosomal protein L18
Best-hit-arabi-symbol: RPL18
Gorai.007G301700.1.D
PCC=0.209, p=0.391
mRNA Profile
Protein Expression Profile
DPA

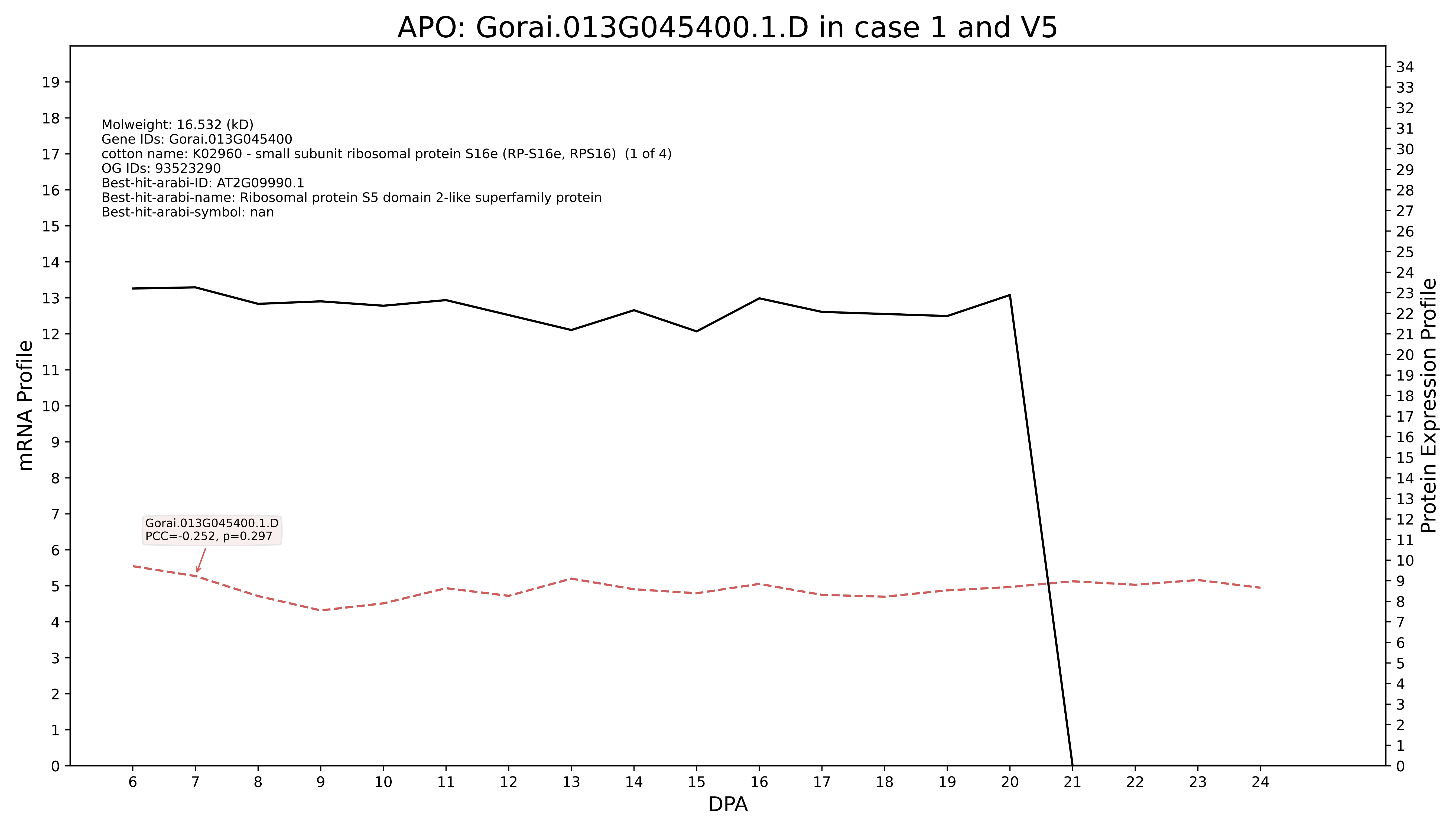
APO: Gorai.013G045400.1.D in case 1 and V5
Molweight: 16.532 (kD)
Gene IDs: Gorai.013G045400
cotton name: K02960 - small subunit ribosomal protein S16e (RP-S16e, RPS16) (1 of 4)
OG IDs: 93523290
Best-hit-arabi-ID: AT2G09990.1
Best-hit-arabi-name: Ribosomal protein S5 domain 2-like superfamily protein
Best-hit-arabi-symbol: nan
Gorai.013G045400.1.D
PCC=-0.252, p=0.297
mRNA Profile
Protein Expression Profile
DPA

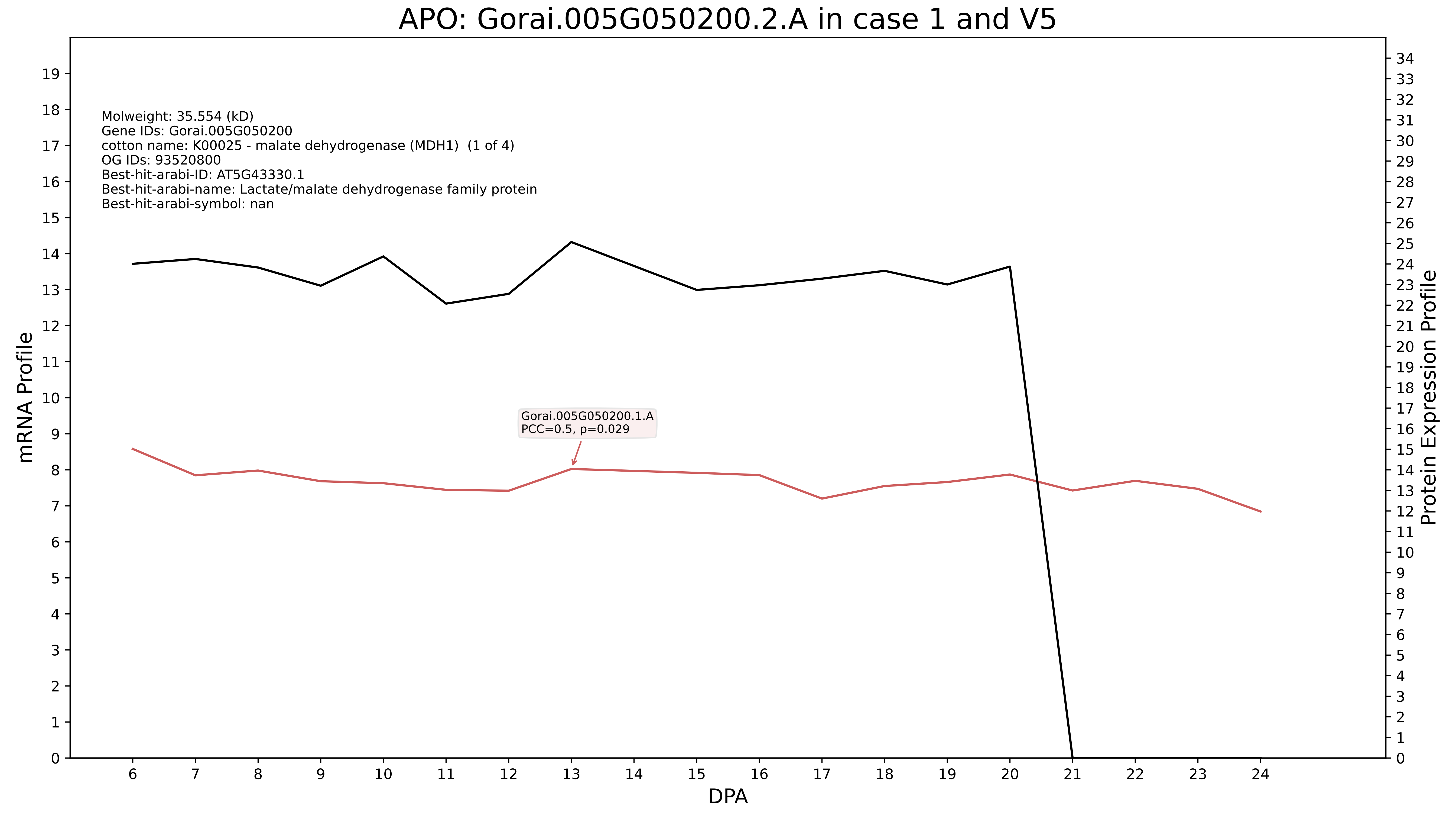
APO: Gorai.005G050200.2.A in case 1 and V5
Molweight: 35.554 (kD)
Gene IDs: Gorai.005G050200
cotton name: K00025 - malate dehydrogenase (MDH1) (1 of 4)
OG IDs: 93520800
Best-hit-arabi-ID: AT5G43330.1
Best-hit-arabi-name: Lactate/malate dehydrogenase family protein
Best-hit-arabi-symbol: nan
Gorai.005G050200.1.A
PCC=0.5, p=0.029
mRNA Profile
Protein Expression Profile
DPA

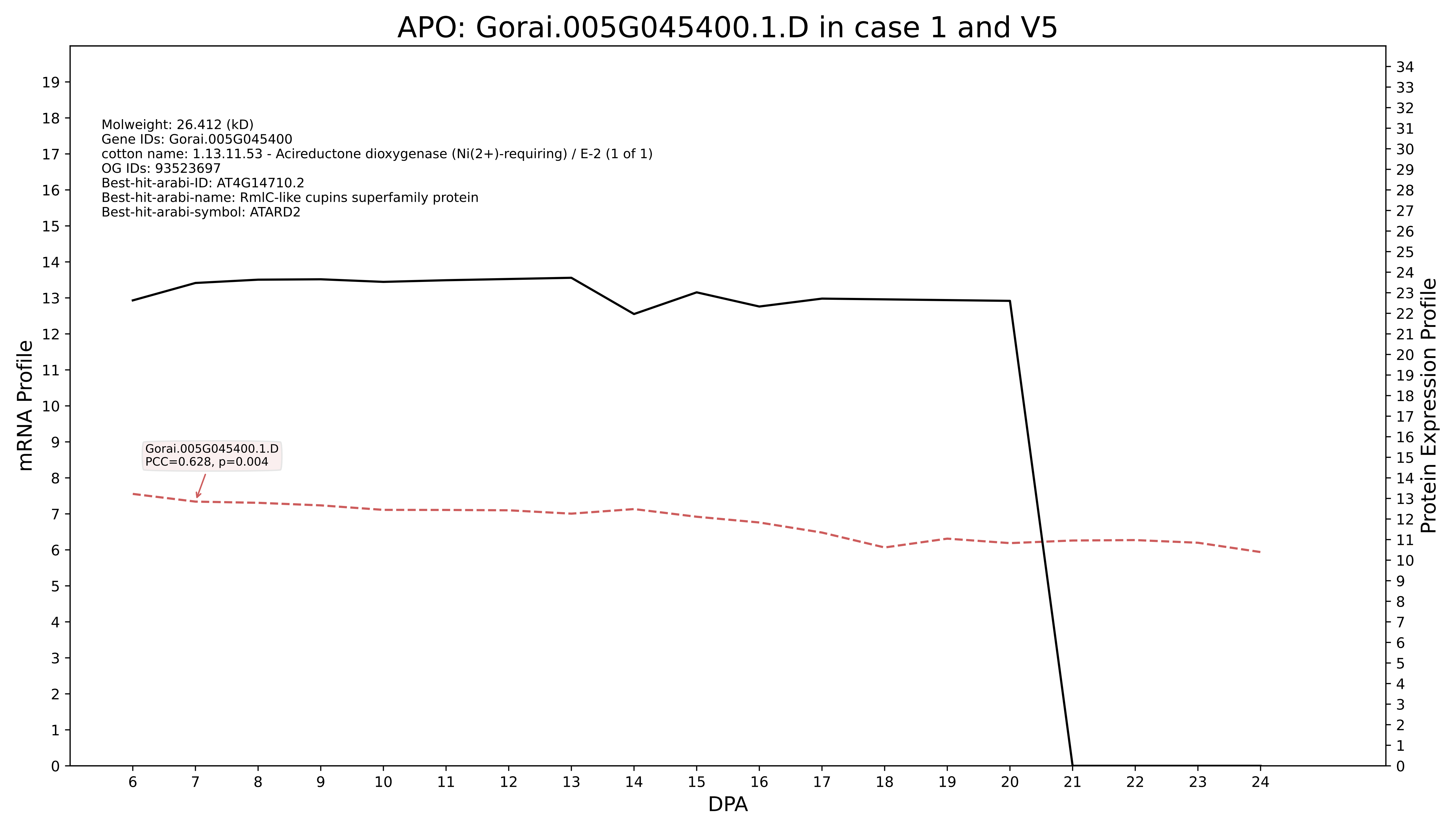
APO: Gorai.005G045400.1.D in case 1 and V5
Molweight: 26.412 (kD)
Gene IDs: Gorai.005G045400
cotton name: 1.13.11.53 - Acireductone dioxygenase (Ni(2+)-requiring) / E-2 (1 of 1)
OG IDs: 93523697
Best-hit-arabi-ID: AT4G14710.2
Best-hit-arabi-name: RmlC-like cupins superfamily protein
Best-hit-arabi-symbol: ATARD2
Gorai.005G045400.1.D
PCC=0.628, p=0.004
mRNA Profile
Protein Expression Profile
DPA
0 1 2 3 4 5 6 7 8 9 10 11 12 13 14 15 16 17 18 19
0 1 2 3 4 5 6 7 8 9 10 11 12 13 14 15 16 17 18 19 20 21 22 23 24 25 26 27 28 29 30 31 32 33 34
6 7 8 9 10 11 12 13 14 15 16 17 18 19 20 21 22 23 24

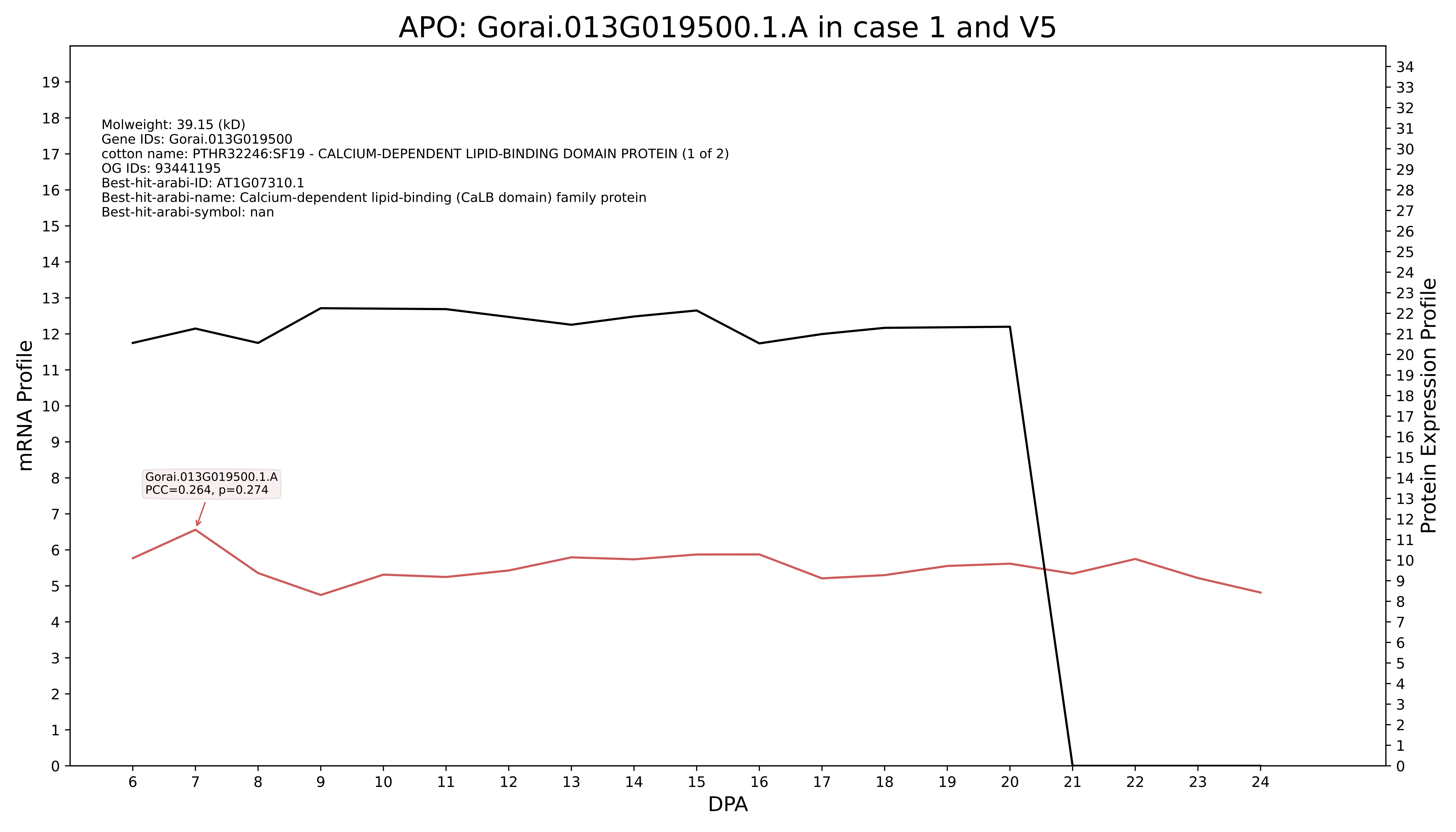

APO: Gorai.013G019500.1.A in case 1 and V5
Molweight: 39.15 (kD)
Gene IDs: Gorai.013G019500
cotton name: PTHR32246:SF19 - CALCIUM-DEPENDENT LIPID-BINDING DOMAIN PROTEIN (1 of 2)
OG IDs: 93441195
Best-hit-arabi-ID: AT1G07310.1
Best-hit-arabi-name: Calcium-dependent lipid-binding (CaLB domain) family protein
Best-hit-arabi-symbol: nan
Gorai.013G019500.1.A
PCC=0.264, p=0.274
mRNA Profile
Protein Expression Profile
DPA

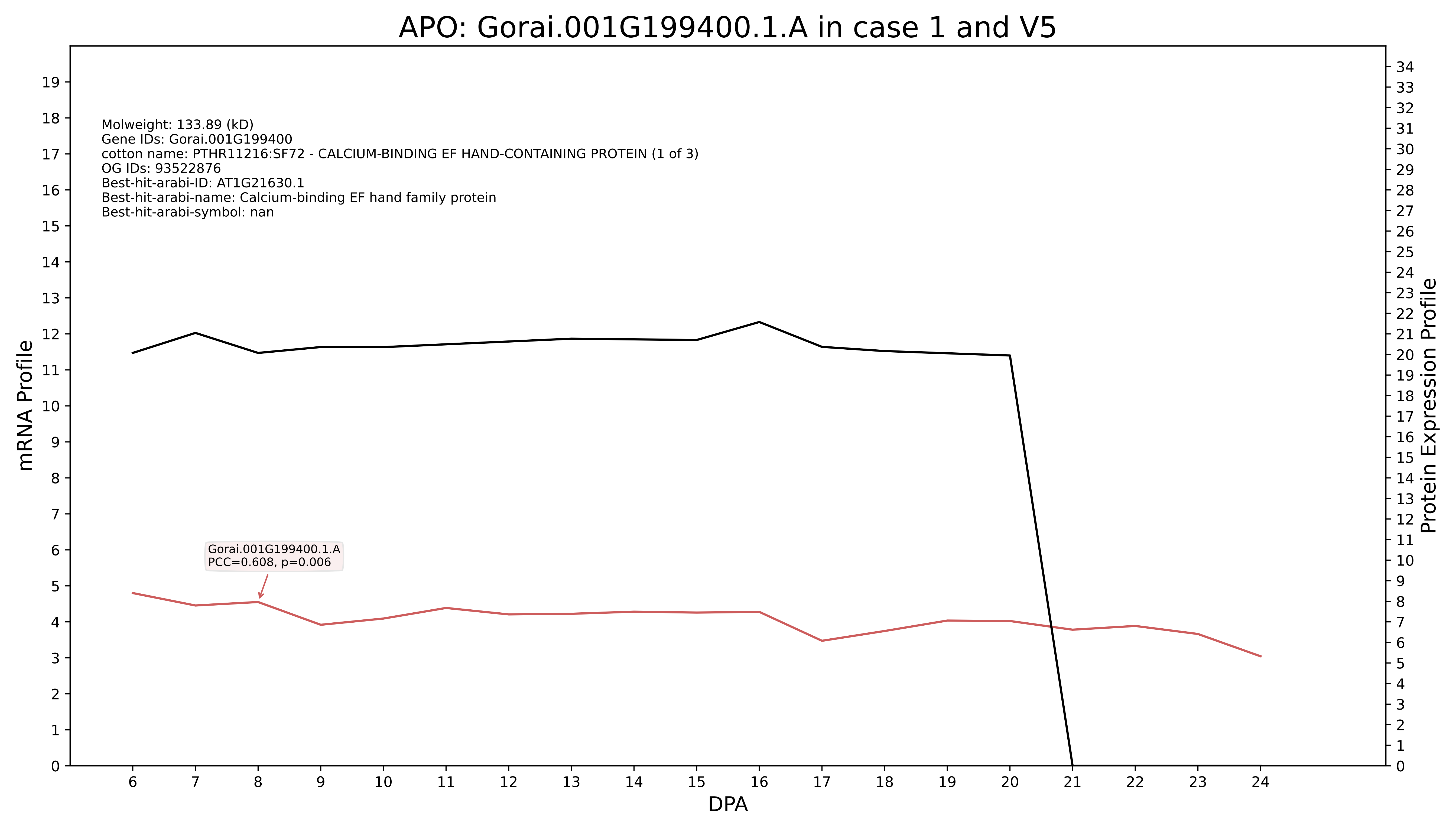
APO: Gorai.001G199400.1.A in case 1 and V5
Molweight: 133.89 (kD)
Gene IDs: Gorai.001G199400
cotton name: PTHR11216:SF72 - CALCIUM-BINDING EF HAND-CONTAINING PROTEIN (1 of 3)
OG IDs: 93522876
Best-hit-arabi-ID: AT1G21630.1
Best-hit-arabi-name: Calcium-binding EF hand family protein
Best-hit-arabi-symbol: nan
Gorai.001G199400.1.A
PCC=0.608, p=0.006
mRNA Profile
Protein Expression Profile
DPA

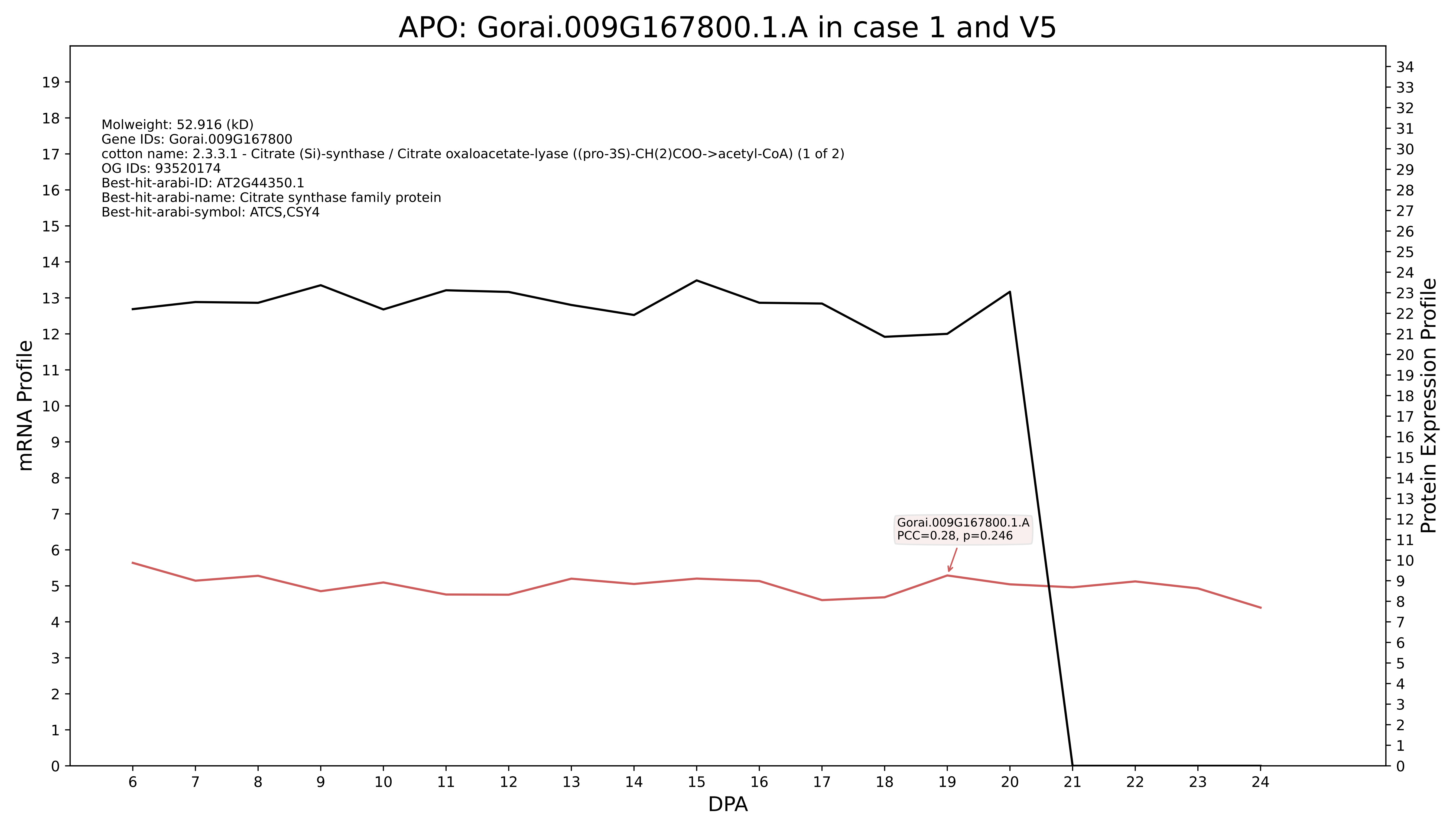
APO: Gorai.009G167800.1.A in case 1 and V5
Molweight: 52.916 (kD)
Gene IDs: Gorai.009G167800
cotton name: 2.3.3.1 - Citrate (Si)-synthase / Citrate oxaloacetate-lyase ((pro-3S)-CH(2)COO->acetyl-CoA) (1 of 2)
OG IDs: 93520174
Best-hit-arabi-ID: AT2G44350.1
Best-hit-arabi-name: Citrate synthase family protein
Best-hit-arabi-symbol: ATCS,CSY4
Gorai.009G167800.1.A
PCC=0.28, p=0.246
mRNA Profile
Protein Expression Profile
DPA

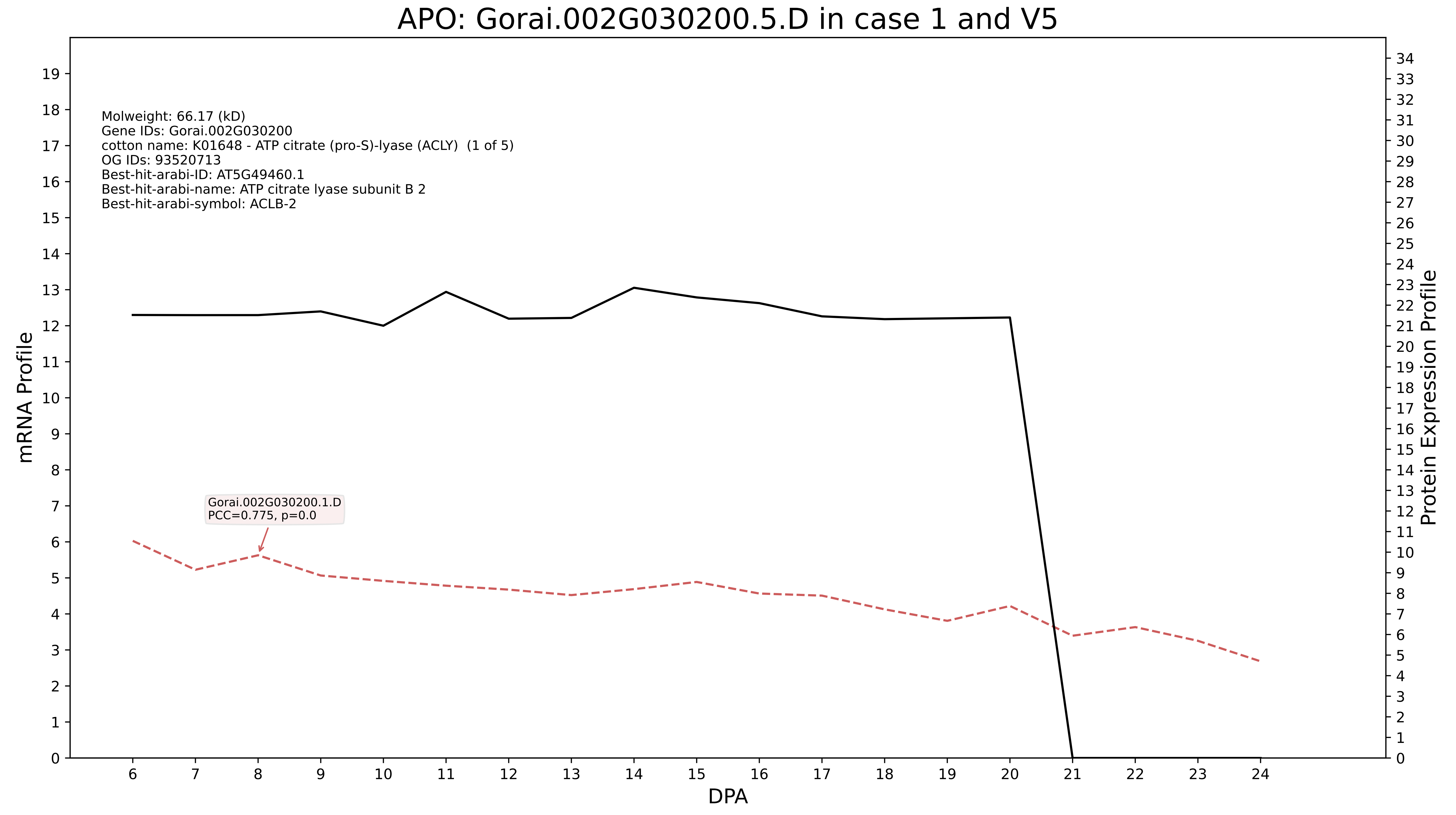
APO: Gorai.002G030200.5.D in case 1 and V5
Molweight: 66.17 (kD)
Gene IDs: Gorai.002G030200
cotton name: K01648 - ATP citrate (pro-S)-lyase (ACLY) (1 of 5)
OG IDs: 93520713
Best-hit-arabi-ID: AT5G49460.1
Best-hit-arabi-name: ATP citrate lyase subunit B 2
Best-hit-arabi-symbol: ACLB-2
Gorai.002G030200.1.D
PCC=0.775, p=0.0
mRNA Profile
Protein Expression Profile
DPA

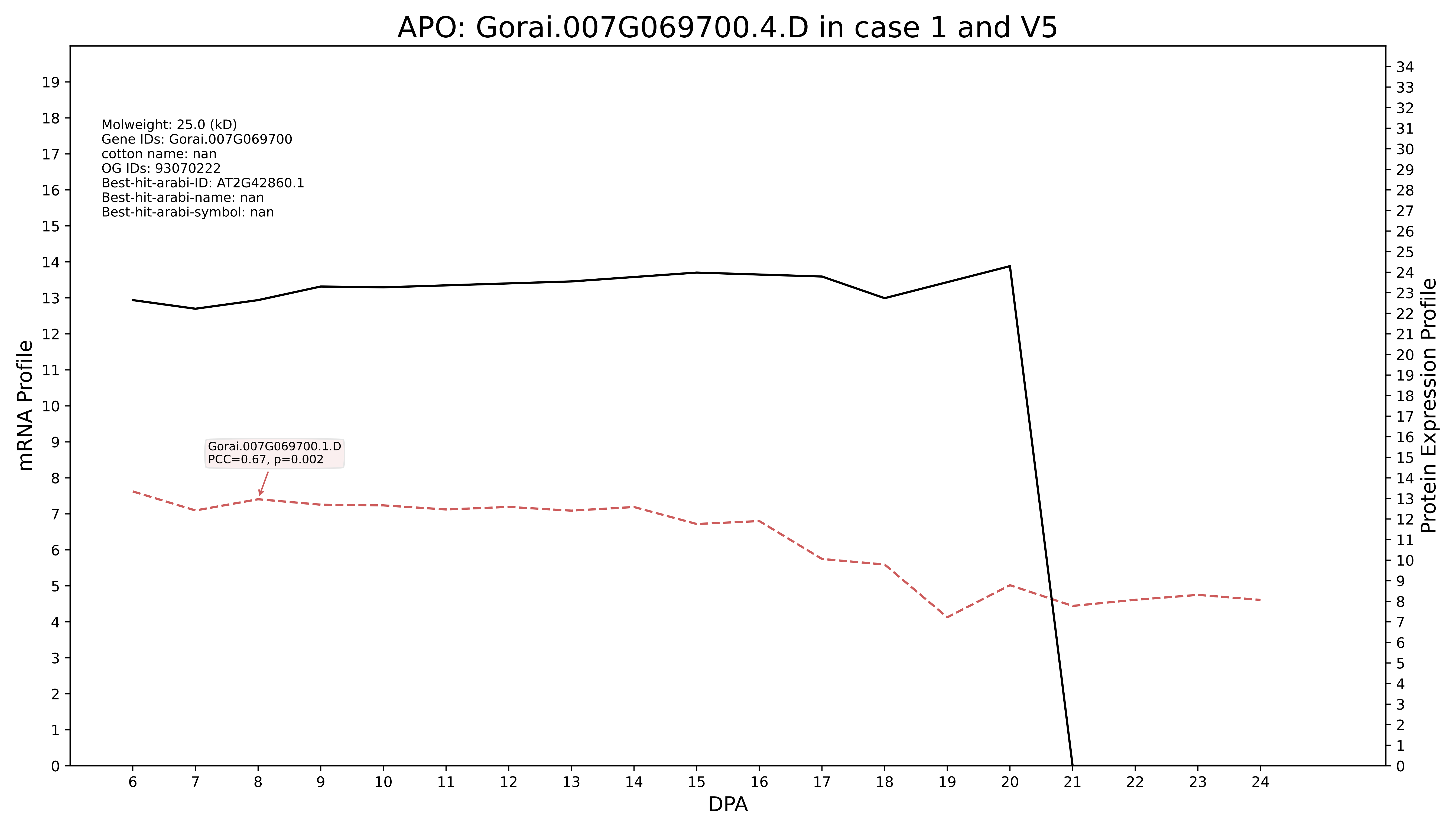
APO: Gorai.007G069700.4.D in case 1 and V5
Molweight: 25.0 (kD)
Gene IDs: Gorai.007G069700
cotton name: nan
OG IDs: 93070222
Best-hit-arabi-ID: AT2G42860.1
Best-hit-arabi-name: nan
Best-hit-arabi-symbol: nan
Gorai.007G069700.1.D
PCC=0.67, p=0.002
mRNA Profile
Protein Expression Profile
DPA

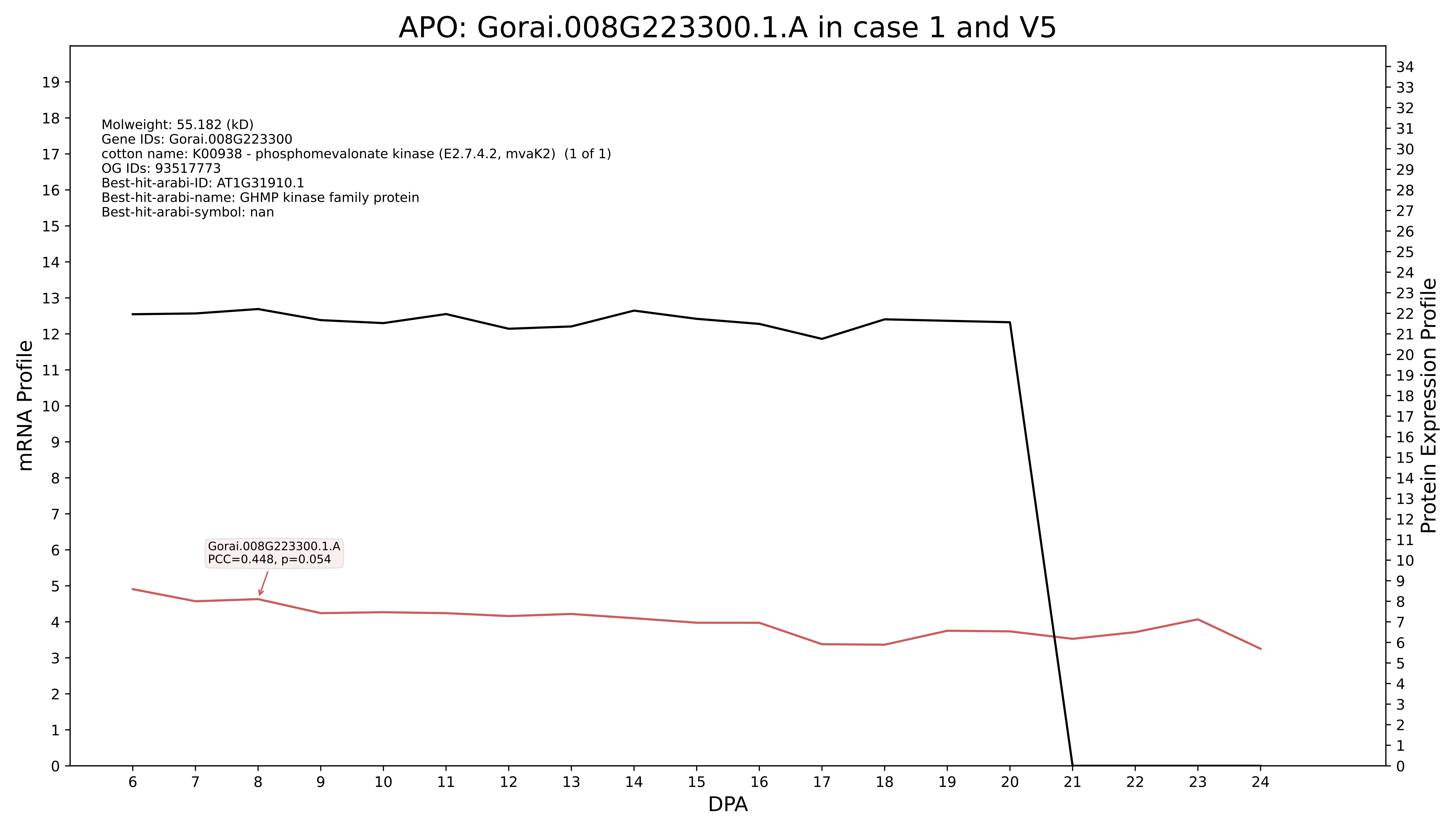
APO: Gorai.008G223300.1.A in case 1 and V5
Molweight: 55.182 (kD)
Gene IDs: Gorai.008G223300
cotton name: K00938 - phosphomevalonate kinase (E2.7.4.2, mvaK2) (1 of 1)
OG IDs: 93517773
Best-hit-arabi-ID: AT1G31910.1
Best-hit-arabi-name: GHMP kinase family protein
Best-hit-arabi-symbol: nan
Gorai.008G223300.1.A
PCC=0.448, p=0.054
mRNA Profile
Protein Expression Profile
DPA

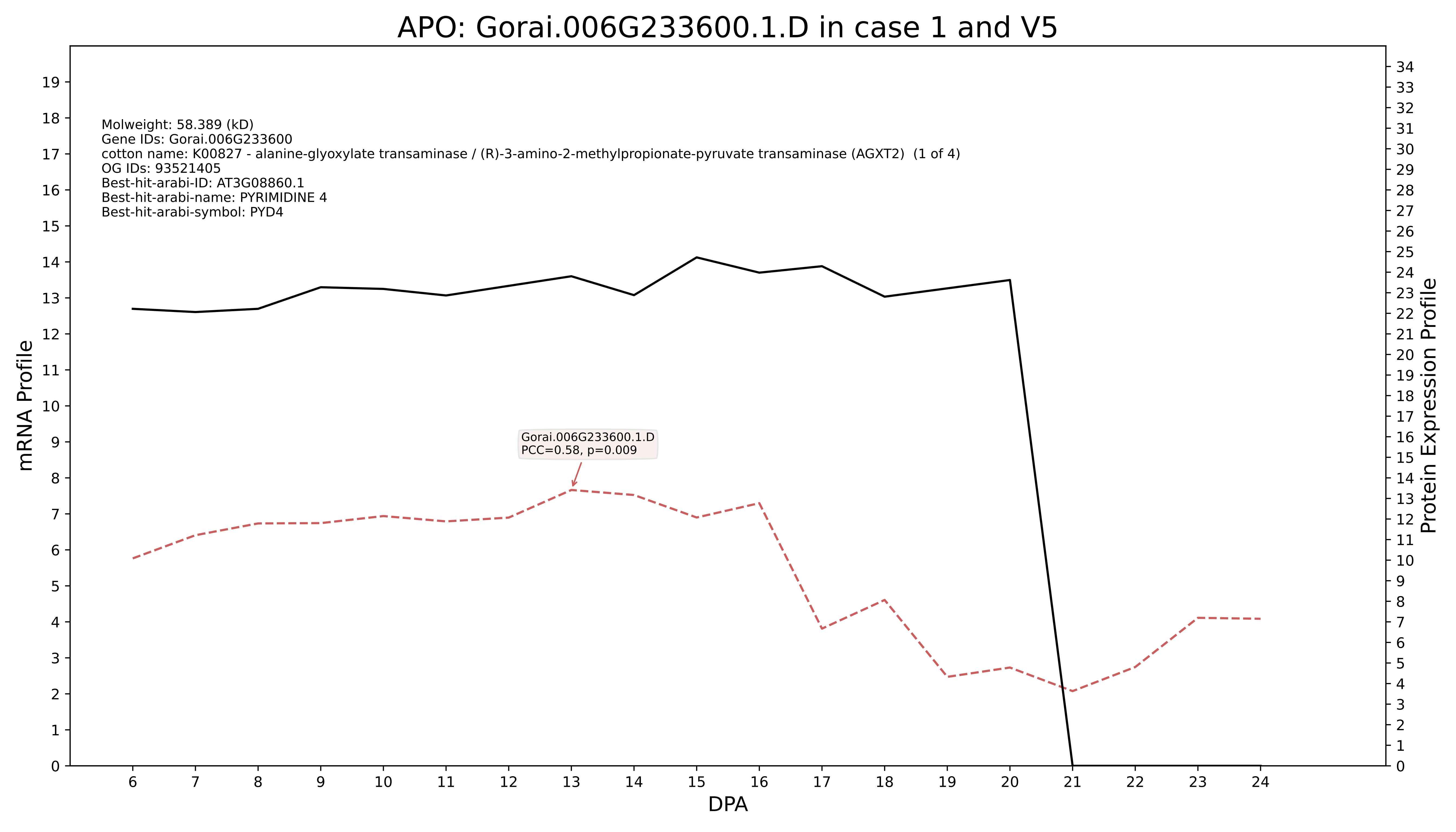
APO: Gorai.006G233600.1.D in case 1 and V5
Molweight: 58.389 (kD)
Gene IDs: Gorai.006G233600
cotton name: K00827 - alanine-glyoxylate transaminase / (R)-3-amino-2-methylpropionate-pyruvate transaminase (AGXT2) (1 of 4)
OG IDs: 93521405
Best-hit-arabi-ID: AT3G08860.1
Best-hit-arabi-name: PYRIMIDINE 4
Best-hit-arabi-symbol: PYD4
Gorai.006G233600.1.D
PCC=0.58, p=0.009
mRNA Profile
Protein Expression Profile
DPA

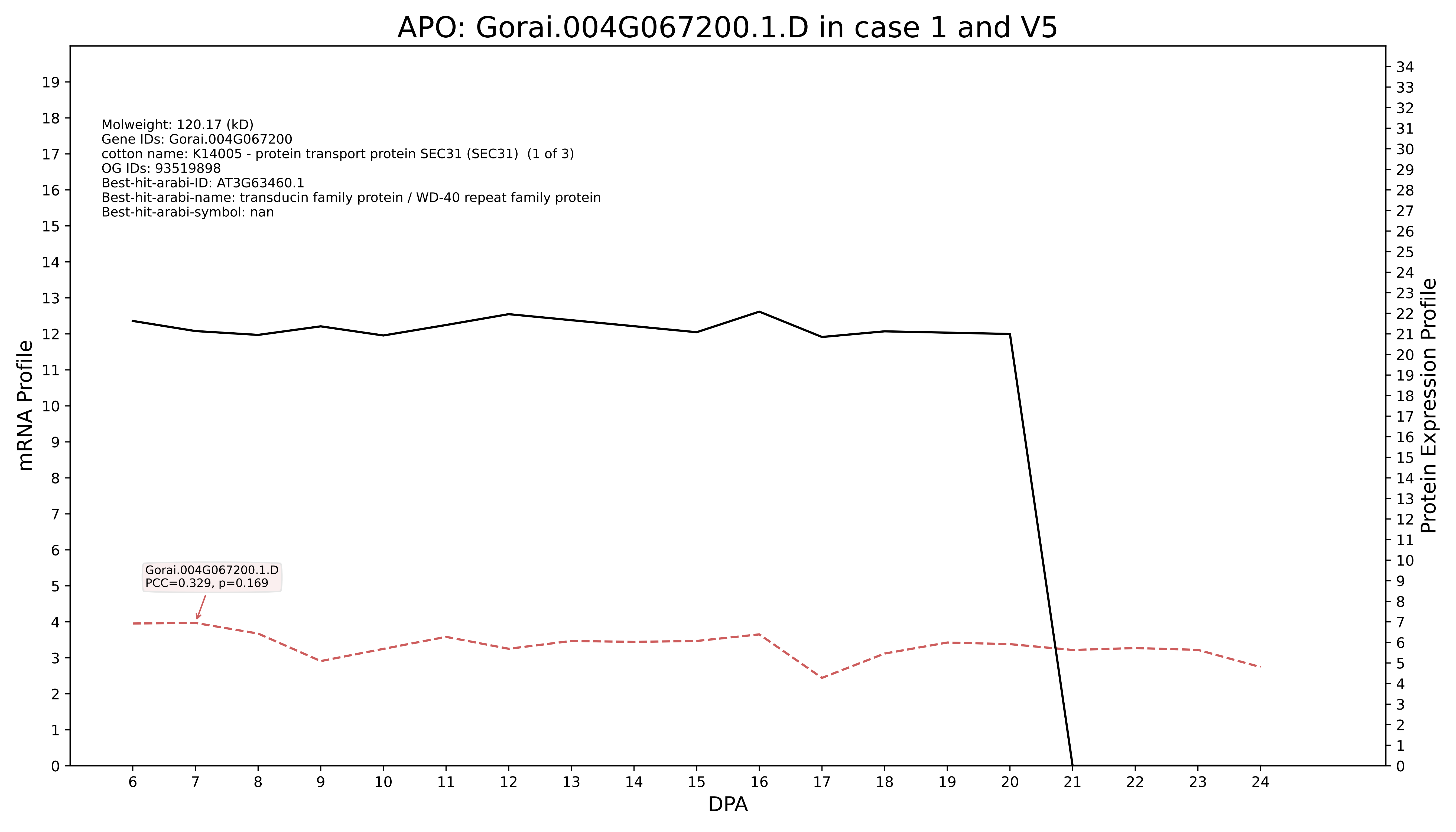
APO: Gorai.004G067200.1.D in case 1 and V5
Molweight: 120.17 (kD)
Gene IDs: Gorai.004G067200
cotton name: K14005 - protein transport protein SEC31 (SEC31) (1 of 3)
OG IDs: 93519898
Best-hit-arabi-ID: AT3G63460.1
Best-hit-arabi-name: transducin family protein / WD-40 repeat family protein
Best-hit-arabi-symbol: nan
Gorai.004G067200.1.D
PCC=0.329, p=0.169
mRNA Profile
Protein Expression Profile
DPA

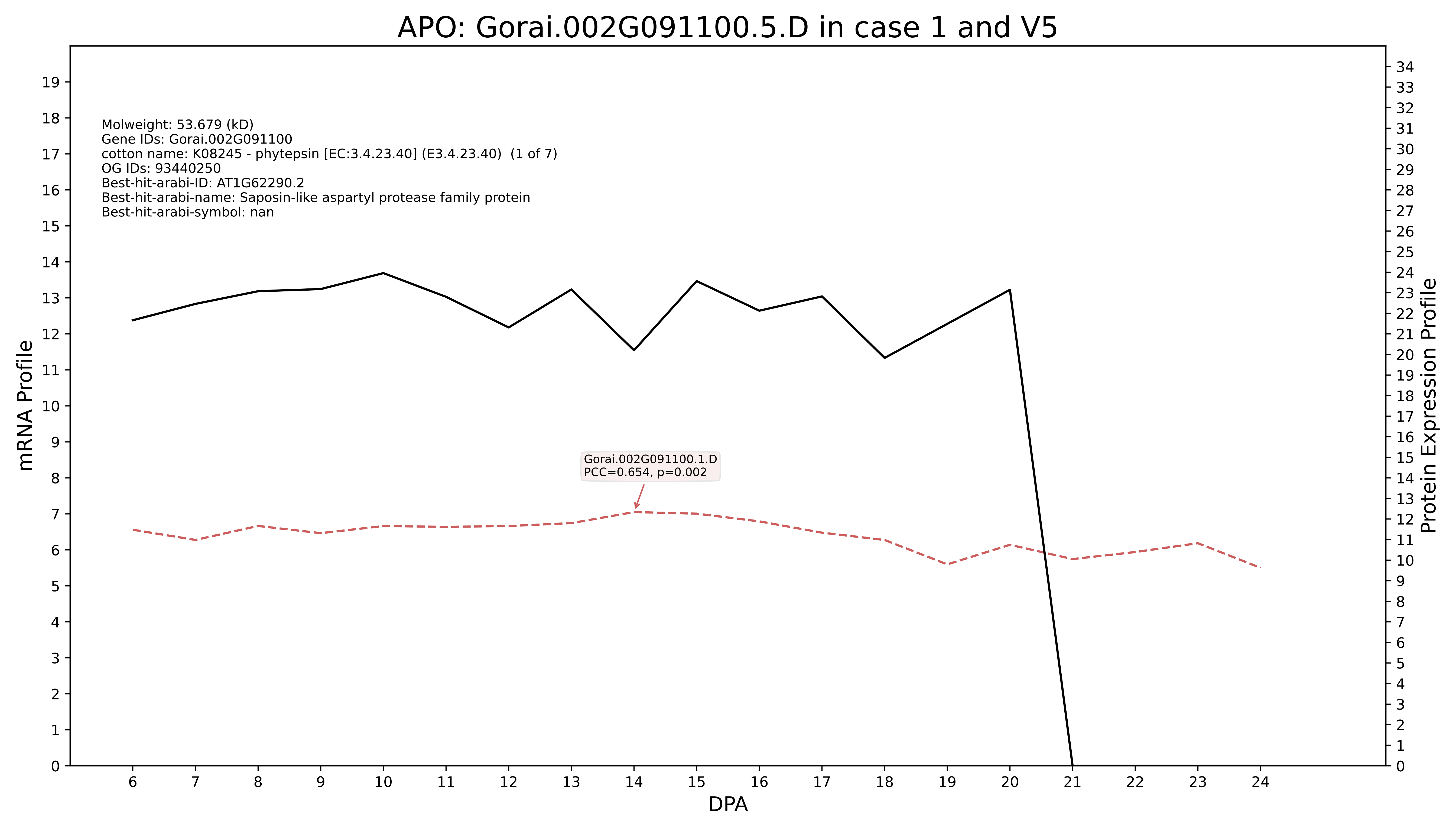

APO: Gorai.002G091100.5.D in case 1 and V5
Molweight: 53.679 (kD)
Gene IDs: Gorai.002G091100
cotton name: K08245 - phytepsin [EC:3.4.23.40] (E3.4.23.40) (1 of 7)
OG IDs: 93440250
Best-hit-arabi-ID: AT1G62290.2
Best-hit-arabi-name: Saposin-like aspartyl protease family protein
Best-hit-arabi-symbol: nan
Gorai.002G091100.1.D
PCC=0.654, p=0.002
mRNA Profile
Protein Expression Profile
DPA

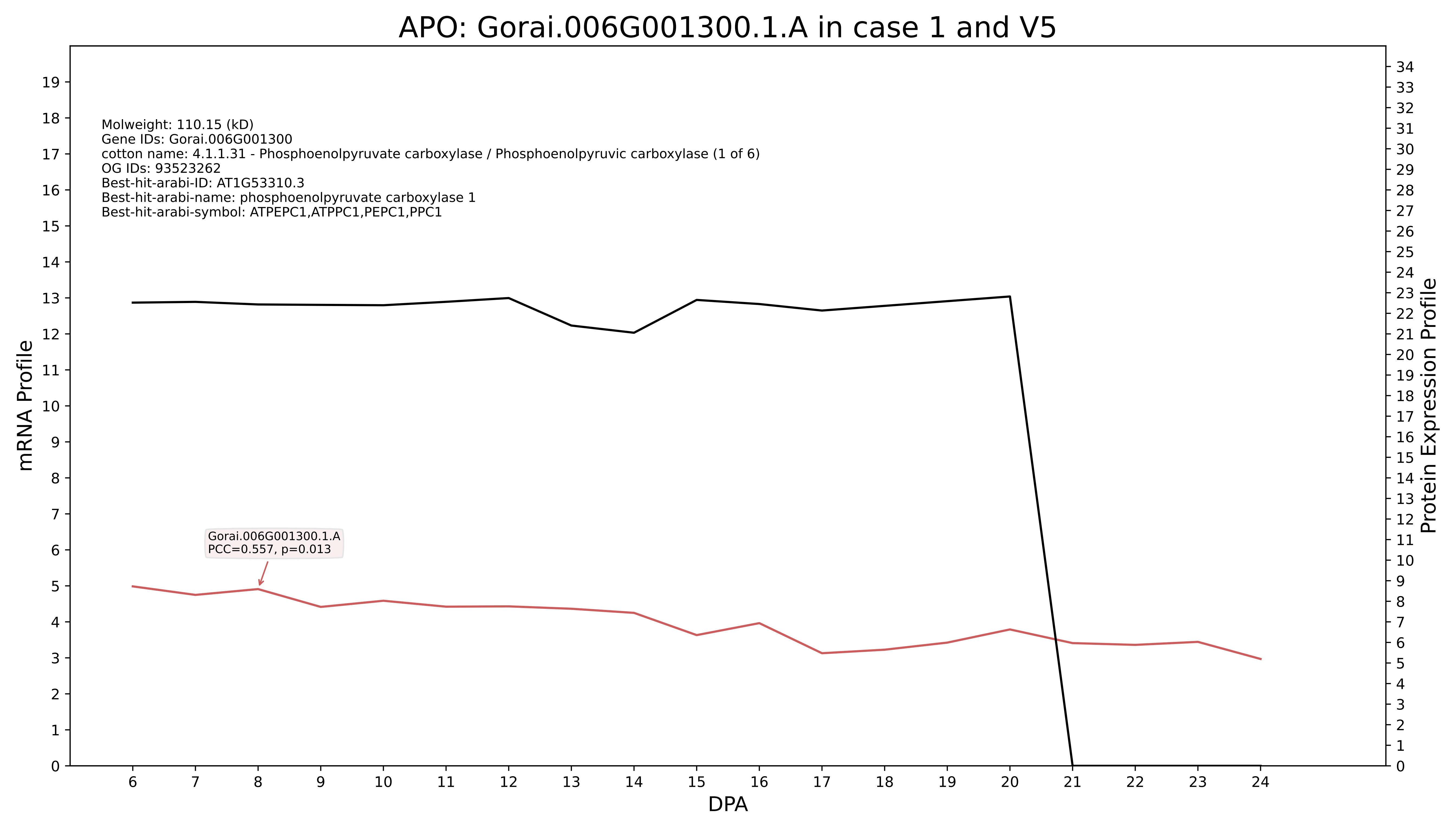
APO: Gorai.006G001300.1.A in case 1 and V5
Molweight: 110.15 (kD)
Gene IDs: Gorai.006G001300
cotton name: 4.1.1.31 - Phosphoenolpyruvate carboxylase / Phosphoenolpyruvic carboxylase (1 of 6)
OG IDs: 93523262
Best-hit-arabi-ID: AT1G53310.3
Best-hit-arabi-name: phosphoenolpyruvate carboxylase 1
Best-hit-arabi-symbol: ATPEPC1,ATPPC1,PEPC1,PPC1
Gorai.006G001300.1.A
PCC=0.557, p=0.013
mRNA Profile
Protein Expression Profile
DPA

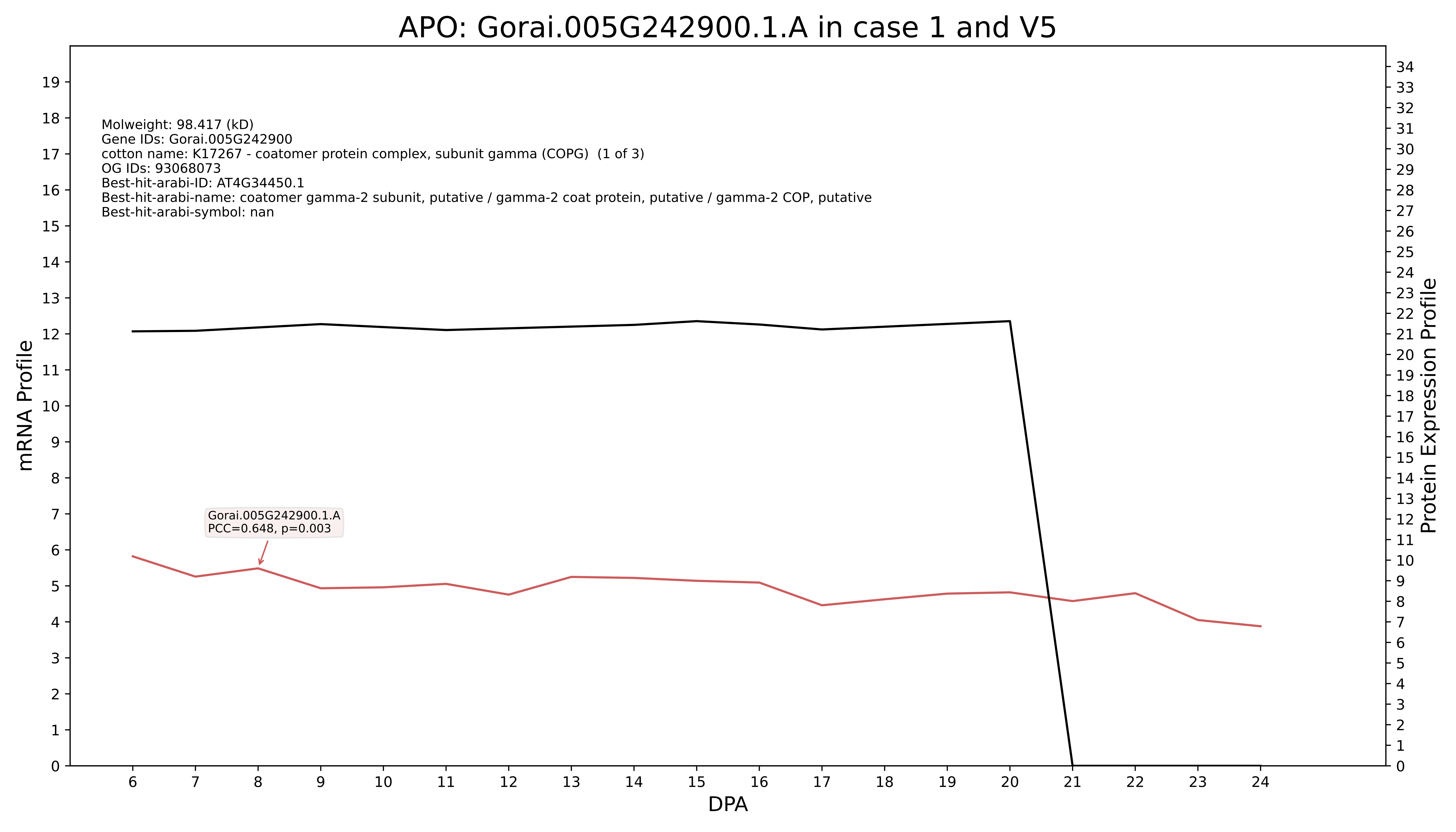

APO: Gorai.005G242900.1.A in case 1 and V5
Molweight: 98.417 (kD)
Gene IDs: Gorai.005G242900
cotton name: K17267 - coatomer protein complex, subunit gamma (COPG) (1 of 3)
OG IDs: 93068073
Best-hit-arabi-ID: AT4G34450.1
Best-hit-arabi-name: coatomer gamma-2 subunit, putative / gamma-2 coat protein, putative / gamma-2 COP, putative
Best-hit-arabi-symbol: nan
Gorai.005G242900.1.A
PCC=0.648, p=0.003
mRNA Profile
Protein Expression Profile
DPA

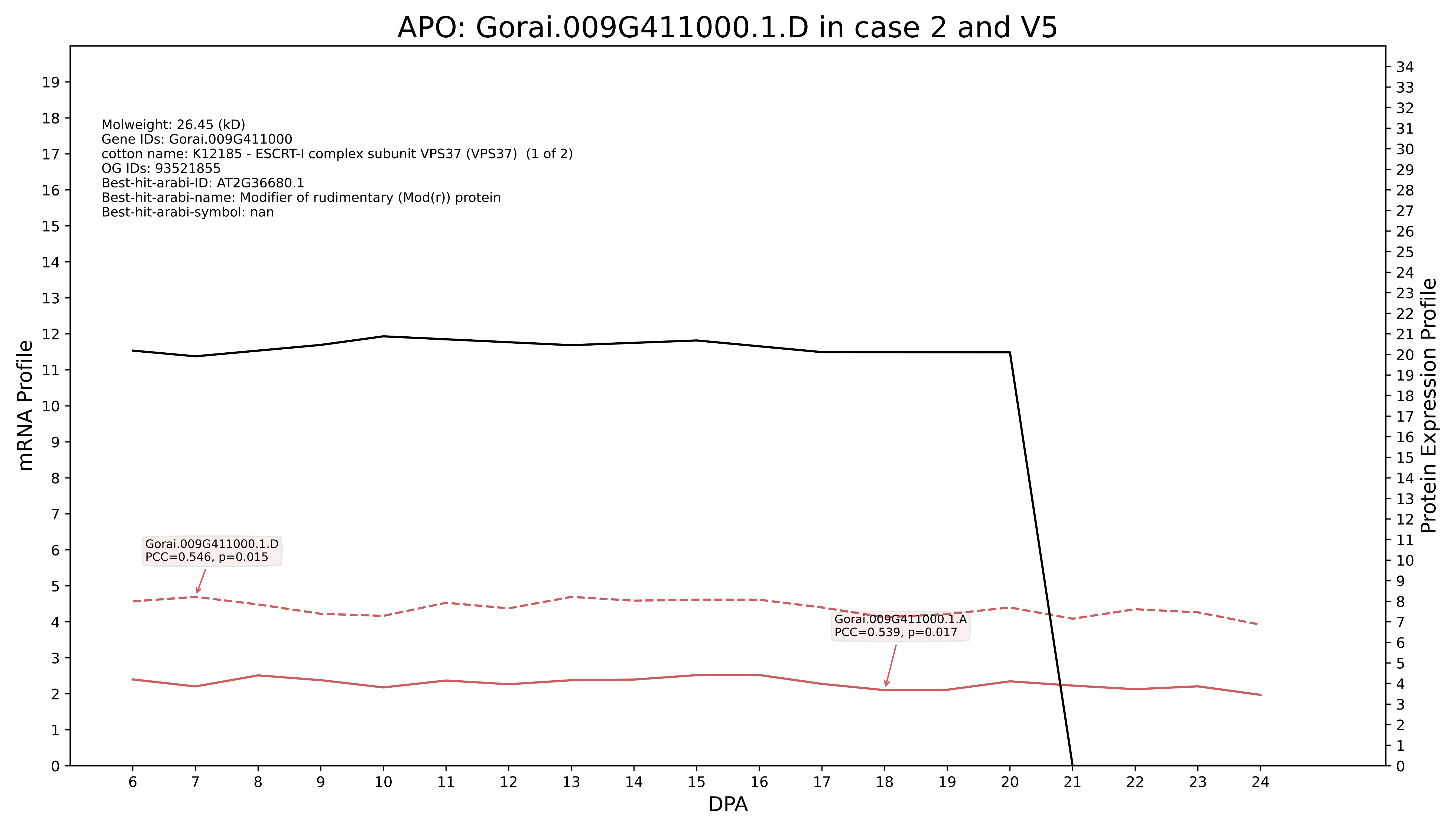

APO: Gorai.009G411000.1.D in case 2 and V5
Molweight: 26.45 (kD)
Gene IDs: Gorai.009G411000
cotton name: K12185 - ESCRT-I complex subunit VPS37 (VPS37) (1 of 2)
OG IDs: 93521855
Best-hit-arabi-ID: AT2G36680.1
Best-hit-arabi-name: Modifier of rudimentary (Mod(r)) protein
Best-hit-arabi-symbol: nan
Gorai.009G411000.1.D
PCC=0.546, p=0.015
Gorai.009G411000.1.A
PCC=0.539, p=0.017
mRNA Profile
Protein Expression Profile
DPA

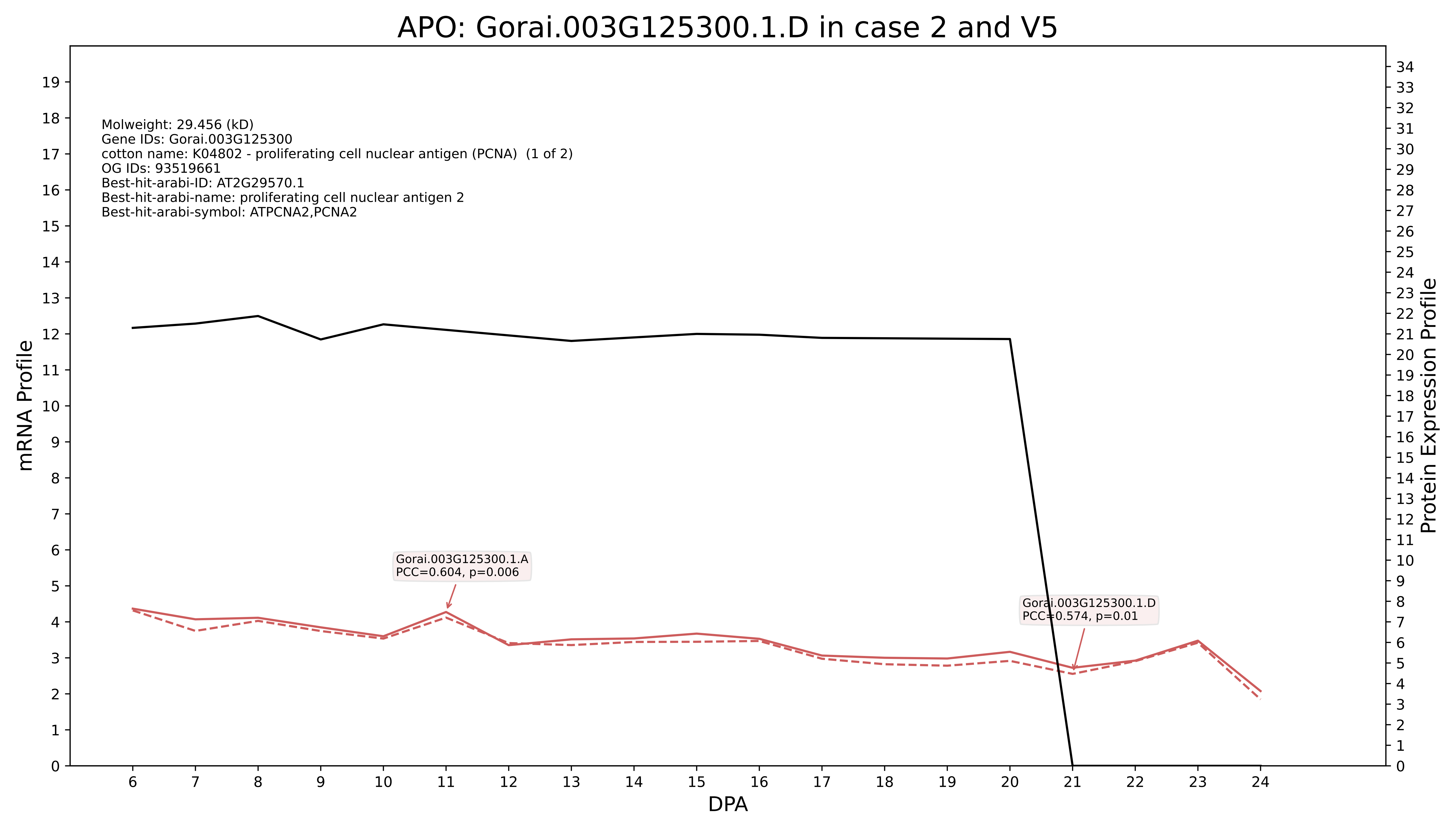
APO: Gorai.003G125300.1.D in case 2 and V5
Molweight: 29.456 (kD)
Gene IDs: Gorai.003G125300
cotton name: K04802 - proliferating cell nuclear antigen (PCNA) (1 of 2)
OG IDs: 93519661
Best-hit-arabi-ID: AT2G29570.1
Best-hit-arabi-name: proliferating cell nuclear antigen 2
Best-hit-arabi-symbol: ATPCNA2,PCNA2
Gorai.003G125300.1.A
PCC=0.604, p=0.006
Gorai.003G125300.1.D
PCC=0.574, p=0.01
mRNA Profile
Protein Expression Profile
DPA

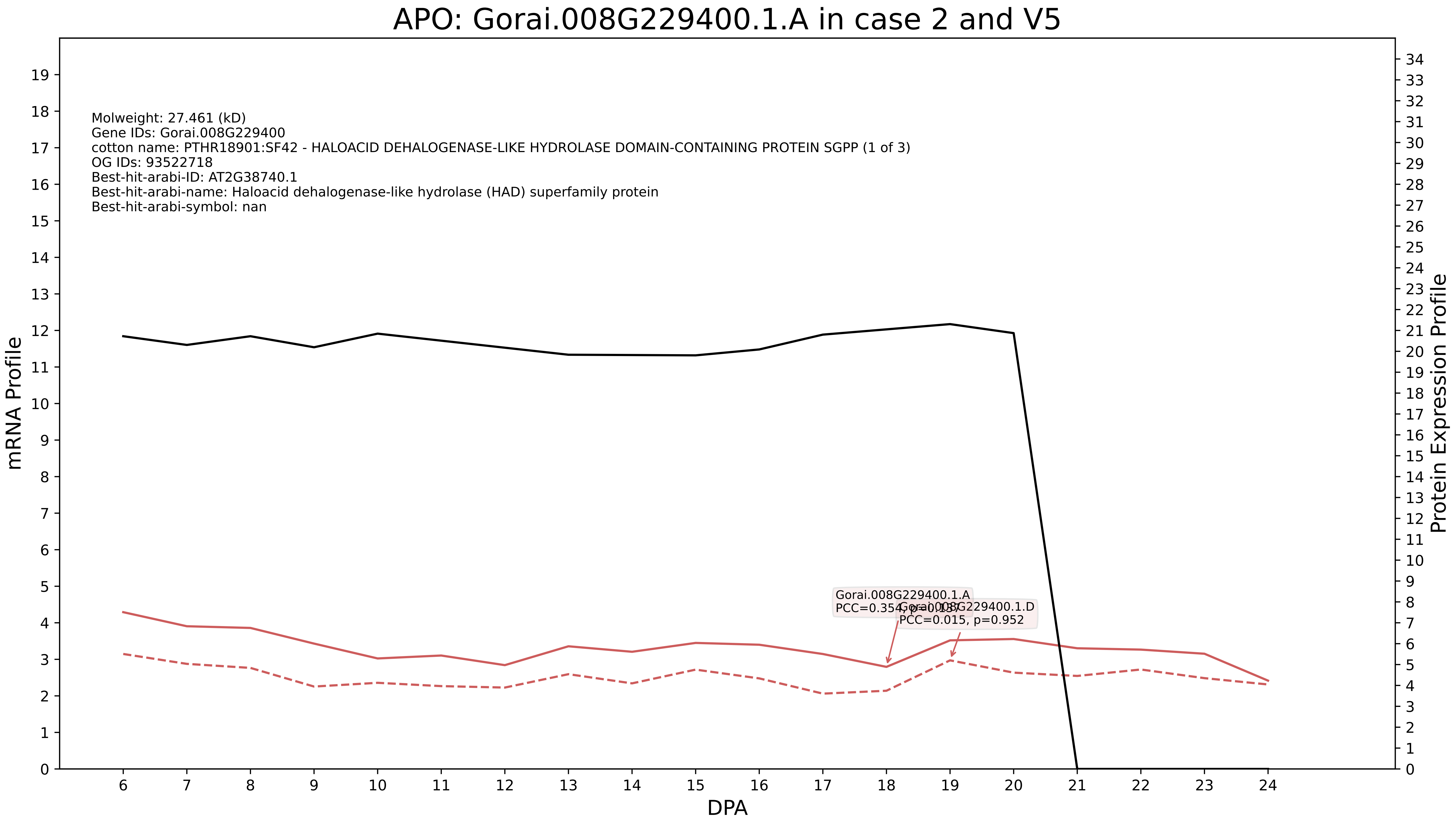
APO: Gorai.008G229400.1.A in case 2 and V5
Molweight: 27.461 (kD)
Gene IDs: Gorai.008G229400
cotton name: PTHR18901:SF42 - HALOACID DEHALOGENASE-LIKE HYDROLASE DOMAIN-CONTAINING PROTEIN SGPP (1 of 3)
OG IDs: 93522718
Best-hit-arabi-ID: AT2G38740.1
Best-hit-arabi-name: Haloacid dehalogenase-like hydrolase (HAD) superfamily protein
Best-hit-arabi-symbol: nan
Gorai.008G229400.1.A
PCC=0.354, p=0.137
Gorai.008G229400.1.D
PCC=0.015, p=0.952
mRNA Profile
Protein Expression Profile
DPA

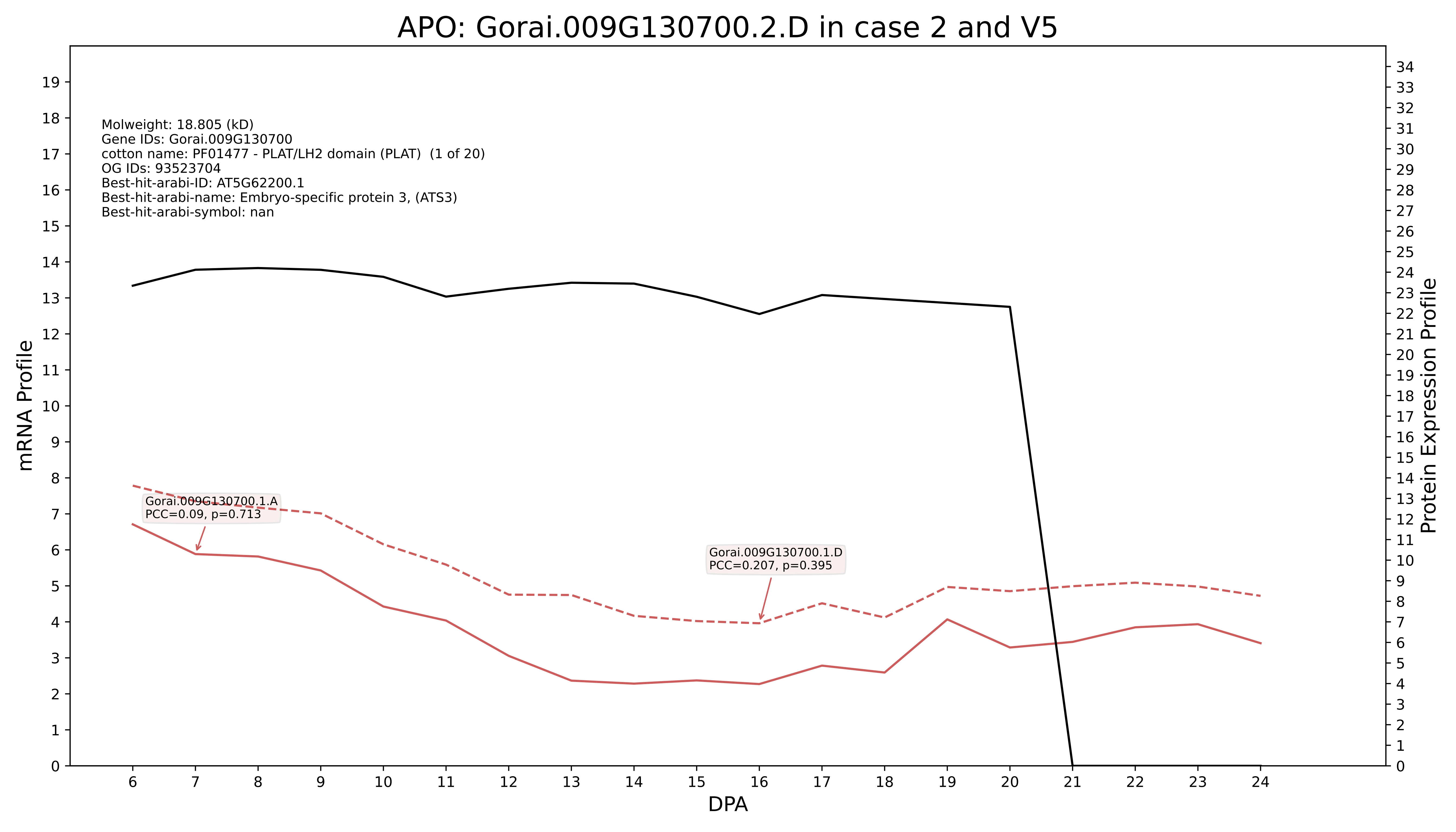
APO: Gorai.009G130700.2.D in case 2 and V5
Molweight: 18.805 (kD)
Gene IDs: Gorai.009G130700
cotton name: PF01477 - PLAT/LH2 domain (PLAT) (1 of 20)
OG IDs: 93523704
Best-hit-arabi-ID: AT5G62200.1
Best-hit-arabi-name: Embryo-specific protein 3, (ATS3)
Best-hit-arabi-symbol: nan
Gorai.009G130700.1.A
PCC=0.09, p=0.713
Gorai.009G130700.1.D
PCC=0.207, p=0.395
mRNA Profile
Protein Expression Profile
DPA

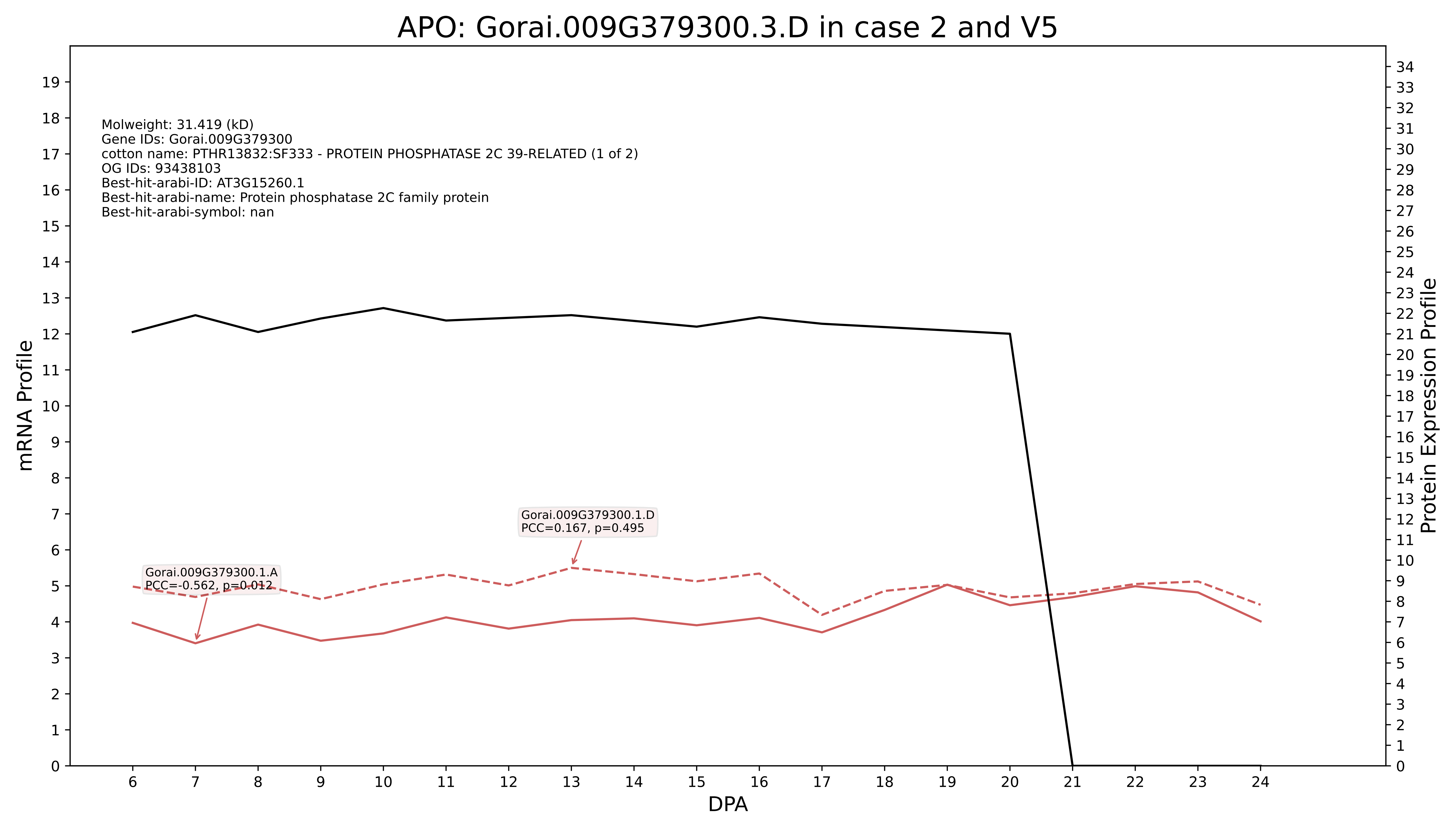
APO: Gorai.009G379300.3.D in case 2 and V5
Molweight: 31.419 (kD)
Gene IDs: Gorai.009G379300
cotton name: PTHR13832:SF333 - PROTEIN PHOSPHATASE 2C 39-RELATED (1 of 2)
OG IDs: 93438103
Best-hit-arabi-ID: AT3G15260.1
Best-hit-arabi-name: Protein phosphatase 2C family protein
Best-hit-arabi-symbol: nan
Gorai.009G379300.1.D
PCC=0.167, p=0.495
Gorai.009G379300.1.A
PCC=-0.562, p=0.012
mRNA Profile
Protein Expression Profile
DPA

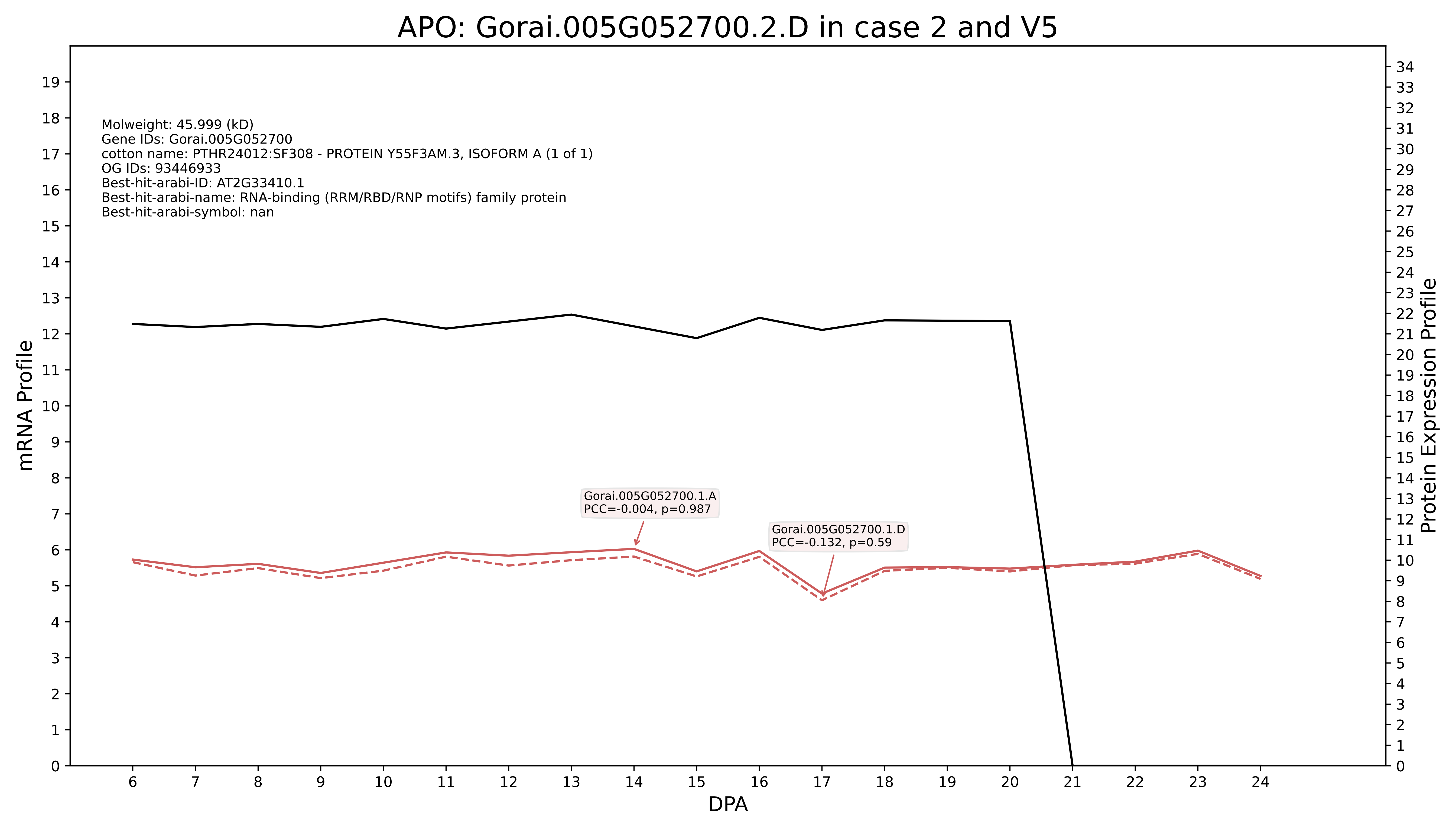

APO: Gorai.005G052700.2.D in case 2 and V5
Molweight: 45.999 (kD)
Gene IDs: Gorai.005G052700
cotton name: PTHR24012:SF308 - PROTEIN Y55F3AM.3, ISOFORM A (1 of 1)
OG IDs: 93446933
Best-hit-arabi-ID: AT2G33410.1
Best-hit-arabi-name: RNA-binding (RRM/RBD/RNP motifs) family protein
Best-hit-arabi-symbol: nan
Gorai.005G052700.1.A
PCC=-0.004, p=0.987
Gorai.005G052700.1.D
PCC=-0.132, p=0.59
mRNA Profile
Protein Expression Profile
DPA

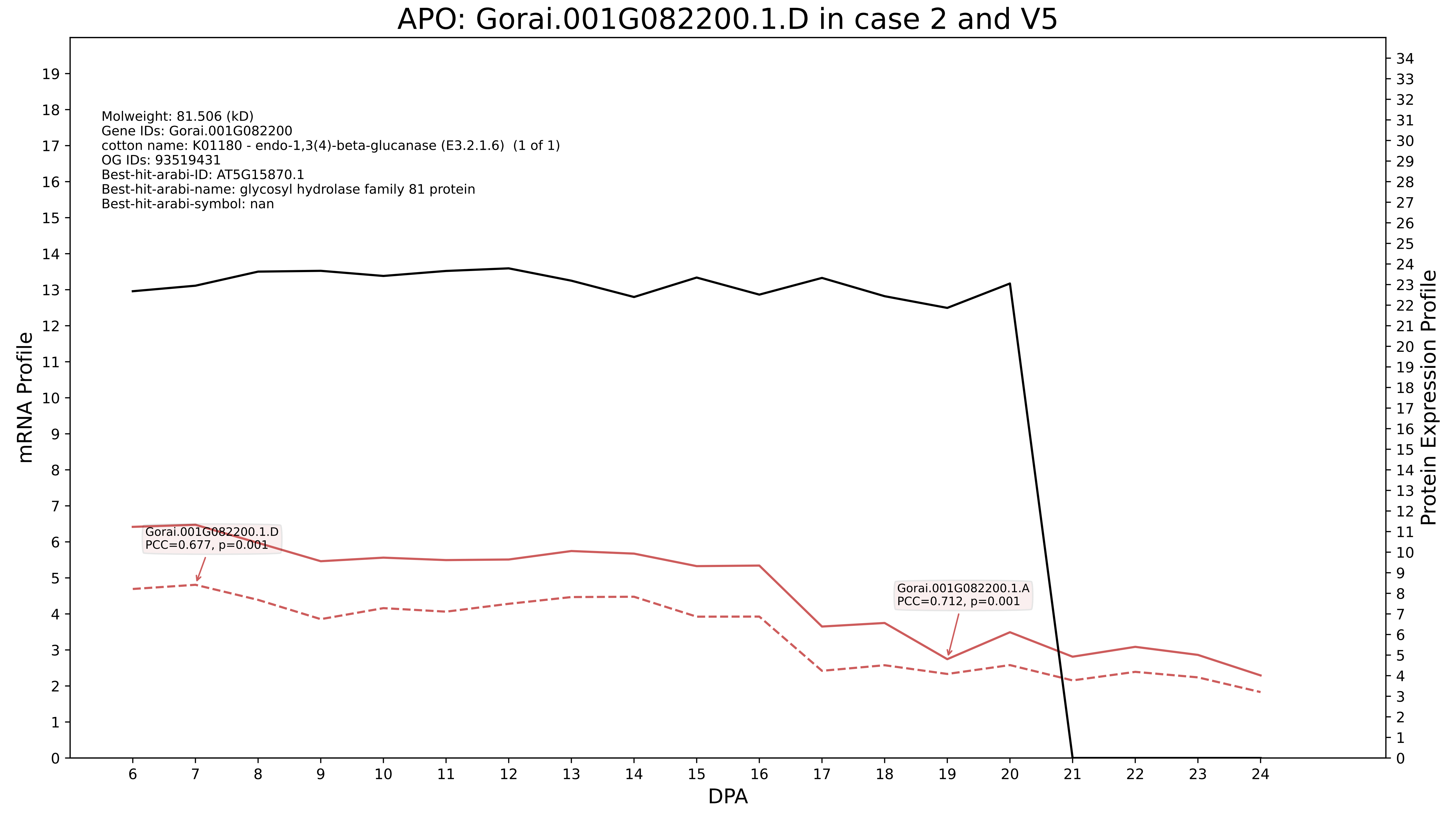
APO: Gorai.001G082200.1.D in case 2 and V5
Molweight: 81.506 (kD)
Gene IDs: Gorai.001G082200
cotton name: K01180 - endo-1,3(4)-beta-glucanase (E3.2.1.6) (1 of 1)
OG IDs: 93519431
Best-hit-arabi-ID: AT5G15870.1
Best-hit-arabi-name: glycosyl hydrolase family 81 protein
Best-hit-arabi-symbol: nan
Gorai.001G082200.1.D
PCC=0.677, p=0.001
Gorai.001G082200.1.A
PCC=0.712, p=0.001
mRNA Profile
Protein Expression Profile
DPA

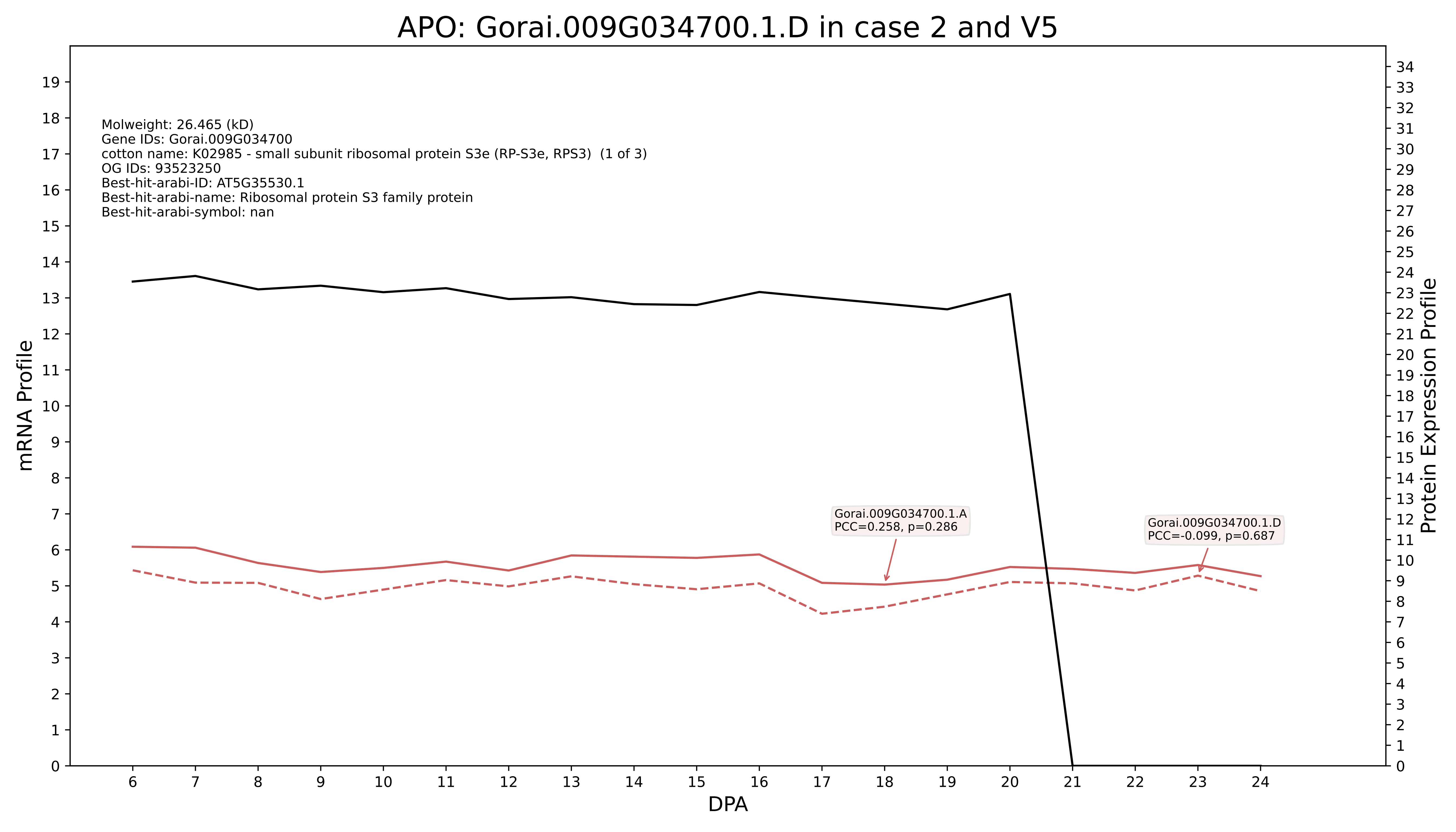

APO: Gorai.009G034700.1.D in case 2 and V5
Molweight: 26.465 (kD)
Gene IDs: Gorai.009G034700
cotton name: K02985 - small subunit ribosomal protein S3e (RP-S3e, RPS3) (1 of 3)
OG IDs: 93523250
Best-hit-arabi-ID: AT5G35530.1
Best-hit-arabi-name: Ribosomal protein S3 family protein
Best-hit-arabi-symbol: nan
Gorai.009G034700.1.A
PCC=0.258, p=0.286
Gorai.009G034700.1.D
PCC=-0.099, p=0.687
mRNA Profile
Protein Expression Profile
DPA

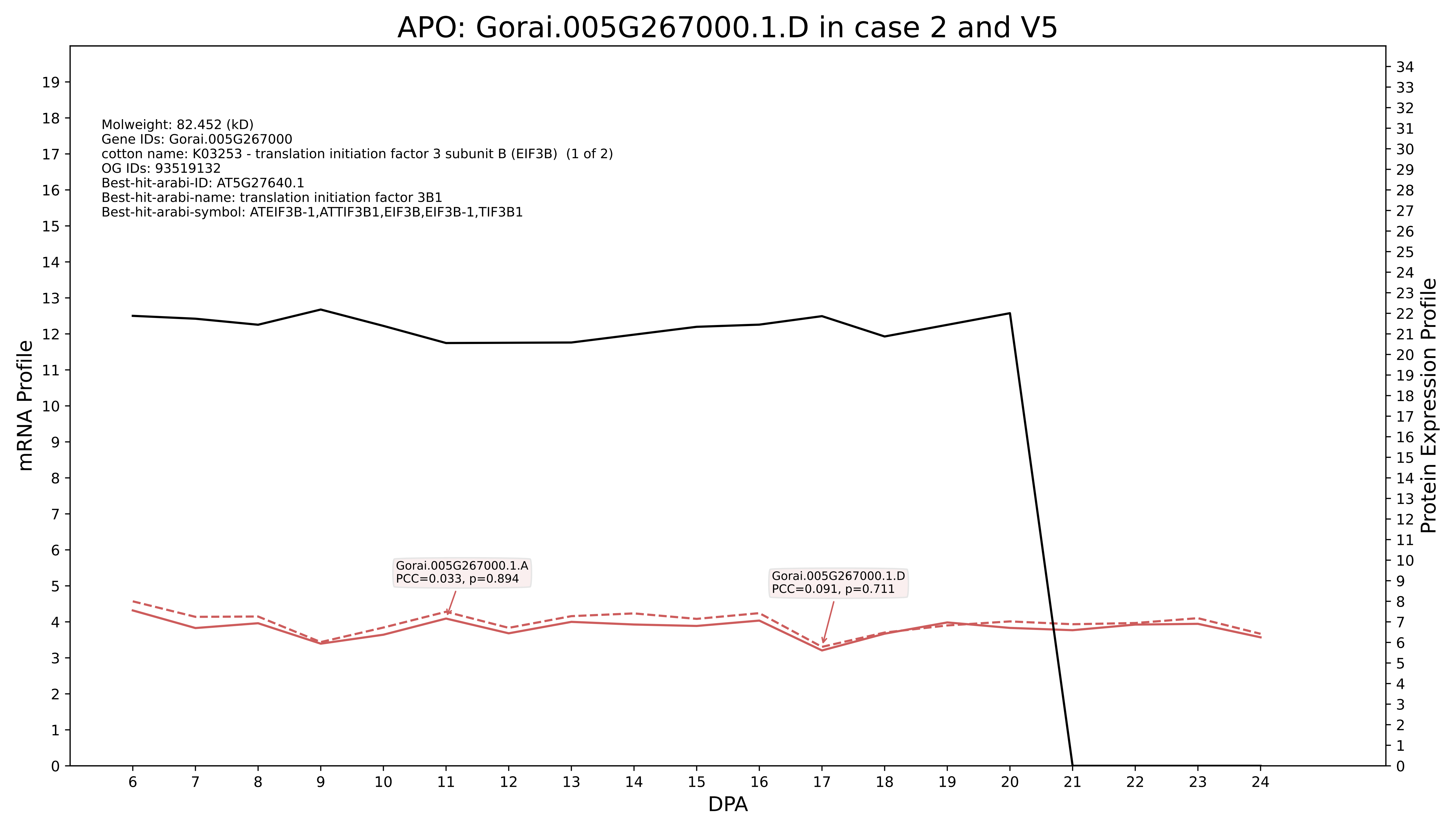
APO: Gorai.005G267000.1.D in case 2 and V5
Molweight: 82.452 (kD)
Gene IDs: Gorai.005G267000
cotton name: K03253 - translation initiation factor 3 subunit B (EIF3B) (1 of 2)
OG IDs: 93519132
Best-hit-arabi-ID: AT5G27640.1
Best-hit-arabi-name: translation initiation factor 3B1
Best-hit-arabi-symbol: ATEIF3B-1,ATTIF3B1,EIF3B,EIF3B-1,TIF3B1
Gorai.005G267000.1.A
PCC=0.033, p=0.894
Gorai.005G267000.1.D
PCC=0.091, p=0.711
mRNA Profile
Protein Expression Profile
DPA
0
1
2
3
4
5
6
7
8
9
10
11
12
13
14
15
16
17
18
19
20
21
22
23
24
25
26
27
28
29
30
31
32
33
34

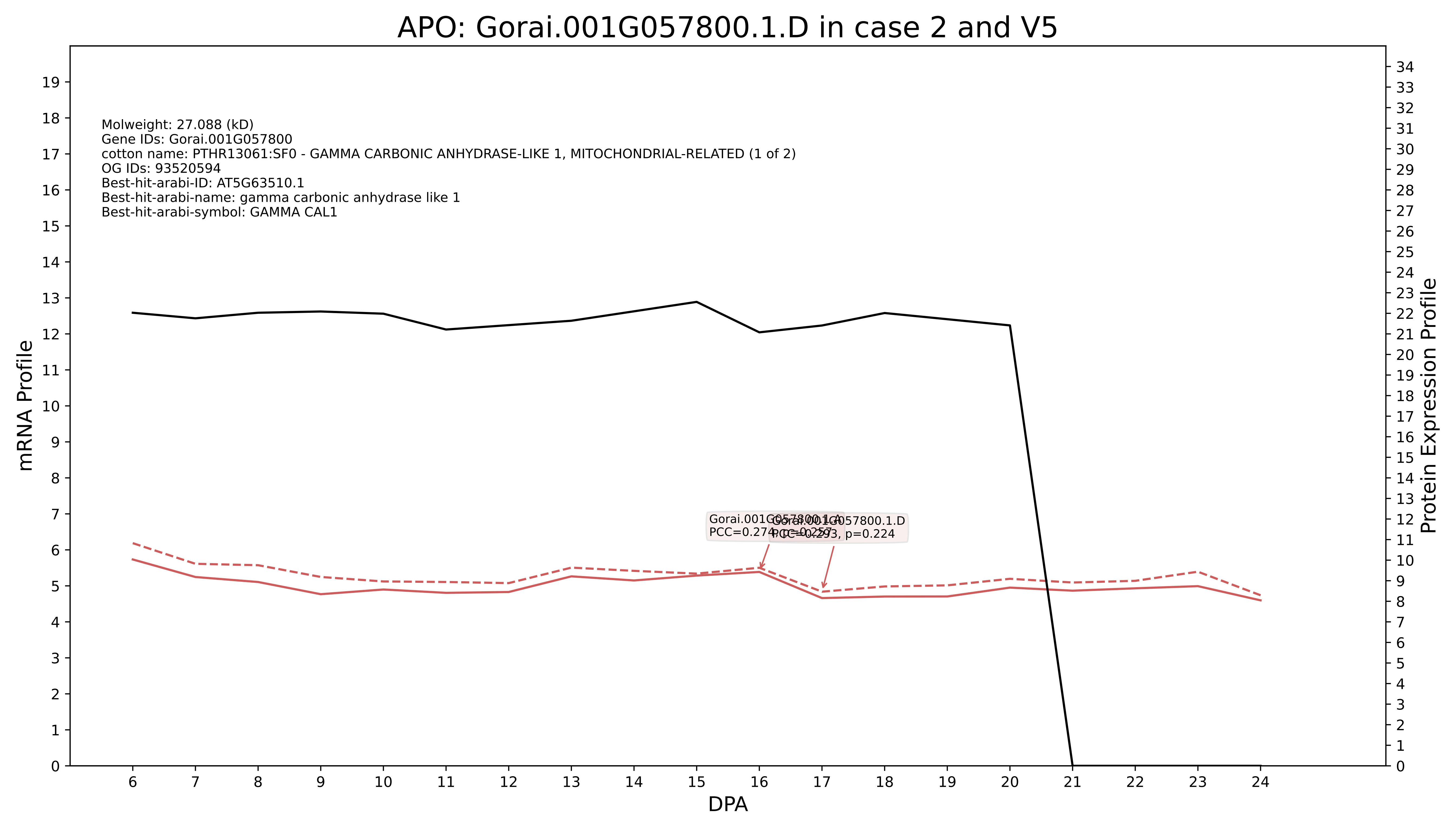
APO: Gorai.001G057800.1.D in case 2 and V5
Molweight: 27.088 (kD)
Gene IDs: Gorai.001G057800
cotton name: PTHR13061:SF0 - GAMMA CARBONIC ANHYDRASE-LIKE 1, MITOCHONDRIAL-RELATED (1 of 2)
OG IDs: 93520594
Best-hit-arabi-ID: AT5G63510.1
Best-hit-arabi-name: gamma carbonic anhydrase like 1
Best-hit-arabi-symbol: GAMMA CAL1
Gorai.001G057800.1.D
PCC=0.293, p=0.224
mRNA Profile
Protein Expression Profile
DPA

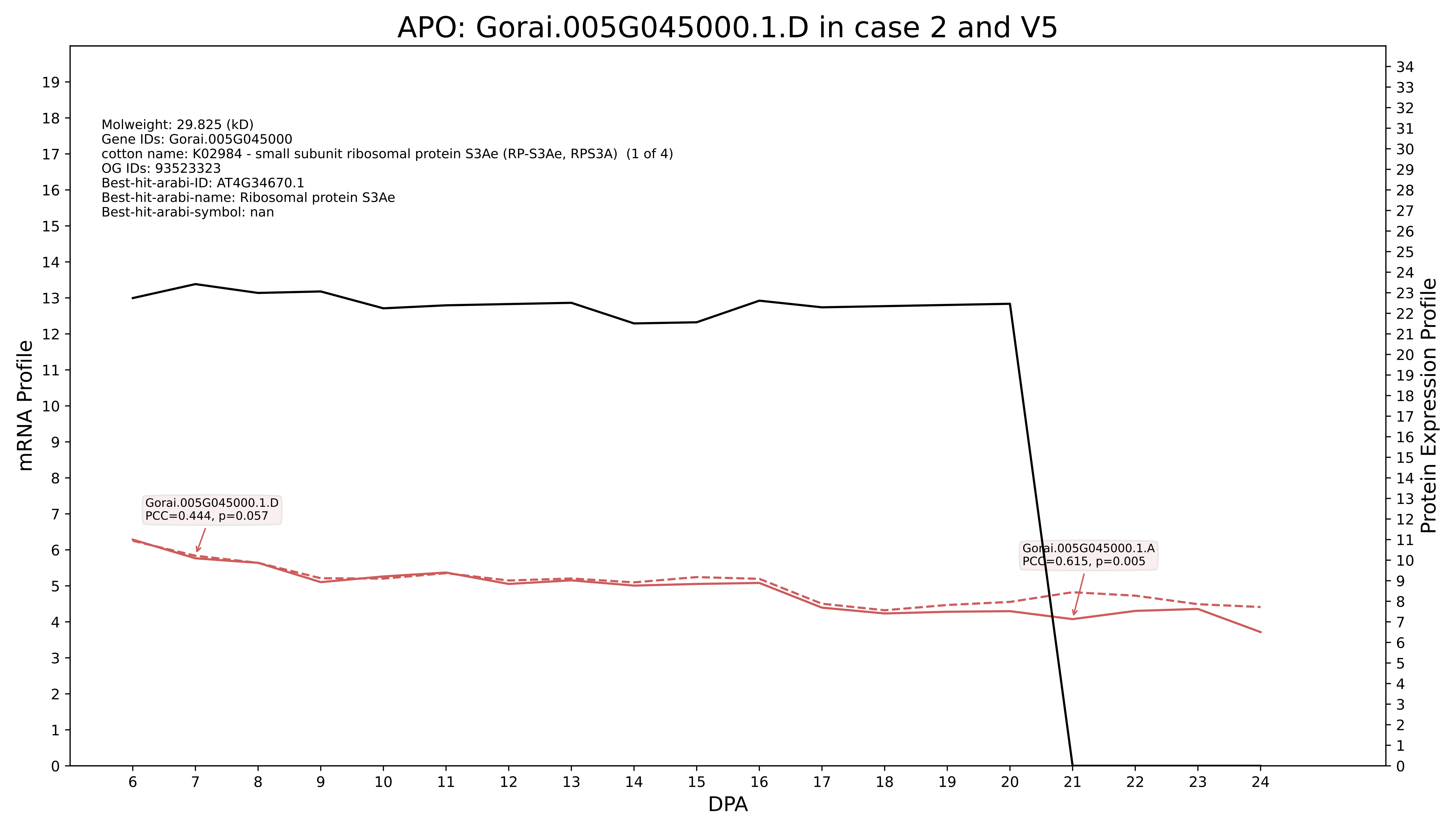
APO: Gorai.005G045000.1.D in case 2 and V5
Molweight: 29.825 (kD)
Gene IDs: Gorai.005G045000
cotton name: K02984 - small subunit ribosomal protein S3Ae (RP-S3Ae, RPS3A) (1 of 4)
OG IDs: 93523323
Best-hit-arabi-ID: AT4G34670.1
Best-hit-arabi-name: Ribosomal protein S3Ae
Best-hit-arabi-symbol: nan
Gorai.005G045000.1.D
PCC=0.444, p=0.057
Gorai.005G045000.1.A
PCC=0.615, p=0.005
mRNA Profile
Protein Expression Profile
DPA

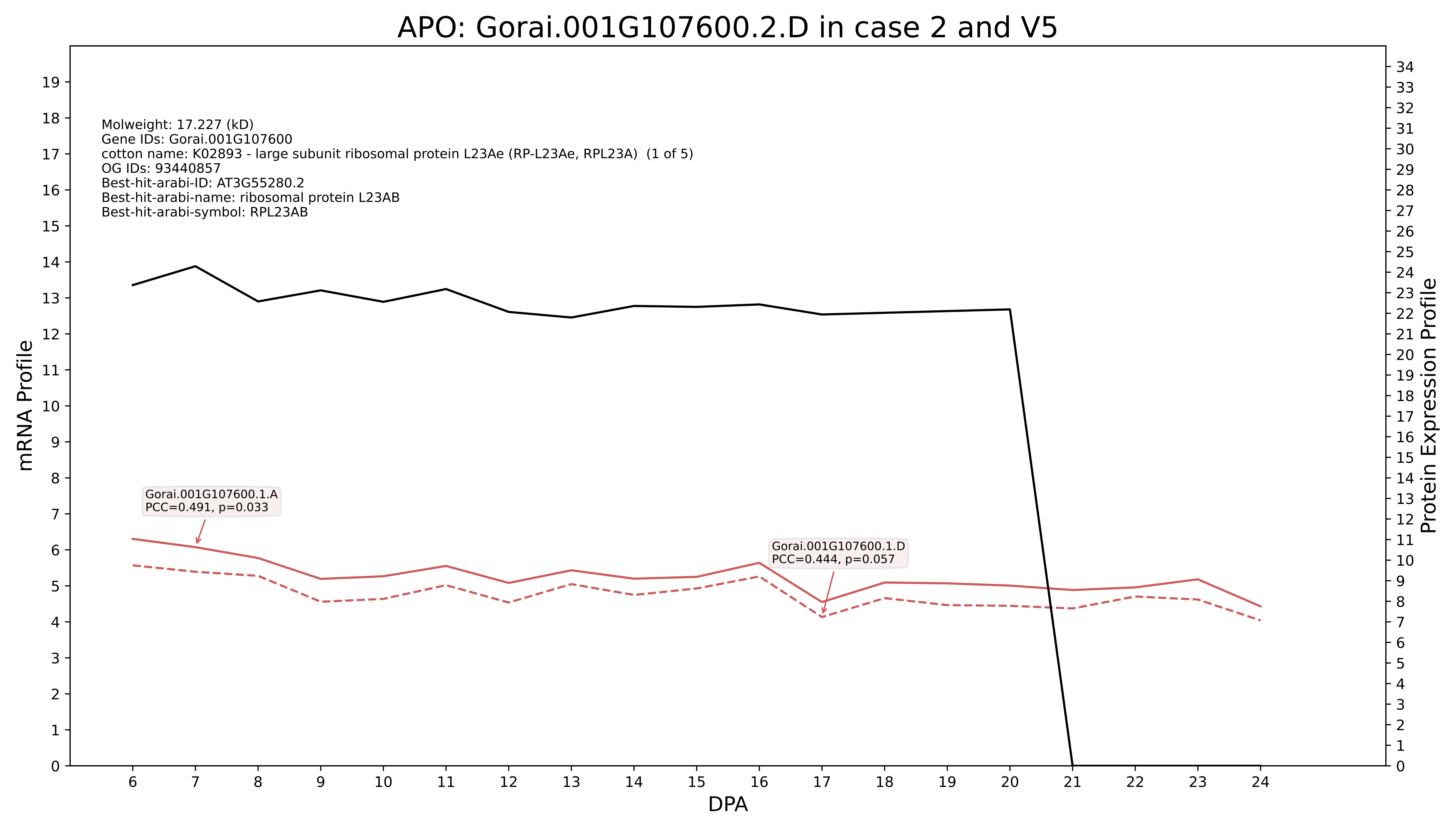

APO: Gorai.001G107600.2.D in case 2 and V5
Molweight: 17.227 (kD)
Gene IDs: Gorai.001G107600
cotton name: K02893 - large subunit ribosomal protein L23Ae (RP-L23Ae, RPL23A) (1 of 5)
OG IDs: 93440857
Best-hit-arabi-ID: AT3G55280.2
Best-hit-arabi-name: ribosomal protein L23AB
Best-hit-arabi-symbol: RPL23AB
Gorai.001G107600.1.A
PCC=0.491, p=0.033
Gorai.001G107600.1.D
PCC=0.444, p=0.057
mRNA Profile
Protein Expression Profile
DPA

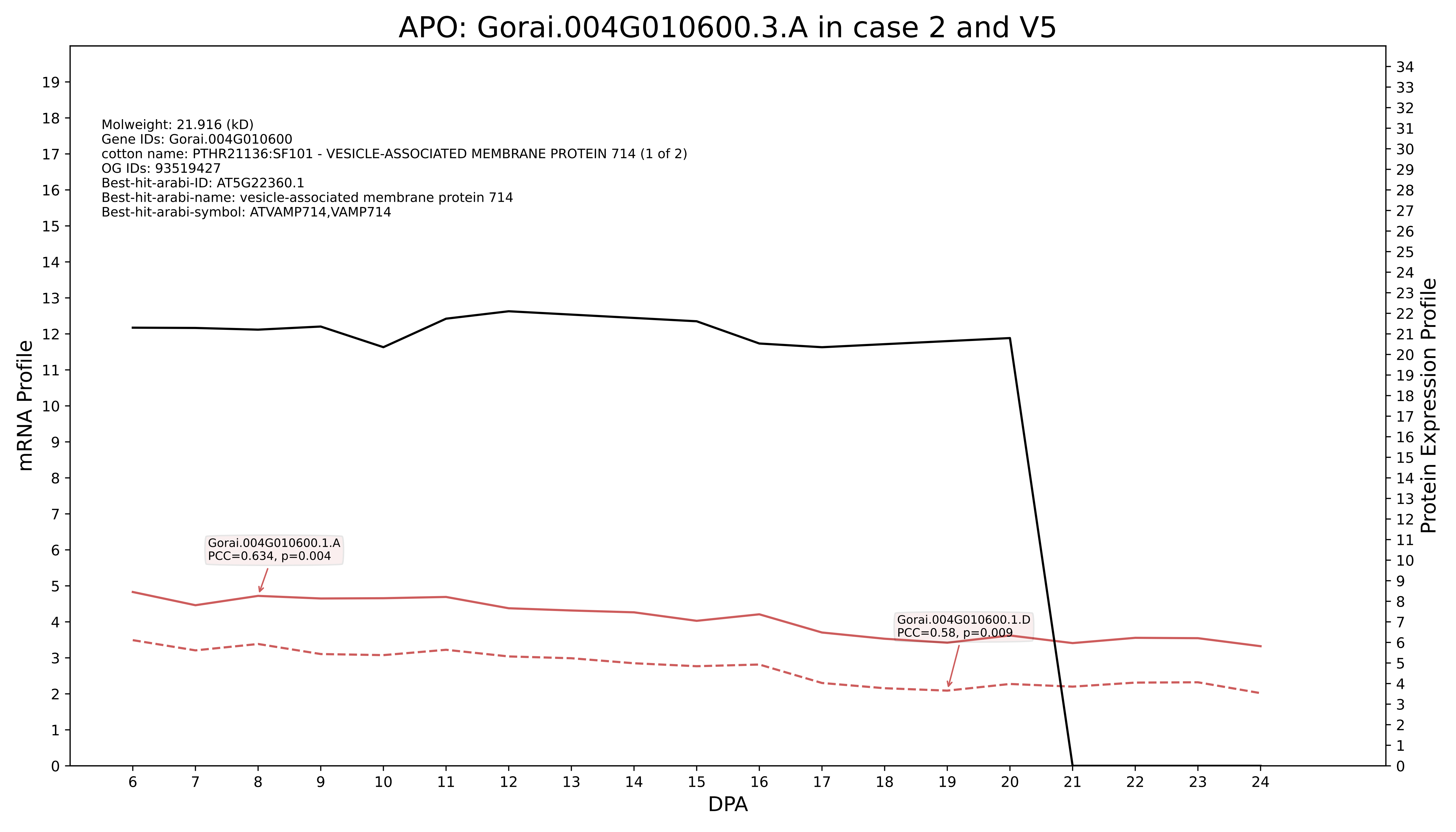

APO: Gorai.004G010600.3.A in case 2 and V5
Molweight: 21.916 (kD)
Gene IDs: Gorai.004G010600
cotton name: PTHR21136:SF101 - VESICLE-ASSOCIATED MEMBRANE PROTEIN 714 (1 of 2)
OG IDs: 93519427
Best-hit-arabi-ID: AT5G22360.1
Best-hit-arabi-name: vesicle-associated membrane protein 714
Best-hit-arabi-symbol: ATVAMP714,VAMP714
Gorai.004G010600.1.A
PCC=0.634, p=0.004
Gorai.004G010600.1.D
PCC=0.58, p=0.009
mRNA Profile
Protein Expression Profile
DPA

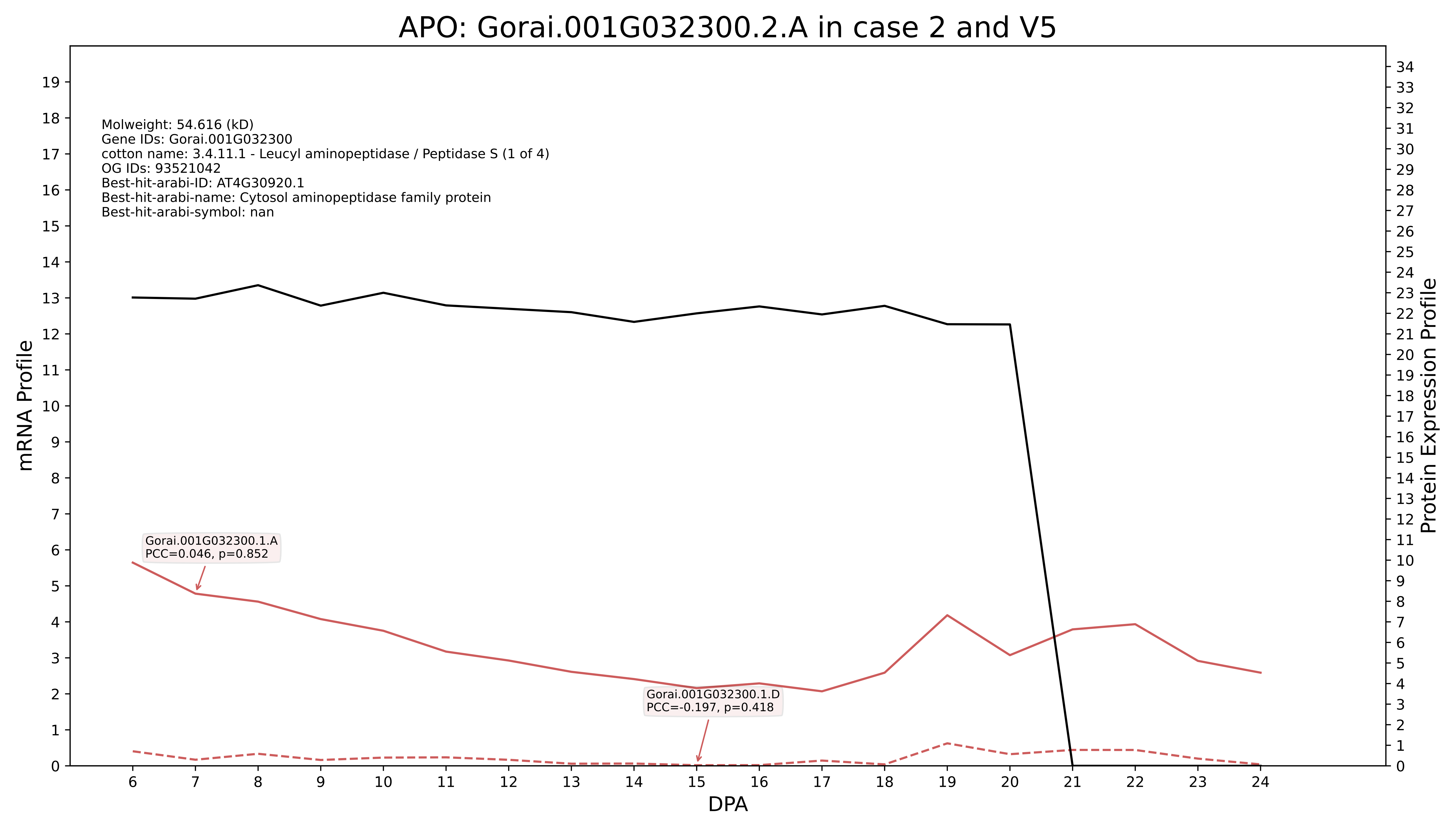
APO: Gorai.001G032300.2.A in case 2 and V5
Molweight: 54.616 (kD)
Gene IDs: Gorai.001G032300
cotton name: 3.4.11.1 - Leucyl aminopeptidase / Peptidase S (1 of 4)
OG IDs: 93521042
Best-hit-arabi-ID: AT4G30920.1
Best-hit-arabi-name: Cytosol aminopeptidase family protein
Best-hit-arabi-symbol: nan
Gorai.001G032300.1.A
PCC=0.046, p=0.852
Gorai.001G032300.1.D
PCC=-0.197, p=0.418
mRNA Profile
Protein Expression Profile
DPA
0
1
2
3
4
5
6
7
8
9
10
11
12
13
14
15
16
17
18
19
20
21
22
23
24
25
26
27
28
29
30
31
32
33
34

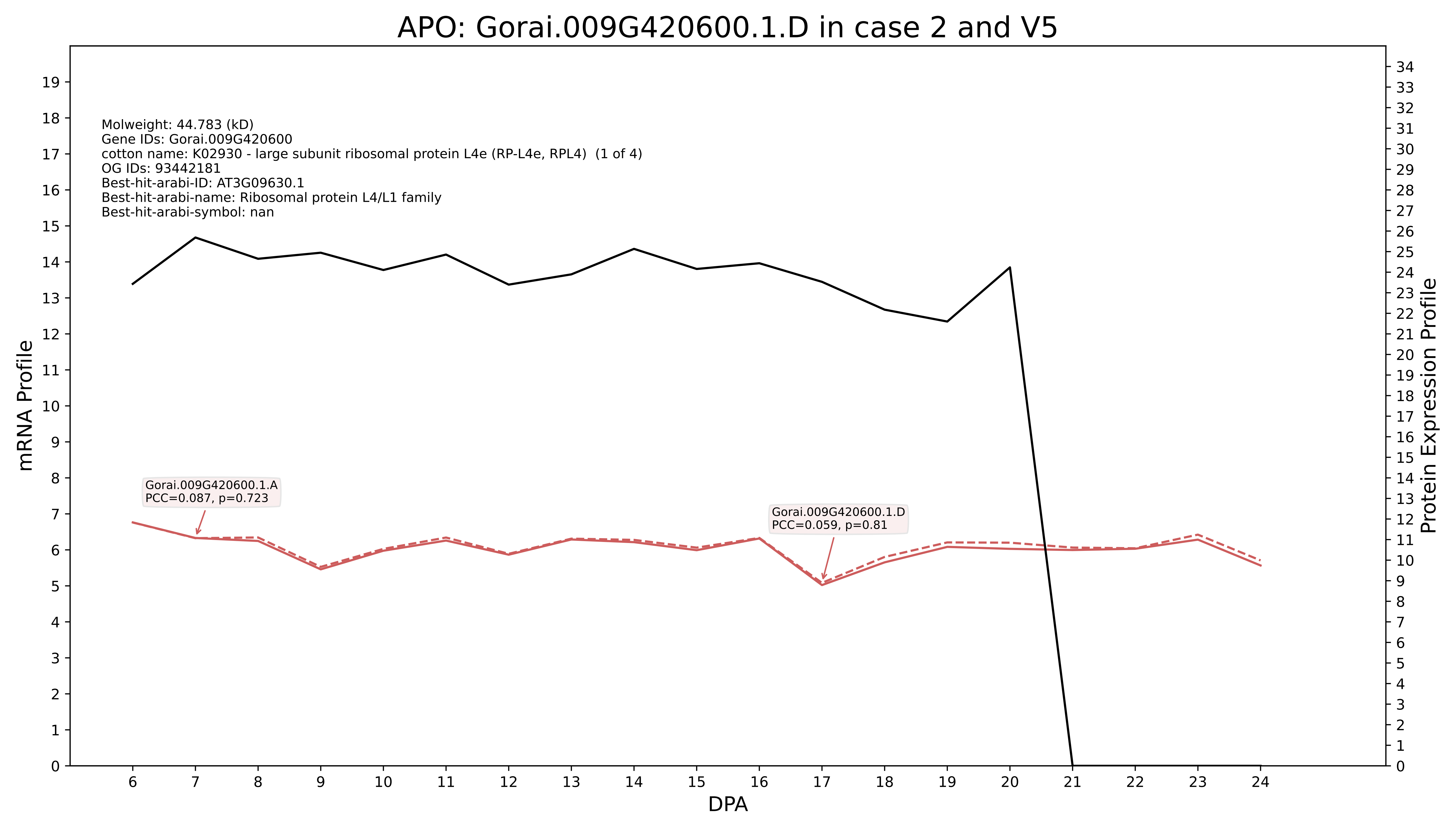

APO: Gorai.009G420600.1.D in case 2 and V5
Molweight: 44.783 (kD)
Gene IDs: Gorai.009G420600
cotton name: K02930 - large subunit ribosomal protein L4e (RP-L4e, RPL4) (1 of 4)
OG IDs: 93442181
Best-hit-arabi-ID: AT3G09630.1
Best-hit-arabi-name: Ribosomal protein L4/L1 family
Best-hit-arabi-symbol: nan
Gorai.009G420600.1.A
PCC=0.087, p=0.723
Gorai.009G420600.1.D
PCC=0.059, p=0.81
mRNA Profile
Protein Expression Profile
DPA

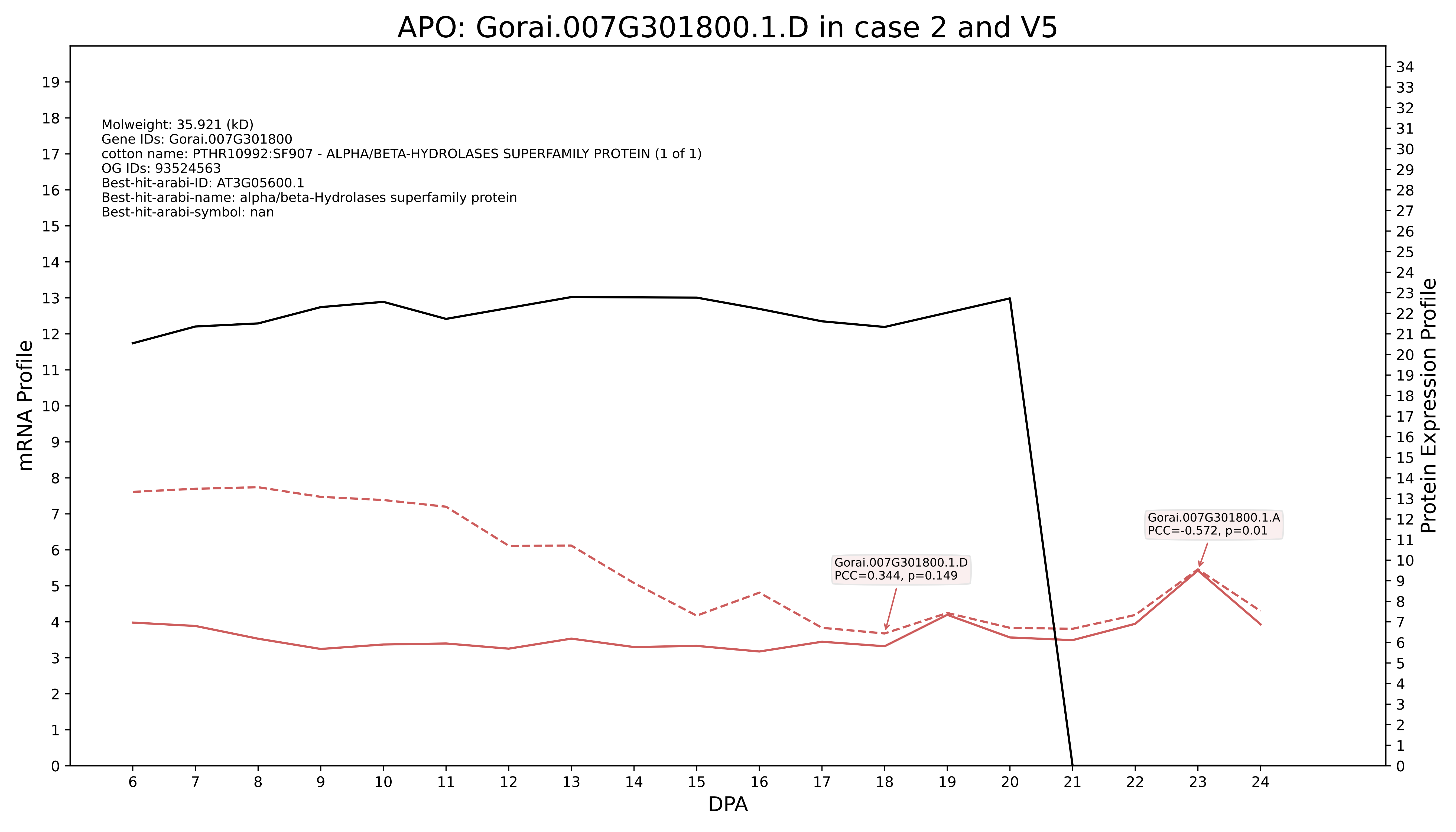
APO: Gorai.007G301800.1.D in case 2 and V5
Molweight: 35.921 (kD)
Gene IDs: Gorai.007G301800
cotton name: PTHR10992:SF907 - ALPHA/BETA-HYDROLASES SUPERFAMILY PROTEIN (1 of 1)
OG IDs: 93524563
Best-hit-arabi-ID: AT3G05600.1
Best-hit-arabi-name: alpha/beta-Hydrolases superfamily protein
Best-hit-arabi-symbol: nan
Gorai.007G301800.1.D
PCC=0.344, p=0.149
Gorai.007G301800.1.A
PCC=-0.572, p=0.01
mRNA Profile
Protein Expression Profile
DPA

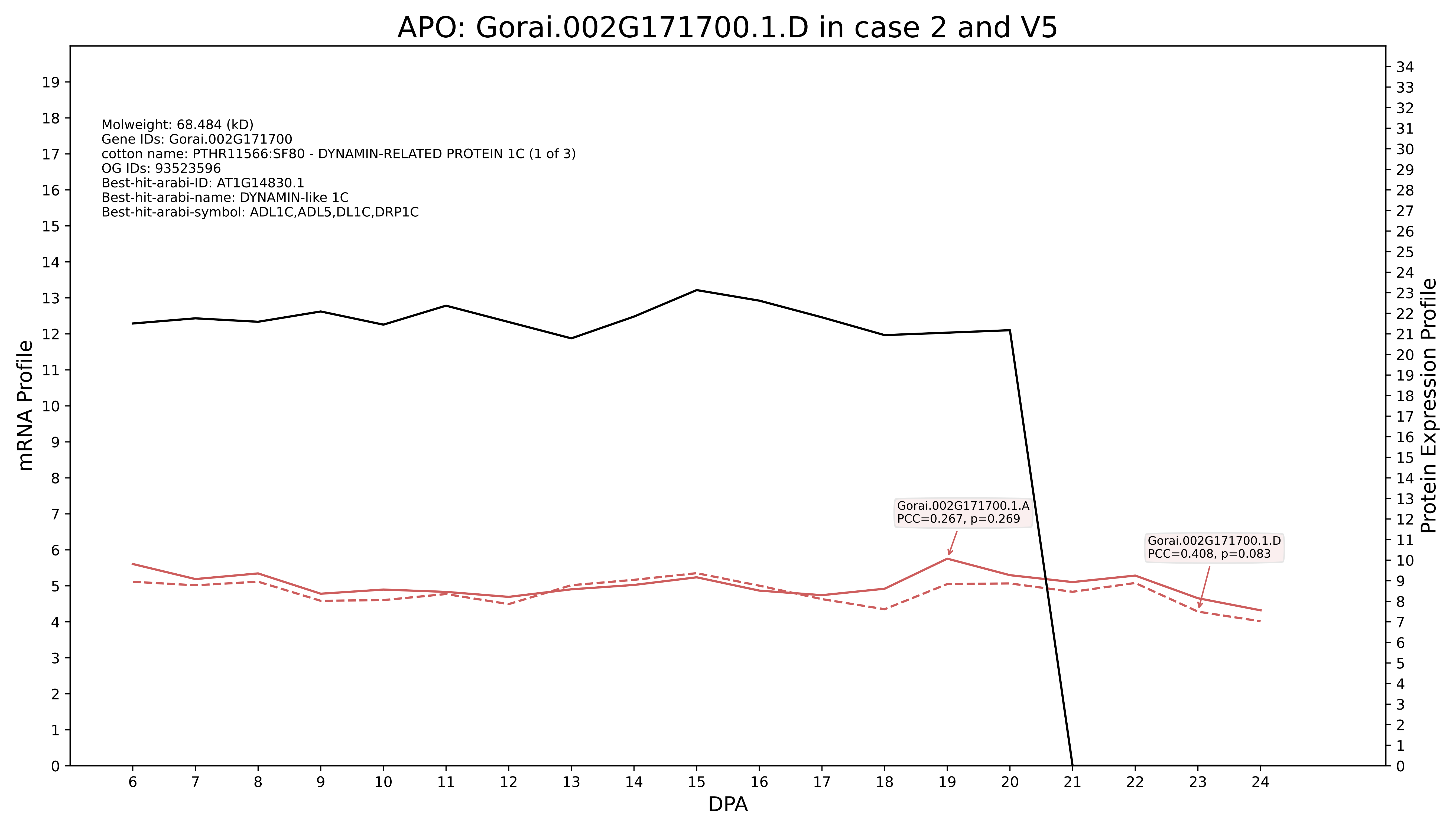
APO: Gorai.002G171700.1.D in case 2 and V5
Molweight: 68.484 (kD)
Gene IDs: Gorai.002G171700
cotton name: PTHR11566:SF80 - DYNAMIN-RELATED PROTEIN 1C (1 of 3)
OG IDs: 93523596
Best-hit-arabi-ID: AT1G14830.1
Best-hit-arabi-name: DYNAMIN-like 1C
Best-hit-arabi-symbol: ADL1C,ADL5,DL1C,DRP1C
Gorai.002G171700.1.A
PCC=0.267, p=0.269
Gorai.002G171700.1.D
PCC=0.408, p=0.083
mRNA Profile
Protein Expression Profile
DPA

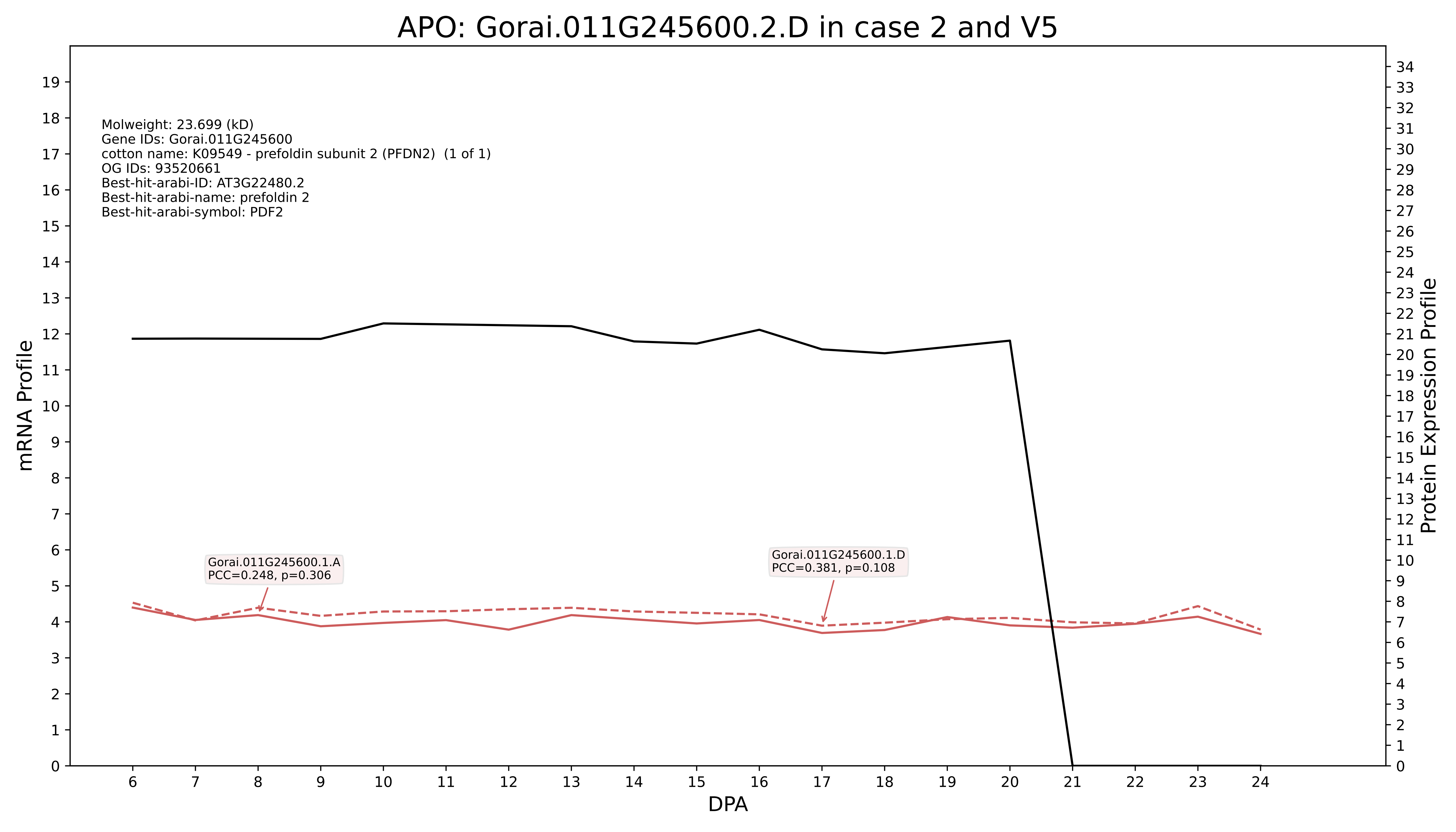

APO: Gorai.011G245600.2.D in case 2 and V5
Molweight: 23.699 (kD)
Gene IDs: Gorai.011G245600
cotton name: K09549 - prefoldin subunit 2 (PFDN2) (1 of 1)
OG IDs: 93520661
Best-hit-arabi-ID: AT3G22480.2
Best-hit-arabi-name: prefoldin 2
Best-hit-arabi-symbol: PDF2
Gorai.011G245600.1.A
PCC=0.248, p=0.306
Gorai.011G245600.1.D
PCC=0.381, p=0.108
mRNA Profile
Protein Expression Profile
DPA

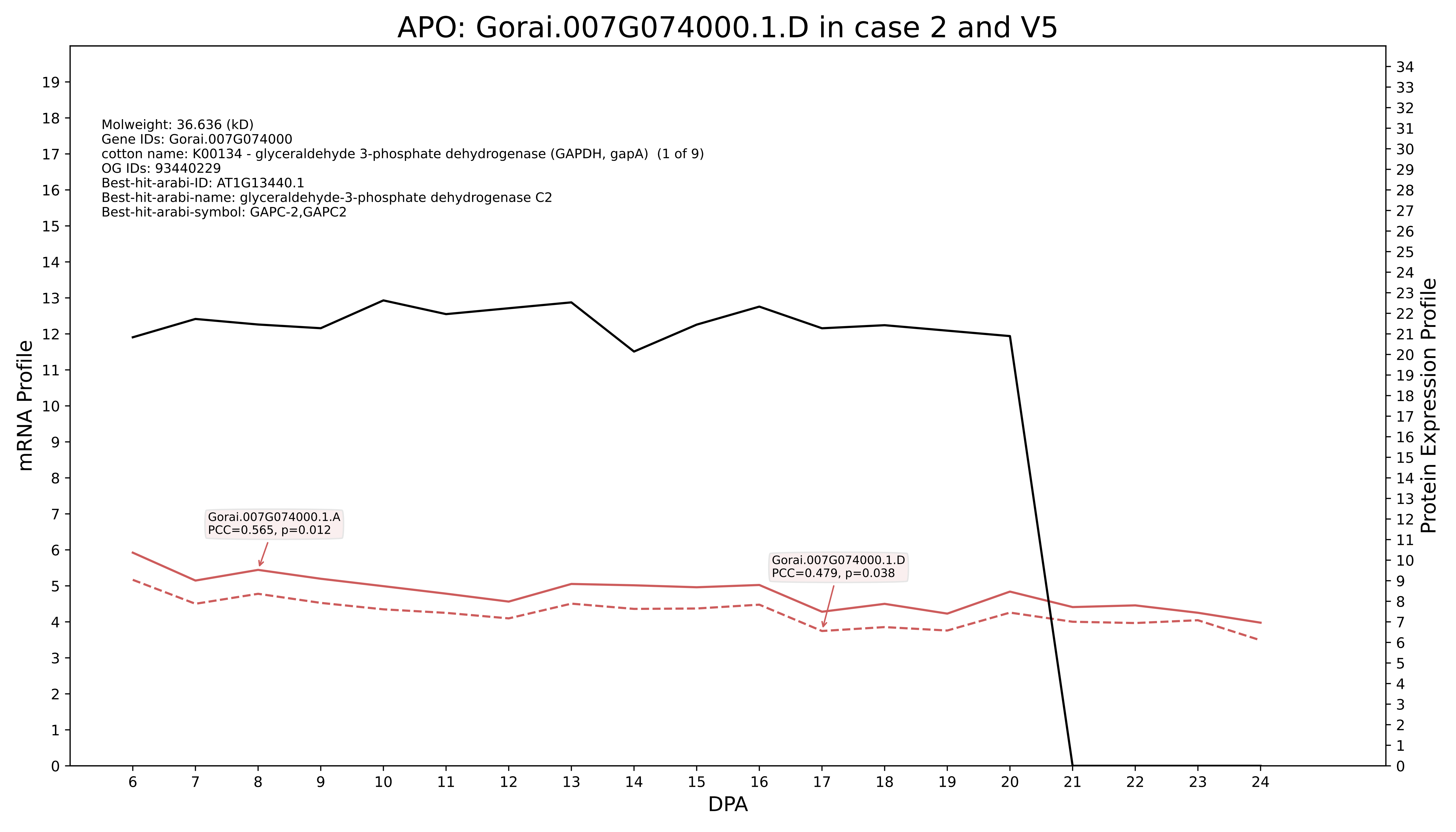

APO: Gorai.007G074000.1.D in case 2 and V5
Molweight: 36.636 (kD)
Gene IDs: Gorai.007G074000
cotton name: K00134 - glyceraldehyde 3-phosphate dehydrogenase (GAPDH, gapA) (1 of 9)
OG IDs: 93440229
Best-hit-arabi-ID: AT1G13440.1
Best-hit-arabi-name: glyceraldehyde-3-phosphate dehydrogenase C2
Best-hit-arabi-symbol: GAPC-2,GAPC2
Gorai.007G074000.1.A
PCC=0.565, p=0.012
Gorai.007G074000.1.D
PCC=0.479, p=0.038
mRNA Profile
Protein Expression Profile
DPA

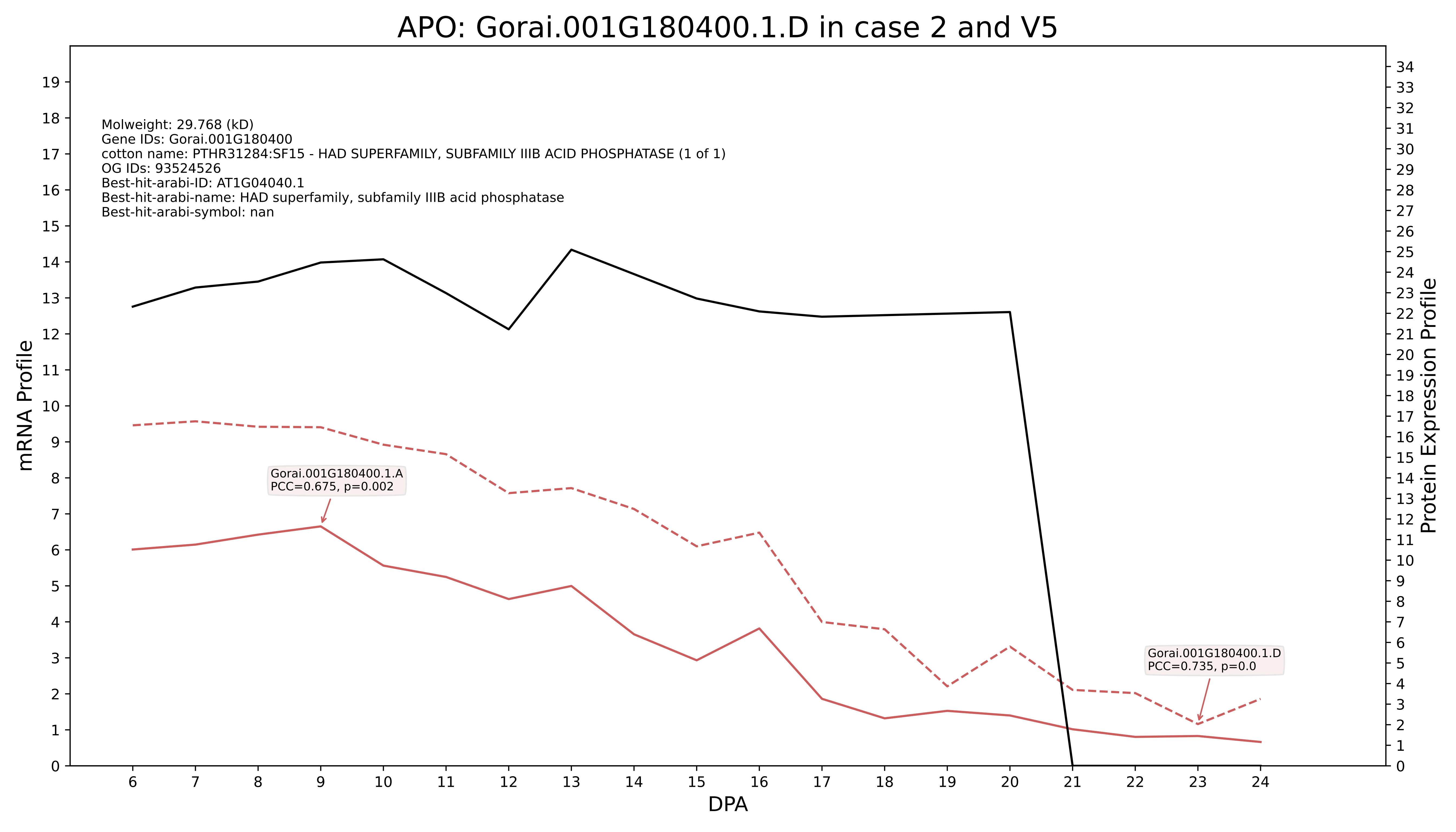

APO: Gorai.001G180400.1.D in case 2 and V5
Molweight: 29.768 (kD)
Gene IDs: Gorai.001G180400
cotton name: PTHR31284:SF15 - HAD SUPERFAMILY, SUBFAMILY IIIB ACID PHOSPHATASE (1 of 1)
OG IDs: 93524526
Best-hit-arabi-ID: AT1G04040.1
Best-hit-arabi-name: HAD superfamily, subfamily IIIB acid phosphatase
Best-hit-arabi-symbol: nan
Gorai.001G180400.1.A
PCC=0.675, p=0.002
Gorai.001G180400.1.D
PCC=0.735, p=0.0
mRNA Profile
Protein Expression Profile
DPA

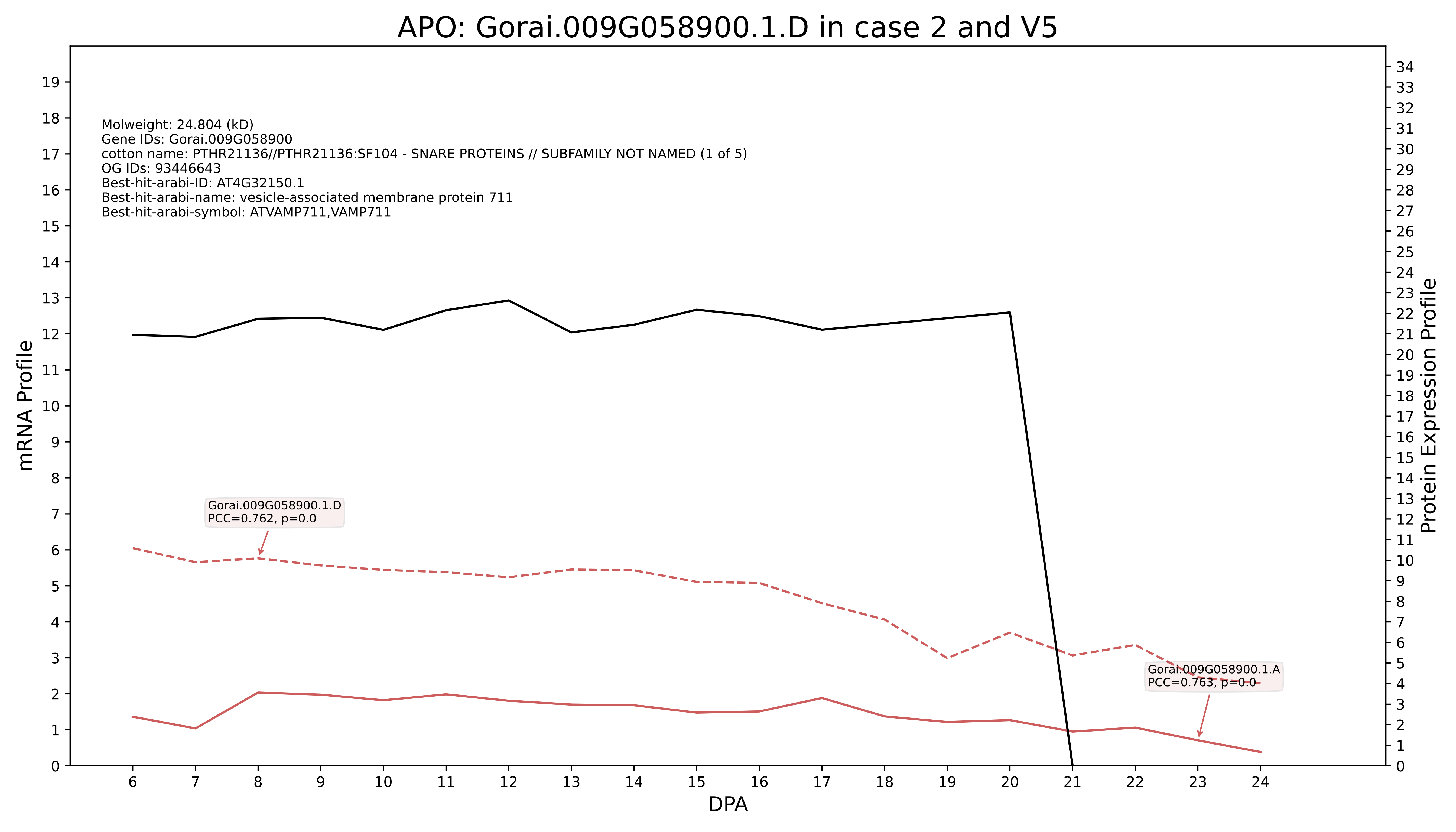
APO: Gorai.009G058900.1.D in case 2 and V5
Molweight: 24.804 (kD)
Gene IDs: Gorai.009G058900
cotton name: PTHR21136//PTHR21136:SF104 - SNARE PROTEINS // SUBFAMILY NOT NAMED (1 of 5)
OG IDs: 93446643
Best-hit-arabi-ID: AT4G32150.1
Best-hit-arabi-name: vesicle-associated membrane protein 711
Best-hit-arabi-symbol: ATVAMP711,VAMP711
Gorai.009G058900.1.D
PCC=0.762, p=0.0
Gorai.009G058900.1.A
PCC=0.763, p=0.0
mRNA Profile
Protein Expression Profile
DPA

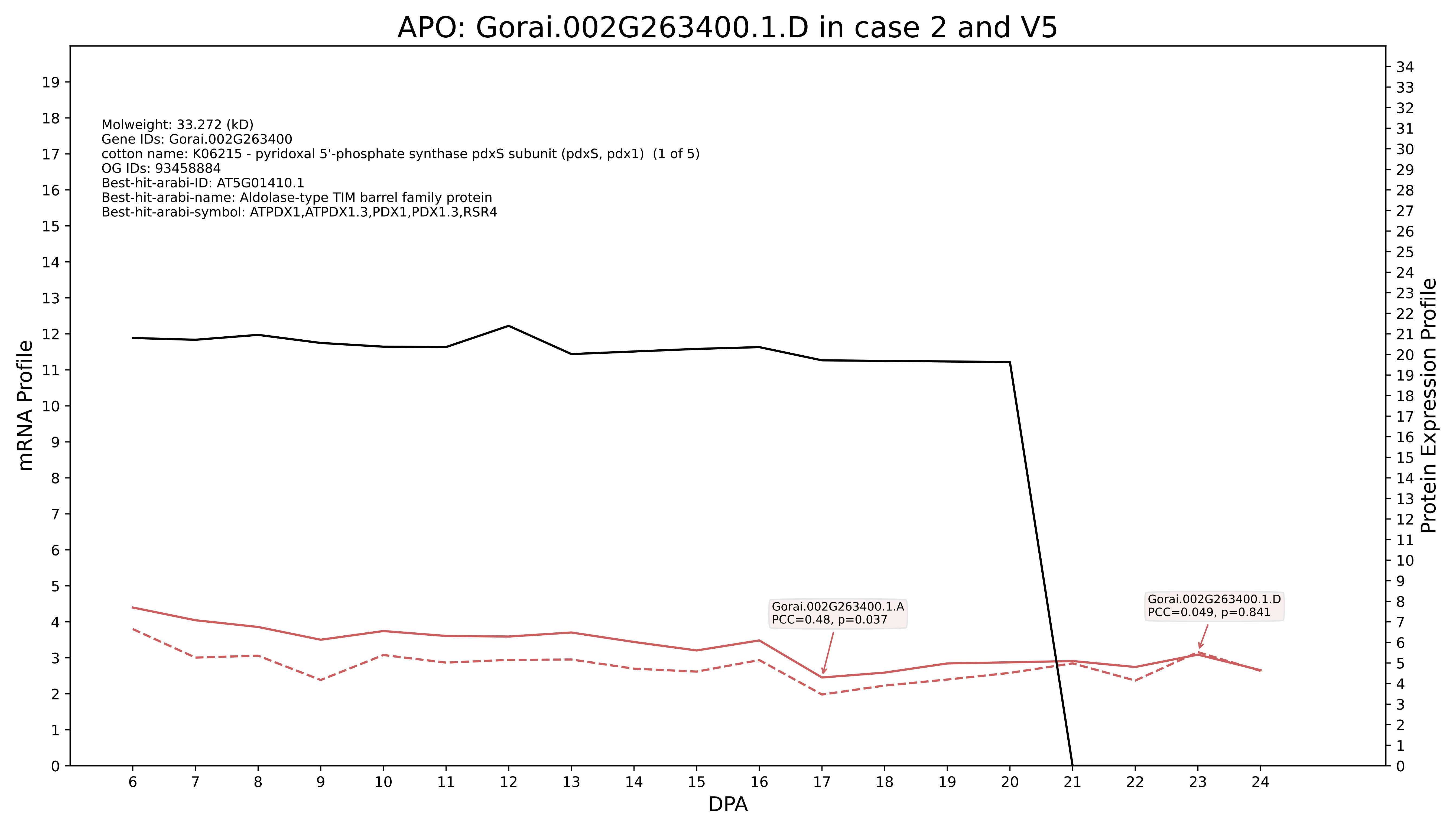
APO: Gorai.002G263400.1.D in case 2 and V5
Molweight: 33.272 (kD)
Gene IDs: Gorai.002G263400
cotton name: K06215 - pyridoxal 5'-phosphate synthase pdxS subunit (pdxS, pdx1) (1 of 5)
OG IDs: 93458884
Best-hit-arabi-ID: AT5G01410.1
Best-hit-arabi-name: Aldolase-type TIM barrel family protein
Best-hit-arabi-symbol: ATPDX1,ATPDX1.3,PDX1,PDX1.3,RSR4
Gorai.002G263400.1.A
PCC=0.48, p=0.037
Gorai.002G263400.1.D
PCC=0.049, p=0.841
mRNA Profile
Protein Expression Profile
DPA

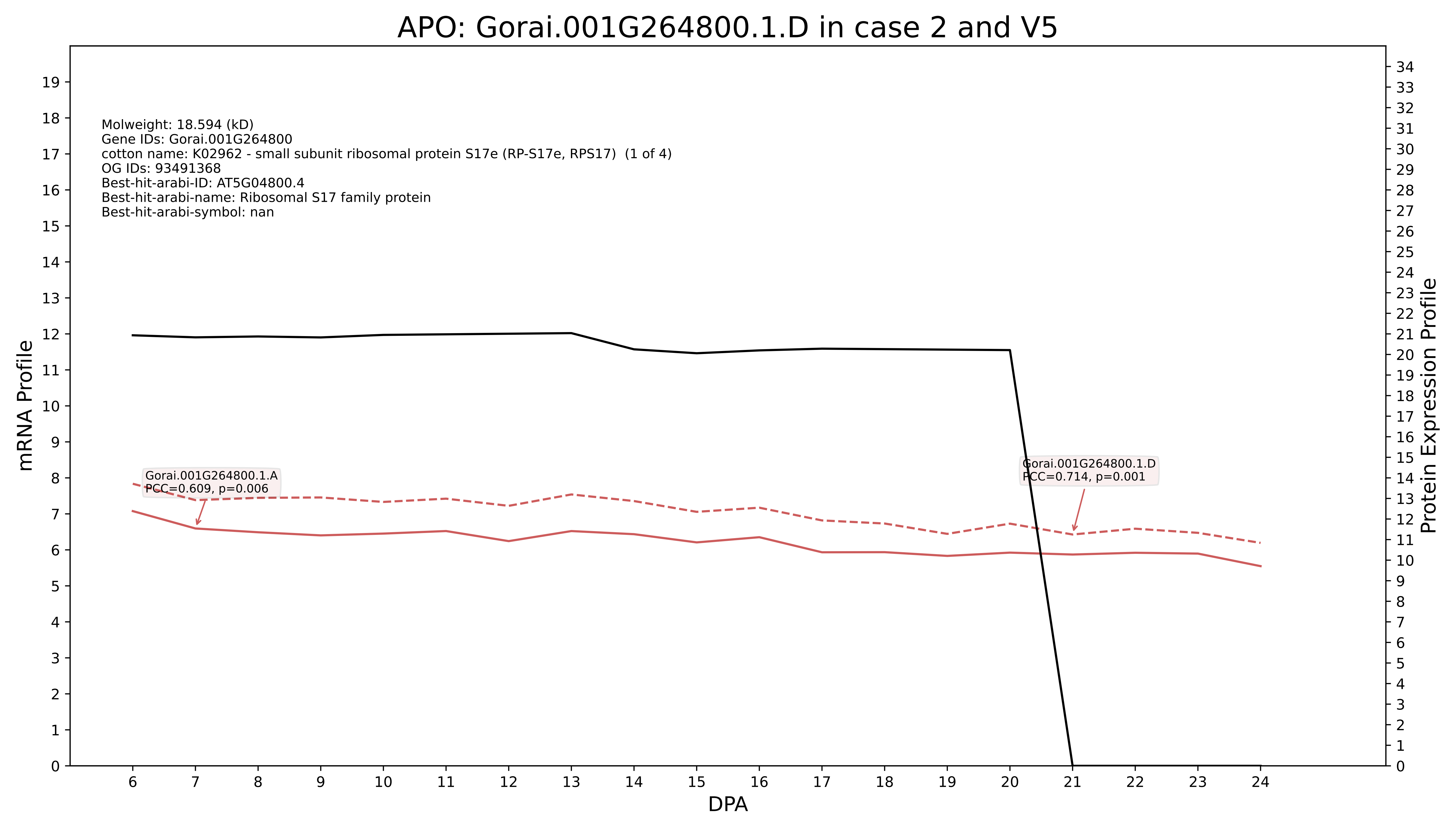

APO: Gorai.001G264800.1.D in case 2 and V5
Molweight: 18.594 (kD)
Gene IDs: Gorai.001G264800
cotton name: K02962 - small subunit ribosomal protein S17e (RP-S17e, RPS17) (1 of 4)
OG IDs: 93491368
Best-hit-arabi-ID: AT5G04800.4
Best-hit-arabi-name: Ribosomal S17 family protein
Best-hit-arabi-symbol: nan
Gorai.001G264800.1.A
PCC=0.609, p=0.006
Gorai.001G264800.1.D
PCC=0.714, p=0.001
mRNA Profile
Protein Expression Profile
DPA

APO: Gorai.001G076300.1.D in case 2 and V5

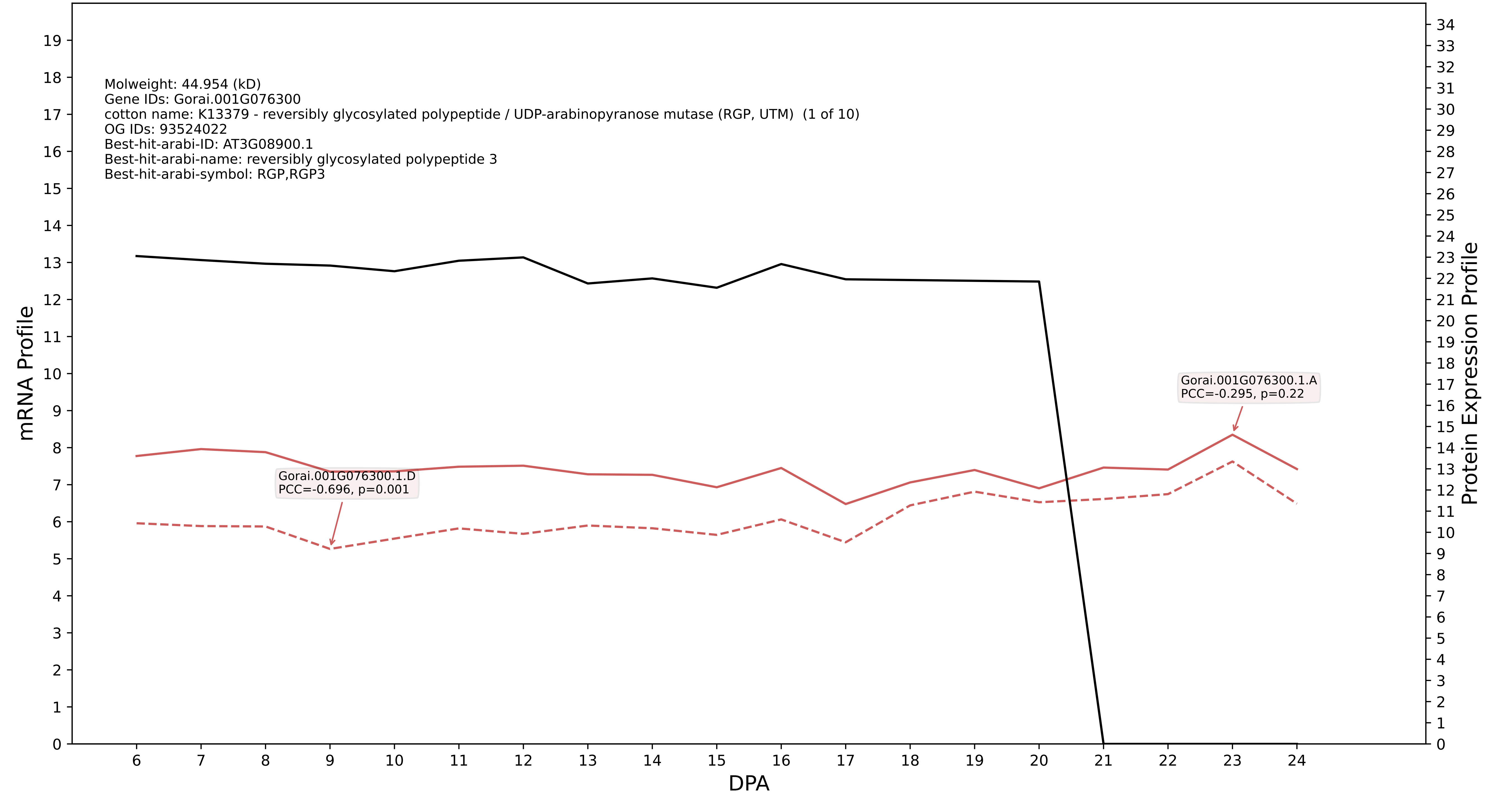

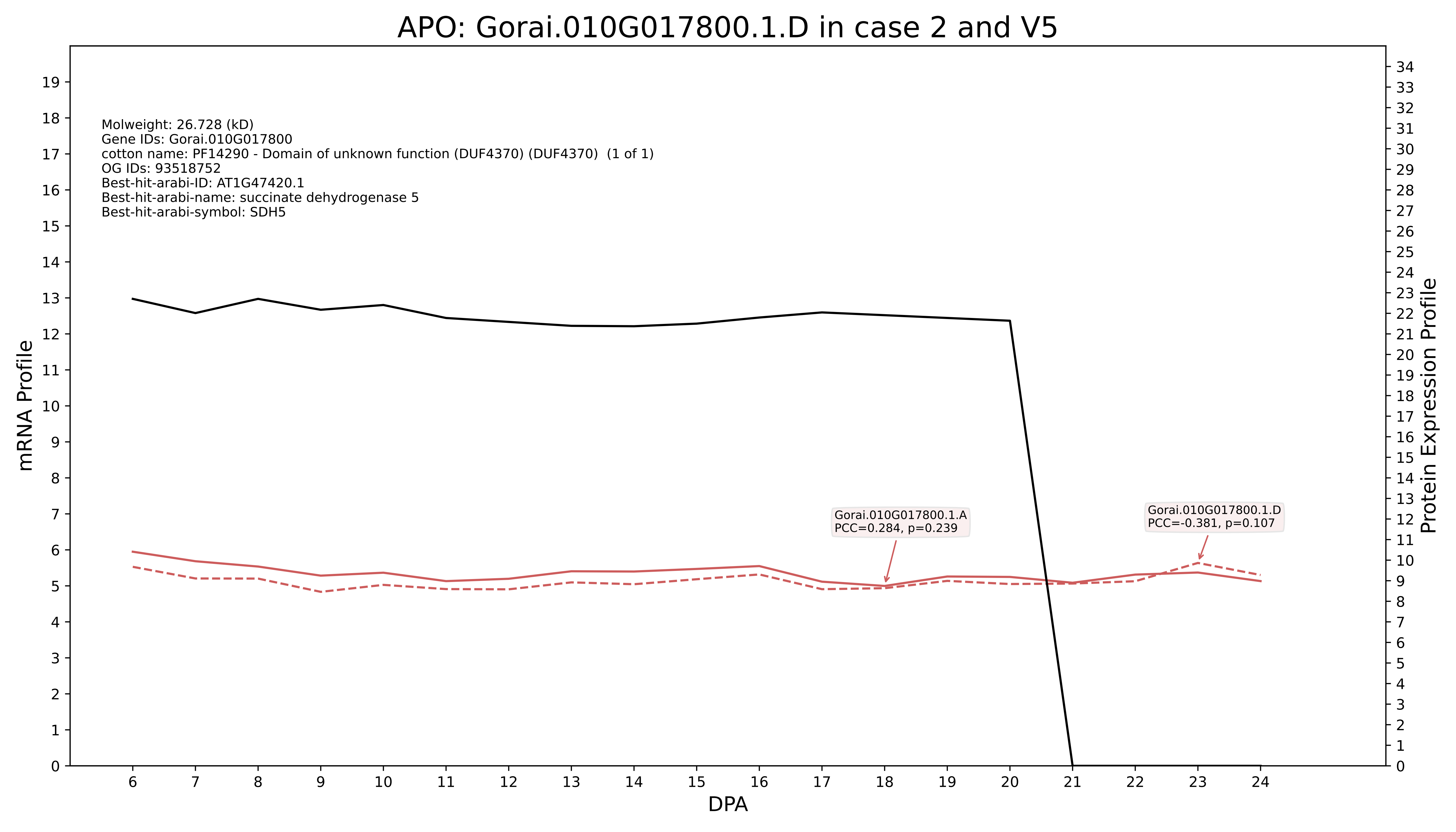
APO: Gorai.010G017800.1.D in case 2 and V5
Molweight: 26.728 (kD)
Gene IDs: Gorai.010G017800
cotton name: PF14290 - Domain of unknown function (DUF4370) (DUF4370) (1 of 1)
OG IDs: 93518752
Best-hit-arabi-ID: AT1G47420.1
Best-hit-arabi-name: succinate dehydrogenase 5
Best-hit-arabi-symbol: SDH5
Gorai.010G017800.1.A
PCC=0.284, p=0.239
Gorai.010G017800.1.D
PCC=-0.381, p=0.107
mRNA Profile
Protein Expression Profile
DPA

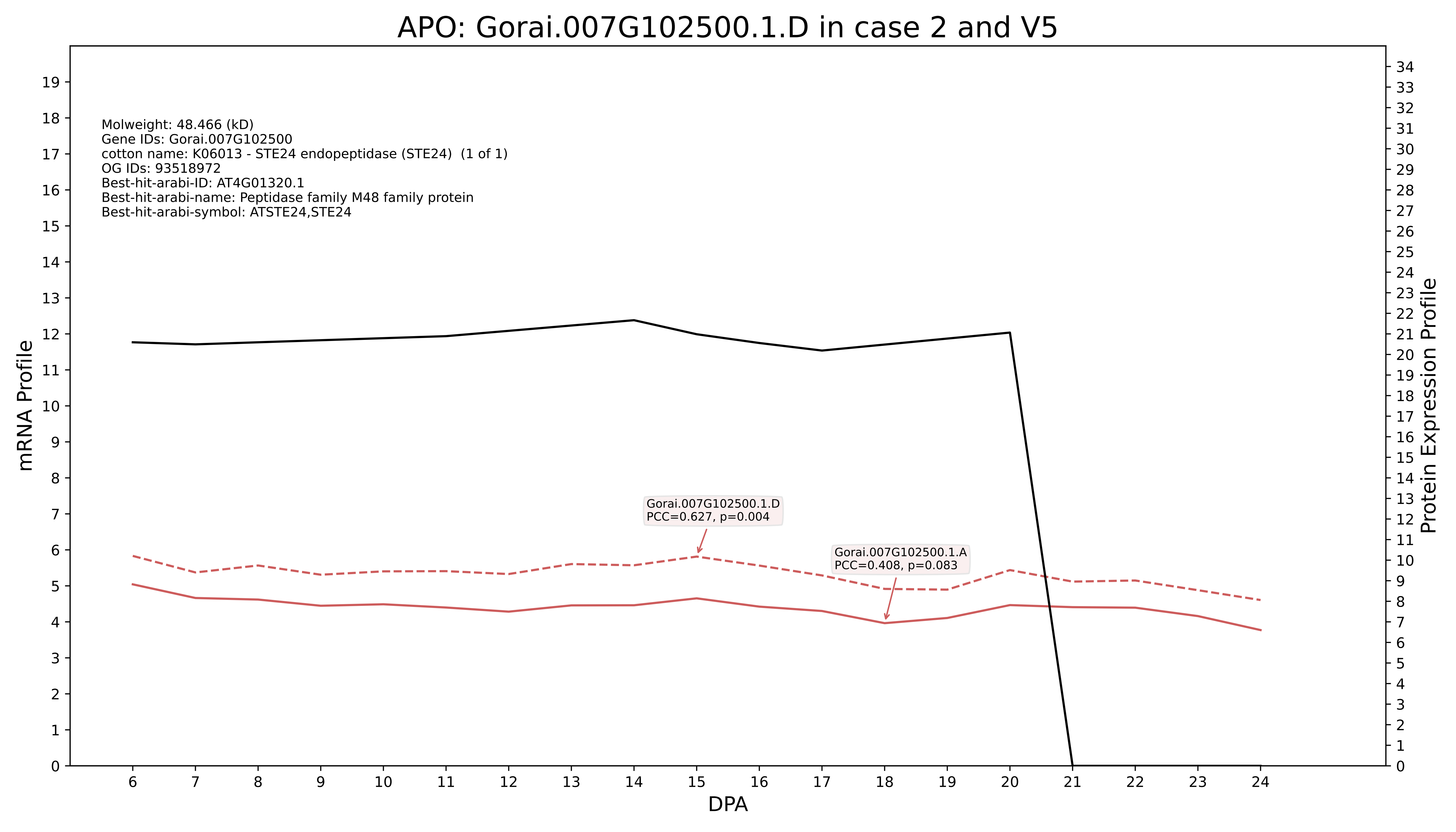
APO: Gorai.007G102500.1.D in case 2 and V5
Molweight: 48.466 (kD)
Gene IDs: Gorai.007G102500
cotton name: K06013 - STE24 endopeptidase (STE24) (1 of 1)
OG IDs: 93518972
Best-hit-arabi-ID: AT4G01320.1
Best-hit-arabi-name: Peptidase family M48 family protein
Best-hit-arabi-symbol: ATSTE24,STE24
Gorai.007G102500.1.D
PCC=0.627, p=0.004
Gorai.007G102500.1.A
PCC=0.408, p=0.083
mRNA Profile
Protein Expression Profile
DPA

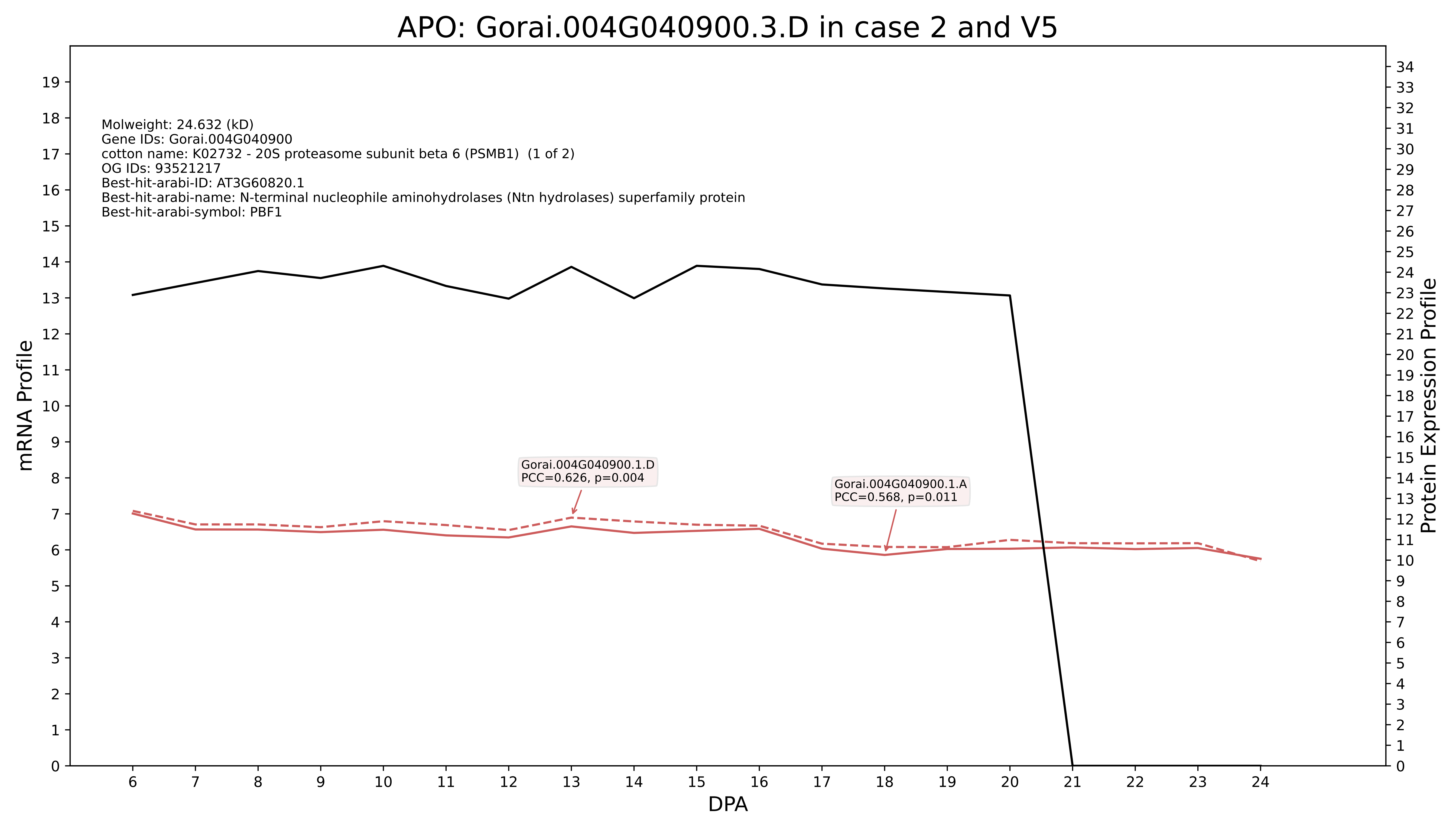
APO: Gorai.004G040900.3.D in case 2 and V5
Molweight: 24.632 (kD)
Gene IDs: Gorai.004G040900
cotton name: K02732 - 20S proteasome subunit beta 6 (PSMB1) (1 of 2)
OG IDs: 93521217
Best-hit-arabi-ID: AT3G60820.1
Best-hit-arabi-name: N-terminal nucleophile aminohydrolases (Ntn hydrolases) superfamily protein
Best-hit-arabi-symbol: PBF1
Gorai.004G040900.1.D
PCC=0.626, p=0.004
Gorai.004G040900.1.A
PCC=0.568, p=0.011
mRNA Profile
Protein Expression Profile
DPA

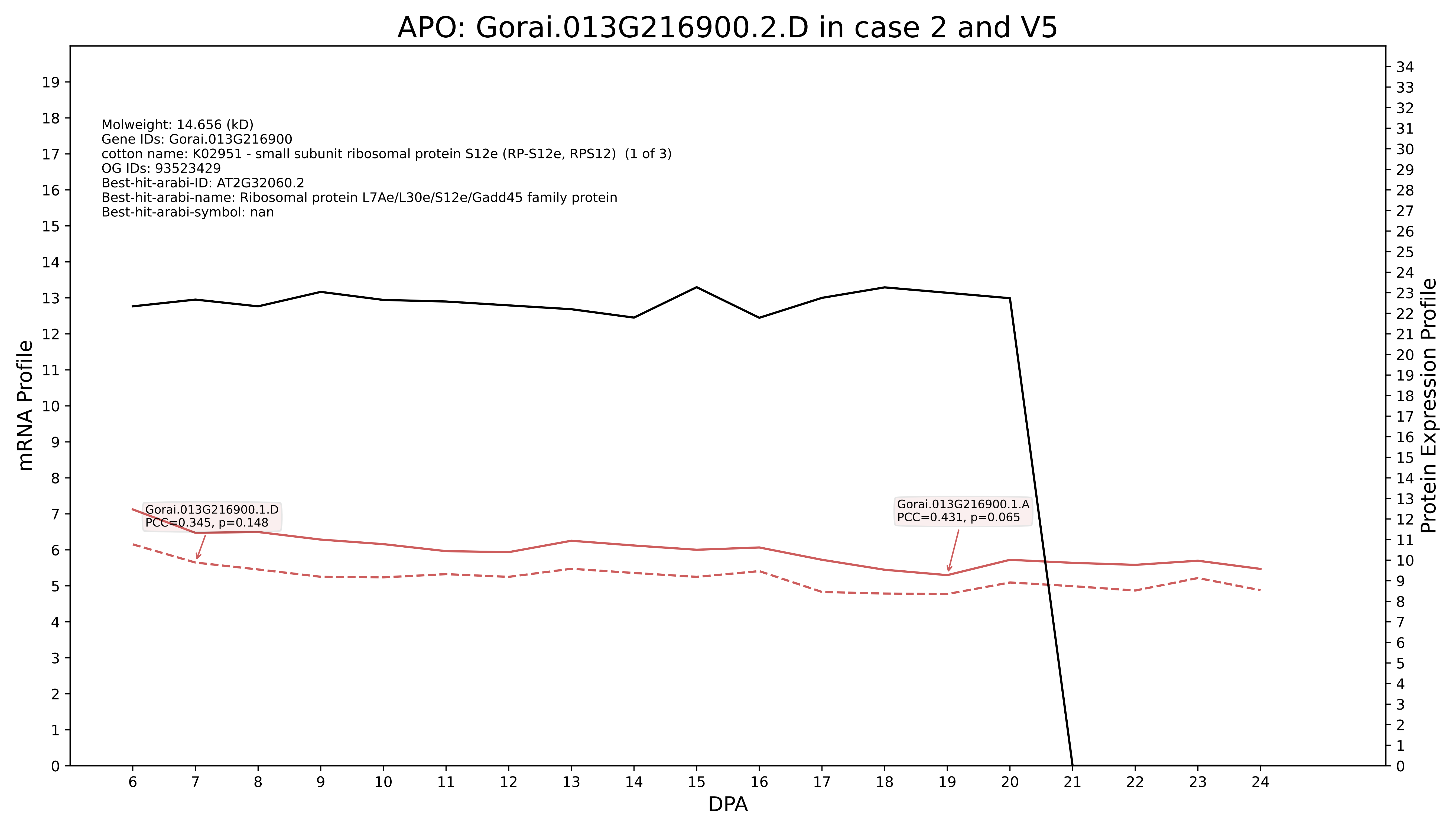
APO: Gorai.013G216900.2.D in case 2 and V5
Molweight: 14.656 (kD)
Gene IDs: Gorai.013G216900
cotton name: K02951 - small subunit ribosomal protein S12e (RP-S12e, RPS12) (1 of 3)
OG IDs: 93523429
Best-hit-arabi-ID: AT2G32060.2
Best-hit-arabi-name: Ribosomal protein L7Ae/L30e/S12e/Gadd45 family protein
Best-hit-arabi-symbol: nan
Gorai.013G216900.1.D
PCC=0.345, p=0.148
Gorai.013G216900.1.A
PCC=0.431, p=0.065
mRNA Profile
Protein Expression Profile
DPA

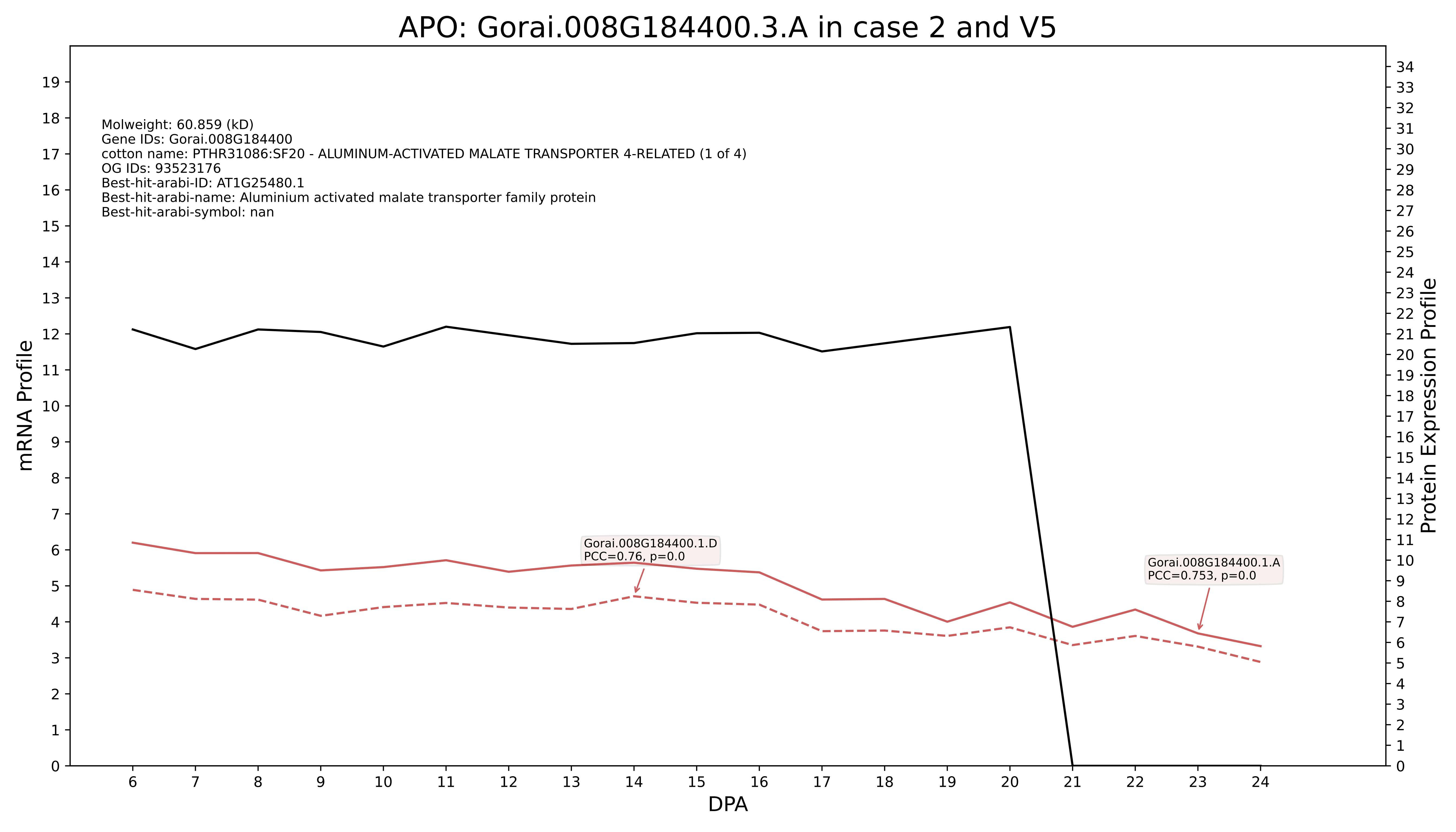
APO: Gorai.008G184400.3.A in case 2 and V5
Molweight: 60.859 (kD)
Gene IDs: Gorai.008G184400
cotton name: PTHR31086:SF20 - ALUMINUM-ACTIVATED MALATE TRANSPORTER 4-RELATED (1 of 4)
OG IDs: 93523176
Best-hit-arabi-ID: AT1G25480.1
Best-hit-arabi-name: Aluminium activated malate transporter family protein
Best-hit-arabi-symbol: nan
Gorai.008G184400.1.D
PCC=0.76, p=0.0
Gorai.008G184400.1.A
PCC=0.753, p=0.0
mRNA Profile
Protein Expression Profile
DPA

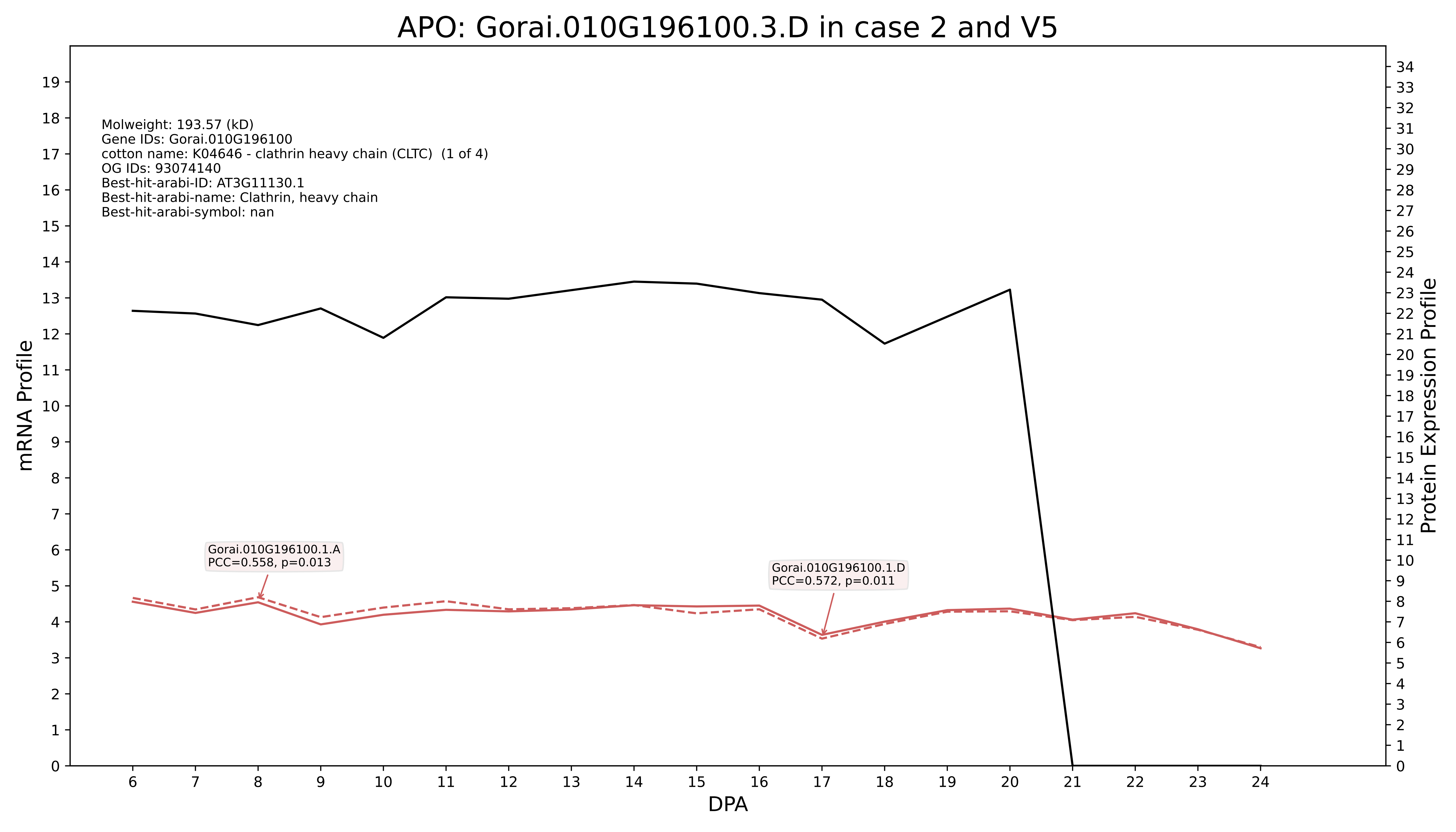

APO: Gorai.010G196100.3.D in case 2 and V5
Molweight: 193.57 (kD)
Gene IDs: Gorai.010G196100
cotton name: K04646 - clathrin heavy chain (CLTC) (1 of 4)
OG IDs: 93074140
Best-hit-arabi-ID: AT3G11130.1
Best-hit-arabi-name: Clathrin, heavy chain
Best-hit-arabi-symbol: nan
Gorai.010G196100.1.A
PCC=0.558, p=0.013
Gorai.010G196100.1.D
PCC=0.572, p=0.011
mRNA Profile
Protein Expression Profile
DPA

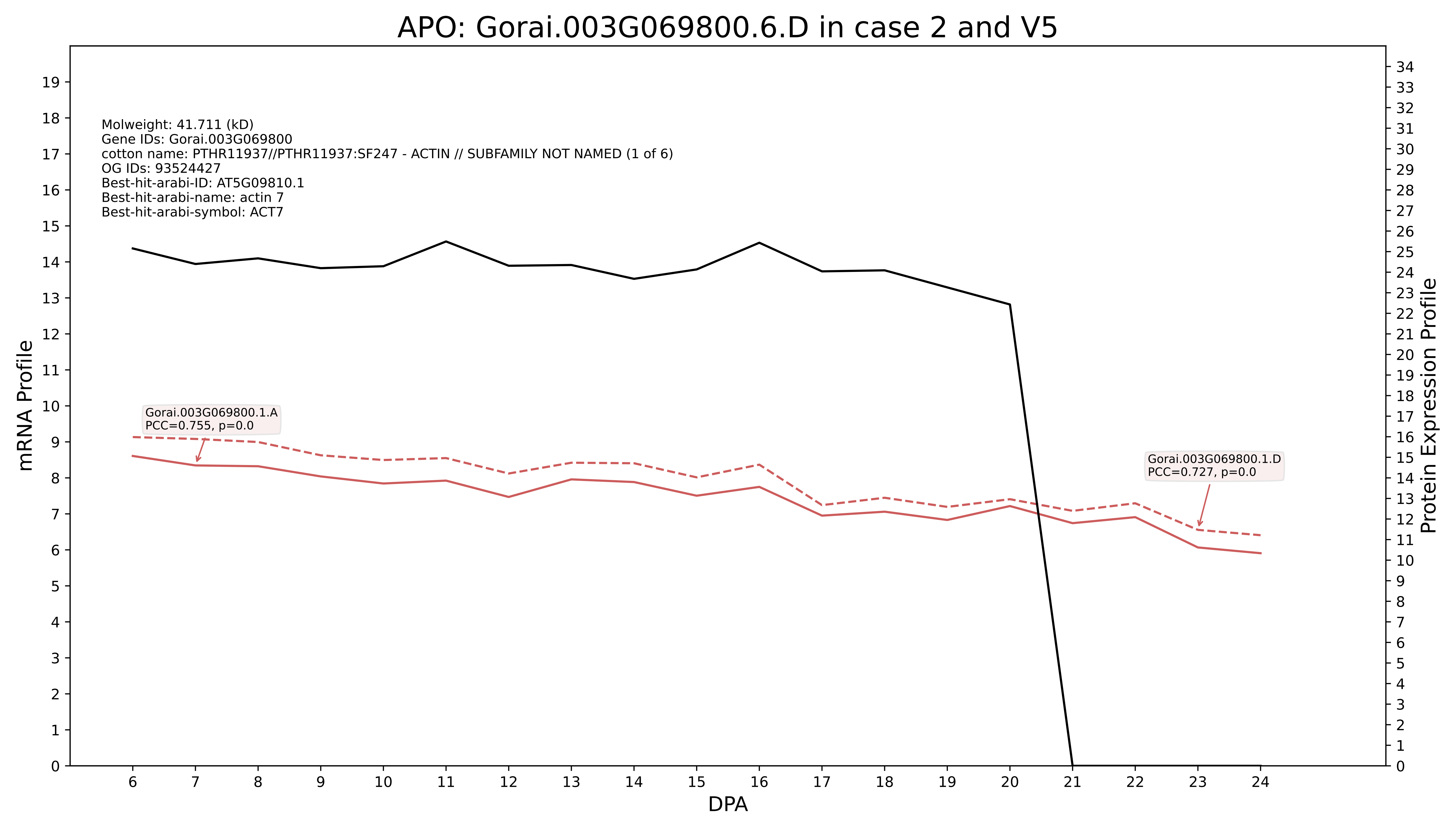
APO: Gorai.003G069800.6.D in case 2 and V5
Molweight: 41.711 (kD)
Gene IDs: Gorai.003G069800
cotton name: PTHR11937//PTHR11937:SF247 - ACTIN // SUBFAMILY NOT NAMED (1 of 6)
OG IDs: 93524427
Best-hit-arabi-ID: AT5G09810.1
Best-hit-arabi-name: actin 7
Best-hit-arabi-symbol: ACT7
Gorai.003G069800.1.A
PCC=0.755, p=0.0
Gorai.003G069800.1.D
PCC=0.727, p=0.0
mRNA Profile
Protein Expression Profile
DPA

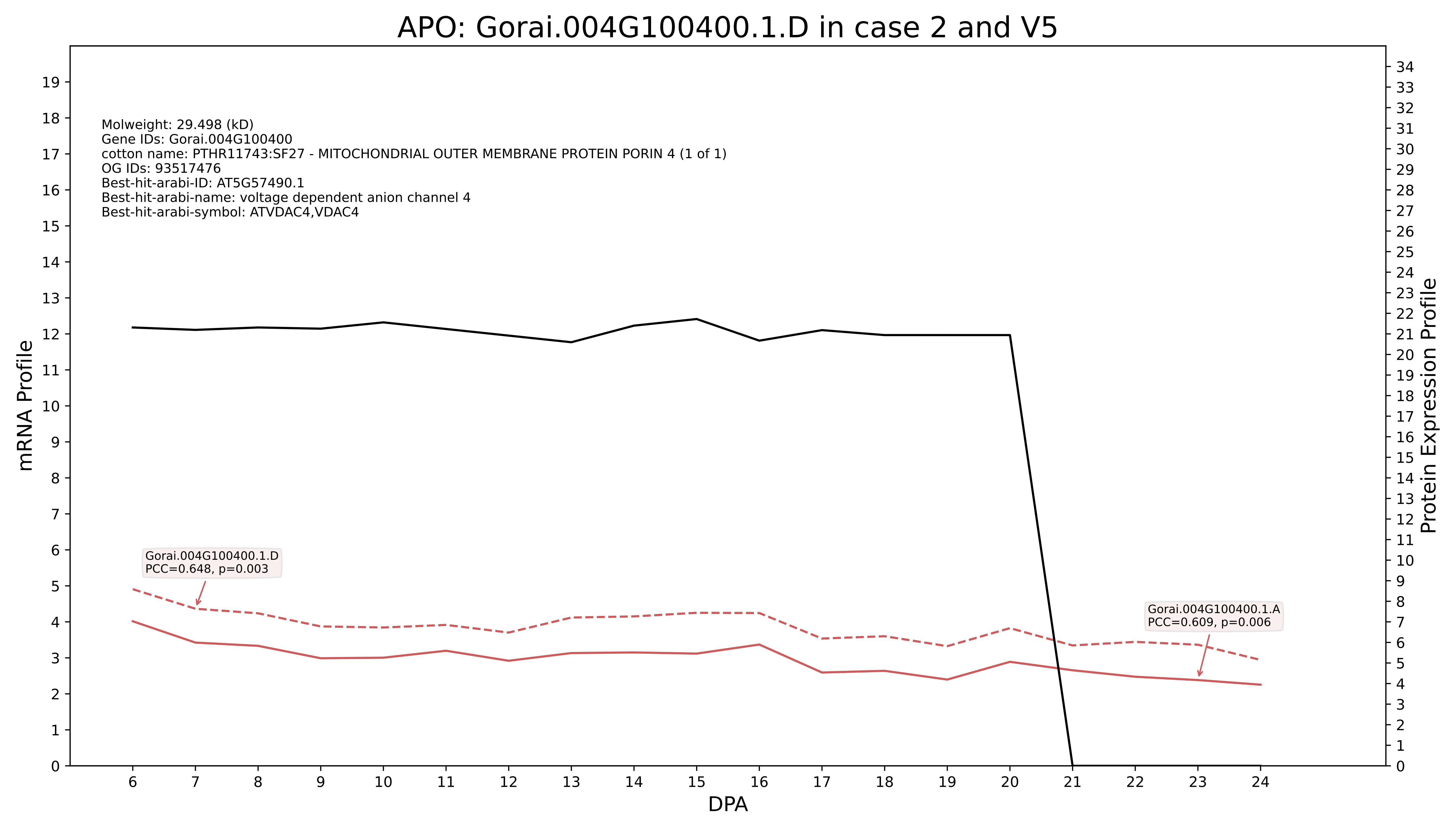

APO: Gorai.004G100400.1.D in case 2 and V5
Molweight: 29.498 (kD)
Gene IDs: Gorai.004G100400
cotton name: PTHR11743:SF27 - MITOCHONDRIAL OUTER MEMBRANE PROTEIN PORIN 4 (1 of 1)
OG IDs: 93517476
Best-hit-arabi-ID: AT5G57490.1
Best-hit-arabi-name: voltage dependent anion channel 4
Best-hit-arabi-symbol: ATVDAC4,VDAC4
Gorai.004G100400.1.D
PCC=0.648, p=0.003
Gorai.004G100400.1.A
PCC=0.609, p=0.006
mRNA Profile
Protein Expression Profile
DPA

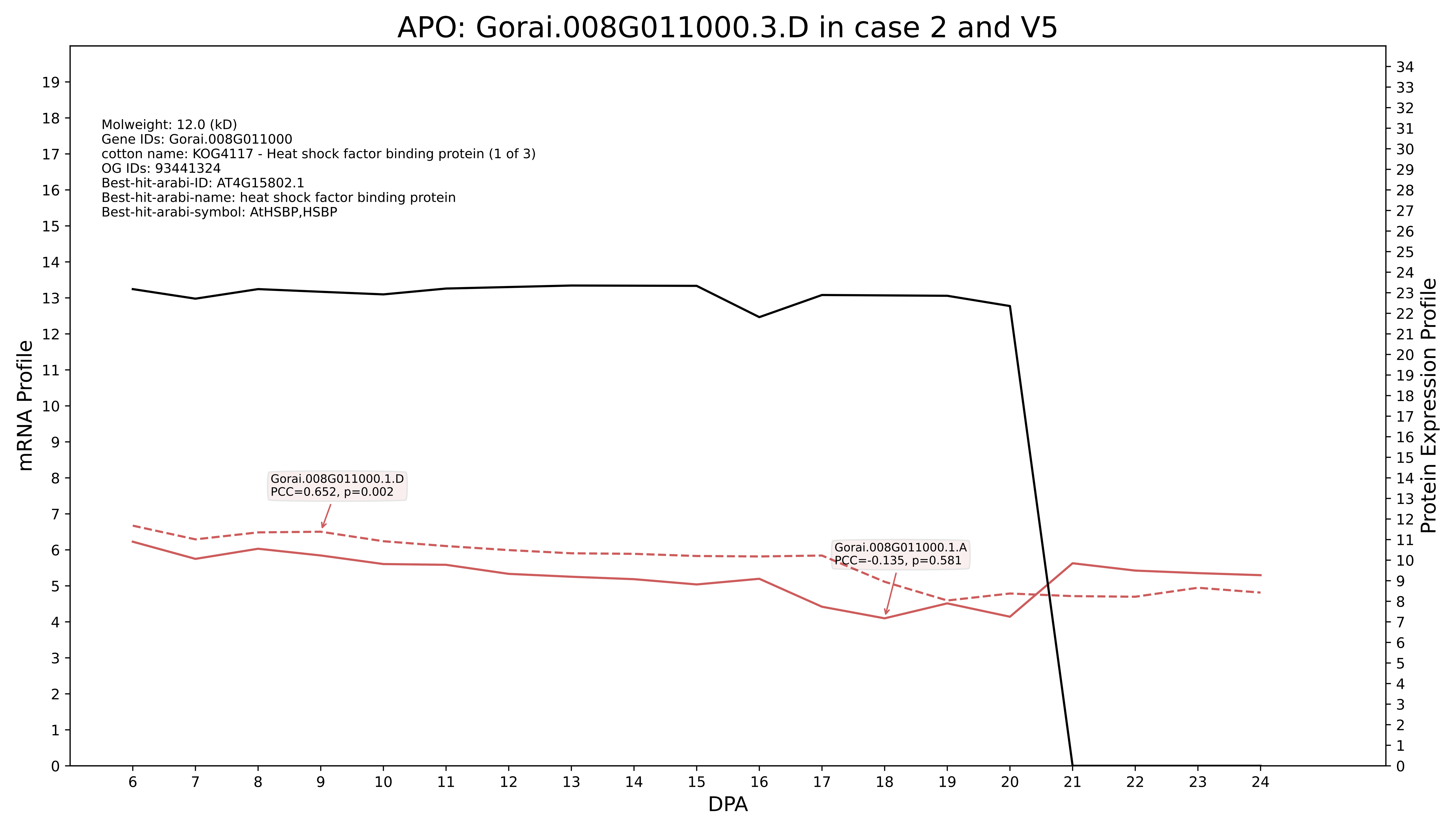
APO: Gorai.008G011000.3.D in case 2 and V5
Molweight: 12.0 (kD)
Gene IDs: Gorai.008G011000
cotton name: KOG4117 - Heat shock factor binding protein (1 of 3)
OG IDs: 93441324
Best-hit-arabi-ID: AT4G15802.1
Best-hit-arabi-name: heat shock factor binding protein
Best-hit-arabi-symbol: AtHSBP,HSBP
Gorai.008G011000.1.D
PCC=0.652, p=0.002
Gorai.008G011000.1.A
PCC=-0.135, p=0.581
mRNA Profile
Protein Expression Profile
DPA

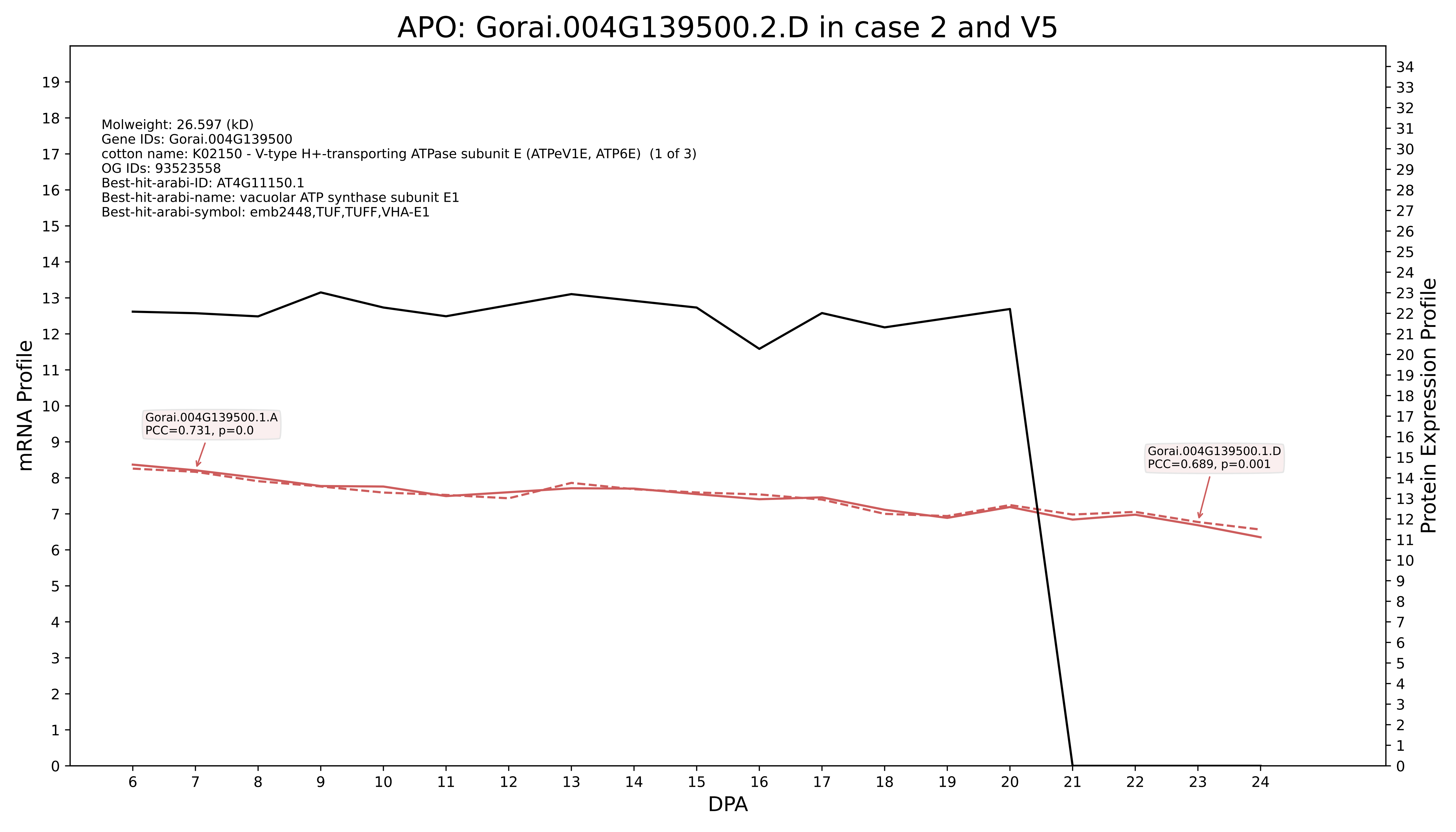
APO: Gorai.004G139500.2.D in case 2 and V5
Molweight: 26.597 (kD)
Gene IDs: Gorai.004G139500
cotton name: K02150 - V-type H+-transporting ATPase subunit E (ATPeV1E, ATP6E) (1 of 3)
OG IDs: 93523558
Best-hit-arabi-ID: AT4G11150.1
Best-hit-arabi-name: vacuolar ATP synthase subunit E1
Best-hit-arabi-symbol: emb2448,TUF,TUFF,VHA-E1
Gorai.004G139500.1.A
PCC=0.731, p=0.0
Gorai.004G139500.1.D
PCC=0.689, p=0.001
mRNA Profile
Protein Expression Profile
DPA

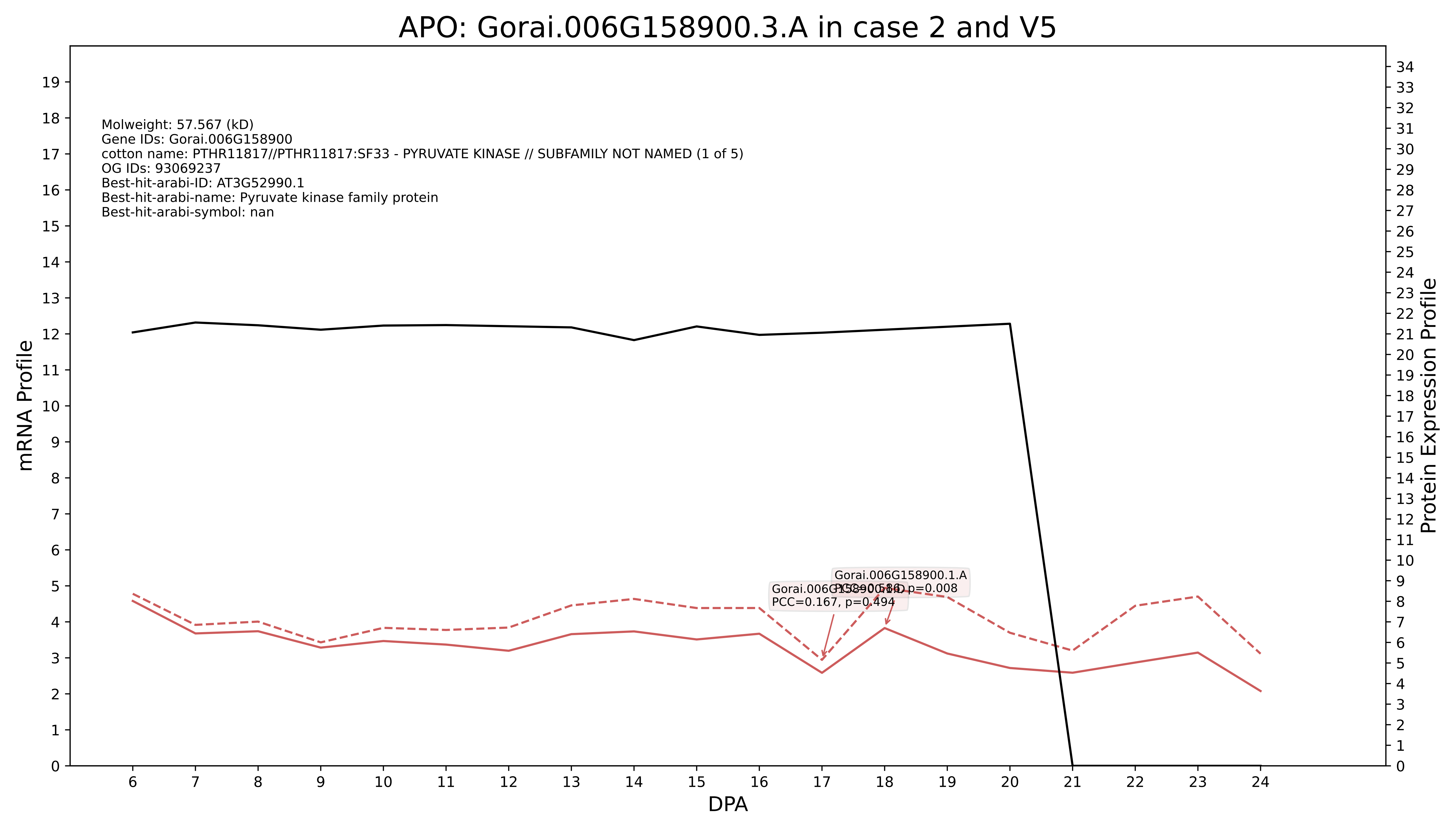
APO: Gorai.006G158900.3.A in case 2 and V5
Molweight: 57.567 (kD)
Gene IDs: Gorai.006G158900
cotton name: PTHR11817//PTHR11817:SF33 - PYRUVATE KINASE // SUBFAMILY NOT NAMED (1 of 5)
OG IDs: 93069237
Best-hit-arabi-ID: AT3G52990.1
Best-hit-arabi-name: Pyruvate kinase family protein
Best-hit-arabi-symbol: nan
Gorai.006G158900.1.A
PCC=0.586, p=0.008
PCC=0.167, p=0.494
mRNA Profile
Protein Expression Profile
DPA

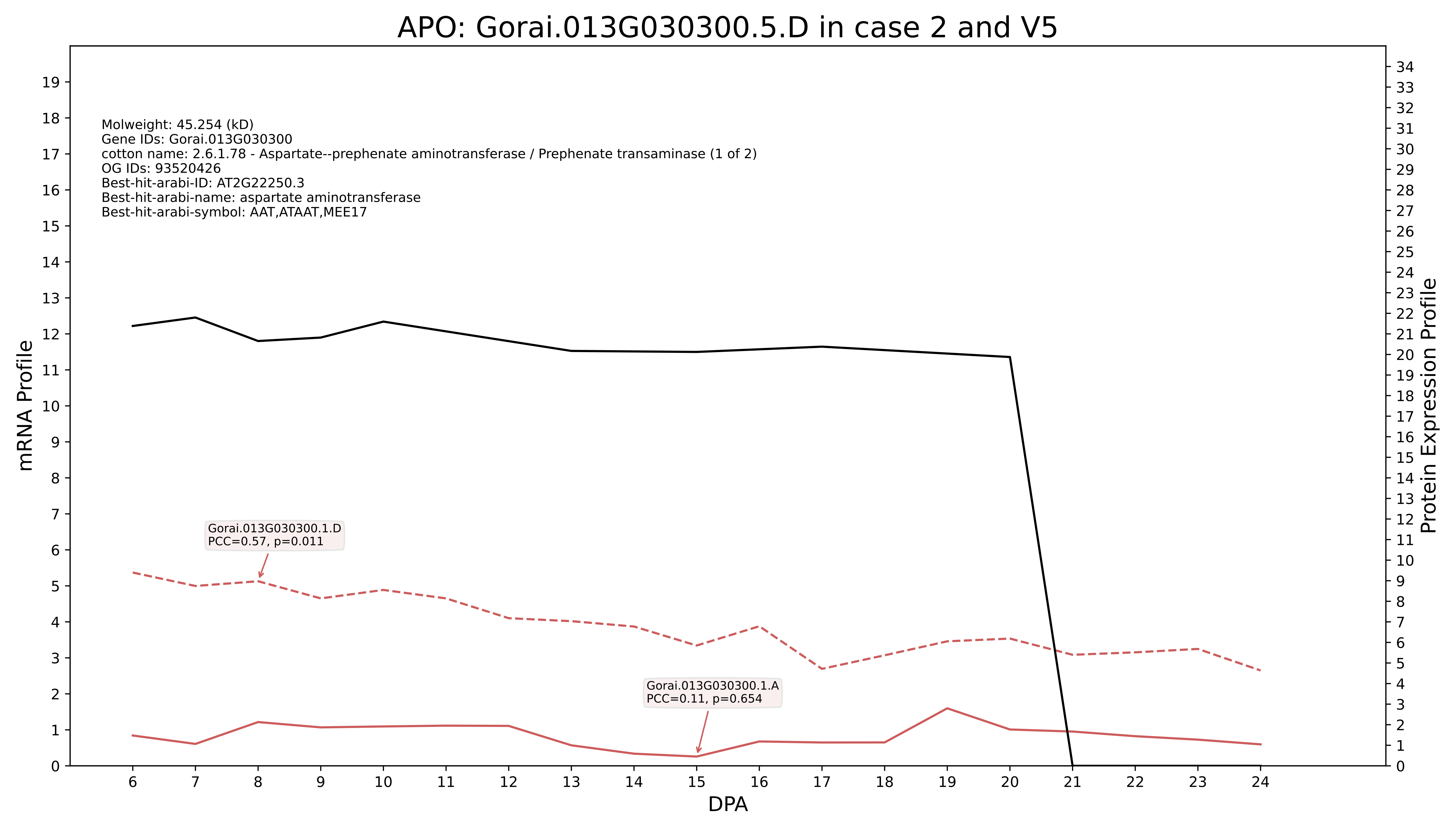

APO: Gorai.013G030300.5.D in case 2 and V5
Molweight: 45.254 (kD)
Gene IDs: Gorai.013G030300
cotton name: 2.6.1.78 - Aspartate--prephenate aminotransferase / Prephenate transaminase (1 of 2)
OG IDs: 93520426
Best-hit-arabi-ID: AT2G22250.3
Best-hit-arabi-name: aspartate aminotransferase
Best-hit-arabi-symbol: AAT,ATAAT,MEE17
Gorai.013G030300.1.D
PCC=0.57, p=0.011
Gorai.013G030300.1.A
PCC=0.11, p=0.654
mRNA Profile
Protein Expression Profile
DPA

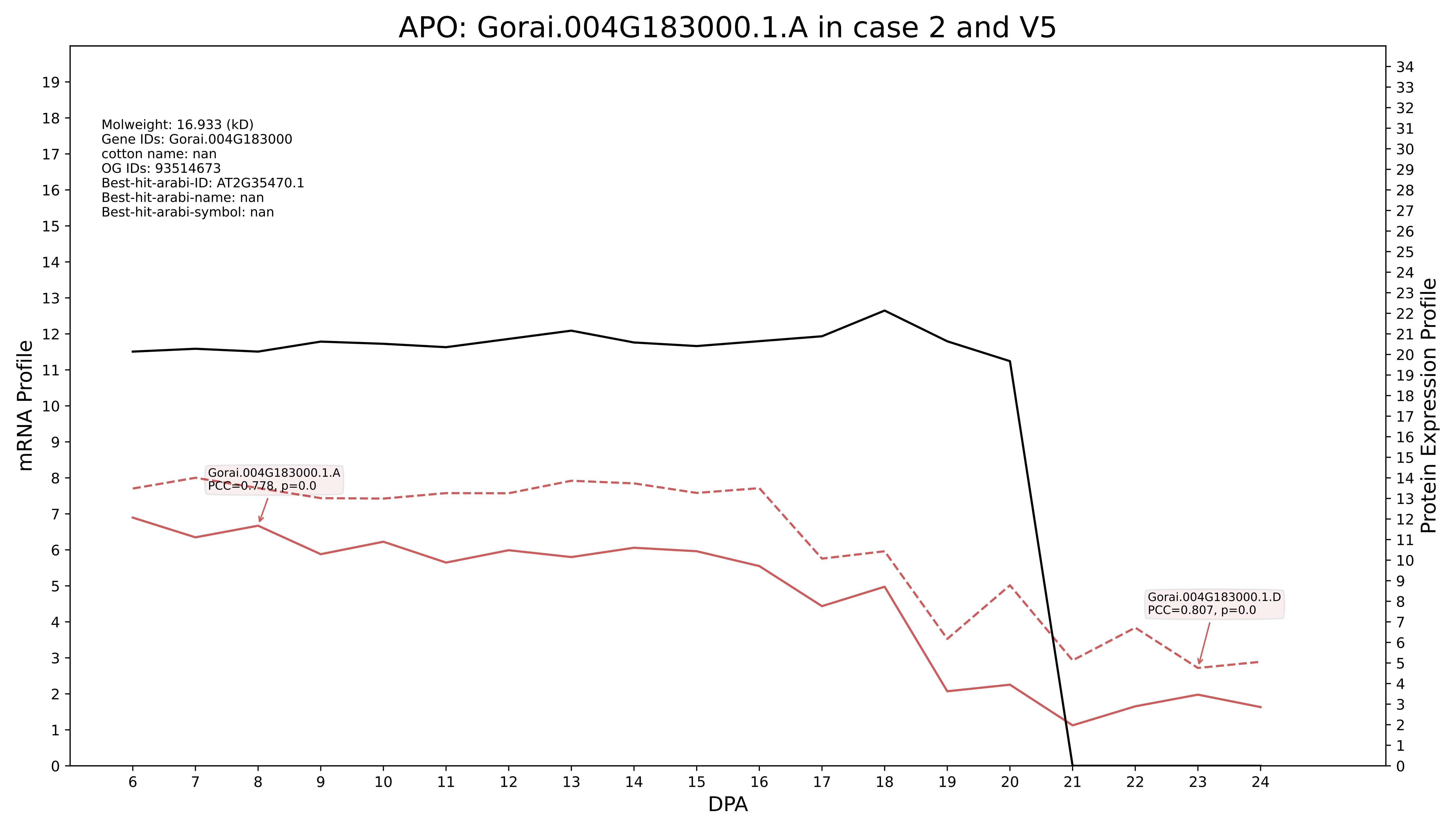
APO: Gorai.004G183000.1.A in case 2 and V5
Molweight: 16.933 (kD)
Gene IDs: Gorai.004G183000
cotton name: nan
OG IDs: 93514673
Best-hit-arabi-ID: AT2G35470.1
Best-hit-arabi-name: nan
Best-hit-arabi-symbol: nan
Gorai.004G183000.1.A
PCC=0.778, p=0.0
Gorai.004G183000.1.D
PCC=0.807, p=0.0
mRNA Profile
Protein Expression Profile
DPA

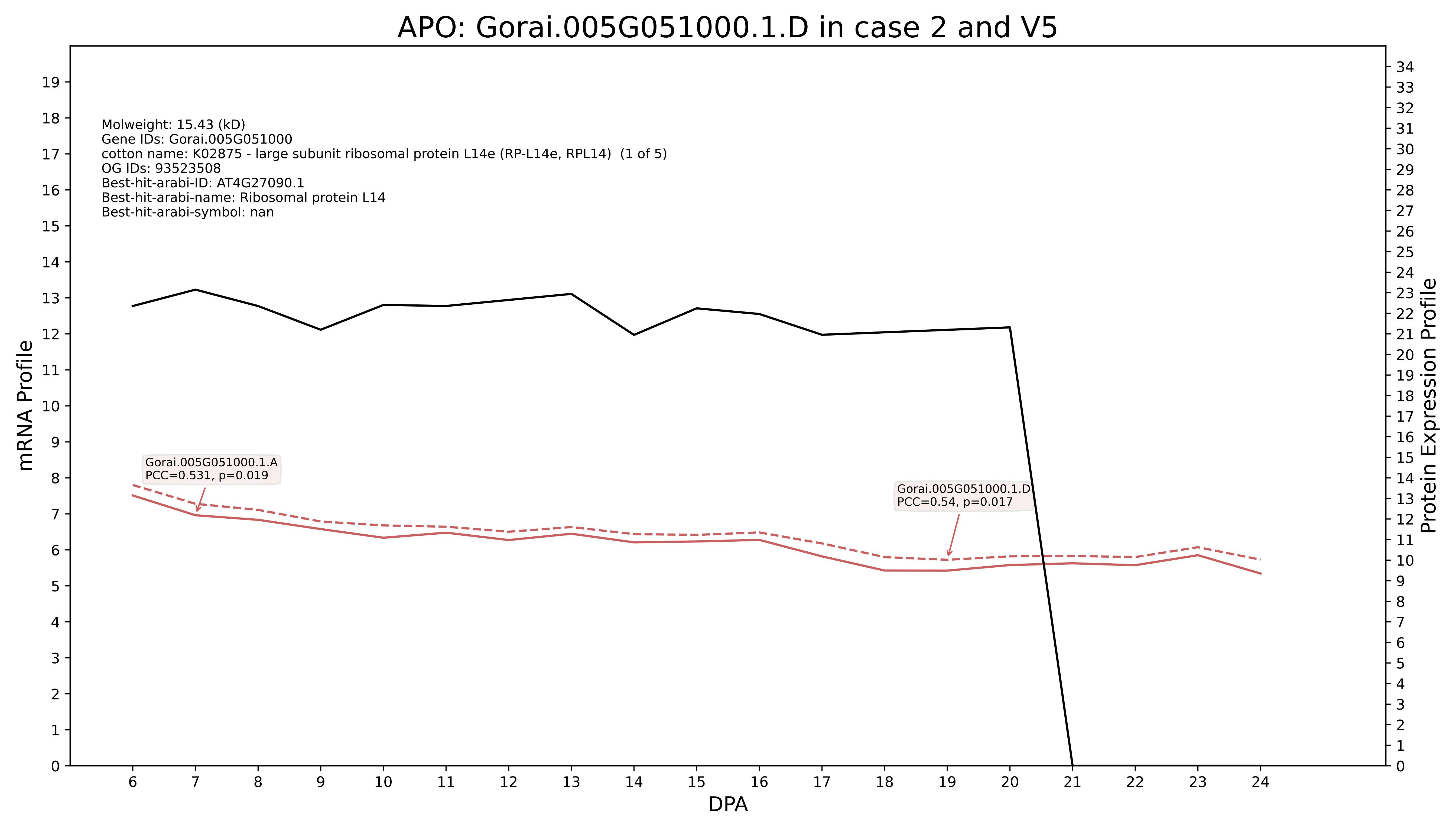

APO: Gorai.005G051000.1.D in case 2 and V5
Molweight: 15.43 (kD)
Gene IDs: Gorai.005G051000
cotton name: K02875 - large subunit ribosomal protein L14e (RP-L14e, RPL14) (1 of 5)
OG IDs: 93523508
Best-hit-arabi-ID: AT4G27090.1
Best-hit-arabi-name: Ribosomal protein L14
Best-hit-arabi-symbol: nan
Gorai.005G051000.1.A
PCC=0.531, p=0.019
Gorai.005G051000.1.D
PCC=0.54, p=0.017
mRNA Profile
Protein Expression Profile
DPA

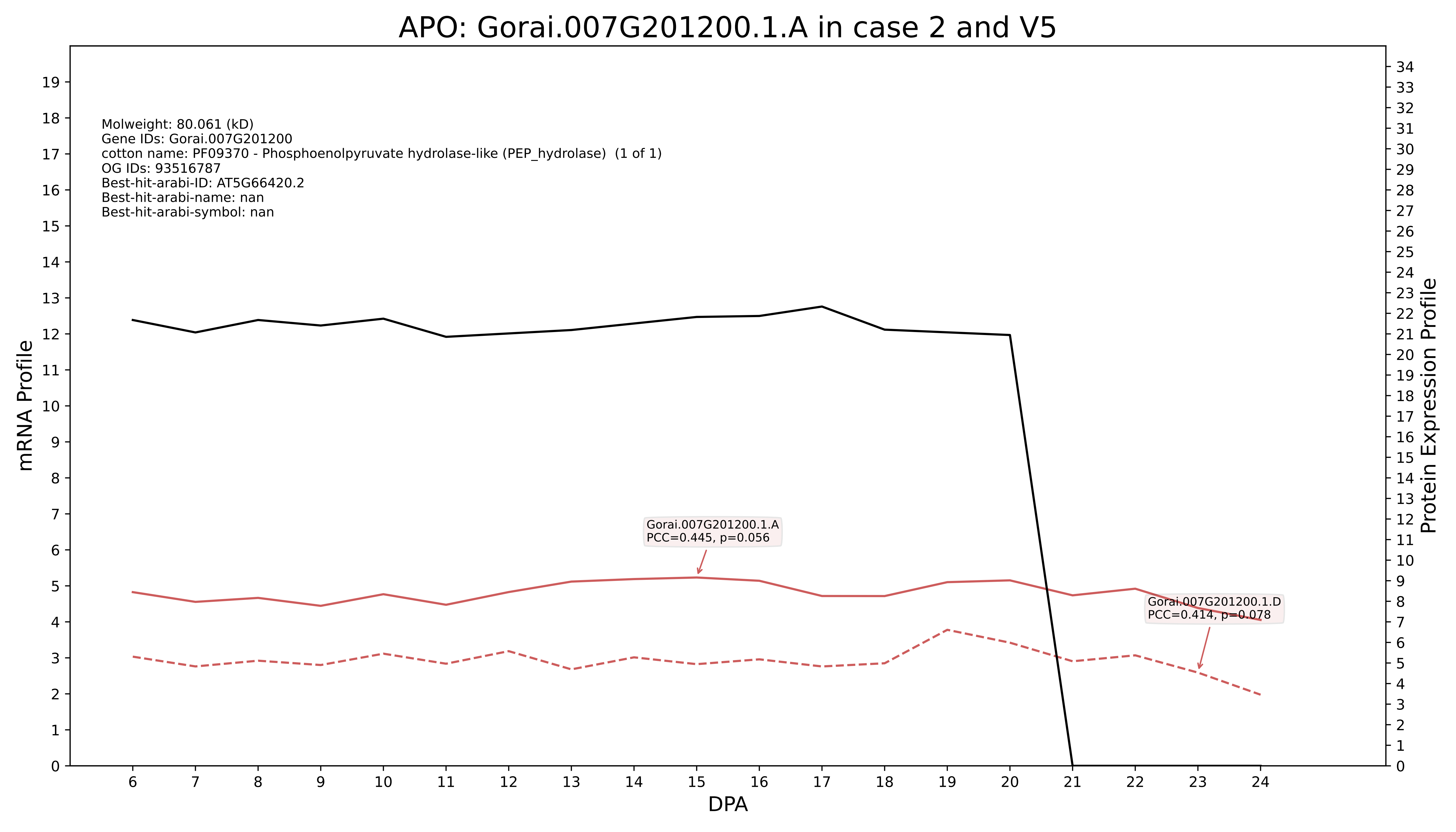
APO: Gorai.007G201200.1.A in case 2 and V5
Molweight: 80.061 (kD)
Gene IDs: Gorai.007G201200
cotton name: PF09370 - Phosphoenolpyruvate hydrolase-like (PEP_hydrolase) (1 of 1)
OG IDs: 93516787
Best-hit-arabi-ID: AT5G66420.2
Best-hit-arabi-name: nan
Best-hit-arabi-symbol: nan
Gorai.007G201200.1.A
PCC=0.445, p=0.056
Gorai.007G201200.1.D
PCC=0.414, p=0.078
mRNA Profile
Protein Expression Profile
DPA

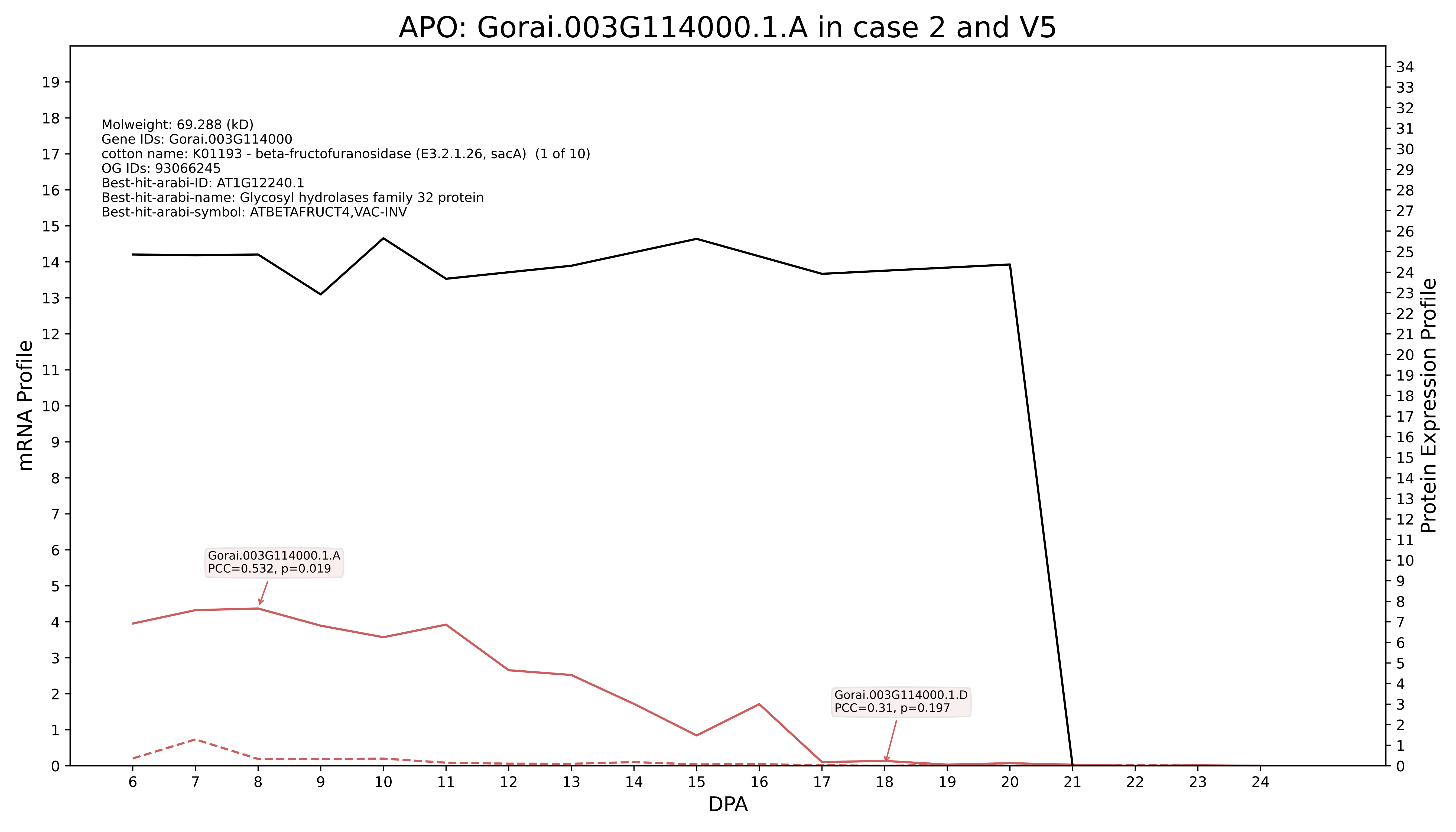

APO: Gorai.003G114000.1.A in case 2 and V5
Molweight: 69.288 (kD)
Gene IDs: Gorai.003G114000
cotton name: K01193 - beta-fructofuranosidase (E3.2.1.26, sacA) (1 of 10)
OG IDs: 93066245
Best-hit-arabi-ID: AT1G12240.1
Best-hit-arabi-name: Glycosyl hydrolases family 32 protein
Best-hit-arabi-symbol: ATBETAFRUCT4,VAC-INV
Gorai.003G114000.1.A
PCC=0.532, p=0.019
Gorai.003G114000.1.D
PCC=0.31, p=0.197
mRNA Profile
Protein Expression Profile
DPA

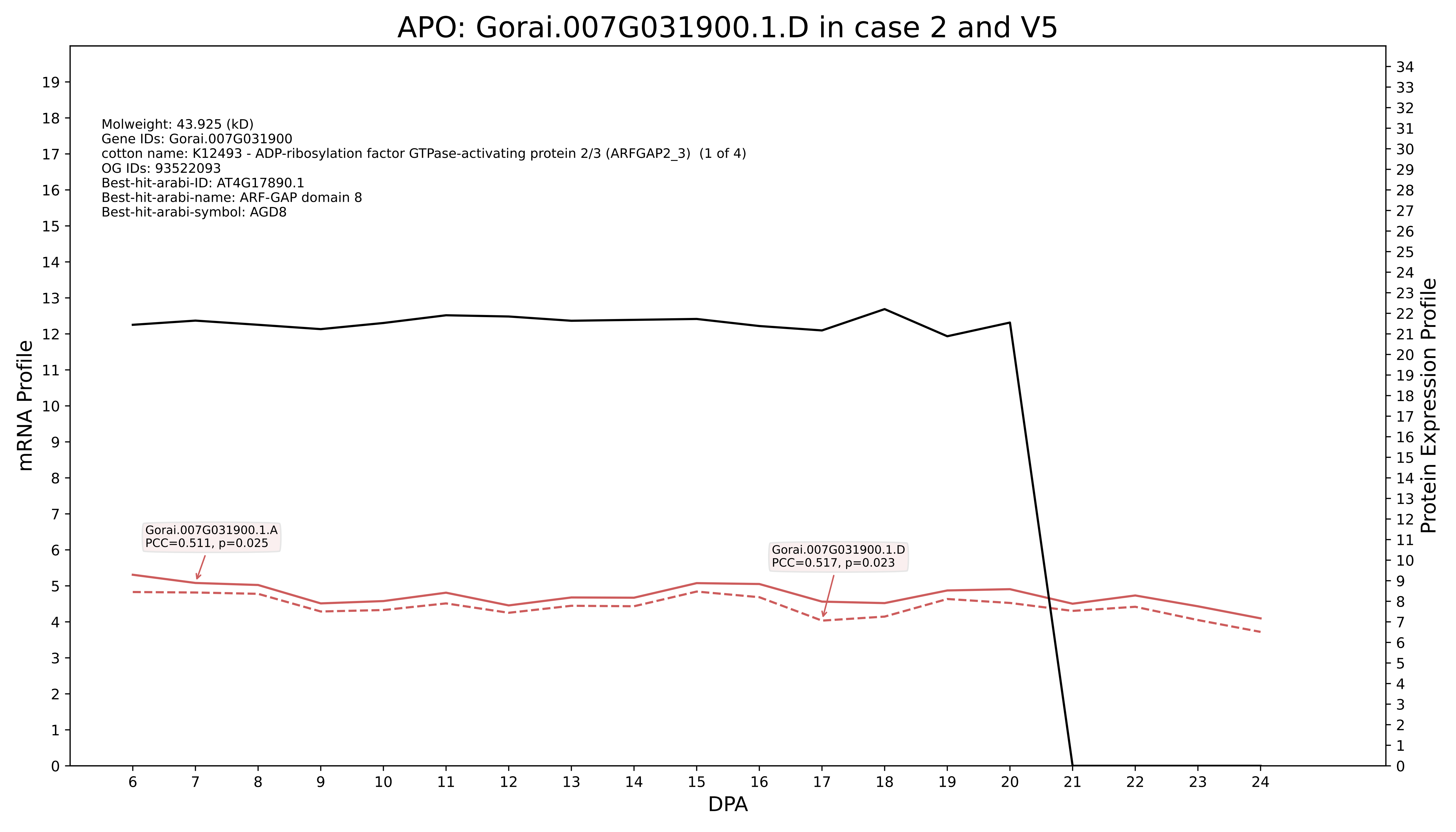
APO: Gorai.007G031900.1.D in case 2 and V5
Molweight: 43.925 (kD)
Gene IDs: Gorai.007G031900
cotton name: K12493 - ADP-ribosylation factor GTPase-activating protein 2/3 (ARFGAP2_3) (1 of 4)
OG IDs: 93522093
Best-hit-arabi-ID: AT4G17890.1
Best-hit-arabi-name: ARF-GAP domain 8
Best-hit-arabi-symbol: AGD8
Gorai.007G031900.1.A
PCC=0.511, p=0.025
Gorai.007G031900.1.D
PCC=0.517, p=0.023
mRNA Profile
Protein Expression Profile
DPA

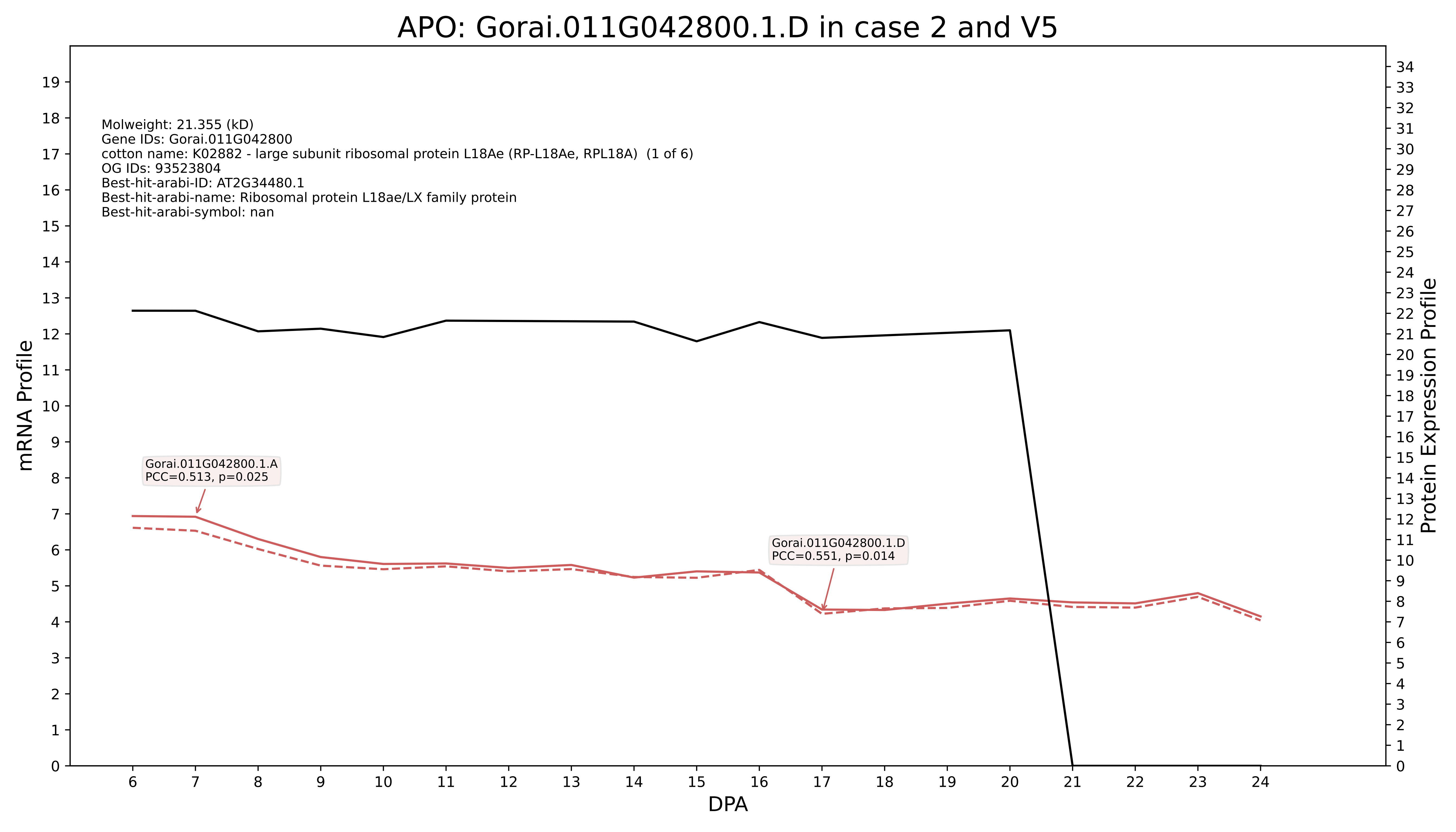

APO: Gorai.011G042800.1.D in case 2 and V5
Molweight: 21.355 (kD)
Gene IDs: Gorai.011G042800
cotton name: K02882 - large subunit ribosomal protein L18Ae (RP-L18Ae, RPL18A) (1 of 6)
OG IDs: 93523804
Best-hit-arabi-ID: AT2G34480.1
Best-hit-arabi-name: Ribosomal protein L18ae/LX family protein
Best-hit-arabi-symbol: nan
Gorai.011G042800.1.A
PCC=0.513, p=0.025
Gorai.011G042800.1.D
PCC=0.551, p=0.014
mRNA Profile
Protein Expression Profile
DPA

APO: Gorai.001G229200.2.D in case 2 and V5

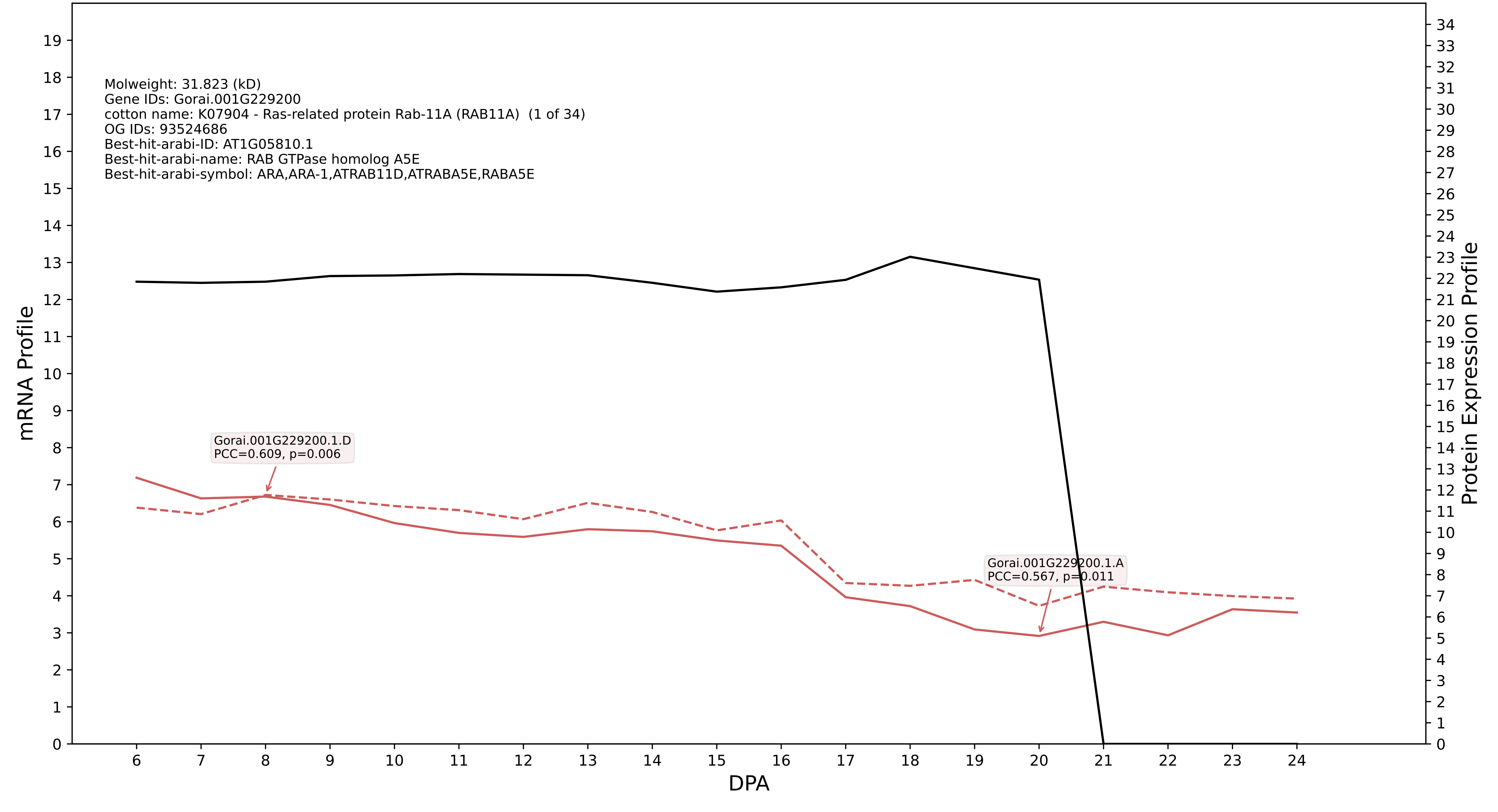

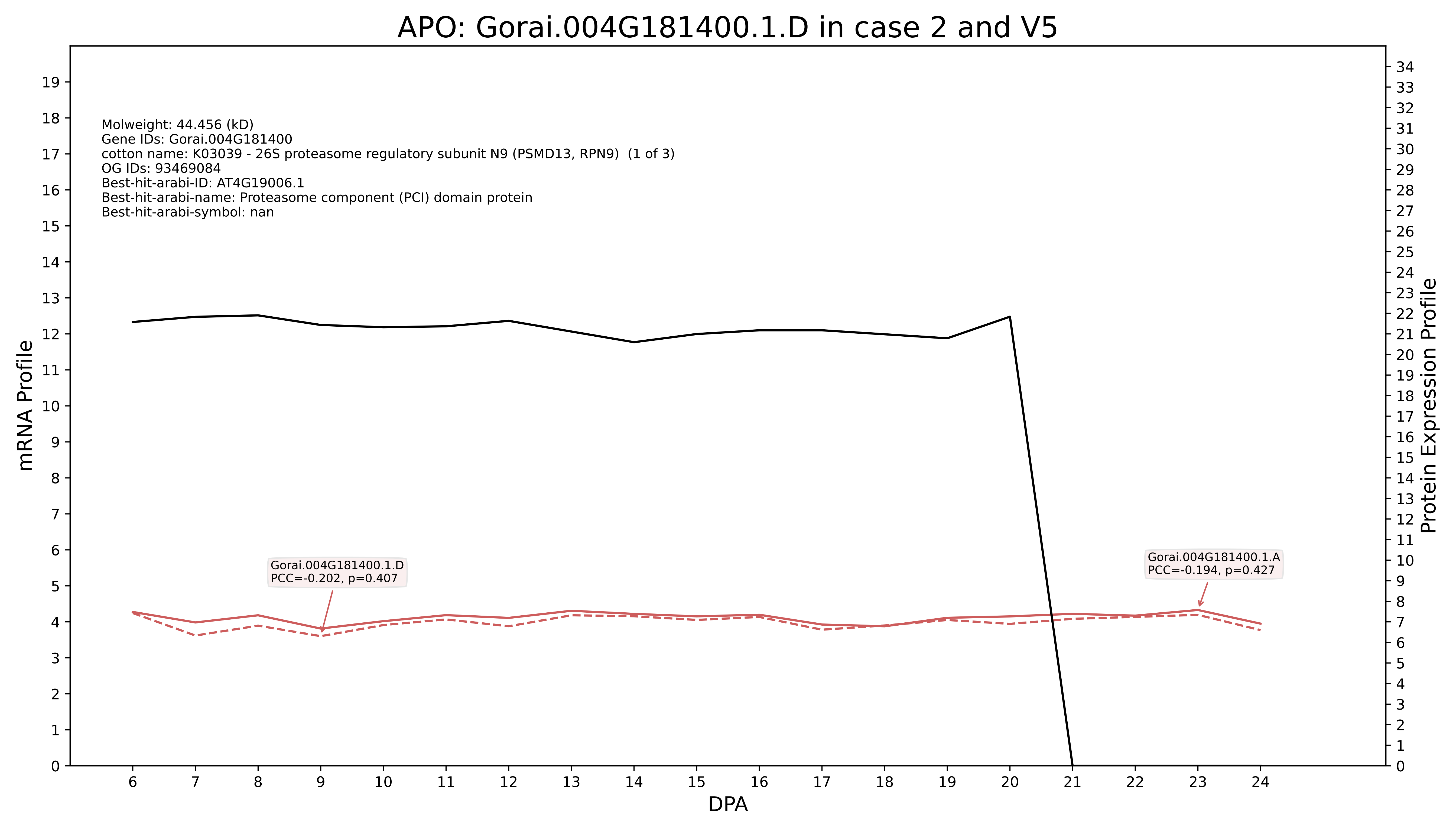
APO: Gorai.004G181400.1.D in case 2 and V5
Molweight: 44.456 (kD)
Gene IDs: Gorai.004G181400
cotton name: K03039 - 26S proteasome regulatory subunit N9 (PSMD13, RPN9) (1 of 3)
OG IDs: 93469084
Best-hit-arabi-ID: AT4G19006.1
Best-hit-arabi-name: Proteasome component (PCI) domain protein
Best-hit-arabi-symbol: nan
Gorai.004G181400.1.D
PCC=-0.202, p=0.407
Gorai.004G181400.1.A
PCC=-0.194, p=0.427
mRNA Profile
Protein Expression Profile
DPA
0
1
2
3
4
5
6
7
8
9
10
11
12
13
14
15
16
17
18
19
20
21
22
23
24
25
26
27
28
29
30
31
32
33
34

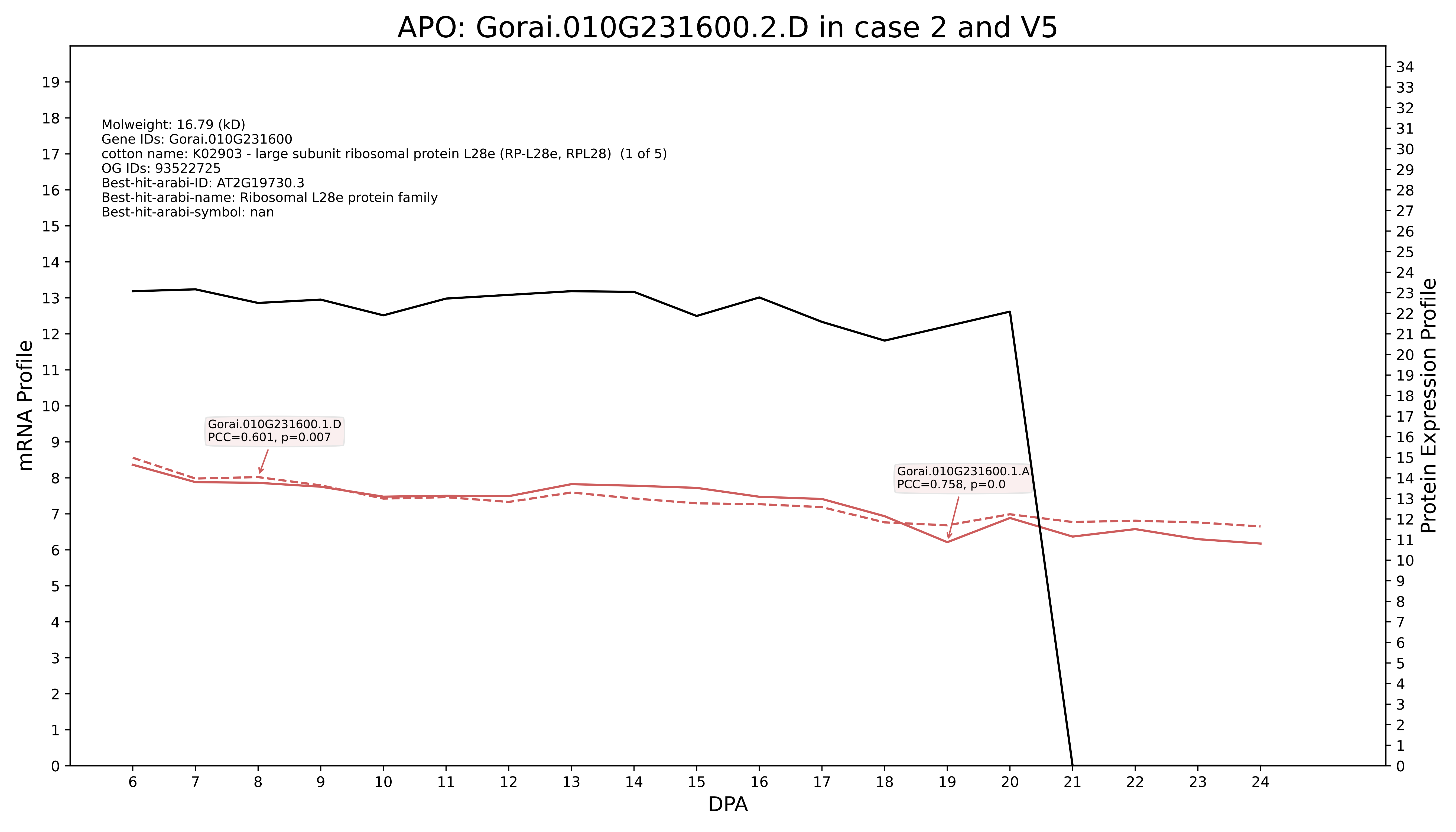

APO: Gorai.010G231600.2.D in case 2 and V5
Molweight: 16.79 (kD)
Gene IDs: Gorai.010G231600
cotton name: K02903 - large subunit ribosomal protein L28e (RP-L28e, RPL28) (1 of 5)
OG IDs: 93522725
Best-hit-arabi-ID: AT2G19730.3
Best-hit-arabi-name: Ribosomal L28e protein family
Best-hit-arabi-symbol: nan
Gorai.010G231600.1.D
PCC=0.601, p=0.007
Gorai.010G231600.1.A
PCC=0.758, p=0.0
mRNA Profile
Protein Expression Profile
DPA

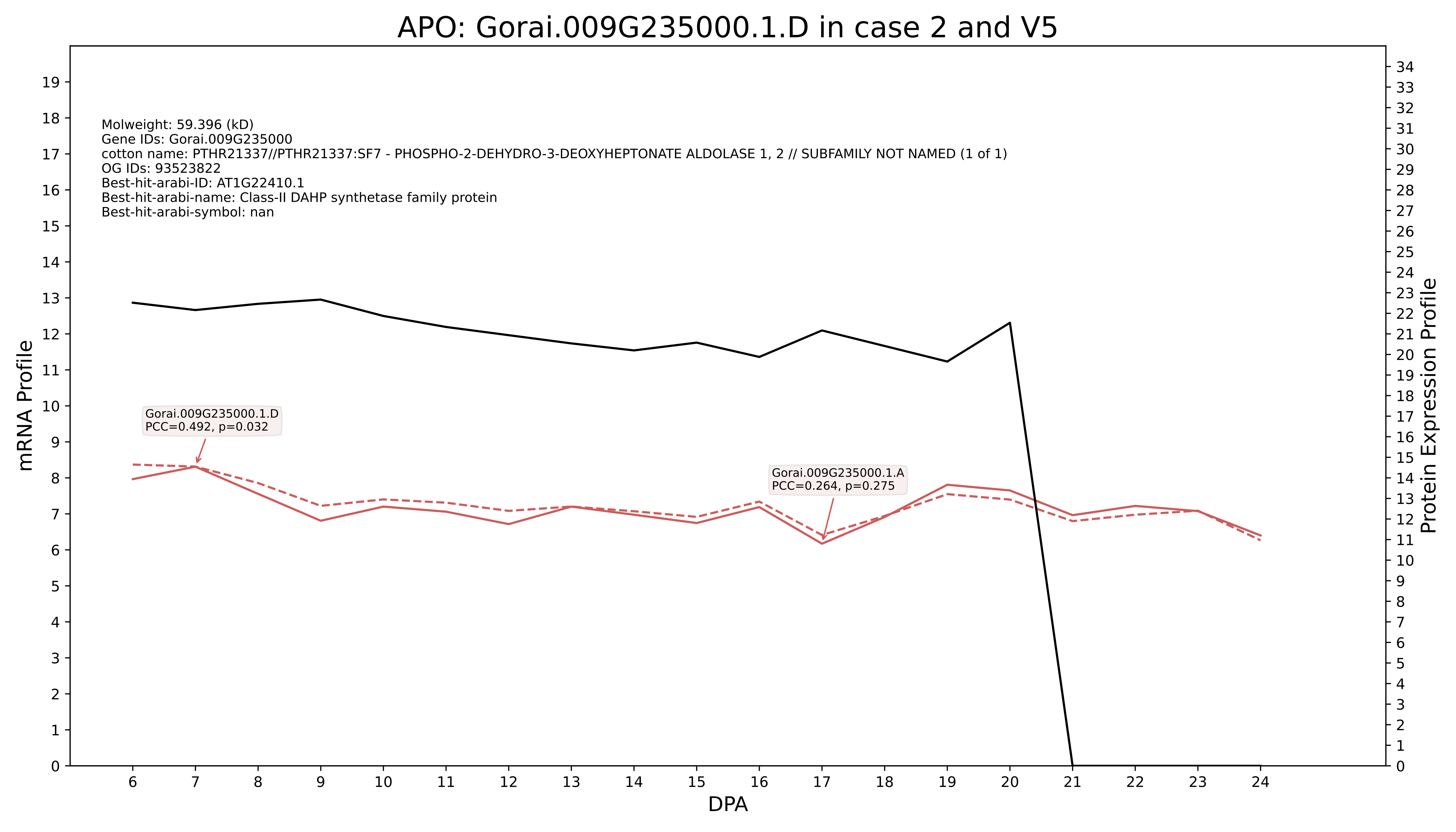
APO: Gorai.009G235000.1.D in case 2 and V5
Molweight: 59.396 (kD)
Gene IDs: Gorai.009G235000
cotton name: PTHR21337//PTHR21337:SF7 - PHOSPHO-2-DEHYDRO-3-DEOXYHEPTONATE ALDOLASE 1, 2 // SUBFAMILY NOT NAMED (1 of 1)
OG IDs: 93523822
Best-hit-arabi-ID: AT1G22410.1
Best-hit-arabi-name: Class-II DAHP synthetase family protein
Best-hit-arabi-symbol: nan
Gorai.009G235000.1.D
PCC=0.492, p=0.032
Gorai.009G235000.1.A
PCC=0.264, p=0.275
mRNA Profile
Protein Expression Profile
DPA

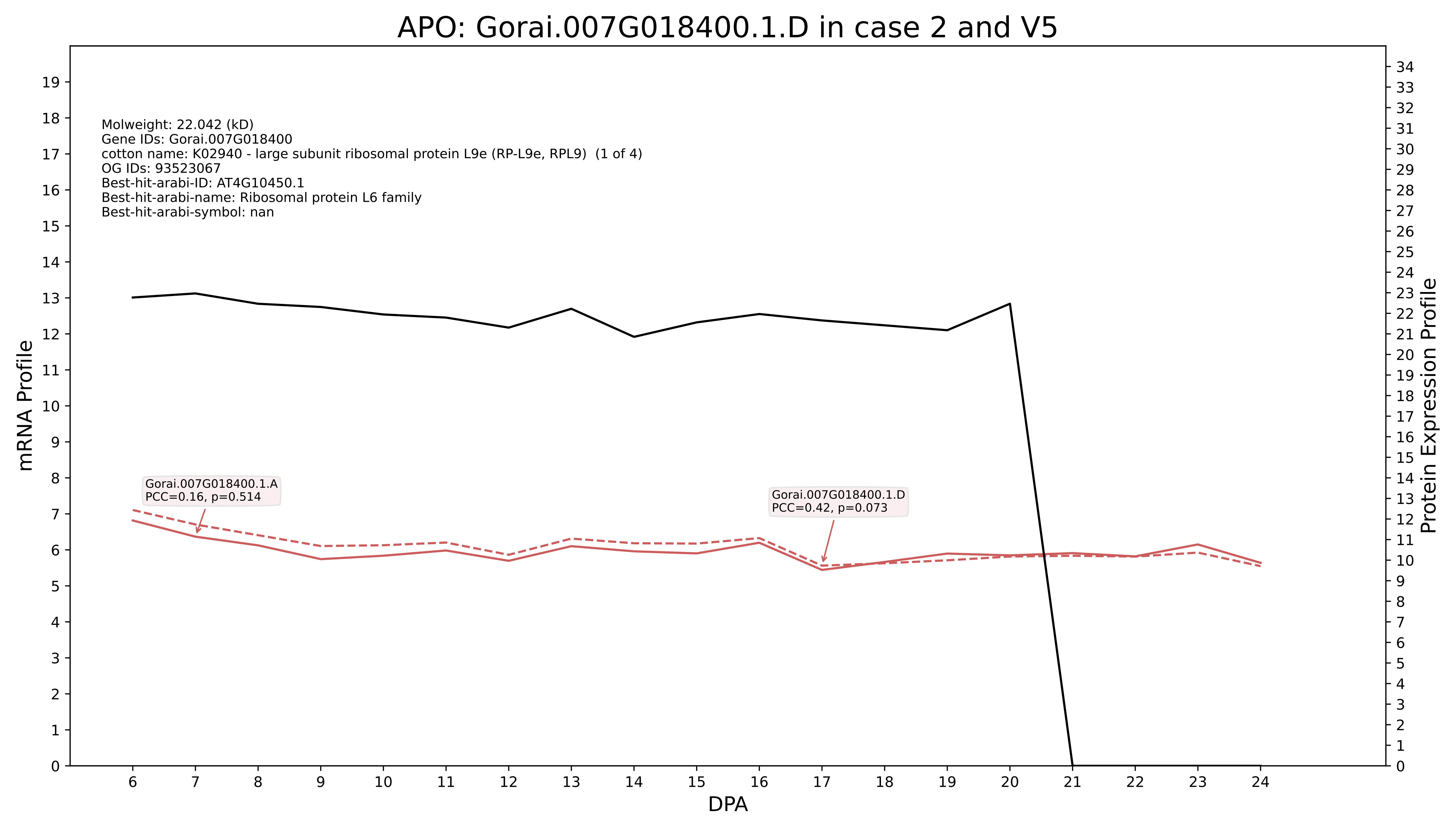
APO: Gorai.007G018400.1.D in case 2 and V5
Molweight: 22.042 (kD)
Gene IDs: Gorai.007G018400
cotton name: K02940 - large subunit ribosomal protein L9e (RP-L9e, RPL9) (1 of 4)
OG IDs: 93523067
Best-hit-arabi-ID: AT4G10450.1
Best-hit-arabi-name: Ribosomal protein L6 family
Best-hit-arabi-symbol: nan
Gorai.007G018400.1.A
PCC=0.16, p=0.514
Gorai.007G018400.1.D
PCC=0.42, p=0.073
mRNA Profile
Protein Expression Profile
DPA
0
1
2
3
4
5
6
7
8
9
10
11
12
13
14
15
16
17
18
19
0
1
2
3
4
5
6
7
8
9
10
11
12
13
14
15
16
17
18
19
20
21
22
23
24
25
26
27
28
29
30
31
32
33
34
6
7
8
9
10
11
12
13
14
15
16
17
18
19
20
21
22
23
24

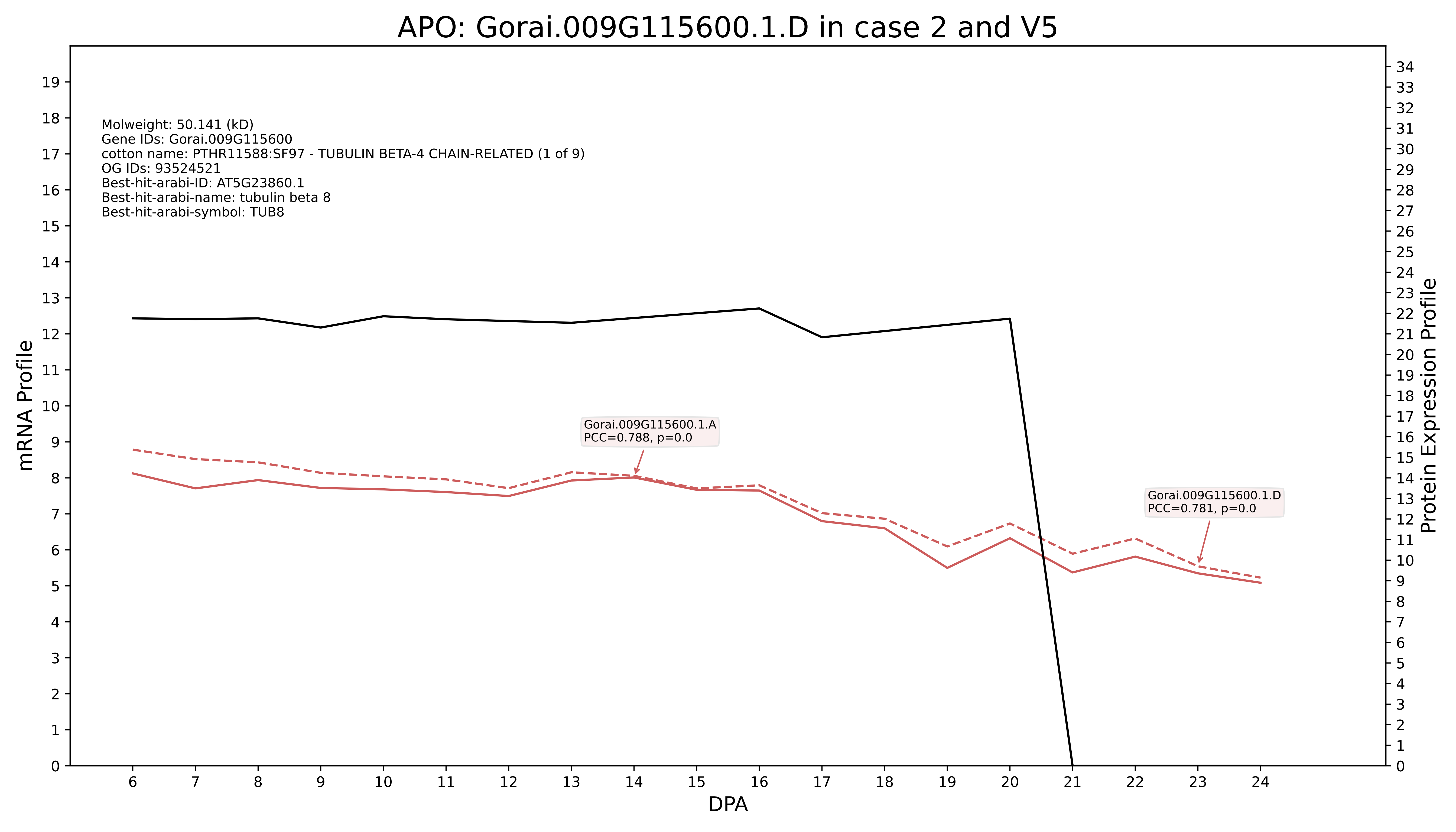

APO: Gorai.009G115600.1.D in case 2 and V5
Molweight: 50.141 (kD)
Gene IDs: Gorai.009G115600
cotton name: PTHR11588:SF97 - TUBULIN BETA-4 CHAIN-RELATED (1 of 9)
OG IDs: 93524521
Best-hit-arabi-ID: AT5G23860.1
Best-hit-arabi-name: tubulin beta 8
Best-hit-arabi-symbol: TUB8
Gorai.009G115600.1.A
PCC=0.788, p=0.0
Gorai.009G115600.1.D
PCC=0.781, p=0.0
mRNA Profile
Protein Expression Profile
DPA

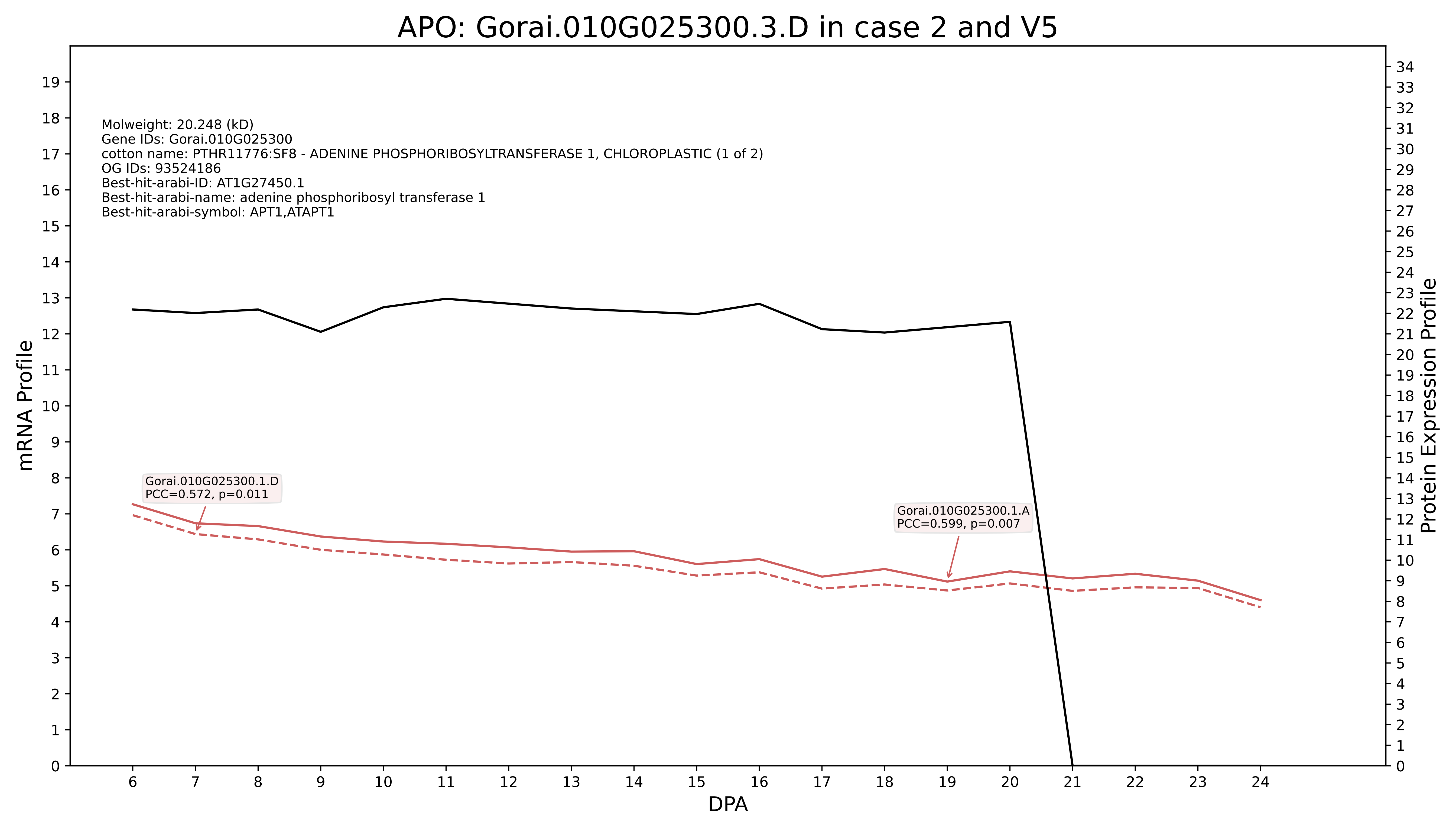
APO: Gorai.010G025300.3.D in case 2 and V5
Molweight: 20.248 (kD)
Gene IDs: Gorai.010G025300
cotton name: PTHR11776:SF8 - ADENINE PHOSPHORIBOSYLTRANSFERASE 1, CHLOROPLASTIC (1 of 2)
OG IDs: 93524186
Best-hit-arabi-ID: AT1G27450.1
Best-hit-arabi-name: adenine phosphoribosyl transferase 1
Best-hit-arabi-symbol: APT1,ATAPT1
Gorai.010G025300.1.D
PCC=0.572, p=0.011
Gorai.010G025300.1.A
PCC=0.599, p=0.007
mRNA Profile
Protein Expression Profile
DPA

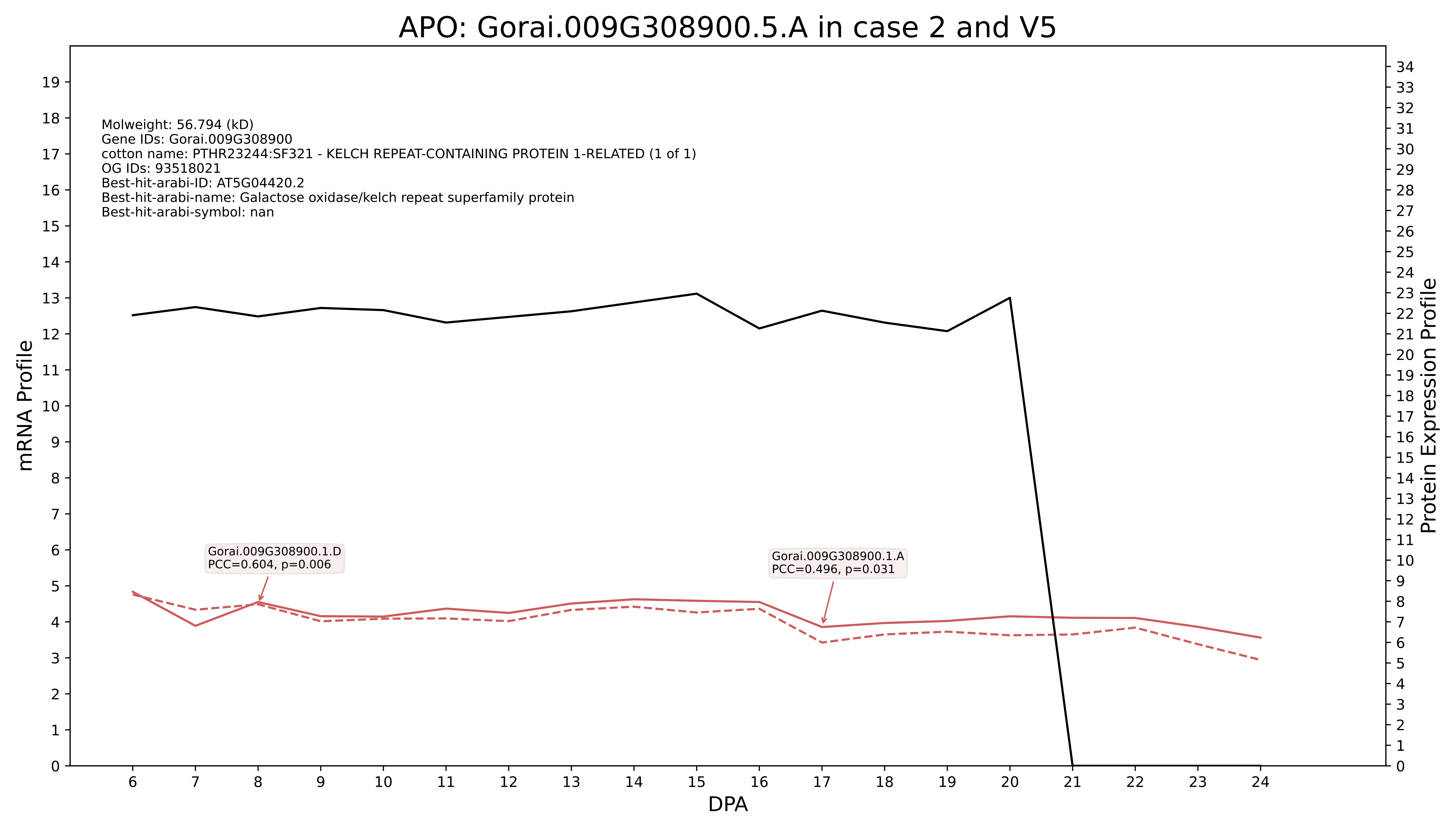

APO: Gorai.009G308900.5.A in case 2 and V5
Molweight: 56.794 (kD)
Gene IDs: Gorai.009G308900
cotton name: PTHR23244:SF321 - KELCH REPEAT-CONTAINING PROTEIN 1-RELATED (1 of 1)
OG IDs: 93518021
Best-hit-arabi-ID: AT5G04420.2
Best-hit-arabi-name: Galactose oxidase/kelch repeat superfamily protein
Best-hit-arabi-symbol: nan
Gorai.009G308900.1.D
PCC=0.604, p=0.006
Gorai.009G308900.1.A
PCC=0.496, p=0.031
mRNA Profile
Protein Expression Profile
DPA

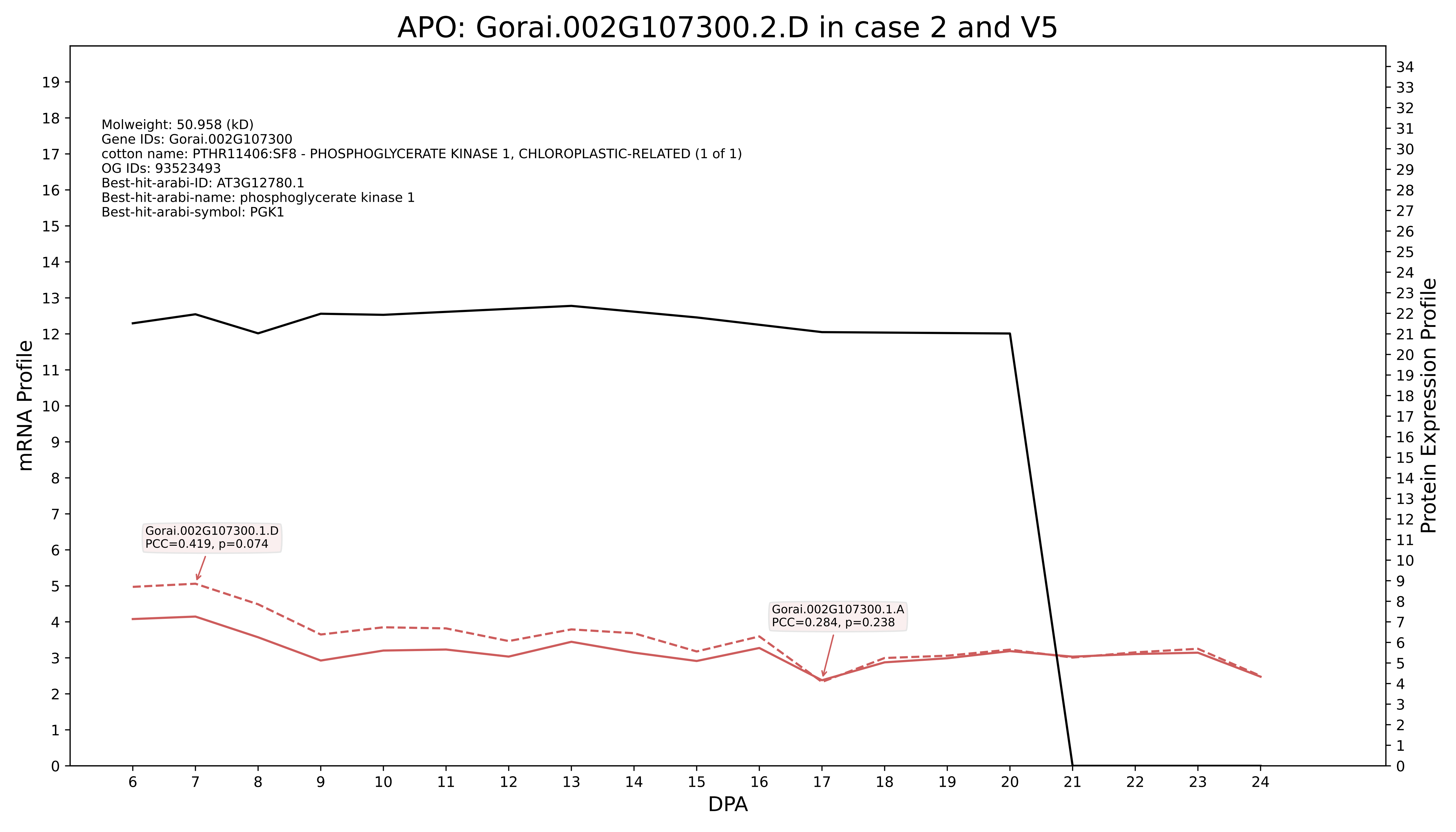
APO: Gorai.002G107300.2.D in case 2 and V5
Molweight: 50.958 (kD)
Gene IDs: Gorai.002G107300
cotton name: PTHR11406:SF8 - PHOSPHOGLYCERATE KINASE 1, CHLOROPLASTIC-RELATED (1 of 1)
OG IDs: 93523493
Best-hit-arabi-ID: AT3G12780.1
Best-hit-arabi-name: phosphoglycerate kinase 1
Best-hit-arabi-symbol: PGK1
Gorai.002G107300.1.D
PCC=0.419, p=0.074
Gorai.002G107300.1.A
PCC=0.284, p=0.238
mRNA Profile
Protein Expression Profile
DPA

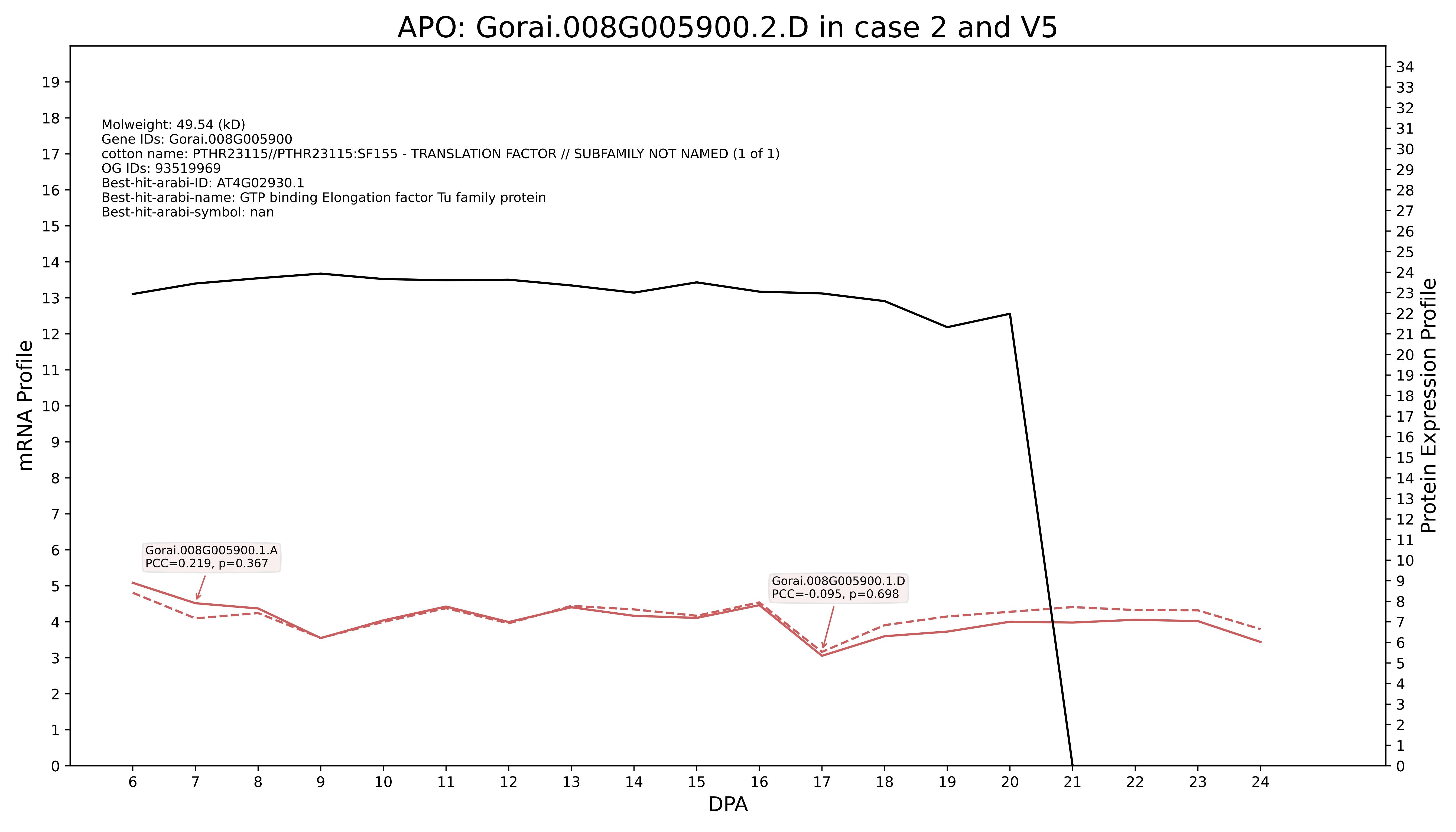
APO: Gorai.008G005900.2.D in case 2 and V5
Molweight: 49.54 (kD)
Gene IDs: Gorai.008G005900
cotton name: PTHR23115//PTHR23115:SF155 - TRANSLATION FACTOR // SUBFAMILY NOT NAMED (1 of 1)
OG IDs: 93519969
Best-hit-arabi-ID: AT4G02930.1
Best-hit-arabi-name: GTP binding Elongation factor Tu family protein
Best-hit-arabi-symbol: nan
Gorai.008G005900.1.A
PCC=0.219, p=0.367
Gorai.008G005900.1.D
PCC=-0.095, p=0.698
mRNA Profile
Protein Expression Profile
DPA

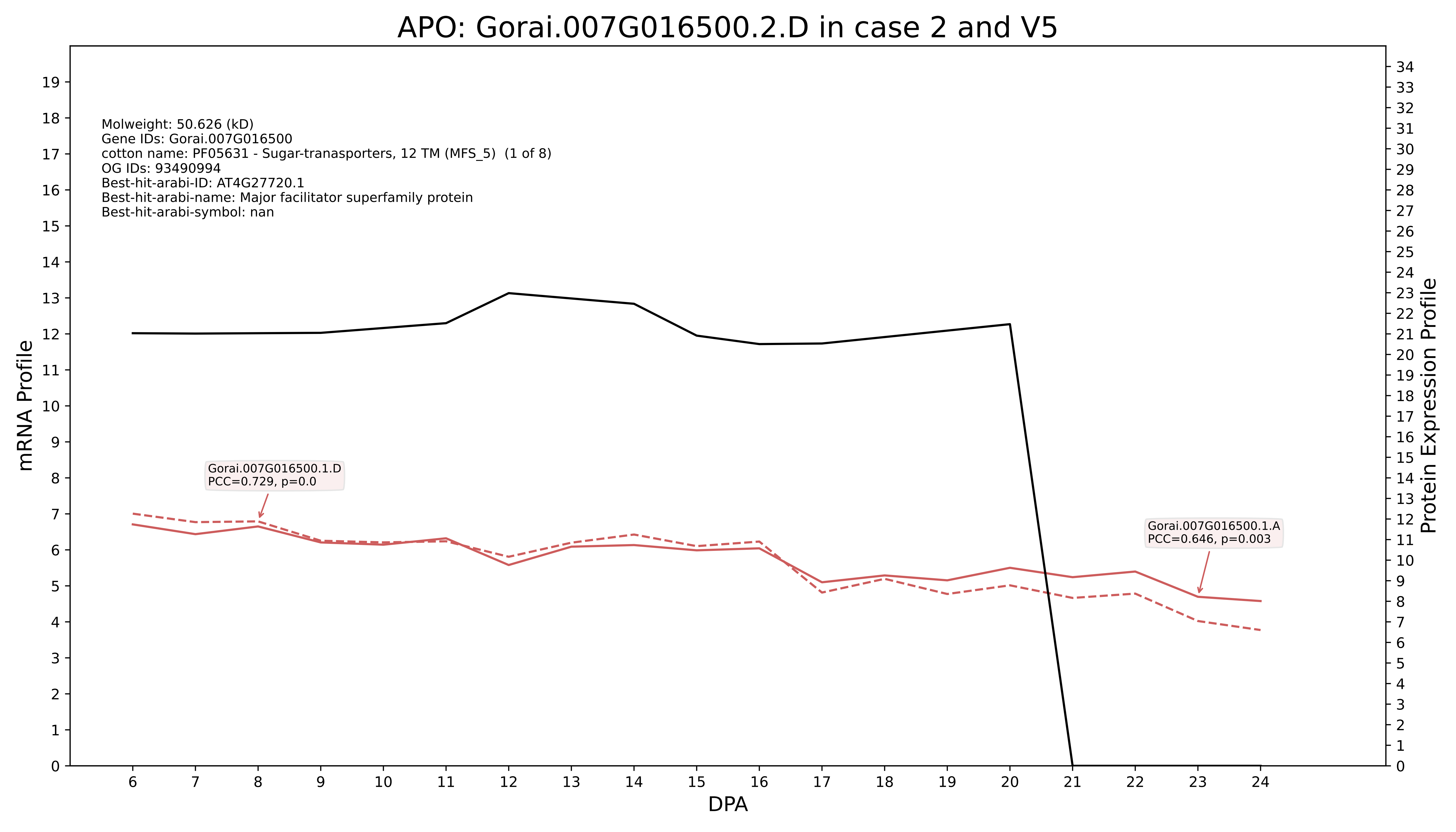

APO: Gorai.007G016500.2.D in case 2 and V5
Molweight: 50.626 (kD)
Gene IDs: Gorai.007G016500
cotton name: PF05631 - Sugar-tranasporters, 12 TM (MFS_5) (1 of 8)
OG IDs: 93490994
Best-hit-arabi-ID: AT4G27720.1
Best-hit-arabi-name: Major facilitator superfamily protein
Best-hit-arabi-symbol: nan
Gorai.007G016500.1.D
PCC=0.729, p=0.0
Gorai.007G016500.1.A
PCC=0.646, p=0.003
mRNA Profile
Protein Expression Profile
DPA
0
1
2
3
4
5
6
7
8
9
10
11
12
13
14
15
16
17
18
19
20
21
22
23
24
25
26
27
28
29
30
31
32
33
34

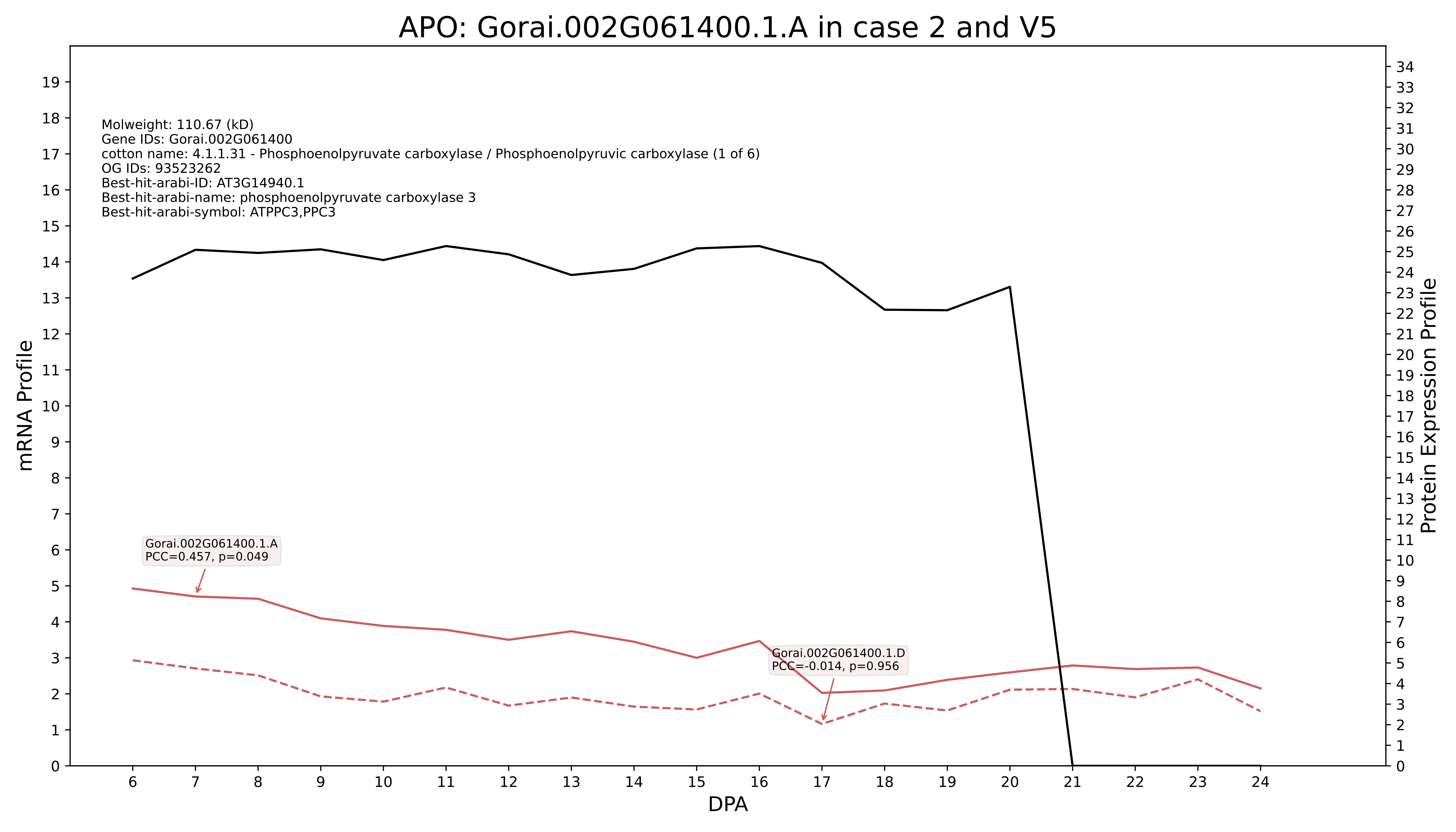

APO: Gorai.002G061400.1.A in case 2 and V5
Molweight: 110.67 (kD)
Gene IDs: Gorai.002G061400
cotton name: 4.1.1.31 - Phosphoenolpyruvate carboxylase / Phosphoenolpyruvic carboxylase (1 of 6)
OG IDs: 93523262
Best-hit-arabi-ID: AT3G14940.1
Best-hit-arabi-name: phosphoenolpyruvate carboxylase 3
Best-hit-arabi-symbol: ATPPC3,PPC3
Gorai.002G061400.1.A
PCC=0.457, p=0.049
Gorai.002G061400.1.D
PCC=-0.014, p=0.956
mRNA Profile
Protein Expression Profile
DPA

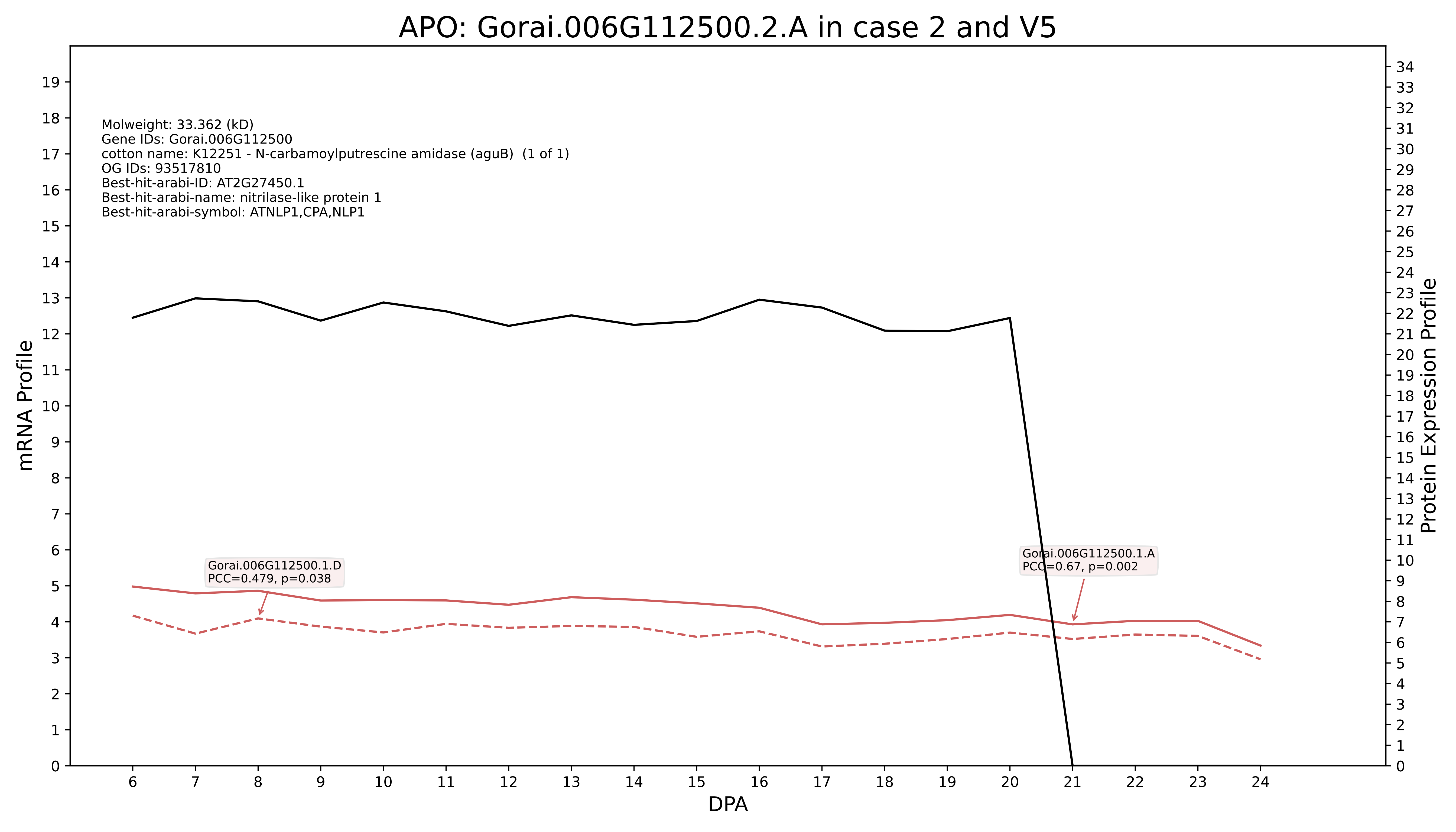

APO: Gorai.006G112500.2.A in case 2 and V5
Molweight: 33.362 (kD)
Gene IDs: Gorai.006G112500
cotton name: K12251 - N-carbamoylputrescine amidase (aguB) (1 of 1)
OG IDs: 93517810
Best-hit-arabi-ID: AT2G27450.1
Best-hit-arabi-name: nitrilase-like protein 1
Best-hit-arabi-symbol: ATNLP1,CPA,NLP1
Gorai.006G112500.1.D
PCC=0.479, p=0.038
Gorai.006G112500.1.A
PCC=0.67, p=0.002
mRNA Profile
Protein Expression Profile
DPA

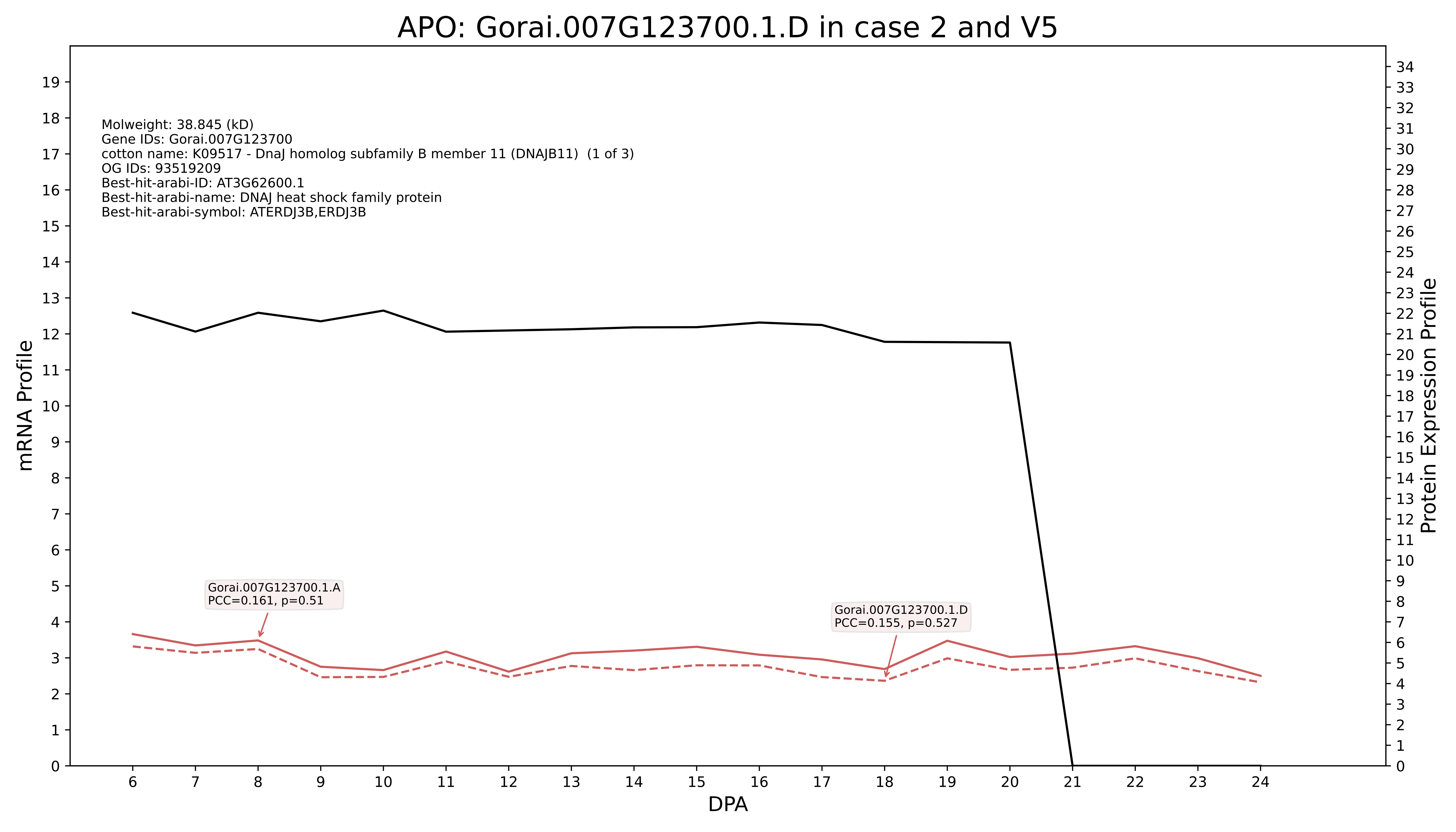
APO: Gorai.007G123700.1.D in case 2 and V5
Molweight: 38.845 (kD)
Gene IDs: Gorai.007G123700
cotton name: K09517 - DnaJ homolog subfamily B member 11 (DNAJB11) (1 of 3)
OG IDs: 93519209
Best-hit-arabi-ID: AT3G62600.1
Best-hit-arabi-name: DNAJ heat shock family protein
Best-hit-arabi-symbol: ATERDJ3B,ERDJ3B
Gorai.007G123700.1.A
PCC=0.161, p=0.51
Gorai.007G123700.1.D
PCC=0.155, p=0.527
mRNA Profile
Protein Expression Profile
DPA

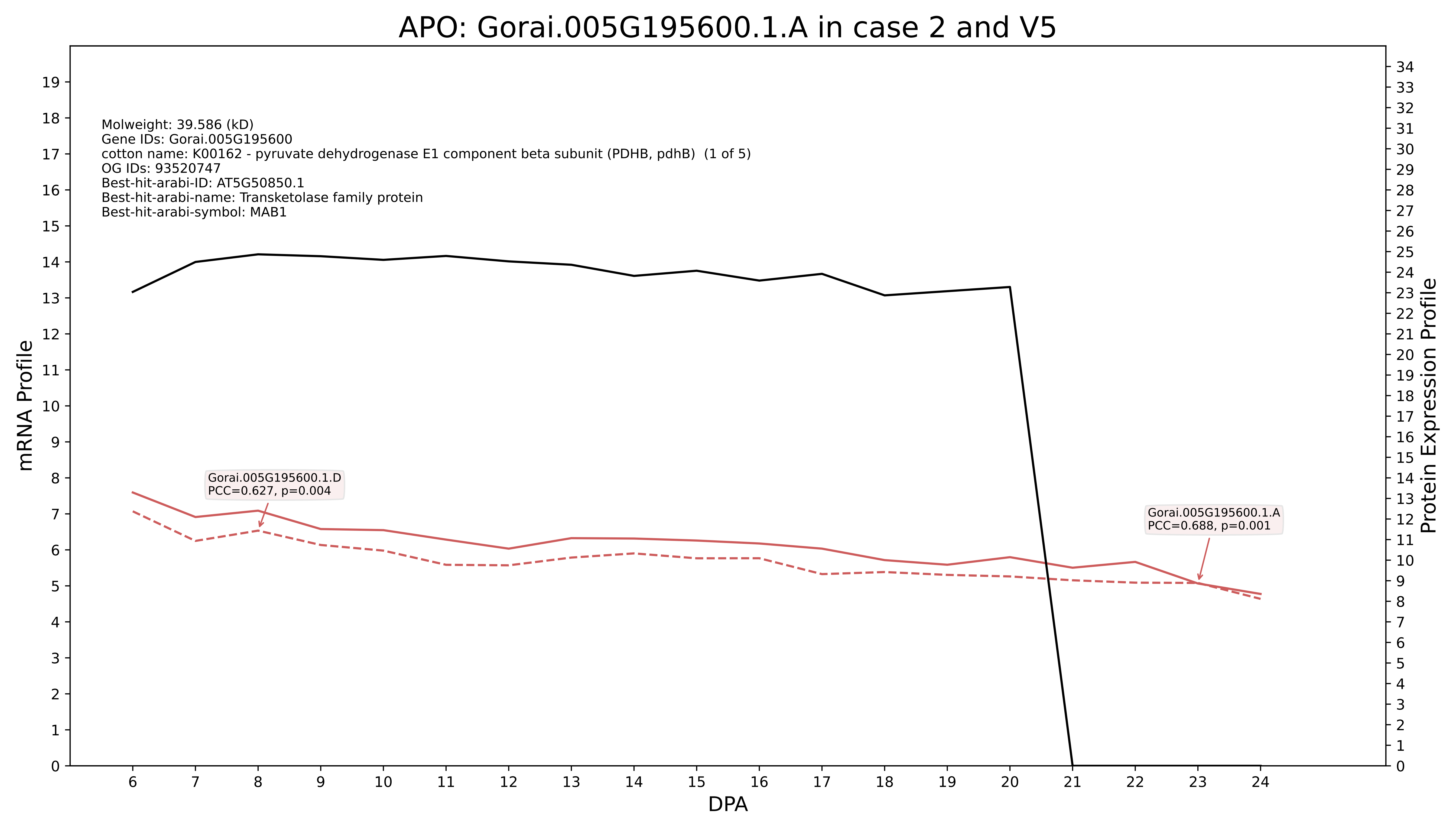
APO: Gorai.005G195600.1.A in case 2 and V5
Molweight: 39.586 (kD)
Gene IDs: Gorai.005G195600
cotton name: K00162 - pyruvate dehydrogenase E1 component beta subunit (PDHB, pdhB) (1 of 5)
OG IDs: 93520747
Best-hit-arabi-ID: AT5G50850.1
Best-hit-arabi-name: Transketolase family protein
Best-hit-arabi-symbol: MAB1
Gorai.005G195600.1.D
PCC=0.627, p=0.004
Gorai.005G195600.1.A
PCC=0.688, p=0.001
mRNA Profile
Protein Expression Profile
DPA
0
1
2
3
4
5
6
7
8
9
10
11
12
13
14
15
16
17
18
19
20
21
22
23
24
25
26
27
28
29
30
31
32
33
34

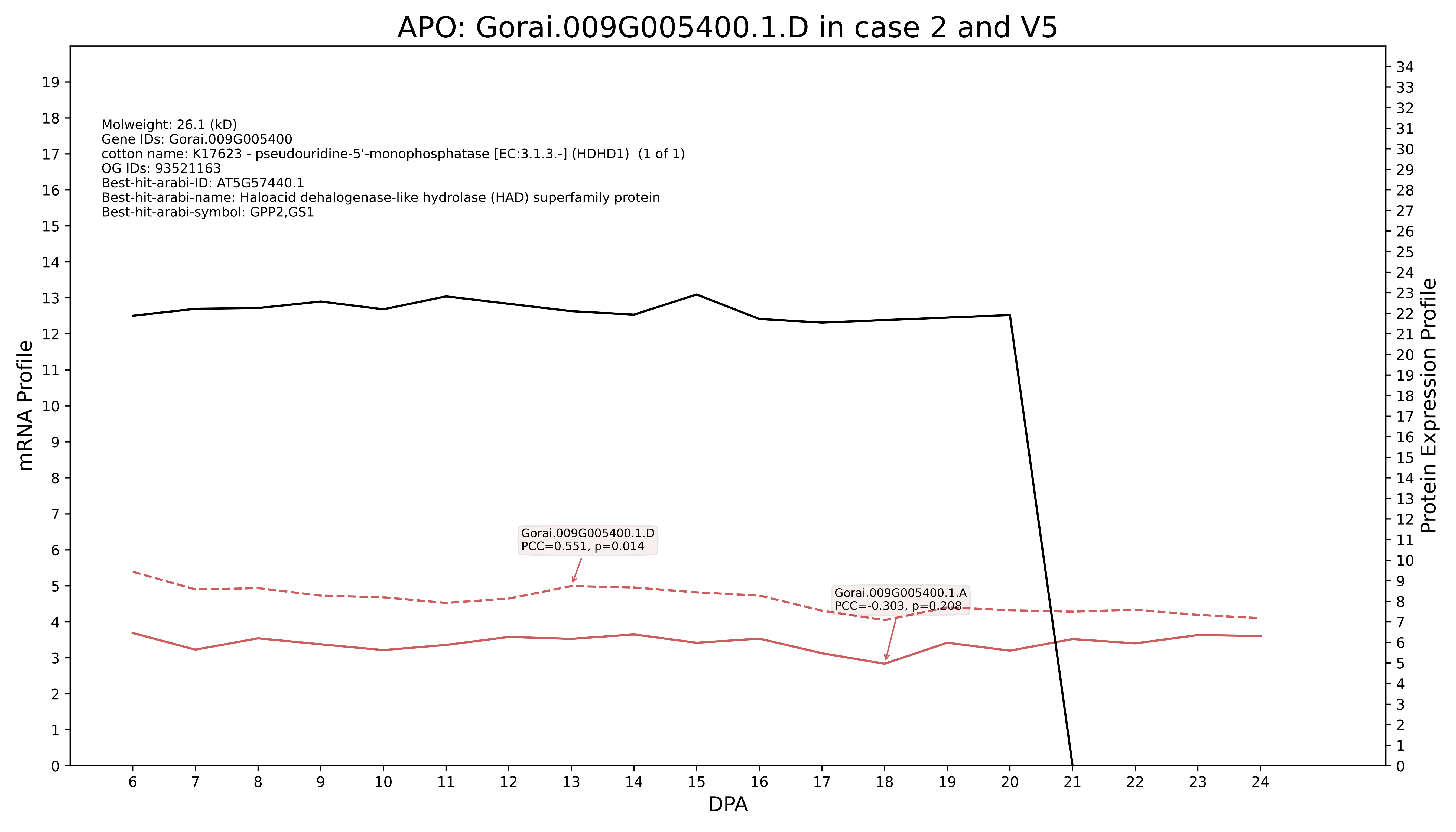

APO: Gorai.009G005400.1.D in case 2 and V5
Molweight: 26.1 (kD)
Gene IDs: Gorai.009G005400
cotton name: K17623 - pseudouridine-5'-monophosphatase [EC:3.1.3.-] (HDHD1) (1 of 1)
OG IDs: 93521163
Best-hit-arabi-ID: AT5G57440.1
Best-hit-arabi-name: Haloacid dehalogenase-like hydrolase (HAD) superfamily protein
Best-hit-arabi-symbol: GPP2,GS1
Gorai.009G005400.1.D
PCC=0.551, p=0.014
Gorai.009G005400.1.A
PCC=-0.303, p=0.208
mRNA Profile
Protein Expression Profile
DPA

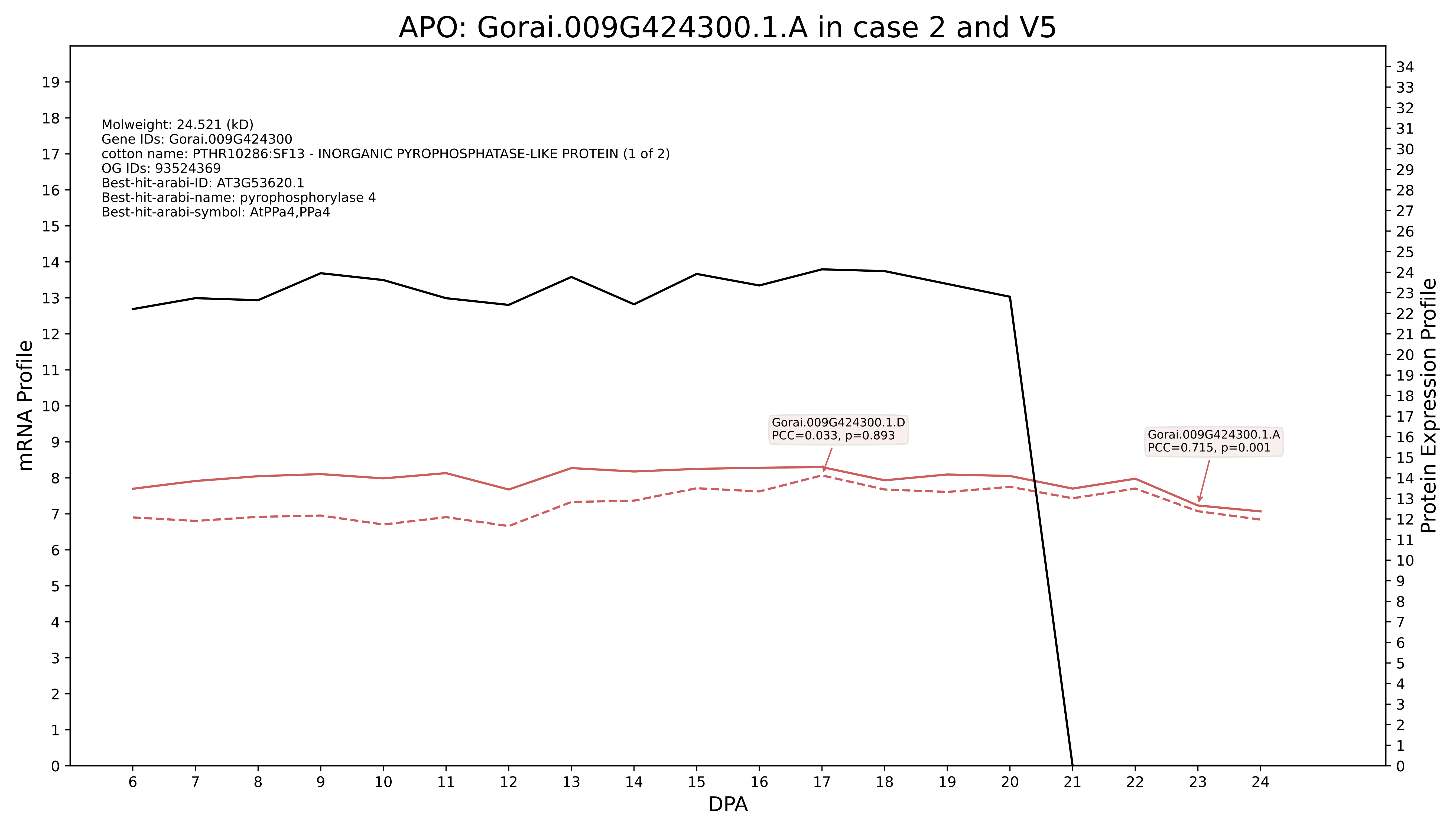
APO: Gorai.009G424300.1.A in case 2 and V5
Molweight: 24.521 (kD)
Gene IDs: Gorai.009G424300
cotton name: PTHR10286:SF13 - INORGANIC PYROPHOSPHATASE-LIKE PROTEIN (1 of 2)
OG IDs: 93524369
Best-hit-arabi-ID: AT3G53620.1
Best-hit-arabi-name: pyrophosphorylase 4
Best-hit-arabi-symbol: AtPPa4,PPa4
Gorai.009G424300.1.D
PCC=0.033, p=0.893
Gorai.009G424300.1.A
PCC=0.715, p=0.001
mRNA Profile
Protein Expression Profile
DPA

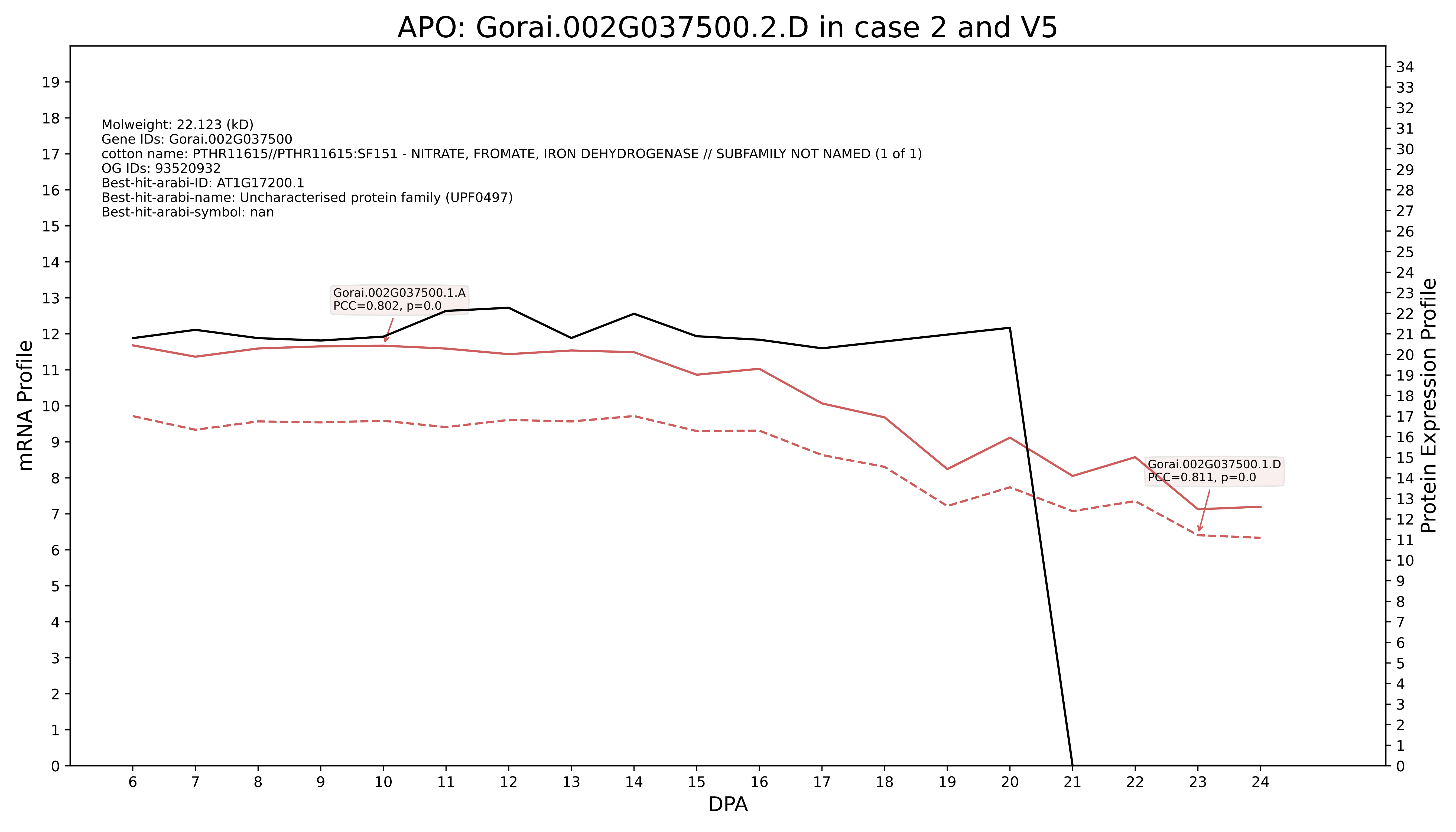
APO: Gorai.002G037500.2.D in case 2 and V5
Molweight: 22.123 (kD)
Gene IDs: Gorai.002G037500
cotton name: PTHR11615//PTHR11615:SF151 - NITRATE, FROMATE, IRON DEHYDROGENASE // SUBFAMILY NOT NAMED (1 of 1)
OG IDs: 93520932
Best-hit-arabi-ID: AT1G17200.1
Best-hit-arabi-name: Uncharacterised protein family (UPF0497)
Best-hit-arabi-symbol: nan
Gorai.002G037500.1.A
PCC=0.802, p=0.0
Gorai.002G037500.1.D
PCC=0.811, p=0.0
mRNA Profile
Protein Expression Profile
DPA

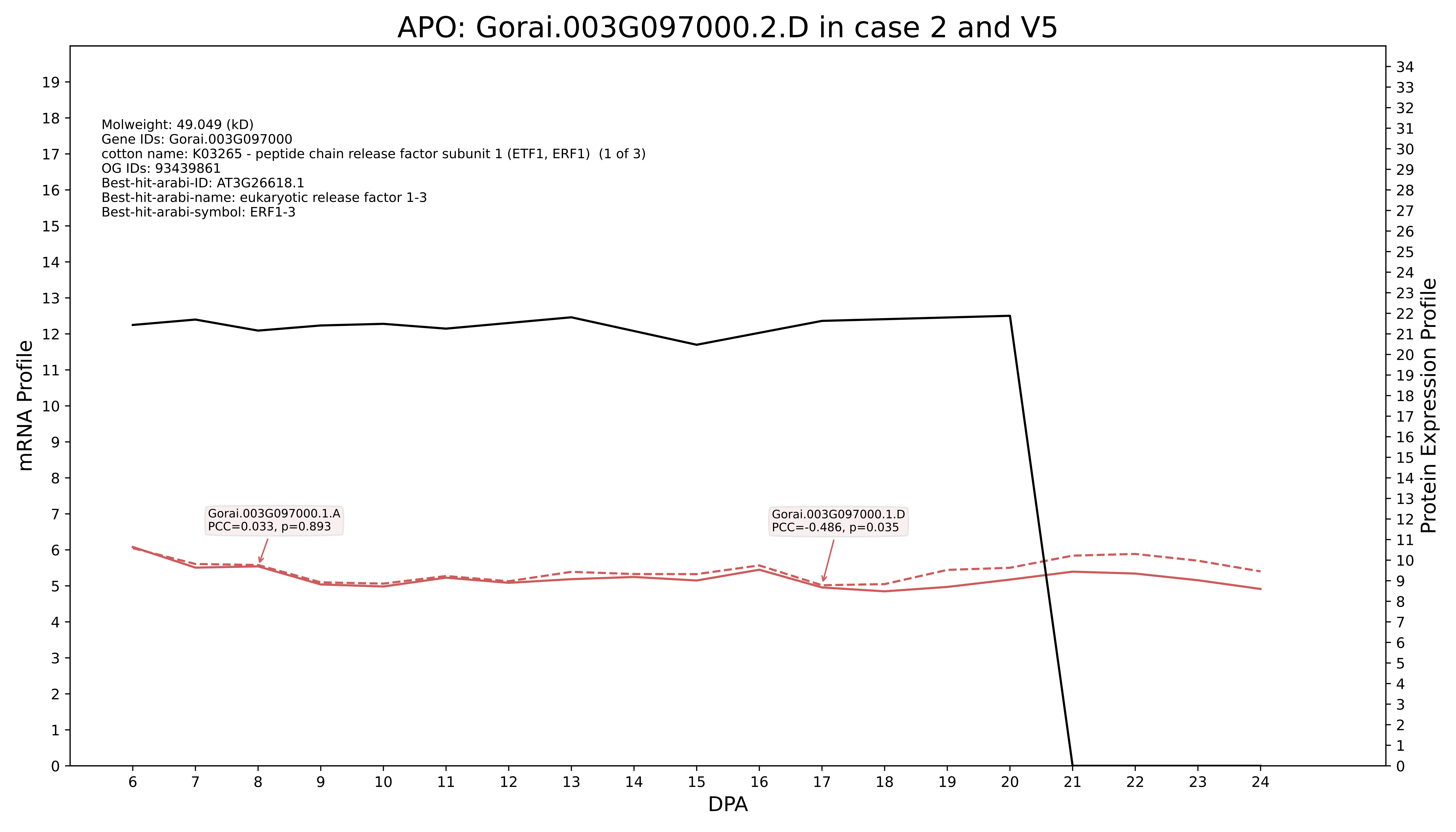

APO: Gorai.003G097000.2.D in case 2 and V5
Molweight: 49.049 (kD)
Gene IDs: Gorai.003G097000
cotton name: K03265 - peptide chain release factor subunit 1 (ETF1, ERF1) (1 of 3)
OG IDs: 93439861
Best-hit-arabi-ID: AT3G26618.1
Best-hit-arabi-name: eukaryotic release factor 1-3
Best-hit-arabi-symbol: ERF1-3
Gorai.003G097000.1.A
PCC=0.033, p=0.893
Gorai.003G097000.1.D
PCC=-0.486, p=0.035
mRNA Profile
Protein Expression Profile
DPA

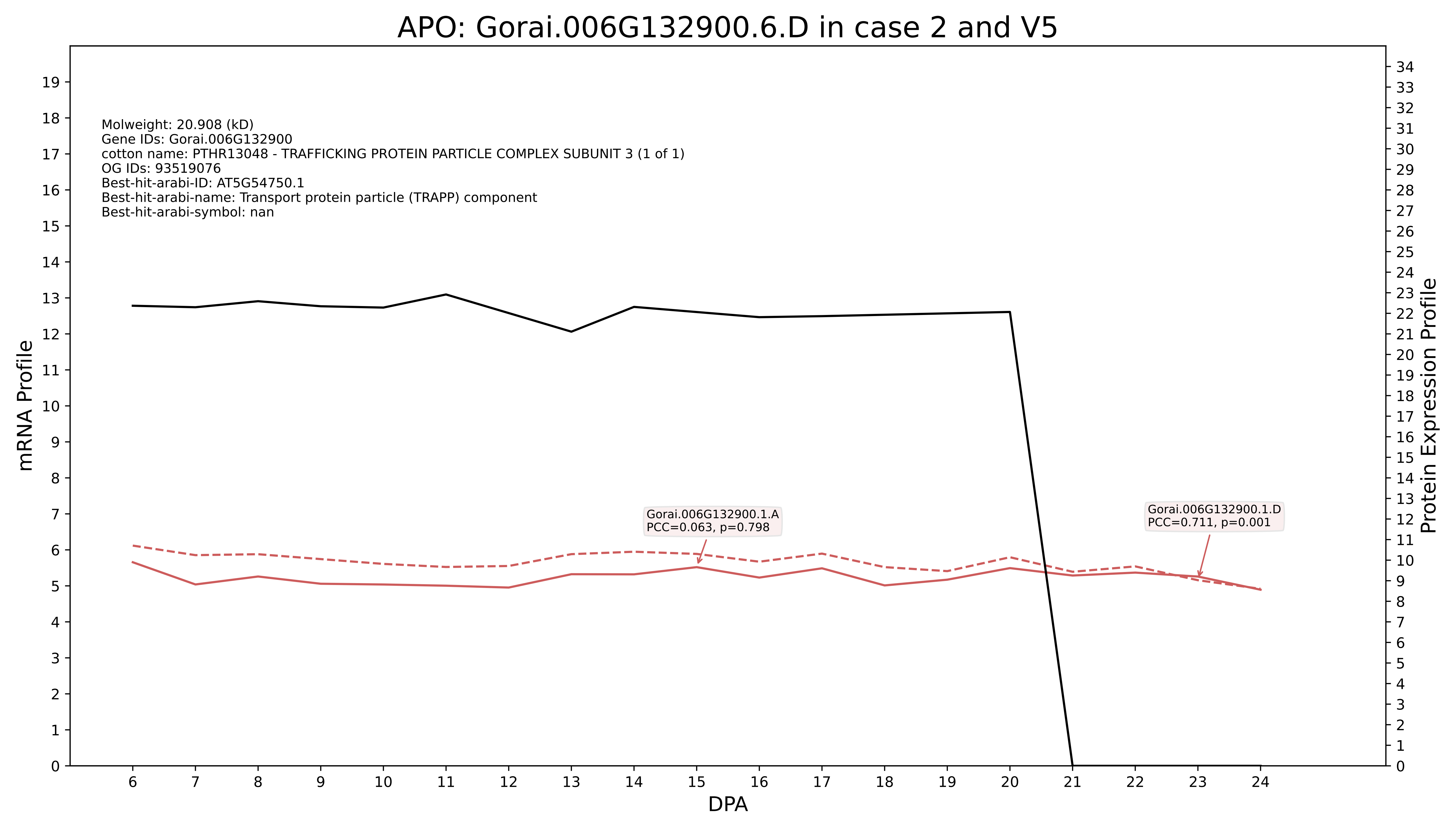
APO: Gorai.006G132900.6.D in case 2 and V5
Molweight: 20.908 (kD)
Gene IDs: Gorai.006G132900
cotton name: PTHR13048 - TRAFFICKING PROTEIN PARTICLE COMPLEX SUBUNIT 3 (1 of 1)
OG IDs: 93519076
Best-hit-arabi-ID: AT5G54750.1
Best-hit-arabi-name: Transport protein particle (TRAPP) component
Best-hit-arabi-symbol: nan
Gorai.006G132900.1.A
PCC=0.063, p=0.798
Gorai.006G132900.1.D
PCC=0.711, p=0.001
mRNA Profile
Protein Expression Profile
DPA

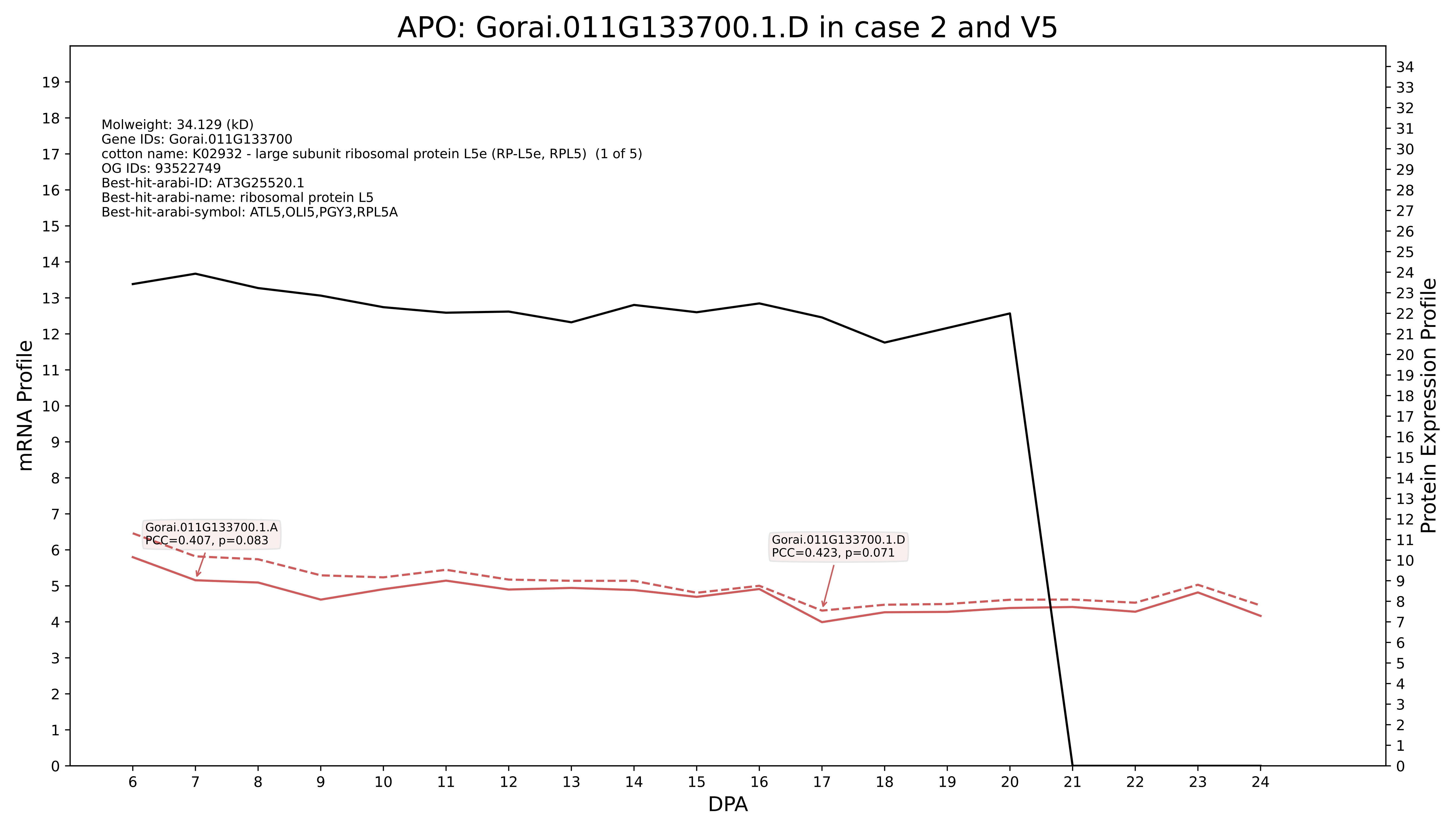
APO: Gorai.011G133700.1.D in case 2 and V5
Molweight: 34.129 (kD)
Gene IDs: Gorai.011G133700
cotton name: K02932 - large subunit ribosomal protein L5e (RP-L5e, RPL5) (1 of 5)
OG IDs: 93522749
Best-hit-arabi-ID: AT3G25520.1
Best-hit-arabi-name: ribosomal protein L5
Best-hit-arabi-symbol: ATL5,OLI5,PGY3,RPL5A
Gorai.011G133700.1.A
PCC=0.407, p=0.083
Gorai.011G133700.1.D
PCC=0.423, p=0.071
mRNA Profile
Protein Expression Profile
DPA

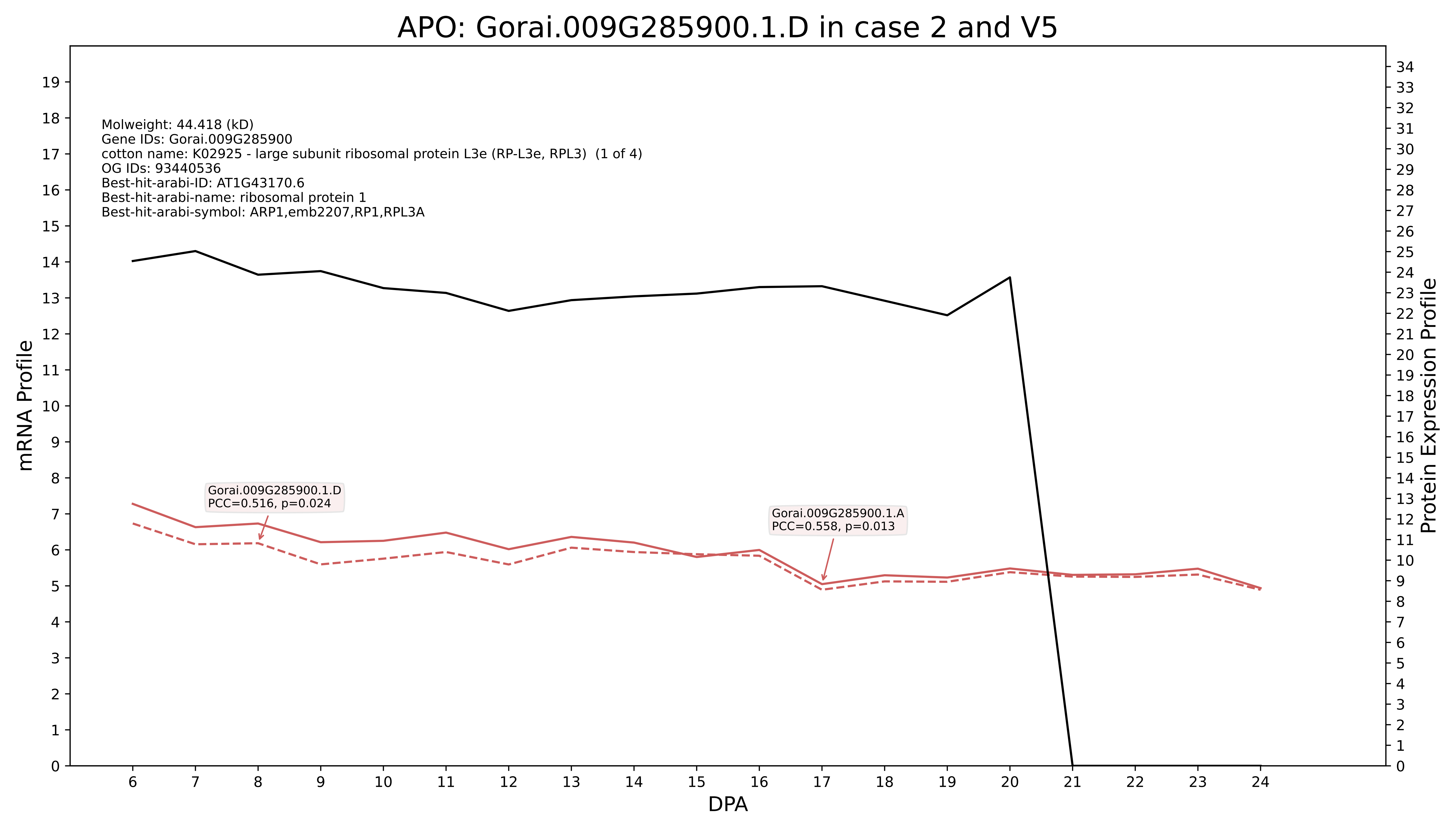
APO: Gorai.009G285900.1.D in case 2 and V5
Molweight: 44.418 (kD)
Gene IDs: Gorai.009G285900
cotton name: K02925 - large subunit ribosomal protein L3e (RP-L3e, RPL3) (1 of 4)
OG IDs: 93440536
Best-hit-arabi-ID: AT1G43170.6
Best-hit-arabi-name: ribosomal protein 1
Best-hit-arabi-symbol: ARP1,emb2207,RP1,RPL3A
Gorai.009G285900.1.D
PCC=0.516, p=0.024
Gorai.009G285900.1.A
PCC=0.558, p=0.013
mRNA Profile
Protein Expression Profile
DPA

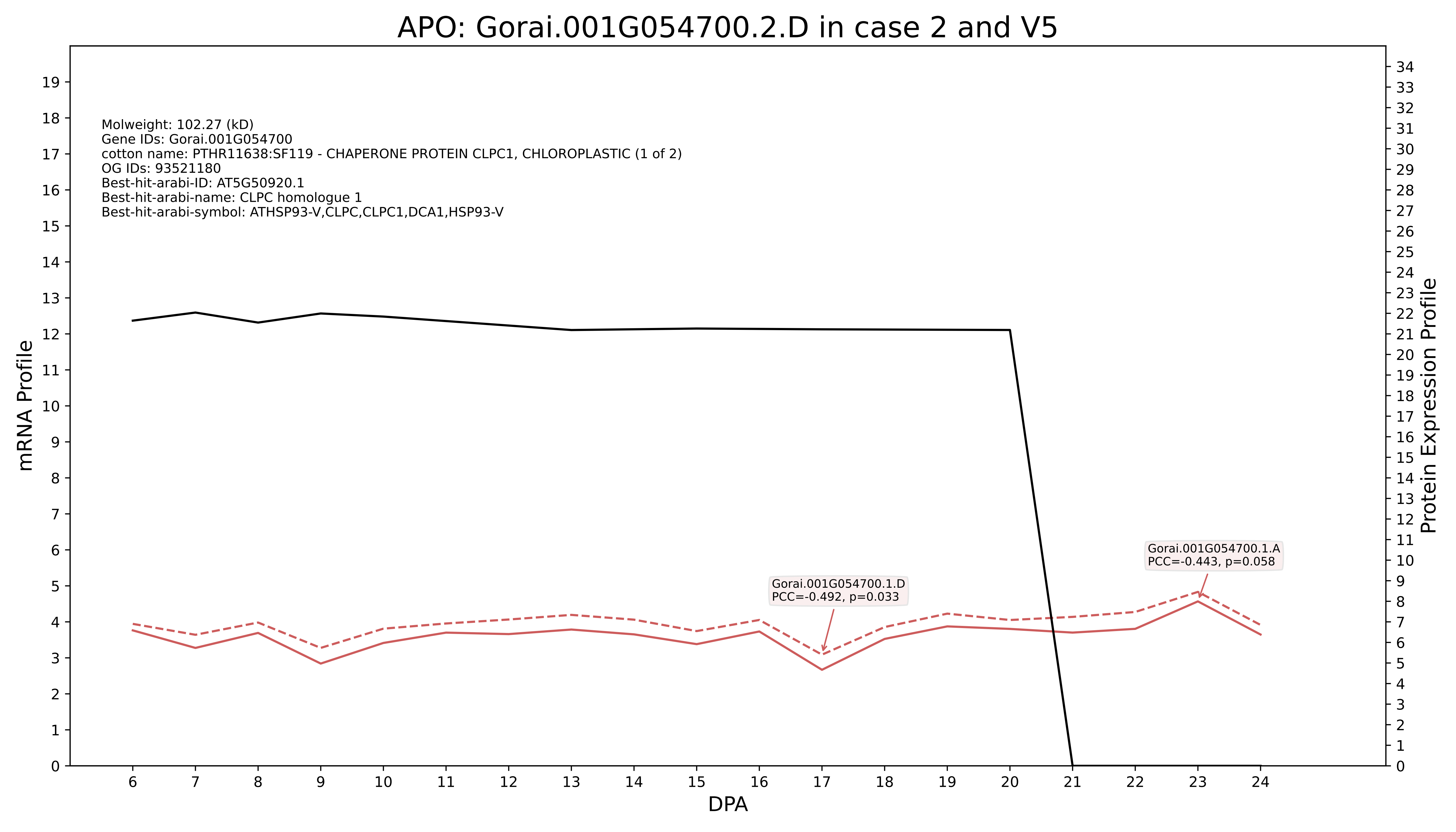

APO: Gorai.001G054700.2.D in case 2 and V5
Molweight: 102.27 (kD)
Gene IDs: Gorai.001G054700
cotton name: PTHR11638:SF119 - CHAPERONE PROTEIN CLPC1, CHLOROPLASTIC (1 of 2)
OG IDs: 93521180
Best-hit-arabi-ID: AT5G50920.1
Best-hit-arabi-name: CLPC homologue 1
Best-hit-arabi-symbol: ATHSP93-V,CLPC,CLPC1,DCA1,HSP93-V
Gorai.001G054700.1.D
PCC=-0.492, p=0.033
Gorai.001G054700.1.A
PCC=-0.443, p=0.058
mRNA Profile
Protein Expression Profile
DPA

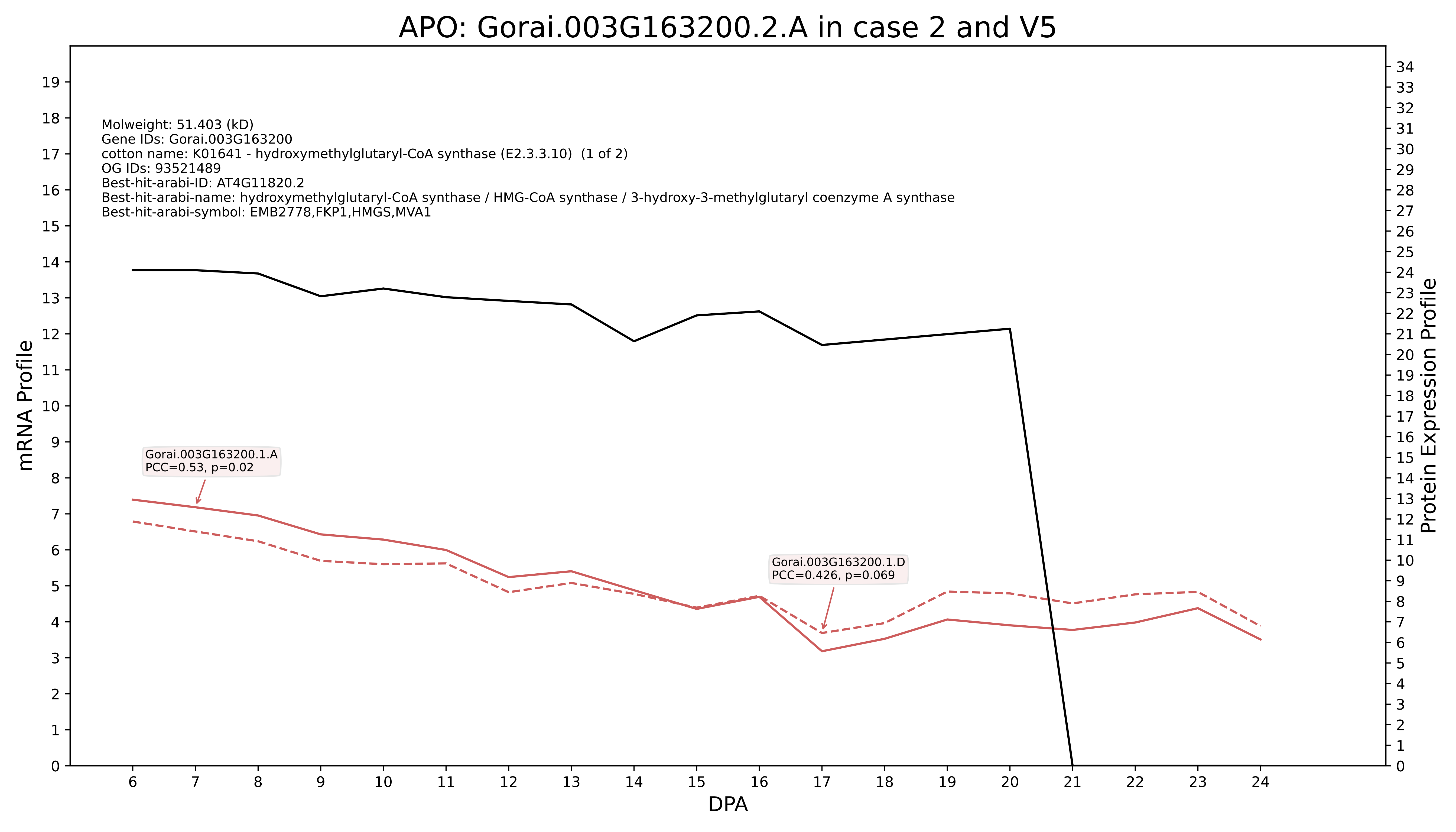
APO: Gorai.003G163200.2.A in case 2 and V5
Molweight: 51.403 (kD)
Gene IDs: Gorai.003G163200
cotton name: K01641 - hydroxymethylglutaryl-CoA synthase (E2.3.3.10) (1 of 2)
OG IDs: 93521489
Best-hit-arabi-ID: AT4G11820.2
Best-hit-arabi-name: hydroxymethylglutaryl-CoA synthase / HMG-CoA synthase / 3-hydroxy-3-methylglutaryl coenzyme A synthase
Best-hit-arabi-symbol: EMB2778,FKP1,HMGS,MVA1
Gorai.003G163200.1.A
PCC=0.53, p=0.02
Gorai.003G163200.1.D
PCC=0.426, p=0.069
mRNA Profile
Protein Expression Profile
DPA

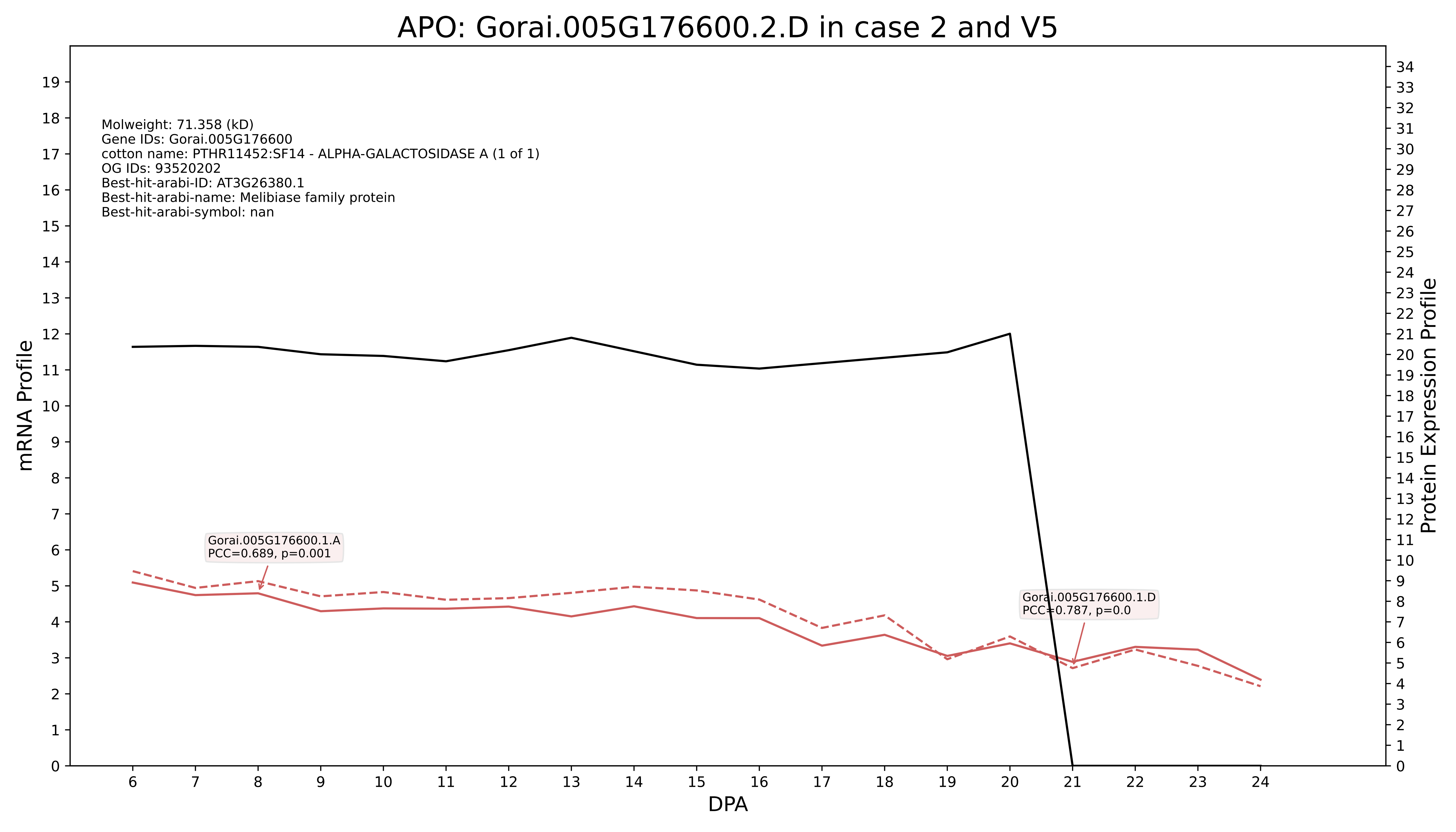

APO: Gorai.005G176600.2.D in case 2 and V5
Molweight: 71.358 (kD)
Gene IDs: Gorai.005G176600
cotton name: PTHR11452:SF14 - ALPHA-GALACTOSIDASE A (1 of 1)
OG IDs: 93520202
Best-hit-arabi-ID: AT3G26380.1
Best-hit-arabi-name: Melibiase family protein
Best-hit-arabi-symbol: nan
Gorai.005G176600.1.A
PCC=0.689, p=0.001
Gorai.005G176600.1.D
PCC=0.787, p=0.0
mRNA Profile
Protein Expression Profile
DPA

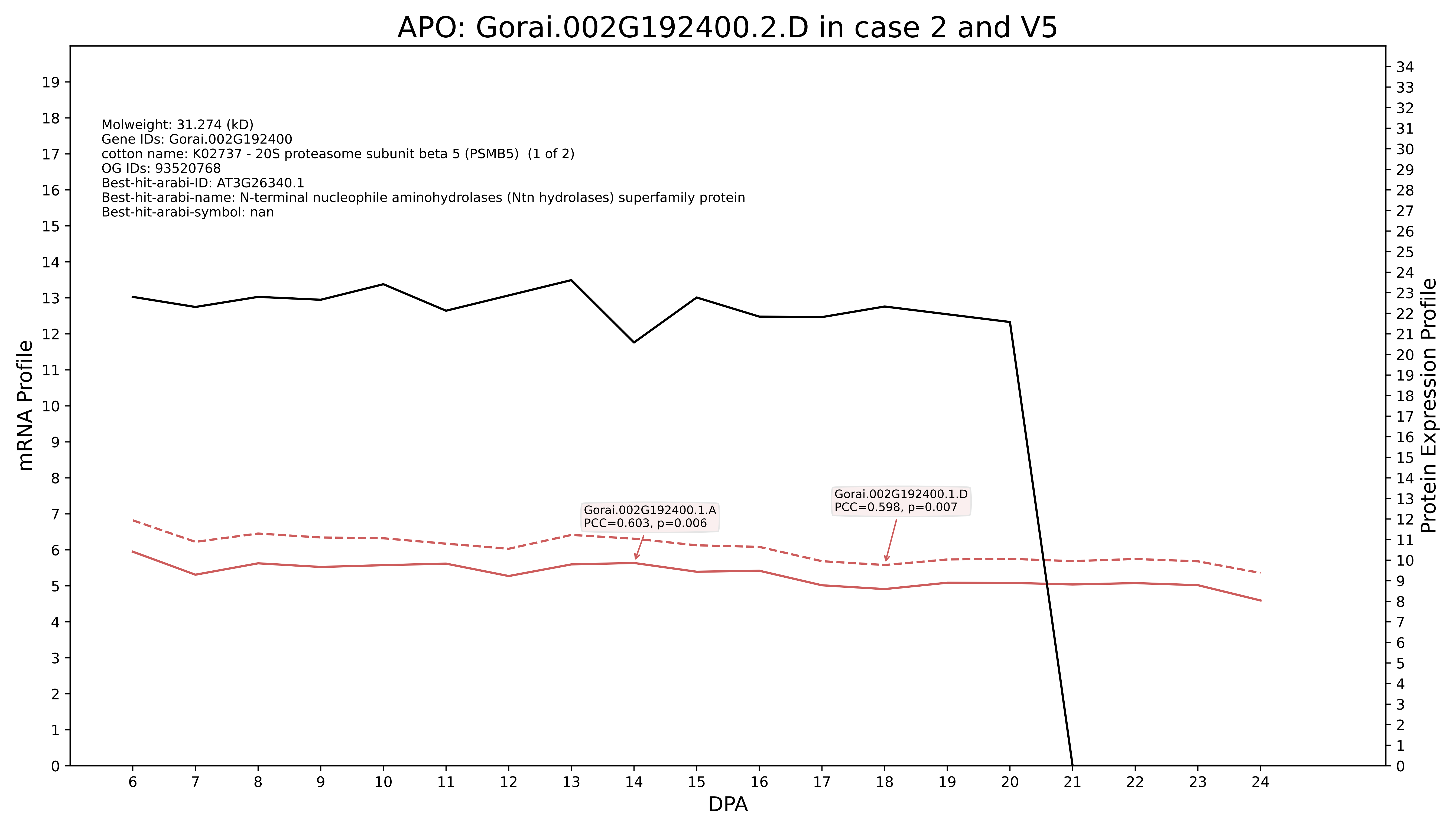
APO: Gorai.002G192400.2.D in case 2 and V5
Molweight: 31.274 (kD)
Gene IDs: Gorai.002G192400
cotton name: K02737 - 20S proteasome subunit beta 5 (PSMB5) (1 of 2)
OG IDs: 93520768
Best-hit-arabi-ID: AT3G26340.1
Best-hit-arabi-name: N-terminal nucleophile aminohydrolases (Ntn hydrolases) superfamily protein
Best-hit-arabi-symbol: nan
Gorai.002G192400.1.A
PCC=0.603, p=0.006
Gorai.002G192400.1.D
PCC=0.598, p=0.007
mRNA Profile
Protein Expression Profile
DPA
0
1
2
3
4
5
6
7
8
9
10
11
12
13
14
15
16
17
18
19
20
21
22
23
24
25
26
27
28
29
30
31
32
33
34

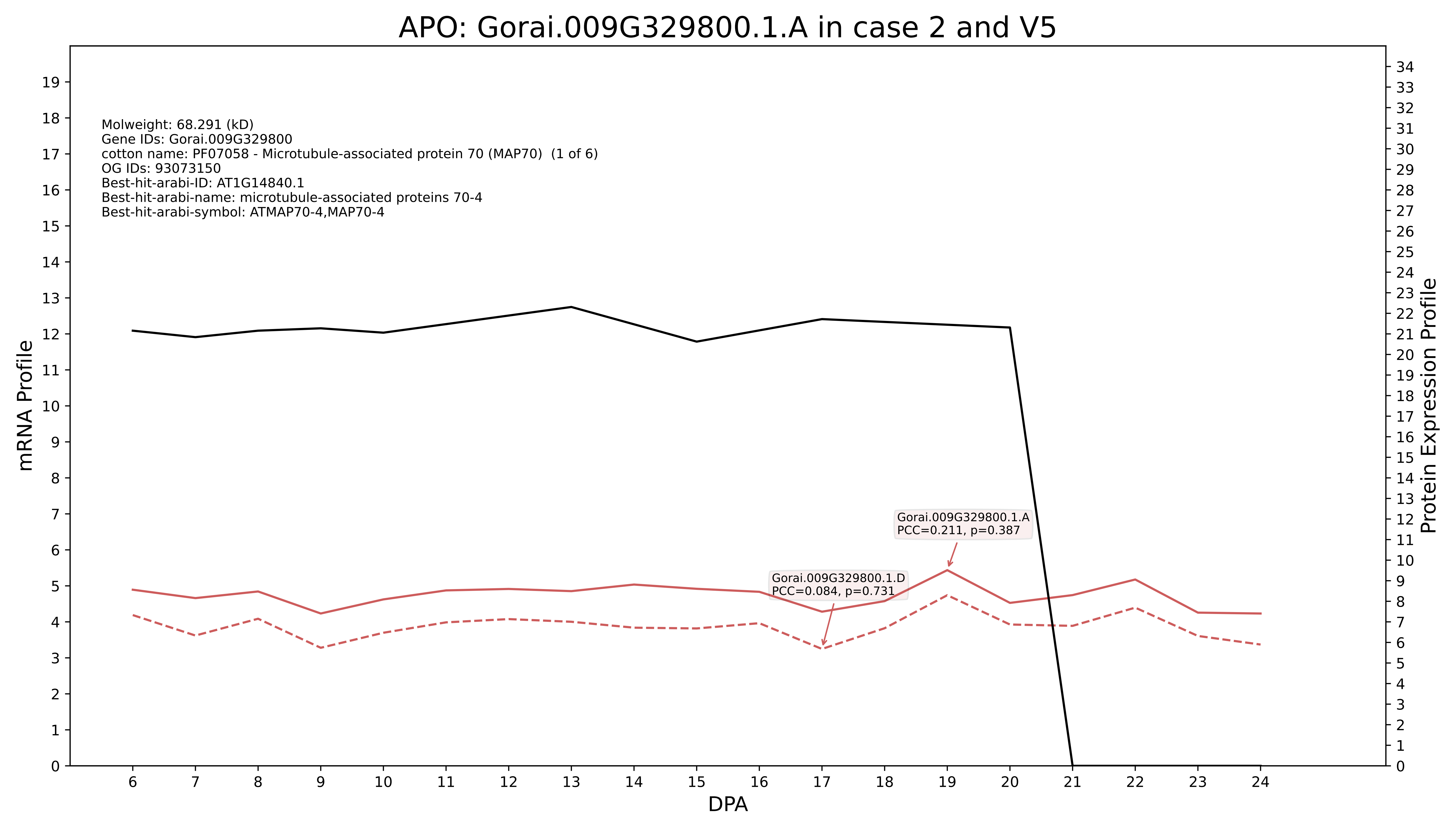

APO: Gorai.009G329800.1.A in case 2 and V5
Molweight: 68.291 (kD)
Gene IDs: Gorai.009G329800
cotton name: PF07058 - Microtubule-associated protein 70 (MAP70) (1 of 6)
OG IDs: 93073150
Best-hit-arabi-ID: AT1G14840.1
Best-hit-arabi-name: microtubule-associated proteins 70-4
Best-hit-arabi-symbol: ATMAP70-4,MAP70-4
Gorai.009G329800.1.A
PCC=0.211, p=0.387
Gorai.009G329800.1.D
PCC=0.084, p=0.731
mRNA Profile
Protein Expression Profile
DPA

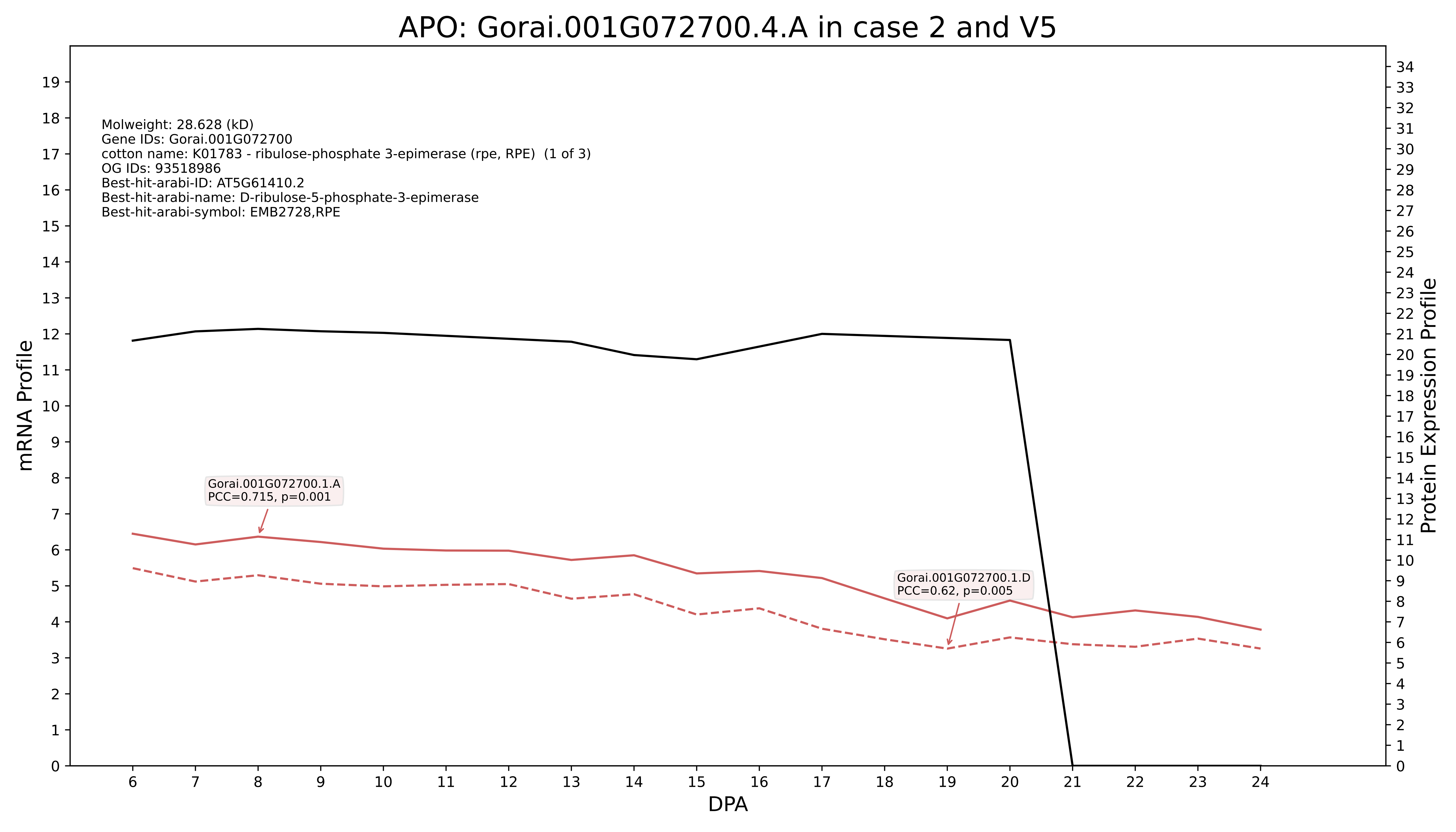
APO: Gorai.001G072700.4.A in case 2 and V5
Molweight: 28.628 (kD)
Gene IDs: Gorai.001G072700
cotton name: K01783 - ribulose-phosphate 3-epimerase (rpe, RPE) (1 of 3)
OG IDs: 93518986
Best-hit-arabi-ID: AT5G61410.2
Best-hit-arabi-name: D-ribulose-5-phosphate-3-epimerase
Best-hit-arabi-symbol: EMB2728,RPE
Gorai.001G072700.1.A
PCC=0.715, p=0.001
Gorai.001G072700.1.D
PCC=0.62, p=0.005
mRNA Profile
Protein Expression Profile
DPA

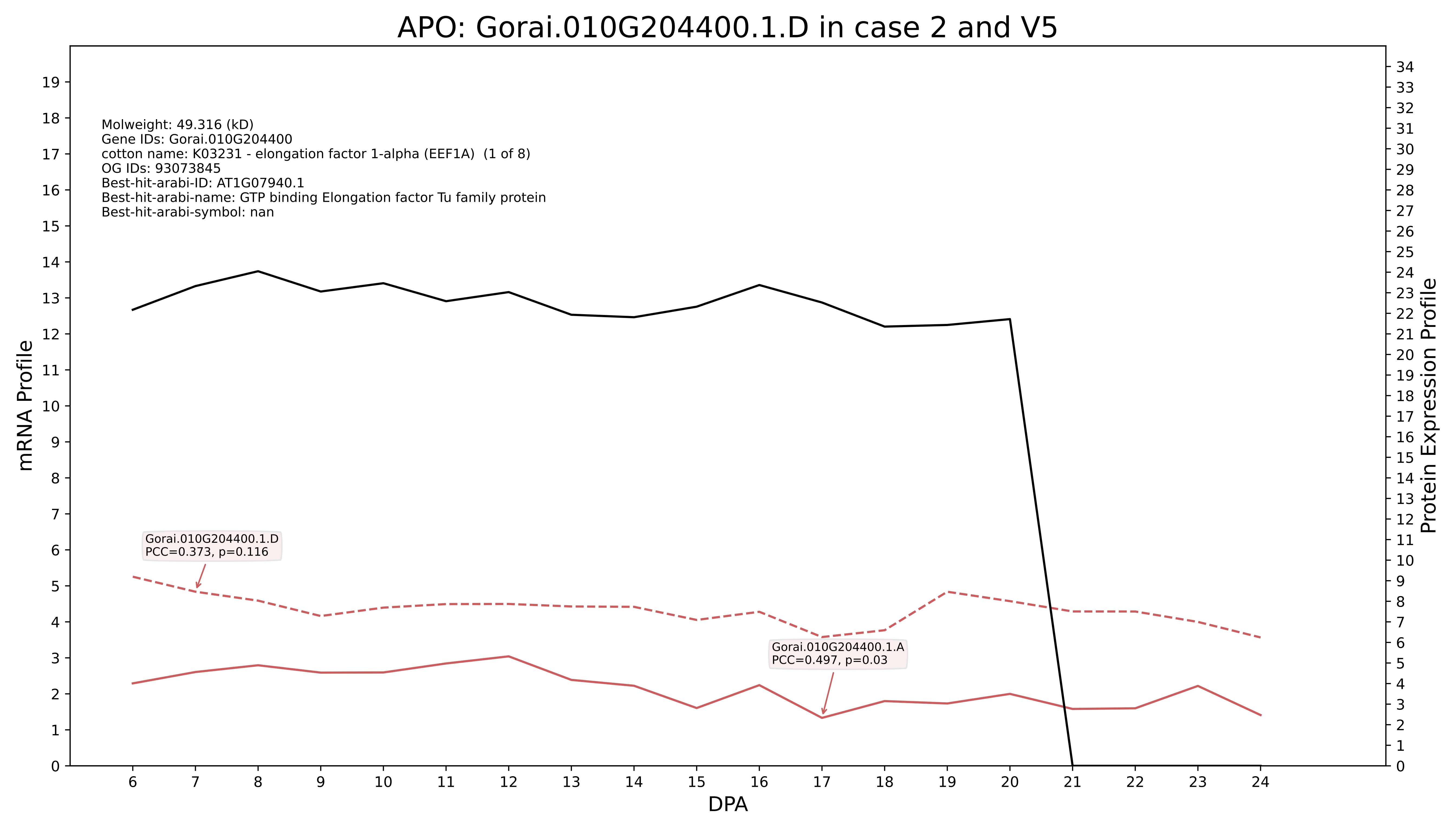

APO: Gorai.010G204400.1.D in case 2 and V5
Molweight: 49.316 (kD)
Gene IDs: Gorai.010G204400
cotton name: K03231 - elongation factor 1-alpha (EEF1A) (1 of 8)
OG IDs: 93073845
Best-hit-arabi-ID: AT1G07940.1
Best-hit-arabi-name: GTP binding Elongation factor Tu family protein
Best-hit-arabi-symbol: nan
Gorai.010G204400.1.D
PCC=0.373, p=0.116
Gorai.010G204400.1.A
PCC=0.497, p=0.03
mRNA Profile
Protein Expression Profile
DPA

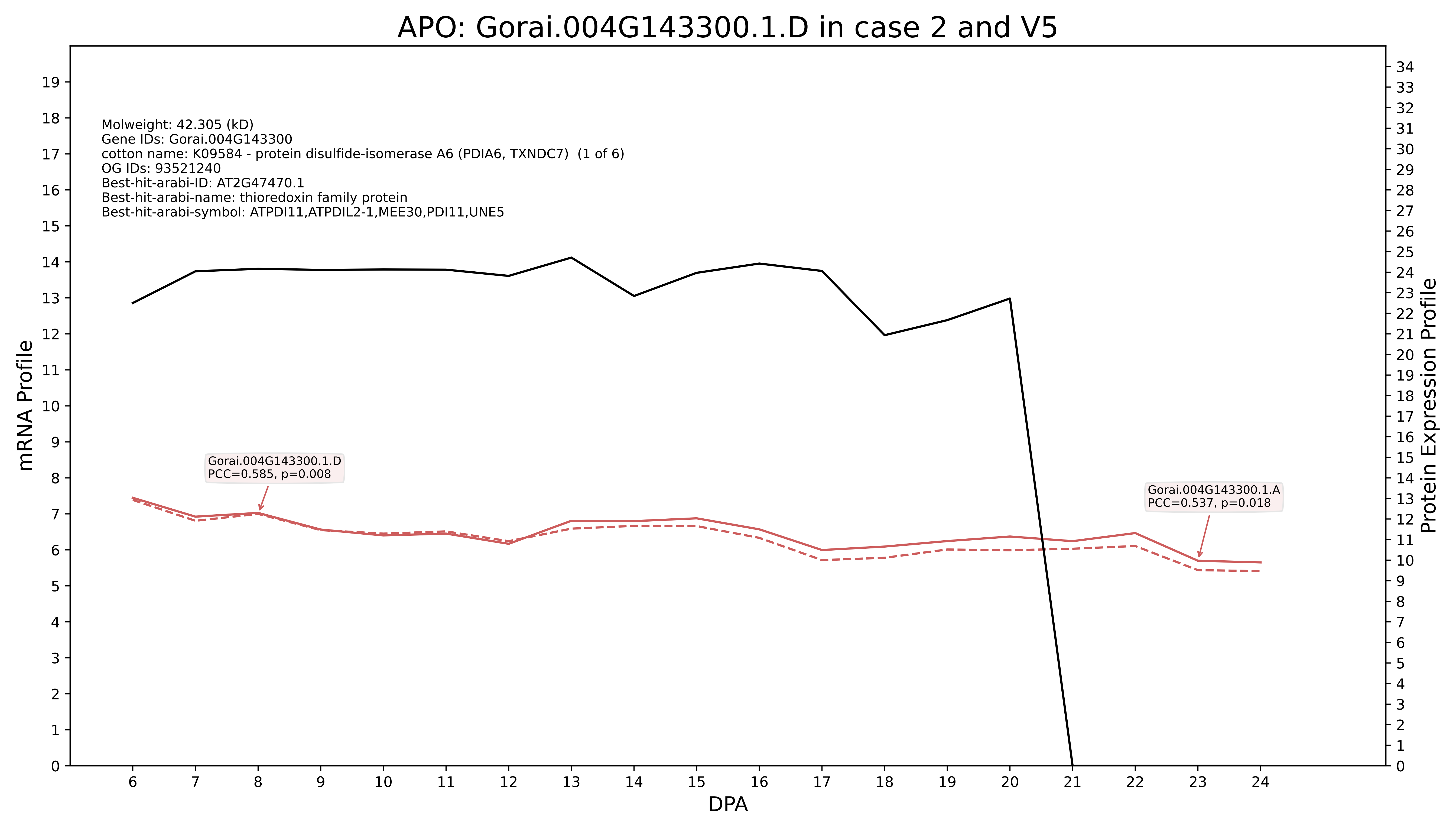
APO: Gorai.004G143300.1.D in case 2 and V5
Molweight: 42.305 (kD)
Gene IDs: Gorai.004G143300
cotton name: K09584 - protein disulfide-isomerase A6 (PDIA6, TXNDC7) (1 of 6)
OG IDs: 93521240
Best-hit-arabi-ID: AT2G47470.1
Best-hit-arabi-name: thioredoxin family protein
Best-hit-arabi-symbol: ATPDI11,ATPDIL2-1,MEE30,PDI11,UNE5
Gorai.004G143300.1.D
PCC=0.585, p=0.008
Gorai.004G143300.1.A
PCC=0.537, p=0.018
mRNA Profile
Protein Expression Profile
DPA

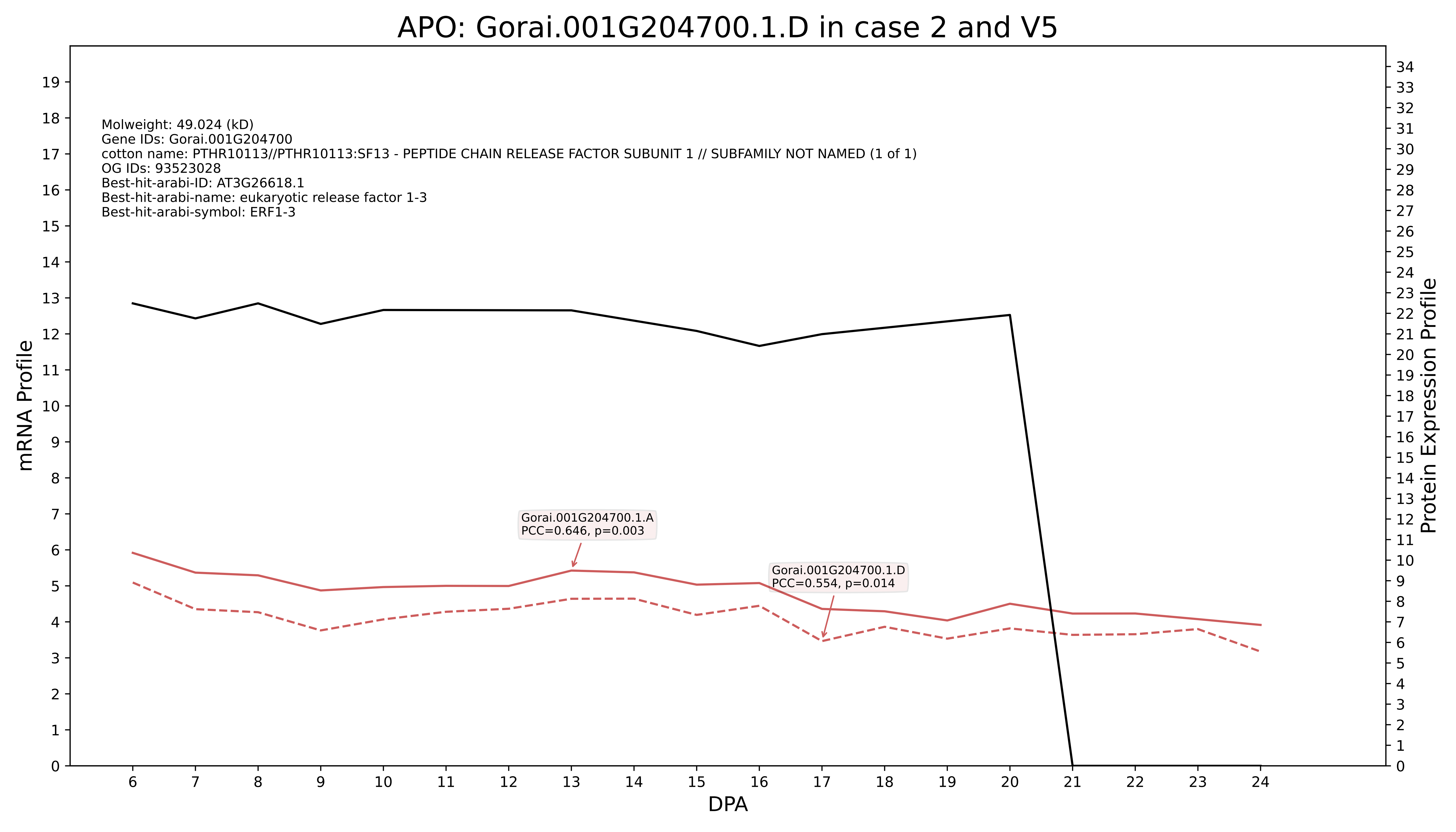

APO: Gorai.001G204700.1.D in case 2 and V5
Molweight: 49.024 (kD)
Gene IDs: Gorai.001G204700
cotton name: PTHR10113//PTHR10113:SF13 - PEPTIDE CHAIN RELEASE FACTOR SUBUNIT 1 // SUBFAMILY NOT NAMED (1 of 1)
OG IDs: 93523028
Best-hit-arabi-ID: AT3G26618.1
Best-hit-arabi-name: eukaryotic release factor 1-3
Best-hit-arabi-symbol: ERF1-3
Gorai.001G204700.1.A
PCC=0.646, p=0.003
Gorai.001G204700.1.D
PCC=0.554, p=0.014
mRNA Profile
Protein Expression Profile
DPA

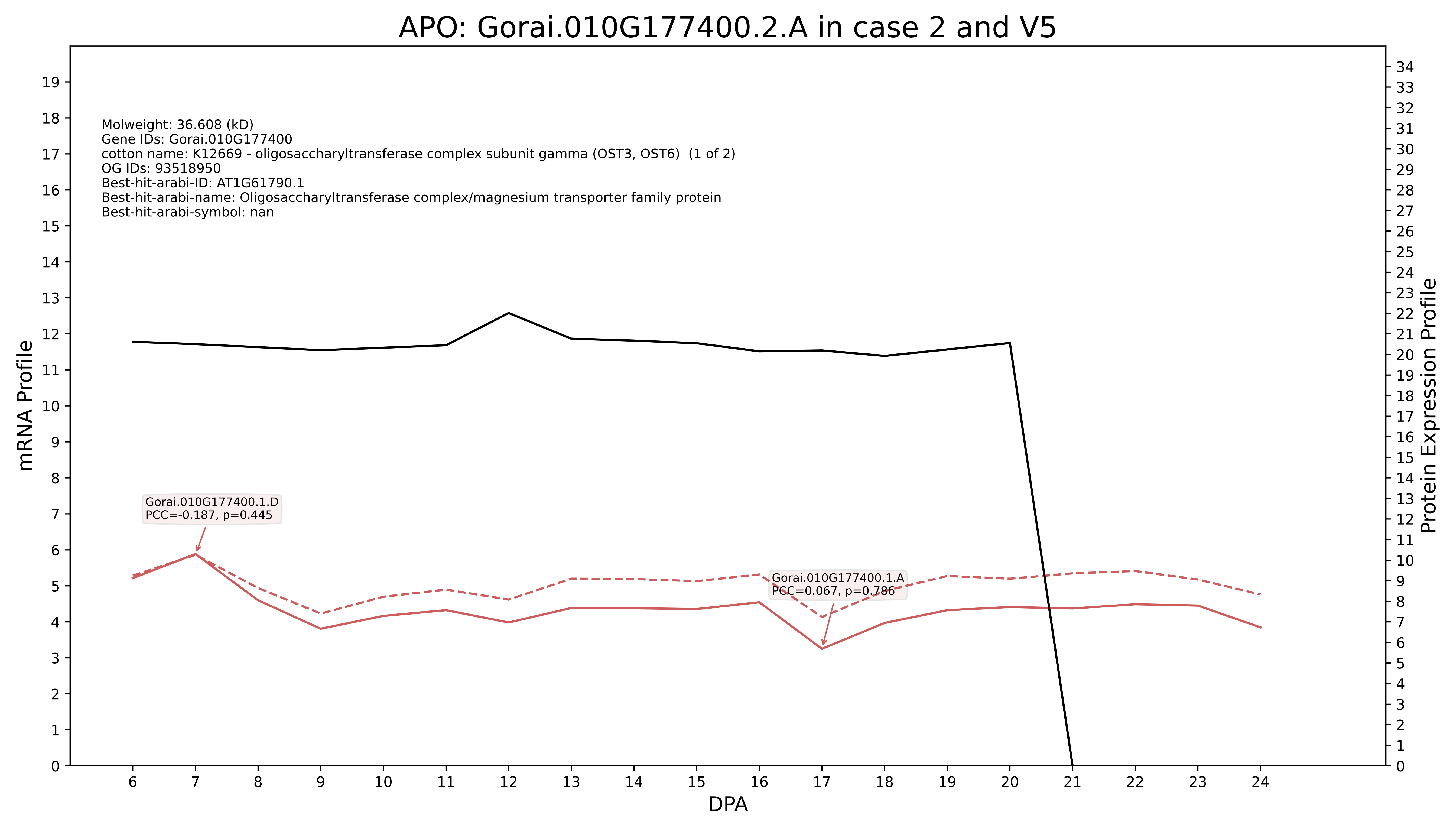

APO: Gorai.010G177400.2.A in case 2 and V5
Molweight: 36.608 (kD)
Gene IDs: Gorai.010G177400
cotton name: K12669 - oligosaccharyltransferase complex subunit gamma (OST3, OST6) (1 of 2)
OG IDs: 93518950
Best-hit-arabi-ID: AT1G61790.1
Best-hit-arabi-name: Oligosaccharyltransferase complex/magnesium transporter family protein
Best-hit-arabi-symbol: nan
Gorai.010G177400.1.D
PCC=-0.187, p=0.445
Gorai.010G177400.1.A
PCC=0.067, p=0.786
mRNA Profile
Protein Expression Profile
DPA

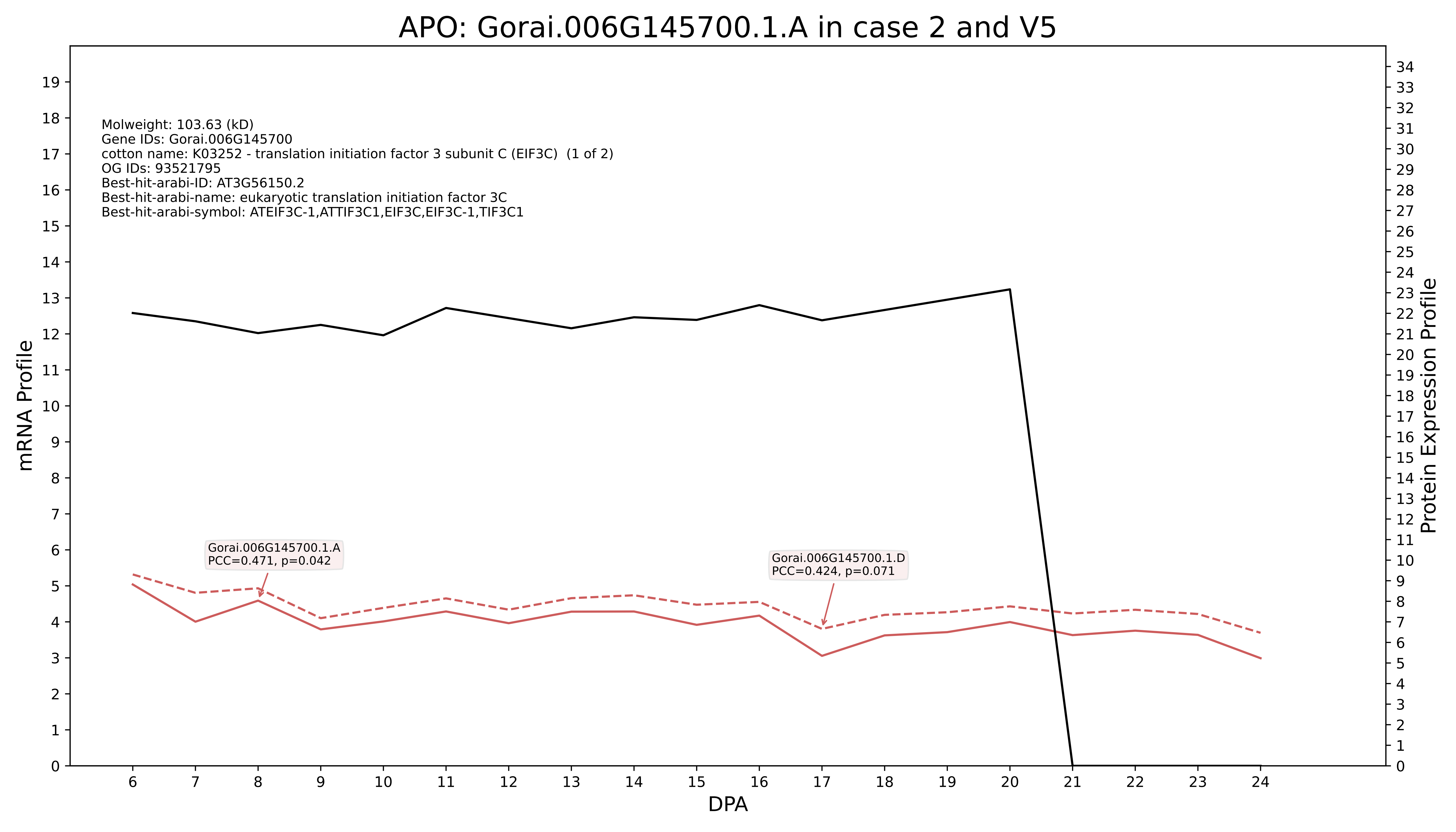
APO: Gorai.006G145700.1.A in case 2 and V5
Molweight: 103.63 (kD)
Gene IDs: Gorai.006G145700
cotton name: K03252 - translation initiation factor 3 subunit C (EIF3C) (1 of 2)
OG IDs: 93521795
Best-hit-arabi-ID: AT3G56150.2
Best-hit-arabi-name: eukaryotic translation initiation factor 3C
Best-hit-arabi-symbol: ATEIF3C-1,ATTIF3C1,EIF3C,EIF3C-1,TIF3C1
Gorai.006G145700.1.A
PCC=0.471, p=0.042
Gorai.006G145700.1.D
PCC=0.424, p=0.071
mRNA Profile
Protein Expression Profile
DPA

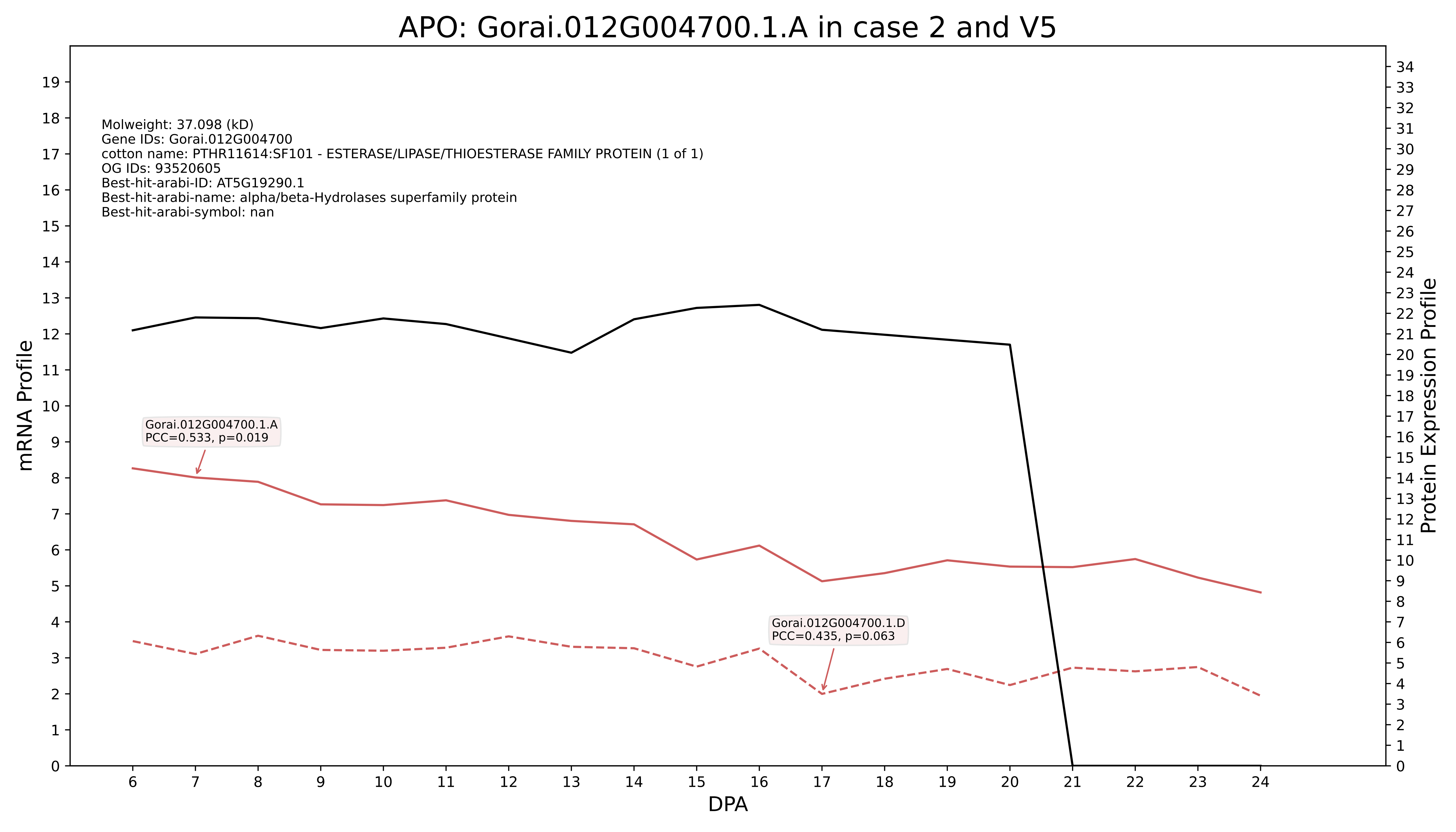

APO: Gorai.012G004700.1.A in case 2 and V5
Molweight: 37.098 (kD)
Gene IDs: Gorai.012G004700
cotton name: PTHR11614:SF101 - ESTERASE/LIPASE/THIOESTERASE FAMILY PROTEIN (1 of 1)
OG IDs: 93520605
Best-hit-arabi-ID: AT5G19290.1
Best-hit-arabi-name: alpha/beta-Hydrolases superfamily protein
Best-hit-arabi-symbol: nan
Gorai.012G004700.1.A
PCC=0.533, p=0.019
Gorai.012G004700.1.D
PCC=0.435, p=0.063
mRNA Profile
Protein Expression Profile
DPA

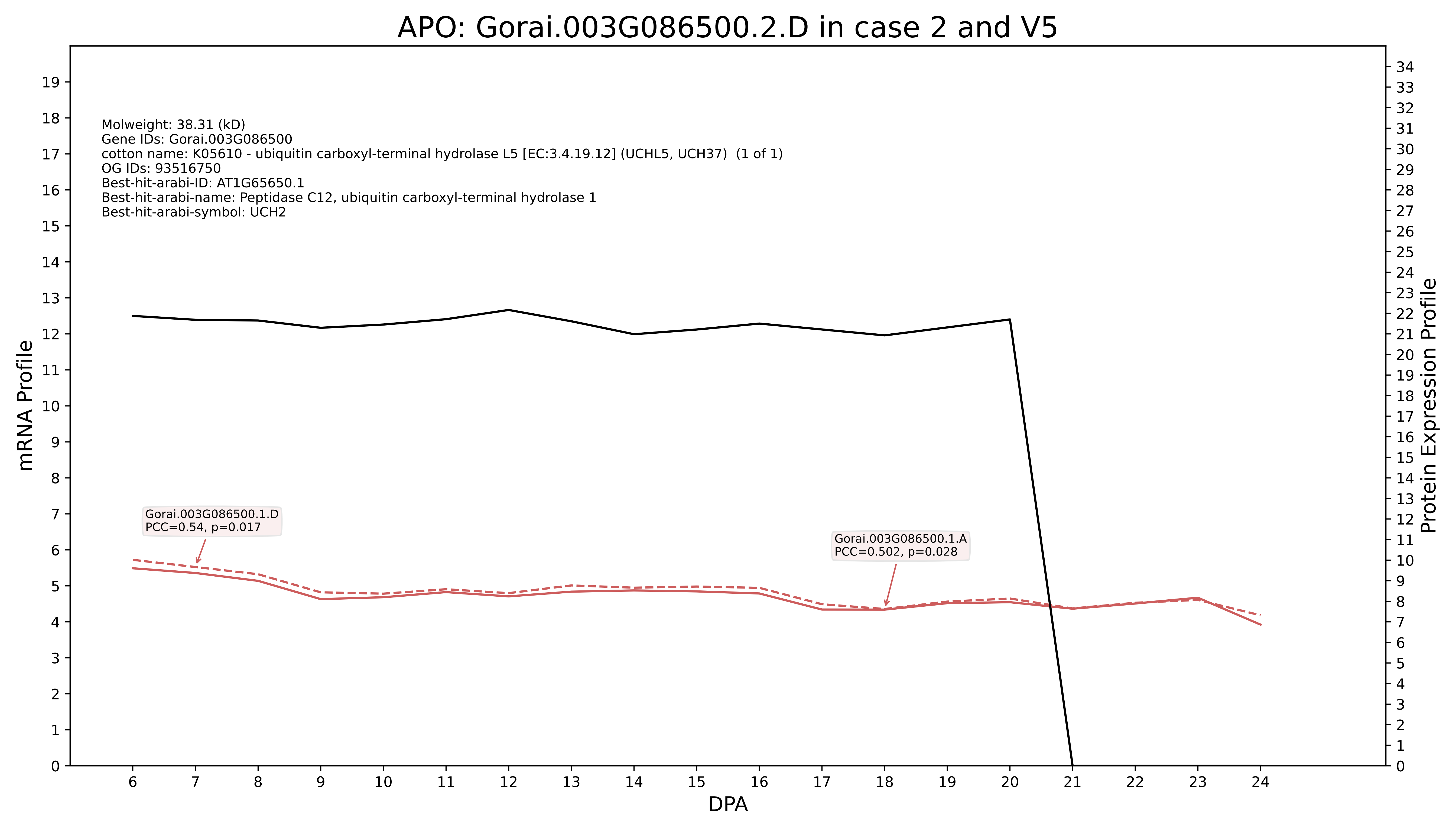
APO: Gorai.003G086500.2.D in case 2 and V5
Molweight: 38.31 (kD)
Gene IDs: Gorai.003G086500
cotton name: K05610 - ubiquitin carboxyl-terminal hydrolase L5 [EC:3.4.19.12] (UCHL5, UCH37) (1 of 1)
OG IDs: 93516750
Best-hit-arabi-ID: AT1G65650.1
Best-hit-arabi-name: Peptidase C12, ubiquitin carboxyl-terminal hydrolase 1
Best-hit-arabi-symbol: UCH2
Gorai.003G086500.1.D
PCC=0.54, p=0.017
Gorai.003G086500.1.A
PCC=0.502, p=0.028
mRNA Profile
Protein Expression Profile
DPA
0
1
2
3
4
5
6
7
8
9
10
11
12
13
14
15
16
17
18
19
20
21
22
23
24
25
26
27
28
29
30
31
32
33
34

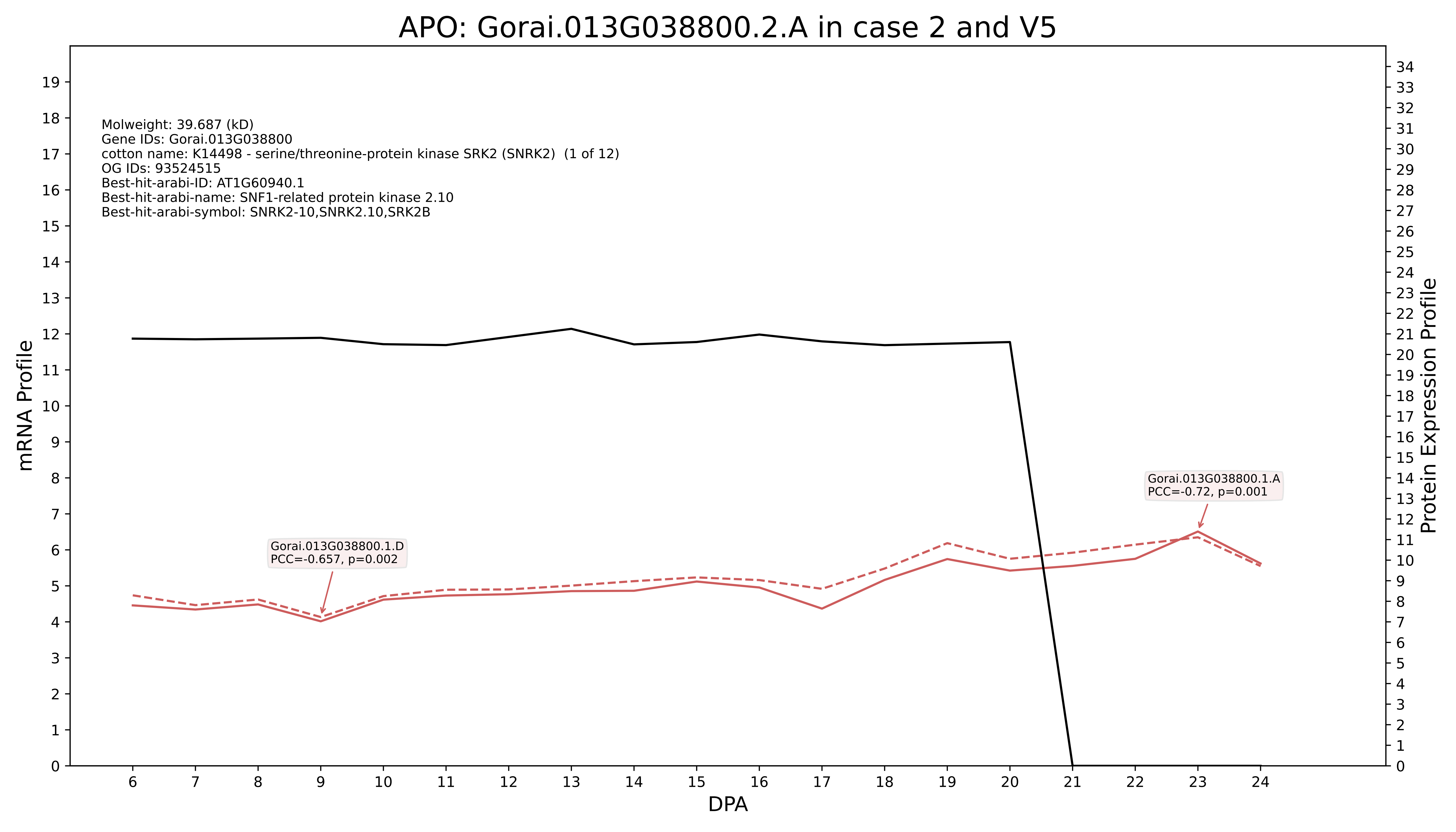

APO: Gorai.013G038800.2.A in case 2 and V5
Molweight: 39.687 (kD)
Gene IDs: Gorai.013G038800
cotton name: K14498 - serine/threonine-protein kinase SRK2 (SNRK2) (1 of 12)
OG IDs: 93524515
Best-hit-arabi-ID: AT1G60940.1
Best-hit-arabi-name: SNF1-related protein kinase 2.10
Best-hit-arabi-symbol: SNRK2-10,SNRK2.10,SRK2B
Gorai.013G038800.1.D
PCC=-0.657, p=0.002
Gorai.013G038800.1.A
PCC=-0.72, p=0.001
mRNA Profile
Protein Expression Profile
DPA

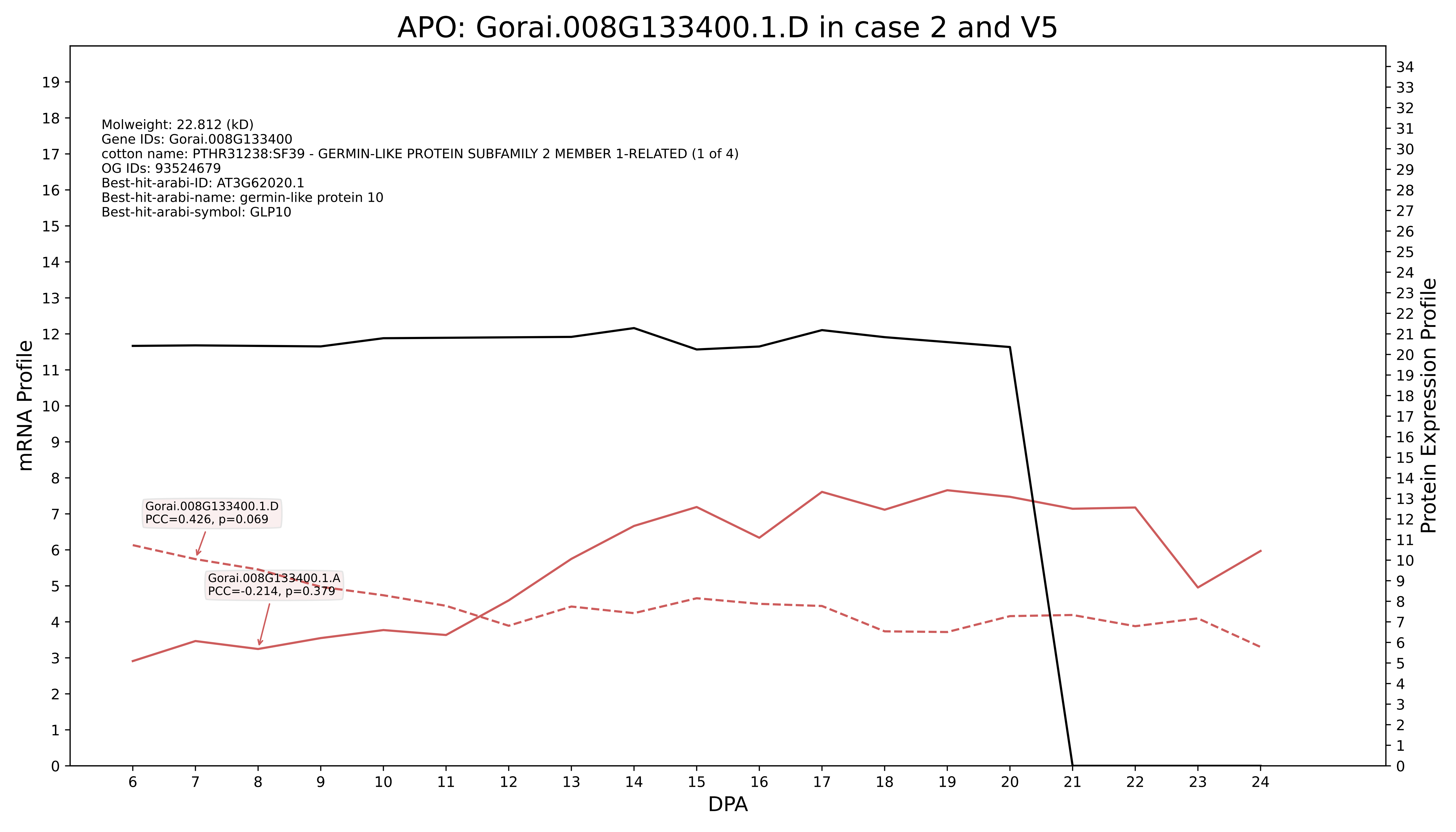
APO: Gorai.008G133400.1.D in case 2 and V5
Molweight: 22.812 (kD)
Gene IDs: Gorai.008G133400
cotton name: PTHR31238:SF39 - GERMIN-LIKE PROTEIN SUBFAMILY 2 MEMBER 1-RELATED (1 of 4)
OG IDs: 93524679
Best-hit-arabi-ID: AT3G62020.1
Best-hit-arabi-name: germin-like protein 10
Best-hit-arabi-symbol: GLP10
Gorai.008G133400.1.D
PCC=0.426, p=0.069
Gorai.008G133400.1.A
PCC=-0.214, p=0.379
mRNA Profile
Protein Expression Profile
DPA
0
1
2
3
4
5
6
7
8
9
10
11
12
13
14
15
16
17
18
19
20
21
22
23
24
25
26
27
28
29
30
31
32
33
34

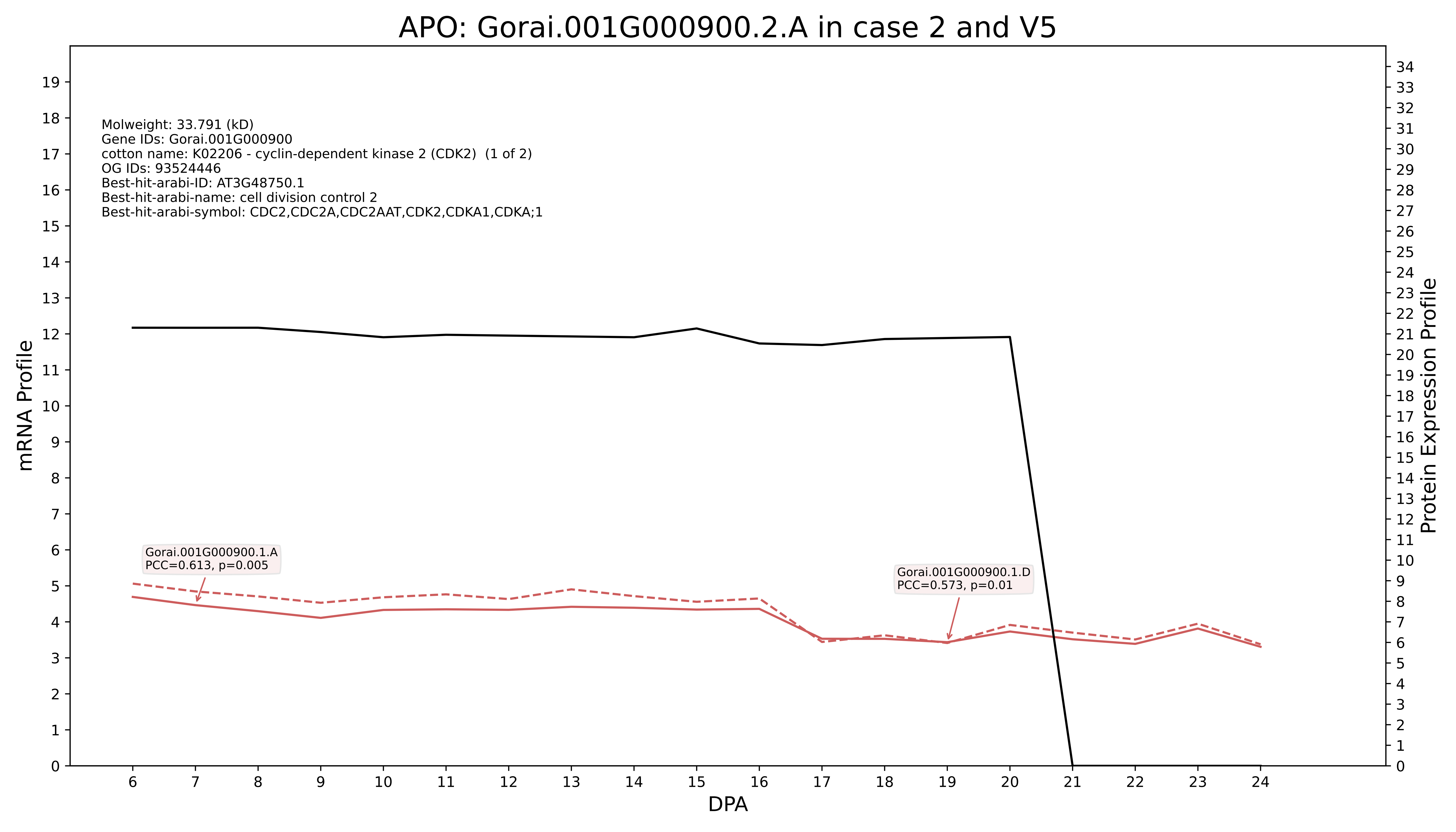

APO: Gorai.001G000900.2.A in case 2 and V5
Molweight: 33.791 (kD)
Gene IDs: Gorai.001G000900
cotton name: K02206 - cyclin-dependent kinase 2 (CDK2) (1 of 2)
OG IDs: 93524446
Best-hit-arabi-ID: AT3G48750.1
Best-hit-arabi-name: cell division control 2
Best-hit-arabi-symbol: CDC2,CDC2A,CDC2AAT,CDK2,CDKA1,CDKA;1
Gorai.001G000900.1.A
PCC=0.613, p=0.005
Gorai.001G000900.1.D
PCC=0.573, p=0.01
mRNA Profile
Protein Expression Profile
DPA

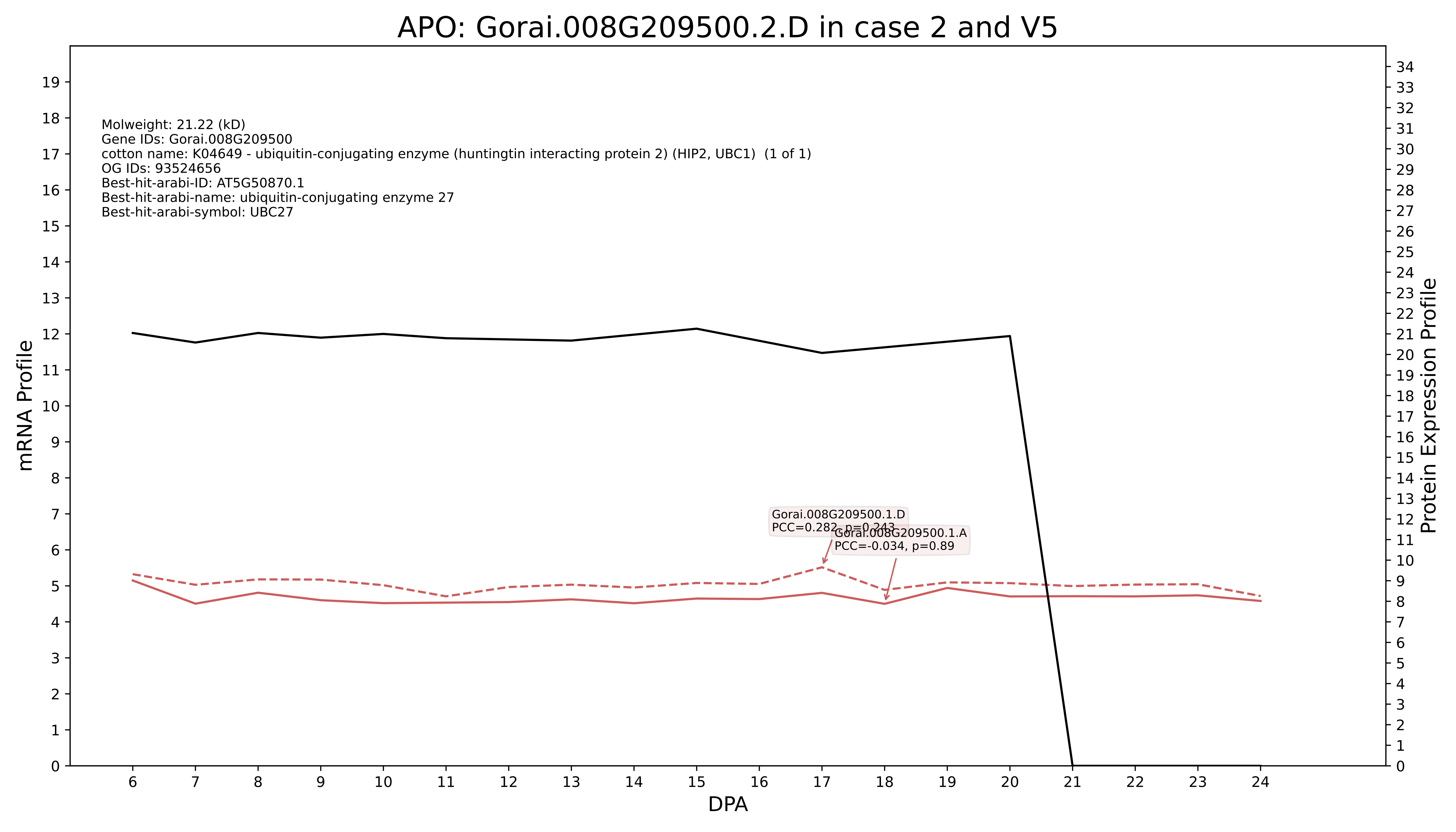

APO: Gorai.008G209500.2.D in case 2 and V5
Molweight: 21.22 (kD)
Gene IDs: Gorai.008G209500
cotton name: K04649 - ubiquitin-conjugating enzyme (huntingtin interacting protein 2) (HIP2, UBC1) (1 of 1)
OG IDs: 93524656
Best-hit-arabi-ID: AT5G50870.1
Best-hit-arabi-name: ubiquitin-conjugating enzyme 27
Best-hit-arabi-symbol: UBC27
Gorai.008G209500.1.D
PCC=0.282, p=0.243
Gorai.008G209500.1.A
PCC=-0.034, p=0.89
mRNA Profile
Protein Expression Profile
DPA

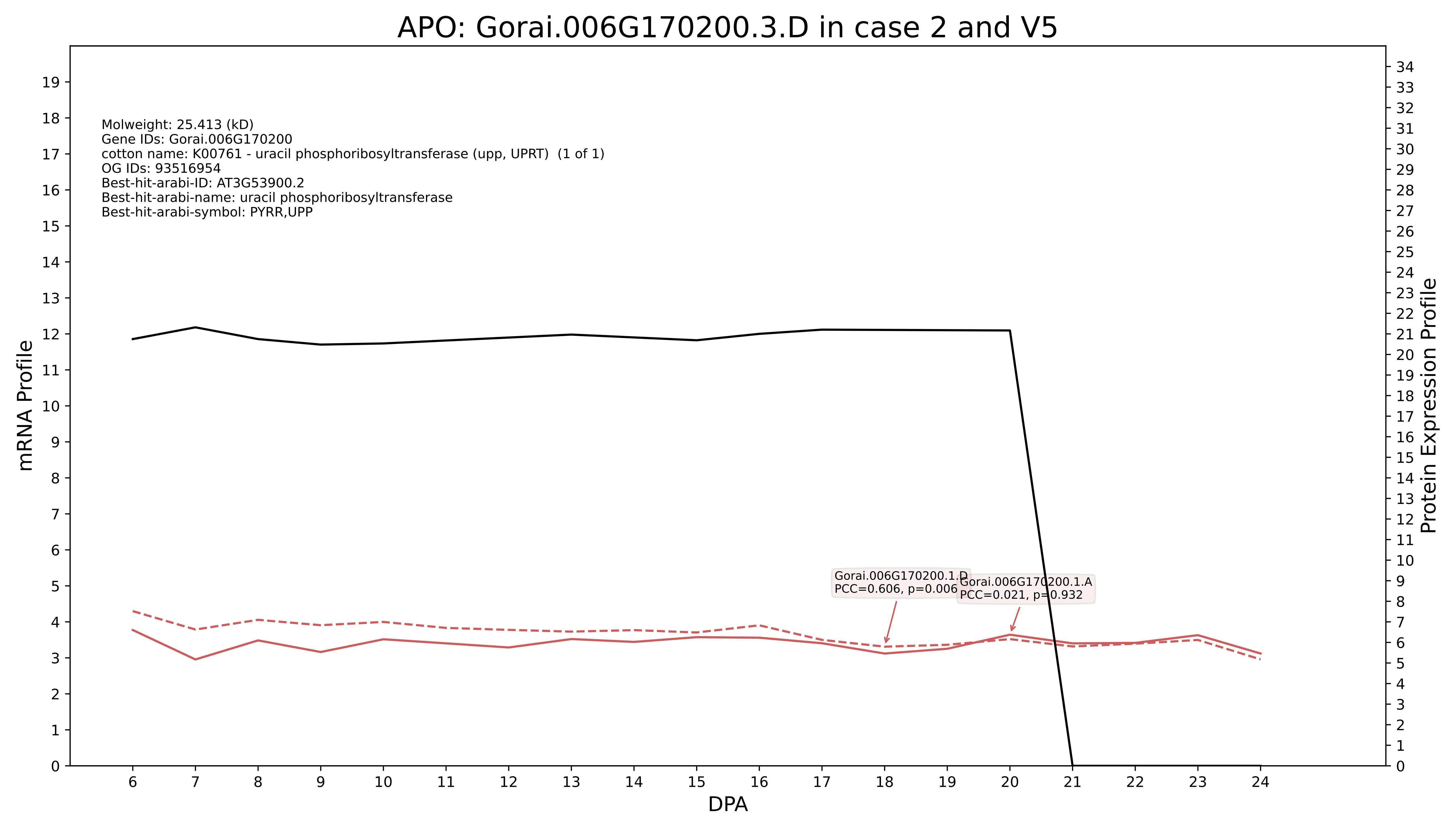
APO: Gorai.006G170200.3.D in case 2 and V5
Molweight: 25.413 (kD)
Gene IDs: Gorai.006G170200
cotton name: K00761 - uracil phosphoribosyltransferase (upp, UPRT) (1 of 1)
OG IDs: 93516954
Best-hit-arabi-ID: AT3G53900.2
Best-hit-arabi-name: uracil phosphoribosyltransferase
Best-hit-arabi-symbol: PYRR,UPP
Gorai.006G170200.1.D
PCC=0.606, p=0.006
Gorai.006G170200.1.A
PCC=0.021, p=0.932
mRNA Profile
Protein Expression Profile
DPA

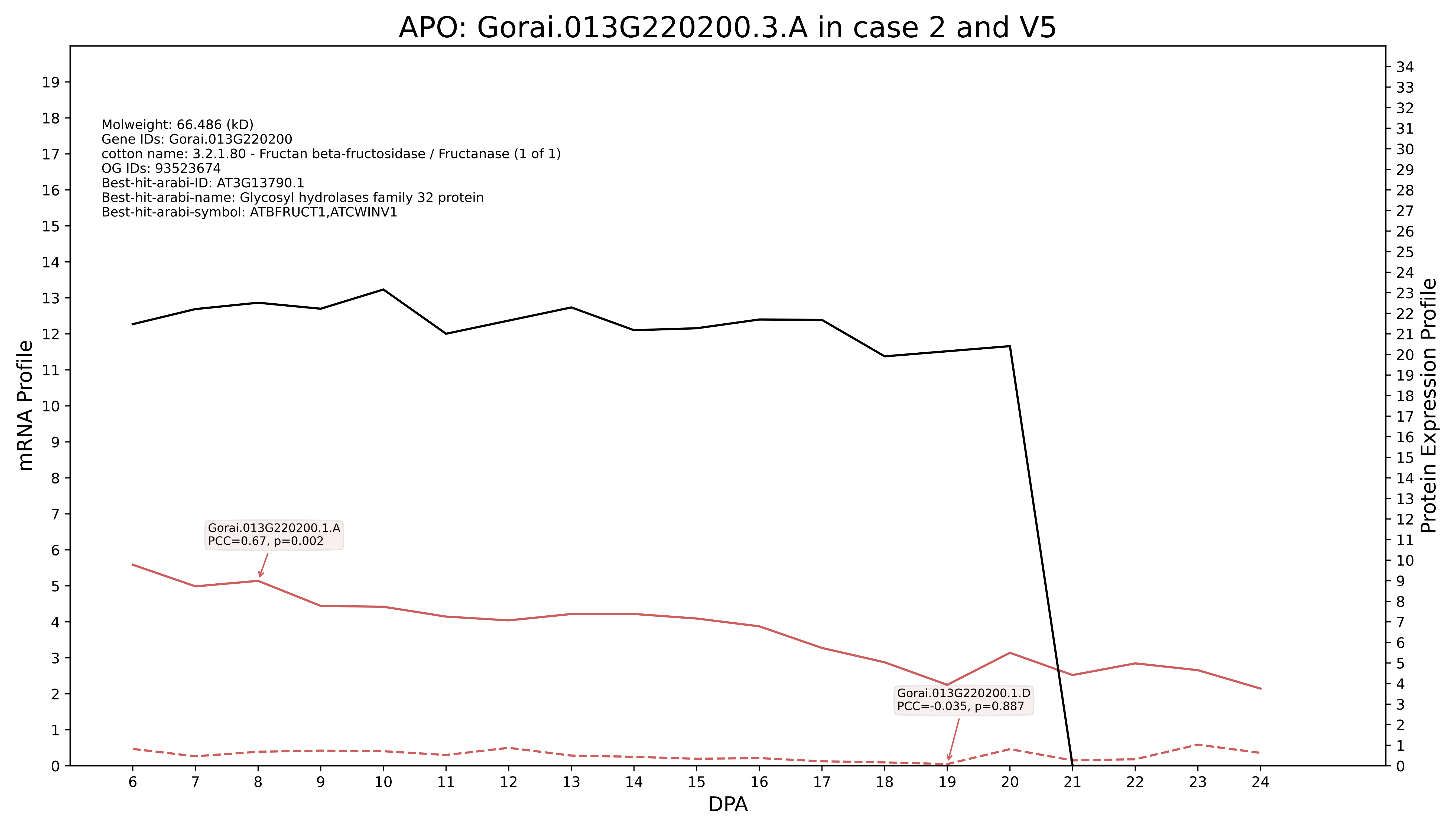
APO: Gorai.013G220200.3.A in case 2 and V5
Molweight: 66.486 (kD)
Gene IDs: Gorai.013G220200
cotton name: 3.2.1.80 - Fructan beta-fructosidase / Fructanase (1 of 1)
OG IDs: 93523674
Best-hit-arabi-ID: AT3G13790.1
Best-hit-arabi-name: Glycosyl hydrolases family 32 protein
Best-hit-arabi-symbol: ATBFRUCT1,ATCWINV1
Gorai.013G220200.1.A
PCC=0.67, p=0.002
Gorai.013G220200.1.D
PCC=-0.035, p=0.887
mRNA Profile
Protein Expression Profile
DPA
0
1
2
3
4
5
6
7
8
9
10
11
12
13
14
15
16
17
18
19
20
21
22
23
24
25
26
27
28
29
30
31
32
33
34

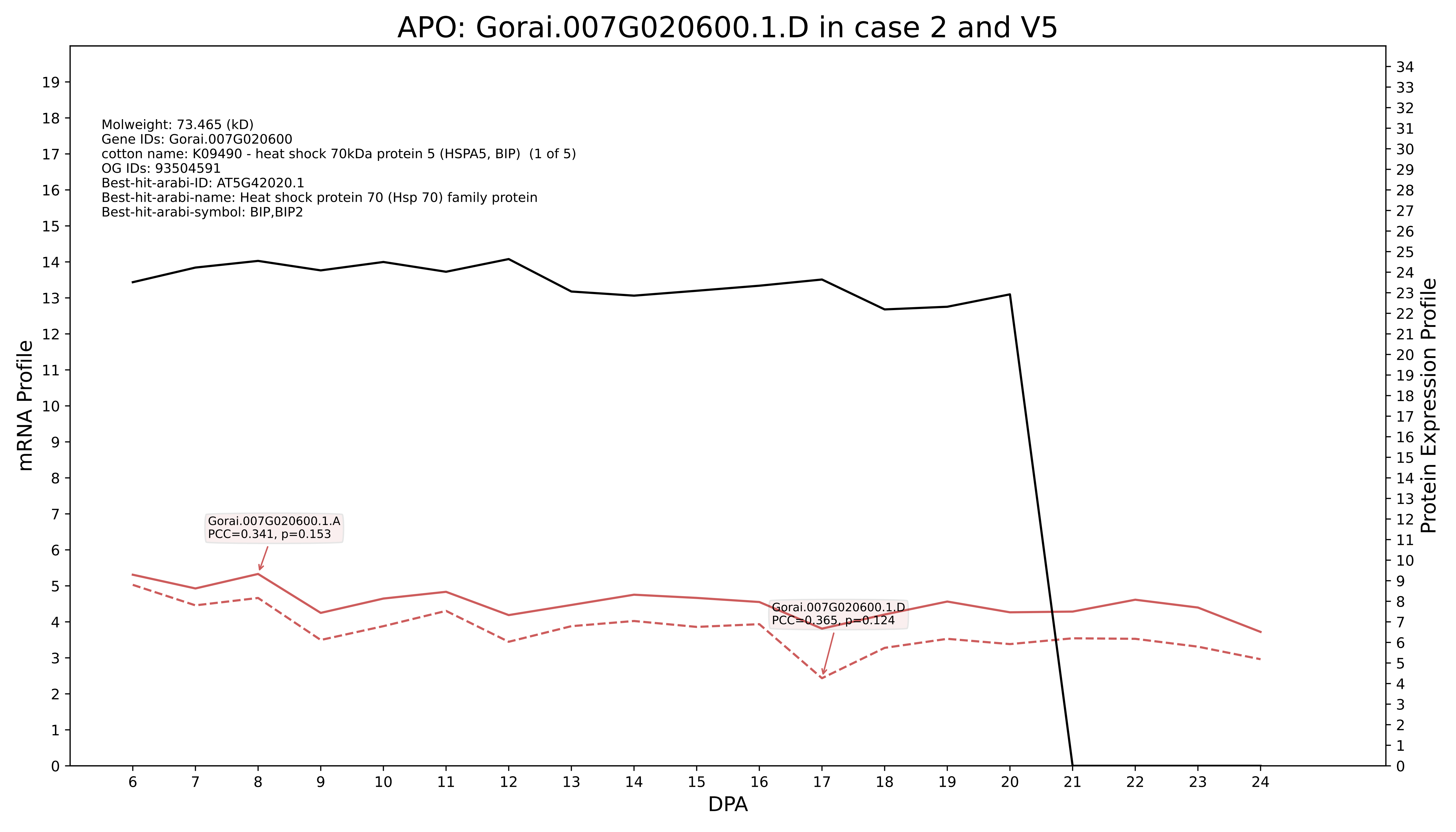
APO: Gorai.007G020600.1.D in case 2 and V5
Molweight: 73.465 (kD)
Gene IDs: Gorai.007G020600
cotton name: K09490 - heat shock 70kDa protein 5 (HSPA5, BIP) (1 of 5)
OG IDs: 93504591
Best-hit-arabi-ID: AT5G42020.1
Best-hit-arabi-name: Heat shock protein 70 (Hsp 70) family protein
Best-hit-arabi-symbol: BIP,BIP2
Gorai.007G020600.1.A
PCC=0.341, p=0.153
Gorai.007G020600.1.D
PCC=0.365, p=0.124
mRNA Profile
Protein Expression Profile
DPA

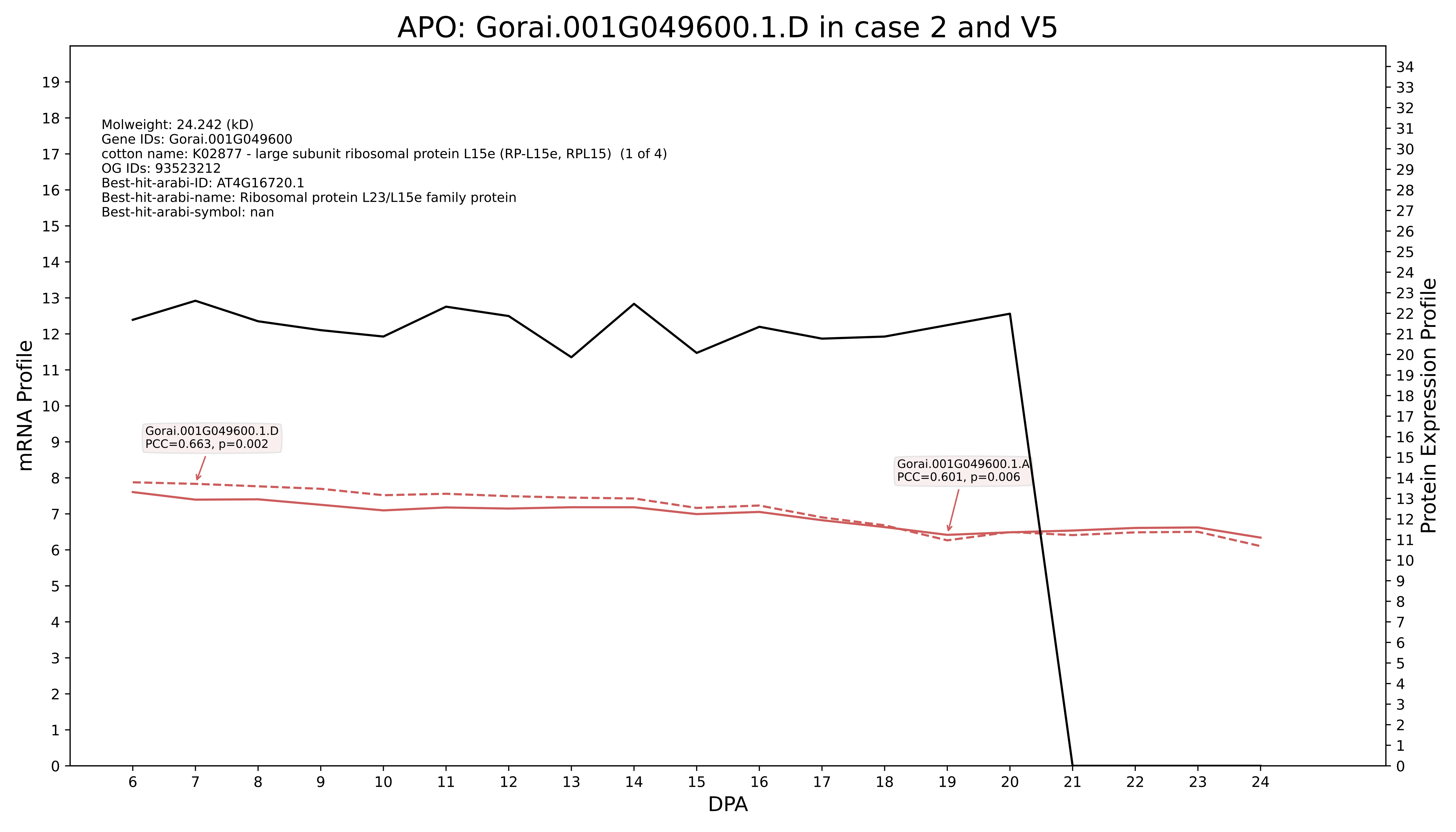
APO: Gorai.001G049600.1.D in case 2 and V5
Molweight: 24.242 (kD)
Gene IDs: Gorai.001G049600
cotton name: K02877 - large subunit ribosomal protein L15e (RP-L15e, RPL15) (1 of 4)
OG IDs: 93523212
Best-hit-arabi-ID: AT4G16720.1
Best-hit-arabi-name: Ribosomal protein L23/L15e family protein
Best-hit-arabi-symbol: nan
Gorai.001G049600.1.D
PCC=0.663, p=0.002
Gorai.001G049600.1.A
PCC=0.601, p=0.006
mRNA Profile
Protein Expression Profile
DPA

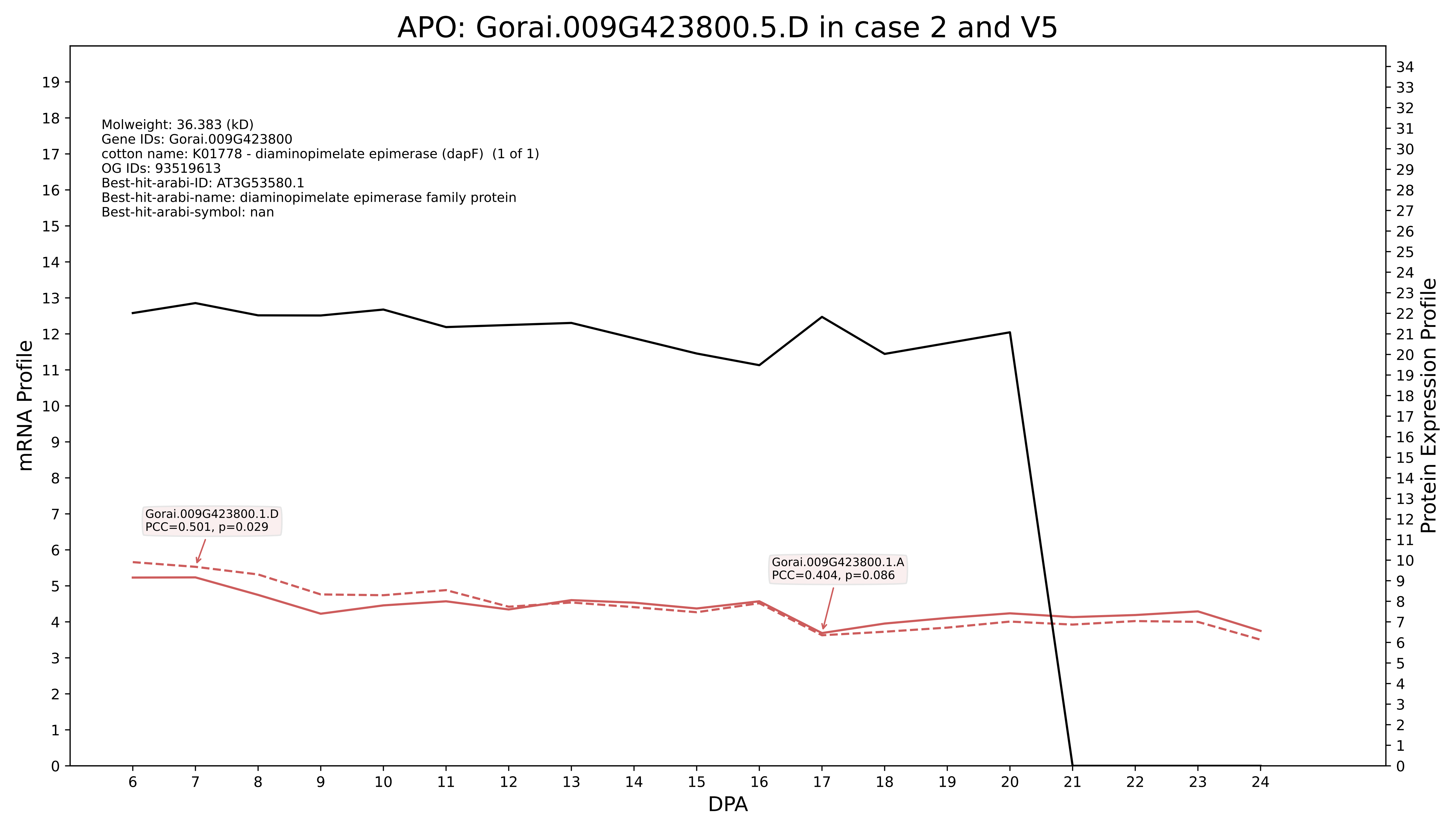
APO: Gorai.009G423800.5.D in case 2 and V5
Molweight: 36.383 (kD)
Gene IDs: Gorai.009G423800
cotton name: K01778 - diaminopimelate epimerase (dapF) (1 of 1)
OG IDs: 93519613
Best-hit-arabi-ID: AT3G53580.1
Best-hit-arabi-name: diaminopimelate epimerase family protein
Best-hit-arabi-symbol: nan
Gorai.009G423800.1.D
PCC=0.501, p=0.029
Gorai.009G423800.1.A
PCC=0.404, p=0.086
mRNA Profile
Protein Expression Profile
DPA

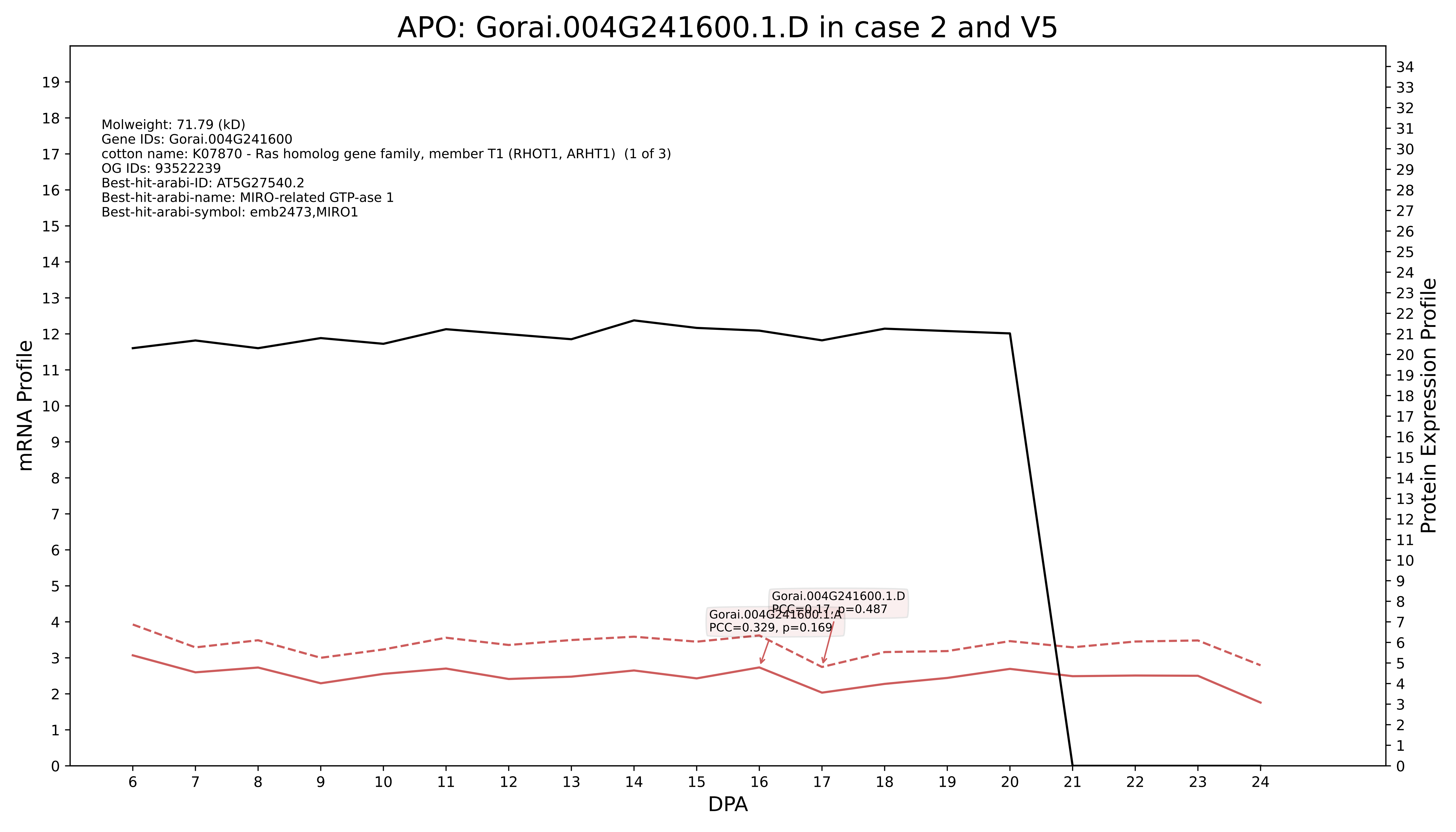
APO: Gorai.004G241600.1.D in case 2 and V5
Molweight: 71.79 (kD)
Gene IDs: Gorai.004G241600
cotton name: K07870 - Ras homolog gene family, member T1 (RHOT1, ARHT1) (1 of 3)
OG IDs: 93522239
Best-hit-arabi-ID: AT5G27540.2
Best-hit-arabi-name: MIRO-related GTP-ase 1
Best-hit-arabi-symbol: emb2473,MIRO1
Gorai.004G241600.1.D
PCC=0.17, p=0.487
Gorai.004G241600.1.A
PCC=0.329, p=0.169
mRNA Profile
Protein Expression Profile
DPA

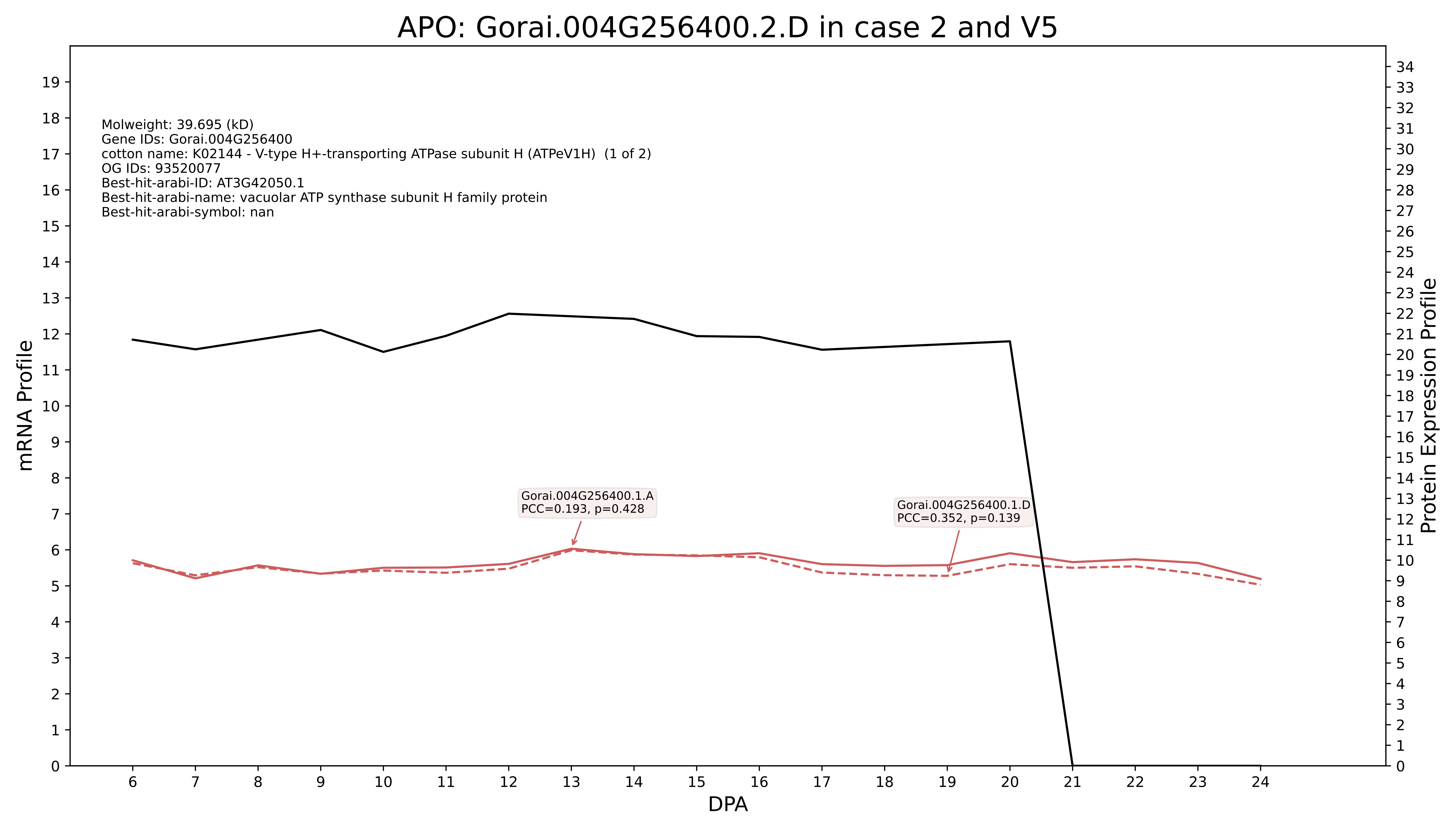
APO: Gorai.004G256400.2.D in case 2 and V5
Molweight: 39.695 (kD)
Gene IDs: Gorai.004G256400
cotton name: K02144 - V-type H+-transporting ATPase subunit H (ATPeV1H) (1 of 2)
OG IDs: 93520077
Best-hit-arabi-ID: AT3G42050.1
Best-hit-arabi-name: vacuolar ATP synthase subunit H family protein
Best-hit-arabi-symbol: nan
Gorai.004G256400.1.A
PCC=0.193, p=0.428
Gorai.004G256400.1.D
PCC=0.352, p=0.139
mRNA Profile
Protein Expression Profile
DPA

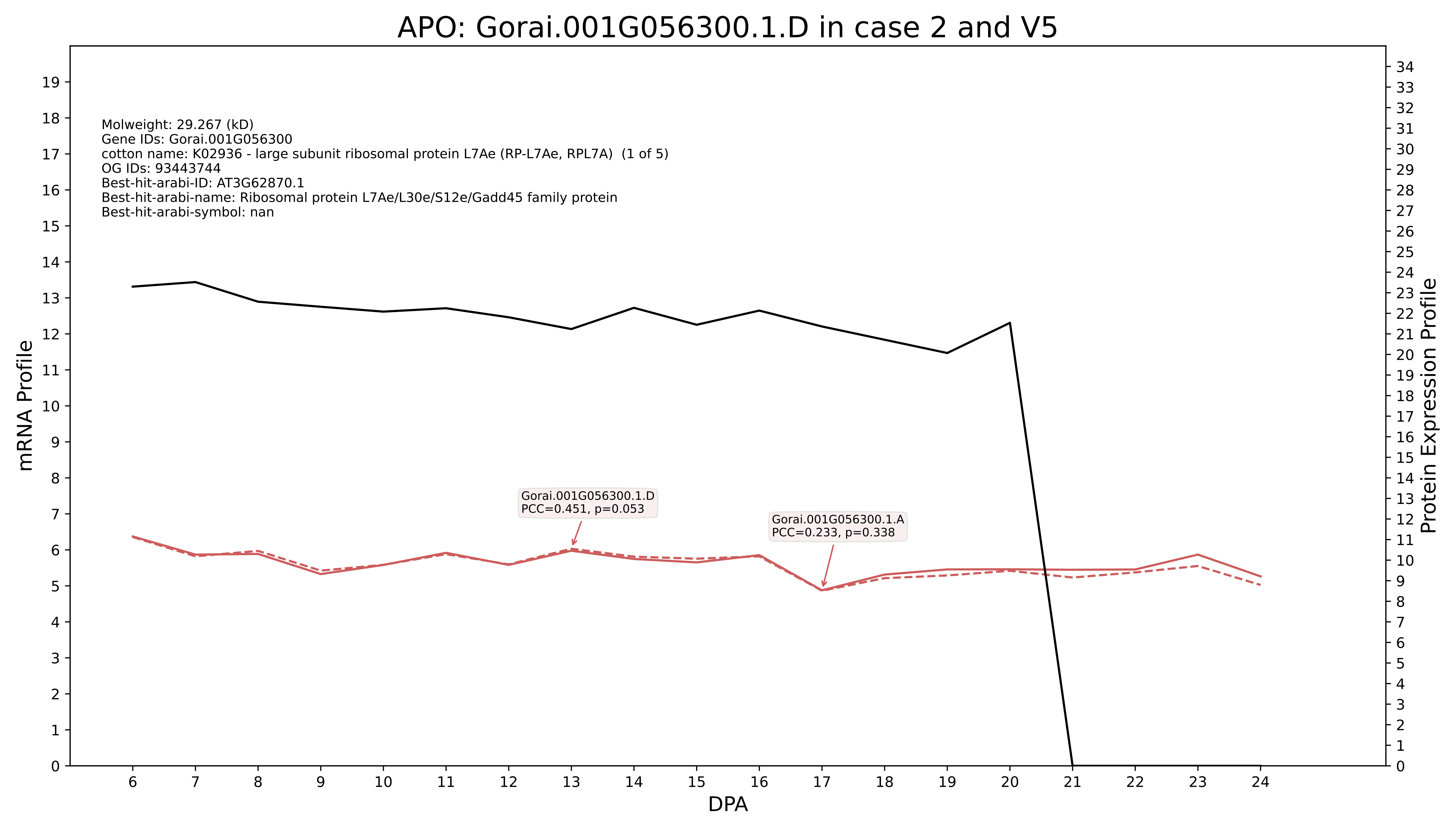

APO: Gorai.001G056300.1.D in case 2 and V5
Molweight: 29.267 (kD)
Gene IDs: Gorai.001G056300
cotton name: K02936 - large subunit ribosomal protein L7Ae (RP-L7Ae, RPL7A) (1 of 5)
OG IDs: 93443744
Best-hit-arabi-ID: AT3G62870.1
Best-hit-arabi-name: Ribosomal protein L7Ae/L30e/S12e/Gadd45 family protein
Best-hit-arabi-symbol: nan
Gorai.001G056300.1.D
PCC=0.451, p=0.053
Gorai.001G056300.1.A
PCC=0.233, p=0.338
mRNA Profile
Protein Expression Profile
DPA

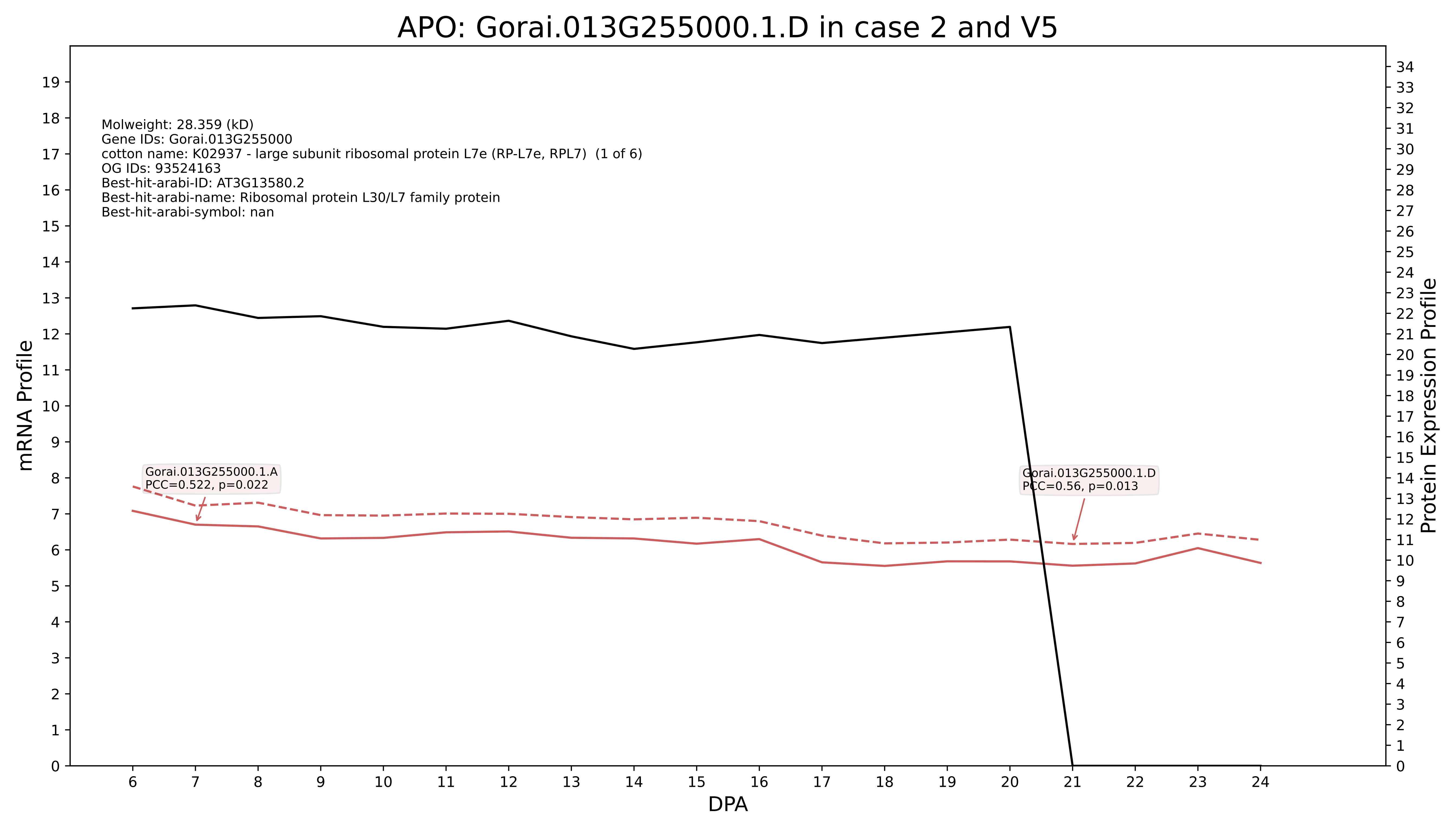
APO: Gorai.013G255000.1.D in case 2 and V5
Molweight: 28.359 (kD)
Gene IDs: Gorai.013G255000
cotton name: K02937 - large subunit ribosomal protein L7e (RP-L7e, RPL7) (1 of 6)
OG IDs: 93524163
Best-hit-arabi-ID: AT3G13580.2
Best-hit-arabi-name: Ribosomal protein L30/L7 family protein
Best-hit-arabi-symbol: nan
Gorai.013G255000.1.A
PCC=0.522, p=0.022
Gorai.013G255000.1.D
PCC=0.56, p=0.013
mRNA Profile
Protein Expression Profile
DPA
6 7 8 9 10 11 12 13 14 15 16 17 18 19 20 21 22 23 24

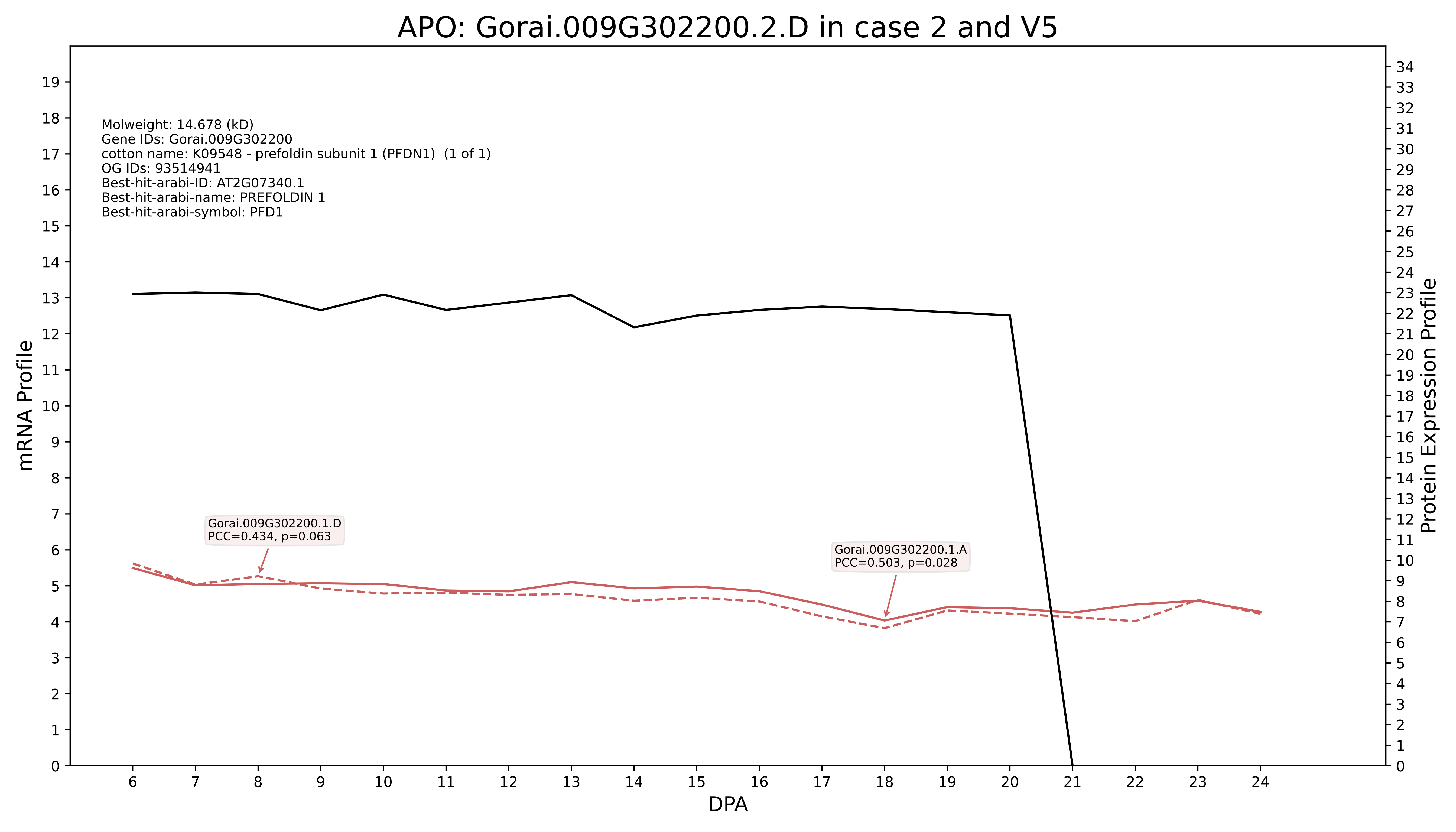
APO: Gorai.009G302200.2.D in case 2 and V5
Molweight: 14.678 (kD)
Gene IDs: Gorai.009G302200
cotton name: K09548 - prefoldin subunit 1 (PFDN1) (1 of 1)
OG IDs: 93514941
Best-hit-arabi-ID: AT2G07340.1
Best-hit-arabi-name: PREFOLDIN 1
Best-hit-arabi-symbol: PFD1
Gorai.009G302200.1.D
PCC=0.434, p=0.063
Gorai.009G302200.1.A
PCC=0.503, p=0.028
mRNA Profile
Protein Expression Profile
DPA

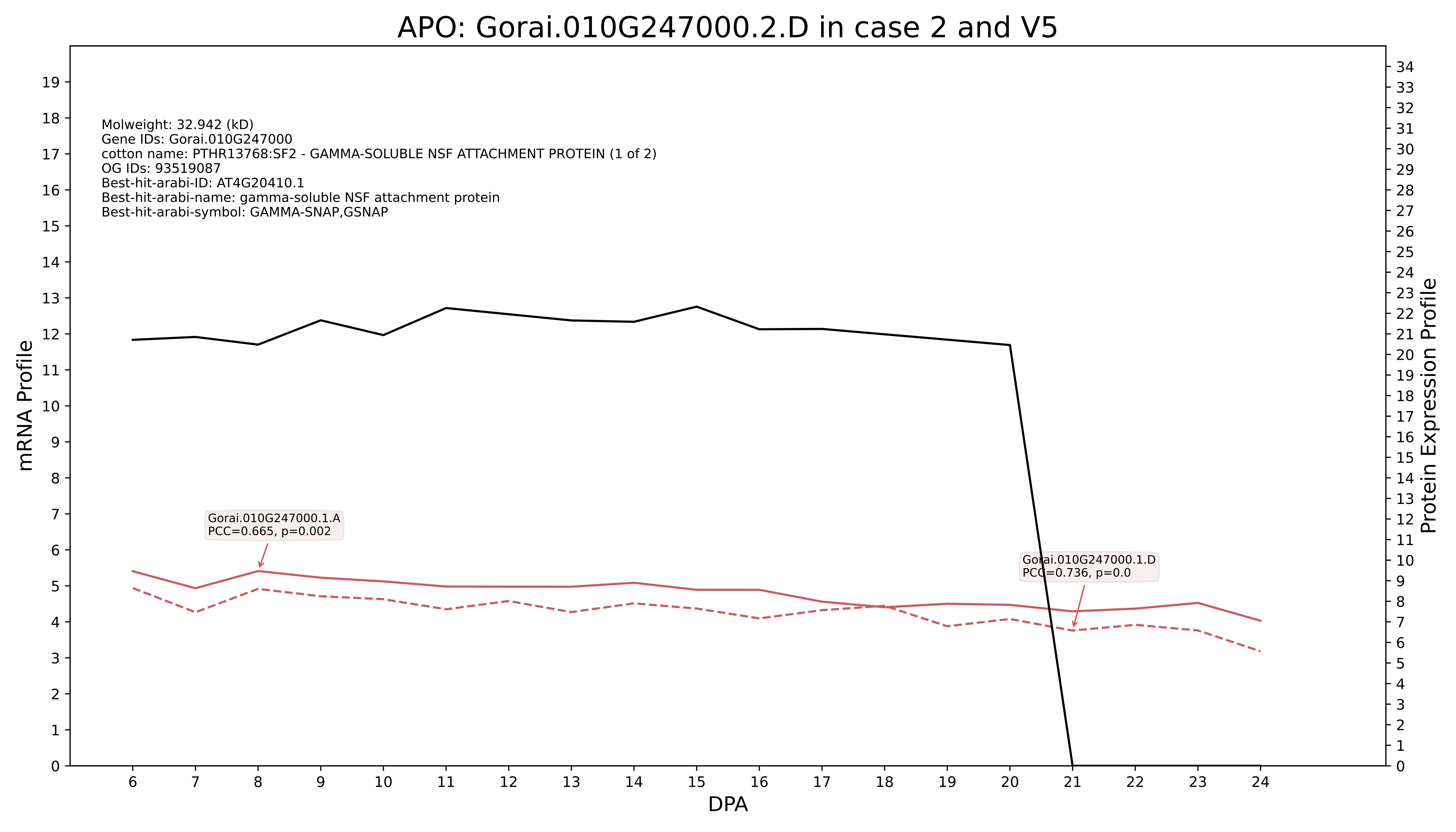

APO: Gorai.010G247000.2.D in case 2 and V5
Molweight: 32.942 (kD)
Gene IDs: Gorai.010G247000
cotton name: PTHR13768:SF2 - GAMMA-SOLUBLE NSF ATTACHMENT PROTEIN (1 of 2)
OG IDs: 93519087
Best-hit-arabi-ID: AT4G20410.1
Best-hit-arabi-name: gamma-soluble NSF attachment protein
Best-hit-arabi-symbol: GAMMA-SNAP,GSNAP
Gorai.010G247000.1.A
PCC=0.665, p=0.002
Gorai.010G247000.1.D
PCC=0.736, p=0.0
mRNA Profile
Protein Expression Profile
DPA

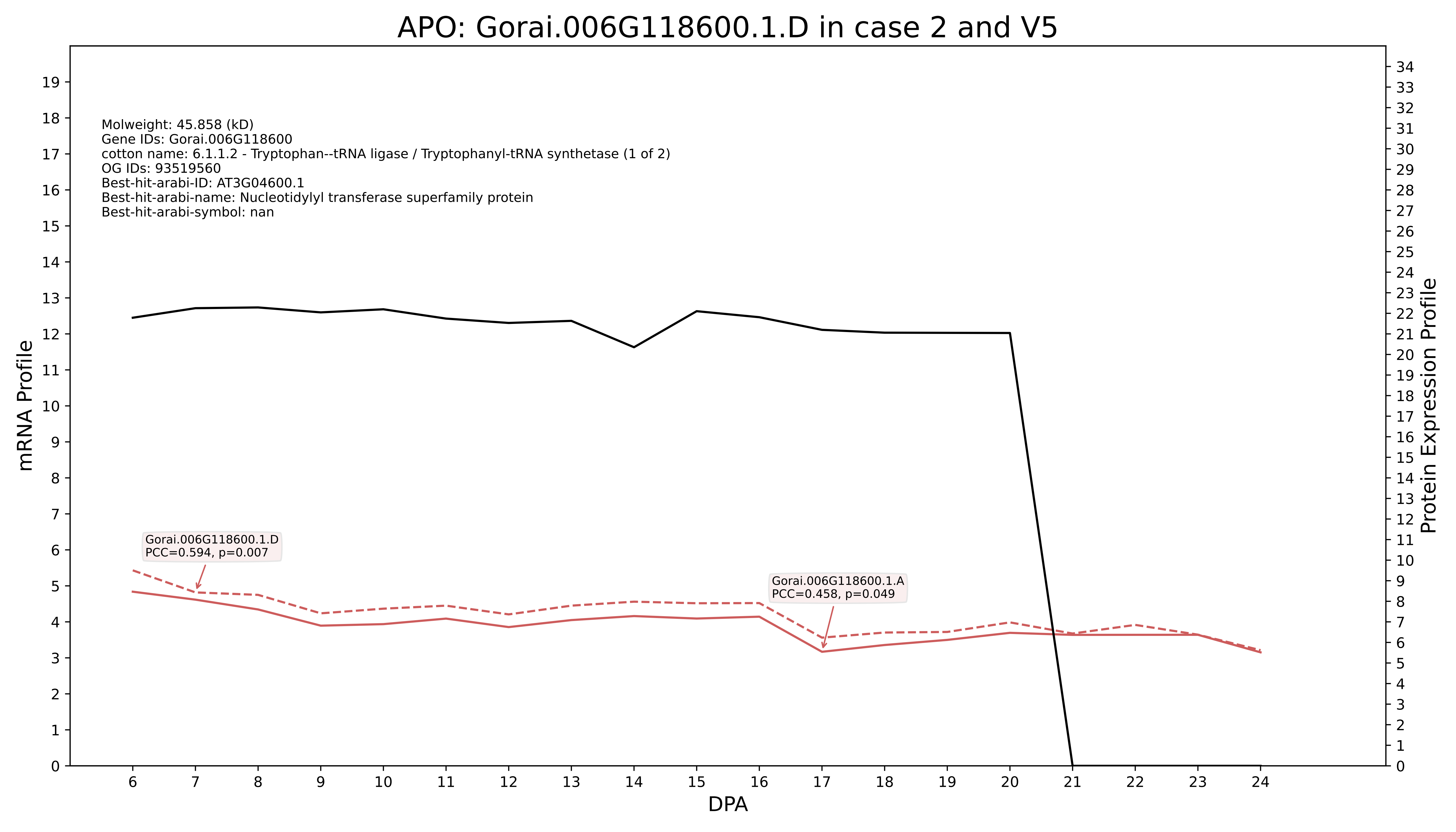
APO: Gorai.006G118600.1.D in case 2 and V5
Molweight: 45.858 (kD)
Gene IDs: Gorai.006G118600
cotton name: 6.1.1.2 - Tryptophan--tRNA ligase / Tryptophanyl-tRNA synthetase (1 of 2)
OG IDs: 93519560
Best-hit-arabi-ID: AT3G04600.1
Best-hit-arabi-name: Nucleotidylyl transferase superfamily protein
Best-hit-arabi-symbol: nan
Gorai.006G118600.1.D
PCC=0.594, p=0.007
Gorai.006G118600.1.A
PCC=0.458, p=0.049
mRNA Profile
Protein Expression Profile
DPA

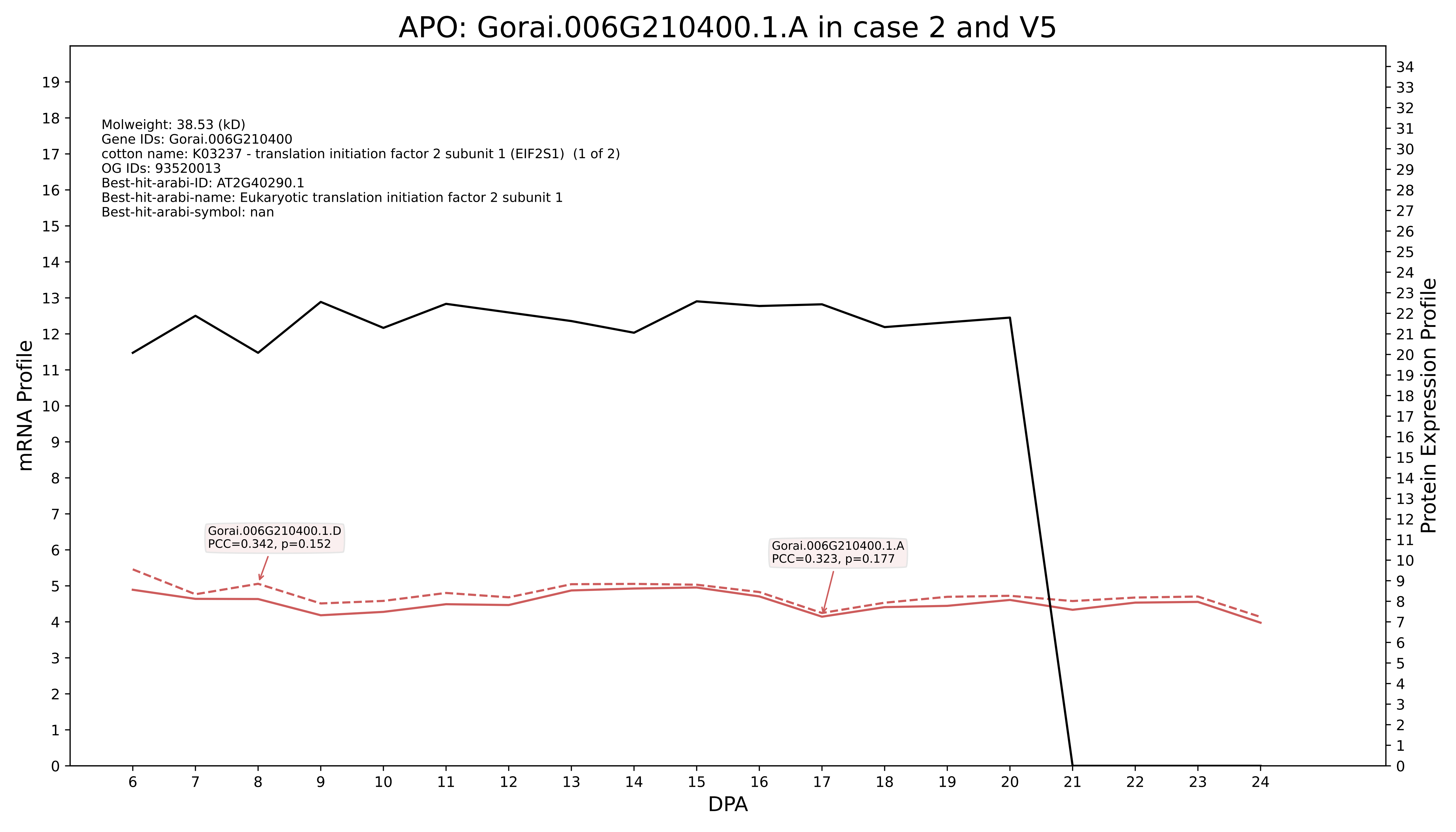

APO: Gorai.006G210400.1.A in case 2 and V5
Molweight: 38.53 (kD)
Gene IDs: Gorai.006G210400
cotton name: K03237 - translation initiation factor 2 subunit 1 (EIF2S1) (1 of 2)
OG IDs: 93520013
Best-hit-arabi-ID: AT2G40290.1
Best-hit-arabi-name: Eukaryotic translation initiation factor 2 subunit 1
Best-hit-arabi-symbol: nan
Gorai.006G210400.1.D
PCC=0.342, p=0.152
Gorai.006G210400.1.A
PCC=0.323, p=0.177
mRNA Profile
Protein Expression Profile
DPA

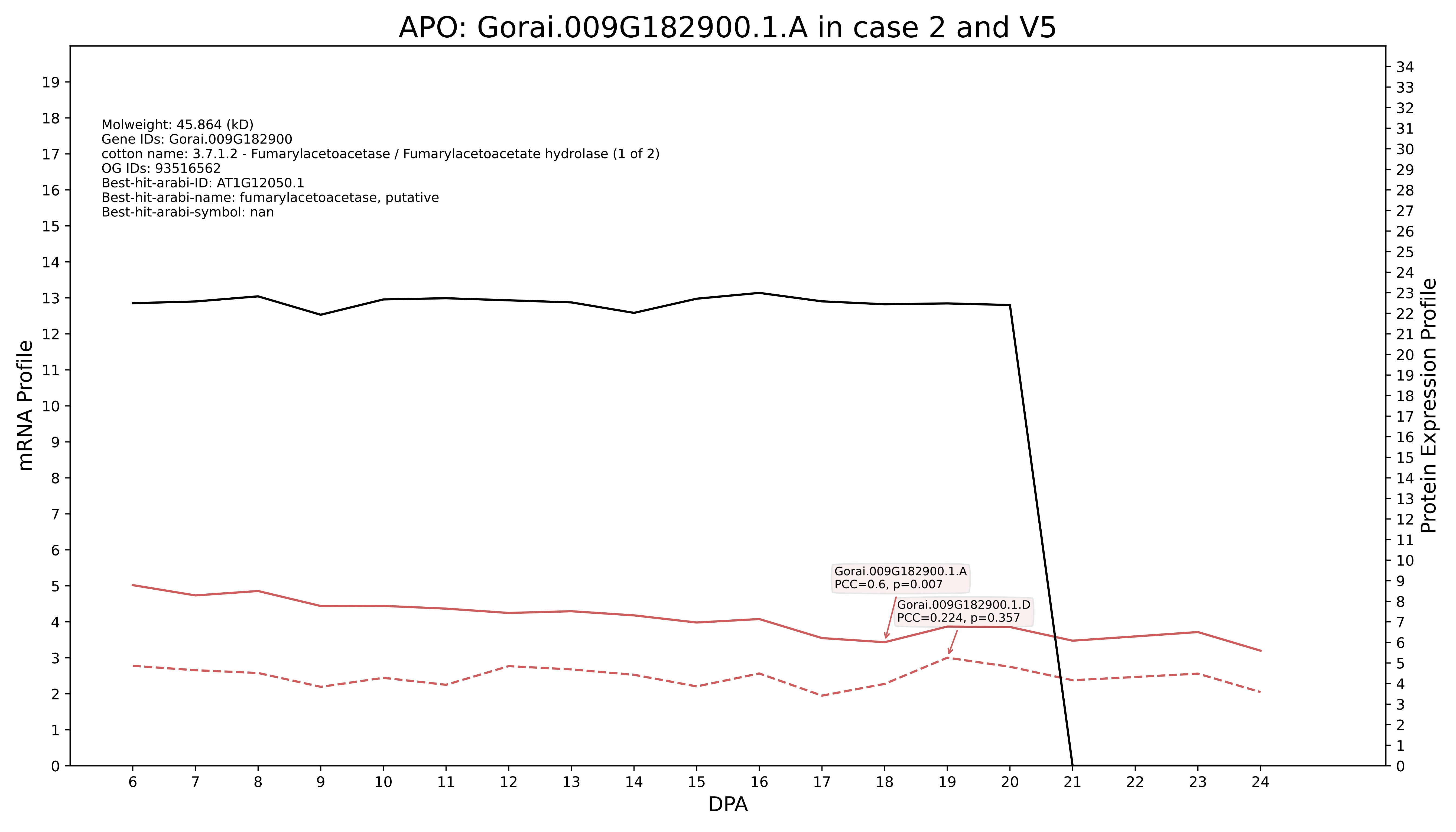
APO: Gorai.009G182900.1.A in case 2 and V5
Molweight: 45.864 (kD)
Gene IDs: Gorai.009G182900
cotton name: 3.7.1.2 - Fumarylacetoacetase / Fumarylacetoacetate hydrolase (1 of 2)
OG IDs: 93516562
Best-hit-arabi-ID: AT1G12050.1
Best-hit-arabi-name: fumarylacetoacetase, putative
Best-hit-arabi-symbol: nan
Gorai.009G182900.1.A
PCC=0.6, p=0.007
Gorai.009G182900.1.D
PCC=0.224, p=0.357
mRNA Profile
Protein Expression Profile
DPA

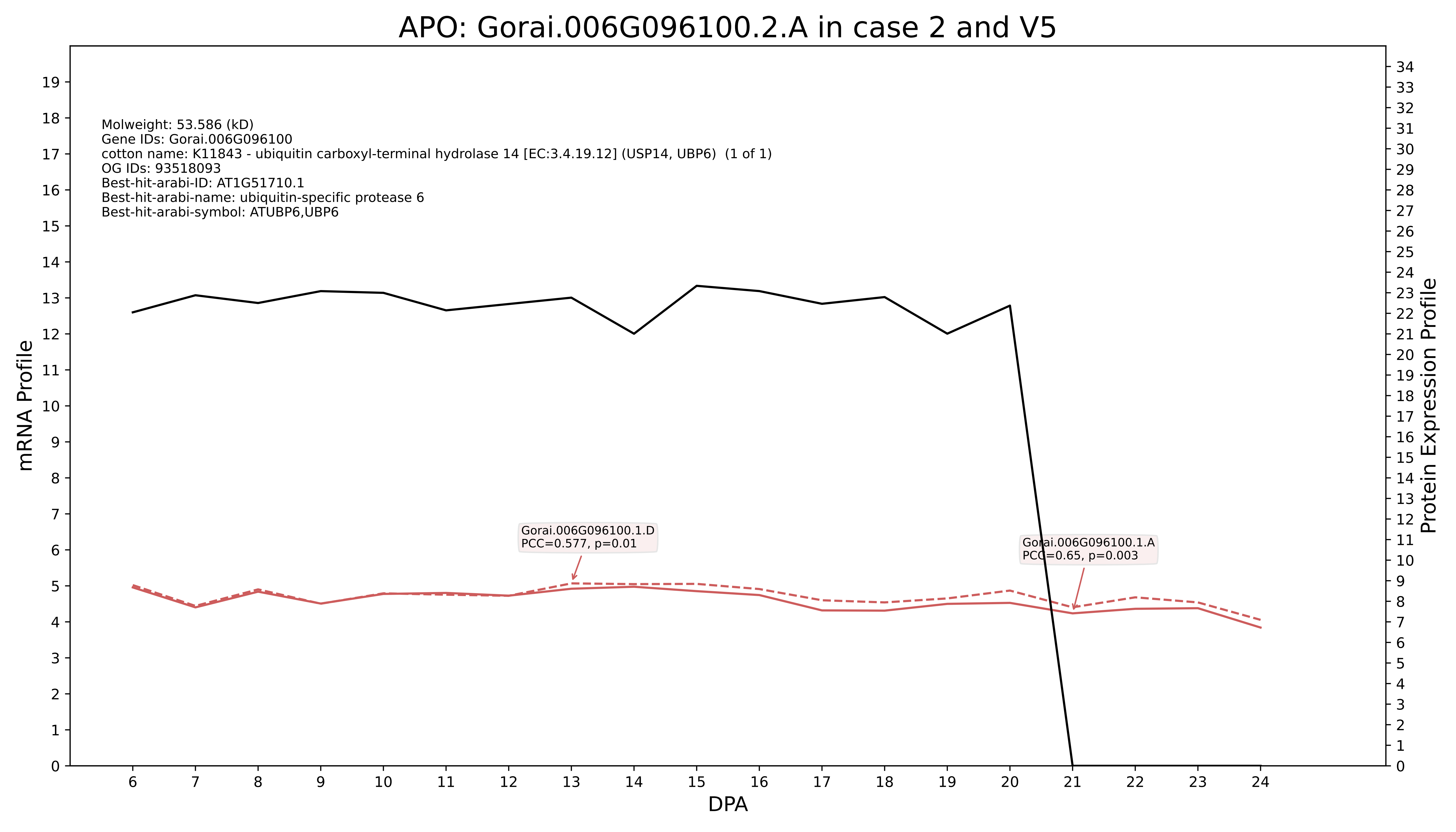
APO: Gorai.006G096100.2.A in case 2 and V5
Molweight: 53.586 (kD)
Gene IDs: Gorai.006G096100
cotton name: K11843 - ubiquitin carboxyl-terminal hydrolase 14 [EC:3.4.19.12] (USP14, UBP6) (1 of 1)
OG IDs: 93518093
Best-hit-arabi-ID: AT1G51710.1
Best-hit-arabi-name: ubiquitin-specific protease 6
Best-hit-arabi-symbol: ATUBP6,UBP6
Gorai.006G096100.1.D
PCC=0.577, p=0.01
Gorai.006G096100.1.A
PCC=0.65, p=0.003
mRNA Profile
Protein Expression Profile
DPA

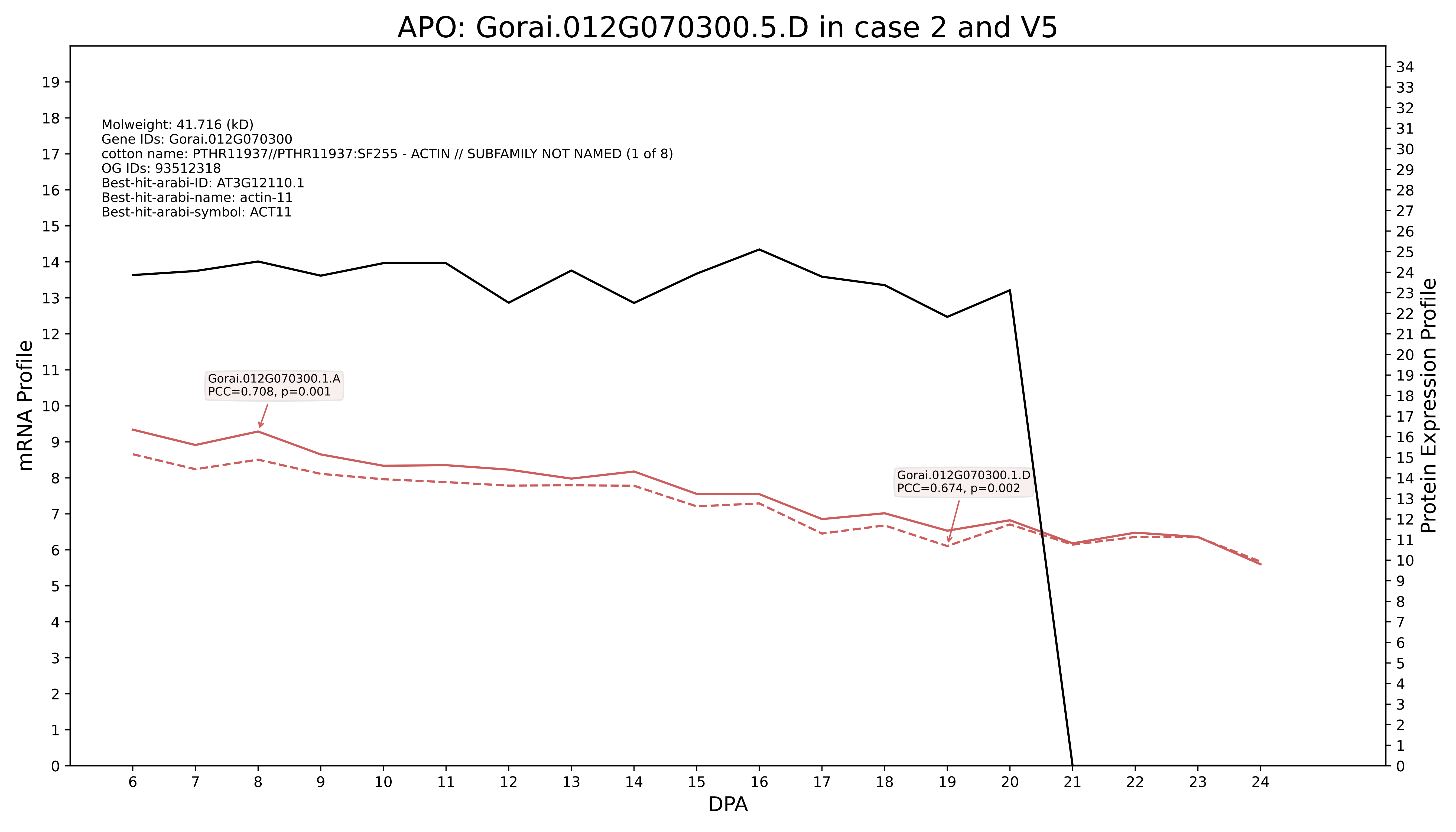
APO: Gorai.012G070300.5.D in case 2 and V5
Molweight: 41.716 (kD)
Gene IDs: Gorai.012G070300
cotton name: PTHR11937//PTHR11937:SF255 - ACTIN // SUBFAMILY NOT NAMED (1 of 8)
OG IDs: 93512318
Best-hit-arabi-ID: AT3G12110.1
Best-hit-arabi-name: actin-11
Best-hit-arabi-symbol: ACT11
Gorai.012G070300.1.A
PCC=0.708, p=0.001
Gorai.012G070300.1.D
PCC=0.674, p=0.002
mRNA Profile
Protein Expression Profile
DPA

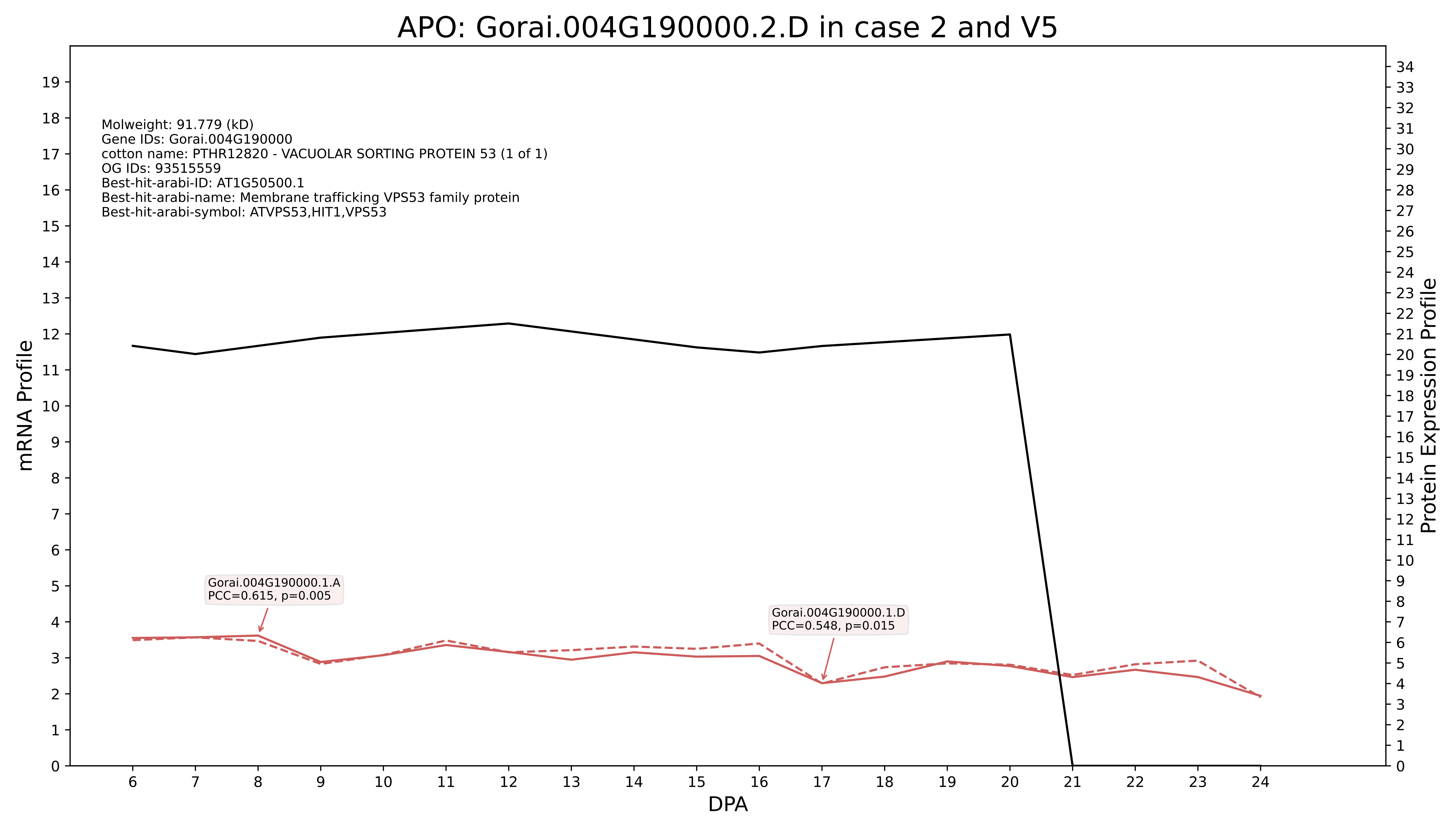

APO: Gorai.004G190000.2.D in case 2 and V5
Molweight: 91.779 (kD)
Gene IDs: Gorai.004G190000
cotton name: PTHR12820 - VACUOLAR SORTING PROTEIN 53 (1 of 1)
OG IDs: 93515559
Best-hit-arabi-ID: AT1G50500.1
Best-hit-arabi-name: Membrane trafficking VPS53 family protein
Best-hit-arabi-symbol: ATVPS53,HIT1,VPS53
Gorai.004G190000.1.A
PCC=0.615, p=0.005
Gorai.004G190000.1.D
PCC=0.548, p=0.015
mRNA Profile
Protein Expression Profile
DPA

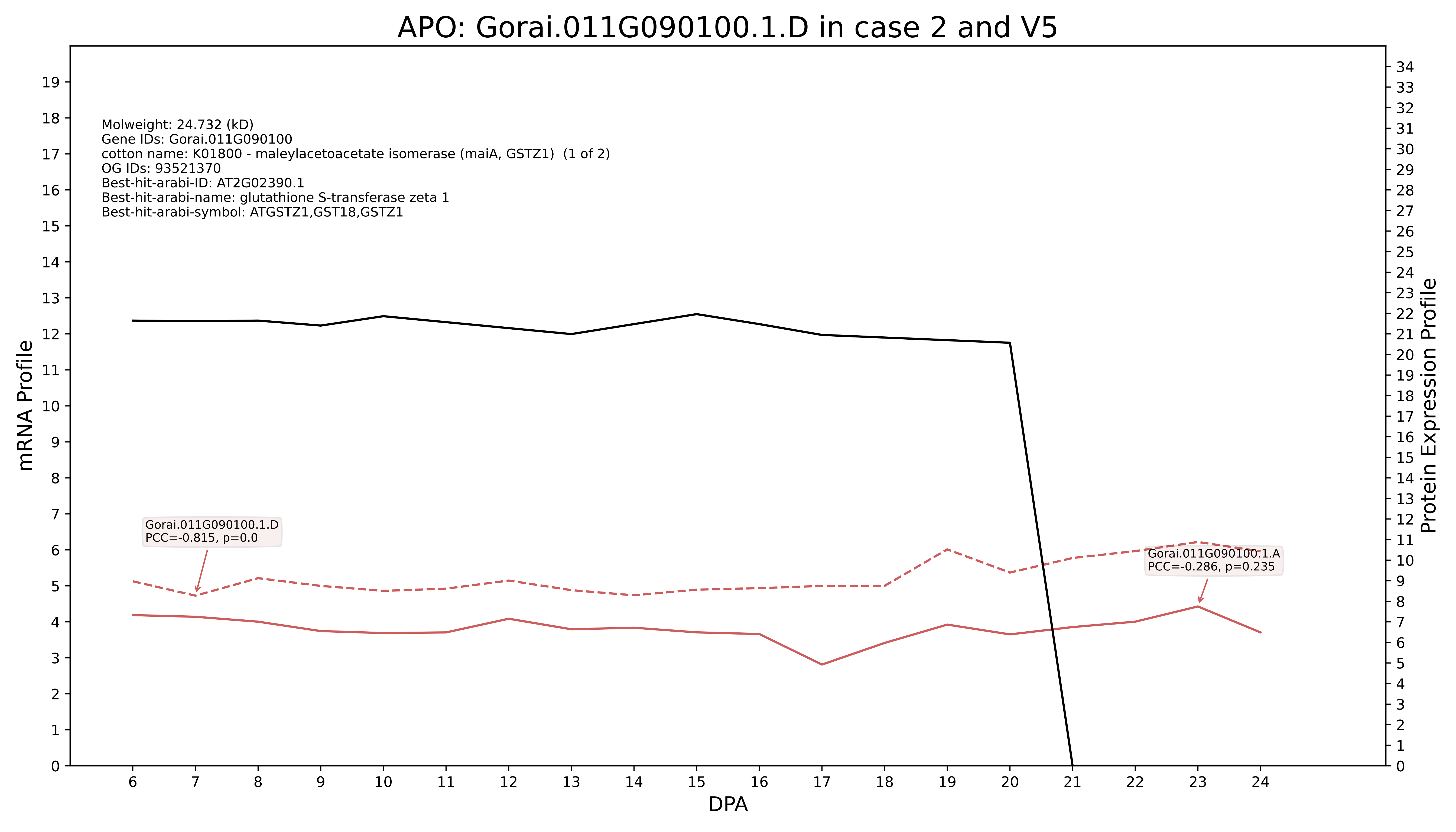
APO: Gorai.011G090100.1.D in case 2 and V5
Molweight: 24.732 (kD)
Gene IDs: Gorai.011G090100
cotton name: K01800 - maleylacetoacetate isomerase (maiA, GSTZ1) (1 of 2)
OG IDs: 93521370
Best-hit-arabi-ID: AT2G02390.1
Best-hit-arabi-name: glutathione S-transferase zeta 1
Best-hit-arabi-symbol: ATGSTZ1,GST18,GSTZ1
Gorai.011G090100.1.D
PCC=-0.815, p=0.0
Gorai.011G090100.1.A
PCC=-0.286, p=0.235
mRNA Profile
Protein Expression Profile
DPA

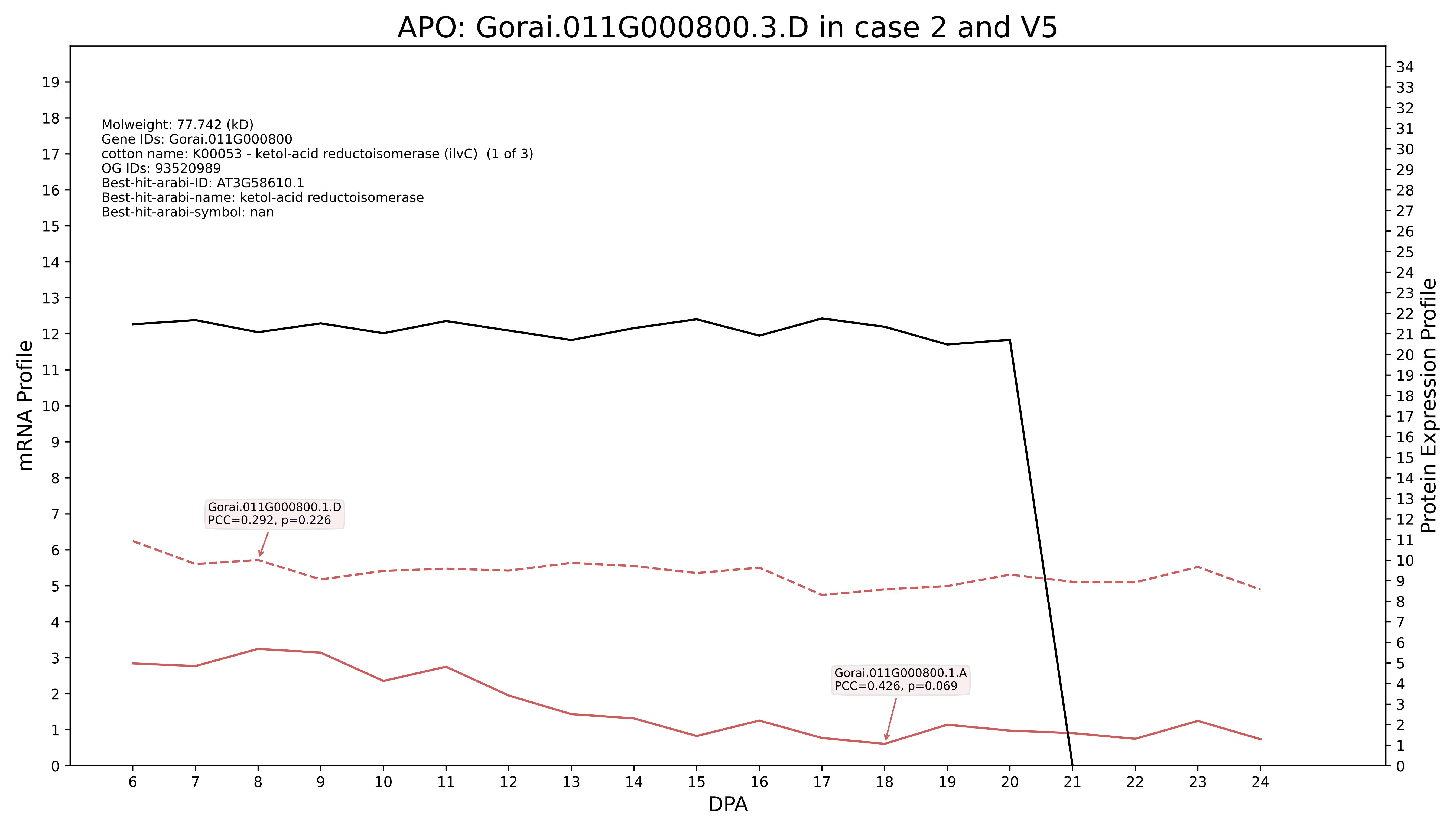

APO: Gorai.011G000800.3.D in case 2 and V5
Molweight: 77.742 (kD)
Gene IDs: Gorai.011G000800
cotton name: K00053 - ketol-acid reductoisomerase (ilvC) (1 of 3)
OG IDs: 93520989
Best-hit-arabi-ID: AT3G58610.1
Best-hit-arabi-name: ketol-acid reductoisomerase
Best-hit-arabi-symbol: nan
Gorai.011G000800.1.D
PCC=0.292, p=0.226
Gorai.011G000800.1.A
PCC=0.426, p=0.069
mRNA Profile
Protein Expression Profile
DPA

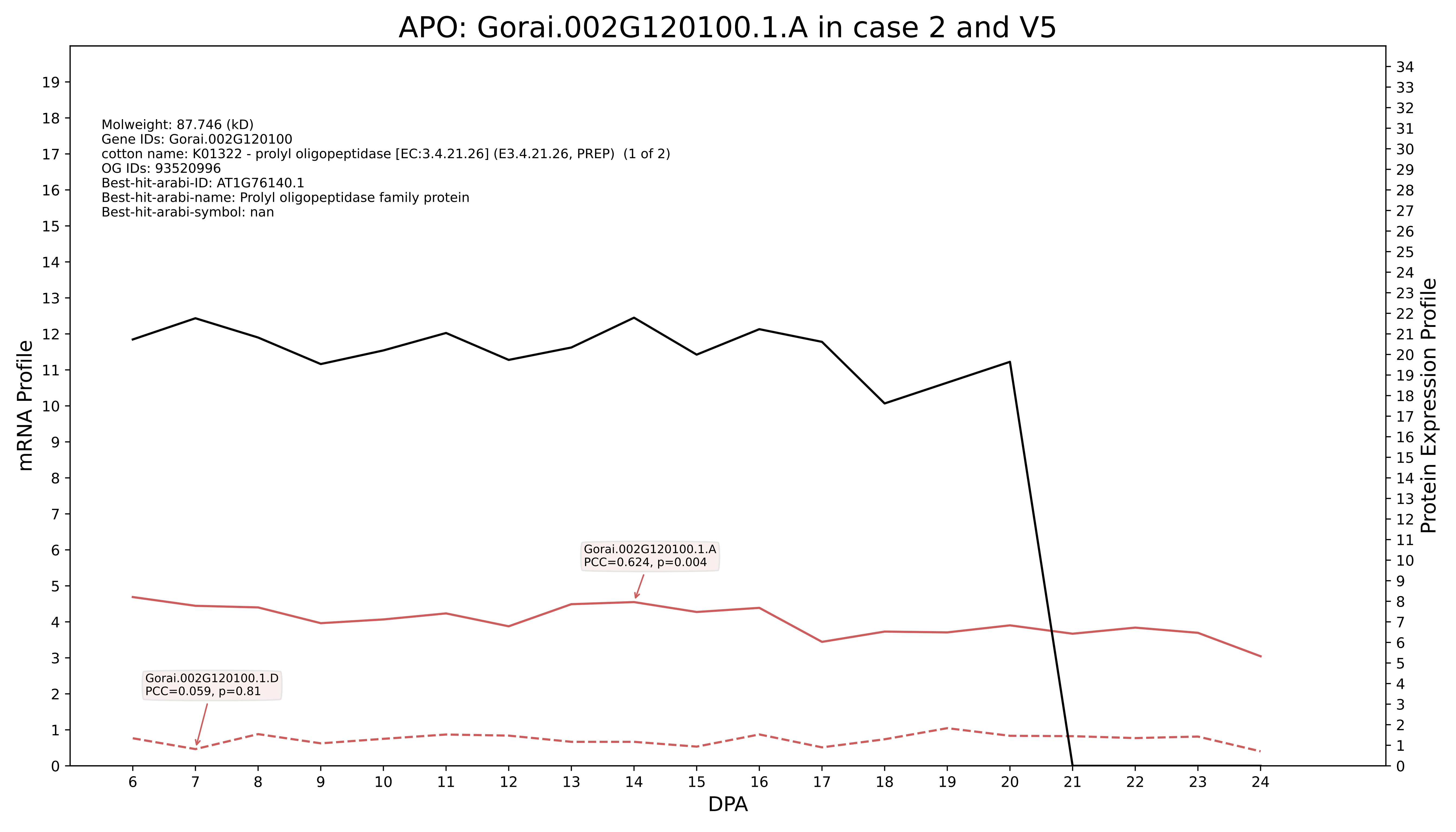
APO: Gorai.002G120100.1.A in case 2 and V5
Molweight: 87.746 (kD)
Gene IDs: Gorai.002G120100
cotton name: K01322 - prolyl oligopeptidase [EC:3.4.21.26] (E3.4.21.26, PREP) (1 of 2)
OG IDs: 93520996
Best-hit-arabi-ID: AT1G76140.1
Best-hit-arabi-name: Prolyl oligopeptidase family protein
Best-hit-arabi-symbol: nan
Gorai.002G120100.1.A
PCC=0.624, p=0.004
Gorai.002G120100.1.D
PCC=0.059, p=0.81
mRNA Profile
Protein Expression Profile
DPA

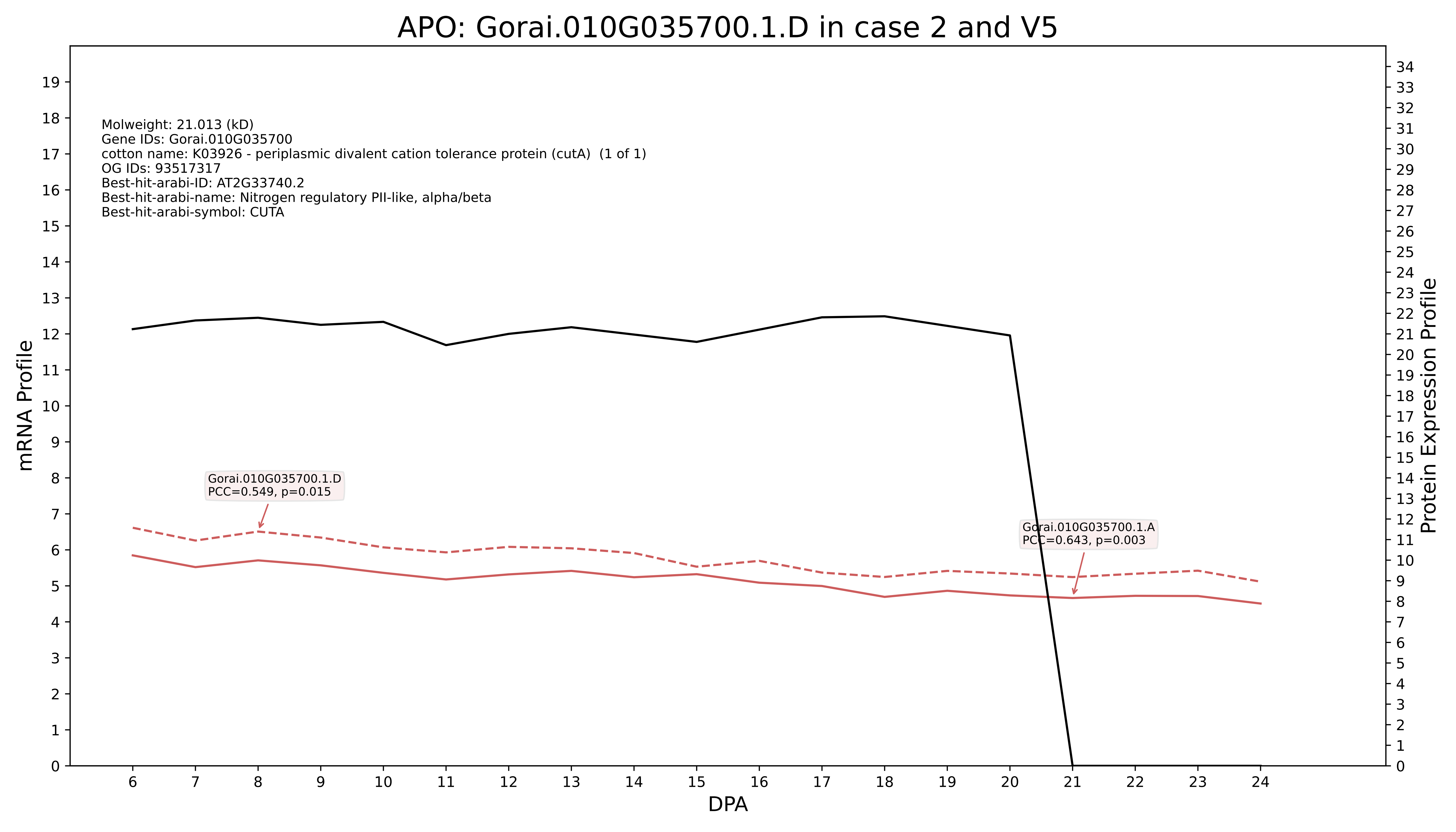

APO: Gorai.010G035700.1.D in case 2 and V5
Molweight: 21.013 (kD)
Gene IDs: Gorai.010G035700
cotton name: K03926 - periplasmic divalent cation tolerance protein (cutA) (1 of 1)
OG IDs: 93517317
Best-hit-arabi-ID: AT2G33740.2
Best-hit-arabi-name: Nitrogen regulatory PII-like, alpha/beta
Best-hit-arabi-symbol: CUTA
Gorai.010G035700.1.D
PCC=0.549, p=0.015
Gorai.010G035700.1.A
PCC=0.643, p=0.003
mRNA Profile
Protein Expression Profile
DPA

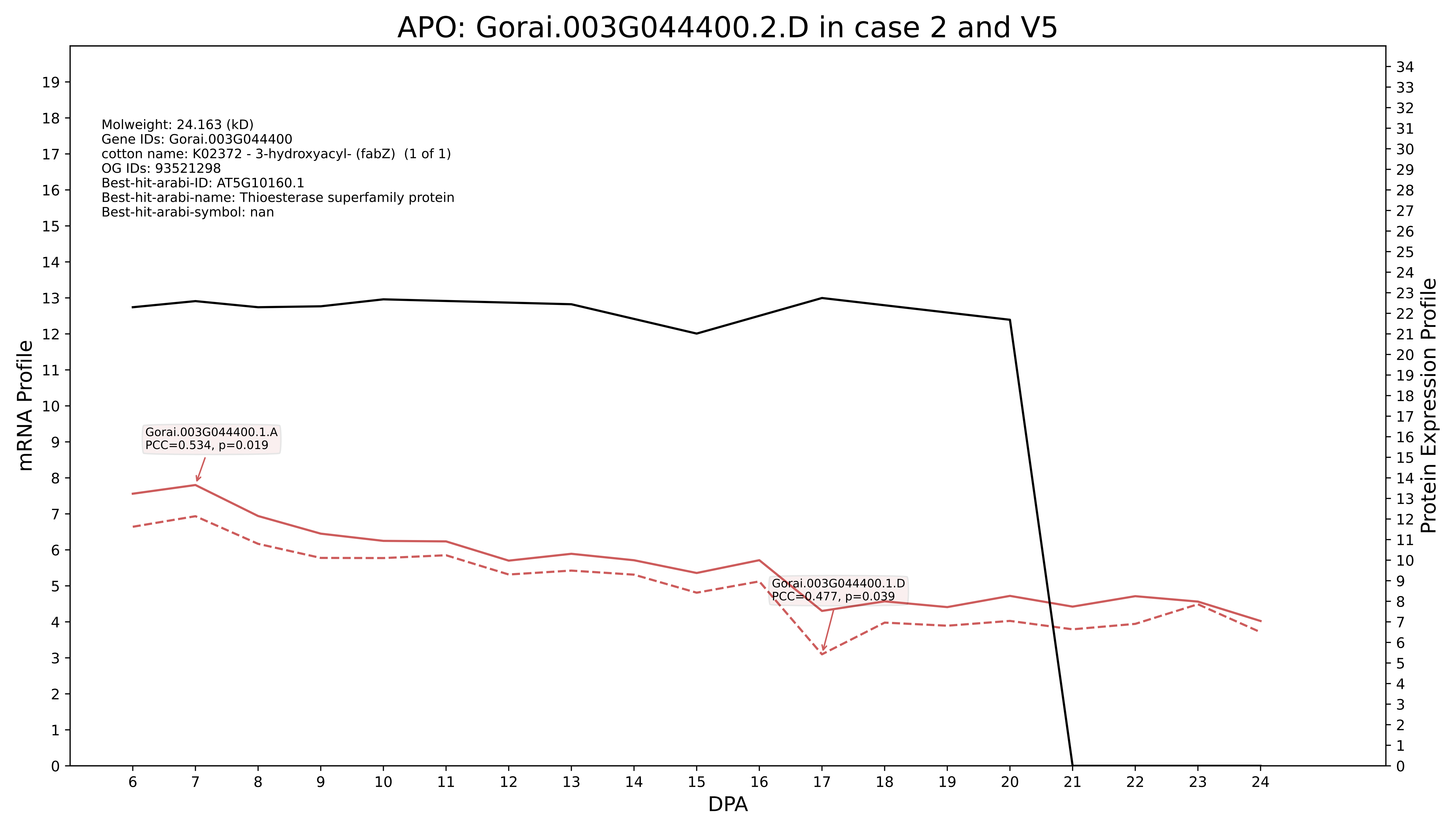

APO: Gorai.003G044400.2.D in case 2 and V5
Molweight: 24.163 (kD)
Gene IDs: Gorai.003G044400
cotton name: K02372 - 3-hydroxyacyl- (fabZ) (1 of 1)
OG IDs: 93521298
Best-hit-arabi-ID: AT5G10160.1
Best-hit-arabi-name: Thioesterase superfamily protein
Best-hit-arabi-symbol: nan
Gorai.003G044400.1.A
PCC=0.534, p=0.019
Gorai.003G044400.1.D
PCC=0.477, p=0.039
mRNA Profile
Protein Expression Profile
DPA

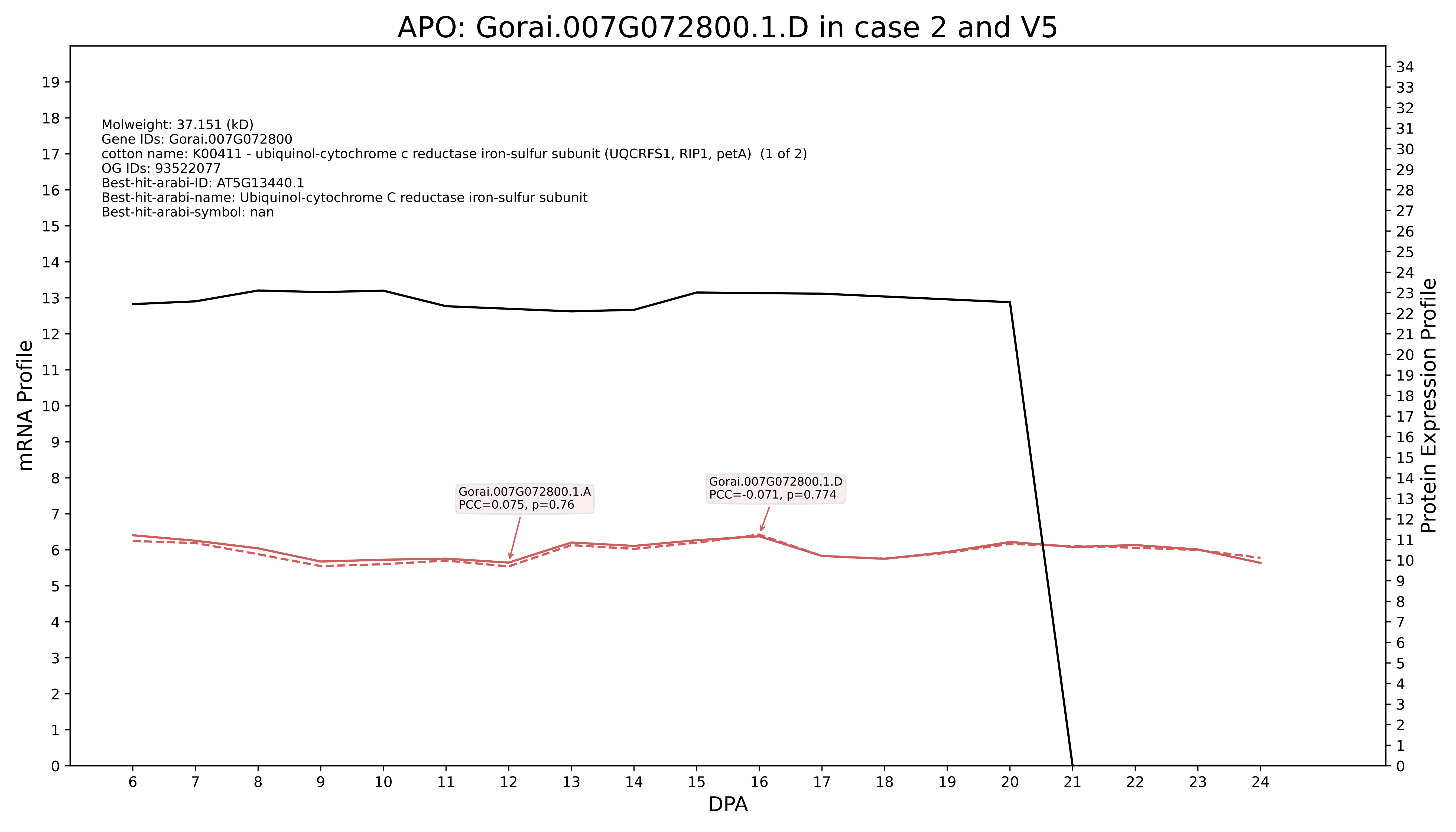
APO: Gorai.007G072800.1.D in case 2 and V5
Molweight: 37.151 (kD)
Gene IDs: Gorai.007G072800
cotton name: K00411 - ubiquinol-cytochrome c reductase iron-sulfur subunit (UQCRFS1, RIP1, petA) (1 of 2)
OG IDs: 93522077
Best-hit-arabi-ID: AT5G13440.1
Best-hit-arabi-name: Ubiquinol-cytochrome C reductase iron-sulfur subunit
Best-hit-arabi-symbol: nan
Gorai.007G072800.1.A
PCC=0.075, p=0.76
Gorai.007G072800.1.D
PCC=-0.071, p=0.774
mRNA Profile
Protein Expression Profile
DPA

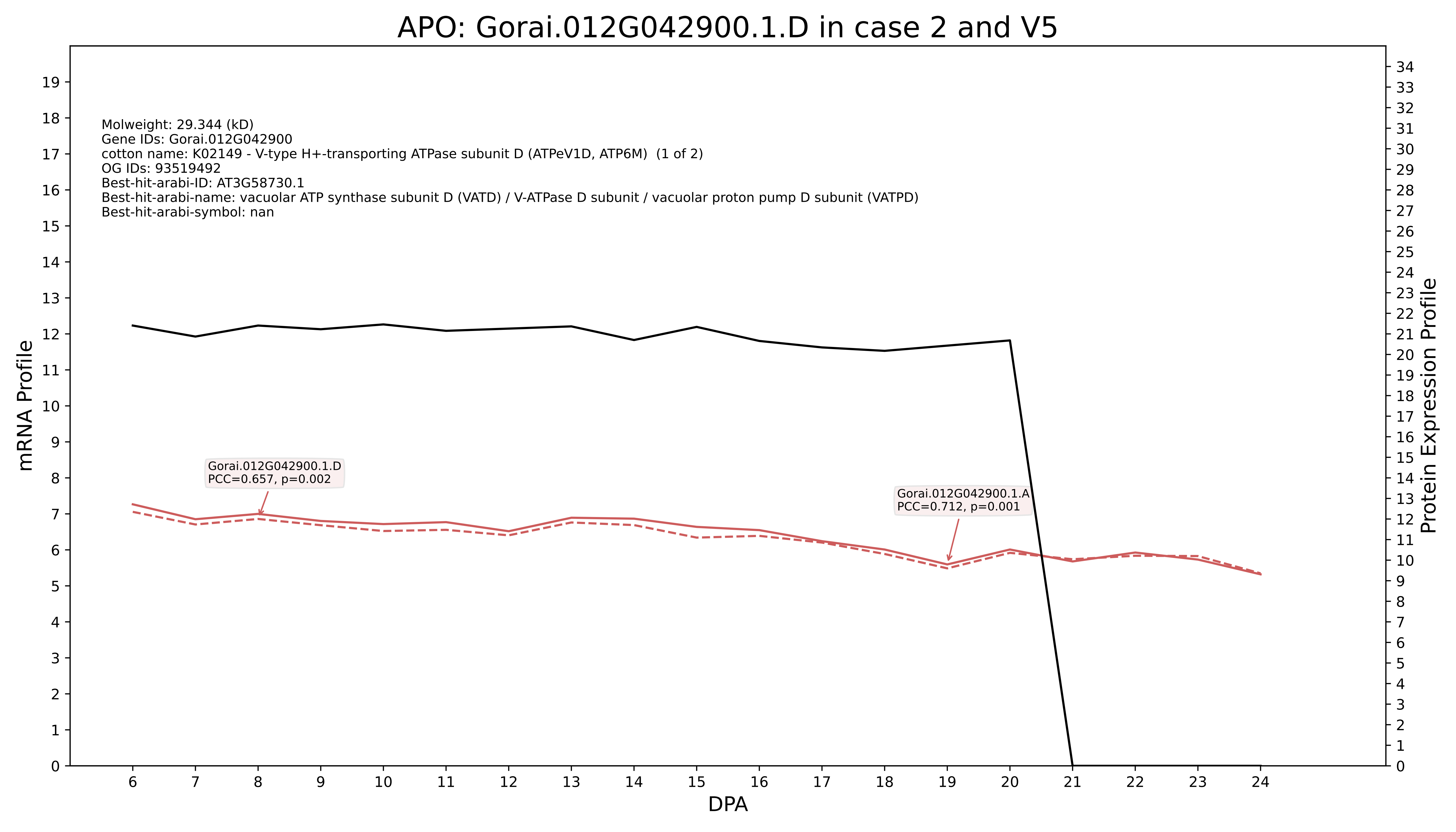
APO: Gorai.012G042900.1.D in case 2 and V5
Molweight: 29.344 (kD)
Gene IDs: Gorai.012G042900
cotton name: K02149 - V-type H+-transporting ATPase subunit D (ATPeV1D, ATP6M) (1 of 2)
OG IDs: 93519492
Best-hit-arabi-ID: AT3G58730.1
Best-hit-arabi-name: vacuolar ATP synthase subunit D (VATD) / V-ATPase D subunit / vacuolar proton pump D subunit (VATPD)
Best-hit-arabi-symbol: nan
Gorai.012G042900.1.D
PCC=0.657, p=0.002
Gorai.012G042900.1.A
PCC=0.712, p=0.001
mRNA Profile
Protein Expression Profile
DPA
0
1
2
3
4
5
6
7
8
9
10
11
12
13
14
15
16
17
18
19
20
21
22
23
24
25
26
27
28
29
30
31
32
33
34

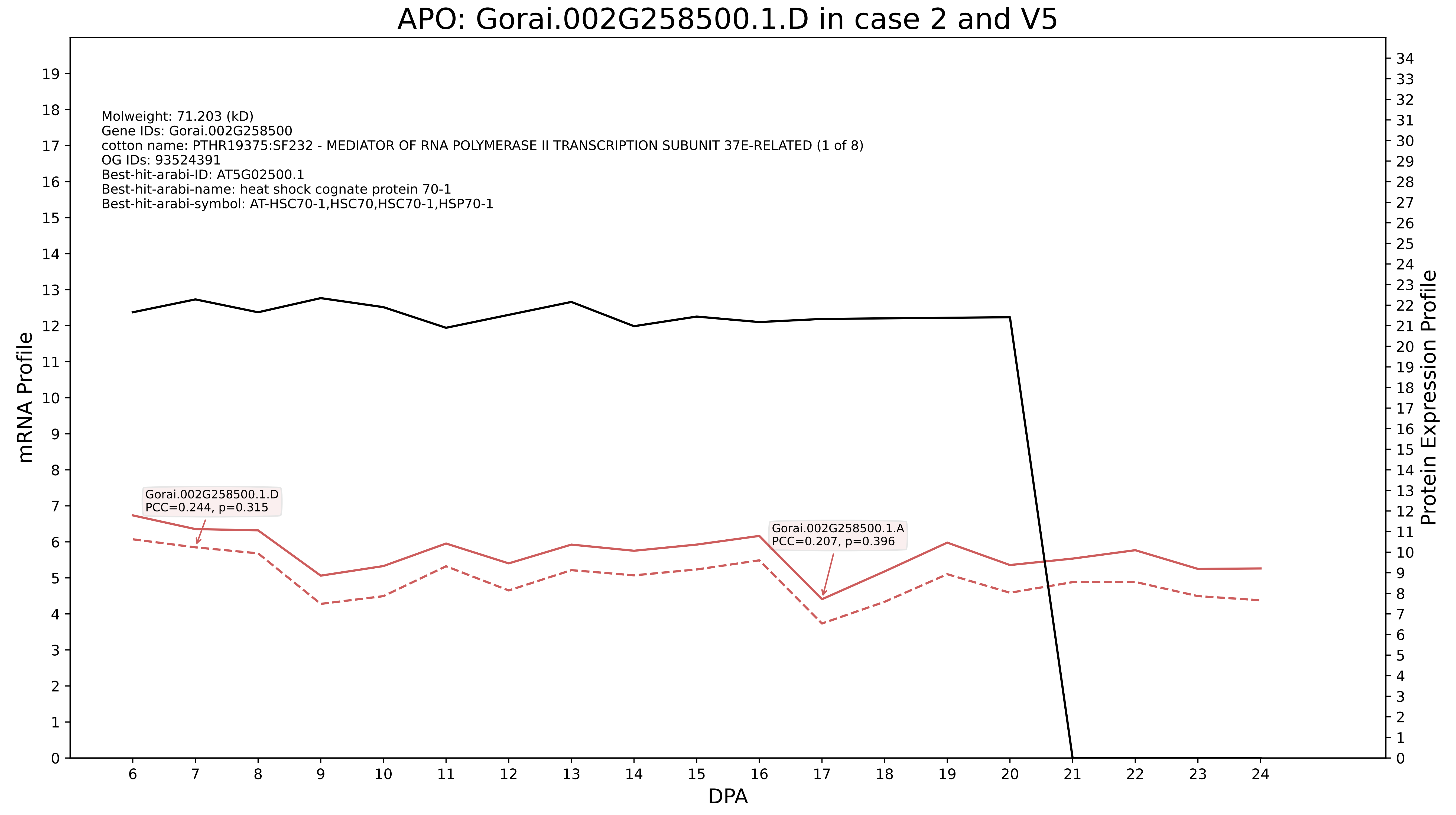
APO: Gorai.002G258500.1.D in case 2 and V5
Molweight: 71.203 (kD)
Gene IDs: Gorai.002G258500
cotton name: PTHR19375:SF232 - MEDIATOR OF RNA POLYMERASE II TRANSCRIPTION SUBUNIT 37E-RELATED (1 of 8)
OG IDs: 93524391
Best-hit-arabi-ID: AT5G02500.1
Best-hit-arabi-name: heat shock cognate protein 70-1
Best-hit-arabi-symbol: AT-HSC70-1,HSC70,HSC70-1,HSP70-1
Gorai.002G258500.1.D
PCC=0.244, p=0.315
Gorai.002G258500.1.A
PCC=0.207, p=0.396
mRNA Profile
Protein Expression Profile
DPA

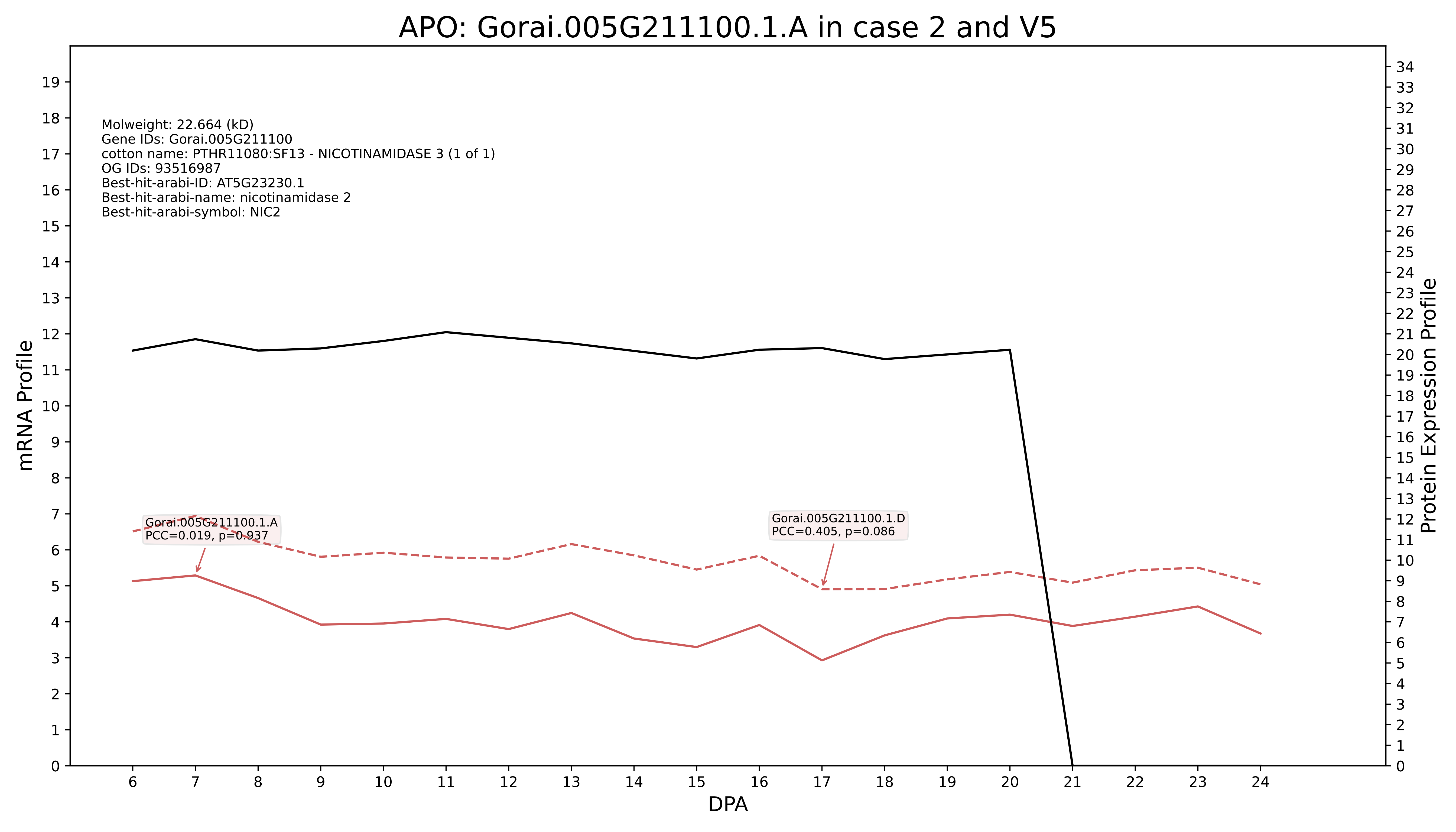

APO: Gorai.005G211100.1.A in case 2 and V5
Molweight: 22.664 (kD)
Gene IDs: Gorai.005G211100
cotton name: PTHR11080:SF13 - NICOTINAMIDASE 3 (1 of 1)
OG IDs: 93516987
Best-hit-arabi-ID: AT5G23230.1
Best-hit-arabi-name: nicotinamidase 2
Best-hit-arabi-symbol: NIC2
Gorai.005G211100.1.A
PCC=0.019, p=0.937
Gorai.005G211100.1.D
PCC=0.405, p=0.086
mRNA Profile
Protein Expression Profile
DPA

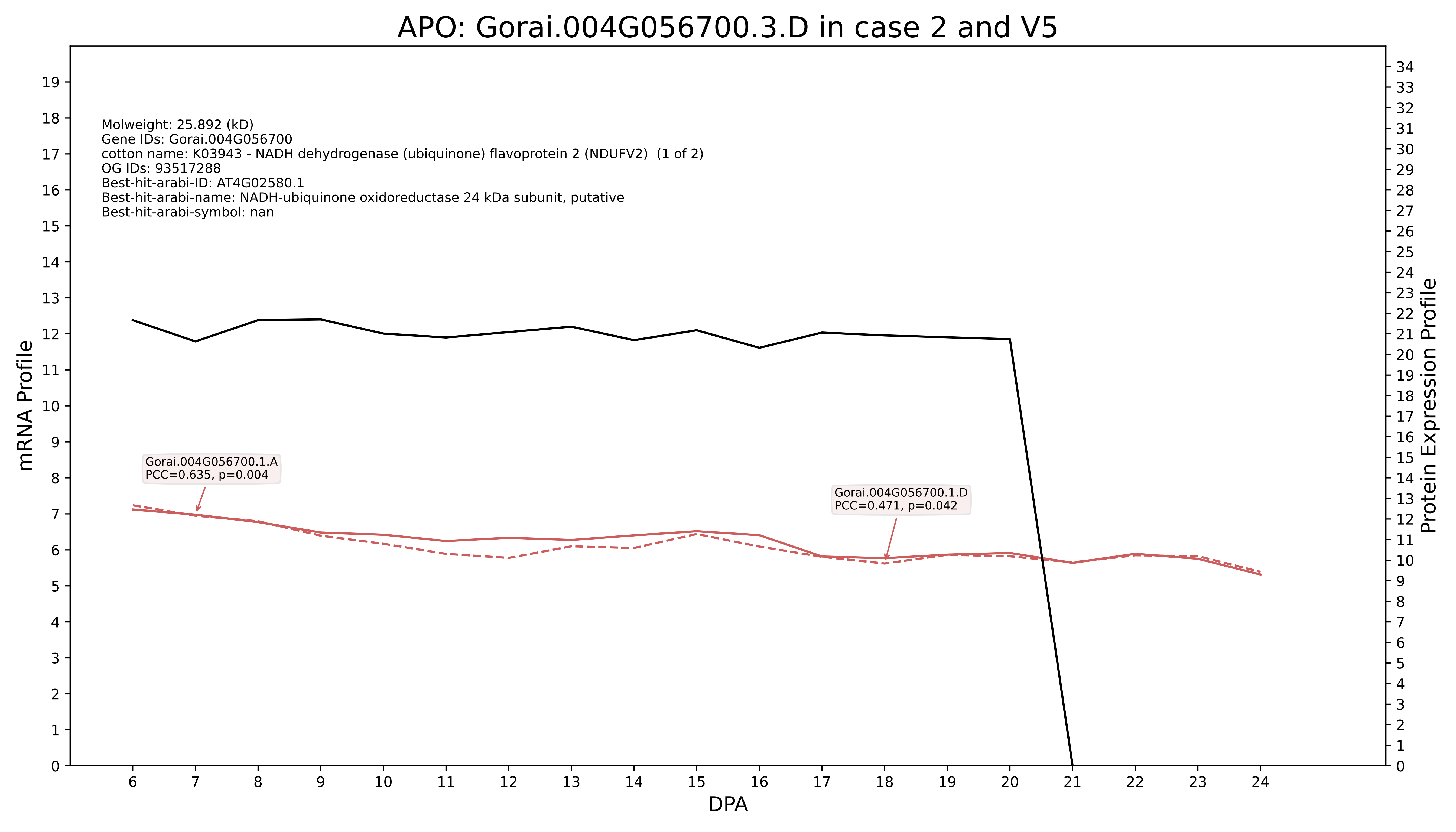

APO: Gorai.004G056700.3.D in case 2 and V5
Molweight: 25.892 (kD)
Gene IDs: Gorai.004G056700
cotton name: K03943 - NADH dehydrogenase (ubiquinone) flavoprotein 2 (NDUFV2) (1 of 2)
OG IDs: 93517288
Best-hit-arabi-ID: AT4G02580.1
Best-hit-arabi-name: NADH-ubiquinone oxidoreductase 24 kDa subunit, putative
Best-hit-arabi-symbol: nan
Gorai.004G056700.1.A
PCC=0.635, p=0.004
Gorai.004G056700.1.D
PCC=0.471, p=0.042
mRNA Profile
Protein Expression Profile
DPA

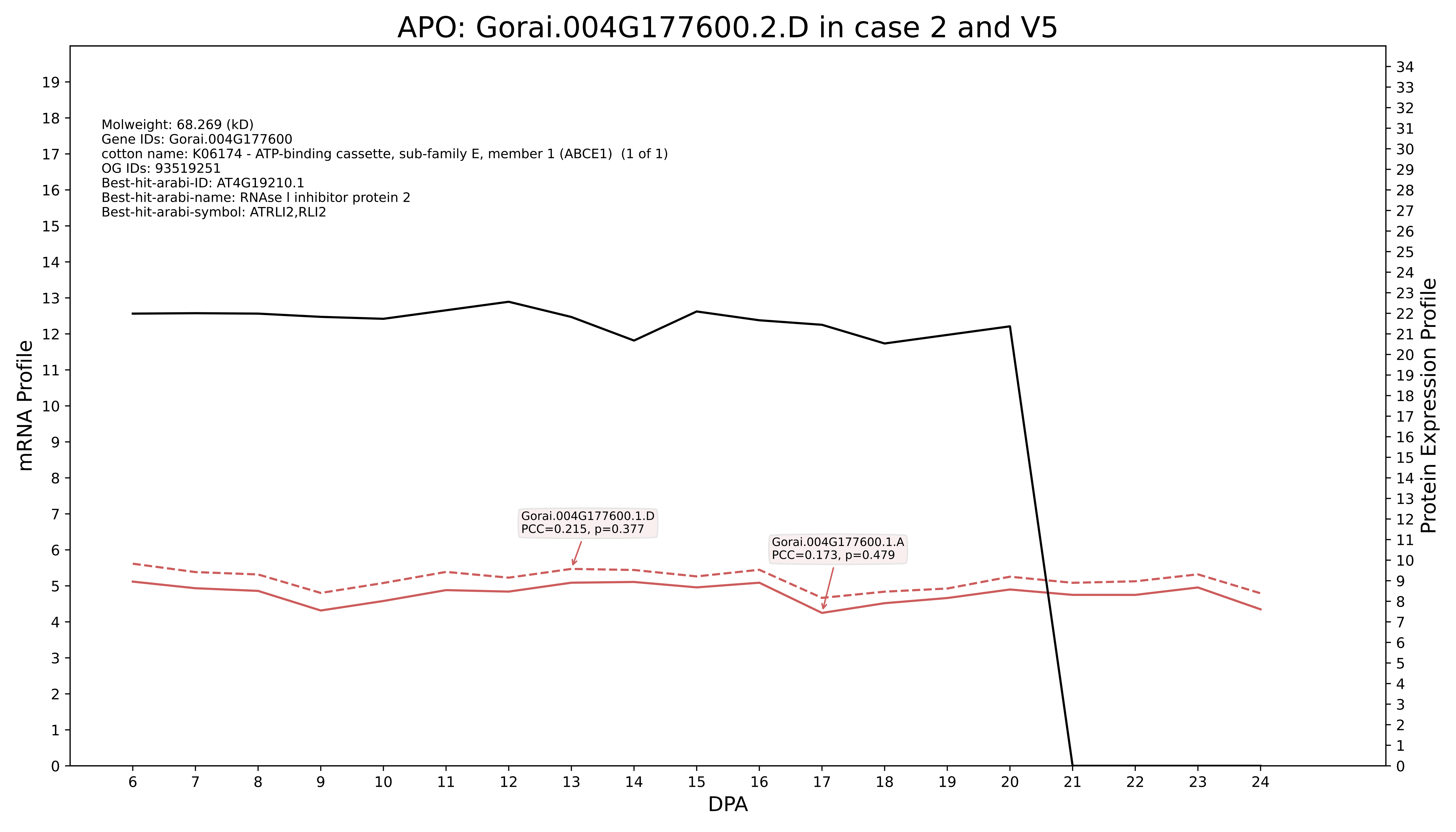

APO: Gorai.004G177600.2.D in case 2 and V5
Molweight: 68.269 (kD)
Gene IDs: Gorai.004G177600
cotton name: K06174 - ATP-binding cassette, sub-family E, member 1 (ABCE1) (1 of 1)
OG IDs: 93519251
Best-hit-arabi-ID: AT4G19210.1
Best-hit-arabi-name: RNAse l inhibitor protein 2
Best-hit-arabi-symbol: ATRLI2,RLI2
Gorai.004G177600.1.D
PCC=0.215, p=0.377
Gorai.004G177600.1.A
PCC=0.173, p=0.479
mRNA Profile
Protein Expression Profile
DPA

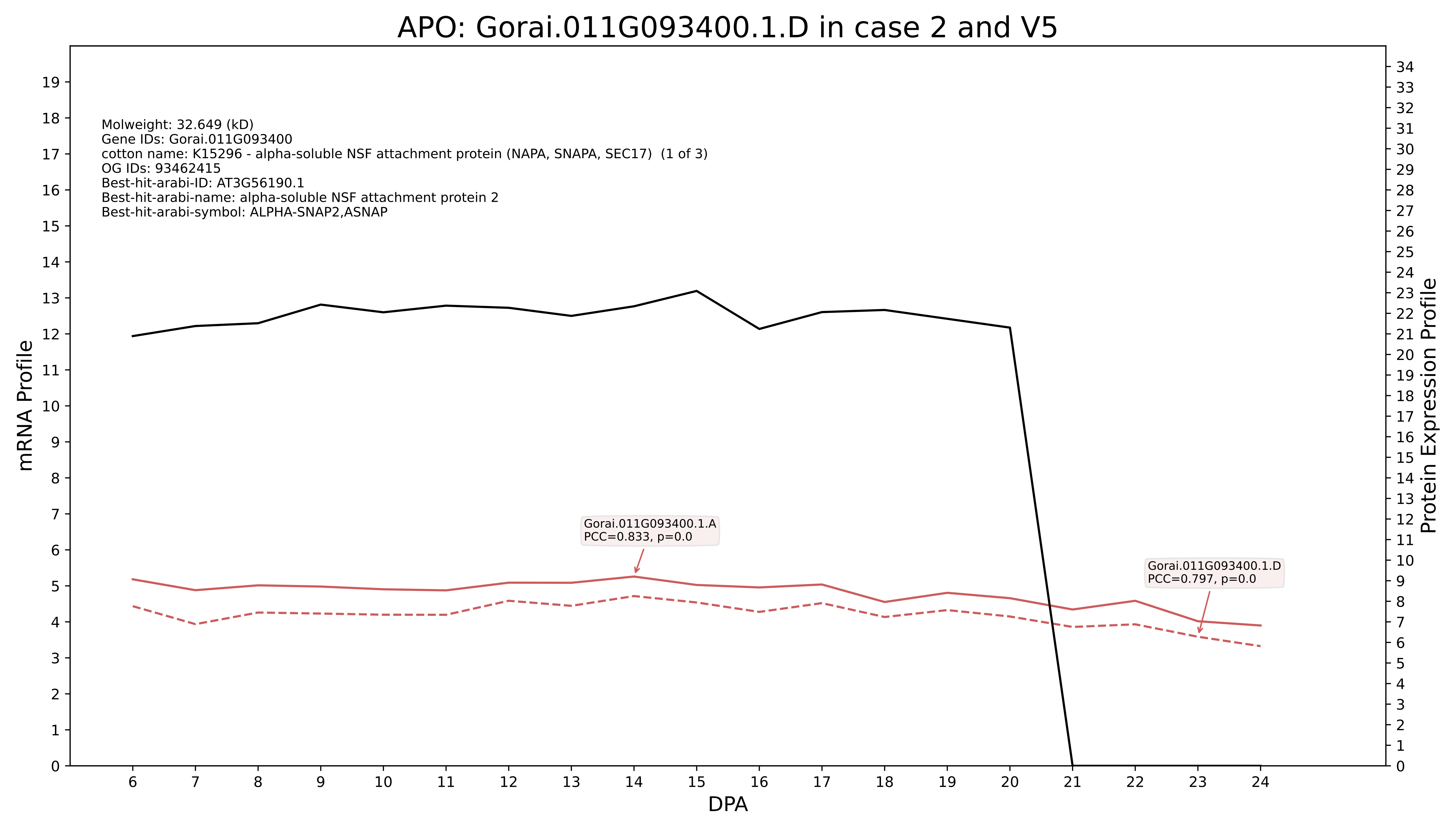
APO: Gorai.011G093400.1.D in case 2 and V5
Molweight: 32.649 (kD)
Gene IDs: Gorai.011G093400
cotton name: K15296 - alpha-soluble NSF attachment protein (NAPA, SNAPA, SEC17) (1 of 3)
OG IDs: 93462415
Best-hit-arabi-ID: AT3G56190.1
Best-hit-arabi-name: alpha-soluble NSF attachment protein 2
Best-hit-arabi-symbol: ALPHA-SNAP2,ASNAP
Gorai.011G093400.1.A
PCC=0.833, p=0.0
Gorai.011G093400.1.D
PCC=0.797, p=0.0
mRNA Profile
Protein Expression Profile
DPA
0
1
2
3
4
5
6
7
8
9
10
11
12
13
14
15
16
17
18
19
20
21
22
23
24
25
26
27
28
29
30
31
32
33
34

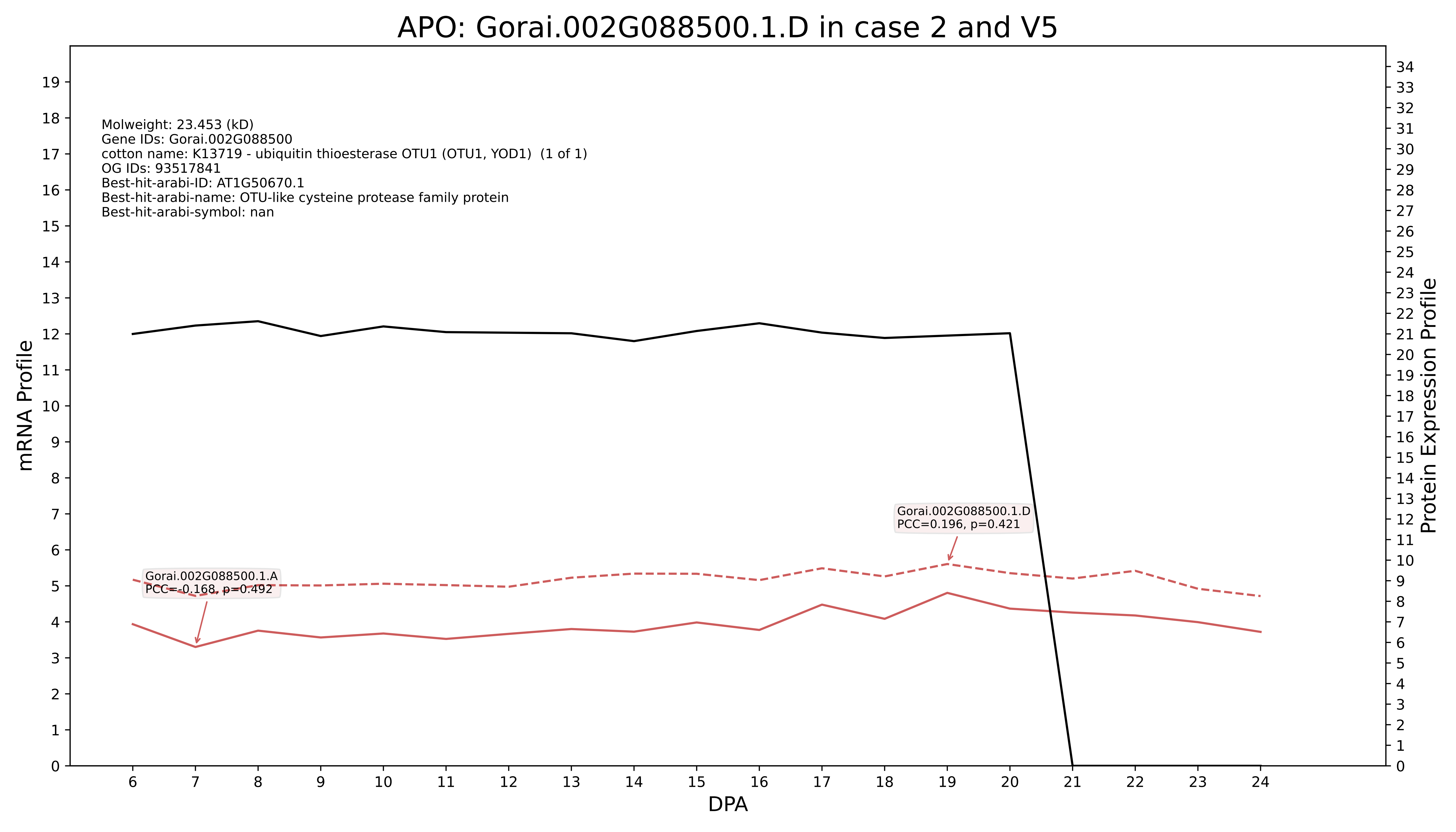
APO: Gorai.002G088500.1.D in case 2 and V5
Molweight: 23.453 (kD)
Gene IDs: Gorai.002G088500
cotton name: K13719 - ubiquitin thioesterase OTU1 (OTU1, YOD1) (1 of 1)
OG IDs: 93517841
Best-hit-arabi-ID: AT1G50670.1
Best-hit-arabi-name: OTU-like cysteine protease family protein
Best-hit-arabi-symbol: nan
Gorai.002G088500.1.D
PCC=0.196, p=0.421
Gorai.002G088500.1.A
PCC=-0.168, p=0.492
mRNA Profile
Protein Expression Profile
DPA

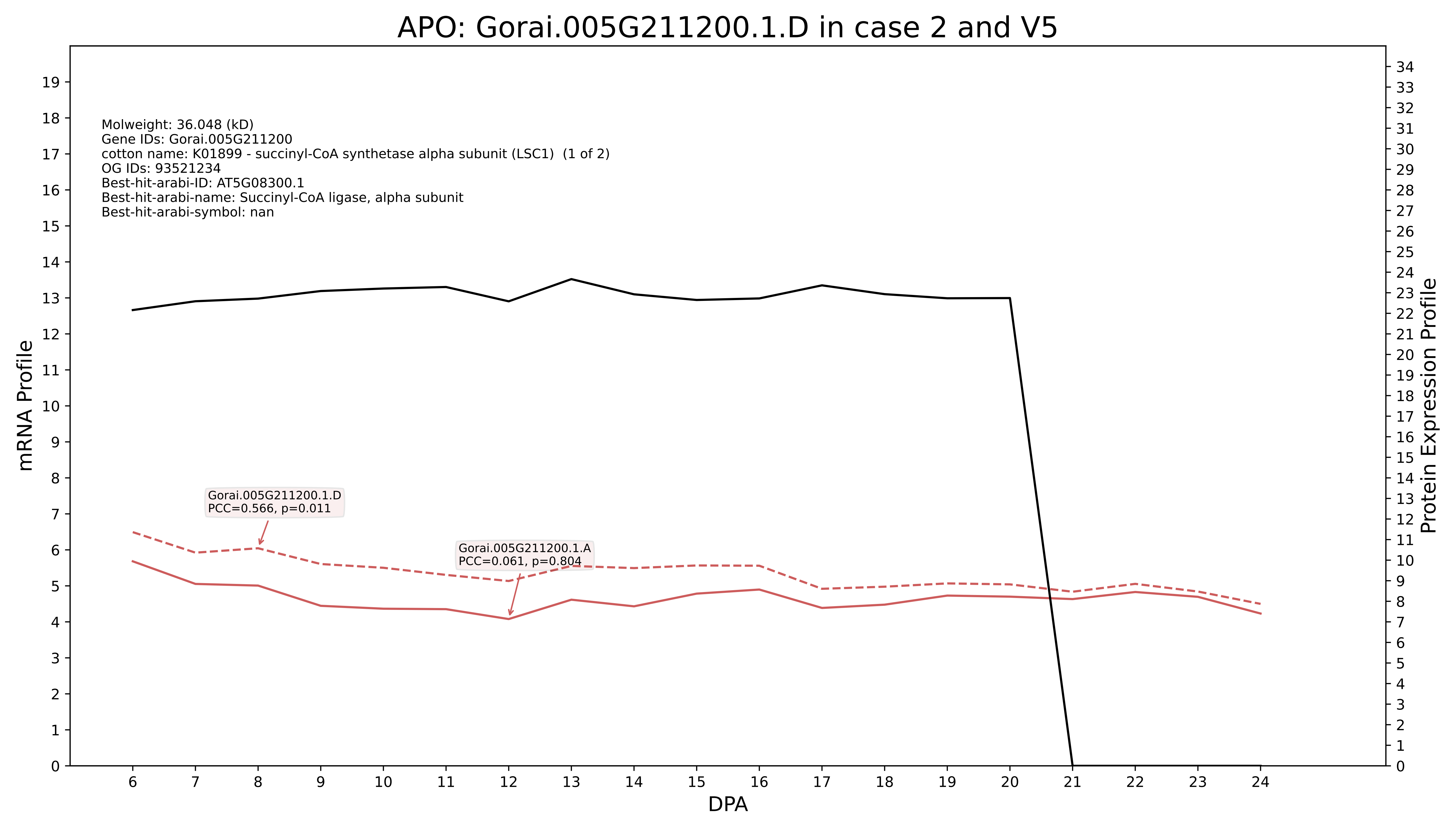

APO: Gorai.005G211200.1.D in case 2 and V5
Molweight: 36.048 (kD)
Gene IDs: Gorai.005G211200
cotton name: K01899 - succinyl-CoA synthetase alpha subunit (LSC1) (1 of 2)
OG IDs: 93521234
Best-hit-arabi-ID: AT5G08300.1
Best-hit-arabi-name: Succinyl-CoA ligase, alpha subunit
Best-hit-arabi-symbol: nan
Gorai.005G211200.1.D
PCC=0.566, p=0.011
Gorai.005G211200.1.A
PCC=0.061, p=0.804
mRNA Profile
Protein Expression Profile
DPA

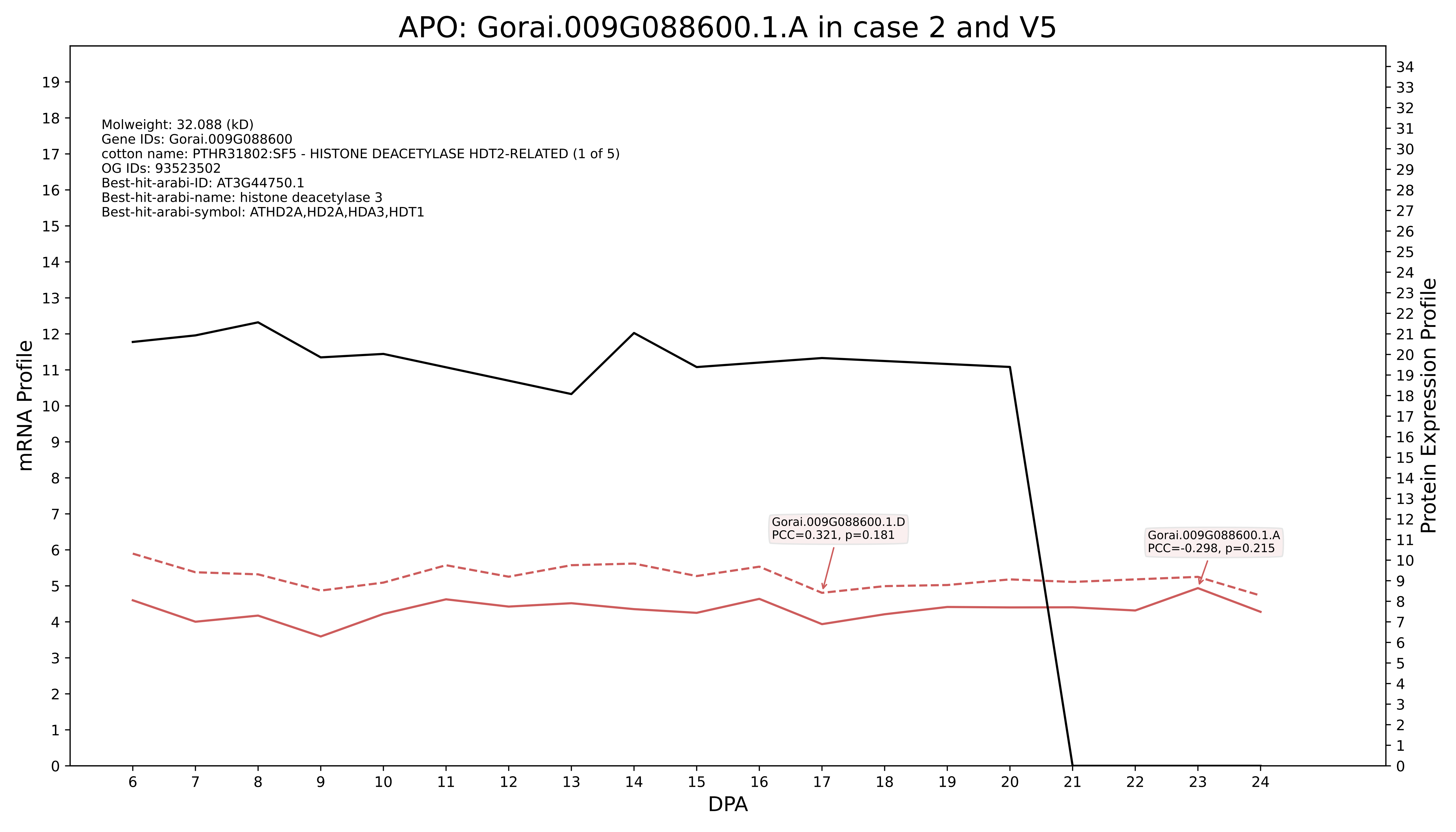
APO: Gorai.009G088600.1.A in case 2 and V5
Molweight: 32.088 (kD)
Gene IDs: Gorai.009G088600
cotton name: PTHR31802:SF5 - HISTONE DEACETYLASE HDT2-RELATED (1 of 5)
OG IDs: 93523502
Best-hit-arabi-ID: AT3G44750.1
Best-hit-arabi-name: histone deacetylase 3
Best-hit-arabi-symbol: ATHD2A,HD2A,HDA3,HDT1
Gorai.009G088600.1.D
PCC=0.321, p=0.181
Gorai.009G088600.1.A
PCC=-0.298, p=0.215
mRNA Profile
Protein Expression Profile
DPA

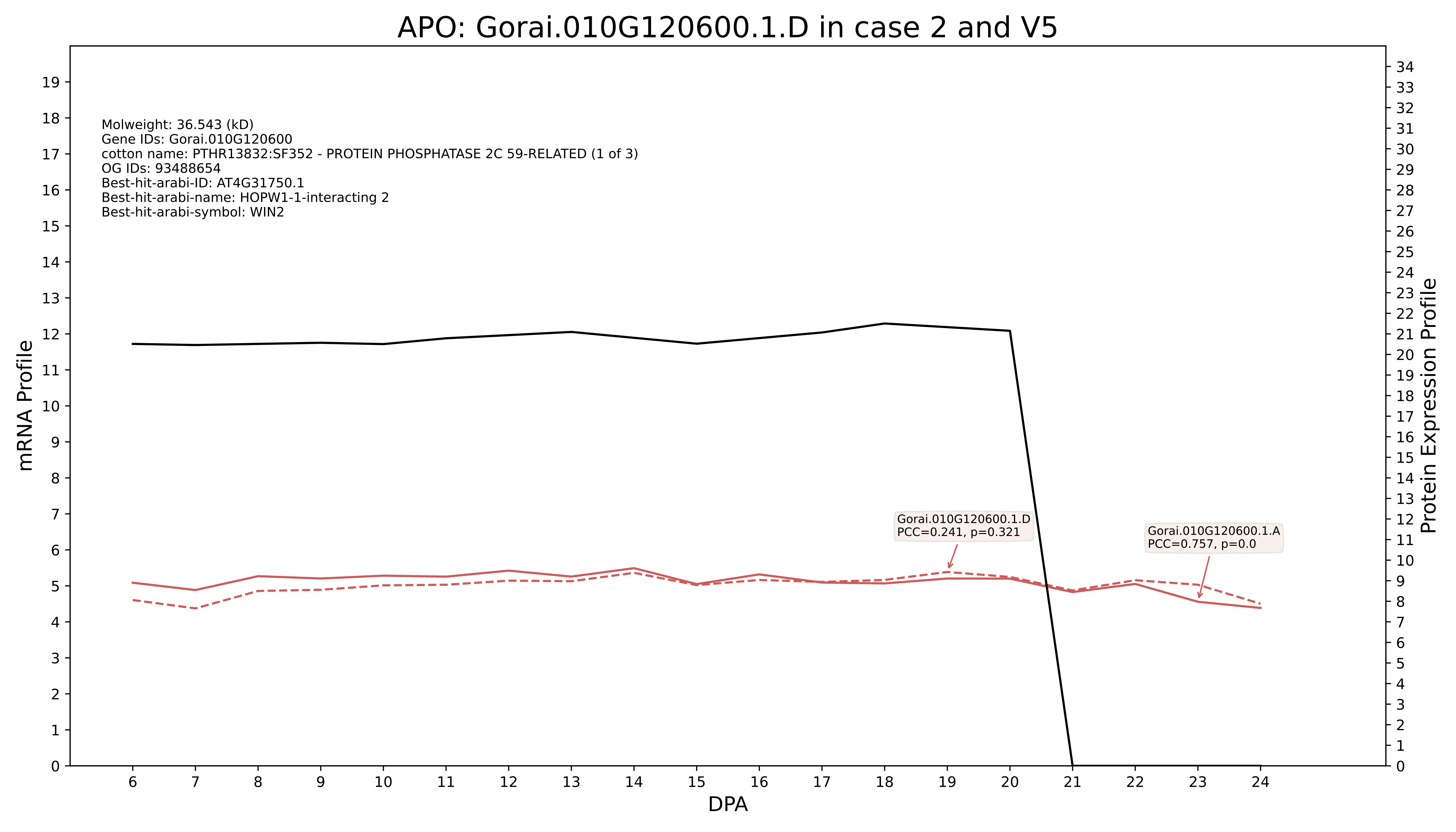
APO: Gorai.010G120600.1.D in case 2 and V5
Molweight: 36.543 (kD)
Gene IDs: Gorai.010G120600
cotton name: PTHR13832:SF352 - PROTEIN PHOSPHATASE 2C 59-RELATED (1 of 3)
OG IDs: 93488654
Best-hit-arabi-ID: AT4G31750.1
Best-hit-arabi-name: HOPW1-1-interacting 2
Best-hit-arabi-symbol: WIN2
Gorai.010G120600.1.D
PCC=0.241, p=0.321
Gorai.010G120600.1.A
PCC=0.757, p=0.0
mRNA Profile
Protein Expression Profile
DPA

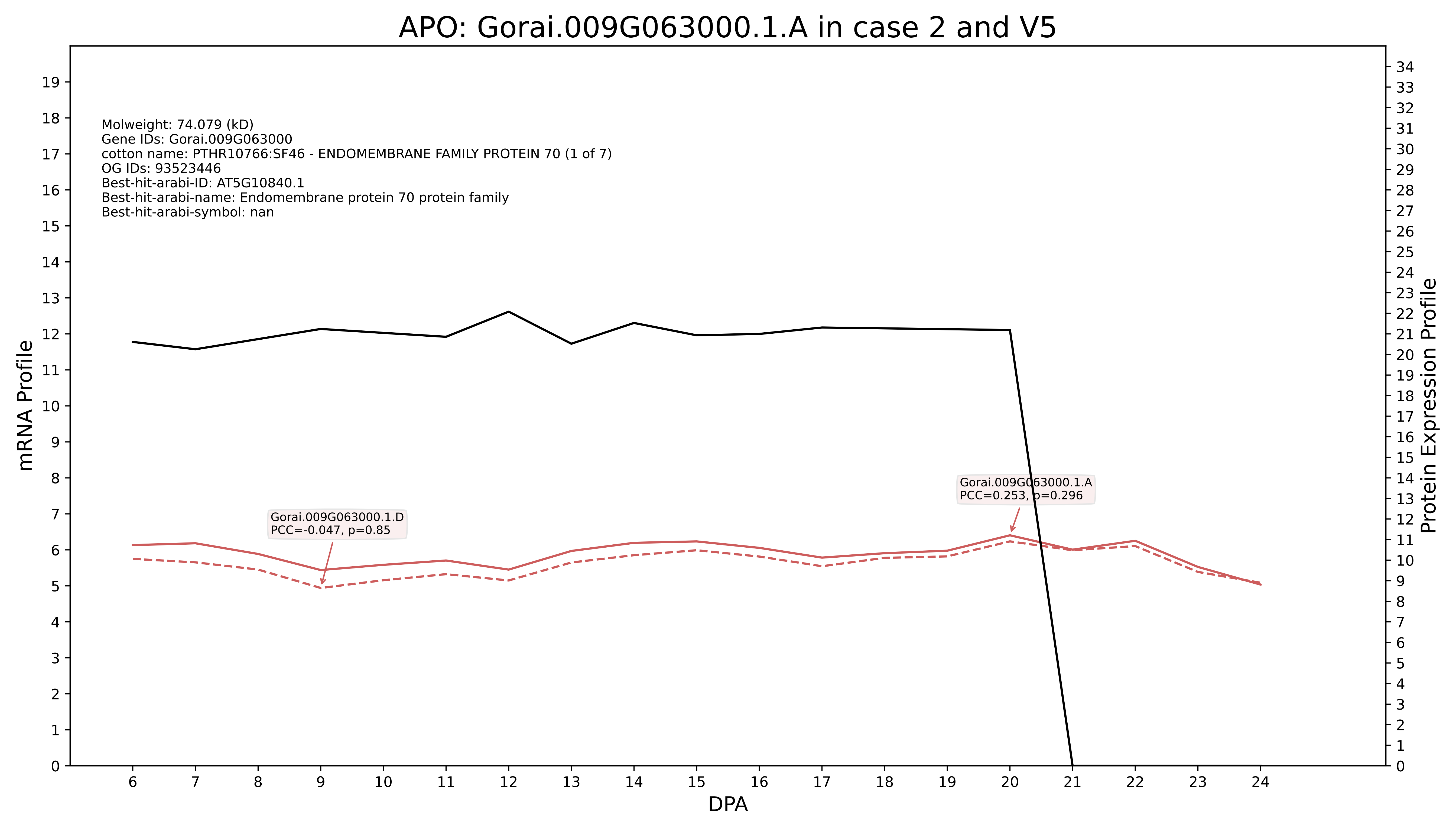
APO: Gorai.009G063000.1.A in case 2 and V5
Molweight: 74.079 (kD)
Gene IDs: Gorai.009G063000
cotton name: PTHR10766:SF46 - ENDOMEMBRANE FAMILY PROTEIN 70 (1 of 7)
OG IDs: 93523446
Best-hit-arabi-ID: AT5G10840.1
Best-hit-arabi-name: Endomembrane protein 70 protein family
Best-hit-arabi-symbol: nan
Gorai.009G063000.1.D
PCC=-0.047, p=0.85
Gorai.009G063000.1.A
PCC=0.253, p=0.296
mRNA Profile
Protein Expression Profile
DPA

APO: Gorai.008G199000.4.D in case 2 and V5

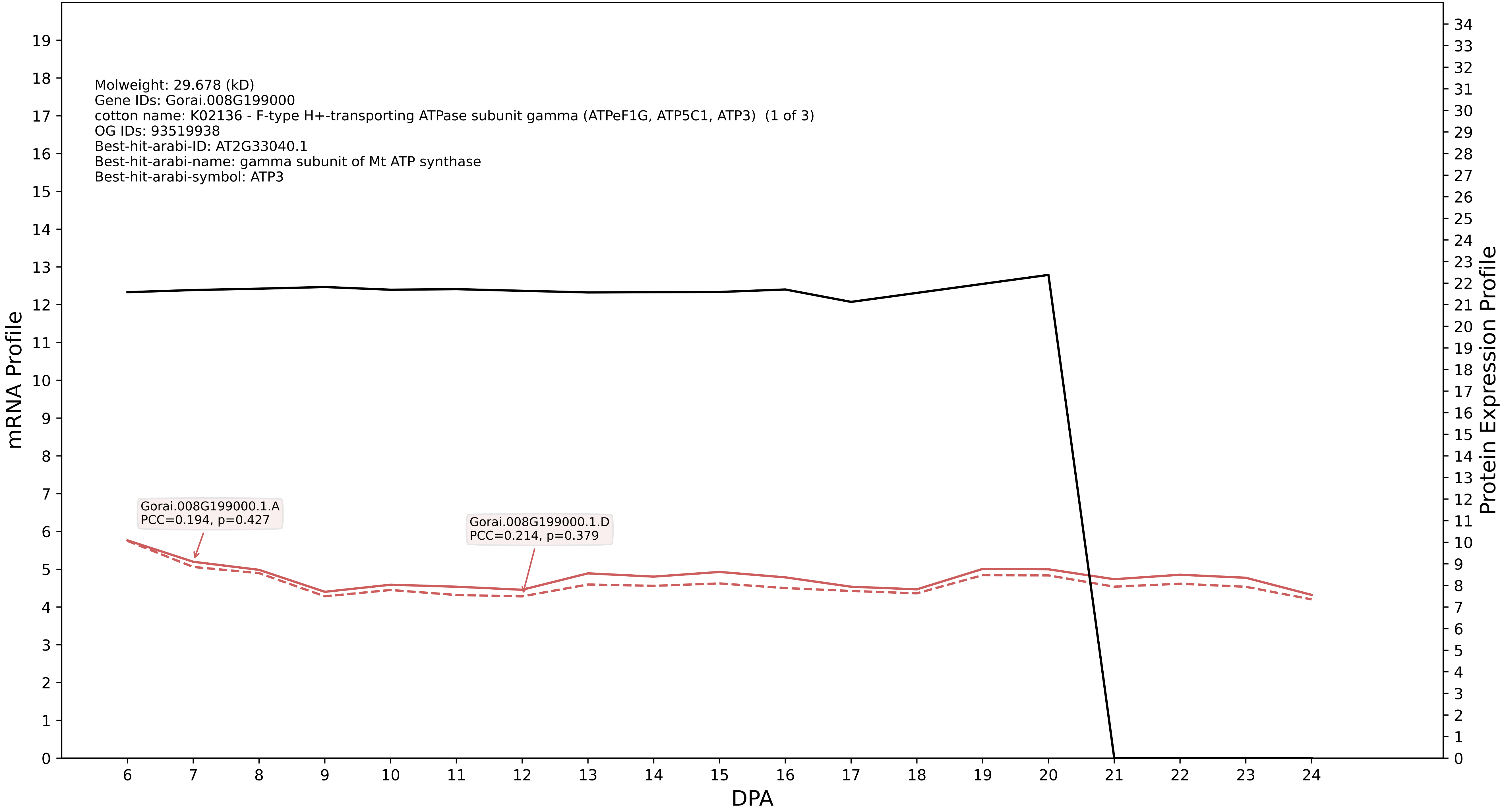

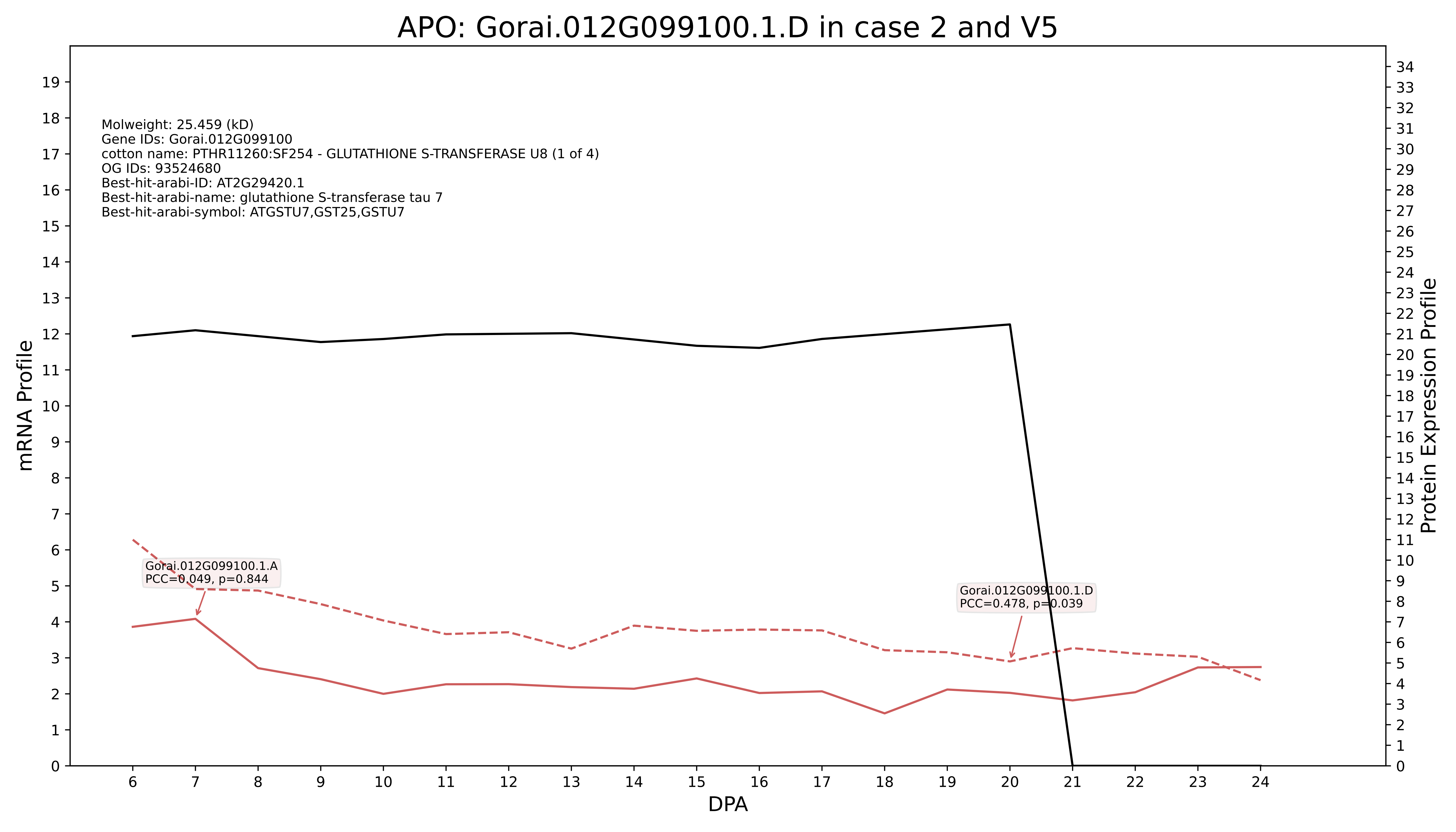
APO: Gorai.012G099100.1.D in case 2 and V5
Molweight: 25.459 (kD)
Gene IDs: Gorai.012G099100
cotton name: PTHR11260:SF254 - GLUTATHIONE S-TRANSFERASE U8 (1 of 4)
OG IDs: 93524680
Best-hit-arabi-ID: AT2G29420.1
Best-hit-arabi-name: glutathione S-transferase tau 7
Best-hit-arabi-symbol: ATGSTU7,GST25,GSTU7
Gorai.012G099100.1.A
PCC=0.049, p=0.844
Gorai.012G099100.1.D
PCC=0.478, p=0.039
mRNA Profile
Protein Expression Profile
DPA

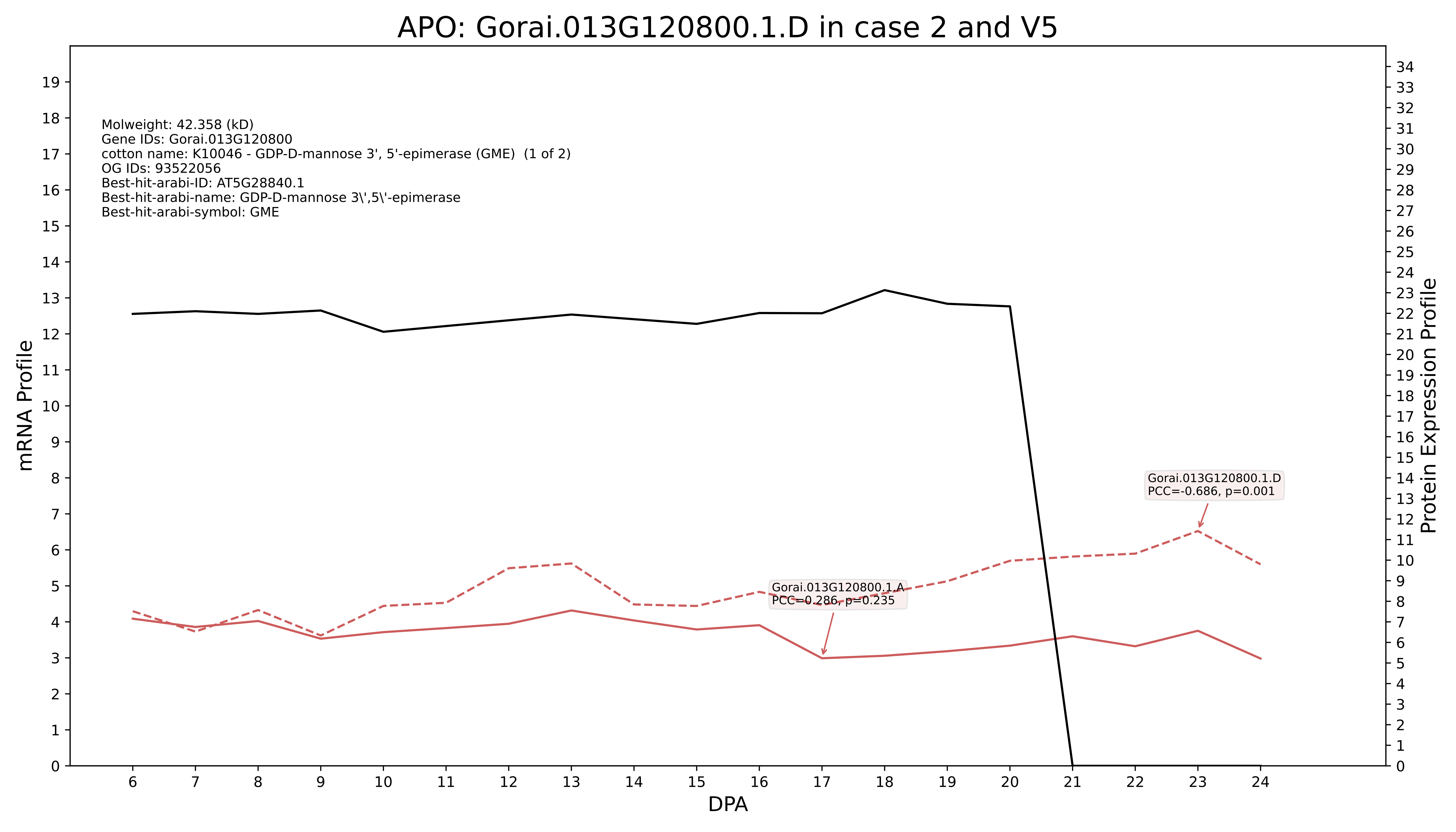
APO: Gorai.013G120800.1.D in case 2 and V5
Molweight: 42.358 (kD)
Gene IDs: Gorai.013G120800
cotton name: K10046 - GDP-D-mannose 3', 5'-epimerase (GME) (1 of 2)
OG IDs: 93522056
Best-hit-arabi-ID: AT5G28840.1
Best-hit-arabi-name: GDP-D-mannose 3\',5\'-epimerase
Best-hit-arabi-symbol: GME
Gorai.013G120800.1.D
PCC=-0.686, p=0.001
Gorai.013G120800.1.A
PCC=0.286, p=0.235
mRNA Profile
Protein Expression Profile
DPA
0
1
2
3
4
5
6
7
8
9
10
11
12
13
14
15
16
17
18
19
20
21
22
23
24
25
26
27
28
29
30
31
32
33
34

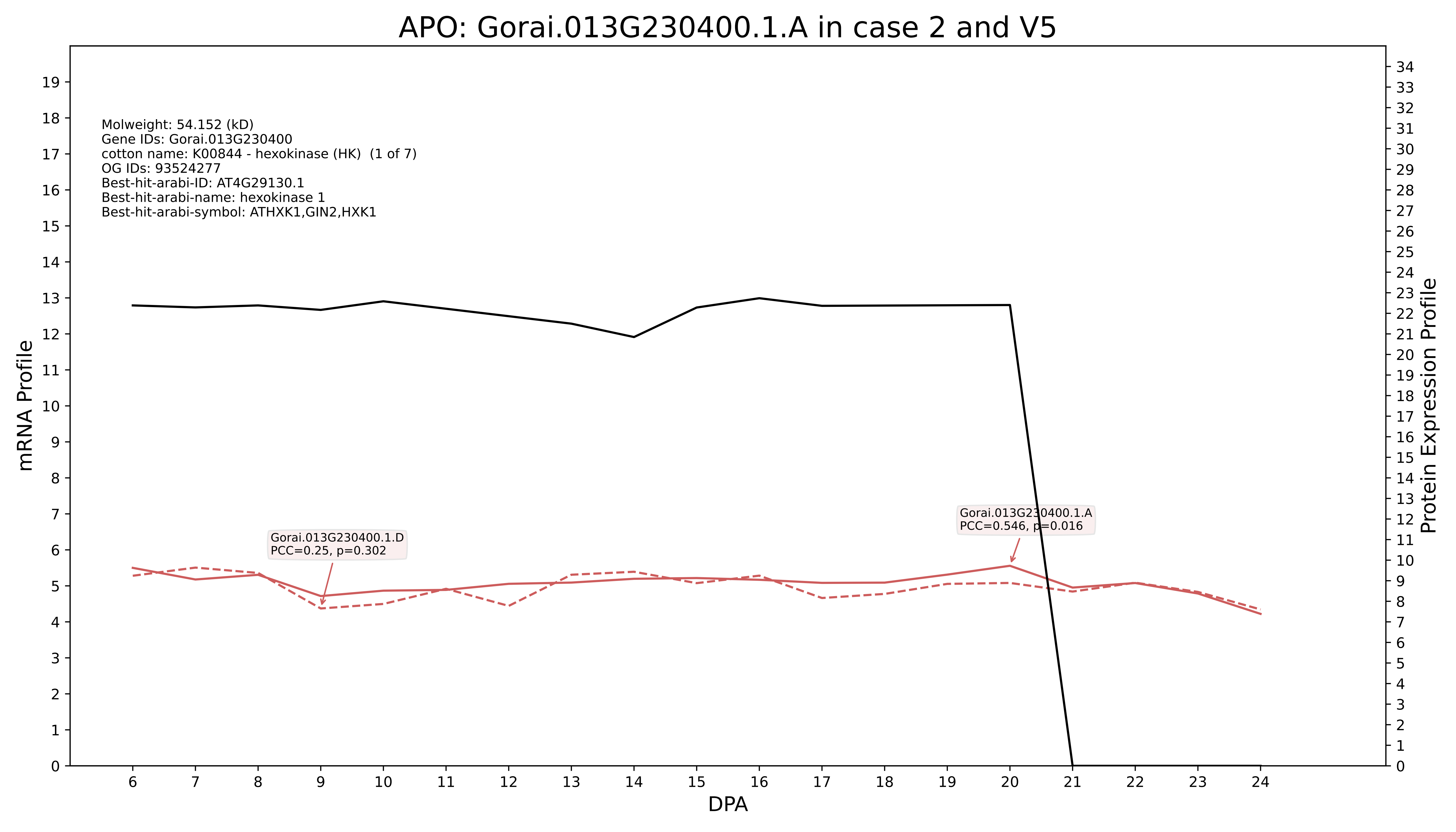

APO: Gorai.013G230400.1.A in case 2 and V5
Molweight: 54.152 (kD)
Gene IDs: Gorai.013G230400
cotton name: K00844 - hexokinase (HK) (1 of 7)
OG IDs: 93524277
Best-hit-arabi-ID: AT4G29130.1
Best-hit-arabi-name: hexokinase 1
Best-hit-arabi-symbol: ATHXK1,GIN2,HXK1
Gorai.013G230400.1.D
PCC=0.25, p=0.302
Gorai.013G230400.1.A
PCC=0.546, p=0.016
mRNA Profile
Protein Expression Profile
DPA

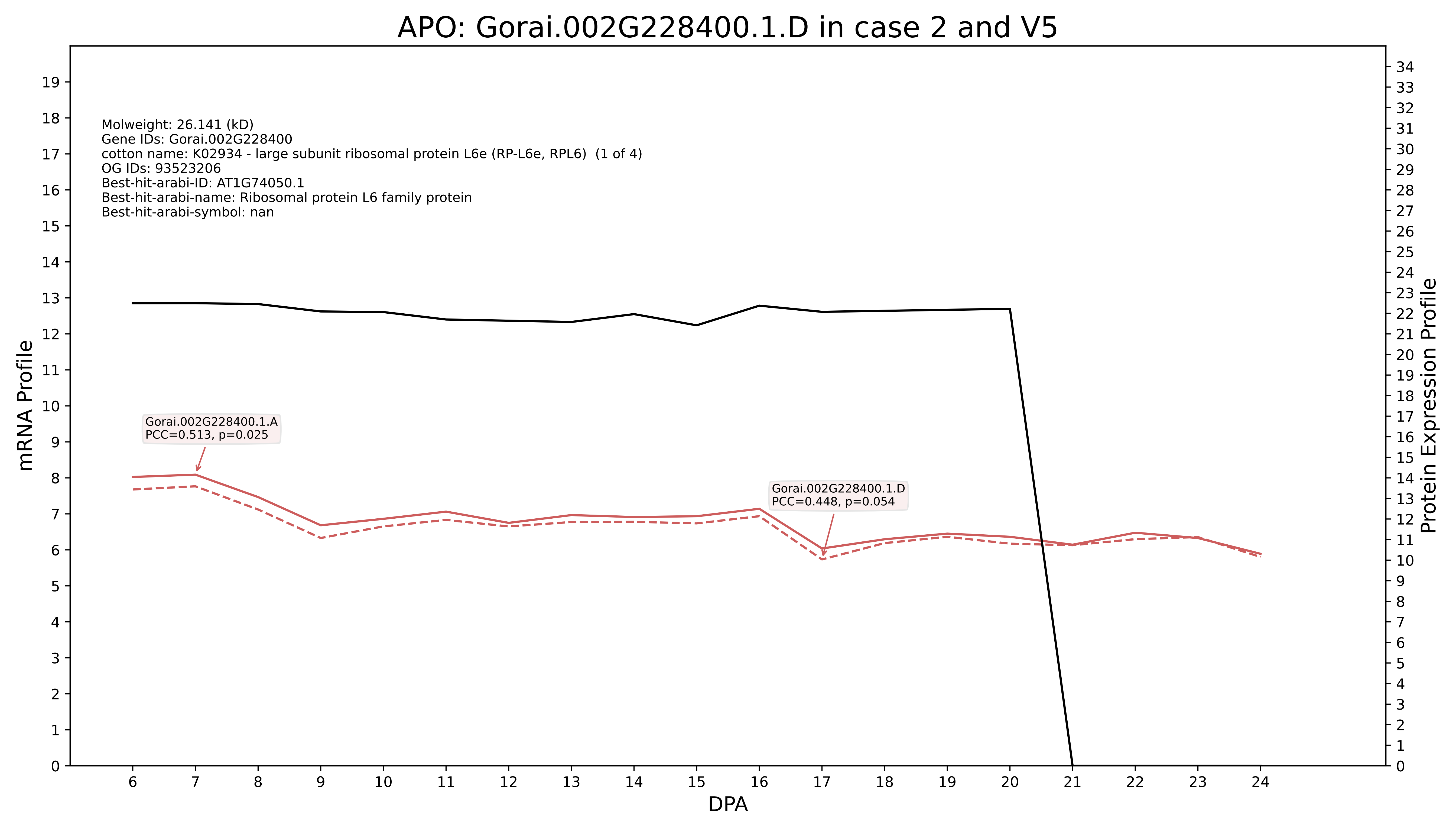

APO: Gorai.002G228400.1.D in case 2 and V5
Molweight: 26.141 (kD)
Gene IDs: Gorai.002G228400
cotton name: K02934 - large subunit ribosomal protein L6e (RP-L6e, RPL6) (1 of 4)
OG IDs: 93523206
Best-hit-arabi-ID: AT1G74050.1
Best-hit-arabi-name: Ribosomal protein L6 family protein
Best-hit-arabi-symbol: nan
Gorai.002G228400.1.A
PCC=0.513, p=0.025
Gorai.002G228400.1.D
PCC=0.448, p=0.054
mRNA Profile
Protein Expression Profile
DPA

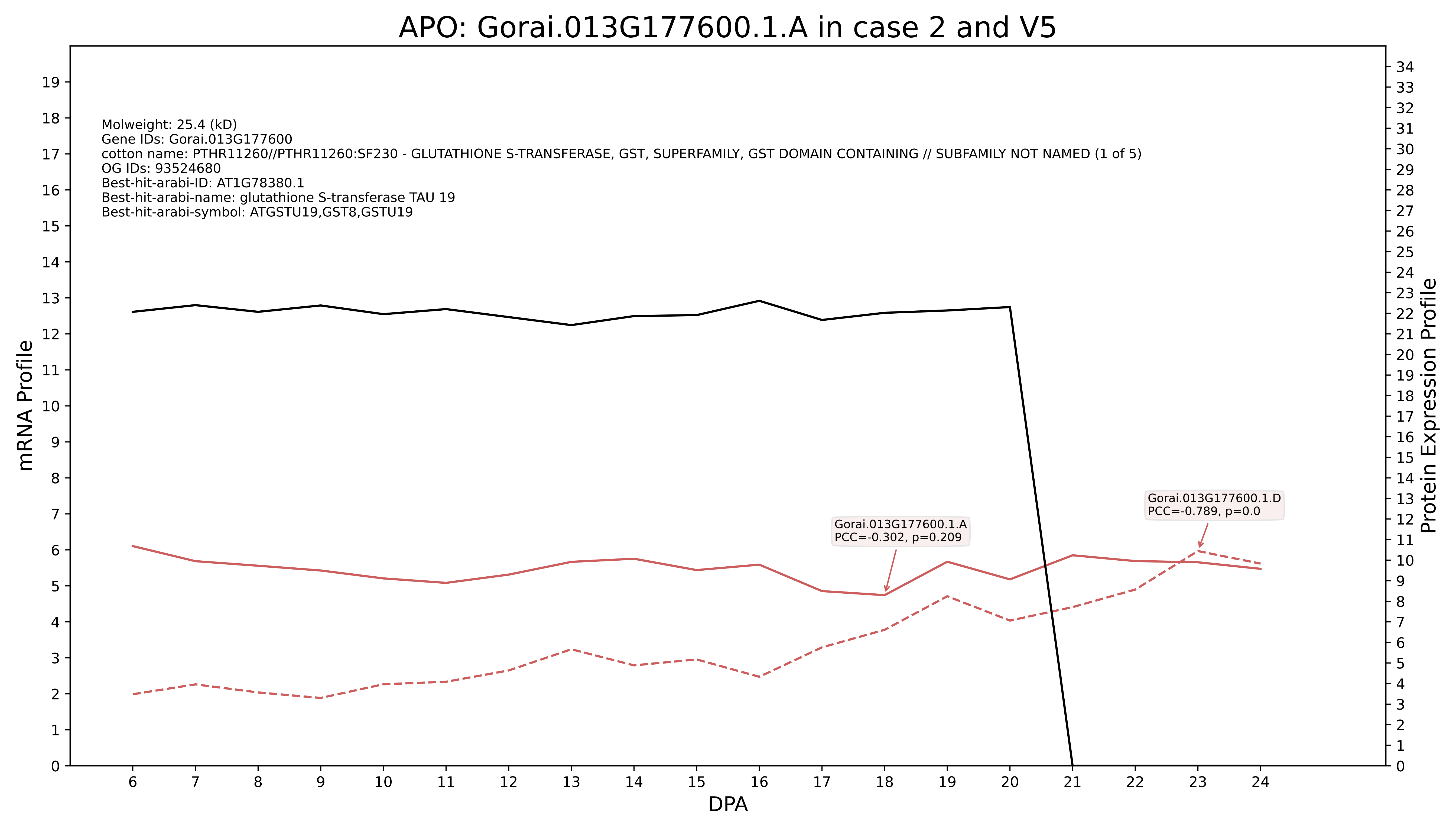

APO: Gorai.013G177600.1.A in case 2 and V5
Molweight: 25.4 (kD)
Gene IDs: Gorai.013G177600
cotton name: PTHR11260//PTHR11260:SF230 - GLUTATHIONE S-TRANSFERASE, GST, SUPERFAMILY, GST DOMAIN CONTAINING // SUBFAMILY NOT NAMED (1 of 5)
OG IDs: 93524680
Best-hit-arabi-ID: AT1G78380.1
Best-hit-arabi-name: glutathione S-transferase TAU 19
Best-hit-arabi-symbol: ATGSTU19,GST8,GSTU19
Gorai.013G177600.1.A
PCC=-0.302, p=0.209
Gorai.013G177600.1.D
PCC=-0.789, p=0.0
mRNA Profile
Protein Expression Profile
DPA

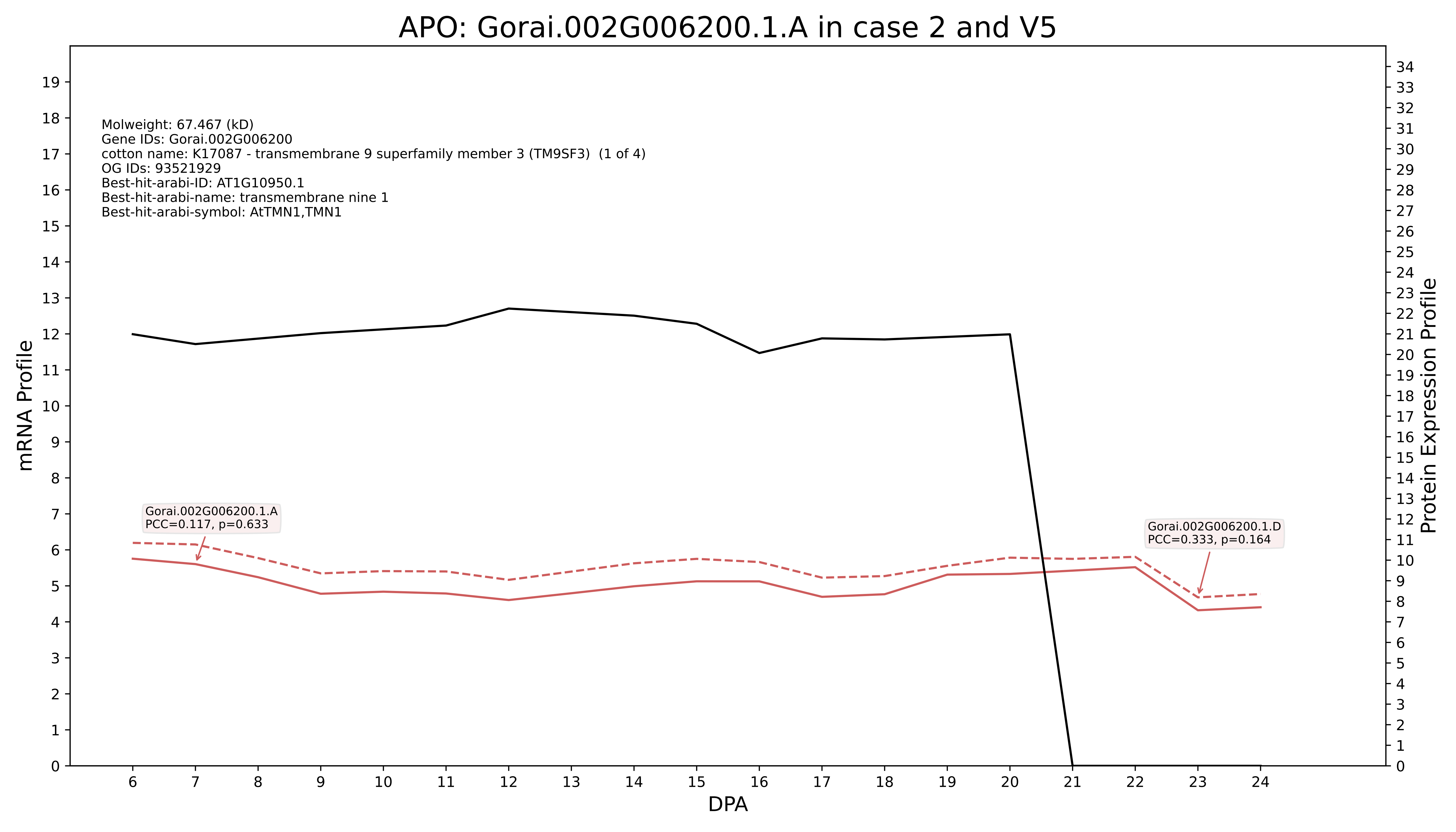
APO: Gorai.002G006200.1.A in case 2 and V5
Molweight: 67.467 (kD)
Gene IDs: Gorai.002G006200
cotton name: K17087 - transmembrane 9 superfamily member 3 (TM9SF3) (1 of 4)
OG IDs: 93521929
Best-hit-arabi-ID: AT1G10950.1
Best-hit-arabi-name: transmembrane nine 1
Best-hit-arabi-symbol: AtTMN1,TMN1
Gorai.002G006200.1.A
PCC=0.117, p=0.633
Gorai.002G006200.1.D
PCC=0.333, p=0.164
mRNA Profile
Protein Expression Profile
DPA

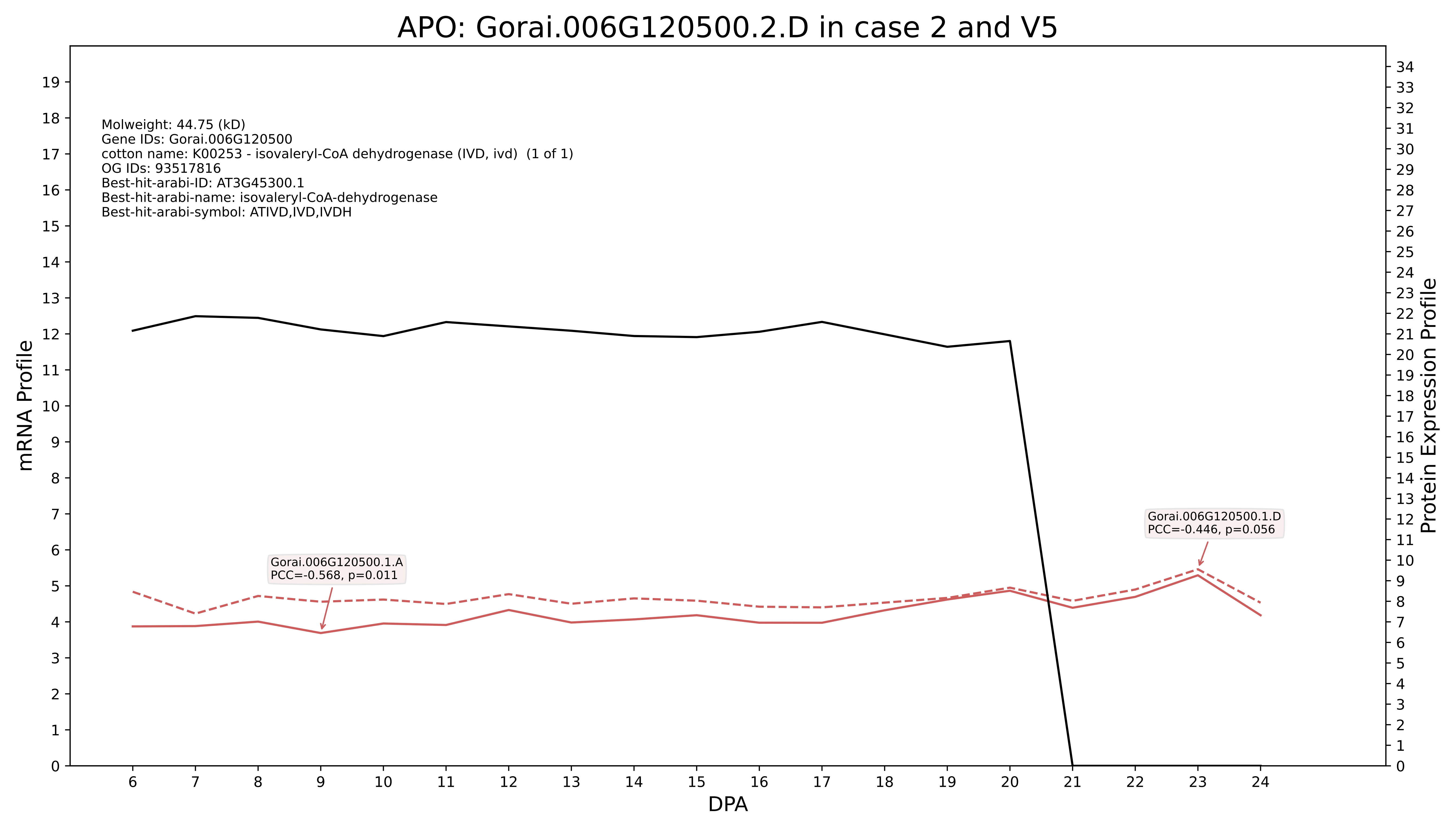
APO: Gorai.006G120500.2.D in case 2 and V5
Molweight: 44.75 (kD)
Gene IDs: Gorai.006G120500
cotton name: K00253 - isovaleryl-CoA dehydrogenase (IVD, ivd) (1 of 1)
OG IDs: 93517816
Best-hit-arabi-ID: AT3G45300.1
Best-hit-arabi-name: isovaleryl-CoA-dehydrogenase
Best-hit-arabi-symbol: ATIVD,IVD,IVDH
Gorai.006G120500.1.A
PCC=-0.568, p=0.011
Gorai.006G120500.1.D
PCC=-0.446, p=0.056
mRNA Profile
Protein Expression Profile
DPA
0
1
2
3
4
5
6
7
8
9
10
11
12
13
14
15
16
17
18
19
20
21
22
23
24
25
26
27
28
29
30
31
32
33
34

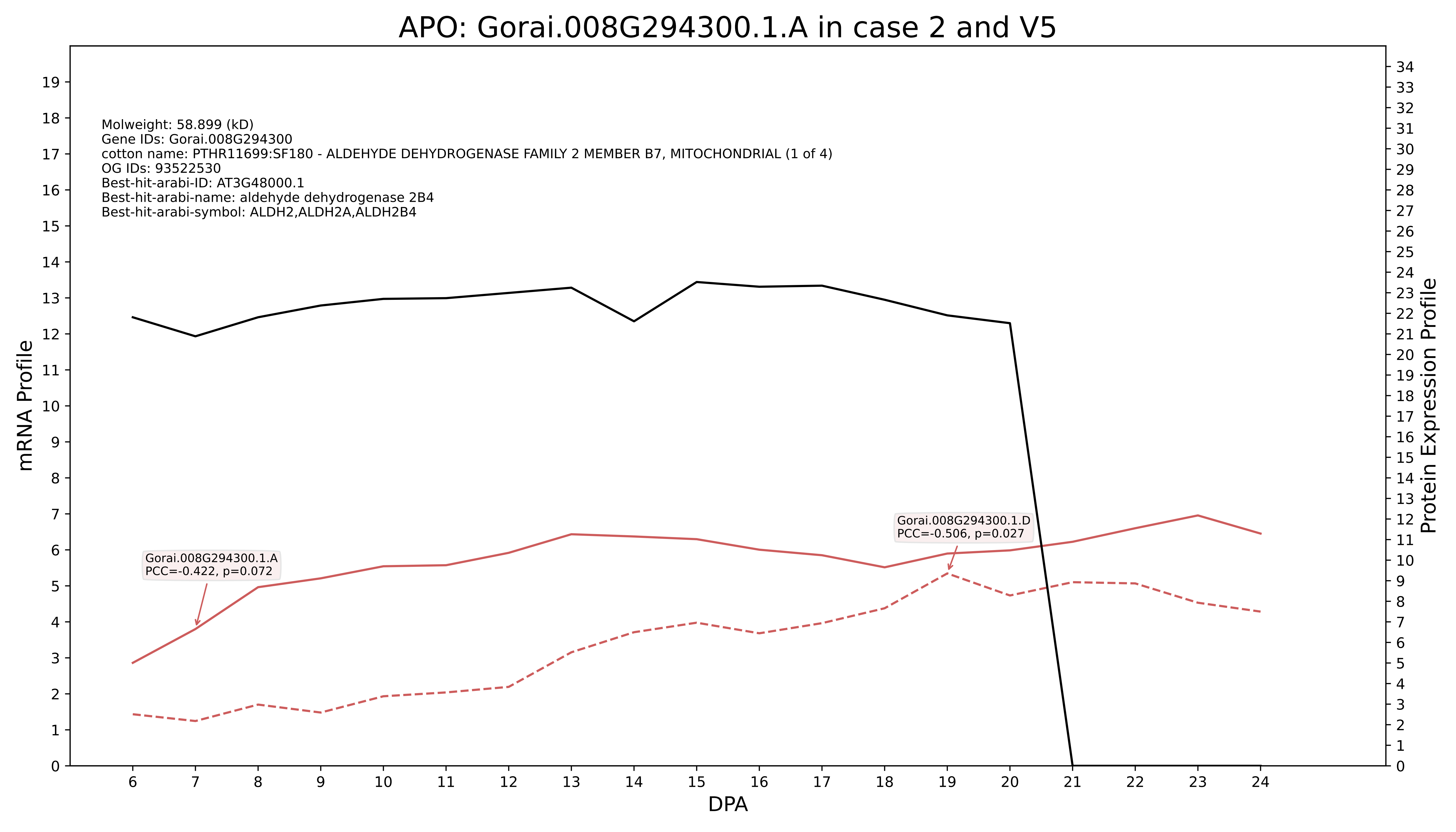
APO: Gorai.008G294300.1.A in case 2 and V5
Molweight: 58.899 (kD)
Gene IDs: Gorai.008G294300
cotton name: PTHR11699:SF180 - ALDEHYDE DEHYDROGENASE FAMILY 2 MEMBER B7, MITOCHONDRIAL (1 of 4)
OG IDs: 93522530
Best-hit-arabi-ID: AT3G48000.1
Best-hit-arabi-name: aldehyde dehydrogenase 2B4
Best-hit-arabi-symbol: ALDH2,ALDH2A,ALDH2B4
Gorai.008G294300.1.A
PCC=-0.422, p=0.072
Gorai.008G294300.1.D
PCC=-0.506, p=0.027
mRNA Profile
Protein Expression Profile
DPA
6
7
8
9
10
11
12
13
14
15
16
17
18
19
20
21
22
23
24

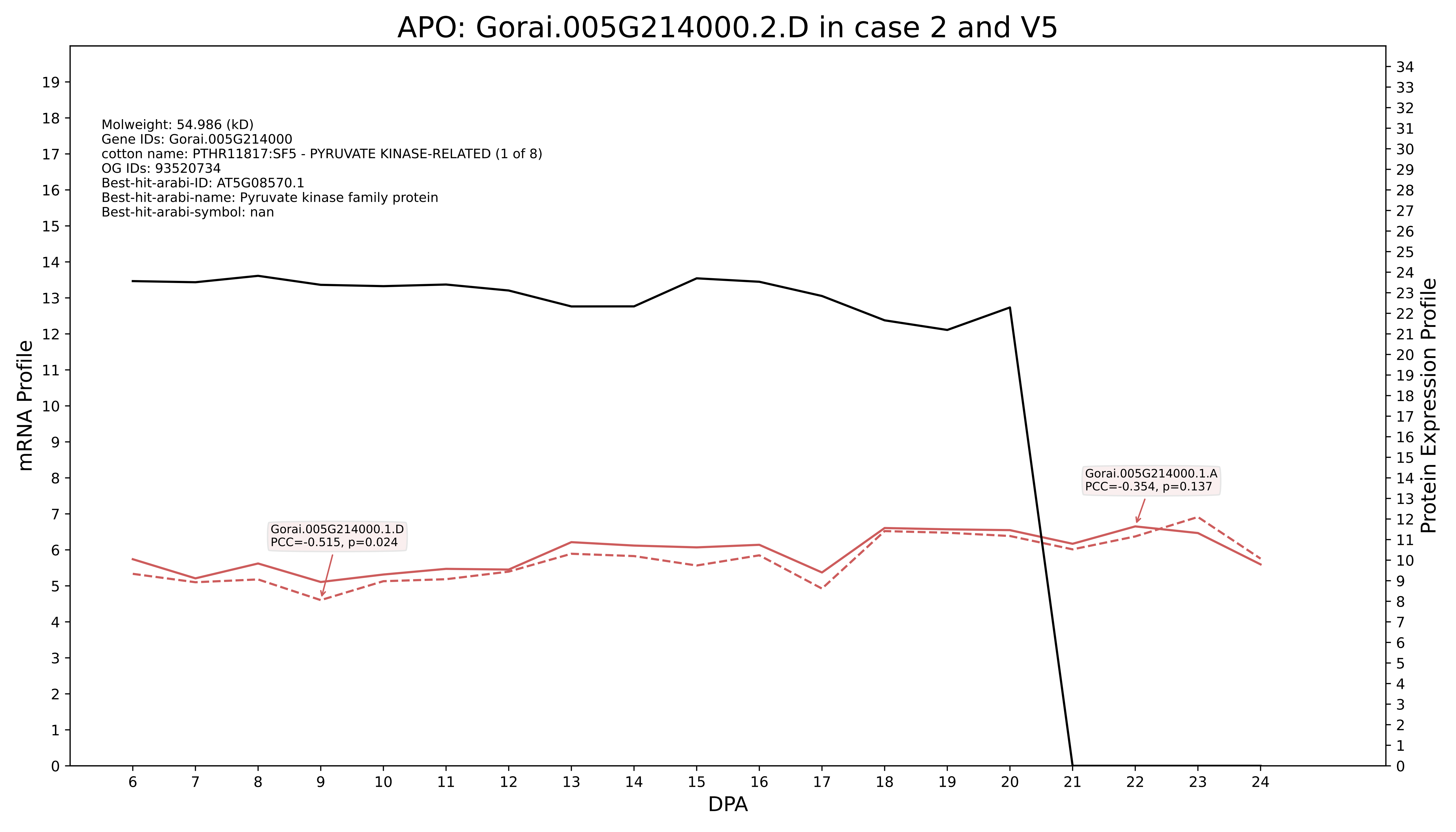
APO: Gorai.005G214000.2.D in case 2 and V5
Molweight: 54.986 (kD)
Gene IDs: Gorai.005G214000
cotton name: PTHR11817:SF5 - PYRUVATE KINASE-RELATED (1 of 8)
OG IDs: 93520734
Best-hit-arabi-ID: AT5G08570.1
Best-hit-arabi-name: Pyruvate kinase family protein
Best-hit-arabi-symbol: nan
Gorai.005G214000.1.D
PCC=-0.515, p=0.024
Gorai.005G214000.1.A
PCC=-0.354, p=0.137
mRNA Profile
Protein Expression Profile
DPA

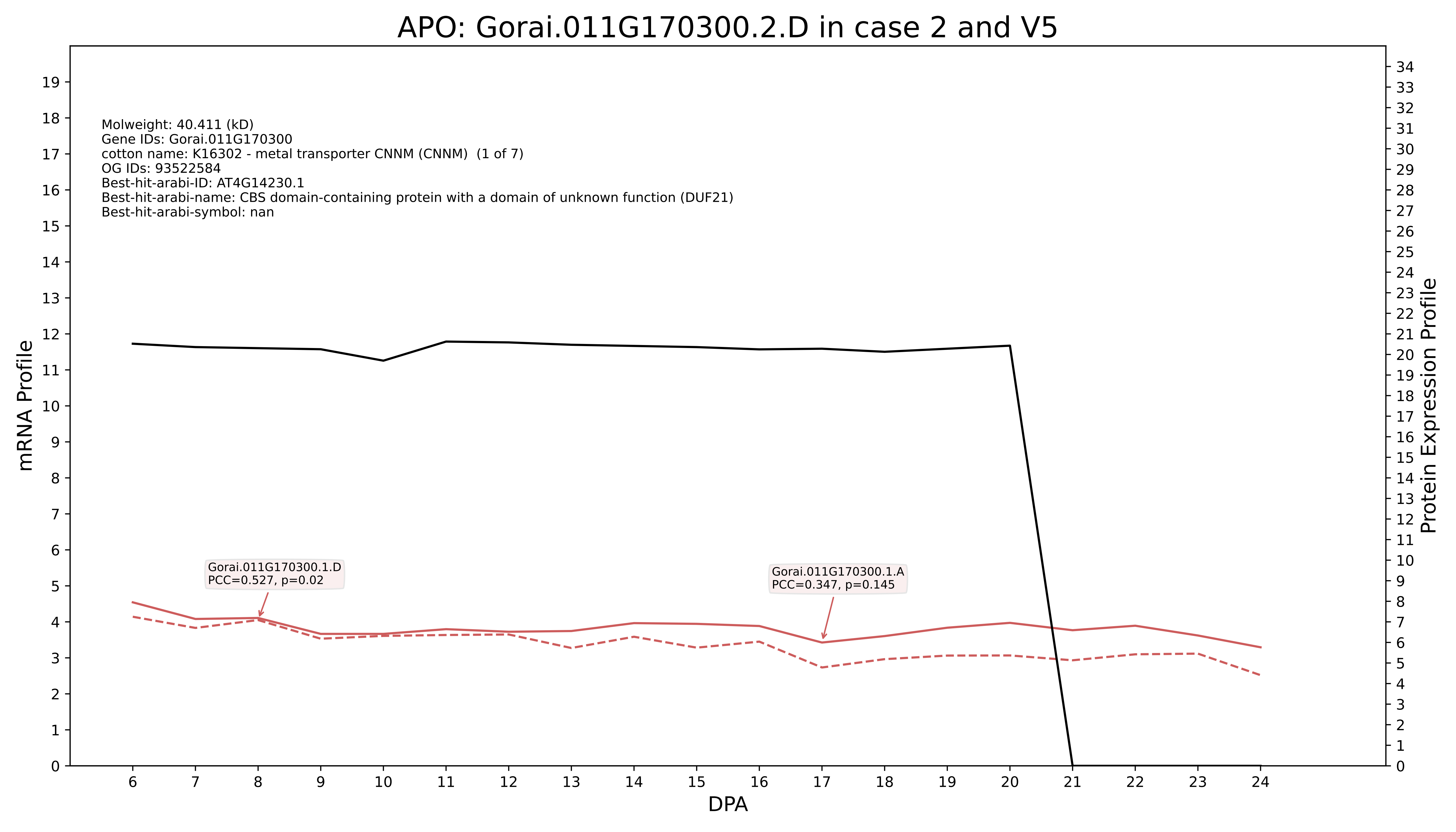

APO: Gorai.011G170300.2.D in case 2 and V5
Molweight: 40.411 (kD)
Gene IDs: Gorai.011G170300
cotton name: K16302 - metal transporter CNNM (CNNM) (1 of 7)
OG IDs: 93522584
Best-hit-arabi-ID: AT4G14230.1
Best-hit-arabi-name: CBS domain-containing protein with a domain of unknown function (DUF21)
Best-hit-arabi-symbol: nan
Gorai.011G170300.1.D
PCC=0.527, p=0.02
Gorai.011G170300.1.A
PCC=0.347, p=0.145
mRNA Profile
Protein Expression Profile
DPA
0
1
2
3
4
5
6
7
8
9
10
11
12
13
14
15
16
17
18
19
20
21
22
23
24
25
26
27
28
29
30
31
32
33
34

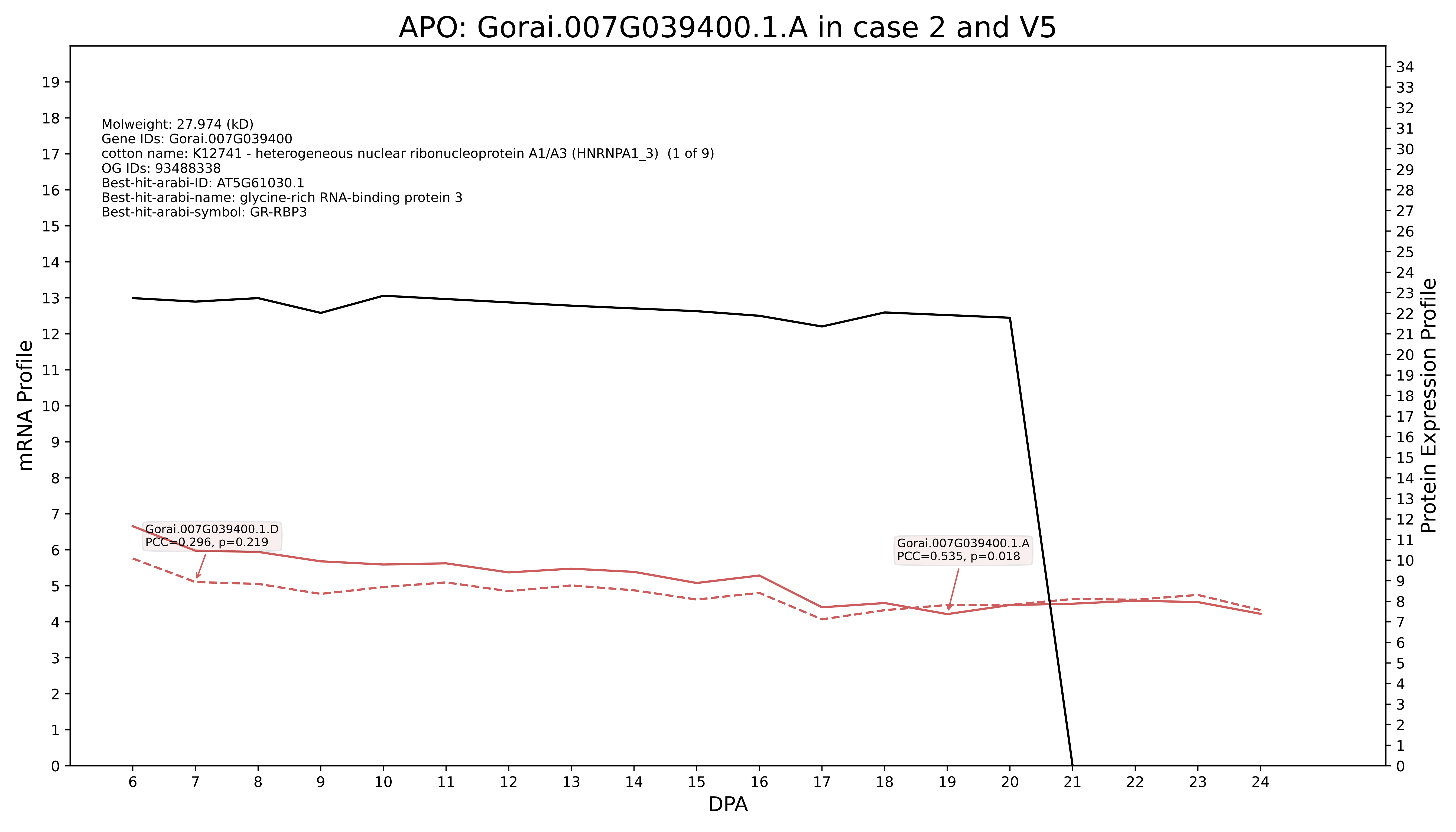

APO: Gorai.007G039400.1.A in case 2 and V5
Molweight: 27.974 (kD)
Gene IDs: Gorai.007G039400
cotton name: K12741 - heterogeneous nuclear ribonucleoprotein A1/A3 (HNRNPA1_3) (1 of 9)
OG IDs: 93488338
Best-hit-arabi-ID: AT5G61030.1
Best-hit-arabi-name: glycine-rich RNA-binding protein 3
Best-hit-arabi-symbol: GR-RBP3
Gorai.007G039400.1.D
PCC=0.296, p=0.219
Gorai.007G039400.1.A
PCC=0.535, p=0.018
mRNA Profile
Protein Expression Profile
DPA

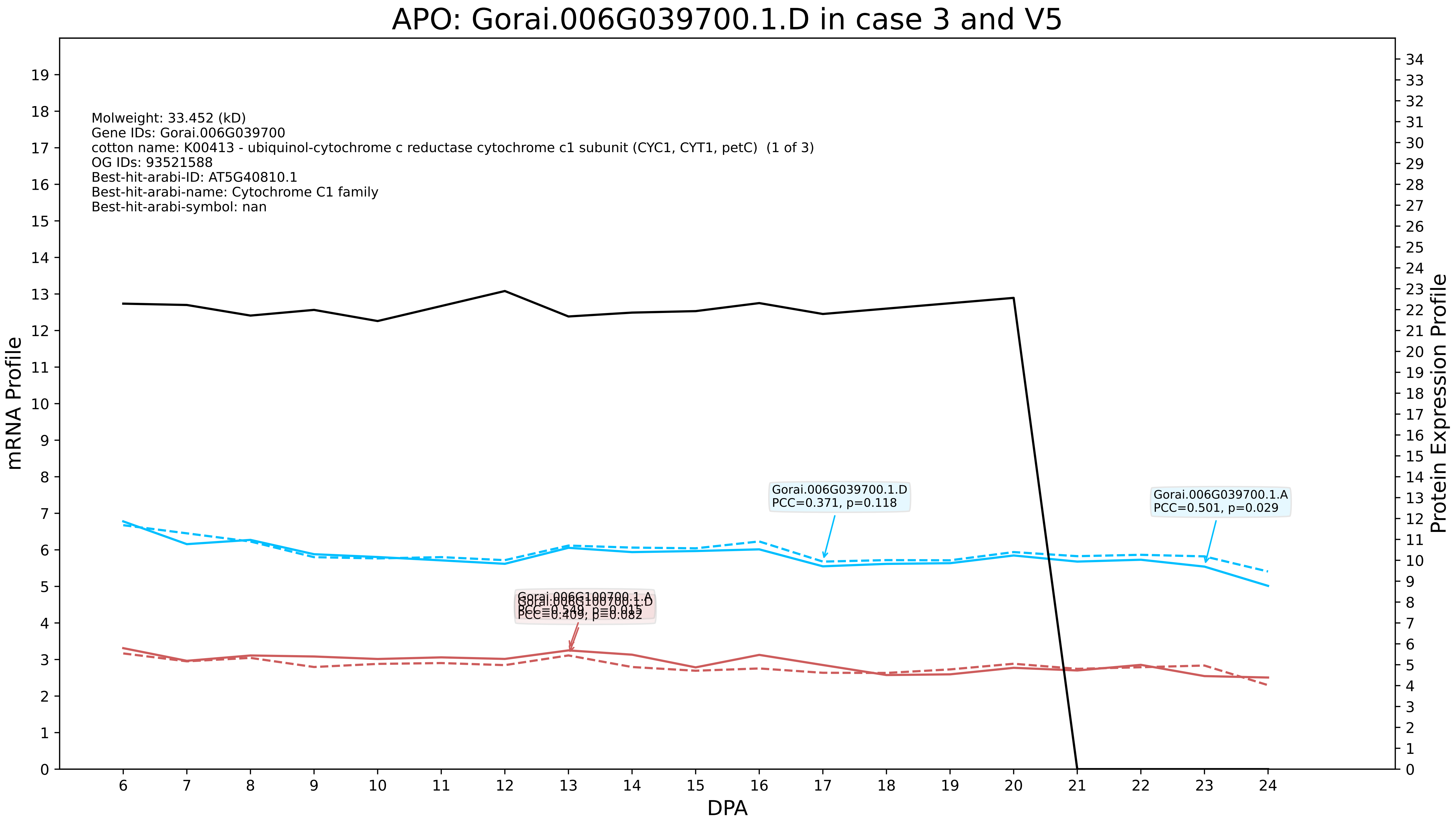
APO: Gorai.006G039700.1.D in case 3 and V5
Molweight: 33.452 (kD)
Gene IDs: Gorai.006G039700
cotton name: K00413 - ubiquinol-cytochrome c reductase cytochrome c1 subunit (CYC1, CYT1, petC) (1 of 3)
OG IDs: 93521588
Best-hit-arabi-ID: AT5G40810.1
Best-hit-arabi-name: Cytochrome C1 family
Best-hit-arabi-symbol: nan
Gorai.006G039700.1.D
PCC=0.371, p=0.118
Gorai.006G039700.1.A
PCC=0.501, p=0.029
Gorai.006G100700.1.A
PCC=0.549, p=0.015
Gorai.006G100700.1.D
PCC=0.409, p=0.082
mRNA Profile
Protein Expression Profile
DPA

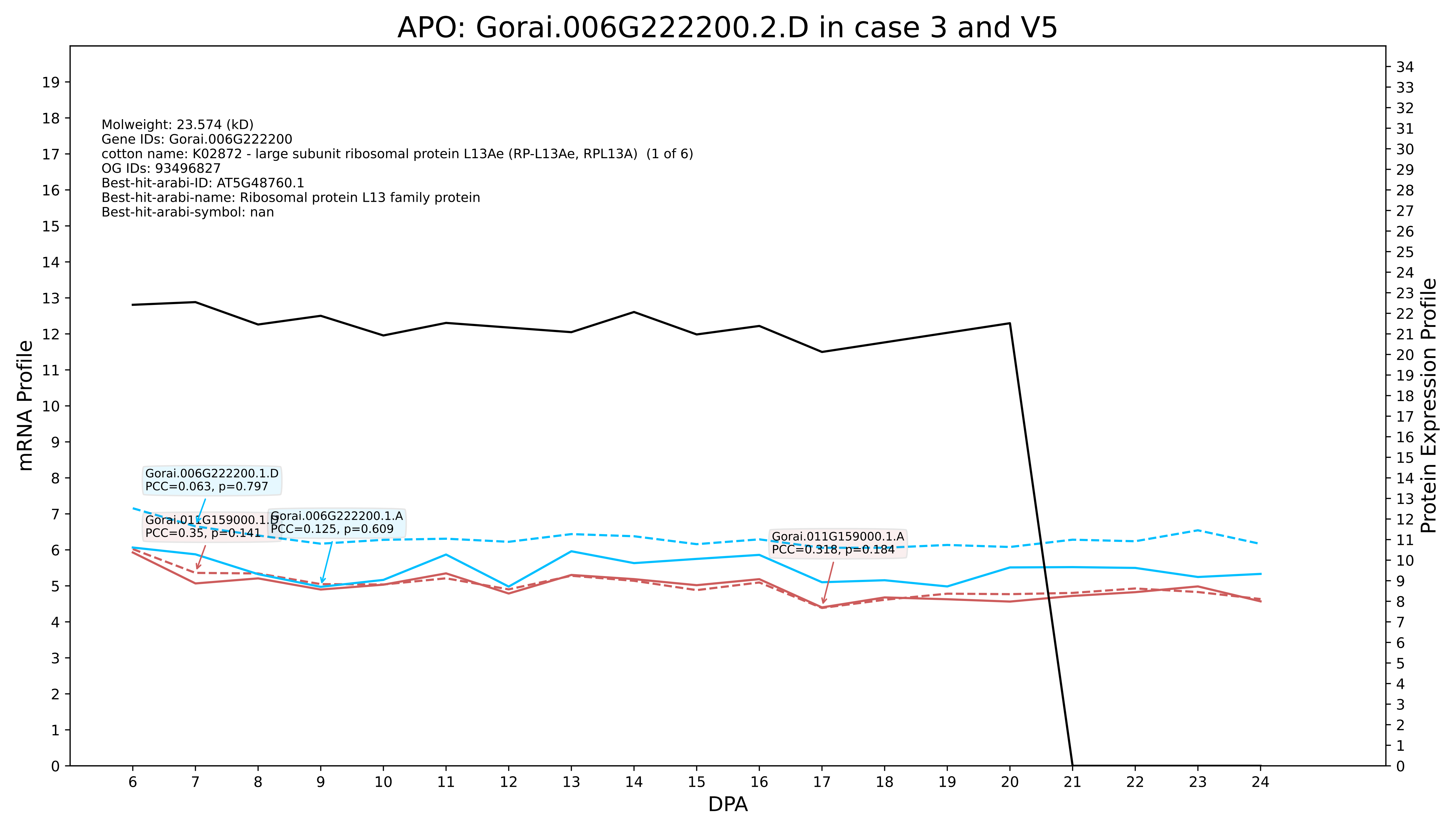
APO: Gorai.006G222200.2.D in case 3 and V5
Molweight: 23.574 (kD)
Gene IDs: Gorai.006G222200
cotton name: K02872 - large subunit ribosomal protein L13Ae (RP-L13Ae, RPL13A) (1 of 6)
OG IDs: 93496827
Best-hit-arabi-ID: AT5G48760.1
Best-hit-arabi-name: Ribosomal protein L13 family protein
Best-hit-arabi-symbol: nan
Gorai.006G222200.1.D
PCC=0.063, p=0.797
Gorai.011G159000.1.D
PCC=0.35, p=0.141
Gorai.006G222200.1.A
PCC=0.125, p=0.609
Gorai.011G159000.1.A
PCC=0.318, p=0.184
mRNA Profile
Protein Expression Profile
DPA

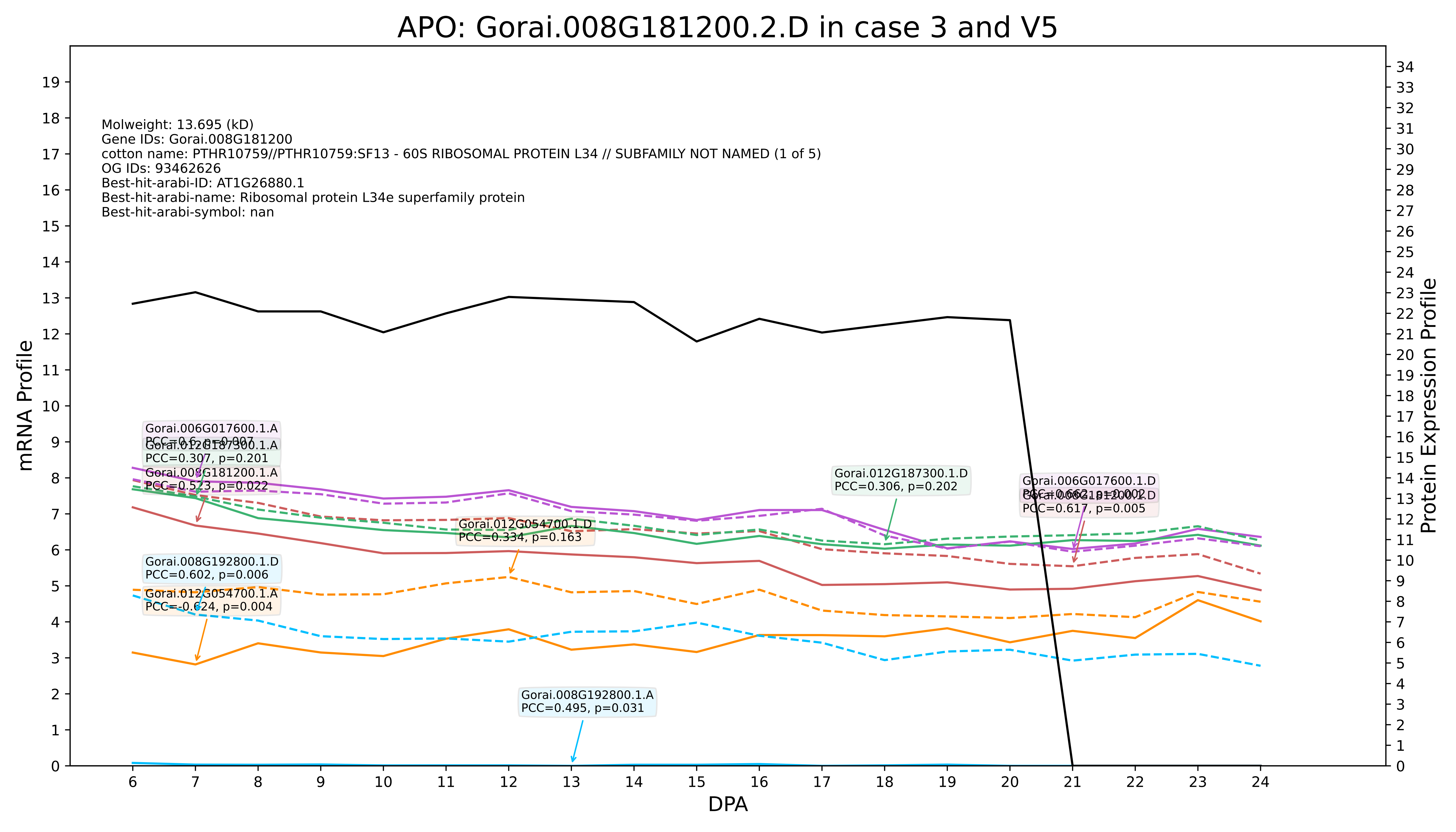

APO: Gorai.008G181200.2.D in case 3 and V5
Molweight: 13.695 (kD)
Gene IDs: Gorai.008G181200
cotton name: PTHR10759//PTHR10759:SF13 - 60S RIBOSOMAL PROTEIN L34 // SUBFAMILY NOT NAMED (1 of 5)
OG IDs: 93462626
Best-hit-arabi-ID: AT1G26880.1
Best-hit-arabi-name: Ribosomal protein L34e superfamily protein
Best-hit-arabi-symbol: nan
Gorai.006G017600.1.A
Gorai.012G187300.1.A
PCC=0.307, p=0.201
Gorai.008G181200.1.A
PCC=0.523, p=0.022
Gorai.012G187300.1.D
PCC=0.306, p=0.202
Gorai.006G017600.1.D
PCC=0.617, p=0.005
Gorai.012G054700.1.D
PCC=0.334, p=0.163
Gorai.008G192800.1.D
PCC=0.602, p=0.006
Gorai.012G054700.1.A
PCC=-0.624, p=0.004
Gorai.008G192800.1.A
PCC=0.495, p=0.031
mRNA Profile
Protein Expression Profile
DPA

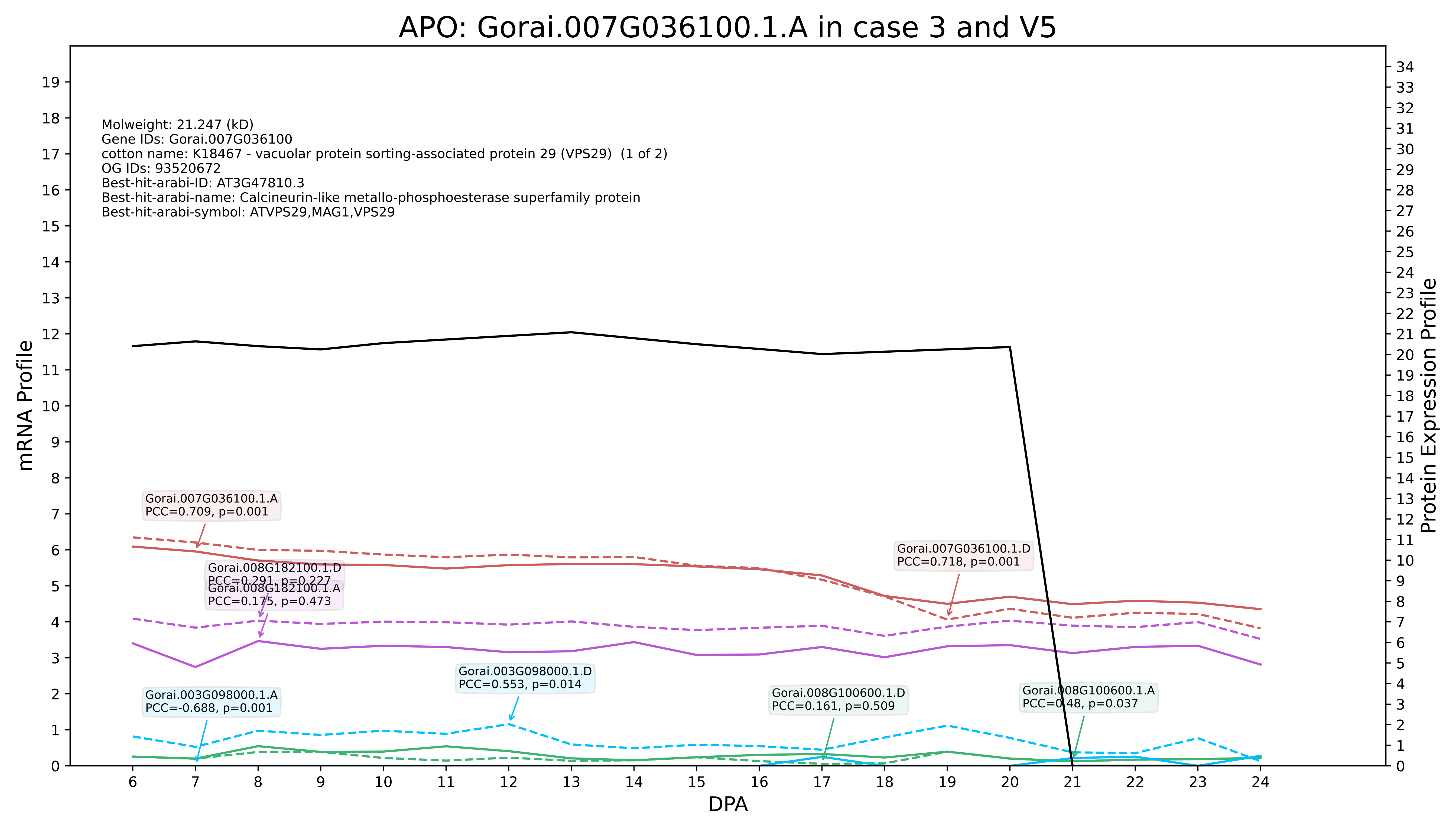
APO: Gorai.007G036100.1.A in case 3 and V5
Molweight: 21.247 (kD)
Gene IDs: Gorai.007G036100
cotton name: K18467 - vacuolar protein sorting-associated protein 29 (VPS29) (1 of 2)
OG IDs: 93520672
Best-hit-arabi-ID: AT3G47810.3
Best-hit-arabi-name: Calcineurin-like metallo-phosphoesterase superfamily protein
Best-hit-arabi-symbol: ATVPS29,MAG1,VPS29
Gorai.007G036100.1.A
PCC=0.709, p=0.001
Gorai.007G036100.1.D
PCC=0.718, p=0.001
Gorai.008G182100.1.D
PCC=0.291, p=0.227
Gorai.008G182100.1.A
PCC=0.175, p=0.473
Gorai.003G098000.1.D
PCC=0.553, p=0.014
Gorai.003G098000.1.A
PCC=-0.688, p=0.001
Gorai.008G100600.1.D
PCC=0.161, p=0.509
Gorai.008G100600.1.A
PCC=0.48, p=0.037
mRNA Profile
Protein Expression Profile
DPA

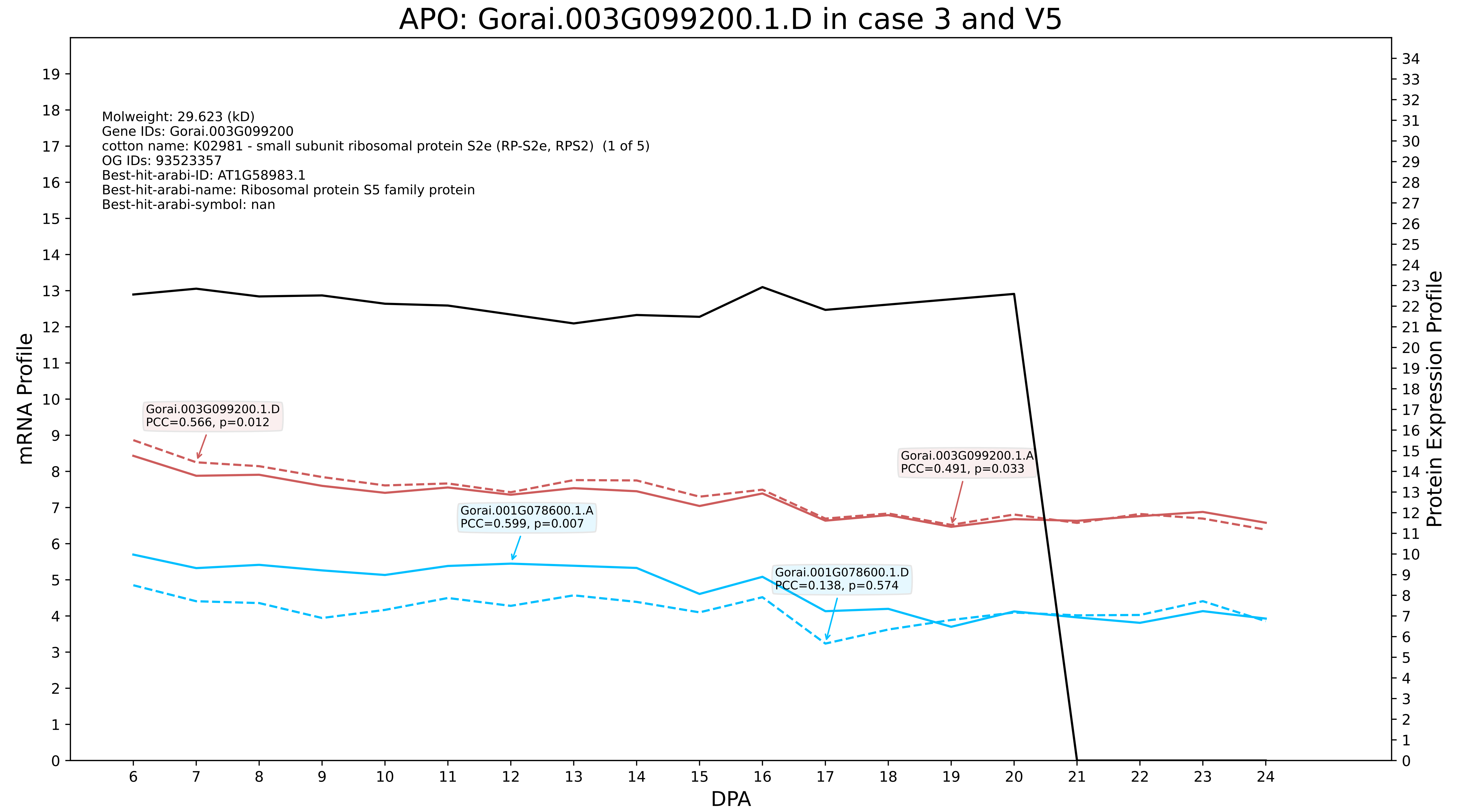
APO: Gorai.003G099200.1.D in case 3 and V5
Molweight: 29.623 (kD)
Gene IDs: Gorai.003G099200
cotton name: K02981 - small subunit ribosomal protein S2e (RP-S2e, RPS2) (1 of 5)
OG IDs: 93523357
Best-hit-arabi-ID: AT1G58983.1
Best-hit-arabi-name: Ribosomal protein S5 family protein
Best-hit-arabi-symbol: nan
Gorai.003G099200.1.D
PCC=0.566, p=0.012
Gorai.003G099200.1.A
PCC=0.491, p=0.033
Gorai.001G078600.1.A
PCC=0.599, p=0.007
Gorai.001G078600.1.D
PCC=0.138, p=0.574
mRNA Profile
Protein Expression Profile
DPA

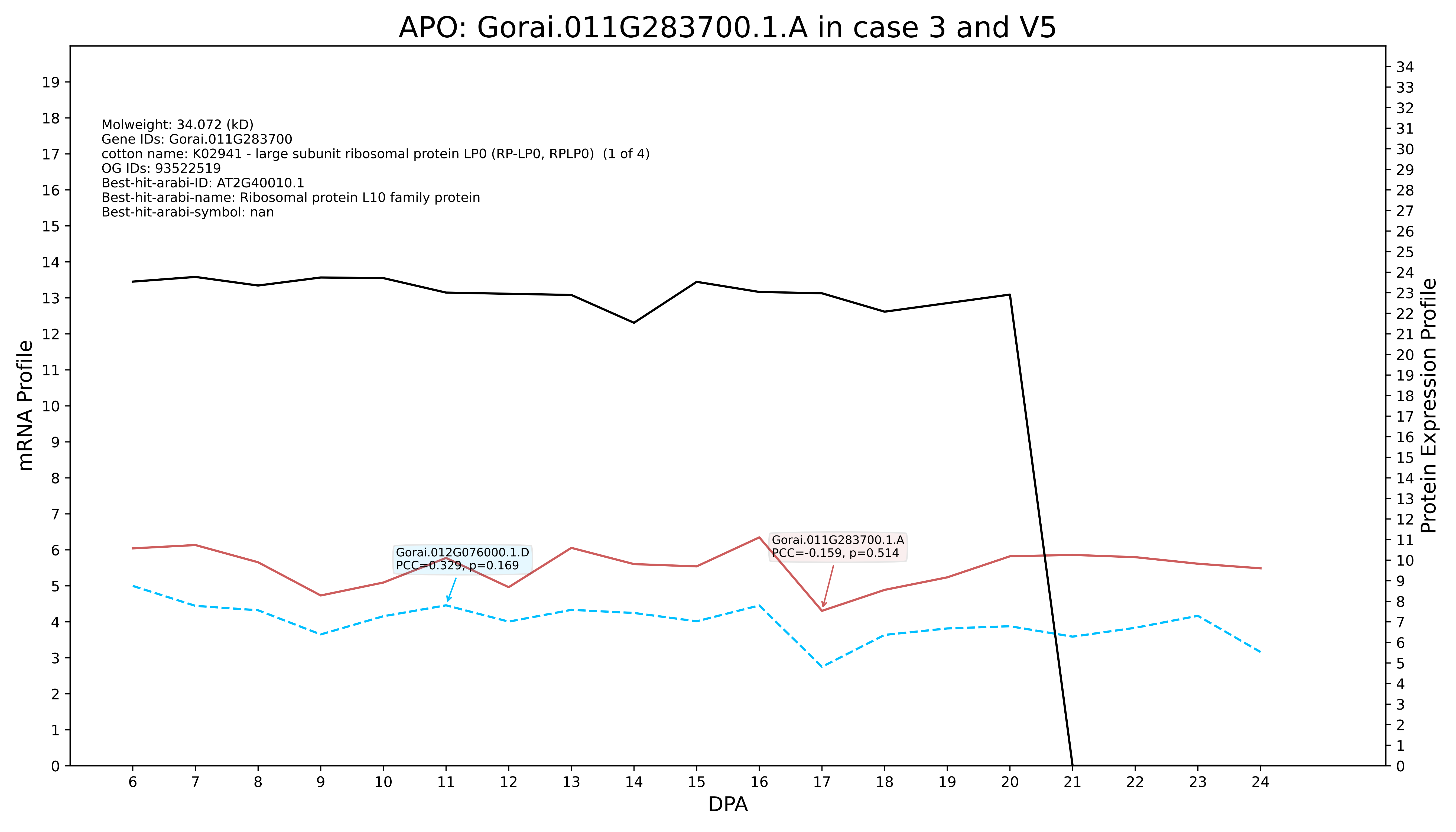
APO: Gorai.011G283700.1.A in case 3 and V5
Molweight: 34.072 (kD)
Gene IDs: Gorai.011G283700
cotton name: K02941 - large subunit ribosomal protein LP0 (RP-LP0, RPLP0) (1 of 4)
OG IDs: 93522519
Best-hit-arabi-ID: AT2G40010.1
Best-hit-arabi-name: Ribosomal protein L10 family protein
Best-hit-arabi-symbol: nan
Gorai.012G076000.1.D
PCC=0.329, p=0.169
Gorai.011G283700.1.A
PCC=-0.159, p=0.514
mRNA Profile
Protein Expression Profile
DPA

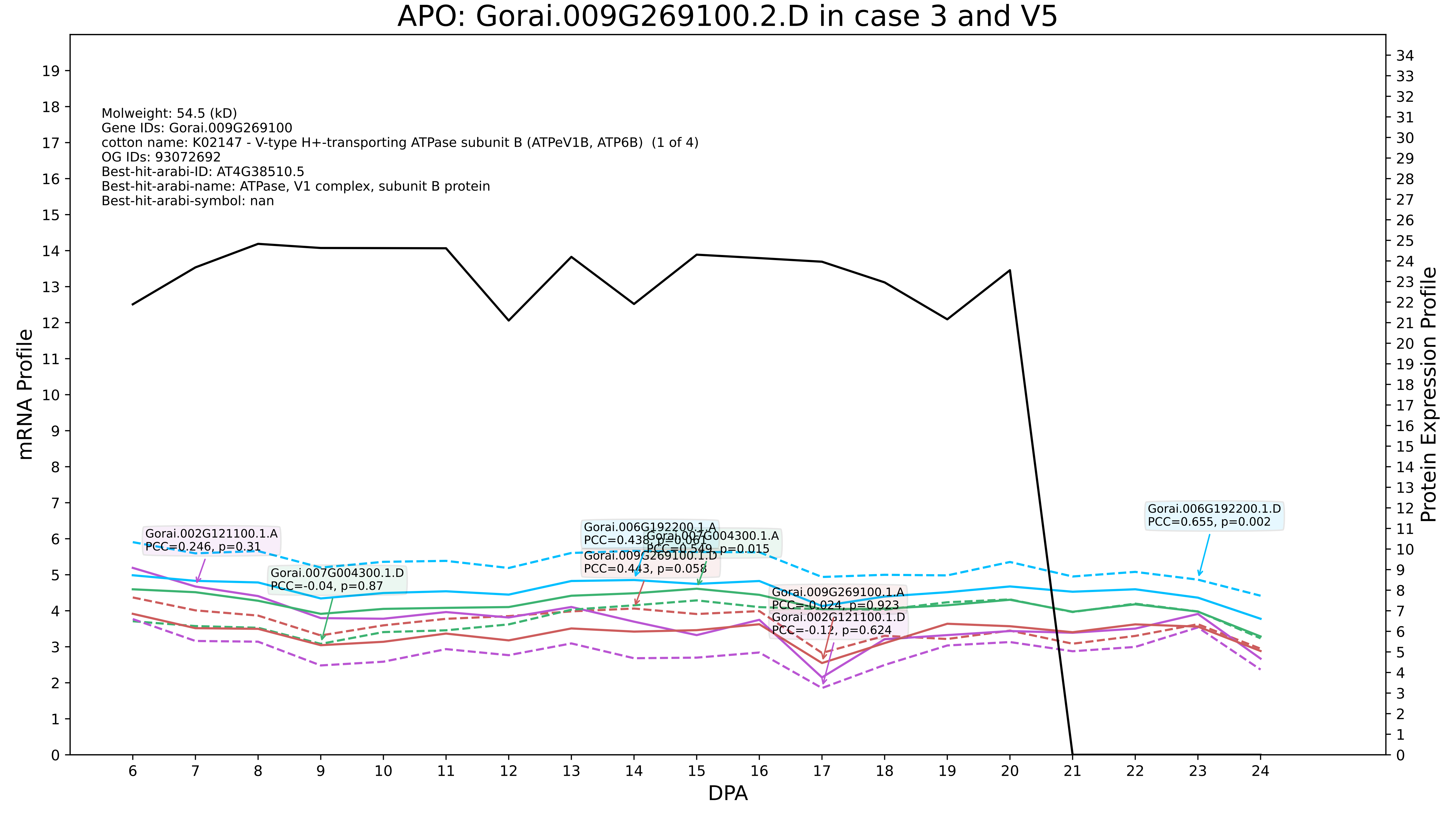

APO: Gorai.009G269100.2.D in case 3 and V5
Molweight: 54.5 (kD)
Gene IDs: Gorai.009G269100
cotton name: K02147 - V-type H+-transporting ATPase subunit B (ATPeV1B, ATP6B) (1 of 4)
OG IDs: 93072692
Best-hit-arabi-ID: AT4G38510.5
Best-hit-arabi-name: ATPase, V1 complex, subunit B protein
Best-hit-arabi-symbol: nan
Gorai.002G121100.1.A
PCC=0.246, p=0.31
Gorai.007G004300.1.D
PCC=-0.04, p=0.87
Gorai.006G192200.1.A
PCC=0.438, p=0.061
Gorai.007G004300.1.A
PCC=0.549, p=0.015
Gorai.009G269100.1.D
PCC=0.443, p=0.058
Gorai.009G269100.1.A
PCC=-0.024, p=0.923
Gorai.002G121100.1.D
PCC=-0.12, p=0.624
Gorai.006G192200.1.D
PCC=0.655, p=0.002
mRNA Profile
Protein Expression Profile
DPA

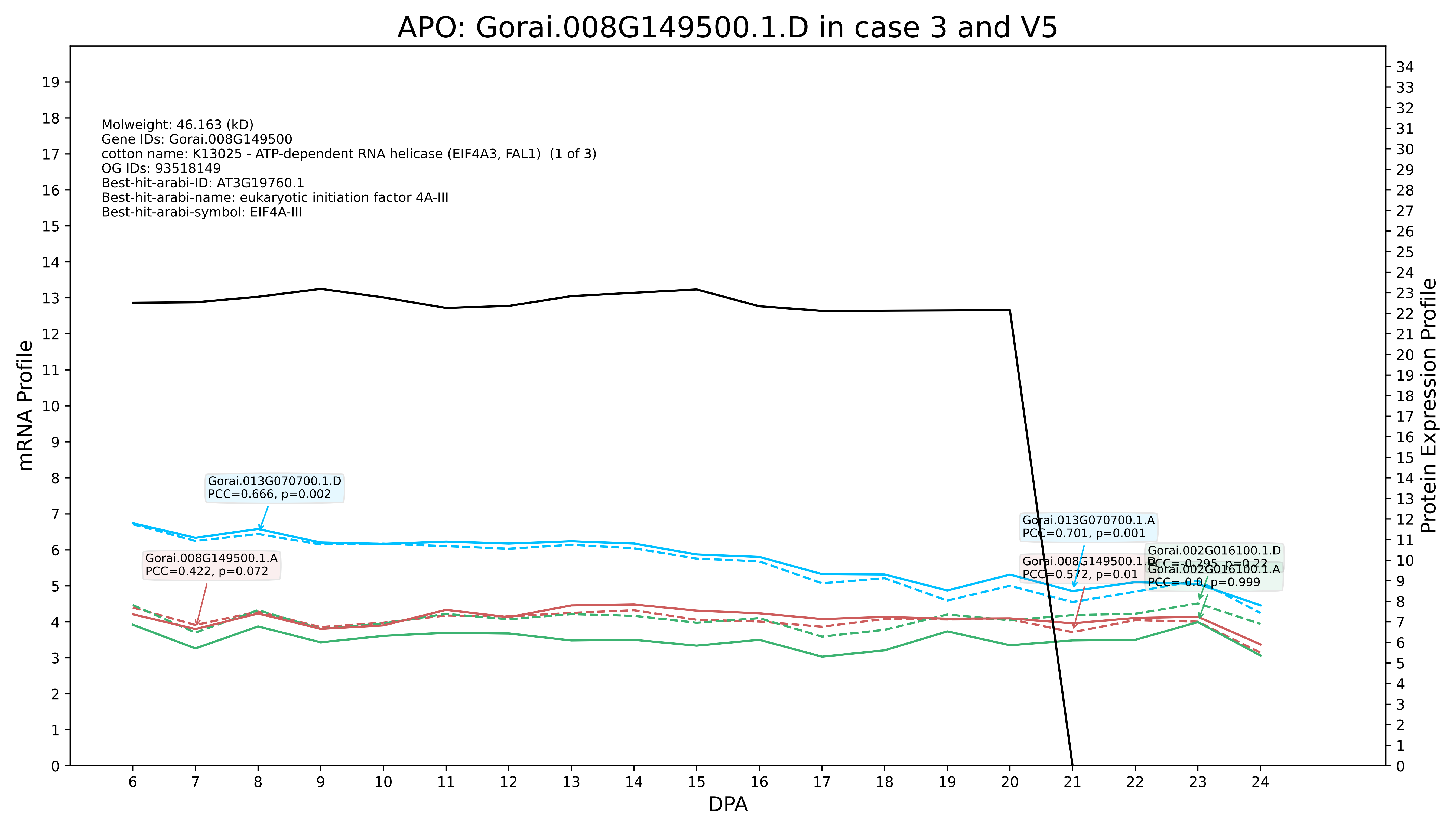

APO: Gorai.008G149500.1.D in case 3 and V5
Molweight: 46.163 (kD)
Gene IDs: Gorai.008G149500
cotton name: K13025 - ATP-dependent RNA helicase (EIF4A3, FAL1) (1 of 3)
OG IDs: 93518149
Best-hit-arabi-ID: AT3G19760.1
Best-hit-arabi-name: eukaryotic initiation factor 4A-III
Best-hit-arabi-symbol: EIF4A-III
Gorai.013G070700.1.D
PCC=0.666, p=0.002
Gorai.008G149500.1.A
PCC=0.422, p=0.072
Gorai.013G070700.1.A
PCC=0.701, p=0.001
Gorai.008G149500.1.D
PCC=0.572, p=0.01
Gorai.002G016100.1.D
PCC=-0.295, p=0.22
Gorai.002G016100.1.A
PCC=-0.0, p=0.999
mRNA Profile
Protein Expression Profile
DPA

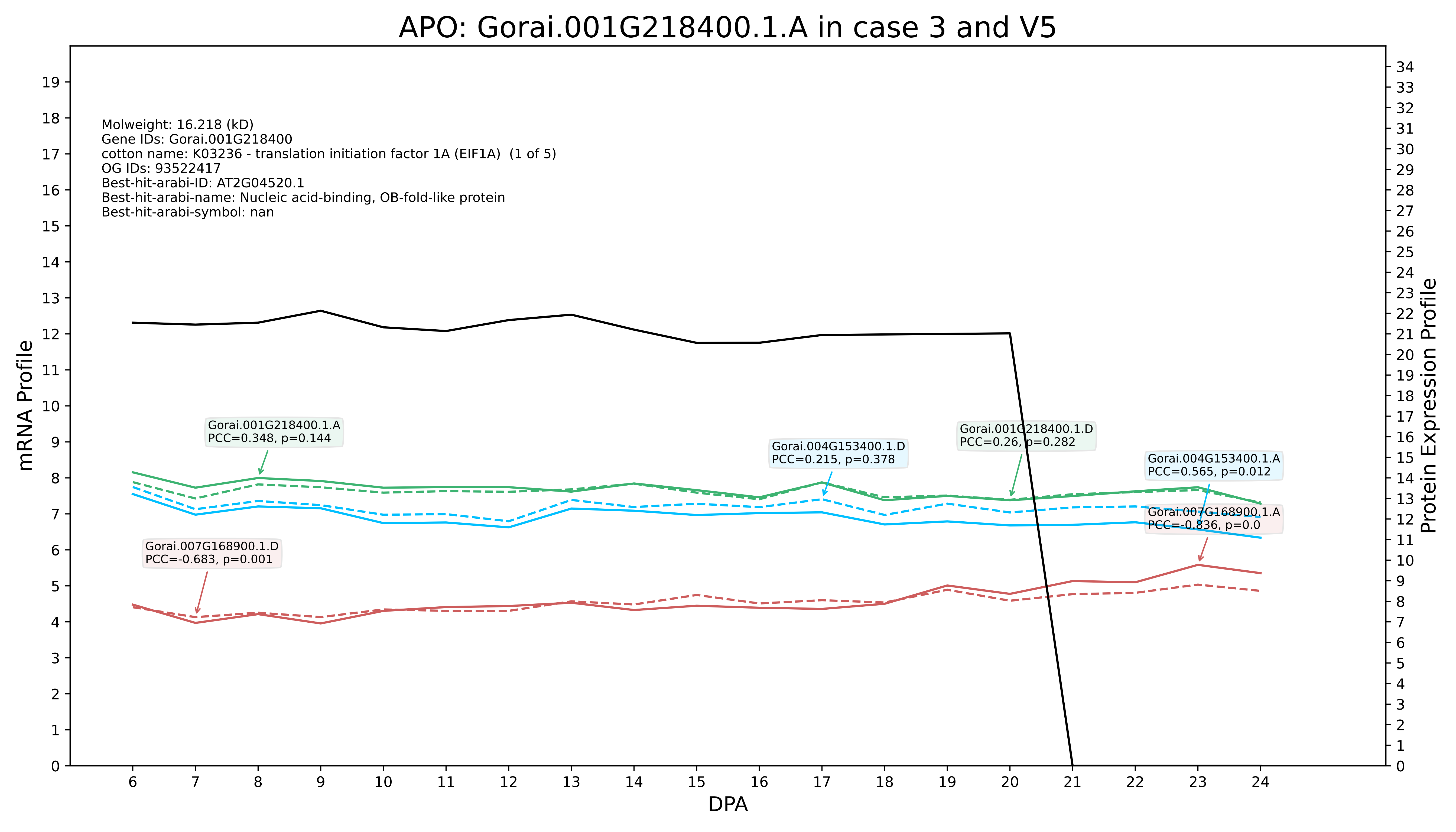
APO: Gorai.001G218400.1.A in case 3 and V5
Molweight: 16.218 (kD)
Gene IDs: Gorai.001G218400
cotton name: K03236 - translation initiation factor 1A (EIF1A) (1 of 5)
OG IDs: 93522417
Best-hit-arabi-ID: AT2G04520.1
Best-hit-arabi-name: Nucleic acid-binding, OB-fold-like protein
Best-hit-arabi-symbol: nan
Gorai.001G218400.1.A
PCC=0.348, p=0.144
Gorai.001G218400.1.D
PCC=0.26, p=0.282
Gorai.004G153400.1.D
PCC=0.215, p=0.378
Gorai.004G153400.1.A
PCC=0.565, p=0.012
Gorai.007G168900.1.A
PCC=-0.836, p=0.0
Gorai.007G168900.1.D
PCC=-0.683, p=0.001
mRNA Profile
Protein Expression Profile
DPA

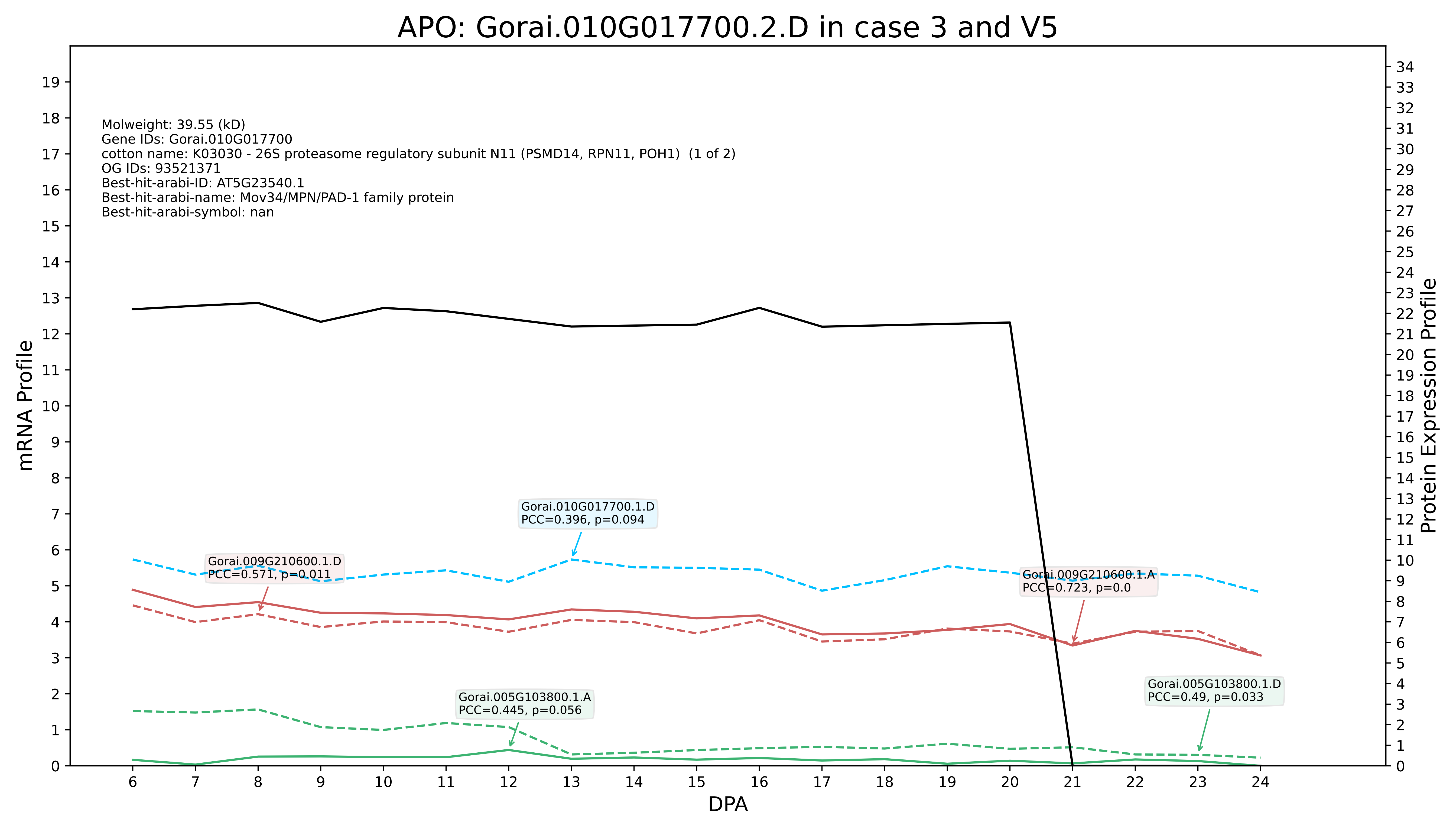
APO: Gorai.010G017700.2.D in case 3 and V5
Molweight: 39.55 (kD)
Gene IDs: Gorai.010G017700
cotton name: K03030 - 26S proteasome regulatory subunit N11 (PSMD14, RPN11, POH1) (1 of 2)
OG IDs: 93521371
Best-hit-arabi-ID: AT5G23540.1
Best-hit-arabi-name: Mov34/MPN/PAD-1 family protein
Best-hit-arabi-symbol: nan
Gorai.010G017700.1.D
PCC=0.396, p=0.094
Gorai.009G210600.1.D
PCC=0.571, p=0.011
Gorai.009G210600.1.A
PCC=0.723, p=0.0
Gorai.005G103800.1.A
PCC=0.445, p=0.056
Gorai.005G103800.1.D
PCC=0.49, p=0.033
mRNA Profile
Protein Expression Profile
DPA

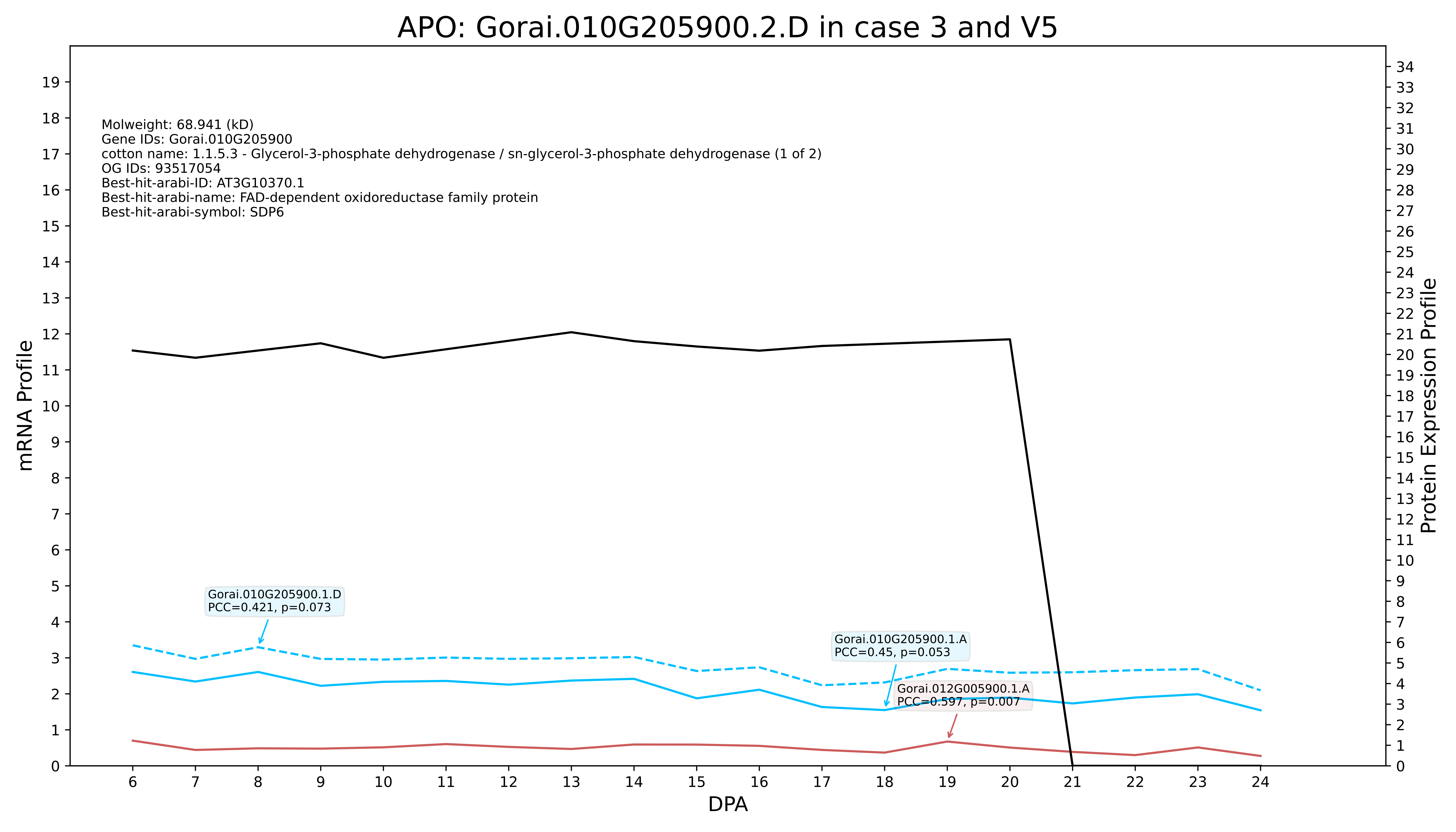
APO: Gorai.010G205900.2.D in case 3 and V5
Molweight: 68.941 (kD)
Gene IDs: Gorai.010G205900
cotton name: 1.1.5.3 - Glycerol-3-phosphate dehydrogenase / sn-glycerol-3-phosphate dehydrogenase (1 of 2)
OG IDs: 93517054
Best-hit-arabi-ID: AT3G10370.1
Best-hit-arabi-name: FAD-dependent oxidoreductase family protein
Best-hit-arabi-symbol: SDP6
Gorai.010G205900.1.D
PCC=0.421, p=0.073
Gorai.010G205900.1.A
PCC=0.45, p=0.053
Gorai.012G005900.1.A
PCC=0.597, p=0.007
mRNA Profile
Protein Expression Profile
DPA

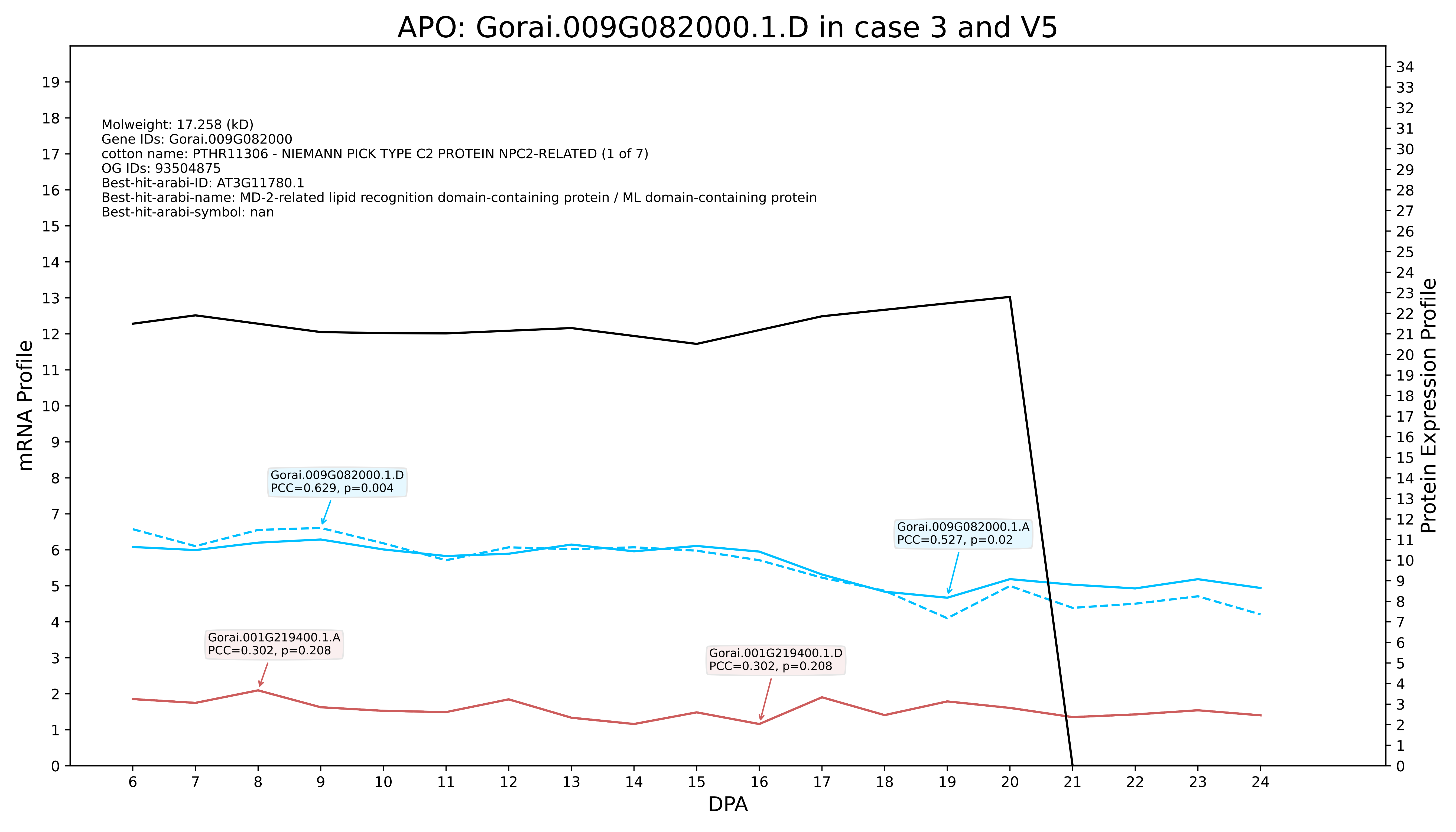
APO: Gorai.009G082000.1.D in case 3 and V5
Molweight: 17.258 (kD)
Gene IDs: Gorai.009G082000
cotton name: PTHR11306 - NIEMANN PICK TYPE C2 PROTEIN NPC2-RELATED (1 of 7)
OG IDs: 93504875
Best-hit-arabi-ID: AT3G11780.1
Best-hit-arabi-name: MD-2-related lipid recognition domain-containing protein / ML domain-containing protein
Best-hit-arabi-symbol: nan
Gorai.009G082000.1.D
PCC=0.629, p=0.004
Gorai.009G082000.1.A
PCC=0.527, p=0.02
Gorai.001G219400.1.A
PCC=0.302, p=0.208
Gorai.001G219400.1.D
PCC=0.302, p=0.208
mRNA Profile
Protein Expression Profile
DPA

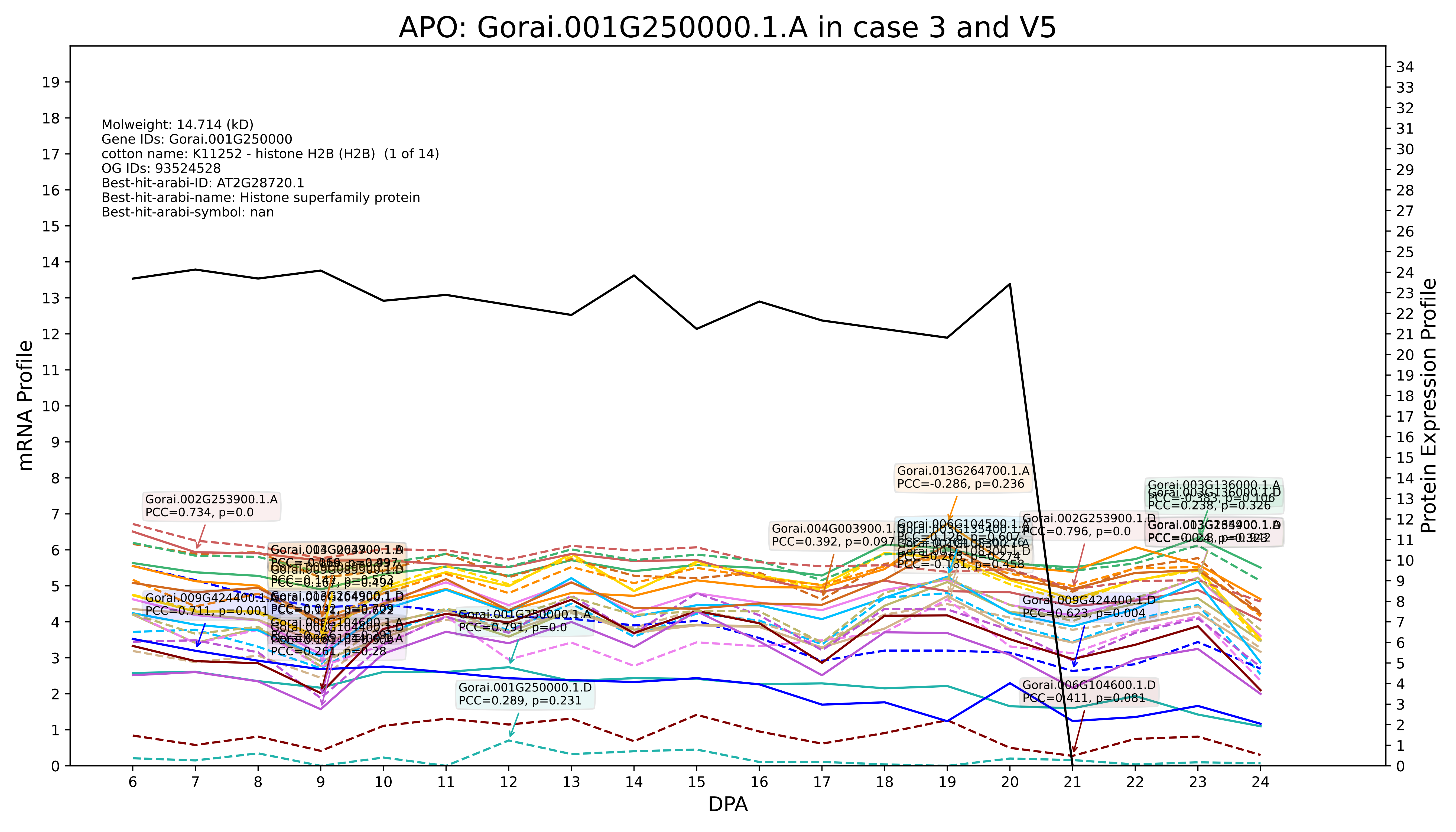

APO: Gorai.001G250000.1.A in case 3 and V5
Molweight: 14.714 (kD)
Gene IDs: Gorai.001G250000
cotton name: K11252 - histone H2B (H2B) (1 of 14)
OG IDs: 93524528
Best-hit-arabi-ID: AT2G28720.1
Best-hit-arabi-name: Histone superfamily protein
Best-hit-arabi-symbol: nan
Gorai.002G253900.1.A
PCC=0.734, p=0.0
Gorai.001G250000.1.A
PCC=0.791, p=0.0
Gorai.001G250000.1.D
PCC=0.289, p=0.231
Gorai.013G264700.1.A
PCC=-0.286, p=0.236
Gorai.004G003900.1.D
PCC=0.392, p=0.097
Gorai.002G253900.1.D
PCC=0.796, p=0.0
Gorai.009G424400.1.D
PCC=0.623, p=0.004
Gorai.006G104600.1.D
PCC=0.411, p=0.081
Gorai.003G136000.1.A
PCC=-0.383, p=0.106
PCC=0.238, p=0.326
Gorai.009G424400.1.A
PCC=0.711, p=0.001
PCC=0.261, p=0.28
mRNA Profile
Protein Expression Profile
DPA

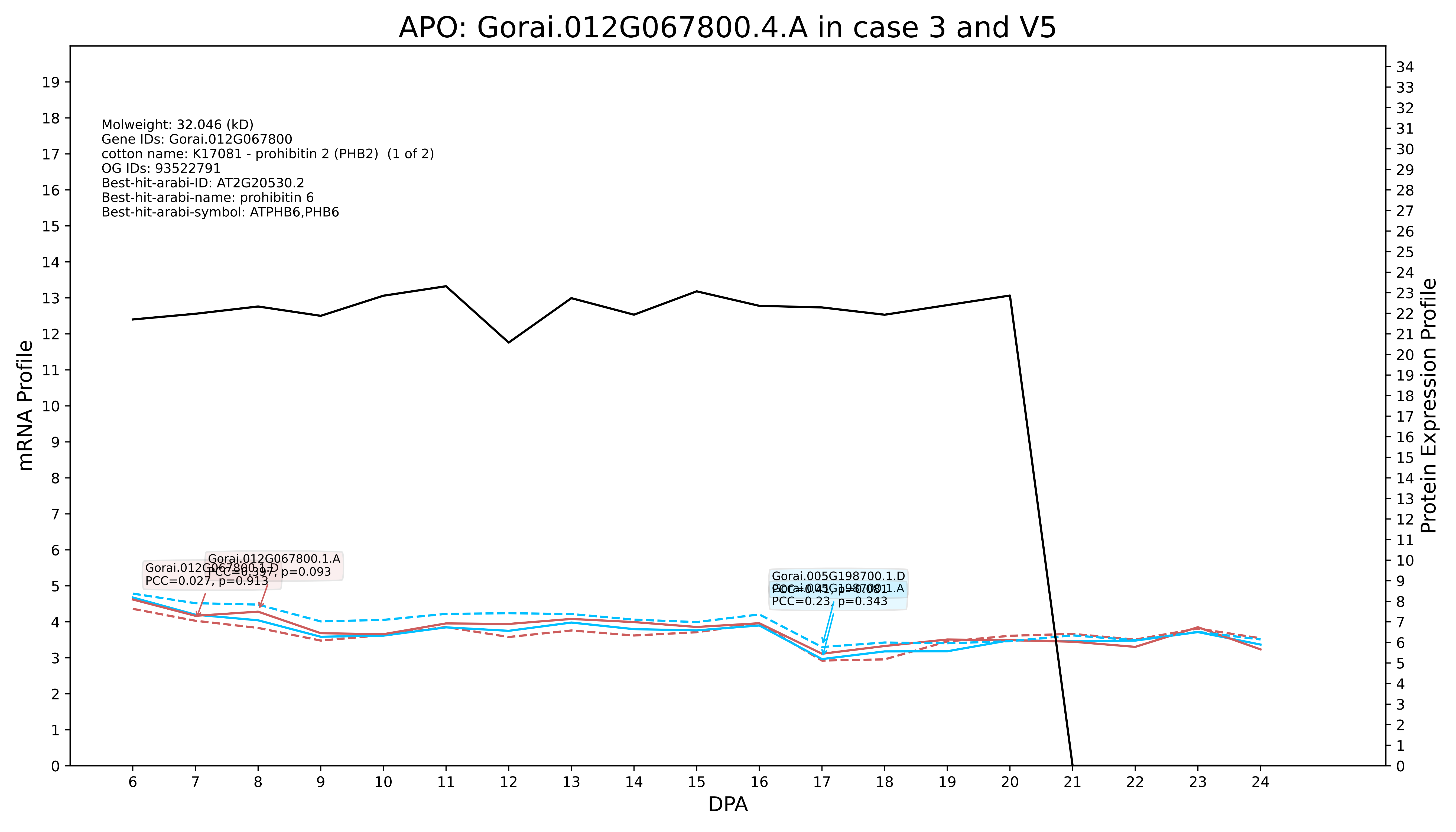

APO: Gorai.012G067800.4.A in case 3 and V5
Molweight: 32.046 (kD)
Gene IDs: Gorai.012G067800
cotton name: K17081 - prohibitin 2 (PHB2) (1 of 2)
OG IDs: 93522791
Best-hit-arabi-ID: AT2G20530.2
Best-hit-arabi-name: prohibitin 6
Best-hit-arabi-symbol: ATPHB6,PHB6
Gorai.012G067800.1.A
PCC=0.397, p=0.093
Gorai.012G067800.1.D
PCC=0.027, p=0.913
Gorai.005G198700.1.D
PCC=0.41, p=0.081
PCC=0.23, p=0.343
mRNA Profile
Protein Expression Profile
DPA

APO: Gorai.008G144500.1.D in case 3 and V5

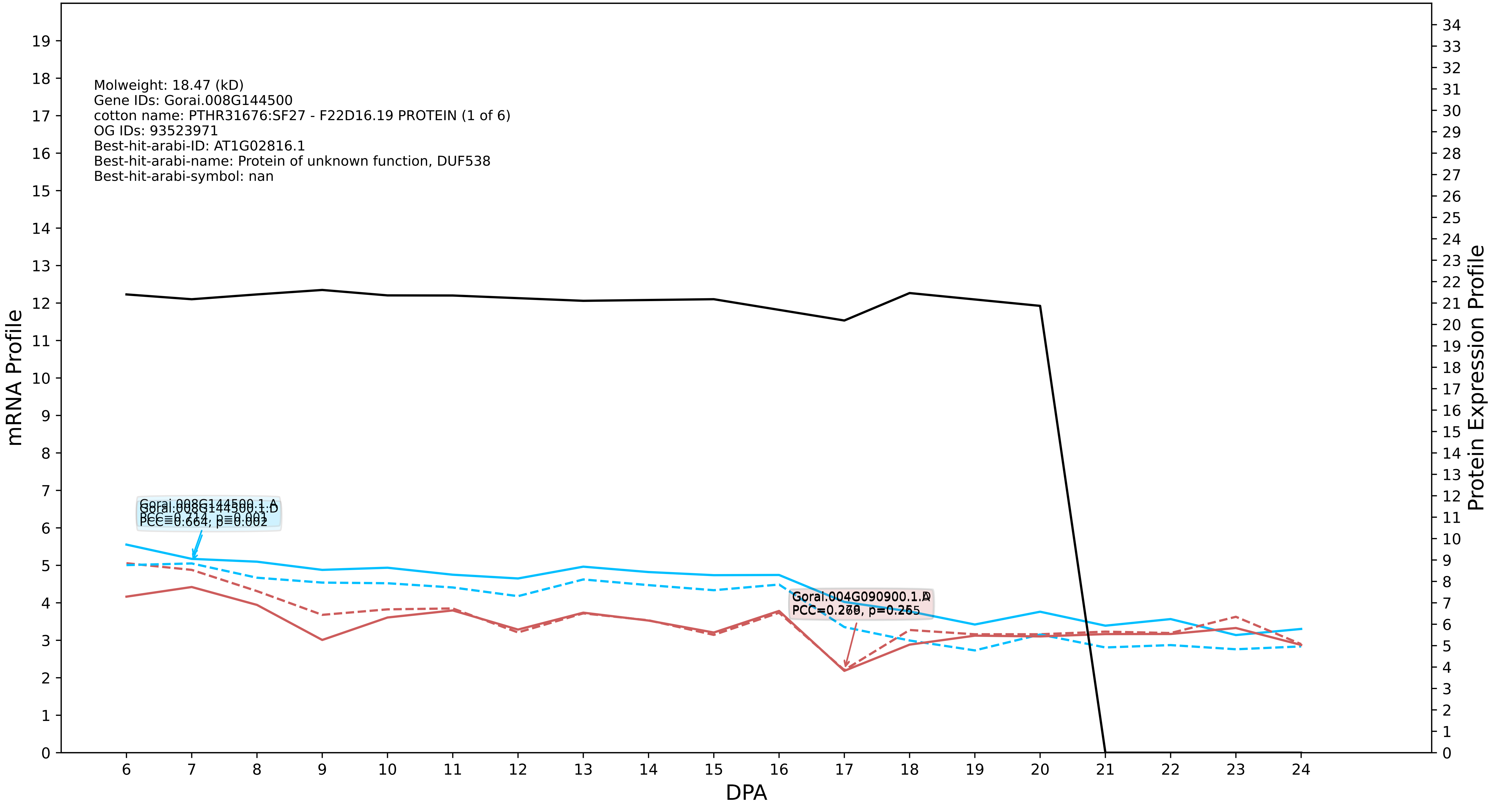

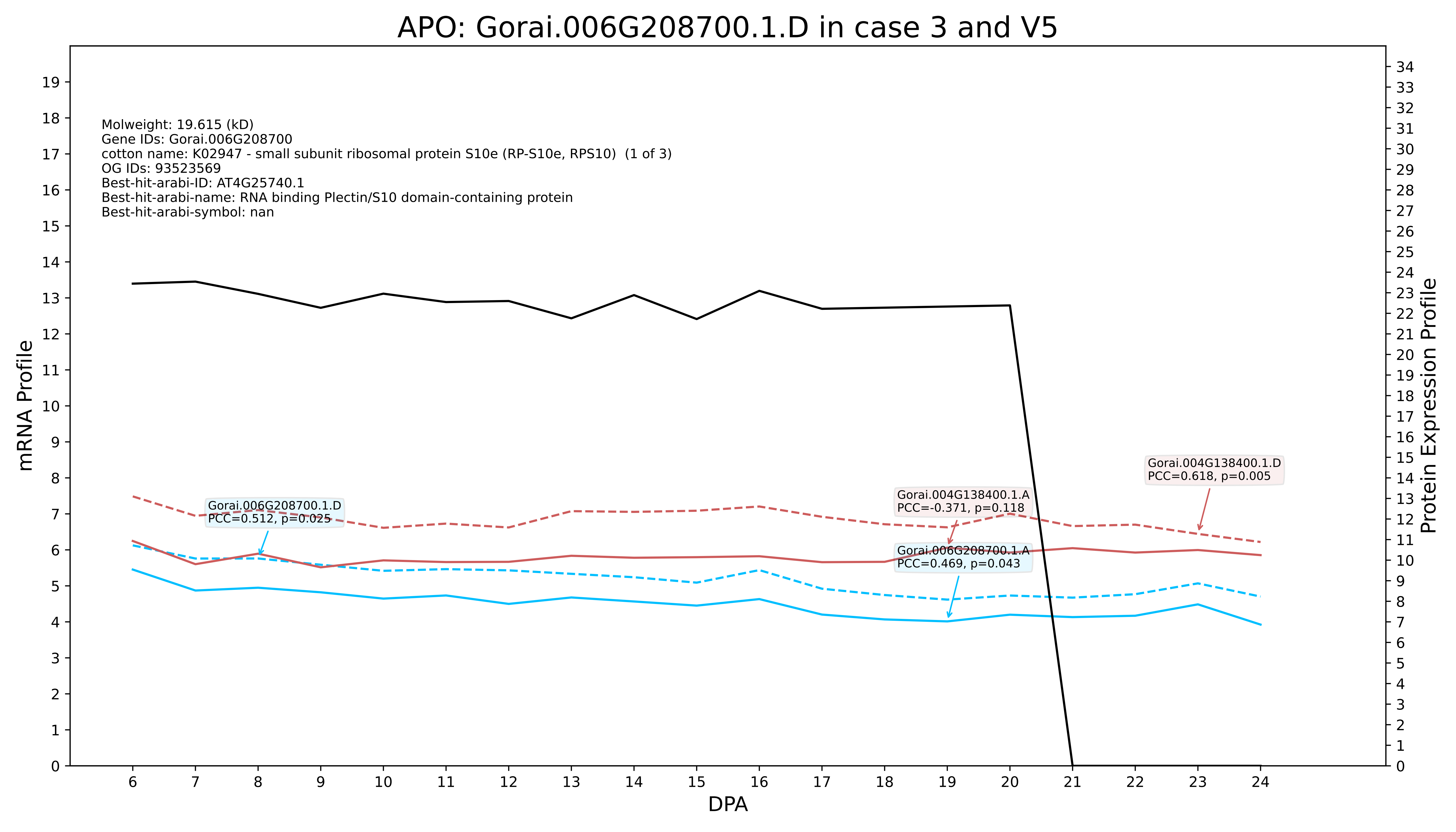

APO: Gorai.006G208700.1.D in case 3 and V5
Molweight: 19.615 (kD)
Gene IDs: Gorai.006G208700
cotton name: K02947 - small subunit ribosomal protein S10e (RP-S10e, RPS10) (1 of 3)
OG IDs: 93523569
Best-hit-arabi-ID: AT4G25740.1
Best-hit-arabi-name: RNA binding Plectin/S10 domain-containing protein
Best-hit-arabi-symbol: nan
Gorai.006G208700.1.D
PCC=0.512, p=0.025
Gorai.004G138400.1.A
PCC=-0.371, p=0.118
Gorai.006G208700.1.A
PCC=0.469, p=0.043
Gorai.004G138400.1.D
PCC=0.618, p=0.005
mRNA Profile
Protein Expression Profile
DPA

APO: Gorai.003G136100.4.D in case 3 and V5

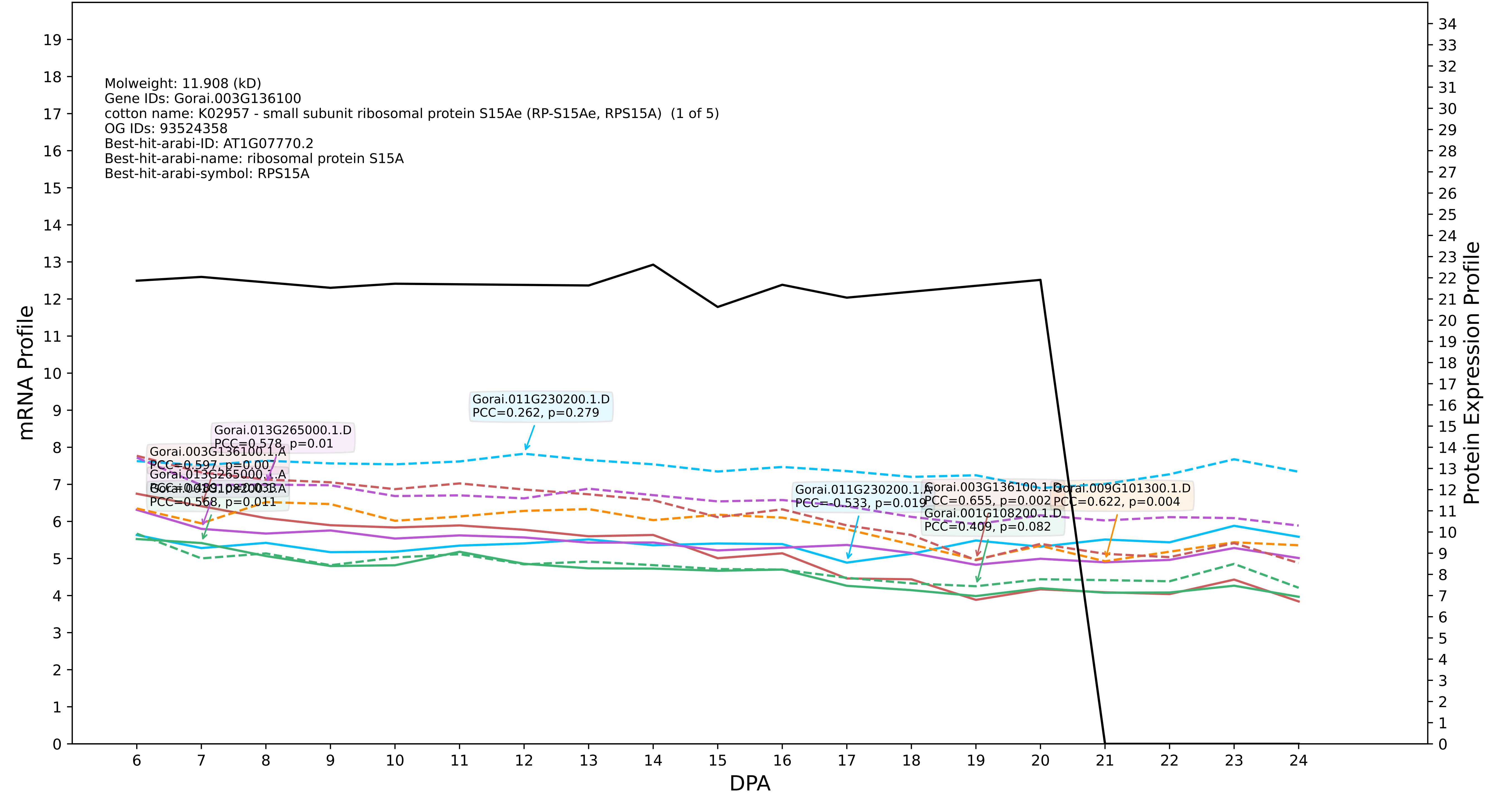

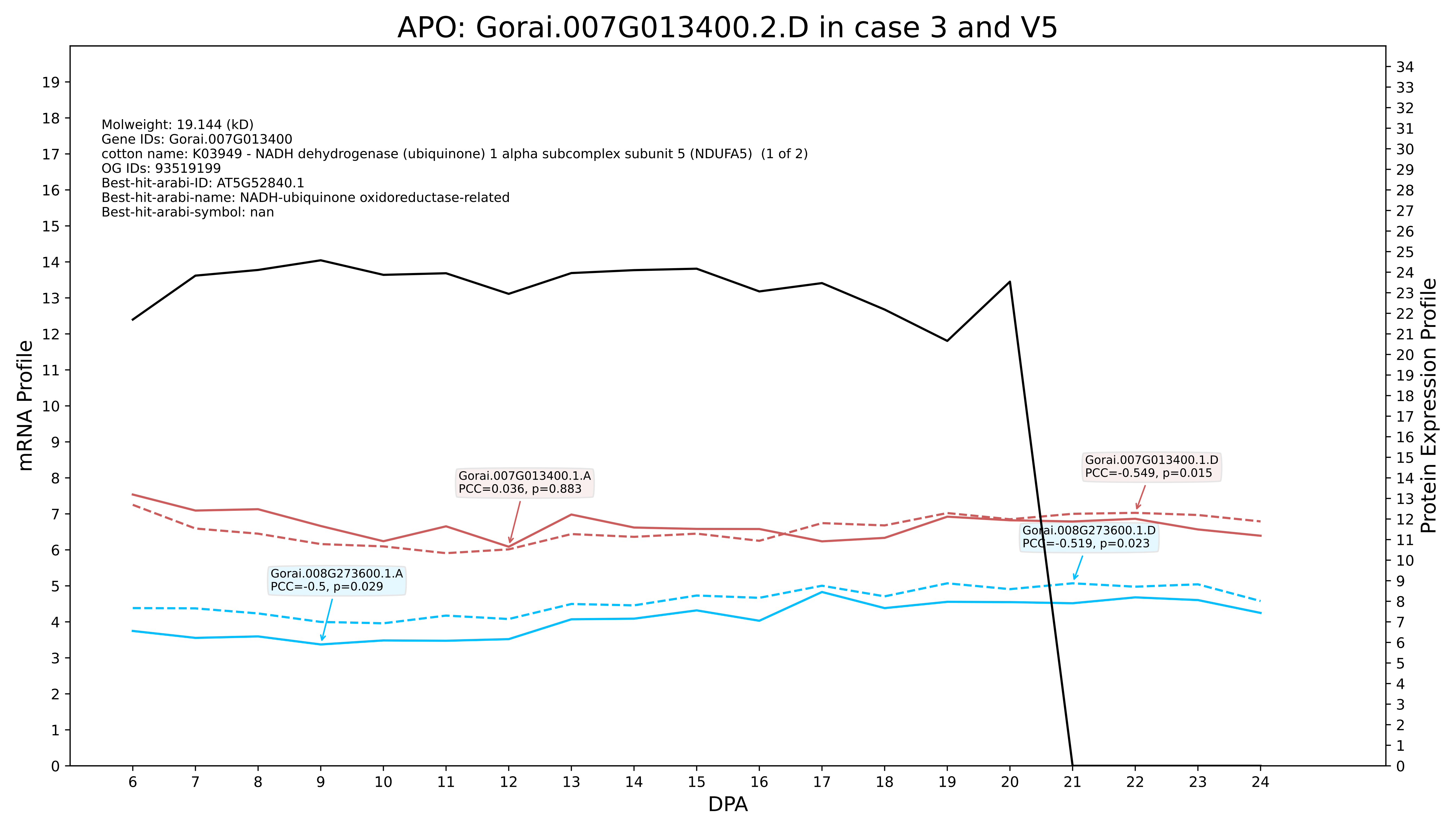
APO: Gorai.007G013400.2.D in case 3 and V5
Molweight: 19.144 (kD)
Gene IDs: Gorai.007G013400
cotton name: K03949 - NADH dehydrogenase (ubiquinone) 1 alpha subcomplex subunit 5 (NDUFA5) (1 of 2)
OG IDs: 93519199
Best-hit-arabi-ID: AT5G52840.1
Best-hit-arabi-name: NADH-ubiquinone oxidoreductase-related
Best-hit-arabi-symbol: nan
Gorai.007G013400.1.A
PCC=0.036, p=0.883
Gorai.007G013400.1.D
PCC=-0.549, p=0.015
Gorai.008G273600.1.D
PCC=-0.519, p=0.023
Gorai.008G273600.1.A
PCC=-0.5, p=0.029
mRNA Profile
Protein Expression Profile
DPA

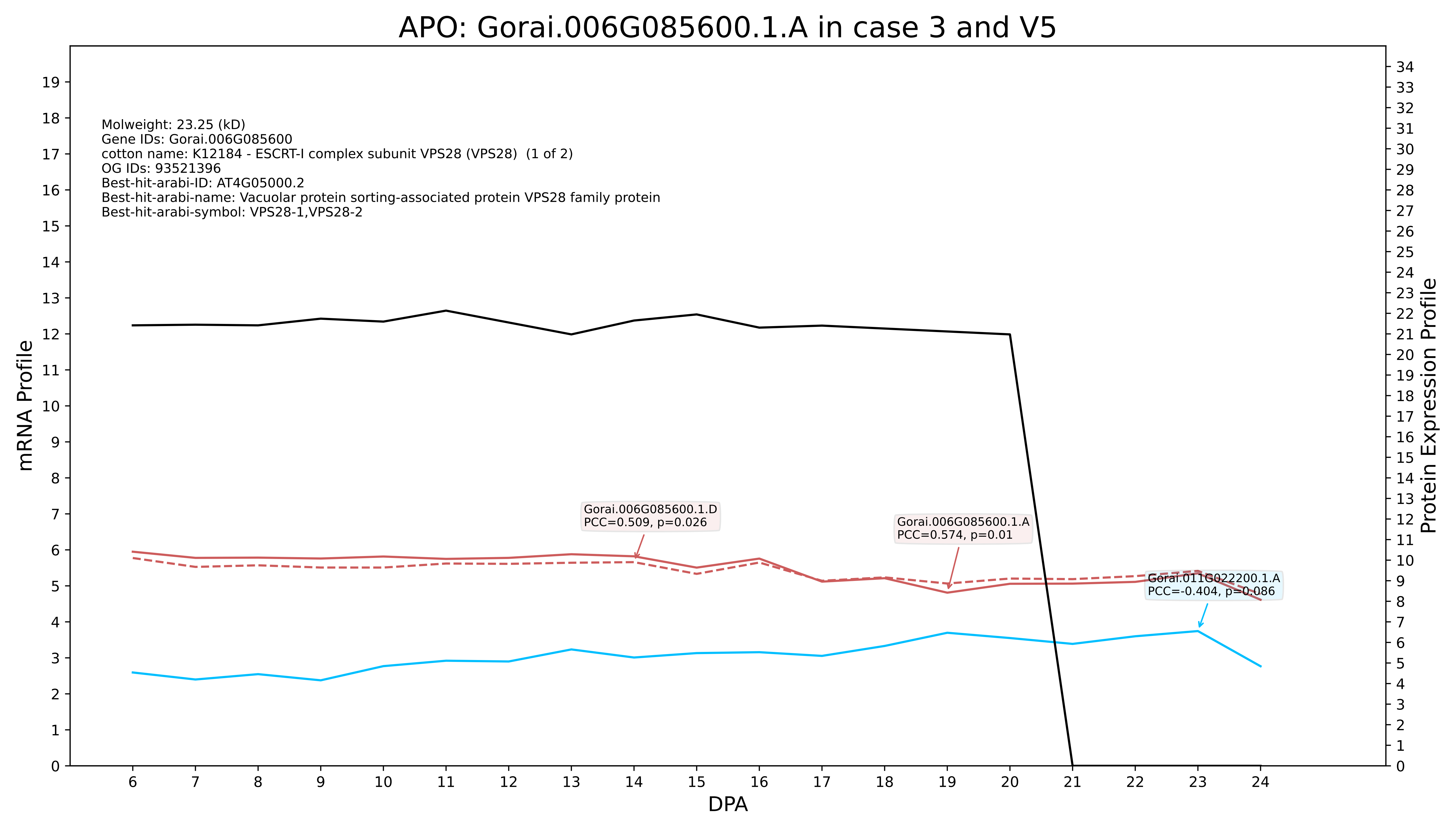
APO: Gorai.006G085600.1.A in case 3 and V5
Molweight: 23.25 (kD)
Gene IDs: Gorai.006G085600
cotton name: K12184 - ESCRT-I complex subunit VPS28 (VPS28) (1 of 2)
OG IDs: 93521396
Best-hit-arabi-ID: AT4G05000.2
Best-hit-arabi-name: Vacuolar protein sorting-associated protein VPS28 family protein
Best-hit-arabi-symbol: VPS28-1,VPS28-2
Gorai.006G085600.1.D
PCC=0.509, p=0.026
Gorai.006G085600.1.A
PCC=0.574, p=0.01
Gorai.011G022200.1.A
PCC=-0.404, p=0.086
mRNA Profile
Protein Expression Profile
DPA

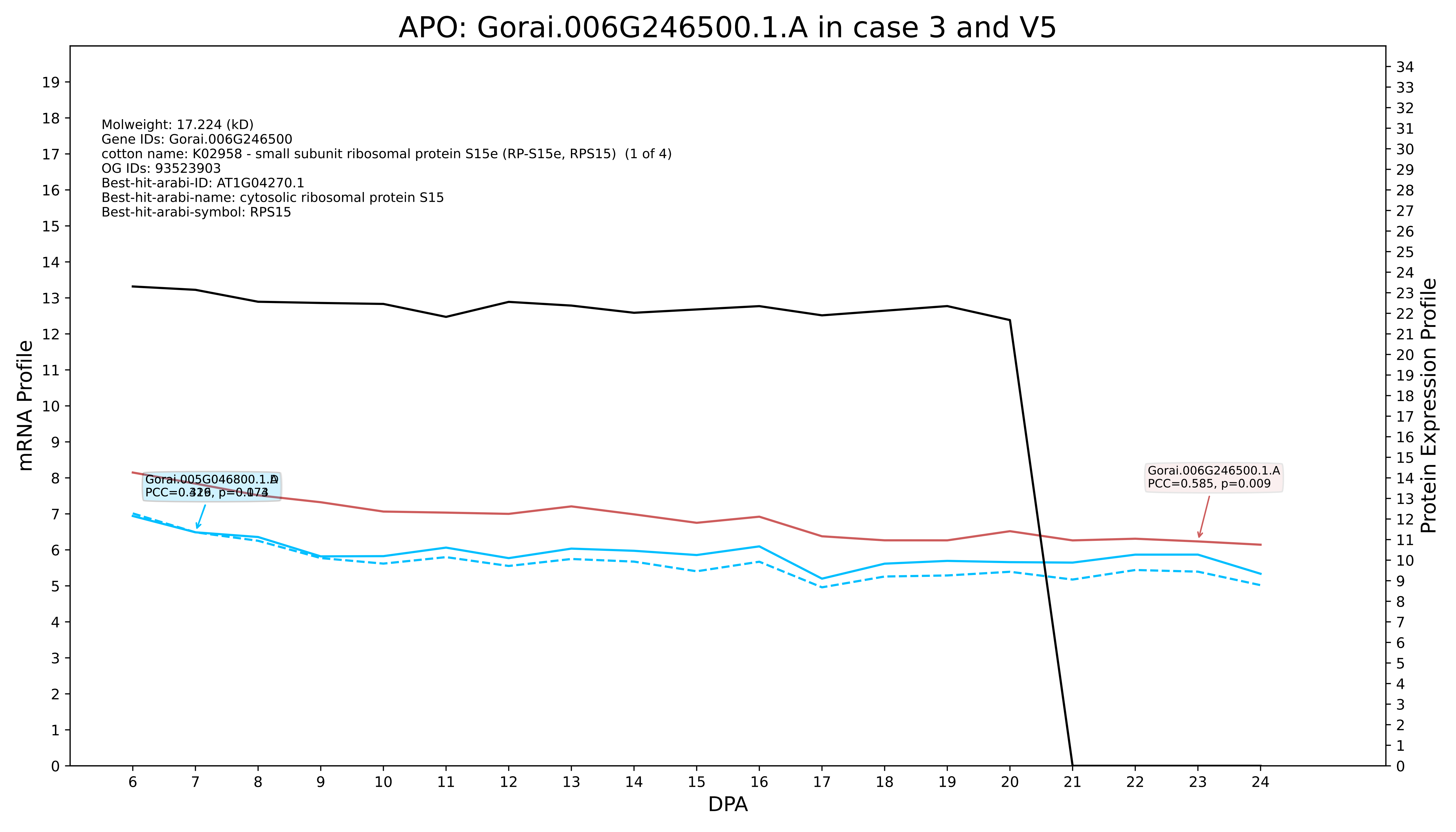
APO: Gorai.006G246500.1.A in case 3 and V5
Molweight: 17.224 (kD)
Gene IDs: Gorai.006G246500
cotton name: K02958 - small subunit ribosomal protein S15e (RP-S15e, RPS15) (1 of 4)
OG IDs: 93523903
Best-hit-arabi-ID: AT1G04270.1
Best-hit-arabi-name: cytosolic ribosomal protein S15
Best-hit-arabi-symbol: RPS15
Gorai.006G246500.1.A
PCC=0.585, p=0.009
mRNA Profile
Protein Expression Profile
DPA

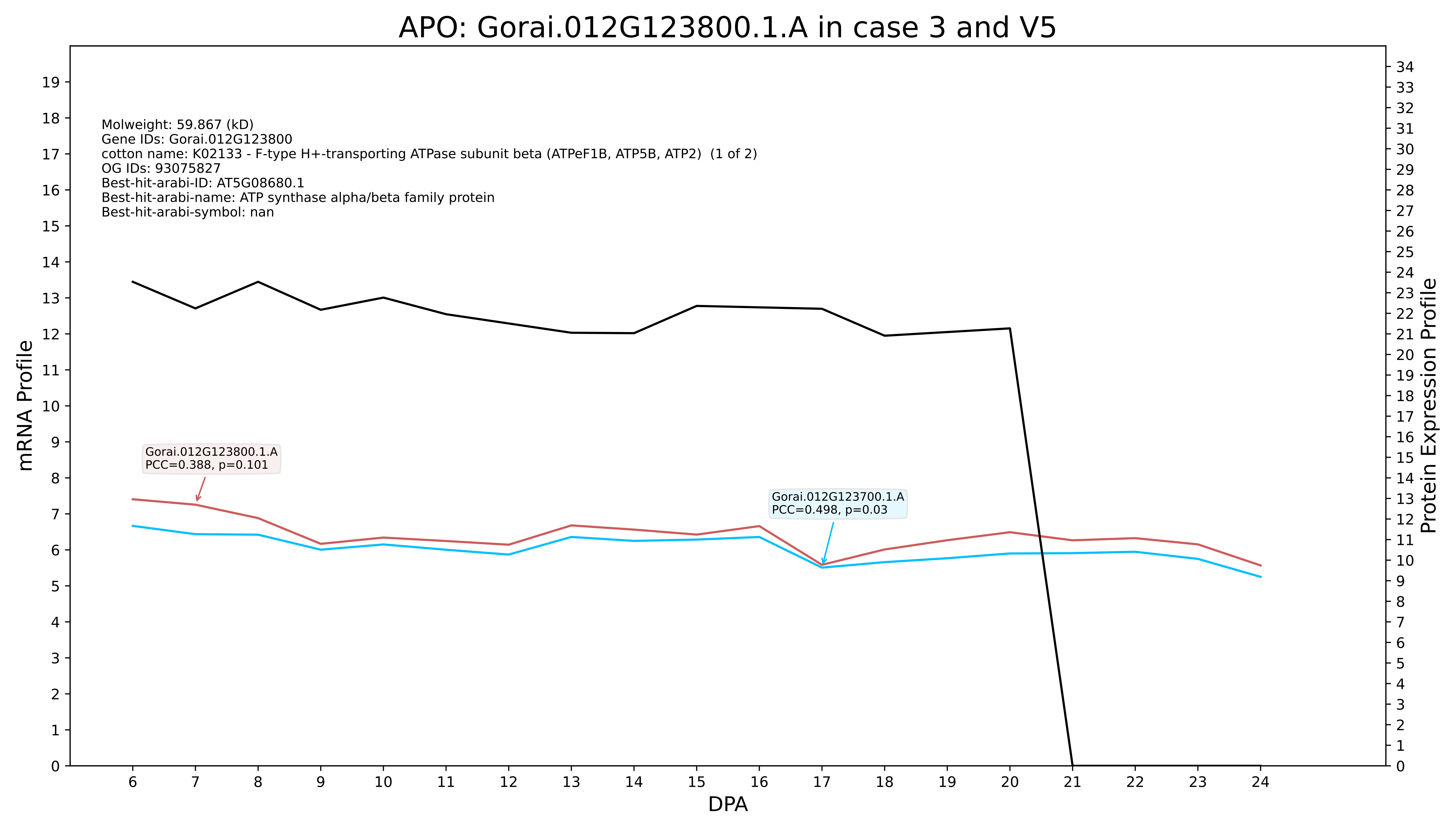

APO: Gorai.012G123800.1.A in case 3 and V5
Molweight: 59.867 (kD)
Gene IDs: Gorai.012G123800
cotton name: K02133 - F-type H+-transporting ATPase subunit beta (ATPeF1B, ATP5B, ATP2) (1 of 2)
OG IDs: 93075827
Best-hit-arabi-ID: AT5G08680.1
Best-hit-arabi-name: ATP synthase alpha/beta family protein
Best-hit-arabi-symbol: nan
Gorai.012G123800.1.A
PCC=0.388, p=0.101
Gorai.012G123700.1.A
PCC=0.498, p=0.03
mRNA Profile
Protein Expression Profile
DPA

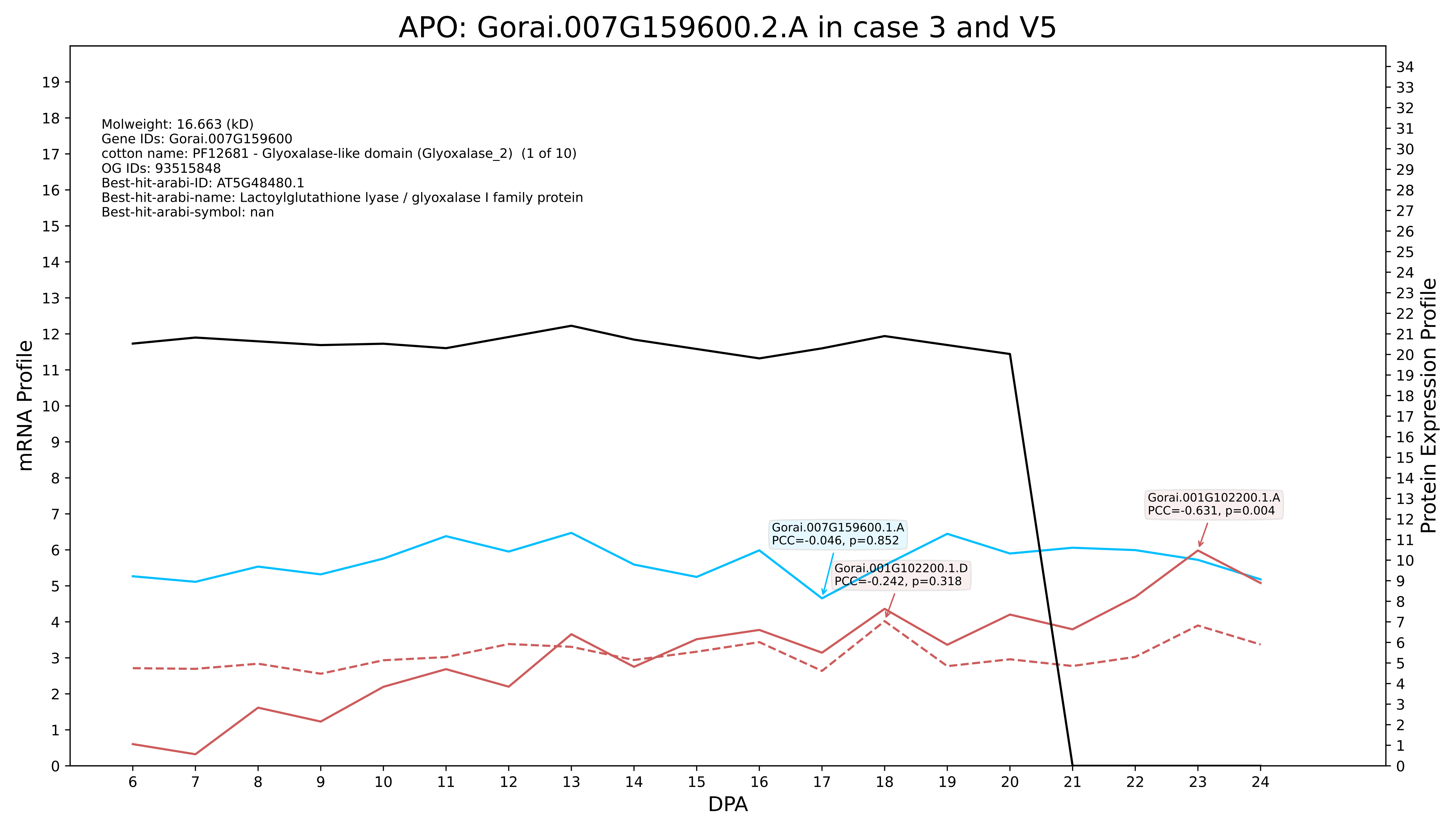

APO: Gorai.007G159600.2.A in case 3 and V5
Molweight: 16.663 (kD)
Gene IDs: Gorai.007G159600
cotton name: PF12681 - Glyoxalase-like domain (Glyoxalase_2) (1 of 10)
OG IDs: 93515848
Best-hit-arabi-ID: AT5G48480.1
Best-hit-arabi-name: Lactoylglutathione lyase / glyoxalase I family protein
Best-hit-arabi-symbol: nan
Gorai.007G159600.1.A
PCC=-0.046, p=0.852
Gorai.001G102200.1.D
PCC=-0.242, p=0.318
Gorai.001G102200.1.A
PCC=-0.631, p=0.004
mRNA Profile
Protein Expression Profile
DPA

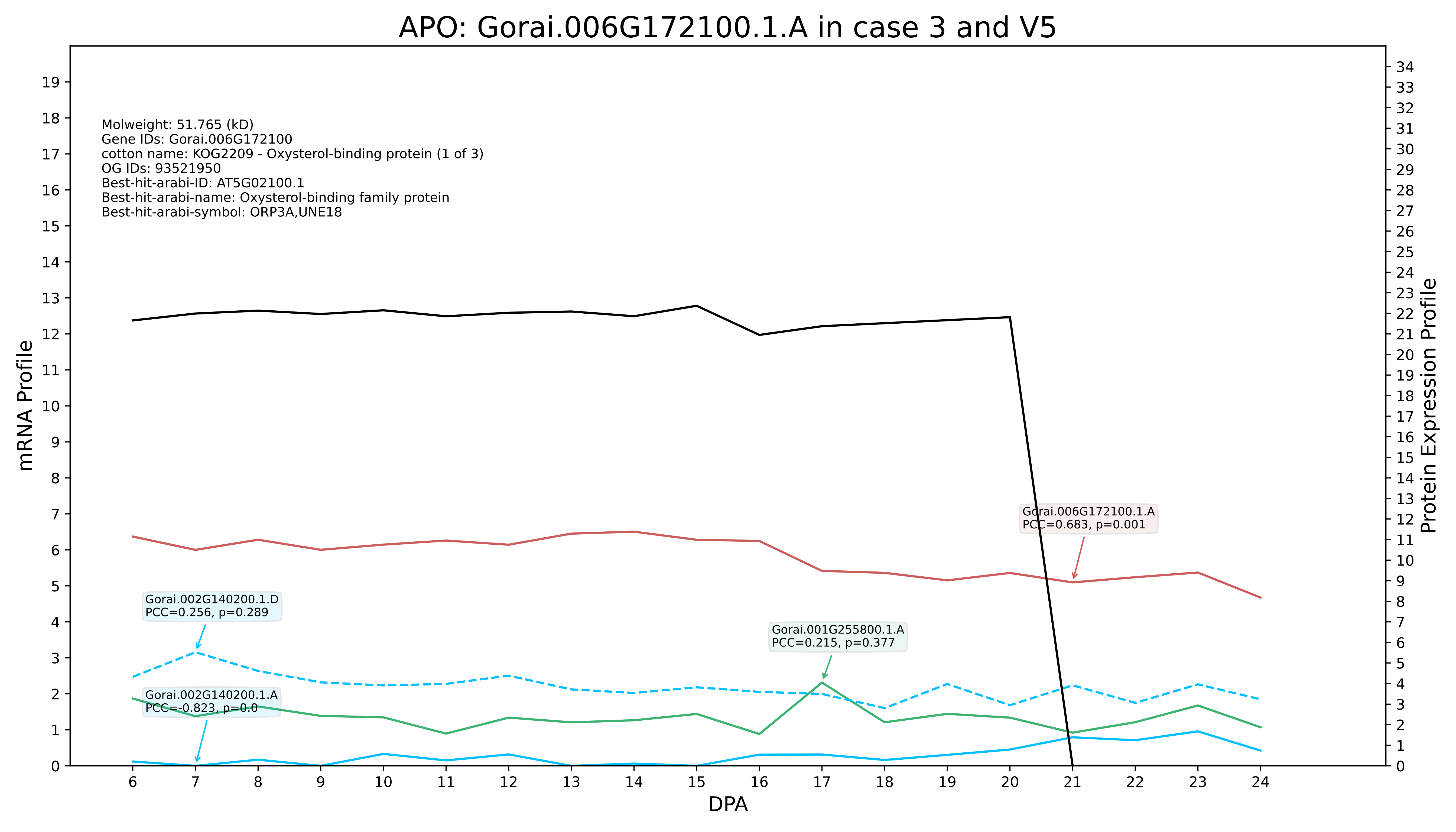

APO: Gorai.006G172100.1.A in case 3 and V5
Molweight: 51.765 (kD)
Gene IDs: Gorai.006G172100
cotton name: KOG2209 - Oxysterol-binding protein (1 of 3)
OG IDs: 93521950
Best-hit-arabi-ID: AT5G02100.1
Best-hit-arabi-name: Oxysterol-binding family protein
Best-hit-arabi-symbol: ORP3A,UNE18
Gorai.006G172100.1.A
PCC=0.683, p=0.001
Gorai.002G140200.1.D
PCC=0.256, p=0.289
Gorai.001G255800.1.A
PCC=0.215, p=0.377
Gorai.002G140200.1.A
PCC=-0.823, p=0.0
mRNA Profile
Protein Expression Profile
DPA

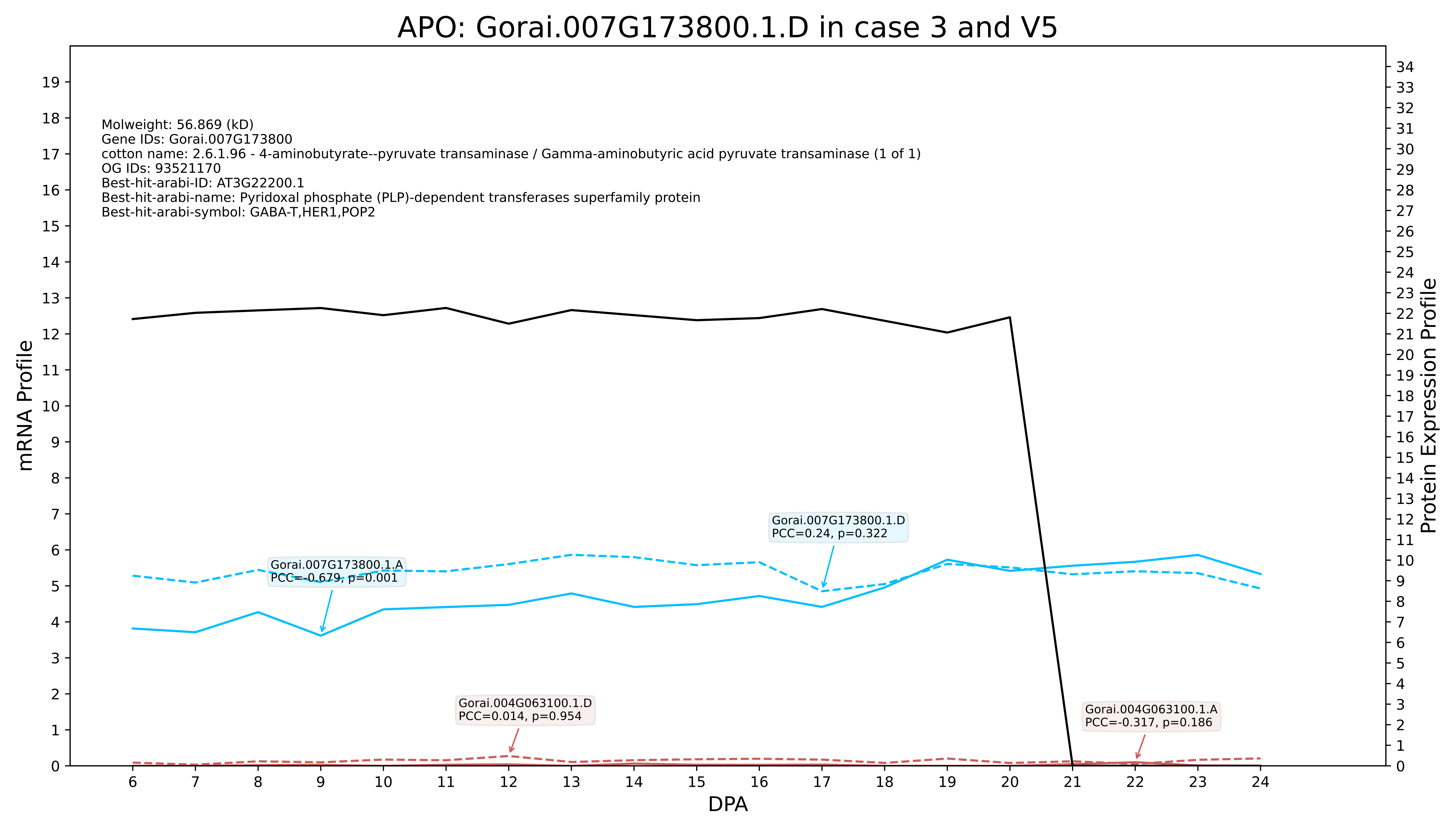
APO: Gorai.007G173800.1.D in case 3 and V5
Molweight: 56.869 (kD)
Gene IDs: Gorai.007G173800
cotton name: 2.6.1.96 - 4-aminobutyrate--pyruvate transaminase / Gamma-aminobutyric acid pyruvate transaminase (1 of 1)
OG IDs: 93521170
Best-hit-arabi-ID: AT3G22200.1
Best-hit-arabi-name: Pyridoxal phosphate (PLP)-dependent transferases superfamily protein
Best-hit-arabi-symbol: GABA-T,HER1,POP2
Gorai.007G173800.1.D
PCC=0.24, p=0.322
Gorai.007G173800.1.A
PCC=-0.679, p=0.001
Gorai.004G063100.1.D
PCC=0.014, p=0.954
Gorai.004G063100.1.A
PCC=-0.317, p=0.186
mRNA Profile
Protein Expression Profile
DPA

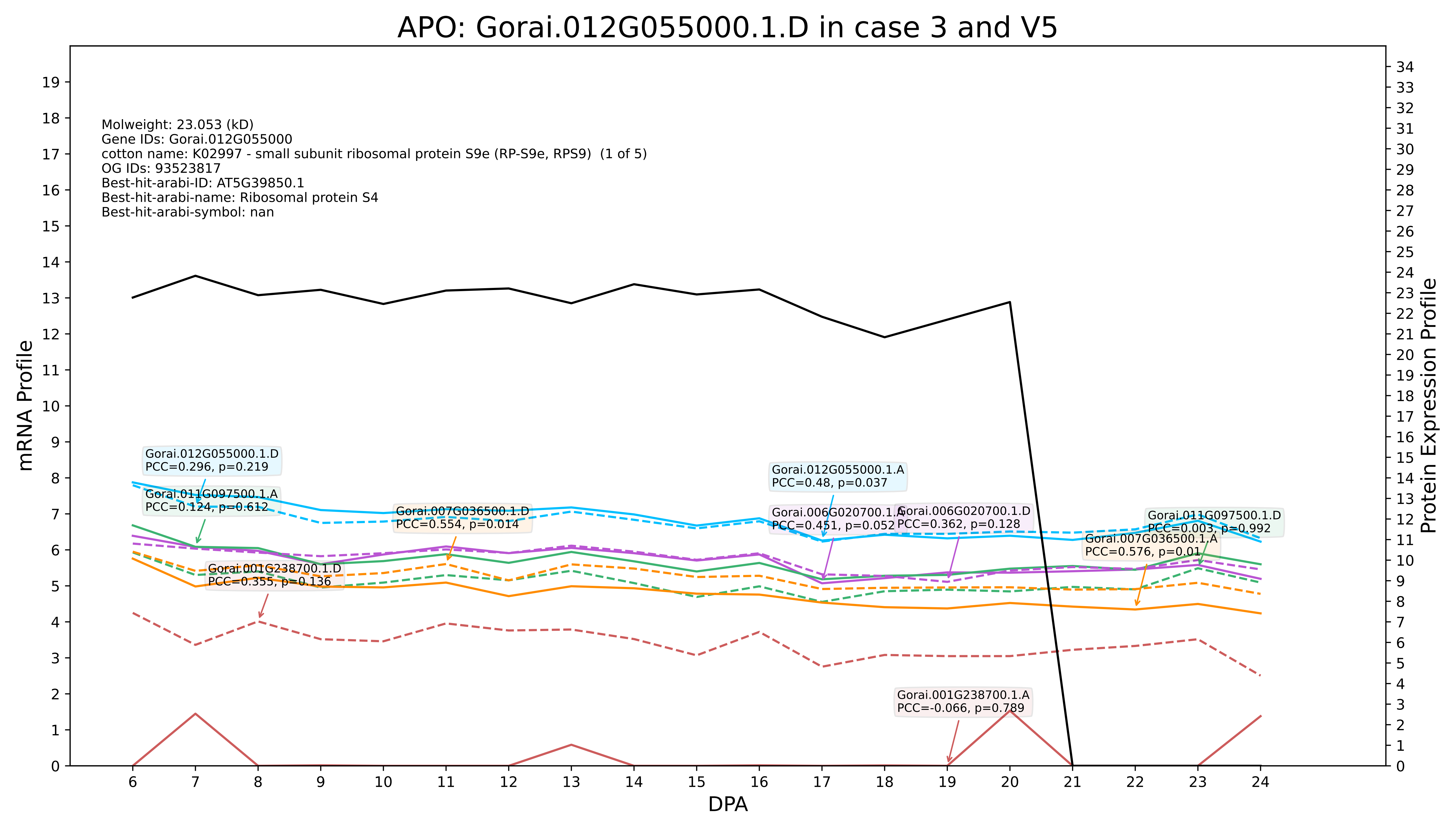
APO: Gorai.012G055000.1.D in case 3 and V5
Molweight: 23.053 (kD)
Gene IDs: Gorai.012G055000
cotton name: K02997 - small subunit ribosomal protein S9e (RP-S9e, RPS9) (1 of 5)
OG IDs: 93523817
Best-hit-arabi-ID: AT5G39850.1
Best-hit-arabi-name: Ribosomal protein S4
Best-hit-arabi-symbol: nan
Gorai.012G055000.1.D
PCC=0.296, p=0.219
Gorai.011G097500.1.A
PCC=0.124, p=0.612
Gorai.007G036500.1.D
PCC=0.554, p=0.014
Gorai.001G238700.1.D
PCC=0.355, p=0.136
Gorai.012G055000.1.A
PCC=0.48, p=0.037
Gorai.006G020700.1.A
PCC=0.451, p=0.052
Gorai.006G020700.1.D
PCC=0.362, p=0.128
Gorai.011G097500.1.D
PCC=0.003, p=0.992
Gorai.007G036500.1.A
PCC=0.576, p=0.01
Gorai.001G238700.1.A
PCC=-0.066, p=0.789
mRNA Profile
Protein Expression Profile
DPA

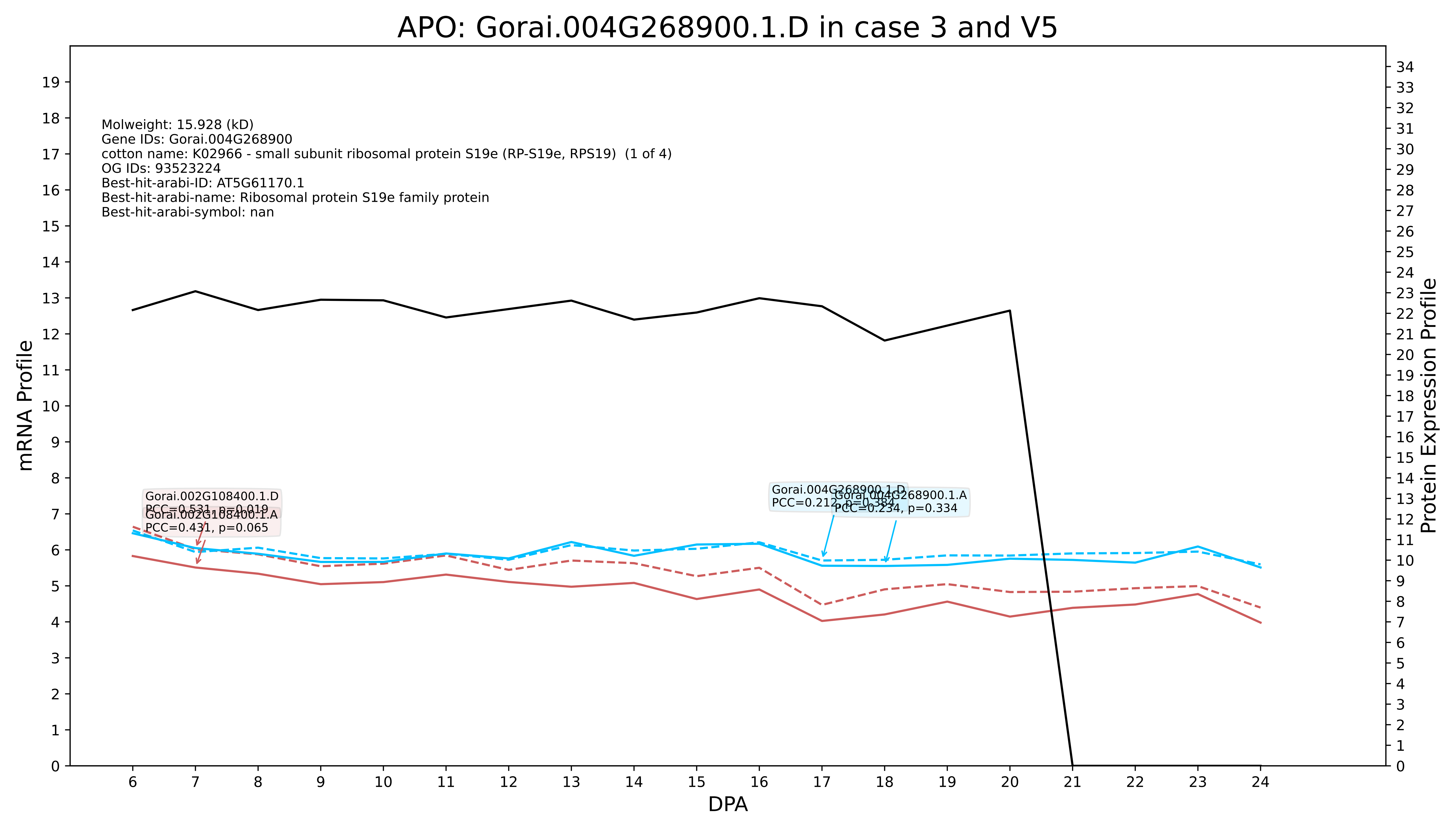
APO: Gorai.004G268900.1.D in case 3 and V5
Molweight: 15.928 (kD)
Gene IDs: Gorai.004G268900
cotton name: K02966 - small subunit ribosomal protein S19e (RP-S19e, RPS19) (1 of 4)
OG IDs: 93523224
Best-hit-arabi-ID: AT5G61170.1
Best-hit-arabi-name: Ribosomal protein S19e family protein
Best-hit-arabi-symbol: nan
Gorai.002G108400.1.D
PCC=0.531, p=0.019
Gorai.002G108400.1.A
PCC=0.431, p=0.065
Gorai.004G268900.1.D
PCC=0.212, p=0.384
Gorai.004G268900.1.A
PCC=0.234, p=0.334
mRNA Profile
Protein Expression Profile
DPA

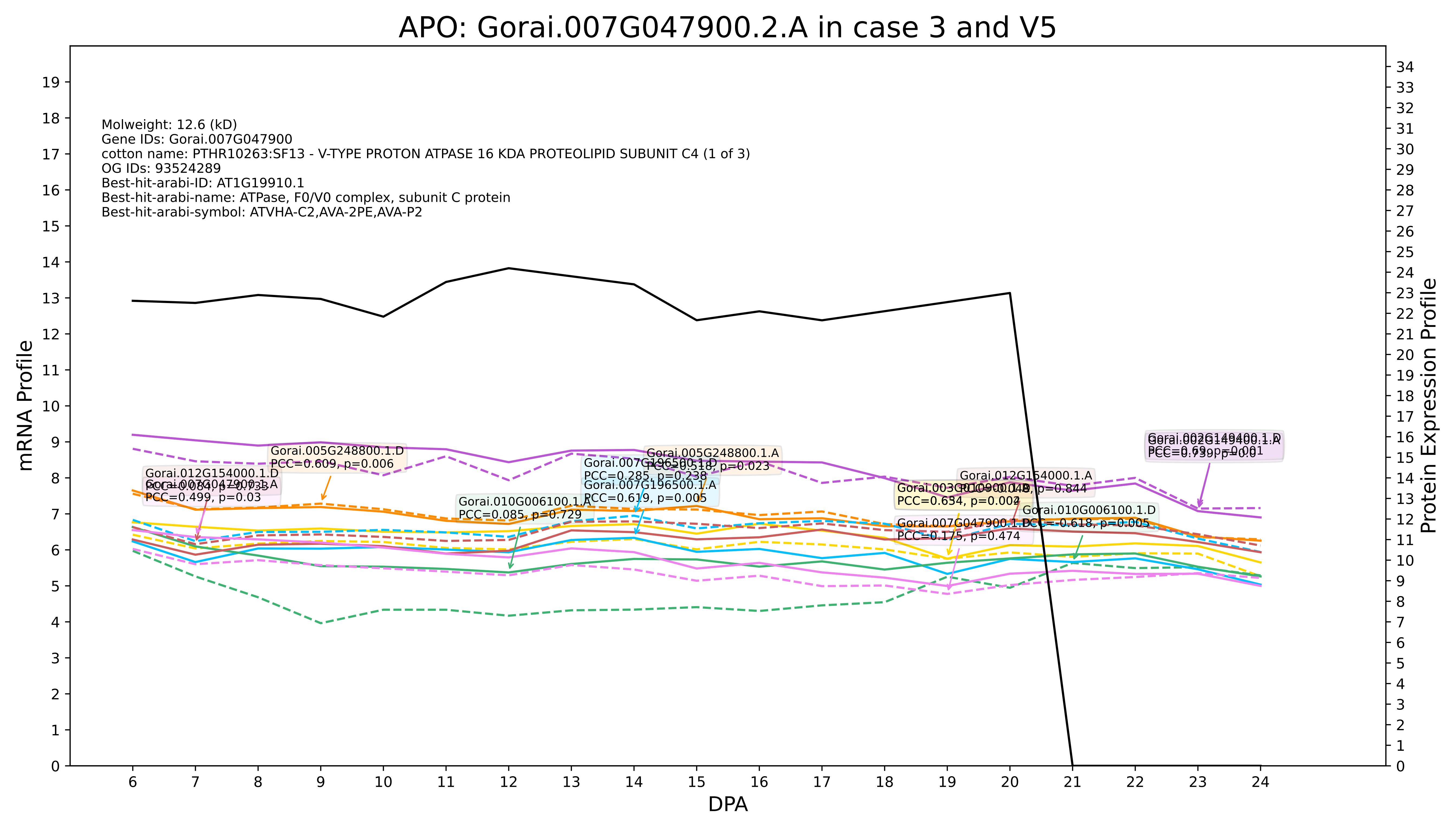

APO: Gorai.007G047900.2.A in case 3 and V5
Molweight: 12.6 (kD)
Gene IDs: Gorai.007G047900
cotton name: PTHR10263:SF13 - V-TYPE PROTON ATPASE 16 KDA PROTEOLIPID SUBUNIT C4 (1 of 3)
OG IDs: 93524289
Best-hit-arabi-ID: AT1G19910.1
Best-hit-arabi-name: ATPase, F0/V0 complex, subunit C protein
Best-hit-arabi-symbol: ATVHA-C2,AVA-2PE,AVA-P2
Gorai.005G248800.1.D
PCC=0.609, p=0.006
Gorai.005G248800.1.A
PCC=0.518, p=0.023
Gorai.010G006100.1.A
PCC=0.085, p=0.729
Gorai.007G196500.1.A
PCC=0.619, p=0.005
Gorai.012G154000.1.A
Gorai.010G006100.1.D
PCC=0.618, p=0.005
PCC=0.634, p=0.004
Gorai.007G047900.1.D
PCC=0.175, p=0.474
PCC=0.499, p=0.03
Gorai.012G154000.1.D
mRNA Profile
Protein Expression Profile
DPA

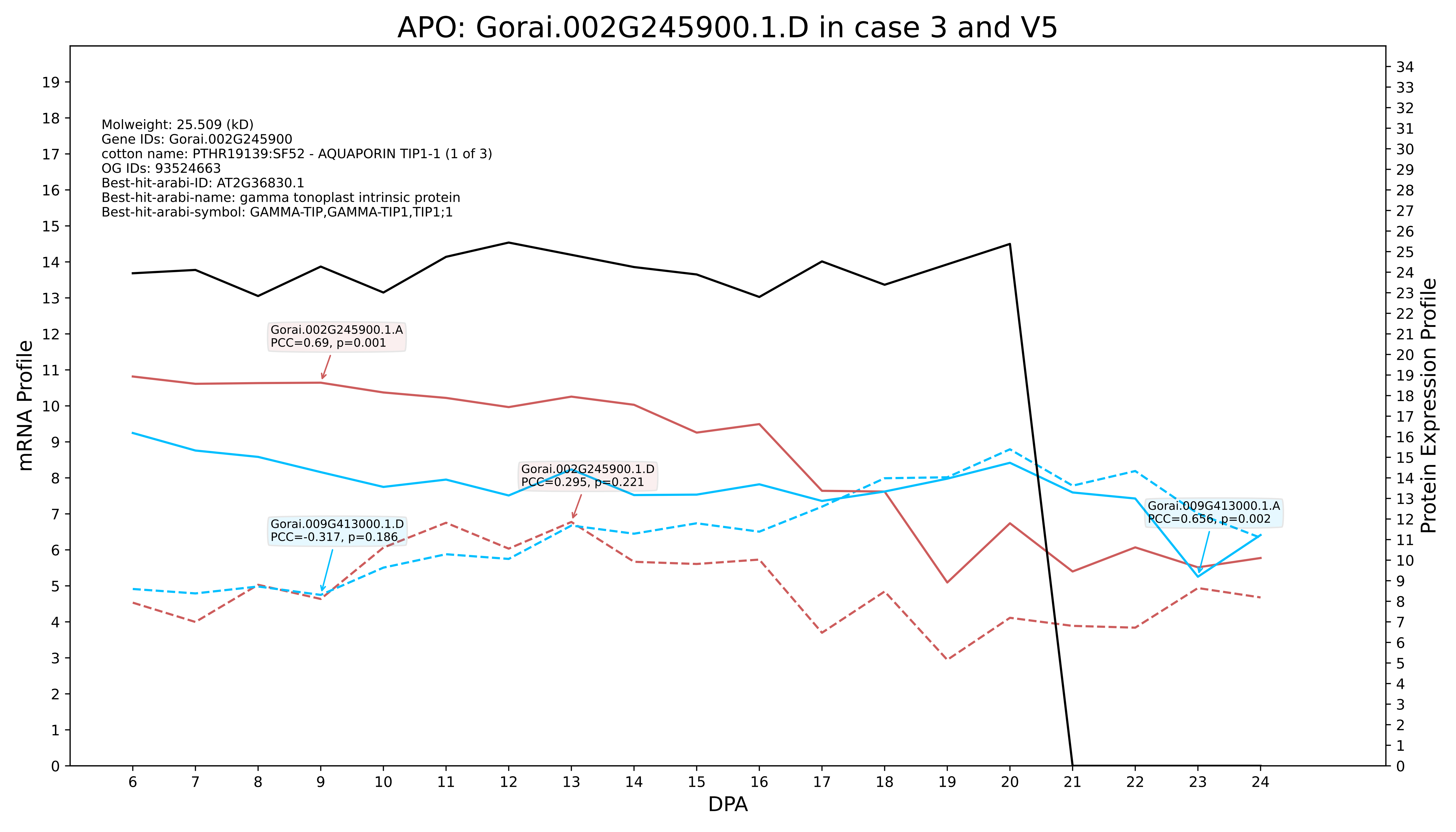
APO: Gorai.002G245900.1.D in case 3 and V5
Molweight: 25.509 (kD)
Gene IDs: Gorai.002G245900
cotton name: PTHR19139:SF52 - AQUAPORIN TIP1-1 (1 of 3)
OG IDs: 93524663
Best-hit-arabi-ID: AT2G36830.1
Best-hit-arabi-name: gamma tonoplast intrinsic protein
Best-hit-arabi-symbol: GAMMA-TIP,GAMMA-TIP1,TIP1;1
Gorai.002G245900.1.A
PCC=0.69, p=0.001
Gorai.002G245900.1.D
PCC=0.295, p=0.221
Gorai.009G413000.1.D
PCC=-0.317, p=0.186
Gorai.009G413000.1.A
PCC=0.656, p=0.002
mRNA Profile
Protein Expression Profile
DPA

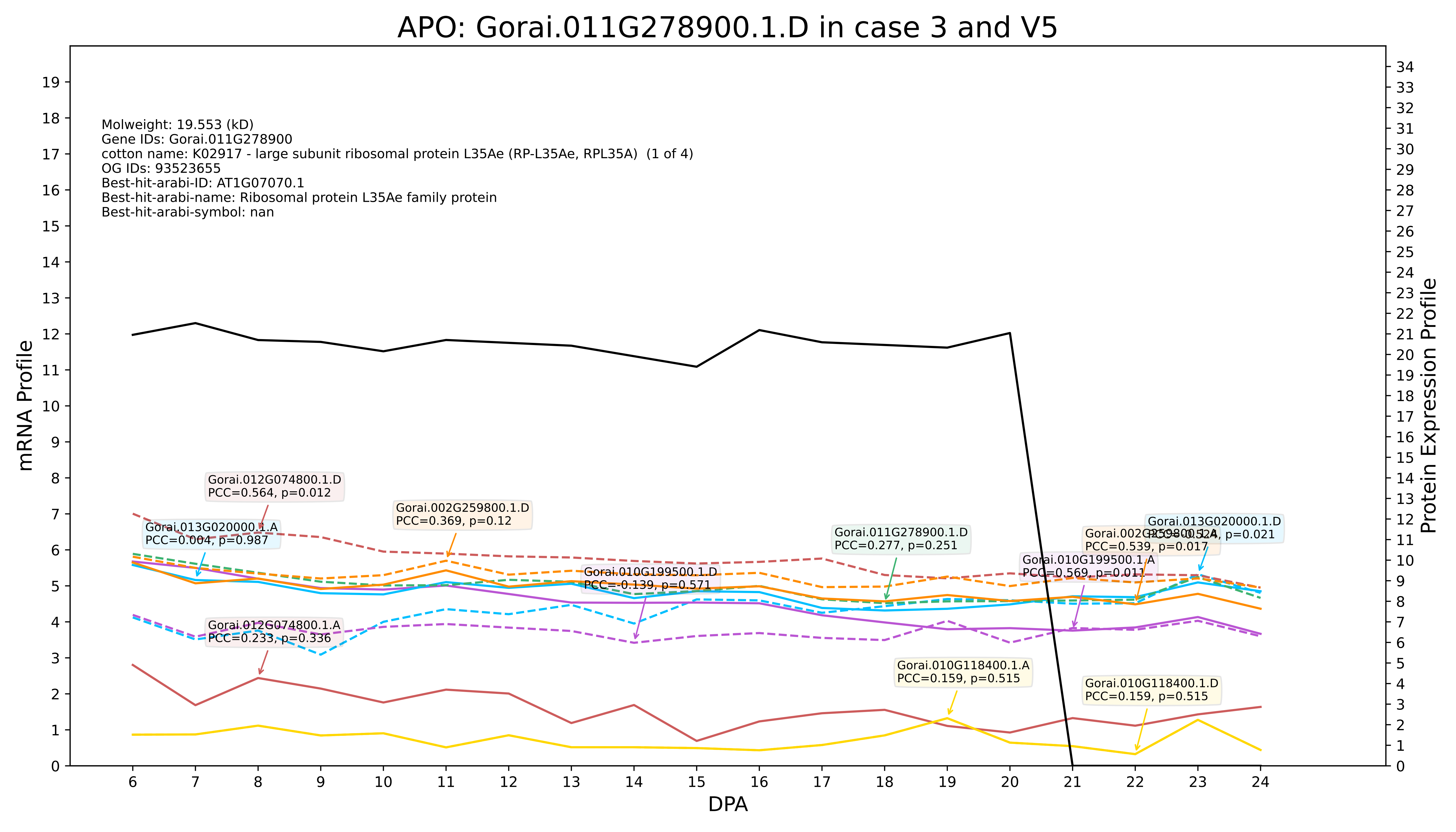
APO: Gorai.011G278900.1.D in case 3 and V5
Molweight: 19.553 (kD)
Gene IDs: Gorai.011G278900
cotton name: K02917 - large subunit ribosomal protein L35Ae (RP-L35Ae, RPL35A) (1 of 4)
OG IDs: 93523655
Best-hit-arabi-ID: AT1G07070.1
Best-hit-arabi-name: Ribosomal protein L35Ae family protein
Best-hit-arabi-symbol: nan
Gorai.012G074800.1.D
PCC=0.564, p=0.012
Gorai.002G259800.1.D
PCC=0.369, p=0.12
Gorai.013G020000.1.A
PCC=0.004, p=0.987
Gorai.011G278900.1.D
PCC=0.277, p=0.251
Gorai.013G020000.1.D
PCC=0.524, p=0.021
Gorai.002G259800.1.A
PCC=0.539, p=0.017
Gorai.010G199500.1.A
PCC=0.569, p=0.011
Gorai.010G199500.1.D
PCC=-0.139, p=0.571
Gorai.012G074800.1.A
PCC=0.233, p=0.336
Gorai.010G118400.1.A
PCC=0.159, p=0.515
Gorai.010G118400.1.D
PCC=0.159, p=0.515
mRNA Profile
Protein Expression Profile
DPA

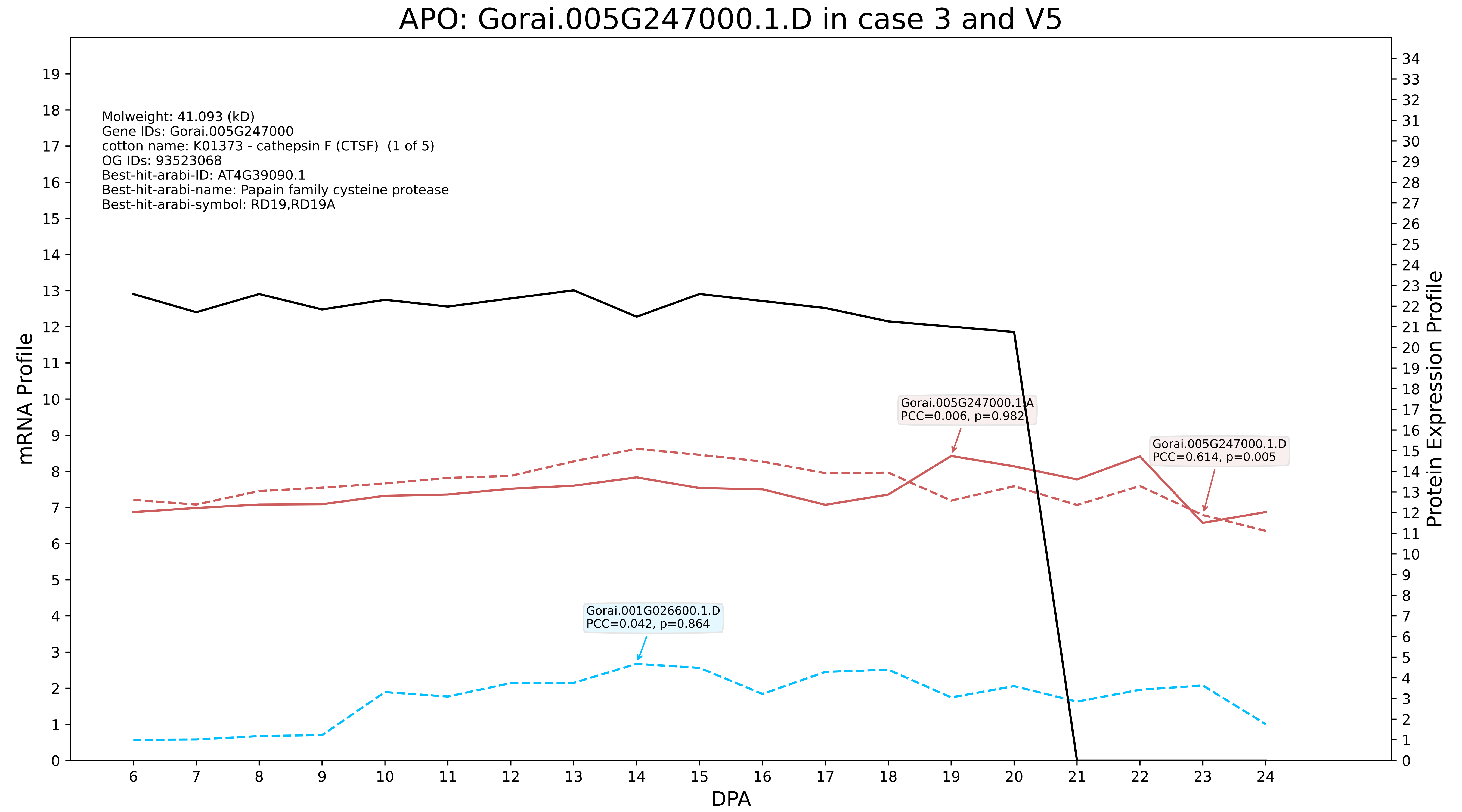
APO: Gorai.005G247000.1.D in case 3 and V5
Molweight: 41.093 (kD)
Gene IDs: Gorai.005G247000
cotton name: K01373 - cathepsin F (CTSF) (1 of 5)
OG IDs: 93523068
Best-hit-arabi-ID: AT4G39090.1
Best-hit-arabi-name: Papain family cysteine protease
Best-hit-arabi-symbol: RD19,RD19A
Gorai.005G247000.1.A
PCC=0.006, p=0.982
Gorai.005G247000.1.D
PCC=0.614, p=0.005
Gorai.001G026600.1.D
PCC=0.042, p=0.864
mRNA Profile
Protein Expression Profile
DPA

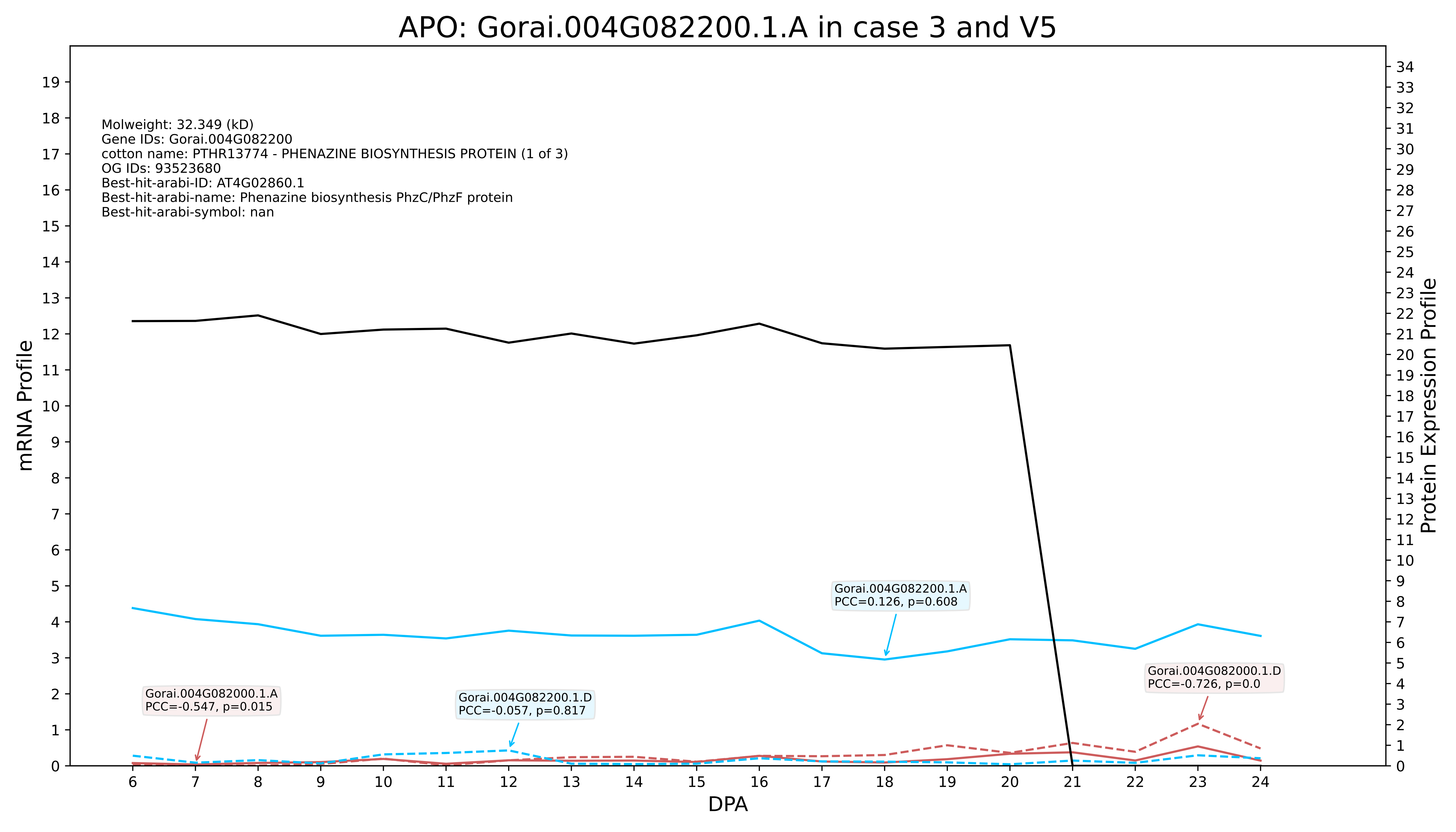
APO: Gorai.004G082200.1.A in case 3 and V5
Molweight: 32.349 (kD)
Gene IDs: Gorai.004G082200
cotton name: PTHR13774 - PHENAZINE BIOSYNTHESIS PROTEIN (1 of 3)
OG IDs: 93523680
Best-hit-arabi-ID: AT4G02860.1
Best-hit-arabi-name: Phenazine biosynthesis PhzC/PhzF protein
Best-hit-arabi-symbol: nan
Gorai.004G082200.1.A
PCC=0.126, p=0.608
Gorai.004G082000.1.D
PCC=-0.726, p=0.0
Gorai.004G082000.1.A
PCC=-0.547, p=0.015
Gorai.004G082200.1.D
PCC=-0.057, p=0.817
mRNA Profile
Protein Expression Profile
DPA

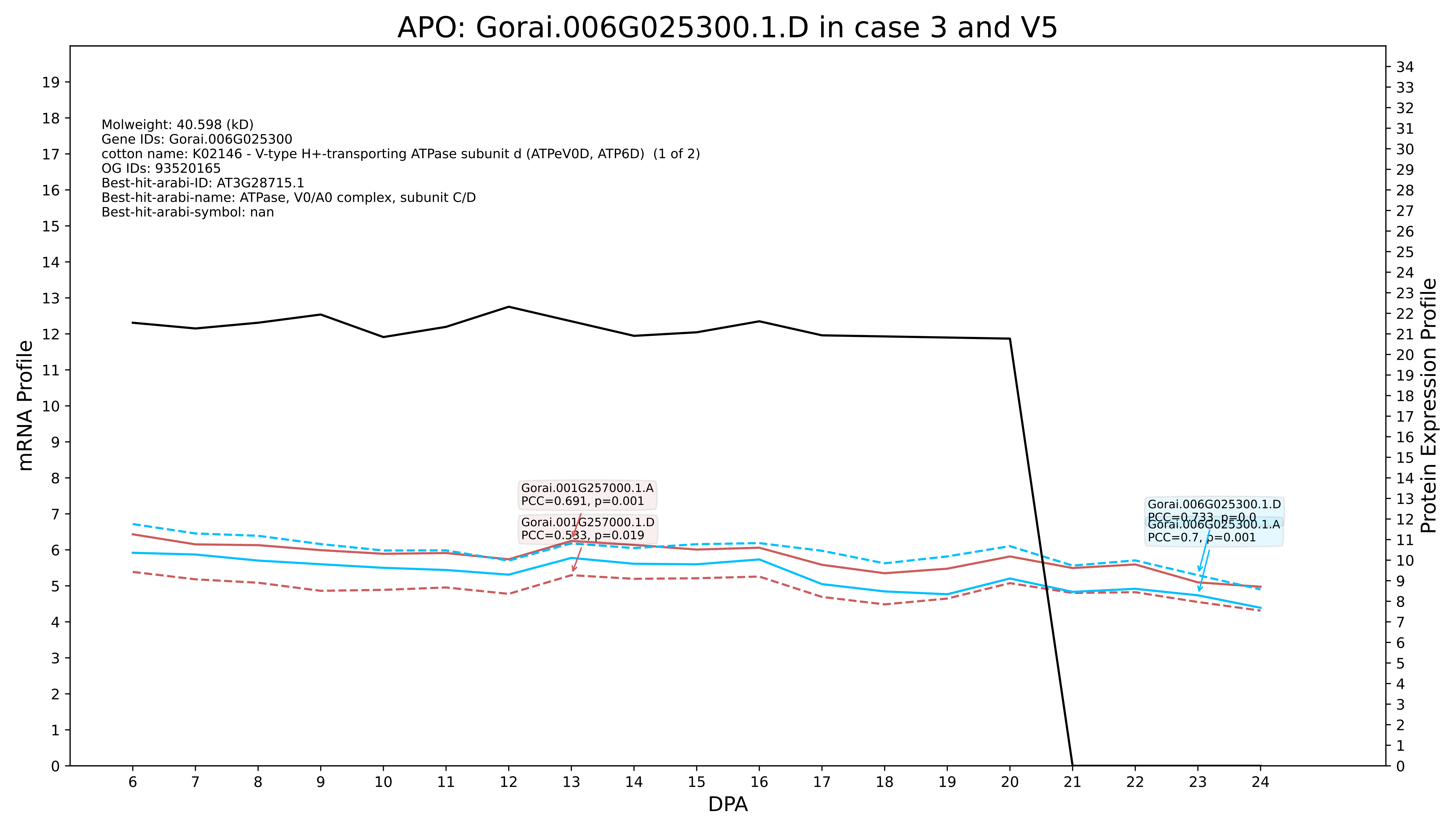

APO: Gorai.006G025300.1.D in case 3 and V5
Molweight: 40.598 (kD)
Gene IDs: Gorai.006G025300
cotton name: K02146 - V-type H+-transporting ATPase subunit d (ATPeV0D, ATP6D) (1 of 2)
OG IDs: 93520165
Best-hit-arabi-ID: AT3G28715.1
Best-hit-arabi-name: ATPase, V0/A0 complex, subunit C/D
Best-hit-arabi-symbol: nan
Gorai.001G257000.1.A
PCC=0.691, p=0.001
Gorai.001G257000.1.D
PCC=0.533, p=0.019
Gorai.006G025300.1.D
PCC=0.733, p=0.0
Gorai.006G025300.1.A
PCC=0.7, p=0.001
mRNA Profile
Protein Expression Profile
DPA

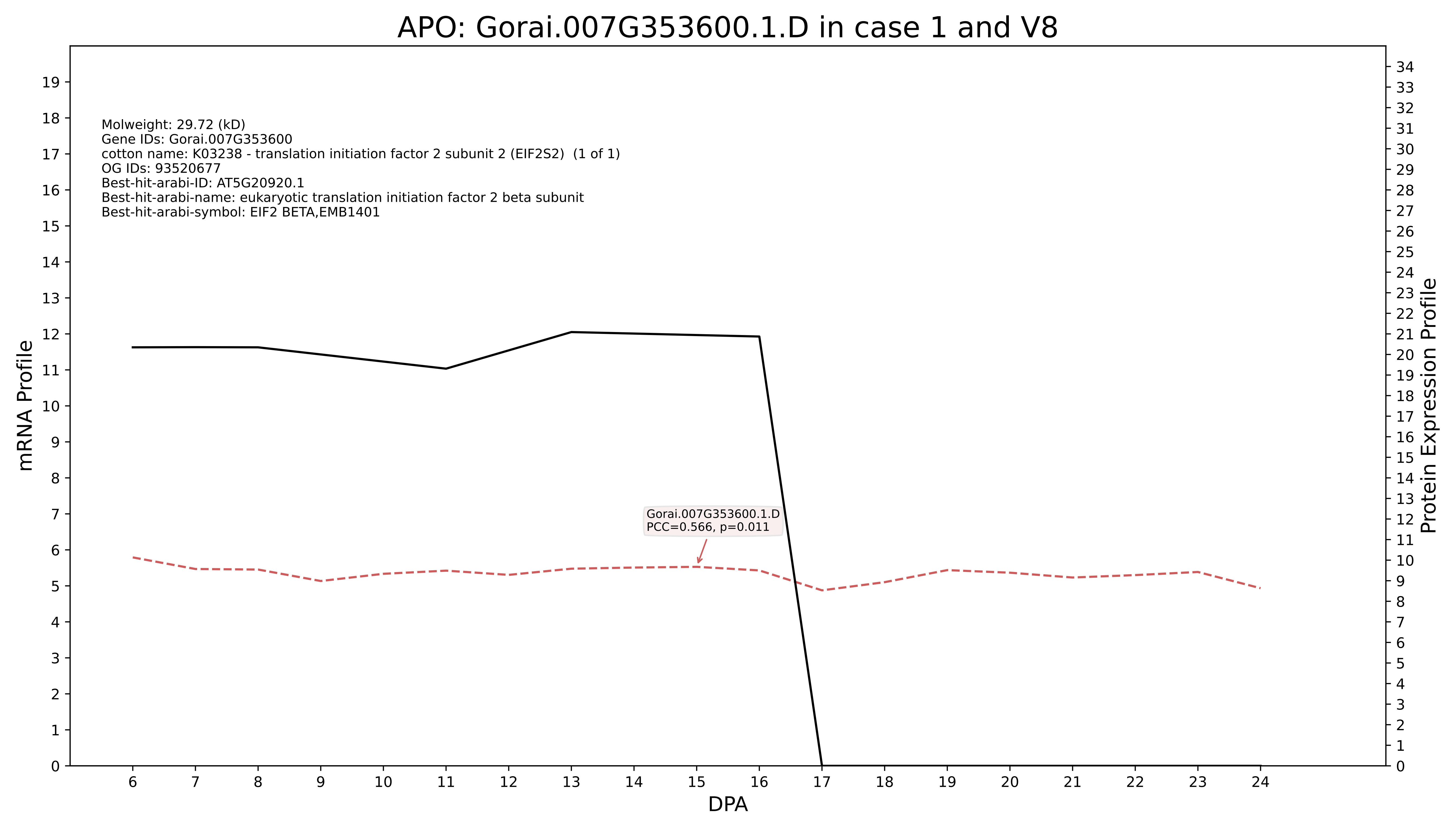
APO: Gorai.007G353600.1.D in case 1 and V8
Molweight: 29.72 (kD)
Gene IDs: Gorai.007G353600
cotton name: K03238 - translation initiation factor 2 subunit 2 (EIF2S2) (1 of 1)
OG IDs: 93520677
Best-hit-arabi-ID: AT5G20920.1
Best-hit-arabi-name: eukaryotic translation initiation factor 2 beta subunit
Best-hit-arabi-symbol: EIF2 BETA,EMB1401
Gorai.007G353600.1.D
PCC=0.566, p=0.011
mRNA Profile
Protein Expression Profile
DPA

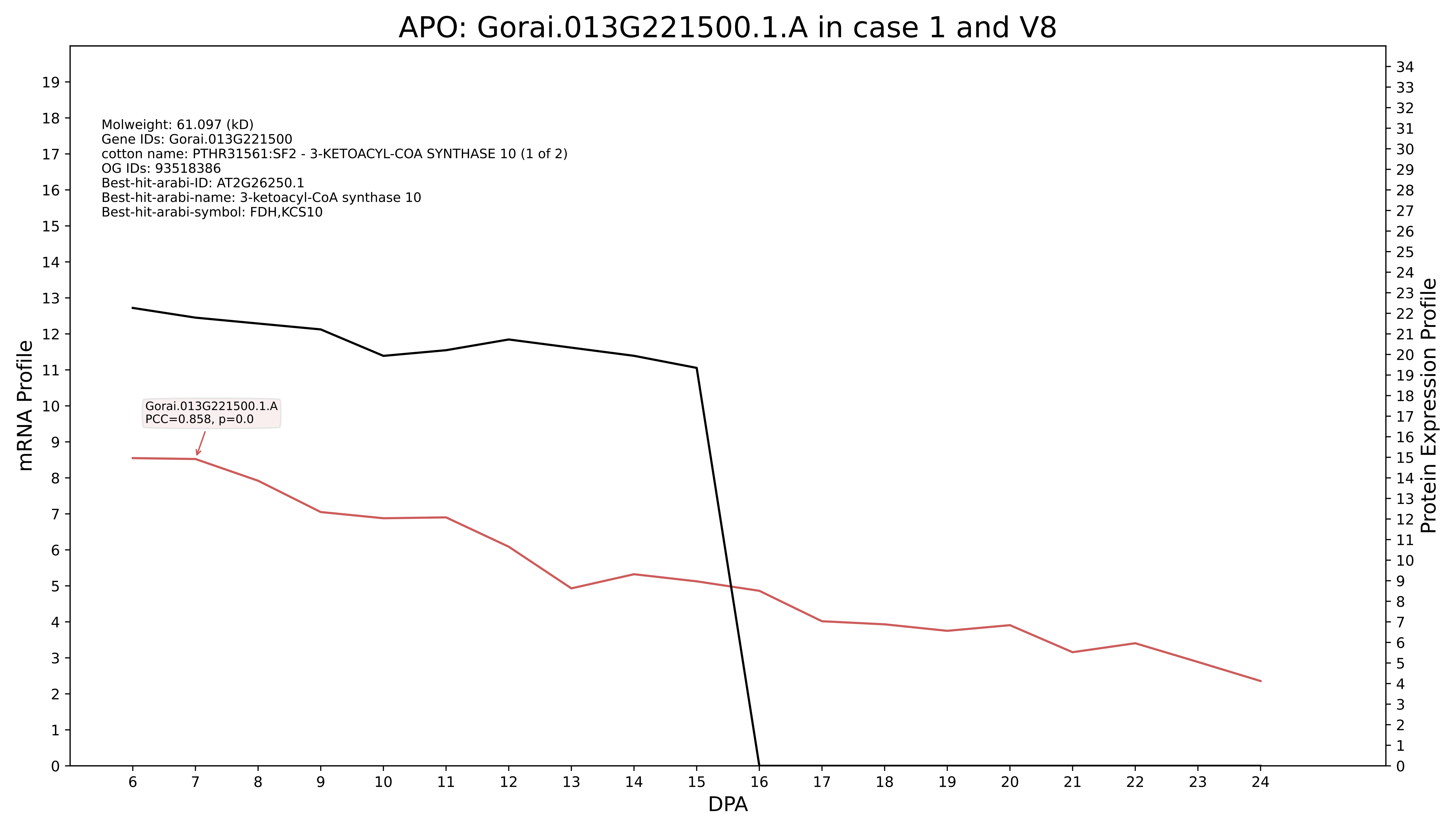
APO: Gorai.013G221500.1.A in case 1 and V8
Molweight: 61.097 (kD)
Gene IDs: Gorai.013G221500
cotton name: PTHR31561:SF2 - 3-KETOACYL-COA SYNTHASE 10 (1 of 2)
OG IDs: 93518386
Best-hit-arabi-ID: AT2G26250.1
Best-hit-arabi-name: 3-ketoacyl-CoA synthase 10
Best-hit-arabi-symbol: FDH,KCS10
Gorai.013G221500.1.A
PCC=0.858, p=0.0
mRNA Profile
Protein Expression Profile
DPA

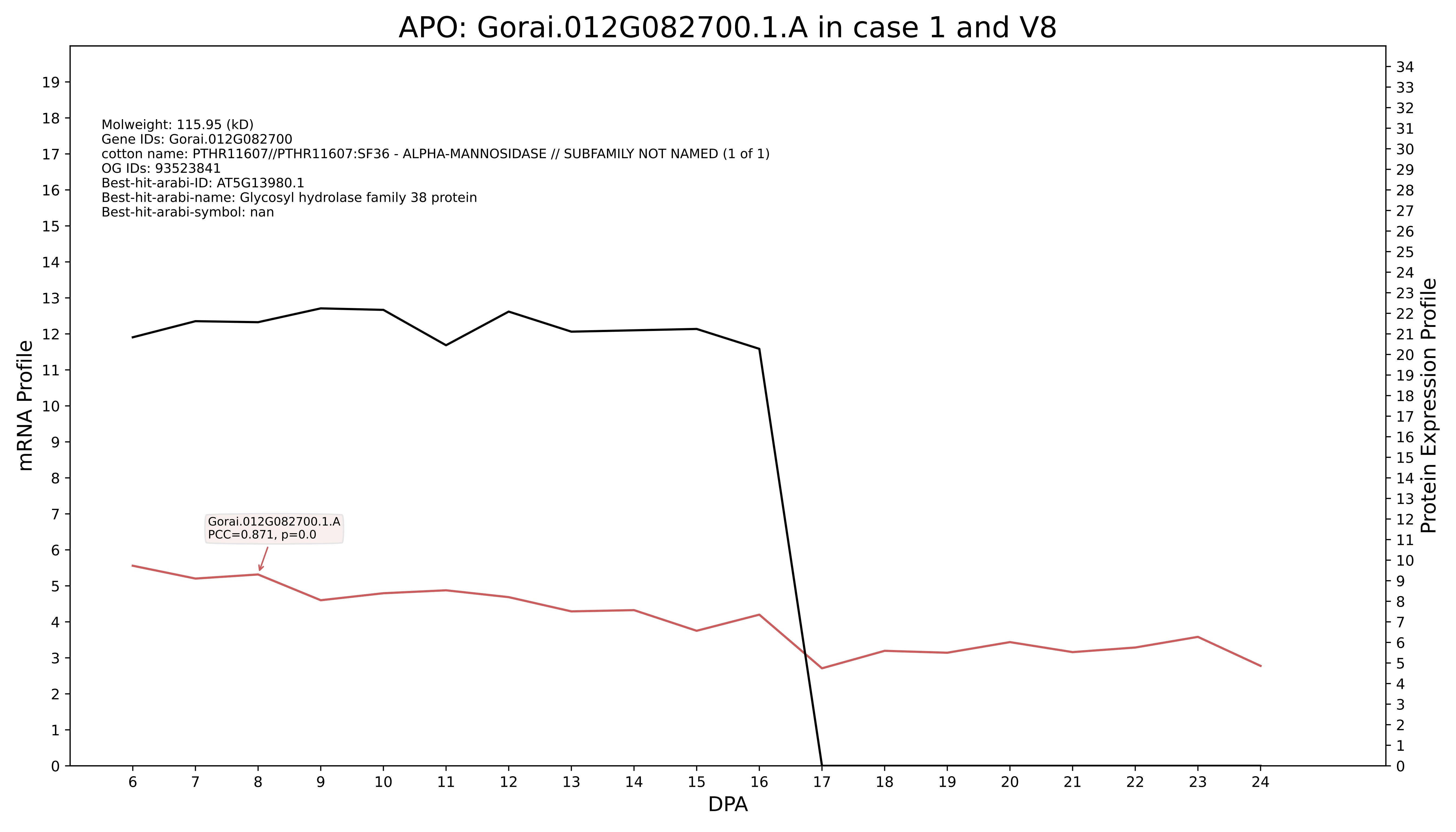
APO: Gorai.012G082700.1.A in case 1 and V8
Molweight: 115.95 (kD)
Gene IDs: Gorai.012G082700
cotton name: PTHR11607//PTHR11607:SF36 - ALPHA-MANNOSIDASE // SUBFAMILY NOT NAMED (1 of 1)
OG IDs: 93523841
Best-hit-arabi-ID: AT5G13980.1
Best-hit-arabi-name: Glycosyl hydrolase family 38 protein
Best-hit-arabi-symbol: nan
Gorai.012G082700.1.A
PCC=0.871, p=0.0
mRNA Profile
Protein Expression Profile
DPA

APO: Gorai.011G117000.1.D in case 1 and V8

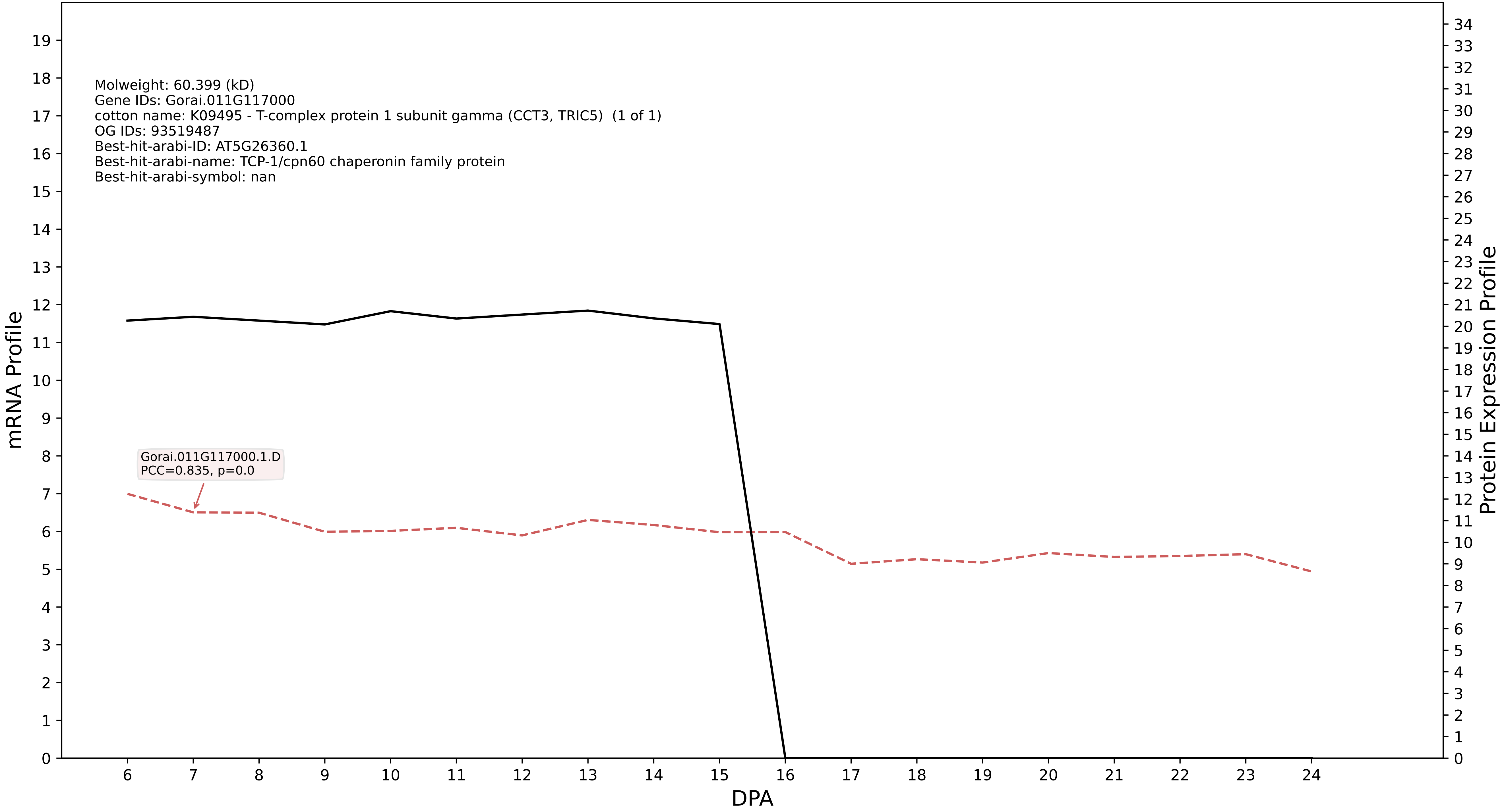

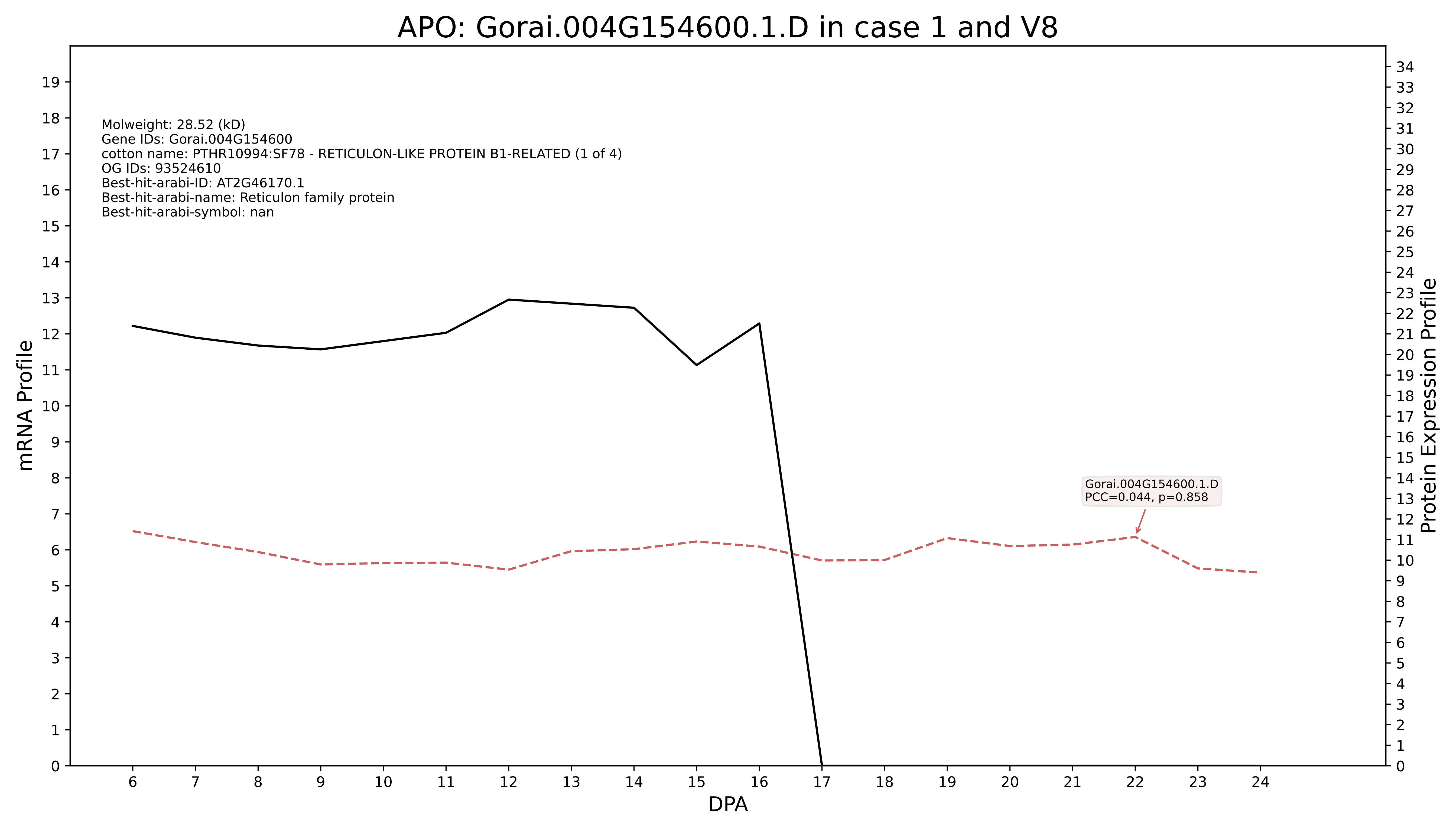

APO: Gorai.004G154600.1.D in case 1 and V8
Molweight: 28.52 (kD)
Gene IDs: Gorai.004G154600
cotton name: PTHR10994:SF78 - RETICULON-LIKE PROTEIN B1-RELATED (1 of 4)
OG IDs: 93524610
Best-hit-arabi-ID: AT2G46170.1
Best-hit-arabi-name: Reticulon family protein
Best-hit-arabi-symbol: nan
Gorai.004G154600.1.D
PCC=0.044, p=0.858
mRNA Profile
Protein Expression Profile
DPA

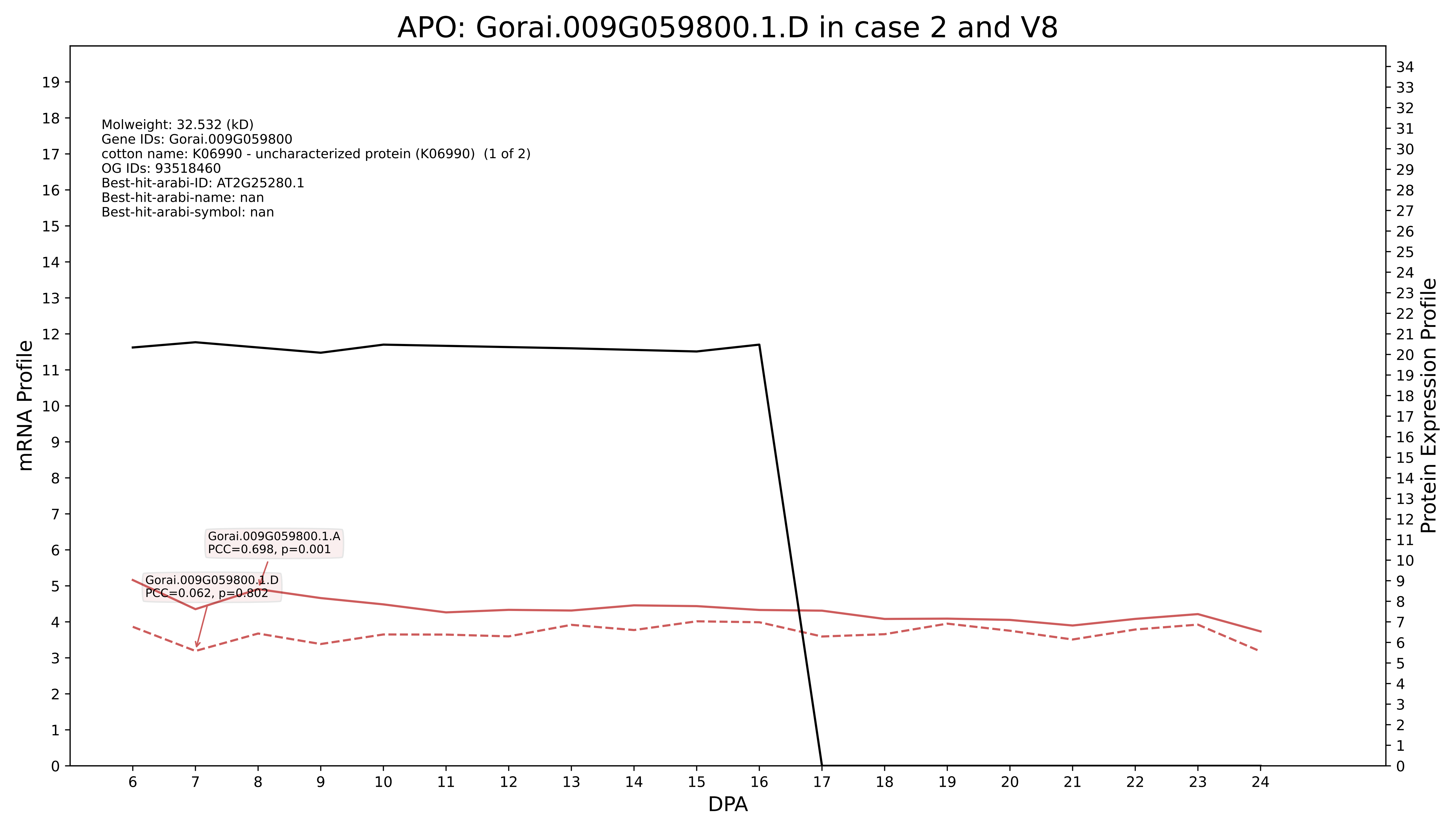

APO: Gorai.009G059800.1.D in case 2 and V8
Molweight: 32.532 (kD)
Gene IDs: Gorai.009G059800
cotton name: K06990 - uncharacterized protein (K06990) (1 of 2)
OG IDs: 93518460
Best-hit-arabi-ID: AT2G25280.1
Best-hit-arabi-name: nan
Best-hit-arabi-symbol: nan
Gorai.009G059800.1.A
PCC=0.698, p=0.001
Gorai.009G059800.1.D
PCC=0.062, p=0.802
mRNA Profile
Protein Expression Profile
DPA

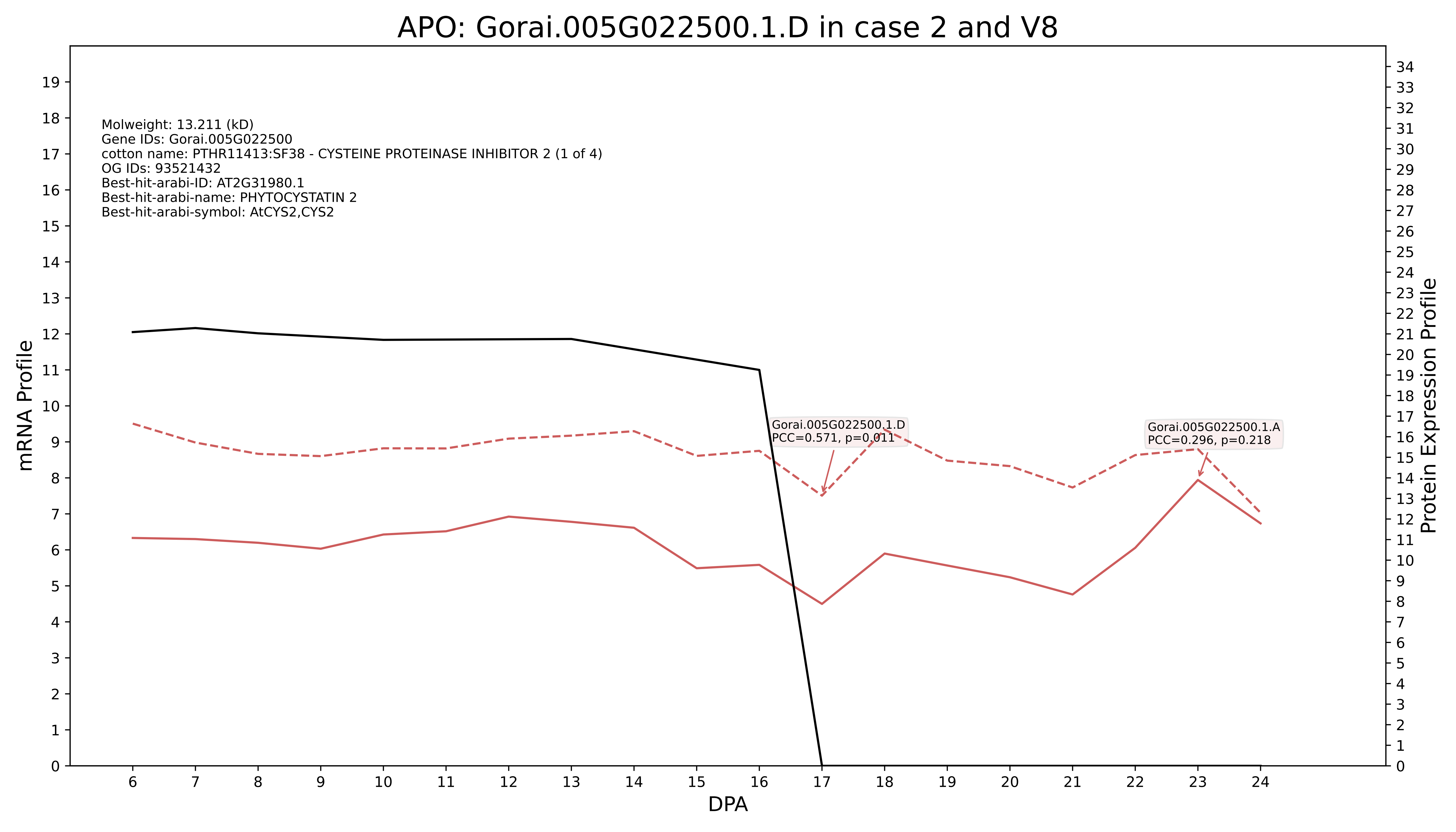

APO: Gorai.005G022500.1.D in case 2 and V8
Molweight: 13.211 (kD)
Gene IDs: Gorai.005G022500
cotton name: PTHR11413:SF38 - CYSTEINE PROTEINASE INHIBITOR 2 (1 of 4)
OG IDs: 93521432
Best-hit-arabi-ID: AT2G31980.1
Best-hit-arabi-name: PHYTOCYSTATIN 2
Best-hit-arabi-symbol: AtCYS2,CYS2
Gorai.005G022500.1.D
PCC=0.571, p=0.011
Gorai.005G022500.1.A
PCC=0.296, p=0.218
mRNA Profile
Protein Expression Profile
DPA

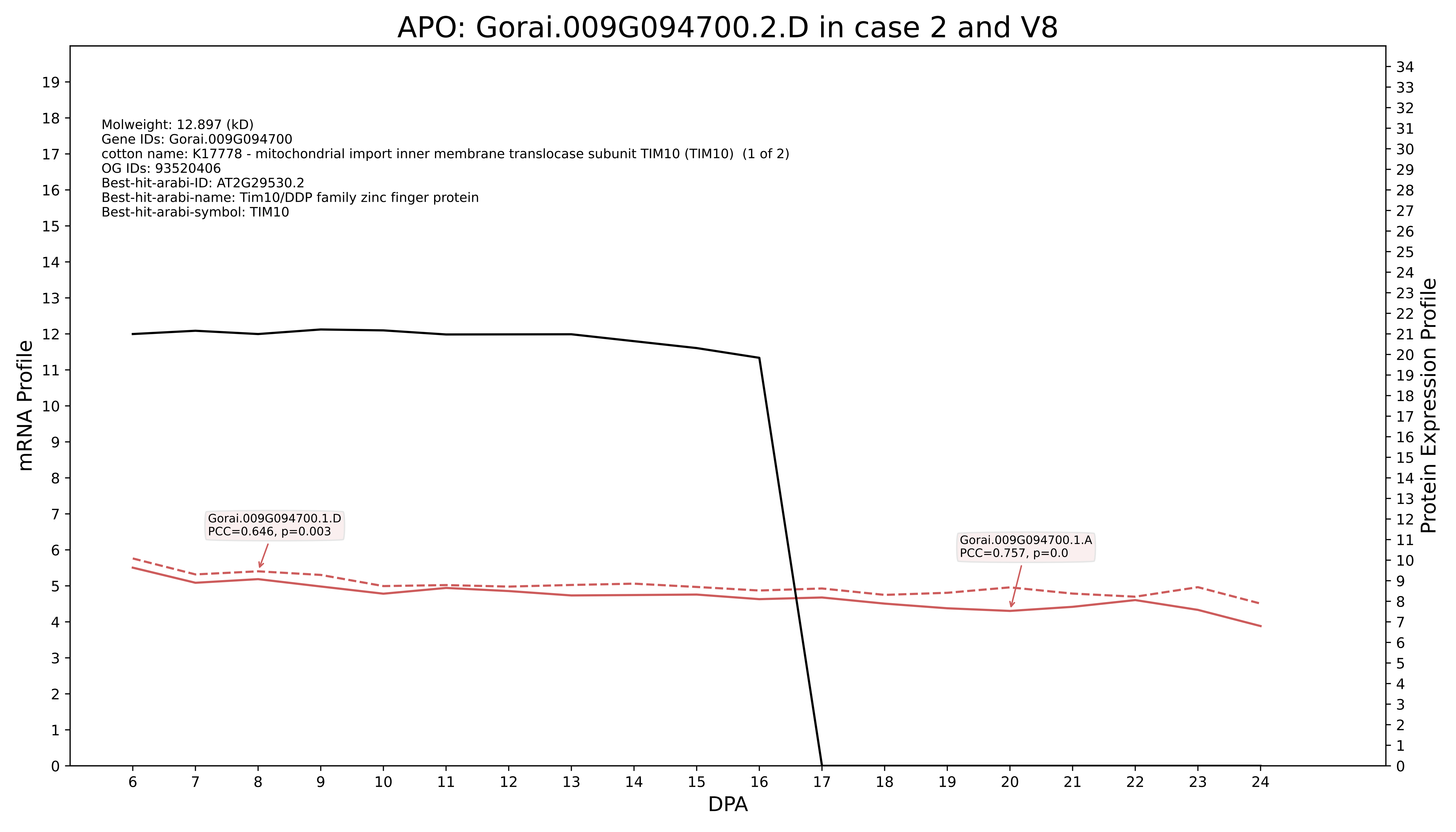

APO: Gorai.009G094700.2.D in case 2 and V8
Molweight: 12.897 (kD)
Gene IDs: Gorai.009G094700
cotton name: K17778 - mitochondrial import inner membrane translocase subunit TIM10 (TIM10) (1 of 2)
OG IDs: 93520406
Best-hit-arabi-ID: AT2G29530.2
Best-hit-arabi-name: Tim10/DDP family zinc finger protein
Best-hit-arabi-symbol: TIM10
Gorai.009G094700.1.D
PCC=0.646, p=0.003
Gorai.009G094700.1.A
PCC=0.757, p=0.0
mRNA Profile
Protein Expression Profile
DPA

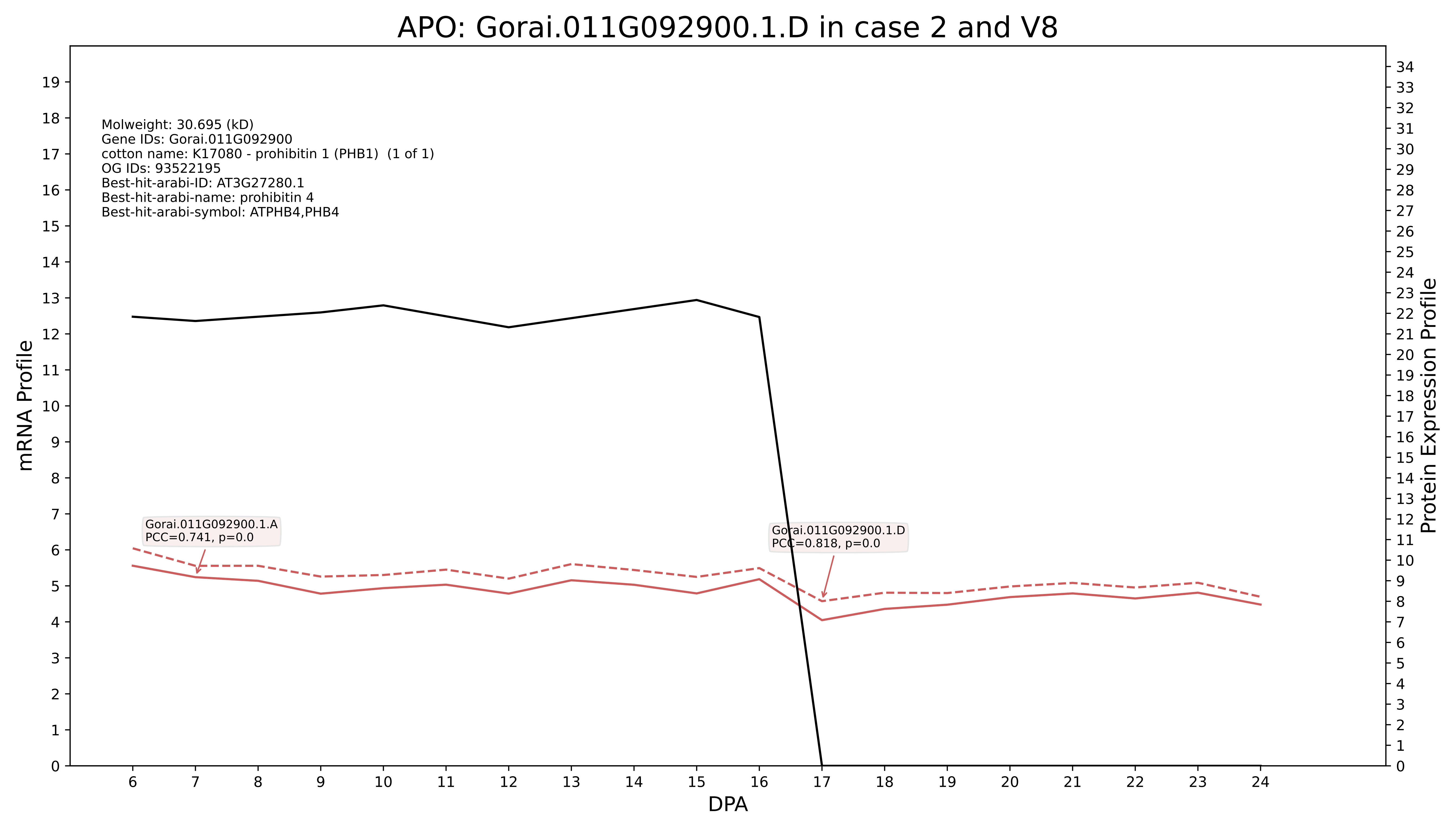
APO: Gorai.011G092900.1.D in case 2 and V8
Molweight: 30.695 (kD)
Gene IDs: Gorai.011G092900
cotton name: K17080 - prohibitin 1 (PHB1) (1 of 1)
OG IDs: 93522195
Best-hit-arabi-ID: AT3G27280.1
Best-hit-arabi-name: prohibitin 4
Best-hit-arabi-symbol: ATPHB4,PHB4
Gorai.011G092900.1.A
PCC=0.741, p=0.0
Gorai.011G092900.1.D
PCC=0.818, p=0.0
mRNA Profile
Protein Expression Profile
DPA

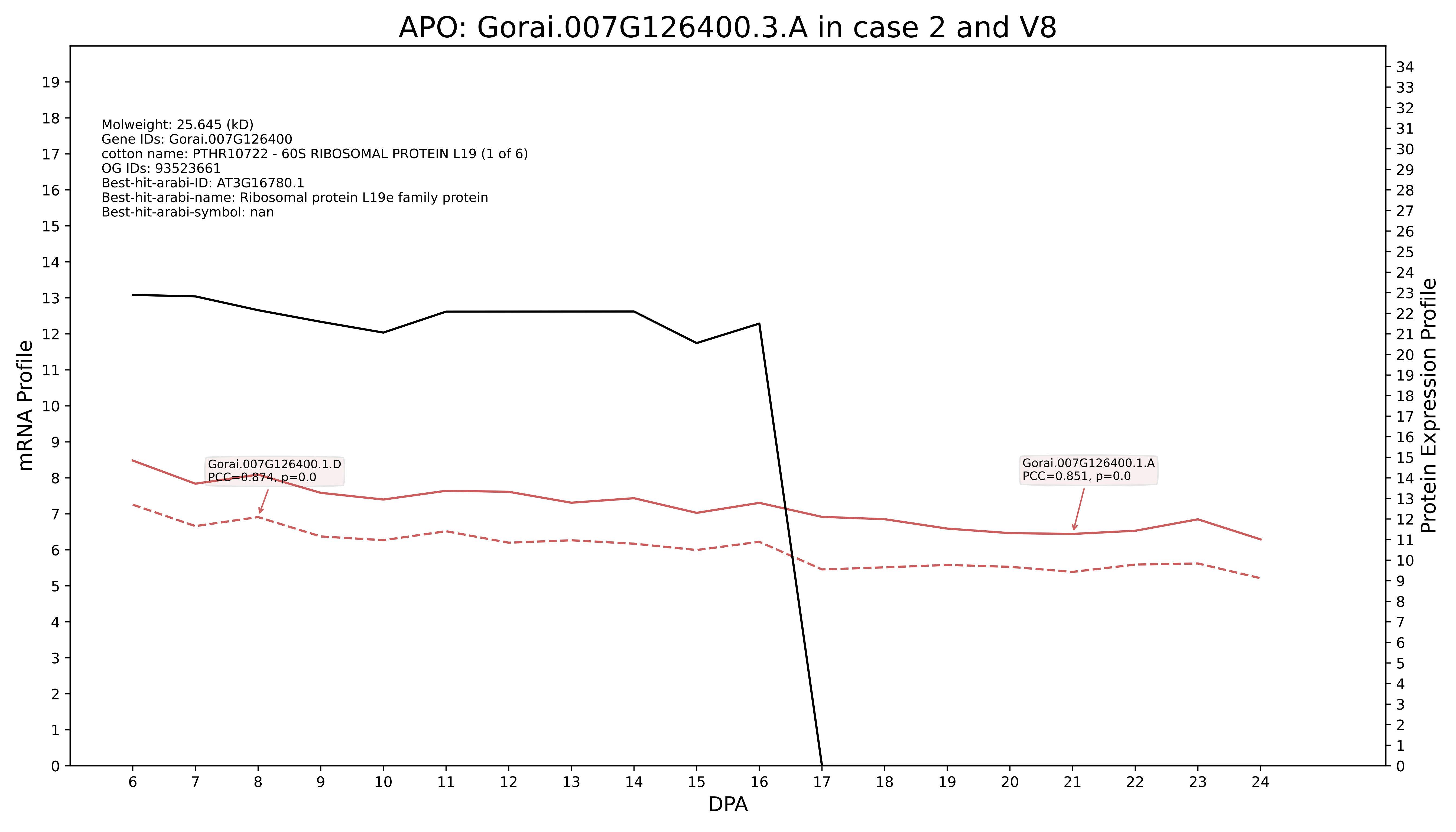
APO: Gorai.007G126400.3.A in case 2 and V8
Molweight: 25.645 (kD)
Gene IDs: Gorai.007G126400
cotton name: PTHR10722 - 60S RIBOSOMAL PROTEIN L19 (1 of 6)
OG IDs: 93523661
Best-hit-arabi-ID: AT3G16780.1
Best-hit-arabi-name: Ribosomal protein L19e family protein
Best-hit-arabi-symbol: nan
Gorai.007G126400.1.D
PCC=0.874, p=0.0
Gorai.007G126400.1.A
PCC=0.851, p=0.0
mRNA Profile
Protein Expression Profile
DPA

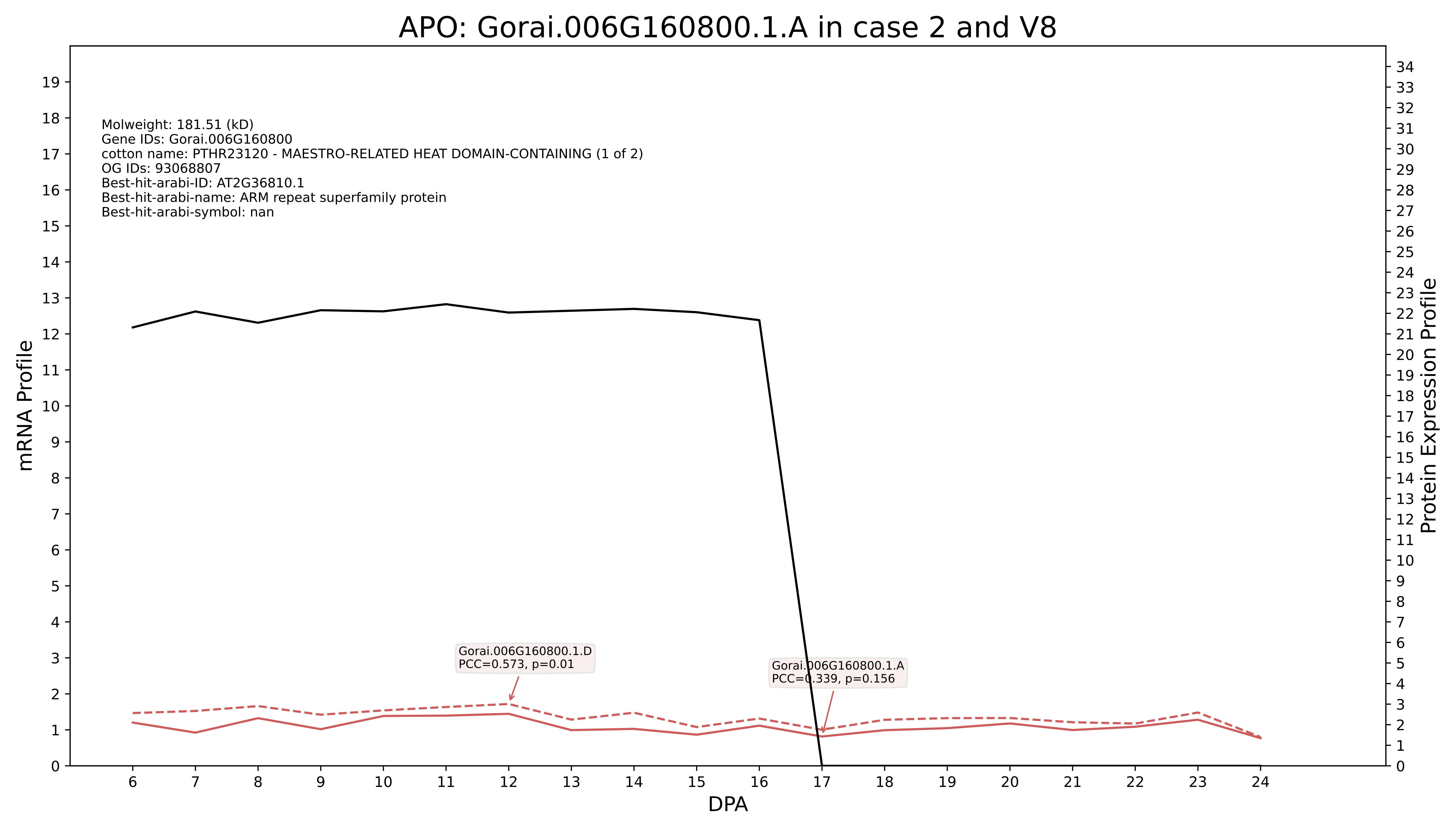
APO: Gorai.006G160800.1.A in case 2 and V8
Molweight: 181.51 (kD)
Gene IDs: Gorai.006G160800
cotton name: PTHR23120 - MAESTRO-RELATED HEAT DOMAIN-CONTAINING (1 of 2)
OG IDs: 93068807
Best-hit-arabi-ID: AT2G36810.1
Best-hit-arabi-name: ARM repeat superfamily protein
Best-hit-arabi-symbol: nan
Gorai.006G160800.1.D
PCC=0.573, p=0.01
Gorai.006G160800.1.A
PCC=0.339, p=0.156
mRNA Profile
Protein Expression Profile
DPA

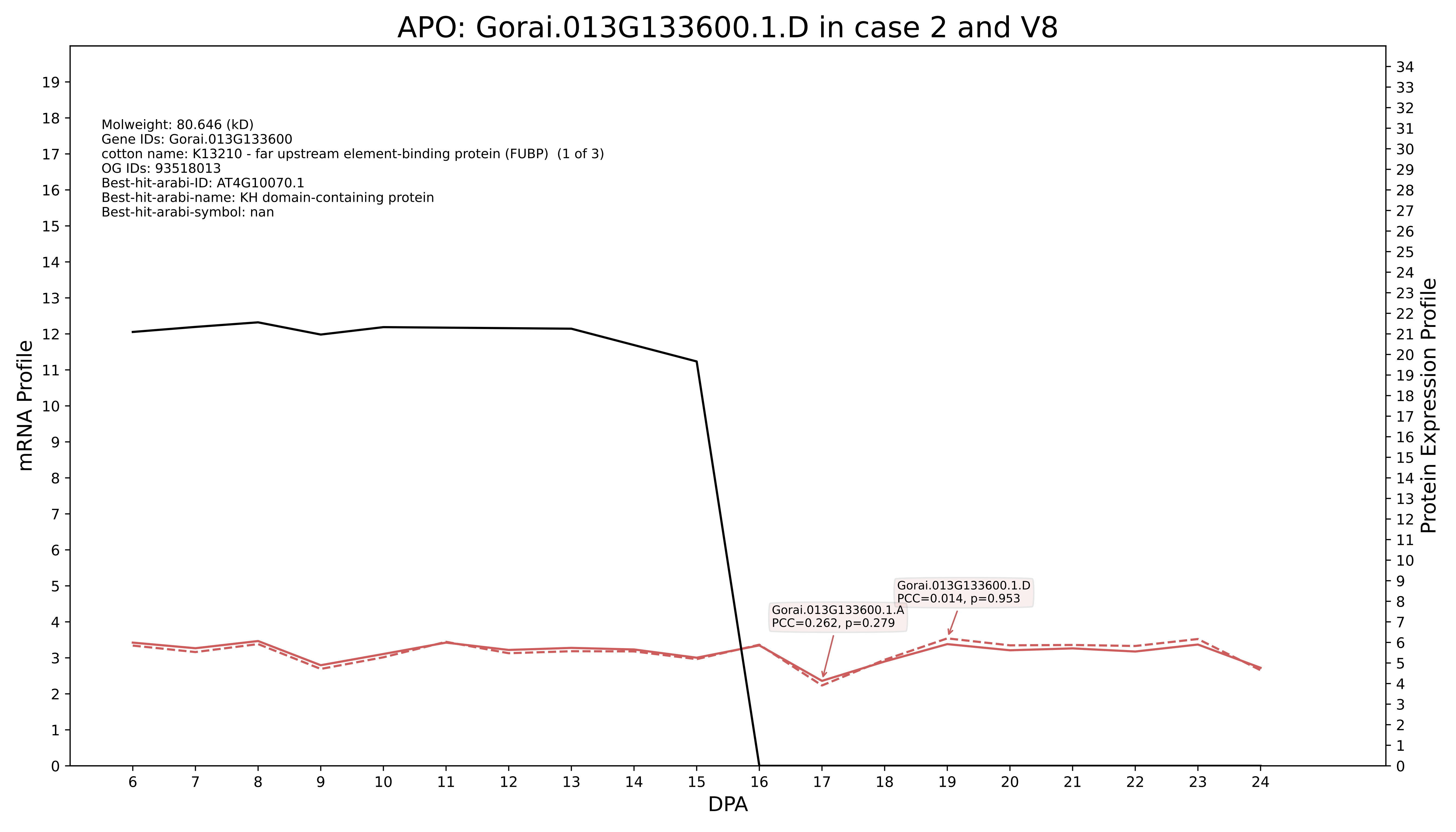
APO: Gorai.013G133600.1.D in case 2 and V8
Molweight: 80.646 (kD)
Gene IDs: Gorai.013G133600
cotton name: K13210 - far upstream element-binding protein (FUBP) (1 of 3)
OG IDs: 93518013
Best-hit-arabi-ID: AT4G10070.1
Best-hit-arabi-name: KH domain-containing protein
Best-hit-arabi-symbol: nan
Gorai.013G133600.1.D
PCC=0.014, p=0.953
Gorai.013G133600.1.A
PCC=0.262, p=0.279
mRNA Profile
Protein Expression Profile
DPA

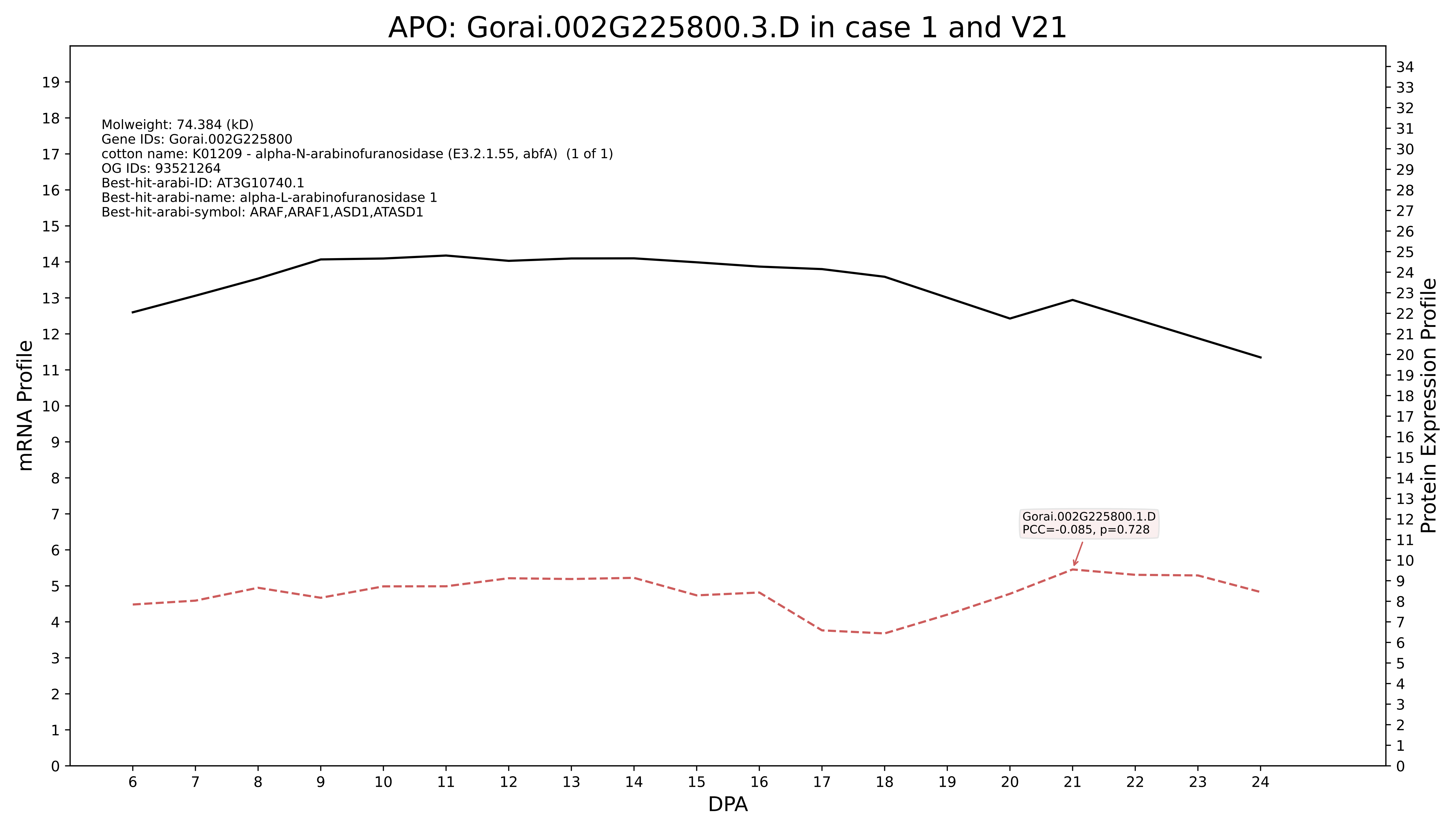

APO: Gorai.002G225800.3.D in case 1 and V21
Molweight: 74.384 (kD)
Gene IDs: Gorai.002G225800
cotton name: K01209 - alpha-N-arabinofuranosidase (E3.2.1.55, abfA) (1 of 1)
OG IDs: 93521264
Best-hit-arabi-ID: AT3G10740.1
Best-hit-arabi-name: alpha-L-arabinofuranosidase 1
Best-hit-arabi-symbol: ARAF,ARAF1,ASD1,ATASD1
Gorai.002G225800.1.D
PCC=-0.085, p=0.728
mRNA Profile
Protein Expression Profile
DPA

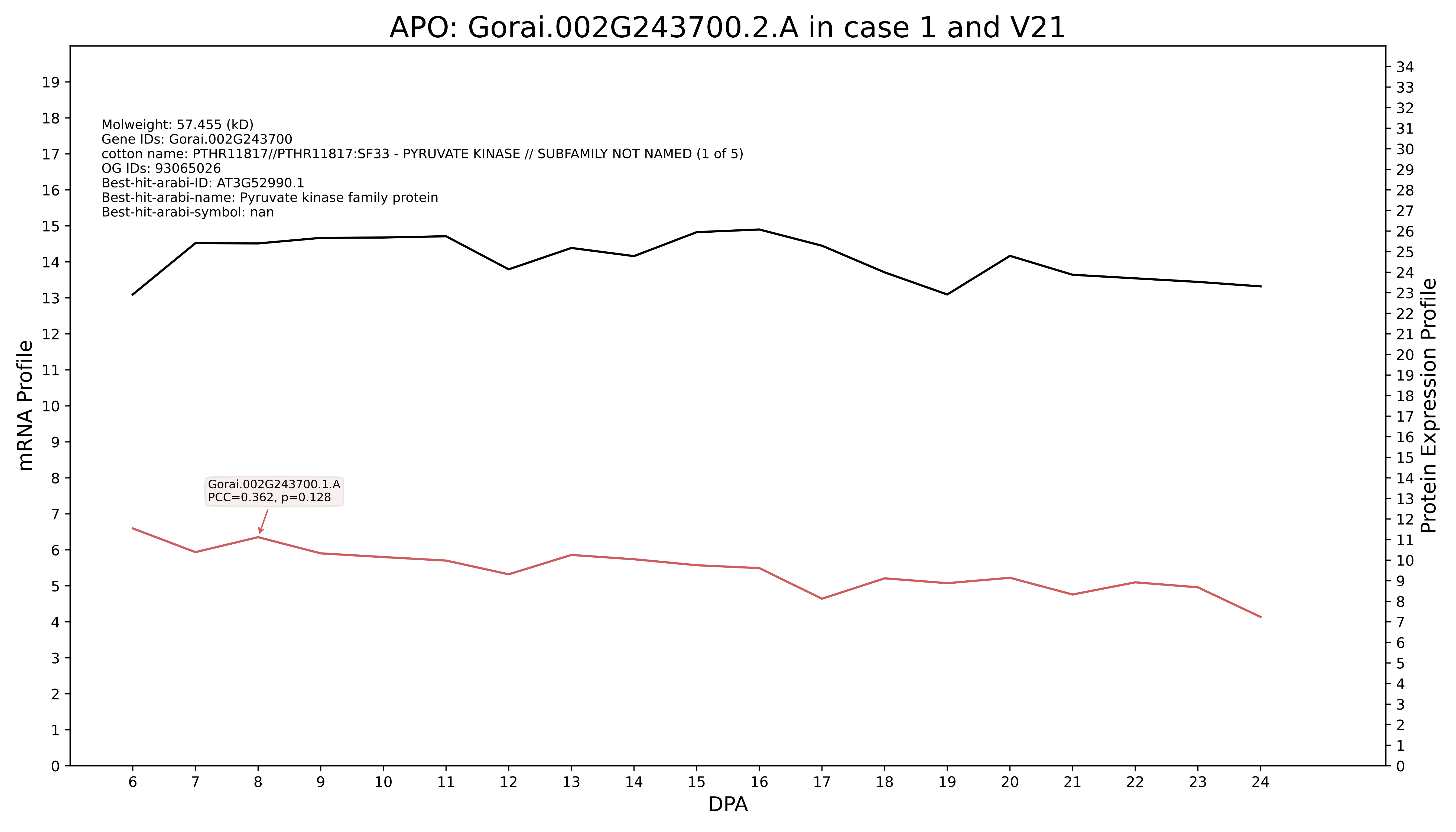
APO: Gorai.002G243700.2.A in case 1 and V21
Molweight: 57.455 (kD)
Gene IDs: Gorai.002G243700
cotton name: PTHR11817//PTHR11817:SF33 - PYRUVATE KINASE // SUBFAMILY NOT NAMED (1 of 5)
OG IDs: 93065026
Best-hit-arabi-ID: AT3G52990.1
Best-hit-arabi-name: Pyruvate kinase family protein
Best-hit-arabi-symbol: nan
Gorai.002G243700.1.A
PCC=0.362, p=0.128
mRNA Profile
Protein Expression Profile
DPA

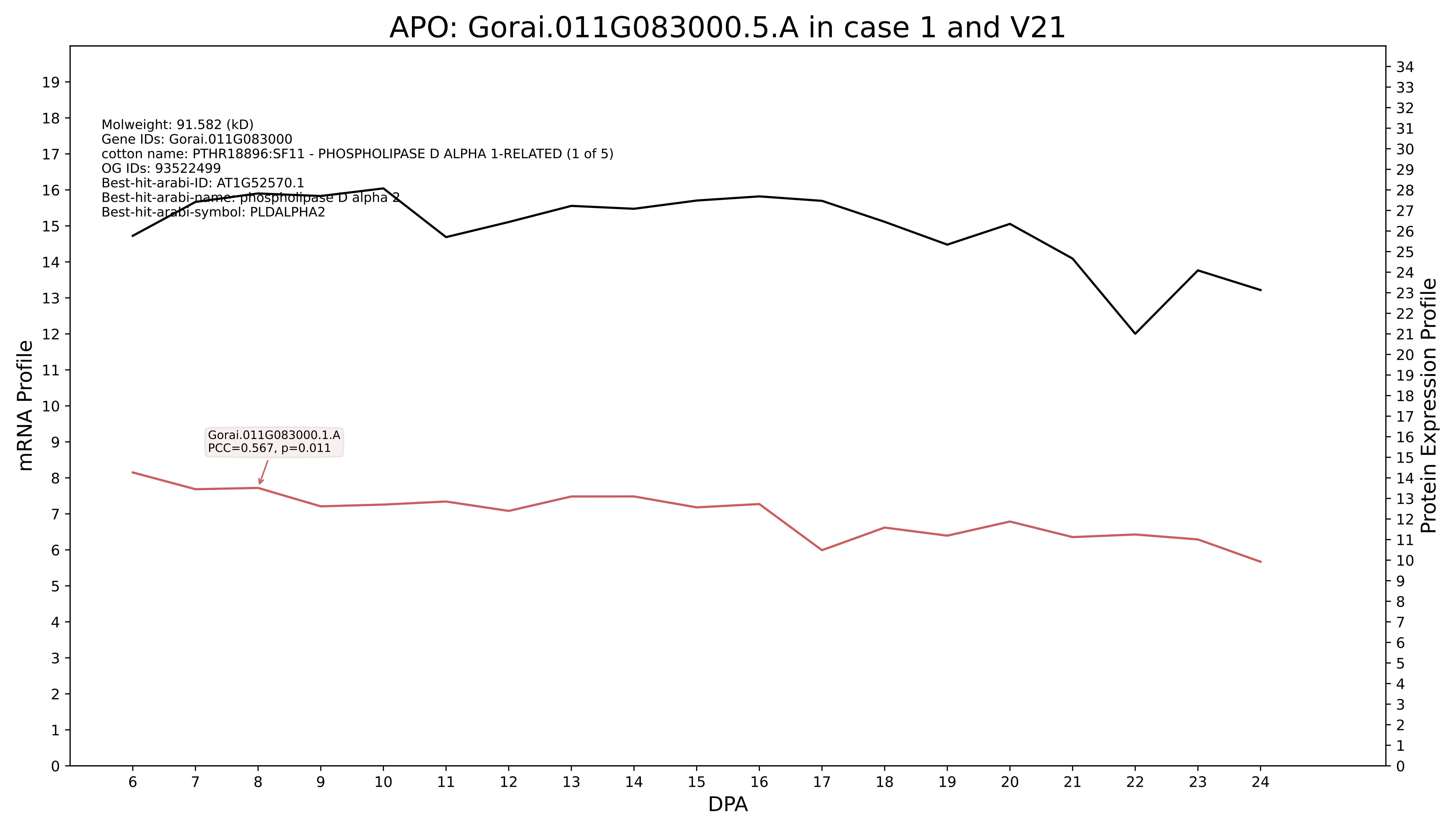

APO: Gorai.011G083000.5.A in case 1 and V21
Molweight: 91.582 (kD)
Gene IDs: Gorai.011G083000
cotton name: PTHR18896:SF11 - PHOSPHOLIPASE D ALPHA 1-RELATED (1 of 5)
OG IDs: 93522499
Best-hit-arabi-ID: AT1G52570.1
Best-hit-arabi-name: phospholipase D alpha 2
Best-hit-arabi-symbol: PLDALPHA2
Gorai.011G083000.1.A
PCC=0.567, p=0.011
mRNA Profile
Protein Expression Profile
DPA

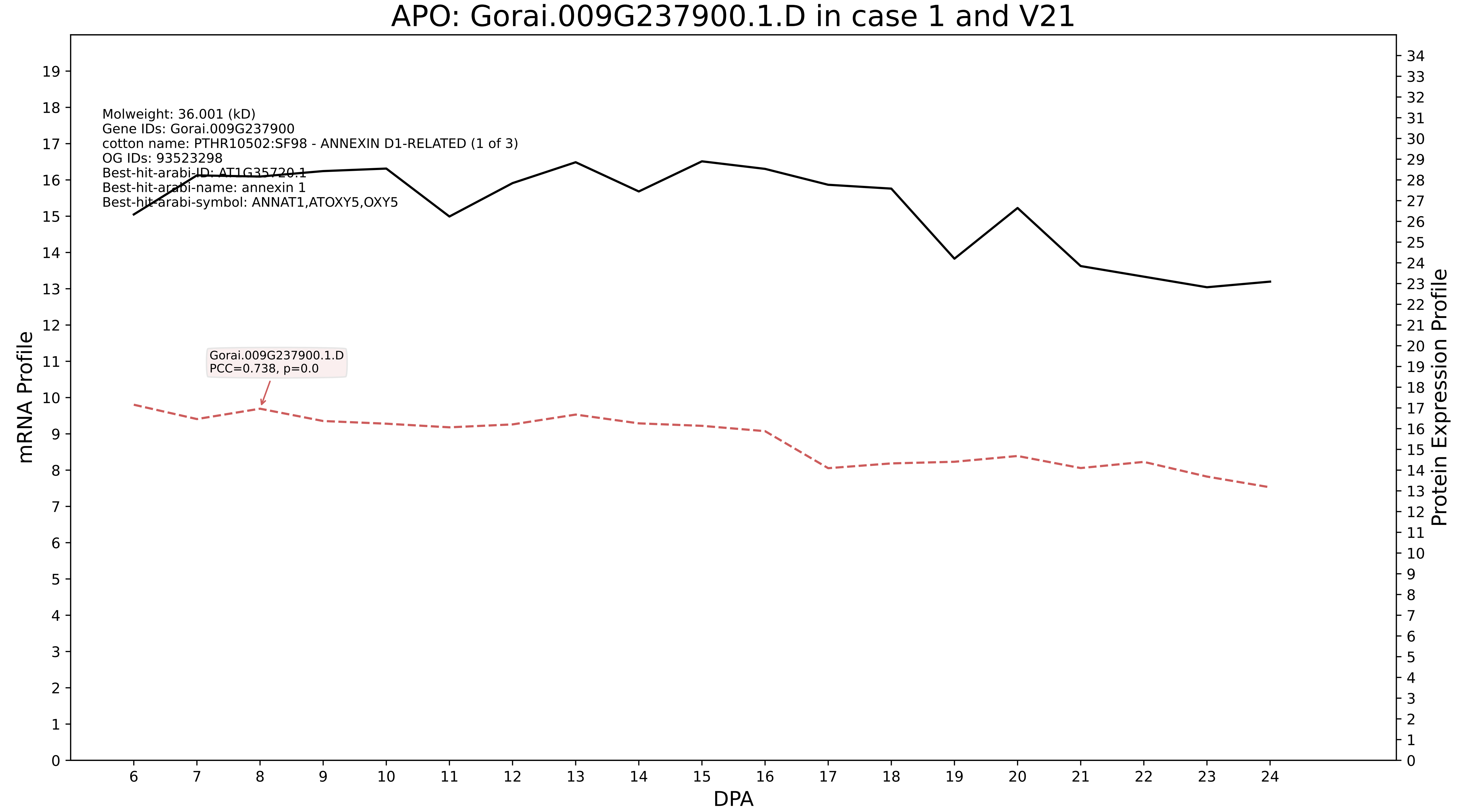
APO: Gorai.009G237900.1.D in case 1 and V21
Molweight: 36.001 (kD)
Gene IDs: Gorai.009G237900
cotton name: PTHR10502:SF98 - ANNEXIN D1-RELATED (1 of 3)
OG IDs: 93523298
Best-hit-arabi-ID: AT1G35720.1
Best-hit-arabi-name: annexin 1
Best-hit-arabi-symbol: ANNAT1,ATOXY5,OXY5
Gorai.009G237900.1.D
PCC=0.738, p=0.0
mRNA Profile
Protein Expression Profile
DPA

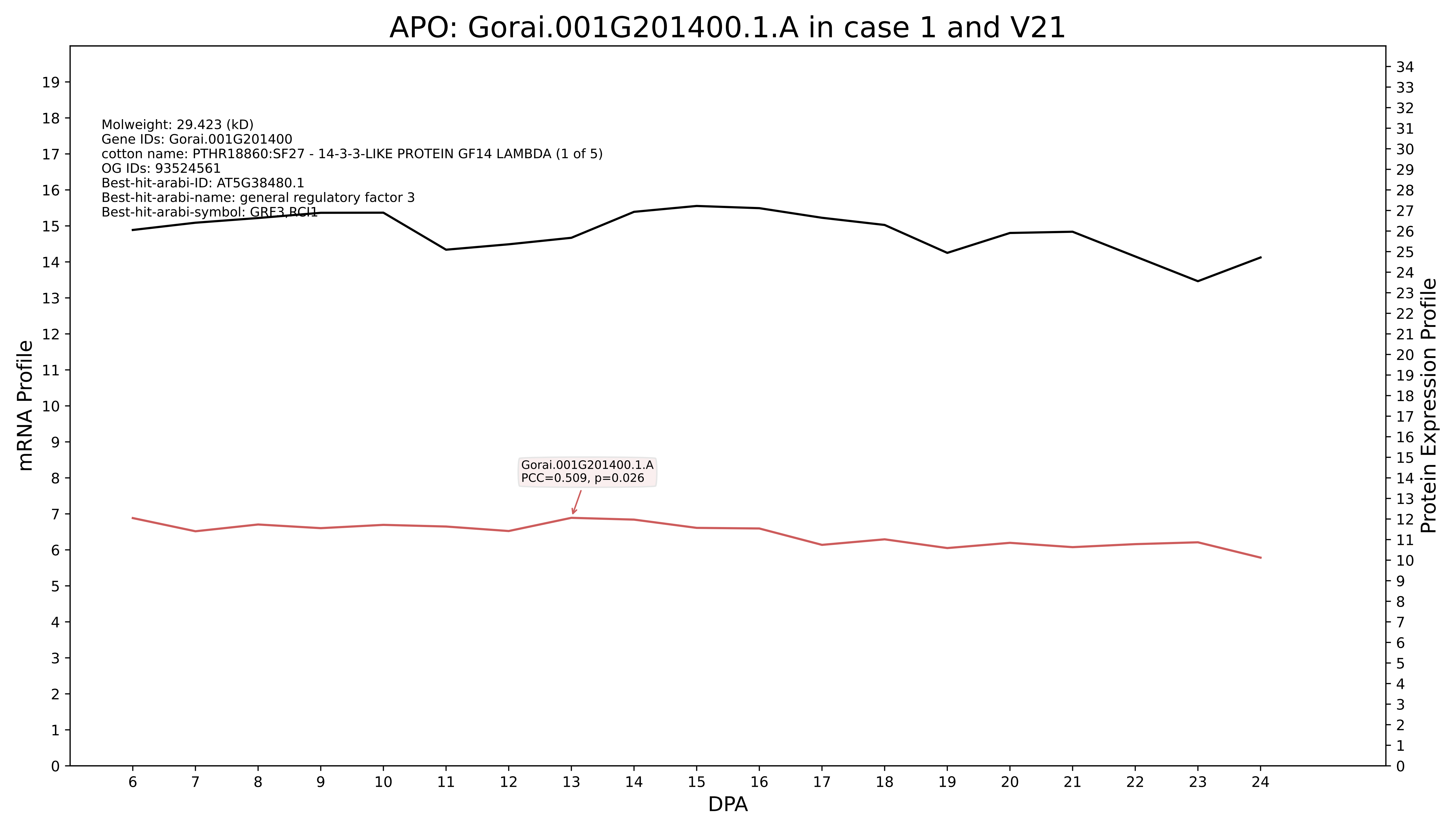
APO: Gorai.001G201400.1.A in case 1 and V21
Molweight: 29.423 (kD)
Gene IDs: Gorai.001G201400
cotton name: PTHR18860:SF27 - 14-3-3-LIKE PROTEIN GF14 LAMBDA (1 of 5)
OG IDs: 93524561
Best-hit-arabi-ID: AT5G38480.1
Best-hit-arabi-name: general regulatory factor 3
Best-hit-arabi-symbol: GRF3,RCI1
Gorai.001G201400.1.A
PCC=0.509, p=0.026
mRNA Profile
Protein Expression Profile
DPA

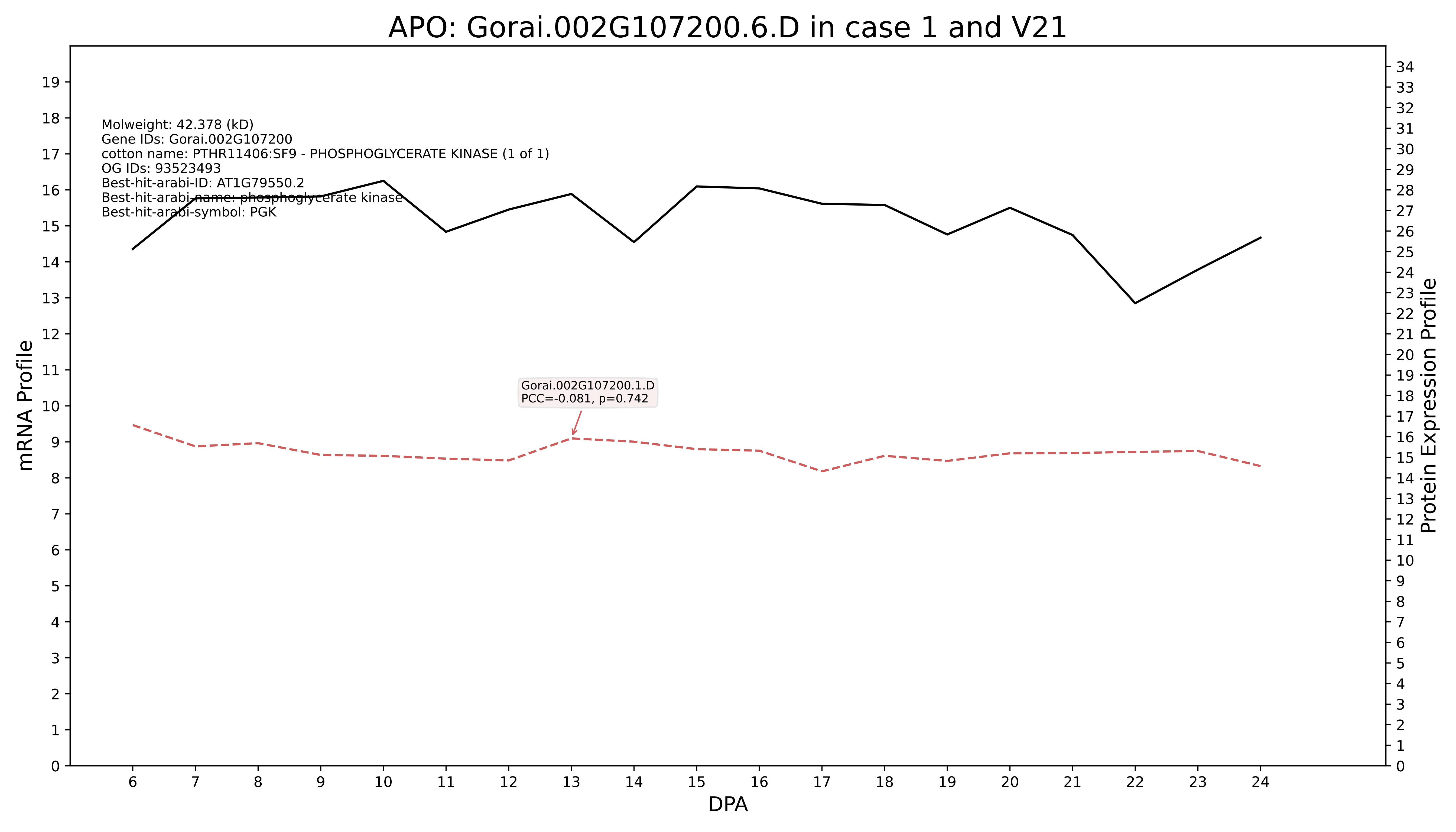
APO: Gorai.002G107200.6.D in case 1 and V21
Molweight: 42.378 (kD)
Gene IDs: Gorai.002G107200
cotton name: PTHR11406:SF9 - PHOSPHOGLYCERATE KINASE (1 of 1)
OG IDs: 93523493
Best-hit-arabi-ID: AT1G79550.2
Best-hit-arabi-name: phosphoglycerate kinase
Best-hit-arabi-symbol: PGK
Gorai.002G107200.1.D
PCC=-0.081, p=0.742
mRNA Profile
Protein Expression Profile
DPA

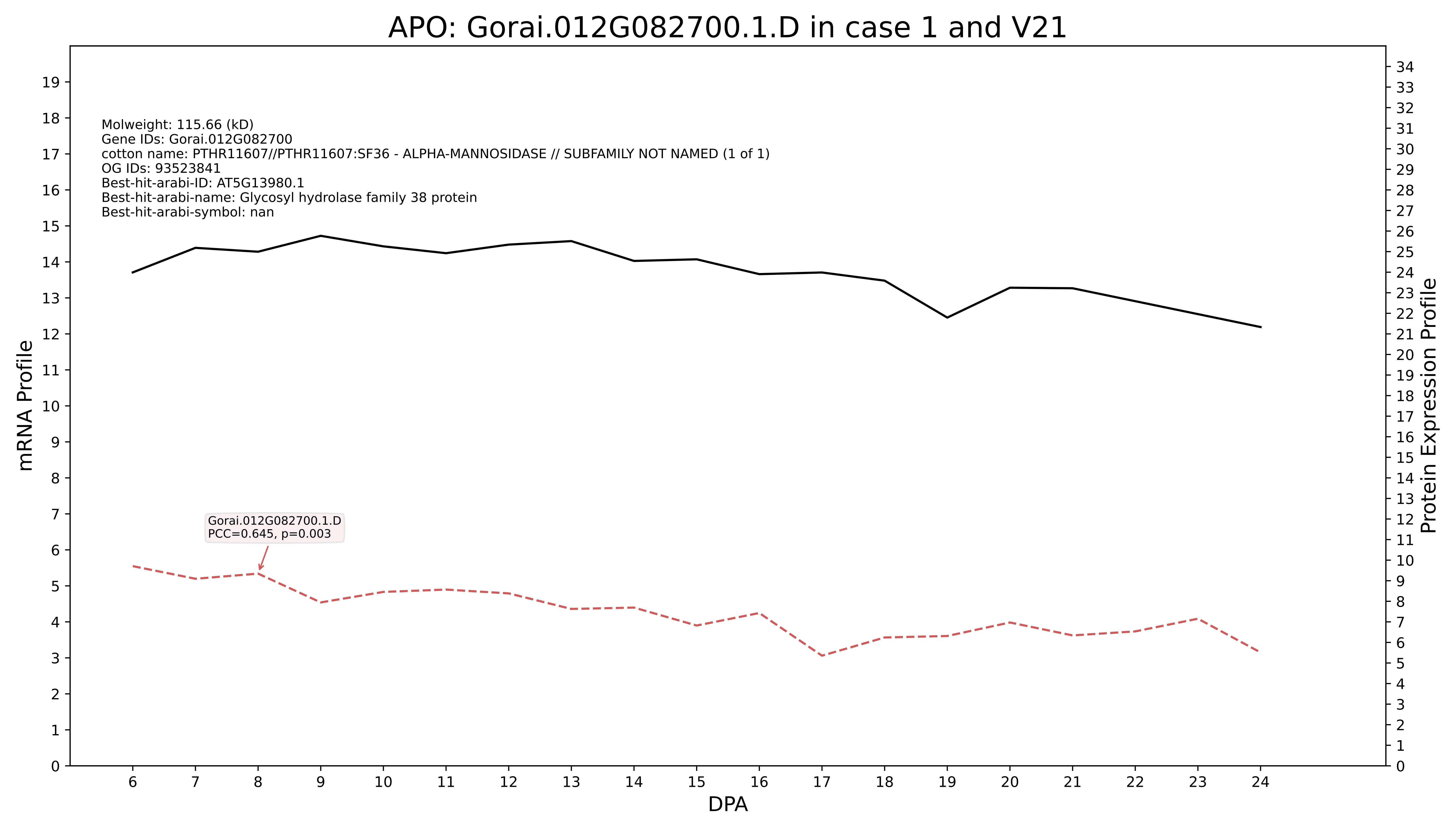
APO: Gorai.012G082700.1.D in case 1 and V21
Molweight: 115.66 (kD)
Gene IDs: Gorai.012G082700
cotton name: PTHR11607//PTHR11607:SF36 - ALPHA-MANNOSIDASE // SUBFAMILY NOT NAMED (1 of 1)
OG IDs: 93523841
Best-hit-arabi-ID: AT5G13980.1
Best-hit-arabi-name: Glycosyl hydrolase family 38 protein
Best-hit-arabi-symbol: nan
Gorai.012G082700.1.D
PCC=0.645, p=0.003
mRNA Profile
Protein Expression Profile
DPA

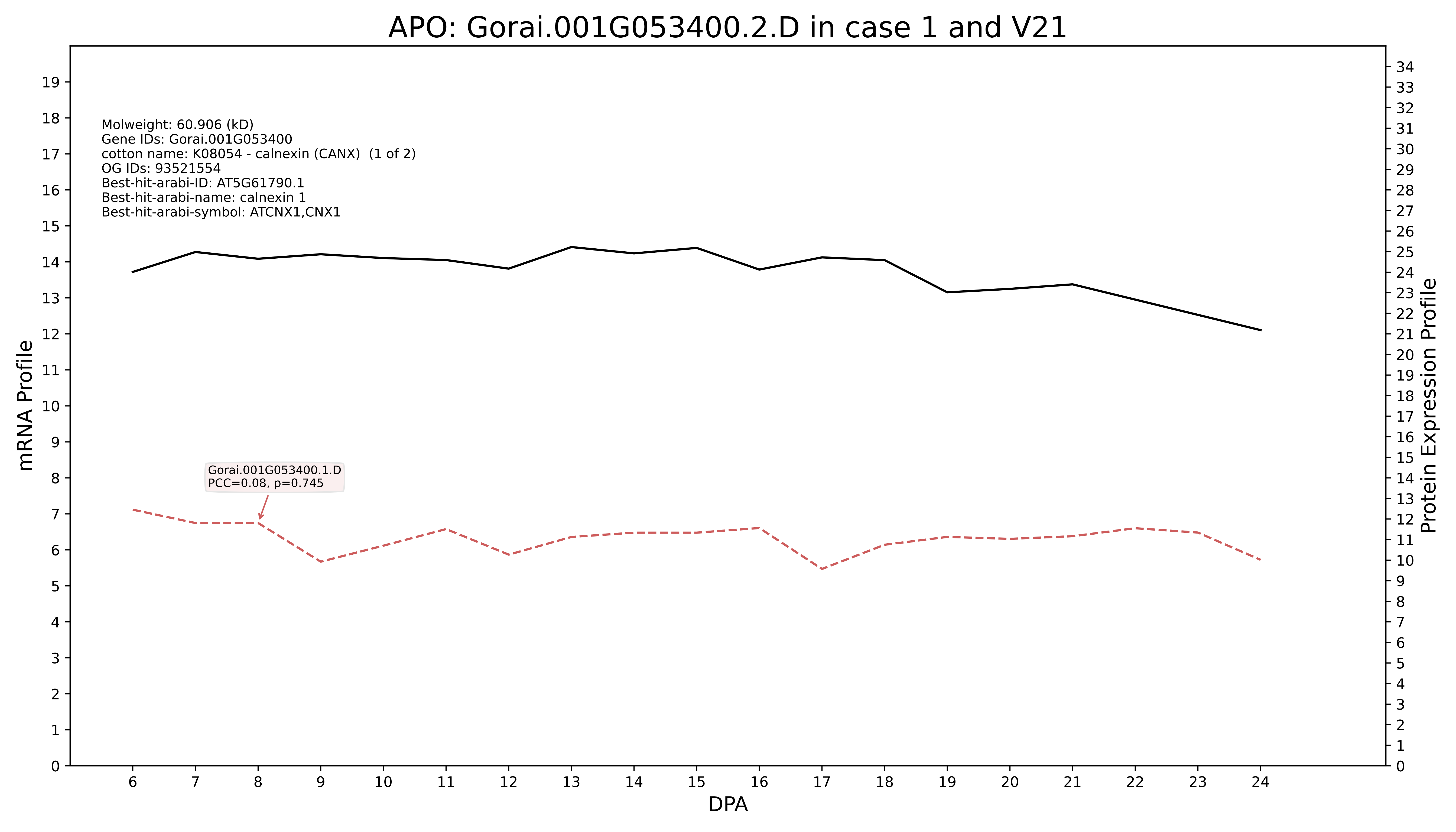
APO: Gorai.001G053400.2.D in case 1 and V21
Molweight: 60.906 (kD)
Gene IDs: Gorai.001G053400
cotton name: K08054 - calnexin (CANX) (1 of 2)
OG IDs: 93521554
Best-hit-arabi-ID: AT5G61790.1
Best-hit-arabi-name: calnexin 1
Best-hit-arabi-symbol: ATCNX1,CNX1
Gorai.001G053400.1.D
PCC=0.08, p=0.745
mRNA Profile
Protein Expression Profile
DPA

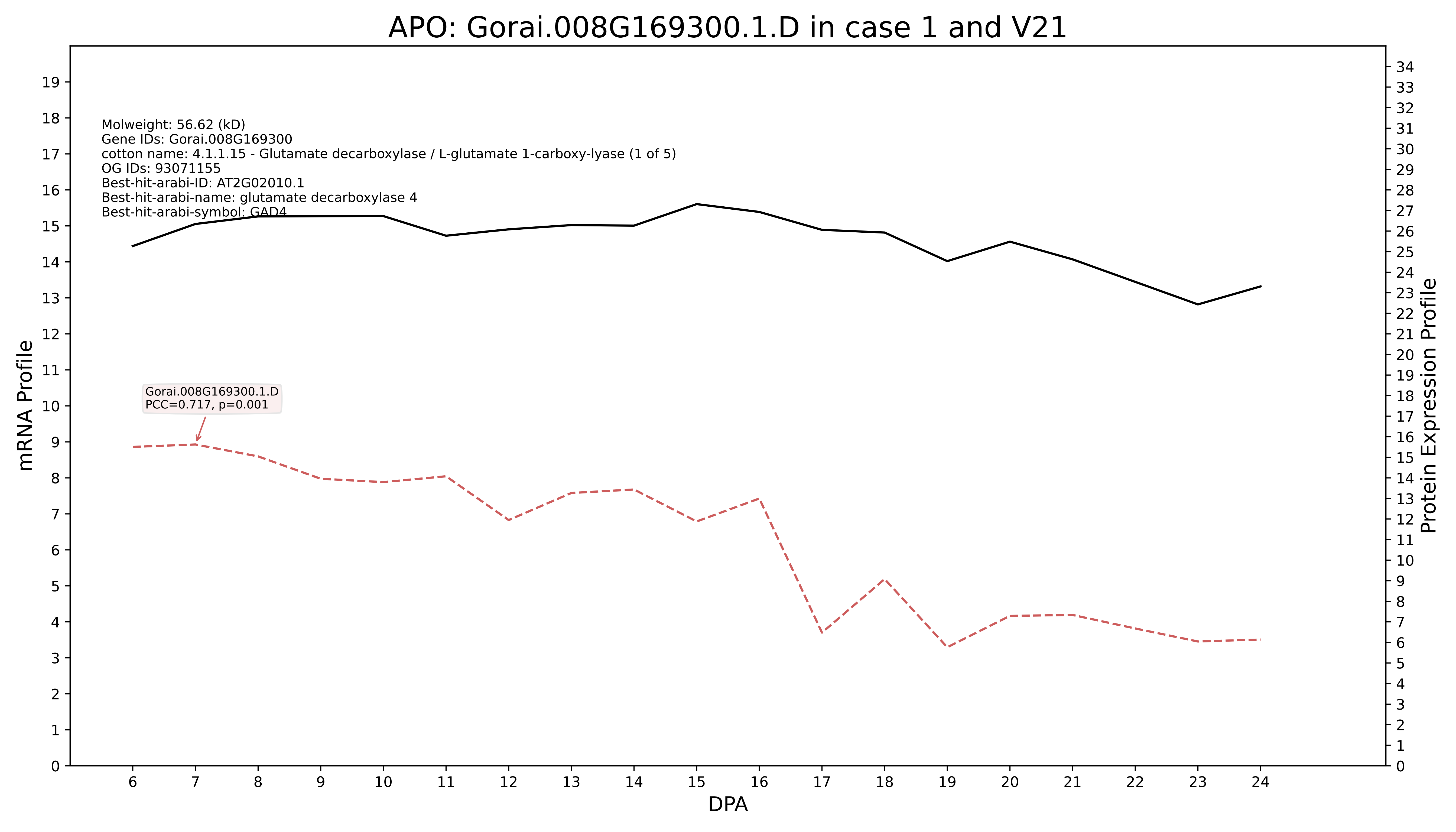
APO: Gorai.008G169300.1.D in case 1 and V21
Molweight: 56.62 (kD)
Gene IDs: Gorai.008G169300
cotton name: 4.1.1.15 - Glutamate decarboxylase / L-glutamate 1-carboxy-lyase (1 of 5)
OG IDs: 93071155
Best-hit-arabi-ID: AT2G02010.1
Best-hit-arabi-name: glutamate decarboxylase 4
Best-hit-arabi-symbol: GAD4
Gorai.008G169300.1.D
PCC=0.717, p=0.001
mRNA Profile
Protein Expression Profile
DPA
0
1
2
3
4
5
6
7
8
9
10
11
12
13
14
15
16
17
18
19
20
21
22
23
24
25
26
27
28
29
30
31
32
33
34

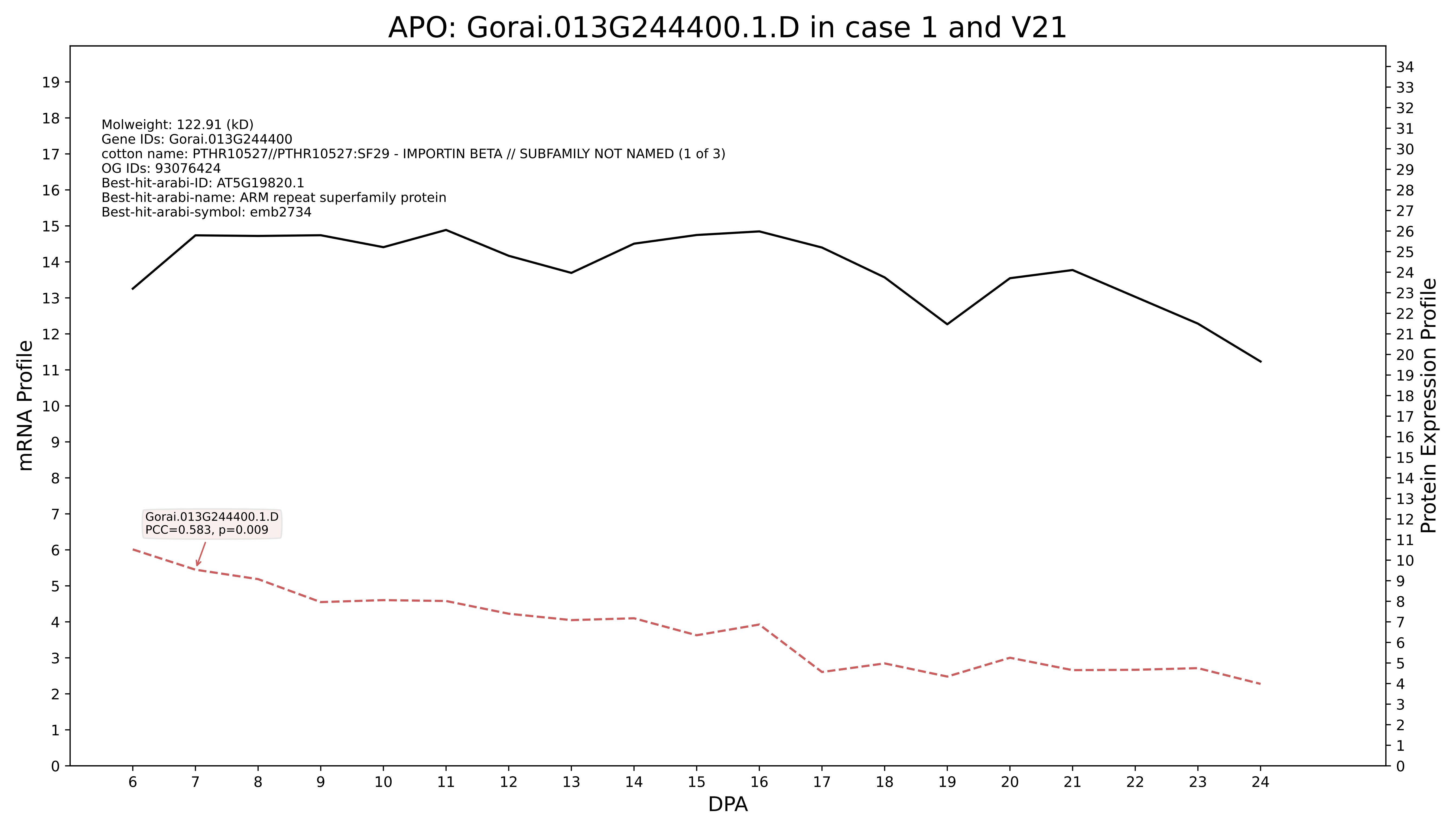

APO: Gorai.013G244400.1.D in case 1 and V21
Molweight: 122.91 (kD)
Gene IDs: Gorai.013G244400
cotton name: PTHR10527//PTHR10527:SF29 - IMPORTIN BETA // SUBFAMILY NOT NAMED (1 of 3)
OG IDs: 93076424
Best-hit-arabi-ID: AT5G19820.1
Best-hit-arabi-name: ARM repeat superfamily protein
Best-hit-arabi-symbol: emb2734
Gorai.013G244400.1.D
PCC=0.583, p=0.009
mRNA Profile
Protein Expression Profile
DPA

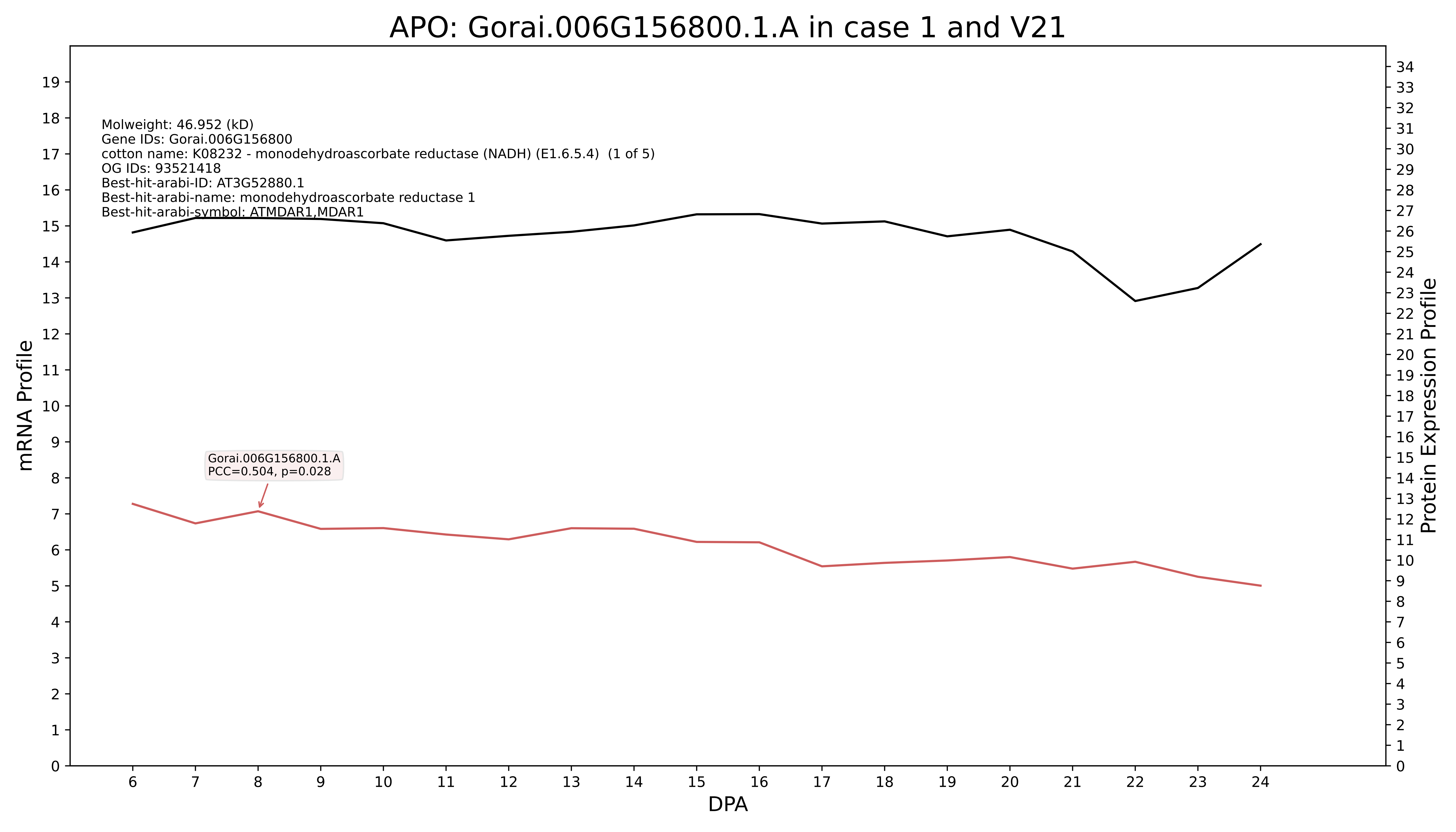
APO: Gorai.006G156800.1.A in case 1 and V21
Molweight: 46.952 (kD)
Gene IDs: Gorai.006G156800
cotton name: K08232 - monodehydroascorbate reductase (NADH) (E1.6.5.4) (1 of 5)
OG IDs: 93521418
Best-hit-arabi-ID: AT3G52880.1
Best-hit-arabi-name: monodehydroascorbate reductase 1
Best-hit-arabi-symbol: ATMDAR1,MDAR1
Gorai.006G156800.1.A
PCC=0.504, p=0.028
mRNA Profile
Protein Expression Profile
DPA

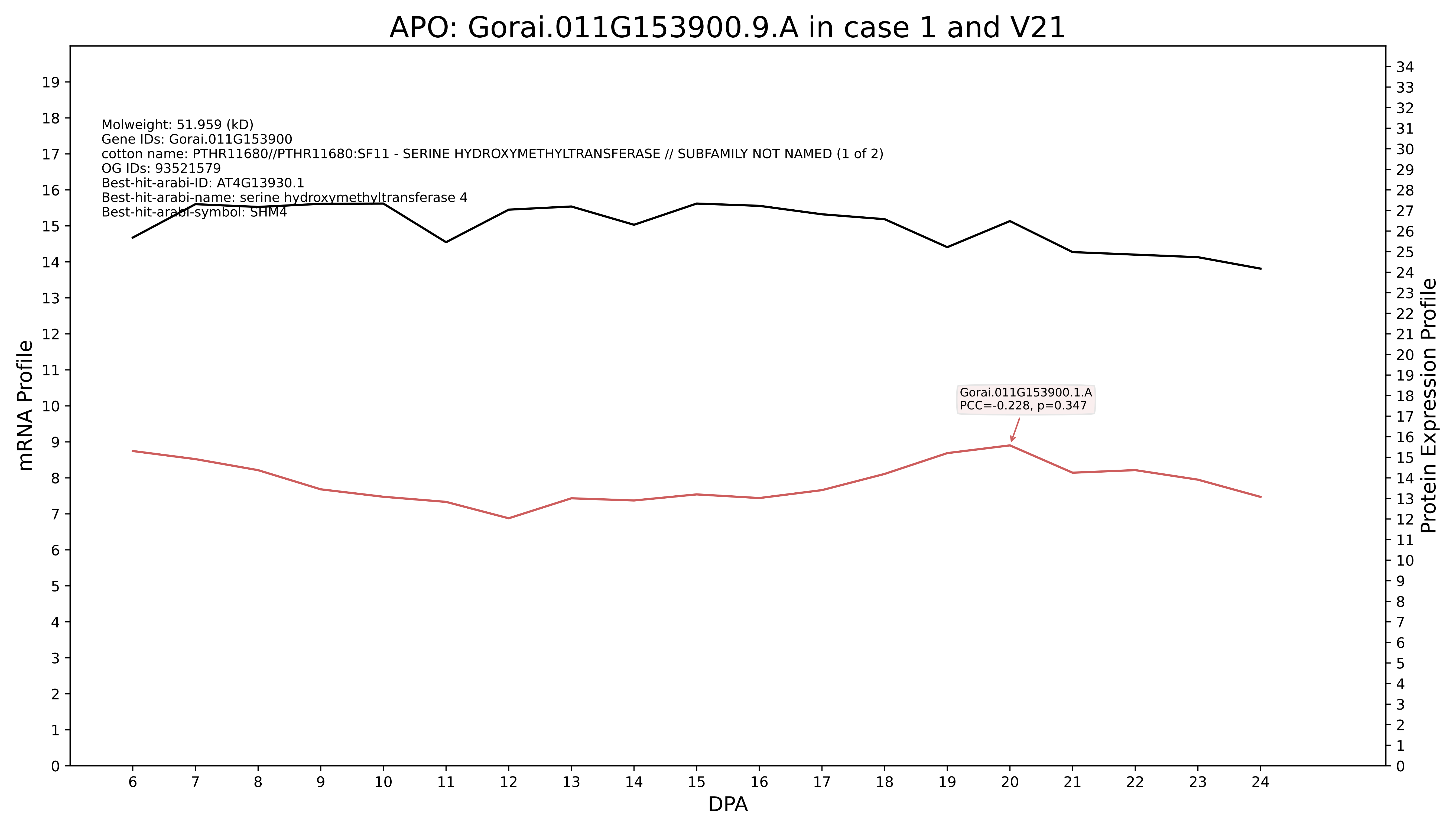

APO: Gorai.011G153900.9.A in case 1 and V21
Molweight: 51.959 (kD)
Gene IDs: Gorai.011G153900
cotton name: PTHR11680//PTHR11680:SF11 - SERINE HYDROXYMETHYLTRANSFERASE // SUBFAMILY NOT NAMED (1 of 2)
OG IDs: 93521579
Best-hit-arabi-ID: AT4G13930.1
Best-hit-arabi-name: serine hydroxymethyltransferase 4
Best-hit-arabi-symbol: SHM4
Gorai.011G153900.1.A
PCC=-0.228, p=0.347
mRNA Profile
Protein Expression Profile
DPA

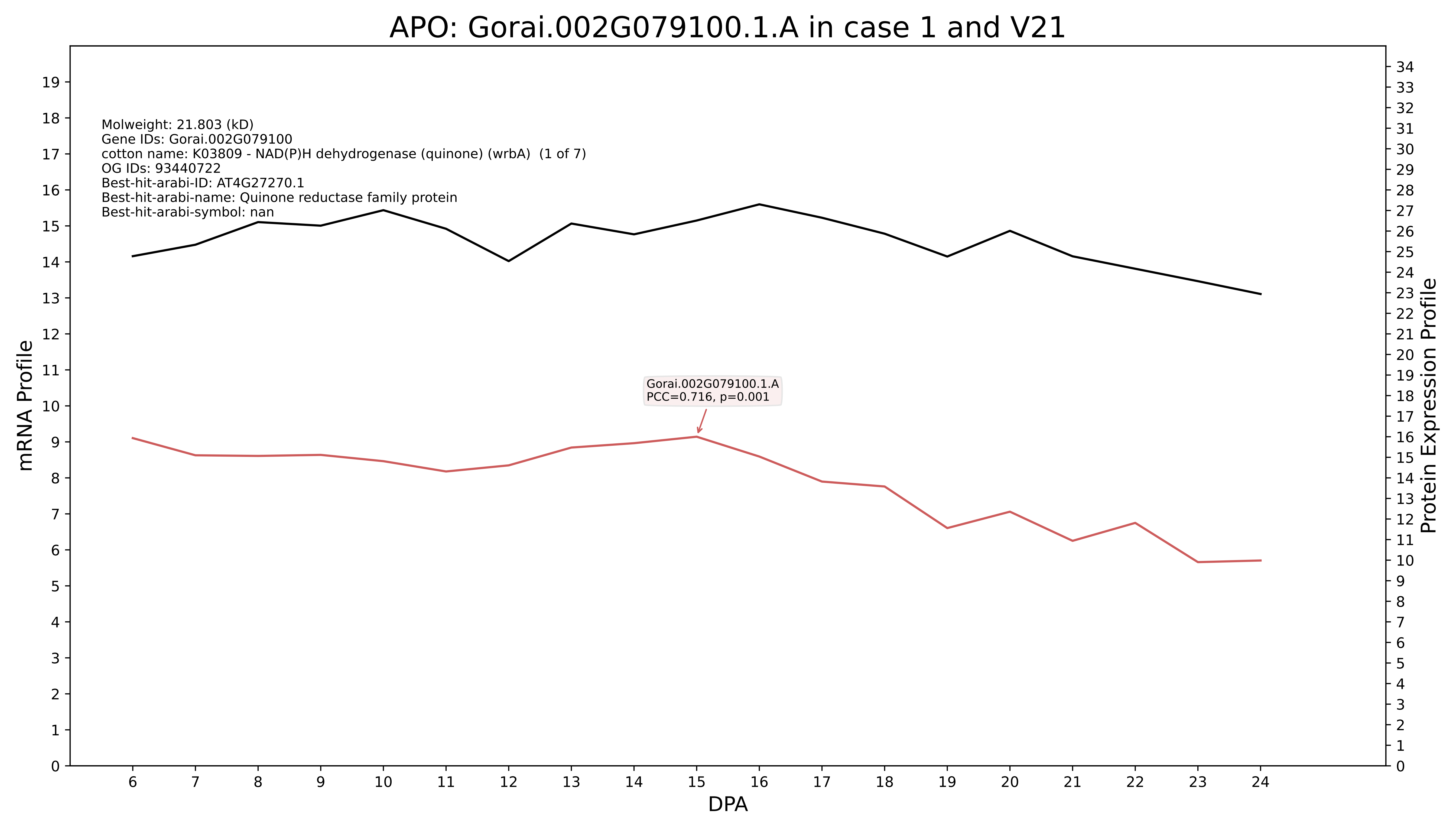

APO: Gorai.002G079100.1.A in case 1 and V21
Molweight: 21.803 (kD)
Gene IDs: Gorai.002G079100
cotton name: K03809 - NAD(P)H dehydrogenase (quinone) (wrbA) (1 of 7)
OG IDs: 93440722
Best-hit-arabi-ID: AT4G27270.1
Best-hit-arabi-name: Quinone reductase family protein
Best-hit-arabi-symbol: nan
Gorai.002G079100.1.A
PCC=0.716, p=0.001
mRNA Profile
Protein Expression Profile
DPA

APO: Gorai.009G038000.6.D in case 1 and V21

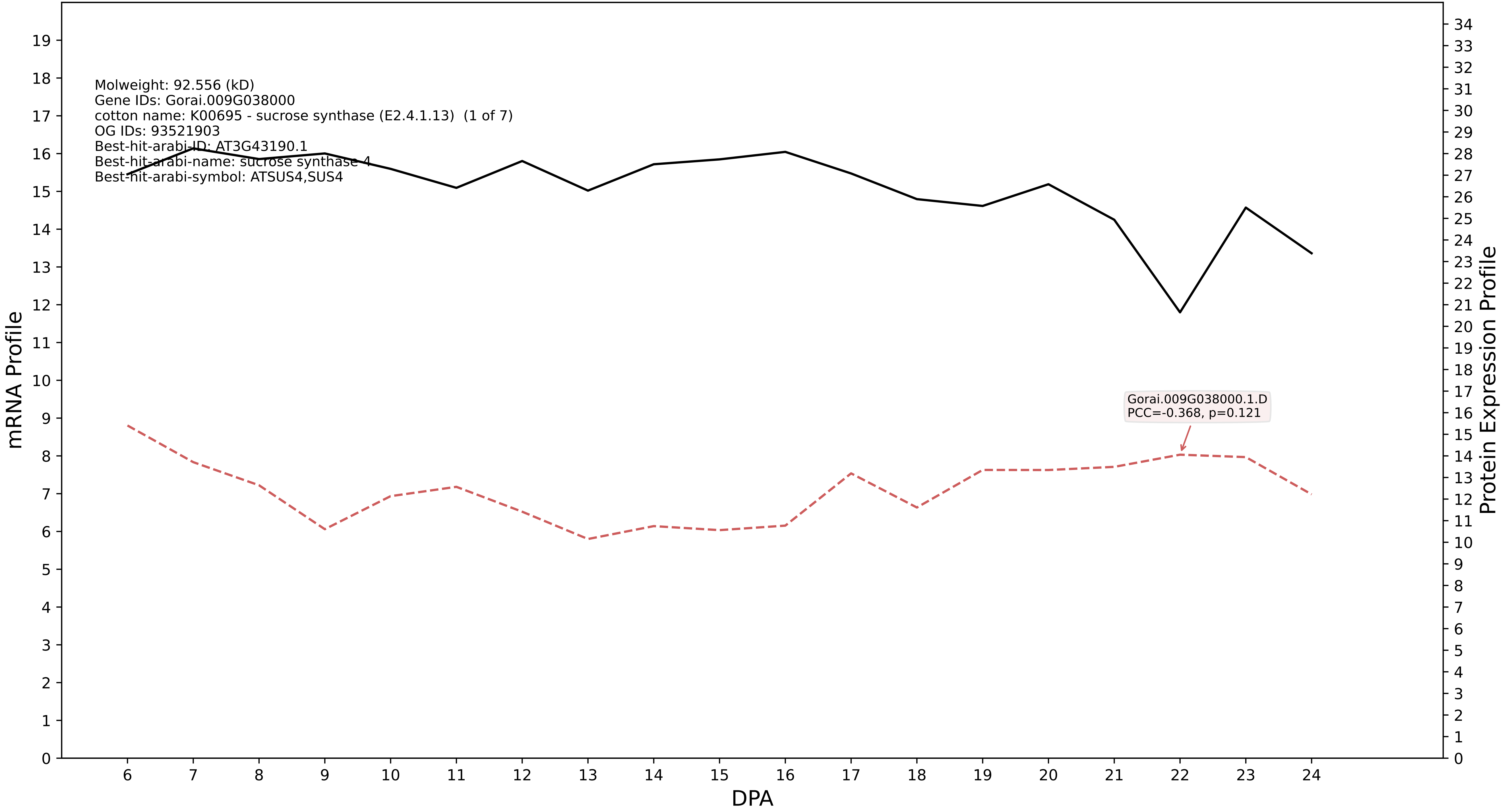

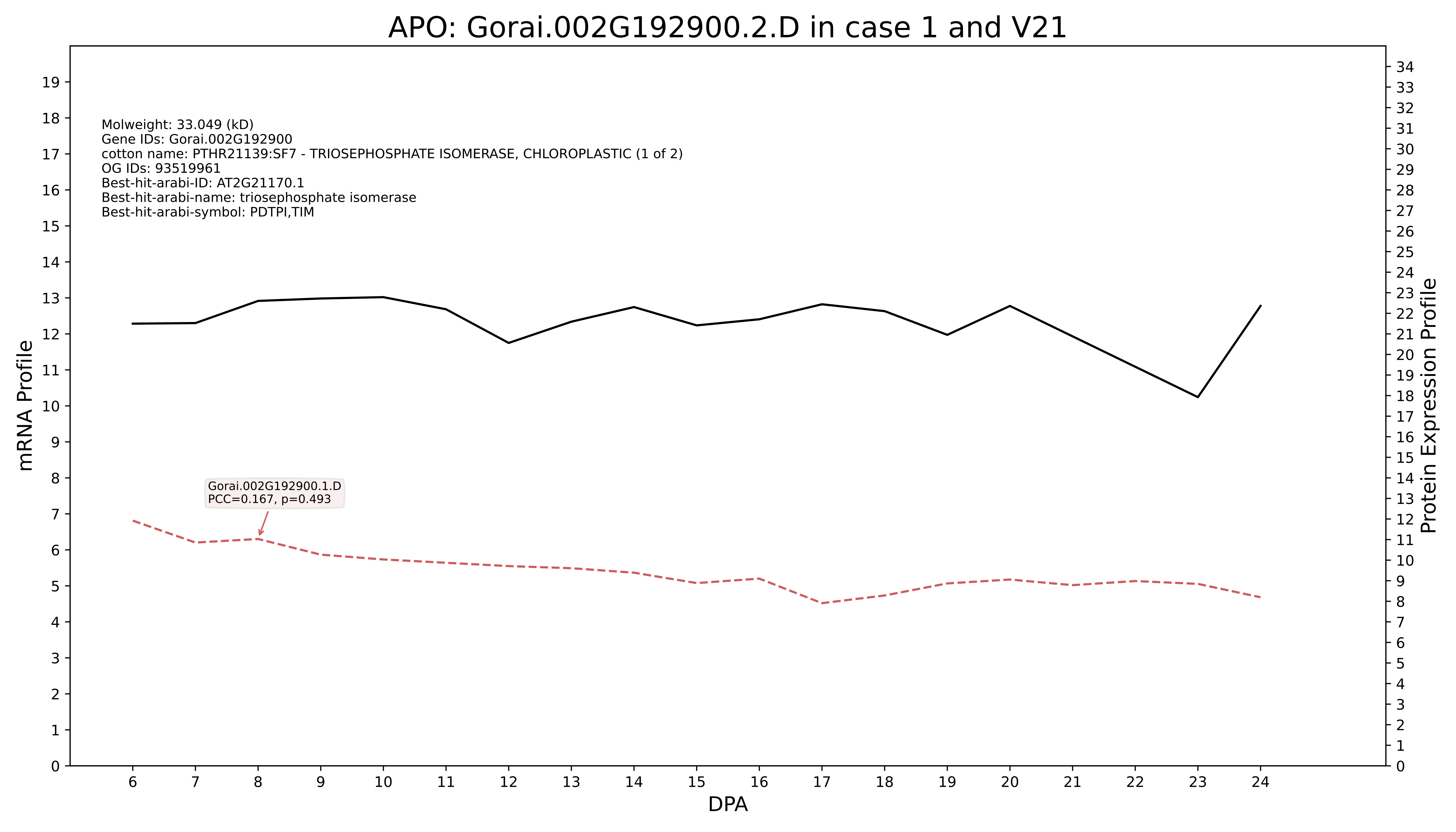
APO: Gorai.002G192900.2.D in case 1 and V21
Molweight: 33.049 (kD)
Gene IDs: Gorai.002G192900
cotton name: PTHR21139:SF7 - TRIOSEPHOSPHATE ISOMERASE, CHLOROPLASTIC (1 of 2)
OG IDs: 93519961
Best-hit-arabi-ID: AT2G21170.1
Best-hit-arabi-name: triosephosphate isomerase
Best-hit-arabi-symbol: PDTPI,TIM
Gorai.002G192900.1.D
PCC=0.167, p=0.493
mRNA Profile
Protein Expression Profile
DPA

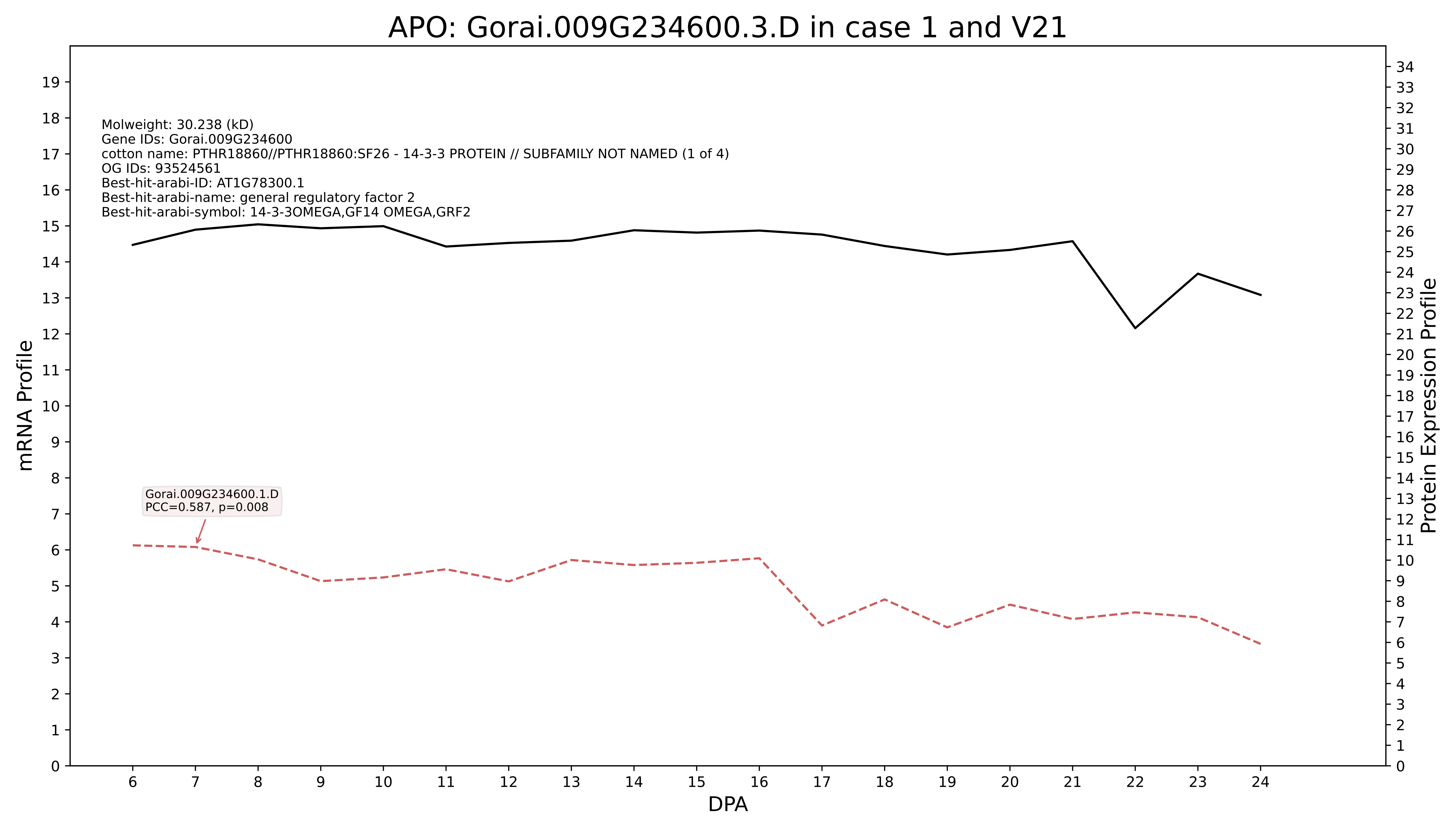
APO: Gorai.009G234600.3.D in case 1 and V21
Molweight: 30.238 (kD)
Gene IDs: Gorai.009G234600
cotton name: PTHR18860//PTHR18860:SF26 - 14-3-3 PROTEIN // SUBFAMILY NOT NAMED (1 of 4)
OG IDs: 93524561
Best-hit-arabi-ID: AT1G78300.1
Best-hit-arabi-name: general regulatory factor 2
Best-hit-arabi-symbol: 14-3-3OMEGA,GF14 OMEGA,GRF2
Gorai.009G234600.1.D
PCC=0.587, p=0.008
mRNA Profile
Protein Expression Profile
DPA

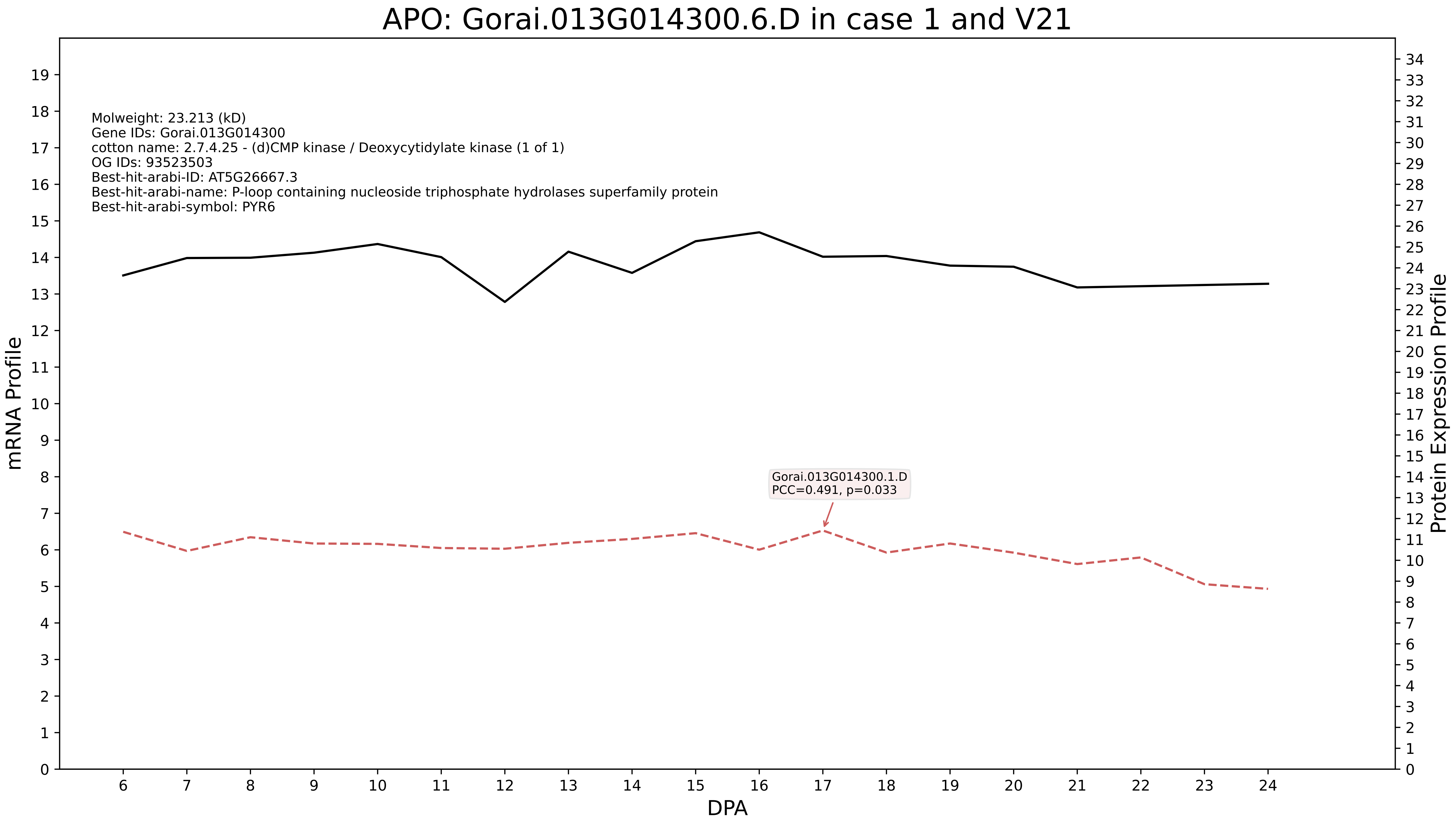
APO: Gorai.013G014300.6.D in case 1 and V21
Molweight: 23.213 (kD)
Gene IDs: Gorai.013G014300
cotton name: 2.7.4.25 - (d)CMP kinase / Deoxycytidylate kinase (1 of 1)
OG IDs: 93523503
Best-hit-arabi-ID: AT5G26667.3
Best-hit-arabi-name: P-loop containing nucleoside triphosphate hydrolases superfamily protein
Best-hit-arabi-symbol: PYR6
Gorai.013G014300.1.D
PCC=0.491, p=0.033
mRNA Profile
Protein Expression Profile
DPA

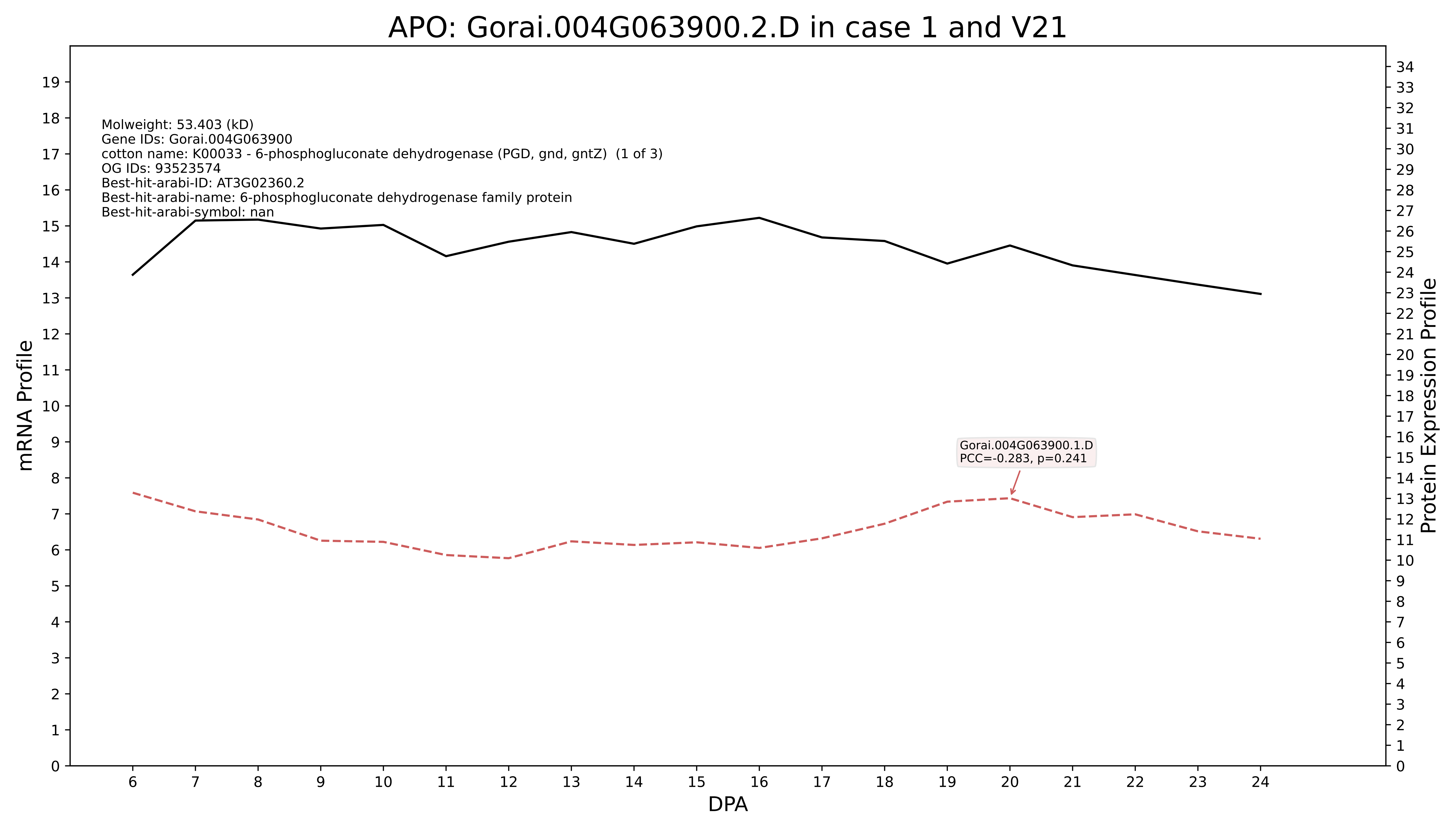

APO: Gorai.004G063900.2.D in case 1 and V21
Molweight: 53.403 (kD)
Gene IDs: Gorai.004G063900
cotton name: K00033 - 6-phosphogluconate dehydrogenase (PGD, gnd, gntZ) (1 of 3)
OG IDs: 93523574
Best-hit-arabi-ID: AT3G02360.2
Best-hit-arabi-name: 6-phosphogluconate dehydrogenase family protein
Best-hit-arabi-symbol: nan
Gorai.004G063900.1.D
PCC=-0.283, p=0.241
mRNA Profile
Protein Expression Profile
DPA

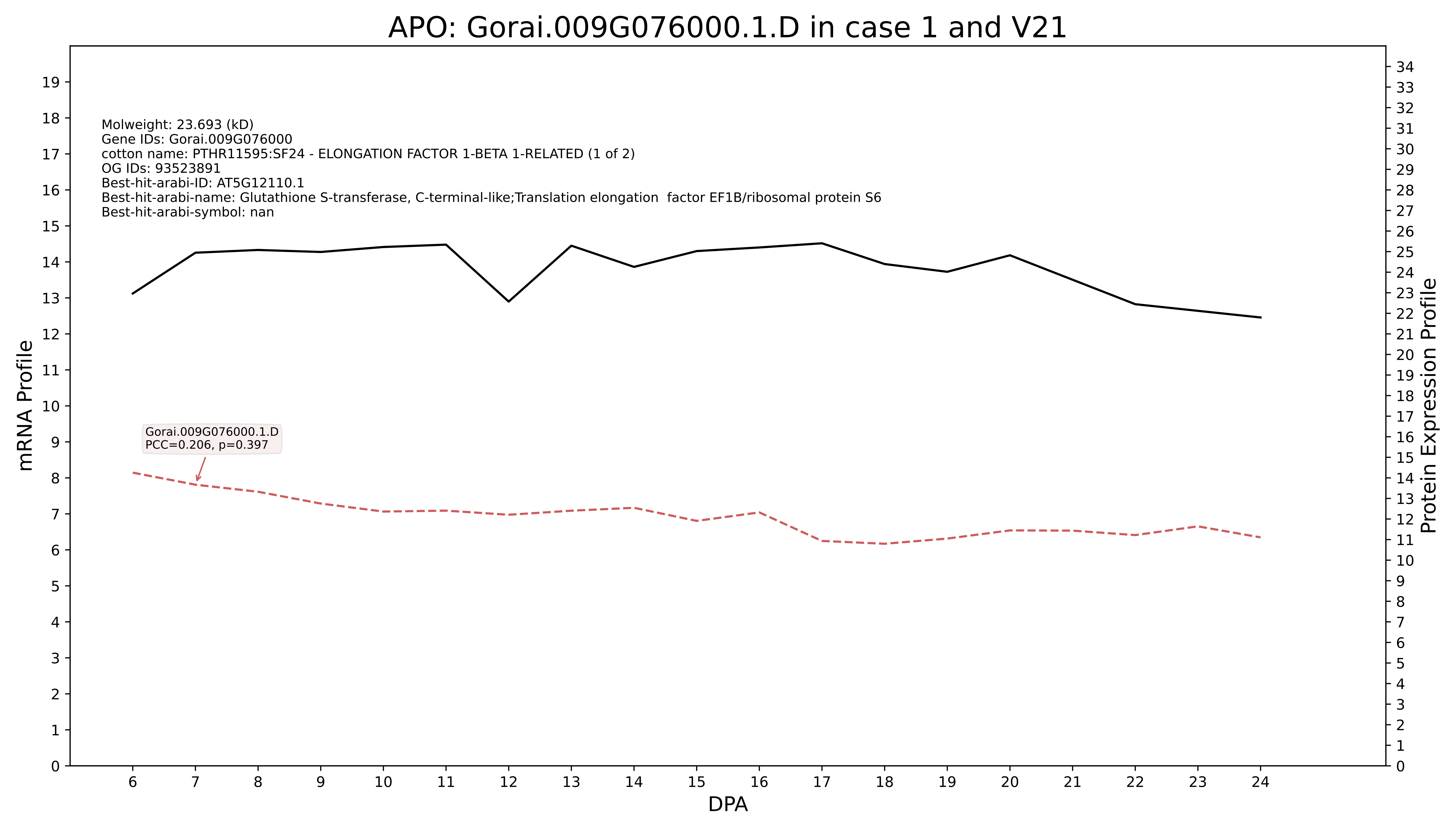
APO: Gorai.009G076000.1.D in case 1 and V21
Molweight: 23.693 (kD)
Gene IDs: Gorai.009G076000
cotton name: PTHR11595:SF24 - ELONGATION FACTOR 1-BETA 1-RELATED (1 of 2)
OG IDs: 93523891
Best-hit-arabi-ID: AT5G12110.1
Best-hit-arabi-name: Glutathione S-transferase, C-terminal-like;Translation elongation factor EF1B/ribosomal protein S6
Best-hit-arabi-symbol: nan
Gorai.009G076000.1.D
PCC=0.206, p=0.397
mRNA Profile
Protein Expression Profile
DPA

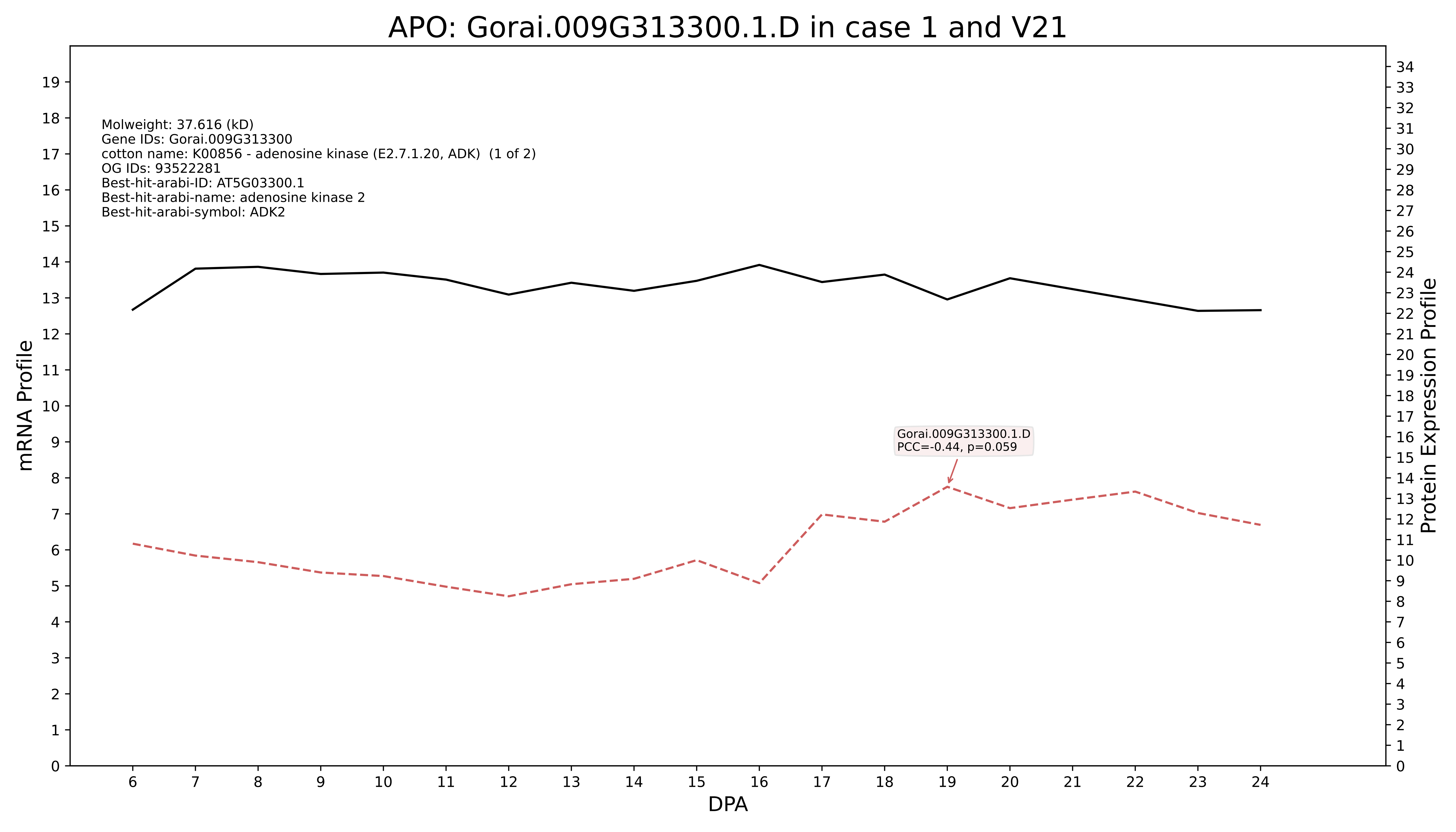

APO: Gorai.009G313300.1.D in case 1 and V21
Molweight: 37.616 (kD)
Gene IDs: Gorai.009G313300
cotton name: K00856 - adenosine kinase (E2.7.1.20, ADK) (1 of 2)
OG IDs: 93522281
Best-hit-arabi-ID: AT5G03300.1
Best-hit-arabi-name: adenosine kinase 2
Best-hit-arabi-symbol: ADK2
Gorai.009G313300.1.D
PCC=-0.44, p=0.059
mRNA Profile
Protein Expression Profile
DPA

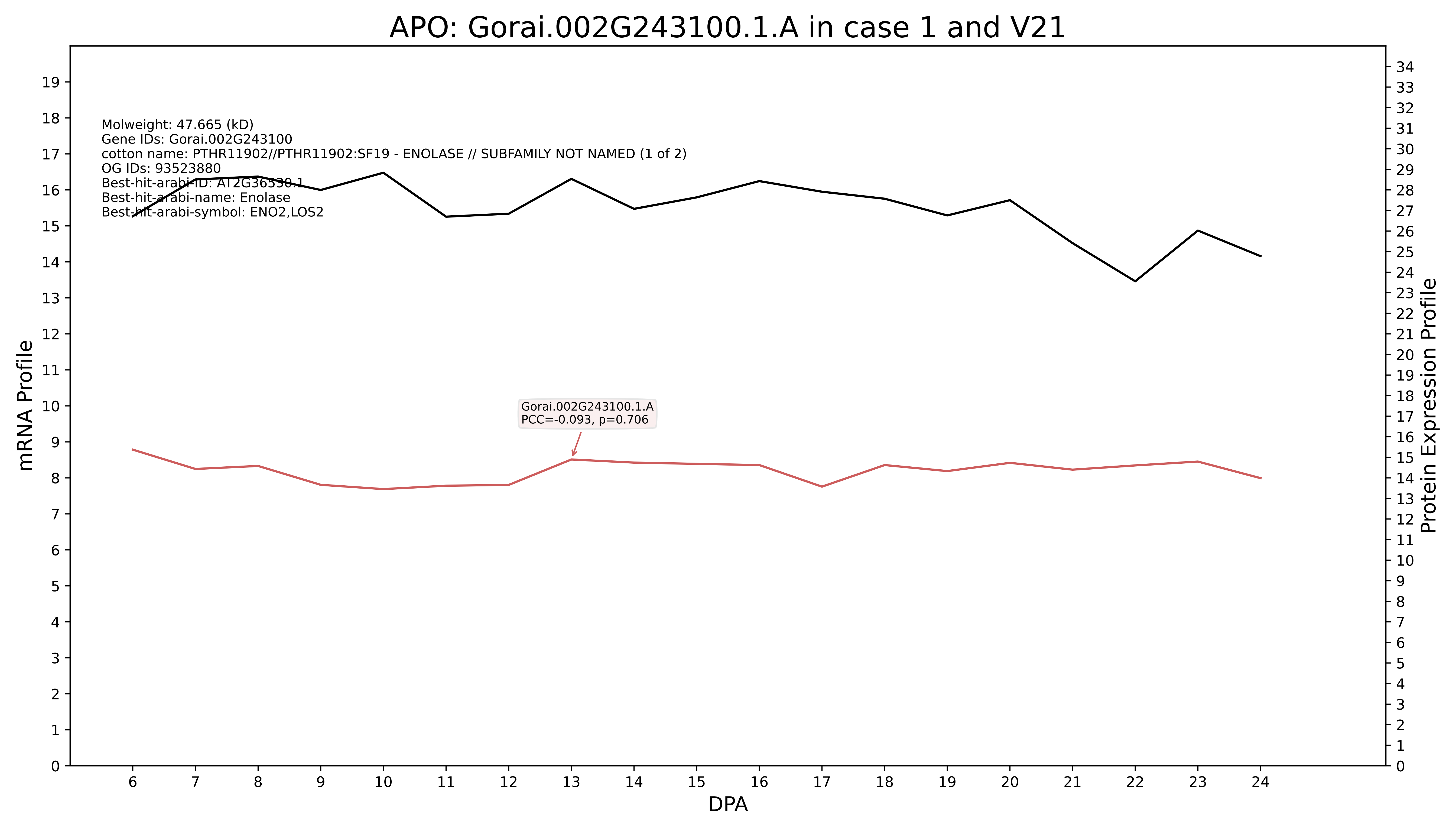

APO: Gorai.002G243100.1.A in case 1 and V21
Molweight: 47.665 (kD)
Gene IDs: Gorai.002G243100
cotton name: PTHR11902//PTHR11902:SF19 - ENOLASE // SUBFAMILY NOT NAMED (1 of 2)
OG IDs: 93523880
Best-hit-arabi-ID: AT2G36530.1
Best-hit-arabi-name: Enolase
Best-hit-arabi-symbol: ENO2,LOS2
Gorai.002G243100.1.A
PCC=-0.093, p=0.706
mRNA Profile
Protein Expression Profile
DPA

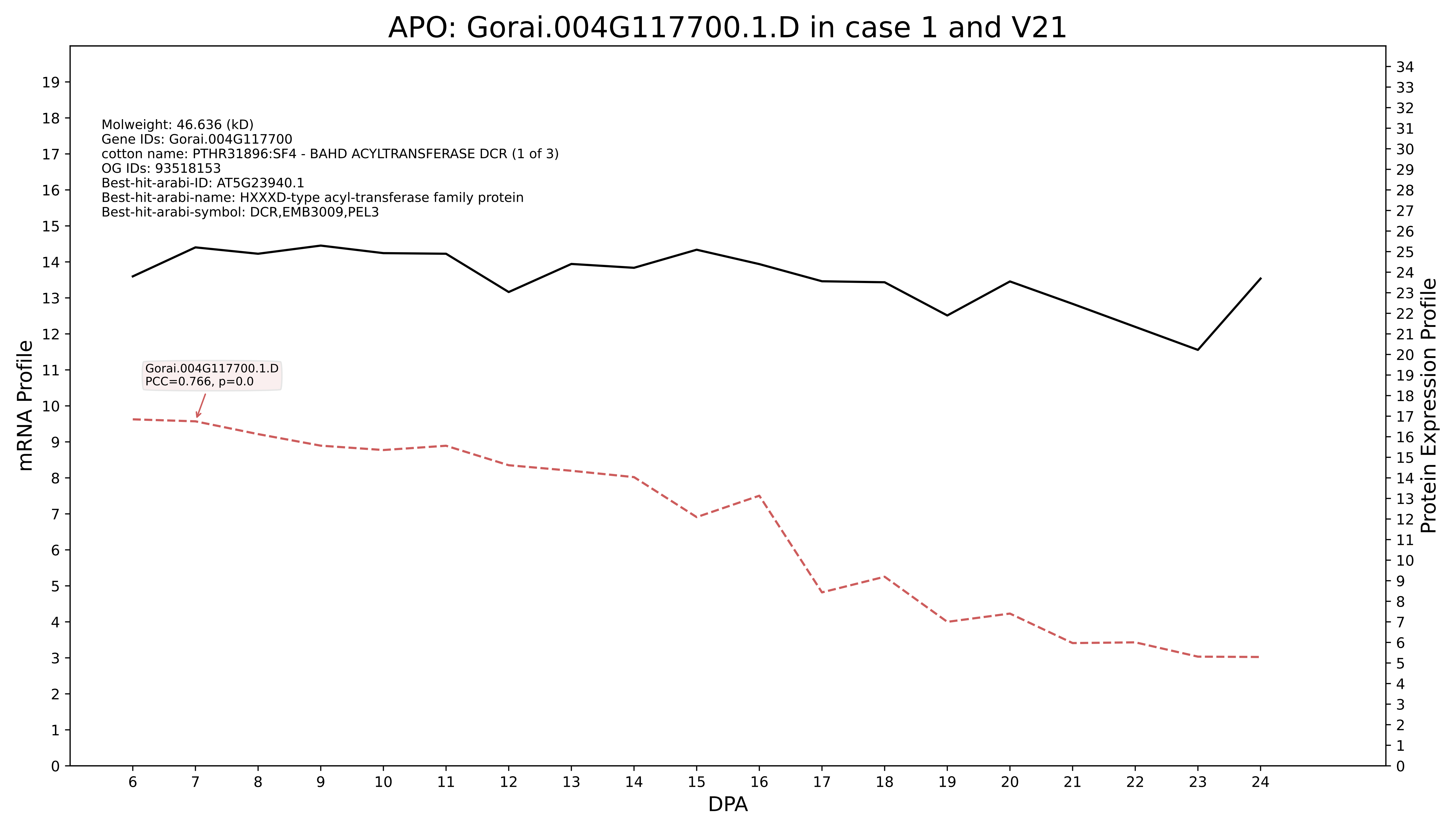

APO: Gorai.004G117700.1.D in case 1 and V21
Molweight: 46.636 (kD)
Gene IDs: Gorai.004G117700
cotton name: PTHR31896:SF4 - BAHD ACYLTRANSFERASE DCR (1 of 3)
OG IDs: 93518153
Best-hit-arabi-ID: AT5G23940.1
Best-hit-arabi-name: HXXXD-type acyl-transferase family protein
Best-hit-arabi-symbol: DCR,EMB3009,PEL3
Gorai.004G117700.1.D
PCC=0.766, p=0.0
mRNA Profile
Protein Expression Profile
DPA

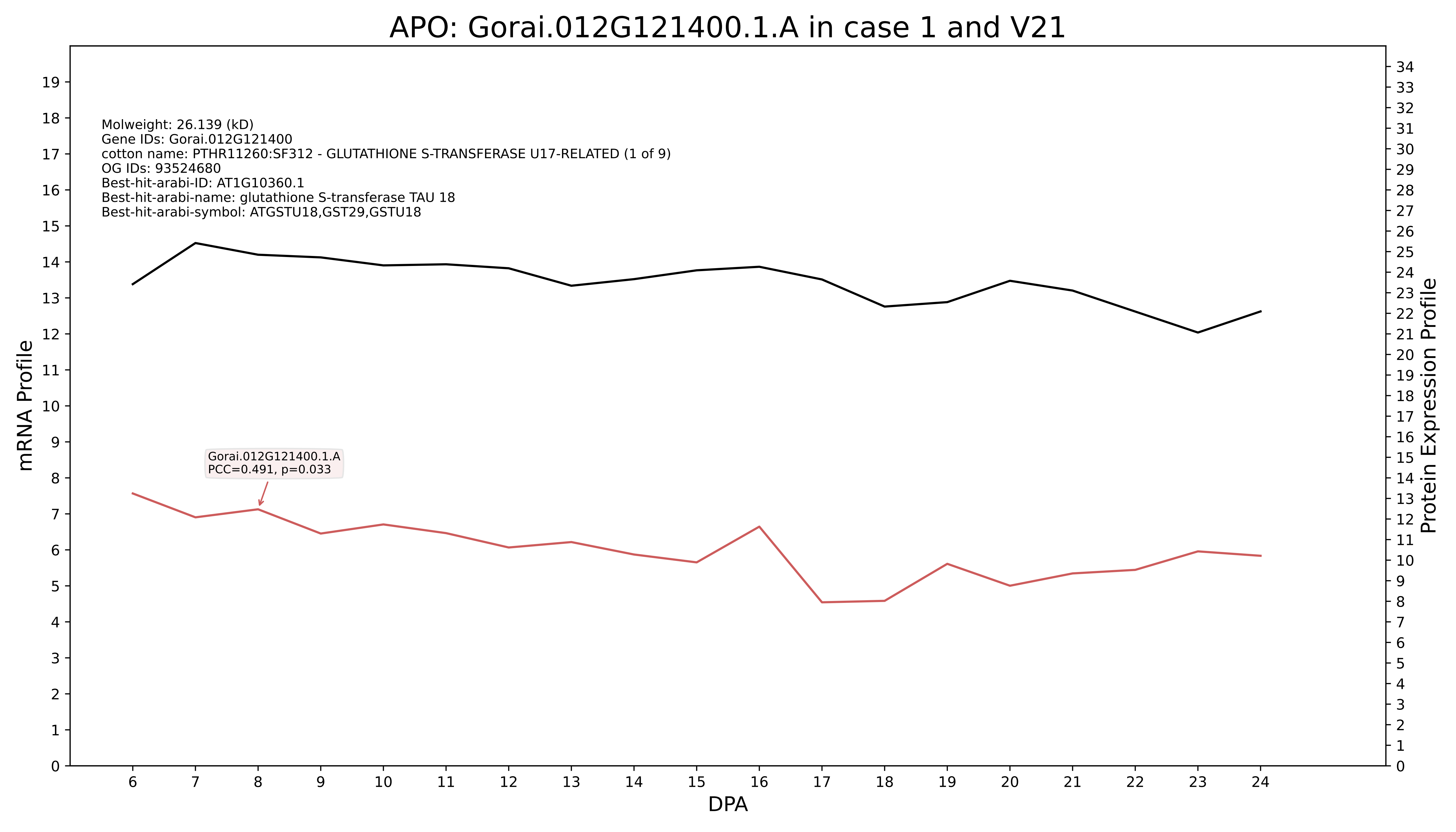

APO: Gorai.012G121400.1.A in case 1 and V21
Molweight: 26.139 (kD)
Gene IDs: Gorai.012G121400
cotton name: PTHR11260:SF312 - GLUTATHIONE S-TRANSFERASE U17-RELATED (1 of 9)
OG IDs: 93524680
Best-hit-arabi-ID: AT1G10360.1
Best-hit-arabi-name: glutathione S-transferase TAU 18
Best-hit-arabi-symbol: ATGSTU18,GST29,GSTU18
Gorai.012G121400.1.A
PCC=0.491, p=0.033
mRNA Profile
Protein Expression Profile
DPA

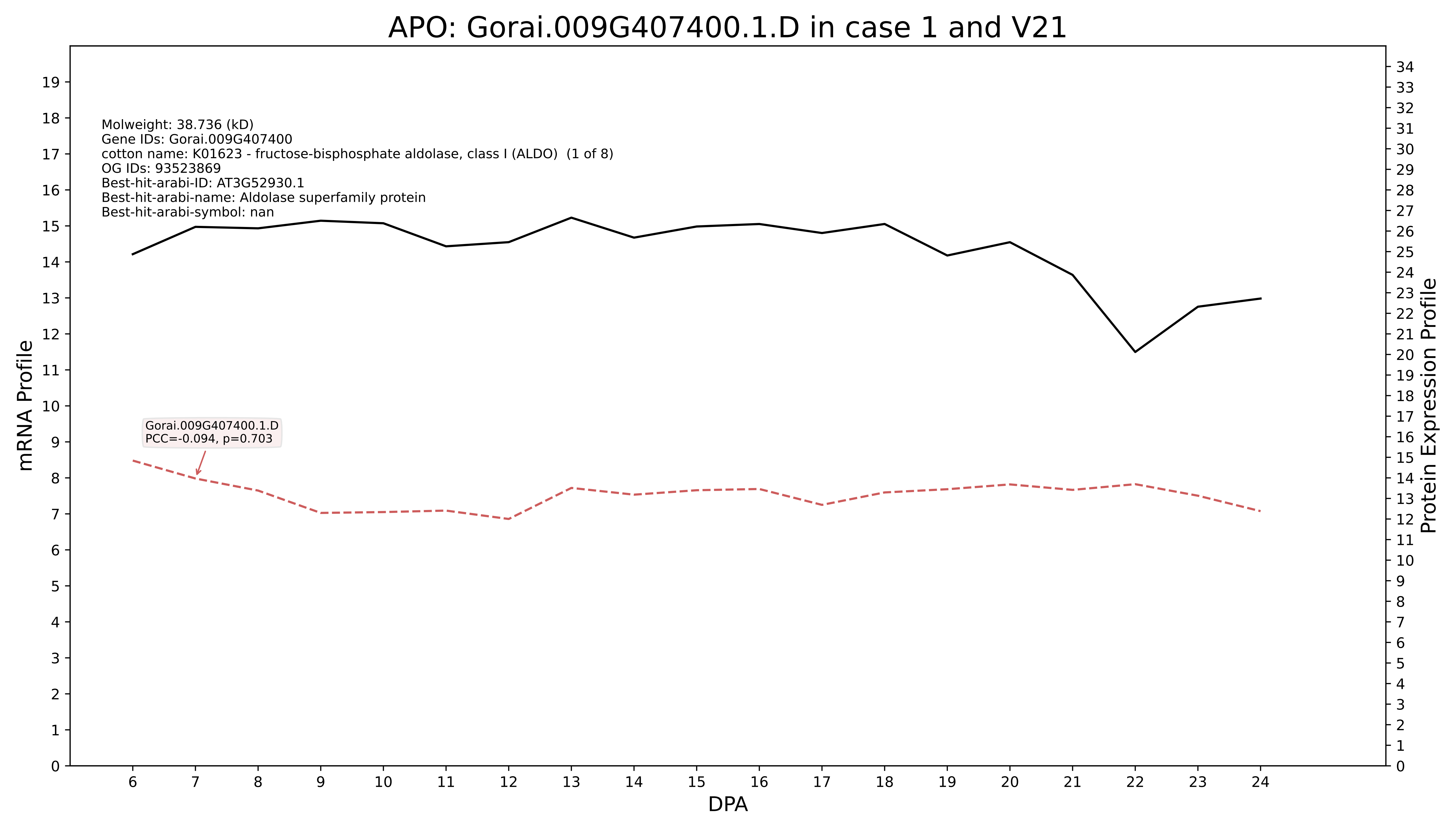
APO: Gorai.009G407400.1.D in case 1 and V21
Molweight: 38.736 (kD)
Gene IDs: Gorai.009G407400
cotton name: K01623 - fructose-bisphosphate aldolase, class I (ALDO) (1 of 8)
OG IDs: 93523869
Best-hit-arabi-ID: AT3G52930.1
Best-hit-arabi-name: Aldolase superfamily protein
Best-hit-arabi-symbol: nan
Gorai.009G407400.1.D
PCC=-0.094, p=0.703
mRNA Profile
Protein Expression Profile
DPA

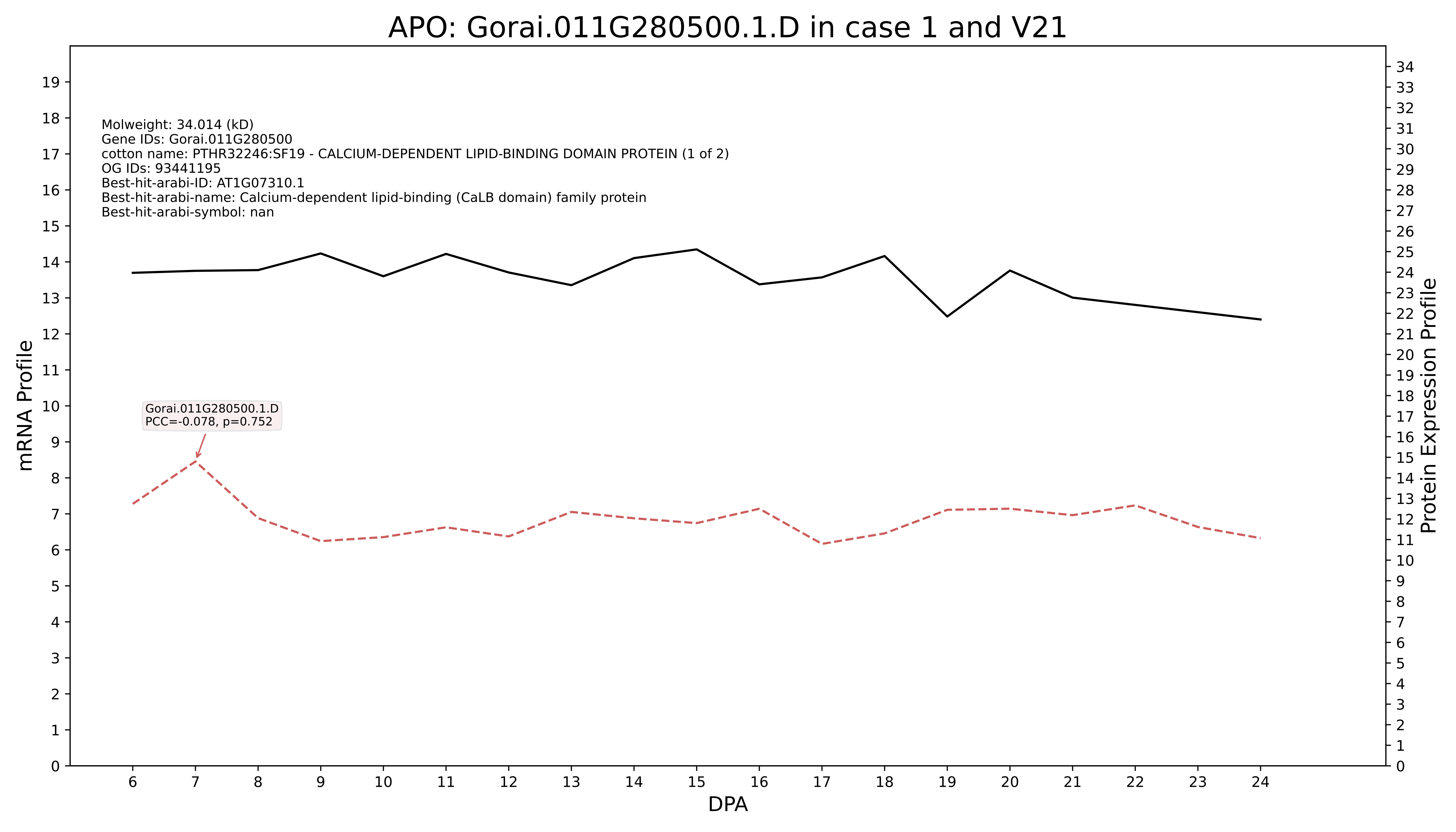

APO: Gorai.011G280500.1.D in case 1 and V21
Molweight: 34.014 (kD)
Gene IDs: Gorai.011G280500
cotton name: PTHR32246:SF19 - CALCIUM-DEPENDENT LIPID-BINDING DOMAIN PROTEIN (1 of 2)
OG IDs: 93441195
Best-hit-arabi-ID: AT1G07310.1
Best-hit-arabi-name: Calcium-dependent lipid-binding (CaLB domain) family protein
Best-hit-arabi-symbol: nan
Gorai.011G280500.1.D
PCC=-0.078, p=0.752
mRNA Profile
Protein Expression Profile
DPA

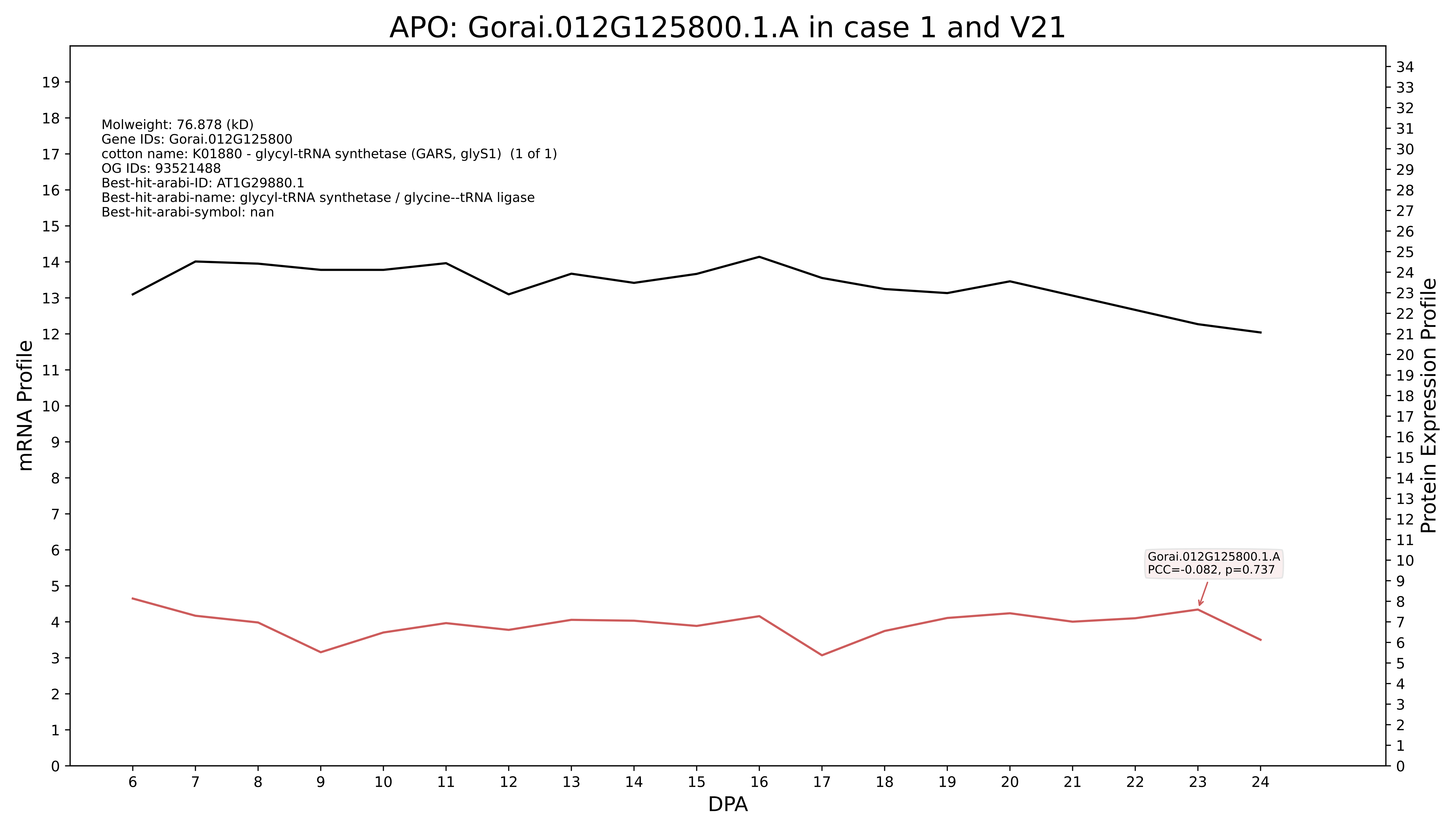
APO: Gorai.012G125800.1.A in case 1 and V21
Molweight: 76.878 (kD)
Gene IDs: Gorai.012G125800
cotton name: K01880 - glycyl-tRNA synthetase (GARS, glyS1) (1 of 1)
OG IDs: 93521488
Best-hit-arabi-ID: AT1G29880.1
Best-hit-arabi-name: glycyl-tRNA synthetase / glycine--tRNA ligase
Best-hit-arabi-symbol: nan
Gorai.012G125800.1.A
PCC=-0.082, p=0.737
mRNA Profile
Protein Expression Profile
DPA
6
7
8
9
10
11
12
13
14
15
16
17
18
19
20
21
22
23
24

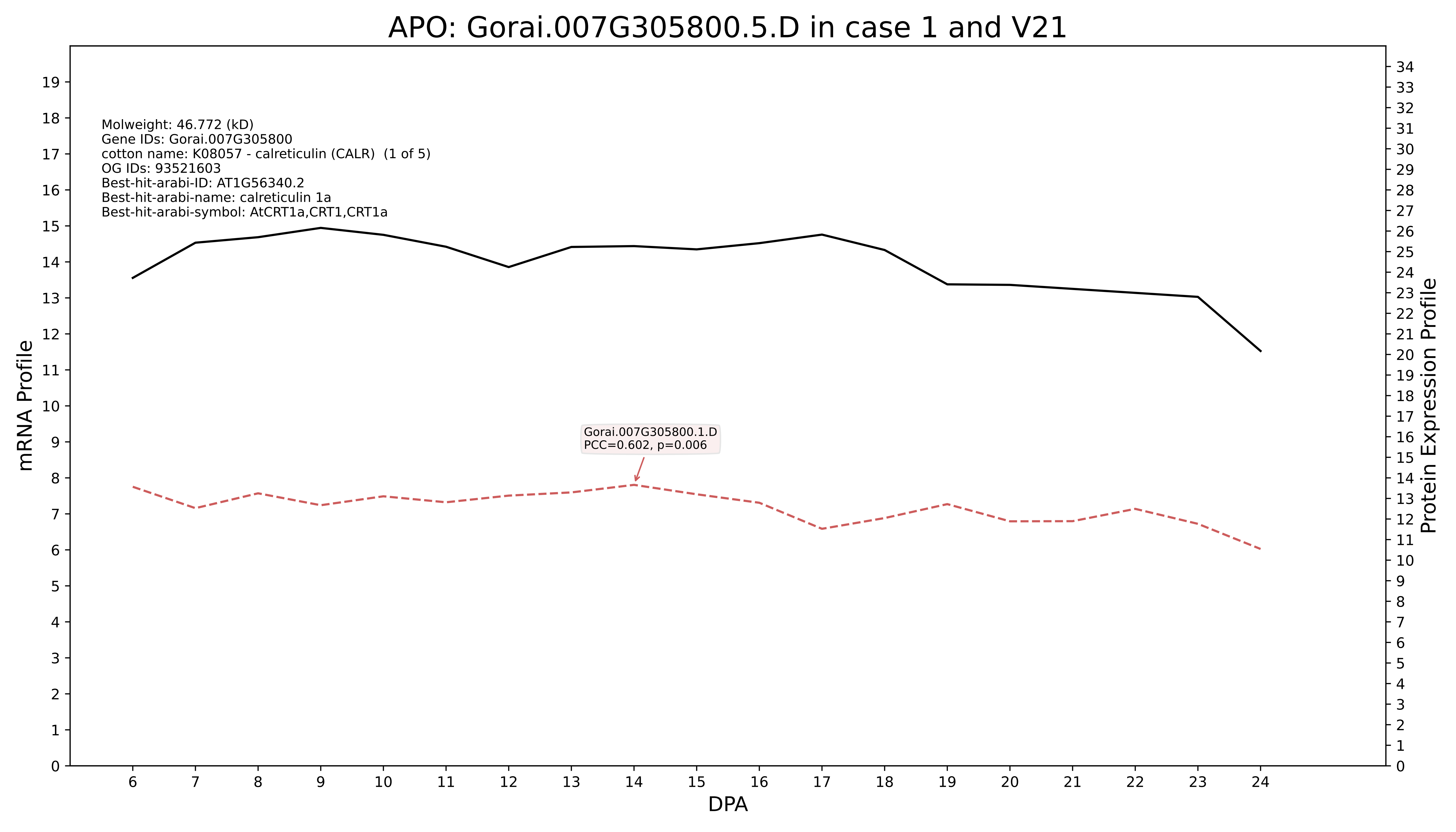

APO: Gorai.007G305800.5.D in case 1 and V21
Molweight: 46.772 (kD)
Gene IDs: Gorai.007G305800
cotton name: K08057 - calreticulin (CALR) (1 of 5)
OG IDs: 93521603
Best-hit-arabi-ID: AT1G56340.2
Best-hit-arabi-name: calreticulin 1a
Best-hit-arabi-symbol: AtCRT1a,CRT1,CRT1a
Gorai.007G305800.1.D
PCC=0.602, p=0.006
mRNA Profile
Protein Expression Profile
DPA

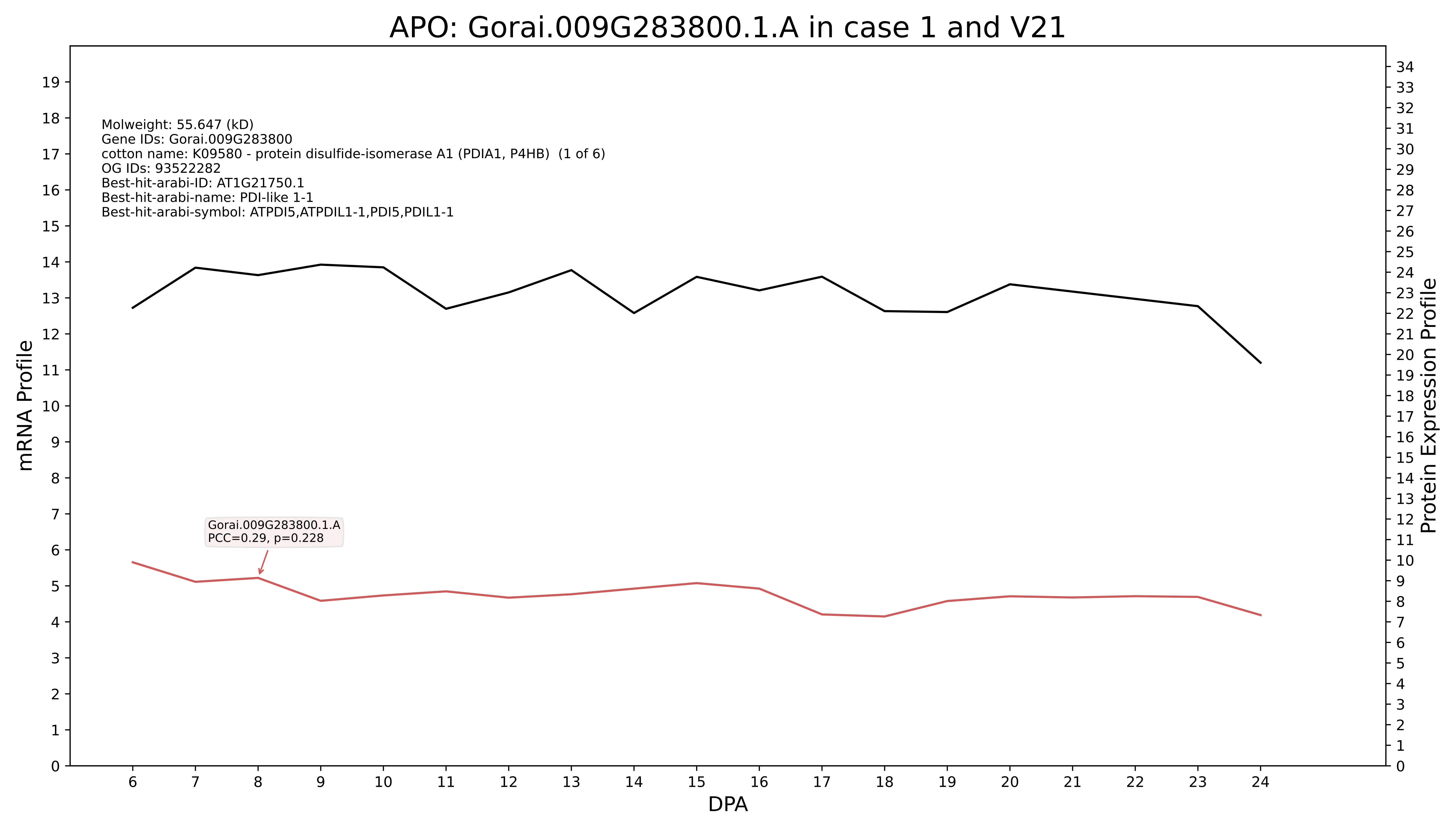
APO: Gorai.009G283800.1.A in case 1 and V21
Molweight: 55.647 (kD)
Gene IDs: Gorai.009G283800
cotton name: K09580 - protein disulfide-isomerase A1 (PDIA1, P4HB) (1 of 6)
OG IDs: 93522282
Best-hit-arabi-ID: AT1G21750.1
Best-hit-arabi-name: PDI-like 1-1
Best-hit-arabi-symbol: ATPDI5,ATPDIL1-1,PDI5,PDIL1-1
Gorai.009G283800.1.A
PCC=0.29, p=0.228
mRNA Profile
Protein Expression Profile
DPA

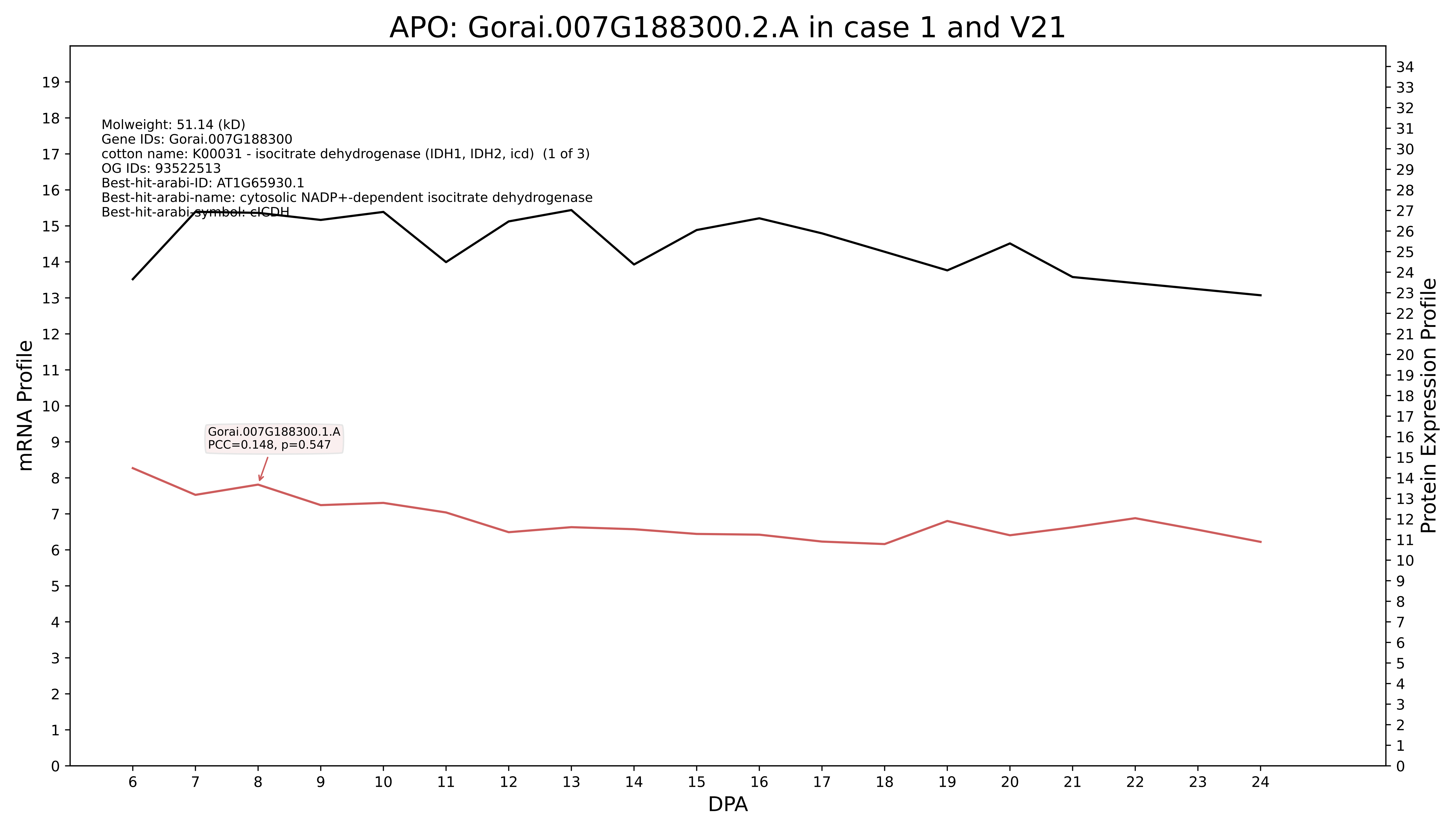
APO: Gorai.007G188300.2.A in case 1 and V21
Molweight: 51.14 (kD)
Gene IDs: Gorai.007G188300
cotton name: K00031 - isocitrate dehydrogenase (IDH1, IDH2, icd) (1 of 3)
OG IDs: 93522513
Best-hit-arabi-ID: AT1G65930.1
Best-hit-arabi-name: cytosolic NADP+-dependent isocitrate dehydrogenase
Best-hit-arabi-symbol: cICDH
Gorai.007G188300.1.A
PCC=0.148, p=0.547
mRNA Profile
Protein Expression Profile
DPA
0
1
2
3
4
5
6
7
8
9
10
11
12
13
14
15
16
17
18
19
20
21
22
23
24
25
26
27
28
29
30
31
32
33
34

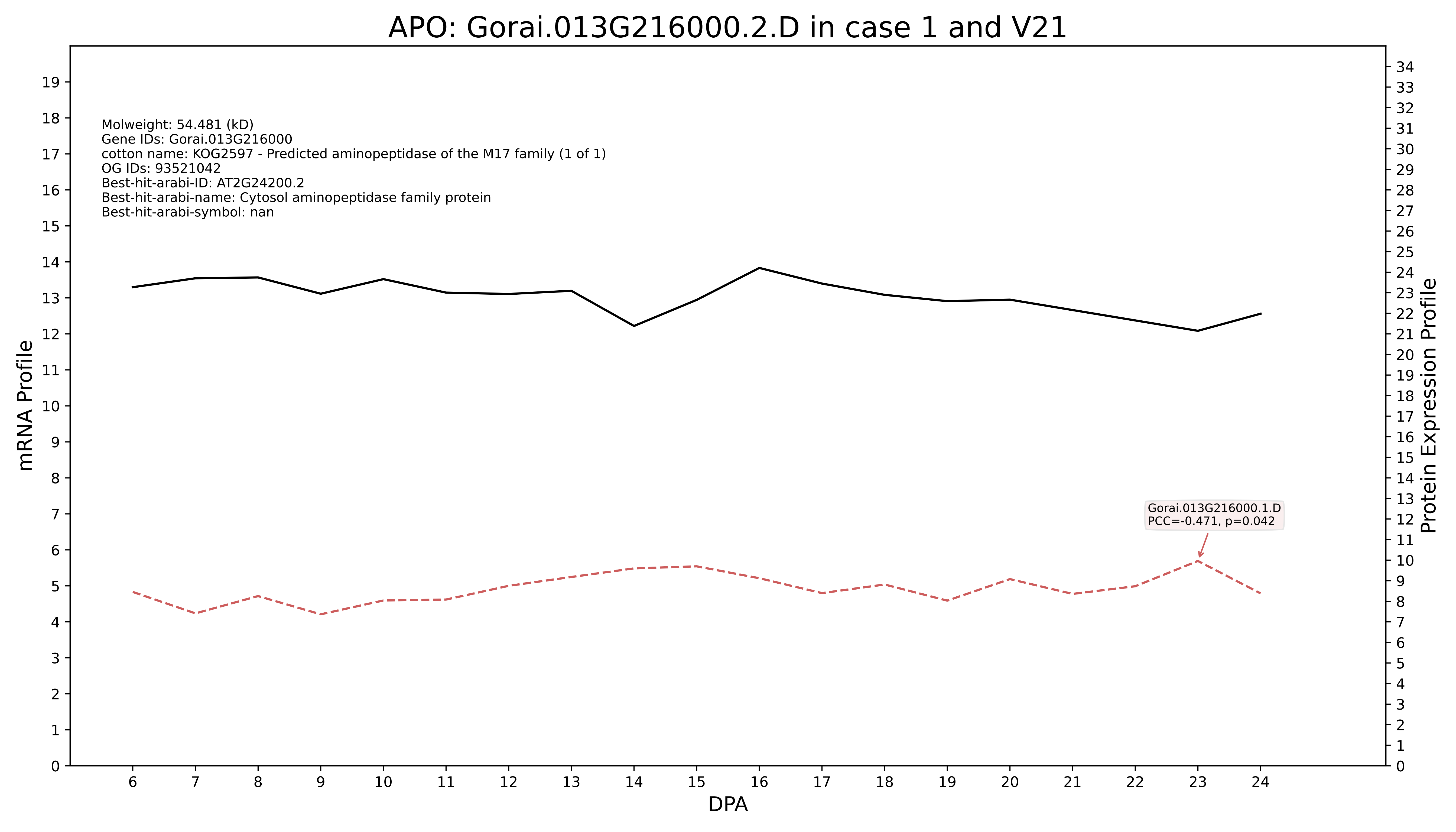
APO: Gorai.013G216000.2.D in case 1 and V21
Molweight: 54.481 (kD)
Gene IDs: Gorai.013G216000
cotton name: KOG2597 - Predicted aminopeptidase of the M17 family (1 of 1)
OG IDs: 93521042
Best-hit-arabi-ID: AT2G24200.2
Best-hit-arabi-name: Cytosol aminopeptidase family protein
Best-hit-arabi-symbol: nan
Gorai.013G216000.1.D
PCC=-0.471, p=0.042
mRNA Profile
Protein Expression Profile
DPA

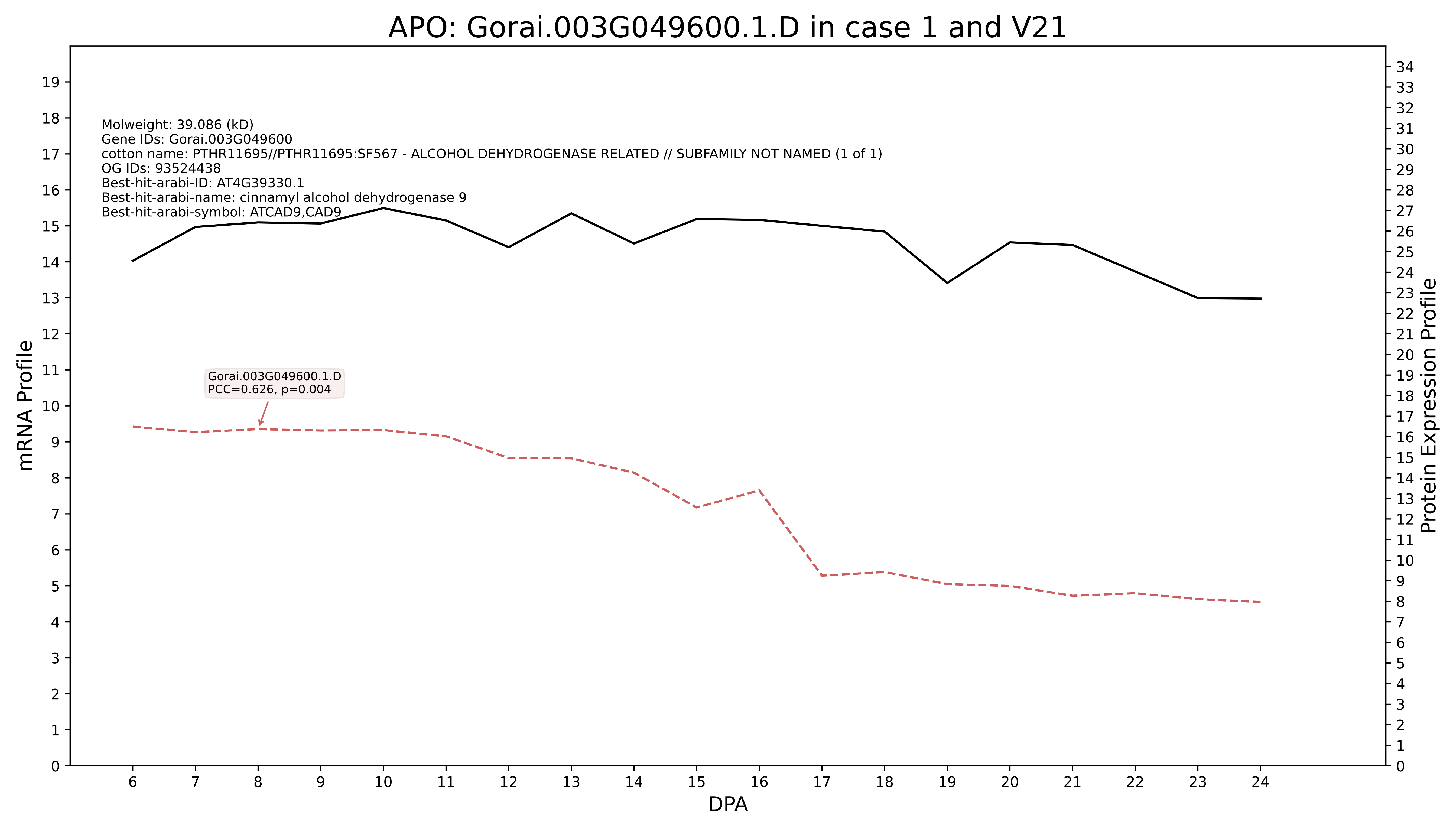

APO: Gorai.003G049600.1.D in case 1 and V21
Molweight: 39.086 (kD)
Gene IDs: Gorai.003G049600
cotton name: PTHR11695//PTHR11695:SF567 - ALCOHOL DEHYDROGENASE RELATED // SUBFAMILY NOT NAMED (1 of 1)
OG IDs: 93524438
Best-hit-arabi-ID: AT4G39330.1
Best-hit-arabi-name: cinnamyl alcohol dehydrogenase 9
Best-hit-arabi-symbol: ATCAD9,CAD9
Gorai.003G049600.1.D
PCC=0.626, p=0.004
mRNA Profile
Protein Expression Profile
DPA

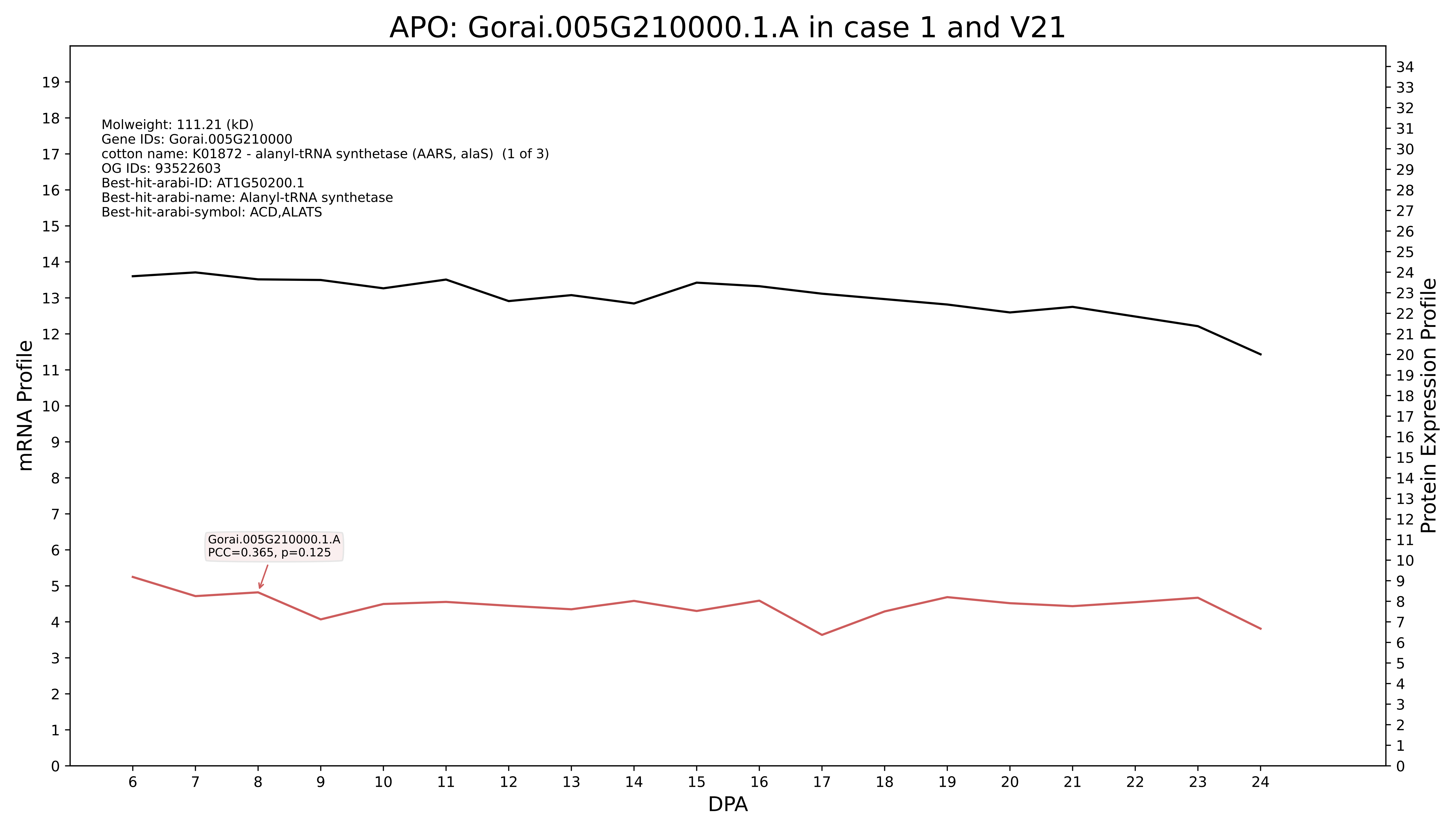

APO: Gorai.005G210000.1.A in case 1 and V21
Molweight: 111.21 (kD)
Gene IDs: Gorai.005G210000
cotton name: K01872 - alanyl-tRNA synthetase (AARS, alaS) (1 of 3)
OG IDs: 93522603
Best-hit-arabi-ID: AT1G50200.1
Best-hit-arabi-name: Alanyl-tRNA synthetase
Best-hit-arabi-symbol: ACD,ALATS
Gorai.005G210000.1.A
PCC=0.365, p=0.125
mRNA Profile
Protein Expression Profile
DPA

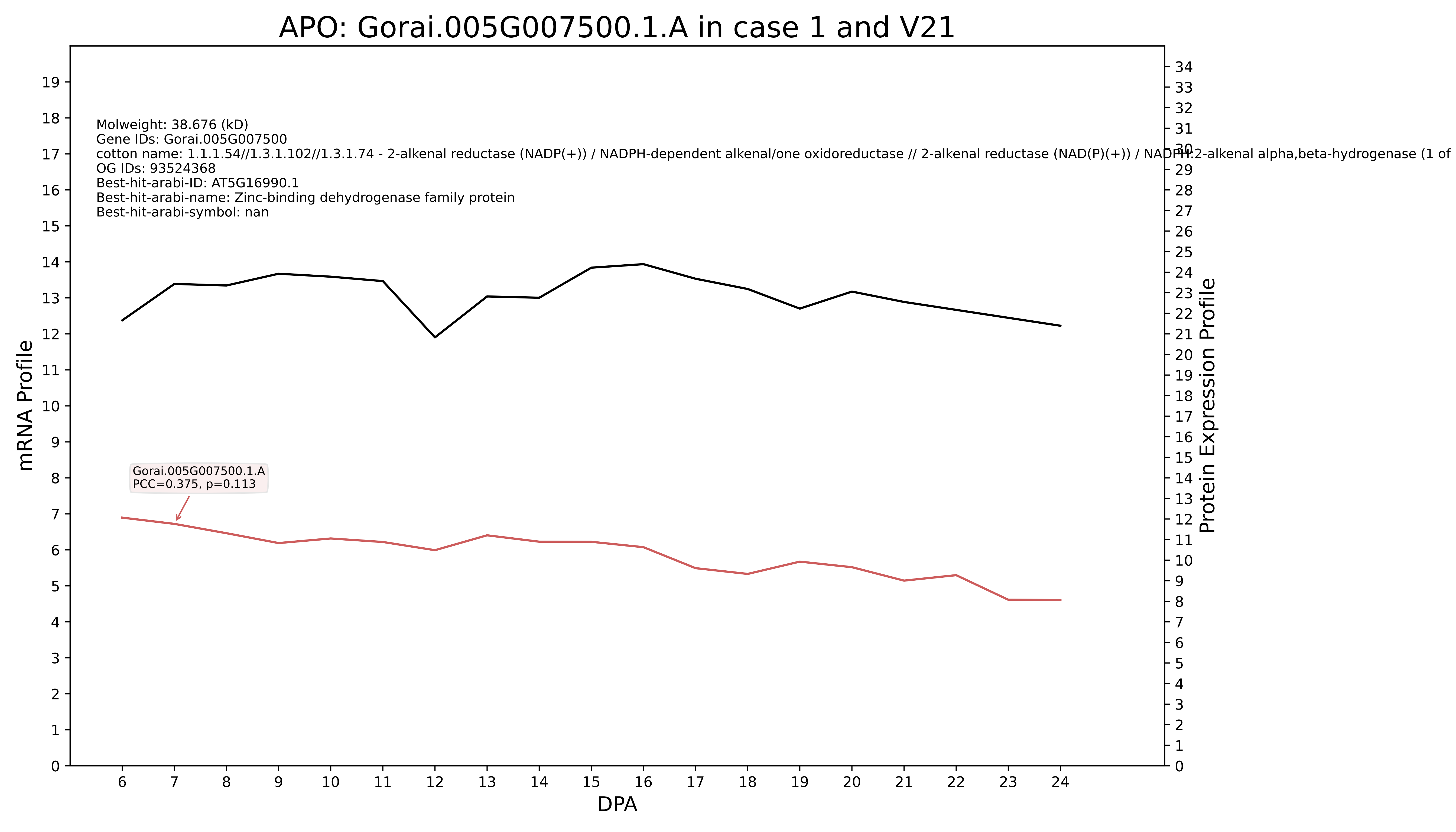
APO: Gorai.005G007500.1.A in case 1 and V21
Molweight: 38.676 (kD)
Gene IDs: Gorai.005G007500
cotton name: 1.1.1.54//1.3.1.102//1.3.1.74 - 2-alkenal reductase (NADP(+)) / NADPH-dependent alkenal/one oxidoreductase // 2-alkenal reductase (NAD(P)(+)) / NADPH:2-alkenal alpha,beta-hydrogenase (1 of
OG IDs: 93524368
Best-hit-arabi-ID: AT5G16990.1
Best-hit-arabi-name: Zinc-binding dehydrogenase family protein
Best-hit-arabi-symbol: nan
Gorai.005G007500.1.A
PCC=0.375, p=0.113
mRNA Profile
Protein Expression Profile
DPA

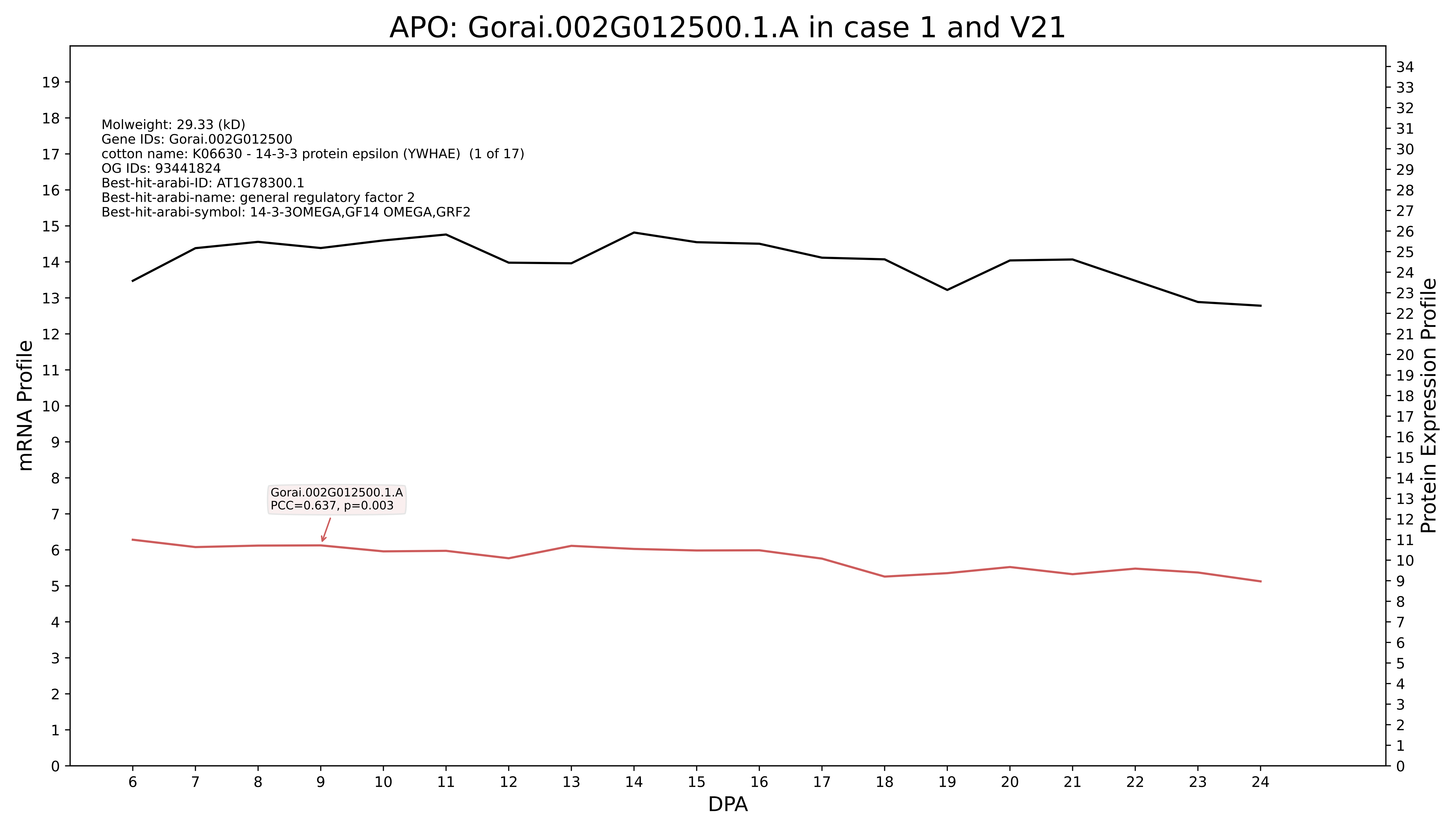

APO: Gorai.002G012500.1.A in case 1 and V21
Molweight: 29.33 (kD)
Gene IDs: Gorai.002G012500
cotton name: K06630 - 14-3-3 protein epsilon (YWHAE) (1 of 17)
OG IDs: 93441824
Best-hit-arabi-ID: AT1G78300.1
Best-hit-arabi-name: general regulatory factor 2
Best-hit-arabi-symbol: 14-3-3OMEGA,GF14 OMEGA,GRF2
Gorai.002G012500.1.A
PCC=0.637, p=0.003
mRNA Profile
Protein Expression Profile
DPA

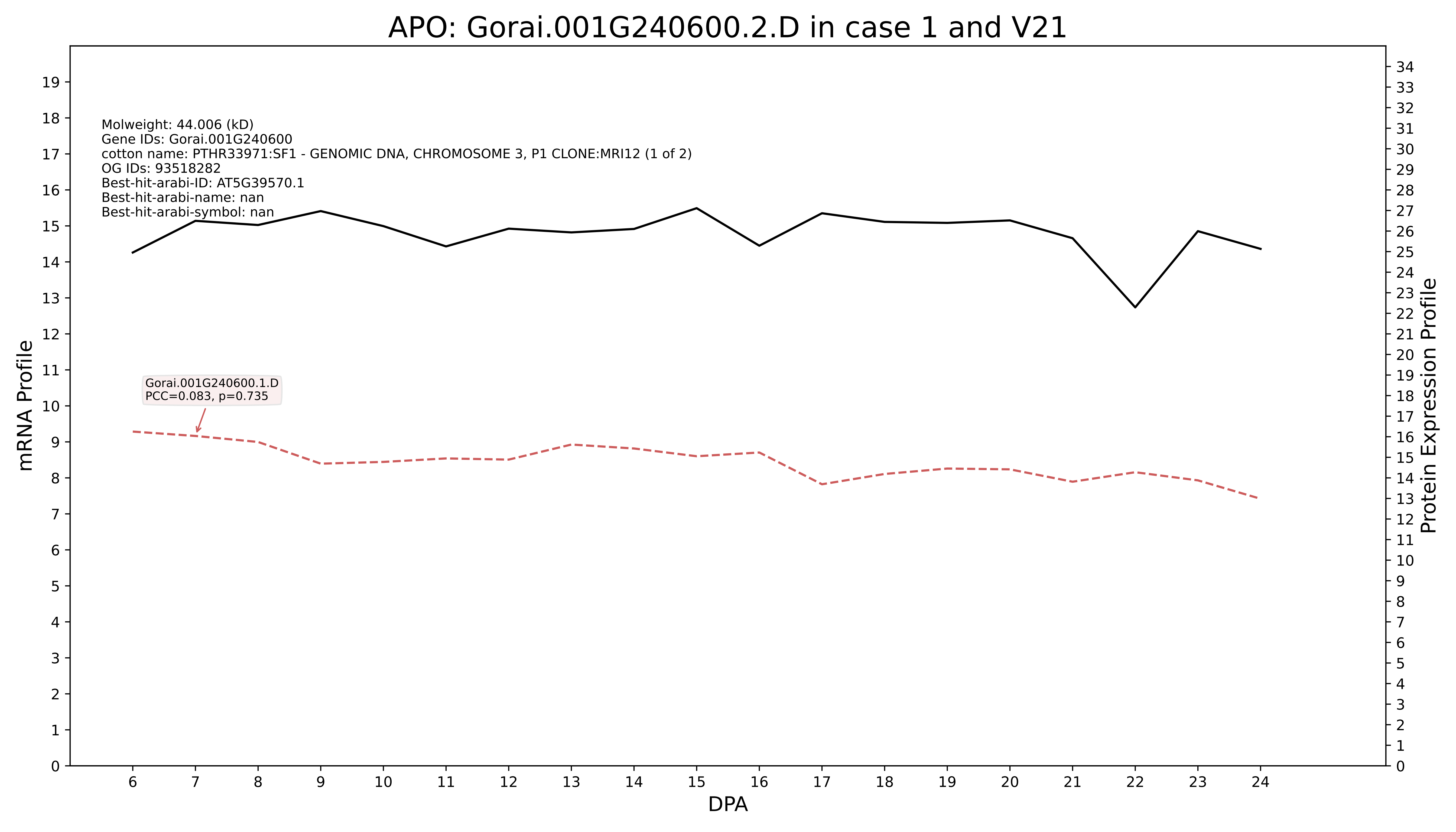

APO: Gorai.001G240600.2.D in case 1 and V21
Molweight: 44.006 (kD)
Gene IDs: Gorai.001G240600
cotton name: PTHR33971:SF1 - GENOMIC DNA, CHROMOSOME 3, P1 CLONE:MRI12 (1 of 2)
OG IDs: 93518282
Best-hit-arabi-ID: AT5G39570.1
Best-hit-arabi-name: nan
Best-hit-arabi-symbol: nan
Gorai.001G240600.1.D
PCC=0.083, p=0.735
mRNA Profile
Protein Expression Profile
DPA

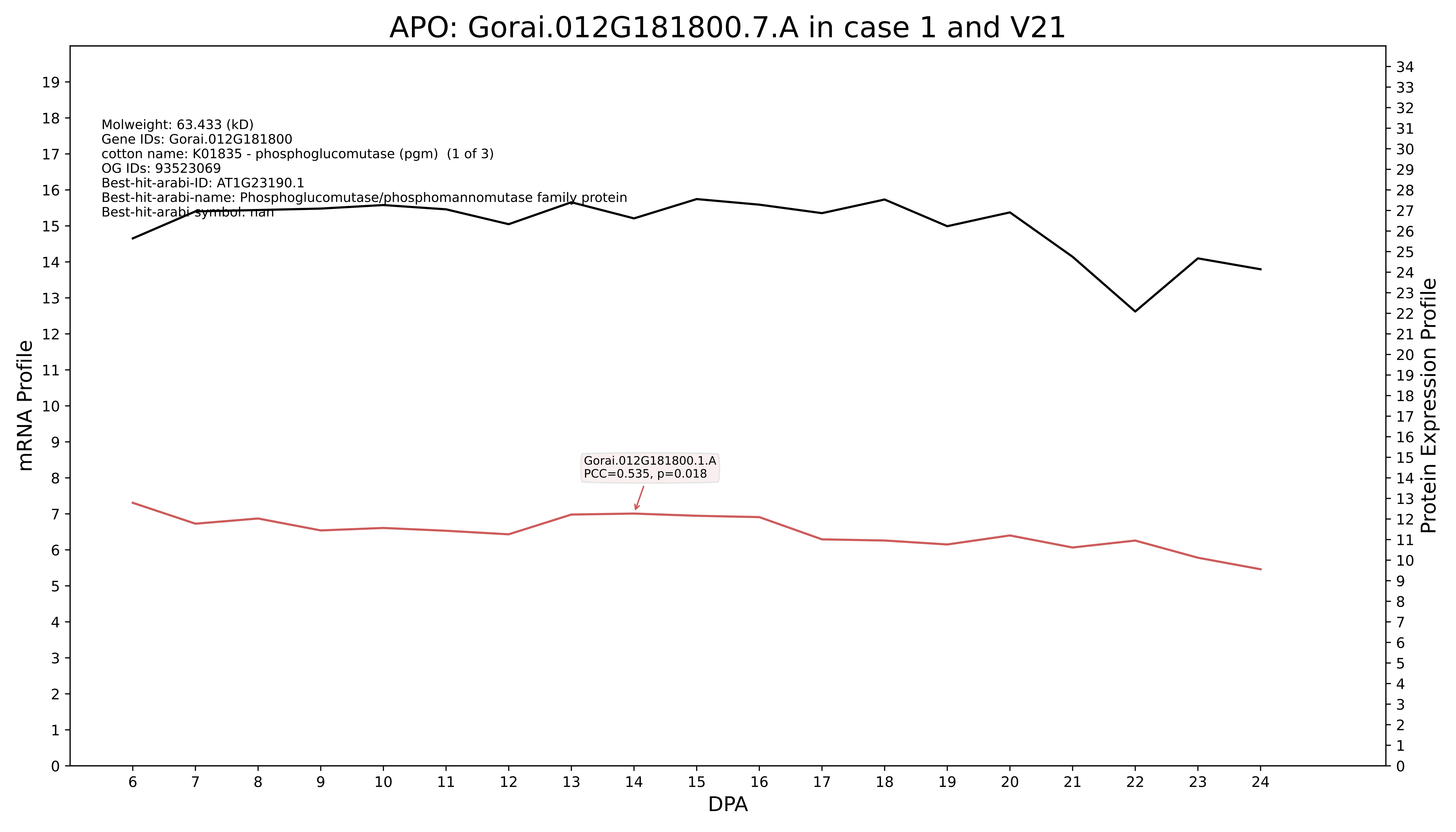
APO: Gorai.012G181800.7.A in case 1 and V21
Molweight: 63.433 (kD)
Gene IDs: Gorai.012G181800
cotton name: K01835 - phosphoglucomutase (pgm) (1 of 3)
OG IDs: 93523069
Best-hit-arabi-ID: AT1G23190.1
Best-hit-arabi-name: Phosphoglucomutase/phosphomannomutase family protein
Best-hit-arabi-symbol: nan
Gorai.012G181800.1.A
PCC=0.535, p=0.018
mRNA Profile
Protein Expression Profile
DPA

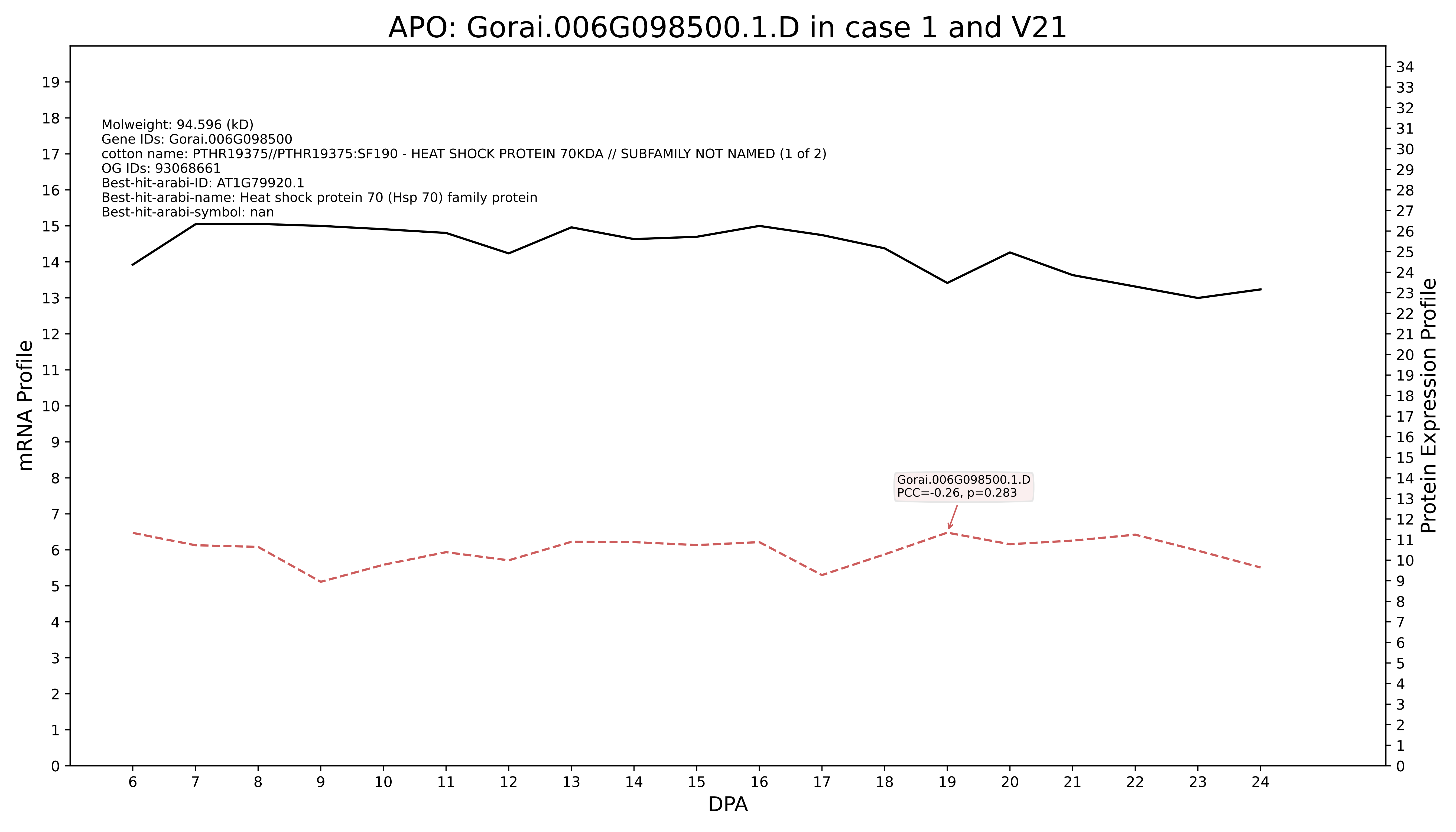
APO: Gorai.006G098500.1.D in case 1 and V21
Molweight: 94.596 (kD)
Gene IDs: Gorai.006G098500
cotton name: PTHR19375//PTHR19375:SF190 - HEAT SHOCK PROTEIN 70KDA // SUBFAMILY NOT NAMED (1 of 2)
OG IDs: 93068661
Best-hit-arabi-ID: AT1G79920.1
Best-hit-arabi-name: Heat shock protein 70 (Hsp 70) family protein
Best-hit-arabi-symbol: nan
Gorai.006G098500.1.D
PCC=-0.26, p=0.283
mRNA Profile
Protein Expression Profile
DPA

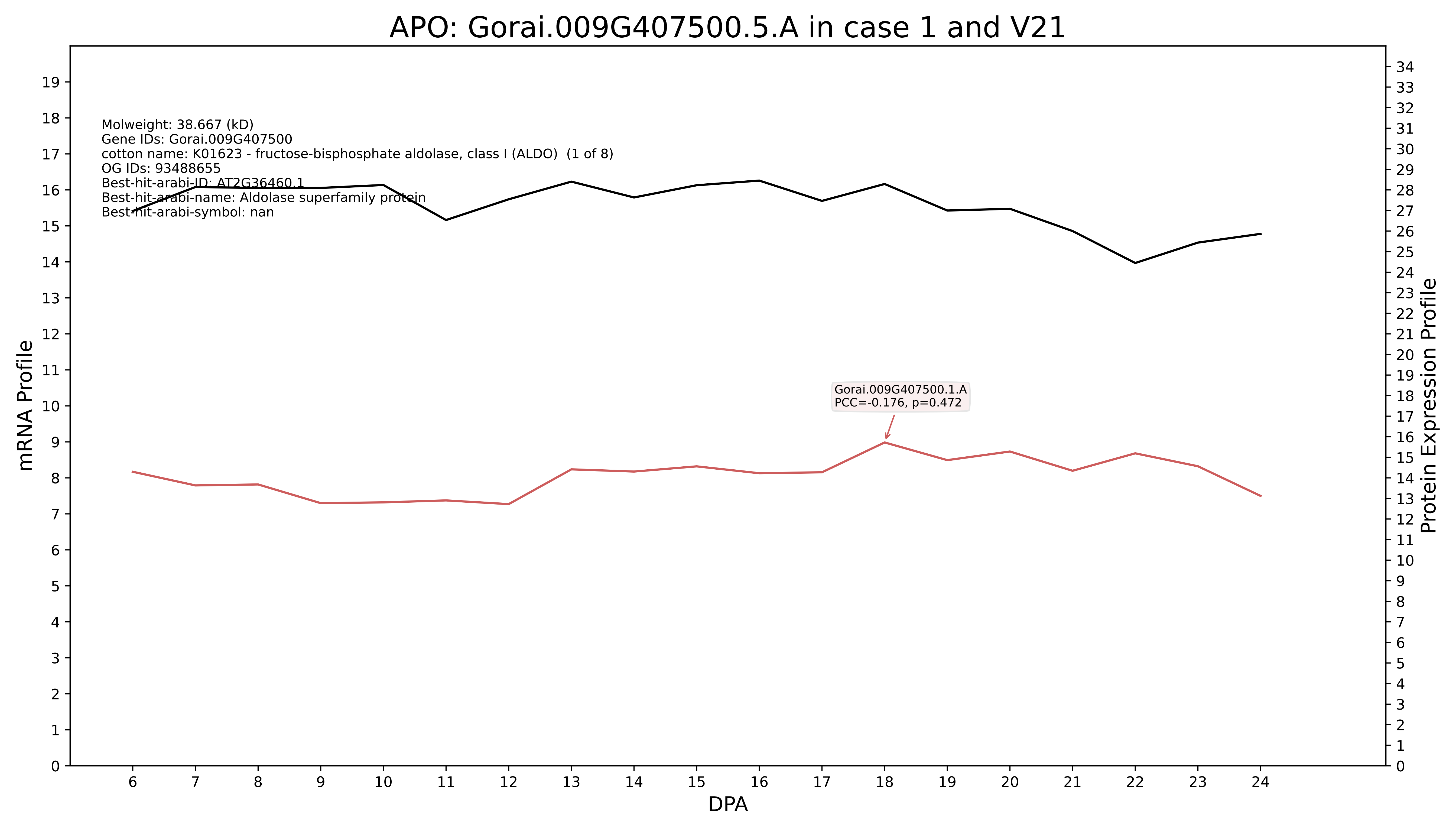
APO: Gorai.009G407500.5.A in case 1 and V21
Molweight: 38.667 (kD)
Gene IDs: Gorai.009G407500
cotton name: K01623 - fructose-bisphosphate aldolase, class I (ALDO) (1 of 8)
OG IDs: 93488655
Best-hit-arabi-ID: AT2G36460.1
Best-hit-arabi-name: Aldolase superfamily protein
Best-hit-arabi-symbol: nan
Gorai.009G407500.1.A
PCC=-0.176, p=0.472
mRNA Profile
Protein Expression Profile
DPA

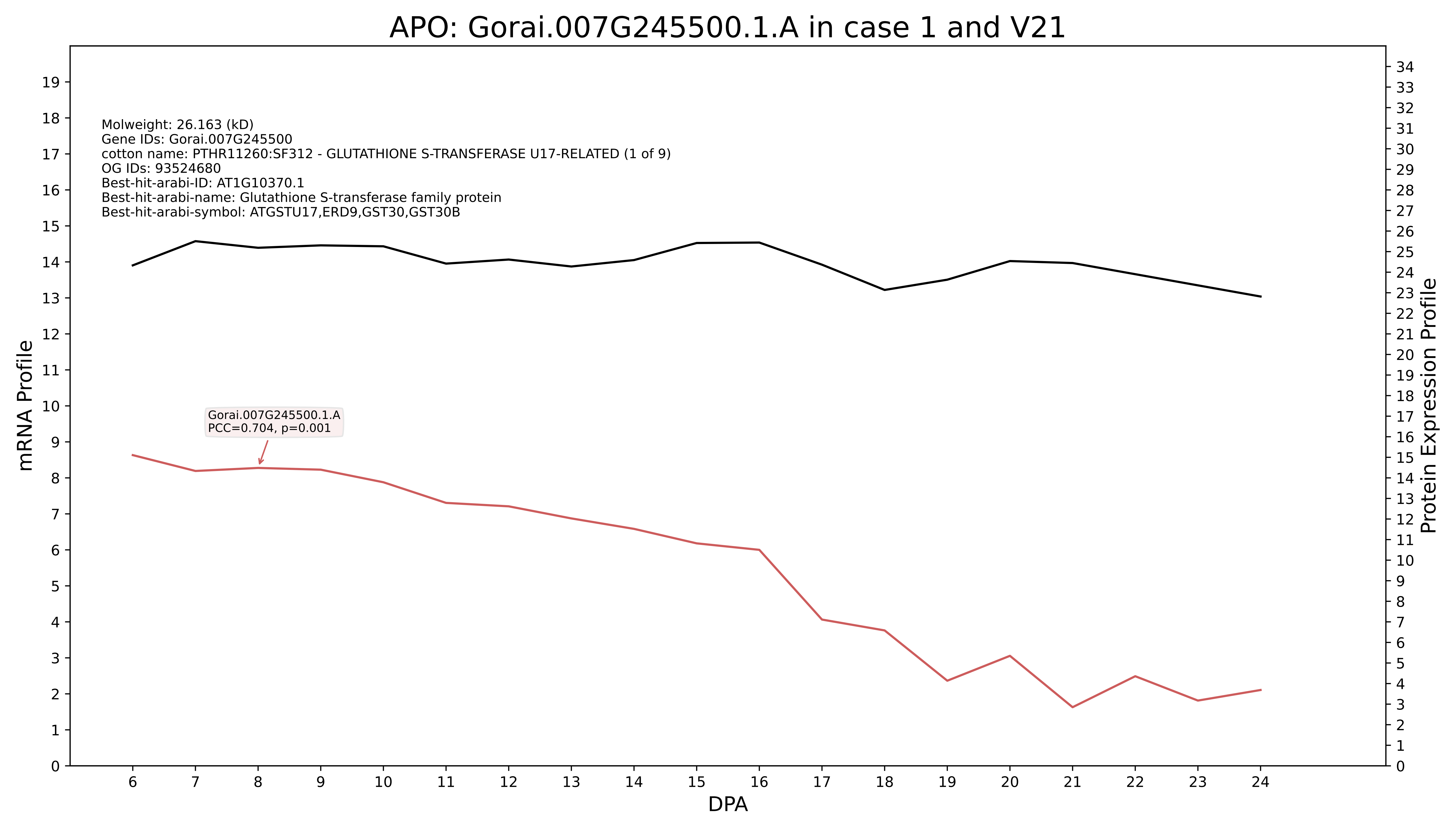

APO: Gorai.007G245500.1.A in case 1 and V21
Molweight: 26.163 (kD)
Gene IDs: Gorai.007G245500
cotton name: PTHR11260:SF312 - GLUTATHIONE S-TRANSFERASE U17-RELATED (1 of 9)
OG IDs: 93524680
Best-hit-arabi-ID: AT1G10370.1
Best-hit-arabi-name: Glutathione S-transferase family protein
Best-hit-arabi-symbol: ATGSTU17,ERD9,GST30,GST30B
Gorai.007G245500.1.A
PCC=0.704, p=0.001
mRNA Profile
Protein Expression Profile
DPA

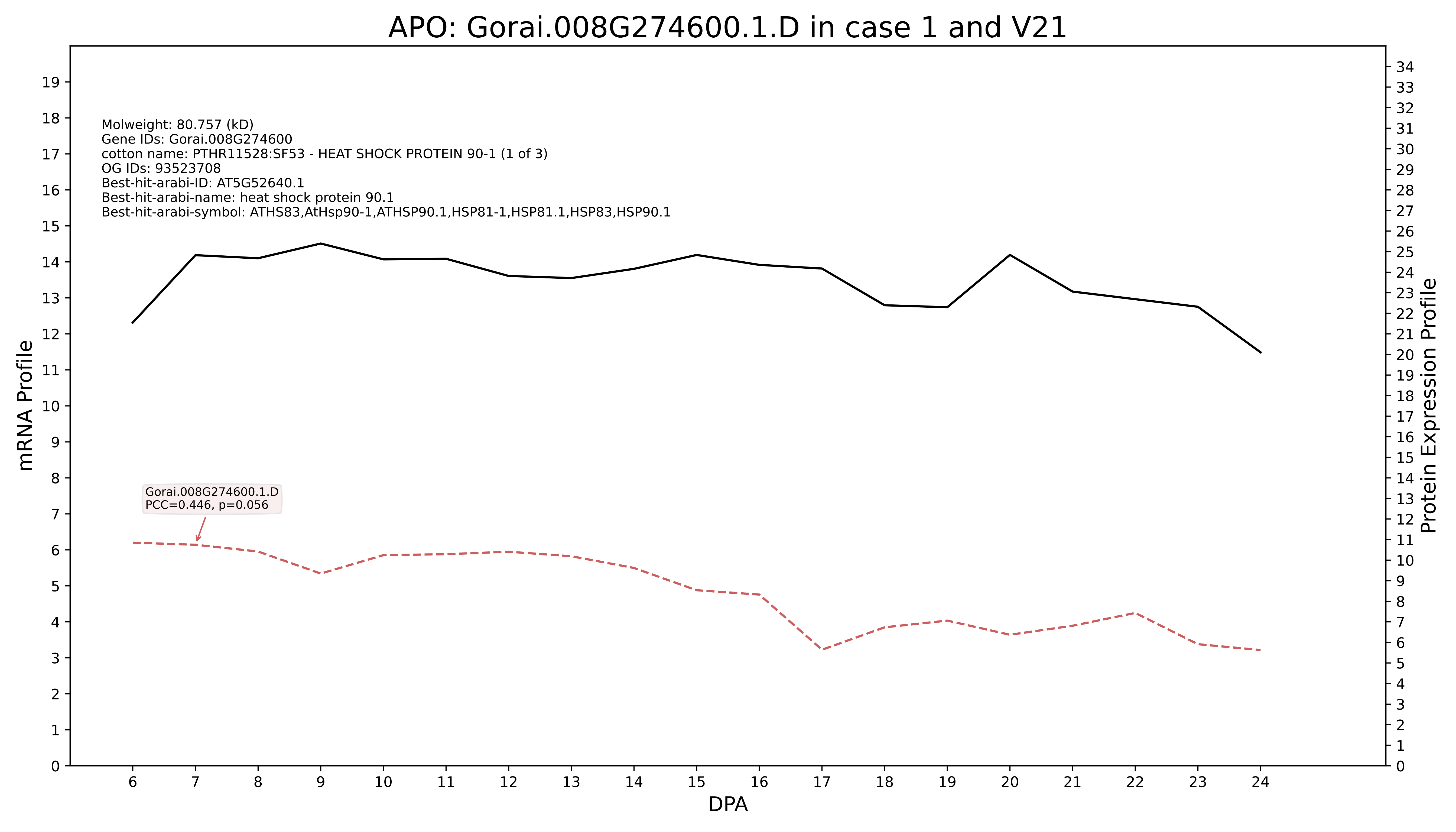
APO: Gorai.008G274600.1.D in case 1 and V21
Molweight: 80.757 (kD)
Gene IDs: Gorai.008G274600
cotton name: PTHR11528:SF53 - HEAT SHOCK PROTEIN 90-1 (1 of 3)
OG IDs: 93523708
Best-hit-arabi-ID: AT5G52640.1
Best-hit-arabi-name: heat shock protein 90.1
Best-hit-arabi-symbol: ATHS83,AtHsp90-1,ATHSP90.1,HSP81-1,HSP81.1,HSP83,HSP90.1
Gorai.008G274600.1.D
PCC=0.446, p=0.056
mRNA Profile
Protein Expression Profile
DPA

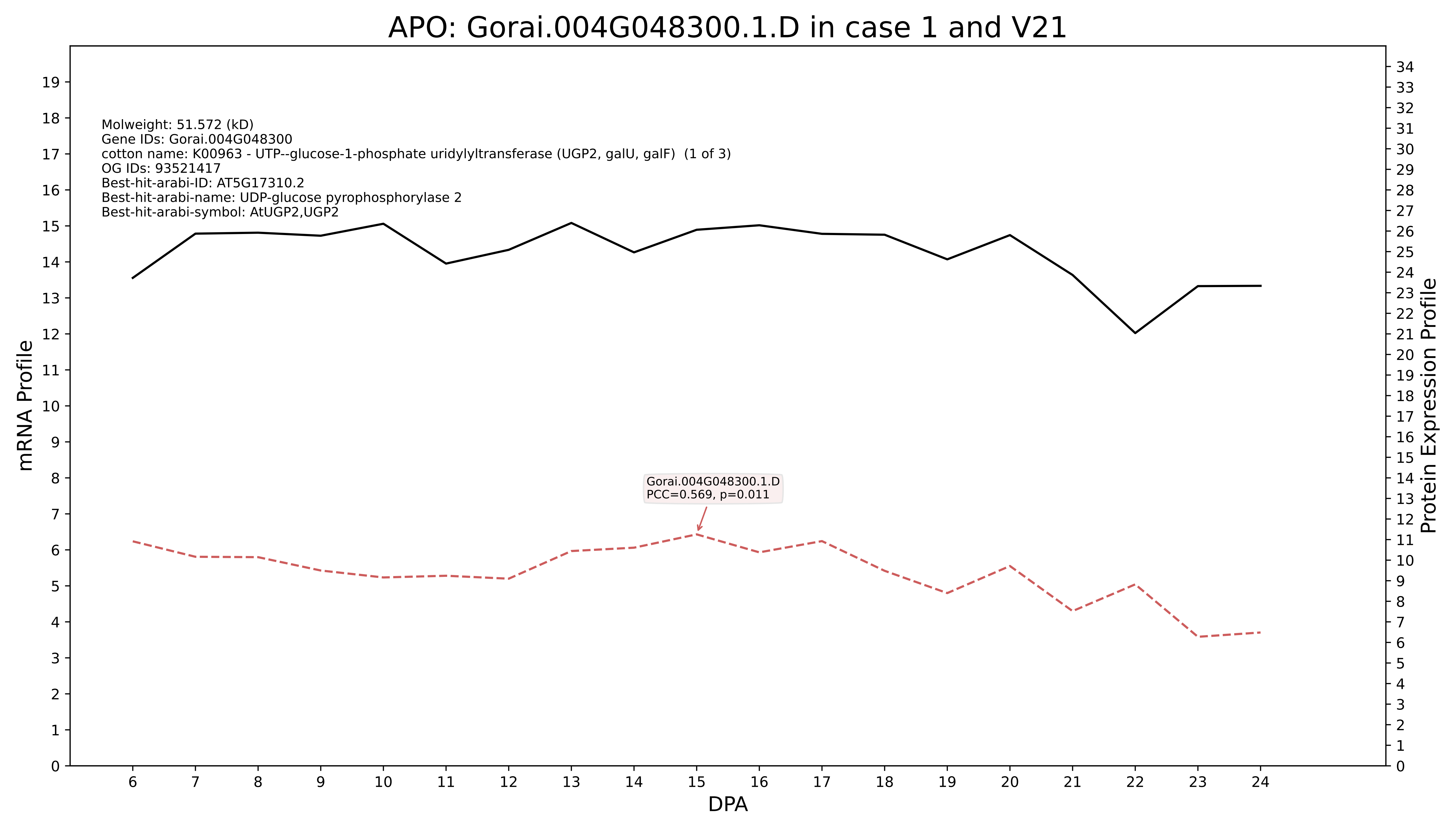

APO: Gorai.004G048300.1.D in case 1 and V21
Molweight: 51.572 (kD)
Gene IDs: Gorai.004G048300
cotton name: K00963 - UTP--glucose-1-phosphate uridylyltransferase (UGP2, galU, galF) (1 of 3)
OG IDs: 93521417
Best-hit-arabi-ID: AT5G17310.2
Best-hit-arabi-name: UDP-glucose pyrophosphorylase 2
Best-hit-arabi-symbol: AtUGP2,UGP2
Gorai.004G048300.1.D
PCC=0.569, p=0.011
mRNA Profile
Protein Expression Profile
DPA

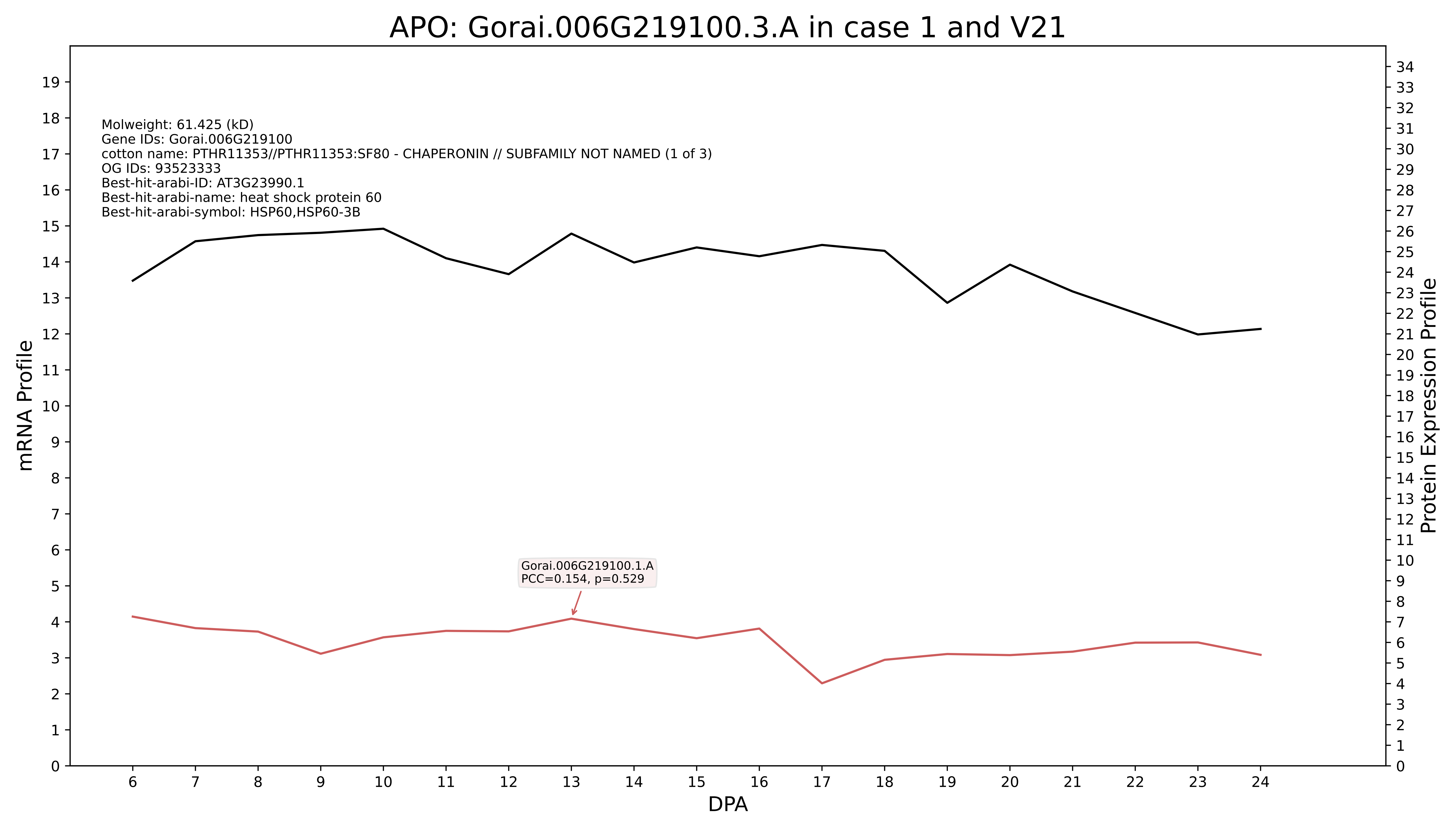

APO: Gorai.006G219100.3.A in case 1 and V21
Molweight: 61.425 (kD)
Gene IDs: Gorai.006G219100
cotton name: PTHR11353//PTHR11353:SF80 - CHAPERONIN // SUBFAMILY NOT NAMED (1 of 3)
OG IDs: 93523333
Best-hit-arabi-ID: AT3G23990.1
Best-hit-arabi-name: heat shock protein 60
Best-hit-arabi-symbol: HSP60,HSP60-3B
Gorai.006G219100.1.A
PCC=0.154, p=0.529
mRNA Profile
Protein Expression Profile
DPA

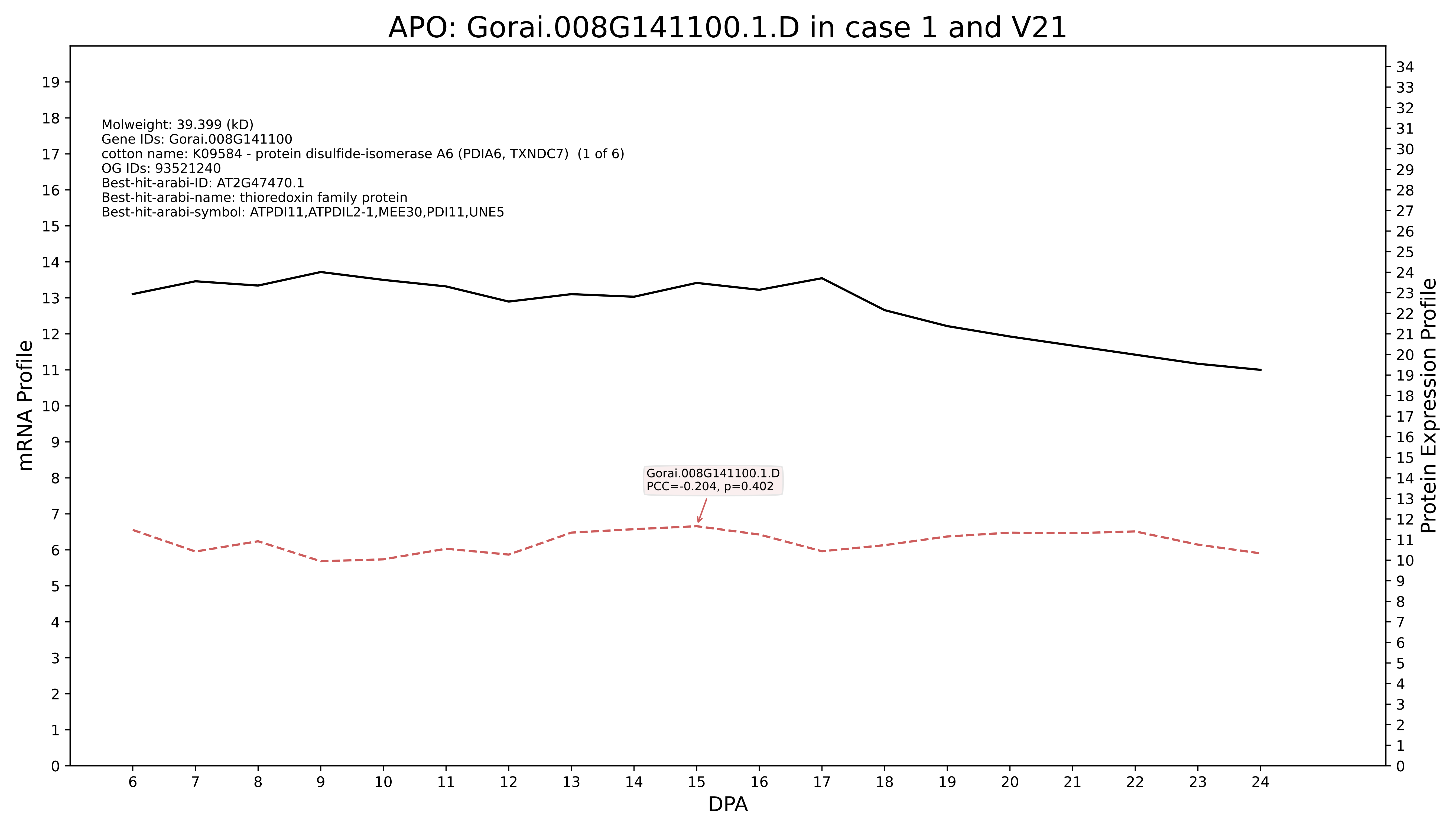
APO: Gorai.008G141100.1.D in case 1 and V21
Molweight: 39.399 (kD)
Gene IDs: Gorai.008G141100
cotton name: K09584 - protein disulfide-isomerase A6 (PDIA6, TXNDC7) (1 of 6)
OG IDs: 93521240
Best-hit-arabi-ID: AT2G47470.1
Best-hit-arabi-name: thioredoxin family protein
Best-hit-arabi-symbol: ATPDI11,ATPDIL2-1,MEE30,PDI11,UNE5
Gorai.008G141100.1.D
PCC=-0.204, p=0.402
mRNA Profile
Protein Expression Profile
DPA

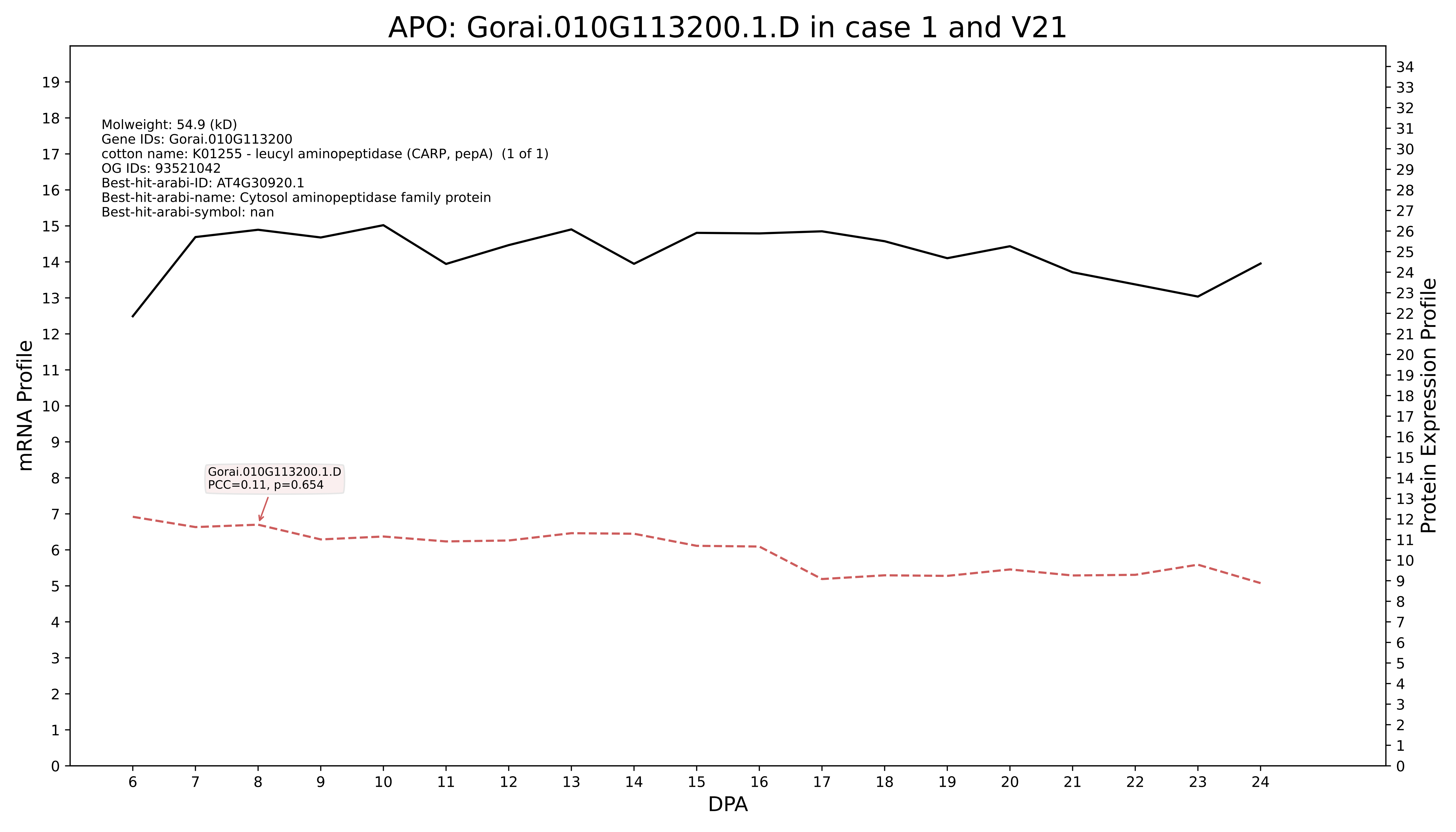
APO: Gorai.010G113200.1.D in case 1 and V21
Molweight: 54.9 (kD)
Gene IDs: Gorai.010G113200
cotton name: K01255 - leucyl aminopeptidase (CARP, pepA) (1 of 1)
OG IDs: 93521042
Best-hit-arabi-ID: AT4G30920.1
Best-hit-arabi-name: Cytosol aminopeptidase family protein
Best-hit-arabi-symbol: nan
Gorai.010G113200.1.D
PCC=0.11, p=0.654
mRNA Profile
Protein Expression Profile
DPA

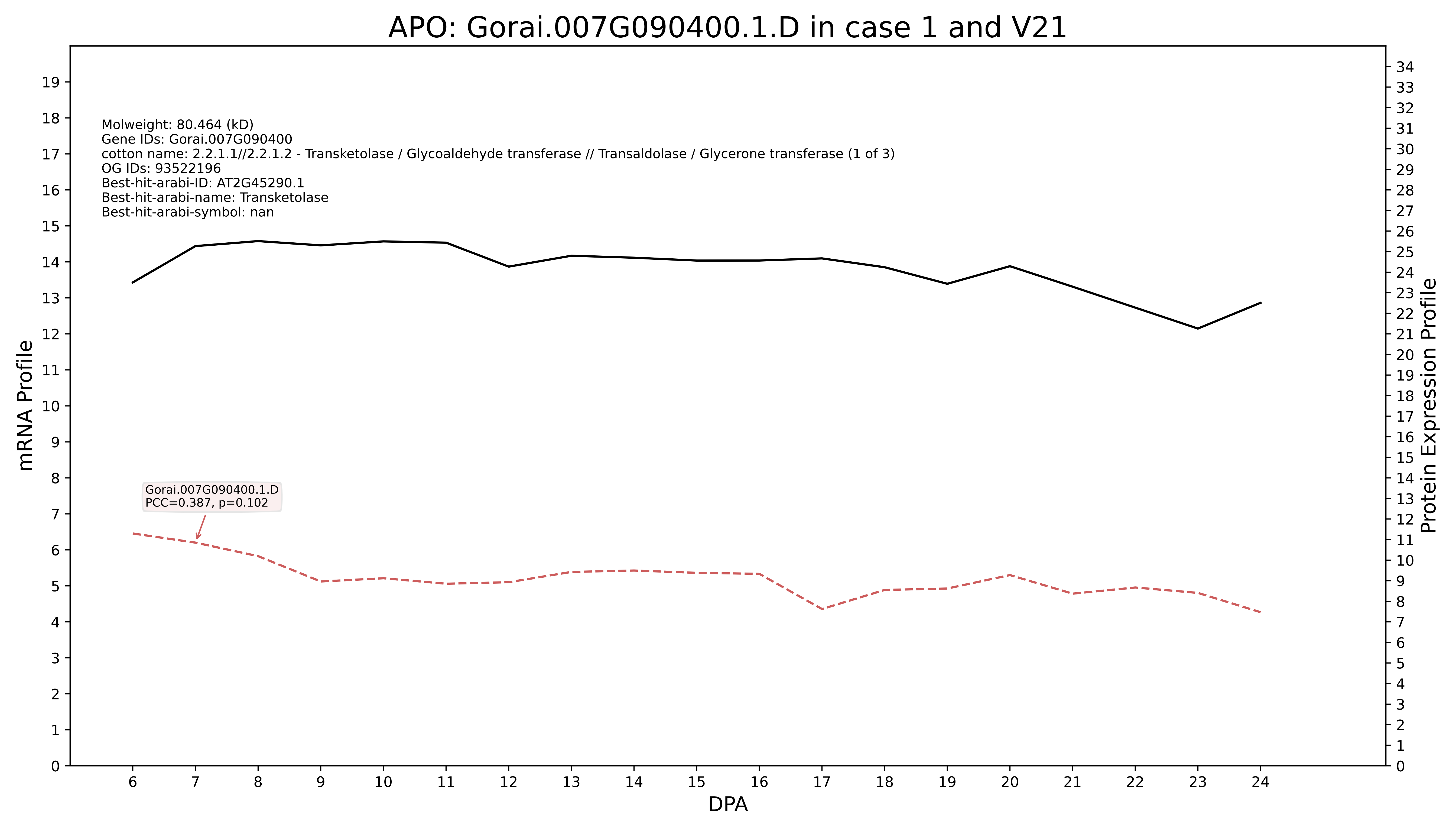

APO: Gorai.007G090400.1.D in case 1 and V21
Molweight: 80.464 (kD)
Gene IDs: Gorai.007G090400
cotton name: 2.2.1.1//2.2.1.2 - Transketolase / Glycoaldehyde transferase // Transaldolase / Glycerone transferase (1 of 3)
OG IDs: 93522196
Best-hit-arabi-ID: AT2G45290.1
Best-hit-arabi-name: Transketolase
Best-hit-arabi-symbol: nan
Gorai.007G090400.1.D
PCC=0.387, p=0.102
mRNA Profile
Protein Expression Profile
DPA

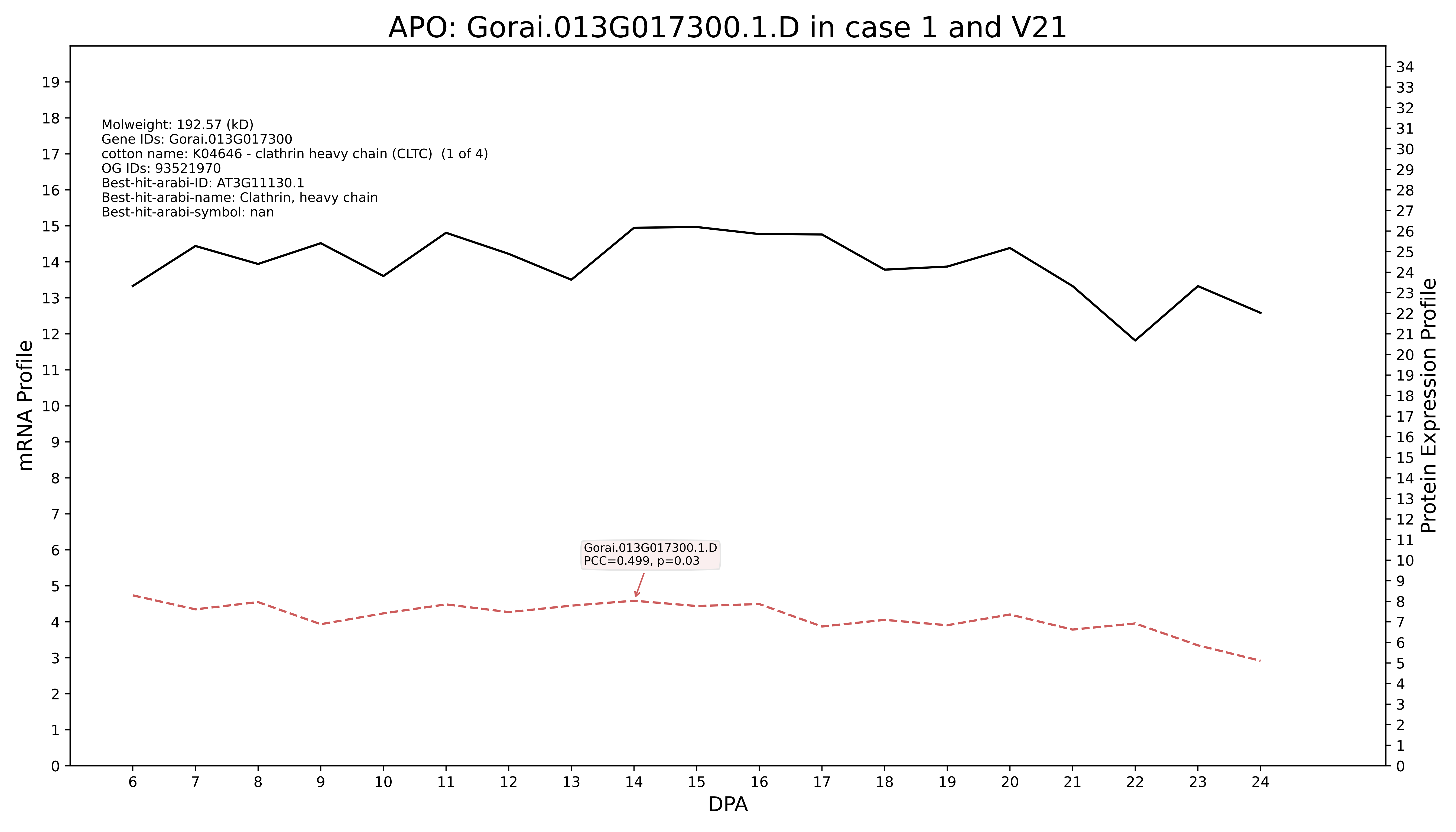
APO: Gorai.013G017300.1.D in case 1 and V21
Molweight: 192.57 (kD)
Gene IDs: Gorai.013G017300
cotton name: K04646 - clathrin heavy chain (CLTC) (1 of 4)
OG IDs: 93521970
Best-hit-arabi-ID: AT3G11130.1
Best-hit-arabi-name: Clathrin, heavy chain
Best-hit-arabi-symbol: nan
Gorai.013G017300.1.D
PCC=0.499, p=0.03
mRNA Profile
Protein Expression Profile
DPA

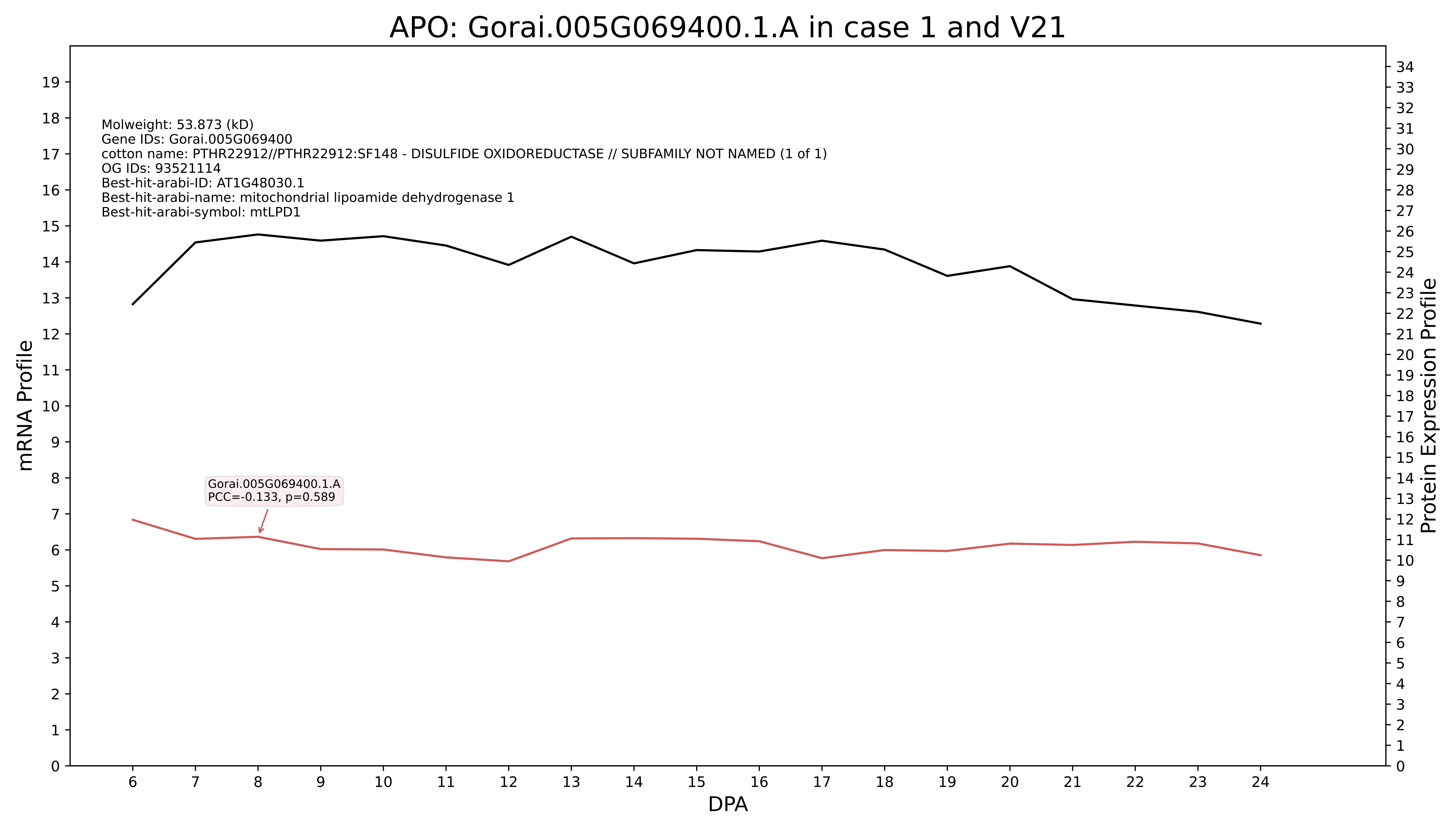

APO: Gorai.005G069400.1.A in case 1 and V21
Molweight: 53.873 (kD)
Gene IDs: Gorai.005G069400
cotton name: PTHR22912//PTHR22912:SF148 - DISULFIDE OXIDOREDUCTASE // SUBFAMILY NOT NAMED (1 of 1)
OG IDs: 93521114
Best-hit-arabi-ID: AT1G48030.1
Best-hit-arabi-name: mitochondrial lipoamide dehydrogenase 1
Best-hit-arabi-symbol: mtLPD1
Gorai.005G069400.1.A
PCC=-0.133, p=0.589
mRNA Profile
Protein Expression Profile
DPA

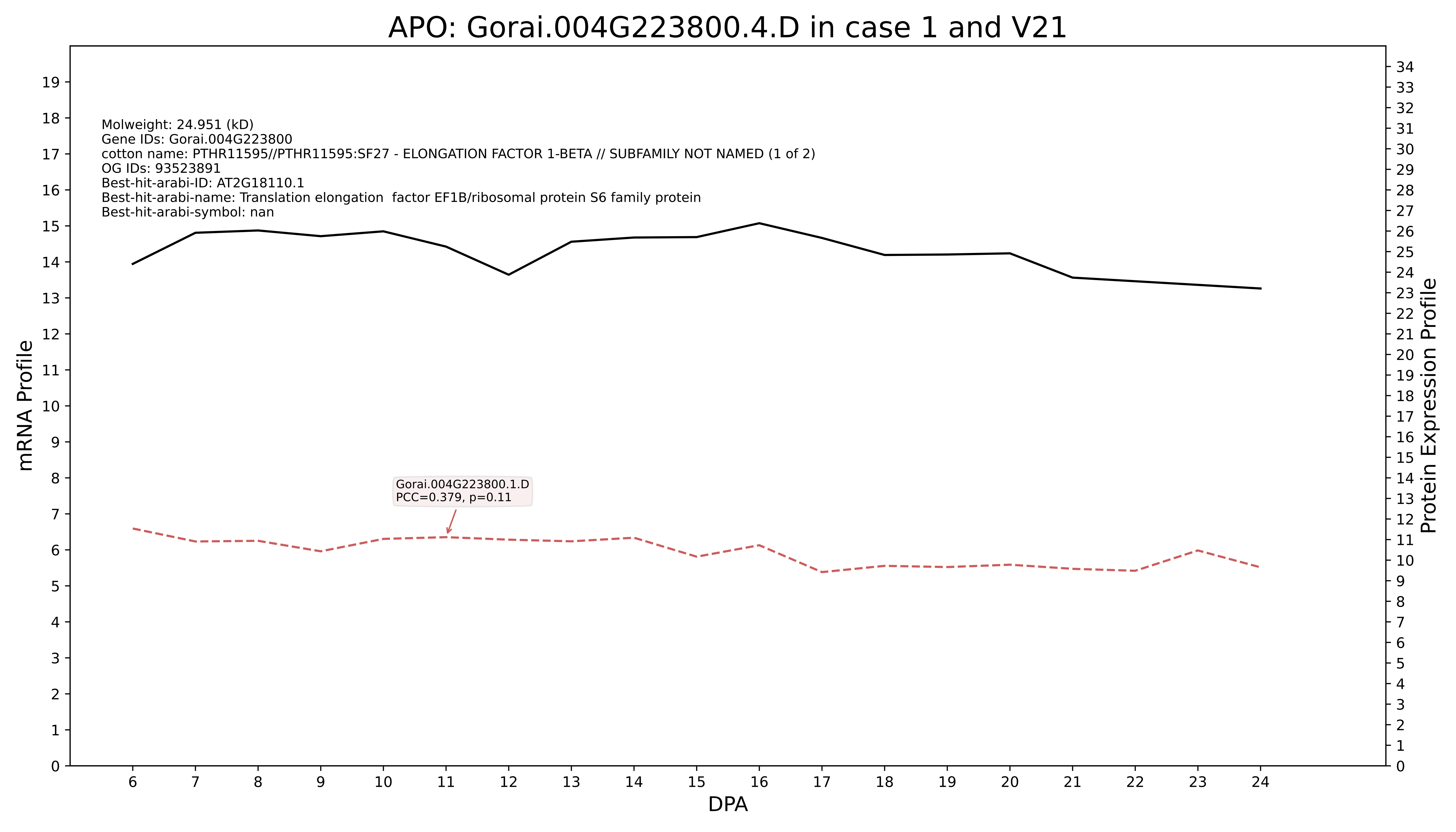

APO: Gorai.004G223800.4.D in case 1 and V21
Molweight: 24.951 (kD)
Gene IDs: Gorai.004G223800
cotton name: PTHR11595//PTHR11595:SF27 - ELONGATION FACTOR 1-BETA // SUBFAMILY NOT NAMED (1 of 2)
OG IDs: 93523891
Best-hit-arabi-ID: AT2G18110.1
Best-hit-arabi-name: Translation elongation factor EF1B/ribosomal protein S6 family protein
Best-hit-arabi-symbol: nan
Gorai.004G223800.1.D
PCC=0.379, p=0.11
mRNA Profile
Protein Expression Profile
DPA

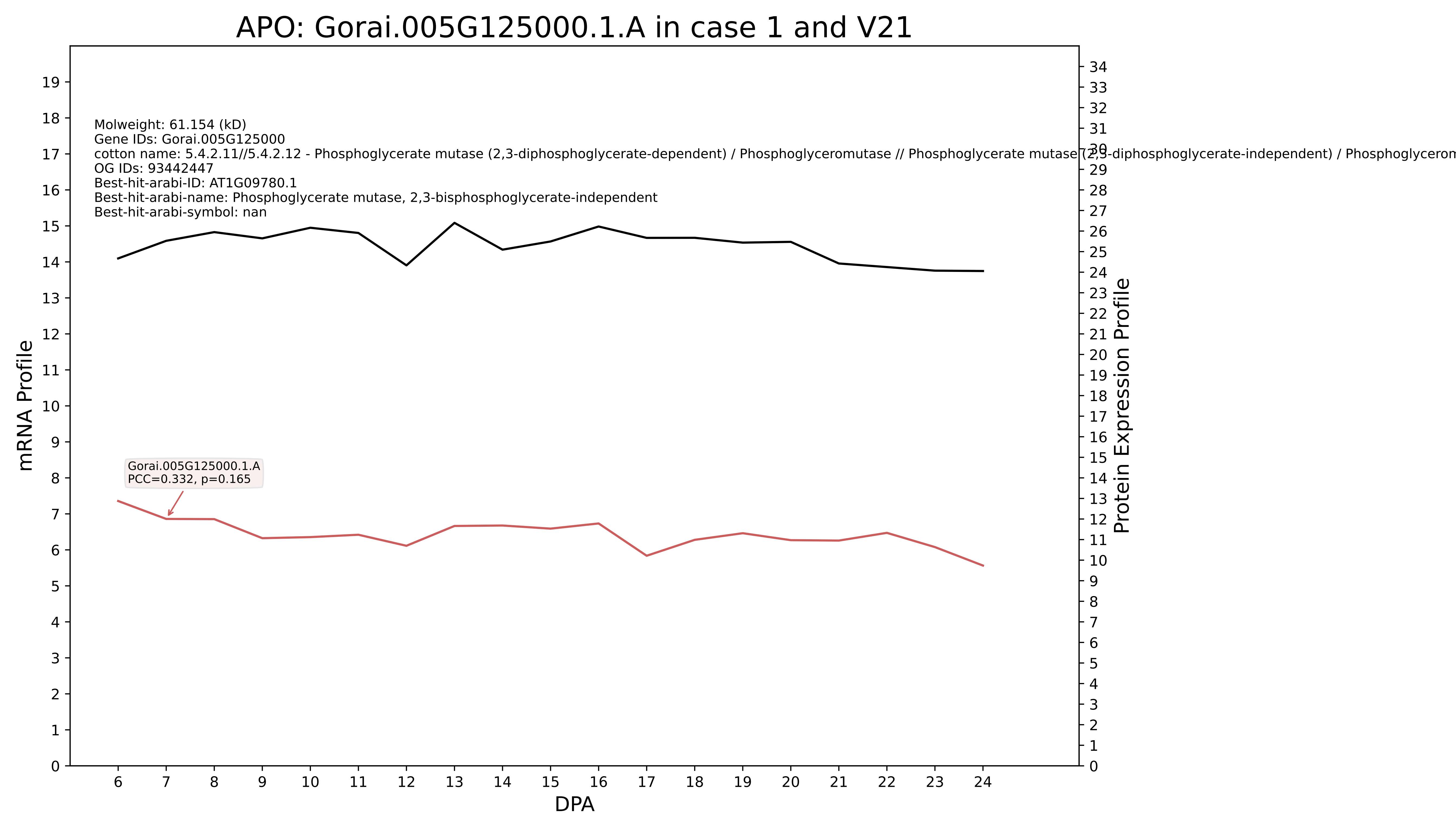
APO: Gorai.005G125000.1.A in case 1 and V21
Molweight: 61.154 (kD)
Gene IDs: Gorai.005G125000
cotton name: 5.4.2.11//5.4.2.12 - Phosphoglycerate mutase (2,3-diphosphoglycerate-dependent) / Phosphoglyceromutase // Phosphoglycerate mutase (2,3-diphosphoglycerate-independent) / Phosphoglyceron
OG IDs: 93442447
Best-hit-arabi-ID: AT1G09780.1
Best-hit-arabi-name: Phosphoglycerate mutase, 2,3-bisphosphoglycerate-independent
Best-hit-arabi-symbol: nan
Gorai.005G125000.1.A
PCC=0.332, p=0.165
mRNA Profile
Protein Expression Profile
DPA

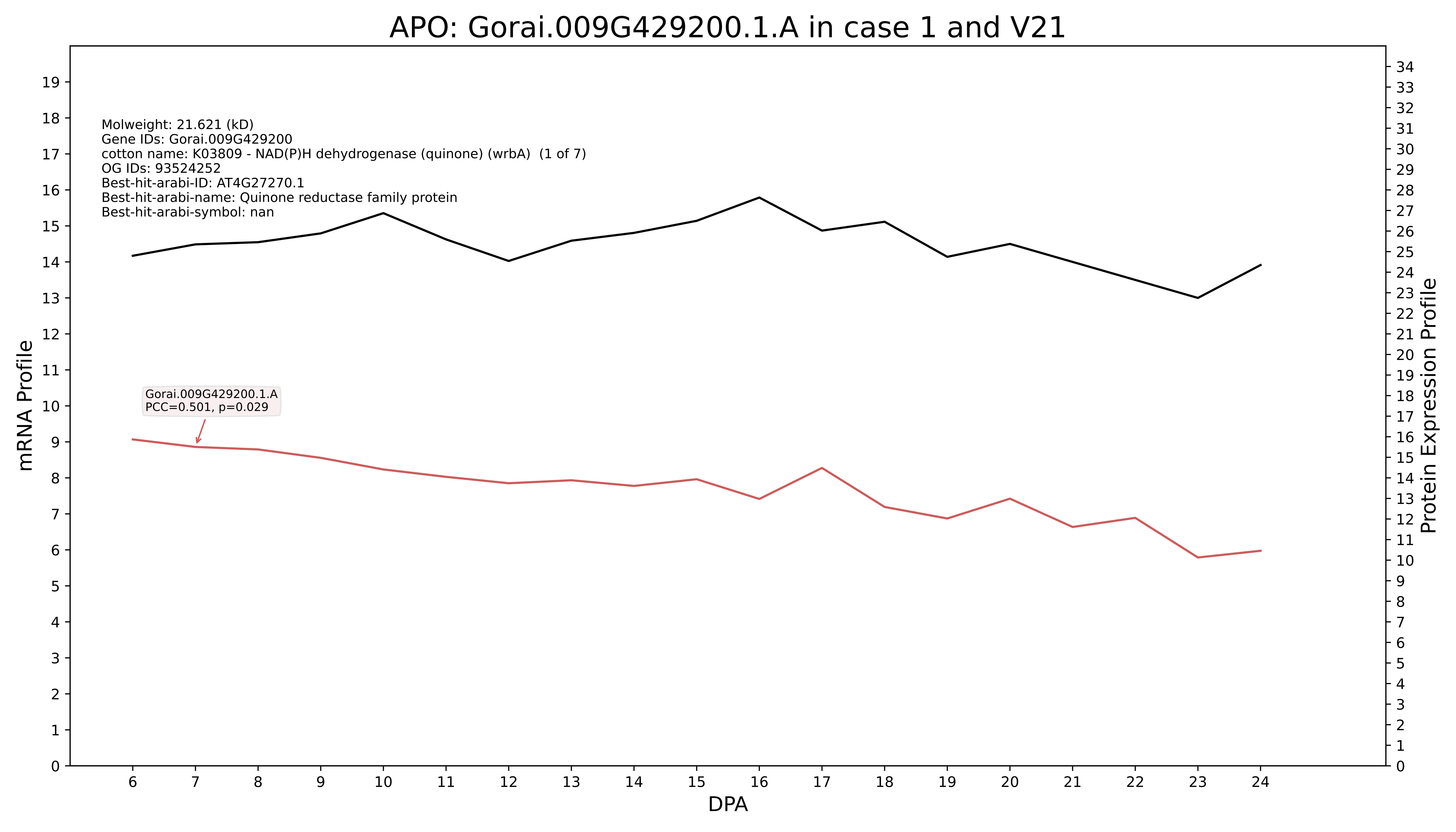

APO: Gorai.009G429200.1.A in case 1 and V21
Molweight: 21.621 (kD)
Gene IDs: Gorai.009G429200
cotton name: K03809 - NAD(P)H dehydrogenase (quinone) (wrbA) (1 of 7)
OG IDs: 93524252
Best-hit-arabi-ID: AT4G27270.1
Best-hit-arabi-name: Quinone reductase family protein
Best-hit-arabi-symbol: nan
Gorai.009G429200.1.A
PCC=0.501, p=0.029
mRNA Profile
Protein Expression Profile
DPA

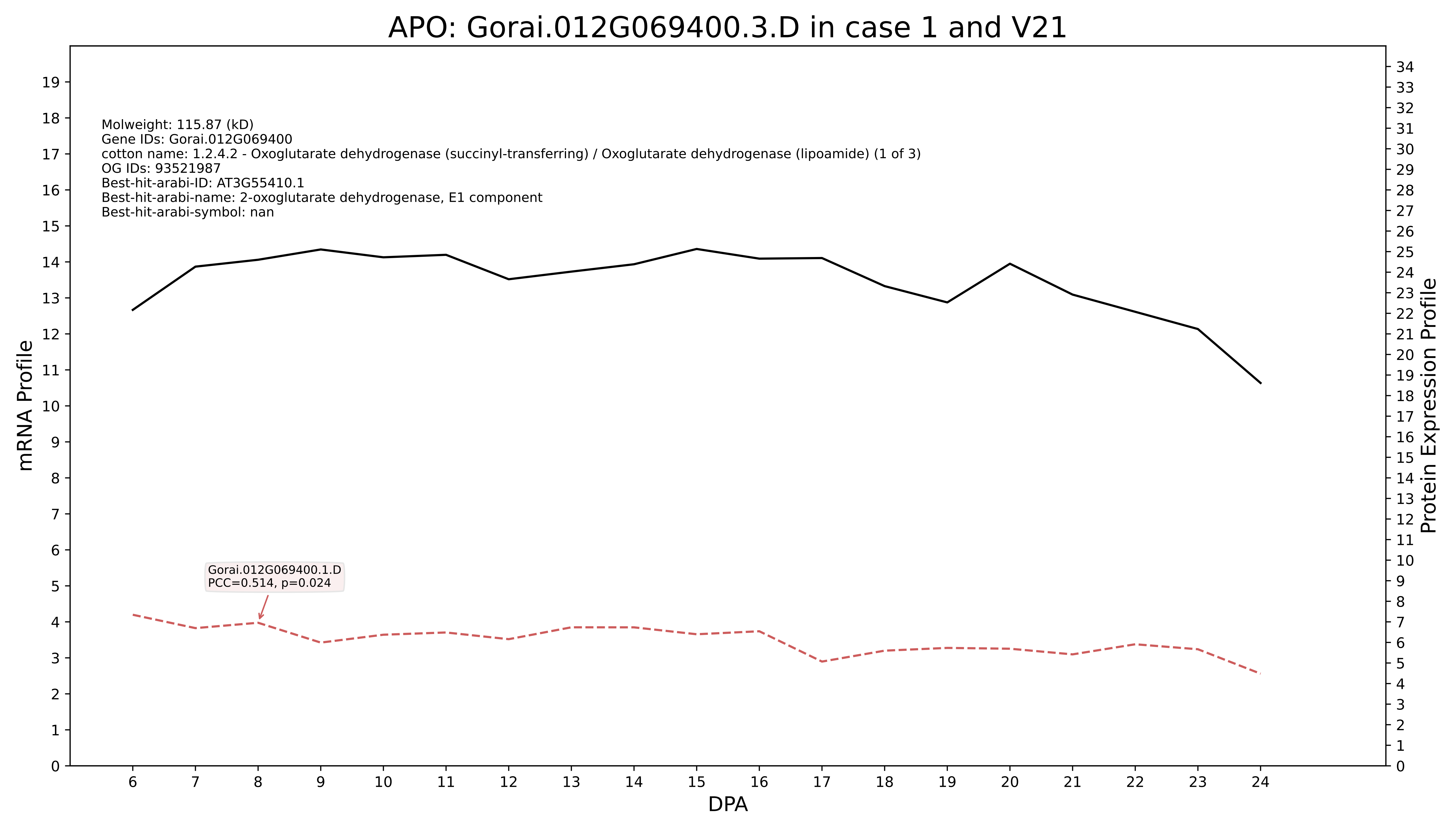
APO: Gorai.012G069400.3.D in case 1 and V21
Molweight: 115.87 (kD)
Gene IDs: Gorai.012G069400
cotton name: 1.2.4.2 - Oxoglutarate dehydrogenase (succinyl-transferring) / Oxoglutarate dehydrogenase (lipoamide) (1 of 3)
OG IDs: 93521987
Best-hit-arabi-ID: AT3G55410.1
Best-hit-arabi-name: 2-oxoglutarate dehydrogenase, E1 component
Best-hit-arabi-symbol: nan
Gorai.012G069400.1.D
PCC=0.514, p=0.024
mRNA Profile
Protein Expression Profile
DPA

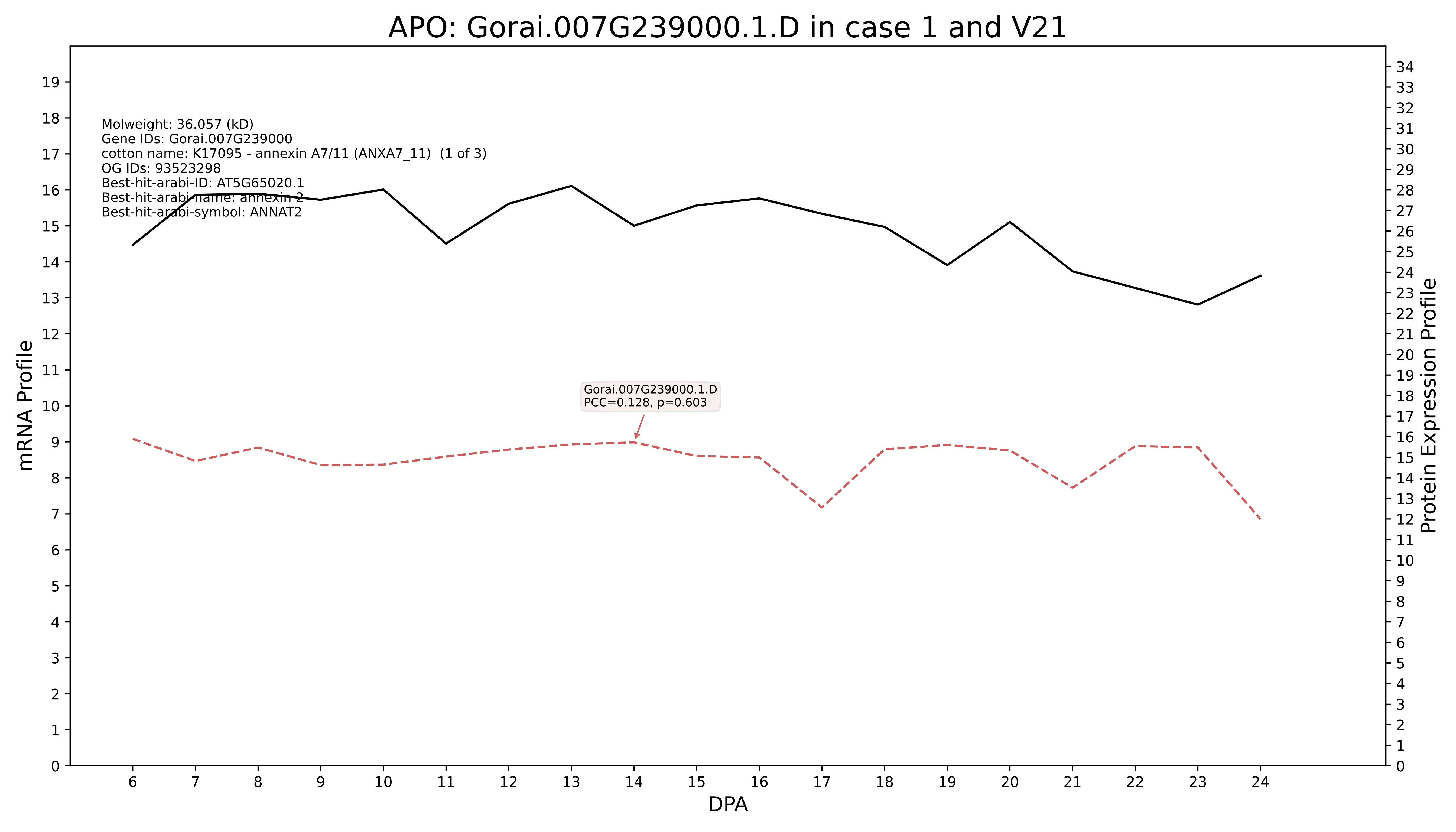

APO: Gorai.007G239000.1.D in case 1 and V21
Molweight: 36.057 (kD)
Gene IDs: Gorai.007G239000
cotton name: K17095 - annexin A7/11 (ANXA7_11) (1 of 3)
OG IDs: 93523298
Best-hit-arabi-ID: AT5G65020.1
Best-hit-arabi-name: annexin 2
Best-hit-arabi-symbol: ANNAT2
Gorai.007G239000.1.D
PCC=0.128, p=0.603
mRNA Profile
Protein Expression Profile
DPA

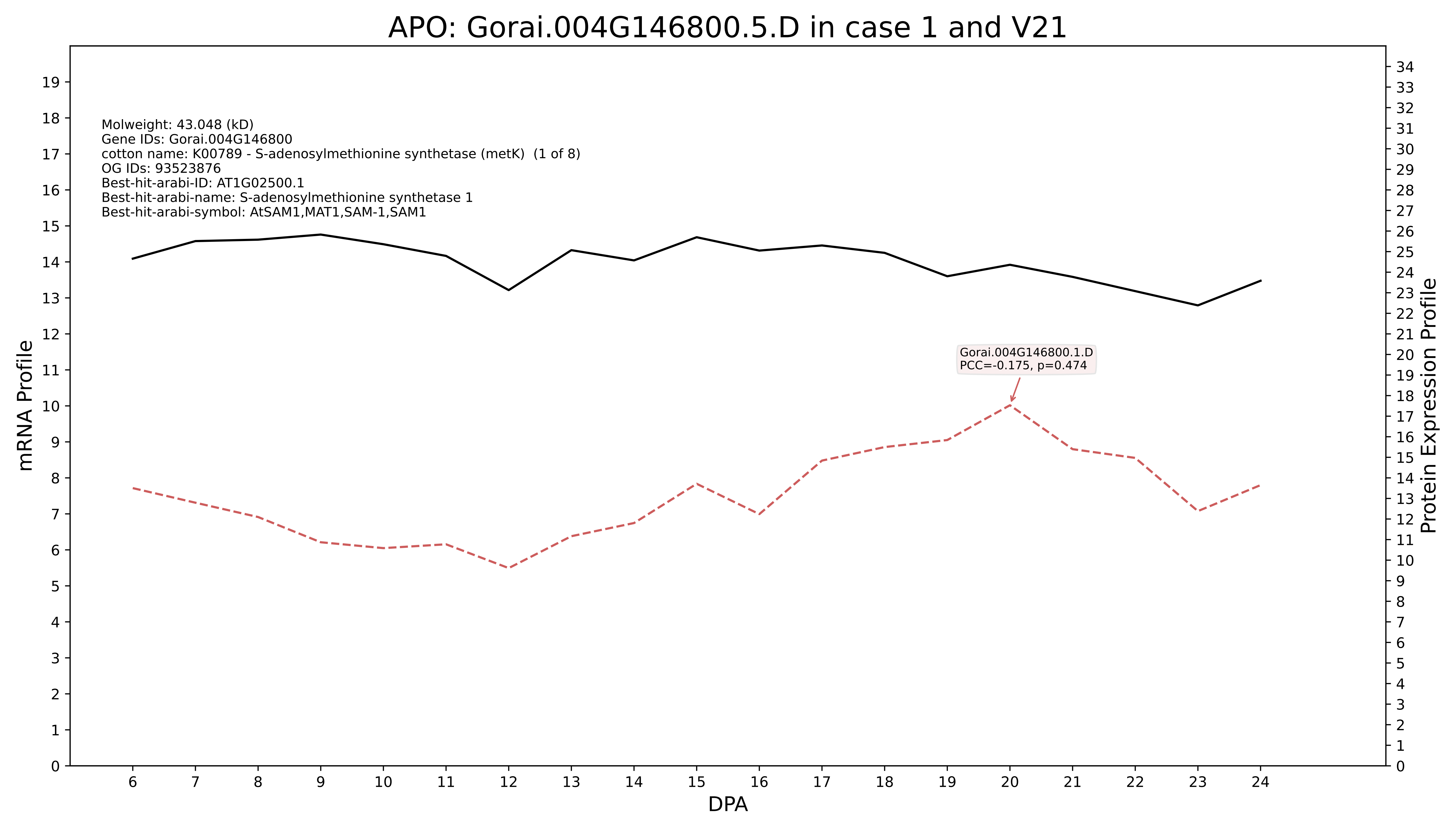
APO: Gorai.004G146800.5.D in case 1 and V21
Molweight: 43.048 (kD)
Gene IDs: Gorai.004G146800
cotton name: K00789 - S-adenosylmethionine synthetase (metK) (1 of 8)
OG IDs: 93523876
Best-hit-arabi-ID: AT1G02500.1
Best-hit-arabi-name: S-adenosylmethionine synthetase 1
Best-hit-arabi-symbol: AtSAM1,MAT1,SAM-1,SAM1
Gorai.004G146800.1.D
PCC=-0.175, p=0.474
mRNA Profile
Protein Expression Profile
DPA

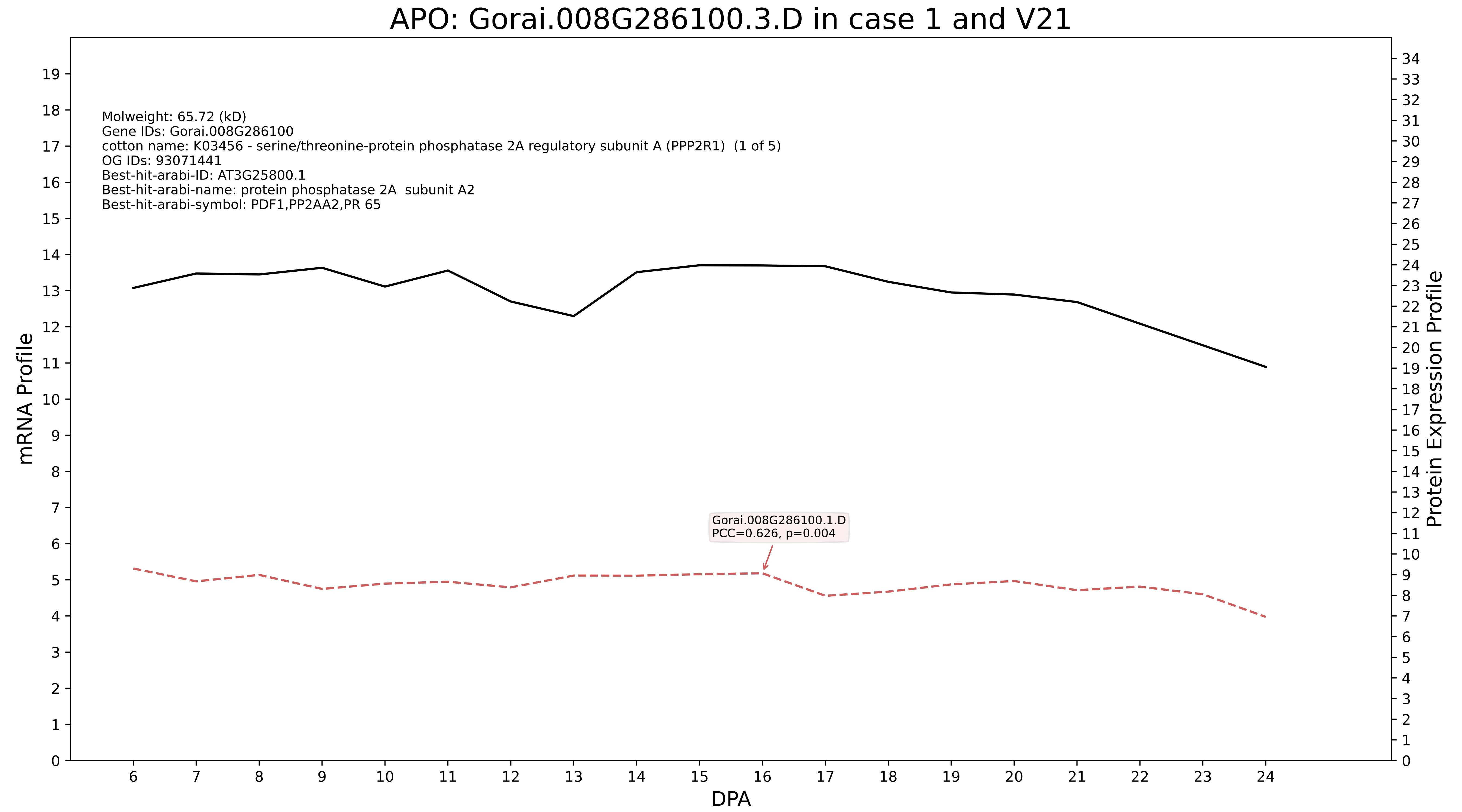

APO: Gorai.008G286100.3.D in case 1 and V21
Molweight: 65.72 (kD)
Gene IDs: Gorai.008G286100
cotton name: K03456 - serine/threonine-protein phosphatase 2A regulatory subunit A (PPP2R1) (1 of 5)
OG IDs: 93071441
Best-hit-arabi-ID: AT3G25800.1
Best-hit-arabi-name: protein phosphatase 2A subunit A2
Best-hit-arabi-symbol: PDF1,PP2AA2,PR 65
Gorai.008G286100.1.D
PCC=0.626, p=0.004
mRNA Profile
Protein Expression Profile
DPA

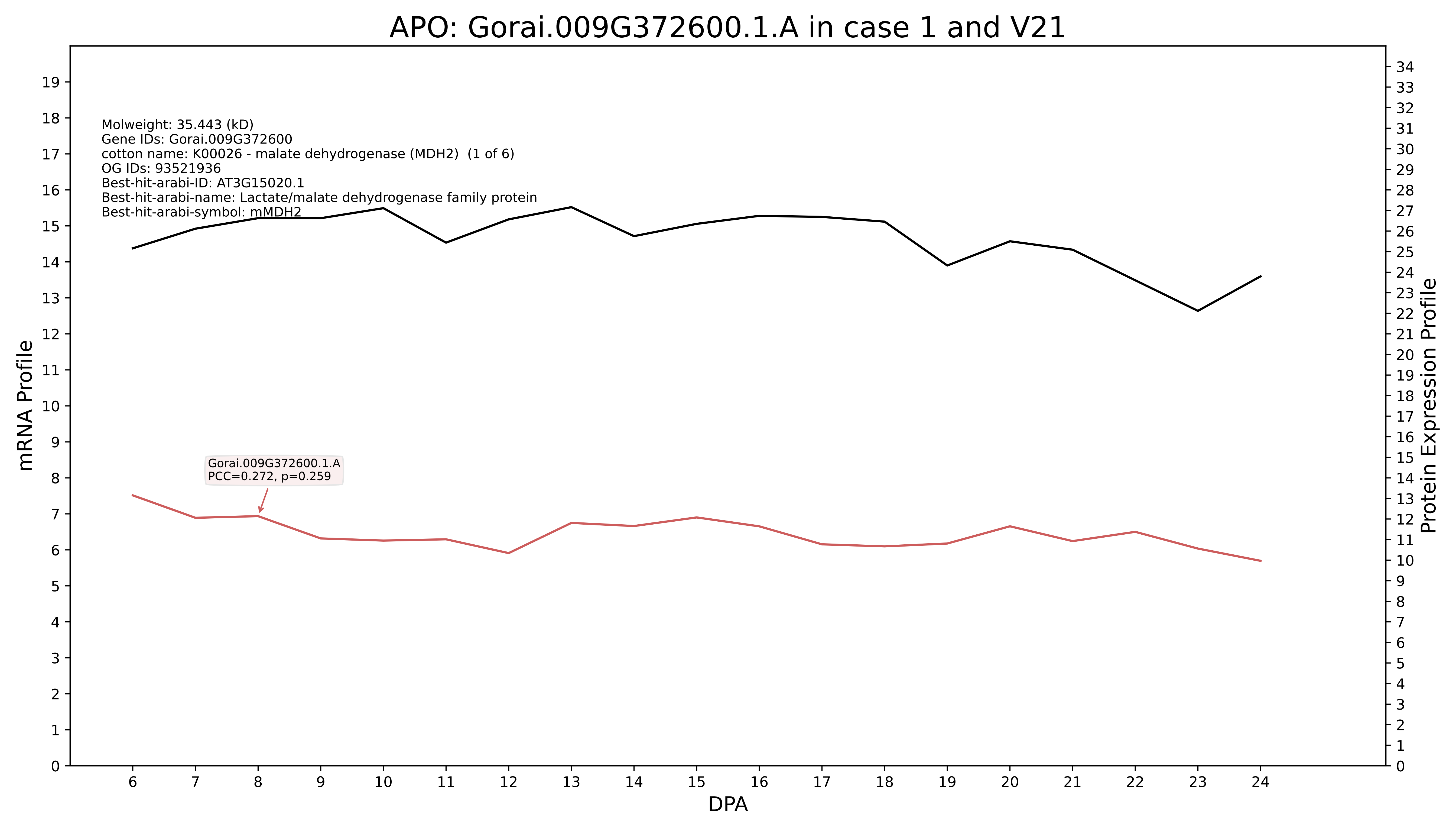

APO: Gorai.009G372600.1.A in case 1 and V21
Molweight: 35.443 (kD)
Gene IDs: Gorai.009G372600
cotton name: K00026 - malate dehydrogenase (MDH2) (1 of 6)
OG IDs: 93521936
Best-hit-arabi-ID: AT3G15020.1
Best-hit-arabi-name: Lactate/malate dehydrogenase family protein
Best-hit-arabi-symbol: mMDH2
Gorai.009G372600.1.A
PCC=0.272, p=0.259
mRNA Profile
Protein Expression Profile
DPA

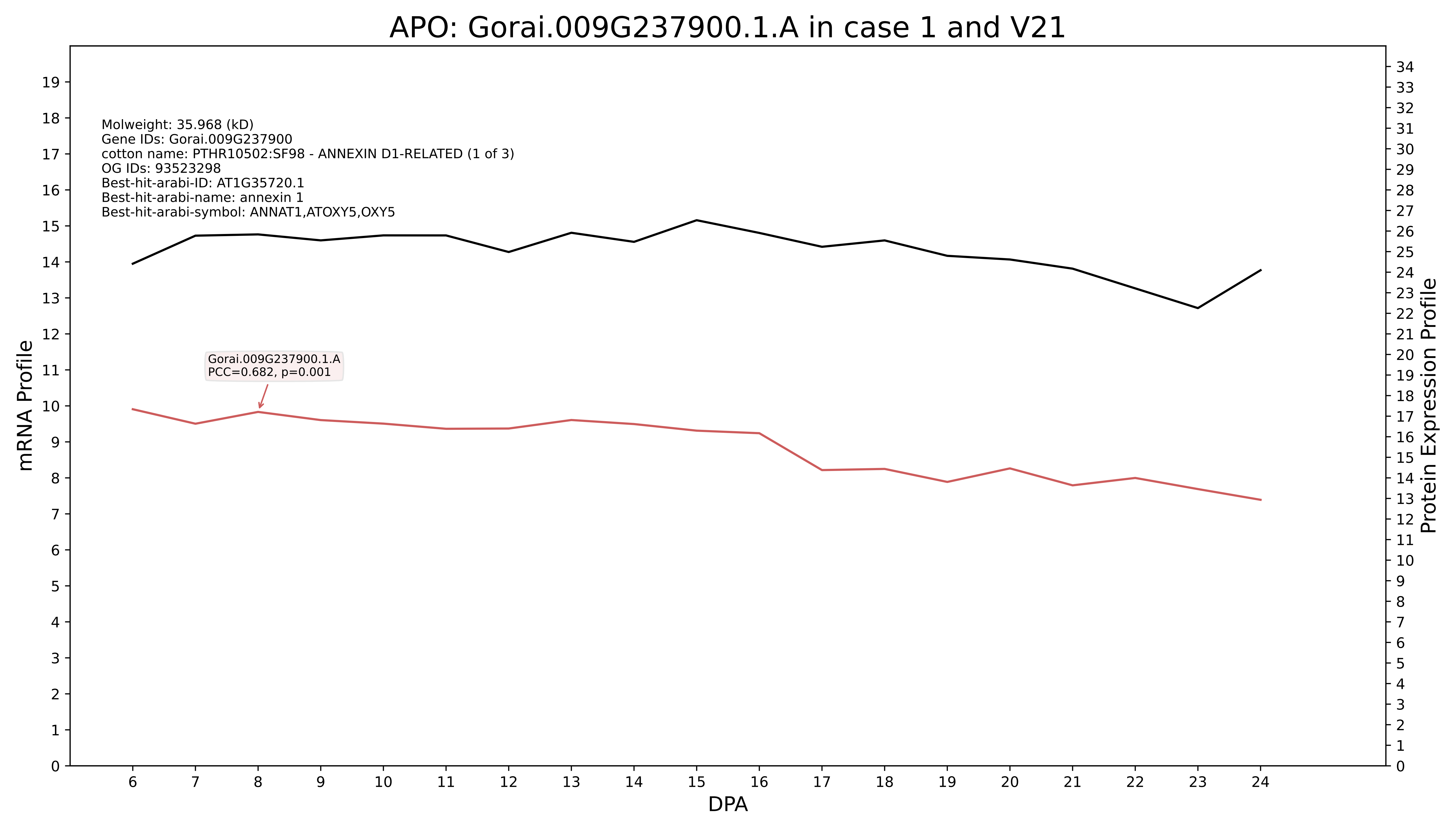

APO: Gorai.009G237900.1.A in case 1 and V21
Molweight: 35.968 (kD)
Gene IDs: Gorai.009G237900
cotton name: PTHR10502:SF98 - ANNEXIN D1-RELATED (1 of 3)
OG IDs: 93523298
Best-hit-arabi-ID: AT1G35720.1
Best-hit-arabi-name: annexin 1
Best-hit-arabi-symbol: ANNAT1,ATOXY5,OXY5
Gorai.009G237900.1.A
PCC=0.682, p=0.001
mRNA Profile
Protein Expression Profile
DPA

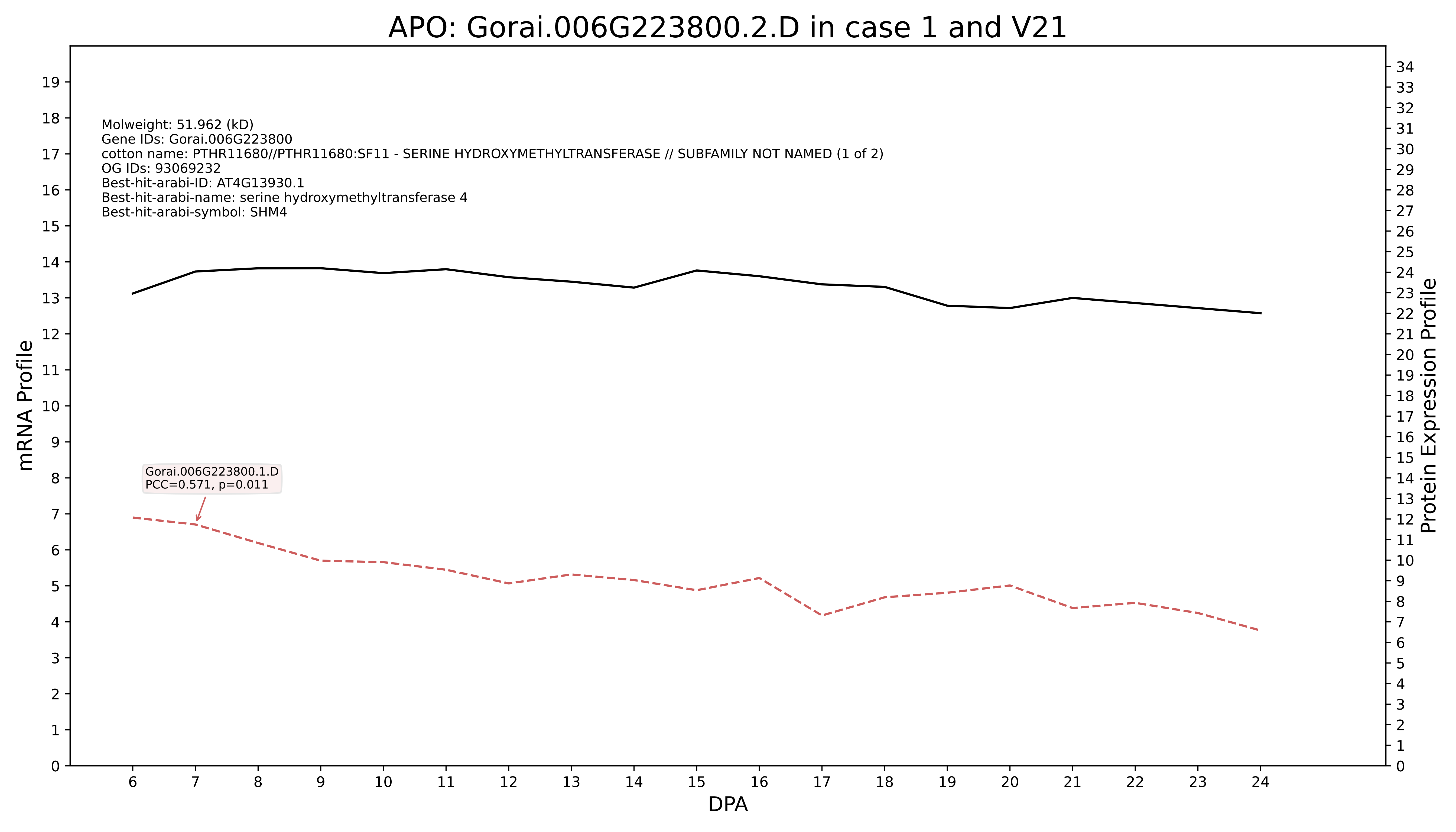
APO: Gorai.006G223800.2.D in case 1 and V21
Molweight: 51.962 (kD)
Gene IDs: Gorai.006G223800
cotton name: PTHR11680//PTHR11680:SF11 - SERINE HYDROXYMETHYLTRANSFERASE // SUBFAMILY NOT NAMED (1 of 2)
OG IDs: 93069232
Best-hit-arabi-ID: AT4G13930.1
Best-hit-arabi-name: serine hydroxymethyltransferase 4
Best-hit-arabi-symbol: SHM4
Gorai.006G223800.1.D
PCC=0.571, p=0.011
mRNA Profile
Protein Expression Profile
DPA

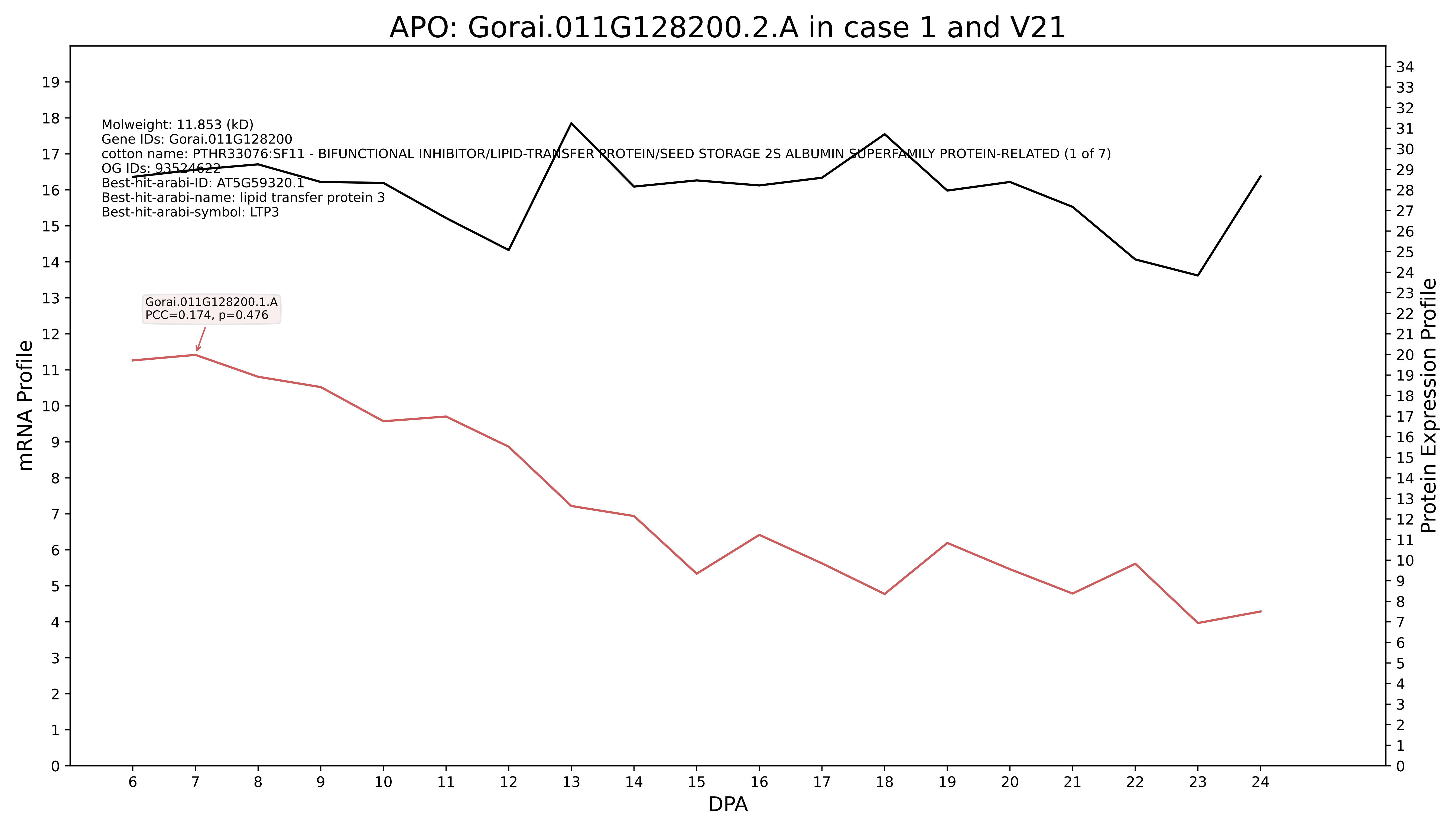
APO: Gorai.011G128200.2.A in case 1 and V21
Molweight: 11.853 (kD)
Gene IDs: Gorai.011G128200
cotton name: PTHR33076:SF11 - BIFUNCTIONAL INHIBITOR/LIPID-TRANSFER PROTEIN/SEED STORAGE 2S ALBUMIN SUPERFAMILY PROTEIN-RELATED (1 of 7)
OG IDs: 93524622
Best-hit-arabi-ID: AT5G59320.1
Best-hit-arabi-name: lipid transfer protein 3
Best-hit-arabi-symbol: LTP3
Gorai.011G128200.1.A
PCC=0.174, p=0.476
mRNA Profile
Protein Expression Profile
DPA

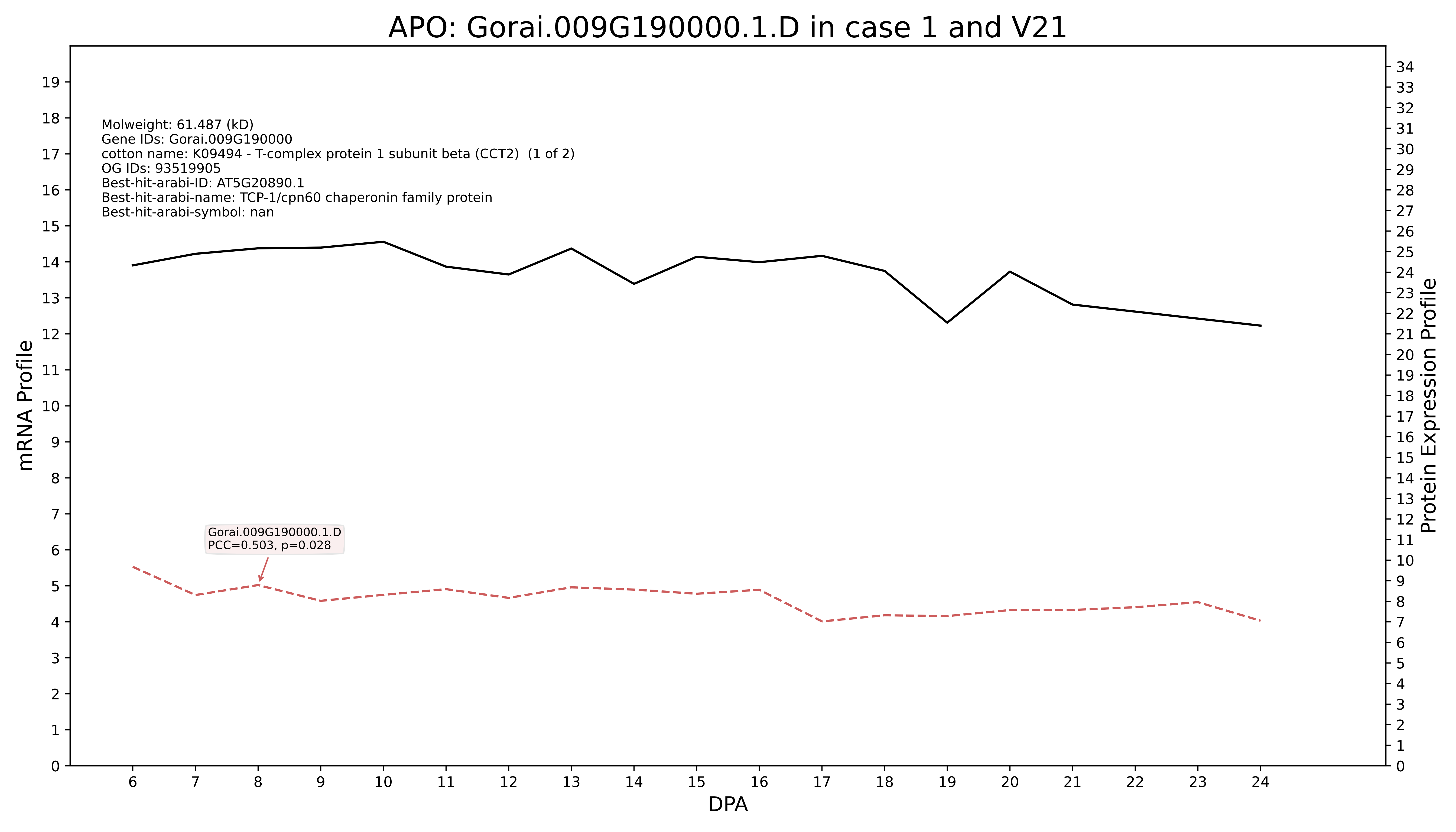

APO: Gorai.009G190000.1.D in case 1 and V21
Molweight: 61.487 (kD)
Gene IDs: Gorai.009G190000
cotton name: K09494 - T-complex protein 1 subunit beta (CCT2) (1 of 2)
OG IDs: 93519905
Best-hit-arabi-ID: AT5G20890.1
Best-hit-arabi-name: TCP-1/cpn60 chaperonin family protein
Best-hit-arabi-symbol: nan
Gorai.009G190000.1.D
PCC=0.503, p=0.028
mRNA Profile
Protein Expression Profile
DPA

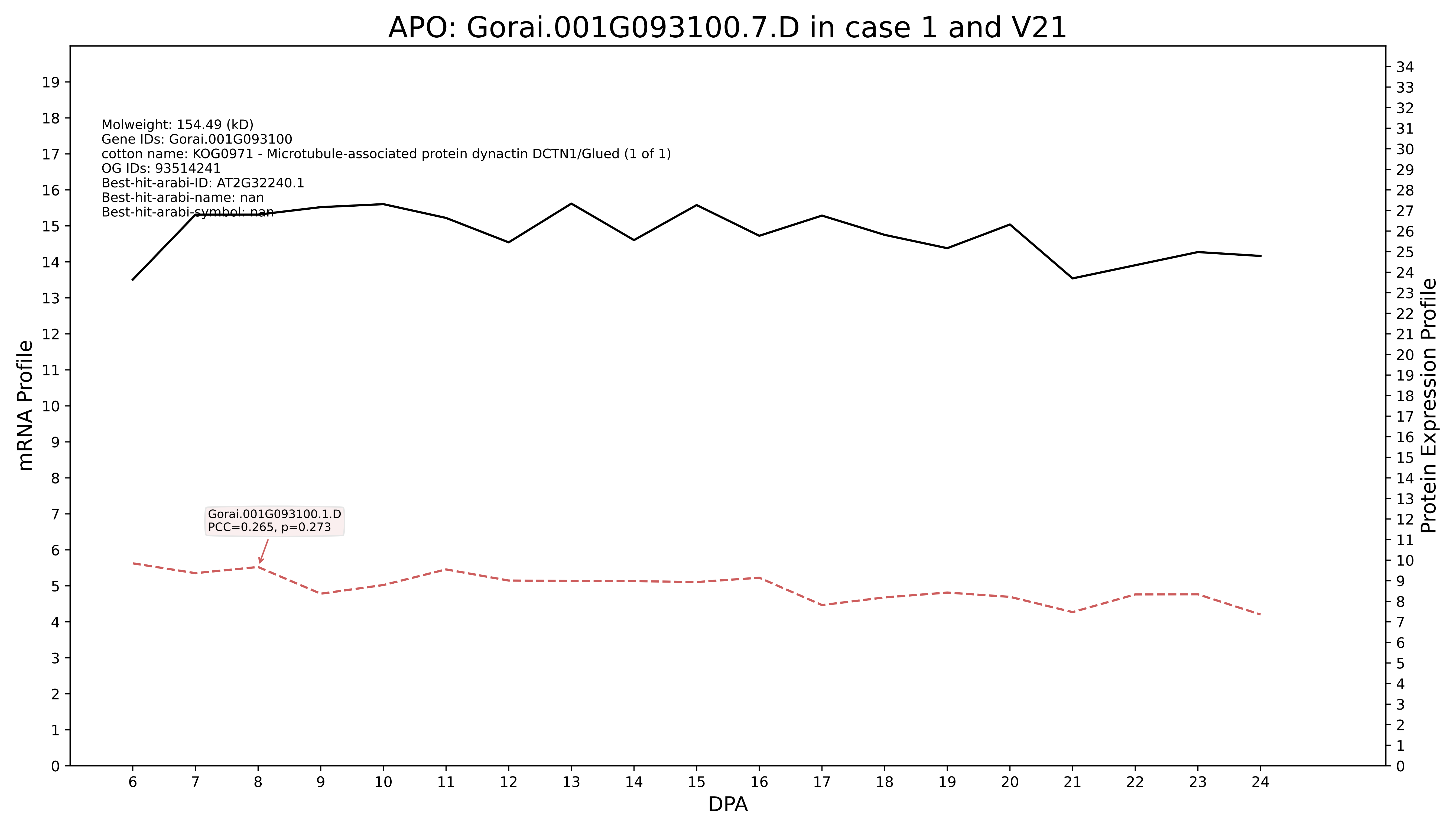

APO: Gorai.001G093100.7.D in case 1 and V21
Molweight: 154.49 (kD)
Gene IDs: Gorai.001G093100
cotton name: KOG0971 - Microtubule-associated protein dynactin DCTN1/Glued (1 of 1)
OG IDs: 93514241
Best-hit-arabi-ID: AT2G32240.1
Best-hit-arabi-name: nan
Best-hit-arabi-symbol: nan
Gorai.001G093100.1.D
PCC=0.265, p=0.273
mRNA Profile
Protein Expression Profile
DPA

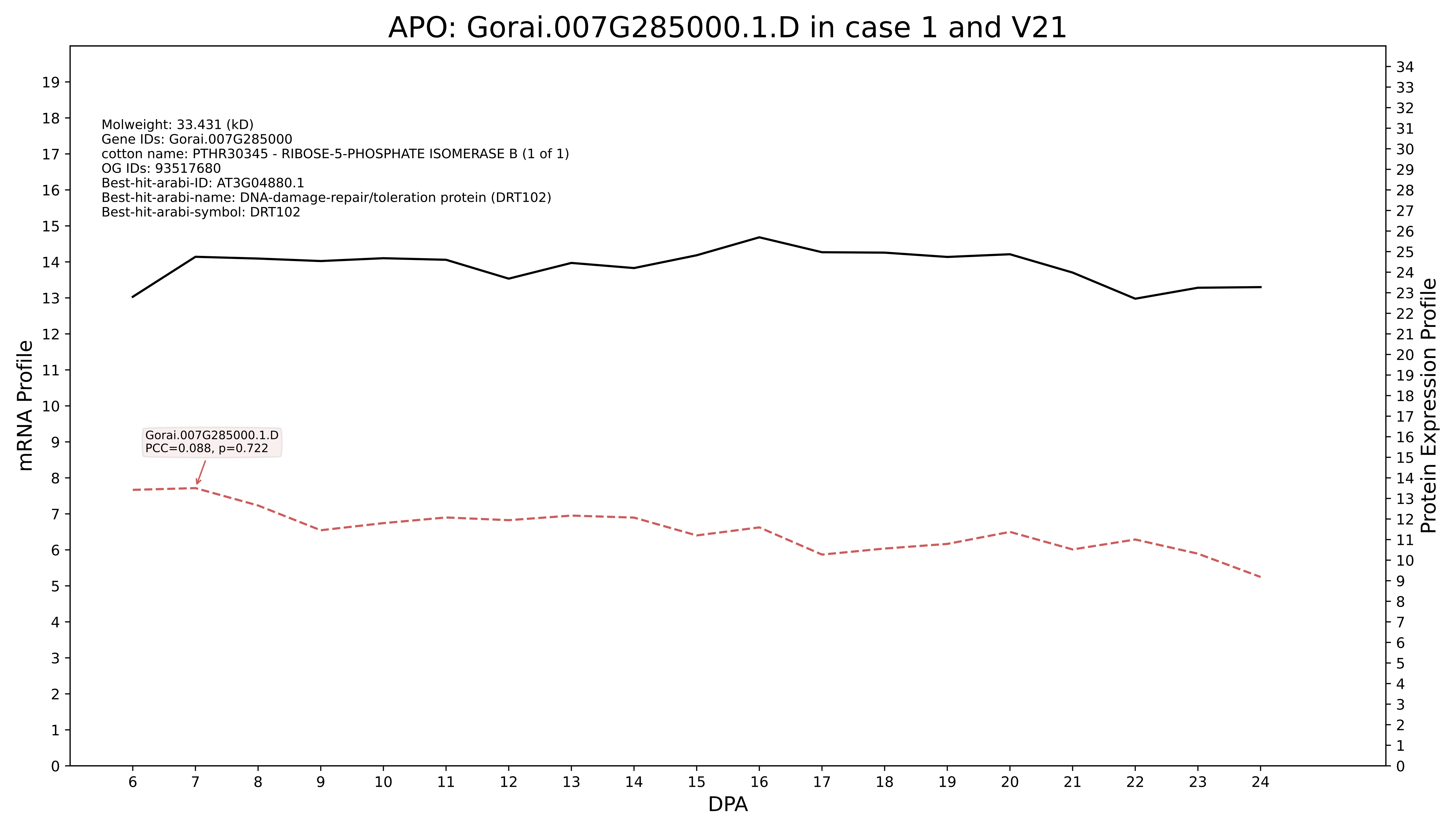

APO: Gorai.007G285000.1.D in case 1 and V21
Molweight: 33.431 (kD)
Gene IDs: Gorai.007G285000
cotton name: PTHR30345 - RIBOSE-5-PHOSPHATE ISOMERASE B (1 of 1)
OG IDs: 93517680
Best-hit-arabi-ID: AT3G04880.1
Best-hit-arabi-name: DNA-damage-repair/toleration protein (DRT102)
Best-hit-arabi-symbol: DRT102
Gorai.007G285000.1.D
PCC=0.088, p=0.722
mRNA Profile
Protein Expression Profile
DPA

APO: Gorai.008G018300.2.A in case 1 and V21

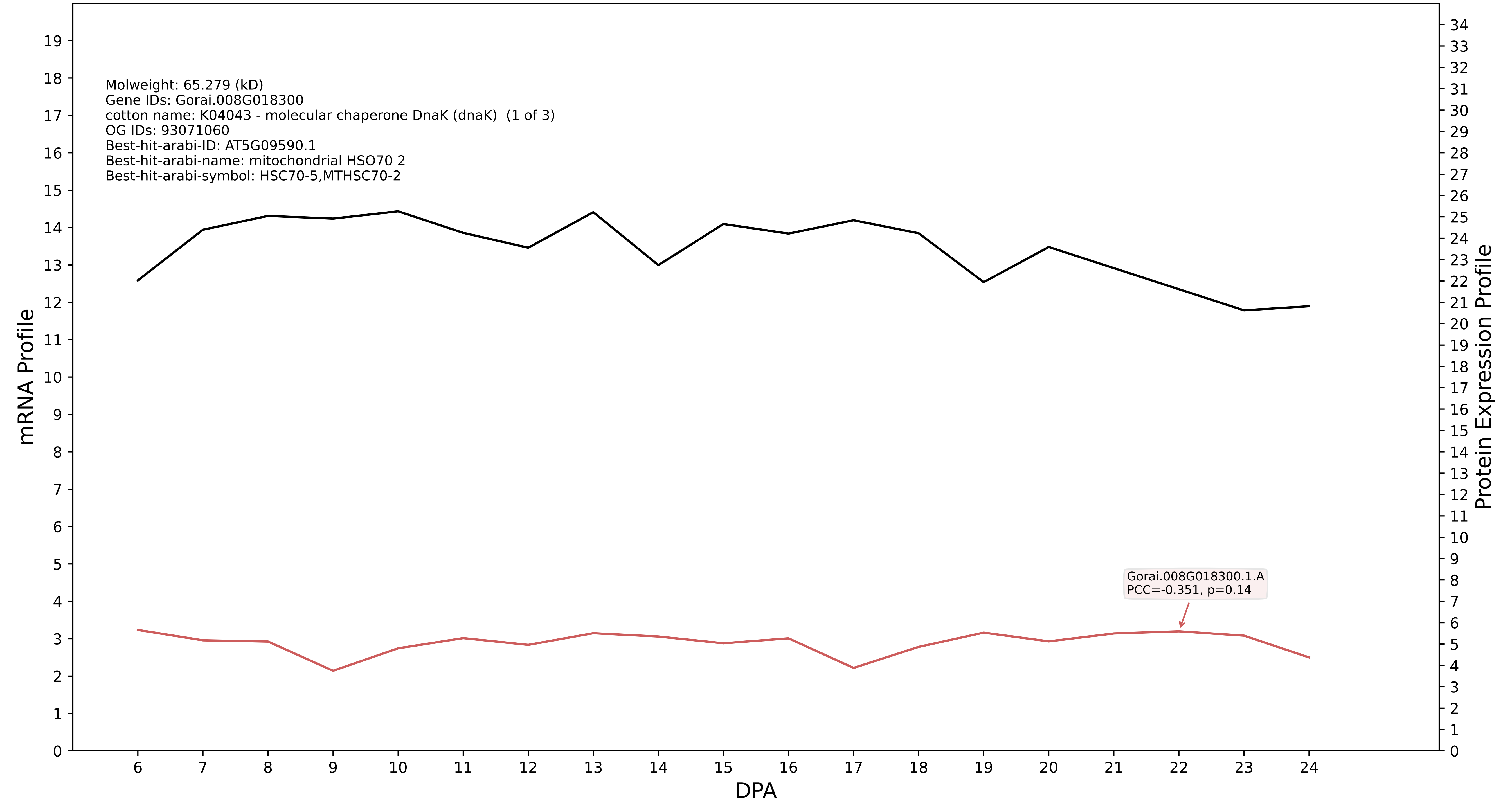

APO: Gorai.009G094600.1.D in case 1 and V21

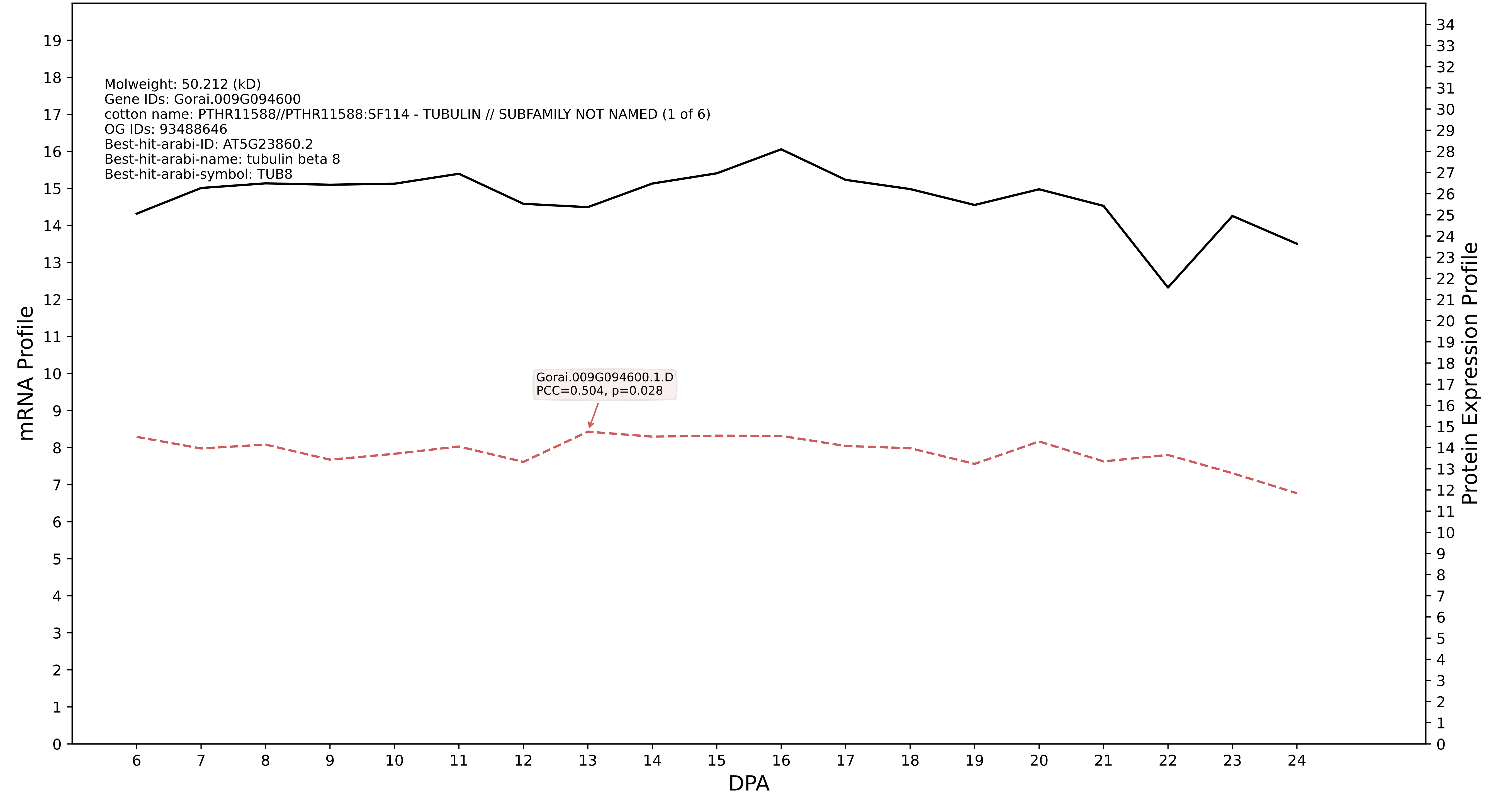

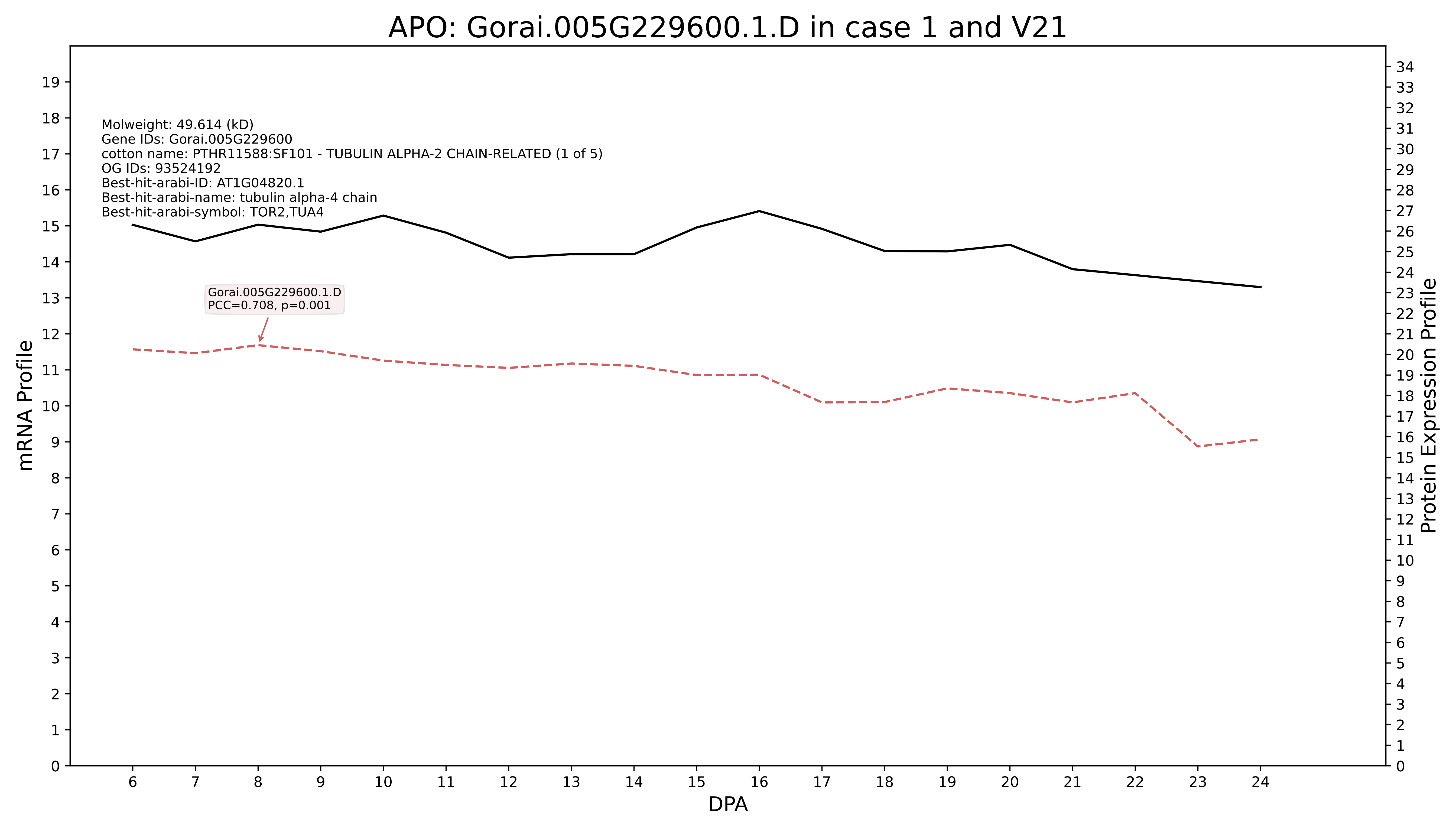
APO: Gorai.005G229600.1.D in case 1 and V21
Molweight: 49.614 (kD)
Gene IDs: Gorai.005G229600
cotton name: PTHR11588:SF101 - TUBULIN ALPHA-2 CHAIN-RELATED (1 of 5)
OG IDs: 93524192
Best-hit-arabi-ID: AT1G04820.1
Best-hit-arabi-name: tubulin alpha-4 chain
Best-hit-arabi-symbol: TOR2,TUA4
Gorai.005G229600.1.D
PCC=0.708, p=0.001
mRNA Profile
Protein Expression Profile
DPA
0
1
2
3
4
5
6
7
8
9
10
11
12
13
14
15
16
17
18
19
20
21
22
23
24
25
26
27
28
29
30
31
32
33
34

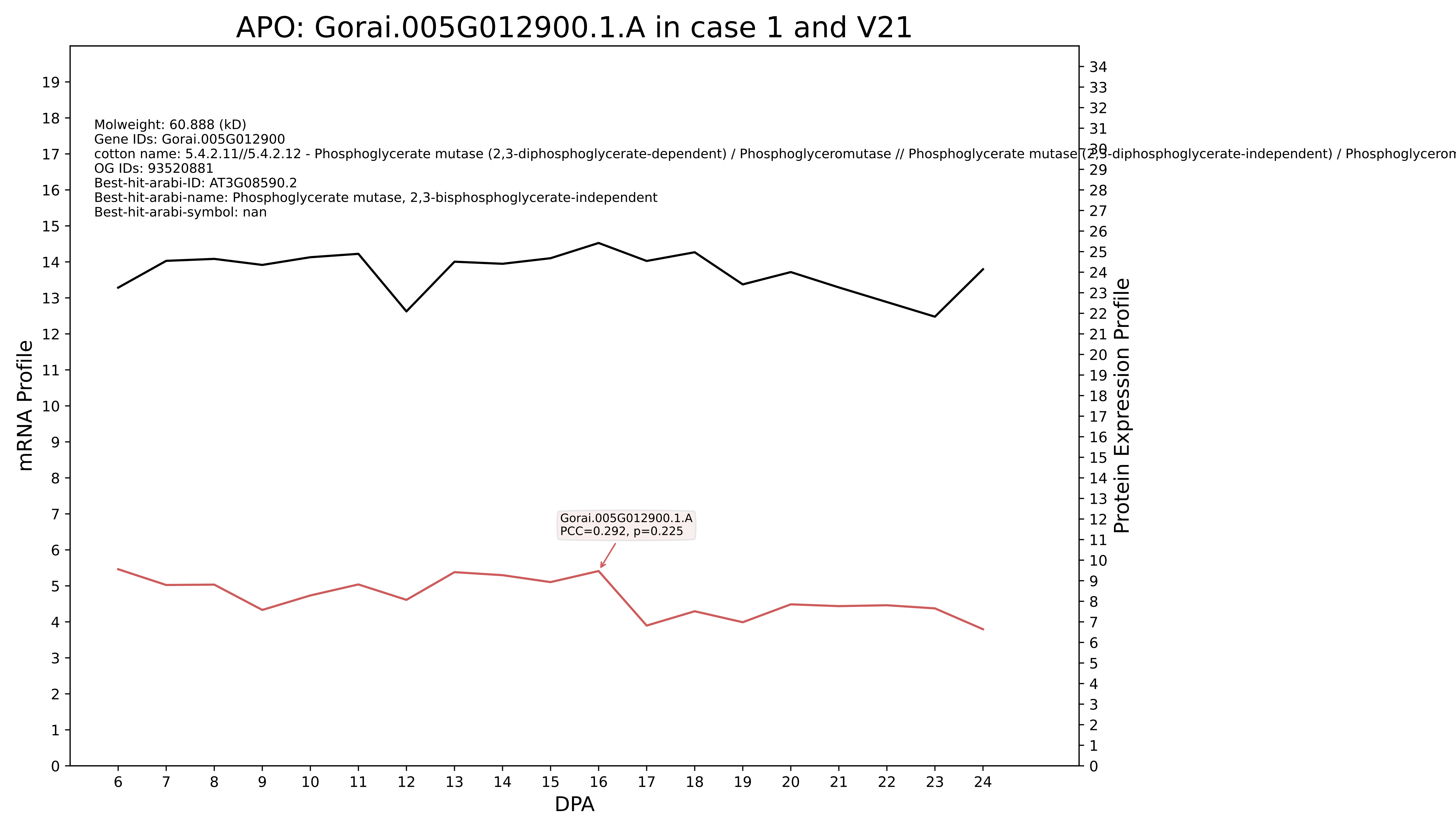
APO: Gorai.005G012900.1.A in case 1 and V21
Molweight: 60.888 (kD)
Gene IDs: Gorai.005G012900
cotton name: 5.4.2.11//5.4.2.12 - Phosphoglycerate mutase (2,3-diphosphoglycerate-dependent) / Phosphoglyceromutase // Phosphoglycerate mutase (2,3-diphosphoglycerate-independent) / Phosphoglycerom
OG IDs: 93520881
Best-hit-arabi-ID: AT3G08590.2
Best-hit-arabi-name: Phosphoglycerate mutase, 2,3-bisphosphoglycerate-independent
Best-hit-arabi-symbol: nan
Gorai.005G012900.1.A
PCC=0.292, p=0.225
mRNA Profile
Protein Expression Profile
DPA

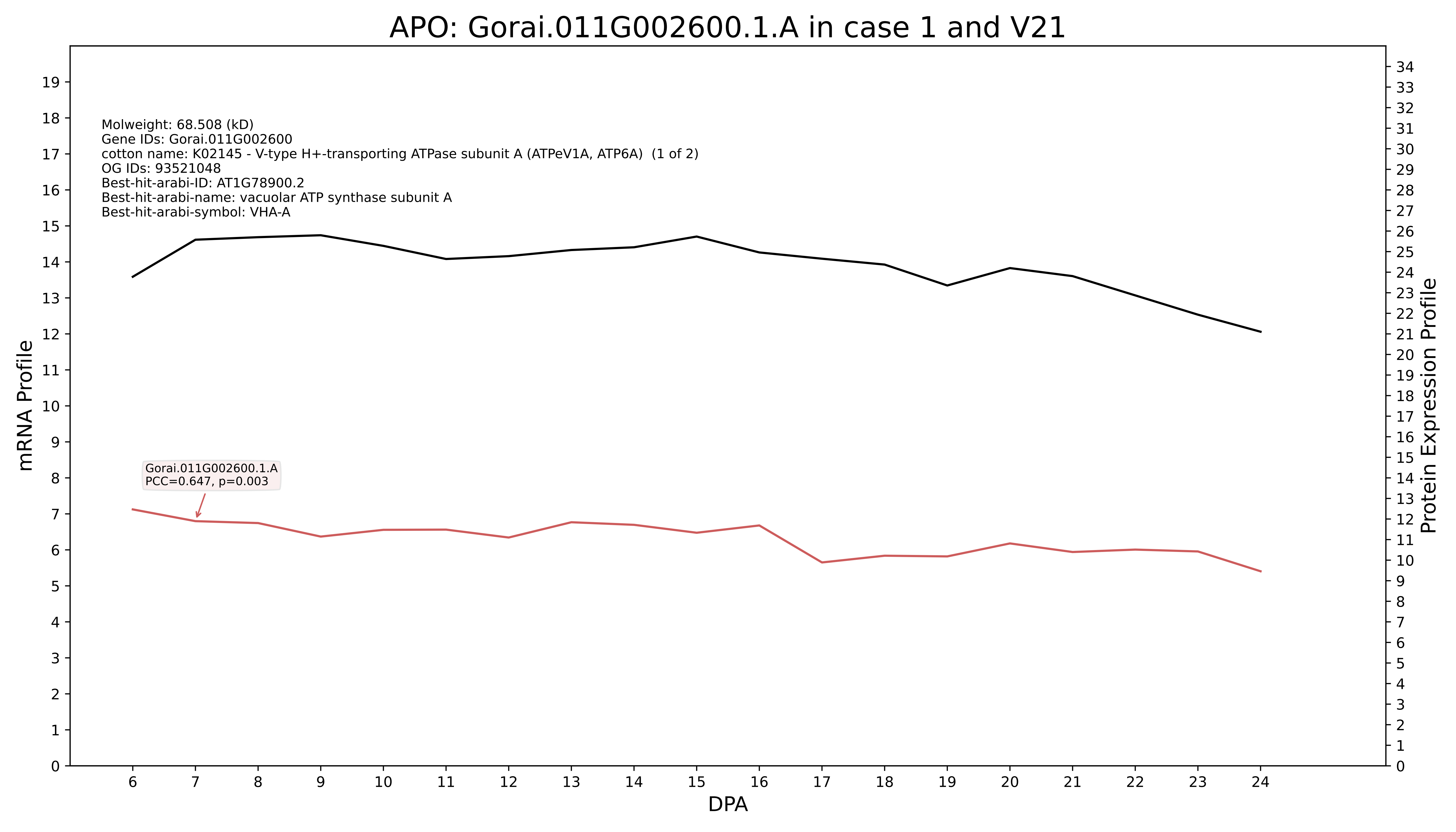
APO: Gorai.011G002600.1.A in case 1 and V21
Molweight: 68.508 (kD)
Gene IDs: Gorai.011G002600
cotton name: K02145 - V-type H+-transporting ATPase subunit A (ATPeV1A, ATP6A) (1 of 2)
OG IDs: 93521048
Best-hit-arabi-ID: AT1G78900.2
Best-hit-arabi-name: vacuolar ATP synthase subunit A
Best-hit-arabi-symbol: VHA-A
Gorai.011G002600.1.A
PCC=0.647, p=0.003
mRNA Profile
Protein Expression Profile
DPA
6
7
8
9
10
11
12
13
14
15
16
17
18
19
20
21
22
23
24

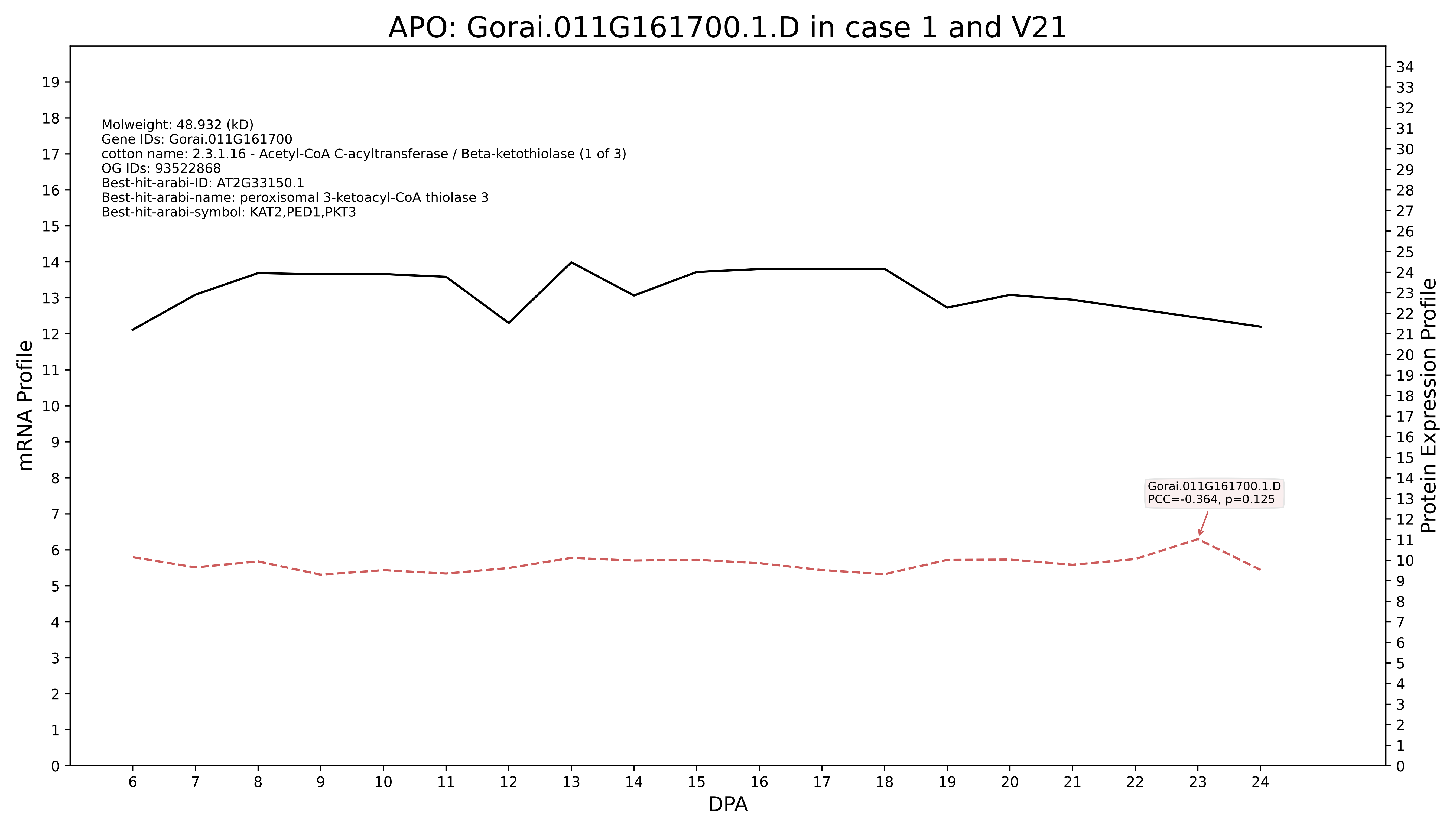
APO: Gorai.011G161700.1.D in case 1 and V21
Molweight: 48.932 (kD)
Gene IDs: Gorai.011G161700
cotton name: 2.3.1.16 - Acetyl-CoA C-acyltransferase / Beta-ketothiolase (1 of 3)
OG IDs: 93522868
Best-hit-arabi-ID: AT2G33150.1
Best-hit-arabi-name: peroxisomal 3-ketoacyl-CoA thiolase 3
Best-hit-arabi-symbol: KAT2,PED1,PKT3
Gorai.011G161700.1.D
PCC=-0.364, p=0.125
mRNA Profile
Protein Expression Profile
DPA

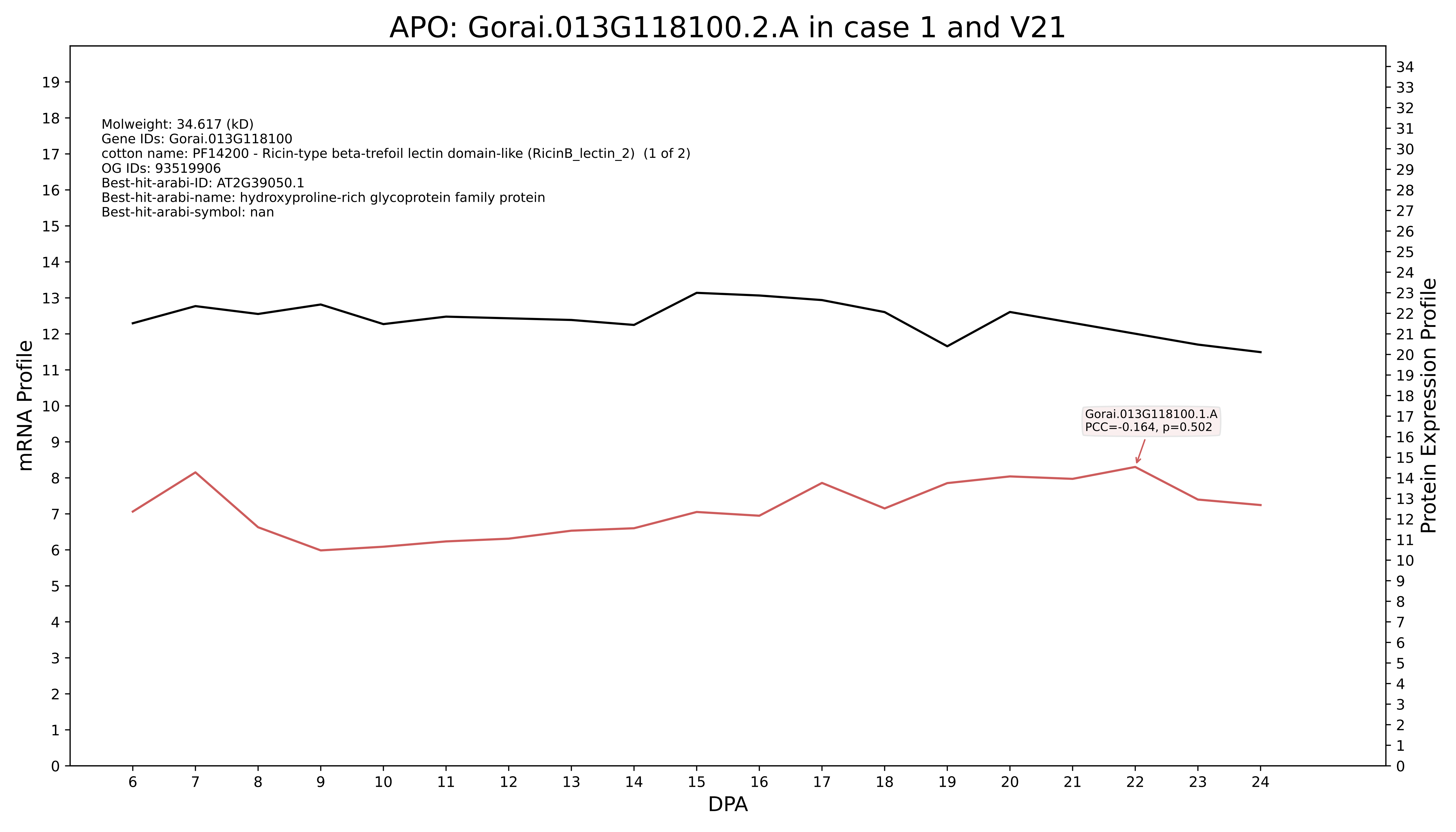
APO: Gorai.013G118100.2.A in case 1 and V21
Molweight: 34.617 (kD)
Gene IDs: Gorai.013G118100
cotton name: PF14200 - Ricin-type beta-trefoil lectin domain-like (RicinB_lectin_2) (1 of 2)
OG IDs: 93519906
Best-hit-arabi-ID: AT2G39050.1
Best-hit-arabi-name: hydroxyproline-rich glycoprotein family protein
Best-hit-arabi-symbol: nan
Gorai.013G118100.1.A
PCC=-0.164, p=0.502
mRNA Profile
Protein Expression Profile
DPA

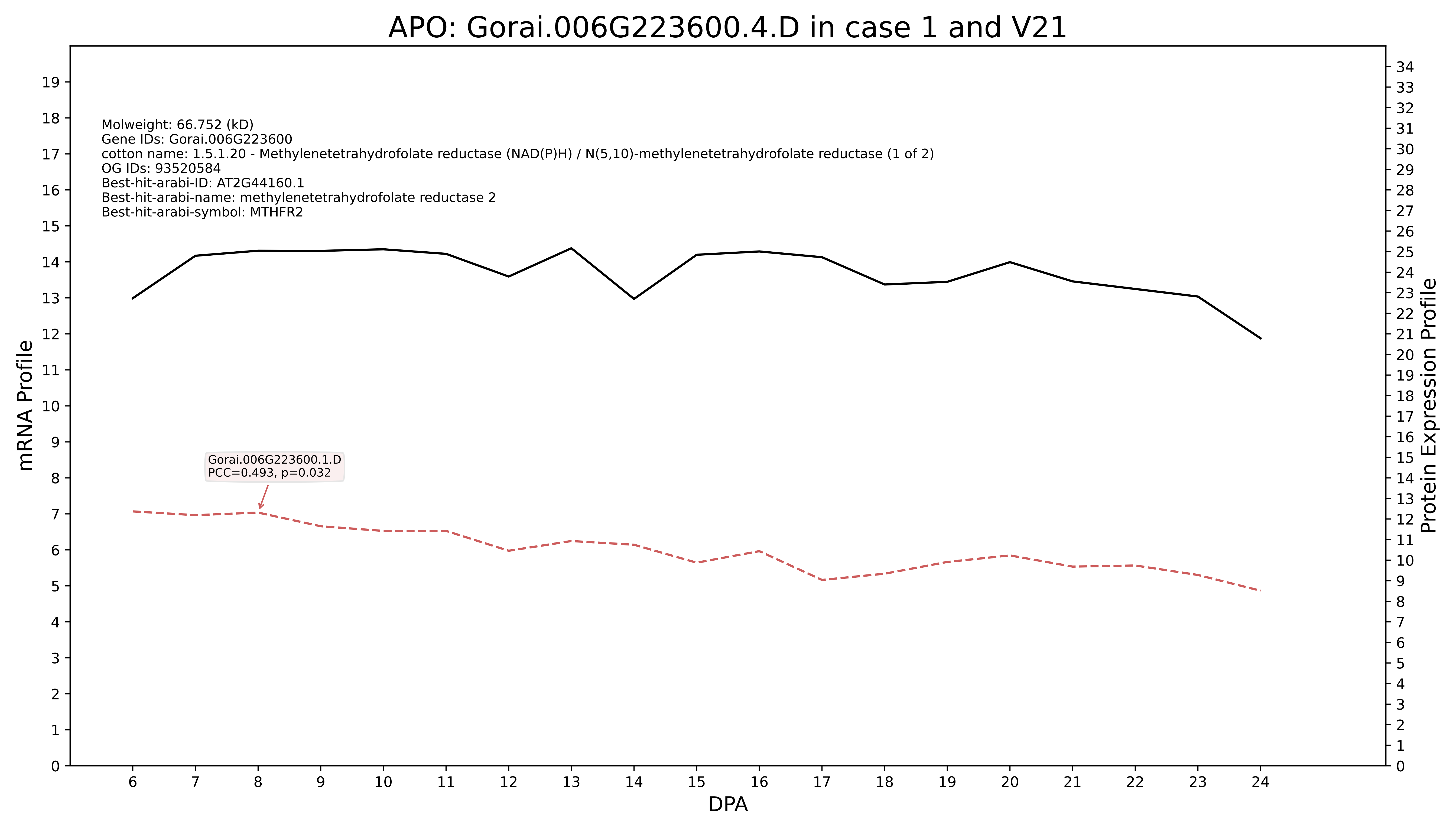

APO: Gorai.006G223600.4.D in case 1 and V21
Molweight: 66.752 (kD)
Gene IDs: Gorai.006G223600
cotton name: 1.5.1.20 - Methylenetetrahydrofolate reductase (NAD(P)H) / N(5,10)-methylenetetrahydrofolate reductase (1 of 2)
OG IDs: 93520584
Best-hit-arabi-ID: AT2G44160.1
Best-hit-arabi-name: methylenetetrahydrofolate reductase 2
Best-hit-arabi-symbol: MTHFR2
Gorai.006G223600.1.D
PCC=0.493, p=0.032
mRNA Profile
Protein Expression Profile
DPA

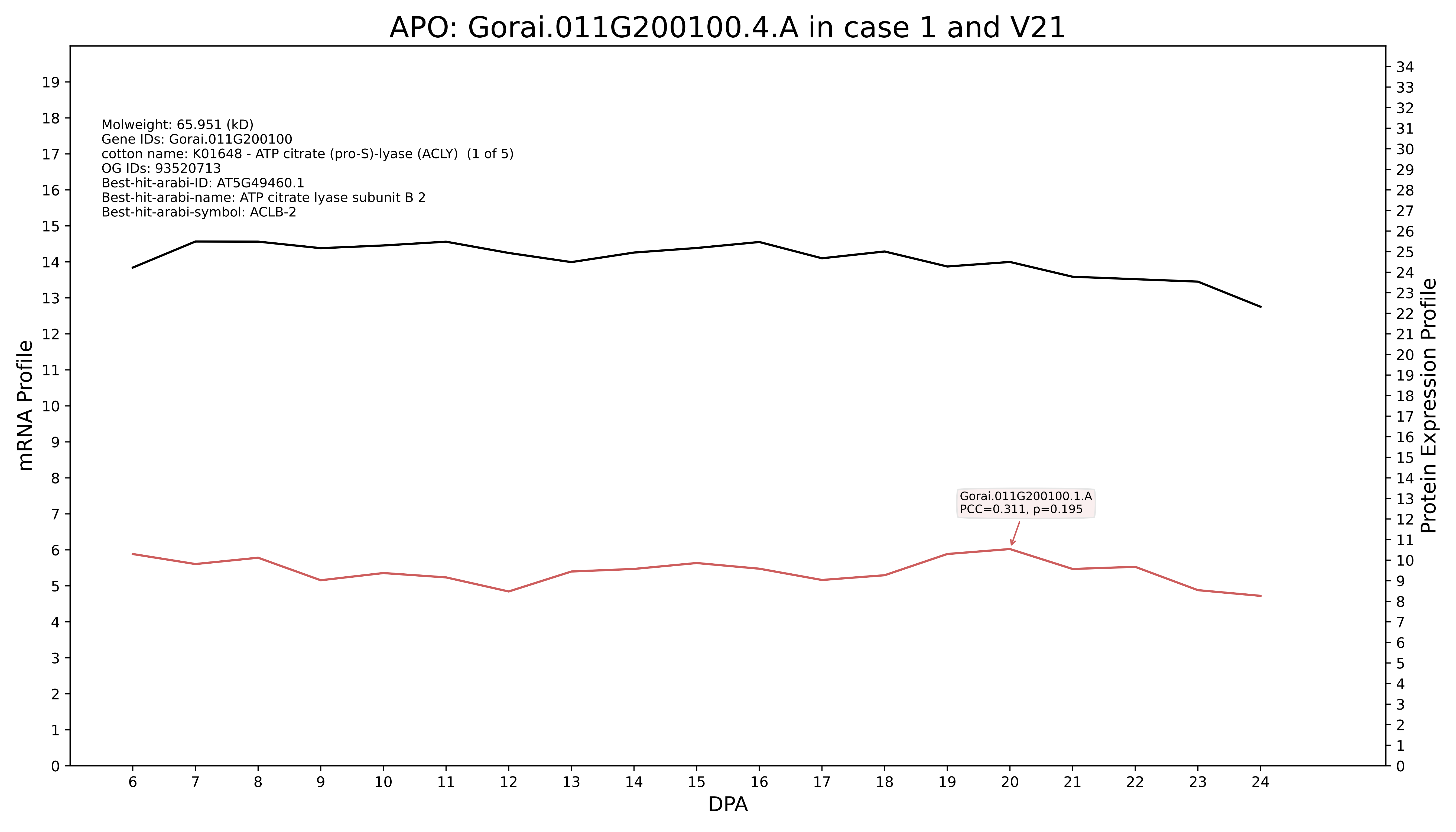
APO: Gorai.011G200100.4.A in case 1 and V21
Molweight: 65.951 (kD)
Gene IDs: Gorai.011G200100
cotton name: K01648 - ATP citrate (pro-S)-lyase (ACLY) (1 of 5)
OG IDs: 93520713
Best-hit-arabi-ID: AT5G49460.1
Best-hit-arabi-name: ATP citrate lyase subunit B 2
Best-hit-arabi-symbol: ACLB-2
Gorai.011G200100.1.A
PCC=0.311, p=0.195
mRNA Profile
Protein Expression Profile
DPA

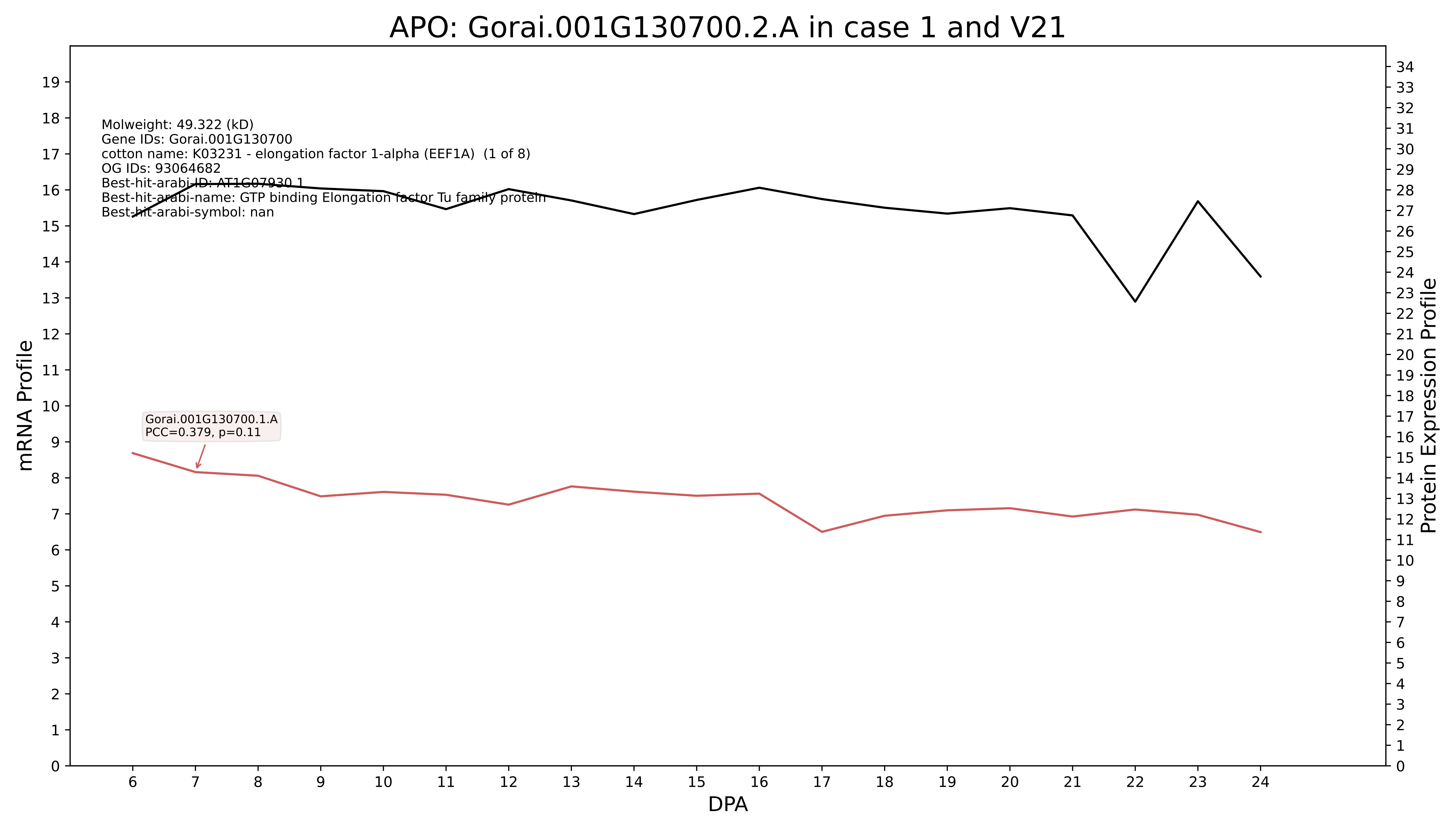
APO: Gorai.001G130700.2.A in case 1 and V21
Molweight: 49.322 (kD)
Gene IDs: Gorai.001G130700
cotton name: K03231 - elongation factor 1-alpha (EEF1A) (1 of 8)
OG IDs: 93064682
Best-hit-arabi-ID: AT1G07930.1
Best-hit-arabi-name: GTP binding Elongation factor Tu family protein
Best-hit-arabi-symbol: nan
Gorai.001G130700.1.A
PCC=0.379, p=0.11
mRNA Profile
Protein Expression Profile
DPA
0
1
2
3
4
5
6
7
8
9
10
11
12
13
14
15
16
17
18
19
20
21
22
23
24
25
26
27
28
29
30
31
32
33
34

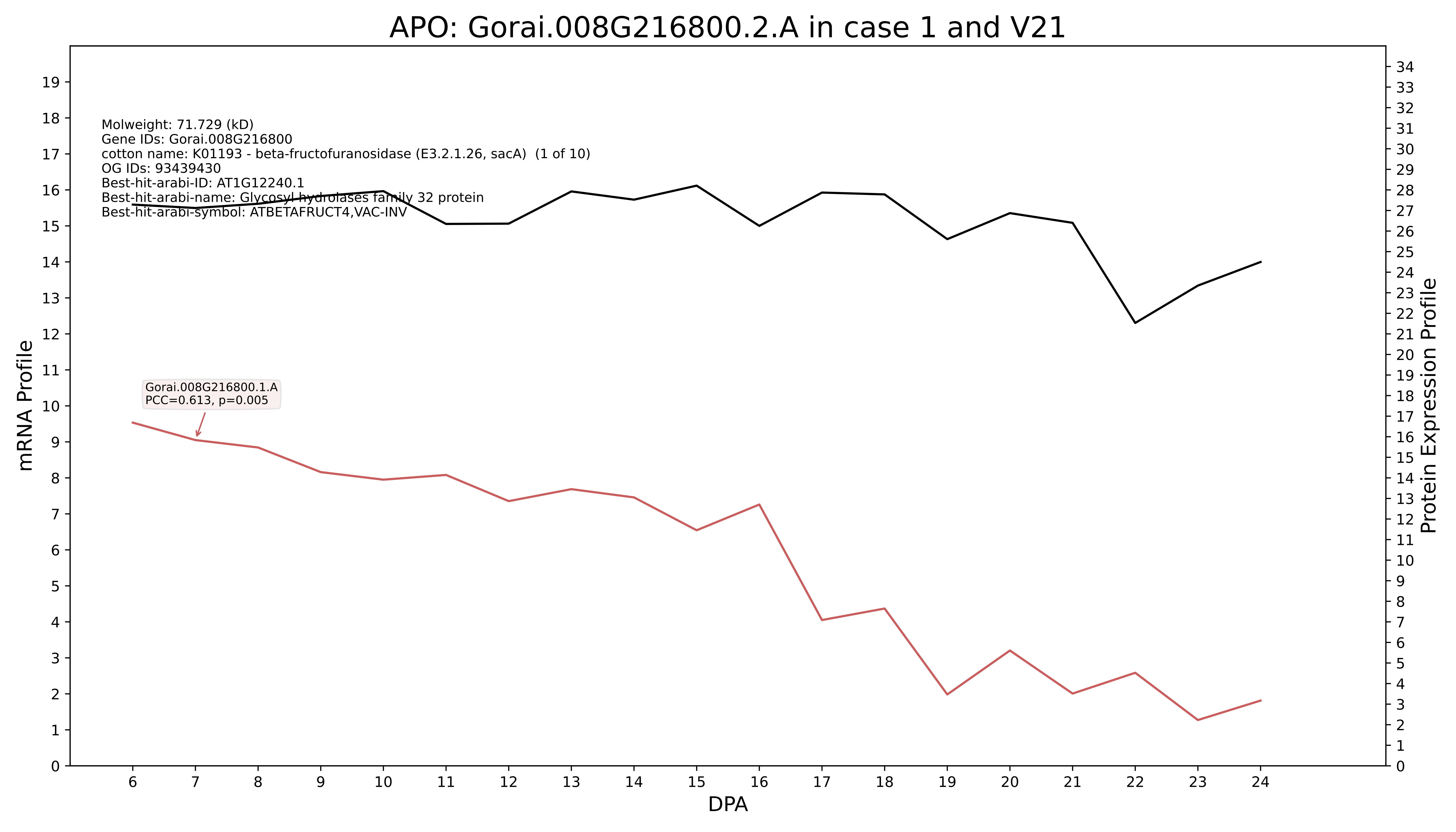
APO: Gorai.008G216800.2.A in case 1 and V21
Molweight: 71.729 (kD)
Gene IDs: Gorai.008G216800
cotton name: K01193 - beta-fructofuranosidase (E3.2.1.26, sacA) (1 of 10)
OG IDs: 93439430
Best-hit-arabi-ID: AT1G12240.1
Best-hit-arabi-name: Glycosyl hydrolases family 32 protein
Best-hit-arabi-symbol: ATBETAFRUCT4,VAC-INV
Gorai.008G216800.1.A
PCC=0.613, p=0.005
mRNA Profile
Protein Expression Profile
DPA

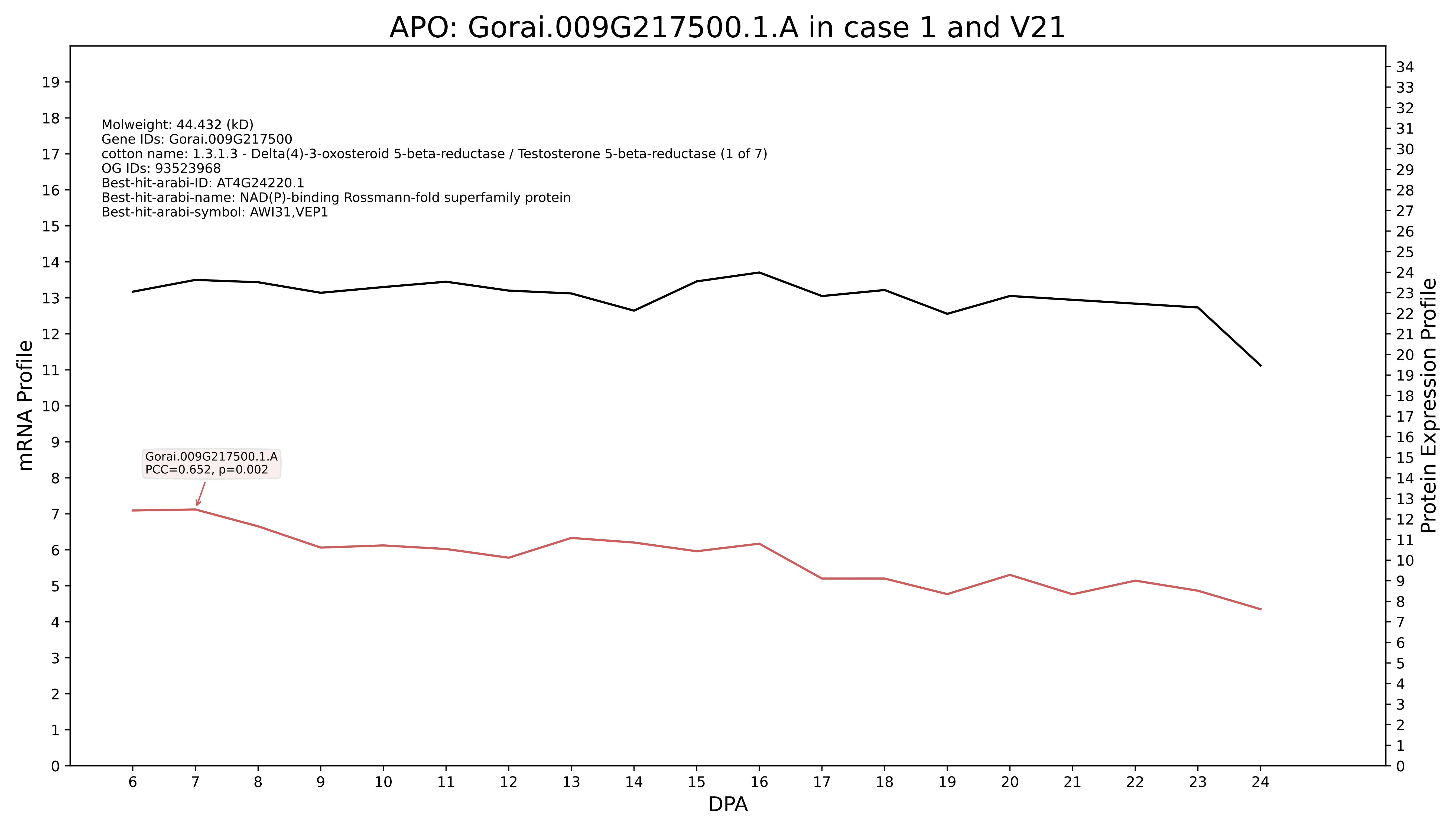
APO: Gorai.009G217500.1.A in case 1 and V21
Molweight: 44.432 (kD)
Gene IDs: Gorai.009G217500
cotton name: 1.3.1.3 - Delta(4)-3-oxosteroid 5-beta-reductase / Testosterone 5-beta-reductase (1 of 7)
OG IDs: 93523968
Best-hit-arabi-ID: AT4G24220.1
Best-hit-arabi-name: NAD(P)-binding Rossmann-fold superfamily protein
Best-hit-arabi-symbol: AWI31,VEP1
Gorai.009G217500.1.A
PCC=0.652, p=0.002
mRNA Profile
Protein Expression Profile
DPA

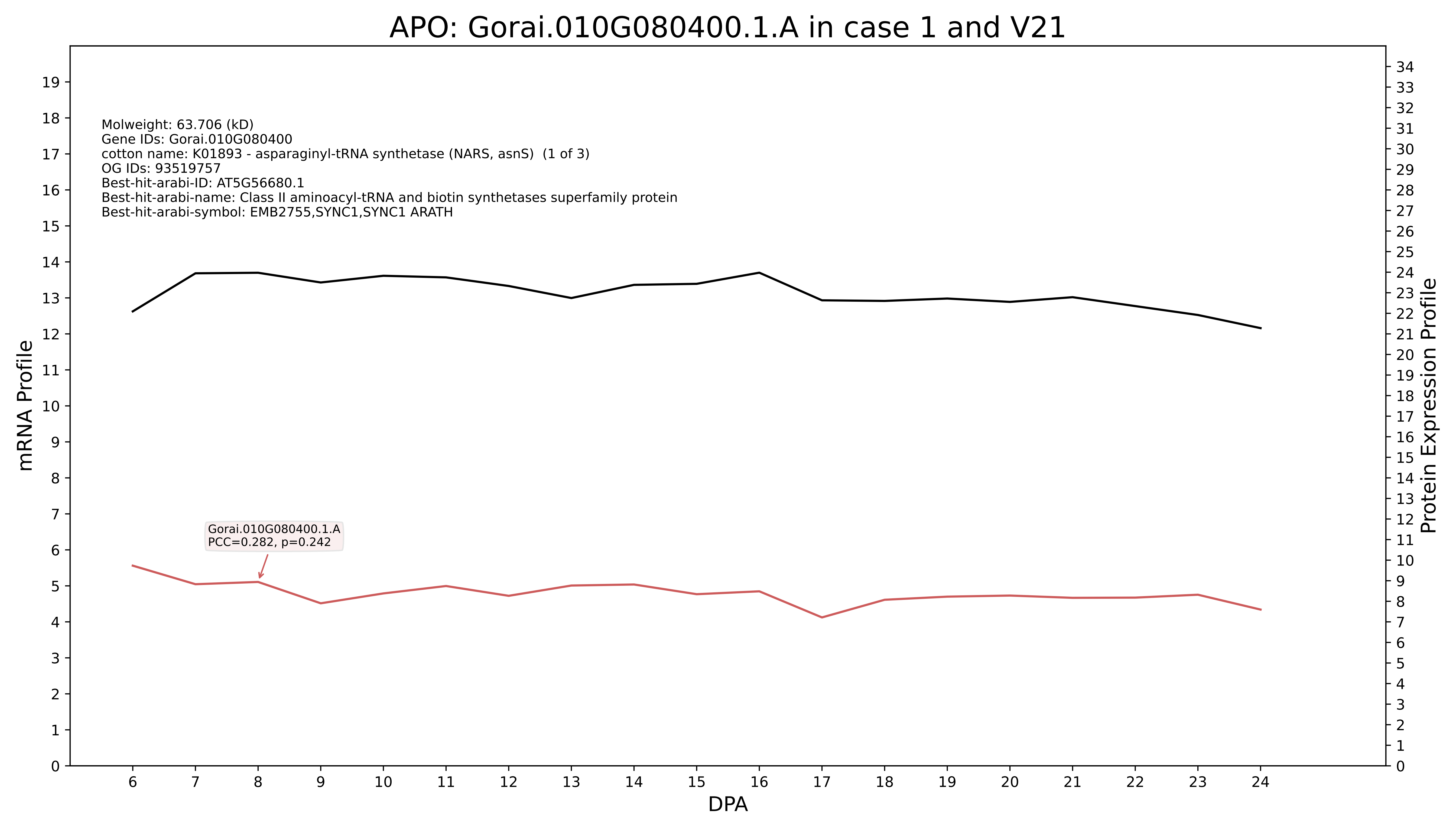

APO: Gorai.010G080400.1.A in case 1 and V21
Molweight: 63.706 (kD)
Gene IDs: Gorai.010G080400
cotton name: K01893 - asparaginyl-tRNA synthetase (NARS, asnS) (1 of 3)
OG IDs: 93519757
Best-hit-arabi-ID: AT5G56680.1
Best-hit-arabi-name: Class II aminoacyl-tRNA and biotin synthetases superfamily protein
Best-hit-arabi-symbol: EMB2755,SYNC1,SYNC1 ARATH
Gorai.010G080400.1.A
PCC=0.282, p=0.242
mRNA Profile
Protein Expression Profile
DPA

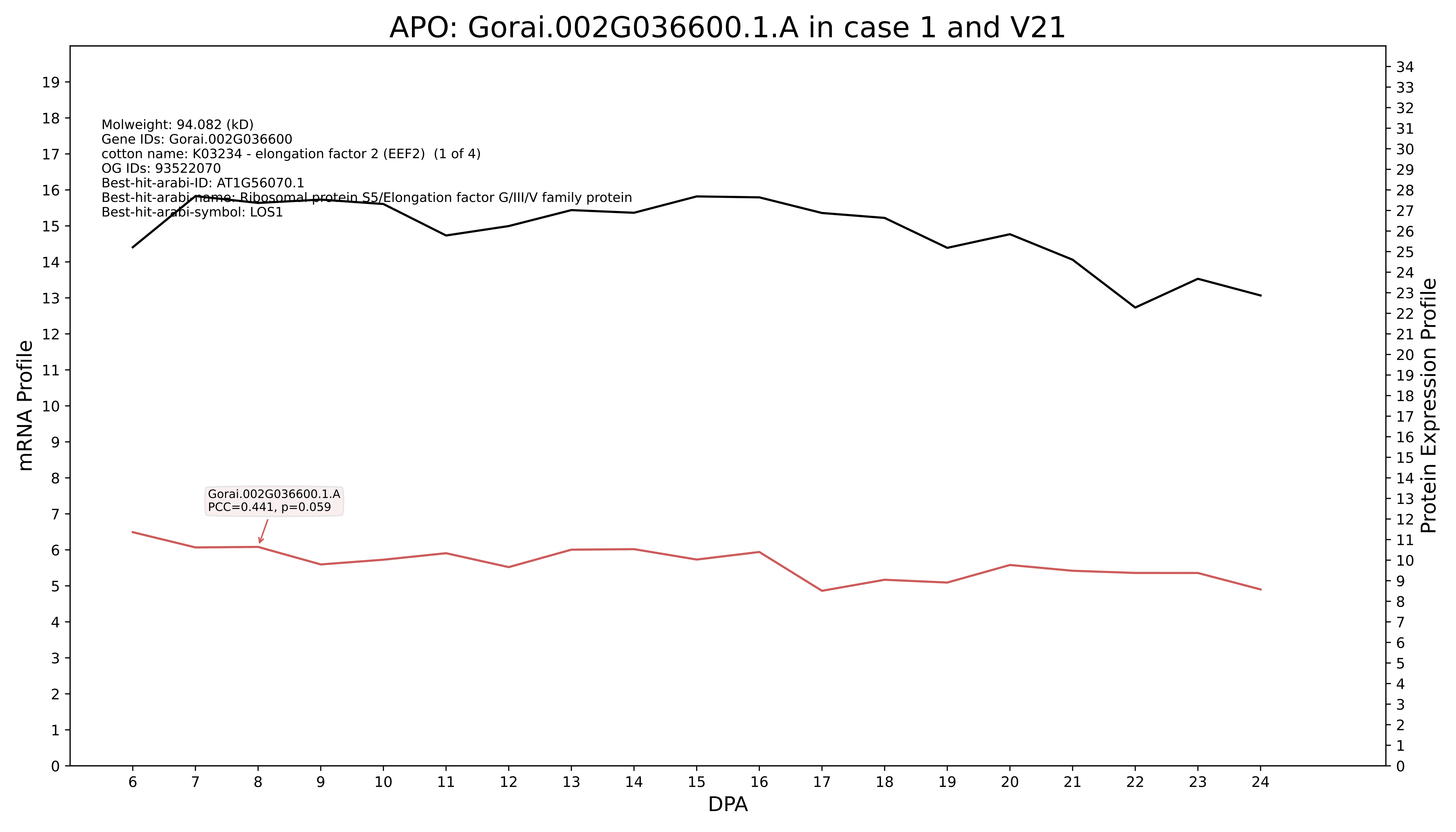

APO: Gorai.002G036600.1.A in case 1 and V21
Molweight: 94.082 (kD)
Gene IDs: Gorai.002G036600
cotton name: K03234 - elongation factor 2 (EEF2) (1 of 4)
OG IDs: 93522070
Best-hit-arabi-ID: AT1G56070.1
Best-hit-arabi-name: Ribosomal protein S5/Elongation factor G/III/V family protein
Best-hit-arabi-symbol: LOS1
Gorai.002G036600.1.A
PCC=0.441, p=0.059
mRNA Profile
Protein Expression Profile
DPA

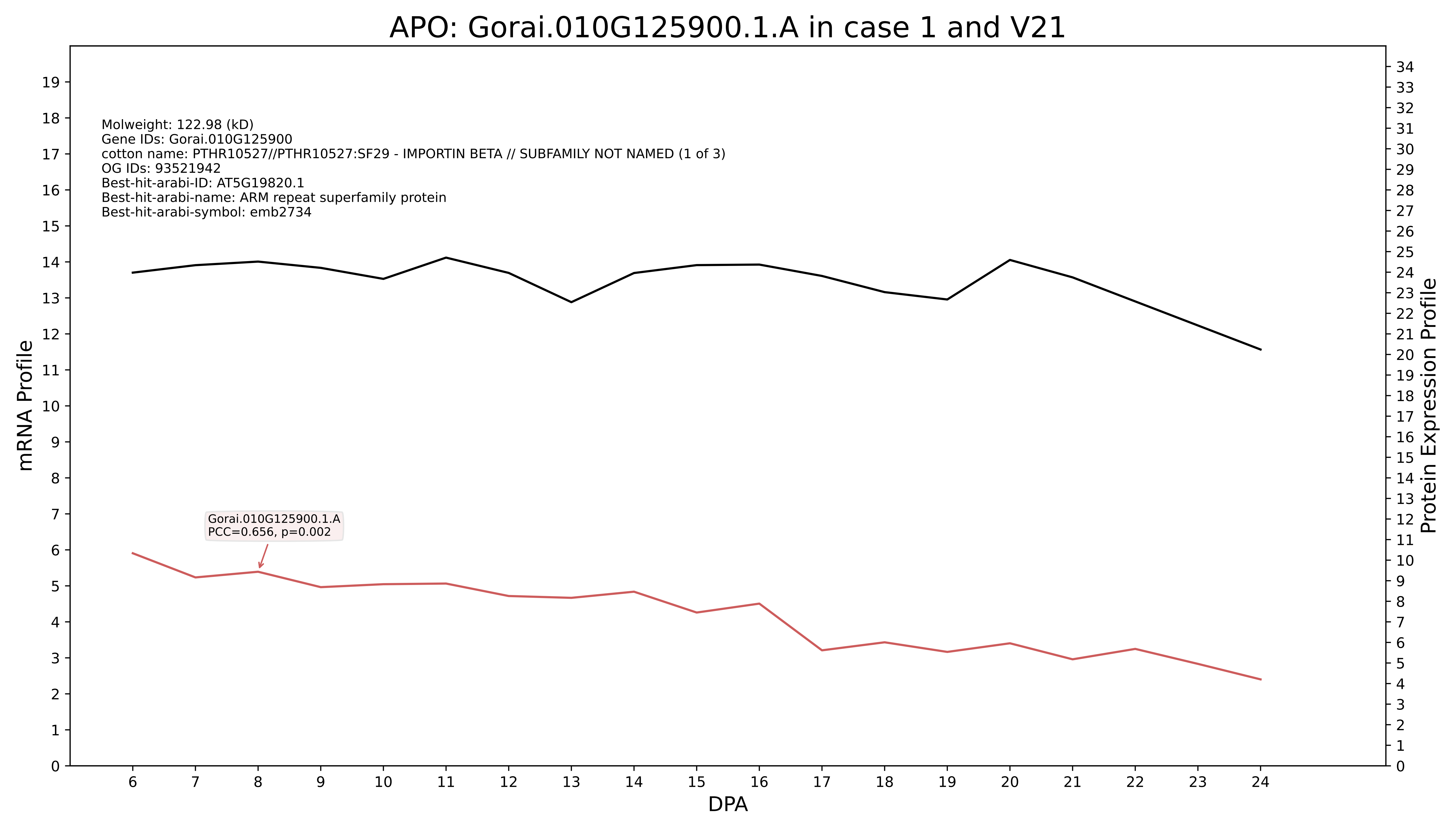
APO: Gorai.010G125900.1.A in case 1 and V21
Molweight: 122.98 (kD)
Gene IDs: Gorai.010G125900
cotton name: PTHR10527//PTHR10527:SF29 - IMPORTIN BETA // SUBFAMILY NOT NAMED (1 of 3)
OG IDs: 93521942
Best-hit-arabi-ID: AT5G19820.1
Best-hit-arabi-name: ARM repeat superfamily protein
Best-hit-arabi-symbol: emb2734
Gorai.010G125900.1.A
PCC=0.656, p=0.002
mRNA Profile
Protein Expression Profile
DPA

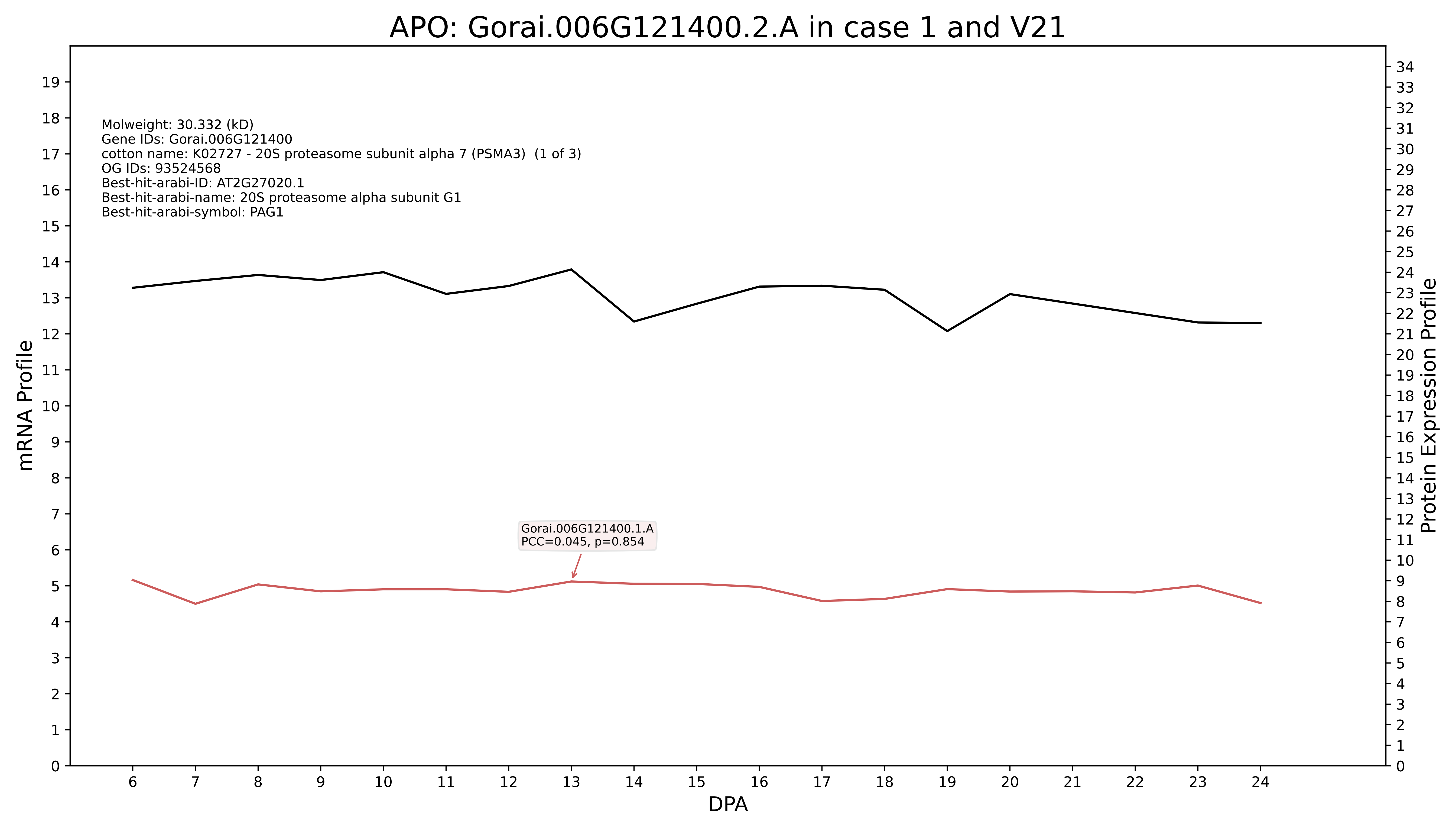
APO: Gorai.006G121400.2.A in case 1 and V21
Molweight: 30.332 (kD)
Gene IDs: Gorai.006G121400
cotton name: K02727 - 20S proteasome subunit alpha 7 (PSMA3) (1 of 3)
OG IDs: 93524568
Best-hit-arabi-ID: AT2G27020.1
Best-hit-arabi-name: 20S proteasome alpha subunit G1
Best-hit-arabi-symbol: PAG1
Gorai.006G121400.1.A
PCC=0.045, p=0.854
mRNA Profile
Protein Expression Profile
DPA

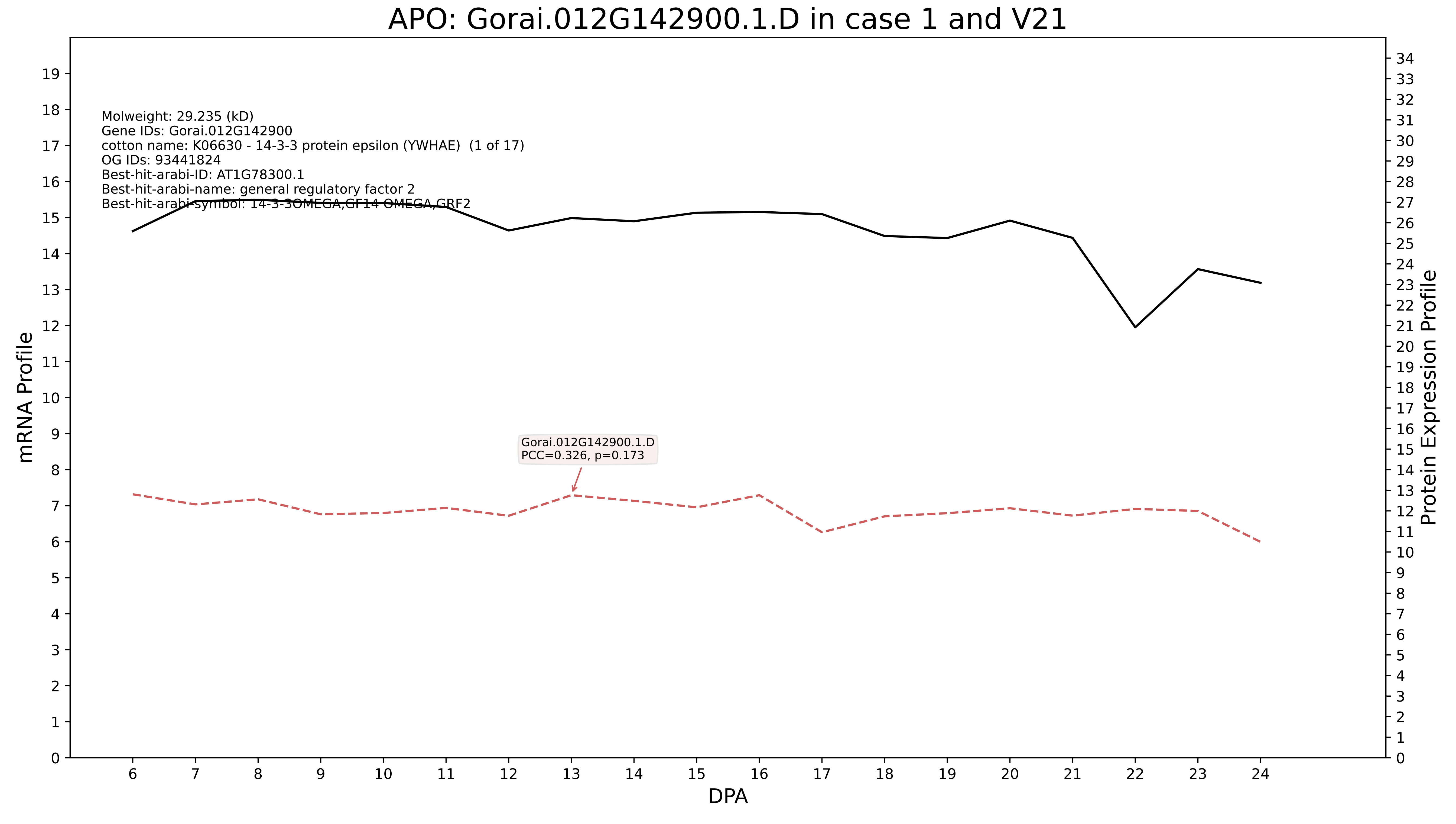

APO: Gorai.012G142900.1.D in case 1 and V21
Molweight: 29.235 (kD)
Gene IDs: Gorai.012G142900
cotton name: K06630 - 14-3-3 protein epsilon (YWHAE) (1 of 17)
OG IDs: 93441824
Best-hit-arabi-ID: AT1G78300.1
Best-hit-arabi-name: general regulatory factor 2
Best-hit-arabi-symbol: 14-3-3OMEGA,GF14 OMEGA,GRF2
Gorai.012G142900.1.D
PCC=0.326, p=0.173
mRNA Profile
Protein Expression Profile
DPA

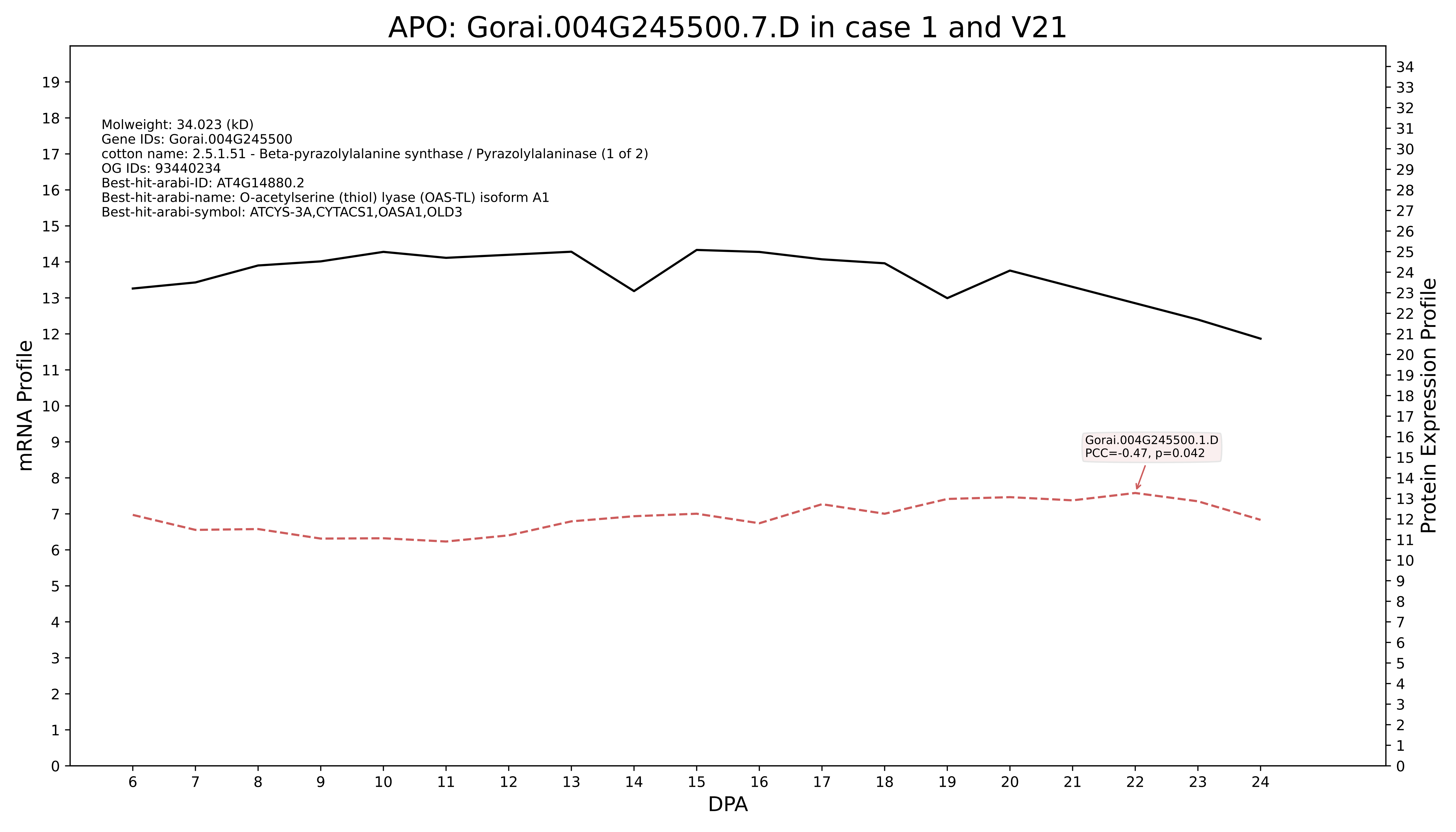

APO: Gorai.004G245500.7.D in case 1 and V21
Molweight: 34.023 (kD)
Gene IDs: Gorai.004G245500
cotton name: 2.5.1.51 - Beta-pyrazolylalanine synthase / Pyrazolylalaninase (1 of 2)
OG IDs: 93440234
Best-hit-arabi-ID: AT4G14880.2
Best-hit-arabi-name: O-acetylserine (thiol) lyase (OAS-TL) isoform A1
Best-hit-arabi-symbol: ATCYS-3A,CYTACS1,OASA1,OLD3
Gorai.004G245500.1.D
PCC=-0.47, p=0.042
mRNA Profile
Protein Expression Profile
DPA

APO: Gorai.009G143100.1.A in case 1 and V21

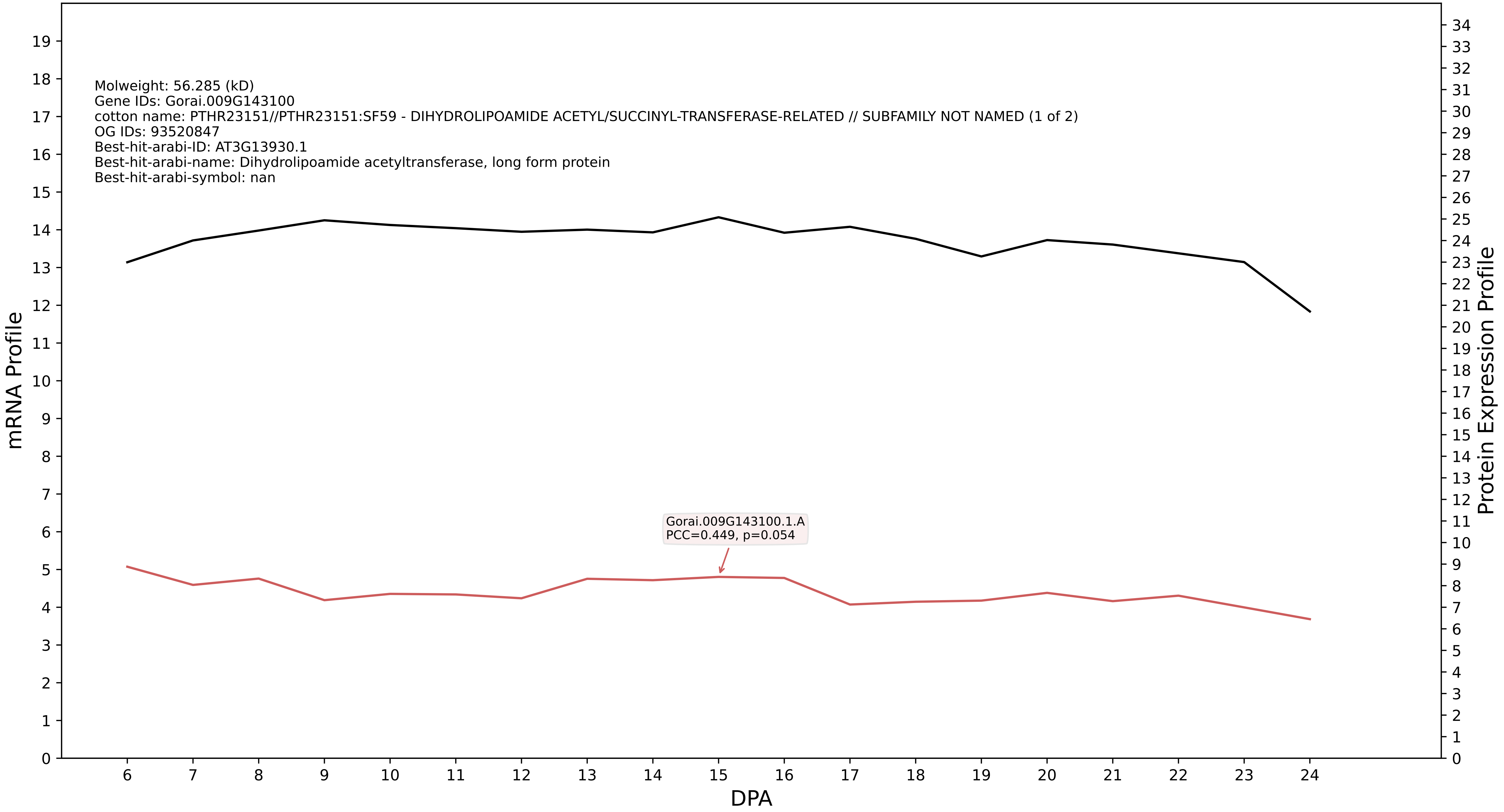

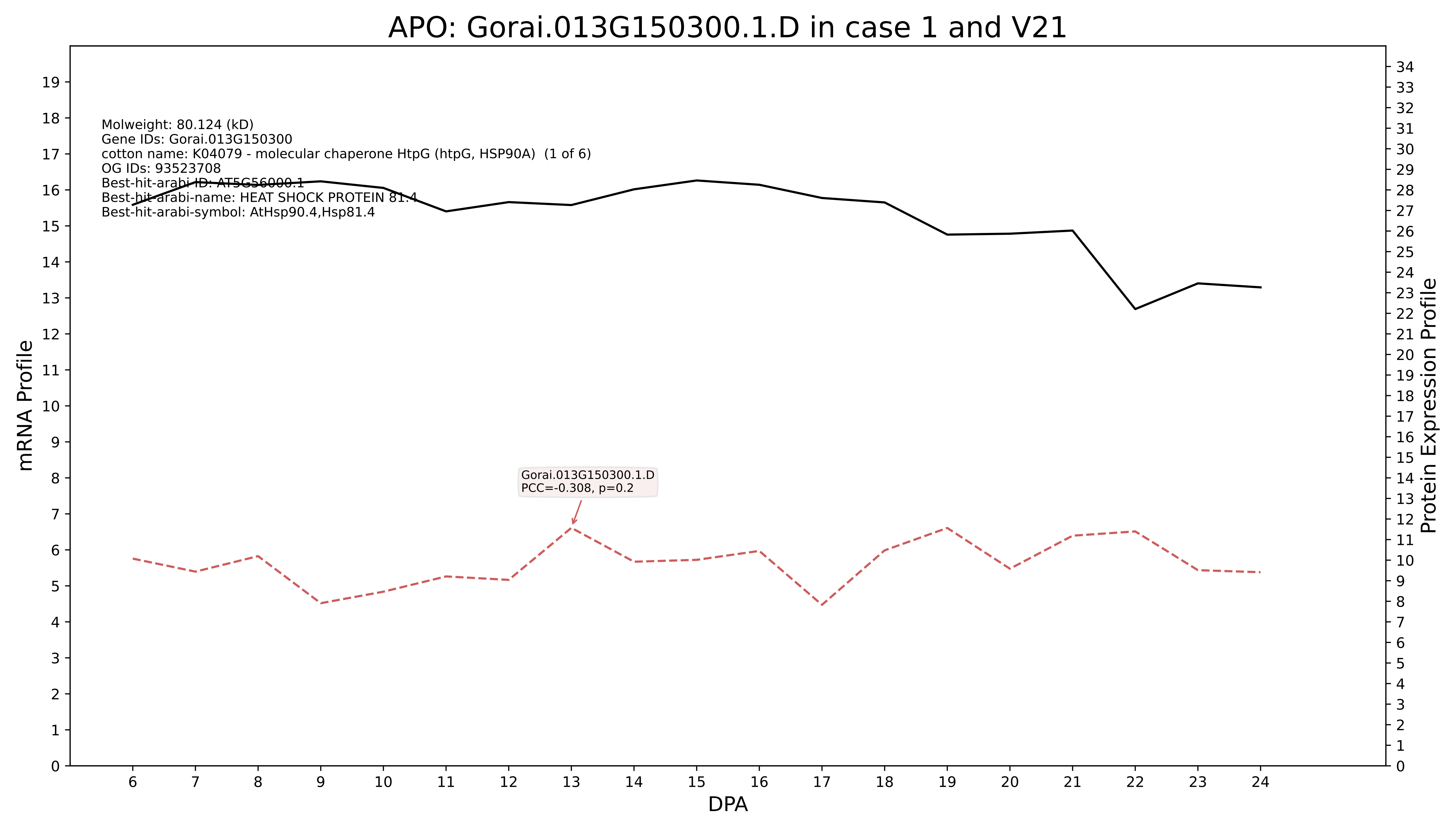
APO: Gorai.013G150300.1.D in case 1 and V21
Molweight: 80.124 (kD)
Gene IDs: Gorai.013G150300
cotton name: K04079 - molecular chaperone HtpG (htpG, HSP90A) (1 of 6)
OG IDs: 93523708
Best-hit-arabi ID: AT5G56000.1
Best-hit-arabi-name: HEAT SHOCK PROTEIN 81.4
Best-hit-arabi-symbol: AtHsp90.4,Hsp81.4
Gorai.013G150300.1.D
PCC=-0.308, p=0.2
mRNA Profile
Protein Expression Profile
DPA

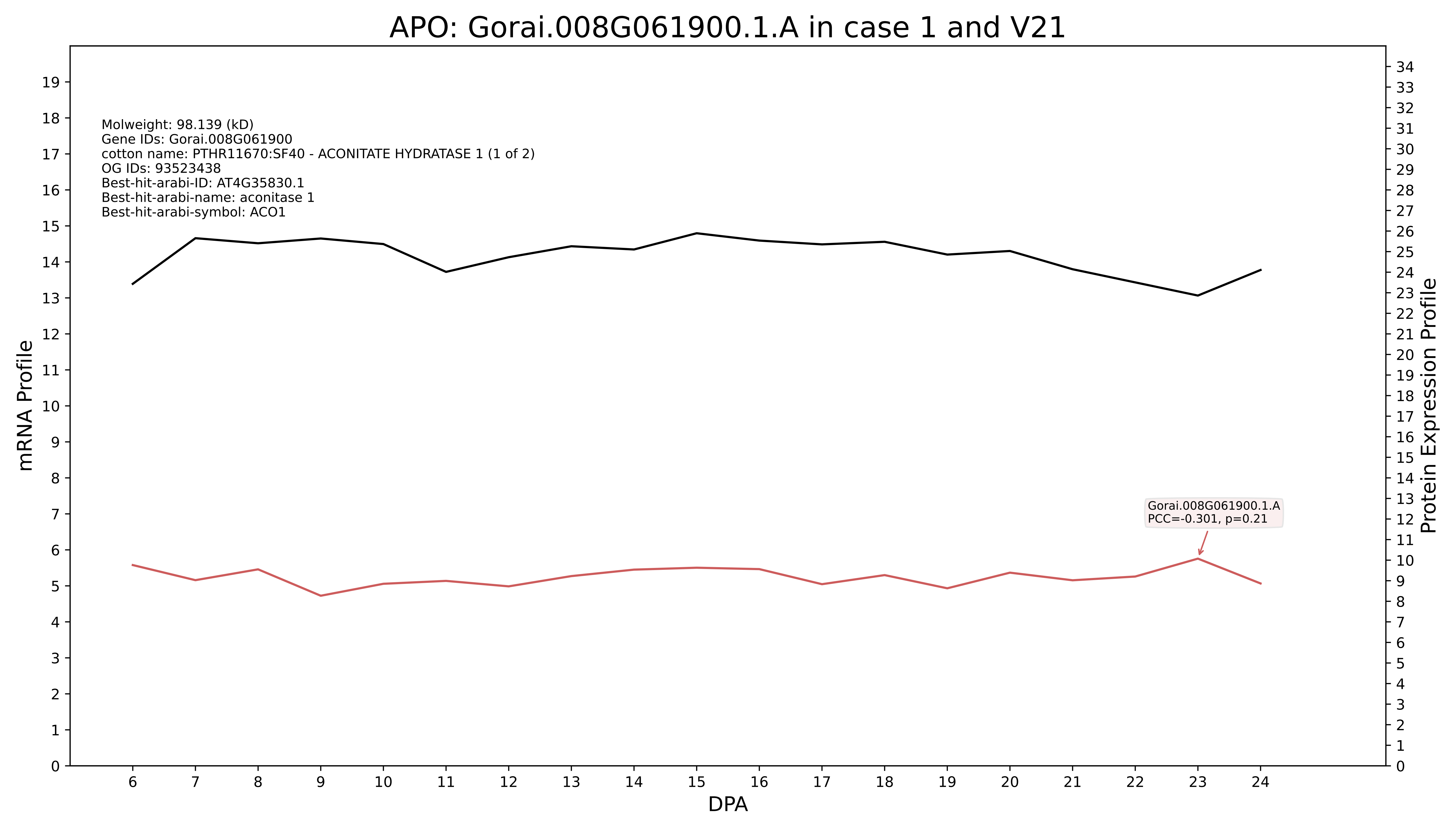
APO: Gorai.008G061900.1.A in case 1 and V21
Molweight: 98.139 (kD)
Gene IDs: Gorai.008G061900
cotton name: PTHR11670:SF40 - ACONITATE HYDRATASE 1 (1 of 2)
OG IDs: 93523438
Best-hit-arabi-ID: AT4G35830.1
Best-hit-arabi-name: aconitase 1
Best-hit-arabi-symbol: ACO1
Gorai.008G061900.1.A
PCC=-0.301, p=0.21
mRNA Profile
Protein Expression Profile
DPA

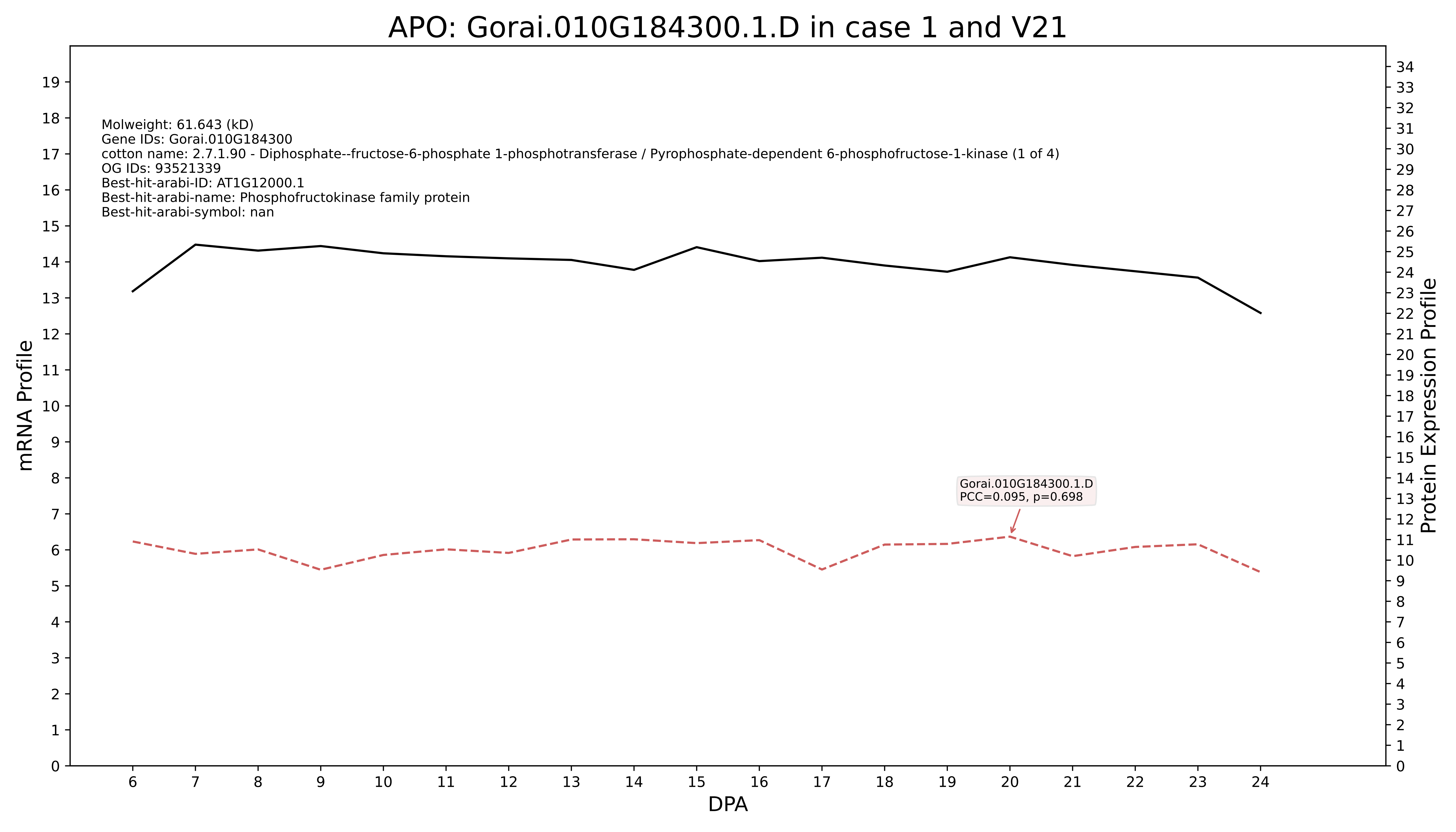

APO: Gorai.010G184300.1.D in case 1 and V21
Molweight: 61.643 (kD)
Gene IDs: Gorai.010G184300
cotton name: 2.7.1.90 - Diphosphate--fructose-6-phosphate 1-phosphotransferase / Pyrophosphate-dependent 6-phosphofructose-1-kinase (1 of 4)
OG IDs: 93521339
Best-hit-arabi-ID: AT1G12000.1
Best-hit-arabi-name: Phosphofructokinase family protein
Best-hit-arabi-symbol: nan
Gorai.010G184300.1.D
PCC=0.095, p=0.698
mRNA Profile
Protein Expression Profile
DPA

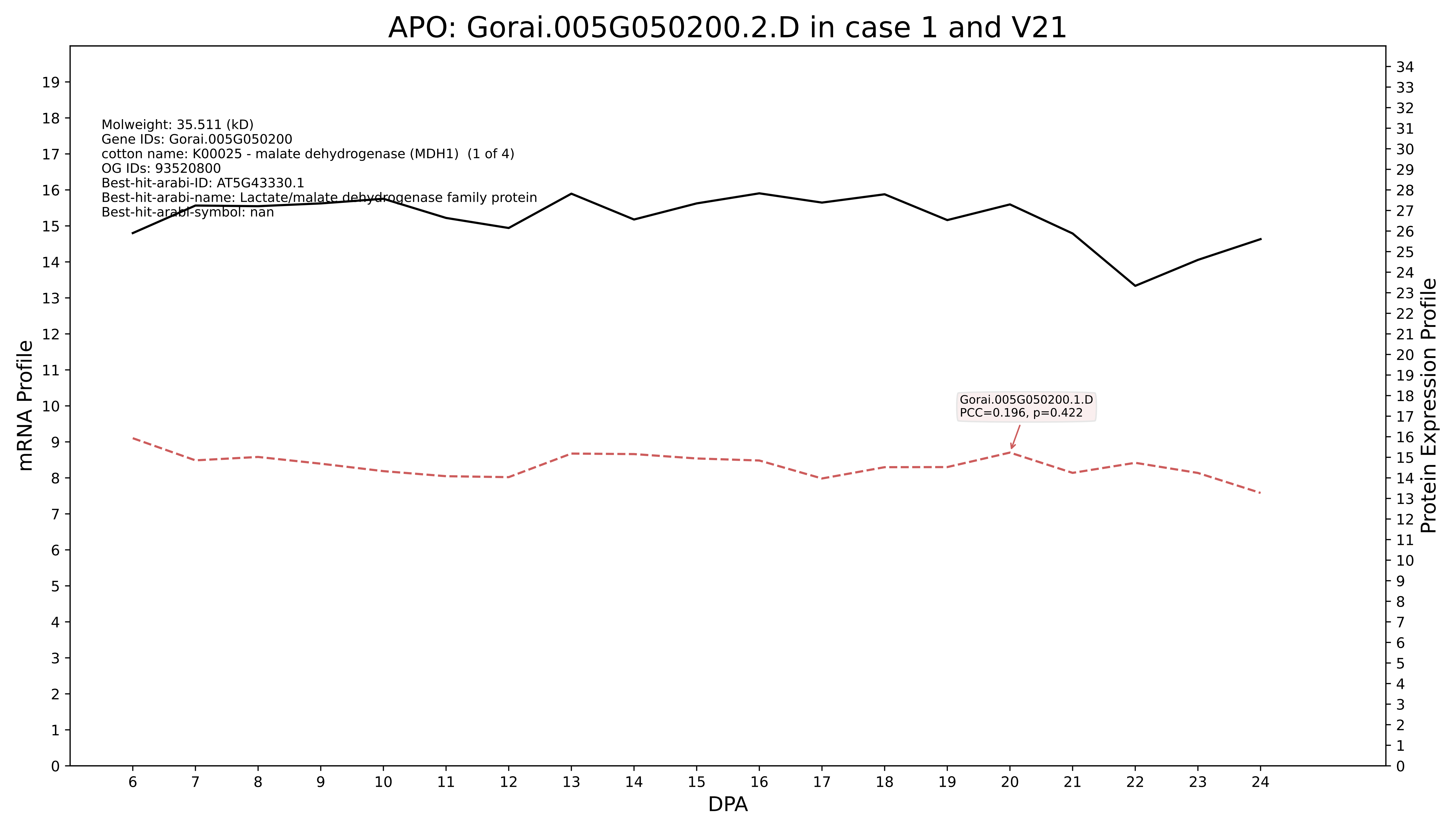
APO: Gorai.005G050200.2.D in case 1 and V21
Molweight: 35.511 (kD)
Gene IDs: Gorai.005G050200
cotton name: K00025 - malate dehydrogenase (MDH1) (1 of 4)
OG IDs: 93520800
Best-hit-arabi-ID: AT5G43330.1
Best-hit-arabi-name: Lactate/malate dehydrogenase family protein
Best-hit-arabi-symbol: nan
Gorai.005G050200.1.D
PCC=0.196, p=0.422
mRNA Profile
Protein Expression Profile
DPA

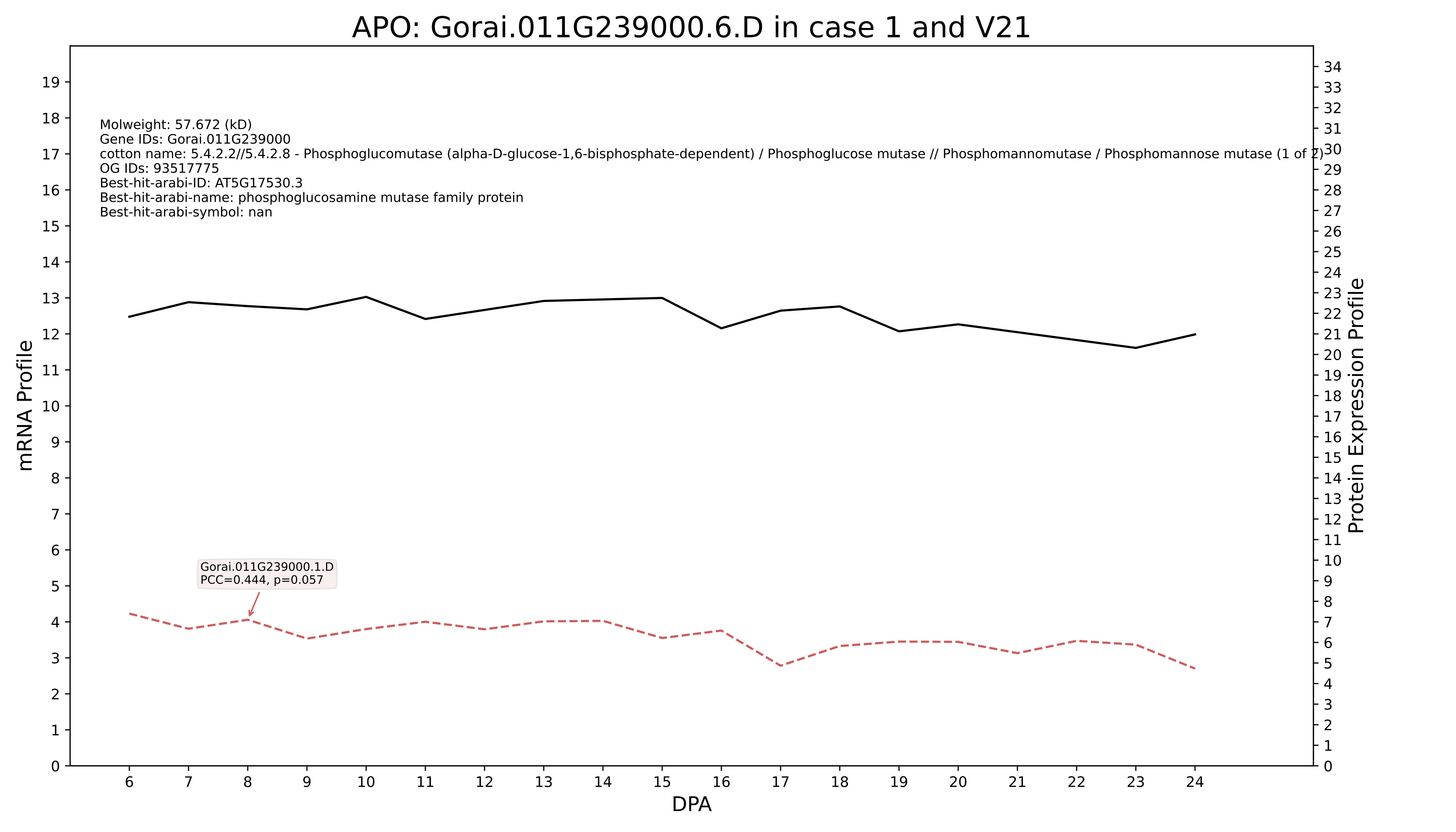

APO: Gorai.011G239000.6.D in case 1 and V21
Molweight: 57.672 (kD)
Gene IDs: Gorai.011G239000
cotton name: 5.4.2.2//5.4.2.8 - Phosphoglucomutase (alpha-D-glucose-1,6-bisphosphate-dependent) / Phosphoglucose mutase // Phosphomannomutase / Phosphomannose mutase (1 of 2)
OG IDs: 93517775
Best-hit-arabi-ID: AT5G17530.3
Best-hit-arabi-name: phosphoglucosamine mutase family protein
Best-hit-arabi-symbol: nan
Gorai.011G239000.1.D
PCC=0.444, p=0.057
mRNA Profile
Protein Expression Profile
DPA

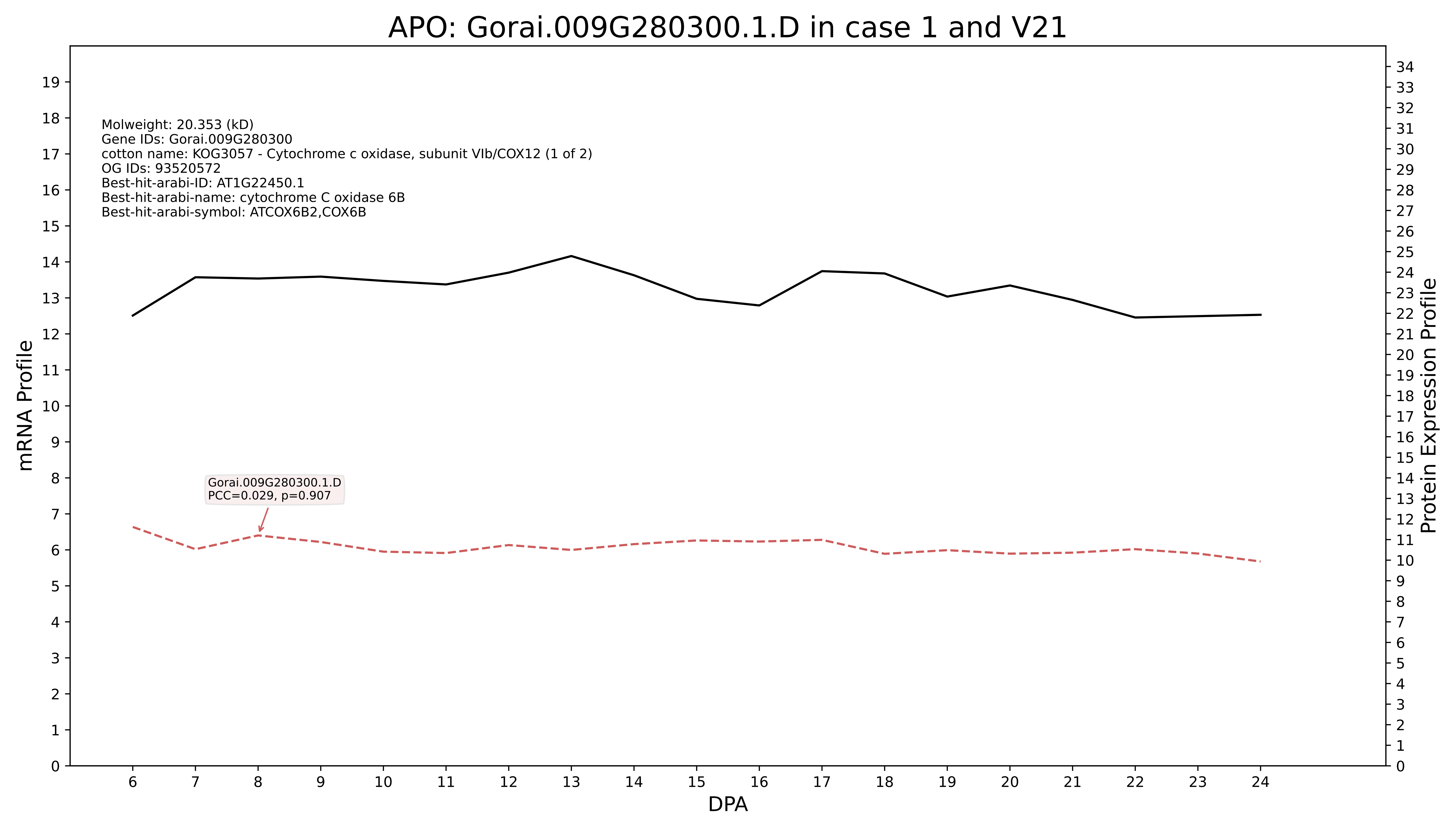
APO: Gorai.009G280300.1.D in case 1 and V21
Molweight: 20.353 (kD)
Gene IDs: Gorai.009G280300
cotton name: KOG3057 - Cytochrome c oxidase, subunit VIb/COX12 (1 of 2)
OG IDs: 93520572
Best-hit-arabi-ID: AT1G22450.1
Best-hit-arabi-name: cytochrome C oxidase 6B
Best-hit-arabi-symbol: ATCOX6B2,COX6B
Gorai.009G280300.1.D
PCC=0.029, p=0.907
mRNA Profile
Protein Expression Profile
DPA

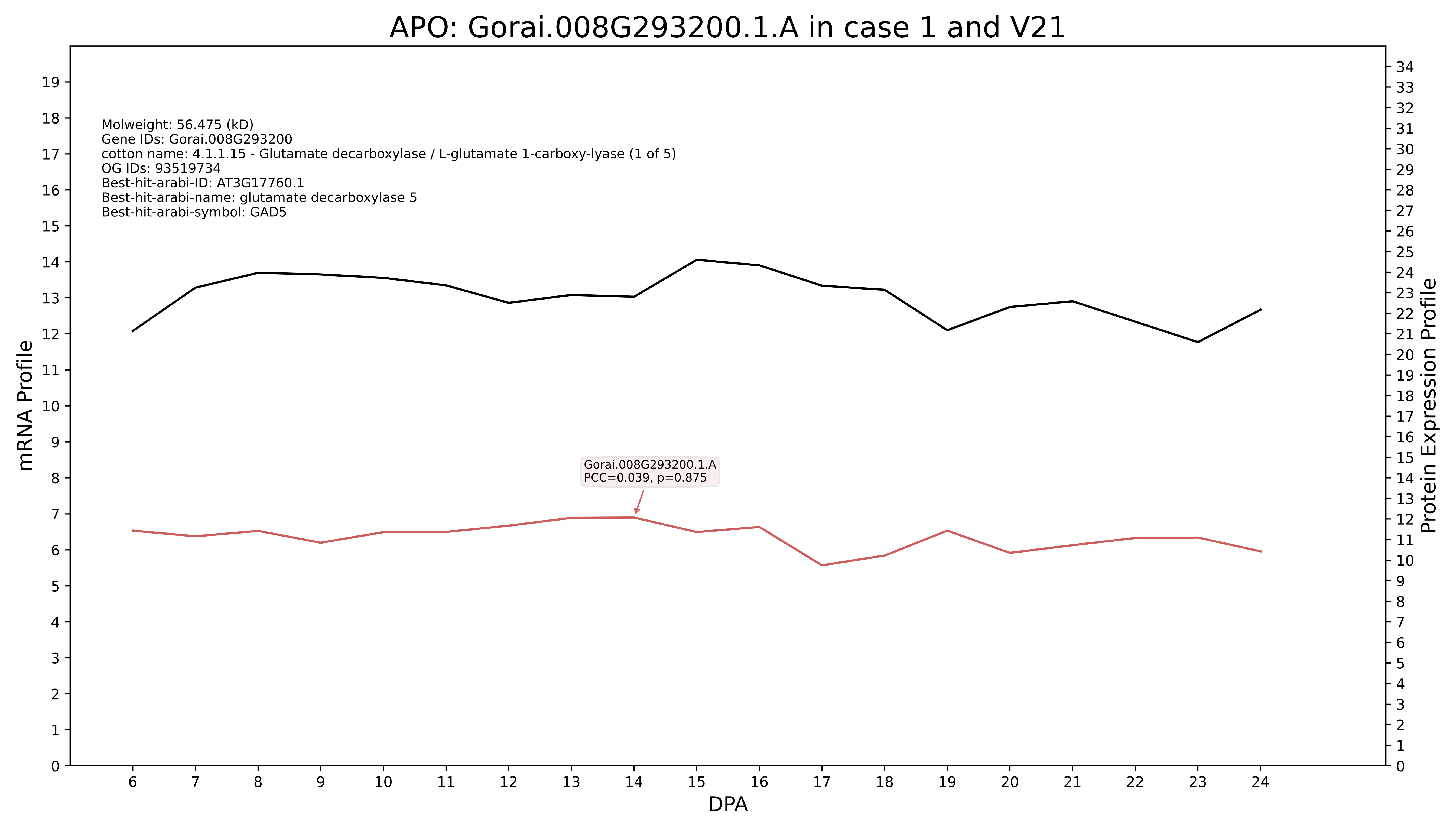
APO: Gorai.008G293200.1.A in case 1 and V21
Molweight: 56.475 (kD)
Gene IDs: Gorai.008G293200
cotton name: 4.1.1.15 - Glutamate decarboxylase / L-glutamate 1-carboxy-lyase (1 of 5)
OG IDs: 93519734
Best-hit-arabi-ID: AT3G17760.1
Best-hit-arabi-name: glutamate decarboxylase 5
Best-hit-arabi-symbol: GAD5
Gorai.008G293200.1.A
PCC=0.039, p=0.875
mRNA Profile
Protein Expression Profile
DPA

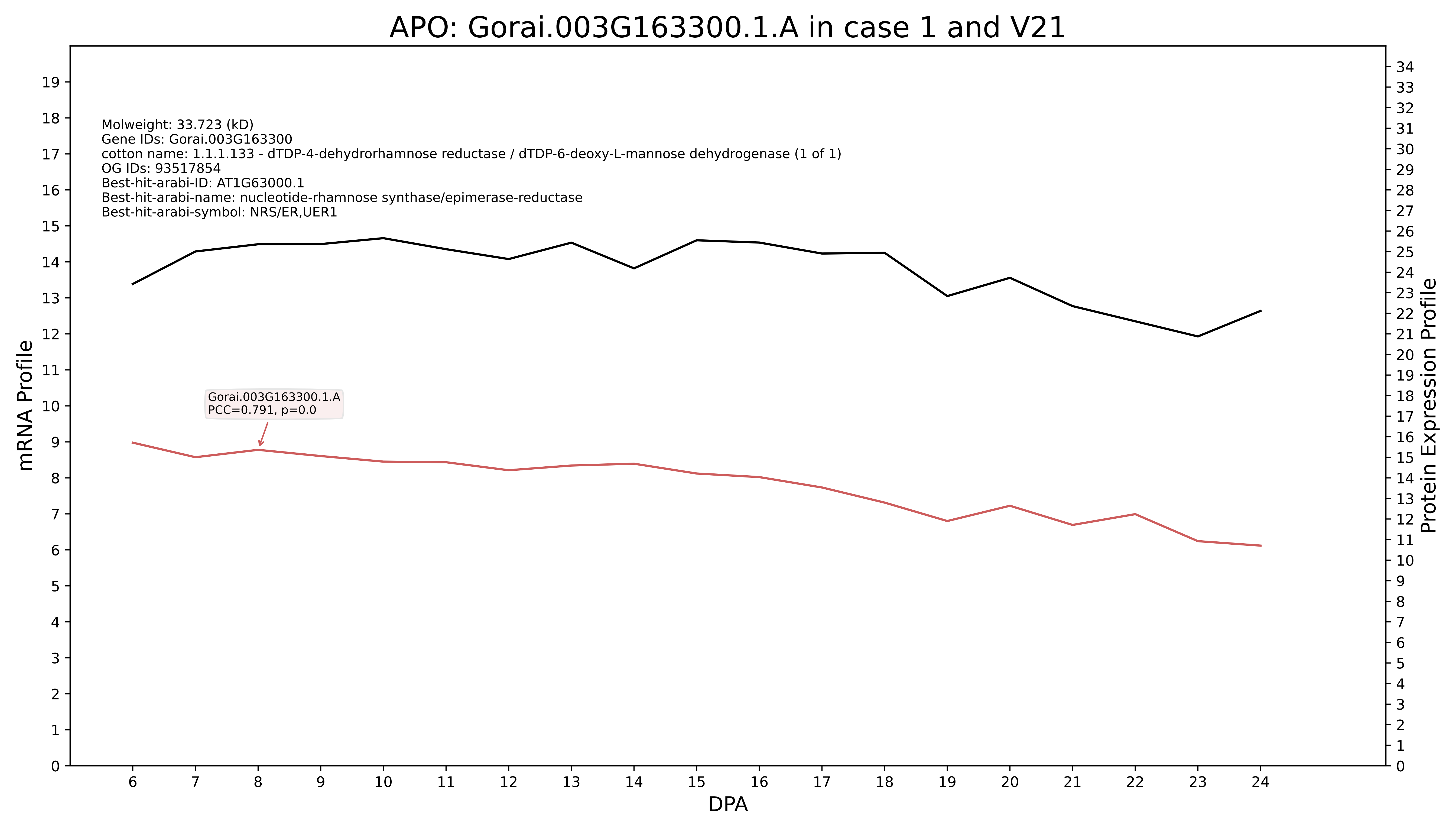
APO: Gorai.003G163300.1.A in case 1 and V21
Molweight: 33.723 (kD)
Gene IDs: Gorai.003G163300
cotton name: 1.1.1.133 - dTDP-4-dehydrorhamnose reductase / dTDP-6-deoxy-L-mannose dehydrogenase (1 of 1)
OG IDs: 93517854
Best-hit-arabi-ID: AT1G63000.1
Best-hit-arabi-name: nucleotide-rhamnose synthase/epimerase-reductase
Best-hit-arabi-symbol: NRS/ER,UER1
Gorai.003G163300.1.A
PCC=0.791, p=0.0
mRNA Profile
Protein Expression Profile
DPA

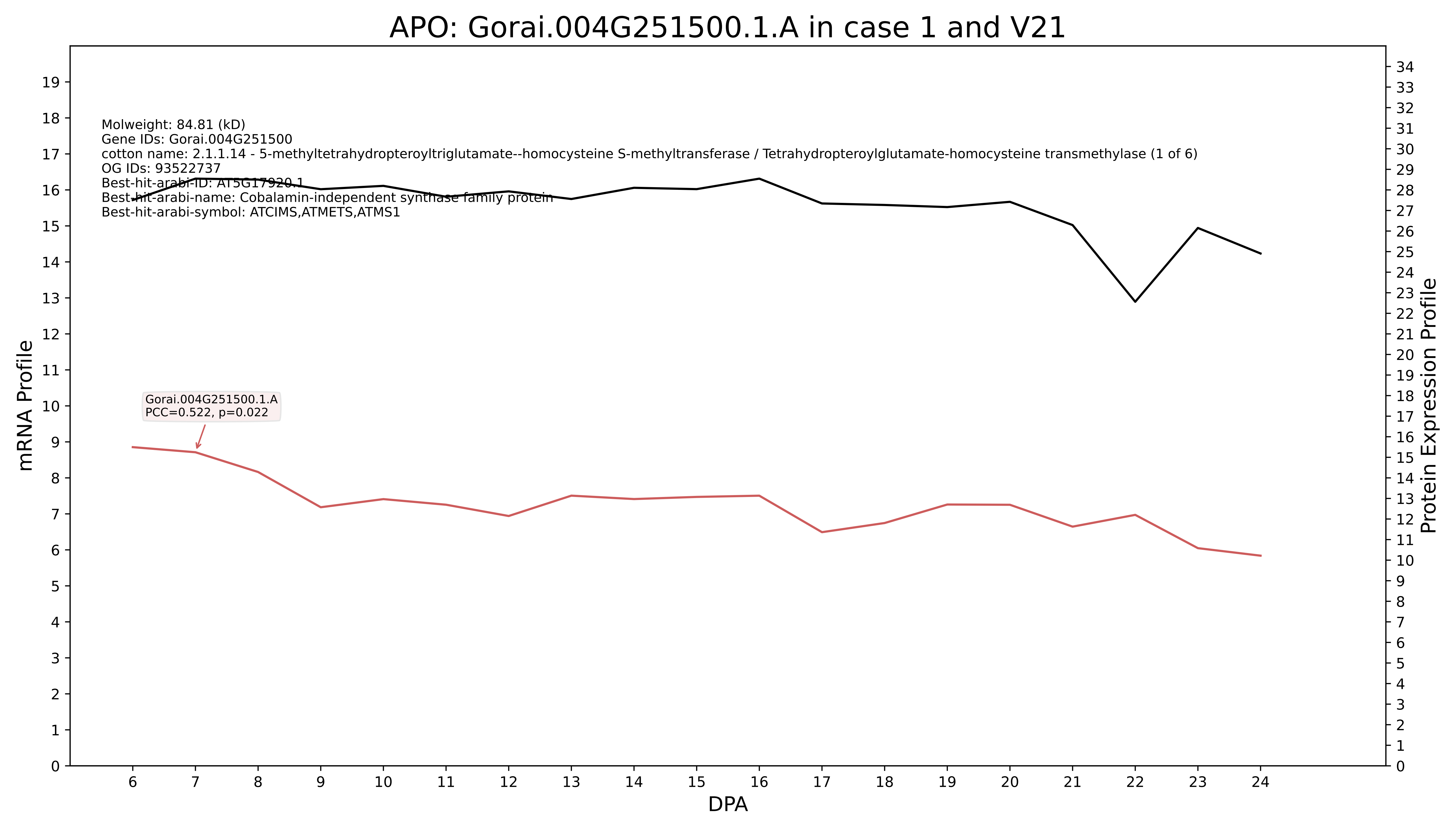

APO: Gorai.004G251500.1.A in case 1 and V21
Molweight: 84.81 (kD)
Gene IDs: Gorai.004G251500
cotton name: 2.1.1.14 - 5-methyltetrahydropteroyltriglutamate--homocysteine S-methyltransferase / Tetrahydropteroylglutamate-homocysteine transmethylase (1 of 6)
OG IDs: 93522737
Best-hit-arabi-ID: AT5G17920.1
Best-hit-arabi-name: Cobalamin-independent synthase family protein
Best-hit-arabi-symbol: ATCIMS,ATMETS,ATMS1
Gorai.004G251500.1.A
PCC=0.522, p=0.022
mRNA Profile
Protein Expression Profile
DPA

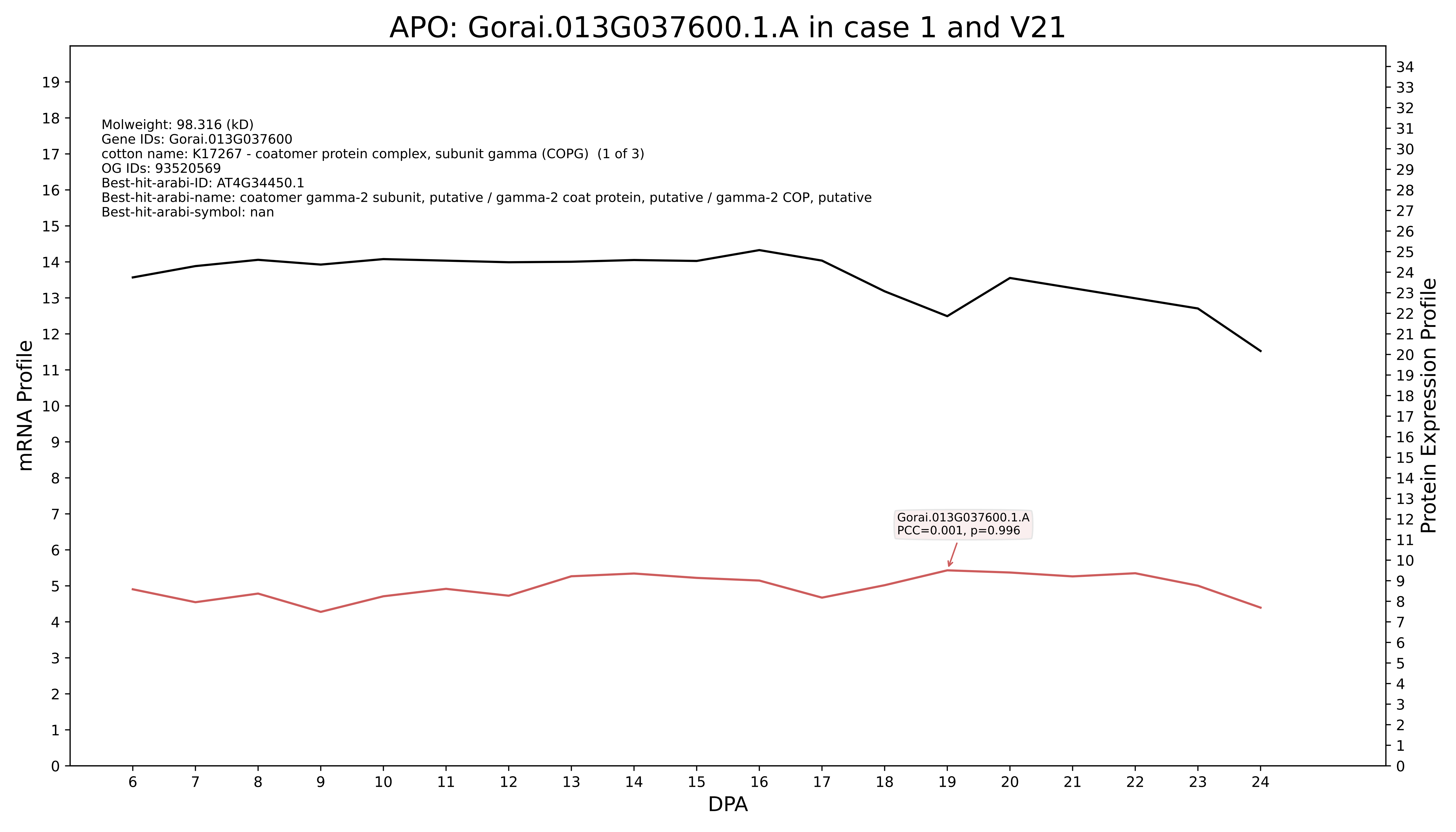
APO: Gorai.013G037600.1.A in case 1 and V21
Molweight: 98.316 (kD)
Gene IDs: Gorai.013G037600
cotton name: K17267 - coatomer protein complex, subunit gamma (COPG) (1 of 3)
OG IDs: 93520569
Best-hit-arabi-ID: AT4G34450.1
Best-hit-arabi-name: coatomer gamma-2 subunit, putative / gamma-2 coat protein, putative / gamma-2 COP, putative
Best-hit-arabi-symbol: nan
Gorai.013G037600.1.A
PCC=0.001, p=0.996
mRNA Profile
Protein Expression Profile
DPA
0 1 2 3 4 5 6 7 8 9 10 11 12 13 14 15 16 17 18 19
0 1 2 3 4 5 6 7 8 9 10 11 12 13 14 15 16 17 18 19 20 21 22 23 24 25 26 27 28 29 30 31 32 33 34
6 7 8 9 10 11 12 13 14 15 16 17 18 19 20 21 22 23 24

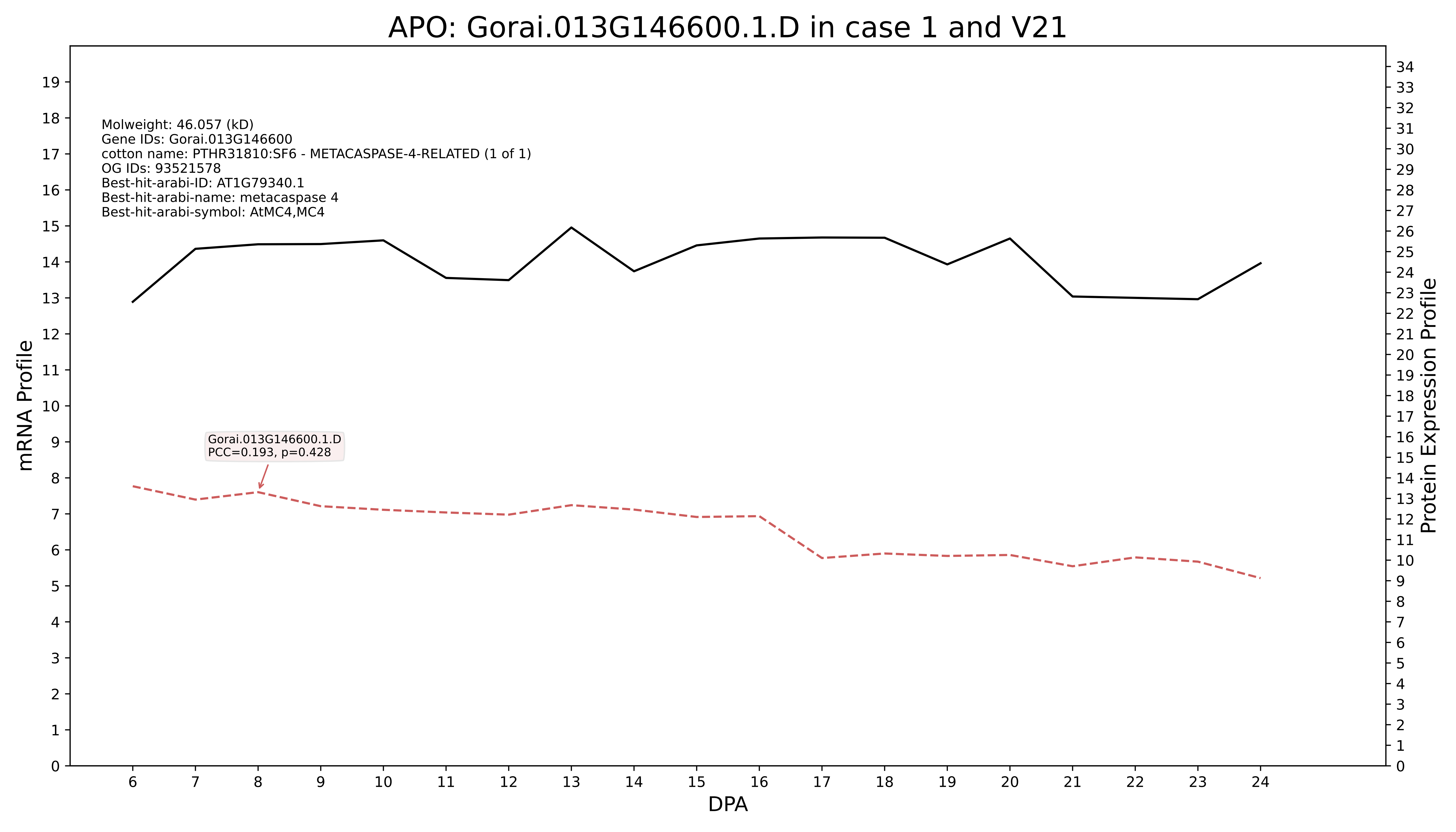
APO: Gorai.013G146600.1.D in case 1 and V21
Molweight: 46.057 (kD)
Gene IDs: Gorai.013G146600
cotton name: PTHR31810:SF6 - METACASPASE-4-RELATED (1 of 1)
OG IDs: 93521578
Best-hit-arabi-ID: AT1G79340.1
Best-hit-arabi-name: metacaspase 4
Best-hit-arabi-symbol: AtMC4,MC4
Gorai.013G146600.1.D
PCC=0.193, p=0.428
mRNA Profile
Protein Expression Profile
DPA

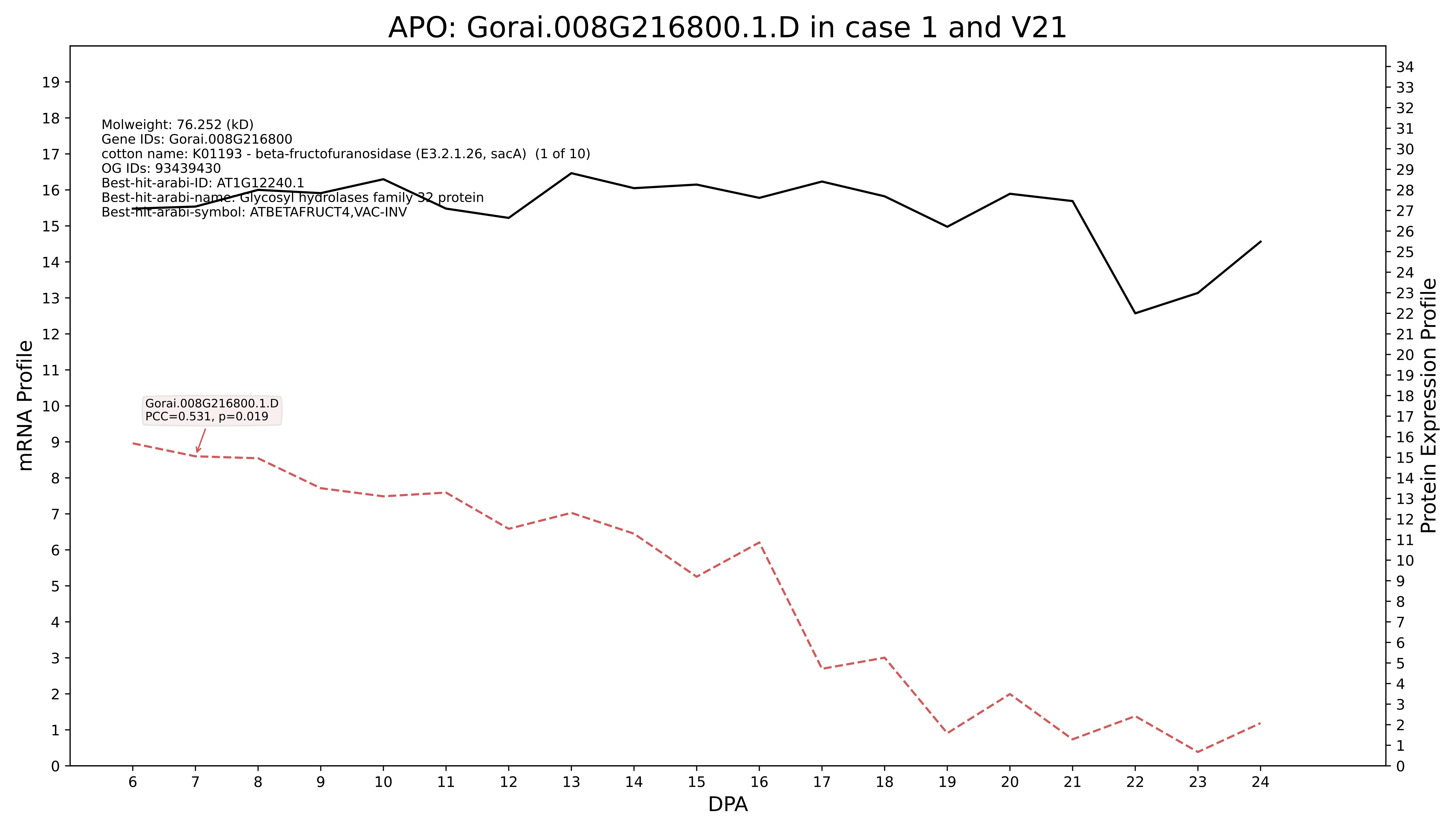
APO: Gorai.008G216800.1.D in case 1 and V21
Molweight: 76.252 (kD)
Gene IDs: Gorai.008G216800
cotton name: K01193 - beta-fructofuranosidase (E3.2.1.26, sacA) (1 of 10)
OG IDs: 93439430
Best-hit-arabi-ID: AT1G12240.1
Best-hit-arabi-name: Glycosyl hydrolases family 32 protein
Best-hit-arabi-symbol: ATBETAFRUCT4,VAC-INV
Gorai.008G216800.1.D
PCC=0.531, p=0.019
mRNA Profile
Protein Expression Profile
DPA

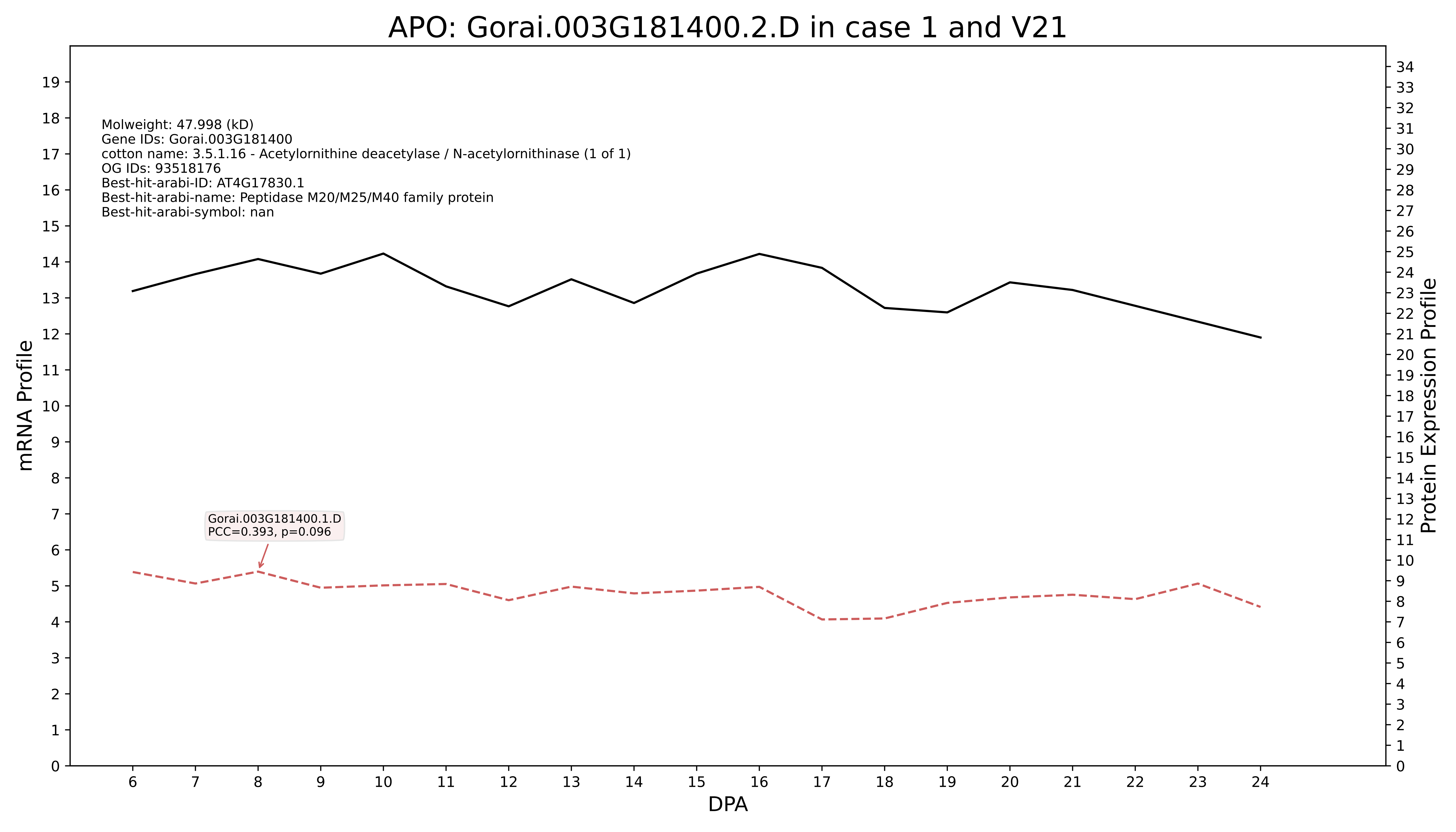

APO: Gorai.003G181400.2.D in case 1 and V21
Molweight: 47.998 (kD)
Gene IDs: Gorai.003G181400
cotton name: 3.5.1.16 - Acetylornithine deacetylase / N-acetylornithinase (1 of 1)
OG IDs: 93518176
Best-hit-arabi-ID: AT4G17830.1
Best-hit-arabi-name: Peptidase M20/M25/M40 family protein
Best-hit-arabi-symbol: nan
Gorai.003G181400.1.D
PCC=0.393, p=0.096
mRNA Profile
Protein Expression Profile
DPA

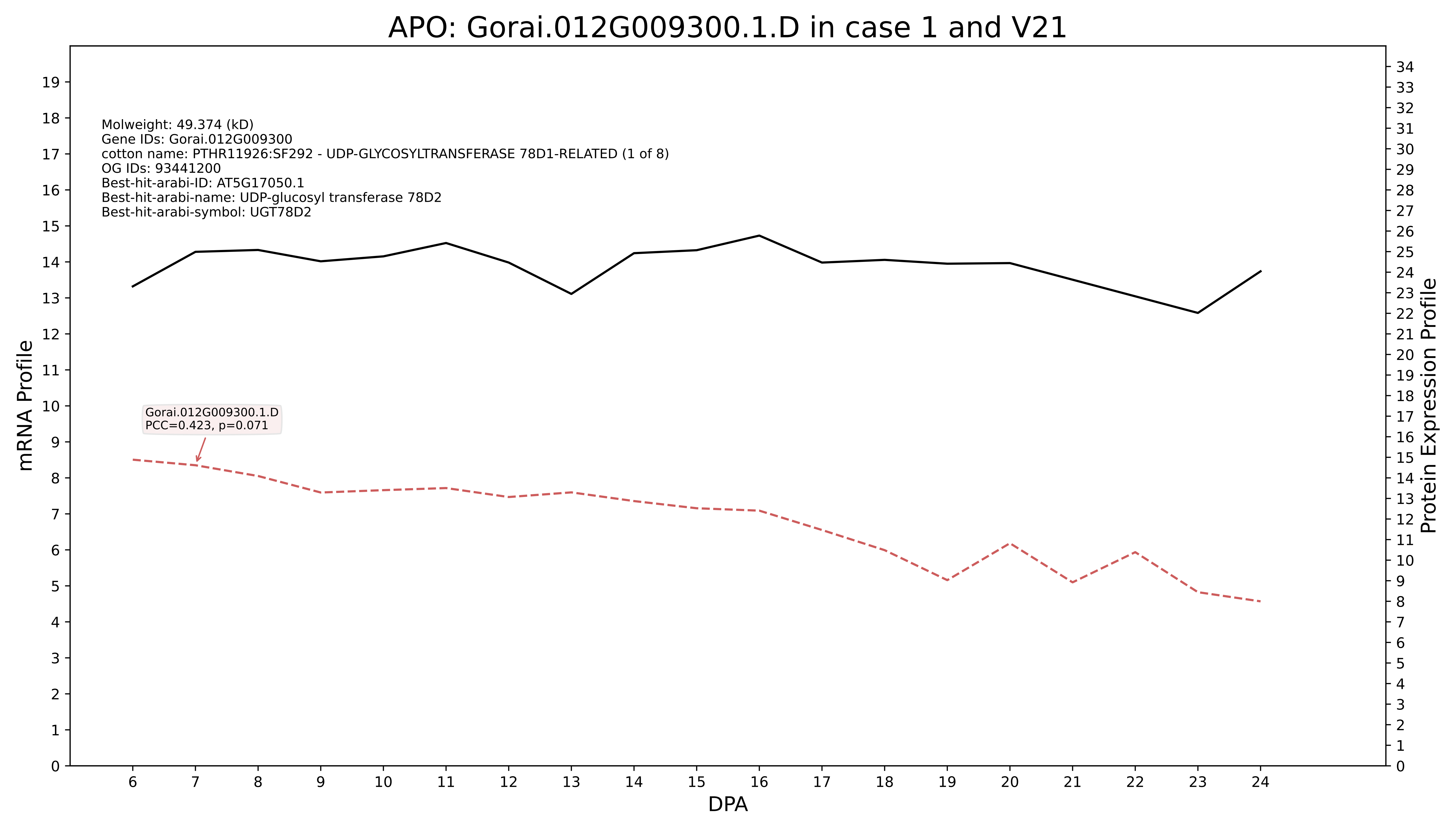
APO: Gorai.012G009300.1.D in case 1 and V21
Molweight: 49.374 (kD)
Gene IDs: Gorai.012G009300
cotton name: PTHR11926:SF292 - UDP-GLYCOSYLTRANSFERASE 78D1-RELATED (1 of 8)
OG IDs: 93441200
Best-hit-arabi-ID: AT5G17050.1
Best-hit-arabi-name: UDP-glucosyl transferase 78D2
Best-hit-arabi-symbol: UGT78D2
Gorai.012G009300.1.D
PCC=0.423, p=0.071
mRNA Profile
Protein Expression Profile
DPA
0
1
2
3
4
5
6
7
8
9
10
11
12
13
14
15
16
17
18
19
20
21
22
23
24
25
26
27
28
29
30
31
32
33
34

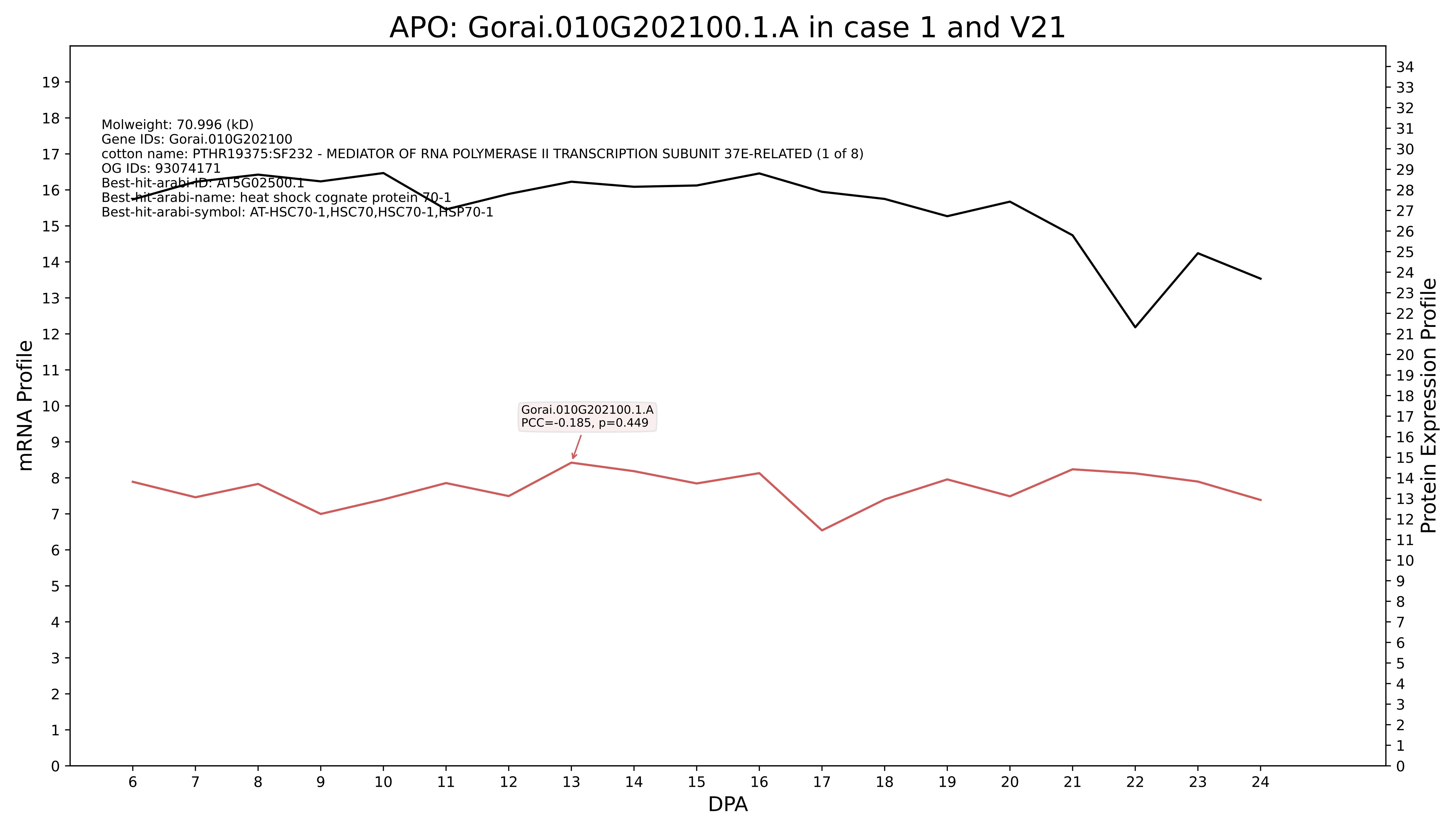

APO: Gorai.010G202100.1.A in case 1 and V21
Molweight: 70.996 (kD)
Gene IDs: Gorai.010G202100
cotton name: PTHR19375:SF232 - MEDIATOR OF RNA POLYMERASE II TRANSCRIPTION SUBUNIT 37E-RELATED (1 of 8)
OG IDs: 93074171
Best-hit-arabi-ID: AT5G02500.1
Best-hit-arabi-name: heat shock cognate protein 70-1
Best-hit-arabi-symbol: AT-HSC70-1,HSC70,HSC70-1,HSP70-1
Gorai.010G202100.1.A
PCC=-0.185, p=0.449
mRNA Profile
Protein Expression Profile
DPA

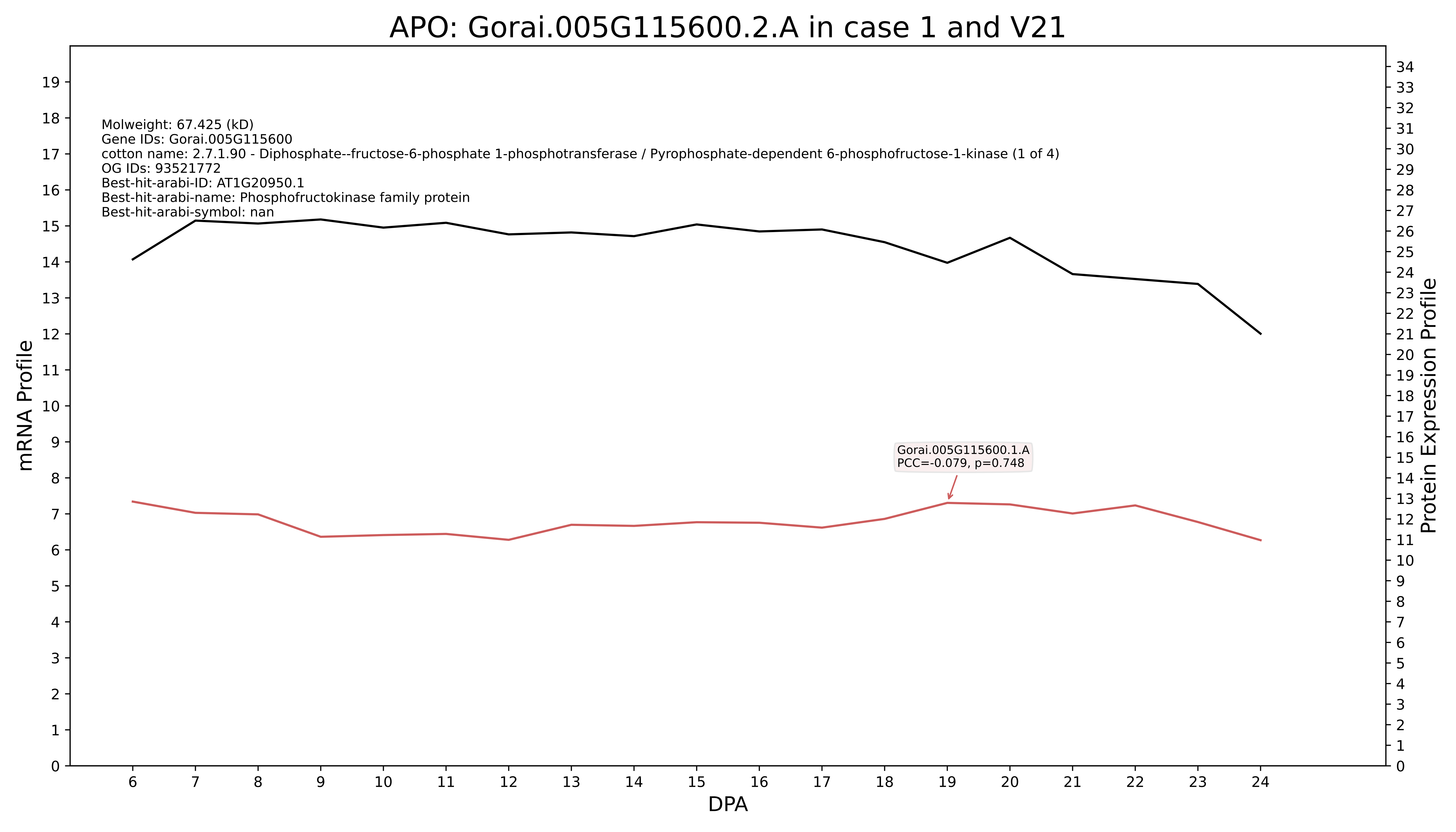
APO: Gorai.005G115600.2.A in case 1 and V21
Molweight: 67.425 (kD)
Gene IDs: Gorai.005G115600
cotton name: 2.7.1.90 - Diphosphate--fructose-6-phosphate 1-phosphotransferase / Pyrophosphate-dependent 6-phosphofructose-1-kinase (1 of 4)
OG IDs: 93521772
Best-hit-arabi-ID: AT1G20950.1
Best-hit-arabi-name: Phosphofructokinase family protein
Best-hit-arabi-symbol: nan
Gorai.005G115600.1.A
PCC=-0.079, p=0.748
mRNA Profile
Protein Expression Profile
DPA

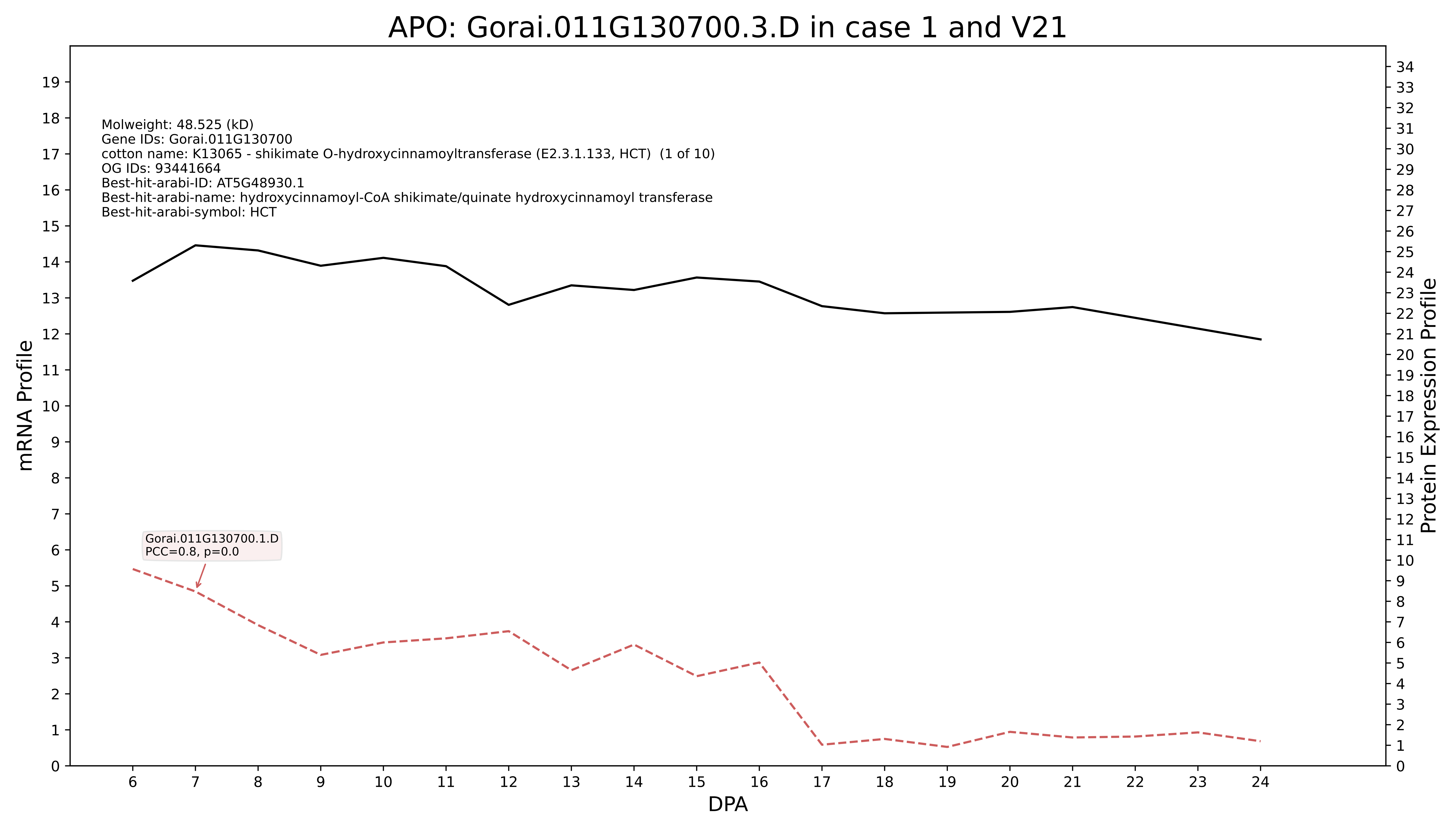

APO: Gorai.011G130700.3.D in case 1 and V21
Molweight: 48.525 (kD)
Gene IDs: Gorai.011G130700
cotton name: K13065 - shikimate O-hydroxycinnamoyltransferase (E2.3.1.133, HCT) (1 of 10)
OG IDs: 93441664
Best-hit-arabi-ID: AT5G48930.1
Best-hit-arabi-name: hydroxycinnamoyl-CoA shikimate/quinate hydroxycinnamoyl transferase
Best-hit-arabi-symbol: HCT
Gorai.011G130700.1.D
PCC=0.8, p=0.0
mRNA Profile
Protein Expression Profile
DPA

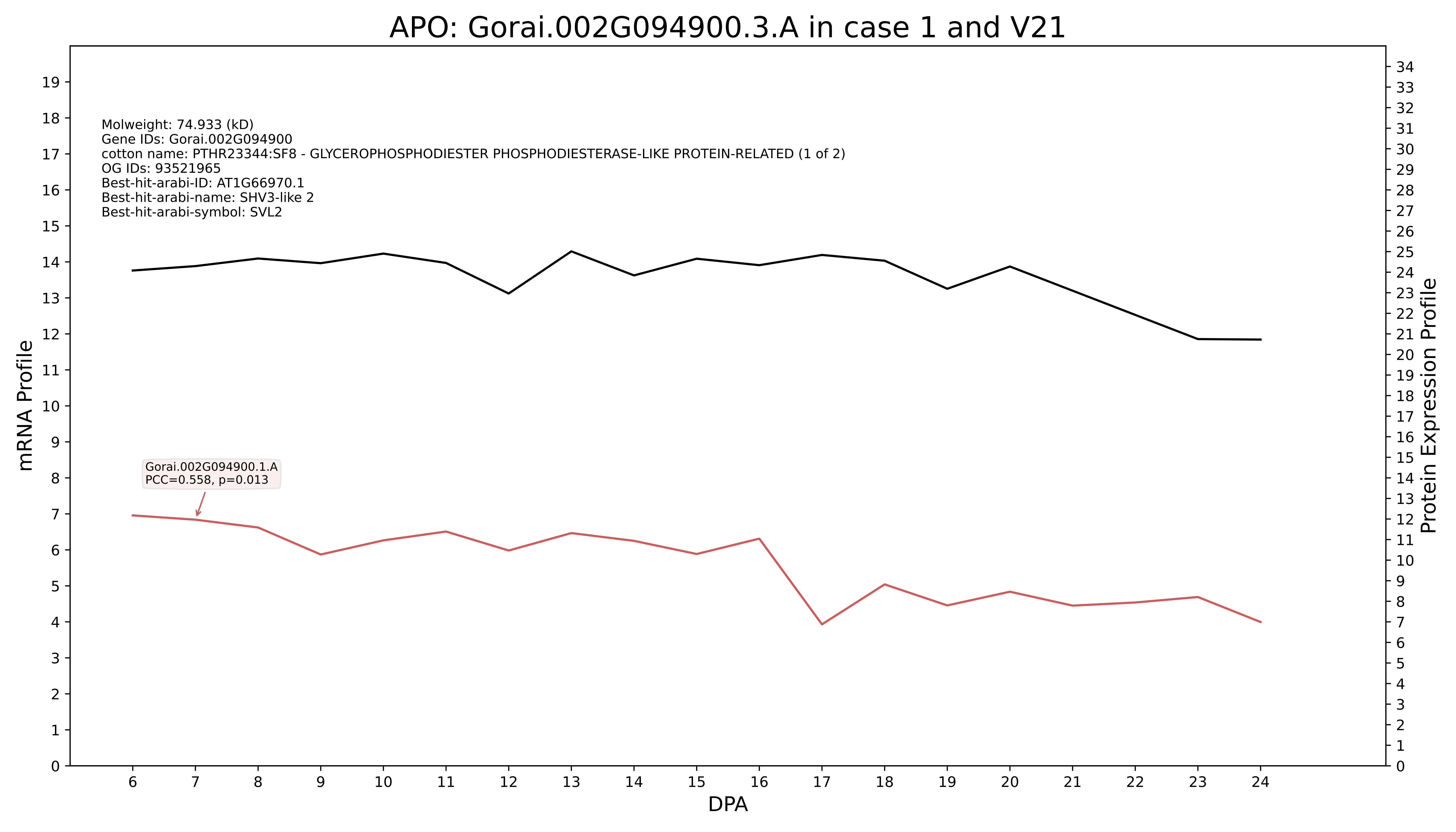
APO: Gorai.002G094900.3.A in case 1 and V21
Molweight: 74.933 (kD)
Gene IDs: Gorai.002G094900
cotton name: PTHR23344:SF8 - GLYCEROPHOSPHODIESTER PHOSPHODIESTERASE-LIKE PROTEIN-RELATED (1 of 2)
OG IDs: 93521965
Best-hit-arabi-ID: AT1G66970.1
Best-hit-arabi-name: SHV3-like 2
Best-hit-arabi-symbol: SVL2
Gorai.002G094900.1.A
PCC=0.558, p=0.013
mRNA Profile
Protein Expression Profile
DPA

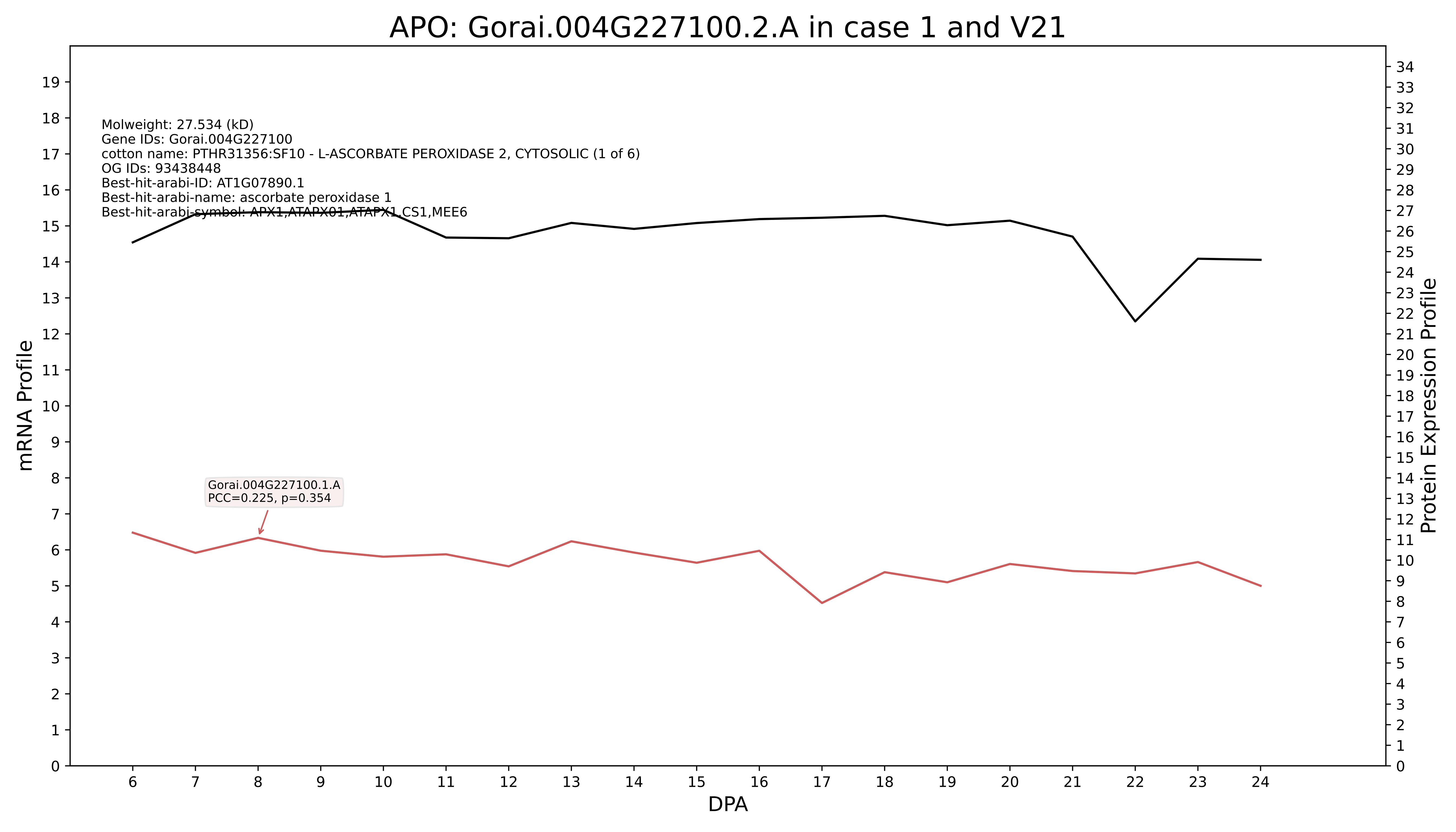
APO: Gorai.004G227100.2.A in case 1 and V21
Molweight: 27.534 (kD)
Gene IDs: Gorai.004G227100
cotton name: PTHR31356:SF10 - L-ASCORBATE PEROXIDASE 2, CYTOSOLIC (1 of 6)
OG IDs: 93438448
Best-hit-arabi-ID: AT1G07890.1
Best-hit-arabi-name: ascorbate peroxidase 1
Best-hit-arabi-symbol: APX1,ATAPX01,ATAPX1,CS1,MEE6
Gorai.004G227100.1.A
PCC=0.225, p=0.354
mRNA Profile
Protein Expression Profile
DPA
0
1
2
3
4
5
6
7
8
9
10
11
12
13
14
15
16
17
18
19
20
21
22
23
24
25
26
27
28
29
30
31
32
33
34

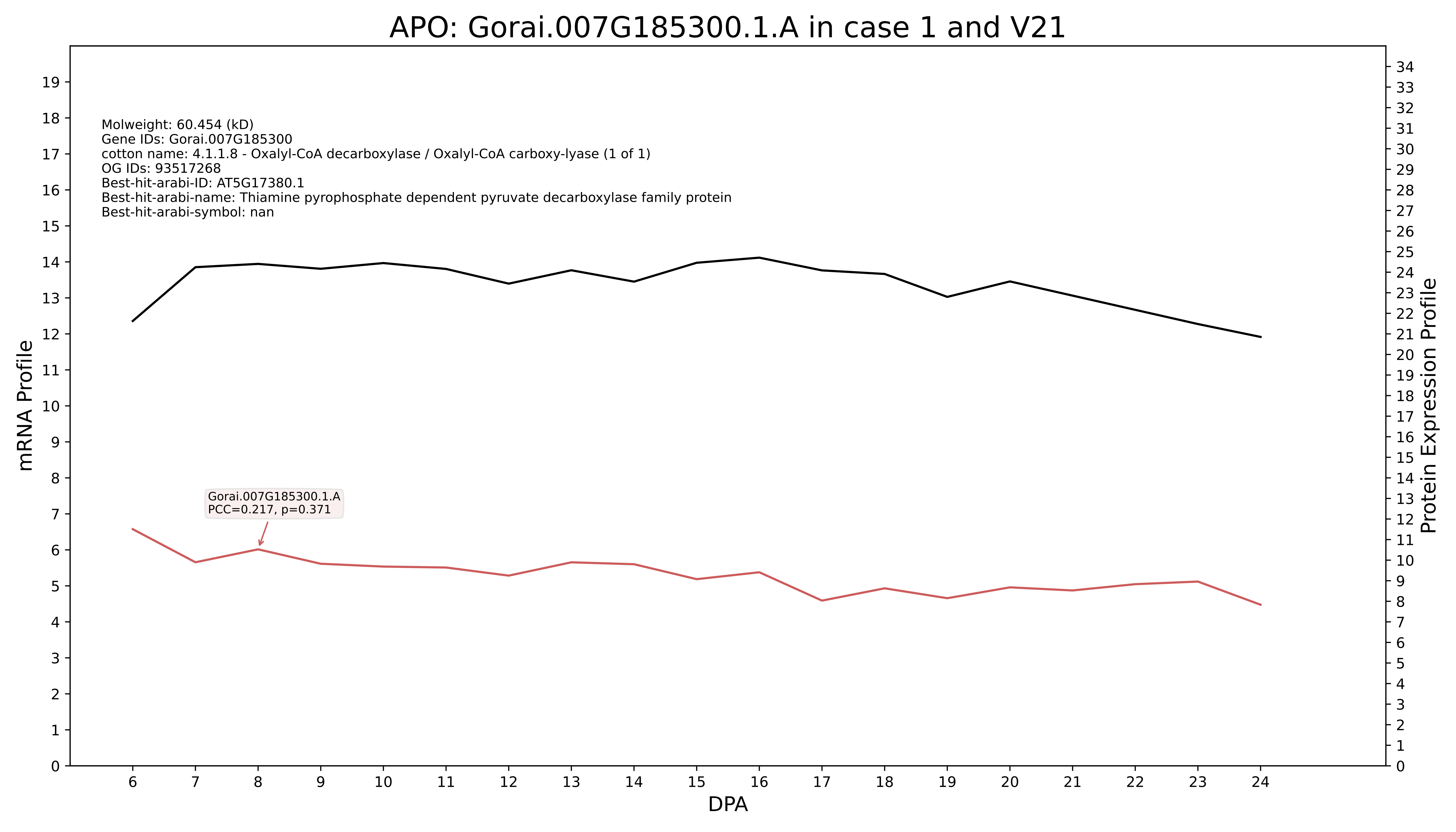
APO: Gorai.007G185300.1.A in case 1 and V21
Molweight: 60.454 (kD)
Gene IDs: Gorai.007G185300
cotton name: 4.1.1.8 - Oxalyl-CoA decarboxylase / Oxalyl-CoA carboxy-lyase (1 of 1)
OG IDs: 93517268
Best-hit-arabi-ID: AT5G17380.1
Best-hit-arabi-name: Thiamine pyrophosphate dependent pyruvate decarboxylase family protein
Best-hit-arabi-symbol: nan
Gorai.007G185300.1.A
PCC=0.217, p=0.371
mRNA Profile
Protein Expression Profile
DPA

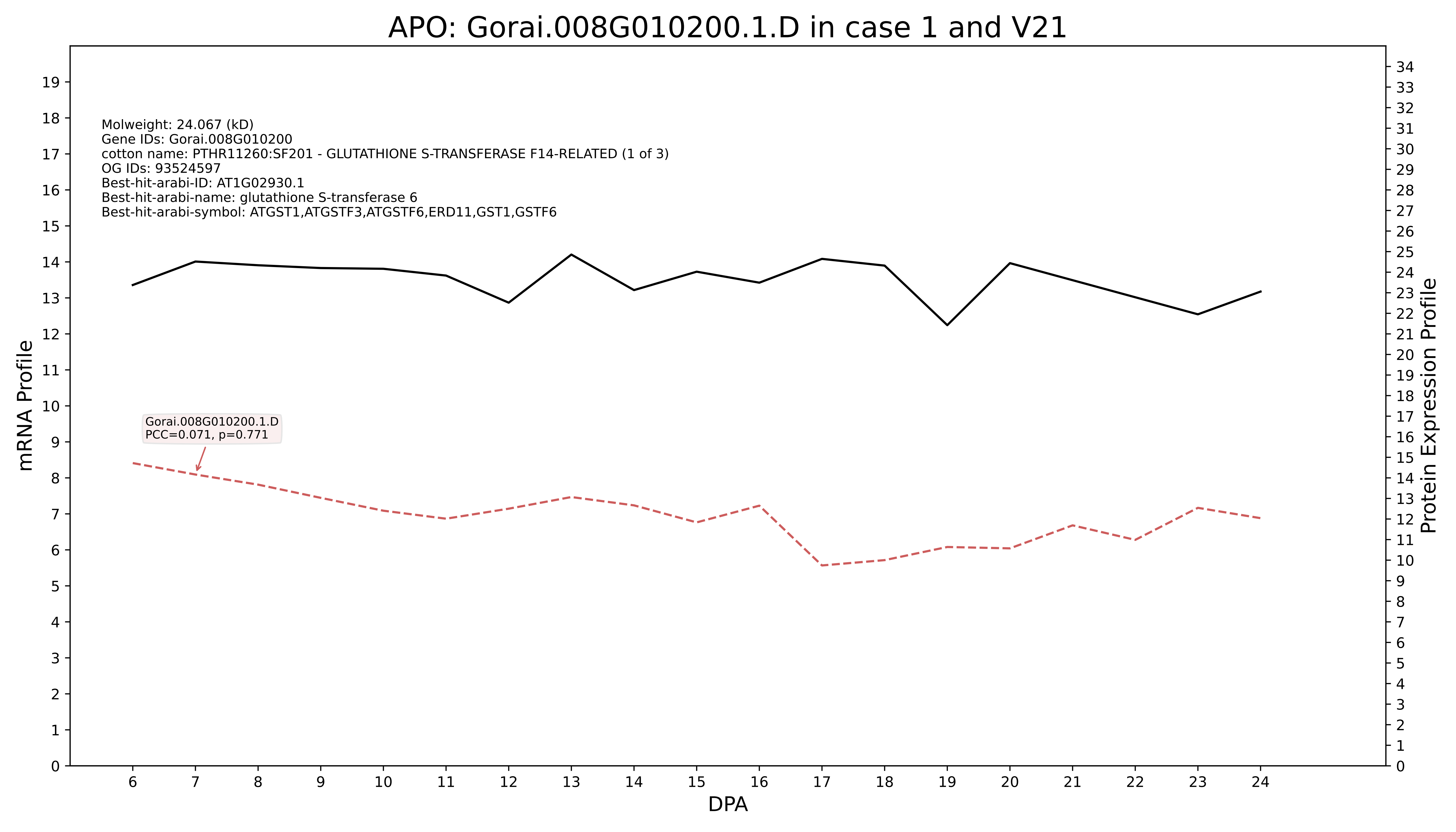

APO: Gorai.008G010200.1.D in case 1 and V21
Molweight: 24.067 (kD)
Gene IDs: Gorai.008G010200
cotton name: PTHR11260:SF201 - GLUTATHIONE S-TRANSFERASE F14-RELATED (1 of 3)
OG IDs: 93524597
Best-hit-arabi-ID: AT1G02930.1
Best-hit-arabi-name: glutathione S-transferase 6
Best-hit-arabi-symbol: ATGST1,ATGSTF3,ATGSTF6,ERD11,GST1,GSTF6
Gorai.008G010200.1.D
PCC=0.071, p=0.771
mRNA Profile
Protein Expression Profile
DPA

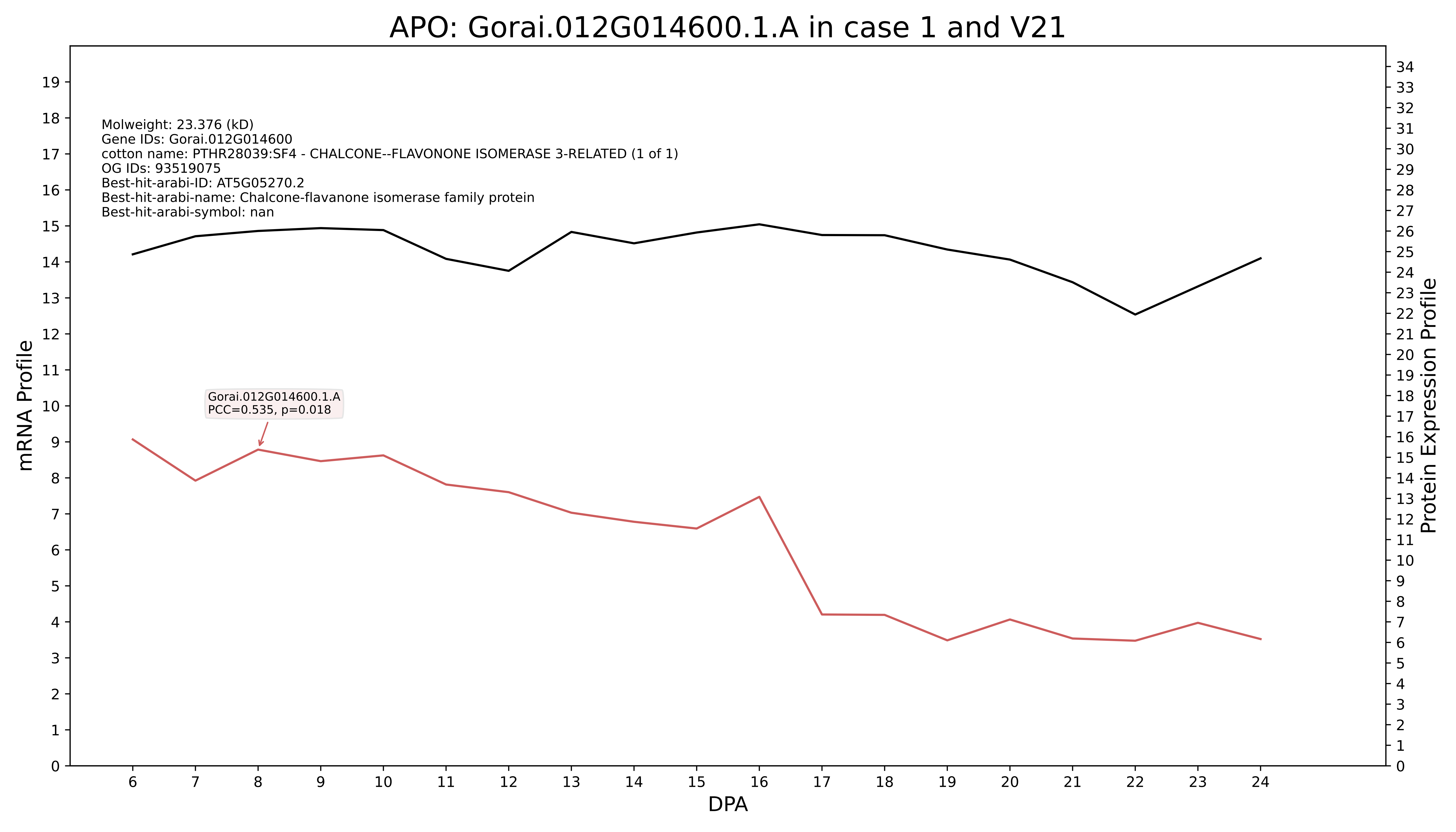
APO: Gorai.012G014600.1.A in case 1 and V21
Molweight: 23.376 (kD)
Gene IDs: Gorai.012G014600
cotton name: PTHR28039:SF4 - CHALCONE--FLAVONONE ISOMERASE 3-RELATED (1 of 1)
OG IDs: 93519075
Best-hit-arabi-ID: AT5G05270.2
Best-hit-arabi-name: Chalcone-flavanone isomerase family protein
Best-hit-arabi-symbol: nan
Gorai.012G014600.1.A
PCC=0.535, p=0.018
mRNA Profile
Protein Expression Profile
DPA
6
7
8
9
10
11
12
13
14
15
16
17
18
19
20
21
22
23
24

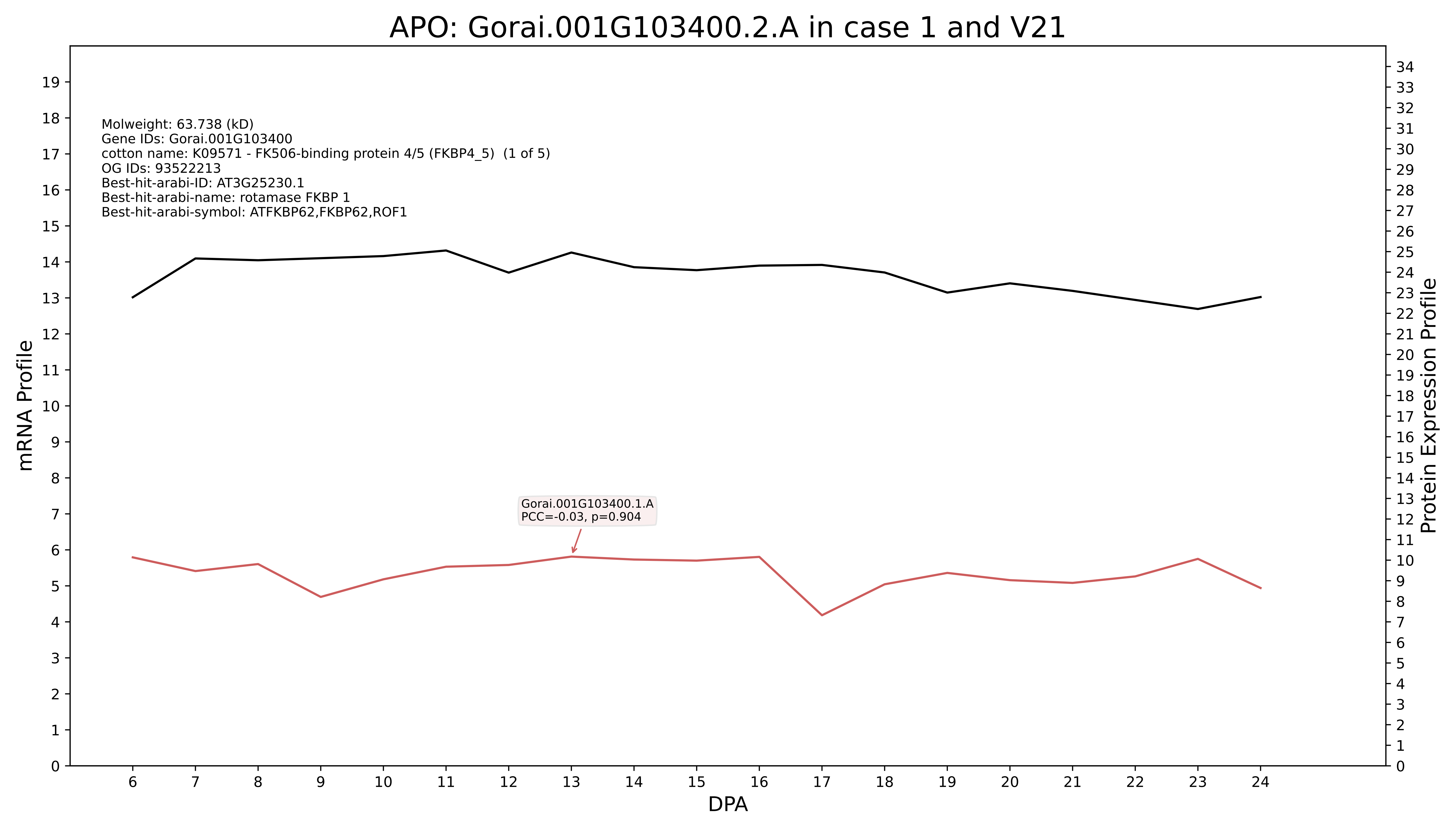
APO: Gorai.001G103400.2.A in case 1 and V21
Molweight: 63.738 (kD)
Gene IDs: Gorai.001G103400
cotton name: K09571 - FK506-binding protein 4/5 (FKBP4_5) (1 of 5)
OG IDs: 93522213
Best-hit-arabi-ID: AT3G25230.1
Best-hit-arabi-name: rotamase FKBP 1
Best-hit-arabi-symbol: ATFKBP62,FKBP62,ROF1
Gorai.001G103400.1.A
PCC=-0.03, p=0.904
mRNA Profile
Protein Expression Profile
DPA

APO: Gorai.007G373300.1.A in case 1 and V21

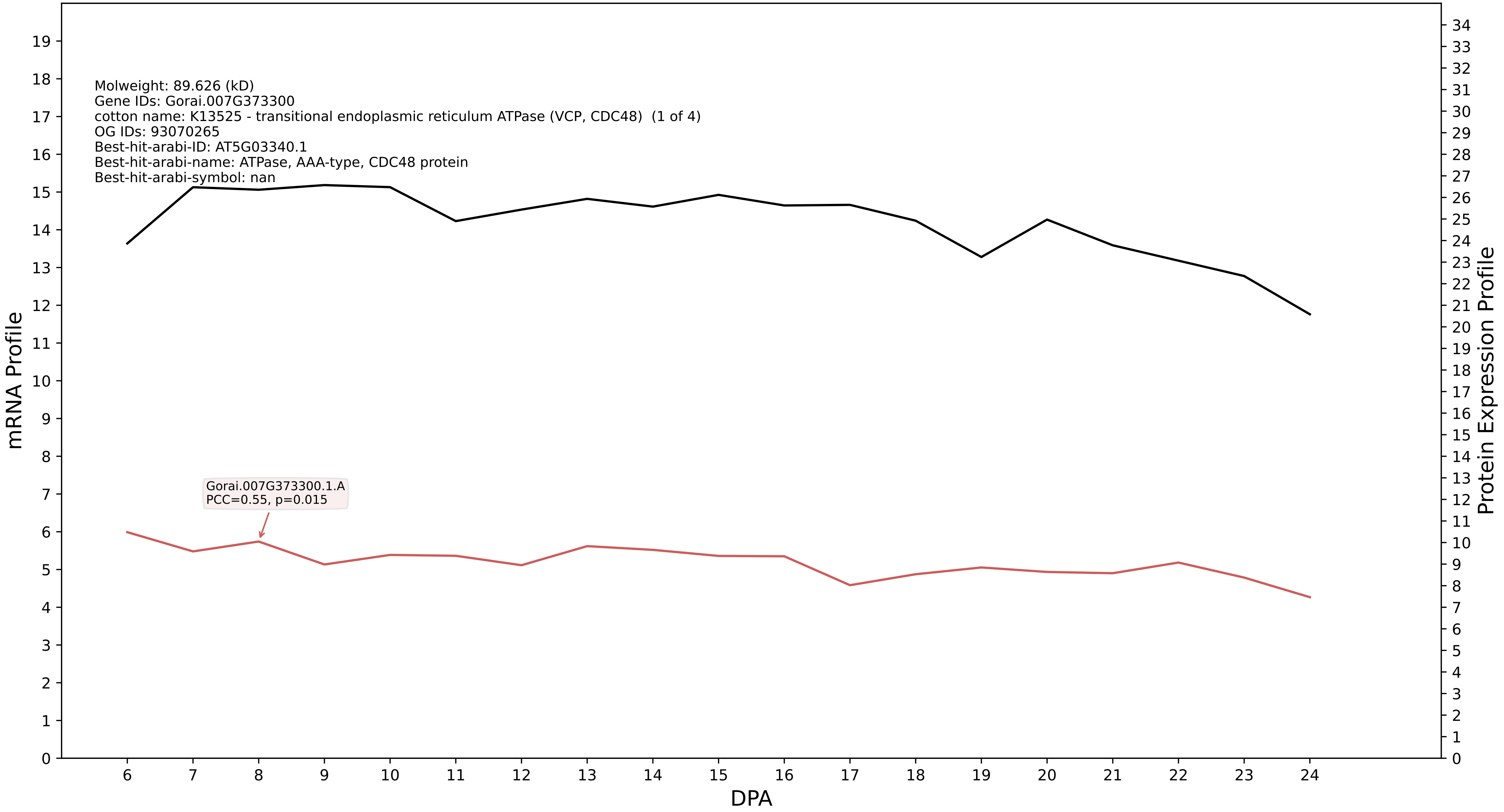

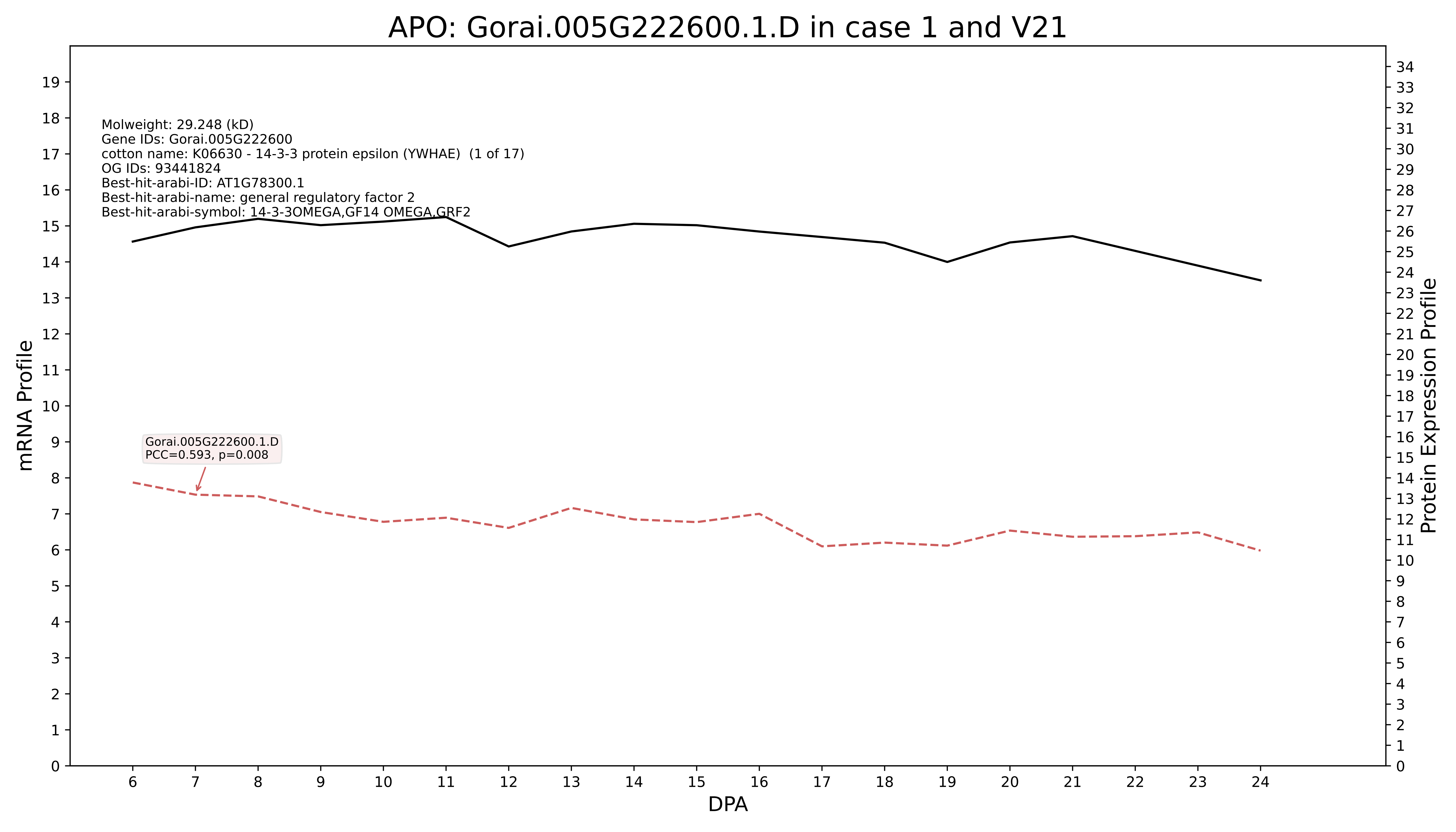
APO: Gorai.005G222600.1.D in case 1 and V21
Molweight: 29.248 (kD)
Gene IDs: Gorai.005G222600
cotton name: K06630 - 14-3-3 protein epsilon (YWHAE) (1 of 17)
OG IDs: 93441824
Best-hit-arabi-ID: AT1G78300.1
Best-hit-arabi-name: general regulatory factor 2
Best-hit-arabi-symbol: 14-3-3OMEGA,GF14 OMEGA,GRF2
Gorai.005G222600.1.D
PCC=0.593, p=0.008
mRNA Profile
Protein Expression Profile
DPA

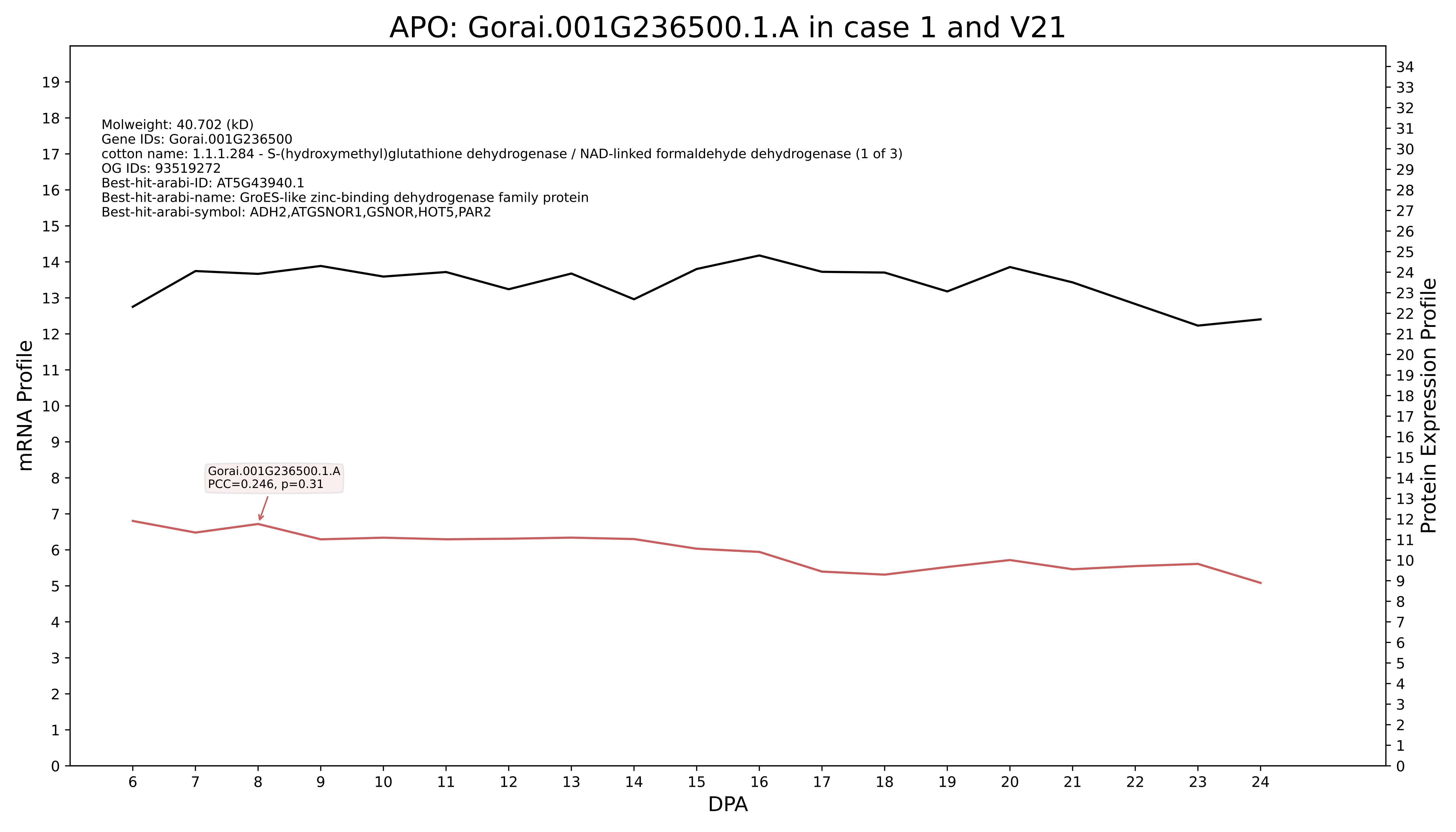
APO: Gorai.001G236500.1.A in case 1 and V21
Molweight: 40.702 (kD)
Gene IDs: Gorai.001G236500
cotton name: 1.1.1.284 - S-(hydroxymethyl)glutathione dehydrogenase / NAD-linked formaldehyde dehydrogenase (1 of 3)
OG IDs: 93519272
Best-hit-arabi-ID: AT5G43940.1
Best-hit-arabi-name: GroES-like zinc-binding dehydrogenase family protein
Best-hit-arabi-symbol: ADH2,ATGSNOR1,GSNOR,HOT5,PAR2
Gorai.001G236500.1.A
PCC=0.246, p=0.31
mRNA Profile
Protein Expression Profile
DPA

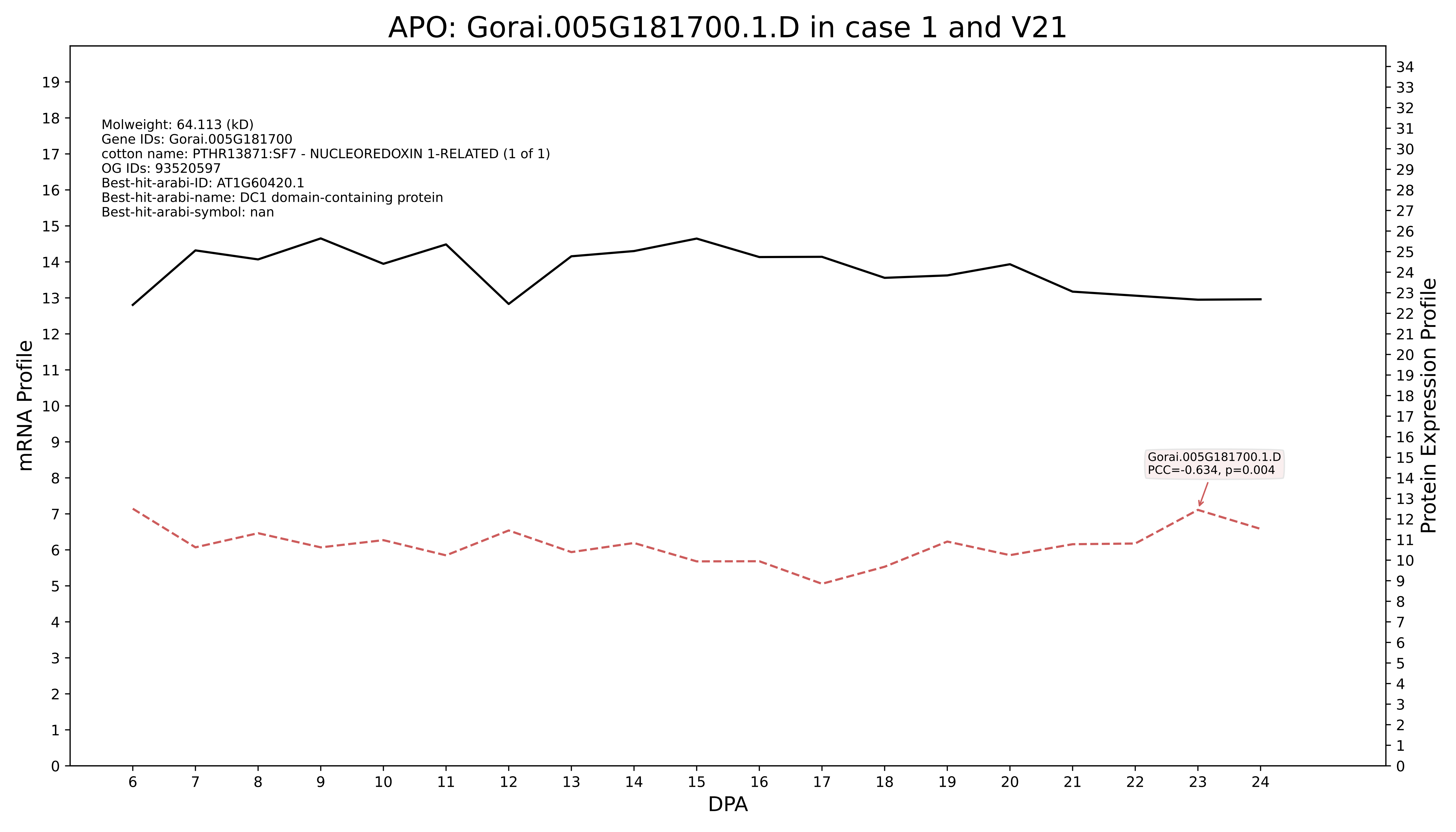
APO: Gorai.005G181700.1.D in case 1 and V21
Molweight: 64.113 (kD)
Gene IDs: Gorai.005G181700
cotton name: PTHR13871:SF7 - NUCLEOREDOXIN 1-RELATED (1 of 1)
OG IDs: 93520597
Best-hit-arabi-ID: AT1G60420.1
Best-hit-arabi-name: DC1 domain-containing protein
Best-hit-arabi-symbol: nan
Gorai.005G181700.1.D
PCC=-0.634, p=0.004
mRNA Profile
Protein Expression Profile
DPA

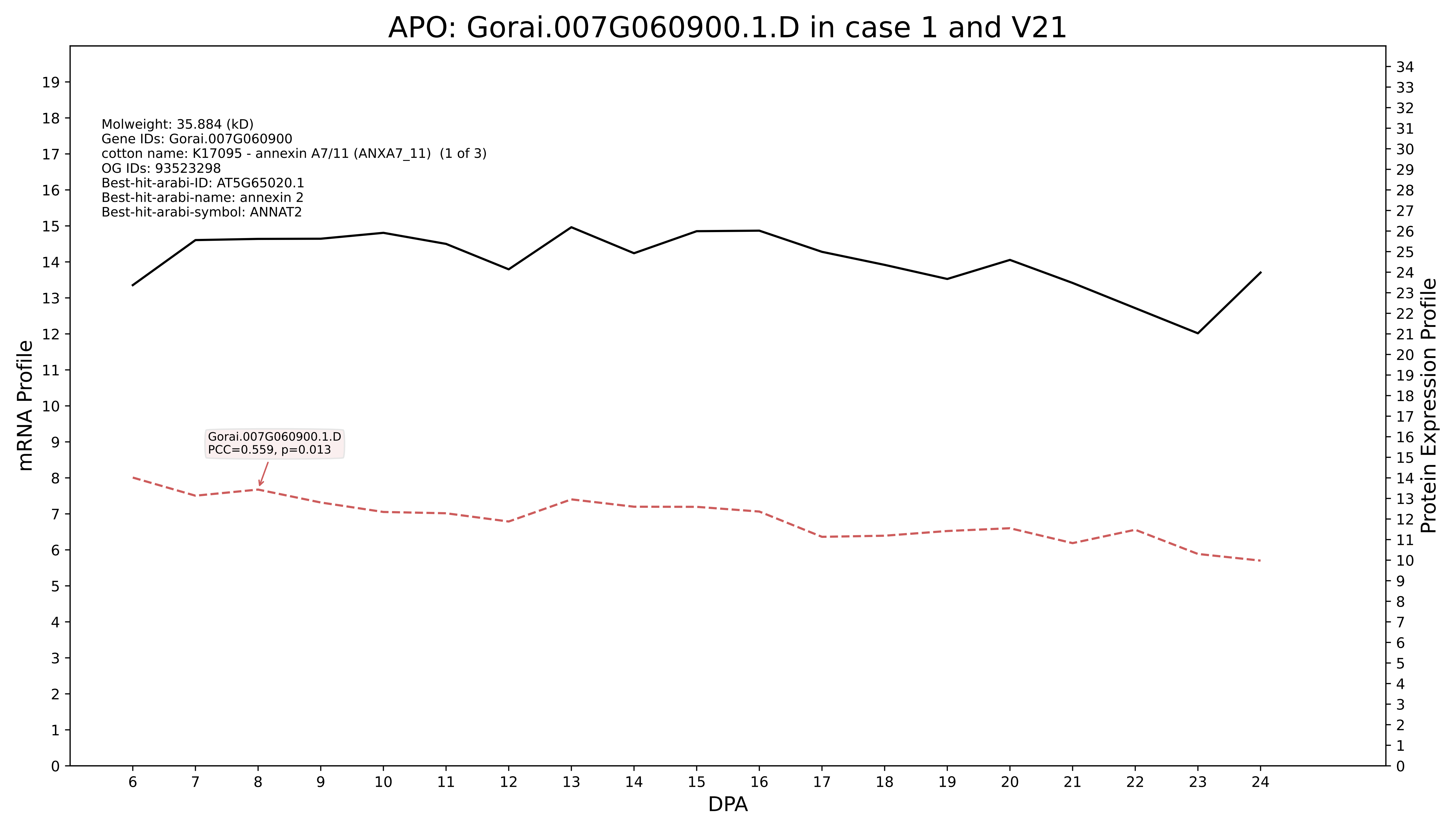

APO: Gorai.007G060900.1.D in case 1 and V21
Molweight: 35.884 (kD)
Gene IDs: Gorai.007G060900
cotton name: K17095 - annexin A7/11 (ANXA7_11) (1 of 3)
OG IDs: 93523298
Best-hit-arabi-ID: AT5G65020.1
Best-hit-arabi-name: annexin 2
Best-hit-arabi-symbol: ANNAT2
Gorai.007G060900.1.D
PCC=0.559, p=0.013
mRNA Profile
Protein Expression Profile
DPA

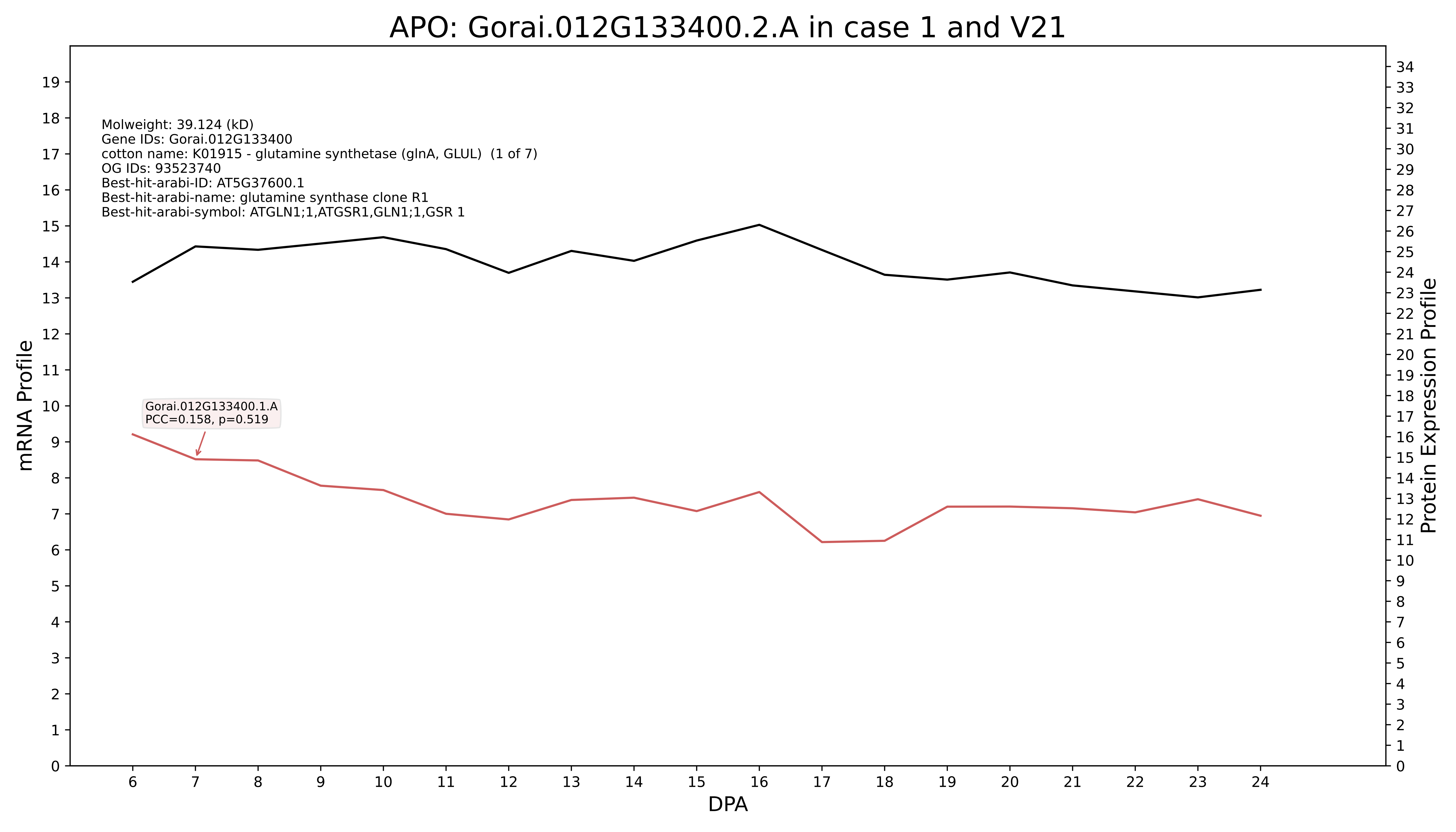
APO: Gorai.012G133400.2.A in case 1 and V21
Molweight: 39.124 (kD)
Gene IDs: Gorai.012G133400
cotton name: K01915 - glutamine synthetase (glnA, GLUL) (1 of 7)
OG IDs: 93523740
Best-hit-arabi-ID: AT5G37600.1
Best-hit-arabi-name: glutamine synthase clone R1
Best-hit-arabi-symbol: ATGLN1;1,ATGSR1,GLN1;1,GSR 1
Gorai.012G133400.1.A
PCC=0.158, p=0.519
mRNA Profile
Protein Expression Profile
DPA

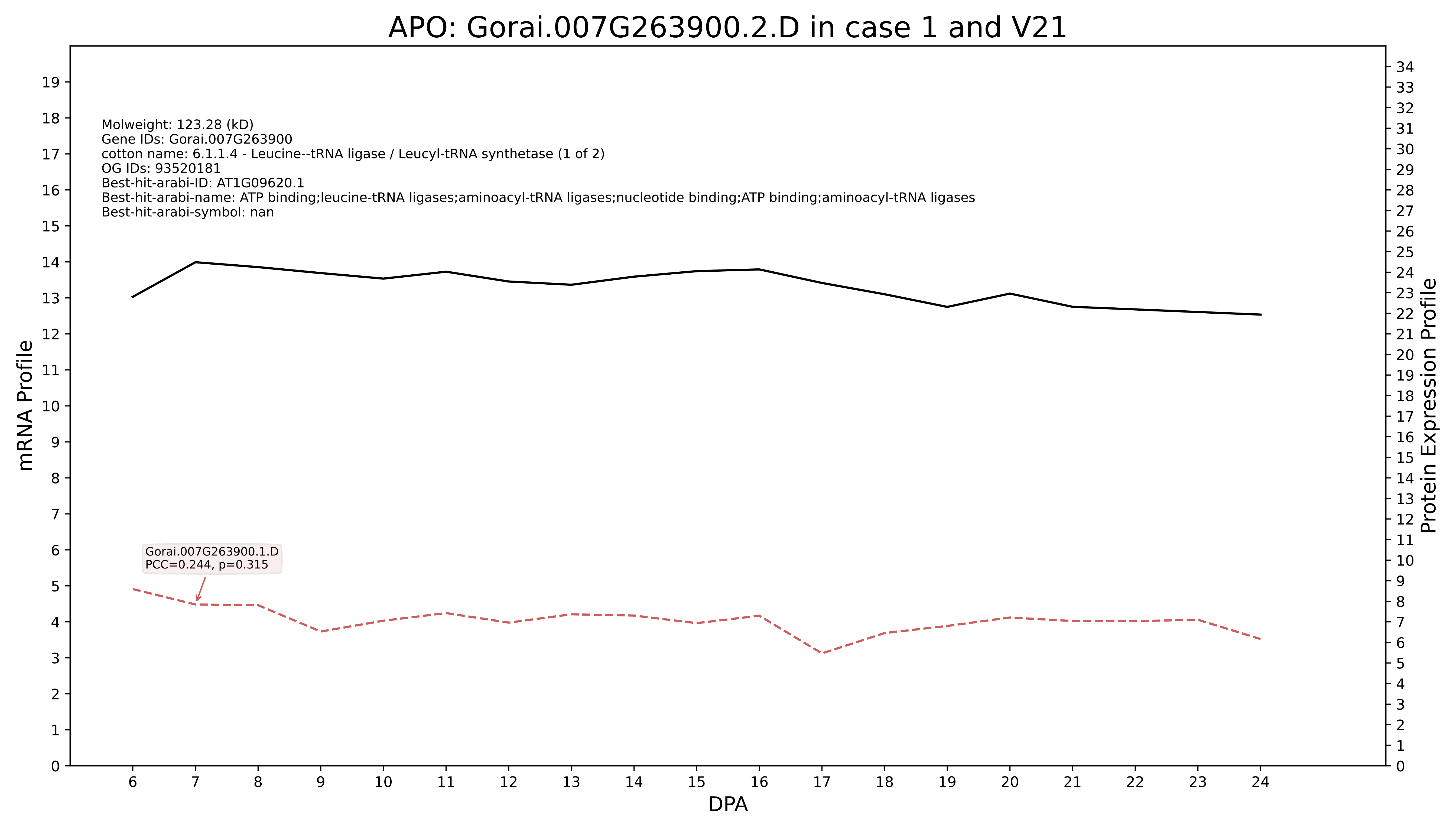

APO: Gorai.007G263900.2.D in case 1 and V21
Molweight: 123.28 (kD)
Gene IDs: Gorai.007G263900
cotton name: 6.1.1.4 - Leucine--tRNA ligase / Leucyl-tRNA synthetase (1 of 2)
OG IDs: 93520181
Best-hit-arabi-ID: AT1G09620.1
Best-hit-arabi-name: ATP binding;leucine-tRNA ligases;aminoacyl-tRNA ligases;nucleotide binding;ATP binding;aminoacyl-tRNA ligases
Best-hit-arabi-symbol: nan
Gorai.007G263900.1.D
PCC=0.244, p=0.315
mRNA Profile
Protein Expression Profile
DPA

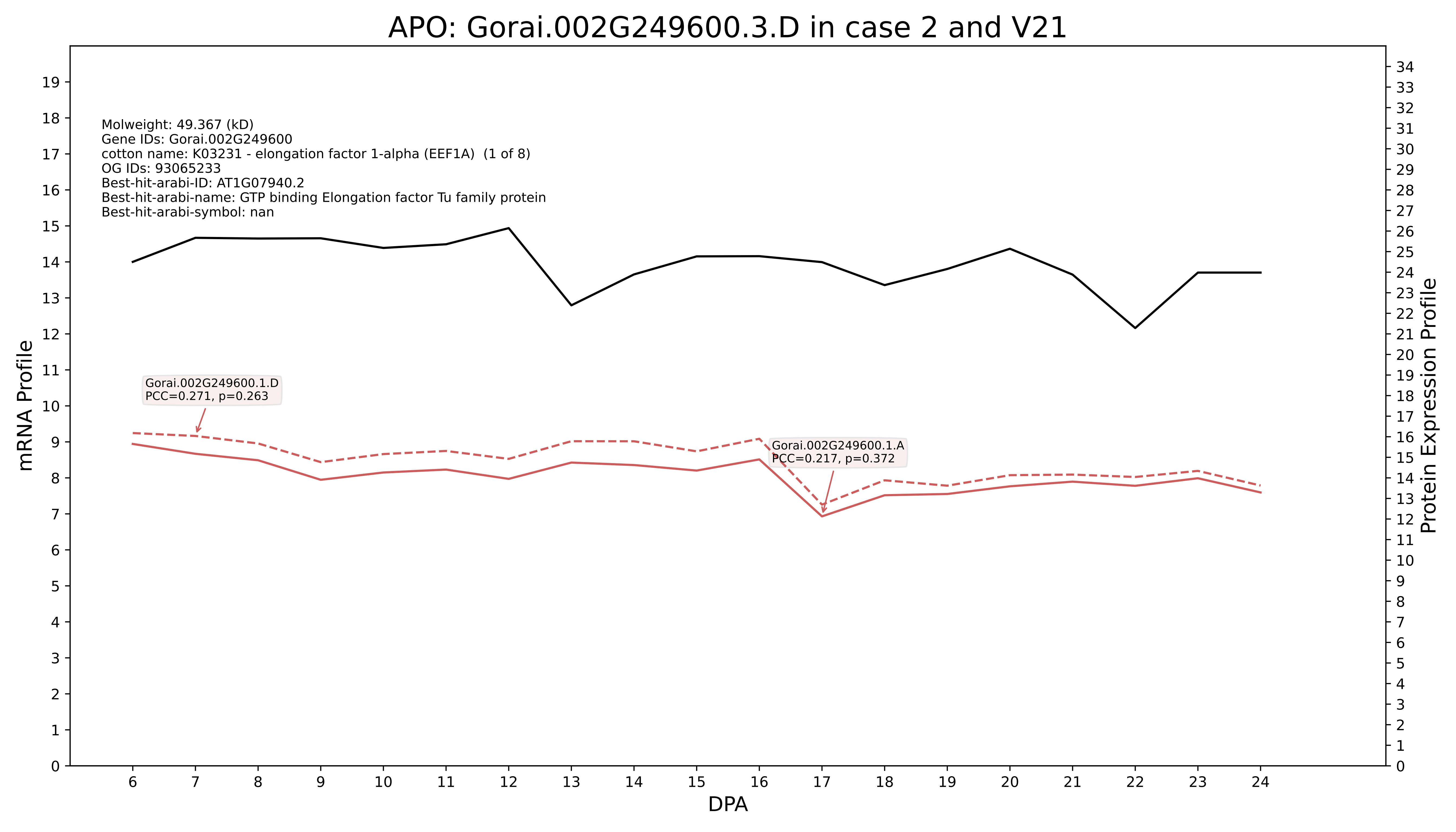

APO: Gorai.002G249600.3.D in case 2 and V21
Molweight: 49.367 (kD)
Gene IDs: Gorai.002G249600
cotton name: K03231 - elongation factor 1-alpha (EEF1A) (1 of 8)
OG IDs: 93065233
Best-hit-arabi-ID: AT1G07940.2
Best-hit-arabi-name: GTP binding Elongation factor Tu family protein
Best-hit-arabi-symbol: nan
Gorai.002G249600.1.D
PCC=0.271, p=0.263
Gorai.002G249600.1.A
PCC=0.217, p=0.372
mRNA Profile
Protein Expression Profile
DPA

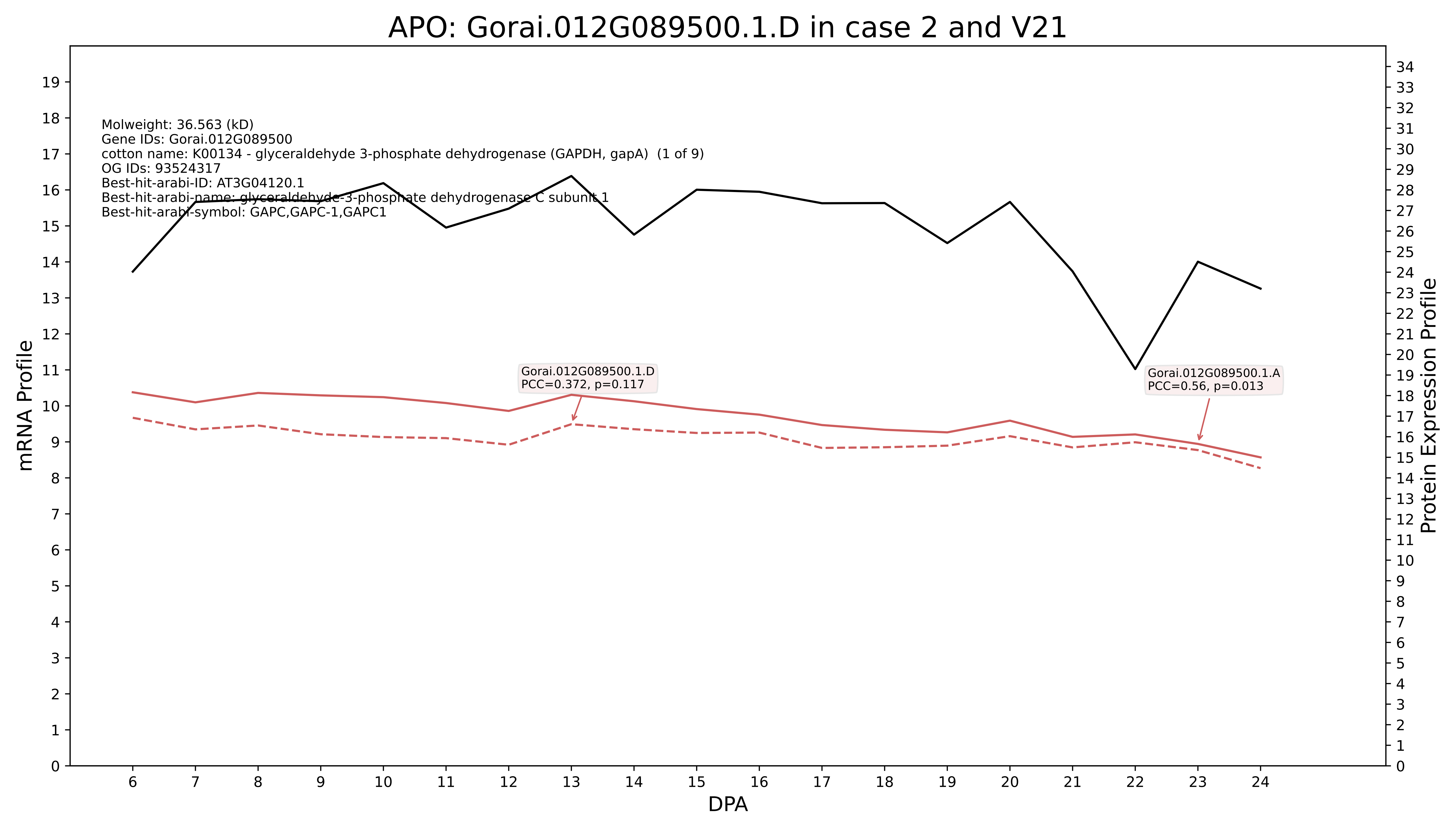

APO: Gorai.012G089500.1.D in case 2 and V21
Molweight: 36.563 (kD)
Gene IDs: Gorai.012G089500
cotton name: K00134 - glyceraldehyde 3-phosphate dehydrogenase (GAPDH, gapA) (1 of 9)
OG IDs: 93524317
Best-hit-arabi-ID: AT3G04120.1
Best-hit-arabi-name: glyceraldehyde-3-phosphate dehydrogenase C subunit 1
Best-hit-arabi-symbol: GAPC,GAPC-1,GAPC1
Gorai.012G089500.1.D
PCC=0.372, p=0.117
Gorai.012G089500.1.A
PCC=0.56, p=0.013
mRNA Profile
Protein Expression Profile
DPA

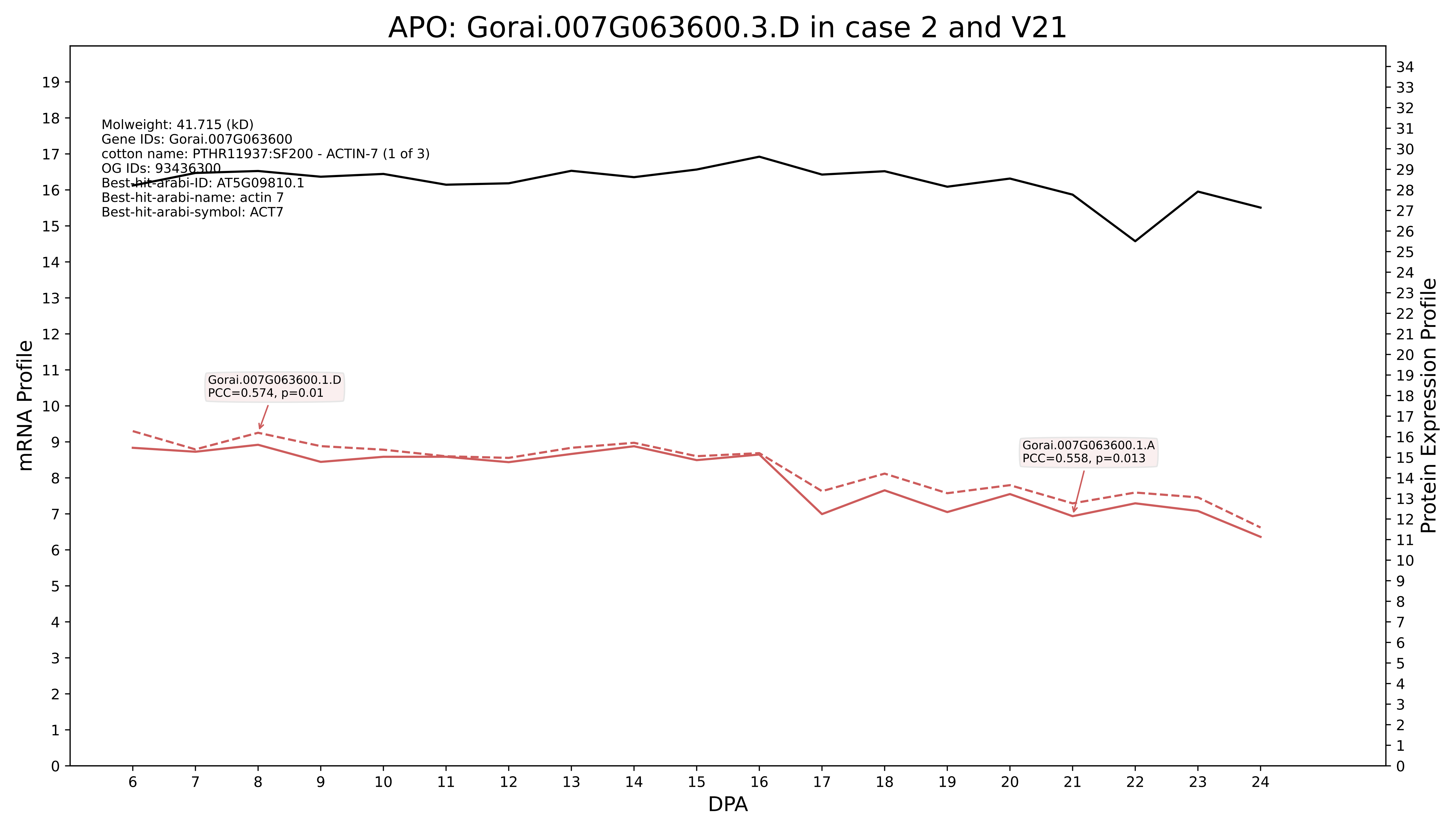
APO: Gorai.007G063600.3.D in case 2 and V21
Molweight: 41.715 (kD)
Gene IDs: Gorai.007G063600
cotton name: PTHR11937:SF200 - ACTIN-7 (1 of 3)
OG IDs: 93436300
Best-hit-arabi-ID: AT5G09810.1
Best-hit-arabi-name: actin 7
Best-hit-arabi-symbol: ACT7
Gorai.007G063600.1.D
PCC=0.574, p=0.01
Gorai.007G063600.1.A
PCC=0.558, p=0.013
mRNA Profile
Protein Expression Profile
DPA

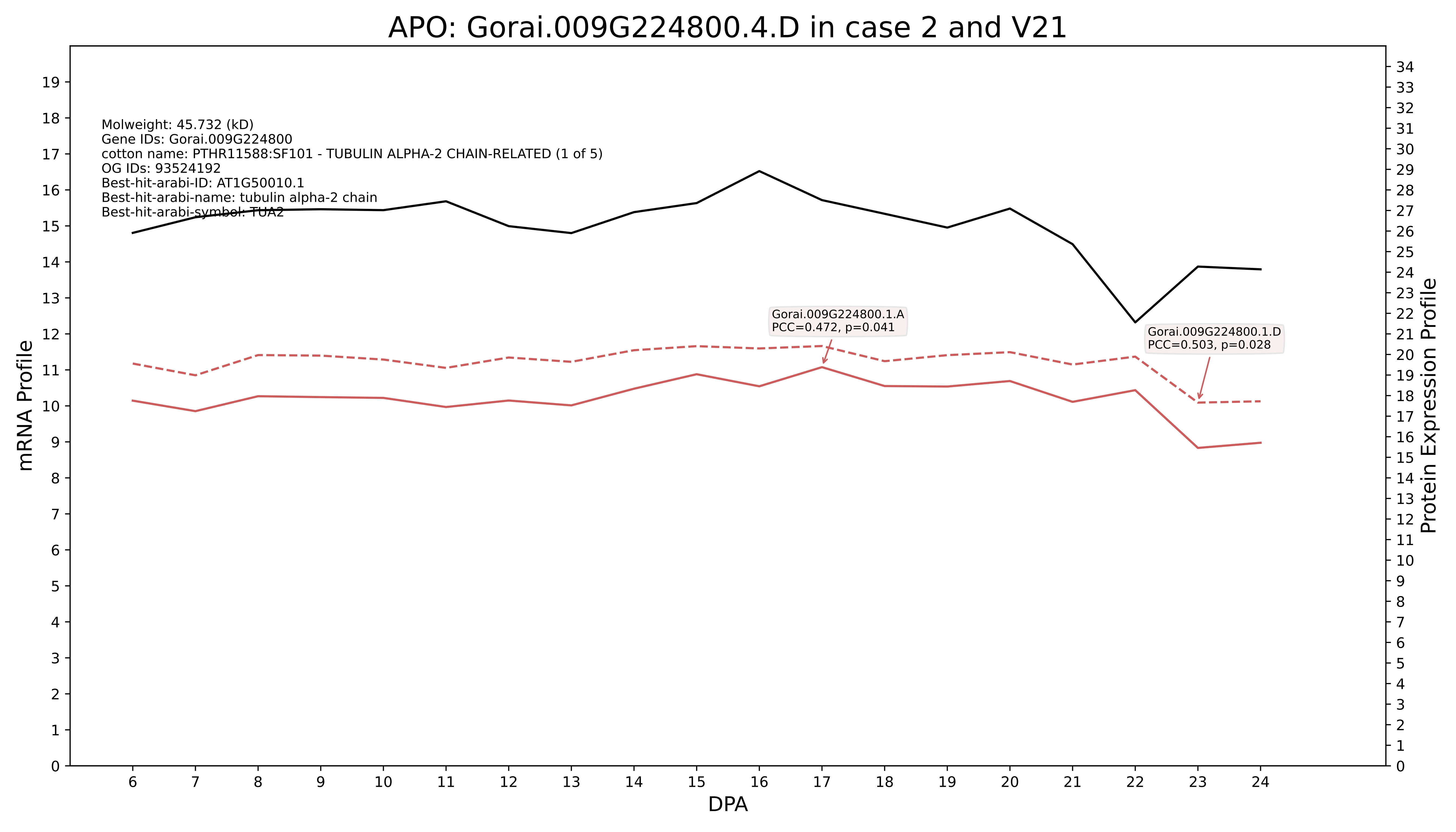
APO: Gorai.009G224800.4.D in case 2 and V21
Molweight: 45.732 (kD)
Gene IDs: Gorai.009G224800
cotton name: PTHR11588:SF101 - TUBULIN ALPHA-2 CHAIN-RELATED (1 of 5)
OG IDs: 93524192
Best-hit-arabi-ID: AT1G50010.1
Best-hit-arabi-name: tubulin alpha-2 chain
Best-hit-arabi-symbol: TUA2
Gorai.009G224800.1.A
PCC=0.472, p=0.041
Gorai.009G224800.1.D
PCC=0.503, p=0.028
mRNA Profile
Protein Expression Profile
DPA

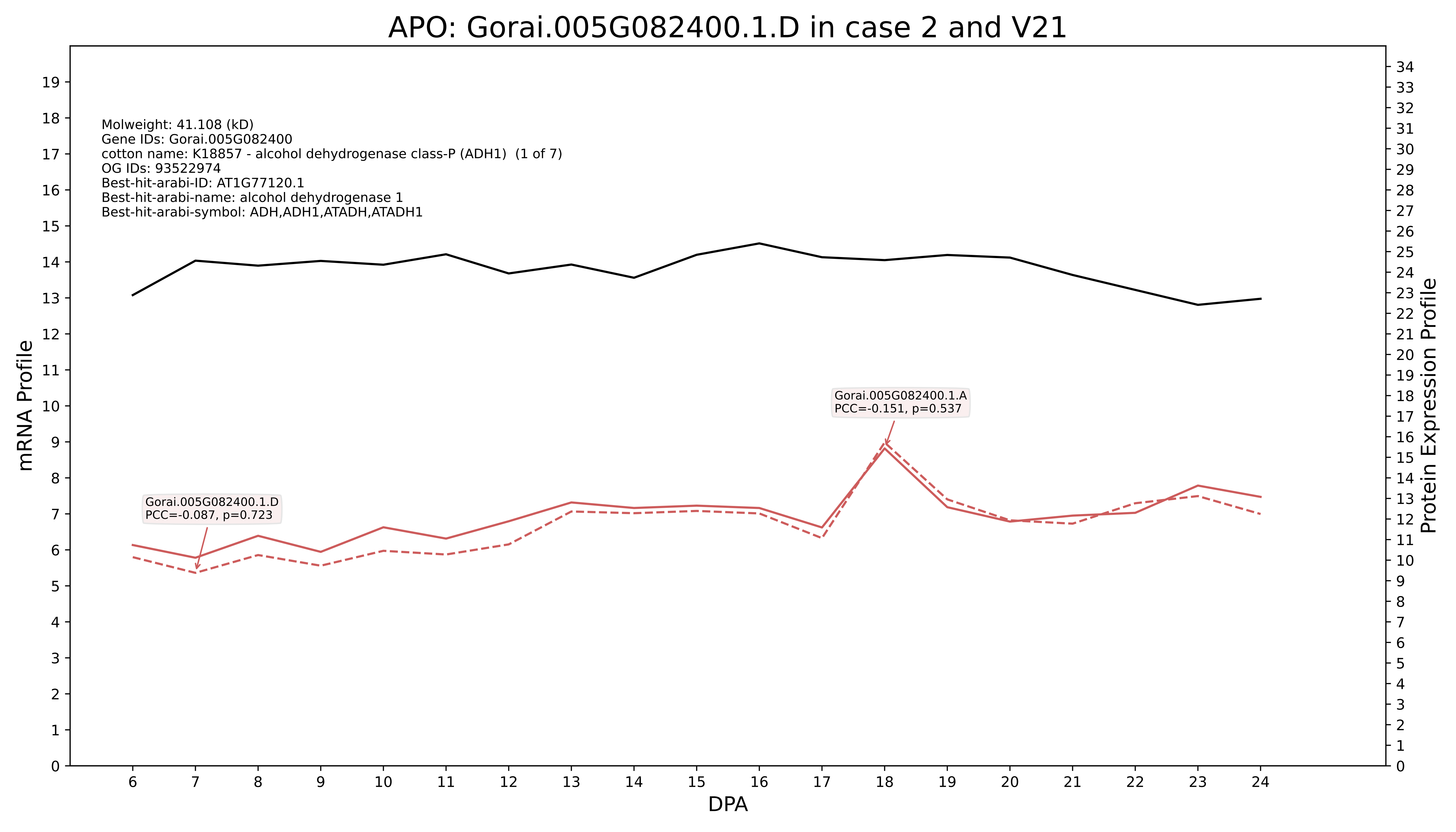

APO: Gorai.005G082400.1.D in case 2 and V21
Molweight: 41.108 (kD)
Gene IDs: Gorai.005G082400
cotton name: K18857 - alcohol dehydrogenase class-P (ADH1) (1 of 7)
OG IDs: 93522974
Best-hit-arabi-ID: AT1G77120.1
Best-hit-arabi-name: alcohol dehydrogenase 1
Best-hit-arabi-symbol: ADH,ADH1,ATADH,ATADH1
Gorai.005G082400.1.A
PCC=-0.151, p=0.537
Gorai.005G082400.1.D
PCC=-0.087, p=0.723
mRNA Profile
Protein Expression Profile
DPA

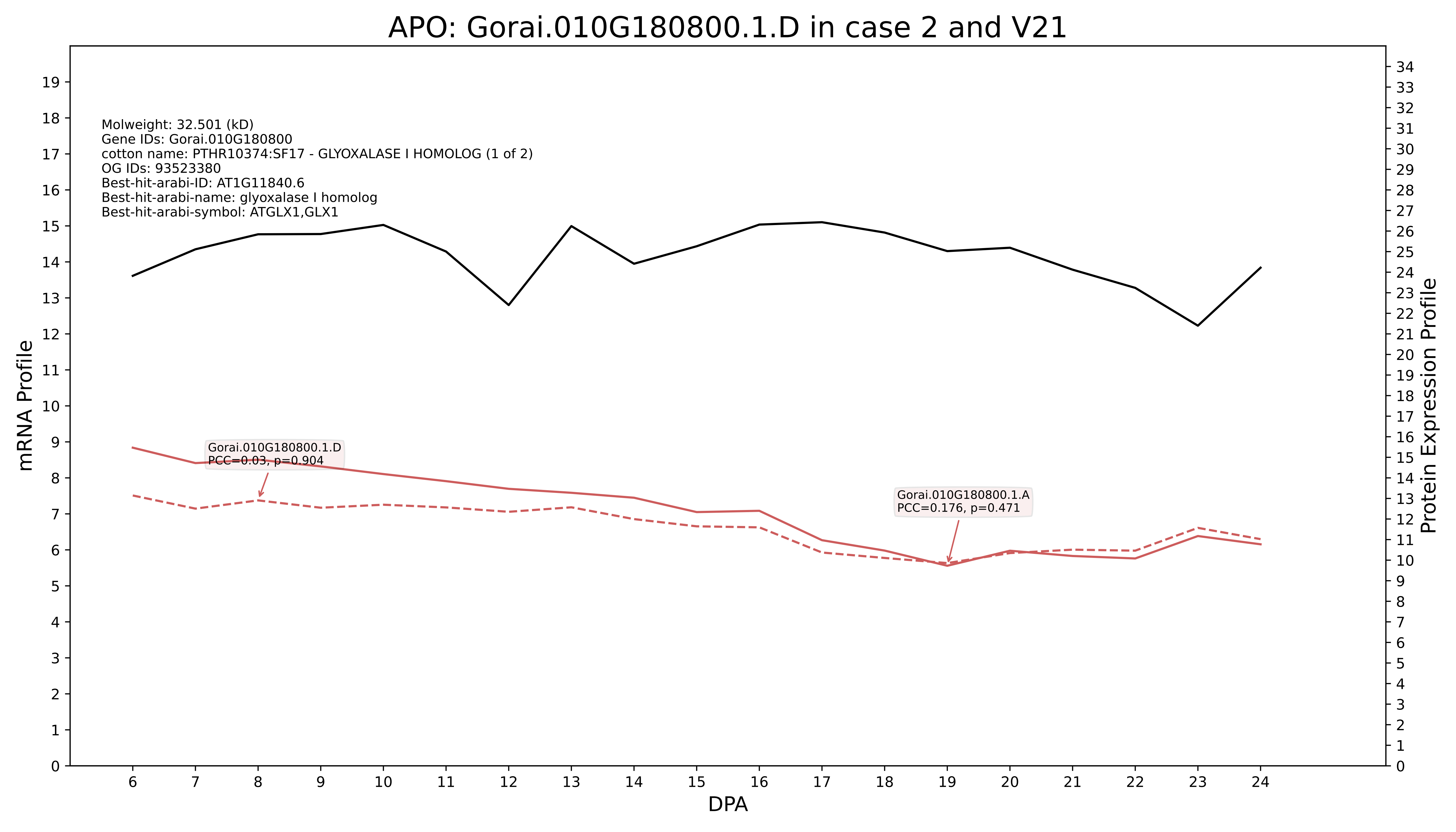
APO: Gorai.010G180800.1.D in case 2 and V21
Molweight: 32.501 (kD)
Gene IDs: Gorai.010G180800
cotton name: PTHR10374:SF17 - GLYOXALASE I HOMOLOG (1 of 2)
OG IDs: 93523380
Best-hit-arabi-ID: AT1G11840.6
Best-hit-arabi-name: glyoxalase I homolog
Best-hit-arabi-symbol: ATGLX1,GLX1
Gorai.010G180800.1.D
PCC=0.03, p=0.904
Gorai.010G180800.1.A
PCC=0.176, p=0.471
mRNA Profile
Protein Expression Profile
DPA
0
1
2
3
4
5
6
7
8
9
10
11
12
13
14
15
16
17
18
19
20
21
22
23
24
25
26
27
28
29
30
31
32
33
34

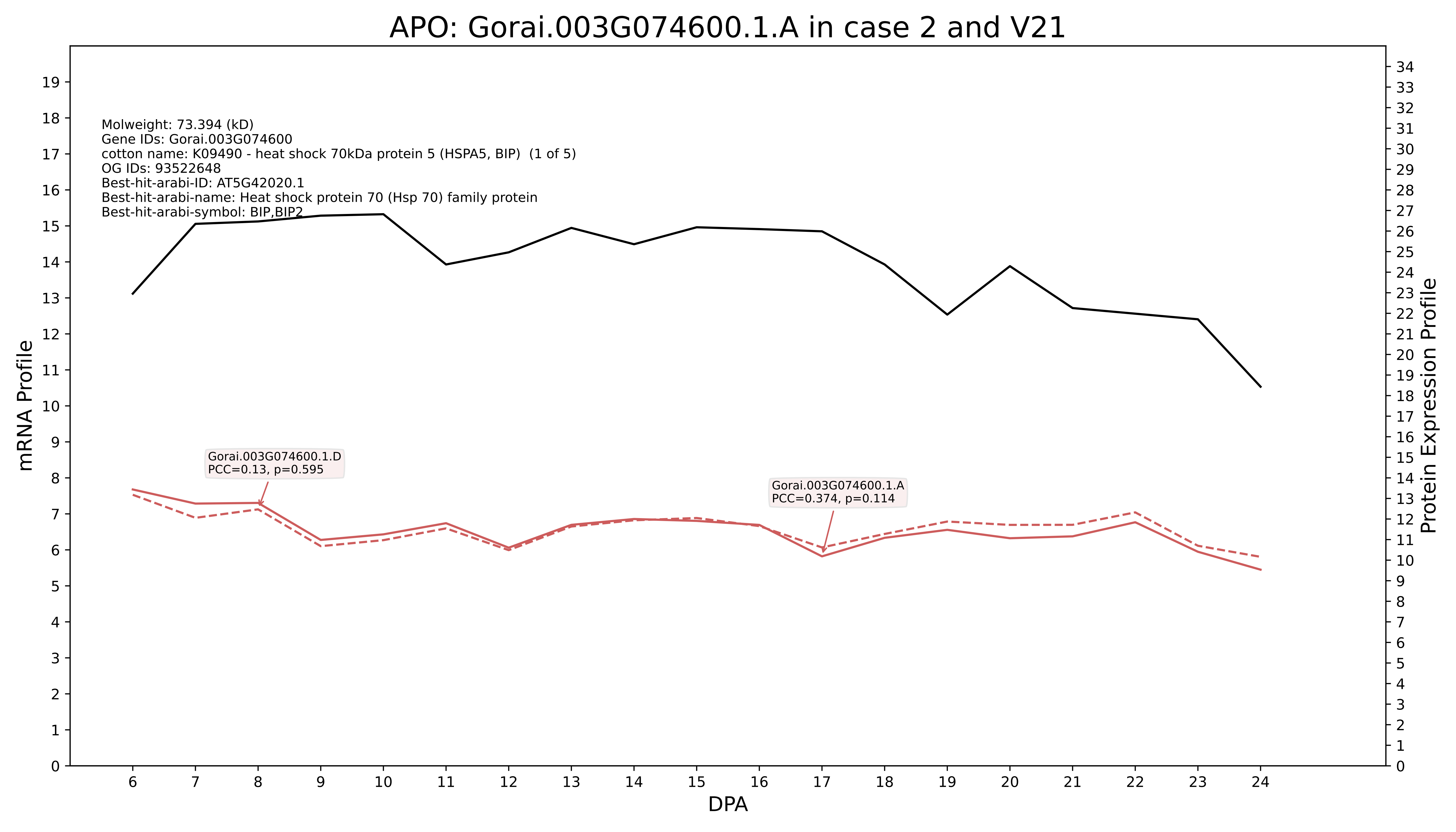
APO: Gorai.003G074600.1.A in case 2 and V21
Molweight: 73.394 (kD)
Gene IDs: Gorai.003G074600
cotton name: K09490 - heat shock 70kDa protein 5 (HSPA5, BIP) (1 of 5)
OG IDs: 93522648
Best-hit-arabi-ID: AT5G42020.1
Best-hit-arabi-name: Heat shock protein 70 (Hsp 70) family protein
Best-hit-arabi-symbol: BIP,BIP2
Gorai.003G074600.1.D
PCC=0.13, p=0.595
Gorai.003G074600.1.A
PCC=0.374, p=0.114
mRNA Profile
Protein Expression Profile
DPA

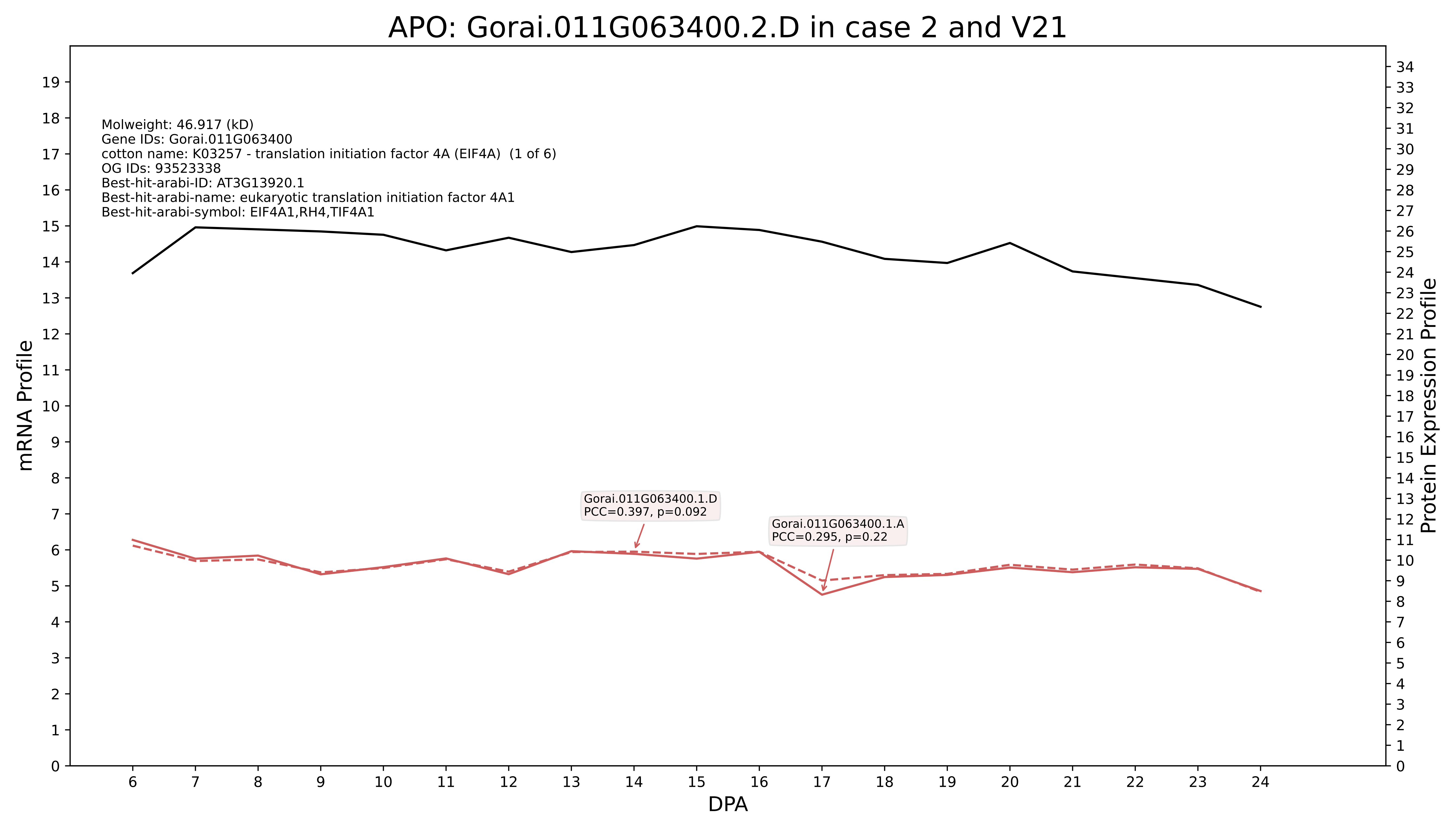

APO: Gorai.011G063400.2.D in case 2 and V21
Molweight: 46.917 (kD)
Gene IDs: Gorai.011G063400
cotton name: K03257 - translation initiation factor 4A (EIF4A) (1 of 6)
OG IDs: 93523338
Best-hit-arabi-ID: AT3G13920.1
Best-hit-arabi-name: eukaryotic translation initiation factor 4A1
Best-hit-arabi-symbol: EIF4A1,RH4,TIF4A1
Gorai.011G063400.1.D
PCC=0.397, p=0.092
Gorai.011G063400.1.A
PCC=0.295, p=0.22
mRNA Profile
Protein Expression Profile
DPA

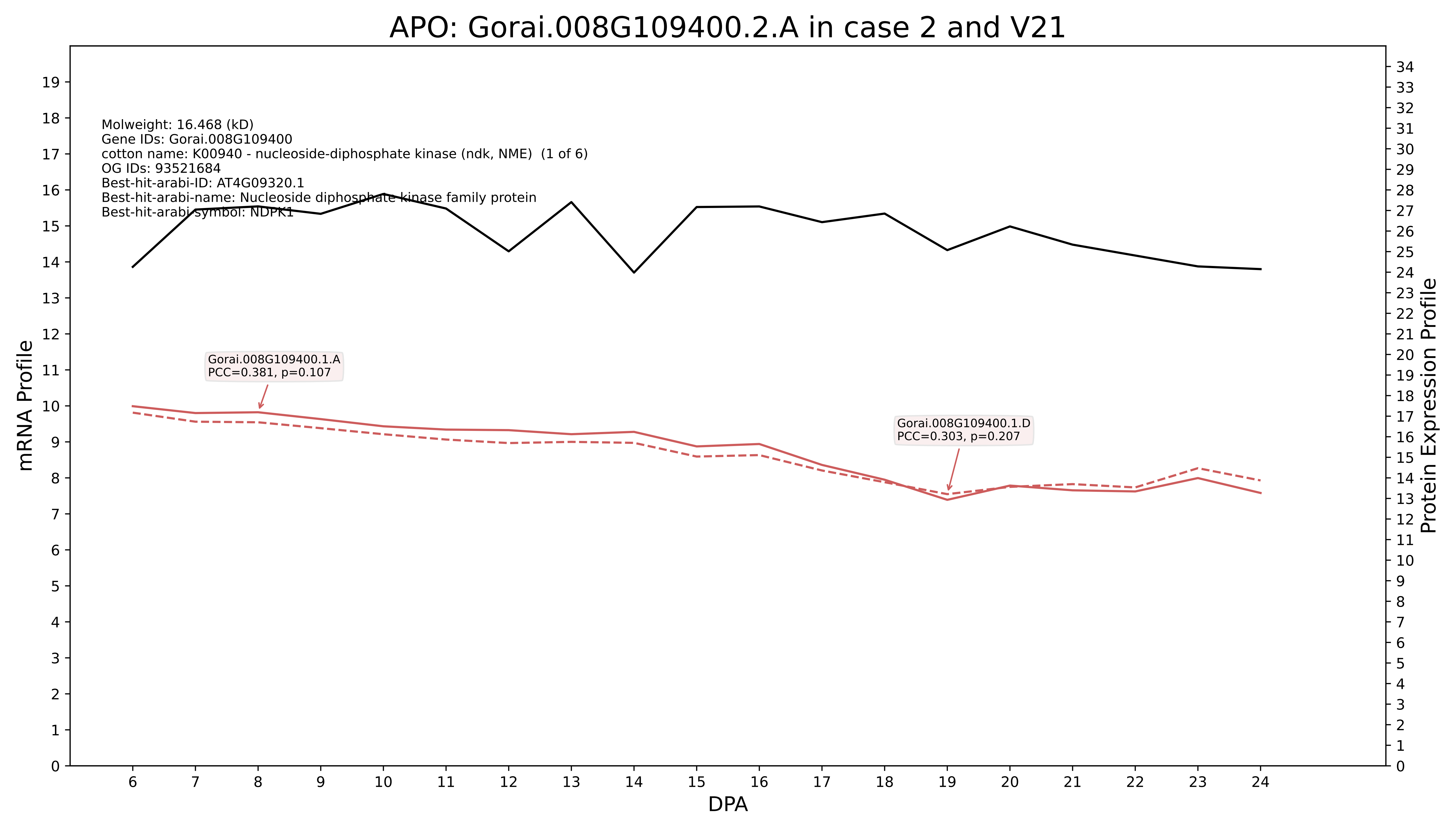
APO: Gorai.008G109400.2.A in case 2 and V21
Molweight: 16.468 (kD)
Gene IDs: Gorai.008G109400
cotton name: K00940 - nucleoside-diphosphate kinase (ndk, NME) (1 of 6)
OG IDs: 93521684
Best-hit-arabi-ID: AT4G09320.1
Best-hit-arabi-name: Nucleoside diphosphate kinase family protein
Best-hit-arabi-symbol: NDPK1
Gorai.008G109400.1.A
PCC=0.381, p=0.107
Gorai.008G109400.1.D
PCC=0.303, p=0.207
mRNA Profile
Protein Expression Profile
DPA

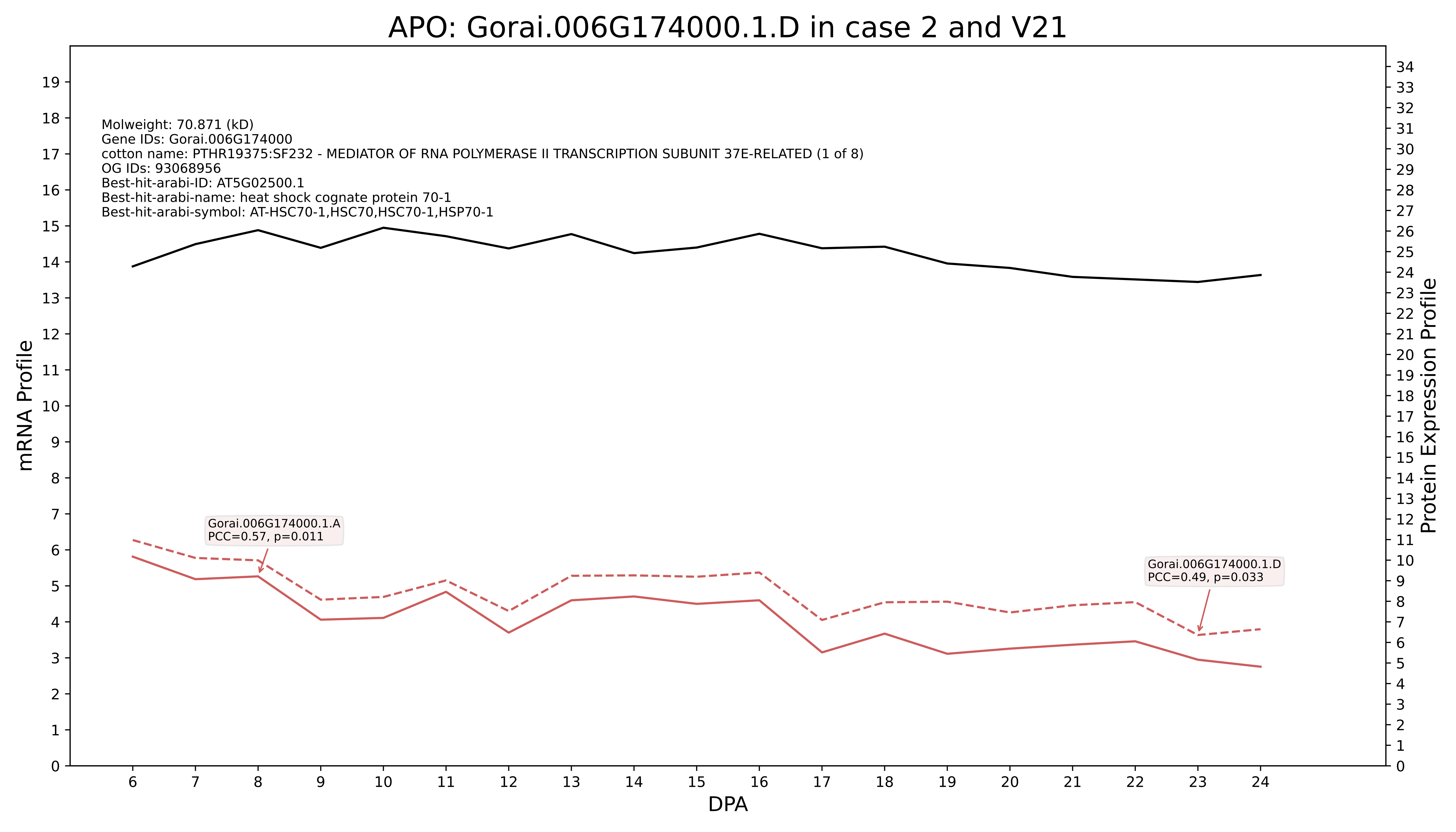
APO: Gorai.006G174000.1.D in case 2 and V21
Molweight: 70.871 (kD)
Gene IDs: Gorai.006G174000
cotton name: PTHR19375:SF232 - MEDIATOR OF RNA POLYMERASE II TRANSCRIPTION SUBUNIT 37E-RELATED (1 of 8)
OG IDs: 93068956
Best-hit-arabi-ID: AT5G02500.1
Best-hit-arabi-name: heat shock cognate protein 70-1
Best-hit-arabi-symbol: AT-HSC70-1,HSC70,HSC70-1,HSP70-1
Gorai.006G174000.1.A
PCC=0.57, p=0.011
Gorai.006G174000.1.D
PCC=0.49, p=0.033
mRNA Profile
Protein Expression Profile
DPA

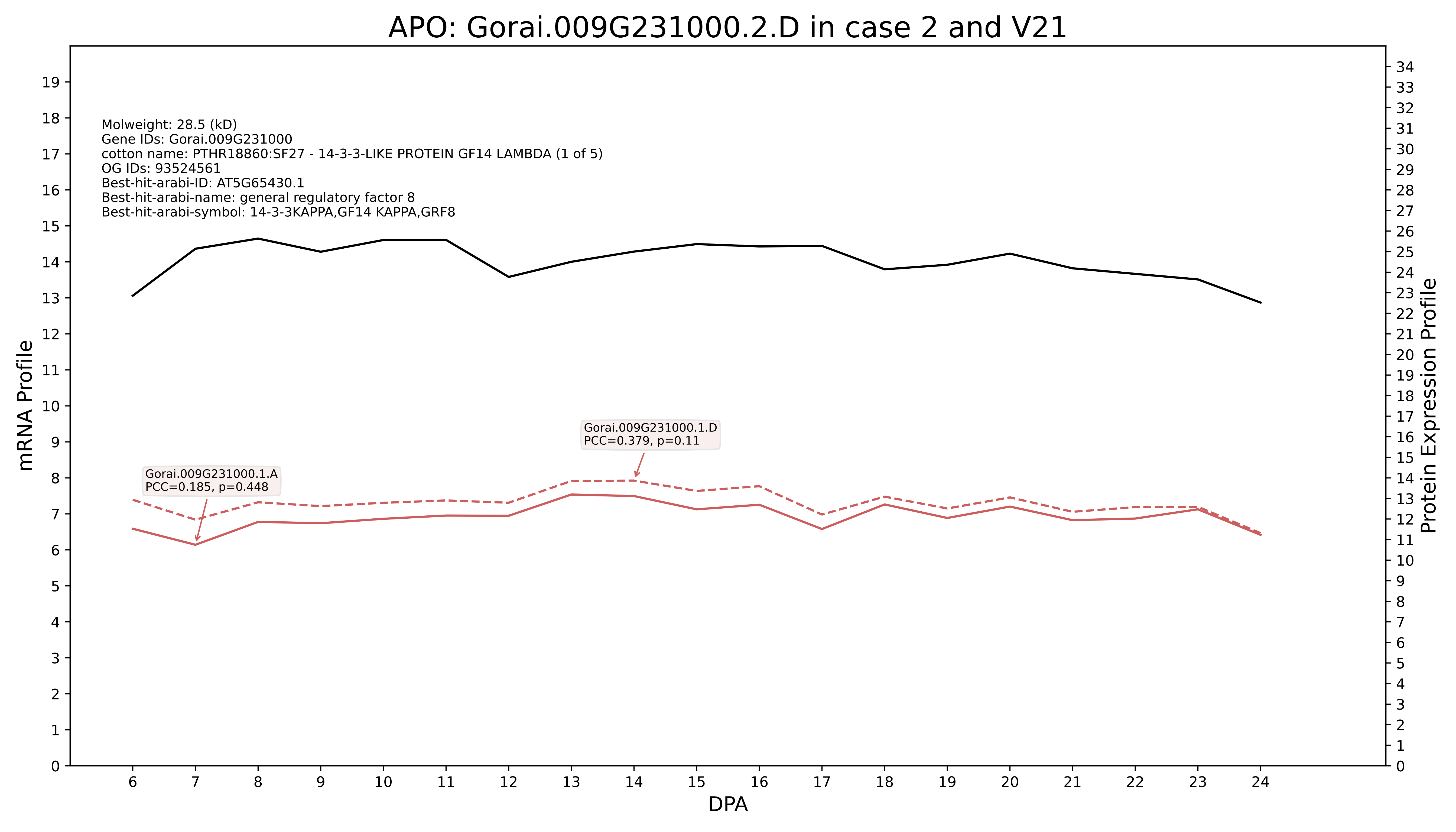

APO: Gorai.009G231000.2.D in case 2 and V21
Molweight: 28.5 (kD)
Gene IDs: Gorai.009G231000
cotton name: PTHR18860:SF27 - 14-3-3-LIKE PROTEIN GF14 LAMBDA (1 of 5)
OG IDs: 93524561
Best-hit-arabi-ID: AT5G65430.1
Best-hit-arabi-name: general regulatory factor 8
Best-hit-arabi-symbol: 14-3-3KAPPA,GF14 KAPPA,GRF8
Gorai.009G231000.1.D
PCC=0.379, p=0.11
Gorai.009G231000.1.A
PCC=0.185, p=0.448
mRNA Profile
Protein Expression Profile
DPA

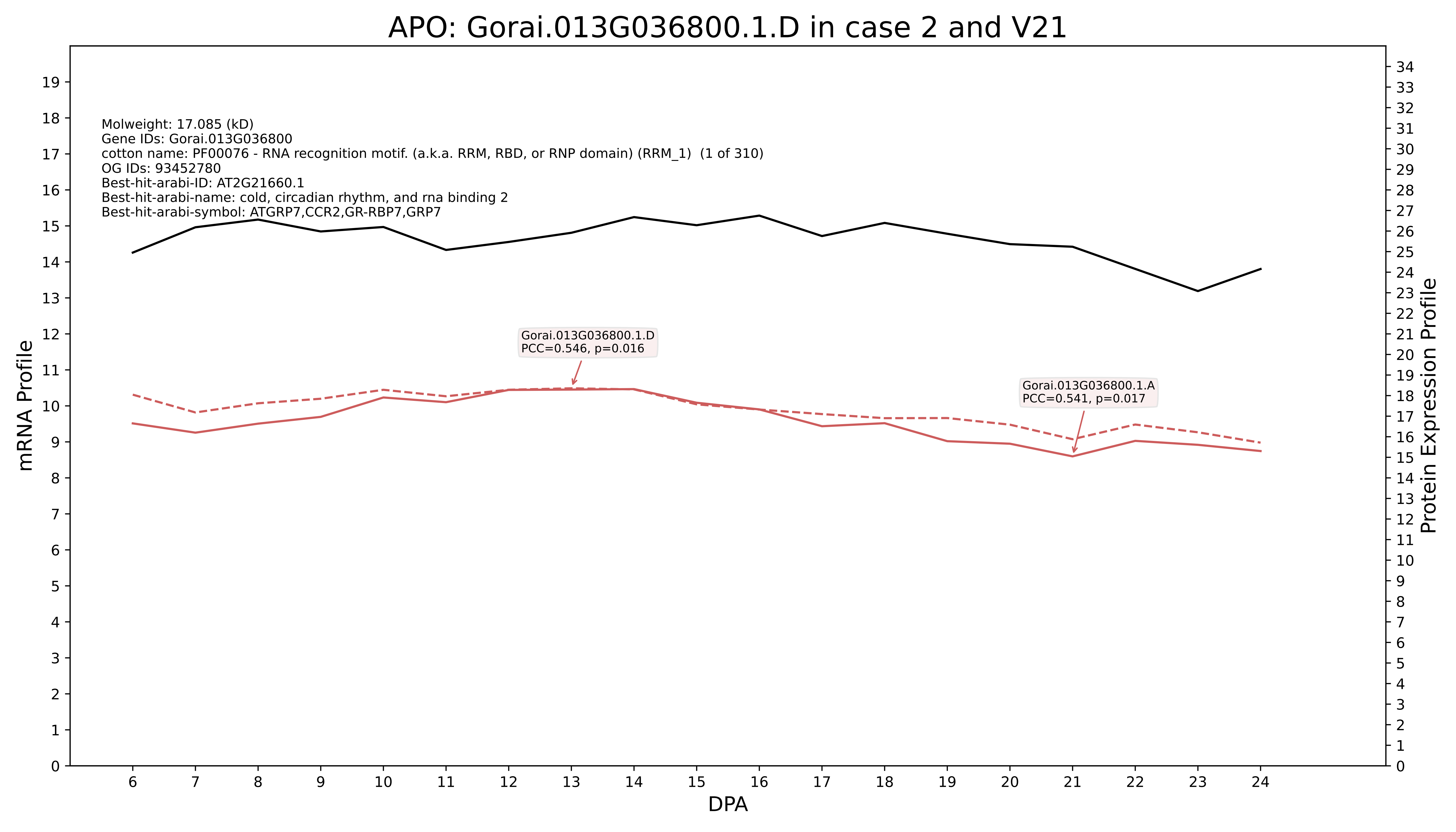

APO: Gorai.013G036800.1.D in case 2 and V21
Molweight: 17.085 (kD)
Gene IDs: Gorai.013G036800
cotton name: PF00076 - RNA recognition motif. (a.k.a. RRM, RBD, or RNP domain) (RRM_1) (1 of 310)
OG IDs: 93452780
Best-hit-arabi-ID: AT2G21660.1
Best-hit-arabi-name: cold, circadian rhythm, and rna binding 2
Best-hit-arabi-symbol: ATGRP7,CCR2,GR-RBP7,GRP7
Gorai.013G036800.1.D
PCC=0.546, p=0.016
Gorai.013G036800.1.A
PCC=0.541, p=0.017
mRNA Profile
Protein Expression Profile
DPA

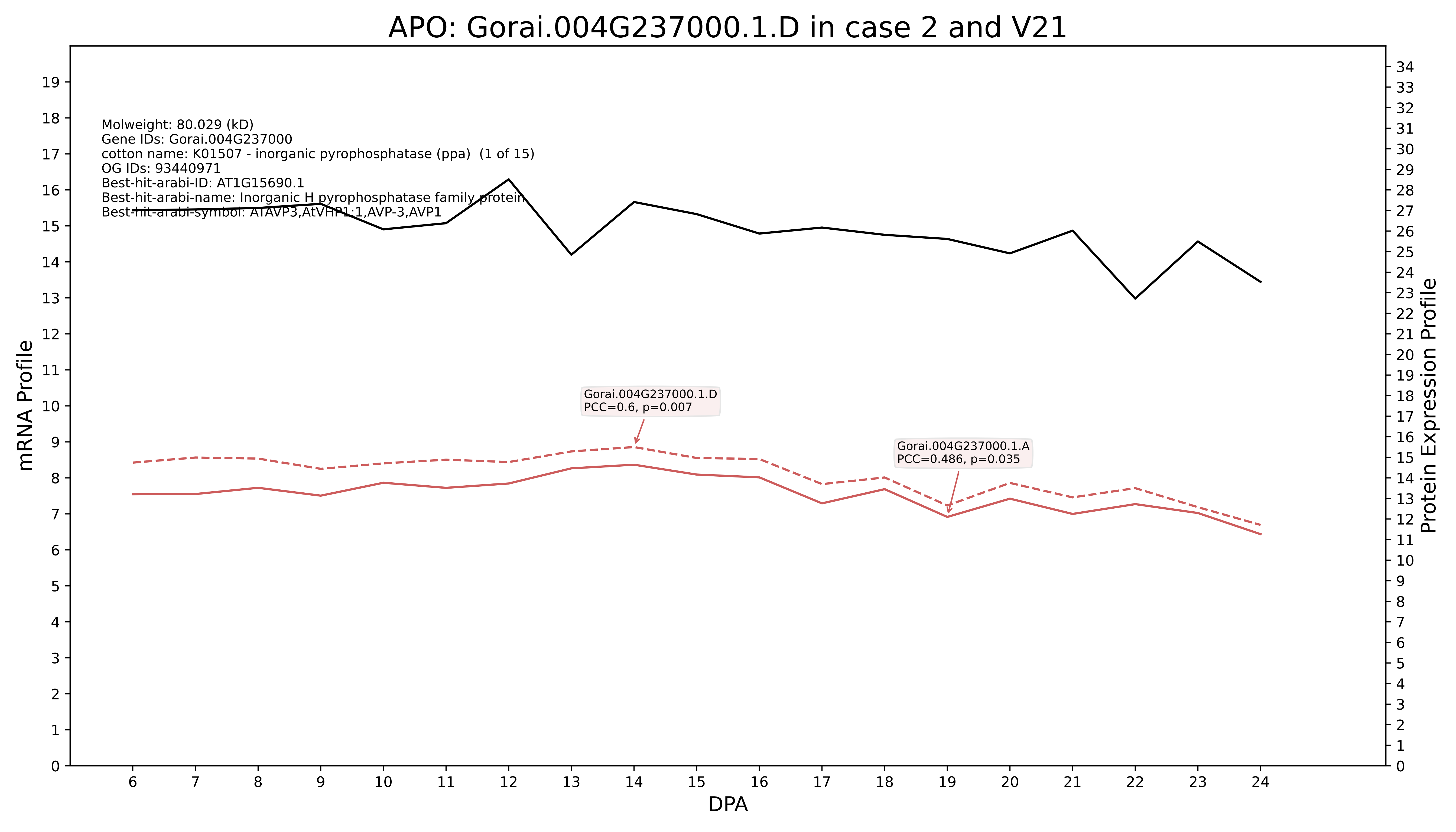
APO: Gorai.004G237000.1.D in case 2 and V21
Molweight: 80.029 (kD)
Gene IDs: Gorai.004G237000
cotton name: K01507 - inorganic pyrophosphatase (ppa) (1 of 15)
OG IDs: 93440971
Best-hit-arabi-ID: AT1G15690.1
Best-hit-arabi-name: Inorganic H pyrophosphatase family protein
Best-hit-arabi-symbol: ATAVP3,AtVHP1;1,AVP-3,AVP1
Gorai.004G237000.1.D
PCC=0.6, p=0.007
Gorai.004G237000.1.A
PCC=0.486, p=0.035
mRNA Profile
Protein Expression Profile
DPA
0
1
2
3
4
5
6
7
8
9
10
11
12
13
14
15
16
17
18
19
20
21
22
23
24
25
26
27
28
29
30
31
32
33
34

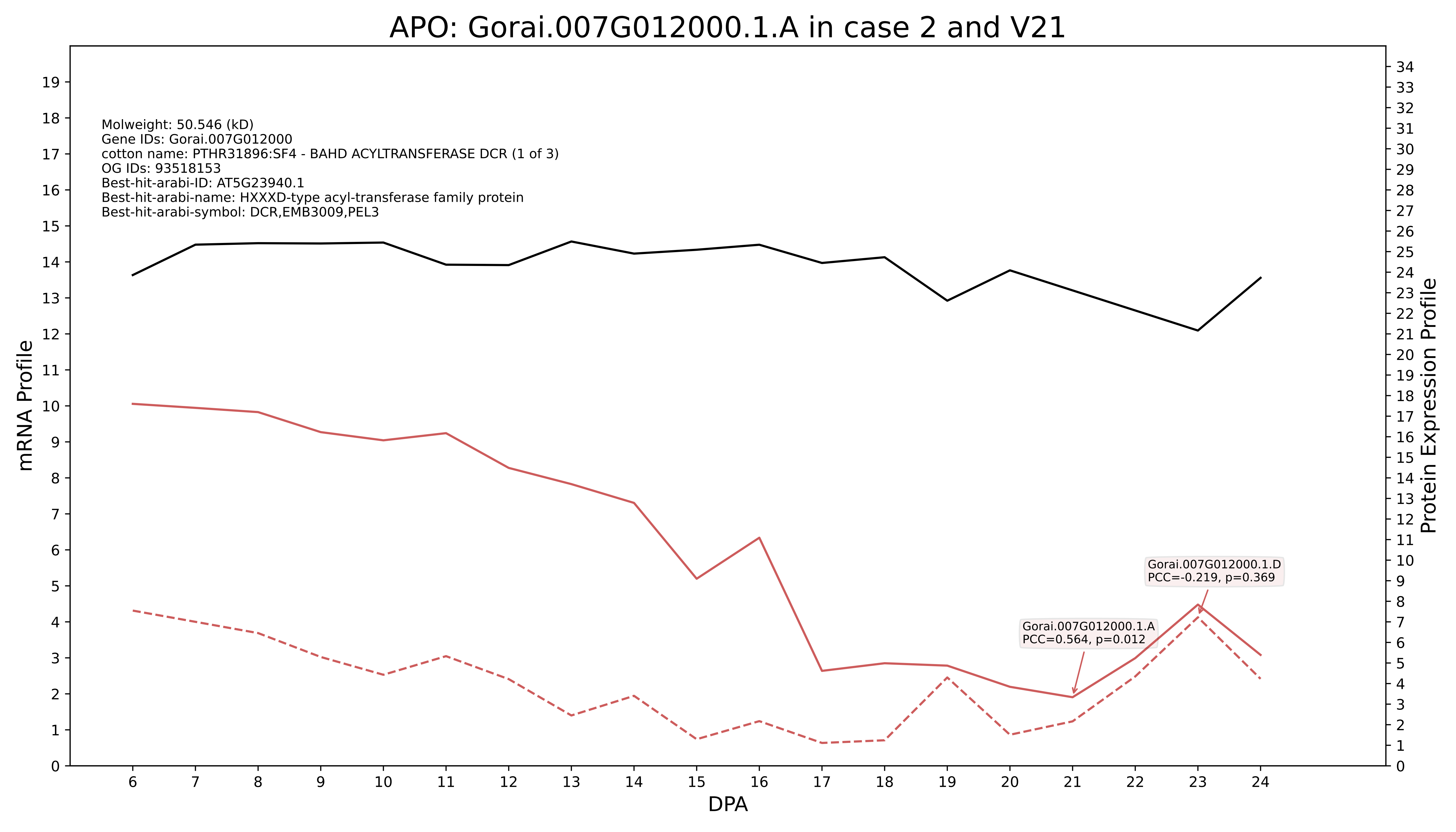

APO: Gorai.007G012000.1.A in case 2 and V21
Molweight: 50.546 (kD)
Gene IDs: Gorai.007G012000
cotton name: PTHR31896:SF4 - BAHD ACYLTRANSFERASE DCR (1 of 3)
OG IDs: 93518153
Best-hit-arabi-ID: AT5G23940.1
Best-hit-arabi-name: HXXXD-type acyl-transferase family protein
Best-hit-arabi-symbol: DCR,EMB3009,PEL3
Gorai.007G012000.1.D
PCC=-0.219, p=0.369
Gorai.007G012000.1.A
PCC=0.564, p=0.012
mRNA Profile
Protein Expression Profile
DPA

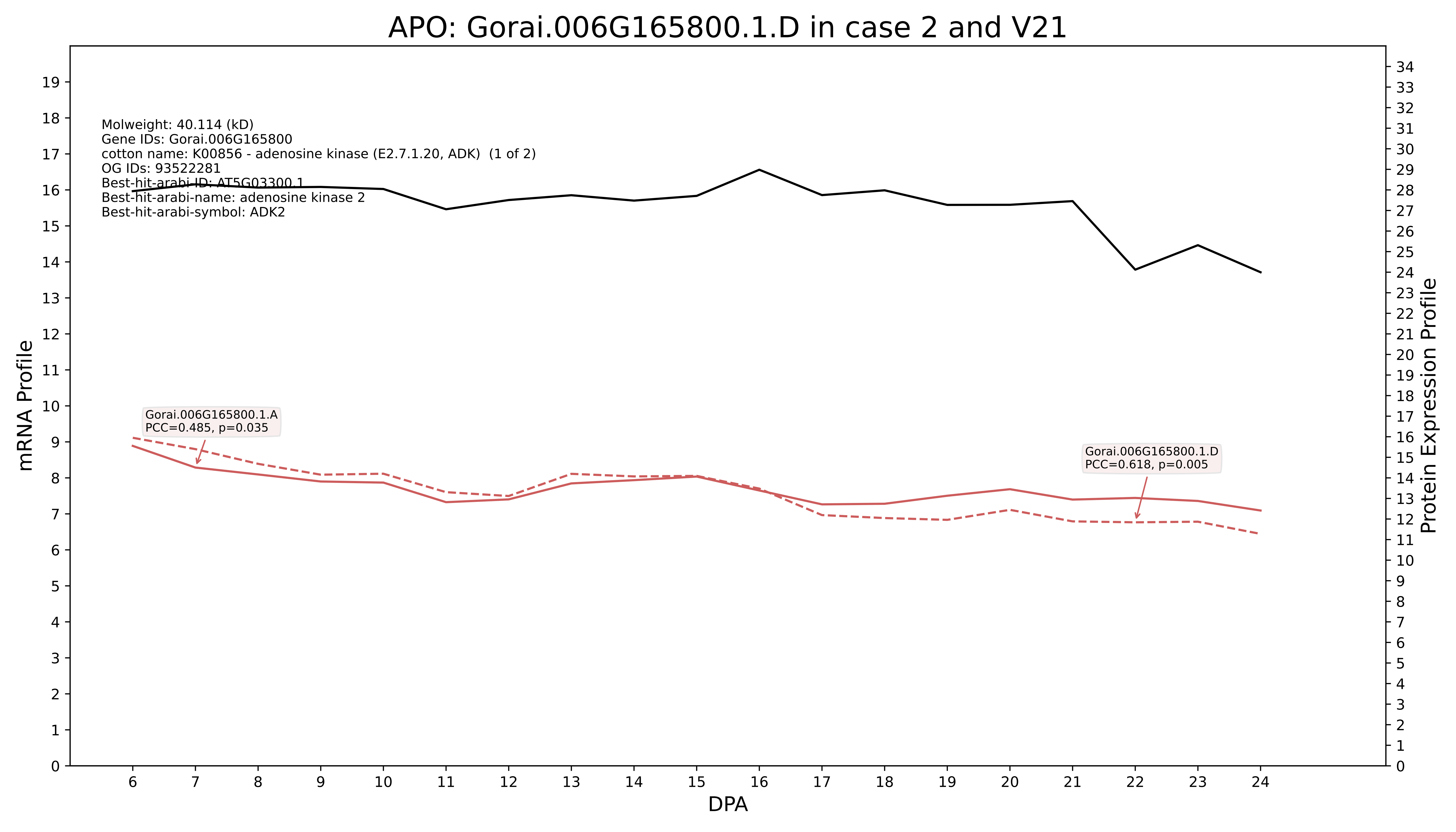

APO: Gorai.006G165800.1.D in case 2 and V21
Molweight: 40.114 (kD)
Gene IDs: Gorai.006G165800
cotton name: K00856 - adenosine kinase (E2.7.1.20, ADK) (1 of 2)
OG IDs: 93522281
Best-hit-arabi-ID: AT5G03300.1
Best-hit-arabi-name: adenosine kinase 2
Best-hit-arabi-symbol: ADK2
Gorai.006G165800.1.A
PCC=0.485, p=0.035
Gorai.006G165800.1.D
PCC=0.618, p=0.005
mRNA Profile
Protein Expression Profile
DPA

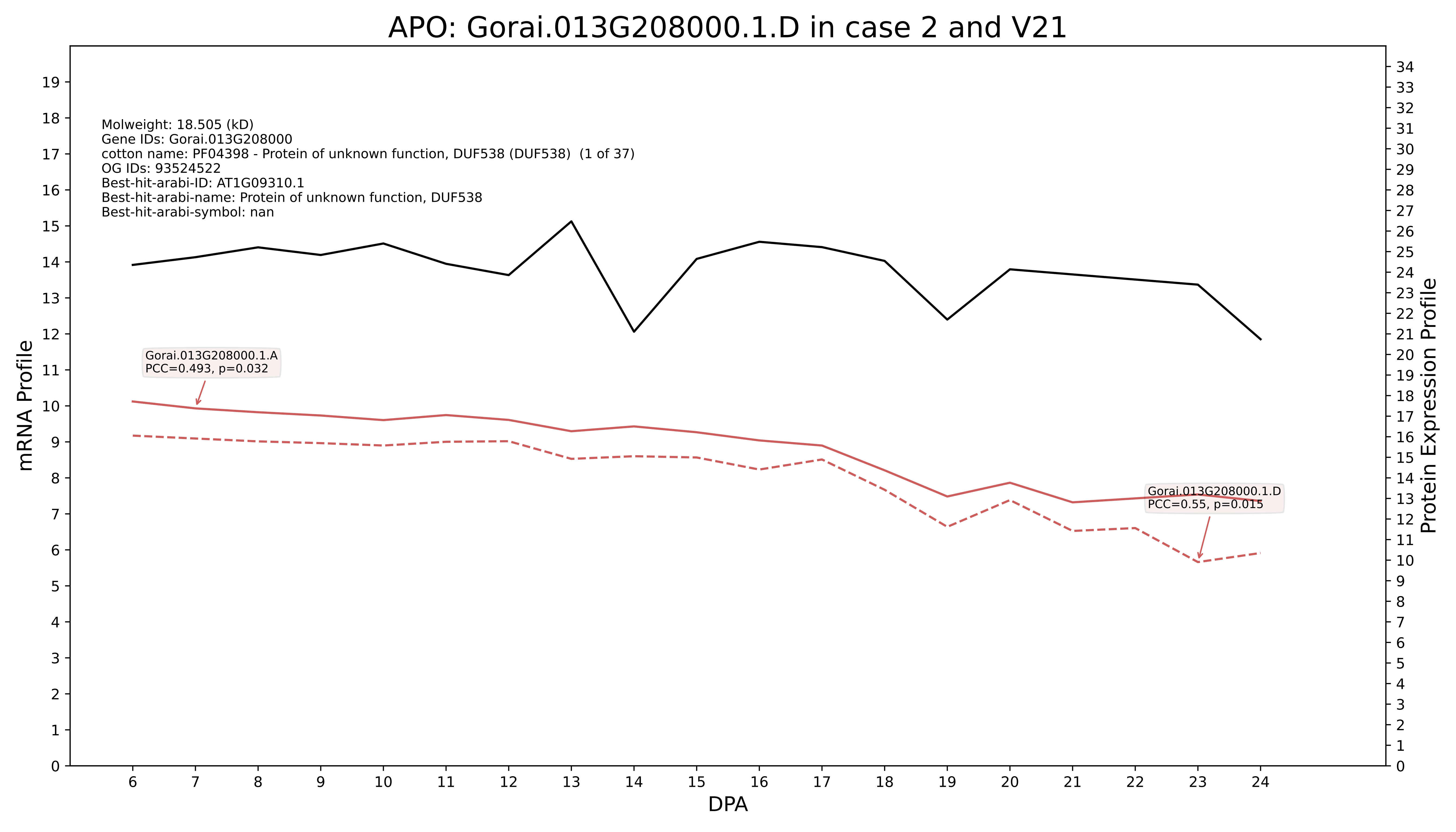

APO: Gorai.013G208000.1.D in case 2 and V21
Molweight: 18.505 (kD)
Gene IDs: Gorai.013G208000
cotton name: PF04398 - Protein of unknown function, DUF538 (DUF538) (1 of 37)
OG IDs: 93524522
Best-hit-arabi-ID: AT1G09310.1
Best-hit-arabi-name: Protein of unknown function, DUF538
Best-hit-arabi-symbol: nan
Gorai.013G208000.1.A
PCC=0.493, p=0.032
Gorai.013G208000.1.D
PCC=0.55, p=0.015
mRNA Profile
Protein Expression Profile
DPA

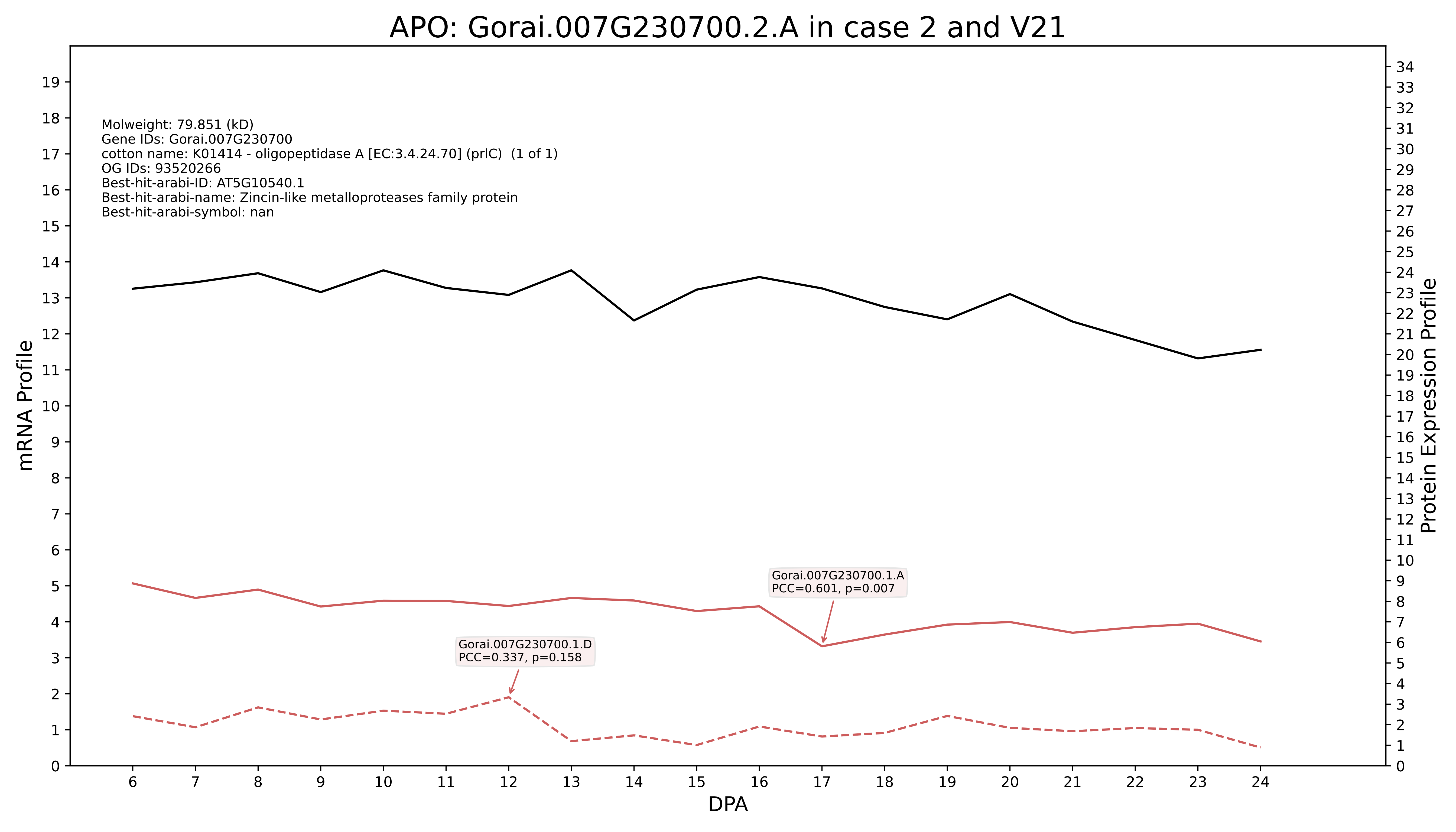
APO: Gorai.007G230700.2.A in case 2 and V21
Molweight: 79.851 (kD)
Gene IDs: Gorai.007G230700
cotton name: K01414 - oligopeptidase A [EC:3.4.24.70] (prlC) (1 of 1)
OG IDs: 93520266
Best-hit-arabi-ID: AT5G10540.1
Best-hit-arabi-name: Zincin-like metalloproteases family protein
Best-hit-arabi-symbol: nan
Gorai.007G230700.1.A
PCC=0.601, p=0.007
Gorai.007G230700.1.D
PCC=0.337, p=0.158
mRNA Profile
Protein Expression Profile
DPA

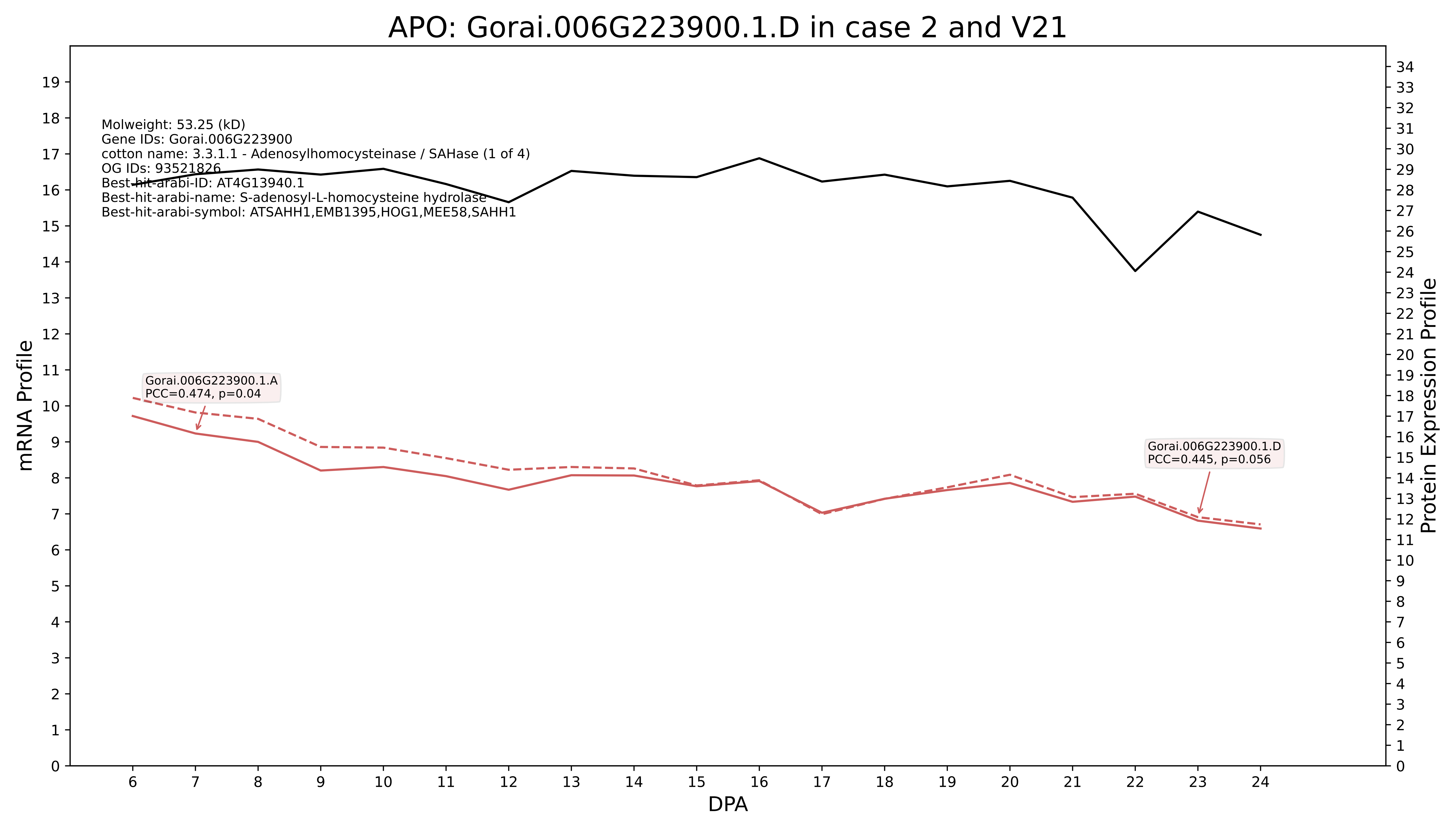
APO: Gorai.006G223900.1.D in case 2 and V21
Molweight: 53.25 (kD)
Gene IDs: Gorai.006G223900
cotton name: 3.3.1.1 - Adenosylhomocysteinase / SAHase (1 of 4)
OG IDs: 93521826
Best-hit-arabi-ID: AT4G13940.1
Best-hit-arabi-name: S-adenosyl-L-homocysteine hydrolase
Best-hit-arabi-symbol: ATSAHH1,EMB1395,HOG1,MEE58,SAHH1
Gorai.006G223900.1.A
PCC=0.474, p=0.04
Gorai.006G223900.1.D
PCC=0.445, p=0.056
mRNA Profile
Protein Expression Profile
DPA

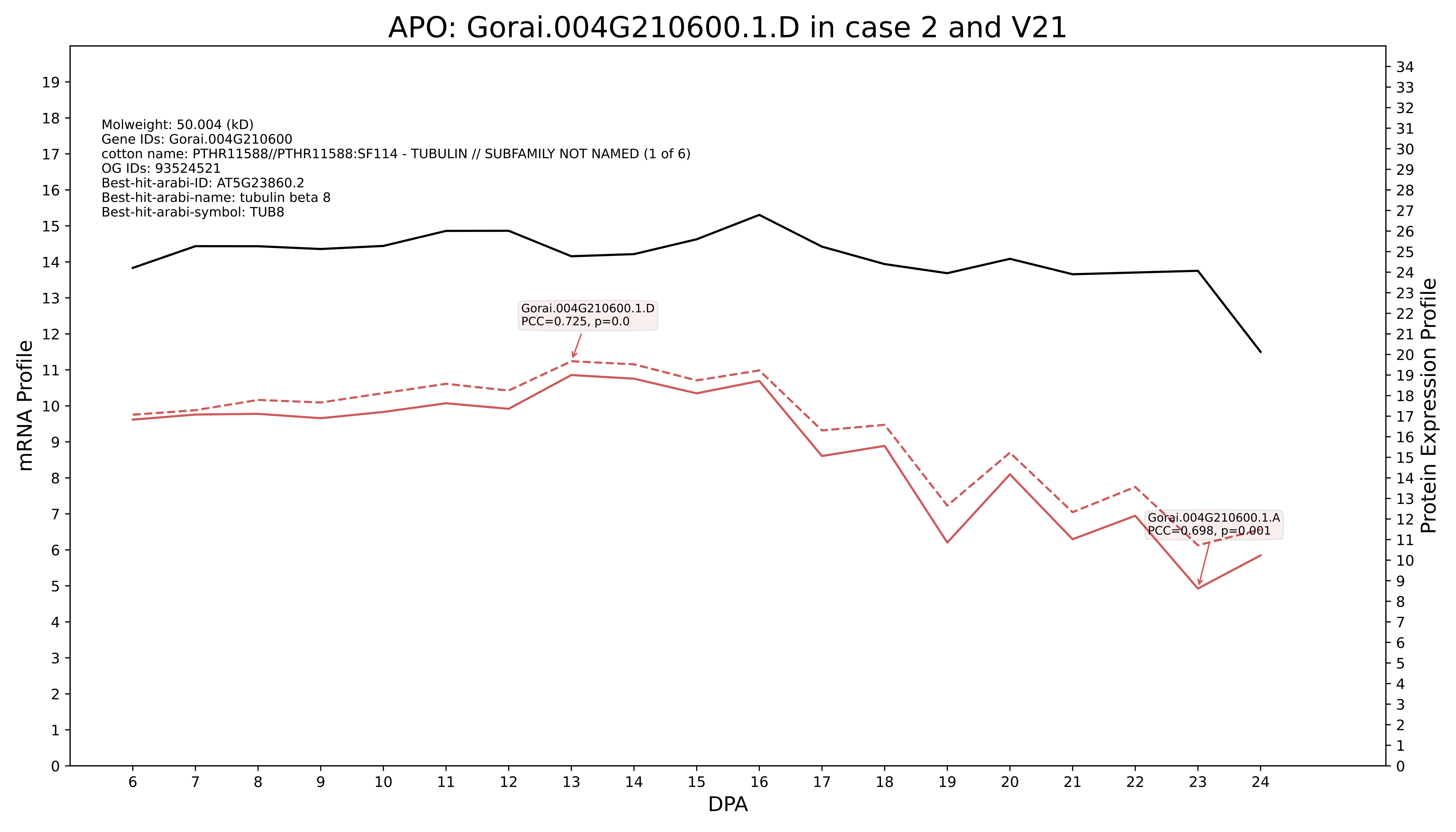

APO: Gorai.004G210600.1.D in case 2 and V21
Molweight: 50.004 (kD)
Gene IDs: Gorai.004G210600
cotton name: PTHR11588//PTHR11588:SF114 - TUBULIN // SUBFAMILY NOT NAMED (1 of 6)
OG IDs: 93524521
Best-hit-arabi-ID: AT5G23860.2
Best-hit-arabi-name: tubulin beta 8
Best-hit-arabi-symbol: TUB8
Gorai.004G210600.1.D
PCC=0.725, p=0.0
Gorai.004G210600.1.A
PCC=0.698, p=0.001
mRNA Profile
Protein Expression Profile
DPA

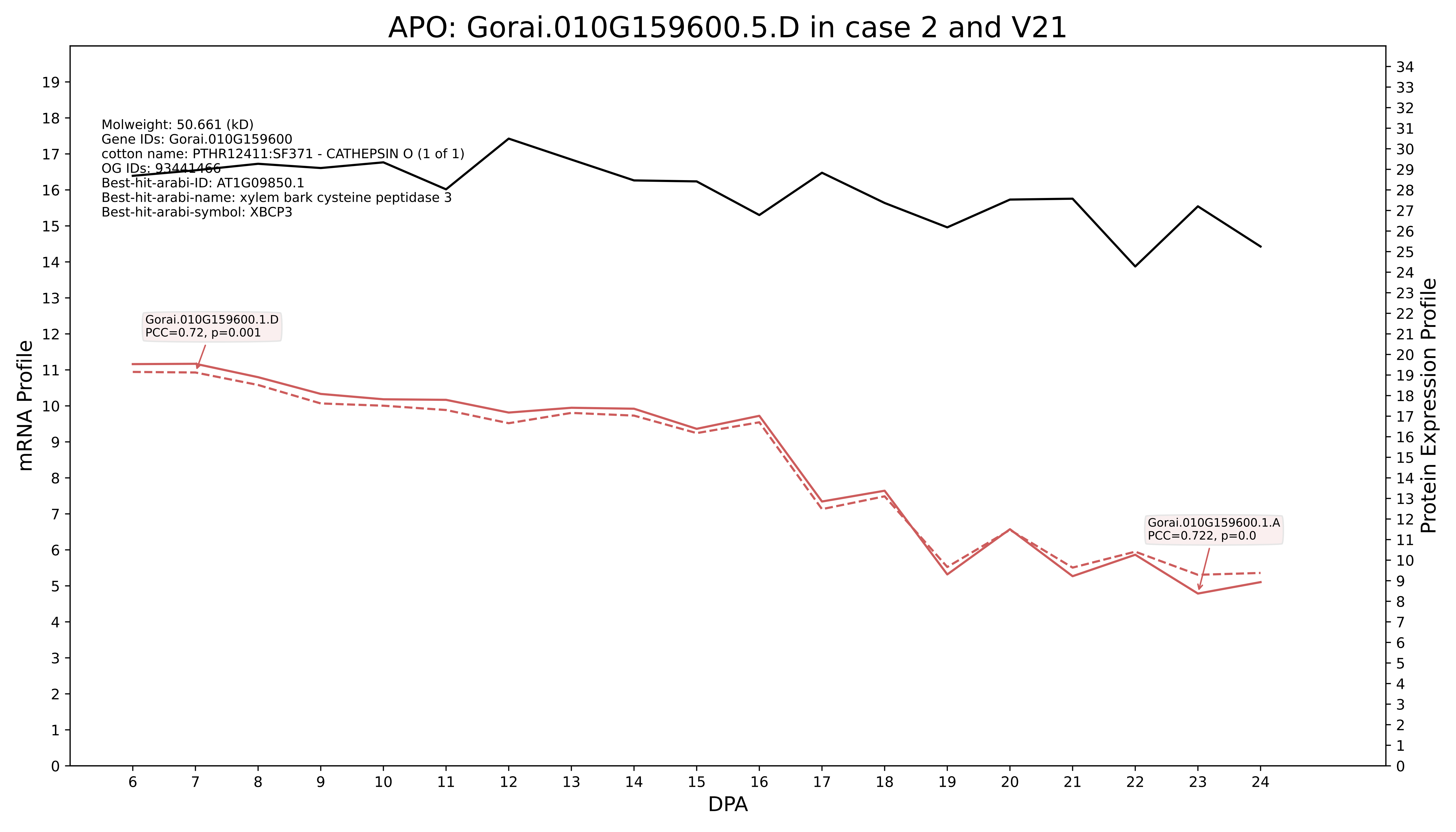

APO: Gorai.010G159600.5.D in case 2 and V21
Molweight: 50.661 (kD)
Gene IDs: Gorai.010G159600
cotton name: PTHR12411:SF371 - CATHEPSIN O (1 of 1)
OG IDs: 93441466
Best-hit-arabi-ID: AT1G09850.1
Best-hit-arabi-name: xylem bark cysteine peptidase 3
Best-hit-arabi-symbol: XBCP3
Gorai.010G159600.1.D
PCC=0.72, p=0.001
Gorai.010G159600.1.A
PCC=0.722, p=0.0
mRNA Profile
Protein Expression Profile
DPA

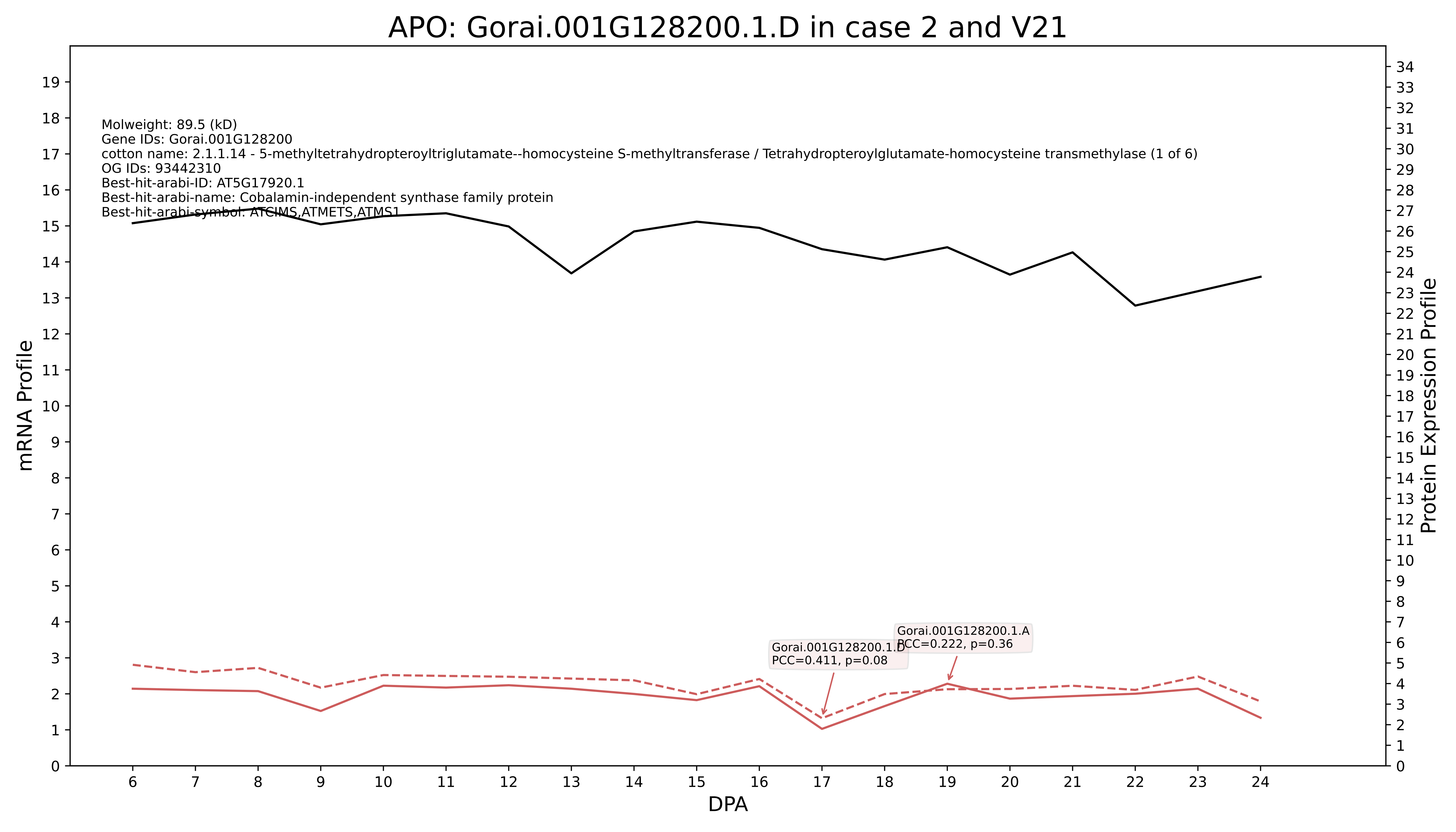

APO: Gorai.001G128200.1.D in case 2 and V21
Molweight: 89.5 (kD)
Gene IDs: Gorai.001G128200
cotton name: 2.1.1.14 - 5-methyltetrahydropteroyltriglutamate--homocysteine S-methyltransferase / Tetrahydropteroylglutamate-homocysteine transmethylase (1 of 6)
OG IDs: 93442310
Best-hit-arabi-ID: AT5G17920.1
Best-hit-arabi-name: Cobalamin-independent synthase family protein
Best-hit-arabi-symbol: ATCIMS,ATMETS,ATMS1
Gorai.001G128200.1.D
PCC=0.411, p=0.08
Gorai.001G128200.1.A
PCC=0.222, p=0.36
mRNA Profile
Protein Expression Profile
DPA

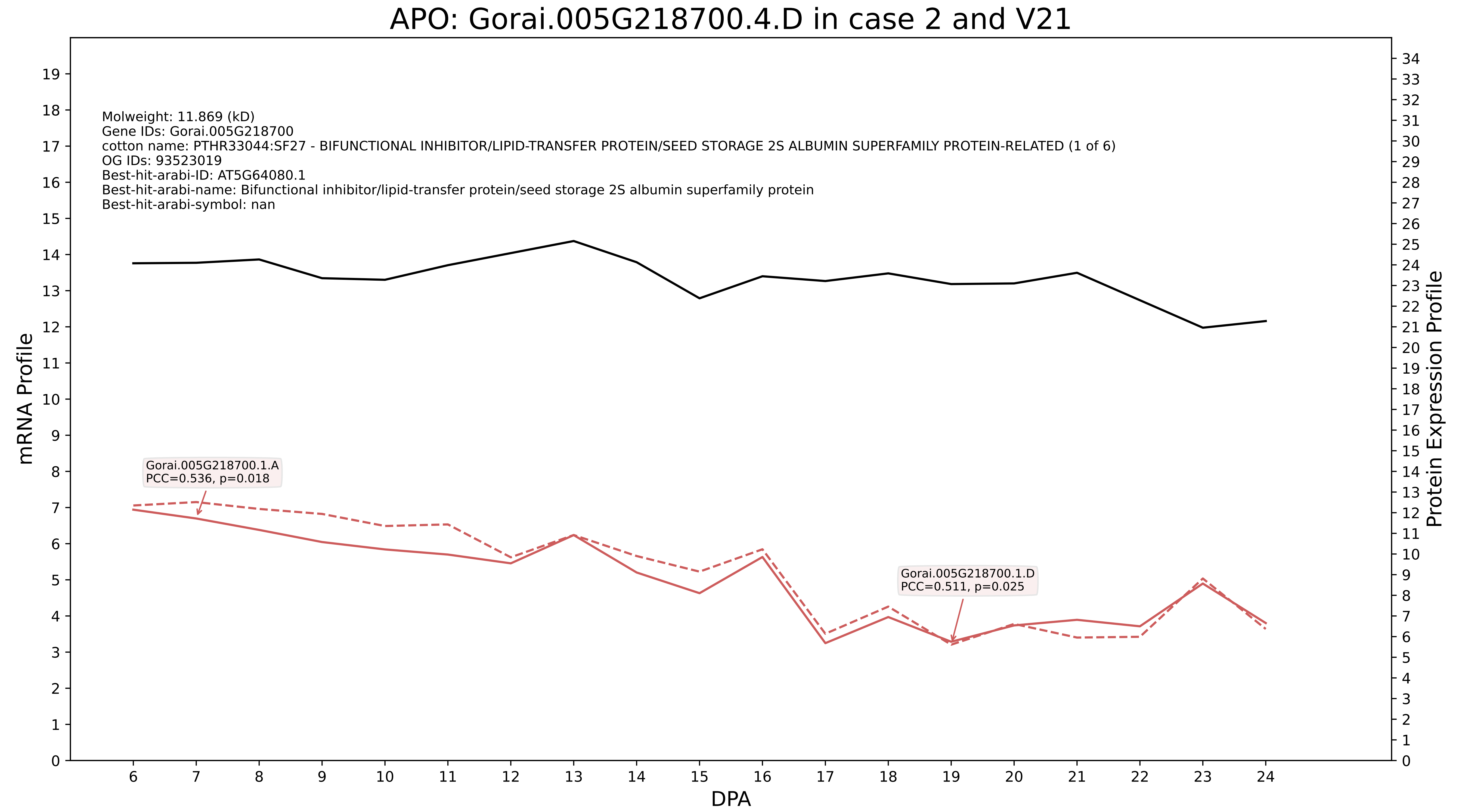

APO: Gorai.005G218700.4.D in case 2 and V21
Molweight: 11.869 (kD)
Gene IDs: Gorai.005G218700
cotton name: PTHR33044:SF27 - BIFUNCTIONAL INHIBITOR/LIPID-TRANSFER PROTEIN/SEED STORAGE 2S ALBUMIN SUPERFAMILY PROTEIN-RELATED (1 of 6)
OG IDs: 93523019
Best-hit-arabi-ID: AT5G64080.1
Best-hit-arabi-name: Bifunctional inhibitor/lipid-transfer protein/seed storage 2S albumin superfamily protein
Best-hit-arabi-symbol: nan
Gorai.005G218700.1.A
PCC=0.536, p=0.018
Gorai.005G218700.1.D
PCC=0.511, p=0.025
mRNA Profile
Protein Expression Profile
DPA

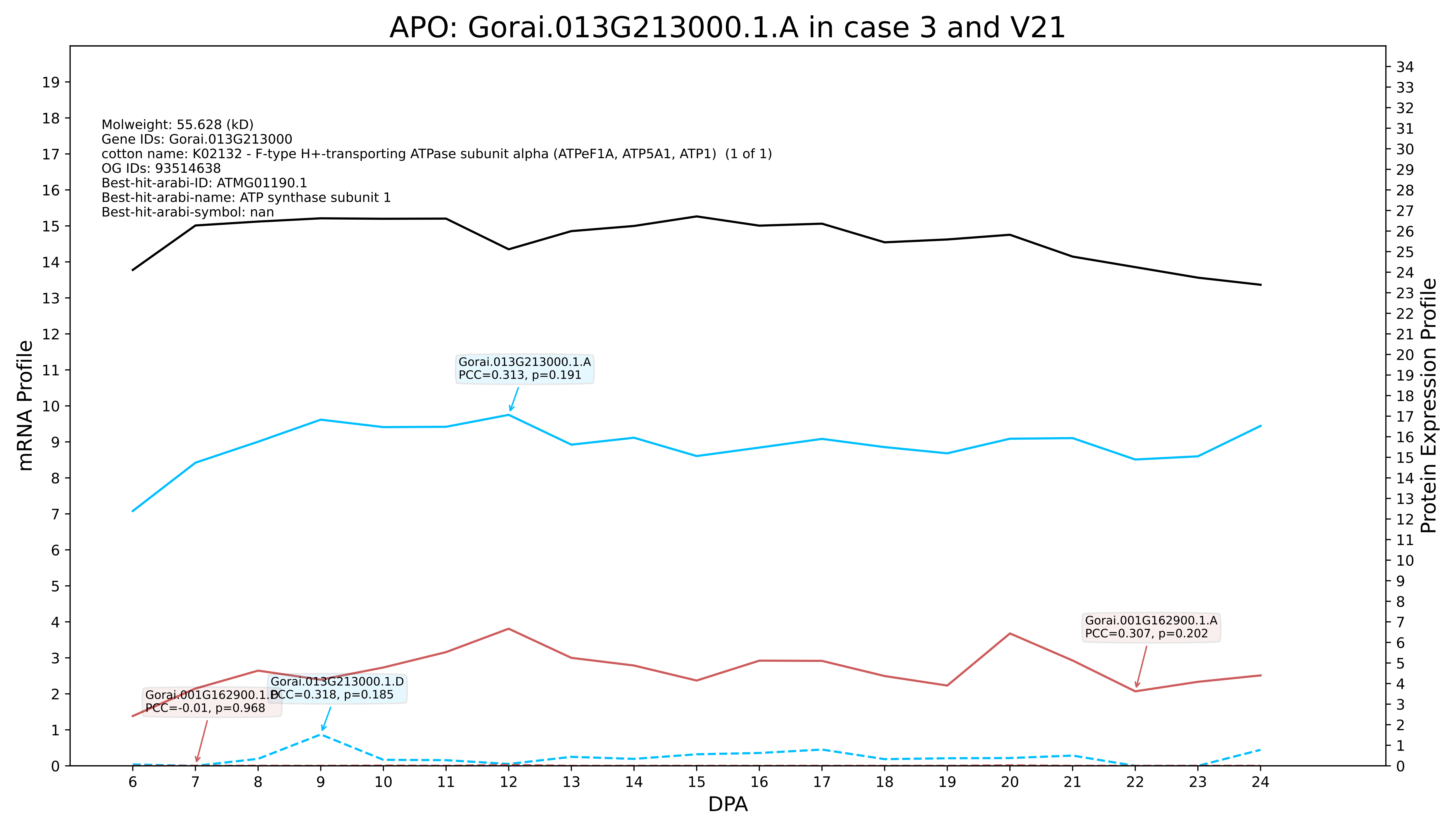
APO: Gorai.013G213000.1.A in case 3 and V21
Molweight: 55.628 (kD)
Gene IDs: Gorai.013G213000
cotton name: K02132 - F-type H+-transporting ATPase subunit alpha (ATPeF1A, ATP5A1, ATP1) (1 of 1)
OG IDs: 93514638
Best-hit-arabi-ID: ATMG01190.1
Best-hit-arabi-name: ATP synthase subunit 1
Best-hit-arabi-symbol: nan
Gorai.013G213000.1.A
PCC=0.313, p=0.191
Gorai.001G162900.1.A
PCC=0.307, p=0.202
Gorai.001G162900.1.D
PCC=-0.01, p=0.968
Gorai.013G213000.1.D
PCC=0.318, p=0.185
mRNA Profile
Protein Expression Profile
DPA

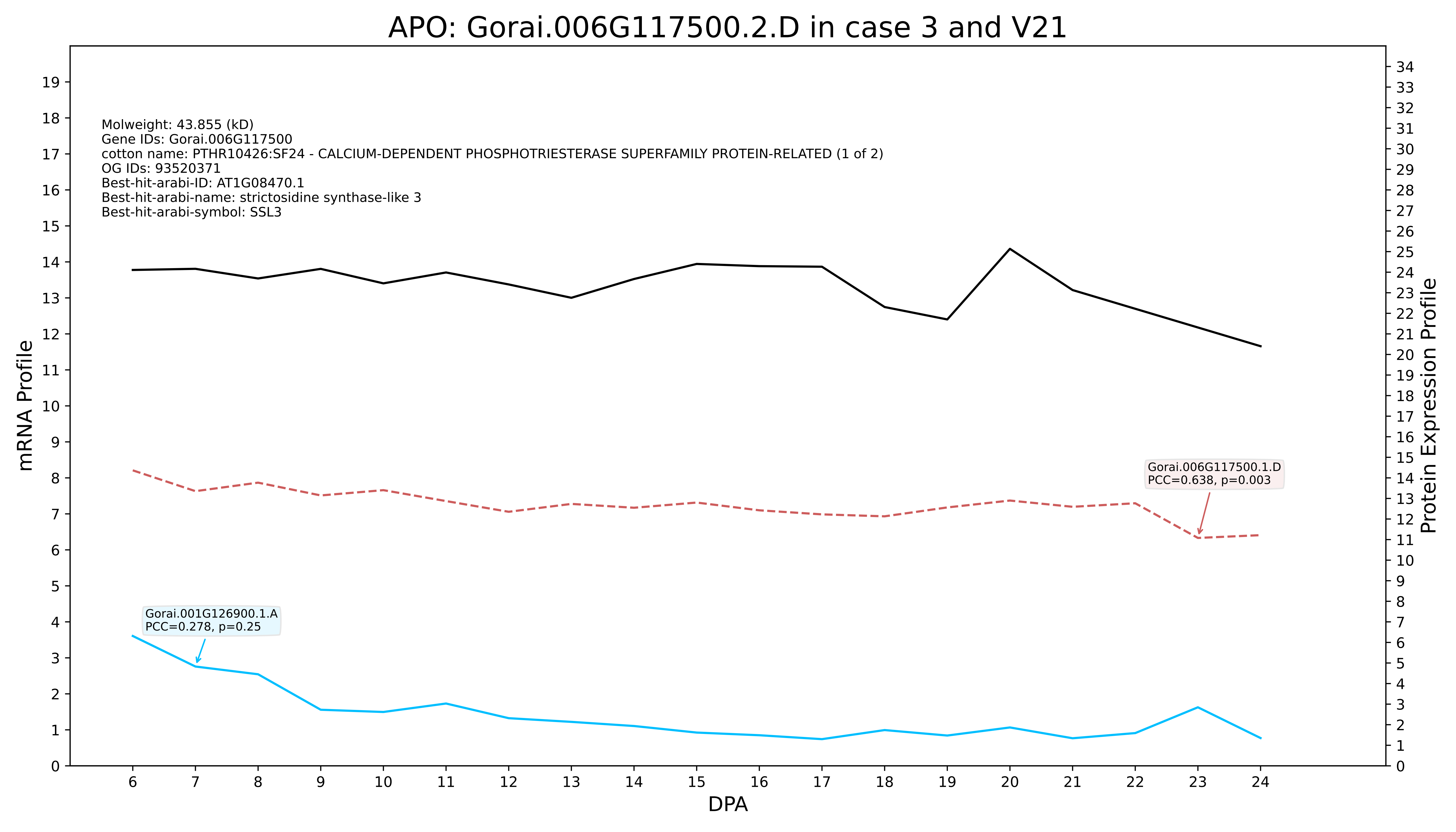
APO: Gorai.006G117500.2.D in case 3 and V21
Molweight: 43.855 (kD)
Gene IDs: Gorai.006G117500
cotton name: PTHR10426:SF24 - CALCIUM-DEPENDENT PHOSPHOTRIESTERASE SUPERFAMILY PROTEIN-RELATED (1 of 2)
OG IDs: 93520371
Best-hit-arabi-ID: AT1G08470.1
Best-hit-arabi-name: strictosidine synthase-like 3
Best-hit-arabi-symbol: SSL3
Gorai.006G117500.1.D
PCC=0.638, p=0.003
Gorai.001G126900.1.A
PCC=0.278, p=0.25
mRNA Profile
Protein Expression Profile
DPA

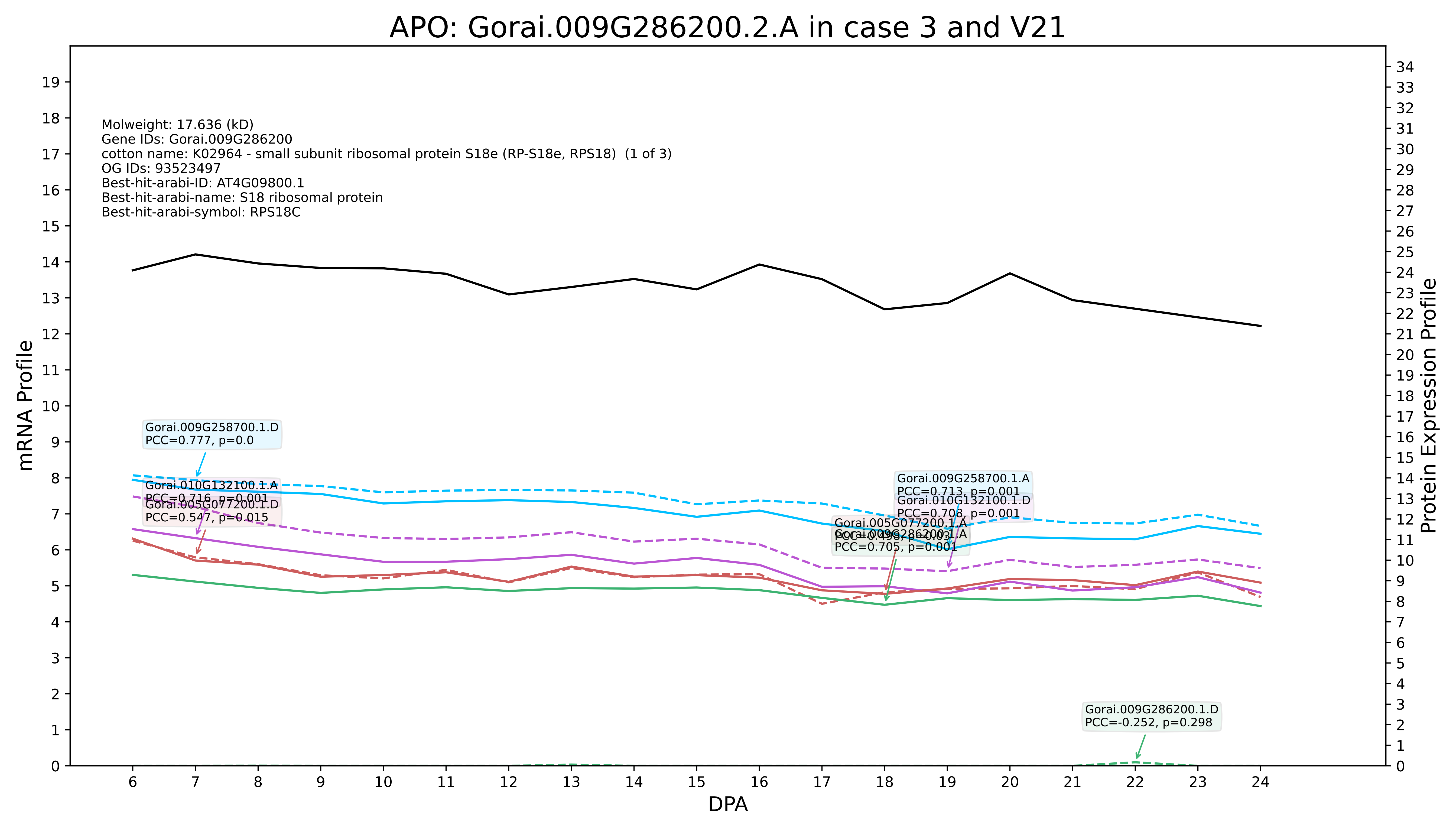

APO: Gorai.009G286200.2.A in case 3 and V21
Molweight: 17.636 (kD)
Gene IDs: Gorai.009G286200
cotton name: K02964 - small subunit ribosomal protein S18e (RP-S18e, RPS18) (1 of 3)
OG IDs: 93523497
Best-hit-arabi-ID: AT4G09800.1
Best-hit-arabi-name: S18 ribosomal protein
Best-hit-arabi-symbol: RPS18C
Gorai.009G258700.1.D
PCC=0.777, p=0.0
Gorai.010G132100.1.A
PCC=0.716, p=0.001
Gorai.005G077200.1.D
PCC=0.547, p=0.015
Gorai.009G258700.1.A
PCC=0.713, p=0.001
Gorai.010G132100.1.D
PCC=0.708, p=0.001
Gorai.005G077200.1.A
Gorai.009G286200.1.A
PCC=0.705, p=0.001
Gorai.009G286200.1.D
PCC=-0.252, p=0.298
mRNA Profile
Protein Expression Profile
DPA

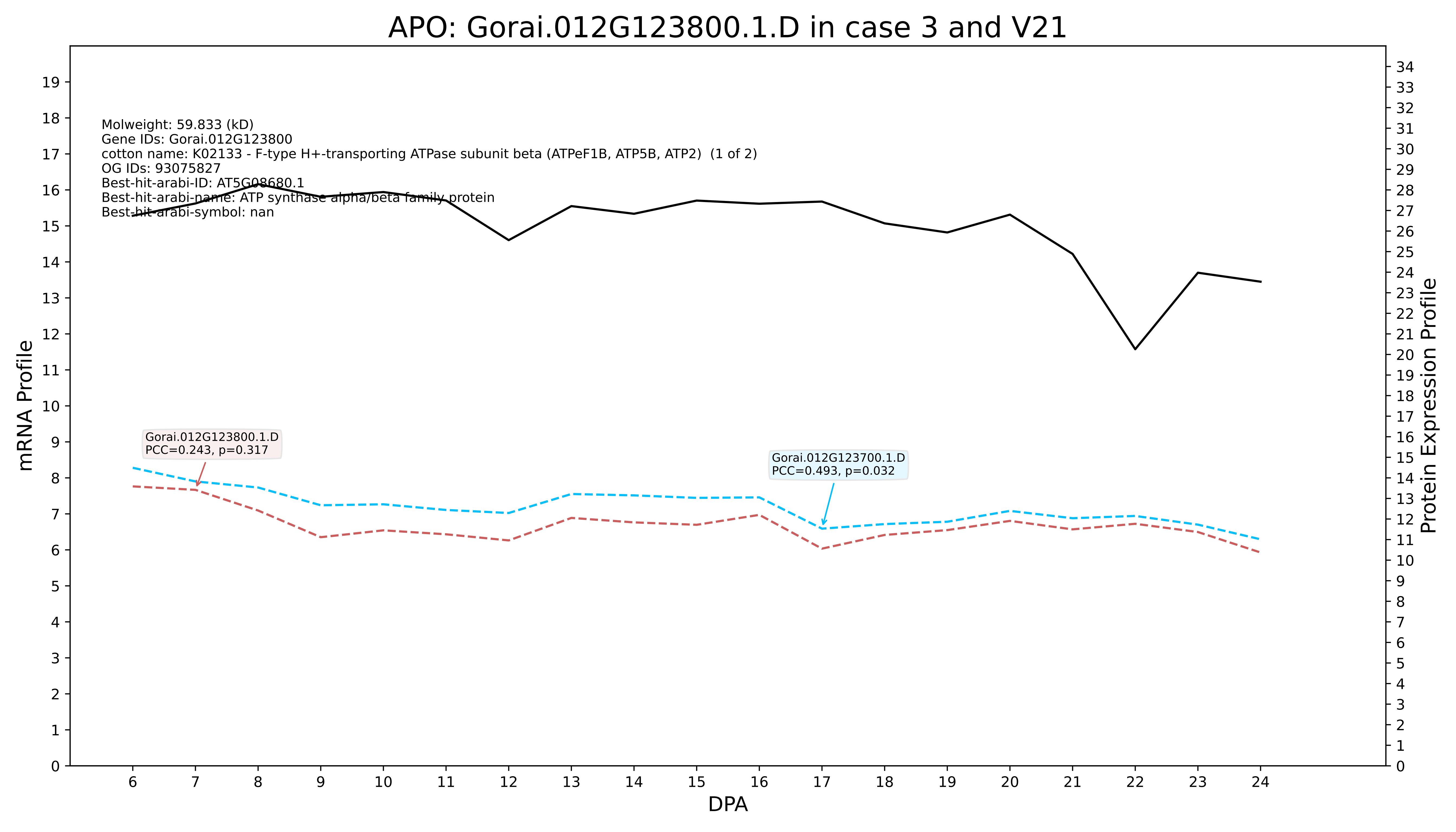

APO: Gorai.012G123800.1.D in case 3 and V21
Molweight: 59.833 (kD)
Gene IDs: Gorai.012G123800
cotton name: K02133 - F-type H+-transporting ATPase subunit beta (ATPeF1B, ATP5B, ATP2) (1 of 2)
OG IDs: 93075827
Best-hit-arabi-ID: AT5G08680.1
Best-hit-arabi-name: ATP synthase alpha/beta family protein
Best-hit-arabi-symbol: nan
Gorai.012G123800.1.D
PCC=0.243, p=0.317
Gorai.012G123700.1.D
PCC=0.493, p=0.032
mRNA Profile
Protein Expression Profile
DPA

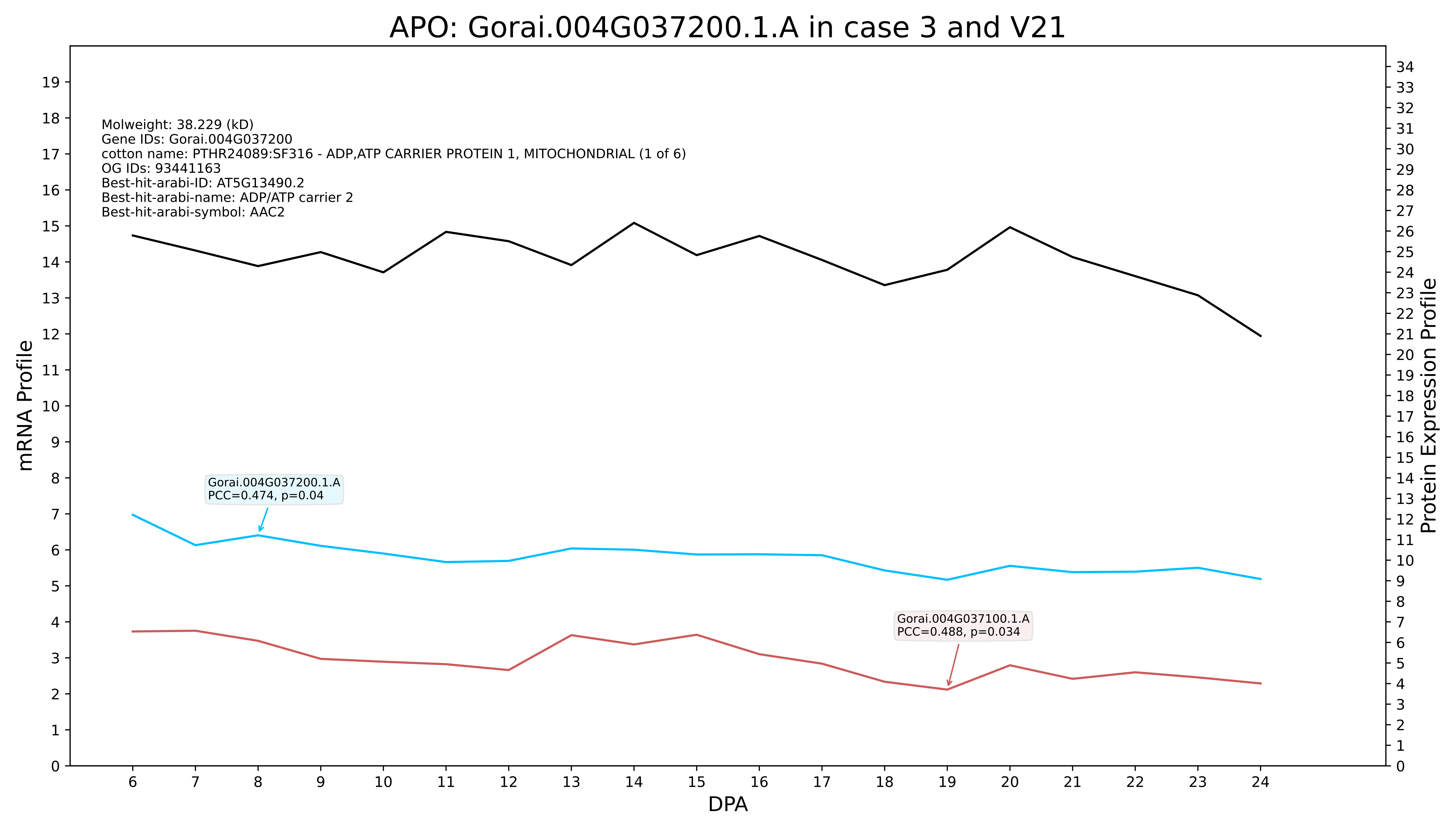

APO: Gorai.004G037200.1.A in case 3 and V21
Molweight: 38.229 (kD)
Gene IDs: Gorai.004G037200
cotton name: PTHR24089:SF316 - ADP,ATP CARRIER PROTEIN 1, MITOCHONDRIAL (1 of 6)
OG IDs: 93441163
Best-hit-arabi-ID: AT5G13490.2
Best-hit-arabi-name: ADP/ATP carrier 2
Best-hit-arabi-symbol: AAC2
Gorai.004G037200.1.A
PCC=0.474, p=0.04
Gorai.004G037100.1.A
PCC=0.488, p=0.034
mRNA Profile
Protein Expression Profile
DPA

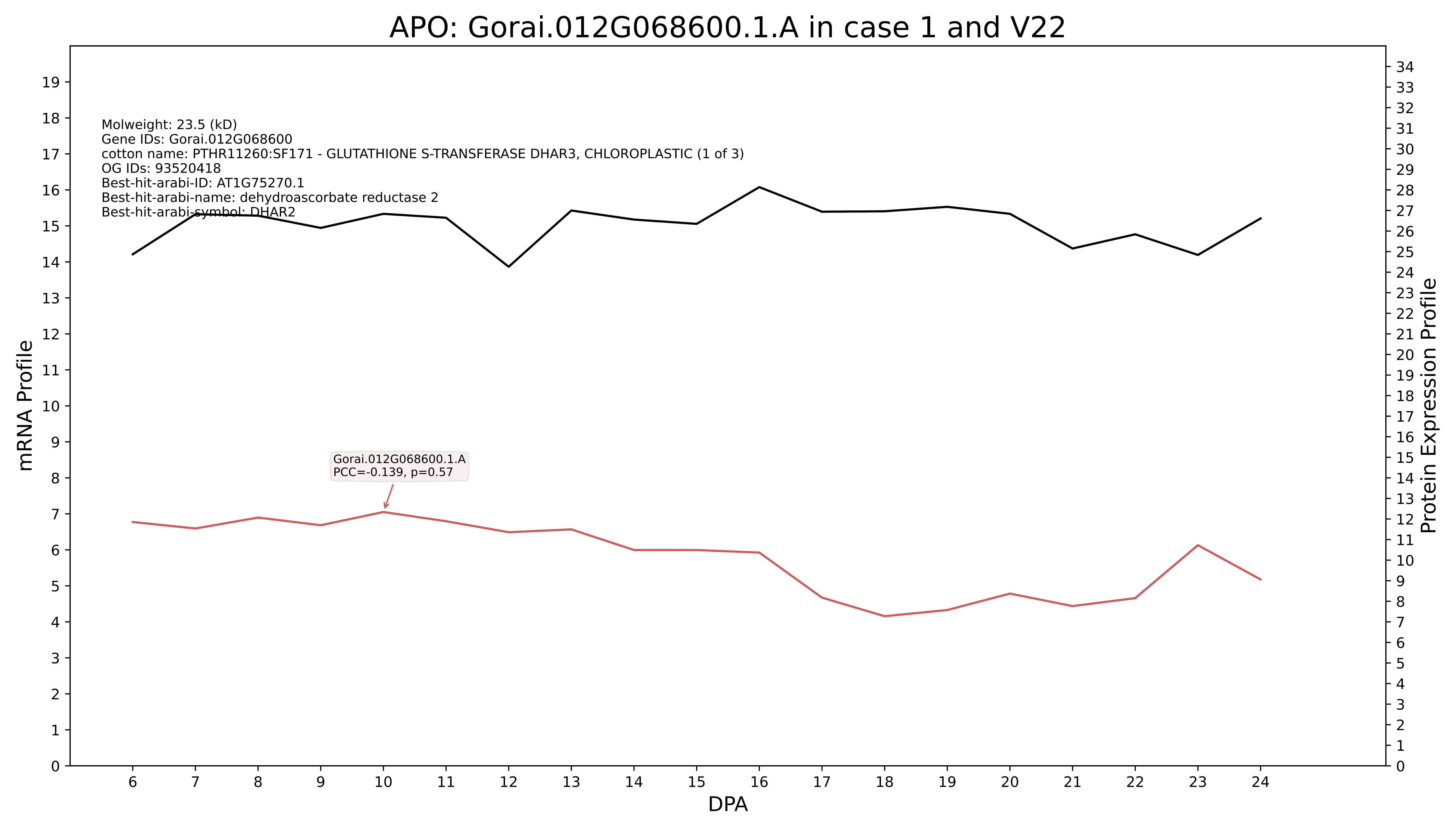

APO: Gorai.012G068600.1.A in case 1 and V22
Molweight: 23.5 (kD)
Gene IDs: Gorai.012G068600
cotton name: PTHR11260:SF171 - GLUTATHIONE S-TRANSFERASE DHAR3, CHLOROPLASTIC (1 of 3)
OG IDs: 93520418
Best-hit-arabi-ID: AT1G75270.1
Best-hit-arabi-name: dehydroascorbate reductase 2
Best-hit-arabi-symbol: DHAR2
Gorai.012G068600.1.A
PCC=-0.139, p=0.57
mRNA Profile
Protein Expression Profile
DPA

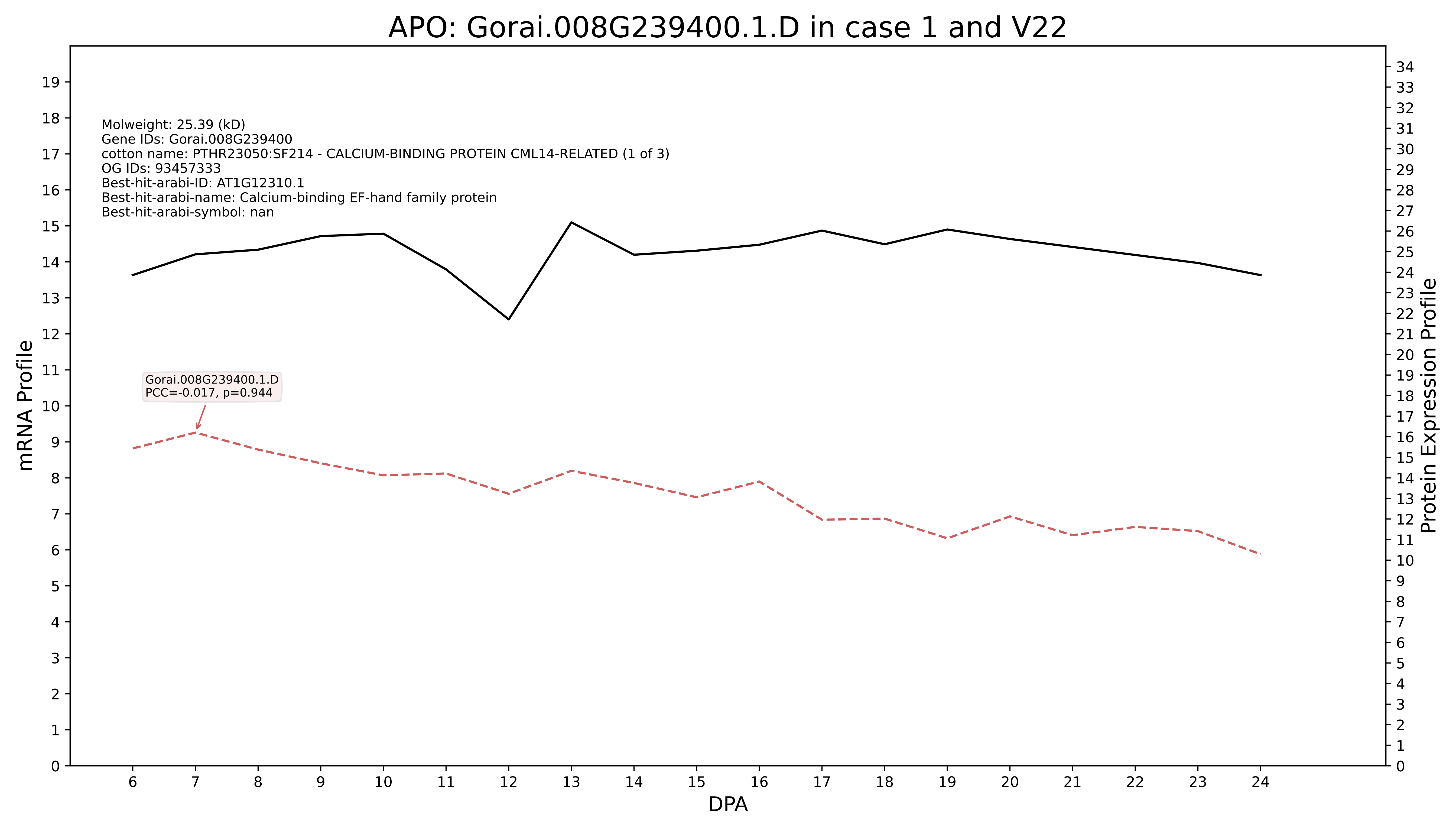

APO: Gorai.008G239400.1.D in case 1 and V22
Molweight: 25.39 (kD)
Gene IDs: Gorai.008G239400
cotton name: PTHR23050:SF214 - CALCIUM-BINDING PROTEIN CML14-RELATED (1 of 3)
OG IDs: 93457333
Best-hit-arabi-ID: AT1G12310.1
Best-hit-arabi-name: Calcium-binding EF-hand family protein
Best-hit-arabi-symbol: nan
Gorai.008G239400.1.D
PCC=-0.017, p=0.944
mRNA Profile
Protein Expression Profile
DPA

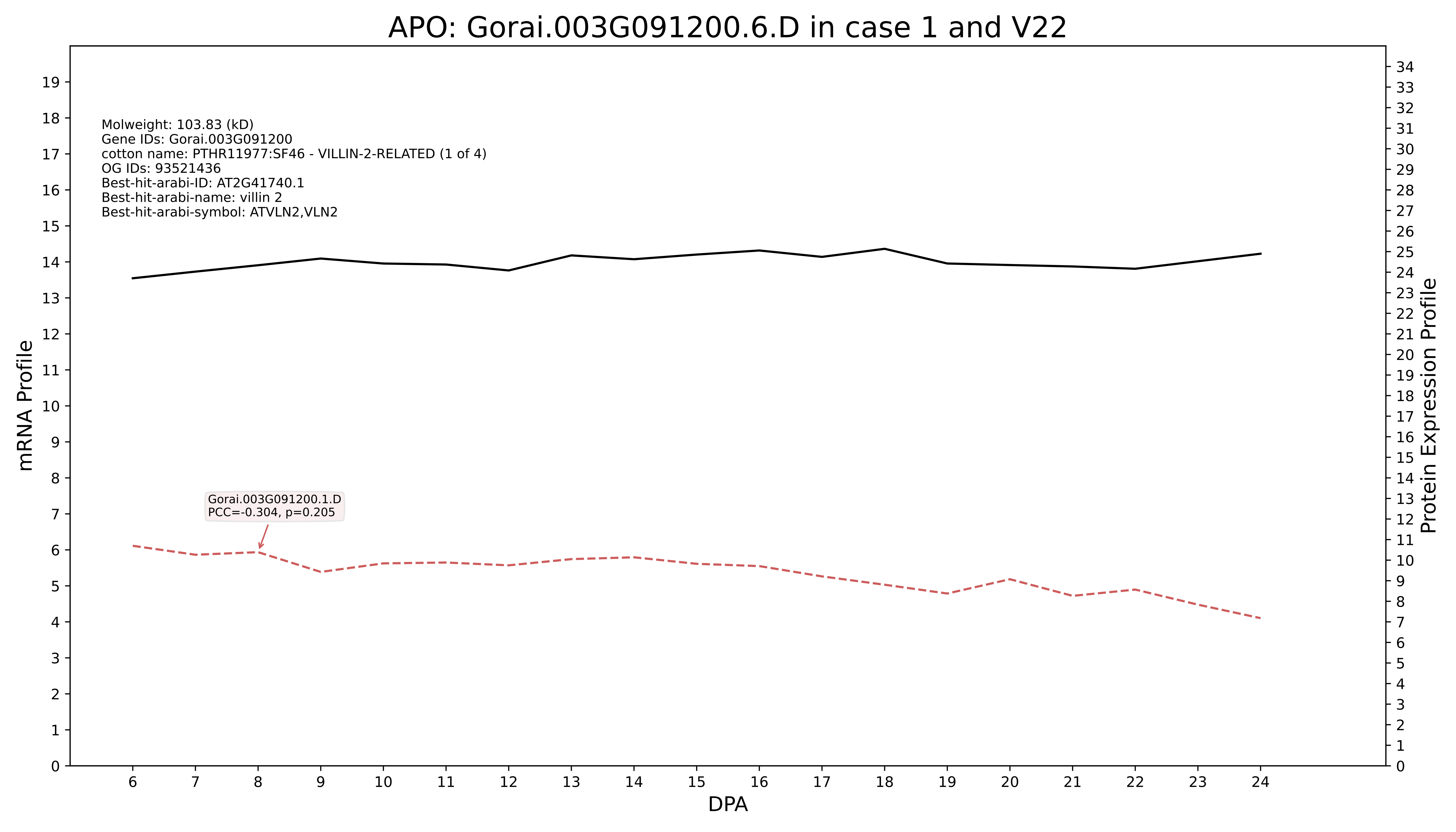

APO: Gorai.003G091200.6.D in case 1 and V22
Molweight: 103.83 (kD)
Gene IDs: Gorai.003G091200
cotton name: PTHR11977:SF46 - VILLIN-2-RELATED (1 of 4)
OG IDs: 93521436
Best-hit-arabi-ID: AT2G41740.1
Best-hit-arabi-name: villin 2
Best-hit-arabi-symbol: ATVLN2,VLN2
Gorai.003G091200.1.D
PCC=-0.304, p=0.205
mRNA Profile
Protein Expression Profile
DPA

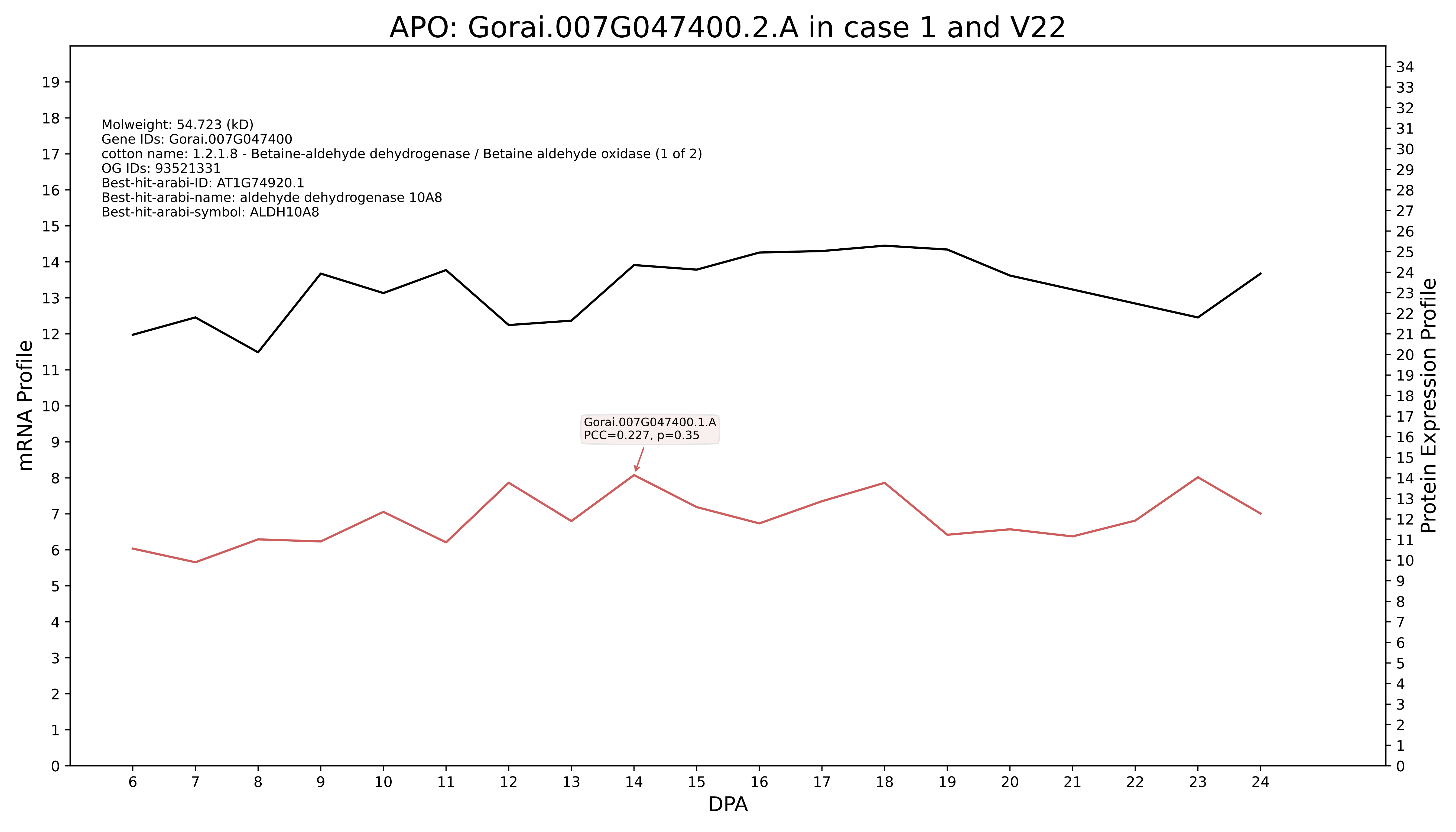
APO: Gorai.007G047400.2.A in case 1 and V22
Molweight: 54.723 (kD)
Gene IDs: Gorai.007G047400
cotton name: 1.2.1.8 - Betaine-aldehyde dehydrogenase / Betaine aldehyde oxidase (1 of 2)
OG IDs: 93521331
Best-hit-arabi-ID: AT1G74920.1
Best-hit-arabi-name: aldehyde dehydrogenase 10A8
Best-hit-arabi-symbol: ALDH10A8
Gorai.007G047400.1.A
PCC=0.227, p=0.35
mRNA Profile
Protein Expression Profile
DPA
0
1
2
3
4
5
6
7
8
9
10
11
12
13
14
15
16
17
18
19
20
21
22
23
24
25
26
27
28
29
30
31
32
33
34

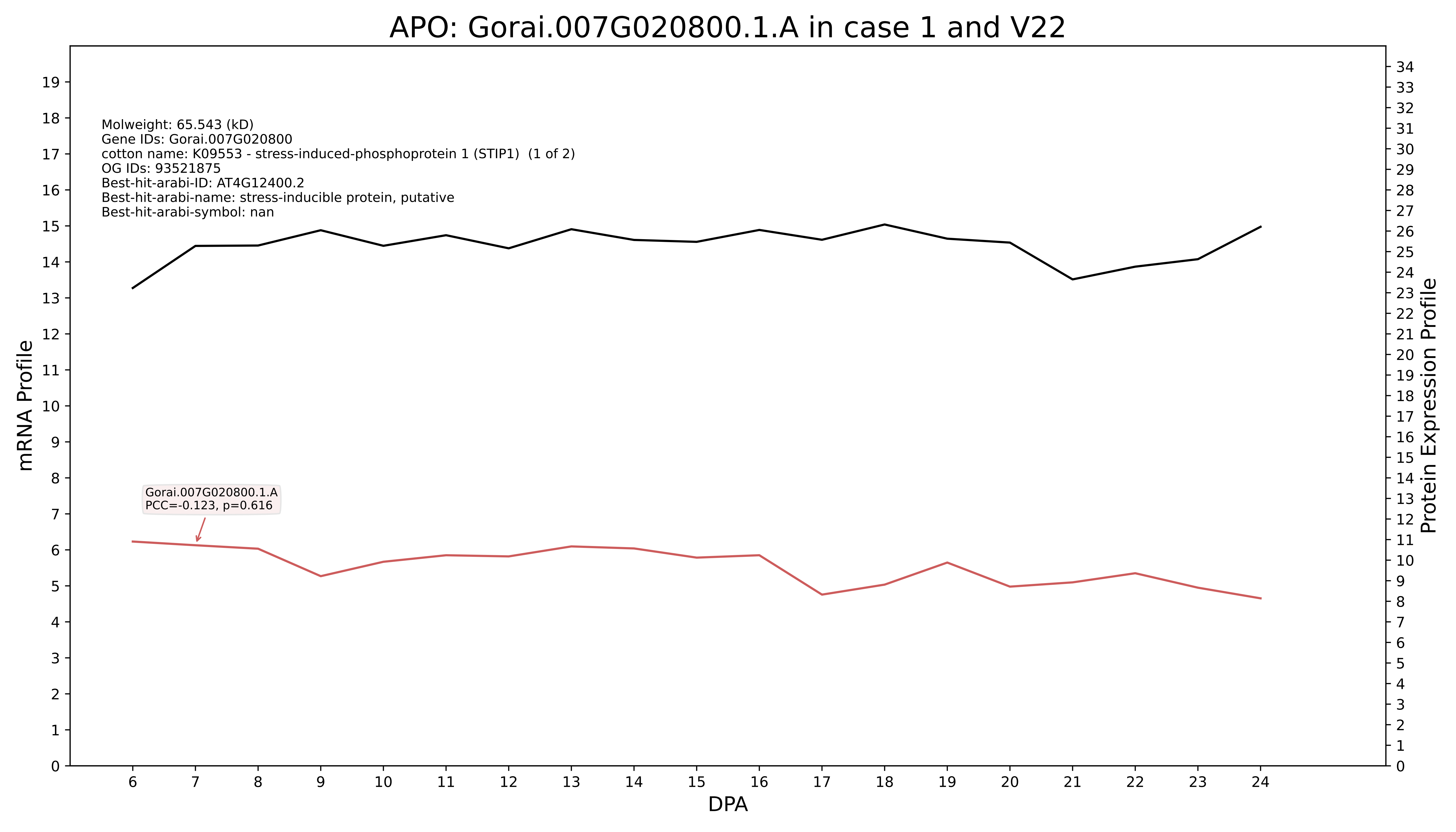
APO: Gorai.007G020800.1.A in case 1 and V22
Molweight: 65.543 (kD)
Gene IDs: Gorai.007G020800
cotton name: K09553 - stress-induced-phosphoprotein 1 (STIP1) (1 of 2)
OG IDs: 93521875
Best-hit-arabi-ID: AT4G12400.2
Best-hit-arabi-name: stress-inducible protein, putative
Best-hit-arabi-symbol: nan
Gorai.007G020800.1.A
PCC=-0.123, p=0.616
mRNA Profile
Protein Expression Profile
DPA
0
1
2
3
4
5
6
7
8
9
10
11
12
13
14
15
16
17
18
19
0
1
2
3
4
5
6
7
8
9
10
11
12
13
14
15
16
17
18
19
20
21
22
23
24
25
26
27
28
29
30
31
32
33
34
6
7
8
9
10
11
12
13
14
15
16
17
18
19
20
21
22
23
24

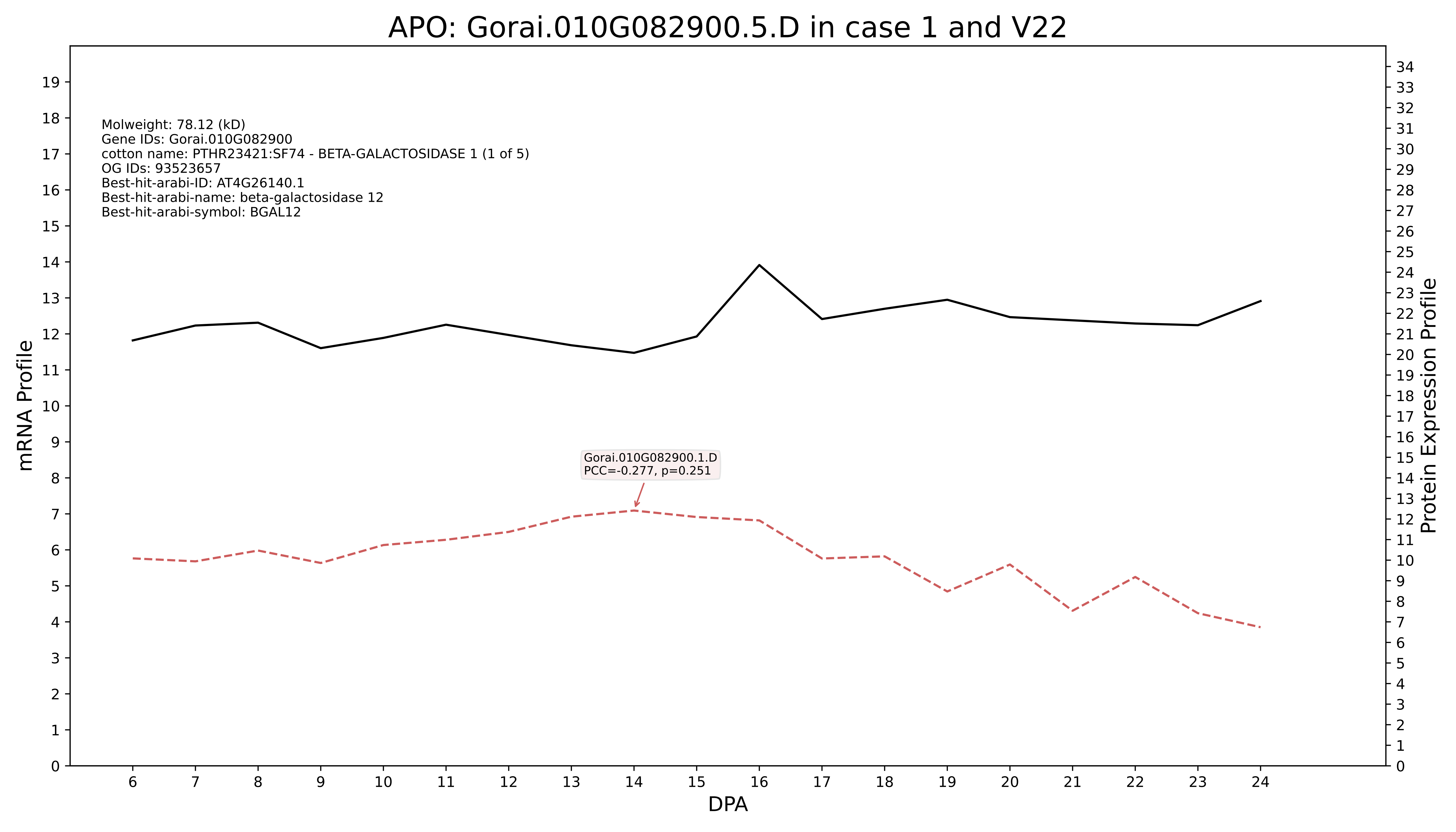
APO: Gorai.010G082900.5.D in case 1 and V22
Molweight: 78.12 (kD)
Gene IDs: Gorai.010G082900
cotton name: PTHR23421:SF74 - BETA-GALACTOSIDASE 1 (1 of 5)
OG IDs: 93523657
Best-hit-arabi-ID: AT4G26140.1
Best-hit-arabi-name: beta-galactosidase 12
Best-hit-arabi-symbol: BGAL12
Gorai.010G082900.1.D
PCC=-0.277, p=0.251
mRNA Profile
Protein Expression Profile
DPA

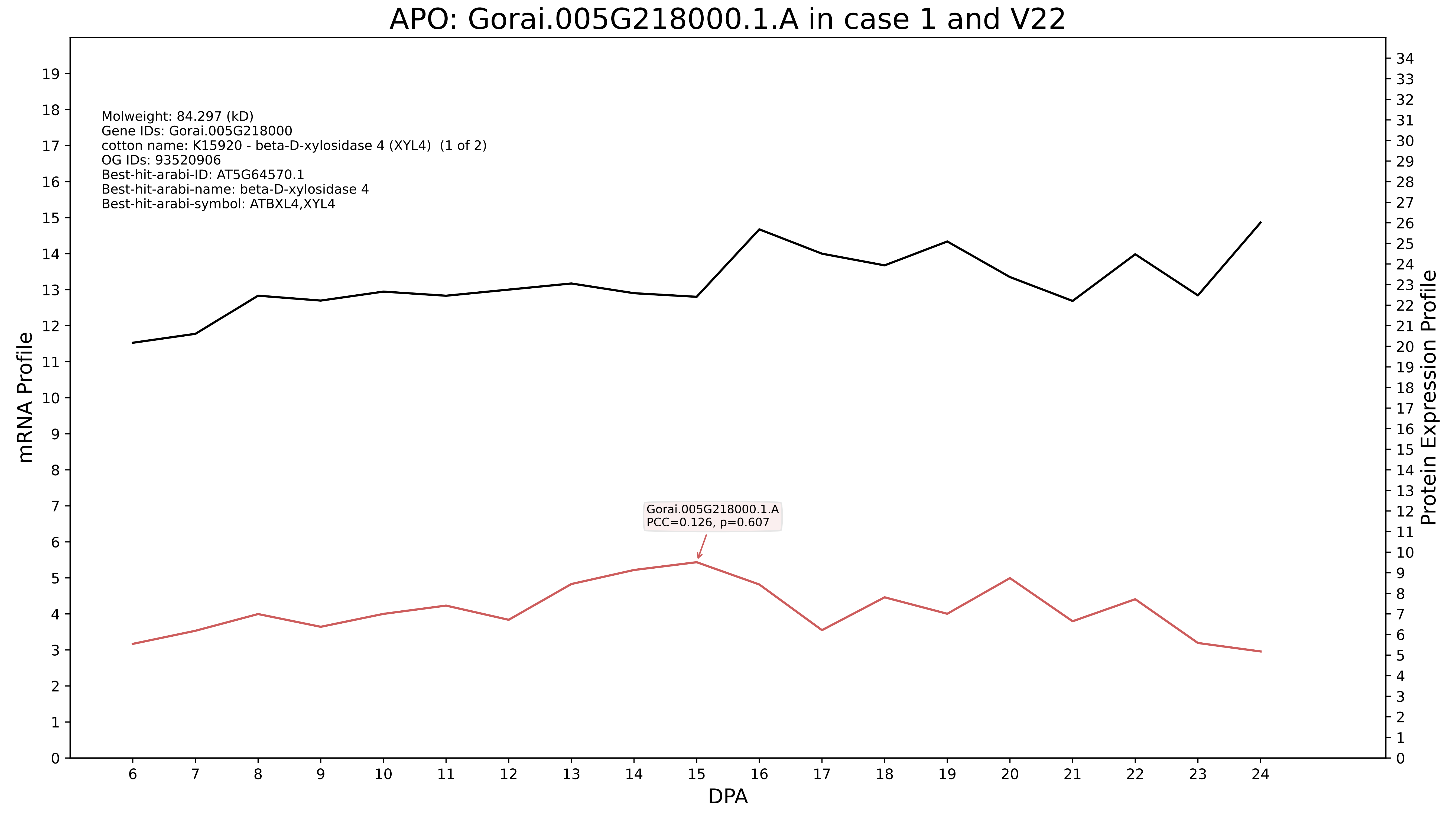
APO: Gorai.005G218000.1.A in case 1 and V22
Molweight: 84.297 (kD)
Gene IDs: Gorai.005G218000
cotton name: K15920 - beta-D-xylosidase 4 (XYL4) (1 of 2)
OG IDs: 93520906
Best-hit-arabi-ID: AT5G64570.1
Best-hit-arabi-name: beta-D-xylosidase 4
Best-hit-arabi-symbol: ATBXL4,XYL4
Gorai.005G218000.1.A
PCC=0.126, p=0.607
mRNA Profile
Protein Expression Profile
DPA

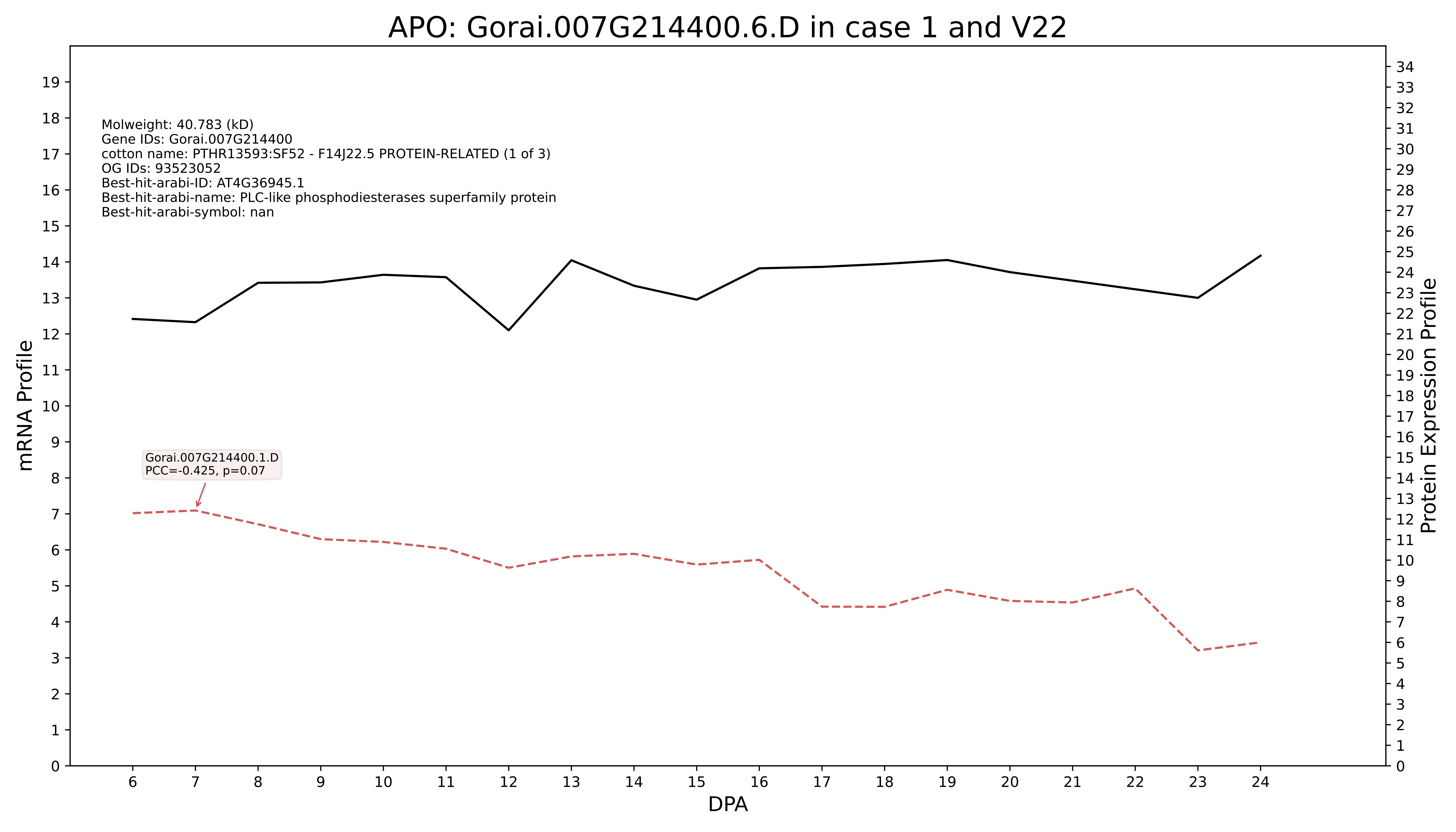

APO: Gorai.007G214400.6.D in case 1 and V22
Molweight: 40.783 (kD)
Gene IDs: Gorai.007G214400
cotton name: PTHR13593:SF52 - F14J22.5 PROTEIN-RELATED (1 of 3)
OG IDs: 93523052
Best-hit-arabi-ID: AT4G36945.1
Best-hit-arabi-name: PLC-like phosphodiesterases superfamily protein
Best-hit-arabi-symbol: nan
Gorai.007G214400.1.D
PCC=-0.425, p=0.07
mRNA Profile
Protein Expression Profile
DPA
0
1
2
3
4
5
6
7
8
9
10
11
12
13
14
15
16
17
18
19
20
21
22
23
24
25
26
27
28
29
30
31
32
33
34

APO: Gorai.006G212500.5.A in case 1 and V22

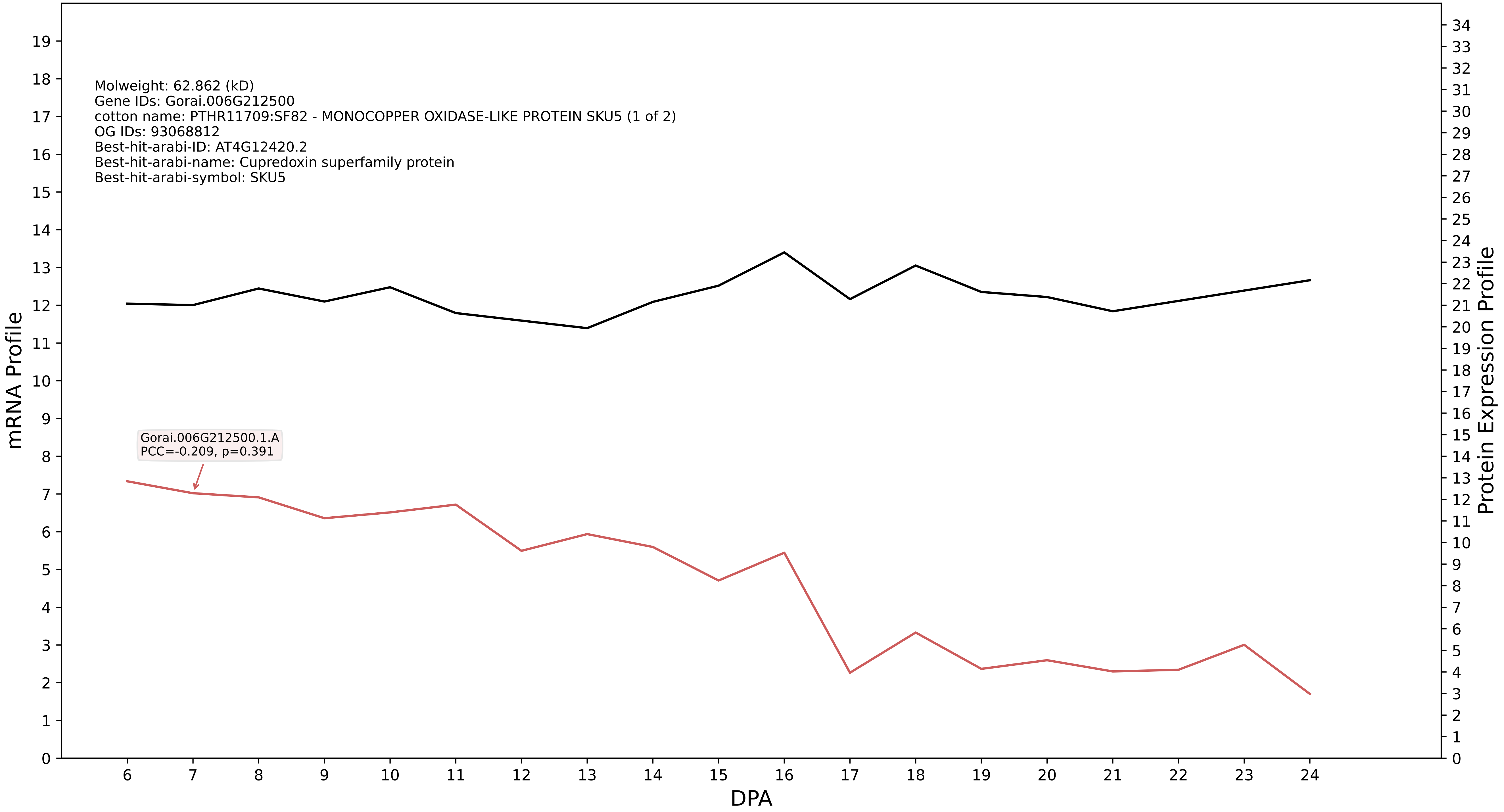

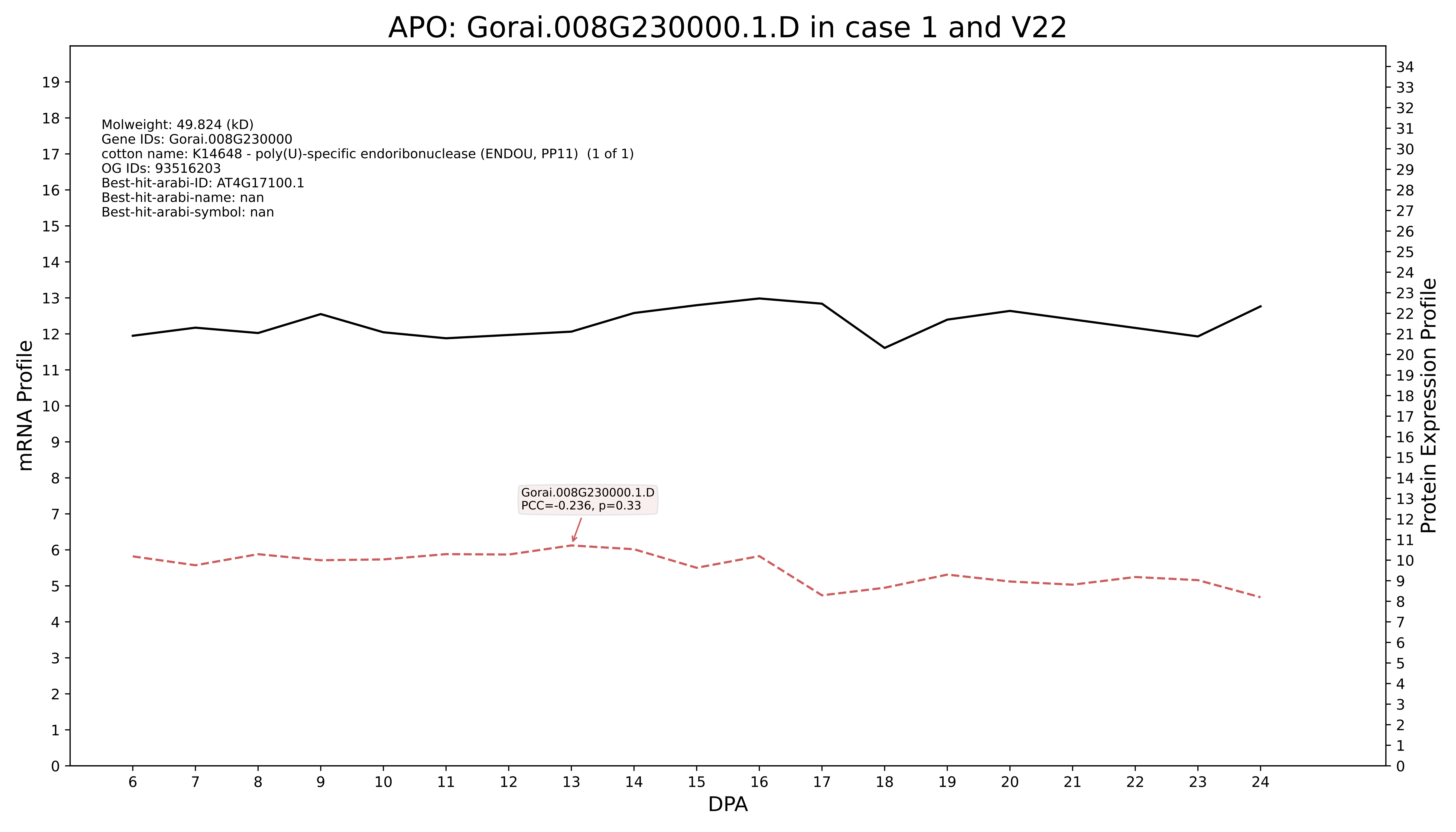

APO: Gorai.008G230000.1.D in case 1 and V22
Molweight: 49.824 (kD)
Gene IDs: Gorai.008G230000
cotton name: K14648 - poly(U)-specific endoribonuclease (ENDOU, PP11) (1 of 1)
OG IDs: 93516203
Best-hit-arabi-ID: AT4G17100.1
Best-hit-arabi-name: nan
Best-hit-arabi-symbol: nan
Gorai.008G230000.1.D
PCC=-0.236, p=0.33
mRNA Profile
Protein Expression Profile
DPA

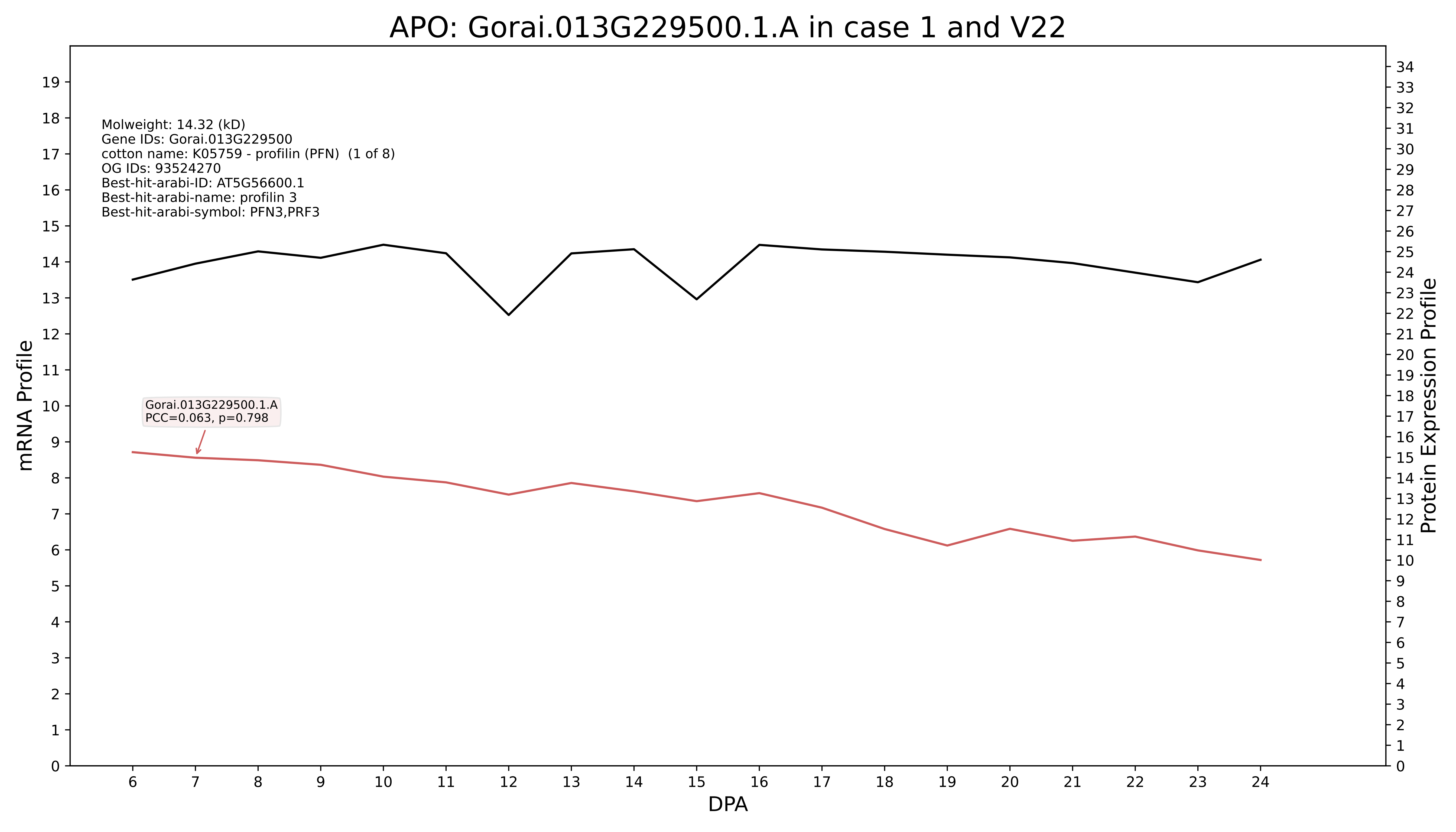
APO: Gorai.013G229500.1.A in case 1 and V22
Molweight: 14.32 (kD)
Gene IDs: Gorai.013G229500
cotton name: K05759 - profilin (PFN) (1 of 8)
OG IDs: 93524270
Best-hit-arabi-ID: AT5G56600.1
Best-hit-arabi-name: profilin 3
Best-hit-arabi-symbol: PFN3,PRF3
Gorai.013G229500.1.A
PCC=0.063, p=0.798
mRNA Profile
Protein Expression Profile
DPA

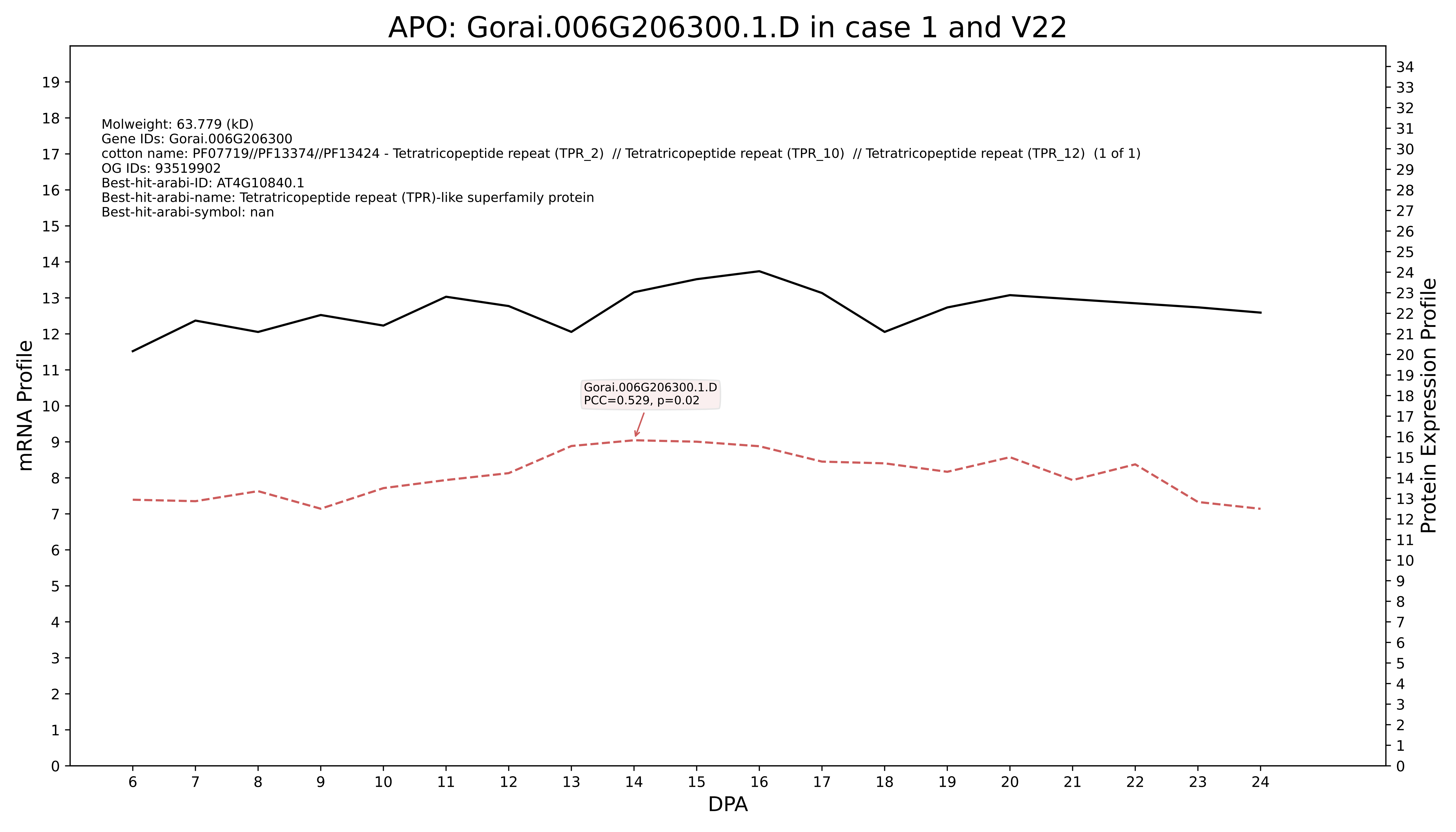
APO: Gorai.006G206300.1.D in case 1 and V22
Molweight: 63.779 (kD)
Gene IDs: Gorai.006G206300
cotton name: PF07719//PF13374//PF13424 - Tetratricopeptide repeat (TPR_2) // Tetratricopeptide repeat (TPR_10) // Tetratricopeptide repeat (TPR_12) (1 of 1)
OG IDs: 93519902
Best-hit-arabi-ID: AT4G10840.1
Best-hit-arabi-name: Tetratricopeptide repeat (TPR)-like superfamily protein
Best-hit-arabi-symbol: nan
Gorai.006G206300.1.D
PCC=0.529, p=0.02
mRNA Profile
Protein Expression Profile
DPA

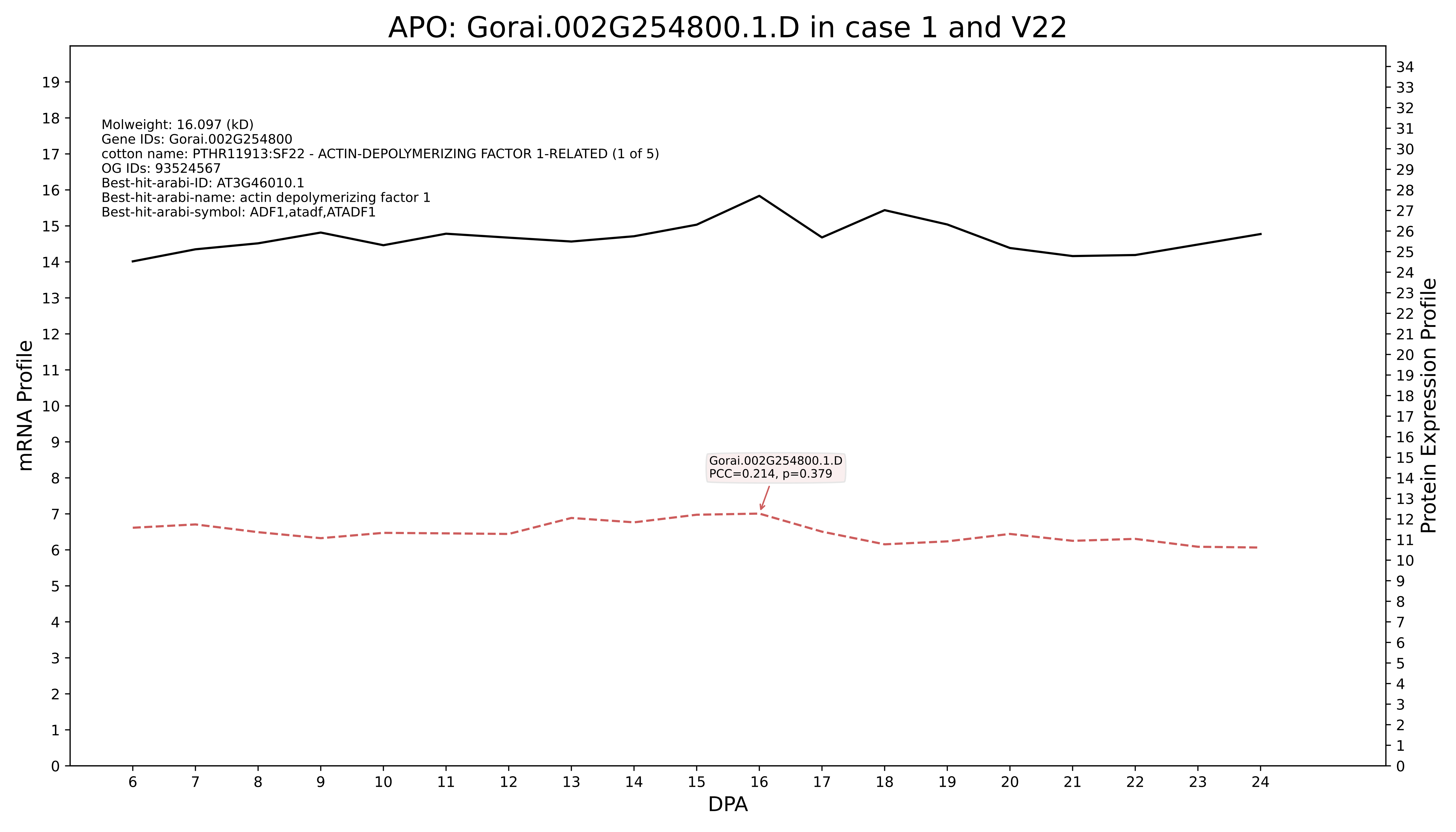
APO: Gorai.002G254800.1.D in case 1 and V22
Molweight: 16.097 (kD)
Gene IDs: Gorai.002G254800
cotton name: PTHR11913:SF22 - ACTIN-DEPOLYMERIZING FACTOR 1-RELATED (1 of 5)
OG IDs: 93524567
Best-hit-arabi-ID: AT3G46010.1
Best-hit-arabi-name: actin depolymerizing factor 1
Best-hit-arabi-symbol: ADF1,atadf,ATADF1
Gorai.002G254800.1.D
PCC=0.214, p=0.379
mRNA Profile
Protein Expression Profile
DPA

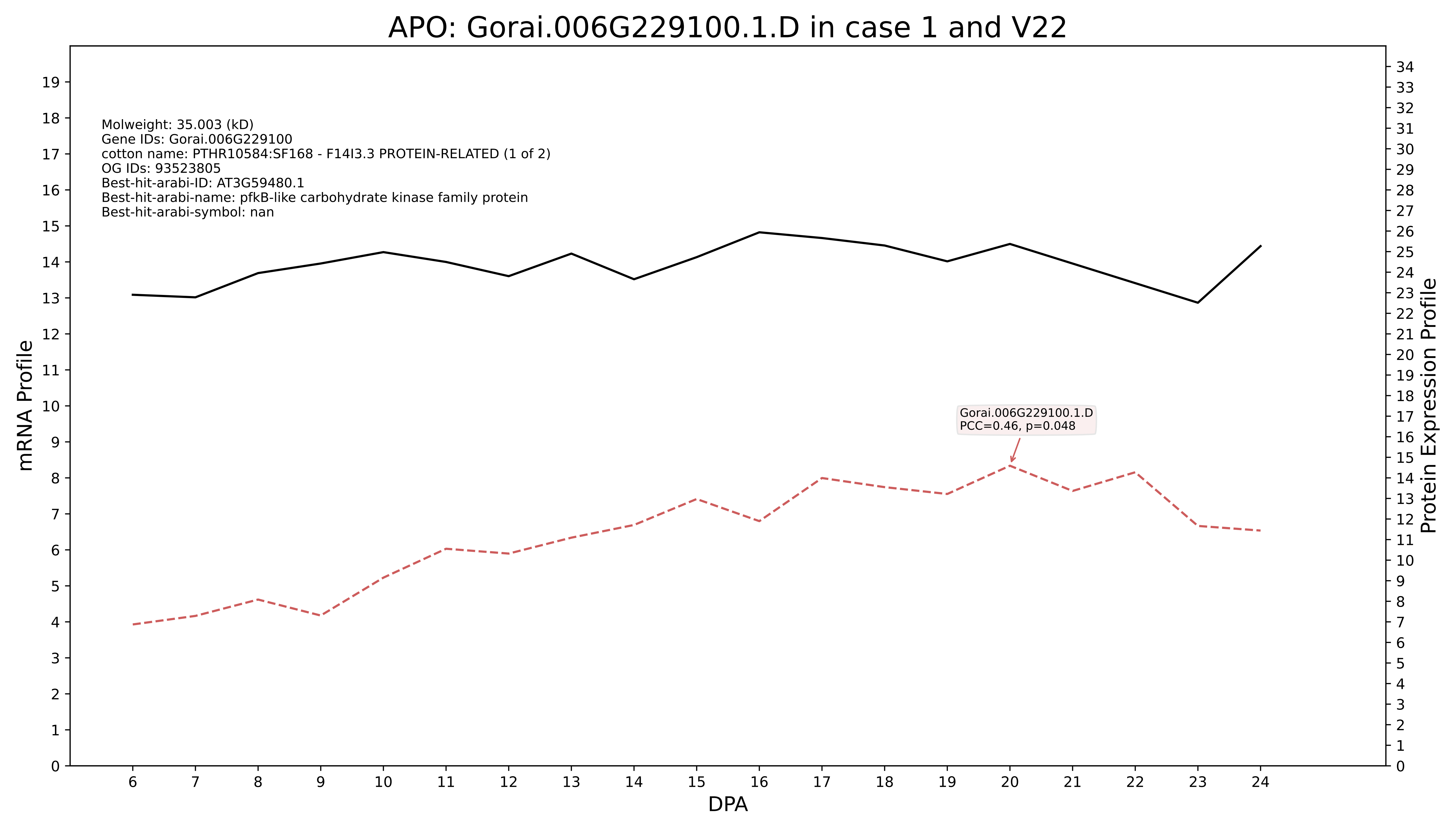
APO: Gorai.006G229100.1.D in case 1 and V22
Molweight: 35.003 (kD)
Gene IDs: Gorai.006G229100
cotton name: PTHR10584:SF168 - F14I3.3 PROTEIN-RELATED (1 of 2)
OG IDs: 93523805
Best-hit-arabi-ID: AT3G59480.1
Best-hit-arabi-name: pfkB-like carbohydrate kinase family protein
Best-hit-arabi-symbol: nan
Gorai.006G229100.1.D
PCC=0.46, p=0.048
mRNA Profile
Protein Expression Profile
DPA
0 1 2 3 4 5 6 7 8 9 10 11 12 13 14 15 16 17 18 19
0 1 2 3 4 5 6 7 8 9 10 11 12 13 14 15 16 17 18 19 20 21 22 23 24 25 26 27 28 29 30 31 32 33 34
6 7 8 9 10 11 12 13 14 15 16 17 18 19 20 21 22 23 24

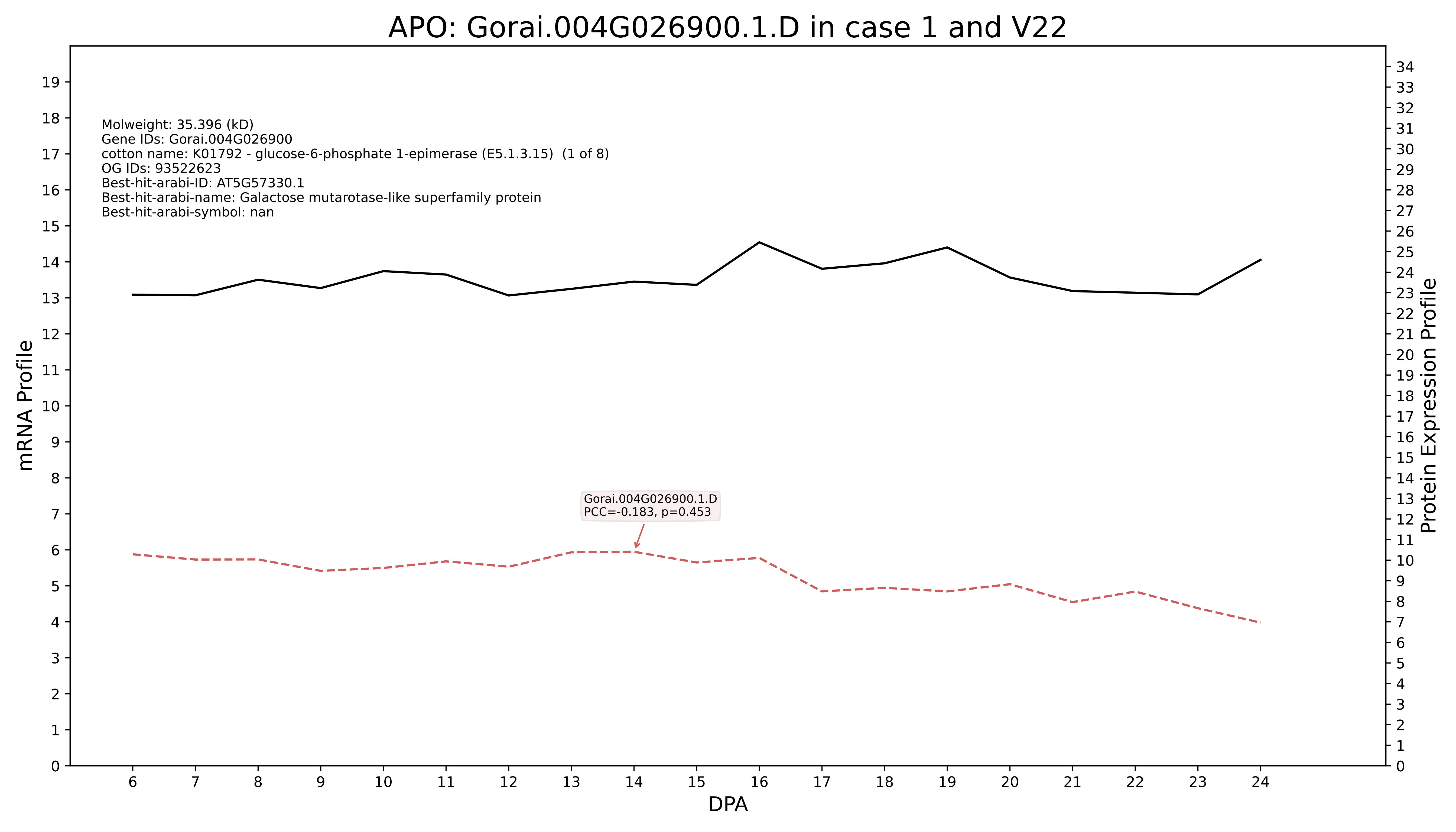

APO: Gorai.004G026900.1.D in case 1 and V22
Molweight: 35.396 (kD)
Gene IDs: Gorai.004G026900
cotton name: K01792 - glucose-6-phosphate 1-epimerase (E5.1.3.15) (1 of 8)
OG IDs: 93522623
Best-hit-arabi-ID: AT5G57330.1
Best-hit-arabi-name: Galactose mutarotase-like superfamily protein
Best-hit-arabi-symbol: nan
Gorai.004G026900.1.D
PCC=-0.183, p=0.453
mRNA Profile
Protein Expression Profile
DPA

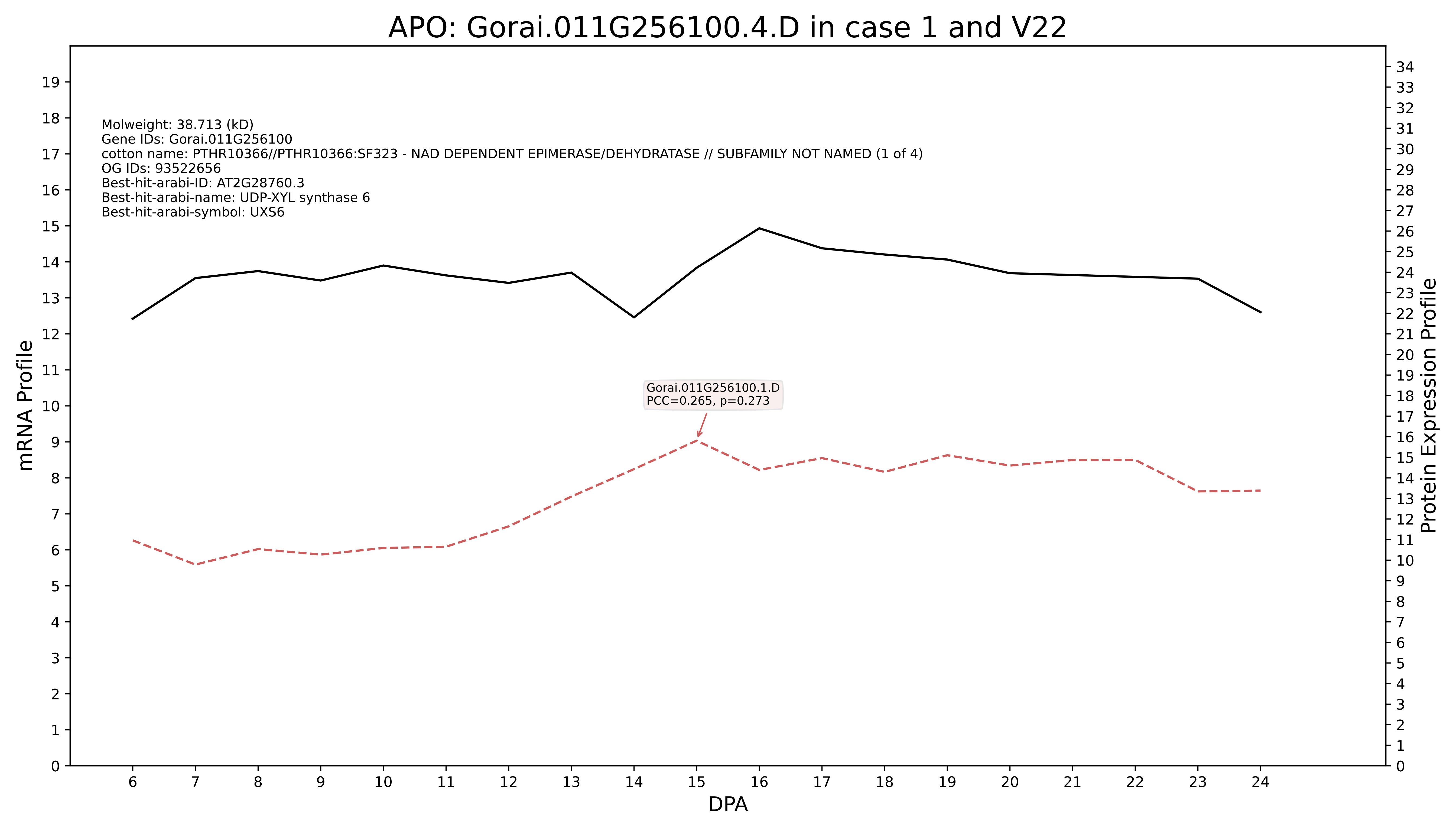
APO: Gorai.011G256100.4.D in case 1 and V22
Molweight: 38.713 (kD)
Gene IDs: Gorai.011G256100
cotton name: PTHR10366//PTHR10366:SF323 - NAD DEPENDENT EPIMERASE/DEHYDRATASE // SUBFAMILY NOT NAMED (1 of 4)
OG IDs: 93522656
Best-hit-arabi-ID: AT2G28760.3
Best-hit-arabi-name: UDP-XYL synthase 6
Best-hit-arabi-symbol: UXS6
Gorai.011G256100.1.D
PCC=0.265, p=0.273
mRNA Profile
Protein Expression Profile
DPA

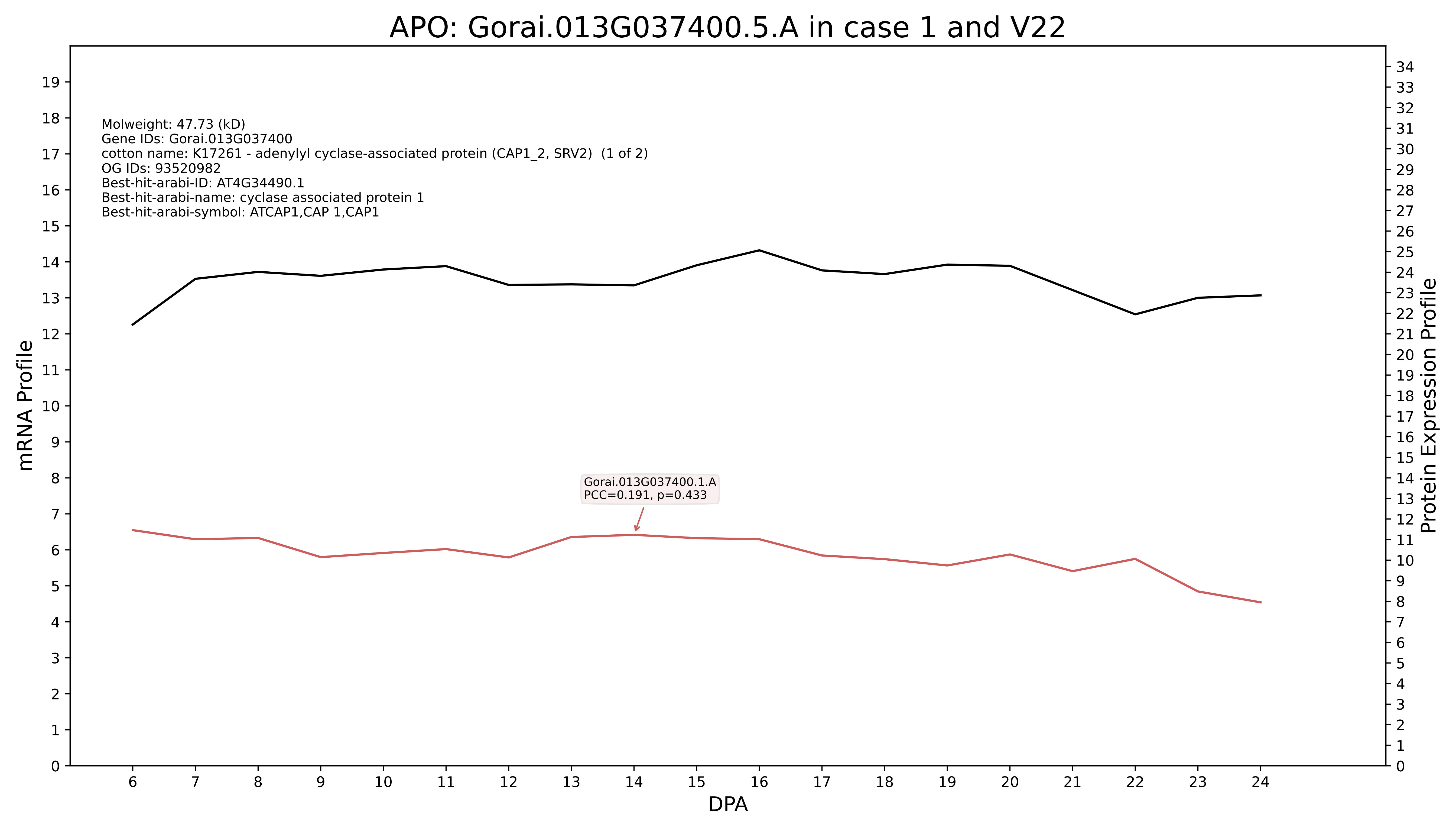

APO: Gorai.013G037400.5.A in case 1 and V22
Molweight: 47.73 (kD)
Gene IDs: Gorai.013G037400
cotton name: K17261 - adenylyl cyclase-associated protein (CAP1_2, SRV2) (1 of 2)
OG IDs: 93520982
Best-hit-arabi-ID: AT4G34490.1
Best-hit-arabi-name: cyclase associated protein 1
Best-hit-arabi-symbol: ATCAP1,CAP 1,CAP1
Gorai.013G037400.1.A
PCC=0.191, p=0.433
mRNA Profile
Protein Expression Profile
DPA

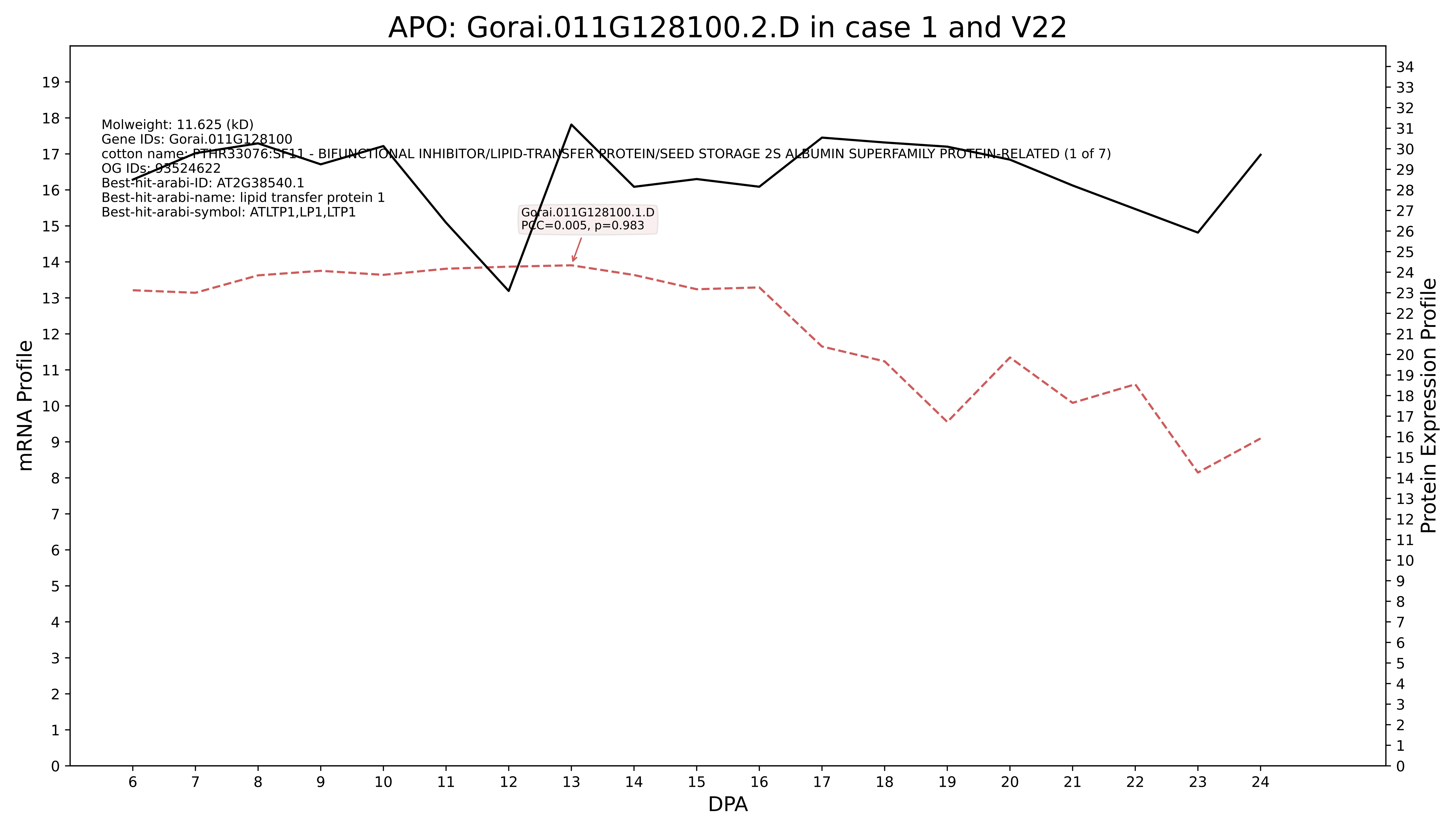
APO: Gorai.011G128100.2.D in case 1 and V22
Molweight: 11.625 (kD)
Gene IDs: Gorai.011G128100
cotton name: PTHR33076:SF11 - BIFUNCTIONAL INHIBITOR/LIPID-TRANSFER PROTEIN/SEED STORAGE 2S ALBUMIN SUPERFAMILY PROTEIN-RELATED (1 of 7)
OG IDs: 93524622
Best-hit-arabi-ID: AT2G38540.1
Best-hit-arabi-name: lipid transfer protein 1
Best-hit-arabi-symbol: ATLTP1,LP1,LTP1
Gorai.011G128100.1.D
PCC=0.005, p=0.983
mRNA Profile
Protein Expression Profile
DPA

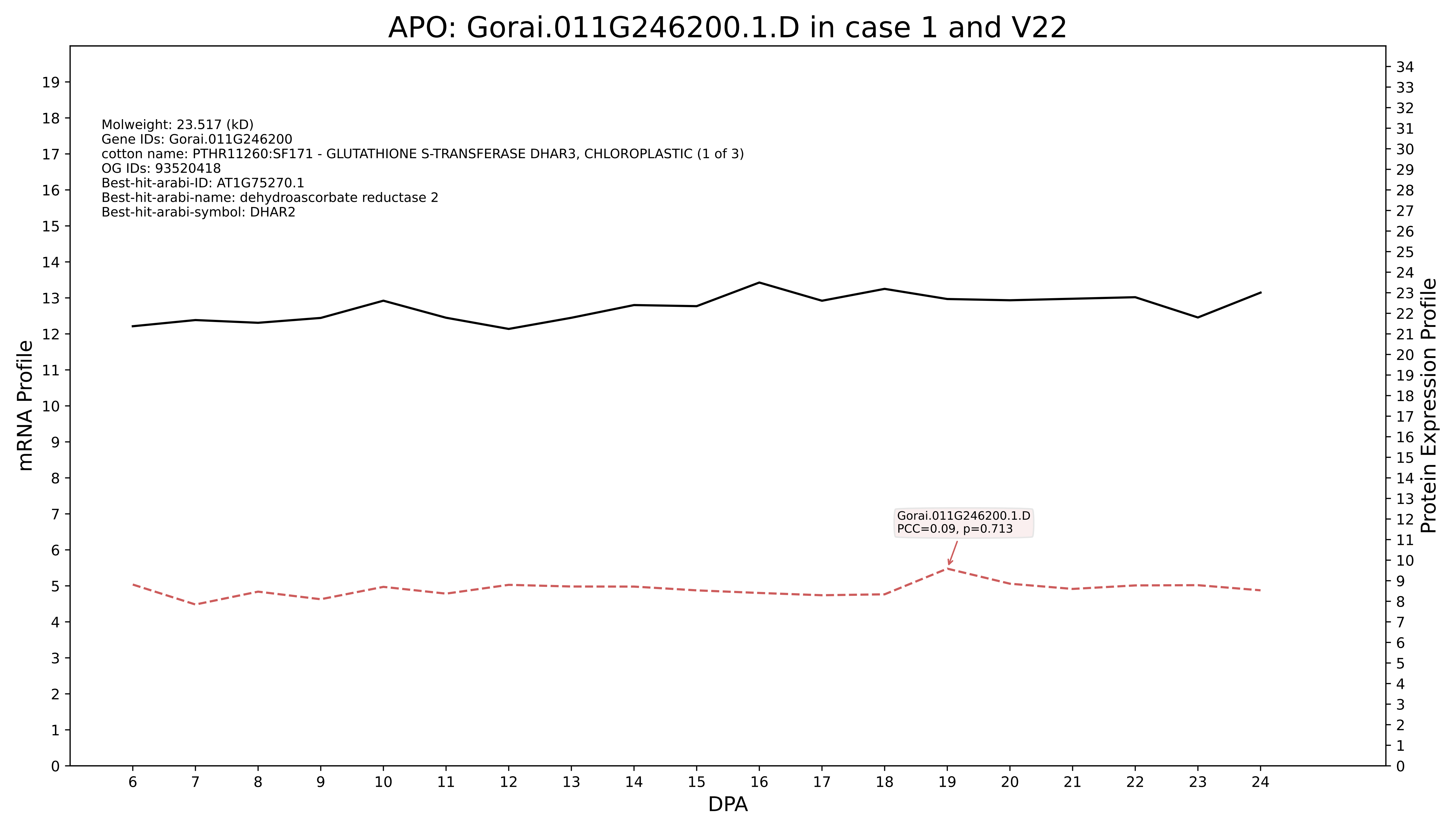
APO: Gorai.011G246200.1.D in case 1 and V22
Molweight: 23.517 (kD)
Gene IDs: Gorai.011G246200
cotton name: PTHR11260:SF171 - GLUTATHIONE S-TRANSFERASE DHAR3, CHLOROPLASTIC (1 of 3)
OG IDs: 93520418
Best-hit-arabi-ID: AT1G75270.1
Best-hit-arabi-name: dehydroascorbate reductase 2
Best-hit-arabi-symbol: DHAR2
Gorai.011G246200.1.D
PCC=0.09, p=0.713
mRNA Profile
Protein Expression Profile
DPA

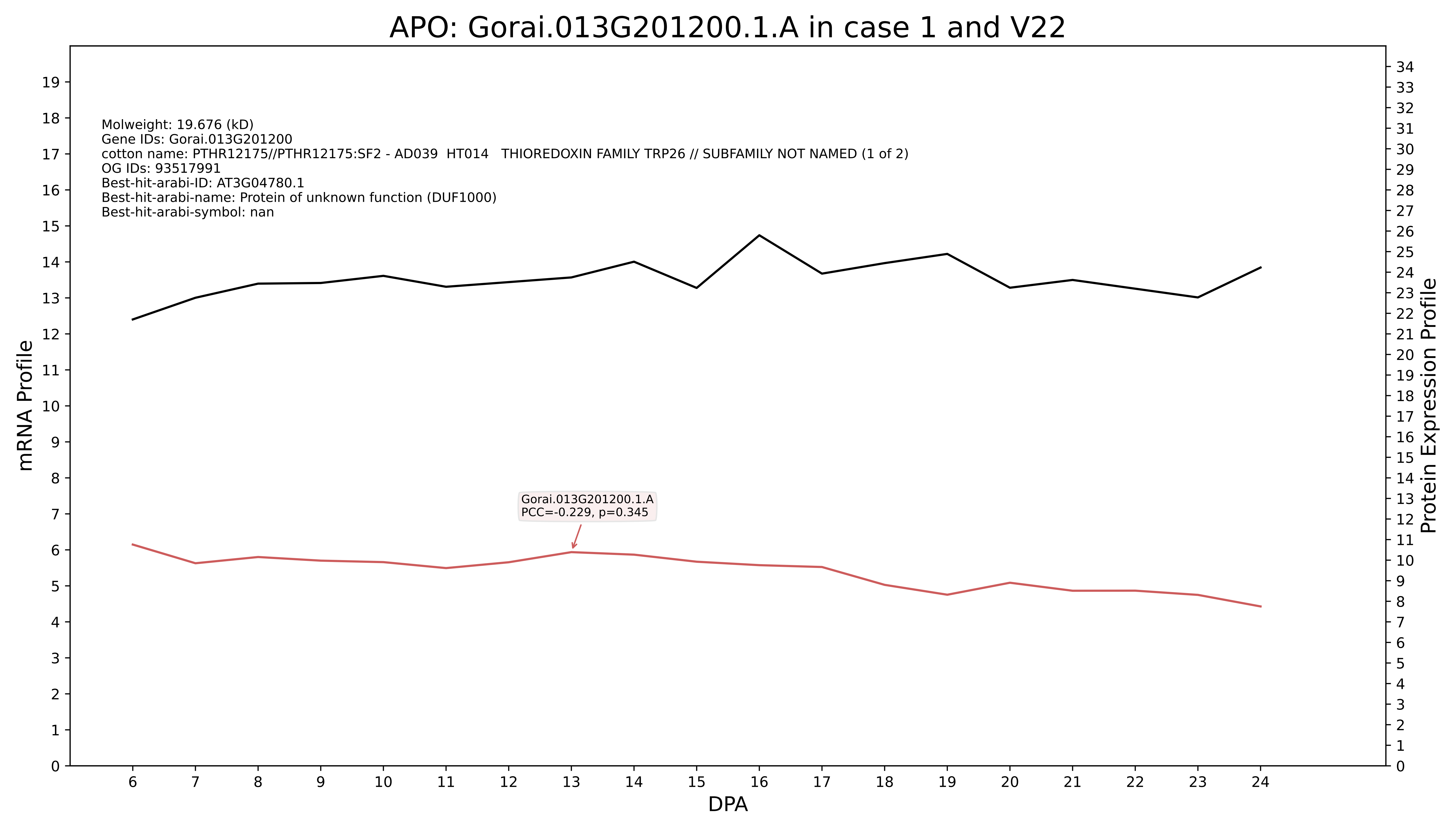

APO: Gorai.013G201200.1.A in case 1 and V22
Molweight: 19.676 (kD)
Gene IDs: Gorai.013G201200
cotton name: PTHR12175//PTHR12175:SF2 - AD039 HT014 THIOREDOXIN FAMILY TRP26 // SUBFAMILY NOT NAMED (1 of 2)
OG IDs: 93517991
Best-hit-arabi-ID: AT3G04780.1
Best-hit-arabi-name: Protein of unknown function (DUF1000)
Best-hit-arabi-symbol: nan
Gorai.013G201200.1.A
PCC=-0.229, p=0.345
mRNA Profile
Protein Expression Profile
DPA

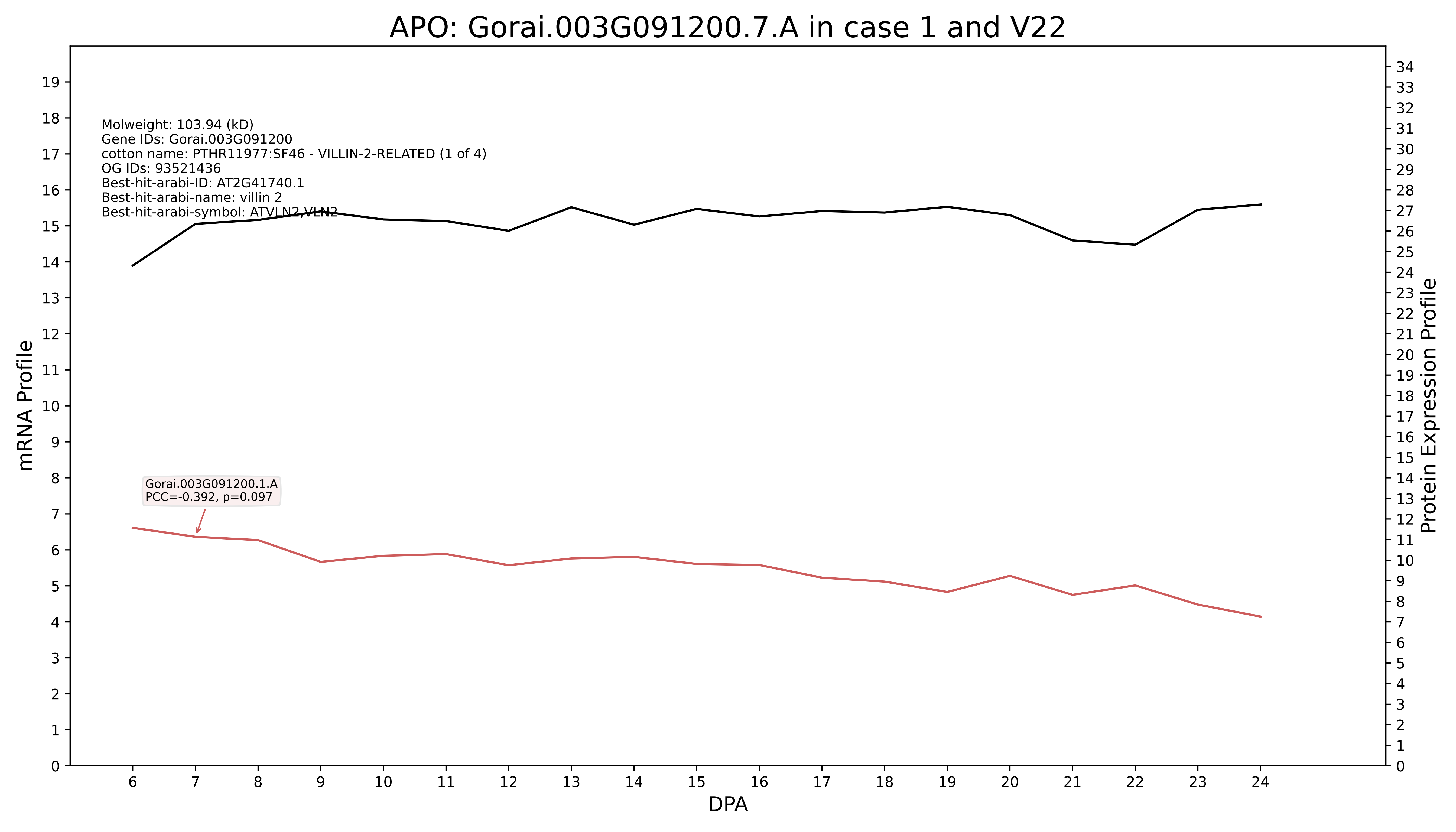

APO: Gorai.003G091200.7.A in case 1 and V22
Molweight: 103.94 (kD)
Gene IDs: Gorai.003G091200
cotton name: PTHR11977:SF46 - VILLIN-2-RELATED (1 of 4)
OG IDs: 93521436
Best-hit-arabi-ID: AT2G41740.1
Best-hit-arabi-name: villin 2
Best-hit-arabi-symbol: ATVLN2,VLN2
Gorai.003G091200.1.A
PCC=-0.392, p=0.097
mRNA Profile
Protein Expression Profile
DPA

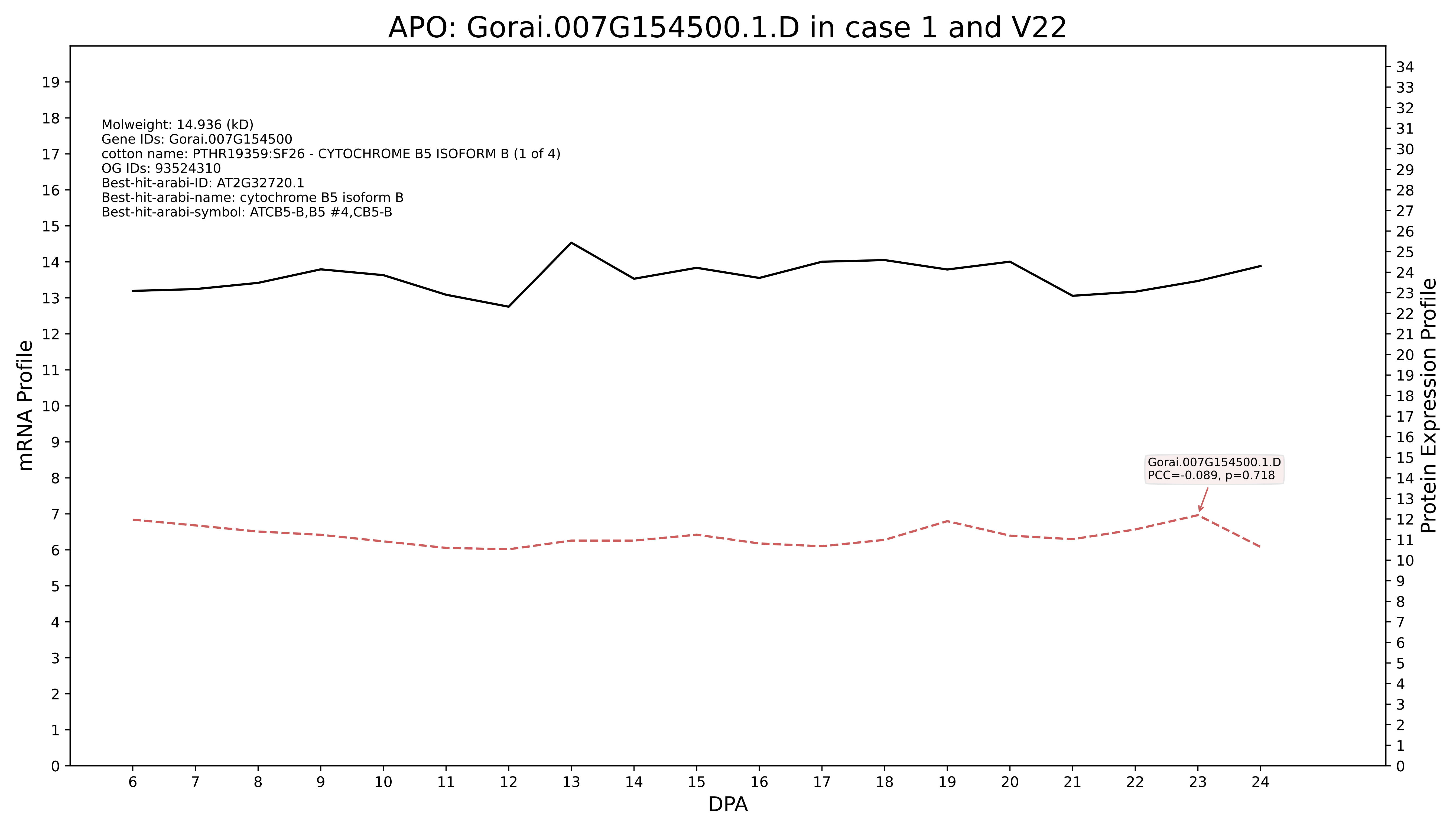

APO: Gorai.007G154500.1.D in case 1 and V22
Molweight: 14.936 (kD)
Gene IDs: Gorai.007G154500
cotton name: PTHR19359:SF26 - CYTOCHROME B5 ISOFORM B (1 of 4)
OG IDs: 93524310
Best-hit-arabi-ID: AT2G32720.1
Best-hit-arabi-name: cytochrome B5 isoform B
Best-hit-arabi-symbol: ATCB5-B,B5 #4,CB5-B
Gorai.007G154500.1.D
PCC=-0.089, p=0.718
mRNA Profile
Protein Expression Profile
DPA

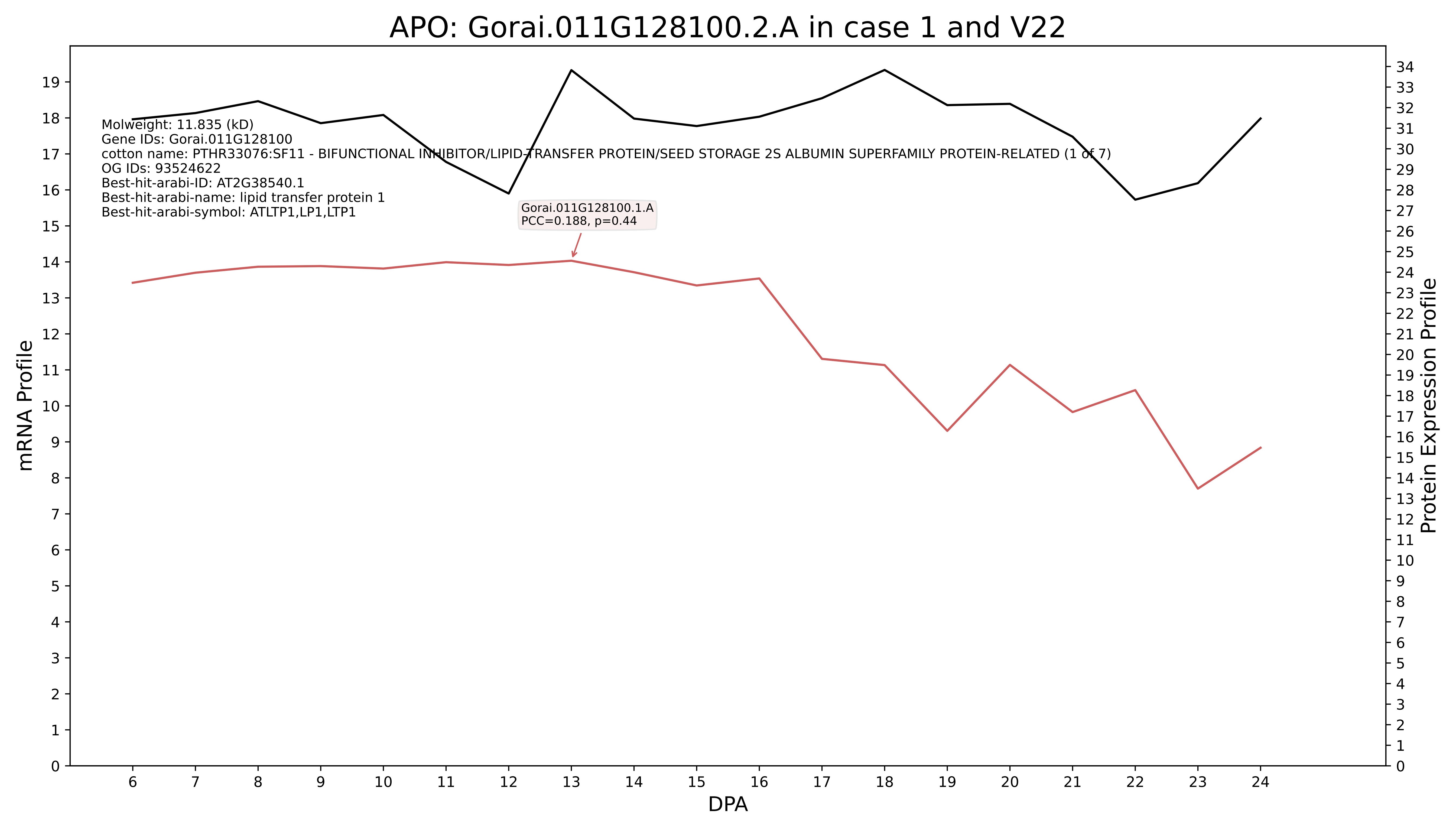
APO: Gorai.011G128100.2.A in case 1 and V22
Molweight: 11.835 (kD)
Gene IDs: Gorai.011G128100
cotton name: PTHR33076:SF11 - BIFUNCTIONAL INHIBITOR/LIPID-TRANSFER PROTEIN/SEED STORAGE 2S ALBUMIN SUPERFAMILY PROTEIN-RELATED (1 of 7)
OG IDs: 93524622
Best-hit-arabi-ID: AT2G38540.1
Best-hit-arabi-name: lipid transfer protein 1
Best-hit-arabi-symbol: ATLTP1,LP1,LTP1
Gorai.011G128100.1.A
PCC=0.188, p=0.44
mRNA Profile
Protein Expression Profile
DPA

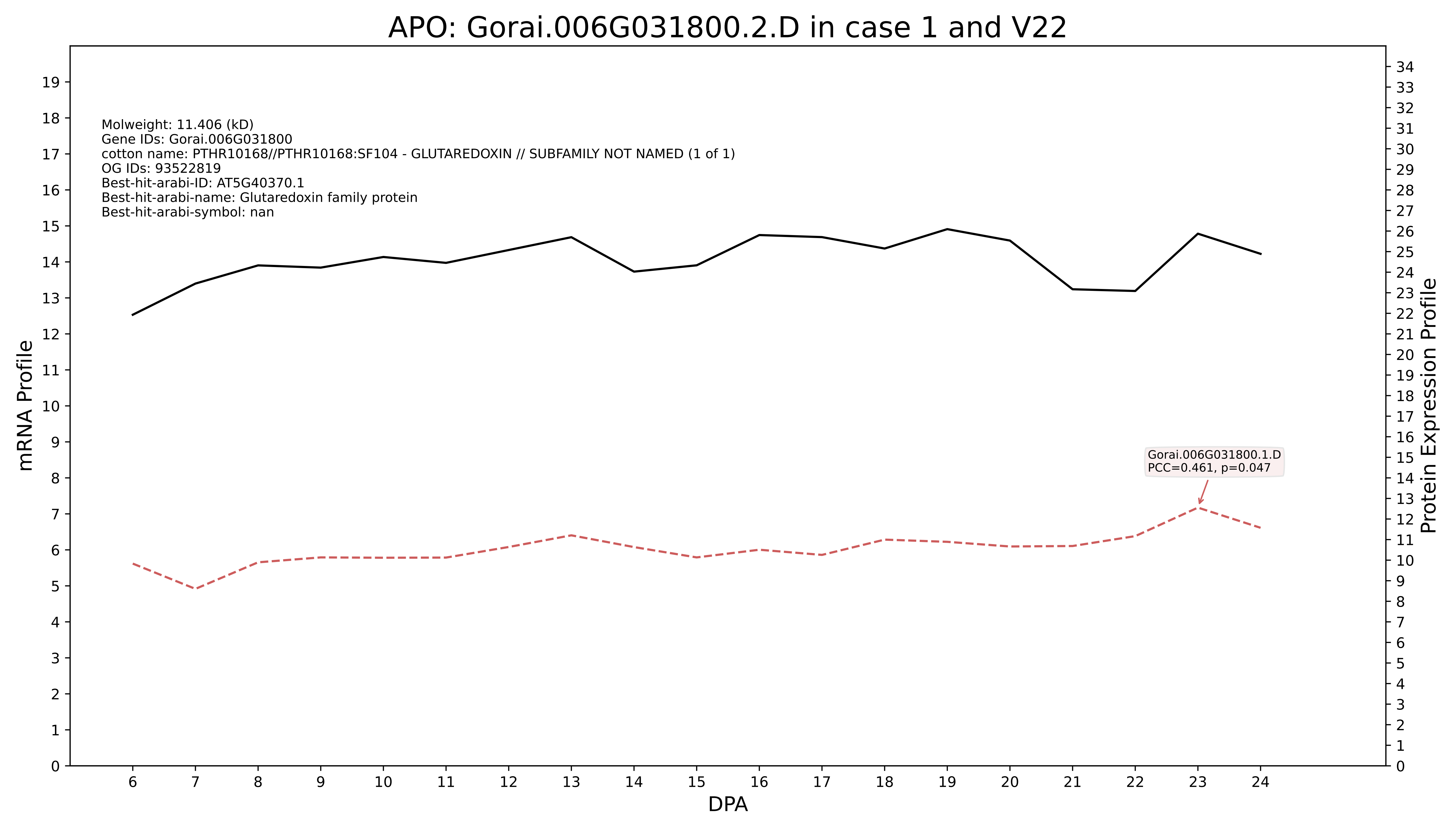

APO: Gorai.006G031800.2.D in case 1 and V22
Molweight: 11.406 (kD)
Gene IDs: Gorai.006G031800
cotton name: PTHR10168//PTHR10168:SF104 - GLUTAREDOXIN // SUBFAMILY NOT NAMED (1 of 1)
OG IDs: 93522819
Best-hit-arabi-ID: AT5G40370.1
Best-hit-arabi-name: Glutaredoxin family protein
Best-hit-arabi-symbol: nan
Gorai.006G031800.1.D
PCC=0.461, p=0.047
mRNA Profile
Protein Expression Profile
DPA

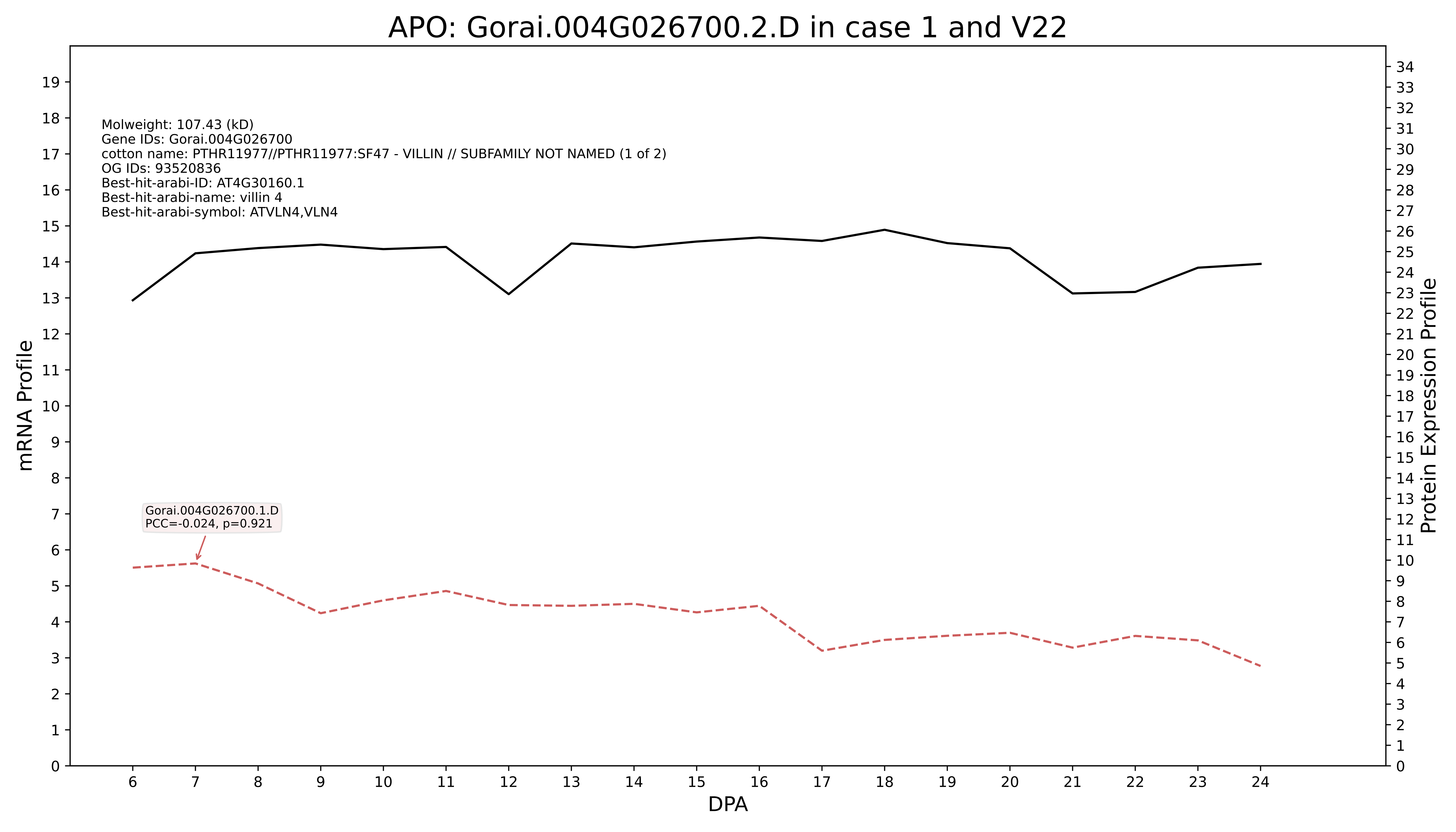
APO: Gorai.004G026700.2.D in case 1 and V22
Molweight: 107.43 (kD)
Gene IDs: Gorai.004G026700
cotton name: PTHR11977//PTHR11977:SF47 - VILLIN // SUBFAMILY NOT NAMED (1 of 2)
OG IDs: 93520836
Best-hit-arabi-ID: AT4G30160.1
Best-hit-arabi-name: villin 4
Best-hit-arabi-symbol: ATVLN4,VLN4
Gorai.004G026700.1.D
PCC=-0.024, p=0.921
mRNA Profile
Protein Expression Profile
DPA

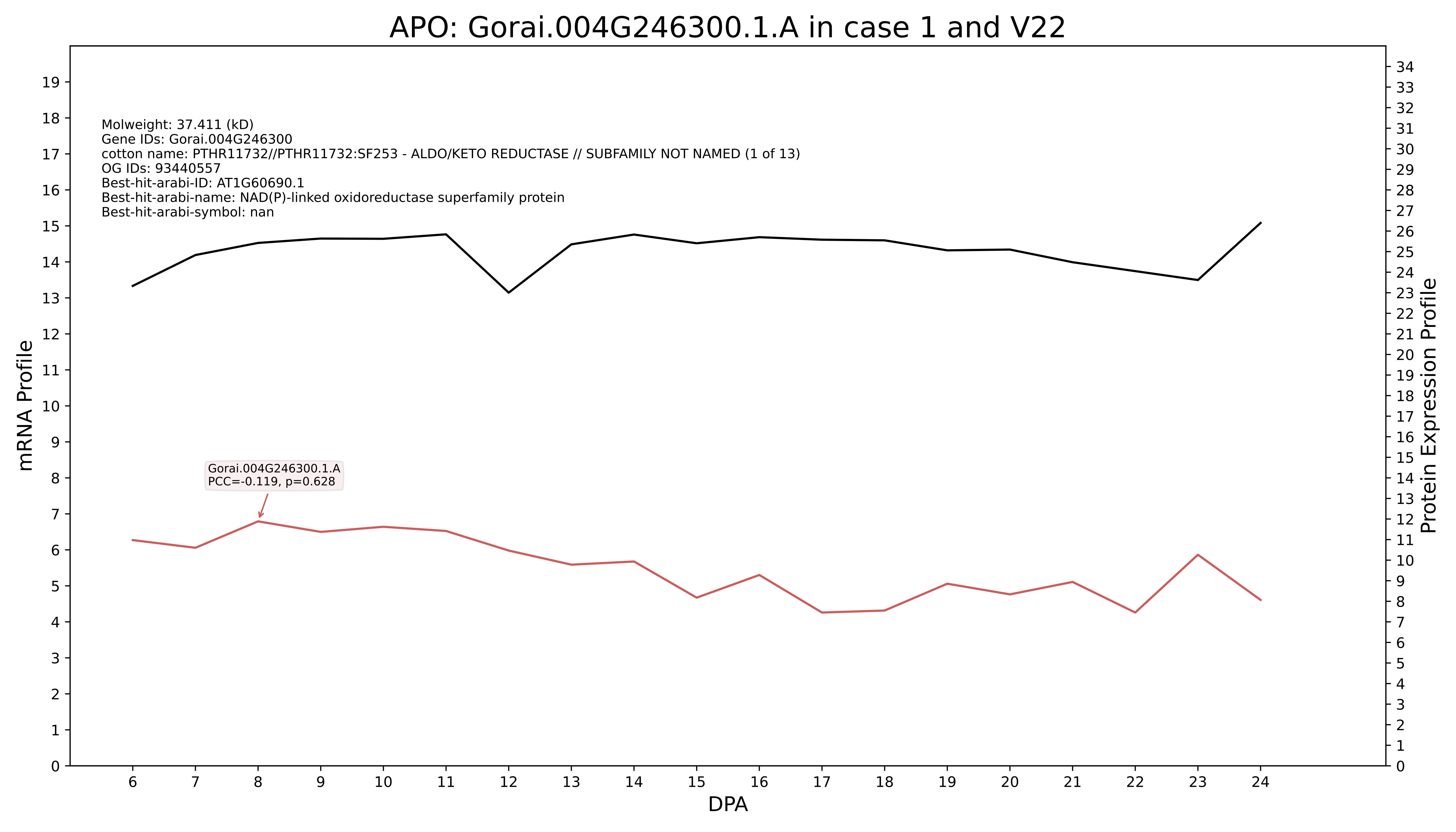
APO: Gorai.004G246300.1.A in case 1 and V22
Molweight: 37.411 (kD)
Gene IDs: Gorai.004G246300
cotton name: PTHR11732//PTHR11732:SF253 - ALDO/KETO REDUCTASE // SUBFAMILY NOT NAMED (1 of 13)
OG IDs: 93440557
Best-hit-arabi-ID: AT1G60690.1
Best-hit-arabi-name: NAD(P)-linked oxidoreductase superfamily protein
Best-hit-arabi-symbol: nan
Gorai.004G246300.1.A
PCC=-0.119, p=0.628
mRNA Profile
Protein Expression Profile
DPA
0
1
2
3
4
5
6
7
8
9
10
11
12
13
14
15
16
17
18
19
20
21
22
23
24
25
26
27
28
29
30
31
32
33
34

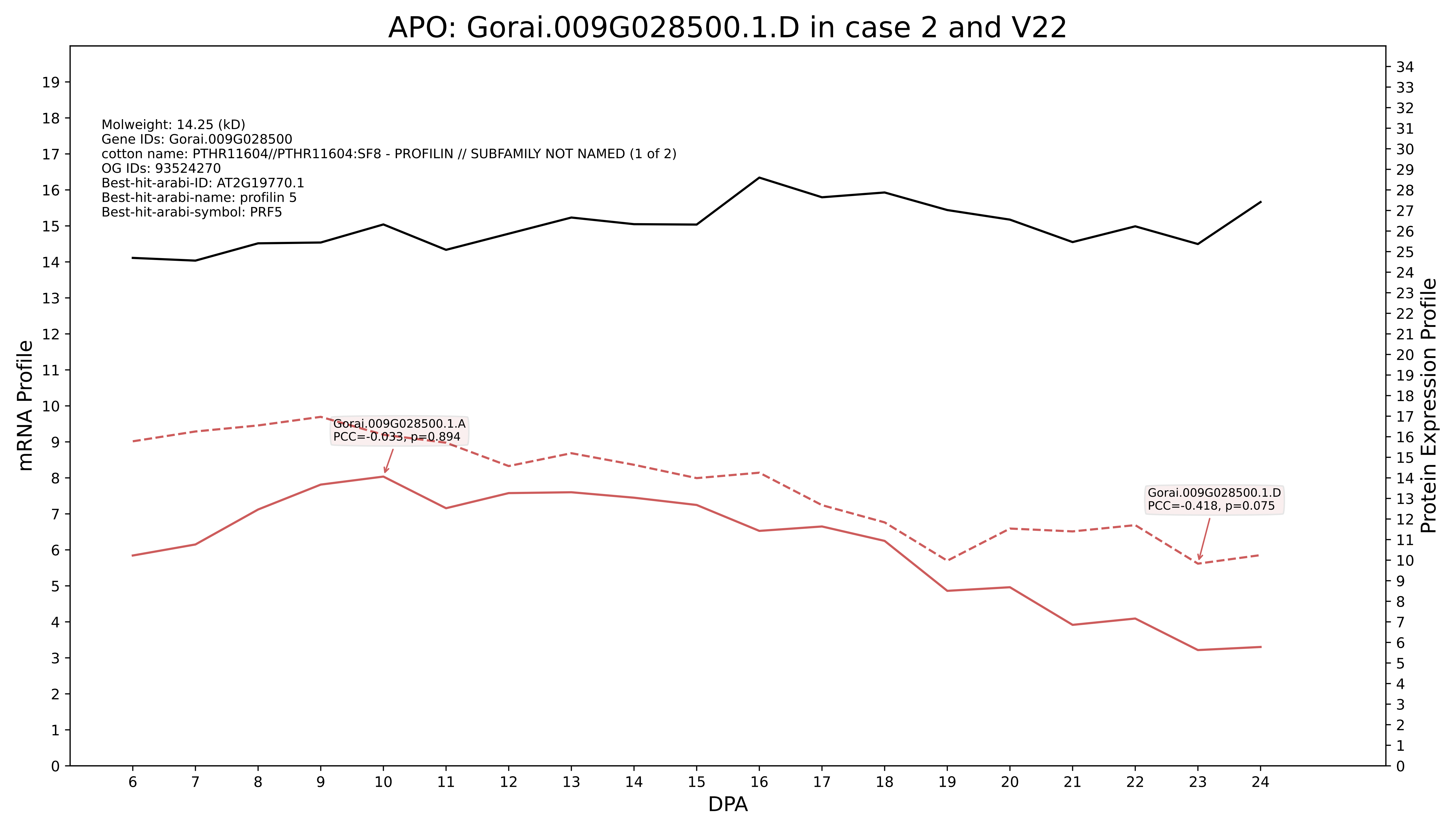
APO: Gorai.009G028500.1.D in case 2 and V22
Molweight: 14.25 (kD)
Gene IDs: Gorai.009G028500
cotton name: PTHR11604//PTHR11604:SF8 - PROFILIN // SUBFAMILY NOT NAMED (1 of 2)
OG IDs: 93524270
Best-hit-arabi-ID: AT2G19770.1
Best-hit-arabi-name: profilin 5
Best-hit-arabi-symbol: PRF5
Gorai.009G028500.1.A
PCC=-0.033, p=0.894
Gorai.009G028500.1.D
PCC=-0.418, p=0.075
mRNA Profile
Protein Expression Profile
DPA

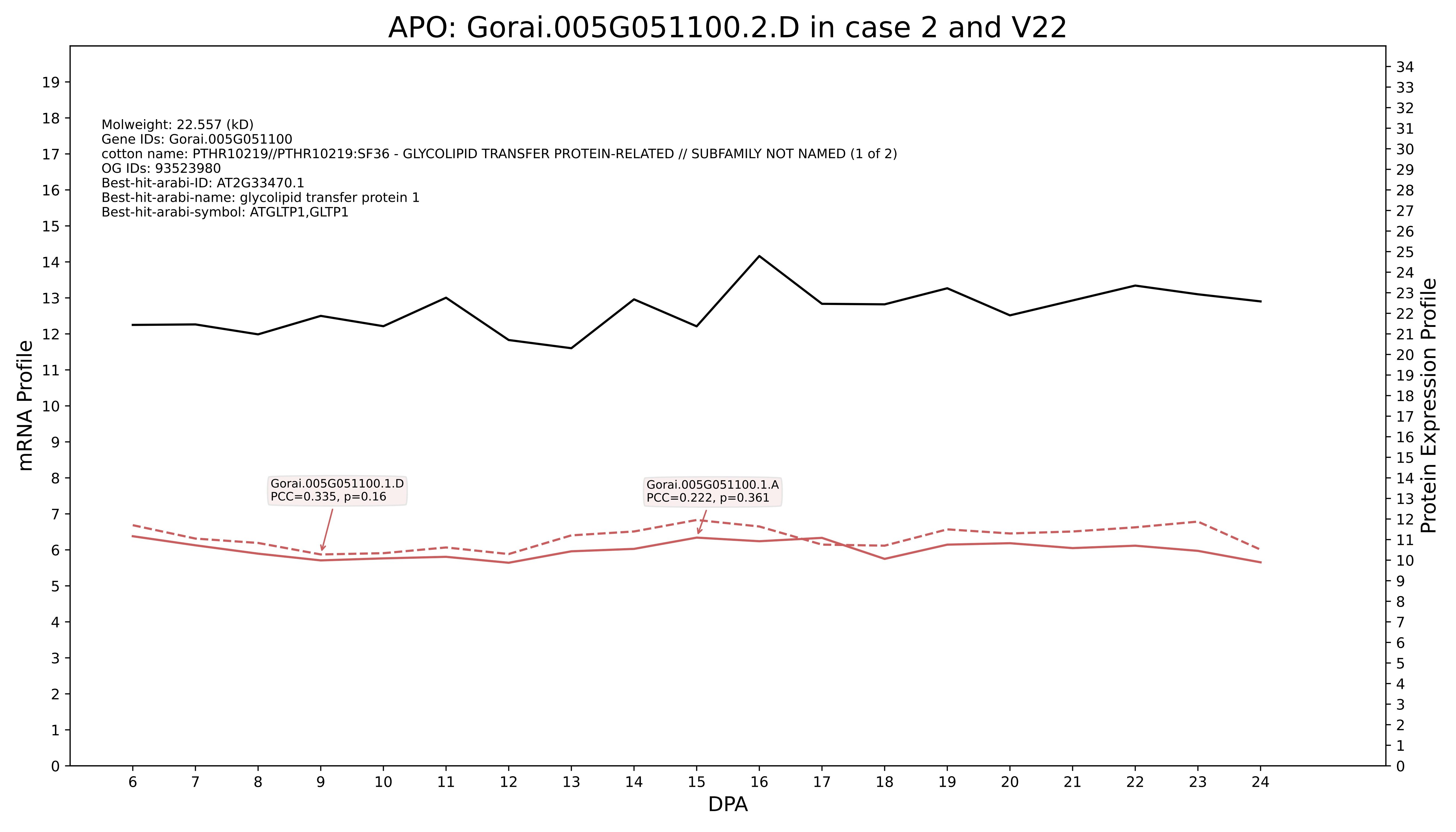

APO: Gorai.005G051100.2.D in case 2 and V22
Molweight: 22.557 (kD)
Gene IDs: Gorai.005G051100
cotton name: PTHR10219//PTHR10219:SF36 - GLYCOLIPID TRANSFER PROTEIN-RELATED // SUBFAMILY NOT NAMED (1 of 2)
OG IDs: 93523980
Best-hit-arabi-ID: AT2G33470.1
Best-hit-arabi-name: glycolipid transfer protein 1
Best-hit-arabi-symbol: ATGLTP1,GLTP1
Gorai.005G051100.1.D
PCC=0.335, p=0.16
Gorai.005G051100.1.A
PCC=0.222, p=0.361
mRNA Profile
Protein Expression Profile
DPA

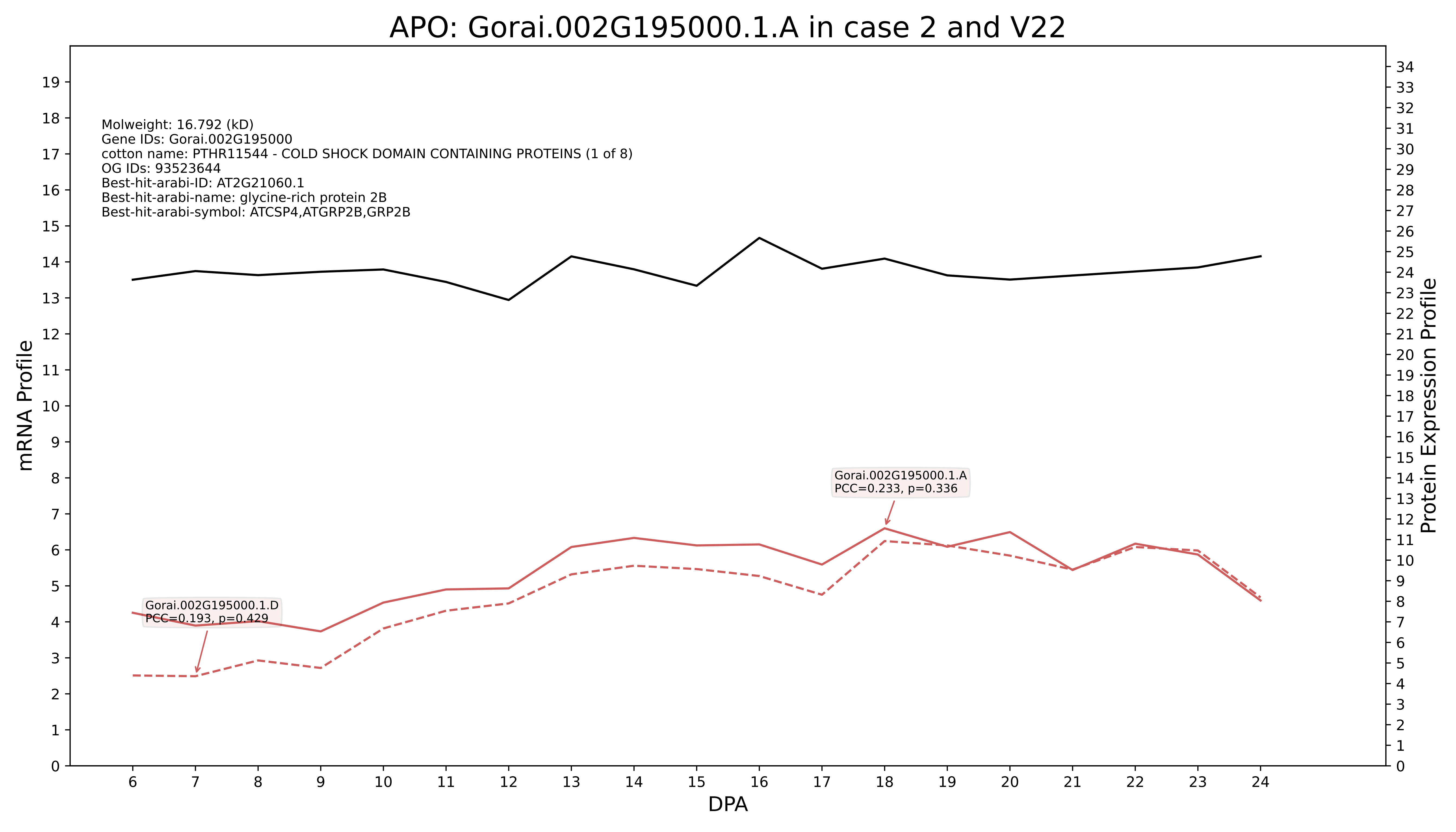
APO: Gorai.002G195000.1.A in case 2 and V22
Molweight: 16.792 (kD)
Gene IDs: Gorai.002G195000
cotton name: PTHR11544 - COLD SHOCK DOMAIN CONTAINING PROTEINS (1 of 8)
OG IDs: 93523644
Best-hit-arabi-ID: AT2G21060.1
Best-hit-arabi-name: glycine-rich protein 2B
Best-hit-arabi-symbol: ATCSP4,ATGRP2B,GRP2B
Gorai.002G195000.1.A
PCC=0.233, p=0.336
Gorai.002G195000.1.D
PCC=0.193, p=0.429
mRNA Profile
Protein Expression Profile
DPA
0
1
2
3
4
5
6
7
8
9
10
11
12
13
14
15
16
17
18
19
20
21
22
23
24
25
26
27
28
29
30
31
32
33
34

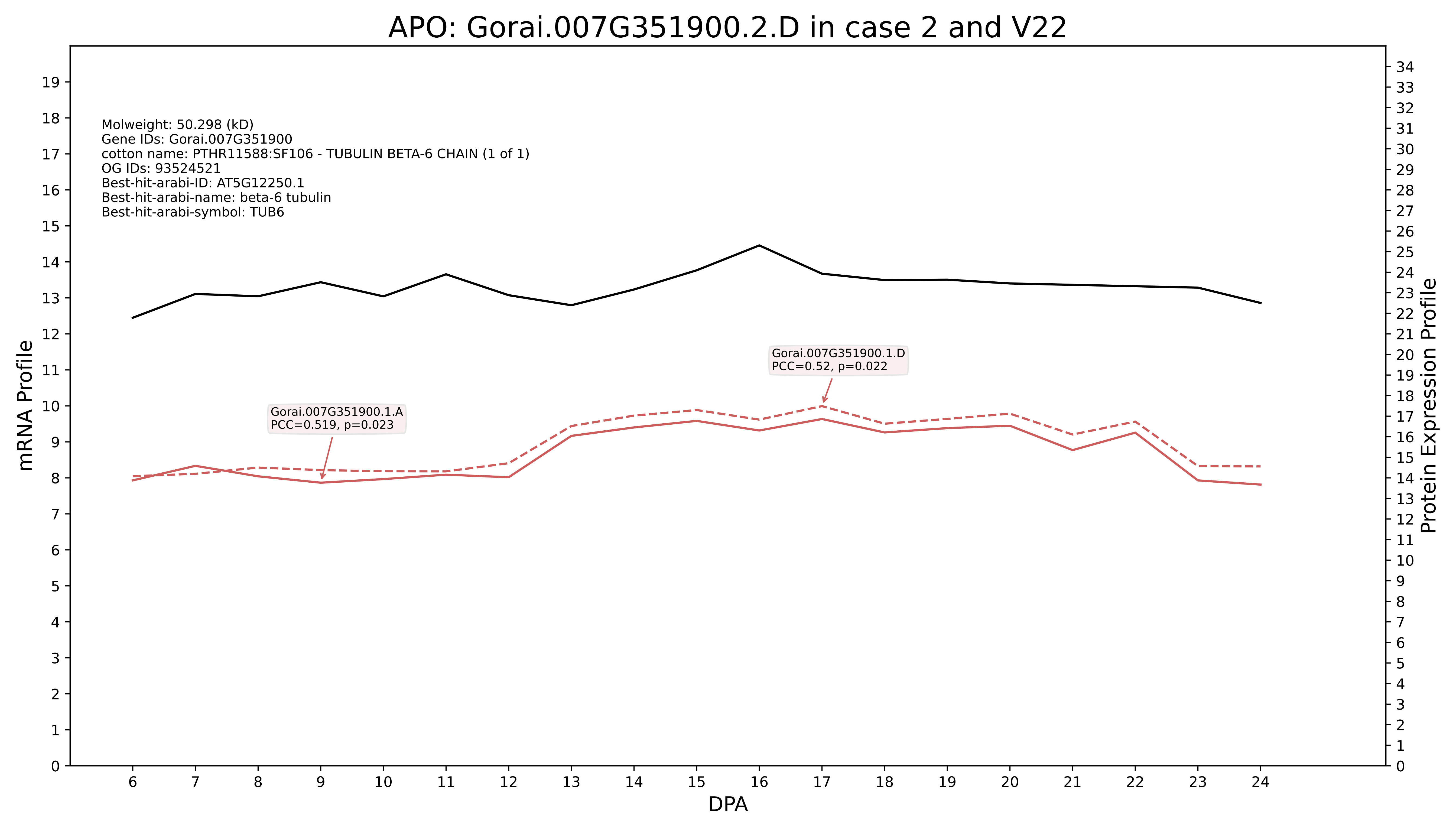

APO: Gorai.007G351900.2.D in case 2 and V22
Molweight: 50.298 (kD)
Gene IDs: Gorai.007G351900
cotton name: PTHR11588:SF106 - TUBULIN BETA-6 CHAIN (1 of 1)
OG IDs: 93524521
Best-hit-arabi-ID: AT5G12250.1
Best-hit-arabi-name: beta-6 tubulin
Best-hit-arabi-symbol: TUB6
Gorai.007G351900.1.D
PCC=0.52, p=0.022
Gorai.007G351900.1.A
PCC=0.519, p=0.023
mRNA Profile
Protein Expression Profile
DPA

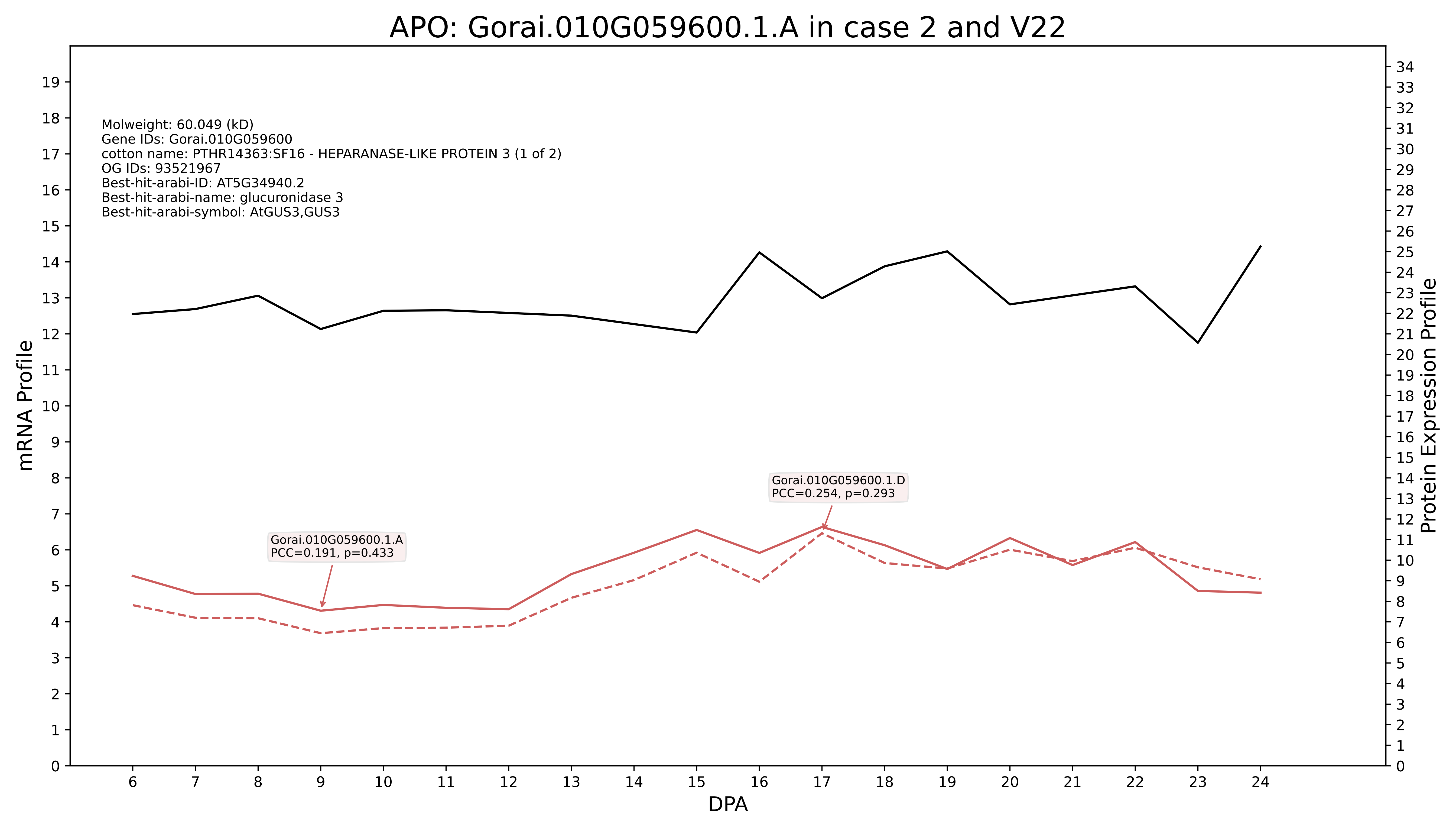

APO: Gorai.010G059600.1.A in case 2 and V22
Molweight: 60.049 (kD)
Gene IDs: Gorai.010G059600
cotton name: PTHR14363:SF16 - HEPARANASE-LIKE PROTEIN 3 (1 of 2)
OG IDs: 93521967
Best-hit-arabi-ID: AT5G34940.2
Best-hit-arabi-name: glucuronidase 3
Best-hit-arabi-symbol: AtGUS3,GUS3
Gorai.010G059600.1.D
PCC=0.254, p=0.293
Gorai.010G059600.1.A
PCC=0.191, p=0.433
mRNA Profile
Protein Expression Profile
DPA

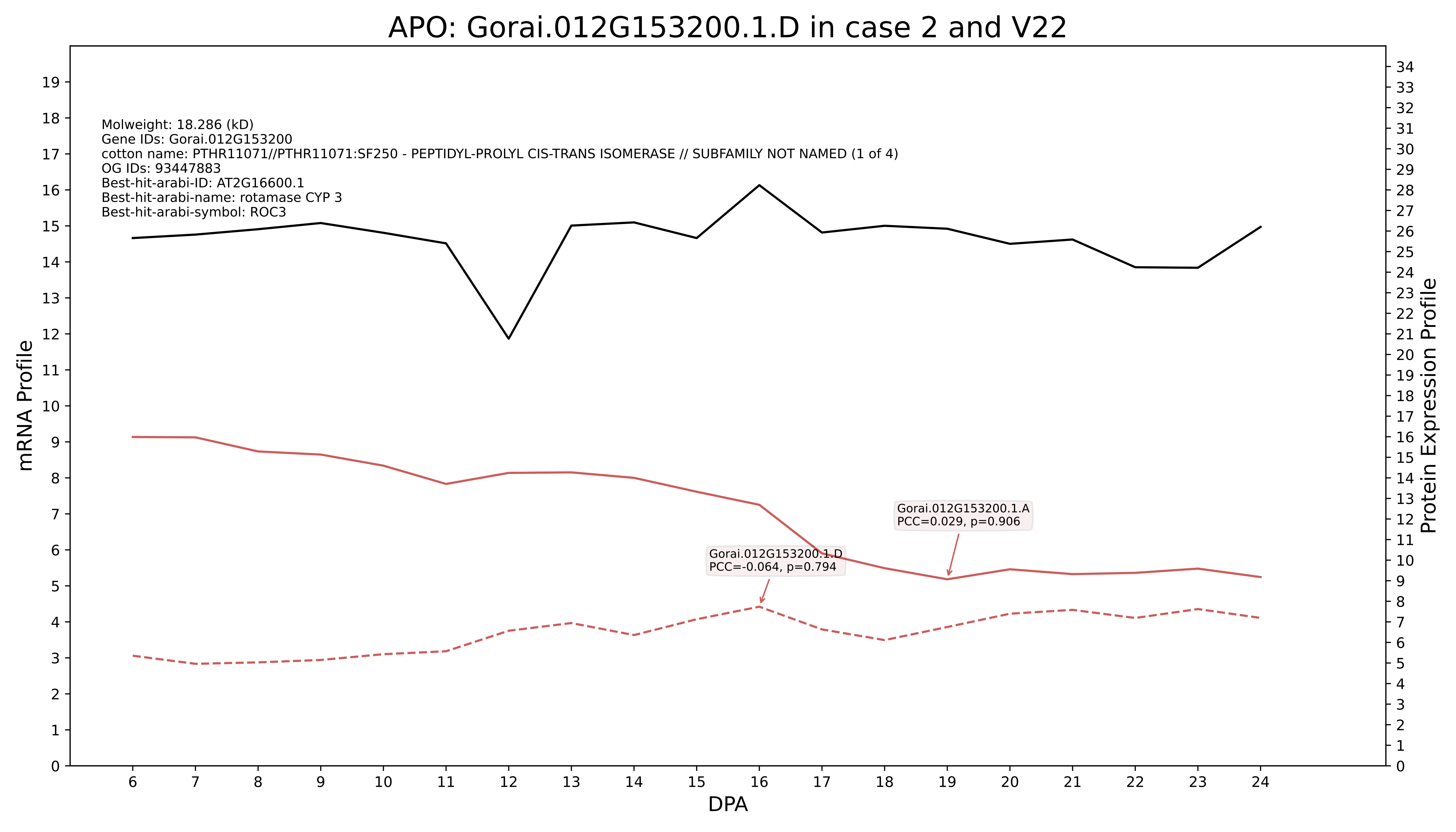
APO: Gorai.012G153200.1.D in case 2 and V22
Molweight: 18.286 (kD)
Gene IDs: Gorai.012G153200
cotton name: PTHR11071//PTHR11071:SF250 - PEPTIDYL-PROLYL CIS-TRANS ISOMERASE // SUBFAMILY NOT NAMED (1 of 4)
OG IDs: 93447883
Best-hit-arabi-ID: AT2G16600.1
Best-hit-arabi-name: rotamase CYP 3
Best-hit-arabi-symbol: ROC3
Gorai.012G153200.1.A
PCC=0.029, p=0.906
Gorai.012G153200.1.D
PCC=-0.064, p=0.794
mRNA Profile
Protein Expression Profile
DPA

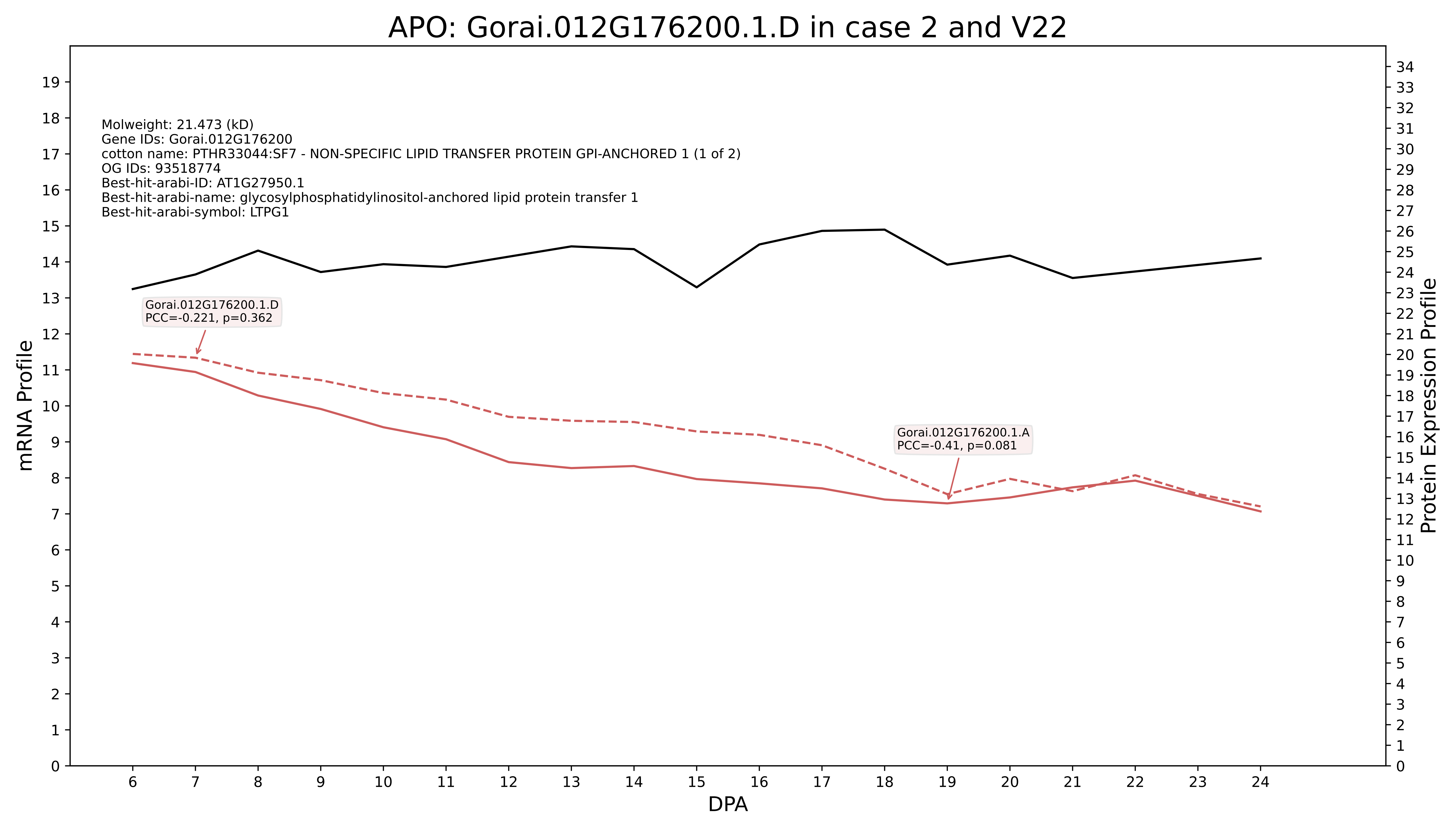
APO: Gorai.012G176200.1.D in case 2 and V22
Molweight: 21.473 (kD)
Gene IDs: Gorai.012G176200
cotton name: PTHR33044:SF7 - NON-SPECIFIC LIPID TRANSFER PROTEIN GPI-ANCHORED 1 (1 of 2)
OG IDs: 93518774
Best-hit-arabi-ID: AT1G27950.1
Best-hit-arabi-name: glycosylphosphatidylinositol-anchored lipid protein transfer 1
Best-hit-arabi-symbol: LTPG1
Gorai.012G176200.1.D
PCC=-0.221, p=0.362
Gorai.012G176200.1.A
PCC=-0.41, p=0.081
mRNA Profile
Protein Expression Profile
DPA

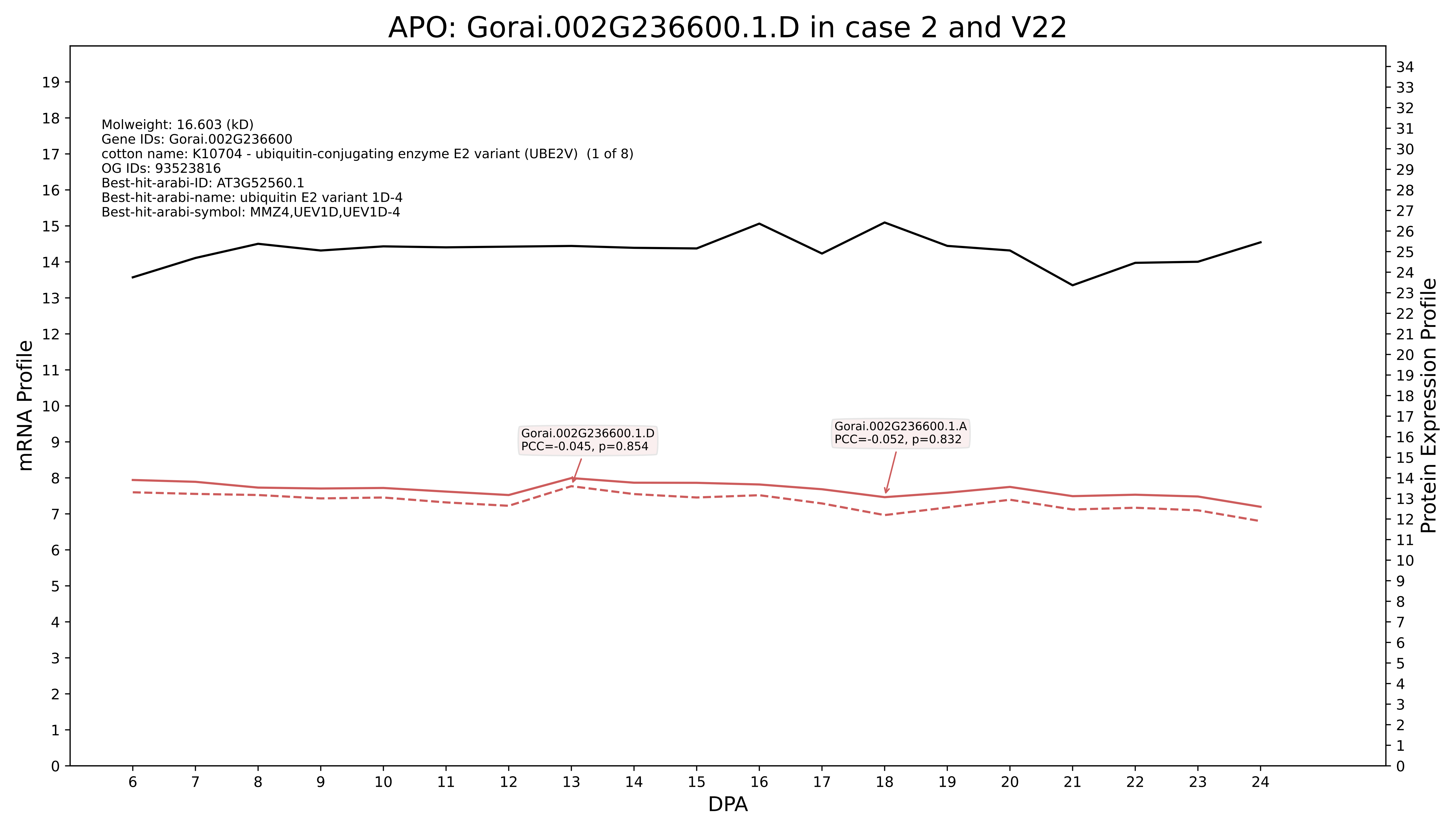
APO: Gorai.002G236600.1.D in case 2 and V22
Molweight: 16.603 (kD)
Gene IDs: Gorai.002G236600
cotton name: K10704 - ubiquitin-conjugating enzyme E2 variant (UBE2V) (1 of 8)
OG IDs: 93523816
Best-hit-arabi-ID: AT3G52560.1
Best-hit-arabi-name: ubiquitin E2 variant 1D-4
Best-hit-arabi-symbol: MMZ4,UEV1D,UEV1D-4
Gorai.002G236600.1.D
PCC=-0.045, p=0.854
Gorai.002G236600.1.A
PCC=-0.052, p=0.832
mRNA Profile
Protein Expression Profile
DPA

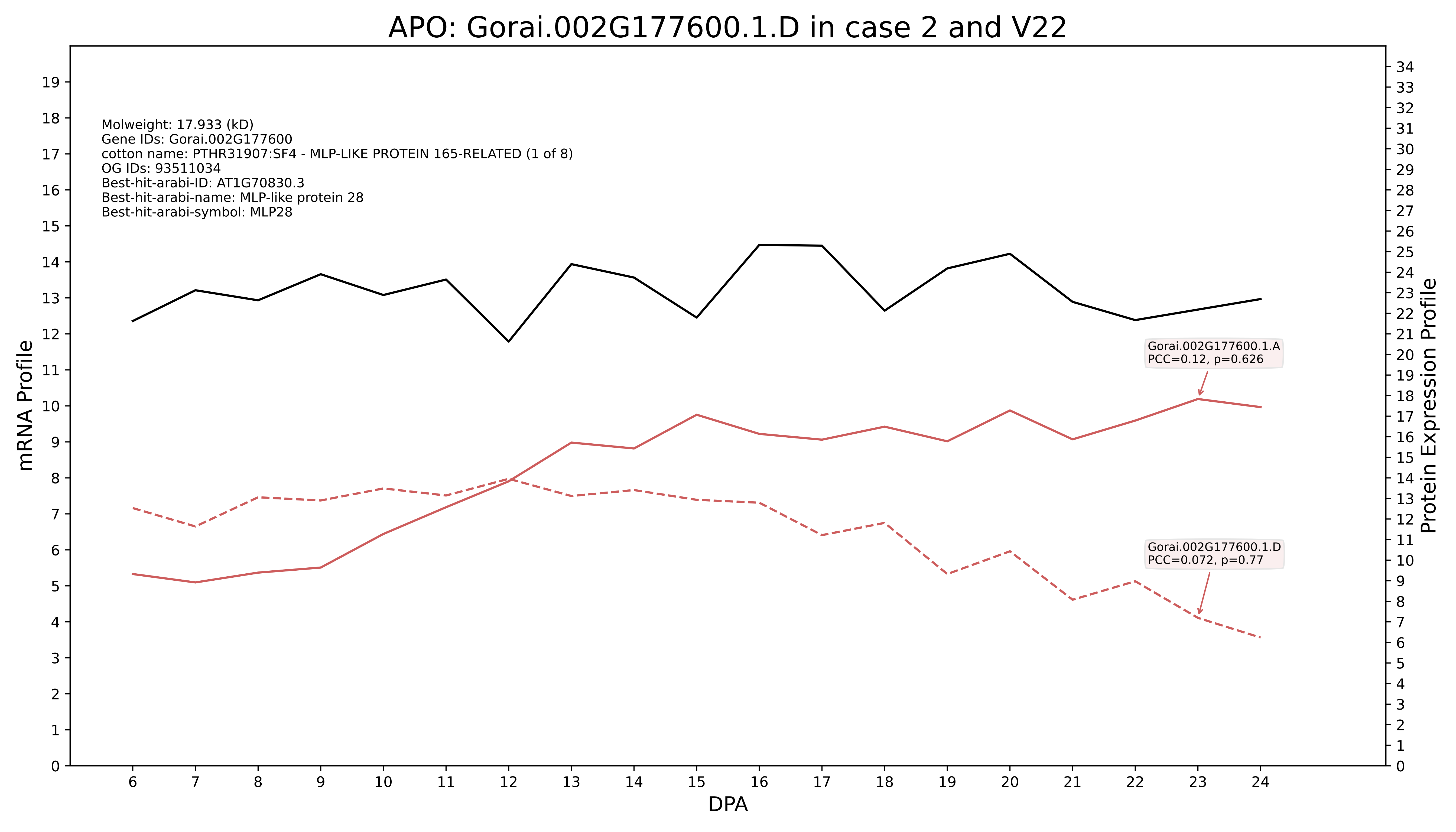

APO: Gorai.002G177600.1.D in case 2 and V22
Molweight: 17.933 (kD)
Gene IDs: Gorai.002G177600
cotton name: PTHR31907:SF4 - MLP-LIKE PROTEIN 165-RELATED (1 of 8)
OG IDs: 93511034
Best-hit-arabi-ID: AT1G70830.3
Best-hit-arabi-name: MLP-like protein 28
Best-hit-arabi-symbol: MLP28
Gorai.002G177600.1.A
PCC=0.12, p=0.626
Gorai.002G177600.1.D
PCC=0.072, p=0.77
mRNA Profile
Protein Expression Profile
DPA

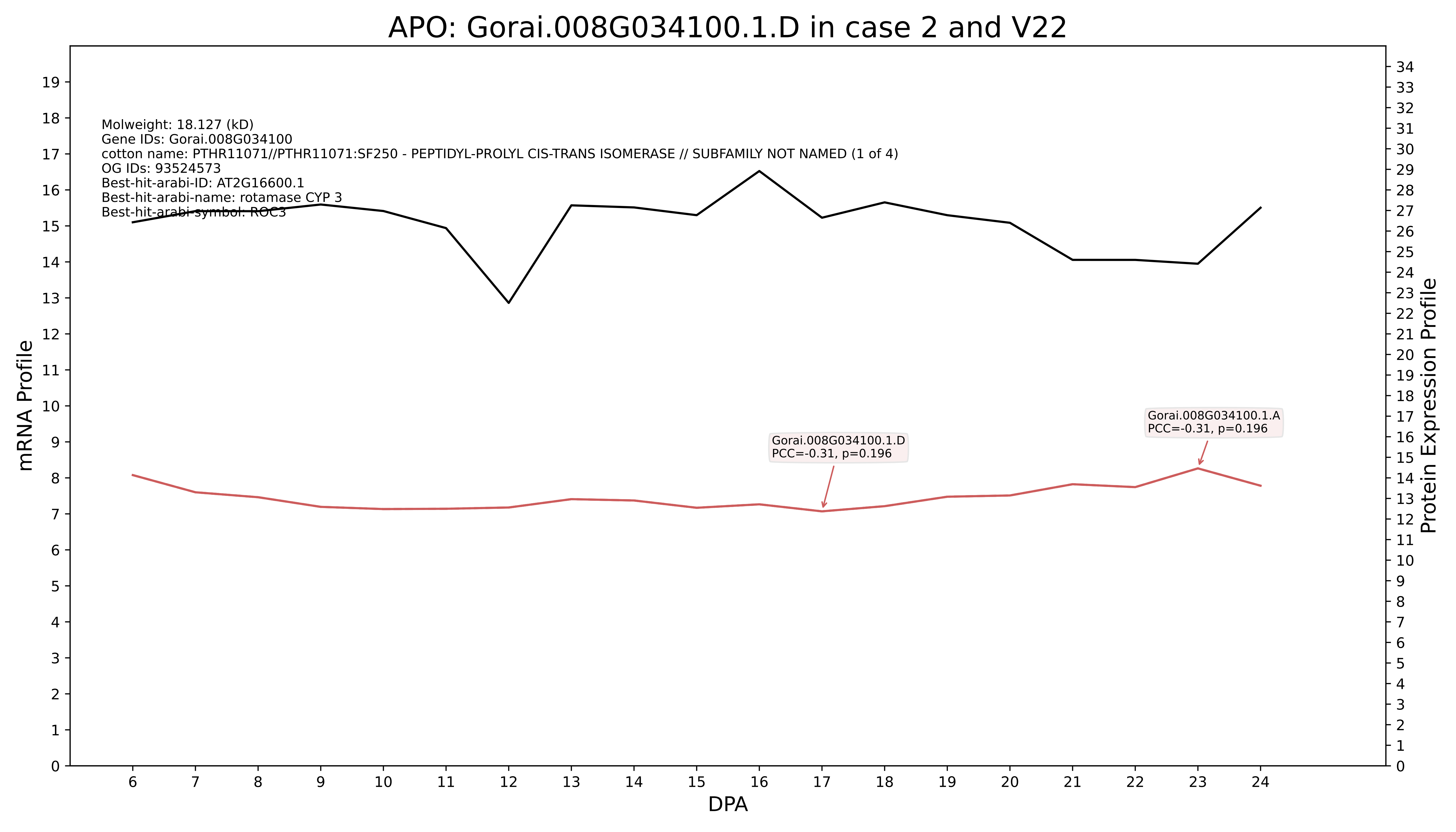
APO: Gorai.008G034100.1.D in case 2 and V22
Molweight: 18.127 (kD)
Gene IDs: Gorai.008G034100
cotton name: PTHR11071//PTHR11071:SF250 - PEPTIDYL-PROLYL CIS-TRANS ISOMERASE // SUBFAMILY NOT NAMED (1 of 4)
OG IDs: 93524573
Best-hit-arabi-ID: AT2G16600.1
Best-hit-arabi-name: rotamase CYP 3
Best-hit-arabi-symbol: ROC3
Gorai.008G034100.1.D
PCC=-0.31, p=0.196
Gorai.008G034100.1.A
PCC=-0.31, p=0.196
mRNA Profile
Protein Expression Profile
DPA

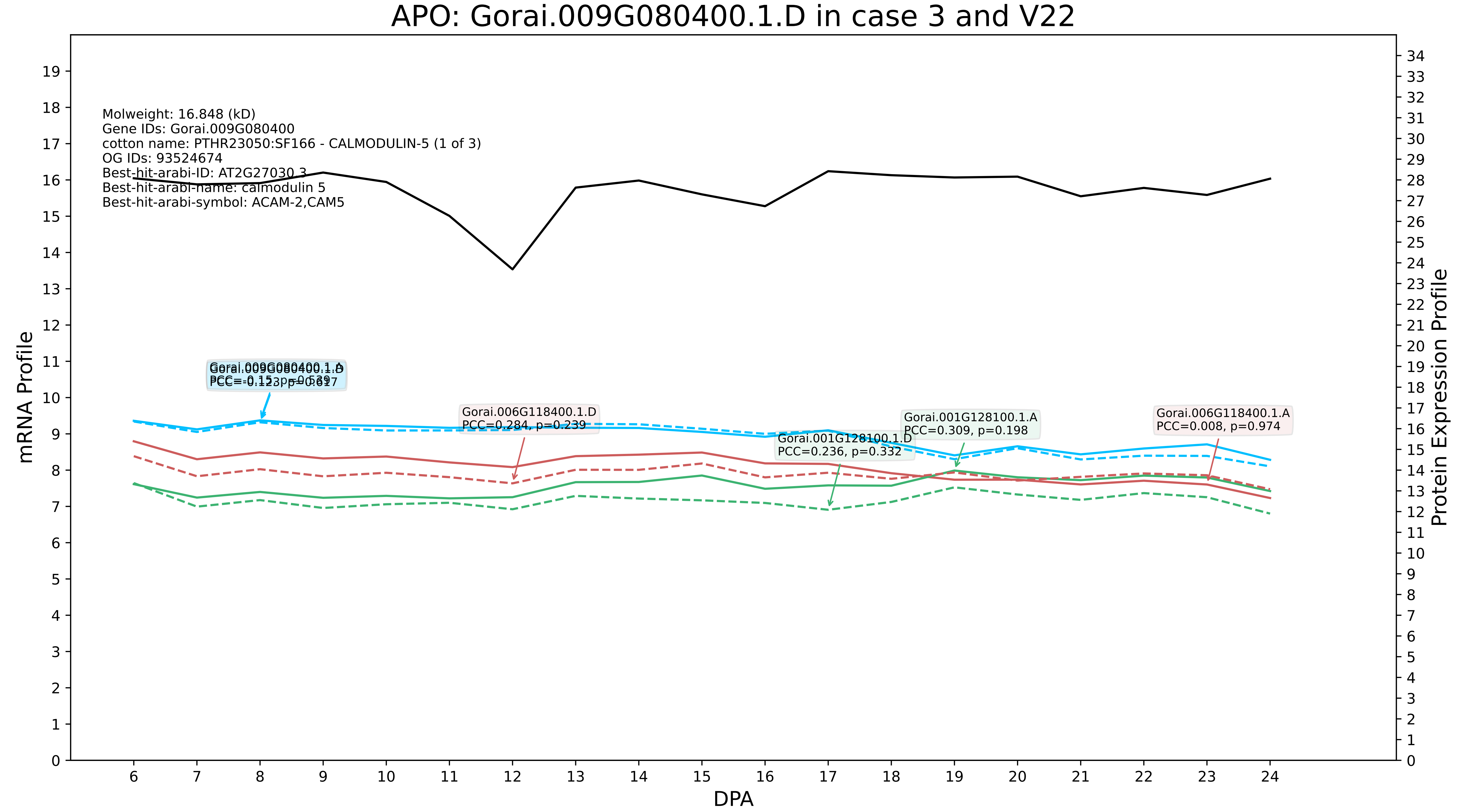

APO: Gorai.009G080400.1.D in case 3 and V22
Molweight: 16.848 (kD)
Gene IDs: Gorai.009G080400
cotton name: PTHR23050:SF166 - CALMODULIN-5 (1 of 3)
OG IDs: 93524674
Best-hit-arabi-ID: AT2G27030.3
Best-hit-arabi-name: calmodulin 5
Best-hit-arabi-symbol: ACAM-2,CAM5
Gorai.006G118400.1.D
PCC=0.284, p=0.239
Gorai.001G128100.1.D
PCC=0.236, p=0.332
Gorai.001G128100.1.A
PCC=0.309, p=0.198
Gorai.006G118400.1.A
PCC=0.008, p=0.974
mRNA Profile
Protein Expression Profile
DPA

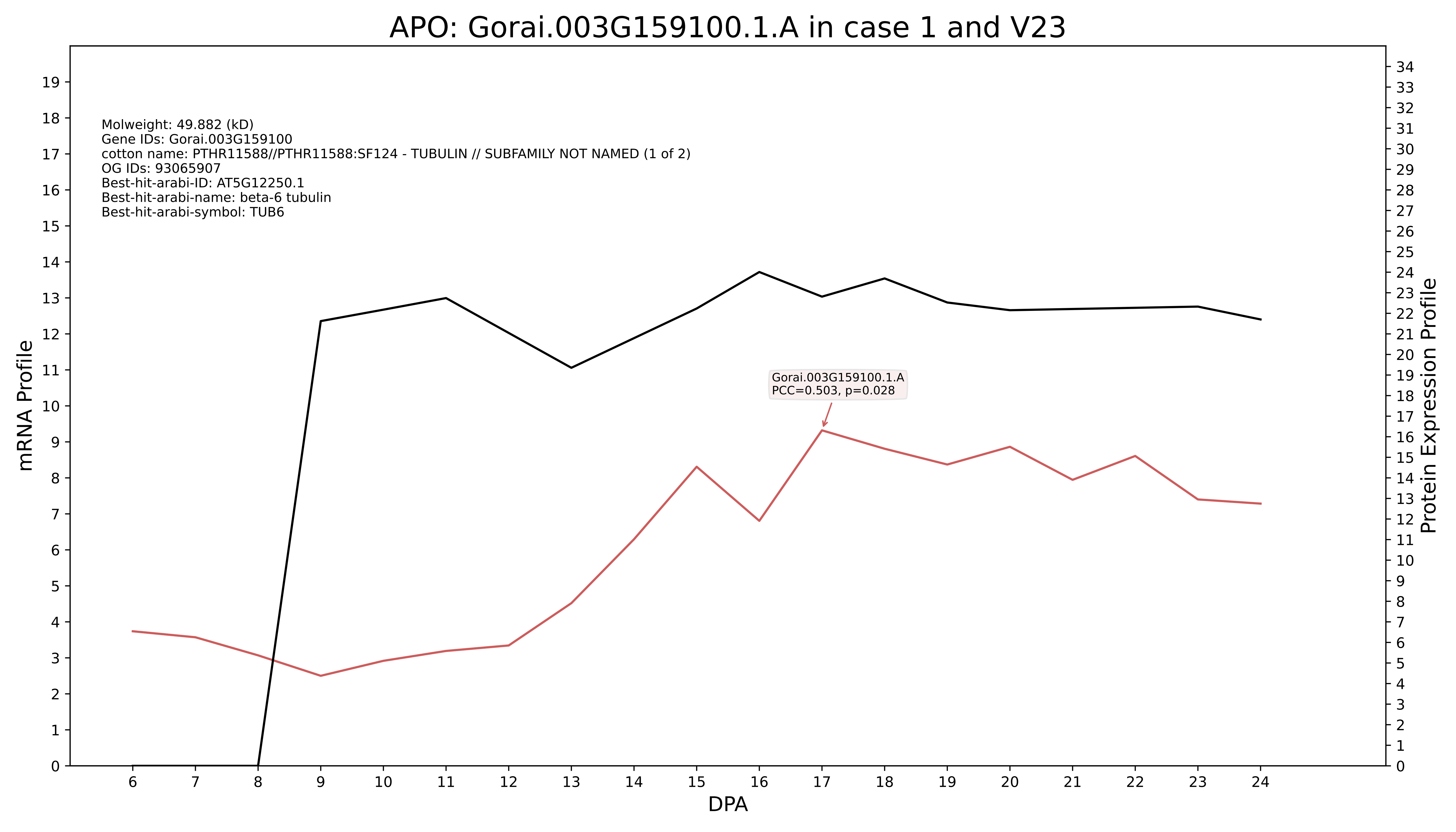
APO: Gorai.003G159100.1.A in case 1 and V23
Molweight: 49.882 (kD)
Gene IDs: Gorai.003G159100
cotton name: PTHR11588//PTHR11588:SF124 - TUBULIN // SUBFAMILY NOT NAMED (1 of 2)
OG IDs: 93065907
Best-hit-arabi-ID: AT5G12250.1
Best-hit-arabi-name: beta-6 tubulin
Best-hit-arabi-symbol: TUB6
Gorai.003G159100.1.A
PCC=0.503, p=0.028
mRNA Profile
Protein Expression Profile
DPA

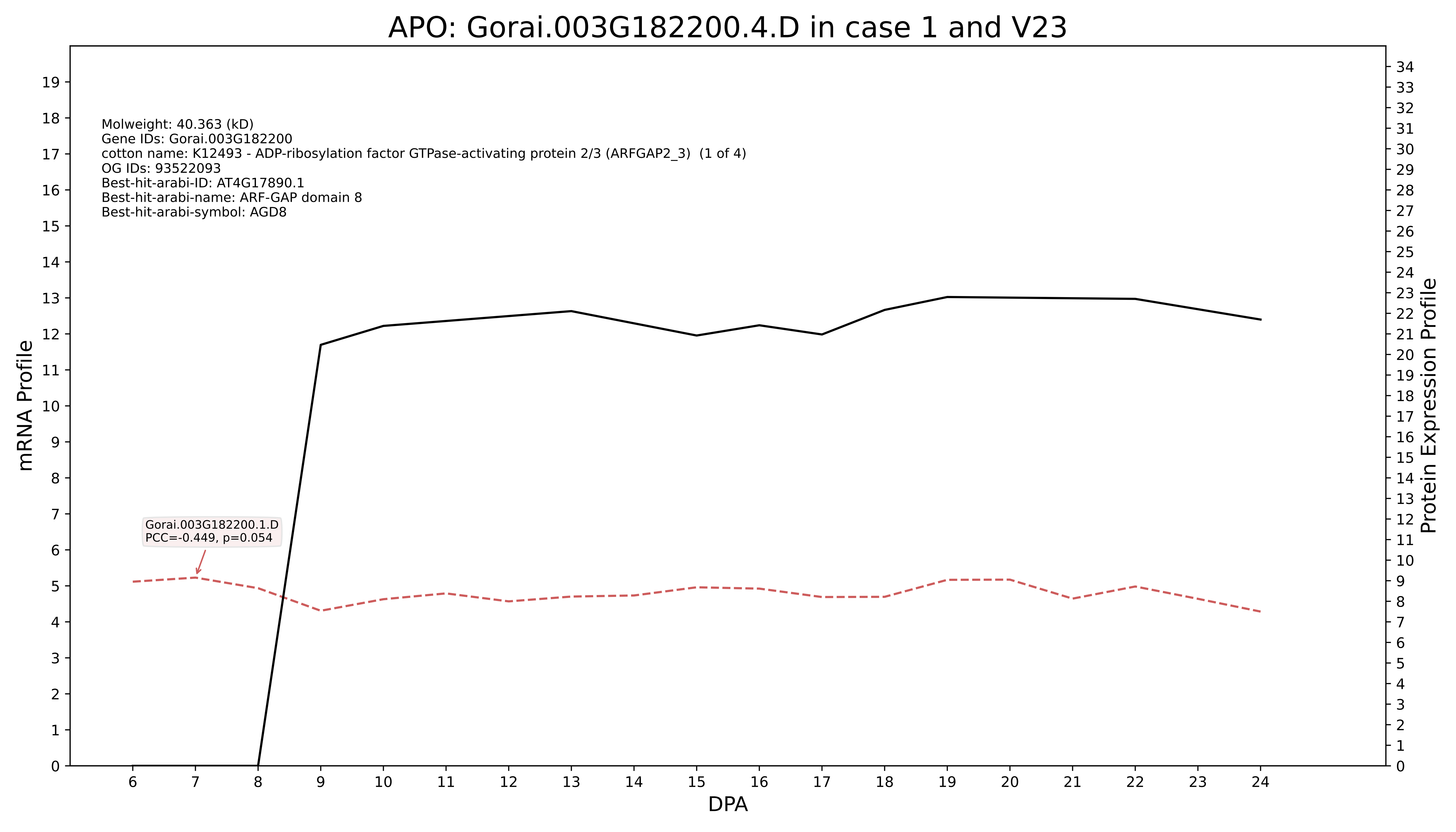
APO: Gorai.003G182200.4.D in case 1 and V23
Molweight: 40.363 (kD)
Gene IDs: Gorai.003G182200
cotton name: K12493 - ADP-ribosylation factor GTPase-activating protein 2/3 (ARFGAP2_3) (1 of 4)
OG IDs: 93522093
Best-hit-arabi-ID: AT4G17890.1
Best-hit-arabi-name: ARF-GAP domain 8
Best-hit-arabi-symbol: AGD8
Gorai.003G182200.1.D
PCC=-0.449, p=0.054
mRNA Profile
Protein Expression Profile
DPA
0
1
2
3
4
5
6
7
8
9
10
11
12
13
14
15
16
17
18
19
20
21
22
23
24
25
26
27
28
29
30
31
32
33
34

APO: Gorai.013G093600.3.A in case 1 and V23

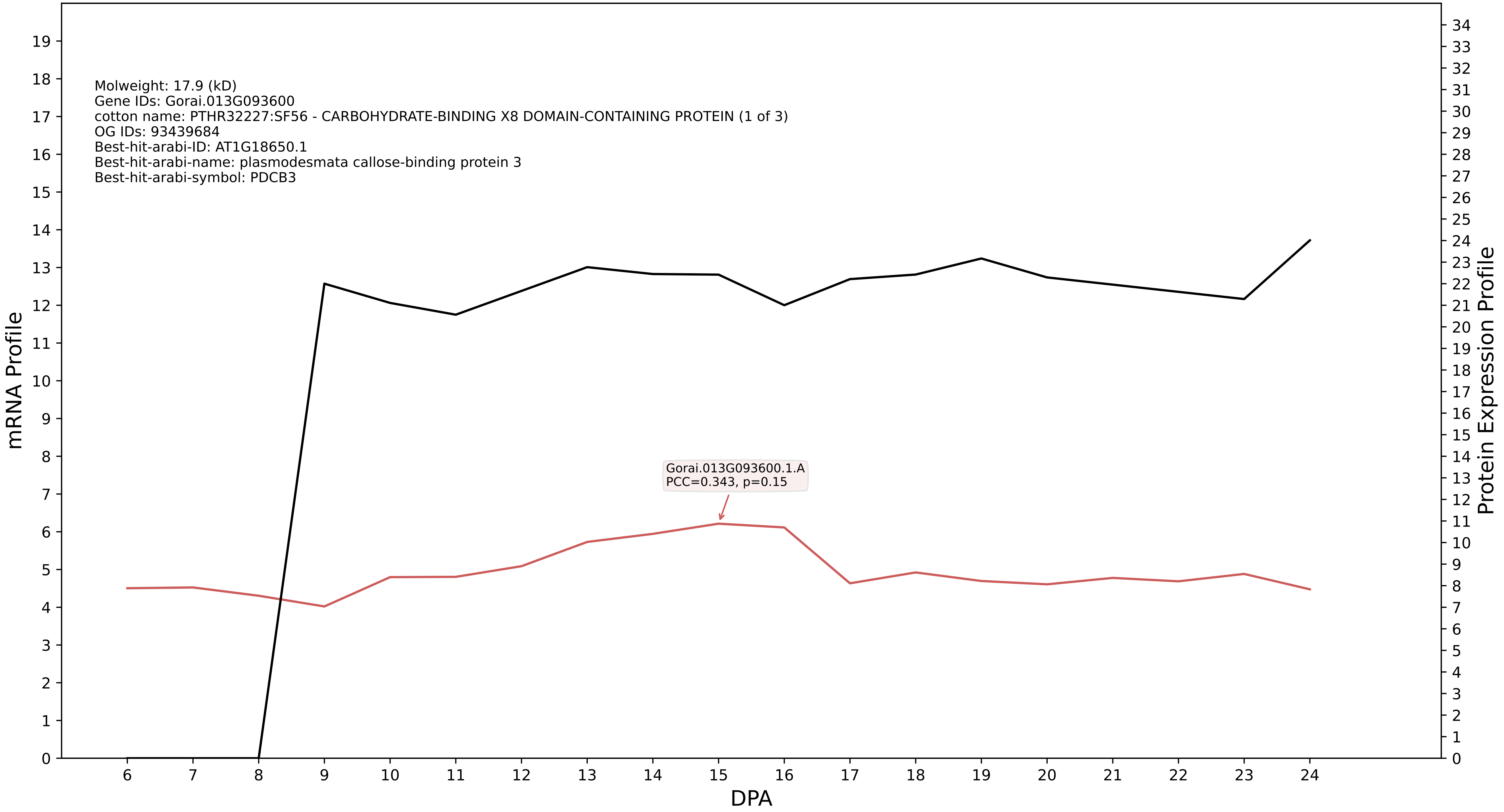

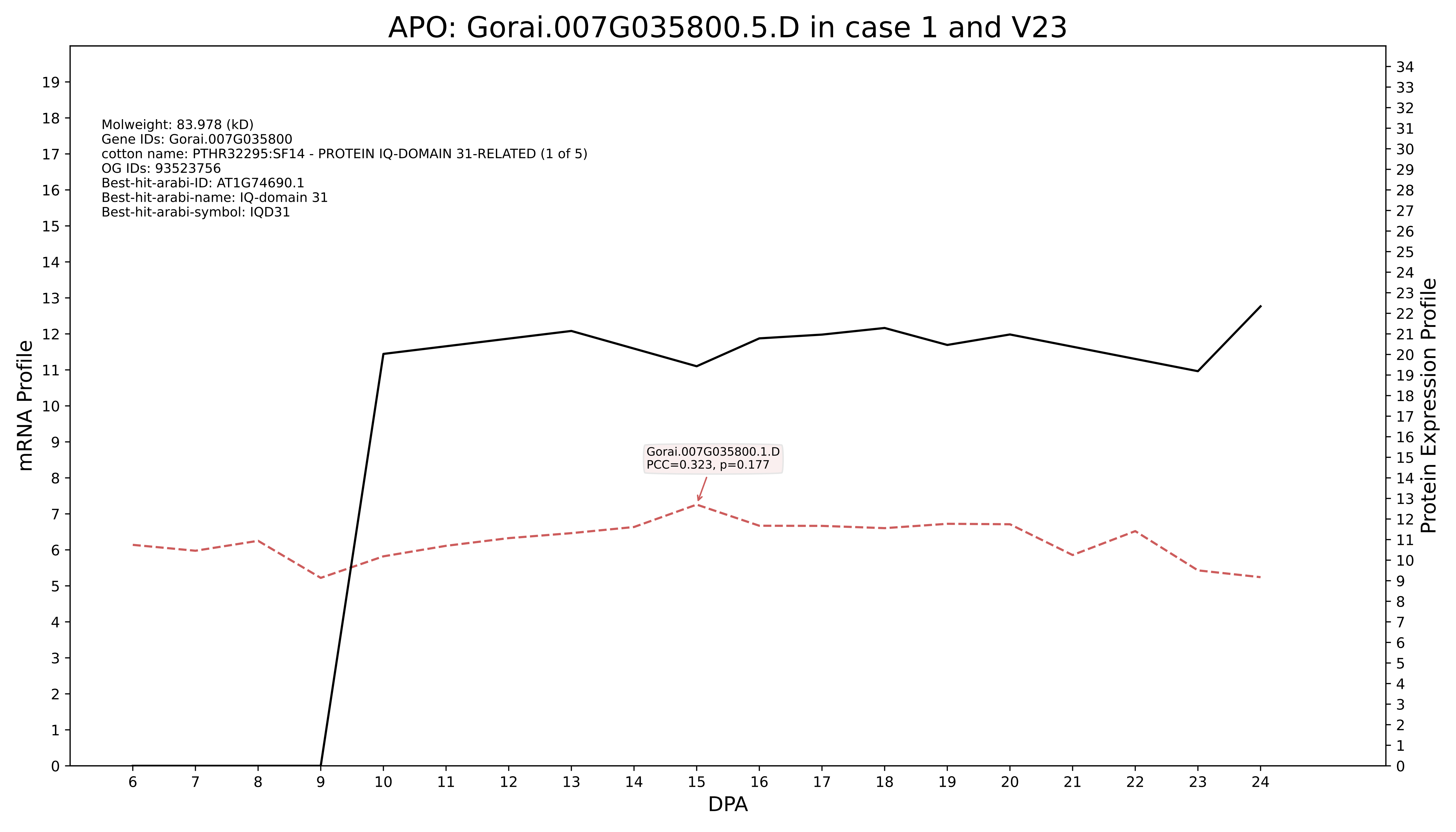
APO: Gorai.007G035800.5.D in case 1 and V23
Molweight: 83.978 (kD)
Gene IDs: Gorai.007G035800
cotton name: PTHR32295:SF14 - PROTEIN IQ-DOMAIN 31-RELATED (1 of 5)
OG IDs: 93523756
Best-hit-arabi-ID: AT1G74690.1
Best-hit-arabi-name: IQ-domain 31
Best-hit-arabi-symbol: IQD31
Gorai.007G035800.1.D
PCC=0.323, p=0.177
mRNA Profile
Protein Expression Profile
DPA

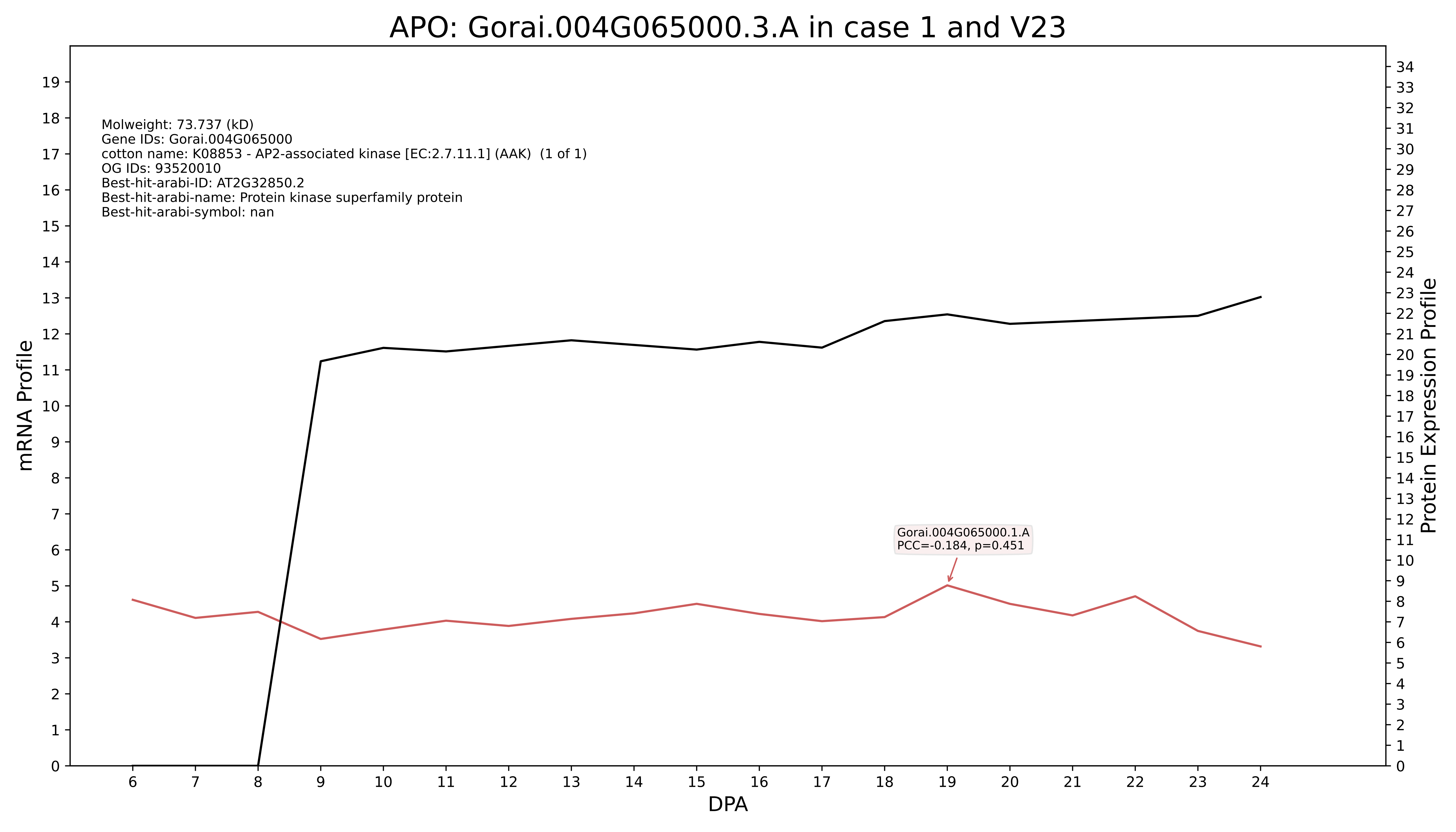

APO: Gorai.004G065000.3.A in case 1 and V23
Molweight: 73.737 (kD)
Gene IDs: Gorai.004G065000
cotton name: K08853 - AP2-associated kinase [EC:2.7.11.1] (AAK) (1 of 1)
OG IDs: 93520010
Best-hit-arabi-ID: AT2G32850.2
Best-hit-arabi-name: Protein kinase superfamily protein
Best-hit-arabi-symbol: nan
Gorai.004G065000.1.A
PCC=-0.184, p=0.451
mRNA Profile
Protein Expression Profile
DPA

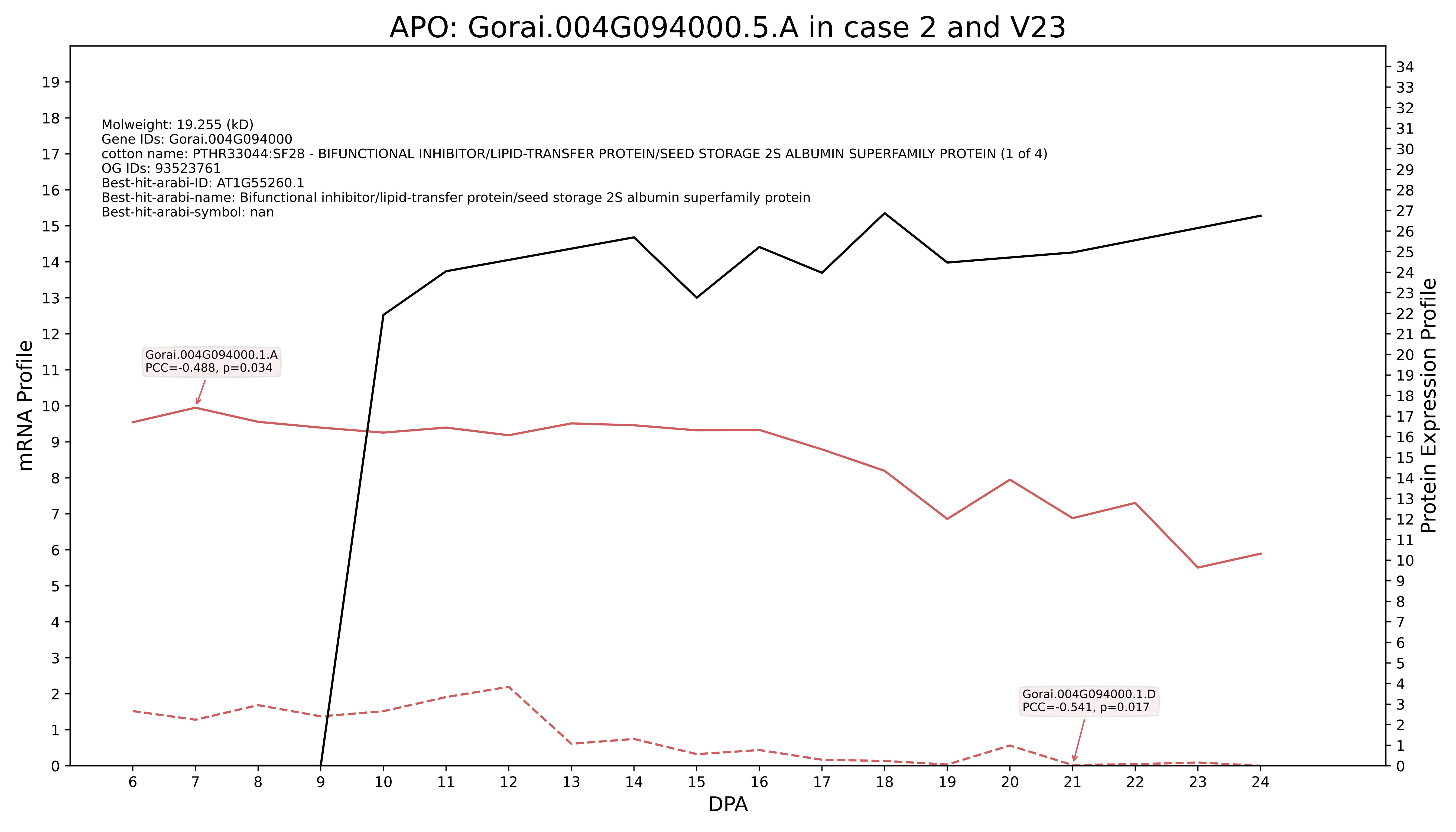
APO: Gorai.004G094000.5.A in case 2 and V23
Molweight: 19.255 (kD)
Gene IDs: Gorai.004G094000
cotton name: PTHR33044:SF28 - BIFUNCTIONAL INHIBITOR/LIPID-TRANSFER PROTEIN/SEED STORAGE 2S ALBUMIN SUPERFAMILY PROTEIN (1 of 4)
OG IDs: 93523761
Best-hit-arabi-ID: AT1G55260.1
Best-hit-arabi-name: Bifunctional inhibitor/lipid-transfer protein/seed storage 2S albumin superfamily protein
Best-hit-arabi-symbol: nan
Gorai.004G094000.1.A
PCC=-0.488, p=0.034
Gorai.004G094000.1.D
PCC=-0.541, p=0.017
mRNA Profile
Protein Expression Profile
DPA

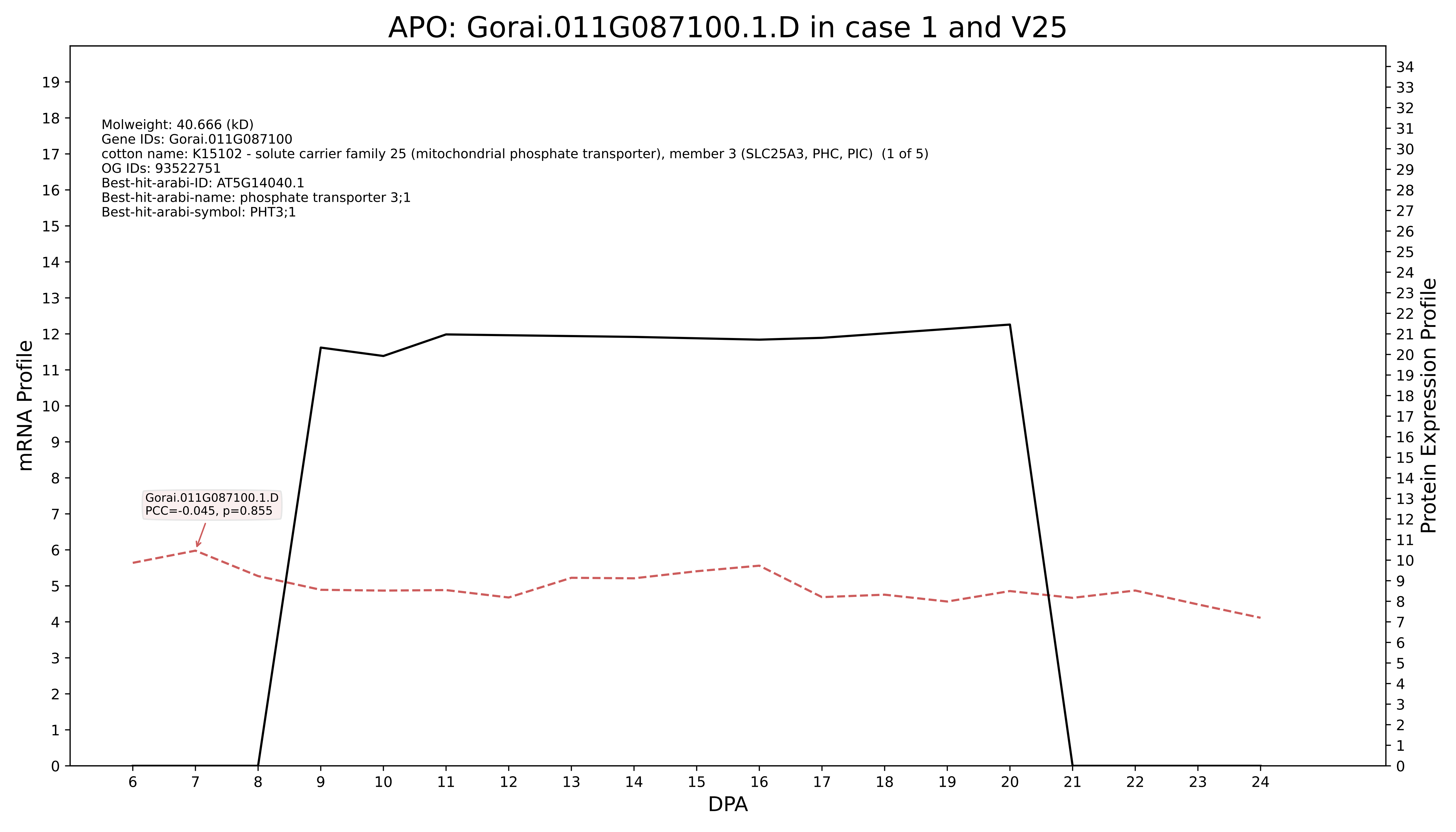
APO: Gorai.011G087100.1.D in case 1 and V25
Molweight: 40.666 (kD)
Gene IDs: Gorai.011G087100
cotton name: K15102 - solute carrier family 25 (mitochondrial phosphate transporter), member 3 (SLC25A3, PHC, PIC) (1 of 5)
OG IDs: 93522751
Best-hit-arabi-ID: AT5G14040.1
Best-hit-arabi-name: phosphate transporter 3;1
Best-hit-arabi-symbol: PHT3;1
Gorai.011G087100.1.D
PCC=-0.045, p=0.855
mRNA Profile
Protein Expression Profile
DPA

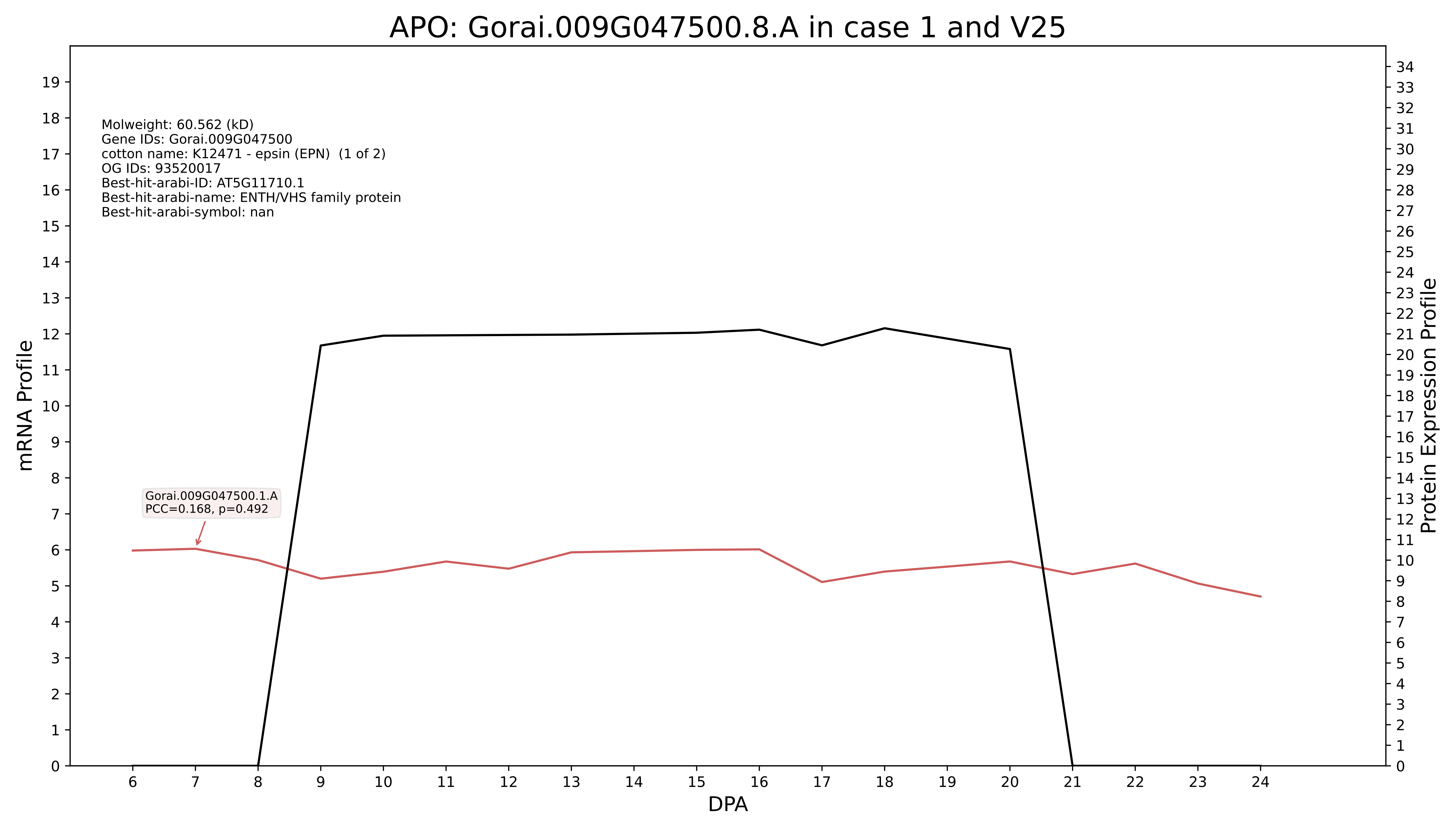
APO: Gorai.009G047500.8.A in case 1 and V25
Molweight: 60.562 (kD)
Gene IDs: Gorai.009G047500
cotton name: K12471 - epsin (EPN) (1 of 2)
OG IDs: 93520017
Best-hit-arabi-ID: AT5G11710.1
Best-hit-arabi-name: ENTH/VHS family protein
Best-hit-arabi-symbol: nan
Gorai.009G047500.1.A
PCC=0.168, p=0.492
mRNA Profile
Protein Expression Profile
DPA

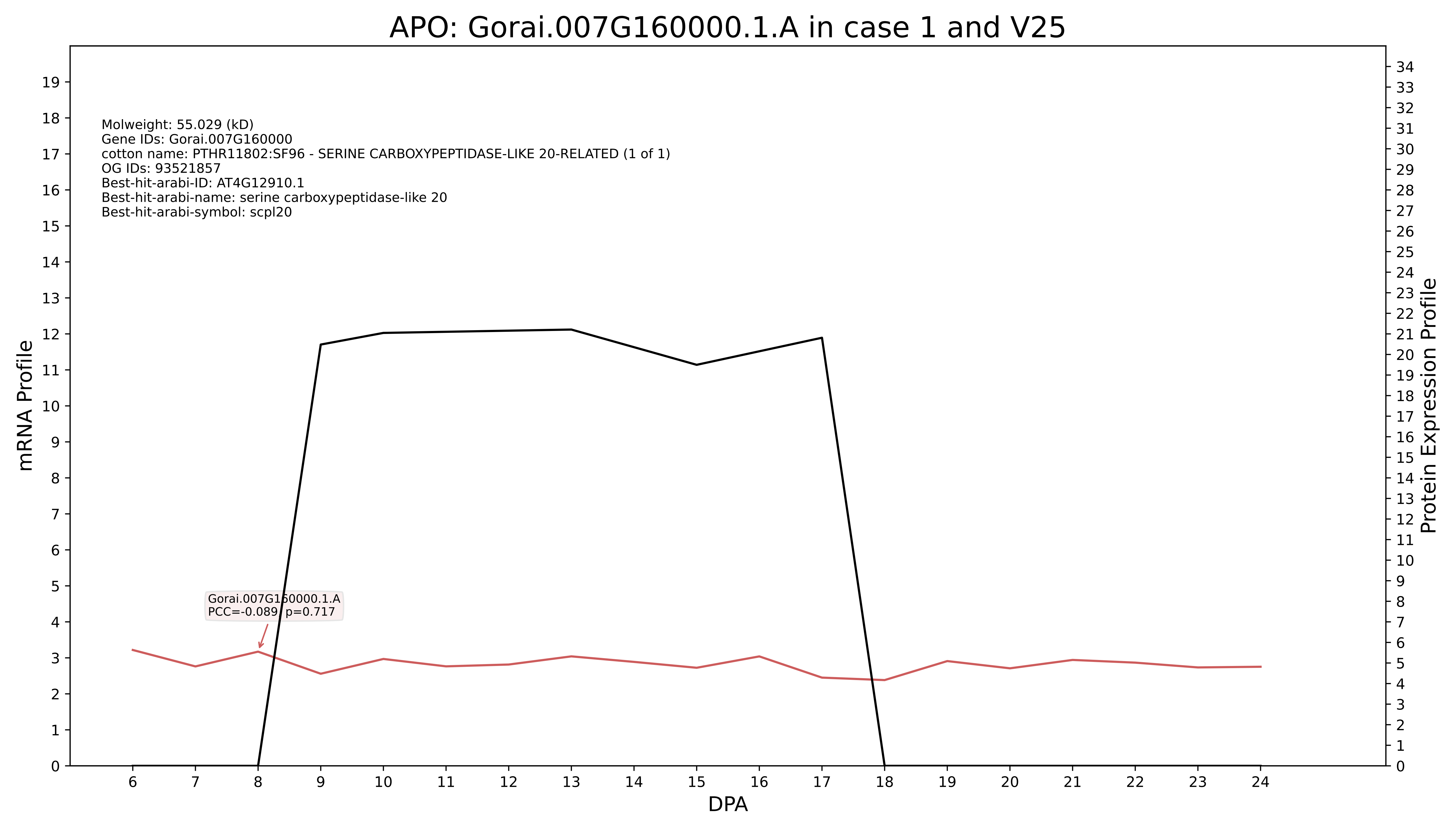
APO: Gorai.007G160000.1.A in case 1 and V25
Molweight: 55.029 (kD)
Gene IDs: Gorai.007G160000
cotton name: PTHR11802:SF96 - SERINE CARBOXYPEPTIDASE-LIKE 20-RELATED (1 of 1)
OG IDs: 93521857
Best-hit-arabi-ID: AT4G12910.1
Best-hit-arabi-name: serine carboxypeptidase-like 20
Best-hit-arabi-symbol: scpl20
Gorai.007G160000.1.A
PCC=-0.089, p=0.717
mRNA Profile
Protein Expression Profile
DPA

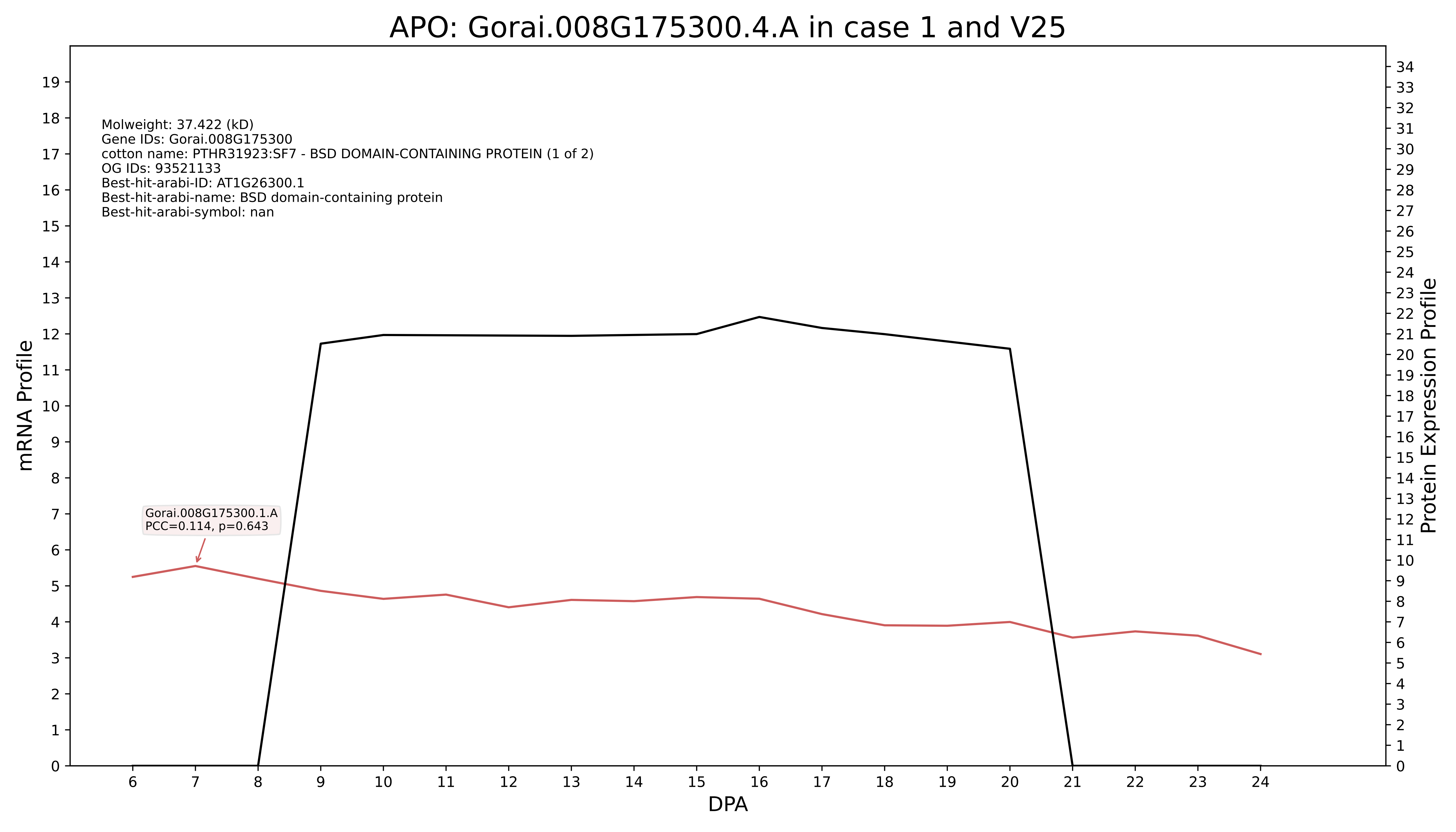
APO: Gorai.008G175300.4.A in case 1 and V25
Molweight: 37.422 (kD)
Gene IDs: Gorai.008G175300
cotton name: PTHR31923:SF7 - BSD DOMAIN-CONTAINING PROTEIN (1 of 2)
OG IDs: 93521133
Best-hit-arabi-ID: AT1G26300.1
Best-hit-arabi-name: BSD domain-containing protein
Best-hit-arabi-symbol: nan
Gorai.008G175300.1.A
PCC=0.114, p=0.643
mRNA Profile
Protein Expression Profile
DPA

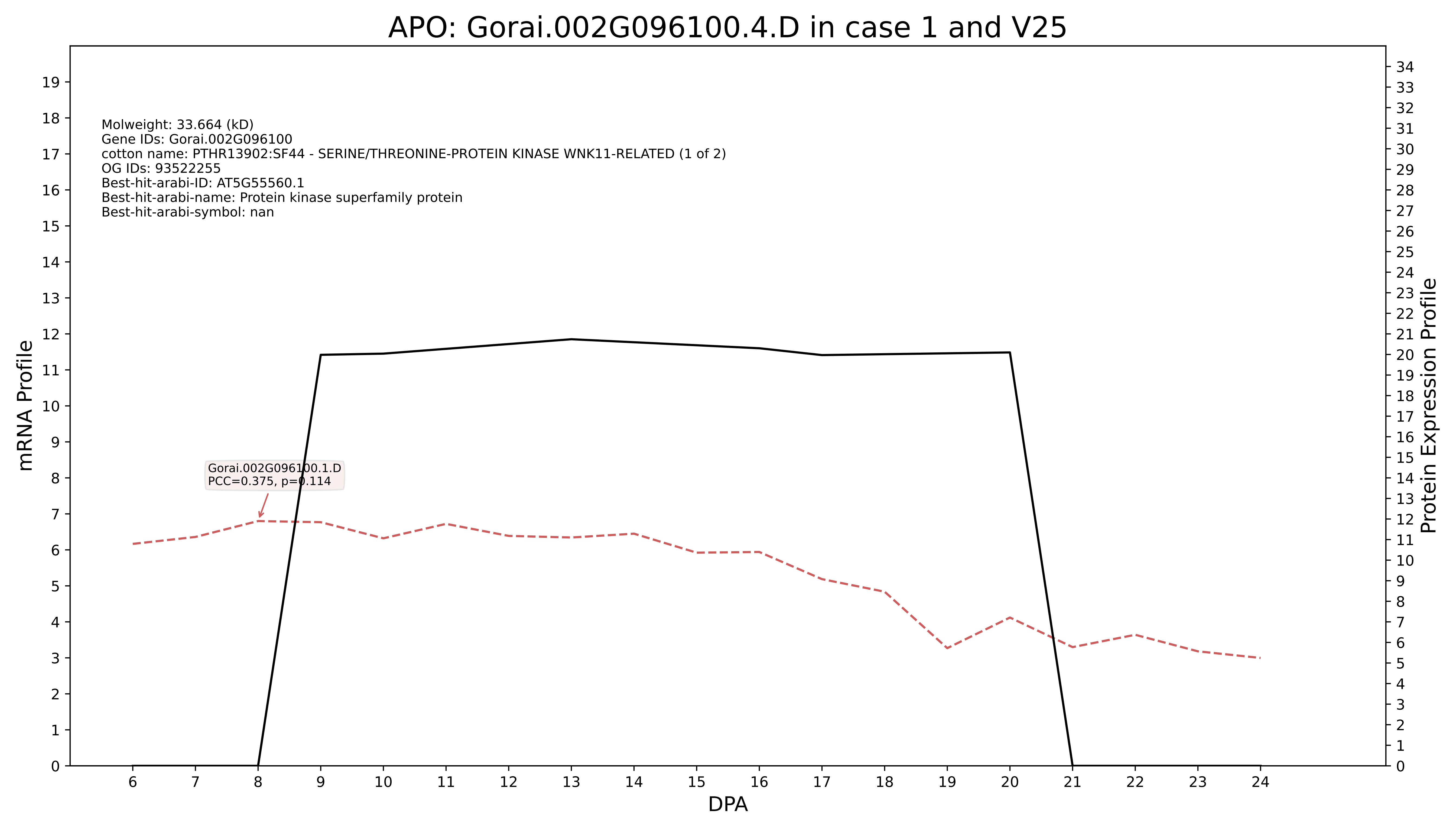

APO: Gorai.002G096100.4.D in case 1 and V25
Molweight: 33.664 (kD)
Gene IDs: Gorai.002G096100
cotton name: PTHR13902:SF44 - SERINE/THREONINE-PROTEIN KINASE WNK11-RELATED (1 of 2)
OG IDs: 93522255
Best-hit-arabi-ID: AT5G55560.1
Best-hit-arabi-name: Protein kinase superfamily protein
Best-hit-arabi-symbol: nan
Gorai.002G096100.1.D
PCC=0.375, p=0.114
mRNA Profile
Protein Expression Profile
DPA
0
1
2
3
4
5
6
7
8
9
10
11
12
13
14
15
16
17
18
19
20
21
22
23
24
25
26
27
28
29
30
31
32
33
34

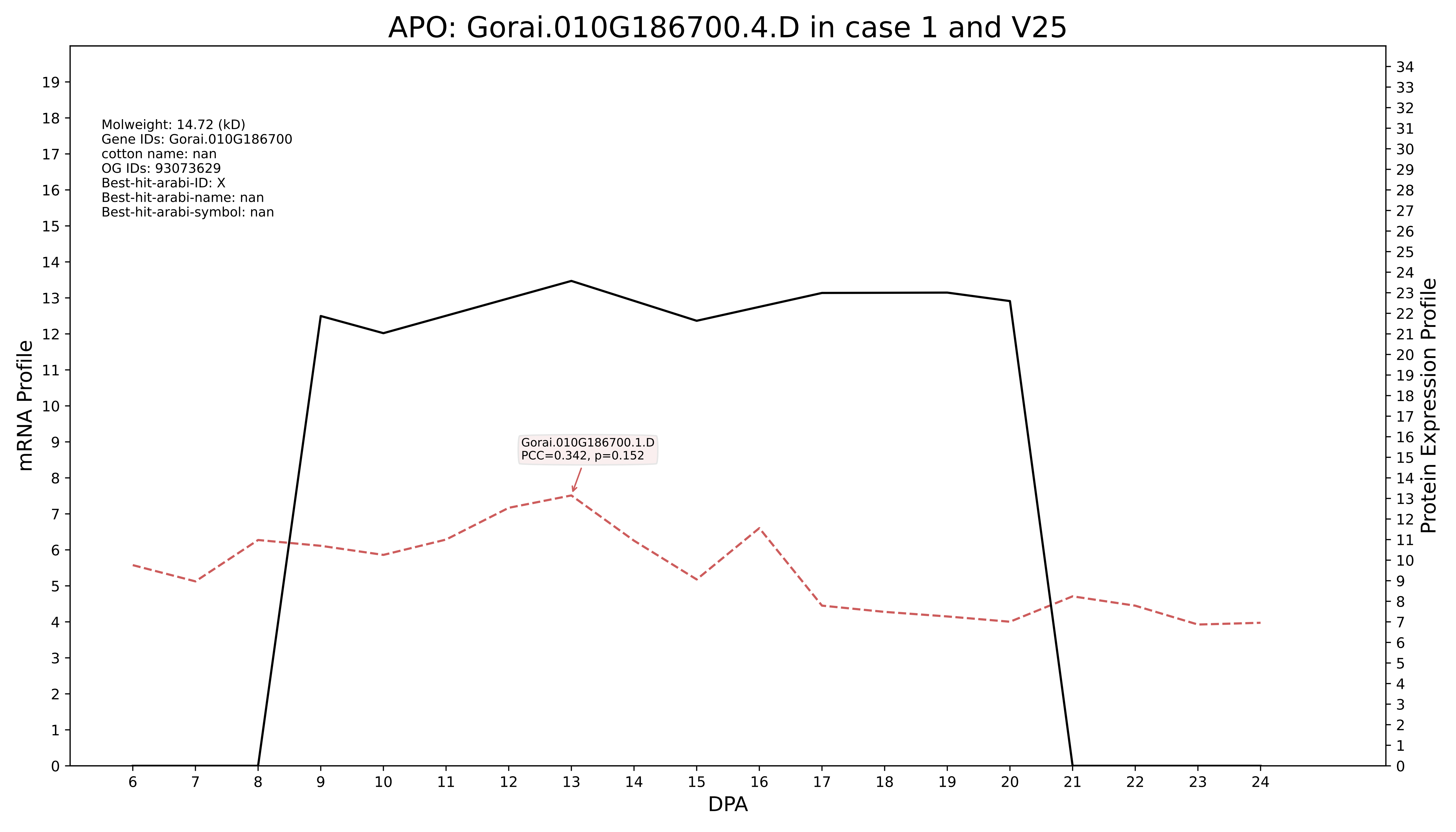
APO: Gorai.010G186700.4.D in case 1 and V25
Molweight: 14.72 (kD)
Gene IDs: Gorai.010G186700
cotton name: nan
OG IDs: 93073629
Best-hit-arabi-ID: X
Best-hit-arabi-name: nan
Best-hit-arabi-symbol: nan
Gorai.010G186700.1.D
PCC=0.342, p=0.152
mRNA Profile
Protein Expression Profile
DPA

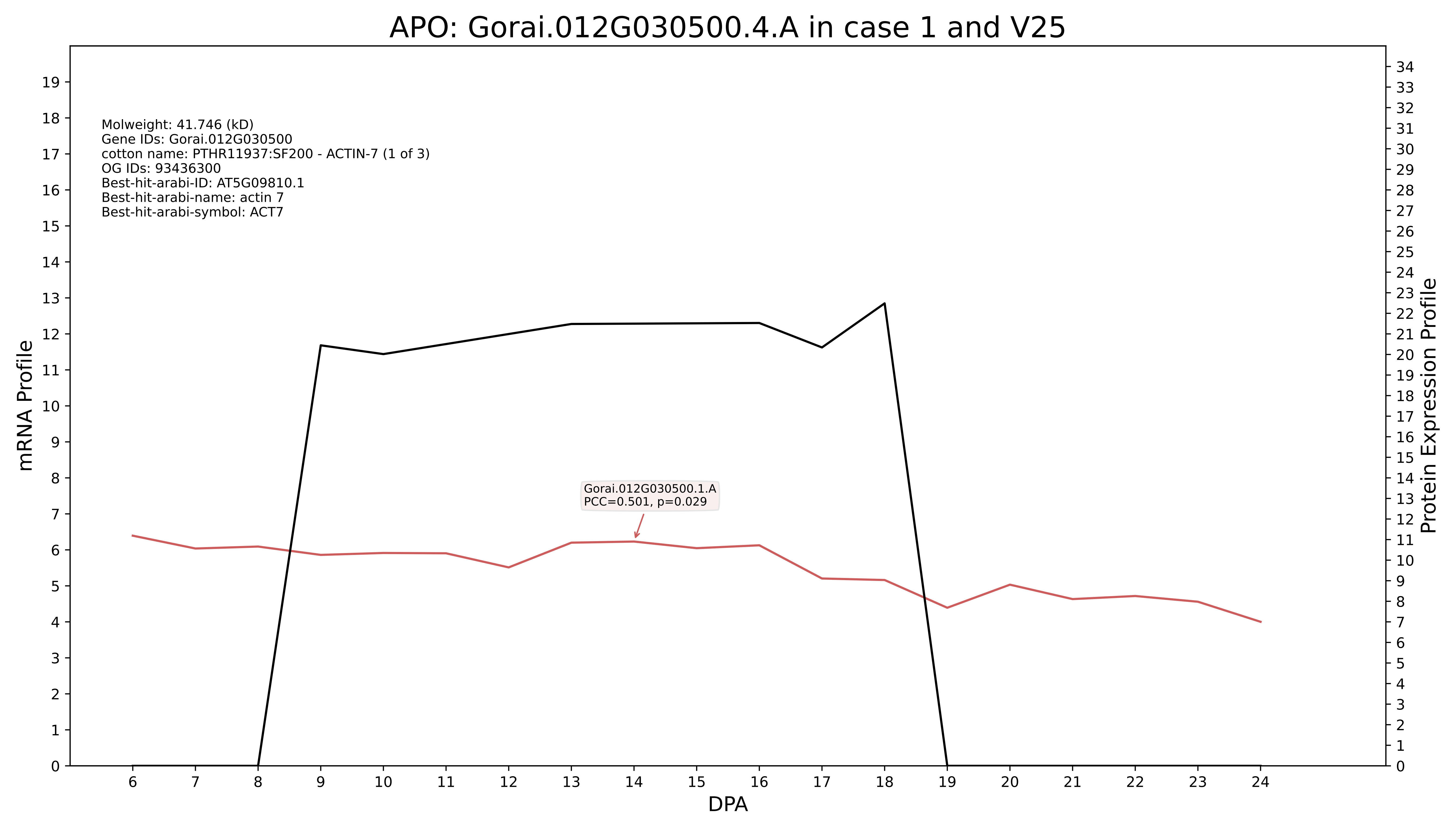

APO: Gorai.012G030500.4.A in case 1 and V25
Molweight: 41.746 (kD)
Gene IDs: Gorai.012G030500
cotton name: PTHR11937:SF200 - ACTIN-7 (1 of 3)
OG IDs: 93436300
Best-hit-arabi-ID: AT5G09810.1
Best-hit-arabi-name: actin 7
Best-hit-arabi-symbol: ACT7
Gorai.012G030500.1.A
PCC=0.501, p=0.029
mRNA Profile
Protein Expression Profile
DPA

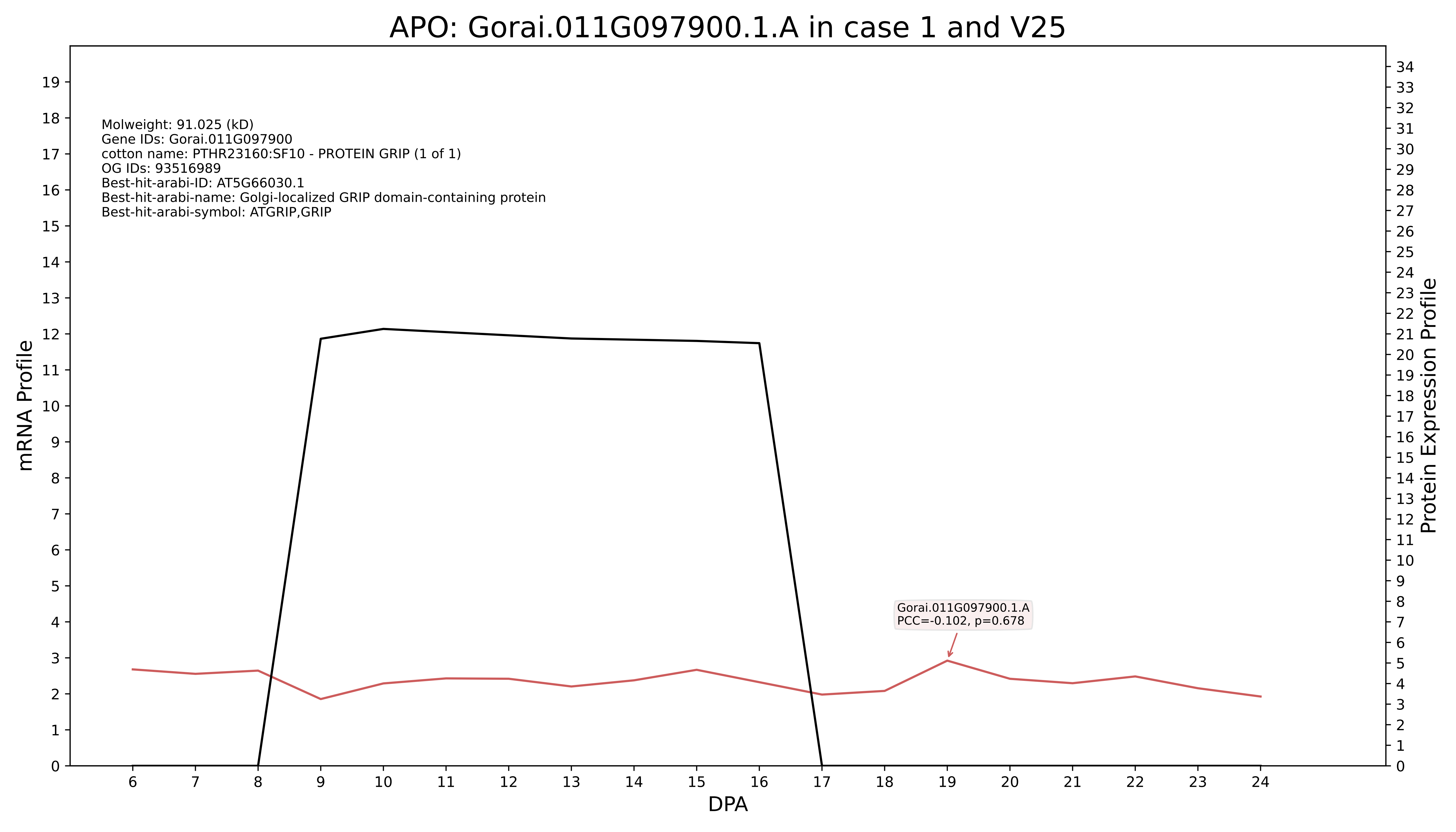
APO: Gorai.011G097900.1.A in case 1 and V25
Molweight: 91.025 (kD)
Gene IDs: Gorai.011G097900
cotton name: PTHR23160:SF10 - PROTEIN GRIP (1 of 1)
OG IDs: 93516989
Best-hit-arabi-ID: AT5G66030.1
Best-hit-arabi-name: Golgi-localized GRIP domain-containing protein
Best-hit-arabi-symbol: ATGRIP,GRIP
Gorai.011G097900.1.A
PCC=-0.102, p=0.678
mRNA Profile
Protein Expression Profile
DPA

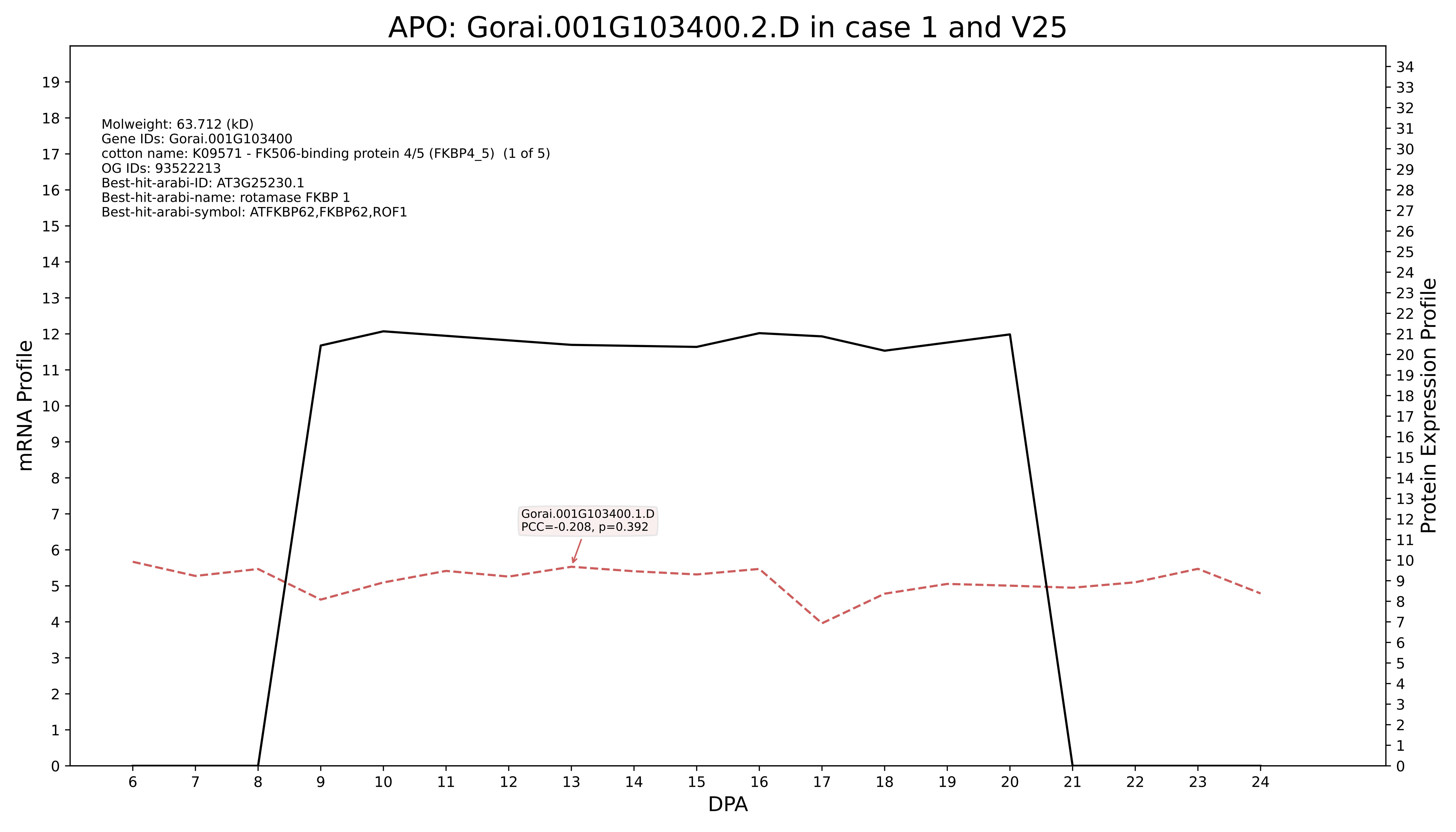
APO: Gorai.001G103400.2.D in case 1 and V25
Molweight: 63.712 (kD)
Gene IDs: Gorai.001G103400
cotton name: K09571 - FK506-binding protein 4/5 (FKBP4_5) (1 of 5)
OG IDs: 93522213
Best-hit-arabi-ID: AT3G25230.1
Best-hit-arabi-name: rotamase FKBP 1
Best-hit-arabi-symbol: ATFKBP62,FKBP62,ROF1
Gorai.001G103400.1.D
PCC=-0.208, p=0.392
mRNA Profile
Protein Expression Profile
DPA

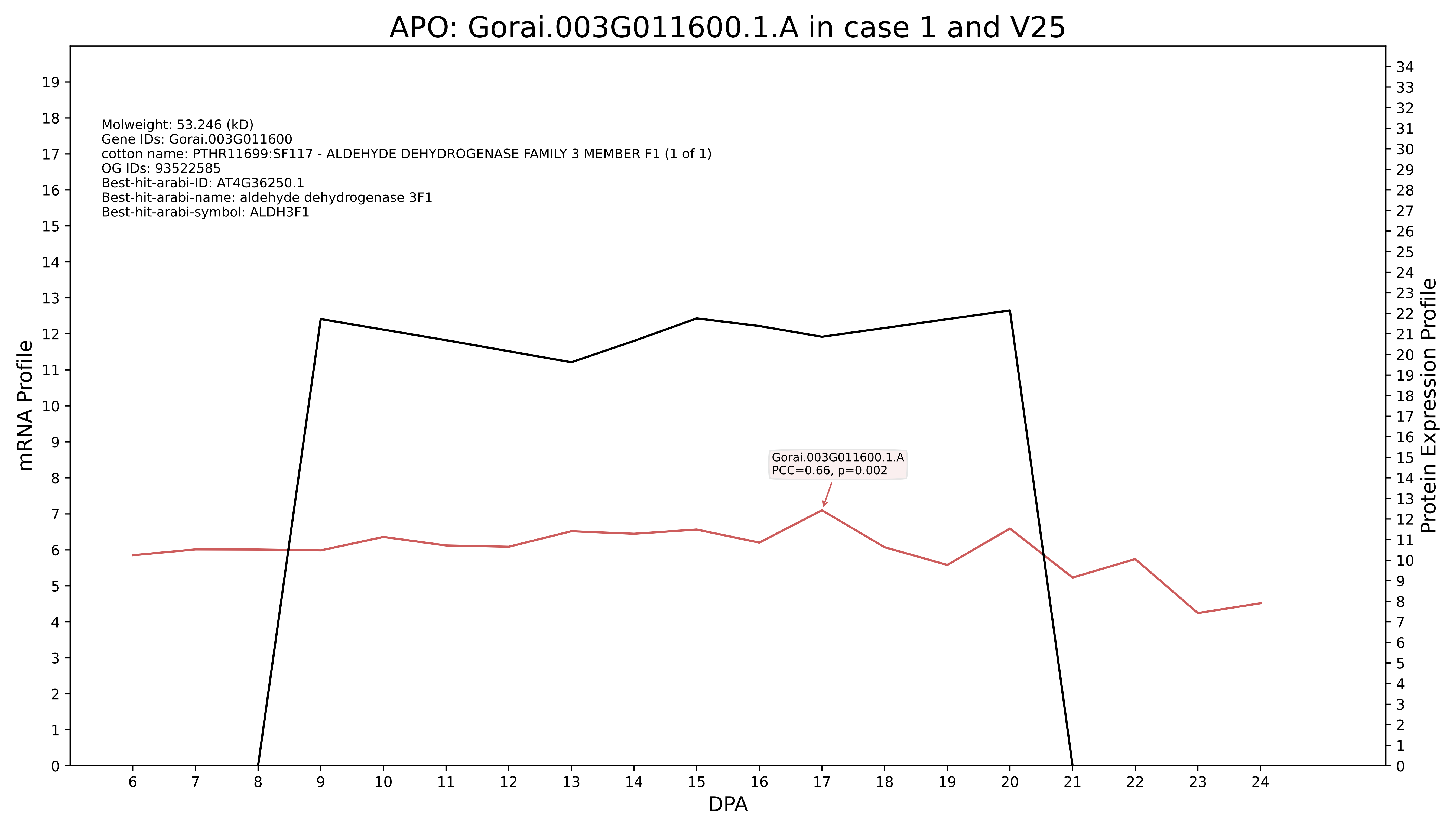
APO: Gorai.003G011600.1.A in case 1 and V25
Molweight: 53.246 (kD)
Gene IDs: Gorai.003G011600
cotton name: PTHR11699:SF117 - ALDEHYDE DEHYDROGENASE FAMILY 3 MEMBER F1 (1 of 1)
OG IDs: 93522585
Best-hit-arabi-ID: AT4G36250.1
Best-hit-arabi-name: aldehyde dehydrogenase 3F1
Best-hit-arabi-symbol: ALDH3F1
Gorai.003G011600.1.A
PCC=0.66, p=0.002
mRNA Profile
Protein Expression Profile
DPA

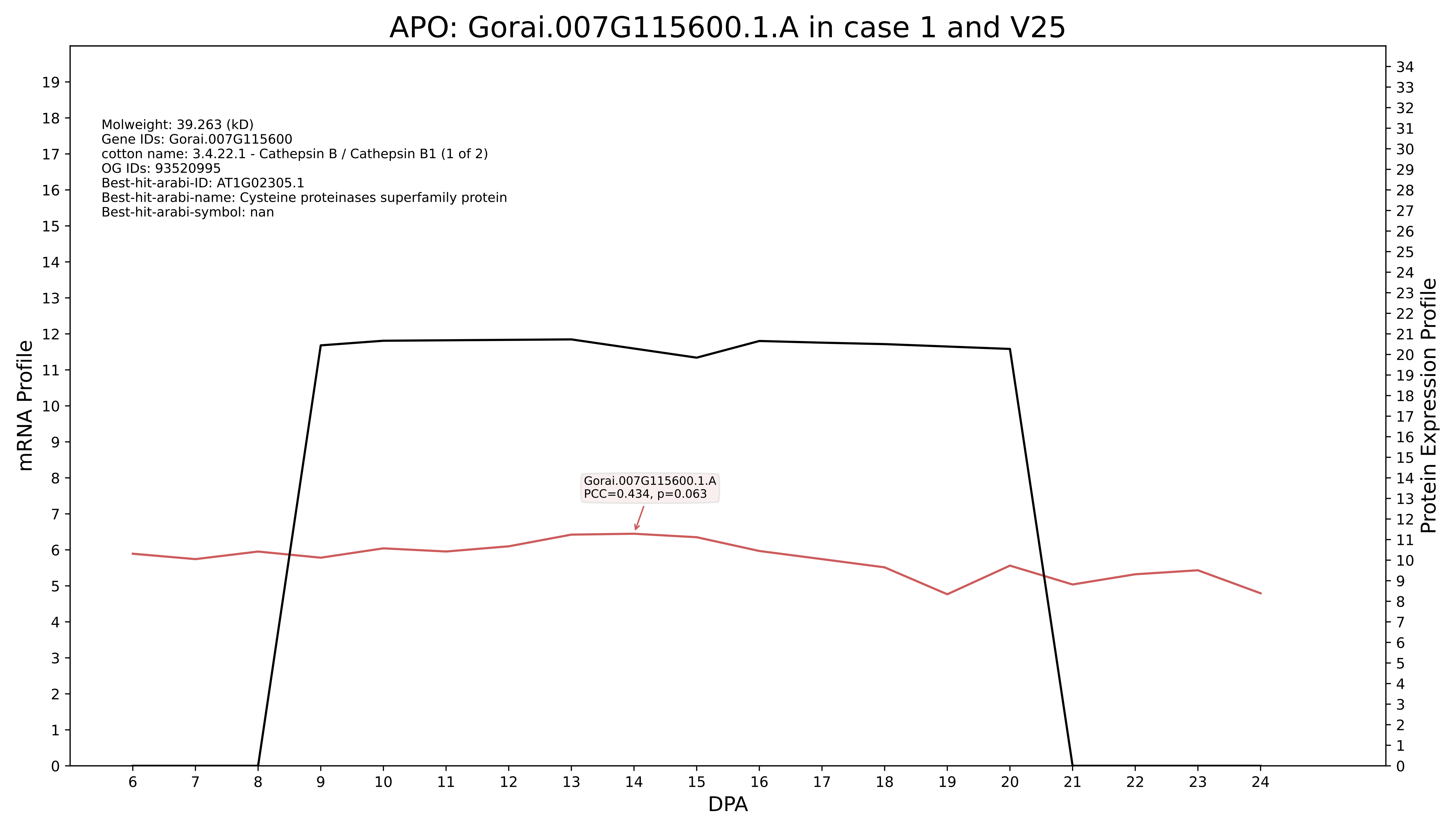

APO: Gorai.007G115600.1.A in case 1 and V25
Molweight: 39.263 (kD)
Gene IDs: Gorai.007G115600
cotton name: 3.4.22.1 - Cathepsin B / Cathepsin B1 (1 of 2)
OG IDs: 93520995
Best-hit-arabi-ID: AT1G02305.1
Best-hit-arabi-name: Cysteine proteinases superfamily protein
Best-hit-arabi-symbol: nan
Gorai.007G115600.1.A
PCC=0.434, p=0.063
mRNA Profile
Protein Expression Profile
DPA

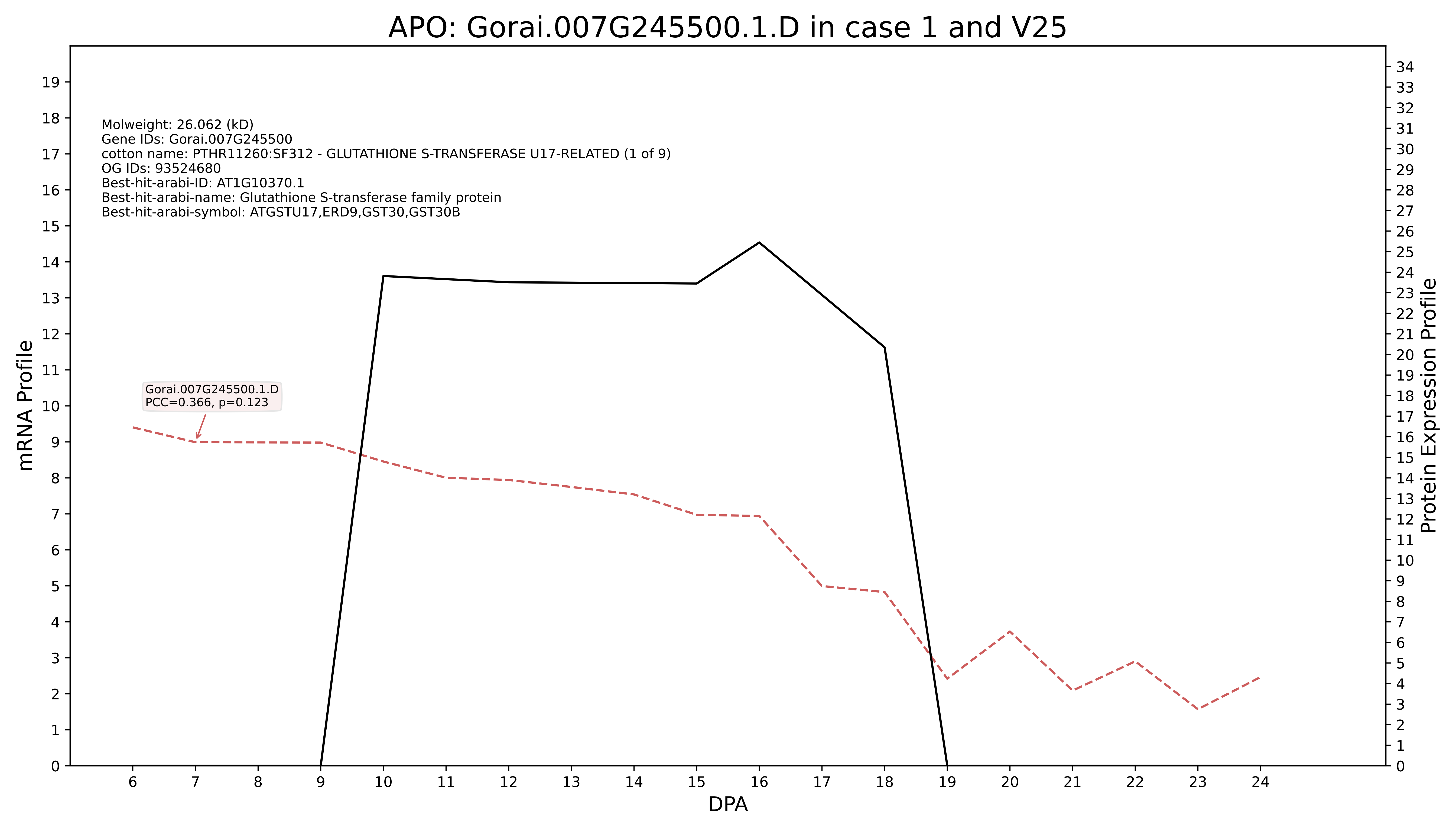
APO: Gorai.007G245500.1.D in case 1 and V25
Molweight: 26.062 (kD)
Gene IDs: Gorai.007G245500
cotton name: PTHR11260:SF312 - GLUTATHIONE S-TRANSFERASE U17-RELATED (1 of 9)
OG IDs: 93524680
Best-hit-arabi-ID: AT1G10370.1
Best-hit-arabi-name: Glutathione S-transferase family protein
Best-hit-arabi-symbol: ATGSTU17,ERD9,GST30,GST30B
Gorai.007G245500.1.D
PCC=0.366, p=0.123
mRNA Profile
Protein Expression Profile
DPA
0
1
2
3
4
5
6
7
8
9
10
11
12
13
14
15
16
17
18
19
6
7
8
9
10
11
12
13
14
15
16
17
18
19
20
21
22
23
24
0
1
2
3
4
5
6
7
8
9
10
11
12
13
14
15
16
17
18
19
20
21
22
23
24
25
26
27
28
29
30
31
32
33
34

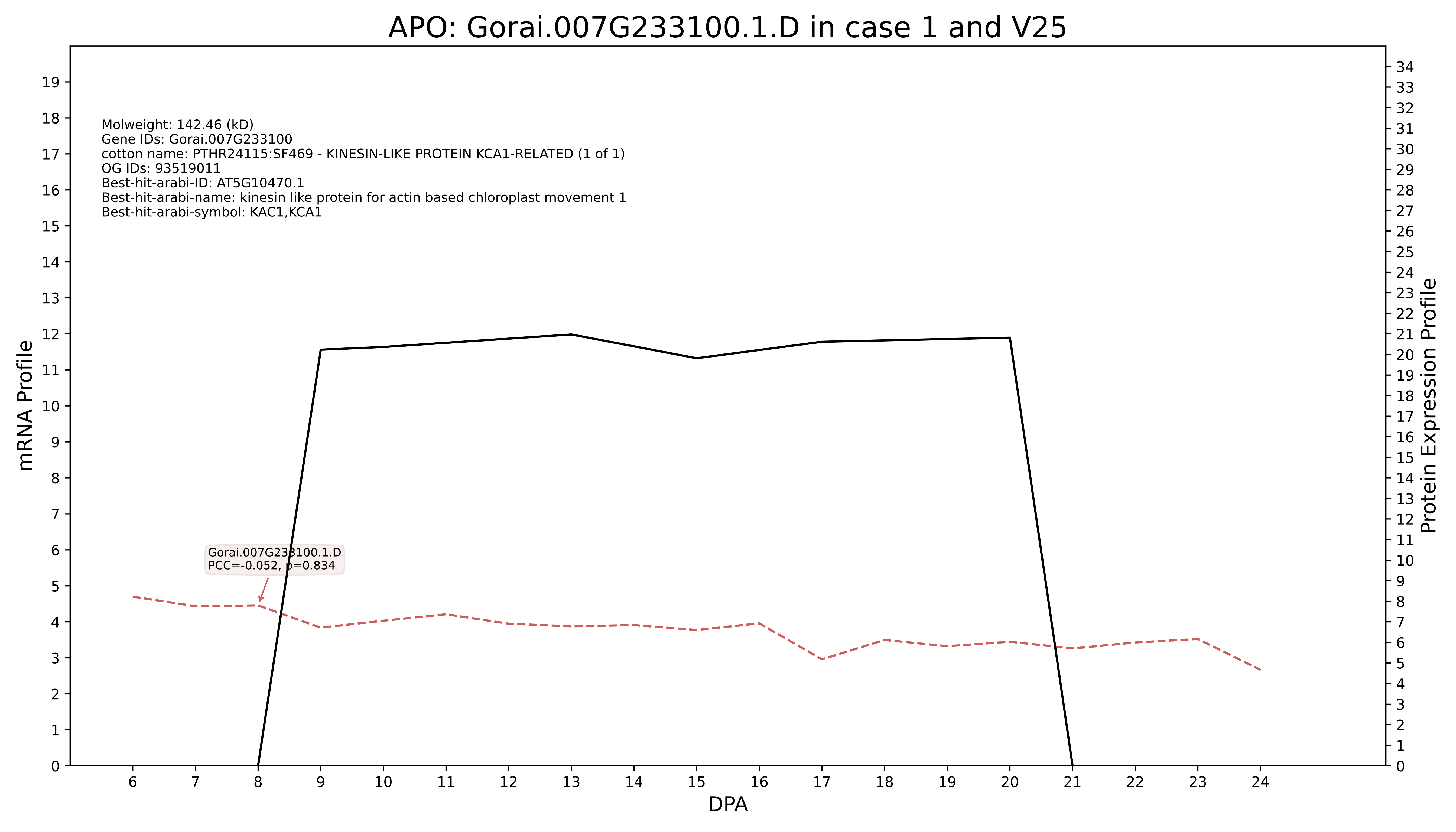
APO: Gorai.007G233100.1.D in case 1 and V25
Molweight: 142.46 (kD)
Gene IDs: Gorai.007G233100
cotton name: PTHR24115:SF469 - KINESIN-LIKE PROTEIN KCA1-RELATED (1 of 1)
OG IDs: 93519011
Best-hit-arabi-ID: AT5G10470.1
Best-hit-arabi-name: kinesin like protein for actin based chloroplast movement 1
Best-hit-arabi-symbol: KAC1,KCA1
Gorai.007G233100.1.D
PCC=-0.052, p=0.834
mRNA Profile
Protein Expression Profile
DPA

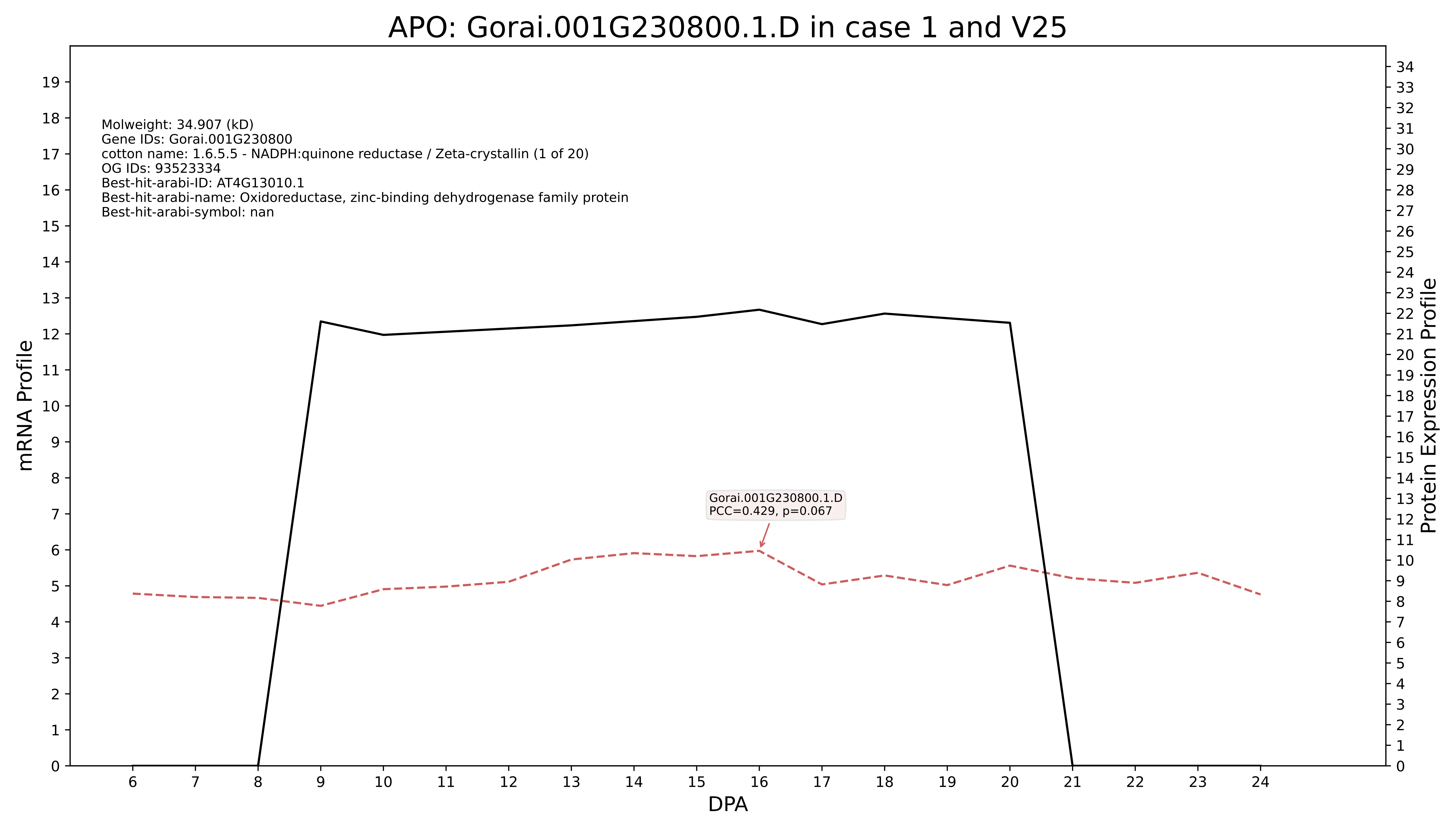
APO: Gorai.001G230800.1.D in case 1 and V25
Molweight: 34.907 (kD)
Gene IDs: Gorai.001G230800
cotton name: 1.6.5.5 - NADPH:quinone reductase / Zeta-crystallin (1 of 20)
OG IDs: 93523334
Best-hit-arabi-ID: AT4G13010.1
Best-hit-arabi-name: Oxidoreductase, zinc-binding dehydrogenase family protein
Best-hit-arabi-symbol: nan
Gorai.001G230800.1.D
PCC=0.429, p=0.067
mRNA Profile
Protein Expression Profile
DPA

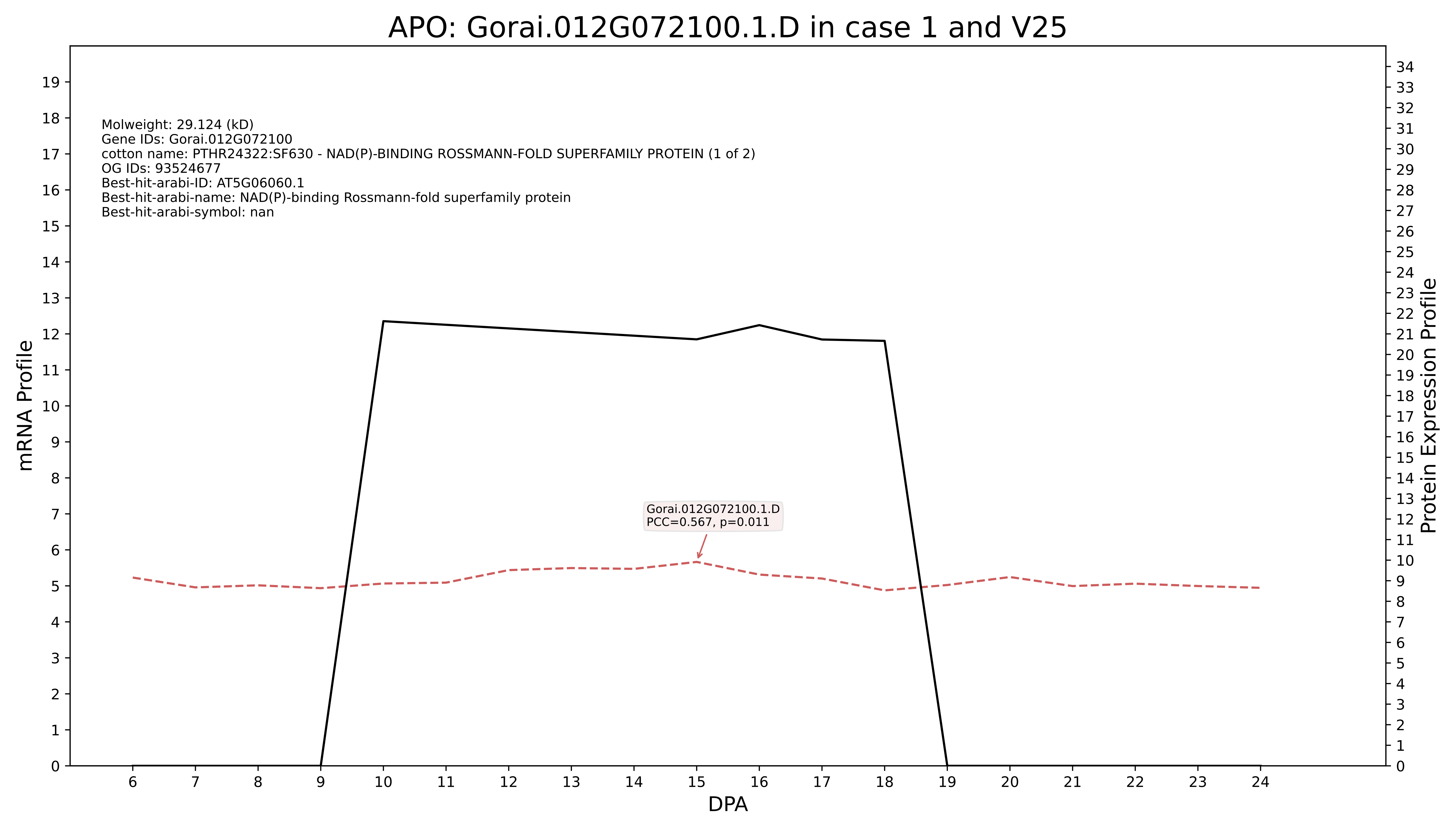
APO: Gorai.012G072100.1.D in case 1 and V25
Molweight: 29.124 (kD)
Gene IDs: Gorai.012G072100
cotton name: PTHR24322:SF630 - NAD(P)-BINDING ROSSMANN-FOLD SUPERFAMILY PROTEIN (1 of 2)
OG IDs: 93524677
Best-hit-arabi-ID: AT5G06060.1
Best-hit-arabi-name: NAD(P)-binding Rossmann-fold superfamily protein
Best-hit-arabi-symbol: nan
Gorai.012G072100.1.D
PCC=0.567, p=0.011
mRNA Profile
Protein Expression Profile
DPA

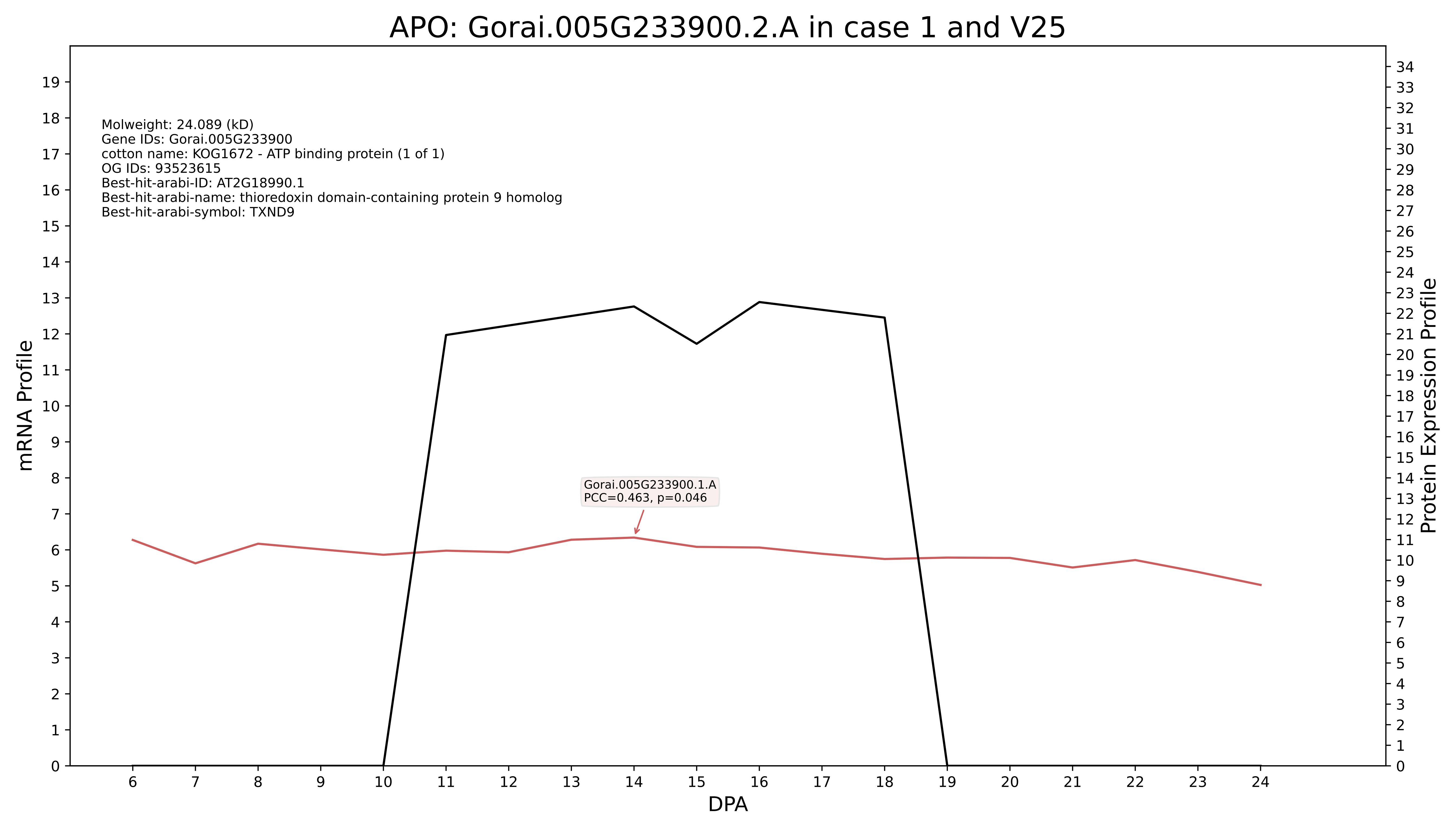

APO: Gorai.005G233900.2.A in case 1 and V25
Molweight: 24.089 (kD)
Gene IDs: Gorai.005G233900
cotton name: KOG1672 - ATP binding protein (1 of 1)
OG IDs: 93523615
Best-hit-arabi-ID: AT2G18990.1
Best-hit-arabi-name: thioredoxin domain-containing protein 9 homolog
Best-hit-arabi-symbol: TXND9
Gorai.005G233900.1.A
PCC=0.463, p=0.046
mRNA Profile
Protein Expression Profile
DPA
0
1
2
3
4
5
6
7
8
9
10
11
12
13
14
15
16
17
18
19
20
21
22
23
24
25
26
27
28
29
30
31
32
33
34

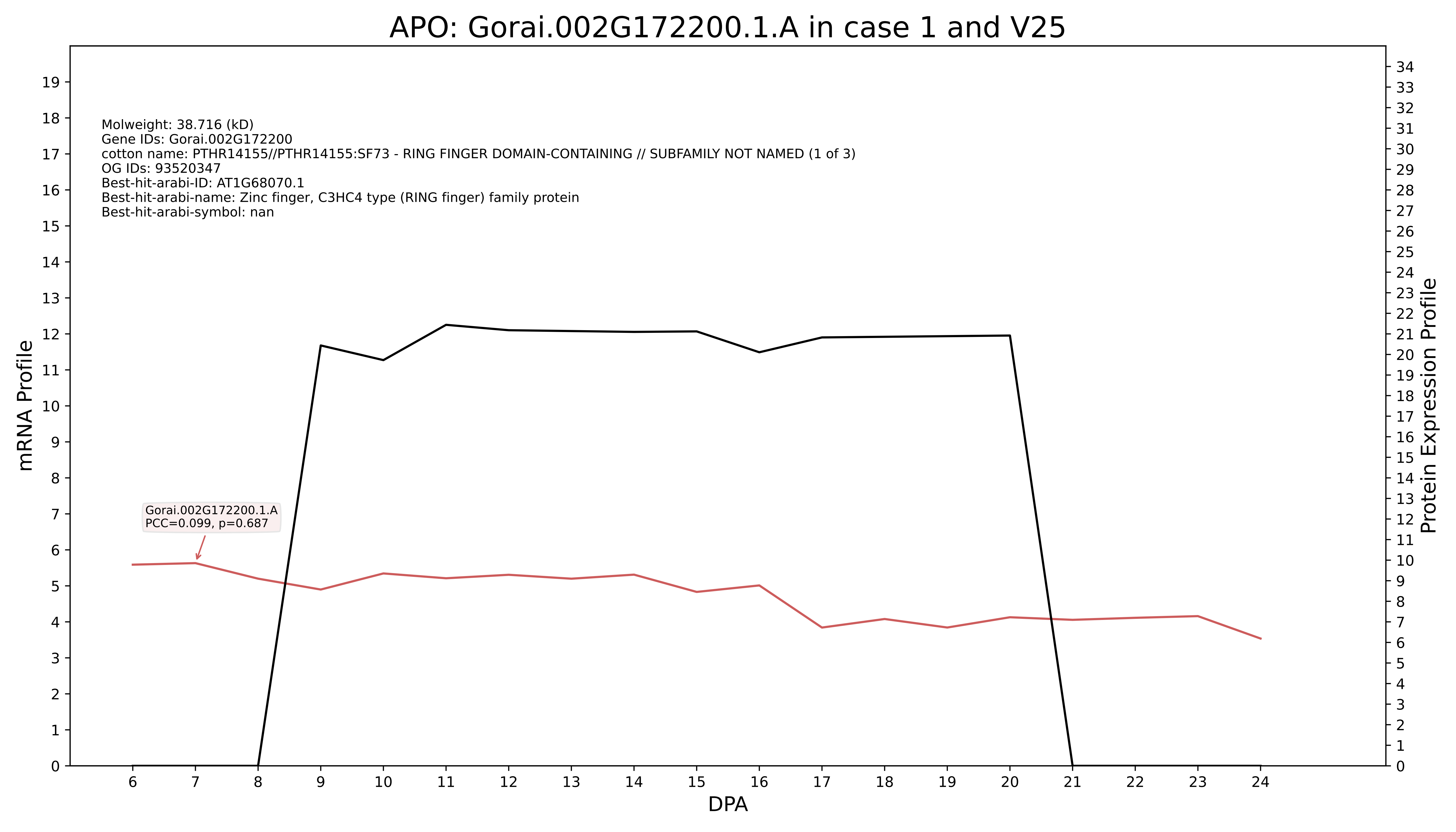
APO: Gorai.002G172200.1.A in case 1 and V25
Molweight: 38.716 (kD)
Gene IDs: Gorai.002G172200
cotton name: PTHR14155//PTHR14155:SF73 - RING FINGER DOMAIN-CONTAINING // SUBFAMILY NOT NAMED (1 of 3)
OG IDs: 93520347
Best-hit-arabi-ID: AT1G68070.1
Best-hit-arabi-name: Zinc finger, C3HC4 type (RING finger) family protein
Best-hit-arabi-symbol: nan
Gorai.002G172200.1.A
PCC=0.099, p=0.687
mRNA Profile
Protein Expression Profile
DPA

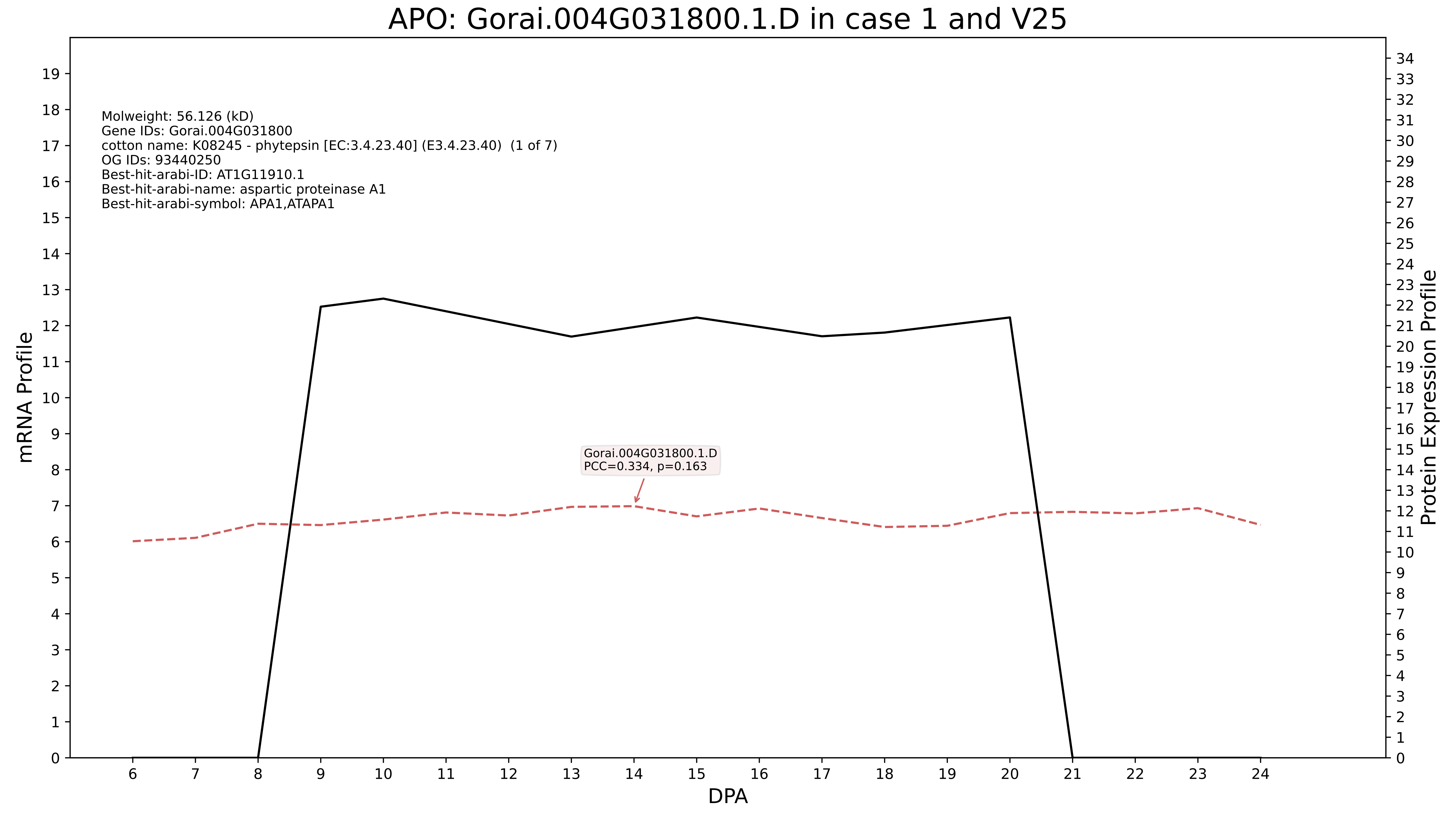

APO: Gorai.004G031800.1.D in case 1 and V25
Molweight: 56.126 (kD)
Gene IDs: Gorai.004G031800
cotton name: K08245 - phytepsin [EC:3.4.23.40] (E3.4.23.40) (1 of 7)
OG IDs: 93440250
Best-hit-arabi-ID: AT1G11910.1
Best-hit-arabi-name: aspartic proteinase A1
Best-hit-arabi-symbol: APA1,ATAPA1
Gorai.004G031800.1.D
PCC=0.334, p=0.163
mRNA Profile
Protein Expression Profile
DPA

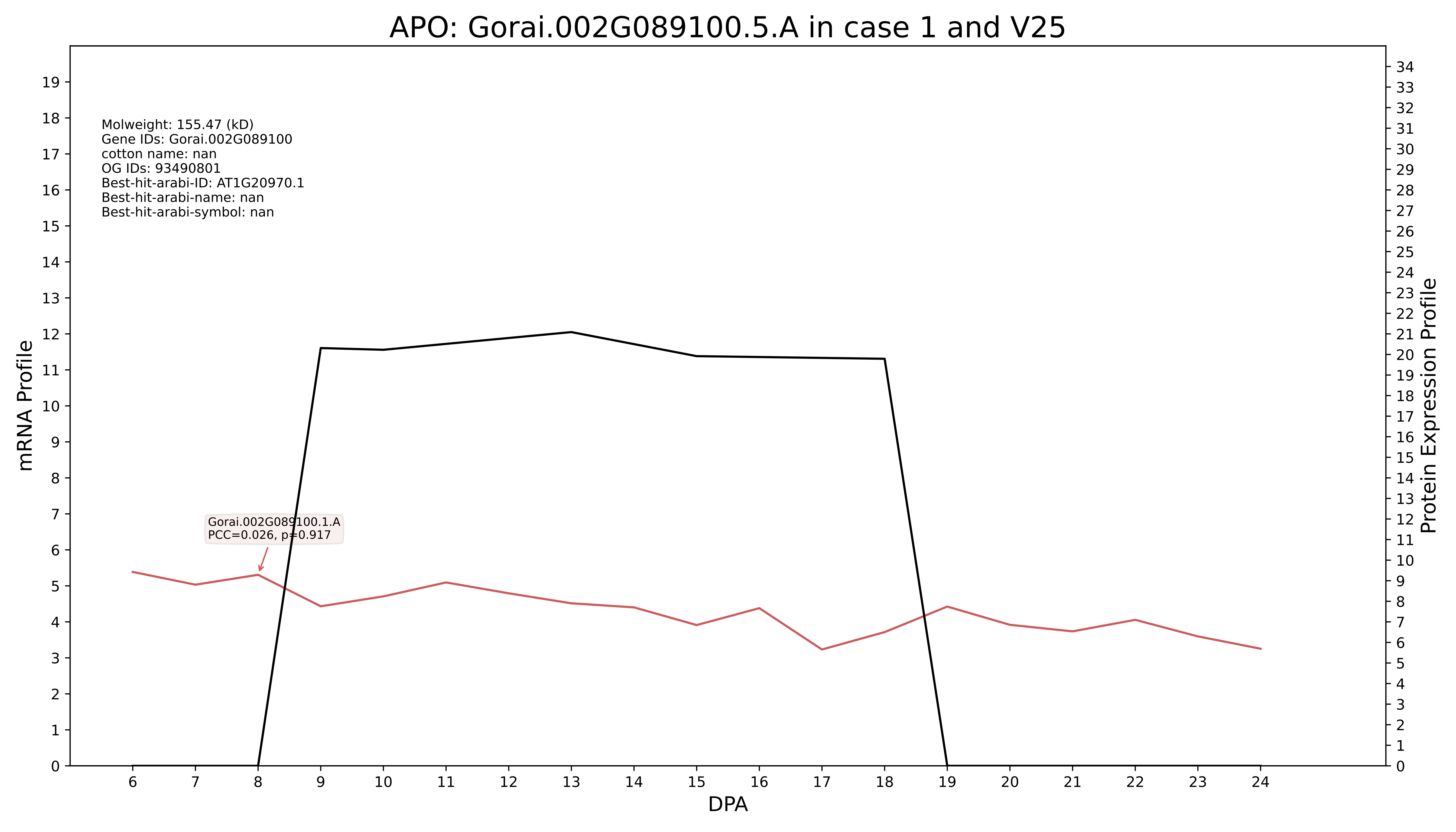

APO: Gorai.002G089100.5.A in case 1 and V25
Molweight: 155.47 (kD)
Gene IDs: Gorai.002G089100
cotton name: nan
OG IDs: 93490801
Best-hit-arabi-ID: AT1G20970.1
Best-hit-arabi-name: nan
Best-hit-arabi-symbol: nan
Gorai.002G089100.1.A
PCC=0.026, p=0.917
mRNA Profile
Protein Expression Profile
DPA

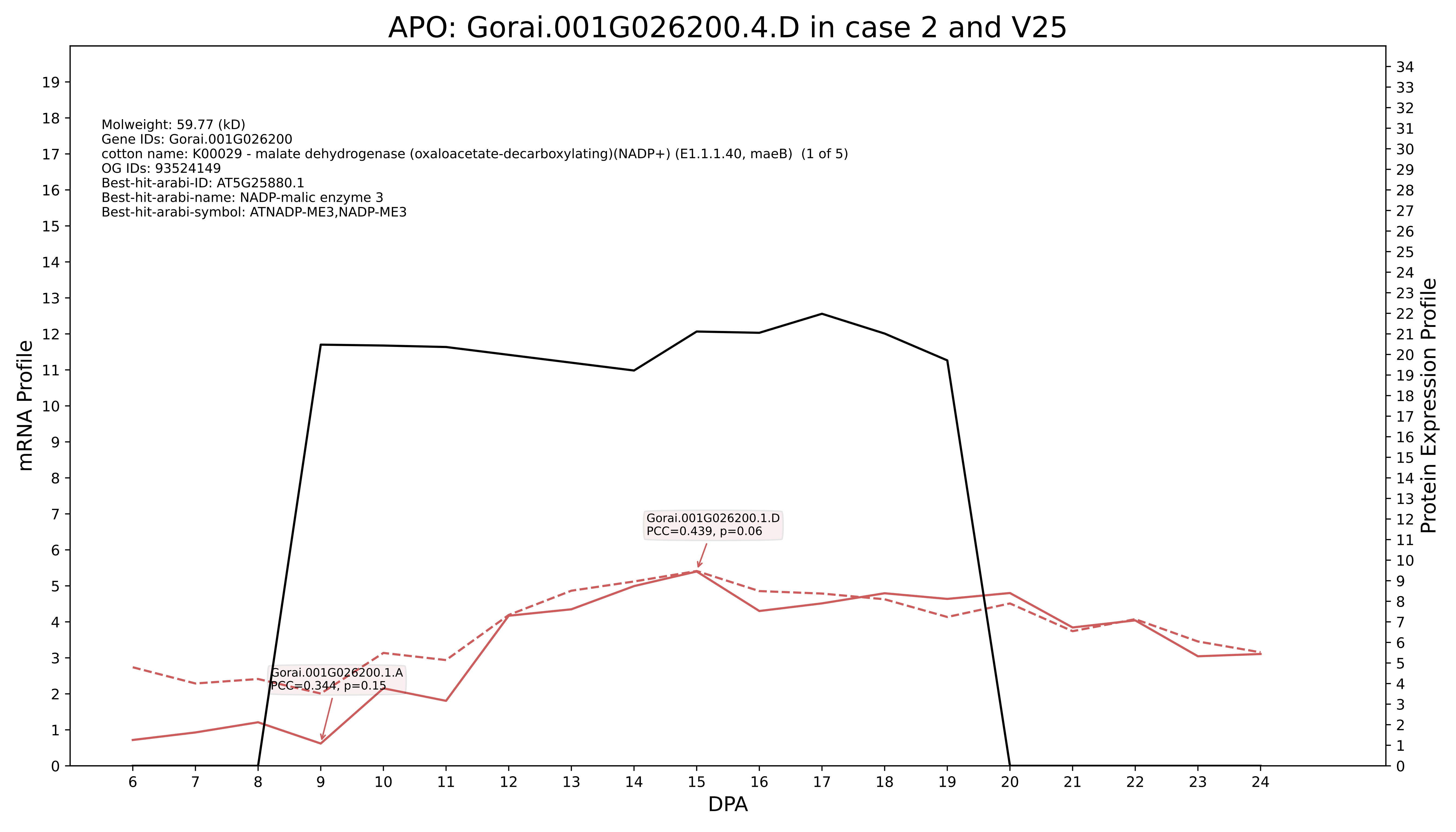

APO: Gorai.001G026200.4.D in case 2 and V25
Molweight: 59.77 (kD)
Gene IDs: Gorai.001G026200
cotton name: K00029 - malate dehydrogenase (oxaloacetate-decarboxylating)(NADP+) (E1.1.1.40, maeB) (1 of 5)
OG IDs: 93524149
Best-hit-arabi-ID: AT5G25880.1
Best-hit-arabi-name: NADP-malic enzyme 3
Best-hit-arabi-symbol: ATNADP-ME3,NADP-ME3
Gorai.001G026200.1.D
PCC=0.439, p=0.06
Gorai.001G026200.1.A
PCC=0.344, p=0.15
mRNA Profile
Protein Expression Profile
DPA

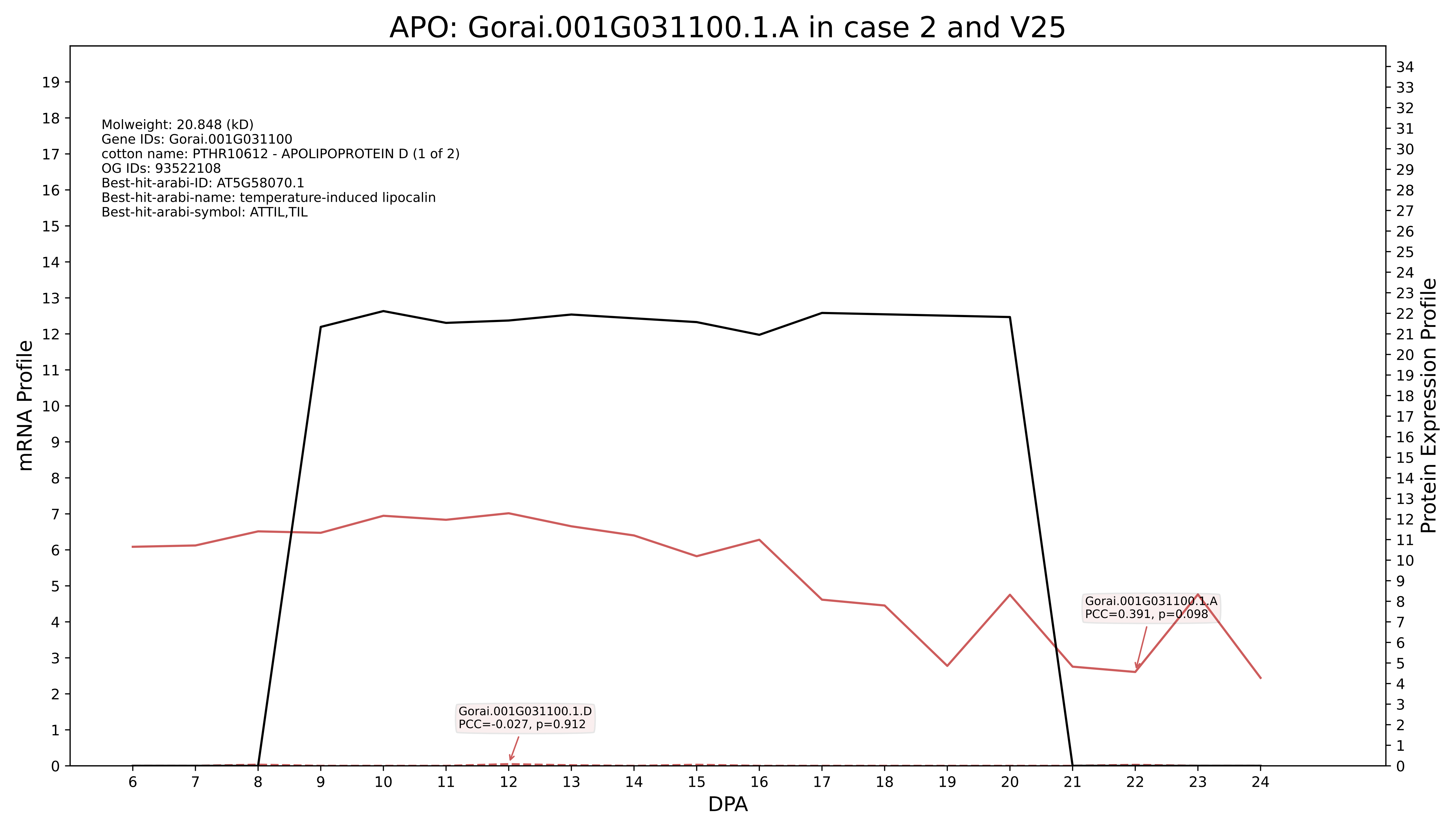

APO: Gorai.001G031100.1.A in case 2 and V25
Molweight: 20.848 (kD)
Gene IDs: Gorai.001G031100
cotton name: PTHR10612 - APOLIPOPROTEIN D (1 of 2)
OG IDs: 93522108
Best-hit-arabi-ID: AT5G58070.1
Best-hit-arabi-name: temperature-induced lipocalin
Best-hit-arabi-symbol: ATTIL,TIL
Gorai.001G031100.1.A
PCC=0.391, p=0.098
Gorai.001G031100.1.D
PCC=-0.027, p=0.912
mRNA Profile
Protein Expression Profile
DPA

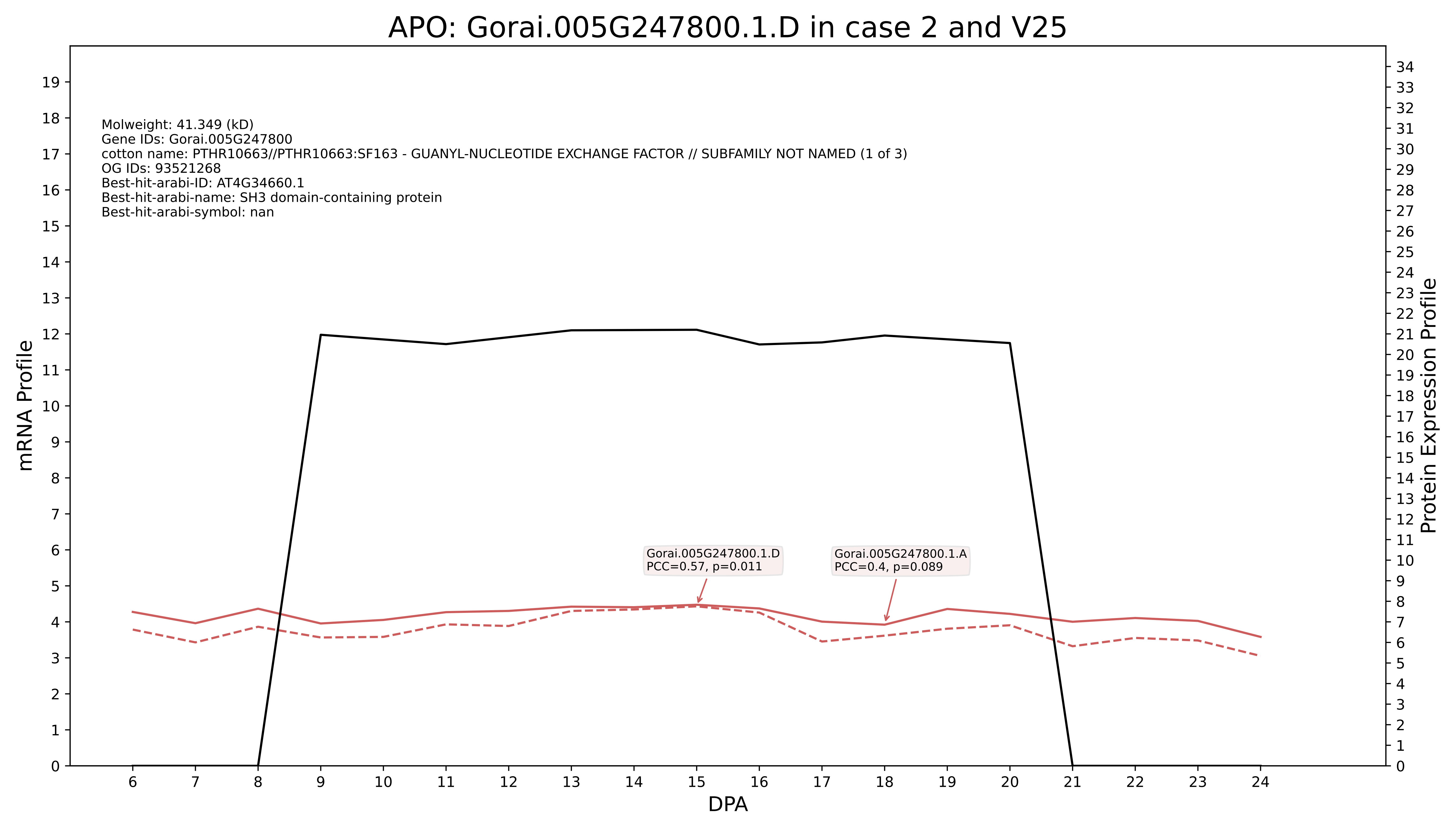
APO: Gorai.005G247800.1.D in case 2 and V25
Molweight: 41.349 (kD)
Gene IDs: Gorai.005G247800
cotton name: PTHR10663//PTHR10663:SF163 - GUANYL-NUCLEOTIDE EXCHANGE FACTOR // SUBFAMILY NOT NAMED (1 of 3)
OG IDs: 93521268
Best-hit-arabi-ID: AT4G34660.1
Best-hit-arabi-name: SH3 domain-containing protein
Best-hit-arabi-symbol: nan
Gorai.005G247800.1.D
PCC=0.57, p=0.011
Gorai.005G247800.1.A
PCC=0.4, p=0.089
mRNA Profile
Protein Expression Profile
DPA
6
7
8
9
10
11
12
13
14
15
16
17
18
19
20
21
22
23
24

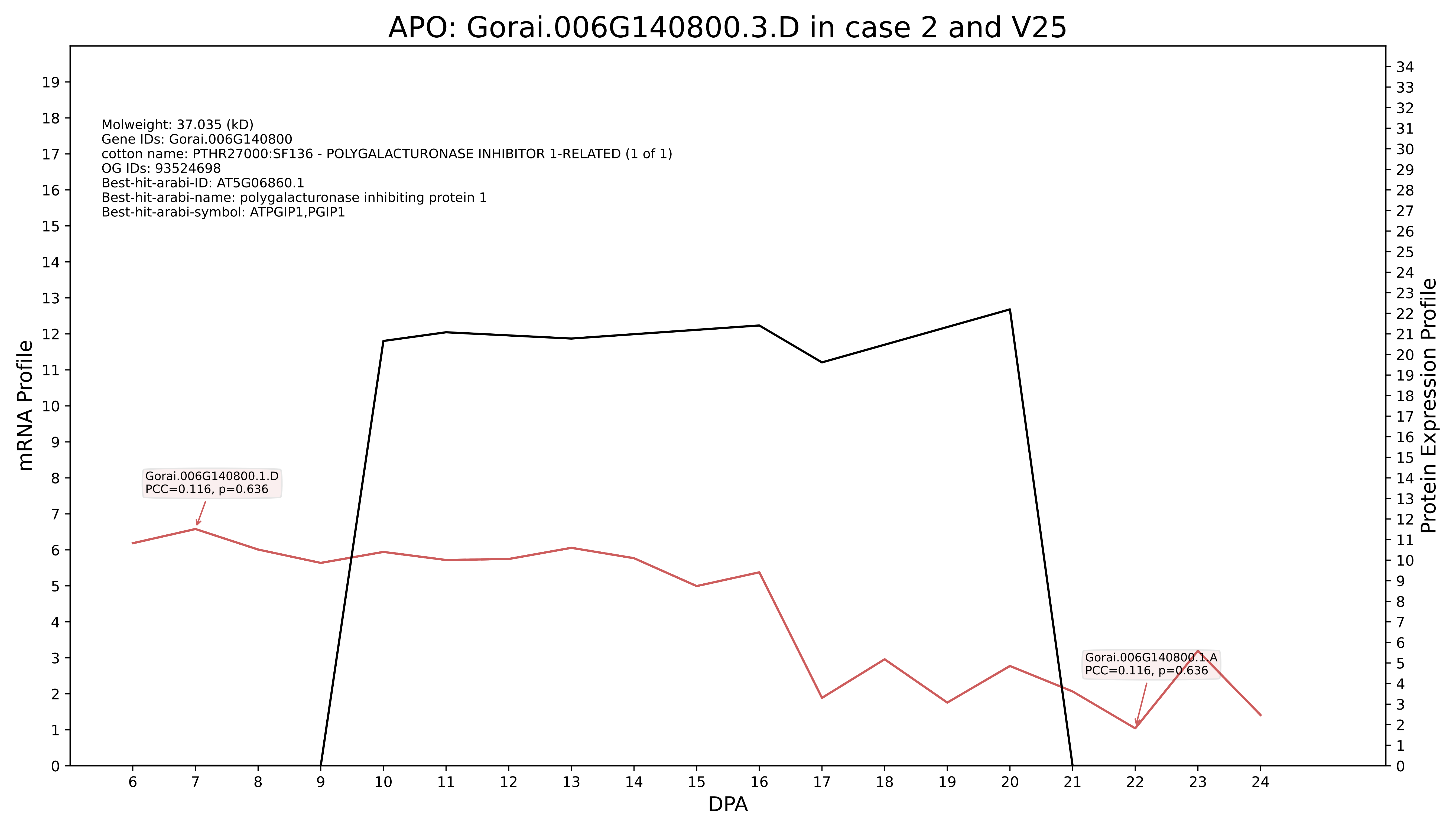

APO: Gorai.006G140800.3.D in case 2 and V25
Molweight: 37.035 (kD)
Gene IDs: Gorai.006G140800
cotton name: PTHR27000:SF136 - POLYGALACTURONASE INHIBITOR 1-RELATED (1 of 1)
OG IDs: 93524698
Best-hit-arabi-ID: AT5G06860.1
Best-hit-arabi-name: polygalacturonase inhibiting protein 1
Best-hit-arabi-symbol: ATPGIP1,PGIP1
Gorai.006G140800.1.D
PCC=0.116, p=0.636
Gorai.006G140800.1.A
PCC=0.116, p=0.636
mRNA Profile
Protein Expression Profile
DPA

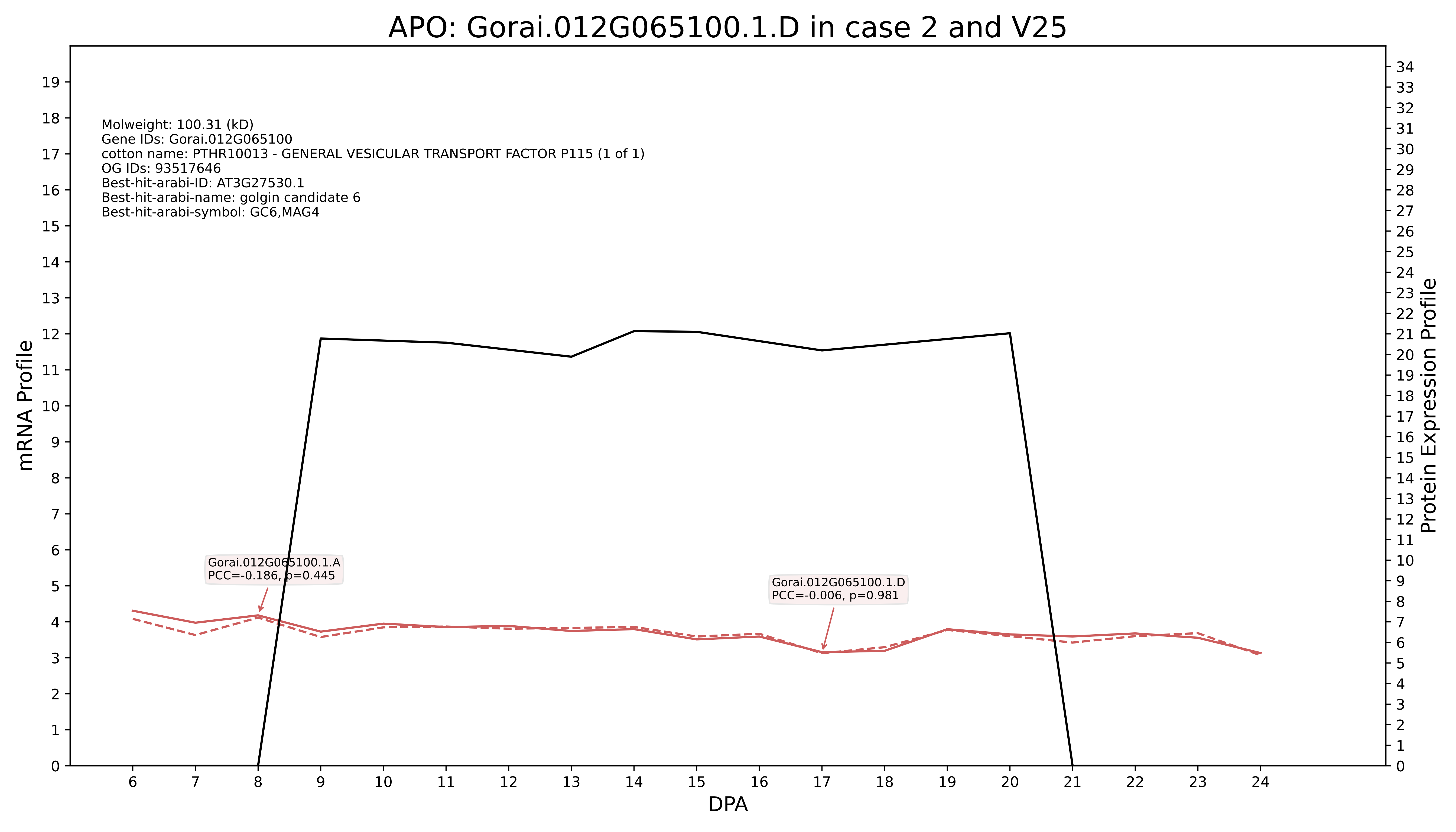

APO: Gorai.012G065100.1.D in case 2 and V25
Molweight: 100.31 (kD)
Gene IDs: Gorai.012G065100
cotton name: PTHR10013 - GENERAL VESICULAR TRANSPORT FACTOR P115 (1 of 1)
OG IDs: 93517646
Best-hit-arabi-ID: AT3G27530.1
Best-hit-arabi-name: golgin candidate 6
Best-hit-arabi-symbol: GC6,MAG4
Gorai.012G065100.1.A
PCC=-0.186, p=0.445
Gorai.012G065100.1.D
PCC=-0.006, p=0.981
mRNA Profile
Protein Expression Profile
DPA

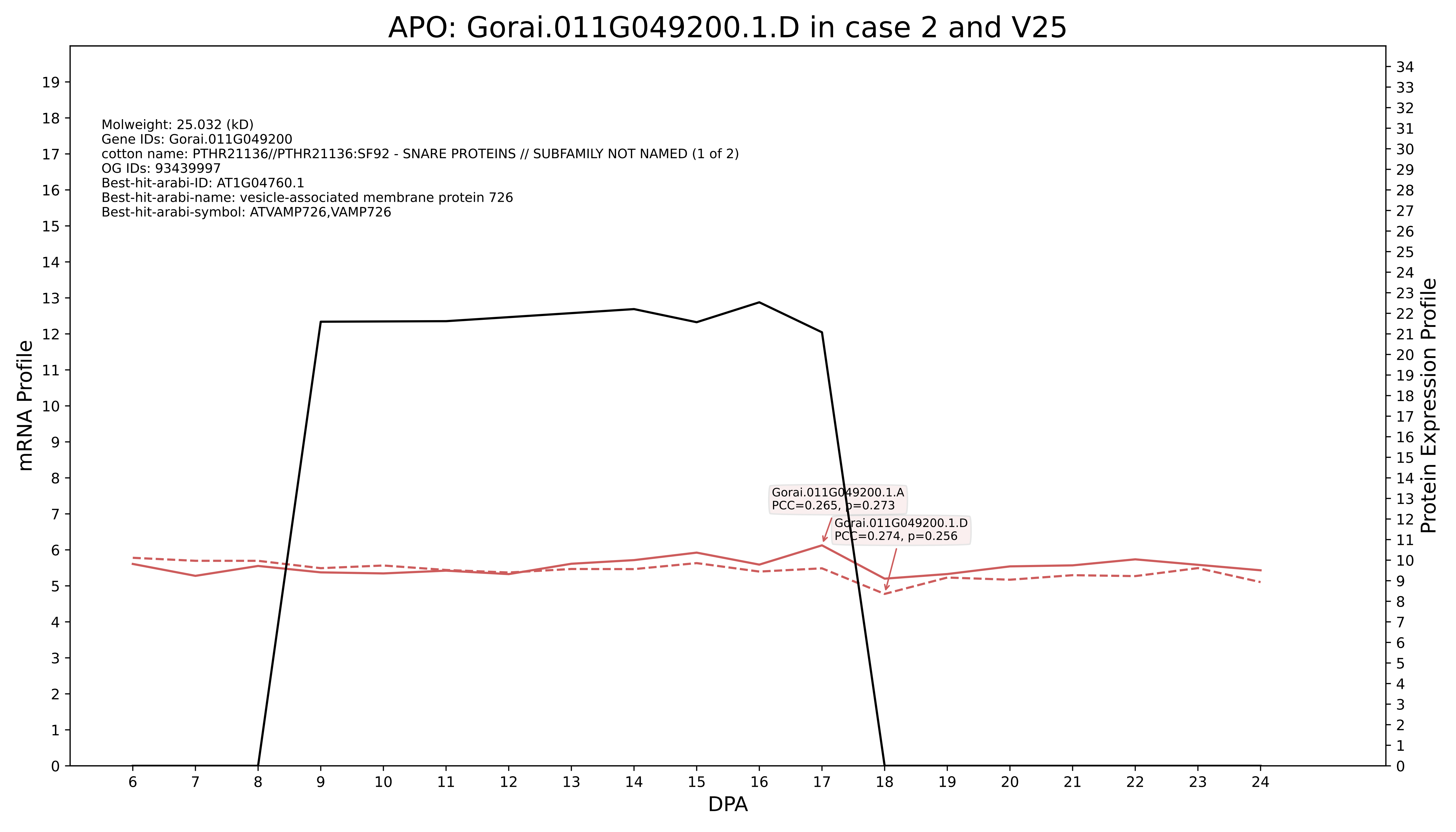
APO: Gorai.011G049200.1.D in case 2 and V25
Molweight: 25.032 (kD)
Gene IDs: Gorai.011G049200
cotton name: PTHR21136//PTHR21136:SF92 - SNARE PROTEINS // SUBFAMILY NOT NAMED (1 of 2)
OG IDs: 93439997
Best-hit-arabi-ID: AT1G04760.1
Best-hit-arabi-name: vesicle-associated membrane protein 726
Best-hit-arabi-symbol: ATVAMP726,VAMP726
Gorai.011G049200.1.A
PCC=0.265, p=0.273
Gorai.011G049200.1.D
PCC=0.274, p=0.256
mRNA Profile
Protein Expression Profile
DPA

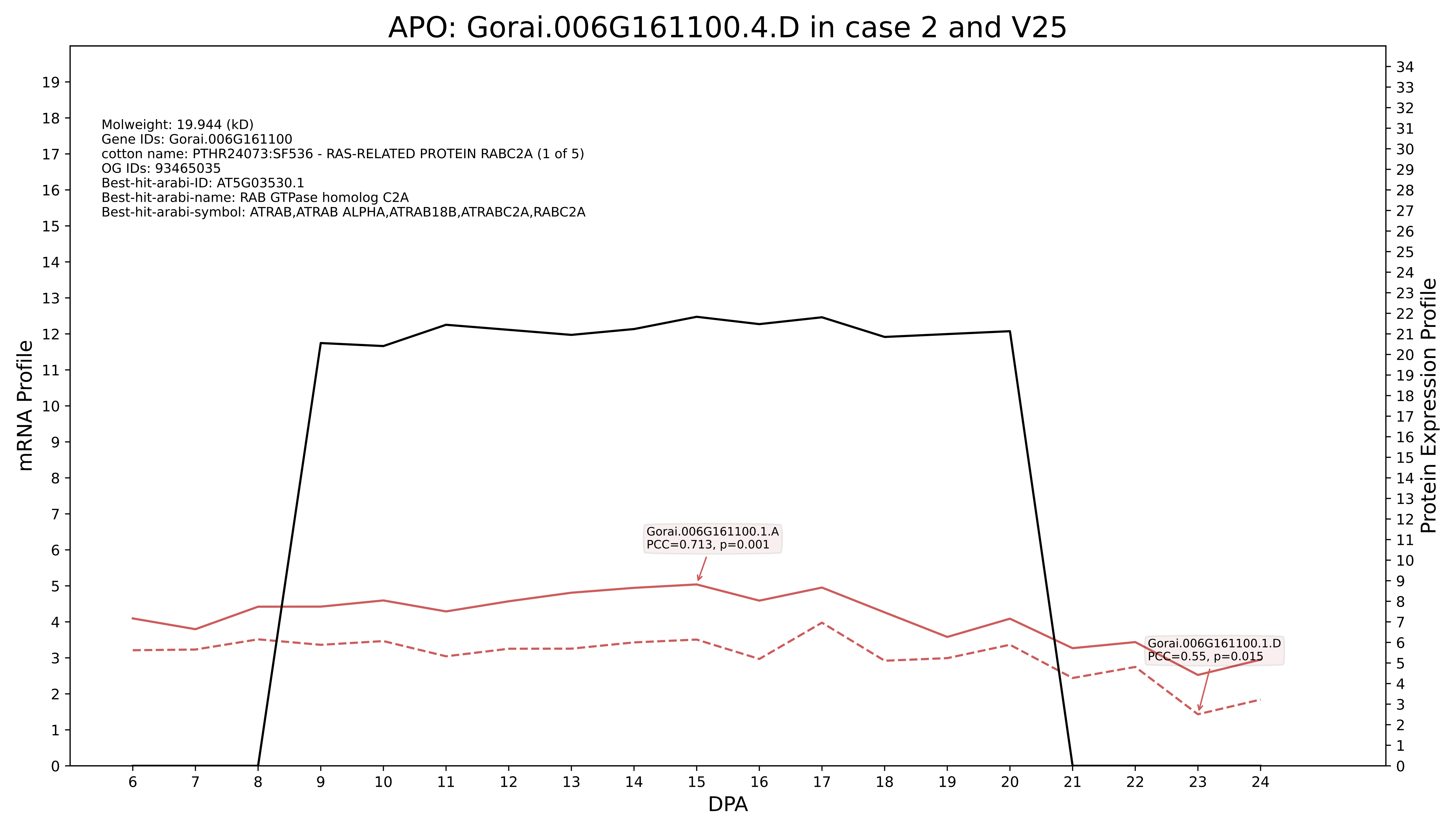

APO: Gorai.006G161100.4.D in case 2 and V25
Molweight: 19.944 (kD)
Gene IDs: Gorai.006G161100
cotton name: PTHR24073:SF536 - RAS-RELATED PROTEIN RABC2A (1 of 5)
OG IDs: 93465035
Best-hit-arabi-ID: AT5G03530.1
Best-hit-arabi-name: RAB GTPase homolog C2A
Best-hit-arabi-symbol: ATRAB,ATRAB ALPHA,ATRAB18B,ATRABC2A,RABC2A
Gorai.006G161100.1.A
PCC=0.713, p=0.001
Gorai.006G161100.1.D
PCC=0.55, p=0.015
mRNA Profile
Protein Expression Profile
DPA

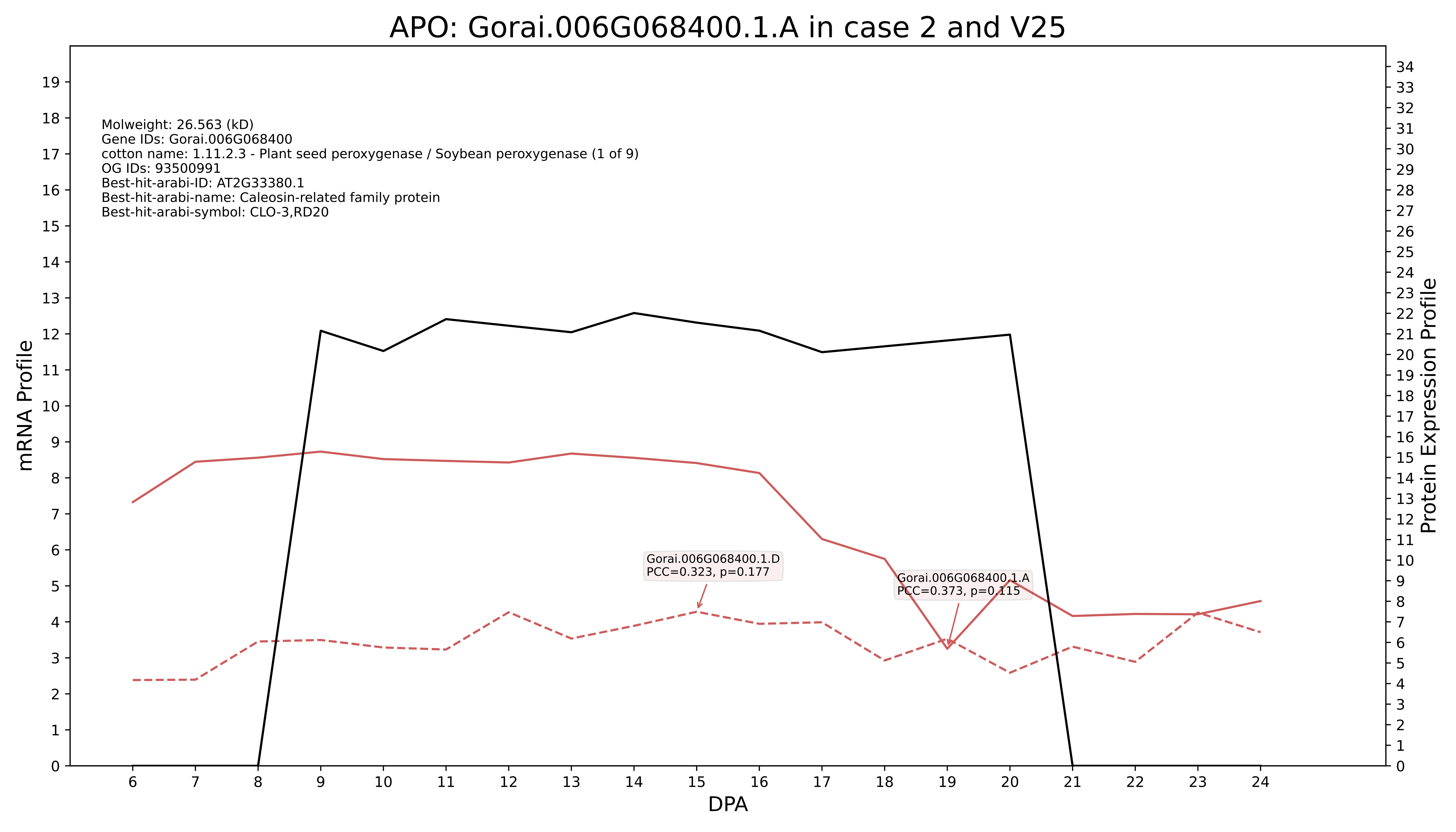
APO: Gorai.006G068400.1.A in case 2 and V25
Molweight: 26.563 (kD)
Gene IDs: Gorai.006G068400
cotton name: 1.11.2.3 - Plant seed peroxygenase / Soybean peroxygenase (1 of 9)
OG IDs: 93500991
Best-hit-arabi-ID: AT2G33380.1
Best-hit-arabi-name: Caleosin-related family protein
Best-hit-arabi-symbol: CLO-3,RD20
Gorai.006G068400.1.D
PCC=0.323, p=0.177
Gorai.006G068400.1.A
PCC=0.373, p=0.115
mRNA Profile
Protein Expression Profile
DPA
0
1
2
3
4
5
6
7
8
9
10
11
12
13
14
15
16
17
18
19
20
21
22
23
24
25
26
27
28
29
30
31
32
33
34

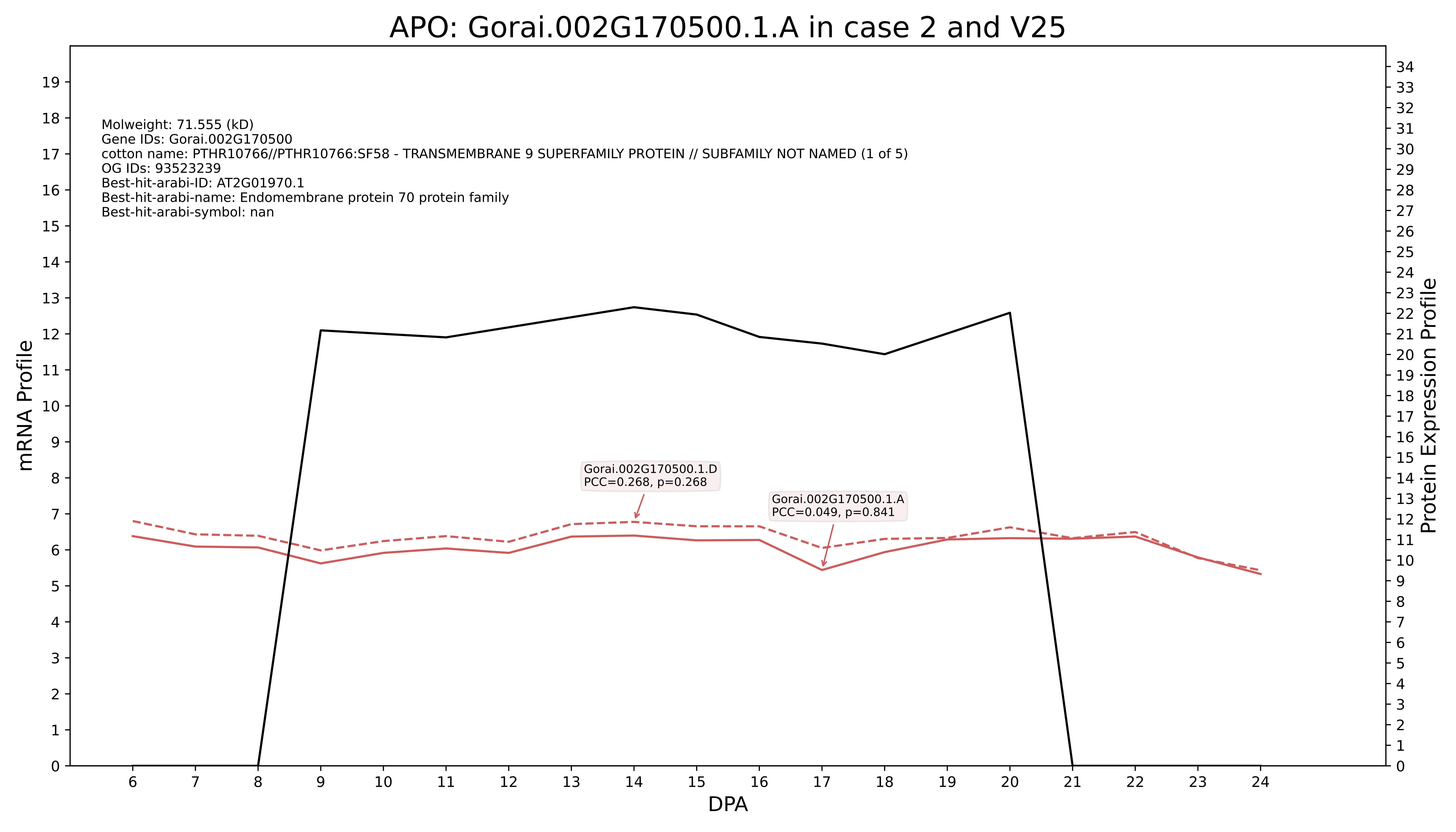

APO: Gorai.002G170500.1.A in case 2 and V25
Molweight: 71.555 (kD)
Gene IDs: Gorai.002G170500
cotton name: PTHR10766//PTHR10766:SF58 - TRANSMEMBRANE 9 SUPERFAMILY PROTEIN // SUBFAMILY NOT NAMED (1 of 5)
OG IDs: 93523239
Best-hit-arabi-ID: AT2G01970.1
Best-hit-arabi-name: Endomembrane protein 70 protein family
Best-hit-arabi-symbol: nan
Gorai.002G170500.1.D
PCC=0.268, p=0.268
Gorai.002G170500.1.A
PCC=0.049, p=0.841
mRNA Profile
Protein Expression Profile
DPA

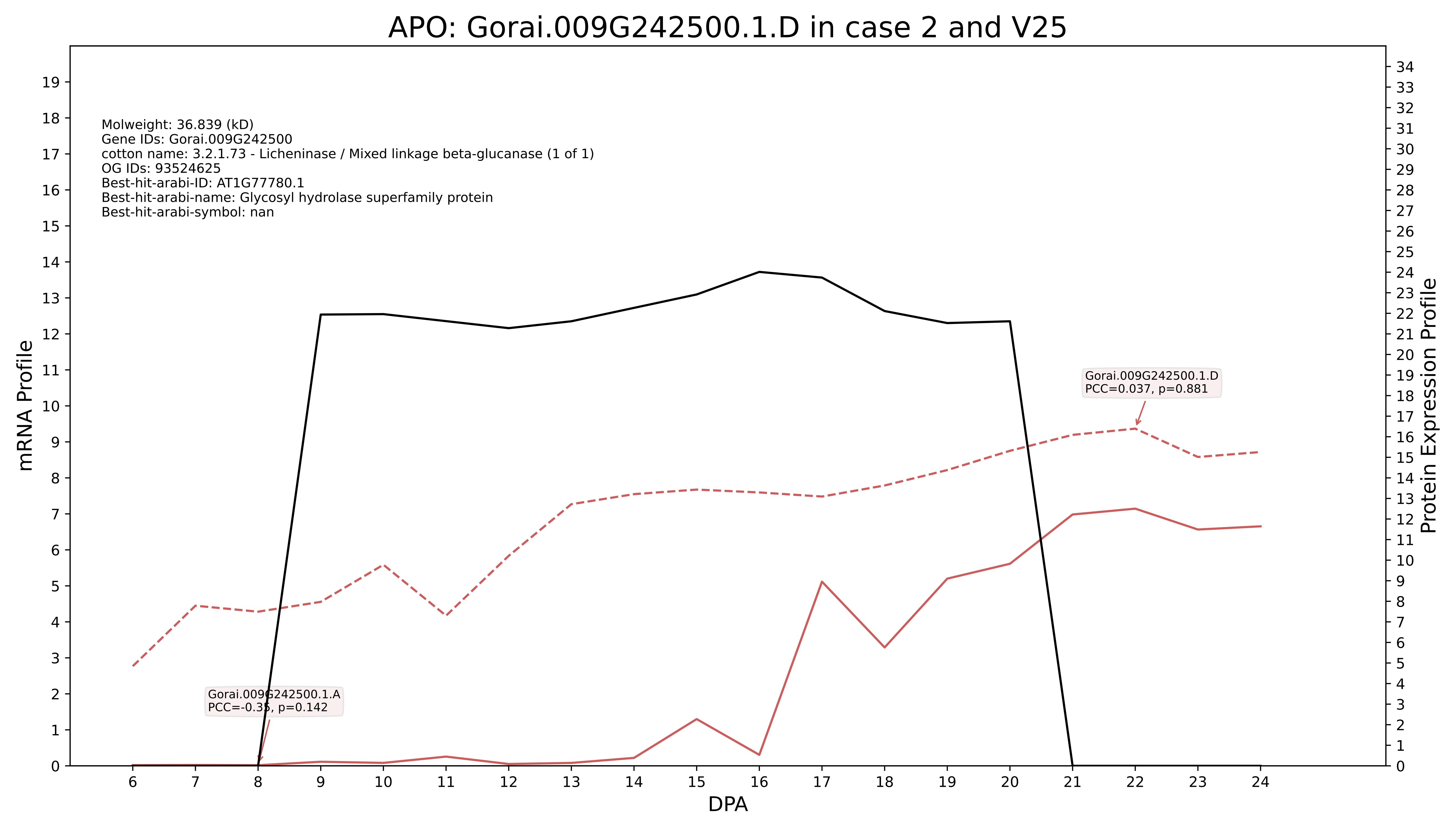
APO: Gorai.009G242500.1.D in case 2 and V25
Molweight: 36.839 (kD)
Gene IDs: Gorai.009G242500
cotton name: 3.2.1.73 - Licheninase / Mixed linkage beta-glucanase (1 of 1)
OG IDs: 93524625
Best-hit-arabi-ID: AT1G77780.1
Best-hit-arabi-name: Glycosyl hydrolase superfamily protein
Best-hit-arabi-symbol: nan
Gorai.009G242500.1.D
PCC=0.037, p=0.881
Gorai.009G242500.1.A
PCC=-0.35, p=0.142
mRNA Profile
Protein Expression Profile
DPA

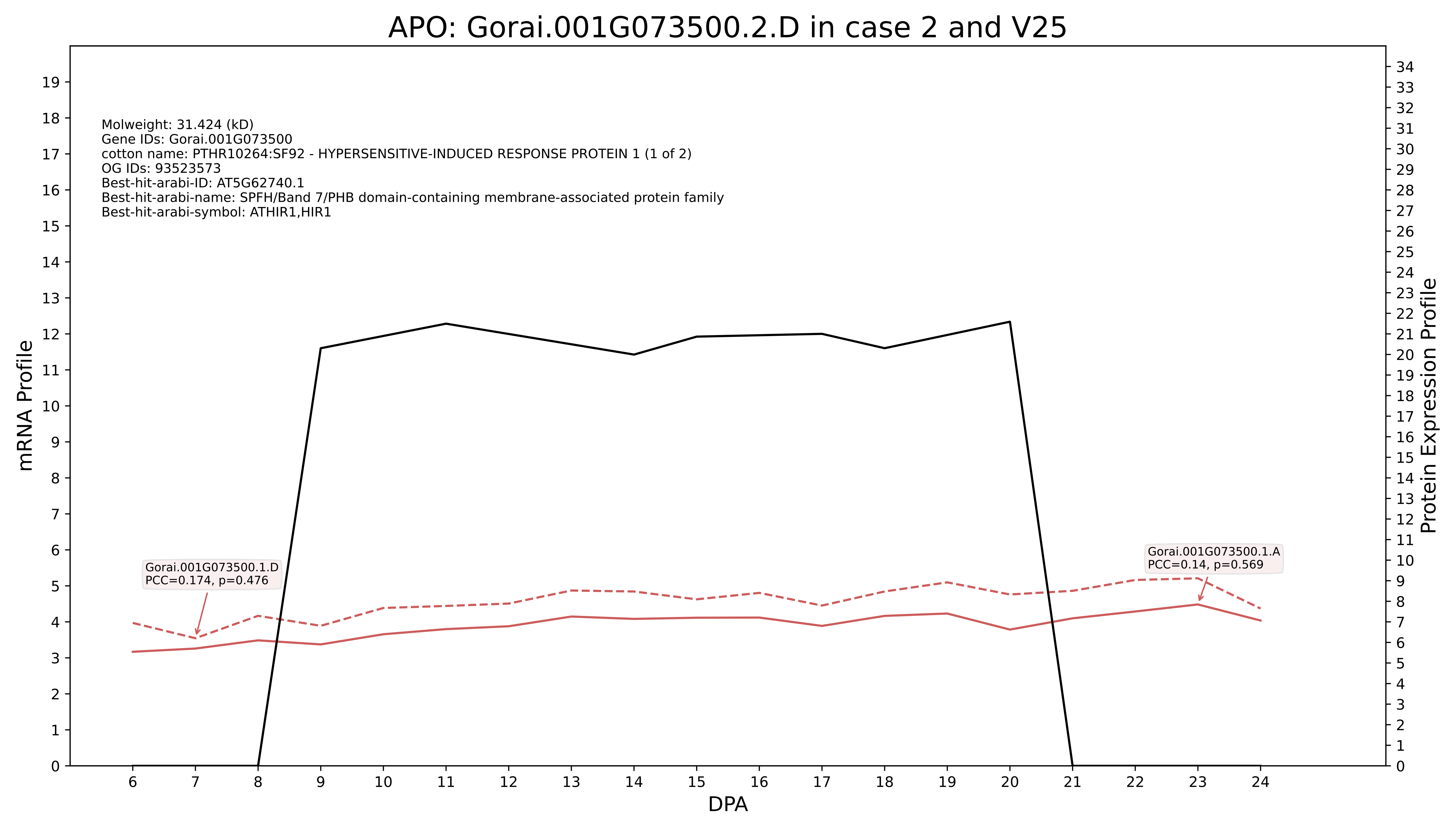
APO: Gorai.001G073500.2.D in case 2 and V25
Molweight: 31.424 (kD)
Gene IDs: Gorai.001G073500
cotton name: PTHR10264:SF92 - HYPERSENSITIVE-INDUCED RESPONSE PROTEIN 1 (1 of 2)
OG IDs: 93523573
Best-hit-arabi-ID: AT5G62740.1
Best-hit-arabi-name: SPFH/Band 7/PHB domain-containing membrane-associated protein family
Best-hit-arabi-symbol: ATHIR1,HIR1
Gorai.001G073500.1.D
PCC=0.174, p=0.476
Gorai.001G073500.1.A
PCC=0.14, p=0.569
mRNA Profile
Protein Expression Profile
DPA
0
1
2
3
4
5
6
7
8
9
10
11
12
13
14
15
16
17
18
19
20
21
22
23
24
25
26
27
28
29
30
31
32
33
34

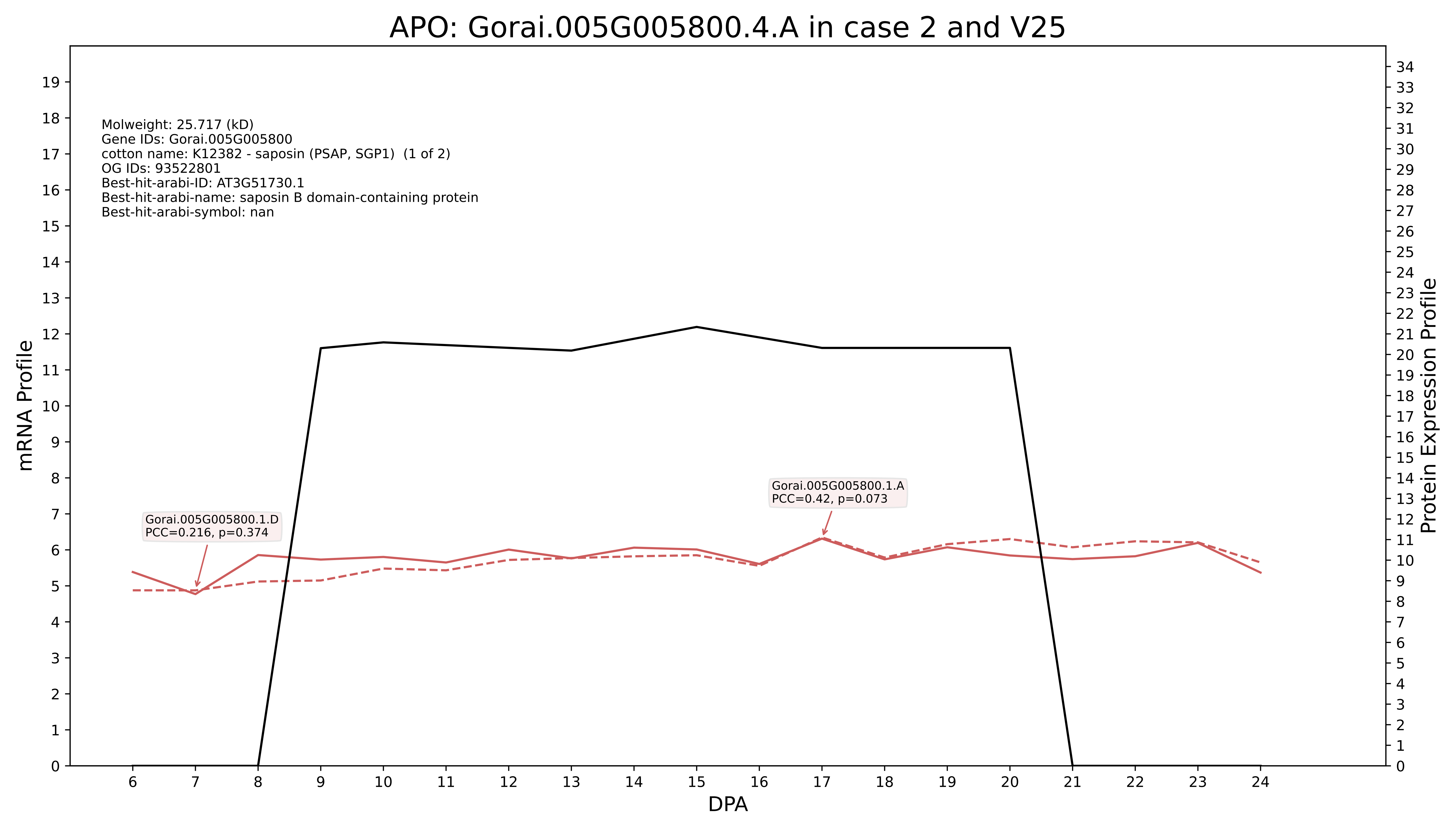

APO: Gorai.005G005800.4.A in case 2 and V25
Molweight: 25.717 (kD)
Gene IDs: Gorai.005G005800
cotton name: K12382 - saposin (PSAP, SGP1) (1 of 2)
OG IDs: 93522801
Best-hit-arabi-ID: AT3G51730.1
Best-hit-arabi-name: saposin B domain-containing protein
Best-hit-arabi-symbol: nan
Gorai.005G005800.1.D
PCC=0.216, p=0.374
Gorai.005G005800.1.A
PCC=0.42, p=0.073
mRNA Profile
Protein Expression Profile
DPA

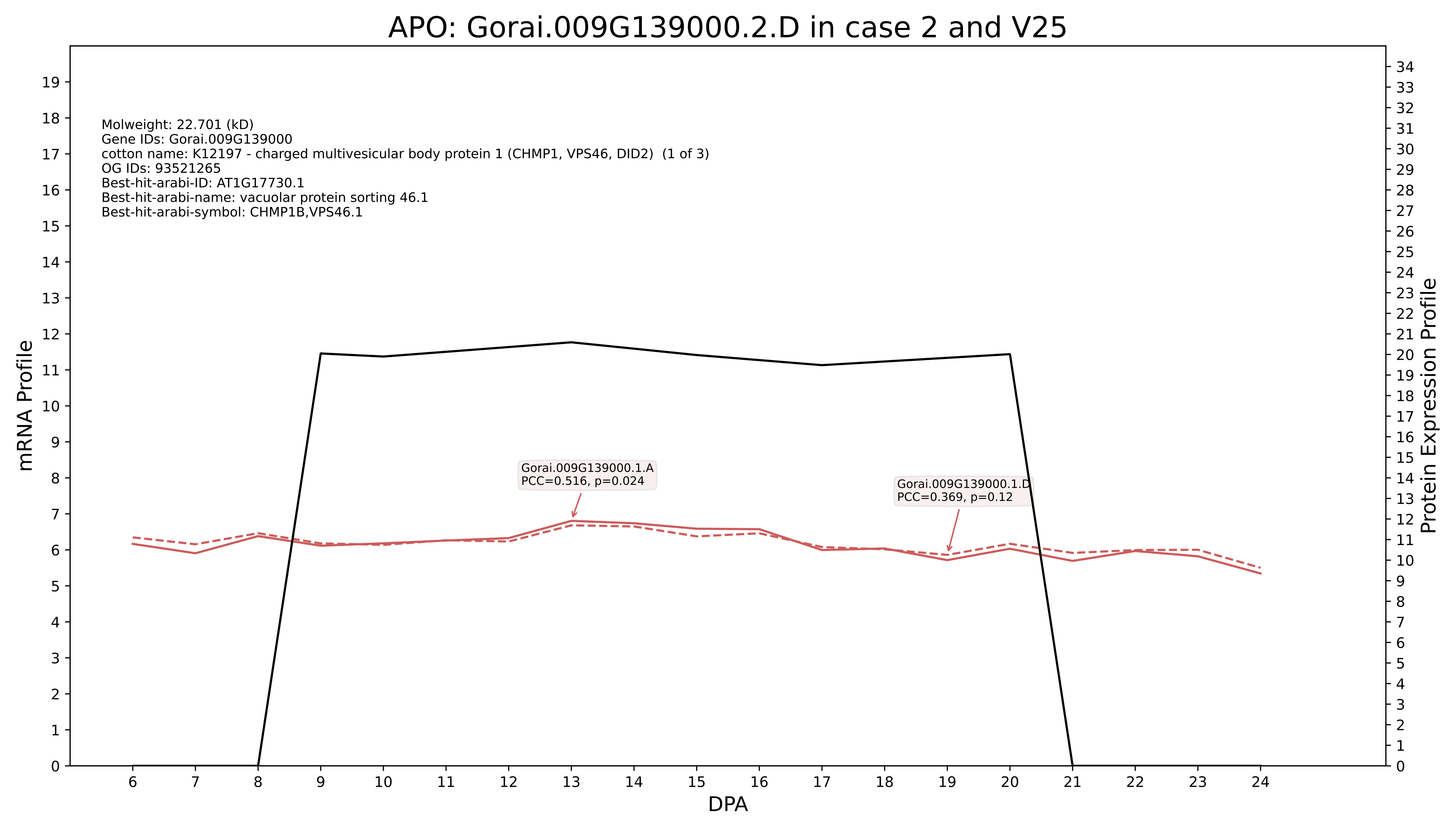
APO: Gorai.009G139000.2.D in case 2 and V25
Molweight: 22.701 (kD)
Gene IDs: Gorai.009G139000
cotton name: K12197 - charged multivesicular body protein 1 (CHMP1, VPS46, DID2) (1 of 3)
OG IDs: 93521265
Best-hit-arabi-ID: AT1G17730.1
Best-hit-arabi-name: vacuolar protein sorting 46.1
Best-hit-arabi-symbol: CHMP1B,VPS46.1
Gorai.009G139000.1.A
PCC=0.516, p=0.024
Gorai.009G139000.1.D
PCC=0.369, p=0.12
mRNA Profile
Protein Expression Profile
DPA

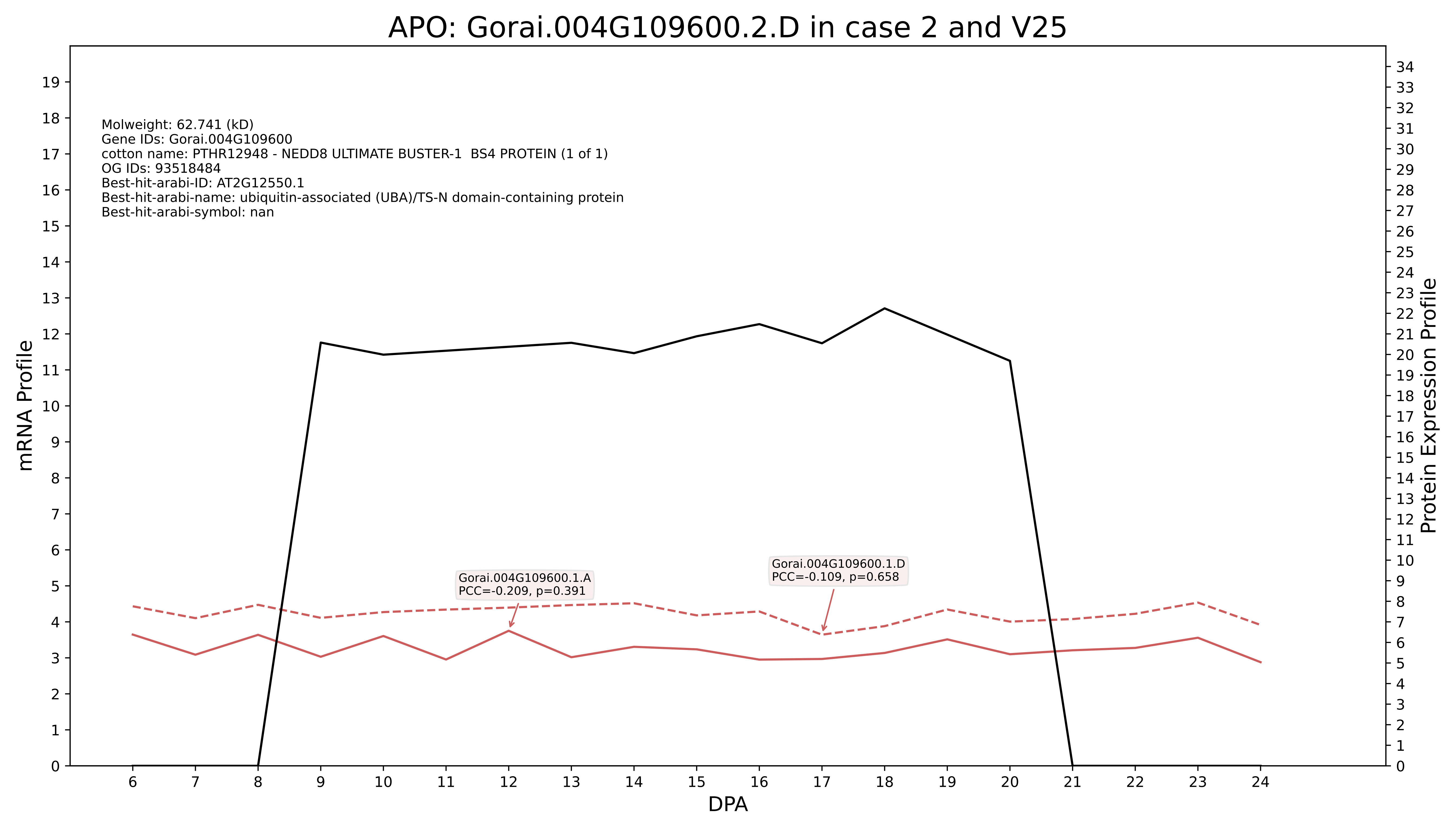

APO: Gorai.004G109600.2.D in case 2 and V25
Molweight: 62.741 (kD)
Gene IDs: Gorai.004G109600
cotton name: PTHR12948 - NEDD8 ULTIMATE BUSTER-1 BS4 PROTEIN (1 of 1)
OG IDs: 93518484
Best-hit-arabi-ID: AT2G12550.1
Best-hit-arabi-name: ubiquitin-associated (UBA)/TS-N domain-containing protein
Best-hit-arabi-symbol: nan
Gorai.004G109600.1.A
PCC=-0.209, p=0.391
Gorai.004G109600.1.D
PCC=-0.109, p=0.658
mRNA Profile
Protein Expression Profile
DPA

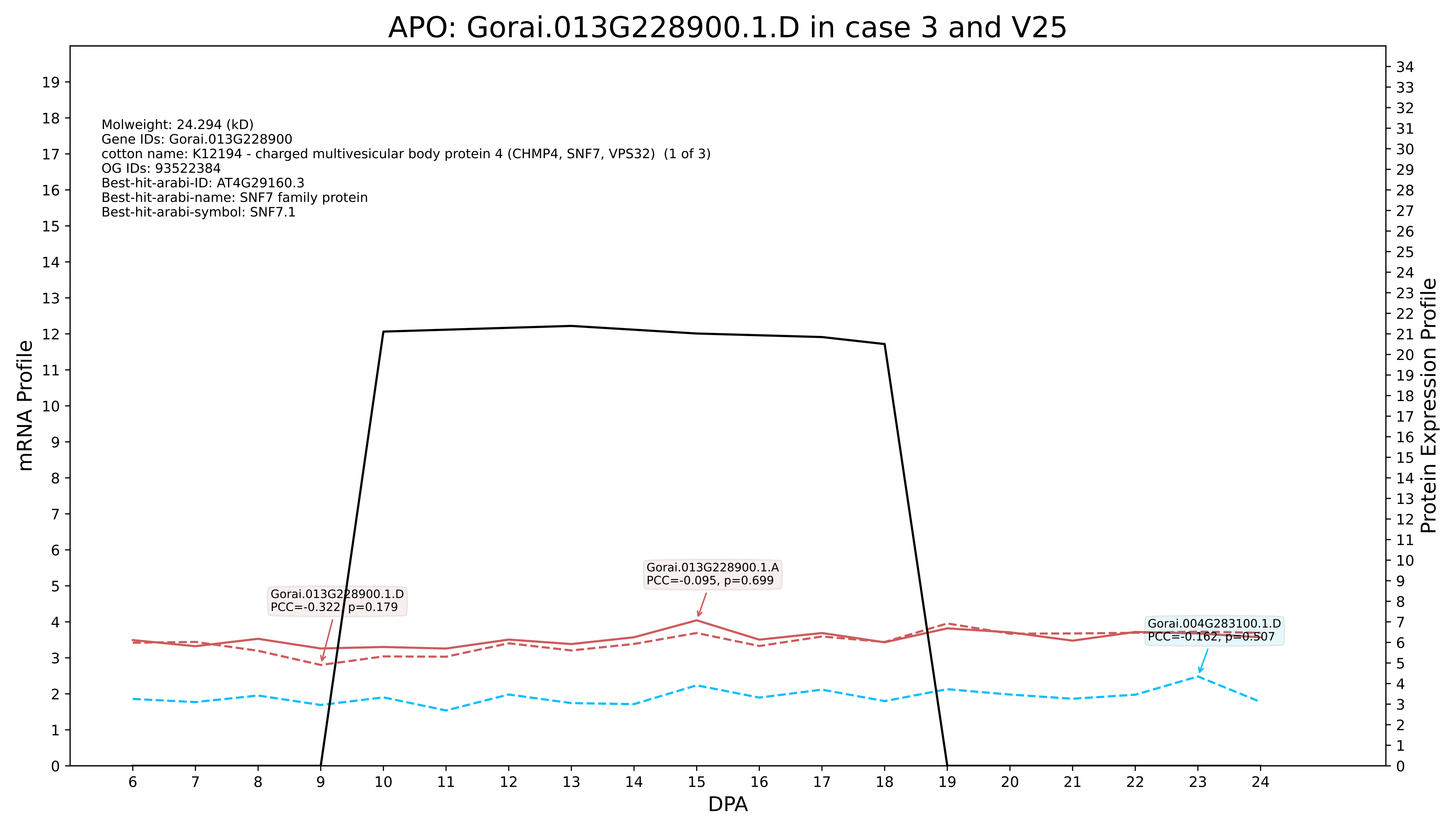

APO: Gorai.013G228900.1.D in case 3 and V25
Molweight: 24.294 (kD)
Gene IDs: Gorai.013G228900
cotton name: K12194 - charged multivesicular body protein 4 (CHMP4, SNF7, VPS32) (1 of 3)
OG IDs: 93522384
Best-hit-arabi-ID: AT4G29160.3
Best-hit-arabi-name: SNF7 family protein
Best-hit-arabi-symbol: SNF7.1
Gorai.013G228900.1.D
PCC=-0.322, p=0.179
Gorai.013G228900.1.A
PCC=-0.095, p=0.699
Gorai.004G283100.1.D
PCC=-0.162, p=0.507
mRNA Profile
Protein Expression Profile
DPA

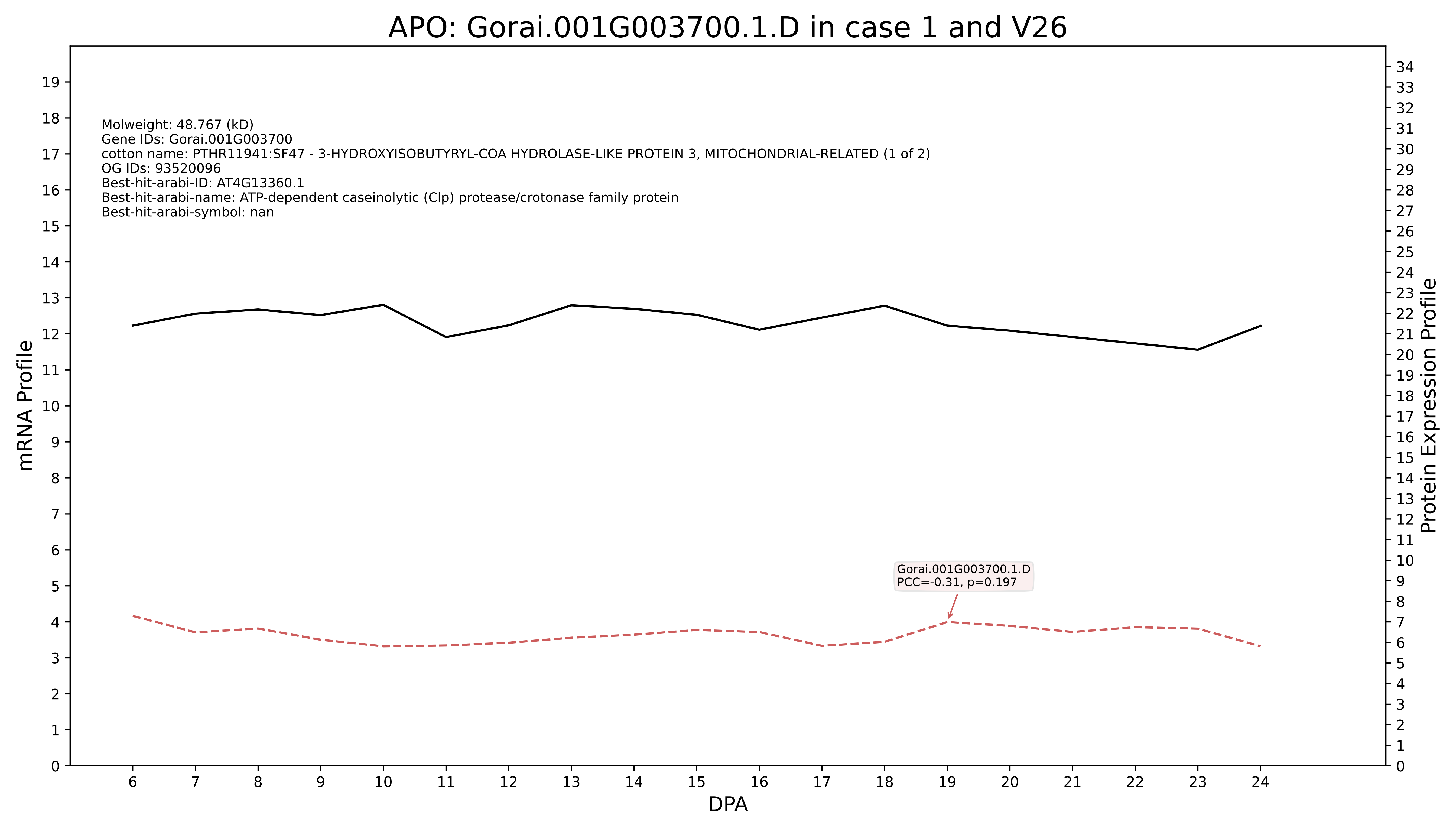

APO: Gorai.001G003700.1.D in case 1 and V26
Molweight: 48.767 (kD)
Gene IDs: Gorai.001G003700
cotton name: PTHR11941:SF47 - 3-HYDROXYISOBUTYRYL-COA HYDROLASE-LIKE PROTEIN 3, MITOCHONDRIAL-RELATED (1 of 2)
OG IDs: 93520096
Best-hit-arabi-ID: AT4G13360.1
Best-hit-arabi-name: ATP-dependent caseinolytic (Clp) protease/crotonase family protein
Best-hit-arabi-symbol: nan
Gorai.001G003700.1.D
PCC=-0.31, p=0.197
mRNA Profile
Protein Expression Profile
DPA

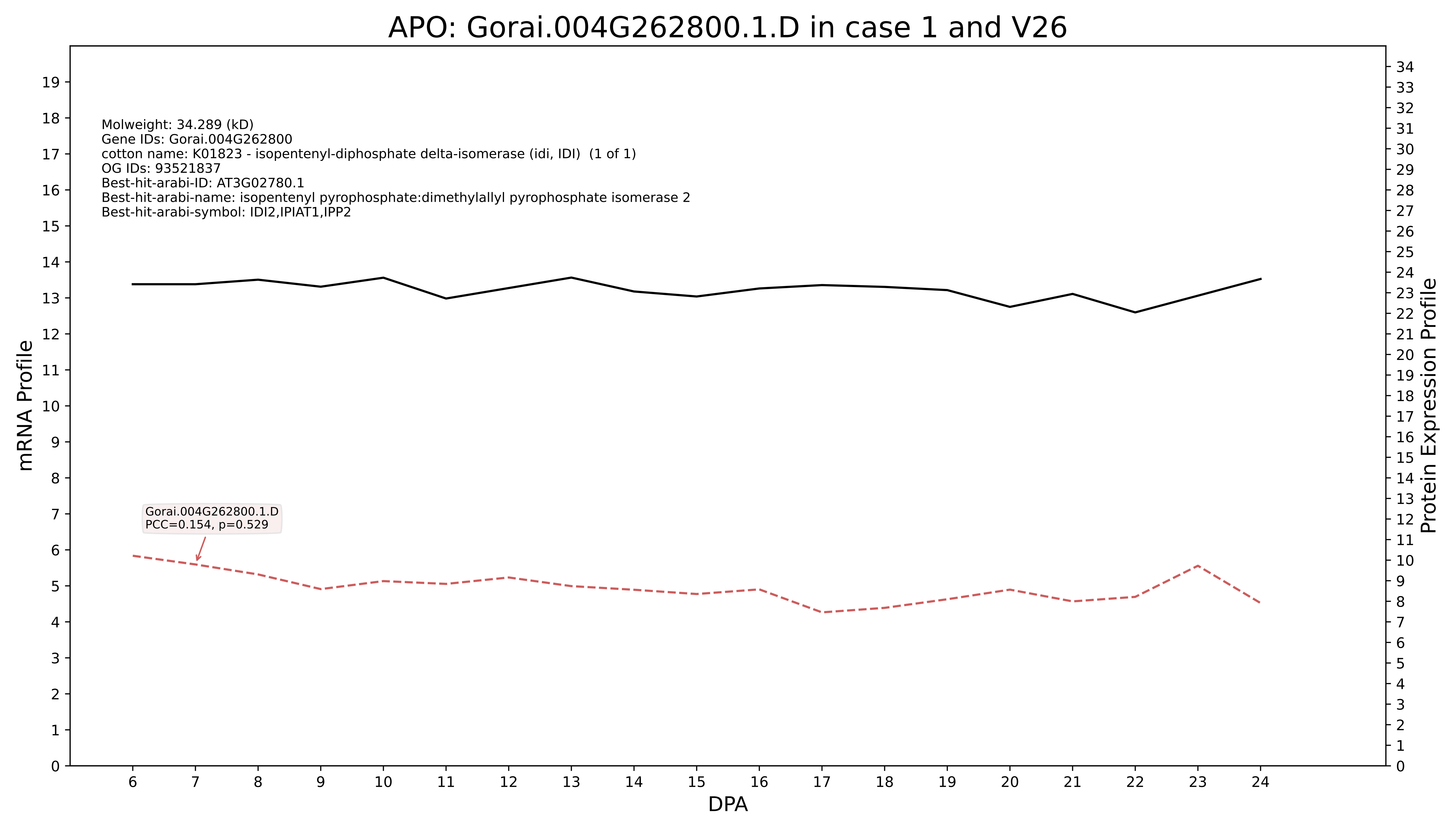
APO: Gorai.004G262800.1.D in case 1 and V26
Molweight: 34.289 (kD)
Gene IDs: Gorai.004G262800
cotton name: K01823 - isopentenyl-diphosphate delta-isomerase (idi, IDI) (1 of 1)
OG IDs: 93521837
Best-hit-arabi-ID: AT3G02780.1
Best-hit-arabi-name: isopentenyl pyrophosphate:dimethylallyl pyrophosphate isomerase 2
Best-hit-arabi-symbol: IDI2,IPIAT1,IPP2
Gorai.004G262800.1.D
PCC=0.154, p=0.529
mRNA Profile
Protein Expression Profile
DPA

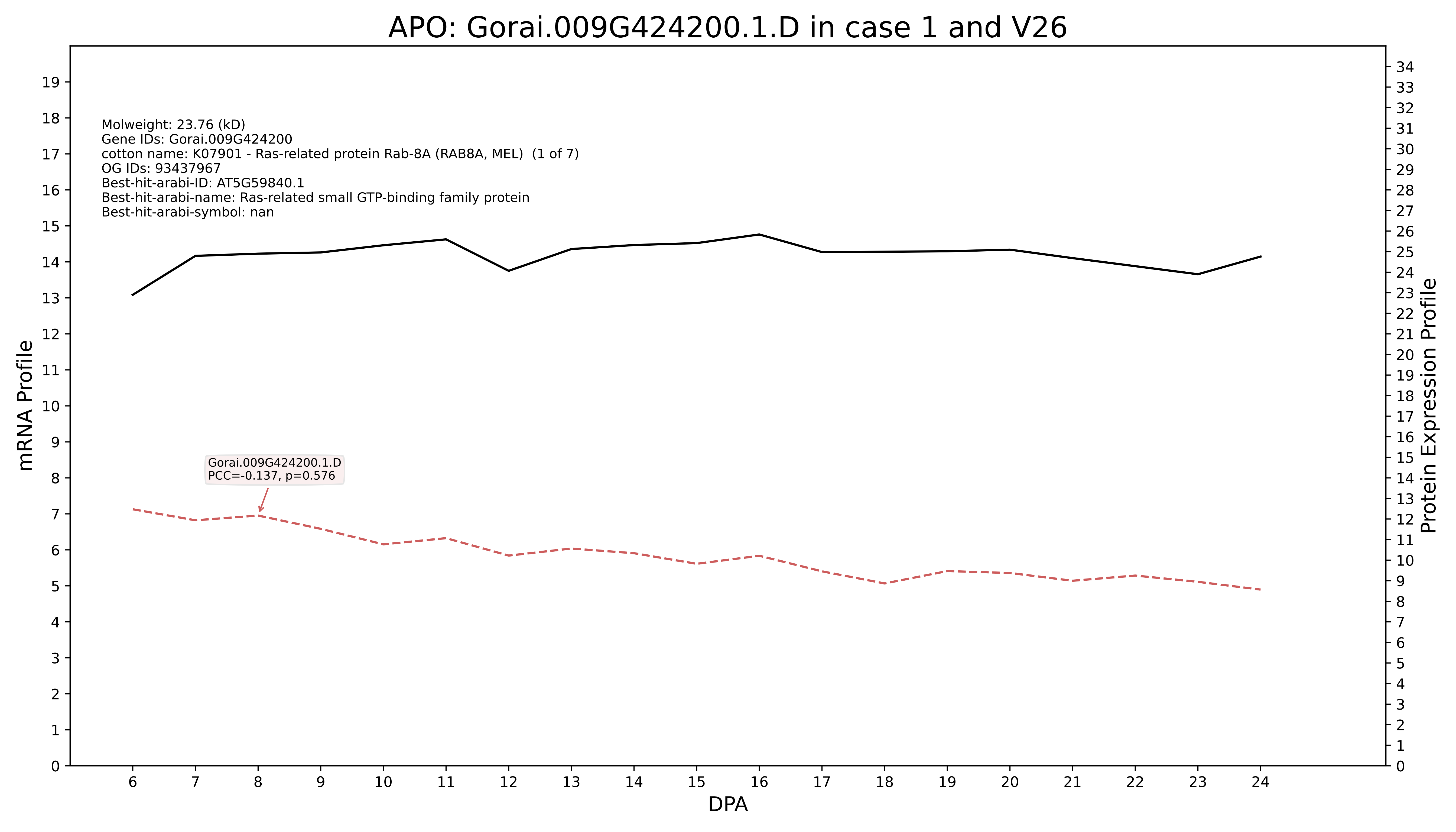
APO: Gorai.009G424200.1.D in case 1 and V26
Molweight: 23.76 (kD)
Gene IDs: Gorai.009G424200
cotton name: K07901 - Ras-related protein Rab-8A (RAB8A, MEL) (1 of 7)
OG IDs: 93437967
Best-hit-arabi-ID: AT5G59840.1
Best-hit-arabi-name: Ras-related small GTP-binding family protein
Best-hit-arabi-symbol: nan
Gorai.009G424200.1.D
PCC=-0.137, p=0.576
mRNA Profile
Protein Expression Profile
DPA
0
1
2
3
4
5
6
7
8
9
10
11
12
13
14
15
16
17
18
19
20
21
22
23
24
25
26
27
28
29
30
31
32
33
34

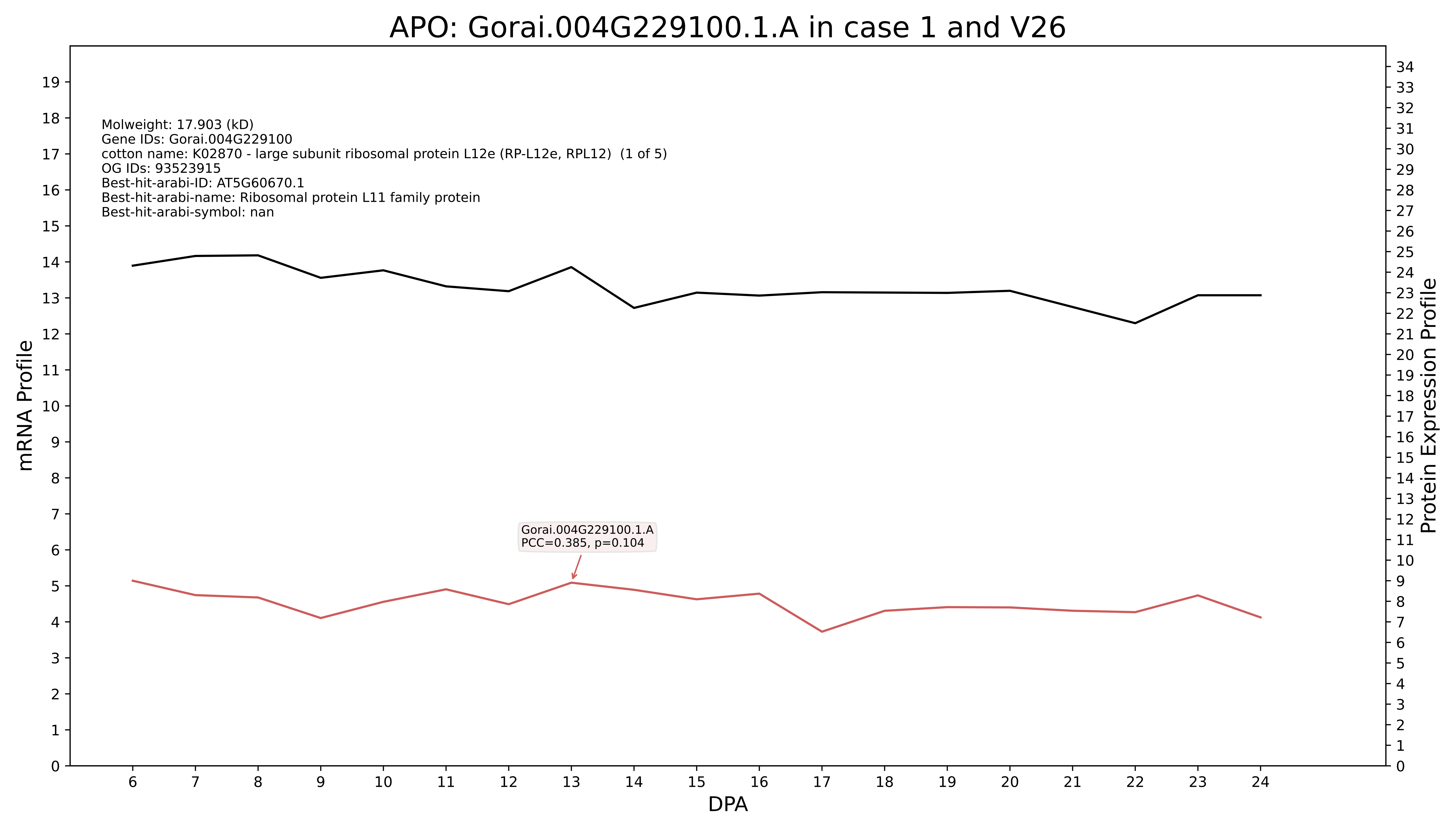
APO: Gorai.004G229100.1.A in case 1 and V26
Molweight: 17.903 (kD)
Gene IDs: Gorai.004G229100
cotton name: K02870 - large subunit ribosomal protein L12e (RP-L12e, RPL12) (1 of 5)
OG IDs: 93523915
Best-hit-arabi-ID: AT5G60670.1
Best-hit-arabi-name: Ribosomal protein L11 family protein
Best-hit-arabi-symbol: nan
Gorai.004G229100.1.A
PCC=0.385, p=0.104
mRNA Profile
Protein Expression Profile
DPA

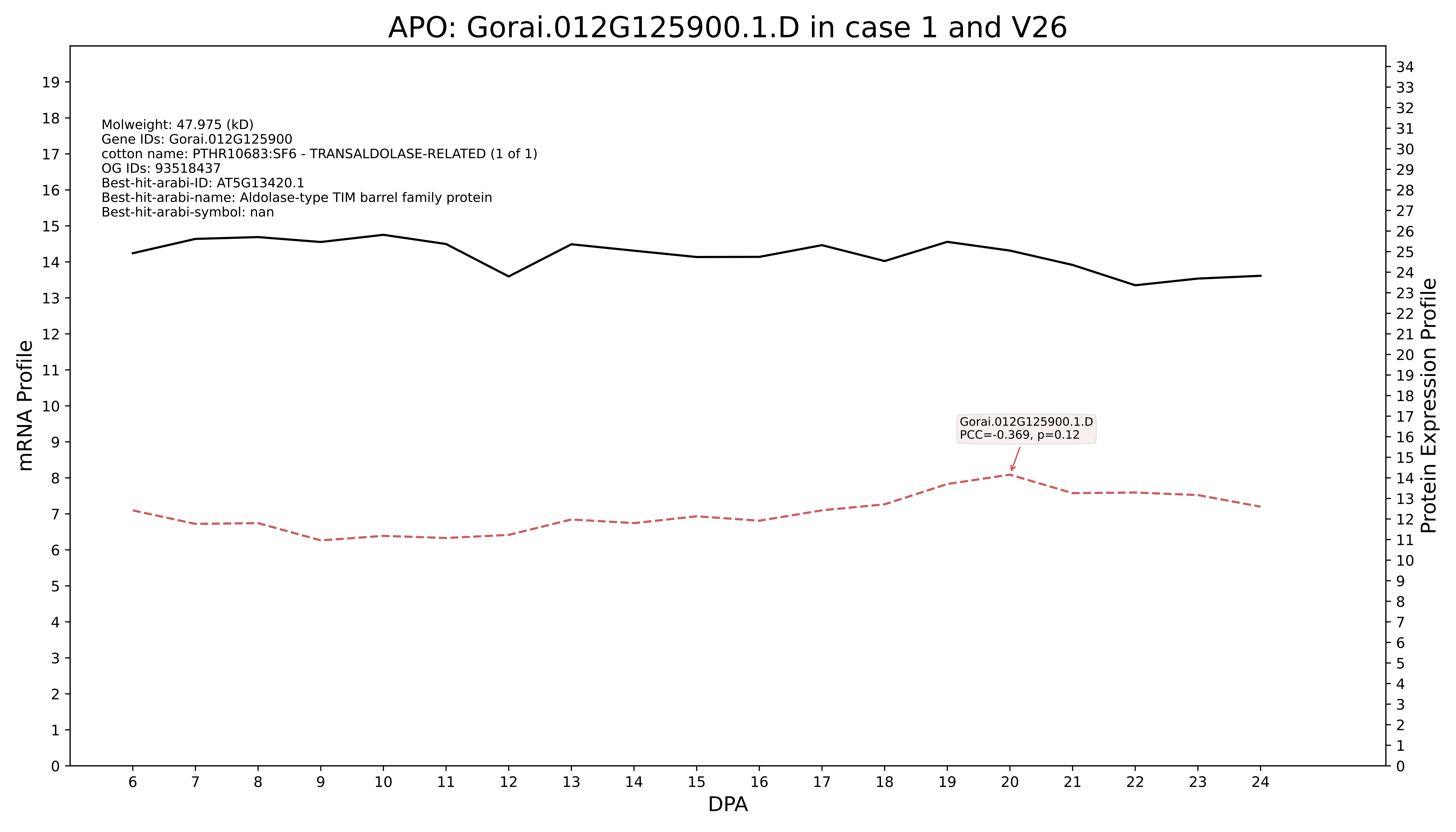

APO: Gorai.012G125900.1.D in case 1 and V26
Molweight: 47.975 (kD)
Gene IDs: Gorai.012G125900
cotton name: PTHR10683:SF6 - TRANSALDOLASE-RELATED (1 of 1)
OG IDs: 93518437
Best-hit-arabi-ID: AT5G13420.1
Best-hit-arabi-name: Aldolase-type TIM barrel family protein
Best-hit-arabi-symbol: nan
Gorai.012G125900.1.D
PCC=-0.369, p=0.12
mRNA Profile
Protein Expression Profile
DPA

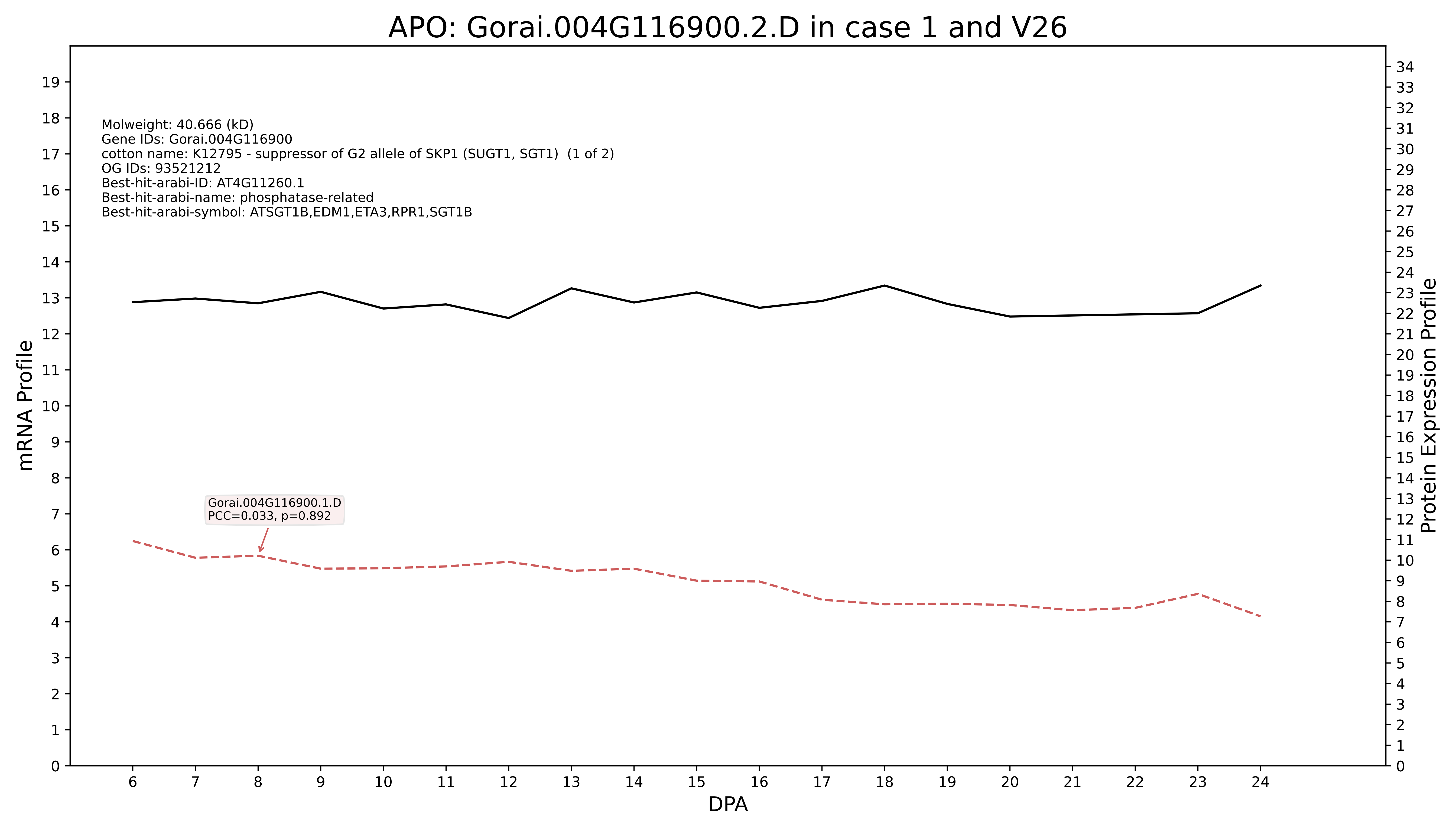
APO: Gorai.004G116900.2.D in case 1 and V26
Molweight: 40.666 (kD)
Gene IDs: Gorai.004G116900
cotton name: K12795 - suppressor of G2 allele of SKP1 (SUGT1, SGT1) (1 of 2)
OG IDs: 93521212
Best-hit-arabi-ID: AT4G11260.1
Best-hit-arabi-name: phosphatase-related
Best-hit-arabi-symbol: ATSGT1B,EDM1,ETA3,RPR1,SGT1B
Gorai.004G116900.1.D
PCC=0.033, p=0.892
mRNA Profile
Protein Expression Profile
DPA

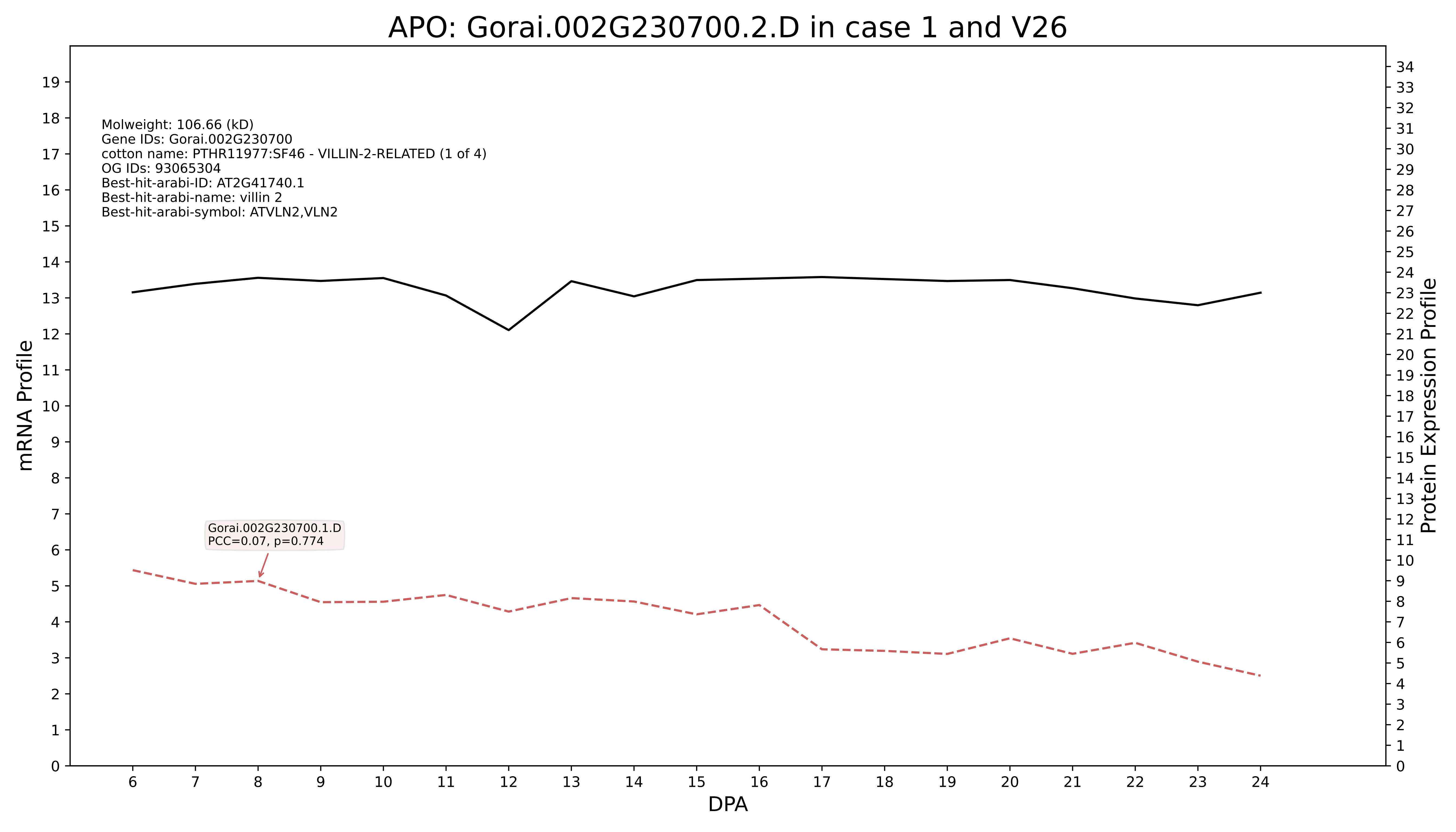
APO: Gorai.002G230700.2.D in case 1 and V26
Molweight: 106.66 (kD)
Gene IDs: Gorai.002G230700
cotton name: PTHR11977:SF46 - VILLIN-2-RELATED (1 of 4)
OG IDs: 93065304
Best-hit-arabi-ID: AT2G41740.1
Best-hit-arabi-name: villin 2
Best-hit-arabi-symbol: ATVLN2,VLN2
Gorai.002G230700.1.D
PCC=0.07, p=0.774
mRNA Profile
Protein Expression Profile
DPA
0
1
2
3
4
5
6
7
8
9
10
11
12
13
14
15
16
17
18
19
20
21
22
23
24
25
26
27
28
29
30
31
32
33
34

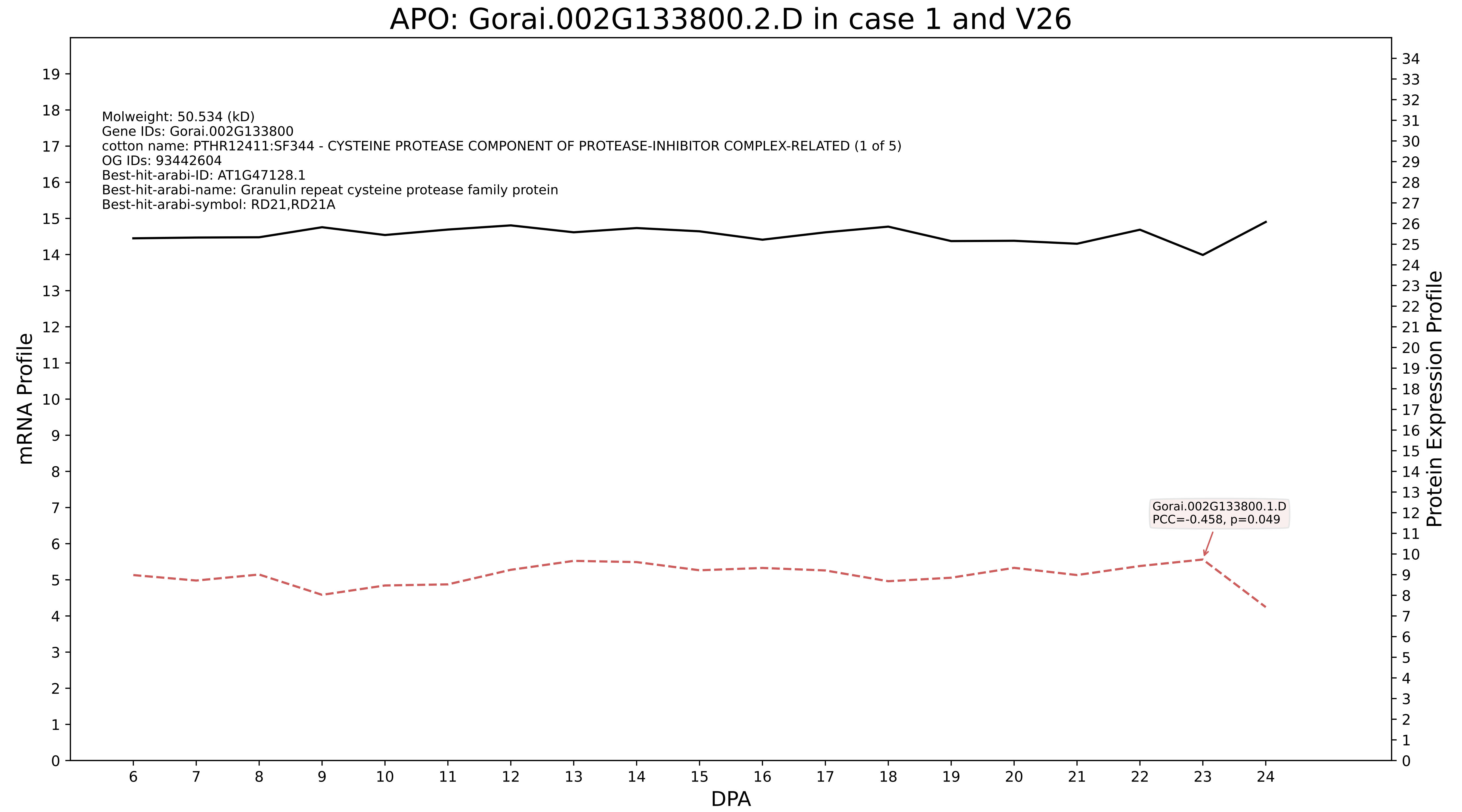
APO: Gorai.002G133800.2.D in case 1 and V26
Molweight: 50.534 (kD)
Gene IDs: Gorai.002G133800
cotton name: PTHR12411:SF344 - CYSTEINE PROTEASE COMPONENT OF PROTEASE-INHIBITOR COMPLEX-RELATED (1 of 5)
OG IDs: 93442604
Best-hit-arabi-ID: AT1G47128.1
Best-hit-arabi-name: Granulin repeat cysteine protease family protein
Best-hit-arabi-symbol: RD21,RD21A
Gorai.002G133800.1.D
PCC=-0.458, p=0.049
mRNA Profile
Protein Expression Profile
DPA

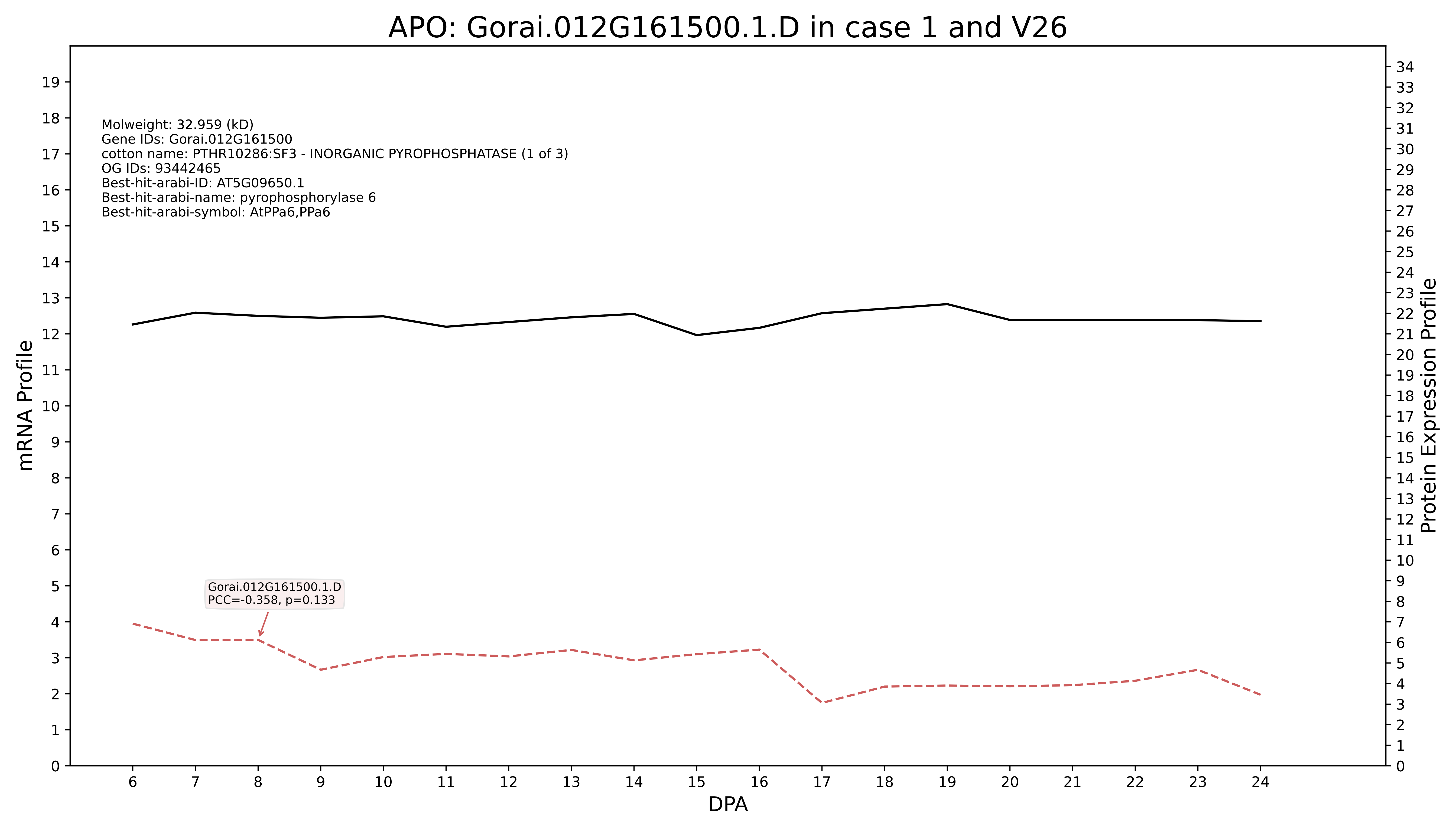
APO: Gorai.012G161500.1.D in case 1 and V26
Molweight: 32.959 (kD)
Gene IDs: Gorai.012G161500
cotton name: PTHR10286:SF3 - INORGANIC PYROPHOSPHATASE (1 of 3)
OG IDs: 93442465
Best-hit-arabi-ID: AT5G09650.1
Best-hit-arabi-name: pyrophosphorylase 6
Best-hit-arabi-symbol: AtPPa6,PPa6
Gorai.012G161500.1.D
PCC=-0.358, p=0.133
mRNA Profile
Protein Expression Profile
DPA

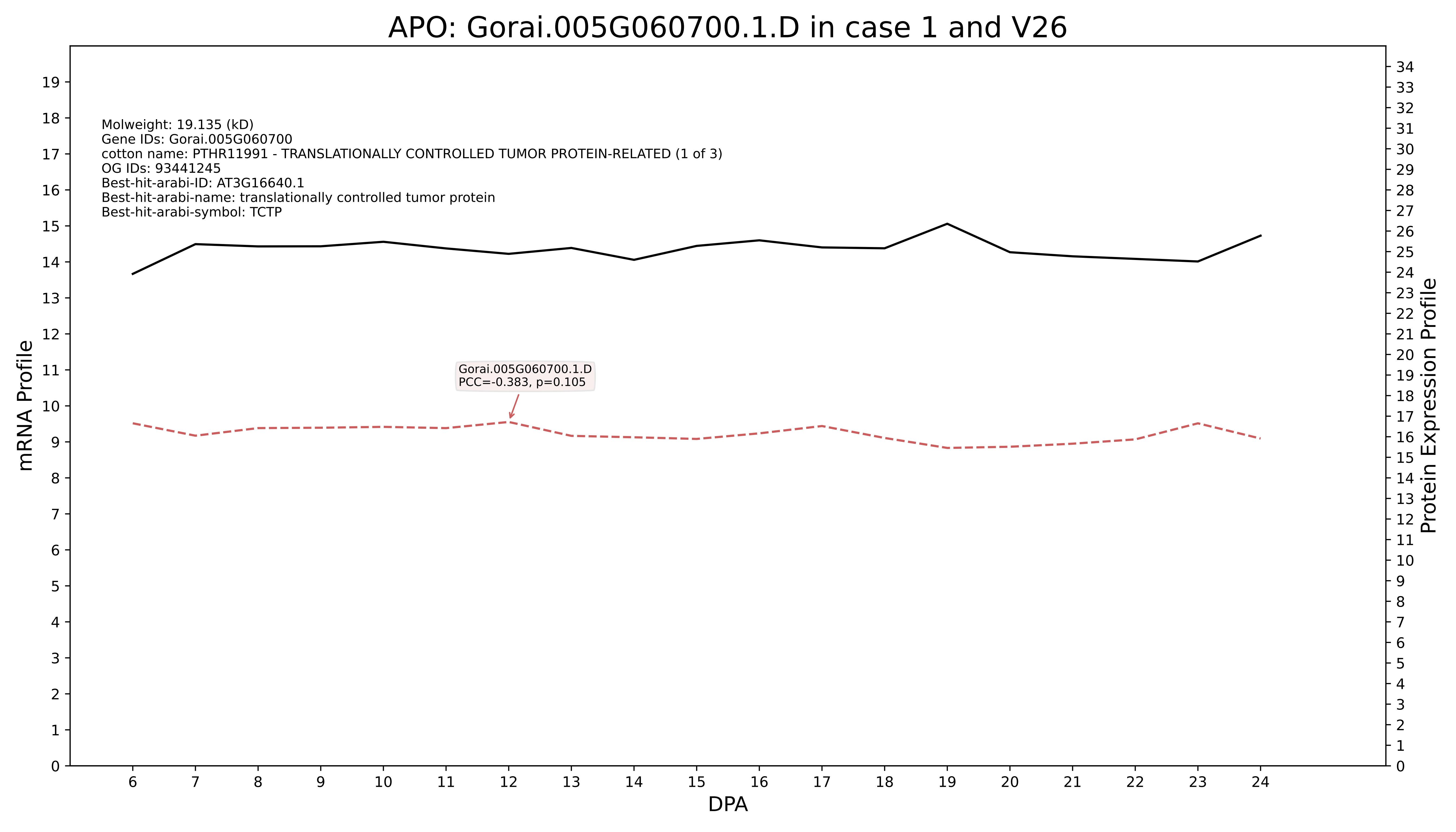
APO: Gorai.005G060700.1.D in case 1 and V26
Molweight: 19.135 (kD)
Gene IDs: Gorai.005G060700
cotton name: PTHR11991 - TRANSLATIONALLY CONTROLLED TUMOR PROTEIN-RELATED (1 of 3)
OG IDs: 93441245
Best-hit-arabi-ID: AT3G16640.1
Best-hit-arabi-name: translationally controlled tumor protein
Best-hit-arabi-symbol: TCTP
Gorai.005G060700.1.D
PCC=-0.383, p=0.105
mRNA Profile
Protein Expression Profile
DPA

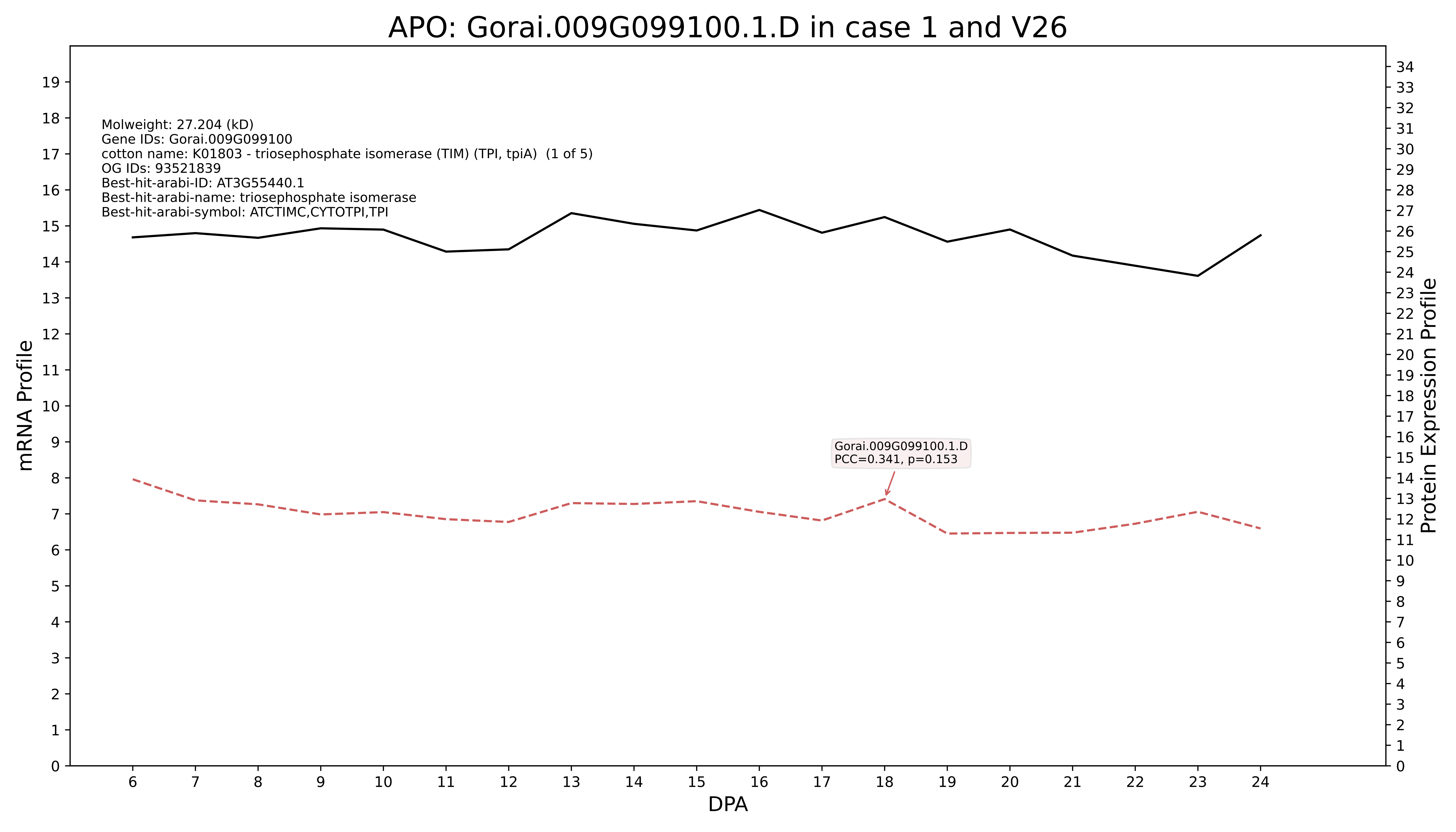
APO: Gorai.009G099100.1.D in case 1 and V26
Molweight: 27.204 (kD)
Gene IDs: Gorai.009G099100
cotton name: K01803 - triosephosphate isomerase (TIM) (TPI, tpiA) (1 of 5)
OG IDs: 93521839
Best-hit-arabi-ID: AT3G55440.1
Best-hit-arabi-name: triosephosphate isomerase
Best-hit-arabi-symbol: ATCTIMC,CYTOTPI,TPI
Gorai.009G099100.1.D
PCC=0.341, p=0.153
mRNA Profile
Protein Expression Profile
DPA

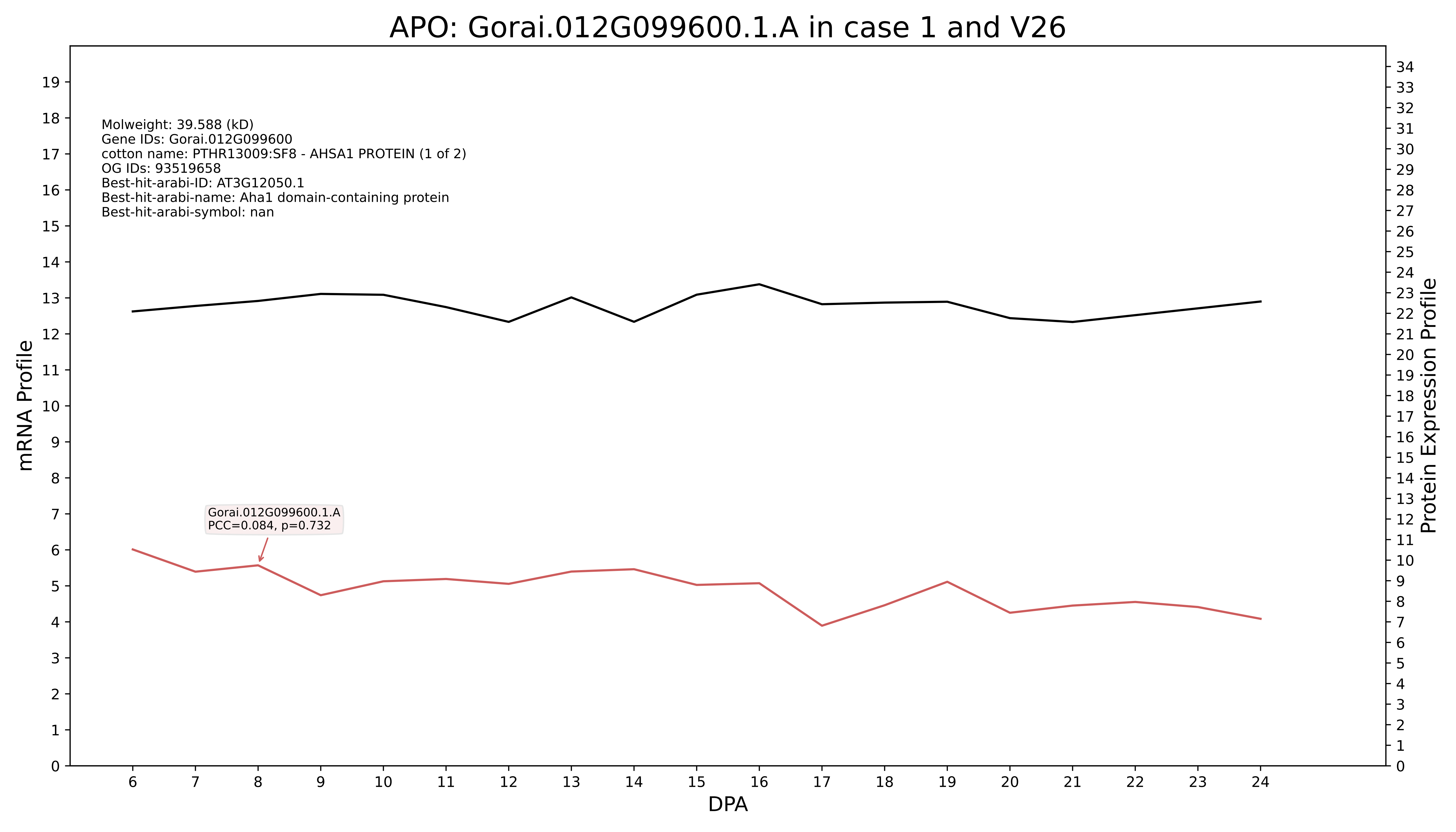
APO: Gorai.012G099600.1.A in case 1 and V26
Molweight: 39.588 (kD)
Gene IDs: Gorai.012G099600
cotton name: PTHR13009:SF8 - AHSA1 PROTEIN (1 of 2)
OG IDs: 93519658
Best-hit-arabi-ID: AT3G12050.1
Best-hit-arabi-name: Aha1 domain-containing protein
Best-hit-arabi-symbol: nan
Gorai.012G099600.1.A
PCC=0.084, p=0.732
mRNA Profile
Protein Expression Profile
DPA

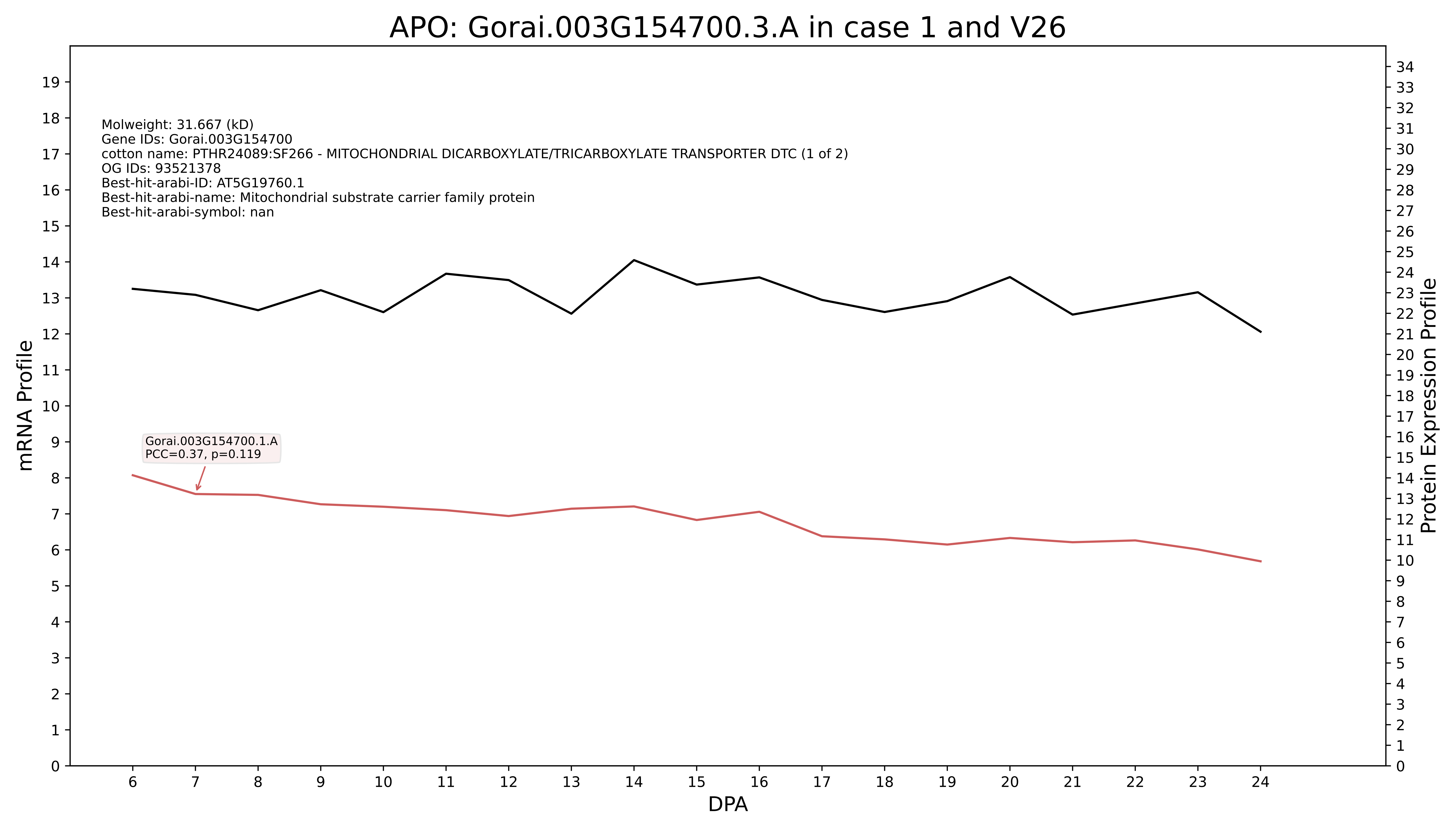

APO: Gorai.003G154700.3.A in case 1 and V26
Molweight: 31.667 (kD)
Gene IDs: Gorai.003G154700
cotton name: PTHR24089:SF266 - MITOCHONDRIAL DICARBOXYLATE/TRICARBOXYLATE TRANSPORTER DTC (1 of 2)
OG IDs: 93521378
Best-hit-arabi-ID: AT5G19760.1
Best-hit-arabi-name: Mitochondrial substrate carrier family protein
Best-hit-arabi-symbol: nan
Gorai.003G154700.1.A
PCC=0.37, p=0.119
mRNA Profile
Protein Expression Profile
DPA

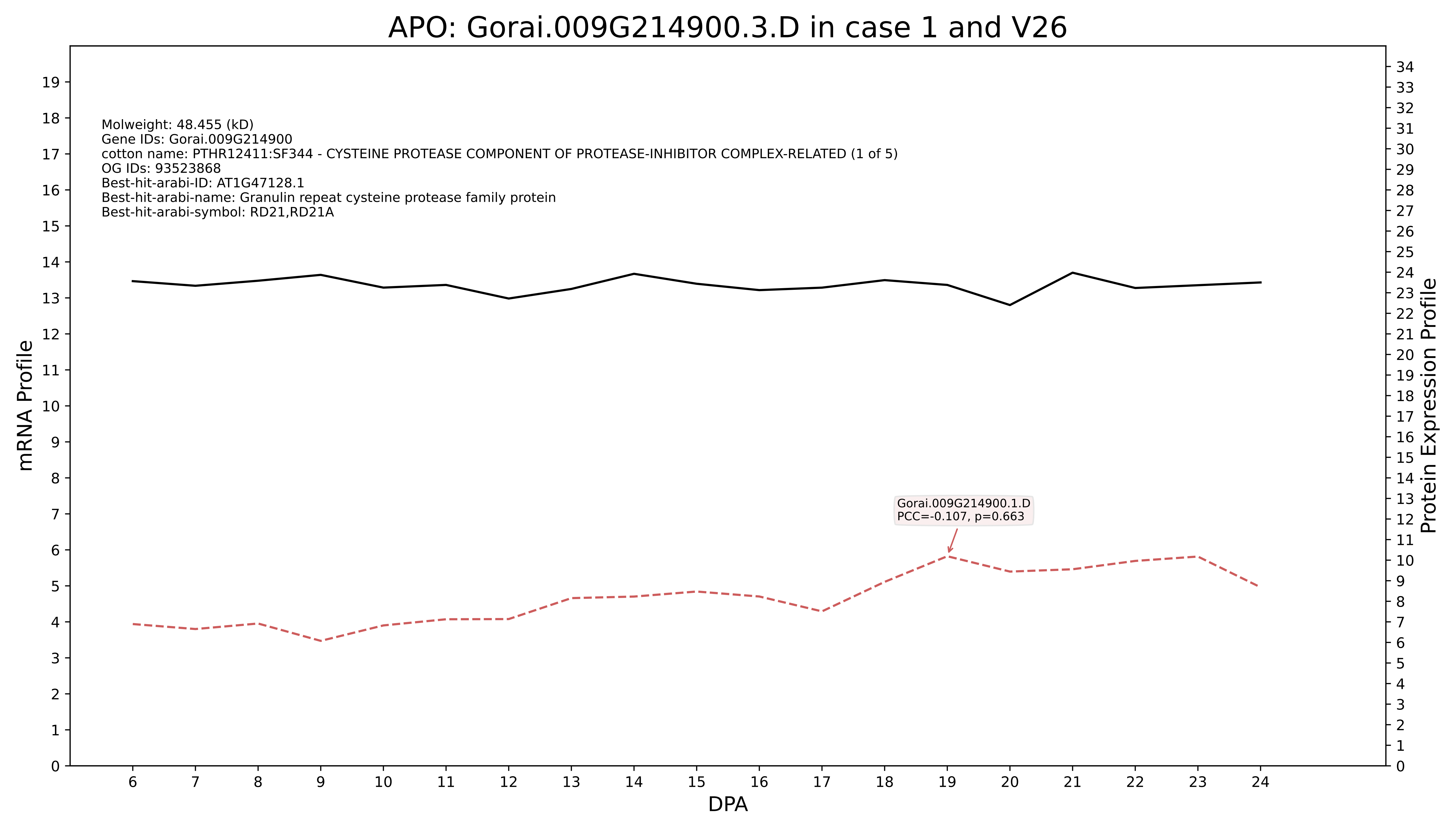
APO: Gorai.009G214900.3.D in case 1 and V26
Molweight: 48.455 (kD)
Gene IDs: Gorai.009G214900
cotton name: PTHR12411:SF344 - CYSTEINE PROTEASE COMPONENT OF PROTEASE-INHIBITOR COMPLEX-RELATED (1 of 5)
OG IDs: 93523868
Best-hit-arabi-ID: AT1G47128.1
Best-hit-arabi-name: Granulin repeat cysteine protease family protein
Best-hit-arabi-symbol: RD21,RD21A
Gorai.009G214900.1.D
PCC=-0.107, p=0.663
mRNA Profile
Protein Expression Profile
DPA

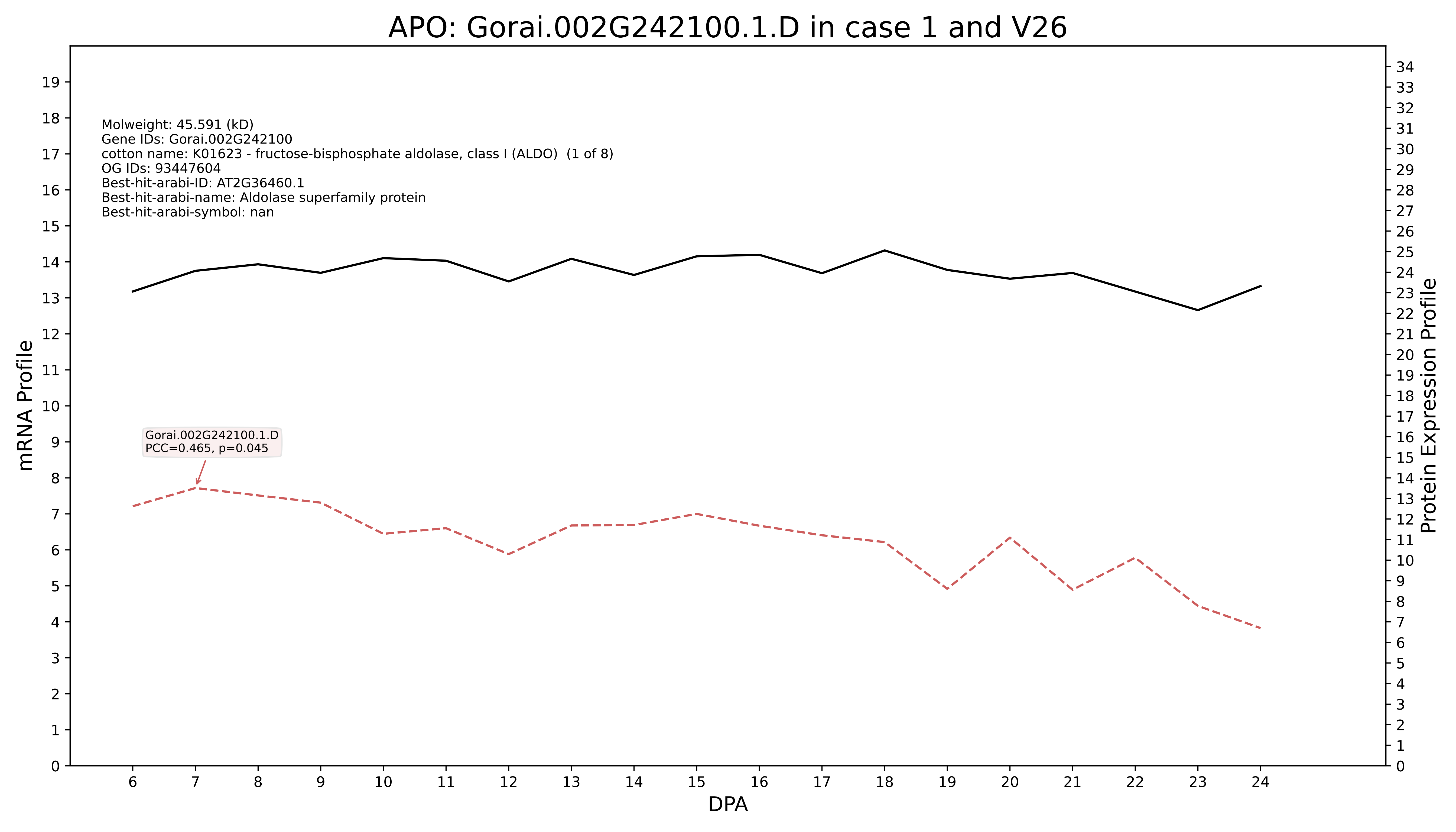
APO: Gorai.002G242100.1.D in case 1 and V26
Molweight: 45.591 (kD)
Gene IDs: Gorai.002G242100
cotton name: K01623 - fructose-bisphosphate aldolase, class I (ALDO) (1 of 8)
OG IDs: 93447604
Best-hit-arabi-ID: AT2G36460.1
Best-hit-arabi-name: Aldolase superfamily protein
Best-hit-arabi-symbol: nan
Gorai.002G242100.1.D
PCC=0.465, p=0.045
mRNA Profile
Protein Expression Profile
DPA

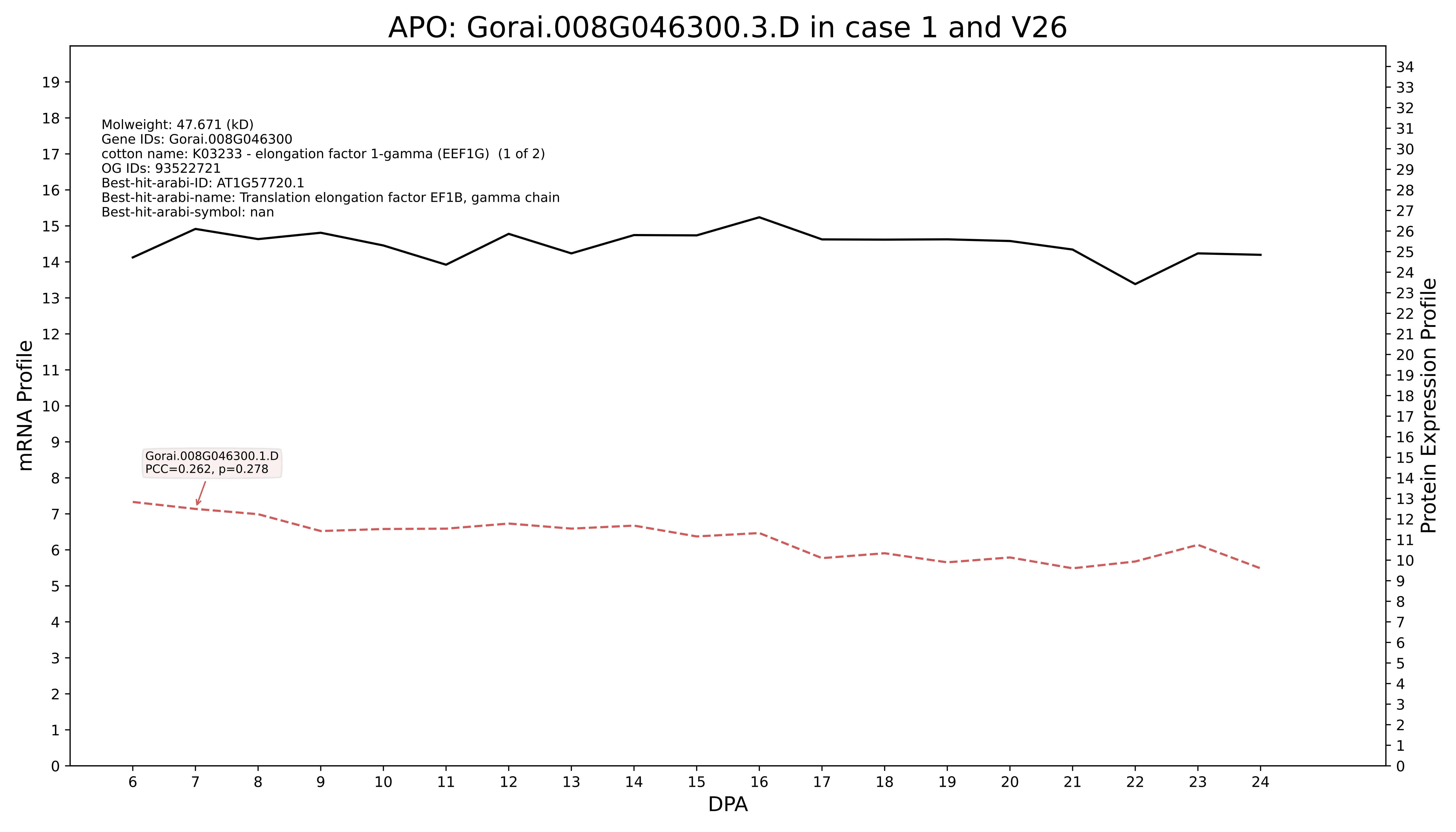

APO: Gorai.008G046300.3.D in case 1 and V26
Molweight: 47.671 (kD)
Gene IDs: Gorai.008G046300
cotton name: K03233 - elongation factor 1-gamma (EEF1G) (1 of 2)
OG IDs: 93522721
Best-hit-arabi-ID: AT1G57720.1
Best-hit-arabi-name: Translation elongation factor EF1B, gamma chain
Best-hit-arabi-symbol: nan
Gorai.008G046300.1.D
PCC=0.262, p=0.278
mRNA Profile
Protein Expression Profile
DPA

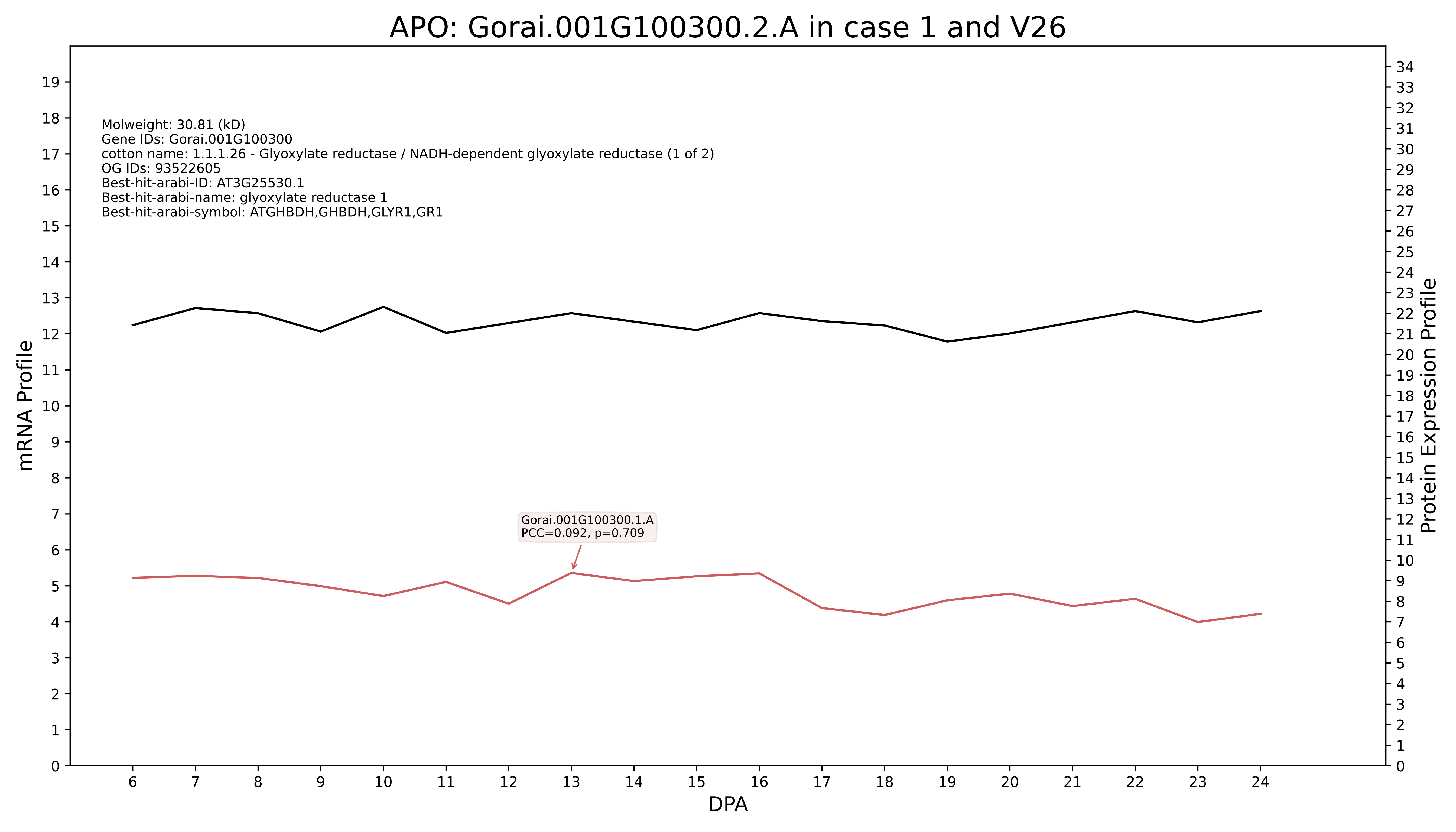

APO: Gorai.001G100300.2.A in case 1 and V26
Molweight: 30.81 (kD)
Gene IDs: Gorai.001G100300
cotton name: 1.1.1.26 - Glyoxylate reductase / NADH-dependent glyoxylate reductase (1 of 2)
OG IDs: 93522605
Best-hit-arabi-ID: AT3G25530.1
Best-hit-arabi-name: glyoxylate reductase 1
Best-hit-arabi-symbol: ATGHBDH,GHBDH,GLYR1,GR1
Gorai.001G100300.1.A
PCC=0.092, p=0.709
mRNA Profile
Protein Expression Profile
DPA

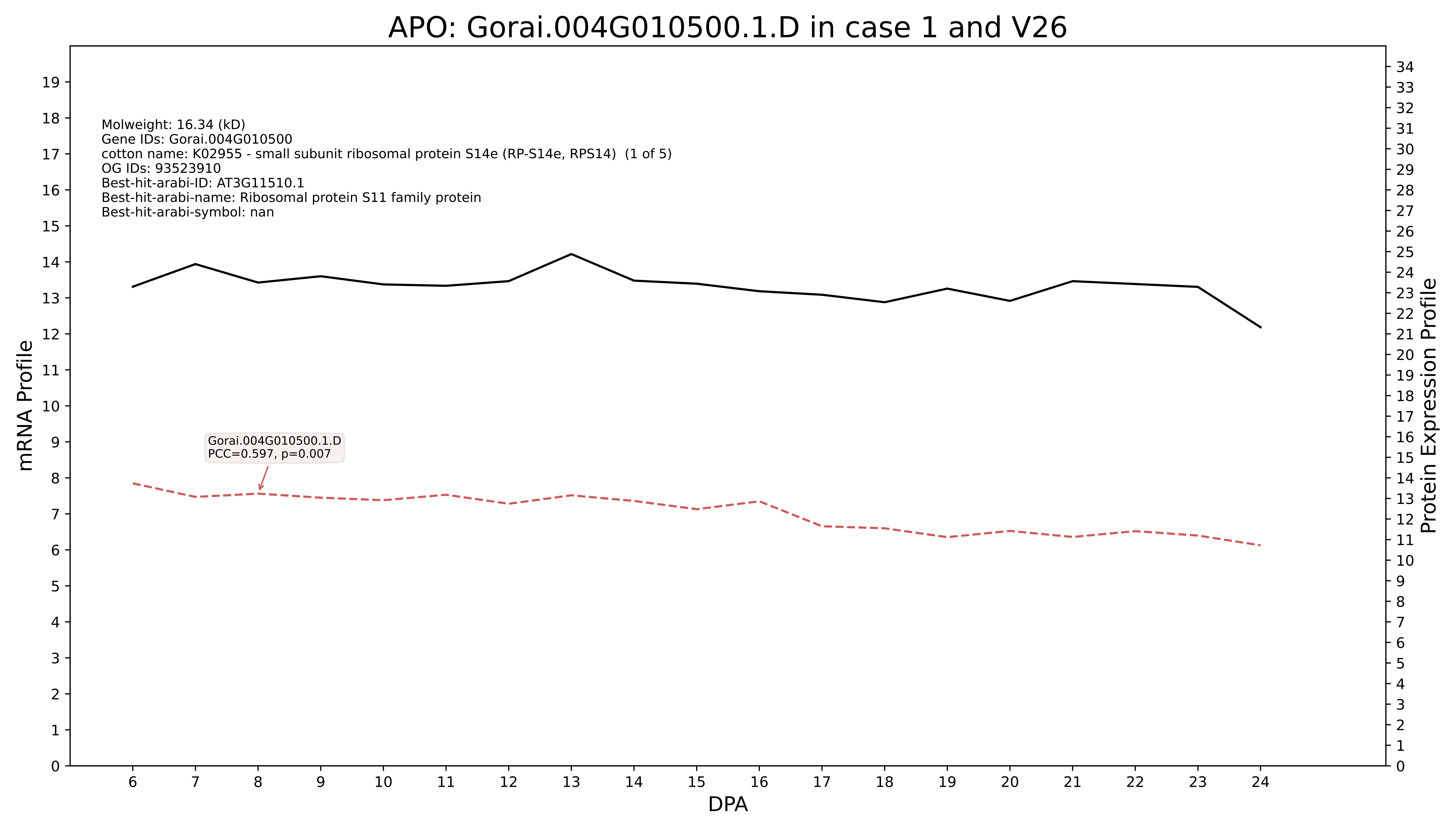

APO: Gorai.004G010500.1.D in case 1 and V26
Molweight: 16.34 (kD)
Gene IDs: Gorai.004G010500
cotton name: K02955 - small subunit ribosomal protein S14e (RP-S14e, RPS14) (1 of 5)
OG IDs: 93523910
Best-hit-arabi-ID: AT3G11510.1
Best-hit-arabi-name: Ribosomal protein S11 family protein
Best-hit-arabi-symbol: nan
Gorai.004G010500.1.D
PCC=0.597, p=0.007
mRNA Profile
Protein Expression Profile
DPA

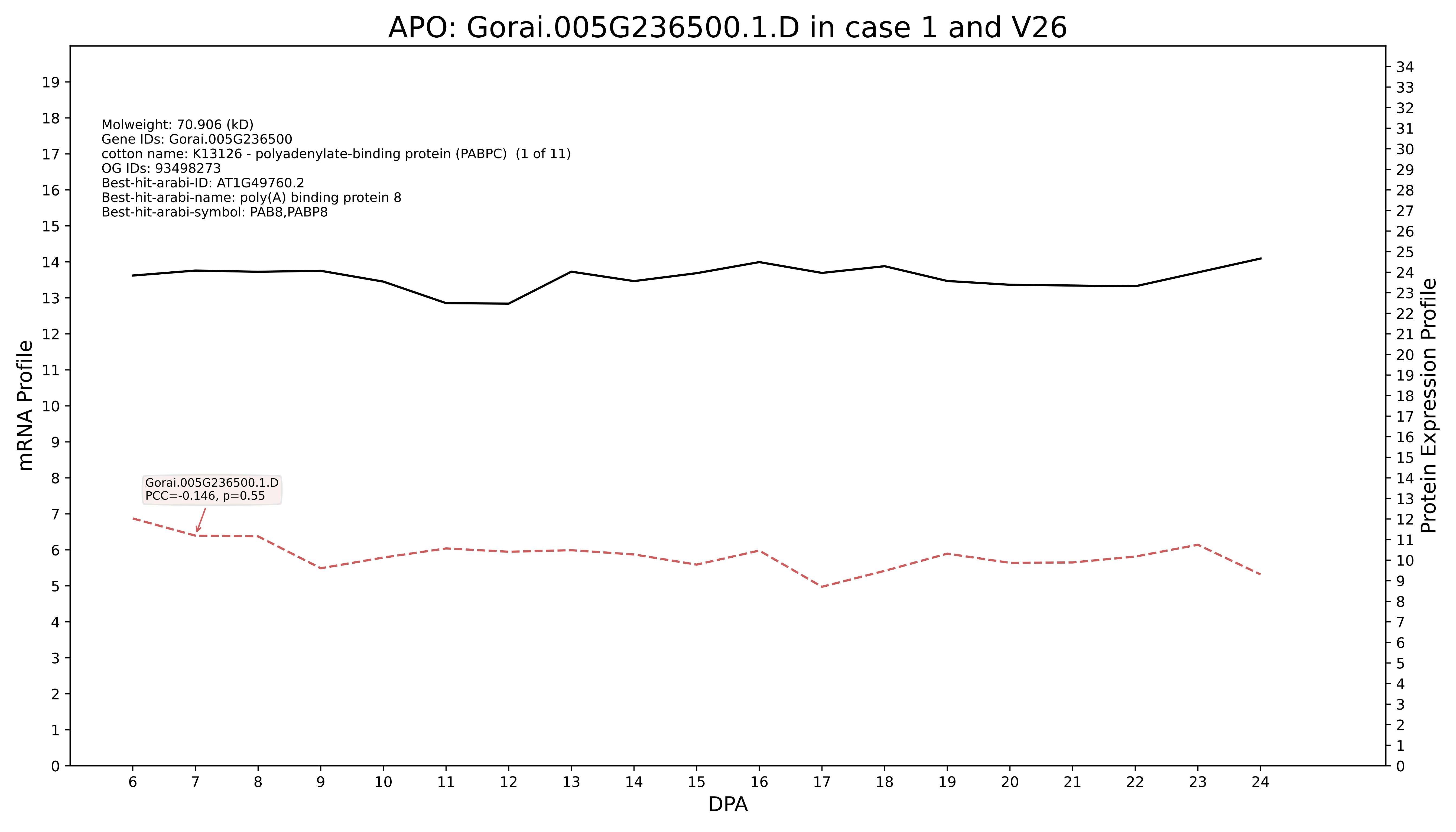

APO: Gorai.005G236500.1.D in case 1 and V26
Molweight: 70.906 (kD)
Gene IDs: Gorai.005G236500
cotton name: K13126 - polyadenylate-binding protein (PABPC) (1 of 11)
OG IDs: 93498273
Best-hit-arabi-ID: AT1G49760.2
Best-hit-arabi-name: poly(A) binding protein 8
Best-hit-arabi-symbol: PAB8,PABP8
Gorai.005G236500.1.D
PCC=-0.146, p=0.55
mRNA Profile
Protein Expression Profile
DPA

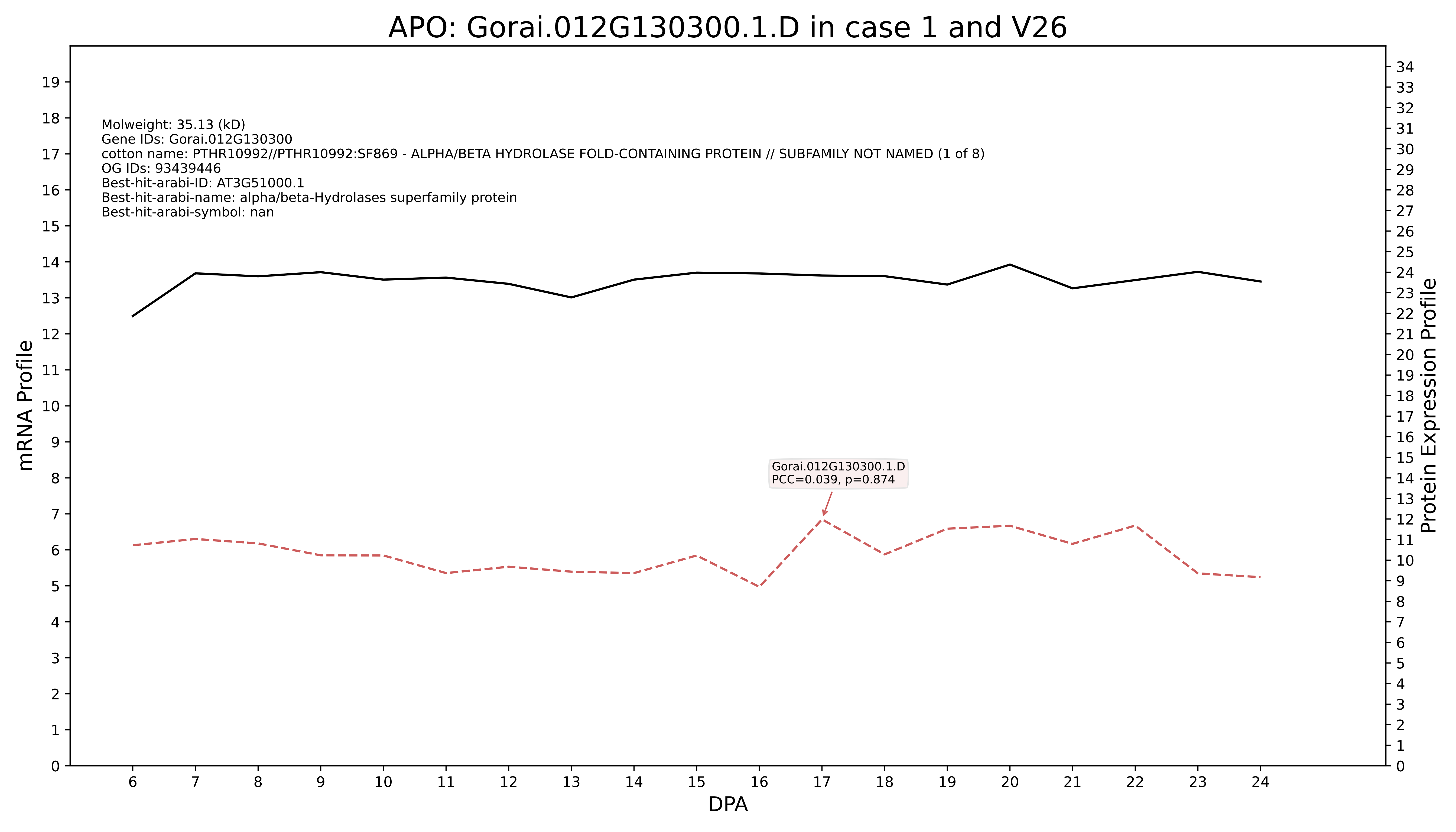

APO: Gorai.012G130300.1.D in case 1 and V26
Molweight: 35.13 (kD)
Gene IDs: Gorai.012G130300
cotton name: PTHR10992//PTHR10992:SF869 - ALPHA/BETA HYDROLASE FOLD-CONTAINING PROTEIN // SUBFAMILY NOT NAMED (1 of 8)
OG IDs: 93439446
Best-hit-arabi-ID: AT3G51000.1
Best-hit-arabi-name: alpha/beta-Hydrolases superfamily protein
Best-hit-arabi-symbol: nan
Gorai.012G130300.1.D
PCC=0.039, p=0.874
mRNA Profile
Protein Expression Profile
DPA

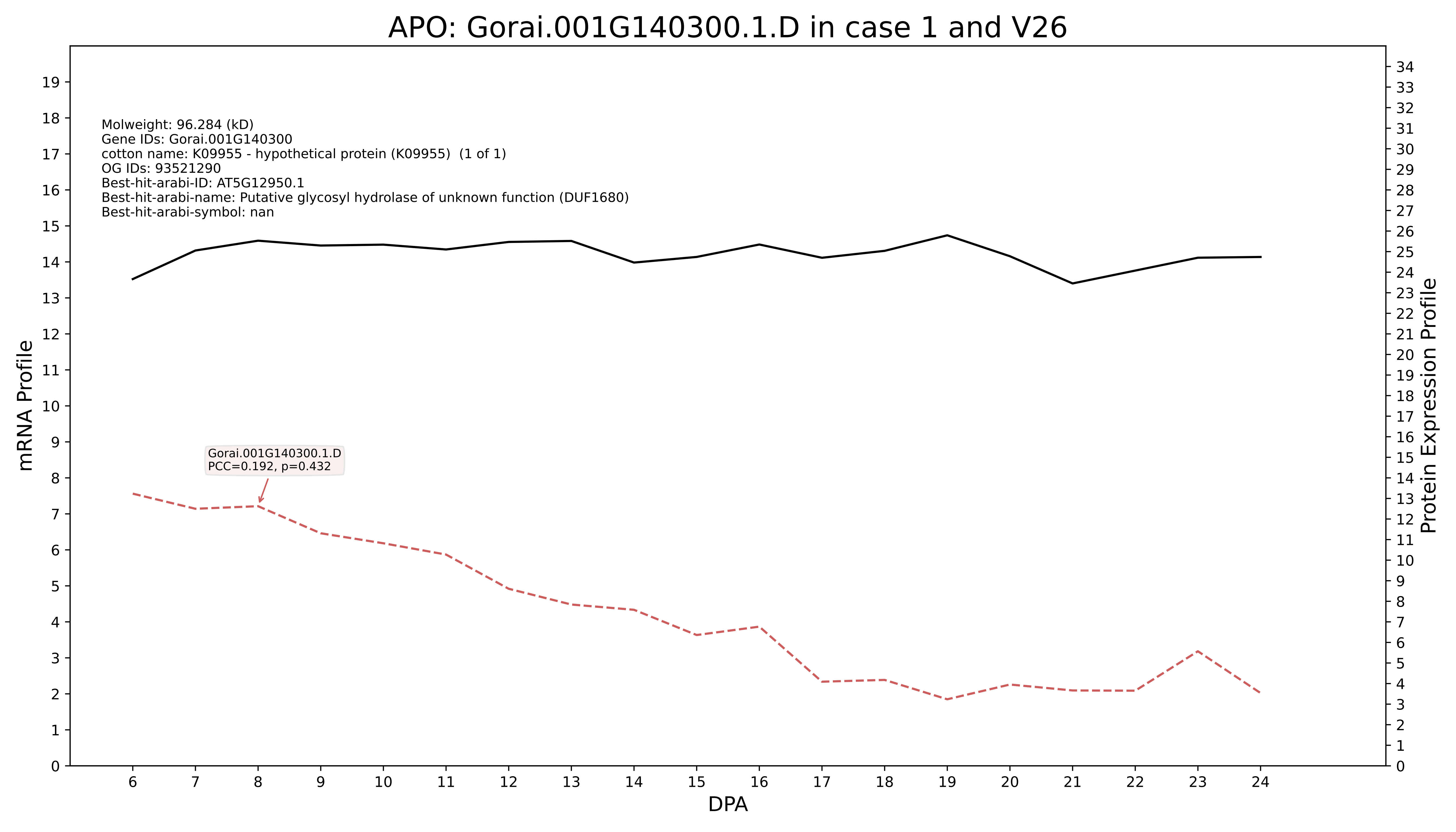

APO: Gorai.001G140300.1.D in case 1 and V26
Molweight: 96.284 (kD)
Gene IDs: Gorai.001G140300
cotton name: K09955 - hypothetical protein (K09955) (1 of 1)
OG IDs: 93521290
Best-hit-arabi-ID: AT5G12950.1
Best-hit-arabi-name: Putative glycosyl hydrolase of unknown function (DUF1680)
Best-hit-arabi-symbol: nan
Gorai.001G140300.1.D
PCC=0.192, p=0.432
mRNA Profile
Protein Expression Profile
DPA

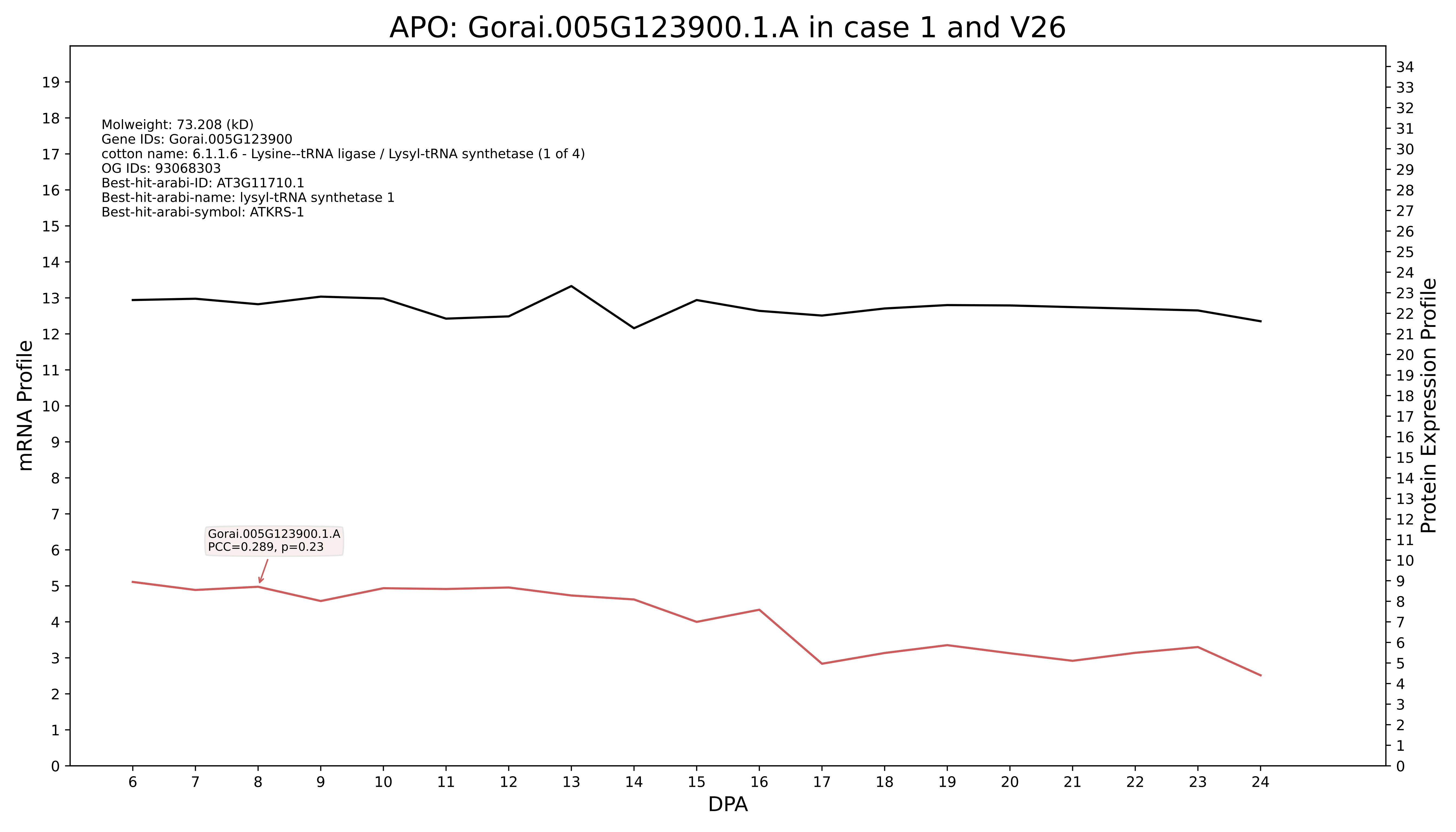
APO: Gorai.005G123900.1.A in case 1 and V26
Molweight: 73.208 (kD)
Gene IDs: Gorai.005G123900
cotton name: 6.1.1.6 - Lysine--tRNA ligase / Lysyl-tRNA synthetase (1 of 4)
OG IDs: 93068303
Best-hit-arabi-ID: AT3G11710.1
Best-hit-arabi-name: lysyl-tRNA synthetase 1
Best-hit-arabi-symbol: ATKRS-1
Gorai.005G123900.1.A
PCC=0.289, p=0.23
mRNA Profile
Protein Expression Profile
DPA

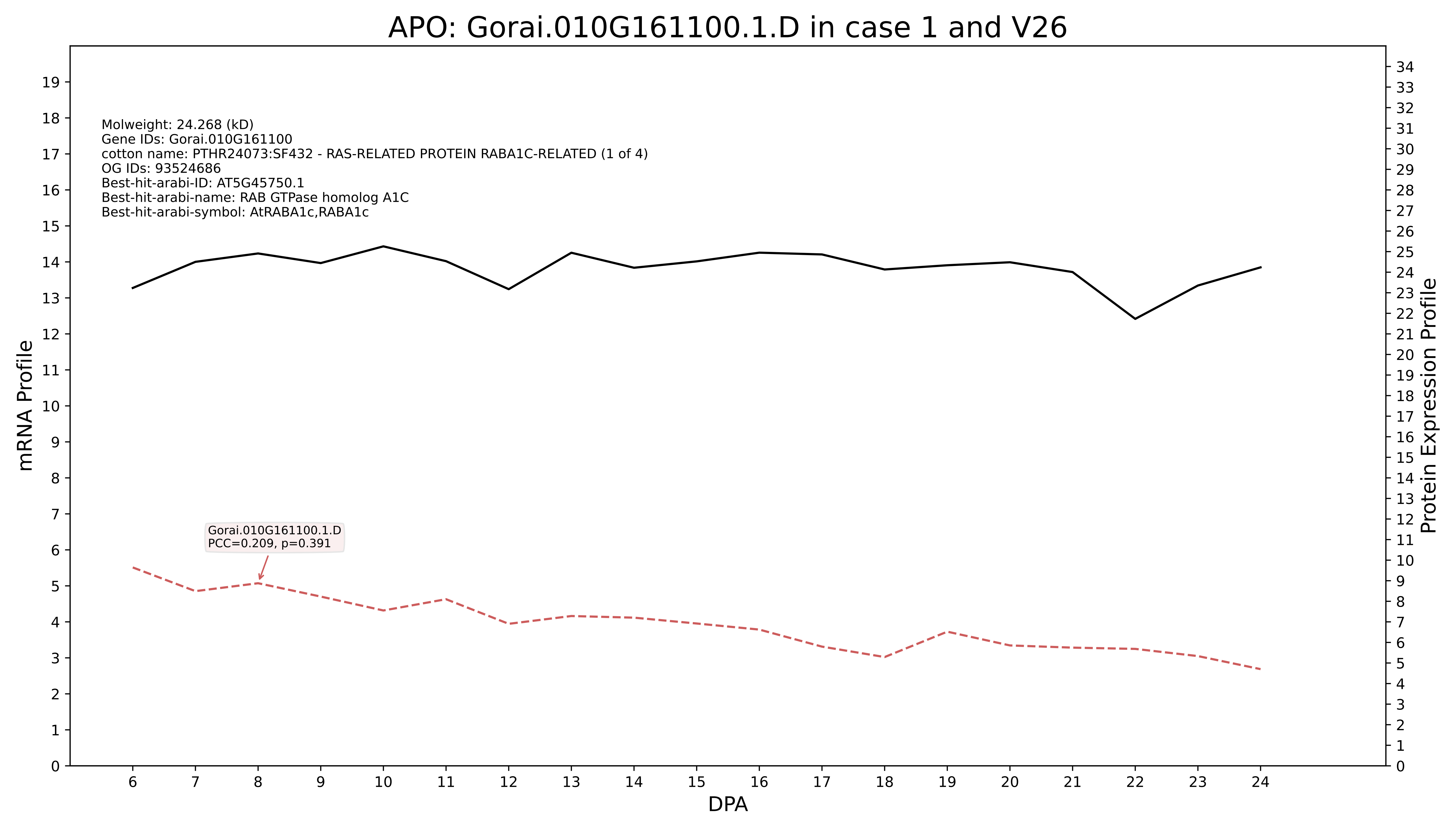

APO: Gorai.010G161100.1.D in case 1 and V26
Molweight: 24.268 (kD)
Gene IDs: Gorai.010G161100
cotton name: PTHR24073:SF432 - RAS-RELATED PROTEIN RABA1C-RELATED (1 of 4)
OG IDs: 93524686
Best-hit-arabi-ID: AT5G45750.1
Best-hit-arabi-name: RAB GTPase homolog A1C
Best-hit-arabi-symbol: AtRABA1c,RABA1c
Gorai.010G161100.1.D
PCC=0.209, p=0.391
mRNA Profile
Protein Expression Profile
DPA

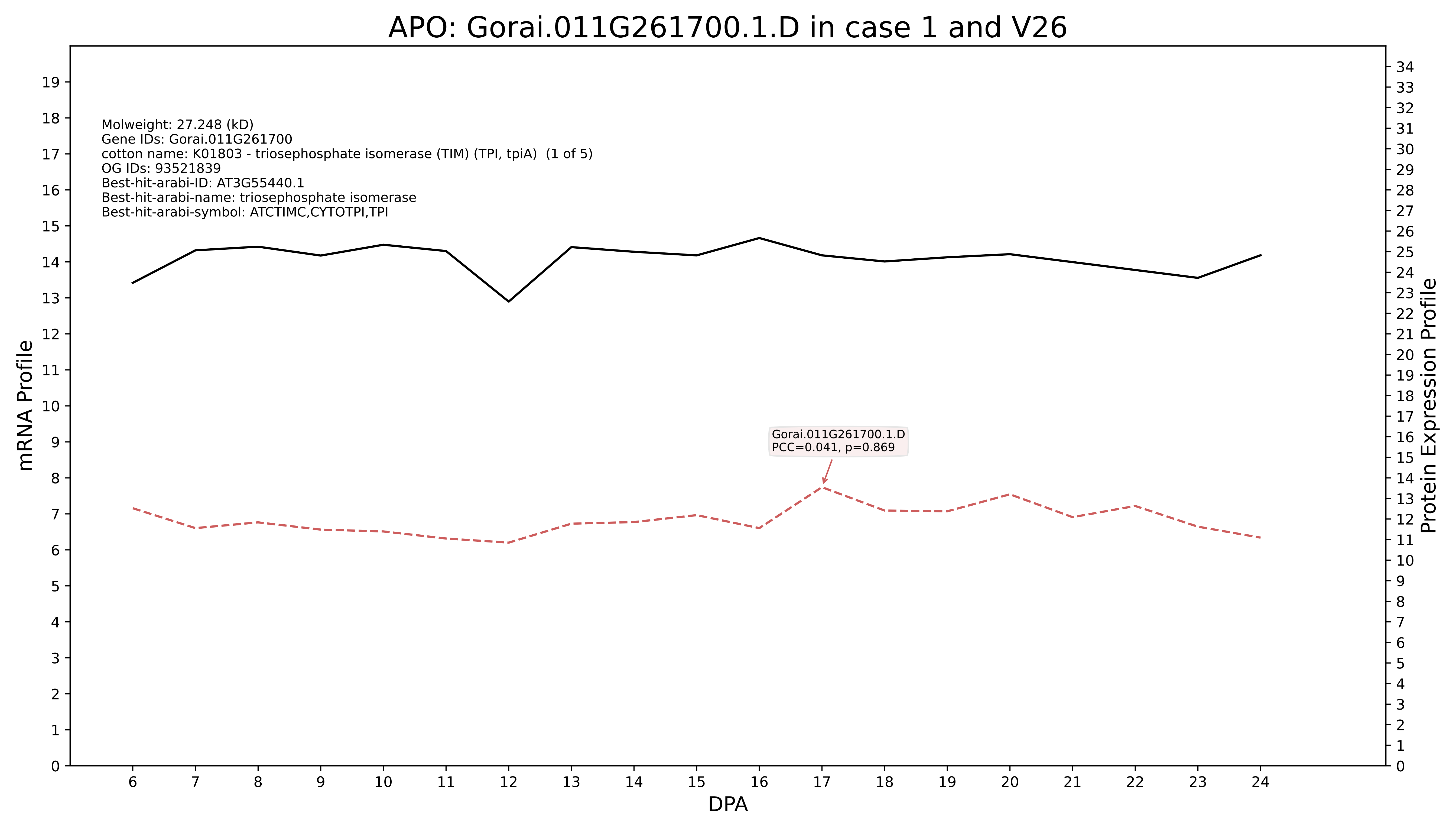
APO: Gorai.011G261700.1.D in case 1 and V26
Molweight: 27.248 (kD)
Gene IDs: Gorai.011G261700
cotton name: K01803 - triosephosphate isomerase (TIM) (TPI, tpiA) (1 of 5)
OG IDs: 93521839
Best-hit-arabi-ID: AT3G55440.1
Best-hit-arabi-name: triosephosphate isomerase
Best-hit-arabi-symbol: ATCTIMC,CYTOTPI,TPI
Gorai.011G261700.1.D
PCC=0.041, p=0.869
mRNA Profile
Protein Expression Profile
DPA

APO: Gorai.005G132500.1.A in case 1 and V26

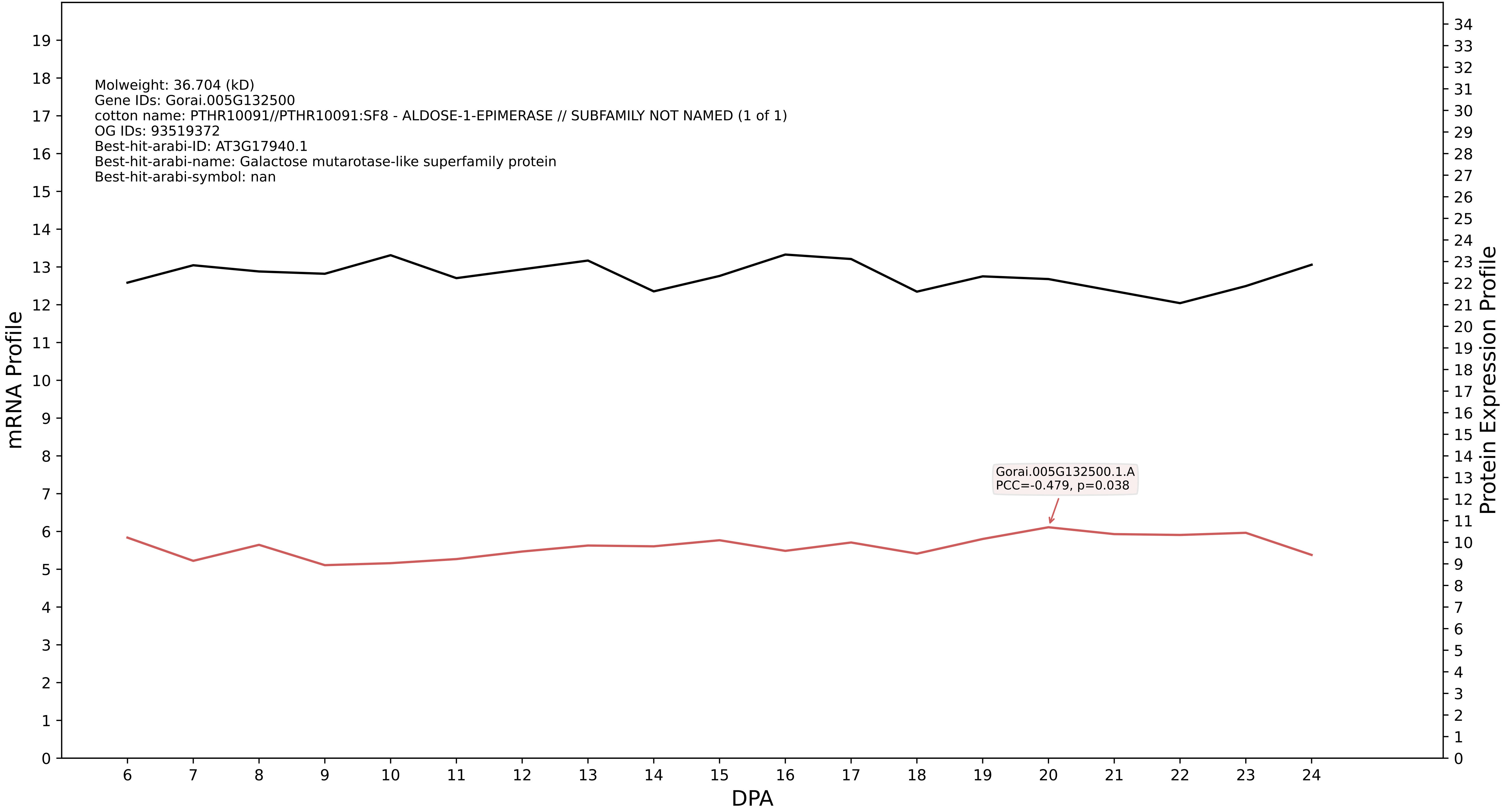

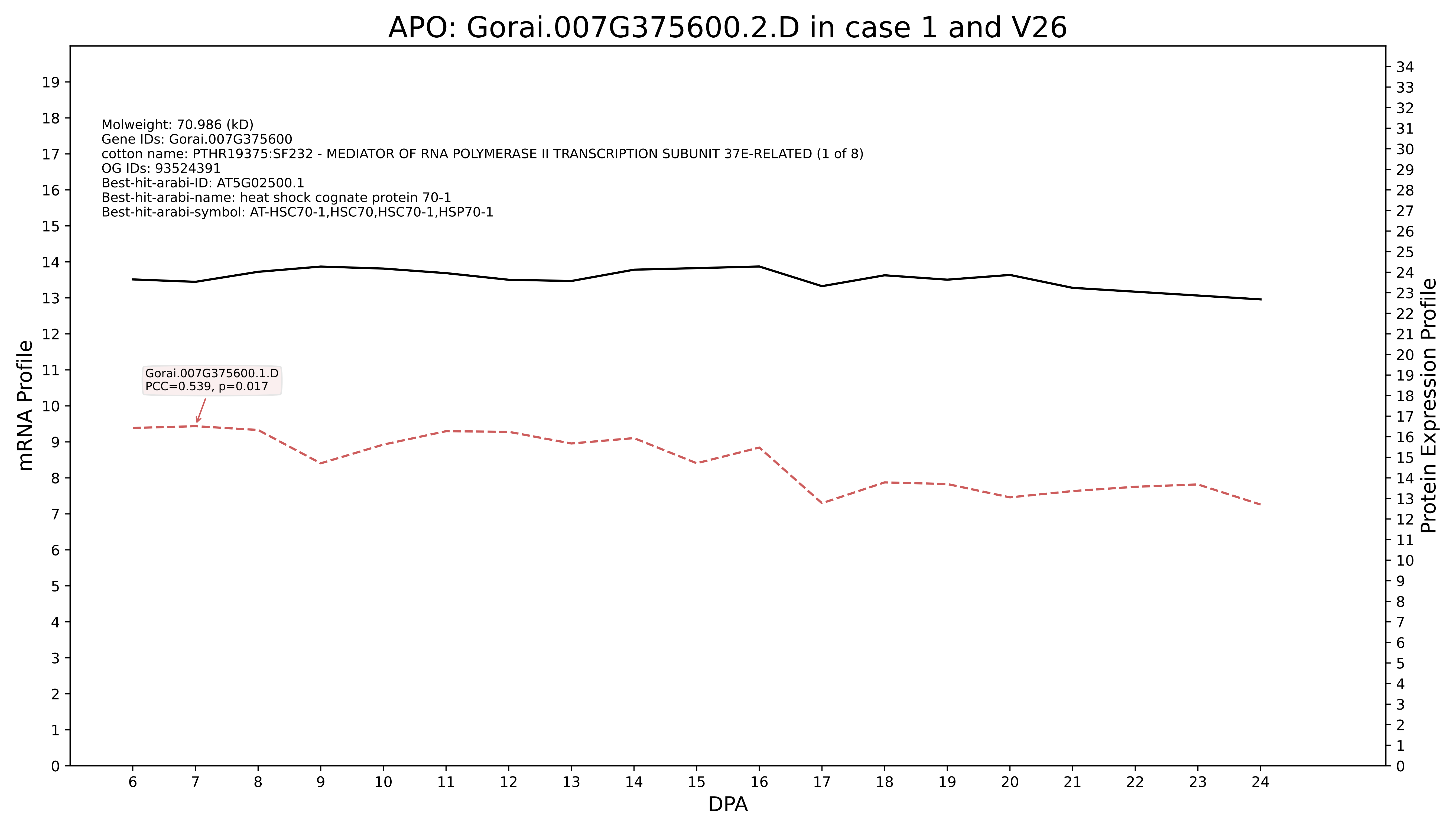
APO: Gorai.007G375600.2.D in case 1 and V26
Molweight: 70.986 (kD)
Gene IDs: Gorai.007G375600
cotton name: PTHR19375:SF232 - MEDIATOR OF RNA POLYMERASE II TRANSCRIPTION SUBUNIT 37E-RELATED (1 of 8)
OG IDs: 93524391
Best-hit-arabi-ID: AT5G02500.1
Best-hit-arabi-name: heat shock cognate protein 70-1
Best-hit-arabi-symbol: AT-HSC70-1,HSC70,HSC70-1,HSP70-1
Gorai.007G375600.1.D
PCC=0.539, p=0.017
mRNA Profile
Protein Expression Profile
DPA

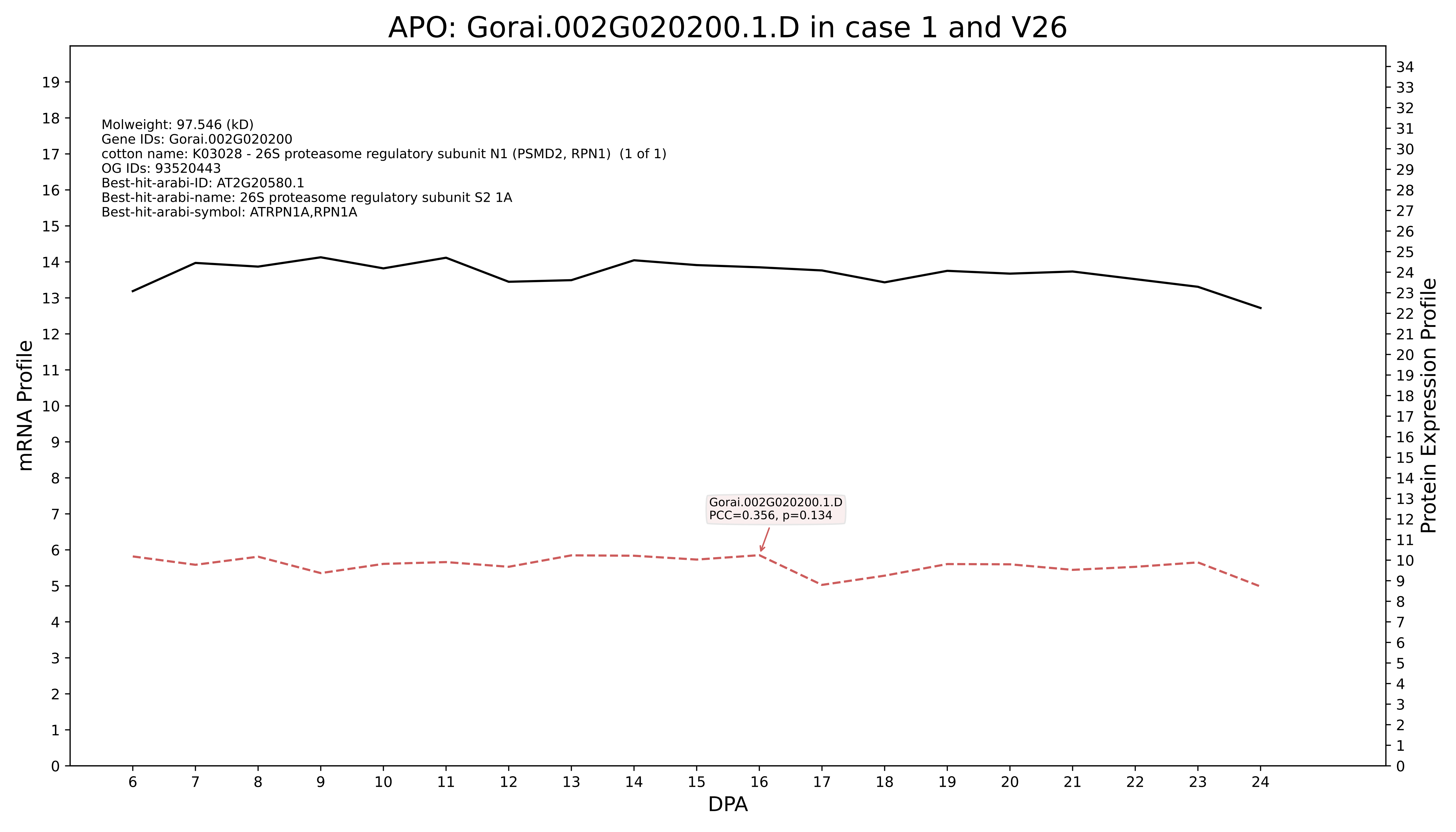
APO: Gorai.002G020200.1.D in case 1 and V26
Molweight: 97.546 (kD)
Gene IDs: Gorai.002G020200
cotton name: K03028 - 26S proteasome regulatory subunit N1 (PSMD2, RPN1) (1 of 1)
OG IDs: 93520443
Best-hit-arabi-ID: AT2G20580.1
Best-hit-arabi-name: 26S proteasome regulatory subunit S2 1A
Best-hit-arabi-symbol: ATRPN1A,RPN1A
Gorai.002G020200.1.D
PCC=0.356, p=0.134
mRNA Profile
Protein Expression Profile
DPA

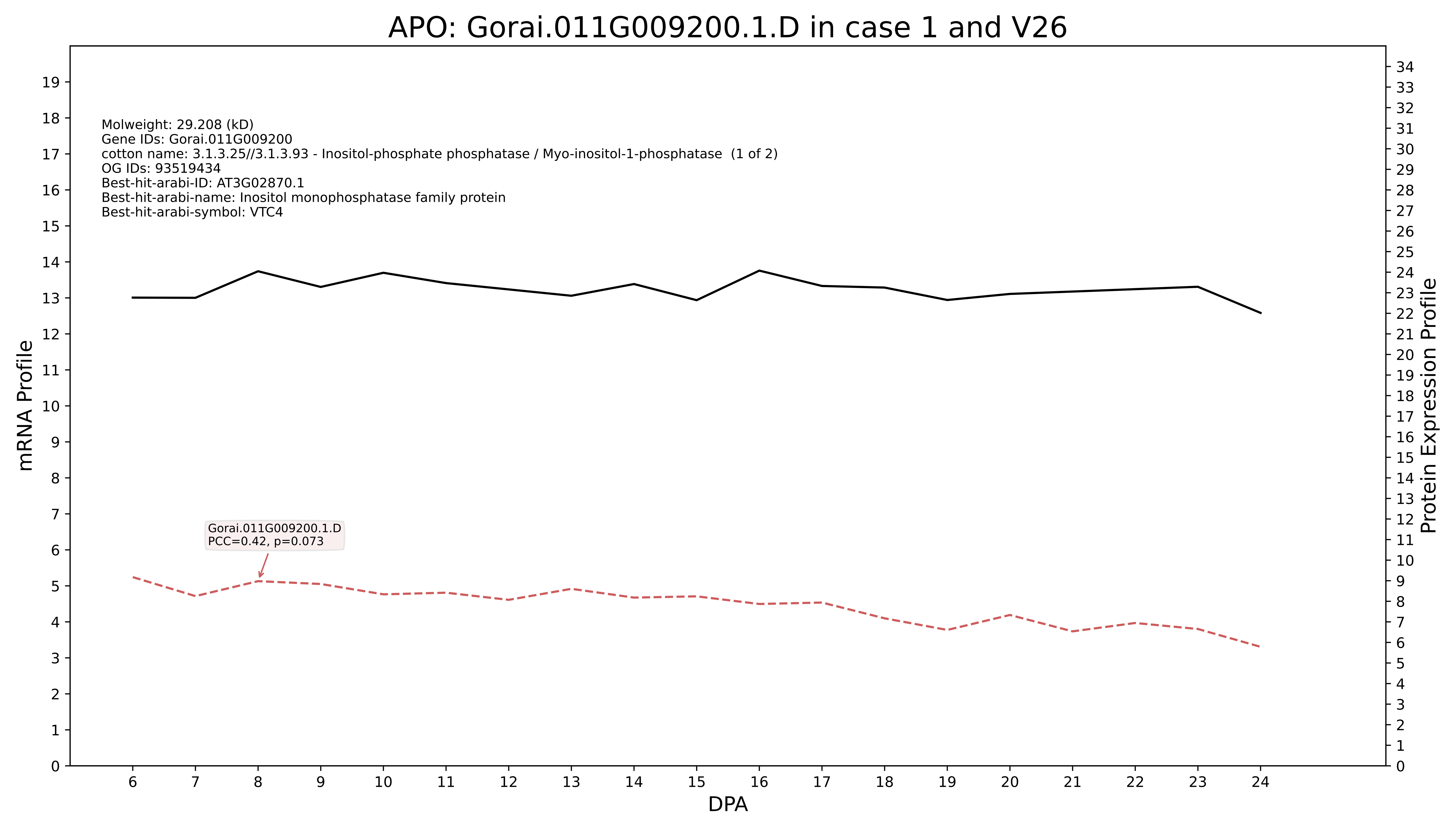
APO: Gorai.011G009200.1.D in case 1 and V26
Molweight: 29.208 (kD)
Gene IDs: Gorai.011G009200
cotton name: 3.1.3.25//3.1.3.93 - Inositol-phosphate phosphatase / Myo-inositol-1-phosphatase (1 of 2)
OG IDs: 93519434
Best-hit-arabi-ID: AT3G02870.1
Best-hit-arabi-name: Inositol monophosphatase family protein
Best-hit-arabi-symbol: VTC4
Gorai.011G009200.1.D
PCC=0.42, p=0.073
mRNA Profile
Protein Expression Profile
DPA

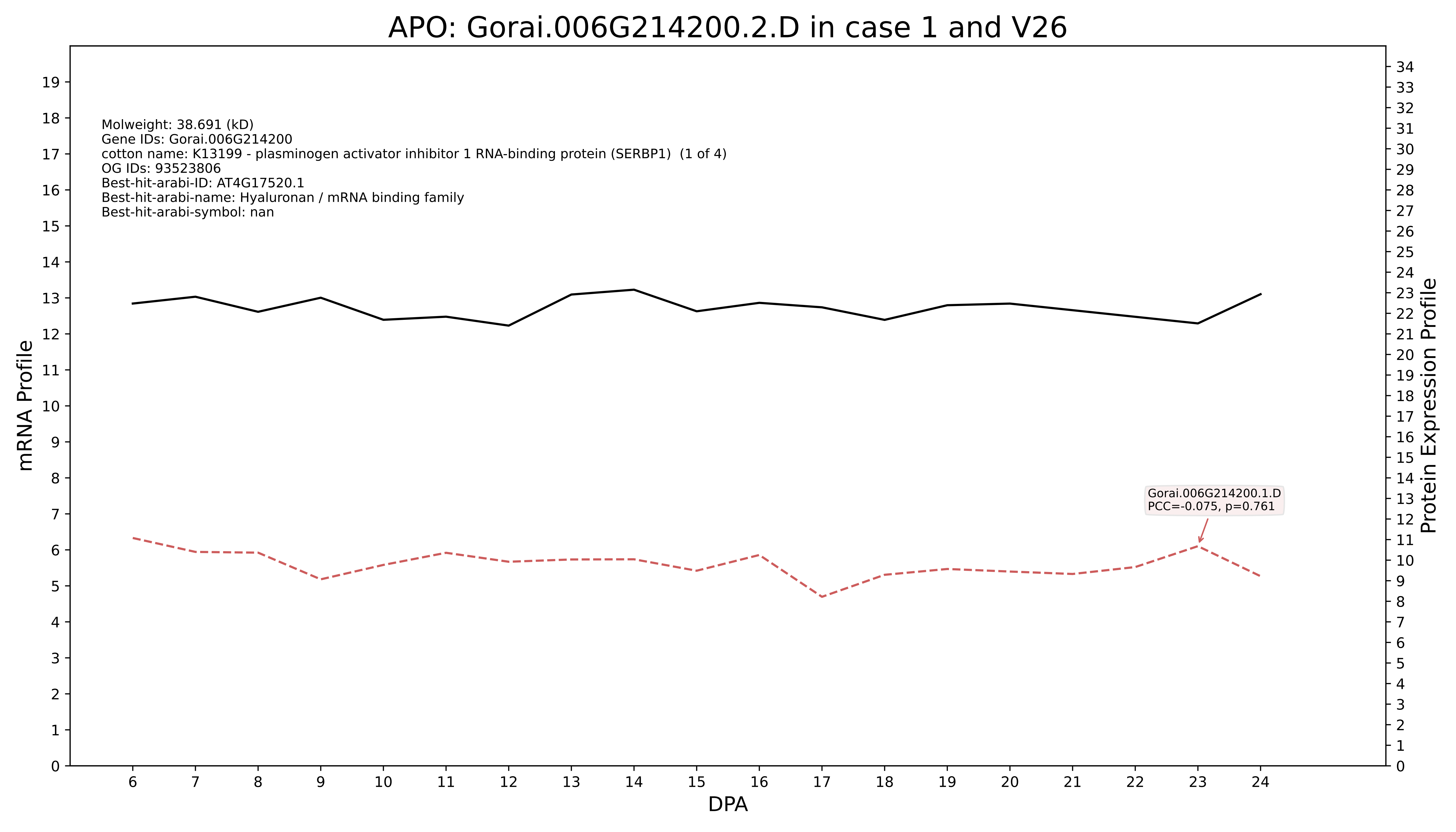

APO: Gorai.006G214200.2.D in case 1 and V26
Molweight: 38.691 (kD)
Gene IDs: Gorai.006G214200
cotton name: K13199 - plasminogen activator inhibitor 1 RNA-binding protein (SERBP1) (1 of 4)
OG IDs: 93523806
Best-hit-arabi-ID: AT4G17520.1
Best-hit-arabi-name: Hyaluronan / mRNA binding family
Best-hit-arabi-symbol: nan
Gorai.006G214200.1.D
PCC=-0.075, p=0.761
mRNA Profile
Protein Expression Profile
DPA

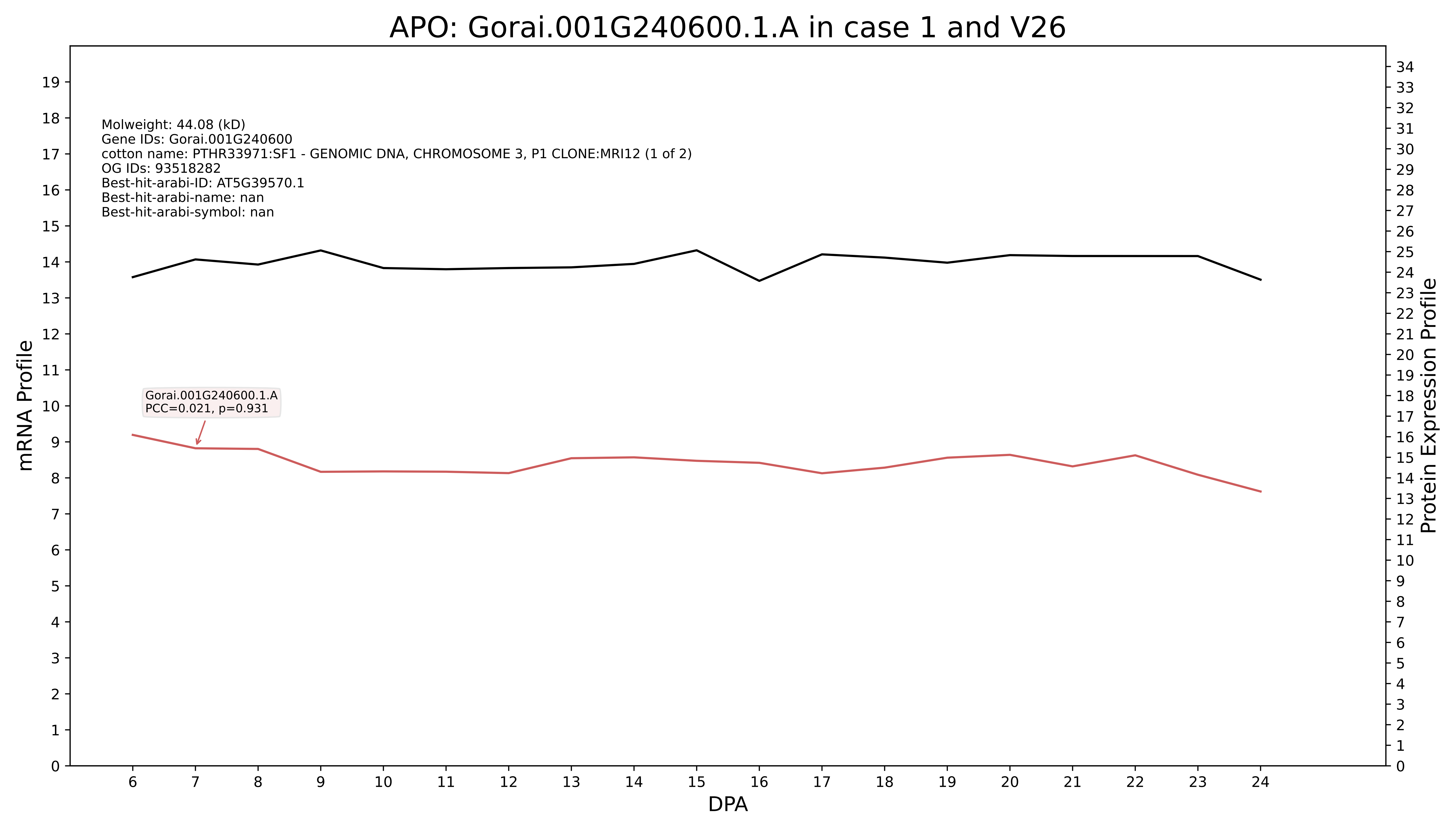
APO: Gorai.001G240600.1.A in case 1 and V26
Molweight: 44.08 (kD)
Gene IDs: Gorai.001G240600
cotton name: PTHR33971:SF1 - GENOMIC DNA, CHROMOSOME 3, P1 CLONE:MRI12 (1 of 2)
OG IDs: 93518282
Best-hit-arabi-ID: AT5G39570.1
Best-hit-arabi-name: nan
Best-hit-arabi-symbol: nan
Gorai.001G240600.1.A
PCC=0.021, p=0.931
mRNA Profile
Protein Expression Profile
DPA

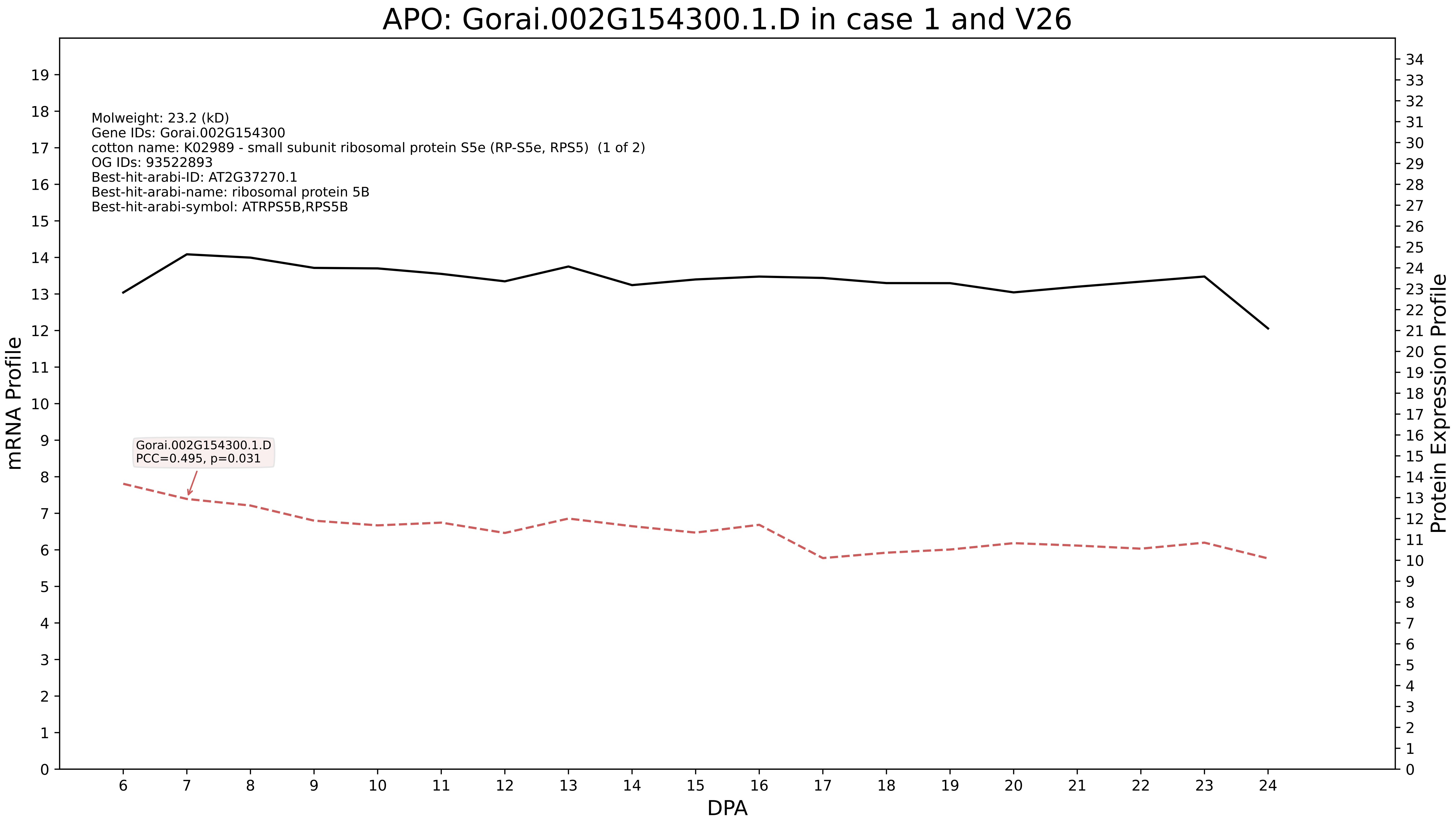
APO: Gorai.002G154300.1.D in case 1 and V26
Molweight: 23.2 (kD)
Gene IDs: Gorai.002G154300
cotton name: K02989 - small subunit ribosomal protein S5e (RP-S5e, RPS5) (1 of 2)
OG IDs: 93522893
Best-hit-arabi-ID: AT2G37270.1
Best-hit-arabi-name: ribosomal protein 5B
Best-hit-arabi-symbol: ATRPS5B,RPS5B
Gorai.002G154300.1.D
PCC=0.495, p=0.031
mRNA Profile
Protein Expression Profile
DPA
0
1
2
3
4
5
6
7
8
9
10
11
12
13
14
15
16
17
18
19
20
21
22
23
24
25
26
27
28
29
30
31
32
33
34

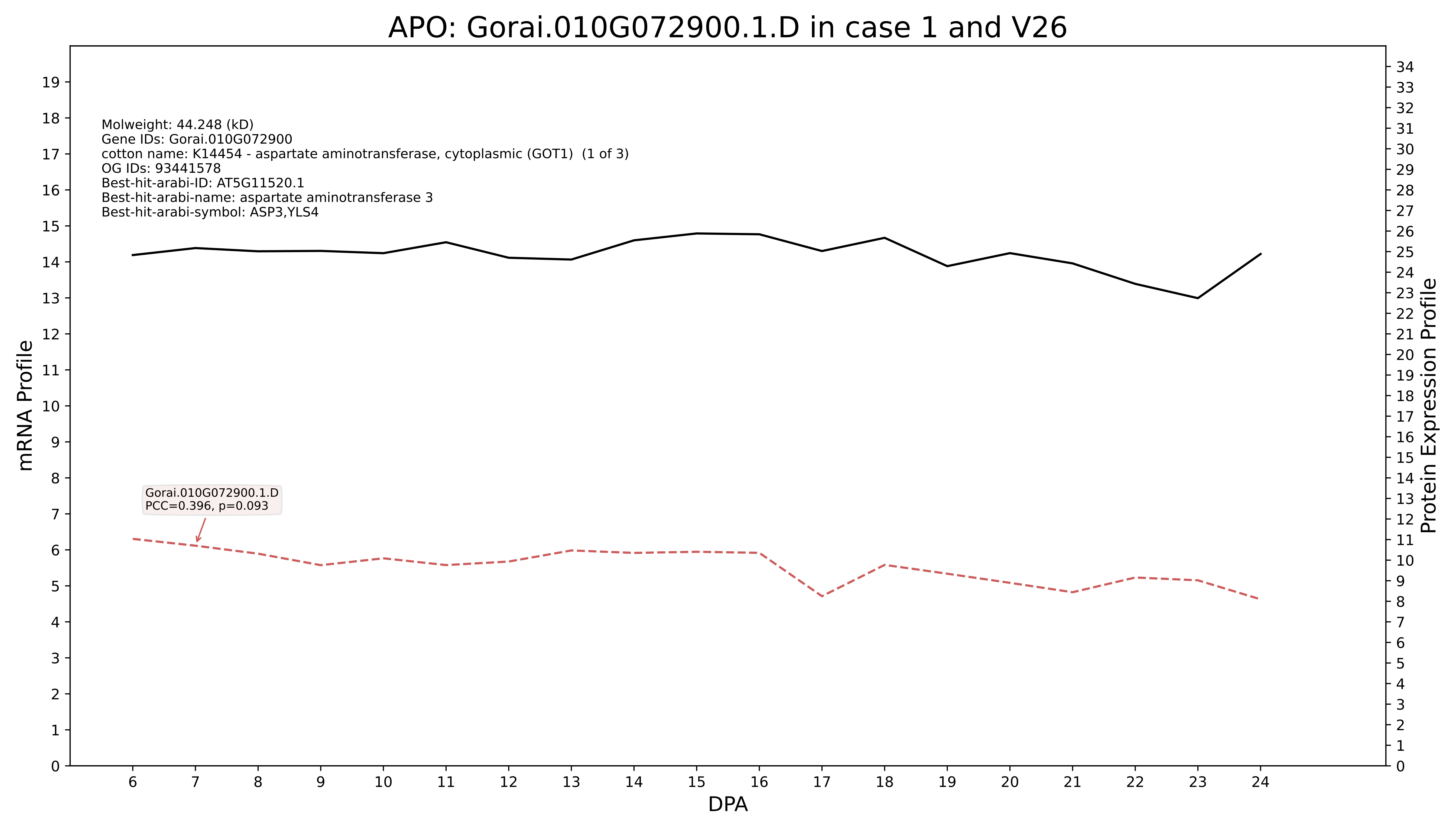
APO: Gorai.010G072900.1.D in case 1 and V26
Molweight: 44.248 (kD)
Gene IDs: Gorai.010G072900
cotton name: K14454 - aspartate aminotransferase, cytoplasmic (GOT1) (1 of 3)
OG IDs: 93441578
Best-hit-arabi-ID: AT5G11520.1
Best-hit-arabi-name: aspartate aminotransferase 3
Best-hit-arabi-symbol: ASP3,YLS4
Gorai.010G072900.1.D
PCC=0.396, p=0.093
mRNA Profile
Protein Expression Profile
DPA

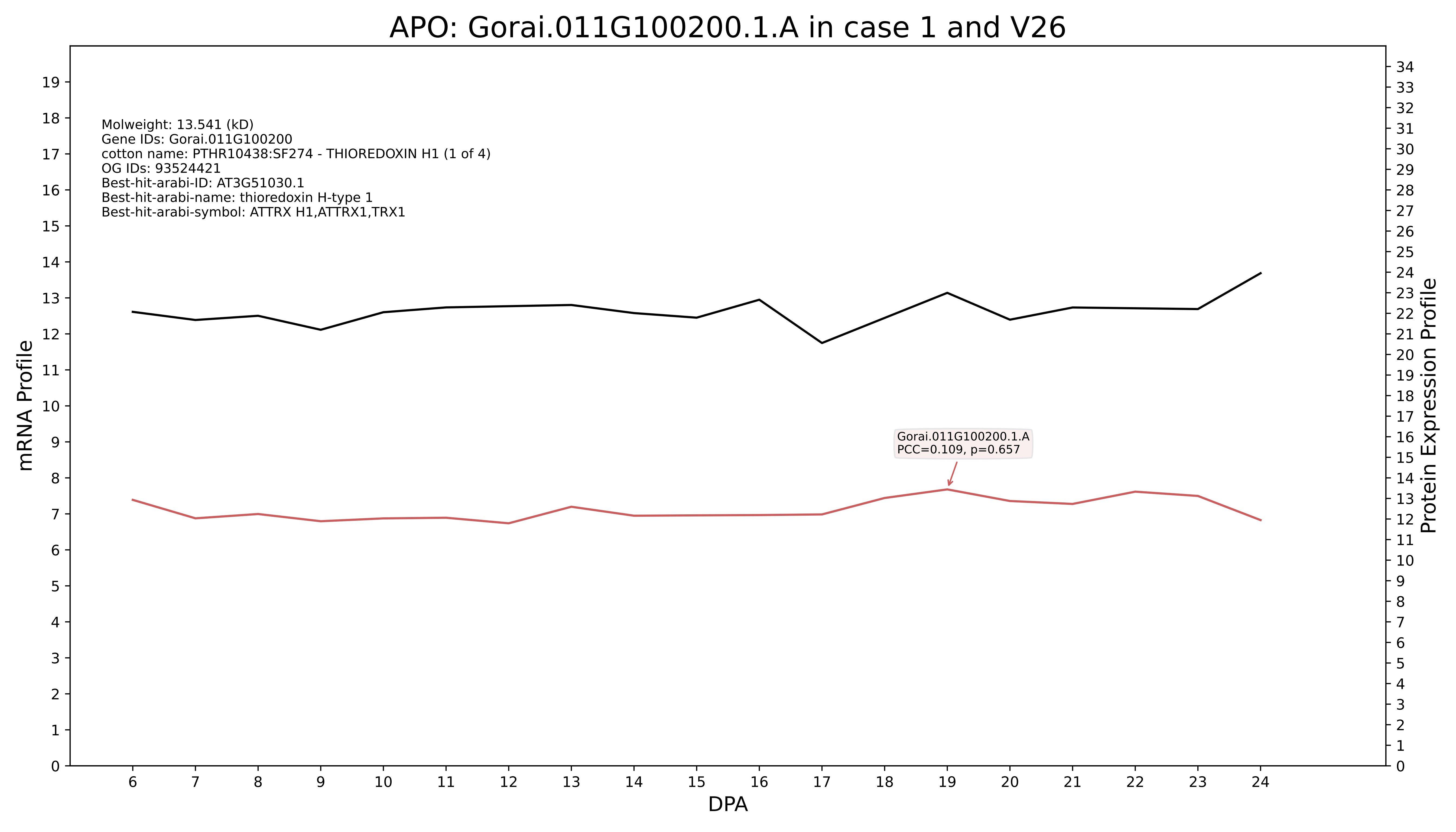
APO: Gorai.011G100200.1.A in case 1 and V26
Molweight: 13.541 (kD)
Gene IDs: Gorai.011G100200
cotton name: PTHR10438:SF274 - THIOREDOXIN H1 (1 of 4)
OG IDs: 93524421
Best-hit-arabi-ID: AT3G51030.1
Best-hit-arabi-name: thioredoxin H-type 1
Best-hit-arabi-symbol: ATTRX H1,ATTRX1,TRX1
Gorai.011G100200.1.A
PCC=0.109, p=0.657
mRNA Profile
Protein Expression Profile
DPA

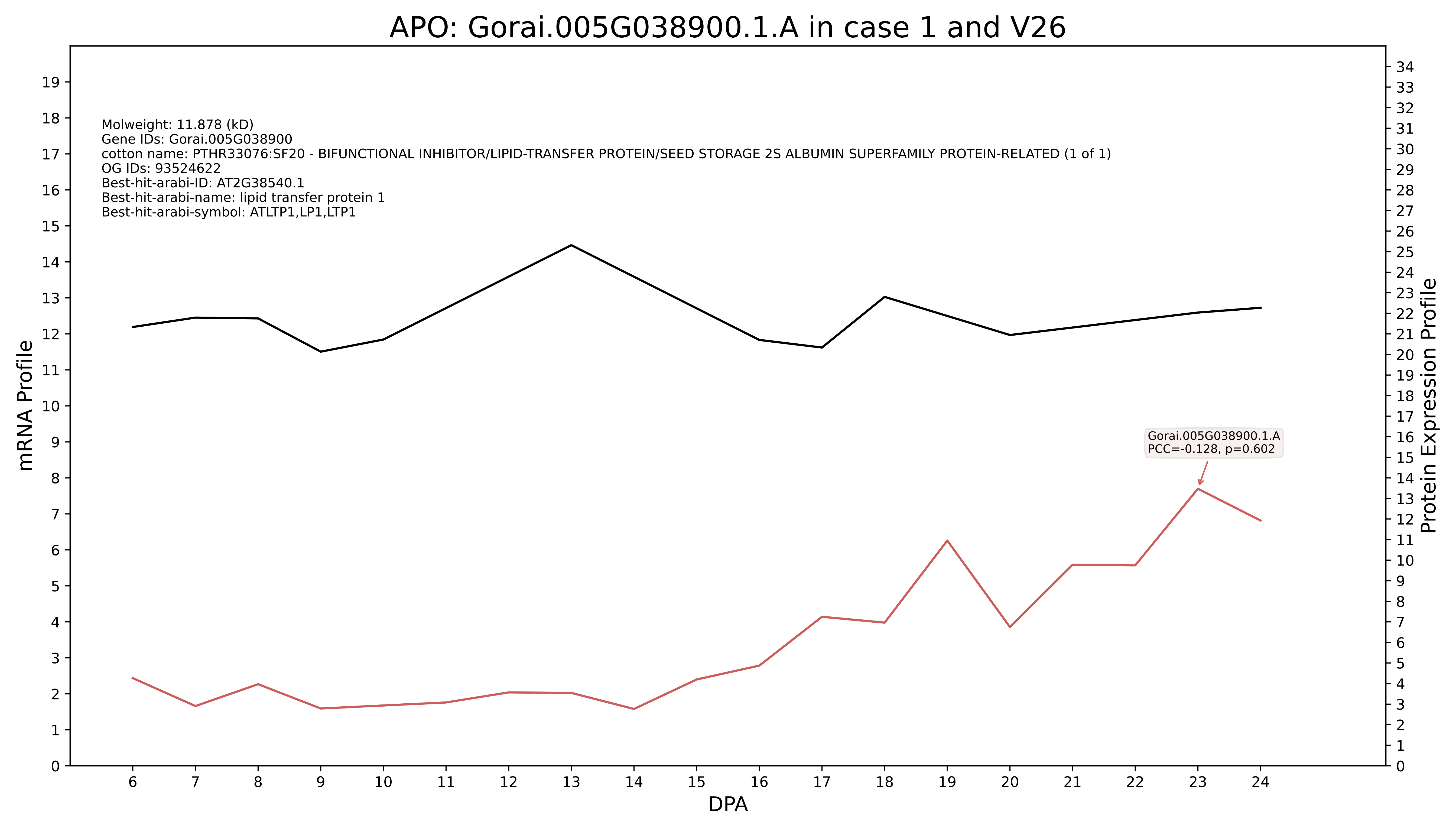

APO: Gorai.005G038900.1.A in case 1 and V26
Molweight: 11.878 (kD)
Gene IDs: Gorai.005G038900
cotton name: PTHR33076:SF20 - BIFUNCTIONAL INHIBITOR/LIPID-TRANSFER PROTEIN/SEED STORAGE 2S ALBUMIN SUPERFAMILY PROTEIN-RELATED (1 of 1)
OG IDs: 93524622
Best-hit-arabi-ID: AT2G38540.1
Best-hit-arabi-name: lipid transfer protein 1
Best-hit-arabi-symbol: ATLTP1,LP1,LTP1
Gorai.005G038900.1.A
PCC=-0.128, p=0.602
mRNA Profile
Protein Expression Profile
DPA
0
1
2
3
4
5
6
7
8
9
10
11
12
13
14
15
16
17
18
19
20
21
22
23
24
25
26
27
28
29
30
31
32
33
34

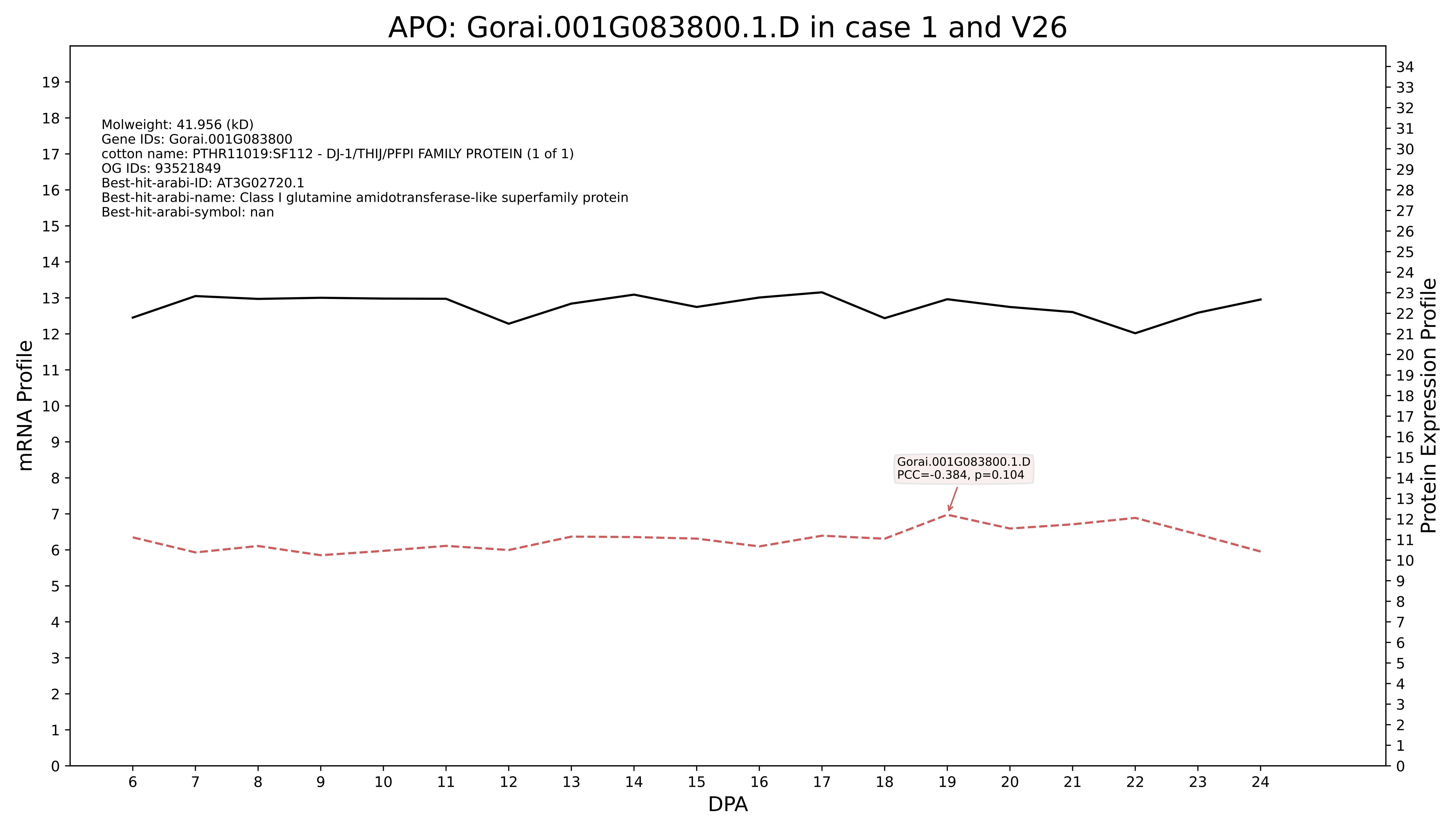
APO: Gorai.001G083800.1.D in case 1 and V26
Molweight: 41.956 (kD)
Gene IDs: Gorai.001G083800
cotton name: PTHR11019:SF112 - DJ-1/THIJ/PFPI FAMILY PROTEIN (1 of 1)
OG IDs: 93521849
Best-hit-arabi-ID: AT3G02720.1
Best-hit-arabi-name: Class I glutamine amidotransferase-like superfamily protein
Best-hit-arabi-symbol: nan
Gorai.001G083800.1.D
PCC=-0.384, p=0.104
mRNA Profile
Protein Expression Profile
DPA

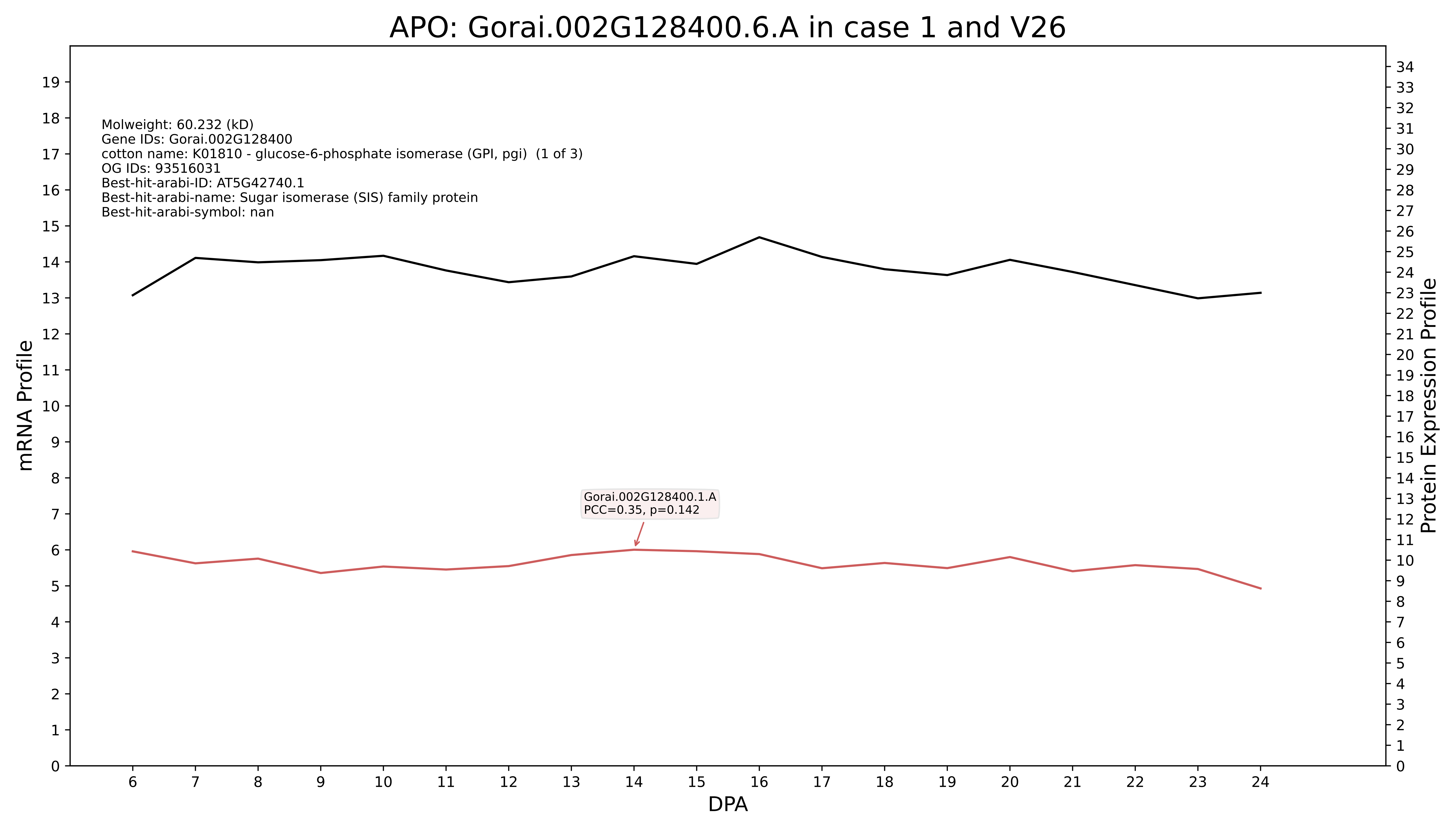
APO: Gorai.002G128400.6.A in case 1 and V26
Molweight: 60.232 (kD)
Gene IDs: Gorai.002G128400
cotton name: K01810 - glucose-6-phosphate isomerase (GPI, pgi) (1 of 3)
OG IDs: 93516031
Best-hit-arabi-ID: AT5G42740.1
Best-hit-arabi-name: Sugar isomerase (SIS) family protein
Best-hit-arabi-symbol: nan
Gorai.002G128400.1.A
PCC=0.35, p=0.142
mRNA Profile
Protein Expression Profile
DPA

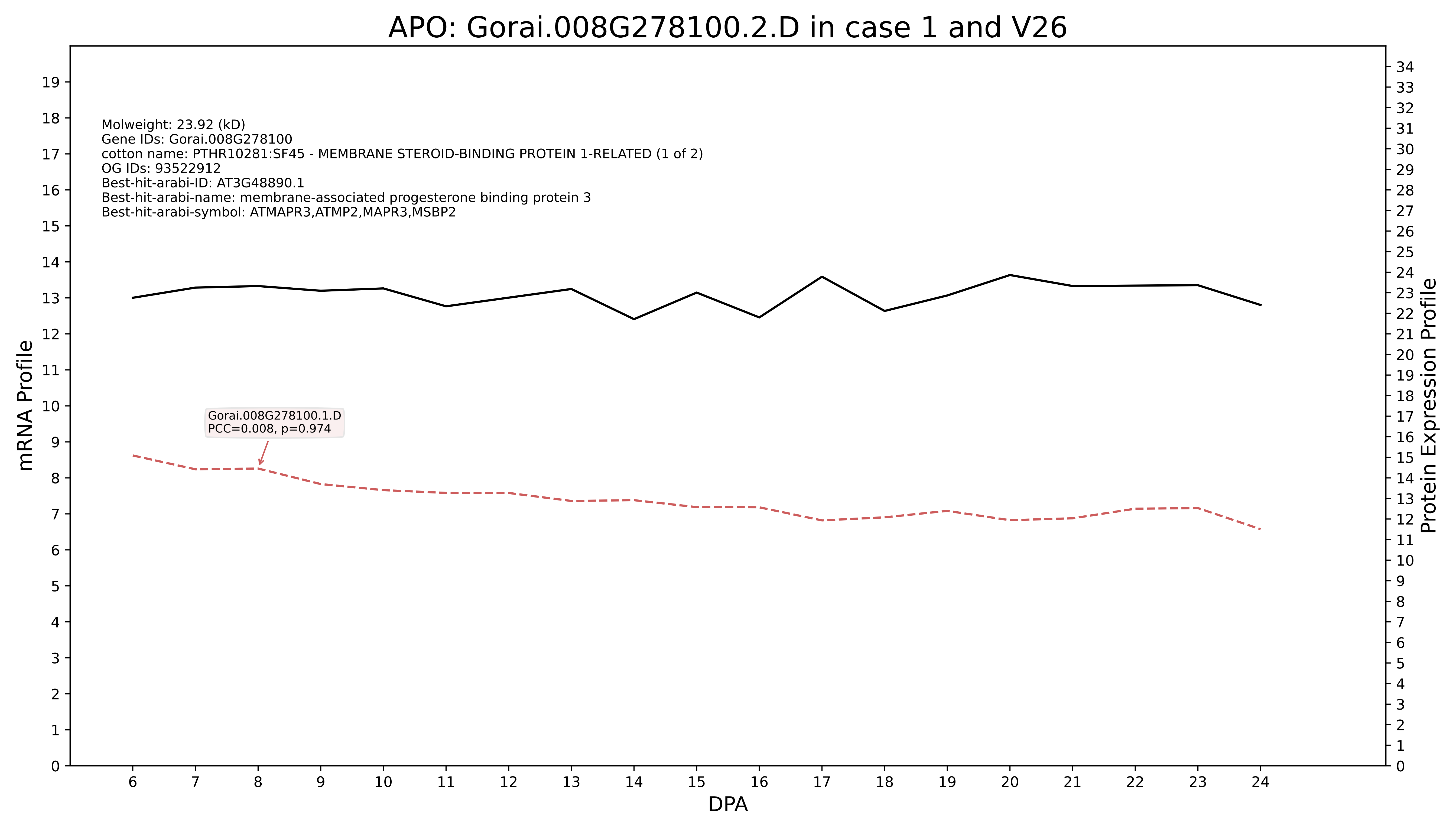
APO: Gorai.008G278100.2.D in case 1 and V26
Molweight: 23.92 (kD)
Gene IDs: Gorai.008G278100
cotton name: PTHR10281:SF45 - MEMBRANE STEROID-BINDING PROTEIN 1-RELATED (1 of 2)
OG IDs: 93522912
Best-hit-arabi-ID: AT3G48890.1
Best-hit-arabi-name: membrane-associated progesterone binding protein 3
Best-hit-arabi-symbol: ATMAPR3,ATMP2,MAPR3,MSBP2
Gorai.008G278100.1.D
PCC=0.008, p=0.974
mRNA Profile
Protein Expression Profile
DPA
0
1
2
3
4
5
6
7
8
9
10
11
12
13
14
15
16
17
18
19
20
21
22
23
24
25
26
27
28
29
30
31
32
33
34

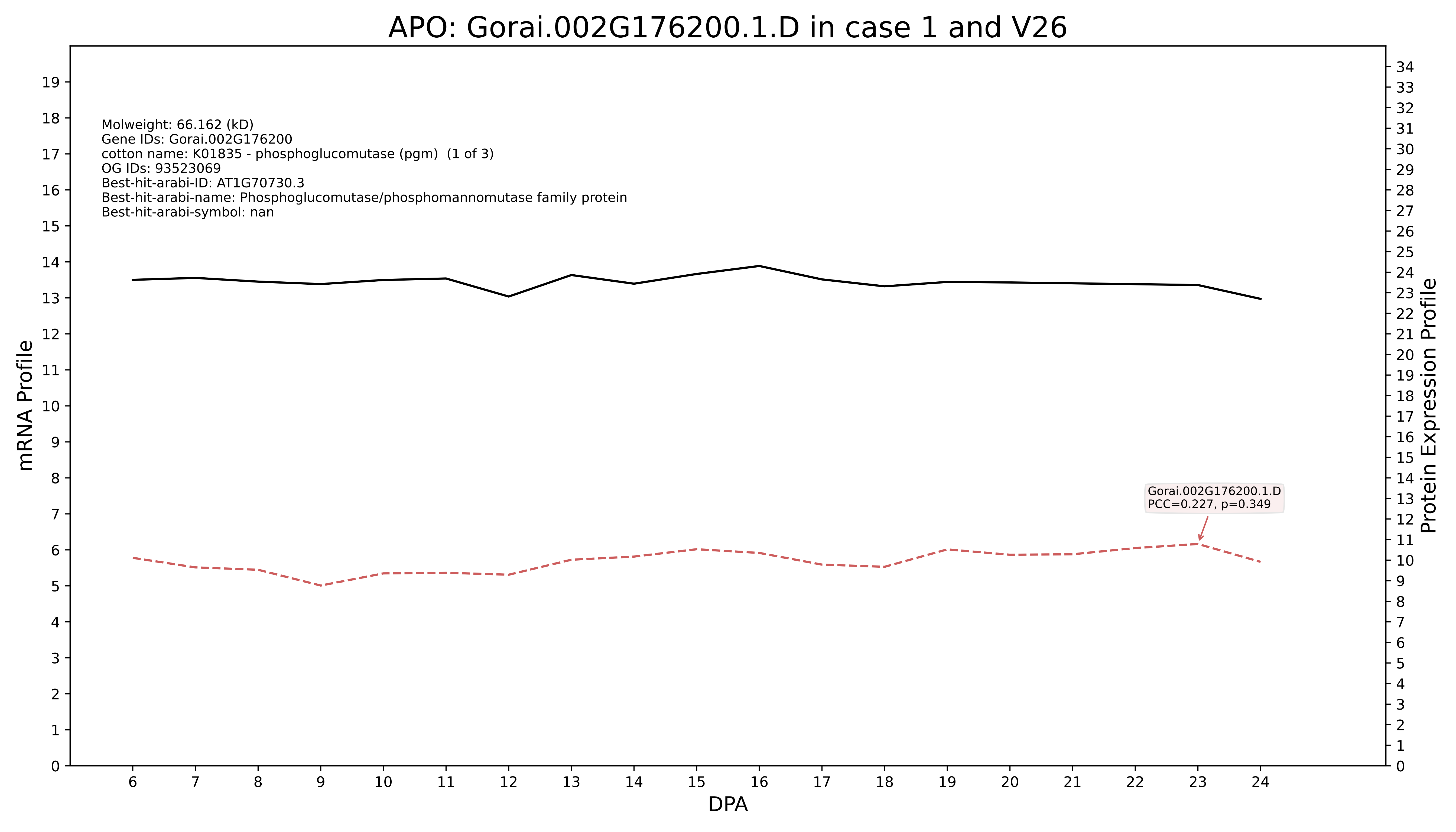

APO: Gorai.002G176200.1.D in case 1 and V26
Molweight: 66.162 (kD)
Gene IDs: Gorai.002G176200
cotton name: K01835 - phosphoglucomutase (pgm) (1 of 3)
OG IDs: 93523069
Best-hit-arabi-ID: AT1G70730.3
Best-hit-arabi-name: Phosphoglucomutase/phosphomannomutase family protein
Best-hit-arabi-symbol: nan
Gorai.002G176200.1.D
PCC=0.227, p=0.349
mRNA Profile
Protein Expression Profile
DPA

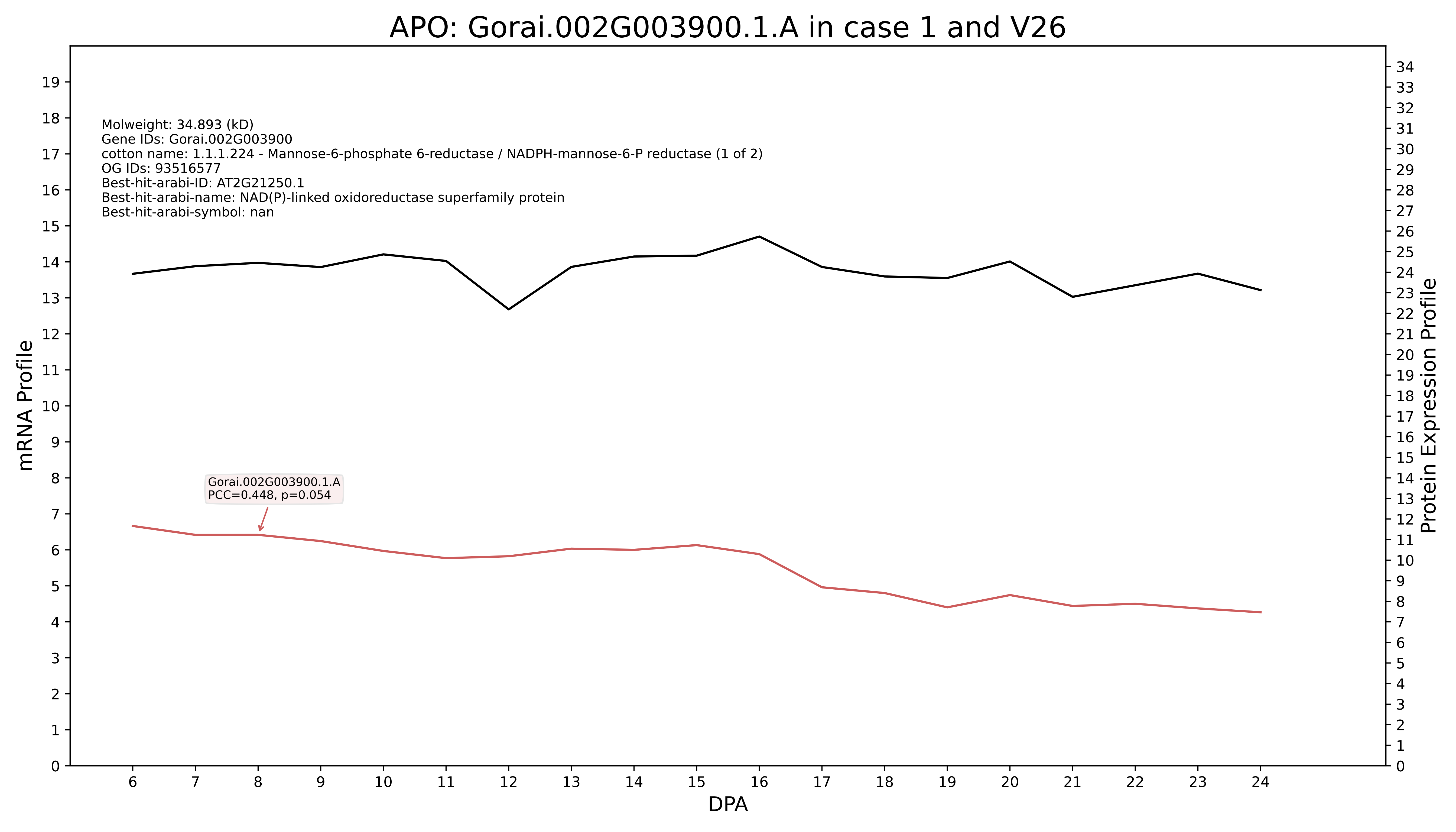
APO: Gorai.002G003900.1.A in case 1 and V26
Molweight: 34.893 (kD)
Gene IDs: Gorai.002G003900
cotton name: 1.1.1.224 - Mannose-6-phosphate 6-reductase / NADPH-mannose-6-P reductase (1 of 2)
OG IDs: 93516577
Best-hit-arabi-ID: AT2G21250.1
Best-hit-arabi-name: NAD(P)-linked oxidoreductase superfamily protein
Best-hit-arabi-symbol: nan
Gorai.002G003900.1.A
PCC=0.448, p=0.054
mRNA Profile
Protein Expression Profile
DPA
0
1
2
3
4
5
6
7
8
9
10
11
12
13
14
15
16
17
18
19
20
21
22
23
24
25
26
27
28
29
30
31
32
33
34

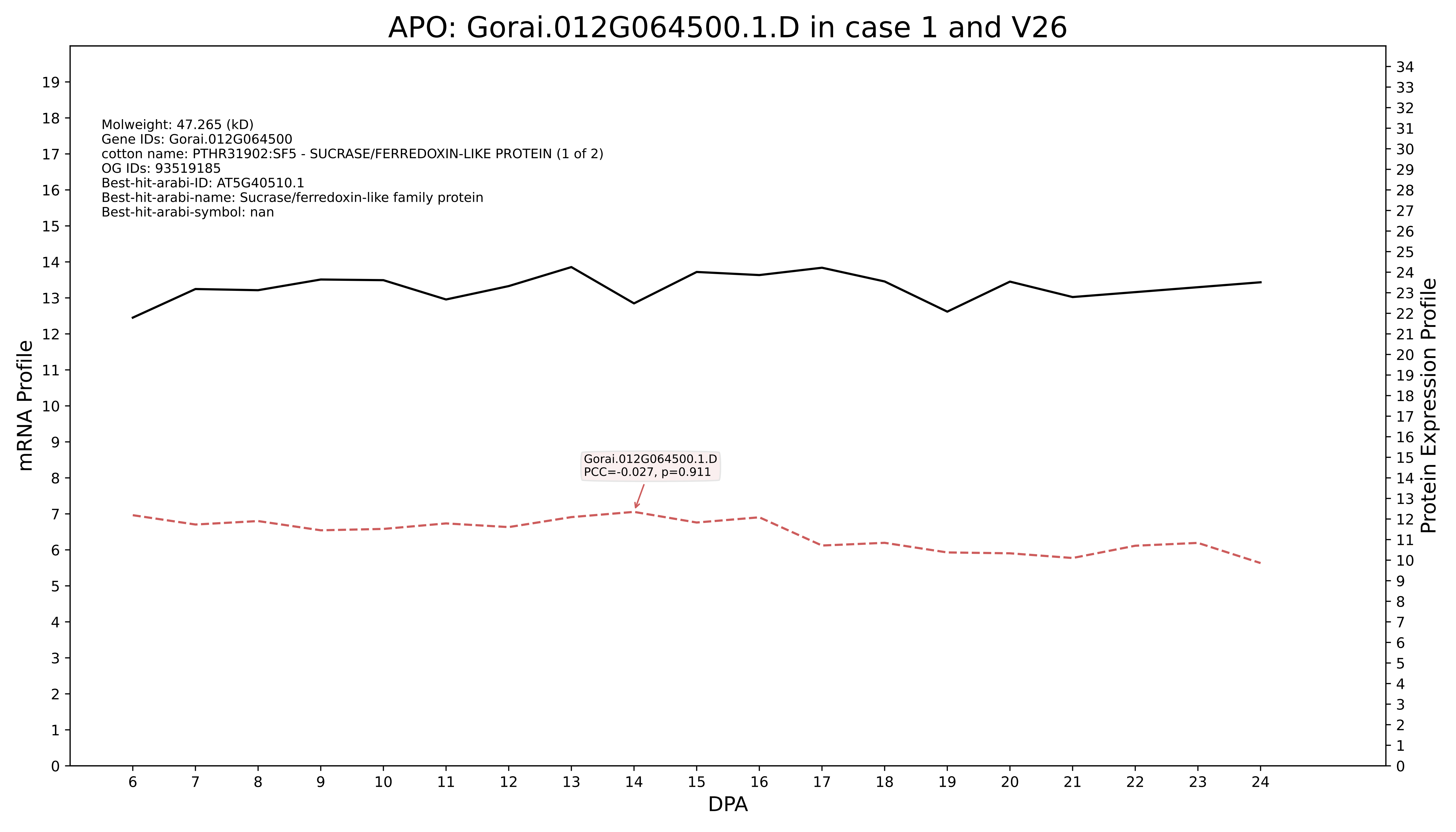
APO: Gorai.012G064500.1.D in case 1 and V26
Molweight: 47.265 (kD)
Gene IDs: Gorai.012G064500
cotton name: PTHR31902:SF5 - SUCRASE/FERREDOXIN-LIKE PROTEIN (1 of 2)
OG IDs: 93519185
Best-hit-arabi-ID: AT5G40510.1
Best-hit-arabi-name: Sucrase/ferredoxin-like family protein
Best-hit-arabi-symbol: nan
Gorai.012G064500.1.D
PCC=-0.027, p=0.911
mRNA Profile
Protein Expression Profile
DPA

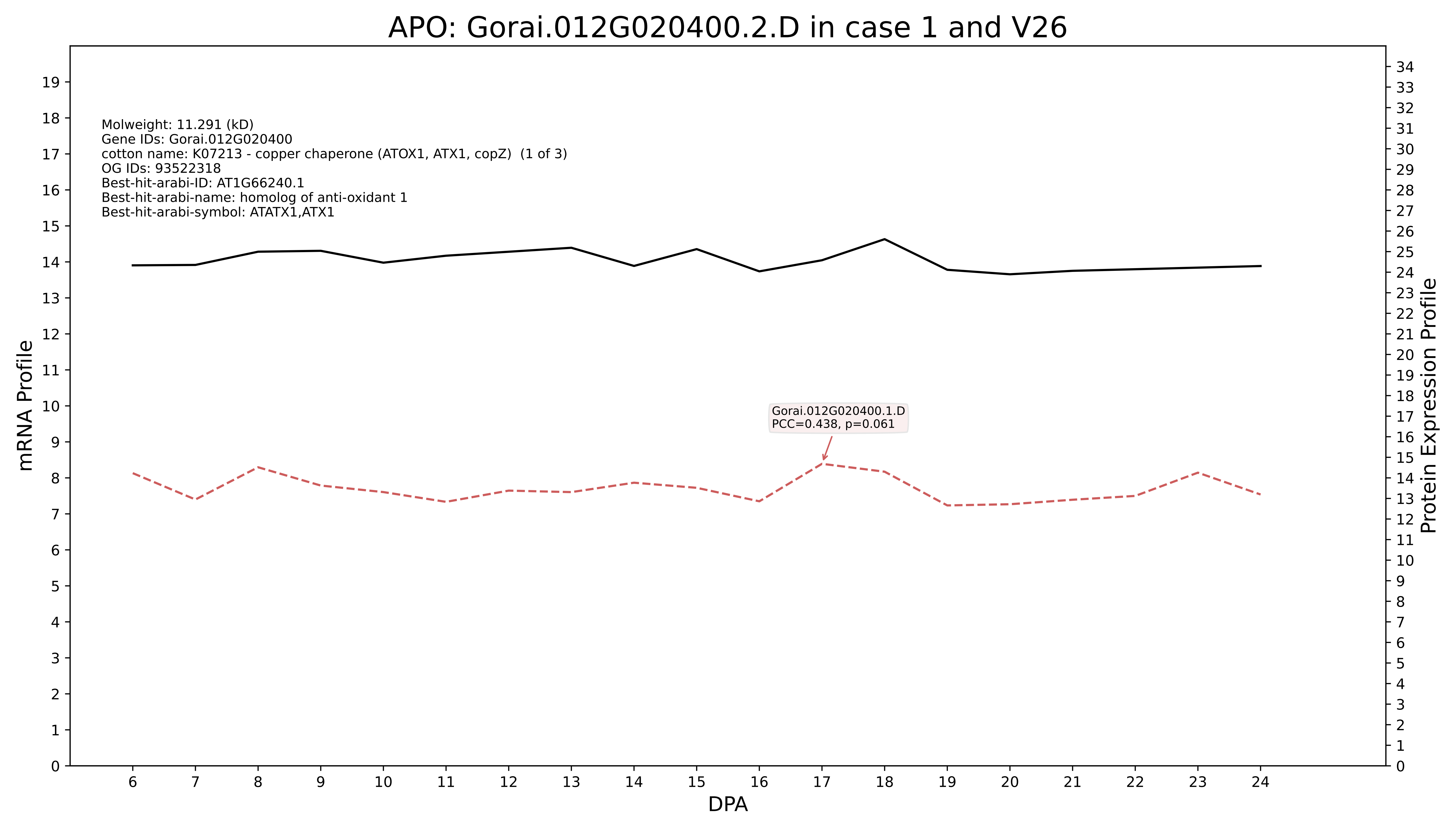
APO: Gorai.012G020400.2.D in case 1 and V26
Molweight: 11.291 (kD)
Gene IDs: Gorai.012G020400
cotton name: K07213 - copper chaperone (ATOX1, ATX1, copZ) (1 of 3)
OG IDs: 93522318
Best-hit-arabi-ID: AT1G66240.1
Best-hit-arabi-name: homolog of anti-oxidant 1
Best-hit-arabi-symbol: ATATX1,ATX1
Gorai.012G020400.1.D
PCC=0.438, p=0.061
mRNA Profile
Protein Expression Profile
DPA

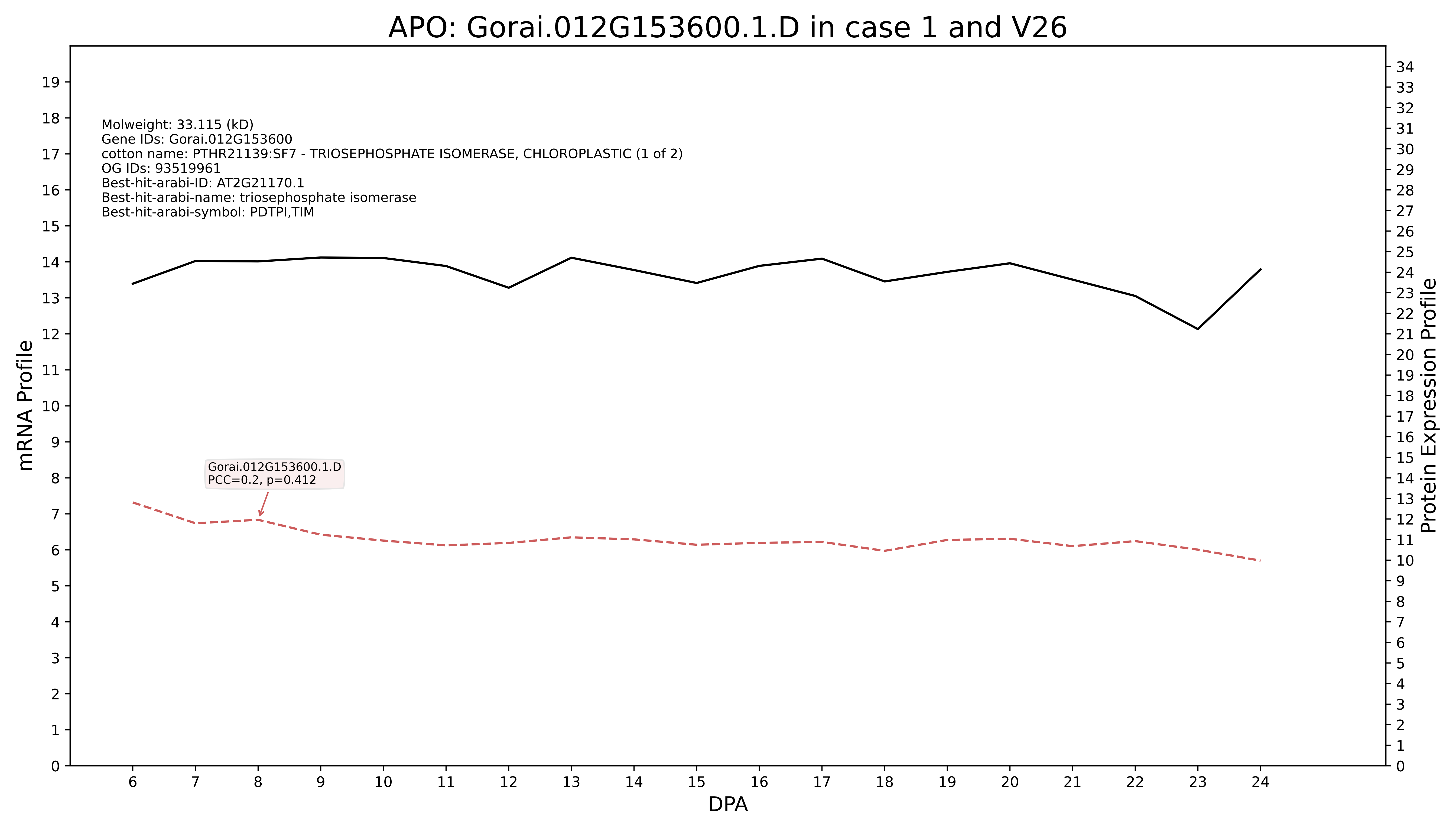
APO: Gorai.012G153600.1.D in case 1 and V26
Molweight: 33.115 (kD)
Gene IDs: Gorai.012G153600
cotton name: PTHR21139:SF7 - TRIOSEPHOSPHATE ISOMERASE, CHLOROPLASTIC (1 of 2)
OG IDs: 93519961
Best-hit-arabi-ID: AT2G21170.1
Best-hit-arabi-name: triosephosphate isomerase
Best-hit-arabi-symbol: PDTPI,TIM
Gorai.012G153600.1.D
PCC=0.2, p=0.412
mRNA Profile
Protein Expression Profile
DPA

APO: Gorai.009G296100.2.A in case 1 and V26

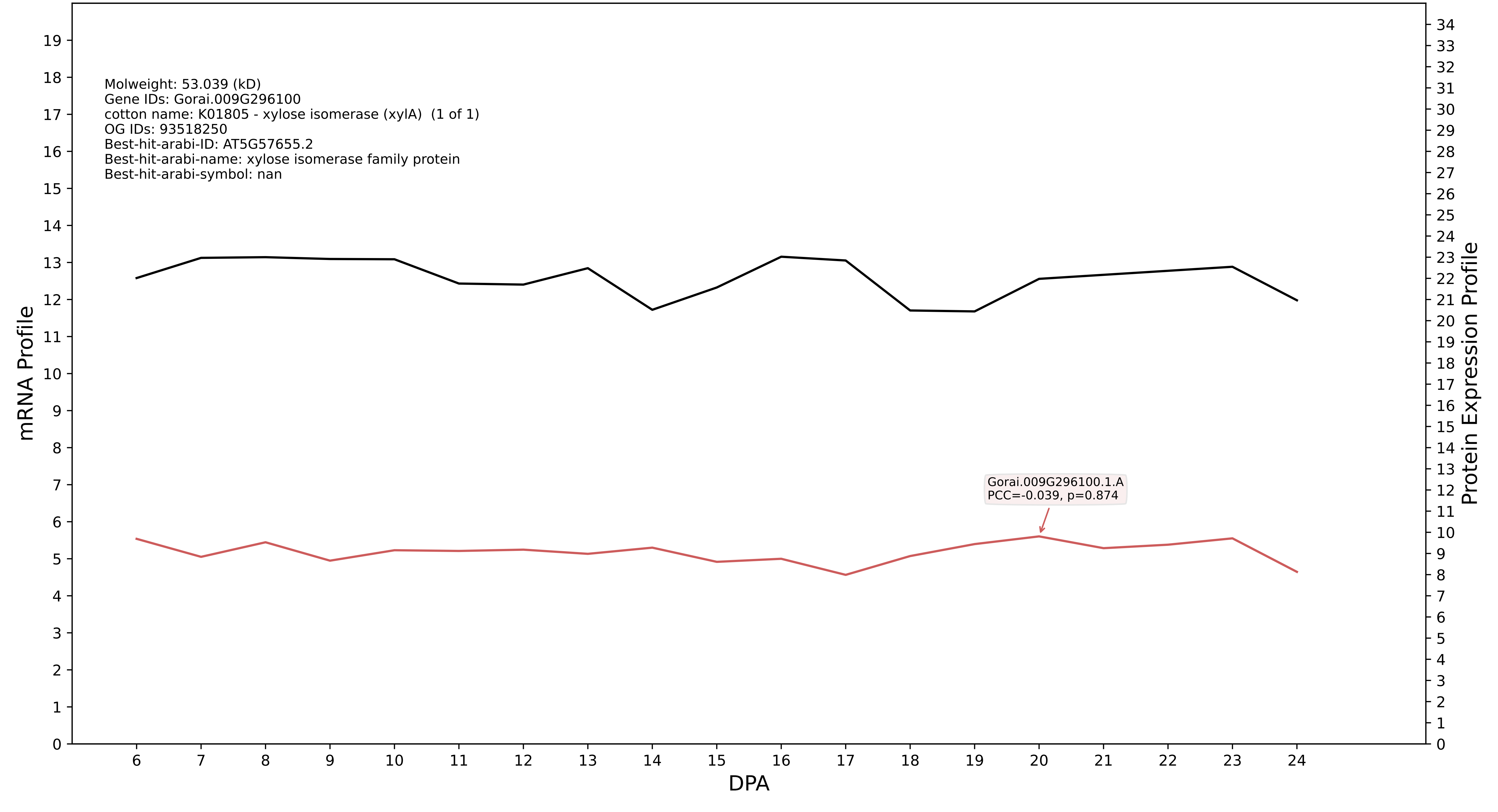

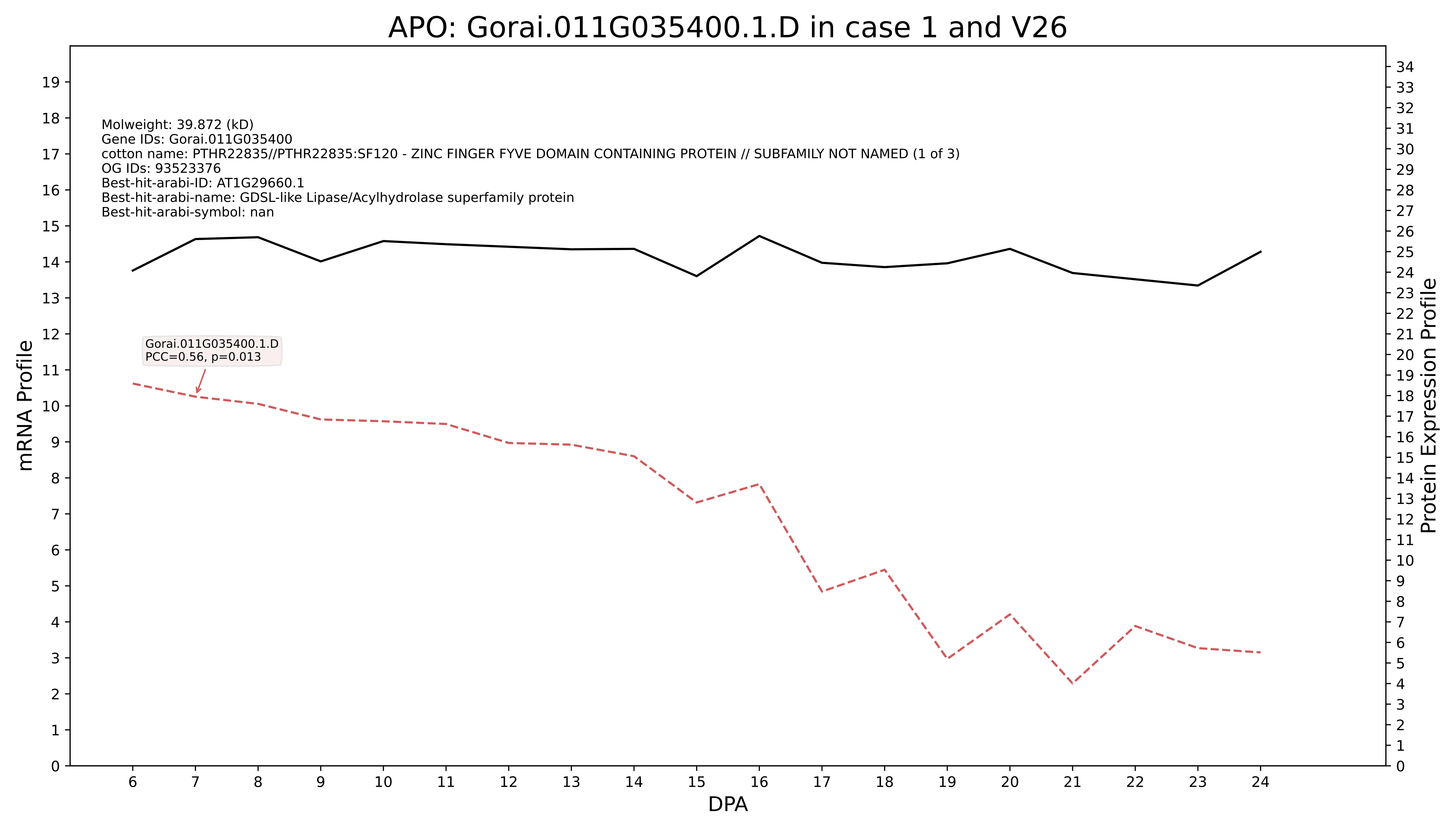
APO: Gorai.011G035400.1.D in case 1 and V26
Molweight: 39.872 (kD)
Gene IDs: Gorai.011G035400
cotton name: PTHR22835//PTHR22835:SF120 - ZINC FINGER FYVE DOMAIN CONTAINING PROTEIN // SUBFAMILY NOT NAMED (1 of 3)
OG IDs: 93523376
Best-hit-arabi-ID: AT1G29660.1
Best-hit-arabi-name: GDSL-like Lipase/Acylhydrolase superfamily protein
Best-hit-arabi-symbol: nan
Gorai.011G035400.1.D
PCC=0.56, p=0.013
mRNA Profile
Protein Expression Profile
DPA

APO: Gorai.002G241300.1.D in case 1 and V26

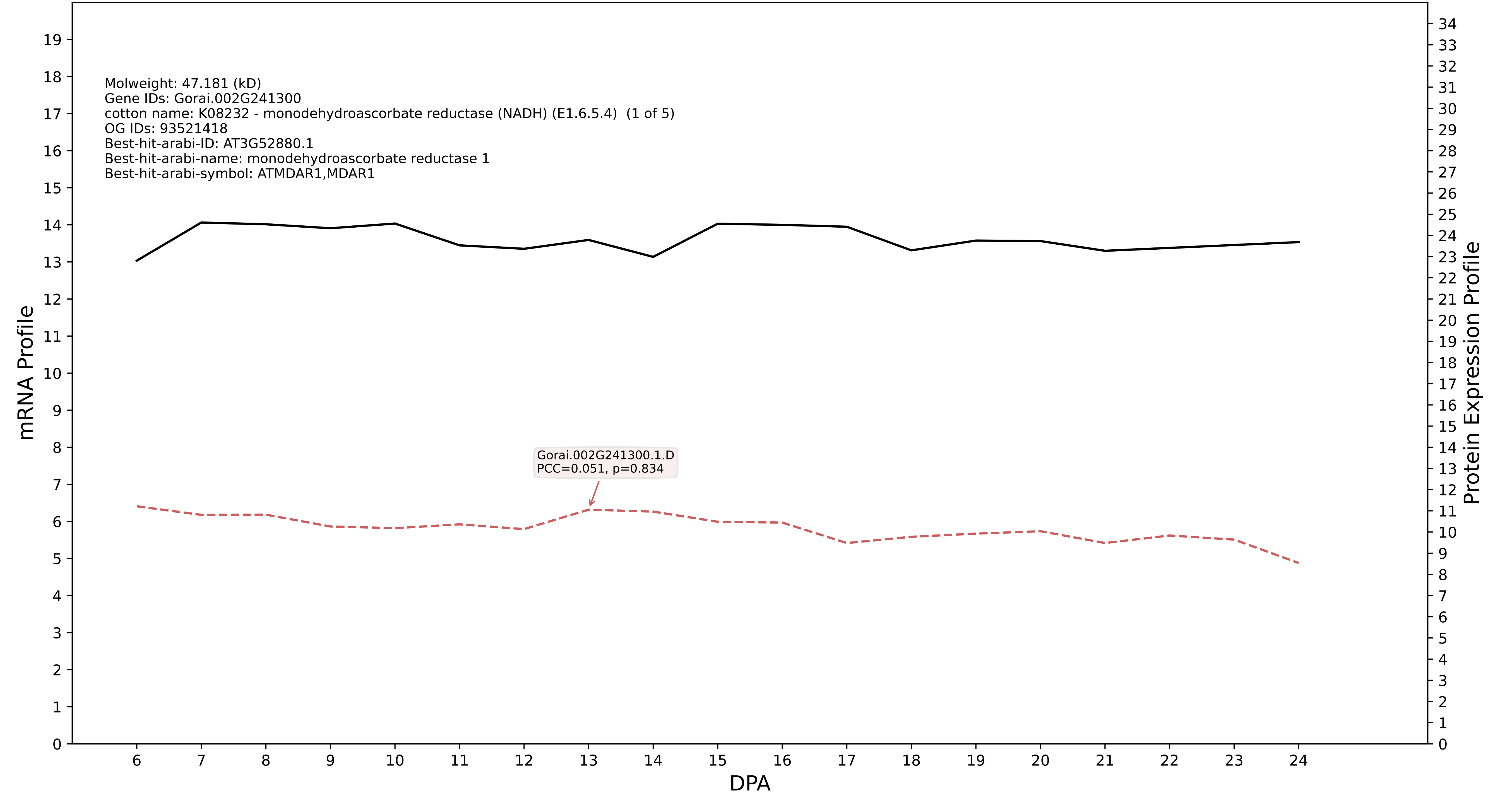

APO: Gorai.009G104500.10.A.p2 in case 1 and V26

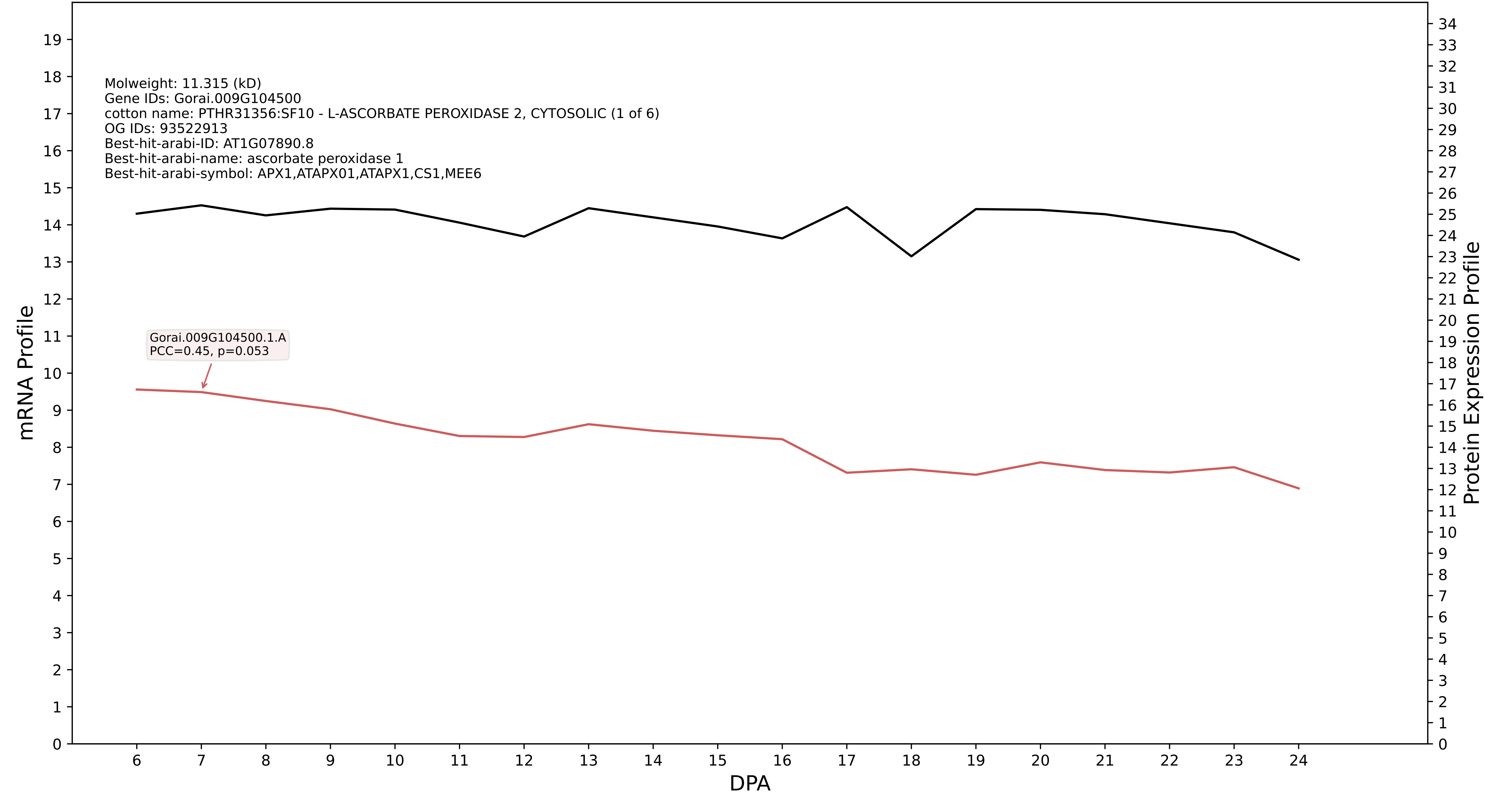

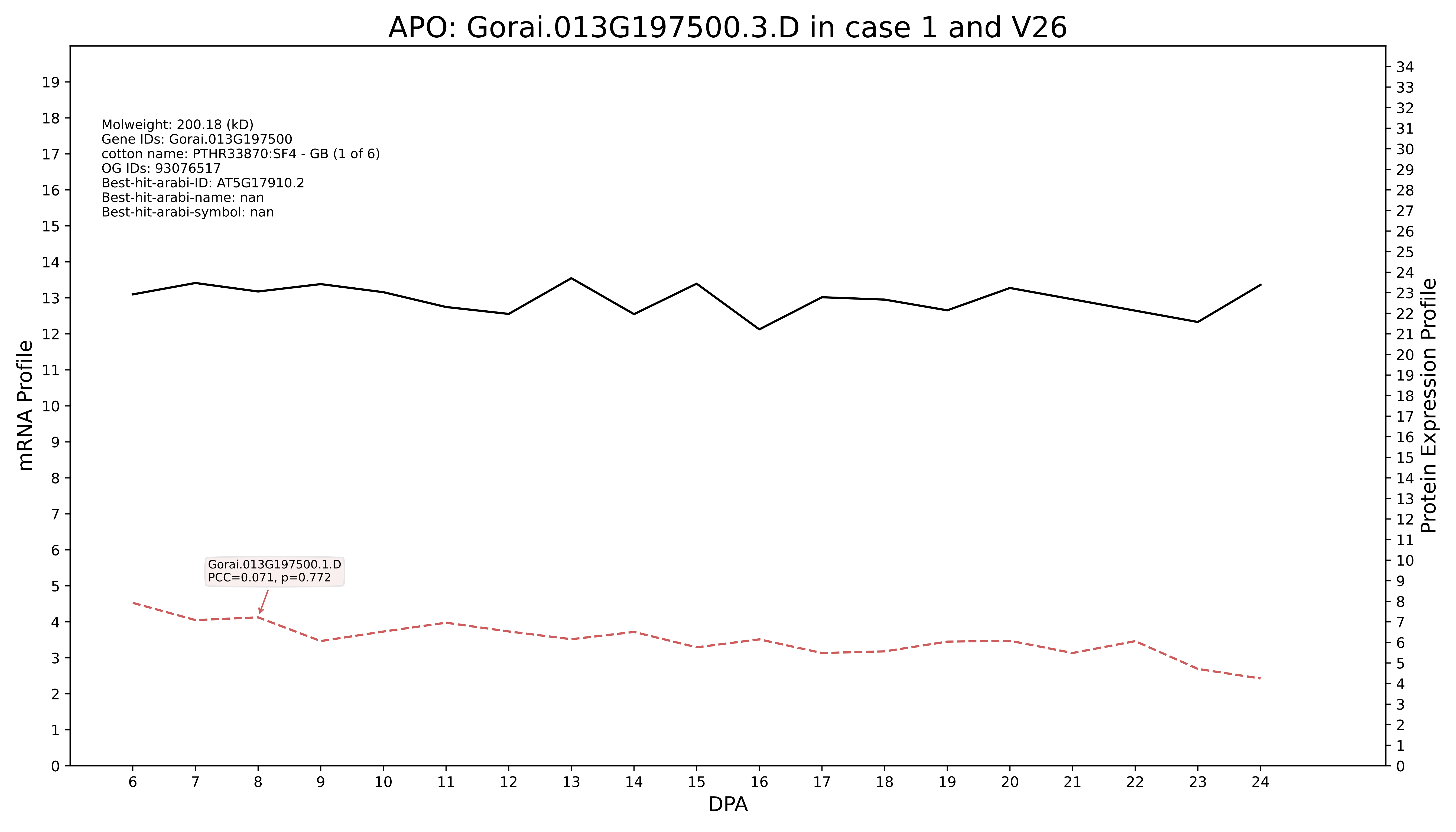
APO: Gorai.013G197500.3.D in case 1 and V26
Molweight: 200.18 (kD)
Gene IDs: Gorai.013G197500
cotton name: PTHR33870:SF4 - GB (1 of 6)
OG IDs: 93076517
Best-hit-arabi-ID: AT5G17910.2
Best-hit-arabi-name: nan
Best-hit-arabi-symbol: nan
Gorai.013G197500.1.D
PCC=0.071, p=0.772
mRNA Profile
Protein Expression Profile
DPA

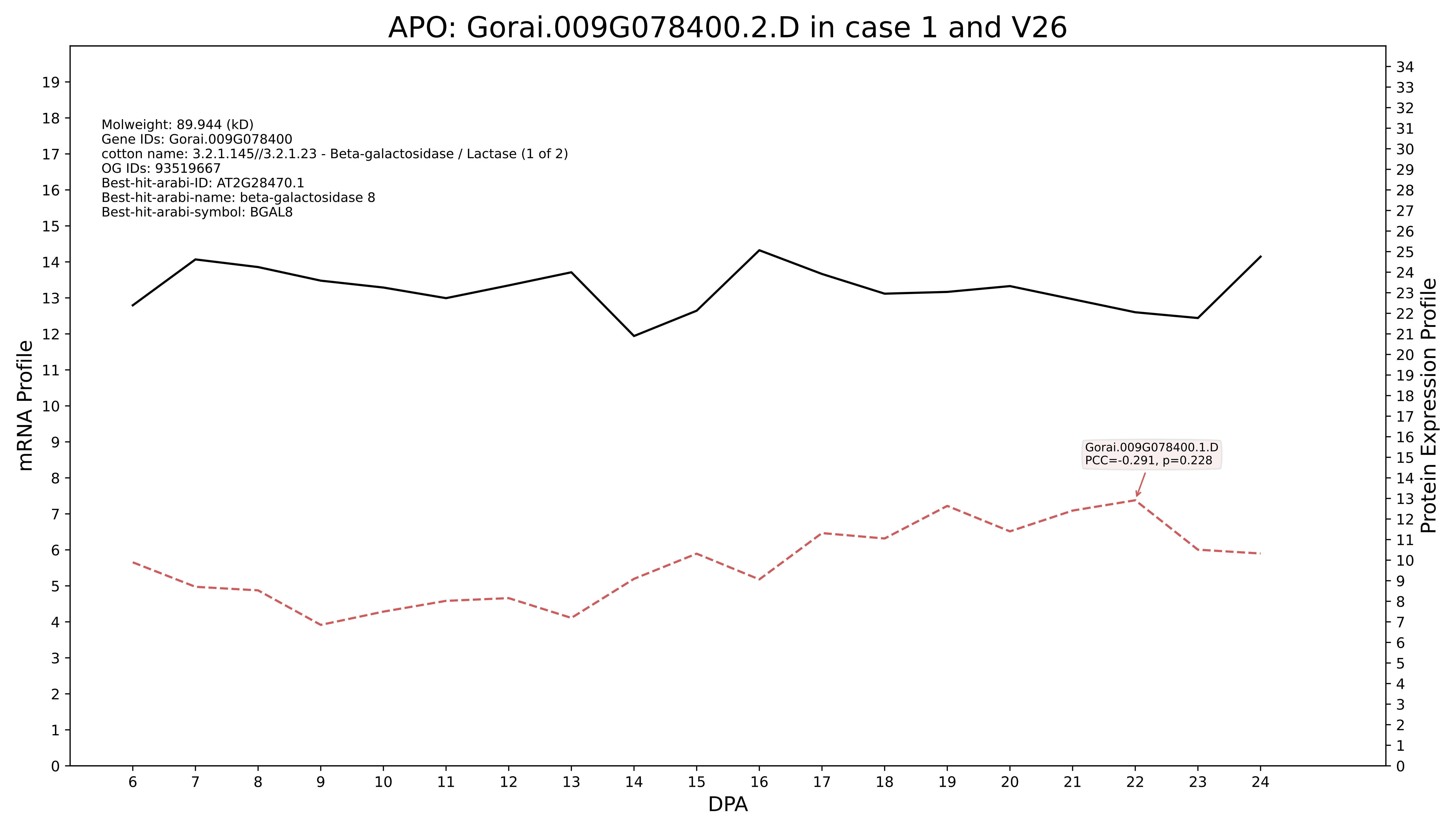
APO: Gorai.009G078400.2.D in case 1 and V26
Molweight: 89.944 (kD)
Gene IDs: Gorai.009G078400
cotton name: 3.2.1.145//3.2.1.23 - Beta-galactosidase / Lactase (1 of 2)
OG IDs: 93519667
Best-hit-arabi-ID: AT2G28470.1
Best-hit-arabi-name: beta-galactosidase 8
Best-hit-arabi-symbol: BGAL8
Gorai.009G078400.1.D
PCC=-0.291, p=0.228
mRNA Profile
Protein Expression Profile
DPA

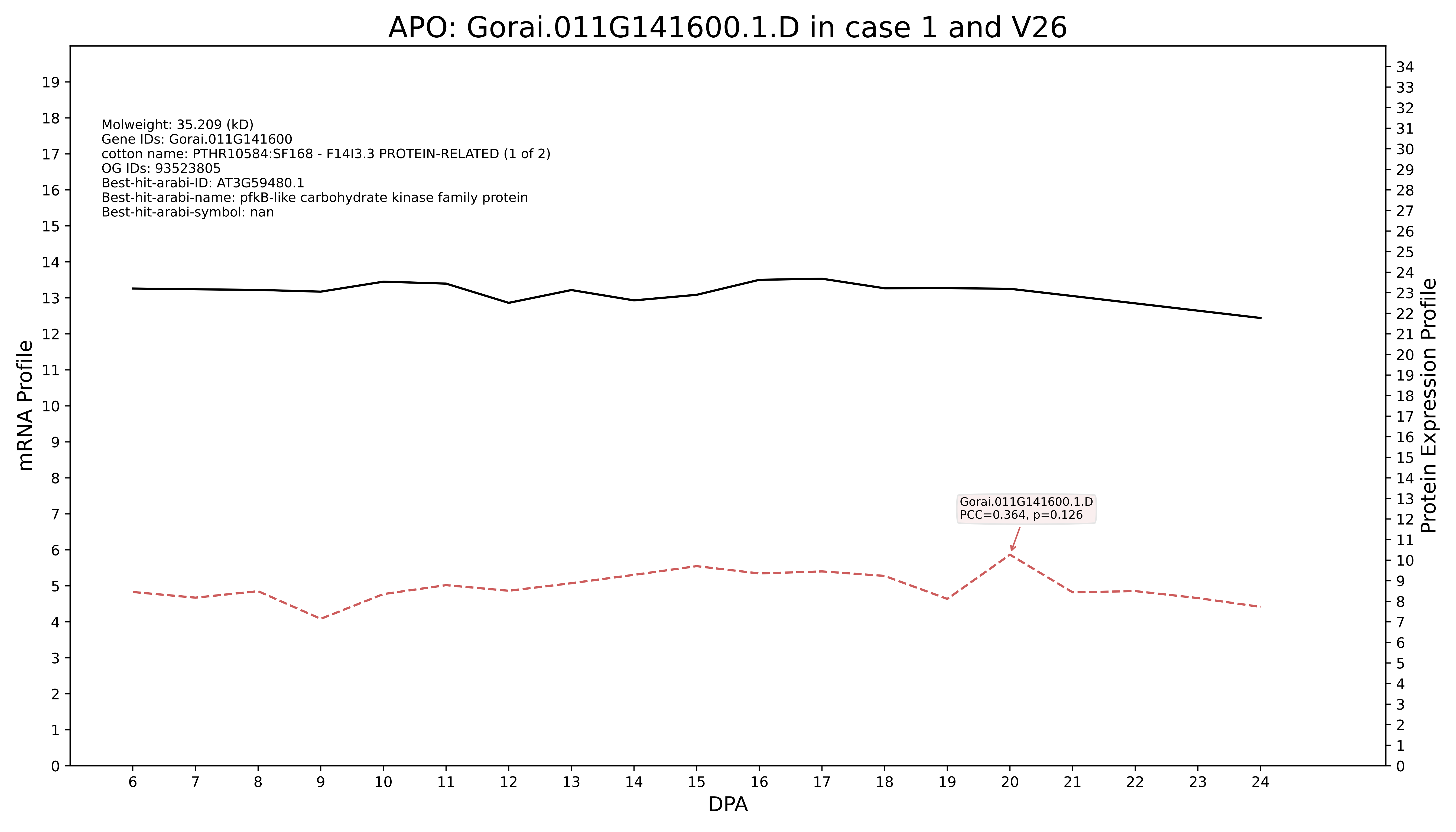
APO: Gorai.011G141600.1.D in case 1 and V26
Molweight: 35.209 (kD)
Gene IDs: Gorai.011G141600
cotton name: PTHR10584:SF168 - F14I3.3 PROTEIN-RELATED (1 of 2)
OG IDs: 93523805
Best-hit-arabi-ID: AT3G59480.1
Best-hit-arabi-name: pfkB-like carbohydrate kinase family protein
Best-hit-arabi-symbol: nan
Gorai.011G141600.1.D
PCC=0.364, p=0.126
mRNA Profile
Protein Expression Profile
DPA

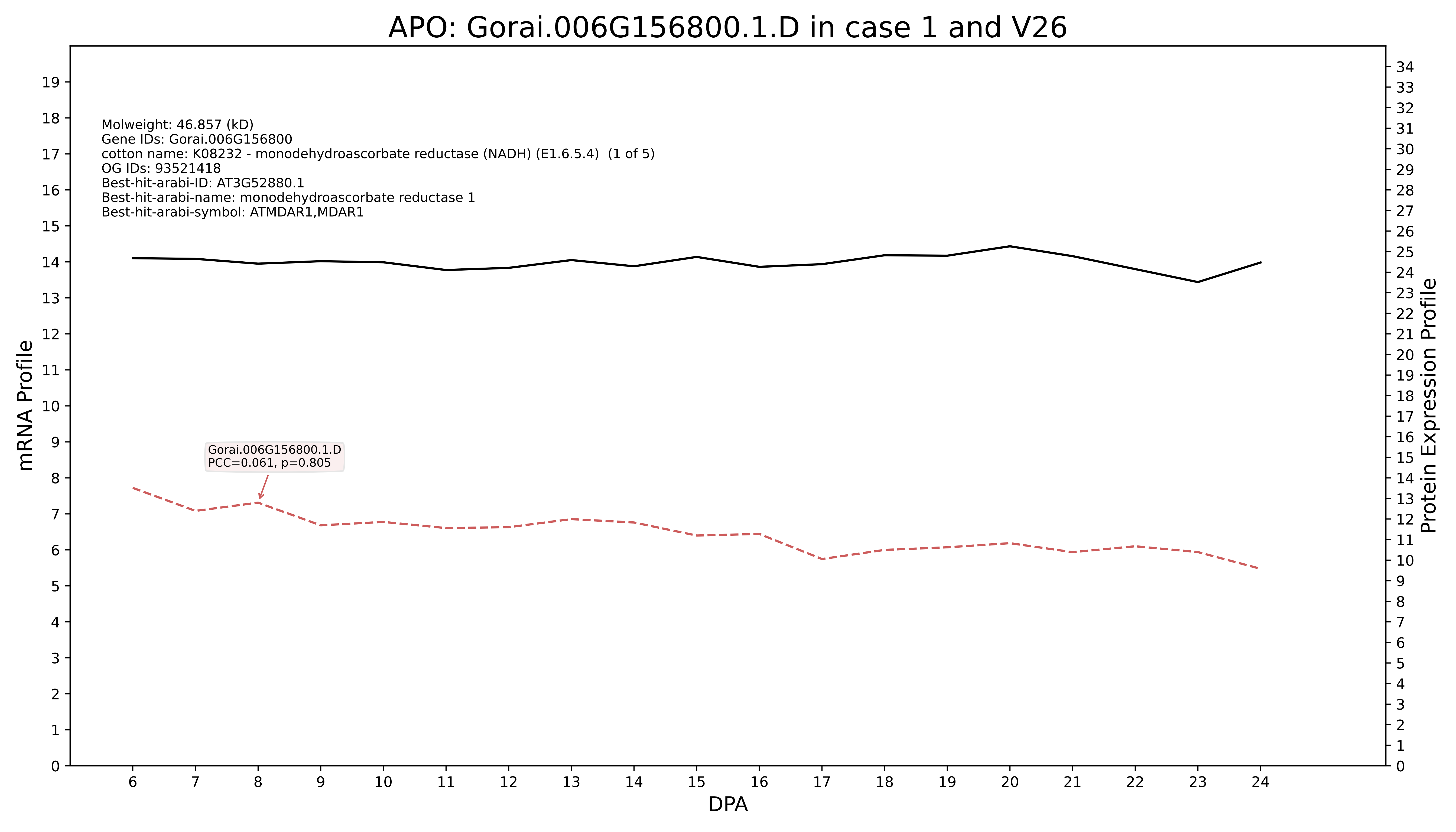
APO: Gorai.006G156800.1.D in case 1 and V26
Molweight: 46.857 (kD)
Gene IDs: Gorai.006G156800
cotton name: K08232 - monodehydroascorbate reductase (NADH) (E1.6.5.4) (1 of 5)
OG IDs: 93521418
Best-hit-arabi-ID: AT3G52880.1
Best-hit-arabi-name: monodehydroascorbate reductase 1
Best-hit-arabi-symbol: ATMDAR1,MDAR1
Gorai.006G156800.1.D
PCC=0.061, p=0.805
mRNA Profile
Protein Expression Profile
DPA

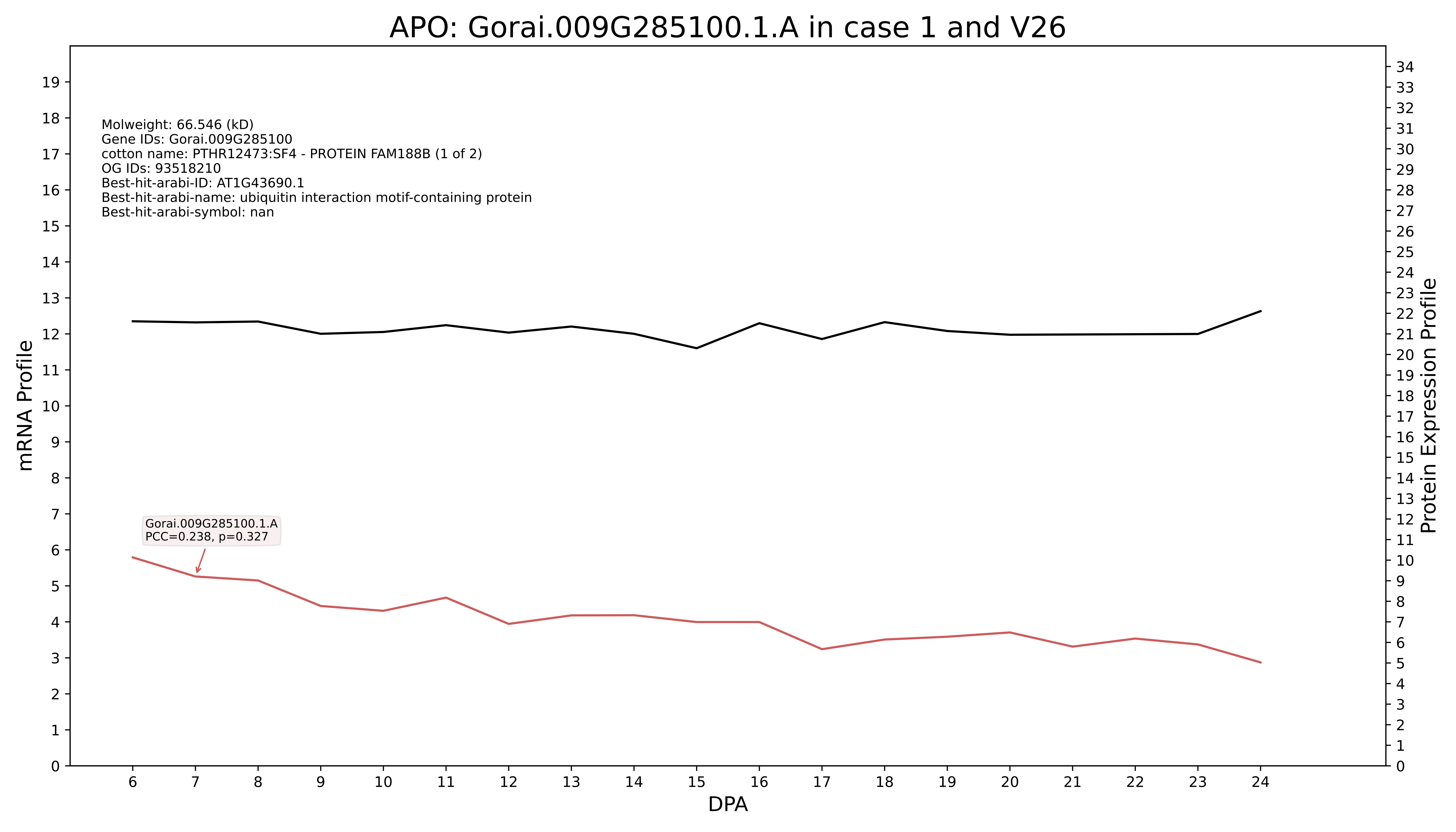
APO: Gorai.009G285100.1.A in case 1 and V26
Molweight: 66.546 (kD)
Gene IDs: Gorai.009G285100
cotton name: PTHR12473:SF4 - PROTEIN FAM188B (1 of 2)
OG IDs: 93518210
Best-hit-arabi-ID: AT1G43690.1
Best-hit-arabi-name: ubiquitin interaction motif-containing protein
Best-hit-arabi-symbol: nan
Gorai.009G285100.1.A
PCC=0.238, p=0.327
mRNA Profile
Protein Expression Profile
DPA

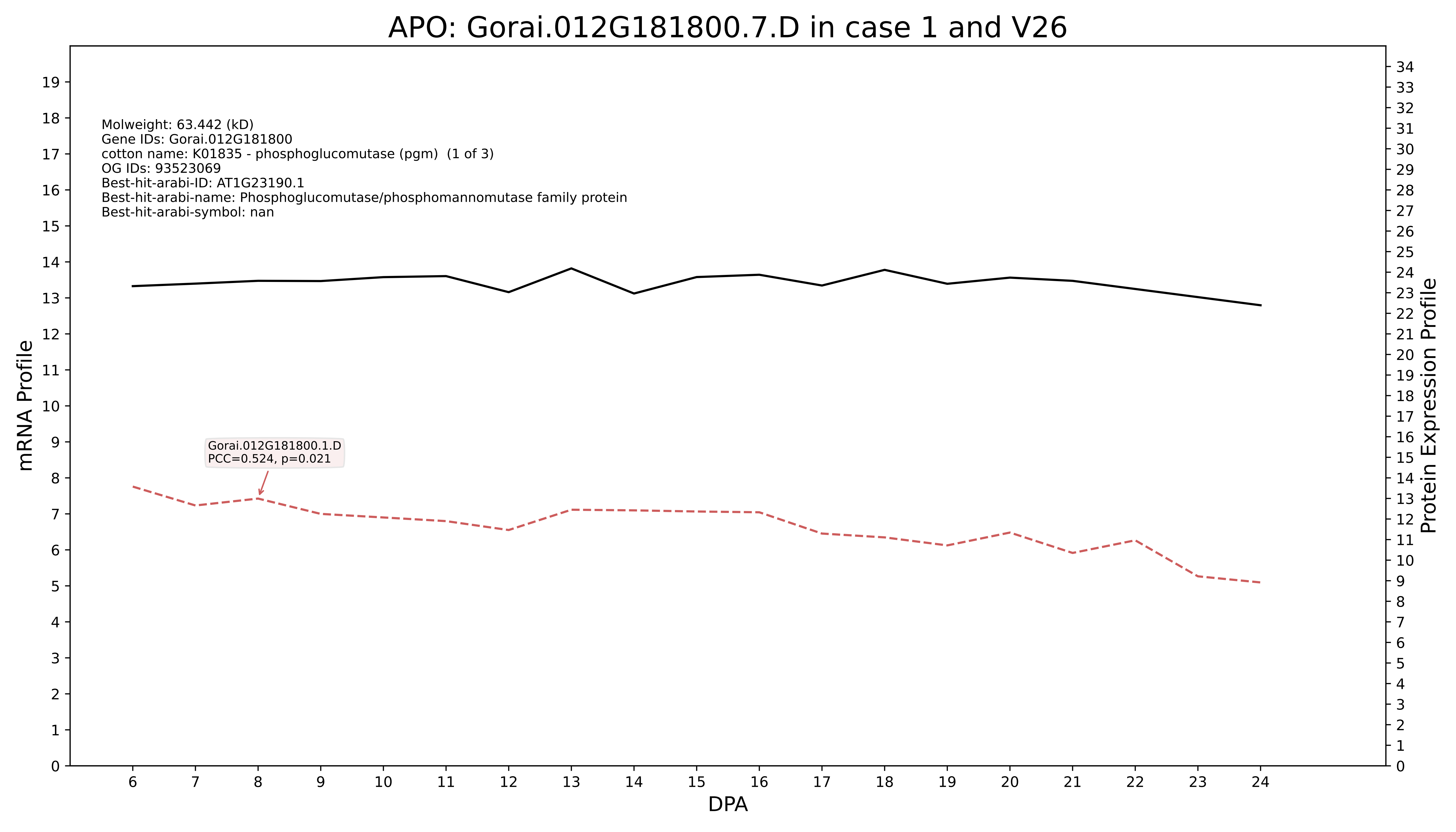
APO: Gorai.012G181800.7.D in case 1 and V26
Molweight: 63.442 (kD)
Gene IDs: Gorai.012G181800
cotton name: K01835 - phosphoglucomutase (pgm) (1 of 3)
OG IDs: 93523069
Best-hit-arabi-ID: AT1G23190.1
Best-hit-arabi-name: Phosphoglucomutase/phosphomannomutase family protein
Best-hit-arabi-symbol: nan
Gorai.012G181800.1.D
PCC=0.524, p=0.021
mRNA Profile
Protein Expression Profile
DPA

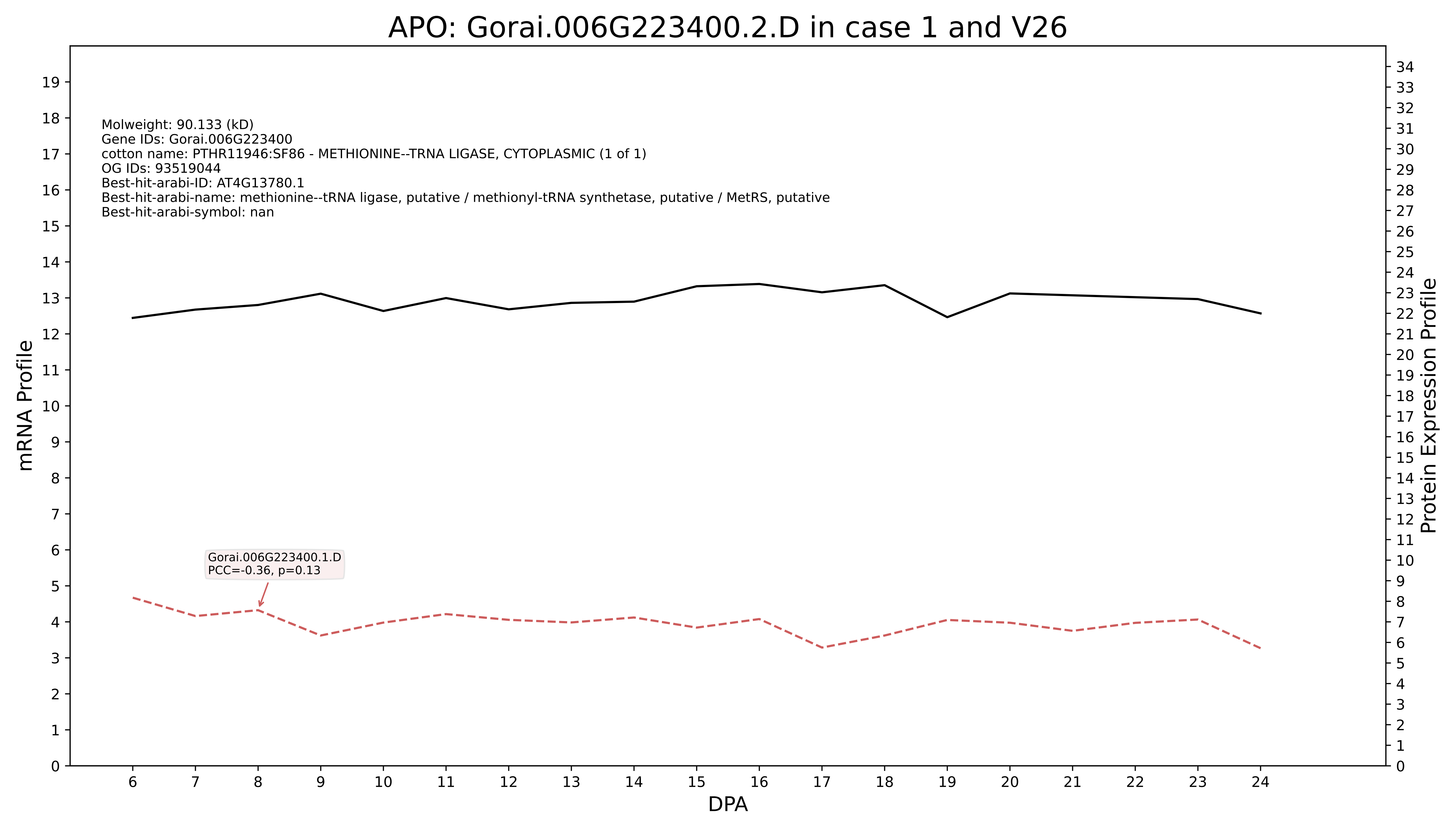
APO: Gorai.006G223400.2.D in case 1 and V26
Molweight: 90.133 (kD)
Gene IDs: Gorai.006G223400
cotton name: PTHR11946:SF86 - METHIONINE--TRNA LIGASE, CYTOPLASMIC (1 of 1)
OG IDs: 93519044
Best-hit-arabi-ID: AT4G13780.1
Best-hit-arabi-name: methionine--tRNA ligase, putative / methionyl-tRNA synthetase, putative / MetRS, putative
Best-hit-arabi-symbol: nan
Gorai.006G223400.1.D
PCC=-0.36, p=0.13
mRNA Profile
Protein Expression Profile
DPA

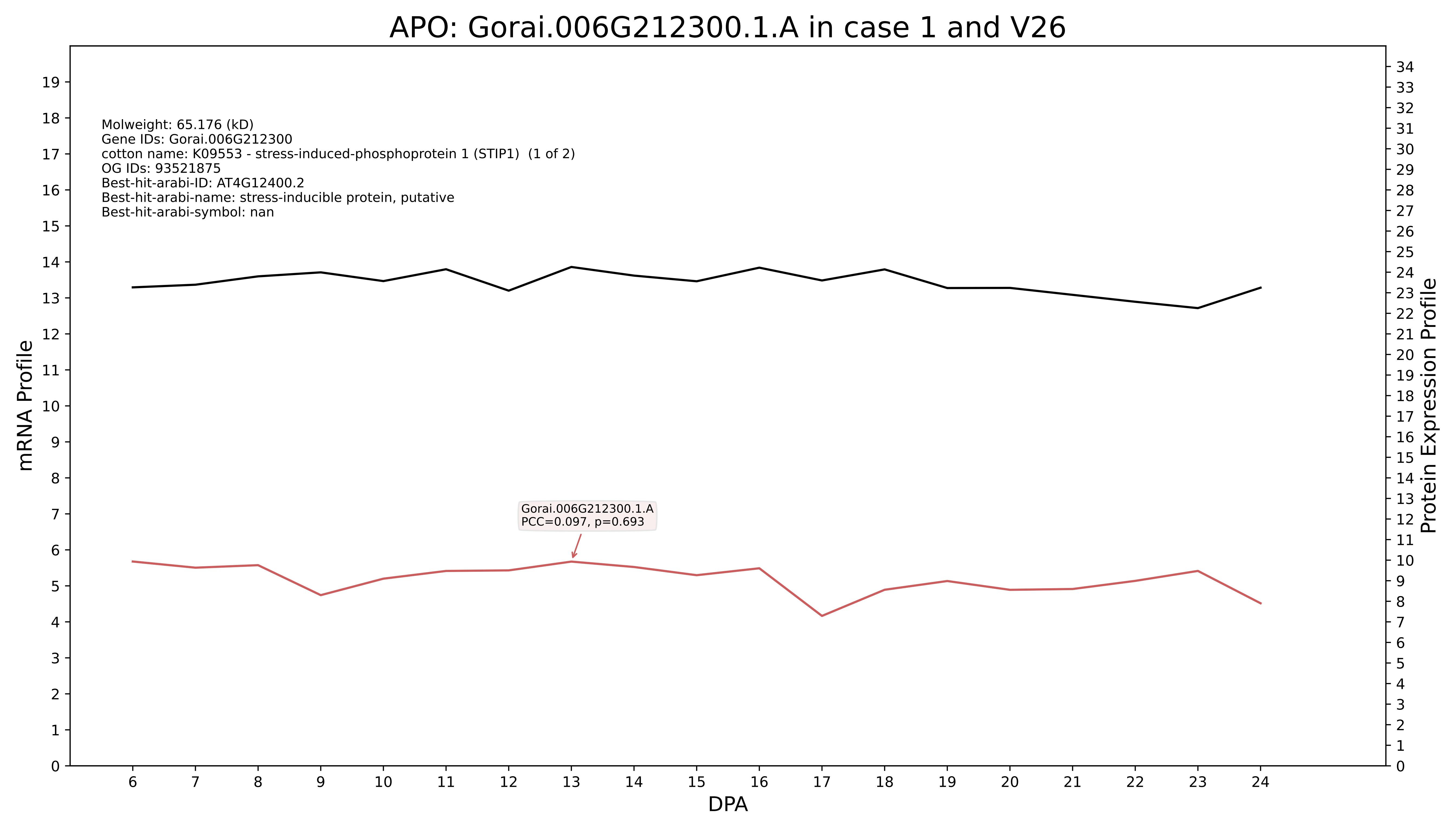
APO: Gorai.006G212300.1.A in case 1 and V26
Molweight: 65.176 (kD)
Gene IDs: Gorai.006G212300
cotton name: K09553 - stress-induced-phosphoprotein 1 (STIP1) (1 of 2)
OG IDs: 93521875
Best-hit-arabi-ID: AT4G12400.2
Best-hit-arabi-name: stress-inducible protein, putative
Best-hit-arabi-symbol: nan
Gorai.006G212300.1.A
PCC=0.097, p=0.693
mRNA Profile
Protein Expression Profile
DPA

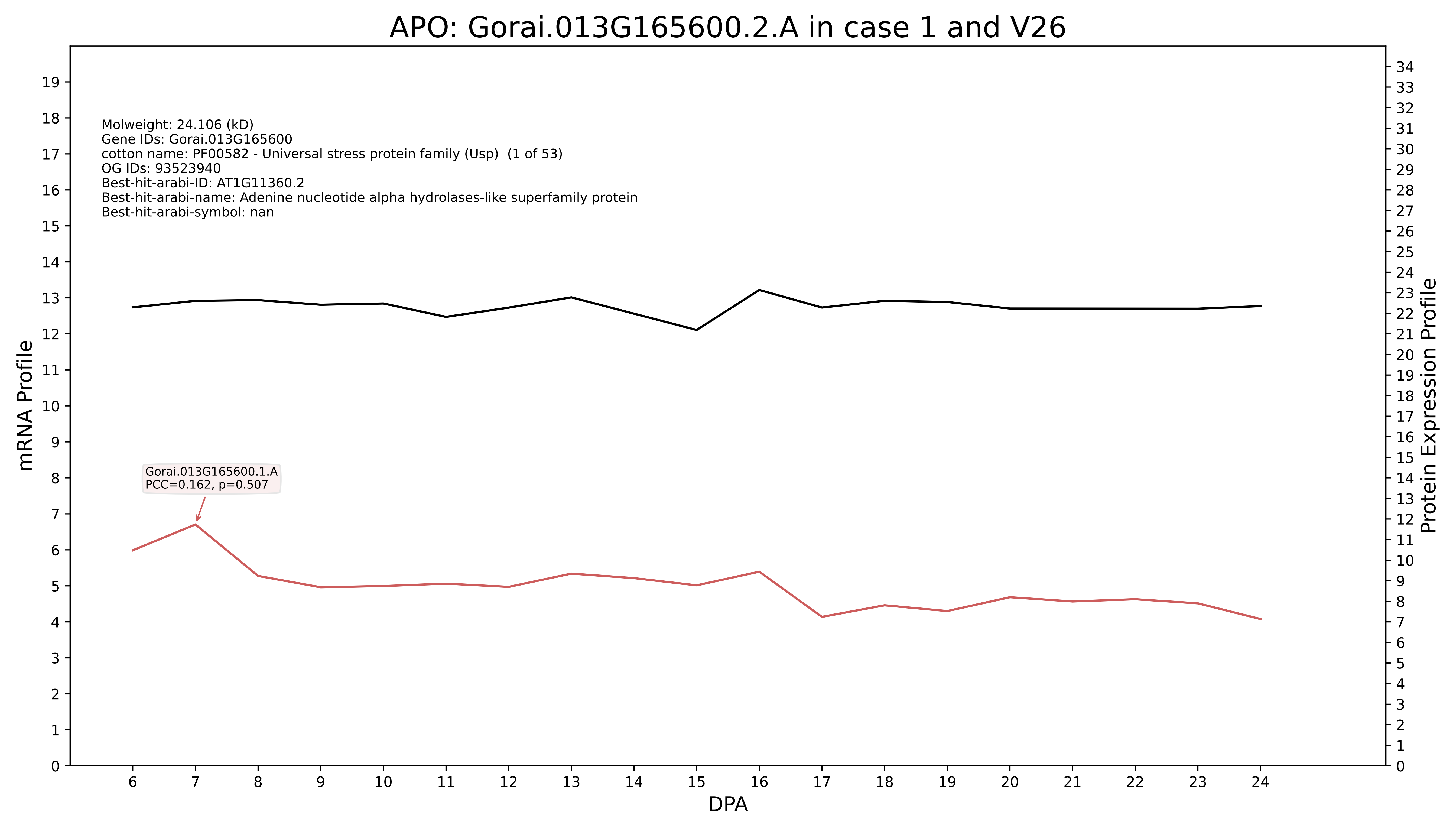
APO: Gorai.013G165600.2.A in case 1 and V26
Molweight: 24.106 (kD)
Gene IDs: Gorai.013G165600
cotton name: PF00582 - Universal stress protein family (Usp) (1 of 53)
OG IDs: 93523940
Best-hit-arabi-ID: AT1G11360.2
Best-hit-arabi-name: Adenine nucleotide alpha hydrolases-like superfamily protein
Best-hit-arabi-symbol: nan
Gorai.013G165600.1.A
PCC=0.162, p=0.507
mRNA Profile
Protein Expression Profile
DPA

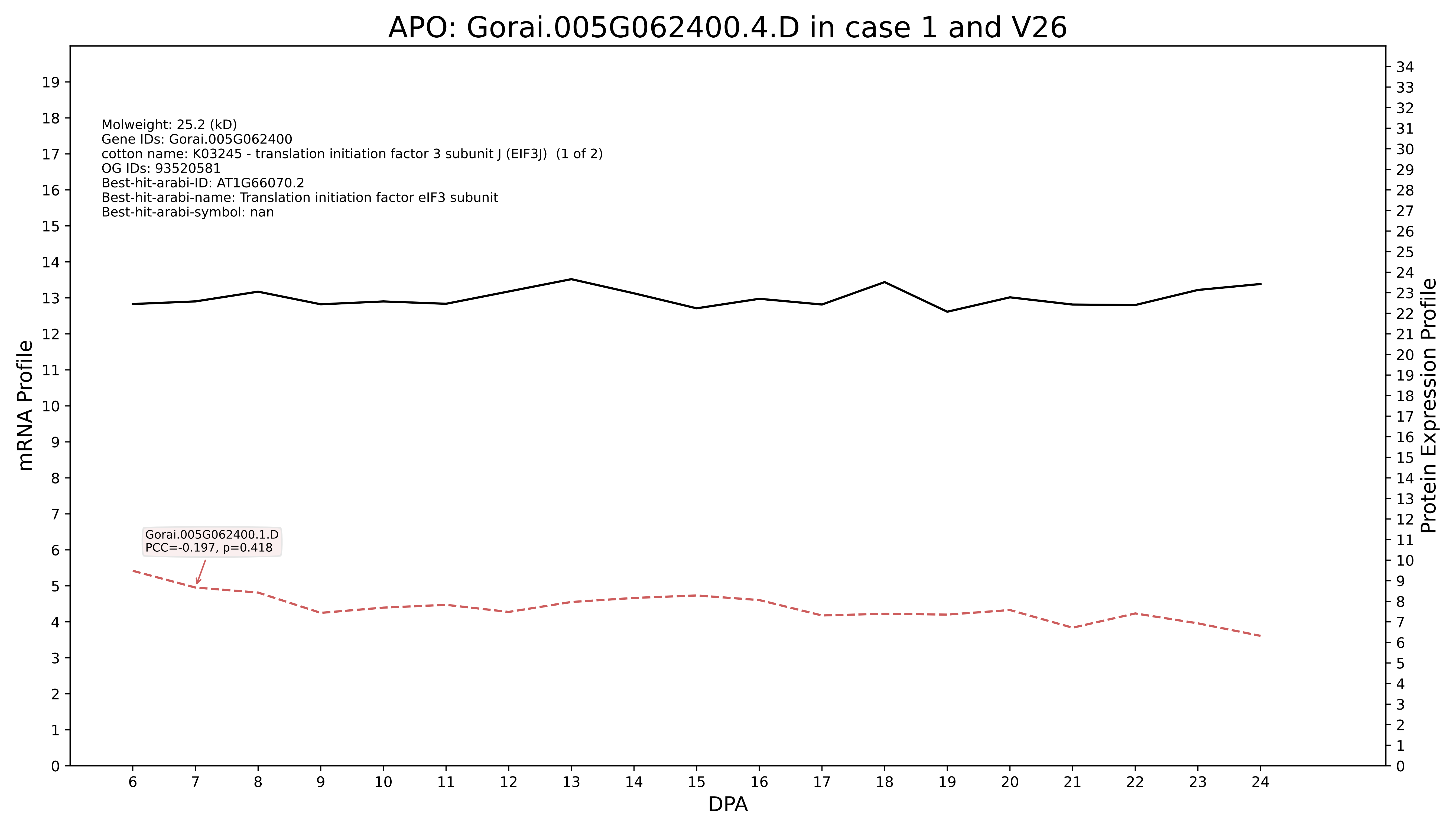

APO: Gorai.005G062400.4.D in case 1 and V26
Molweight: 25.2 (kD)
Gene IDs: Gorai.005G062400
cotton name: K03245 - translation initiation factor 3 subunit J (EIF3J) (1 of 2)
OG IDs: 93520581
Best-hit-arabi-ID: AT1G66070.2
Best-hit-arabi-name: Translation initiation factor eIF3 subunit
Best-hit-arabi-symbol: nan
Gorai.005G062400.1.D
PCC=-0.197, p=0.418
mRNA Profile
Protein Expression Profile
DPA

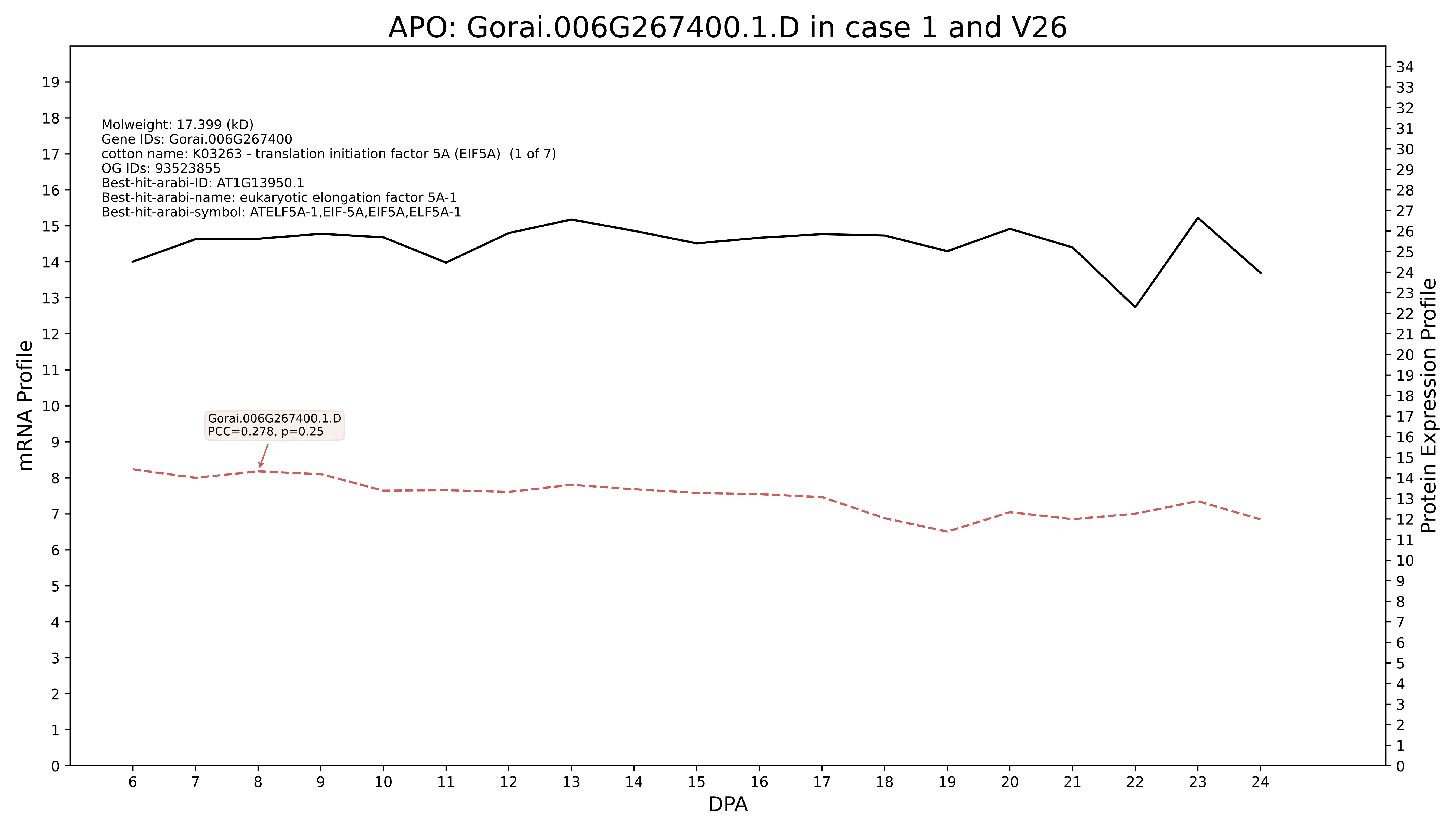
APO: Gorai.006G267400.1.D in case 1 and V26
Molweight: 17.399 (kD)
Gene IDs: Gorai.006G267400
cotton name: K03263 - translation initiation factor 5A (EIF5A) (1 of 7)
OG IDs: 93523855
Best-hit-arabi-ID: AT1G13950.1
Best-hit-arabi-name: eukaryotic elongation factor 5A-1
Best-hit-arabi-symbol: ATELF5A-1,EIF-5A,EIF5A,ELF5A-1
Gorai.006G267400.1.D
PCC=0.278, p=0.25
mRNA Profile
Protein Expression Profile
DPA
0
1
2
3
4
5
6
7
8
9
10
11
12
13
14
15
16
17
18
19
20
21
22
23
24
25
26
27
28
29
30
31
32
33
34

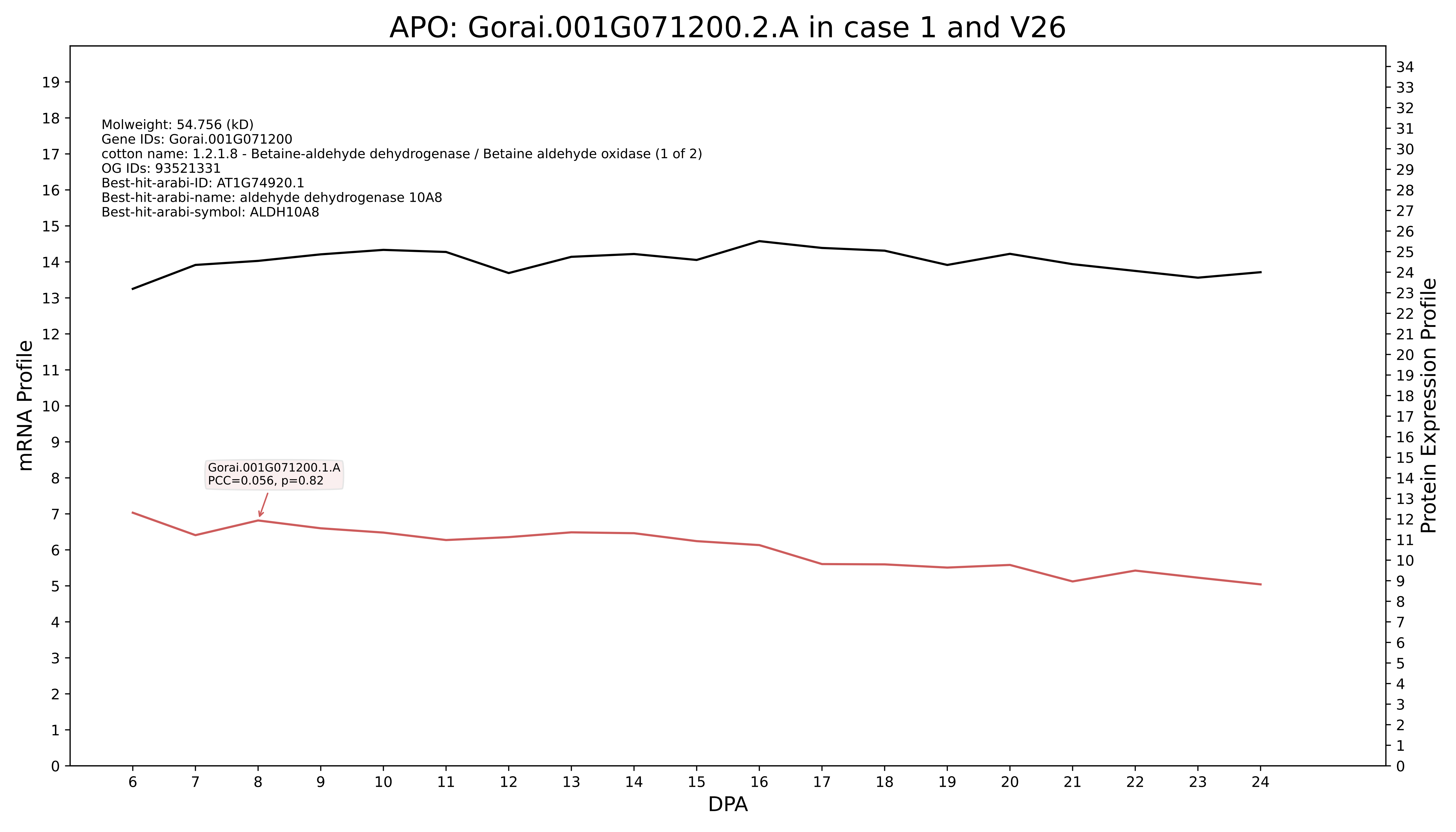
APO: Gorai.001G071200.2.A in case 1 and V26
Molweight: 54.756 (kD)
Gene IDs: Gorai.001G071200
cotton name: 1.2.1.8 - Betaine-aldehyde dehydrogenase / Betaine aldehyde oxidase (1 of 2)
OG IDs: 93521331
Best-hit-arabi-ID: AT1G74920.1
Best-hit-arabi-name: aldehyde dehydrogenase 10A8
Best-hit-arabi-symbol: ALDH10A8
Gorai.001G071200.1.A
PCC=0.056, p=0.82
mRNA Profile
Protein Expression Profile
DPA

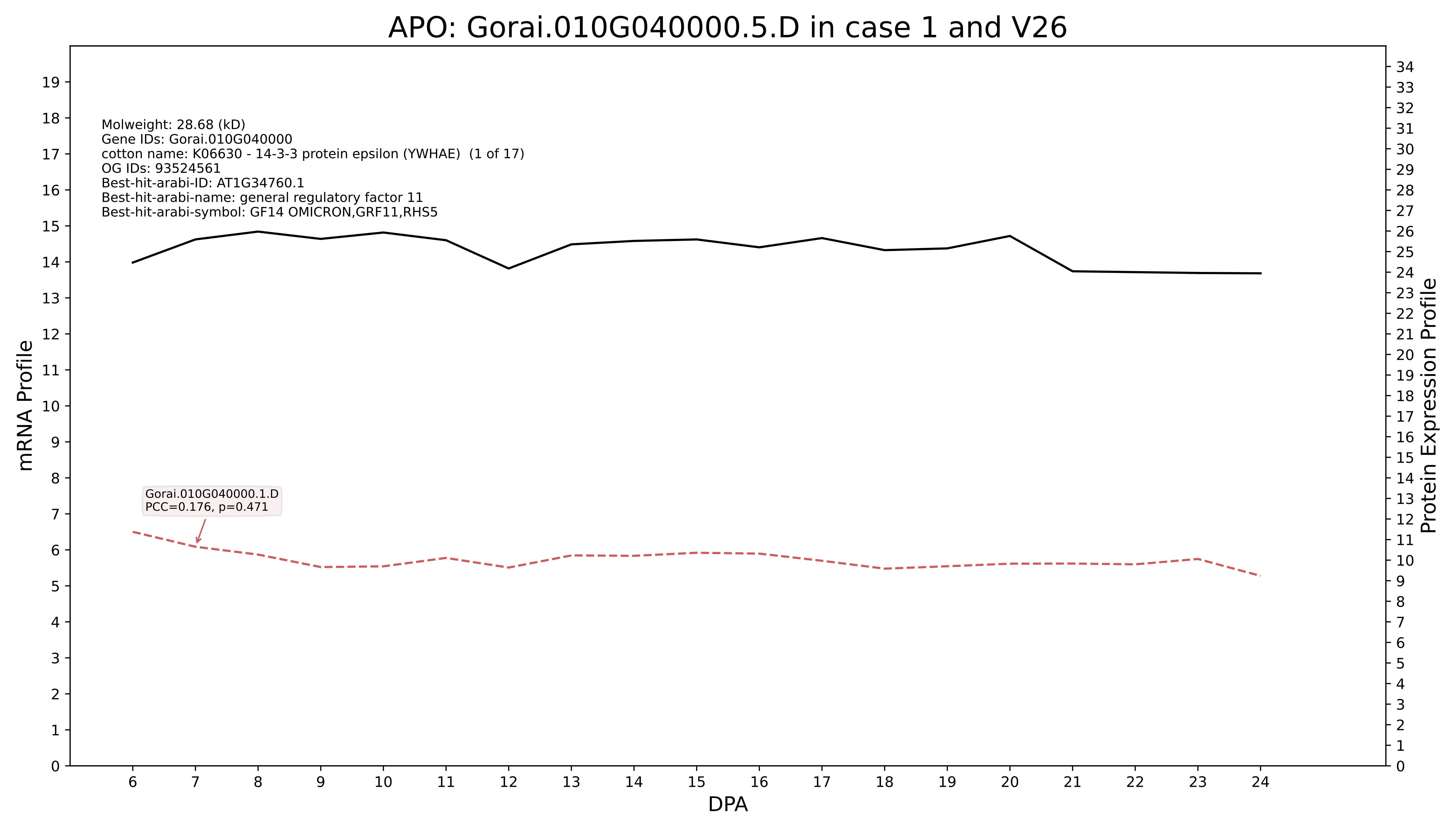
APO: Gorai.010G040000.5.D in case 1 and V26
Molweight: 28.68 (kD)
Gene IDs: Gorai.010G040000
cotton name: K06630 - 14-3-3 protein epsilon (YWHAE) (1 of 17)
OG IDs: 93524561
Best-hit-arabi-ID: AT1G34760.1
Best-hit-arabi-name: general regulatory factor 11
Best-hit-arabi-symbol: GF14 OMICRON,GRF11,RHS5
Gorai.010G040000.1.D
PCC=0.176, p=0.471
mRNA Profile
Protein Expression Profile
DPA

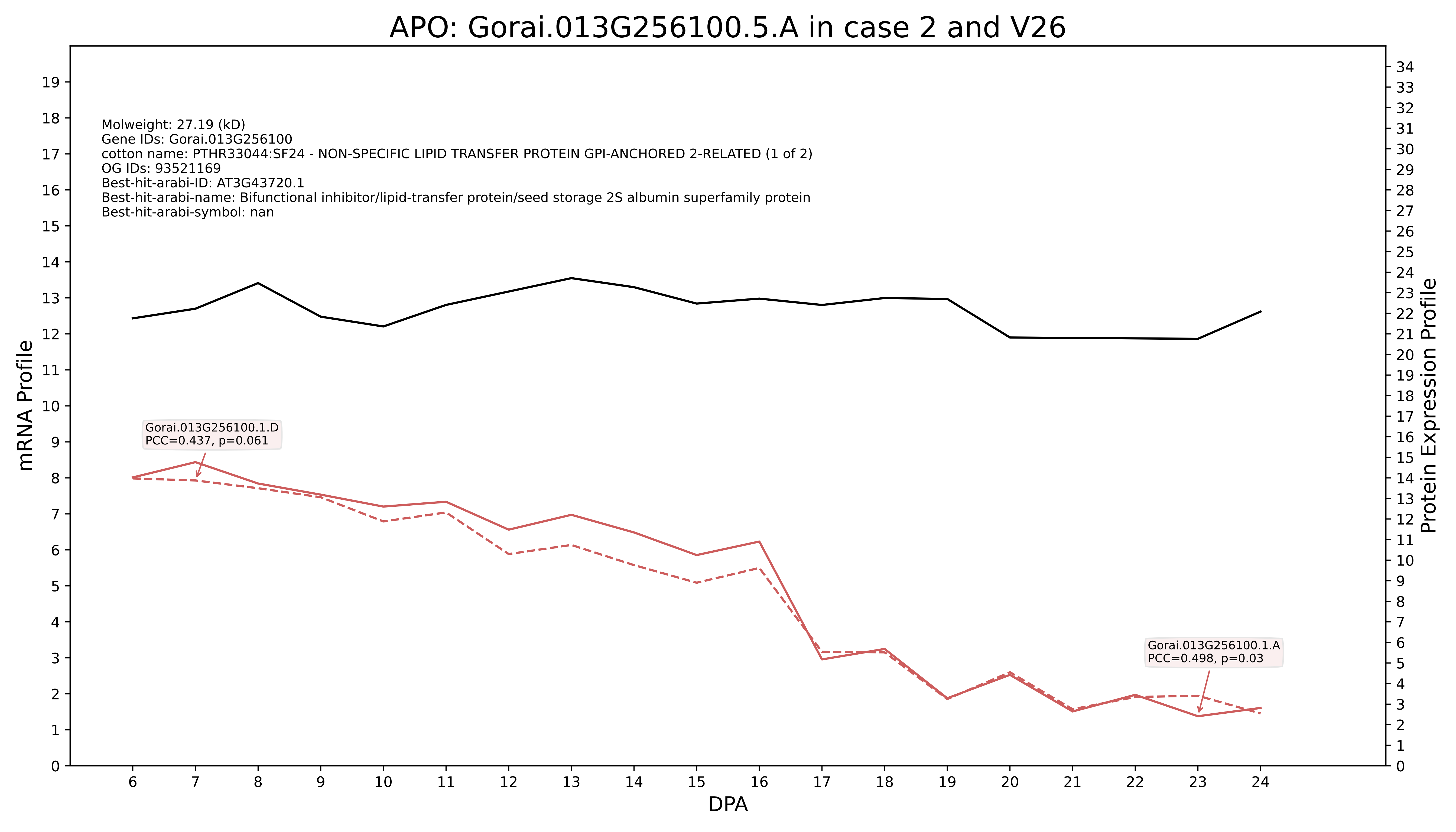

APO: Gorai.013G256100.5.A in case 2 and V26
Molweight: 27.19 (kD)
Gene IDs: Gorai.013G256100
cotton name: PTHR33044:SF24 - NON-SPECIFIC LIPID TRANSFER PROTEIN GPI-ANCHORED 2-RELATED (1 of 2)
OG IDs: 93521169
Best-hit-arabi-ID: AT3G43720.1
Best-hit-arabi-name: Bifunctional inhibitor/lipid-transfer protein/seed storage 2S albumin superfamily protein
Best-hit-arabi-symbol: nan
Gorai.013G256100.1.D
PCC=0.437, p=0.061
Gorai.013G256100.1.A
PCC=0.498, p=0.03
mRNA Profile
Protein Expression Profile
DPA

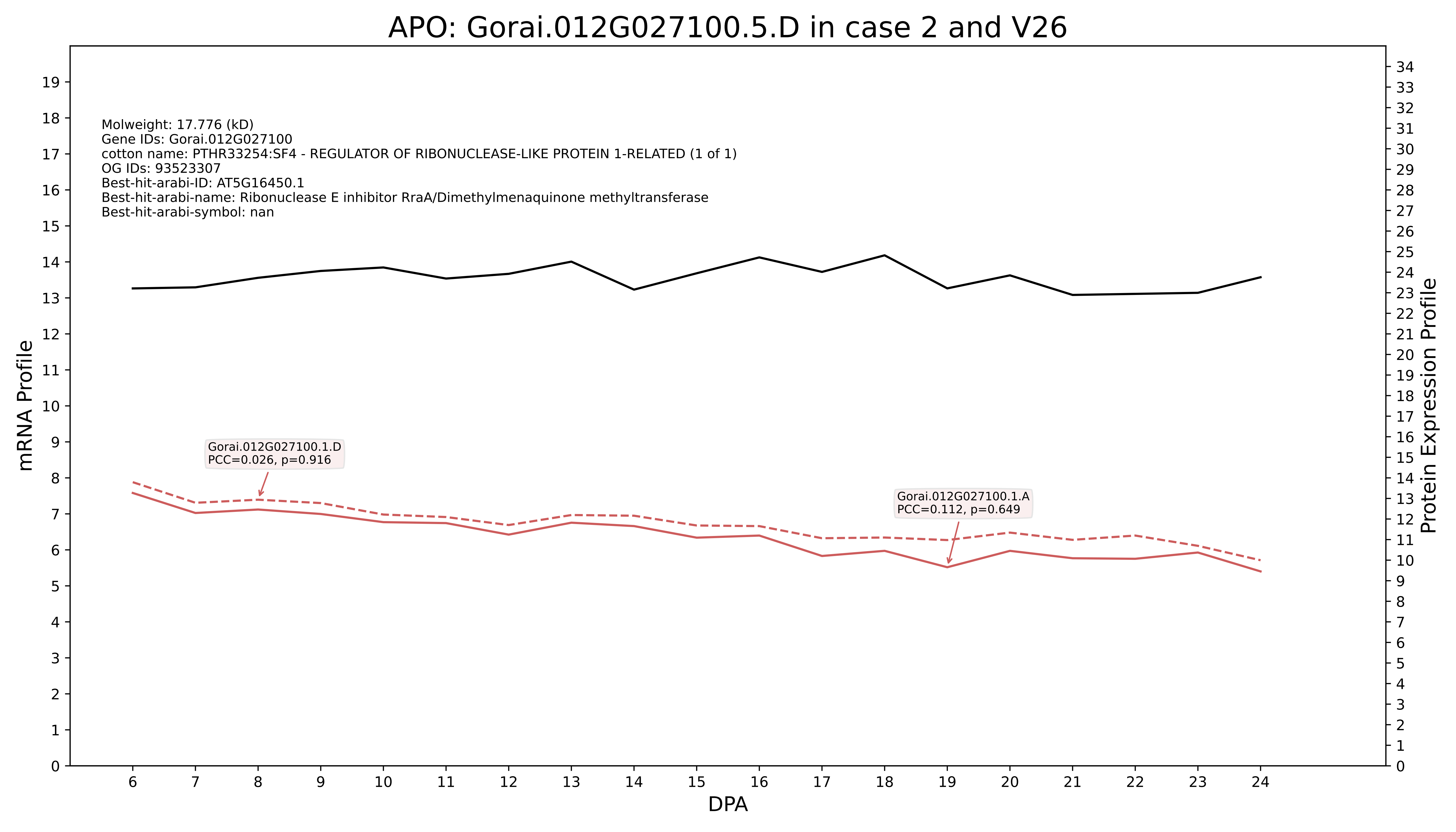

APO: Gorai.012G027100.5.D in case 2 and V26
Molweight: 17.776 (kD)
Gene IDs: Gorai.012G027100
cotton name: PTHR33254:SF4 - REGULATOR OF RIBONUCLEASE-LIKE PROTEIN 1-RELATED (1 of 1)
OG IDs: 93523307
Best-hit-arabi-ID: AT5G16450.1
Best-hit-arabi-name: Ribonuclease E inhibitor RraA/Dimethylmenaquinone methyltransferase
Best-hit-arabi-symbol: nan
Gorai.012G027100.1.D
PCC=0.026, p=0.916
Gorai.012G027100.1.A
PCC=0.112, p=0.649
mRNA Profile
Protein Expression Profile
DPA

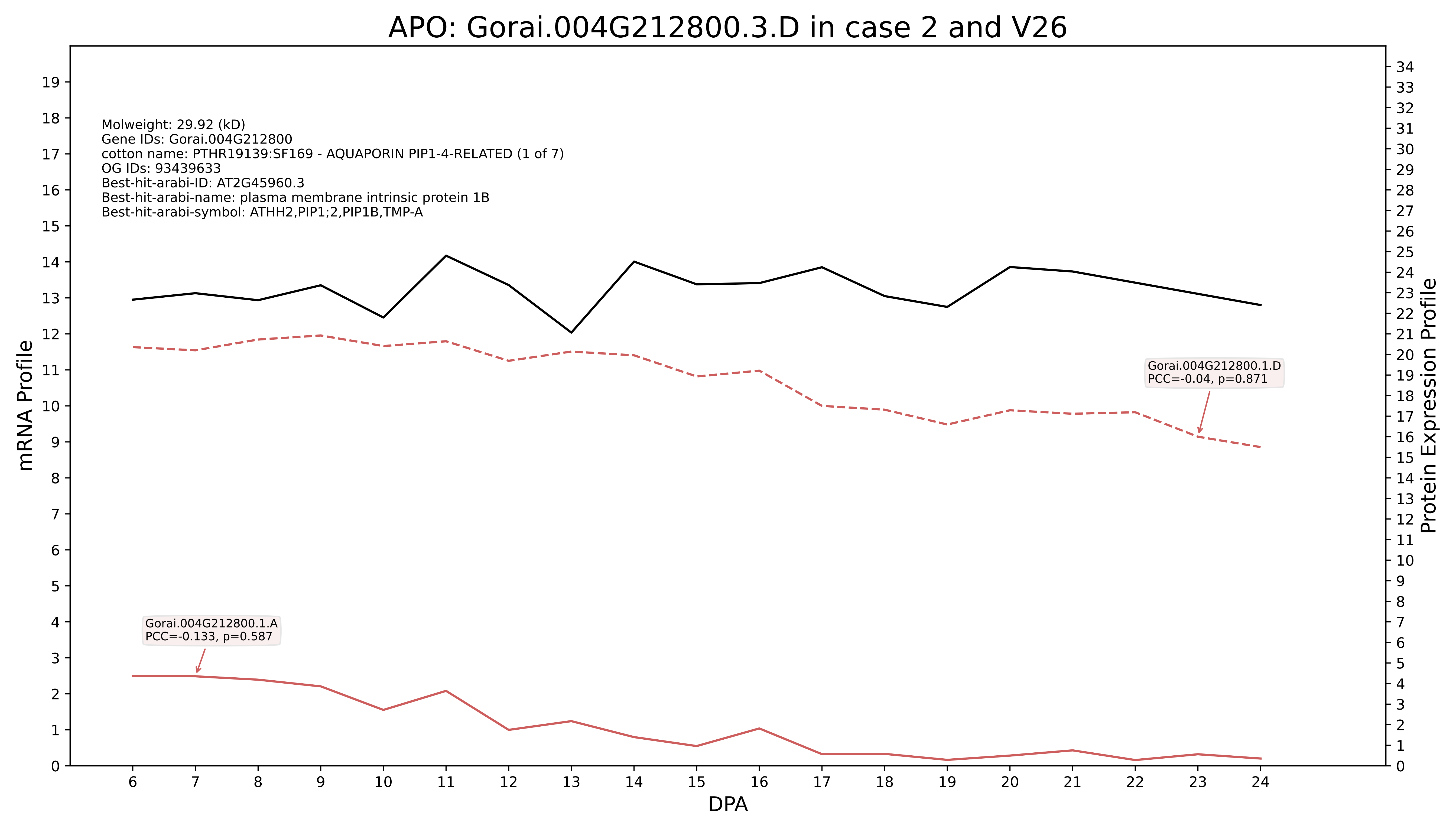

APO: Gorai.004G212800.3.D in case 2 and V26
Molweight: 29.92 (kD)
Gene IDs: Gorai.004G212800
cotton name: PTHR19139:SF169 - AQUAPORIN PIP1-4-RELATED (1 of 7)
OG IDs: 93439633
Best-hit-arabi-ID: AT2G45960.3
Best-hit-arabi-name: plasma membrane intrinsic protein 1B
Best-hit-arabi-symbol: ATHH2,PIP1;2,PIP1B,TMP-A
Gorai.004G212800.1.D
PCC=-0.04, p=0.871
Gorai.004G212800.1.A
PCC=-0.133, p=0.587
mRNA Profile
Protein Expression Profile
DPA

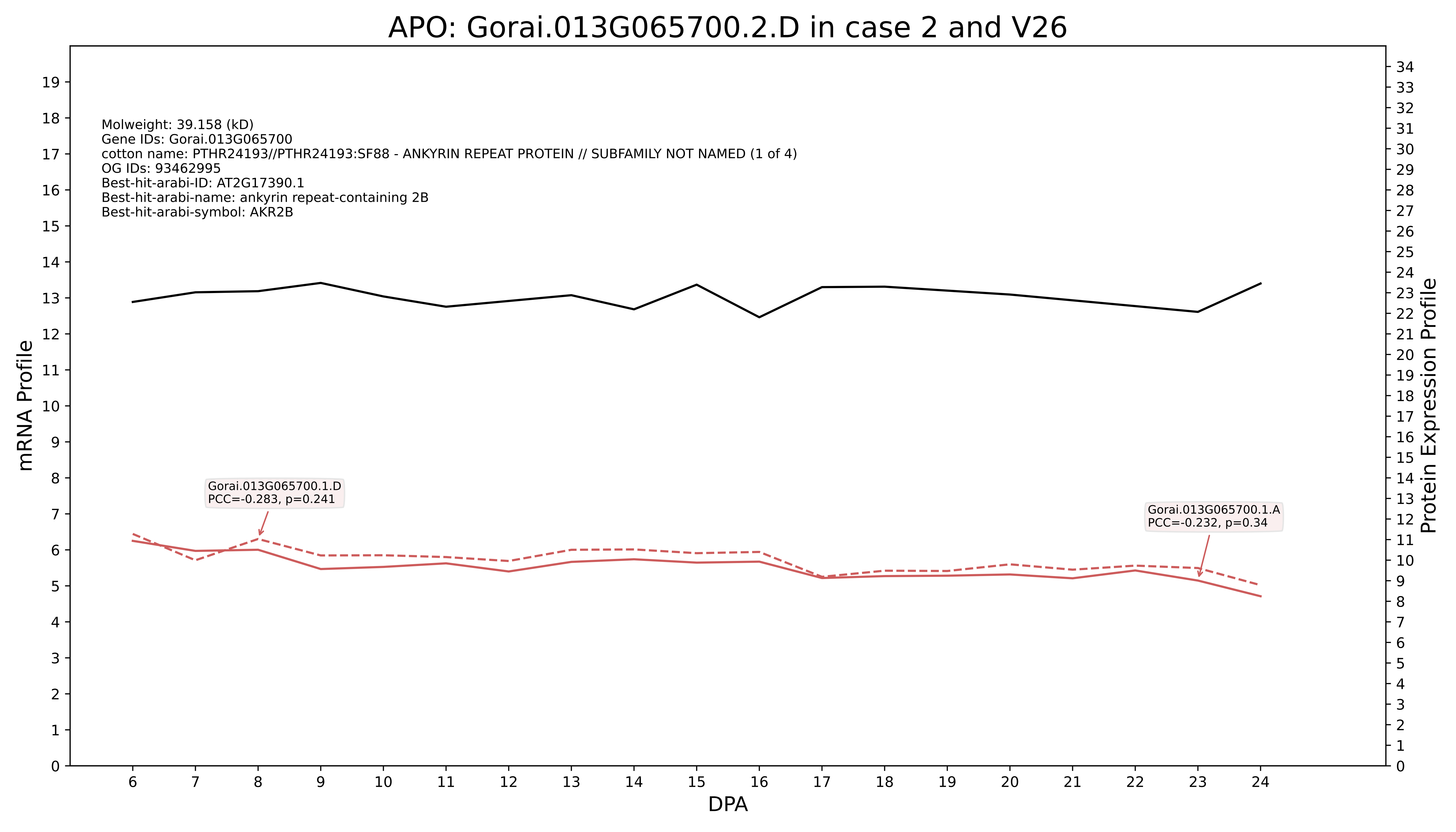
APO: Gorai.013G065700.2.D in case 2 and V26
Molweight: 39.158 (kD)
Gene IDs: Gorai.013G065700
cotton name: PTHR24193//PTHR24193:SF88 - ANKYRIN REPEAT PROTEIN // SUBFAMILY NOT NAMED (1 of 4)
OG IDs: 93462995
Best-hit-arabi-ID: AT2G17390.1
Best-hit-arabi-name: ankyrin repeat-containing 2B
Best-hit-arabi-symbol: AKR2B
Gorai.013G065700.1.D
PCC=-0.283, p=0.241
Gorai.013G065700.1.A
PCC=-0.232, p=0.34
mRNA Profile
Protein Expression Profile
DPA
0
1
2
3
4
5
6
7
8
9
10
11
12
13
14
15
16
17
18
19
20
21
22
23
24
25
26
27
28
29
30
31
32
33
34

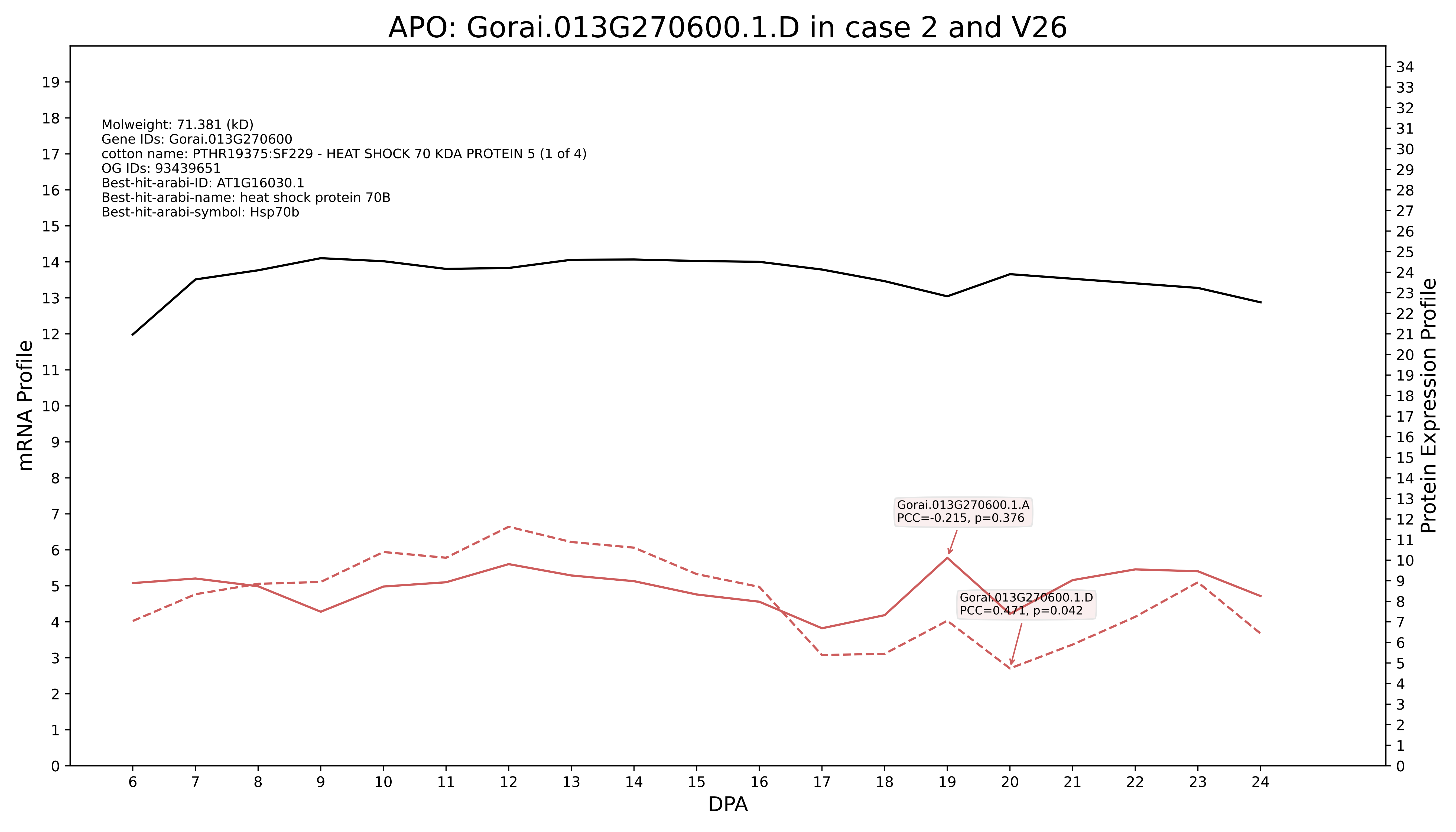
APO: Gorai.013G270600.1.D in case 2 and V26
Molweight: 71.381 (kD)
Gene IDs: Gorai.013G270600
cotton name: PTHR19375:SF229 - HEAT SHOCK 70 KDA PROTEIN 5 (1 of 4)
OG IDs: 93439651
Best-hit-arabi-ID: AT1G16030.1
Best-hit-arabi-name: heat shock protein 70B
Best-hit-arabi-symbol: Hsp70b
Gorai.013G270600.1.A
PCC=-0.215, p=0.376
Gorai.013G270600.1.D
PCC=0.471, p=0.042
mRNA Profile
Protein Expression Profile
DPA

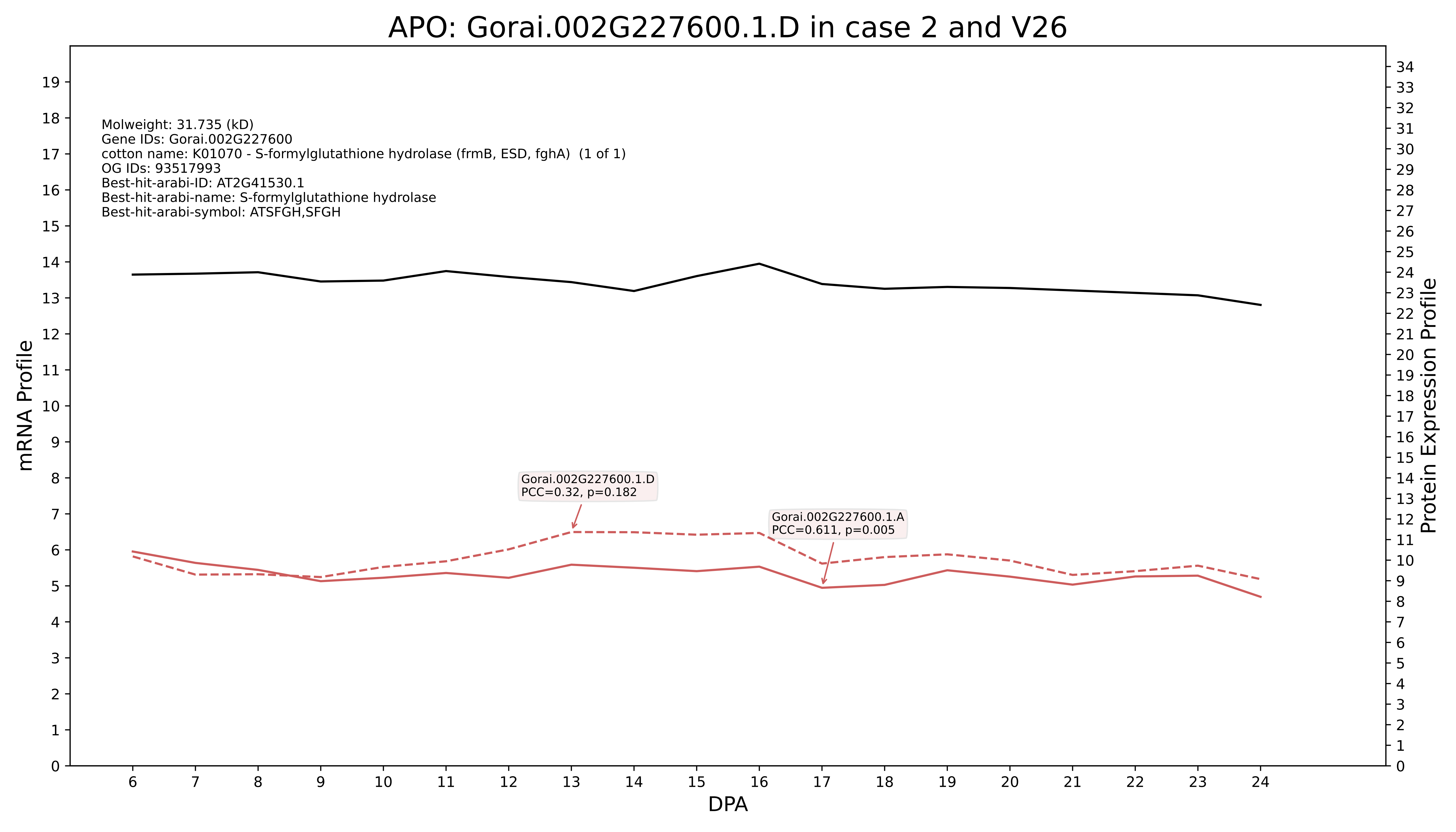

APO: Gorai.002G227600.1.D in case 2 and V26
Molweight: 31.735 (kD)
Gene IDs: Gorai.002G227600
cotton name: K01070 - S-formylglutathione hydrolase (frmB, ESD, fghA) (1 of 1)
OG IDs: 93517993
Best-hit-arabi-ID: AT2G41530.1
Best-hit-arabi-name: S-formylglutathione hydrolase
Best-hit-arabi-symbol: ATSFGH,SFGH
Gorai.002G227600.1.D
PCC=0.32, p=0.182
Gorai.002G227600.1.A
PCC=0.611, p=0.005
mRNA Profile
Protein Expression Profile
DPA

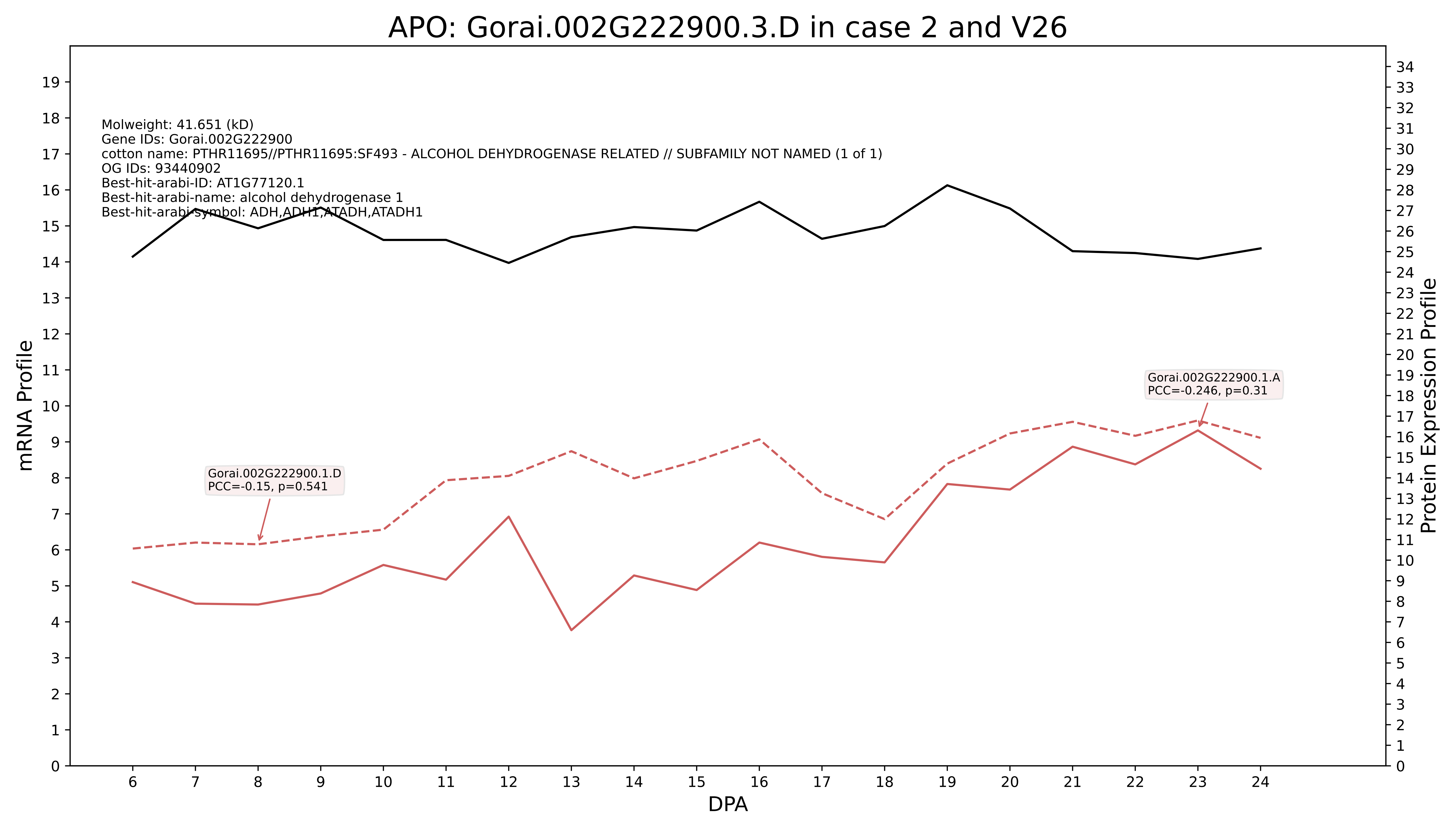

APO: Gorai.002G222900.3.D in case 2 and V26
Molweight: 41.651 (kD)
Gene IDs: Gorai.002G222900
cotton name: PTHR11695//PTHR11695:SF493 - ALCOHOL DEHYDROGENASE RELATED // SUBFAMILY NOT NAMED (1 of 1)
OG IDs: 93440902
Best-hit-arabi-ID: AT1G77120.1
Best-hit-arabi-name: alcohol dehydrogenase 1
Best-hit-arabi-symbol: ADH,ADH1,ATADH,ATADH1
Gorai.002G222900.1.A
PCC=-0.246, p=0.31
Gorai.002G222900.1.D
PCC=-0.15, p=0.541
mRNA Profile
Protein Expression Profile
DPA

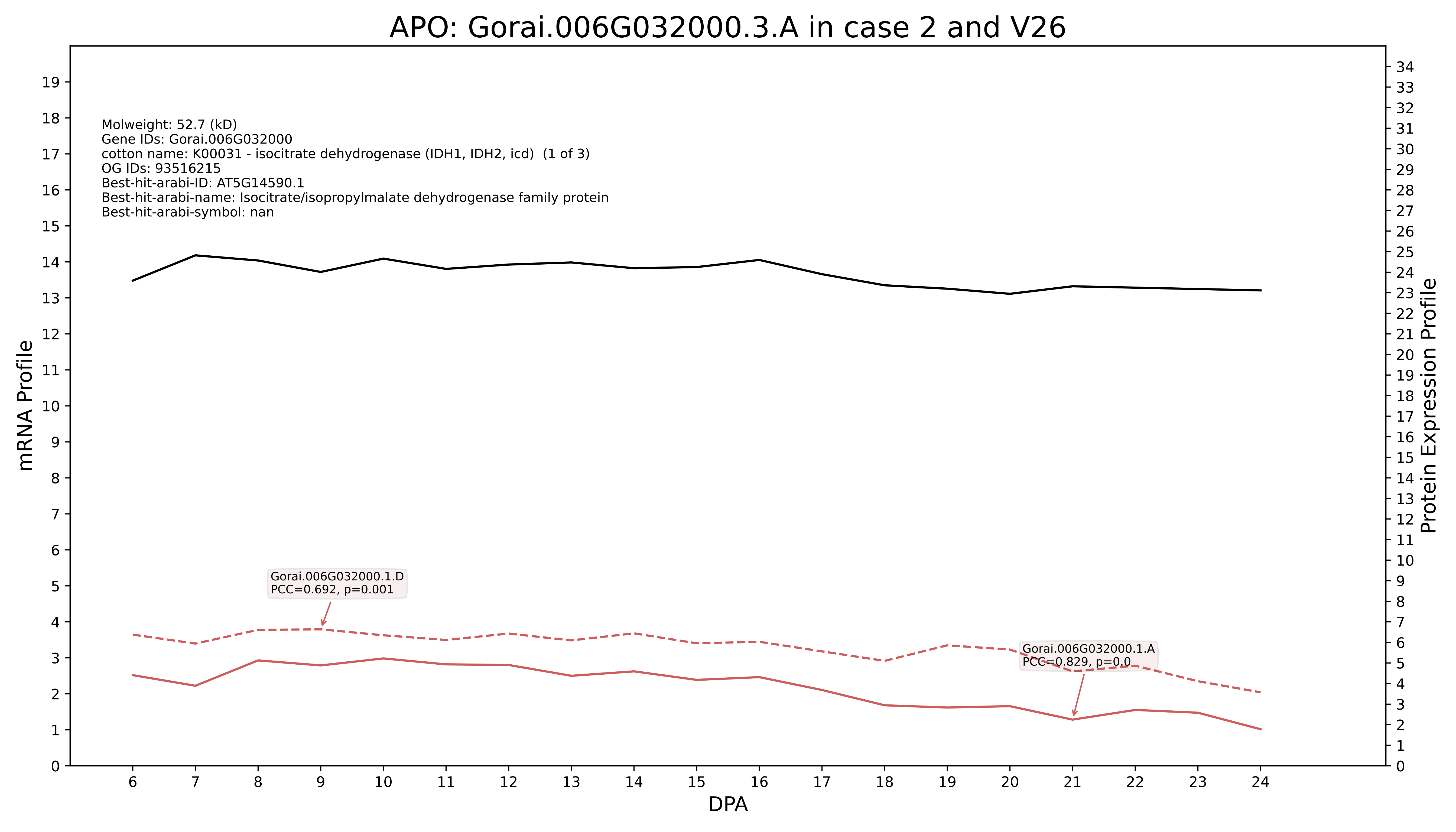
APO: Gorai.006G032000.3.A in case 2 and V26
Molweight: 52.7 (kD)
Gene IDs: Gorai.006G032000
cotton name: K00031 - isocitrate dehydrogenase (IDH1, IDH2, icd) (1 of 3)
OG IDs: 93516215
Best-hit-arabi-ID: AT5G14590.1
Best-hit-arabi-name: Isocitrate/isopropylmalate dehydrogenase family protein
Best-hit-arabi-symbol: nan
Gorai.006G032000.1.D
PCC=0.692, p=0.001
Gorai.006G032000.1.A
PCC=0.829, p=0.0
mRNA Profile
Protein Expression Profile
DPA

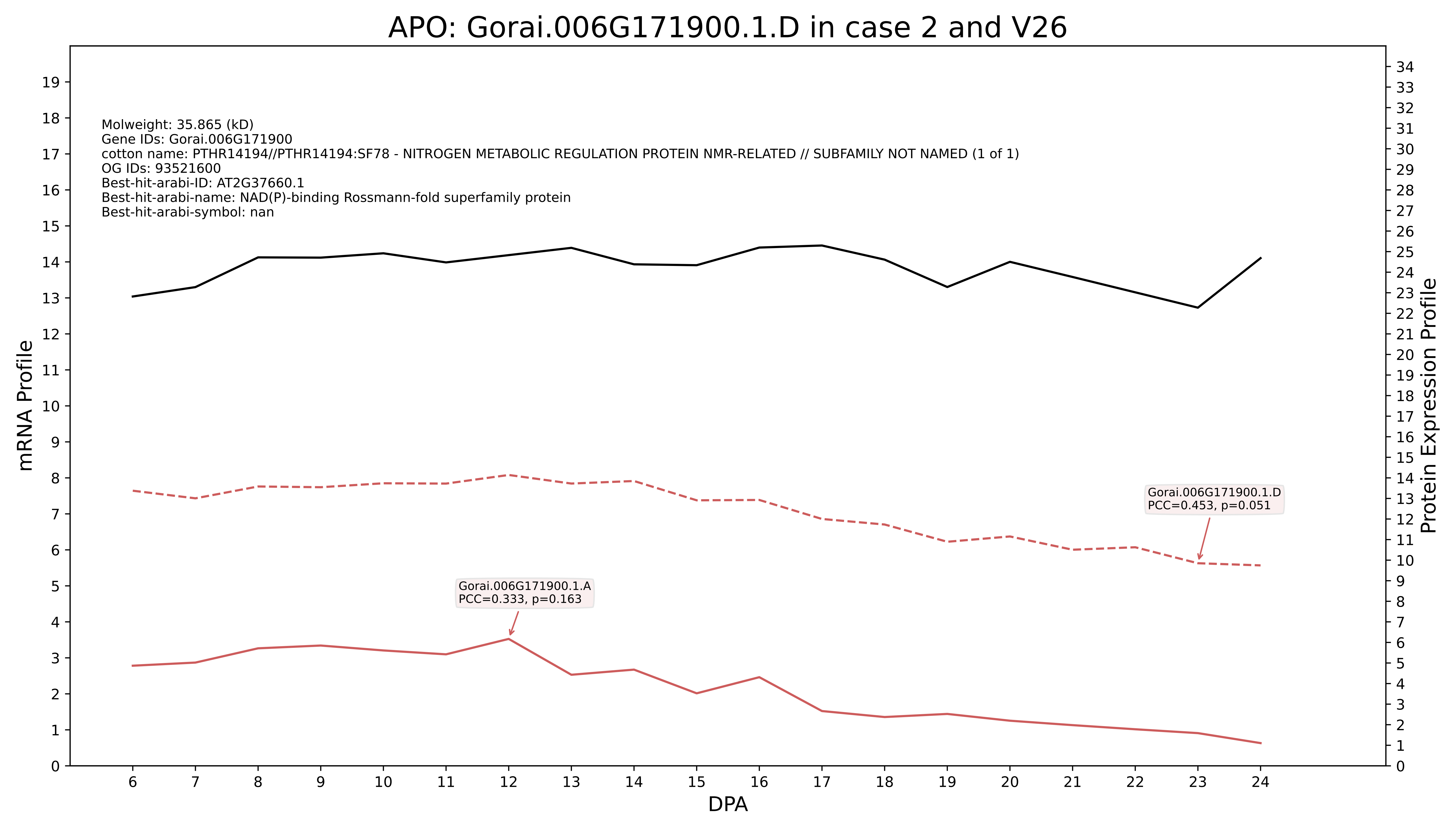

APO: Gorai.006G171900.1.D in case 2 and V26
Molweight: 35.865 (kD)
Gene IDs: Gorai.006G171900
cotton name: PTHR14194//PTHR14194:SF78 - NITROGEN METABOLIC REGULATION PROTEIN NMR-RELATED // SUBFAMILY NOT NAMED (1 of 1)
OG IDs: 93521600
Best-hit-arabi-ID: AT2G37660.1
Best-hit-arabi-name: NAD(P)-binding Rossmann-fold superfamily protein
Best-hit-arabi-symbol: nan
Gorai.006G171900.1.D
PCC=0.453, p=0.051
Gorai.006G171900.1.A
PCC=0.333, p=0.163
mRNA Profile
Protein Expression Profile
DPA

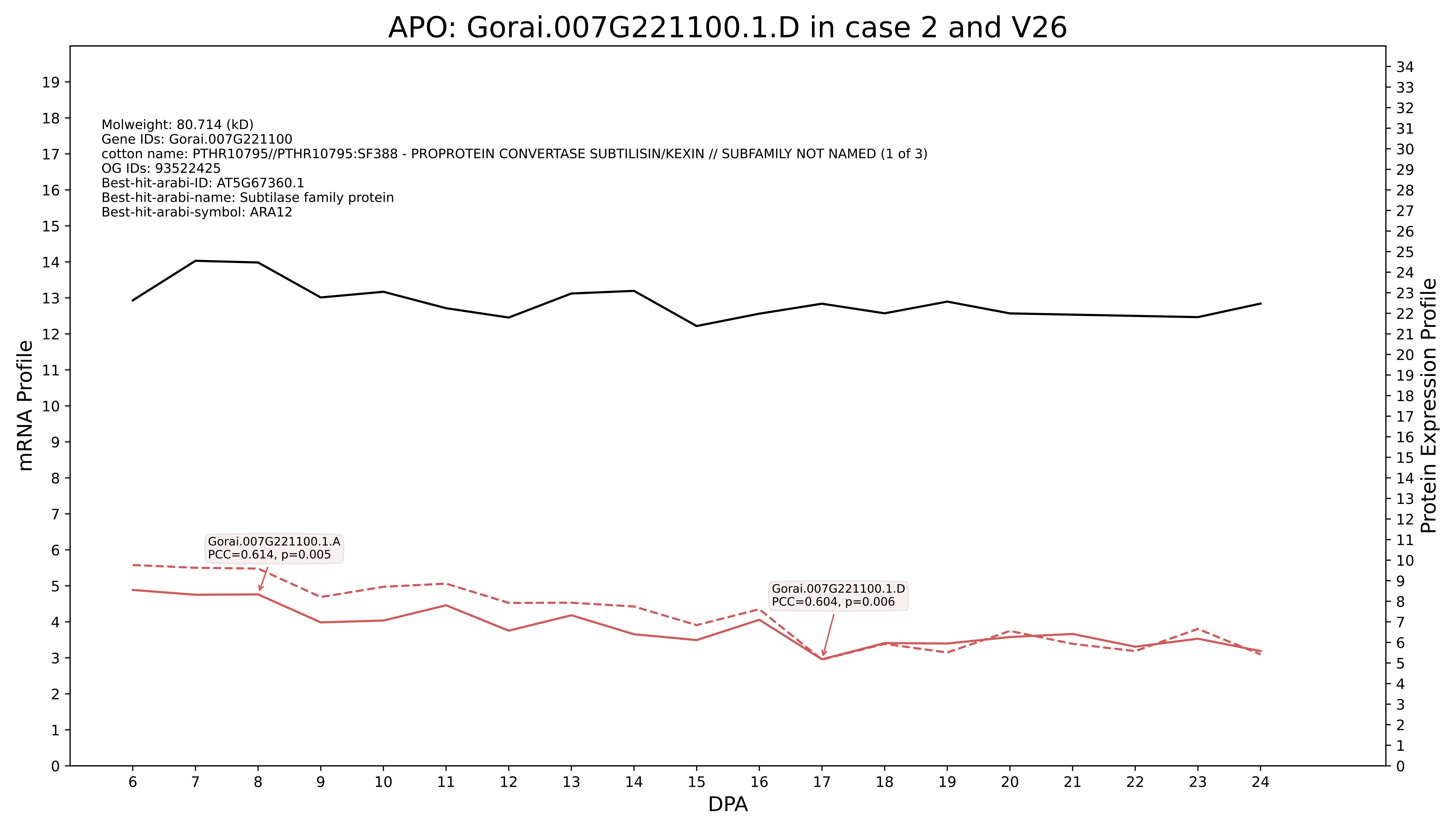
APO: Gorai.007G221100.1.D in case 2 and V26
Molweight: 80.714 (kD)
Gene IDs: Gorai.007G221100
cotton name: PTHR10795//PTHR10795:SF388 - PROPROTEIN CONVERTASE SUBTILISIN/KEXIN // SUBFAMILY NOT NAMED (1 of 3)
OG IDs: 93522425
Best-hit-arabi-ID: AT5G67360.1
Best-hit-arabi-name: Subtilase family protein
Best-hit-arabi-symbol: ARA12
Gorai.007G221100.1.A
PCC=0.614, p=0.005
Gorai.007G221100.1.D
PCC=0.604, p=0.006
mRNA Profile
Protein Expression Profile
DPA

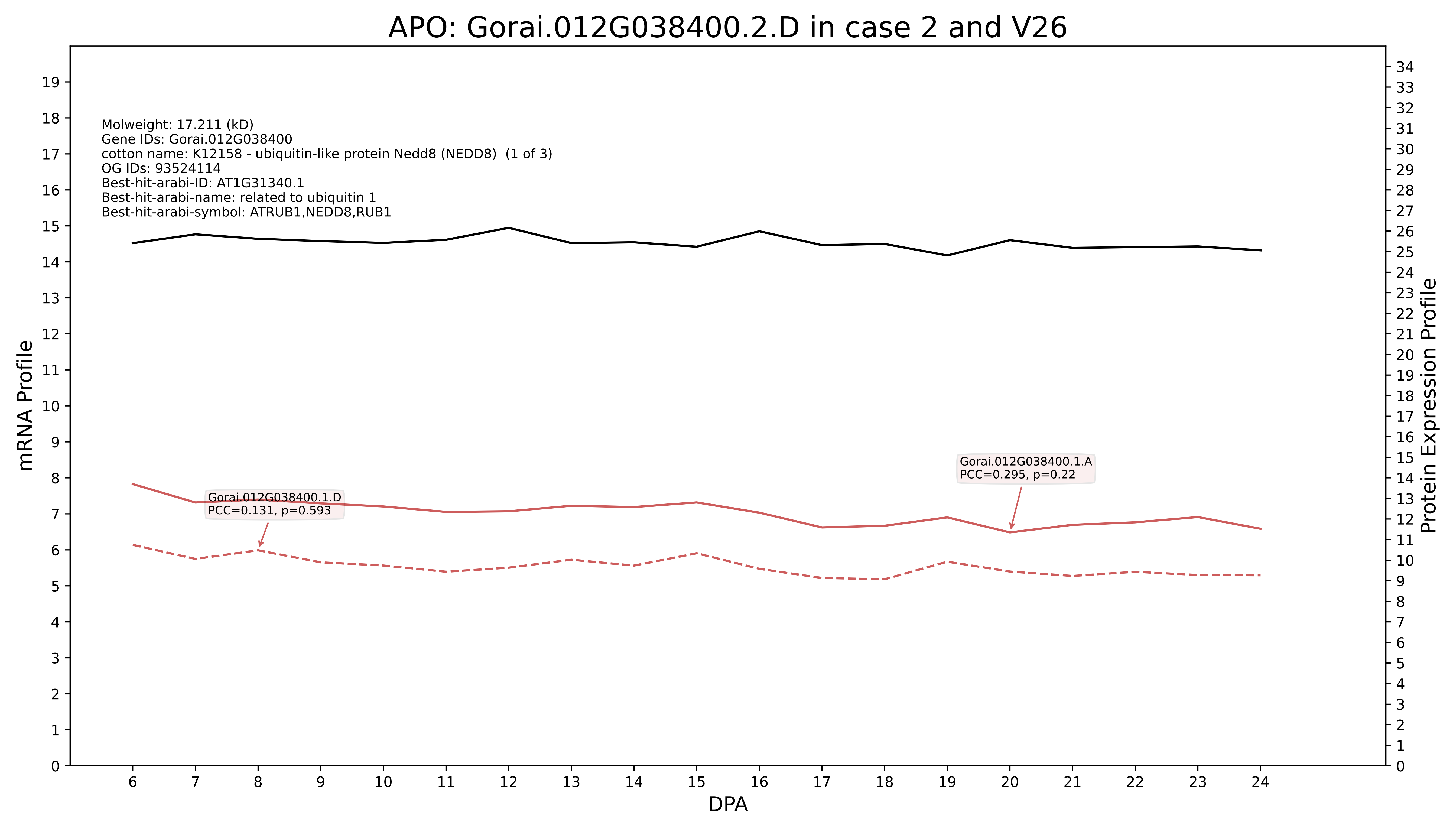

APO: Gorai.012G038400.2.D in case 2 and V26
Molweight: 17.211 (kD)
Gene IDs: Gorai.012G038400
cotton name: K12158 - ubiquitin-like protein Nedd8 (NEDD8) (1 of 3)
OG IDs: 93524114
Best-hit-arabi-ID: AT1G31340.1
Best-hit-arabi-name: related to ubiquitin 1
Best-hit-arabi-symbol: ATRUB1,NEDD8,RUB1
Gorai.012G038400.1.D
PCC=0.131, p=0.593
Gorai.012G038400.1.A
PCC=0.295, p=0.22
mRNA Profile
Protein Expression Profile
DPA

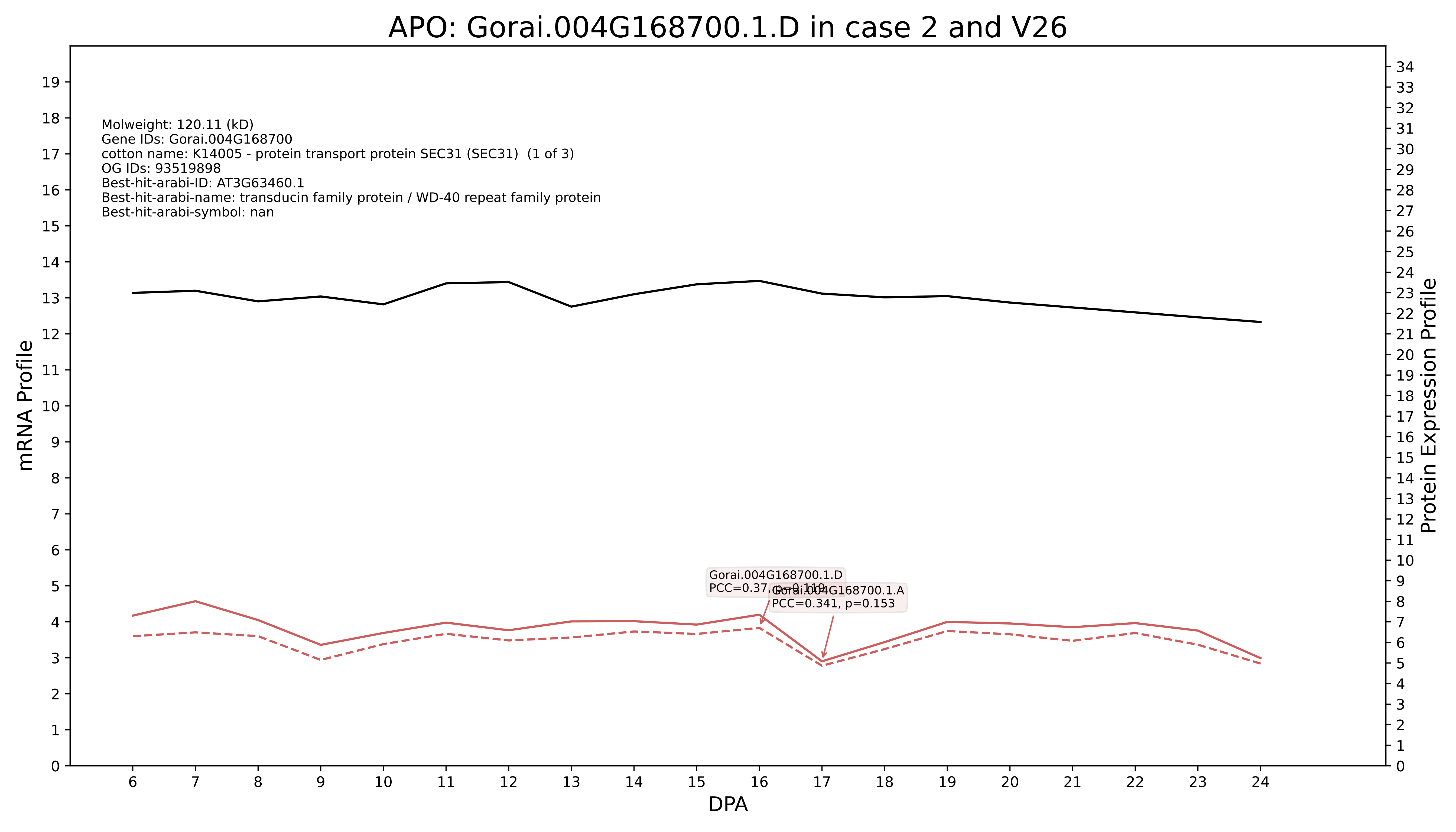

APO: Gorai.004G168700.1.D in case 2 and V26
Molweight: 120.11 (kD)
Gene IDs: Gorai.004G168700
cotton name: K14005 - protein transport protein SEC31 (SEC31) (1 of 3)
OG IDs: 93519898
Best-hit-arabi-ID: AT3G63460.1
Best-hit-arabi-name: transducin family protein / WD-40 repeat family protein
Best-hit-arabi-symbol: nan
Gorai.004G168700.1.D
PCC=0.37, p=0.119
Gorai.004G168700.1.A
PCC=0.341, p=0.153
mRNA Profile
Protein Expression Profile
DPA

APO: Gorai.005G184600.1.D in case 2 and V26

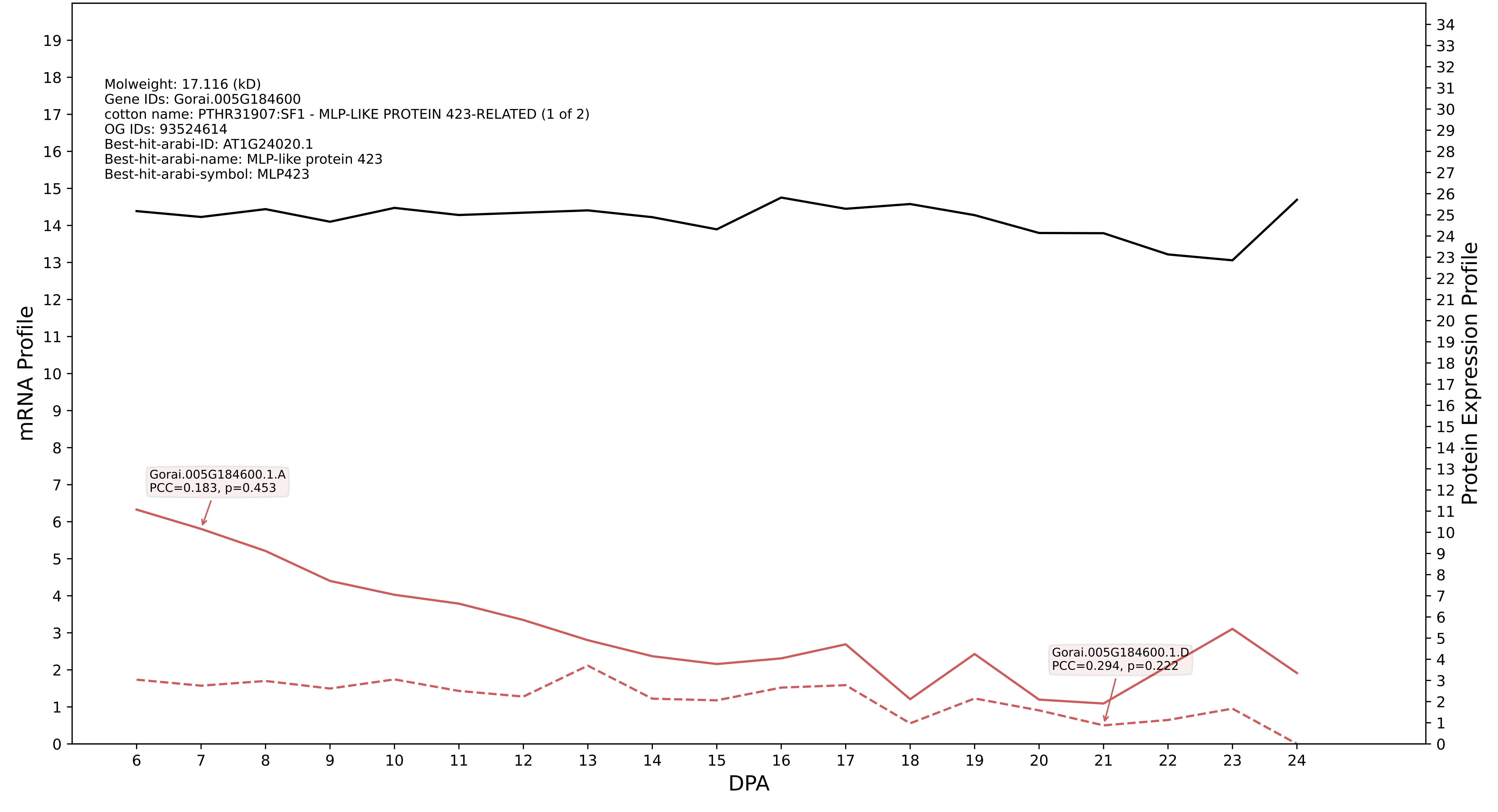

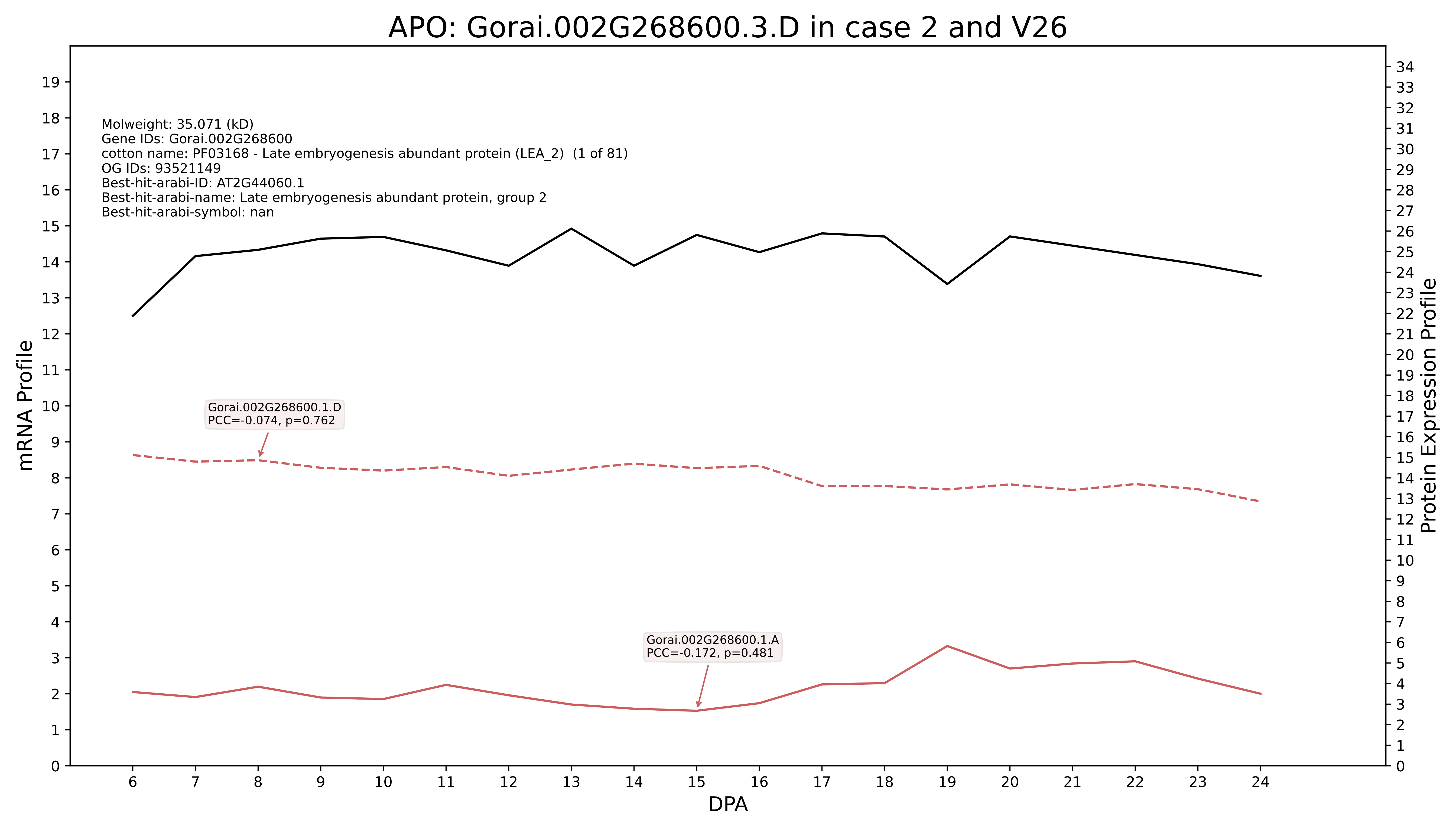
APO: Gorai.002G268600.3.D in case 2 and V26
Molweight: 35.071 (kD)
Gene IDs: Gorai.002G268600
cotton name: PF03168 - Late embryogenesis abundant protein (LEA_2) (1 of 81)
OG IDs: 93521149
Best-hit-arabi-ID: AT2G44060.1
Best-hit-arabi-name: Late embryogenesis abundant protein, group 2
Best-hit-arabi-symbol: nan
Gorai.002G268600.1.D
PCC=-0.074, p=0.762
Gorai.002G268600.1.A
PCC=-0.172, p=0.481
mRNA Profile
Protein Expression Profile
DPA

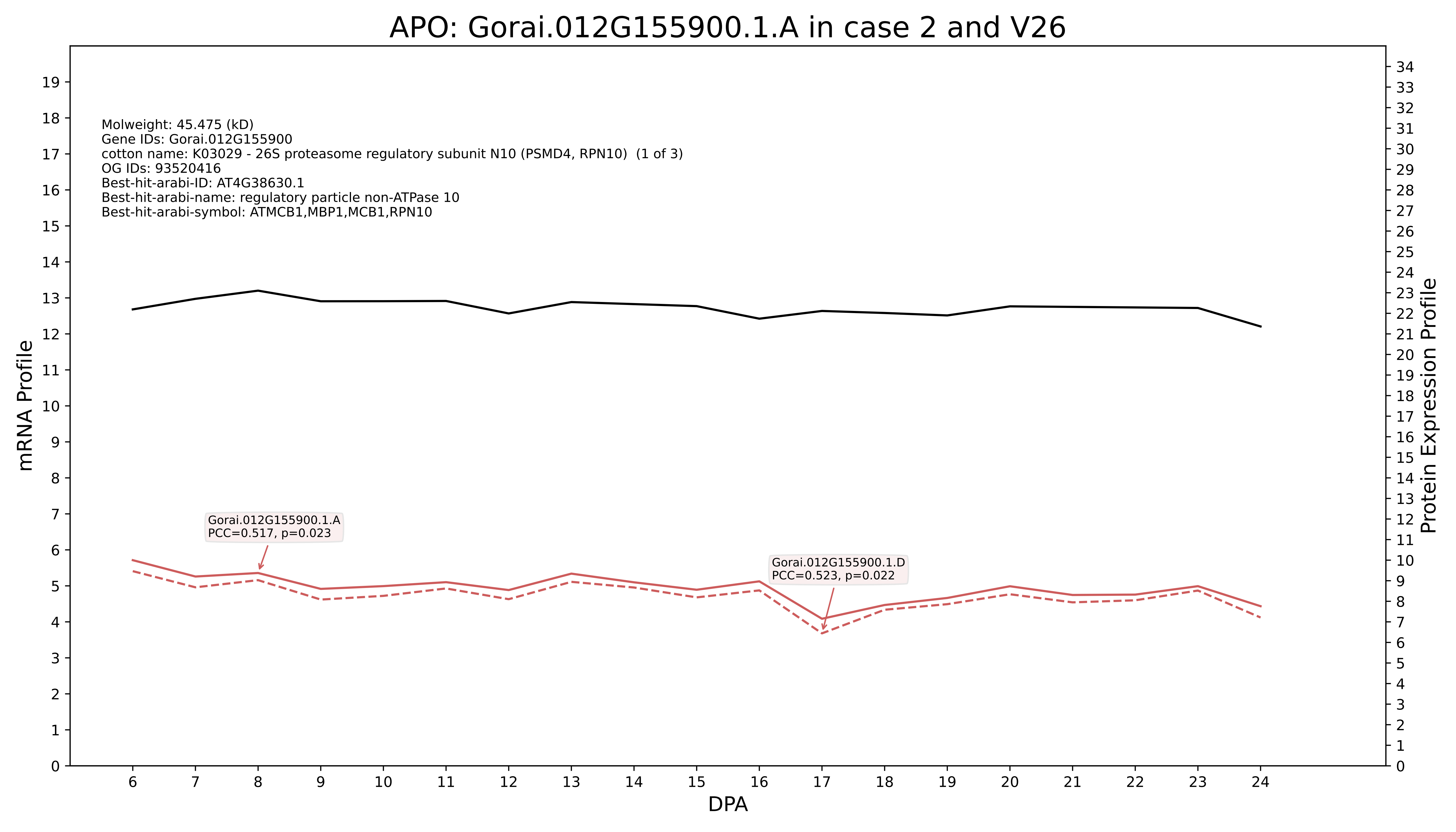
APO: Gorai.012G155900.1.A in case 2 and V26
Molweight: 45.475 (kD)
Gene IDs: Gorai.012G155900
cotton name: K03029 - 26S proteasome regulatory subunit N10 (PSMD4, RPN10) (1 of 3)
OG IDs: 93520416
Best-hit-arabi-ID: AT4G38630.1
Best-hit-arabi-name: regulatory particle non-ATPase 10
Best-hit-arabi-symbol: ATMCB1,MBP1,MCB1,RPN10
Gorai.012G155900.1.A
PCC=0.517, p=0.023
Gorai.012G155900.1.D
PCC=0.523, p=0.022
mRNA Profile
Protein Expression Profile
DPA

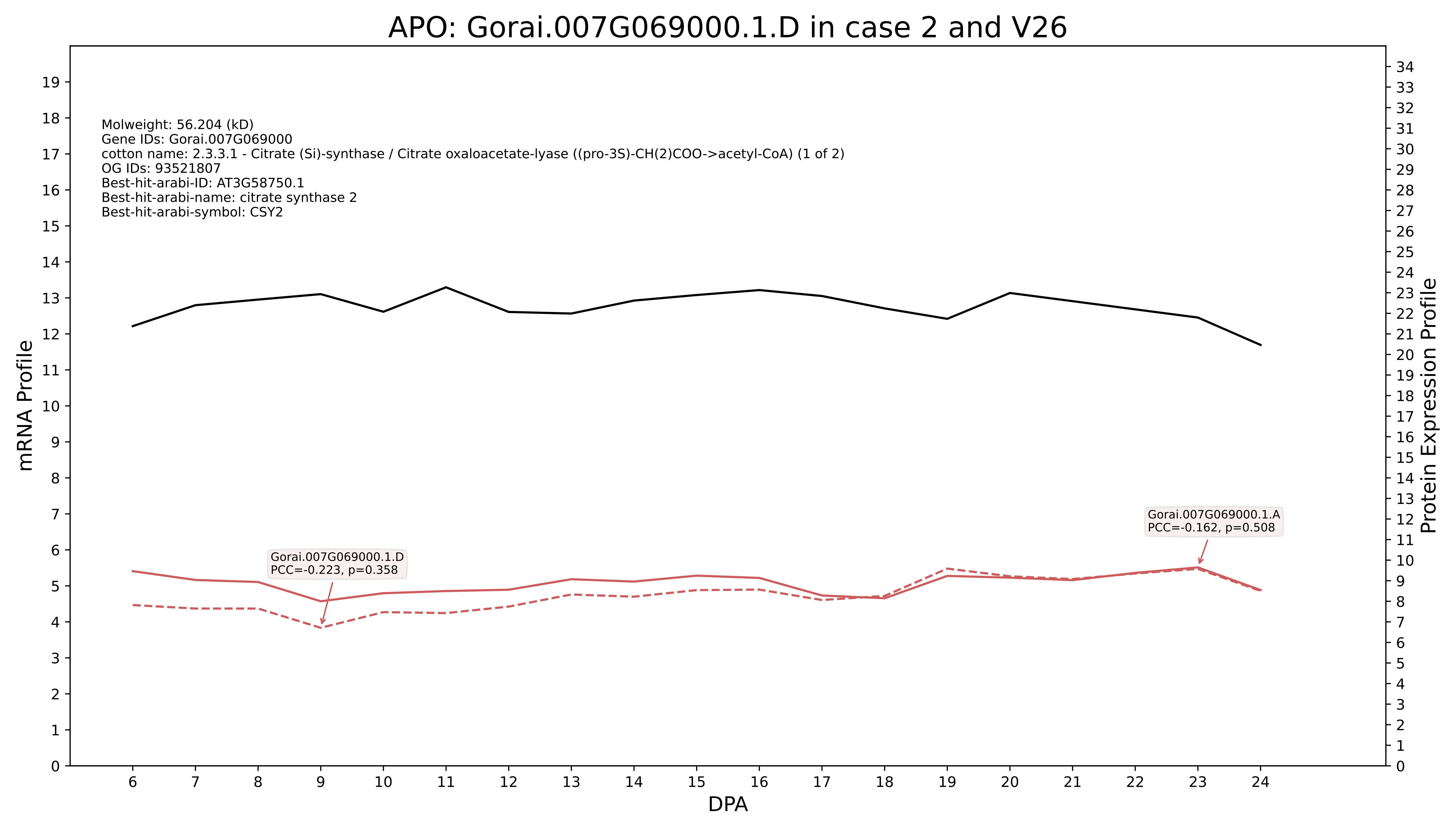
APO: Gorai.007G069000.1.D in case 2 and V26
Molweight: 56.204 (kD)
Gene IDs: Gorai.007G069000
cotton name: 2.3.3.1 - Citrate (Si)-synthase / Citrate oxaloacetate-lyase ((pro-3S)-CH(2)COO->acetyl-CoA) (1 of 2)
OG IDs: 93521807
Best-hit-arabi-ID: AT3G58750.1
Best-hit-arabi-name: citrate synthase 2
Best-hit-arabi-symbol: CSY2
Gorai.007G069000.1.D
PCC=-0.223, p=0.358
Gorai.007G069000.1.A
PCC=-0.162, p=0.508
mRNA Profile
Protein Expression Profile
DPA
0
1
2
3
4
5
6
7
8
9
10
11
12
13
14
15
16
17
18
19
20
21
22
23
24
25
26
27
28
29
30
31
32
33
34

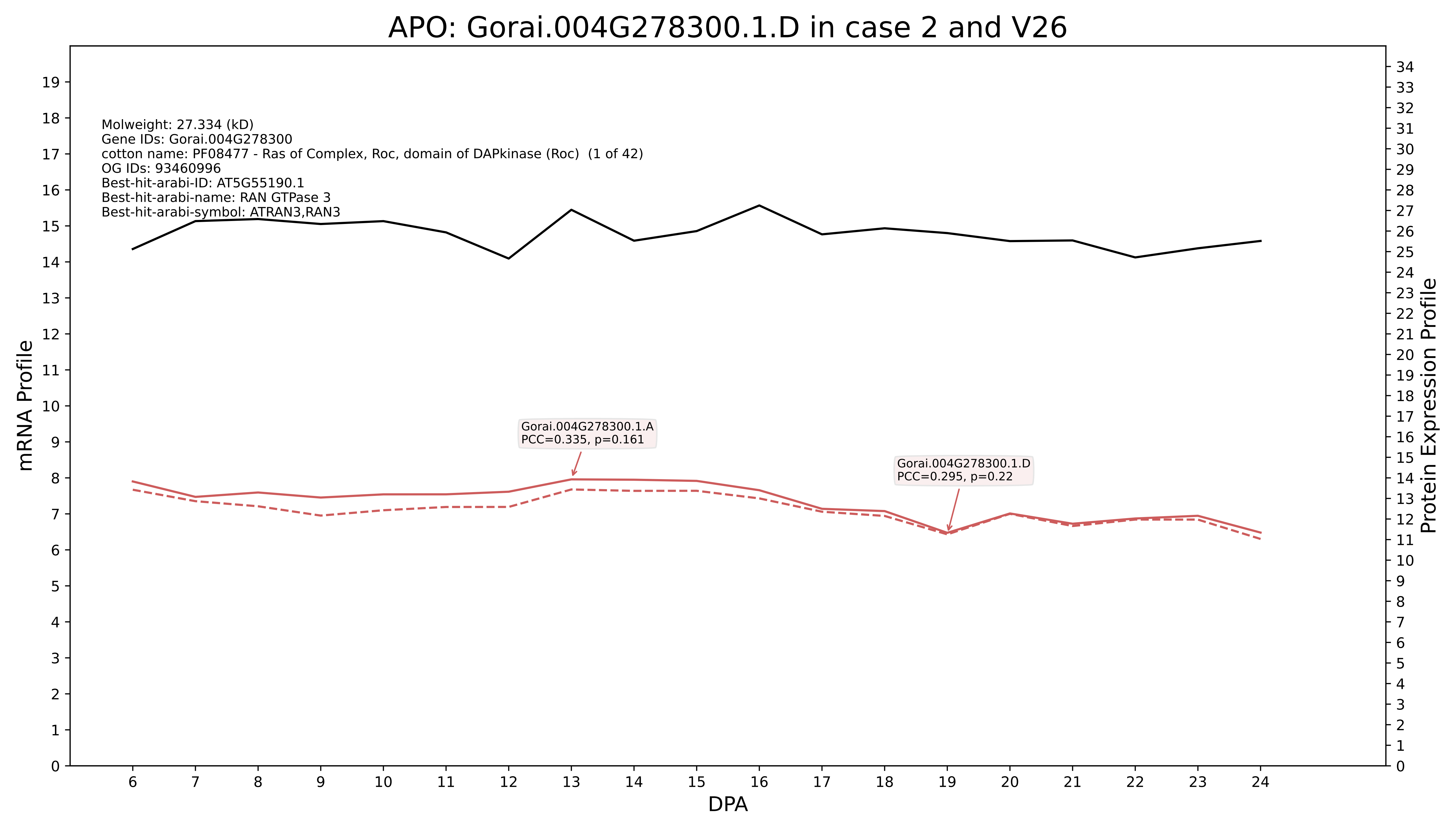

APO: Gorai.004G278300.1.D in case 2 and V26
Molweight: 27.334 (kD)
Gene IDs: Gorai.004G278300
cotton name: PF08477 - Ras of Complex, Roc, domain of DAPkinase (Roc) (1 of 42)
OG IDs: 93460996
Best-hit-arabi-ID: AT5G55190.1
Best-hit-arabi-name: RAN GTPase 3
Best-hit-arabi-symbol: ATRAN3,RAN3
Gorai.004G278300.1.A
PCC=0.335, p=0.161
Gorai.004G278300.1.D
PCC=0.295, p=0.22
mRNA Profile
Protein Expression Profile
DPA

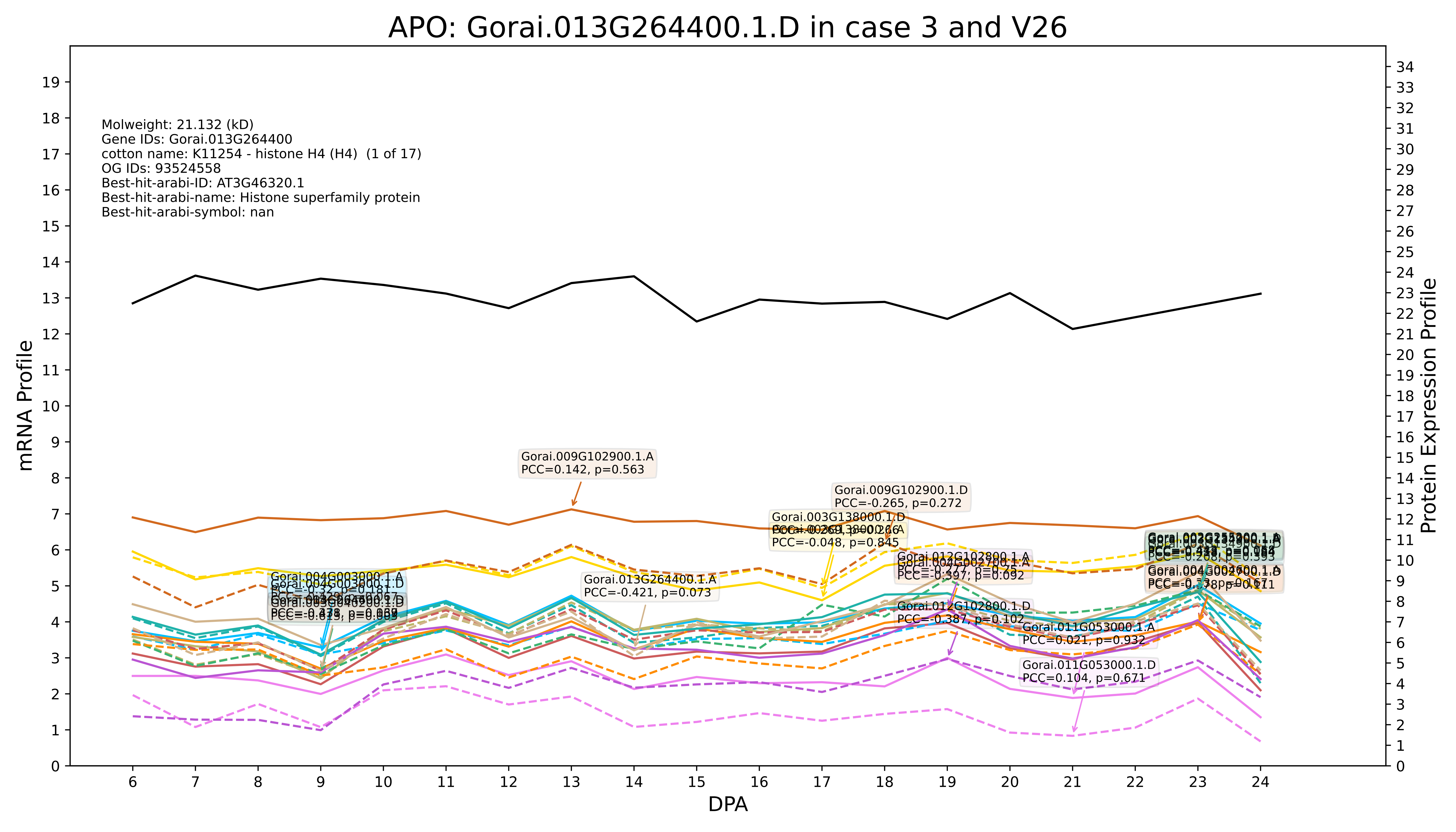

APO: Gorai.013G264400.1.D in case 3 and V26
Molweight: 21.132 (kD)
Gene IDs: Gorai.013G264400
cotton name: K11254 - histone H4 (H4) (1 of 17)
OG IDs: 93524558
Best-hit-arabi-ID: AT3G46320.1
Best-hit-arabi-name: Histone superfamily protein
Best-hit-arabi-symbol: nan
Gorai.009G102900.1.A
PCC=0.142, p=0.563
Gorai.009G102900.1.D
PCC=-0.265, p=0.272
Gorai.003G138000.1.D
PCC=-0.048, p=0.845
Gorai.013G264400.1.A
PCC=-0.421, p=0.073
Gorai.004G003000.1.A
Gorai.012G102800.1.A
Gorai.012G102800.1.D
PCC=-0.387, p=0.102
Gorai.011G053000.1.A
PCC=0.021, p=0.932
Gorai.011G053000.1.D
PCC=0.104, p=0.671
mRNA Profile
Protein Expression Profile
DPA

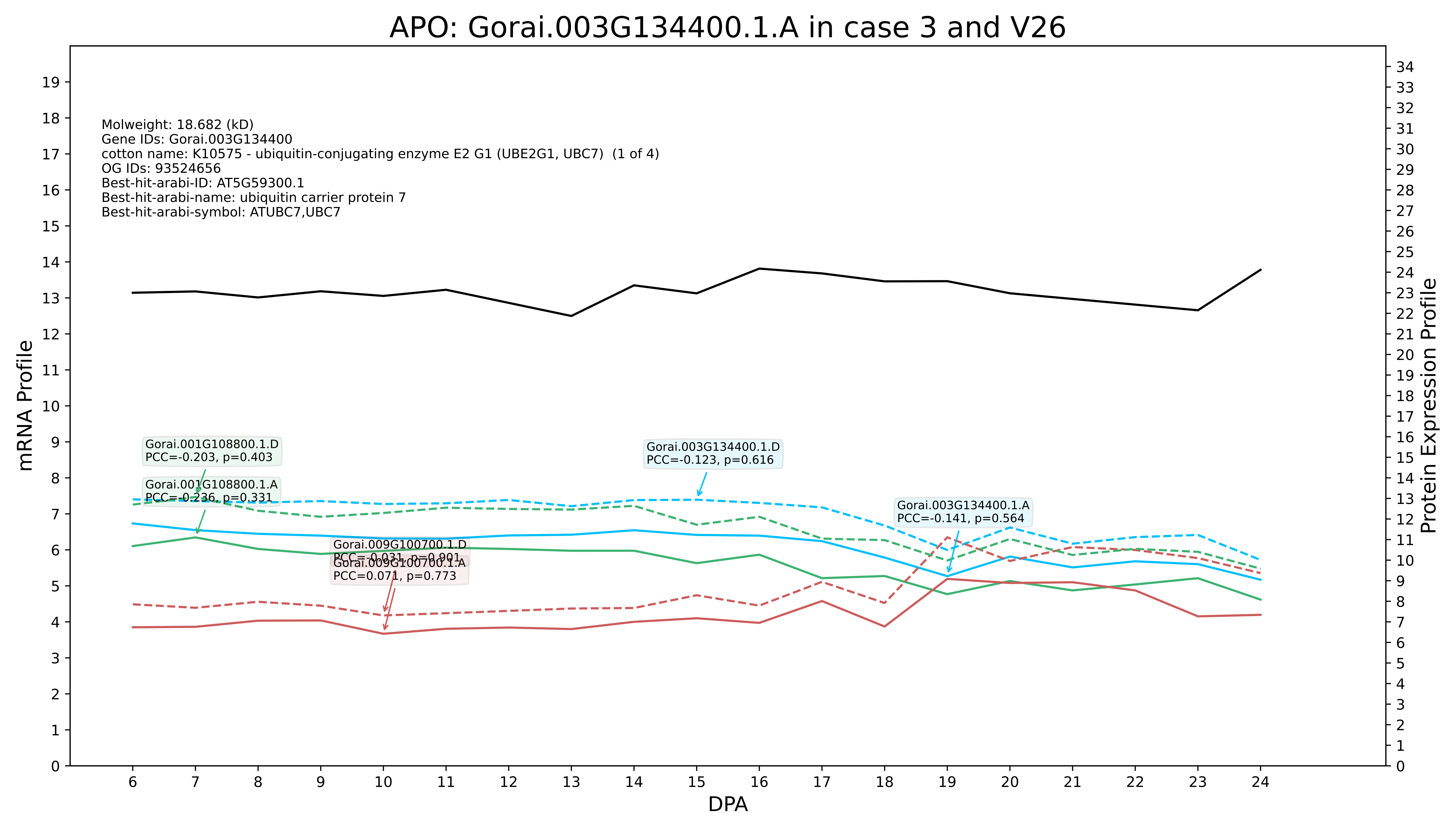

APO: Gorai.003G134400.1.A in case 3 and V26
Molweight: 18.682 (kD)
Gene IDs: Gorai.003G134400
cotton name: K10575 - ubiquitin-conjugating enzyme E2 G1 (UBE2G1, UBC7) (1 of 4)
OG IDs: 93524656
Best-hit-arabi-ID: AT5G59300.1
Best-hit-arabi-name: ubiquitin carrier protein 7
Best-hit-arabi-symbol: ATUBC7,UBC7
Gorai.001G108800.1.D
PCC=-0.203, p=0.403
Gorai.001G108800.1.A
PCC=-0.236, p=0.331
Gorai.003G134400.1.D
PCC=-0.123, p=0.616
Gorai.003G134400.1.A
PCC=-0.141, p=0.564
Gorai.009G100700.1.D
PCC=-0.031, p=0.901
Gorai.009G100700.1.A
PCC=0.071, p=0.773
mRNA Profile
Protein Expression Profile
DPA

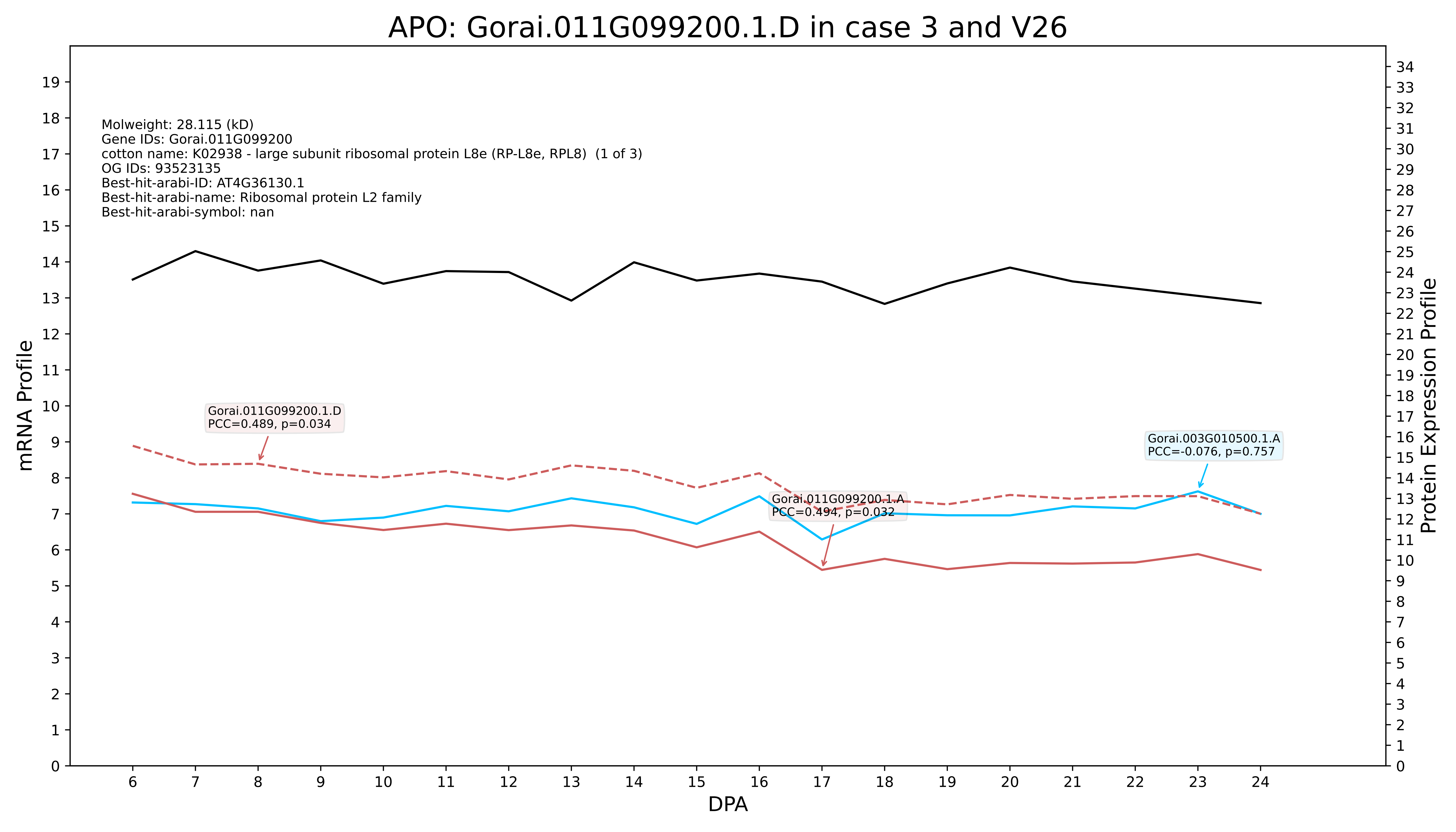
APO: Gorai.011G099200.1.D in case 3 and V26
Molweight: 28.115 (kD)
Gene IDs: Gorai.011G099200
cotton name: K02938 - large subunit ribosomal protein L8e (RP-L8e, RPL8) (1 of 3)
OG IDs: 93523135
Best-hit-arabi-ID: AT4G36130.1
Best-hit-arabi-name: Ribosomal protein L2 family
Best-hit-arabi-symbol: nan
Gorai.011G099200.1.D
PCC=0.489, p=0.034
Gorai.011G099200.1.A
PCC=0.494, p=0.032
Gorai.003G010500.1.A
PCC=-0.076, p=0.757
mRNA Profile
Protein Expression Profile
DPA
0
1
2
3
4
5
6
7
8
9
10
11
12
13
14
15
16
17
18
19
20
21
22
23
24
25
26
27
28
29
30
31
32
33
34

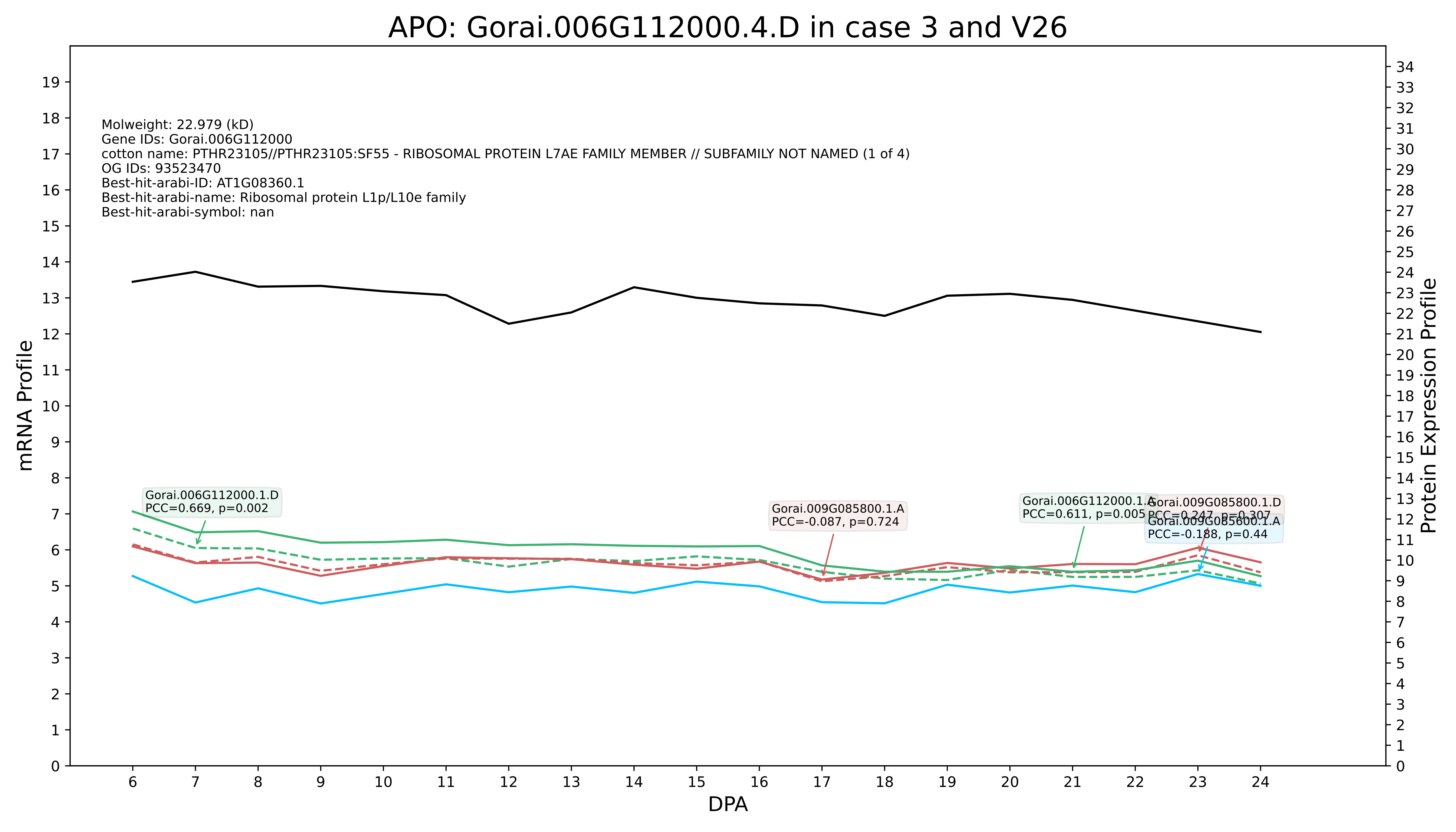
APO: Gorai.006G112000.4.D in case 3 and V26
Molweight: 22.979 (kD)
Gene IDs: Gorai.006G112000
cotton name: PTHR23105//PTHR23105:SF55 - RIBOSOMAL PROTEIN L7AE FAMILY MEMBER // SUBFAMILY NOT NAMED (1 of 4)
OG IDs: 93523470
Best-hit-arabi-ID: AT1G08360.1
Best-hit-arabi-name: Ribosomal protein L1p/L10e family
Best-hit-arabi-symbol: nan
Gorai.006G112000.1.D
PCC=0.669, p=0.002
Gorai.009G085800.1.A
PCC=-0.087, p=0.724
Gorai.006G112000.1.A
PCC=0.611, p=0.005
Gorai.009G085800.1.D
PCC=0.247, p=0.307
Gorai.009G085600.1.A
PCC=-0.188, p=0.44
mRNA Profile
Protein Expression Profile
DPA

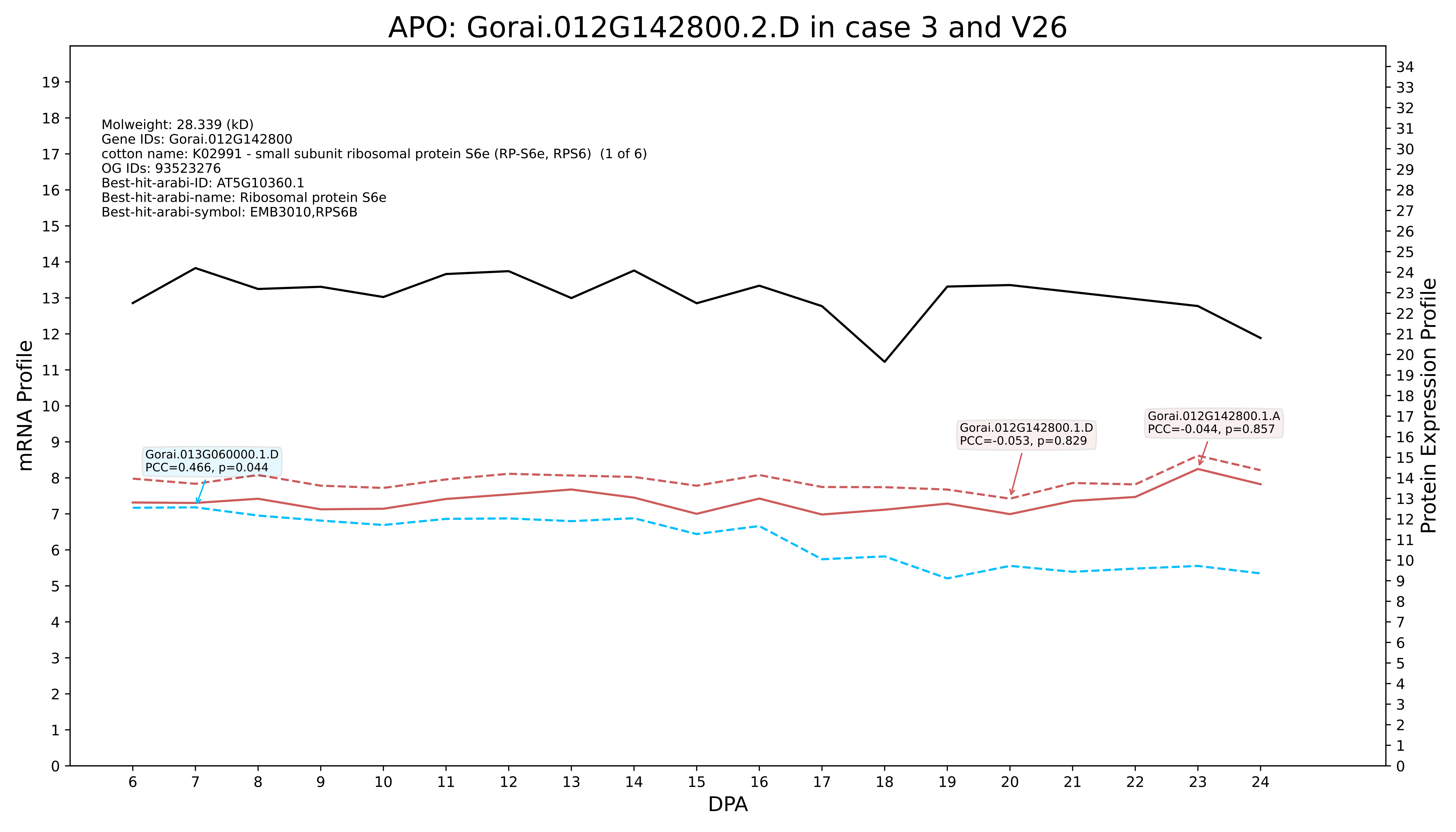
APO: Gorai.012G142800.2.D in case 3 and V26
Molweight: 28.339 (kD)
Gene IDs: Gorai.012G142800
cotton name: K02991 - small subunit ribosomal protein S6e (RP-S6e, RPS6) (1 of 6)
OG IDs: 93523276
Best-hit-arabi-ID: AT5G10360.1
Best-hit-arabi-name: Ribosomal protein S6e
Best-hit-arabi-symbol: EMB3010,RPS6B
Gorai.013G060000.1.D
PCC=0.466, p=0.044
Gorai.012G142800.1.D
PCC=-0.053, p=0.829
Gorai.012G142800.1.A
PCC=-0.044, p=0.857
mRNA Profile
Protein Expression Profile
DPA

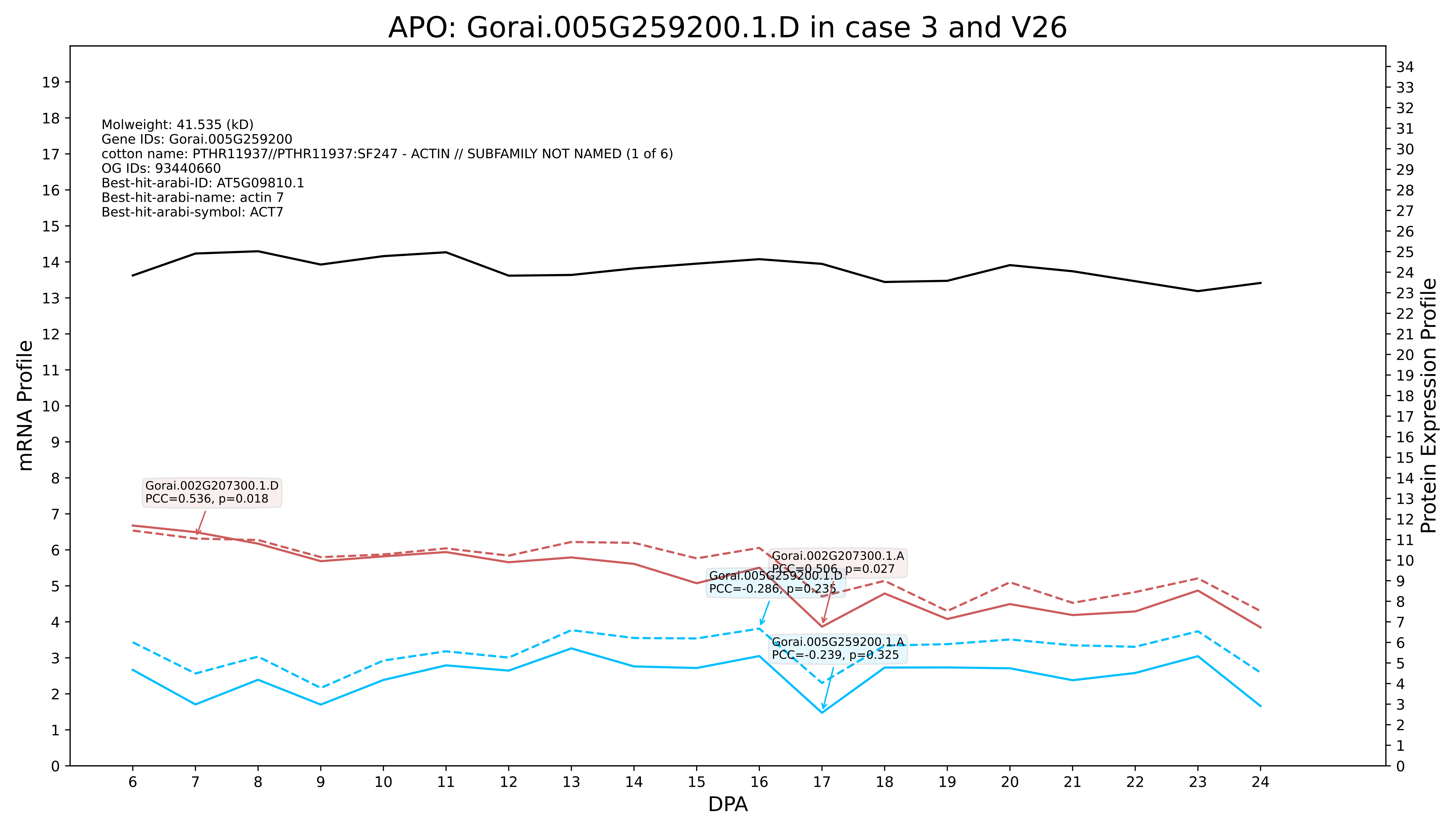

APO: Gorai.005G259200.1.D in case 3 and V26
Molweight: 41.535 (kD)
Gene IDs: Gorai.005G259200
cotton name: PTHR11937//PTHR11937:SF247 - ACTIN // SUBFAMILY NOT NAMED (1 of 6)
OG IDs: 93440660
Best-hit-arabi-ID: AT5G09810.1
Best-hit-arabi-name: actin 7
Best-hit-arabi-symbol: ACT7
Gorai.002G207300.1.D
PCC=0.536, p=0.018
Gorai.002G207300.1.A
PCC=0.506, p=0.027
Gorai.005G259200.1.D
PCC=-0.286, p=0.235
Gorai.005G259200.1.A
PCC=-0.239, p=0.325
mRNA Profile
Protein Expression Profile
DPA

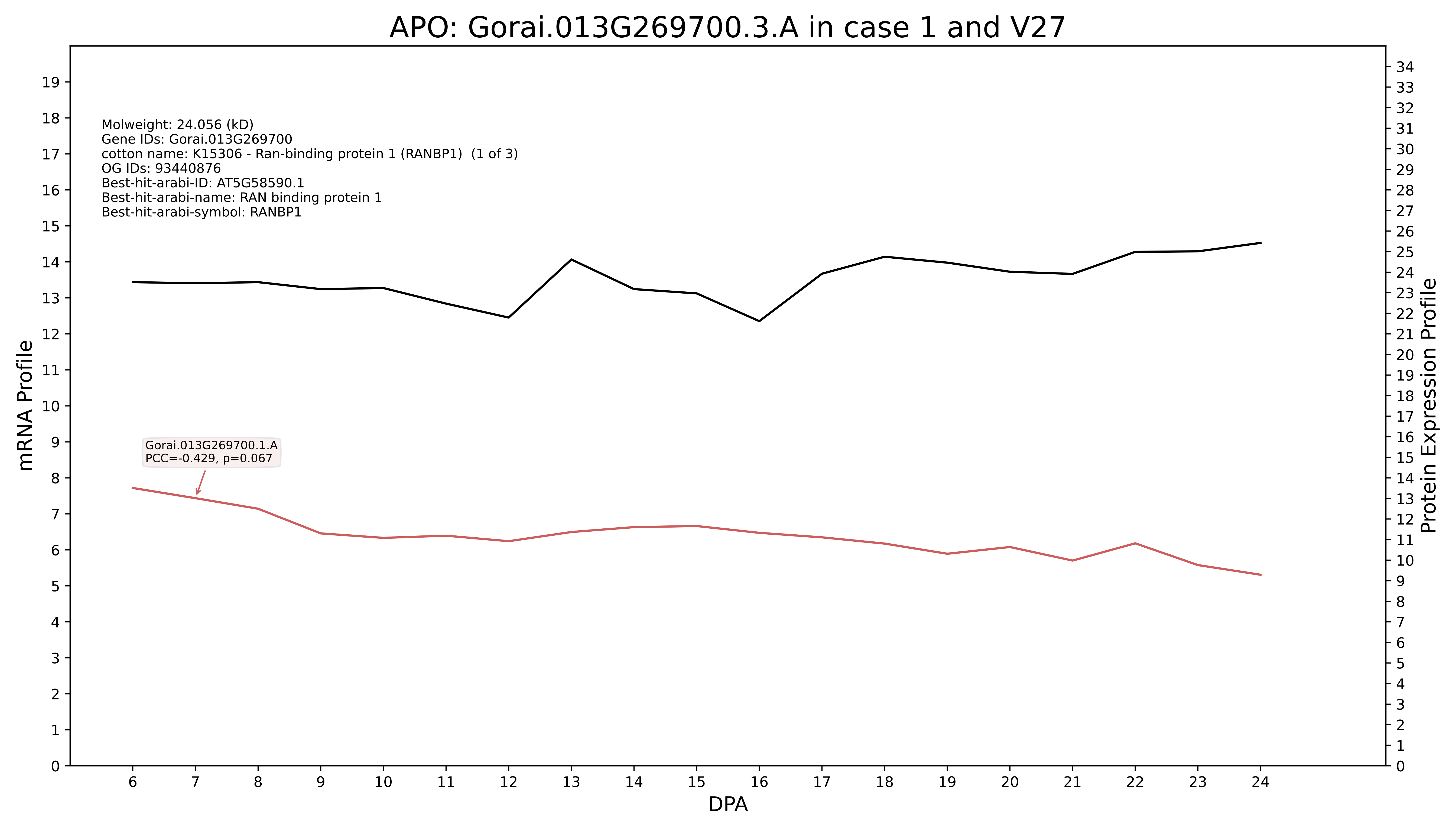
APO: Gorai.013G269700.3.A in case 1 and V27
Molweight: 24.056 (kD)
Gene IDs: Gorai.013G269700
cotton name: K15306 - Ran-binding protein 1 (RANBP1) (1 of 3)
OG IDs: 93440876
Best-hit-arabi-ID: AT5G58590.1
Best-hit-arabi-name: RAN binding protein 1
Best-hit-arabi-symbol: RANBP1
Gorai.013G269700.1.A
PCC=-0.429, p=0.067
mRNA Profile
Protein Expression Profile
DPA

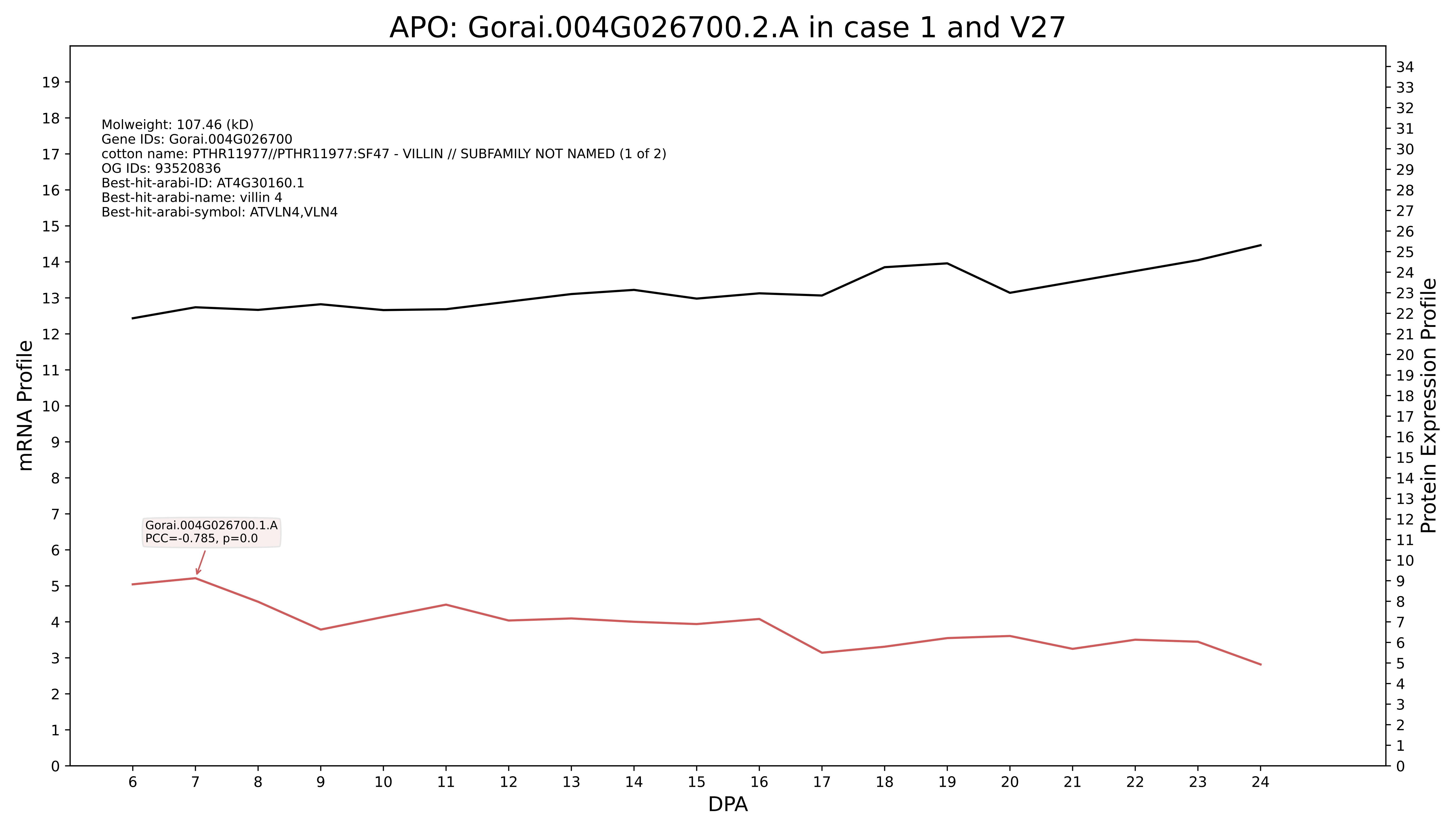

APO: Gorai.004G026700.2.A in case 1 and V27
Molweight: 107.46 (kD)
Gene IDs: Gorai.004G026700
cotton name: PTHR11977//PTHR11977:SF47 - VILLIN // SUBFAMILY NOT NAMED (1 of 2)
OG IDs: 93520836
Best-hit-arabi-ID: AT4G30160.1
Best-hit-arabi-name: villin 4
Best-hit-arabi-symbol: ATVLN4,VLN4
Gorai.004G026700.1.A
PCC=-0.785, p=0.0
mRNA Profile
Protein Expression Profile
DPA

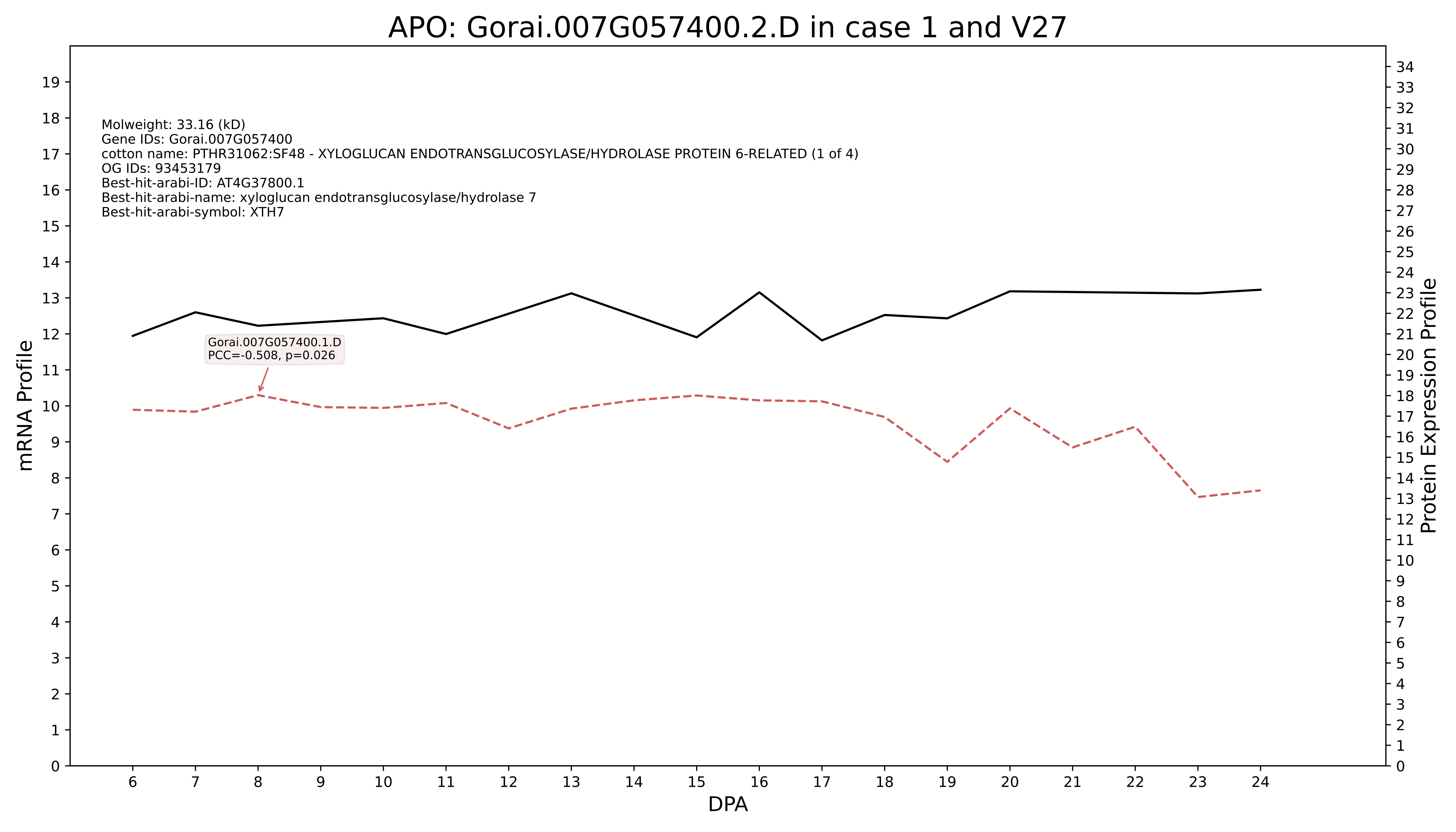
APO: Gorai.007G057400.2.D in case 1 and V27
Molweight: 33.16 (kD)
Gene IDs: Gorai.007G057400
cotton name: PTHR31062:SF48 - XYLOGLUCAN ENDOTRANSGLUCOSYLASE/HYDROLASE PROTEIN 6-RELATED (1 of 4)
OG IDs: 93453179
Best-hit-arabi-ID: AT4G37800.1
Best-hit-arabi-name: xyloglucan endotransglucosylase/hydrolase 7
Best-hit-arabi-symbol: XTH7
Gorai.007G057400.1.D
PCC=-0.508, p=0.026
mRNA Profile
Protein Expression Profile
DPA

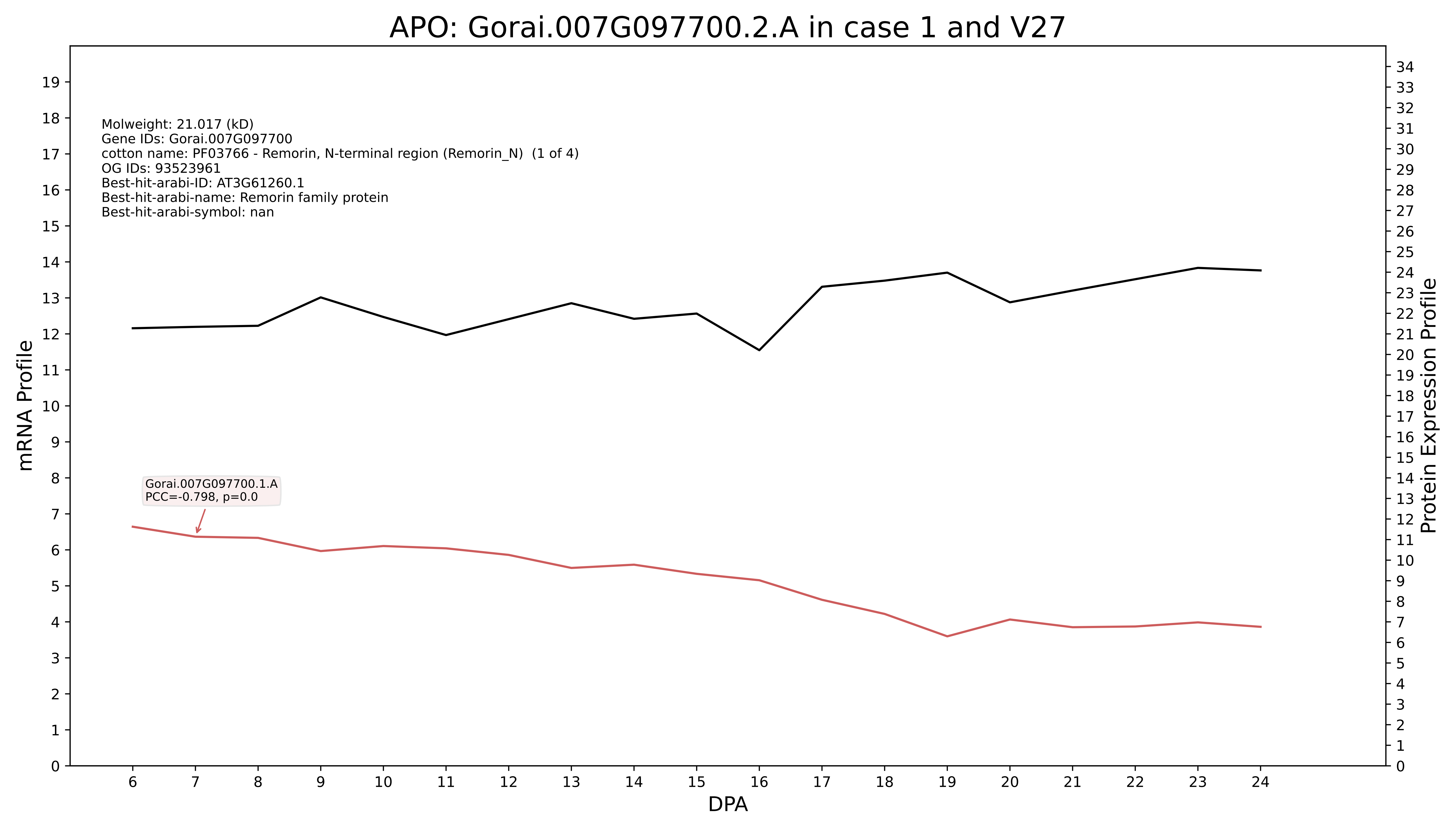
APO: Gorai.007G097700.2.A in case 1 and V27
Molweight: 21.017 (kD)
Gene IDs: Gorai.007G097700
cotton name: PF03766 - Remorin, N-terminal region (Remorin_N) (1 of 4)
OG IDs: 93523961
Best-hit-arabi-ID: AT3G61260.1
Best-hit-arabi-name: Remorin family protein
Best-hit-arabi-symbol: nan
Gorai.007G097700.1.A
PCC=-0.798, p=0.0
mRNA Profile
Protein Expression Profile
DPA

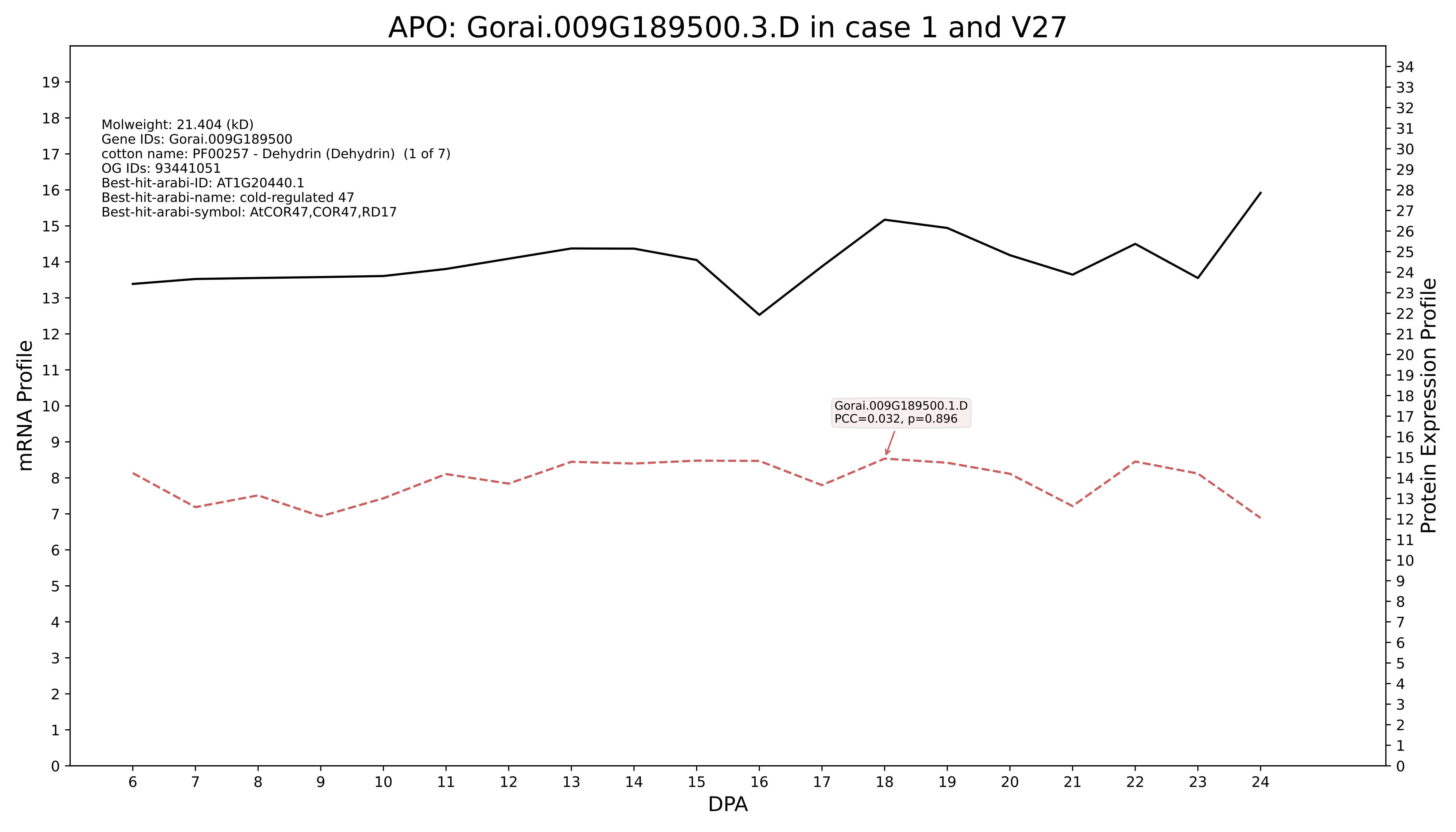
APO: Gorai.009G189500.3.D in case 1 and V27
Molweight: 21.404 (kD)
Gene IDs: Gorai.009G189500
cotton name: PF00257 - Dehydrin (Dehydrin) (1 of 7)
OG IDs: 93441051
Best-hit-arabi-ID: AT1G20440.1
Best-hit-arabi-name: cold-regulated 47
Best-hit-arabi-symbol: AtCOR47,COR47,RD17
Gorai.009G189500.1.D
PCC=0.032, p=0.896
mRNA Profile
Protein Expression Profile
DPA

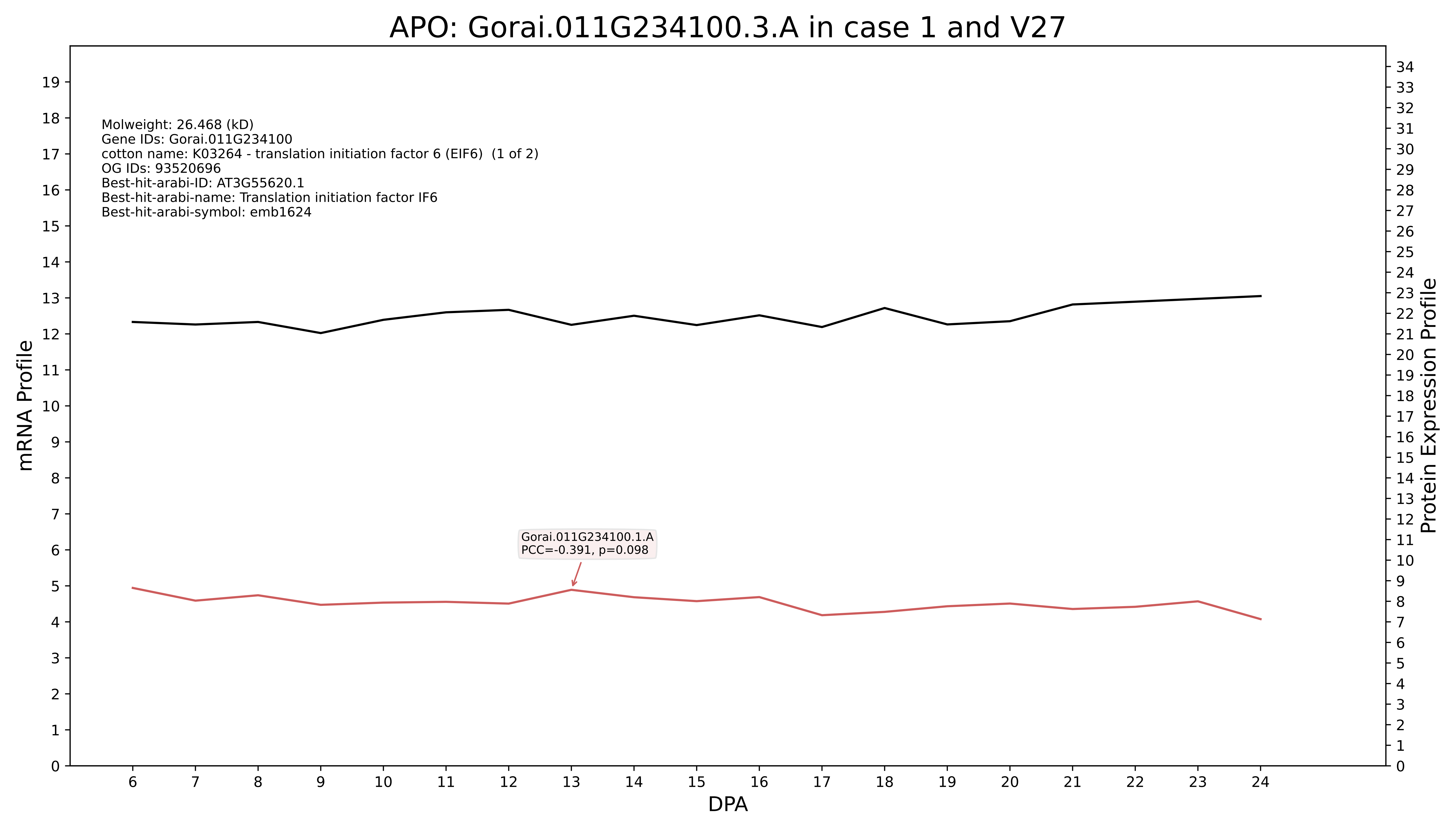

APO: Gorai.011G234100.3.A in case 1 and V27
Molweight: 26.468 (kD)
Gene IDs: Gorai.011G234100
cotton name: K03264 - translation initiation factor 6 (EIF6) (1 of 2)
OG IDs: 93520696
Best-hit-arabi-ID: AT3G55620.1
Best-hit-arabi-name: Translation initiation factor IF6
Best-hit-arabi-symbol: emb1624
Gorai.011G234100.1.A
PCC=-0.391, p=0.098
mRNA Profile
Protein Expression Profile
DPA

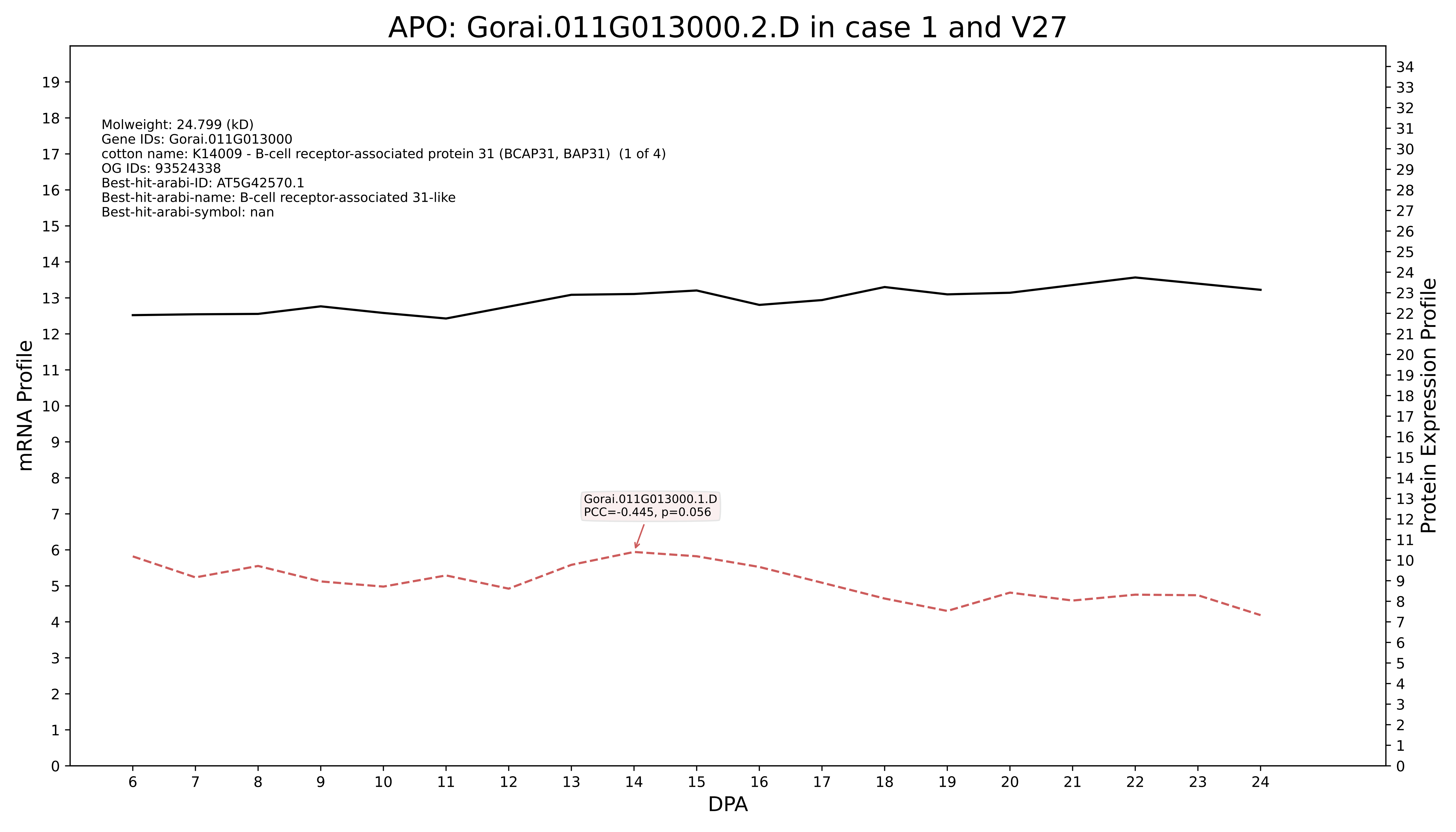
APO: Gorai.011G013000.2.D in case 1 and V27
Molweight: 24.799 (kD)
Gene IDs: Gorai.011G013000
cotton name: K14009 - B-cell receptor-associated protein 31 (BCAP31, BAP31) (1 of 4)
OG IDs: 93524338
Best-hit-arabi-ID: AT5G42570.1
Best-hit-arabi-name: B-cell receptor-associated 31-like
Best-hit-arabi-symbol: nan
Gorai.011G013000.1.D
PCC=-0.445, p=0.056
mRNA Profile
Protein Expression Profile
DPA

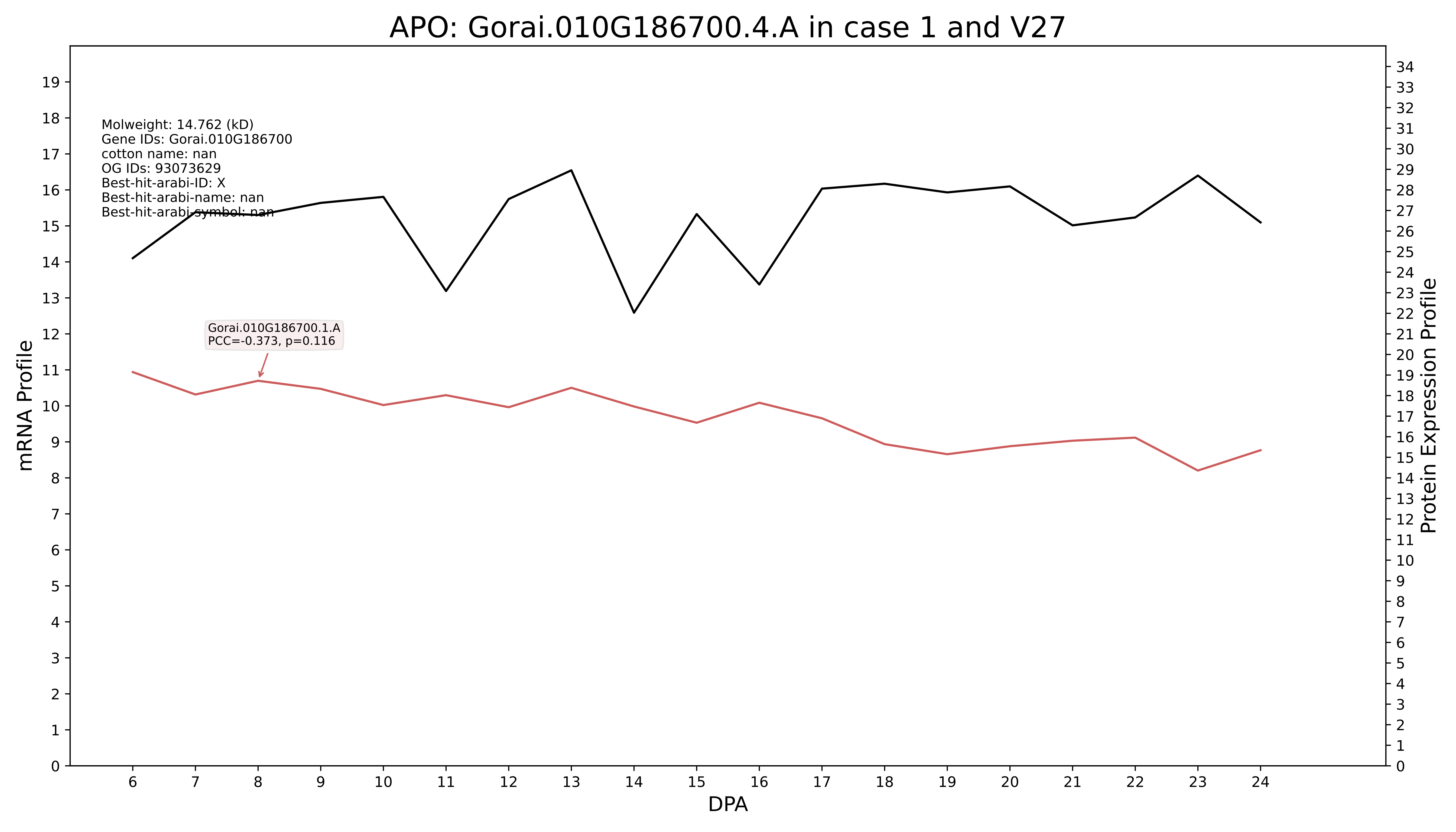

APO: Gorai.010G186700.4.A in case 1 and V27
Molweight: 14.762 (kD)
Gene IDs: Gorai.010G186700
cotton name: nan
OG IDs: 93073629
Best-hit-arabi-ID: X
Best-hit-arabi-name: nan
Best-hit-arabi-symbol: nan
Gorai.010G186700.1.A
PCC=-0.373, p=0.116
mRNA Profile
Protein Expression Profile
DPA
0 1 2 3 4 5 6 7 8 9 10 11 12 13 14 15 16 17 18 19
6 7 8 9 10 11 12 13 14 15 16 17 18 19 20 21 22 23 24
0 1 2 3 4 5 6 7 8 9 10 11 12 13 14 15 16 17 18 19 20 21 22 23 24 25 26 27 28 29 30 31 32 33 34

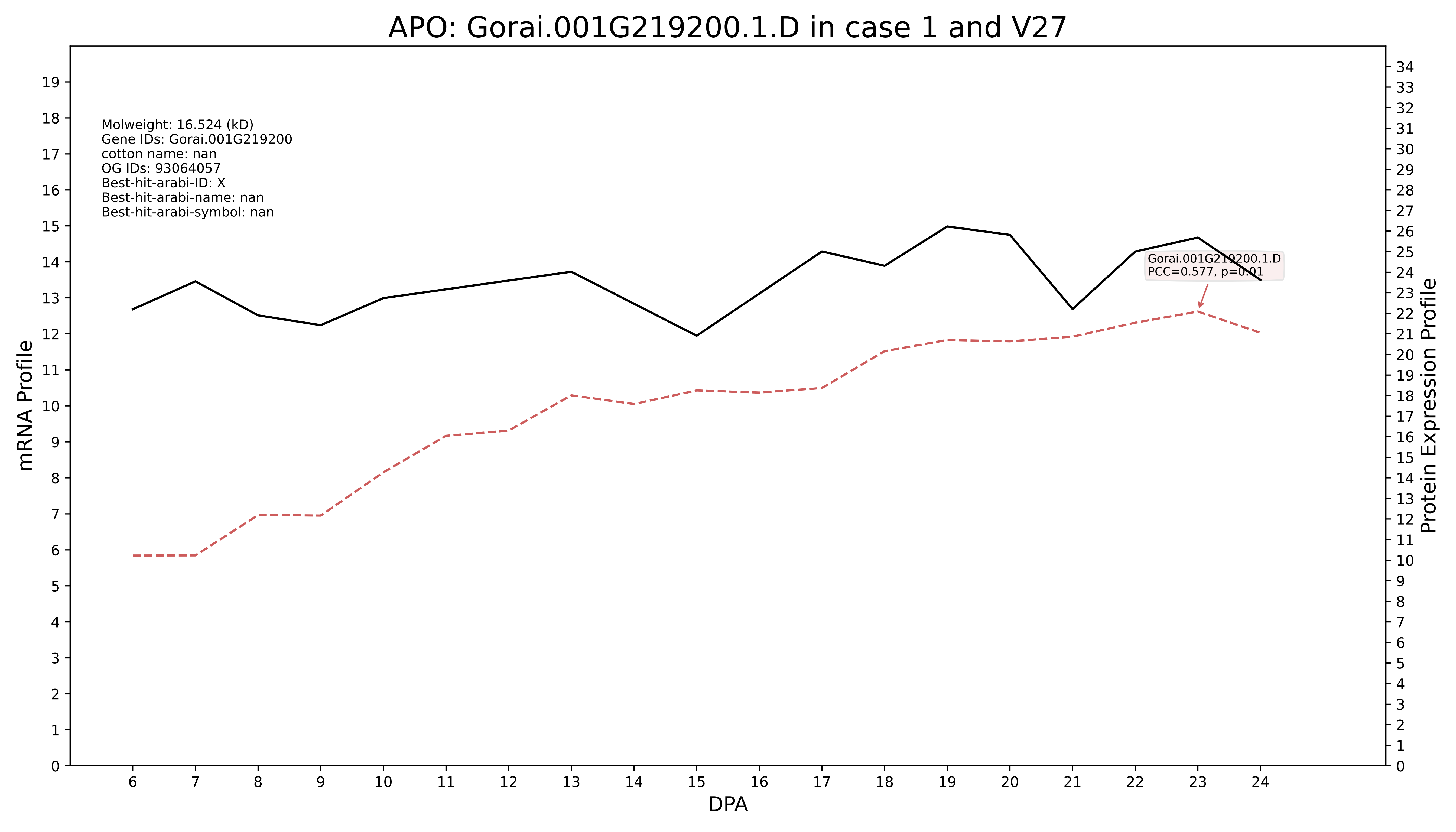
APO: Gorai.001G219200.1.D in case 1 and V27
Molweight: 16.524 (kD)
Gene IDs: Gorai.001G219200
cotton name: nan
OG IDs: 93064057
Best-hit-arabi-ID: X
Best-hit-arabi-name: nan
Best-hit-arabi-symbol: nan
Gorai.001G219200.1.D
PCC=0.577, p=0.01
mRNA Profile
Protein Expression Profile
DPA

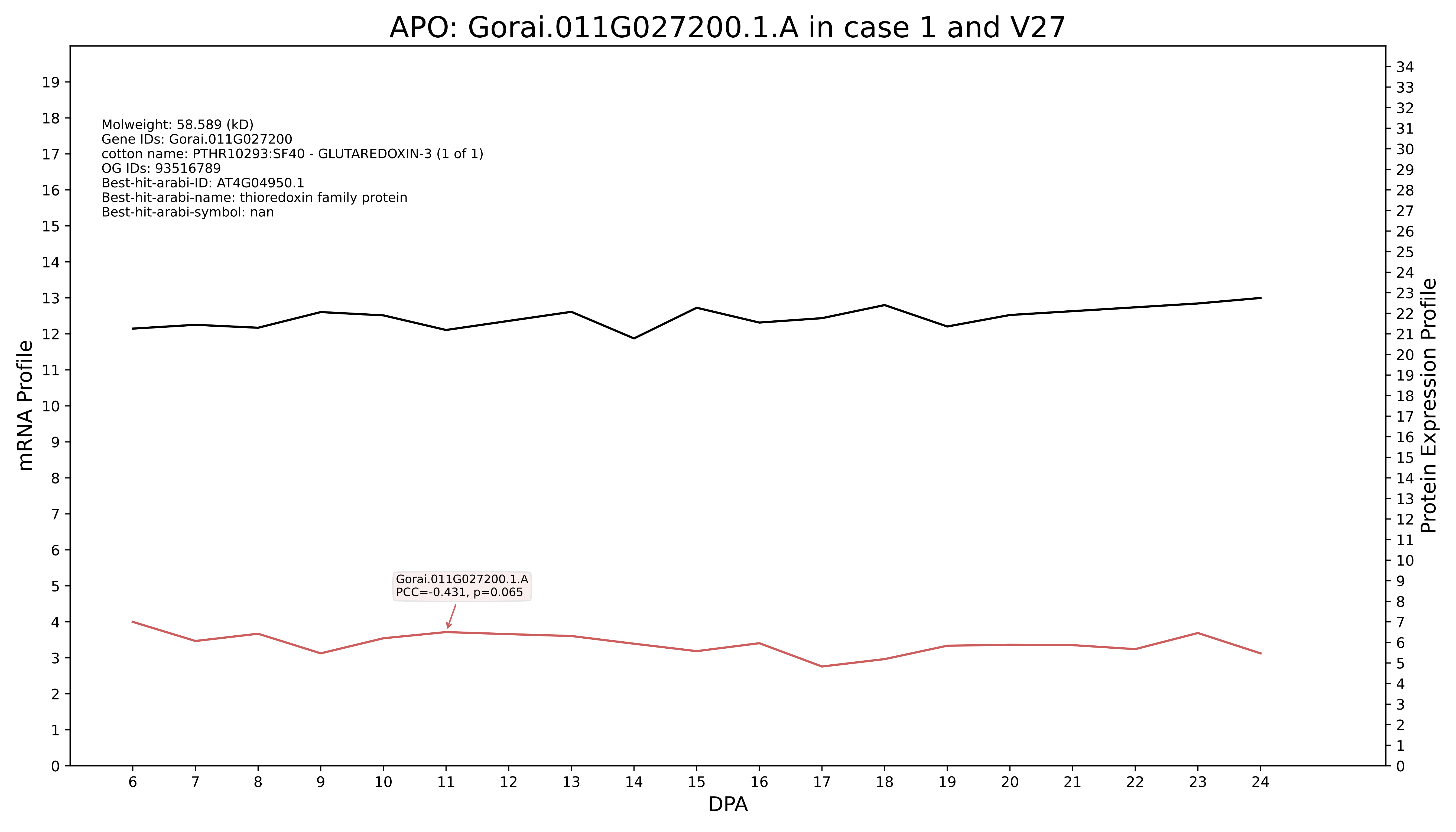

APO: Gorai.011G027200.1.A in case 1 and V27
Molweight: 58.589 (kD)
Gene IDs: Gorai.011G027200
cotton name: PTHR10293:SF40 - GLUTAREDOXIN-3 (1 of 1)
OG IDs: 93516789
Best-hit-arabi-ID: AT4G04950.1
Best-hit-arabi-name: thioredoxin family protein
Best-hit-arabi-symbol: nan
Gorai.011G027200.1.A
PCC=-0.431, p=0.065
mRNA Profile
Protein Expression Profile
DPA

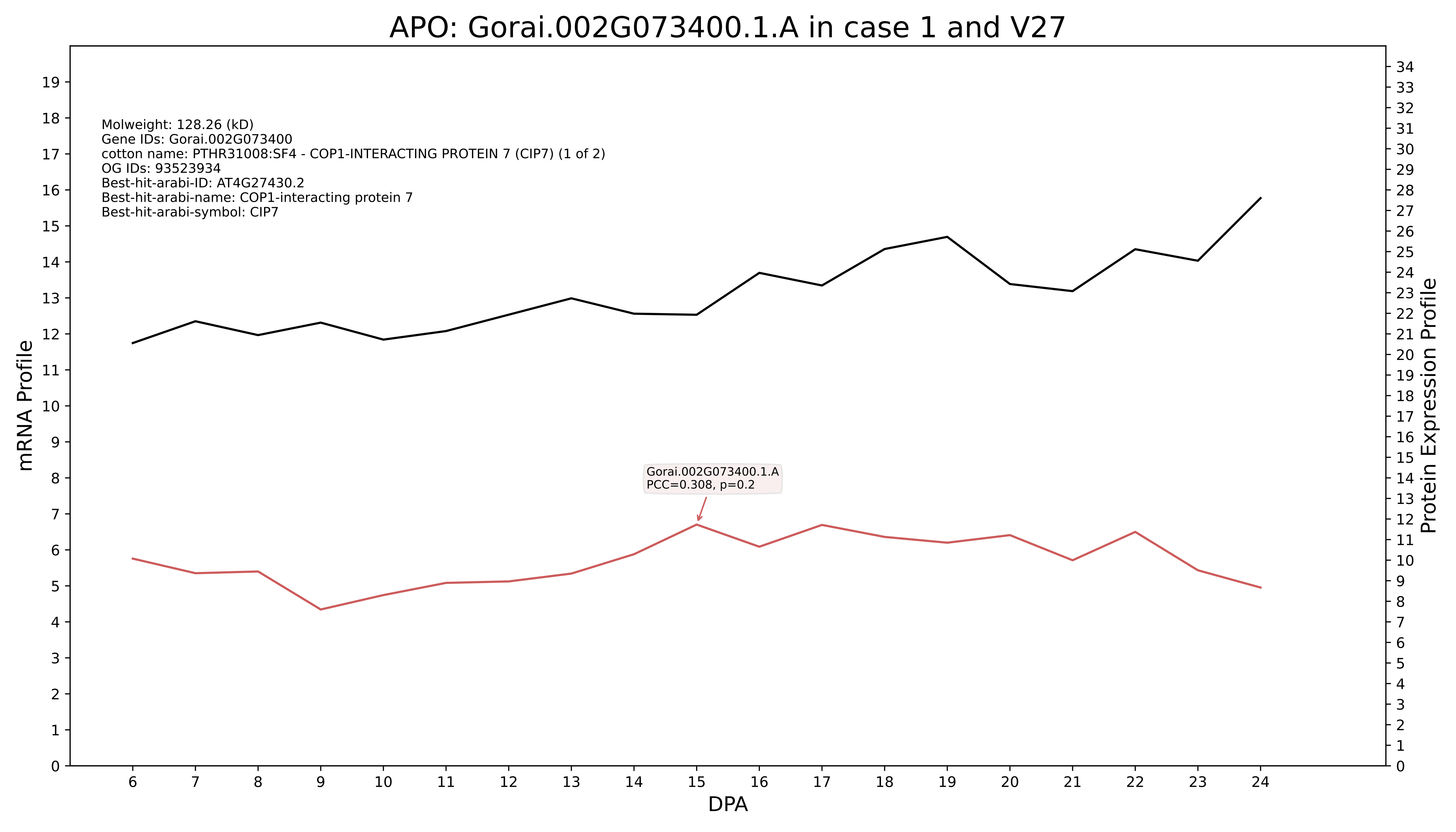
APO: Gorai.002G073400.1.A in case 1 and V27
Molweight: 128.26 (kD)
Gene IDs: Gorai.002G073400
cotton name: PTHR31008:SF4 - COP1-INTERACTING PROTEIN 7 (CIP7) (1 of 2)
OG IDs: 93523934
Best-hit-arabi-ID: AT4G27430.2
Best-hit-arabi-name: COP1-interacting protein 7
Best-hit-arabi-symbol: CIP7
Gorai.002G073400.1.A
PCC=0.308, p=0.2
mRNA Profile
Protein Expression Profile
DPA

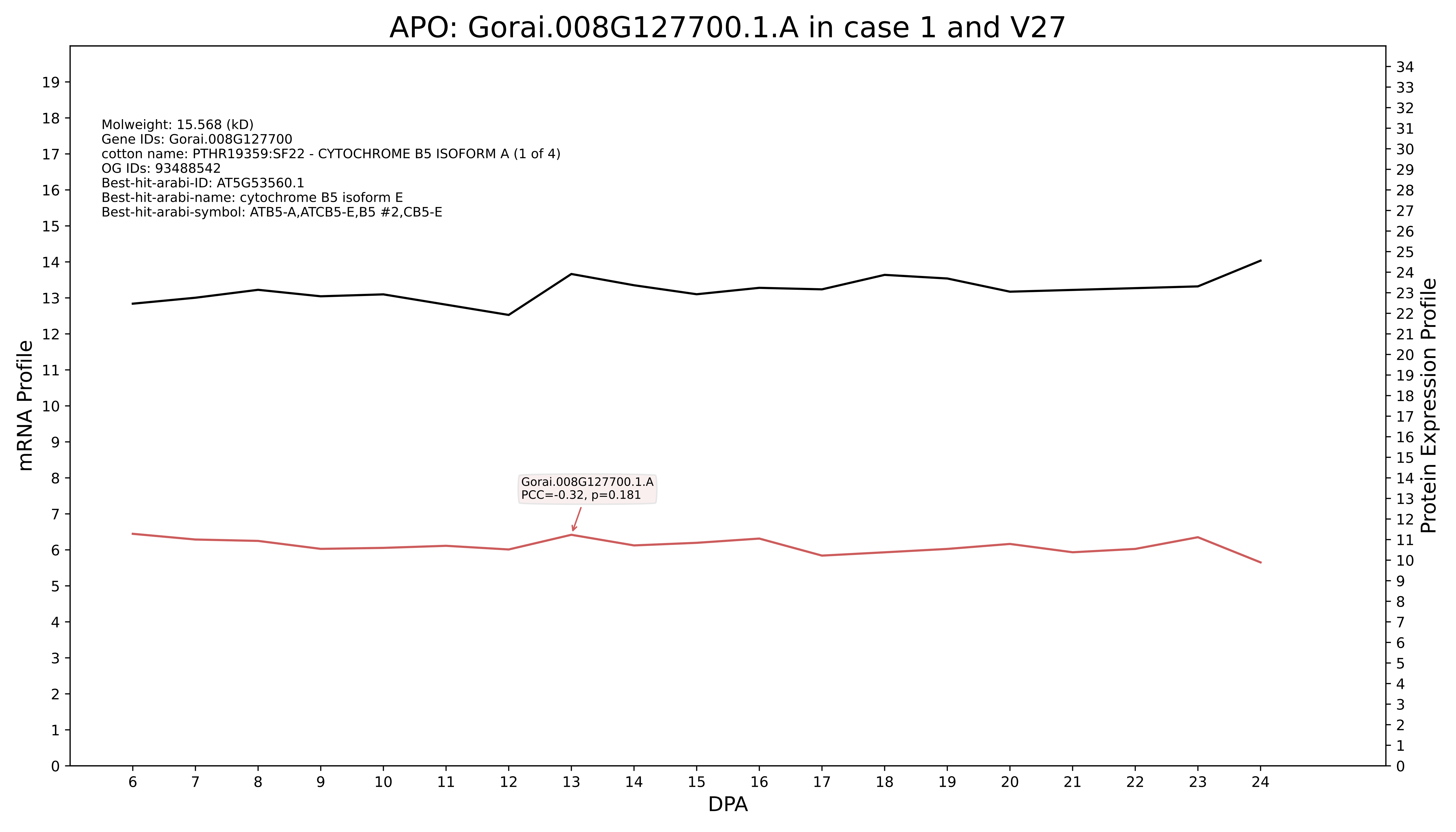
APO: Gorai.008G127700.1.A in case 1 and V27
Molweight: 15.568 (kD)
Gene IDs: Gorai.008G127700
cotton name: PTHR19359:SF22 - CYTOCHROME B5 ISOFORM A (1 of 4)
OG IDs: 93488542
Best-hit-arabi-ID: AT5G53560.1
Best-hit-arabi-name: cytochrome B5 isoform E
Best-hit-arabi-symbol: ATB5-A,ATCB5-E,B5 #2,CB5-E
Gorai.008G127700.1.A
PCC=-0.32, p=0.181
mRNA Profile
Protein Expression Profile
DPA

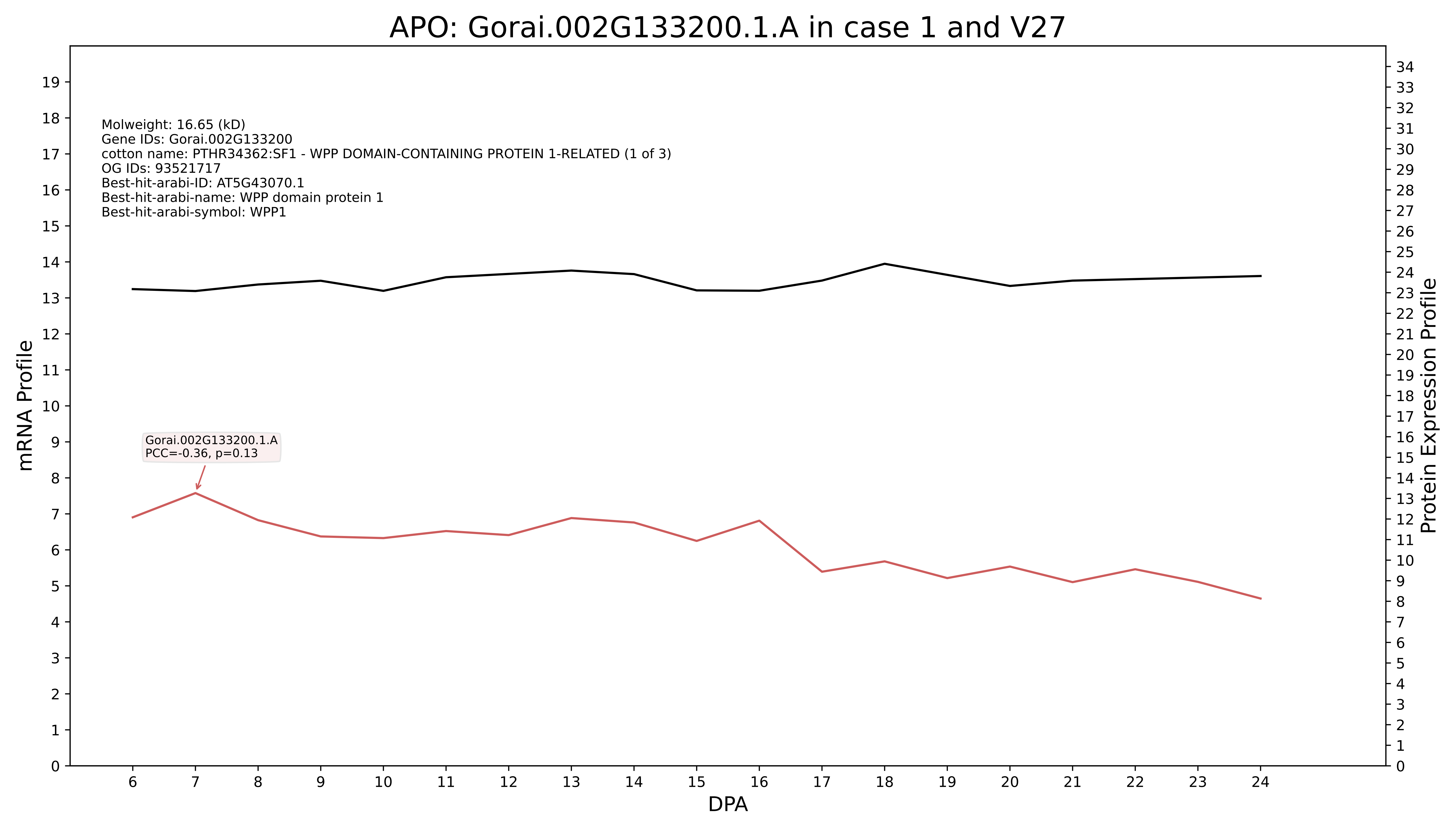
APO: Gorai.002G133200.1.A in case 1 and V27
Molweight: 16.65 (kD)
Gene IDs: Gorai.002G133200
cotton name: PTHR34362:SF1 - WPP DOMAIN-CONTAINING PROTEIN 1-RELATED (1 of 3)
OG IDs: 93521717
Best-hit-arabi-ID: AT5G43070.1
Best-hit-arabi-name: WPP domain protein 1
Best-hit-arabi-symbol: WPP1
Gorai.002G133200.1.A
PCC=-0.36, p=0.13
mRNA Profile
Protein Expression Profile
DPA

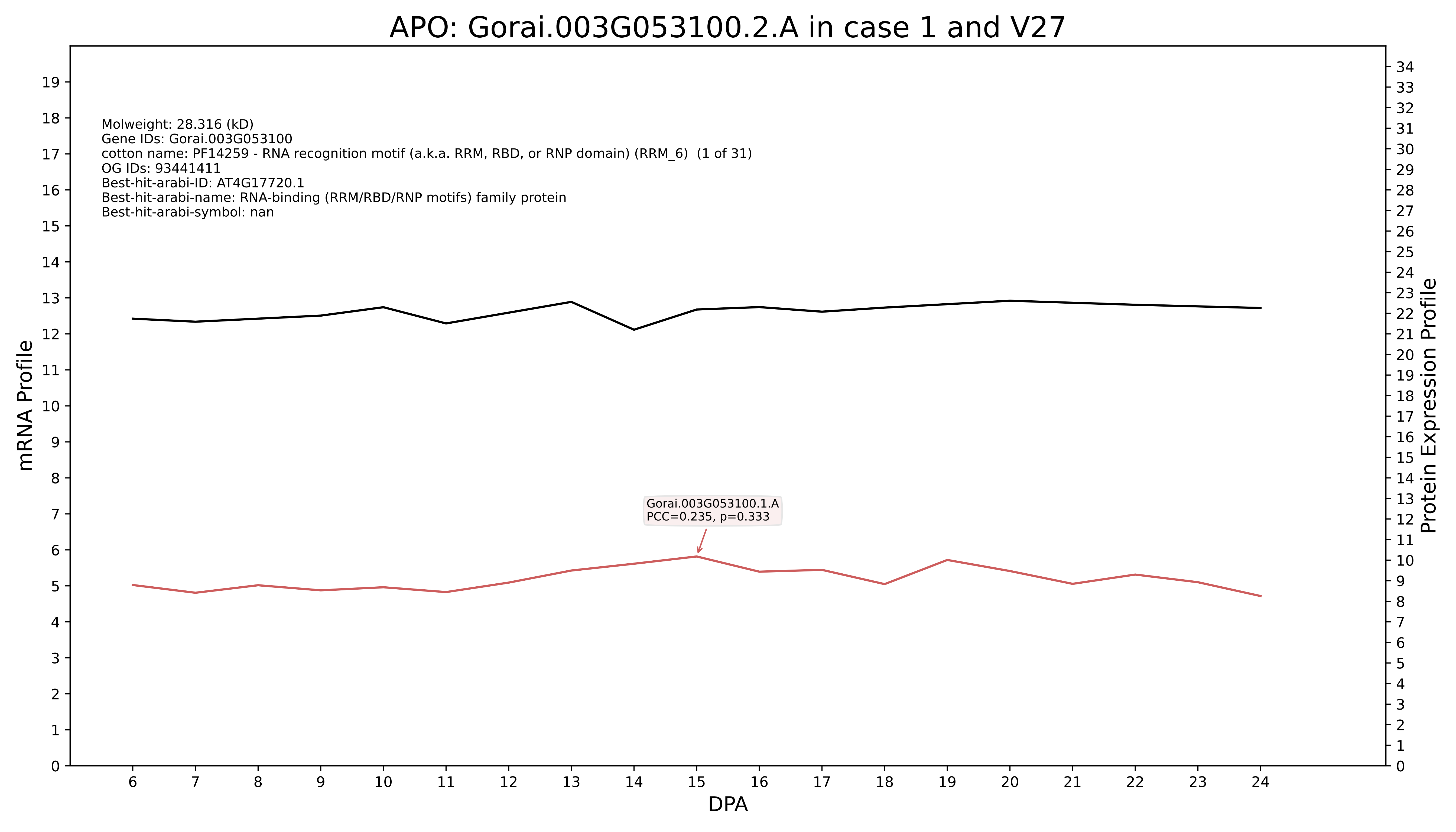

APO: Gorai.003G053100.2.A in case 1 and V27
Molweight: 28.316 (kD)
Gene IDs: Gorai.003G053100
cotton name: PF14259 - RNA recognition motif (a.k.a. RRM, RBD, or RNP domain) (RRM_6) (1 of 31)
OG IDs: 93441411
Best-hit-arabi-ID: AT4G17720.1
Best-hit-arabi-name: RNA-binding (RRM/RBD/RNP motifs) family protein
Best-hit-arabi-symbol: nan
Gorai.003G053100.1.A
PCC=0.235, p=0.333
mRNA Profile
Protein Expression Profile
DPA

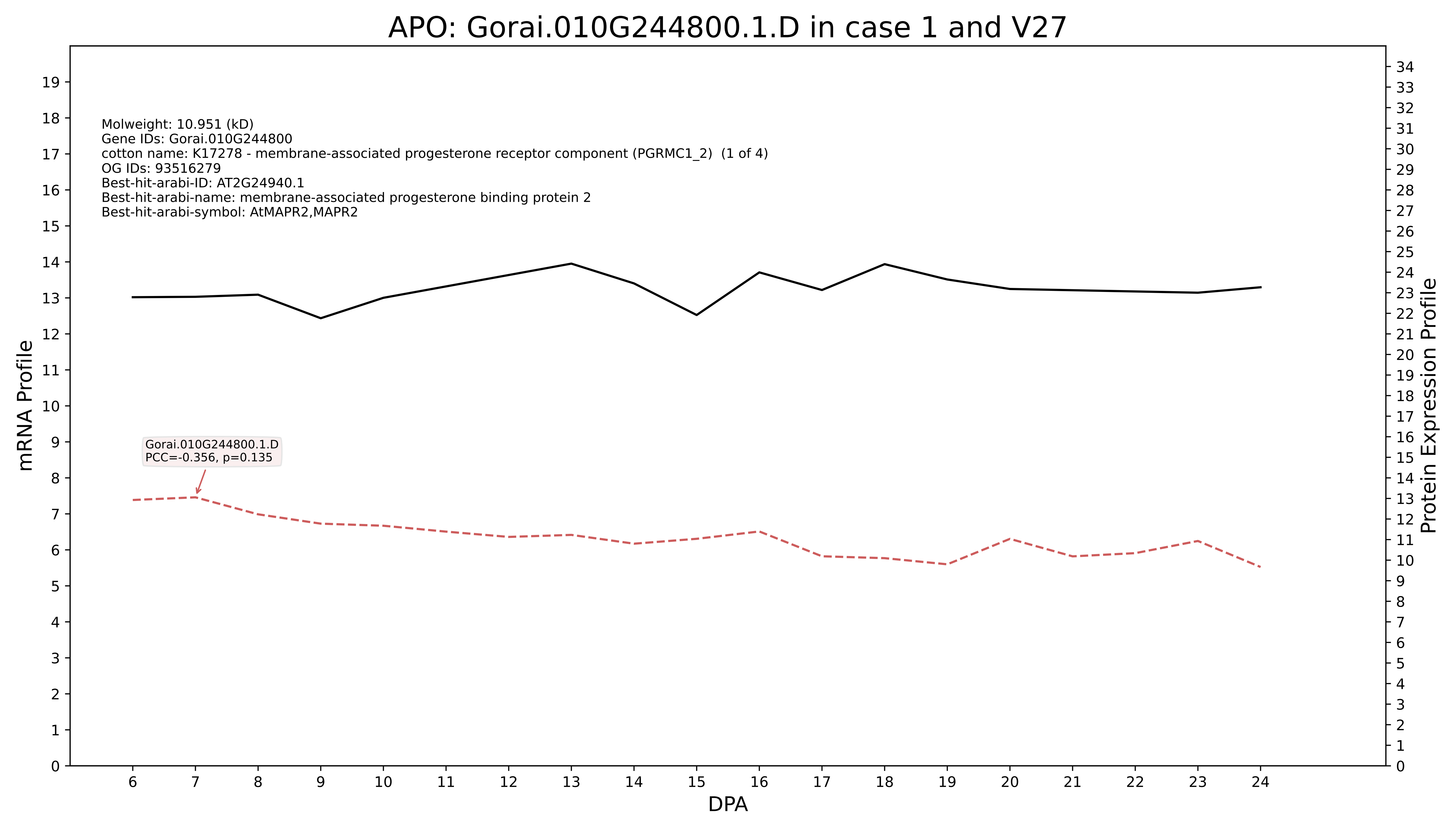

APO: Gorai.010G244800.1.D in case 1 and V27
Molweight: 10.951 (kD)
Gene IDs: Gorai.010G244800
cotton name: K17278 - membrane-associated progesterone receptor component (PGRMC1_2) (1 of 4)
OG IDs: 93516279
Best-hit-arabi-ID: AT2G24940.1
Best-hit-arabi-name: membrane-associated progesterone binding protein 2
Best-hit-arabi-symbol: AtMAPR2,MAPR2
Gorai.010G244800.1.D
PCC=-0.356, p=0.135
mRNA Profile
Protein Expression Profile
DPA

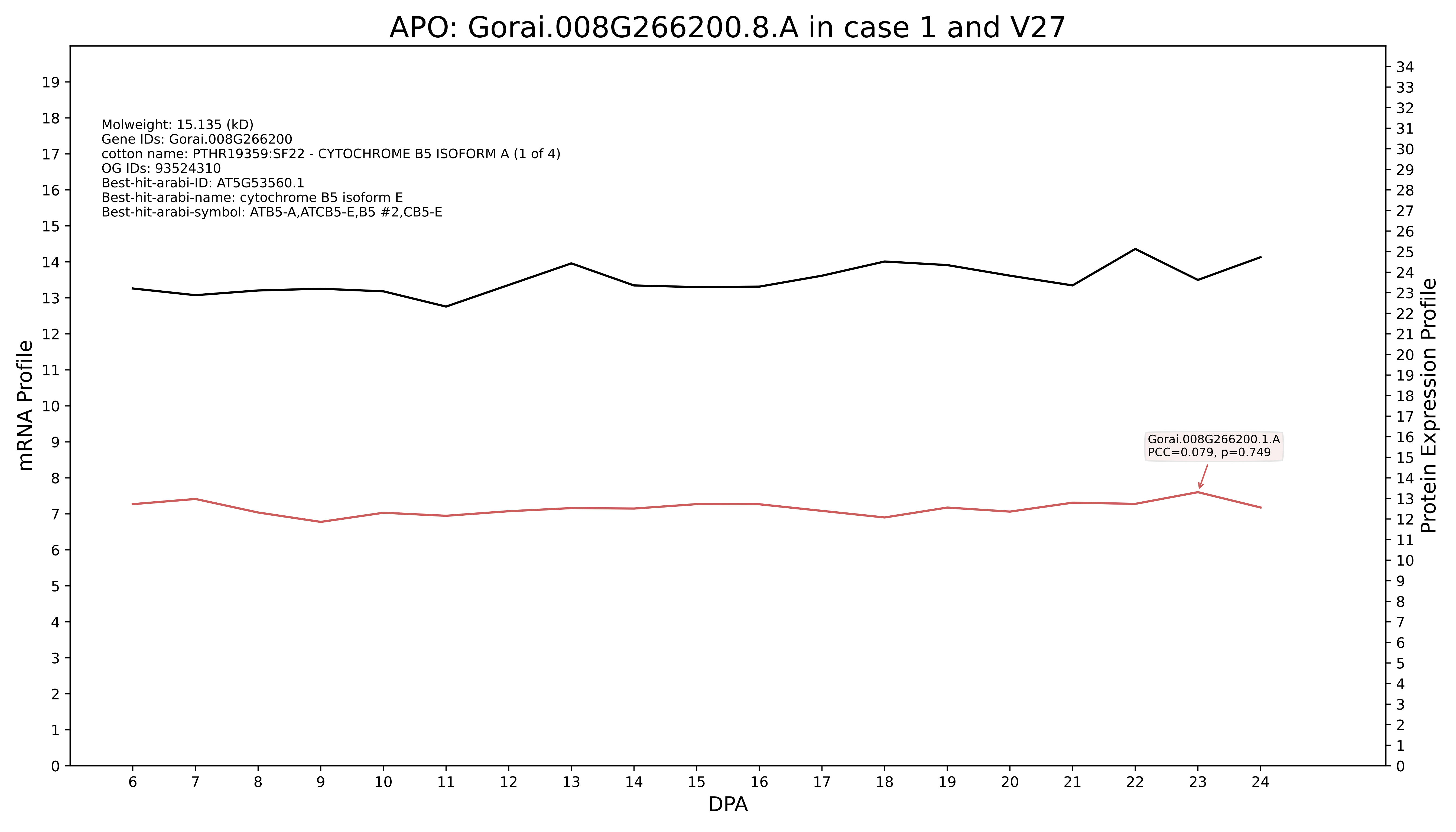
APO: Gorai.008G266200.8.A in case 1 and V27
Molweight: 15.135 (kD)
Gene IDs: Gorai.008G266200
cotton name: PTHR19359:SF22 - CYTOCHROME B5 ISOFORM A (1 of 4)
OG IDs: 93524310
Best-hit-arabi-ID: AT5G53560.1
Best-hit-arabi-name: cytochrome B5 isoform E
Best-hit-arabi-symbol: ATB5-A,ATCB5-E,B5 #2,CB5-E
Gorai.008G266200.1.A
PCC=0.079, p=0.749
mRNA Profile
Protein Expression Profile
DPA
0
1
2
3
4
5
6
7
8
9
10
11
12
13
14
15
16
17
18
19
20
21
22
23
24
25
26
27
28
29
30
31
32
33
34

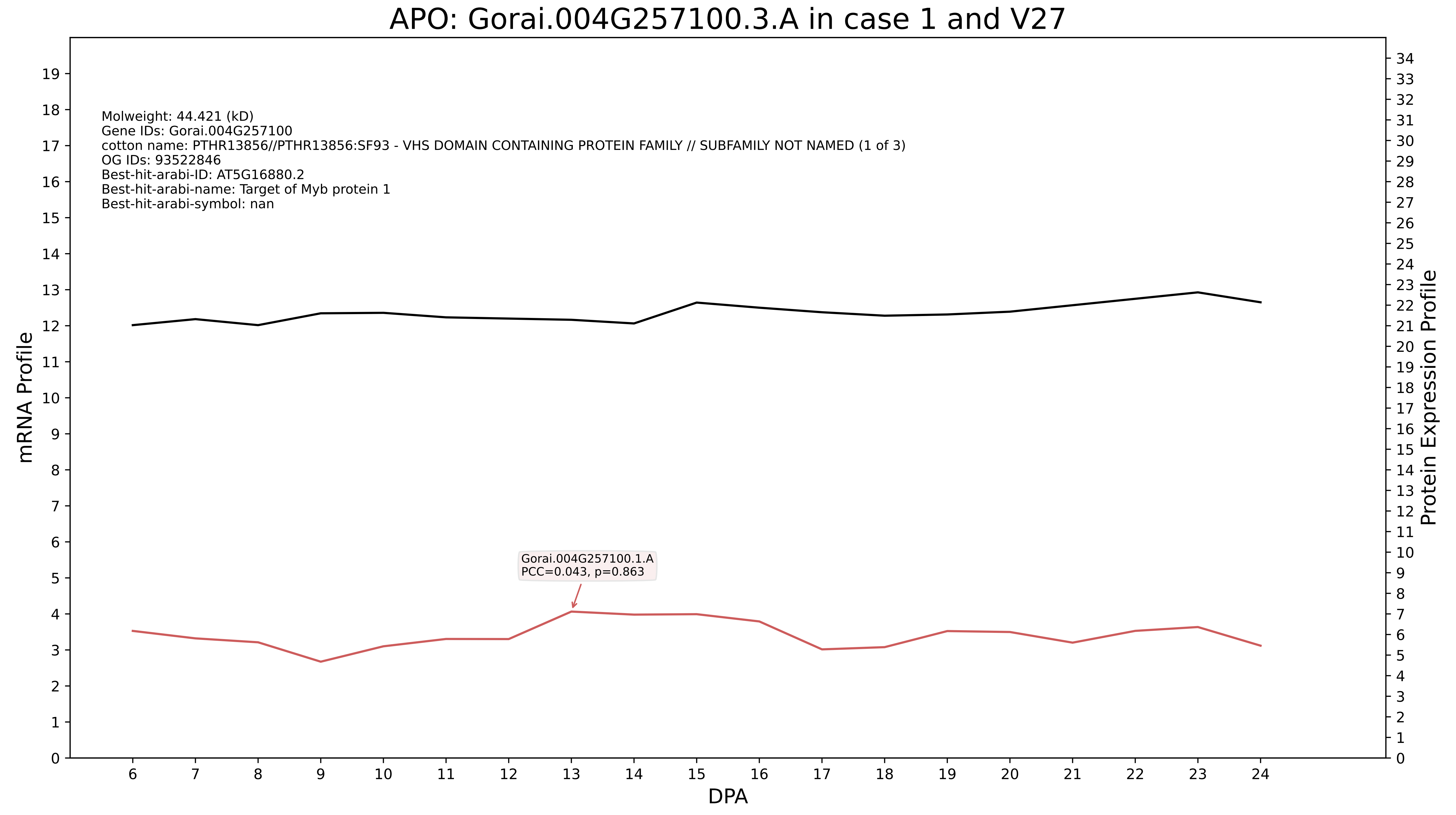
APO: Gorai.004G257100.3.A in case 1 and V27
Molweight: 44.421 (kD)
Gene IDs: Gorai.004G257100
cotton name: PTHR13856//PTHR13856:SF93 - VHS DOMAIN CONTAINING PROTEIN FAMILY // SUBFAMILY NOT NAMED (1 of 3)
OG IDs: 93522846
Best-hit-arabi-ID: AT5G16880.2
Best-hit-arabi-name: Target of Myb protein 1
Best-hit-arabi-symbol: nan
Gorai.004G257100.1.A
PCC=0.043, p=0.863
mRNA Profile
Protein Expression Profile
DPA
0
1
2
3
4
5
6
7
8
9
10
11
12
13
14
15
16
17
18
19
20
21
22
23
24
25
26
27
28
29
30
31
32
33
34

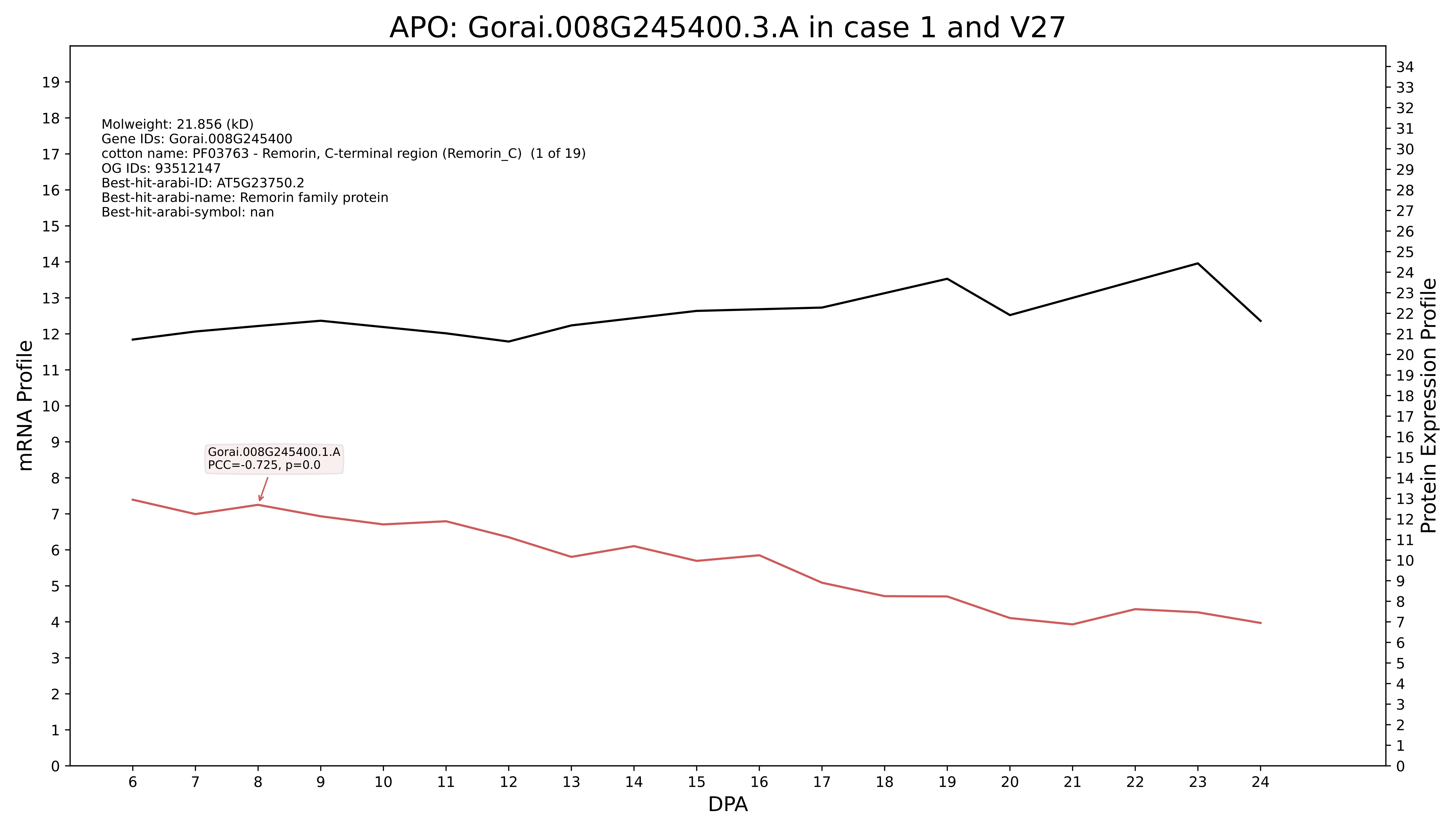
APO: Gorai.008G245400.3.A in case 1 and V27
Molweight: 21.856 (kD)
Gene IDs: Gorai.008G245400
cotton name: PF03763 - Remorin, C-terminal region (Remorin_C) (1 of 19)
OG IDs: 93512147
Best-hit-arabi-ID: AT5G23750.2
Best-hit-arabi-name: Remorin family protein
Best-hit-arabi-symbol: nan
Gorai.008G245400.1.A
PCC=-0.725, p=0.0
mRNA Profile
Protein Expression Profile
DPA

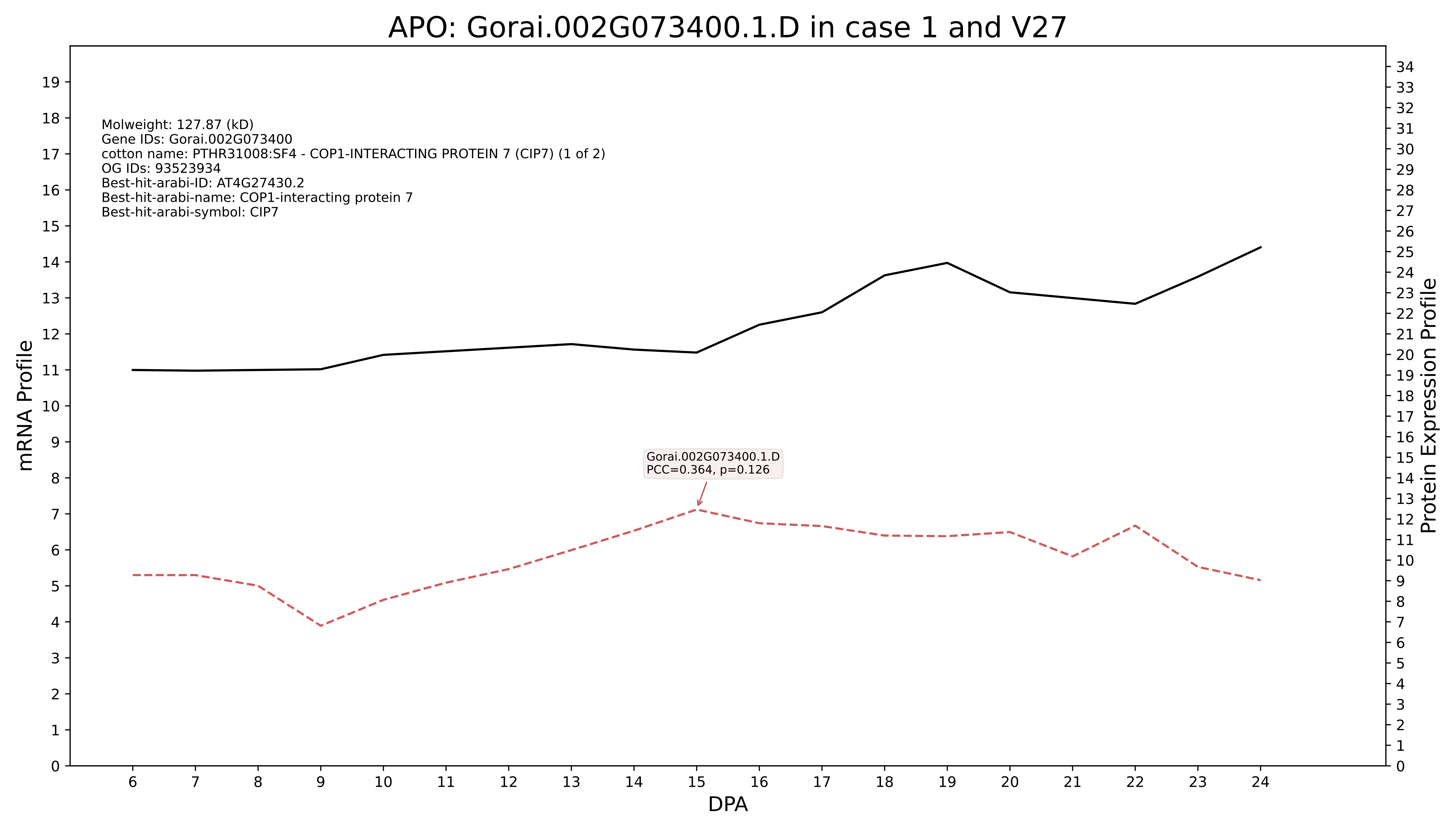
APO: Gorai.002G073400.1.D in case 1 and V27
Molweight: 127.87 (kD)
Gene IDs: Gorai.002G073400
cotton name: PTHR31008:SF4 - COP1-INTERACTING PROTEIN 7 (CIP7) (1 of 2)
OG IDs: 93523934
Best-hit-arabi-ID: AT4G27430.2
Best-hit-arabi-name: COP1-interacting protein 7
Best-hit-arabi-symbol: CIP7
Gorai.002G073400.1.D
PCC=0.364, p=0.126
mRNA Profile
Protein Expression Profile
DPA

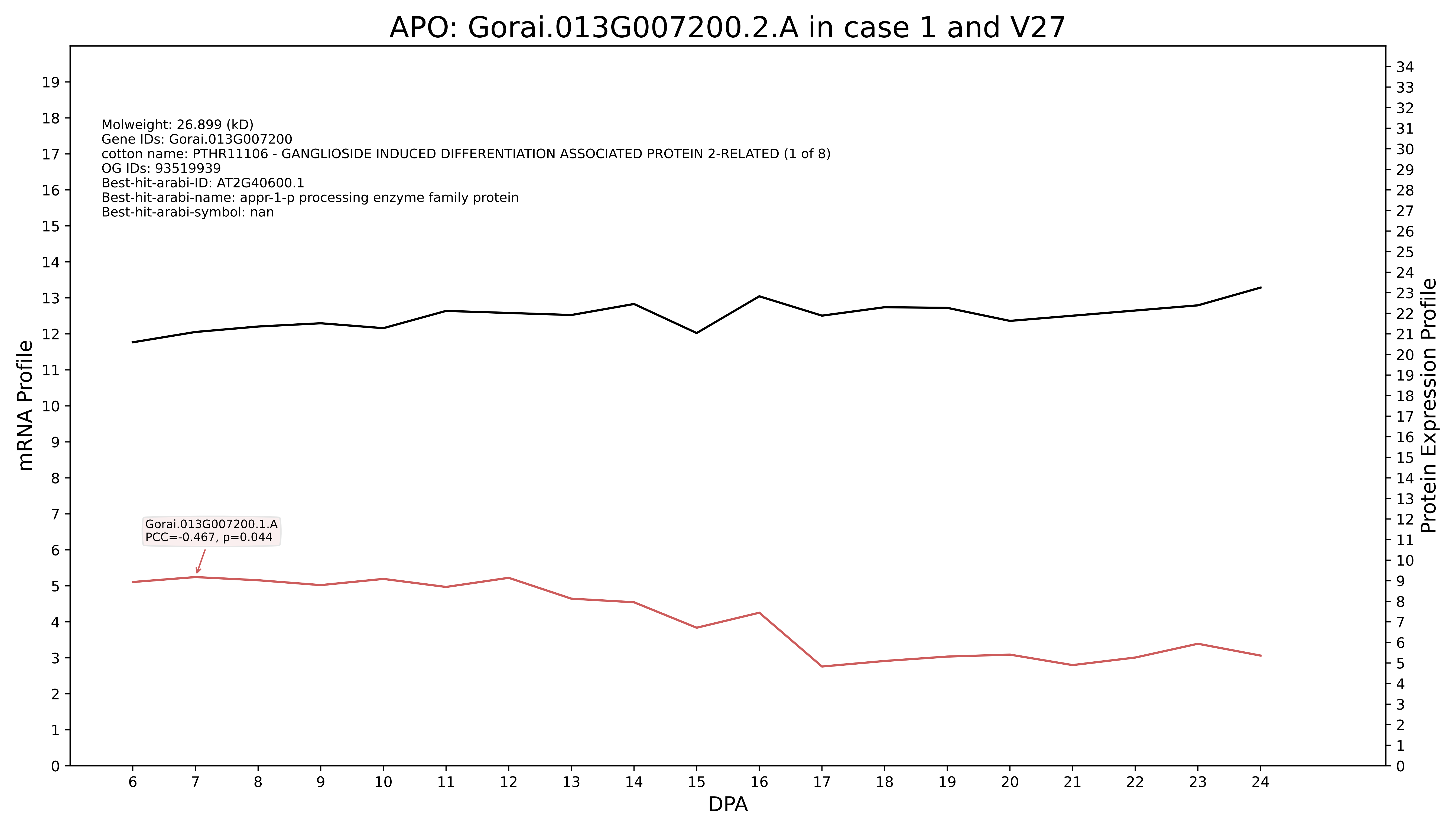
APO: Gorai.013G007200.2.A in case 1 and V27
Molweight: 26.899 (kD)
Gene IDs: Gorai.013G007200
cotton name: PTHR11106 - GANGLIOSIDE INDUCED DIFFERENTIATION ASSOCIATED PROTEIN 2-RELATED (1 of 8)
OG IDs: 93519939
Best-hit-arabi-ID: AT2G40600.1
Best-hit-arabi-name: appr-1-p processing enzyme family protein
Best-hit-arabi-symbol: nan
Gorai.013G007200.1.A
PCC=-0.467, p=0.044
mRNA Profile
Protein Expression Profile
DPA

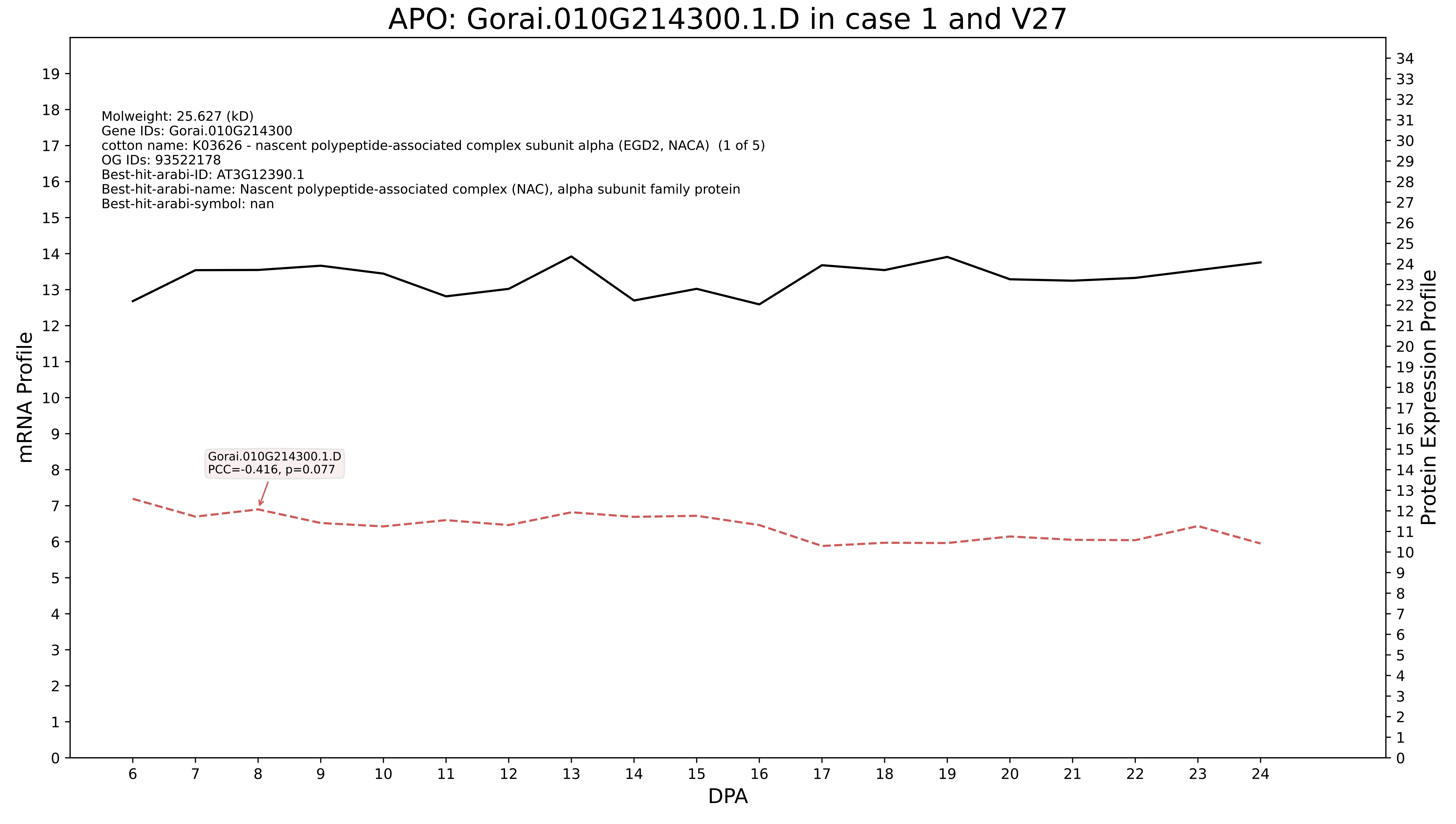
APO: Gorai.010G214300.1.D in case 1 and V27
Molweight: 25.627 (kD)
Gene IDs: Gorai.010G214300
cotton name: K03626 - nascent polypeptide-associated complex subunit alpha (EGD2, NACA) (1 of 5)
OG IDs: 93522178
Best-hit-arabi-ID: AT3G12390.1
Best-hit-arabi-name: Nascent polypeptide-associated complex (NAC), alpha subunit family protein
Best-hit-arabi-symbol: nan
Gorai.010G214300.1.D
PCC=-0.416, p=0.077
mRNA Profile
Protein Expression Profile
DPA

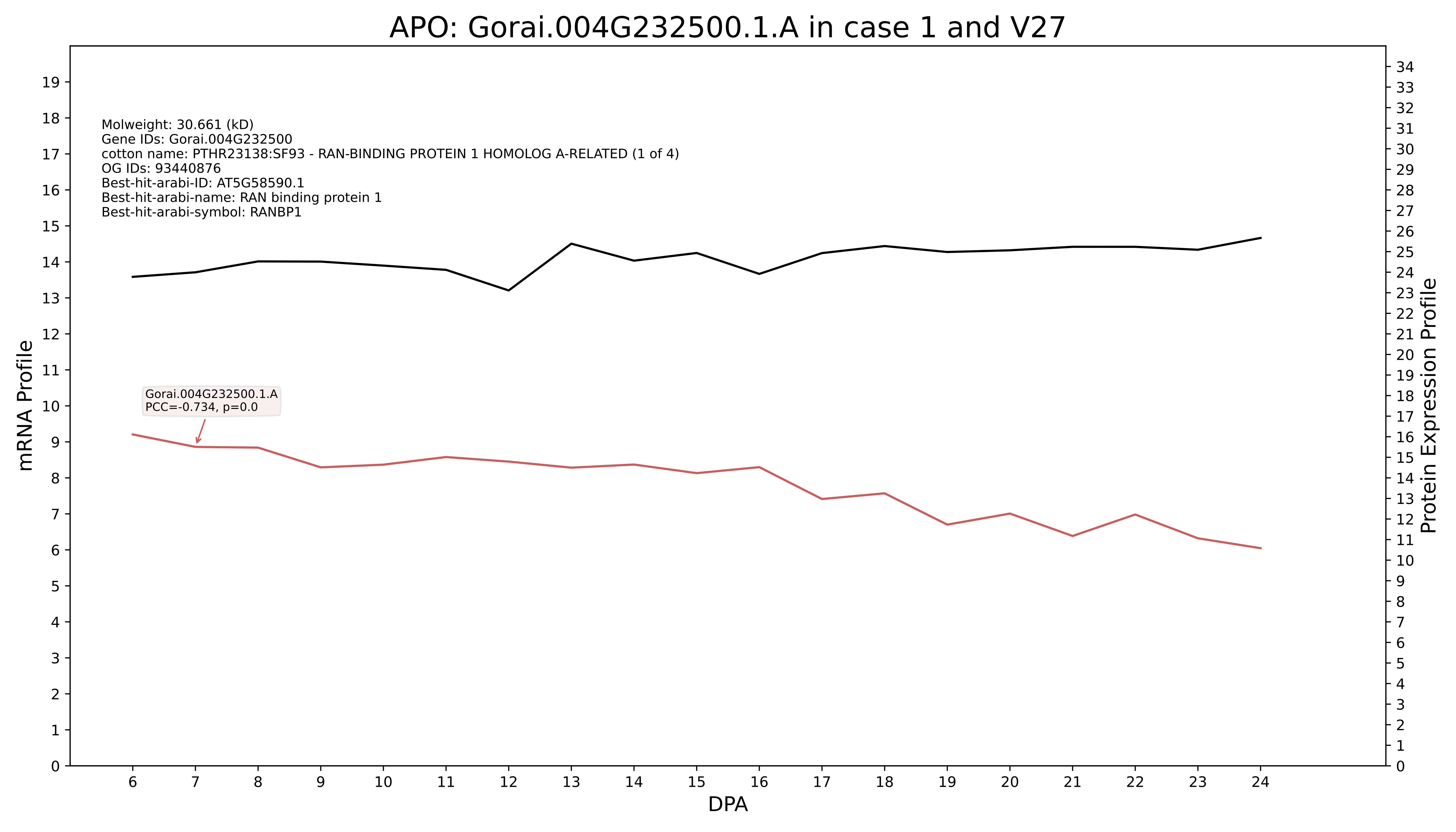
APO: Gorai.004G232500.1.A in case 1 and V27
Molweight: 30.661 (kD)
Gene IDs: Gorai.004G232500
cotton name: PTHR23138:SF93 - RAN-BINDING PROTEIN 1 HOMOLOG A-RELATED (1 of 4)
OG IDs: 93440876
Best-hit-arabi-ID: AT5G58590.1
Best-hit-arabi-name: RAN binding protein 1
Best-hit-arabi-symbol: RANBP1
Gorai.004G232500.1.A
PCC=-0.734, p=0.0
mRNA Profile
Protein Expression Profile
DPA

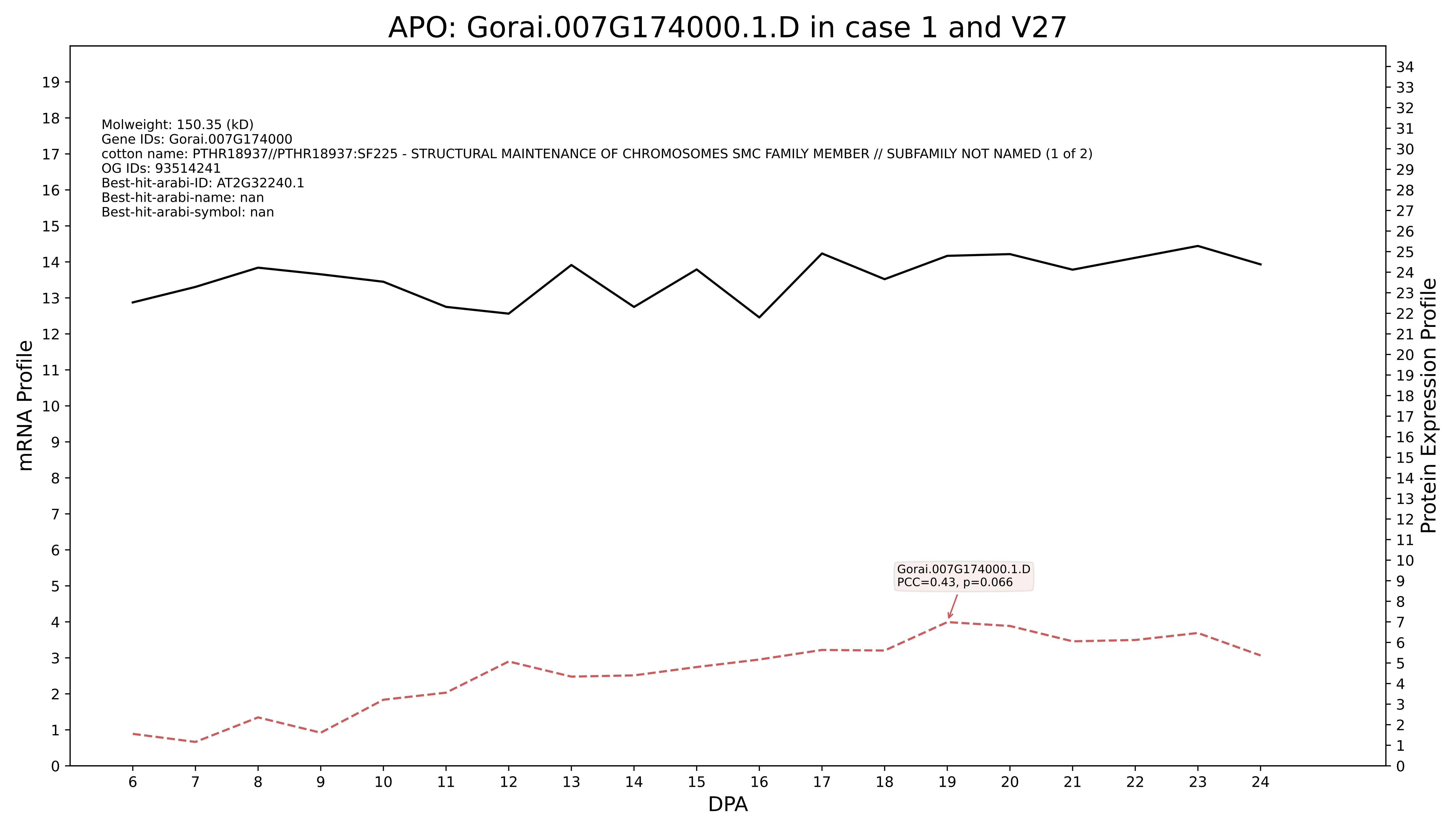
APO: Gorai.007G174000.1.D in case 1 and V27
Molweight: 150.35 (kD)
Gene IDs: Gorai.007G174000
cotton name: PTHR18937//PTHR18937:SF225 - STRUCTURAL MAINTENANCE OF CHROMOSOMES SMC FAMILY MEMBER // SUBFAMILY NOT NAMED (1 of 2)
OG IDs: 93514241
Best-hit-arabi-ID: AT2G32240.1
Best-hit-arabi-name: nan
Best-hit-arabi-symbol: nan
Gorai.007G174000.1.D
PCC=0.43, p=0.066
mRNA Profile
Protein Expression Profile
DPA

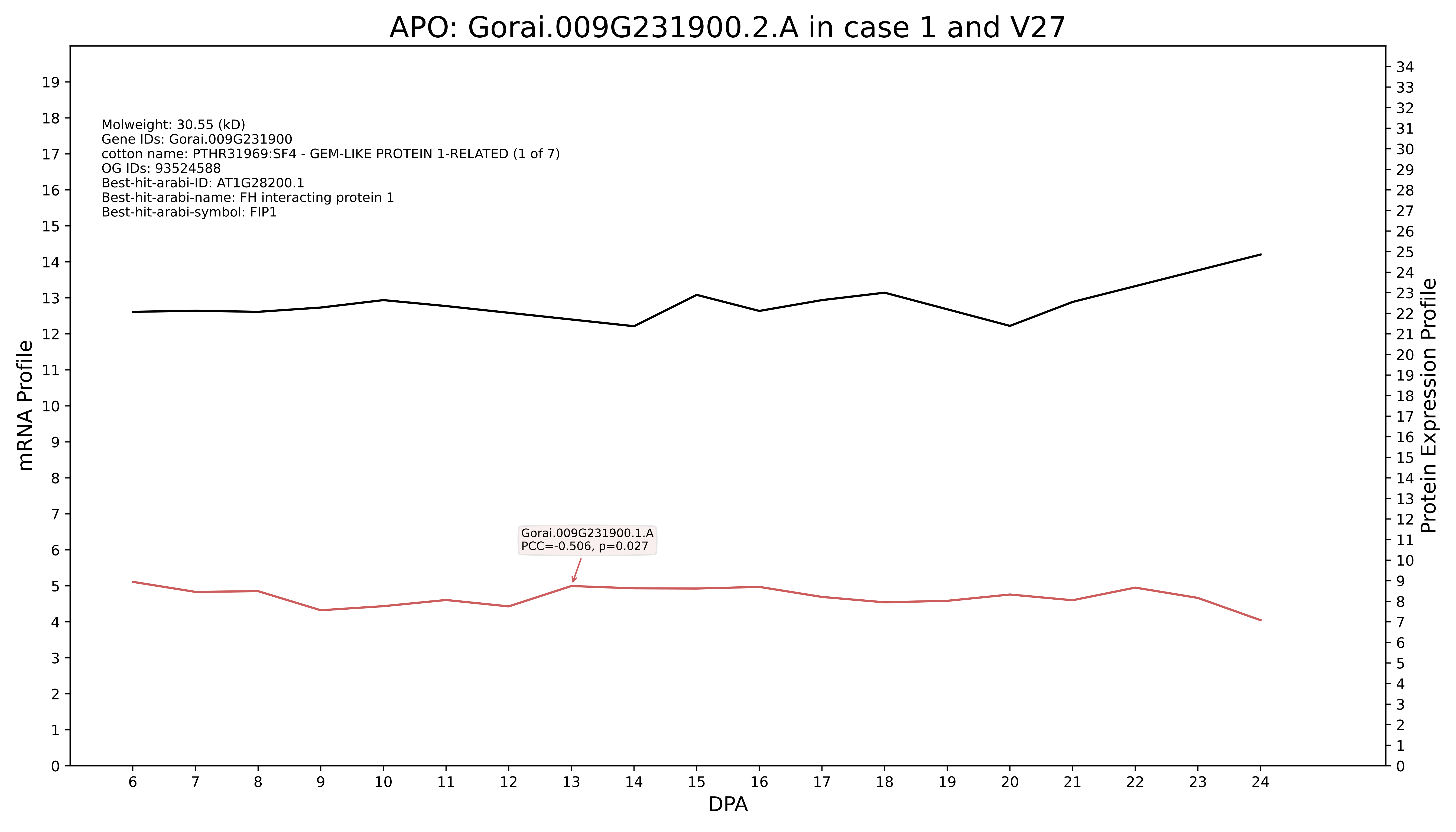
APO: Gorai.009G231900.2.A in case 1 and V27
Molweight: 30.55 (kD)
Gene IDs: Gorai.009G231900
cotton name: PTHR31969:SF4 - GEM-LIKE PROTEIN 1-RELATED (1 of 7)
OG IDs: 93524588
Best-hit-arabi-ID: AT1G28200.1
Best-hit-arabi-name: FH interacting protein 1
Best-hit-arabi-symbol: FIP1
Gorai.009G231900.1.A
PCC=-0.506, p=0.027
mRNA Profile
Protein Expression Profile
DPA

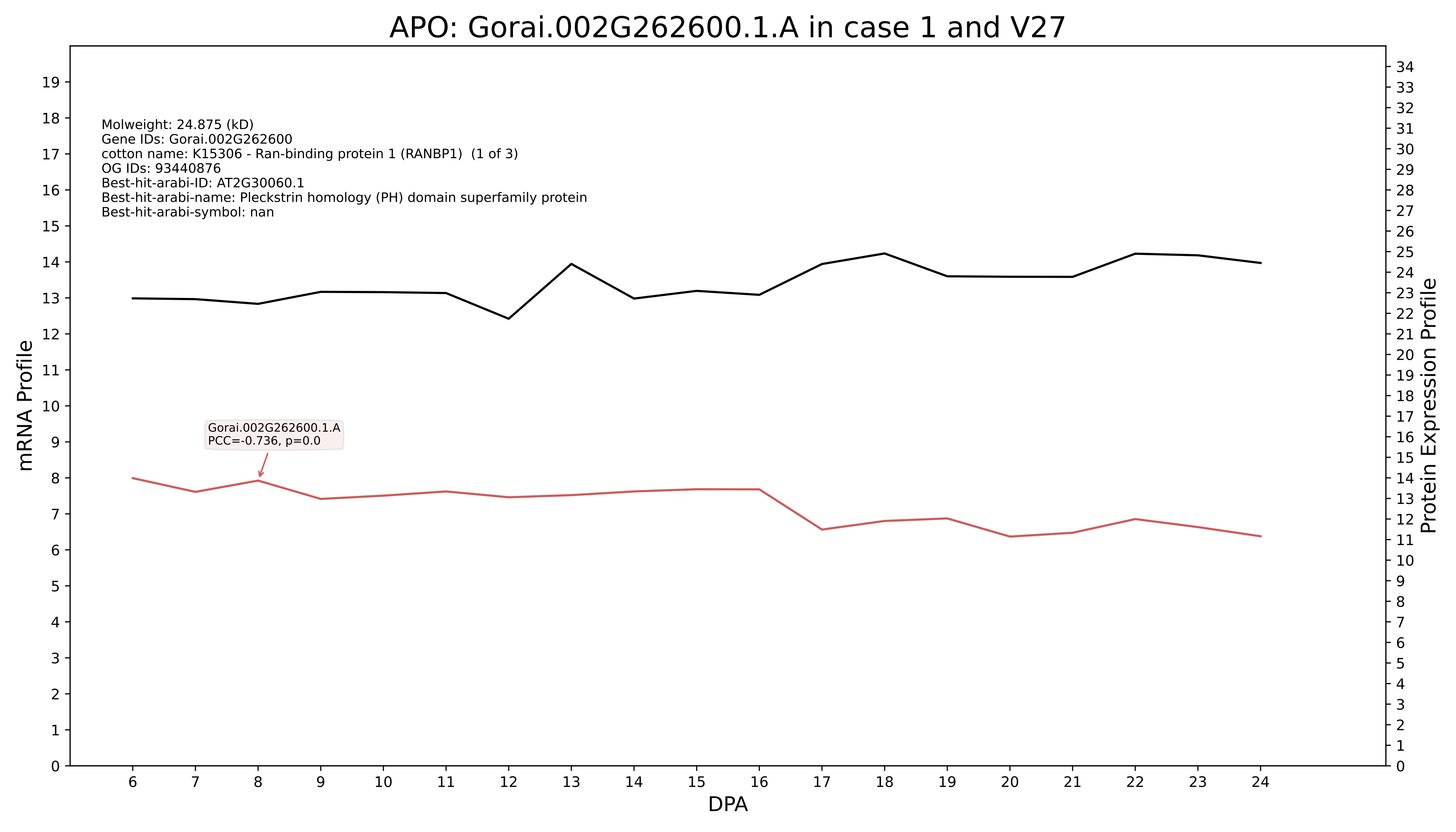
APO: Gorai.002G262600.1.A in case 1 and V27
Molweight: 24.875 (kD)
Gene IDs: Gorai.002G262600
cotton name: K15306 - Ran-binding protein 1 (RANBP1) (1 of 3)
OG IDs: 93440876
Best-hit-arabi-ID: AT2G30060.1
Best-hit-arabi-name: Pleckstrin homology (PH) domain superfamily protein
Best-hit-arabi-symbol: nan
Gorai.002G262600.1.A
PCC=-0.736, p=0.0
mRNA Profile
Protein Expression Profile
DPA

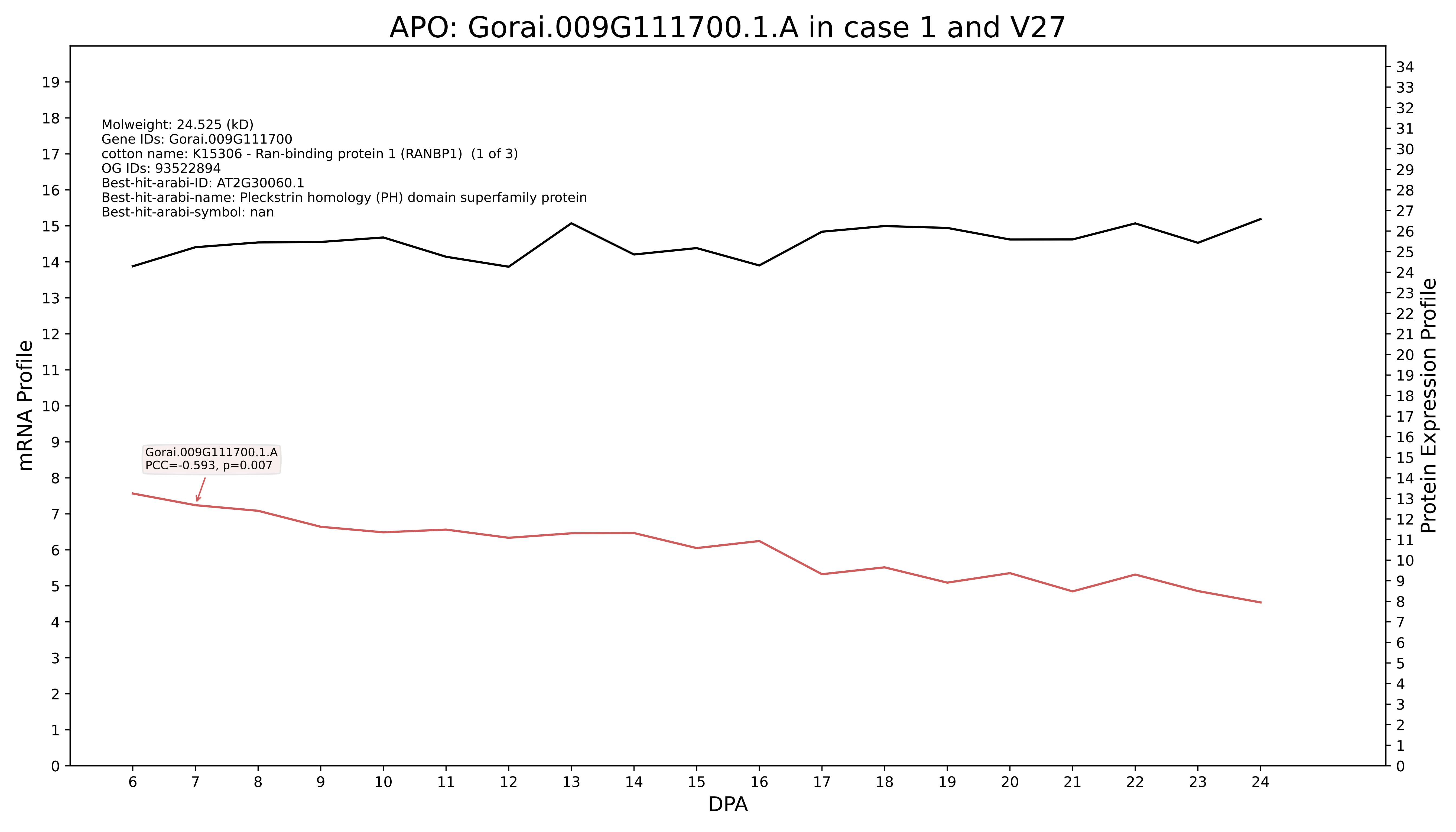

APO: Gorai.009G111700.1.A in case 1 and V27
Molweight: 24.525 (kD)
Gene IDs: Gorai.009G111700
cotton name: K15306 - Ran-binding protein 1 (RANBP1) (1 of 3)
OG IDs: 93522894
Best-hit-arabi-ID: AT2G30060.1
Best-hit-arabi-name: Pleckstrin homology (PH) domain superfamily protein
Best-hit-arabi-symbol: nan
Gorai.009G111700.1.A
PCC=-0.593, p=0.007
mRNA Profile
Protein Expression Profile
DPA

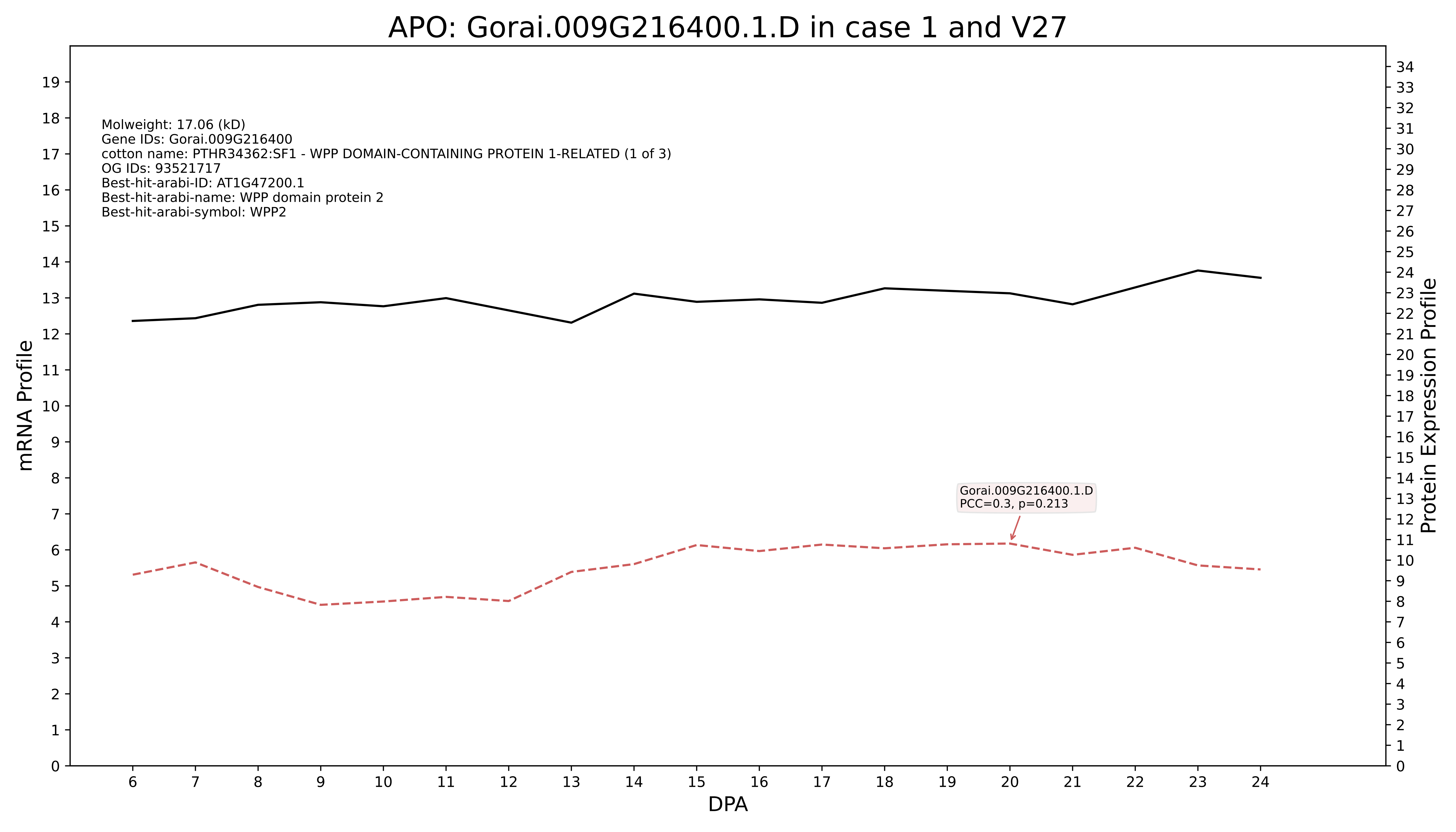

APO: Gorai.009G216400.1.D in case 1 and V27
Molweight: 17.06 (kD)
Gene IDs: Gorai.009G216400
cotton name: PTHR34362:SF1 - WPP DOMAIN-CONTAINING PROTEIN 1-RELATED (1 of 3)
OG IDs: 93521717
Best-hit-arabi-ID: AT1G47200.1
Best-hit-arabi-name: WPP domain protein 2
Best-hit-arabi-symbol: WPP2
Gorai.009G216400.1.D
PCC=0.3, p=0.213
mRNA Profile
Protein Expression Profile
DPA

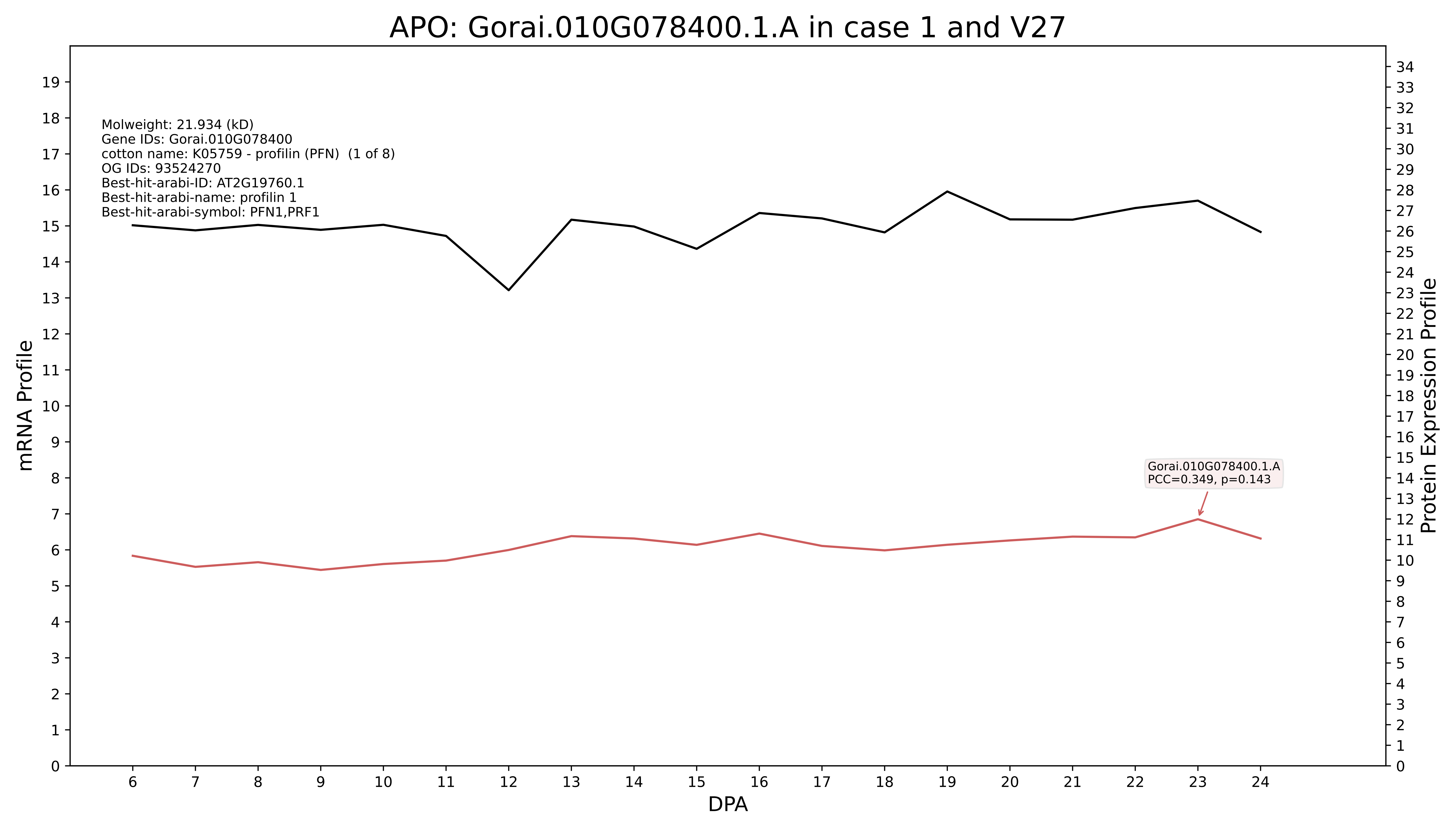
APO: Gorai.010G078400.1.A in case 1 and V27
Molweight: 21.934 (kD)
Gene IDs: Gorai.010G078400
cotton name: K05759 - profilin (PFN) (1 of 8)
OG IDs: 93524270
Best-hit-arabi-ID: AT2G19760.1
Best-hit-arabi-name: profilin 1
Best-hit-arabi-symbol: PFN1,PRF1
Gorai.010G078400.1.A
PCC=0.349, p=0.143
mRNA Profile
Protein Expression Profile
DPA

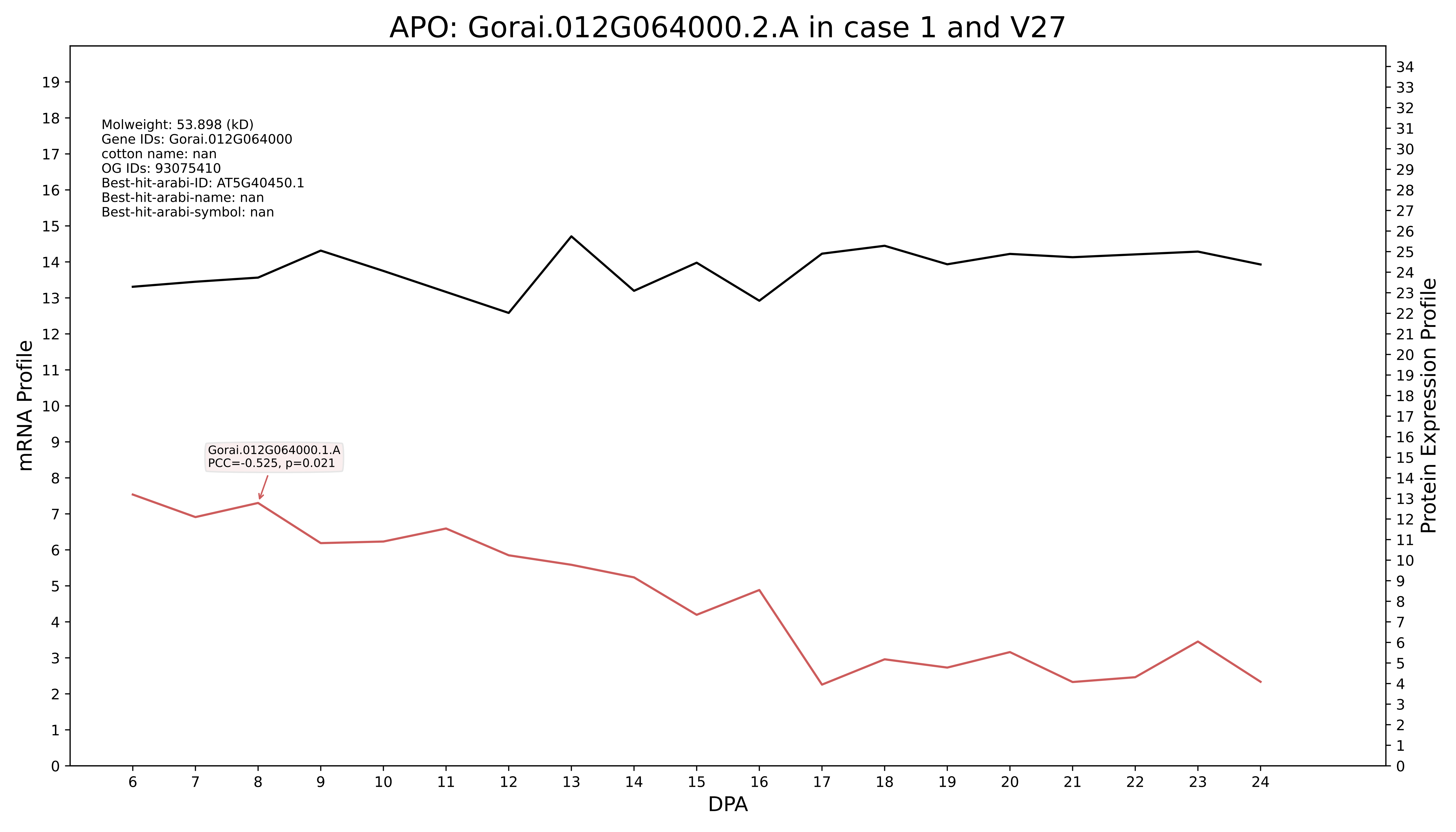
APO: Gorai.012G064000.2.A in case 1 and V27
Molweight: 53.898 (kD)
Gene IDs: Gorai.012G064000
cotton name: nan
OG IDs: 93075410
Best-hit-arabi-ID: AT5G40450.1
Best-hit-arabi-name: nan
Best-hit-arabi-symbol: nan
Gorai.012G064000.1.A
PCC=-0.525, p=0.021
mRNA Profile
Protein Expression Profile
DPA
0
1
2
3
4
5
6
7
8
9
10
11
12
13
14
15
16
17
18
19
20
21
22
23
24
25
26
27
28
29
30
31
32
33
34

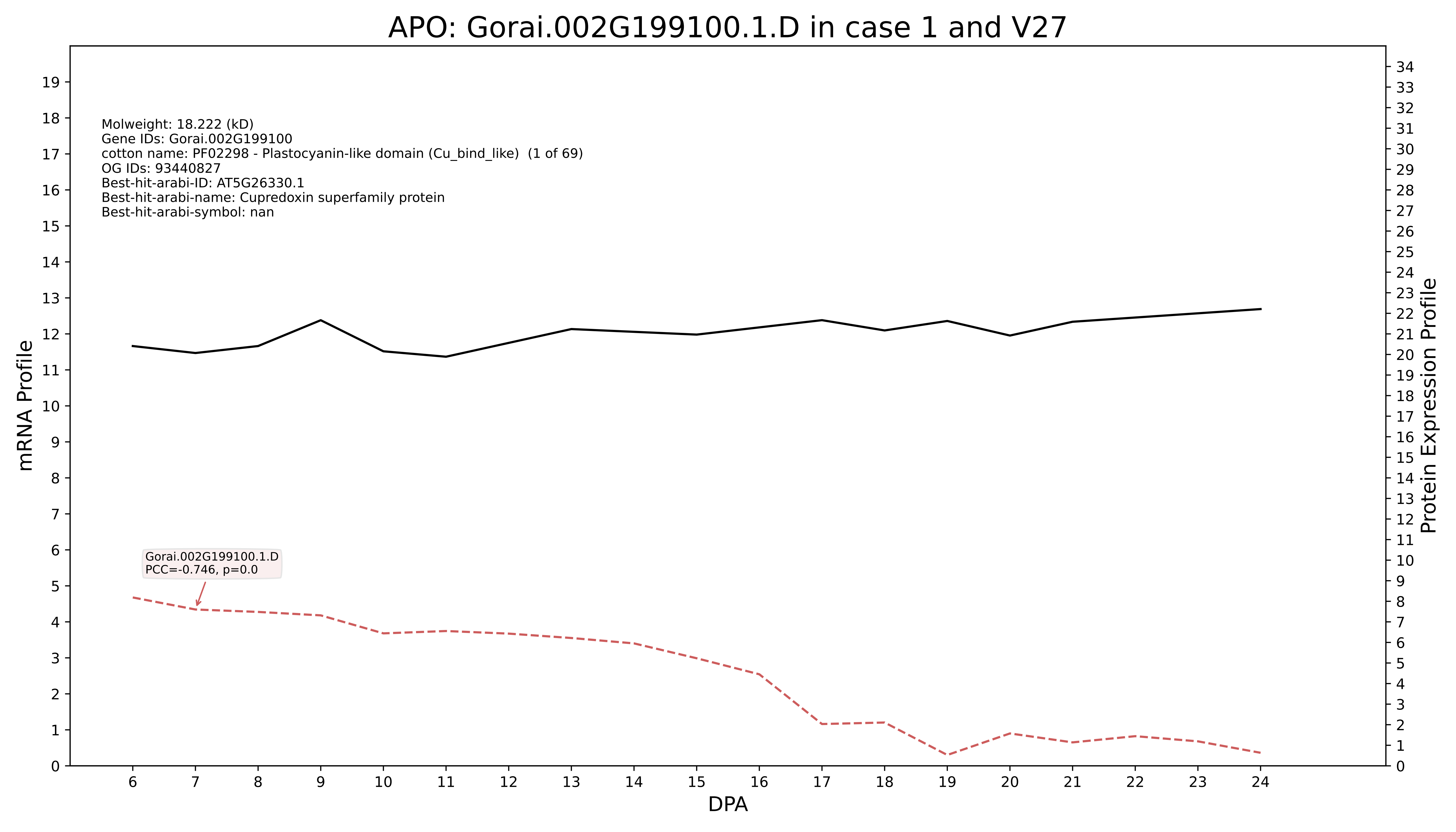

APO: Gorai.002G199100.1.D in case 1 and V27
Molweight: 18.222 (kD)
Gene IDs: Gorai.002G199100
cotton name: PF02298 - Plastocyanin-like domain (Cu_bind_like) (1 of 69)
OG IDs: 93440827
Best-hit-arabi-ID: AT5G26330.1
Best-hit-arabi-name: Cupredoxin superfamily protein
Best-hit-arabi-symbol: nan
Gorai.002G199100.1.D
PCC=-0.746, p=0.0
mRNA Profile
Protein Expression Profile
DPA

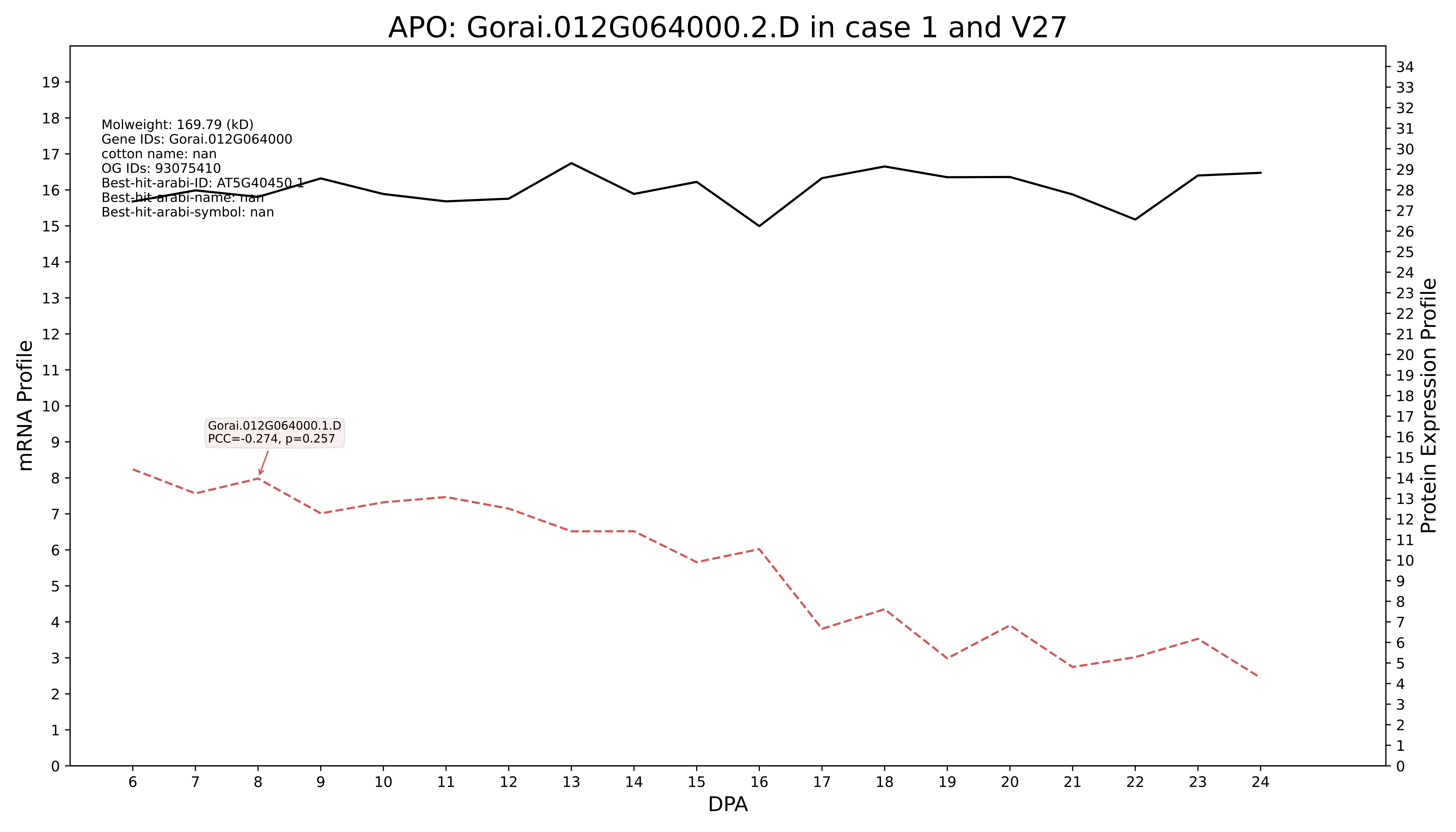

APO: Gorai.012G064000.2.D in case 1 and V27
Molweight: 169.79 (kD)
Gene IDs: Gorai.012G064000
cotton name: nan
OG IDs: 93075410
Best-hit-arabi-ID: AT5G40450.1
Best-hit-arabi-name: nan
Best-hit-arabi-symbol: nan
Gorai.012G064000.1.D
PCC=-0.274, p=0.257
mRNA Profile
Protein Expression Profile
DPA

APO: Gorai.003G182200.1.A in case 1 and V27

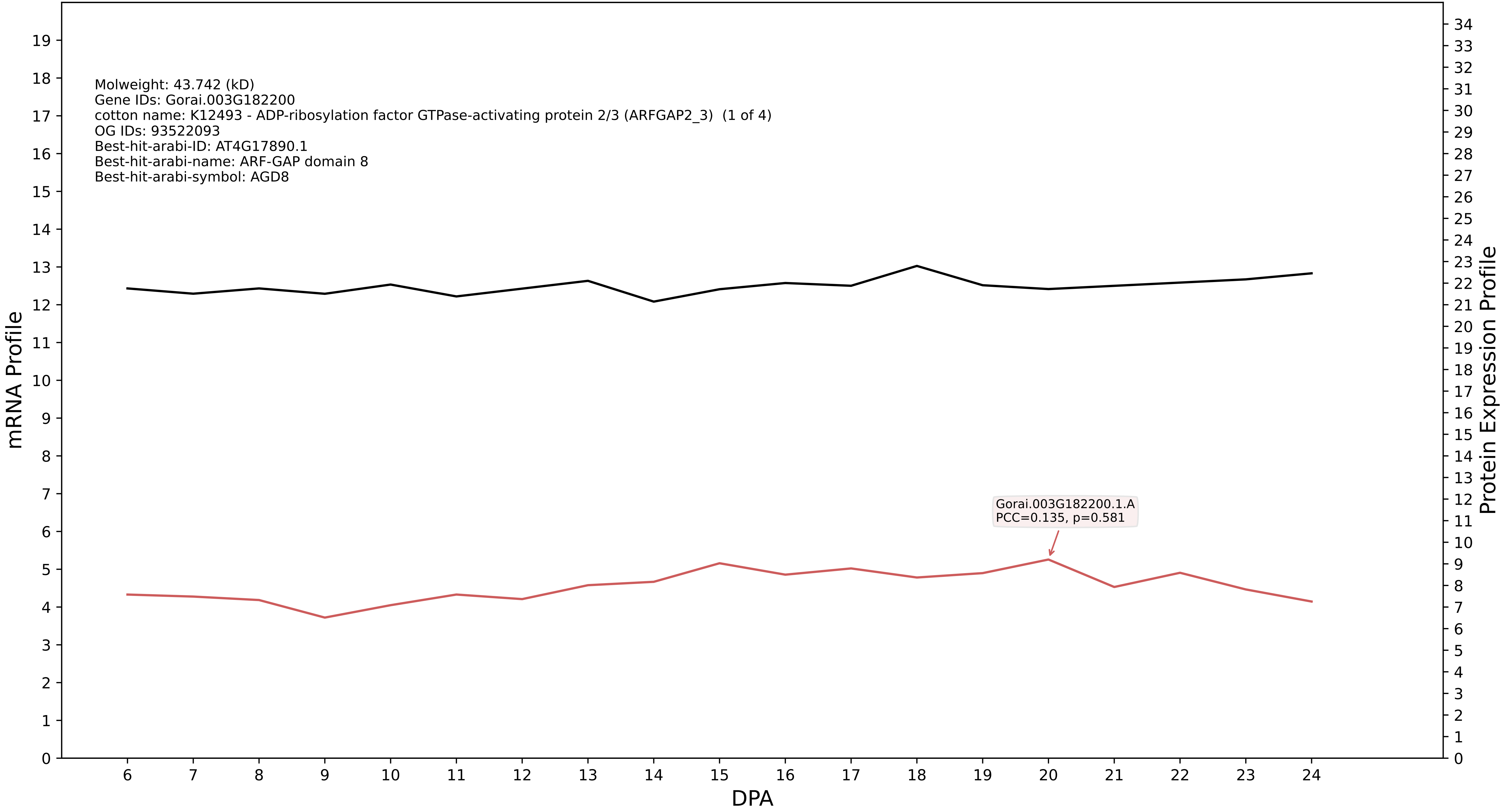

APO: Gorai.009G159200.2.D in case 1 and V27

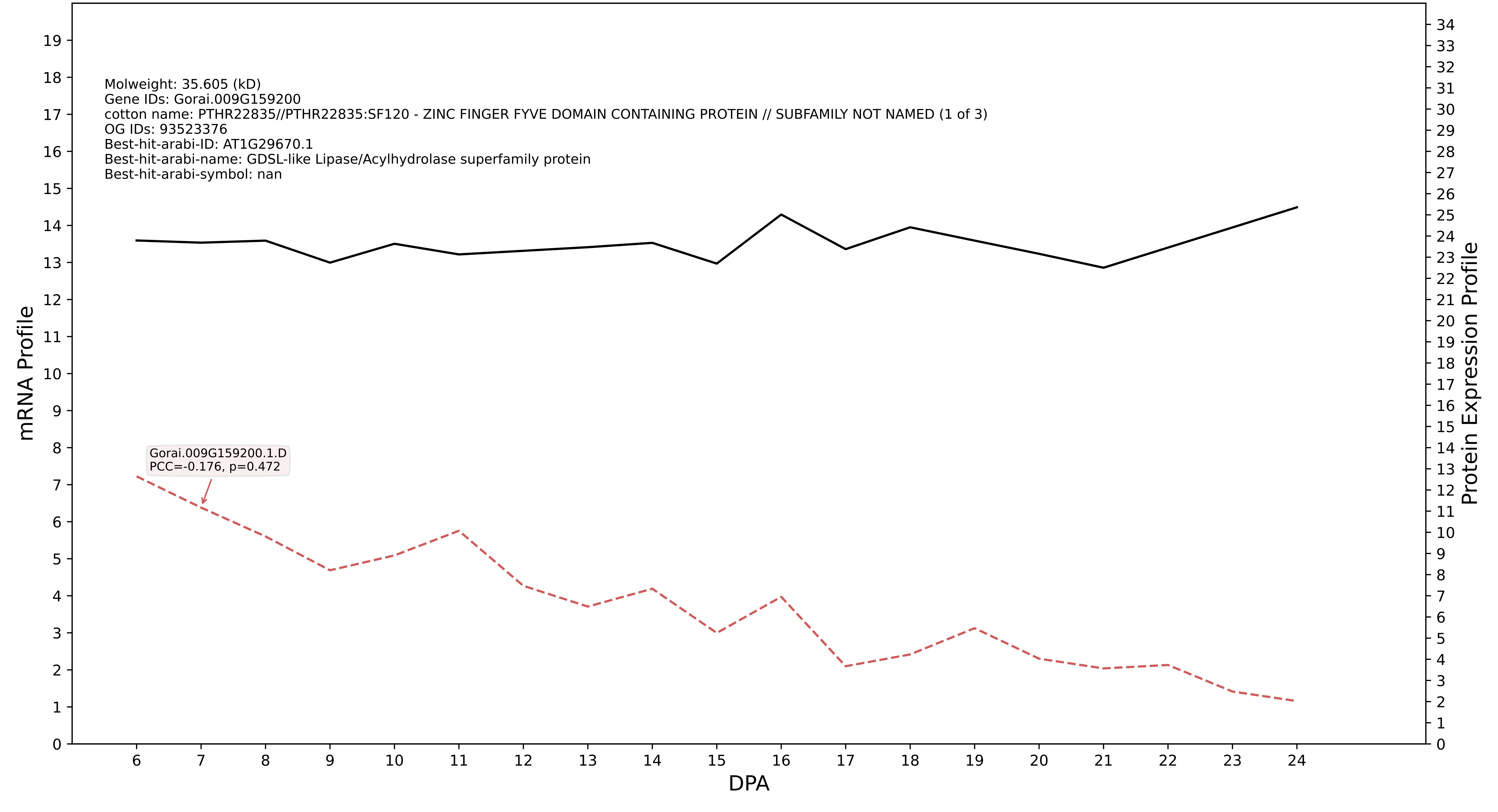

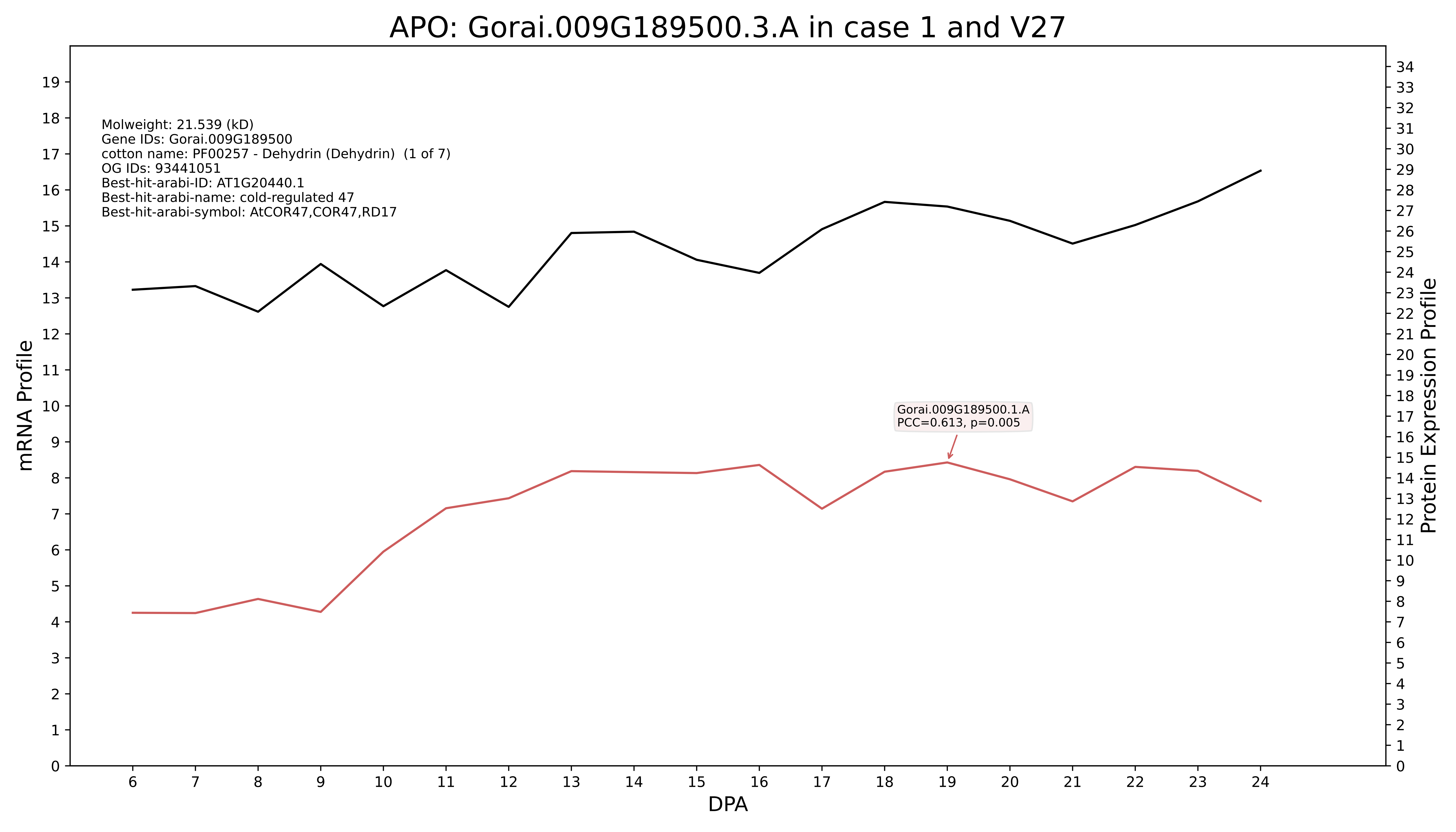
APO: Gorai.009G189500.3.A in case 1 and V27
Molweight: 21.539 (kD)
Gene IDs: Gorai.009G189500
cotton name: PF00257 - Dehydrin (Dehydrin) (1 of 7)
OG IDs: 93441051
Best-hit-arabi-ID: AT1G20440.1
Best-hit-arabi-name: cold-regulated 47
Best-hit-arabi-symbol: AtCOR47,COR47,RD17
Gorai.009G189500.1.A
PCC=0.613, p=0.005
mRNA Profile
Protein Expression Profile
DPA

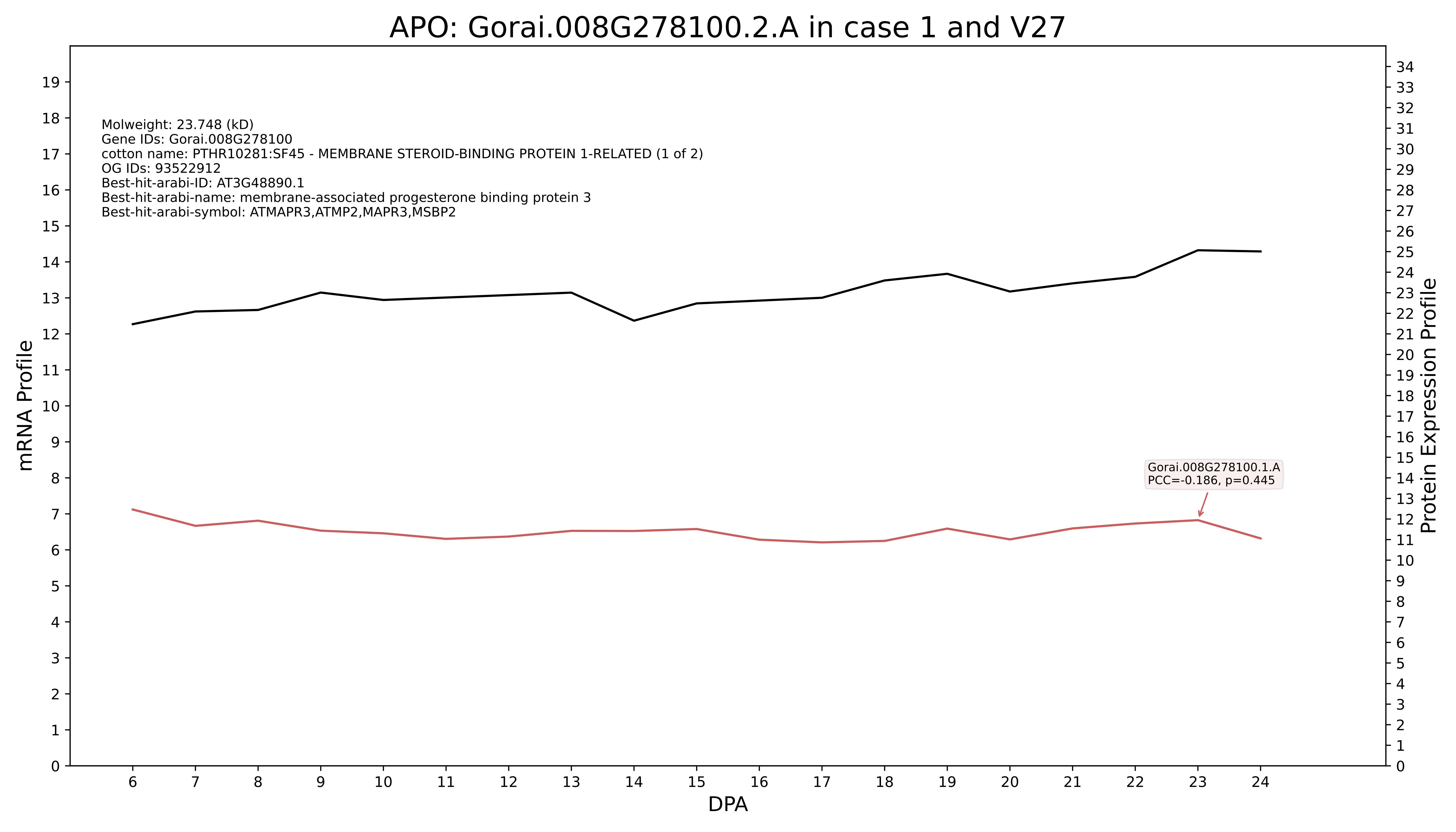

APO: Gorai.008G278100.2.A in case 1 and V27
Molweight: 23.748 (kD)
Gene IDs: Gorai.008G278100
cotton name: PTHR10281:SF45 - MEMBRANE STEROID-BINDING PROTEIN 1-RELATED (1 of 2)
OG IDs: 93522912
Best-hit-arabi-ID: AT3G48890.1
Best-hit-arabi-name: membrane-associated progesterone binding protein 3
Best-hit-arabi-symbol: ATMAPR3,ATMP2,MAPR3,MSBP2
Gorai.008G278100.1.A
PCC=-0.186, p=0.445
mRNA Profile
Protein Expression Profile
DPA

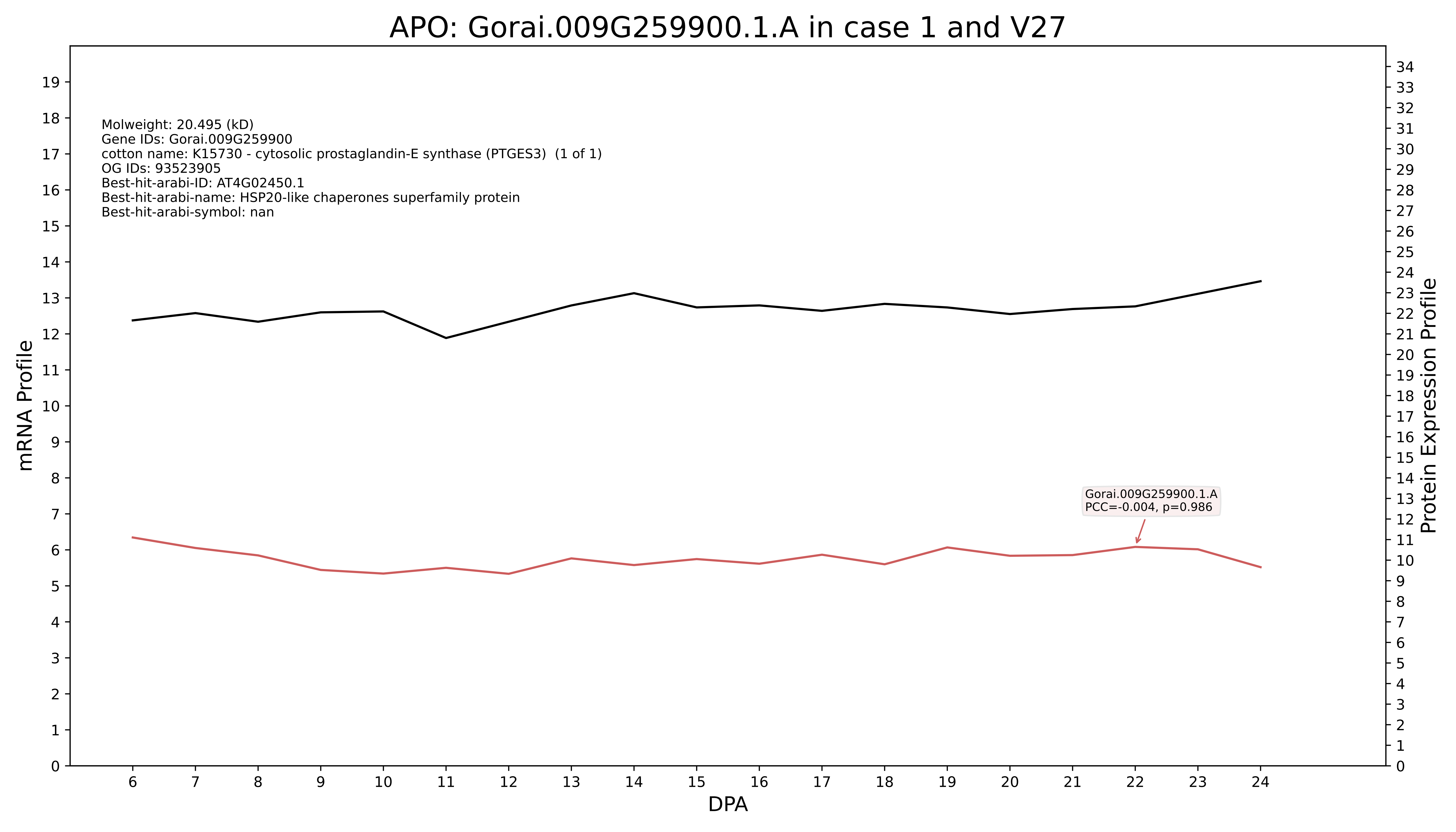
APO: Gorai.009G259900.1.A in case 1 and V27
Molweight: 20.495 (kD)
Gene IDs: Gorai.009G259900
cotton name: K15730 - cytosolic prostaglandin-E synthase (PTGES3) (1 of 1)
OG IDs: 93523905
Best-hit-arabi-ID: AT4G02450.1
Best-hit-arabi-name: HSP20-like chaperones superfamily protein
Best-hit-arabi-symbol: nan
Gorai.009G259900.1.A
PCC=-0.004, p=0.986
mRNA Profile
Protein Expression Profile
DPA

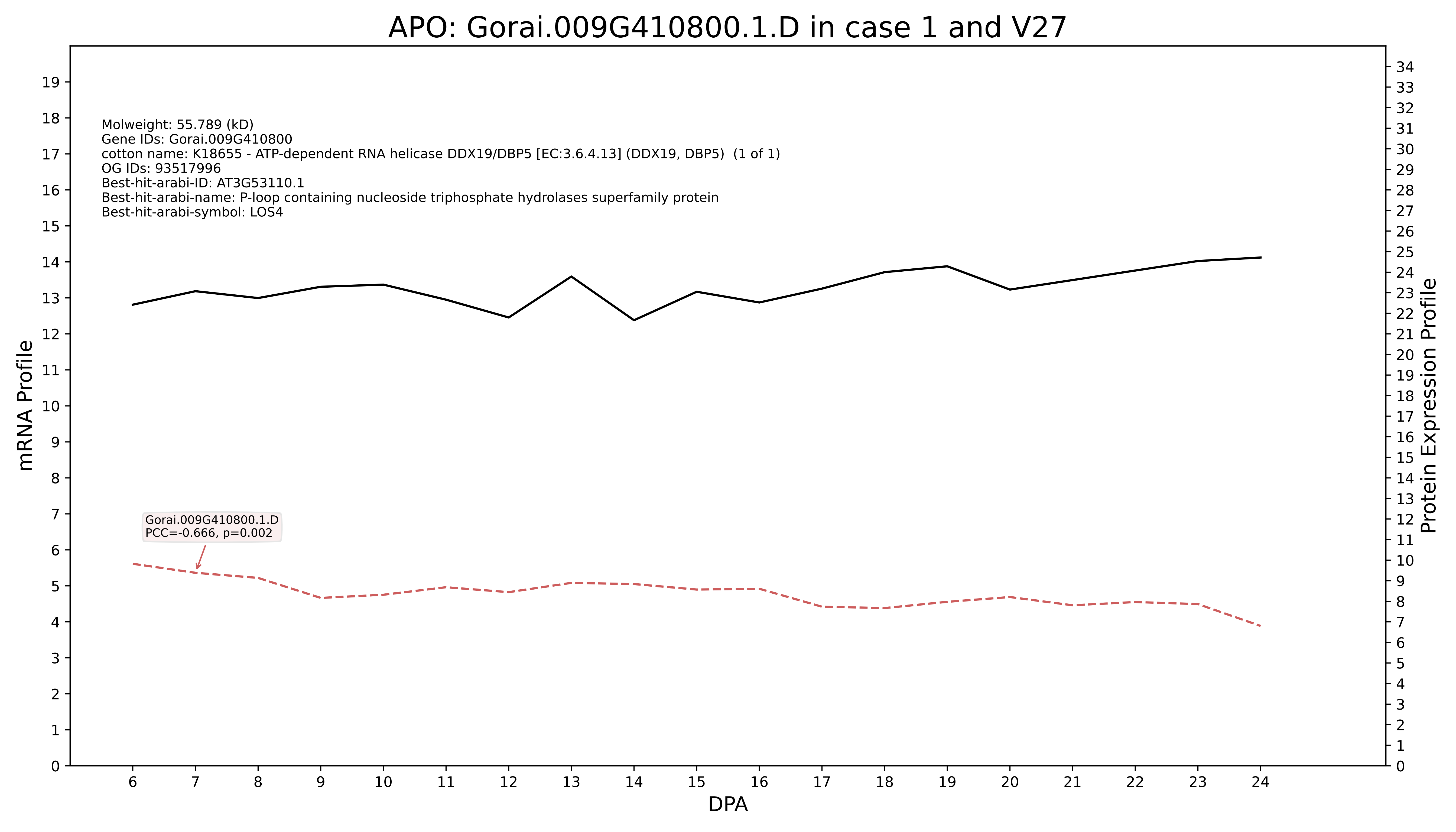

APO: Gorai.009G410800.1.D in case 1 and V27
Molweight: 55.789 (kD)
Gene IDs: Gorai.009G410800
cotton name: K18655 - ATP-dependent RNA helicase DDX19/DBP5 [EC:3.6.4.13] (DDX19, DBP5) (1 of 1)
OG IDs: 93517996
Best-hit-arabi-ID: AT3G53110.1
Best-hit-arabi-name: P-loop containing nucleoside triphosphate hydrolases superfamily protein
Best-hit-arabi-symbol: LOS4
Gorai.009G410800.1.D
PCC=-0.666, p=0.002
mRNA Profile
Protein Expression Profile
DPA

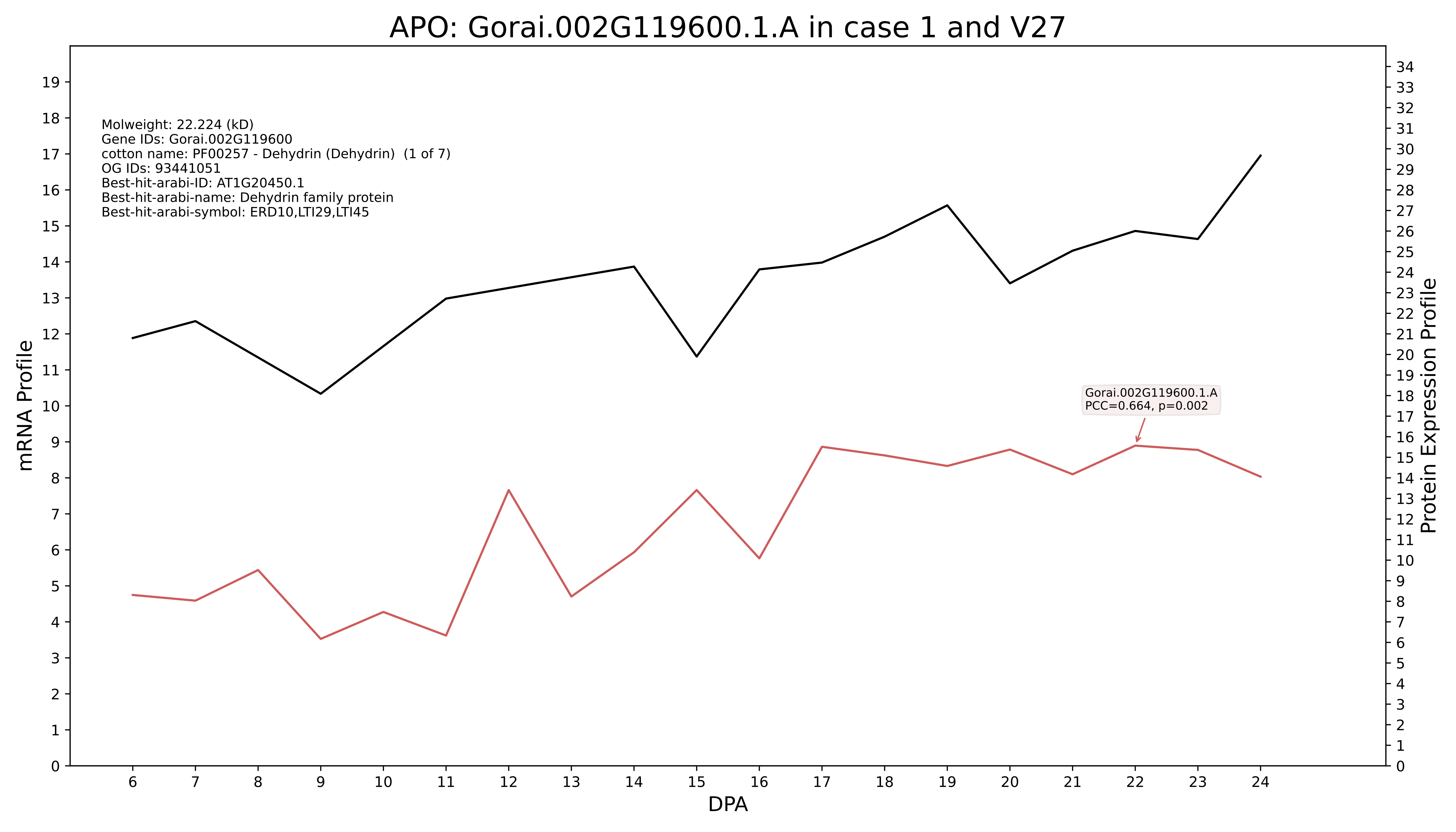
APO: Gorai.002G119600.1.A in case 1 and V27
Molweight: 22.224 (kD)
Gene IDs: Gorai.002G119600
cotton name: PF00257 - Dehydrin (Dehydrin) (1 of 7)
OG IDs: 93441051
Best-hit-arabi-ID: AT1G20450.1
Best-hit-arabi-name: Dehydrin family protein
Best-hit-arabi-symbol: ERD10,LTI29,LTI45
Gorai.002G119600.1.A
PCC=0.664, p=0.002
mRNA Profile
Protein Expression Profile
DPA

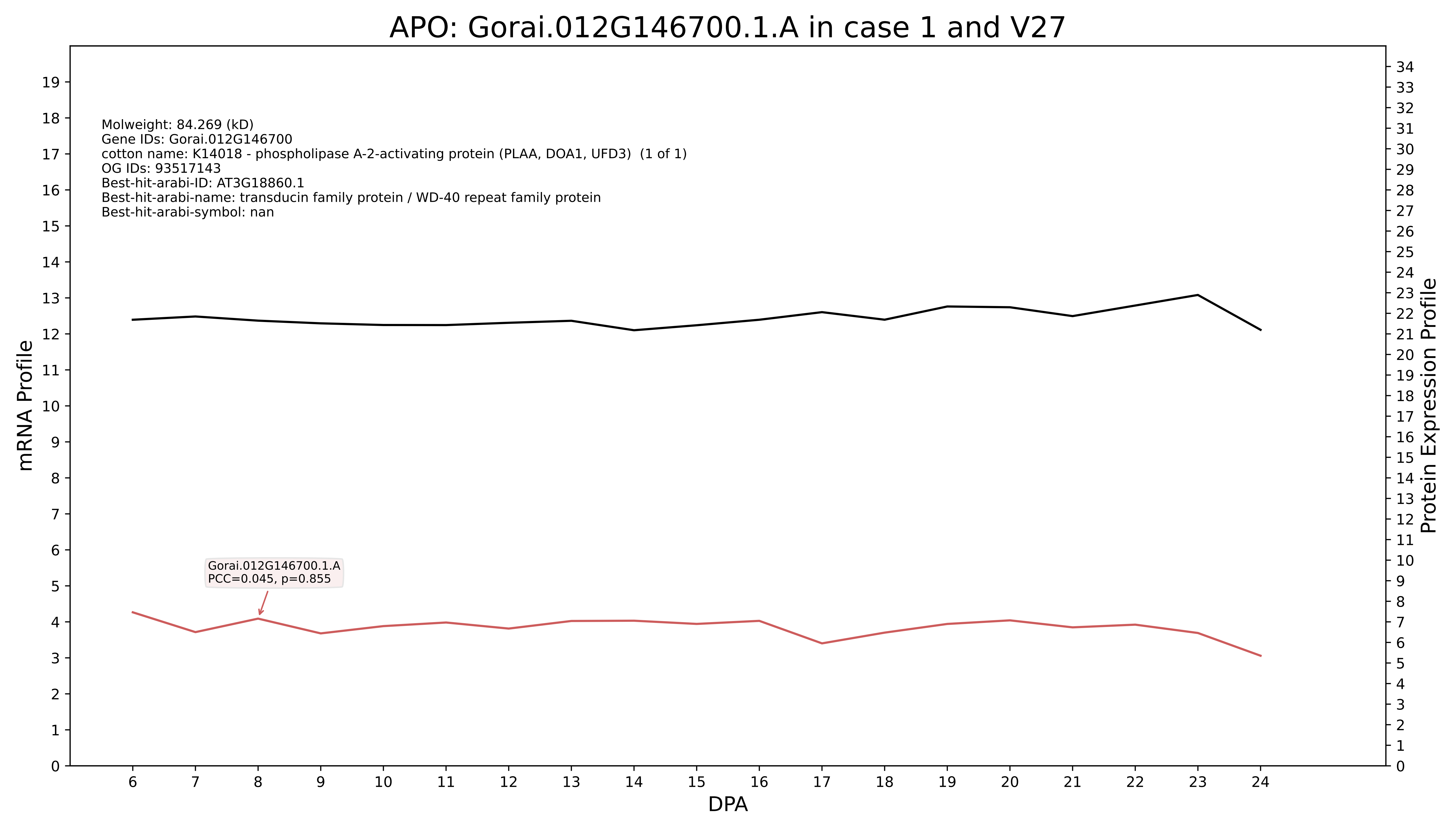
APO: Gorai.012G146700.1.A in case 1 and V27
Molweight: 84.269 (kD)
Gene IDs: Gorai.012G146700
cotton name: K14018 - phospholipase A-2-activating protein (PLAA, DOA1, UFD3) (1 of 1)
OG IDs: 93517143
Best-hit-arabi-ID: AT3G18860.1
Best-hit-arabi-name: transducin family protein / WD-40 repeat family protein
Best-hit-arabi-symbol: nan
Gorai.012G146700.1.A
PCC=0.045, p=0.855
mRNA Profile
Protein Expression Profile
DPA

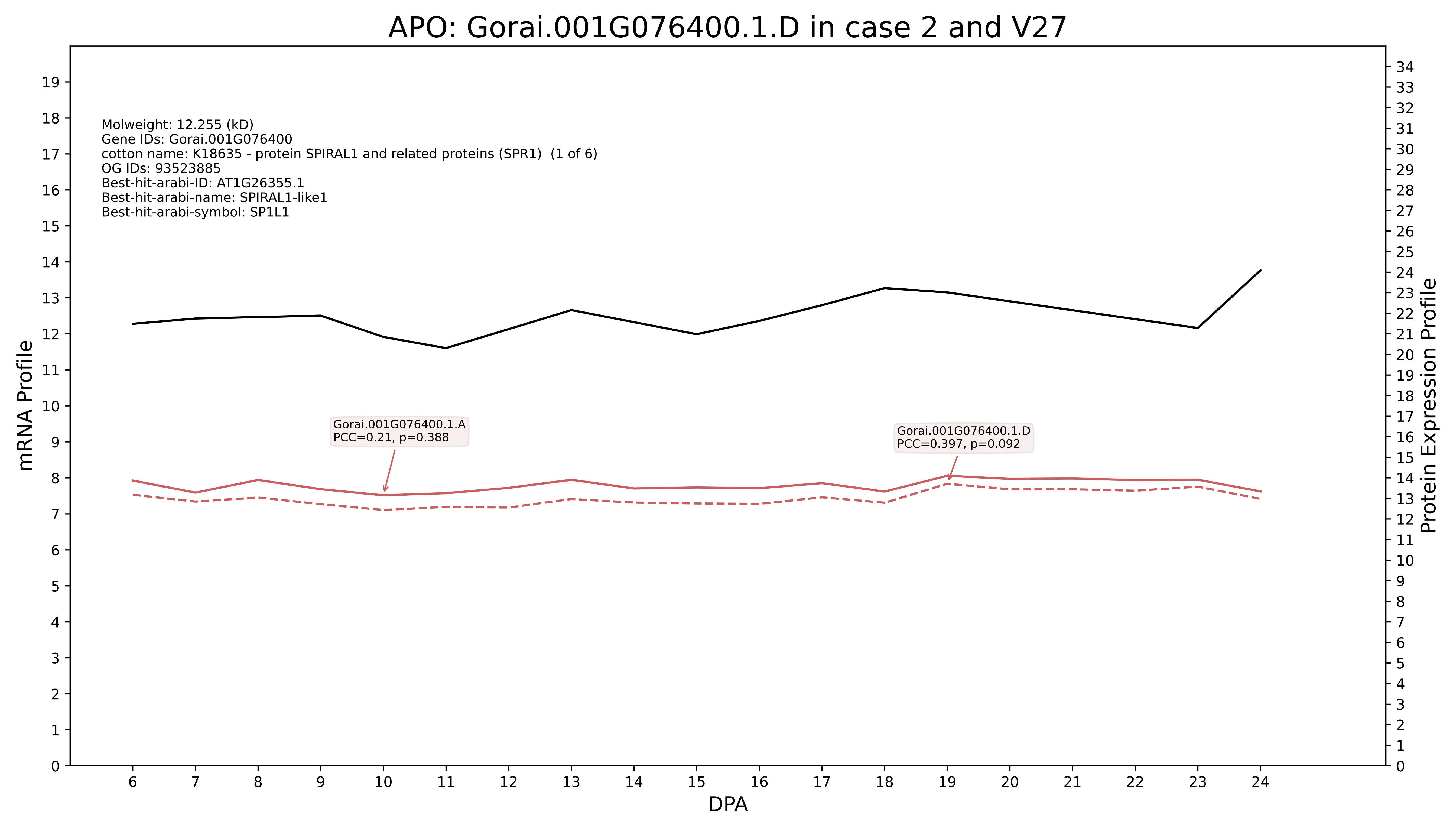
APO: Gorai.001G076400.1.D in case 2 and V27
Molweight: 12.255 (kD)
Gene IDs: Gorai.001G076400
cotton name: K18635 - protein SPIRAL1 and related proteins (SPR1) (1 of 6)
OG IDs: 93523885
Best-hit-arabi-ID: AT1G26355.1
Best-hit-arabi-name: SPIRAL1-like1
Best-hit-arabi-symbol: SP1L1
Gorai.001G076400.1.A
PCC=0.21, p=0.388
Gorai.001G076400.1.D
PCC=0.397, p=0.092
mRNA Profile
Protein Expression Profile
DPA

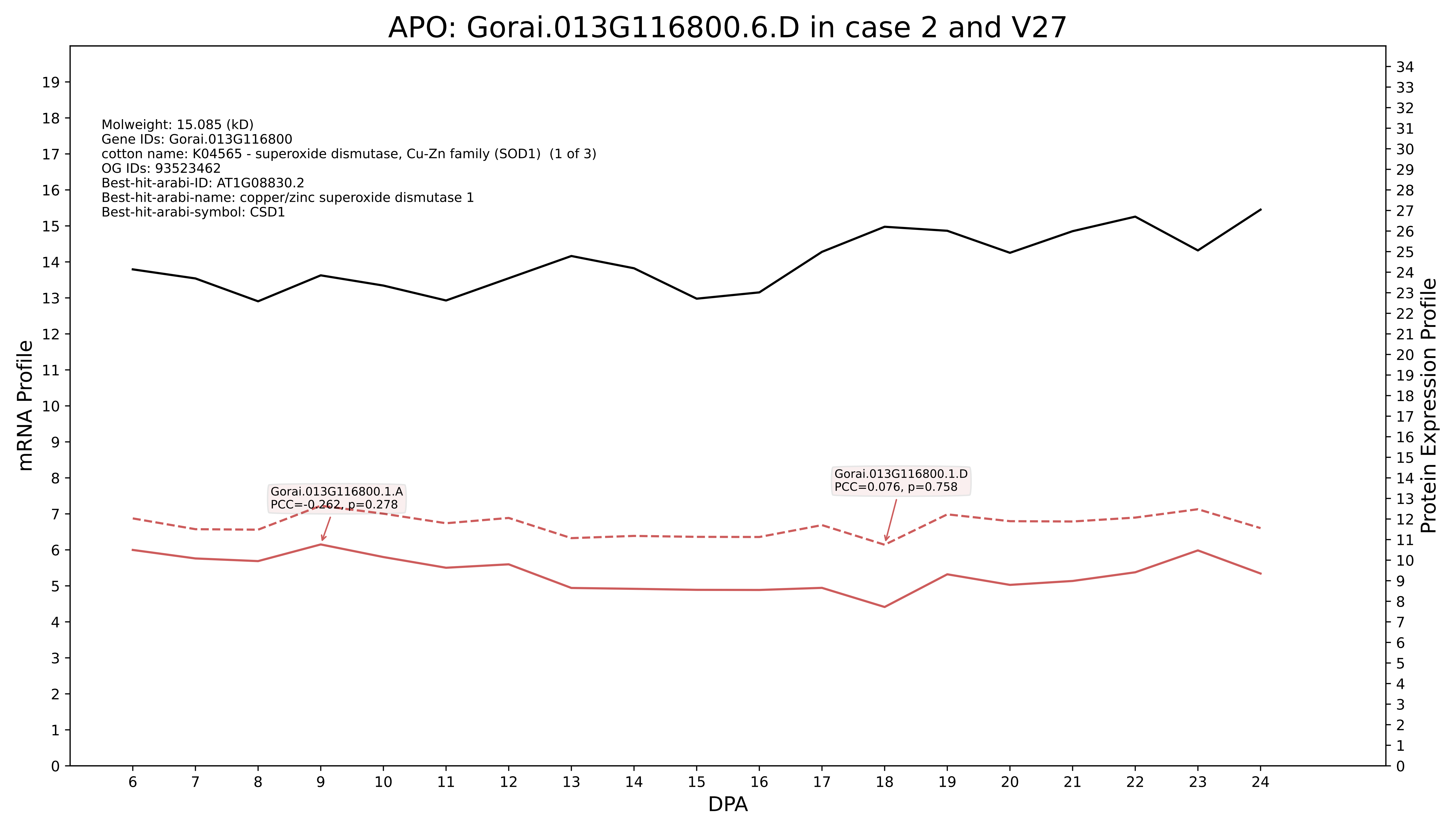
APO: Gorai.013G116800.6.D in case 2 and V27
Molweight: 15.085 (kD)
Gene IDs: Gorai.013G116800
cotton name: K04565 - superoxide dismutase, Cu-Zn family (SOD1) (1 of 3)
OG IDs: 93523462
Best-hit-arabi-ID: AT1G08830.2
Best-hit-arabi-name: copper/zinc superoxide dismutase 1
Best-hit-arabi-symbol: CSD1
Gorai.013G116800.1.A
PCC=-0.262, p=0.278
Gorai.013G116800.1.D
PCC=0.076, p=0.758
mRNA Profile
Protein Expression Profile
DPA

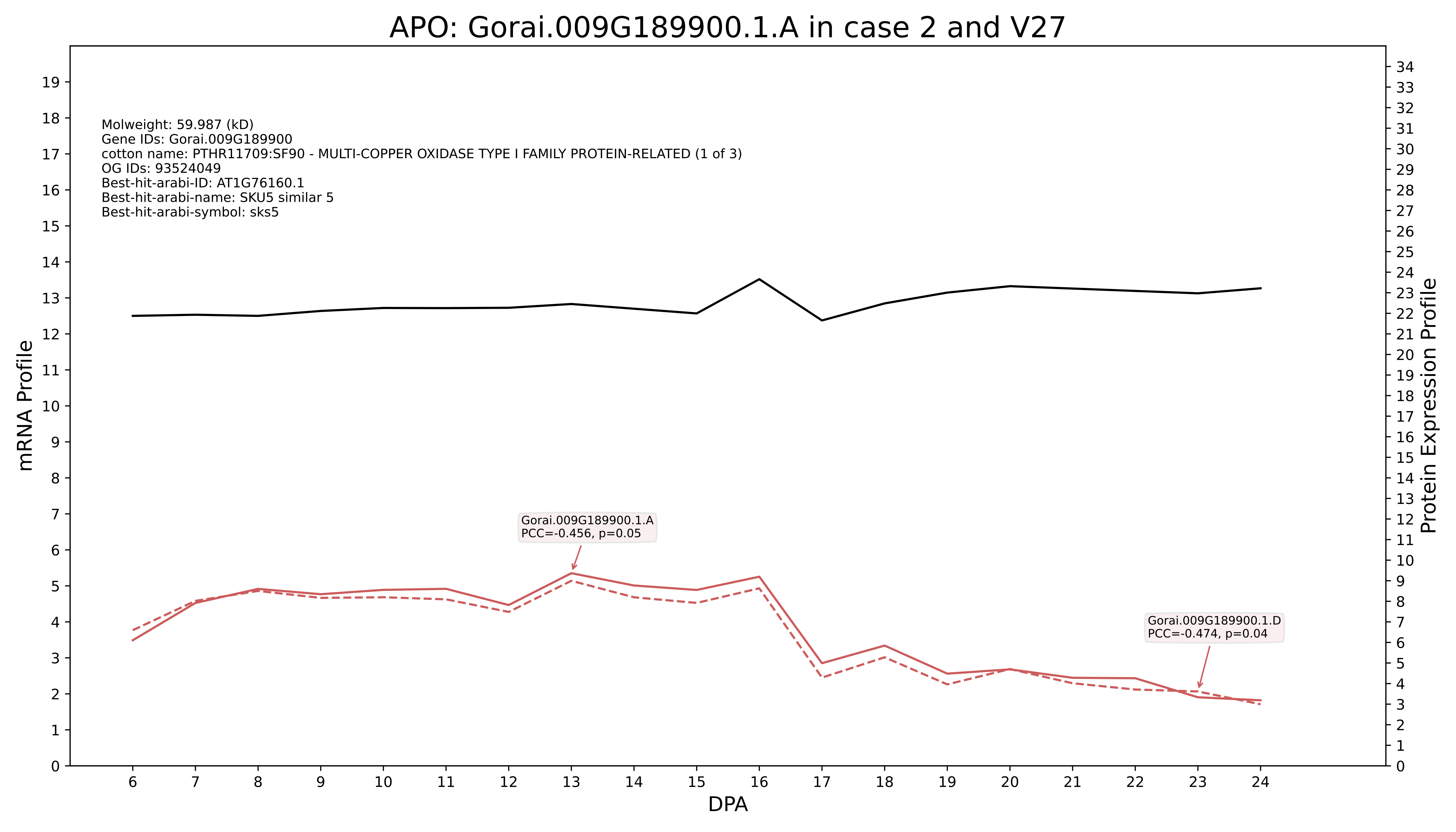
APO: Gorai.009G189900.1.A in case 2 and V27
Molweight: 59.987 (kD)
Gene IDs: Gorai.009G189900
cotton name: PTHR11709:SF90 - MULTI-COPPER OXIDASE TYPE I FAMILY PROTEIN-RELATED (1 of 3)
OG IDs: 93524049
Best-hit-arabi-ID: AT1G76160.1
Best-hit-arabi-name: SKU5 similar 5
Best-hit-arabi-symbol: sks5
Gorai.009G189900.1.A
PCC=-0.456, p=0.05
Gorai.009G189900.1.D
PCC=-0.474, p=0.04
mRNA Profile
Protein Expression Profile
DPA

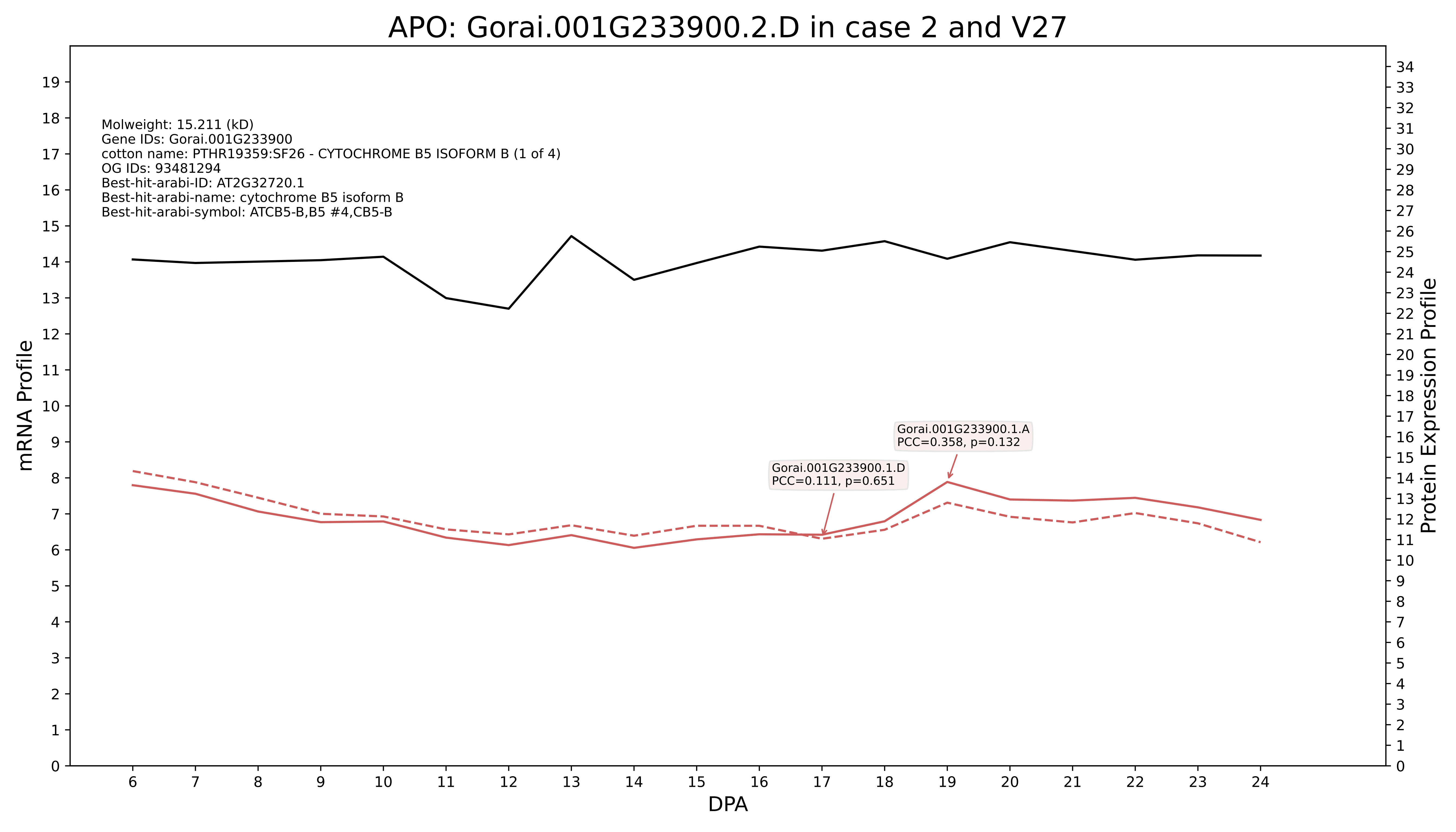
APO: Gorai.001G233900.2.D in case 2 and V27
Molweight: 15.211 (kD)
Gene IDs: Gorai.001G233900
cotton name: PTHR19359:SF26 - CYTOCHROME B5 ISOFORM B (1 of 4)
OG IDs: 93481294
Best-hit-arabi-ID: AT2G32720.1
Best-hit-arabi-name: cytochrome B5 isoform B
Best-hit-arabi-symbol: ATCB5-B,B5 #4,CB5-B
Gorai.001G233900.1.A
PCC=0.358, p=0.132
Gorai.001G233900.1.D
PCC=0.111, p=0.651
mRNA Profile
Protein Expression Profile
DPA

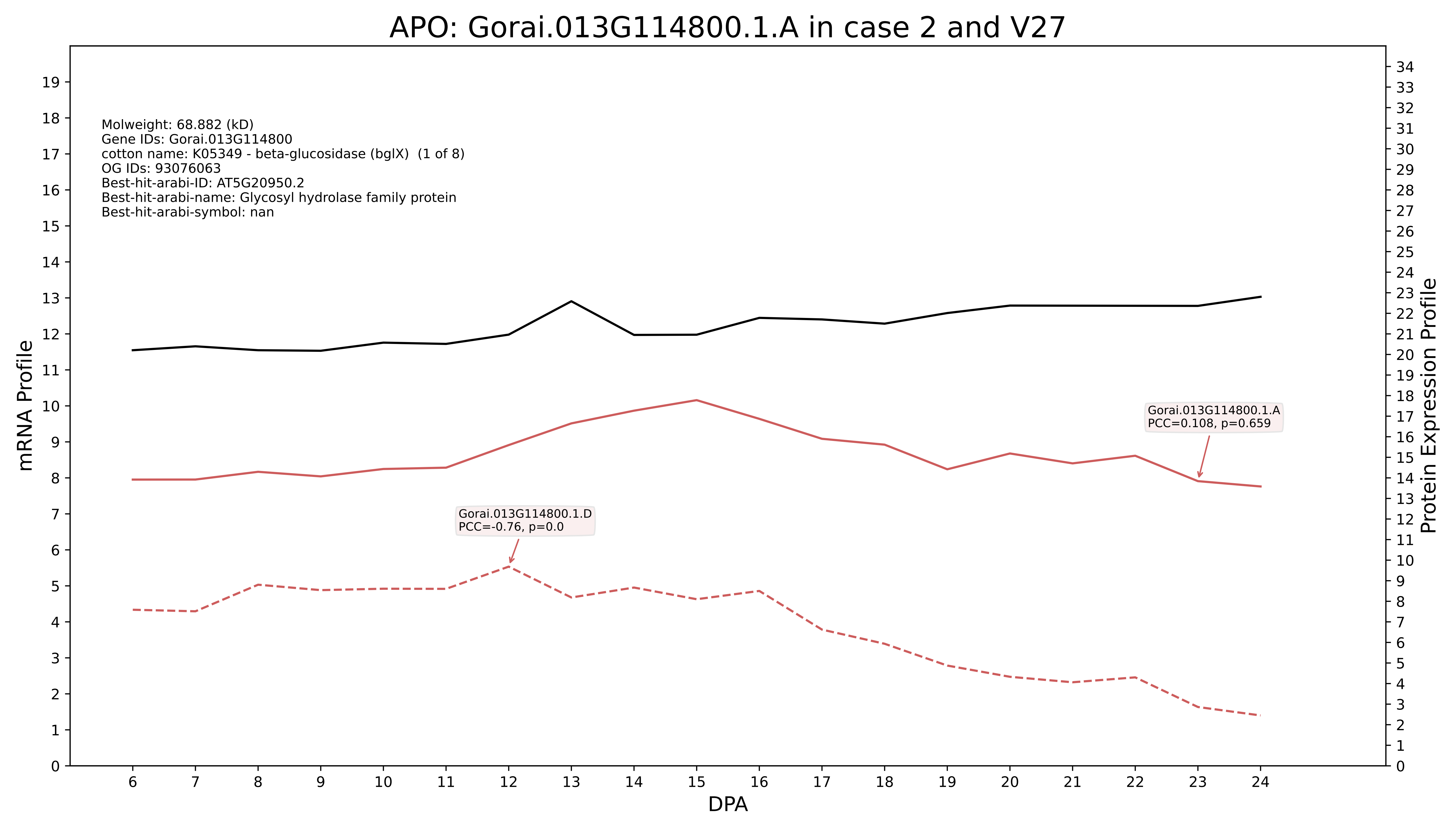

APO: Gorai.013G114800.1.A in case 2 and V27
Molweight: 68.882 (kD)
Gene IDs: Gorai.013G114800
cotton name: K05349 - beta-glucosidase (bglX) (1 of 8)
OG IDs: 93076063
Best-hit-arabi-ID: AT5G20950.2
Best-hit-arabi-name: Glycosyl hydrolase family protein
Best-hit-arabi-symbol: nan
Gorai.013G114800.1.A
PCC=0.108, p=0.659
Gorai.013G114800.1.D
PCC=-0.76, p=0.0
mRNA Profile
Protein Expression Profile
DPA

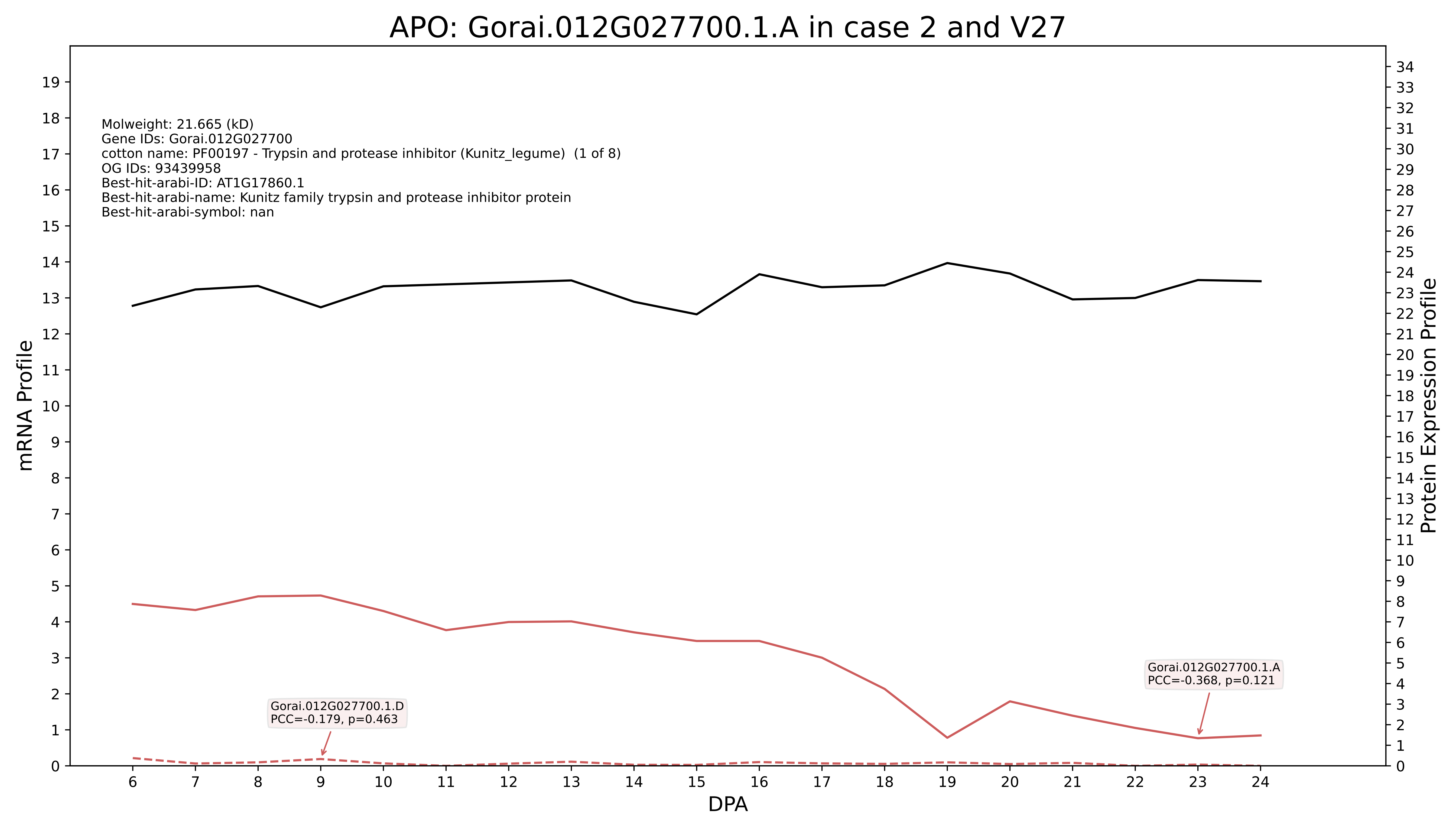
APO: Gorai.012G027700.1.A in case 2 and V27
Molweight: 21.665 (kD)
Gene IDs: Gorai.012G027700
cotton name: PF00197 - Trypsin and protease inhibitor (Kunitz_legume) (1 of 8)
OG IDs: 93439958
Best-hit-arabi-ID: AT1G17860.1
Best-hit-arabi-name: Kunitz family trypsin and protease inhibitor protein
Best-hit-arabi-symbol: nan
Gorai.012G027700.1.D
PCC=-0.179, p=0.463
Gorai.012G027700.1.A
PCC=-0.368, p=0.121
mRNA Profile
Protein Expression Profile
DPA

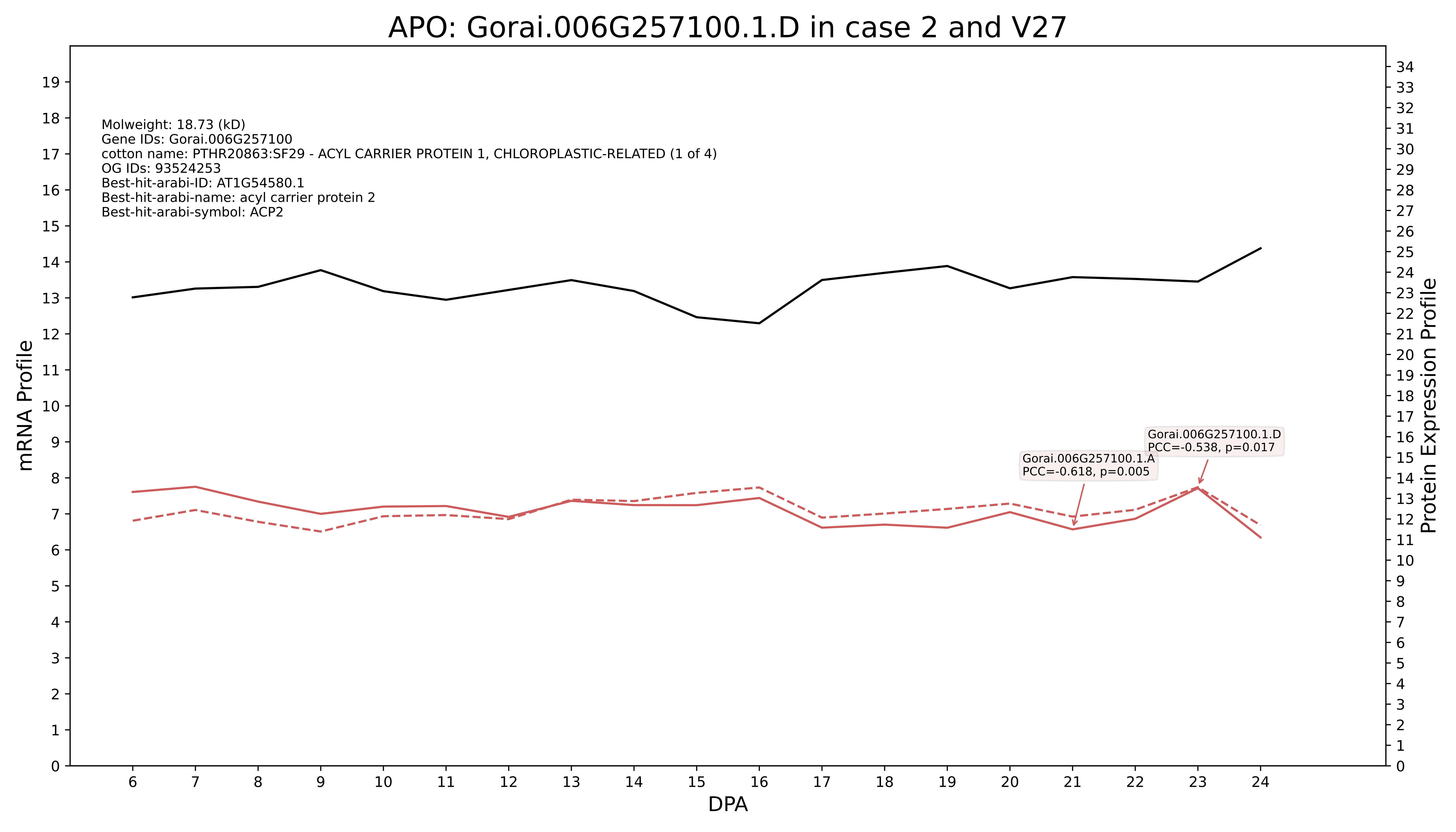
APO: Gorai.006G257100.1.D in case 2 and V27
Molweight: 18.73 (kD)
Gene IDs: Gorai.006G257100
cotton name: PTHR20863:SF29 - ACYL CARRIER PROTEIN 1, CHLOROPLASTIC-RELATED (1 of 4)
OG IDs: 93524253
Best-hit-arabi-ID: AT1G54580.1
Best-hit-arabi-name: acyl carrier protein 2
Best-hit-arabi-symbol: ACP2
Gorai.006G257100.1.A
PCC=-0.618, p=0.005
Gorai.006G257100.1.D
PCC=-0.538, p=0.017
mRNA Profile
Protein Expression Profile
DPA
0
1
2
3
4
5
6
7
8
9
10
11
12
13
14
15
16
17
18
19
20
21
22
23
24
25
26
27
28
29
30
31
32
33
34

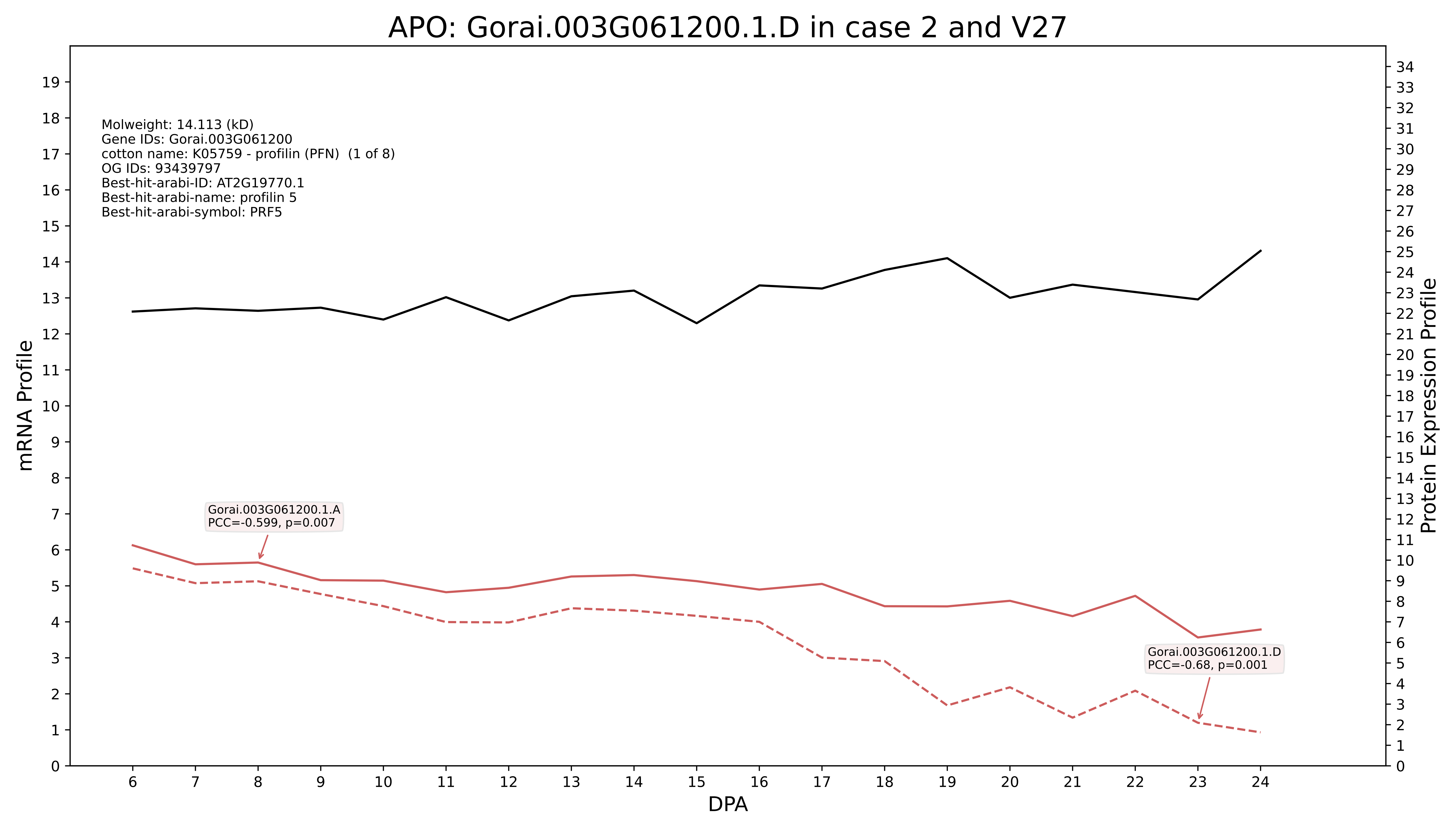
APO: Gorai.003G061200.1.D in case 2 and V27
Molweight: 14.113 (kD)
Gene IDs: Gorai.003G061200
cotton name: K05759 - profilin (PFN) (1 of 8)
OG IDs: 93439797
Best-hit-arabi-ID: AT2G19770.1
Best-hit-arabi-name: profilin 5
Best-hit-arabi-symbol: PRF5
Gorai.003G061200.1.A
PCC=-0.599, p=0.007
Gorai.003G061200.1.D
PCC=-0.68, p=0.001
mRNA Profile
Protein Expression Profile
DPA

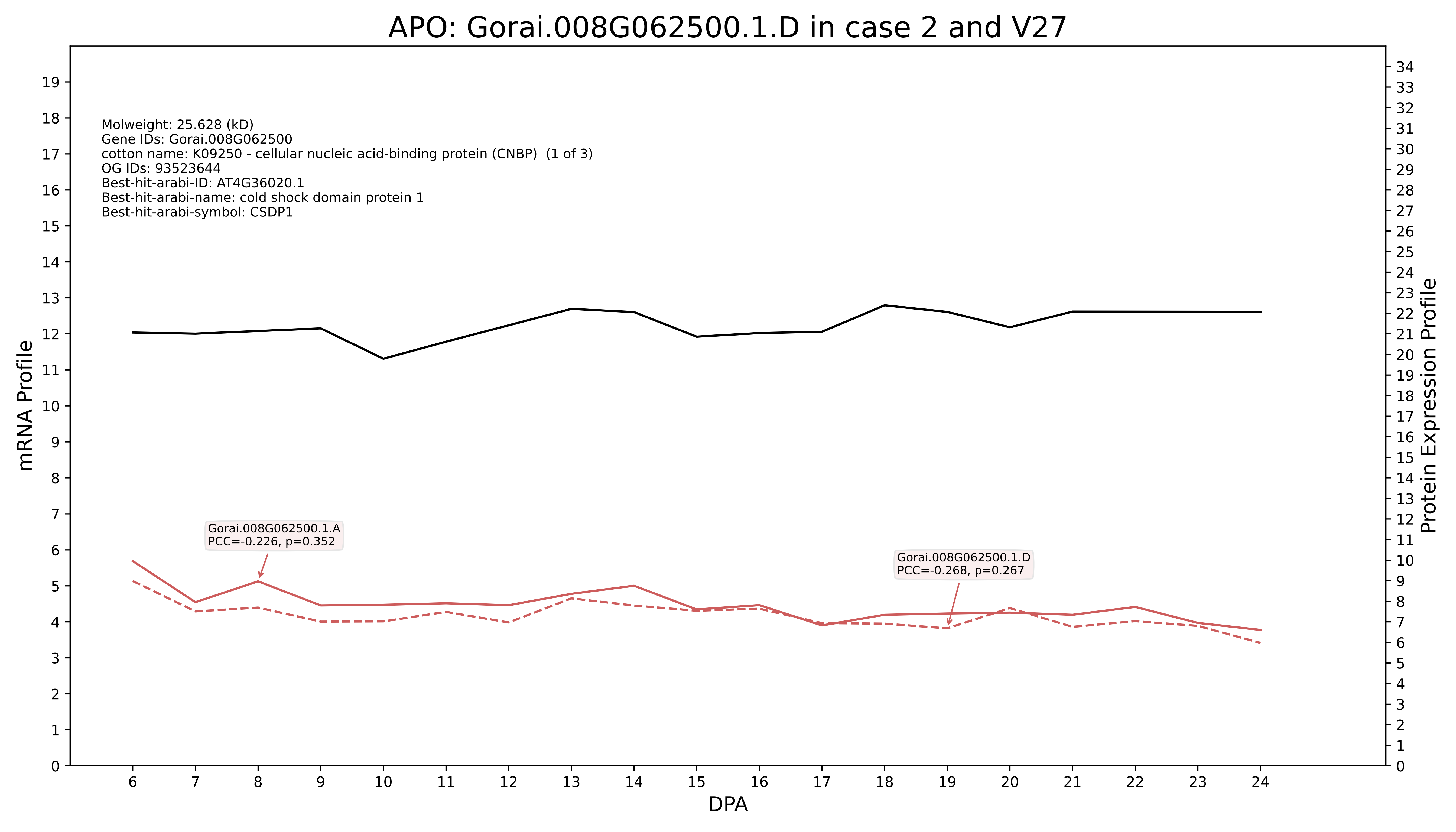
APO: Gorai.008G062500.1.D in case 2 and V27
Molweight: 25.628 (kD)
Gene IDs: Gorai.008G062500
cotton name: K09250 - cellular nucleic acid-binding protein (CNBP) (1 of 3)
OG IDs: 93523644
Best-hit-arabi-ID: AT4G36020.1
Best-hit-arabi-name: cold shock domain protein 1
Best-hit-arabi-symbol: CSDP1
Gorai.008G062500.1.A
PCC=-0.226, p=0.352
Gorai.008G062500.1.D
PCC=-0.268, p=0.267
mRNA Profile
Protein Expression Profile
DPA

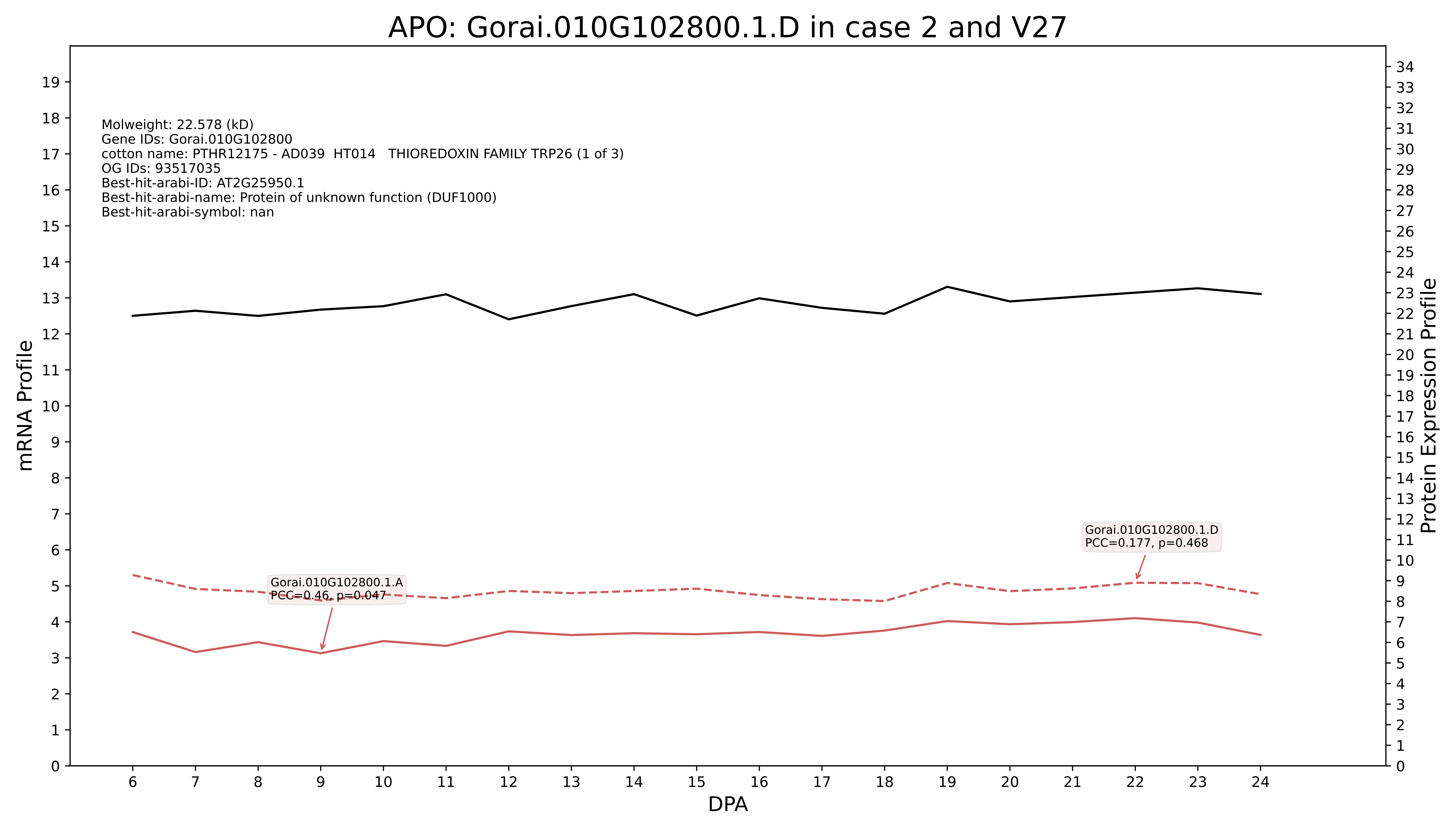
APO: Gorai.010G102800.1.D in case 2 and V27
Molweight: 22.578 (kD)
Gene IDs: Gorai.010G102800
cotton name: PTHR12175 - AD039 HT014 THIOREDOXIN FAMILY TRP26 (1 of 3)
OG IDs: 93517035
Best-hit-arabi-ID: AT2G25950.1
Best-hit-arabi-name: Protein of unknown function (DUF1000)
Best-hit-arabi-symbol: nan
Gorai.010G102800.1.A
PCC=0.46, p=0.047
Gorai.010G102800.1.D
PCC=0.177, p=0.468
mRNA Profile
Protein Expression Profile
DPA

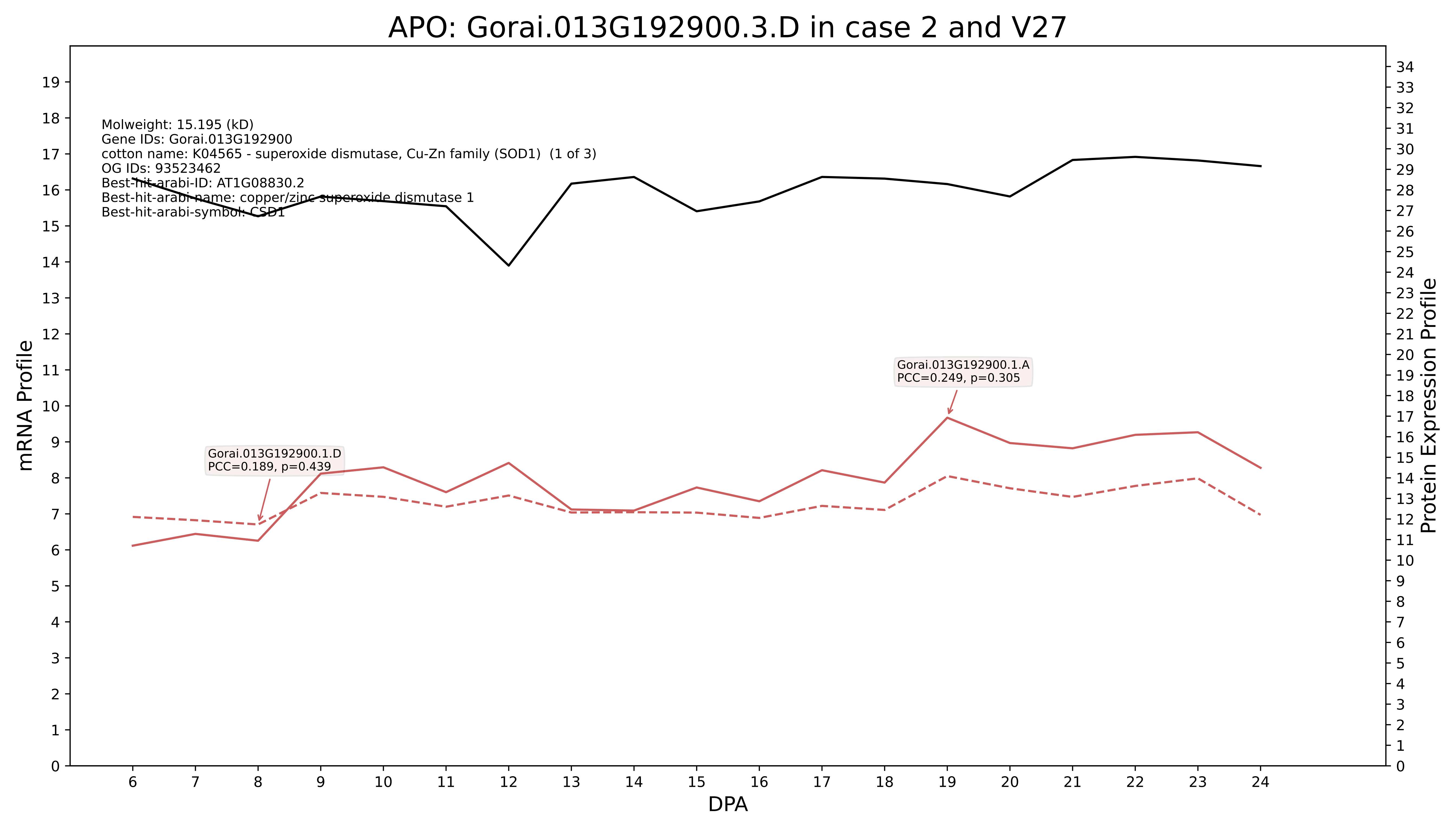

APO: Gorai.013G192900.3.D in case 2 and V27
Molweight: 15.195 (kD)
Gene IDs: Gorai.013G192900
cotton name: K04565 - superoxide dismutase, Cu-Zn family (SOD1) (1 of 3)
OG IDs: 93523462
Best-hit-arabi-ID: AT1G08830.2
Best-hit-arabi-name: copper/zinc superoxide dismutase 1
Best-hit-arabi-symbol: CSD1
Gorai.013G192900.1.A
PCC=0.249, p=0.305
Gorai.013G192900.1.D
PCC=0.189, p=0.439
mRNA Profile
Protein Expression Profile
DPA

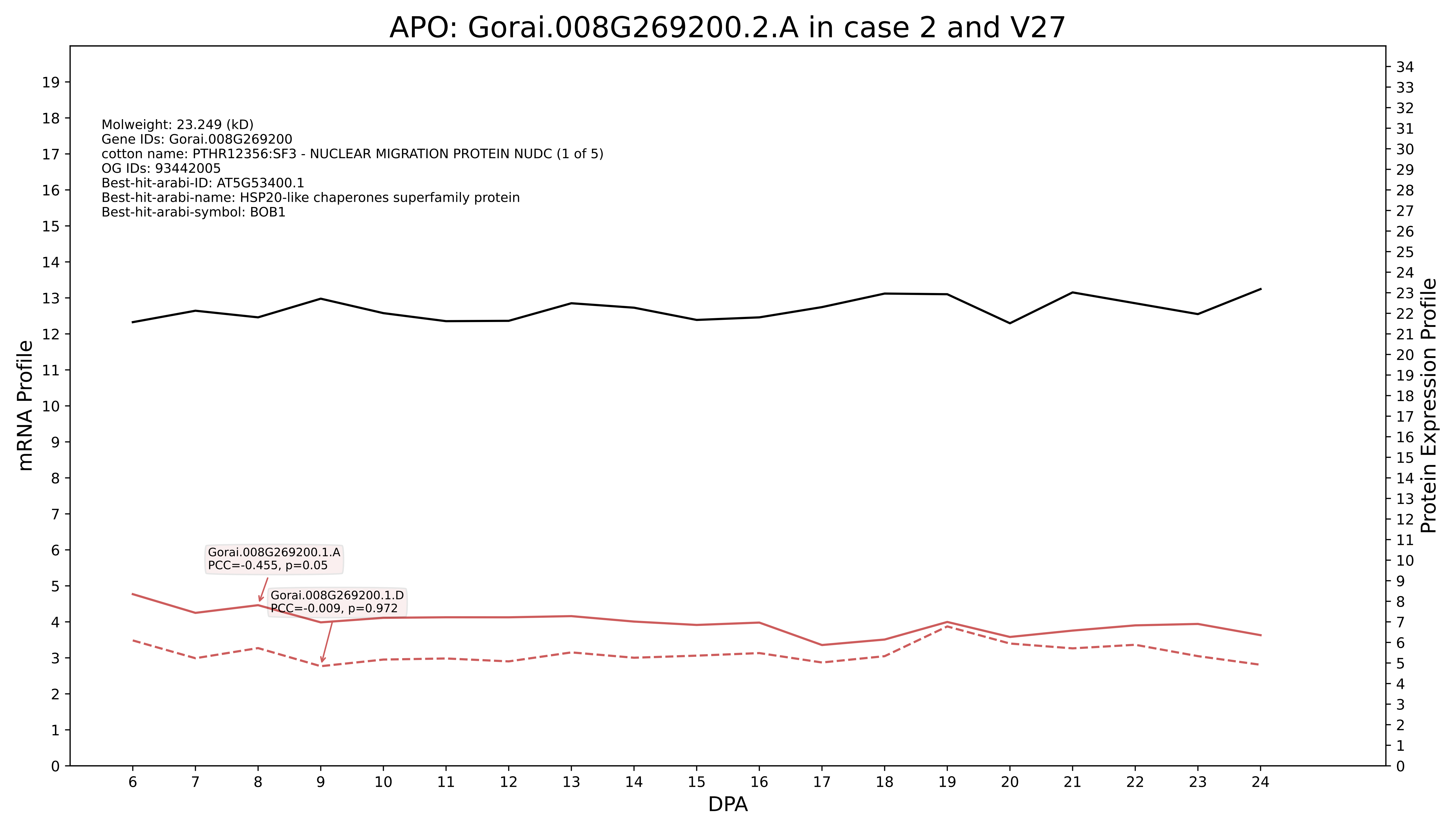

APO: Gorai.008G269200.2.A in case 2 and V27
Molweight: 23.249 (kD)
Gene IDs: Gorai.008G269200
cotton name: PTHR12356:SF3 - NUCLEAR MIGRATION PROTEIN NUDC (1 of 5)
OG IDs: 93442005
Best-hit-arabi-ID: AT5G53400.1
Best-hit-arabi-name: HSP20-like chaperones superfamily protein
Best-hit-arabi-symbol: BOB1
Gorai.008G269200.1.A
PCC=-0.455, p=0.05
Gorai.008G269200.1.D
PCC=-0.009, p=0.972
mRNA Profile
Protein Expression Profile
DPA

APO: Gorai.013G195300.1.D in case 2 and V27

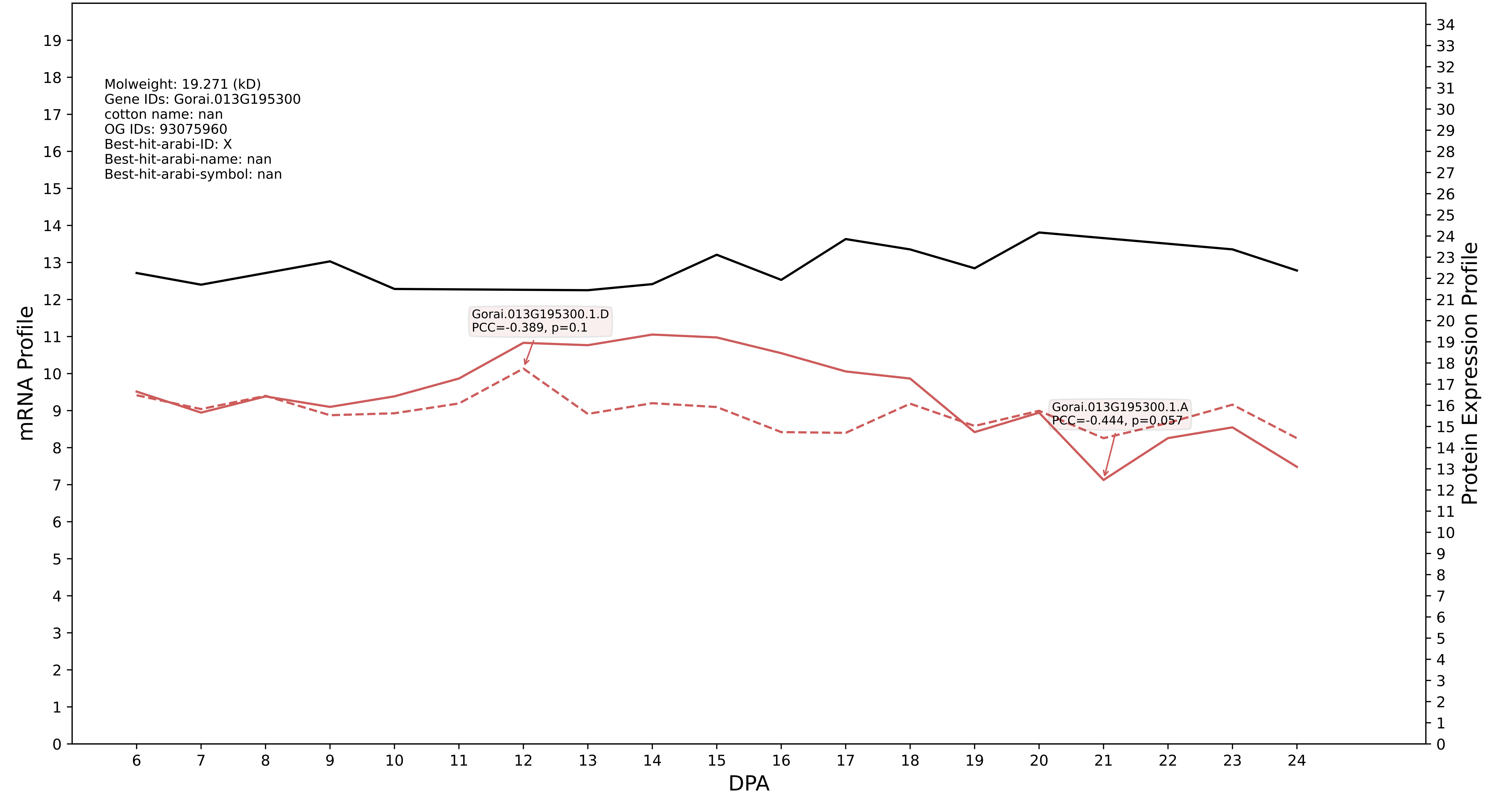

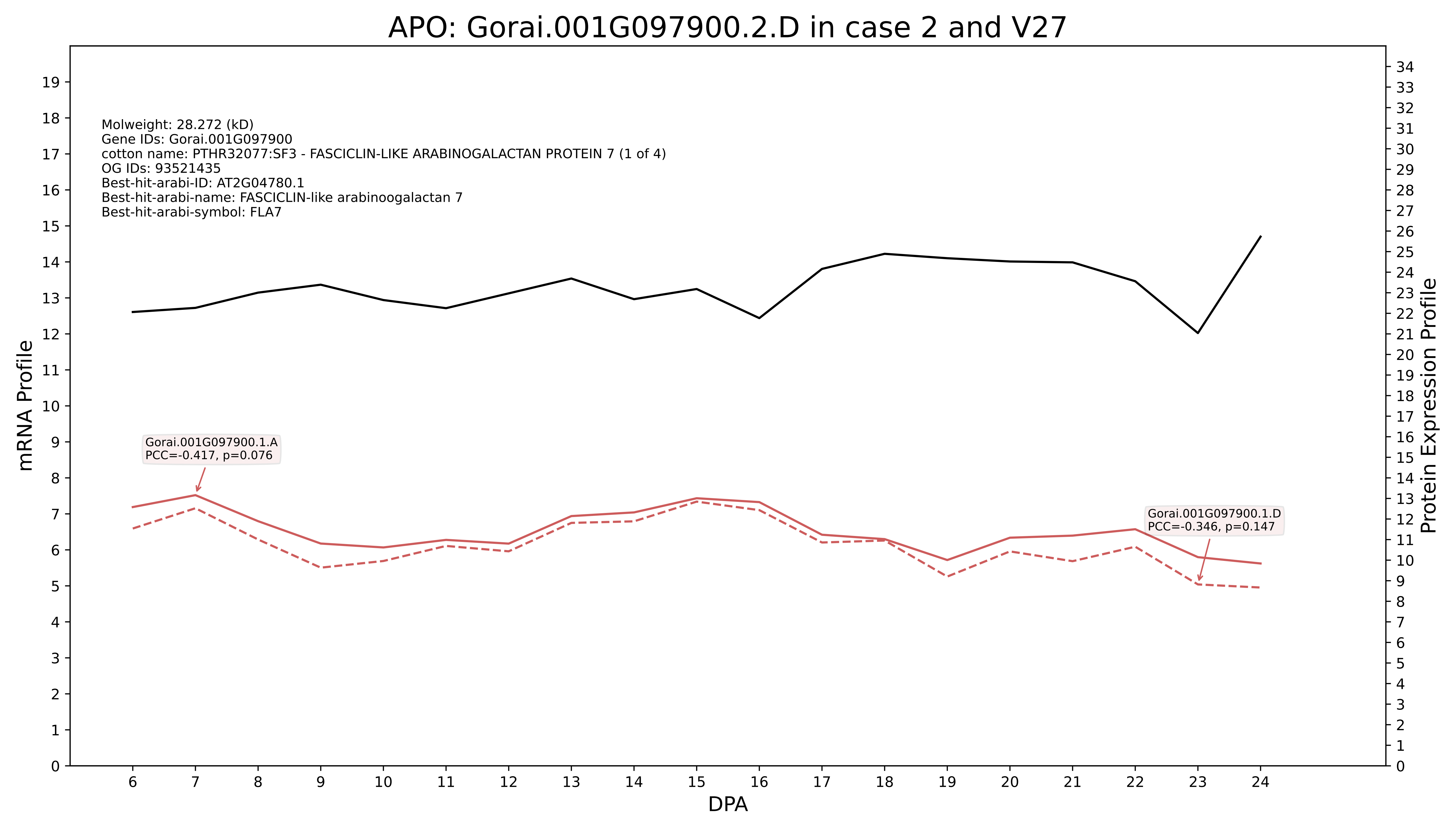
APO: Gorai.001G097900.2.D in case 2 and V27
Molweight: 28.272 (kD)
Gene IDs: Gorai.001G097900
cotton name: PTHR32077:SF3 - FASCICLIN-LIKE ARABINOGALACTAN PROTEIN 7 (1 of 4)
OG IDs: 93521435
Best-hit-arabi-ID: AT2G04780.1
Best-hit-arabi-name: FASCICLIN-like arabinoogalactan 7
Best-hit-arabi-symbol: FLA7
Gorai.001G097900.1.A
PCC=-0.417, p=0.076
Gorai.001G097900.1.D
PCC=-0.346, p=0.147
mRNA Profile
Protein Expression Profile
DPA
0
1
2
3
4
5
6
7
8
9
10
11
12
13
14
15
16
17
18
19
20
21
22
23
24
25
26
27
28
29
30
31
32
33
34

APO: Gorai.004G058800.1.D in case 2 and V27

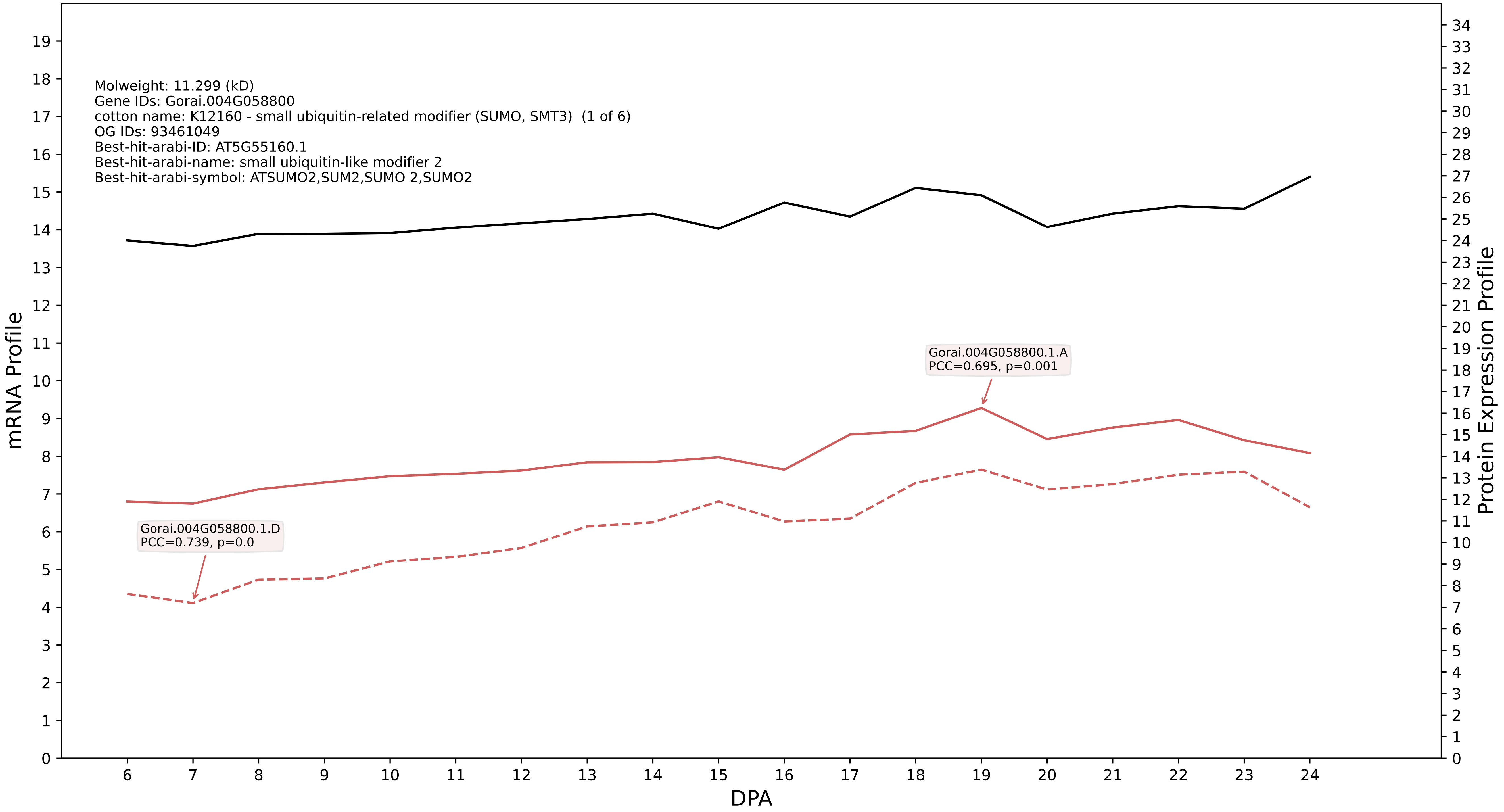

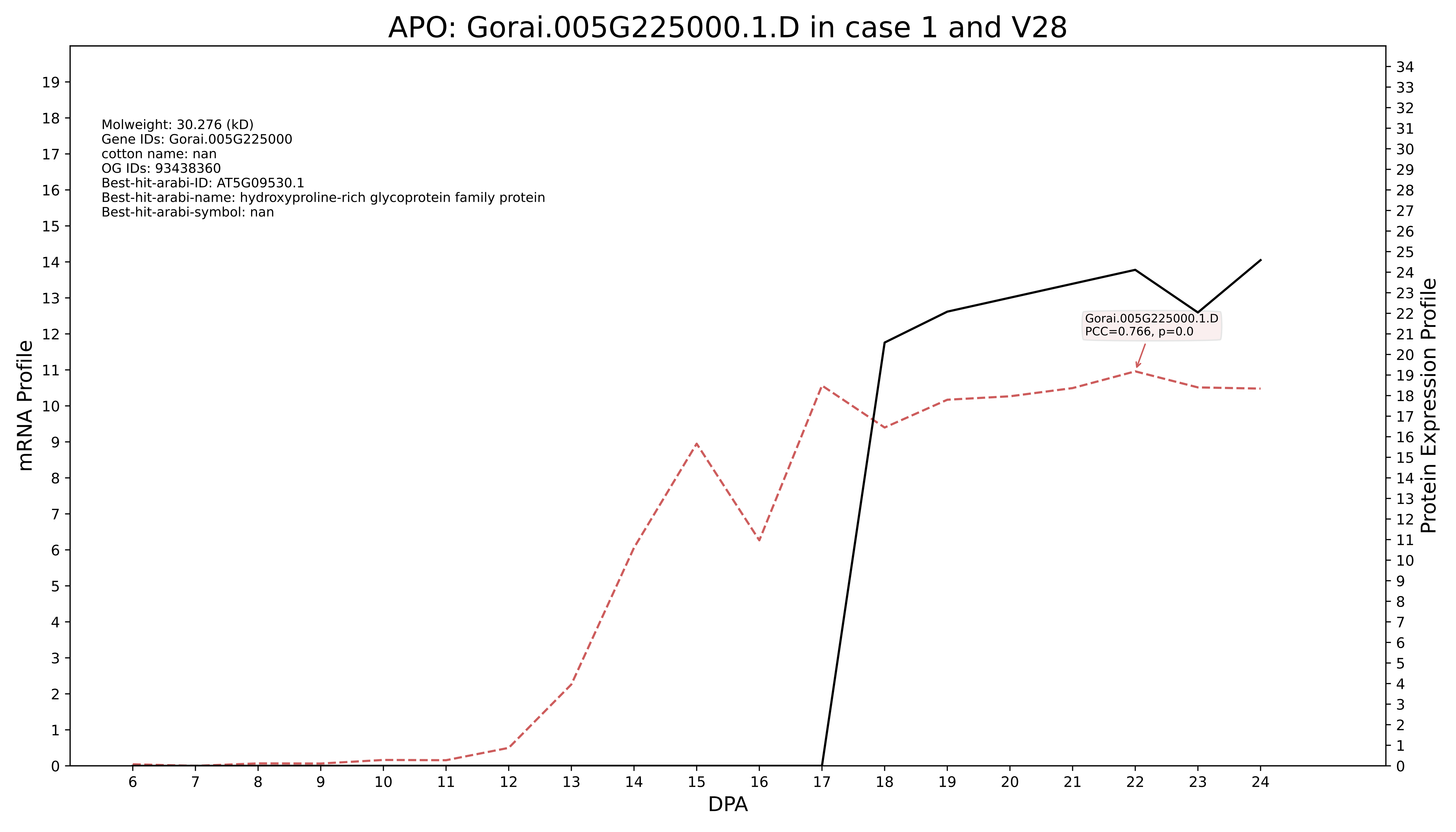

APO: Gorai.005G225000.1.D in case 1 and V28
Molweight: 30.276 (kD)
Gene IDs: Gorai.005G225000
cotton name: nan
OG IDs: 93438360
Best-hit-arabi-ID: AT5G09530.1
Best-hit-arabi-name: hydroxyproline-rich glycoprotein family protein
Best-hit-arabi-symbol: nan
Gorai.005G225000.1.D
PCC=0.766, p=0.0
mRNA Profile
Protein Expression Profile
DPA

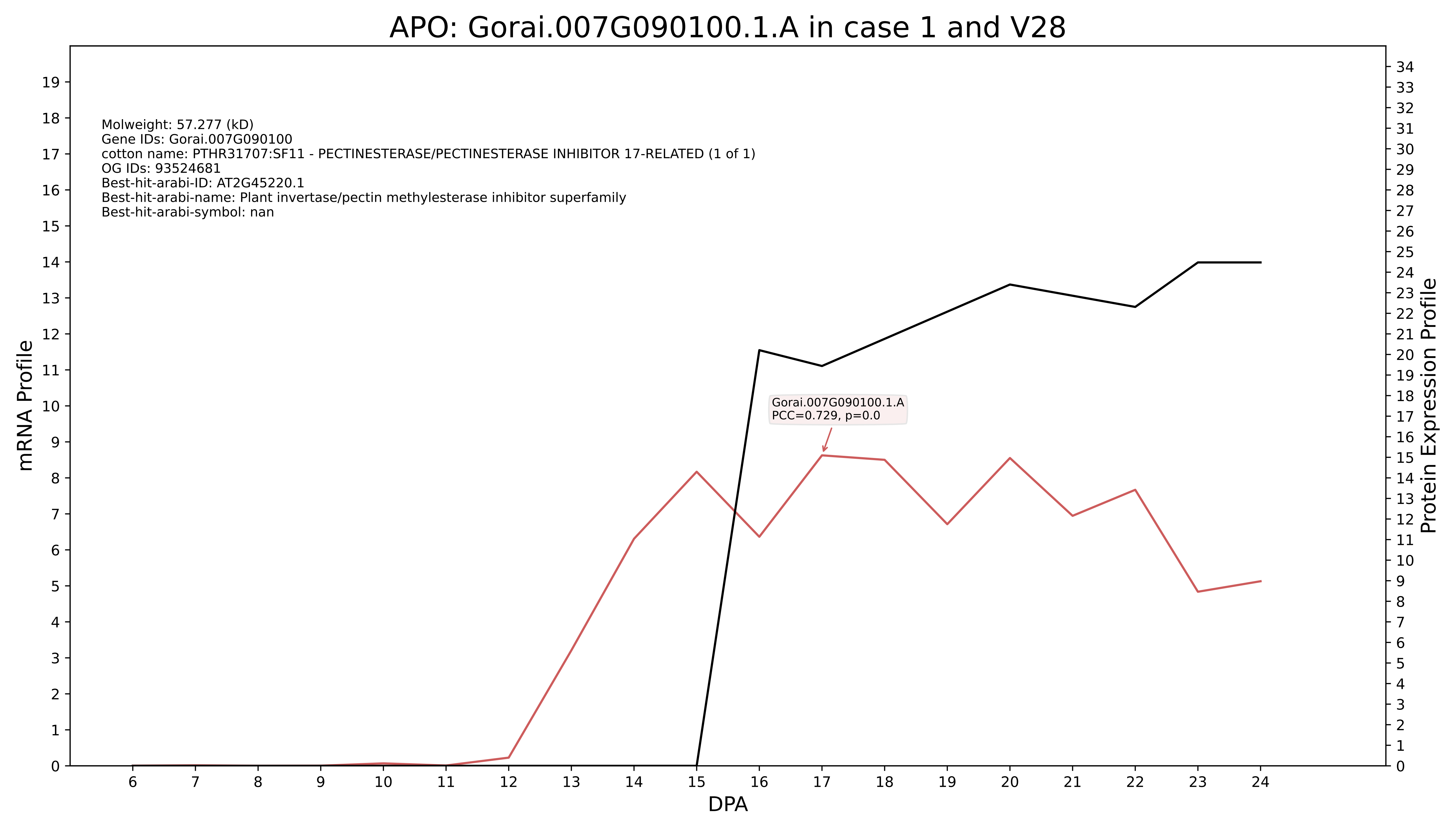

APO: Gorai.007G090100.1.A in case 1 and V28
Molweight: 57.277 (kD)
Gene IDs: Gorai.007G090100
cotton name: PTHR31707:SF11 - PECTINESTERASE/PECTINESTERASE INHIBITOR 17-RELATED (1 of 1)
OG IDs: 93524681
Best-hit-arabi-ID: AT2G45220.1
Best-hit-arabi-name: Plant invertase/pectin methylesterase inhibitor superfamily
Best-hit-arabi-symbol: nan
Gorai.007G090100.1.A
PCC=0.729, p=0.0
mRNA Profile
Protein Expression Profile
DPA

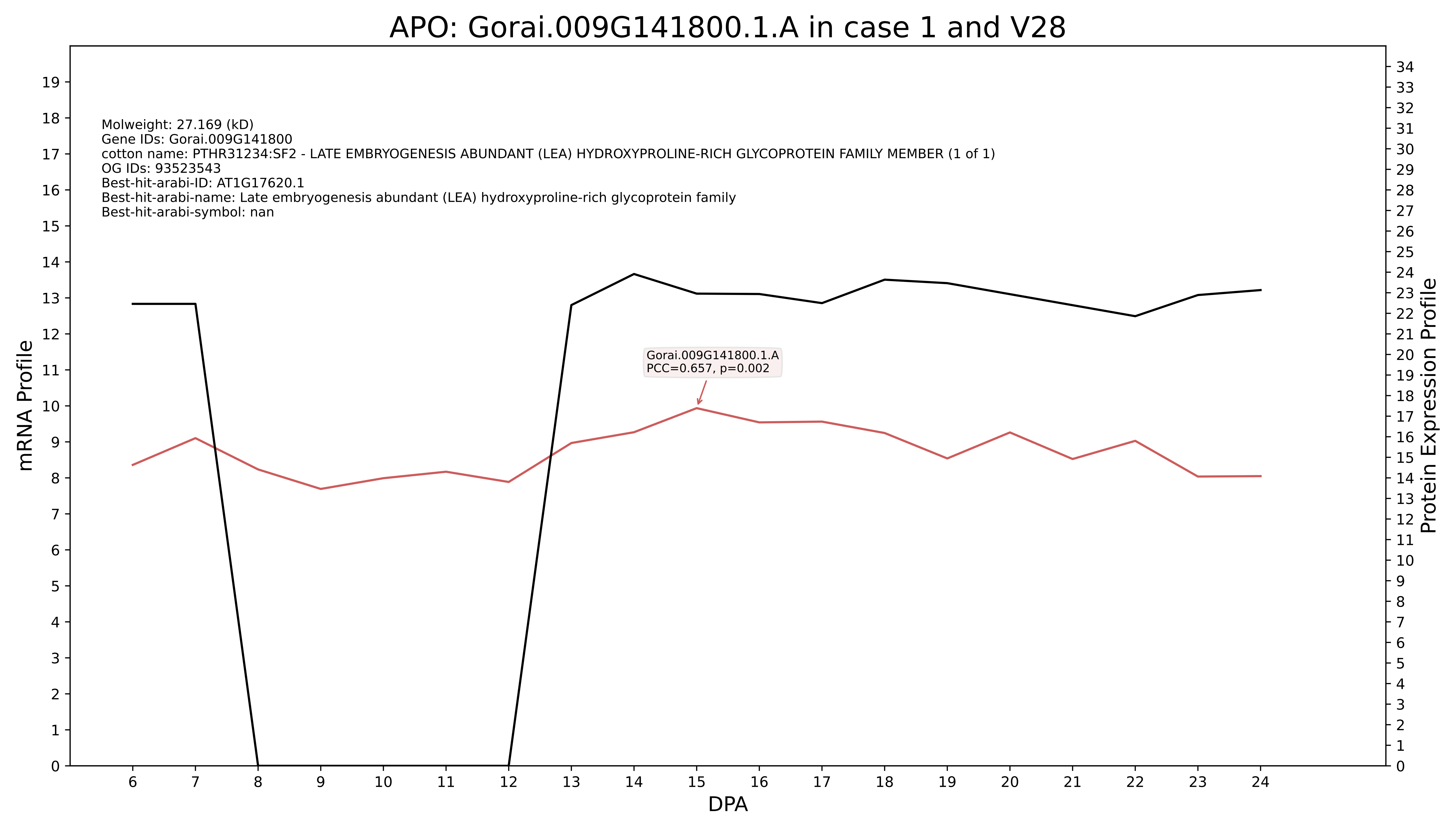
APO: Gorai.009G141800.1.A in case 1 and V28
Molweight: 27.169 (kD)
Gene IDs: Gorai.009G141800
cotton name: PTHR31234:SF2 - LATE EMBRYOGENESIS ABUNDANT (LEA) HYDROXYPROLINE-RICH GLYCOPROTEIN FAMILY MEMBER (1 of 1)
OG IDs: 93523543
Best-hit-arabi-ID: AT1G17620.1
Best-hit-arabi-name: Late embryogenesis abundant (LEA) hydroxyproline-rich glycoprotein family
Best-hit-arabi-symbol: nan
Gorai.009G141800.1.A
PCC=0.657, p=0.002
mRNA Profile
Protein Expression Profile
DPA
0
1
2
3
4
5
6
7
8
9
10
11
12
13
14
15
16
17
18
19
20
21
22
23
24
25
26
27
28
29
30
31
32
33
34

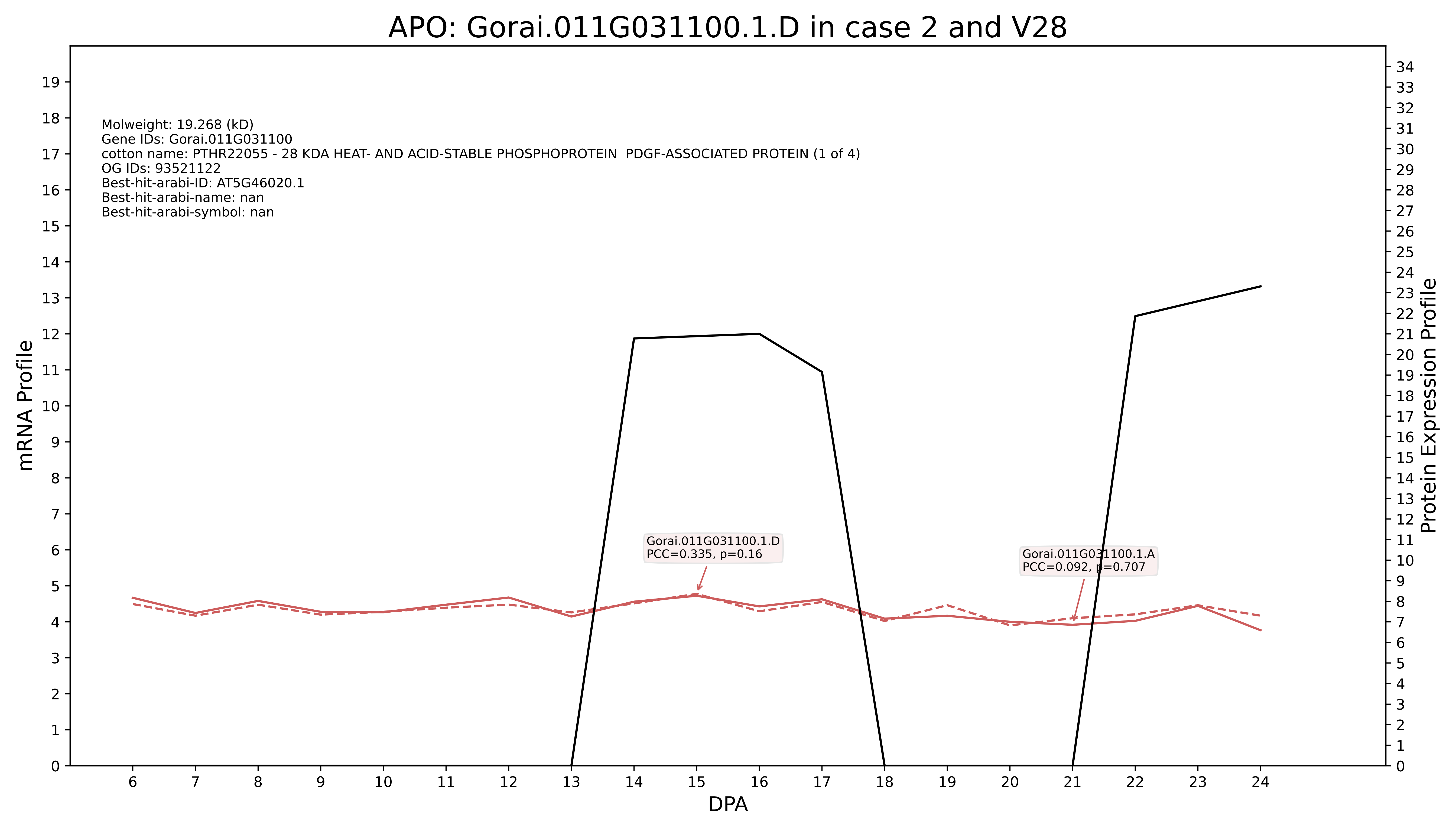

APO: Gorai.011G031100.1.D in case 2 and V28
Molweight: 19.268 (kD)
Gene IDs: Gorai.011G031100
cotton name: PTHR22055 - 28 KDA HEAT- AND ACID-STABLE PHOSPHOPROTEIN PDGF-ASSOCIATED PROTEIN (1 of 4)
OG IDs: 93521122
Best-hit-arabi-ID: AT5G46020.1
Best-hit-arabi-name: nan
Best-hit-arabi-symbol: nan
Gorai.011G031100.1.D
PCC=0.335, p=0.16
Gorai.011G031100.1.A
PCC=0.092, p=0.707
mRNA Profile
Protein Expression Profile
DPA

Supplementary Figure S11. Profiles of paired mRNAs and proteins (P200).

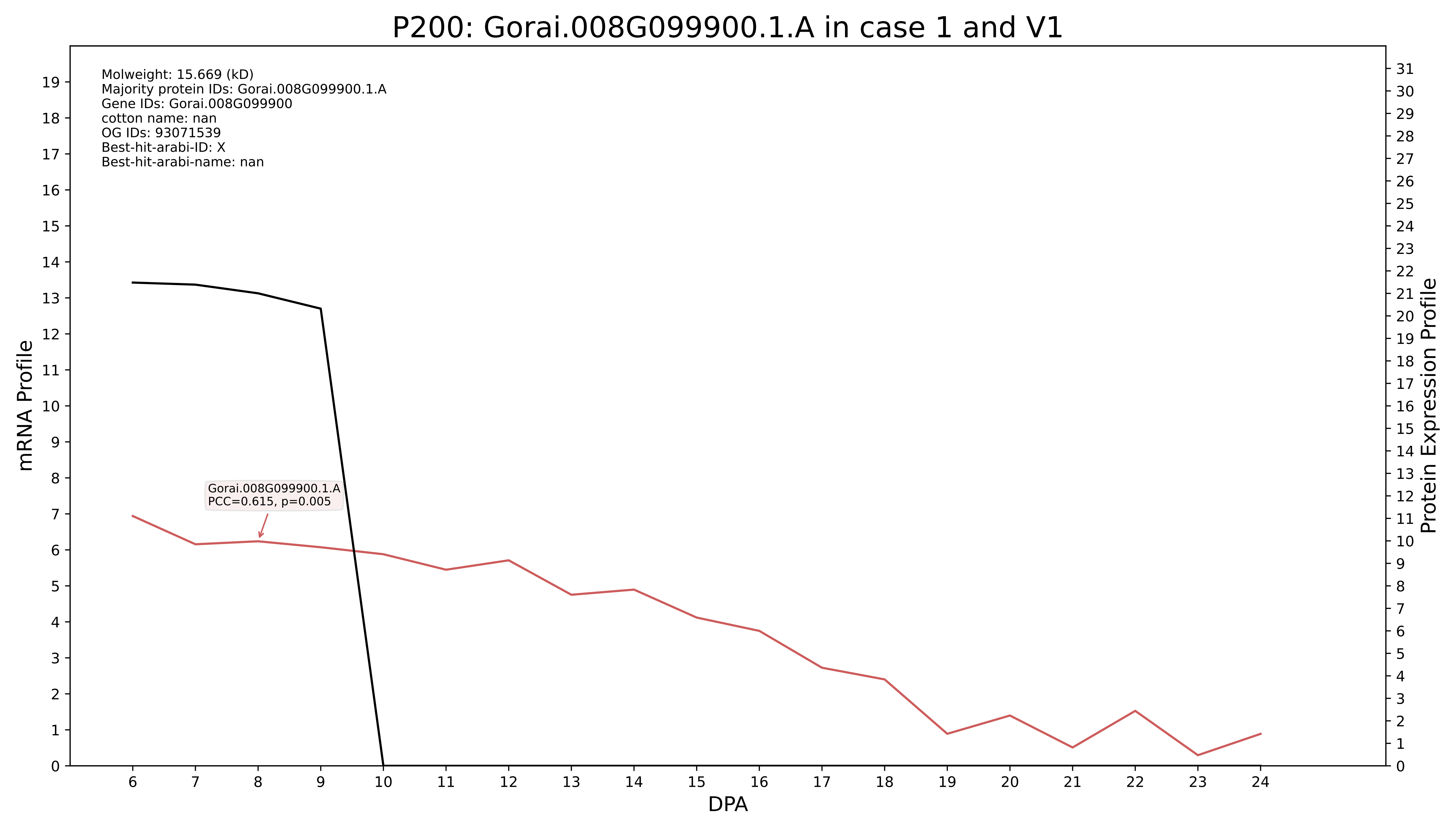
P200: Gorai.008G099900.1.A in case 1 and V1
Molweight: 15.669 (kD)
Majority protein IDs: Gorai.008G099900.1.A
Gene IDs: Gorai.008G099900
cotton name: nan
OG IDs: 93071539
Best-hit-arabi-ID: X
Best-hit-arabi-name: nan
Gorai.008G099900.1.A
PCC=0.615, p=0.005
mRNA Profile
Protein Expression Profile
DPA

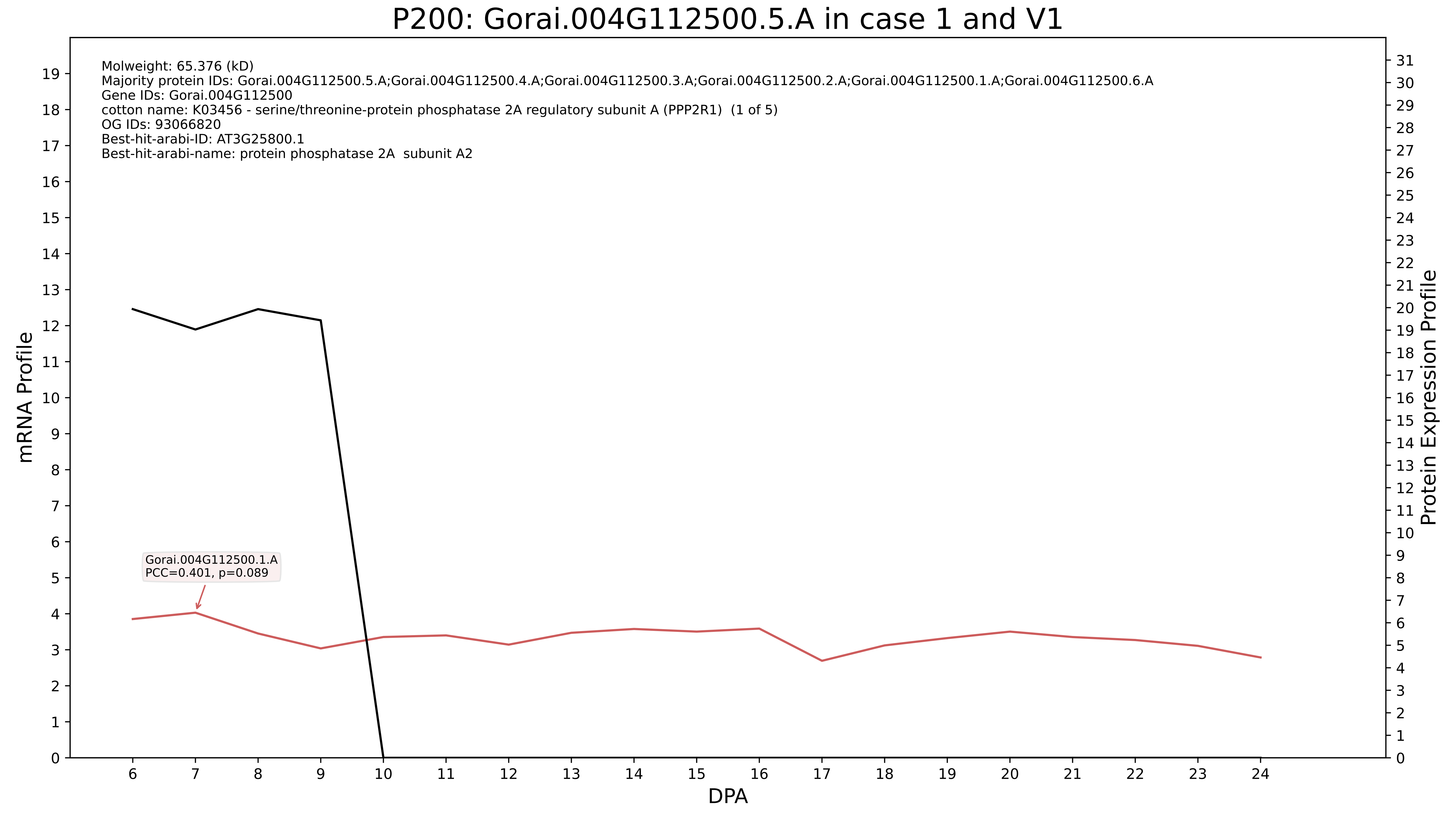

P200: Gorai.004G112500.5.A in case 1 and V1
Molweight: 65.376 (kD)
Majority protein IDs: Gorai.004G112500.5.A;Gorai.004G112500.4.A;Gorai.004G112500.3.A;Gorai.004G112500.2.A;Gorai.004G112500.1.A;Gorai.004G112500.6.A
Gene IDs: Gorai.004G112500
cotton name: K03456 - serine/threonine-protein phosphatase 2A regulatory subunit A (PPP2R1) (1 of 5)
OG IDs: 93066820
Best-hit-arabi-ID: AT3G25800.1
Best-hit-arabi-name: protein phosphatase 2A subunit A2
Gorai.004G112500.1.A
PCC=0.401, p=0.089
mRNA Profile
Protein Expression Profile
DPA

P200: Gorai.006G252900.3.A in case 1 and V1

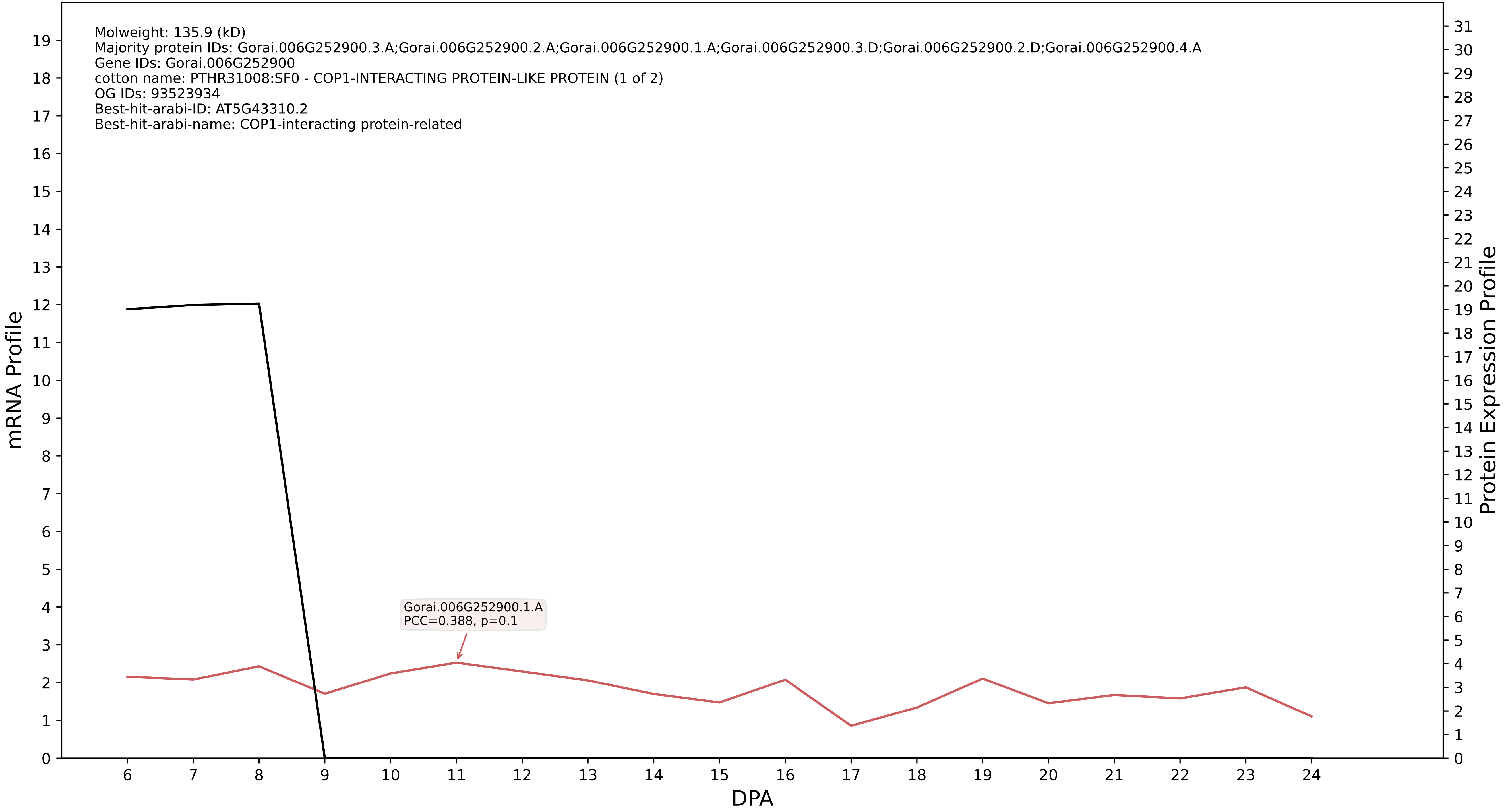

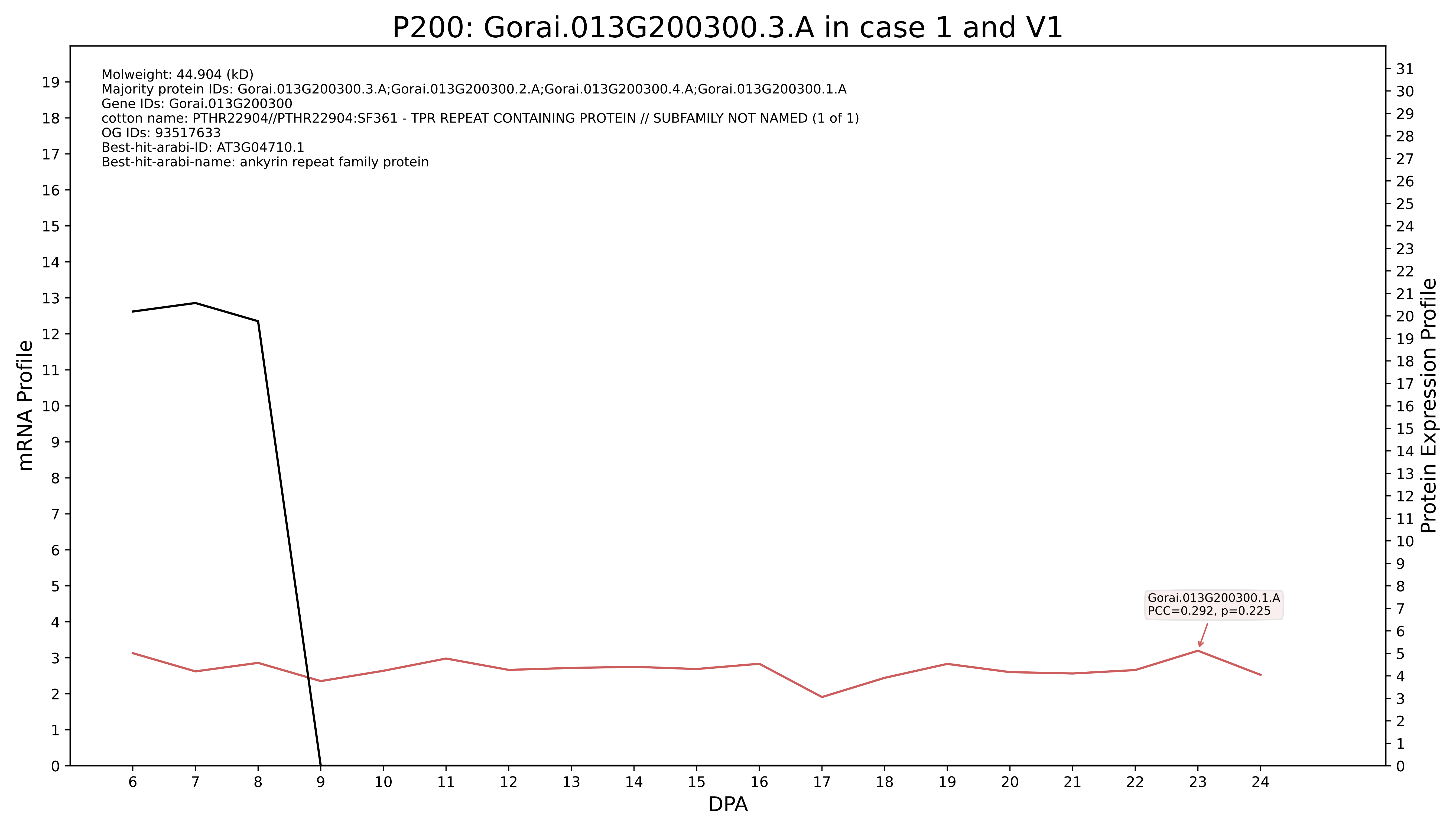
P200: Gorai.013G200300.3.A in case 1 and V1
Molweight: 44.904 (kD)
Majority protein IDs: Gorai.013G200300.3.A;Gorai.013G200300.2.A;Gorai.013G200300.4.A;Gorai.013G200300.1.A
Gene IDs: Gorai.013G200300
cotton name: PTHR22904//PTHR22904:SF361 - TPR REPEAT CONTAINING PROTEIN // SUBFAMILY NOT NAMED (1 of 1)
OG IDs: 93517633
Best-hit-arabi-ID: AT3G04710.1
Best-hit-arabi-name: ankyrin repeat family protein
Gorai.013G200300.1.A
PCC=0.292, p=0.225
mRNA Profile
Protein Expression Profile
DPA

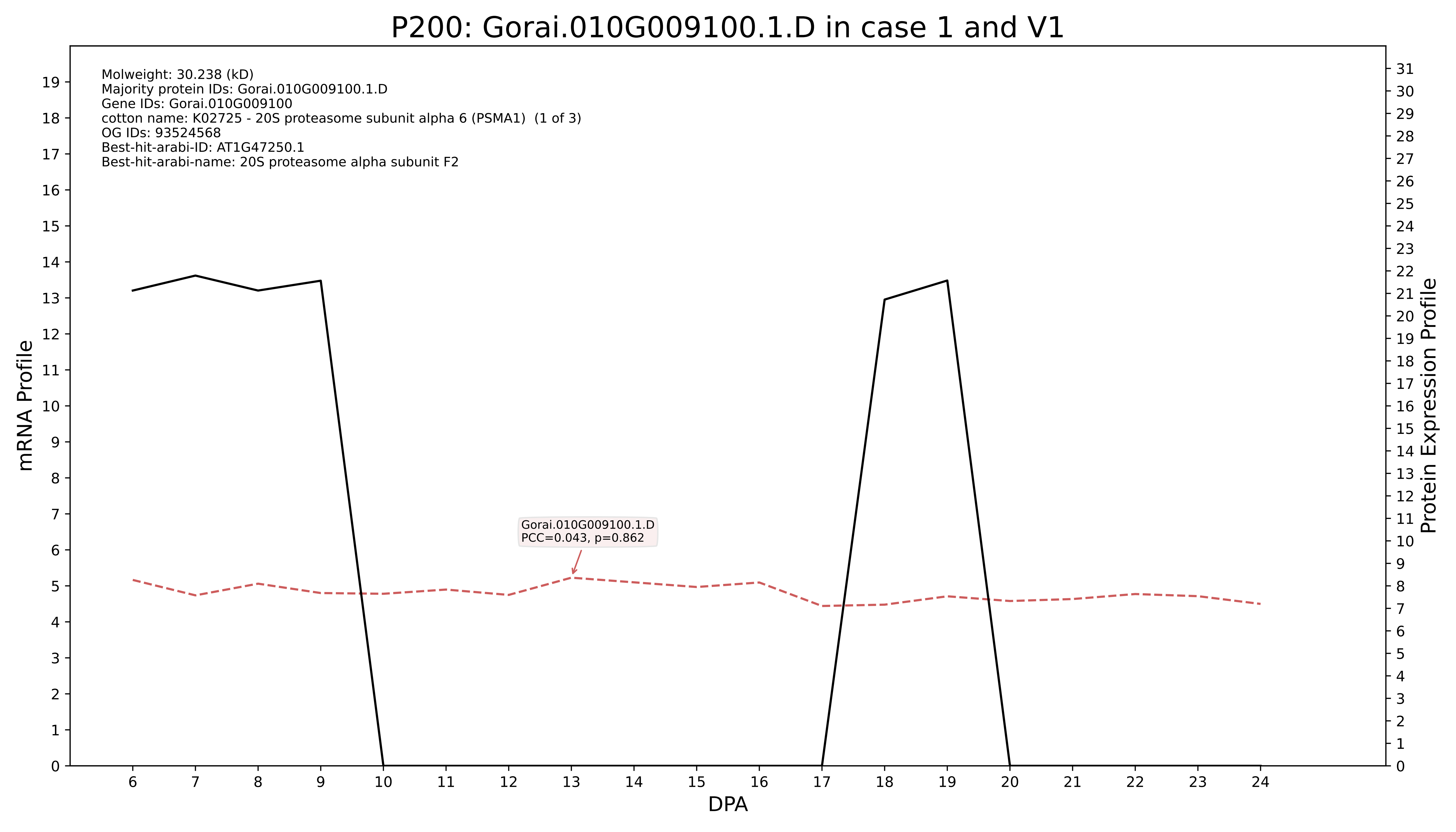
P200: Gorai.010G009100.1.D in case 1 and V1
Molweight: 30.238 (kD)
Majority protein IDs: Gorai.010G009100.1.D
Gene IDs: Gorai.010G009100
cotton name: K02725 - 20S proteasome subunit alpha 6 (PSMA1) (1 of 3)
OG IDs: 93524568
Best-hit-arabi-ID: AT1G47250.1
Best-hit-arabi-name: 20S proteasome alpha subunit F2
Gorai.010G009100.1.D
PCC=0.043, p=0.862
mRNA Profile
Protein Expression Profile
DPA

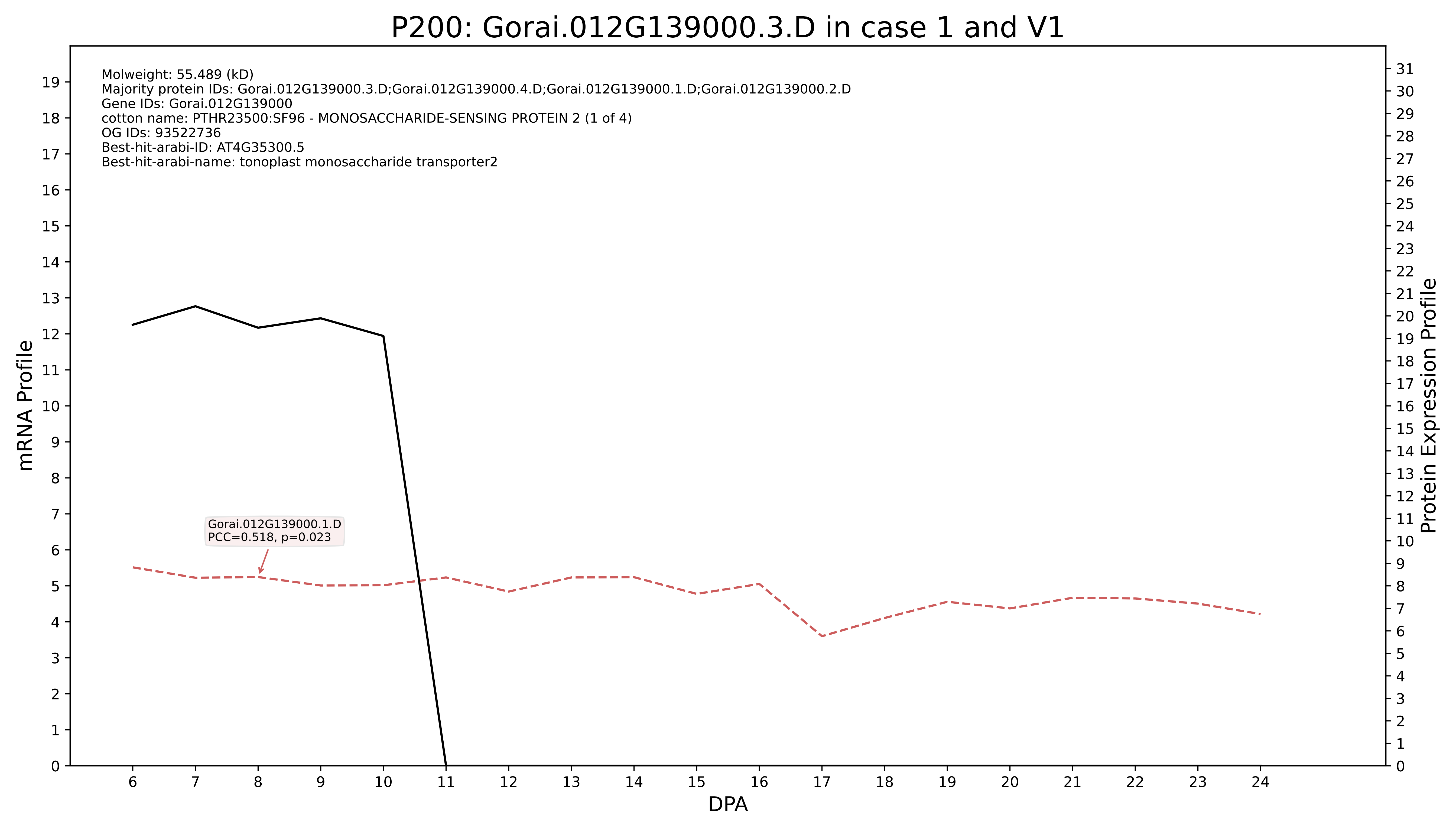
P200: Gorai.012G139000.3.D in case 1 and V1
Molweight: 55.489 (kD)
Majority protein IDs: Gorai.012G139000.3.D;Gorai.012G139000.4.D;Gorai.012G139000.1.D;Gorai.012G139000.2.D
Gene IDs: Gorai.012G139000
cotton name: PTHR23500:SF96 - MONOSACCHARIDE-SENSING PROTEIN 2 (1 of 4)
OG IDs: 93522736
Best-hit-arabi-ID: AT4G35300.5
Best-hit-arabi-name: tonoplast monosaccharide transporter2
Gorai.012G139000.1.D
PCC=0.518, p=0.023
mRNA Profile
Protein Expression Profile
DPA

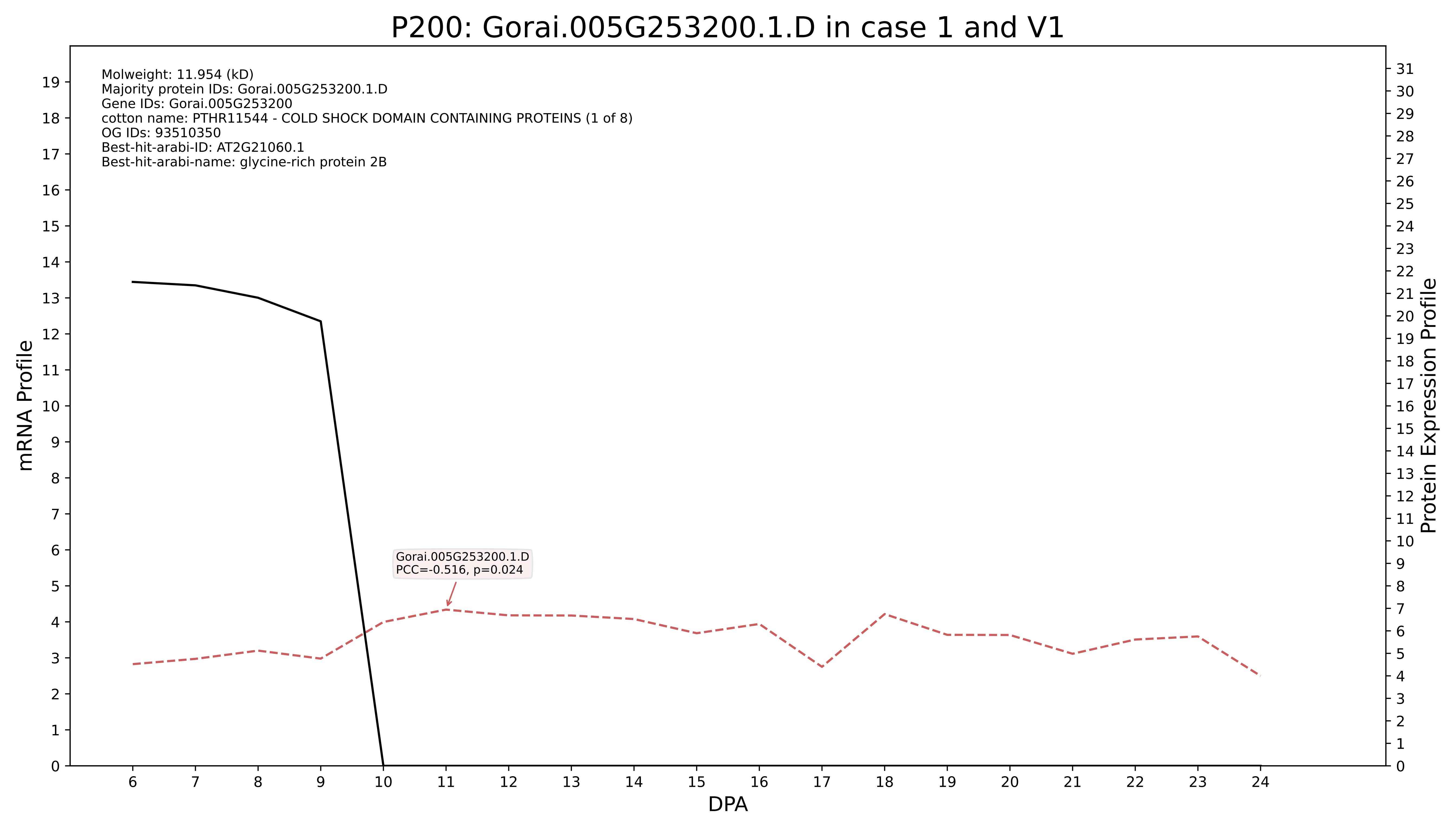
P200: Gorai.005G253200.1.D in case 1 and V1
Molweight: 11.954 (kD)
Majority protein IDs: Gorai.005G253200.1.D
Gene IDs: Gorai.005G253200
cotton name: PTHR11544 - COLD SHOCK DOMAIN CONTAINING PROTEINS (1 of 8)
OG IDs: 93510350
Best-hit-arabi-ID: AT2G21060.1
Best-hit-arabi-name: glycine-rich protein 2B
Gorai.005G253200.1.D
PCC=-0.516, p=0.024
mRNA Profile
Protein Expression Profile
DPA

P200: Gorai.002G245700.2.A in case 1 and V1

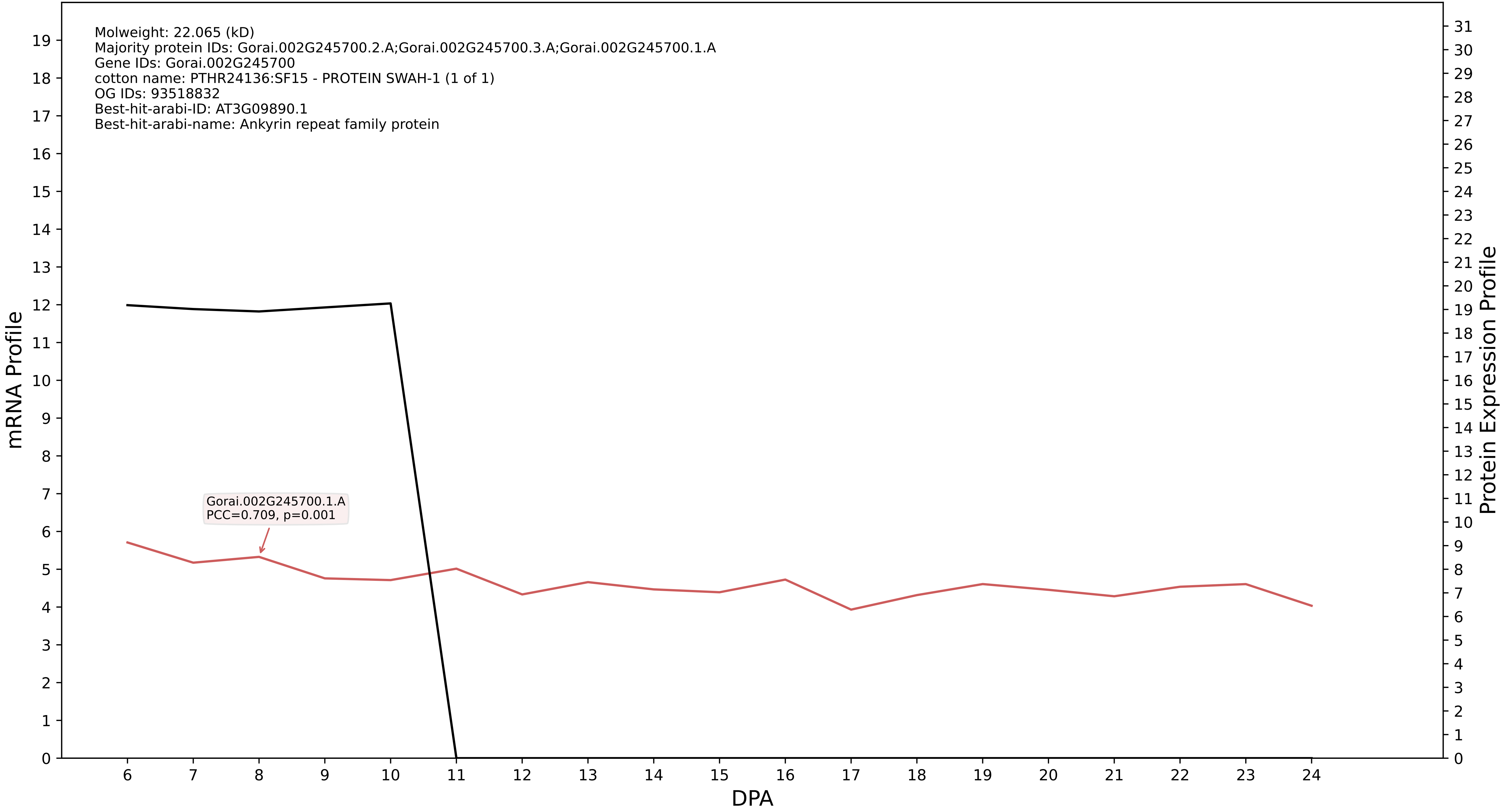

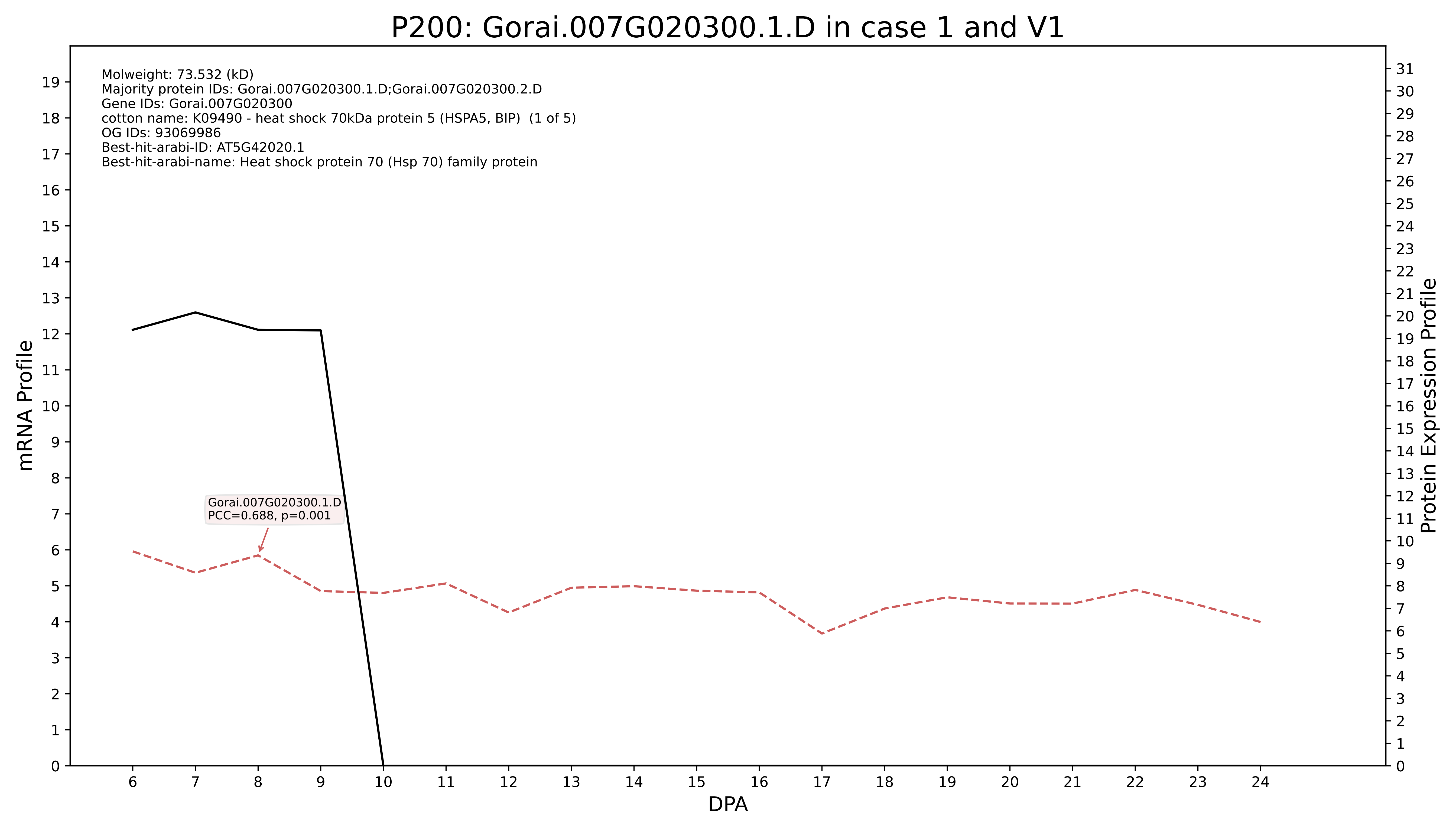

P200: Gorai.007G020300.1.D in case 1 and V1
Molweight: 73.532 (kD)
Majority protein IDs: Gorai.007G020300.1.D;Gorai.007G020300.2.D
Gene IDs: Gorai.007G020300
cotton name: K09490 - heat shock 70kDa protein 5 (HSPA5, BIP) (1 of 5)
OG IDs: 93069986
Best-hit-arabi-ID: AT5G42020.1
Best-hit-arabi-name: Heat shock protein 70 (Hsp 70) family protein
Gorai.007G020300.1.D
PCC=0.688, p=0.001
mRNA Profile
Protein Expression Profile
DPA

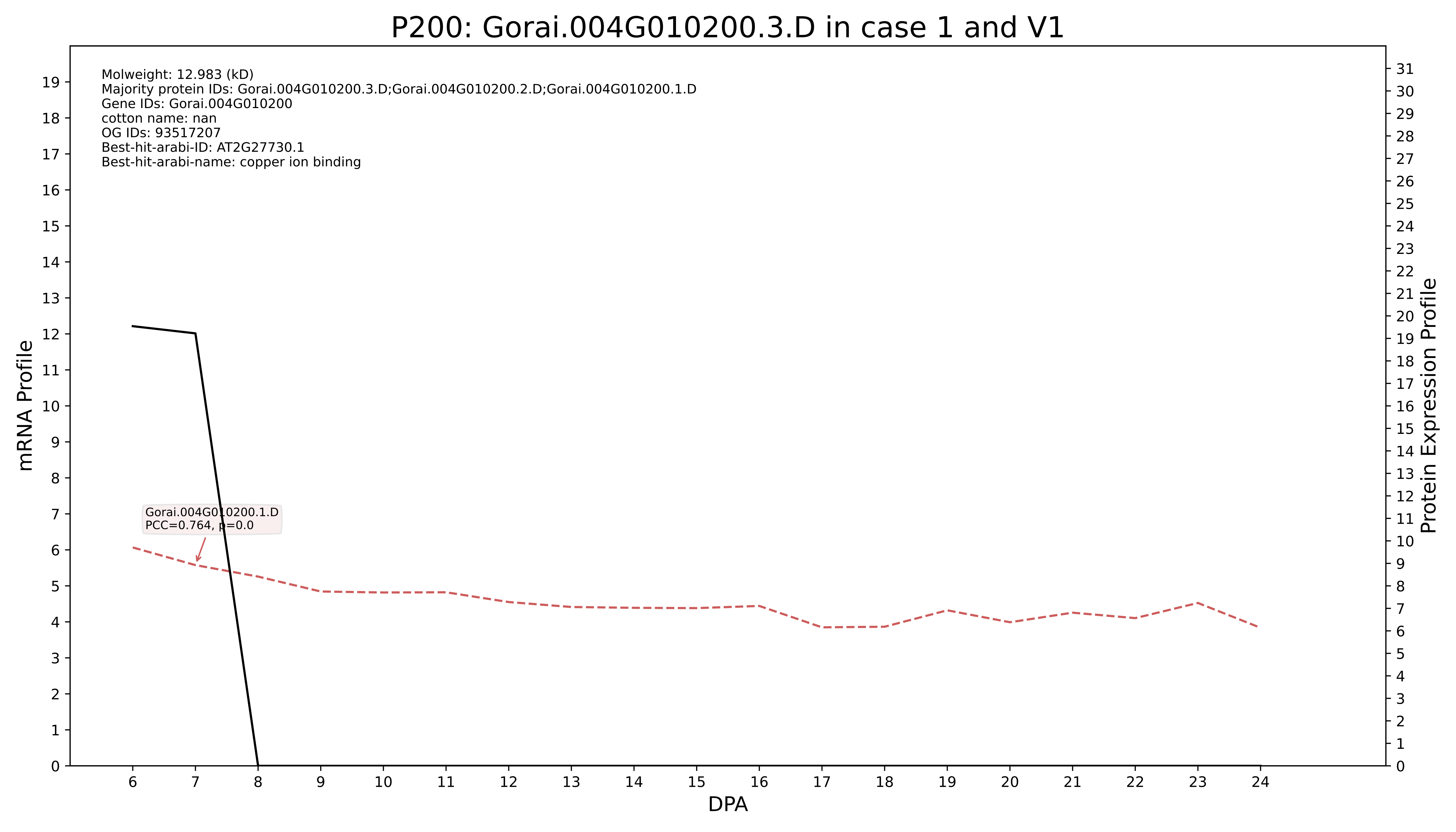
P200: Gorai.004G010200.3.D in case 1 and V1
Molweight: 12.983 (kD)
Majority protein IDs: Gorai.004G010200.3.D;Gorai.004G010200.2.D;Gorai.004G010200.1.D
Gene IDs: Gorai.004G010200
cotton name: nan
OG IDs: 93517207
Best-hit-arabi-ID: AT2G27730.1
Best-hit-arabi-name: copper ion binding
Gorai.004G010200.1.D
PCC=0.764, p=0.0
mRNA Profile
Protein Expression Profile
DPA

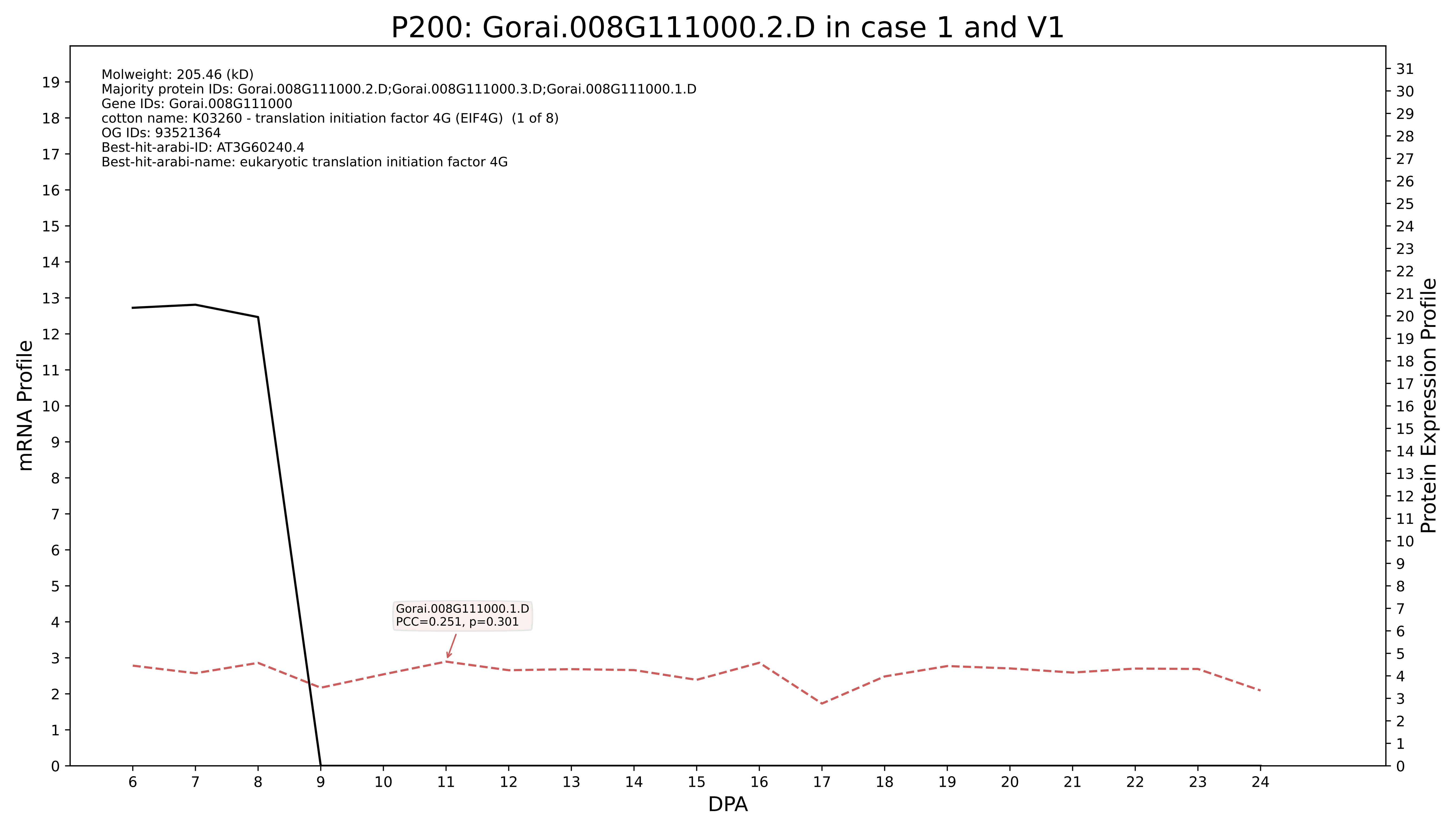
P200: Gorai.008G111000.2.D in case 1 and V1
Molweight: 205.46 (kD)
Majority protein IDs: Gorai.008G111000.2.D;Gorai.008G111000.3.D;Gorai.008G111000.1.D
Gene IDs: Gorai.008G111000
cotton name: K03260 - translation initiation factor 4G (EIF4G) (1 of 8)
OG IDs: 93521364
Best-hit-arabi-ID: AT3G60240.4
Best-hit-arabi-name: eukaryotic translation initiation factor 4G
Gorai.008G111000.1.D
PCC=0.251, p=0.301
mRNA Profile
Protein Expression Profile
DPA

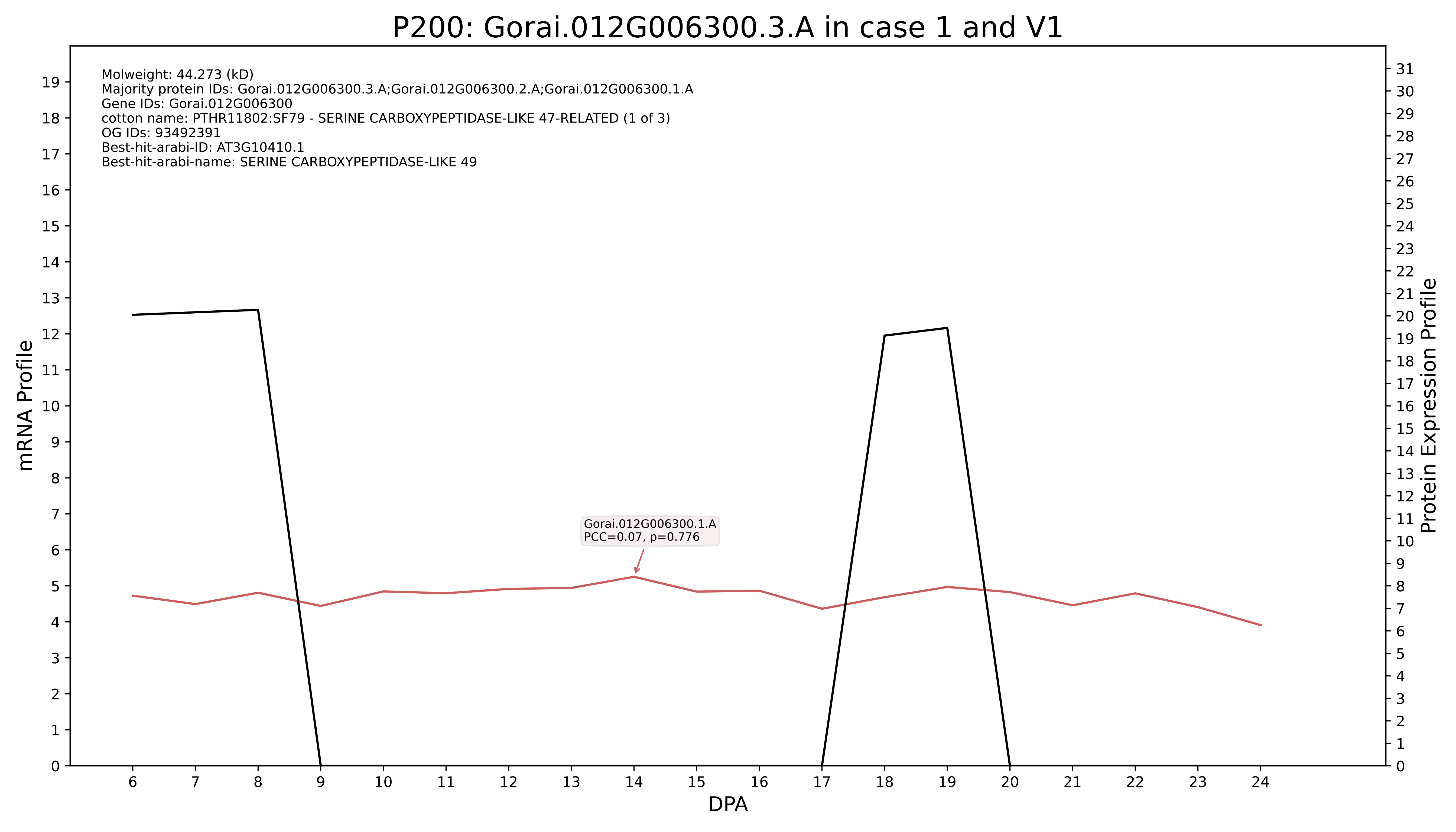
P200: Gorai.012G006300.3.A in case 1 and V1
Molweight: 44.273 (kD)
Majority protein IDs: Gorai.012G006300.3.A;Gorai.012G006300.2.A;Gorai.012G006300.1.A
Gene IDs: Gorai.012G006300
cotton name: PTHR11802:SF79 - SERINE CARBOXYPEPTIDASE-LIKE 47-RELATED (1 of 3)
OG IDs: 93492391
Best-hit-arabi-ID: AT3G10410.1
Best-hit-arabi-name: SERINE CARBOXYPEPTIDASE-LIKE 49
Gorai.012G006300.1.A
PCC=0.07, p=0.776
mRNA Profile
Protein Expression Profile
DPA

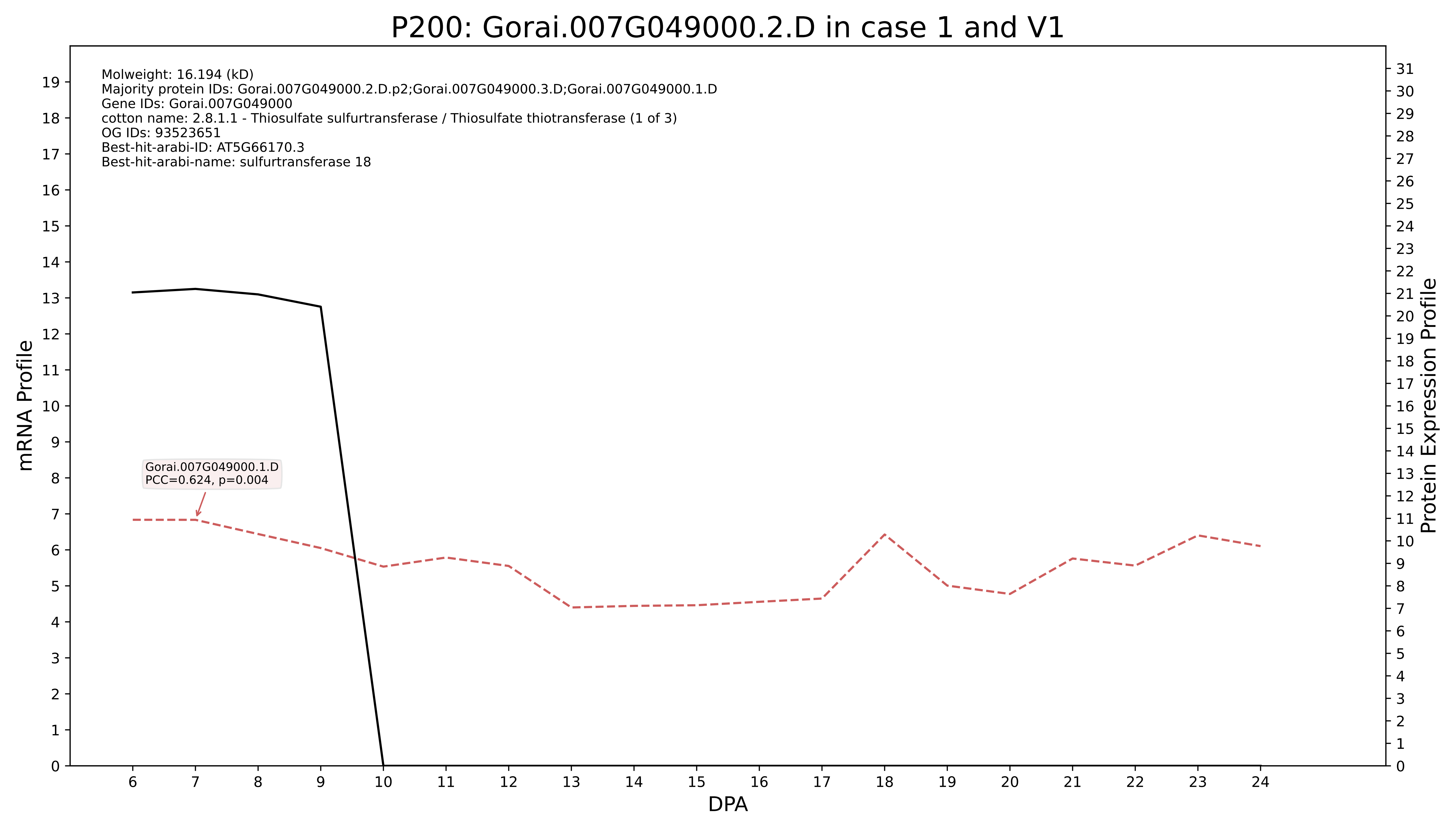

P200: Gorai.007G049000.2.D in case 1 and V1
Molweight: 16.194 (kD)
Majority protein IDs: Gorai.007G049000.2.D.p2;Gorai.007G049000.3.D;Gorai.007G049000.1.D
Gene IDs: Gorai.007G049000
cotton name: 2.8.1.1 - Thiosulfate sulfurtransferase / Thiosulfate thiotransferase (1 of 3)
OG IDs: 93523651
Best-hit-arabi-ID: AT5G66170.3
Best-hit-arabi-name: sulfurtransferase 18
Gorai.007G049000.1.D
PCC=0.624, p=0.004
mRNA Profile
Protein Expression Profile
DPA

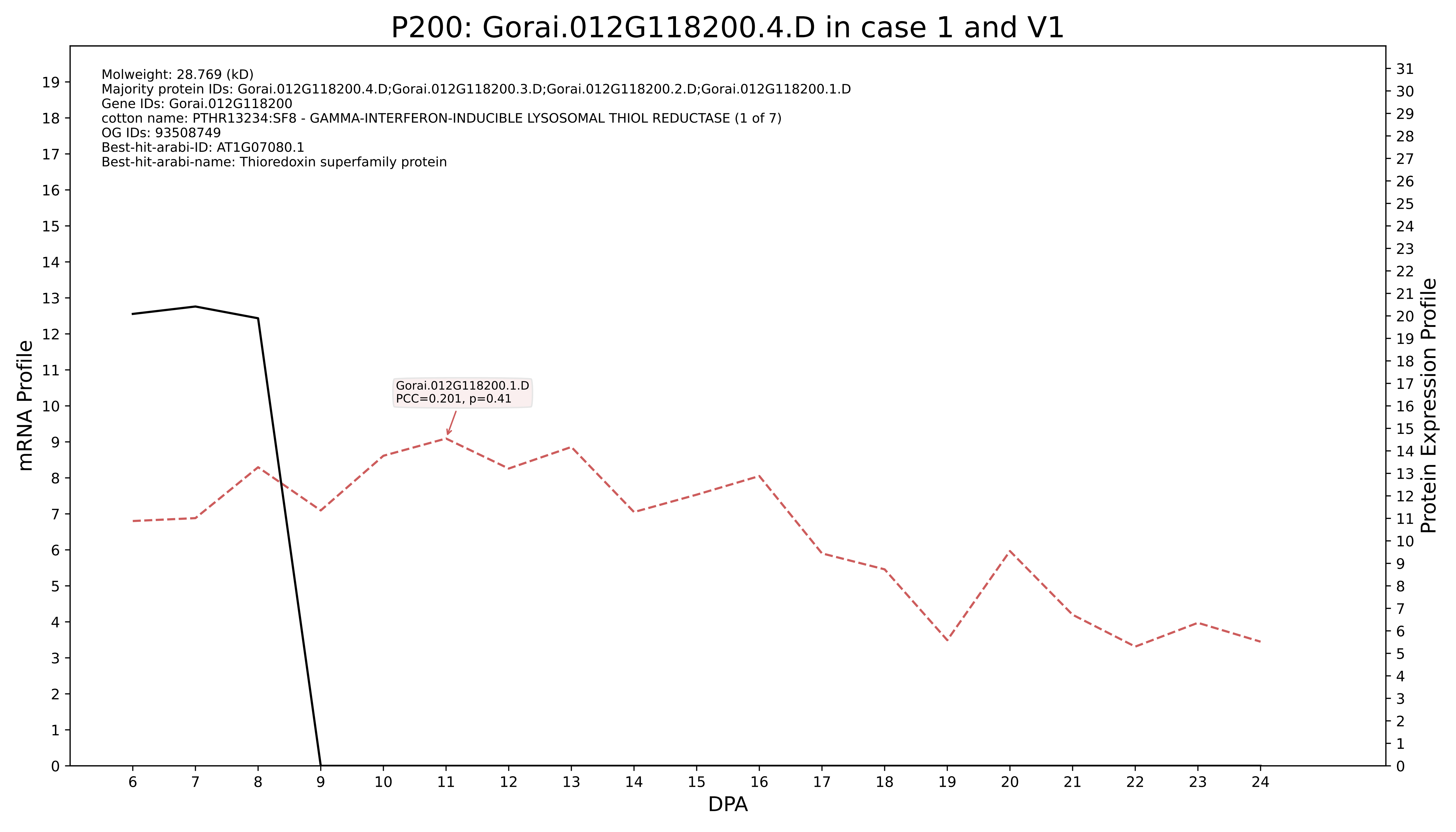
P200: Gorai.012G118200.4.D in case 1 and V1
Molweight: 28.769 (kD)
Majority protein IDs: Gorai.012G118200.4.D;Gorai.012G118200.3.D;Gorai.012G118200.2.D;Gorai.012G118200.1.D
Gene IDs: Gorai.012G118200
cotton name: PTHR13234:SF8 - GAMMA-INTERFERON-INDUCIBLE LYSOSOMAL THIOL REDUCTASE (1 of 7)
OG IDs: 93508749
Best-hit-arabi-ID: AT1G07080.1
Best-hit-arabi-name: Thioredoxin superfamily protein
Gorai.012G118200.1.D
PCC=0.201, p=0.41
mRNA Profile
Protein Expression Profile
DPA

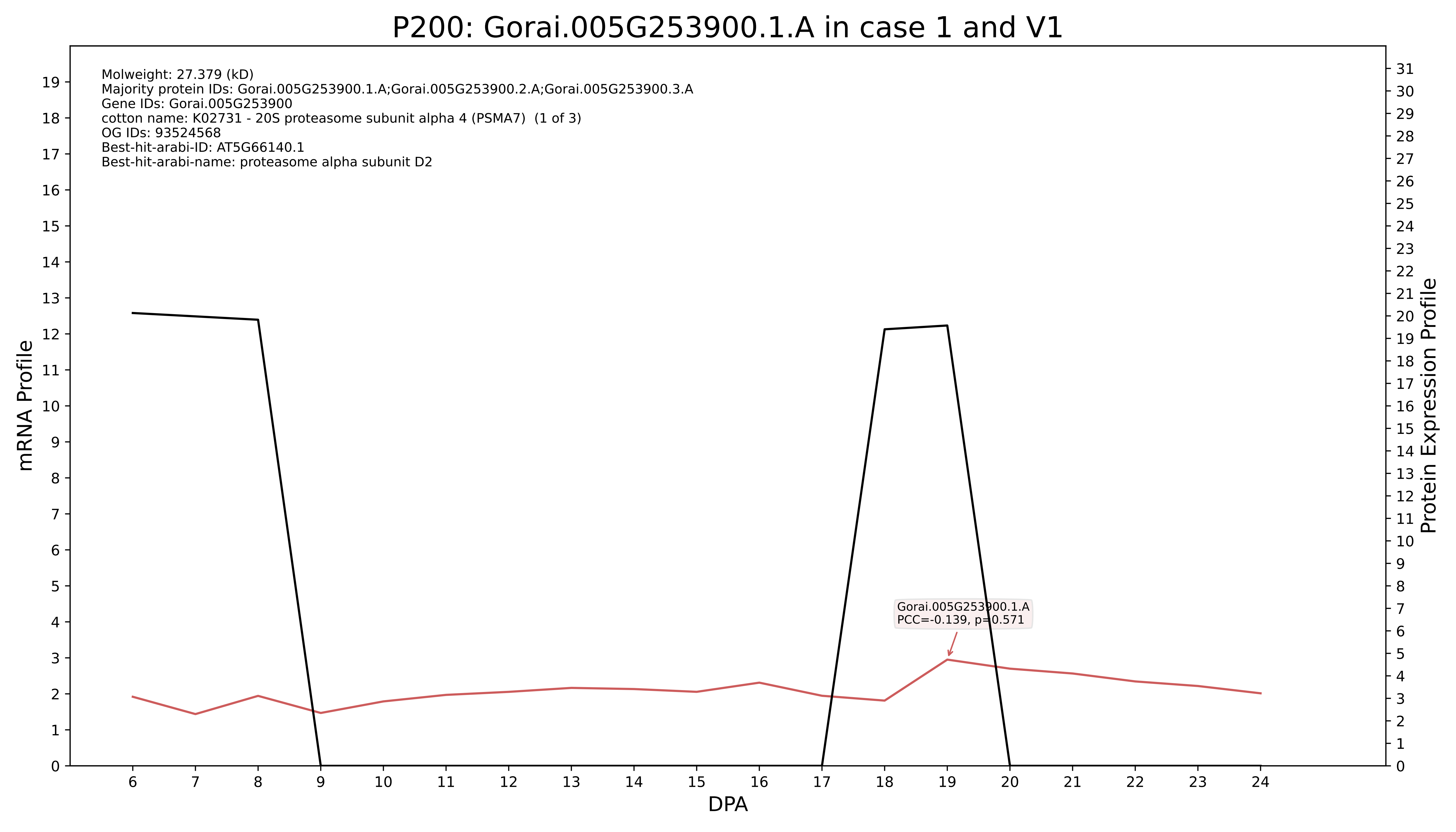
P200: Gorai.005G253900.1.A in case 1 and V1
Molweight: 27.379 (kD)
Majority protein IDs: Gorai.005G253900.1.A;Gorai.005G253900.2.A;Gorai.005G253900.3.A
Gene IDs: Gorai.005G253900
cotton name: K02731 - 20S proteasome subunit alpha 4 (PSMA7) (1 of 3)
OG IDs: 93524568
Best-hit-arabi-ID: AT5G66140.1
Best-hit-arabi-name: proteasome alpha subunit D2
Gorai.005G253900.1.A
PCC=-0.139, p=0.571
mRNA Profile
Protein Expression Profile
DPA

P200: Gorai.004G249700.1.A in case 2 and V1

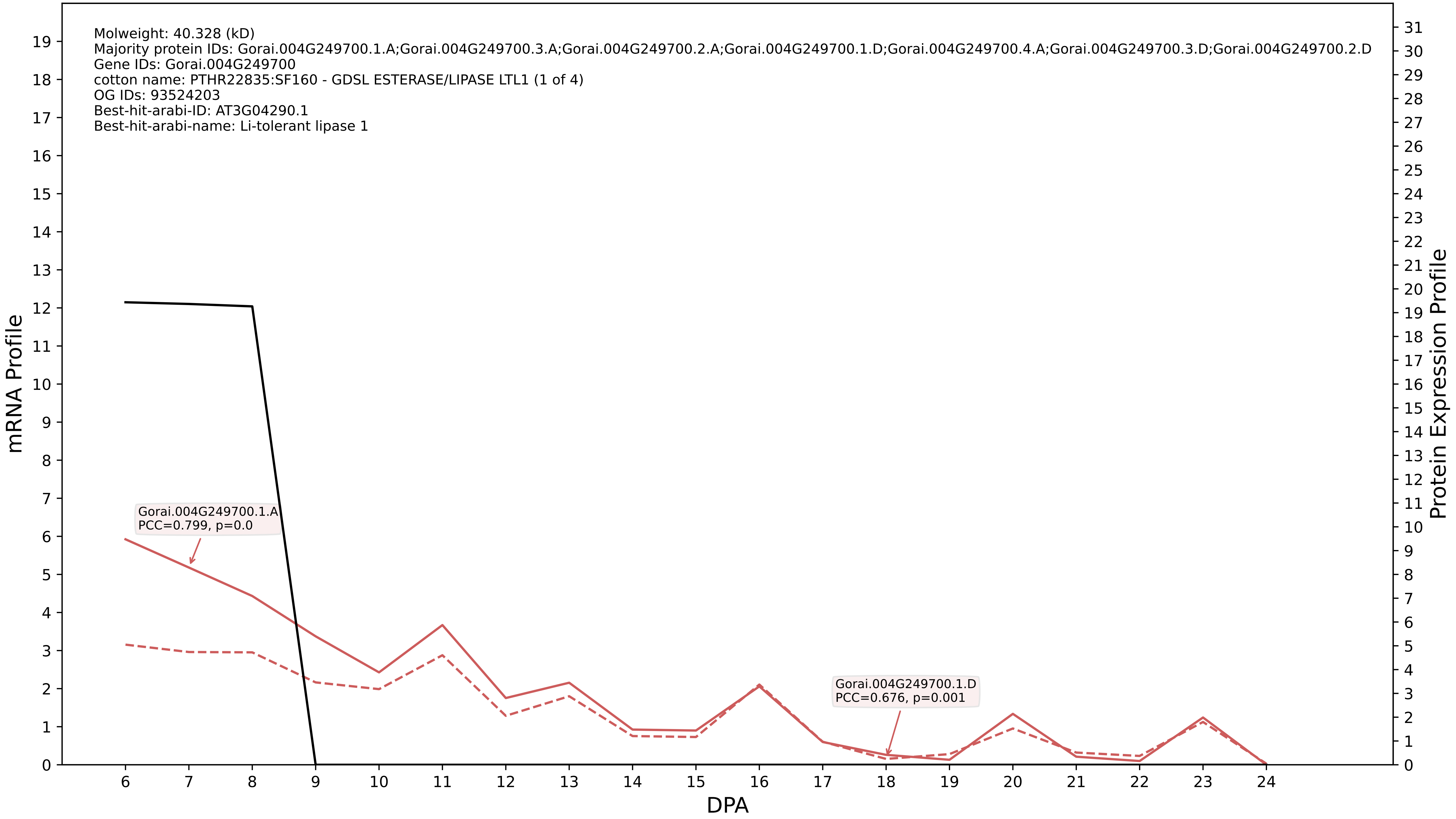

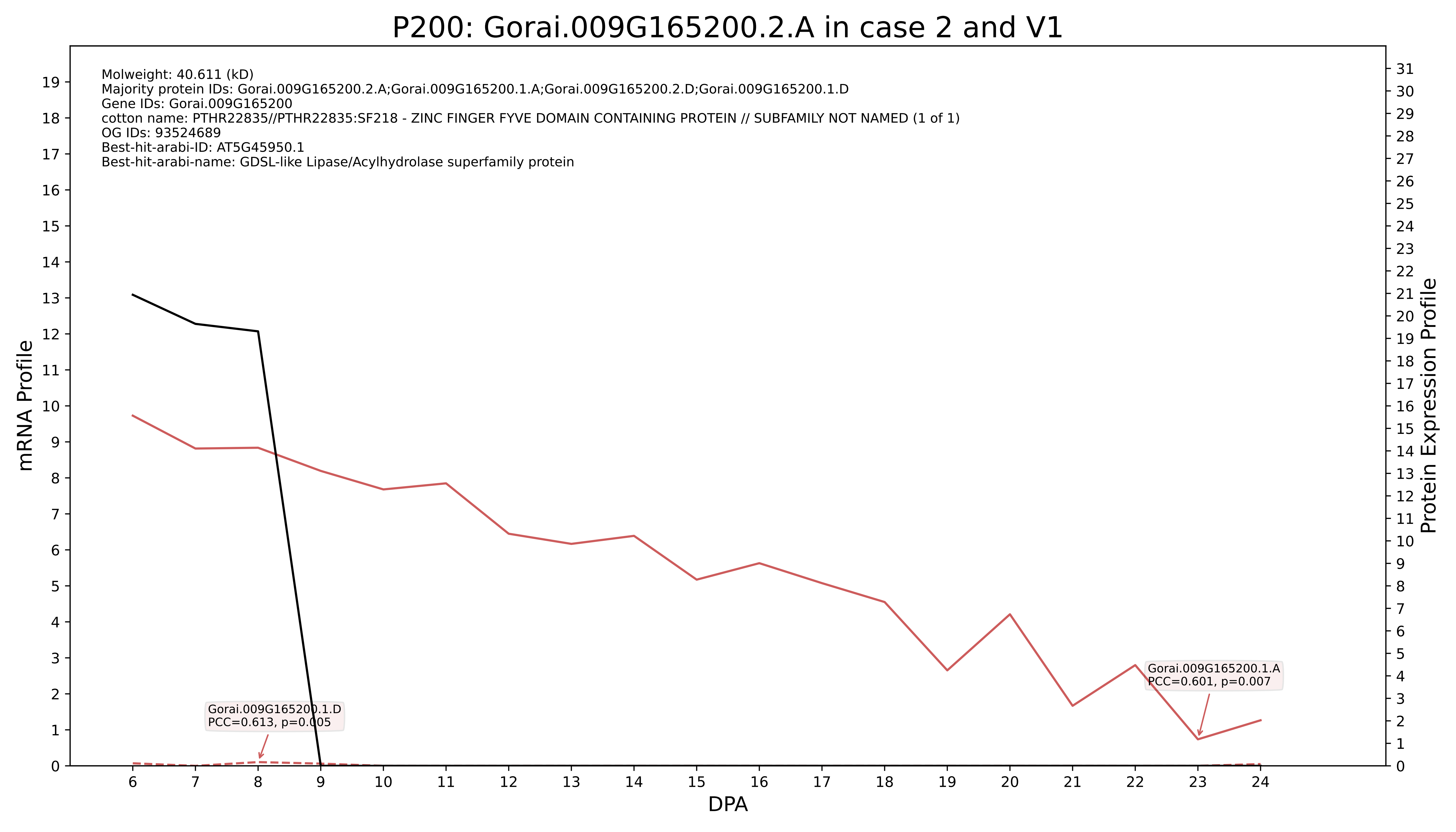
P200: Gorai.009G165200.2.A in case 2 and V1
Molweight: 40.611 (kD)
Majority protein IDs: Gorai.009G165200.2.A;Gorai.009G165200.1.A;Gorai.009G165200.2.D;Gorai.009G165200.1.D
Gene IDs: Gorai.009G165200
cotton name: PTHR22835//PTHR22835:SF218 - ZINC FINGER FYVE DOMAIN CONTAINING PROTEIN // SUBFAMILY NOT NAMED (1 of 1)
OG IDs: 93524689
Best-hit-arabi-ID: AT5G45950.1
Best-hit-arabi-name: GDSL-like Lipase/Acylhydrolase superfamily protein
Gorai.009G165200.1.D
PCC=0.613, p=0.005
Gorai.009G165200.1.A
PCC=0.601, p=0.007
mRNA Profile
Protein Expression Profile
DPA

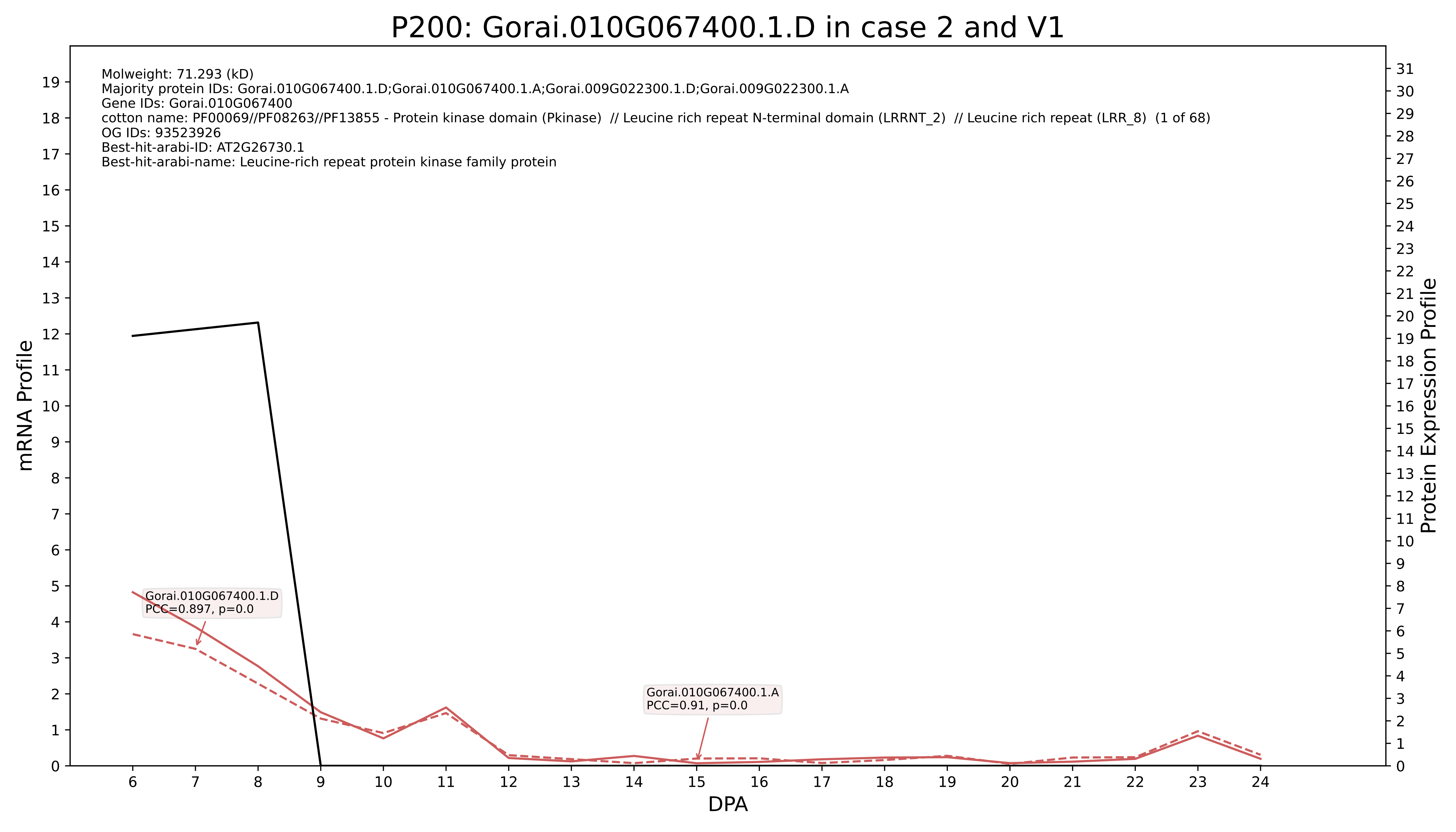
P200: Gorai.010G067400.1.D in case 2 and V1
Molweight: 71.293 (kD)
Majority protein IDs: Gorai.010G067400.1.D;Gorai.010G067400.1.A;Gorai.009G022300.1.D;Gorai.009G022300.1.A
Gene IDs: Gorai.010G067400
cotton name: PF00069//PF08263//PF13855 - Protein kinase domain (Pkinase) // Leucine rich repeat N-terminal domain (LRRNT_2) // Leucine rich repeat (LRR_8) (1 of 68)
OG IDs: 93523926
Best-hit-arabi-ID: AT2G26730.1
Best-hit-arabi-name: Leucine-rich repeat protein kinase family protein
Gorai.010G067400.1.D
PCC=0.897, p=0.0
Gorai.010G067400.1.A
PCC=0.91, p=0.0
mRNA Profile
Protein Expression Profile
DPA
0
1
2
3
4
5
6
7
8
9
10
11
12
13
14
15
16
17
18
19
20
21
22
23
24
25
26
27
28
29
30
31

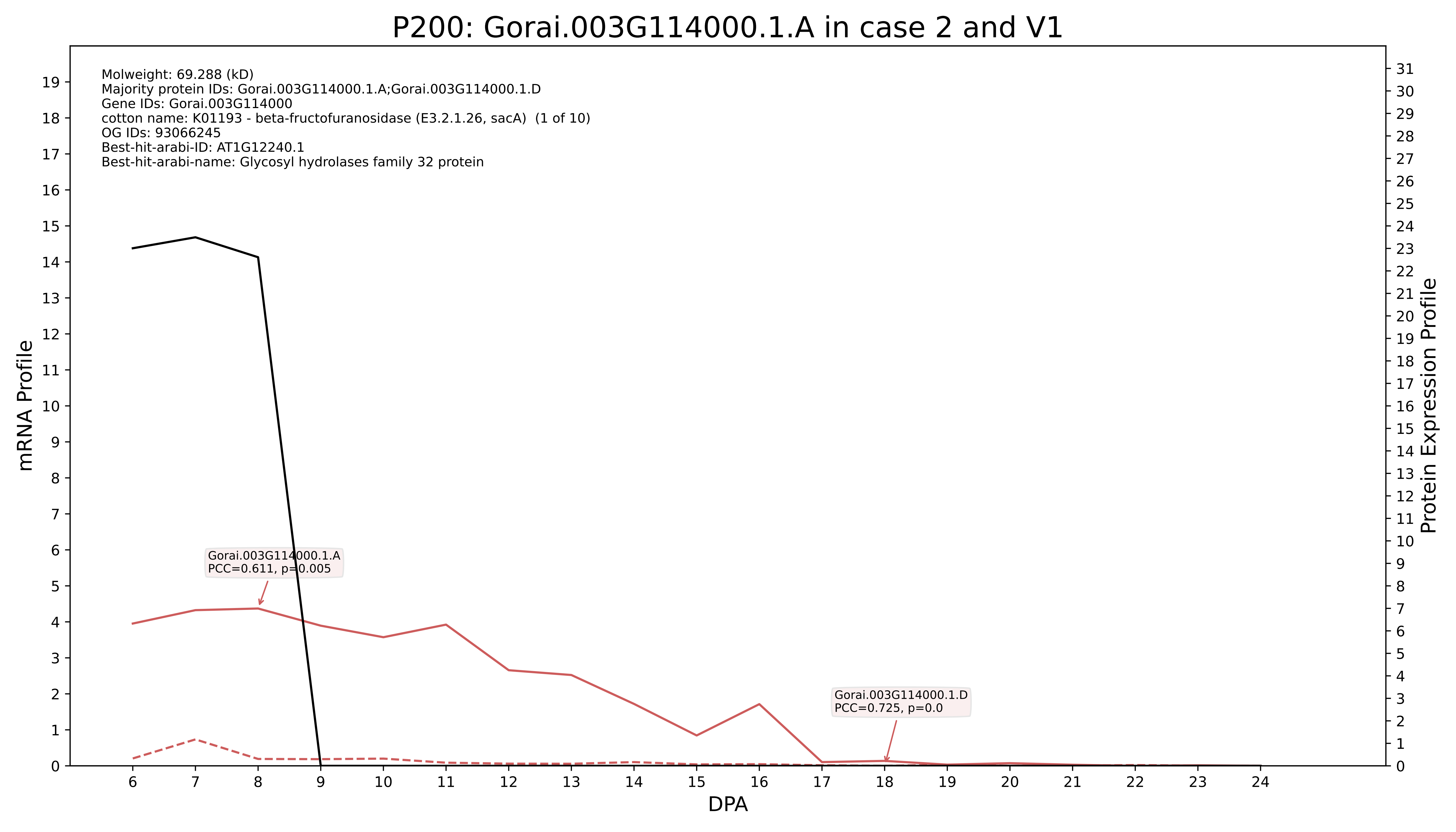
P200: Gorai.003G114000.1.A in case 2 and V1
Molweight: 69.288 (kD)
Majority protein IDs: Gorai.003G114000.1.A;Gorai.003G114000.1.D
Gene IDs: Gorai.003G114000
cotton name: K01193 - beta-fructofuranosidase (E3.2.1.26, sacA) (1 of 10)
OG IDs: 93066245
Best-hit-arabi-ID: AT1G12240.1
Best-hit-arabi-name: Glycosyl hydrolases family 32 protein
Gorai.003G114000.1.A
PCC=0.611, p=0.005
Gorai.003G114000.1.D
PCC=0.725, p=0.0
mRNA Profile
Protein Expression Profile
DPA

P200: Gorai.006G256500.1.A in case 2 and V1

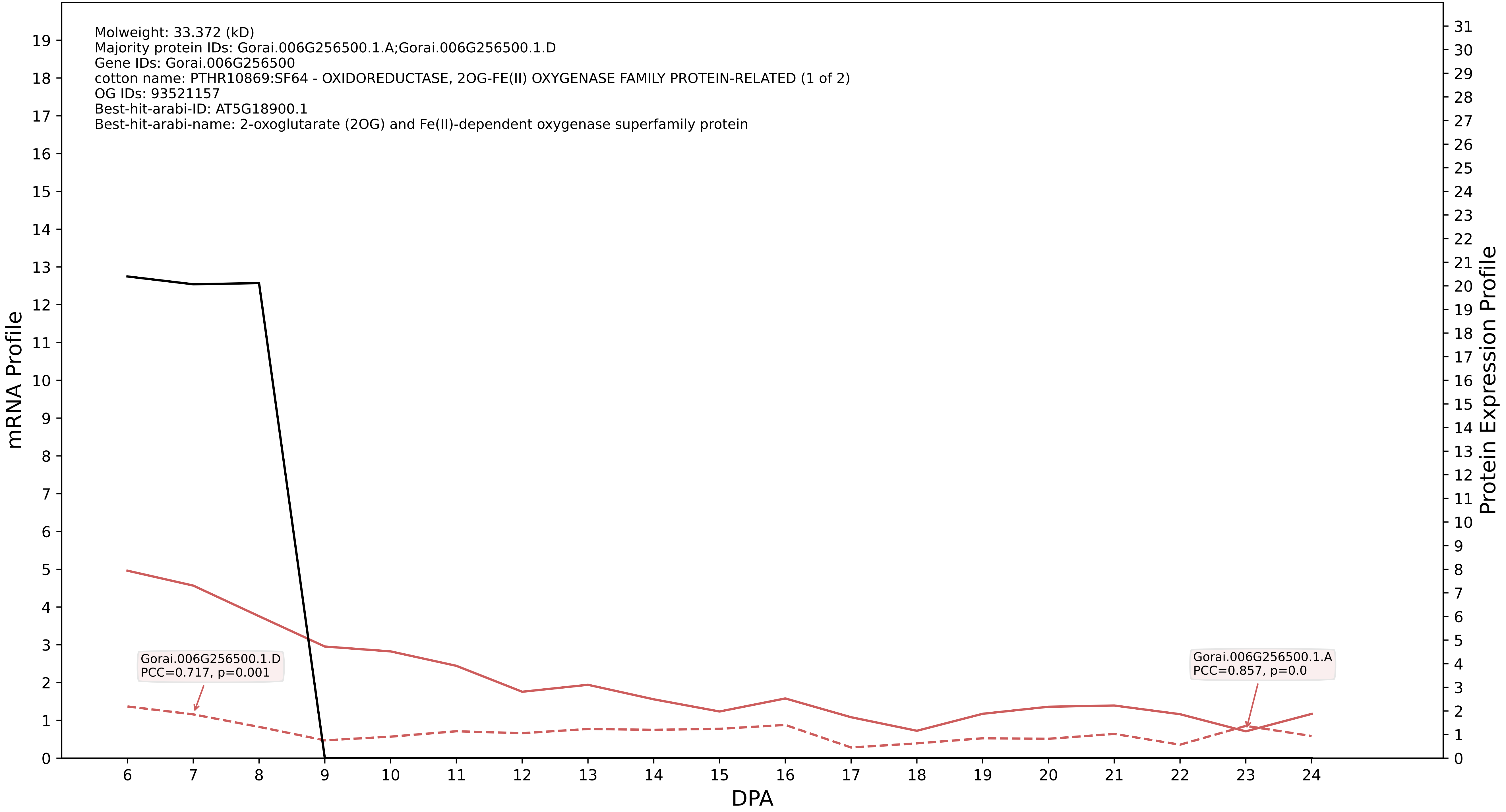

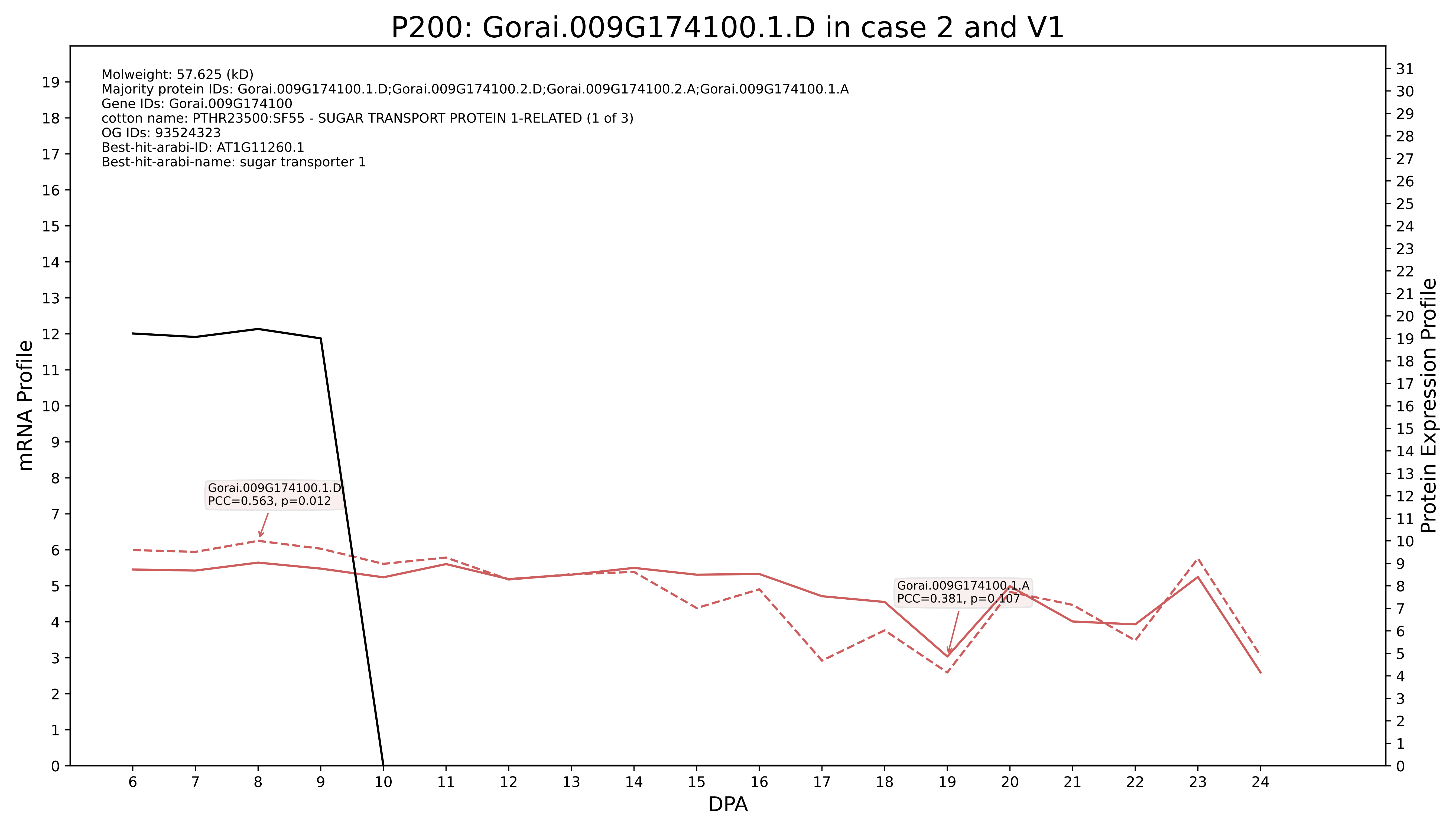
P200: Gorai.009G174100.1.D in case 2 and V1
Molweight: 57.625 (kD)
Majority protein IDs: Gorai.009G174100.1.D;Gorai.009G174100.2.D;Gorai.009G174100.2.A;Gorai.009G174100.1.A
Gene IDs: Gorai.009G174100
cotton name: PTHR23500:SF55 - SUGAR TRANSPORT PROTEIN 1-RELATED (1 of 3)
OG IDs: 93524323
Best-hit-arabi-ID: AT1G11260.1
Best-hit-arabi-name: sugar transporter 1
Gorai.009G174100.1.D
PCC=0.563, p=0.012
Gorai.009G174100.1.A
PCC=0.381, p=0.107
mRNA Profile
Protein Expression Profile
DPA

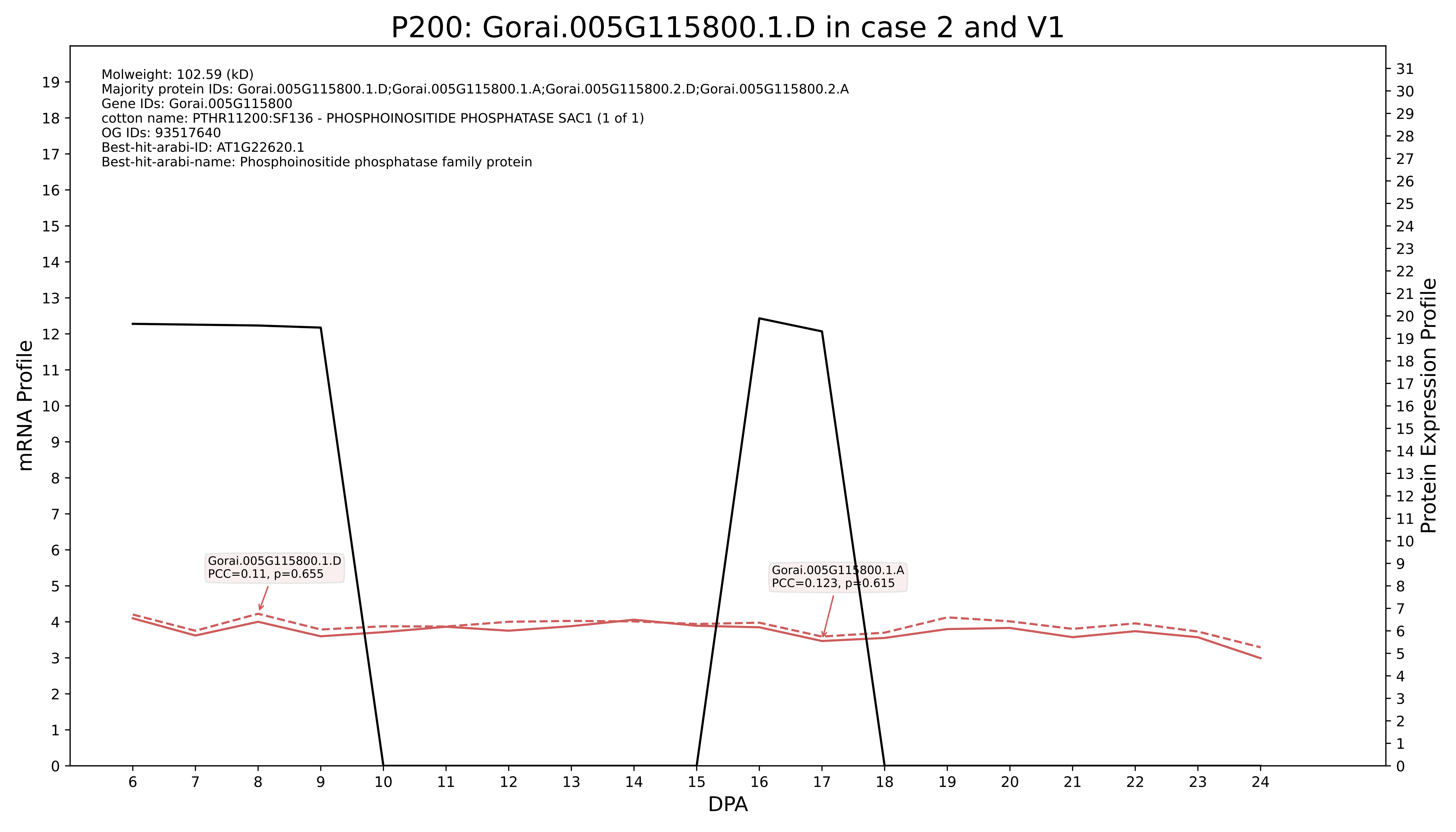
P200: Gorai.005G115800.1.D in case 2 and V1
Molweight: 102.59 (kD)
Majority protein IDs: Gorai.005G115800.1.D;Gorai.005G115800.1.A;Gorai.005G115800.2.D;Gorai.005G115800.2.A
Gene IDs: Gorai.005G115800
cotton name: PTHR11200:SF136 - PHOSPHOINOSITIDE PHOSPHATASE SAC1 (1 of 1)
OG IDs: 93517640
Best-hit-arabi-ID: AT1G22620.1
Best-hit-arabi-name: Phosphoinositide phosphatase family protein
Gorai.005G115800.1.D
PCC=0.11, p=0.655
Gorai.005G115800.1.A
PCC=0.123, p=0.615
mRNA Profile
Protein Expression Profile
DPA

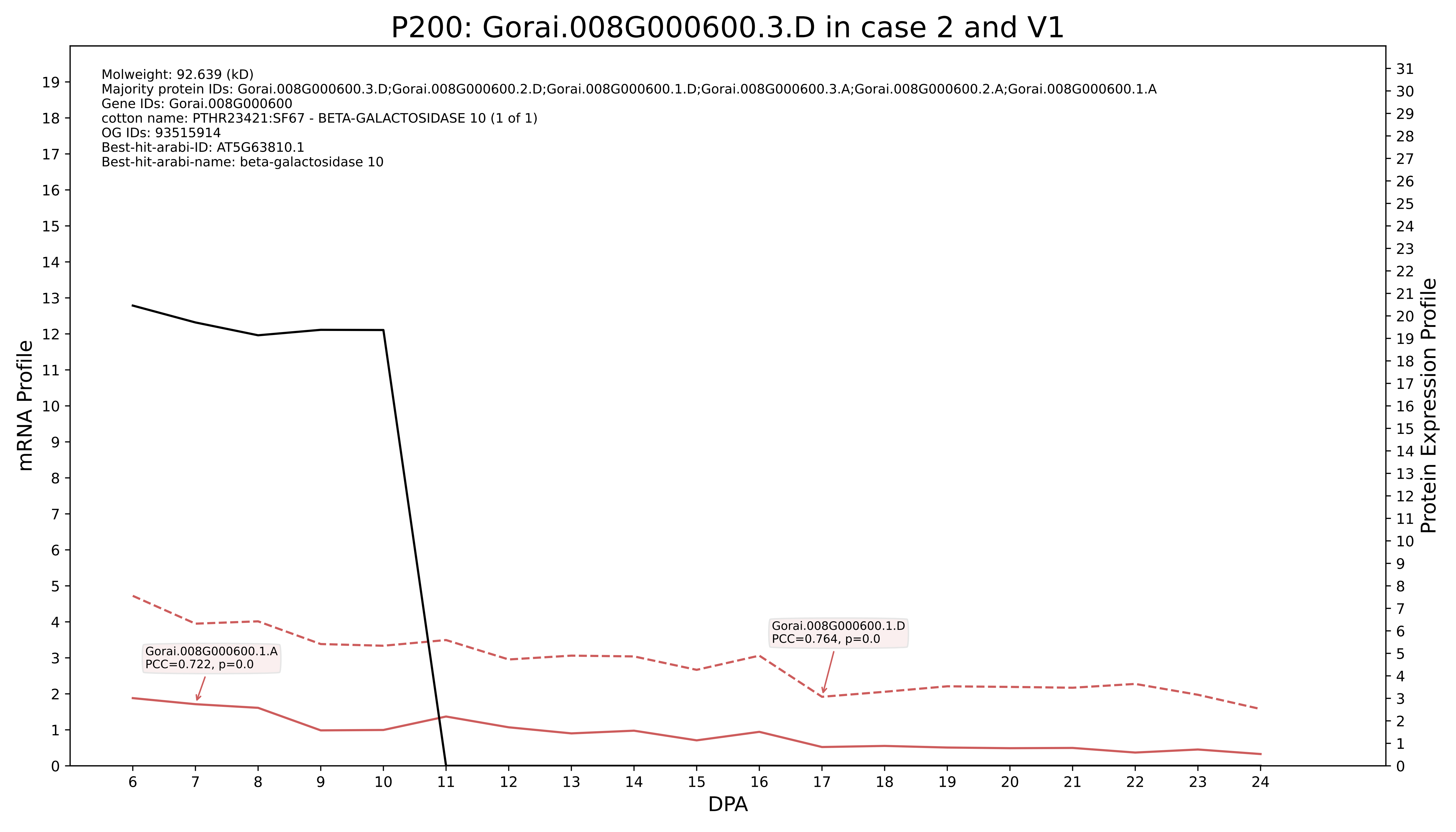
P200: Gorai.008G000600.3.D in case 2 and V1
Molweight: 92.639 (kD)
Majority protein IDs: Gorai.008G000600.3.D;Gorai.008G000600.2.D;Gorai.008G000600.1.D;Gorai.008G000600.3.A;Gorai.008G000600.2.A;Gorai.008G000600.1.A
Gene IDs: Gorai.008G000600
cotton name: PTHR23421:SF67 - BETA-GALACTOSIDASE 10 (1 of 1)
OG IDs: 93515914
Best-hit-arabi-ID: AT5G63810.1
Best-hit-arabi-name: beta-galactosidase 10
Gorai.008G000600.1.A
PCC=0.722, p=0.0
Gorai.008G000600.1.D
PCC=0.764, p=0.0
mRNA Profile
Protein Expression Profile
DPA

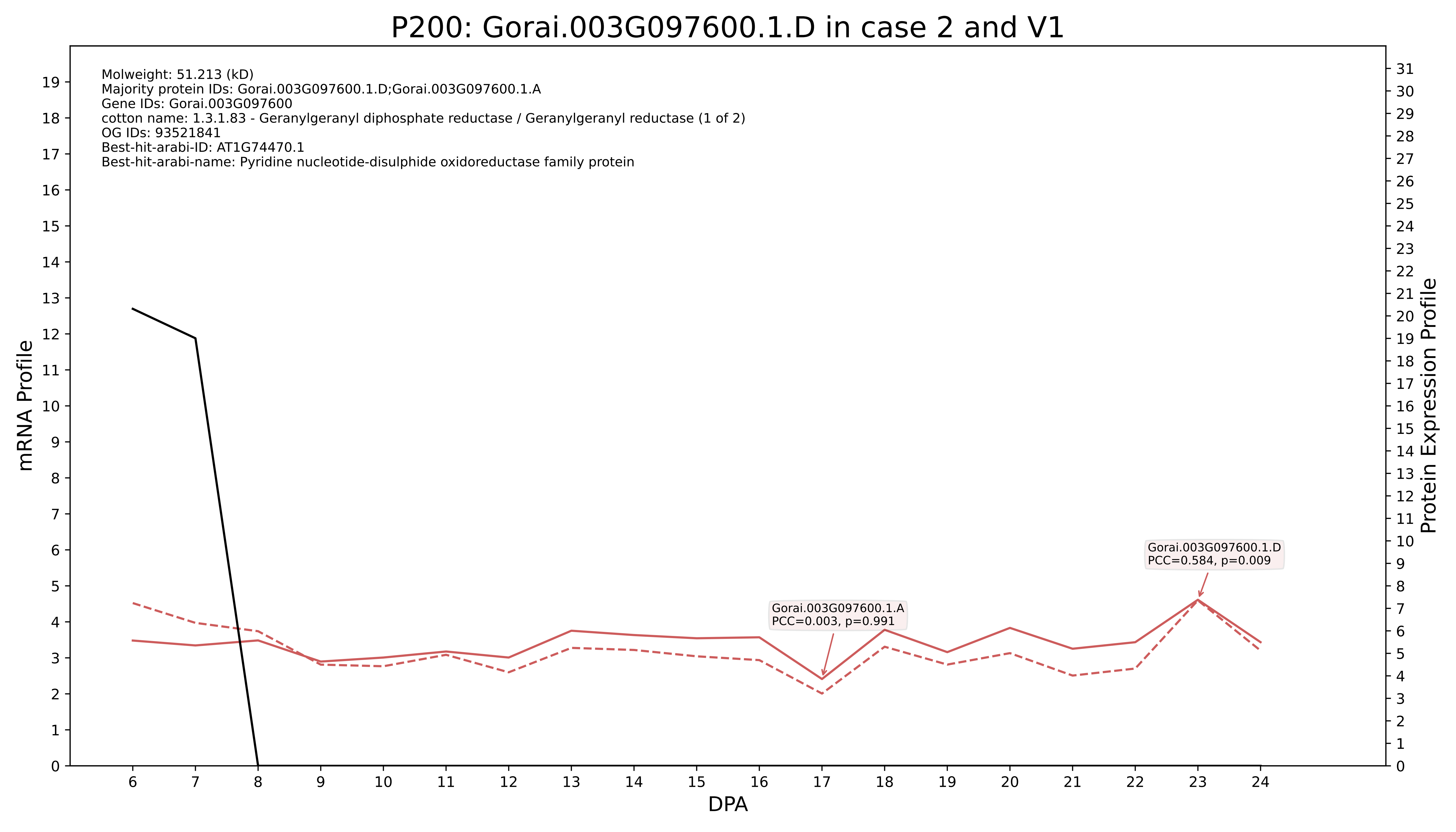

P200: Gorai.003G097600.1.D in case 2 and V1
Molweight: 51.213 (kD)
Majority protein IDs: Gorai.003G097600.1.D;Gorai.003G097600.1.A
Gene IDs: Gorai.003G097600
cotton name: 1.3.1.83 - Geranylgeranyl diphosphate reductase / Geranylgeranyl reductase (1 of 2)
OG IDs: 93521841
Best-hit-arabi-ID: AT1G74470.1
Best-hit-arabi-name: Pyridine nucleotide-disulphide oxidoreductase family protein
Gorai.003G097600.1.D
PCC=0.584, p=0.009
Gorai.003G097600.1.A
PCC=0.003, p=0.991
mRNA Profile
Protein Expression Profile
DPA

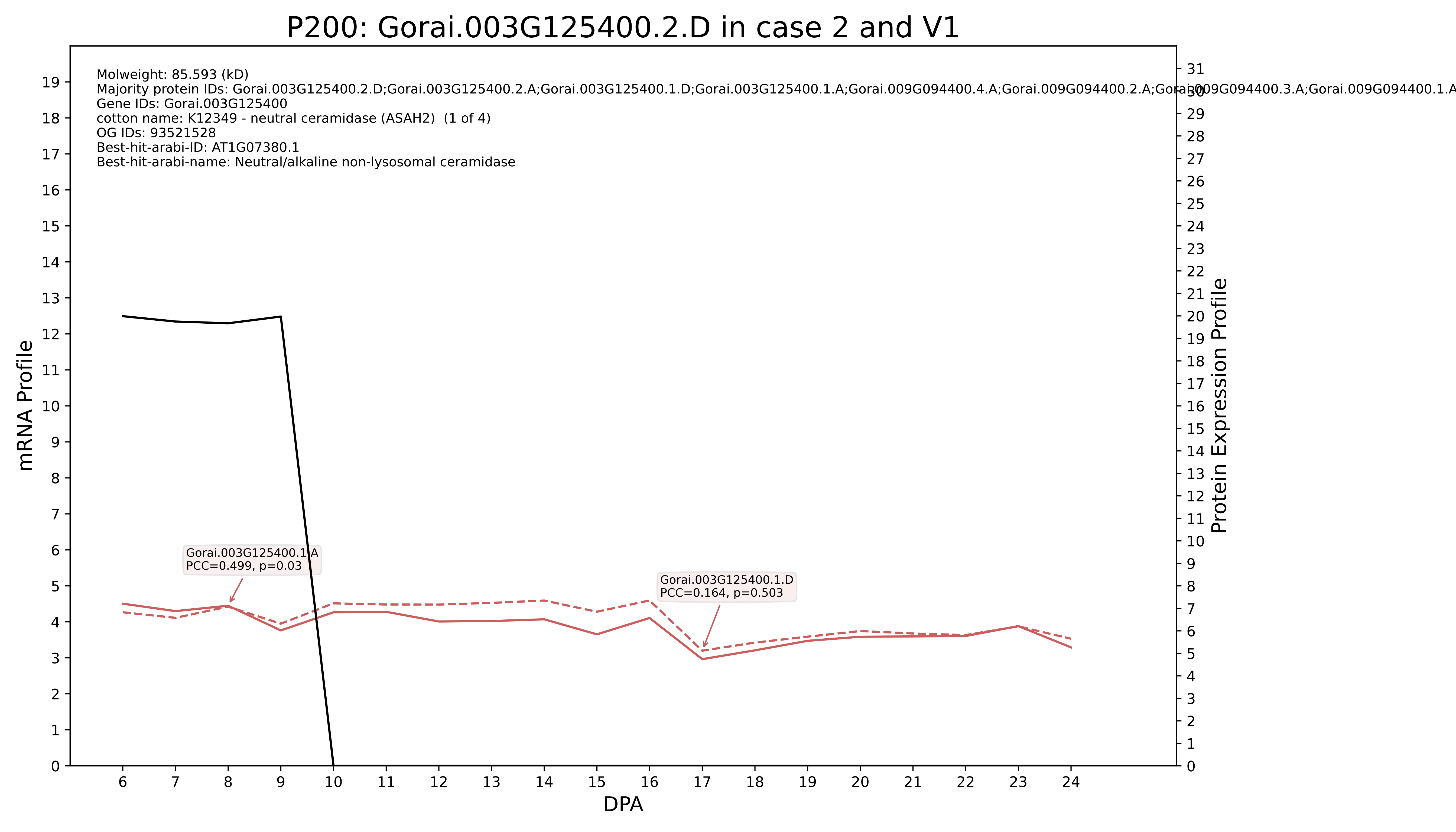
P200: Gorai.003G125400.2.D in case 2 and V1
Molweight: 85.593 (kD)
Majority protein IDs: Gorai.003G125400.2.D;Gorai.003G125400.2.A;Gorai.003G125400.1.D;Gorai.003G125400.1.A;Gorai.009G094400.4.A;Gorai.009G094400.2.A;Gorai.009G094400.3.A;Gorai.009G094400.1.A
Gene IDs: Gorai.003G125400
cotton name: K12349 - neutral ceramidase (ASAH2) (1 of 4)
OG IDs: 93521528
Best-hit-arabi-ID: AT1G07380.1
Best-hit-arabi-name: Neutral/alkaline non-lysosomal ceramidase
Gorai.003G125400.1.A
PCC=0.499, p=0.03
Gorai.003G125400.1.D
PCC=0.164, p=0.503
mRNA Profile
Protein Expression Profile
DPA

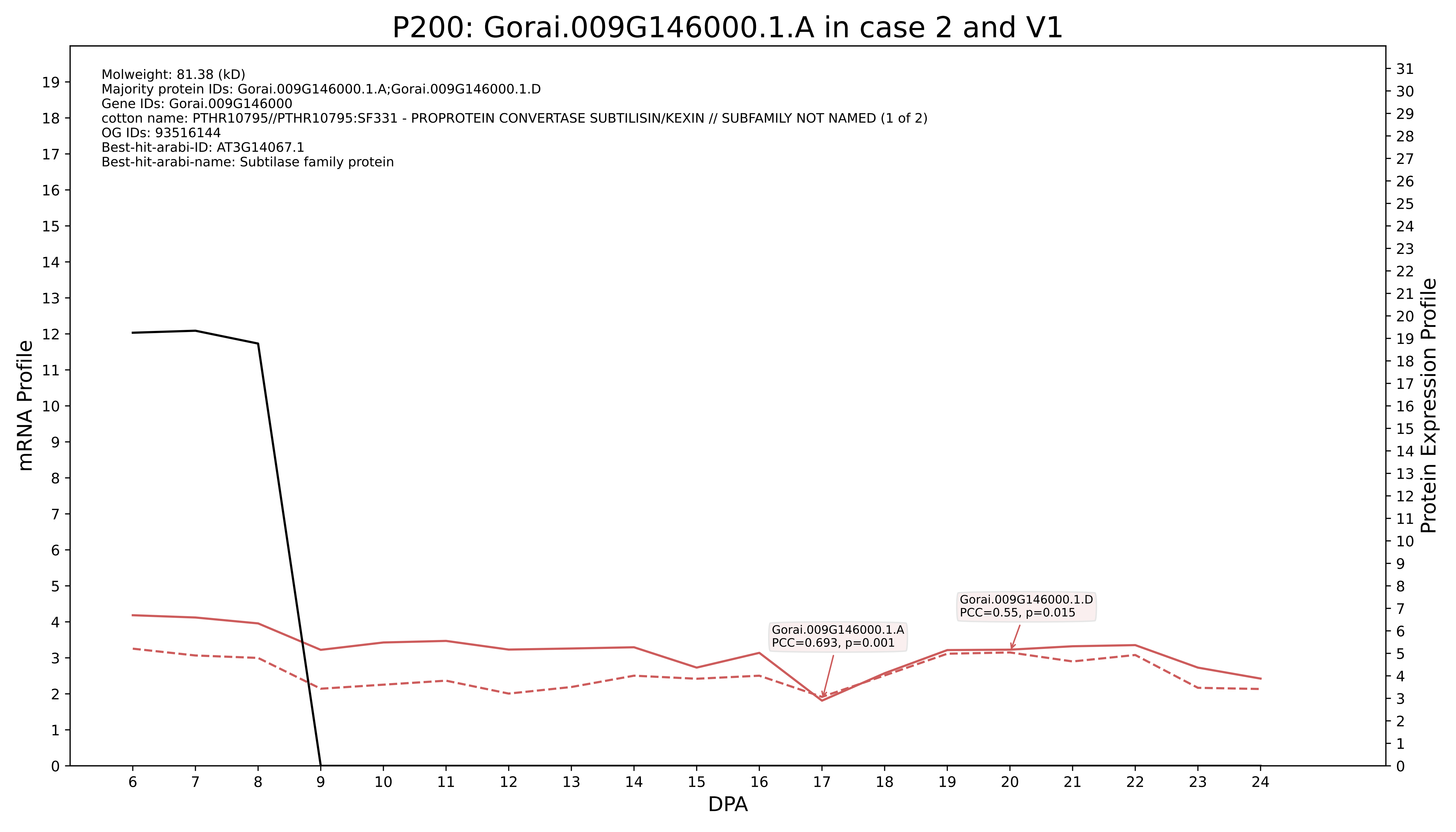

P200: Gorai.009G146000.1.A in case 2 and V1
Molweight: 81.38 (kD)
Majority protein IDs: Gorai.009G146000.1.A;Gorai.009G146000.1.D
Gene IDs: Gorai.009G146000
cotton name: PTHR10795//PTHR10795:SF331 - PROPROTEIN CONVERTASE SUBTILISIN/KEXIN // SUBFAMILY NOT NAMED (1 of 2)
OG IDs: 93516144
Best-hit-arabi-ID: AT3G14067.1
Best-hit-arabi-name: Subtilase family protein
Gorai.009G146000.1.A
PCC=0.693, p=0.001
Gorai.009G146000.1.D
PCC=0.55, p=0.015
mRNA Profile
Protein Expression Profile
DPA

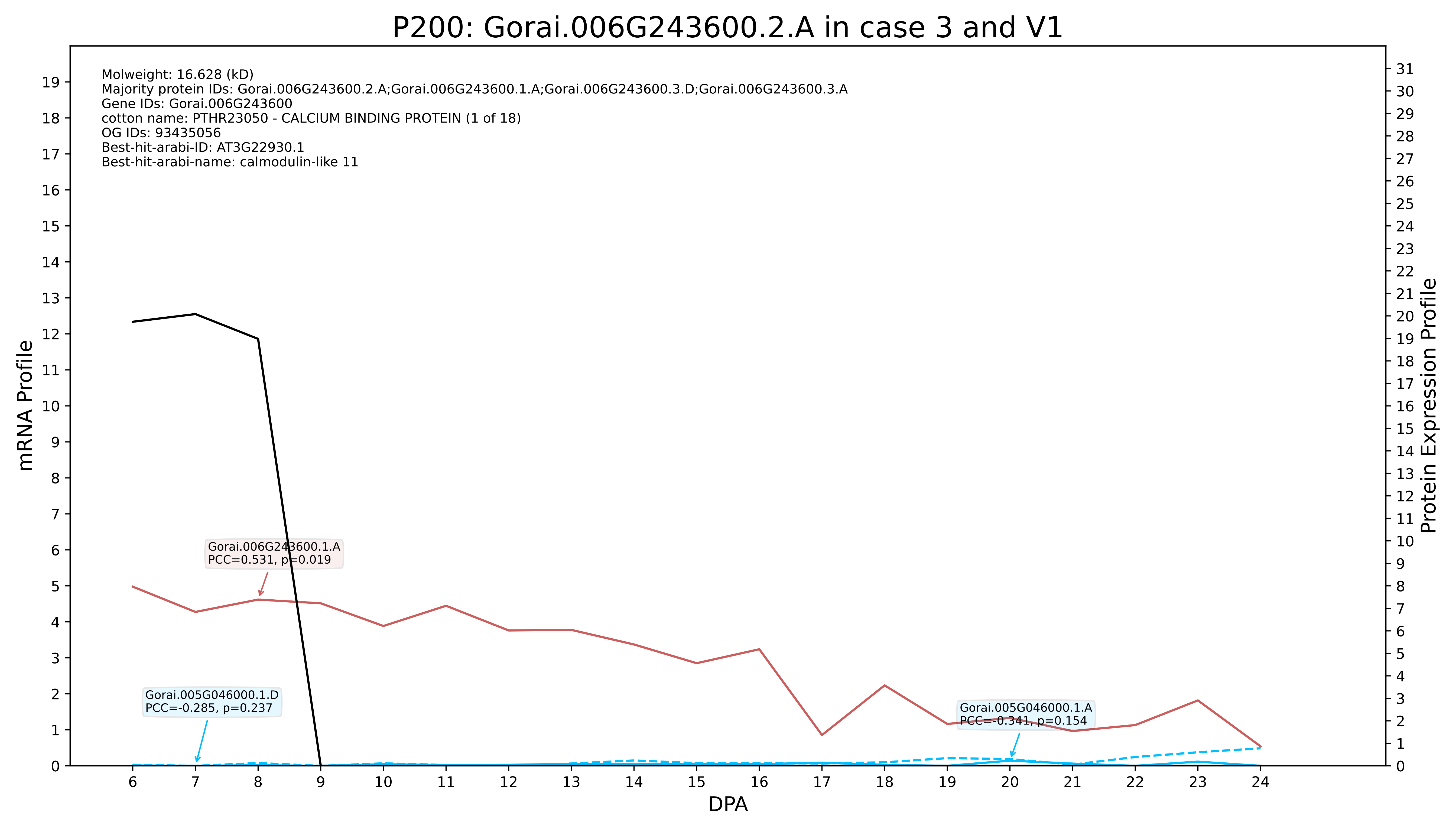
P200: Gorai.006G243600.2.A in case 3 and V1
Molweight: 16.628 (kD)
Majority protein IDs: Gorai.006G243600.2.A;Gorai.006G243600.1.A;Gorai.006G243600.3.D;Gorai.006G243600.3.A
Gene IDs: Gorai.006G243600
cotton name: PTHR23050 - CALCIUM BINDING PROTEIN (1 of 18)
OG IDs: 93435056
Best-hit-arabi-ID: AT3G22930.1
Best-hit-arabi-name: calmodulin-like 11
Gorai.006G243600.1.A
PCC=0.531, p=0.019
Gorai.005G046000.1.D
PCC=-0.285, p=0.237
Gorai.005G046000.1.A
PCC=-0.341, p=0.154
mRNA Profile
Protein Expression Profile
DPA

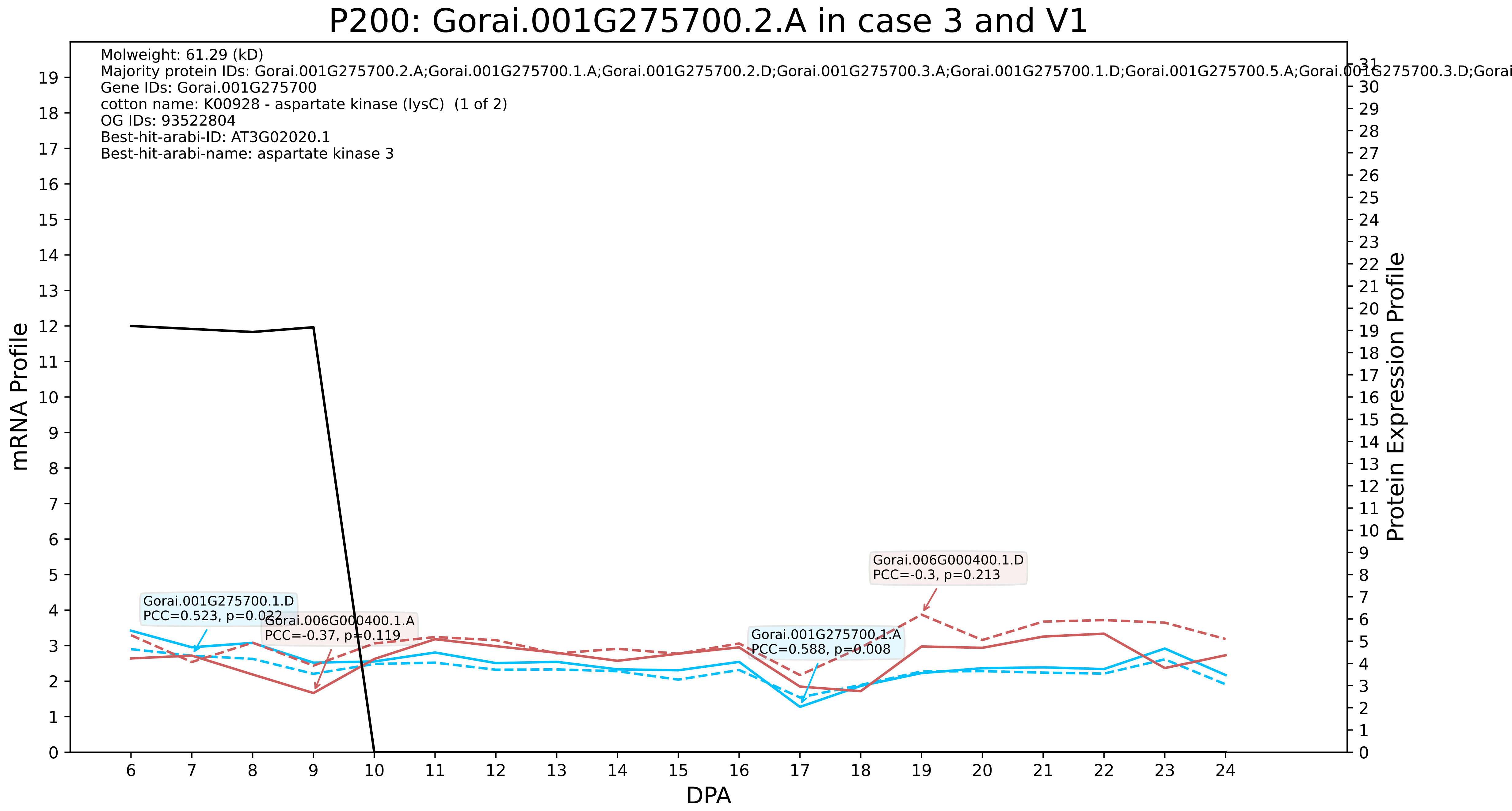

P200: Gorai.001G275700.2.A in case 3 and V1
Molweight: 61.29 (kD)
Majority protein IDs: Gorai.001G275700.2.A;Gorai.001G275700.1.A;Gorai.001G275700.2.D;Gorai.001G275700.3.A;Gorai.001G275700.1.D;Gorai.001G275700.5.A;Gorai.001G275700.3.D;Gorai
Gene IDs: Gorai.001G275700
cotton name: K00928 - aspartate kinase (lysC) (1 of 2)
OG IDs: 93522804
Best-hit-arabi-ID: AT3G02020.1
Best-hit-arabi-name: aspartate kinase 3
Gorai.001G275700.1.D
PCC=0.523, p=0.022
Gorai.006G000400.1.A
PCC=-0.37, p=0.119
Gorai.001G275700.1.A
PCC=0.588, p=0.008
Gorai.006G000400.1.D
PCC=-0.3, p=0.213
mRNA Profile
Protein Expression Profile
DPA

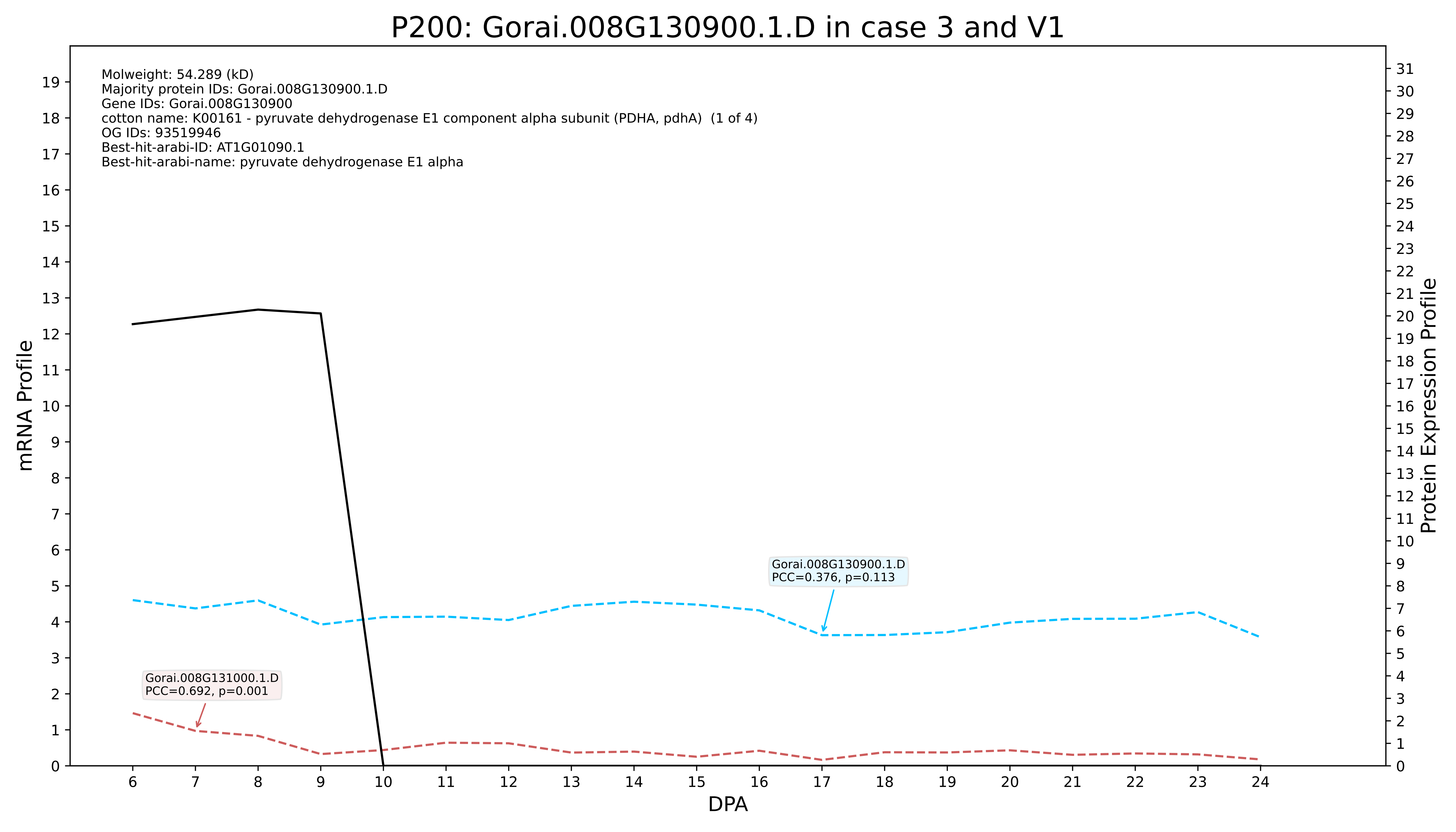

P200: Gorai.008G130900.1.D in case 3 and V1
Molweight: 54.289 (kD)
Majority protein IDs: Gorai.008G130900.1.D
Gene IDs: Gorai.008G130900
cotton name: K00161 - pyruvate dehydrogenase E1 component alpha subunit (PDHA, pdhA) (1 of 4)
OG IDs: 93519946
Best-hit-arabi-ID: AT1G01090.1
Best-hit-arabi-name: pyruvate dehydrogenase E1 alpha
Gorai.008G130900.1.D
PCC=0.376, p=0.113
Gorai.008G131000.1.D
PCC=0.692, p=0.001
mRNA Profile
Protein Expression Profile
DPA

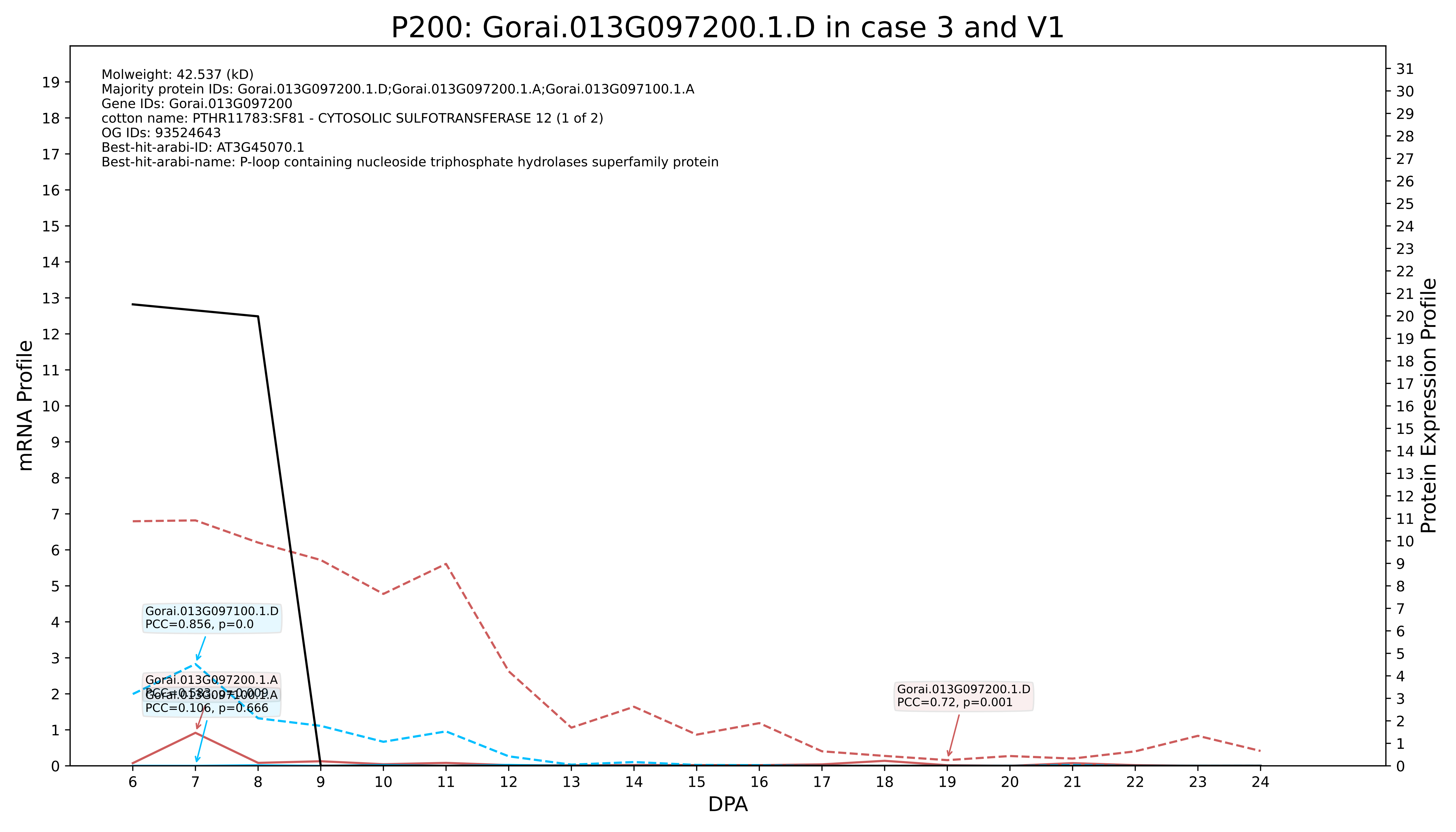

P200: Gorai.013G097200.1.D in case 3 and V1
Molweight: 42.537 (kD)
Majority protein IDs: Gorai.013G097200.1.D;Gorai.013G097200.1.A;Gorai.013G097100.1.A
Gene IDs: Gorai.013G097200
cotton name: PTHR11783:SF81 - CYTOSOLIC SULFOTRANSFERASE 12 (1 of 2)
OG IDs: 93524643
Best-hit-arabi-ID: AT3G45070.1
Best-hit-arabi-name: P-loop containing nucleoside triphosphate hydrolases superfamily protein
Gorai.013G097100.1.D
PCC=0.856, p=0.0
Gorai.013G097200.1.A
Gorai.013G097100.1.A
PCC=0.106, p=0.666
Gorai.013G097200.1.D
PCC=0.72, p=0.001
mRNA Profile
Protein Expression Profile
DPA

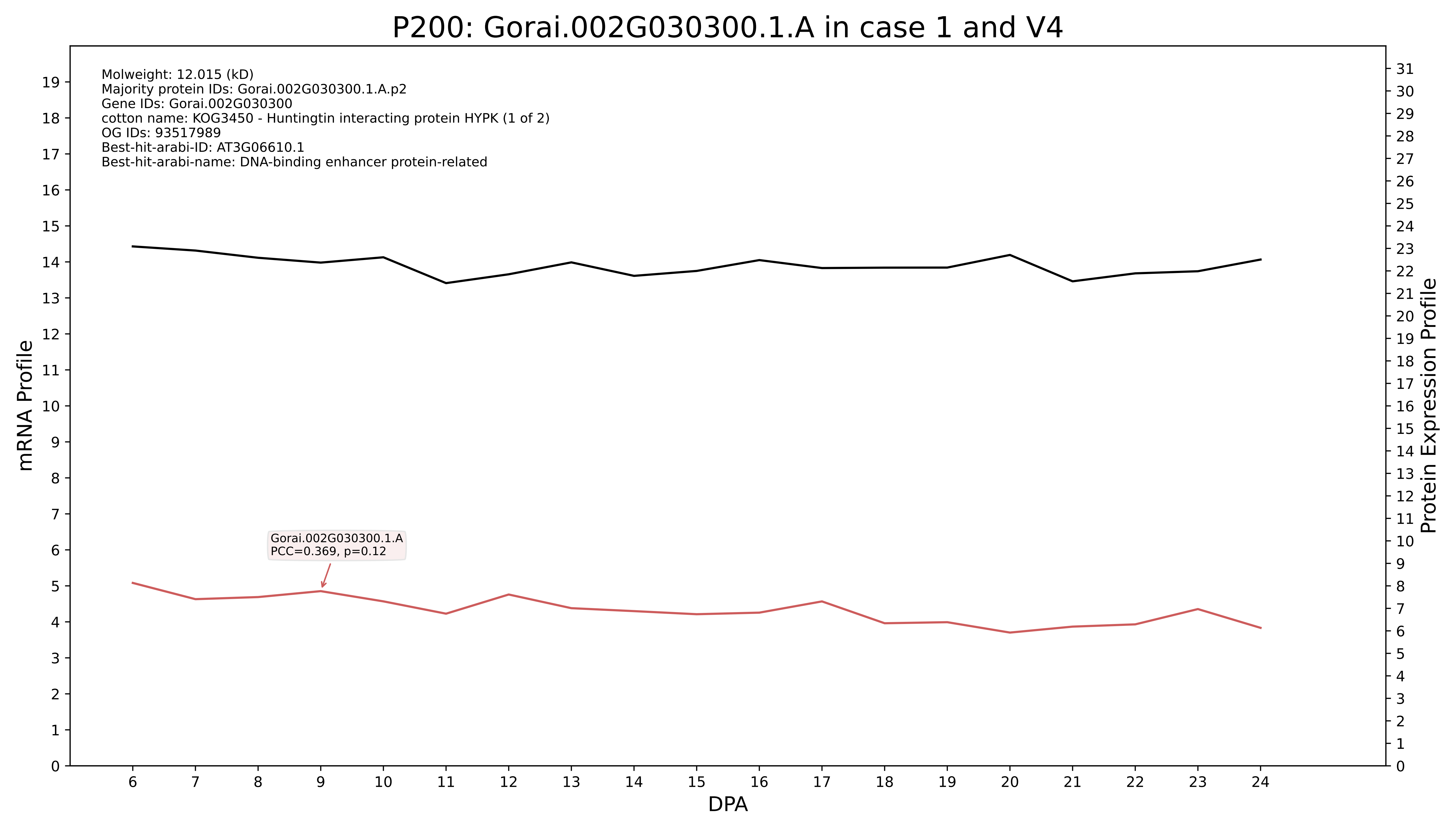
P200: Gorai.002G030300.1.A in case 1 and V4
Molweight: 12.015 (kD)
Majority protein IDs: Gorai.002G030300.1.A.p2
Gene IDs: Gorai.002G030300
cotton name: KOG3450 - Huntingtin interacting protein HYPK (1 of 2)
OG IDs: 93517989
Best-hit-arabi-ID: AT3G06610.1
Best-hit-arabi-name: DNA-binding enhancer protein-related
Gorai.002G030300.1.A
PCC=0.369, p=0.12
mRNA Profile
Protein Expression Profile
DPA
0
1
2
3
4
5
6
7
8
9
10
11
12
13
14
15
16
17
18
19
20
21
22
23
24
25
26
27
28
29
30
31

P200: Gorai.013G197500.3.A in case 1 and V4

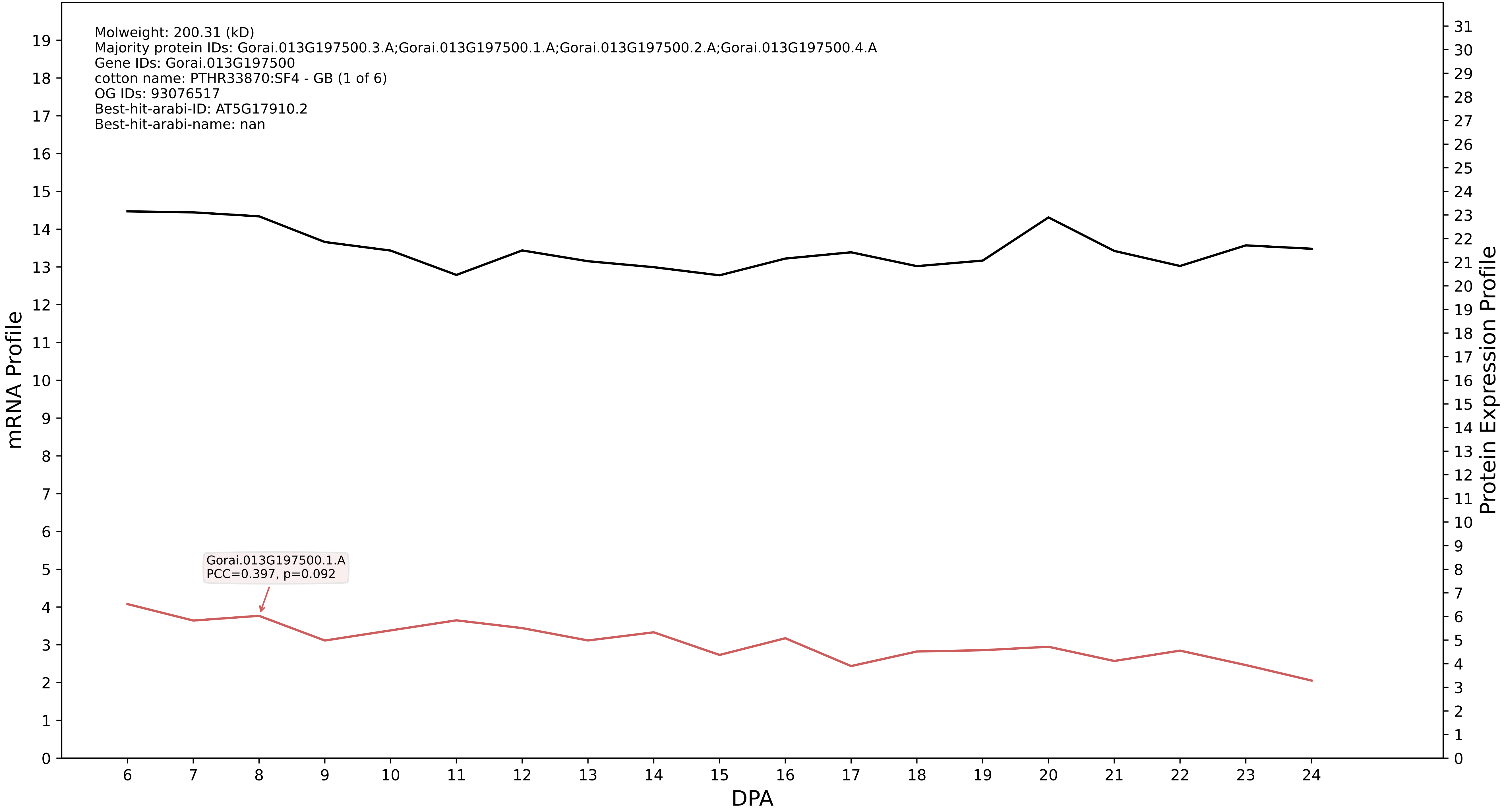

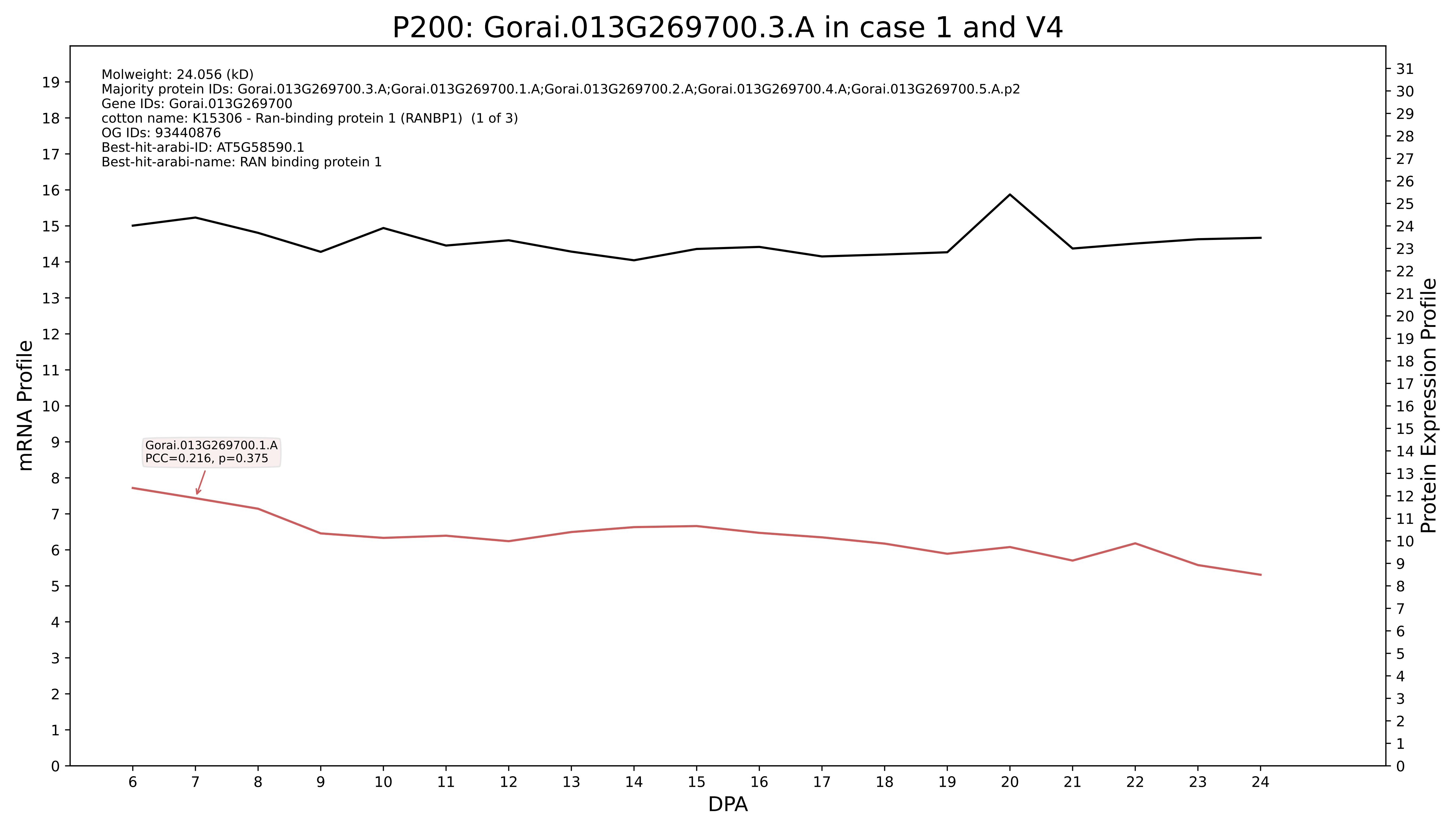

P200: Gorai.013G269700.3.A in case 1 and V4
Molweight: 24.056 (kD)
Majority protein IDs: Gorai.013G269700.3.A;Gorai.013G269700.1.A;Gorai.013G269700.2.A;Gorai.013G269700.4.A;Gorai.013G269700.5.A.p2
Gene IDs: Gorai.013G269700
cotton name: K15306 - Ran-binding protein 1 (RANBP1) (1 of 3)
OG IDs: 93440876
Best-hit-arabi-ID: AT5G58590.1
Best-hit-arabi-name: RAN binding protein 1
Gorai.013G269700.1.A
PCC=0.216, p=0.375
mRNA Profile
Protein Expression Profile
DPA

P200: Gorai.013G268400.1.A in case 1 and V4

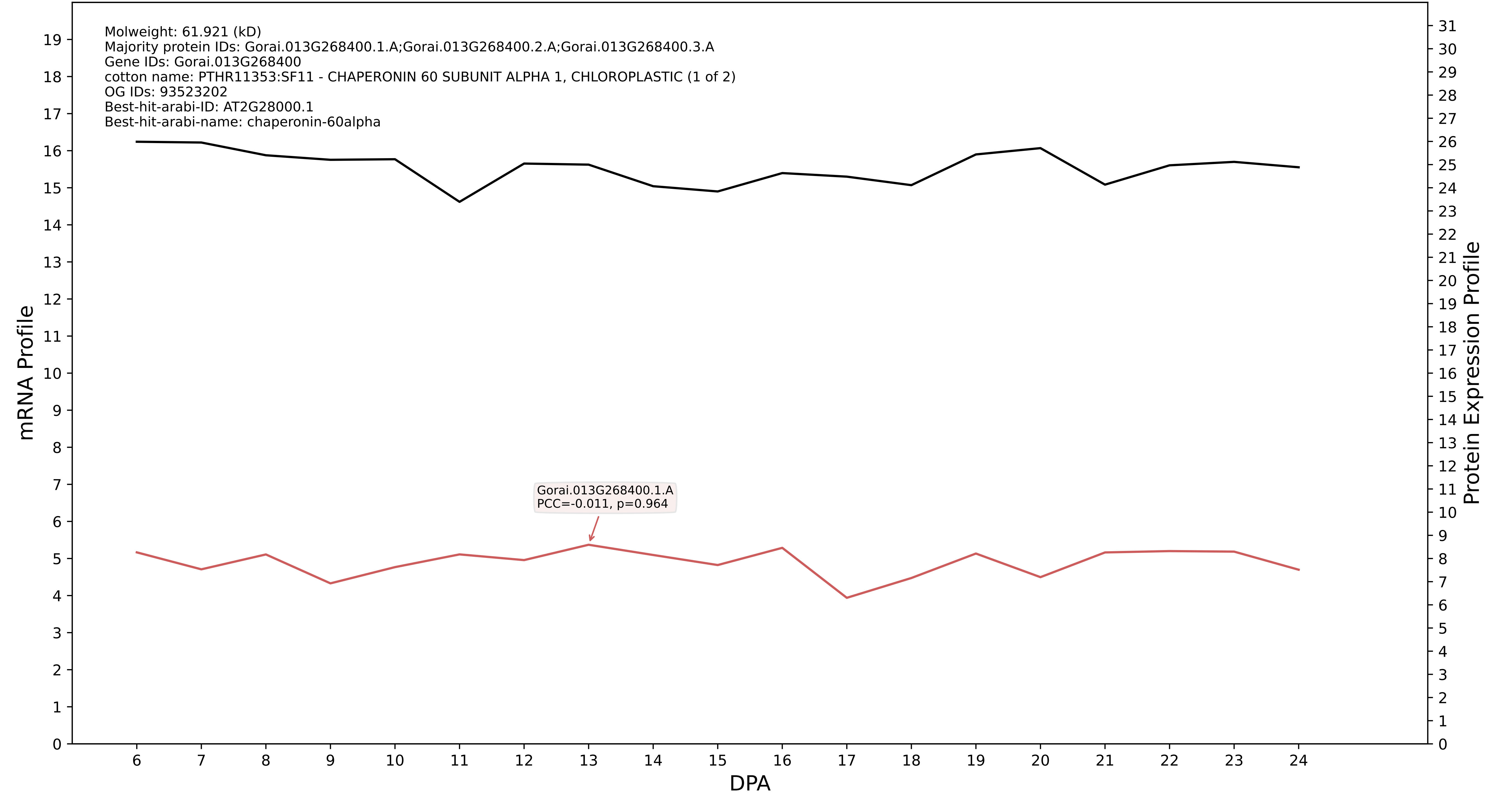

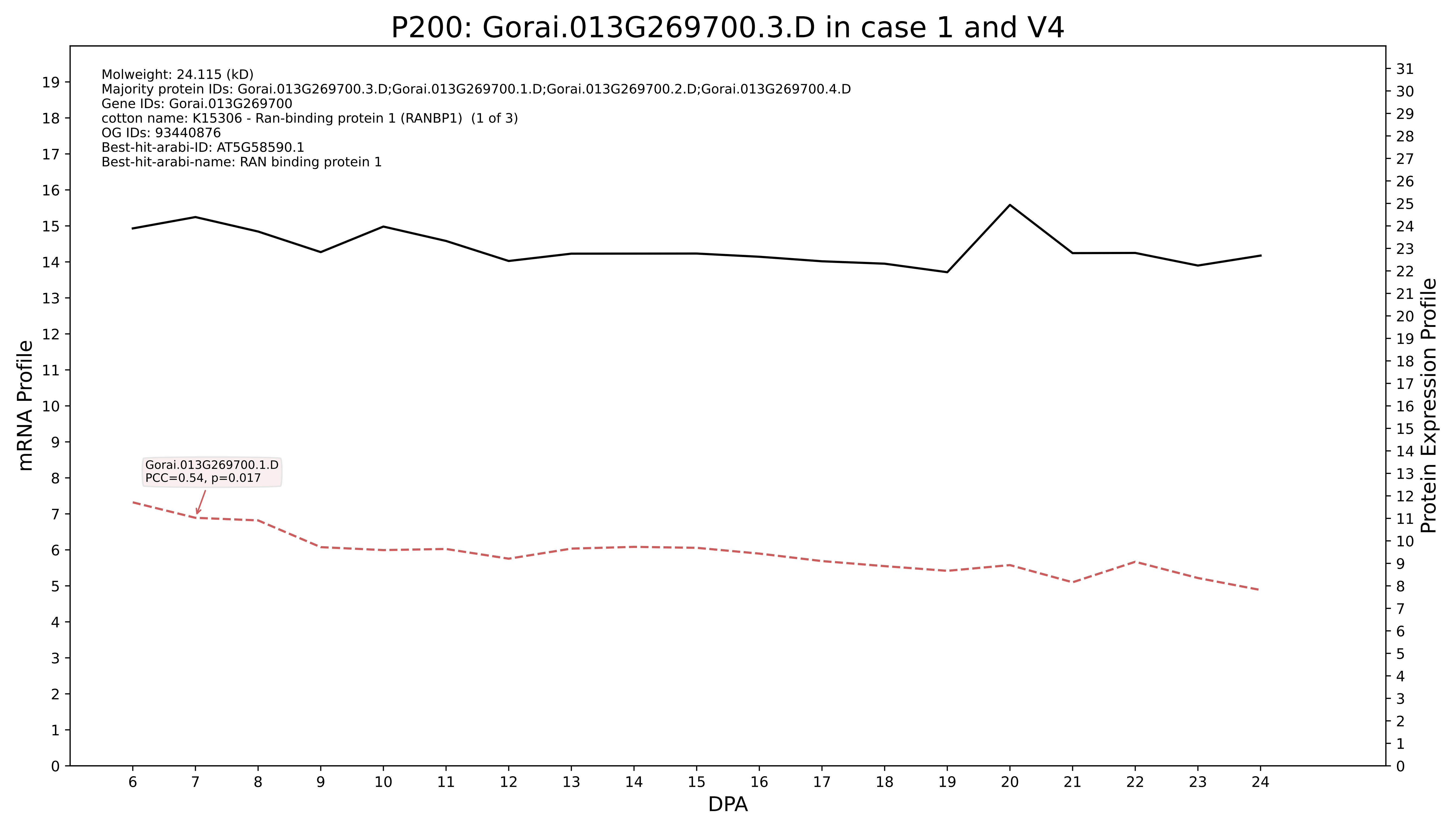
P200: Gorai.013G269700.3.D in case 1 and V4
Molweight: 24.115 (kD)
Majority protein IDs: Gorai.013G269700.3.D;Gorai.013G269700.1.D;Gorai.013G269700.2.D;Gorai.013G269700.4.D
Gene IDs: Gorai.013G269700
cotton name: K15306 - Ran-binding protein 1 (RANBP1) (1 of 3)
OG IDs: 93440876
Best-hit-arabi-ID: AT5G58590.1
Best-hit-arabi-name: RAN binding protein 1
Gorai.013G269700.1.D
PCC=0.54, p=0.017
mRNA Profile
Protein Expression Profile
DPA

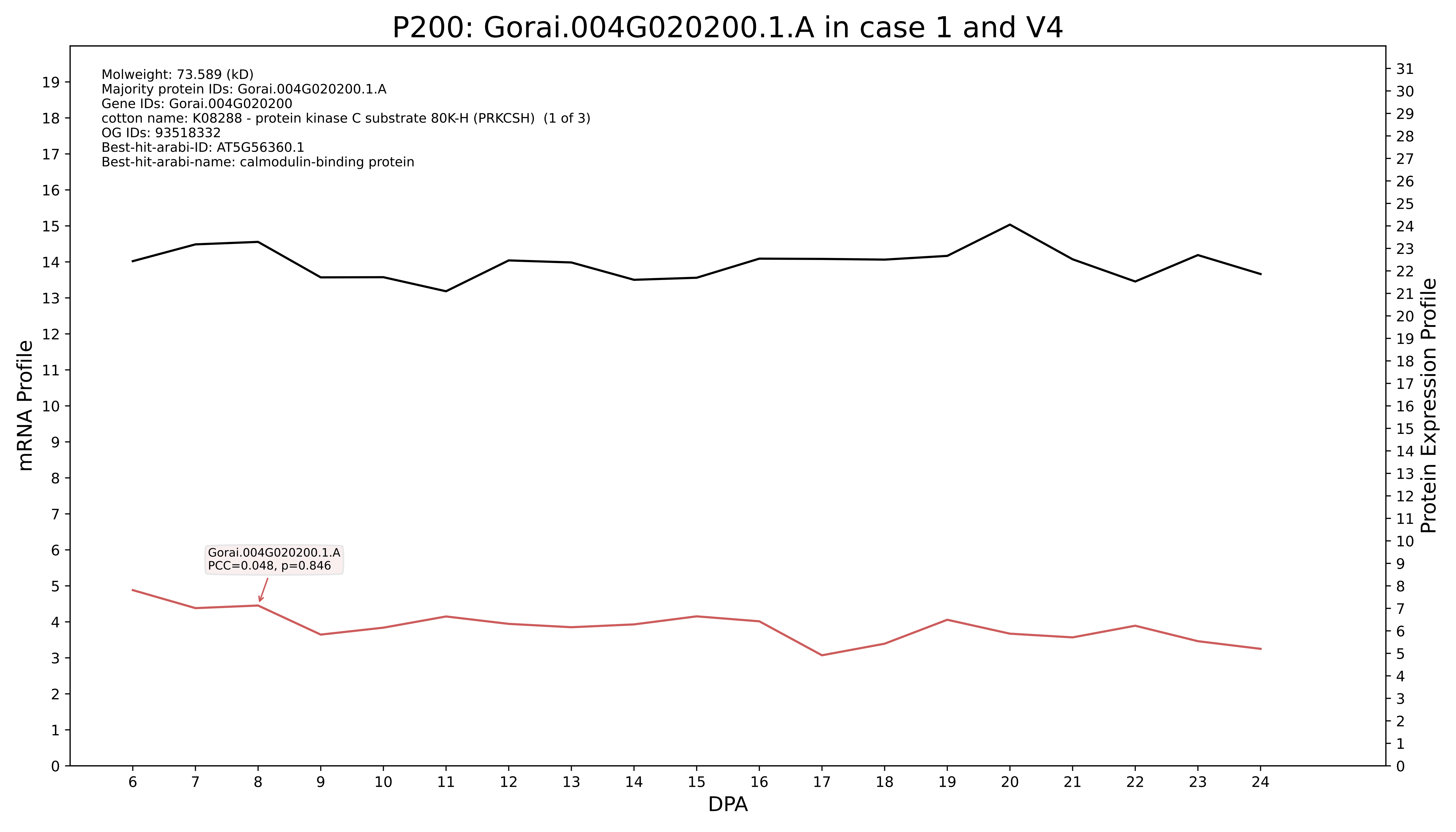
P200: Gorai.004G020200.1.A in case 1 and V4
Molweight: 73.589 (kD)
Majority protein IDs: Gorai.004G020200.1.A
Gene IDs: Gorai.004G020200
cotton name: K08288 - protein kinase C substrate 80K-H (PRKCSH) (1 of 3)
OG IDs: 93518332
Best-hit-arabi-ID: AT5G56360.1
Best-hit-arabi-name: calmodulin-binding protein
Gorai.004G020200.1.A
PCC=0.048, p=0.846
mRNA Profile
Protein Expression Profile
DPA

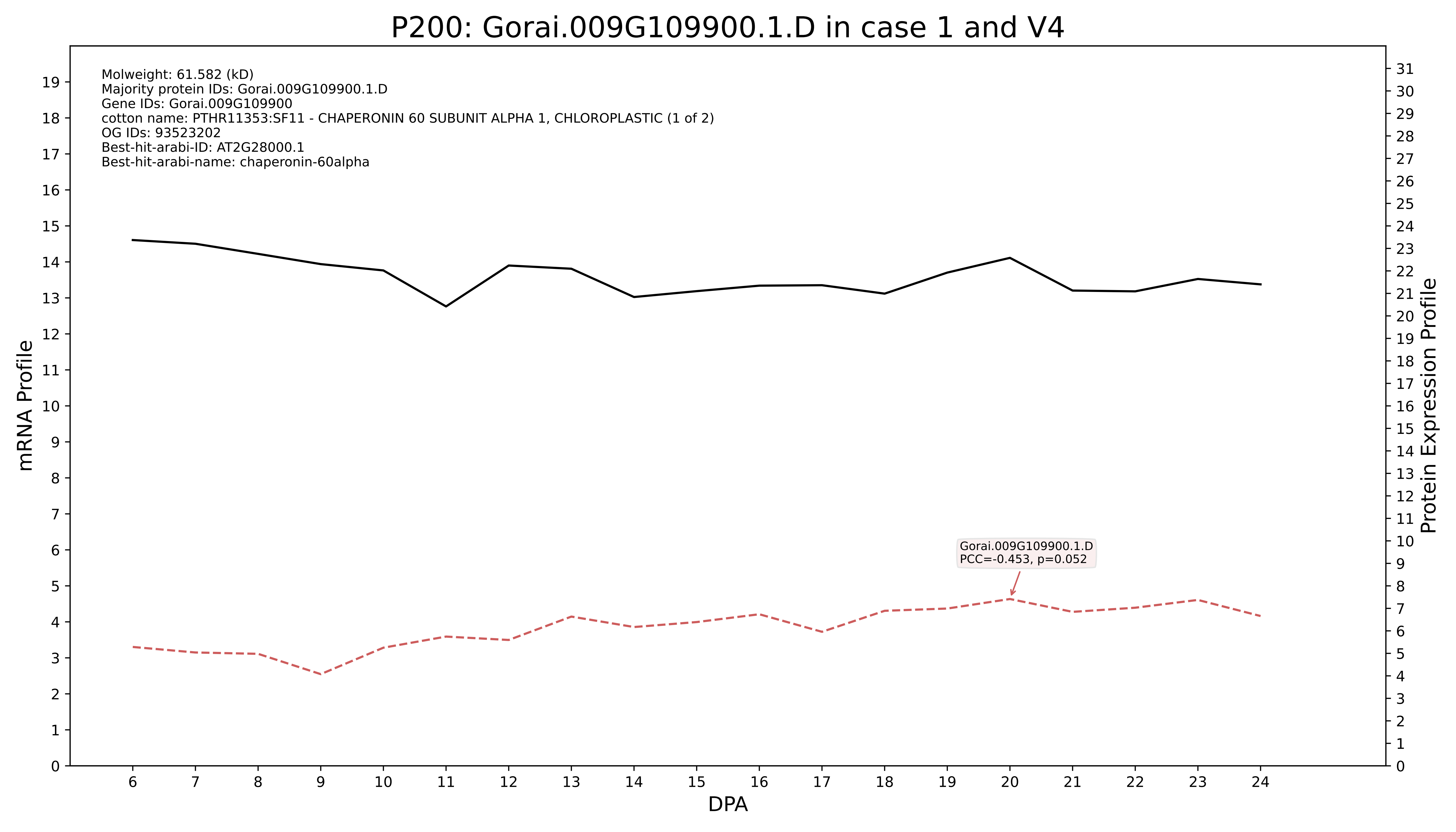
P200: Gorai.009G109900.1.D in case 1 and V4
Molweight: 61.582 (kD)
Majority protein IDs: Gorai.009G109900.1.D
Gene IDs: Gorai.009G109900
cotton name: PTHR11353:SF11 - CHAPERONIN 60 SUBUNIT ALPHA 1, CHLOROPLASTIC (1 of 2)
OG IDs: 93523202
Best-hit-arabi-ID: AT2G28000.1
Best-hit-arabi-name: chaperonin-60alpha
Gorai.009G109900.1.D
PCC=-0.453, p=0.052
mRNA Profile
Protein Expression Profile
DPA

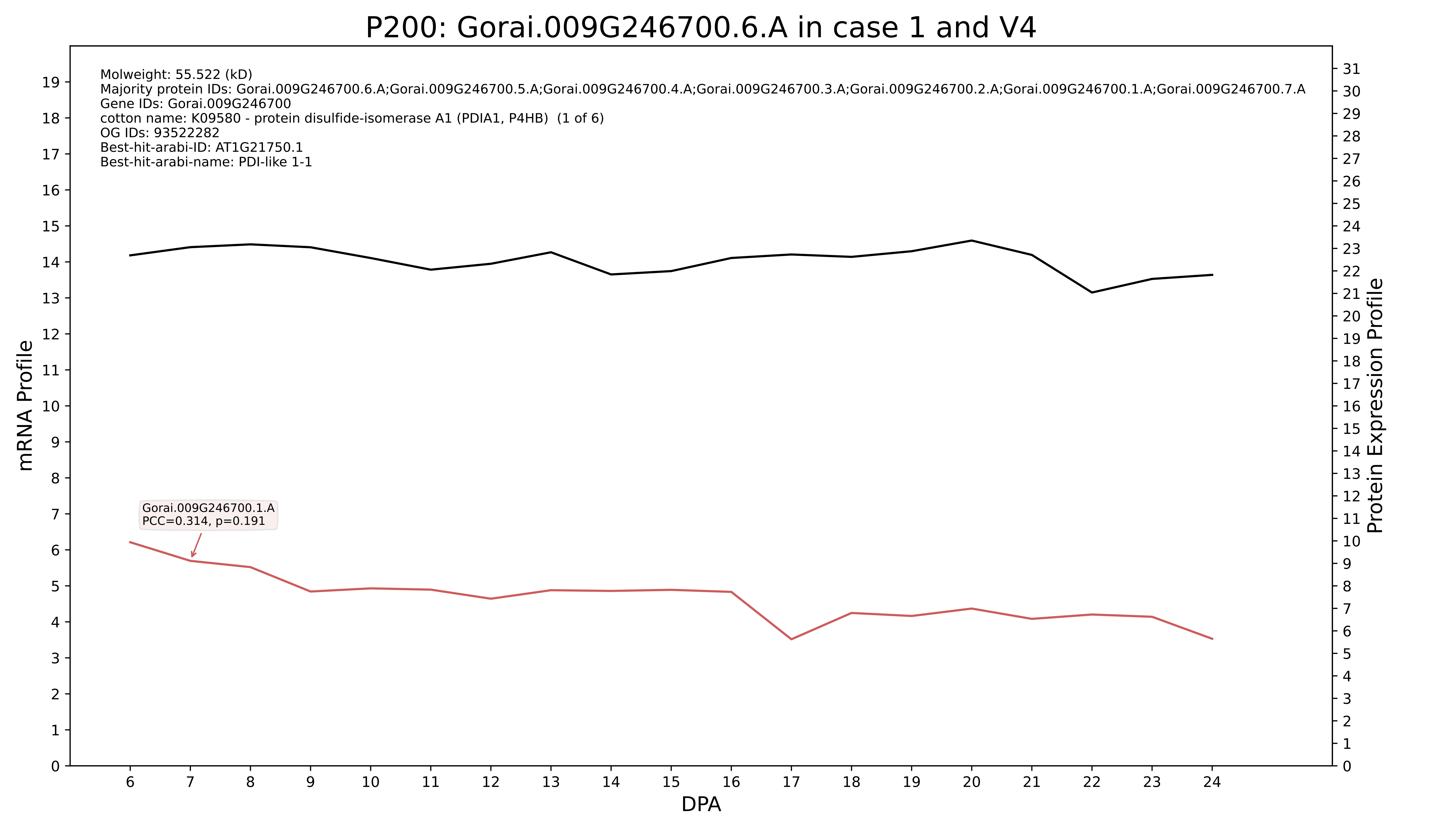

P200: Gorai.009G246700.6.A in case 1 and V4
Molweight: 55.522 (kD)
Majority protein IDs: Gorai.009G246700.6.A;Gorai.009G246700.5.A;Gorai.009G246700.4.A;Gorai.009G246700.3.A;Gorai.009G246700.2.A;Gorai.009G246700.1.A;Gorai.009G246700.7.A
Gene IDs: Gorai.009G246700
cotton name: K09580 - protein disulfide-isomerase A1 (PDIA1, P4HB) (1 of 6)
OG IDs: 93522282
Best-hit-arabi-ID: AT1G21750.1
Best-hit-arabi-name: PDI-like 1-1
Gorai.009G246700.1.A
PCC=0.314, p=0.191
mRNA Profile
Protein Expression Profile
DPA

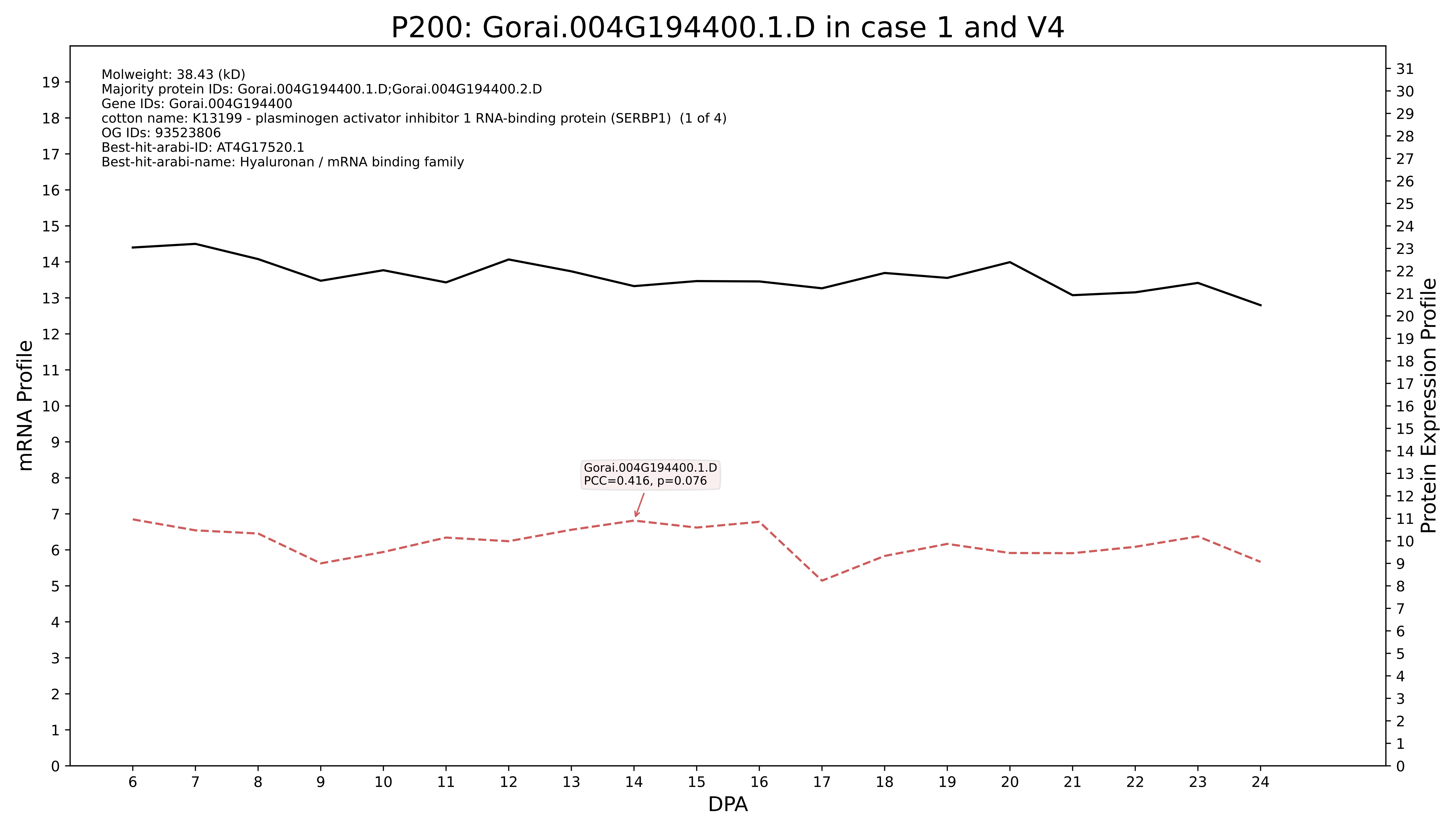
P200: Gorai.004G194400.1.D in case 1 and V4
Molweight: 38.43 (kD)
Majority protein IDs: Gorai.004G194400.1.D;Gorai.004G194400.2.D
Gene IDs: Gorai.004G194400
cotton name: K13199 - plasminogen activator inhibitor 1 RNA-binding protein (SERBP1) (1 of 4)
OG IDs: 93523806
Best-hit-arabi-ID: AT4G17520.1
Best-hit-arabi-name: Hyaluronan / mRNA binding family
Gorai.004G194400.1.D
PCC=0.416, p=0.076
mRNA Profile
Protein Expression Profile
DPA

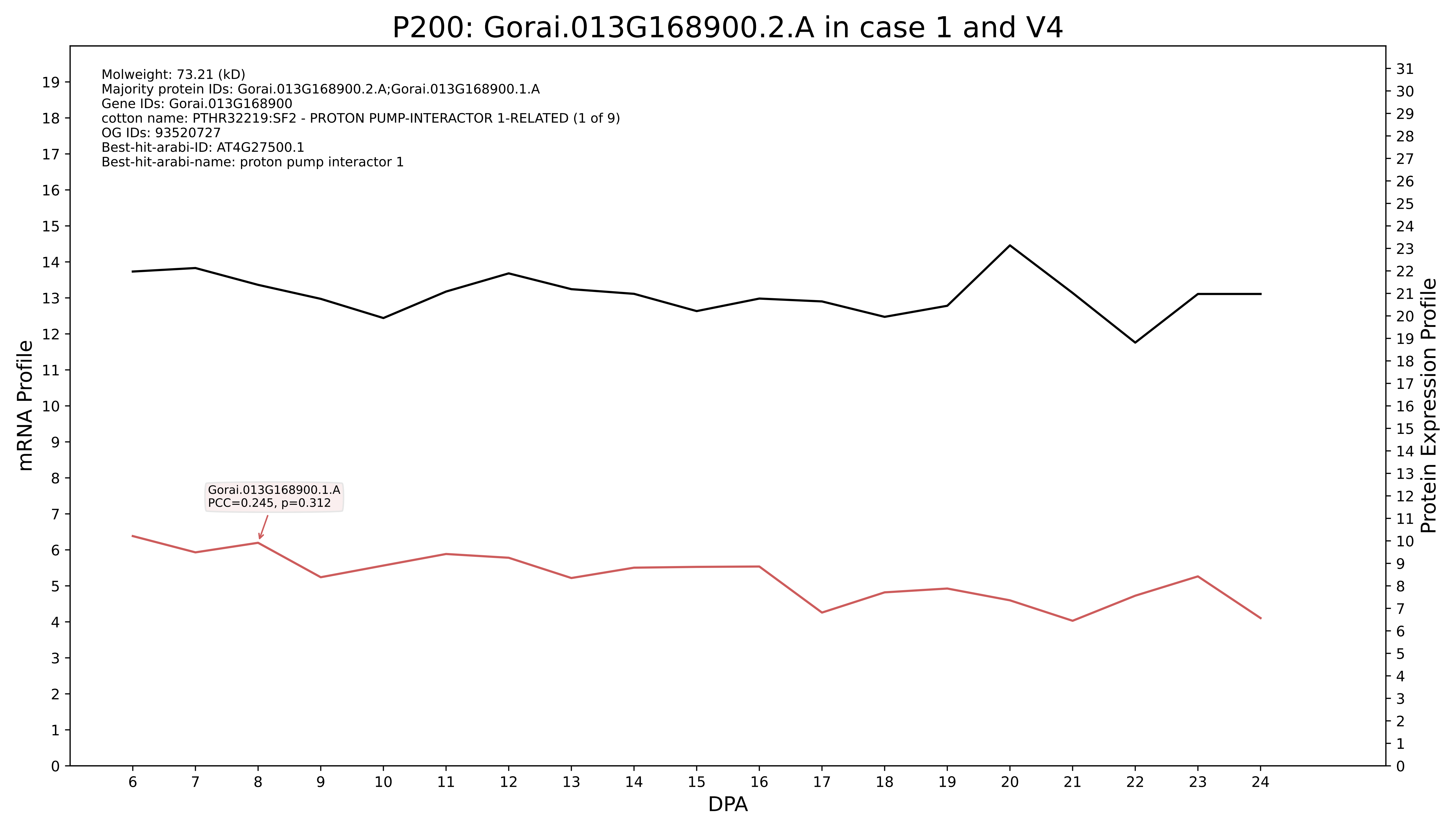

P200: Gorai.013G168900.2.A in case 1 and V4
Molweight: 73.21 (kD)
Majority protein IDs: Gorai.013G168900.2.A;Gorai.013G168900.1.A
Gene IDs: Gorai.013G168900
cotton name: PTHR32219:SF2 - PROTON PUMP-INTERACTOR 1-RELATED (1 of 9)
OG IDs: 93520727
Best-hit-arabi-ID: AT4G27500.1
Best-hit-arabi-name: proton pump interactor 1
Gorai.013G168900.1.A
PCC=0.245, p=0.312
mRNA Profile
Protein Expression Profile
DPA

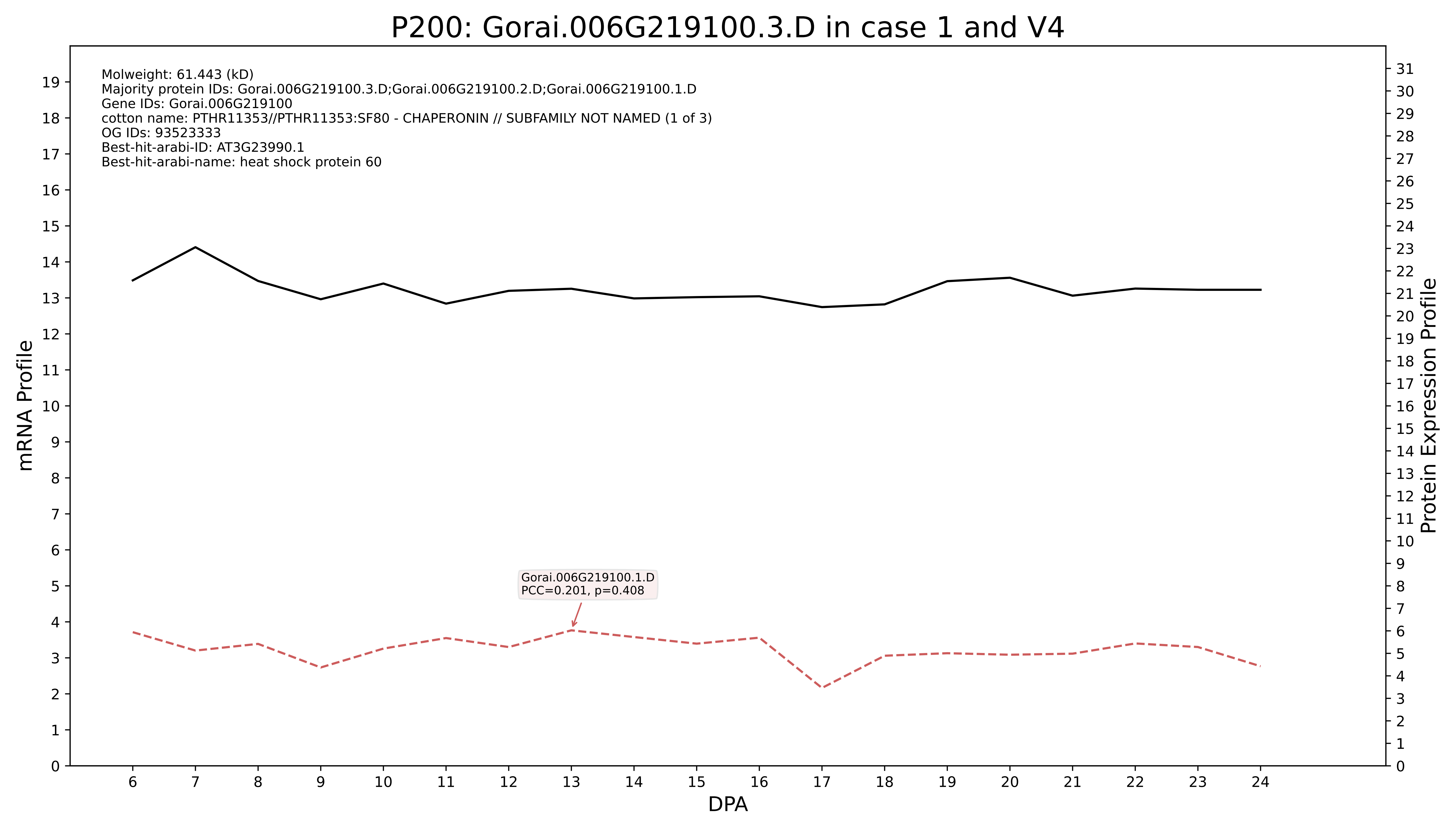

P200: Gorai.006G219100.3.D in case 1 and V4
Molweight: 61.443 (kD)
Majority protein IDs: Gorai.006G219100.3.D;Gorai.006G219100.2.D;Gorai.006G219100.1.D
Gene IDs: Gorai.006G219100
cotton name: PTHR11353//PTHR11353:SF80 - CHAPERONIN // SUBFAMILY NOT NAMED (1 of 3)
OG IDs: 93523333
Best-hit-arabi-ID: AT3G23990.1
Best-hit-arabi-name: heat shock protein 60
Gorai.006G219100.1.D
PCC=0.201, p=0.408
mRNA Profile
Protein Expression Profile
DPA

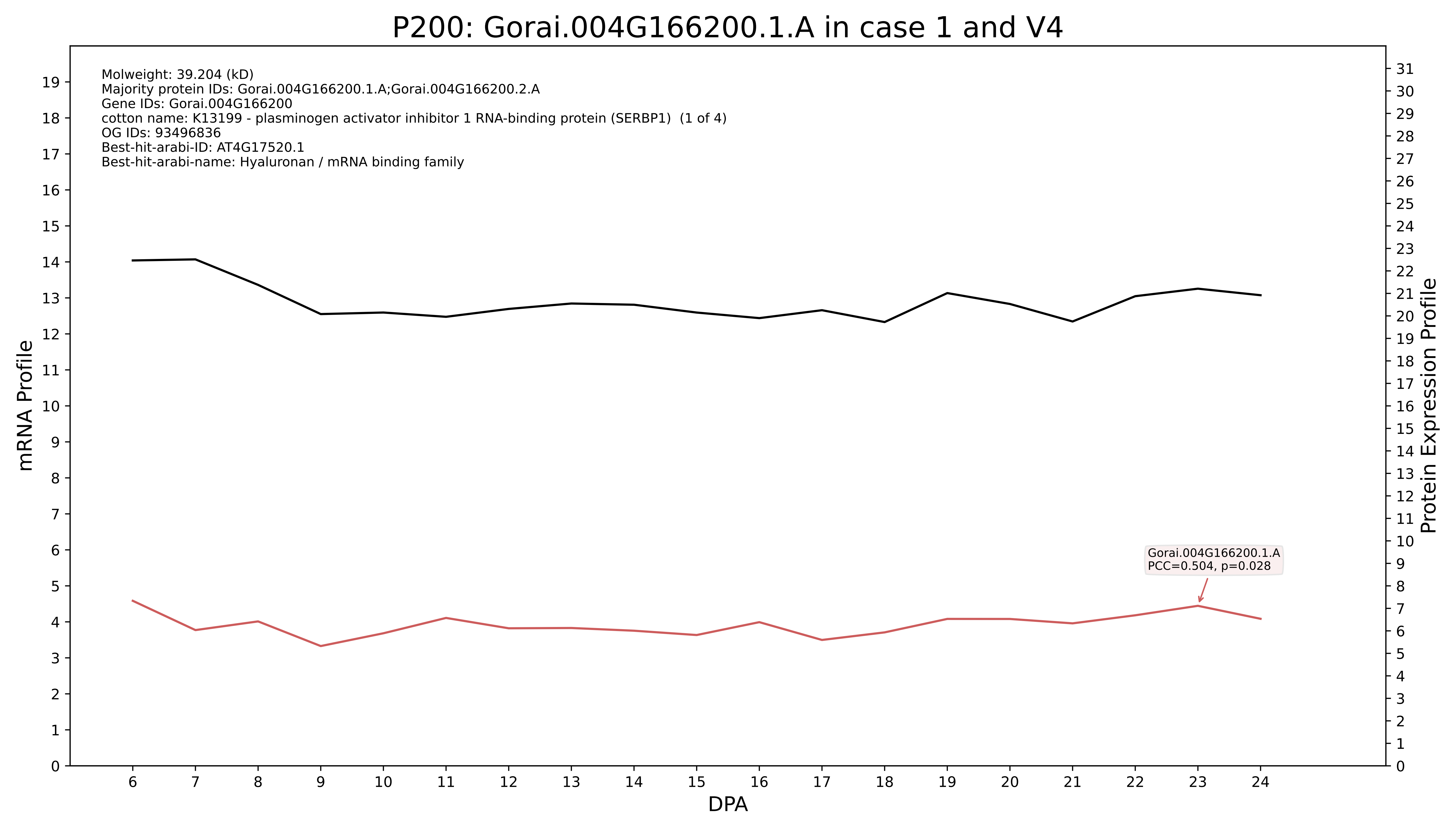
P200: Gorai.004G166200.1.A in case 1 and V4
Molweight: 39.204 (kD)
Majority protein IDs: Gorai.004G166200.1.A;Gorai.004G166200.2.A
Gene IDs: Gorai.004G166200
cotton name: K13199 - plasminogen activator inhibitor 1 RNA-binding protein (SERBP1) (1 of 4)
OG IDs: 93496836
Best-hit-arabi-ID: AT4G17520.1
Best-hit-arabi-name: Hyaluronan / mRNA binding family
Gorai.004G166200.1.A
PCC=0.504, p=0.028
mRNA Profile
Protein Expression Profile
DPA
0
1
2
3
4
5
6
7
8
9
10
11
12
13
14
15
16
17
18
19
20
21
22
23
24
25
26
27
28
29
30
31

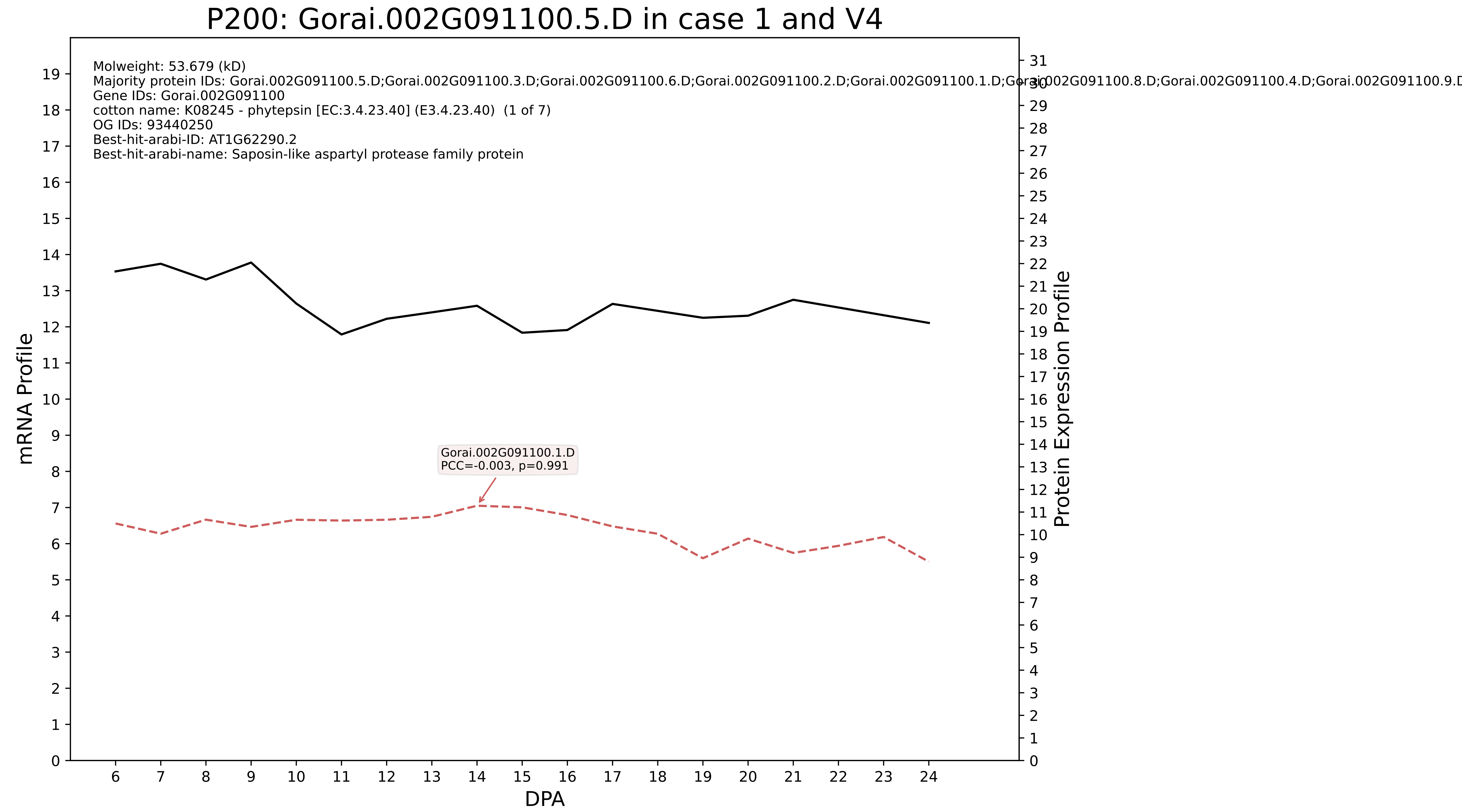

P200: Gorai.002G091100.5.D in case 1 and V4
Molweight: 53.679 (kD)
Majority protein IDs: Gorai.002G091100.5.D;Gorai.002G091100.3.D;Gorai.002G091100.6.D;Gorai.002G091100.2.D;Gorai.002G091100.1.D;Gorai.002G091100.8.D;Gorai.002G091100.4.D;Gorai.002G091100.9.D
Gene IDs: Gorai.002G091100
cotton name: K08245 - phytepsin [EC:3.4.23.40] (E3.4.23.40) (1 of 7)
OG IDs: 93440250
Best-hit-arabi-ID: AT1G62290.2
Best-hit-arabi-name: Saposin-like aspartyl protease family protein
Gorai.002G091100.1.D
PCC=-0.003, p=0.991
mRNA Profile
Protein Expression Profile
DPA

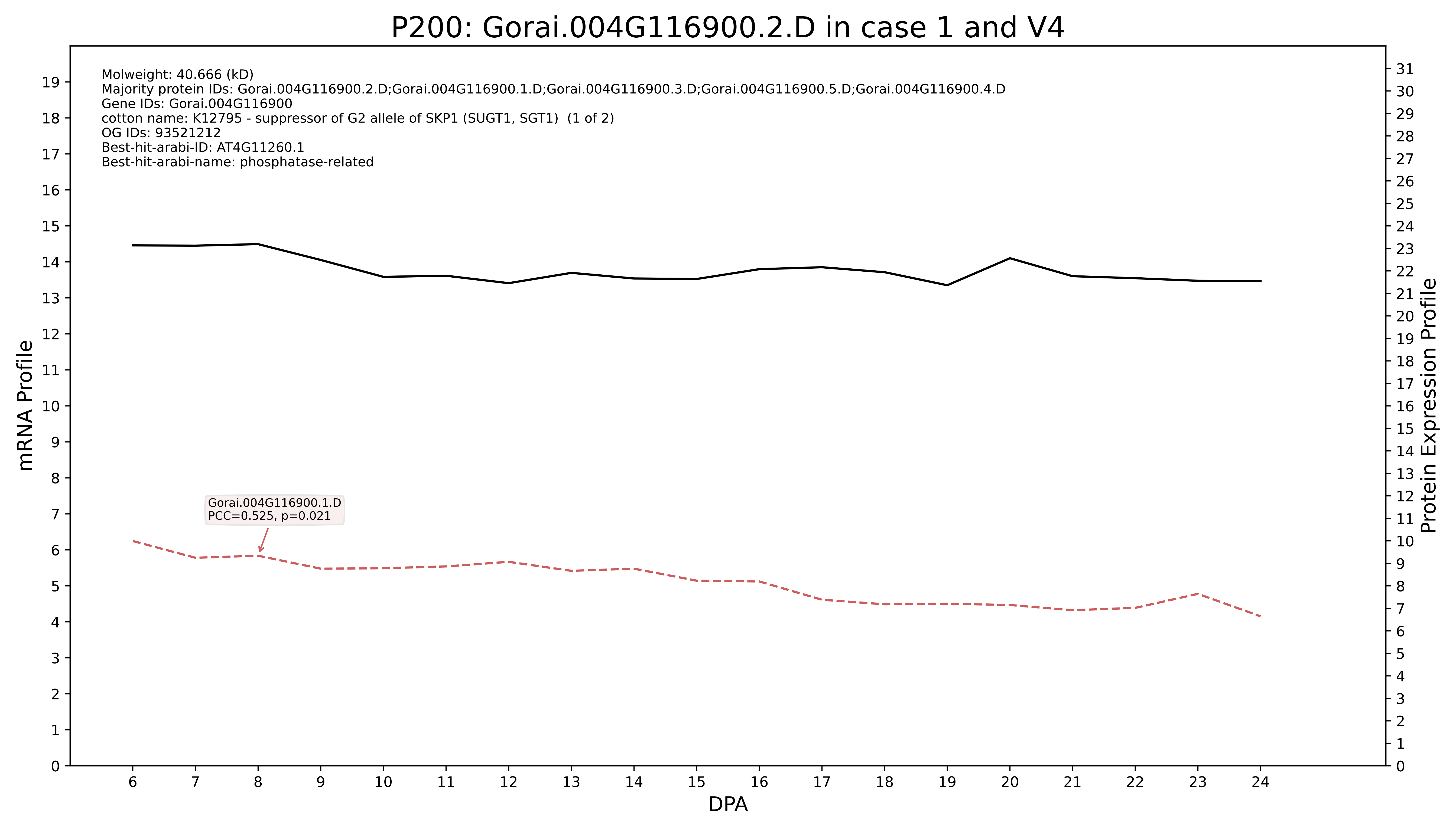
P200: Gorai.004G116900.2.D in case 1 and V4
Molweight: 40.666 (kD)
Majority protein IDs: Gorai.004G116900.2.D;Gorai.004G116900.1.D;Gorai.004G116900.3.D;Gorai.004G116900.5.D;Gorai.004G116900.4.D
Gene IDs: Gorai.004G116900
cotton name: K12795 - suppressor of G2 allele of SKP1 (SUGT1, SGT1) (1 of 2)
OG IDs: 93521212
Best-hit-arabi-ID: AT4G11260.1
Best-hit-arabi-name: phosphatase-related
Gorai.004G116900.1.D
PCC=0.525, p=0.021
mRNA Profile
Protein Expression Profile
DPA

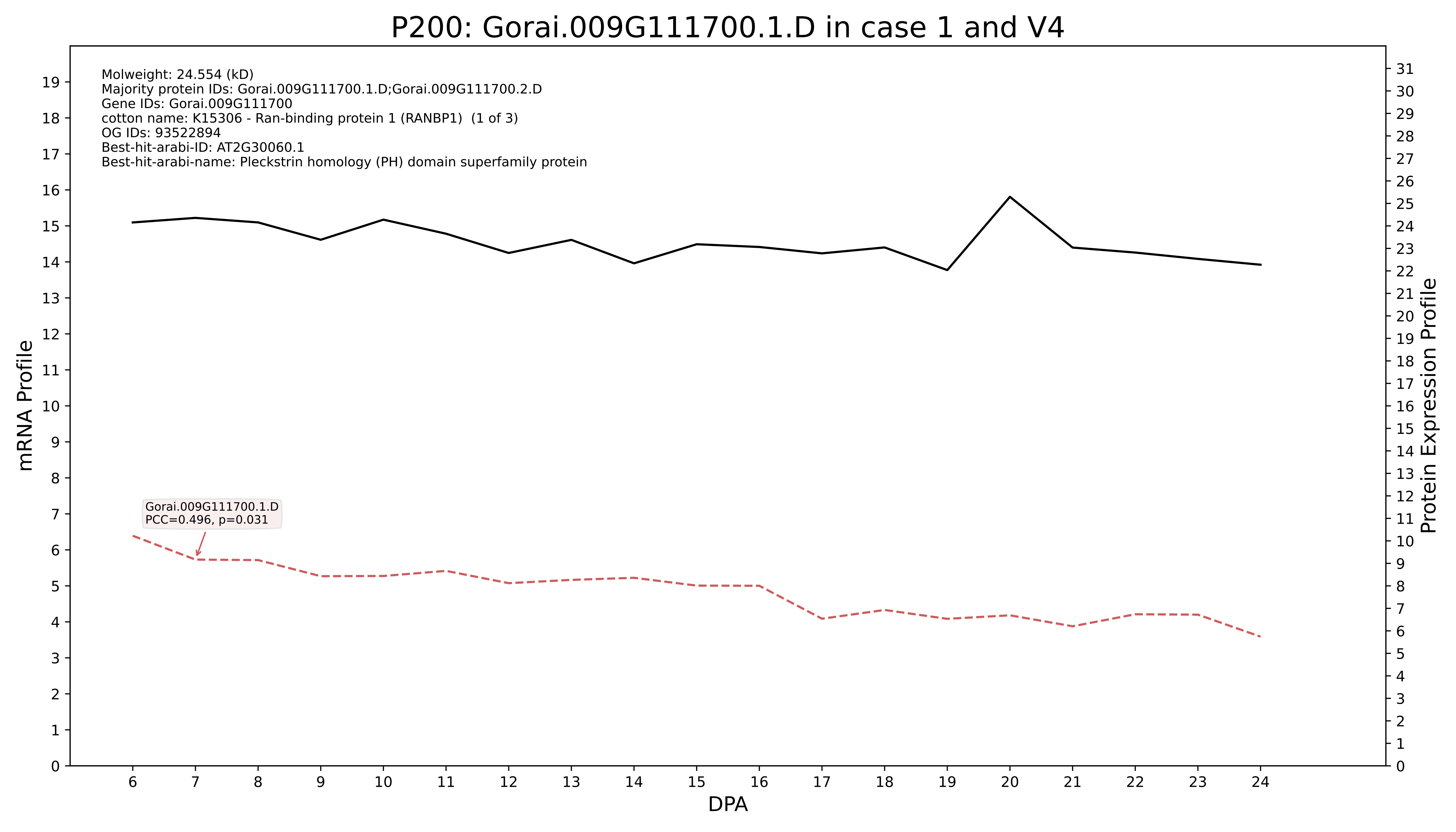

P200: Gorai.009G111700.1.D in case 1 and V4
Molweight: 24.554 (kD)
Majority protein IDs: Gorai.009G111700.1.D;Gorai.009G111700.2.D
Gene IDs: Gorai.009G111700
cotton name: K15306 - Ran-binding protein 1 (RANBP1) (1 of 3)
OG IDs: 93522894
Best-hit-arabi-ID: AT2G30060.1
Best-hit-arabi-name: Pleckstrin homology (PH) domain superfamily protein
Gorai.009G111700.1.D
PCC=0.496, p=0.031
mRNA Profile
Protein Expression Profile
DPA

P200: Gorai.012G082700.1.D in case 1 and V4

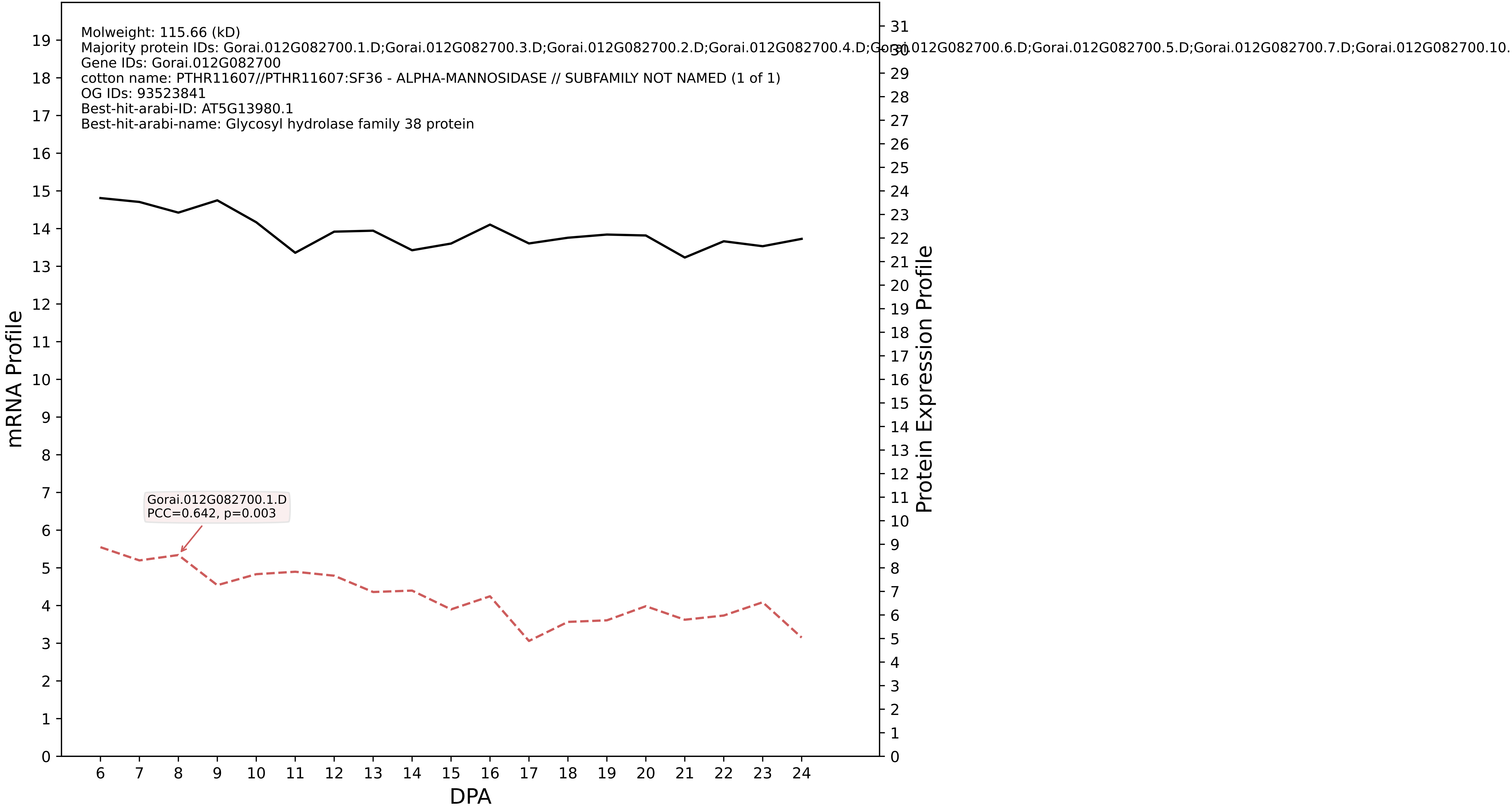

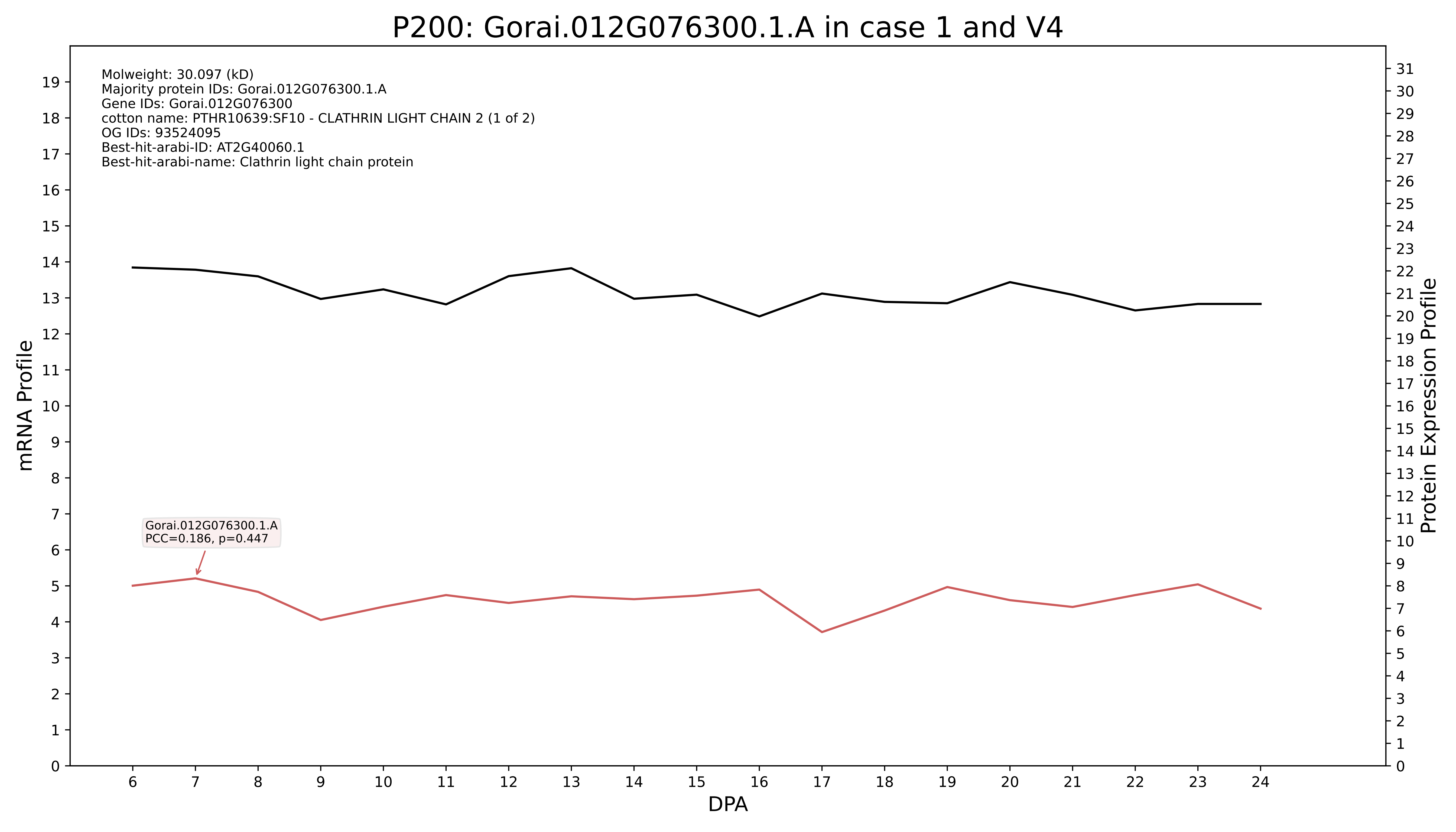

P200: Gorai.012G076300.1.A in case 1 and V4
Molweight: 30.097 (kD)
Majority protein IDs: Gorai.012G076300.1.A
Gene IDs: Gorai.012G076300
cotton name: PTHR10639:SF10 - CLATHRIN LIGHT CHAIN 2 (1 of 2)
OG IDs: 93524095
Best-hit-arabi-ID: AT2G40060.1
Best-hit-arabi-name: Clathrin light chain protein
Gorai.012G076300.1.A
PCC=0.186, p=0.447
mRNA Profile
Protein Expression Profile
DPA

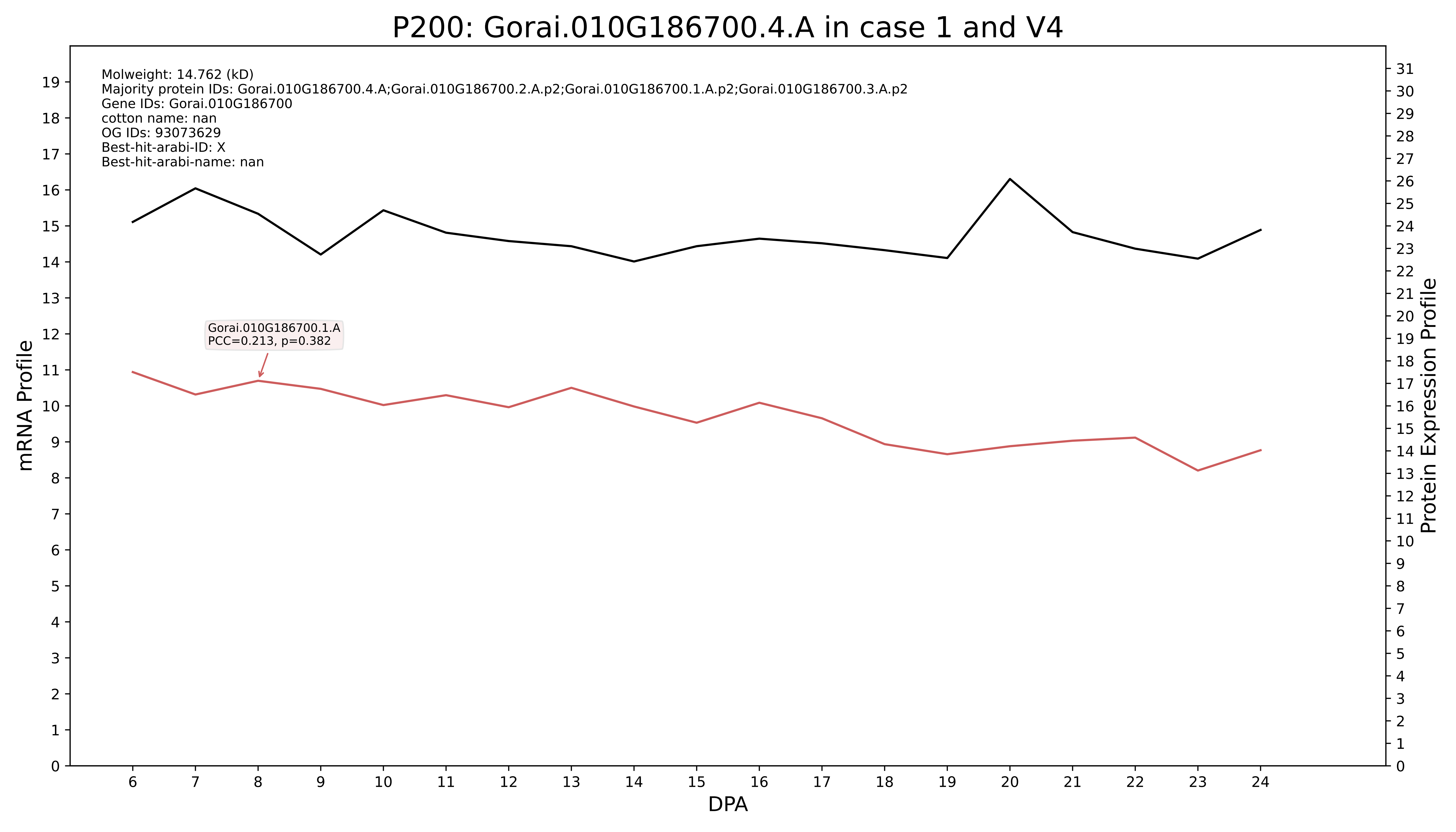
P200: Gorai.010G186700.4.A in case 1 and V4
Molweight: 14.762 (kD)
Majority protein IDs: Gorai.010G186700.4.A;Gorai.010G186700.2.A.p2;Gorai.010G186700.1.A.p2;Gorai.010G186700.3.A.p2
Gene IDs: Gorai.010G186700
cotton name: nan
OG IDs: 93073629
Best-hit-arabi-ID: X
Best-hit-arabi-name: nan
Gorai.010G186700.1.A
PCC=0.213, p=0.382
mRNA Profile
Protein Expression Profile
DPA

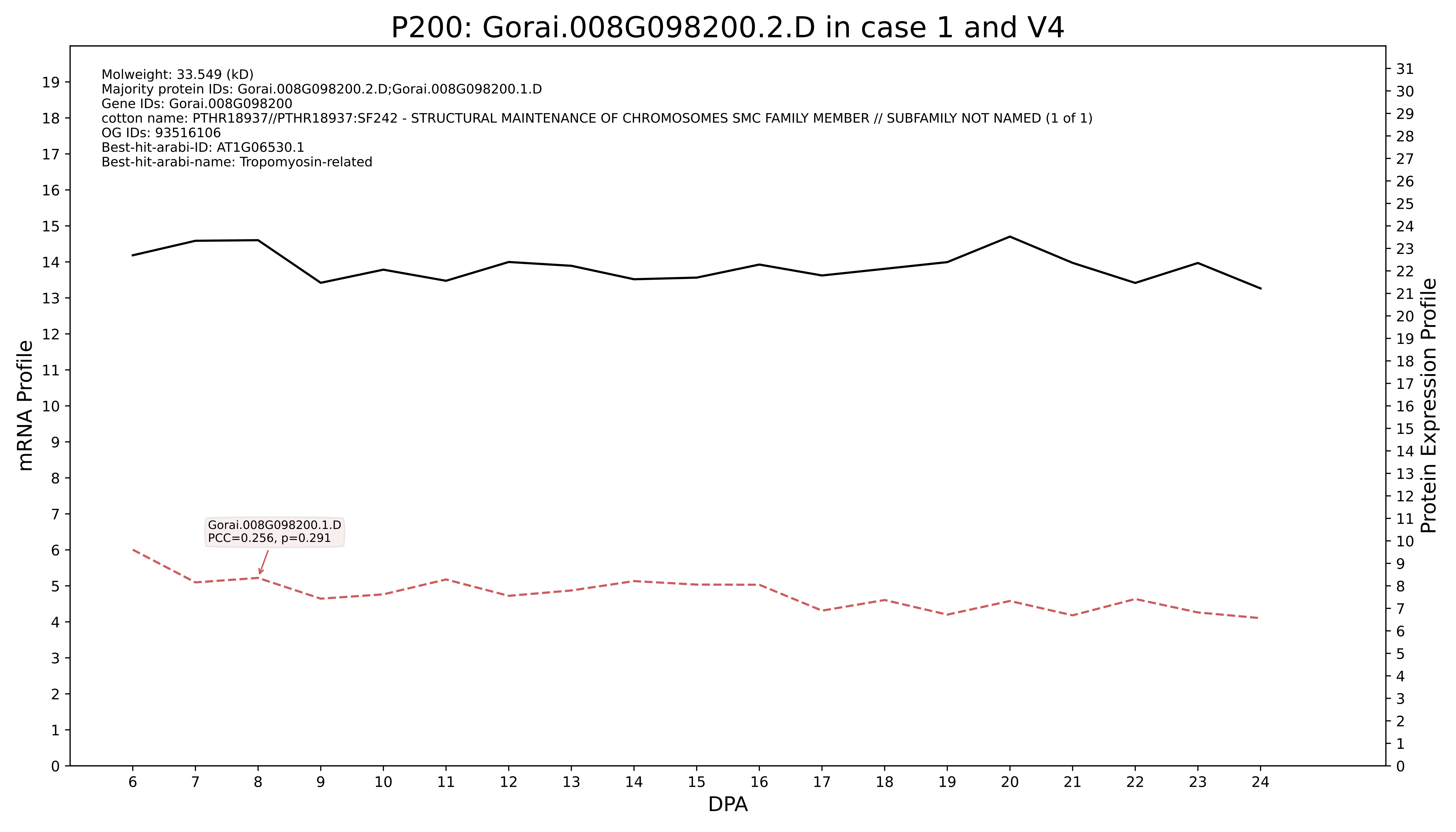

P200: Gorai.008G098200.2.D in case 1 and V4
Molweight: 33.549 (kD)
Majority protein IDs: Gorai.008G098200.2.D;Gorai.008G098200.1.D
Gene IDs: Gorai.008G098200
cotton name: PTHR18937//PTHR18937:SF242 - STRUCTURAL MAINTENANCE OF CHROMOSOMES SMC FAMILY MEMBER // SUBFAMILY NOT NAMED (1 of 1)
OG IDs: 93516106
Best-hit-arabi-ID: AT1G06530.1
Best-hit-arabi-name: Tropomyosin-related
Gorai.008G098200.1.D
PCC=0.256, p=0.291
mRNA Profile
Protein Expression Profile
DPA

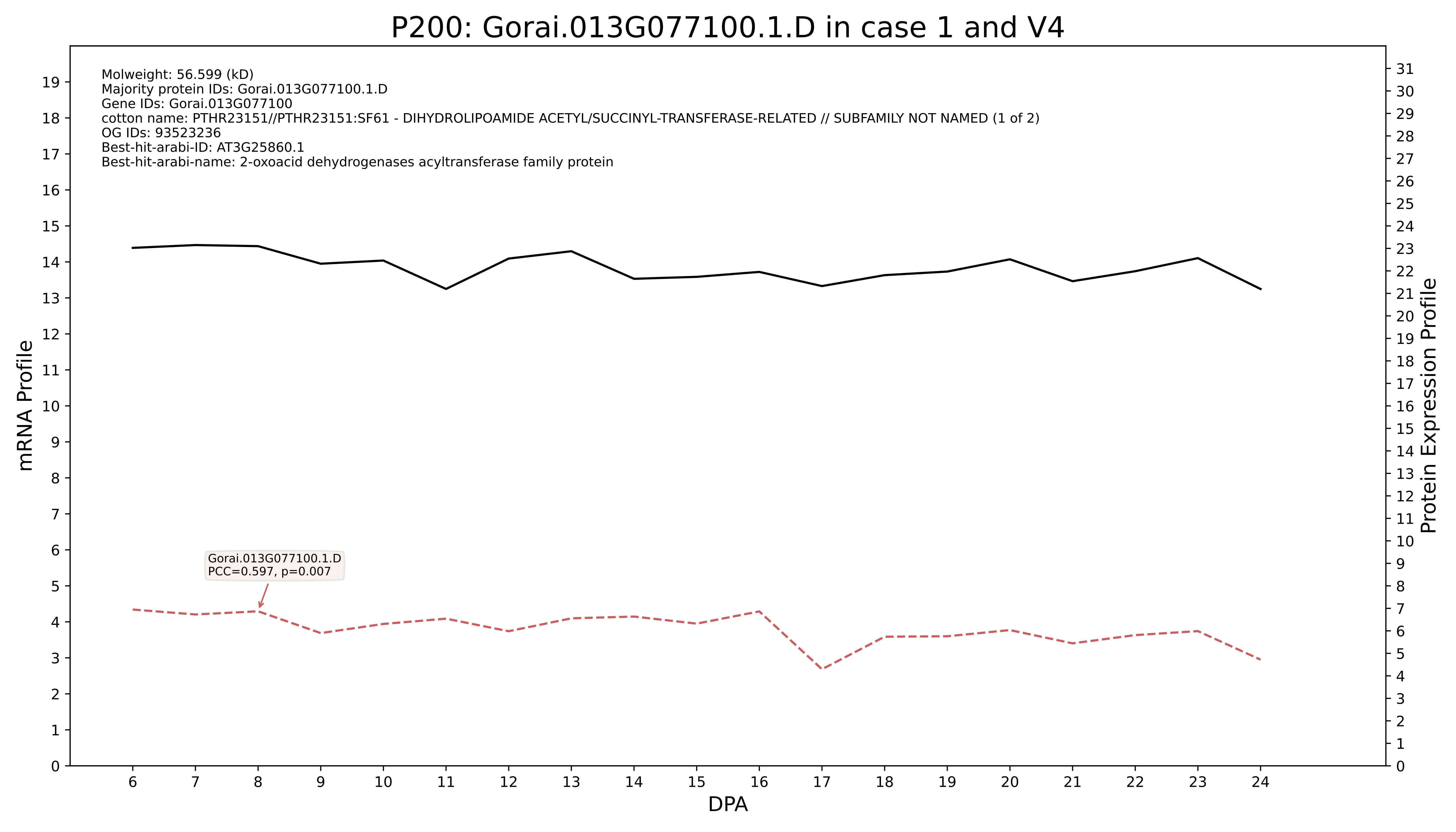
P200: Gorai.013G077100.1.D in case 1 and V4
Molweight: 56.599 (kD)
Majority protein IDs: Gorai.013G077100.1.D
Gene IDs: Gorai.013G077100
cotton name: PTHR23151//PTHR23151:SF61 - DIHYDROLIPOAMIDE ACETYL/SUCCINYL-TRANSFERASE-RELATED // SUBFAMILY NOT NAMED (1 of 2)
OG IDs: 93523236
Best-hit-arabi-ID: AT3G25860.1
Best-hit-arabi-name: 2-oxoacid dehydrogenases acyltransferase family protein
Gorai.013G077100.1.D
PCC=0.597, p=0.007
mRNA Profile
Protein Expression Profile
DPA

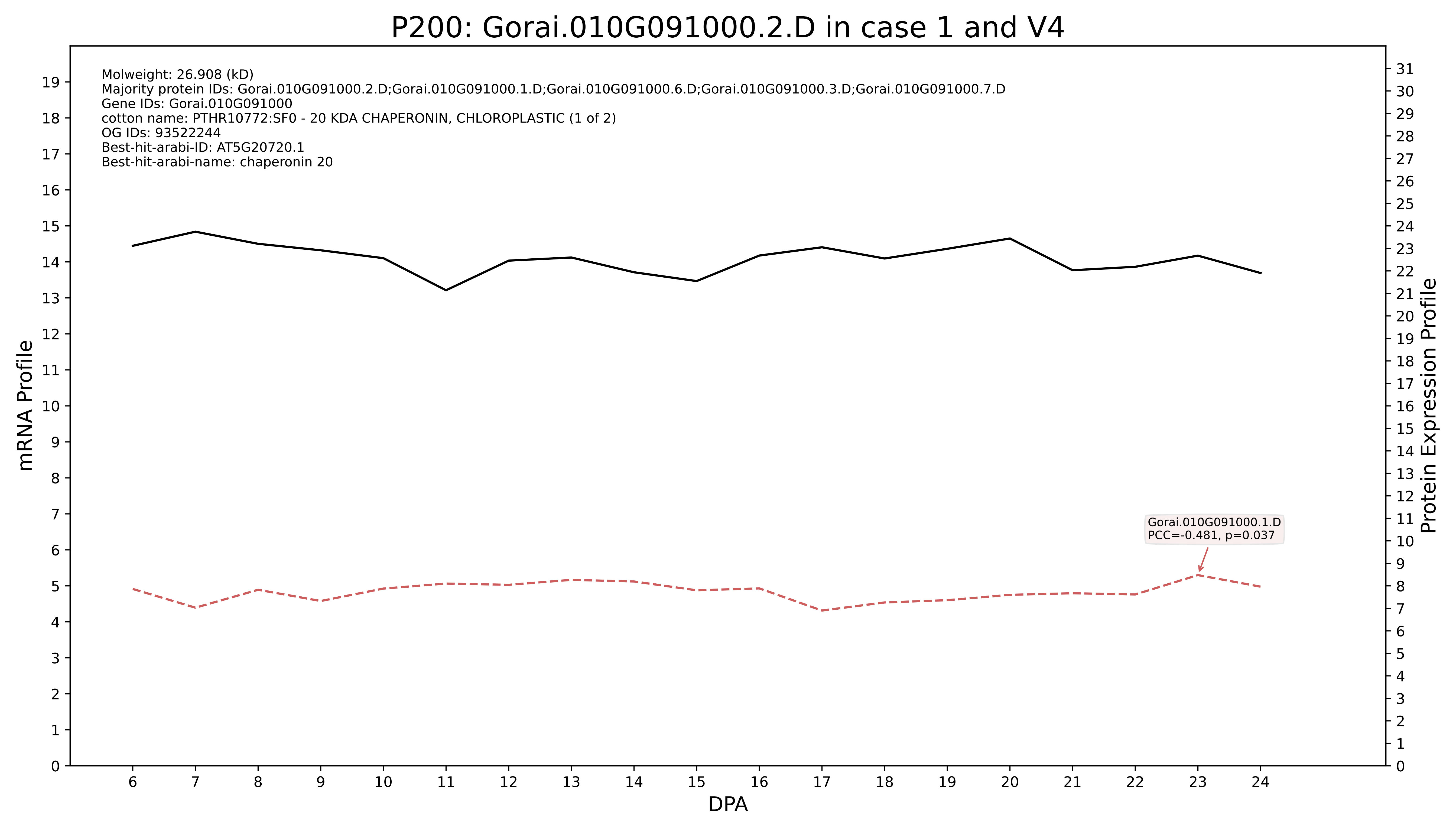
P200: Gorai.010G091000.2.D in case 1 and V4
Molweight: 26.908 (kD)
Majority protein IDs: Gorai.010G091000.2.D;Gorai.010G091000.1.D;Gorai.010G091000.6.D;Gorai.010G091000.3.D;Gorai.010G091000.7.D
Gene IDs: Gorai.010G091000
cotton name: PTHR10772:SF0 - 20 KDA CHAPERONIN, CHLOROPLASTIC (1 of 2)
OG IDs: 93522244
Best-hit-arabi-ID: AT5G20720.1
Best-hit-arabi-name: chaperonin 20
Gorai.010G091000.1.D
PCC=-0.481, p=0.037
mRNA Profile
Protein Expression Profile
DPA
0
1
2
3
4
5
6
7
8
9
10
11
12
13
14
15
16
17
18
19
20
21
22
23
24
25
26
27
28
29
30
31

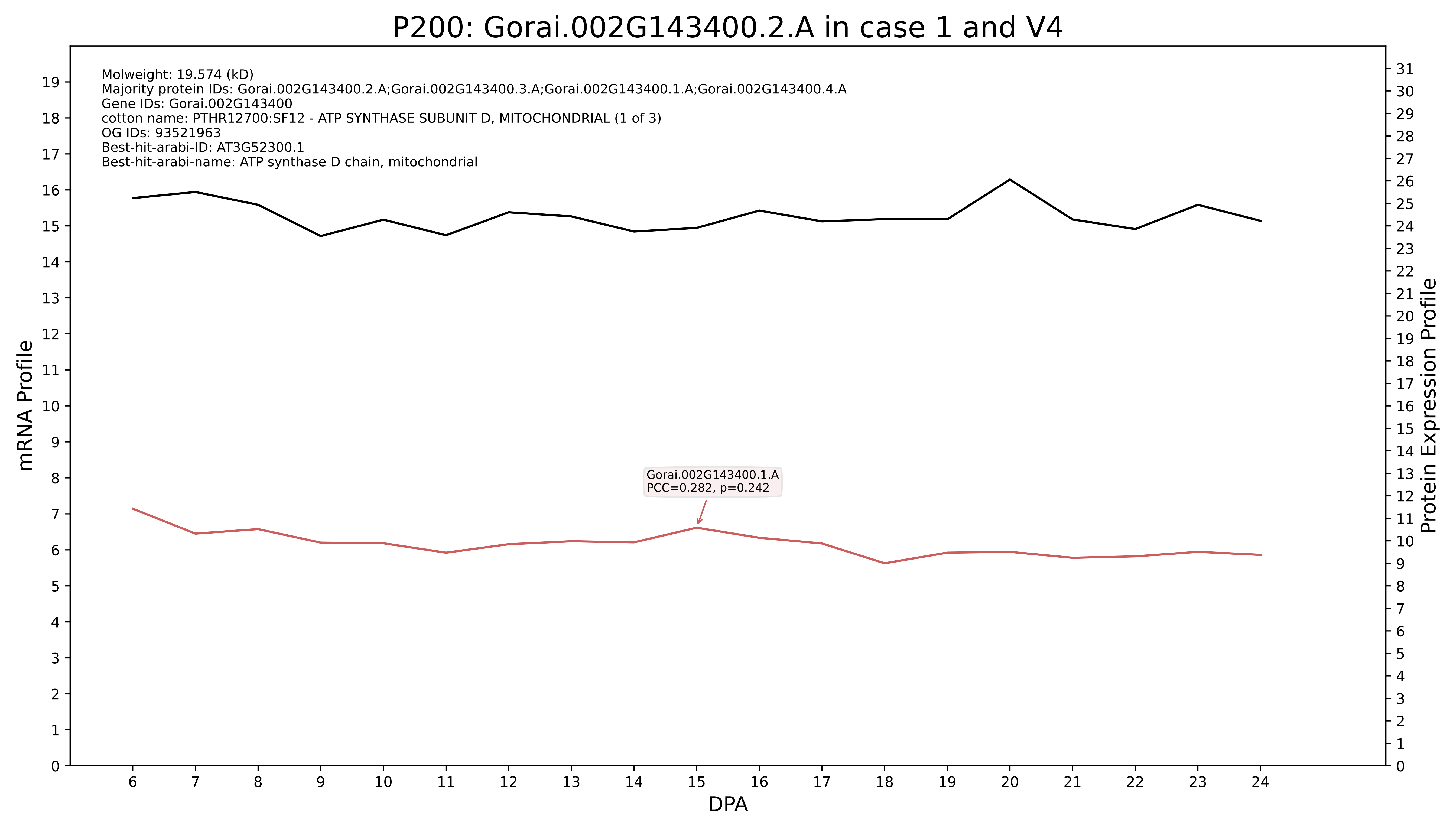

P200: Gorai.002G143400.2.A in case 1 and V4
Molweight: 19.574 (kD)
Majority protein IDs: Gorai.002G143400.2.A;Gorai.002G143400.3.A;Gorai.002G143400.1.A;Gorai.002G143400.4.A
Gene IDs: Gorai.002G143400
cotton name: PTHR12700:SF12 - ATP SYNTHASE SUBUNIT D, MITOCHONDRIAL (1 of 3)
OG IDs: 93521963
Best-hit-arabi-ID: AT3G52300.1
Best-hit-arabi-name: ATP synthase D chain, mitochondrial
Gorai.002G143400.1.A
PCC=0.282, p=0.242
mRNA Profile
Protein Expression Profile
DPA

P200: Gorai.006G232200.1.A in case 1 and V4

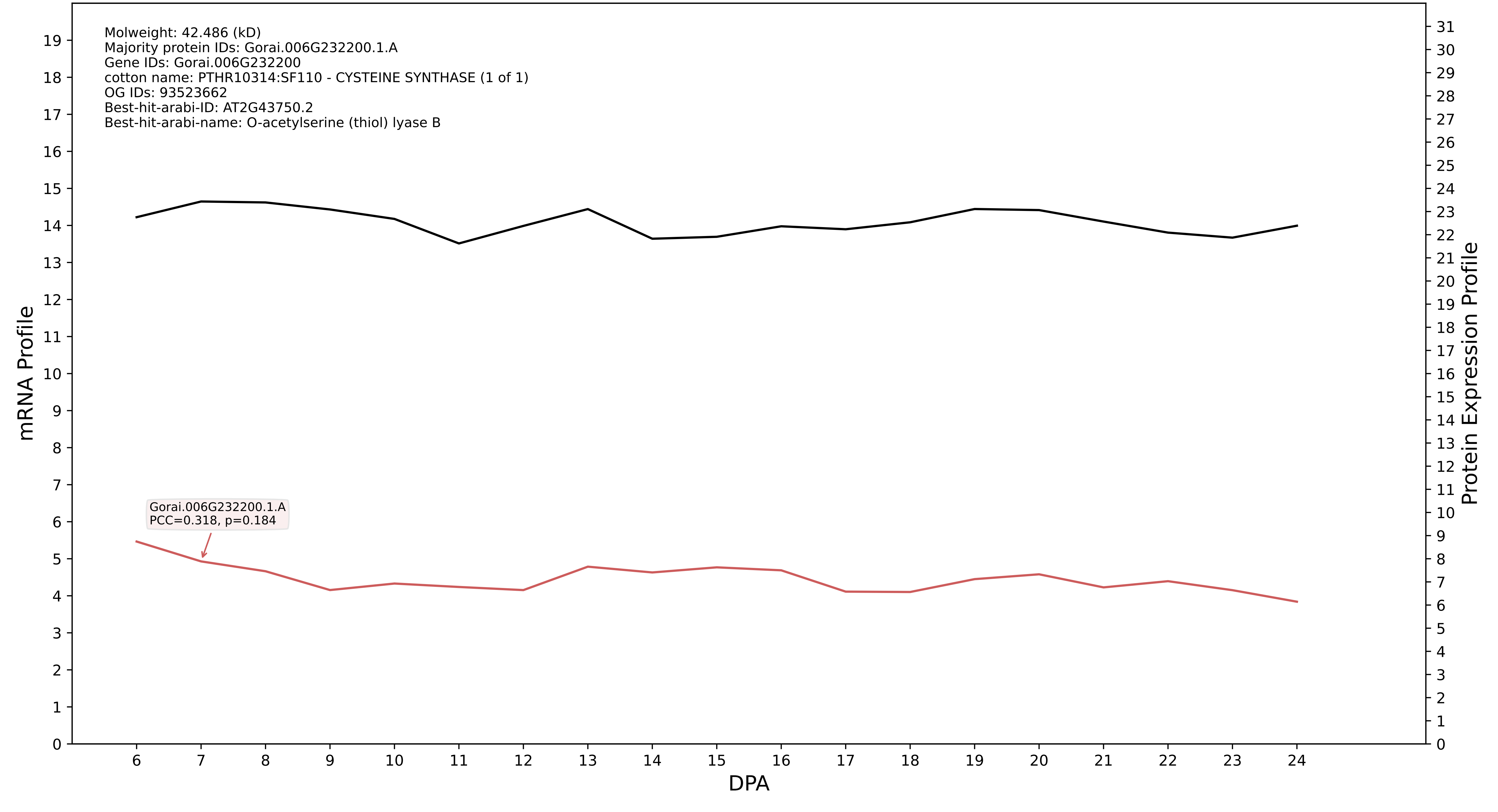

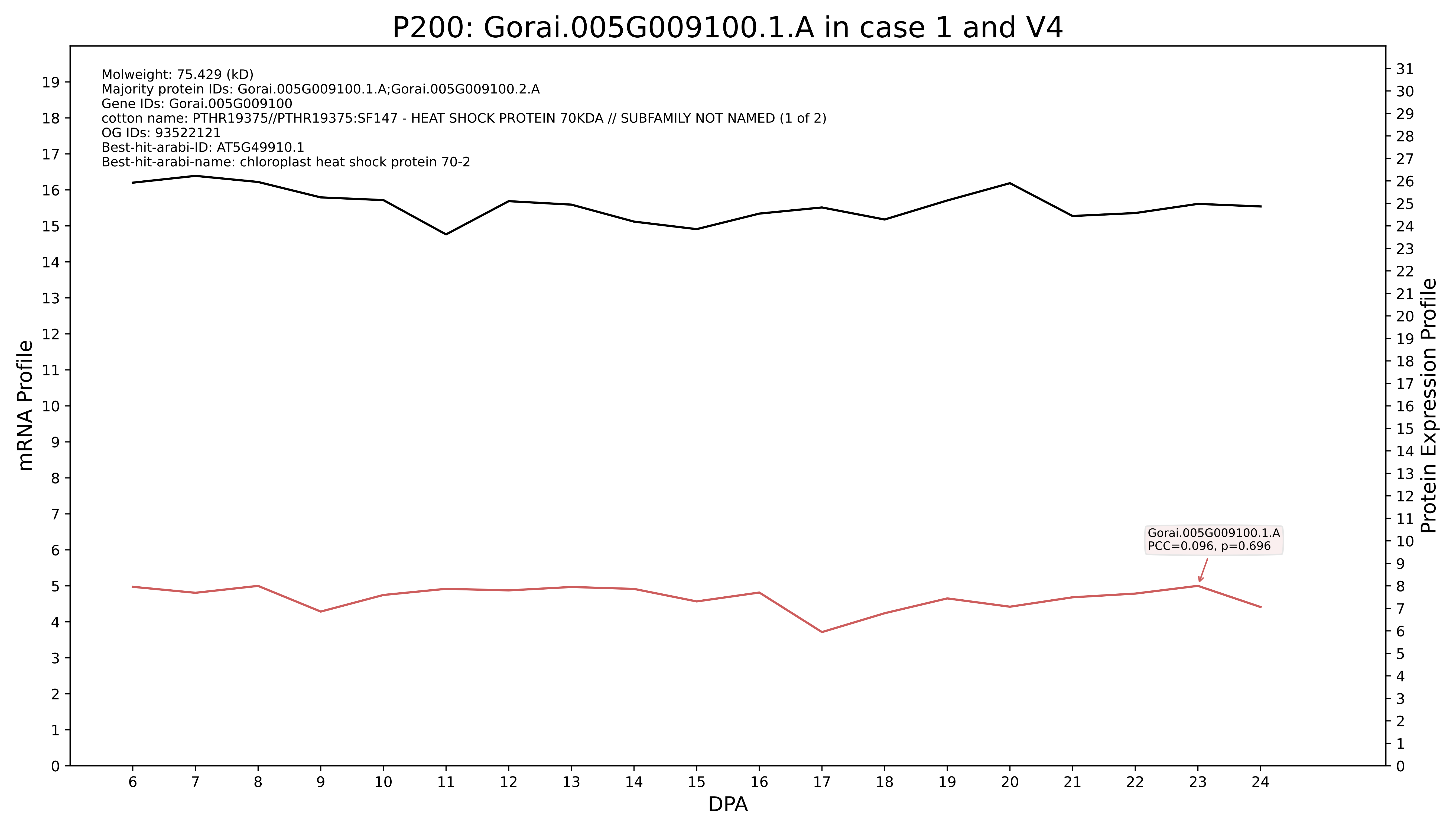
P200: Gorai.005G009100.1.A in case 1 and V4
Molweight: 75.429 (kD)
Majority protein IDs: Gorai.005G009100.1.A;Gorai.005G009100.2.A
Gene IDs: Gorai.005G009100
cotton name: PTHR19375//PTHR19375:SF147 - HEAT SHOCK PROTEIN 70KDA // SUBFAMILY NOT NAMED (1 of 2)
OG IDs: 93522121
Best-hit-arabi-ID: AT5G49910.1
Best-hit-arabi-name: chloroplast heat shock protein 70-2
Gorai.005G009100.1.A
PCC=0.096, p=0.696
mRNA Profile
Protein Expression Profile
DPA

P200: Gorai.004G228300.1.A in case 1 and V4

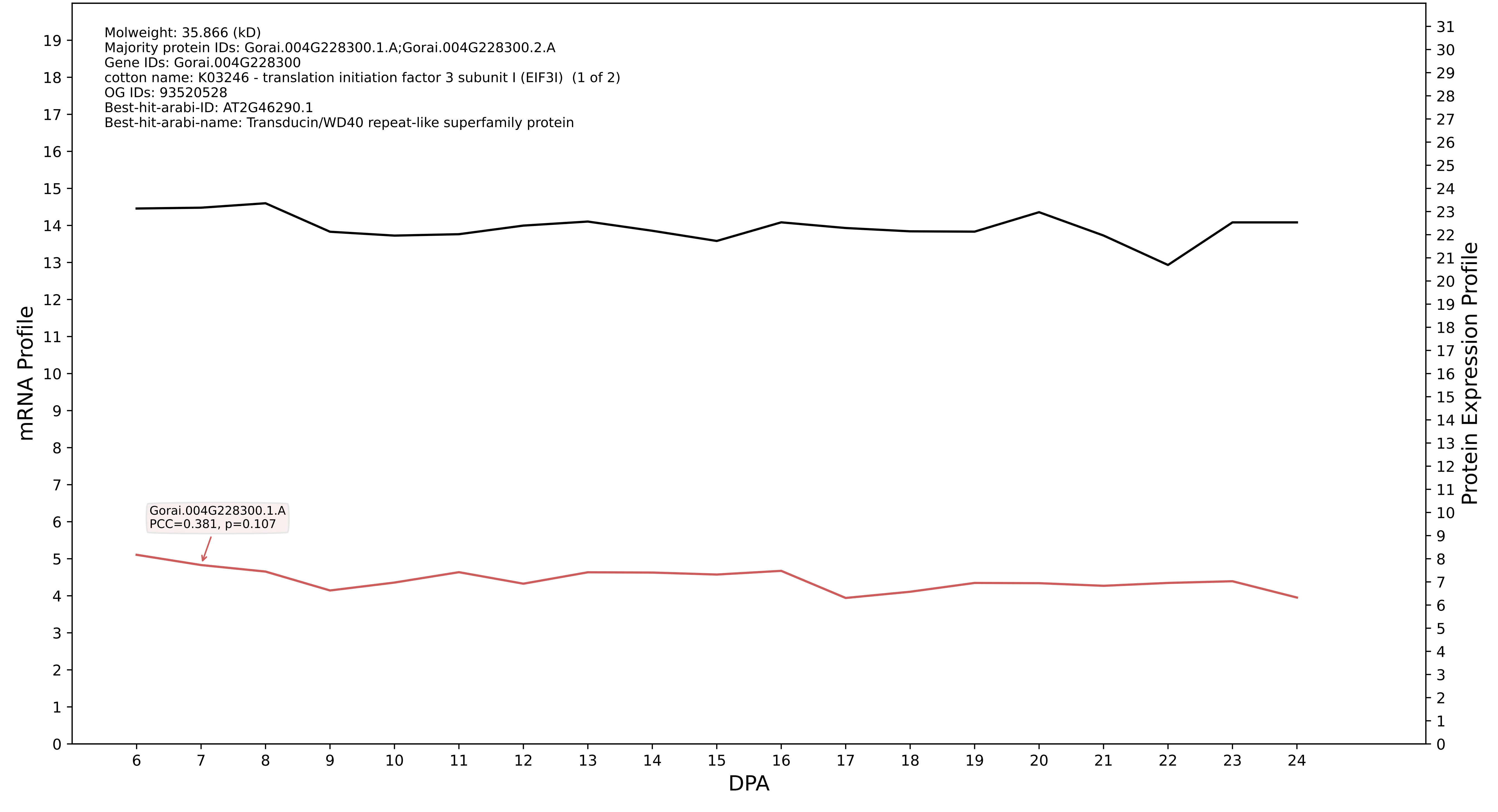

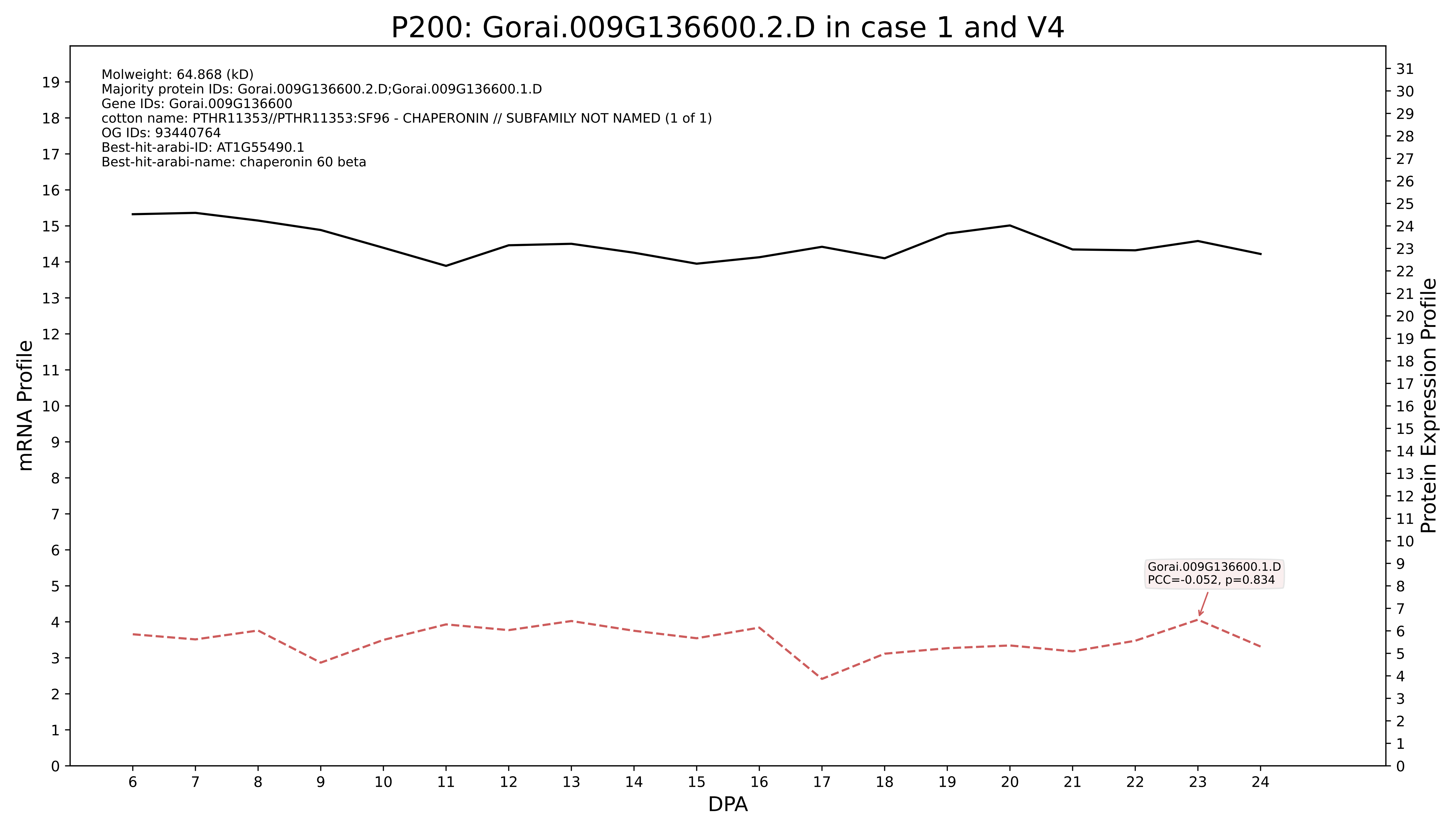
P200: Gorai.009G136600.2.D in case 1 and V4
Molweight: 64.868 (kD)
Majority protein IDs: Gorai.009G136600.2.D;Gorai.009G136600.1.D
Gene IDs: Gorai.009G136600
cotton name: PTHR11353//PTHR11353:SF96 - CHAPERONIN // SUBFAMILY NOT NAMED (1 of 1)
OG IDs: 93440764
Best-hit-arabi-ID: AT1G55490.1
Best-hit-arabi-name: chaperonin 60 beta
Gorai.009G136600.1.D
PCC=-0.052, p=0.834
mRNA Profile
Protein Expression Profile
DPA

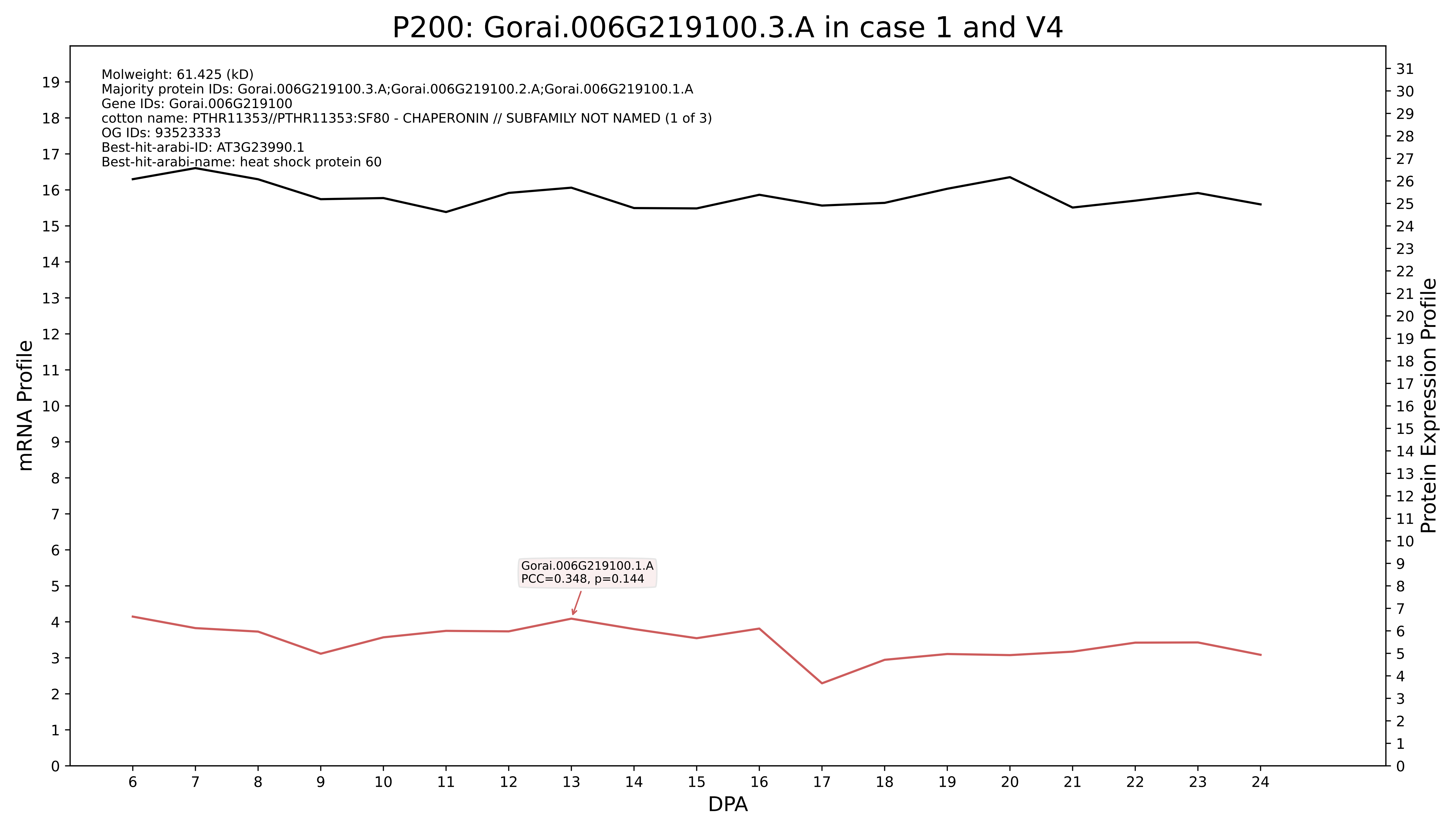
P200: Gorai.006G219100.3.A in case 1 and V4
Molweight: 61.425 (kD)
Majority protein IDs: Gorai.006G219100.3.A;Gorai.006G219100.2.A;Gorai.006G219100.1.A
Gene IDs: Gorai.006G219100
cotton name: PTHR11353//PTHR11353:SF80 - CHAPERONIN // SUBFAMILY NOT NAMED (1 of 3)
OG IDs: 93523333
Best-hit-arabi-ID: AT3G23990.1
Best-hit-arabi-name: heat shock protein 60
Gorai.006G219100.1.A
PCC=0.348, p=0.144
mRNA Profile
Protein Expression Profile
DPA

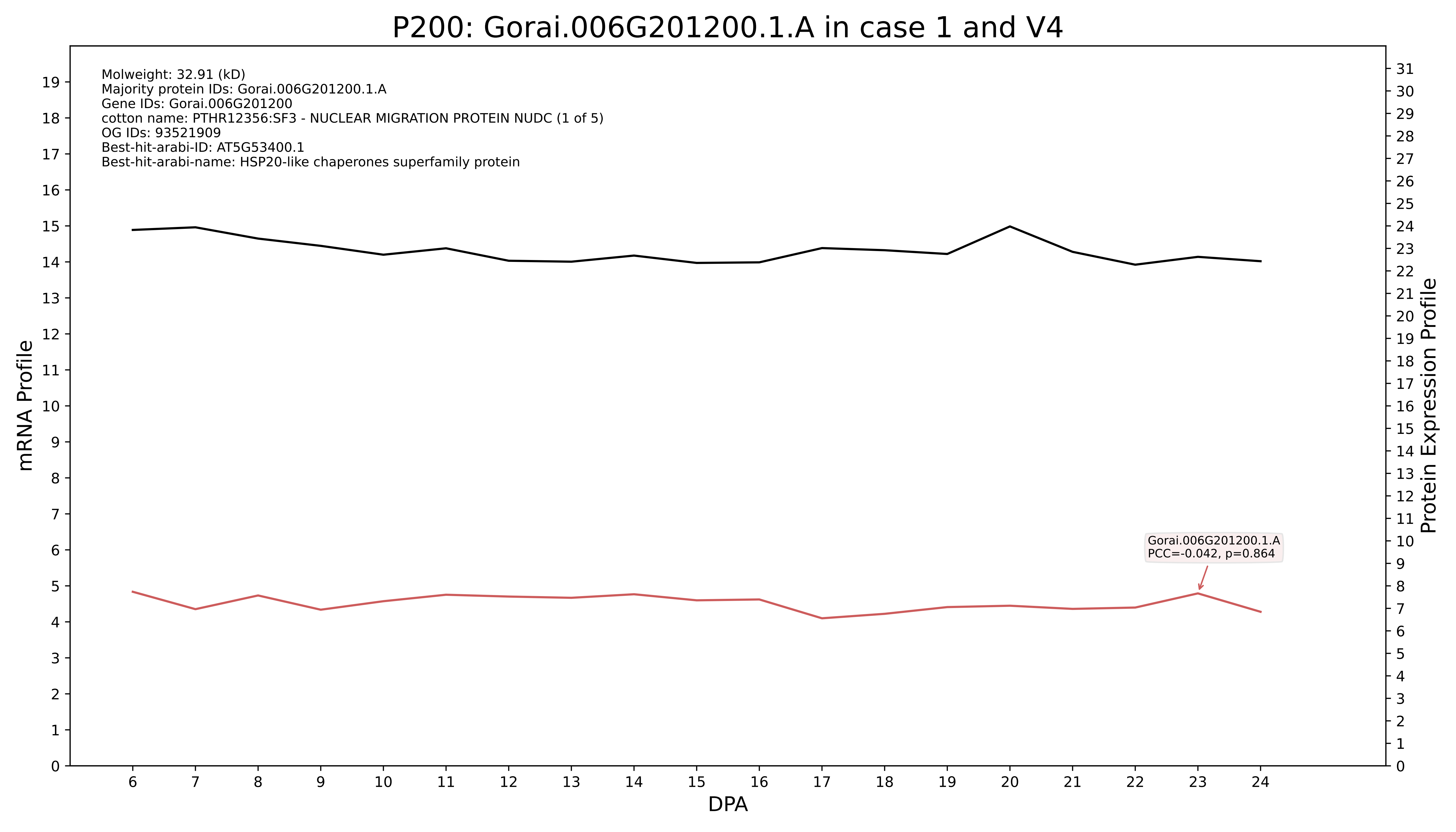
P200: Gorai.006G201200.1.A in case 1 and V4
Molweight: 32.91 (kD)
Majority protein IDs: Gorai.006G201200.1.A
Gene IDs: Gorai.006G201200
cotton name: PTHR12356:SF3 - NUCLEAR MIGRATION PROTEIN NUDC (1 of 5)
OG IDs: 93521909
Best-hit-arabi-ID: AT5G53400.1
Best-hit-arabi-name: HSP20-like chaperones superfamily protein
Gorai.006G201200.1.A
PCC=-0.042, p=0.864
mRNA Profile
Protein Expression Profile
DPA
0
1
2
3
4
5
6
7
8
9
10
11
12
13
14
15
16
17
18
19
20
21
22
23
24
25
26
27
28
29
30
31

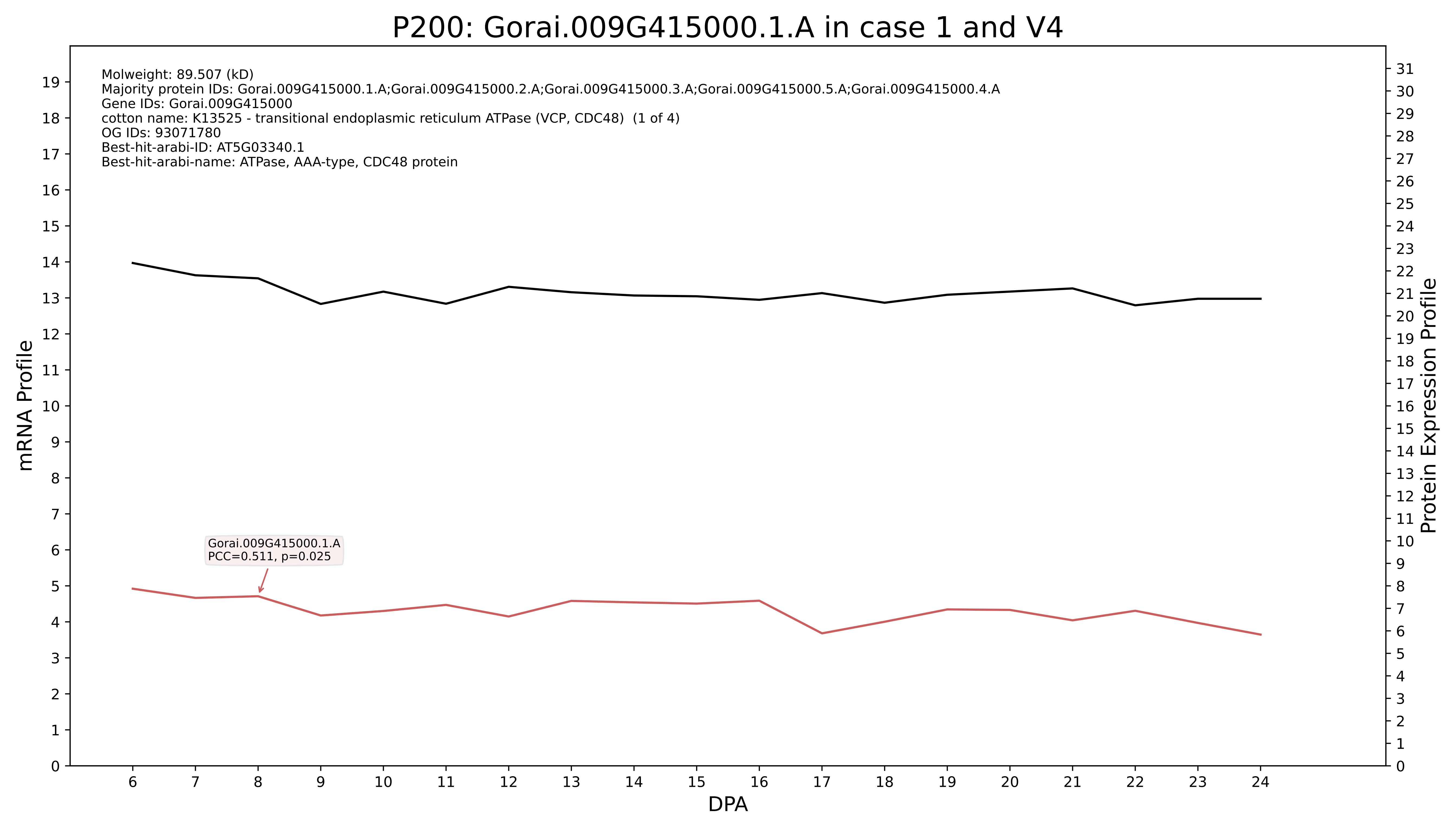

P200: Gorai.009G415000.1.A in case 1 and V4
Molweight: 89.507 (kD)
Majority protein IDs: Gorai.009G415000.1.A;Gorai.009G415000.2.A;Gorai.009G415000.3.A;Gorai.009G415000.5.A;Gorai.009G415000.4.A
Gene IDs: Gorai.009G415000
cotton name: K13525 - transitional endoplasmic reticulum ATPase (VCP, CDC48) (1 of 4)
OG IDs: 93071780
Best-hit-arabi-ID: AT5G03340.1
Best-hit-arabi-name: ATPase, AAA-type, CDC48 protein
Gorai.009G415000.1.A
PCC=0.511, p=0.025
mRNA Profile
Protein Expression Profile
DPA

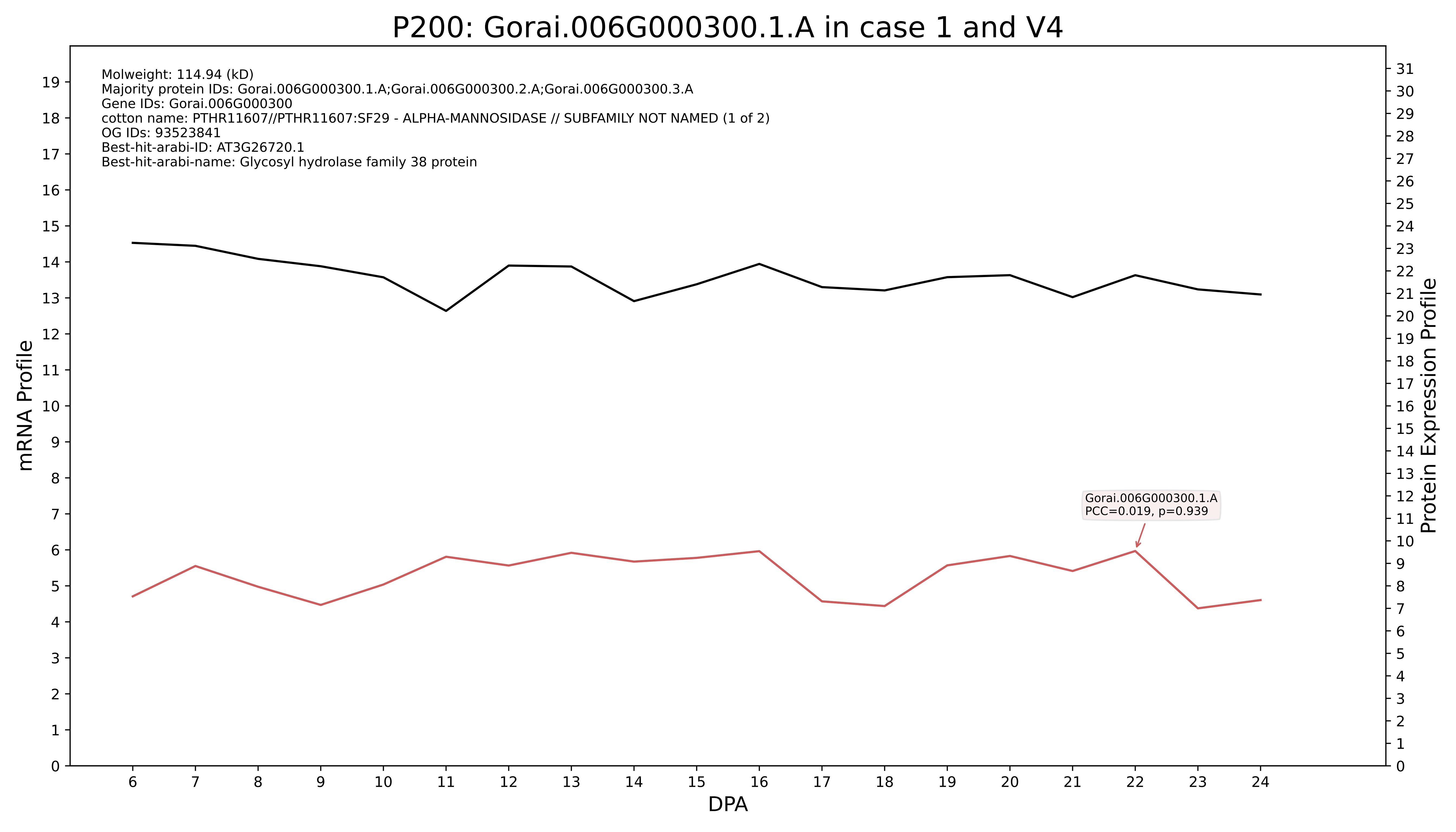
P200: Gorai.006G000300.1.A in case 1 and V4
Molweight: 114.94 (kD)
Majority protein IDs: Gorai.006G000300.1.A;Gorai.006G000300.2.A;Gorai.006G000300.3.A
Gene IDs: Gorai.006G000300
cotton name: PTHR11607//PTHR11607:SF29 - ALPHA-MANNOSIDASE // SUBFAMILY NOT NAMED (1 of 2)
OG IDs: 93523841
Best-hit-arabi-ID: AT3G26720.1
Best-hit-arabi-name: Glycosyl hydrolase family 38 protein
Gorai.006G000300.1.A
PCC=0.019, p=0.939
mRNA Profile
Protein Expression Profile
DPA

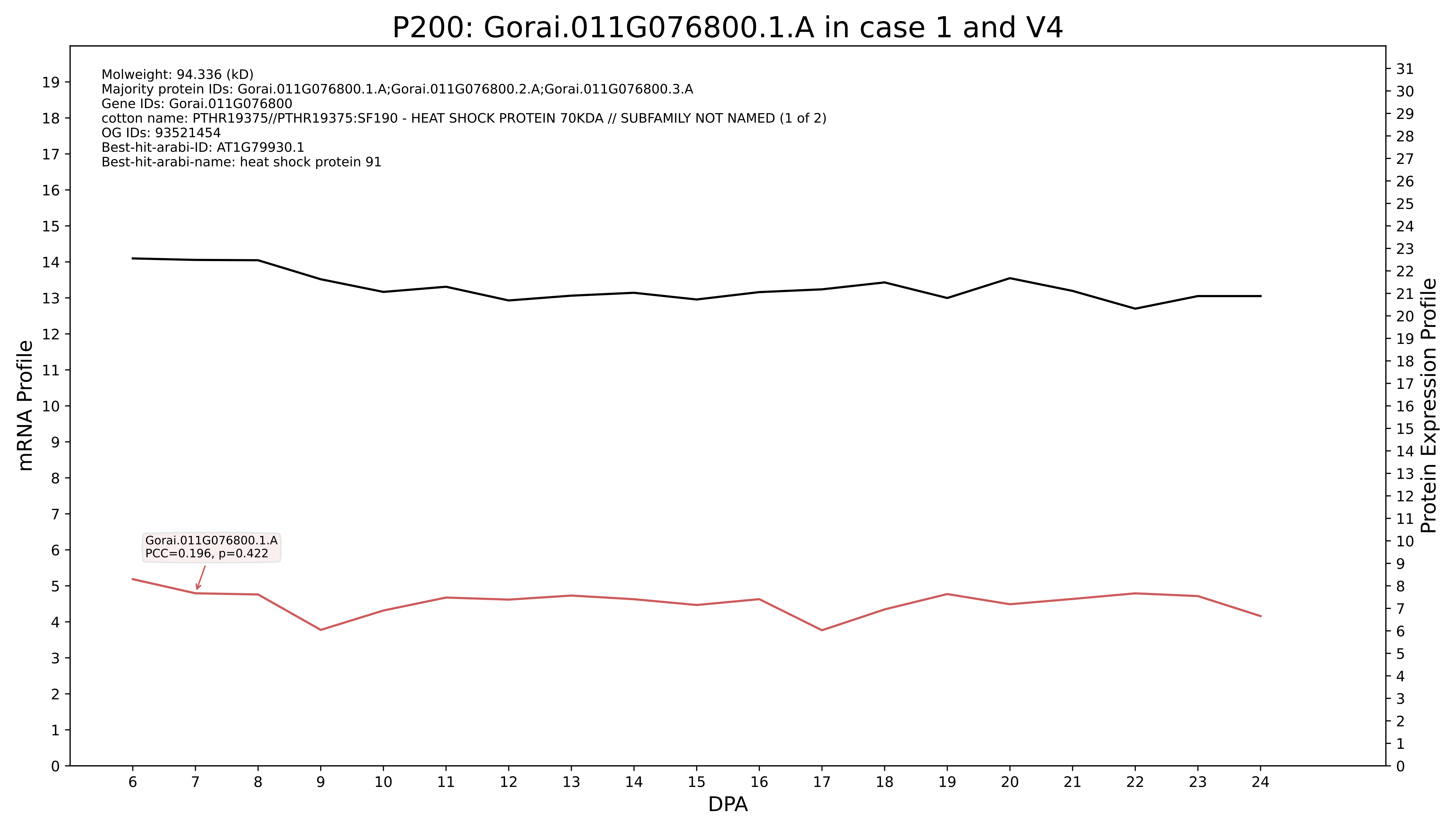
P200: Gorai.011G076800.1.A in case 1 and V4
Molweight: 94.336 (kD)
Majority protein IDs: Gorai.011G076800.1.A;Gorai.011G076800.2.A;Gorai.011G076800.3.A
Gene IDs: Gorai.011G076800
cotton name: PTHR19375//PTHR19375:SF190 - HEAT SHOCK PROTEIN 70KDA // SUBFAMILY NOT NAMED (1 of 2)
OG IDs: 93521454
Best-hit-arabi-ID: AT1G79930.1
Best-hit-arabi-name: heat shock protein 91
Gorai.011G076800.1.A
PCC=0.196, p=0.422
mRNA Profile
Protein Expression Profile
DPA
0
1
2
3
4
5
6
7
8
9
10
11
12
13
14
15
16
17
18
19
20
21
22
23
24
25
26
27
28
29
30
31

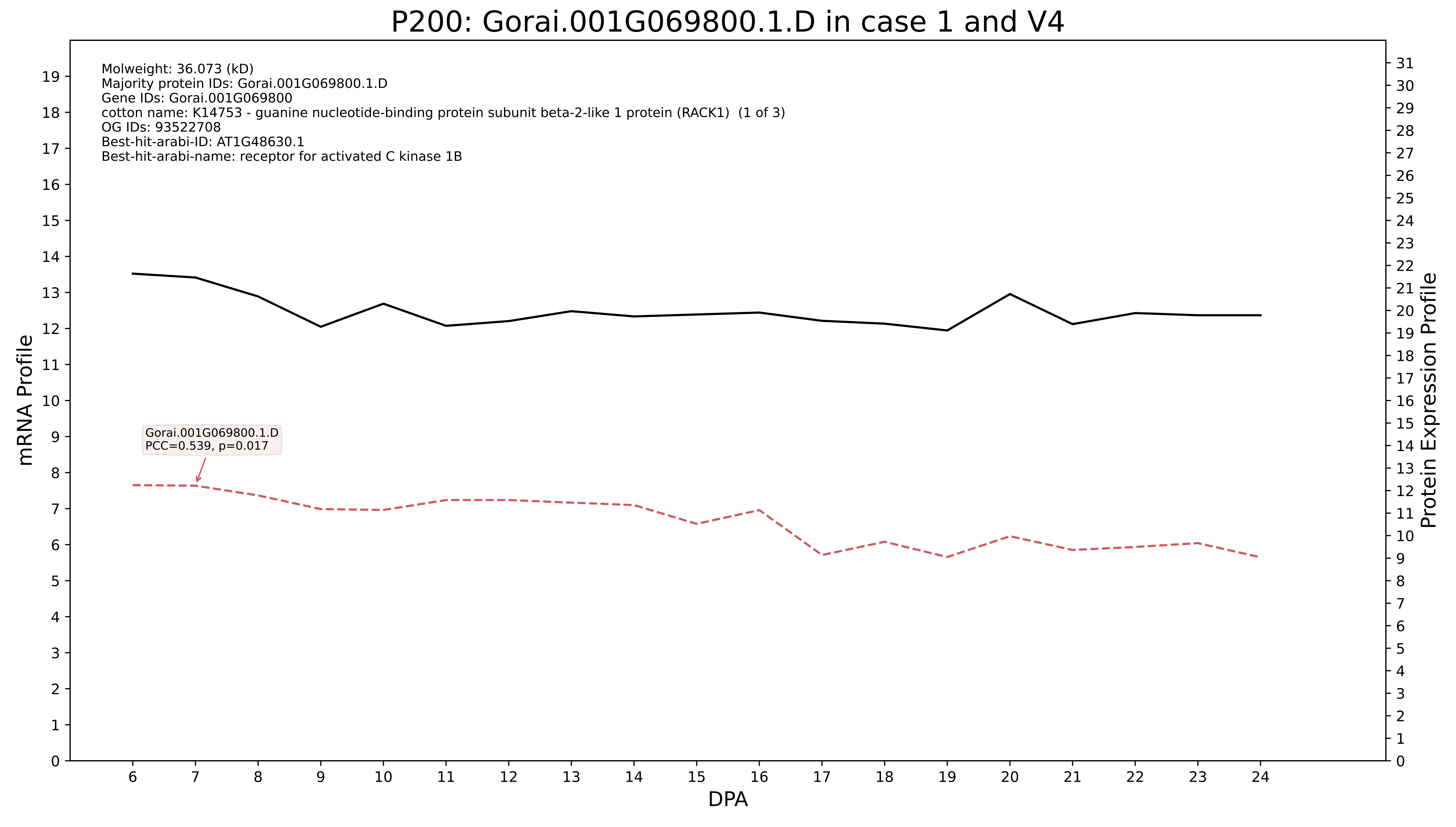
P200: Gorai.001G069800.1.D in case 1 and V4
Molweight: 36.073 (kD)
Majority protein IDs: Gorai.001G069800.1.D
Gene IDs: Gorai.001G069800
cotton name: K14753 - guanine nucleotide-binding protein subunit beta-2-like 1 protein (RACK1) (1 of 3)
OG IDs: 93522708
Best-hit-arabi-ID: AT1G48630.1
Best-hit-arabi-name: receptor for activated C kinase 1B
Gorai.001G069800.1.D
PCC=0.539, p=0.017
mRNA Profile
Protein Expression Profile
DPA

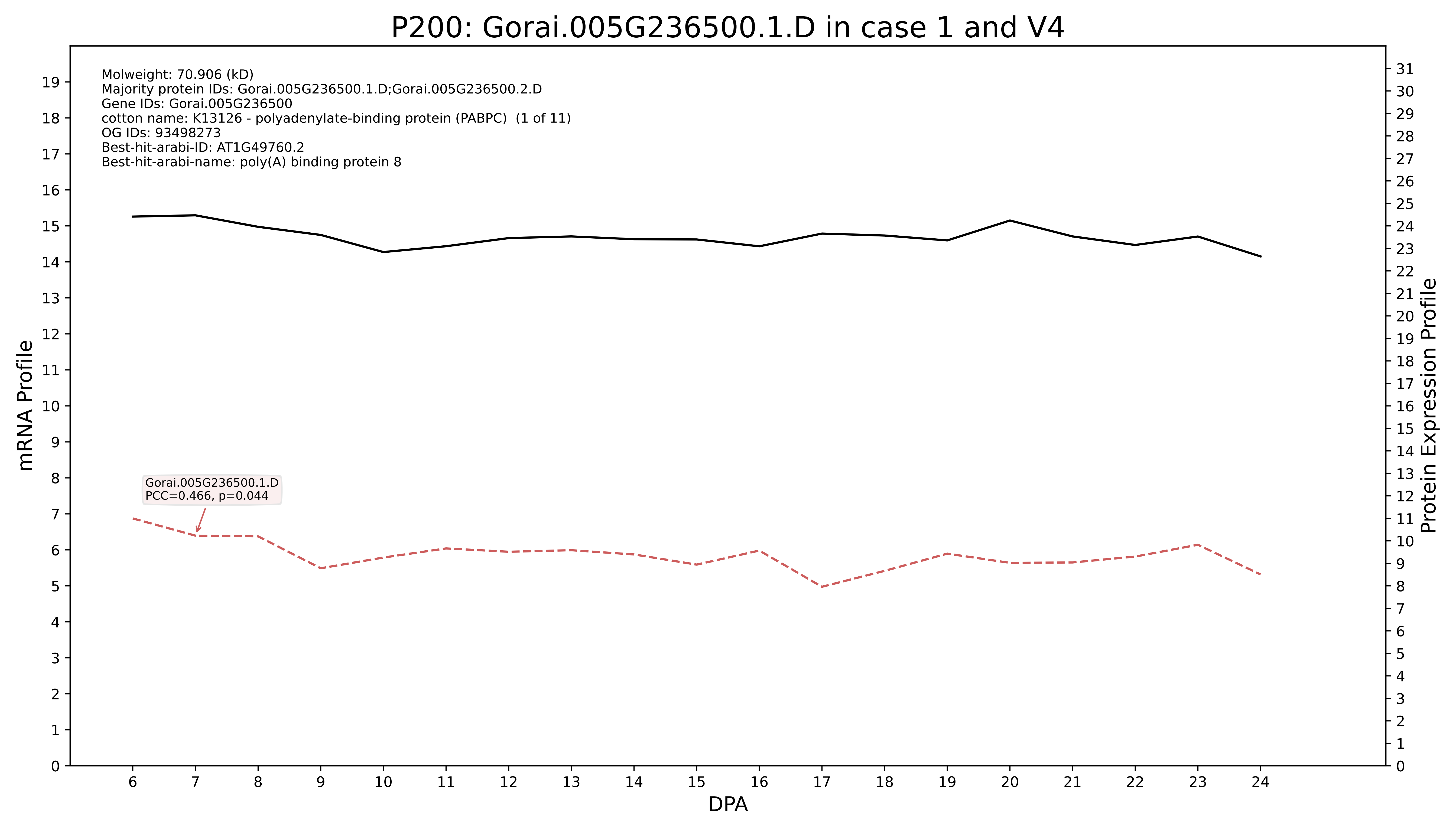

P200: Gorai.005G236500.1.D in case 1 and V4
Molweight: 70.906 (kD)
Majority protein IDs: Gorai.005G236500.1.D;Gorai.005G236500.2.D
Gene IDs: Gorai.005G236500
cotton name: K13126 - polyadenylate-binding protein (PABPC) (1 of 11)
OG IDs: 93498273
Best-hit-arabi-ID: AT1G49760.2
Best-hit-arabi-name: poly(A) binding protein 8
Gorai.005G236500.1.D
PCC=0.466, p=0.044
mRNA Profile
Protein Expression Profile
DPA

P200: Gorai.001G199400.1.A in case 1 and V4

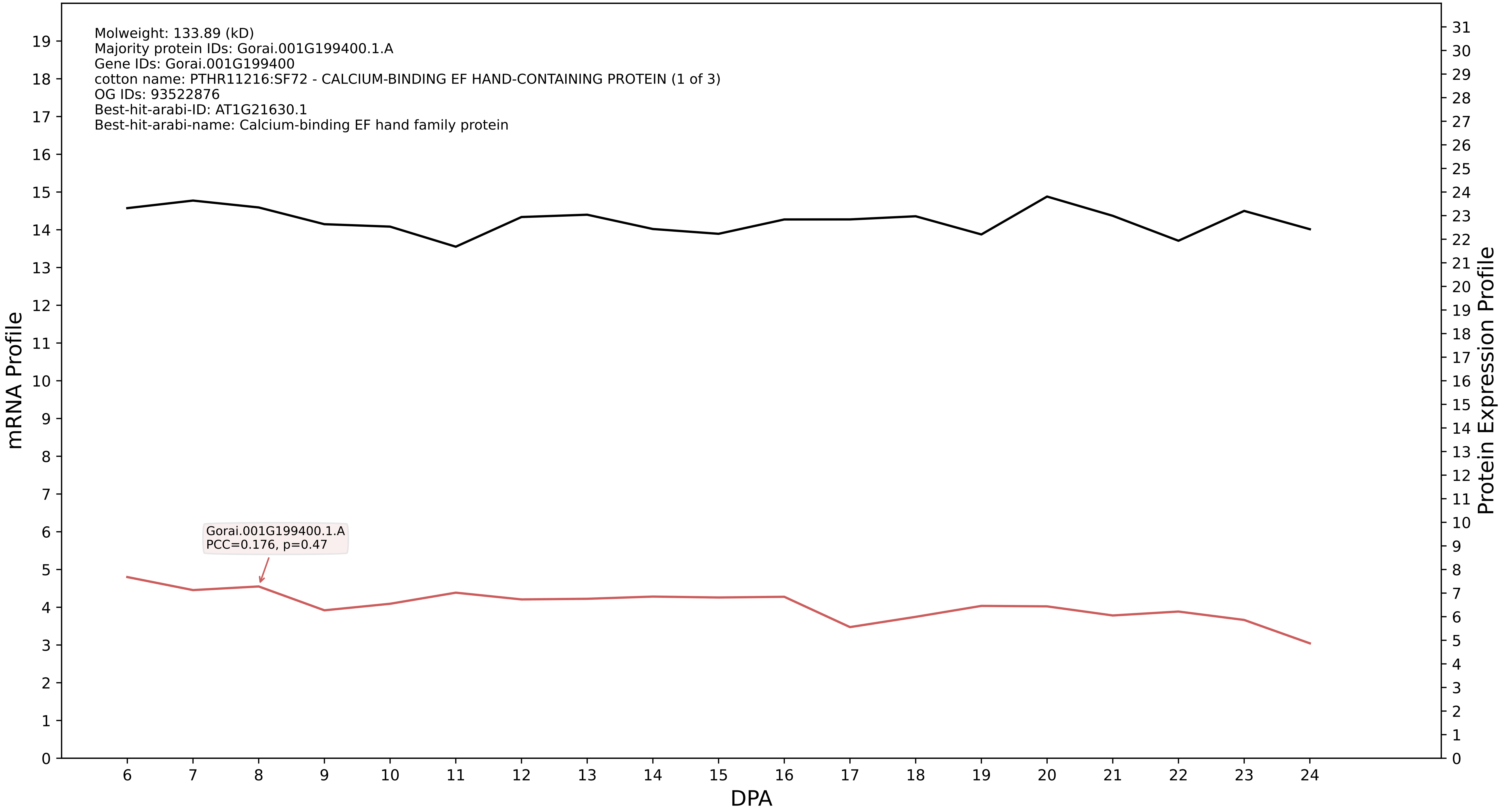

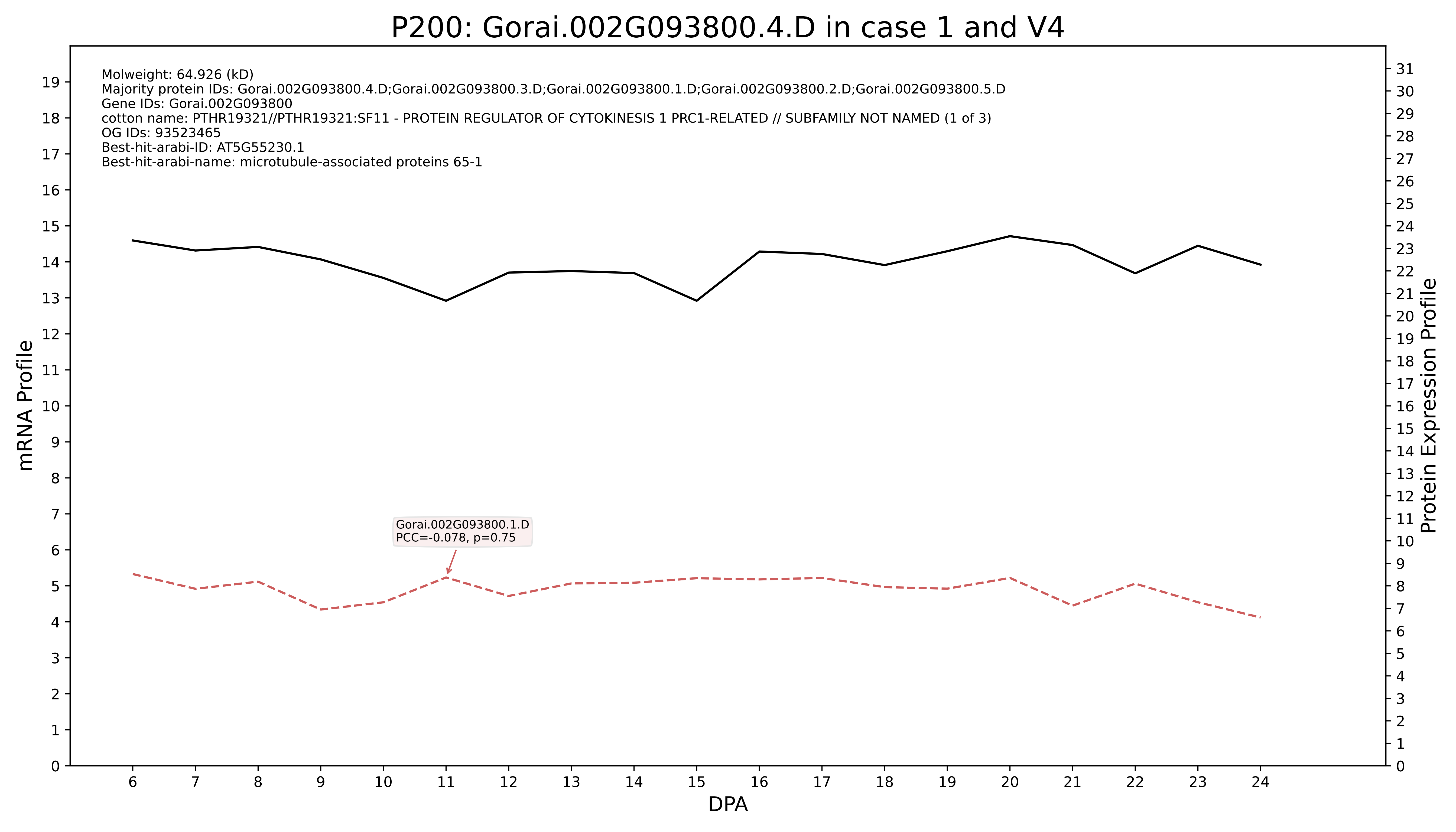
P200: Gorai.002G093800.4.D in case 1 and V4
Molweight: 64.926 (kD)
Majority protein IDs: Gorai.002G093800.4.D;Gorai.002G093800.3.D;Gorai.002G093800.1.D;Gorai.002G093800.2.D;Gorai.002G093800.5.D
Gene IDs: Gorai.002G093800
cotton name: PTHR19321//PTHR19321:SF11 - PROTEIN REGULATOR OF CYTOKINESIS 1 PRC1-RELATED // SUBFAMILY NOT NAMED (1 of 3)
OG IDs: 93523465
Best-hit-arabi-ID: AT5G55230.1
Best-hit-arabi-name: microtubule-associated proteins 65-1
Gorai.002G093800.1.D
PCC=-0.078, p=0.75
mRNA Profile
Protein Expression Profile
DPA

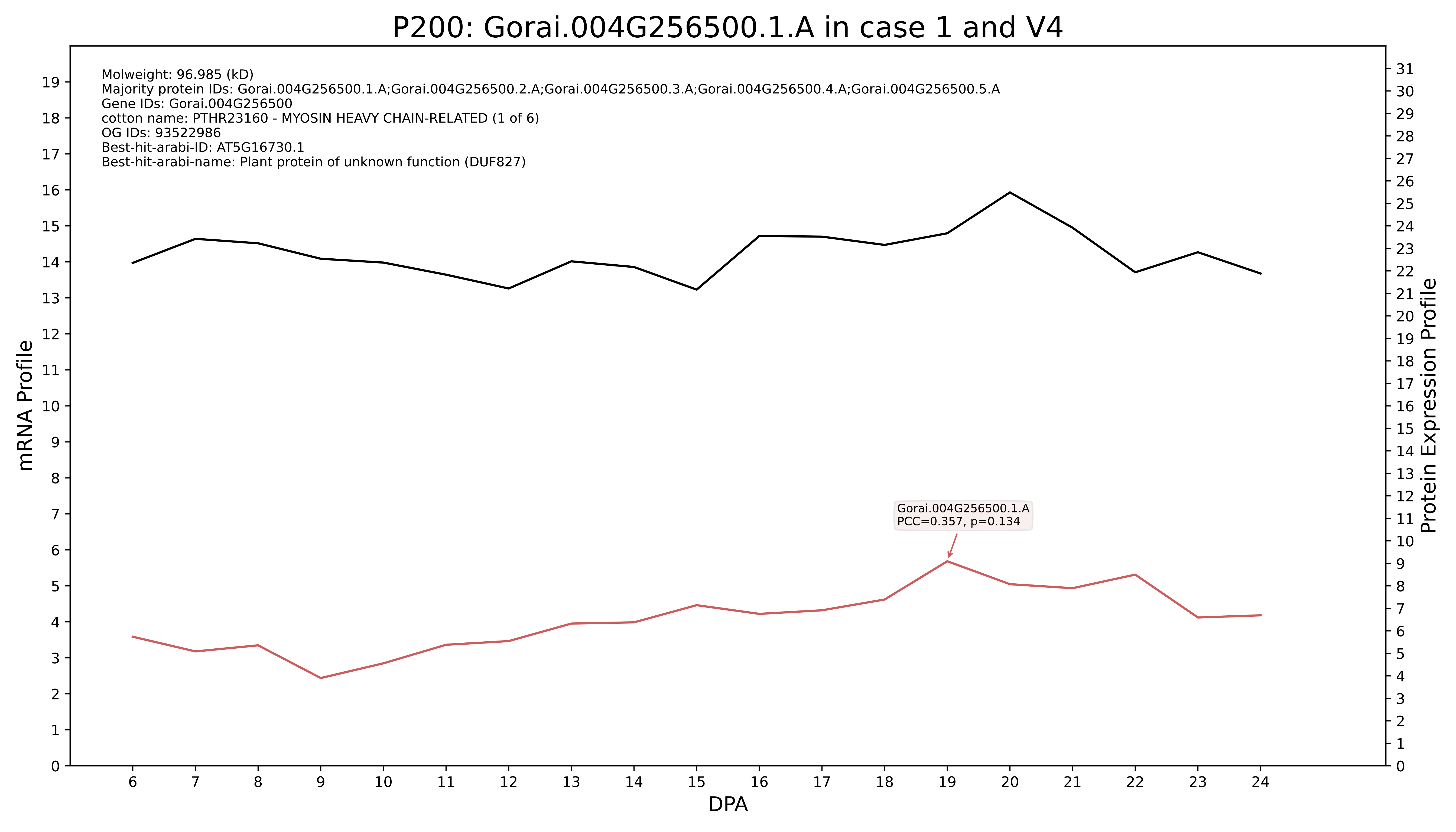
P200: Gorai.004G256500.1.A in case 1 and V4
Molweight: 96.985 (kD)
Majority protein IDs: Gorai.004G256500.1.A;Gorai.004G256500.2.A;Gorai.004G256500.3.A;Gorai.004G256500.4.A;Gorai.004G256500.5.A
Gene IDs: Gorai.004G256500
cotton name: PTHR23160 - MYOSIN HEAVY CHAIN-RELATED (1 of 6)
OG IDs: 93522986
Best-hit-arabi-ID: AT5G16730.1
Best-hit-arabi-name: Plant protein of unknown function (DUF827)
Gorai.004G256500.1.A
PCC=0.357, p=0.134
mRNA Profile
Protein Expression Profile
DPA

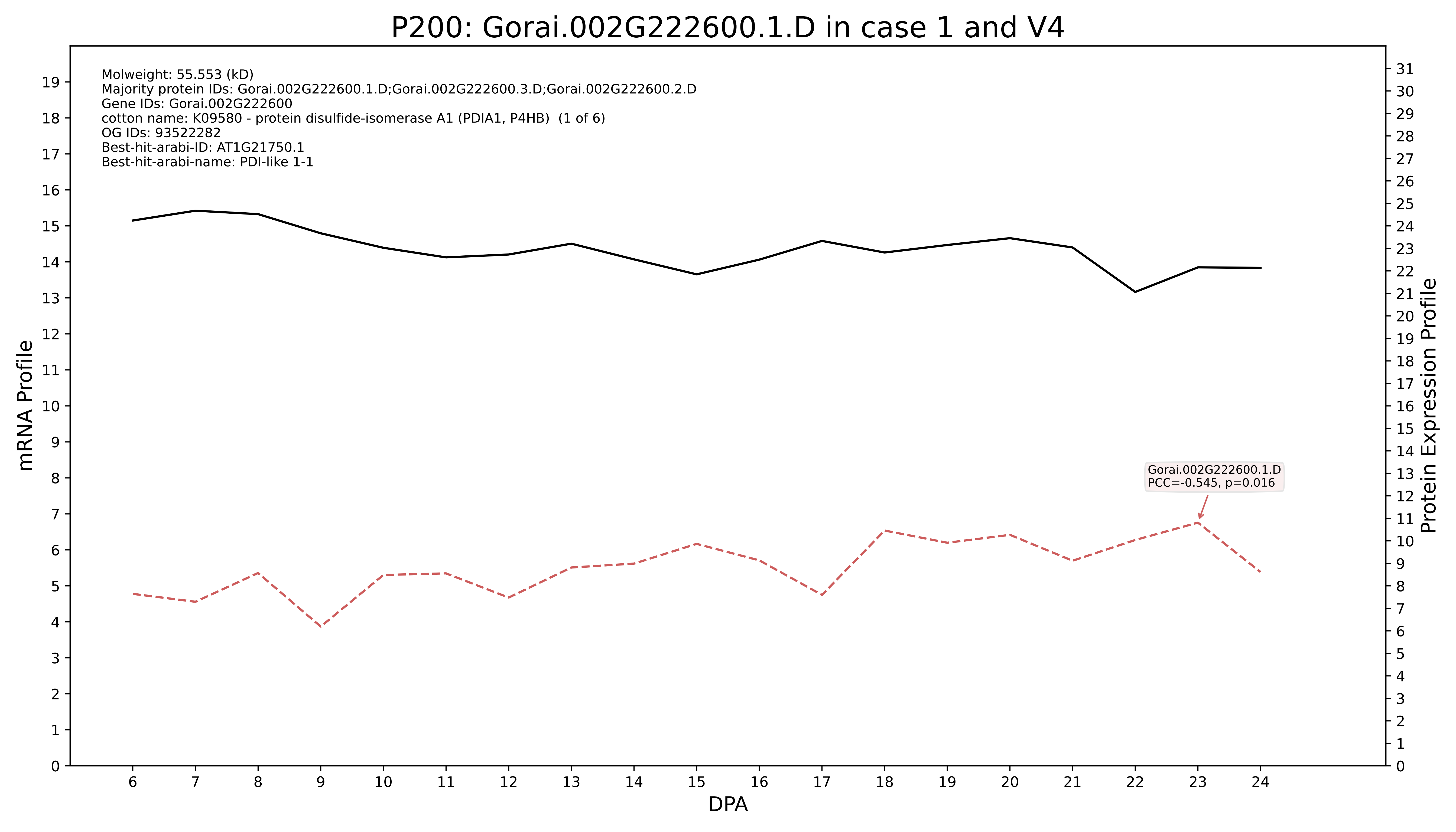
P200: Gorai.002G222600.1.D in case 1 and V4
Molweight: 55.553 (kD)
Majority protein IDs: Gorai.002G222600.1.D;Gorai.002G222600.3.D;Gorai.002G222600.2.D
Gene IDs: Gorai.002G222600
cotton name: K09580 - protein disulfide-isomerase A1 (PDIA1, P4HB) (1 of 6)
OG IDs: 93522282
Best-hit-arabi-ID: AT1G21750.1
Best-hit-arabi-name: PDI-like 1-1
Gorai.002G222600.1.D
PCC=-0.545, p=0.016
mRNA Profile
Protein Expression Profile
DPA

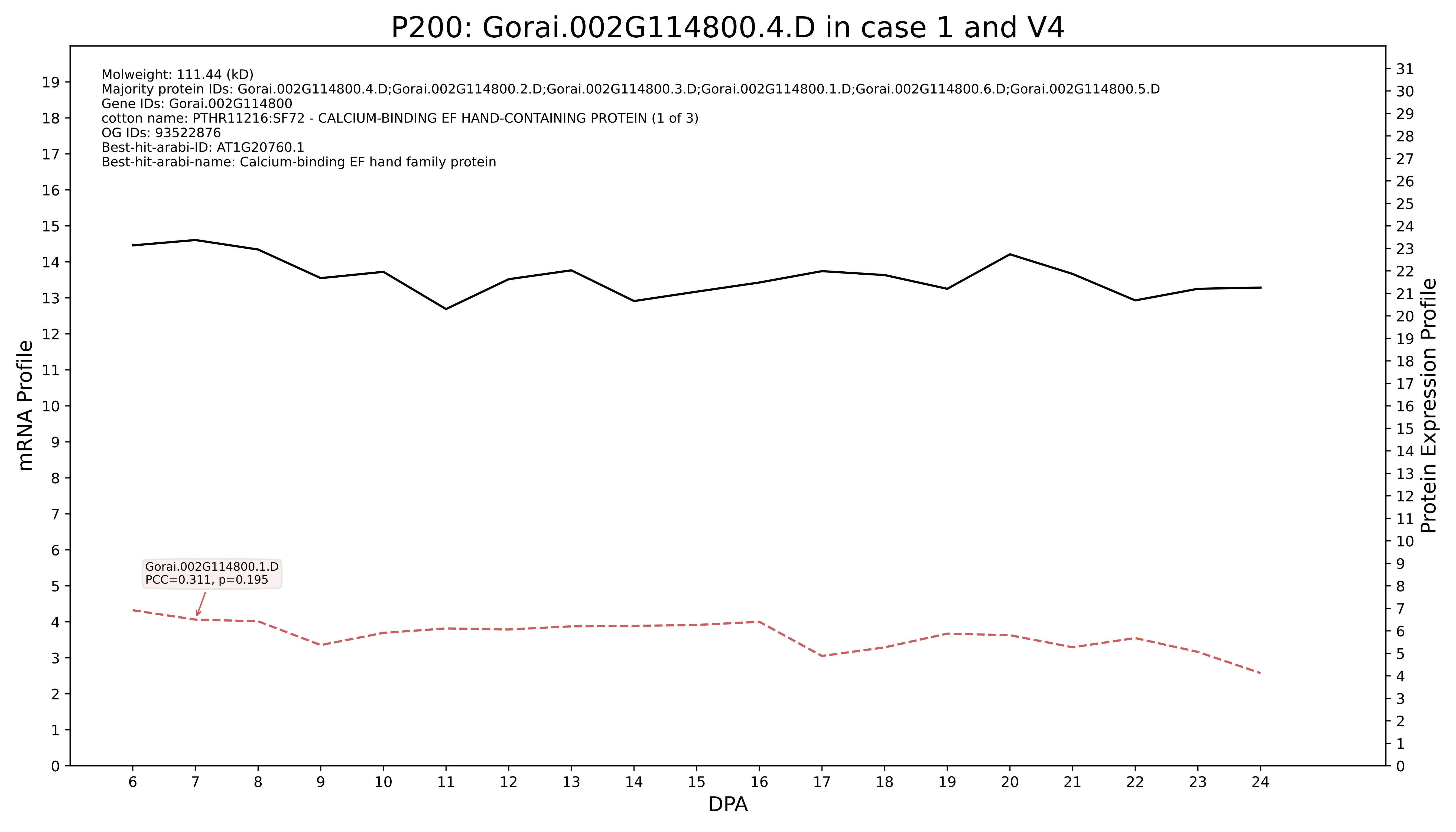
P200: Gorai.002G114800.4.D in case 1 and V4
Molweight: 111.44 (kD)
Majority protein IDs: Gorai.002G114800.4.D;Gorai.002G114800.2.D;Gorai.002G114800.3.D;Gorai.002G114800.1.D;Gorai.002G114800.6.D;Gorai.002G114800.5.D
Gene IDs: Gorai.002G114800
cotton name: PTHR11216:SF72 - CALCIUM-BINDING EF HAND-CONTAINING PROTEIN (1 of 3)
OG IDs: 93522876
Best-hit-arabi-ID: AT1G20760.1
Best-hit-arabi-name: Calcium-binding EF hand family protein
Gorai.002G114800.1.D
PCC=0.311, p=0.195
mRNA Profile
Protein Expression Profile
DPA

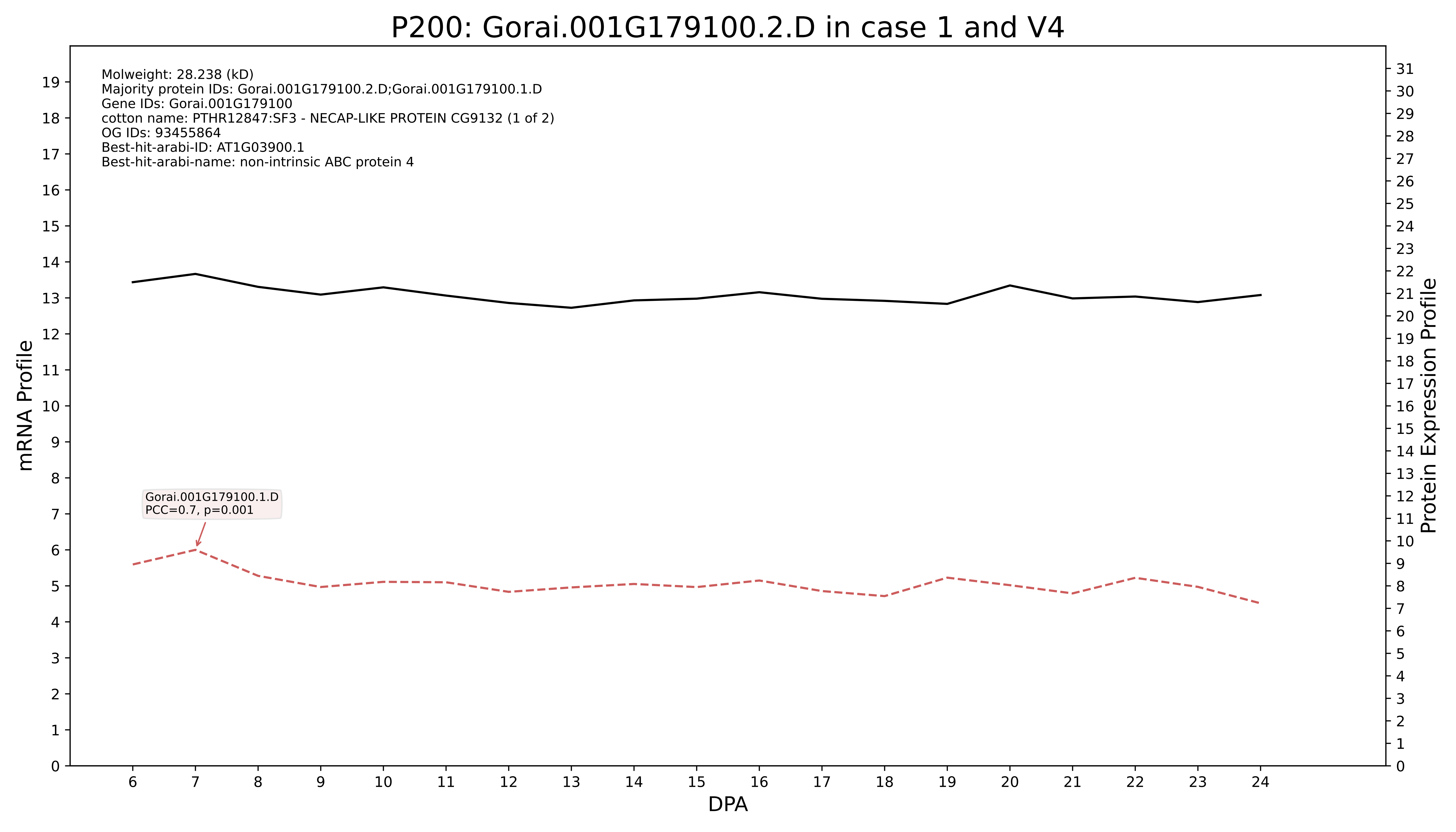
P200: Gorai.001G179100.2.D in case 1 and V4
Molweight: 28.238 (kD)
Majority protein IDs: Gorai.001G179100.2.D;Gorai.001G179100.1.D
Gene IDs: Gorai.001G179100
cotton name: PTHR12847:SF3 - NECAP-LIKE PROTEIN CG9132 (1 of 2)
OG IDs: 93455864
Best-hit-arabi-ID: AT1G03900.1
Best-hit-arabi-name: non-intrinsic ABC protein 4
Gorai.001G179100.1.D
PCC=0.7, p=0.001
mRNA Profile
Protein Expression Profile
DPA

P200: Gorai.006G214200.2.D in case 1 and V4

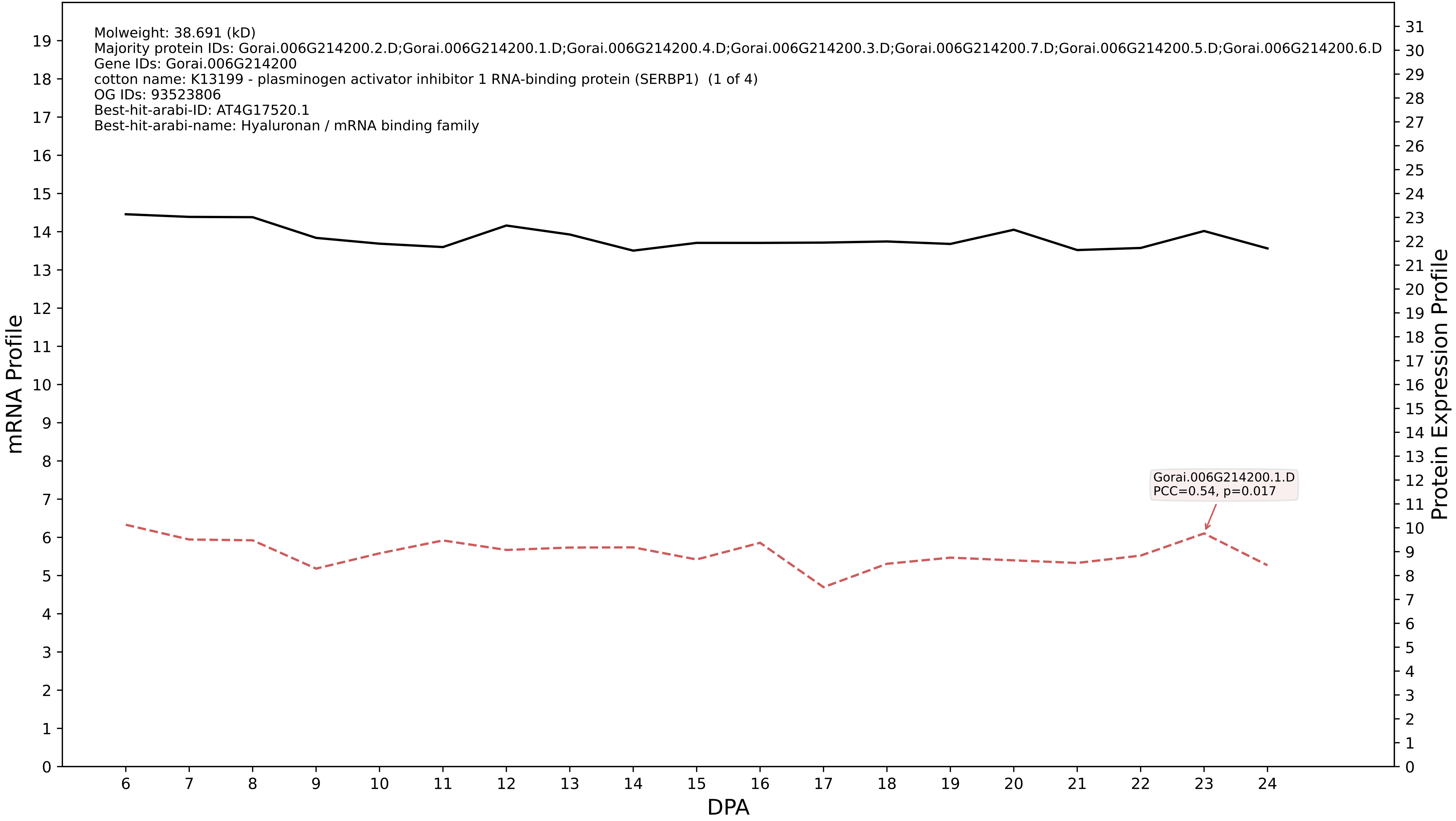

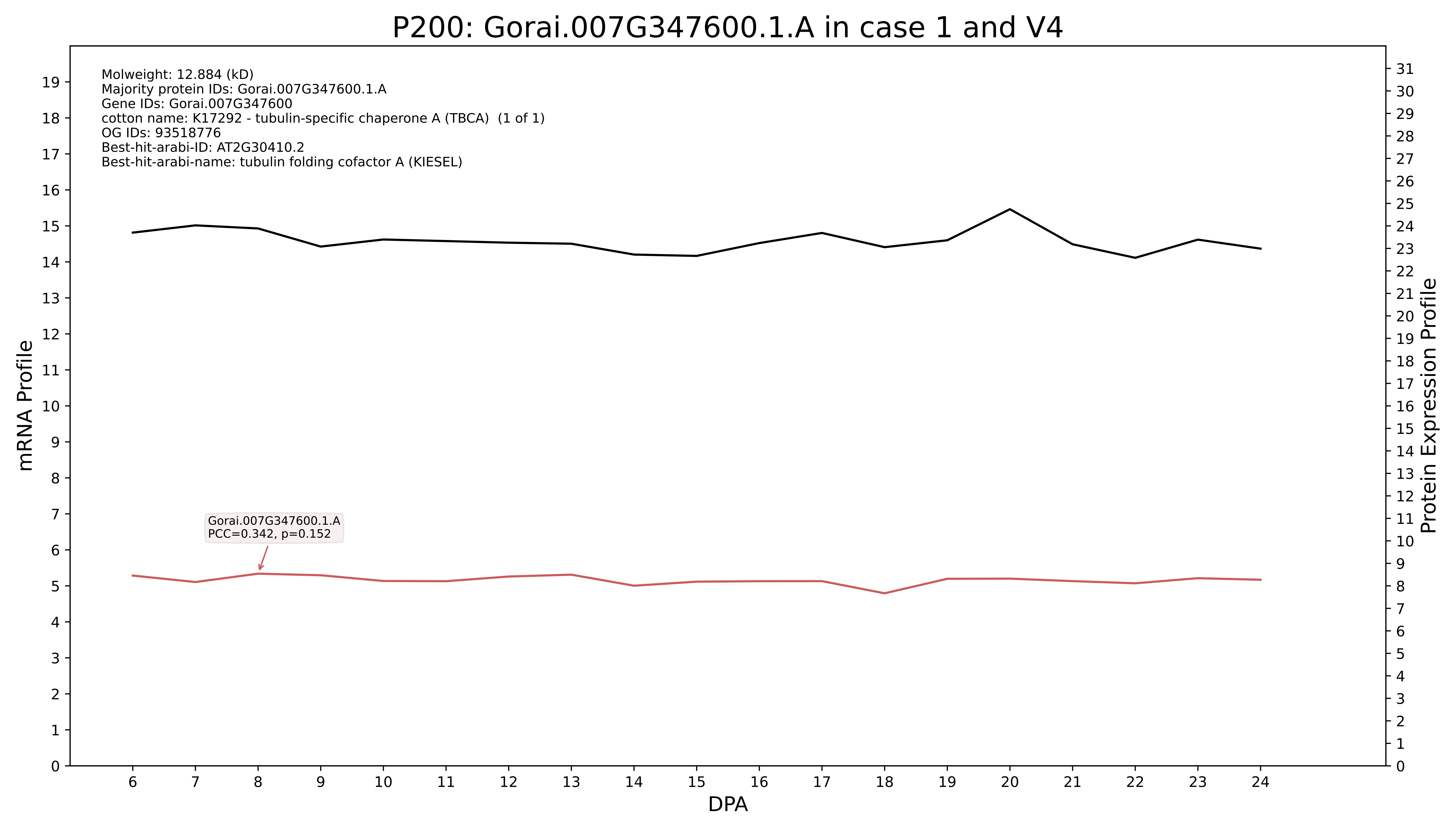
P200: Gorai.007G347600.1.A in case 1 and V4
Molweight: 12.884 (kD)
Majority protein IDs: Gorai.007G347600.1.A
Gene IDs: Gorai.007G347600
cotton name: K17292 - tubulin-specific chaperone A (TBCA) (1 of 1)
OG IDs: 93518776
Best-hit-arabi-ID: AT2G30410.2
Best-hit-arabi-name: tubulin folding cofactor A (KIESEL)
Gorai.007G347600.1.A
PCC=0.342, p=0.152
mRNA Profile
Protein Expression Profile
DPA

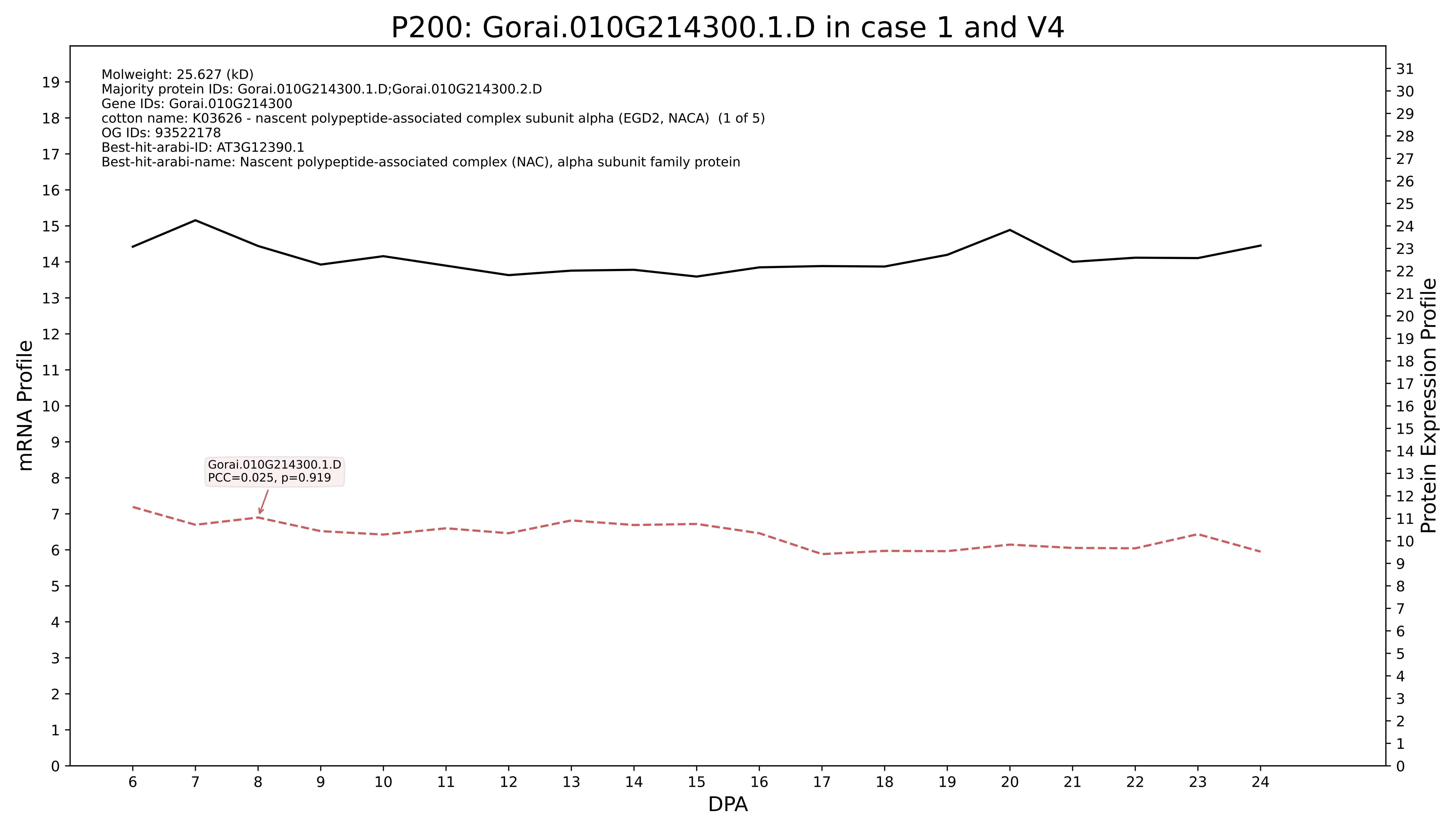
P200: Gorai.010G214300.1.D in case 1 and V4
Molweight: 25.627 (kD)
Majority protein IDs: Gorai.010G214300.1.D;Gorai.010G214300.2.D
Gene IDs: Gorai.010G214300
cotton name: K03626 - nascent polypeptide-associated complex subunit alpha (EGD2, NACA) (1 of 5)
OG IDs: 93522178
Best-hit-arabi-ID: AT3G12390.1
Best-hit-arabi-name: Nascent polypeptide-associated complex (NAC), alpha subunit family protein
Gorai.010G214300.1.D
PCC=0.025, p=0.919
mRNA Profile
Protein Expression Profile
DPA
0
1
2
3
4
5
6
7
8
9
10
11
12
13
14
15
16
17
18
19
20
21
22
23
24
25
26
27
28
29
30
31

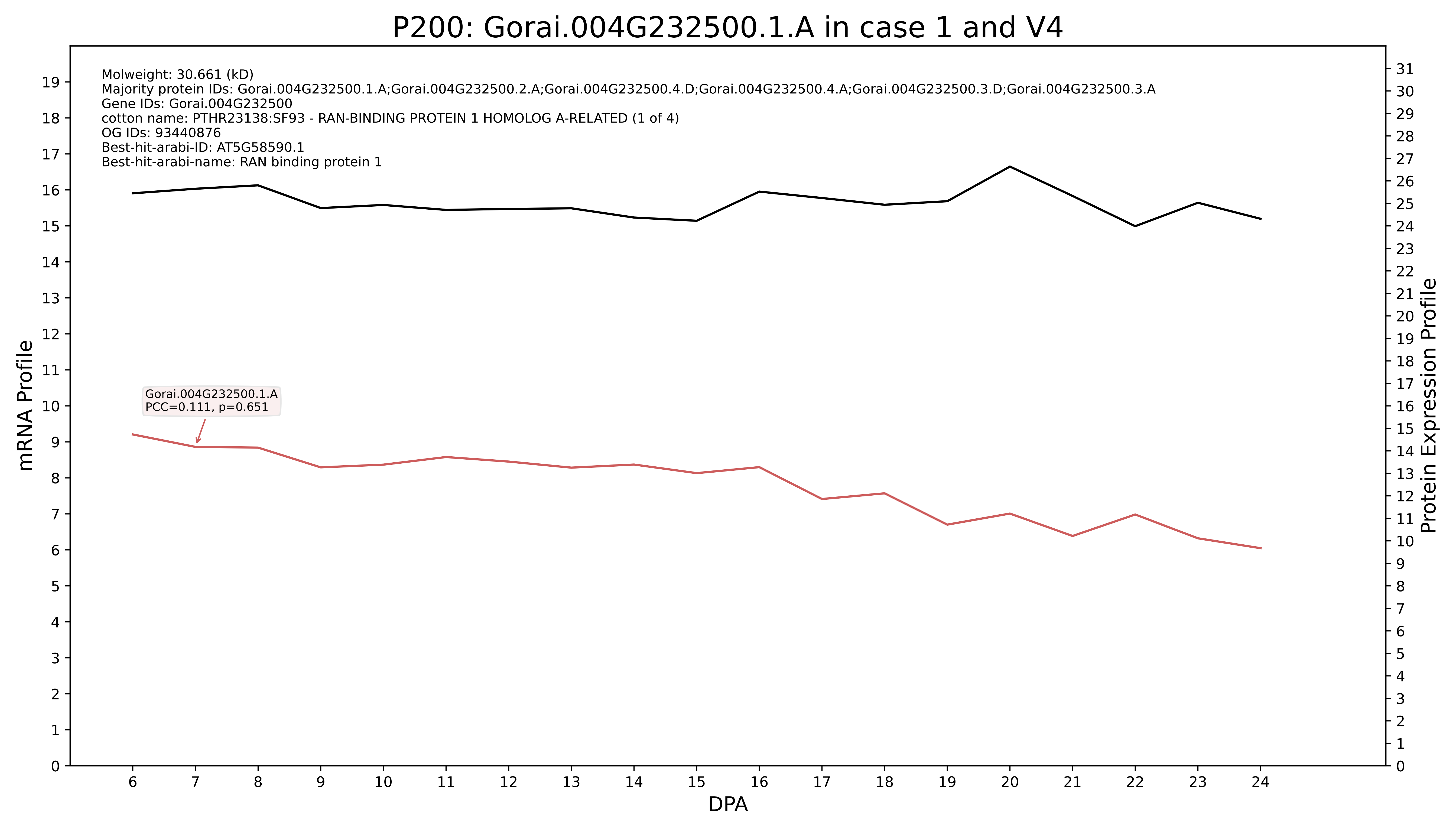
P200: Gorai.004G232500.1.A in case 1 and V4
Molweight: 30.661 (kD)
Majority protein IDs: Gorai.004G232500.1.A;Gorai.004G232500.2.A;Gorai.004G232500.4.D;Gorai.004G232500.4.A;Gorai.004G232500.3.D;Gorai.004G232500.3.A
Gene IDs: Gorai.004G232500
cotton name: PTHR23138:SF93 - RAN-BINDING PROTEIN 1 HOMOLOG A-RELATED (1 of 4)
OG IDs: 93440876
Best-hit-arabi-ID: AT5G58590.1
Best-hit-arabi-name: RAN binding protein 1
Gorai.004G232500.1.A
PCC=0.111, p=0.651
mRNA Profile
Protein Expression Profile
DPA

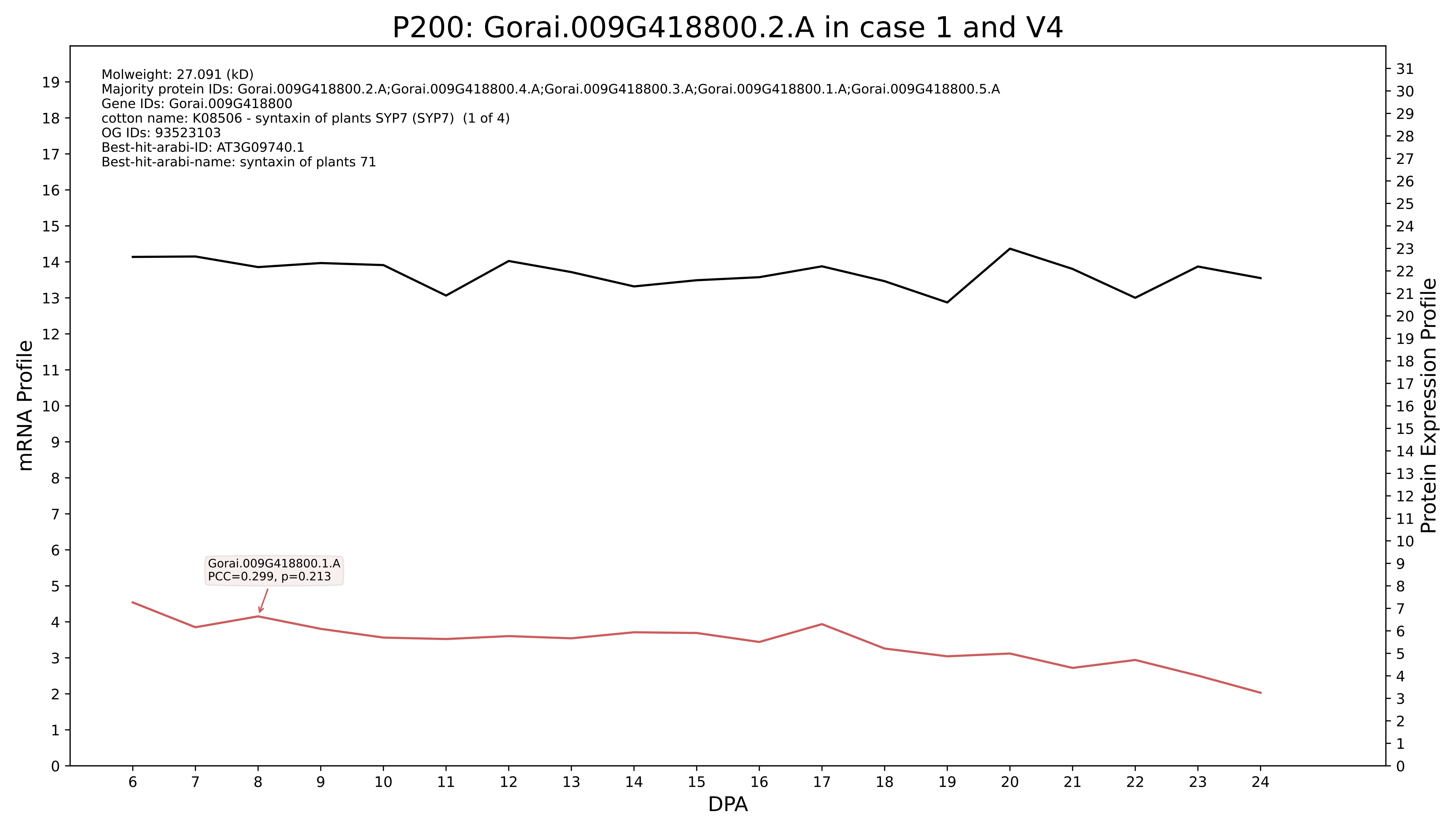
P200: Gorai.009G418800.2.A in case 1 and V4
Molweight: 27.091 (kD)
Majority protein IDs: Gorai.009G418800.2.A;Gorai.009G418800.4.A;Gorai.009G418800.3.A;Gorai.009G418800.1.A;Gorai.009G418800.5.A
Gene IDs: Gorai.009G418800
cotton name: K08506 - syntaxin of plants SYP7 (SYP7) (1 of 4)
OG IDs: 93523103
Best-hit-arabi-ID: AT3G09740.1
Best-hit-arabi-name: syntaxin of plants 71
Gorai.009G418800.1.A
PCC=0.299, p=0.213
mRNA Profile
Protein Expression Profile
DPA

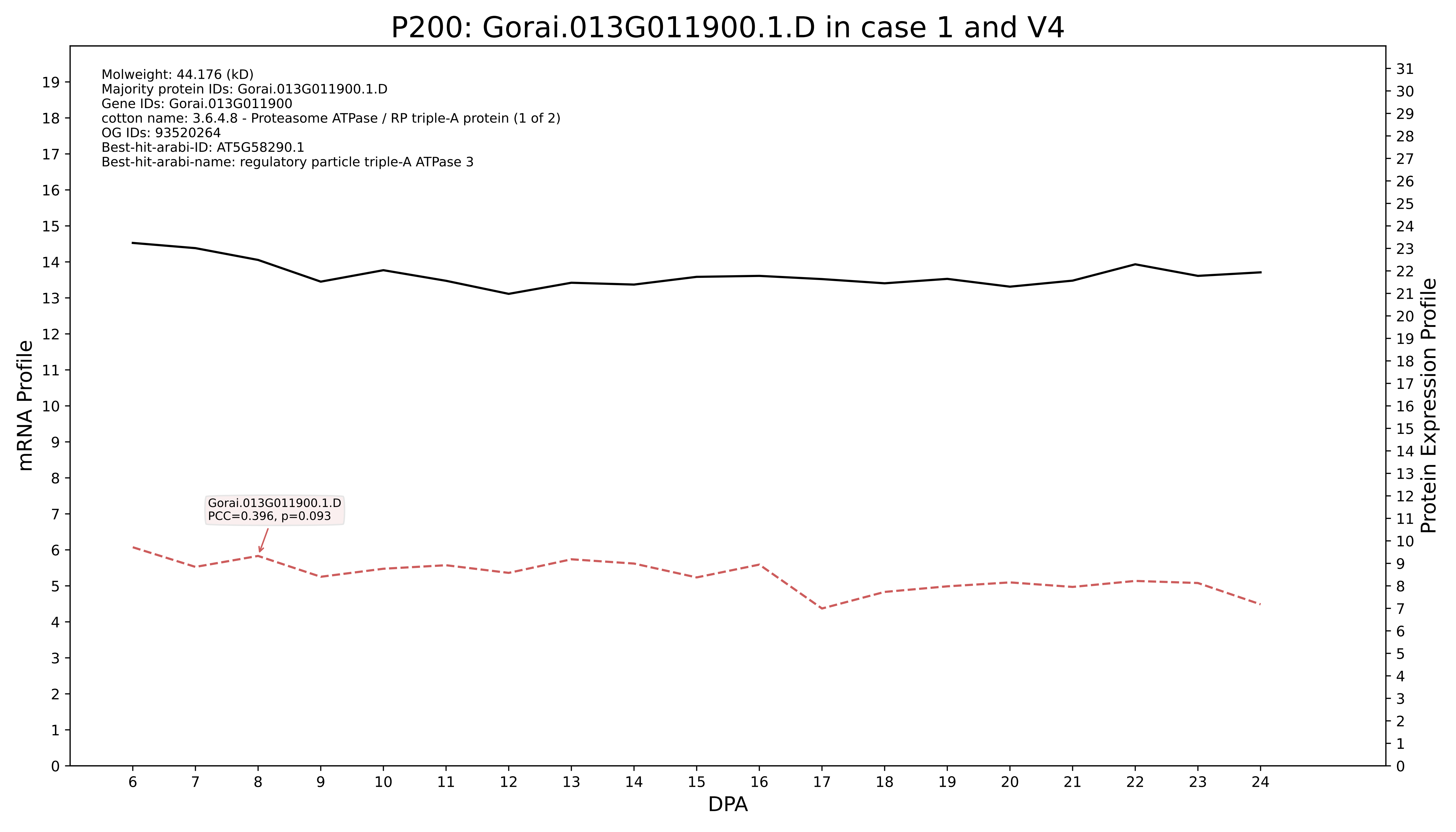

P200: Gorai.013G011900.1.D in case 1 and V4
Molweight: 44.176 (kD)
Majority protein IDs: Gorai.013G011900.1.D
Gene IDs: Gorai.013G011900
cotton name: 3.6.4.8 - Proteasome ATPase / RP triple-A protein (1 of 2)
OG IDs: 93520264
Best-hit-arabi-ID: AT5G58290.1
Best-hit-arabi-name: regulatory particle triple-A ATPase 3
Gorai.013G011900.1.D
PCC=0.396, p=0.093
mRNA Profile
Protein Expression Profile
DPA

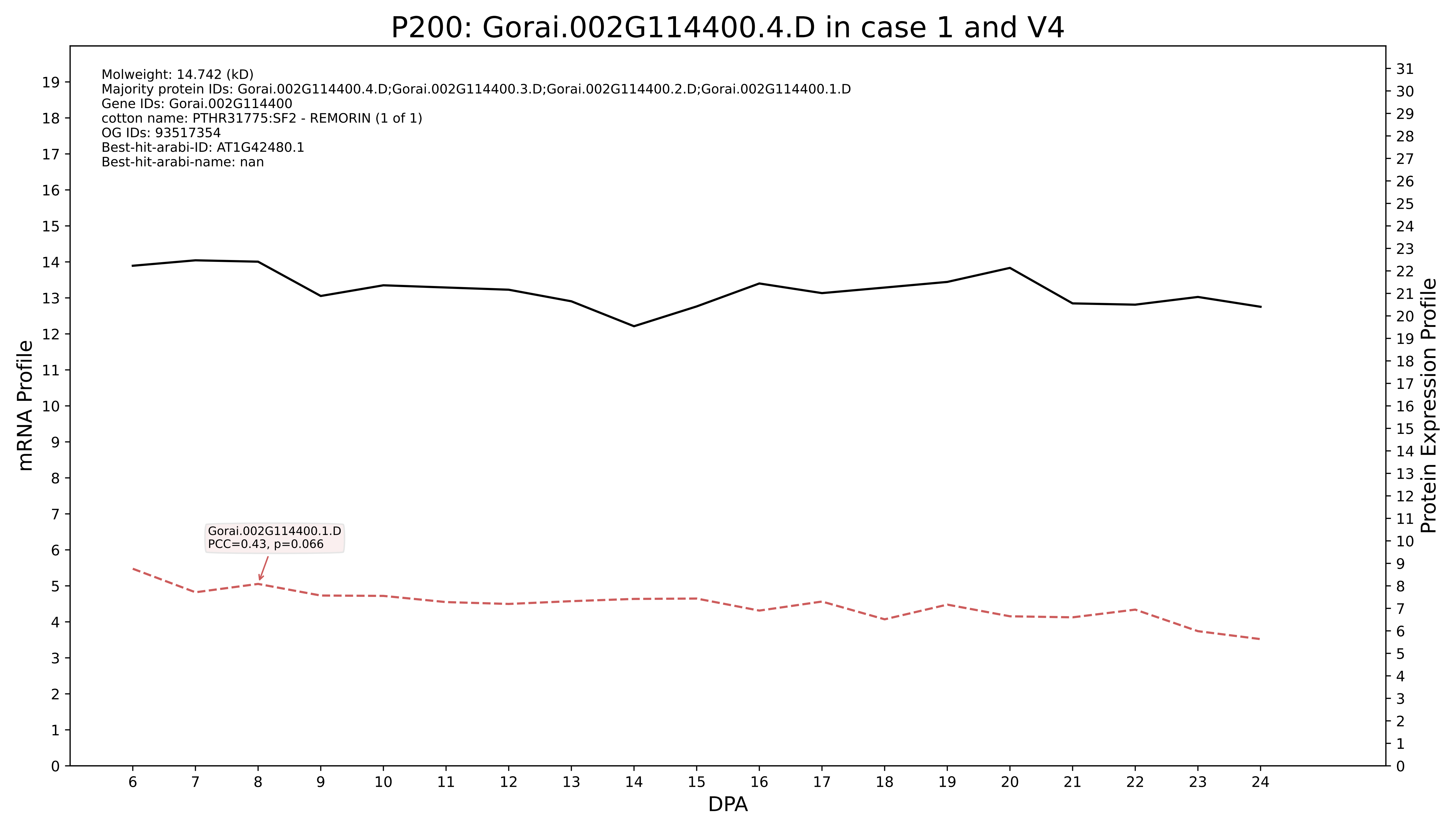
P200: Gorai.002G114400.4.D in case 1 and V4
Molweight: 14.742 (kD)
Majority protein IDs: Gorai.002G114400.4.D;Gorai.002G114400.3.D;Gorai.002G114400.2.D;Gorai.002G114400.1.D
Gene IDs: Gorai.002G114400
cotton name: PTHR31775:SF2 - REMORIN (1 of 1)
OG IDs: 93517354
Best-hit-arabi-ID: AT1G42480.1
Best-hit-arabi-name: nan
Gorai.002G114400.1.D
PCC=0.43, p=0.066
mRNA Profile
Protein Expression Profile
DPA
0
1
2
3
4
5
6
7
8
9
10
11
12
13
14
15
16
17
18
19
20
21
22
23
24
25
26
27
28
29
30
31

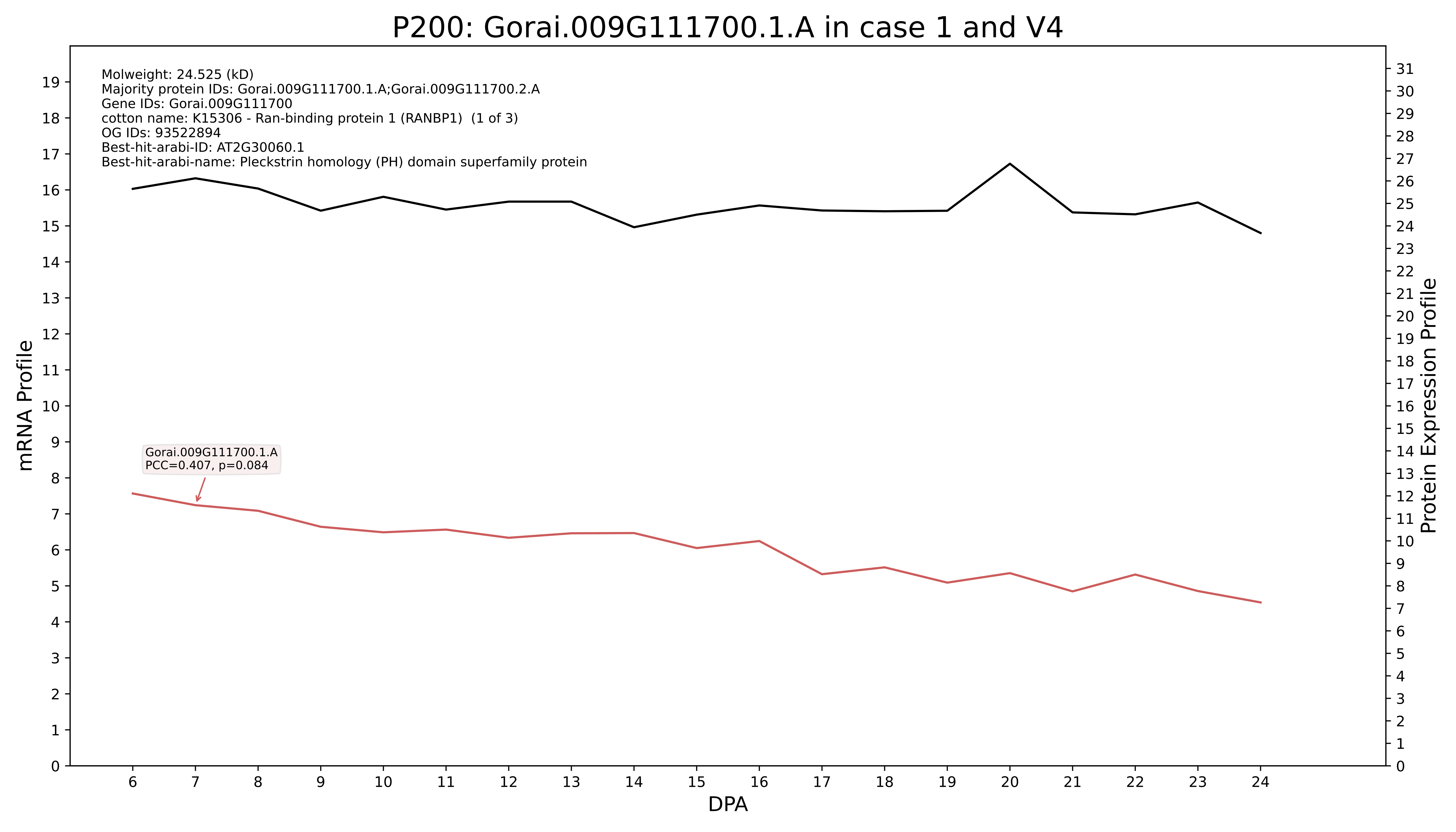
P200: Gorai.009G111700.1.A in case 1 and V4
Molweight: 24.525 (kD)
Majority protein IDs: Gorai.009G111700.1.A;Gorai.009G111700.2.A
Gene IDs: Gorai.009G111700
cotton name: K15306 - Ran-binding protein 1 (RANBP1) (1 of 3)
OG IDs: 93522894
Best-hit-arabi-ID: AT2G30060.1
Best-hit-arabi-name: Pleckstrin homology (PH) domain superfamily protein
Gorai.009G111700.1.A
PCC=0.407, p=0.084
mRNA Profile
Protein Expression Profile
DPA

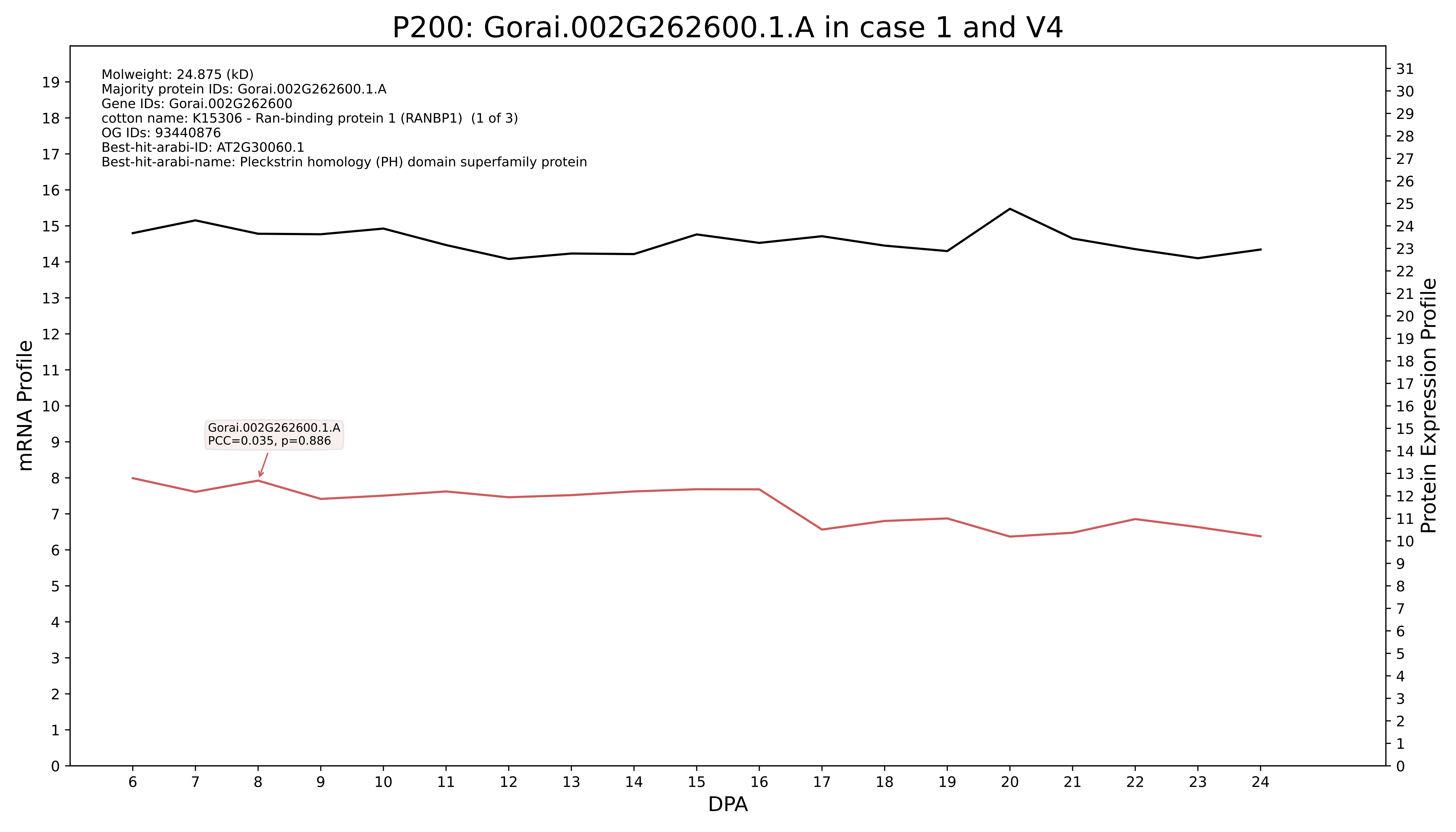
P200: Gorai.002G262600.1.A in case 1 and V4
Molweight: 24.875 (kD)
Majority protein IDs: Gorai.002G262600.1.A
Gene IDs: Gorai.002G262600
cotton name: K15306 - Ran-binding protein 1 (RANBP1) (1 of 3)
OG IDs: 93440876
Best-hit-arabi-ID: AT2G30060.1
Best-hit-arabi-name: Pleckstrin homology (PH) domain superfamily protein
Gorai.002G262600.1.A
PCC=0.035, p=0.886
mRNA Profile
Protein Expression Profile
DPA

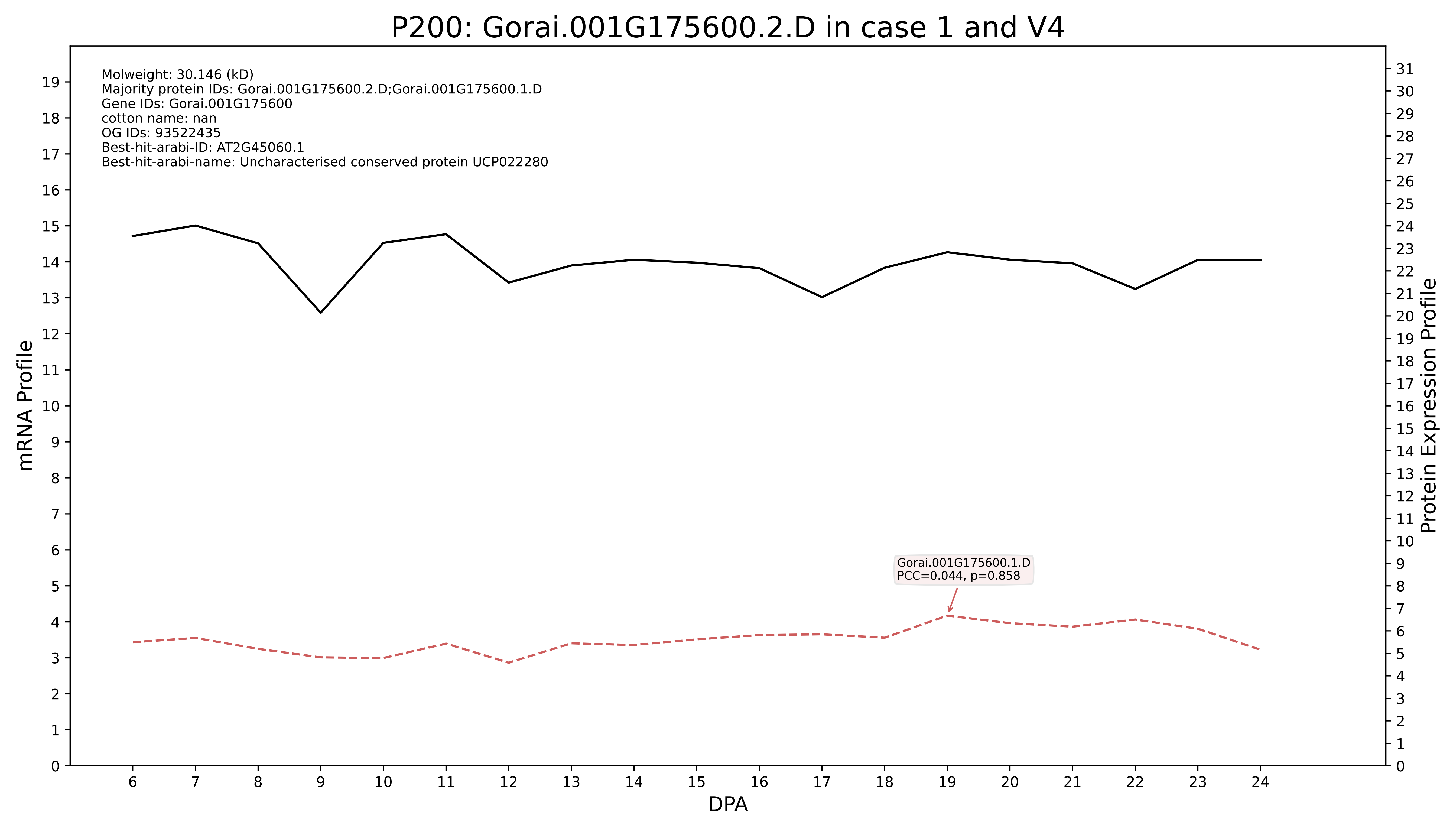

P200: Gorai.001G175600.2.D in case 1 and V4
Molweight: 30.146 (kD)
Majority protein IDs: Gorai.001G175600.2.D;Gorai.001G175600.1.D
Gene IDs: Gorai.001G175600
cotton name: nan
OG IDs: 93522435
Best-hit-arabi-ID: AT2G45060.1
Best-hit-arabi-name: Uncharacterised conserved protein UCP022280
Gorai.001G175600.1.D
PCC=0.044, p=0.858
mRNA Profile
Protein Expression Profile
DPA

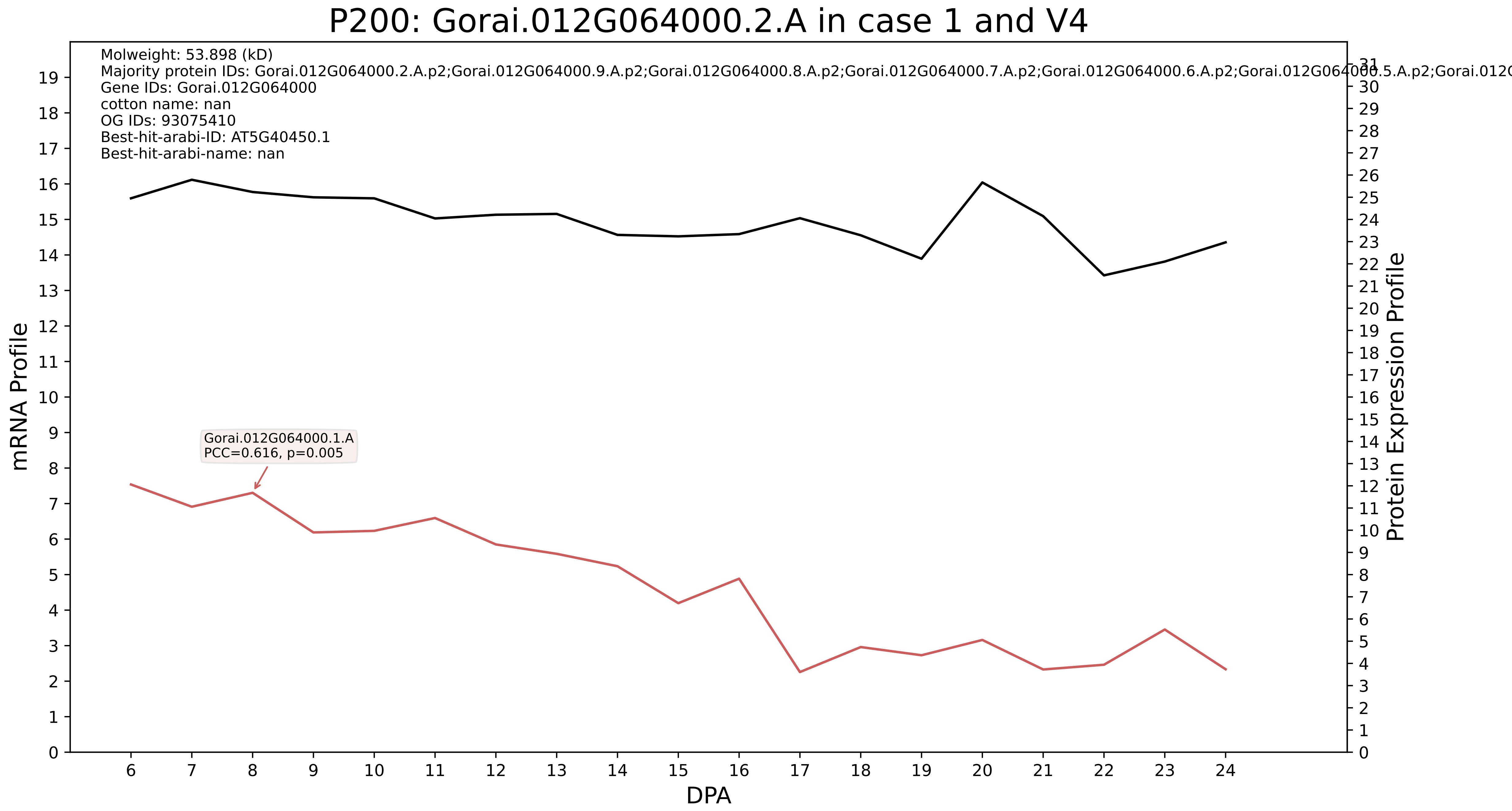

P200: Gorai.012G064000.2.A in case 1 and V4
Molweight: 53.898 (kD)
Majority protein IDs: Gorai.012G064000.2.A.p2;Gorai.012G064000.9.A.p2;Gorai.012G064000.8.A.p2;Gorai.012G064000.7.A.p2;Gorai.012G064000.6.A.p2;Gorai.012G064000.5.A.p2;Gorai.012
Gene IDs: Gorai.012G064000
cotton name: nan
OG IDs: 93075410
Best-hit-arabi-ID: AT5G40450.1
Best-hit-arabi-name: nan
Gorai.012G064000.1.A
PCC=0.616, p=0.005
mRNA Profile
Protein Expression Profile
DPA

P200: Gorai.001G016900.1.D in case 1 and V4

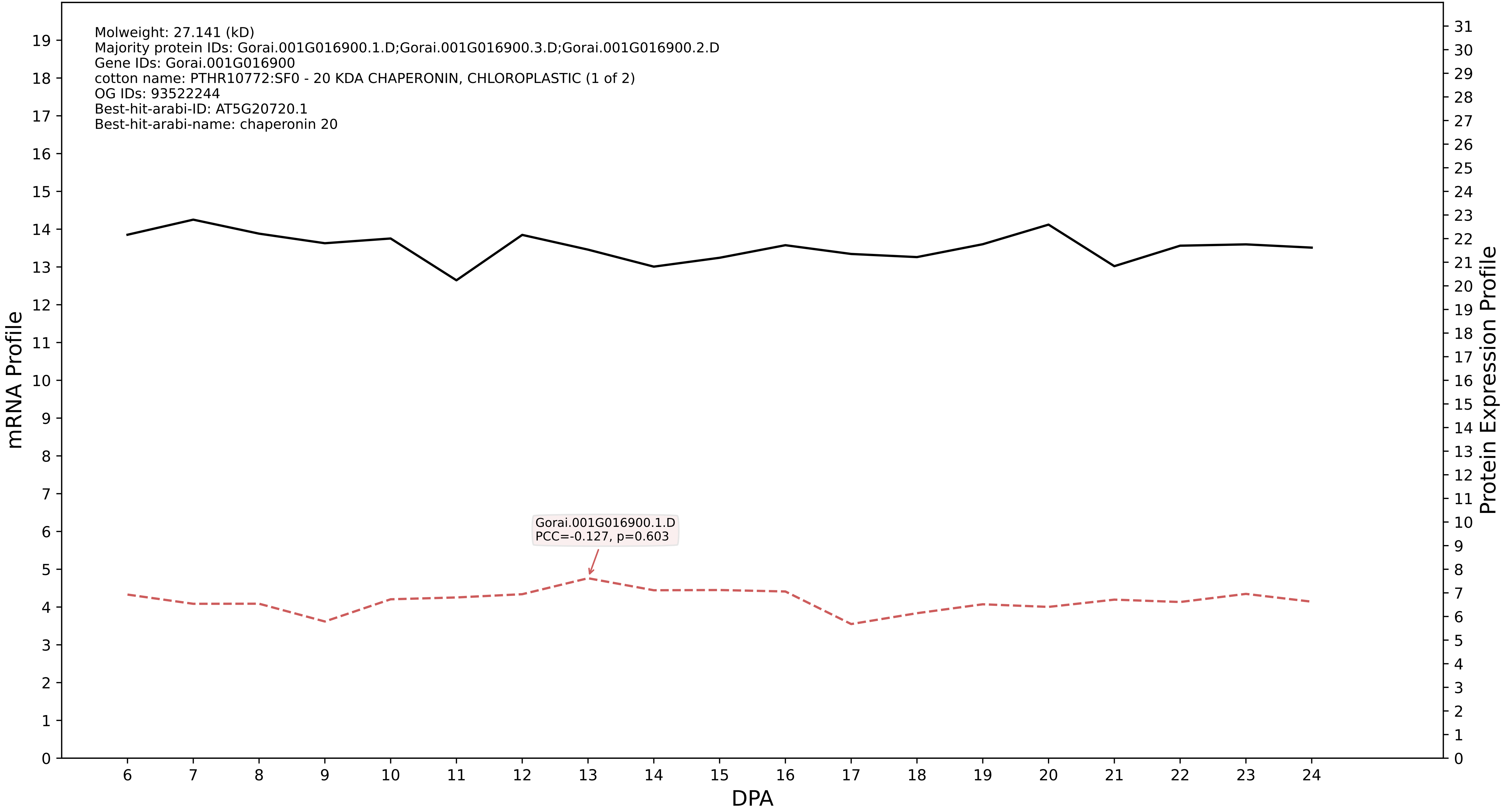

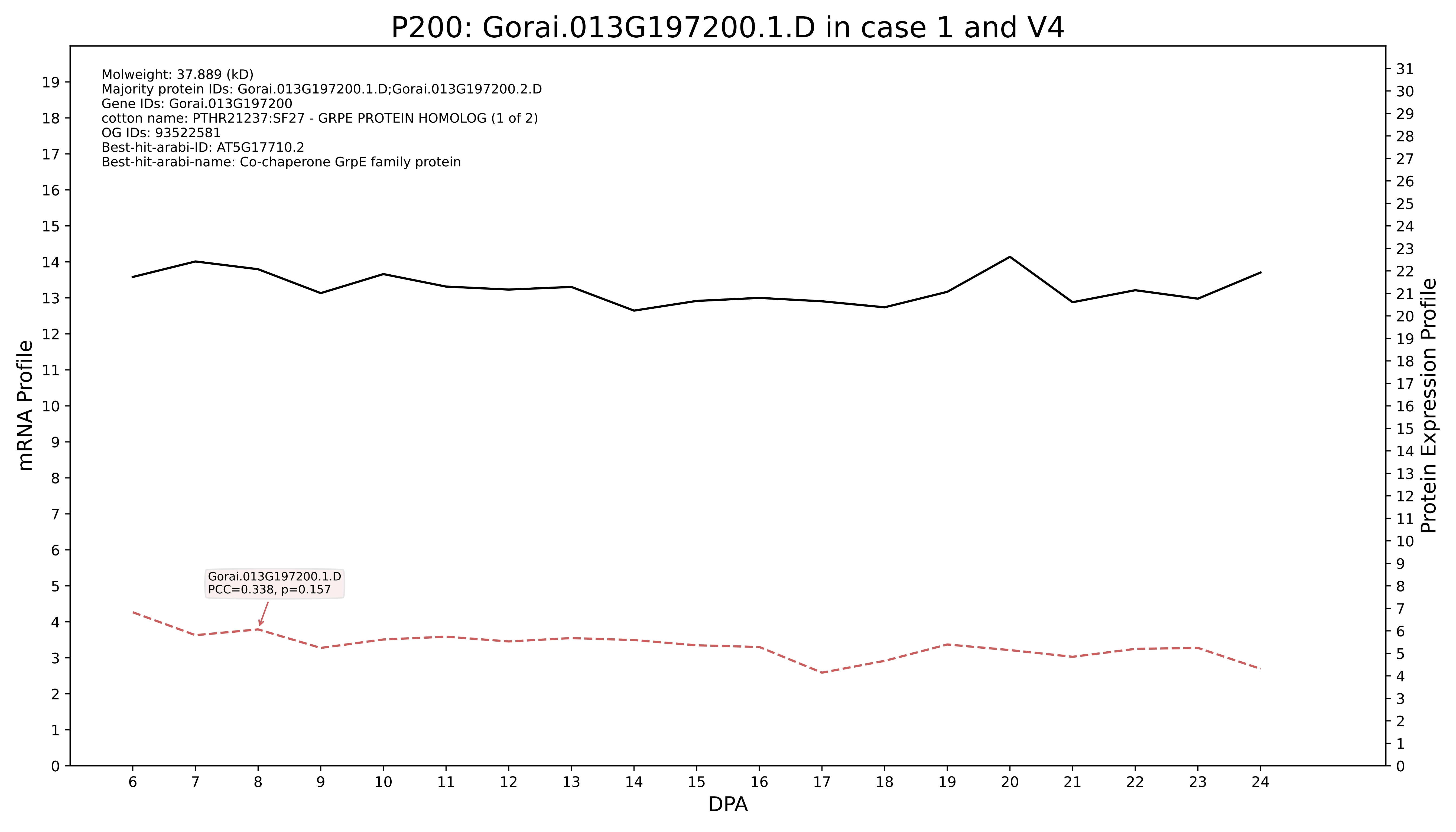
P200: Gorai.013G197200.1.D in case 1 and V4
Molweight: 37.889 (kD)
Majority protein IDs: Gorai.013G197200.1.D;Gorai.013G197200.2.D
Gene IDs: Gorai.013G197200
cotton name: PTHR21237:SF27 - GRPE PROTEIN HOMOLOG (1 of 2)
OG IDs: 93522581
Best-hit-arabi-ID: AT5G17710.2
Best-hit-arabi-name: Co-chaperone GrpE family protein
Gorai.013G197200.1.D
PCC=0.338, p=0.157
mRNA Profile
Protein Expression Profile
DPA

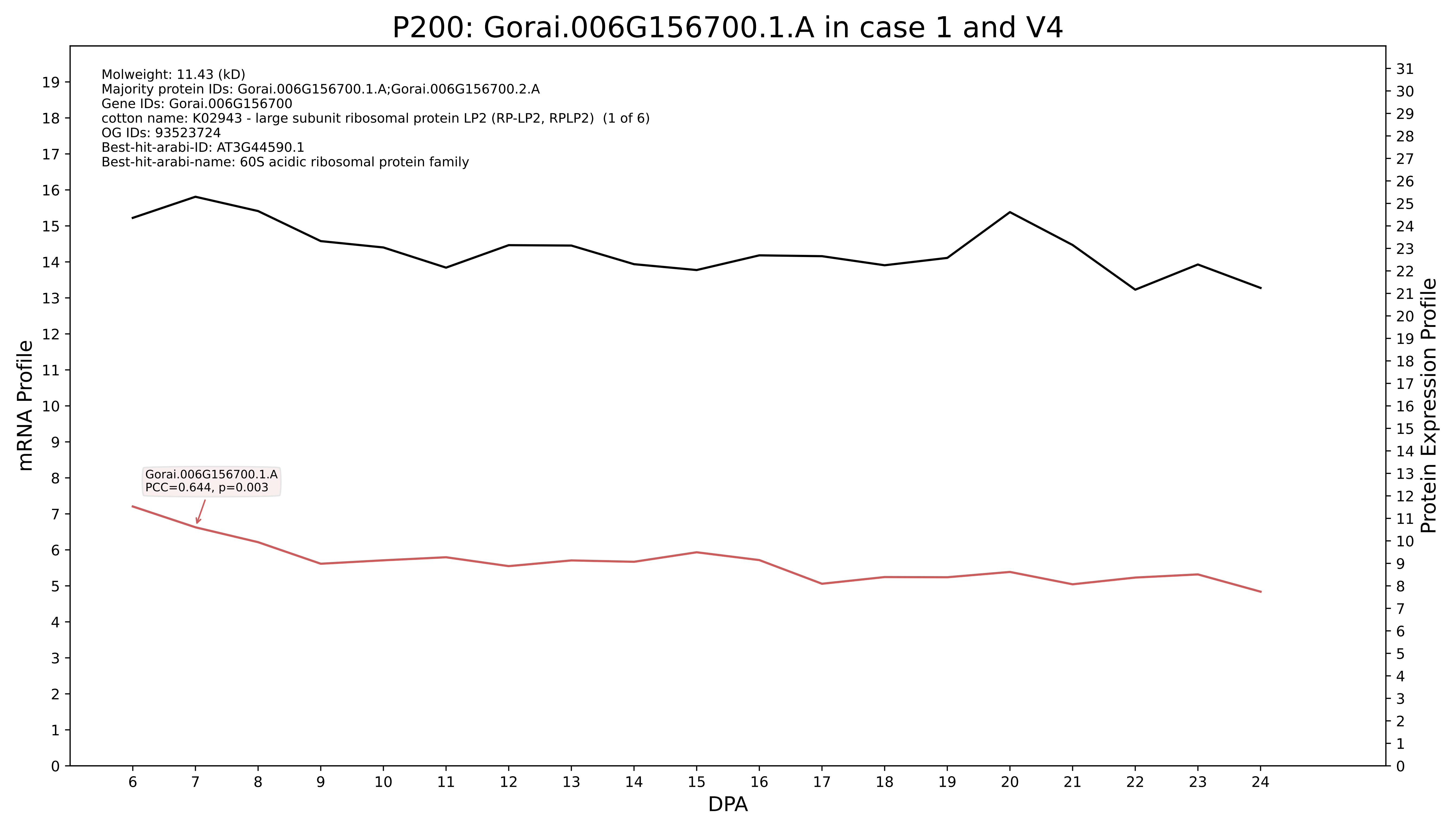
P200: Gorai.006G156700.1.A in case 1 and V4
Molweight: 11.43 (kD)
Majority protein IDs: Gorai.006G156700.1.A;Gorai.006G156700.2.A
Gene IDs: Gorai.006G156700
cotton name: K02943 - large subunit ribosomal protein LP2 (RP-LP2, RPLP2) (1 of 6)
OG IDs: 93523724
Best-hit-arabi-ID: AT3G44590.1
Best-hit-arabi-name: 60S acidic ribosomal protein family
Gorai.006G156700.1.A
PCC=0.644, p=0.003
mRNA Profile
Protein Expression Profile
DPA

P200: Gorai.001G093100.7.A in case 1 and V4

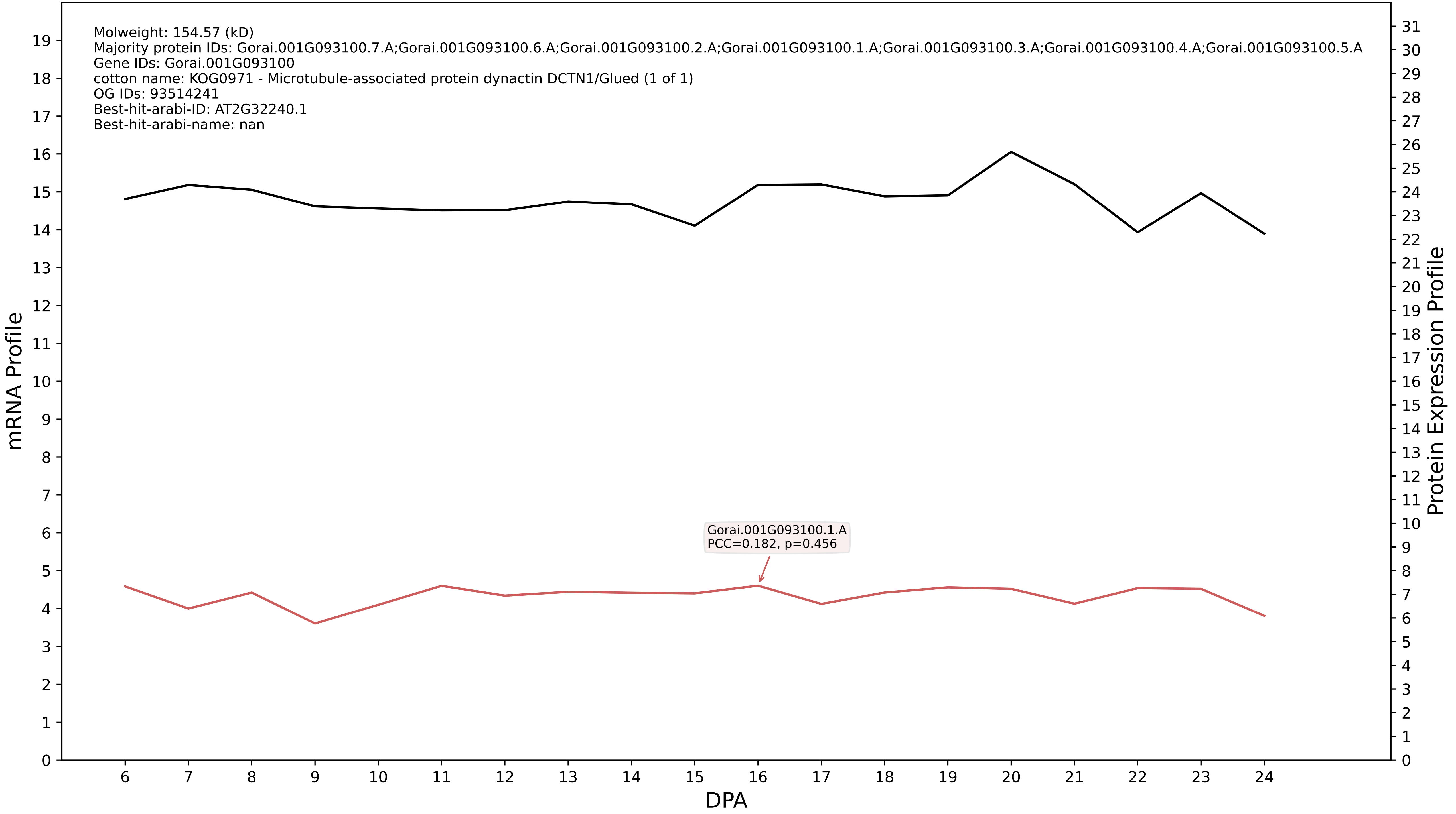

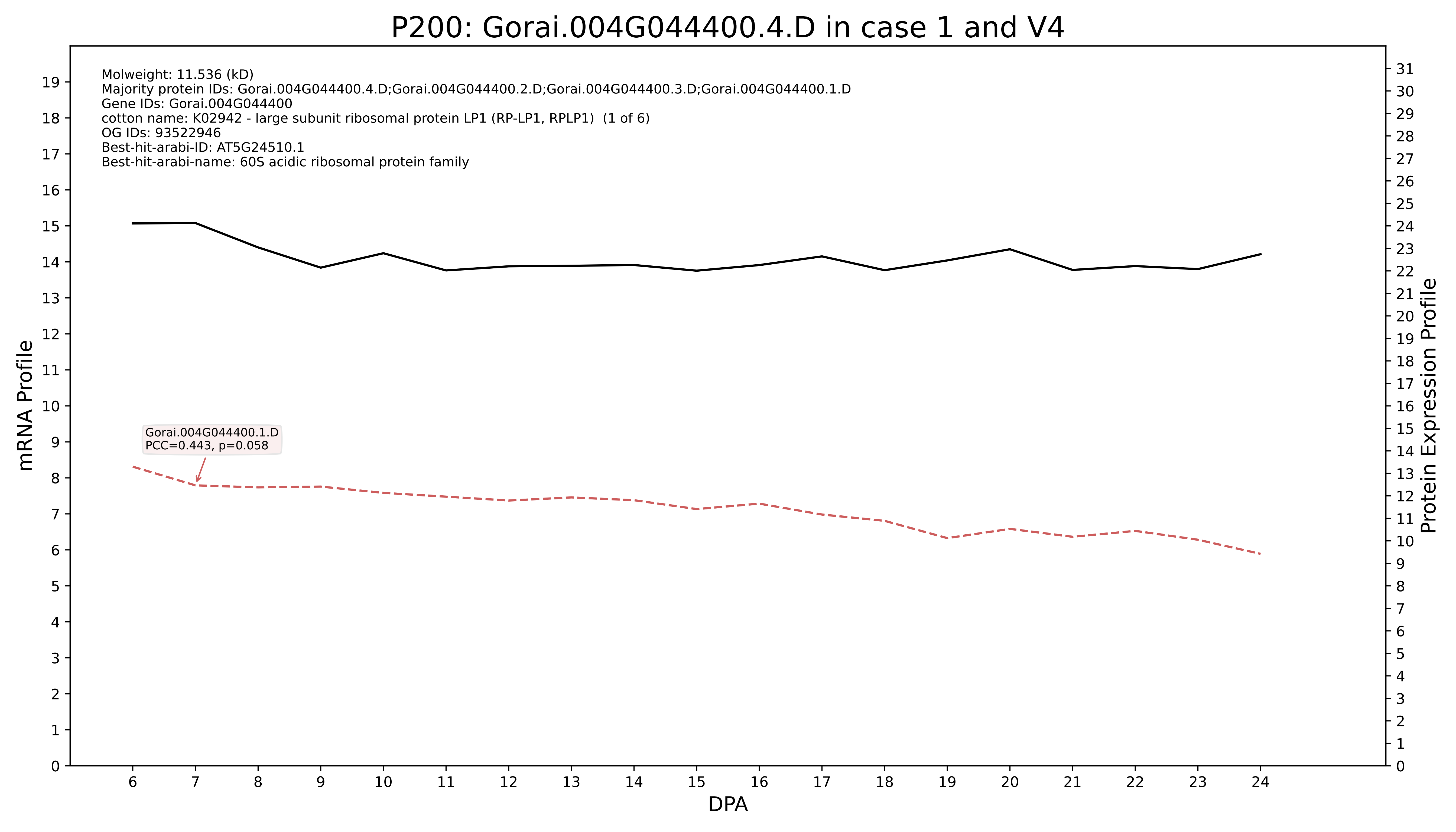
P200: Gorai.004G044400.4.D in case 1 and V4
Molweight: 11.536 (kD)
Majority protein IDs: Gorai.004G044400.4.D;Gorai.004G044400.2.D;Gorai.004G044400.3.D;Gorai.004G044400.1.D
Gene IDs: Gorai.004G044400
cotton name: K02942 - large subunit ribosomal protein LP1 (RP-LP1, RPLP1) (1 of 6)
OG IDs: 93522946
Best-hit-arabi-ID: AT5G24510.1
Best-hit-arabi-name: 60S acidic ribosomal protein family
Gorai.004G044400.1.D
PCC=0.443, p=0.058
mRNA Profile
Protein Expression Profile
DPA
0
1
2
3
4
5
6
7
8
9
10
11
12
13
14
15
16
17
18
19
20
21
22
23
24
25
26
27
28
29
30
31

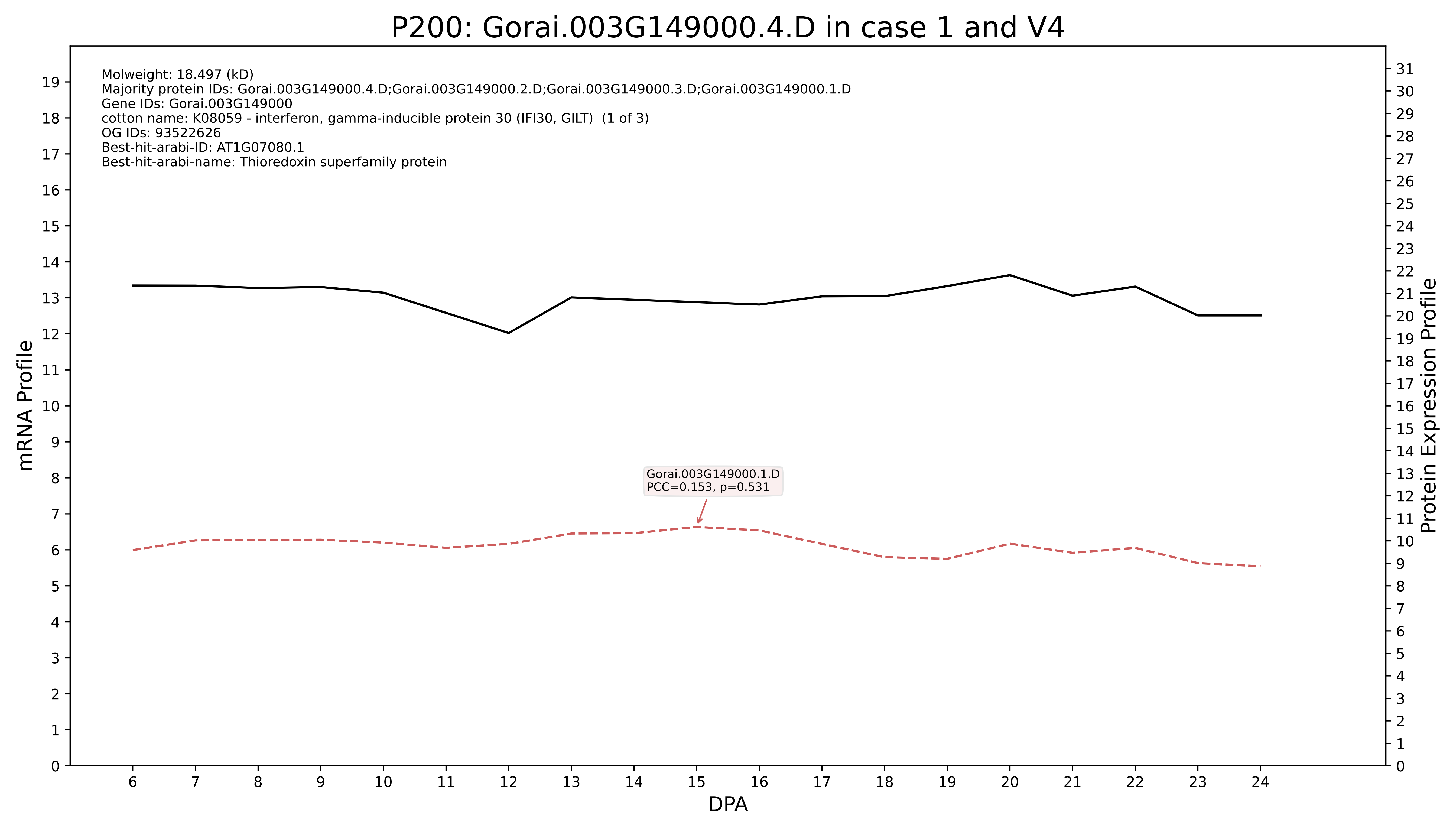
P200: Gorai.003G149000.4.D in case 1 and V4
Molweight: 18.497 (kD)
Majority protein IDs: Gorai.003G149000.4.D;Gorai.003G149000.2.D;Gorai.003G149000.3.D;Gorai.003G149000.1.D
Gene IDs: Gorai.003G149000
cotton name: K08059 - interferon, gamma-inducible protein 30 (IFI30, GILT) (1 of 3)
OG IDs: 93522626
Best-hit-arabi-ID: AT1G07080.1
Best-hit-arabi-name: Thioredoxin superfamily protein
Gorai.003G149000.1.D
PCC=0.153, p=0.531
mRNA Profile
Protein Expression Profile
DPA

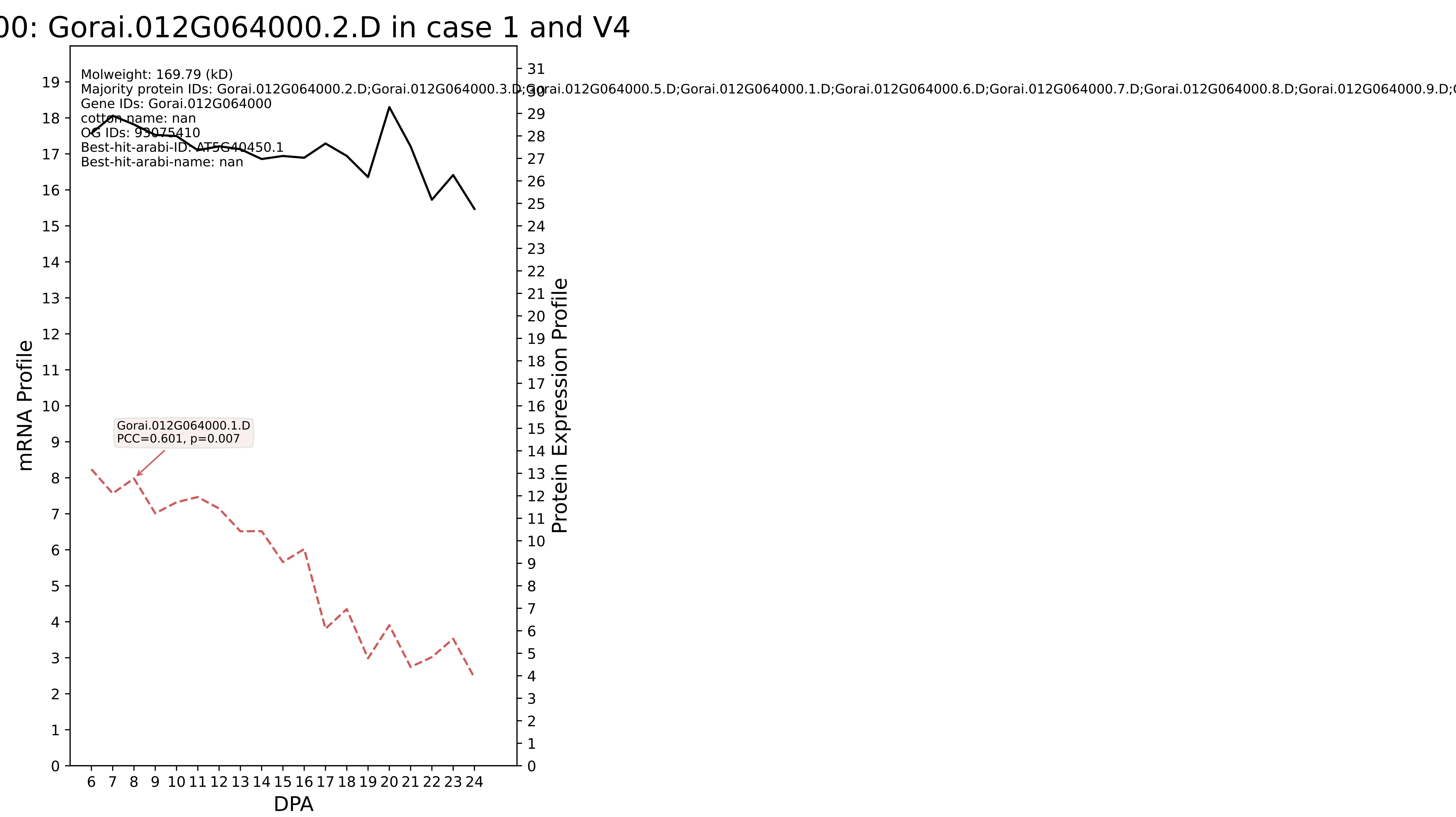
00: Gorai.012G064000.2.D in case 1 and V4
Molweight: 169.79 (kD)
Majority protein IDs: Gorai.012G064000.2.D;Gorai.012G064000.3.D;Gorai.012G064000.5.D;Gorai.012G064000.1.D;Gorai.012G064000.6.D;Gorai.012G064000.7.D;Gorai.012G064000.8.D;Gorai.012G064000.9.D;G
Gene IDs: Gorai.012G064000
cotton-name: nan
OG IDs: 93075410
Best-hit-arabi-ID: AT5G40450.1
Best-hit-arabi-name: nan
Gorai.012G064000.1.D
PCC=0.601, p=0.007
mRNA Profile
Protein Expression Profile
DPA

P200: Gorai.008G078300.2.A in case 1 and V4

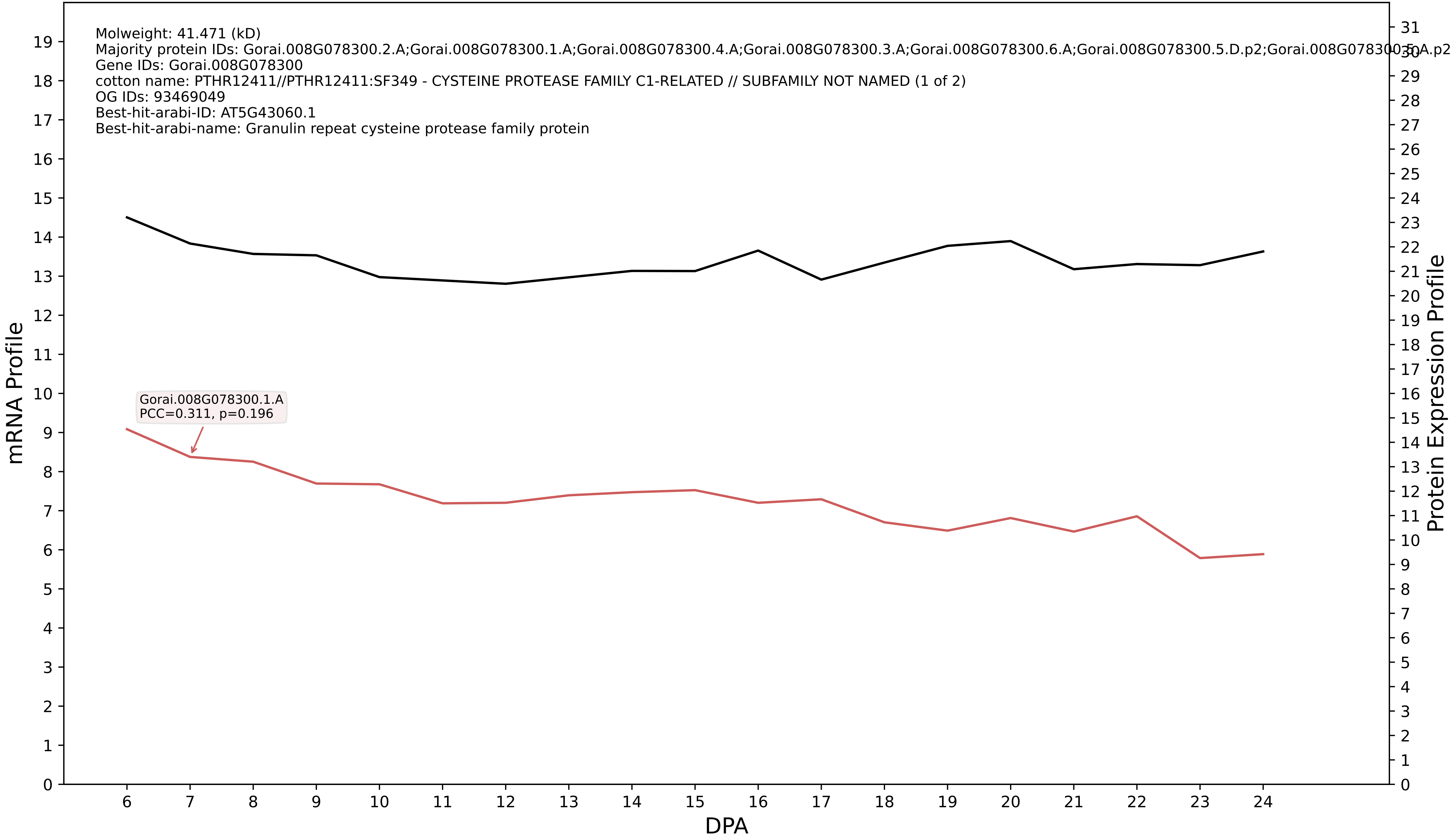

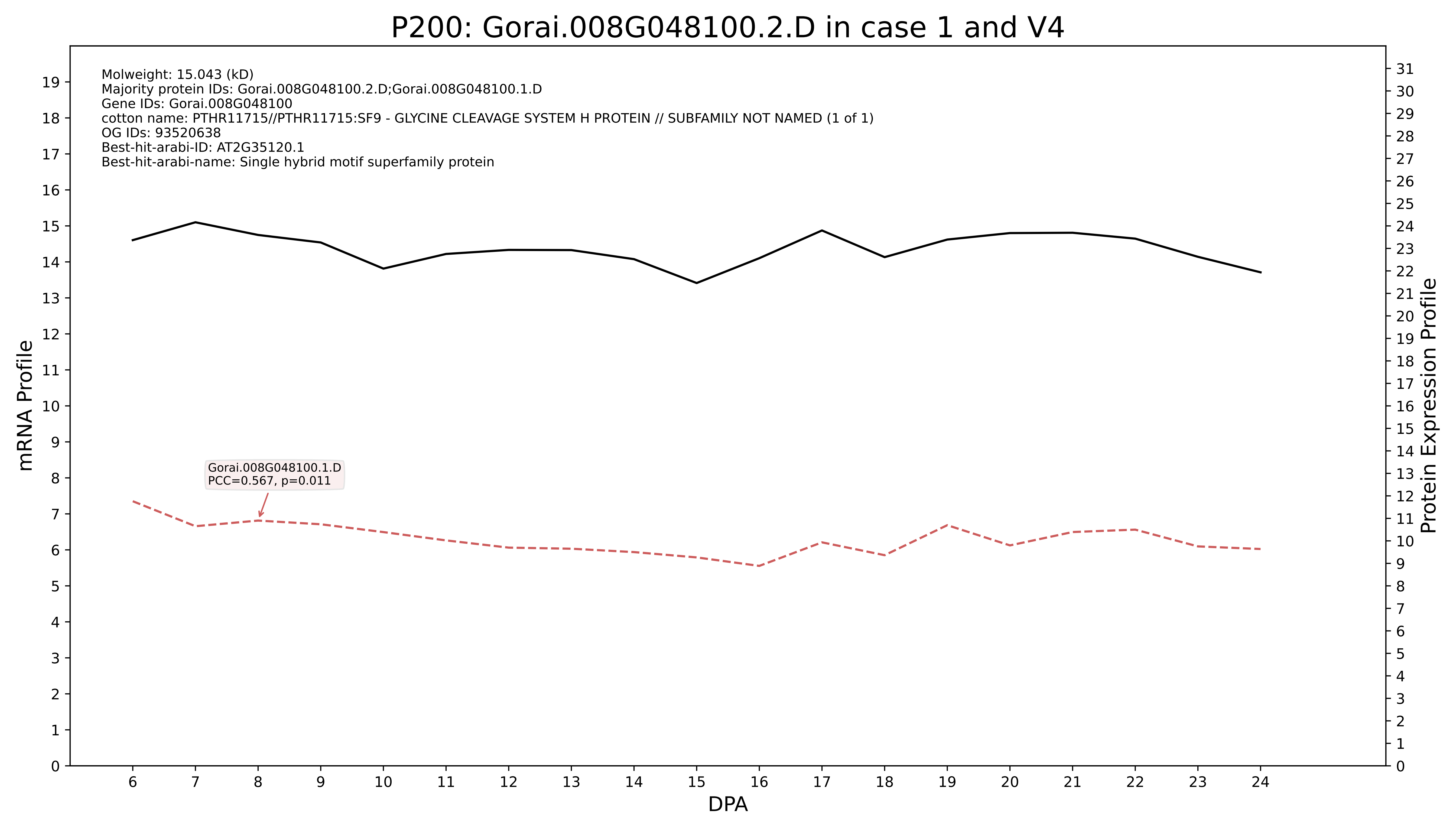
P200: Gorai.008G048100.2.D in case 1 and V4
Molweight: 15.043 (kD)
Majority protein IDs: Gorai.008G048100.2.D;Gorai.008G048100.1.D
Gene IDs: Gorai.008G048100
cotton name: PTHR11715//PTHR11715:SF9 - GLYCINE CLEAVAGE SYSTEM H PROTEIN // SUBFAMILY NOT NAMED (1 of 1)
OG IDs: 93520638
Best-hit-arabi-ID: AT2G35120.1
Best-hit-arabi-name: Single hybrid motif superfamily protein
Gorai.008G048100.1.D
PCC=0.567, p=0.011
mRNA Profile
Protein Expression Profile
DPA

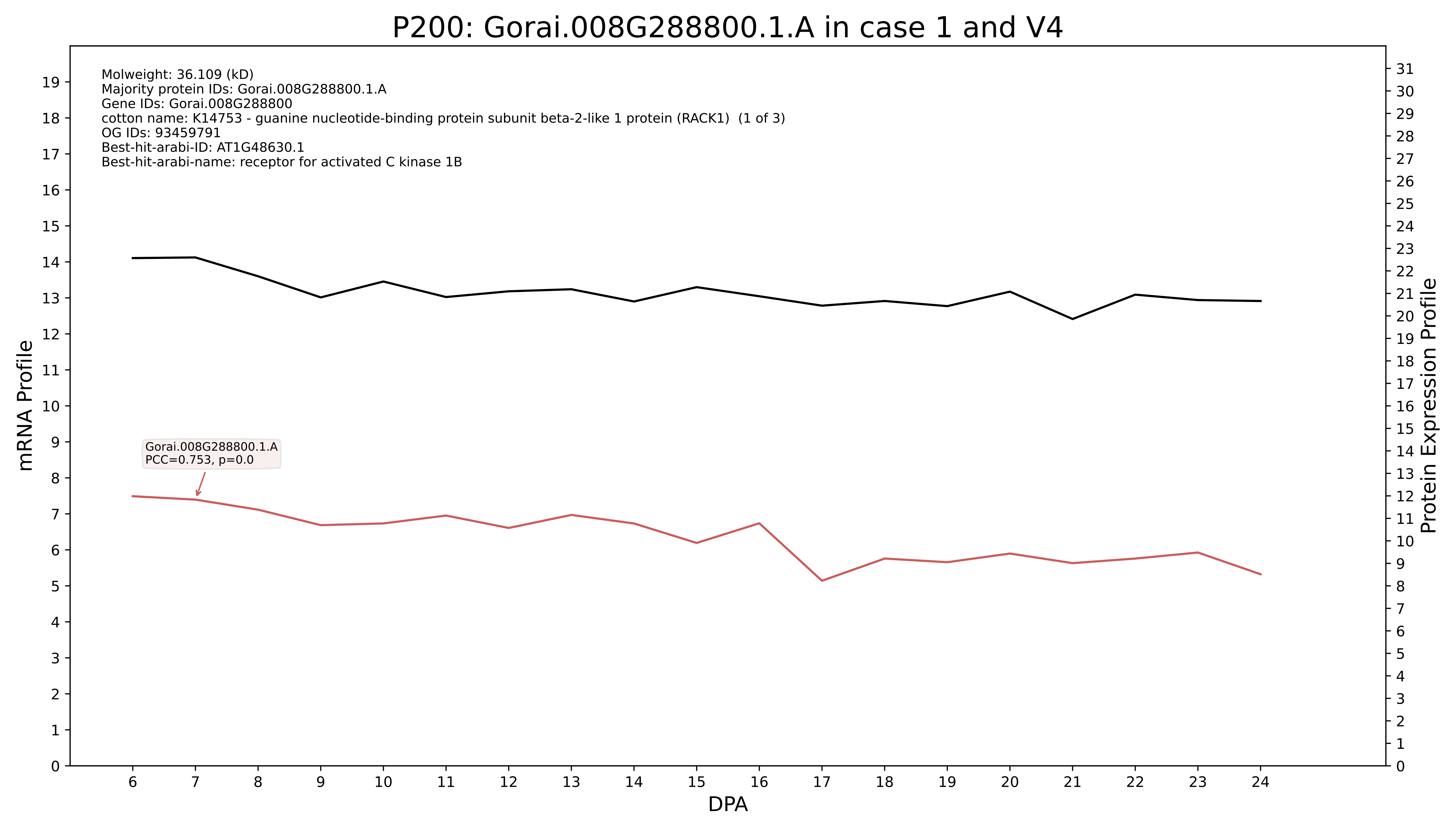
P200: Gorai.008G288800.1.A in case 1 and V4
Molweight: 36.109 (kD)
Majority protein IDs: Gorai.008G288800.1.A
Gene IDs: Gorai.008G288800
cotton name: K14753 - guanine nucleotide-binding protein subunit beta-2-like 1 protein (RACK1) (1 of 3)
OG IDs: 93459791
Best-hit-arabi-ID: AT1G48630.1
Best-hit-arabi-name: receptor for activated C kinase 1B
Gorai.008G288800.1.A
PCC=0.753, p=0.0
mRNA Profile
Protein Expression Profile
DPA
0
1
2
3
4
5
6
7
8
9
10
11
12
13
14
15
16
17
18
19
20
21
22
23
24
25
26
27
28
29
30
31

P200: Gorai.009G087300.1.D in case 1 and V4

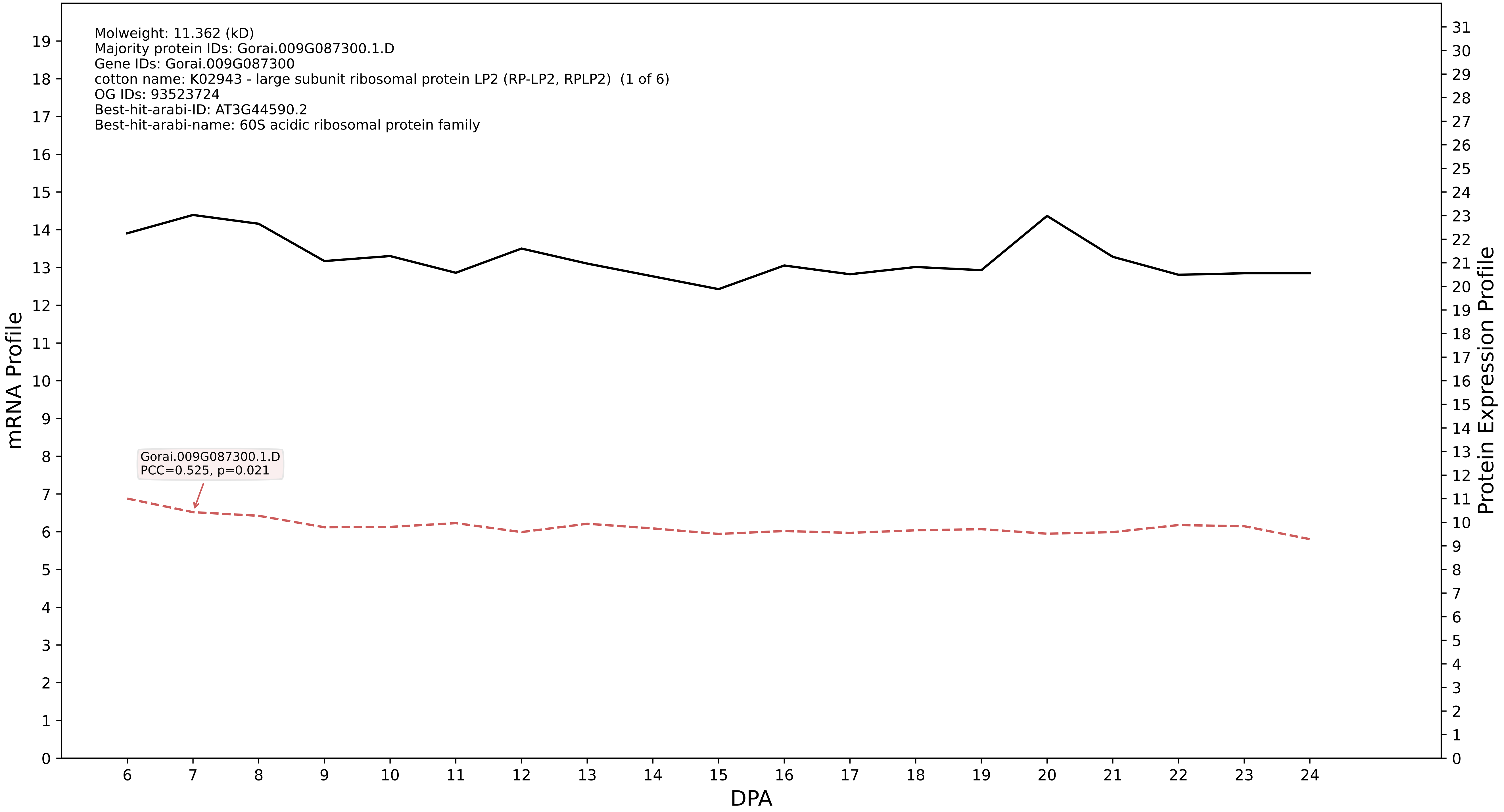

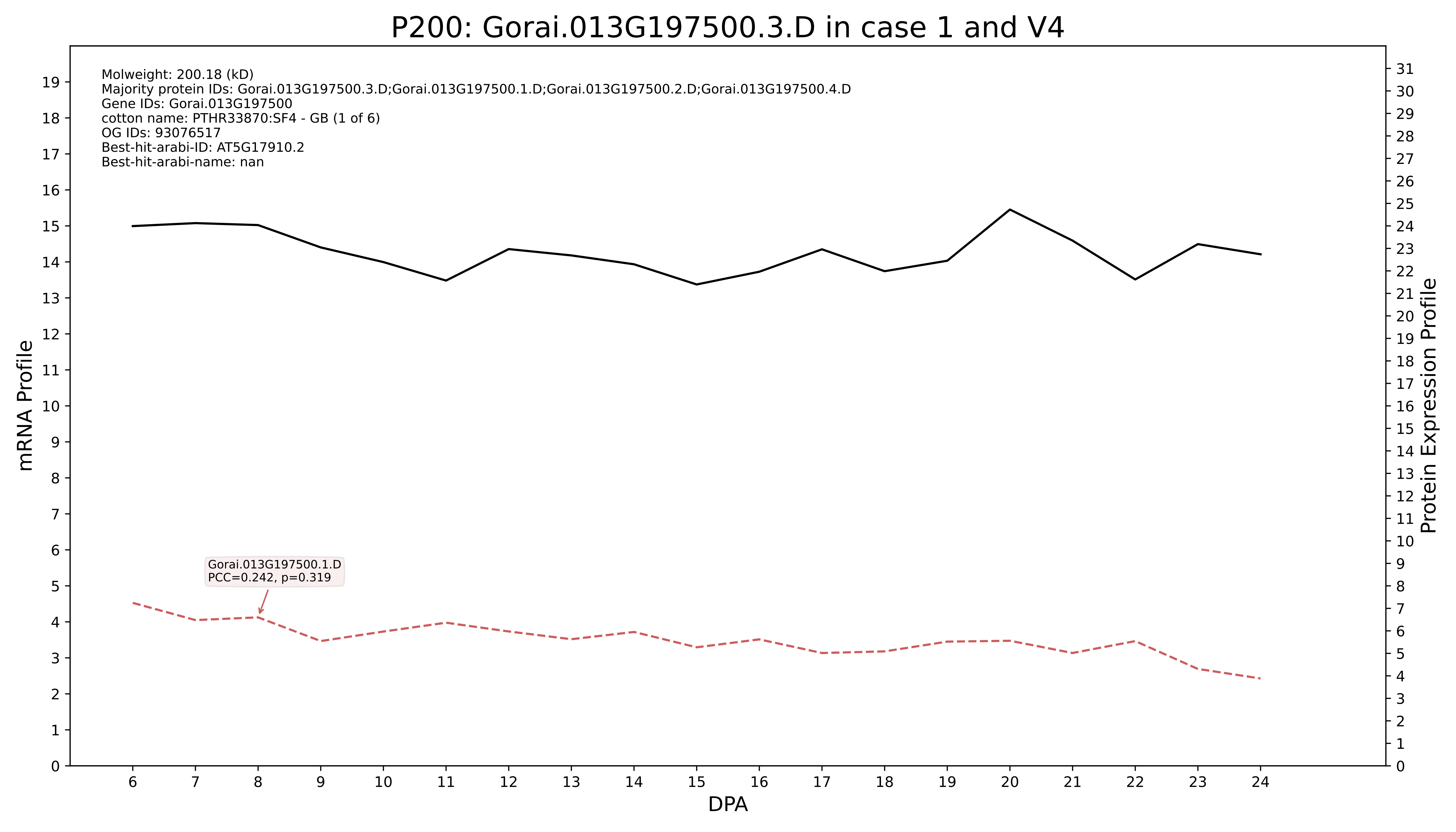
P200: Gorai.013G197500.3.D in case 1 and V4
Molweight: 200.18 (kD)
Majority protein IDs: Gorai.013G197500.3.D;Gorai.013G197500.1.D;Gorai.013G197500.2.D;Gorai.013G197500.4.D
Gene IDs: Gorai.013G197500
cotton name: PTHR33870:SF4 - GB (1 of 6)
OG IDs: 93076517
Best-hit-arabi-ID: AT5G17910.2
Best-hit-arabi-name: nan
Gorai.013G197500.1.D
PCC=0.242, p=0.319
mRNA Profile
Protein Expression Profile
DPA

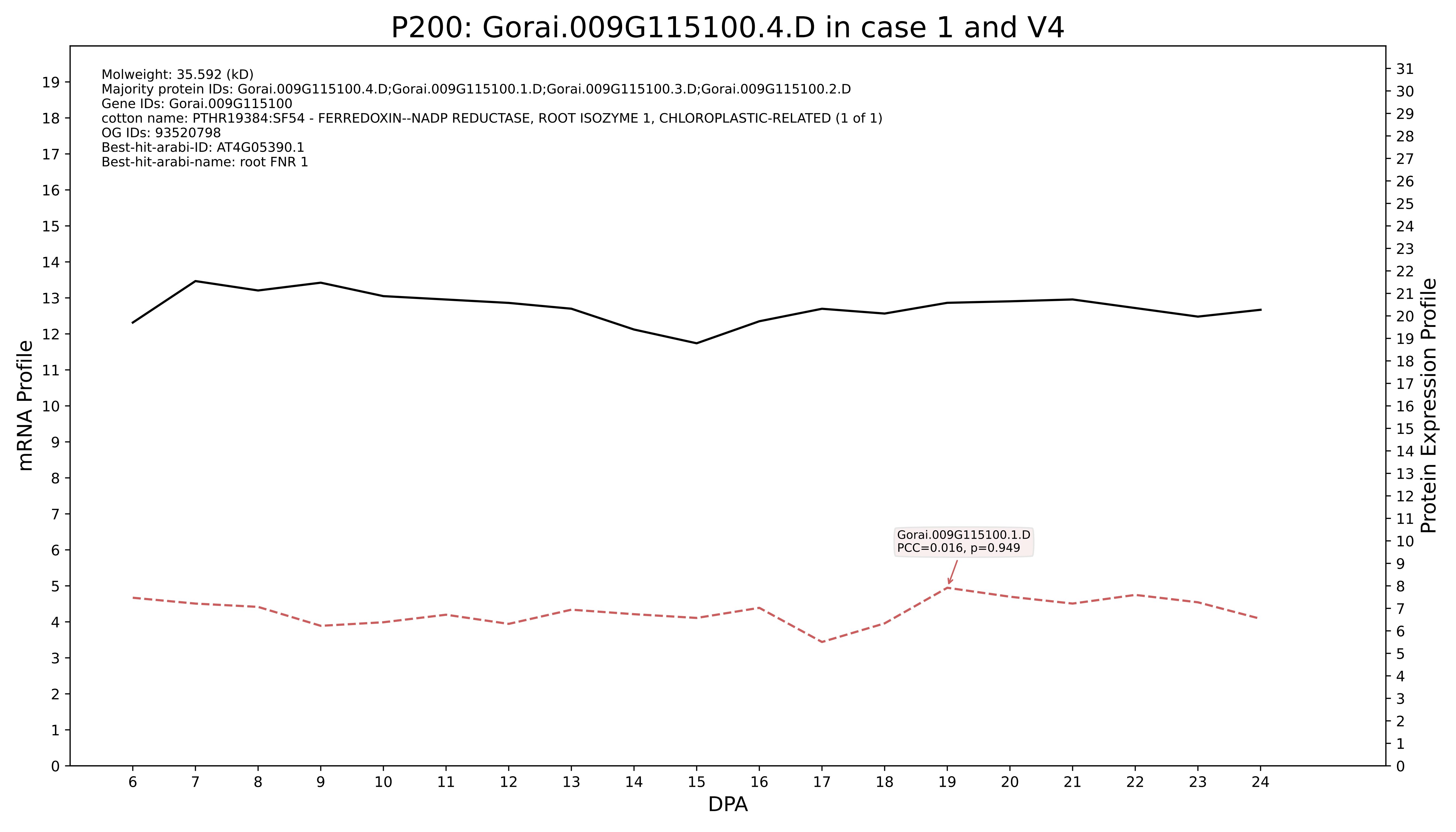
P200: Gorai.009G115100.4.D in case 1 and V4
Molweight: 35.592 (kD)
Majority protein IDs: Gorai.009G115100.4.D;Gorai.009G115100.1.D;Gorai.009G115100.3.D;Gorai.009G115100.2.D
Gene IDs: Gorai.009G115100
cotton name: PTHR19384:SF54 - FERREDOXIN--NADP REDUCTASE, ROOT ISOZYME 1, CHLOROPLASTIC-RELATED (1 of 1)
OG IDs: 93520798
Best-hit-arabi-ID: AT4G05390.1
Best-hit-arabi-name: root FNR 1
Gorai.009G115100.1.D
PCC=0.016, p=0.949
mRNA Profile
Protein Expression Profile
DPA

P200: Gorai.013G076500.1.A in case 1 and V4

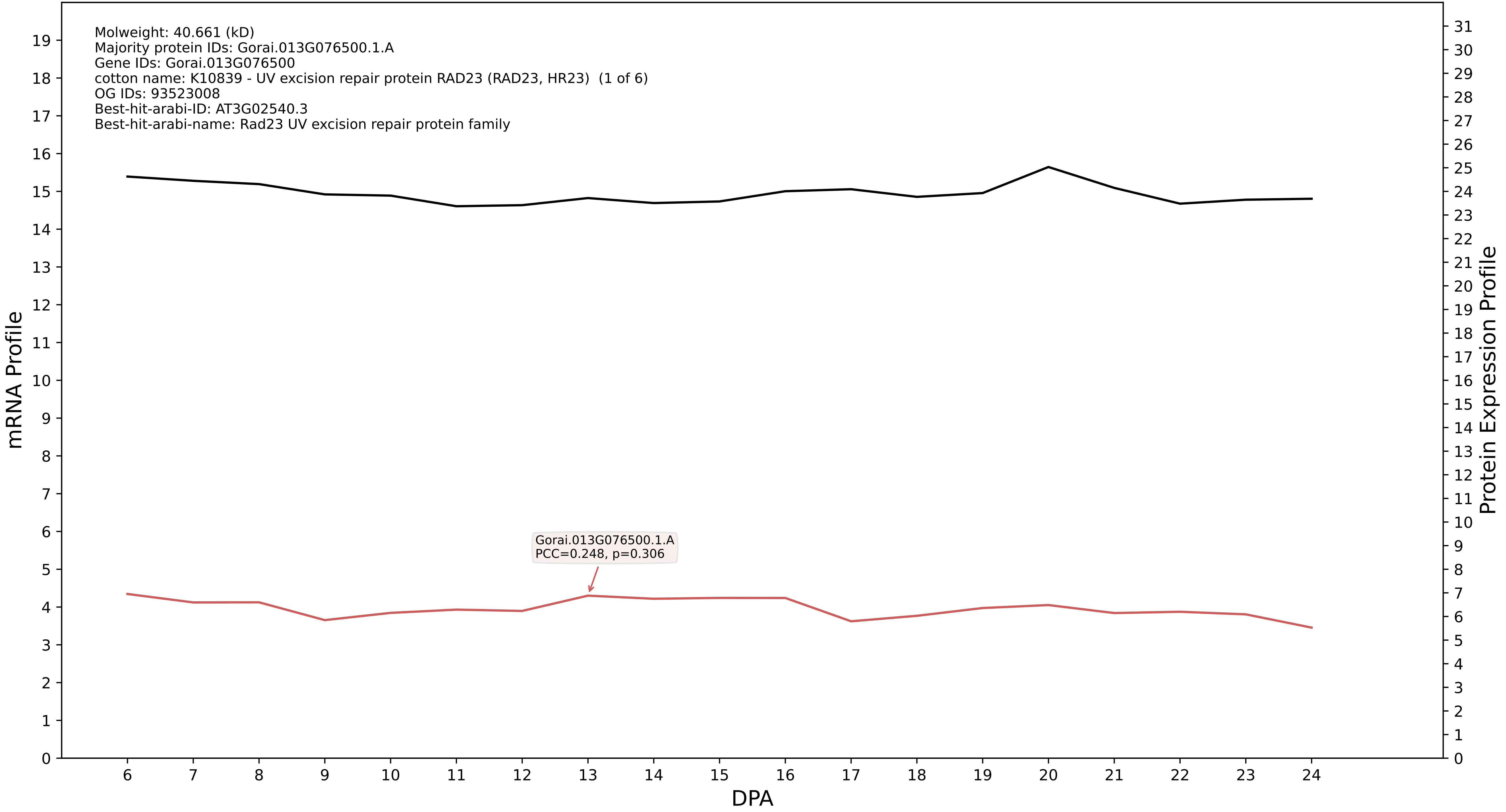

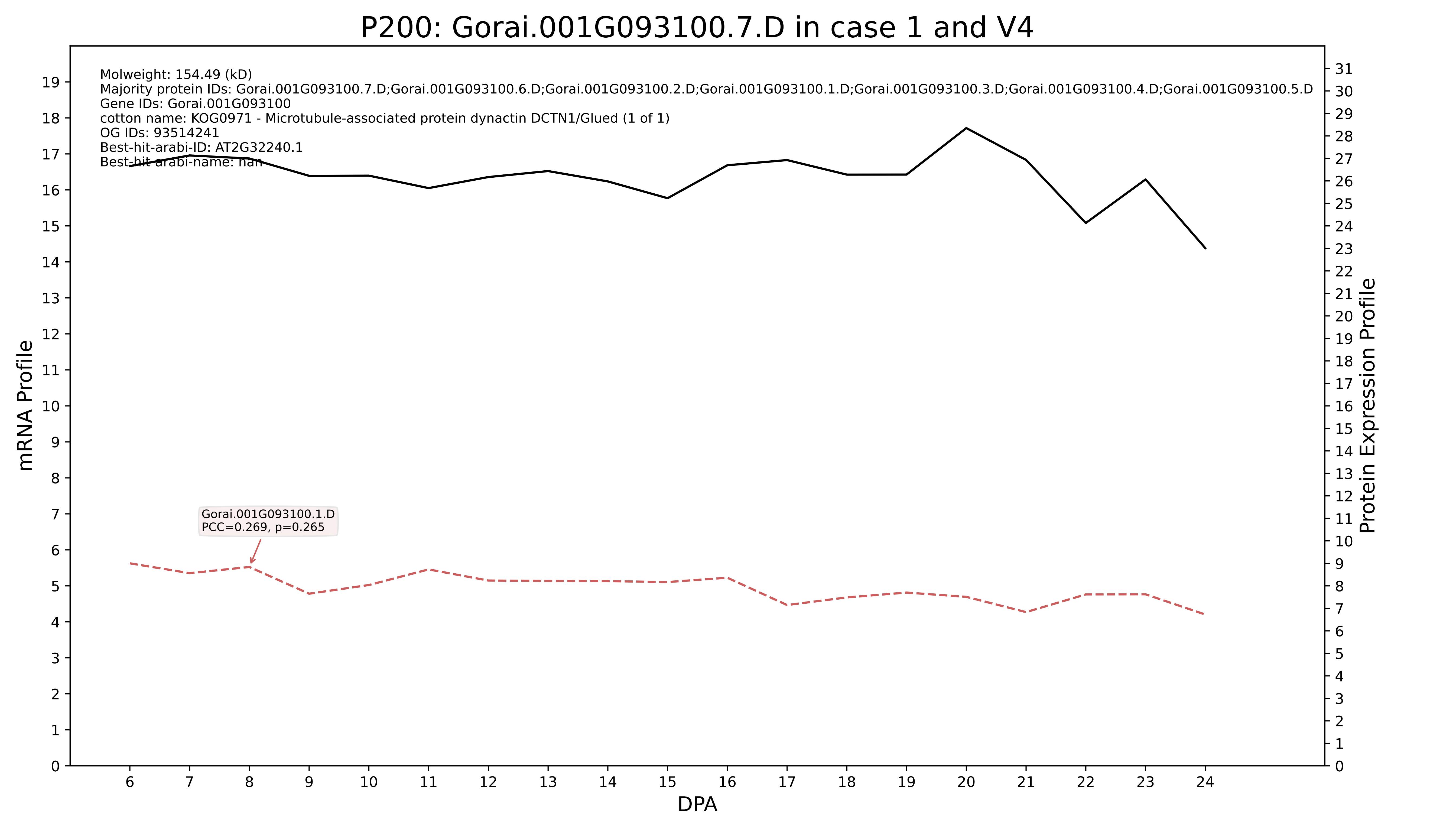
P200: Gorai.001G093100.7.D in case 1 and V4
Molweight: 154.49 (kD)
Majority protein IDs: Gorai.001G093100.7.D;Gorai.001G093100.6.D;Gorai.001G093100.2.D;Gorai.001G093100.1.D;Gorai.001G093100.3.D;Gorai.001G093100.4.D;Gorai.001G093100.5.D
Gene IDs: Gorai.001G093100
cotton name: KOG0971 - Microtubule-associated protein dynactin DCTN1/Glued (1 of 1)
OG IDs: 93514241
Best-hit-arabi-ID: AT2G32240.1
Best-hit-arabi-name: nan
Gorai.001G093100.1.D
PCC=0.269, p=0.265
mRNA Profile
Protein Expression Profile
DPA

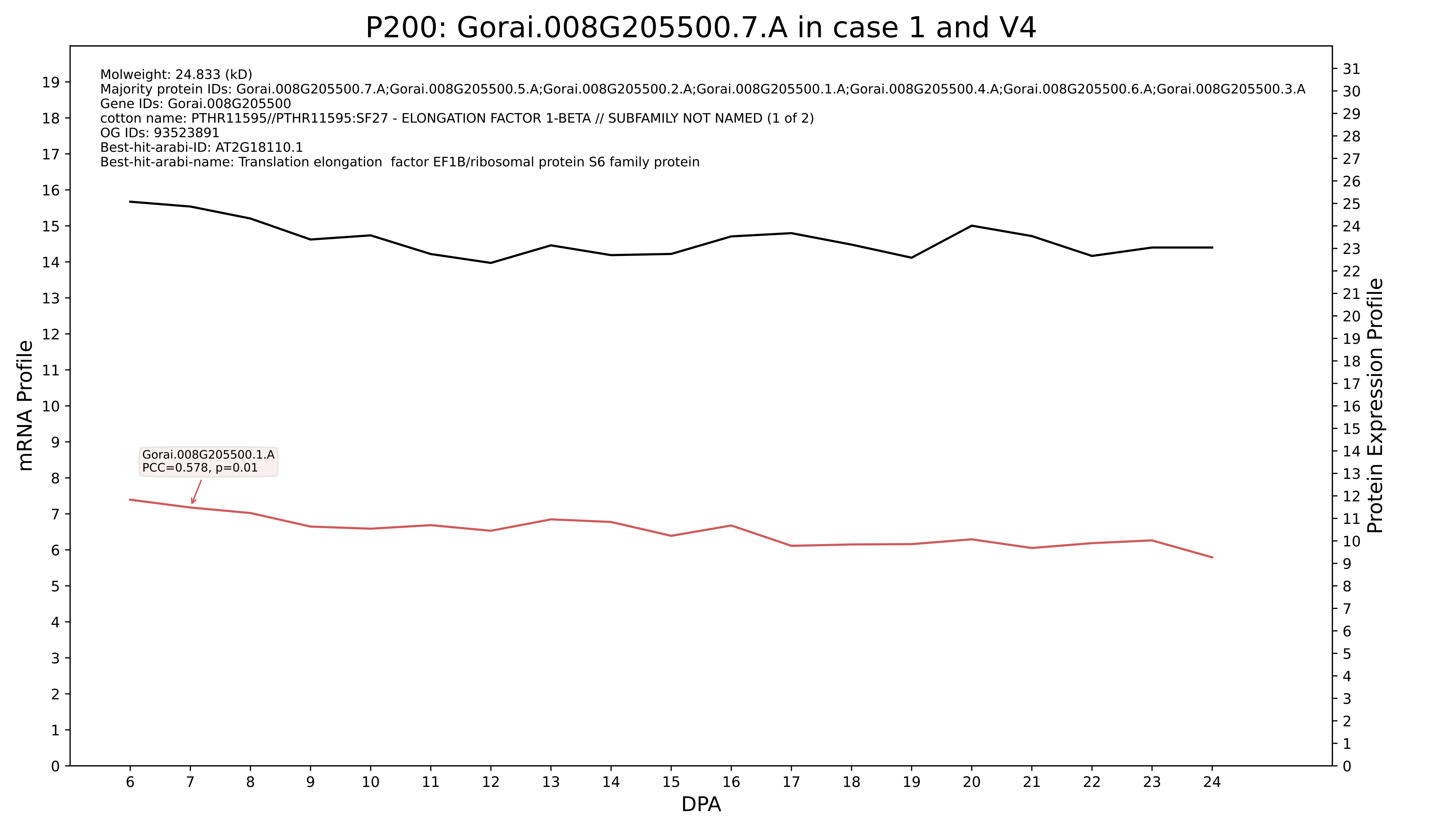
P200: Gorai.008G205500.7.A in case 1 and V4
Molweight: 24.833 (kD)
Majority protein IDs: Gorai.008G205500.7.A;Gorai.008G205500.5.A;Gorai.008G205500.2.A;Gorai.008G205500.1.A;Gorai.008G205500.4.A;Gorai.008G205500.6.A;Gorai.008G205500.3.A
Gene IDs: Gorai.008G205500
cotton name: PTHR11595//PTHR11595:SF27 - ELONGATION FACTOR 1-BETA // SUBFAMILY NOT NAMED (1 of 2)
OG IDs: 93523891
Best-hit-arabi-ID: AT2G18110.1
Best-hit-arabi-name: Translation elongation factor EF1B/ribosomal protein S6 family protein
Gorai.008G205500.1.A
PCC=0.578, p=0.01
mRNA Profile
Protein Expression Profile
DPA

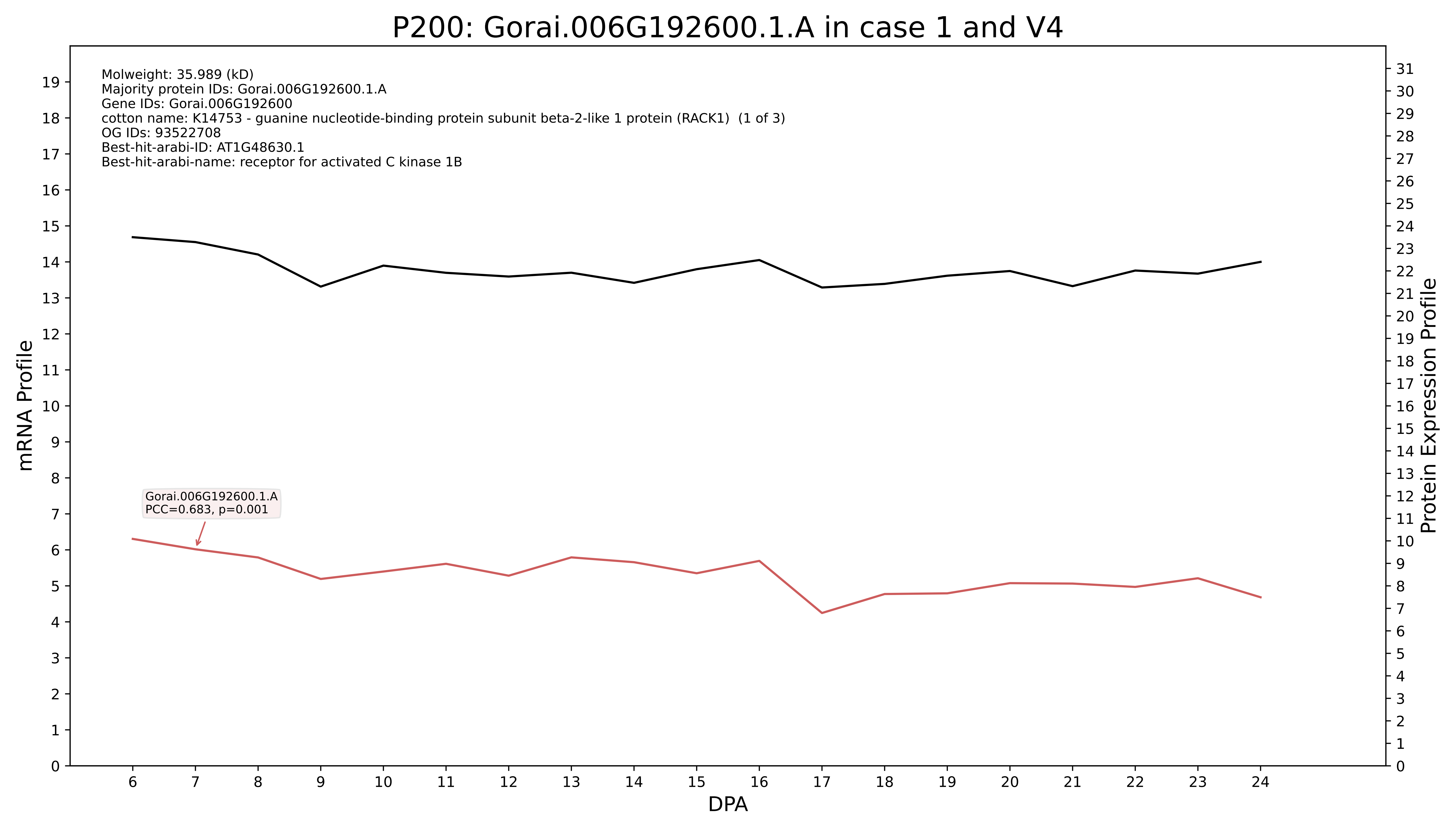

P200: Gorai.006G192600.1.A in case 1 and V4
Molweight: 35.989 (kD)
Majority protein IDs: Gorai.006G192600.1.A
Gene IDs: Gorai.006G192600
cotton name: K14753 - guanine nucleotide-binding protein subunit beta-2-like 1 protein (RACK1) (1 of 3)
OG IDs: 93522708
Best-hit-arabi-ID: AT1G48630.1
Best-hit-arabi-name: receptor for activated C kinase 1B
Gorai.006G192600.1.A
PCC=0.683, p=0.001
mRNA Profile
Protein Expression Profile
DPA

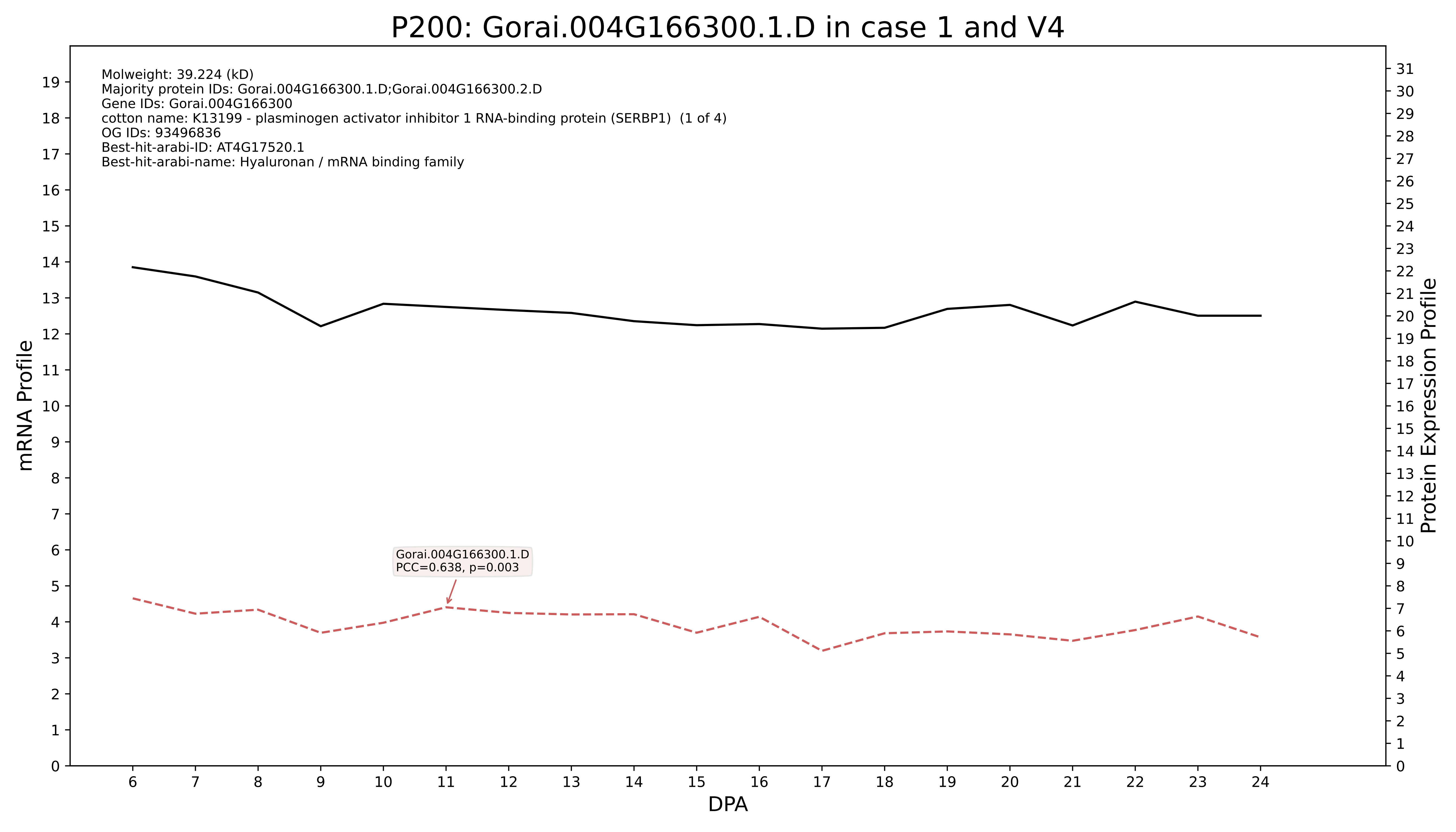
P200: Gorai.004G166300.1.D in case 1 and V4
Molweight: 39.224 (kD)
Majority protein IDs: Gorai.004G166300.1.D;Gorai.004G166300.2.D
Gene IDs: Gorai.004G166300
cotton name: K13199 - plasminogen activator inhibitor 1 RNA-binding protein (SERBP1) (1 of 4)
OG IDs: 93496836
Best-hit-arabi-ID: AT4G17520.1
Best-hit-arabi-name: Hyaluronan / mRNA binding family
Gorai.004G166300.1.D
PCC=0.638, p=0.003
mRNA Profile
Protein Expression Profile
DPA

P200: Gorai.008G273400.1.D in case 1 and V4

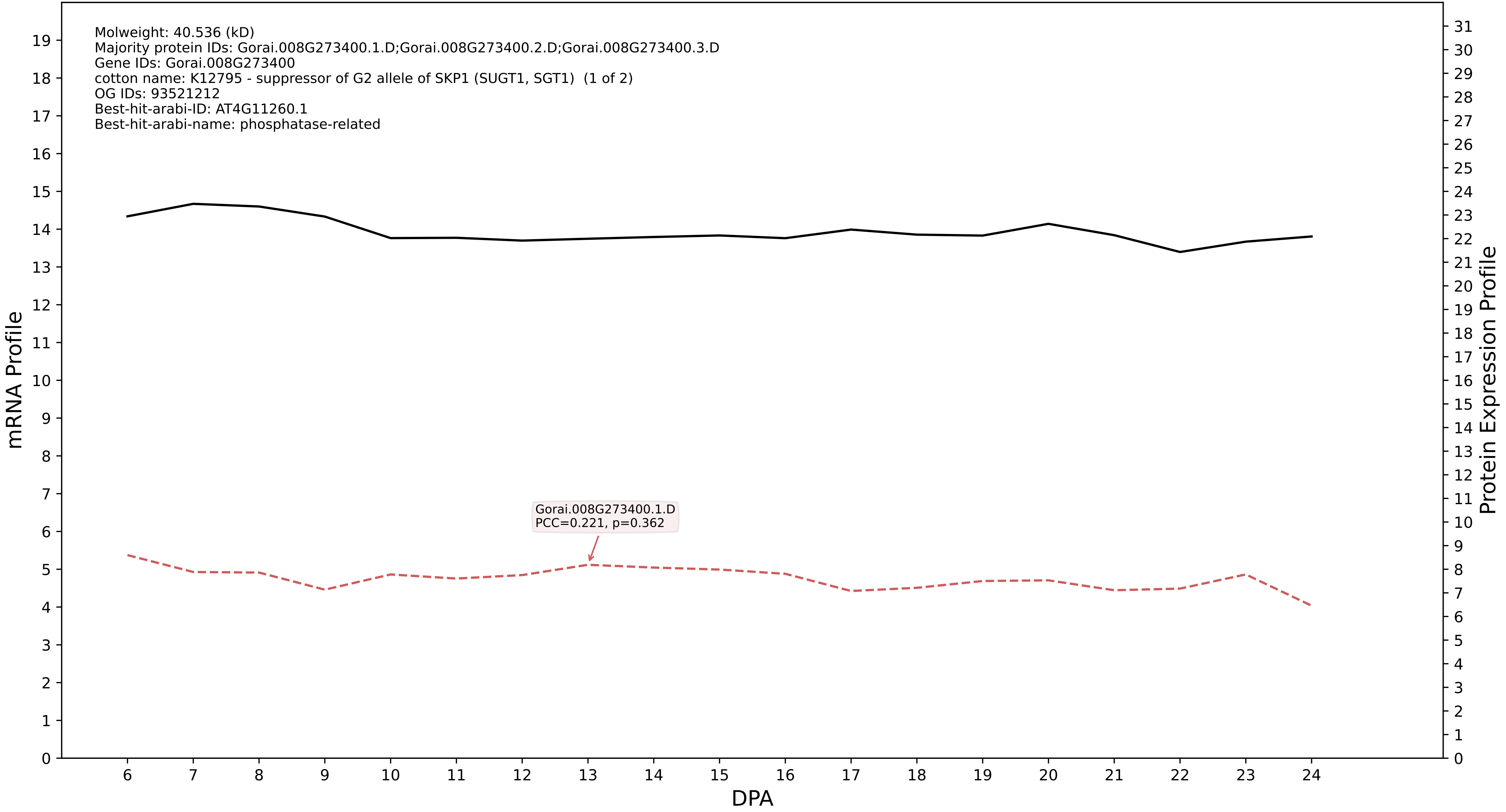

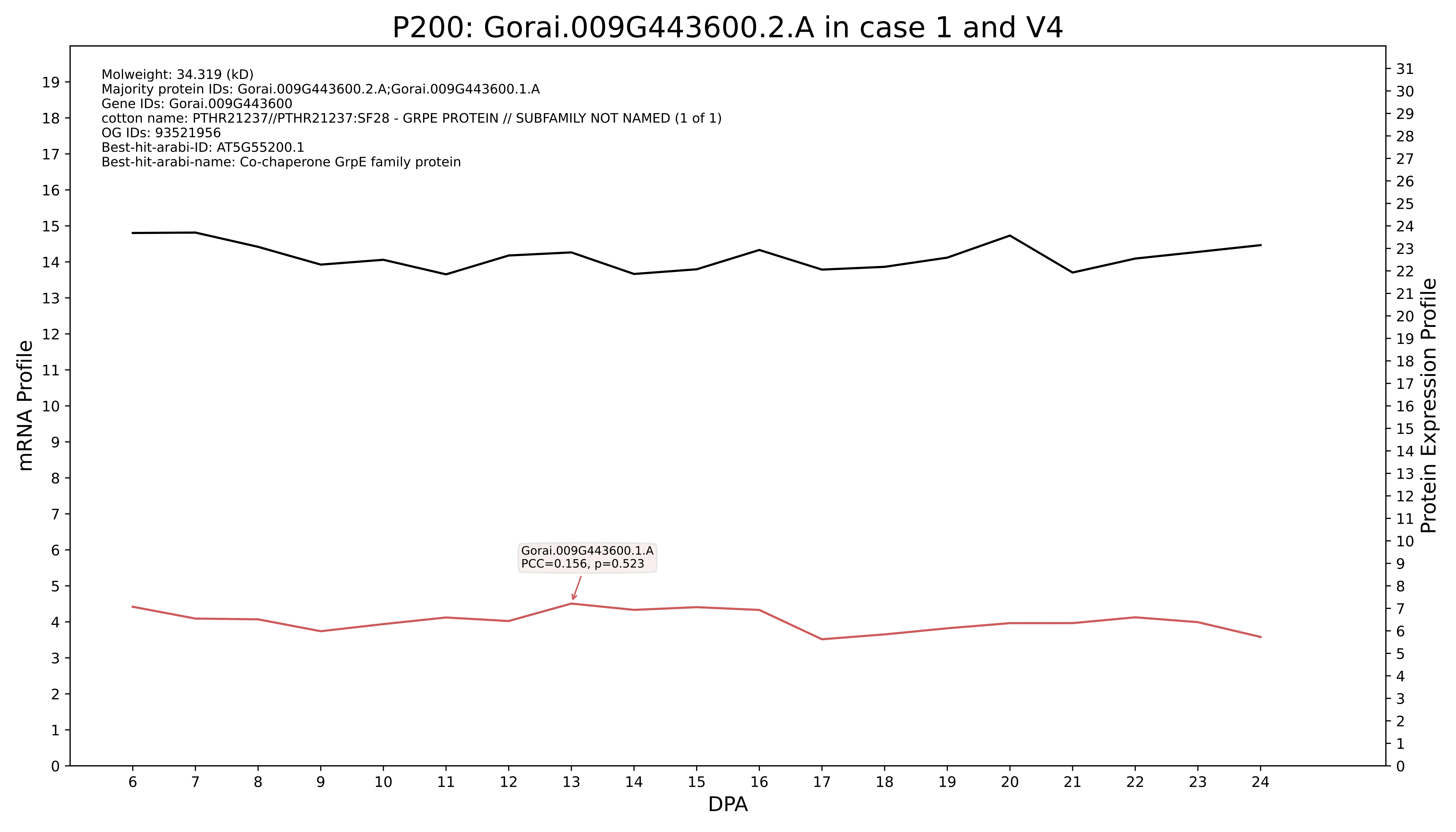

P200: Gorai.009G443600.2.A in case 1 and V4
Molweight: 34.319 (kD)
Majority protein IDs: Gorai.009G443600.2.A;Gorai.009G443600.1.A
Gene IDs: Gorai.009G443600
cotton name: PTHR21237//PTHR21237:SF28 - GRPE PROTEIN // SUBFAMILY NOT NAMED (1 of 1)
OG IDs: 93521956
Best-hit-arabi-ID: AT5G55200.1
Best-hit-arabi-name: Co-chaperone GrpE family protein
Gorai.009G443600.1.A
PCC=0.156, p=0.523
mRNA Profile
Protein Expression Profile
DPA

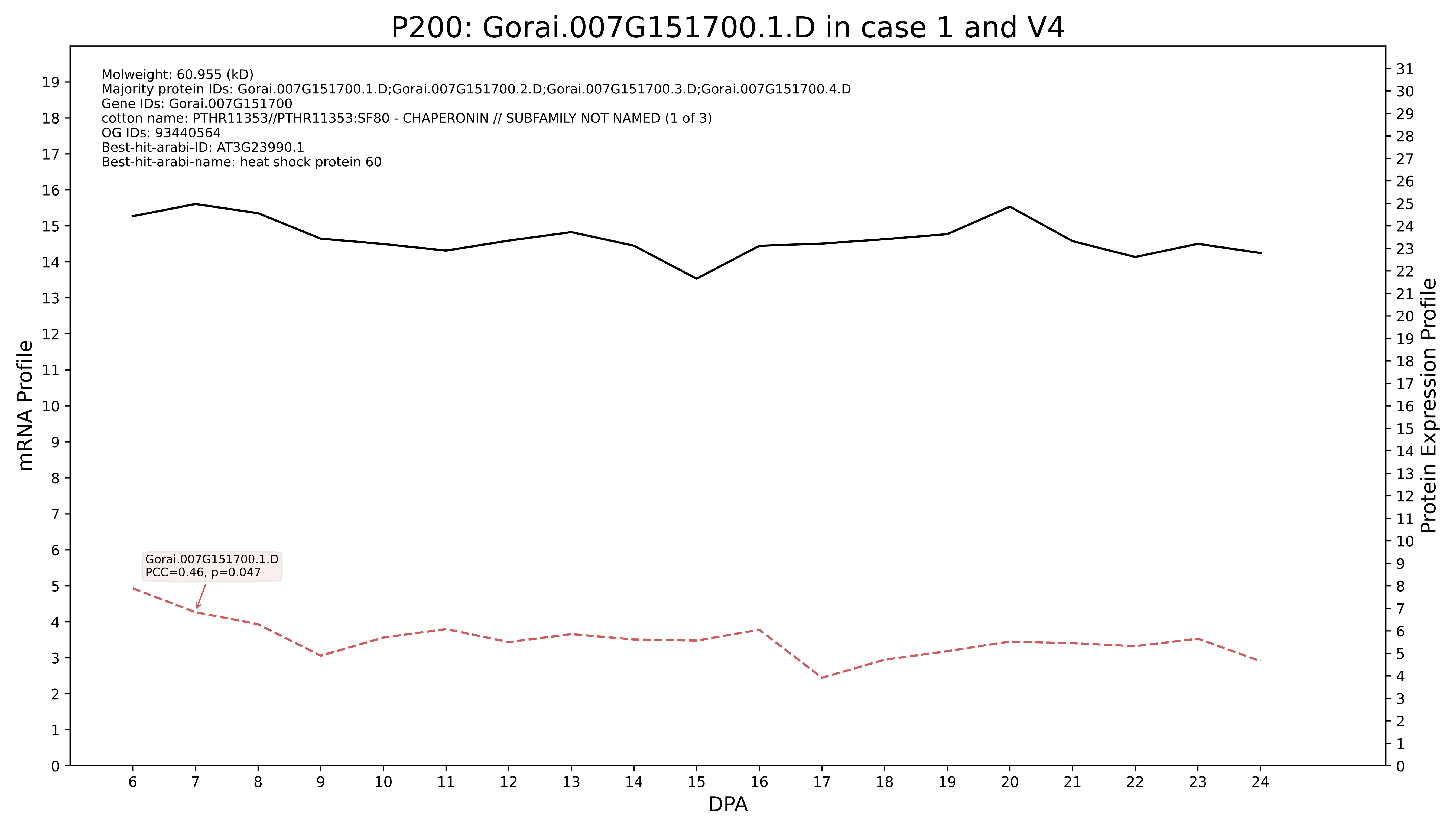
P200: Gorai.007G151700.1.D in case 1 and V4
Molweight: 60.955 (kD)
Majority protein IDs: Gorai.007G151700.1.D;Gorai.007G151700.2.D;Gorai.007G151700.3.D;Gorai.007G151700.4.D
Gene IDs: Gorai.007G151700
cotton name: PTHR11353//PTHR11353:SF80 - CHAPERONIN // SUBFAMILY NOT NAMED (1 of 3)
OG IDs: 93440564
Best-hit-arabi-ID: AT3G23990.1
Best-hit-arabi-name: heat shock protein 60
Gorai.007G151700.1.D
PCC=0.46, p=0.047
mRNA Profile
Protein Expression Profile
DPA

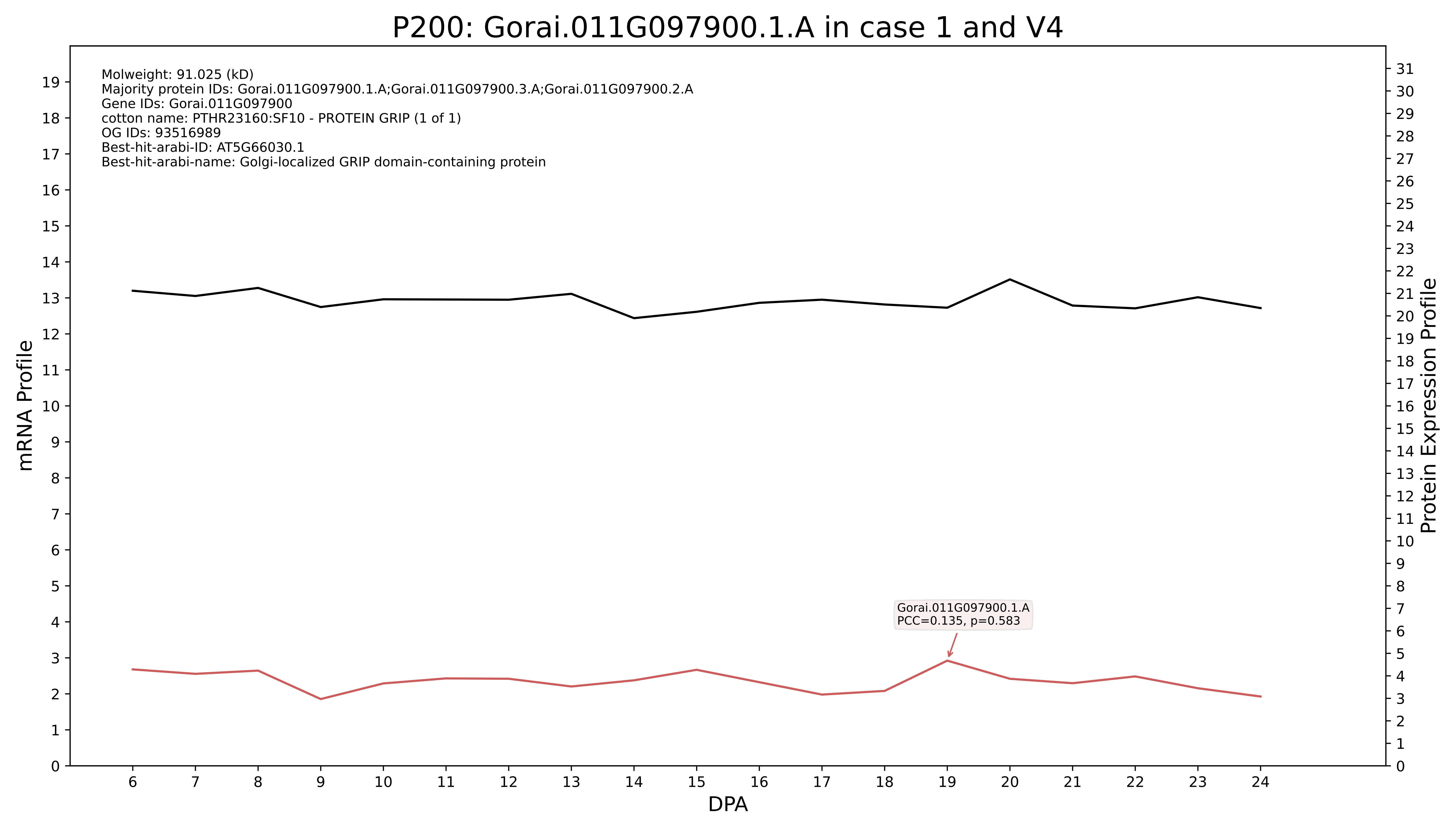

P200: Gorai.011G097900.1.A in case 1 and V4
Molweight: 91.025 (kD)
Majority protein IDs: Gorai.011G097900.1.A;Gorai.011G097900.3.A;Gorai.011G097900.2.A
Gene IDs: Gorai.011G097900
cotton name: PTHR23160:SF10 - PROTEIN GRIP (1 of 1)
OG IDs: 93516989
Best-hit-arabi-ID: AT5G66030.1
Best-hit-arabi-name: Golgi-localized GRIP domain-containing protein
Gorai.011G097900.1.A
PCC=0.135, p=0.583
mRNA Profile
Protein Expression Profile
DPA

P200: Gorai.004G040900.3.D in case 2 and V4

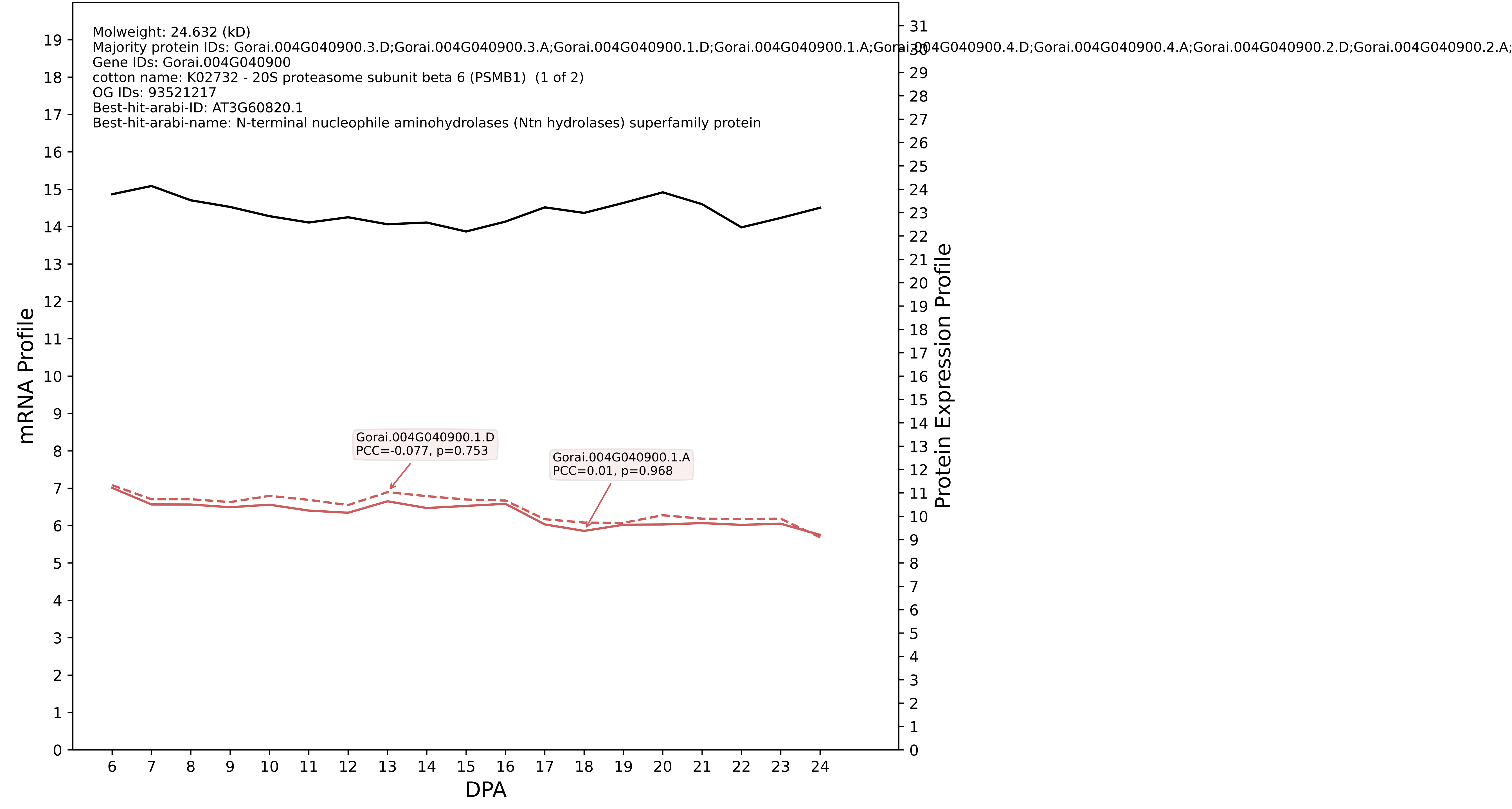

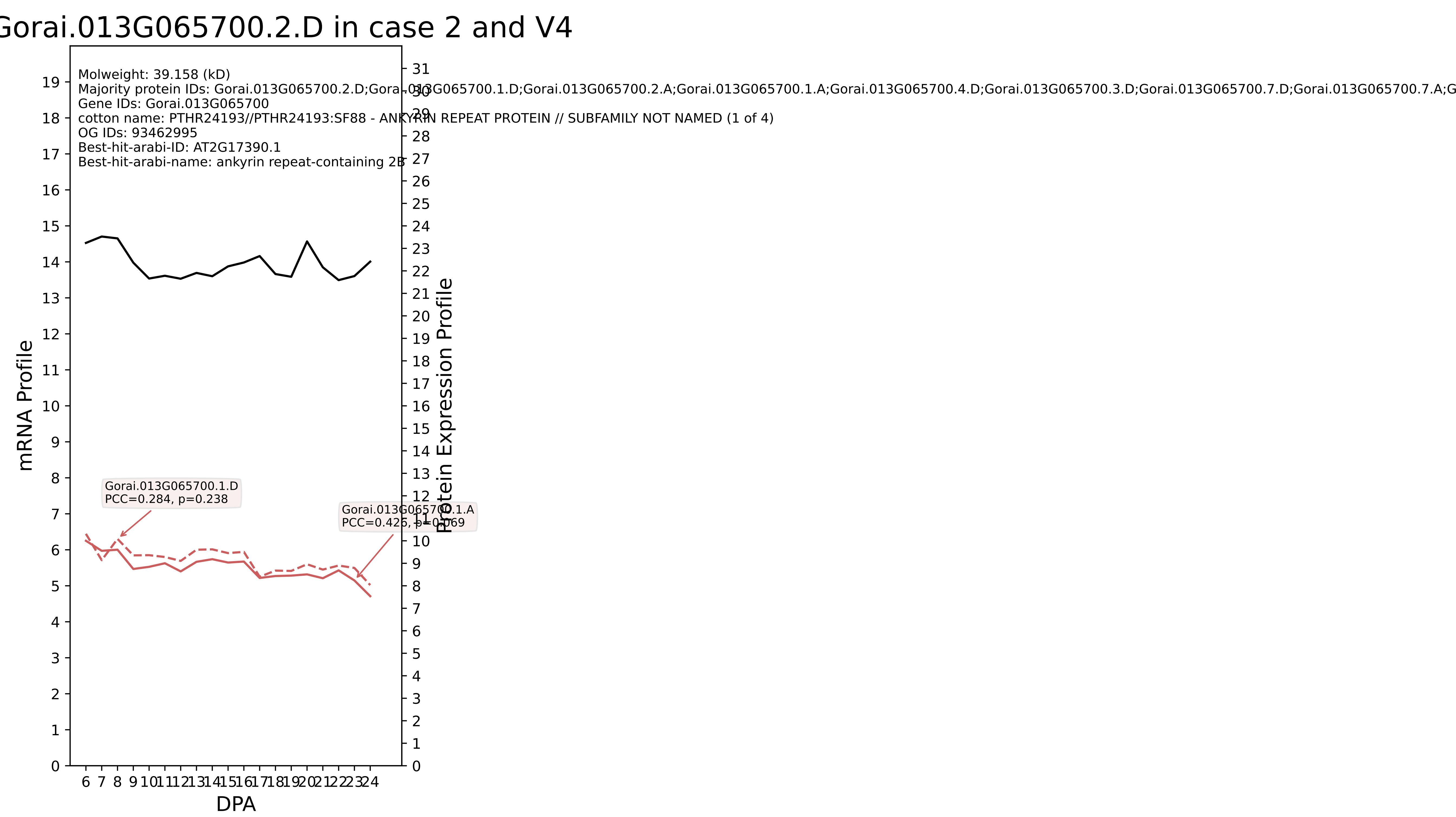
Gorai.013G065700.2.D in case 2 and V4
Molweight: 39.158 (kD)
Majority protein IDs: Gorai.013G065700.2.D;Gorai.013G065700.1.D;Gorai.013G065700.2.A;Gorai.013G065700.1.A;Gorai.013G065700.4.D;Gorai.013G065700.3.D;Gorai.013G065700.7.D;Gorai.013G065700.7.A;G
Gene IDs: Gorai.013G065700
cotton name: PTHR24193//PTHR24193:SF88 - ANKYRIN REPEAT PROTEIN // SUBFAMILY NOT NAMED (1 of 4)
OG IDs: 93462995
Best-hit-arabi-ID: AT2G17390.1
Best-hit-arabi-name: ankyrin repeat-containing 2B
Gorai.013G065700.1.D
PCC=0.284, p=0.238
Gorai.013G065700.1.A
PCC=0.426, p=0.069
mRNA Profile
Protein Expression Profile
DPA

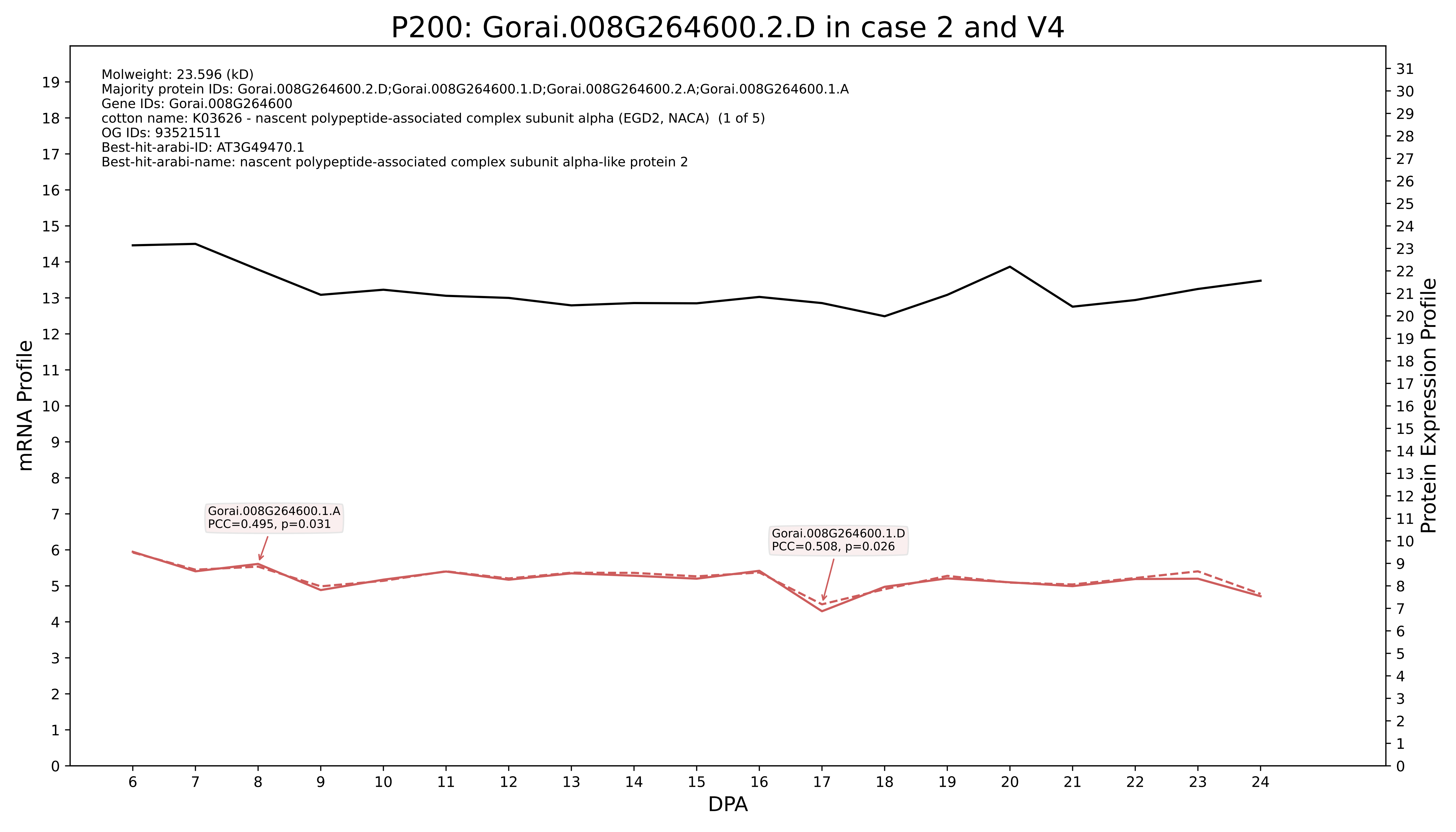
P200: Gorai.008G264600.2.D in case 2 and V4
Molweight: 23.596 (kD)
Majority protein IDs: Gorai.008G264600.2.D;Gorai.008G264600.1.D;Gorai.008G264600.2.A;Gorai.008G264600.1.A
Gene IDs: Gorai.008G264600
cotton name: K03626 - nascent polypeptide-associated complex subunit alpha (EGD2, NACA) (1 of 5)
OG IDs: 93521511
Best-hit-arabi-ID: AT3G49470.1
Best-hit-arabi-name: nascent polypeptide-associated complex subunit alpha-like protein 2
Gorai.008G264600.1.A
PCC=0.495, p=0.031
Gorai.008G264600.1.D
PCC=0.508, p=0.026
mRNA Profile
Protein Expression Profile
DPA

P200: Gorai.009G130700.2.D in case 2 and V4

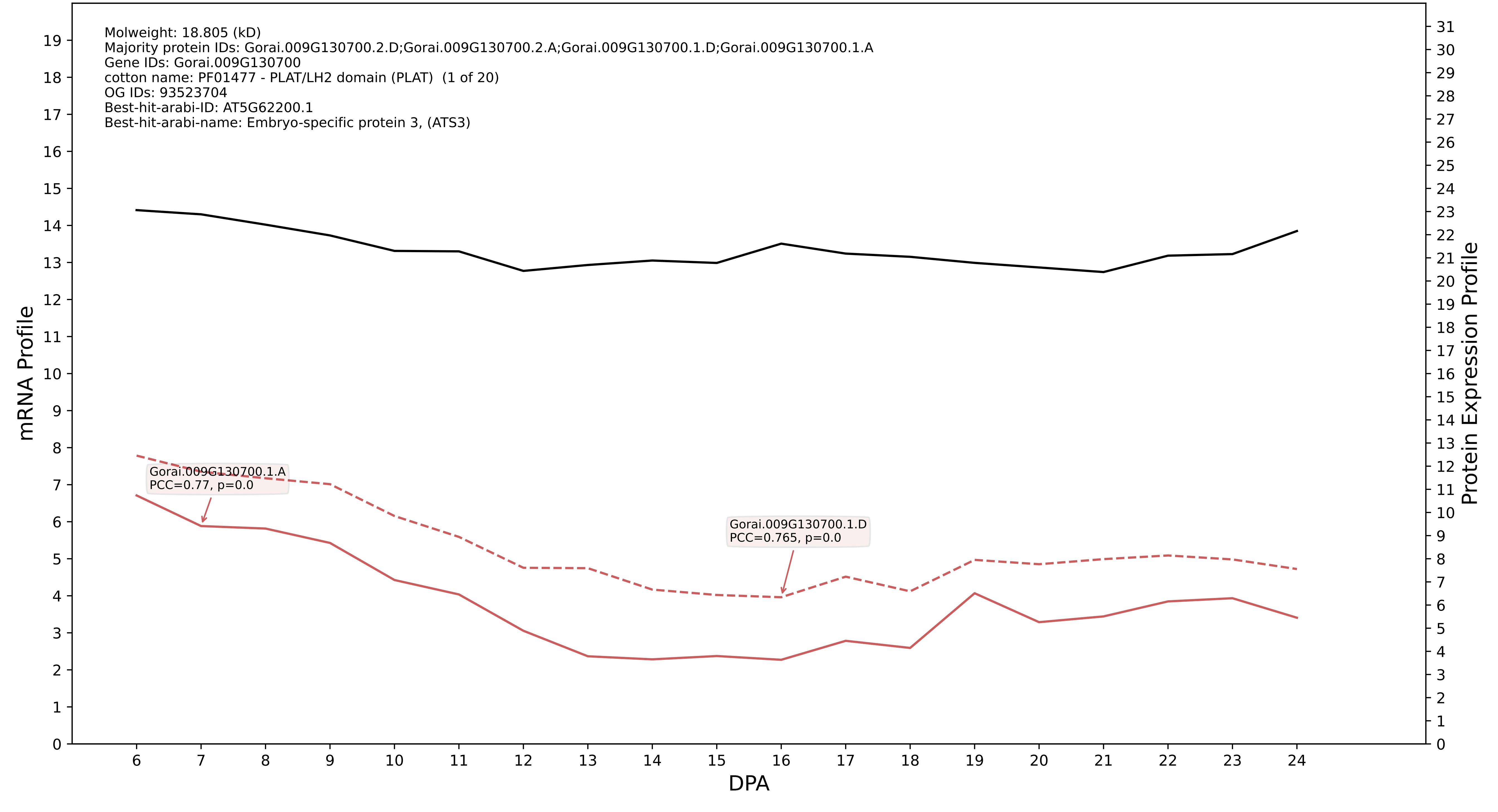

P200: Gorai.009G182500.1.D in case 2 and V4

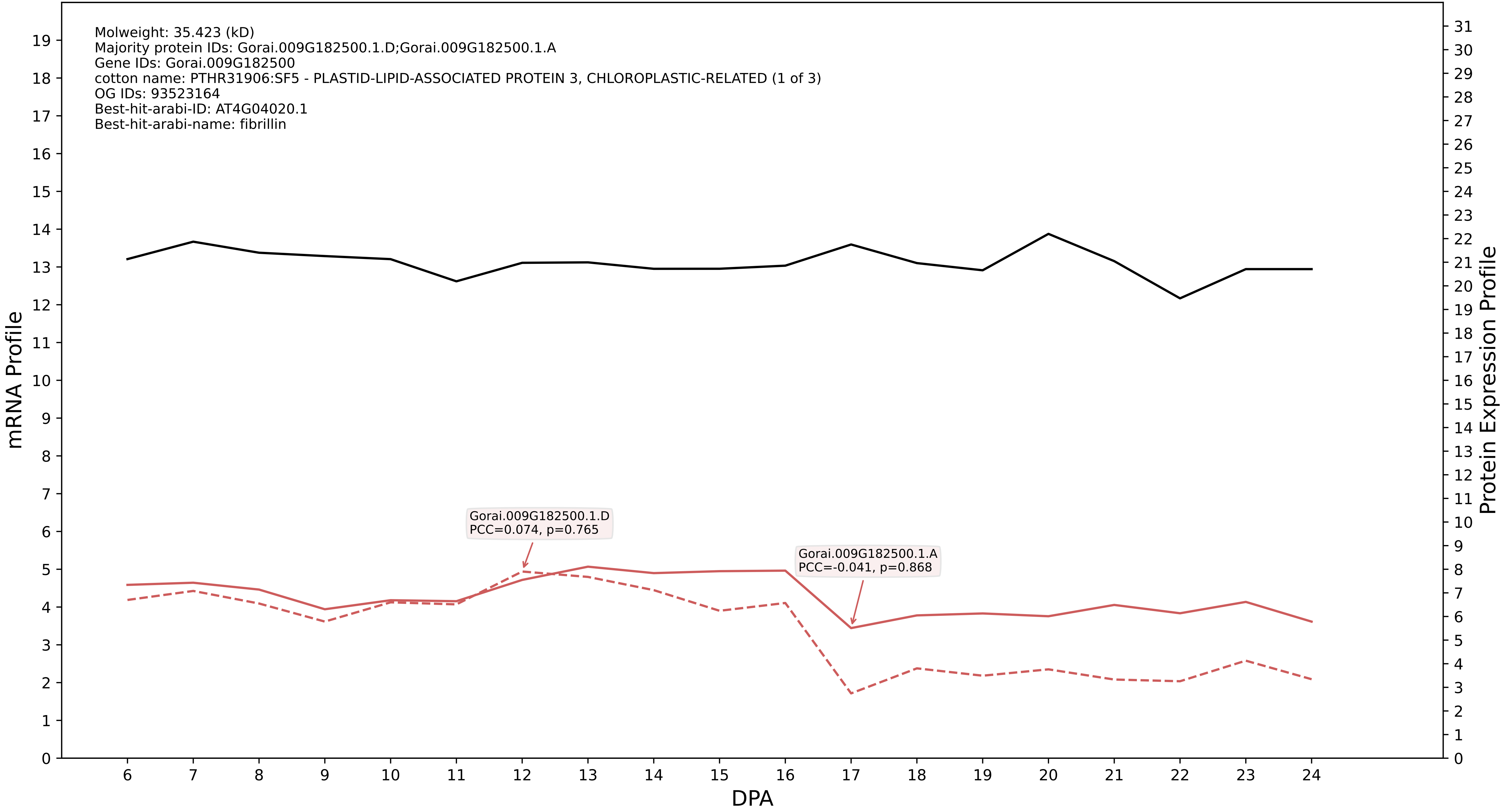

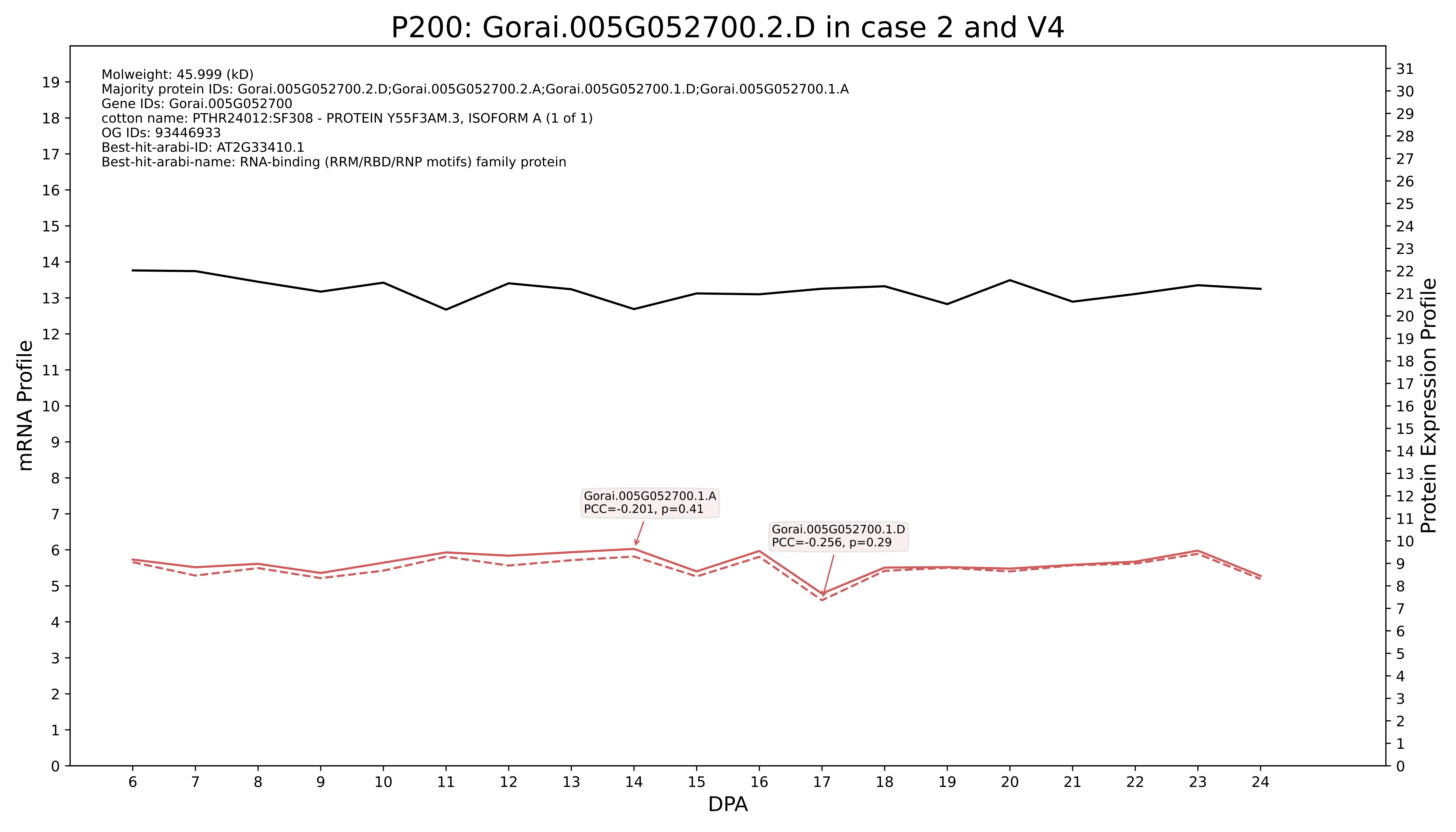

P200: Gorai.005G052700.2.D in case 2 and V4
Molweight: 45.999 (kD)
Majority protein IDs: Gorai.005G052700.2.D;Gorai.005G052700.2.A;Gorai.005G052700.1.D;Gorai.005G052700.1.A
Gene IDs: Gorai.005G052700
cotton name: PTHR24012:SF308 - PROTEIN Y55F3AM.3, ISOFORM A (1 of 1)
OG IDs: 93446933
Best-hit-arabi-ID: AT2G33410.1
Best-hit-arabi-name: RNA-binding (RRM/RBD/RNP motifs) family protein
Gorai.005G052700.1.A
PCC=-0.201, p=0.41
Gorai.005G052700.1.D
PCC=-0.256, p=0.29
mRNA Profile
Protein Expression Profile
DPA

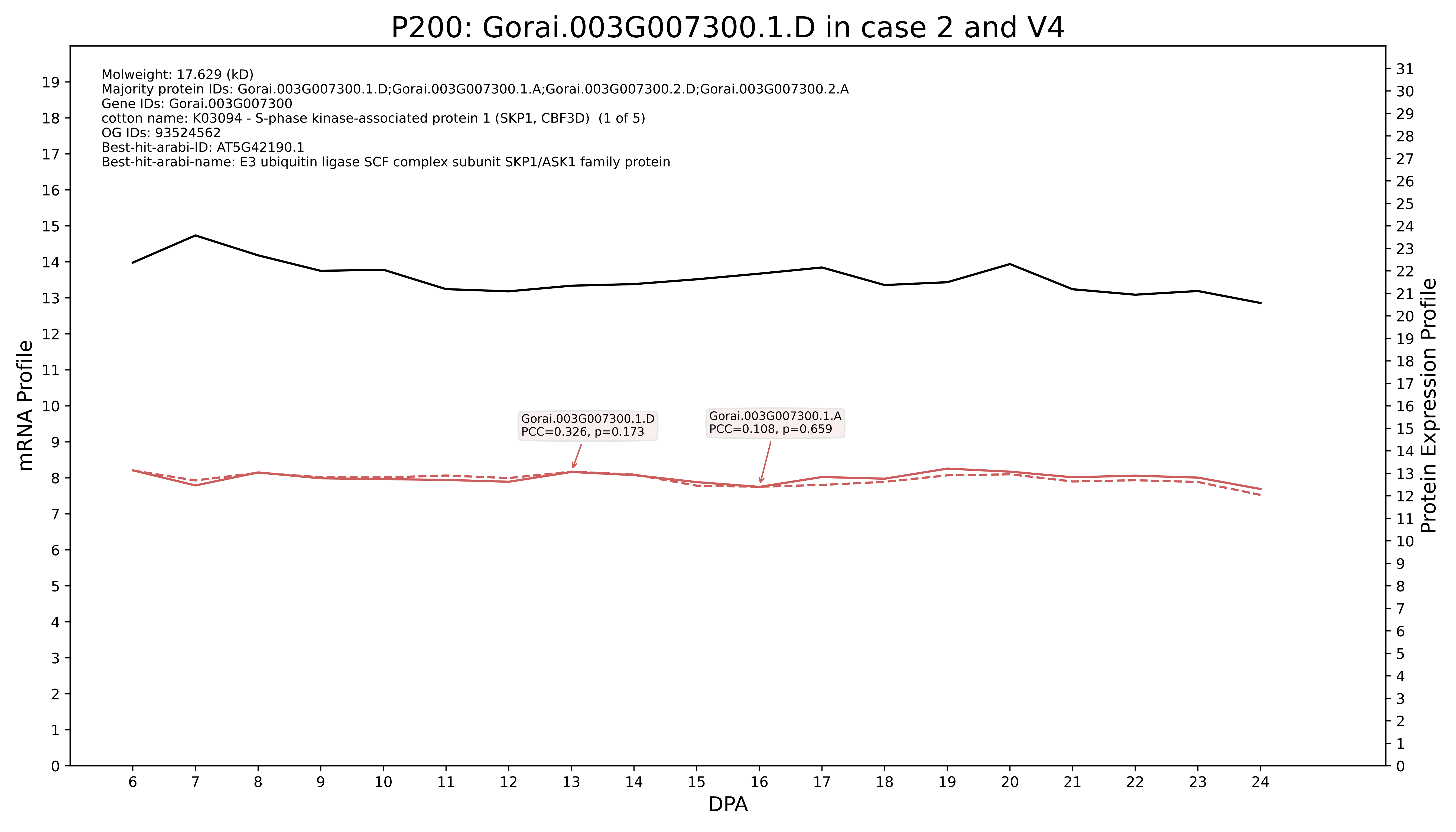
P200: Gorai.003G007300.1.D in case 2 and V4
Molweight: 17.629 (kD)
Majority protein IDs: Gorai.003G007300.1.D;Gorai.003G007300.1.A;Gorai.003G007300.2.D;Gorai.003G007300.2.A
Gene IDs: Gorai.003G007300
cotton name: K03094 - S-phase kinase-associated protein 1 (SKP1, CBF3D) (1 of 5)
OG IDs: 93524562
Best-hit-arabi-ID: AT5G42190.1
Best-hit-arabi-name: E3 ubiquitin ligase SCF complex subunit SKP1/ASK1 family protein
Gorai.003G007300.1.D
PCC=0.326, p=0.173
Gorai.003G007300.1.A
PCC=0.108, p=0.659
mRNA Profile
Protein Expression Profile
DPA

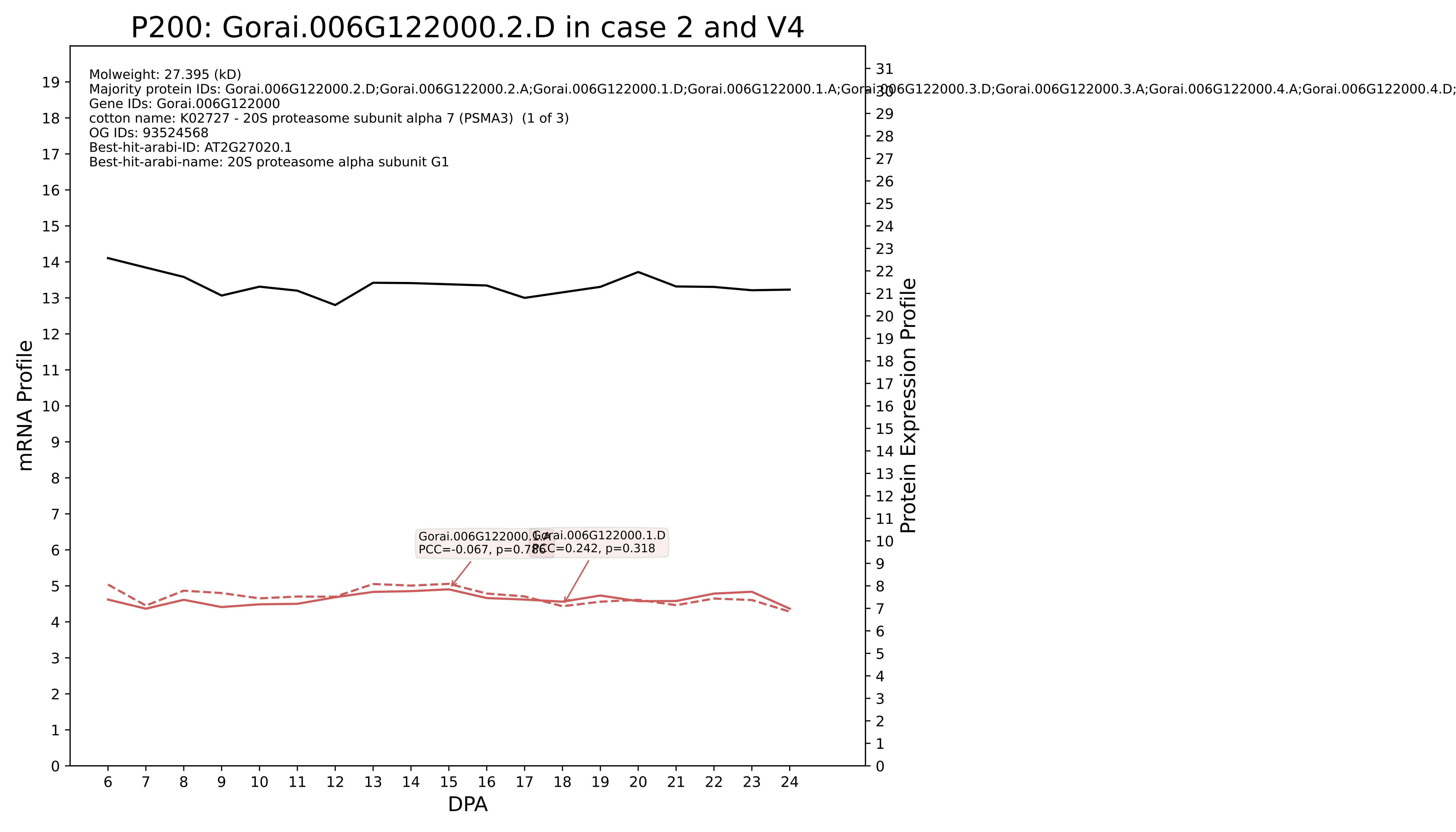
P200: Gorai.006G122000.2.D in case 2 and V4
Molweight: 27.395 (kD)
Majority protein IDs: Gorai.006G122000.2.D;Gorai.006G122000.2.A;Gorai.006G122000.1.D;Gorai.006G122000.1.A;Gorai.006G122000.3.D;Gorai.006G122000.3.A;Gorai.006G122000.4.A;Gorai.006G122000.4.D;
Gene IDs: Gorai.006G122000
cotton name: K02727 - 20S proteasome subunit alpha 7 (PSMA3) (1 of 3)
OG IDs: 93524568
Best-hit-arabi-ID: AT2G27020.1
Best-hit-arabi-name: 20S proteasome alpha subunit G1
Gorai.006G122000.1.A
PCC=-0.067, p=0.786
Gorai.006G122000.1.D
PCC=0.242, p=0.318
mRNA Profile
Protein Expression Profile
DPA

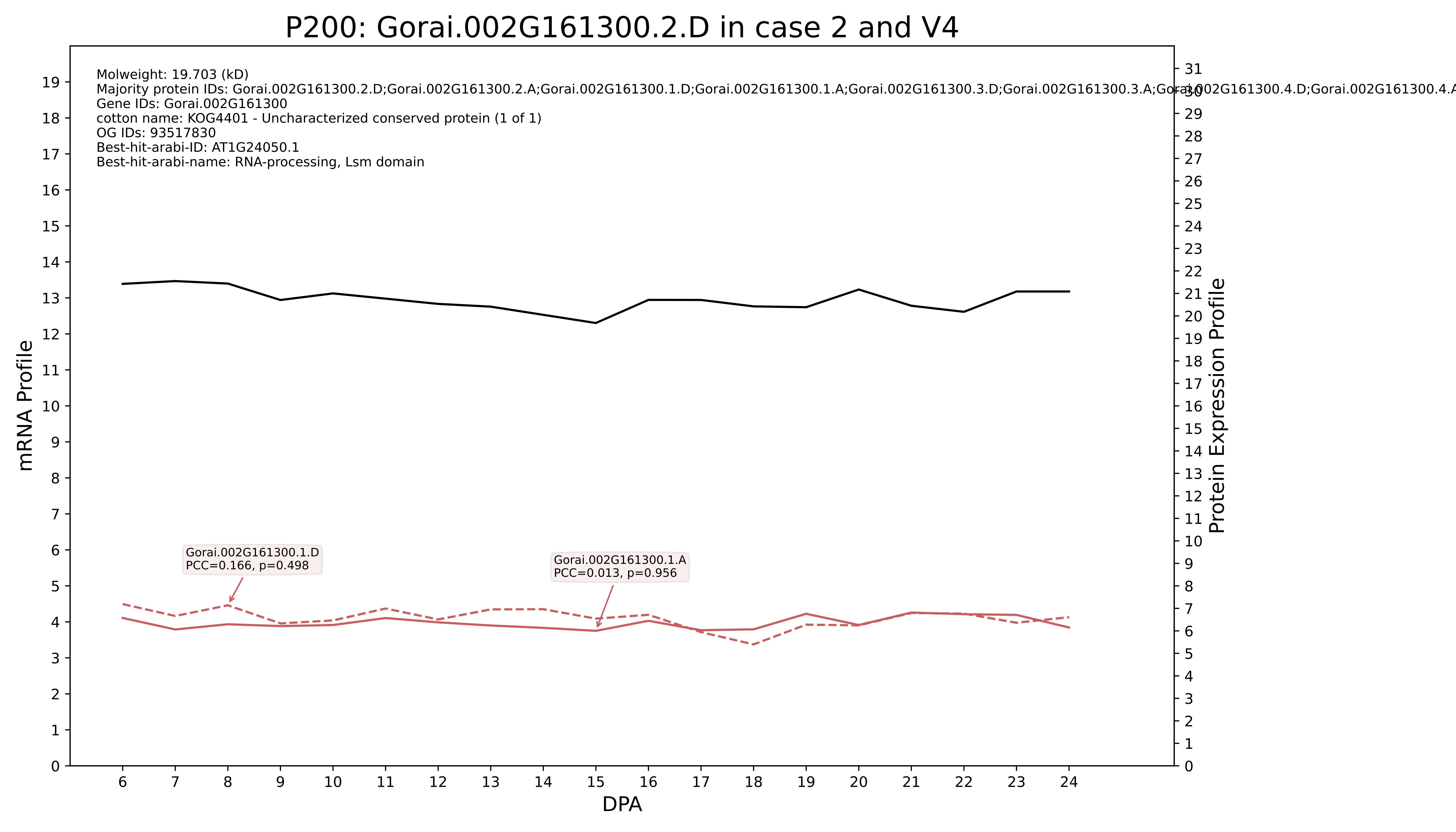
P200: Gorai.002G161300.2.D in case 2 and V4
Molweight: 19.703 (kD)
Majority protein IDs: Gorai.002G161300.2.D;Gorai.002G161300.2.A;Gorai.002G161300.1.D;Gorai.002G161300.1.A;Gorai.002G161300.3.D;Gorai.002G161300.3.A;Gorai.002G161300.4.D;Gorai.002G161300.4.A
Gene IDs: Gorai.002G161300
cotton name: KOG4401 - Uncharacterized conserved protein (1 of 1)
OG IDs: 93517830
Best-hit-arabi-ID: AT1G24050.1
Best-hit-arabi-name: RNA-processing, Lsm domain
Gorai.002G161300.1.D
PCC=0.166, p=0.498
Gorai.002G161300.1.A
PCC=0.013, p=0.956
mRNA Profile
Protein Expression Profile
DPA

P200: Gorai.002G192400.2.D in case 2 and V4

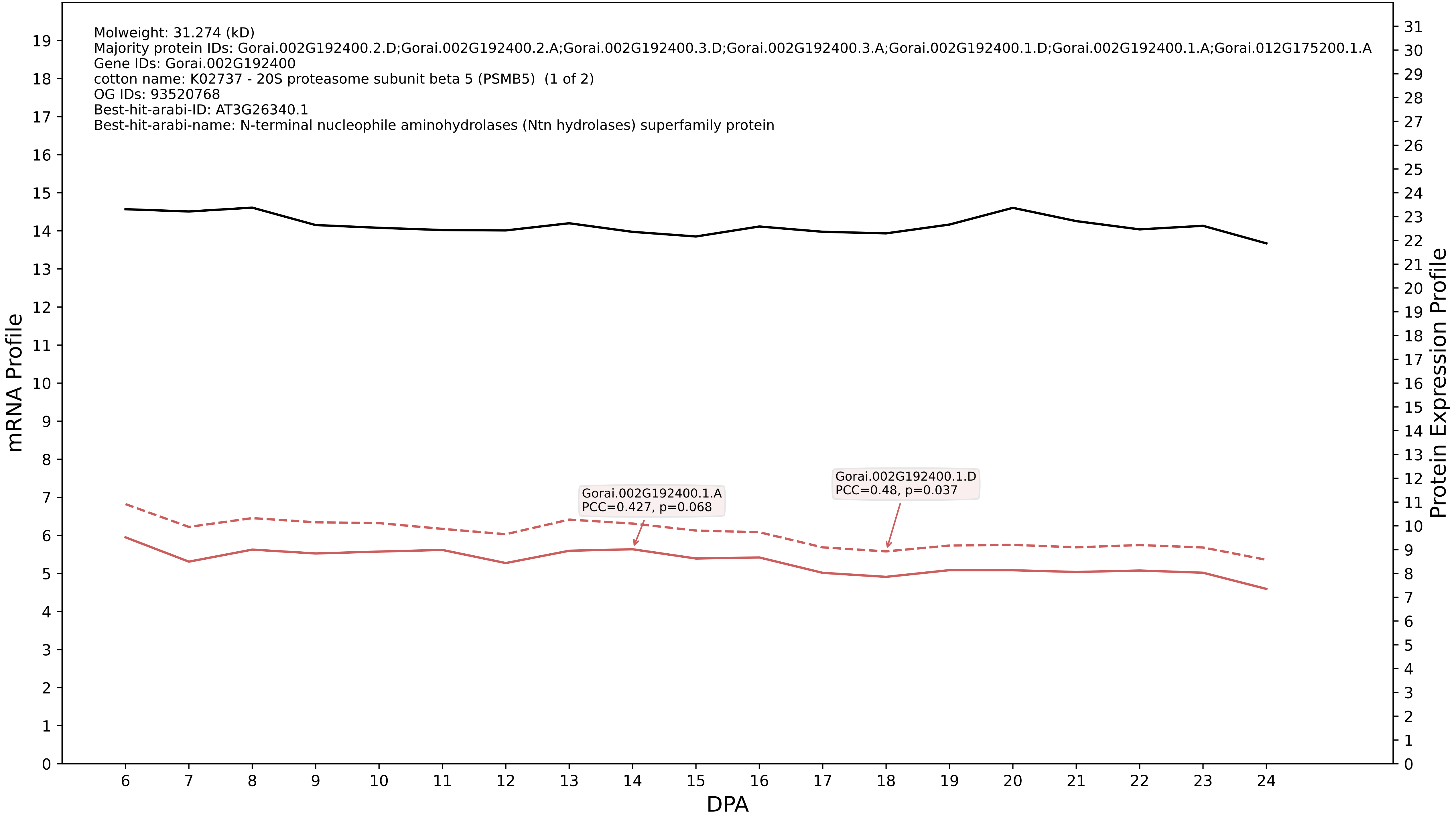

P200: Gorai.013G000500.1.D in case 2 and V4

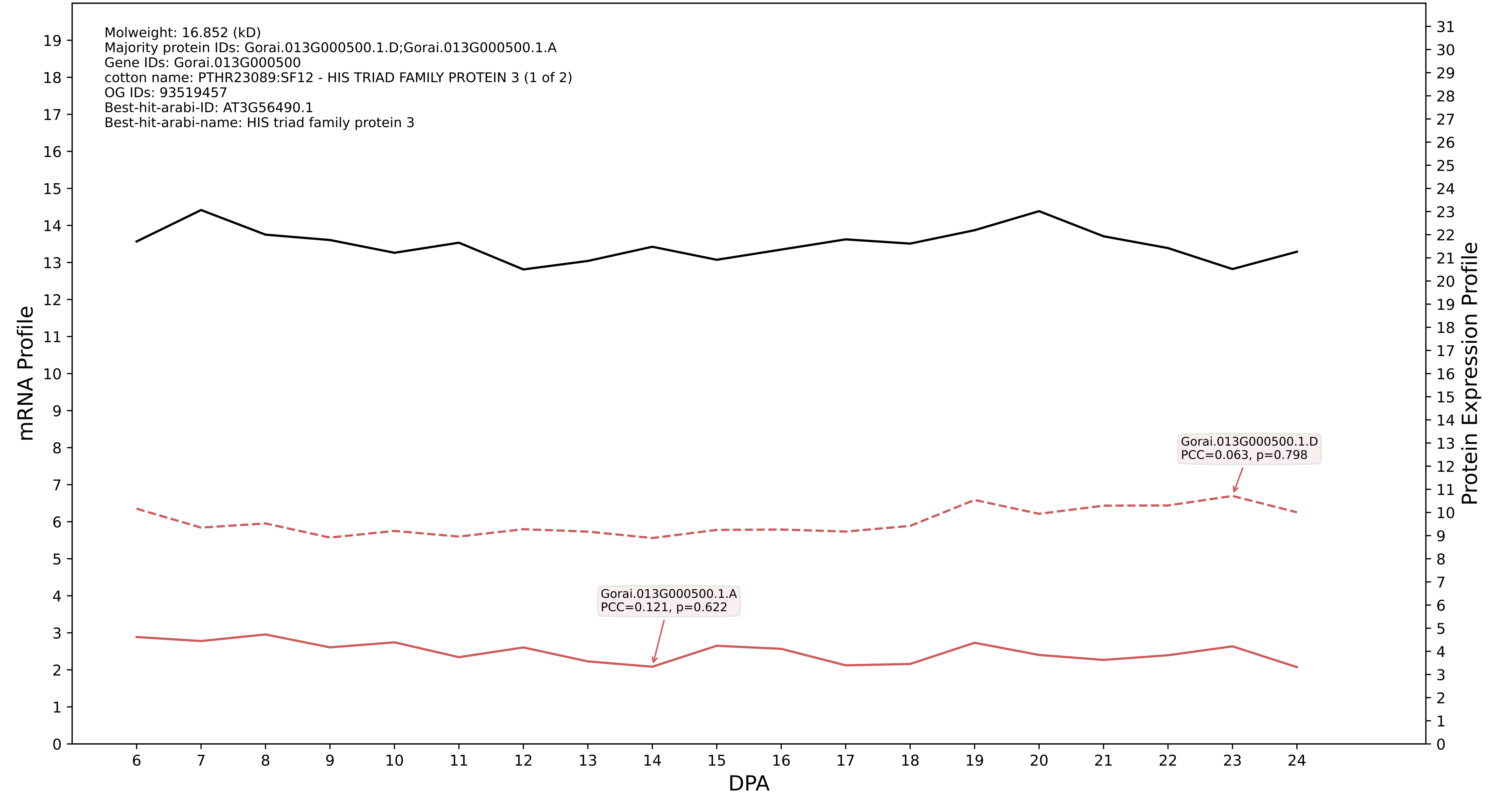

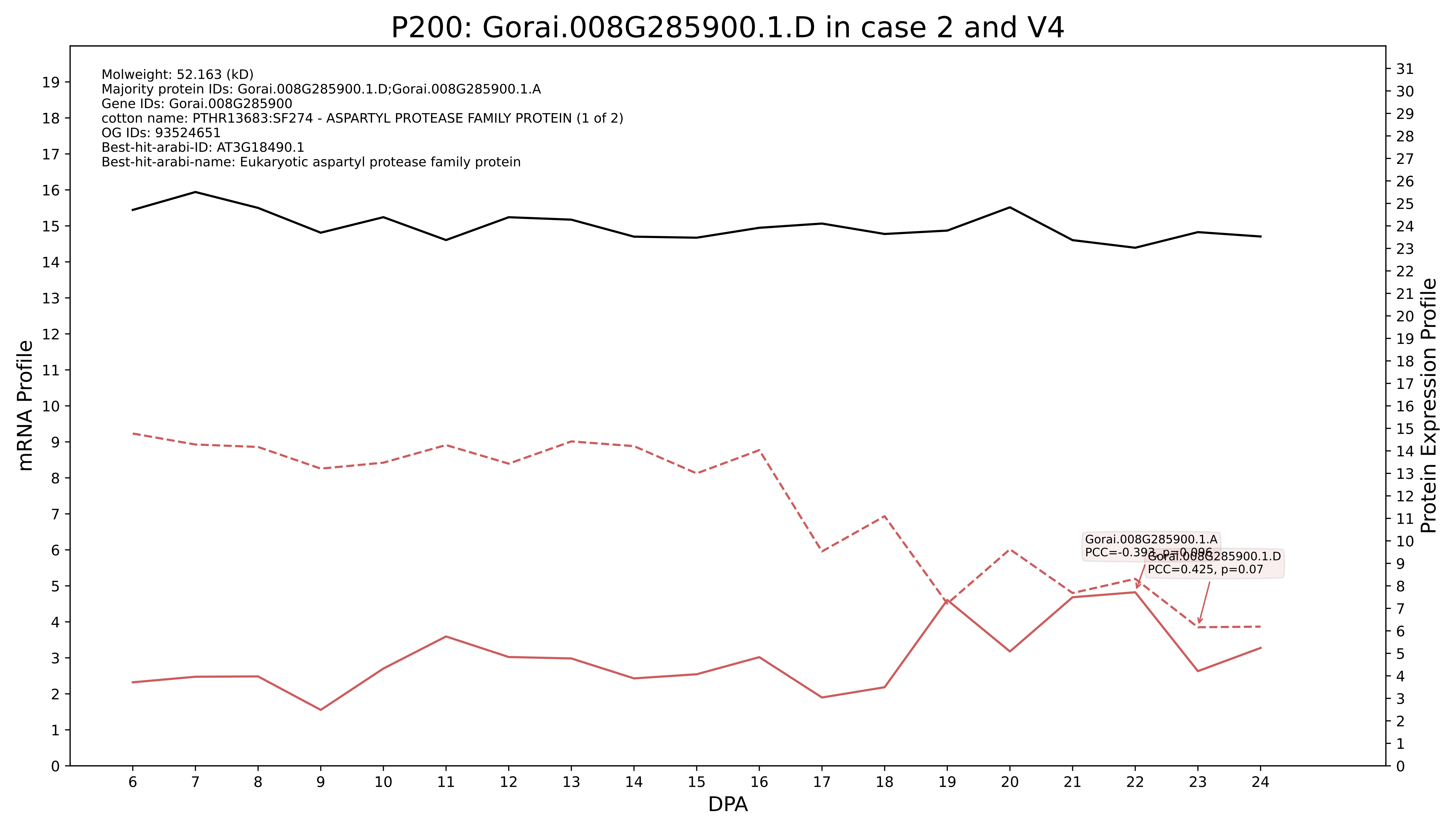

P200: Gorai.008G285900.1.D in case 2 and V4
Molweight: 52.163 (kD)
Majority protein IDs: Gorai.008G285900.1.D;Gorai.008G285900.1.A
Gene IDs: Gorai.008G285900
cotton name: PTHR13683:SF274 - ASPARTYL PROTEASE FAMILY PROTEIN (1 of 2)
OG IDs: 93524651
Best-hit-arabi-ID: AT3G18490.1
Best-hit-arabi-name: Eukaryotic aspartyl protease family protein
Gorai.008G285900.1.A
PCC=-0.393, p=0.096
Gorai.008G285900.1.D
PCC=0.425, p=0.07
mRNA Profile
Protein Expression Profile
DPA

200: Gorai.009G329800.1.A in case 2 and V4

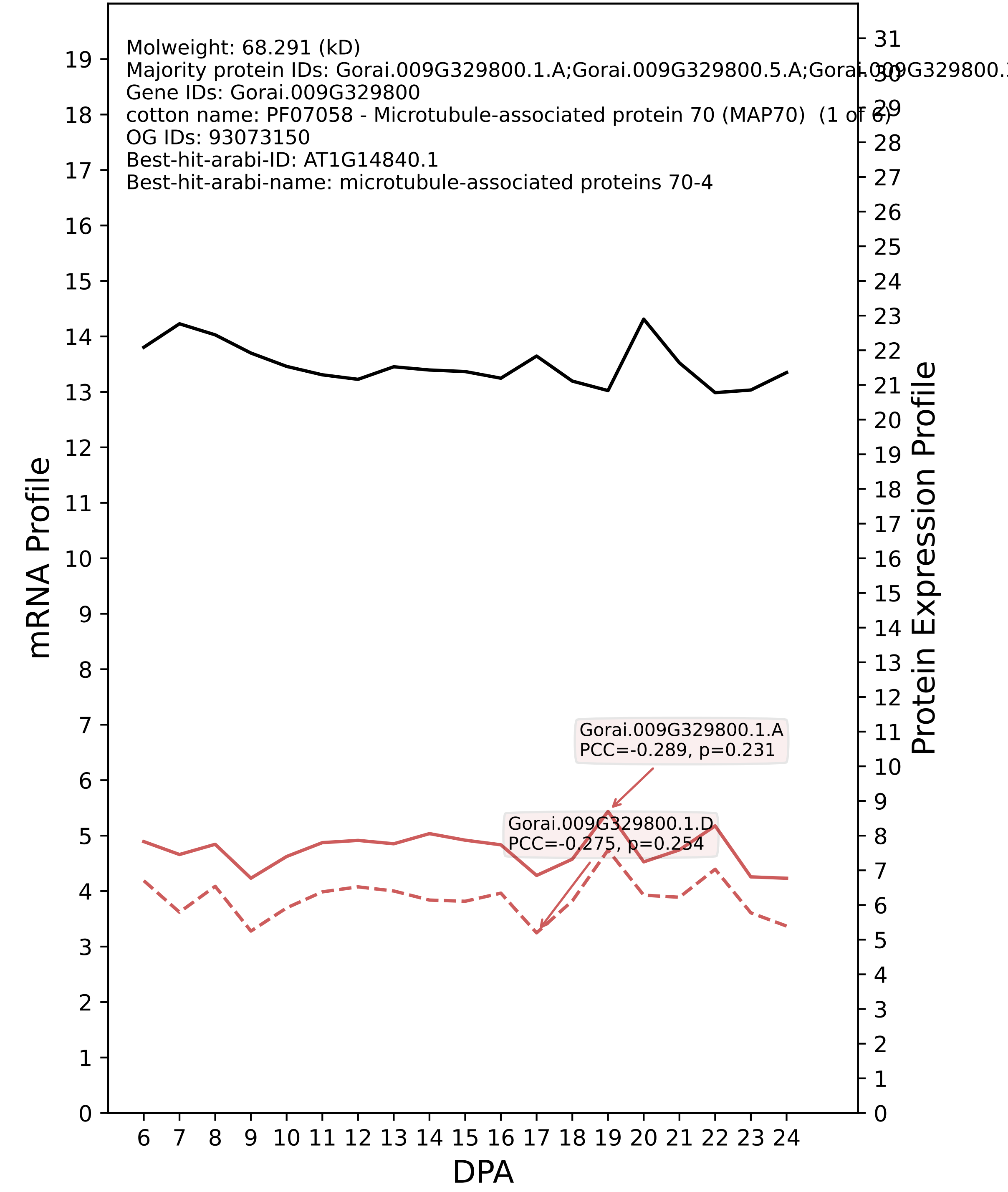

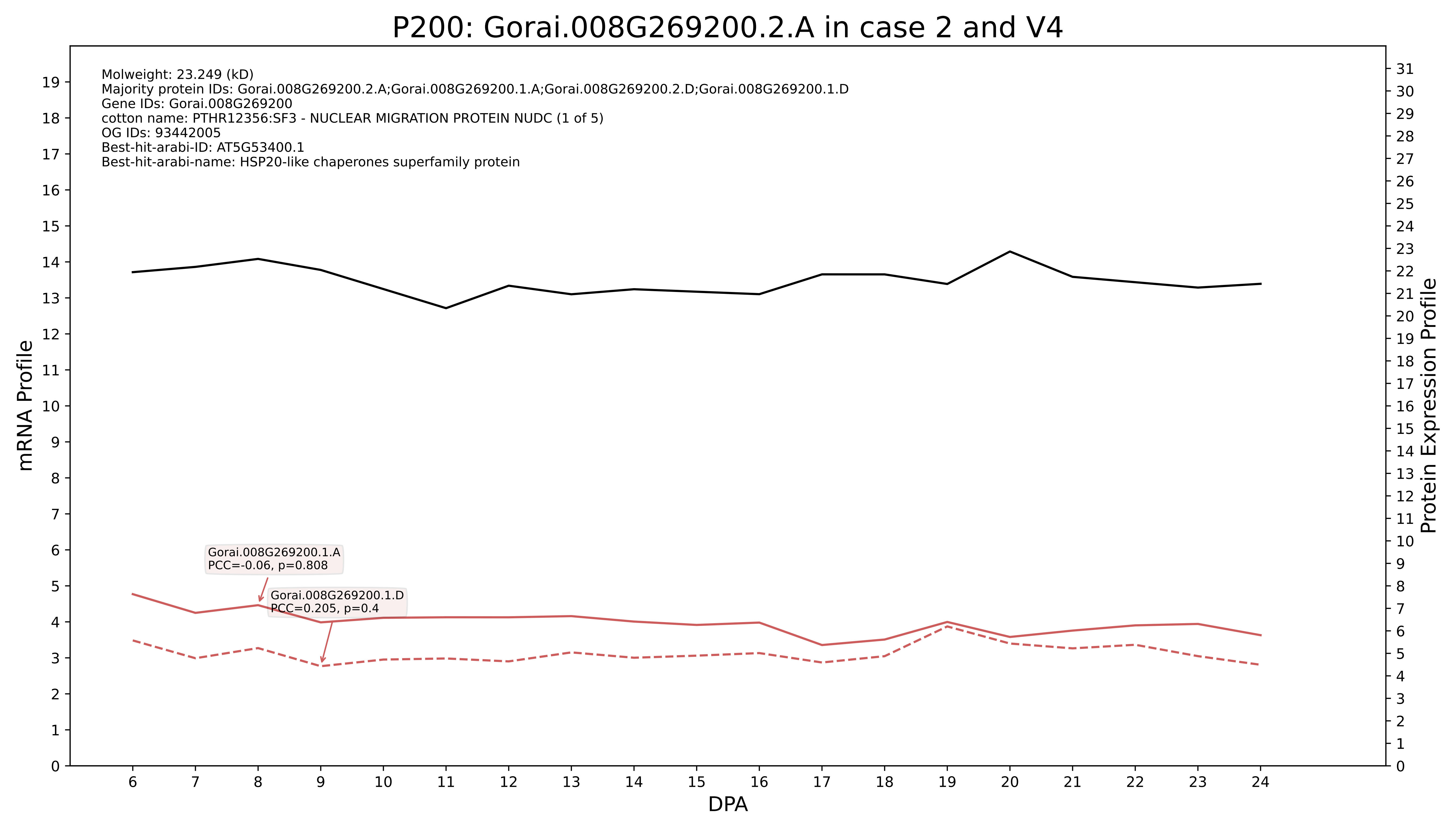

P200: Gorai.008G269200.2.A in case 2 and V4
Molweight: 23.249 (kD)
Majority protein IDs: Gorai.008G269200.2.A;Gorai.008G269200.1.A;Gorai.008G269200.2.D;Gorai.008G269200.1.D
Gene IDs: Gorai.008G269200
cotton name: PTHR12356:SF3 - NUCLEAR MIGRATION PROTEIN NUDC (1 of 5)
OG IDs: 93442005
Best-hit-arabi-ID: AT5G53400.1
Best-hit-arabi-name: HSP20-like chaperones superfamily protein
Gorai.008G269200.1.A
PCC=-0.06, p=0.808
Gorai.008G269200.1.D
PCC=0.205, p=0.4
mRNA Profile
Protein Expression Profile
DPA

P200: Gorai.007G299700.3.D in case 2 and V4

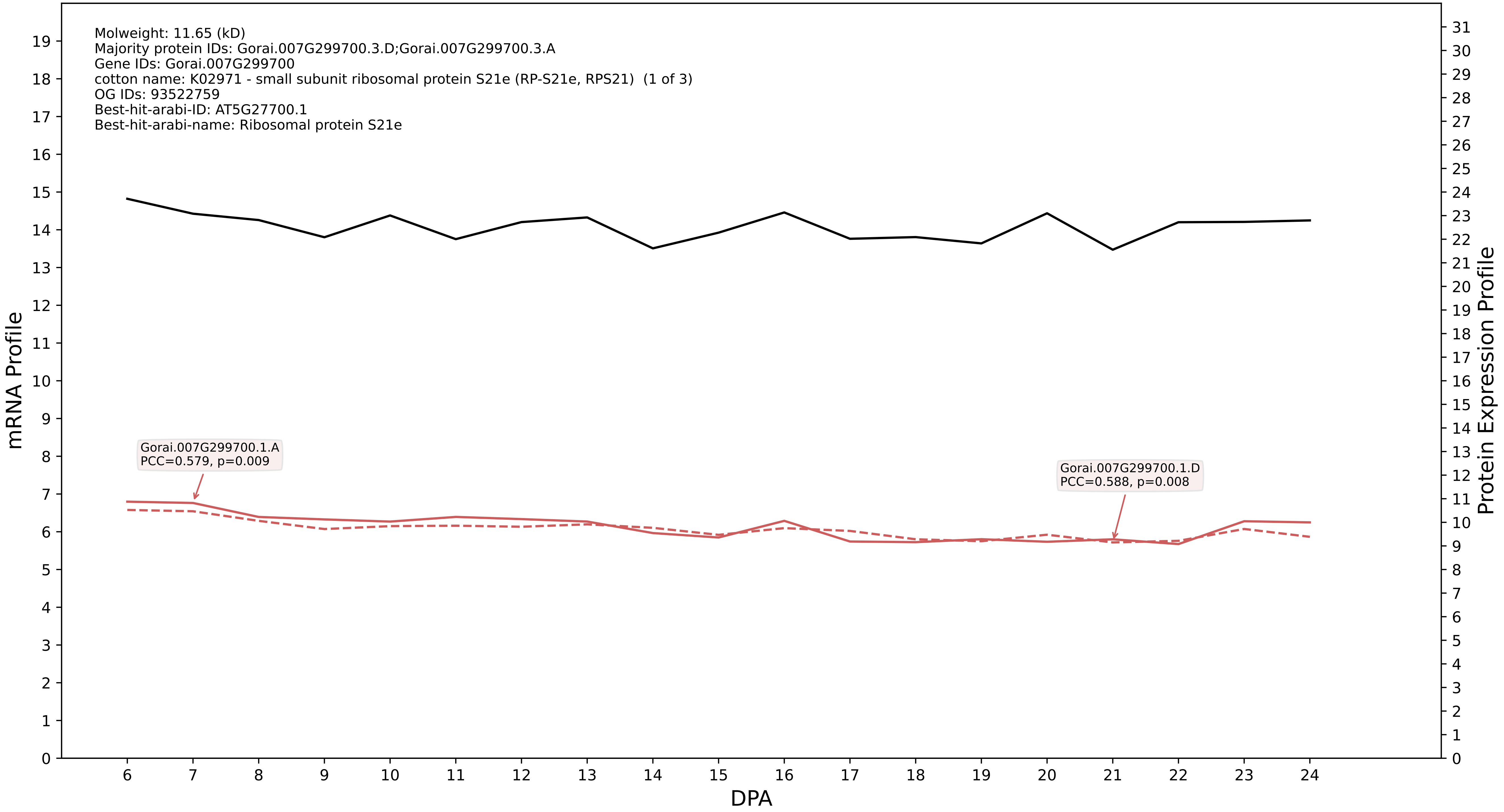

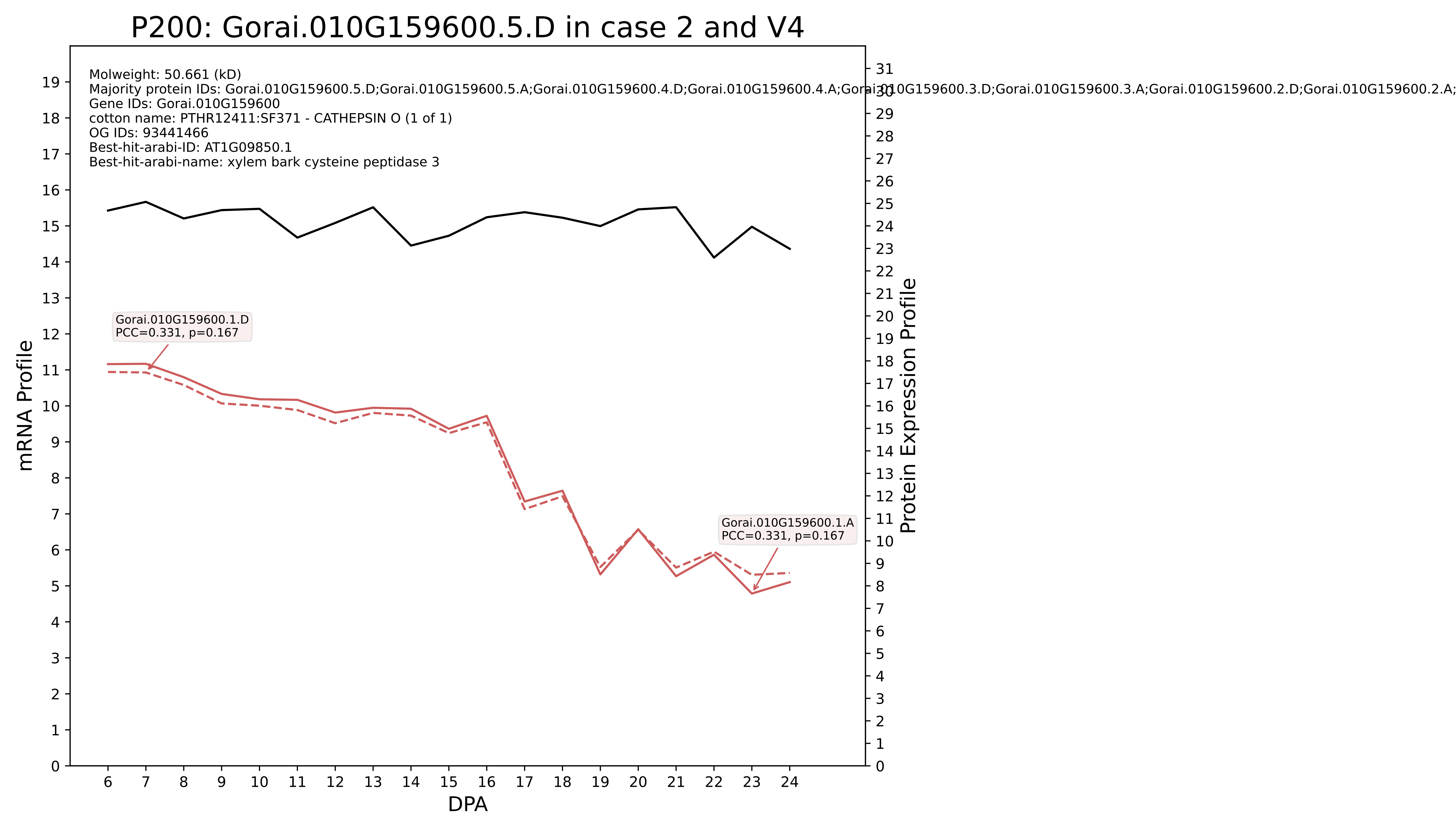
P200: Gorai.010G159600.5.D in case 2 and V4
Molweight: 50.661 (kD)
Majority protein IDs: Gorai.010G159600.5.D;Gorai.010G159600.5.A;Gorai.010G159600.4.D;Gorai.010G159600.4.A;Gorai.010G159600.3.D;Gorai.010G159600.3.A;Gorai.010G159600.2.D;Gorai.010G159600.2.A;
Gene IDs: Gorai.010G159600
cotton name: PTHR12411:SF371 - CATHEPSIN O (1 of 1)
OG IDs: 93441466
Best-hit-arabi-ID: AT1G09850.1
Best-hit-arabi-name: xylem bark cysteine peptidase 3
Gorai.010G159600.1.D
PCC=0.331, p=0.167
Gorai.010G159600.1.A
PCC=0.331, p=0.167
mRNA Profile
Protein Expression Profile
DPA

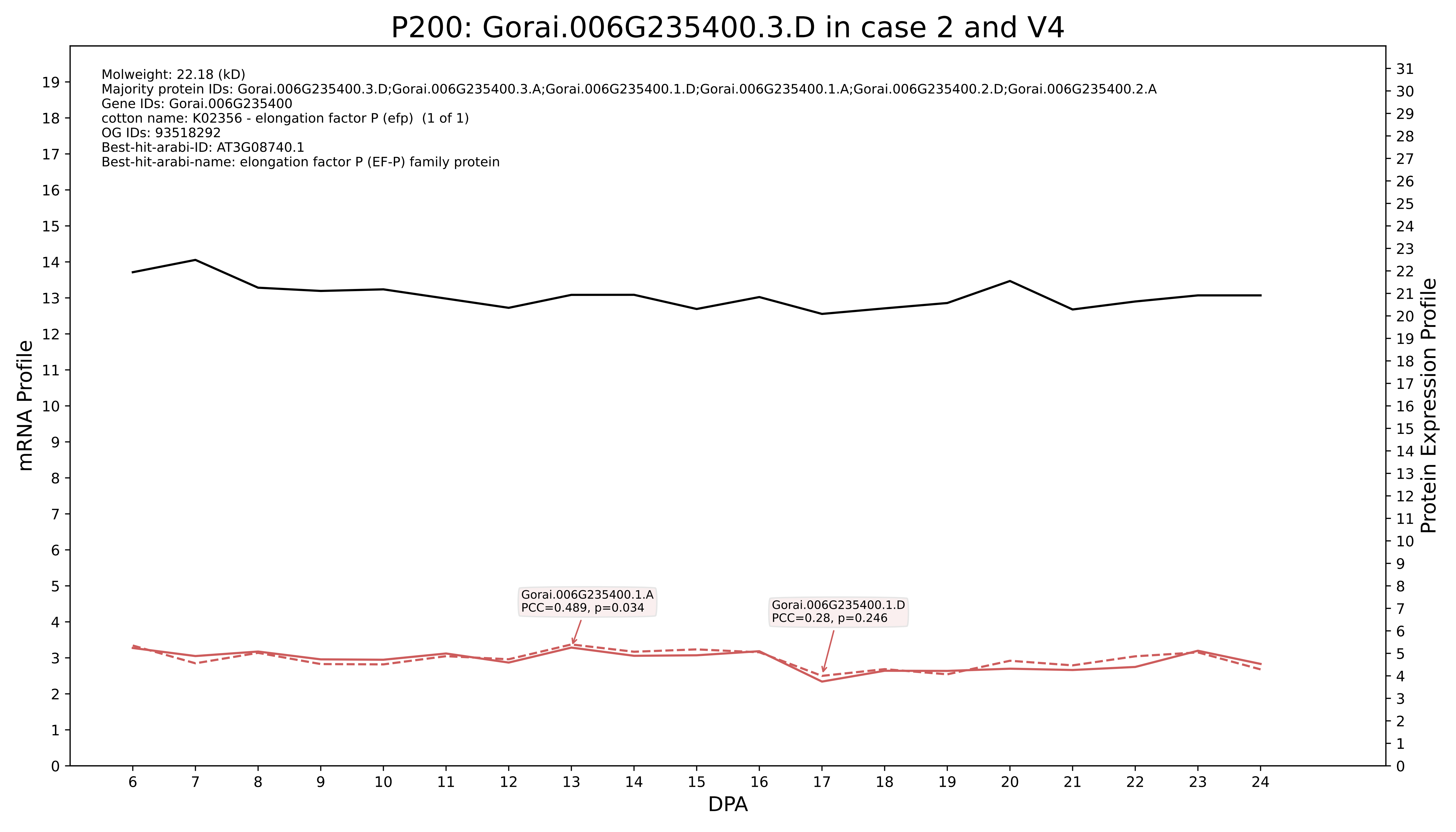

P200: Gorai.006G235400.3.D in case 2 and V4
Molweight: 22.18 (kD)
Majority protein IDs: Gorai.006G235400.3.D;Gorai.006G235400.3.A;Gorai.006G235400.1.D;Gorai.006G235400.1.A;Gorai.006G235400.2.D;Gorai.006G235400.2.A
Gene IDs: Gorai.006G235400
cotton name: K02356 - elongation factor P (efp) (1 of 1)
OG IDs: 93518292
Best-hit-arabi-ID: AT3G08740.1
Best-hit-arabi-name: elongation factor P (EF-P) family protein
Gorai.006G235400.1.A
PCC=0.489, p=0.034
Gorai.006G235400.1.D
PCC=0.28, p=0.246
mRNA Profile
Protein Expression Profile
DPA

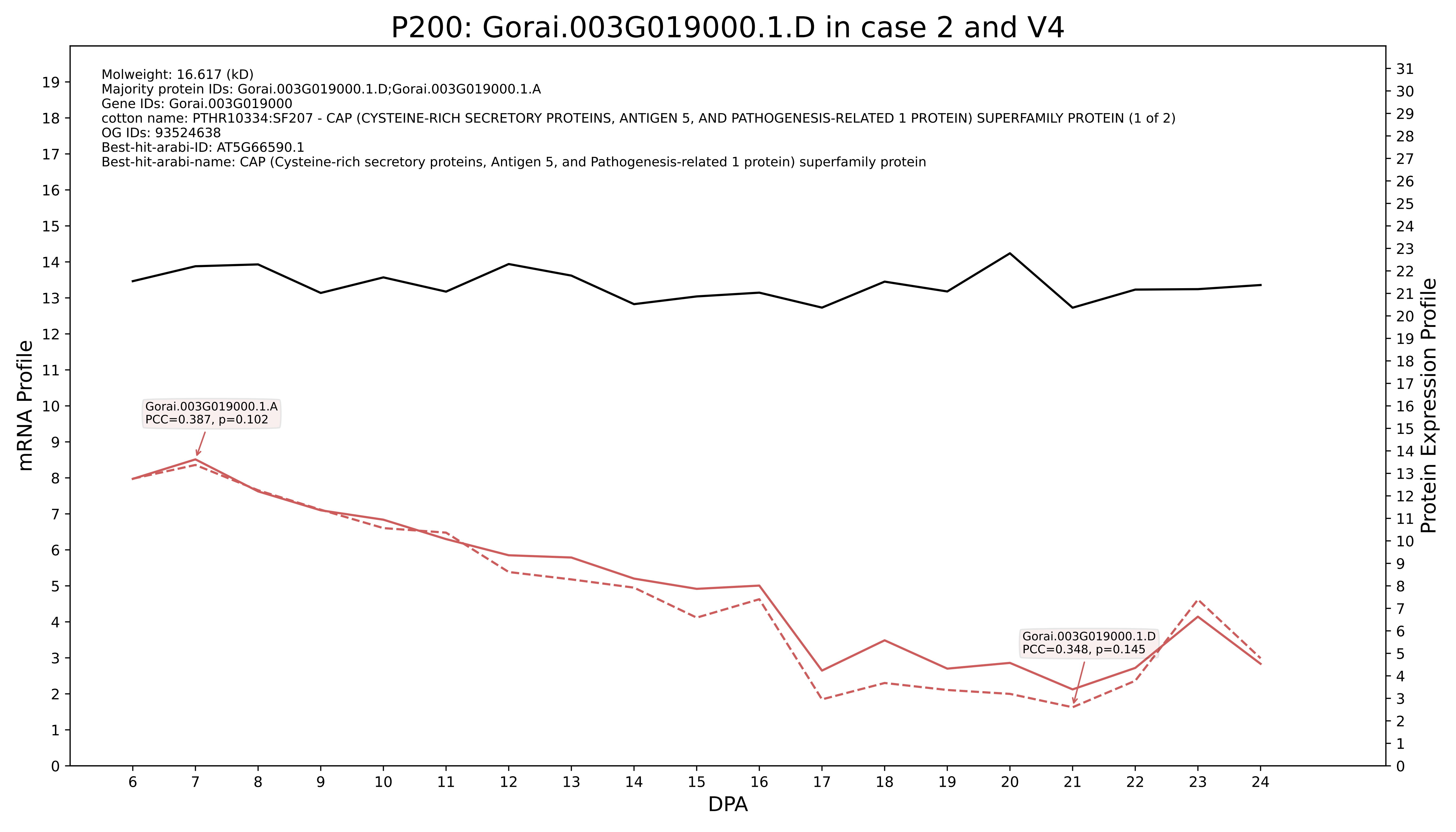
P200: Gorai.003G019000.1.D in case 2 and V4
Molweight: 16.617 (kD)
Majority protein IDs: Gorai.003G019000.1.D;Gorai.003G019000.1.A
Gene IDs: Gorai.003G019000
cotton name: PTHR10334:SF207 - CAP (CYSTEINE-RICH SECRETORY PROTEINS, ANTIGEN 5, AND PATHOGENESIS-RELATED 1 PROTEIN) SUPERFAMILY PROTEIN (1 of 2)
OG IDs: 93524638
Best-hit-arabi-ID: AT5G66590.1
Best-hit-arabi-name: CAP (Cysteine-rich secretory proteins, Antigen 5, and Pathogenesis-related 1 protein) superfamily protein
Gorai.003G019000.1.A
PCC=0.387, p=0.102
Gorai.003G019000.1.D
PCC=0.348, p=0.145
mRNA Profile
Protein Expression Profile
DPA

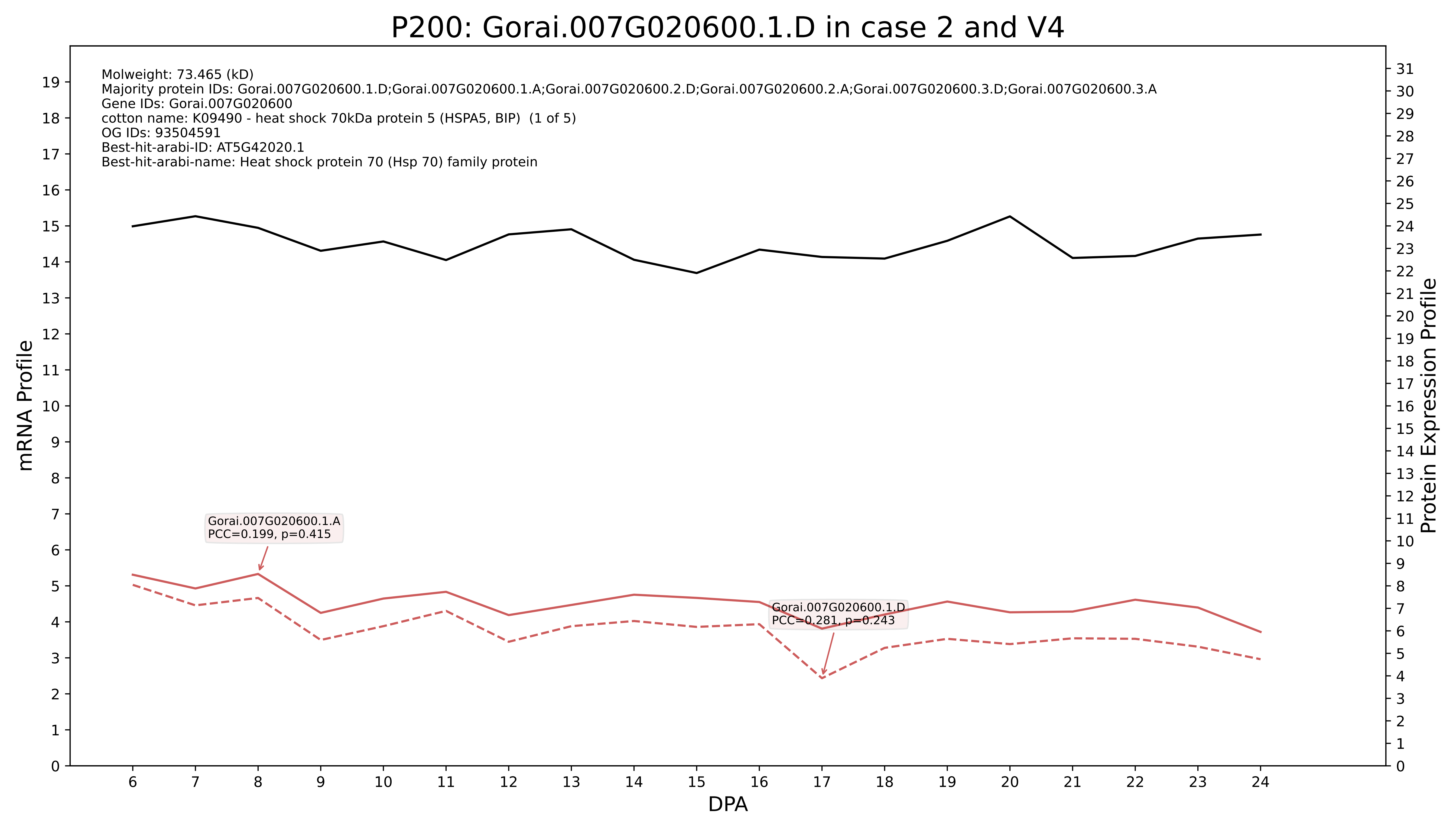
P200: Gorai.007G020600.1.D in case 2 and V4
Molweight: 73.465 (kD)
Majority protein IDs: Gorai.007G020600.1.D;Gorai.007G020600.1.A;Gorai.007G020600.2.D;Gorai.007G020600.2.A;Gorai.007G020600.3.D;Gorai.007G020600.3.A
Gene IDs: Gorai.007G020600
cotton name: K09490 - heat shock 70kDa protein 5 (HSPA5, BIP) (1 of 5)
OG IDs: 93504591
Best-hit-arabi-ID: AT5G42020.1
Best-hit-arabi-name: Heat shock protein 70 (Hsp 70) family protein
Gorai.007G020600.1.A
PCC=0.199, p=0.415
Gorai.007G020600.1.D
PCC=0.281, p=0.243
mRNA Profile
Protein Expression Profile
DPA

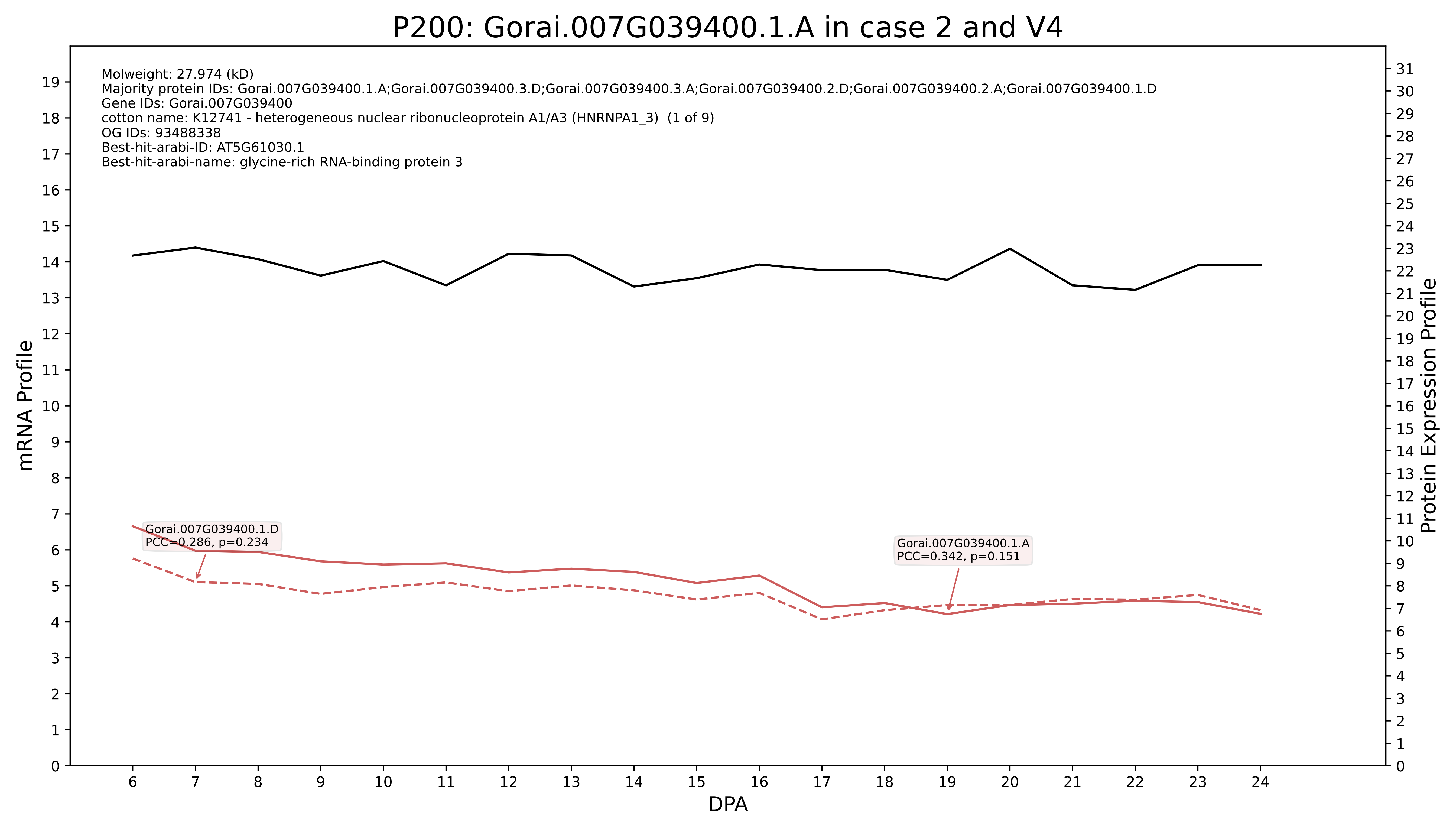
P200: Gorai.007G039400.1.A in case 2 and V4
Molweight: 27.974 (kD)
Majority protein IDs: Gorai.007G039400.1.A;Gorai.007G039400.3.D;Gorai.007G039400.3.A;Gorai.007G039400.2.D;Gorai.007G039400.2.A;Gorai.007G039400.1.D
Gene IDs: Gorai.007G039400
cotton name: K12741 - heterogeneous nuclear ribonucleoprotein A1/A3 (HNRNPA1_3) (1 of 9)
OG IDs: 93488338
Best-hit-arabi-ID: AT5G61030.1
Best-hit-arabi-name: glycine-rich RNA-binding protein 3
Gorai.007G039400.1.D
PCC=0.286, p=0.234
Gorai.007G039400.1.A
PCC=0.342, p=0.151
mRNA Profile
Protein Expression Profile
DPA

P200: Gorai.002G054400.1.D in case 2 and V4

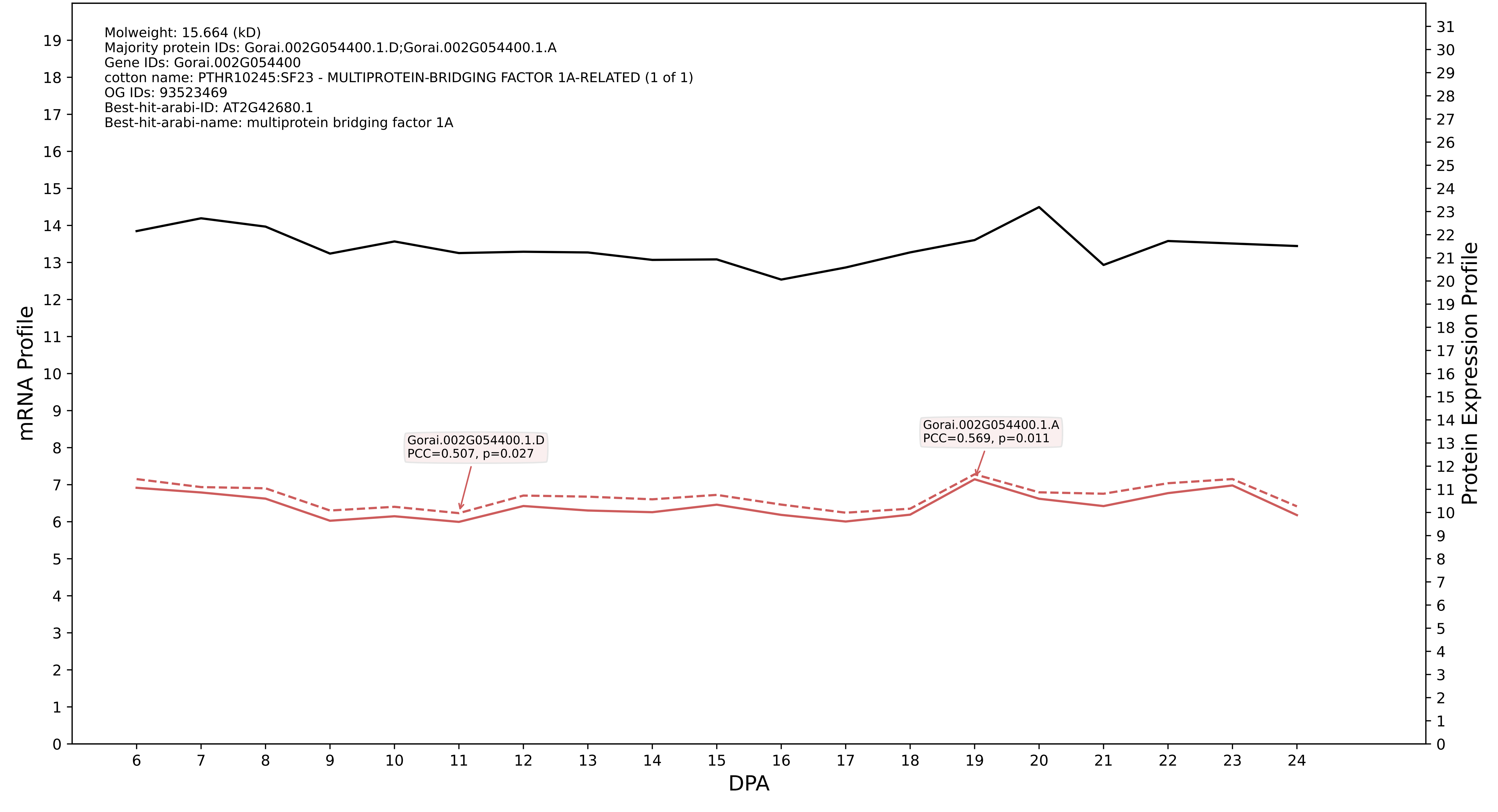

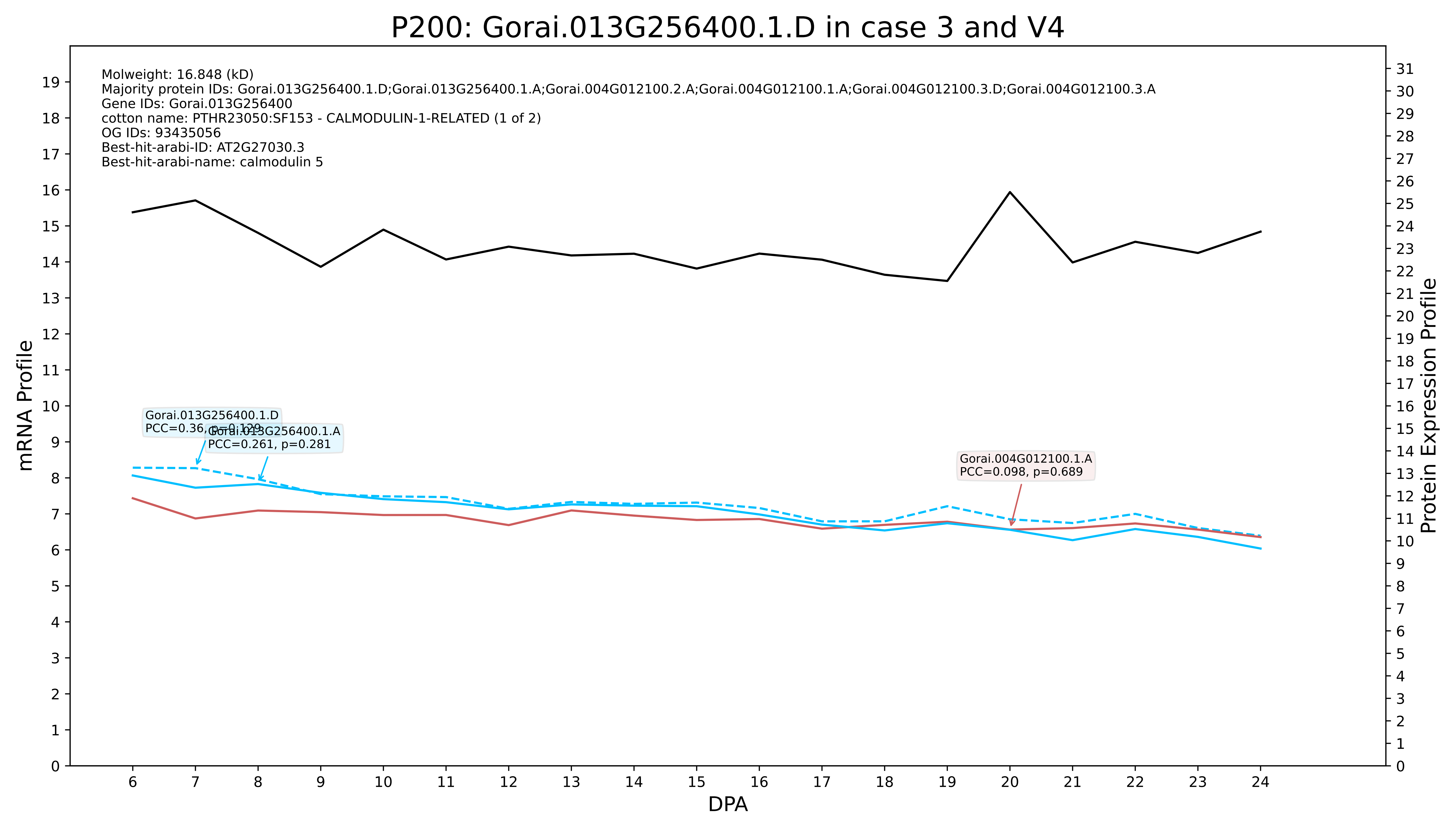
P200: Gorai.013G256400.1.D in case 3 and V4
Molweight: 16.848 (kD)
Majority protein IDs: Gorai.013G256400.1.D;Gorai.013G256400.1.A;Gorai.004G012100.2.A;Gorai.004G012100.1.A;Gorai.004G012100.3.D;Gorai.004G012100.3.A
Gene IDs: Gorai.013G256400
cotton name: PTHR23050:SF153 - CALMODULIN-1-RELATED (1 of 2)
OG IDs: 93435056
Best-hit-arabi-ID: AT2G27030.3
Best-hit-arabi-name: calmodulin 5
Gorai.013G256400.1.D
PCC=0.36, p=0.129
Gorai.013G256400.1.A
PCC=0.261, p=0.281
Gorai.004G012100.1.A
PCC=0.098, p=0.689
mRNA Profile
Protein Expression Profile
DPA

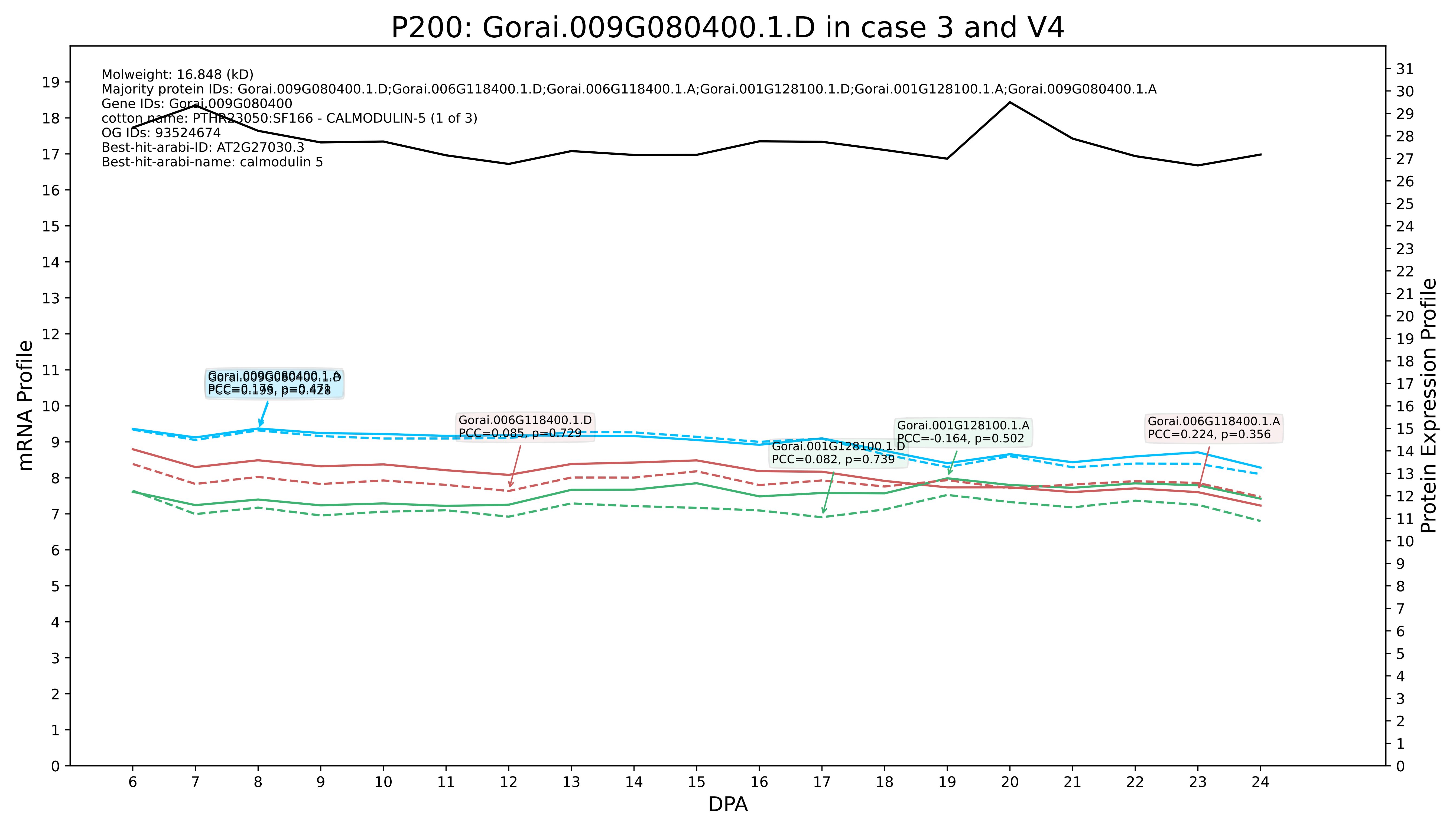

P200: Gorai.009G080400.1.D in case 3 and V4
Molweight: 16.848 (kD)
Majority protein IDs: Gorai.009G080400.1.D;Gorai.006G118400.1.D;Gorai.006G118400.1.A;Gorai.001G128100.1.D;Gorai.001G128100.1.A;Gorai.009G080400.1.A
Gene IDs: Gorai.009G080400
cotton name: PTHR23050:SF166 - CALMODULIN-5 (1 of 3)
OG IDs: 93524674
Best-hit-arabi-ID: AT2G27030.3
Best-hit-arabi-name: calmodulin 5
Gorai.009G080400.1.A
Gorai.009G080400.1.D
PCC=0.176, p=0.471
PCC=0.193, p=0.428
Gorai.006G118400.1.D
PCC=0.085, p=0.729
Gorai.001G128100.1.D
PCC=0.082, p=0.739
Gorai.001G128100.1.A
PCC=-0.164, p=0.502
Gorai.006G118400.1.A
PCC=0.224, p=0.356
mRNA Profile
Protein Expression Profile
DPA

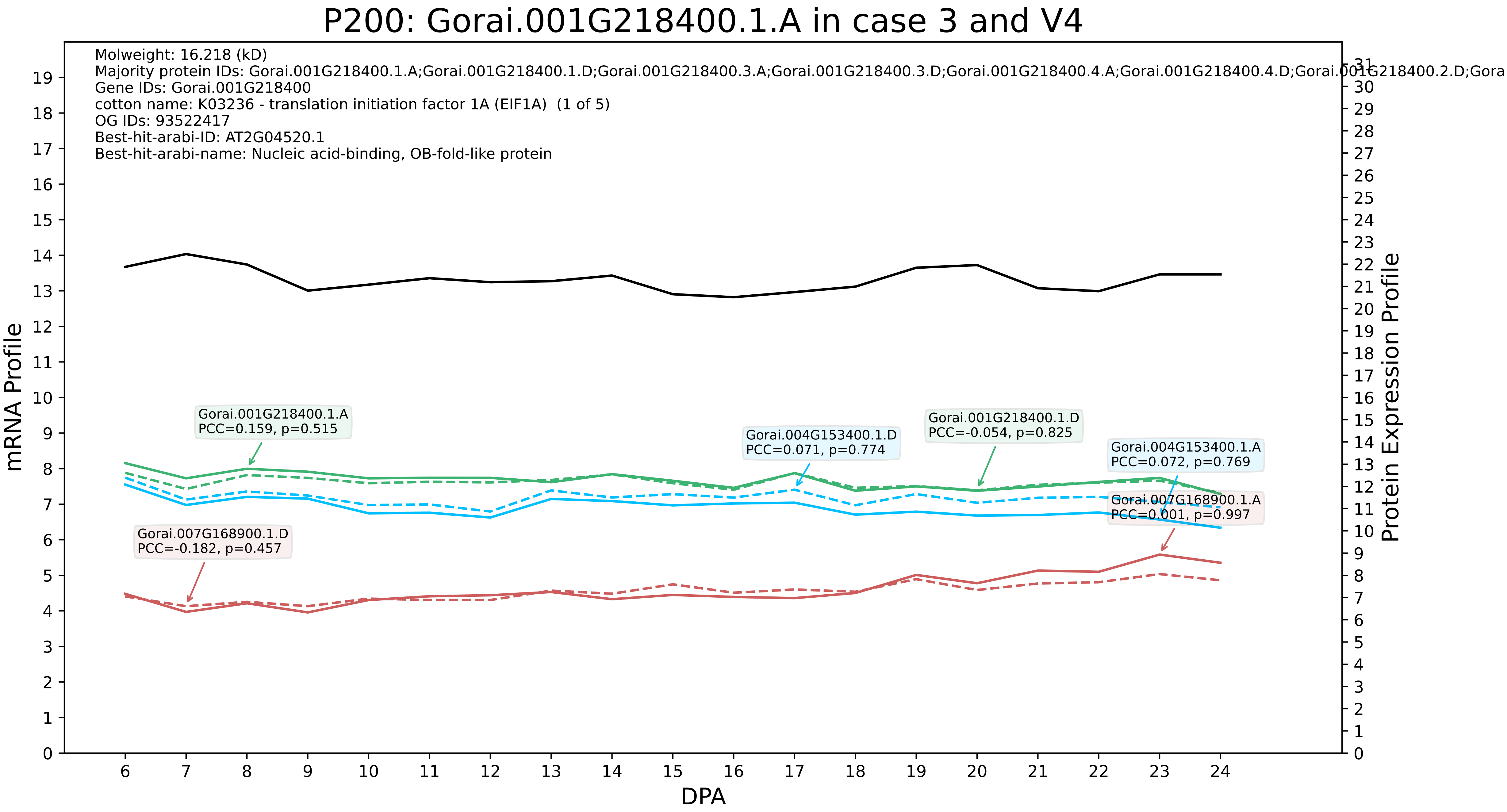

P200: Gorai.001G218400.1.A in case 3 and V4
Molweight: 16.218 (kD)
Majority protein IDs: Gorai.001G218400.1.A;Gorai.001G218400.1.D;Gorai.001G218400.3.A;Gorai.001G218400.3.D;Gorai.001G218400.4.A;Gorai.001G218400.4.D;Gorai.001G218400.2.D;Gorai
Gene IDs: Gorai.001G218400
cotton name: K03236 - translation initiation factor 1A (EIF1A) (1 of 5)
OG IDs: 93522417
Best-hit-arabi-ID: AT2G04520.1
Best-hit-arabi-name: Nucleic acid-binding, OB-fold-like protein
Gorai.001G218400.1.A
PCC=0.159, p=0.515
Gorai.004G153400.1.D
PCC=0.071, p=0.774
Gorai.001G218400.1.D
PCC=-0.054, p=0.825
Gorai.004G153400.1.A
PCC=0.072, p=0.769
Gorai.007G168900.1.A
PCC=0.001, p=0.997
Gorai.007G168900.1.D
PCC=-0.182, p=0.457
mRNA Profile
Protein Expression Profile
DPA

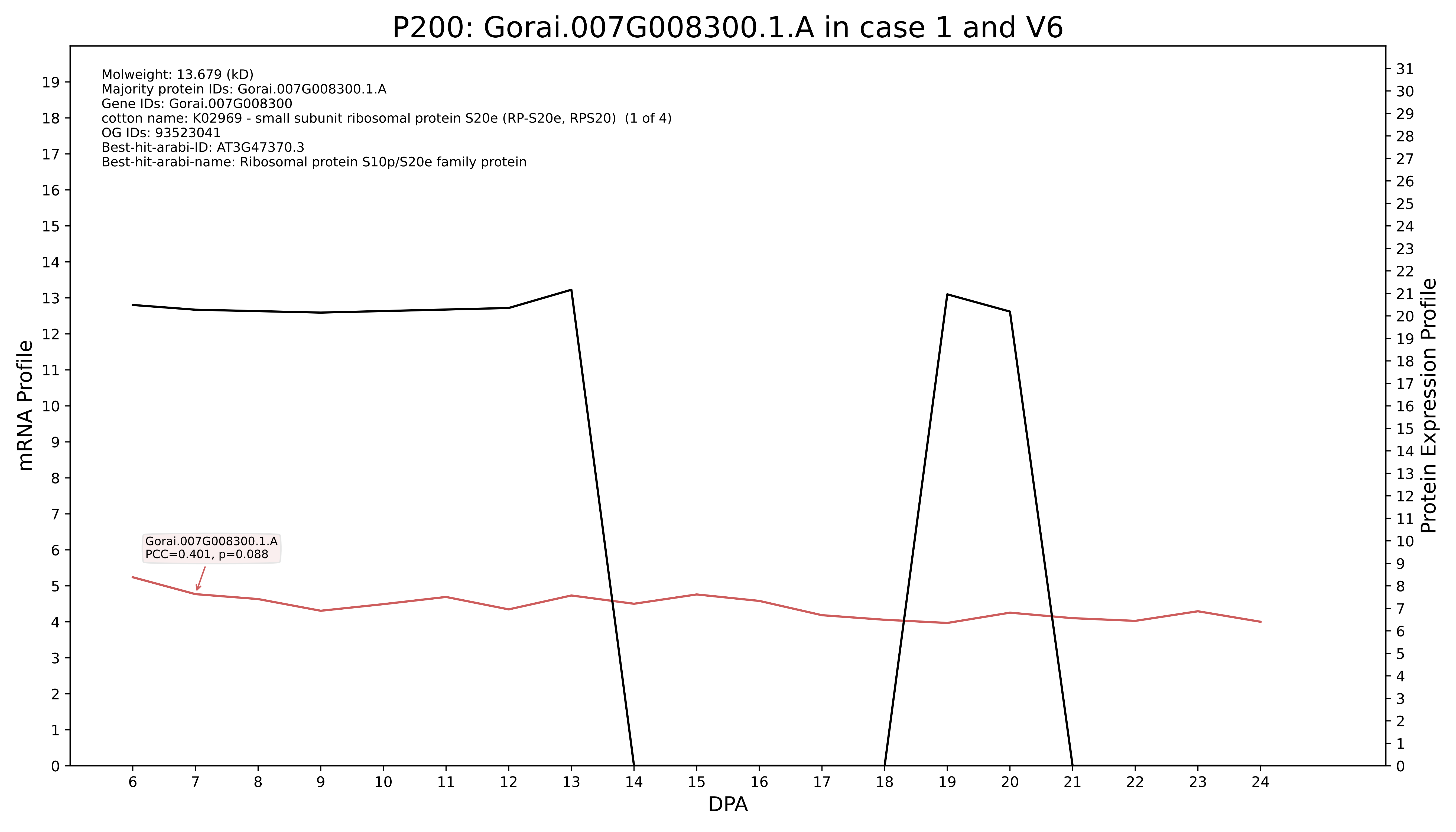

P200: Gorai.007G008300.1.A in case 1 and V6
Molweight: 13.679 (kD)
Majority protein IDs: Gorai.007G008300.1.A
Gene IDs: Gorai.007G008300
cotton name: K02969 - small subunit ribosomal protein S20e (RP-S20e, RPS20) (1 of 4)
OG IDs: 93523041
Best-hit-arabi-ID: AT3G47370.3
Best-hit-arabi-name: Ribosomal protein S10p/S20e family protein
Gorai.007G008300.1.A
PCC=0.401, p=0.088
mRNA Profile
Protein Expression Profile
DPA

P200: Gorai.007G300000.1.D in case 1 and V6

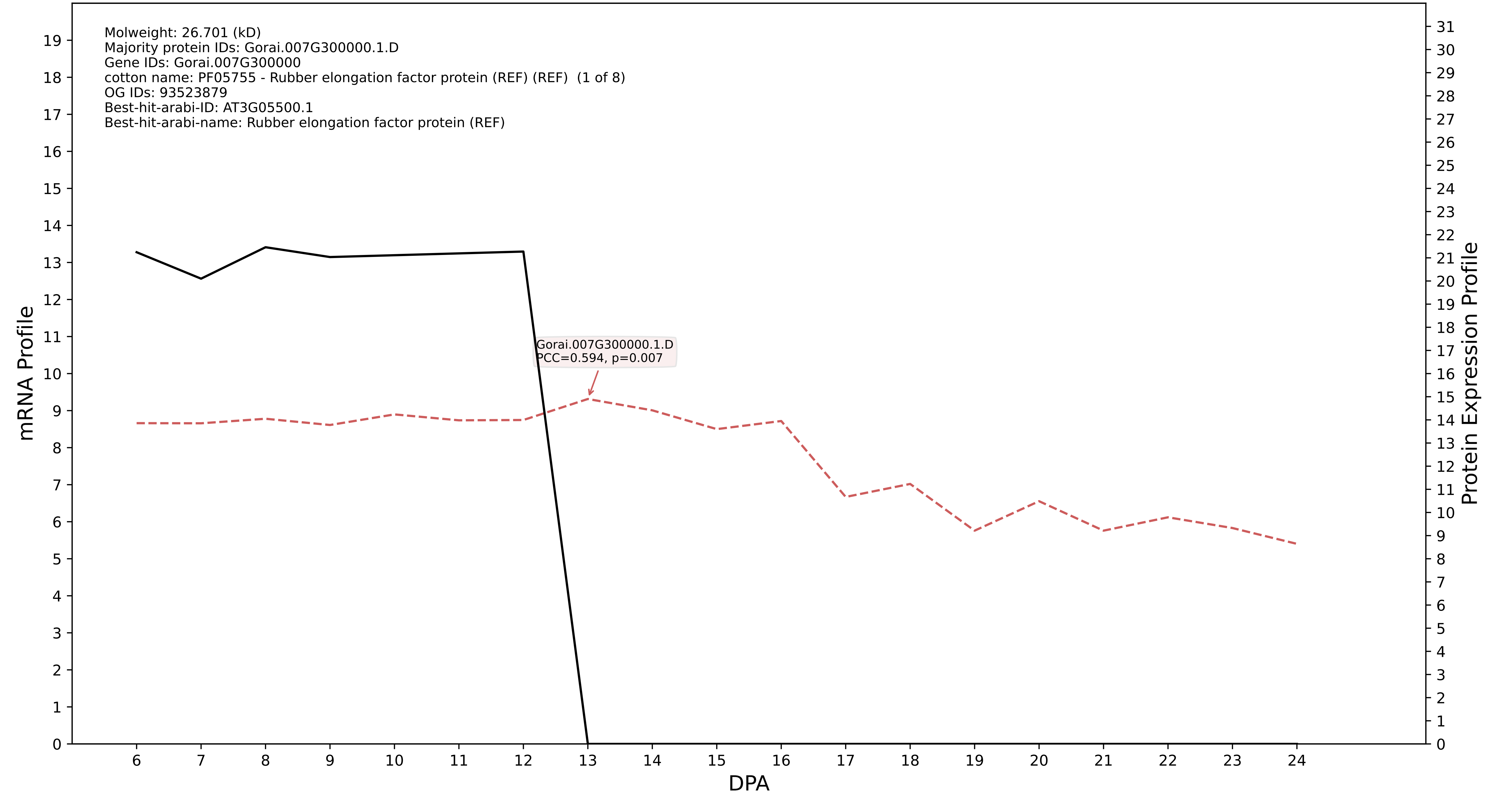

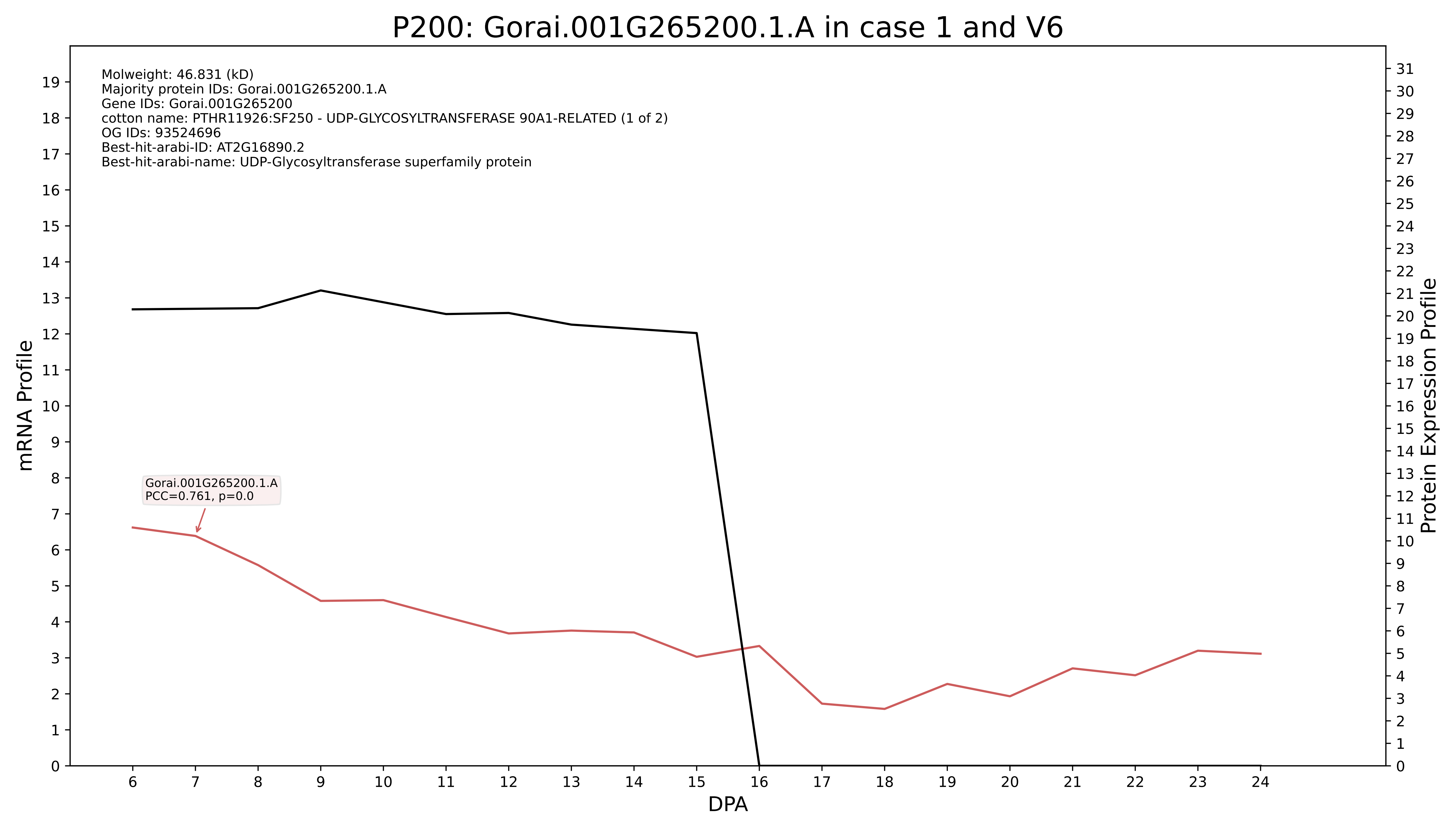
P200: Gorai.001G265200.1.A in case 1 and V6
Molweight: 46.831 (kD)
Majority protein IDs: Gorai.001G265200.1.A
Gene IDs: Gorai.001G265200
cotton name: PTHR11926:SF250 - UDP-GLYCOSYLTRANSFERASE 90A1-RELATED (1 of 2)
OG IDs: 93524696
Best-hit-arabi-ID: AT2G16890.2
Best-hit-arabi-name: UDP-Glycosyltransferase superfamily protein
Gorai.001G265200.1.A
PCC=0.761, p=0.0
mRNA Profile
Protein Expression Profile
DPA

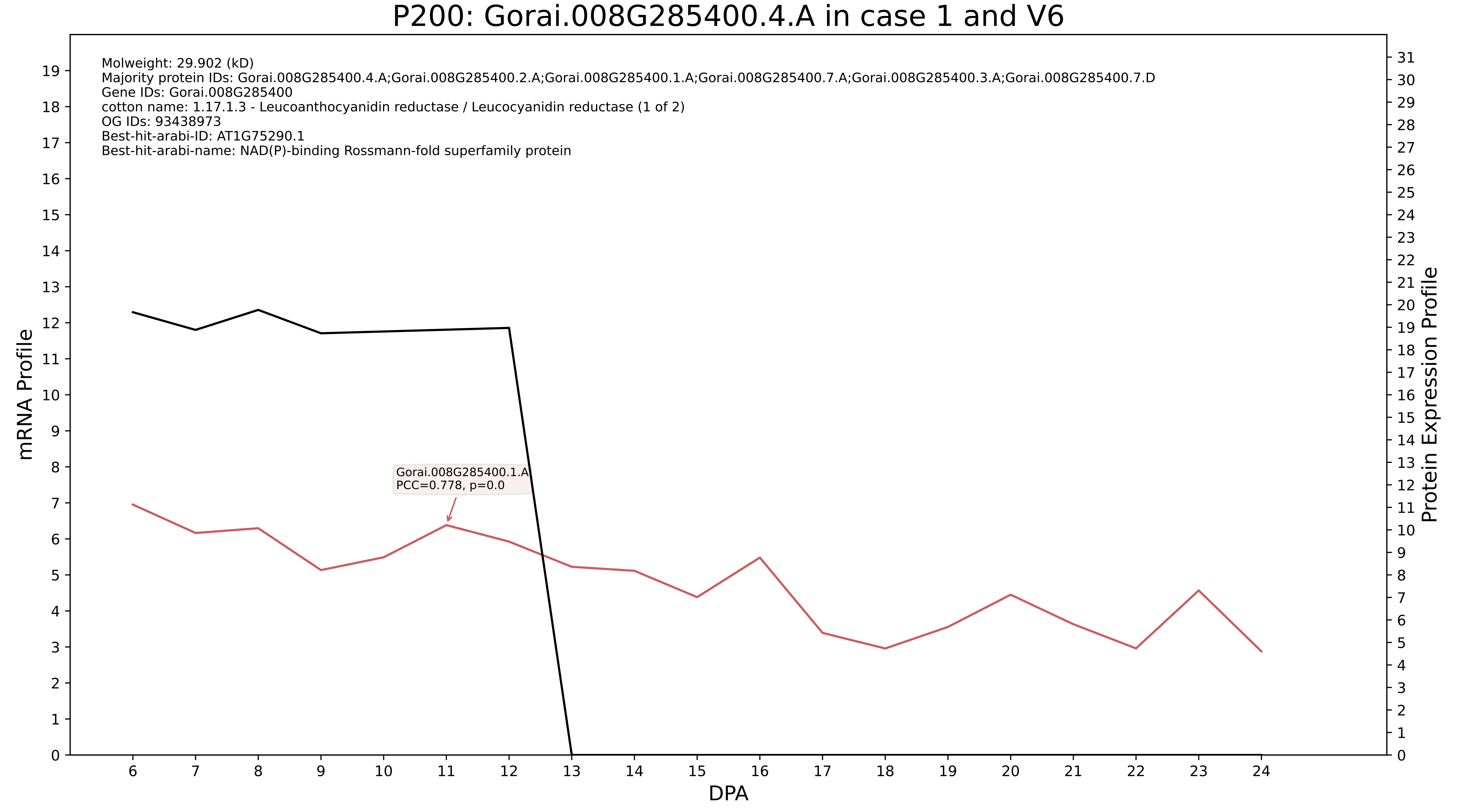
P200: Gorai.008G285400.4.A in case 1 and V6
Molweight: 29.902 (kD)
Majority protein IDs: Gorai.008G285400.4.A;Gorai.008G285400.2.A;Gorai.008G285400.1.A;Gorai.008G285400.7.A;Gorai.008G285400.3.A;Gorai.008G285400.7.D
Gene IDs: Gorai.008G285400
cotton name: 1.17.1.3 - Leucoanthocyanidin reductase / Leucocyanidin reductase (1 of 2)
OG IDs: 93438973
Best-hit-arabi-ID: AT1G75290.1
Best-hit-arabi-name: NAD(P)-binding Rossmann-fold superfamily protein
Gorai.008G285400.1.A
PCC=0.778, p=0.0
mRNA Profile
Protein Expression Profile
DPA

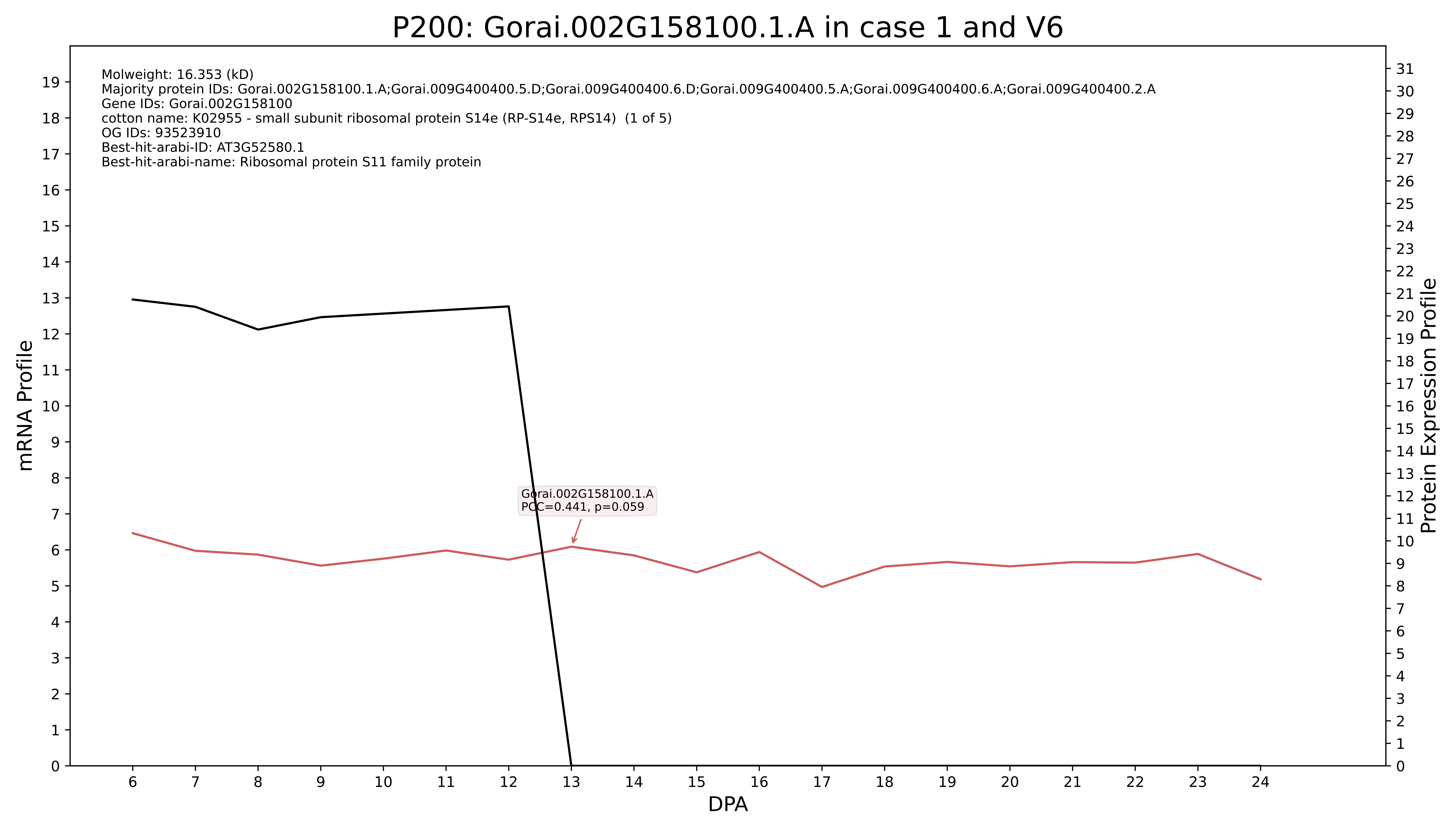
P200: Gorai.002G158100.1.A in case 1 and V6
Molweight: 16.353 (kD)
Majority protein IDs: Gorai.002G158100.1.A;Gorai.009G400400.5.D;Gorai.009G400400.6.D;Gorai.009G400400.5.A;Gorai.009G400400.6.A;Gorai.009G400400.2.A
Gene IDs: Gorai.002G158100
cotton name: K02955 - small subunit ribosomal protein S14e (RP-S14e, RPS14) (1 of 5)
OG IDs: 93523910
Best-hit-arabi-ID: AT3G52580.1
Best-hit-arabi-name: Ribosomal protein S11 family protein
Gorai.002G158100.1.A
PCC=0.441, p=0.059
mRNA Profile
Protein Expression Profile
DPA

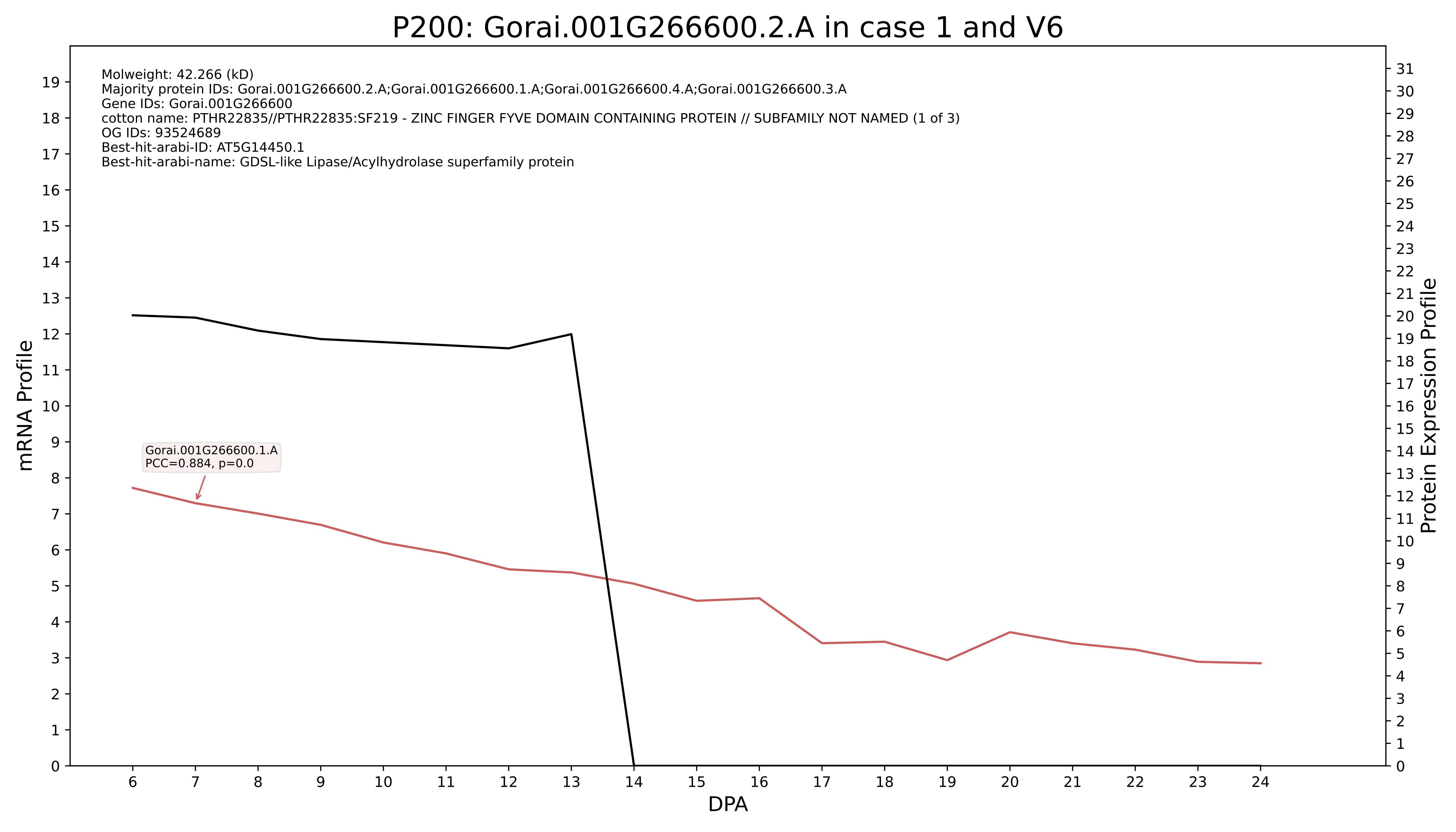
P200: Gorai.001G266600.2.A in case 1 and V6
Molweight: 42.266 (kD)
Majority protein IDs: Gorai.001G266600.2.A;Gorai.001G266600.1.A;Gorai.001G266600.4.A;Gorai.001G266600.3.A
Gene IDs: Gorai.001G266600
cotton name: PTHR22835//PTHR22835:SF219 - ZINC FINGER FYVE DOMAIN CONTAINING PROTEIN // SUBFAMILY NOT NAMED (1 of 3)
OG IDs: 93524689
Best-hit-arabi-ID: AT5G14450.1
Best-hit-arabi-name: GDSL-like Lipase/Acylhydrolase superfamily protein
Gorai.001G266600.1.A
PCC=0.884, p=0.0
mRNA Profile
Protein Expression Profile
DPA

P200: Gorai.003G058100.3.D in case 1 and V6

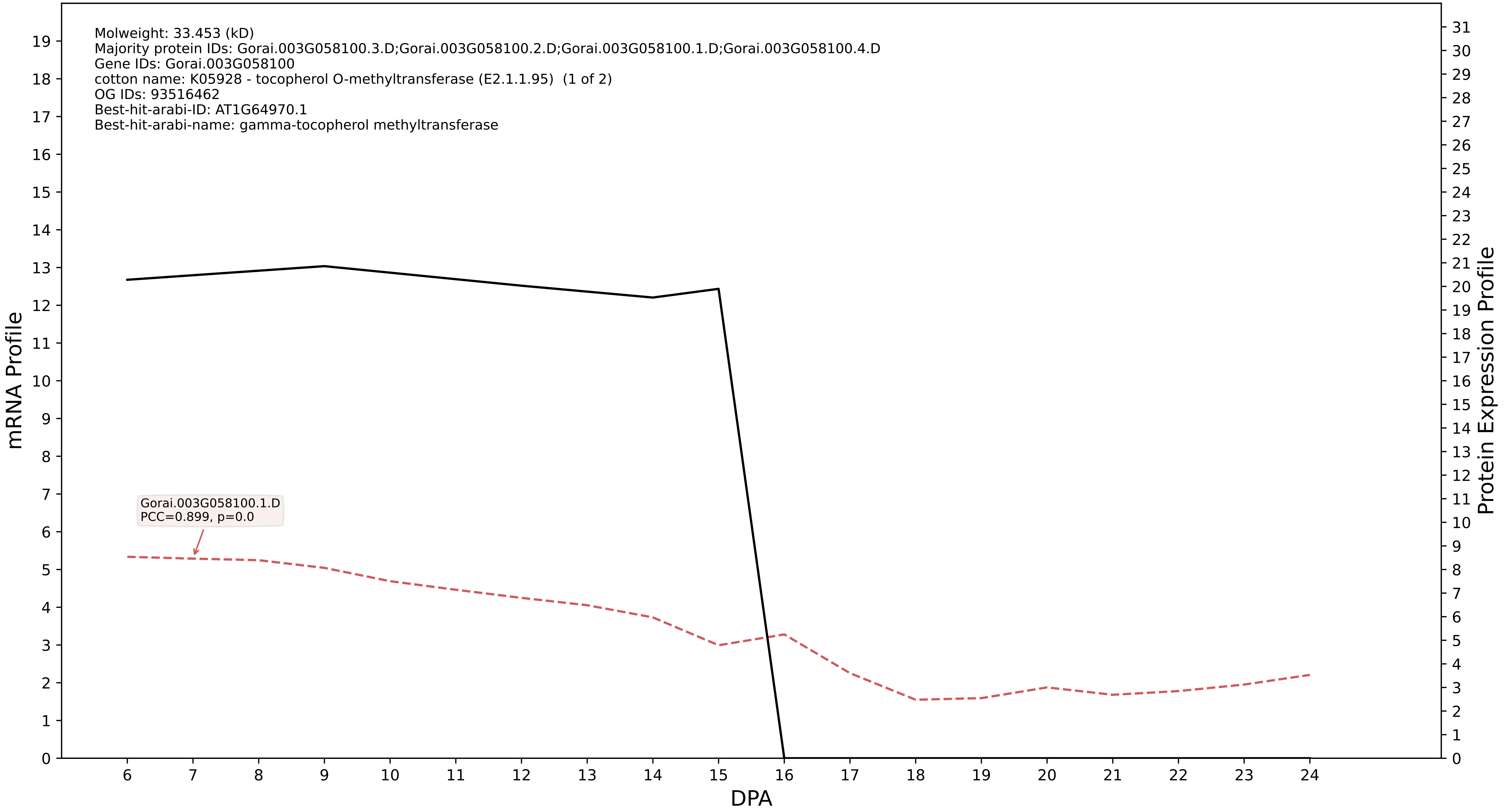

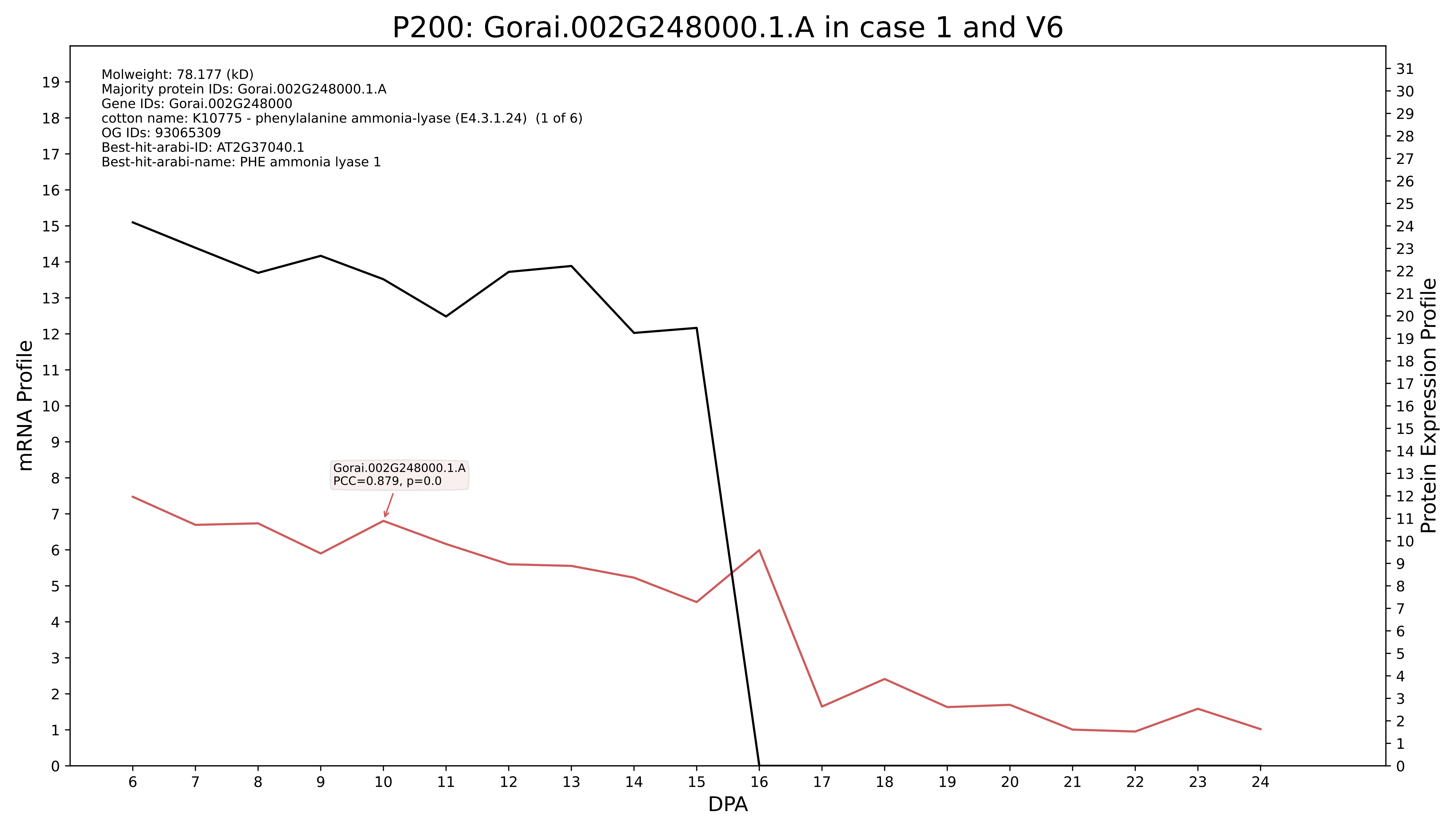

P200: Gorai.002G248000.1.A in case 1 and V6
Molweight: 78.177 (kD)
Majority protein IDs: Gorai.002G248000.1.A
Gene IDs: Gorai.002G248000
cotton name: K10775 - phenylalanine ammonia-lyase (E4.3.1.24) (1 of 6)
OG IDs: 93065309
Best-hit-arabi-ID: AT2G37040.1
Best-hit-arabi-name: PHE ammonia lyase 1
Gorai.002G248000.1.A
PCC=0.879, p=0.0
mRNA Profile
Protein Expression Profile
DPA

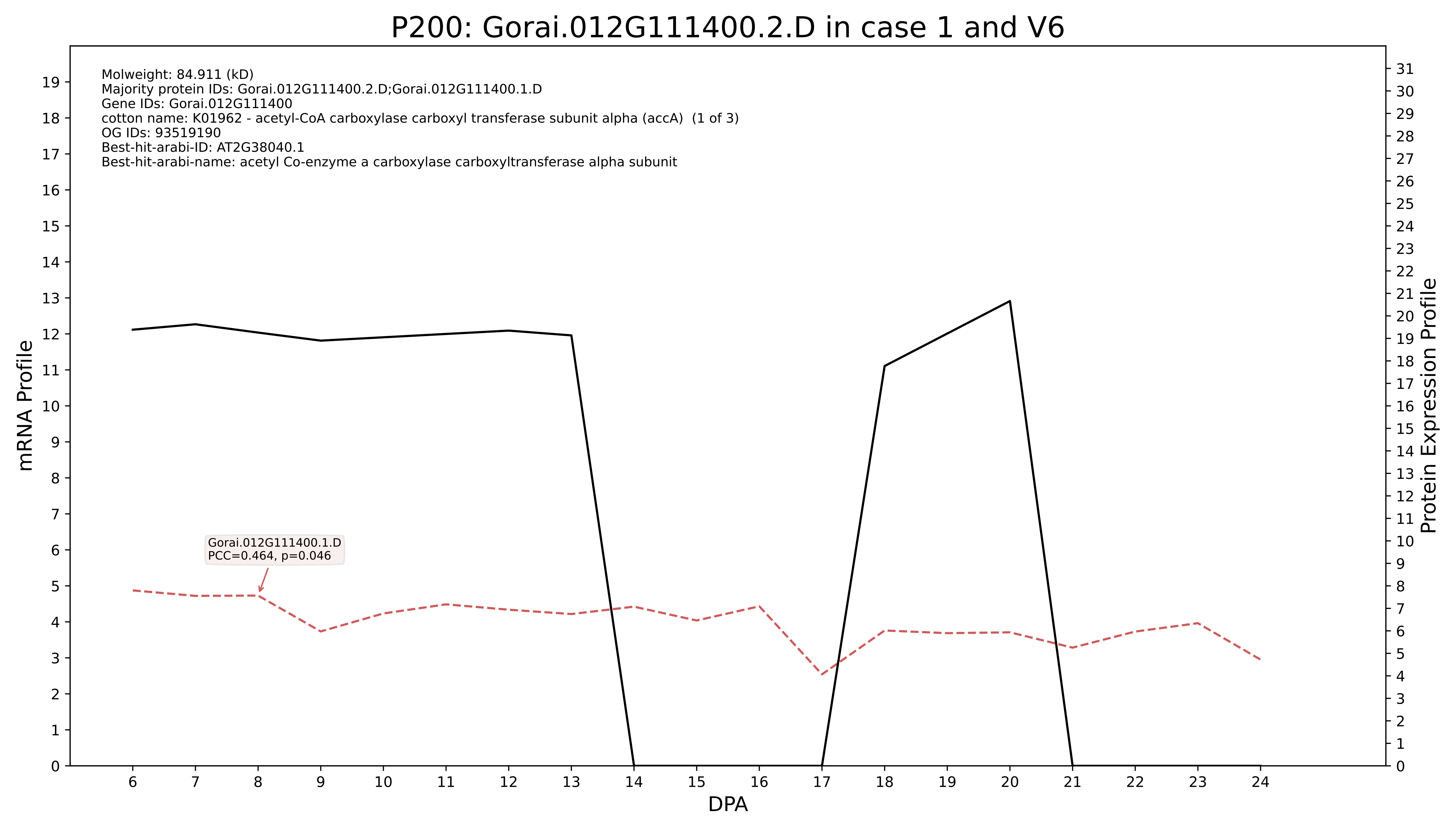
P200: Gorai.012G111400.2.D in case 1 and V6
Molweight: 84.911 (kD)
Majority protein IDs: Gorai.012G111400.2.D;Gorai.012G111400.1.D
Gene IDs: Gorai.012G111400
cotton name: K01962 - acetyl-CoA carboxylase carboxyl transferase subunit alpha (accA) (1 of 3)
OG IDs: 93519190
Best-hit-arabi-ID: AT2G38040.1
Best-hit-arabi-name: acetyl Co-enzyme a carboxylase carboxyltransferase alpha subunit
Gorai.012G111400.1.D
PCC=0.464, p=0.046
mRNA Profile
Protein Expression Profile
DPA

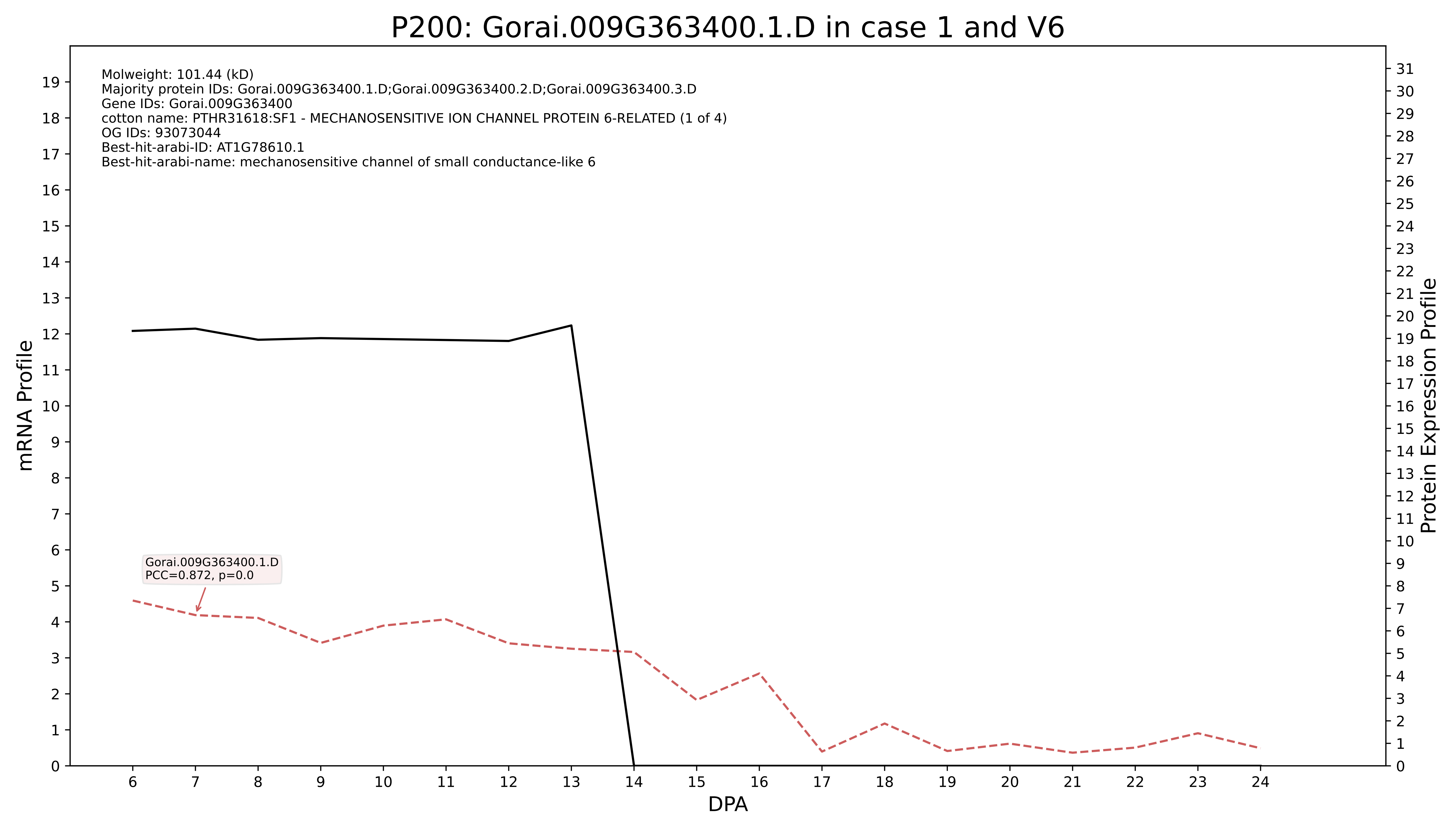
P200: Gorai.009G363400.1.D in case 1 and V6
Molweight: 101.44 (kD)
Majority protein IDs: Gorai.009G363400.1.D;Gorai.009G363400.2.D;Gorai.009G363400.3.D
Gene IDs: Gorai.009G363400
cotton name: PTHR31618:SF1 - MECHANOSENSITIVE ION CHANNEL PROTEIN 6-RELATED (1 of 4)
OG IDs: 93073044
Best-hit-arabi-ID: AT1G78610.1
Best-hit-arabi-name: mechanosensitive channel of small conductance-like 6
Gorai.009G363400.1.D
PCC=0.872, p=0.0
mRNA Profile
Protein Expression Profile
DPA

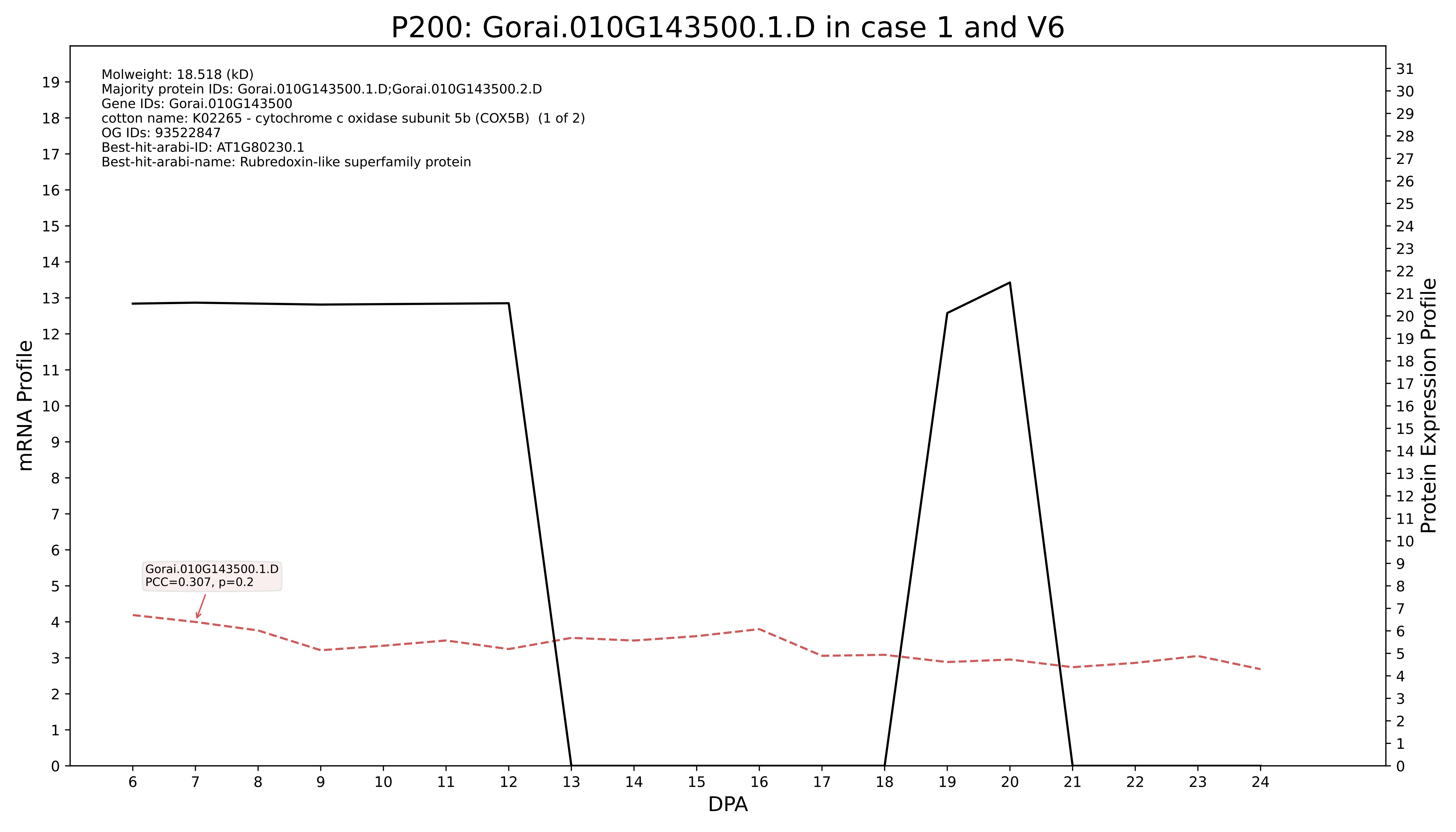
P200: Gorai.010G143500.1.D in case 1 and V6
Molweight: 18.518 (kD)
Majority protein IDs: Gorai.010G143500.1.D;Gorai.010G143500.2.D
Gene IDs: Gorai.010G143500
cotton name: K02265 - cytochrome c oxidase subunit 5b (COX5B) (1 of 2)
OG IDs: 93522847
Best-hit-arabi-ID: AT1G80230.1
Best-hit-arabi-name: Rubredoxin-like superfamily protein
Gorai.010G143500.1.D
PCC=0.307, p=0.2
mRNA Profile
Protein Expression Profile
DPA

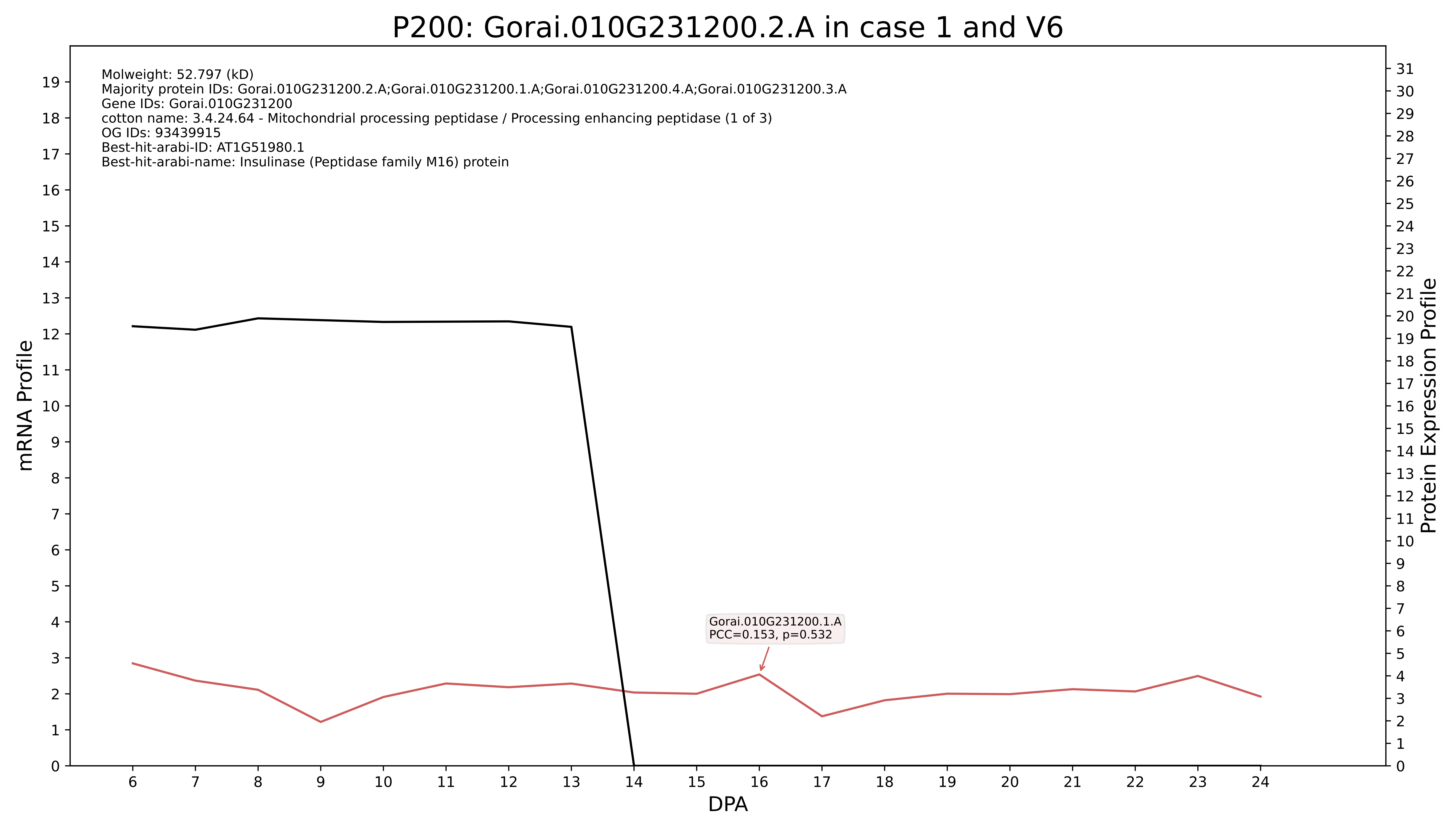
P200: Gorai.010G231200.2.A in case 1 and V6
Molweight: 52.797 (kD)
Majority protein IDs: Gorai.010G231200.2.A;Gorai.010G231200.1.A;Gorai.010G231200.4.A;Gorai.010G231200.3.A
Gene IDs: Gorai.010G231200
cotton name: 3.4.24.64 - Mitochondrial processing peptidase / Processing enhancing peptidase (1 of 3)
OG IDs: 93439915
Best-hit-arabi-ID: AT1G51980.1
Best-hit-arabi-name: Insulinase (Peptidase family M16) protein
Gorai.010G231200.1.A
PCC=0.153, p=0.532
mRNA Profile
Protein Expression Profile
DPA

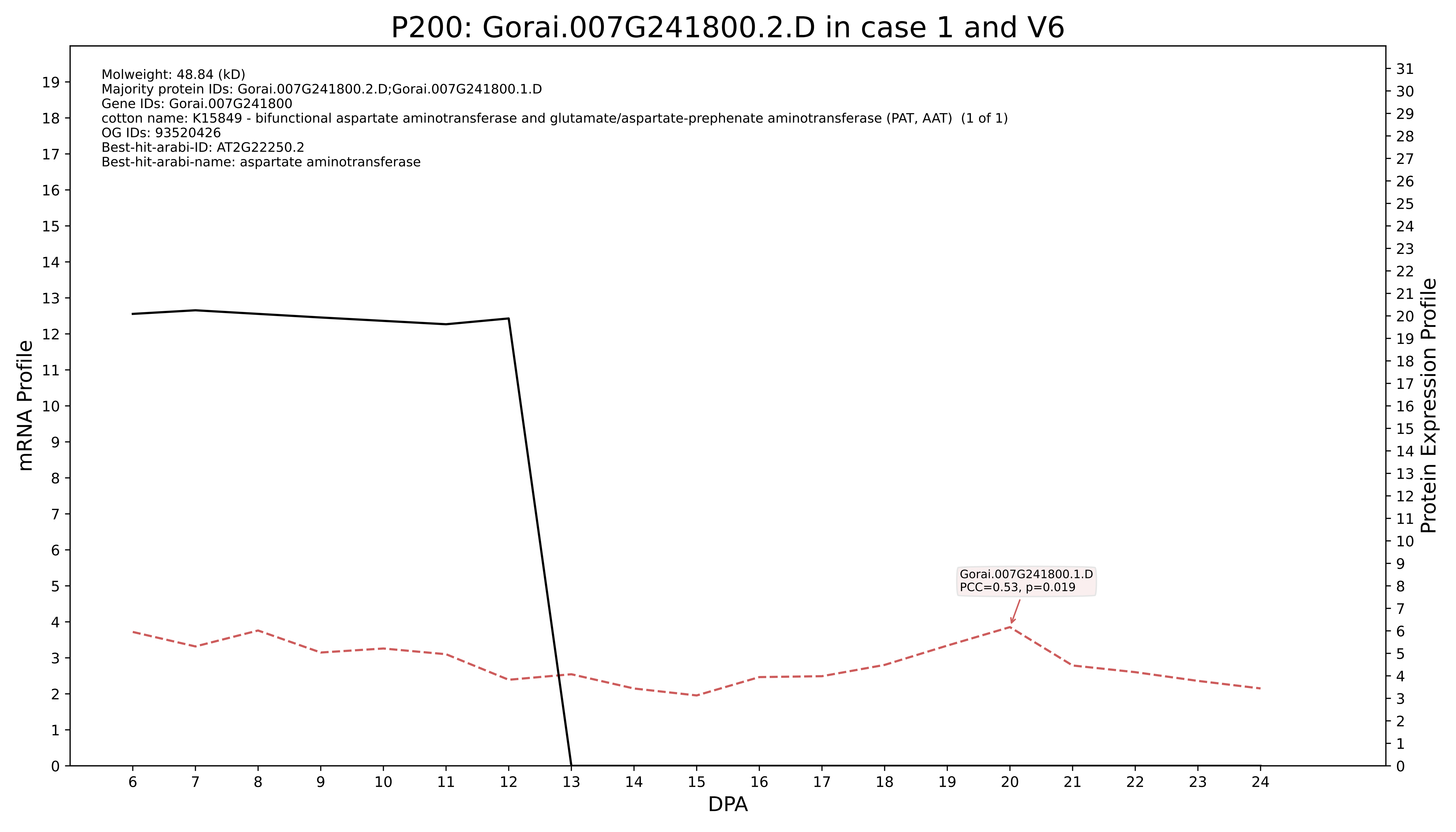
P200: Gorai.007G241800.2.D in case 1 and V6
Molweight: 48.84 (kD)
Majority protein IDs: Gorai.007G241800.2.D;Gorai.007G241800.1.D
Gene IDs: Gorai.007G241800
cotton name: K15849 - bifunctional aspartate aminotransferase and glutamate/aspartate-prephenate aminotransferase (PAT, AAT) (1 of 1)
OG IDs: 93520426
Best-hit-arabi-ID: AT2G22250.2
Best-hit-arabi-name: aspartate aminotransferase
Gorai.007G241800.1.D
PCC=0.53, p=0.019
mRNA Profile
Protein Expression Profile
DPA

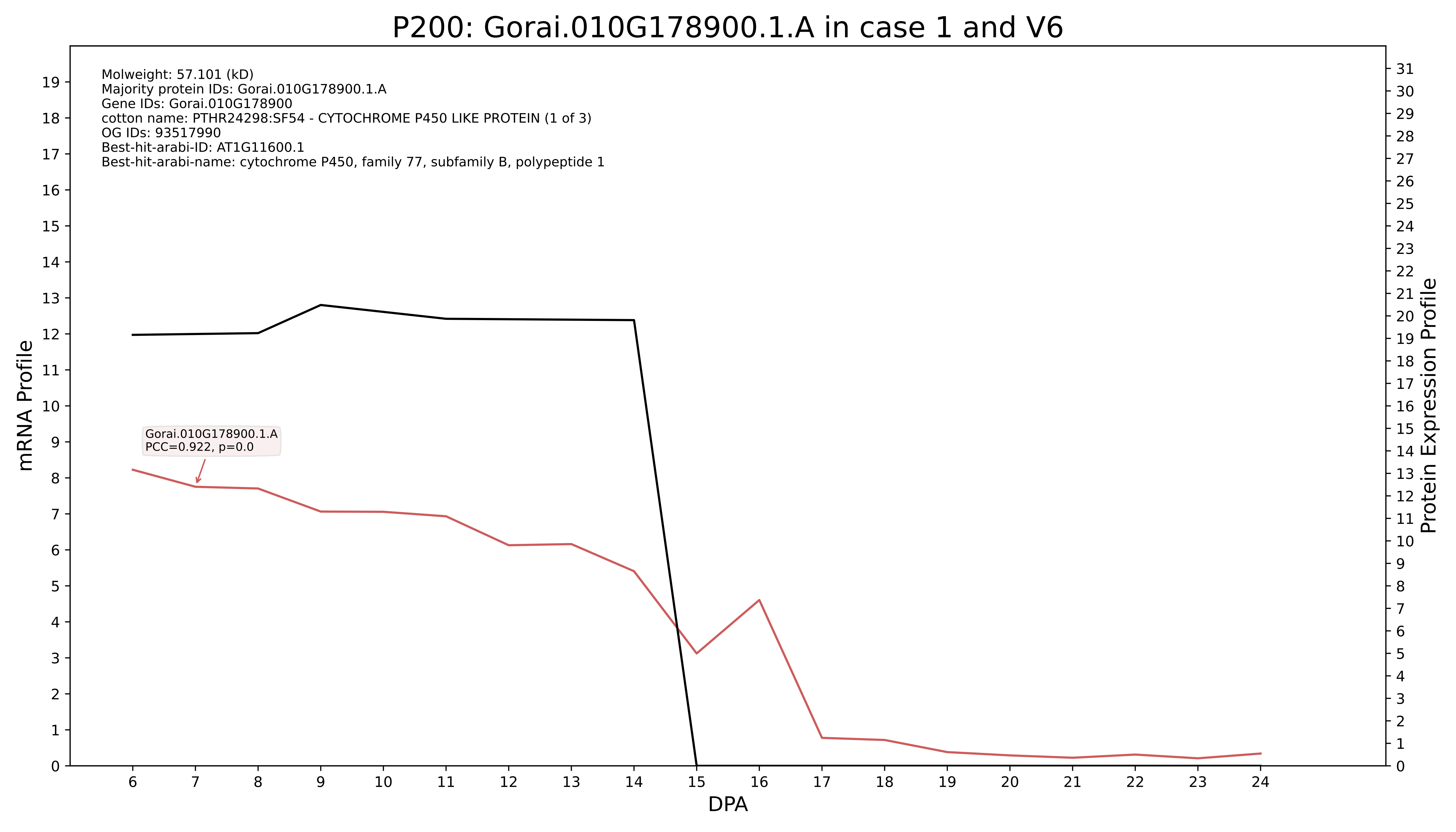
P200: Gorai.010G178900.1.A in case 1 and V6
Molweight: 57.101 (kD)
Majority protein IDs: Gorai.010G178900.1.A
Gene IDs: Gorai.010G178900
cotton name: PTHR24298:SF54 - CYTOCHROME P450 LIKE PROTEIN (1 of 3)
OG IDs: 93517990
Best-hit-arabi-ID: AT1G11600.1
Best-hit-arabi-name: cytochrome P450, family 77, subfamily B, polypeptide 1
Gorai.010G178900.1.A
PCC=0.922, p=0.0
mRNA Profile
Protein Expression Profile
DPA

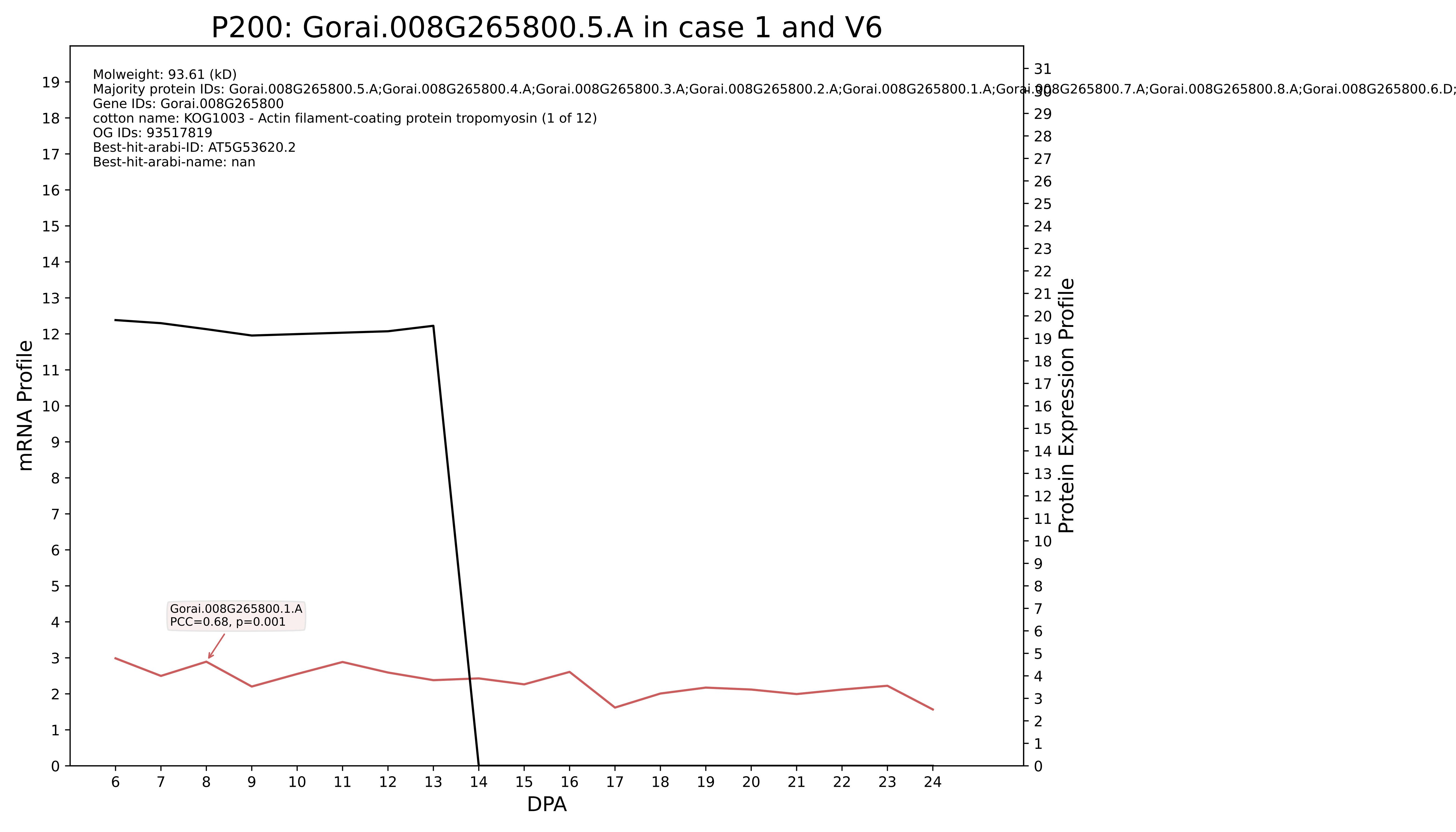

P200: Gorai.008G265800.5.A in case 1 and V6
Molweight: 93.61 (kD)
Majority protein IDs: Gorai.008G265800.5.A;Gorai.008G265800.4.A;Gorai.008G265800.3.A;Gorai.008G265800.2.A;Gorai.008G265800.1.A;Gorai.008G265800.7.A;Gorai.008G265800.8.A;Gorai.008G265800.6.D;
Gene IDs: Gorai.008G265800
cotton name: KOG1003 - Actin filament-coating protein tropomyosin (1 of 12)
OG IDs: 93517819
Best-hit-arabi-ID: AT5G53620.2
Best-hit-arabi-name: nan
Gorai.008G265800.1.A
PCC=0.68, p=0.001
mRNA Profile
Protein Expression Profile
DPA

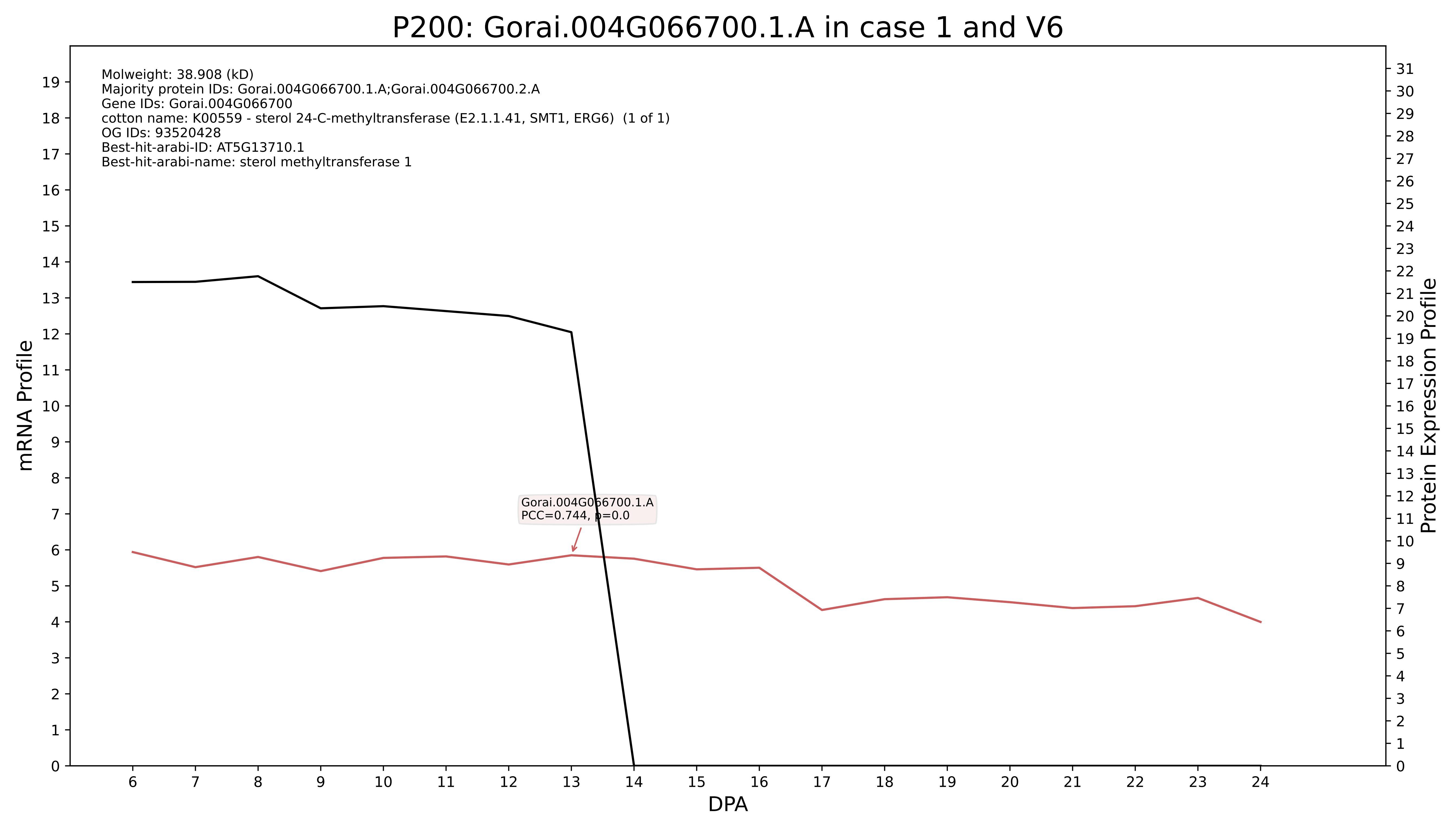
P200: Gorai.004G066700.1.A in case 1 and V6
Molweight: 38.908 (kD)
Majority protein IDs: Gorai.004G066700.1.A;Gorai.004G066700.2.A
Gene IDs: Gorai.004G066700
cotton name: K00559 - sterol 24-C-methyltransferase (E2.1.1.41, SMT1, ERG6) (1 of 1)
OG IDs: 93520428
Best-hit-arabi-ID: AT5G13710.1
Best-hit-arabi-name: sterol methyltransferase 1
Gorai.004G066700.1.A
PCC=0.744, p=0.0
mRNA Profile
Protein Expression Profile
DPA

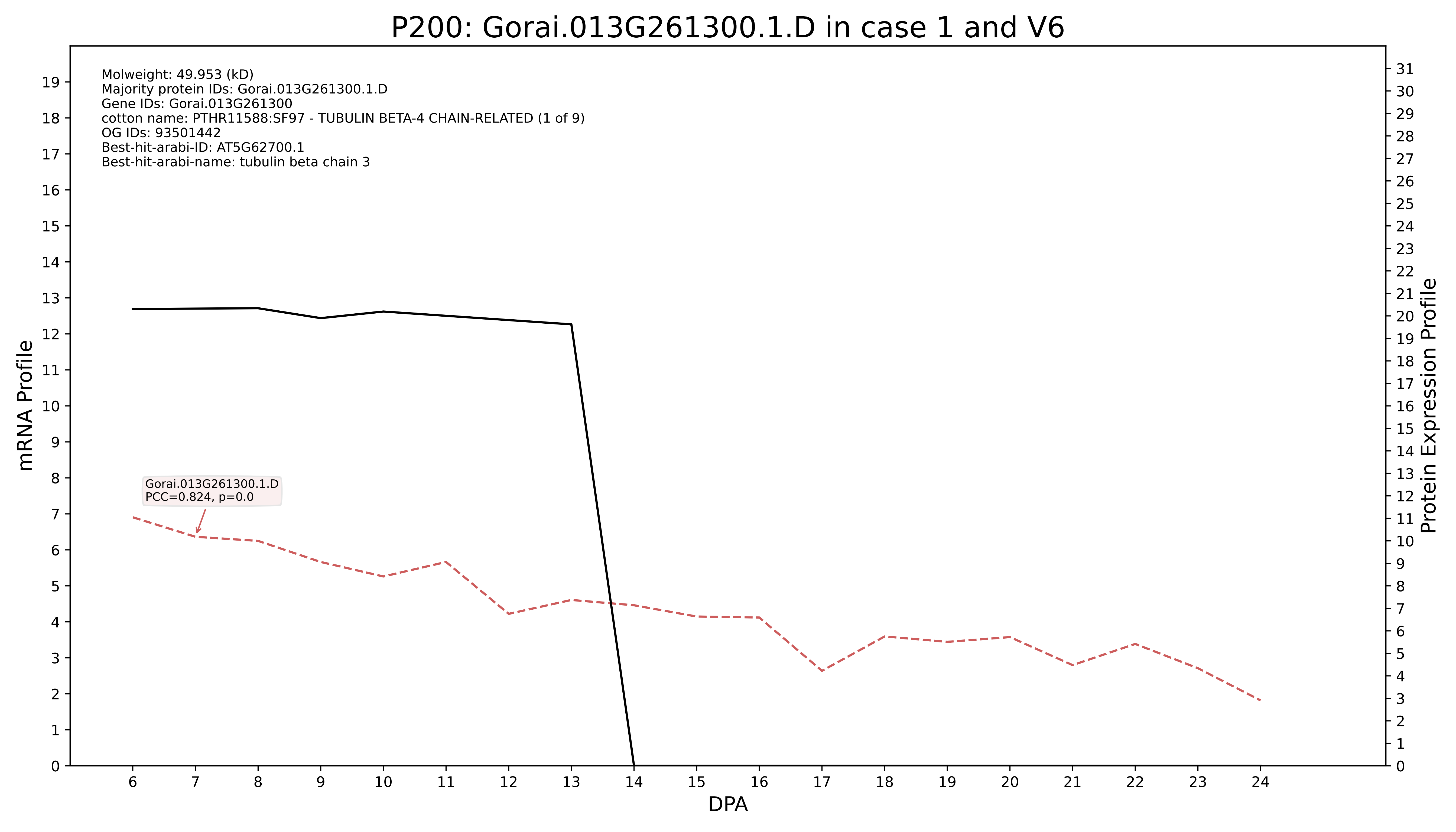

P200: Gorai.013G261300.1.D in case 1 and V6
Molweight: 49.953 (kD)
Majority protein IDs: Gorai.013G261300.1.D
Gene IDs: Gorai.013G261300
cotton name: PTHR11588:SF97 - TUBULIN BETA-4 CHAIN-RELATED (1 of 9)
OG IDs: 93501442
Best-hit-arabi-ID: AT5G62700.1
Best-hit-arabi-name: tubulin beta chain 3
Gorai.013G261300.1.D
PCC=0.824, p=0.0
mRNA Profile
Protein Expression Profile
DPA

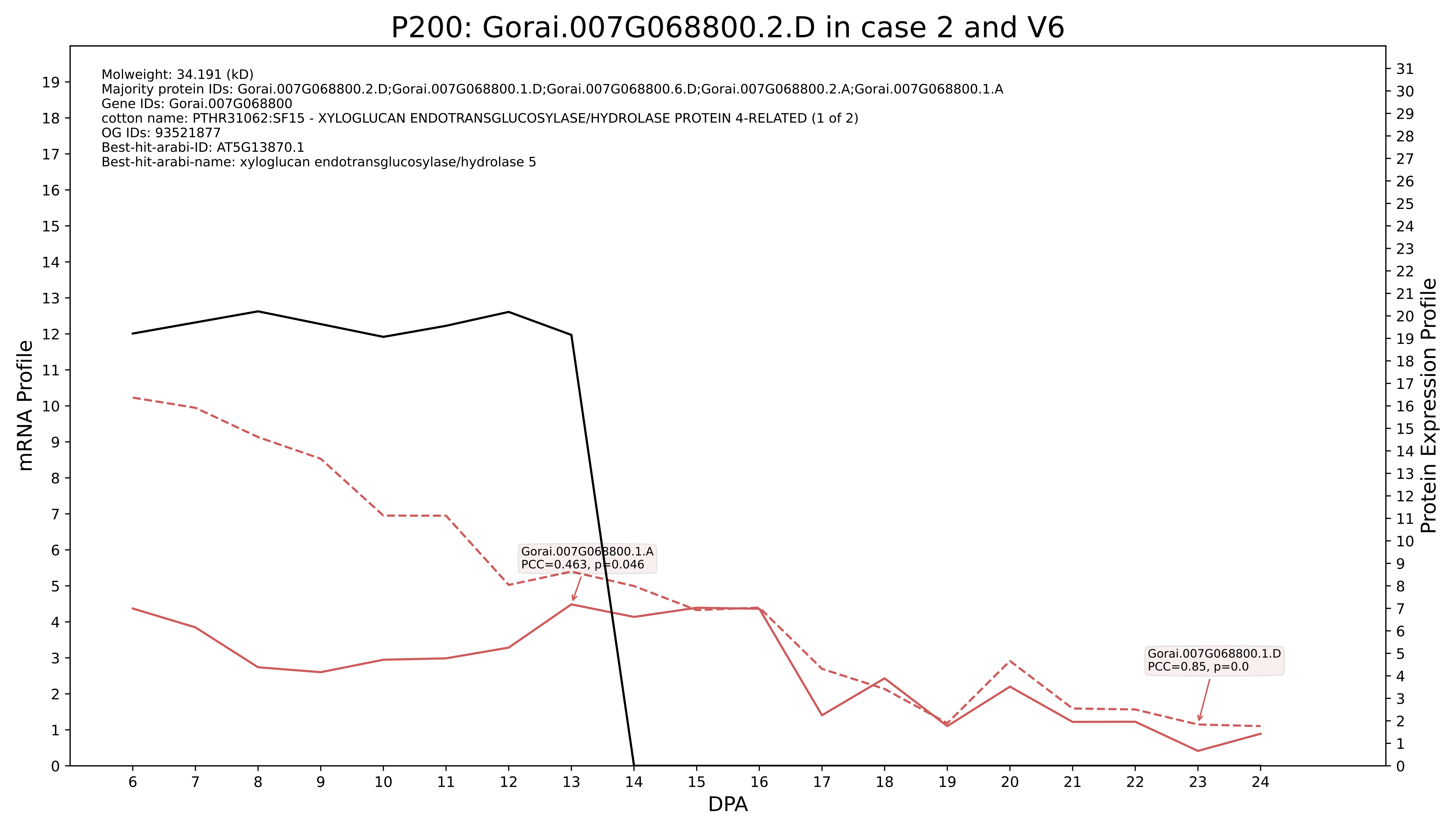
P200: Gorai.007G068800.2.D in case 2 and V6
Molweight: 34.191 (kD)
Majority protein IDs: Gorai.007G068800.2.D;Gorai.007G068800.1.D;Gorai.007G068800.6.D;Gorai.007G068800.2.A;Gorai.007G068800.1.A
Gene IDs: Gorai.007G068800
cotton name: PTHR31062:SF15 - XYLOGLUCAN ENDOTRANSGLUCOSYLASE/HYDROLASE PROTEIN 4-RELATED (1 of 2)
OG IDs: 93521877
Best-hit-arabi-ID: AT5G13870.1
Best-hit-arabi-name: xyloglucan endotransglucosylase/hydrolase 5
Gorai.007G068800.1.A
PCC=0.463, p=0.046
Gorai.007G068800.1.D
PCC=0.85, p=0.0
mRNA Profile
Protein Expression Profile
DPA

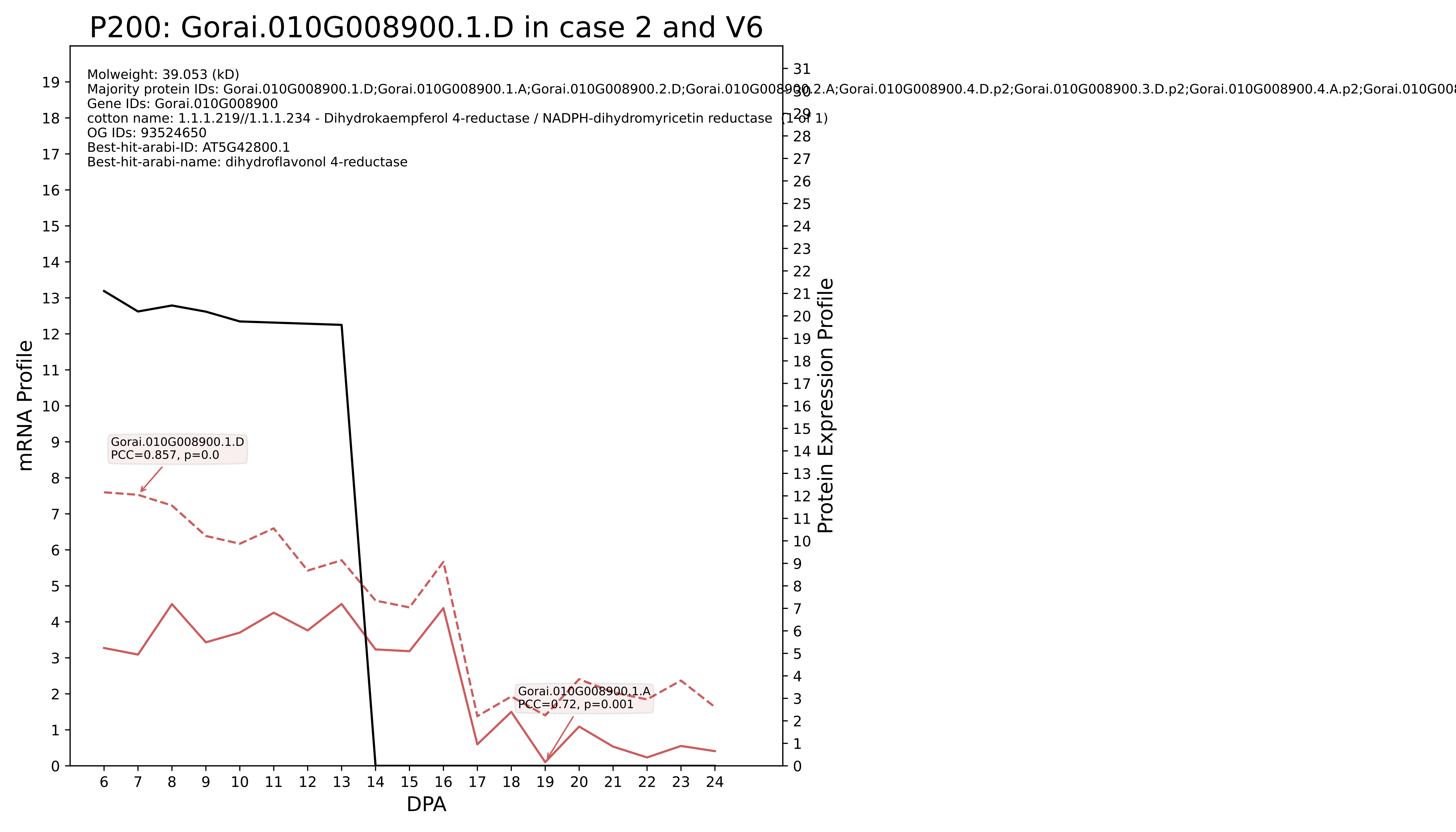
P200: Gorai.010G008900.1.D in case 2 and V6
Molweight: 39.053 (kD)
Majority protein IDs: Gorai.010G008900.1.D;Gorai.010G008900.1.A;Gorai.010G008900.2.D;Gorai.010G008900.2.A;Gorai.010G008900.4.D.p2;Gorai.010G008900.3.D.p2;Gorai.010G008900.4.A.p2;Gorai.010G008
Gene IDs: Gorai.010G008900
cotton name: 1.1.1.219//1.1.1.234 - Dihydrokaempferol 4-reductase / NADPH-dihydromyricetin reductase (1 of 1)
OG IDs: 93524650
Best-hit-arabi-ID: AT5G42800.1
Best-hit-arabi-name: dihydroflavonol 4-reductase
Gorai.010G008900.1.D
PCC=0.857, p=0.0
Gorai.010G008900.1.A
PCC=0.72, p=0.001
mRNA Profile
Protein Expression Profile
DPA

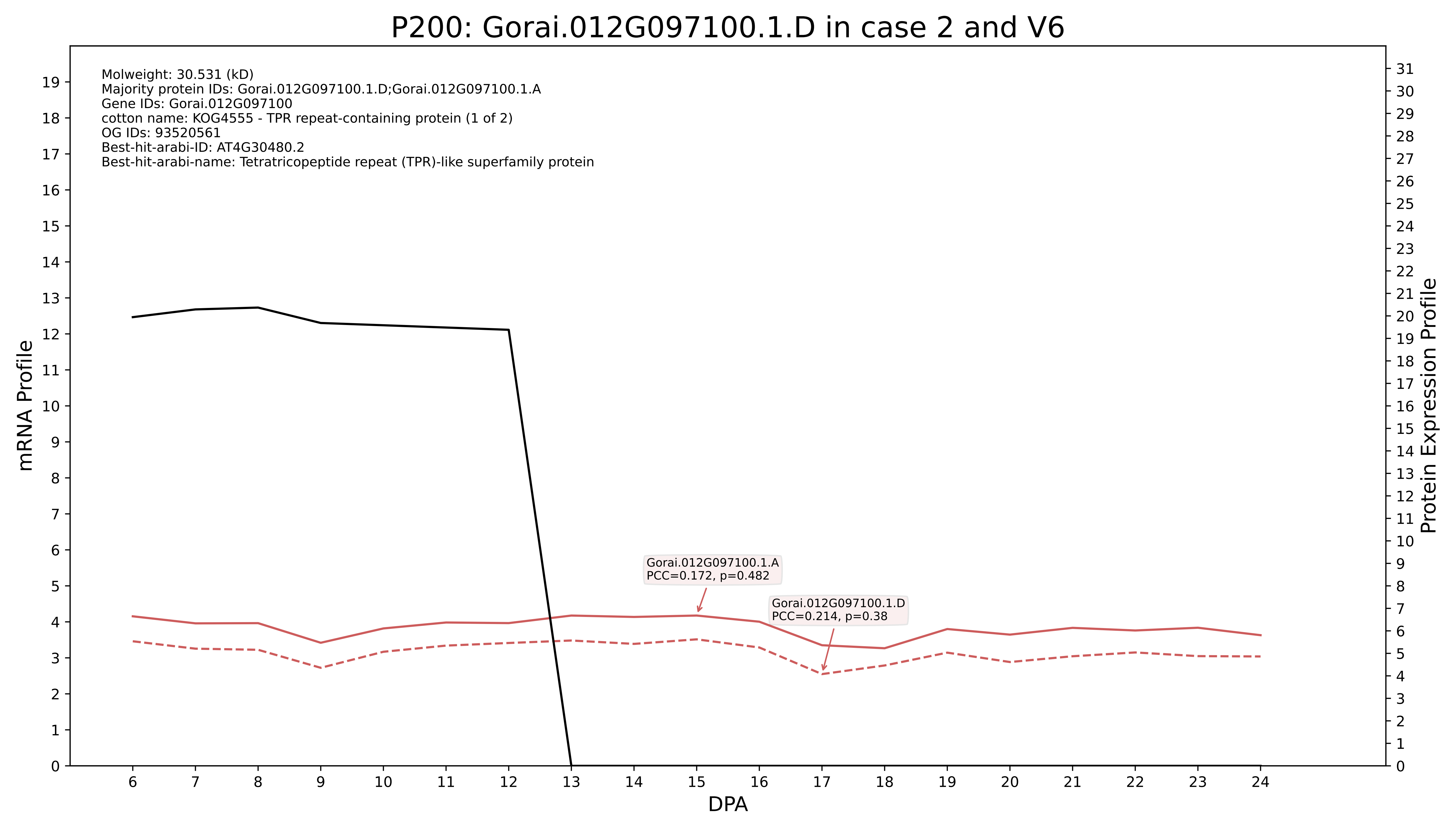

P200: Gorai.012G097100.1.D in case 2 and V6
Molweight: 30.531 (kD)
Majority protein IDs: Gorai.012G097100.1.D;Gorai.012G097100.1.A
Gene IDs: Gorai.012G097100
cotton name: KOG4555 - TPR repeat-containing protein (1 of 2)
OG IDs: 93520561
Best-hit-arabi-ID: AT4G30480.2
Best-hit-arabi-name: Tetratricopeptide repeat (TPR)-like superfamily protein
Gorai.012G097100.1.A
PCC=0.172, p=0.482
Gorai.012G097100.1.D
PCC=0.214, p=0.38
mRNA Profile
Protein Expression Profile
DPA

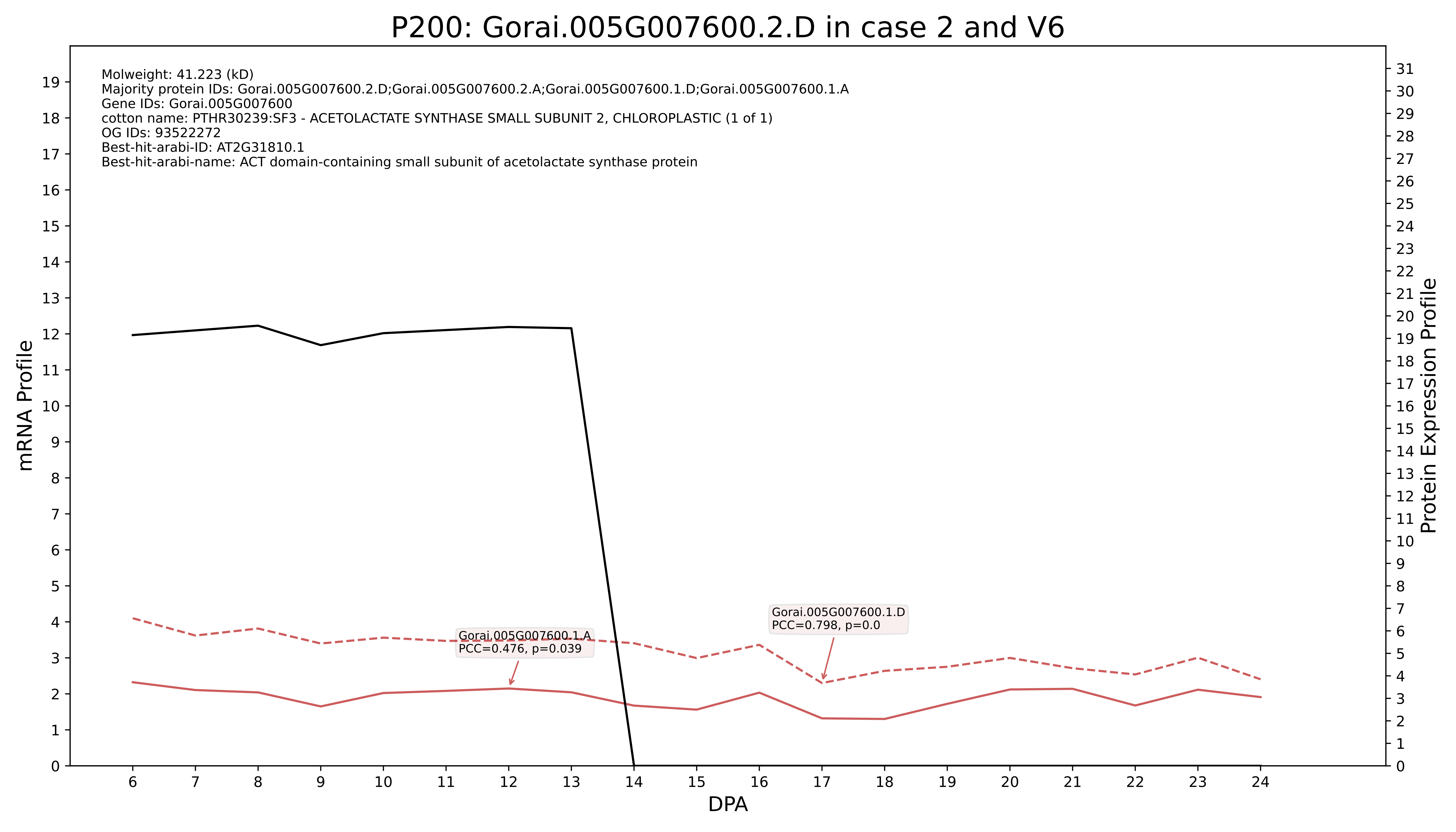
P200: Gorai.005G007600.2.D in case 2 and V6
Molweight: 41.223 (kD)
Majority protein IDs: Gorai.005G007600.2.D;Gorai.005G007600.2.A;Gorai.005G007600.1.D;Gorai.005G007600.1.A
Gene IDs: Gorai.005G007600
cotton name: PTHR30239:SF3 - ACETOLACTATE SYNTHASE SMALL SUBUNIT 2, CHLOROPLASTIC (1 of 1)
OG IDs: 93522272
Best-hit-arabi-ID: AT2G31810.1
Best-hit-arabi-name: ACT domain-containing small subunit of acetolactate synthase protein
Gorai.005G007600.1.A
PCC=0.476, p=0.039
Gorai.005G007600.1.D
PCC=0.798, p=0.0
mRNA Profile
Protein Expression Profile
DPA

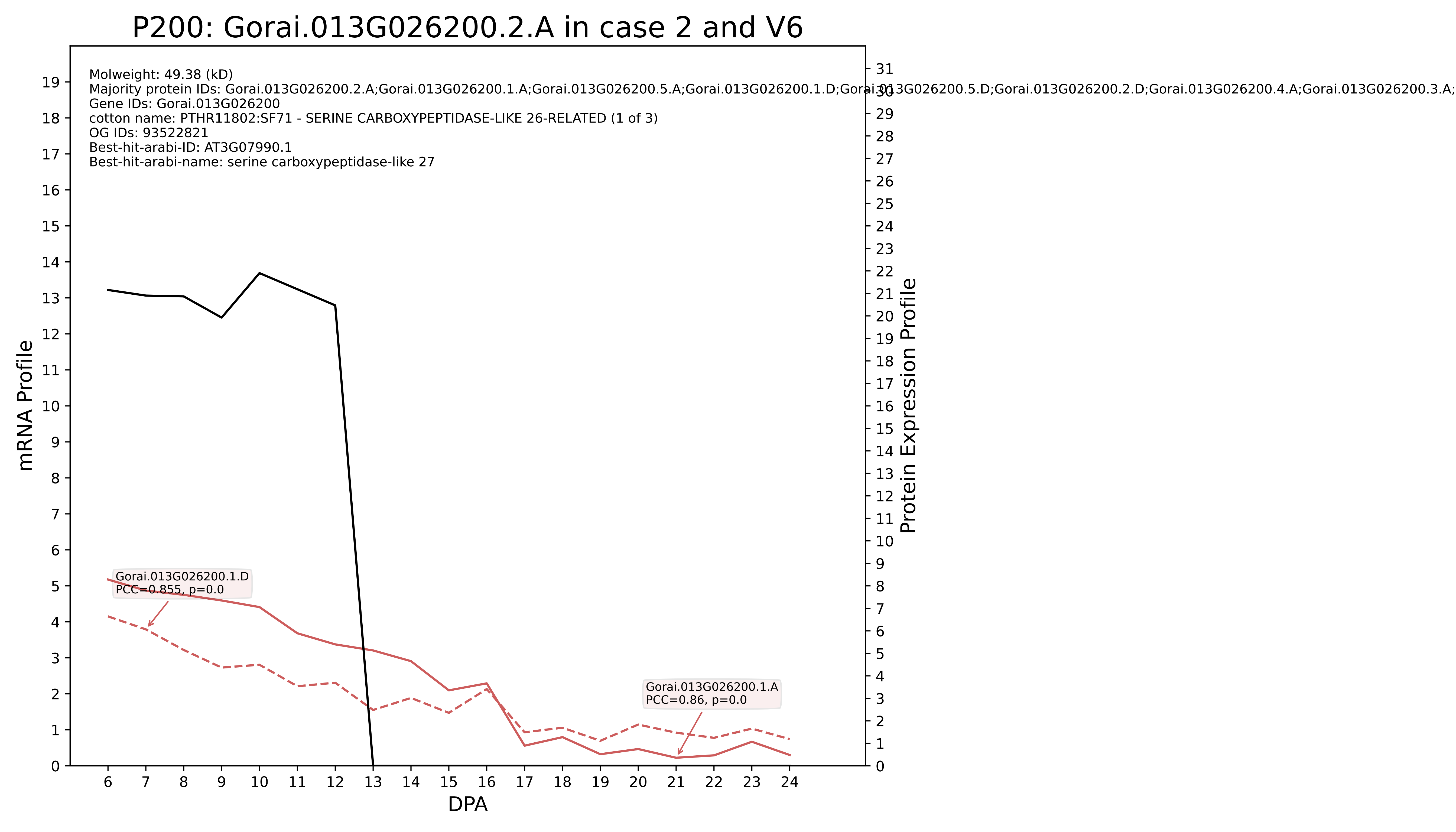
P200: Gorai.013G026200.2.A in case 2 and V6
Molweight: 49.38 (kD)
Majority protein IDs: Gorai.013G026200.2.A;Gorai.013G026200.1.A;Gorai.013G026200.5.A;Gorai.013G026200.1.D;Gorai.013G026200.5.D;Gorai.013G026200.2.D;Gorai.013G026200.4.A;Gorai.013G026200.3.A;
Gene IDs: Gorai.013G026200
cotton name: PTHR11802:SF71 - SERINE CARBOXYPEPTIDASE-LIKE 26-RELATED (1 of 3)
OG IDs: 93522821
Best-hit-arabi-ID: AT3G07990.1
Best-hit-arabi-name: serine carboxypeptidase-like 27
Gorai.013G026200.1.D
PCC=0.855, p=0.0
Gorai.013G026200.1.A
PCC=0.86, p=0.0
mRNA Profile
Protein Expression Profile
DPA

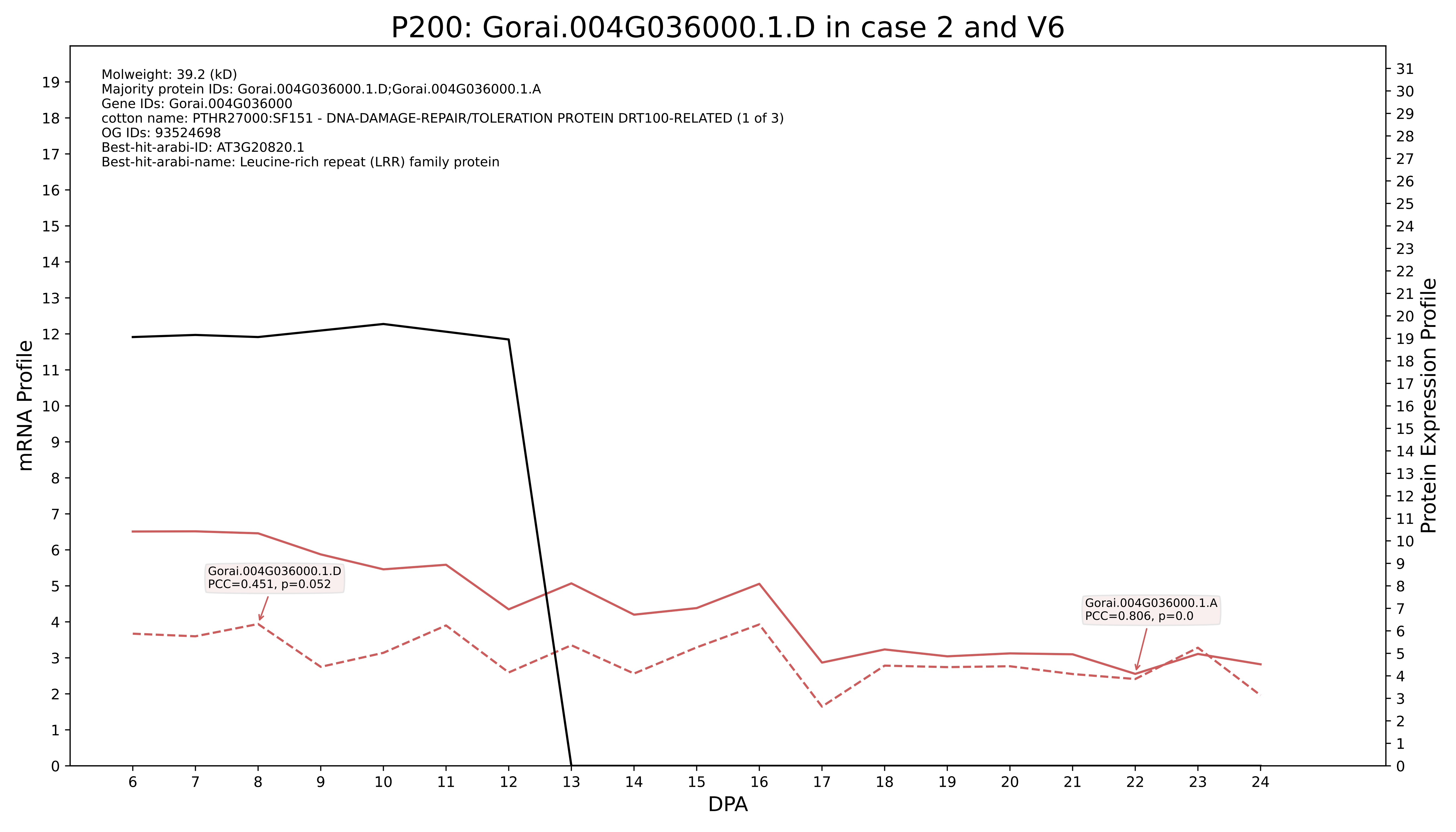
P200: Gorai.004G036000.1.D in case 2 and V6
Molweight: 39.2 (kD)
Majority protein IDs: Gorai.004G036000.1.D;Gorai.004G036000.1.A
Gene IDs: Gorai.004G036000
cotton name: PTHR27000:SF151 - DNA-DAMAGE-REPAIR/TOLERATION PROTEIN DRT100-RELATED (1 of 3)
OG IDs: 93524698
Best-hit-arabi-ID: AT3G20820.1
Best-hit-arabi-name: Leucine-rich repeat (LRR) family protein
Gorai.004G036000.1.D
PCC=0.451, p=0.052
Gorai.004G036000.1.A
PCC=0.806, p=0.0
mRNA Profile
Protein Expression Profile
DPA

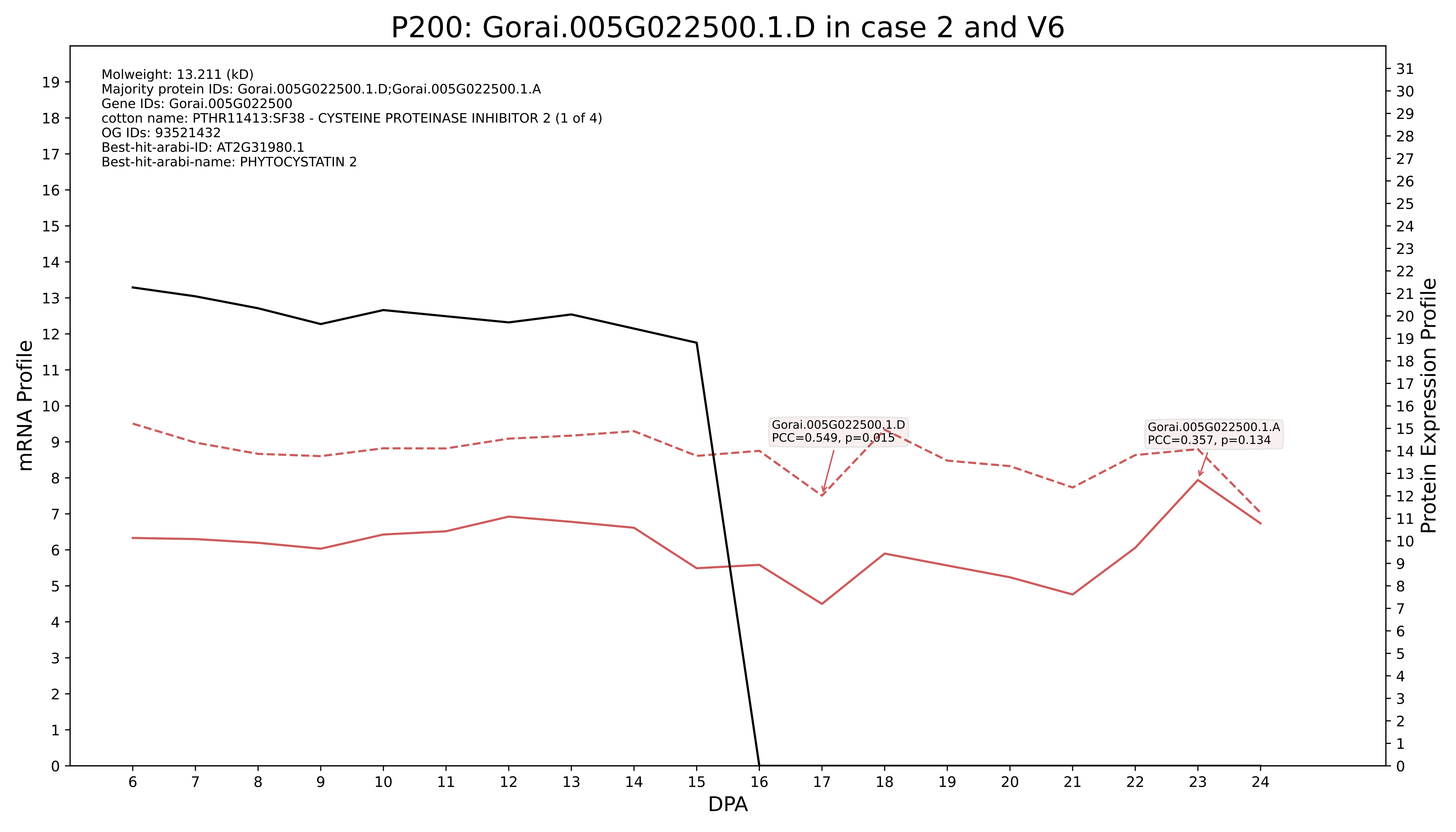
P200: Gorai.005G022500.1.D in case 2 and V6
Molweight: 13.211 (kD)
Majority protein IDs: Gorai.005G022500.1.D;Gorai.005G022500.1.A
Gene IDs: Gorai.005G022500
cotton name: PTHR11413:SF38 - CYSTEINE PROTEINASE INHIBITOR 2 (1 of 4)
OG IDs: 93521432
Best-hit-arabi-ID: AT2G31980.1
Best-hit-arabi-name: PHYTOCYSTATIN 2
Gorai.005G022500.1.D
PCC=0.549, p=0.015
Gorai.005G022500.1.A
PCC=0.357, p=0.134
mRNA Profile
Protein Expression Profile
DPA
0
1
2
3
4
5
6
7
8
9
10
11
12
13
14
15
16
17
18
19
20
21
22
23
24
25
26
27
28
29
30
31

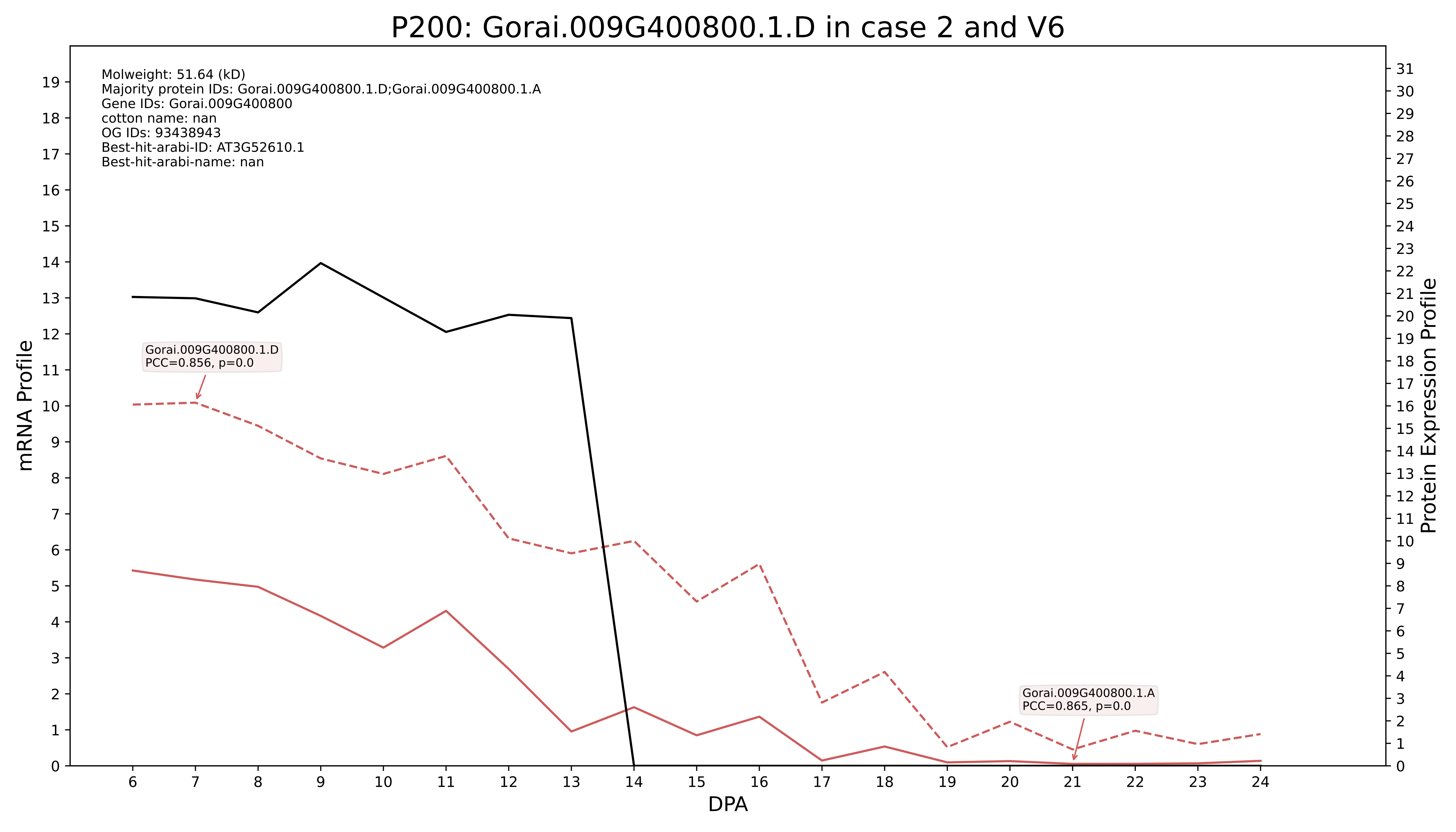
P200: Gorai.009G400800.1.D in case 2 and V6
Molweight: 51.64 (kD)
Majority protein IDs: Gorai.009G400800.1.D;Gorai.009G400800.1.A
Gene IDs: Gorai.009G400800
cotton name: nan
OG IDs: 93438943
Best-hit-arabi-ID: AT3G52610.1
Best-hit-arabi-name: nan
Gorai.009G400800.1.D
PCC=0.856, p=0.0
Gorai.009G400800.1.A
PCC=0.865, p=0.0
mRNA Profile
Protein Expression Profile
DPA

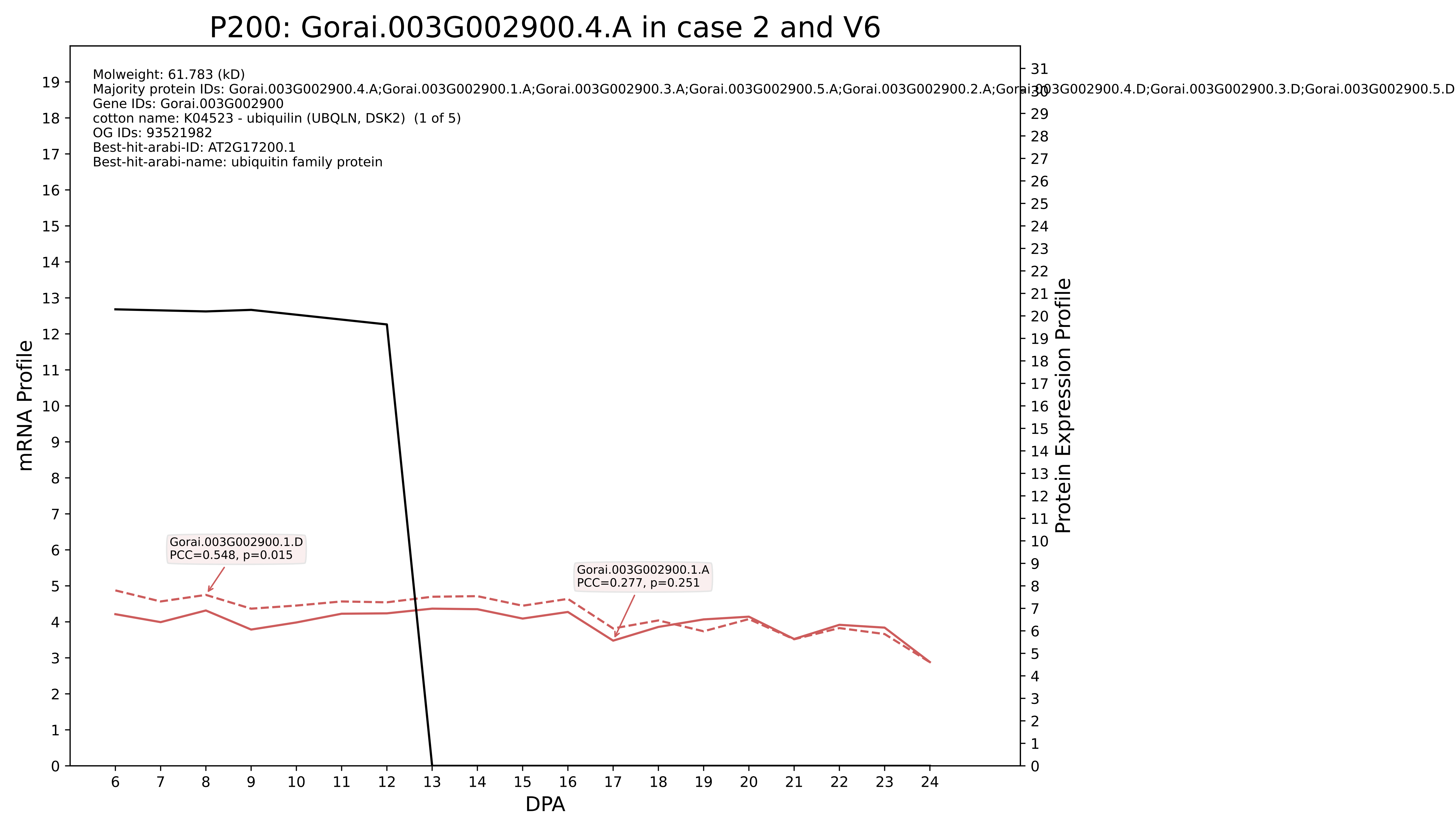
P200: Gorai.003G002900.4.A in case 2 and V6
Molweight: 61.783 (kD)
Majority protein IDs: Gorai.003G002900.4.A;Gorai.003G002900.1.A;Gorai.003G002900.3.A;Gorai.003G002900.5.A;Gorai.003G002900.2.A;Gorai.003G002900.4.D;Gorai.003G002900.3.D;Gorai.003G002900.5.D
Gene IDs: Gorai.003G002900
cotton name: K04523 - ubiquilin (UBQLN, DSK2) (1 of 5)
OG IDs: 93521982
Best-hit-arabi-ID: AT2G17200.1
Best-hit-arabi-name: ubiquitin family protein
Gorai.003G002900.1.D
PCC=0.548, p=0.015
Gorai.003G002900.1.A
PCC=0.277, p=0.251
mRNA Profile
Protein Expression Profile
DPA

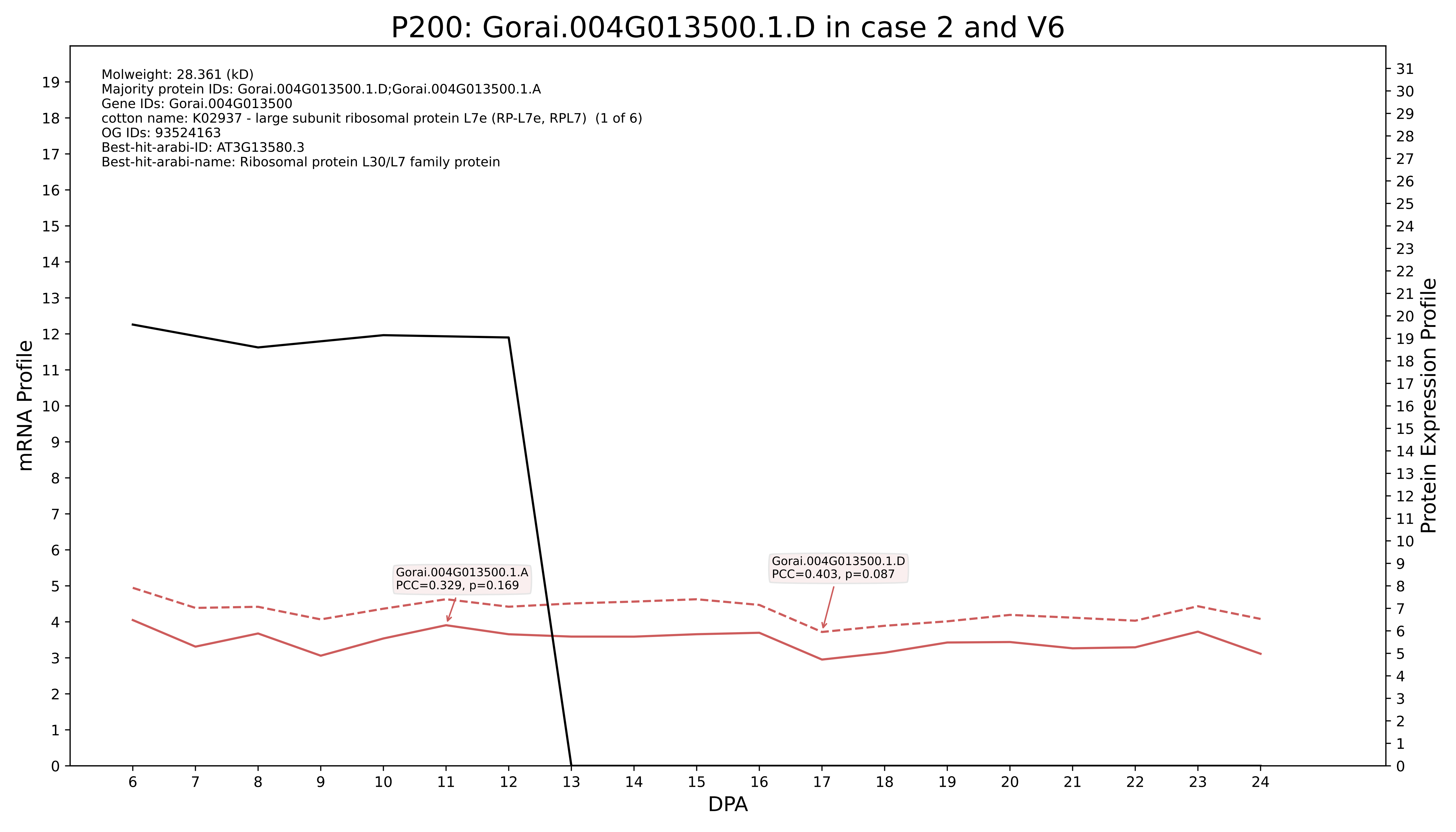

P200: Gorai.004G013500.1.D in case 2 and V6
Molweight: 28.361 (kD)
Majority protein IDs: Gorai.004G013500.1.D;Gorai.004G013500.1.A
Gene IDs: Gorai.004G013500
cotton name: K02937 - large subunit ribosomal protein L7e (RP-L7e, RPL7) (1 of 6)
OG IDs: 93524163
Best-hit-arabi-ID: AT3G13580.3
Best-hit-arabi-name: Ribosomal protein L30/L7 family protein
Gorai.004G013500.1.A
PCC=0.329, p=0.169
Gorai.004G013500.1.D
PCC=0.403, p=0.087
mRNA Profile
Protein Expression Profile
DPA

P200: Gorai.004G271900.1.D in case 2 and V6

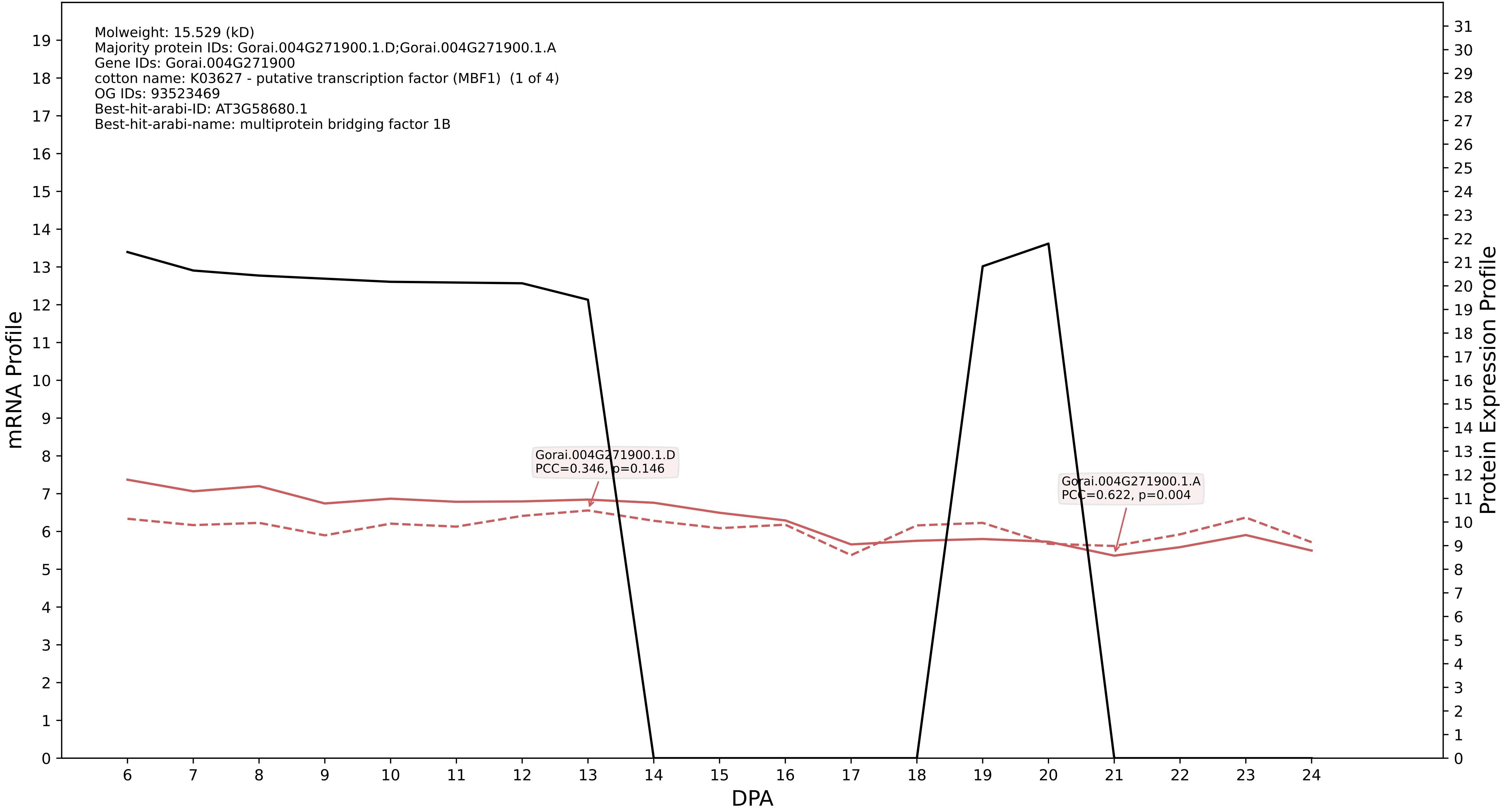

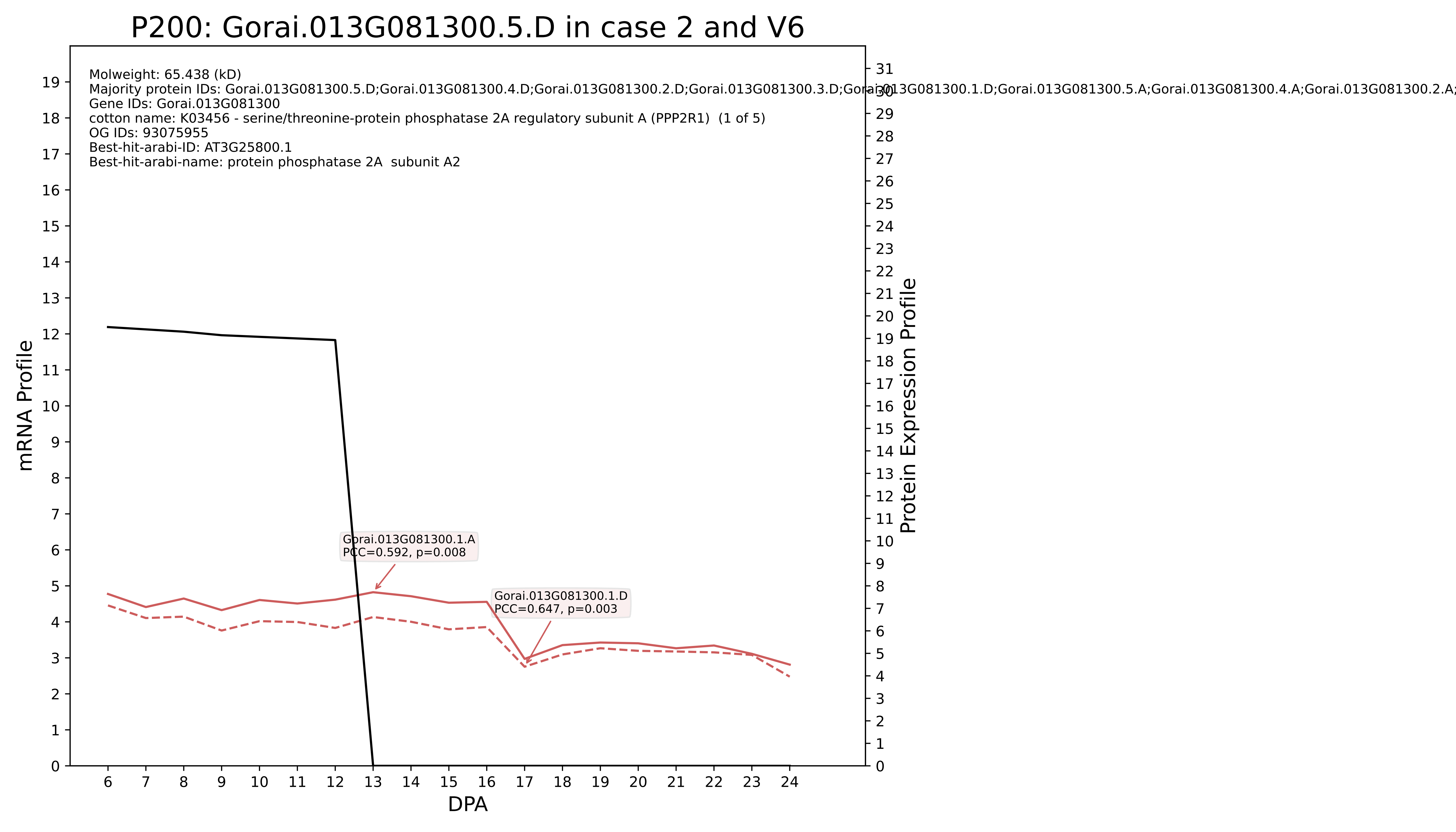
P200: Gorai.013G081300.5.D in case 2 and V6
Molweight: 65.438 (kD)
Majority protein IDs: Gorai.013G081300.5.D;Gorai.013G081300.4.D;Gorai.013G081300.2.D;Gorai.013G081300.3.D;Gorai.013G081300.1.D;Gorai.013G081300.5.A;Gorai.013G081300.4.A;Gorai.013G081300.2.A;
Gene IDs: Gorai.013G081300
cotton name: K03456 - serine/threonine-protein phosphatase 2A regulatory subunit A (PPP2R1) (1 of 5)
OG IDs: 93075955
Best-hit-arabi-ID: AT3G25800.1
Best-hit-arabi-name: protein phosphatase 2A subunit A2
Gorai.013G081300.1.A
PCC=0.592, p=0.008
Gorai.013G081300.1.D
PCC=0.647, p=0.003
mRNA Profile
Protein Expression Profile
DPA

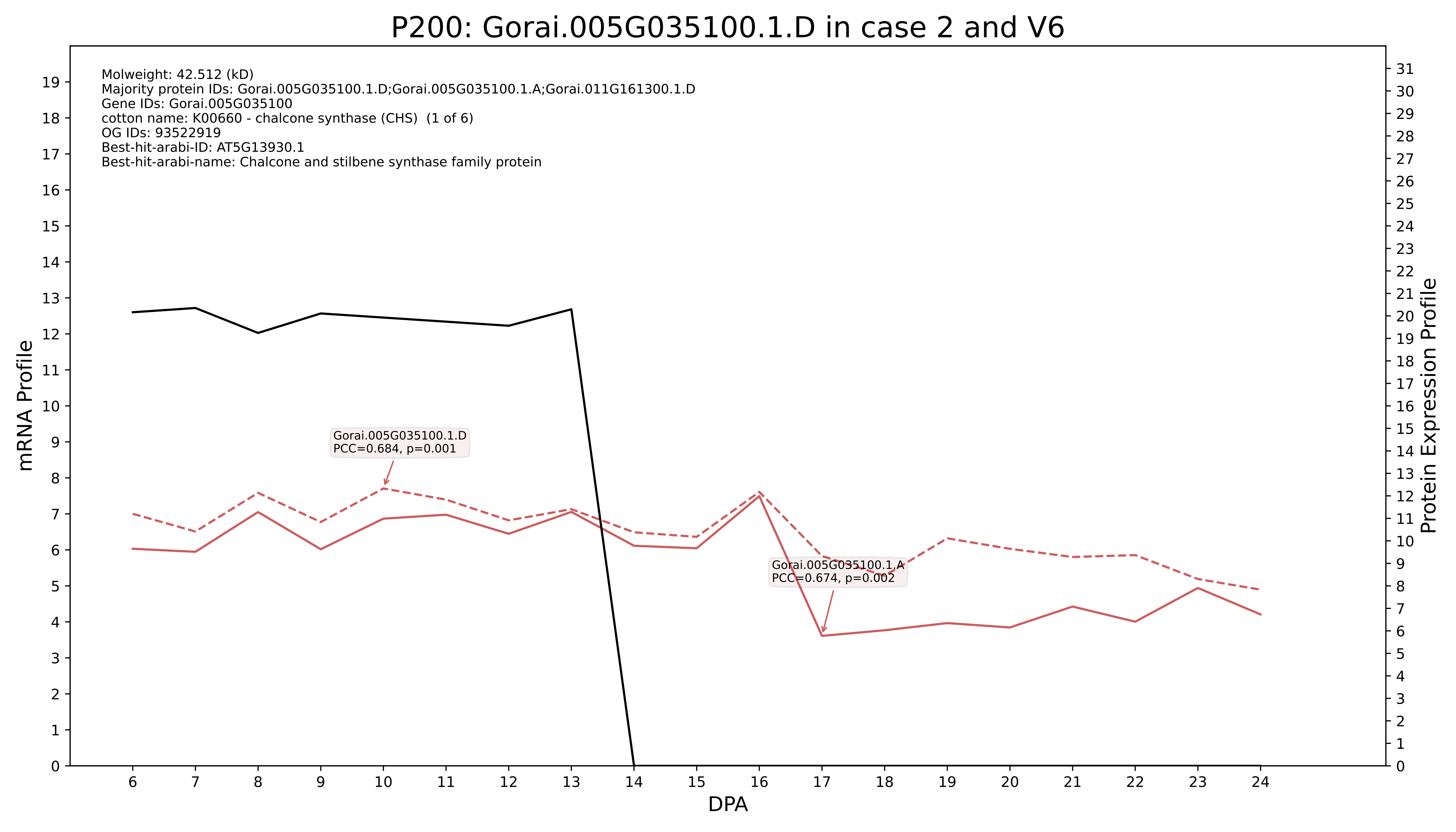
P200: Gorai.005G035100.1.D in case 2 and V6
Molweight: 42.512 (kD)
Majority protein IDs: Gorai.005G035100.1.D;Gorai.005G035100.1.A;Gorai.011G161300.1.D
Gene IDs: Gorai.005G035100
cotton name: K00660 - chalcone synthase (CHS) (1 of 6)
OG IDs: 93522919
Best-hit-arabi-ID: AT5G13930.1
Best-hit-arabi-name: Chalcone and stilbene synthase family protein
Gorai.005G035100.1.D
PCC=0.684, p=0.001
Gorai.005G035100.1.A
PCC=0.674, p=0.002
mRNA Profile
Protein Expression Profile
DPA

200: Gorai.002G103600.2.D in case 2 and V6

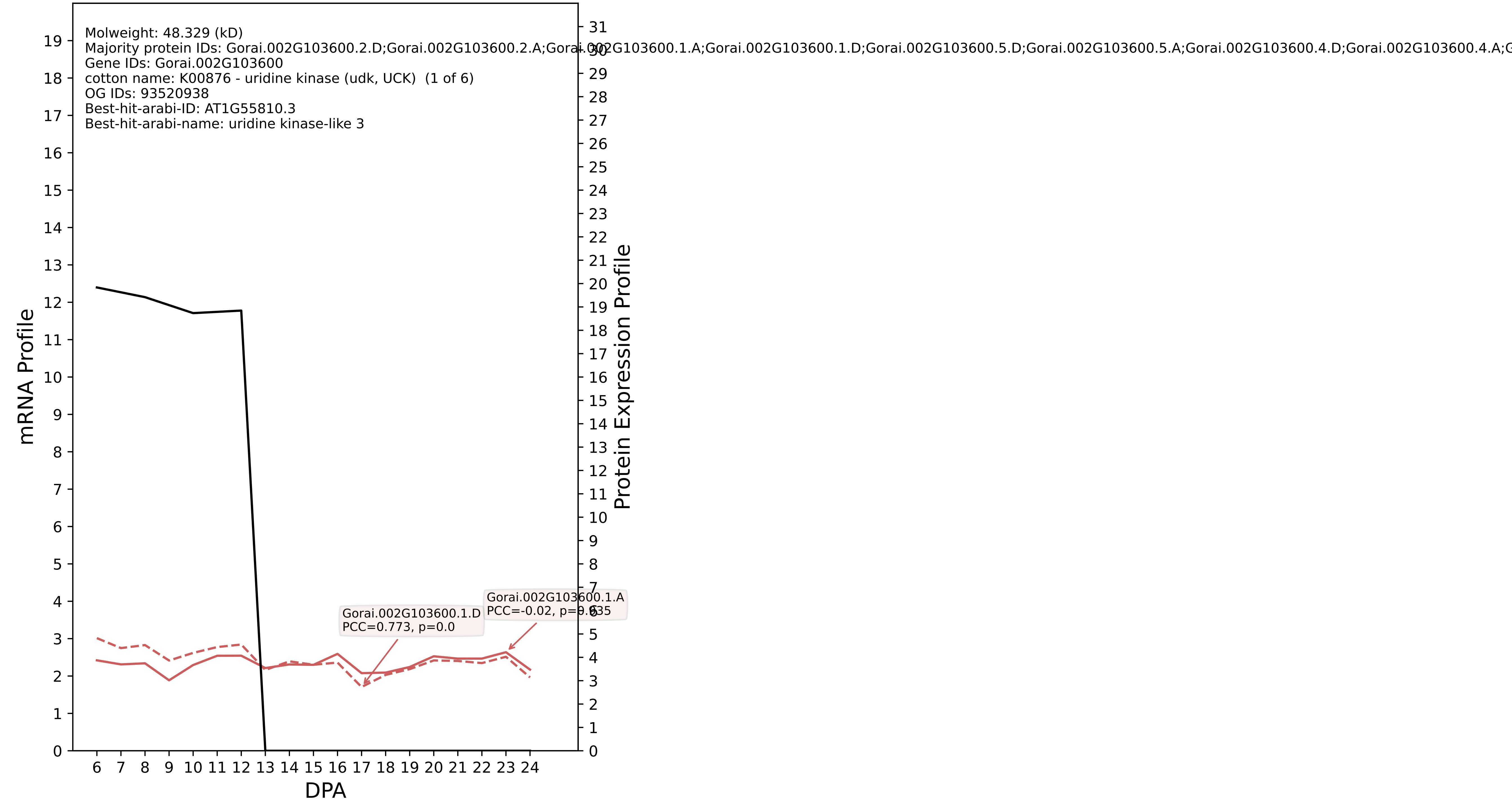

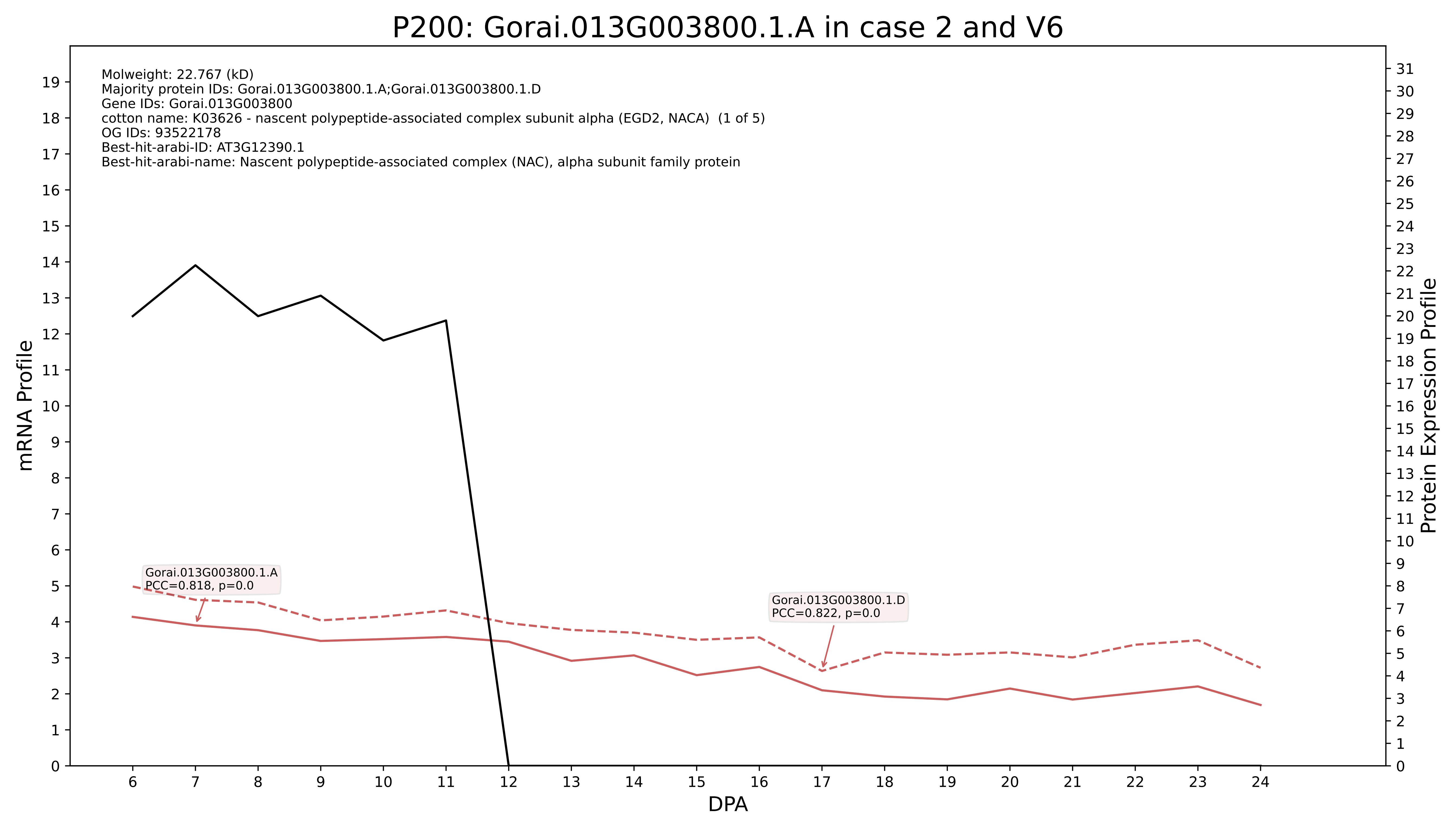

P200: Gorai.013G003800.1.A in case 2 and V6
Molweight: 22.767 (kD)
Majority protein IDs: Gorai.013G003800.1.A;Gorai.013G003800.1.D
Gene IDs: Gorai.013G003800
cotton name: K03626 - nascent polypeptide-associated complex subunit alpha (EGD2, NACA) (1 of 5)
OG IDs: 93522178
Best-hit-arabi-ID: AT3G12390.1
Best-hit-arabi-name: Nascent polypeptide-associated complex (NAC), alpha subunit family protein
Gorai.013G003800.1.A
PCC=0.818, p=0.0
Gorai.013G003800.1.D
PCC=0.822, p=0.0
mRNA Profile
Protein Expression Profile
DPA

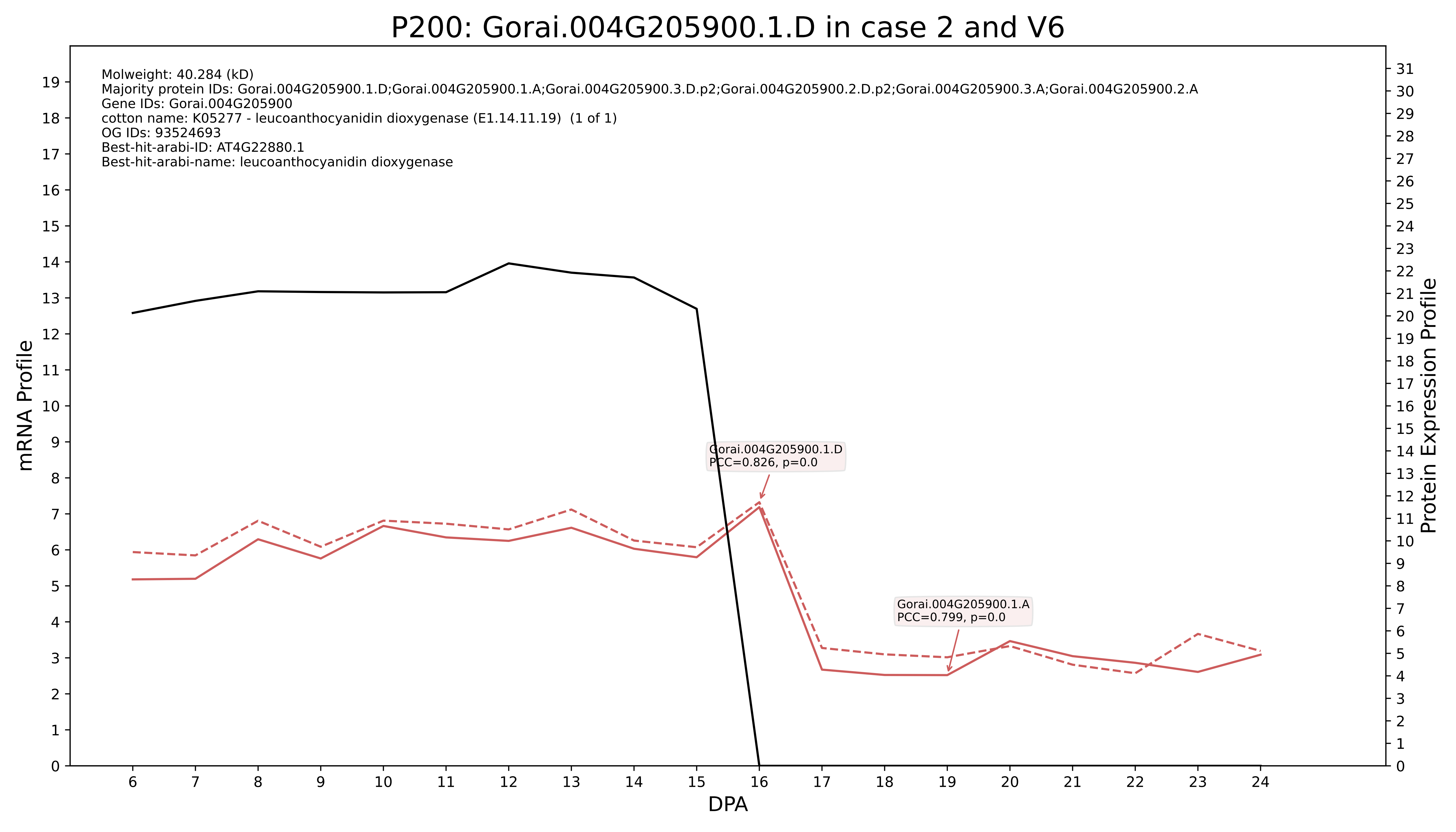
P200: Gorai.004G205900.1.D in case 2 and V6
Molweight: 40.284 (kD)
Majority protein IDs: Gorai.004G205900.1.D;Gorai.004G205900.1.A;Gorai.004G205900.3.D.p2;Gorai.004G205900.2.D.p2;Gorai.004G205900.3.A;Gorai.004G205900.2.A
Gene IDs: Gorai.004G205900
cotton name: K05277 - leucoanthocyanidin dioxygenase (E1.14.11.19) (1 of 1)
OG IDs: 93524693
Best-hit-arabi-ID: AT4G22880.1
Best-hit-arabi-name: leucoanthocyanidin dioxygenase
Gorai.004G205900.1.D
PCC=0.826, p=0.0
Gorai.004G205900.1.A
PCC=0.799, p=0.0
mRNA Profile
Protein Expression Profile
DPA

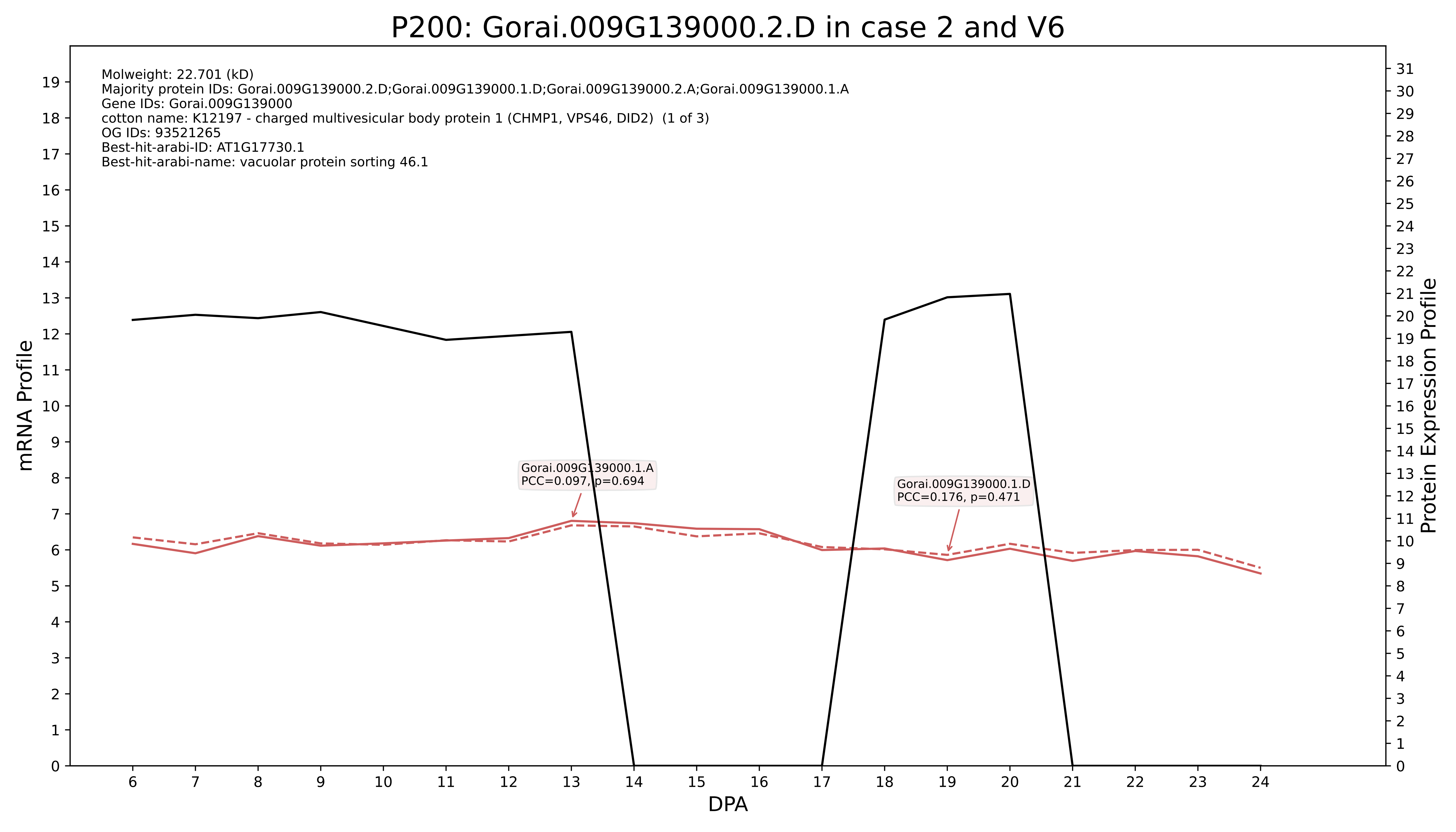
P200: Gorai.009G139000.2.D in case 2 and V6
Molweight: 22.701 (kD)
Majority protein IDs: Gorai.009G139000.2.D;Gorai.009G139000.1.D;Gorai.009G139000.2.A;Gorai.009G139000.1.A
Gene IDs: Gorai.009G139000
cotton name: K12197 - charged multivesicular body protein 1 (CHMP1, VPS46, DID2) (1 of 3)
OG IDs: 93521265
Best-hit-arabi-ID: AT1G17730.1
Best-hit-arabi-name: vacuolar protein sorting 46.1
Gorai.009G139000.1.A
PCC=0.097, p=0.694
Gorai.009G139000.1.D
PCC=0.176, p=0.471
mRNA Profile
Protein Expression Profile
DPA

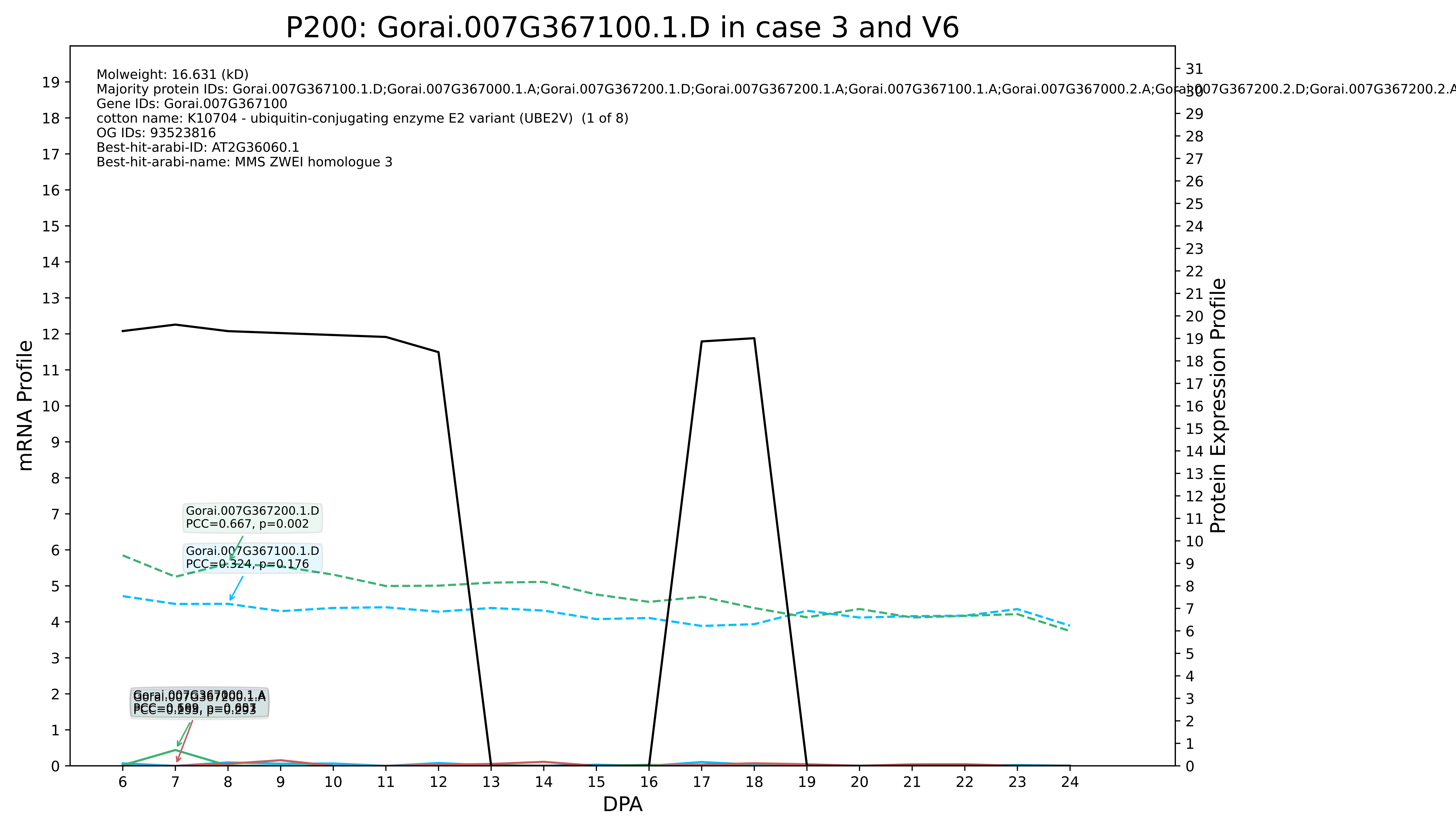
P200: Gorai.007G367100.1.D in case 3 and V6
Molweight: 16.631 (kD)
Majority protein IDs: Gorai.007G367100.1.D;Gorai.007G367000.1.A;Gorai.007G367200.1.D;Gorai.007G367200.1.A;Gorai.007G367100.1.A;Gorai.007G367000.2.A;Gorai.007G367200.2.D;Gorai.007G367200.2.A
Gene IDs: Gorai.007G367100
cotton name: K10704 - ubiquitin-conjugating enzyme E2 variant (UBE2V) (1 of 8)
OG IDs: 93523816
Best-hit-arabi-ID: AT2G36060.1
Best-hit-arabi-name: MMS ZWEI homologue 3
Gorai.007G367200.1.D
PCC=0.667, p=0.002
Gorai.007G367100.1.D
PCC=0.324, p=0.176
mRNA Profile
Protein Expression Profile
DPA

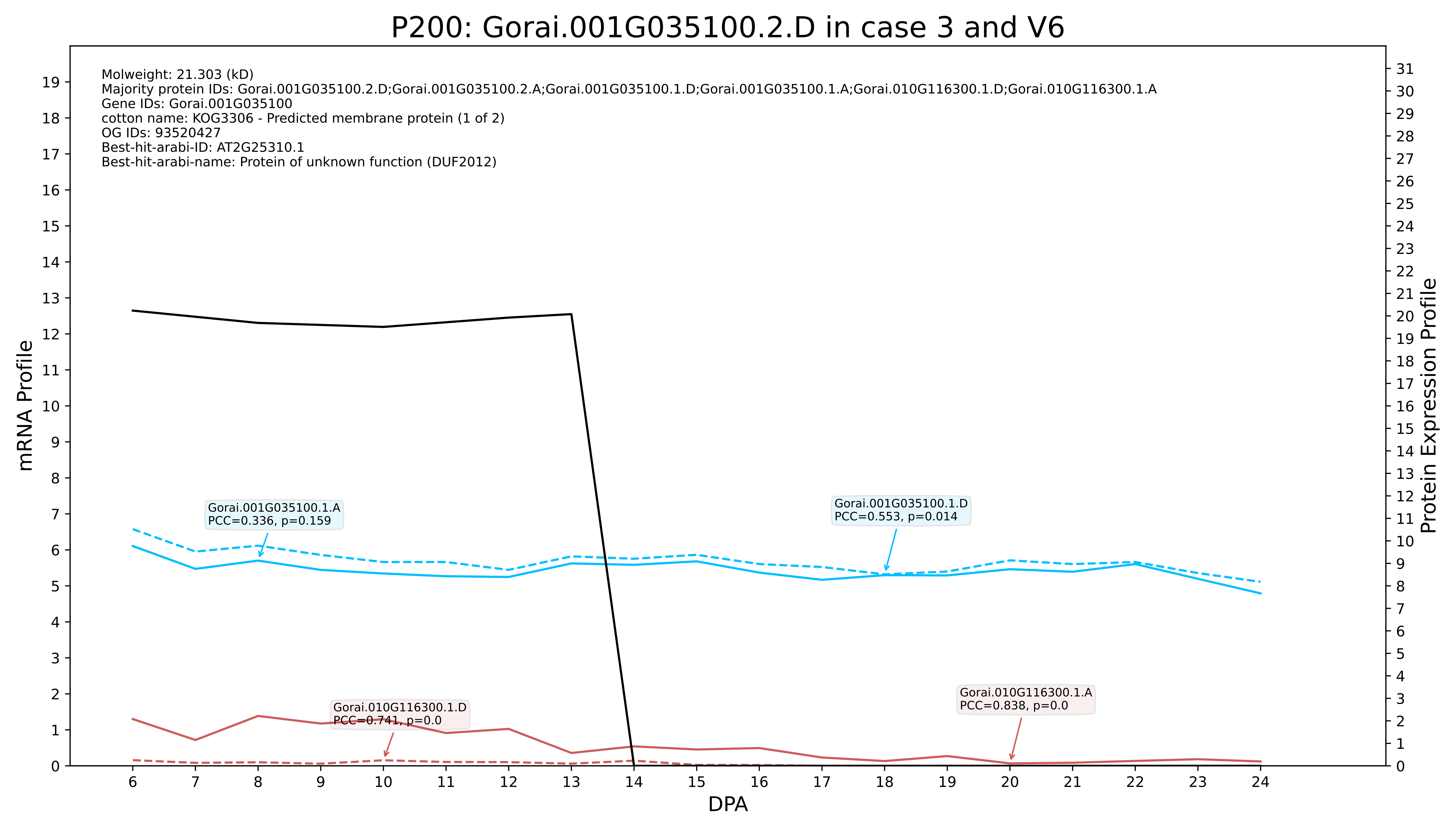
P200: Gorai.001G035100.2.D in case 3 and V6
Molweight: 21.303 (kD)
Majority protein IDs: Gorai.001G035100.2.D;Gorai.001G035100.2.A;Gorai.001G035100.1.D;Gorai.001G035100.1.A;Gorai.010G116300.1.D;Gorai.010G116300.1.A
Gene IDs: Gorai.001G035100
cotton name: KOG3306 - Predicted membrane protein (1 of 2)
OG IDs: 93520427
Best-hit-arabi-ID: AT2G25310.1
Best-hit-arabi-name: Protein of unknown function (DUF2012)
Gorai.001G035100.1.A
PCC=0.336, p=0.159
Gorai.001G035100.1.D
PCC=0.553, p=0.014
Gorai.010G116300.1.D
PCC=0.741, p=0.0
Gorai.010G116300.1.A
PCC=0.838, p=0.0
mRNA Profile
Protein Expression Profile
DPA
0
1
2
3
4
5
6
7
8
9
10
11
12
13
14
15
16
17
18
19
20
21
22
23
24
25
26
27
28
29
30
31

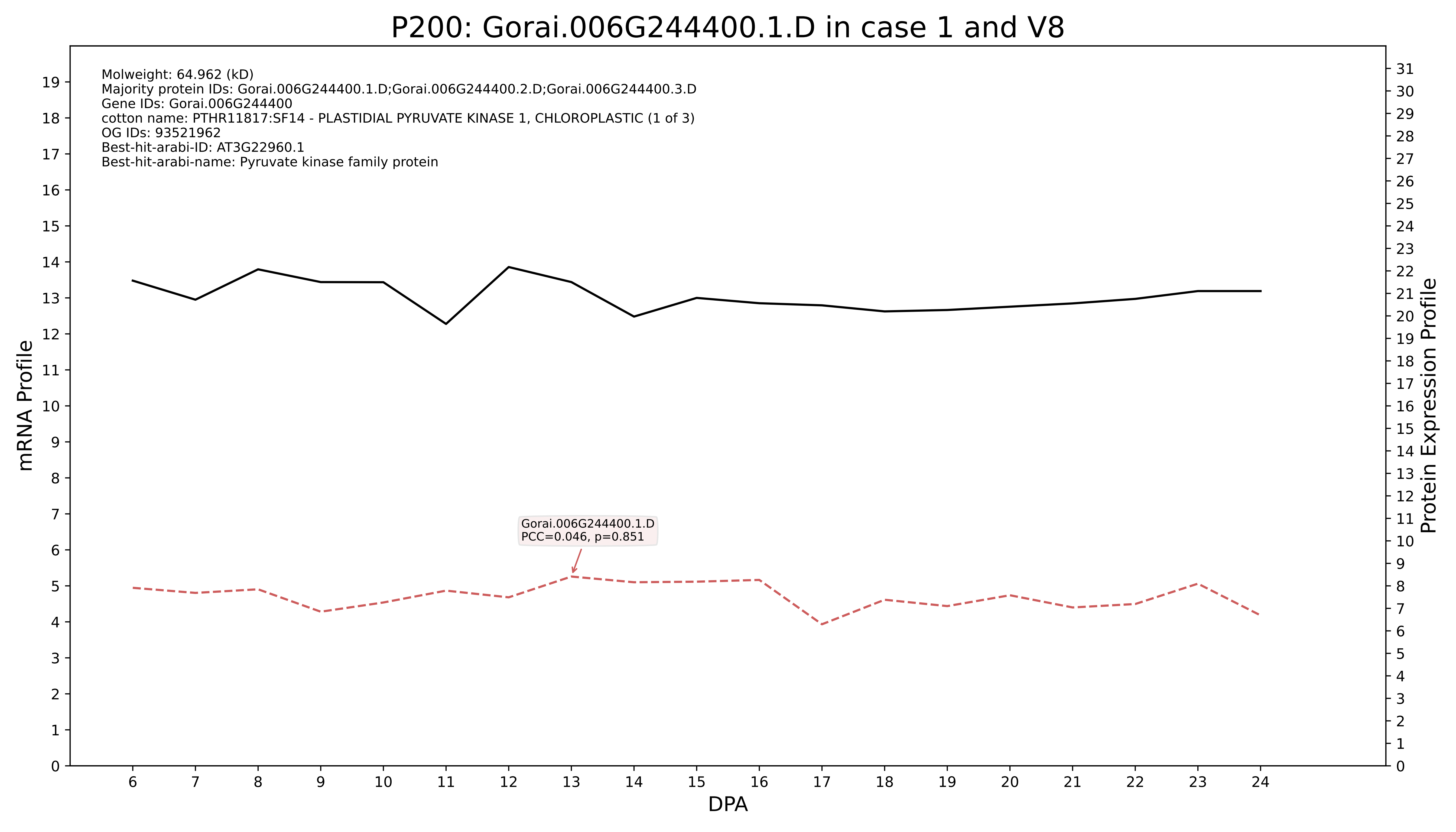
P200: Gorai.006G244400.1.D in case 1 and V8
Molweight: 64.962 (kD)
Majority protein IDs: Gorai.006G244400.1.D;Gorai.006G244400.2.D;Gorai.006G244400.3.D
Gene IDs: Gorai.006G244400
cotton name: PTHR11817:SF14 - PLASTIDIAL PYRUVATE KINASE 1, CHLOROPLASTIC (1 of 3)
OG IDs: 93521962
Best-hit-arabi-ID: AT3G22960.1
Best-hit-arabi-name: Pyruvate kinase family protein
Gorai.006G244400.1.D
PCC=0.046, p=0.851
mRNA Profile
Protein Expression Profile
DPA

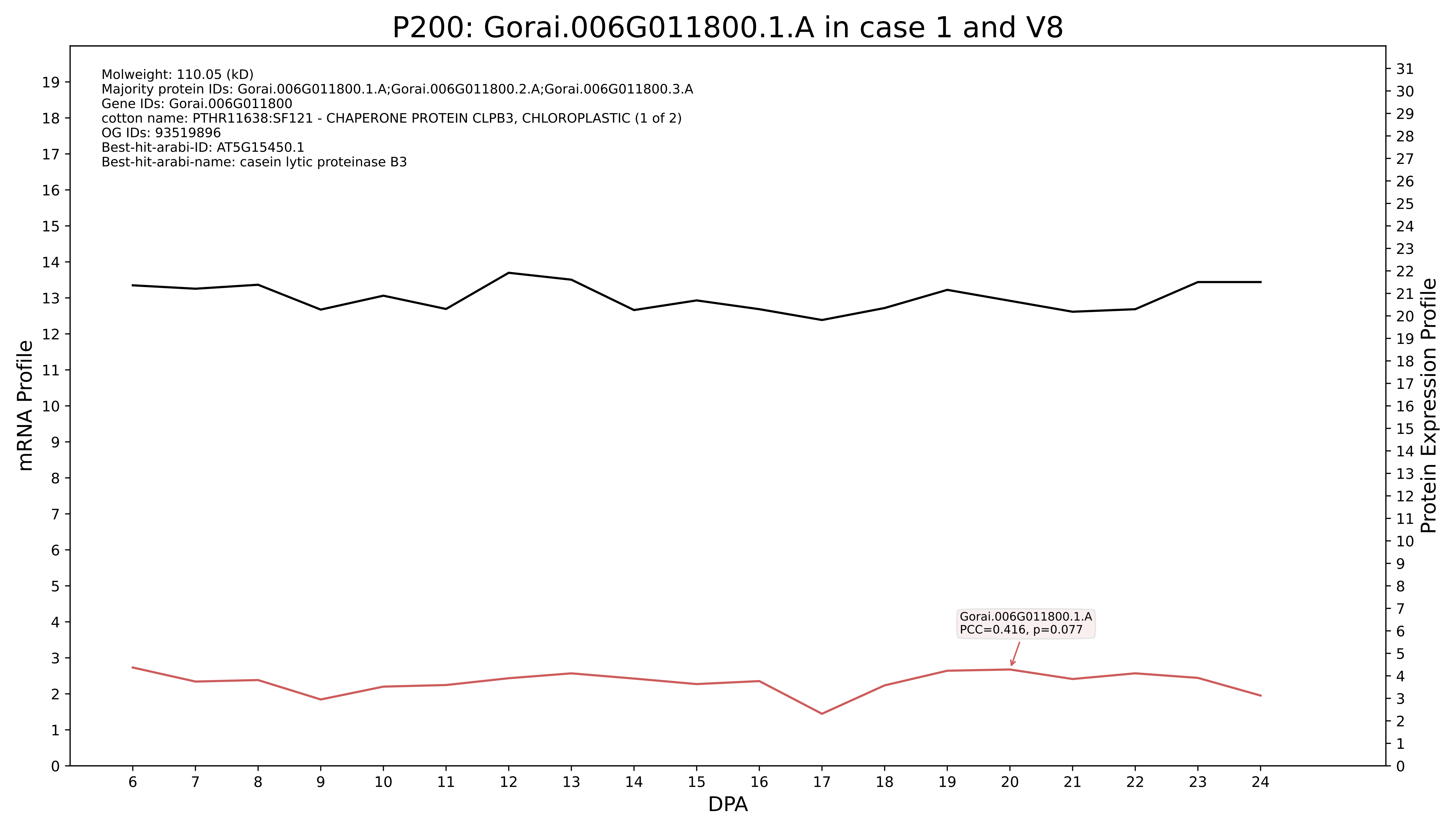
P200: Gorai.006G011800.1.A in case 1 and V8
Molweight: 110.05 (kD)
Majority protein IDs: Gorai.006G011800.1.A;Gorai.006G011800.2.A;Gorai.006G011800.3.A
Gene IDs: Gorai.006G011800
cotton name: PTHR11638:SF121 - CHAPERONE PROTEIN CLPB3, CHLOROPLASTIC (1 of 2)
OG IDs: 93519896
Best-hit-arabi-ID: AT5G15450.1
Best-hit-arabi-name: casein lytic proteinase B3
Gorai.006G011800.1.A
PCC=0.416, p=0.077
mRNA Profile
Protein Expression Profile
DPA

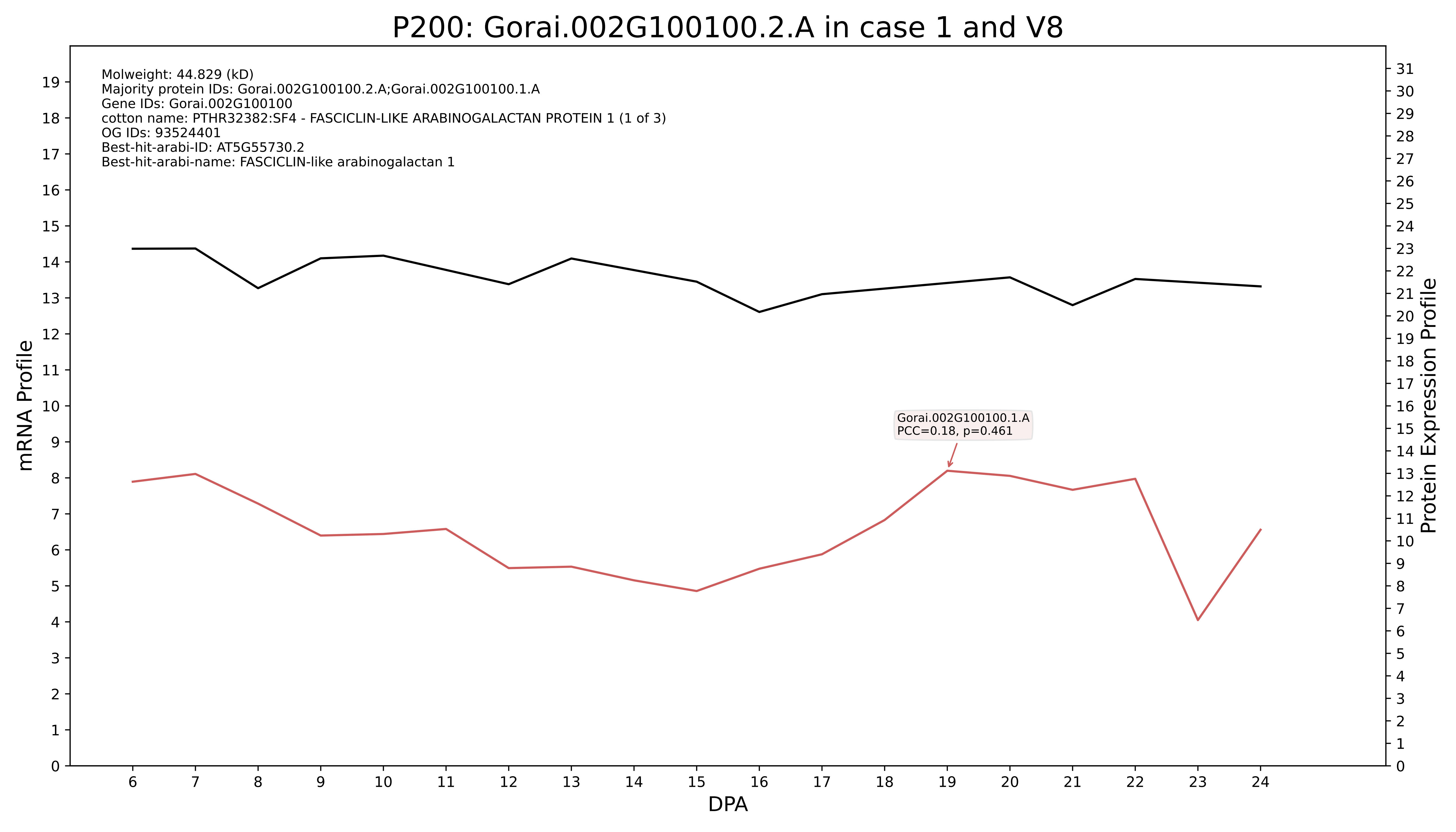
P200: Gorai.002G100100.2.A in case 1 and V8
Molweight: 44.829 (kD)
Majority protein IDs: Gorai.002G100100.2.A;Gorai.002G100100.1.A
Gene IDs: Gorai.002G100100
cotton name: PTHR32382:SF4 - FASCICLIN-LIKE ARABINOGALACTAN PROTEIN 1 (1 of 3)
OG IDs: 93524401
Best-hit-arabi-ID: AT5G55730.2
Best-hit-arabi-name: FASCICLIN-like arabinogalactan 1
Gorai.002G100100.1.A
PCC=0.18, p=0.461
mRNA Profile
Protein Expression Profile
DPA

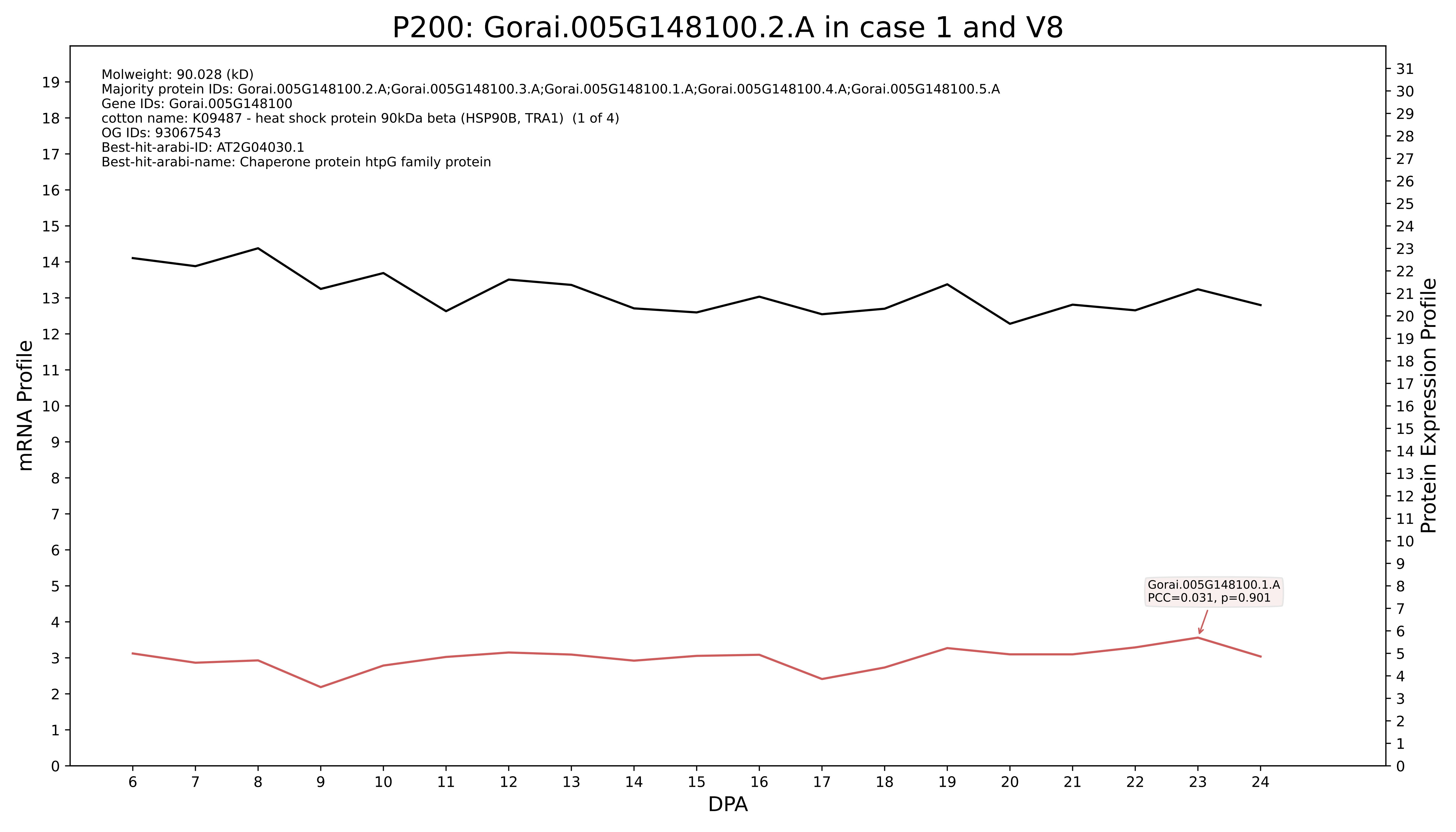

P200: Gorai.005G148100.2.A in case 1 and V8
Molweight: 90.028 (kD)
Majority protein IDs: Gorai.005G148100.2.A;Gorai.005G148100.3.A;Gorai.005G148100.1.A;Gorai.005G148100.4.A;Gorai.005G148100.5.A
Gene IDs: Gorai.005G148100
cotton name: K09487 - heat shock protein 90kDa beta (HSP90B, TRA1) (1 of 4)
OG IDs: 93067543
Best-hit-arabi-ID: AT2G04030.1
Best-hit-arabi-name: Chaperone protein htpG family protein
Gorai.005G148100.1.A
PCC=0.031, p=0.901
mRNA Profile
Protein Expression Profile
DPA

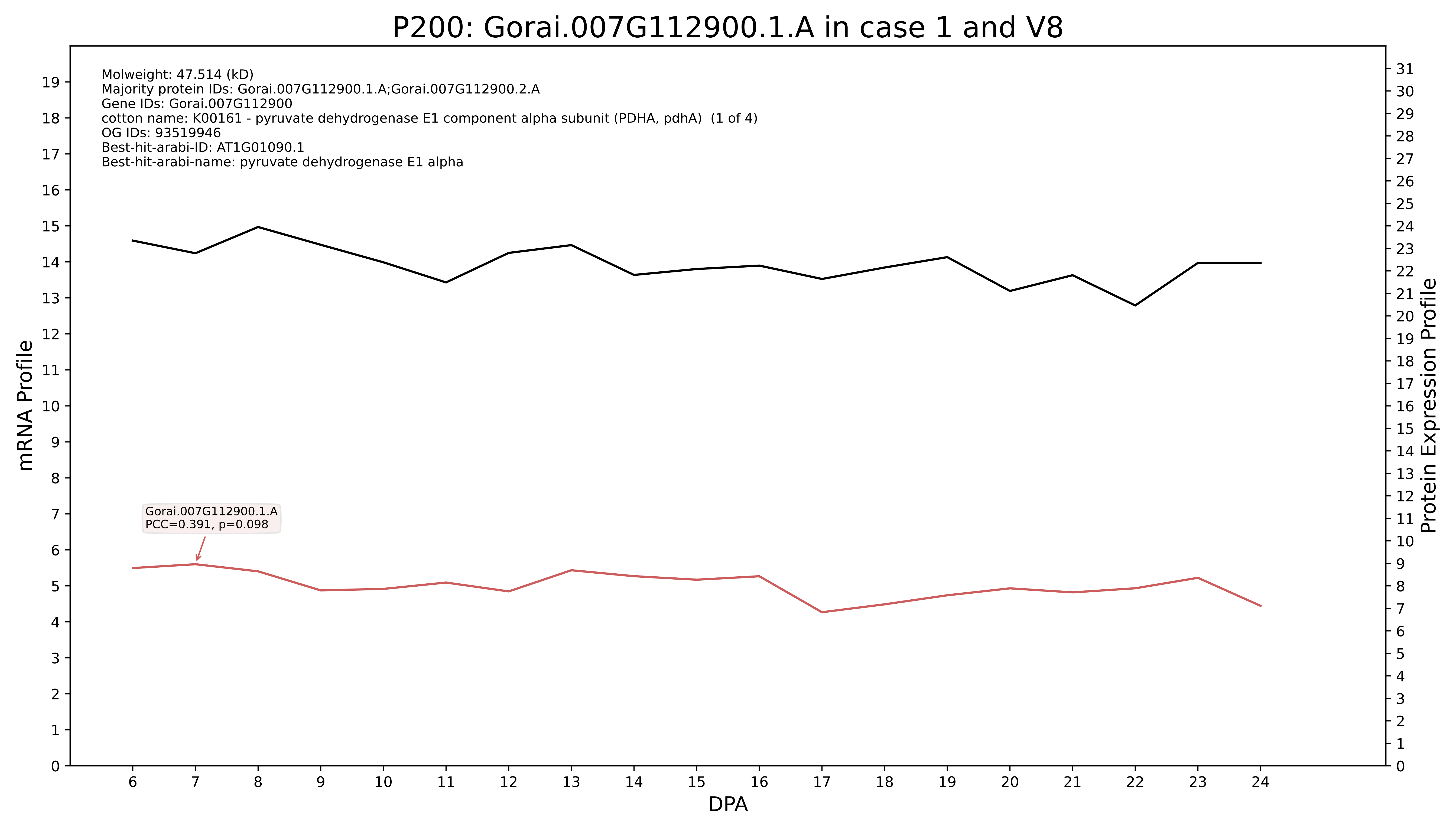
P200: Gorai.007G112900.1.A in case 1 and V8
Molweight: 47.514 (kD)
Majority protein IDs: Gorai.007G112900.1.A;Gorai.007G112900.2.A
Gene IDs: Gorai.007G112900
cotton name: K00161 - pyruvate dehydrogenase E1 component alpha subunit (PDHA, pdhA) (1 of 4)
OG IDs: 93519946
Best-hit-arabi-ID: AT1G01090.1
Best-hit-arabi-name: pyruvate dehydrogenase E1 alpha
Gorai.007G112900.1.A
PCC=0.391, p=0.098
mRNA Profile
Protein Expression Profile
DPA
0
1
2
3
4
5
6
7
8
9
10
11
12
13
14
15
16
17
18
19
20
21
22
23
24
25
26
27
28
29
30
31

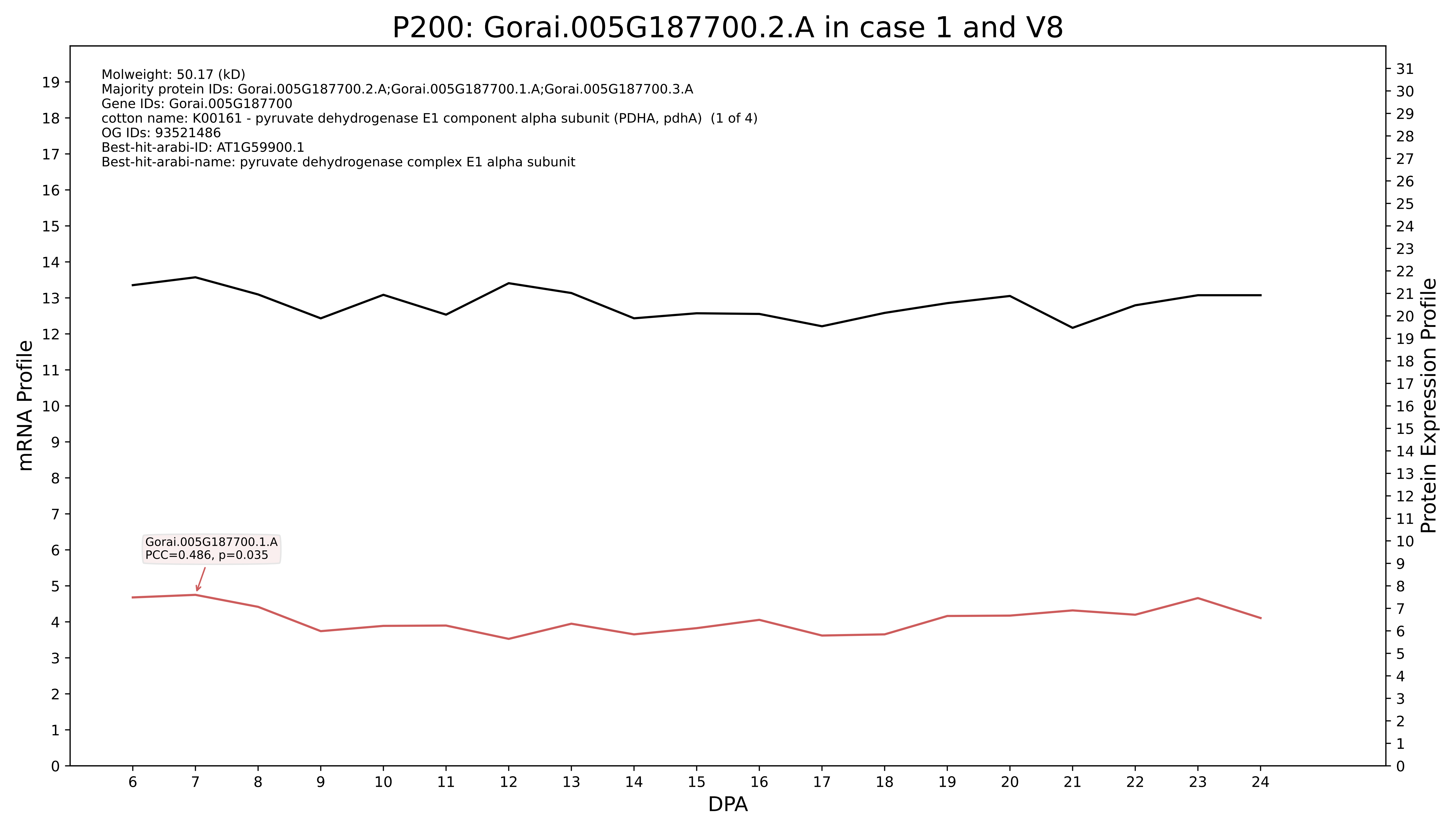
P200: Gorai.005G187700.2.A in case 1 and V8
Molweight: 50.17 (kD)
Majority protein IDs: Gorai.005G187700.2.A;Gorai.005G187700.1.A;Gorai.005G187700.3.A
Gene IDs: Gorai.005G187700
cotton name: K00161 - pyruvate dehydrogenase E1 component alpha subunit (PDHA, pdhA) (1 of 4)
OG IDs: 93521486
Best-hit-arabi-ID: AT1G59900.1
Best-hit-arabi-name: pyruvate dehydrogenase complex E1 alpha subunit
Gorai.005G187700.1.A
PCC=0.486, p=0.035
mRNA Profile
Protein Expression Profile
DPA

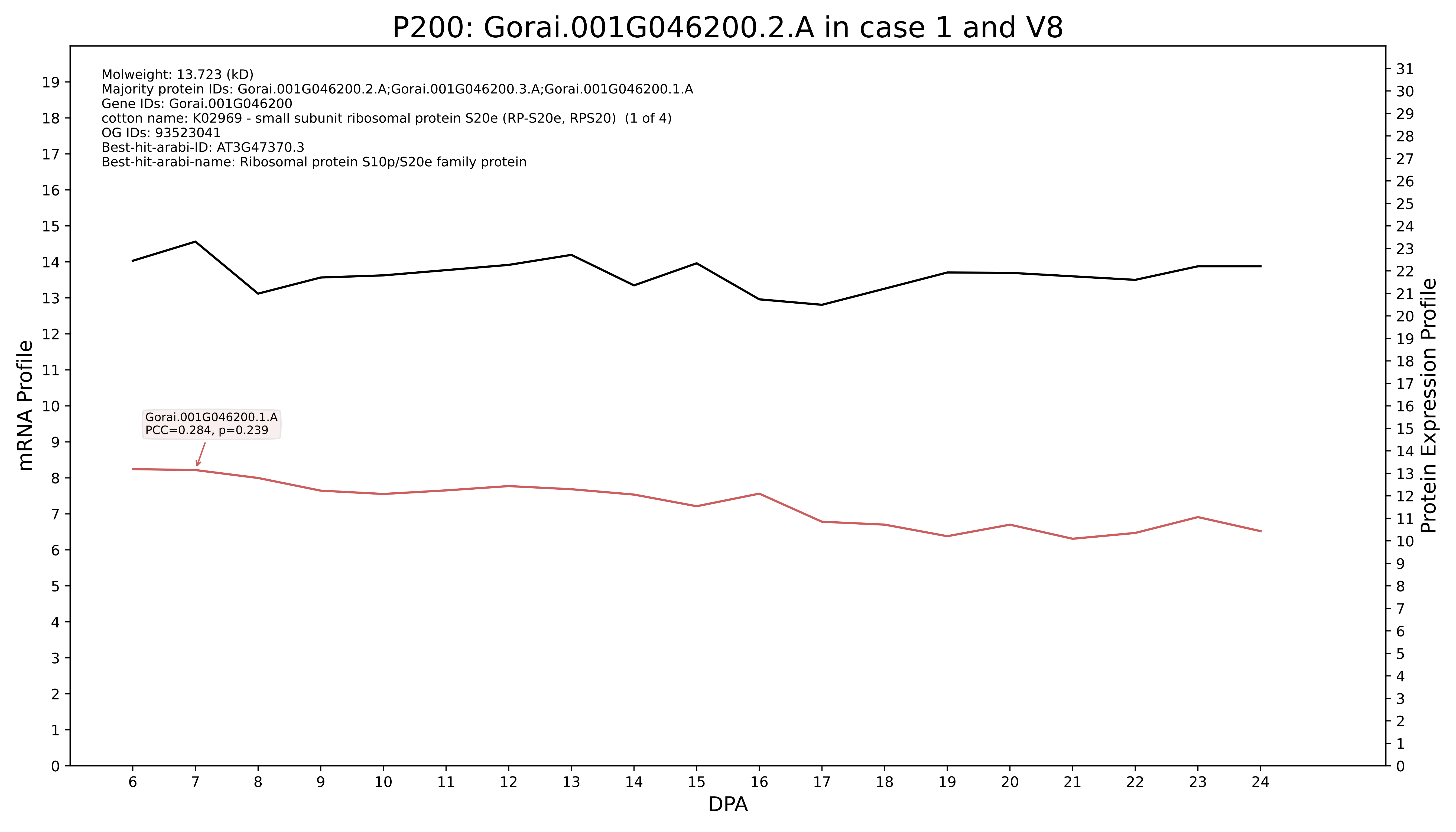
P200: Gorai.001G046200.2.A in case 1 and V8
Molweight: 13.723 (kD)
Majority protein IDs: Gorai.001G046200.2.A;Gorai.001G046200.3.A;Gorai.001G046200.1.A
Gene IDs: Gorai.001G046200
cotton name: K02969 - small subunit ribosomal protein S20e (RP-S20e, RPS20) (1 of 4)
OG IDs: 93523041
Best-hit-arabi-ID: AT3G47370.3
Best-hit-arabi-name: Ribosomal protein S10p/S20e family protein
Gorai.001G046200.1.A
PCC=0.284, p=0.239
mRNA Profile
Protein Expression Profile
DPA
0
1
2
3
4
5
6
7
8
9
10
11
12
13
14
15
16
17
18
19
20
21
22
23
24
25
26
27
28
29
30
31

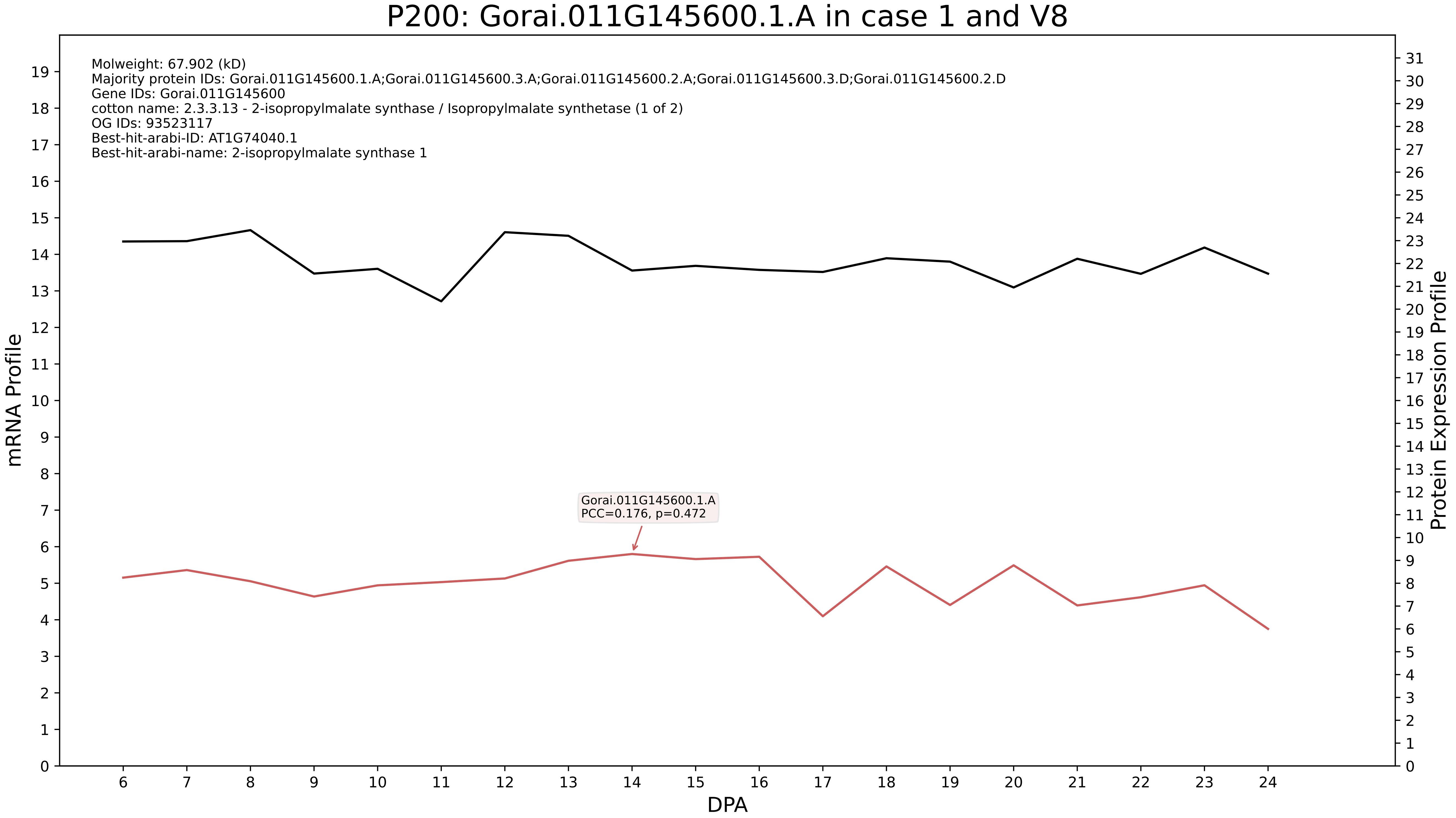
P200: Gorai.011G145600.1.A in case 1 and V8
Molweight: 67.902 (kD)
Majority protein IDs: Gorai.011G145600.1.A;Gorai.011G145600.3.A;Gorai.011G145600.2.A;Gorai.011G145600.3.D;Gorai.011G145600.2.D
Gene IDs: Gorai.011G145600
cotton name: 2.3.3.13 - 2-isopropylmalate synthase / Isopropylmalate synthetase (1 of 2)
OG IDs: 93523117
Best-hit-arabi-ID: AT1G74040.1
Best-hit-arabi-name: 2-isopropylmalate synthase 1
Gorai.011G145600.1.A
PCC=0.176, p=0.472
mRNA Profile
Protein Expression Profile
DPA

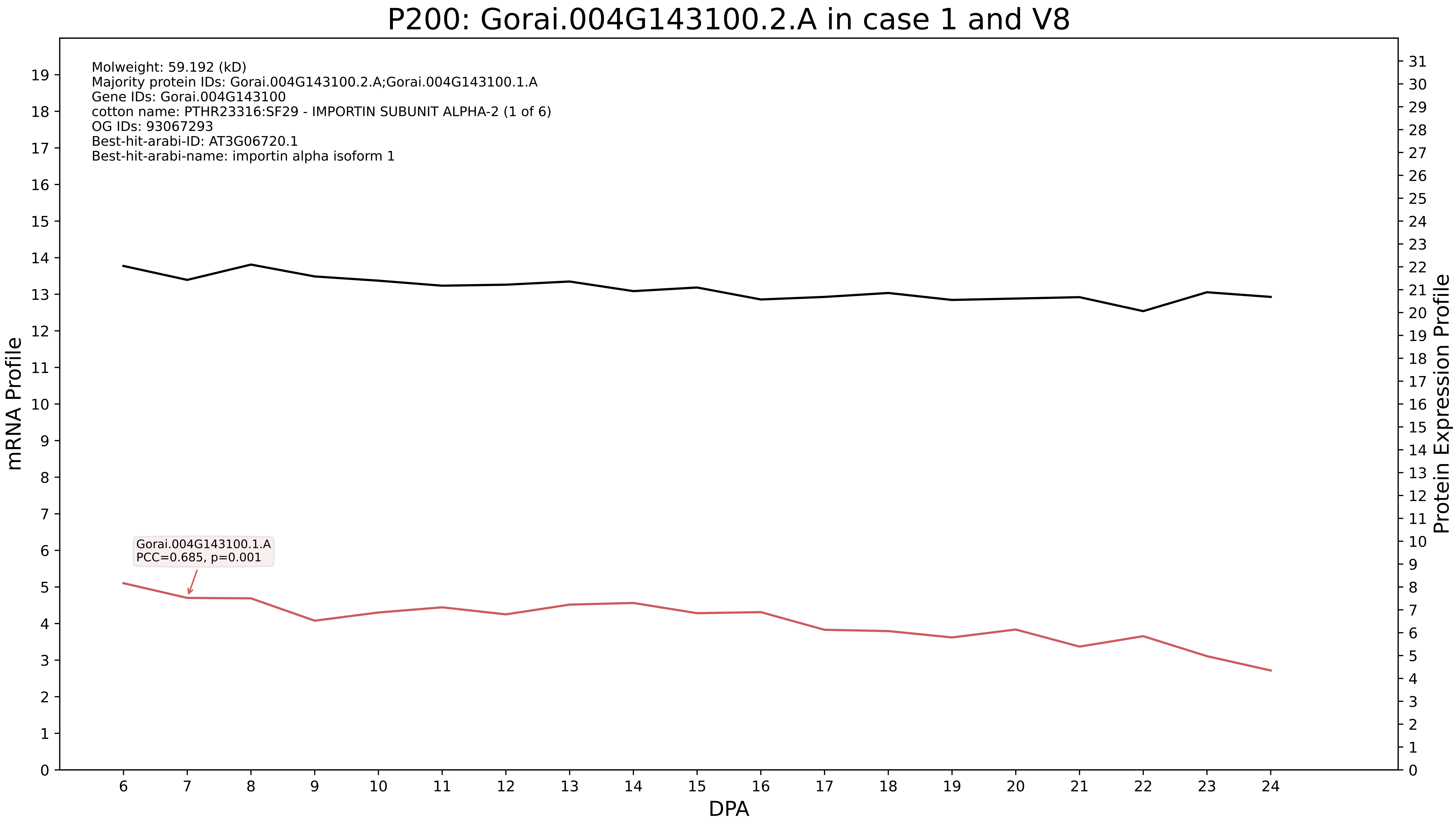
P200: Gorai.004G143100.2.A in case 1 and V8
Molweight: 59.192 (kD)
Majority protein IDs: Gorai.004G143100.2.A;Gorai.004G143100.1.A
Gene IDs: Gorai.004G143100
cotton name: PTHR23316:SF29 - IMPORTIN SUBUNIT ALPHA-2 (1 of 6)
OG IDs: 93067293
Best-hit-arabi-ID: AT3G06720.1
Best-hit-arabi-name: importin alpha isoform 1
Gorai.004G143100.1.A
PCC=0.685, p=0.001
mRNA Profile
Protein Expression Profile
DPA
0
1
2
3
4
5
6
7
8
9
10
11
12
13
14
15
16
17
18
19
20
21
22
23
24
25
26
27
28
29
30
31

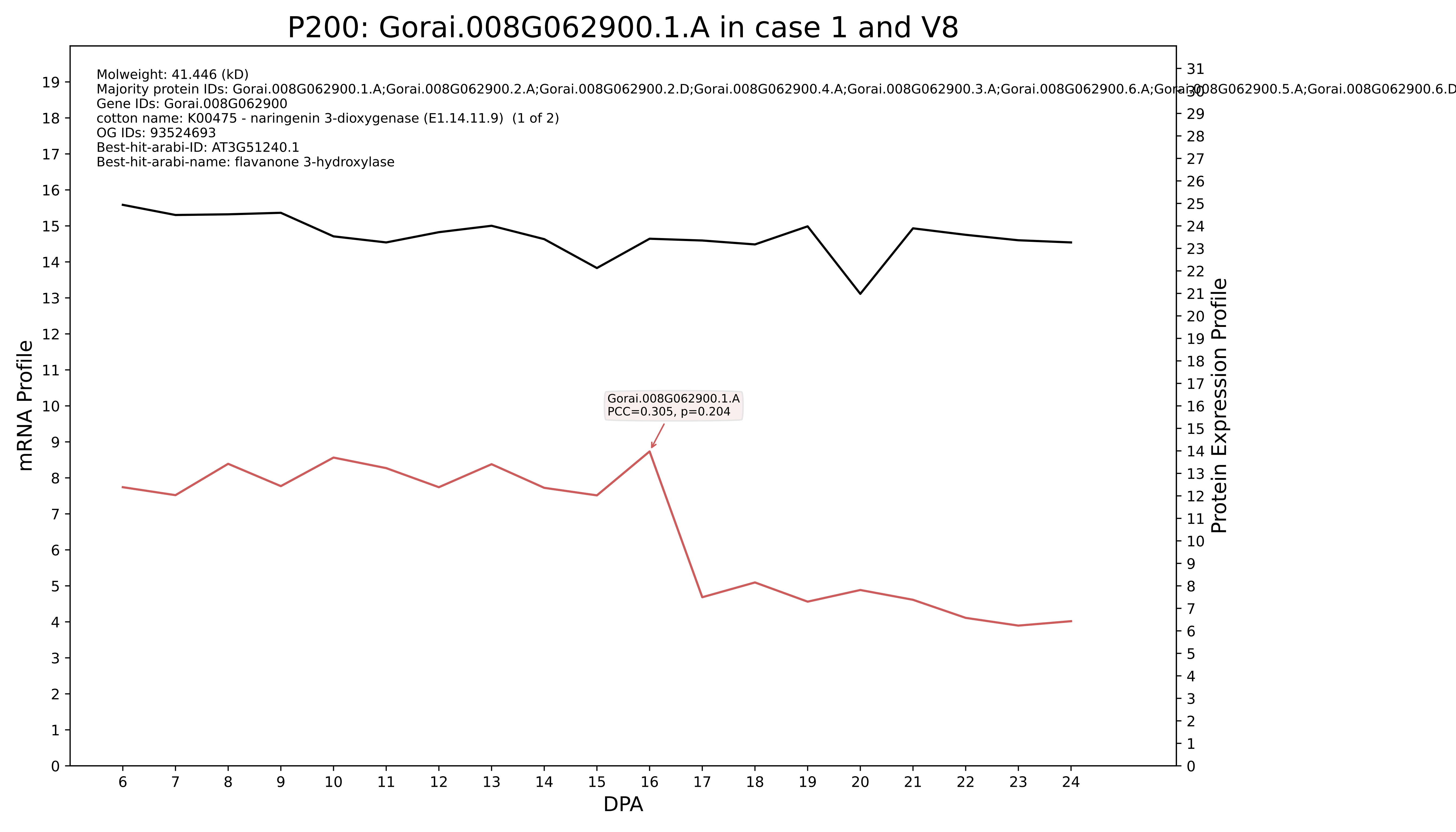
P200: Gorai.008G062900.1.A in case 1 and V8
Molweight: 41.446 (kD)
Majority protein IDs: Gorai.008G062900.1.A;Gorai.008G062900.2.A;Gorai.008G062900.2.D;Gorai.008G062900.4.A;Gorai.008G062900.3.A;Gorai.008G062900.6.A;Gorai.008G062900.5.A;Gorai.008G062900.6.D
Gene IDs: Gorai.008G062900
cotton name: K00475 - naringenin 3-dioxygenase (E1.14.11.9) (1 of 2)
OG IDs: 93524693
Best-hit-arabi-ID: AT3G51240.1
Best-hit-arabi-name: flavanone 3-hydroxylase
Gorai.008G062900.1.A
PCC=0.305, p=0.204
mRNA Profile
Protein Expression Profile
DPA

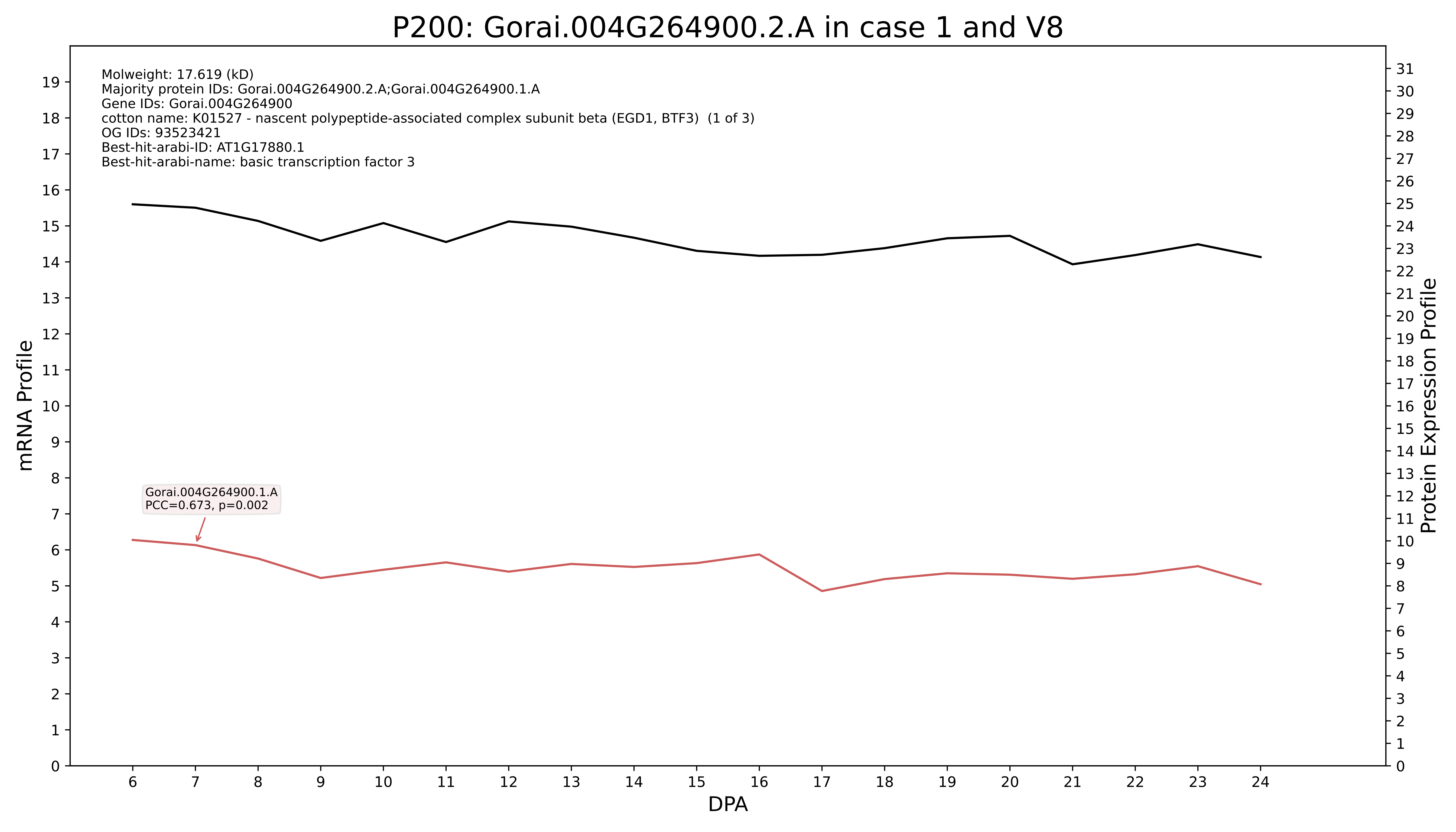
P200: Gorai.004G264900.2.A in case 1 and V8
Molweight: 17.619 (kD)
Majority protein IDs: Gorai.004G264900.2.A;Gorai.004G264900.1.A
Gene IDs: Gorai.004G264900
cotton name: K01527 - nascent polypeptide-associated complex subunit beta (EGD1, BTF3) (1 of 3)
OG IDs: 93523421
Best-hit-arabi-ID: AT1G17880.1
Best-hit-arabi-name: basic transcription factor 3
Gorai.004G264900.1.A
PCC=0.673, p=0.002
mRNA Profile
Protein Expression Profile
DPA

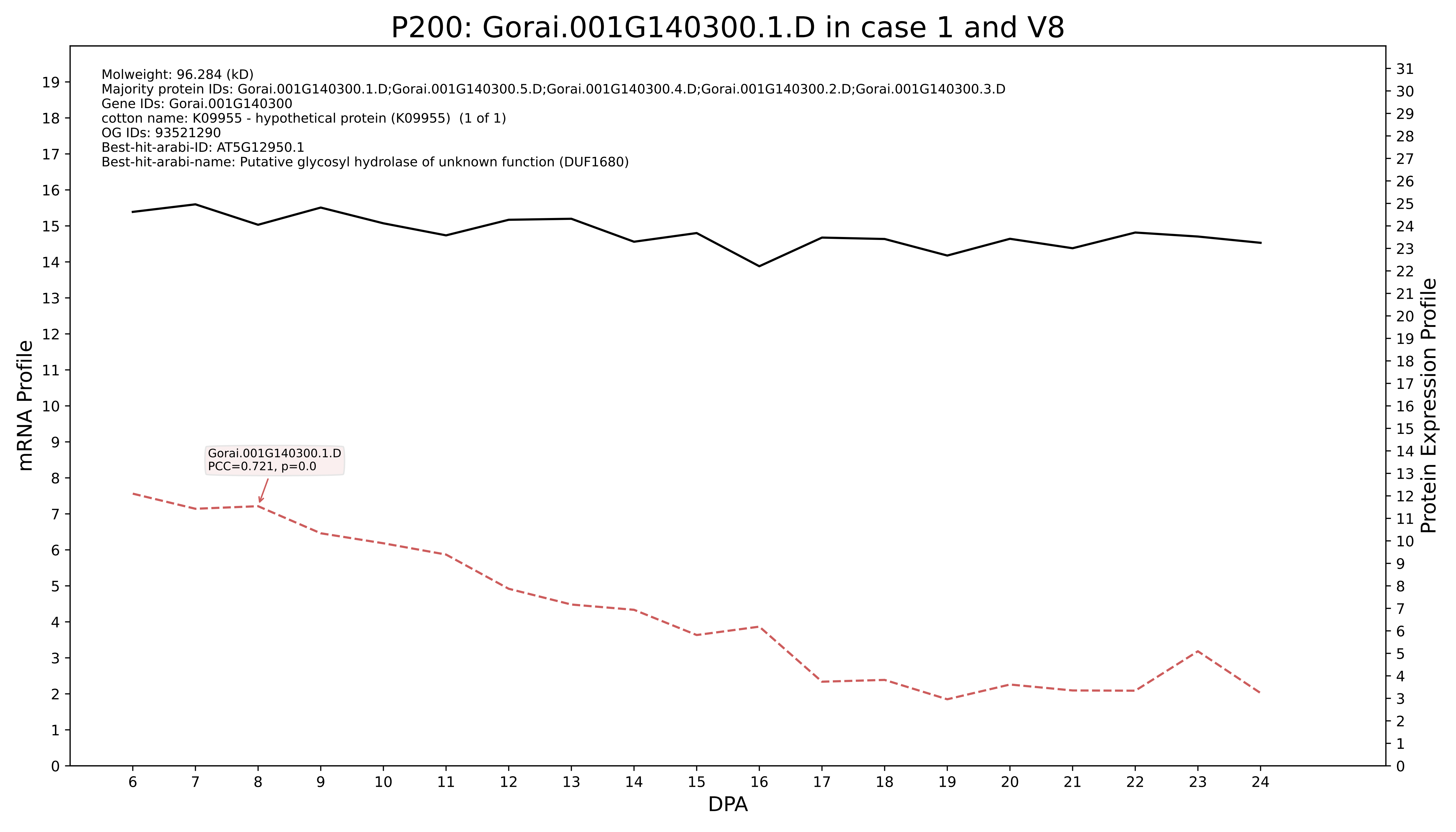
P200: Gorai.001G140300.1.D in case 1 and V8
Molweight: 96.284 (kD)
Majority protein IDs: Gorai.001G140300.1.D;Gorai.001G140300.5.D;Gorai.001G140300.4.D;Gorai.001G140300.2.D;Gorai.001G140300.3.D
Gene IDs: Gorai.001G140300
cotton name: K09955 - hypothetical protein (K09955) (1 of 1)
OG IDs: 93521290
Best-hit-arabi-ID: AT5G12950.1
Best-hit-arabi-name: Putative glycosyl hydrolase of unknown function (DUF1680)
Gorai.001G140300.1.D
PCC=0.721, p=0.0
mRNA Profile
Protein Expression Profile
DPA

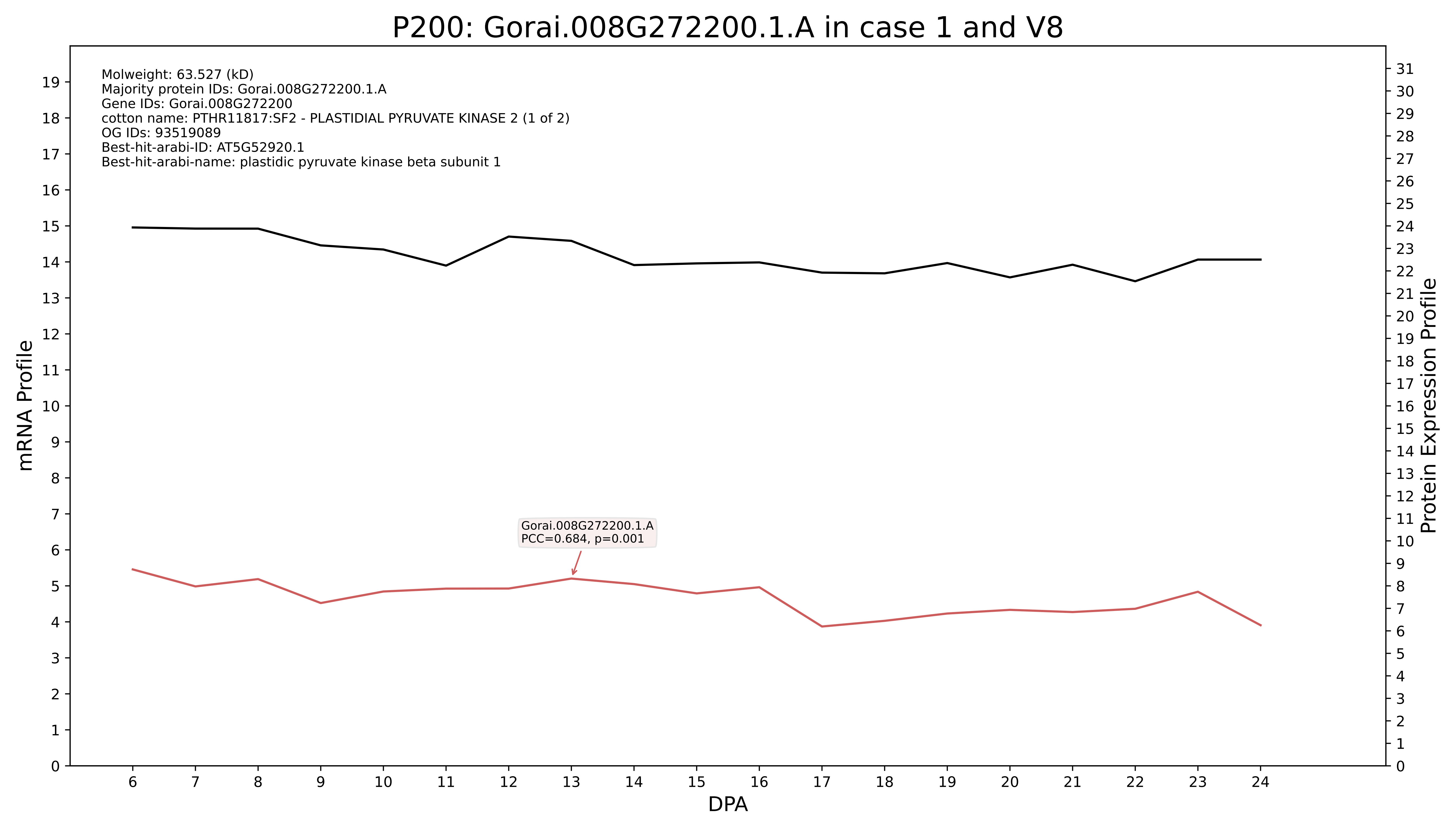

P200: Gorai.008G272200.1.A in case 1 and V8
Molweight: 63.527 (kD)
Majority protein IDs: Gorai.008G272200.1.A
Gene IDs: Gorai.008G272200
cotton name: PTHR11817:SF2 - PLASTIDIAL PYRUVATE KINASE 2 (1 of 2)
OG IDs: 93519089
Best-hit-arabi-ID: AT5G52920.1
Best-hit-arabi-name: plastidic pyruvate kinase beta subunit 1
Gorai.008G272200.1.A
PCC=0.684, p=0.001
mRNA Profile
Protein Expression Profile
DPA

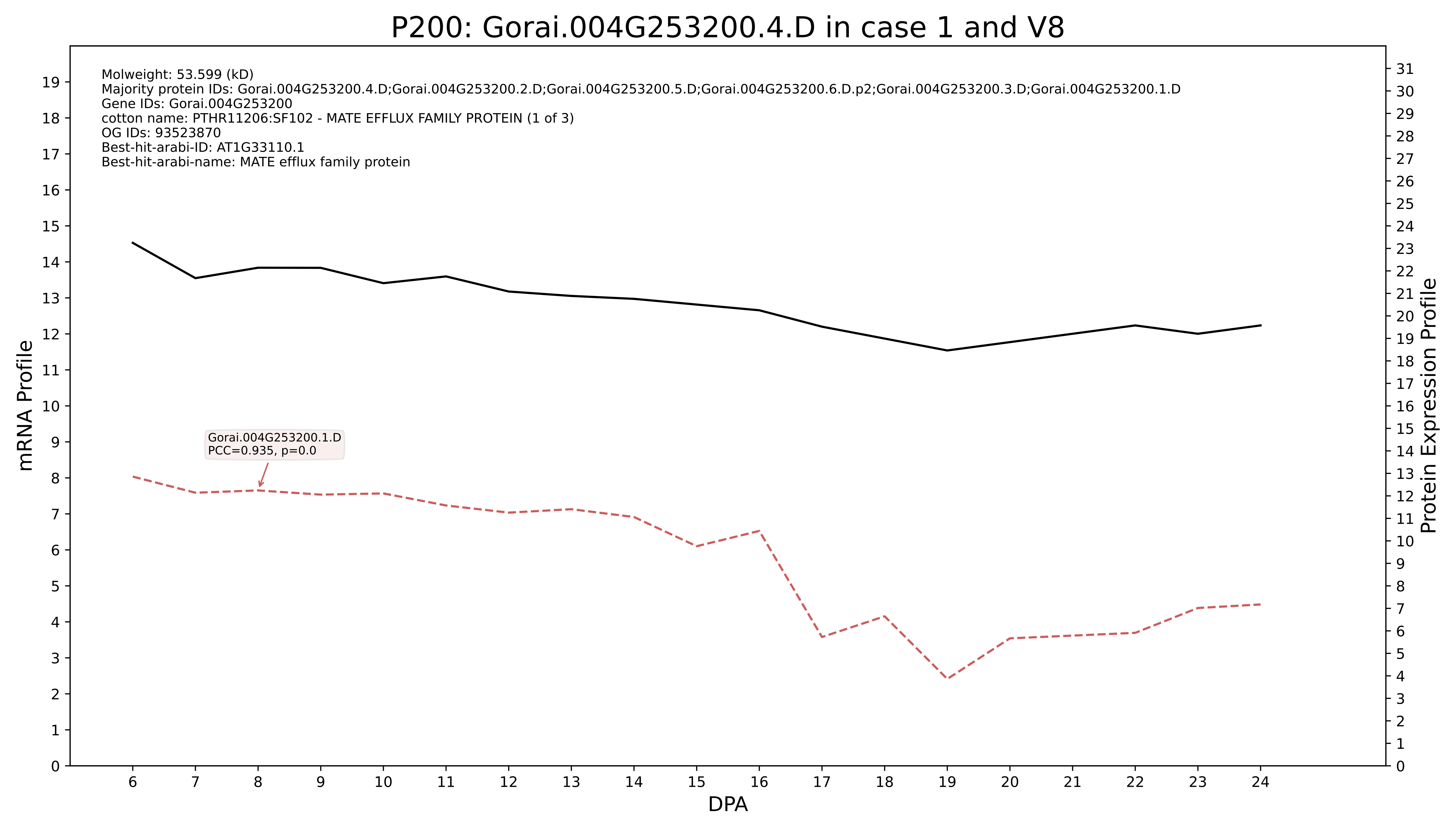
P200: Gorai.004G253200.4.D in case 1 and V8
Molweight: 53.599 (kD)
Majority protein IDs: Gorai.004G253200.4.D;Gorai.004G253200.2.D;Gorai.004G253200.5.D;Gorai.004G253200.6.D.p2;Gorai.004G253200.3.D;Gorai.004G253200.1.D
Gene IDs: Gorai.004G253200
cotton name: PTHR11206:SF102 - MATE EFFLUX FAMILY PROTEIN (1 of 3)
OG IDs: 93523870
Best-hit-arabi-ID: AT1G33110.1
Best-hit-arabi-name: MATE efflux family protein
Gorai.004G253200.1.D
PCC=0.935, p=0.0
mRNA Profile
Protein Expression Profile
DPA
6
7
8
9
10
11
12
13
14
15
16
17
18
19
20
21
22
23
24

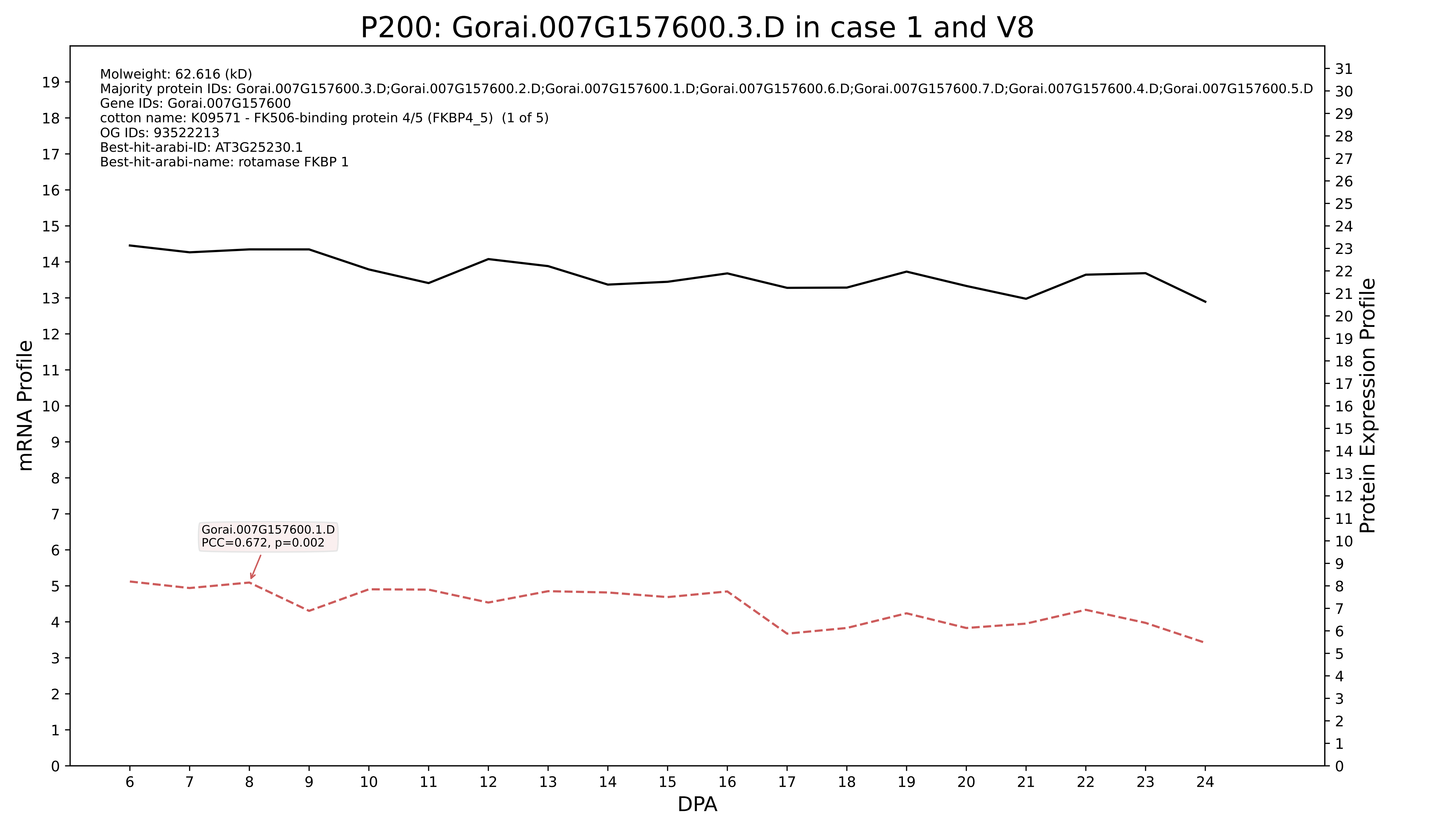

P200: Gorai.007G157600.3.D in case 1 and V8
Molweight: 62.616 (kD)
Majority protein IDs: Gorai.007G157600.3.D;Gorai.007G157600.2.D;Gorai.007G157600.1.D;Gorai.007G157600.6.D;Gorai.007G157600.7.D;Gorai.007G157600.4.D;Gorai.007G157600.5.D
Gene IDs: Gorai.007G157600
cotton name: K09571 - FK506-binding protein 4/5 (FKBP4_5) (1 of 5)
OG IDs: 93522213
Best-hit-arabi-ID: AT3G25230.1
Best-hit-arabi-name: rotamase FKBP 1
Gorai.007G157600.1.D
PCC=0.672, p=0.002
mRNA Profile
Protein Expression Profile
DPA

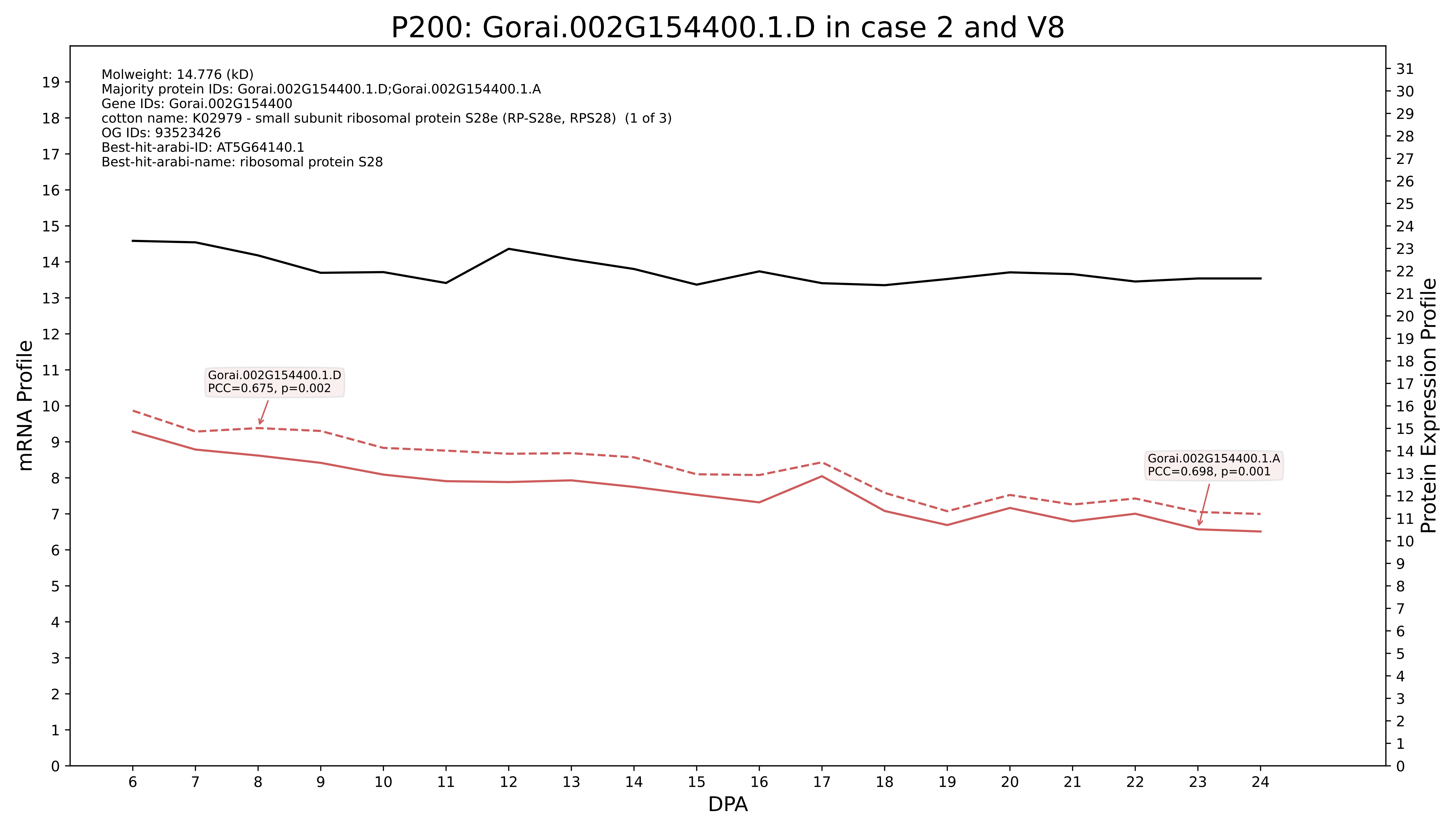
P200: Gorai.002G154400.1.D in case 2 and V8
Molweight: 14.776 (kD)
Majority protein IDs: Gorai.002G154400.1.D;Gorai.002G154400.1.A
Gene IDs: Gorai.002G154400
cotton name: K02979 - small subunit ribosomal protein S28e (RP-S28e, RPS28) (1 of 3)
OG IDs: 93523426
Best-hit-arabi-ID: AT5G64140.1
Best-hit-arabi-name: ribosomal protein S28
Gorai.002G154400.1.D
PCC=0.675, p=0.002
Gorai.002G154400.1.A
PCC=0.698, p=0.001
mRNA Profile
Protein Expression Profile
DPA

P200: Gorai.003G069800.7.D in case 2 and V8

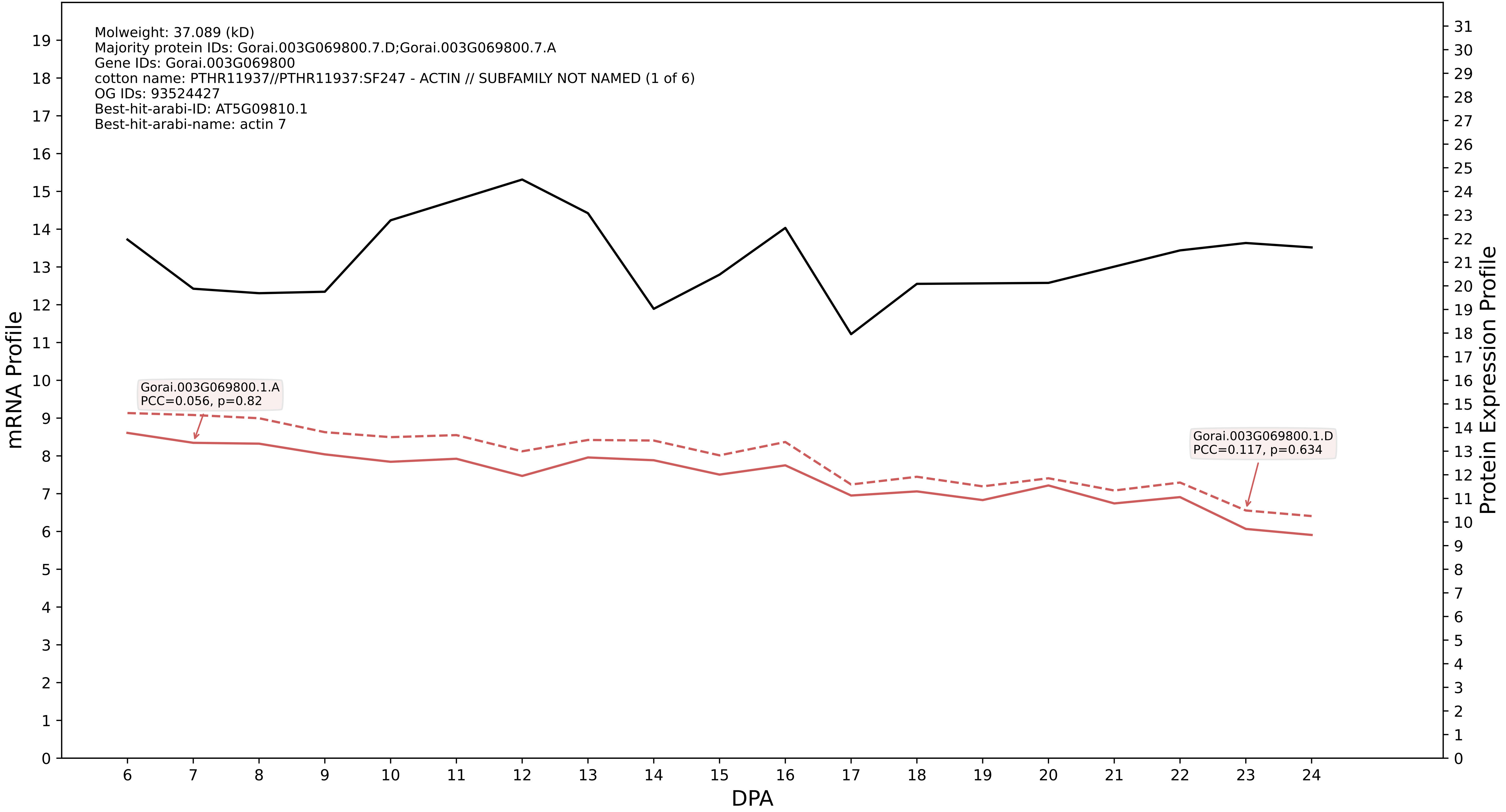

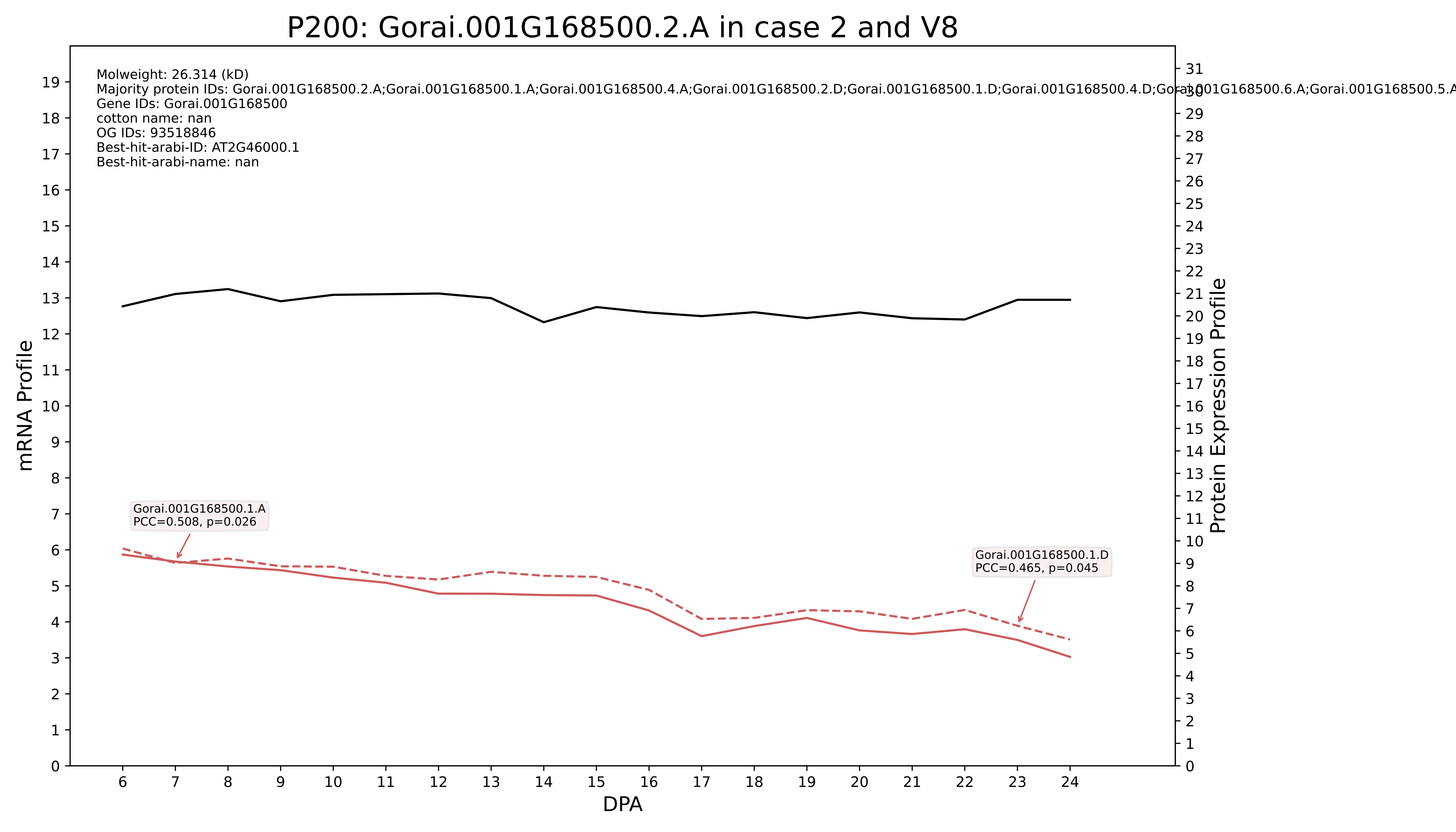
P200: Gorai.001G168500.2.A in case 2 and V8
Molweight: 26.314 (kD)
Majority protein IDs: Gorai.001G168500.2.A;Gorai.001G168500.1.A;Gorai.001G168500.4.A;Gorai.001G168500.2.D;Gorai.001G168500.1.D;Gorai.001G168500.4.D;Gorai.001G168500.6.A;Gorai.001G168500.5.A
Gene IDs: Gorai.001G168500
cotton name: nan
OG IDs: 93518846
Best-hit-arabi-ID: AT2G46000.1
Best-hit-arabi-name: nan
Gorai.001G168500.1.A
PCC=0.508, p=0.026
Gorai.001G168500.1.D
PCC=0.465, p=0.045
mRNA Profile
Protein Expression Profile
DPA

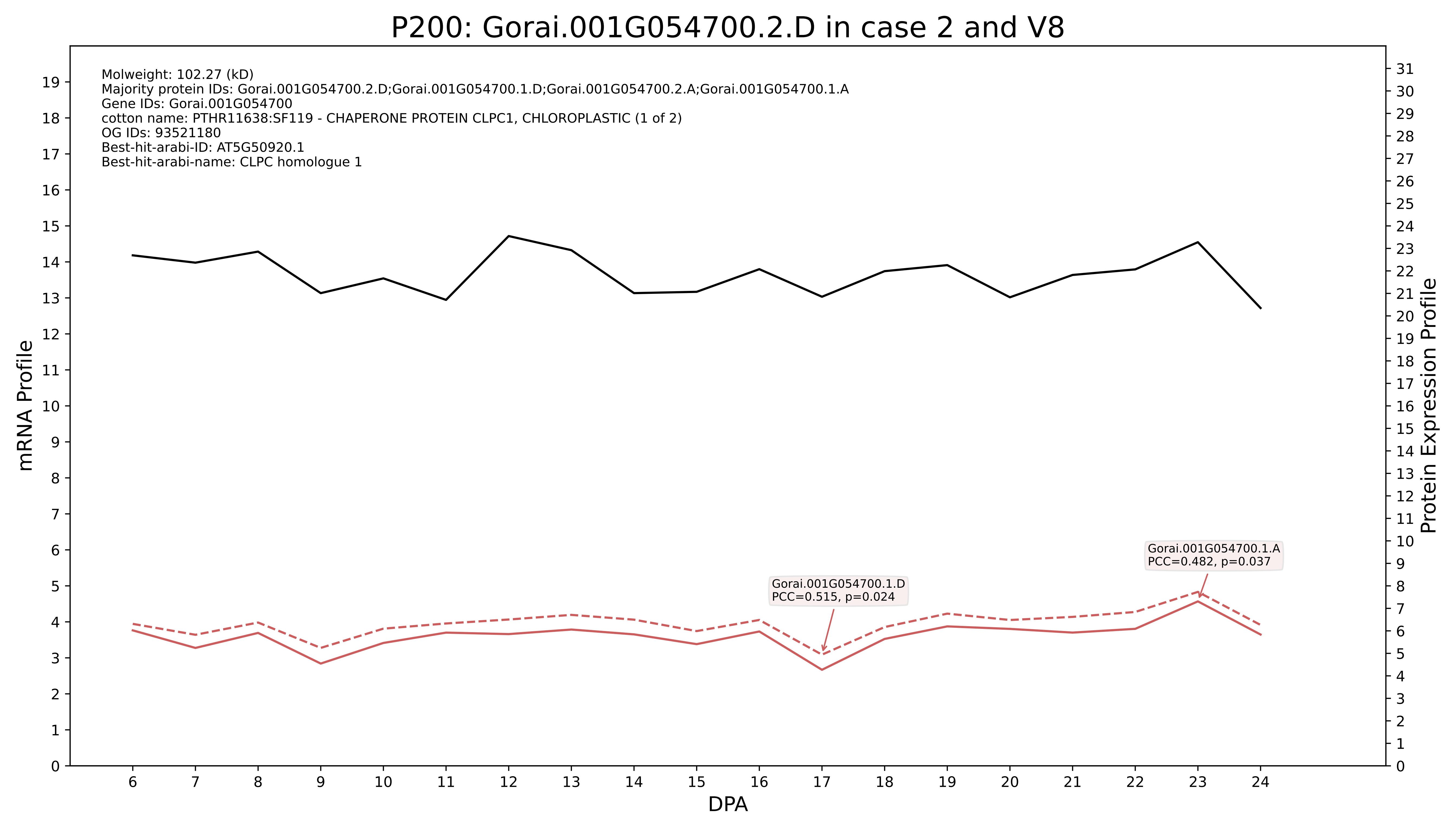

P200: Gorai.001G054700.2.D in case 2 and V8
Molweight: 102.27 (kD)
Majority protein IDs: Gorai.001G054700.2.D;Gorai.001G054700.1.D;Gorai.001G054700.2.A;Gorai.001G054700.1.A
Gene IDs: Gorai.001G054700
cotton name: PTHR11638:SF119 - CHAPERONE PROTEIN CLPC1, CHLOROPLASTIC (1 of 2)
OG IDs: 93521180
Best-hit-arabi-ID: AT5G50920.1
Best-hit-arabi-name: CLPC homologue 1
Gorai.001G054700.1.D
PCC=0.515, p=0.024
Gorai.001G054700.1.A
PCC=0.482, p=0.037
mRNA Profile
Protein Expression Profile
DPA

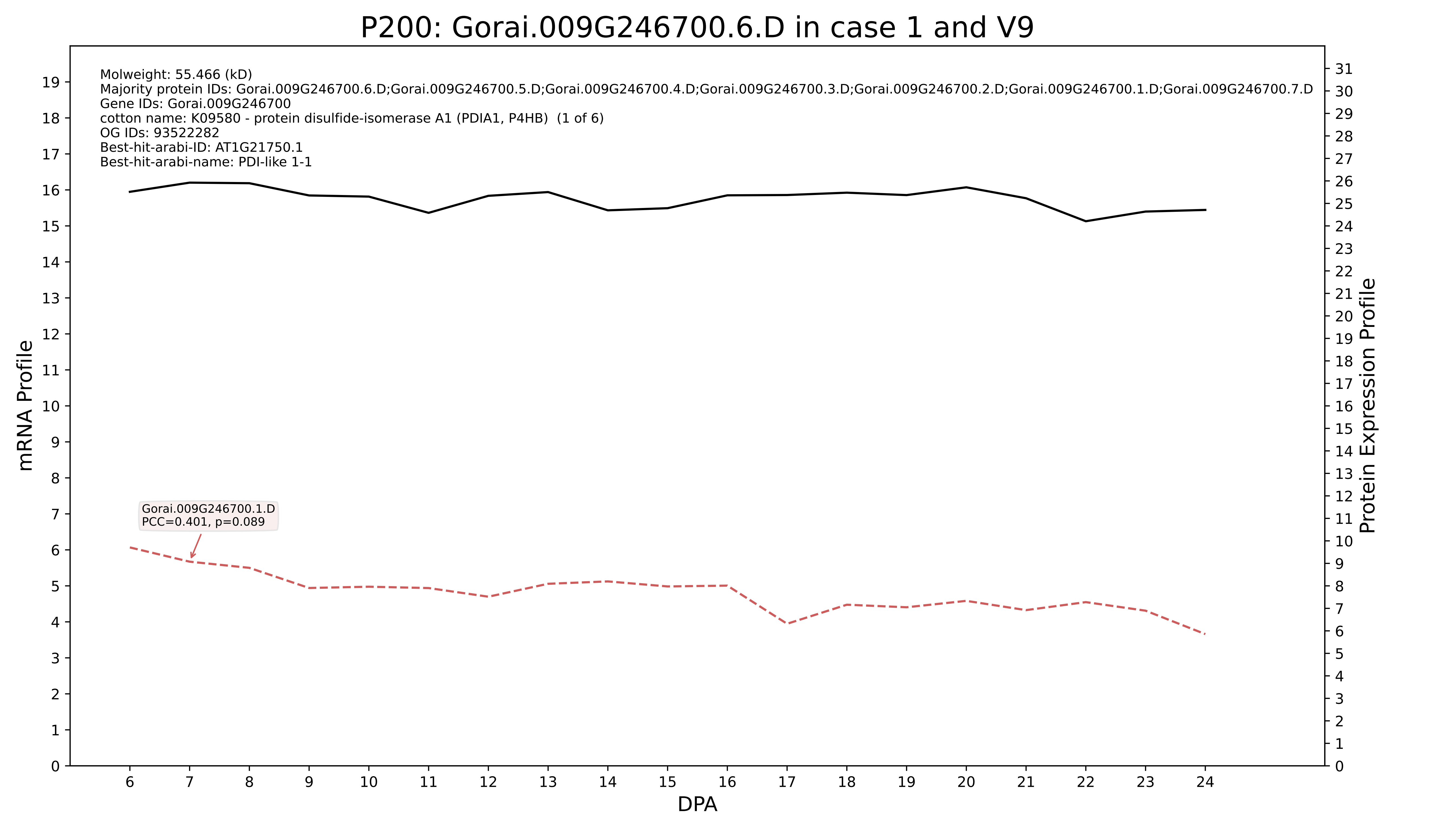

P200: Gorai.009G246700.6.D in case 1 and V9
Molweight: 55.466 (kD)
Majority protein IDs: Gorai.009G246700.6.D;Gorai.009G246700.5.D;Gorai.009G246700.4.D;Gorai.009G246700.3.D;Gorai.009G246700.2.D;Gorai.009G246700.1.D;Gorai.009G246700.7.D
Gene IDs: Gorai.009G246700
cotton name: K09580 - protein disulfide-isomerase A1 (PDIA1, P4HB) (1 of 6)
OG IDs: 93522282
Best-hit-arabi-ID: AT1G21750.1
Best-hit-arabi-name: PDI-like 1-1
Gorai.009G246700.1.D
PCC=0.401, p=0.089
mRNA Profile
Protein Expression Profile
DPA

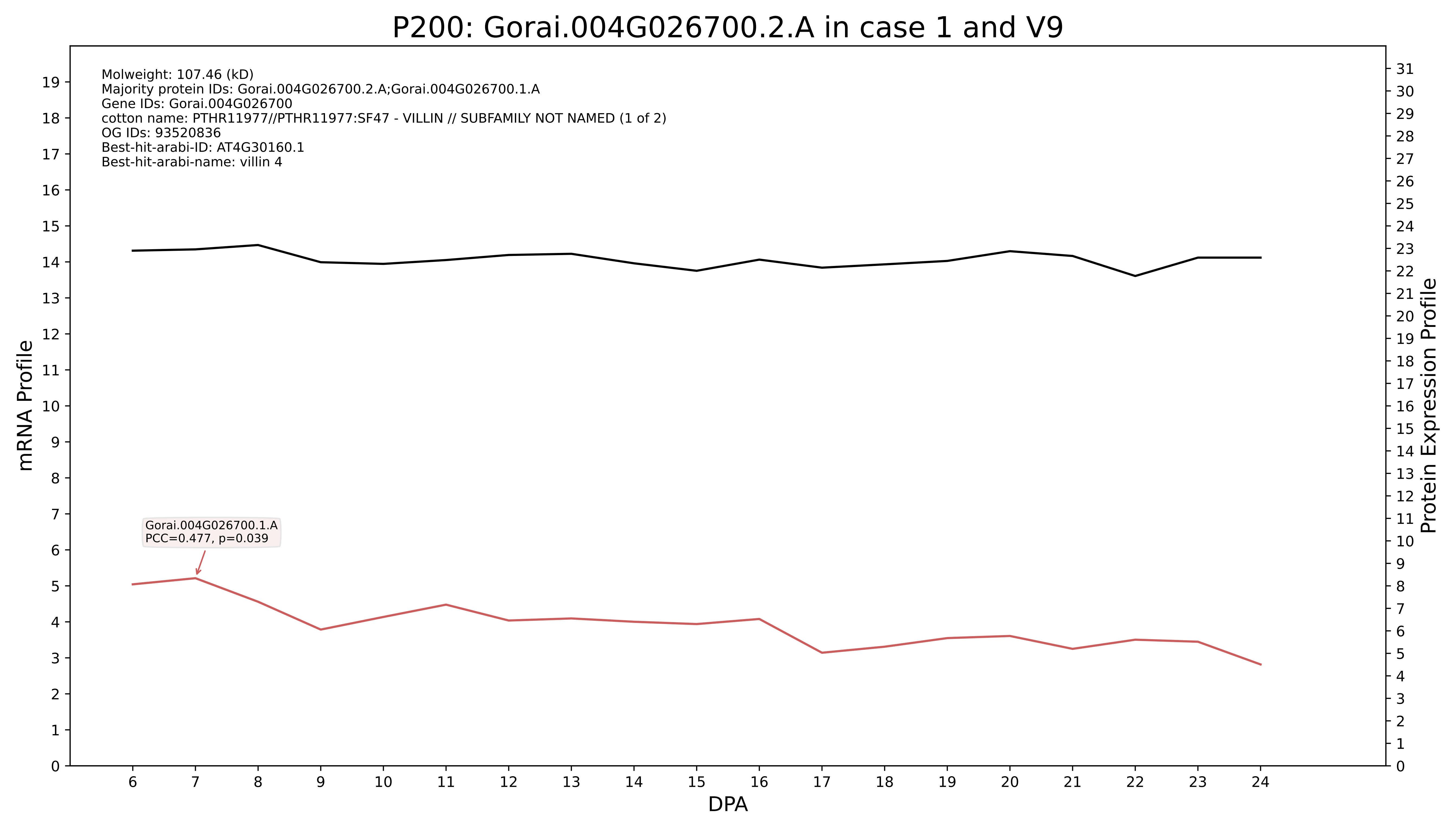

P200: Gorai.004G026700.2.A in case 1 and V9
Molweight: 107.46 (kD)
Majority protein IDs: Gorai.004G026700.2.A;Gorai.004G026700.1.A
Gene IDs: Gorai.004G026700
cotton name: PTHR11977//PTHR11977:SF47 - VILLIN // SUBFAMILY NOT NAMED (1 of 2)
OG IDs: 93520836
Best-hit-arabi-ID: AT4G30160.1
Best-hit-arabi-name: villin 4
Gorai.004G026700.1.A
PCC=0.477, p=0.039
mRNA Profile
Protein Expression Profile
DPA

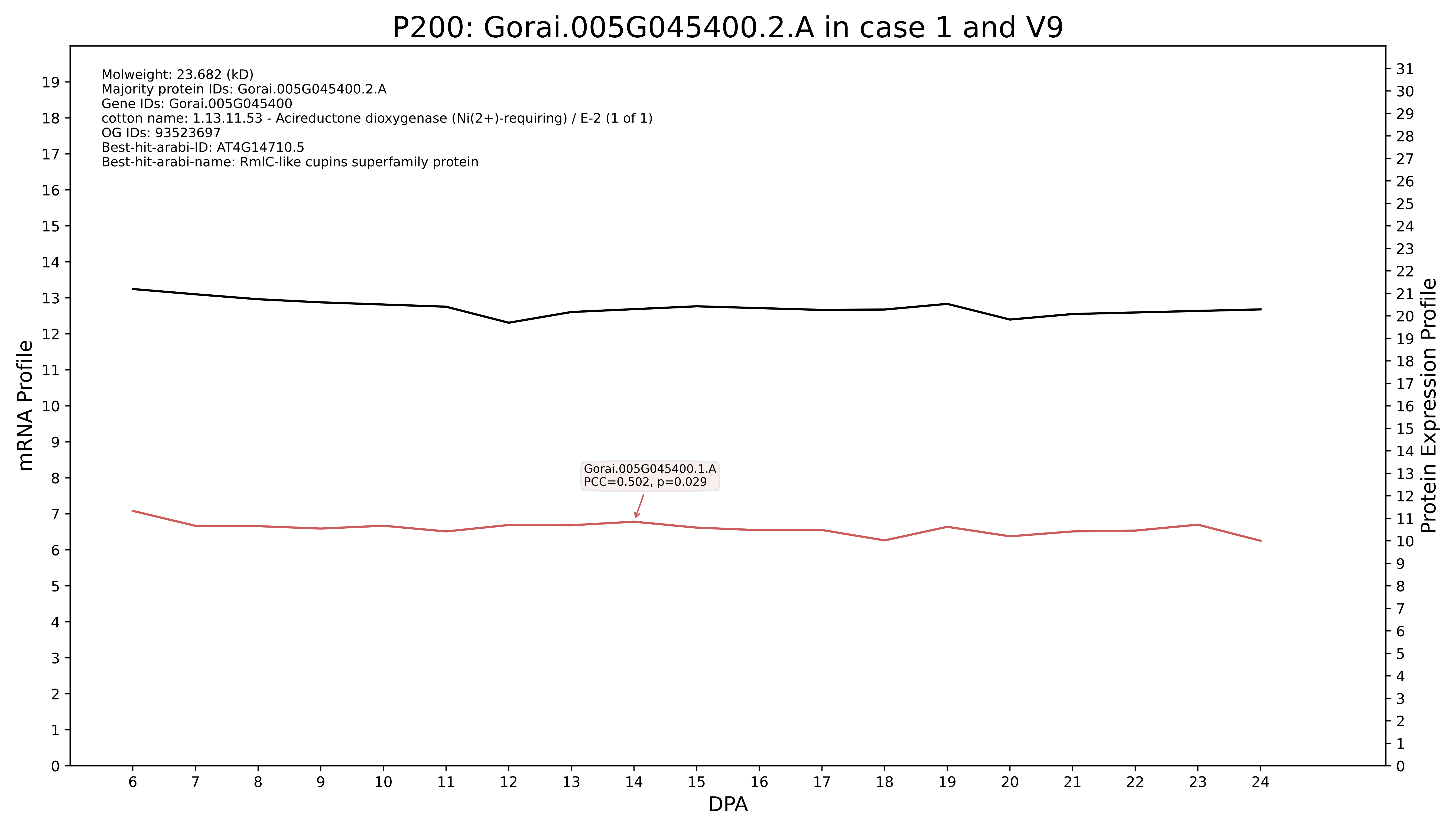

P200: Gorai.005G045400.2.A in case 1 and V9
Molweight: 23.682 (kD)
Majority protein IDs: Gorai.005G045400.2.A
Gene IDs: Gorai.005G045400
cotton name: 1.13.11.53 - Acireductone dioxygenase (Ni(2+)-requiring) / E-2 (1 of 1)
OG IDs: 93523697
Best-hit-arabi-ID: AT4G14710.5
Best-hit-arabi-name: RmlC-like cupins superfamily protein
Gorai.005G045400.1.A
PCC=0.502, p=0.029
mRNA Profile
Protein Expression Profile
DPA

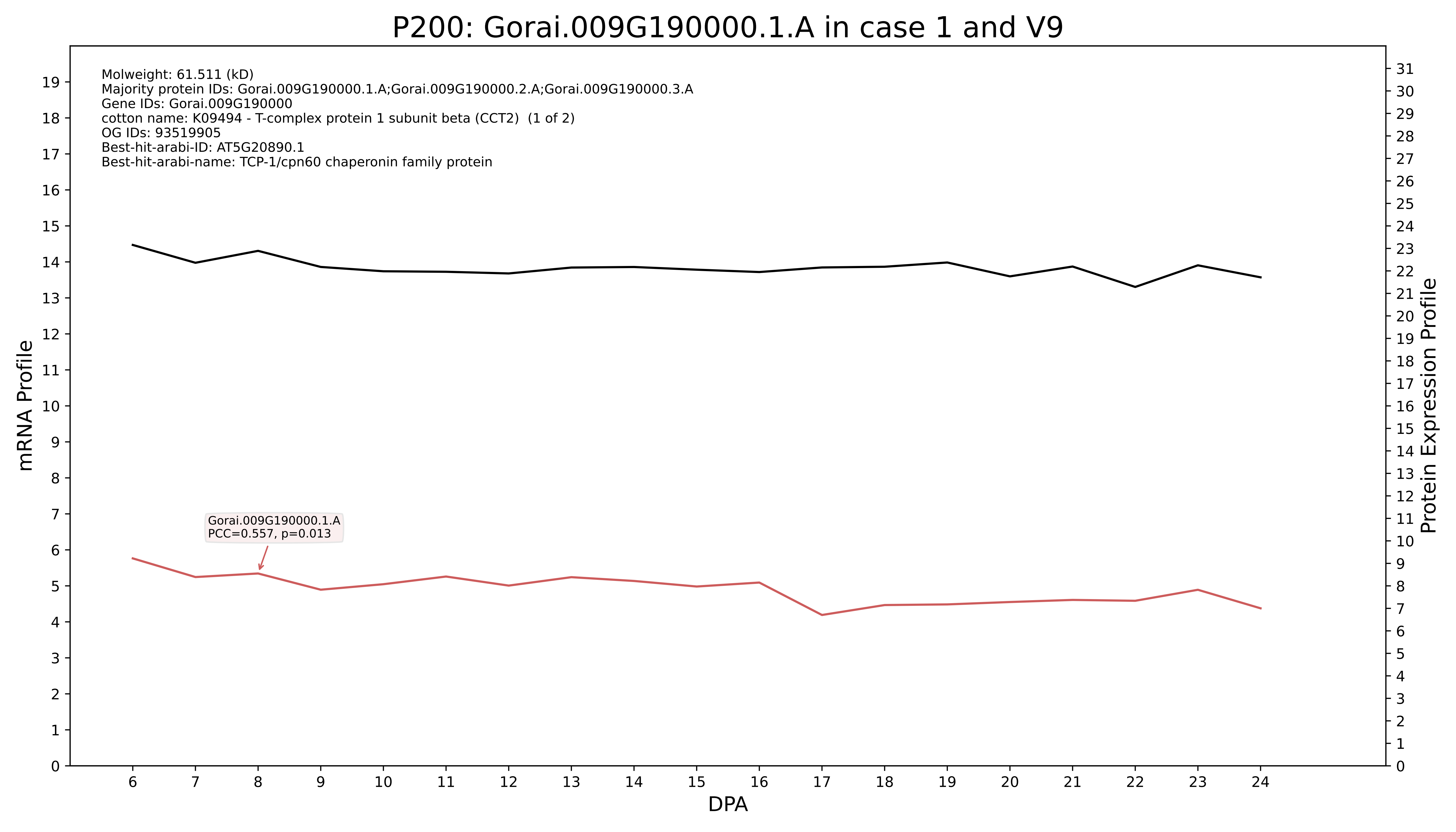

P200: Gorai.009G190000.1.A in case 1 and V9
Molweight: 61.511 (kD)
Majority protein IDs: Gorai.009G190000.1.A;Gorai.009G190000.2.A;Gorai.009G190000.3.A
Gene IDs: Gorai.009G190000
cotton name: K09494 - T-complex protein 1 subunit beta (CCT2) (1 of 2)
OG IDs: 93519905
Best-hit-arabi-ID: AT5G20890.1
Best-hit-arabi-name: TCP-1/cpn60 chaperonin family protein
Gorai.009G190000.1.A
PCC=0.557, p=0.013
mRNA Profile
Protein Expression Profile
DPA

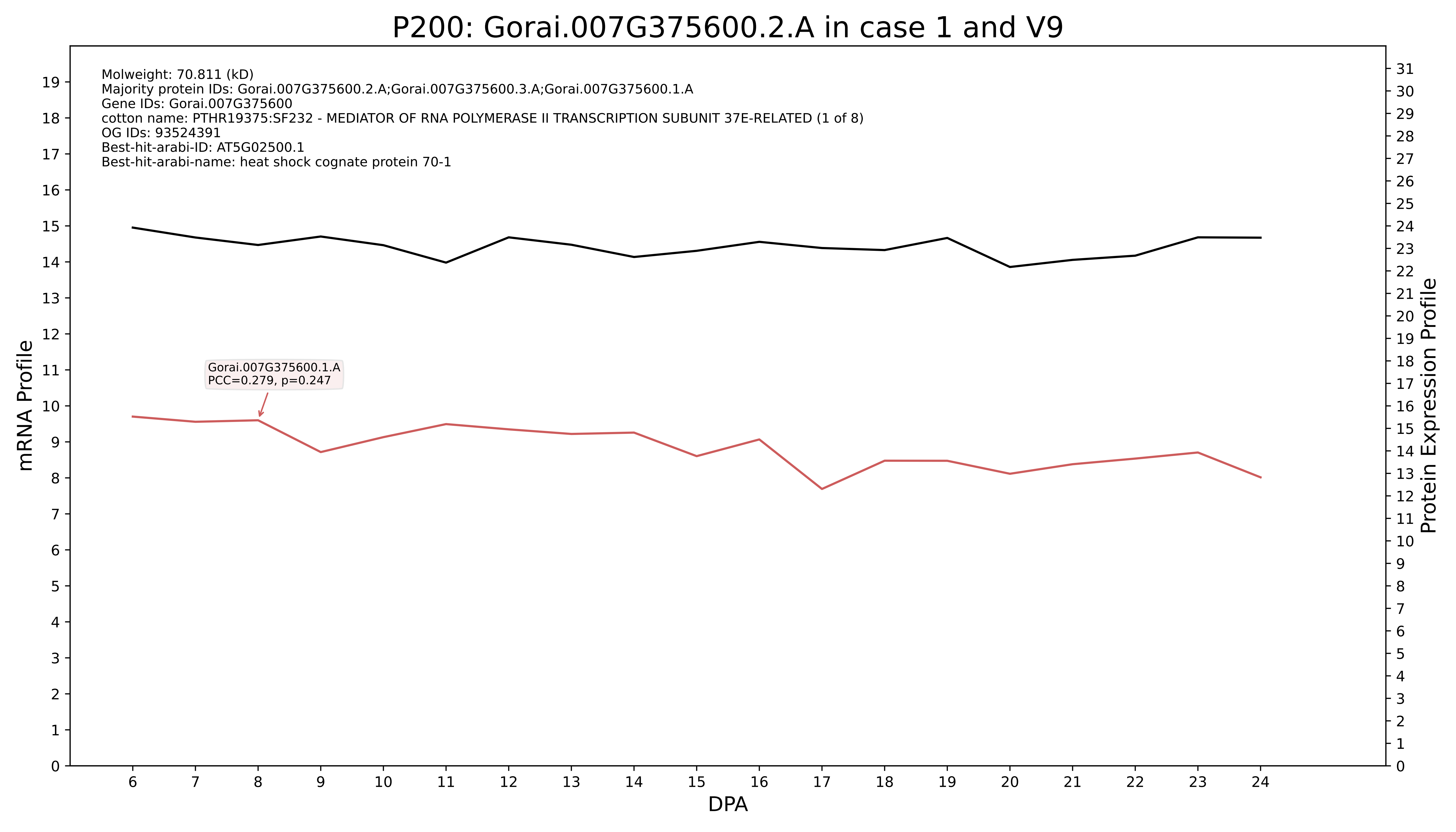
P200: Gorai.007G375600.2.A in case 1 and V9
Molweight: 70.811 (kD)
Majority protein IDs: Gorai.007G375600.2.A;Gorai.007G375600.3.A;Gorai.007G375600.1.A
Gene IDs: Gorai.007G375600
cotton name: PTHR19375:SF232 - MEDIATOR OF RNA POLYMERASE II TRANSCRIPTION SUBUNIT 37E-RELATED (1 of 8)
OG IDs: 93524391
Best-hit-arabi-ID: AT5G02500.1
Best-hit-arabi-name: heat shock cognate protein 70-1
Gorai.007G375600.1.A
PCC=0.279, p=0.247
mRNA Profile
Protein Expression Profile
DPA

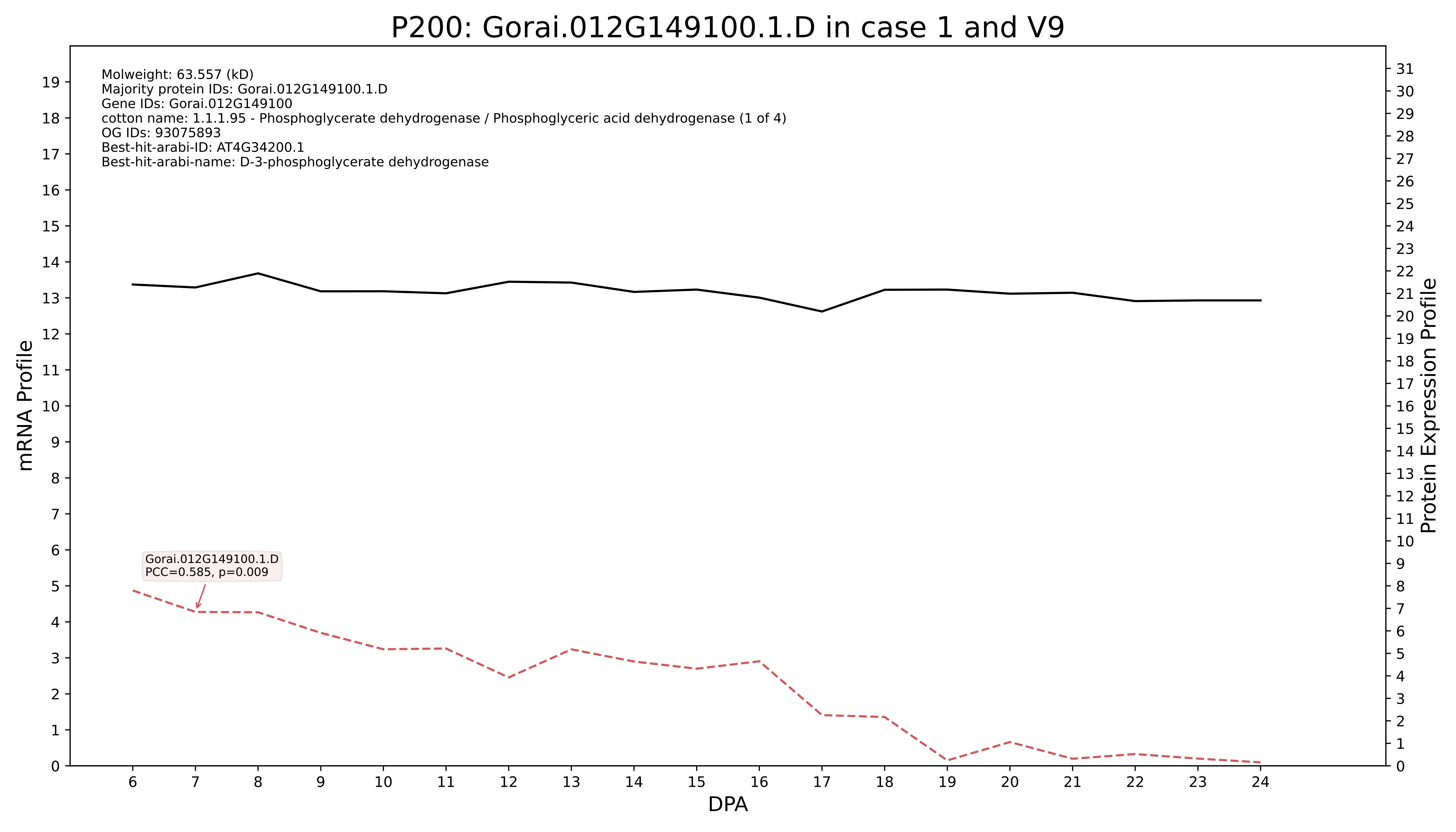

P200: Gorai.012G149100.1.D in case 1 and V9
Molweight: 63.557 (kD)
Majority protein IDs: Gorai.012G149100.1.D
Gene IDs: Gorai.012G149100
cotton name: 1.1.1.95 - Phosphoglycerate dehydrogenase / Phosphoglyceric acid dehydrogenase (1 of 4)
OG IDs: 93075893
Best-hit-arabi-ID: AT4G34200.1
Best-hit-arabi-name: D-3-phosphoglycerate dehydrogenase
Gorai.012G149100.1.D
PCC=0.585, p=0.009
mRNA Profile
Protein Expression Profile
DPA

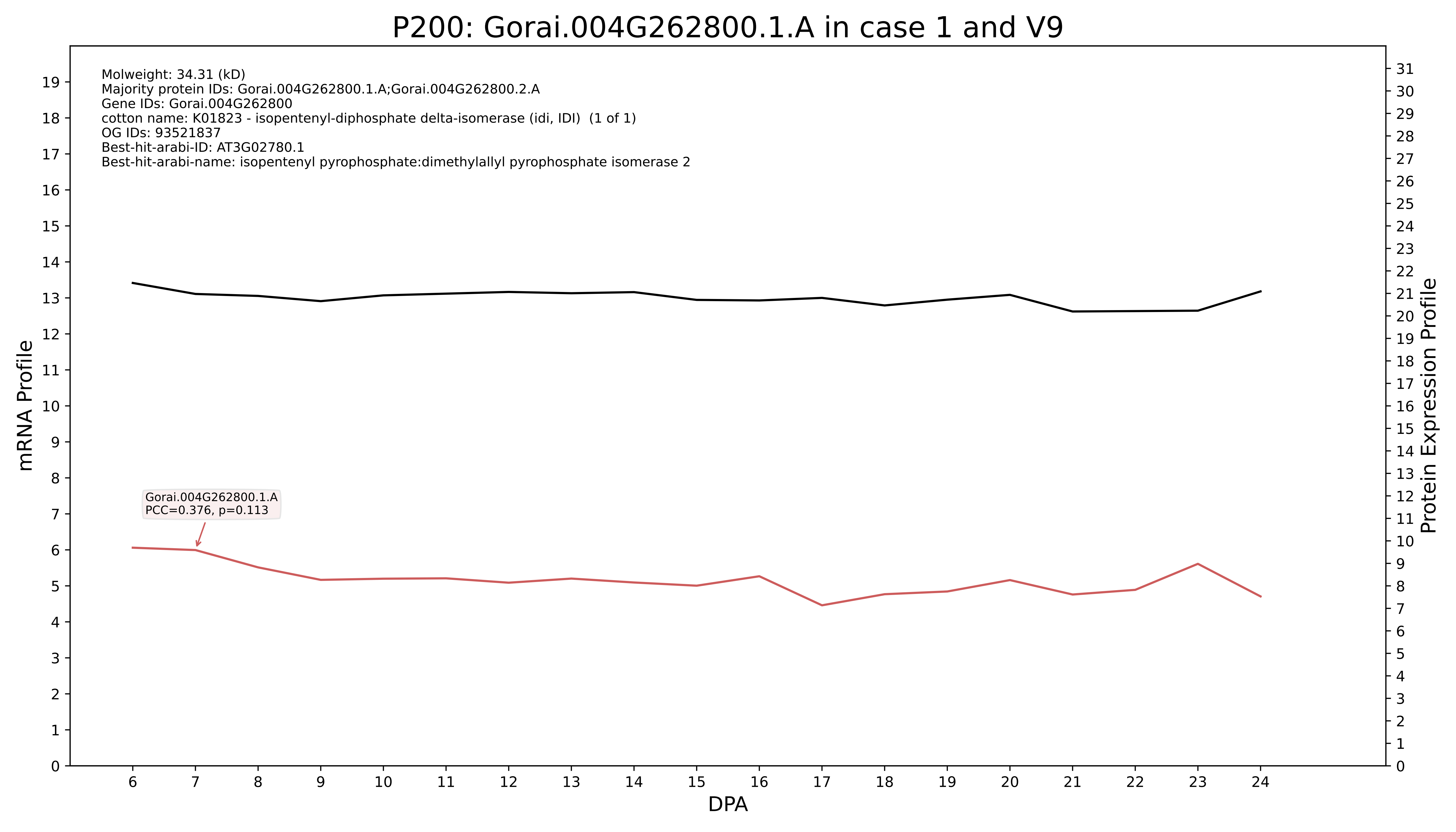
P200: Gorai.004G262800.1.A in case 1 and V9
Molweight: 34.31 (kD)
Majority protein IDs: Gorai.004G262800.1.A;Gorai.004G262800.2.A
Gene IDs: Gorai.004G262800
cotton name: K01823 - isopentenyl-diphosphate delta-isomerase (idi, IDI) (1 of 1)
OG IDs: 93521837
Best-hit-arabi-ID: AT3G02780.1
Best-hit-arabi-name: isopentenyl pyrophosphate:dimethylallyl pyrophosphate isomerase 2
Gorai.004G262800.1.A
PCC=0.376, p=0.113
mRNA Profile
Protein Expression Profile
DPA
0
1
2
3
4
5
6
7
8
9
10
11
12
13
14
15
16
17
18
19
20
21
22
23
24
25
26
27
28
29
30
31

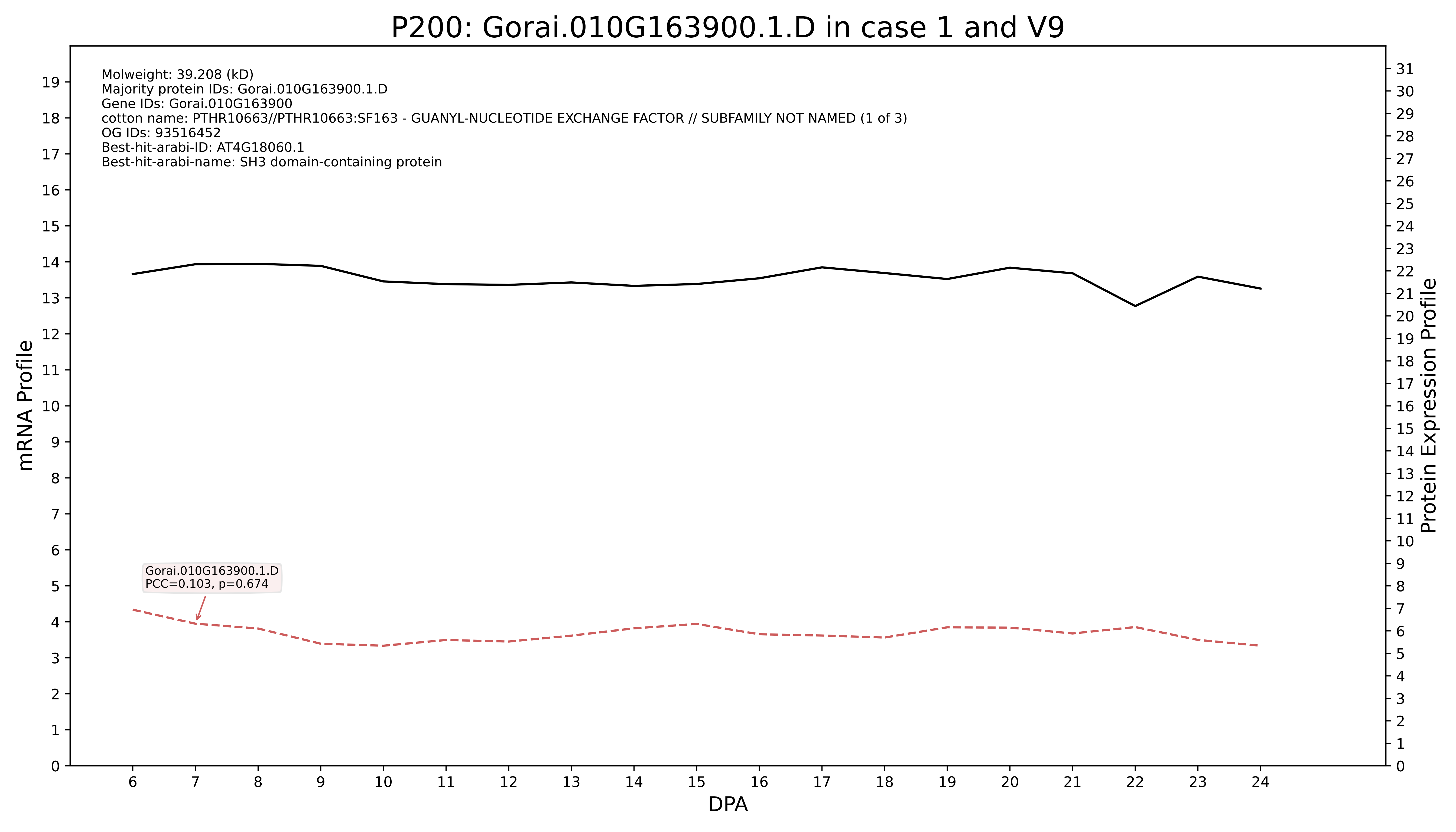
P200: Gorai.010G163900.1.D in case 1 and V9
Molweight: 39.208 (kD)
Majority protein IDs: Gorai.010G163900.1.D
Gene IDs: Gorai.010G163900
cotton name: PTHR10663//PTHR10663:SF163 - GUANYL-NUCLEOTIDE EXCHANGE FACTOR // SUBFAMILY NOT NAMED (1 of 3)
OG IDs: 93516452
Best-hit-arabi-ID: AT4G18060.1
Best-hit-arabi-name: SH3 domain-containing protein
Gorai.010G163900.1.D
PCC=0.103, p=0.674
mRNA Profile
Protein Expression Profile
DPA

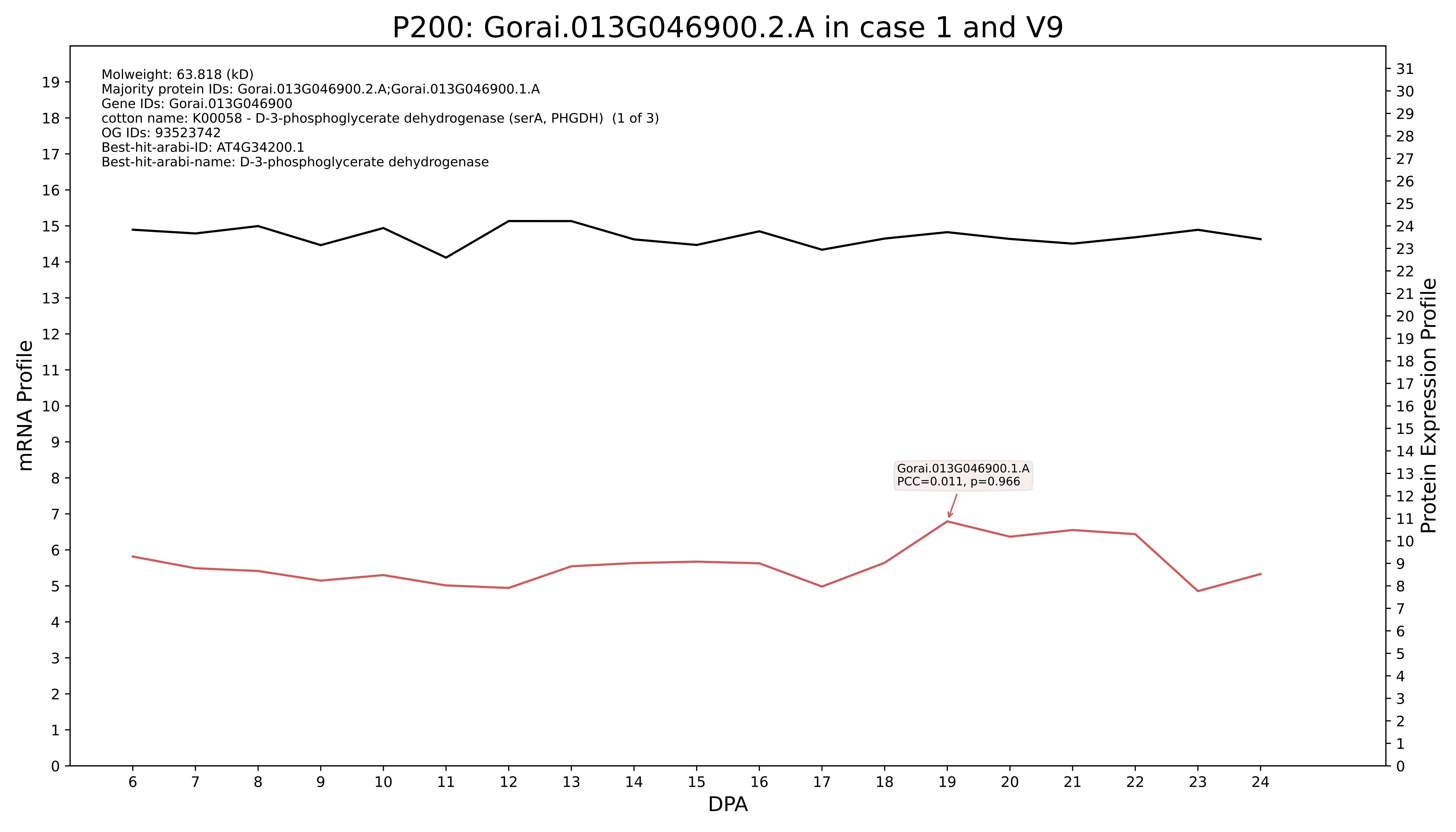
P200: Gorai.013G046900.2.A in case 1 and V9
Molweight: 63.818 (kD)
Majority protein IDs: Gorai.013G046900.2.A;Gorai.013G046900.1.A
Gene IDs: Gorai.013G046900
cotton name: K00058 - D-3-phosphoglycerate dehydrogenase (serA, PHGDH) (1 of 3)
OG IDs: 93523742
Best-hit-arabi-ID: AT4G34200.1
Best-hit-arabi-name: D-3-phosphoglycerate dehydrogenase
Gorai.013G046900.1.A
PCC=0.011, p=0.966
mRNA Profile
Protein Expression Profile
DPA

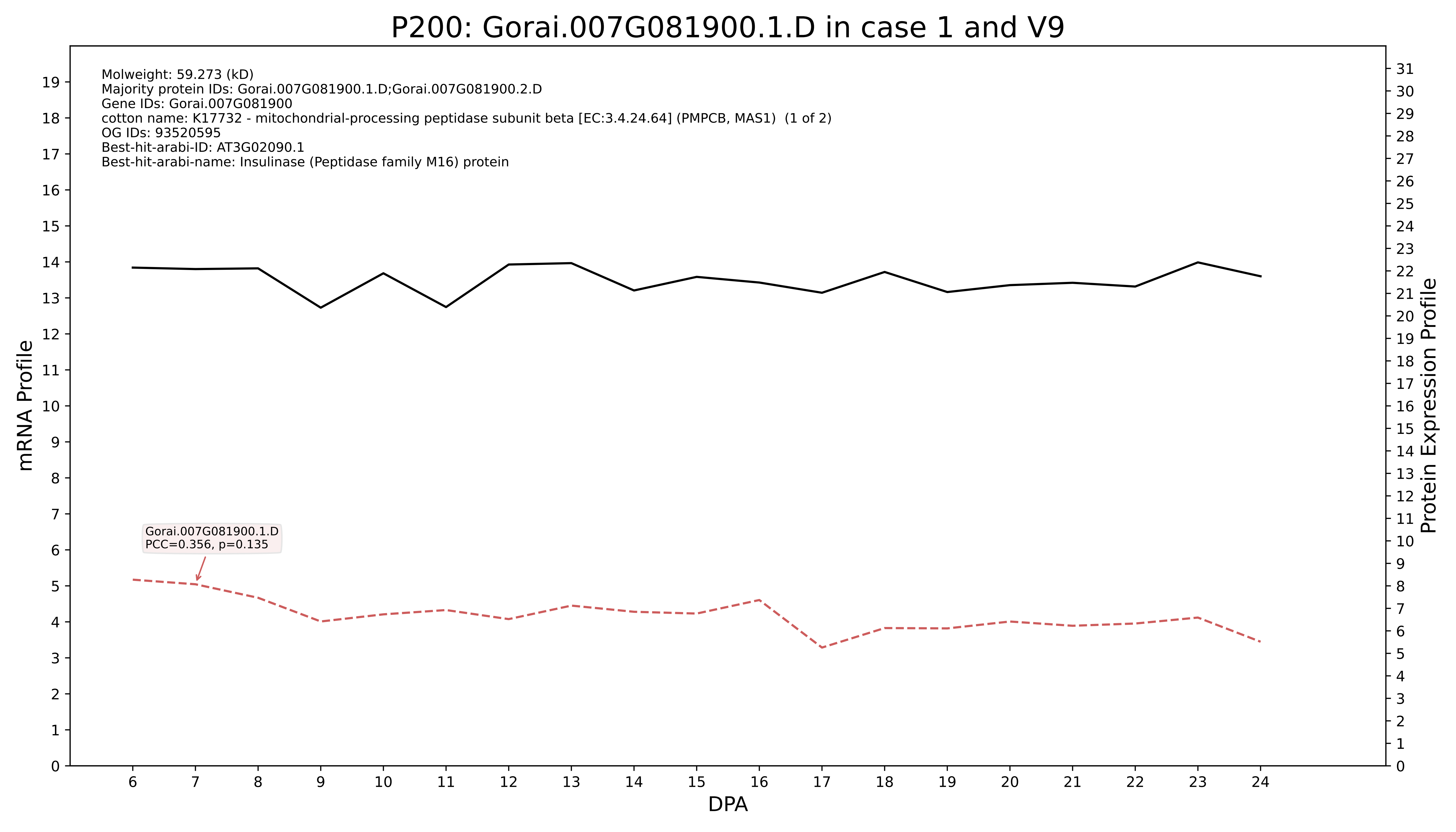
P200: Gorai.007G081900.1.D in case 1 and V9
Molweight: 59.273 (kD)
Majority protein IDs: Gorai.007G081900.1.D;Gorai.007G081900.2.D
Gene IDs: Gorai.007G081900
cotton name: K17732 - mitochondrial-processing peptidase subunit beta [EC:3.4.24.64] (PMPCB, MAS1) (1 of 2)
OG IDs: 93520595
Best-hit-arabi-ID: AT3G02090.1
Best-hit-arabi-name: Insulinase (Peptidase family M16) protein
Gorai.007G081900.1.D
PCC=0.356, p=0.135
mRNA Profile
Protein Expression Profile
DPA

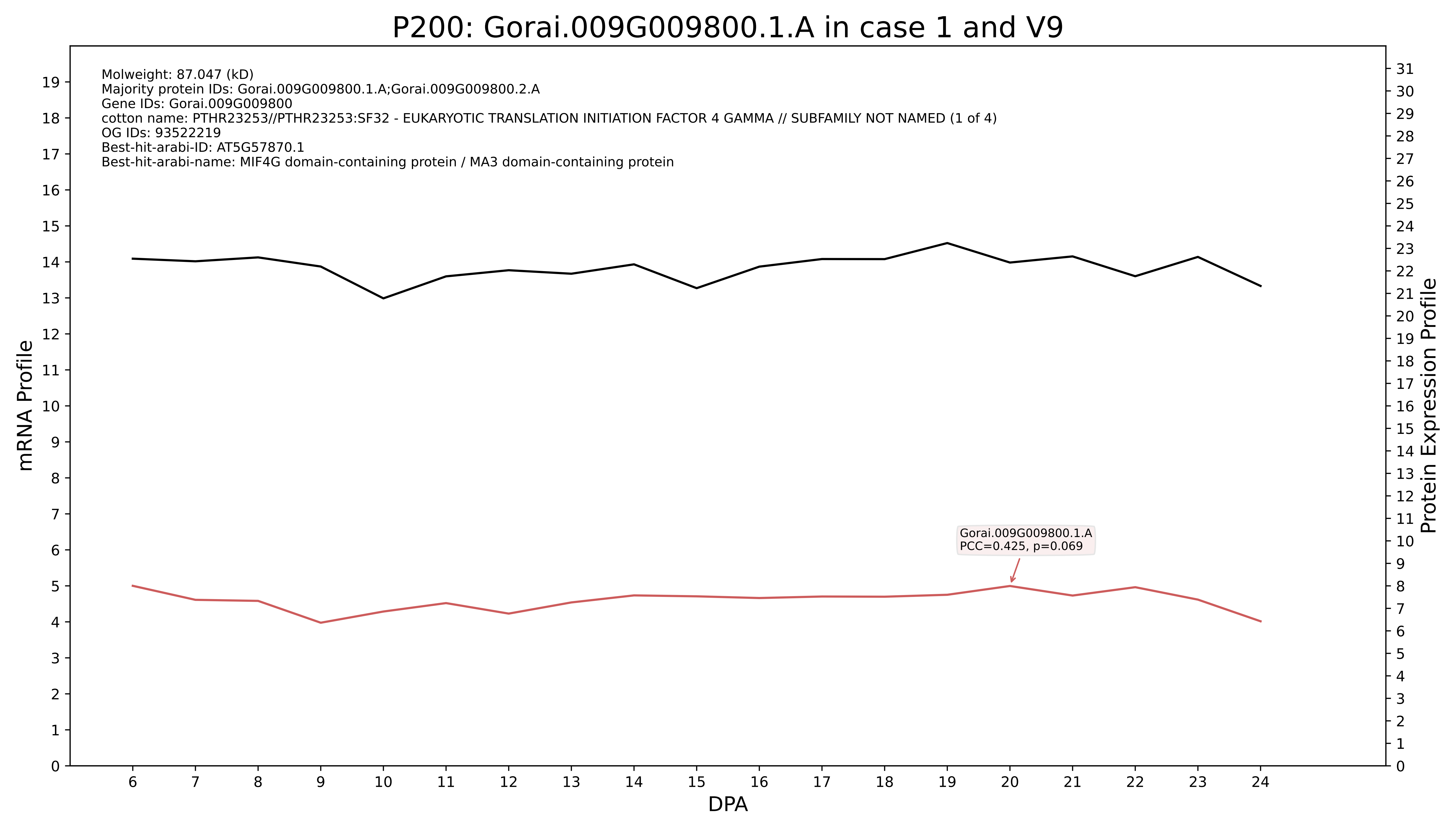

P200: Gorai.009G009800.1.A in case 1 and V9
Molweight: 87.047 (kD)
Majority protein IDs: Gorai.009G009800.1.A;Gorai.009G009800.2.A
Gene IDs: Gorai.009G009800
cotton name: PTHR23253//PTHR23253:SF32 - EUKARYOTIC TRANSLATION INITIATION FACTOR 4 GAMMA // SUBFAMILY NOT NAMED (1 of 4)
OG IDs: 93522219
Best-hit-arabi-ID: AT5G57870.1
Best-hit-arabi-name: MIF4G domain-containing protein / MA3 domain-containing protein
Gorai.009G009800.1.A
PCC=0.425, p=0.069
mRNA Profile
Protein Expression Profile
DPA

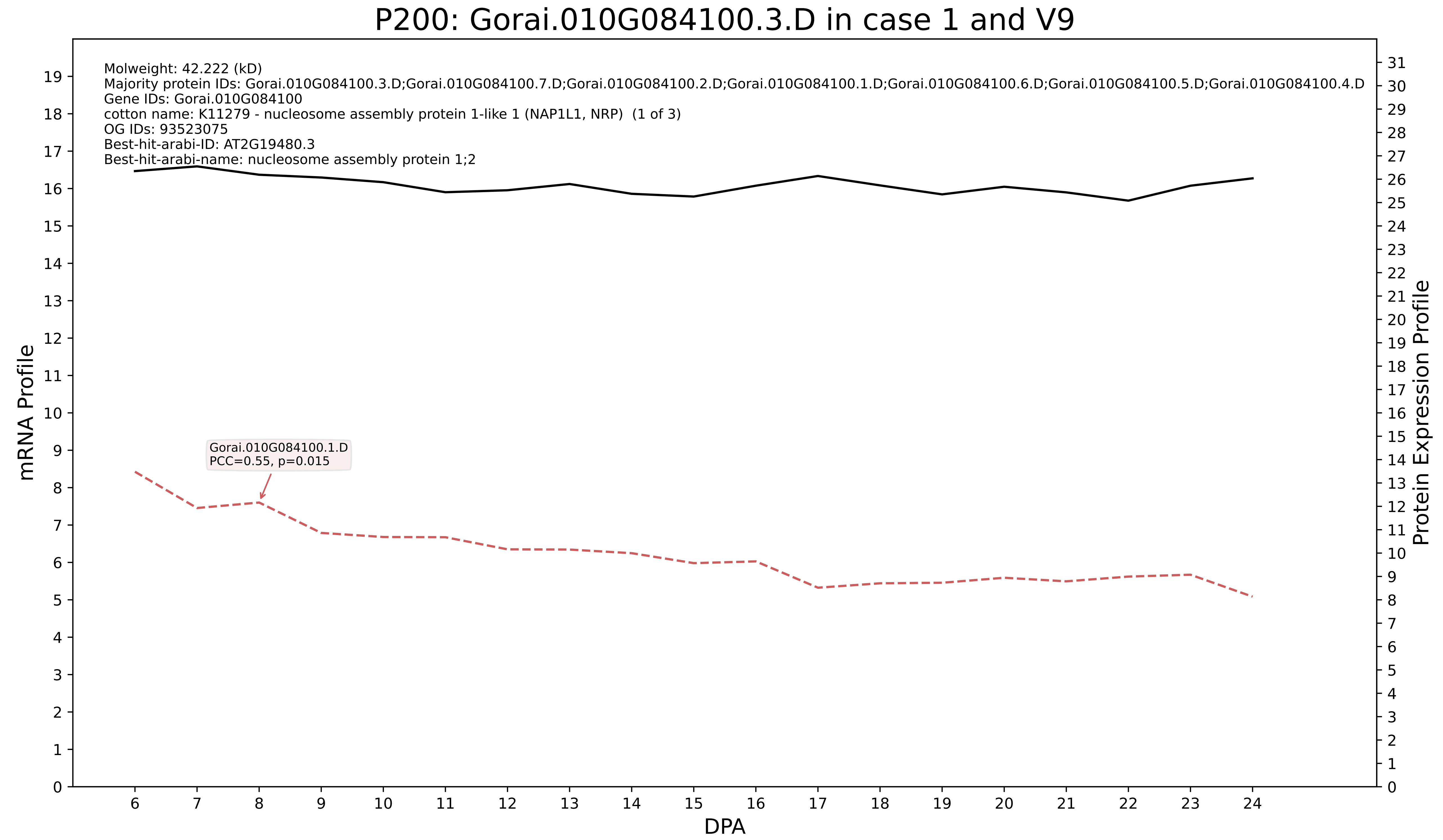

P200: Gorai.010G084100.3.D in case 1 and V9
Molweight: 42.222 (kD)
Majority protein IDs: Gorai.010G084100.3.D;Gorai.010G084100.7.D;Gorai.010G084100.2.D;Gorai.010G084100.1.D;Gorai.010G084100.6.D;Gorai.010G084100.5.D;Gorai.010G084100.4.D
Gene IDs: Gorai.010G084100
cotton name: K11279 - nucleosome assembly protein 1-like 1 (NAP1L1, NRP) (1 of 3)
OG IDs: 93523075
Best-hit-arabi-ID: AT2G19480.3
Best-hit-arabi-name: nucleosome assembly protein 1;2
Gorai.010G084100.1.D
PCC=0.55, p=0.015
mRNA Profile
Protein Expression Profile
DPA

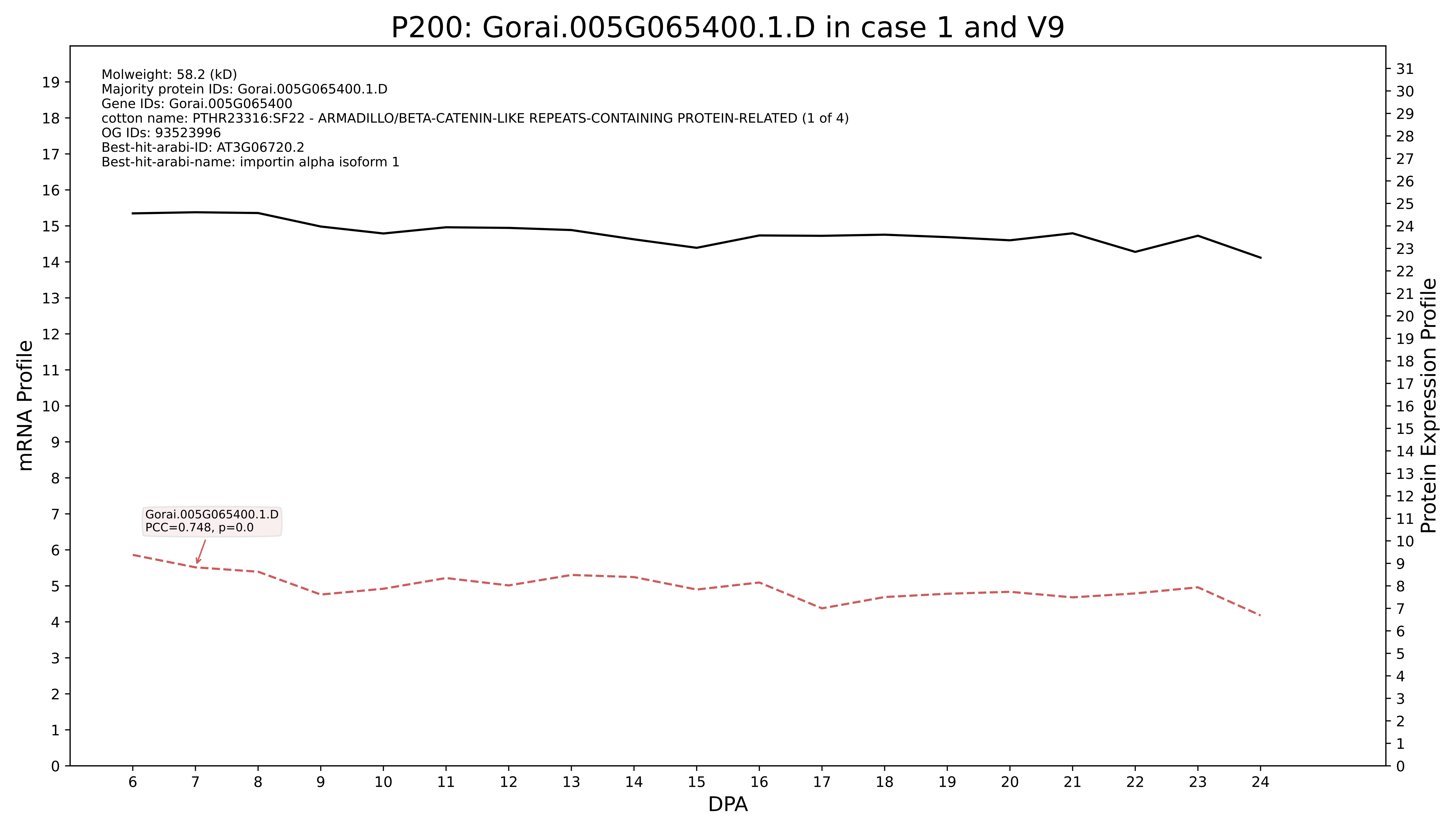
P200: Gorai.005G065400.1.D in case 1 and V9
Molweight: 58.2 (kD)
Majority protein IDs: Gorai.005G065400.1.D
Gene IDs: Gorai.005G065400
cotton name: PTHR23316:SF22 - ARMADILLO/BETA-CATENIN-LIKE REPEATS-CONTAINING PROTEIN-RELATED (1 of 4)
OG IDs: 93523996
Best-hit-arabi-ID: AT3G06720.2
Best-hit-arabi-name: importin alpha isoform 1
Gorai.005G065400.1.D
PCC=0.748, p=0.0
mRNA Profile
Protein Expression Profile
DPA
0
1
2
3
4
5
6
7
8
9
10
11
12
13
14
15
16
17
18
19
20
21
22
23
24
25
26
27
28
29
30
31

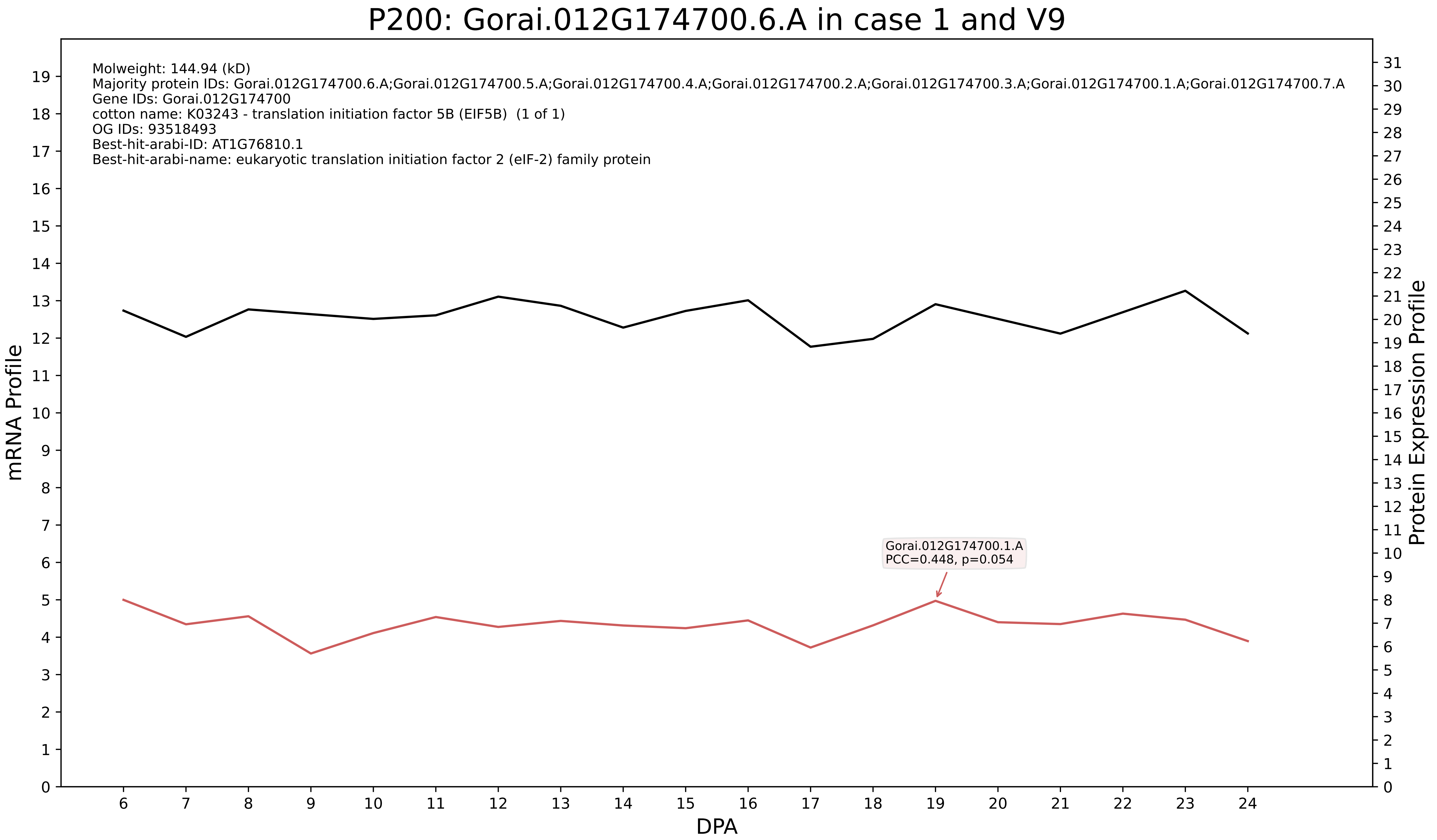
P200: Gorai.012G174700.6.A in case 1 and V9
Molweight: 144.94 (kD)
Majority protein IDs: Gorai.012G174700.6.A;Gorai.012G174700.5.A;Gorai.012G174700.4.A;Gorai.012G174700.2.A;Gorai.012G174700.3.A;Gorai.012G174700.1.A;Gorai.012G174700.7.A
Gene IDs: Gorai.012G174700
cotton name: K03243 - translation initiation factor 5B (EIF5B) (1 of 1)
OG IDs: 93518493
Best-hit-arabi-ID: AT1G76810.1
Best-hit-arabi-name: eukaryotic translation initiation factor 2 (eIF-2) family protein
Gorai.012G174700.1.A
PCC=0.448, p=0.054
mRNA Profile
Protein Expression Profile
DPA

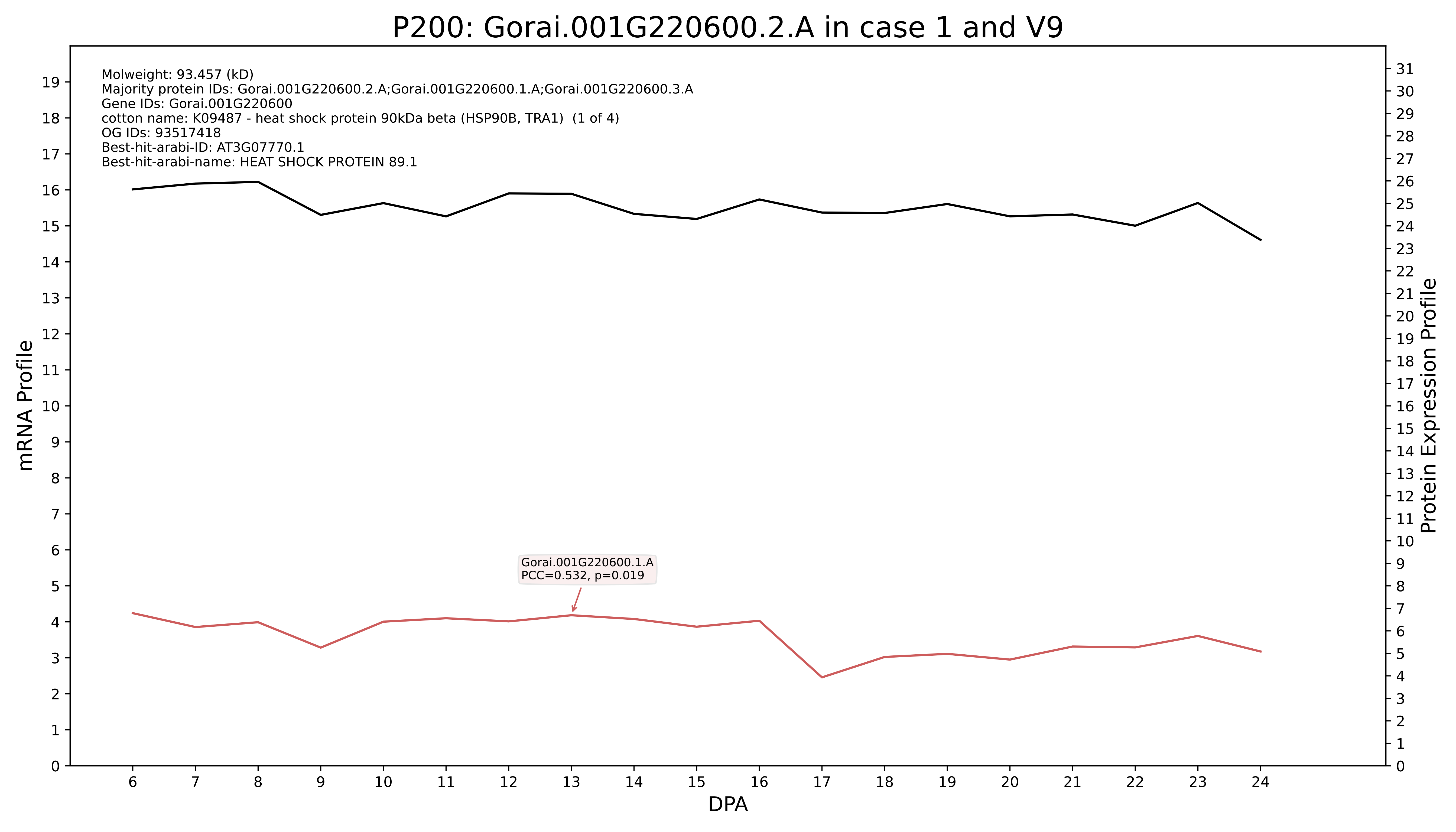
P200: Gorai.001G220600.2.A in case 1 and V9
Molweight: 93.457 (kD)
Majority protein IDs: Gorai.001G220600.2.A;Gorai.001G220600.1.A;Gorai.001G220600.3.A
Gene IDs: Gorai.001G220600
cotton name: K09487 - heat shock protein 90kDa beta (HSP90B, TRA1) (1 of 4)
OG IDs: 93517418
Best-hit-arabi-ID: AT3G07770.1
Best-hit-arabi-name: HEAT SHOCK PROTEIN 89.1
Gorai.001G220600.1.A
PCC=0.532, p=0.019
mRNA Profile
Protein Expression Profile
DPA

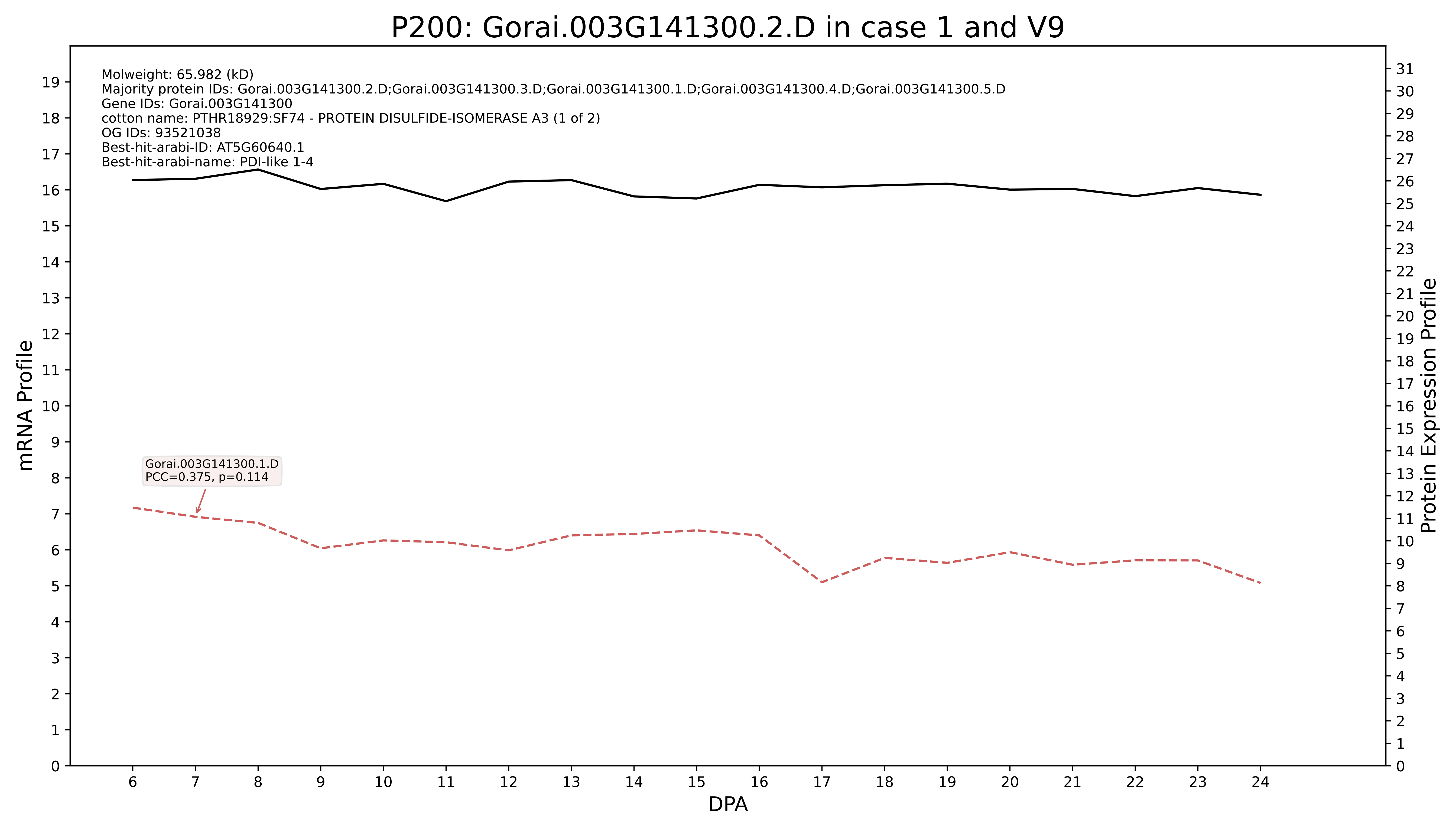
P200: Gorai.003G141300.2.D in case 1 and V9
Molweight: 65.982 (kD)
Majority protein IDs: Gorai.003G141300.2.D;Gorai.003G141300.3.D;Gorai.003G141300.1.D;Gorai.003G141300.4.D;Gorai.003G141300.5.D
Gene IDs: Gorai.003G141300
cotton name: PTHR18929:SF74 - PROTEIN DISULFIDE-ISOMERASE A3 (1 of 2)
OG IDs: 93521038
Best-hit-arabi-ID: AT5G60640.1
Best-hit-arabi-name: PDI-like 1-4
Gorai.003G141300.1.D
PCC=0.375, p=0.114
mRNA Profile
Protein Expression Profile
DPA
0
1
2
3
4
5
6
7
8
9
10
11
12
13
14
15
16
17
18
19
20
21
22
23
24
25
26
27
28
29
30
31

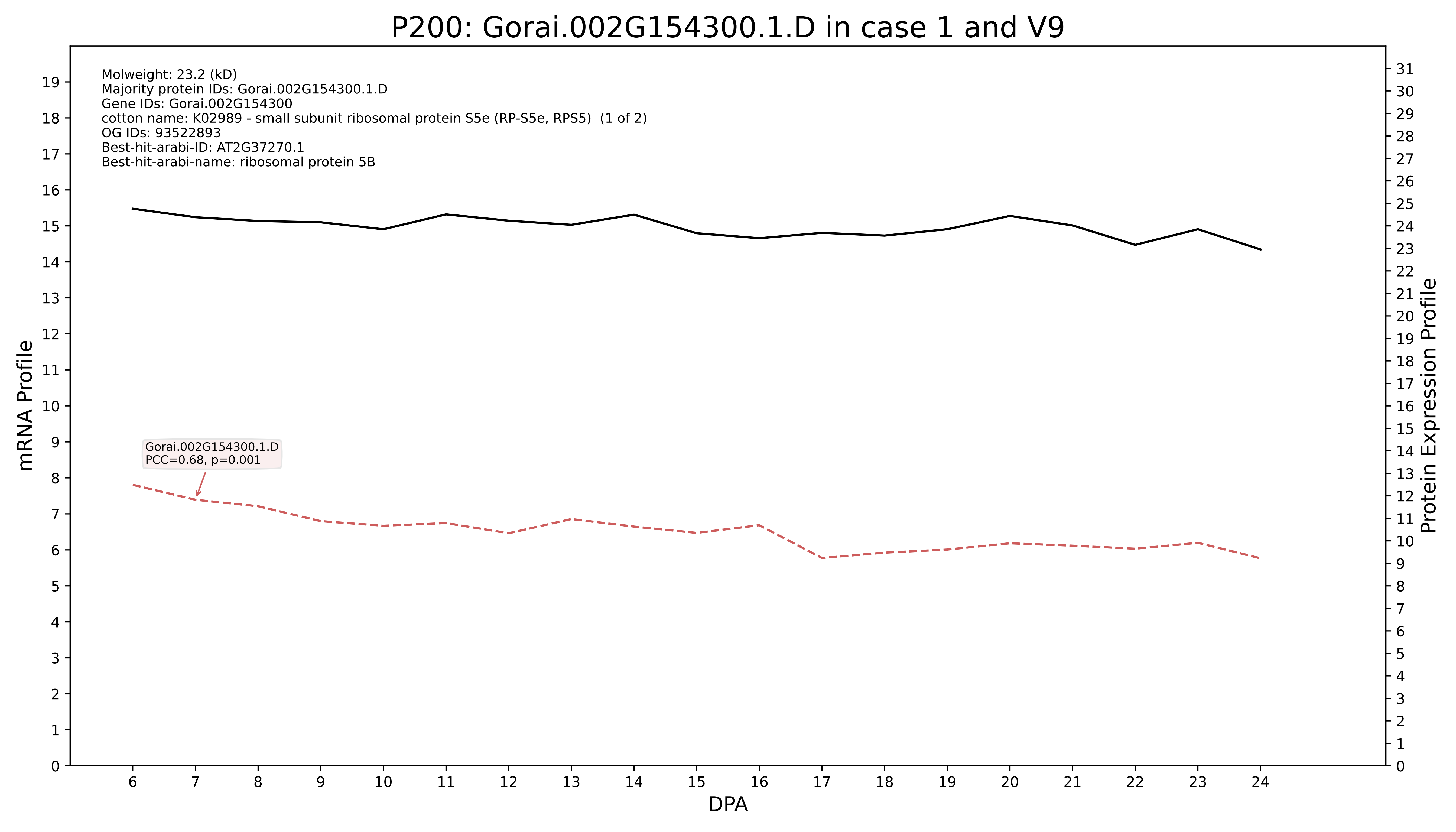
P200: Gorai.002G154300.1.D in case 1 and V9
Molweight: 23.2 (kD)
Majority protein IDs: Gorai.002G154300.1.D
Gene IDs: Gorai.002G154300
cotton name: K02989 - small subunit ribosomal protein S5e (RP-S5e, RPS5) (1 of 2)
OG IDs: 93522893
Best-hit-arabi-ID: AT2G37270.1
Best-hit-arabi-name: ribosomal protein 5B
Gorai.002G154300.1.D
PCC=0.68, p=0.001
mRNA Profile
Protein Expression Profile
DPA

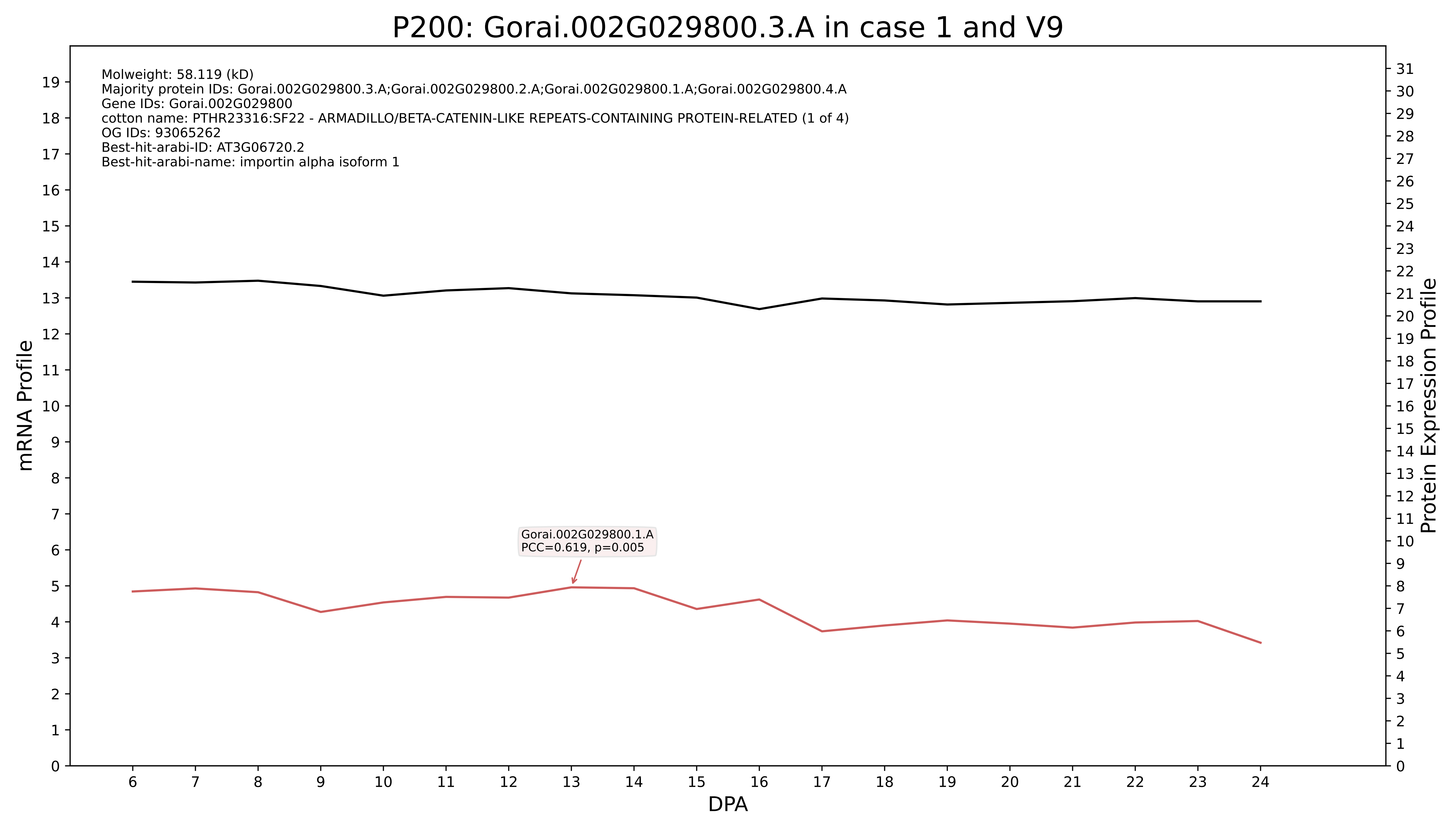
P200: Gorai.002G029800.3.A in case 1 and V9
Molweight: 58.119 (kD)
Majority protein IDs: Gorai.002G029800.3.A;Gorai.002G029800.2.A;Gorai.002G029800.1.A;Gorai.002G029800.4.A
Gene IDs: Gorai.002G029800
cotton name: PTHR23316:SF22 - ARMADILLO/BETA-CATENIN-LIKE REPEATS-CONTAINING PROTEIN-RELATED (1 of 4)
OG IDs: 93065262
Best-hit-arabi-ID: AT3G06720.2
Best-hit-arabi-name: importin alpha isoform 1
Gorai.002G029800.1.A
PCC=0.619, p=0.005
mRNA Profile
Protein Expression Profile
DPA

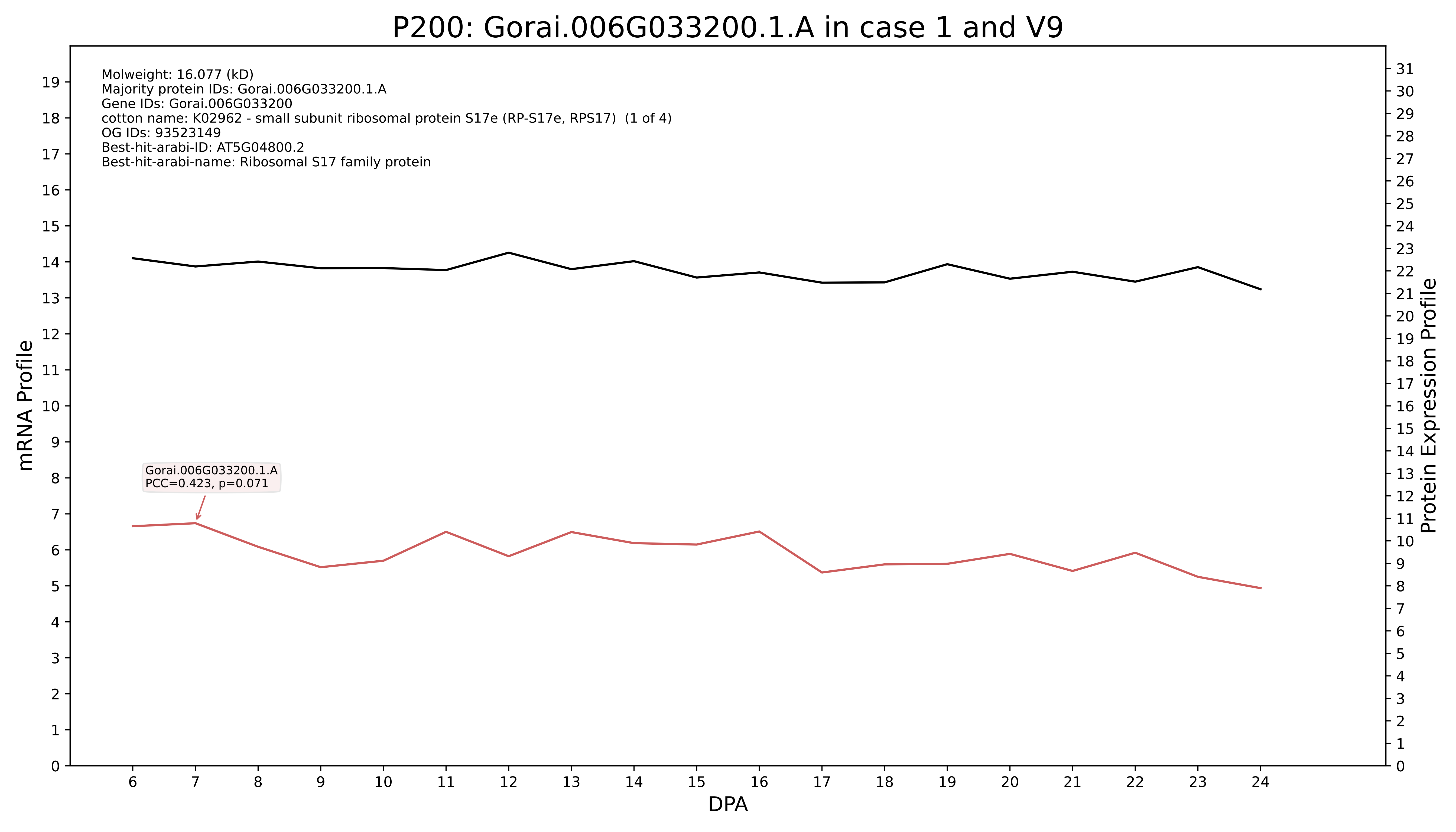
P200: Gorai.006G033200.1.A in case 1 and V9
Molweight: 16.077 (kD)
Majority protein IDs: Gorai.006G033200.1.A
Gene IDs: Gorai.006G033200
cotton name: K02962 - small subunit ribosomal protein S17e (RP-S17e, RPS17) (1 of 4)
OG IDs: 93523149
Best-hit-arabi-ID: AT5G04800.2
Best-hit-arabi-name: Ribosomal S17 family protein
Gorai.006G033200.1.A
PCC=0.423, p=0.071
mRNA Profile
Protein Expression Profile
DPA

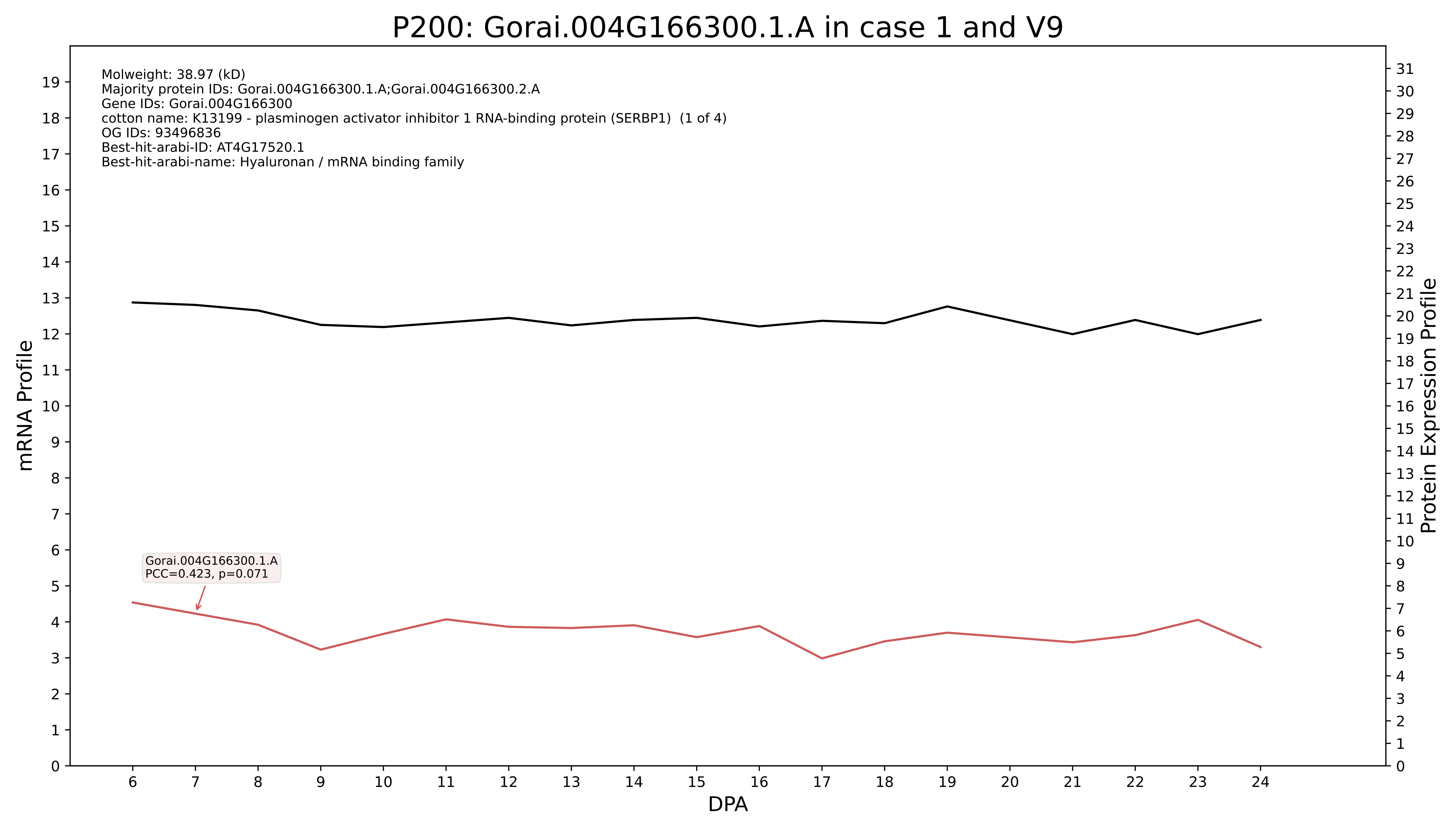
P200: Gorai.004G166300.1.A in case 1 and V9
Molweight: 38.97 (kD)
Majority protein IDs: Gorai.004G166300.1.A;Gorai.004G166300.2.A
Gene IDs: Gorai.004G166300
cotton name: K13199 - plasminogen activator inhibitor 1 RNA-binding protein (SERBP1) (1 of 4)
OG IDs: 93496836
Best-hit-arabi-ID: AT4G17520.1
Best-hit-arabi-name: Hyaluronan / mRNA binding family
Gorai.004G166300.1.A
PCC=0.423, p=0.071
mRNA Profile
Protein Expression Profile
DPA
0 1 2 3 4 5 6 7 8 9 10 11 12 13 14 15 16 17 18 19
0 1 2 3 4 5 6 7 8 9 10 11 12 13 14 15 16 17 18 19 20 21 22 23 24 25 26 27 28 29 30 31
6 7 8 9 10 11 12 13 14 15 16 17 18 19 20 21 22 23 24

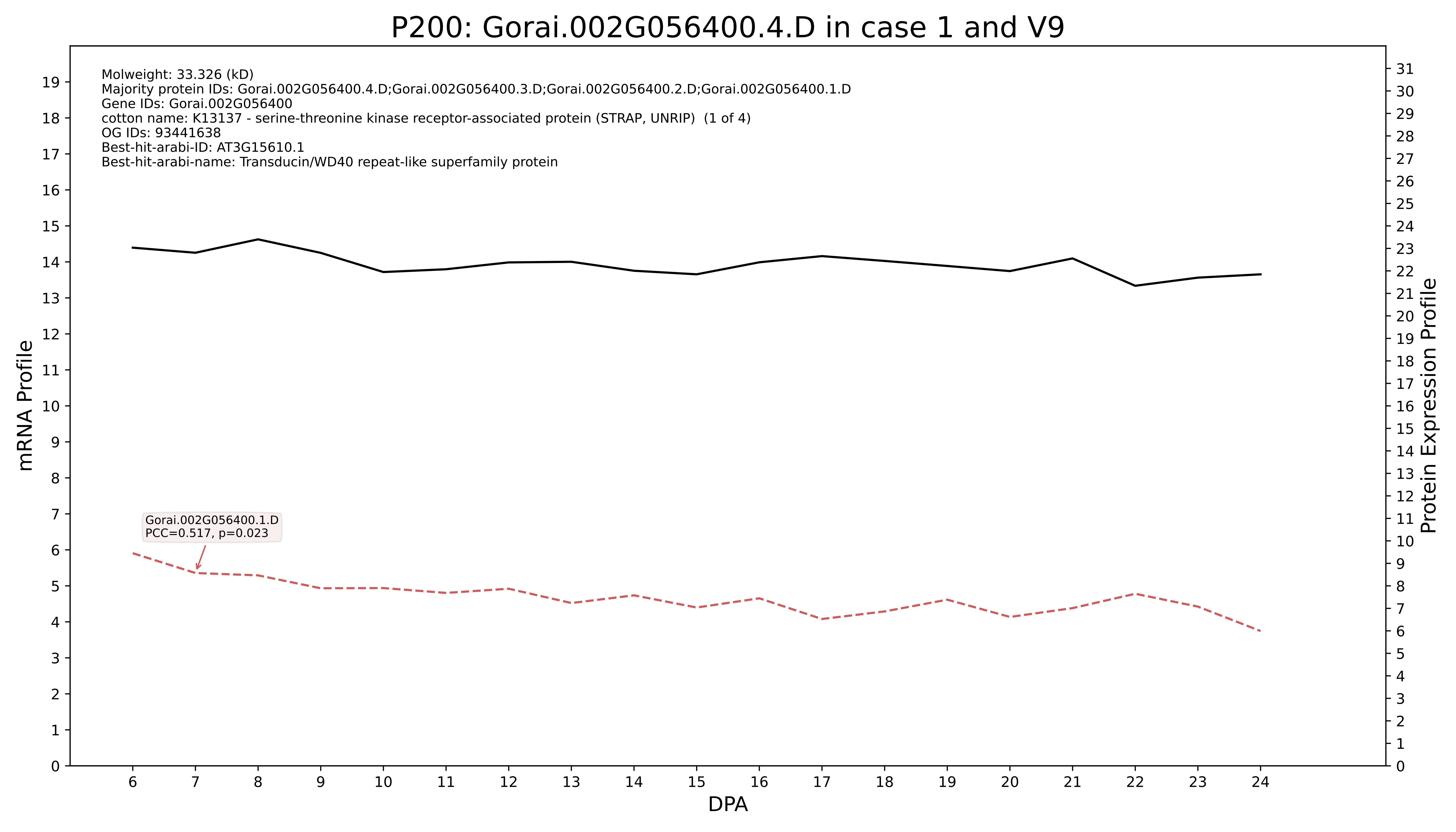
P200: Gorai.002G056400.4.D in case 1 and V9
Molweight: 33.326 (kD)
Majority protein IDs: Gorai.002G056400.4.D;Gorai.002G056400.3.D;Gorai.002G056400.2.D;Gorai.002G056400.1.D
Gene IDs: Gorai.002G056400
cotton name: K13137 - serine-threonine kinase receptor-associated protein (STRAP, UNRIP) (1 of 4)
OG IDs: 93441638
Best-hit-arabi-ID: AT3G15610.1
Best-hit-arabi-name: Transducin/WD40 repeat-like superfamily protein
Gorai.002G056400.1.D
PCC=0.517, p=0.023
mRNA Profile
Protein Expression Profile
DPA

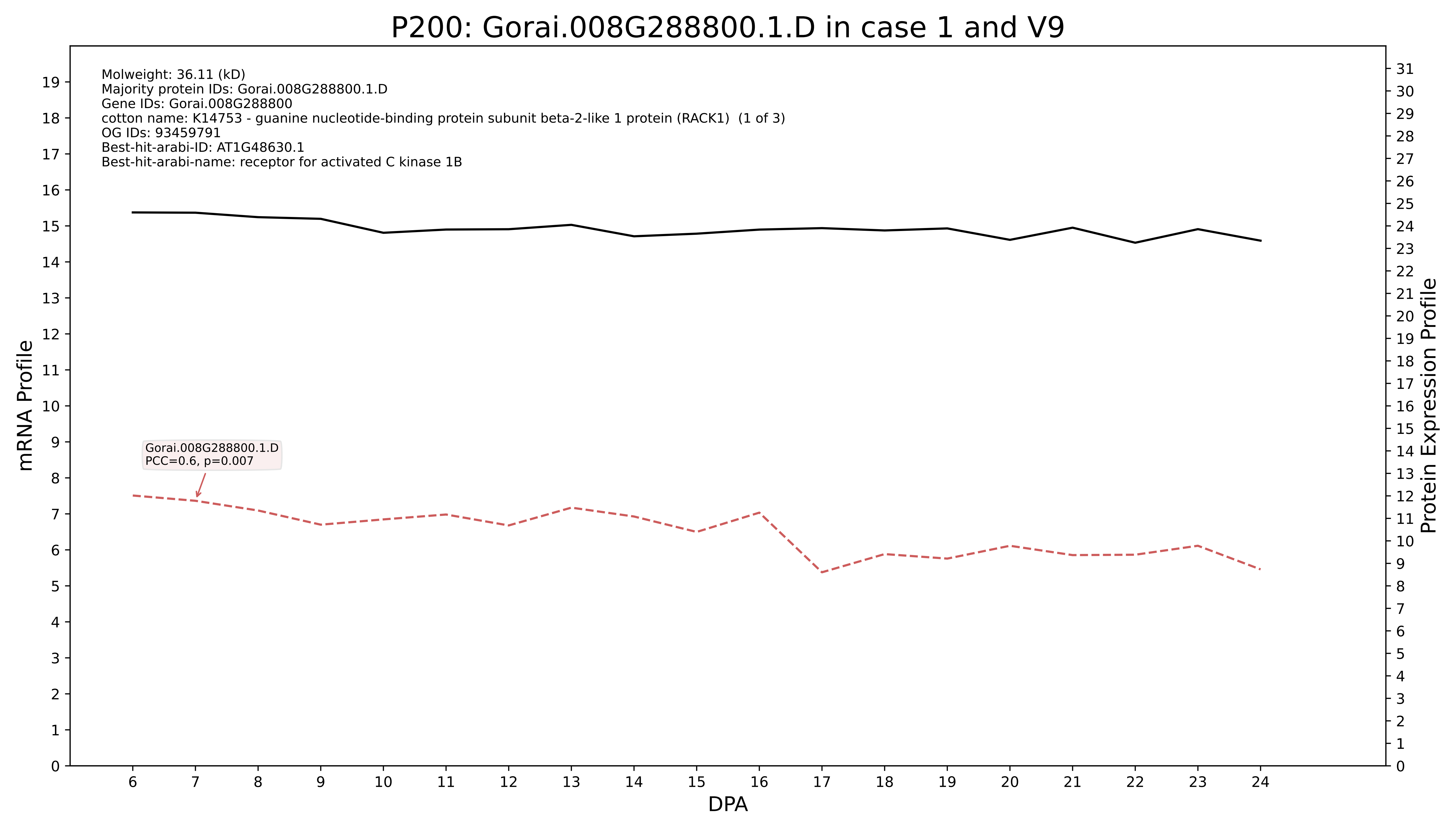
P200: Gorai.008G288800.1.D in case 1 and V9
Molweight: 36.11 (kD)
Majority protein IDs: Gorai.008G288800.1.D
Gene IDs: Gorai.008G288800
cotton name: K14753 - guanine nucleotide-binding protein subunit beta-2-like 1 protein (RACK1) (1 of 3)
OG IDs: 93459791
Best-hit-arabi-ID: AT1G48630.1
Best-hit-arabi-name: receptor for activated C kinase 1B
Gorai.008G288800.1.D
PCC=0.6, p=0.007
mRNA Profile
Protein Expression Profile
DPA

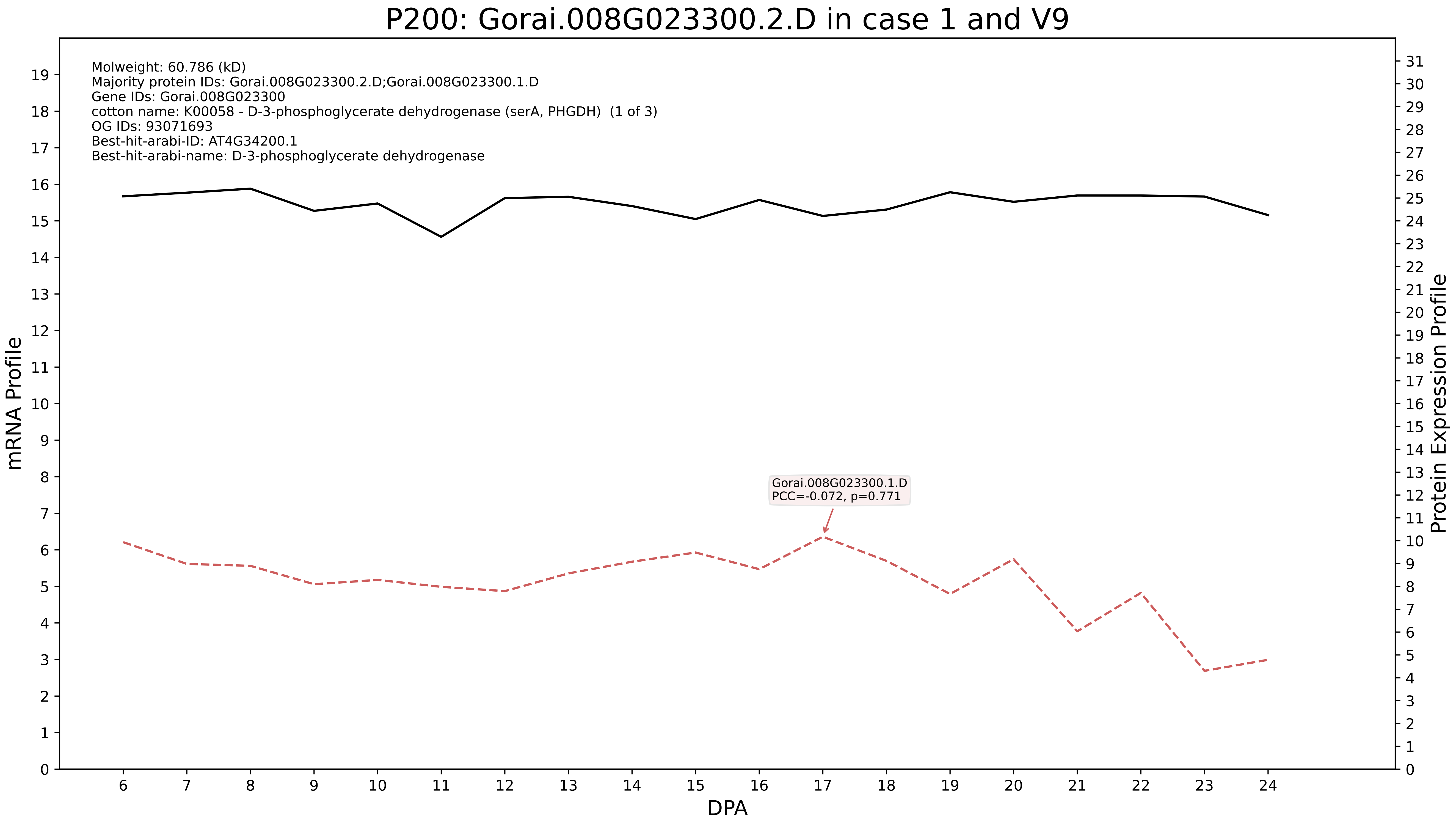
P200: Gorai.008G023300.2.D in case 1 and V9
Molweight: 60.786 (kD)
Majority protein IDs: Gorai.008G023300.2.D;Gorai.008G023300.1.D
Gene IDs: Gorai.008G023300
cotton name: K00058 - D-3-phosphoglycerate dehydrogenase (serA, PHGDH) (1 of 3)
OG IDs: 93071693
Best-hit-arabi-ID: AT4G34200.1
Best-hit-arabi-name: D-3-phosphoglycerate dehydrogenase
Gorai.008G023300.1.D
PCC=-0.072, p=0.771
mRNA Profile
Protein Expression Profile
DPA

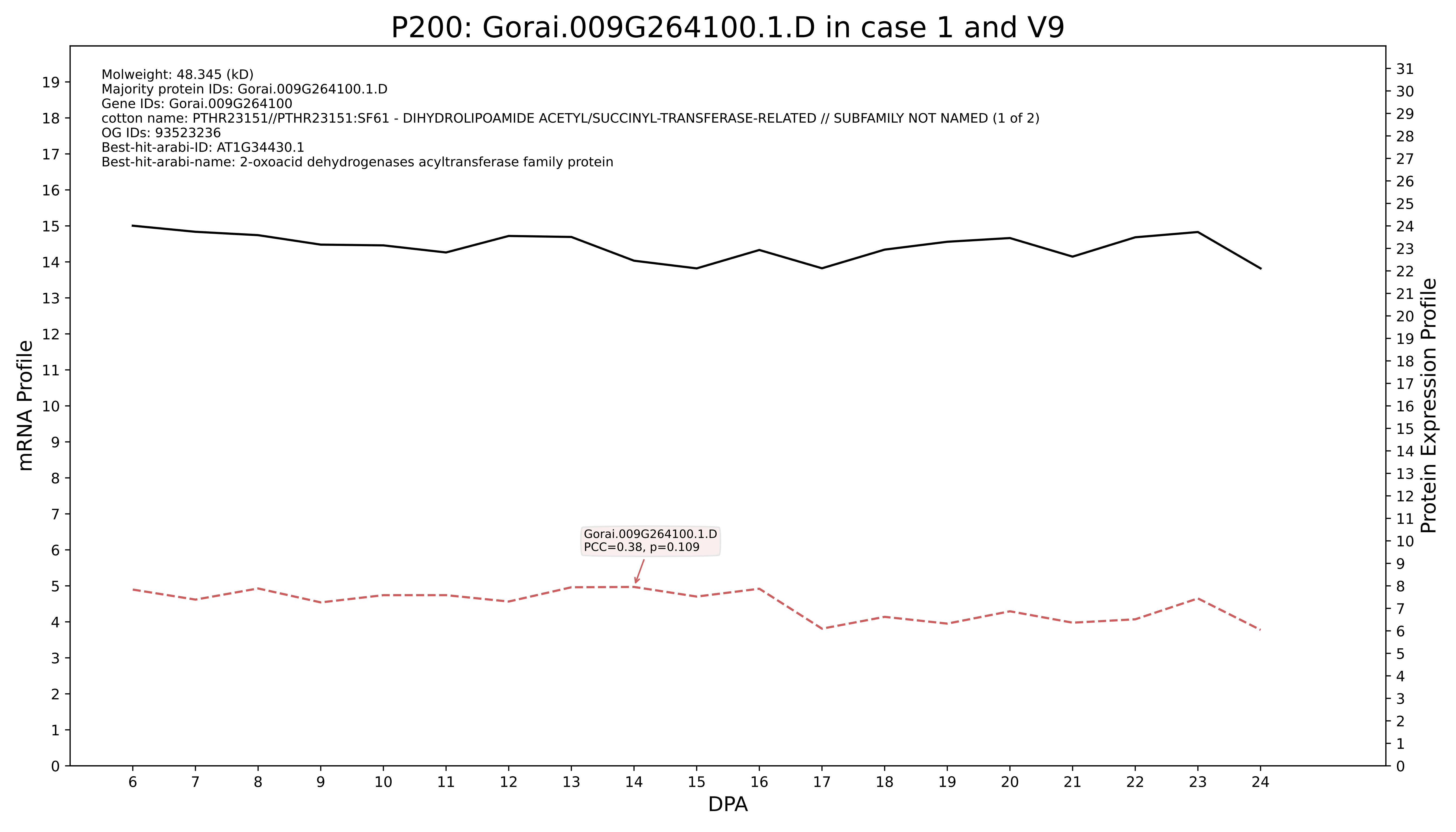
P200: Gorai.009G264100.1.D in case 1 and V9
Molweight: 48.345 (kD)
Majority protein IDs: Gorai.009G264100.1.D
Gene IDs: Gorai.009G264100
cotton name: PTHR23151//PTHR23151:SF61 - DIHYDROLIPOAMIDE ACETYL/SUCCINYL-TRANSFERASE-RELATED // SUBFAMILY NOT NAMED (1 of 2)
OG IDs: 93523236
Best-hit-arabi-ID: AT1G34430.1
Best-hit-arabi-name: 2-oxoacid dehydrogenases acyltransferase family protein
Gorai.009G264100.1.D
PCC=0.38, p=0.109
mRNA Profile
Protein Expression Profile
DPA

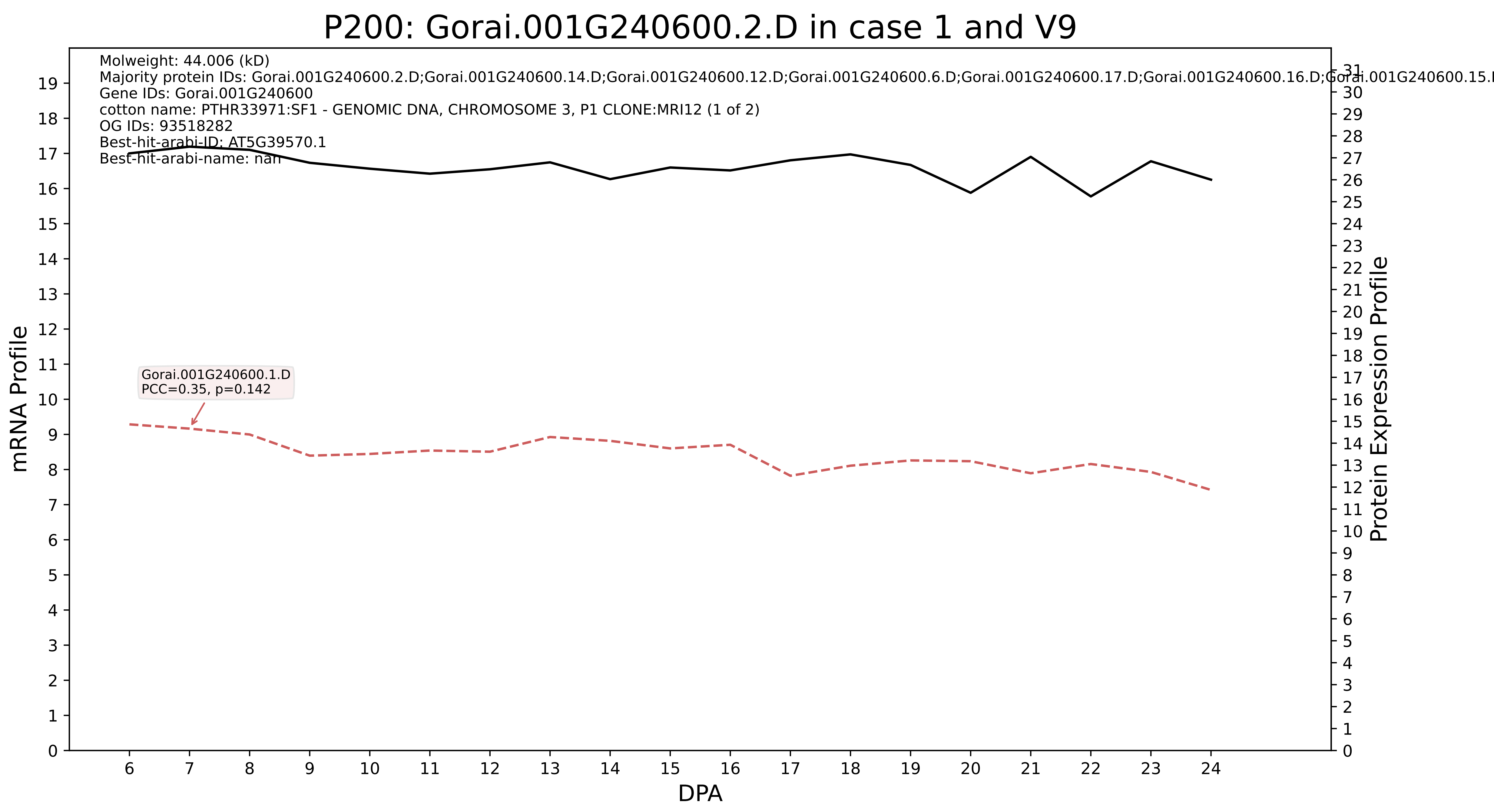

P200: Gorai.001G240600.2.D in case 1 and V9
Molweight: 44.006 (kD)
Majority protein IDs: Gorai.001G240600.2.D;Gorai.001G240600.14.D;Gorai.001G240600.12.D;Gorai.001G240600.6.D;Gorai.001G240600.17.D;Gorai.001G240600.16.D;Gorai.001G240600.15.
Gene IDs: Gorai.001G240600
cotton name: PTHR33971:SF1 - GENOMIC DNA, CHROMOSOME 3, P1 CLONE:MRI12 (1 of 2)
OG IDs: 93518282
Best-hit-arabi-ID: AT5G39570.1
Best-hit-arabi-name: nan
Gorai.001G240600.1.D
PCC=0.35, p=0.142
mRNA Profile
Protein Expression Profile
DPA

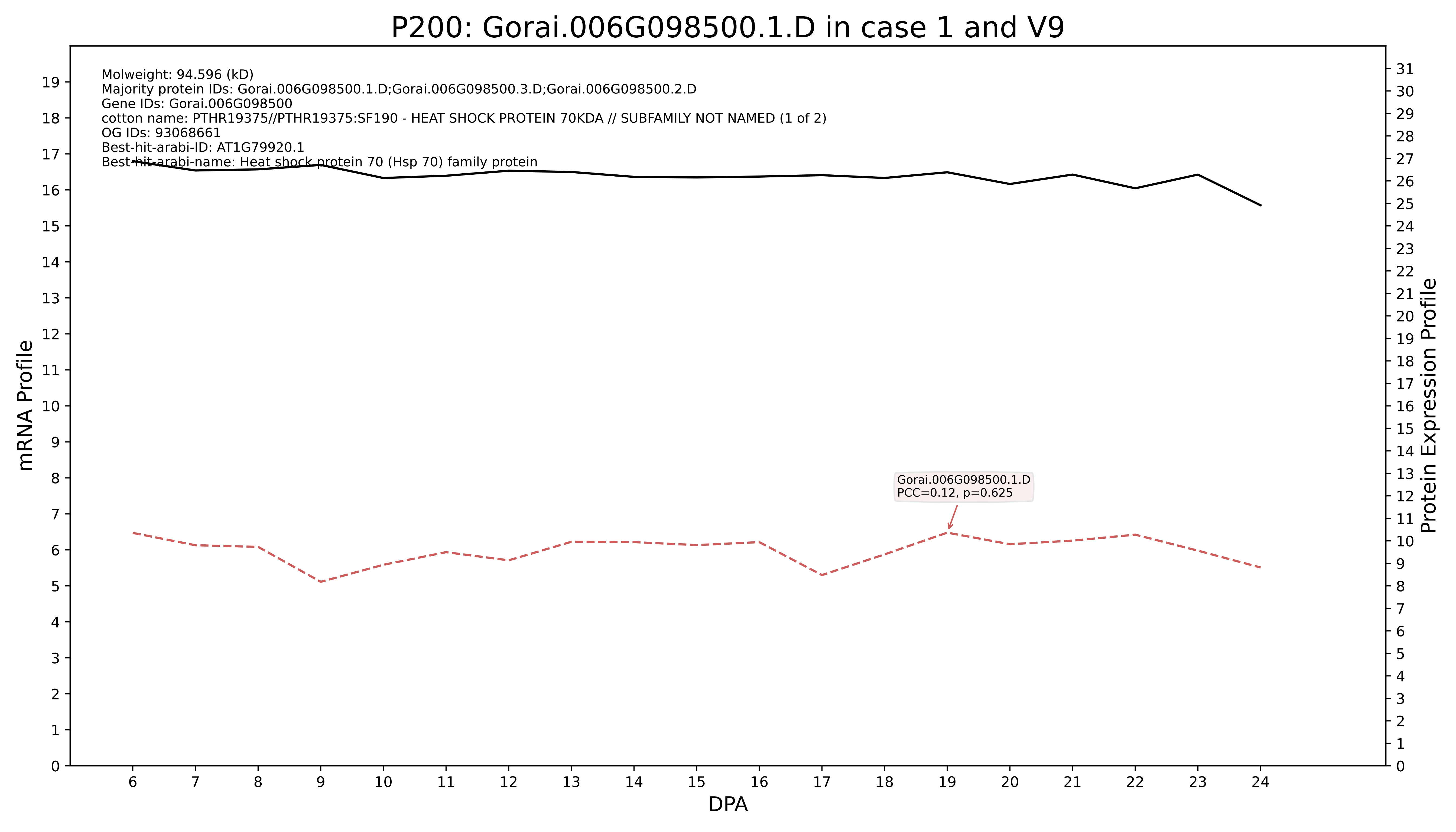

P200: Gorai.006G098500.1.D in case 1 and V9
Molweight: 94.596 (kD)
Majority protein IDs: Gorai.006G098500.1.D;Gorai.006G098500.3.D;Gorai.006G098500.2.D
Gene IDs: Gorai.006G098500
cotton name: PTHR19375//PTHR19375:SF190 - HEAT SHOCK PROTEIN 70KDA // SUBFAMILY NOT NAMED (1 of 2)
OG IDs: 93068661
Best-hit-arabi-ID: AT1G79920.1
Best-hit-arabi-name: Heat shock protein 70 (Hsp 70) family protein
Gorai.006G098500.1.D
PCC=0.12, p=0.625
mRNA Profile
Protein Expression Profile
DPA

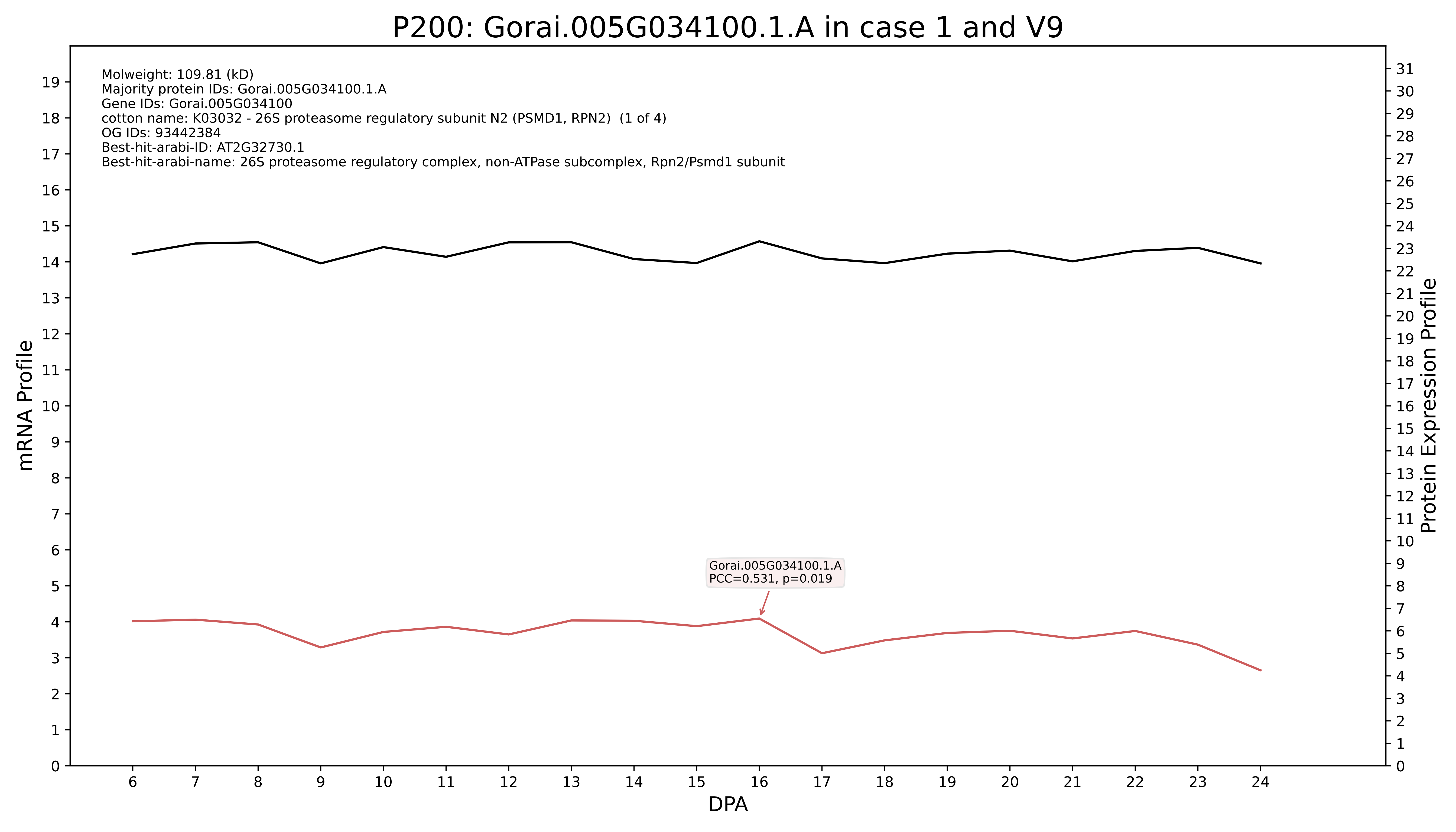
P200: Gorai.005G034100.1.A in case 1 and V9
Molweight: 109.81 (kD)
Majority protein IDs: Gorai.005G034100.1.A
Gene IDs: Gorai.005G034100
cotton name: K03032 - 26S proteasome regulatory subunit N2 (PSMD1, RPN2) (1 of 4)
OG IDs: 93442384
Best-hit-arabi-ID: AT2G32730.1
Best-hit-arabi-name: 26S proteasome regulatory complex, non-ATPase subcomplex, Rpn2/Psmd1 subunit
Gorai.005G034100.1.A
PCC=0.531, p=0.019
mRNA Profile
Protein Expression Profile
DPA

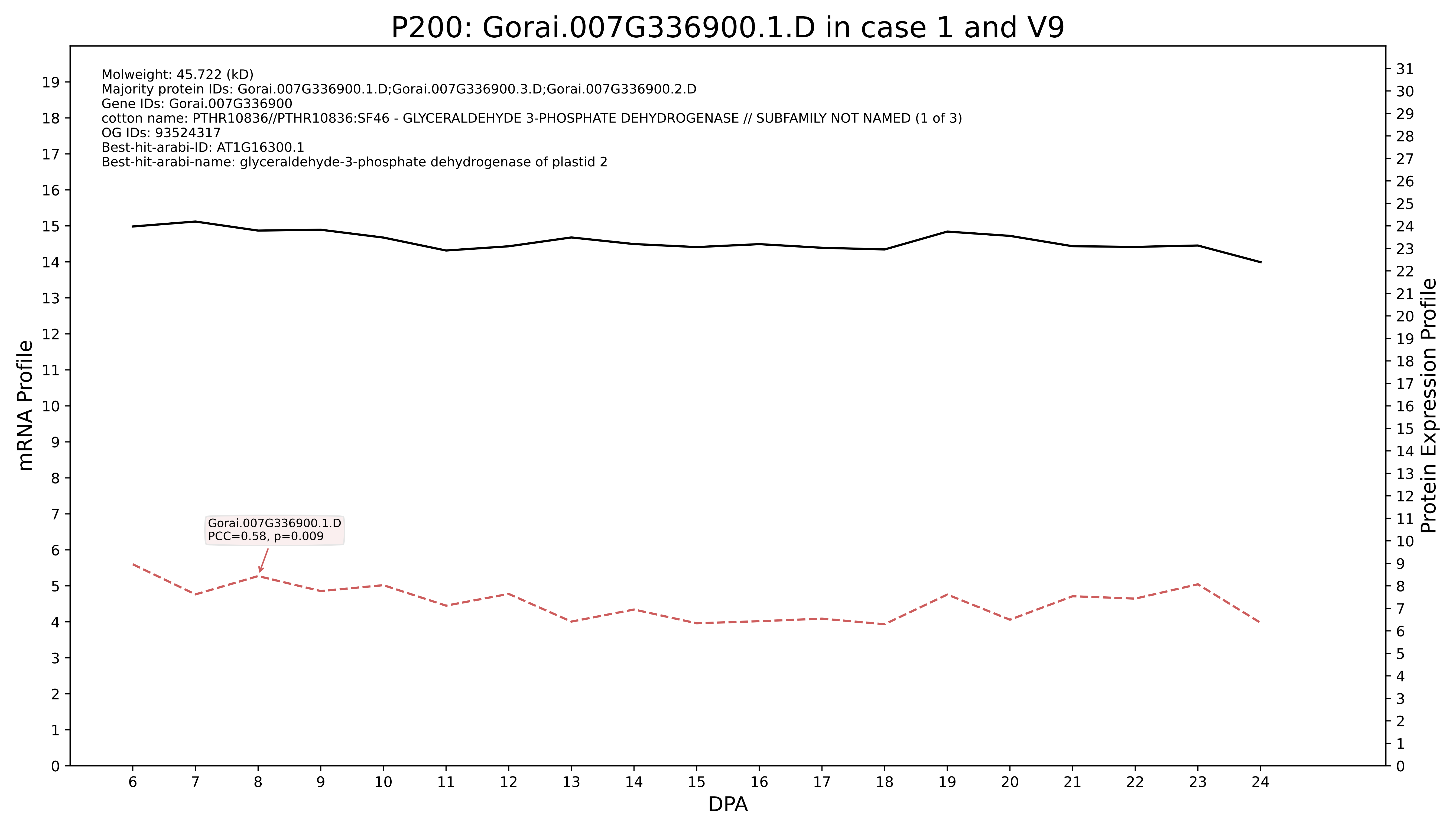
P200: Gorai.007G336900.1.D in case 1 and V9
Molweight: 45.722 (kD)
Majority protein IDs: Gorai.007G336900.1.D;Gorai.007G336900.3.D;Gorai.007G336900.2.D
Gene IDs: Gorai.007G336900
cotton name: PTHR10836//PTHR10836:SF46 - GLYCERALDEHYDE 3-PHOSPHATE DEHYDROGENASE // SUBFAMILY NOT NAMED (1 of 3)
OG IDs: 93524317
Best-hit-arabi-ID: AT1G16300.1
Best-hit-arabi-name: glyceraldehyde-3-phosphate dehydrogenase of plastid 2
Gorai.007G336900.1.D
PCC=0.58, p=0.009
mRNA Profile
Protein Expression Profile
DPA

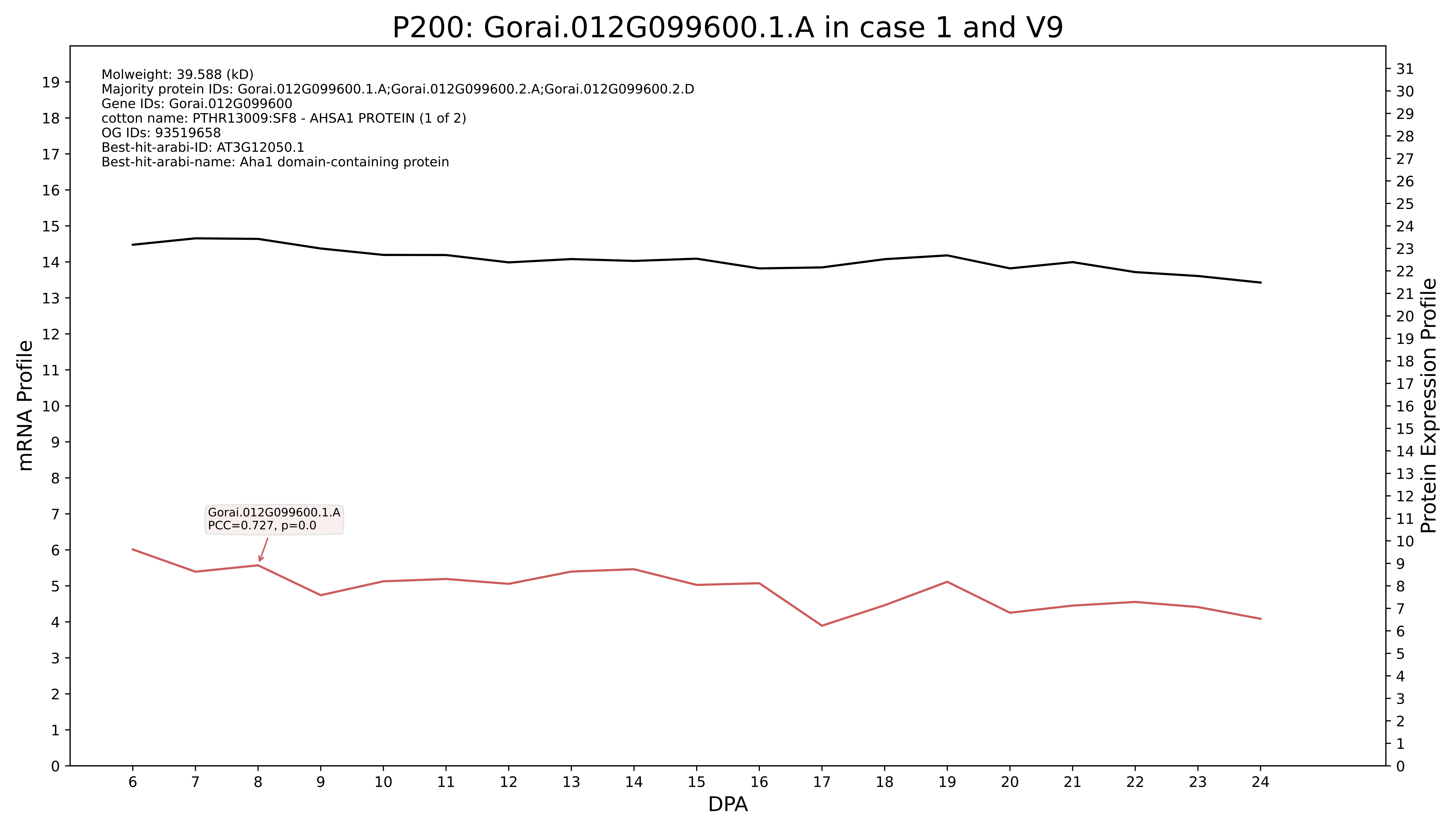

P200: Gorai.012G099600.1.A in case 1 and V9
Molweight: 39.588 (kD)
Majority protein IDs: Gorai.012G099600.1.A;Gorai.012G099600.2.A;Gorai.012G099600.2.D
Gene IDs: Gorai.012G099600
cotton name: PTHR13009:SF8 - AHSA1 PROTEIN (1 of 2)
OG IDs: 93519658
Best-hit-arabi-ID: AT3G12050.1
Best-hit-arabi-name: Aha1 domain-containing protein
Gorai.012G099600.1.A
PCC=0.727, p=0.0
mRNA Profile
Protein Expression Profile
DPA

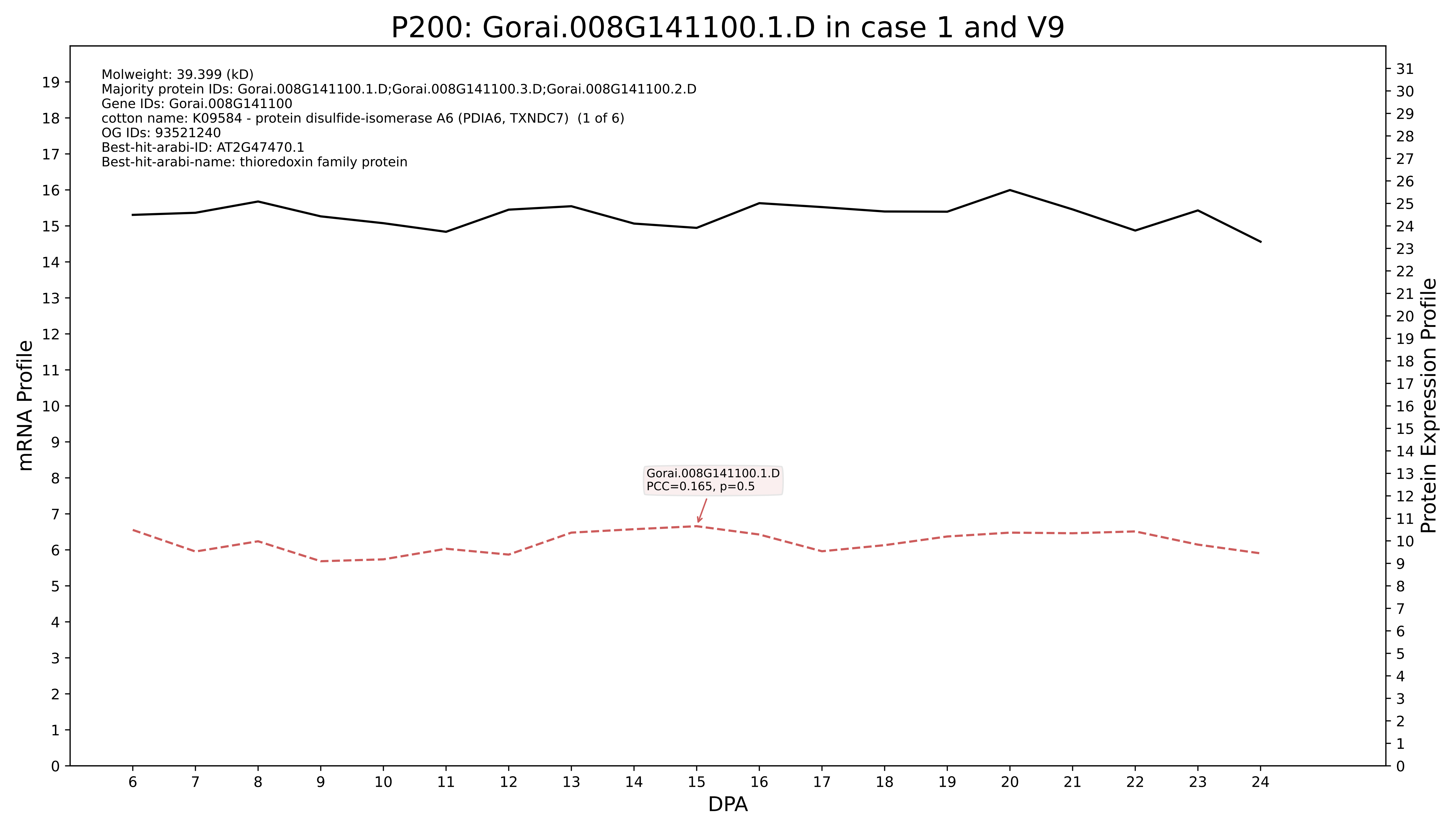
P200: Gorai.008G141100.1.D in case 1 and V9
Molweight: 39.399 (kD)
Majority protein IDs: Gorai.008G141100.1.D;Gorai.008G141100.3.D;Gorai.008G141100.2.D
Gene IDs: Gorai.008G141100
cotton name: K09584 - protein disulfide-isomerase A6 (PDIA6, TXNDC7) (1 of 6)
OG IDs: 93521240
Best-hit-arabi-ID: AT2G47470.1
Best-hit-arabi-name: thioredoxin family protein
Gorai.008G141100.1.D
PCC=0.165, p=0.5
mRNA Profile
Protein Expression Profile
DPA

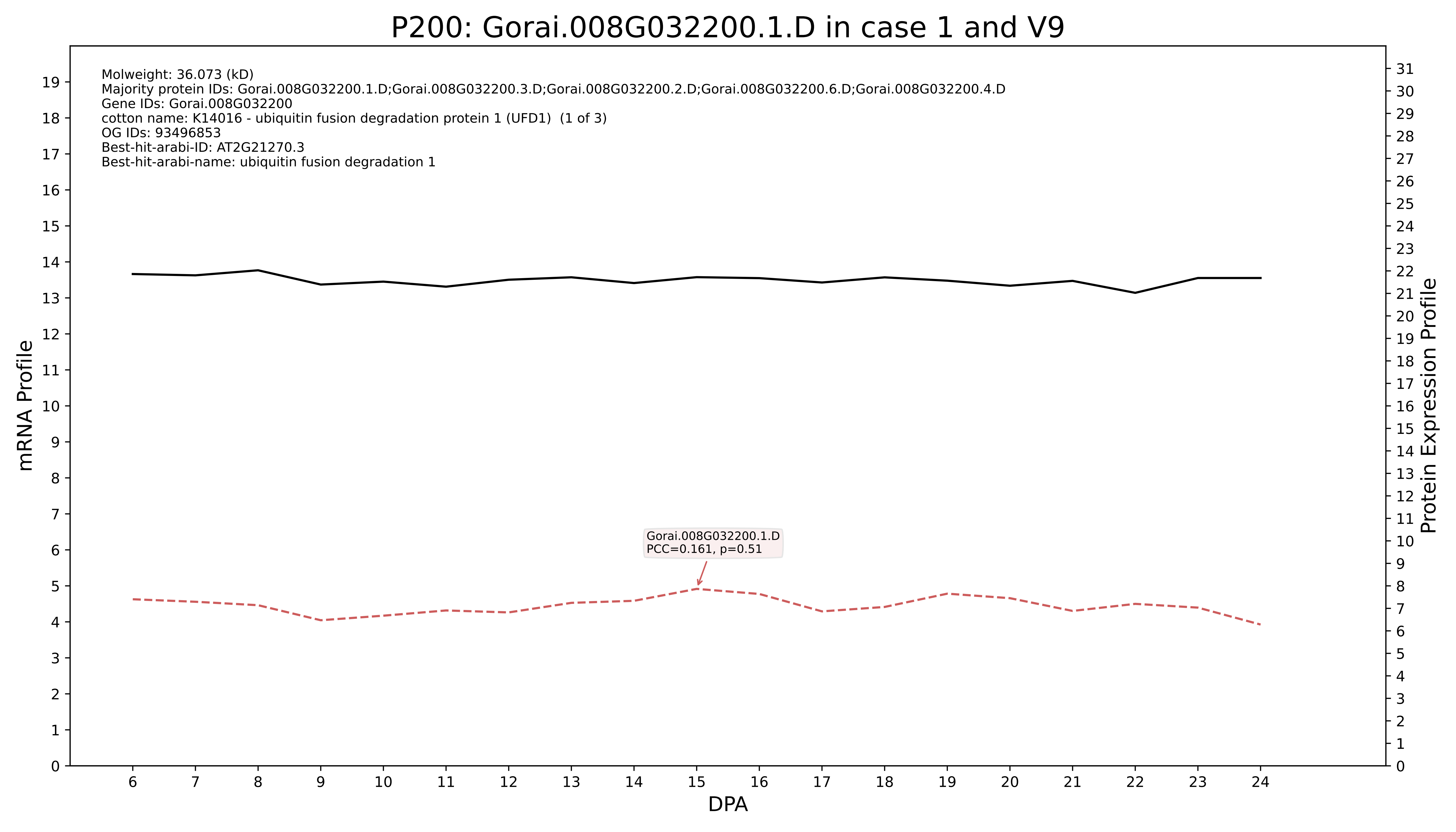
P200: Gorai.008G032200.1.D in case 1 and V9
Molweight: 36.073 (kD)
Majority protein IDs: Gorai.008G032200.1.D;Gorai.008G032200.3.D;Gorai.008G032200.2.D;Gorai.008G032200.6.D;Gorai.008G032200.4.D
Gene IDs: Gorai.008G032200
cotton name: K14016 - ubiquitin fusion degradation protein 1 (UFD1) (1 of 3)
OG IDs: 93496853
Best-hit-arabi-ID: AT2G21270.3
Best-hit-arabi-name: ubiquitin fusion degradation 1
Gorai.008G032200.1.D
PCC=0.161, p=0.51
mRNA Profile
Protein Expression Profile
DPA

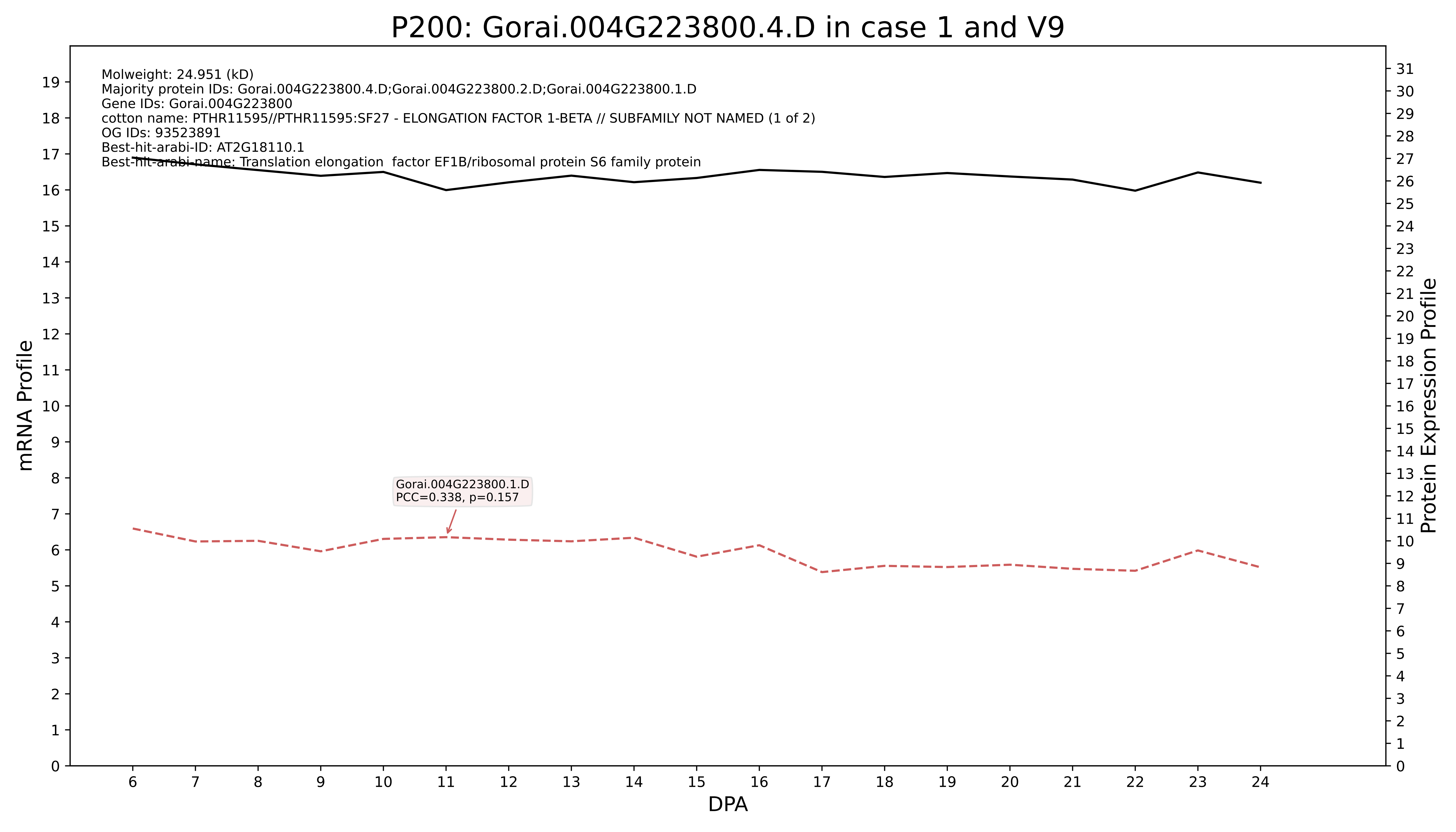
P200: Gorai.004G223800.4.D in case 1 and V9
Molweight: 24.951 (kD)
Majority protein IDs: Gorai.004G223800.4.D;Gorai.004G223800.2.D;Gorai.004G223800.1.D
Gene IDs: Gorai.004G223800
cotton name: PTHR11595//PTHR11595:SF27 - ELONGATION FACTOR 1-BETA // SUBFAMILY NOT NAMED (1 of 2)
OG IDs: 93523891
Best-hit-arabi-ID: AT2G18110.1
Best-hit-arabi-name: Translation elongation factor EF1B/ribosomal protein S6 family protein
Gorai.004G223800.1.D
PCC=0.338, p=0.157
mRNA Profile
Protein Expression Profile
DPA

P200: Gorai.009G032000.8.D in case 1 and V9

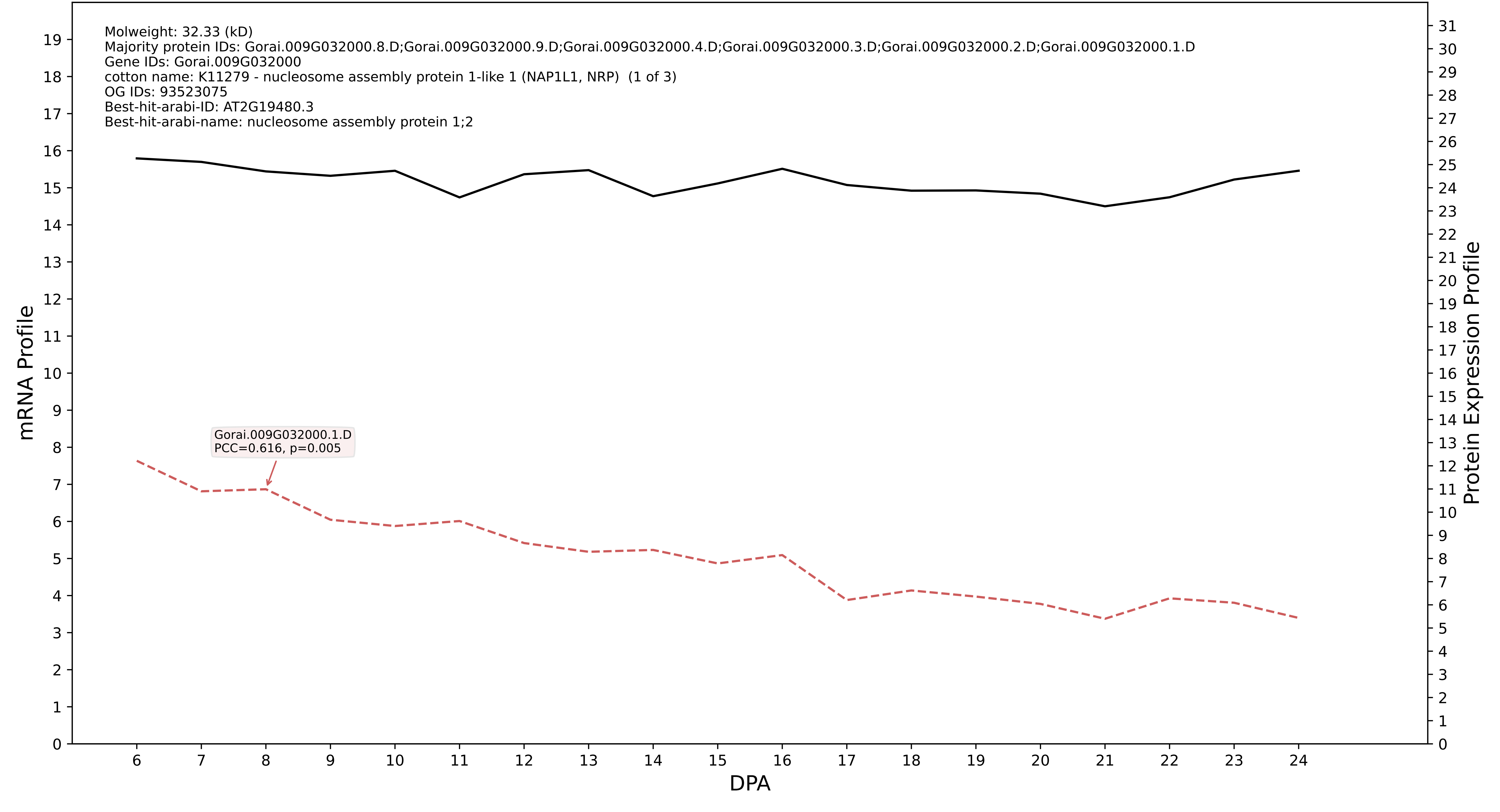

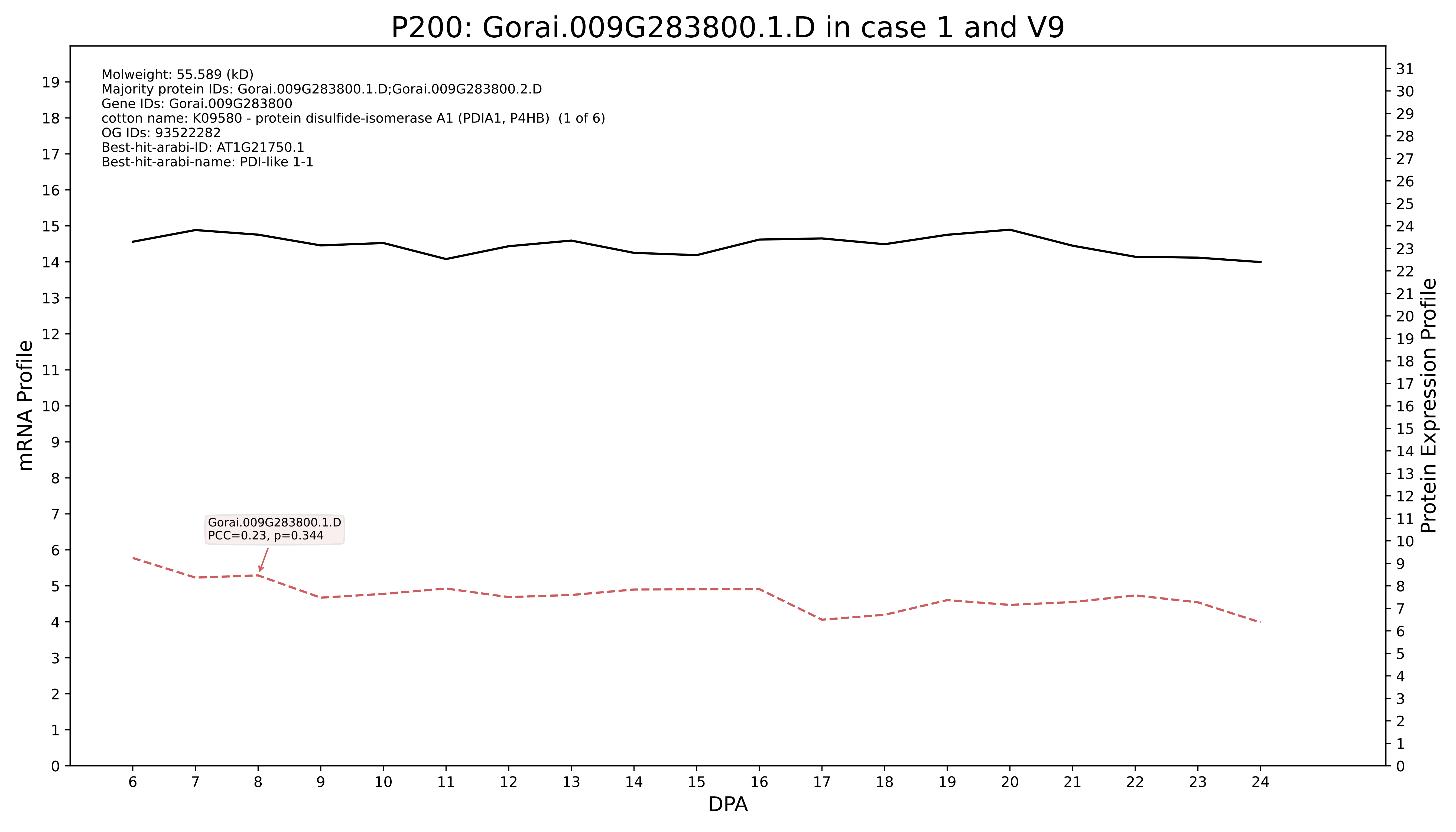
P200: Gorai.009G283800.1.D in case 1 and V9
Molweight: 55.589 (kD)
Majority protein IDs: Gorai.009G283800.1.D;Gorai.009G283800.2.D
Gene IDs: Gorai.009G283800
cotton name: K09580 - protein disulfide-isomerase A1 (PDIA1, P4HB) (1 of 6)
OG IDs: 93522282
Best-hit-arabi-ID: AT1G21750.1
Best-hit-arabi-name: PDI-like 1-1
Gorai.009G283800.1.D
PCC=0.23, p=0.344
mRNA Profile
Protein Expression Profile
DPA

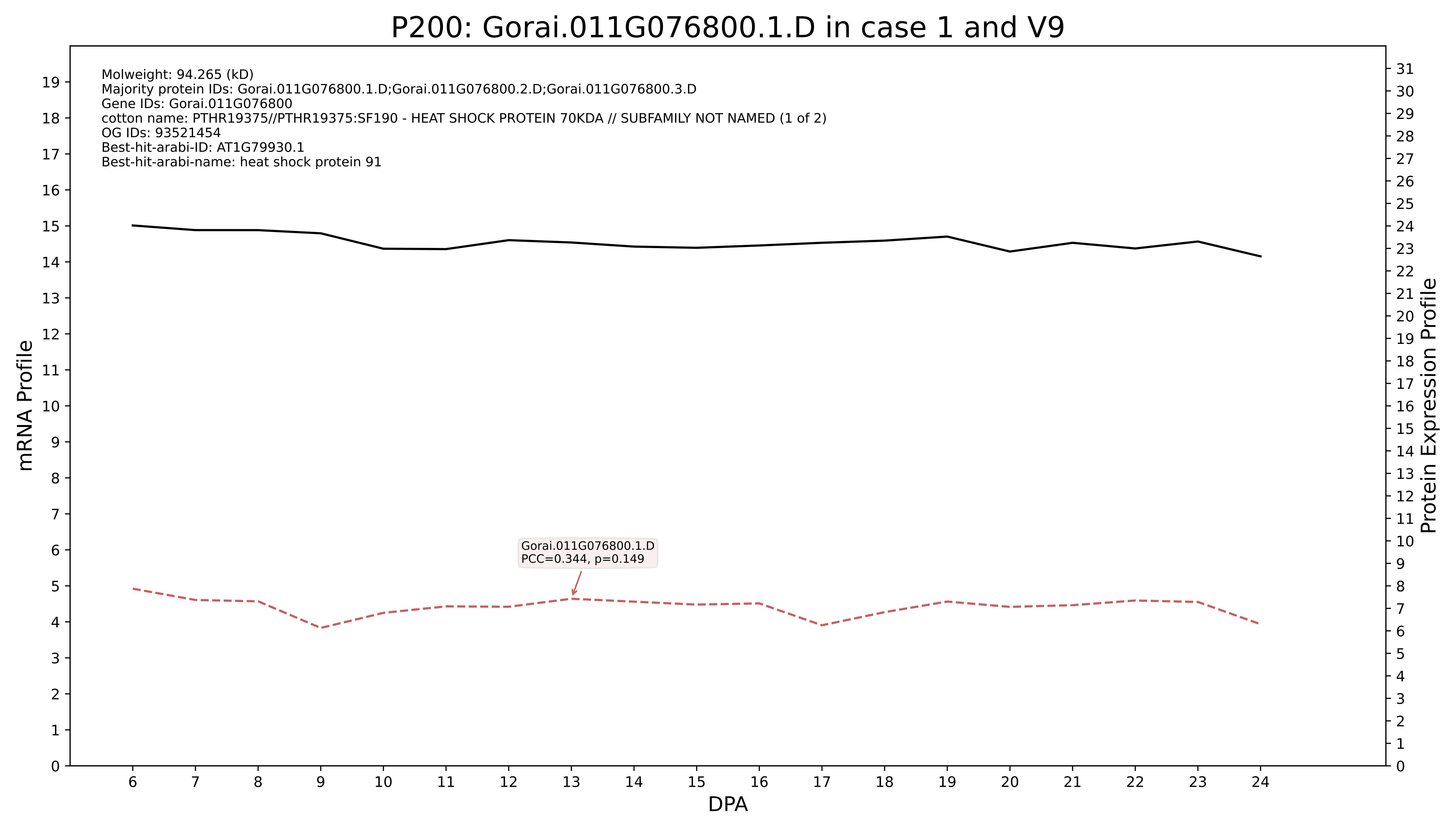
P200: Gorai.011G076800.1.D in case 1 and V9
Molweight: 94.265 (kD)
Majority protein IDs: Gorai.011G076800.1.D;Gorai.011G076800.2.D;Gorai.011G076800.3.D
Gene IDs: Gorai.011G076800
cotton name: PTHR19375//PTHR19375:SF190 - HEAT SHOCK PROTEIN 70KDA // SUBFAMILY NOT NAMED (1 of 2)
OG IDs: 93521454
Best-hit-arabi-ID: AT1G79930.1
Best-hit-arabi-name: heat shock protein 91
Gorai.011G076800.1.D
PCC=0.344, p=0.149
mRNA Profile
Protein Expression Profile
DPA

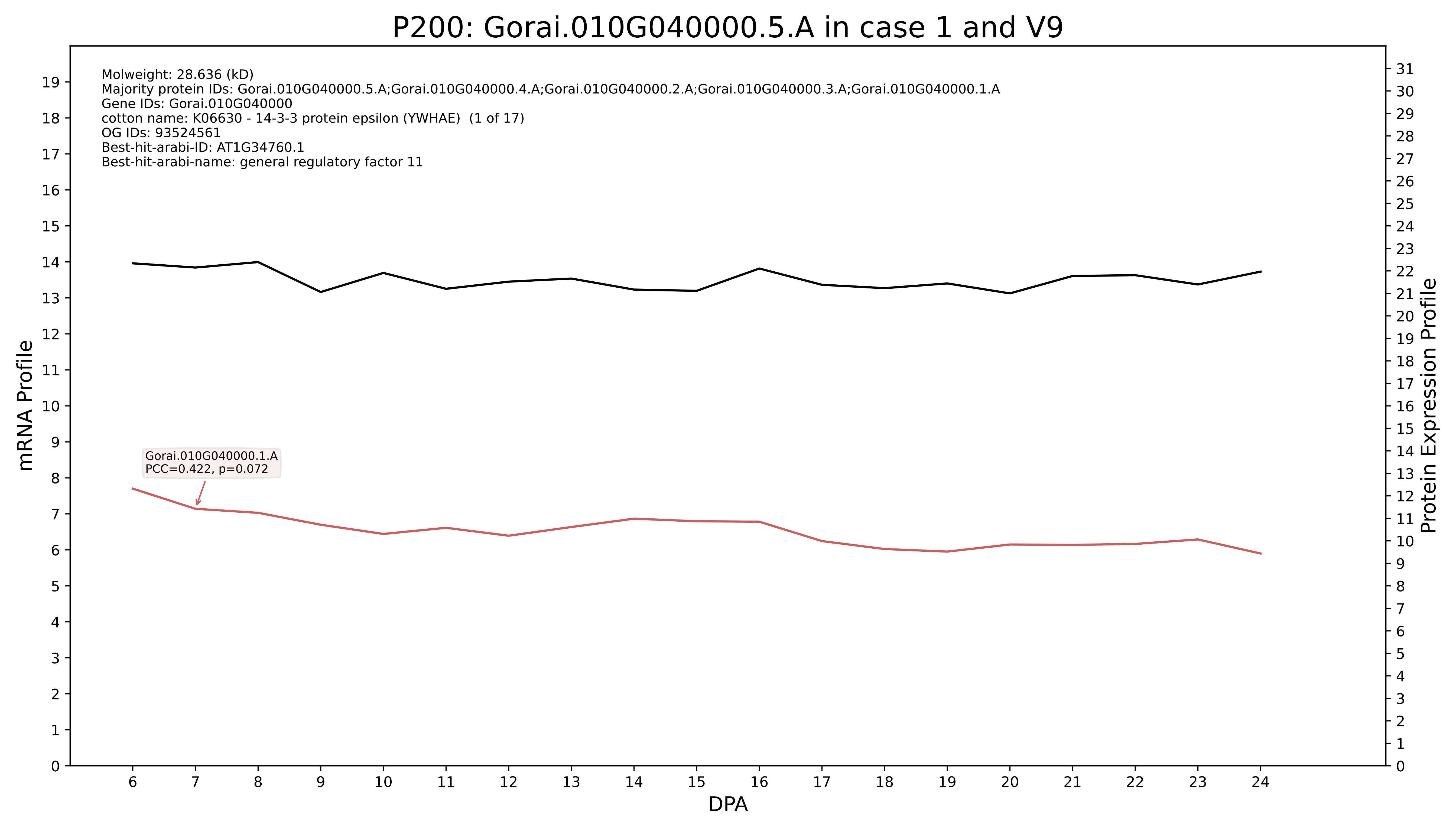

P200: Gorai.010G040000.5.A in case 1 and V9
Molweight: 28.636 (kD)
Majority protein IDs: Gorai.010G040000.5.A;Gorai.010G040000.4.A;Gorai.010G040000.2.A;Gorai.010G040000.3.A;Gorai.010G040000.1.A
Gene IDs: Gorai.010G040000
cotton name: K06630 - 14-3-3 protein epsilon (YWHAE) (1 of 17)
OG IDs: 93524561
Best-hit-arabi-ID: AT1G34760.1
Best-hit-arabi-name: general regulatory factor 11
Gorai.010G040000.1.A
PCC=0.422, p=0.072
mRNA Profile
Protein Expression Profile
DPA

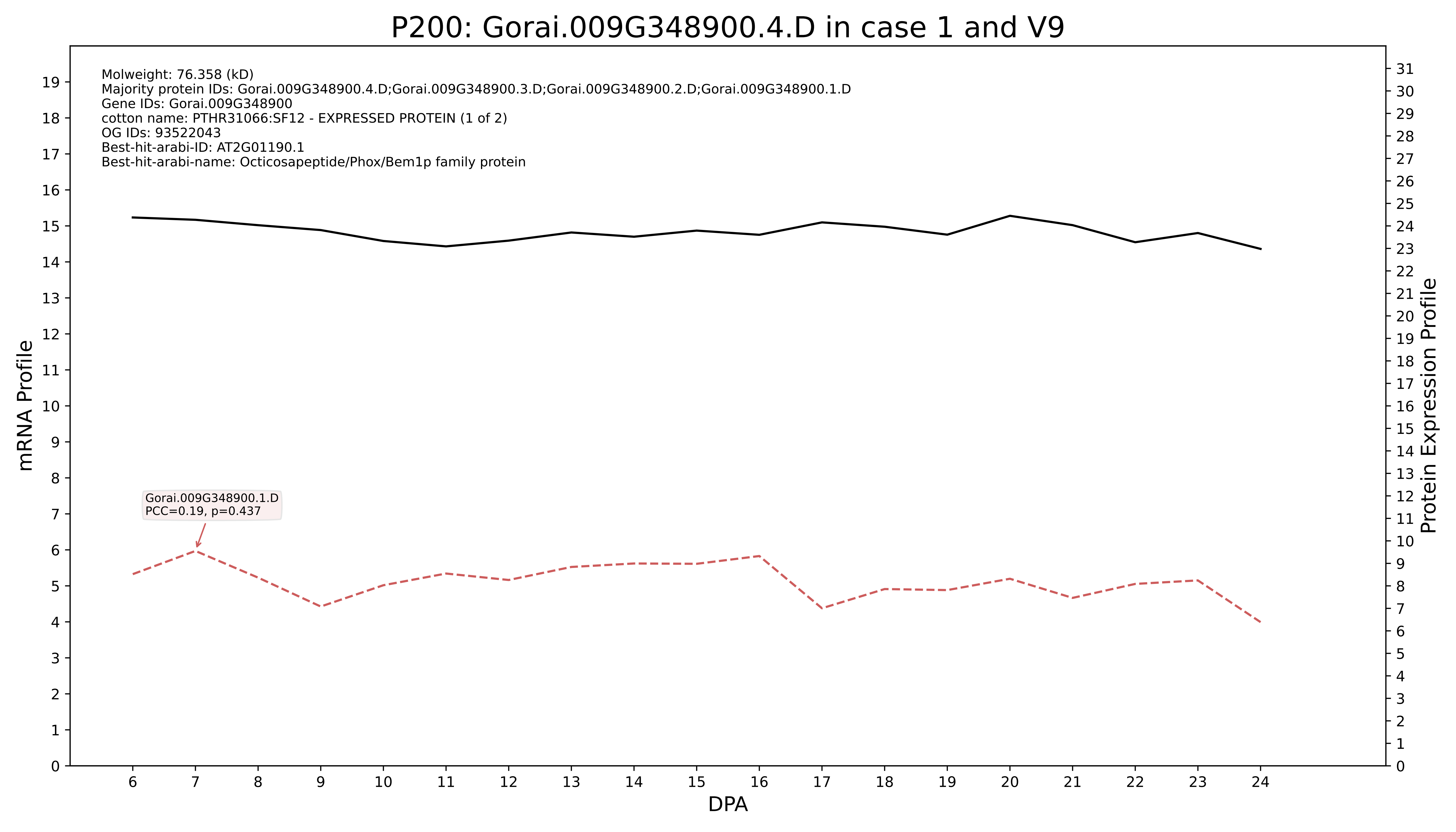

P200: Gorai.009G348900.4.D in case 1 and V9
Molweight: 76.358 (kD)
Majority protein IDs: Gorai.009G348900.4.D;Gorai.009G348900.3.D;Gorai.009G348900.2.D;Gorai.009G348900.1.D
Gene IDs: Gorai.009G348900
cotton name: PTHR31066:SF12 - EXPRESSED PROTEIN (1 of 2)
OG IDs: 93522043
Best-hit-arabi-ID: AT2G01190.1
Best-hit-arabi-name: Octicosapeptide/Phox/Bem1p family protein
Gorai.009G348900.1.D
PCC=0.19, p=0.437
mRNA Profile
Protein Expression Profile
DPA

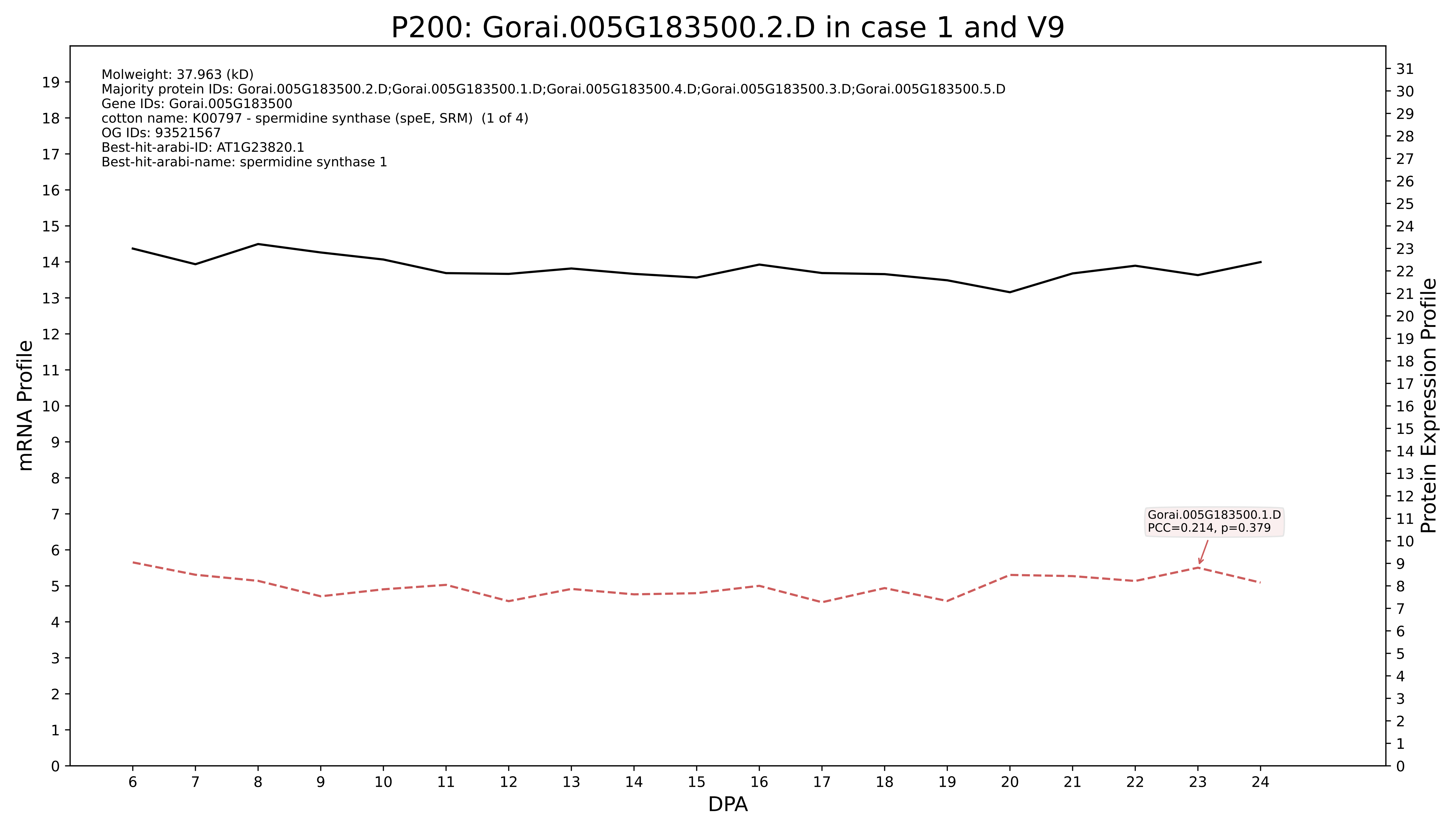
P200: Gorai.005G183500.2.D in case 1 and V9
Molweight: 37.963 (kD)
Majority protein IDs: Gorai.005G183500.2.D;Gorai.005G183500.1.D;Gorai.005G183500.4.D;Gorai.005G183500.3.D;Gorai.005G183500.5.D
Gene IDs: Gorai.005G183500
cotton name: K00797 - spermidine synthase (speE, SRM) (1 of 4)
OG IDs: 93521567
Best-hit-arabi-ID: AT1G23820.1
Best-hit-arabi-name: spermidine synthase 1
Gorai.005G183500.1.D
PCC=0.214, p=0.379
mRNA Profile
Protein Expression Profile
DPA

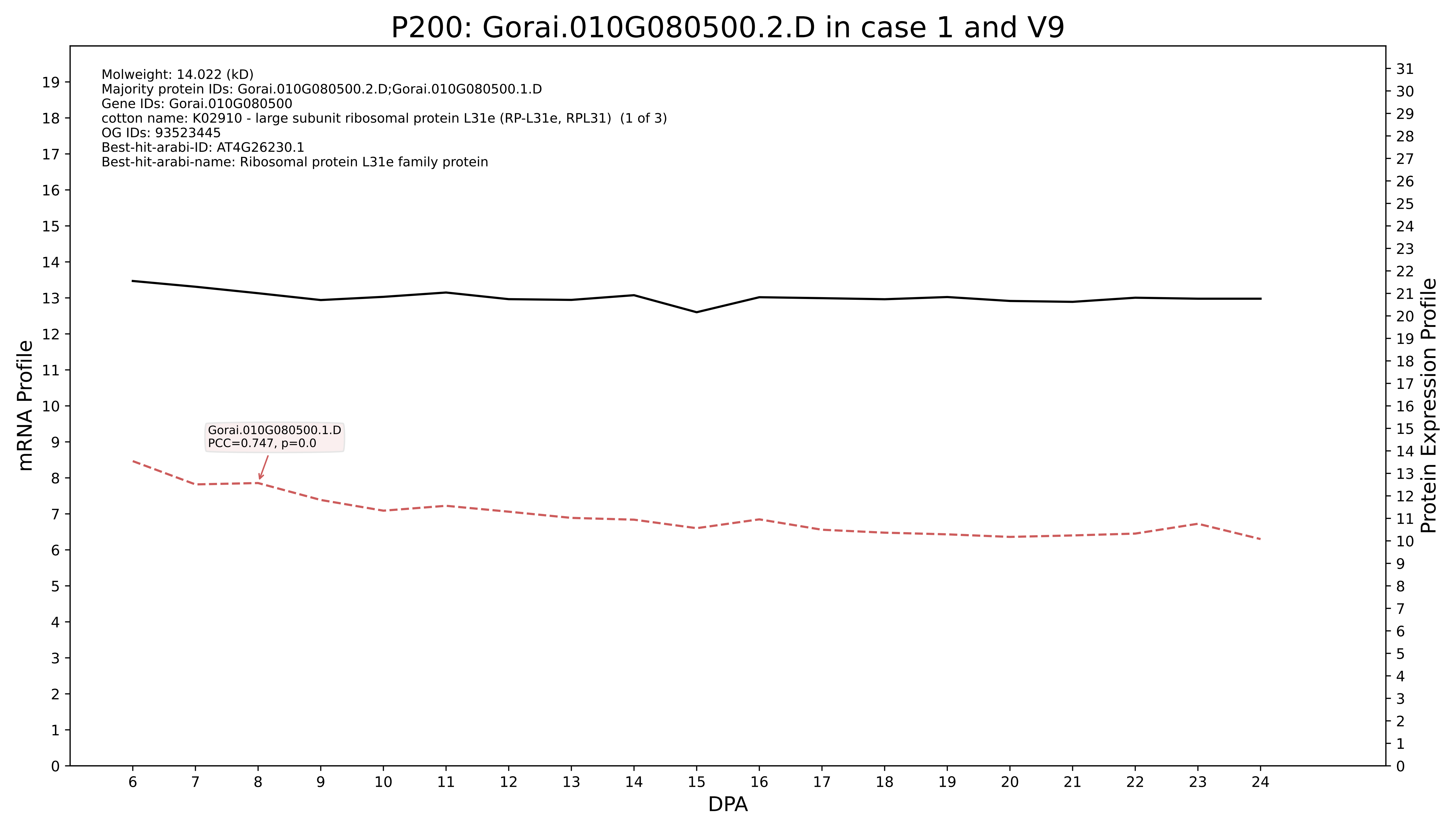
P200: Gorai.010G080500.2.D in case 1 and V9
Molweight: 14.022 (kD)
Majority protein IDs: Gorai.010G080500.2.D;Gorai.010G080500.1.D
Gene IDs: Gorai.010G080500
cotton name: K02910 - large subunit ribosomal protein L31e (RP-L31e, RPL31) (1 of 3)
OG IDs: 93523445
Best-hit-arabi-ID: AT4G26230.1
Best-hit-arabi-name: Ribosomal protein L31e family protein
Gorai.010G080500.1.D
PCC=0.747, p=0.0
mRNA Profile
Protein Expression Profile
DPA

P200: Gorai.007G288800.1.D in case 1 and V9

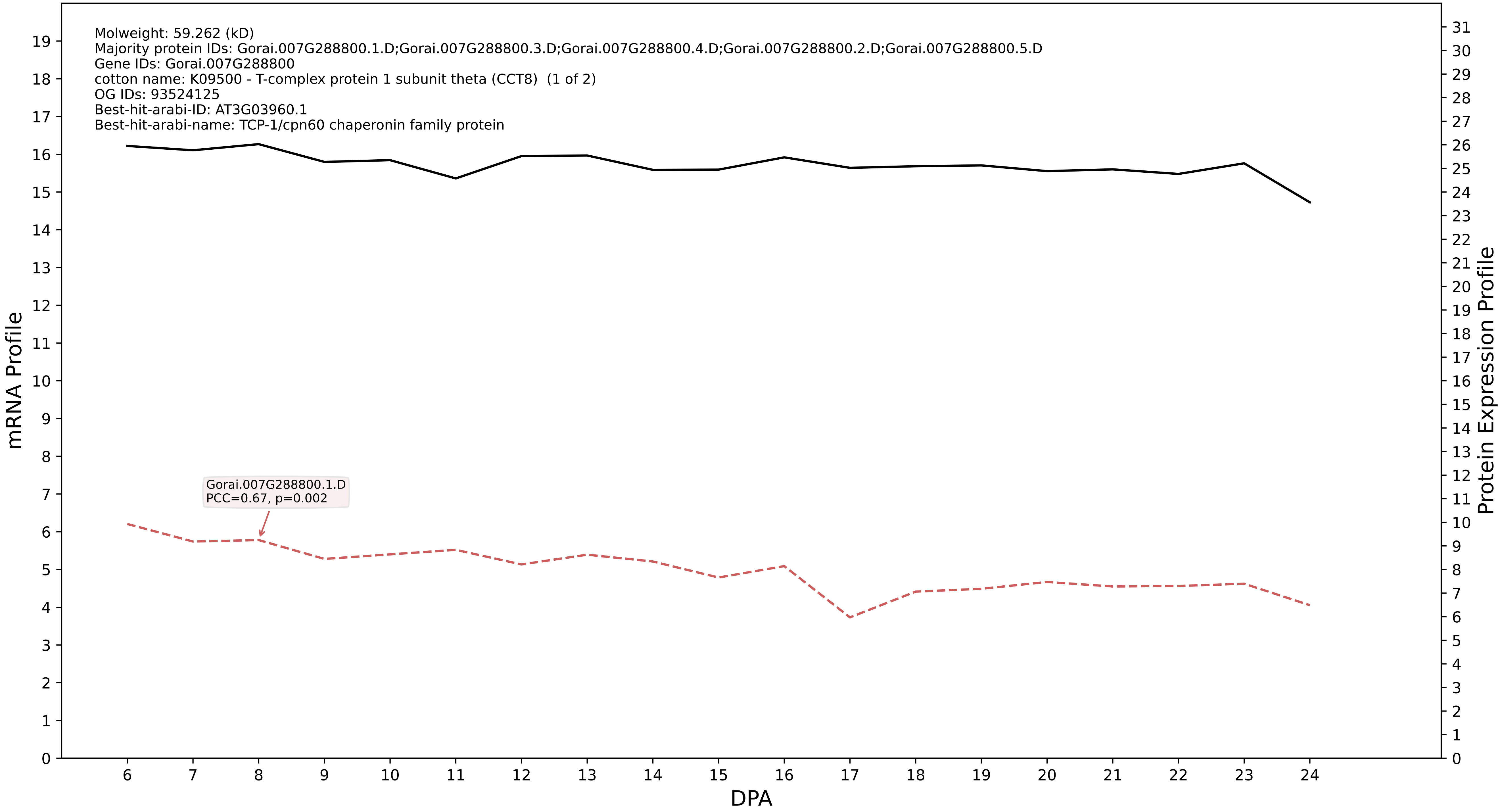

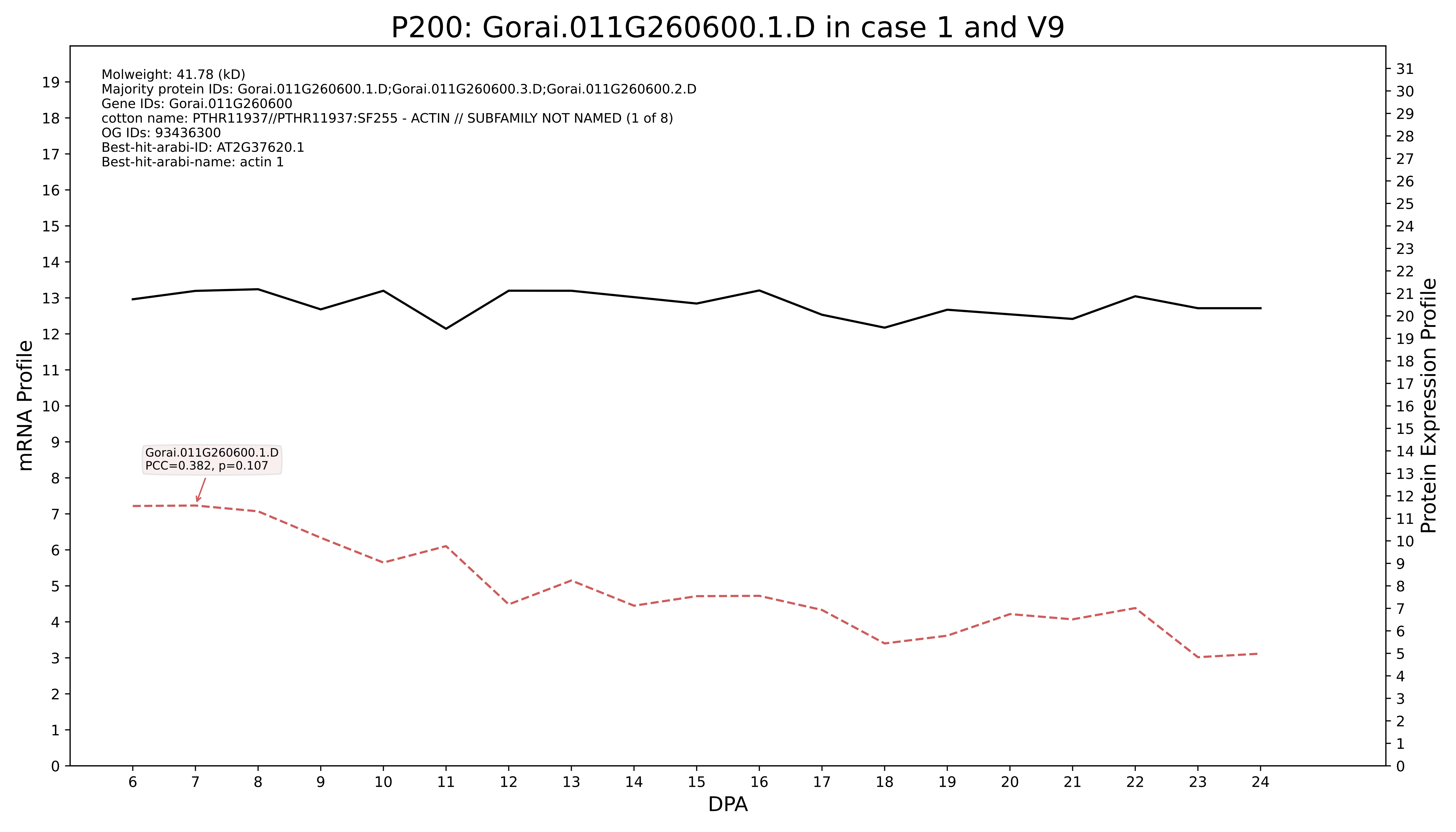
P200: Gorai.011G260600.1.D in case 1 and V9
Molweight: 41.78 (kD)
Majority protein IDs: Gorai.011G260600.1.D;Gorai.011G260600.3.D;Gorai.011G260600.2.D
Gene IDs: Gorai.011G260600
cotton name: PTHR11937//PTHR11937:SF255 - ACTIN // SUBFAMILY NOT NAMED (1 of 8)
OG IDs: 93436300
Best-hit-arabi-ID: AT2G37620.1
Best-hit-arabi-name: actin 1
Gorai.011G260600.1.D
PCC=0.382, p=0.107
mRNA Profile
Protein Expression Profile
DPA

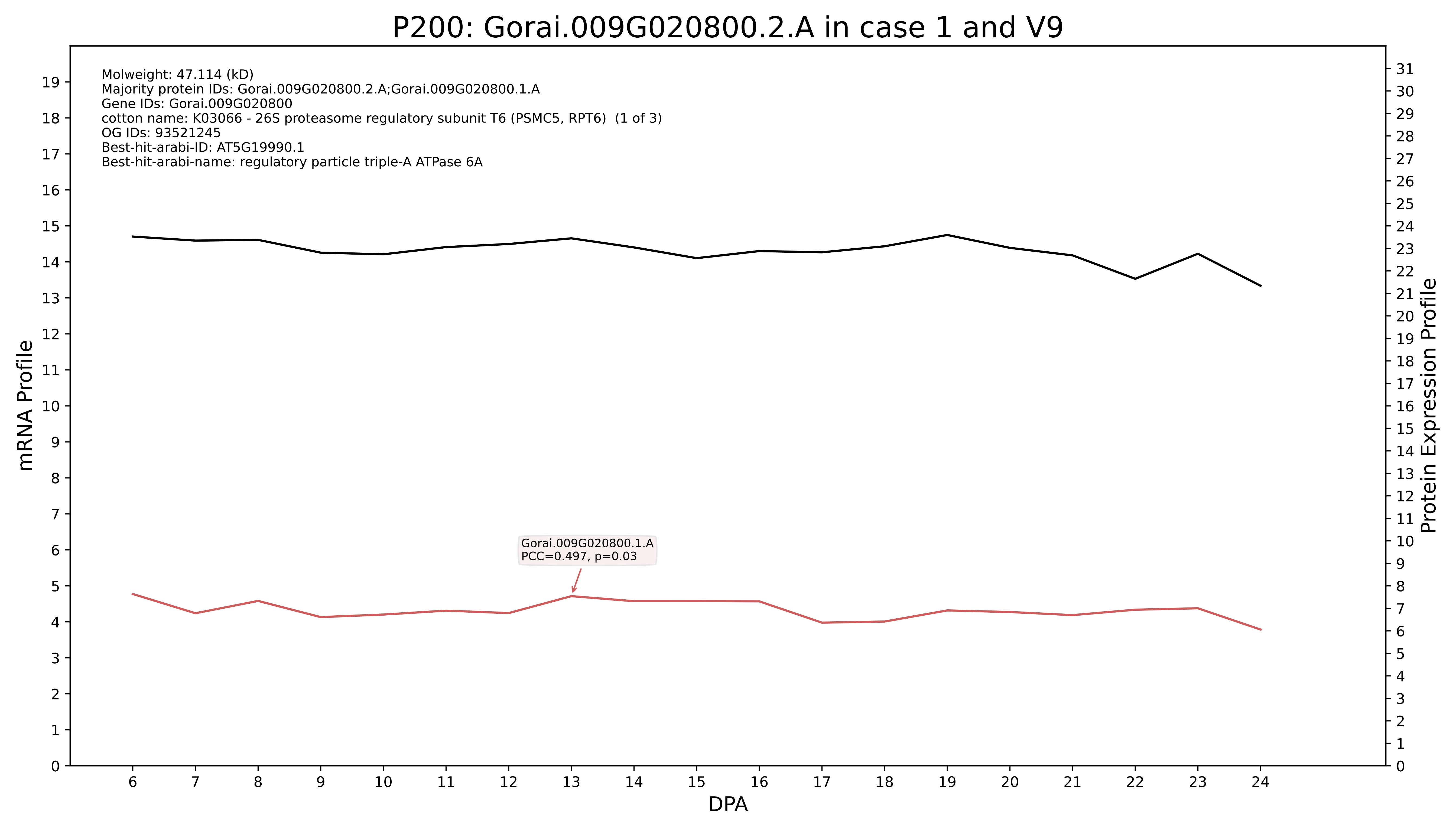
P200: Gorai.009G020800.2.A in case 1 and V9
Molweight: 47.114 (kD)
Majority protein IDs: Gorai.009G020800.2.A;Gorai.009G020800.1.A
Gene IDs: Gorai.009G020800
cotton name: K03066 - 26S proteasome regulatory subunit T6 (PSMC5, RPT6) (1 of 3)
OG IDs: 93521245
Best-hit-arabi-ID: AT5G19990.1
Best-hit-arabi-name: regulatory particle triple-A ATPase 6A
Gorai.009G020800.1.A
PCC=0.497, p=0.03
mRNA Profile
Protein Expression Profile
DPA

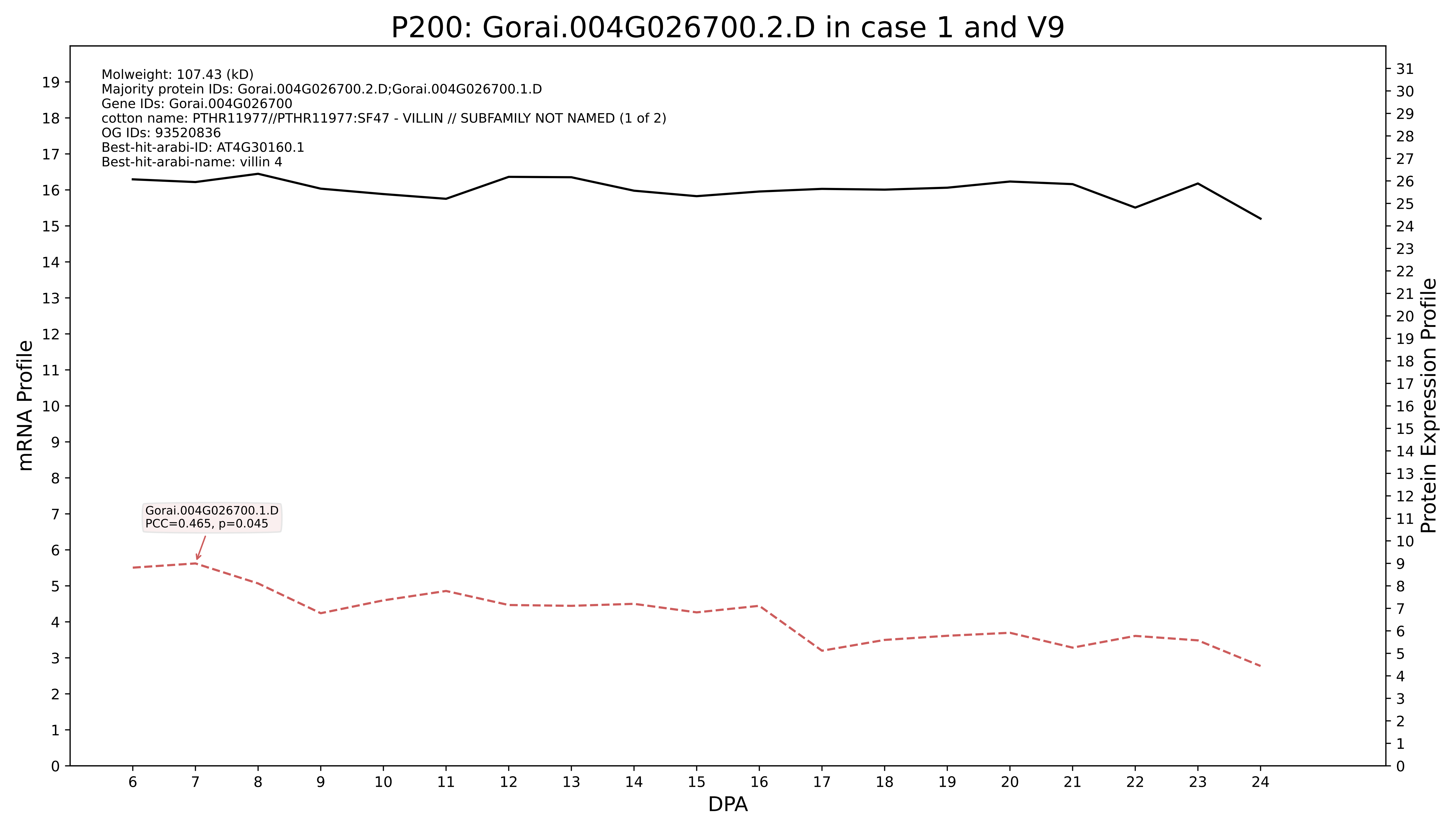
P200: Gorai.004G026700.2.D in case 1 and V9
Molweight: 107.43 (kD)
Majority protein IDs: Gorai.004G026700.2.D;Gorai.004G026700.1.D
Gene IDs: Gorai.004G026700
cotton name: PTHR11977//PTHR11977:SF47 - VILLIN // SUBFAMILY NOT NAMED (1 of 2)
OG IDs: 93520836
Best-hit-arabi-ID: AT4G30160.1
Best-hit-arabi-name: villin 4
Gorai.004G026700.1.D
PCC=0.465, p=0.045
mRNA Profile
Protein Expression Profile
DPA
0
1
2
3
4
5
6
7
8
9
10
11
12
13
14
15
16
17
18
19
20
21
22
23
24
25
26
27
28
29
30
31

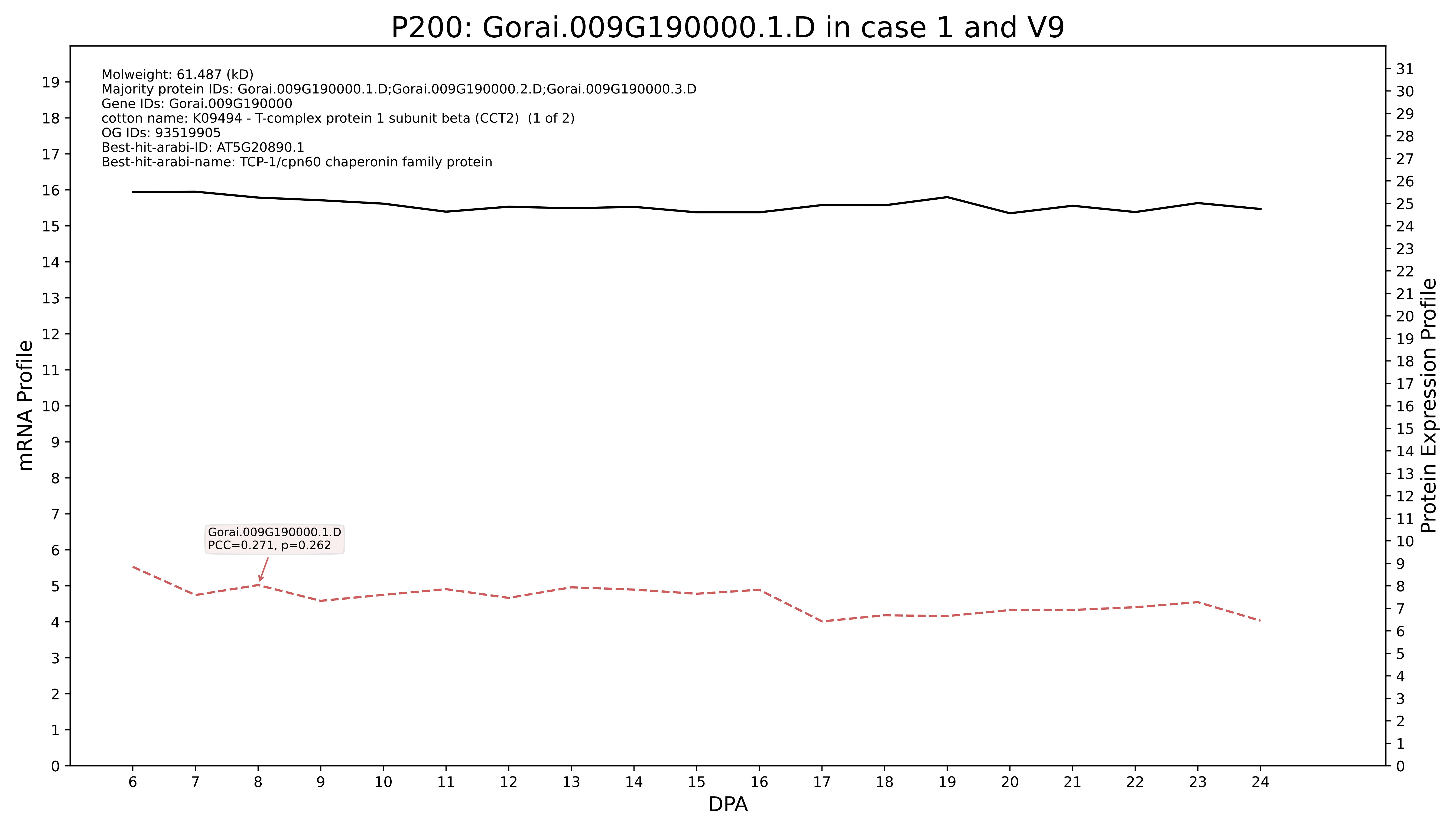
P200: Gorai.009G190000.1.D in case 1 and V9
Molweight: 61.487 (kD)
Majority protein IDs: Gorai.009G190000.1.D;Gorai.009G190000.2.D;Gorai.009G190000.3.D
Gene IDs: Gorai.009G190000
cotton name: K09494 - T-complex protein 1 subunit beta (CCT2) (1 of 2)
OG IDs: 93519905
Best-hit-arabi-ID: AT5G20890.1
Best-hit-arabi-name: TCP-1/cpn60 chaperonin family protein
Gorai.009G190000.1.D
PCC=0.271, p=0.262
mRNA Profile
Protein Expression Profile
DPA

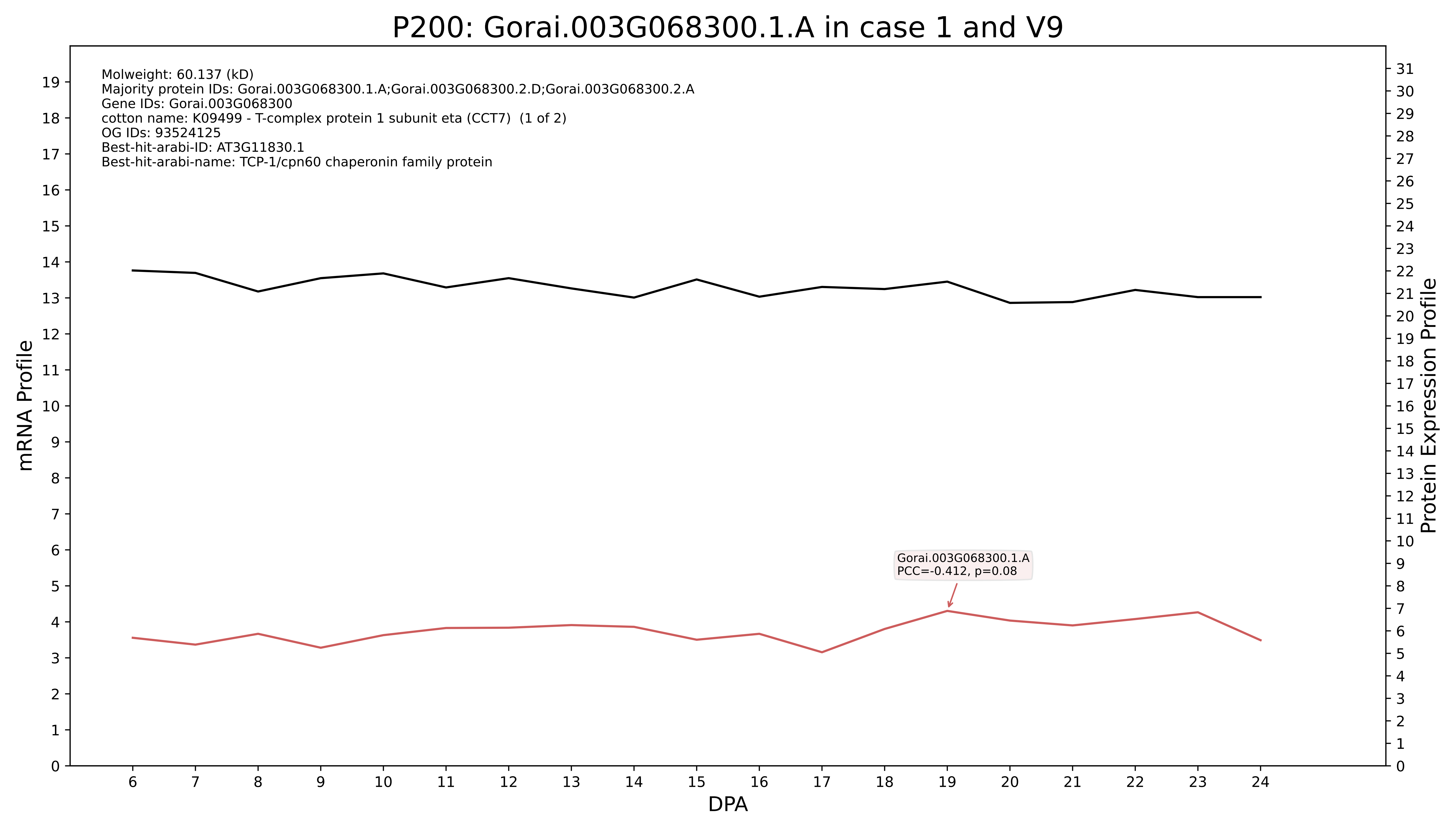
P200: Gorai.003G068300.1.A in case 1 and V9
Molweight: 60.137 (kD)
Majority protein IDs: Gorai.003G068300.1.A;Gorai.003G068300.2.D;Gorai.003G068300.2.A
Gene IDs: Gorai.003G068300
cotton name: K09499 - T-complex protein 1 subunit eta (CCT7) (1 of 2)
OG IDs: 93524125
Best-hit-arabi-ID: AT3G11830.1
Best-hit-arabi-name: TCP-1/cpn60 chaperonin family protein
Gorai.003G068300.1.A
PCC=-0.412, p=0.08
mRNA Profile
Protein Expression Profile
DPA
0
1
2
3
4
5
6
7
8
9
10
11
12
13
14
15
16
17
18
19
20
21
22
23
24
25
26
27
28
29
30
31

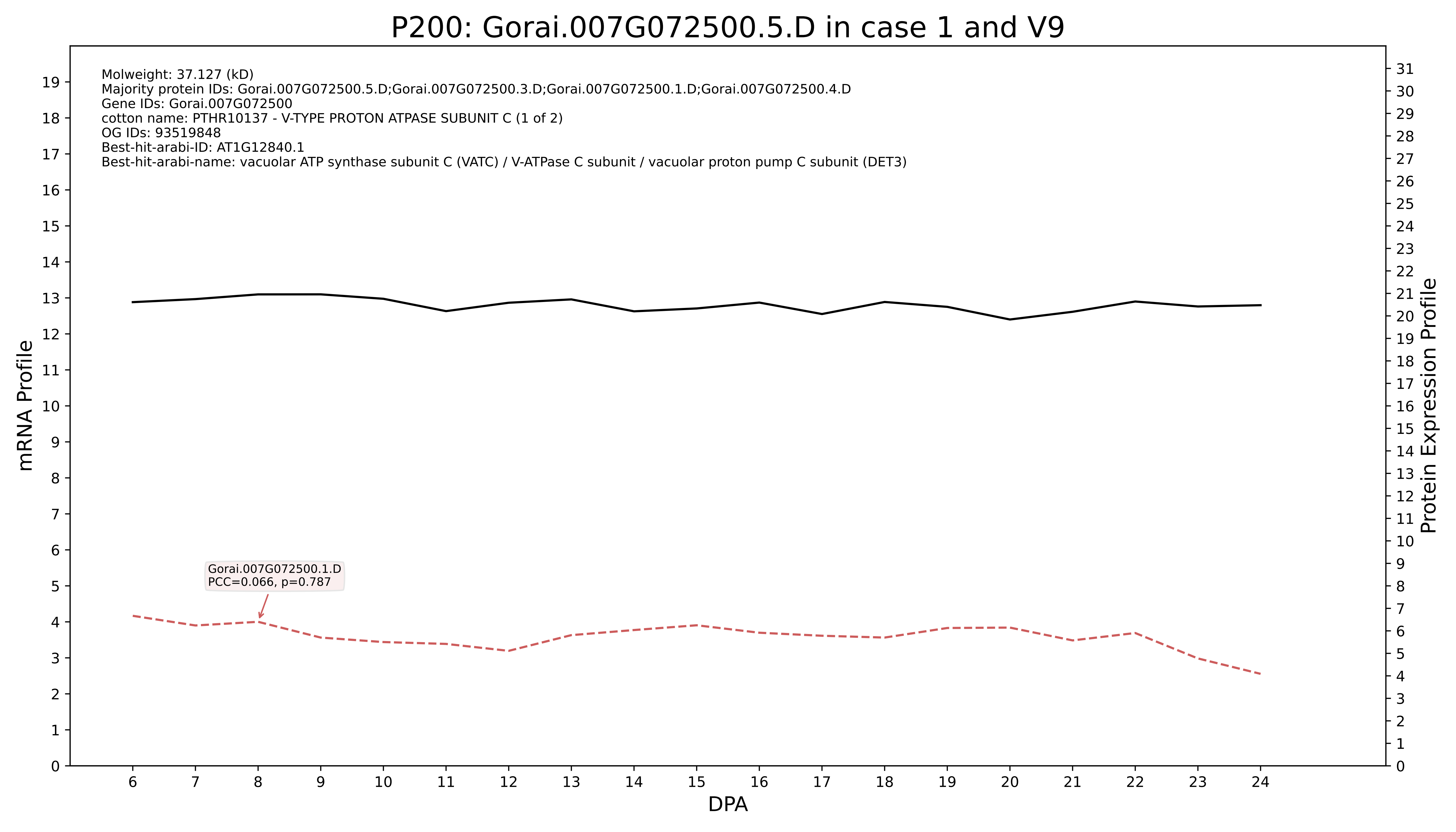

P200: Gorai.007G072500.5.D in case 1 and V9
Molweight: 37.127 (kD)
Majority protein IDs: Gorai.007G072500.5.D;Gorai.007G072500.3.D;Gorai.007G072500.1.D;Gorai.007G072500.4.D
Gene IDs: Gorai.007G072500
cotton name: PTHR10137 - V-TYPE PROTON ATPASE SUBUNIT C (1 of 2)
OG IDs: 93519848
Best-hit-arabi-ID: AT1G12840.1
Best-hit-arabi-name: vacuolar ATP synthase subunit C (VATC) / V-ATPase C subunit / vacuolar proton pump C subunit (DET3)
Gorai.007G072500.1.D
PCC=0.066, p=0.787
mRNA Profile
Protein Expression Profile
DPA

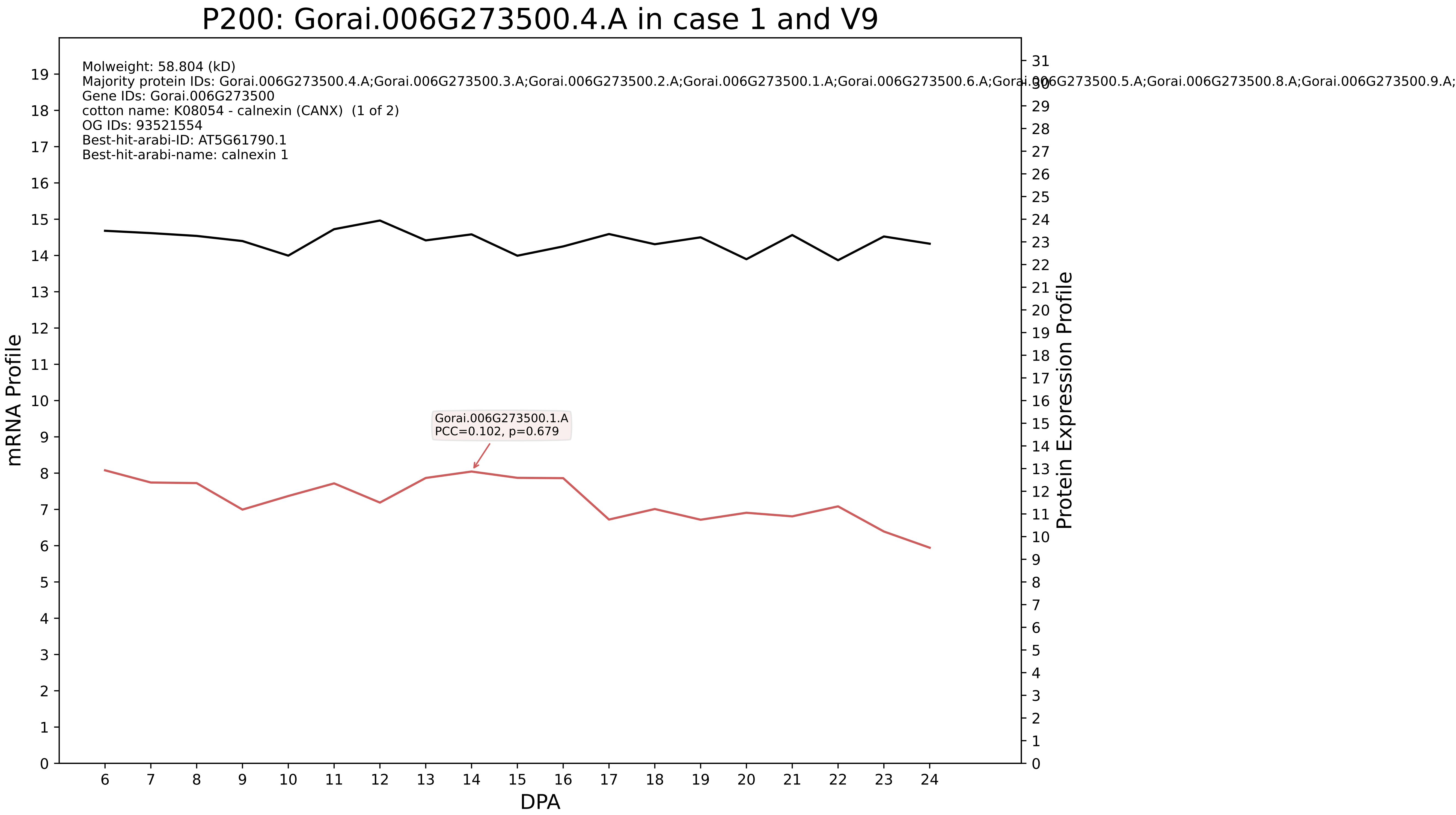
P200: Gorai.006G273500.4.A in case 1 and V9
Molweight: 58.804 (kD)
Majority protein IDs: Gorai.006G273500.4.A;Gorai.006G273500.3.A;Gorai.006G273500.2.A;Gorai.006G273500.1.A;Gorai.006G273500.6.A;Gorai.006G273500.5.A;Gorai.006G273500.8.A;Gorai.006G273500.9.A;
Gene IDs: Gorai.006G273500
cotton name: K08054 - calnexin (CANX) (1 of 2)
OG IDs: 93521554
Best-hit-arabi-ID: AT5G61790.1
Best-hit-arabi-name: calnexin 1
Gorai.006G273500.1.A
PCC=0.102, p=0.679
mRNA Profile
Protein Expression Profile
DPA

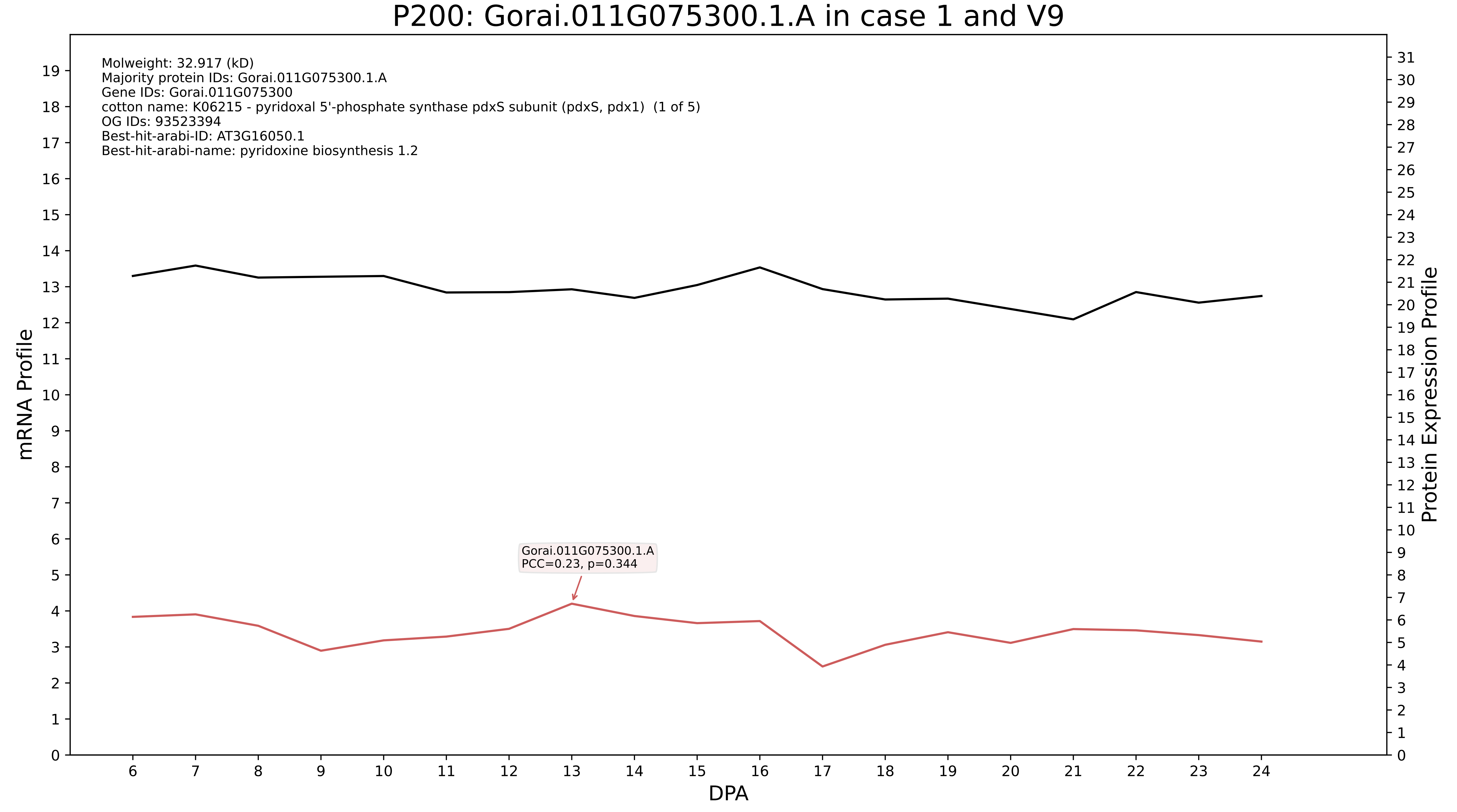
P200: Gorai.011G075300.1.A in case 1 and V9
Molweight: 32.917 (kD)
Majority protein IDs: Gorai.011G075300.1.A
Gene IDs: Gorai.011G075300
cotton name: K06215 - pyridoxal 5'-phosphate synthase pdxS subunit (pdxS, pdx1) (1 of 5)
OG IDs: 93523394
Best-hit-arabi-ID: AT3G16050.1
Best-hit-arabi-name: pyridoxine biosynthesis 1.2
Gorai.011G075300.1.A
PCC=0.23, p=0.344
mRNA Profile
Protein Expression Profile
DPA

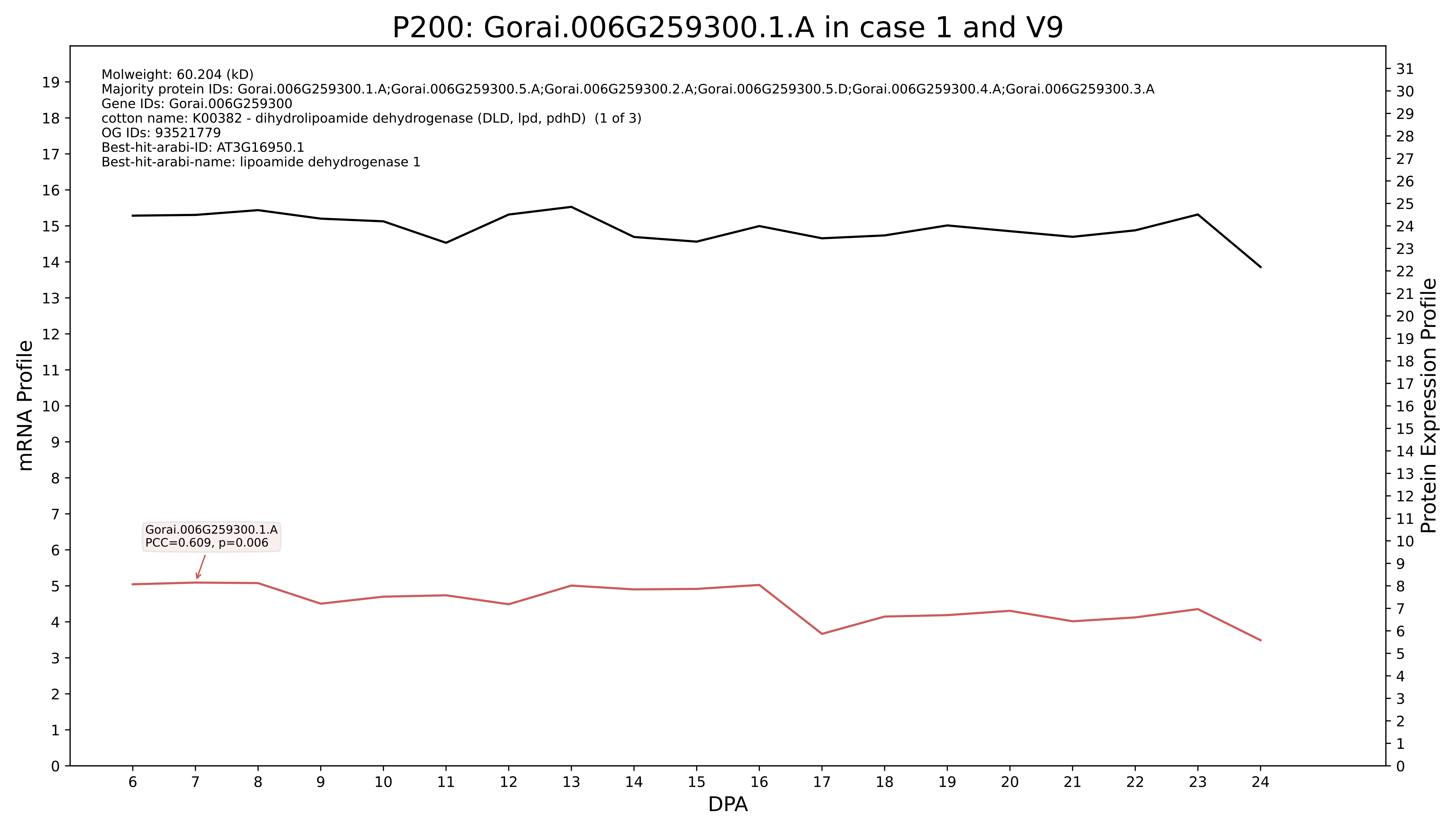
P200: Gorai.006G259300.1.A in case 1 and V9
Molweight: 60.204 (kD)
Majority protein IDs: Gorai.006G259300.1.A;Gorai.006G259300.5.A;Gorai.006G259300.2.A;Gorai.006G259300.5.D;Gorai.006G259300.4.A;Gorai.006G259300.3.A
Gene IDs: Gorai.006G259300
cotton name: K00382 - dihydrolipoamide dehydrogenase (DLD, lpd, pdhD) (1 of 3)
OG IDs: 93521779
Best-hit-arabi-ID: AT3G16950.1
Best-hit-arabi-name: lipoamide dehydrogenase 1
Gorai.006G259300.1.A
PCC=0.609, p=0.006
mRNA Profile
Protein Expression Profile
DPA
0
1
2
3
4
5
6
7
8
9
10
11
12
13
14
15
16
17
18
19
0
1
2
3
4
5
6
7
8
9
10
11
12
13
14
15
16
17
18
19
20
21
22
23
24
25
26
27
28
29
30
31
6
7
8
9
10
11
12
13
14
15
16
17
18
19
20
21
22
23
24

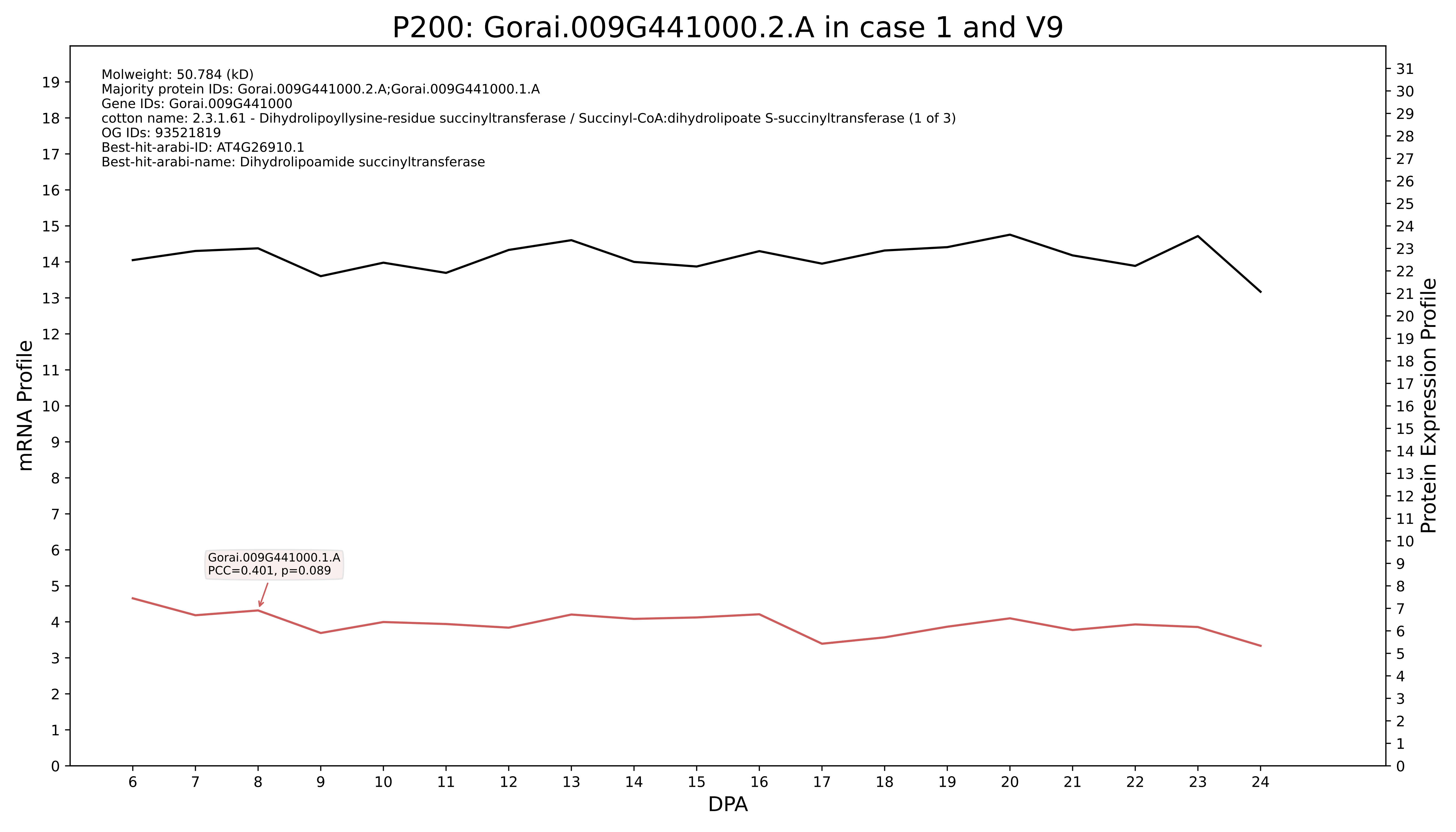

P200: Gorai.009G441000.2.A in case 1 and V9
Molweight: 50.784 (kD)
Majority protein IDs: Gorai.009G441000.2.A;Gorai.009G441000.1.A
Gene IDs: Gorai.009G441000
cotton name: 2.3.1.61 - Dihydrolipoyllysine-residue succinyltransferase / Succinyl-CoA:dihydrolipoate S-succinyltransferase (1 of 3)
OG IDs: 93521819
Best-hit-arabi-ID: AT4G26910.1
Best-hit-arabi-name: Dihydrolipoamide succinyltransferase
Gorai.009G441000.1.A
PCC=0.401, p=0.089
mRNA Profile
Protein Expression Profile
DPA

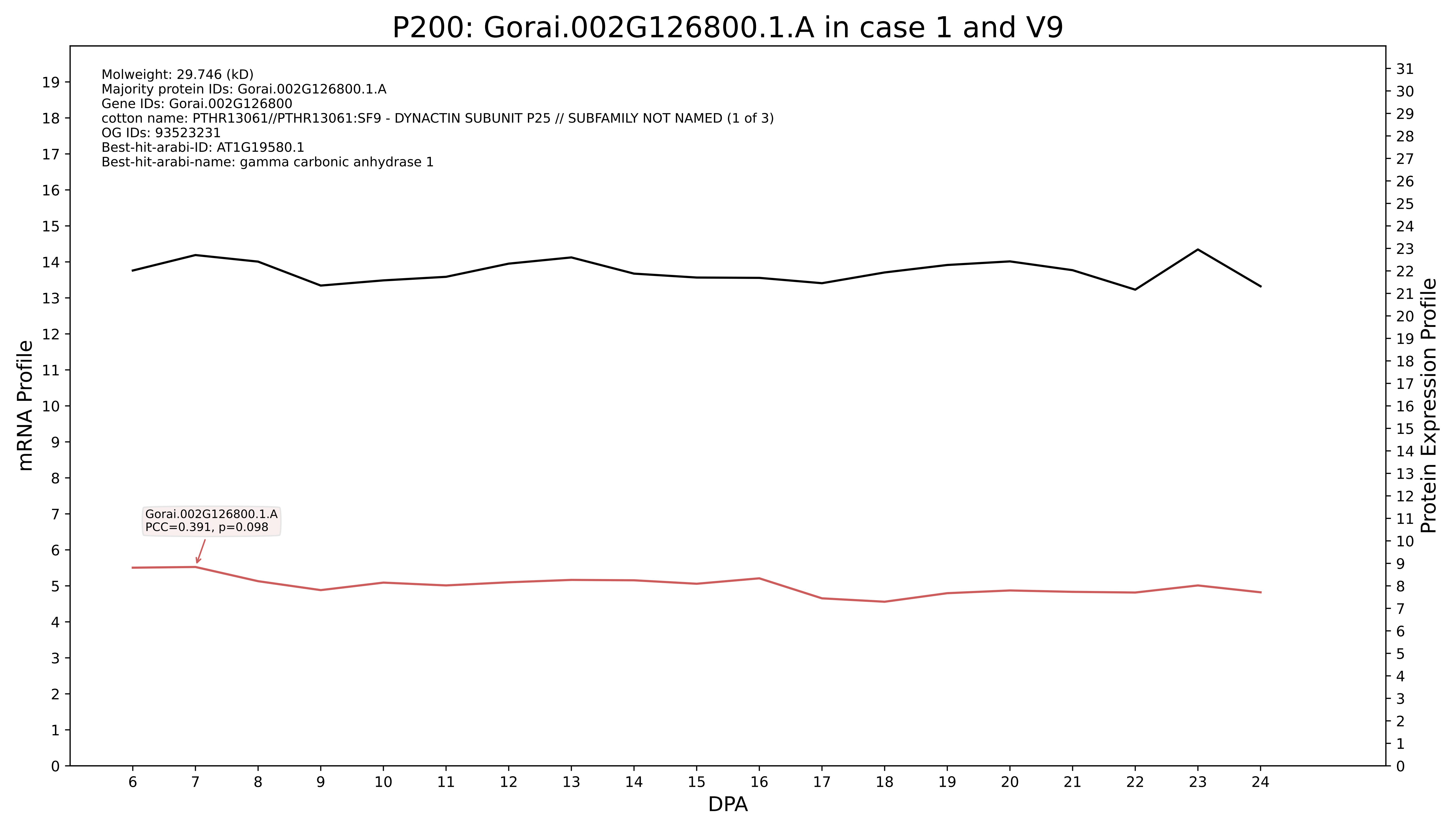
P200: Gorai.002G126800.1.A in case 1 and V9
Molweight: 29.746 (kD)
Majority protein IDs: Gorai.002G126800.1.A
Gene IDs: Gorai.002G126800
cotton name: PTHR13061//PTHR13061:SF9 - DYNACTIN SUBUNIT P25 // SUBFAMILY NOT NAMED (1 of 3)
OG IDs: 93523231
Best-hit-arabi-ID: AT1G19580.1
Best-hit-arabi-name: gamma carbonic anhydrase 1
Gorai.002G126800.1.A
PCC=0.391, p=0.098
mRNA Profile
Protein Expression Profile
DPA

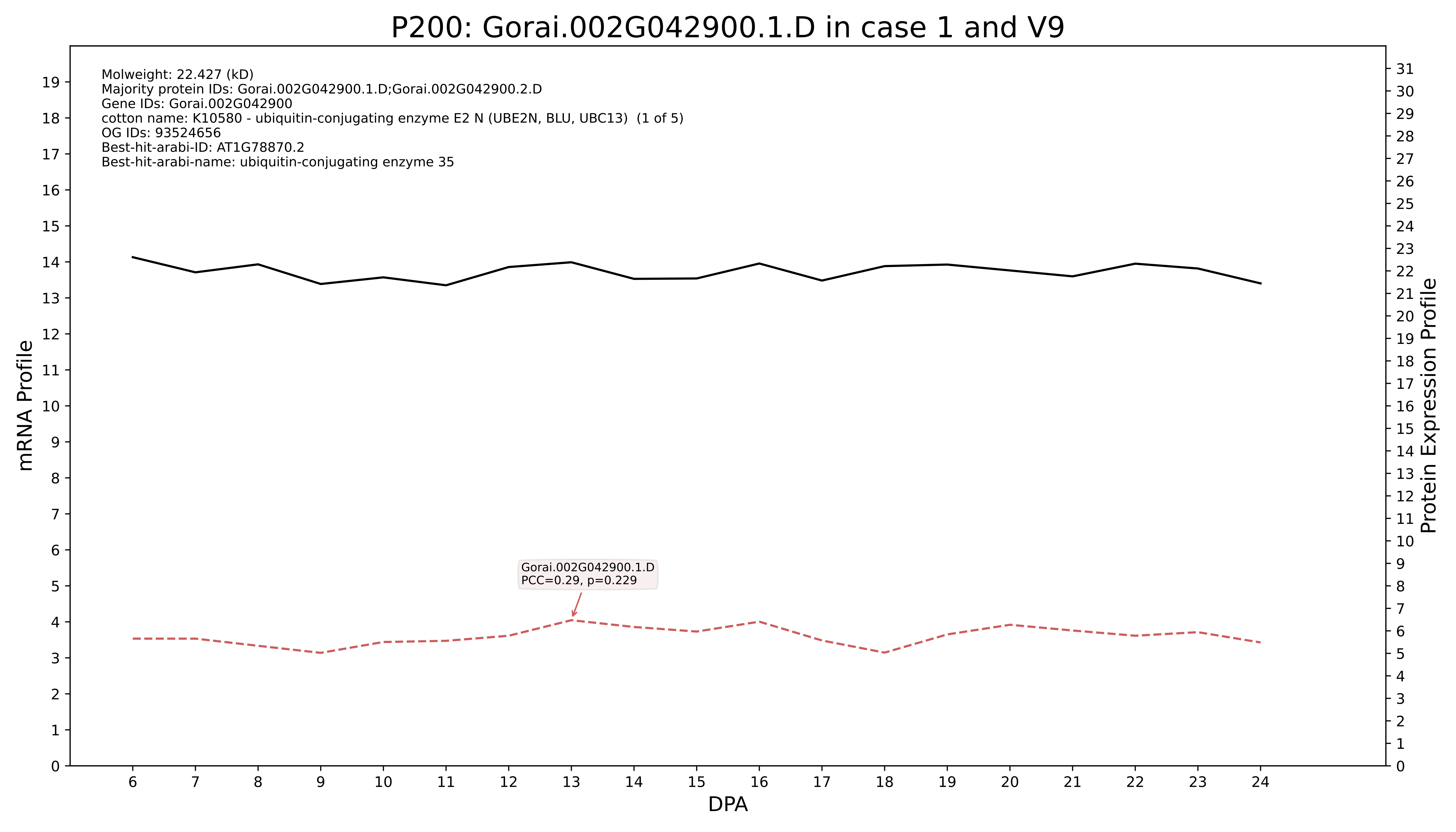
P200: Gorai.002G042900.1.D in case 1 and V9
Molweight: 22.427 (kD)
Majority protein IDs: Gorai.002G042900.1.D;Gorai.002G042900.2.D
Gene IDs: Gorai.002G042900
cotton name: K10580 - ubiquitin-conjugating enzyme E2 N (UBE2N, BLU, UBC13) (1 of 5)
OG IDs: 93524656
Best-hit-arabi-ID: AT1G78870.2
Best-hit-arabi-name: ubiquitin-conjugating enzyme 35
Gorai.002G042900.1.D
PCC=0.29, p=0.229
mRNA Profile
Protein Expression Profile
DPA

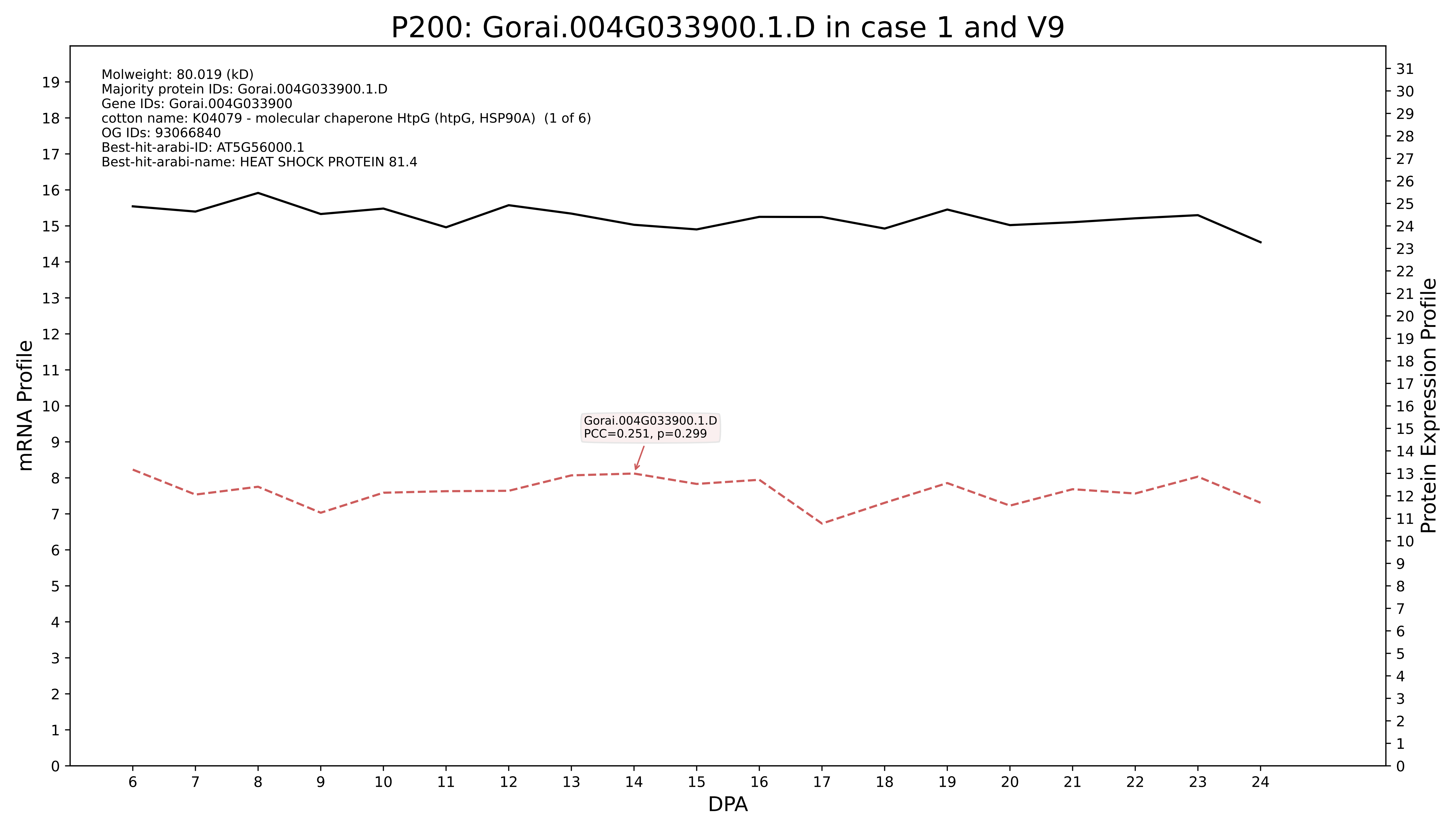
P200: Gorai.004G033900.1.D in case 1 and V9
Molweight: 80.019 (kD)
Majority protein IDs: Gorai.004G033900.1.D
Gene IDs: Gorai.004G033900
cotton name: K04079 - molecular chaperone HtpG (htpG, HSP90A) (1 of 6)
OG IDs: 93066840
Best-hit-arabi-ID: AT5G56000.1
Best-hit-arabi-name: HEAT SHOCK PROTEIN 81.4
Gorai.004G033900.1.D
PCC=0.251, p=0.299
mRNA Profile
Protein Expression Profile
DPA

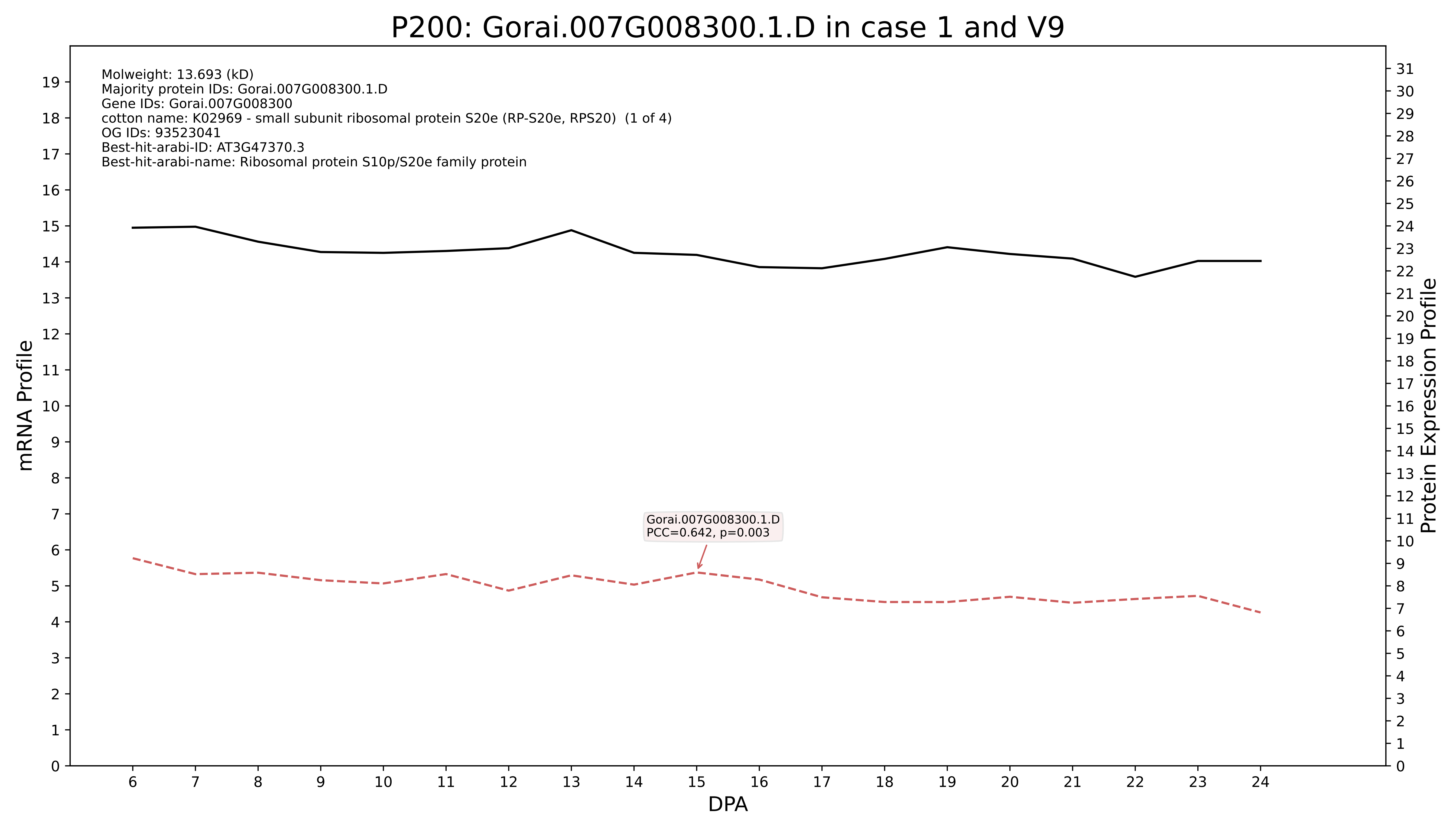
P200: Gorai.007G008300.1.D in case 1 and V9
Molweight: 13.693 (kD)
Majority protein IDs: Gorai.007G008300.1.D
Gene IDs: Gorai.007G008300
cotton name: K02969 - small subunit ribosomal protein S20e (RP-S20e, RPS20) (1 of 4)
OG IDs: 93523041
Best-hit-arabi-ID: AT3G47370.3
Best-hit-arabi-name: Ribosomal protein S10p/S20e family protein
Gorai.007G008300.1.D
PCC=0.642, p=0.003
mRNA Profile
Protein Expression Profile
DPA
0
1
2
3
4
5
6
7
8
9
10
11
12
13
14
15
16
17
18
19
20
21
22
23
24
25
26
27
28
29
30
31

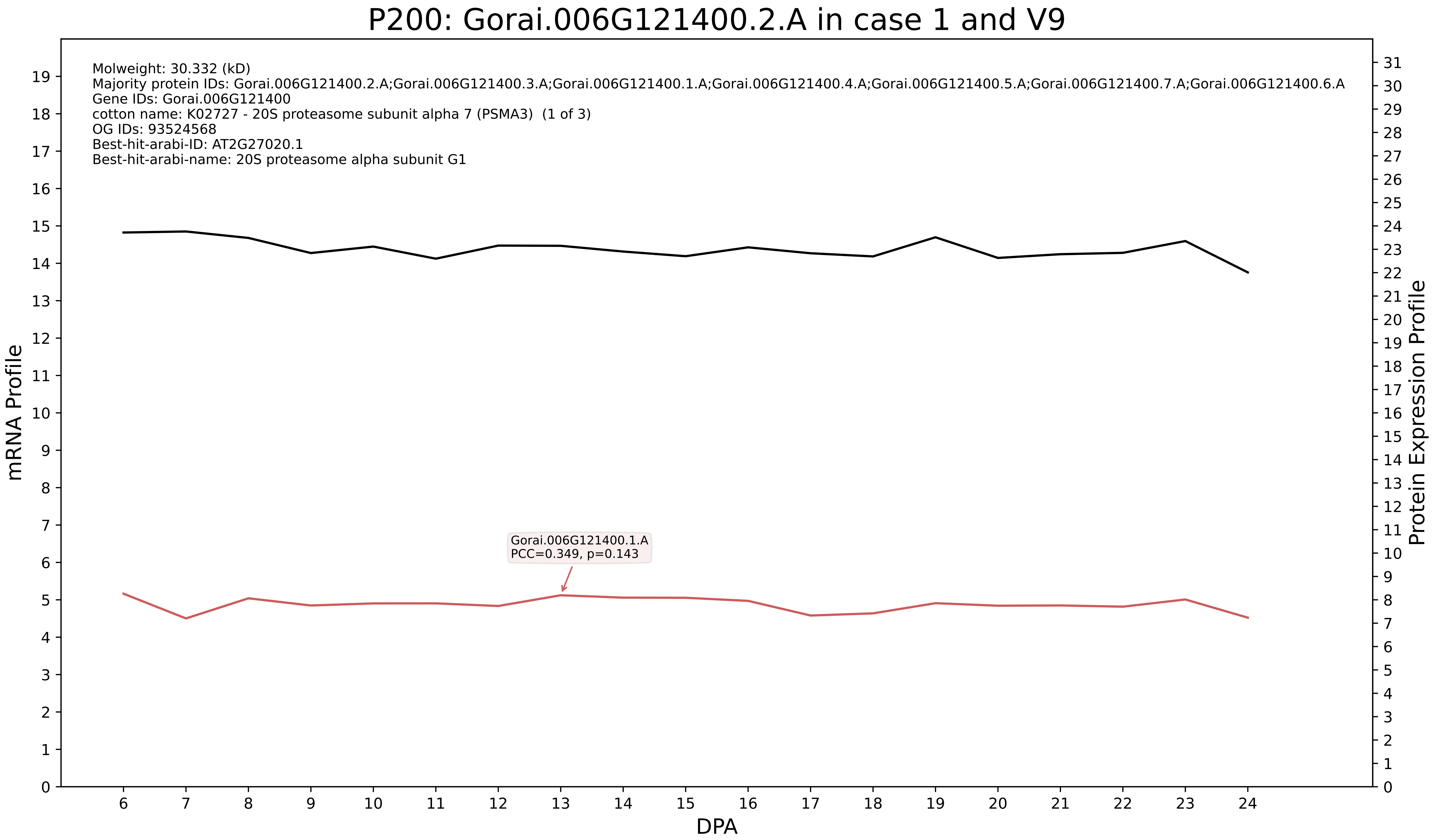
P200: Gorai.006G121400.2.A in case 1 and V9
Molweight: 30.332 (kD)
Majority protein IDs: Gorai.006G121400.2.A;Gorai.006G121400.3.A;Gorai.006G121400.1.A;Gorai.006G121400.4.A;Gorai.006G121400.5.A;Gorai.006G121400.7.A;Gorai.006G121400.6.A
Gene IDs: Gorai.006G121400
cotton name: K02727 - 20S proteasome subunit alpha 7 (PSMA3) (1 of 3)
OG IDs: 93524568
Best-hit-arabi-ID: AT2G27020.1
Best-hit-arabi-name: 20S proteasome alpha subunit G1
Gorai.006G121400.1.A
PCC=0.349, p=0.143
mRNA Profile
Protein Expression Profile
DPA

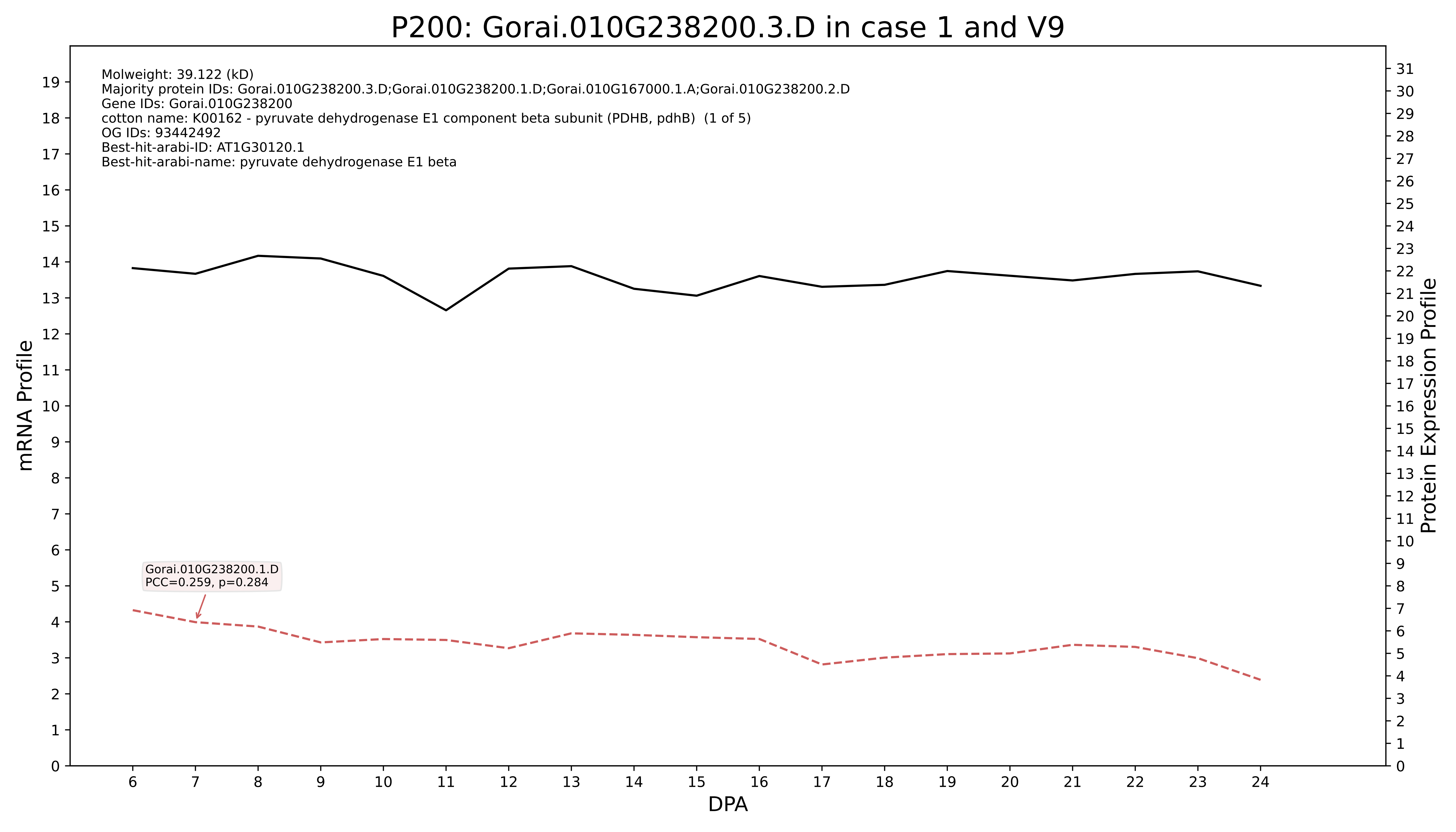
P200: Gorai.010G238200.3.D in case 1 and V9
Molweight: 39.122 (kD)
Majority protein IDs: Gorai.010G238200.3.D;Gorai.010G238200.1.D;Gorai.010G167000.1.A;Gorai.010G238200.2.D
Gene IDs: Gorai.010G238200
cotton name: K00162 - pyruvate dehydrogenase E1 component beta subunit (PDHB, pdhB) (1 of 5)
OG IDs: 93442492
Best-hit-arabi-ID: AT1G30120.1
Best-hit-arabi-name: pyruvate dehydrogenase E1 beta
Gorai.010G238200.1.D
PCC=0.259, p=0.284
mRNA Profile
Protein Expression Profile
DPA

P200: Gorai.003G141300.2.A in case 1 and V9

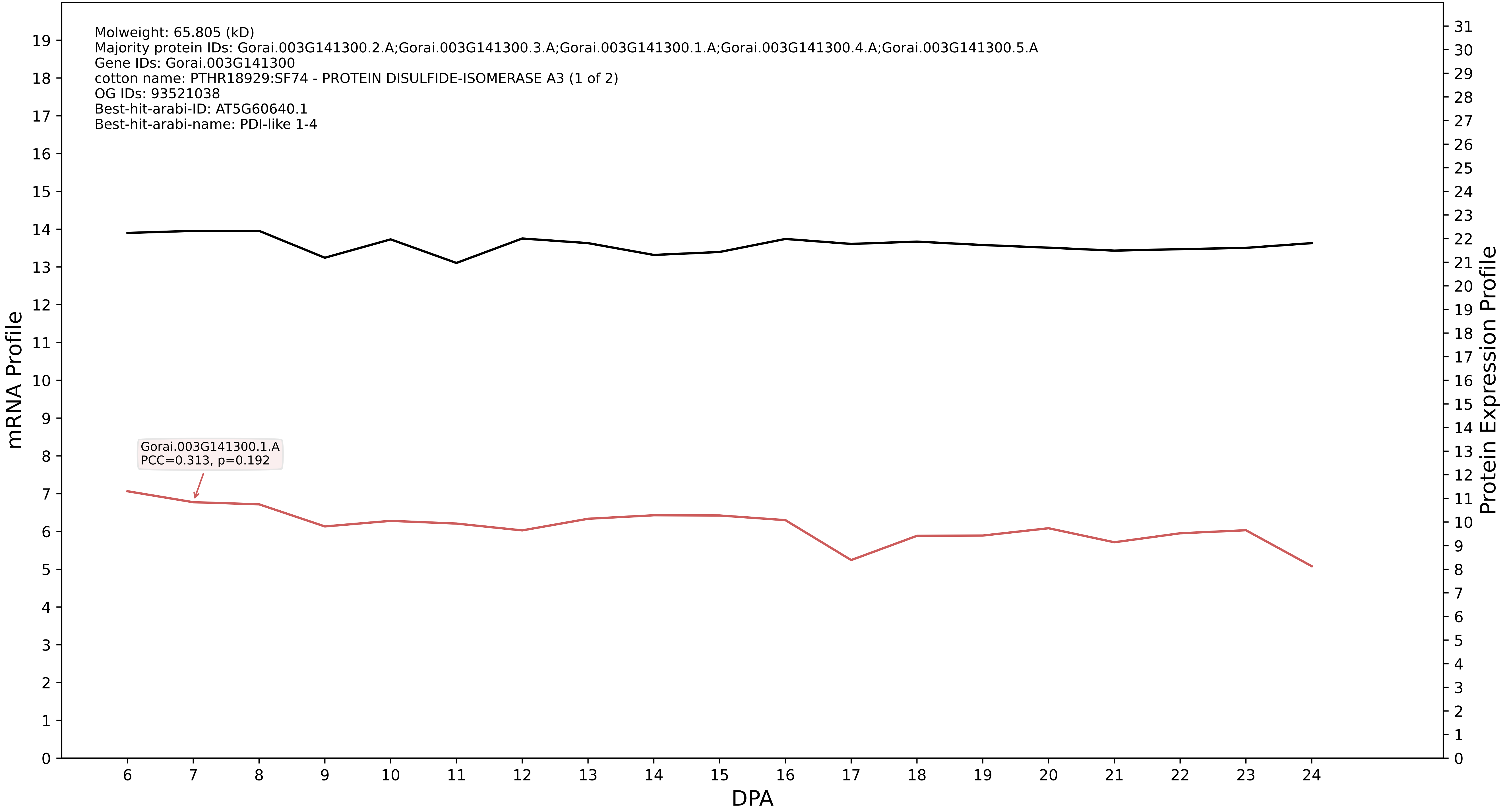

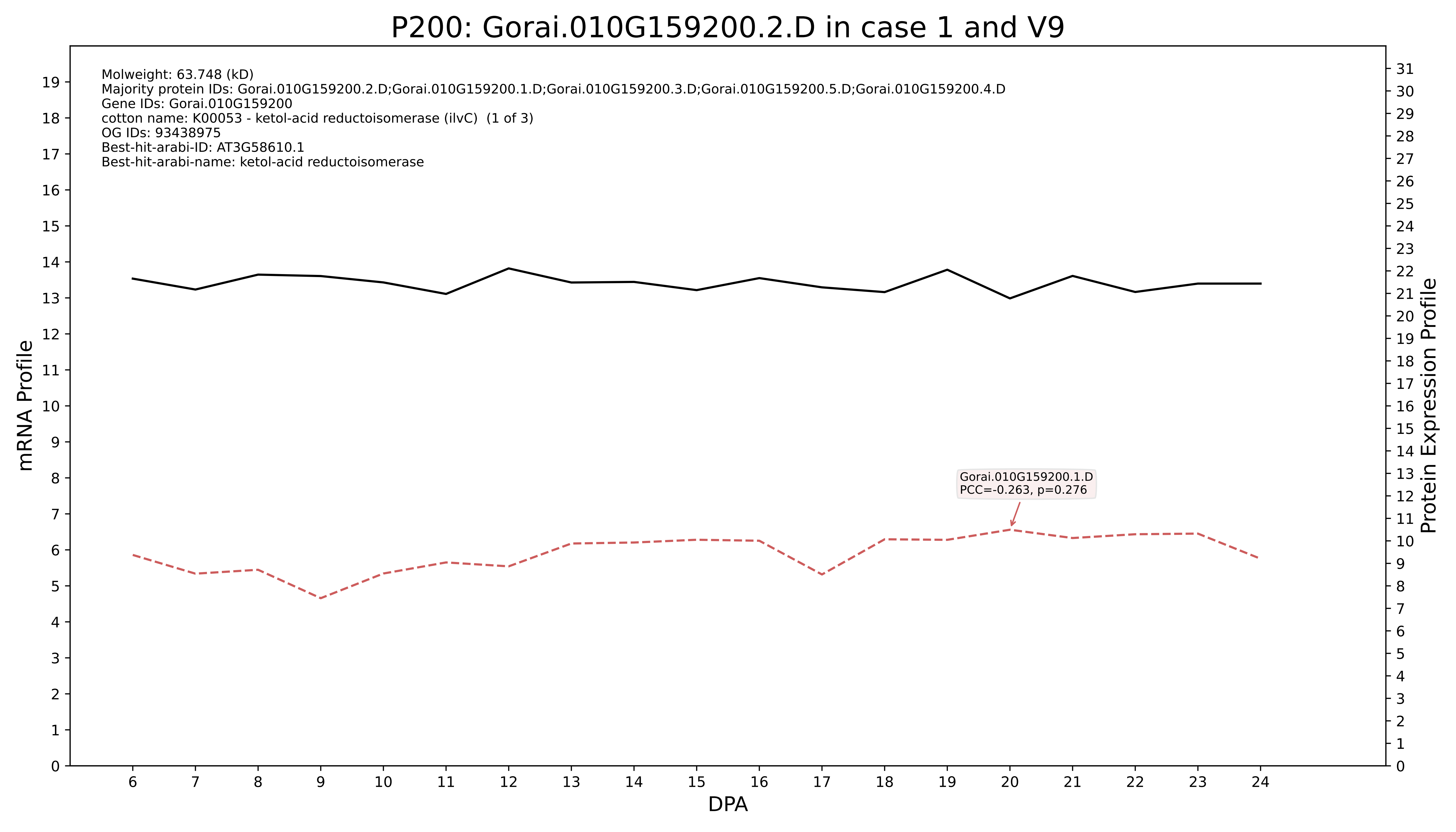
P200: Gorai.010G159200.2.D in case 1 and V9
Molweight: 63.748 (kD)
Majority protein IDs: Gorai.010G159200.2.D;Gorai.010G159200.1.D;Gorai.010G159200.3.D;Gorai.010G159200.5.D;Gorai.010G159200.4.D
Gene IDs: Gorai.010G159200
cotton name: K00053 - ketol-acid reductoisomerase (ilvC) (1 of 3)
OG IDs: 93438975
Best-hit-arabi-ID: AT3G58610.1
Best-hit-arabi-name: ketol-acid reductoisomerase
Gorai.010G159200.1.D
PCC=-0.263, p=0.276
mRNA Profile
Protein Expression Profile
DPA

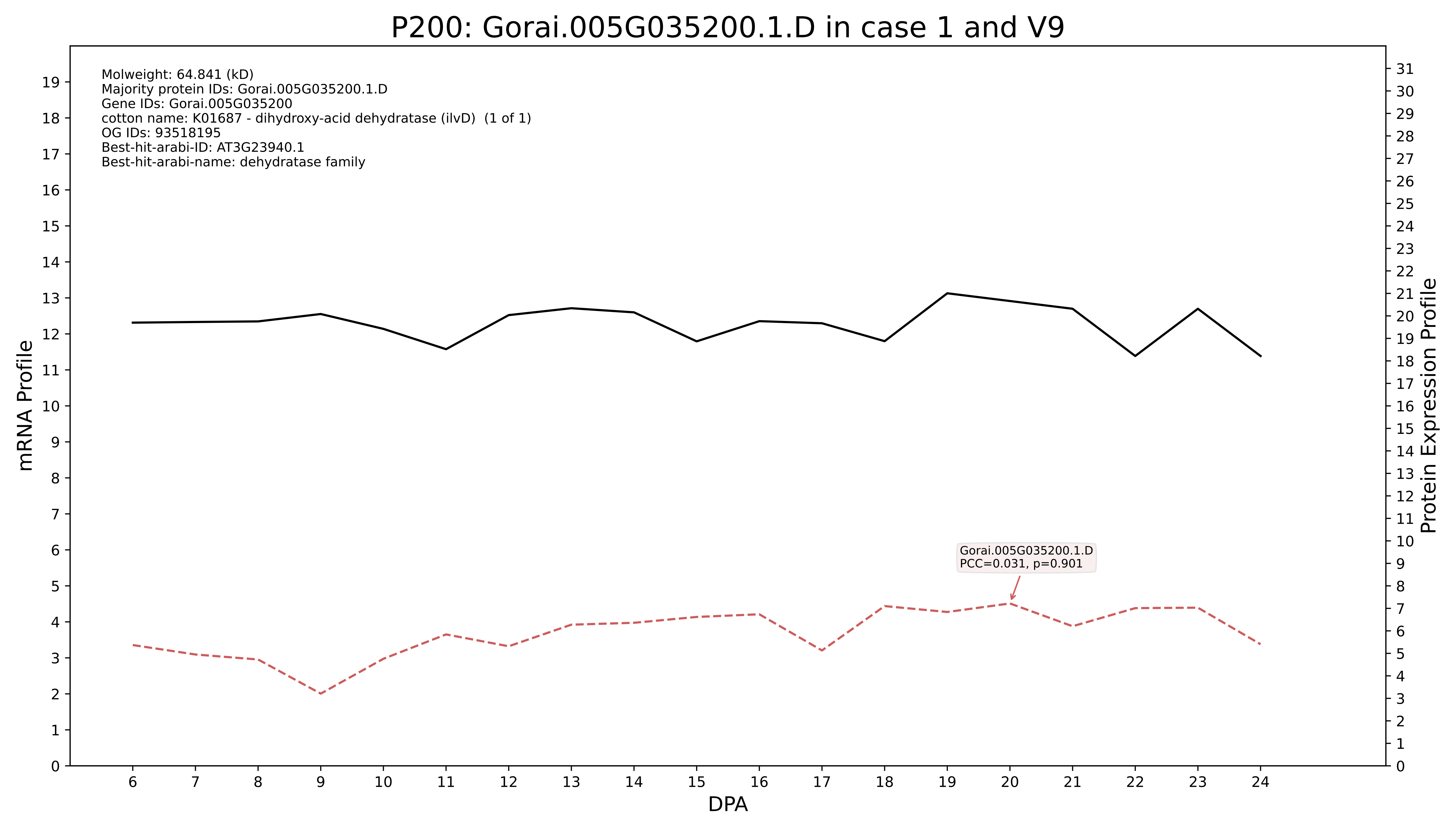

P200: Gorai.005G035200.1.D in case 1 and V9
Molweight: 64.841 (kD)
Majority protein IDs: Gorai.005G035200.1.D
Gene IDs: Gorai.005G035200
cotton name: K01687 - dihydroxy-acid dehydratase (ilvD) (1 of 1)
OG IDs: 93518195
Best-hit-arabi-ID: AT3G23940.1
Best-hit-arabi-name: dehydratase family
Gorai.005G035200.1.D
PCC=0.031, p=0.901
mRNA Profile
Protein Expression Profile
DPA

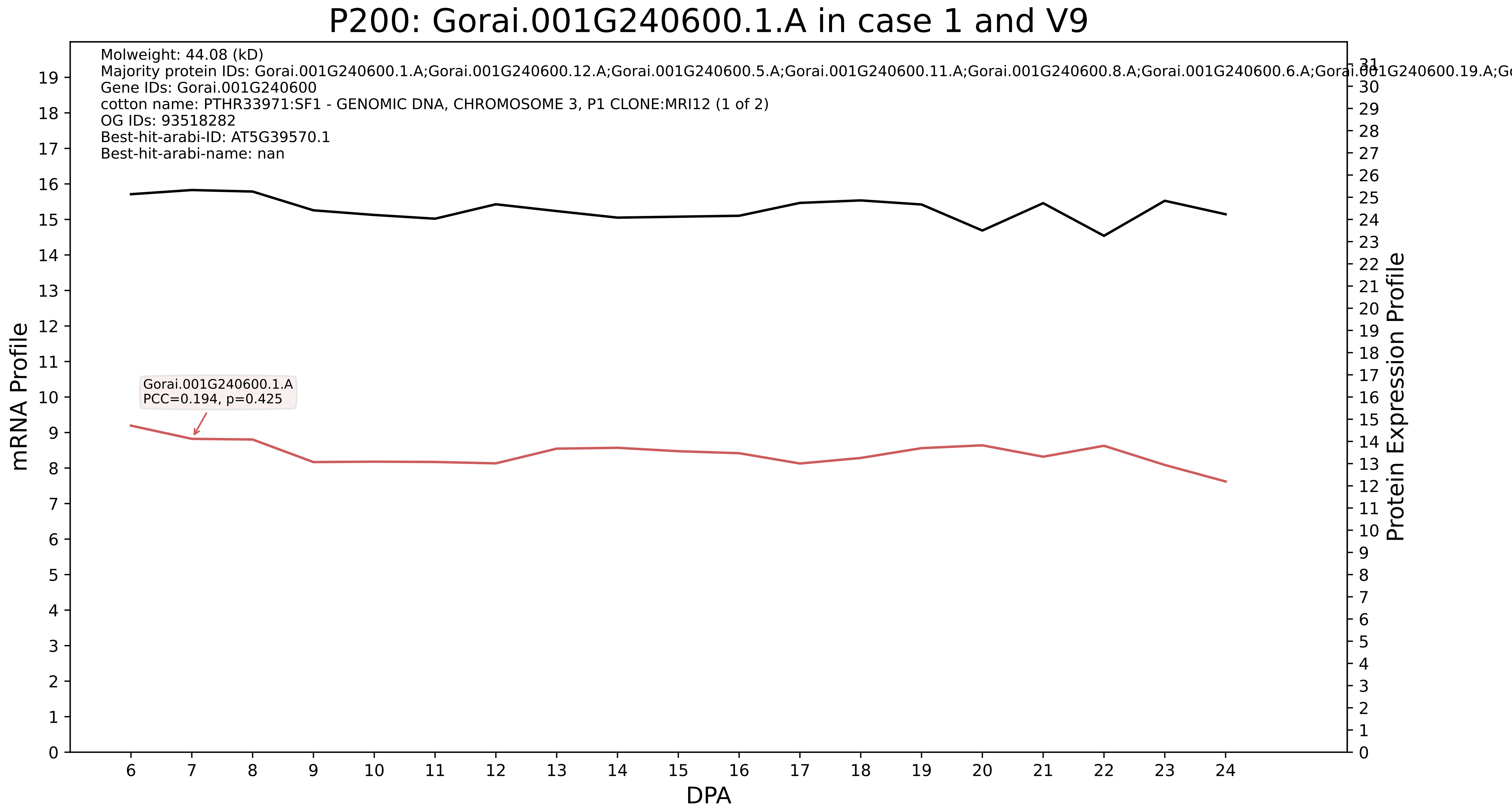
P200: Gorai.001G240600.1.A in case 1 and V9
Molweight: 44.08 (kD)
Majority protein IDs: Gorai.001G240600.1.A;Gorai.001G240600.12.A;Gorai.001G240600.5.A;Gorai.001G240600.11.A;Gorai.001G240600.8.A;Gorai.001G240600.6.A;Gorai.001G240600.19.A;G
Gene IDs: Gorai.001G240600
cotton name: PTHR33971:SF1 - GENOMIC DNA, CHROMOSOME 3, P1 CLONE:MRI12 (1 of 2)
OG IDs: 93518282
Best-hit-arabi-ID: AT5G39570.1
Best-hit-arabi-name: nan
Gorai.001G240600.1.A
PCC=0.194, p=0.425
mRNA Profile
Protein Expression Profile
DPA

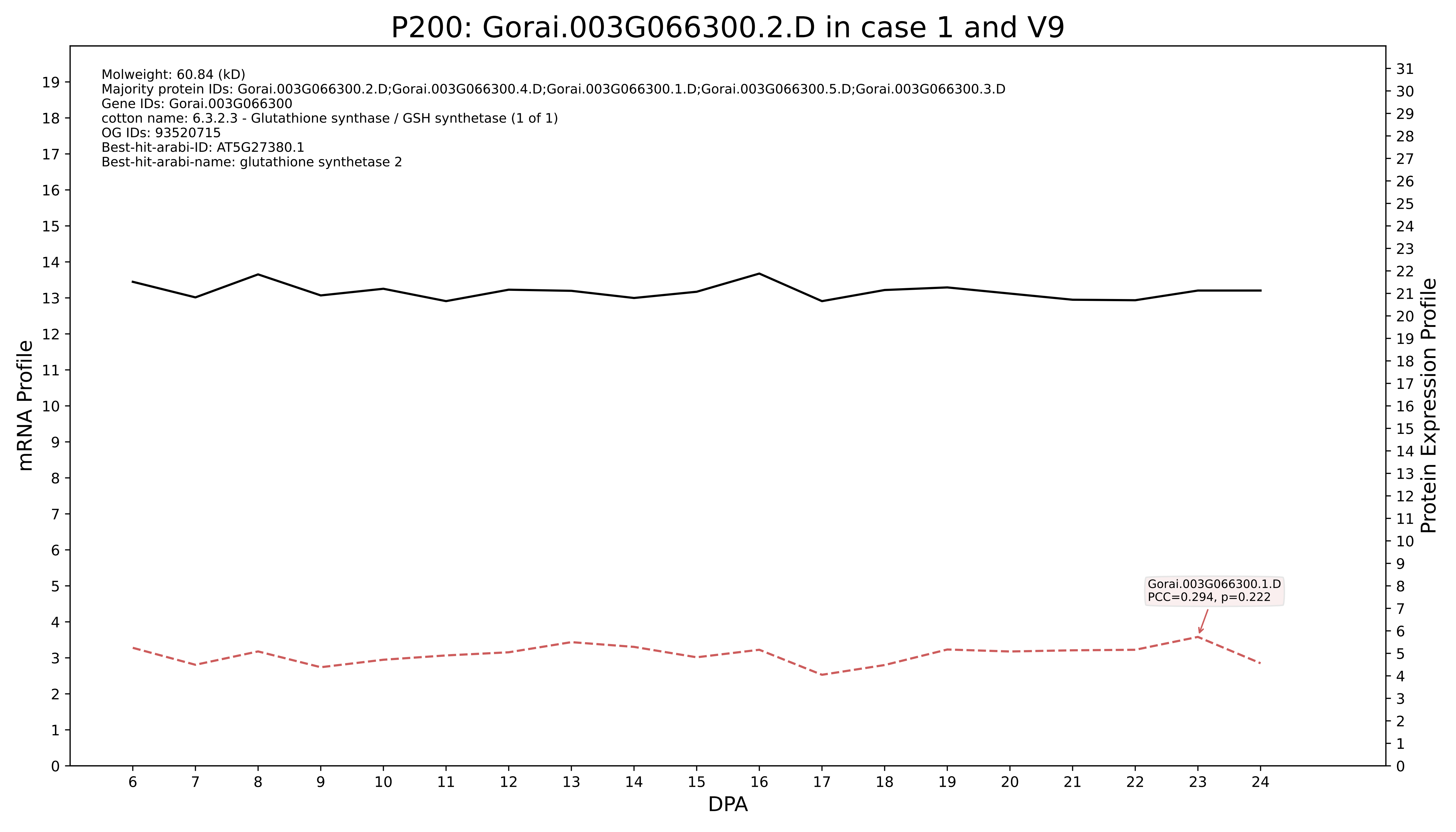
P200: Gorai.003G066300.2.D in case 1 and V9
Molweight: 60.84 (kD)
Majority protein IDs: Gorai.003G066300.2.D;Gorai.003G066300.4.D;Gorai.003G066300.1.D;Gorai.003G066300.5.D;Gorai.003G066300.3.D
Gene IDs: Gorai.003G066300
cotton name: 6.3.2.3 - Glutathione synthase / GSH synthetase (1 of 1)
OG IDs: 93520715
Best-hit-arabi-ID: AT5G27380.1
Best-hit-arabi-name: glutathione synthetase 2
Gorai.003G066300.1.D
PCC=0.294, p=0.222
mRNA Profile
Protein Expression Profile
DPA

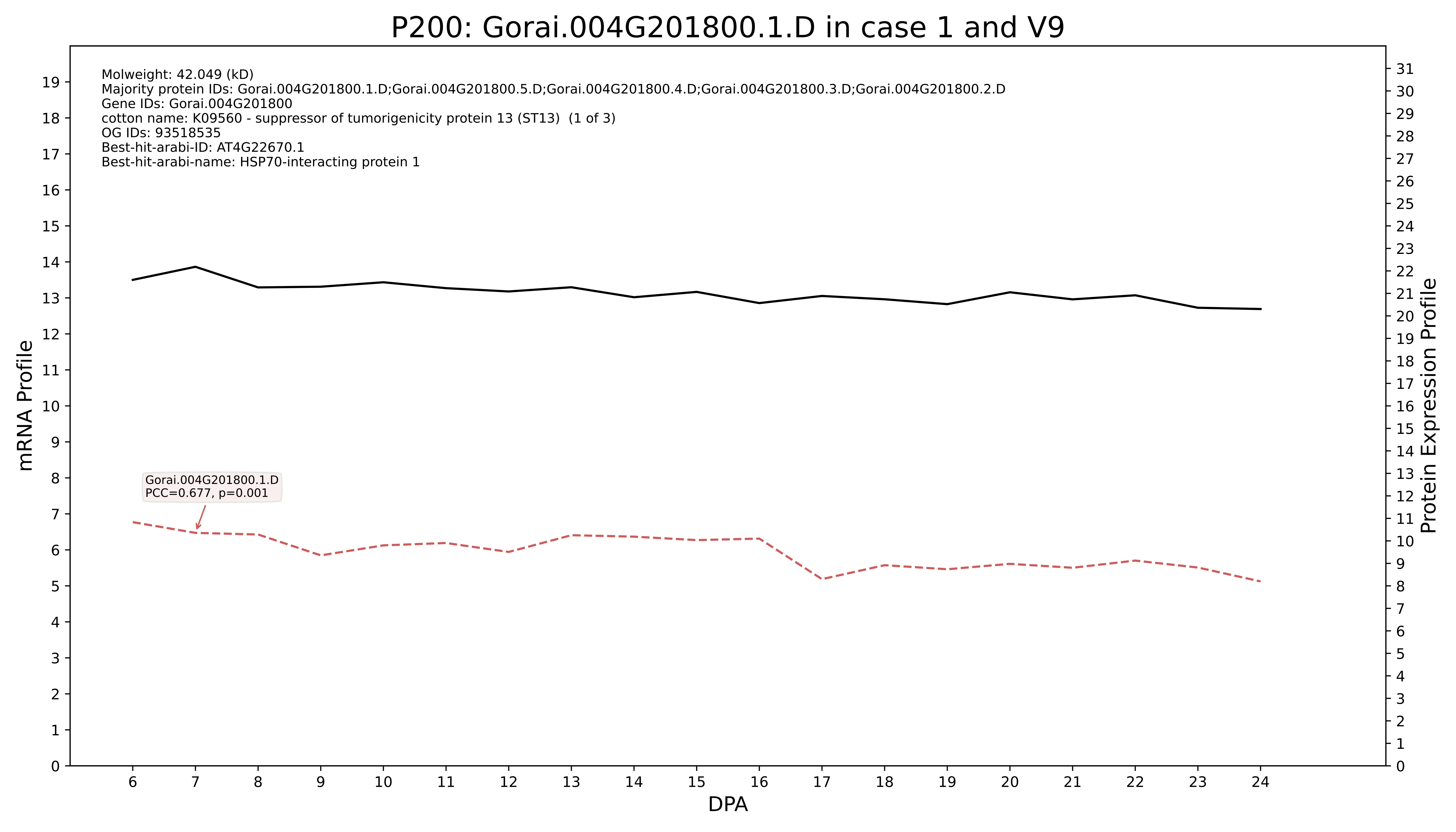
P200: Gorai.004G201800.1.D in case 1 and V9
Molweight: 42.049 (kD)
Majority protein IDs: Gorai.004G201800.1.D;Gorai.004G201800.5.D;Gorai.004G201800.4.D;Gorai.004G201800.3.D;Gorai.004G201800.2.D
Gene IDs: Gorai.004G201800
cotton name: K09560 - suppressor of tumorigenicity protein 13 (ST13) (1 of 3)
OG IDs: 93518535
Best-hit-arabi-ID: AT4G22670.1
Best-hit-arabi-name: HSP70-interacting protein 1
Gorai.004G201800.1.D
PCC=0.677, p=0.001
mRNA Profile
Protein Expression Profile
DPA

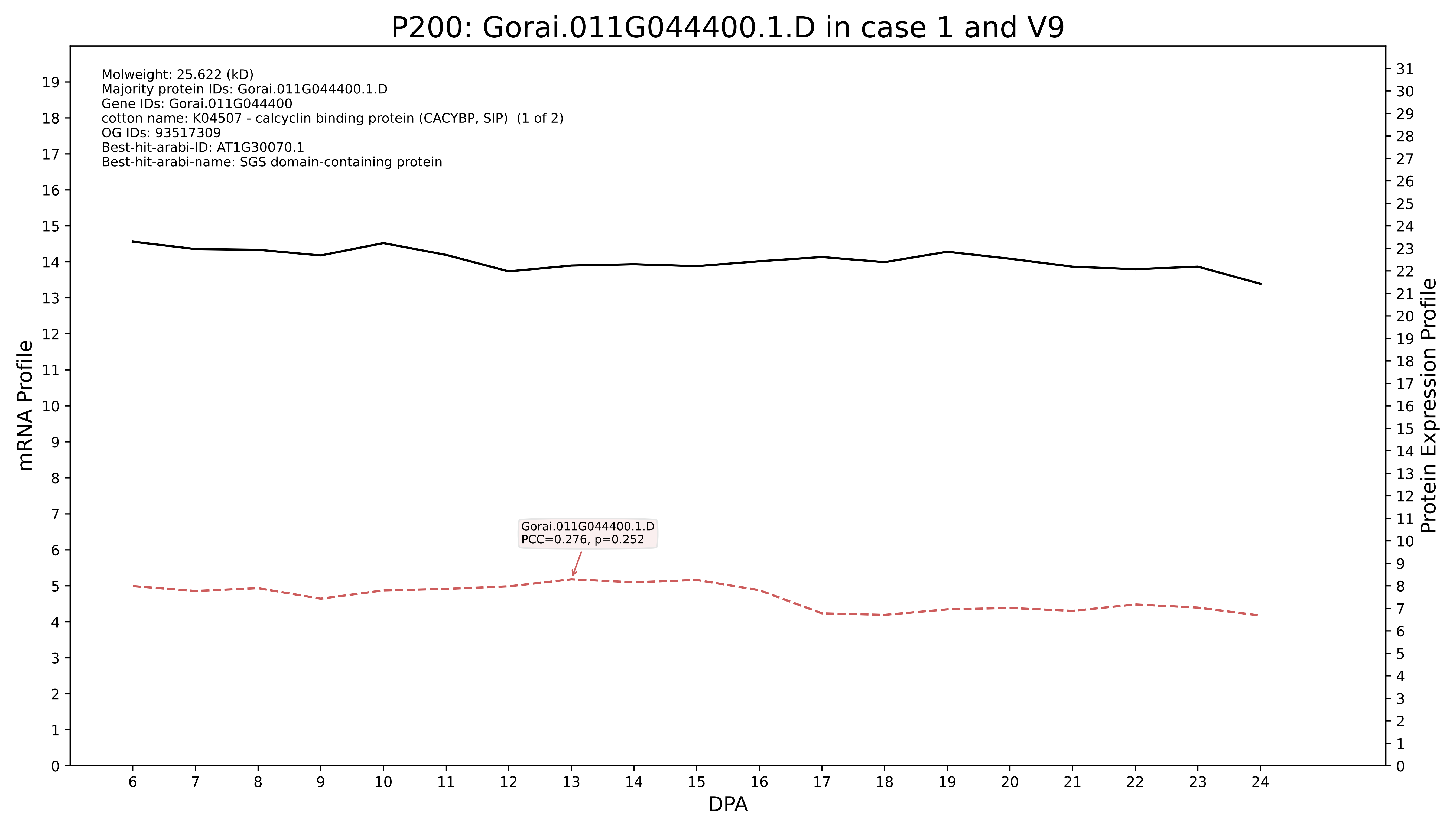
P200: Gorai.011G044400.1.D in case 1 and V9
Molweight: 25.622 (kD)
Majority protein IDs: Gorai.011G044400.1.D
Gene IDs: Gorai.011G044400
cotton name: K04507 - calcyclin binding protein (CACYBP, SIP) (1 of 2)
OG IDs: 93517309
Best-hit-arabi-ID: AT1G30070.1
Best-hit-arabi-name: SGS domain-containing protein
Gorai.011G044400.1.D
PCC=0.276, p=0.252
mRNA Profile
Protein Expression Profile
DPA

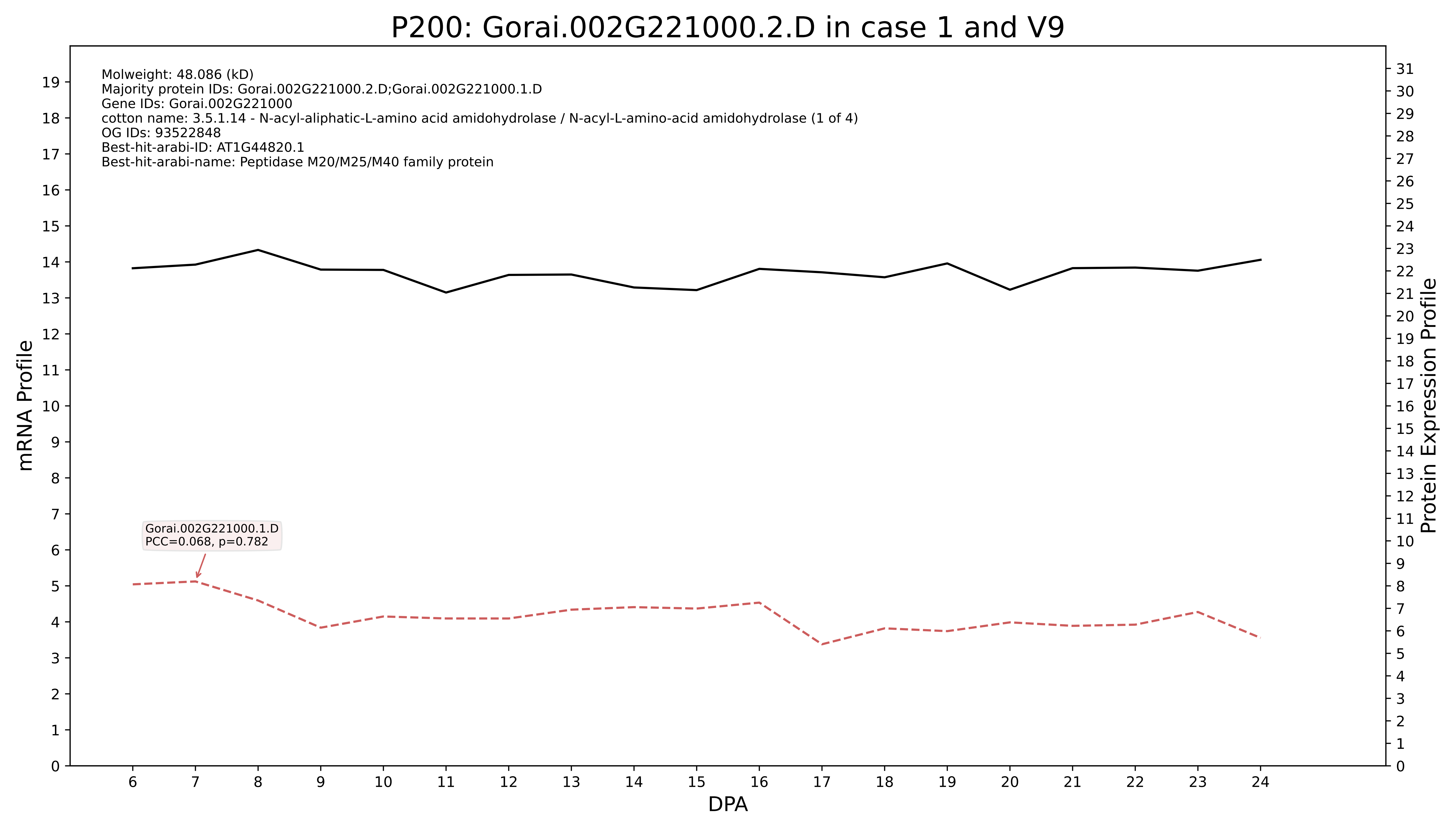
P200: Gorai.002G221000.2.D in case 1 and V9
Molweight: 48.086 (kD)
Majority protein IDs: Gorai.002G221000.2.D;Gorai.002G221000.1.D
Gene IDs: Gorai.002G221000
cotton name: 3.5.1.14 - N-acyl-aliphatic-L-amino acid amidohydrolase / N-acyl-L-amino-acid amidohydrolase (1 of 4)
OG IDs: 93522848
Best-hit-arabi-ID: AT1G44820.1
Best-hit-arabi-name: Peptidase M20/M25/M40 family protein
Gorai.002G221000.1.D
PCC=0.068, p=0.782
mRNA Profile
Protein Expression Profile
DPA

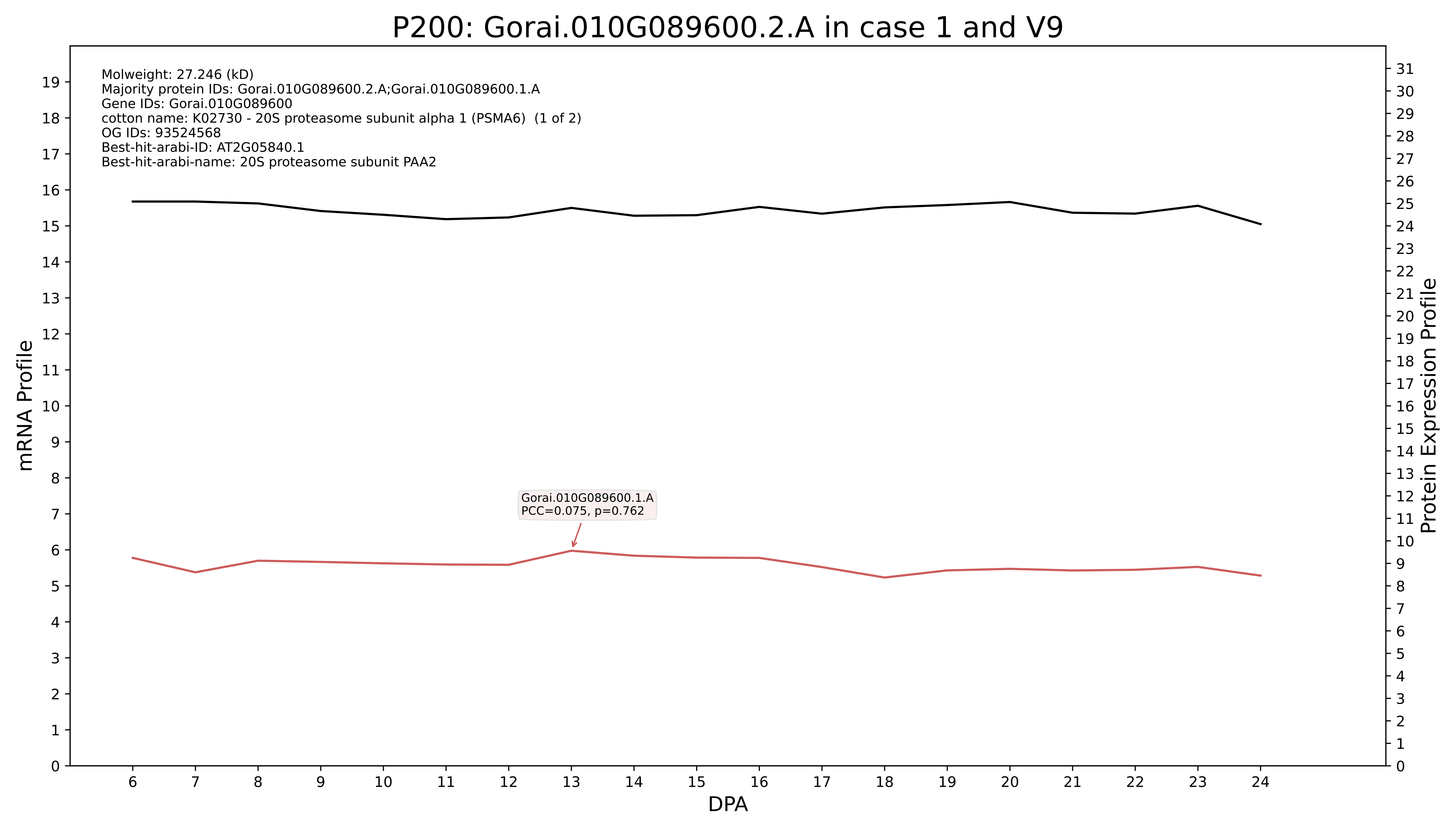
P200: Gorai.010G089600.2.A in case 1 and V9
Molweight: 27.246 (kD)
Majority protein IDs: Gorai.010G089600.2.A;Gorai.010G089600.1.A
Gene IDs: Gorai.010G089600
cotton name: K02730 - 20S proteasome subunit alpha 1 (PSMA6) (1 of 2)
OG IDs: 93524568
Best-hit-arabi-ID: AT2G05840.1
Best-hit-arabi-name: 20S proteasome subunit PAA2
Gorai.010G089600.1.A
PCC=0.075, p=0.762
mRNA Profile
Protein Expression Profile
DPA

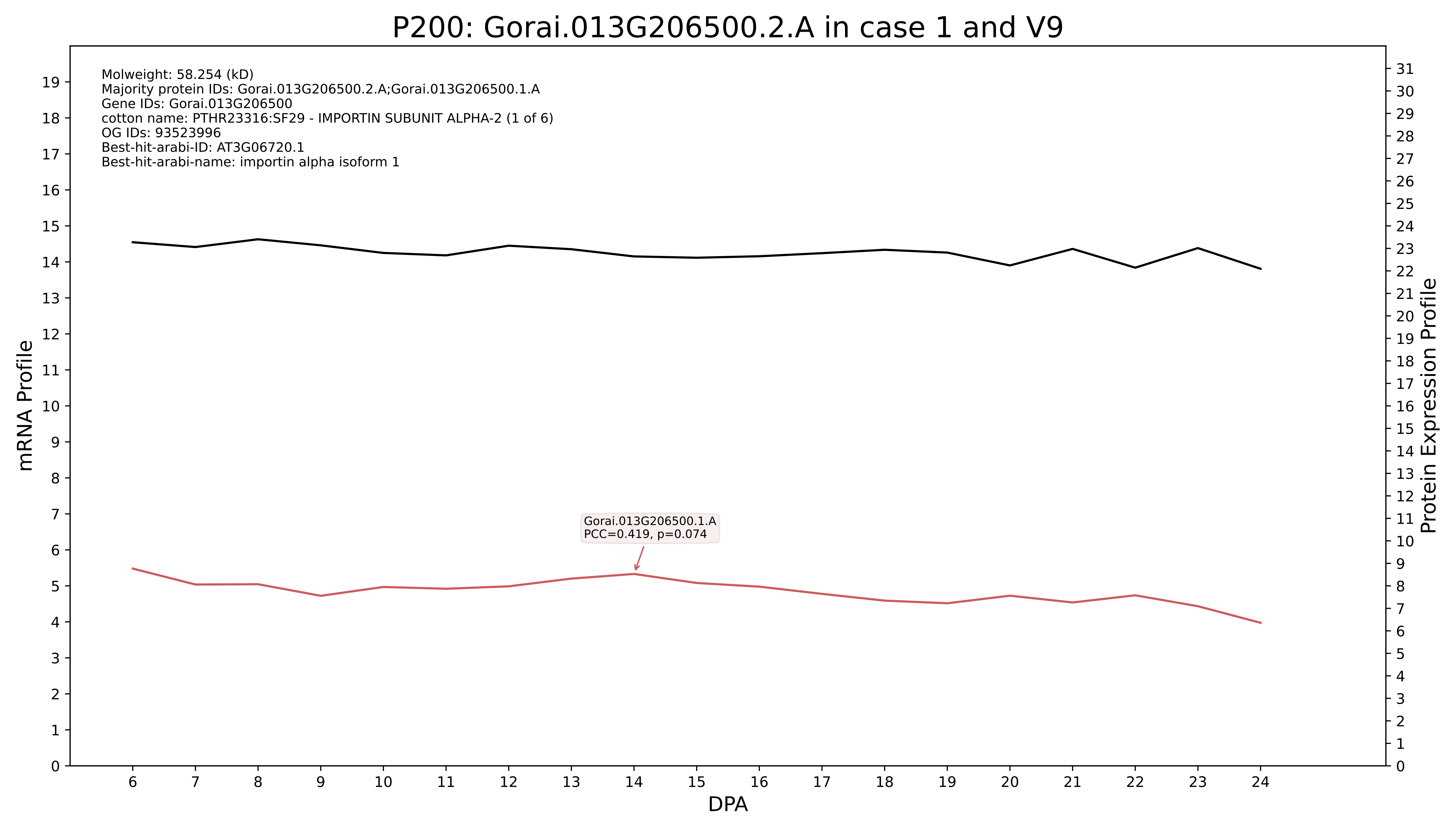
P200: Gorai.013G206500.2.A in case 1 and V9
Molweight: 58.254 (kD)
Majority protein IDs: Gorai.013G206500.2.A;Gorai.013G206500.1.A
Gene IDs: Gorai.013G206500
cotton name: PTHR23316:SF29 - IMPORTIN SUBUNIT ALPHA-2 (1 of 6)
OG IDs: 93523996
Best-hit-arabi-ID: AT3G06720.1
Best-hit-arabi-name: importin alpha isoform 1
Gorai.013G206500.1.A
PCC=0.419, p=0.074
mRNA Profile
Protein Expression Profile
DPA
0
1
2
3
4
5
6
7
8
9
10
11
12
13
14
15
16
17
18
19
20
21
22
23
24
25
26
27
28
29
30
31

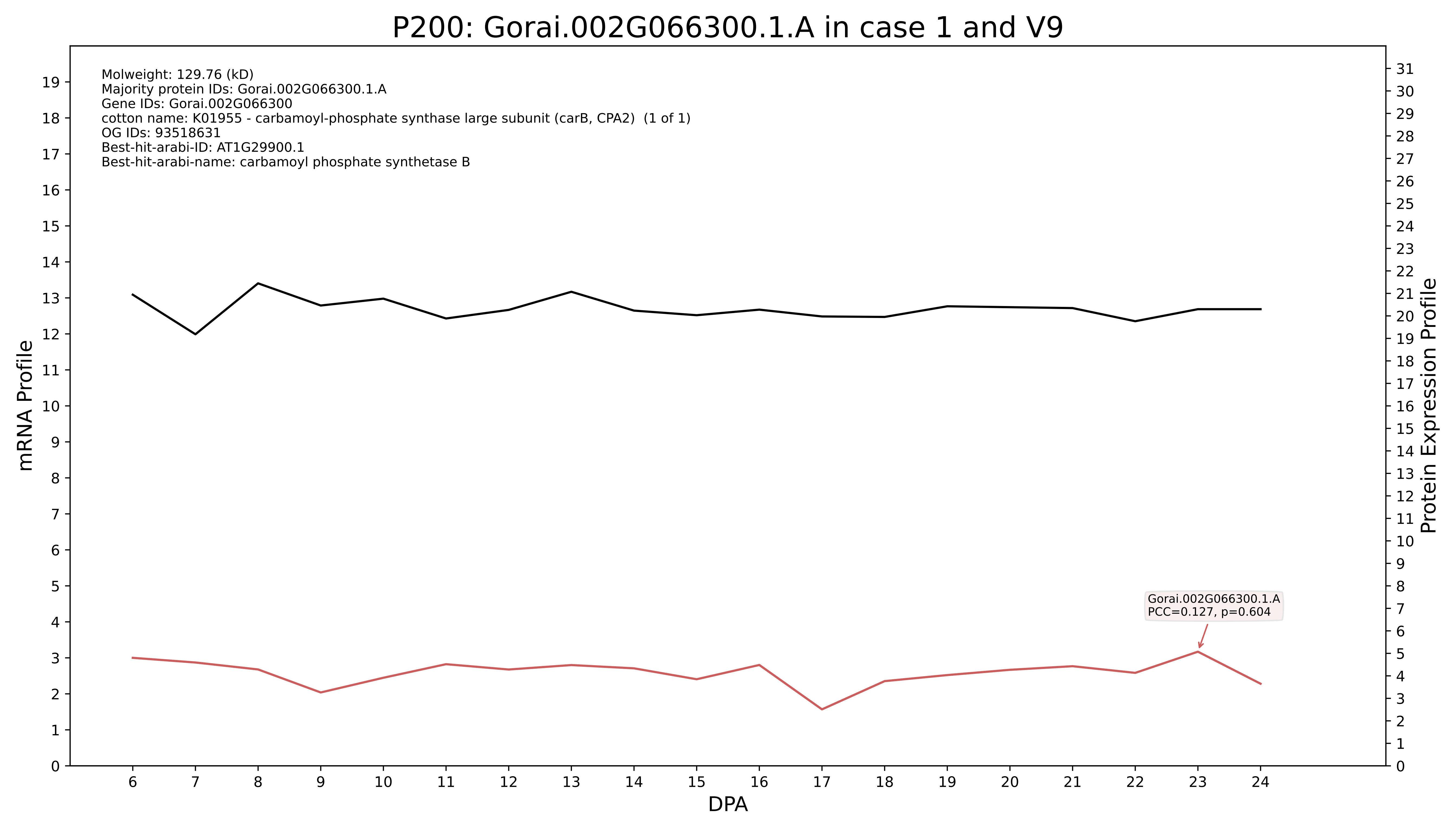
P200: Gorai.002G066300.1.A in case 1 and V9
Molweight: 129.76 (kD)
Majority protein IDs: Gorai.002G066300.1.A
Gene IDs: Gorai.002G066300
cotton name: K01955 - carbamoyl-phosphate synthase large subunit (carB, CPA2) (1 of 1)
OG IDs: 93518631
Best-hit-arabi-ID: AT1G29900.1
Best-hit-arabi-name: carbamoyl phosphate synthetase B
Gorai.002G066300.1.A
PCC=0.127, p=0.604
mRNA Profile
Protein Expression Profile
DPA

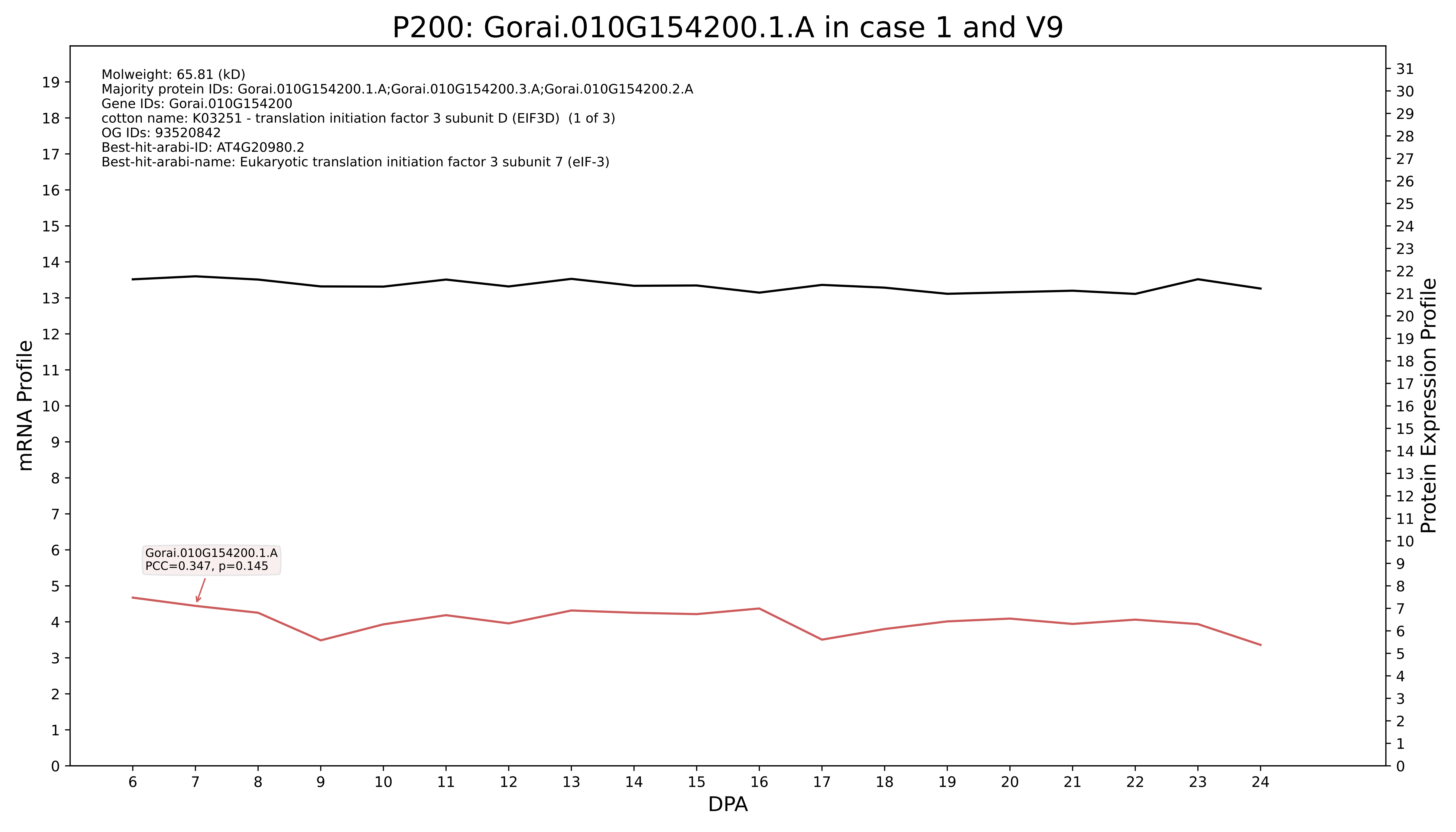
P200: Gorai.010G154200.1.A in case 1 and V9
Molweight: 65.81 (kD)
Majority protein IDs: Gorai.010G154200.1.A;Gorai.010G154200.3.A;Gorai.010G154200.2.A
Gene IDs: Gorai.010G154200
cotton name: K03251 - translation initiation factor 3 subunit D (EIF3D) (1 of 3)
OG IDs: 93520842
Best-hit-arabi-ID: AT4G20980.2
Best-hit-arabi-name: Eukaryotic translation initiation factor 3 subunit 7 (eIF-3)
Gorai.010G154200.1.A
PCC=0.347, p=0.145
mRNA Profile
Protein Expression Profile
DPA

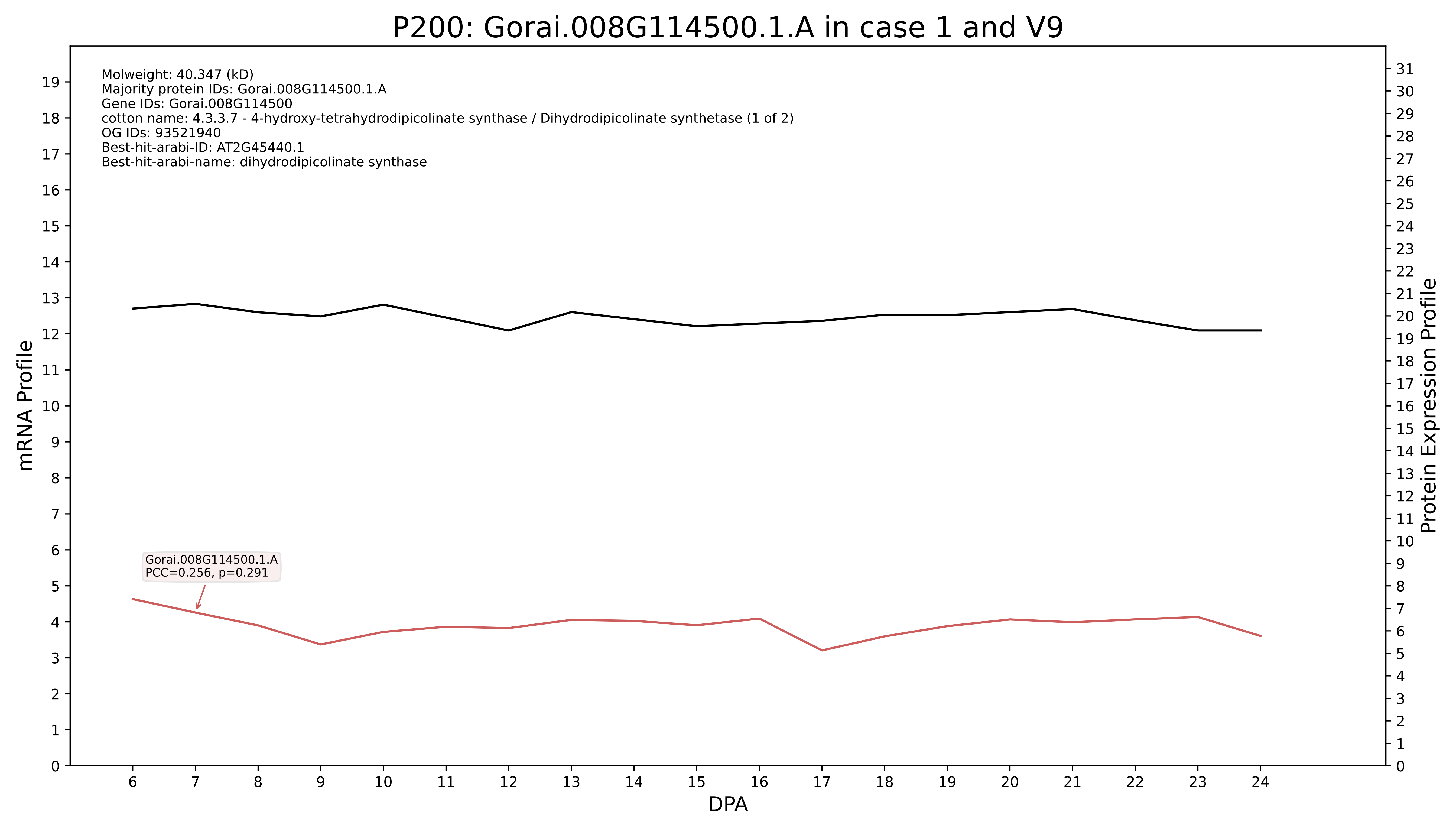
P200: Gorai.008G114500.1.A in case 1 and V9
Molweight: 40.347 (kD)
Majority protein IDs: Gorai.008G114500.1.A
Gene IDs: Gorai.008G114500
cotton name: 4.3.3.7 - 4-hydroxy-tetrahydrodipicolinate synthase / Dihydrodipicolinate synthetase (1 of 2)
OG IDs: 93521940
Best-hit-arabi-ID: AT2G45440.1
Best-hit-arabi-name: dihydrodipicolinate synthase
Gorai.008G114500.1.A
PCC=0.256, p=0.291
mRNA Profile
Protein Expression Profile
DPA

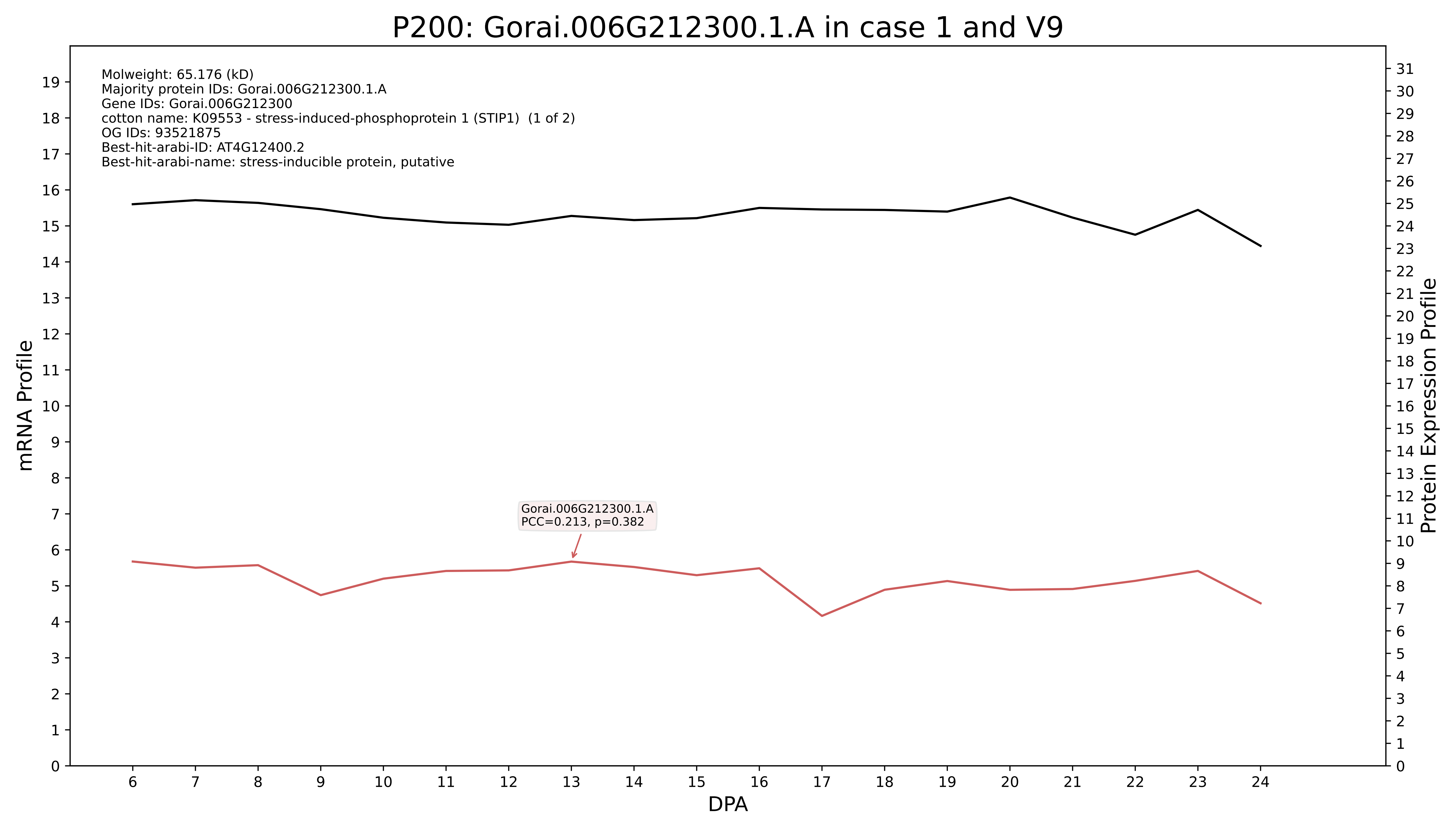

P200: Gorai.006G212300.1.A in case 1 and V9
Molweight: 65.176 (kD)
Majority protein IDs: Gorai.006G212300.1.A
Gene IDs: Gorai.006G212300
cotton name: K09553 - stress-induced-phosphoprotein 1 (STIP1) (1 of 2)
OG IDs: 93521875
Best-hit-arabi-ID: AT4G12400.2
Best-hit-arabi-name: stress-inducible protein, putative
Gorai.006G212300.1.A
PCC=0.213, p=0.382
mRNA Profile
Protein Expression Profile
DPA

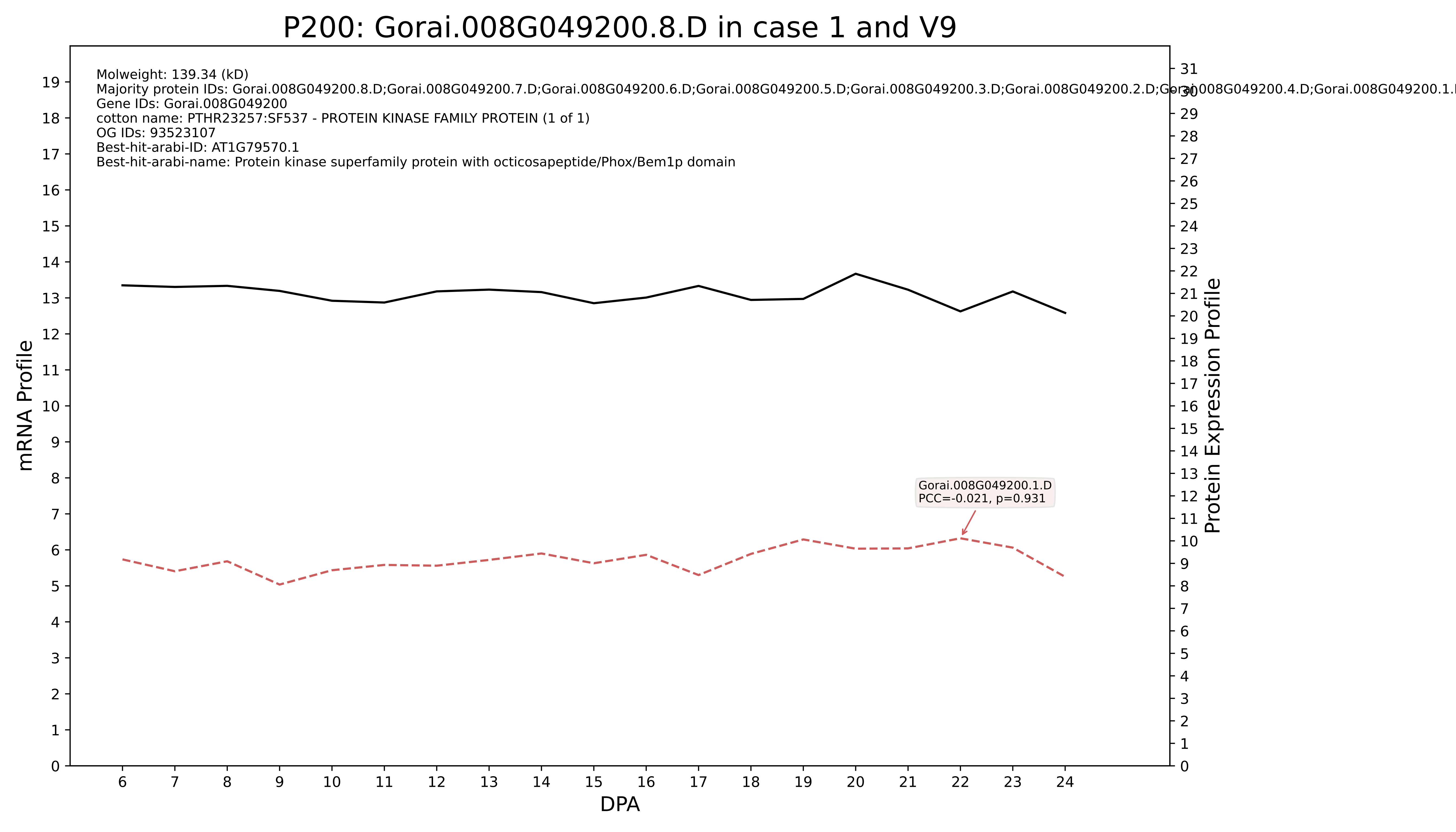
P200: Gorai.008G049200.8.D in case 1 and V9
Molweight: 139.34 (kD)
Majority protein IDs: Gorai.008G049200.8.D;Gorai.008G049200.7.D;Gorai.008G049200.6.D;Gorai.008G049200.5.D;Gorai.008G049200.3.D;Gorai.008G049200.2.D;Gorai.008G049200.4.D;Gorai.008G049200.1.D
Gene IDs: Gorai.008G049200
cotton name: PTHR23257:SF537 - PROTEIN KINASE FAMILY PROTEIN (1 of 1)
OG IDs: 93523107
Best-hit-arabi-ID: AT1G79570.1
Best-hit-arabi-name: Protein kinase superfamily protein with octicosapeptide/Phox/Bem1p domain
Gorai.008G049200.1.D
PCC=-0.021, p=0.931
mRNA Profile
Protein Expression Profile
DPA

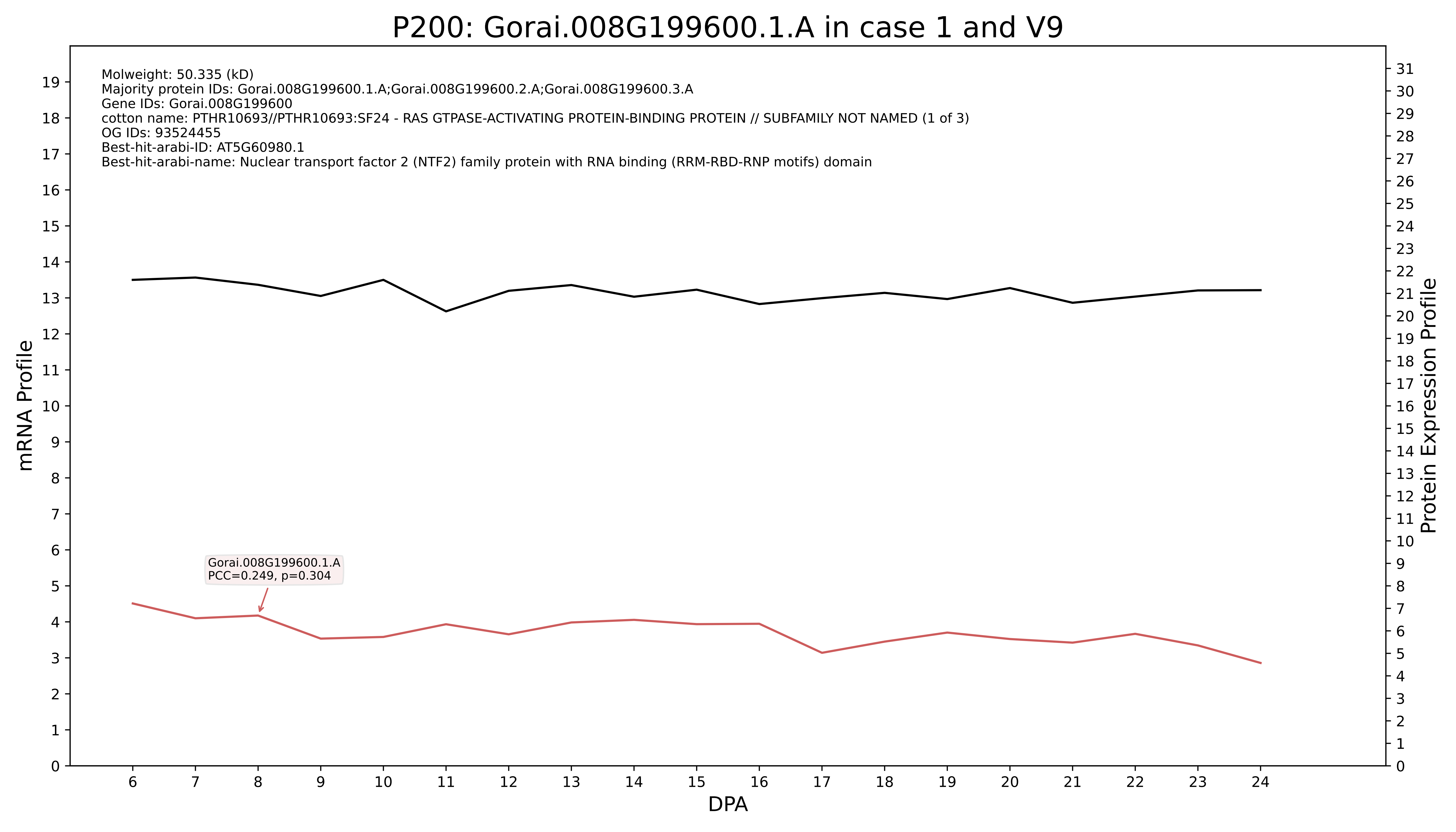

P200: Gorai.008G199600.1.A in case 1 and V9
Molweight: 50.335 (kD)
Majority protein IDs: Gorai.008G199600.1.A;Gorai.008G199600.2.A;Gorai.008G199600.3.A
Gene IDs: Gorai.008G199600
cotton name: PTHR10693//PTHR10693:SF24 - RAS GTPASE-ACTIVATING PROTEIN-BINDING PROTEIN // SUBFAMILY NOT NAMED (1 of 3)
OG IDs: 93524455
Best-hit-arabi-ID: AT5G60980.1
Best-hit-arabi-name: Nuclear transport factor 2 (NTF2) family protein with RNA binding (RRM-RBD-RNP motifs) domain
Gorai.008G199600.1.A
PCC=0.249, p=0.304
mRNA Profile
Protein Expression Profile
DPA

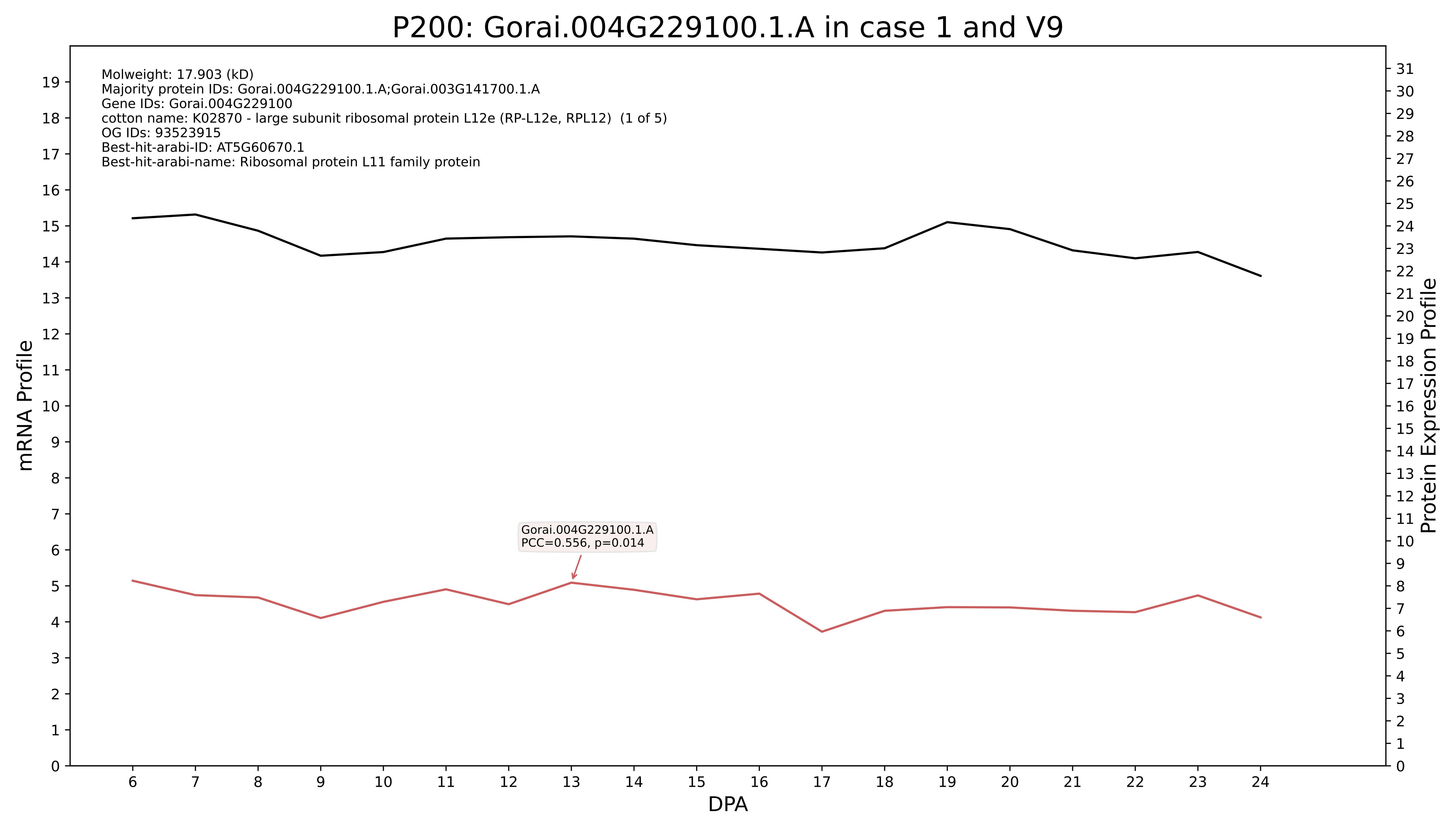
P200: Gorai.004G229100.1.A in case 1 and V9
Molweight: 17.903 (kD)
Majority protein IDs: Gorai.004G229100.1.A;Gorai.003G141700.1.A
Gene IDs: Gorai.004G229100
cotton name: K02870 - large subunit ribosomal protein L12e (RP-L12e, RPL12) (1 of 5)
OG IDs: 93523915
Best-hit-arabi-ID: AT5G60670.1
Best-hit-arabi-name: Ribosomal protein L11 family protein
Gorai.004G229100.1.A
PCC=0.556, p=0.014
mRNA Profile
Protein Expression Profile
DPA

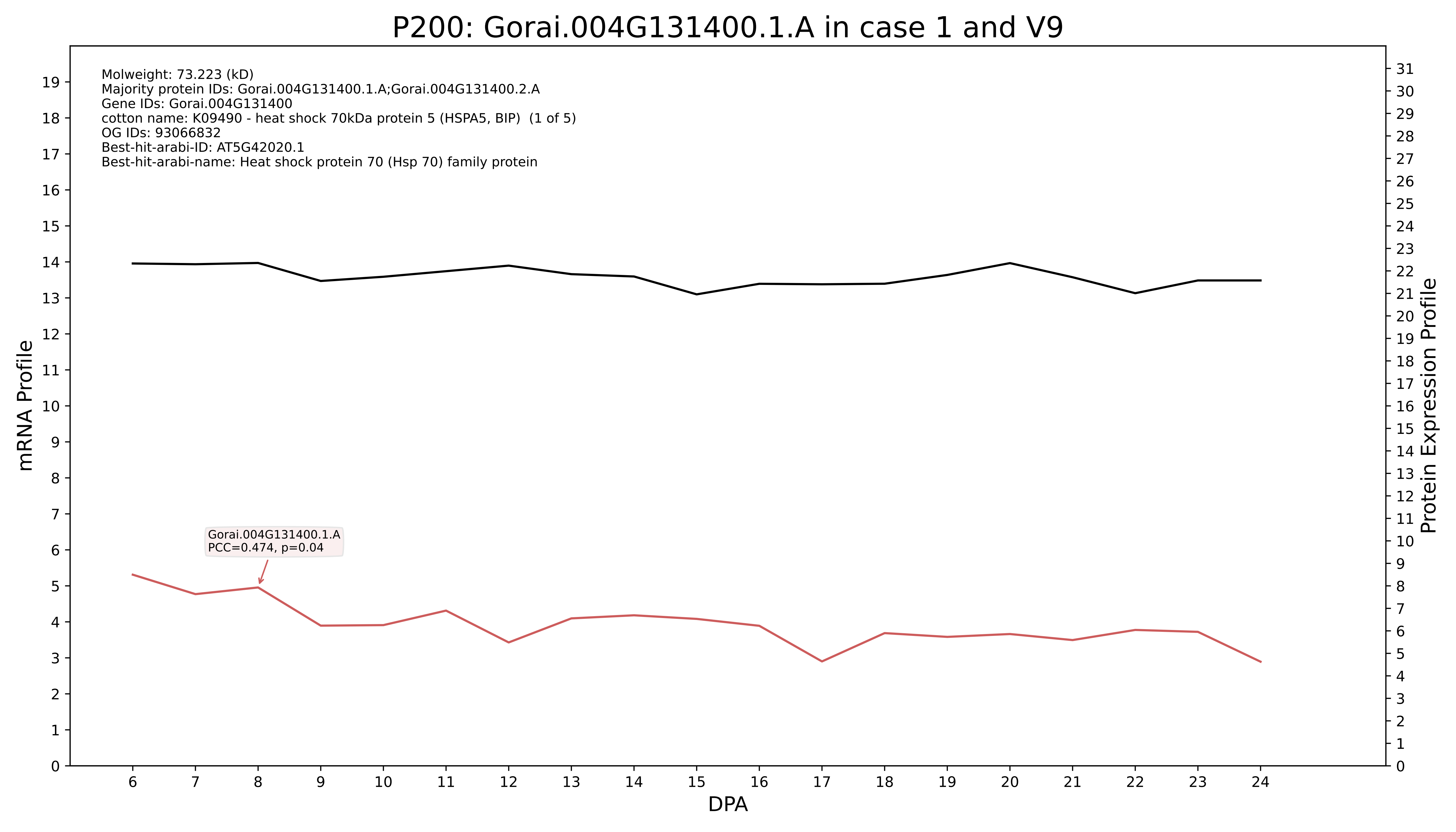
P200: Gorai.004G131400.1.A in case 1 and V9
Molweight: 73.223 (kD)
Majority protein IDs: Gorai.004G131400.1.A;Gorai.004G131400.2.A
Gene IDs: Gorai.004G131400
cotton name: K09490 - heat shock 70kDa protein 5 (HSPA5, BIP) (1 of 5)
OG IDs: 93066832
Best-hit-arabi-ID: AT5G42020.1
Best-hit-arabi-name: Heat shock protein 70 (Hsp 70) family protein
Gorai.004G131400.1.A
PCC=0.474, p=0.04
mRNA Profile
Protein Expression Profile
DPA

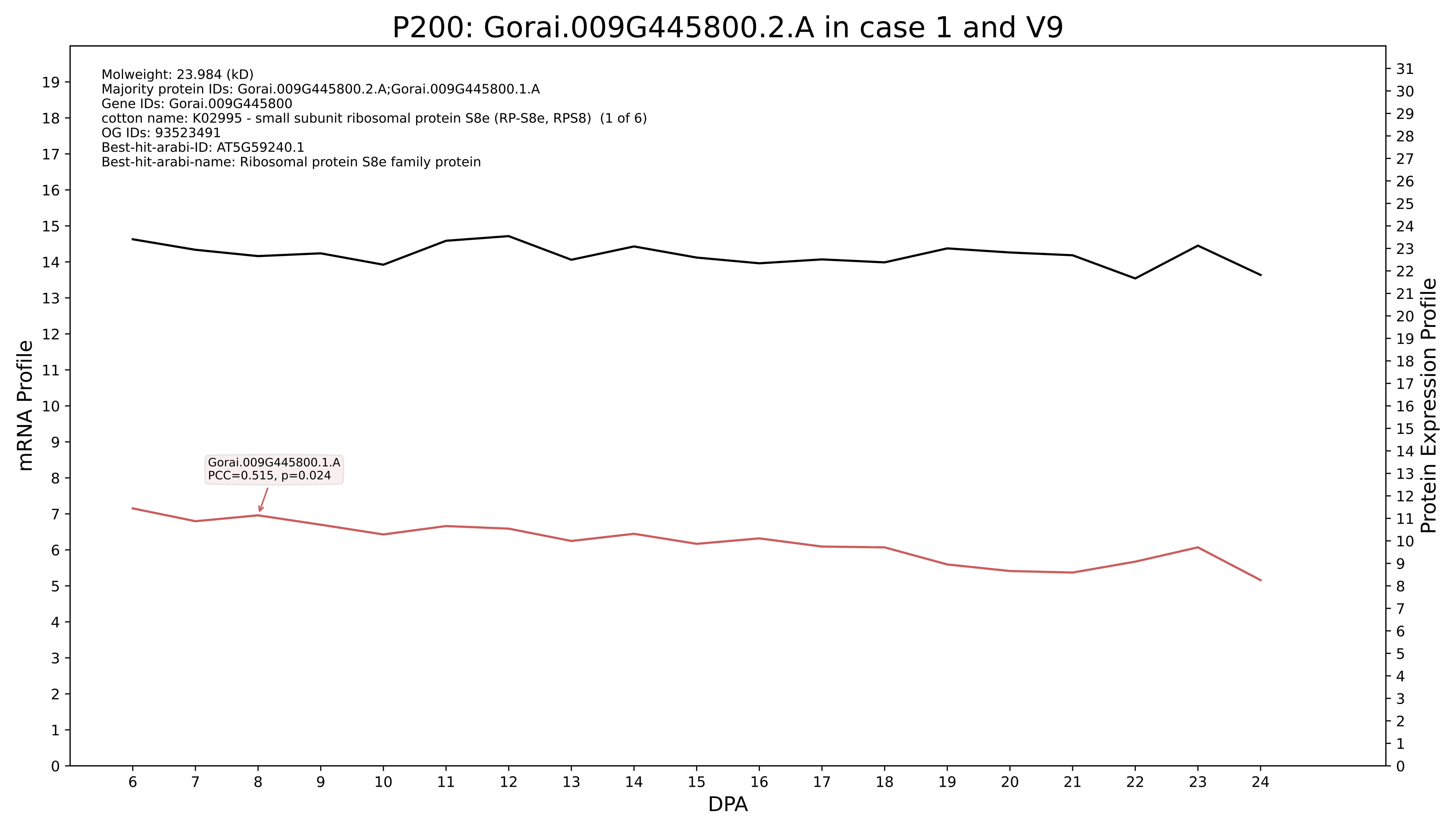
P200: Gorai.009G445800.2.A in case 1 and V9
Molweight: 23.984 (kD)
Majority protein IDs: Gorai.009G445800.2.A;Gorai.009G445800.1.A
Gene IDs: Gorai.009G445800
cotton name: K02995 - small subunit ribosomal protein S8e (RP-S8e, RPS8) (1 of 6)
OG IDs: 93523491
Best-hit-arabi-ID: AT5G59240.1
Best-hit-arabi-name: Ribosomal protein S8e family protein
Gorai.009G445800.1.A
PCC=0.515, p=0.024
mRNA Profile
Protein Expression Profile
DPA
0
1
2
3
4
5
6
7
8
9
10
11
12
13
14
15
16
17
18
19
20
21
22
23
24
25
26
27
28
29
30
31

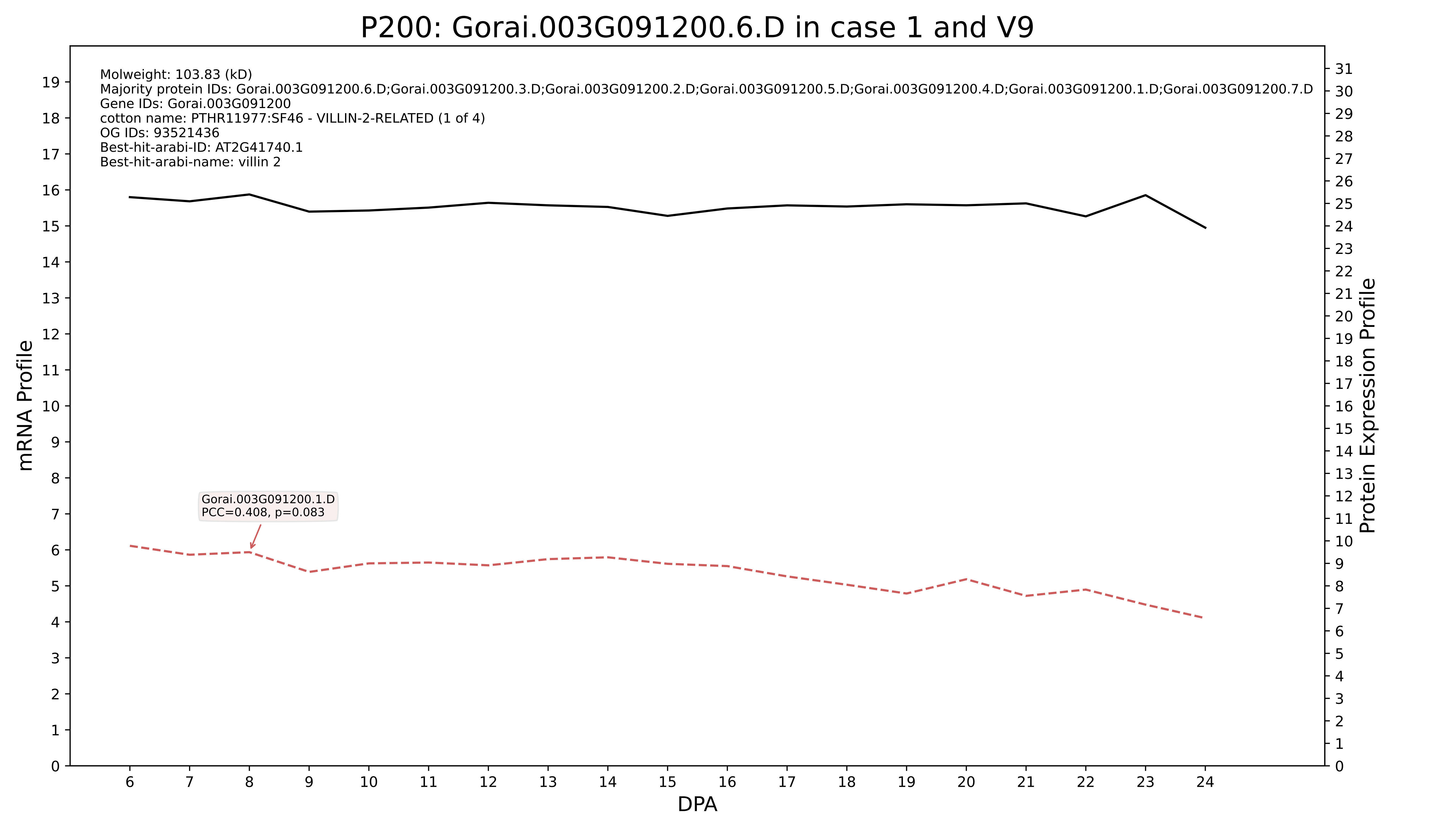
P200: Gorai.003G091200.6.D in case 1 and V9
Molweight: 103.83 (kD)
Majority protein IDs: Gorai.003G091200.6.D;Gorai.003G091200.3.D;Gorai.003G091200.2.D;Gorai.003G091200.5.D;Gorai.003G091200.4.D;Gorai.003G091200.1.D;Gorai.003G091200.7.D
Gene IDs: Gorai.003G091200
cotton name: PTHR11977:SF46 - VILLIN-2-RELATED (1 of 4)
OG IDs: 93521436
Best-hit-arabi-ID: AT2G41740.1
Best-hit-arabi-name: villin 2
Gorai.003G091200.1.D
PCC=0.408, p=0.083
mRNA Profile
Protein Expression Profile
DPA

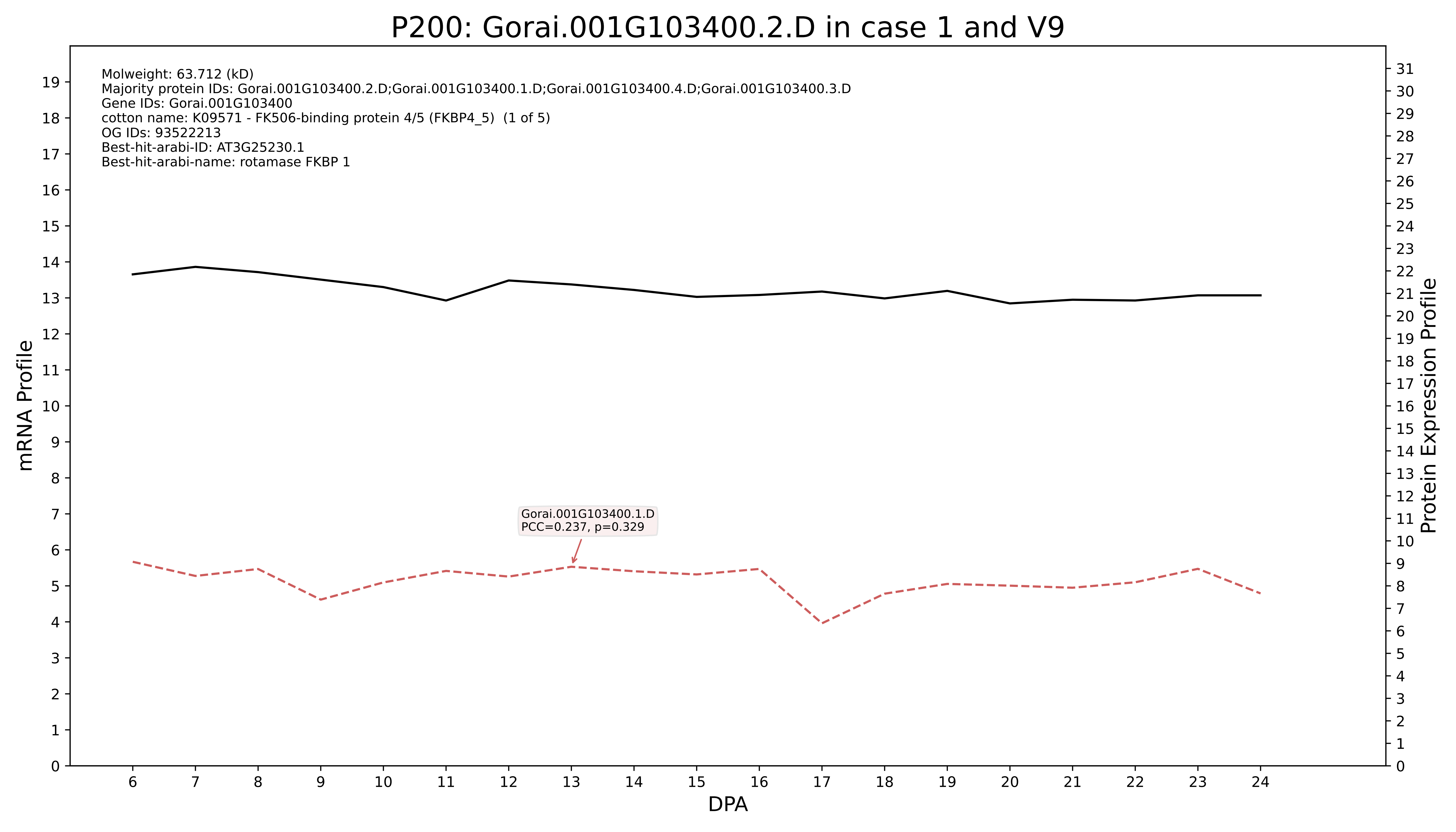

P200: Gorai.001G103400.2.D in case 1 and V9
Molweight: 63.712 (kD)
Majority protein IDs: Gorai.001G103400.2.D;Gorai.001G103400.1.D;Gorai.001G103400.4.D;Gorai.001G103400.3.D
Gene IDs: Gorai.001G103400
cotton name: K09571 - FK506-binding protein 4/5 (FKBP4_5) (1 of 5)
OG IDs: 93522213
Best-hit-arabi-ID: AT3G25230.1
Best-hit-arabi-name: rotamase FKBP 1
Gorai.001G103400.1.D
PCC=0.237, p=0.329
mRNA Profile
Protein Expression Profile
DPA

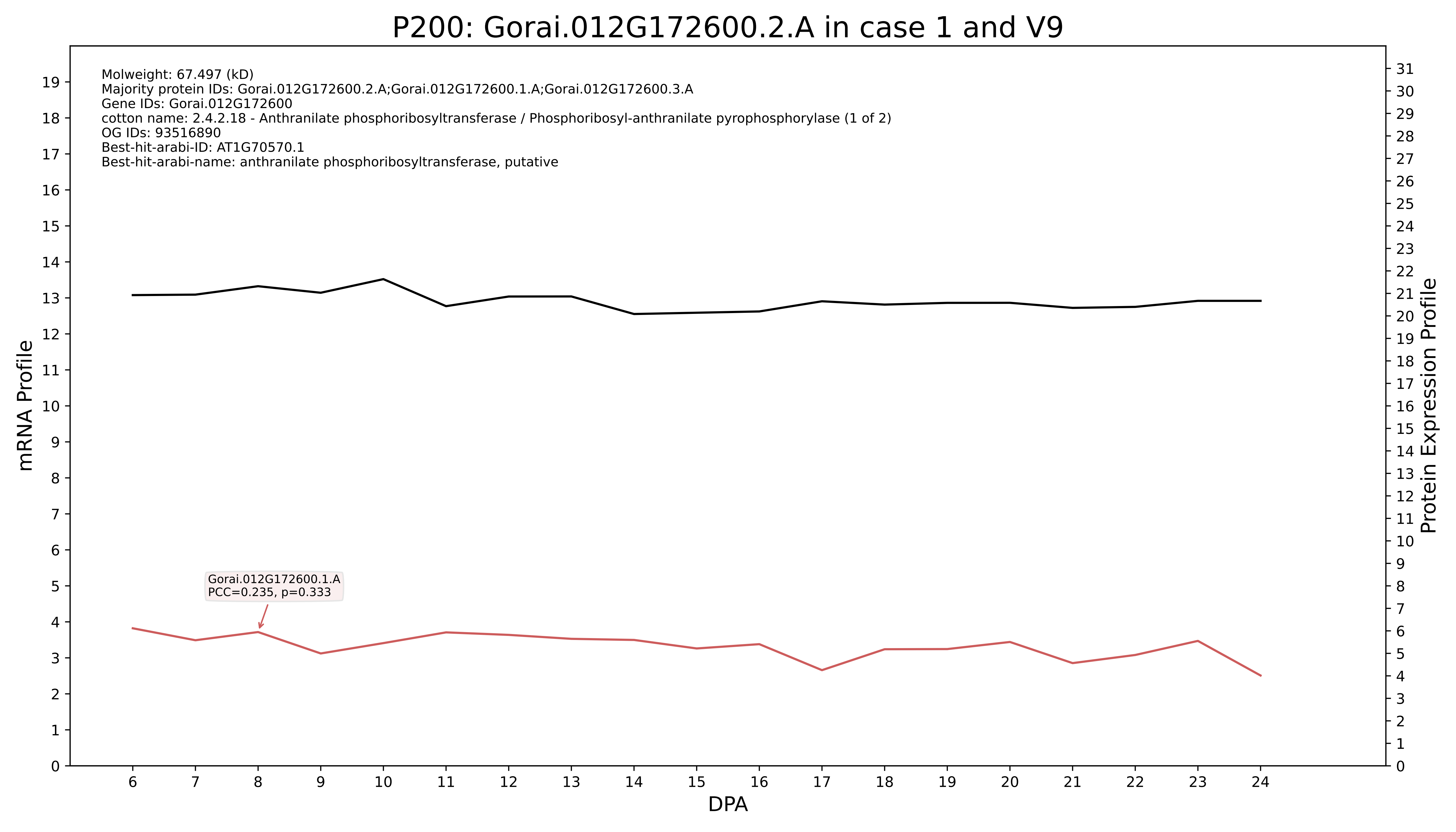
P200: Gorai.012G172600.2.A in case 1 and V9
Molweight: 67.497 (kD)
Majority protein IDs: Gorai.012G172600.2.A;Gorai.012G172600.1.A;Gorai.012G172600.3.A
Gene IDs: Gorai.012G172600
cotton name: 2.4.2.18 - Anthranilate phosphoribosyltransferase / Phosphoribosyl-anthranilate pyrophosphorylase (1 of 2)
OG IDs: 93516890
Best-hit-arabi-ID: AT1G70570.1
Best-hit-arabi-name: anthranilate phosphoribosyltransferase, putative
Gorai.012G172600.1.A
PCC=0.235, p=0.333
mRNA Profile
Protein Expression Profile
DPA

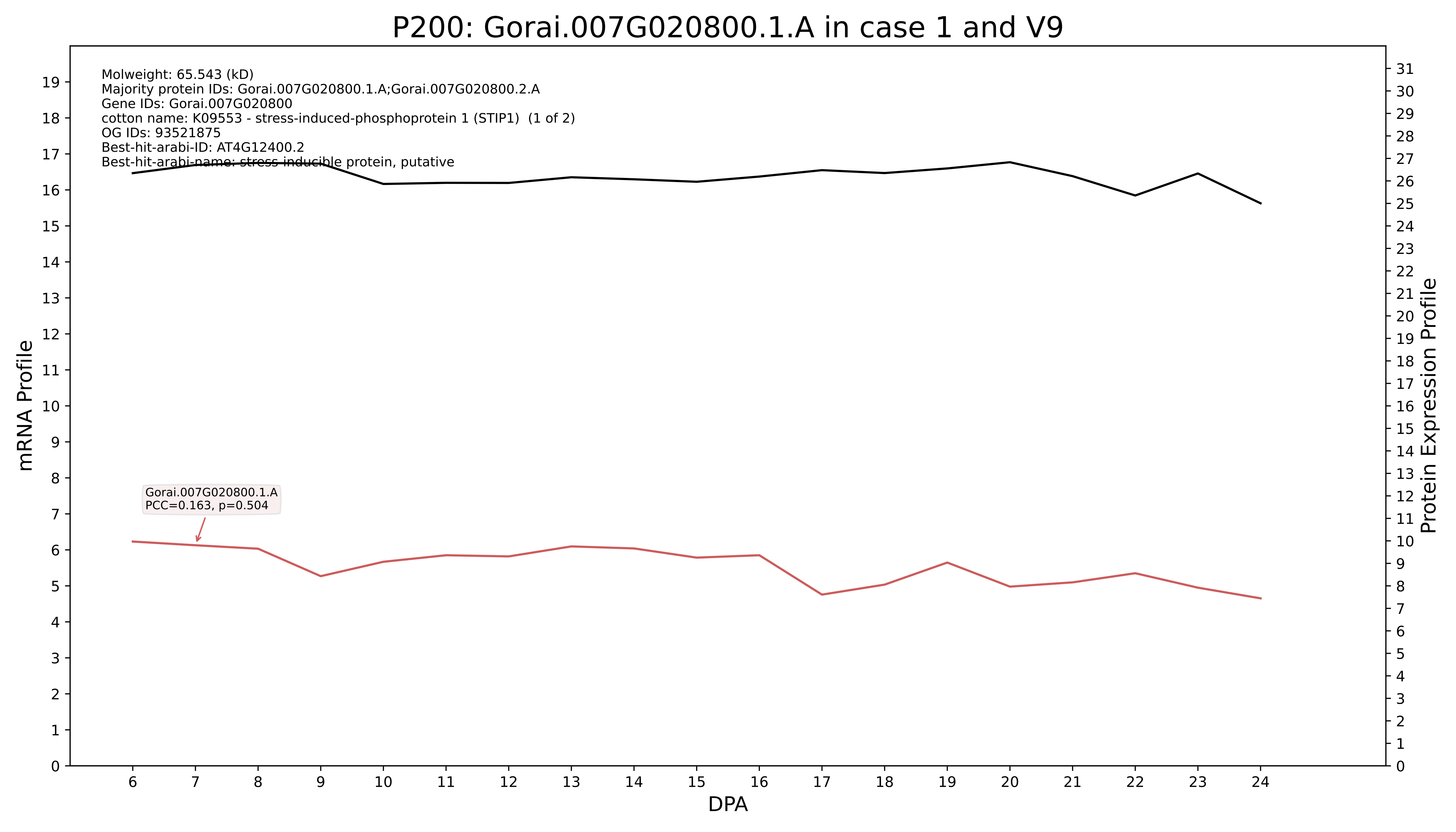

P200: Gorai.007G020800.1.A in case 1 and V9
Molweight: 65.543 (kD)
Majority protein IDs: Gorai.007G020800.1.A;Gorai.007G020800.2.A
Gene IDs: Gorai.007G020800
cotton name: K09553 - stress-induced-phosphoprotein 1 (STIP1) (1 of 2)
OG IDs: 93521875
Best-hit-arabi-ID: AT4G12400.2
Best-hit-arabi-name: stress-inducible protein, putative
Gorai.007G020800.1.A
PCC=0.163, p=0.504
mRNA Profile
Protein Expression Profile
DPA

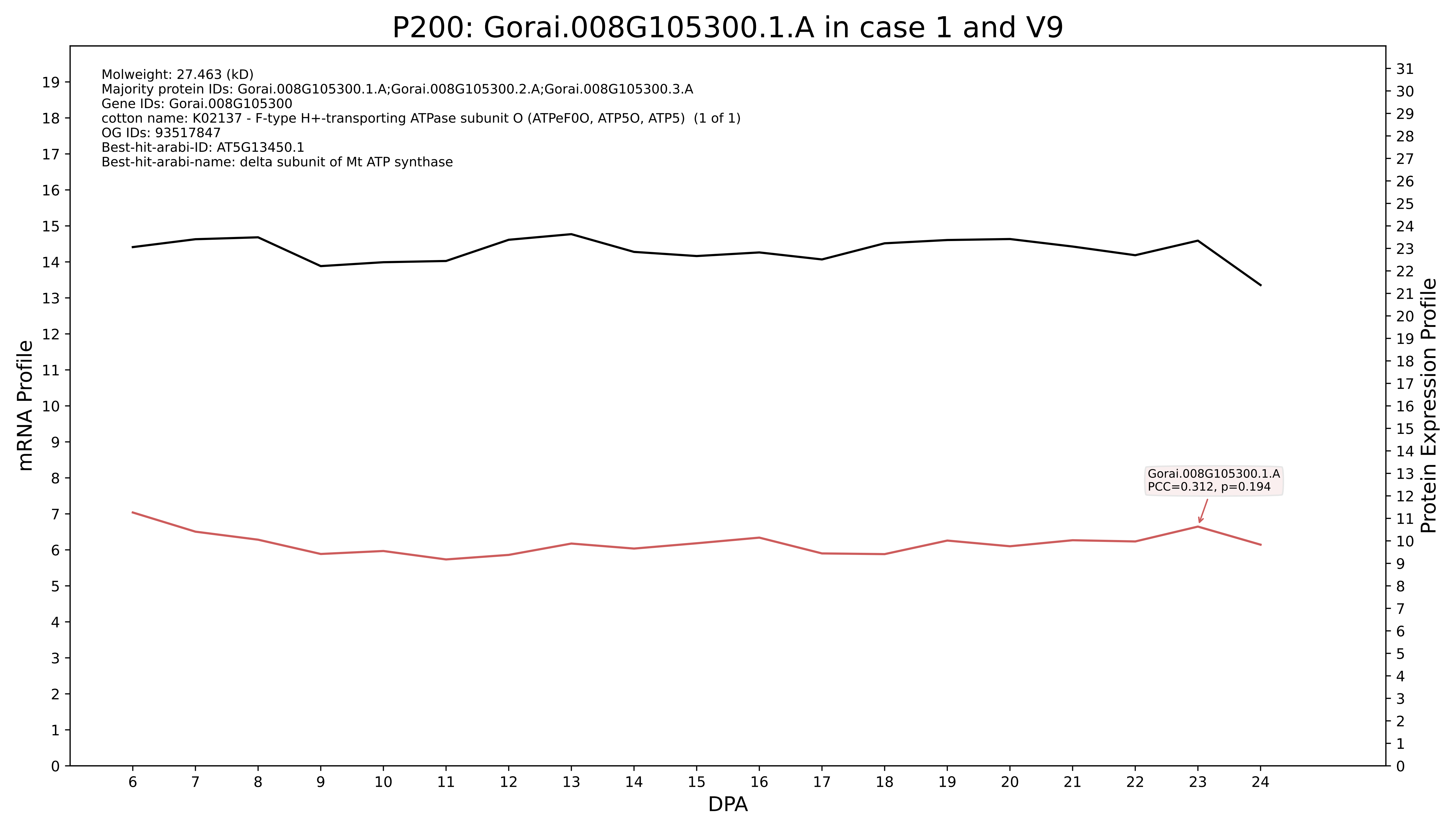

P200: Gorai.008G105300.1.A in case 1 and V9
Molweight: 27.463 (kD)
Majority protein IDs: Gorai.008G105300.1.A;Gorai.008G105300.2.A;Gorai.008G105300.3.A
Gene IDs: Gorai.008G105300
cotton name: K02137 - F-type H+-transporting ATPase subunit O (ATPeF0O, ATP5O, ATP5) (1 of 1)
OG IDs: 93517847
Best-hit-arabi-ID: AT5G13450.1
Best-hit-arabi-name: delta subunit of Mt ATP synthase
Gorai.008G105300.1.A
PCC=0.312, p=0.194
mRNA Profile
Protein Expression Profile
DPA

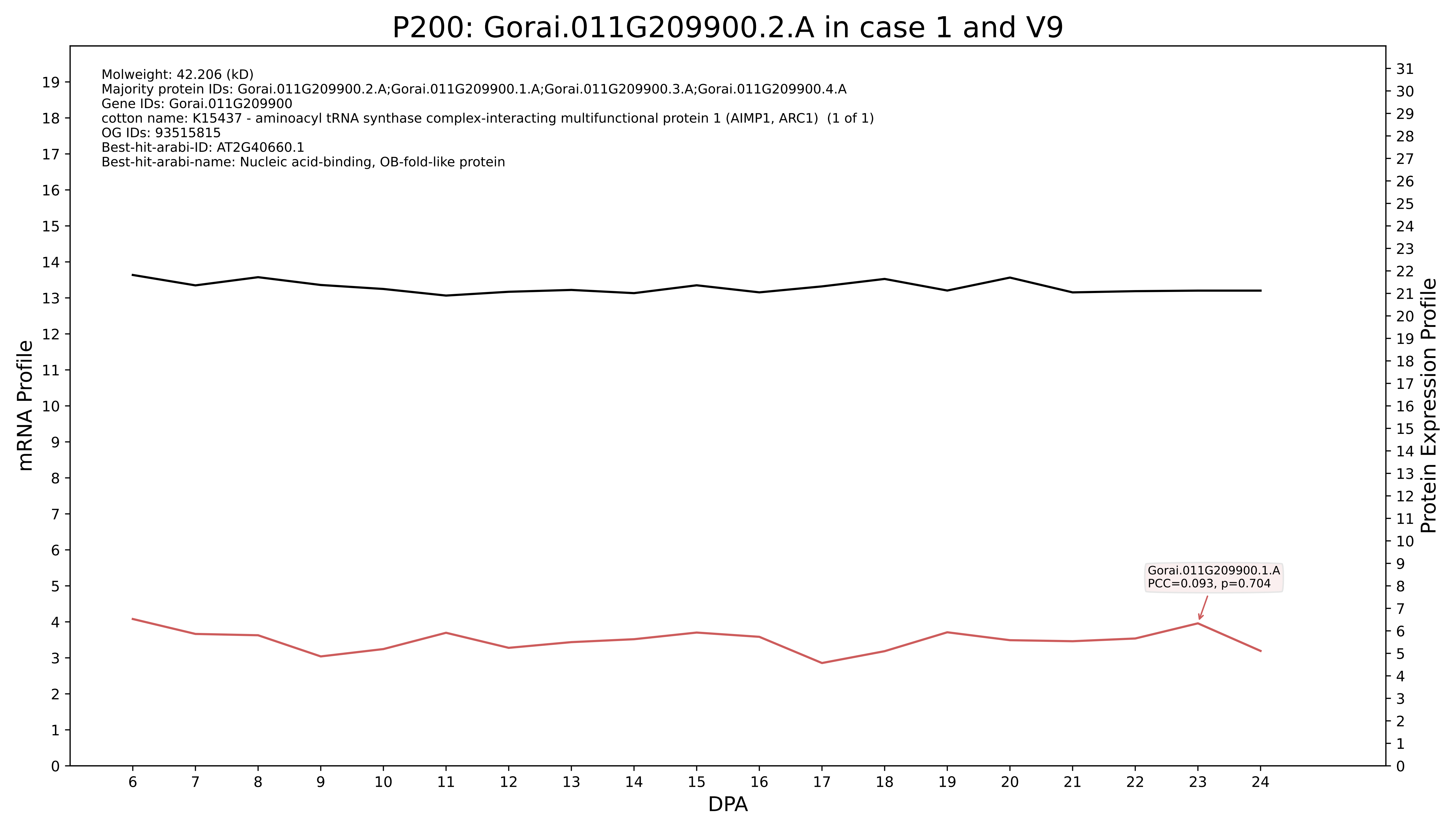
P200: Gorai.011G209900.2.A in case 1 and V9
Molweight: 42.206 (kD)
Majority protein IDs: Gorai.011G209900.2.A;Gorai.011G209900.1.A;Gorai.011G209900.3.A;Gorai.011G209900.4.A
Gene IDs: Gorai.011G209900
cotton name: K15437 - aminoacyl tRNA synthase complex-interacting multifunctional protein 1 (AIMP1, ARC1) (1 of 1)
OG IDs: 93515815
Best-hit-arabi-ID: AT2G40660.1
Best-hit-arabi-name: Nucleic acid-binding, OB-fold-like protein
Gorai.011G209900.1.A
PCC=0.093, p=0.704
mRNA Profile
Protein Expression Profile
DPA
0
1
2
3
4
5
6
7
8
9
10
11
12
13
14
15
16
17
18
19
20
21
22
23
24
25
26
27
28
29
30
31

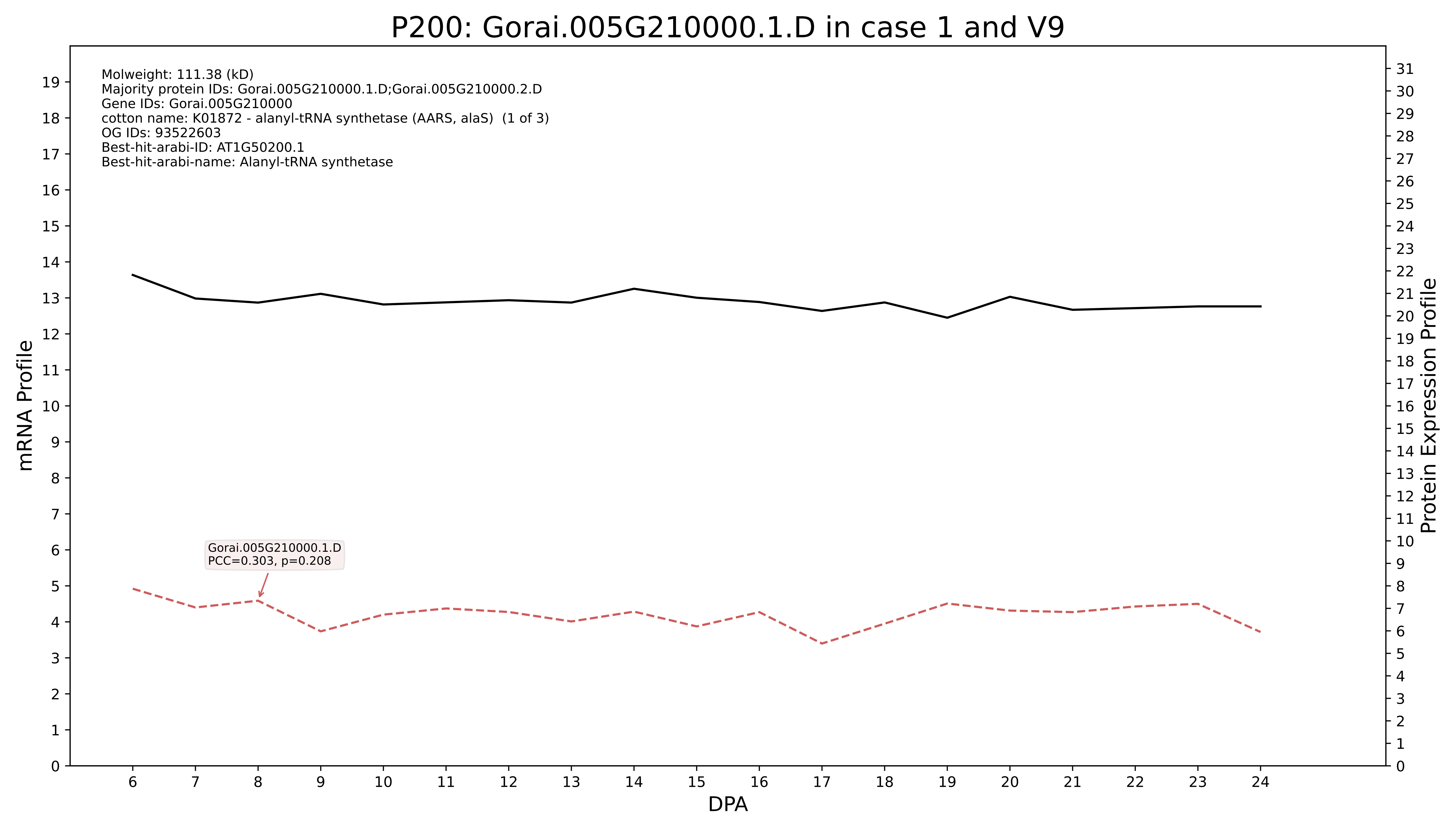
P200: Gorai.005G210000.1.D in case 1 and V9
Molweight: 111.38 (kD)
Majority protein IDs: Gorai.005G210000.1.D;Gorai.005G210000.2.D
Gene IDs: Gorai.005G210000
cotton name: K01872 - alanyl-tRNA synthetase (AARS, alaS) (1 of 3)
OG IDs: 93522603
Best-hit-arabi-ID: AT1G50200.1
Best-hit-arabi-name: Alanyl-tRNA synthetase
Gorai.005G210000.1.D
PCC=0.303, p=0.208
mRNA Profile
Protein Expression Profile
DPA

P200: Gorai.001G103400.2.A in case 1 and V9

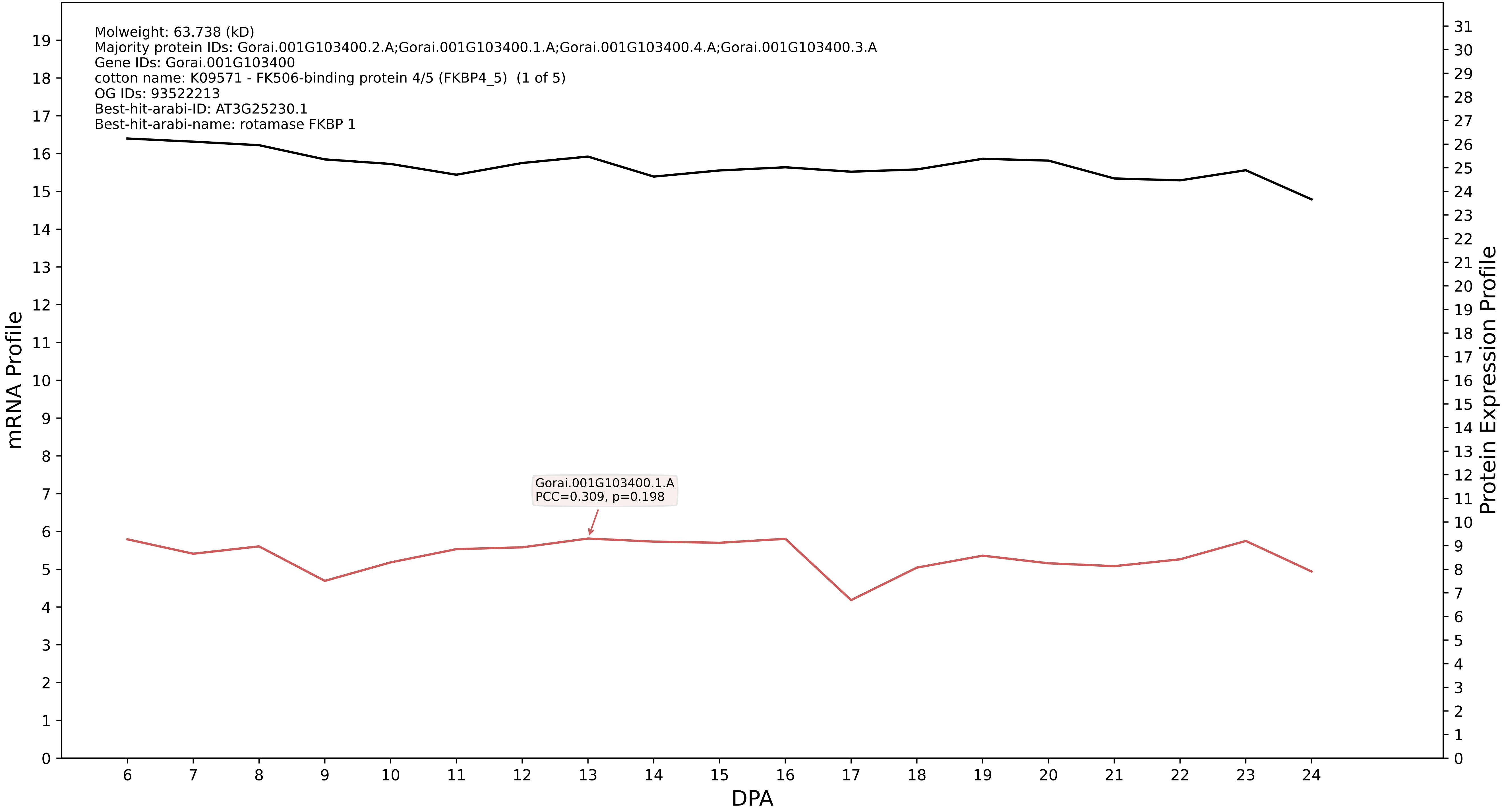

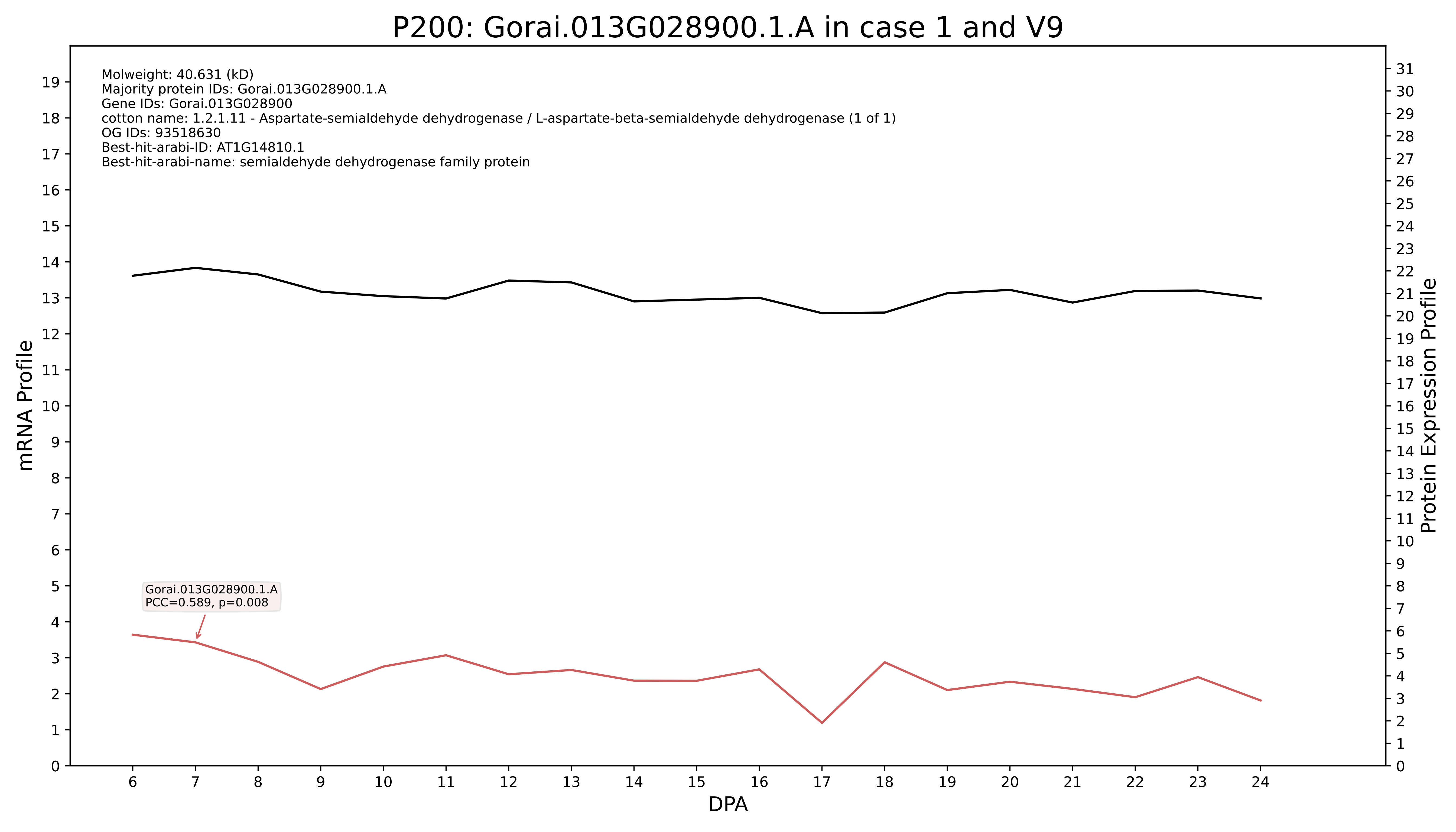
P200: Gorai.013G028900.1.A in case 1 and V9
Molweight: 40.631 (kD)
Majority protein IDs: Gorai.013G028900.1.A
Gene IDs: Gorai.013G028900
cotton name: 1.2.1.11 - Aspartate-semialdehyde dehydrogenase / L-aspartate-beta-semialdehyde dehydrogenase (1 of 1)
OG IDs: 93518630
Best-hit-arabi-ID: AT1G14810.1
Best-hit-arabi-name: semialdehyde dehydrogenase family protein
Gorai.013G028900.1.A
PCC=0.589, p=0.008
mRNA Profile
Protein Expression Profile
DPA

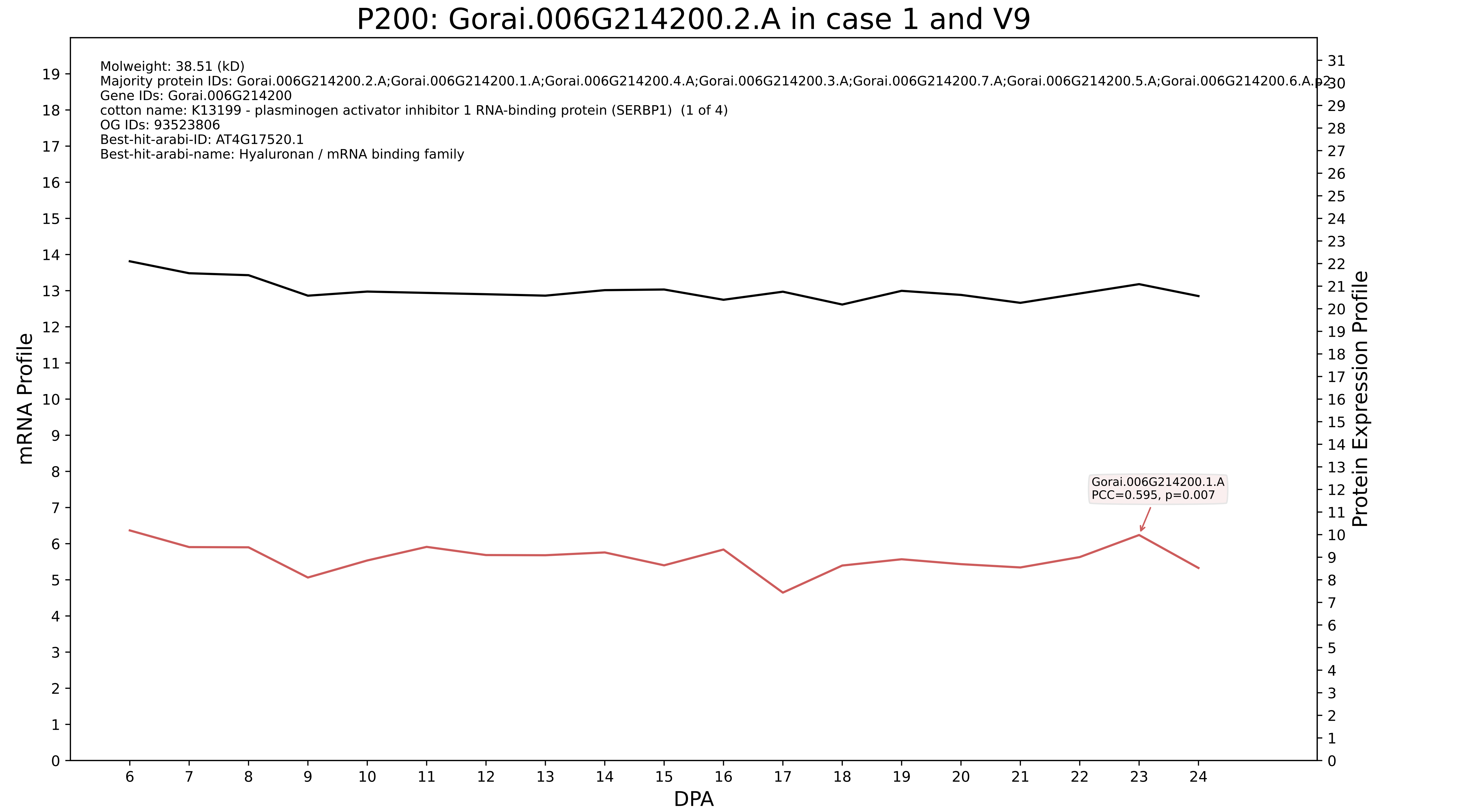
P200: Gorai.006G214200.2.A in case 1 and V9
Molweight: 38.51 (kD)
Majority protein IDs: Gorai.006G214200.2.A;Gorai.006G214200.1.A;Gorai.006G214200.4.A;Gorai.006G214200.3.A;Gorai.006G214200.7.A;Gorai.006G214200.5.A;Gorai.006G214200.6.A;p2
Gene IDs: Gorai.006G214200
cotton name: K13199 - plasminogen activator inhibitor 1 RNA-binding protein (SERBP1) (1 of 4)
OG IDs: 93523806
Best-hit-arabi-ID: AT4G17520.1
Best-hit-arabi-name: Hyaluronan / mRNA binding family
Gorai.006G214200.1.A
PCC=0.595, p=0.007
mRNA Profile
Protein Expression Profile
DPA

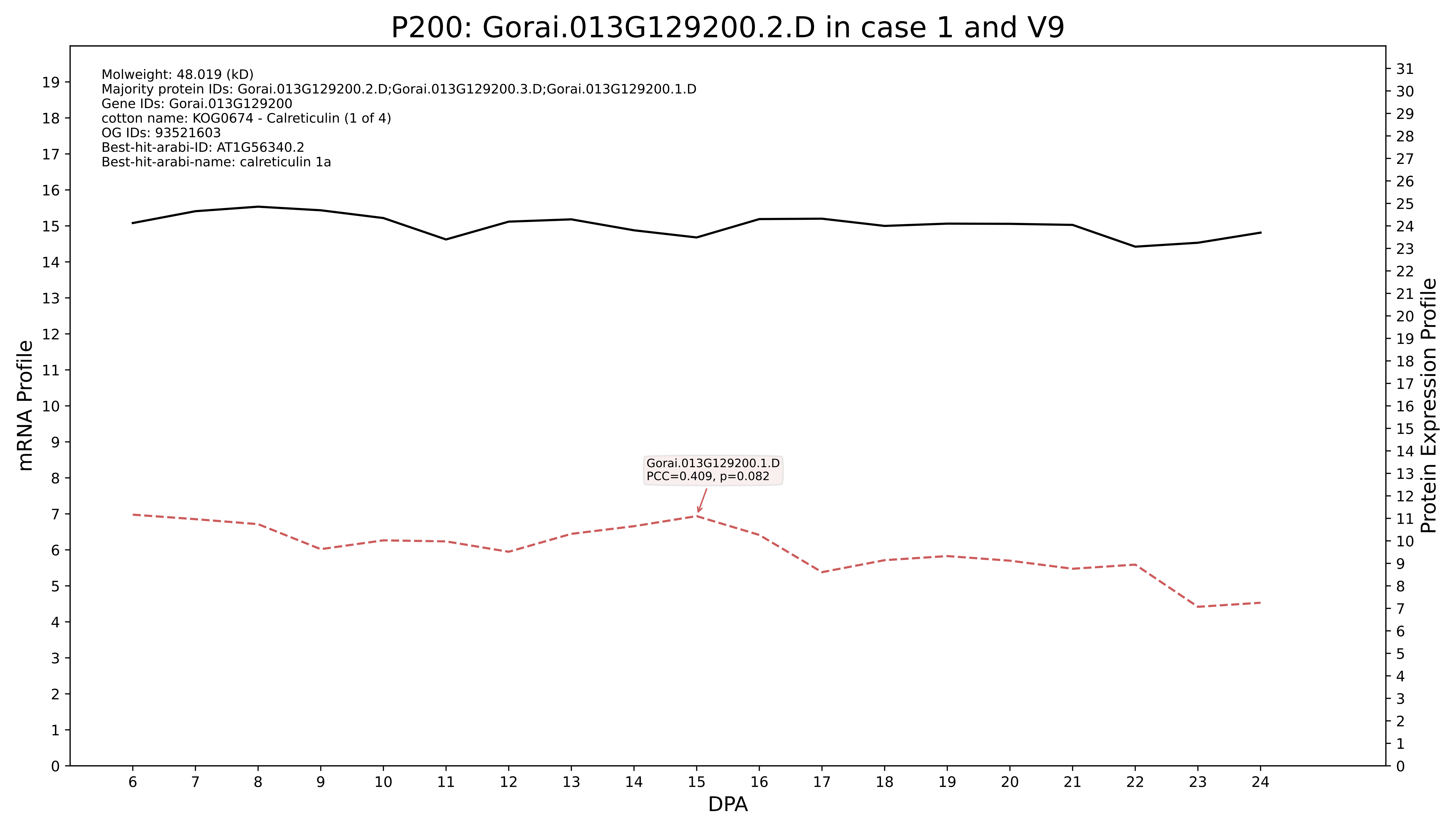
P200: Gorai.013G129200.2.D in case 1 and V9
Molweight: 48.019 (kD)
Majority protein IDs: Gorai.013G129200.2.D;Gorai.013G129200.3.D;Gorai.013G129200.1.D
Gene IDs: Gorai.013G129200
cotton name: KOG0674 - Calreticulin (1 of 4)
OG IDs: 93521603
Best-hit-arabi-ID: AT1G56340.2
Best-hit-arabi-name: calreticulin 1a
Gorai.013G129200.1.D
PCC=0.409, p=0.082
mRNA Profile
Protein Expression Profile
DPA

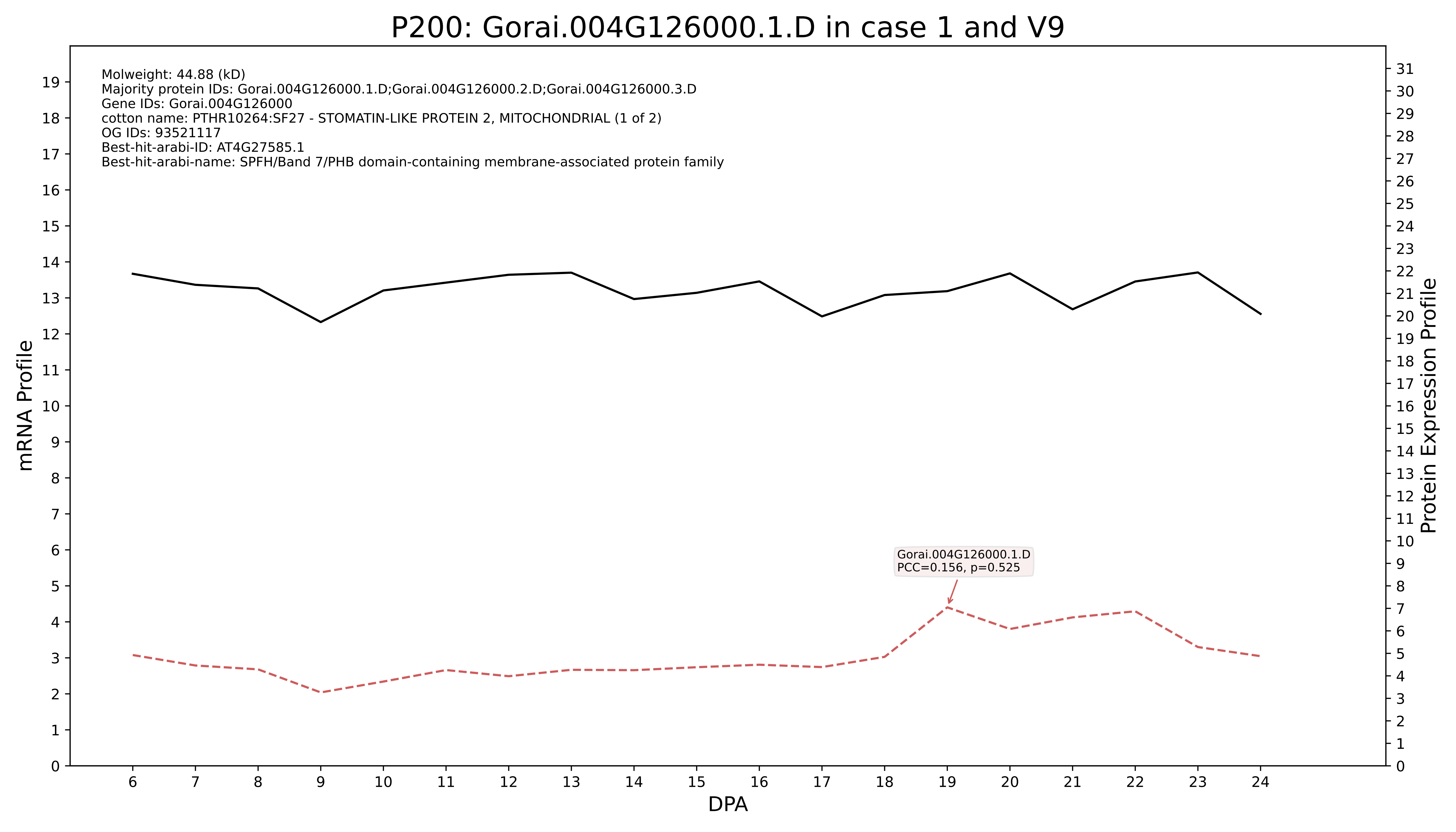

P200: Gorai.004G126000.1.D in case 1 and V9
Molweight: 44.88 (kD)
Majority protein IDs: Gorai.004G126000.1.D;Gorai.004G126000.2.D;Gorai.004G126000.3.D
Gene IDs: Gorai.004G126000
cotton name: PTHR10264:SF27 - STOMATIN-LIKE PROTEIN 2, MITOCHONDRIAL (1 of 2)
OG IDs: 93521117
Best-hit-arabi-ID: AT4G27585.1
Best-hit-arabi-name: SPFH/Band 7/PHB domain-containing membrane-associated protein family
Gorai.004G126000.1.D
PCC=0.156, p=0.525
mRNA Profile
Protein Expression Profile
DPA

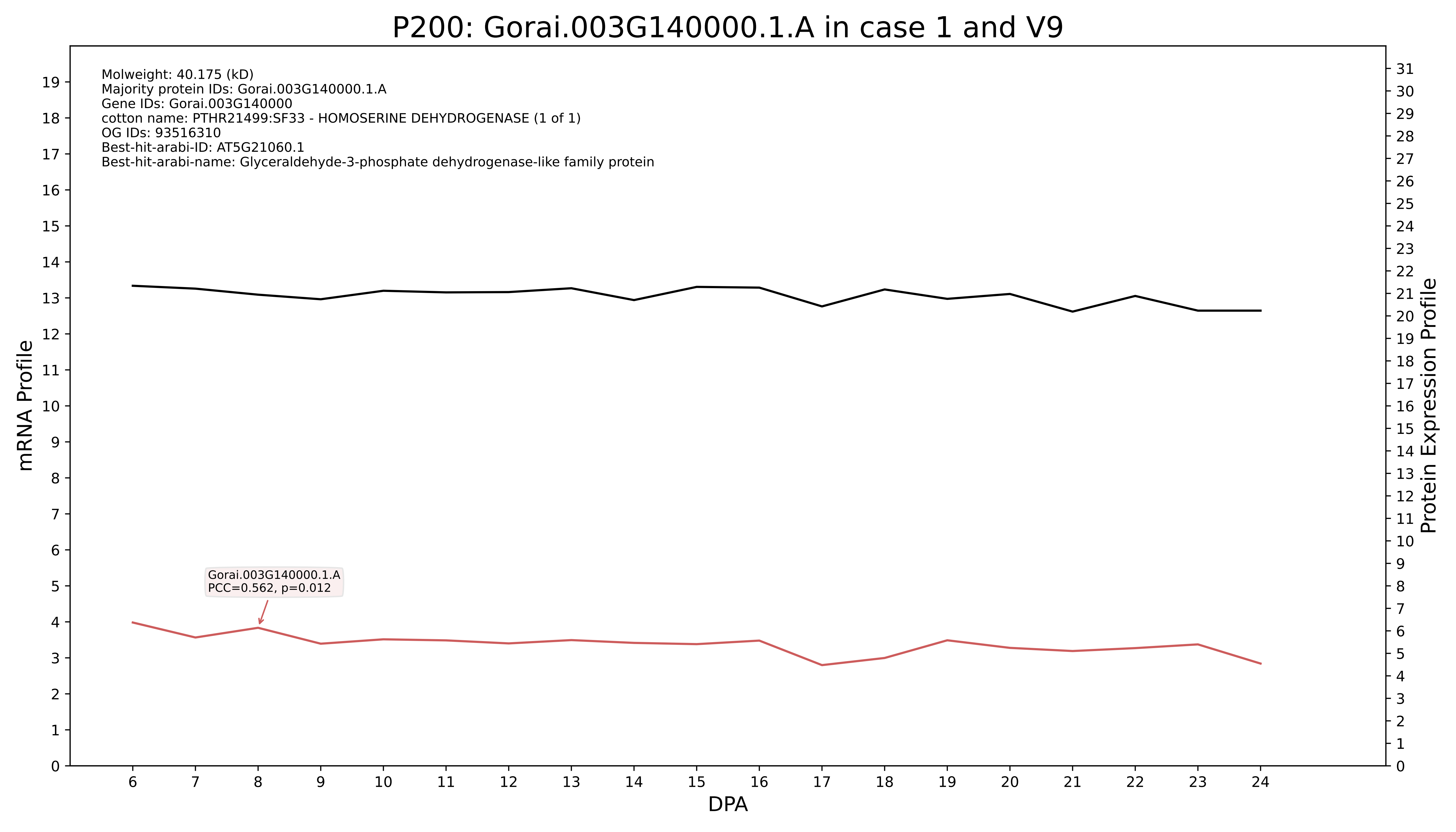
P200: Gorai.003G140000.1.A in case 1 and V9
Molweight: 40.175 (kD)
Majority protein IDs: Gorai.003G140000.1.A
Gene IDs: Gorai.003G140000
cotton name: PTHR21499:SF33 - HOMOSERINE DEHYDROGENASE (1 of 1)
OG IDs: 93516310
Best-hit-arabi-ID: AT5G21060.1
Best-hit-arabi-name: Glyceraldehyde-3-phosphate dehydrogenase-like family protein
Gorai.003G140000.1.A
PCC=0.562, p=0.012
mRNA Profile
Protein Expression Profile
DPA

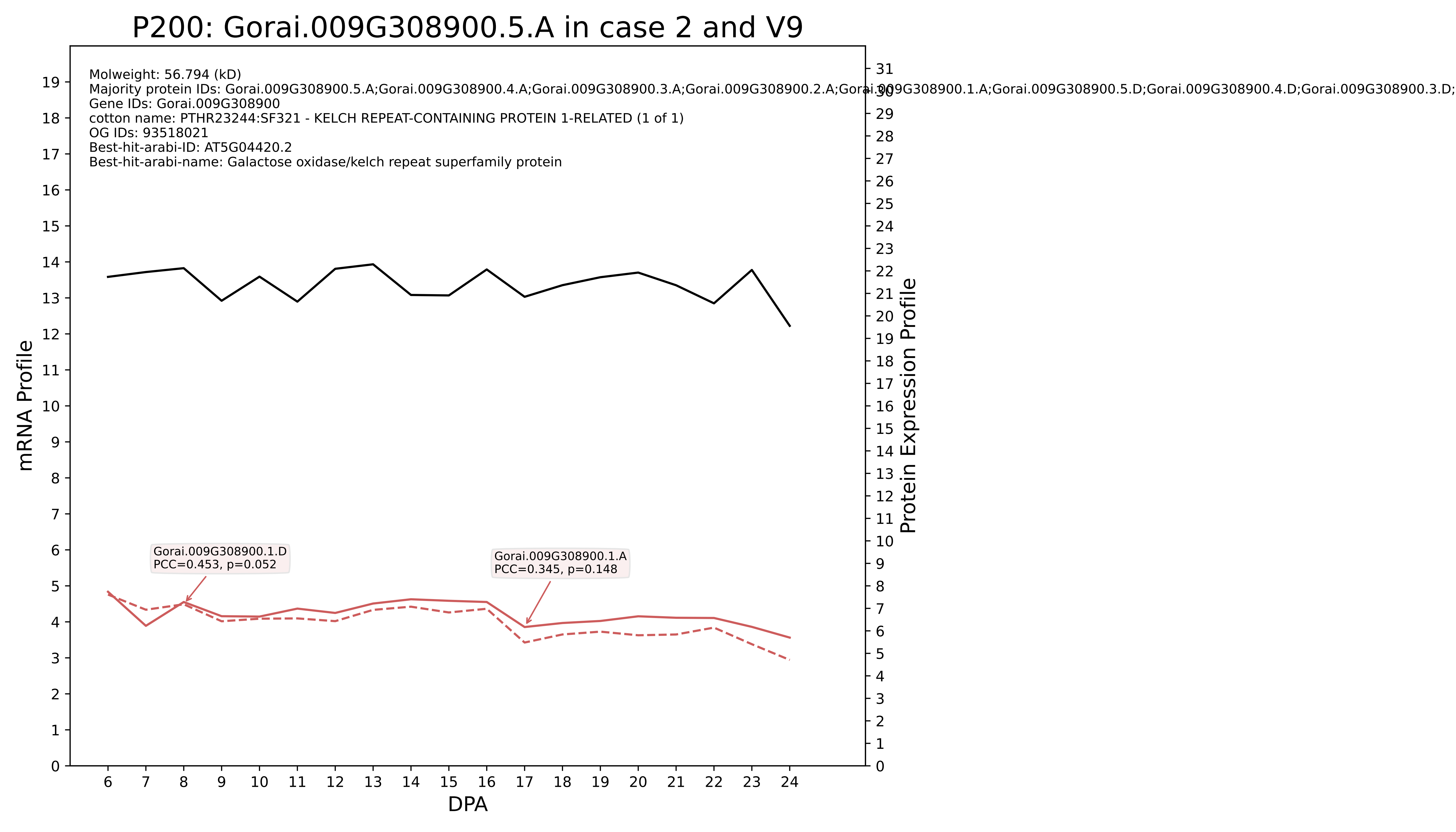
P200: Gorai.009G308900.5.A in case 2 and V9
Molweight: 56.794 (kD)
Majority protein IDs: Gorai.009G308900.5.A;Gorai.009G308900.4.A;Gorai.009G308900.3.A;Gorai.009G308900.2.A;Gorai.009G308900.1.A;Gorai.009G308900.5.D;Gorai.009G308900.4.D;Gorai.009G308900.3.D;
Gene IDs: Gorai.009G308900
cotton name: PTHR23244:SF321 - KELCH REPEAT-CONTAINING PROTEIN 1-RELATED (1 of 1)
OG IDs: 93518021
Best-hit-arabi-ID: AT5G04420.2
Best-hit-arabi-name: Galactose oxidase/kelch repeat superfamily protein
Gorai.009G308900.1.D
PCC=0.453, p=0.052
Gorai.009G308900.1.A
PCC=0.345, p=0.148
mRNA Profile
Protein Expression Profile
DPA

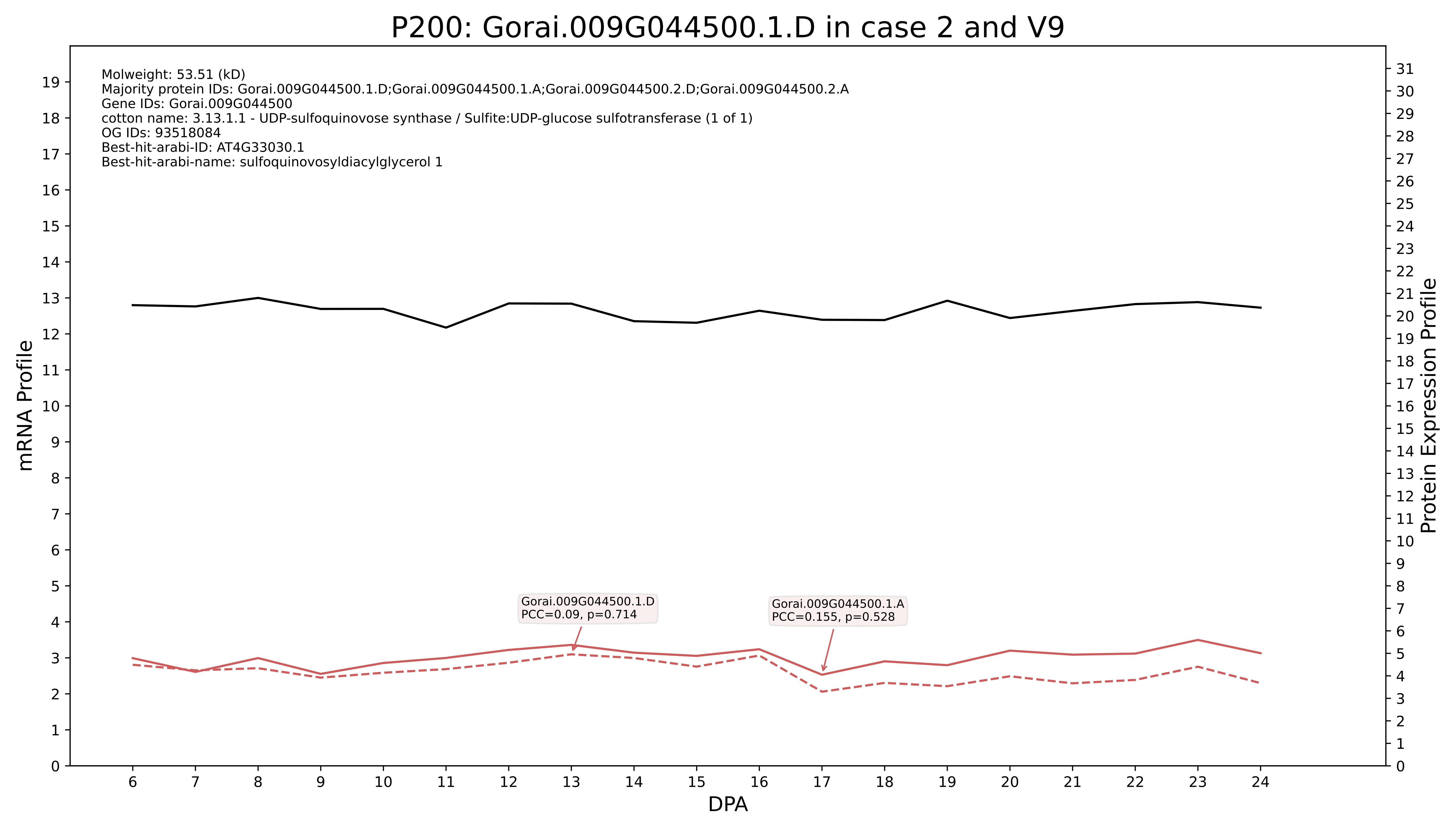
P200: Gorai.009G044500.1.D in case 2 and V9
Molweight: 53.51 (kD)
Majority protein IDs: Gorai.009G044500.1.D;Gorai.009G044500.1.A;Gorai.009G044500.2.D;Gorai.009G044500.2.A
Gene IDs: Gorai.009G044500
cotton name: 3.13.1.1 - UDP-sulfoquinovose synthase / Sulfite:UDP-glucose sulfotransferase (1 of 1)
OG IDs: 93518084
Best-hit-arabi-ID: AT4G33030.1
Best-hit-arabi-name: sulfoquinovosyldiacylglycerol 1
Gorai.009G044500.1.D
PCC=0.09, p=0.714
Gorai.009G044500.1.A
PCC=0.155, p=0.528
mRNA Profile
Protein Expression Profile
DPA
0
1
2
3
4
5
6
7
8
9
10
11
12
13
14
15
16
17
18
19
20
21
22
23
24
25
26
27
28
29
30
31

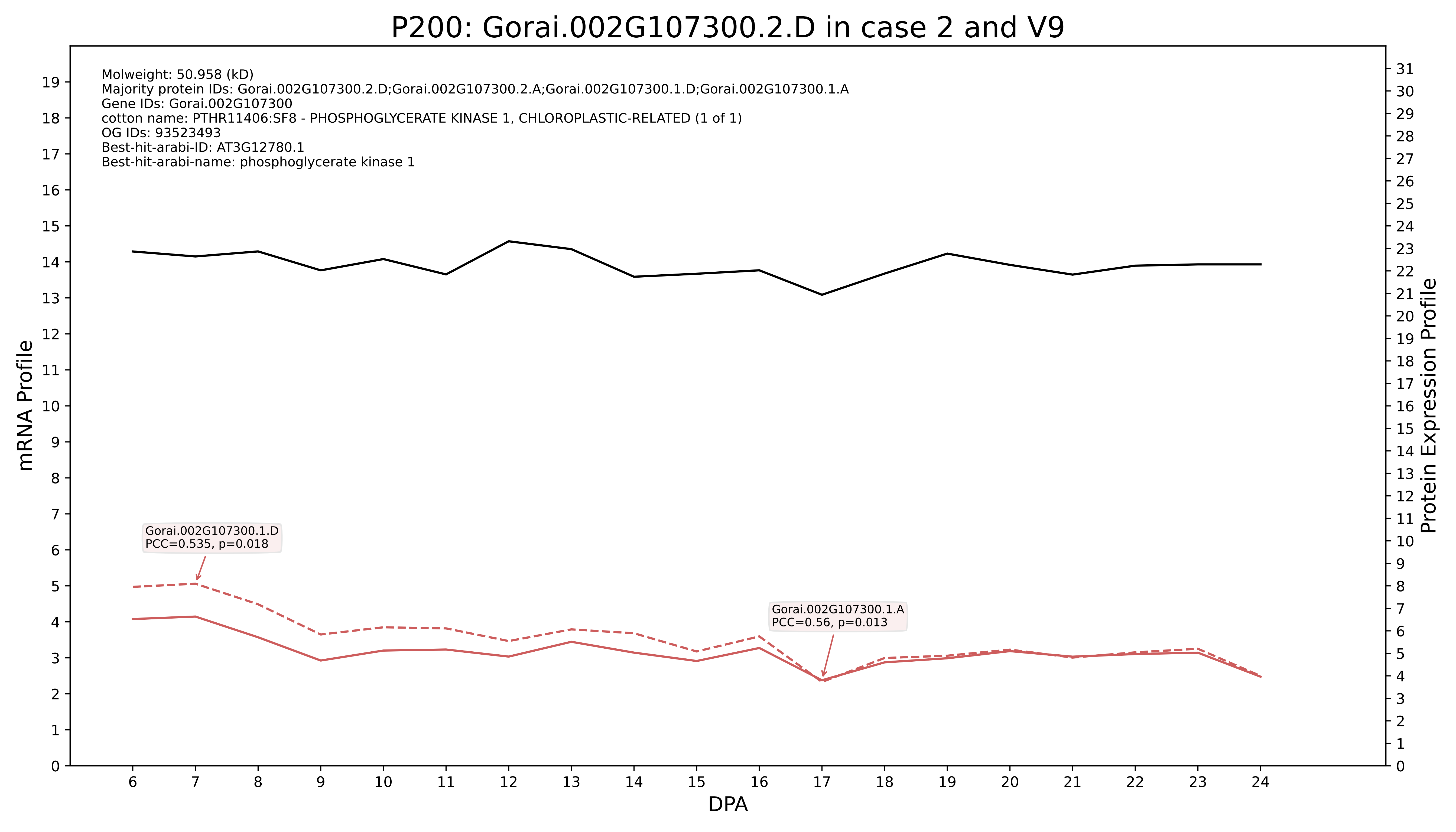
P200: Gorai.002G107300.2.D in case 2 and V9
Molweight: 50.958 (kD)
Majority protein IDs: Gorai.002G107300.2.D;Gorai.002G107300.2.A;Gorai.002G107300.1.D;Gorai.002G107300.1.A
Gene IDs: Gorai.002G107300
cotton name: PTHR11406:SF8 - PHOSPHOGLYCERATE KINASE 1, CHLOROPLASTIC-RELATED (1 of 1)
OG IDs: 93523493
Best-hit-arabi-ID: AT3G12780.1
Best-hit-arabi-name: phosphoglycerate kinase 1
Gorai.002G107300.1.D
PCC=0.535, p=0.018
Gorai.002G107300.1.A
PCC=0.56, p=0.013
mRNA Profile
Protein Expression Profile
DPA
6
7
8
9
10
11
12
13
14
15
16
17
18
19
20
21
22
23
24

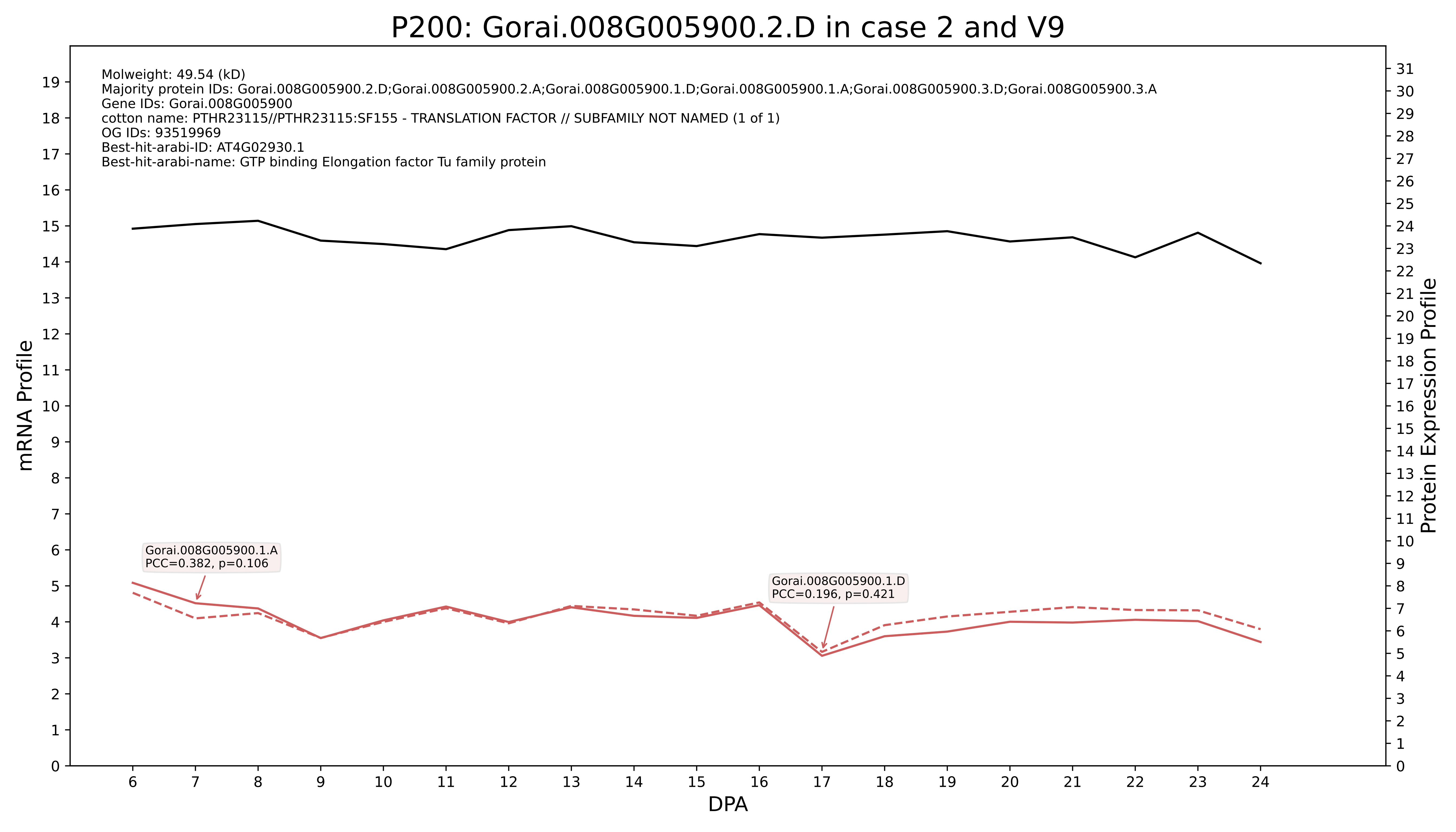
P200: Gorai.008G005900.2.D in case 2 and V9
Molweight: 49.54 (kD)
Majority protein IDs: Gorai.008G005900.2.D;Gorai.008G005900.2.A;Gorai.008G005900.1.D;Gorai.008G005900.1.A;Gorai.008G005900.3.D;Gorai.008G005900.3.A
Gene IDs: Gorai.008G005900
cotton name: PTHR23115//PTHR23115:SF155 - TRANSLATION FACTOR // SUBFAMILY NOT NAMED (1 of 1)
OG IDs: 93519969
Best-hit-arabi-ID: AT4G02930.1
Best-hit-arabi-name: GTP binding Elongation factor Tu family protein
Gorai.008G005900.1.A
PCC=0.382, p=0.106
Gorai.008G005900.1.D
PCC=0.196, p=0.421
mRNA Profile
Protein Expression Profile
DPA

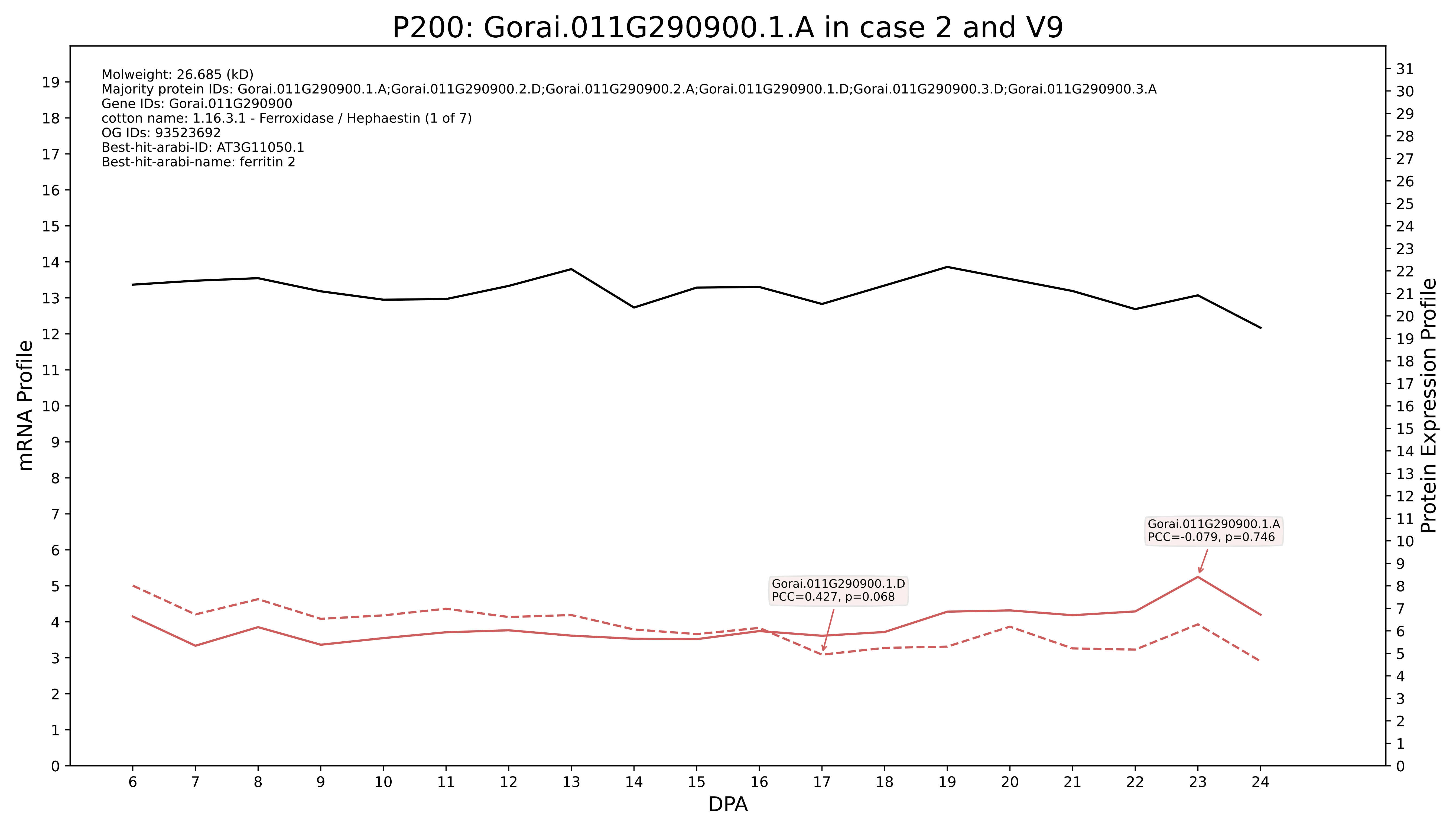
P200: Gorai.011G290900.1.A in case 2 and V9
Molweight: 26.685 (kD)
Majority protein IDs: Gorai.011G290900.1.A;Gorai.011G290900.2.D;Gorai.011G290900.2.A;Gorai.011G290900.1.D;Gorai.011G290900.3.D;Gorai.011G290900.3.A
Gene IDs: Gorai.011G290900
cotton name: 1.16.3.1 - Ferroxidase / Hephaestin (1 of 7)
OG IDs: 93523692
Best-hit-arabi-ID: AT3G11050.1
Best-hit-arabi-name: ferritin 2
Gorai.011G290900.1.A
PCC=-0.079, p=0.746
Gorai.011G290900.1.D
PCC=0.427, p=0.068
mRNA Profile
Protein Expression Profile
DPA

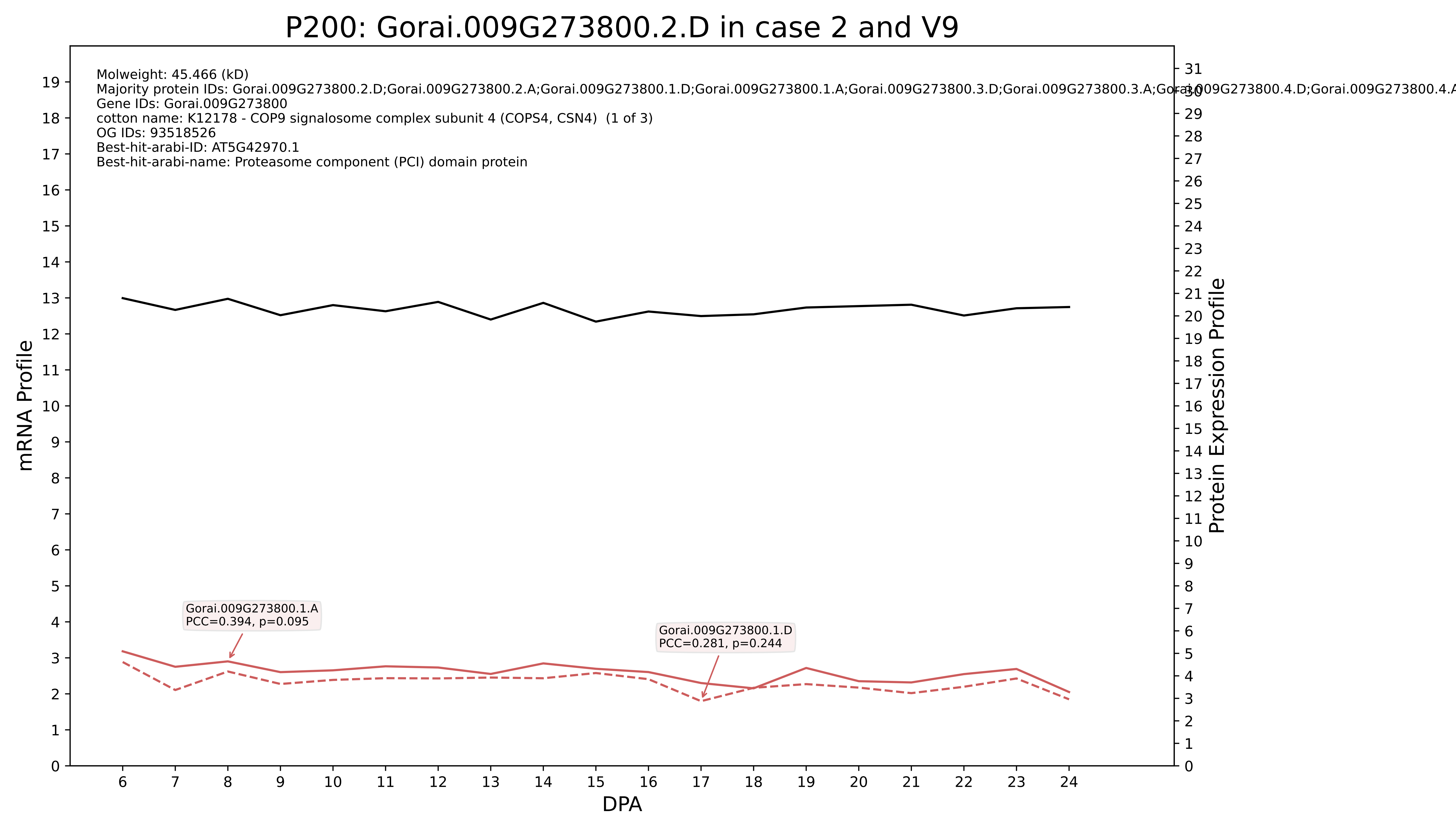
P200: Gorai.009G273800.2.D in case 2 and V9
Molweight: 45.466 (kD)
Majority protein IDs: Gorai.009G273800.2.D;Gorai.009G273800.2.A;Gorai.009G273800.1.D;Gorai.009G273800.1.A;Gorai.009G273800.3.D;Gorai.009G273800.3.A;Gorai.009G273800.4.D;Gorai.009G273800.4.A
Gene IDs: Gorai.009G273800
cotton name: K12178 - COP9 signalosome complex subunit 4 (COPS4, CSN4) (1 of 3)
OG IDs: 93518526
Best-hit-arabi-ID: AT5G42970.1
Best-hit-arabi-name: Proteasome component (PCI) domain protein
Gorai.009G273800.1.A
PCC=0.394, p=0.095
Gorai.009G273800.1.D
PCC=0.281, p=0.244
mRNA Profile
Protein Expression Profile
DPA

009G379300.3.D in case 2 and V9

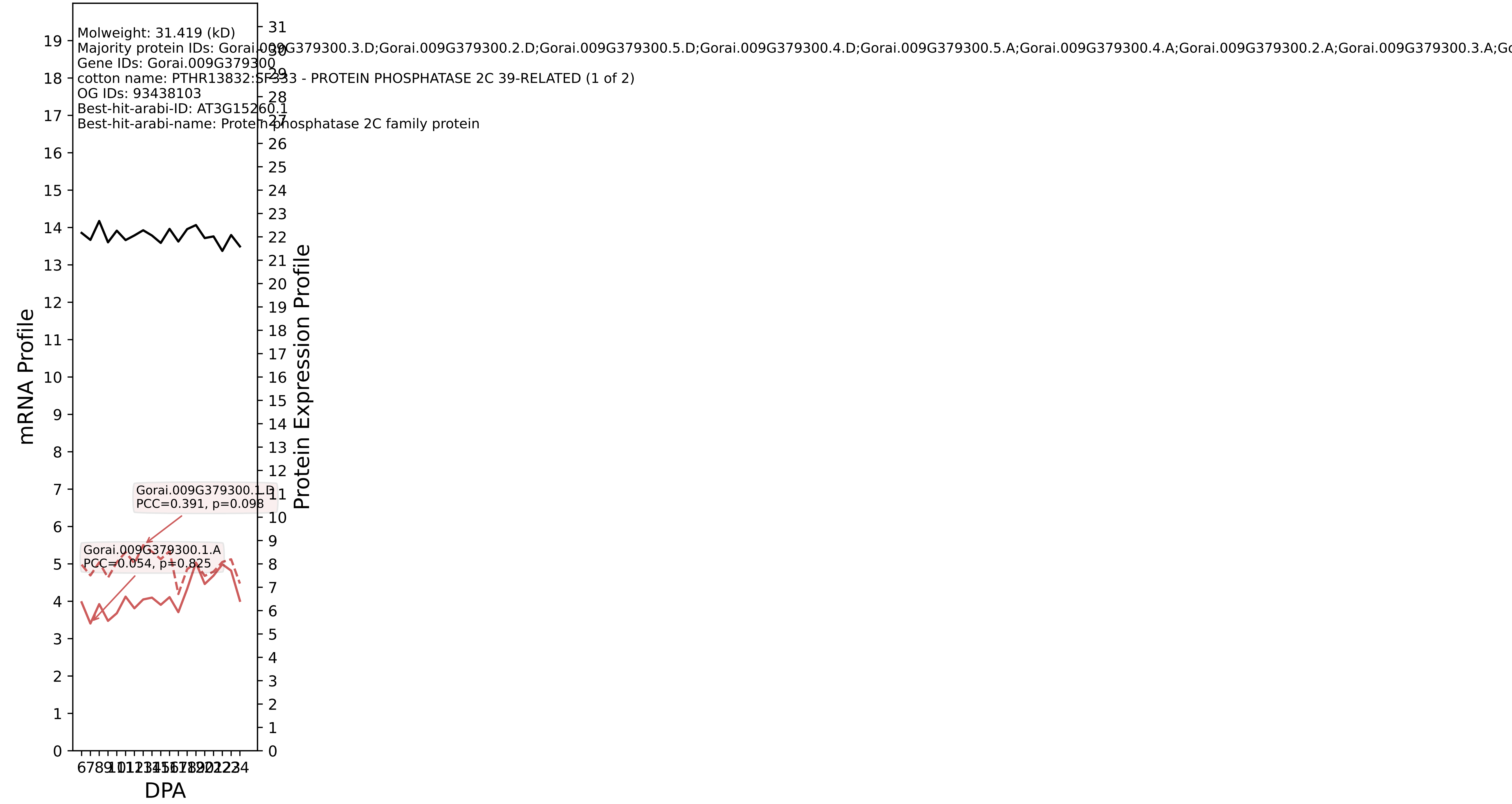

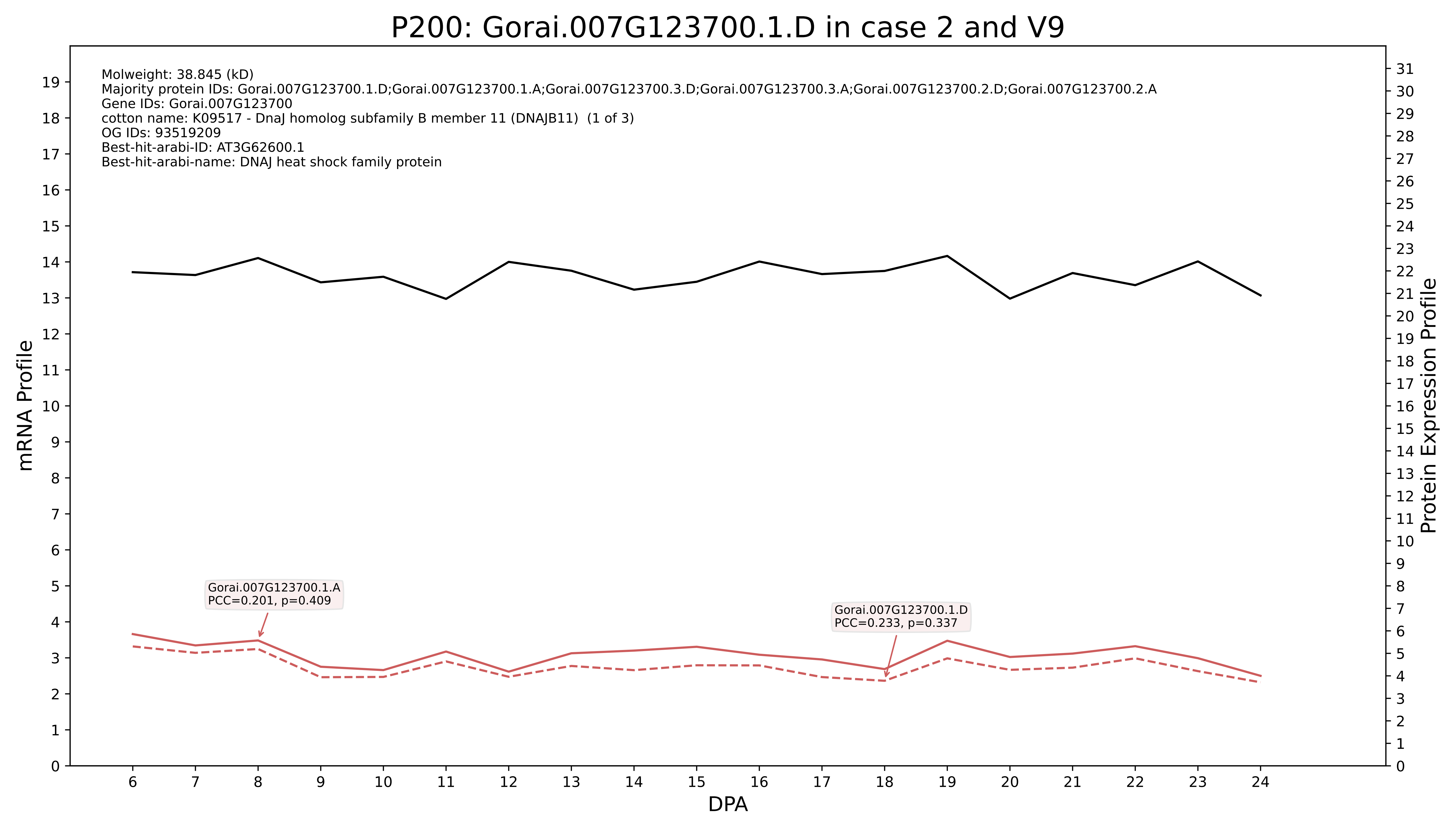

P200: Gorai.007G123700.1.D in case 2 and V9
Molweight: 38.845 (kD)
Majority protein IDs: Gorai.007G123700.1.D;Gorai.007G123700.1.A;Gorai.007G123700.3.D;Gorai.007G123700.3.A;Gorai.007G123700.2.D;Gorai.007G123700.2.A
Gene IDs: Gorai.007G123700
cotton name: K09517 - DnaJ homolog subfamily B member 11 (DNAJB11) (1 of 3)
OG IDs: 93519209
Best-hit-arabi-ID: AT3G62600.1
Best-hit-arabi-name: DNAJ heat shock family protein
Gorai.007G123700.1.A
PCC=0.201, p=0.409
Gorai.007G123700.1.D
PCC=0.233, p=0.337
mRNA Profile
Protein Expression Profile
DPA

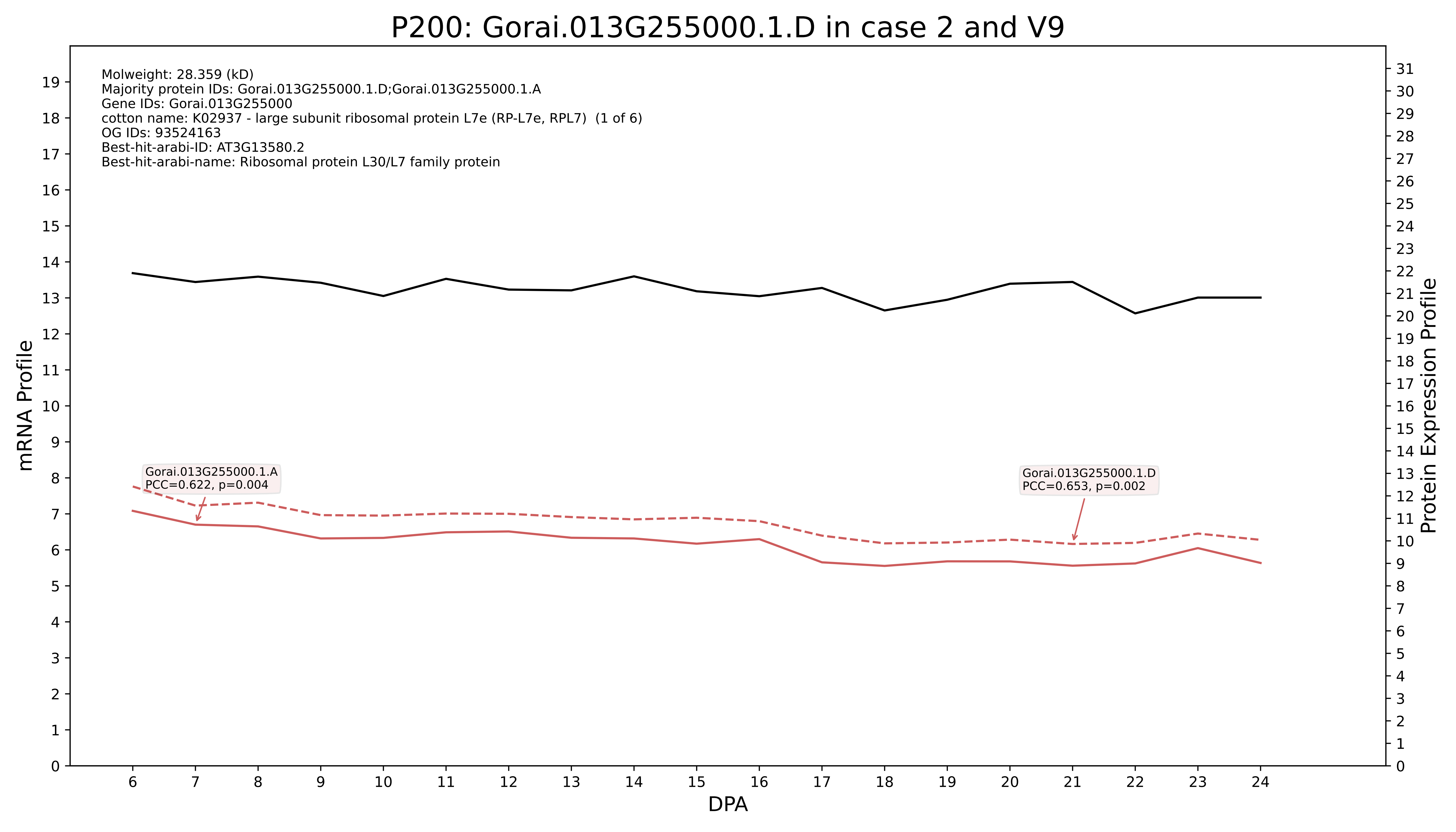

P200: Gorai.013G255000.1.D in case 2 and V9
Molweight: 28.359 (kD)
Majority protein IDs: Gorai.013G255000.1.D;Gorai.013G255000.1.A
Gene IDs: Gorai.013G255000
cotton name: K02937 - large subunit ribosomal protein L7e (RP-L7e, RPL7) (1 of 6)
OG IDs: 93524163
Best-hit-arabi-ID: AT3G13580.2
Best-hit-arabi-name: Ribosomal protein L30/L7 family protein
Gorai.013G255000.1.A
PCC=0.622, p=0.004
Gorai.013G255000.1.D
PCC=0.653, p=0.002
mRNA Profile
Protein Expression Profile
DPA

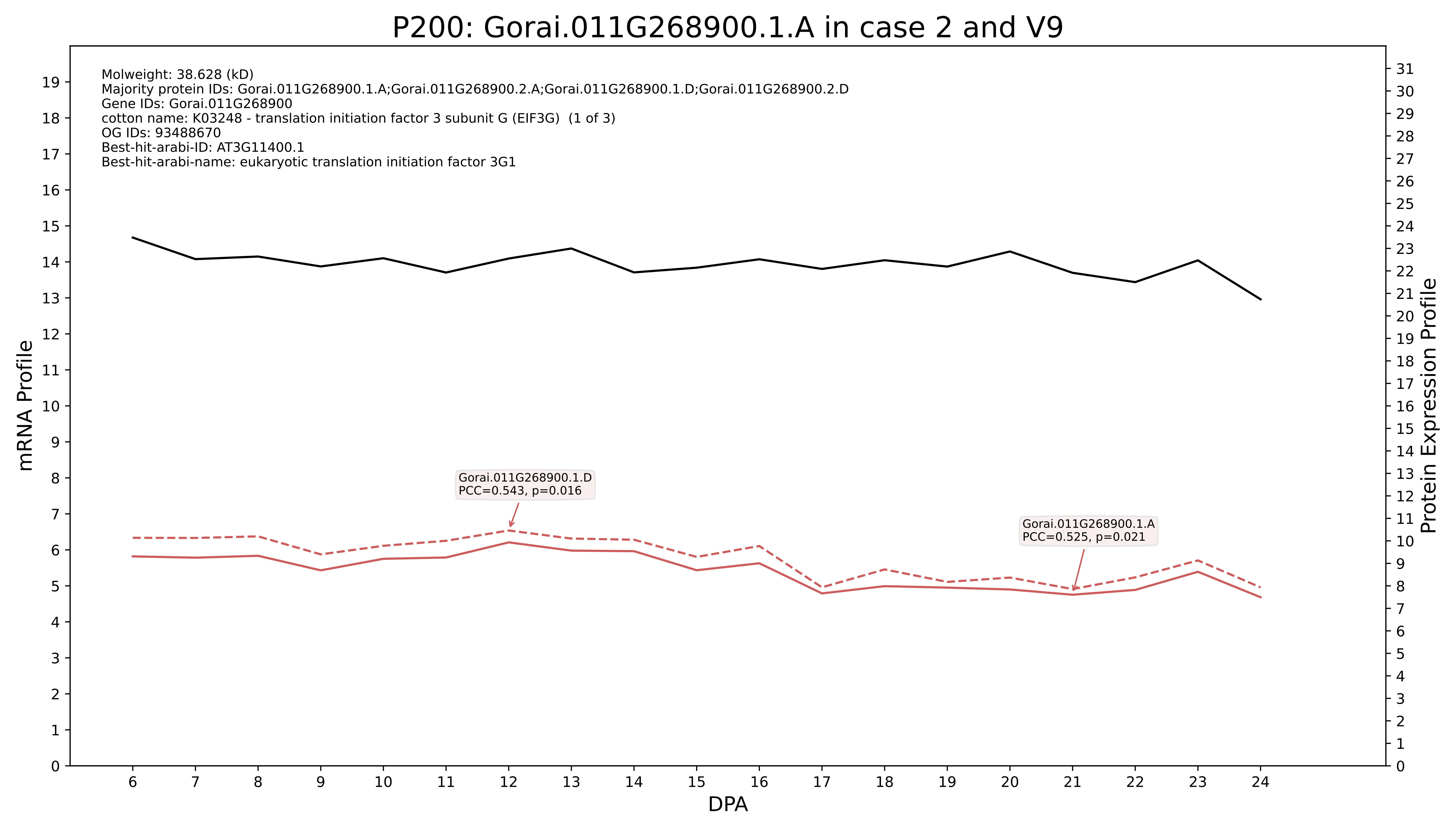
P200: Gorai.011G268900.1.A in case 2 and V9
Molweight: 38.628 (kD)
Majority protein IDs: Gorai.011G268900.1.A;Gorai.011G268900.2.A;Gorai.011G268900.1.D;Gorai.011G268900.2.D
Gene IDs: Gorai.011G268900
cotton name: K03248 - translation initiation factor 3 subunit G (EIF3G) (1 of 3)
OG IDs: 93488670
Best-hit-arabi-ID: AT3G11400.1
Best-hit-arabi-name: eukaryotic translation initiation factor 3G1
Gorai.011G268900.1.D
PCC=0.543, p=0.016
Gorai.011G268900.1.A
PCC=0.525, p=0.021
mRNA Profile
Protein Expression Profile
DPA

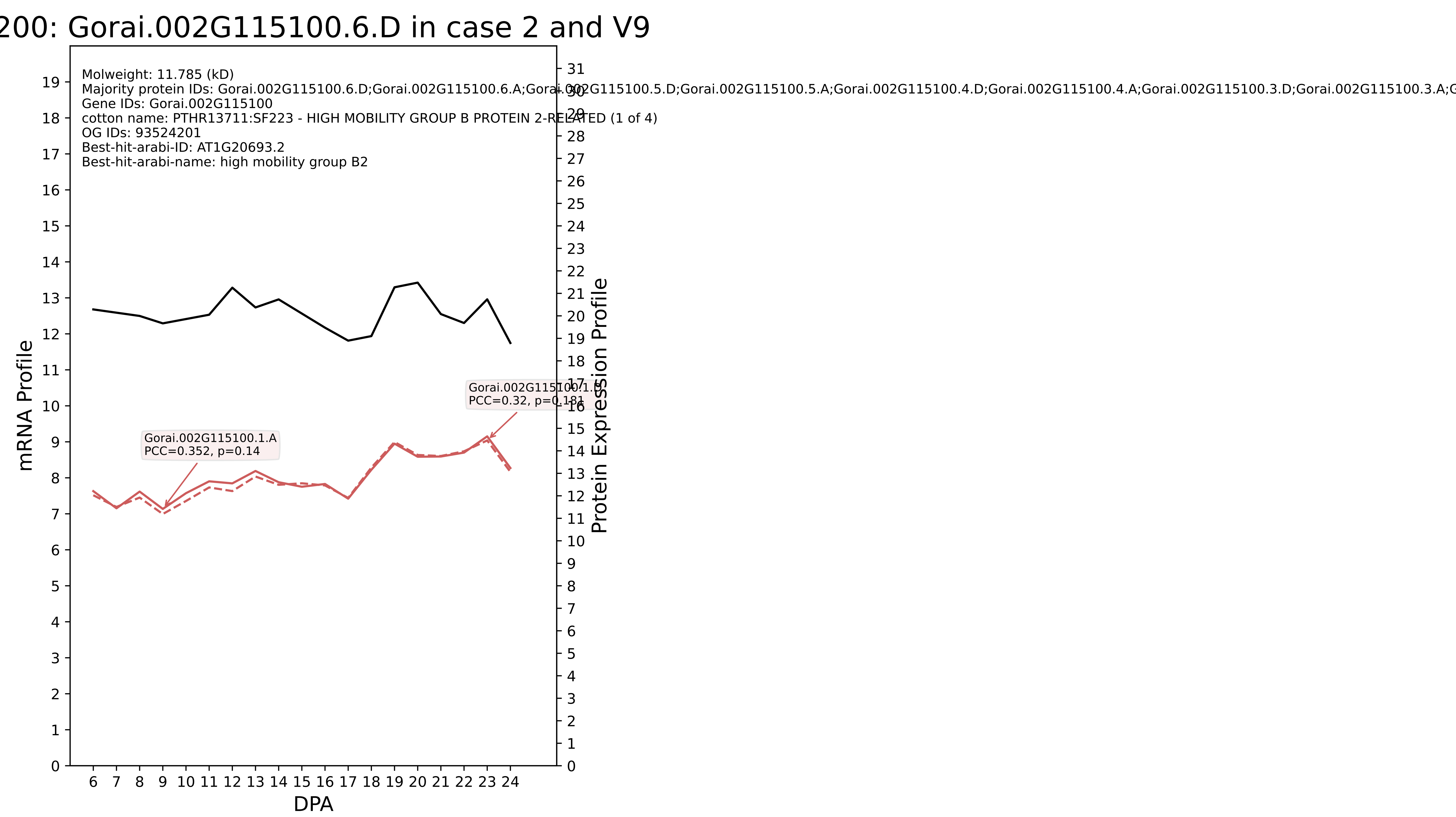
200: Gorai.002G115100.6.D in case 2 and V9
Molweight: 11.785 (kD)
Majority protein IDs: Gorai.002G115100.6.D;Gorai.002G115100.6.A;Gorai.002G115100.5.D;Gorai.002G115100.5.A;Gorai.002G115100.4.D;Gorai.002G115100.4.A;Gorai.002G115100.3.D;Gorai.002G115100.3.A;G
Gene IDs: Gorai.002G115100
cotton name: PTHR13711:SF223 - HIGH MOBILITY GROUP B PROTEIN 2-RELATED (1 of 4)
OG IDs: 93524201
Best-hit-arabi-ID: AT1G20693.2
Best-hit-arabi-name: high mobility group B2
Gorai.002G115100.1.D
PCC=0.32, p=0.181
Gorai.002G115100.1.A
PCC=0.352, p=0.14
mRNA Profile
Protein Expression Profile
DPA

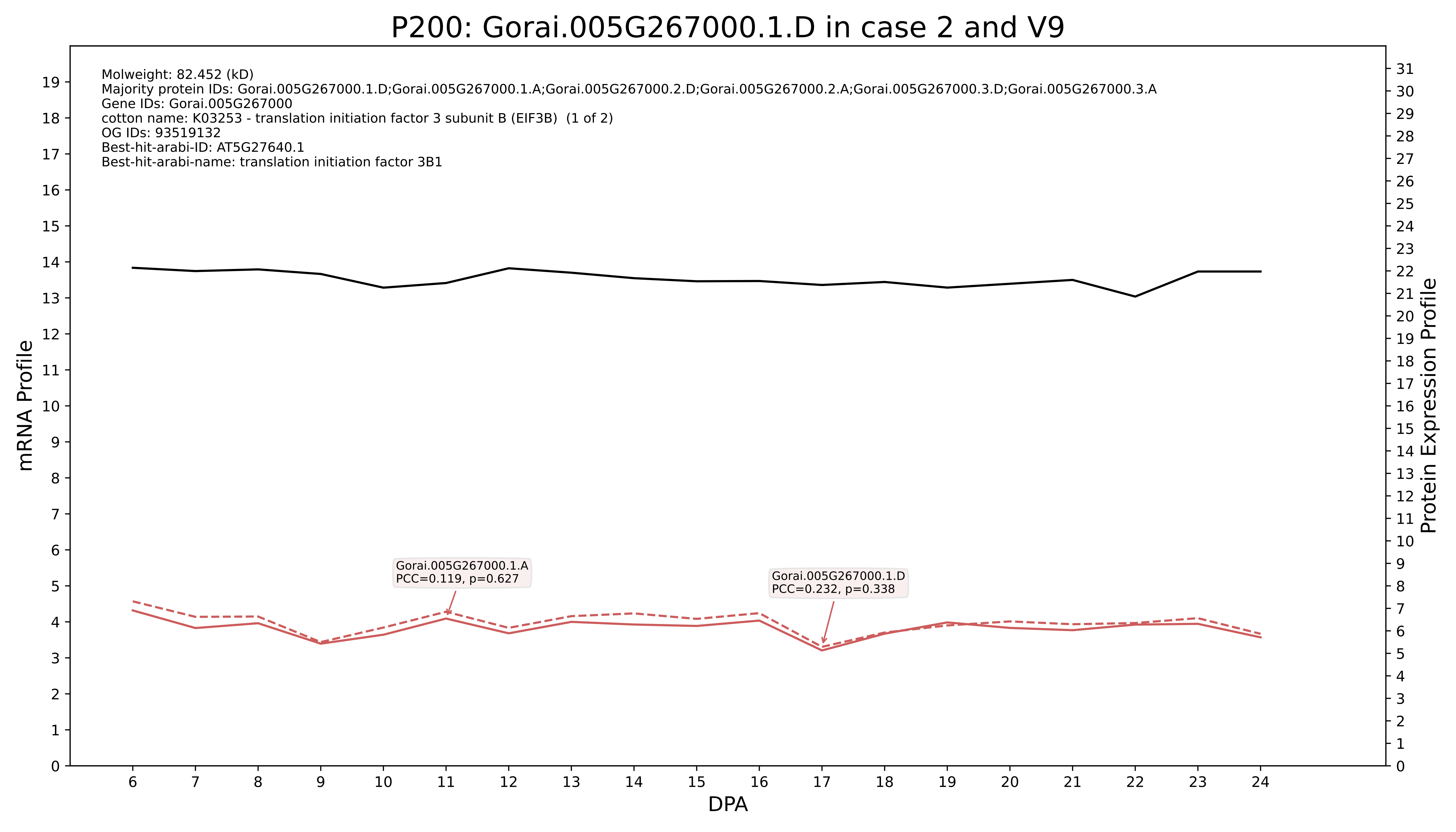
P200: Gorai.005G267000.1.D in case 2 and V9
Molweight: 82.452 (kD)
Majority protein IDs: Gorai.005G267000.1.D;Gorai.005G267000.1.A;Gorai.005G267000.2.D;Gorai.005G267000.2.A;Gorai.005G267000.3.D;Gorai.005G267000.3.A
Gene IDs: Gorai.005G267000
cotton name: K03253 - translation initiation factor 3 subunit B (EIF3B) (1 of 2)
OG IDs: 93519132
Best-hit-arabi-ID: AT5G27640.1
Best-hit-arabi-name: translation initiation factor 3B1
Gorai.005G267000.1.A
PCC=0.119, p=0.627
Gorai.005G267000.1.D
PCC=0.232, p=0.338
mRNA Profile
Protein Expression Profile
DPA

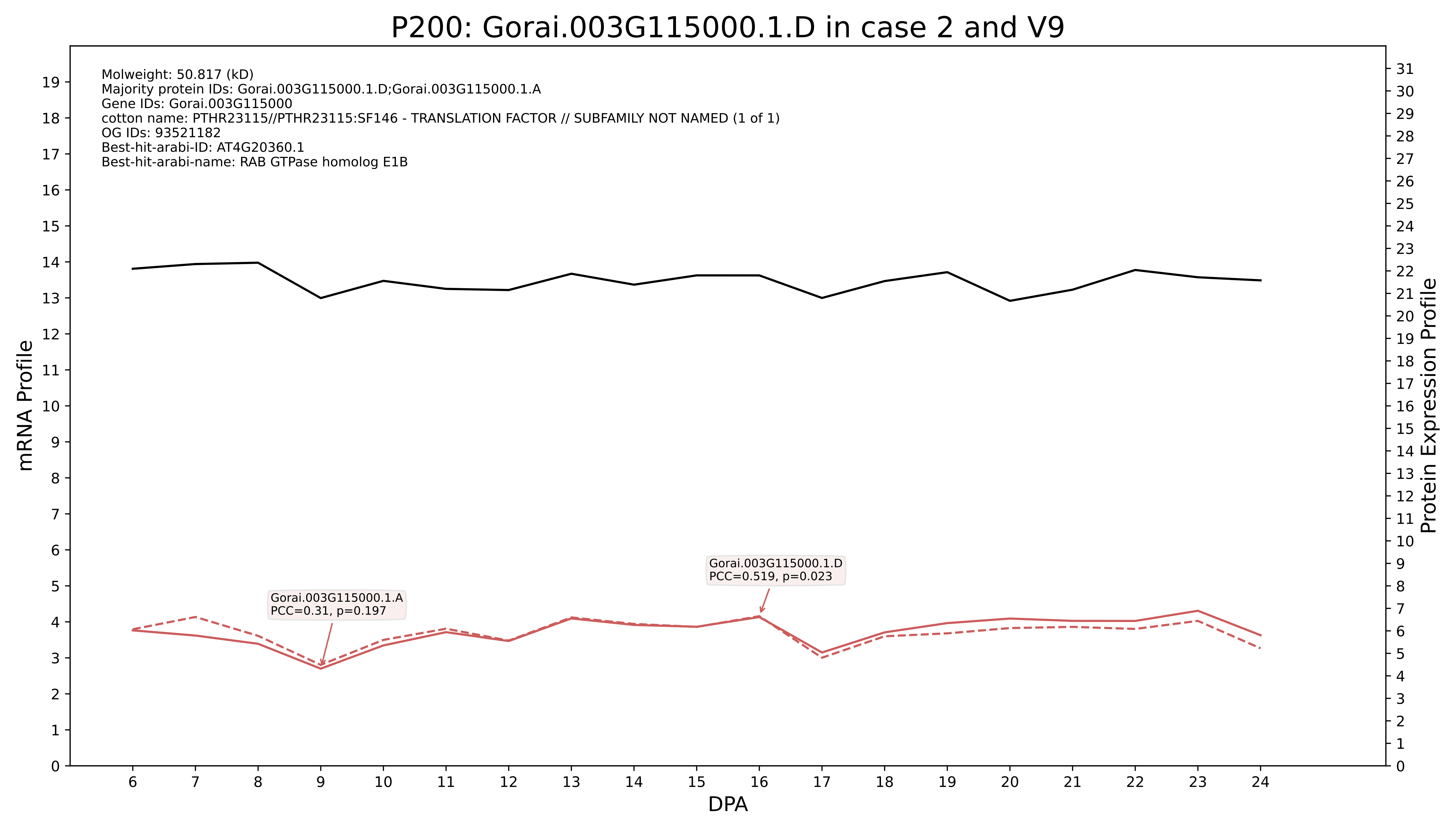
P200: Gorai.003G115000.1.D in case 2 and V9
Molweight: 50.817 (kD)
Majority protein IDs: Gorai.003G115000.1.D;Gorai.003G115000.1.A
Gene IDs: Gorai.003G115000
cotton name: PTHR23115//PTHR23115:SF146 - TRANSLATION FACTOR // SUBFAMILY NOT NAMED (1 of 1)
OG IDs: 93521182
Best-hit-arabi-ID: AT4G20360.1
Best-hit-arabi-name: RAB GTPase homolog E1B
Gorai.003G115000.1.A
PCC=0.31, p=0.197
Gorai.003G115000.1.D
PCC=0.519, p=0.023
mRNA Profile
Protein Expression Profile
DPA

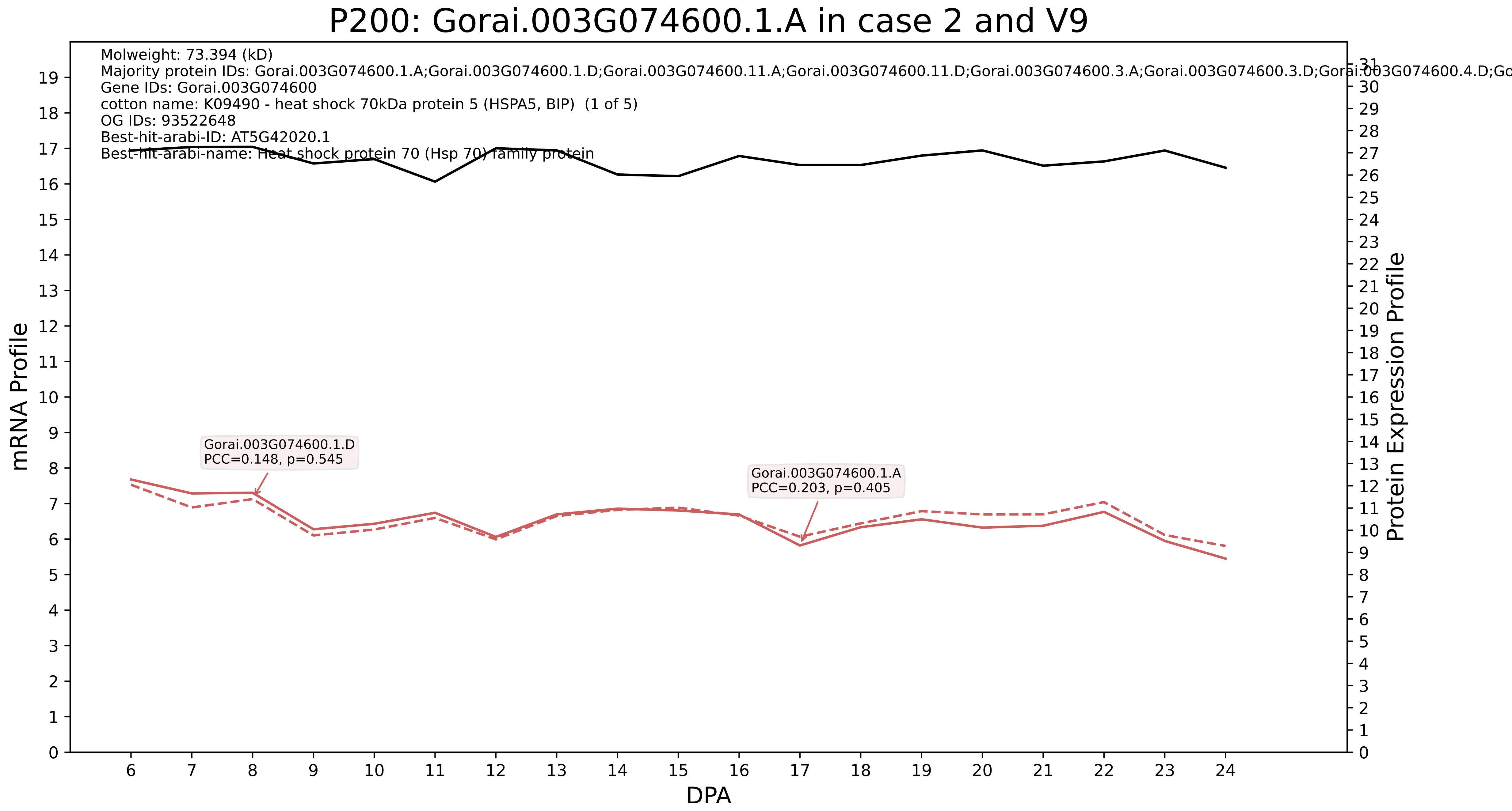
P200: Gorai.003G074600.1.A in case 2 and V9
Molweight: 73.394 (kD)
Majority protein IDs: Gorai.003G074600.1.A;Gorai.003G074600.1.D;Gorai.003G074600.11.A;Gorai.003G074600.11.D;Gorai.003G074600.3.A;Gorai.003G074600.3.D;Gorai.003G074600.4.D;Go
Gene IDs: Gorai.003G074600
cotton name: K09490 - heat shock 70kDa protein 5 (HSPA5, BIP) (1 of 5)
OG IDs: 93522648
Best-hit-arabi-ID: AT5G42020.1
Best-hit-arabi-name: Heat shock protein 70 (Hsp 70) family protein
Gorai.003G074600.1.D
PCC=0.148, p=0.545
Gorai.003G074600.1.A
PCC=0.203, p=0.405
mRNA Profile
Protein Expression Profile
DPA

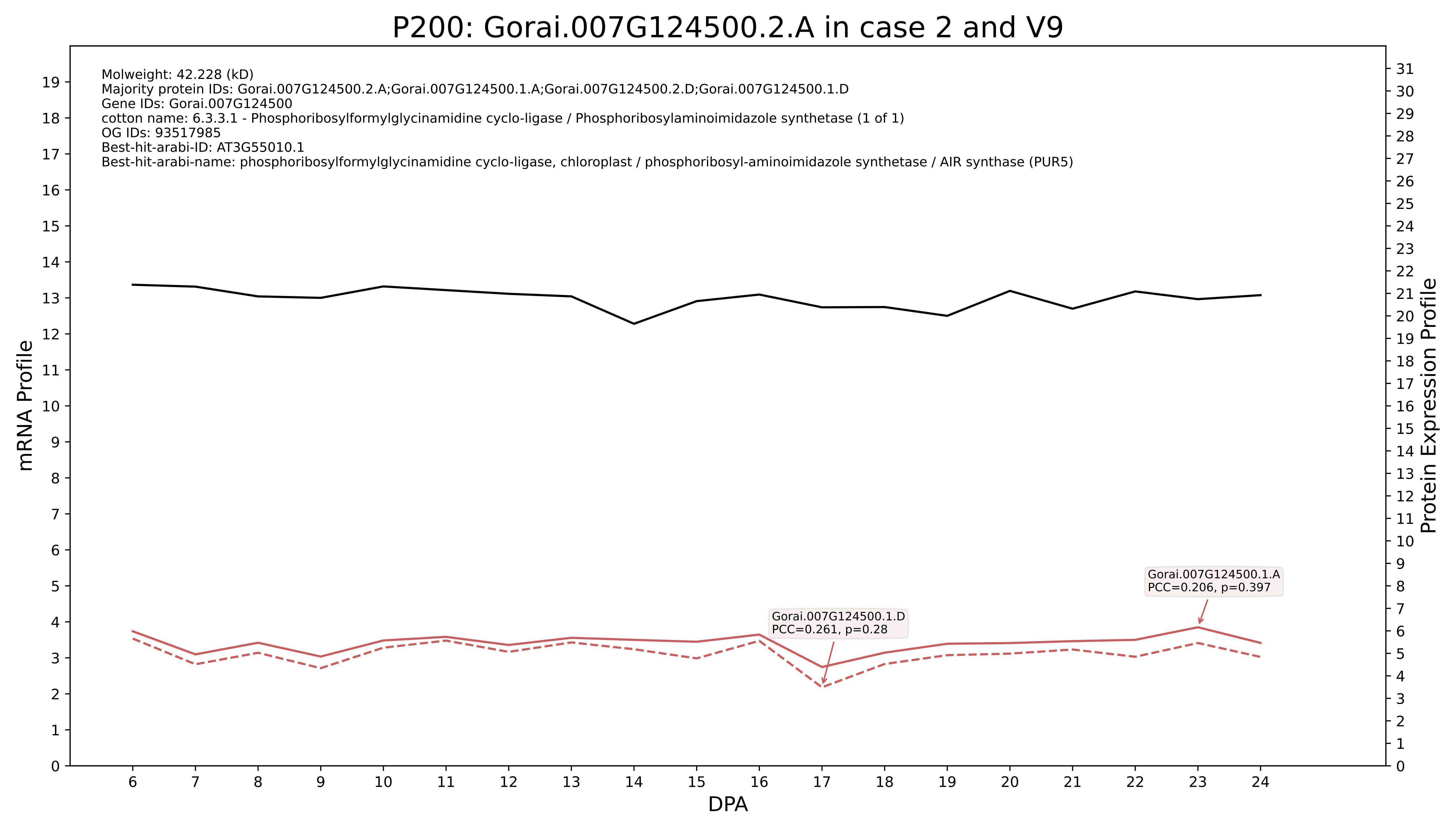
P200: Gorai.007G124500.2.A in case 2 and V9
Molweight: 42.228 (kD)
Majority protein IDs: Gorai.007G124500.2.A;Gorai.007G124500.1.A;Gorai.007G124500.2.D;Gorai.007G124500.1.D
Gene IDs: Gorai.007G124500
cotton name: 6.3.3.1 - Phosphoribosylformylglycinamidine cyclo-ligase / Phosphoribosylaminoimidazole synthetase (1 of 1)
OG IDs: 93517985
Best-hit-arabi-ID: AT3G55010.1
Best-hit-arabi-name: phosphoribosylformylglycinamidine cyclo-ligase, chloroplast / phosphoribosyl-aminoimidazole synthetase / AIR synthase (PUR5)
Gorai.007G124500.1.A
PCC=0.206, p=0.397
Gorai.007G124500.1.D
PCC=0.261, p=0.28
mRNA Profile
Protein Expression Profile
DPA

200: Gorai.008G030600.3.D in case 2 and V9

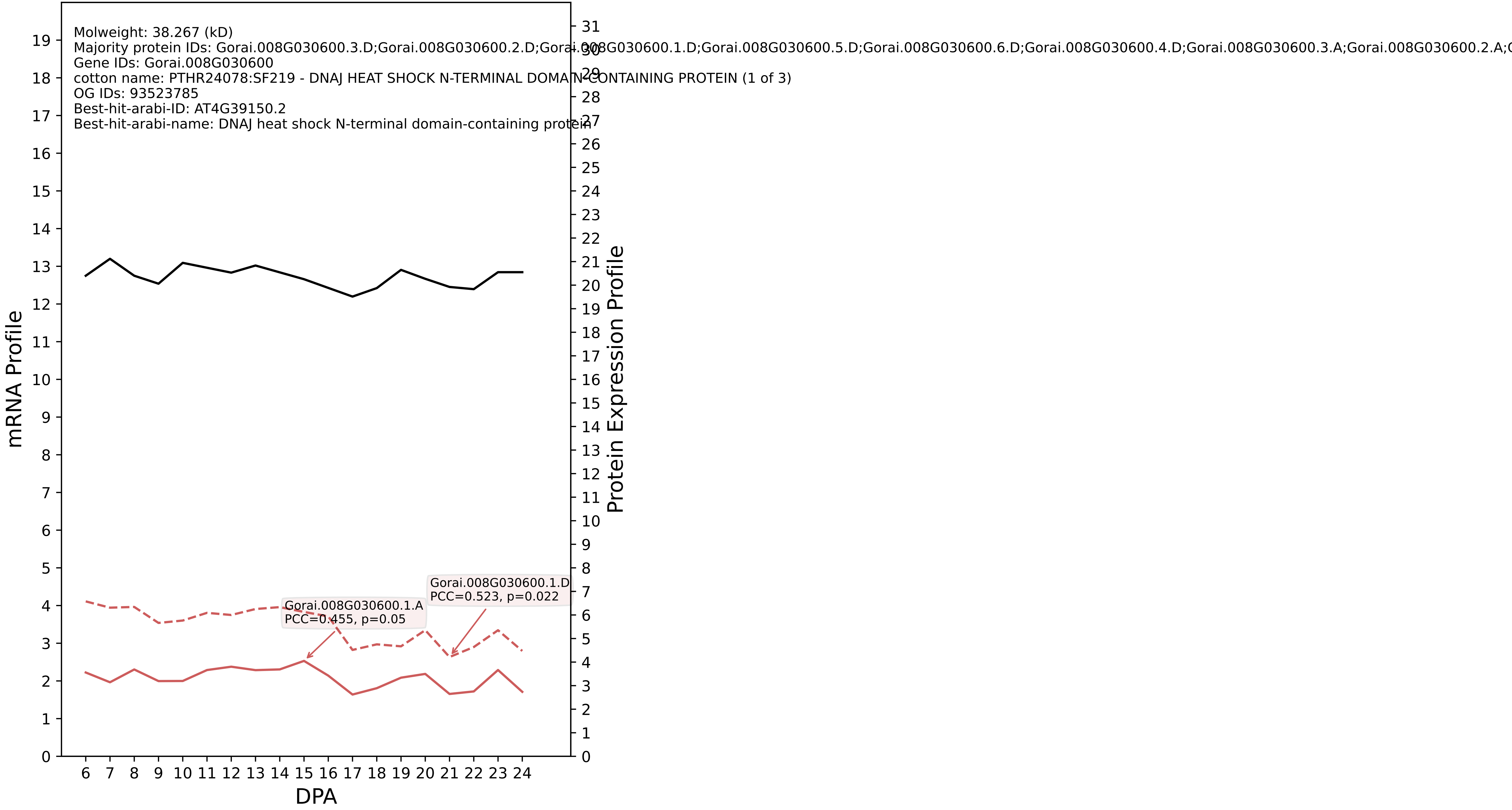

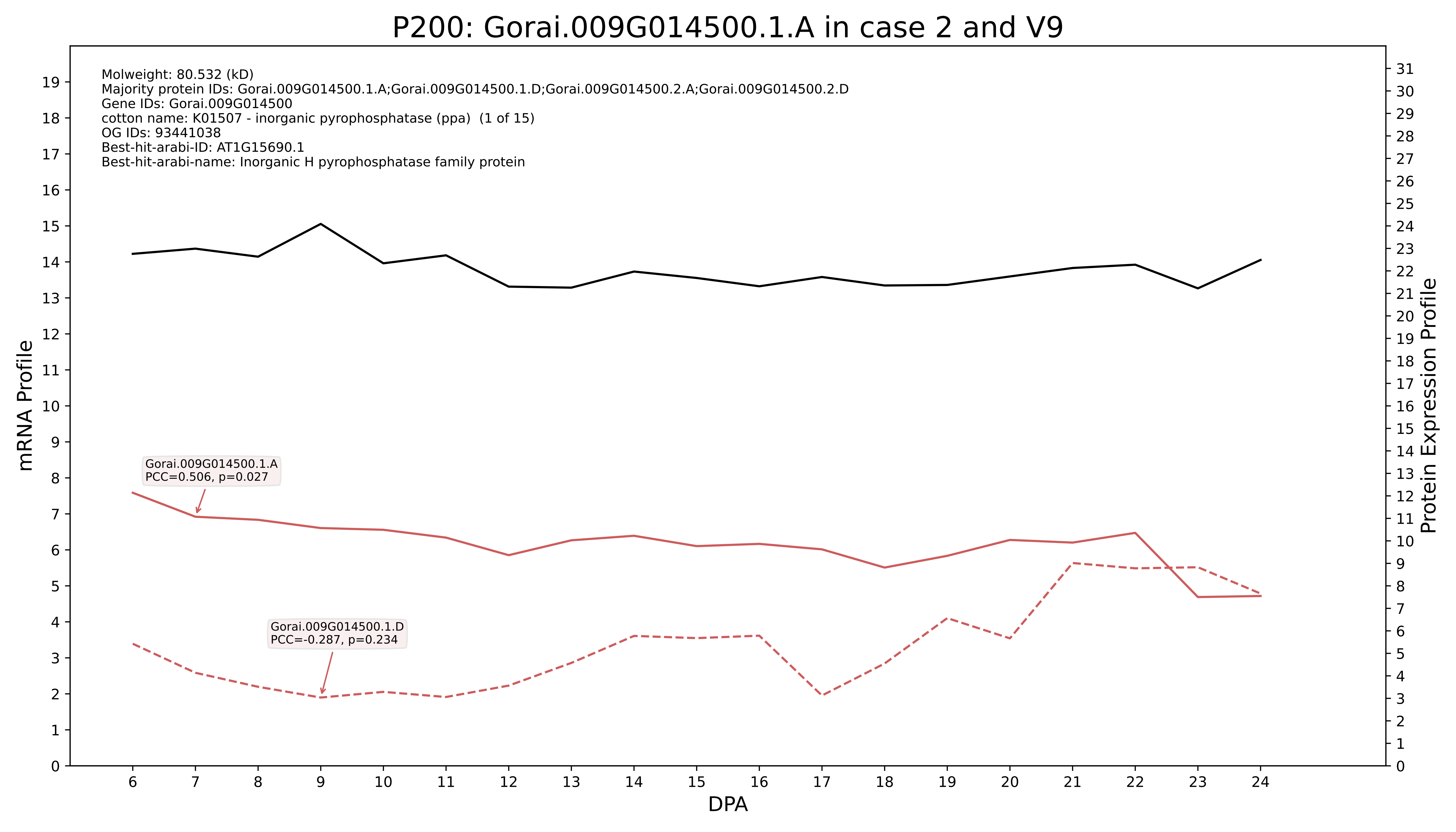
P200: Gorai.009G014500.1.A in case 2 and V9
Molweight: 80.532 (kD)
Majority protein IDs: Gorai.009G014500.1.A;Gorai.009G014500.1.D;Gorai.009G014500.2.A;Gorai.009G014500.2.D
Gene IDs: Gorai.009G014500
cotton name: K01507 - inorganic pyrophosphatase (ppa) (1 of 15)
OG IDs: 93441038
Best-hit-arabi-ID: AT1G15690.1
Best-hit-arabi-name: Inorganic H pyrophosphatase family protein
Gorai.009G014500.1.A
PCC=0.506, p=0.027
Gorai.009G014500.1.D
PCC=-0.287, p=0.234
mRNA Profile
Protein Expression Profile
DPA
0
1
2
3
4
5
6
7
8
9
10
11
12
13
14
15
16
17
18
19
20
21
22
23
24
25
26
27
28
29
30
31

P200: Gorai.001G057800.1.D in case 2 and V9

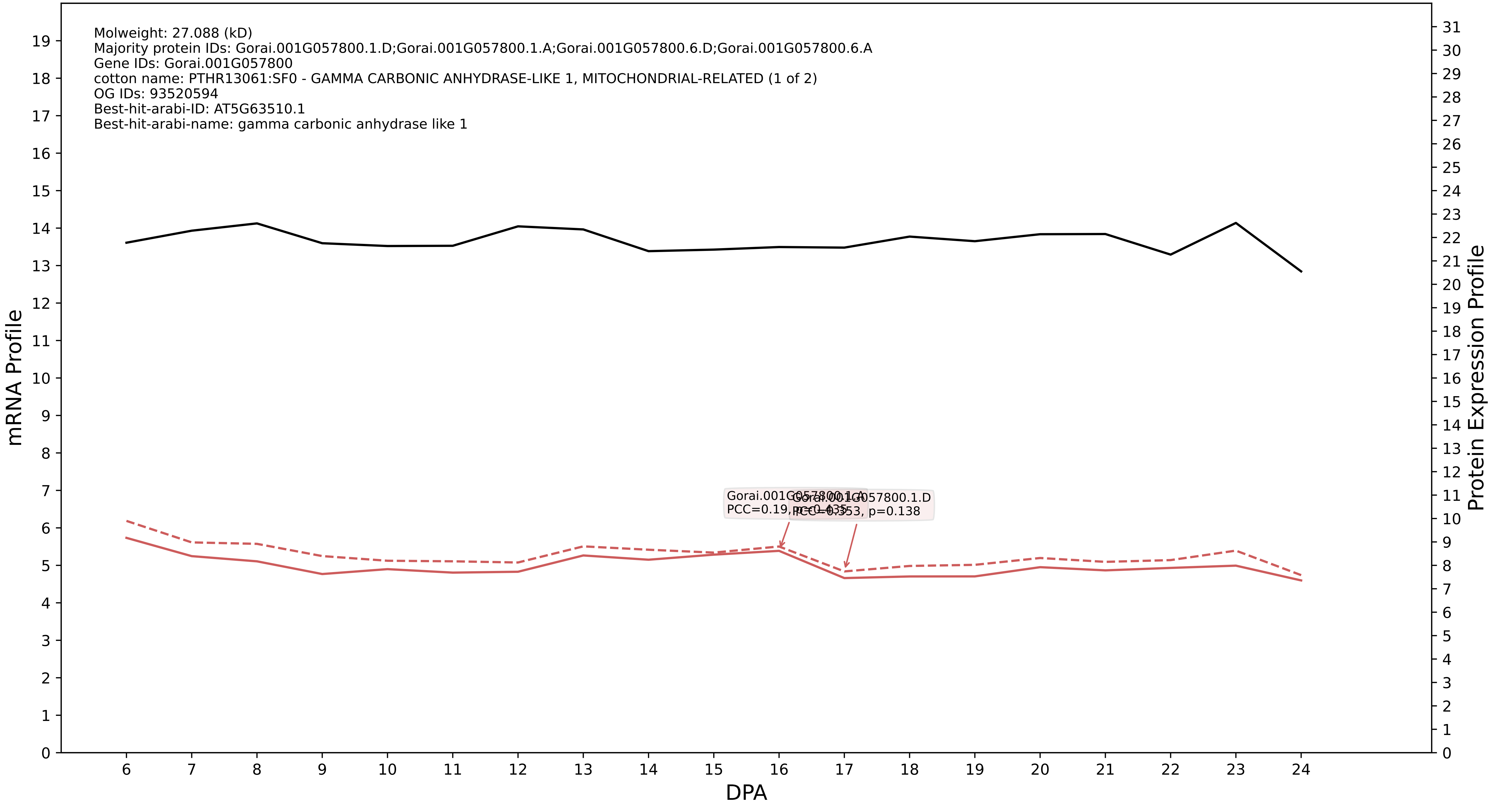

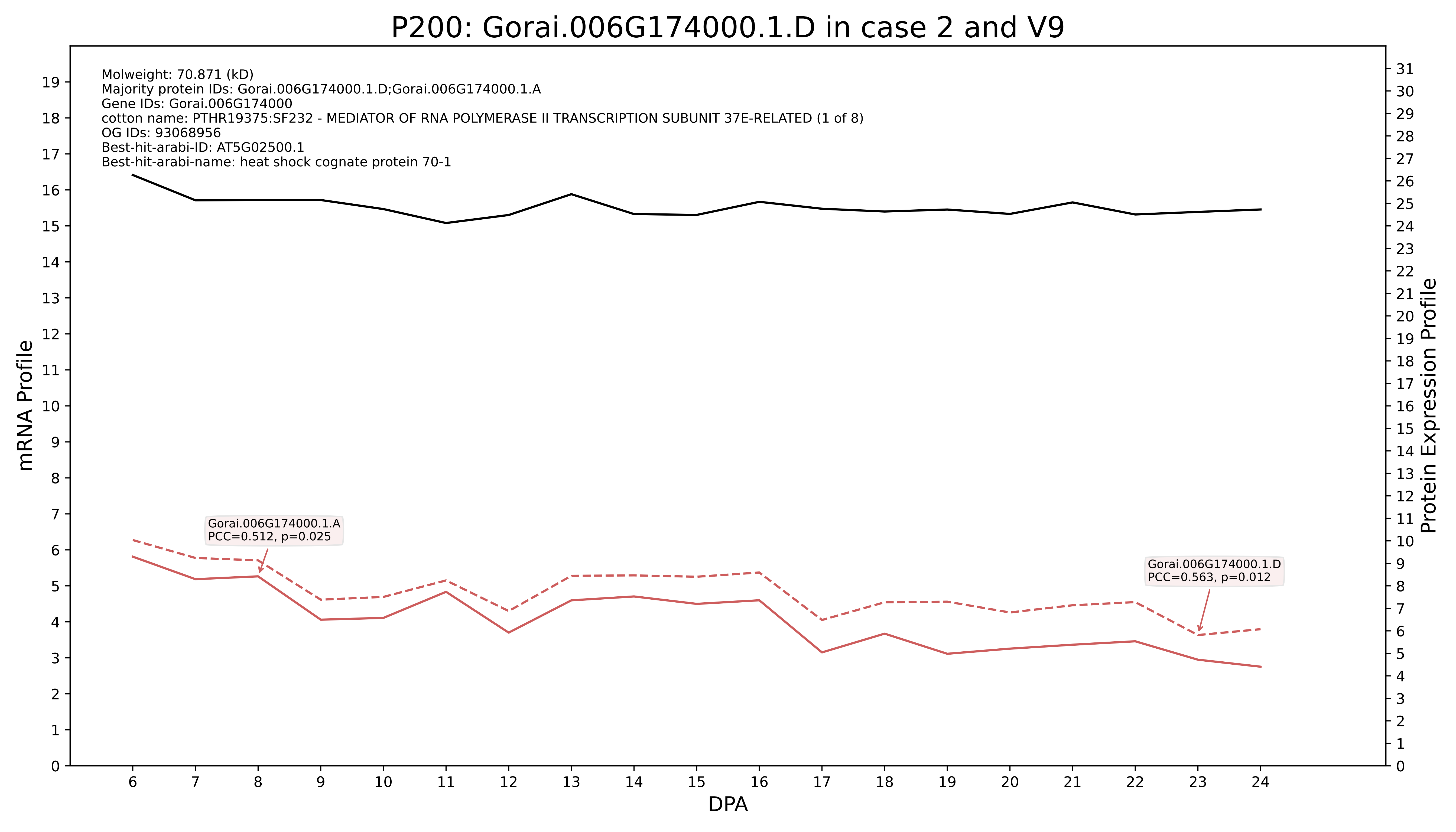

P200: Gorai.006G174000.1.D in case 2 and V9
Molweight: 70.871 (kD)
Majority protein IDs: Gorai.006G174000.1.D;Gorai.006G174000.1.A
Gene IDs: Gorai.006G174000
cotton name: PTHR19375:SF232 - MEDIATOR OF RNA POLYMERASE II TRANSCRIPTION SUBUNIT 37E-RELATED (1 of 8)
OG IDs: 93068956
Best-hit-arabi-ID: AT5G02500.1
Best-hit-arabi-name: heat shock cognate protein 70-1
Gorai.006G174000.1.A
PCC=0.512, p=0.025
Gorai.006G174000.1.D
PCC=0.563, p=0.012
mRNA Profile
Protein Expression Profile
DPA

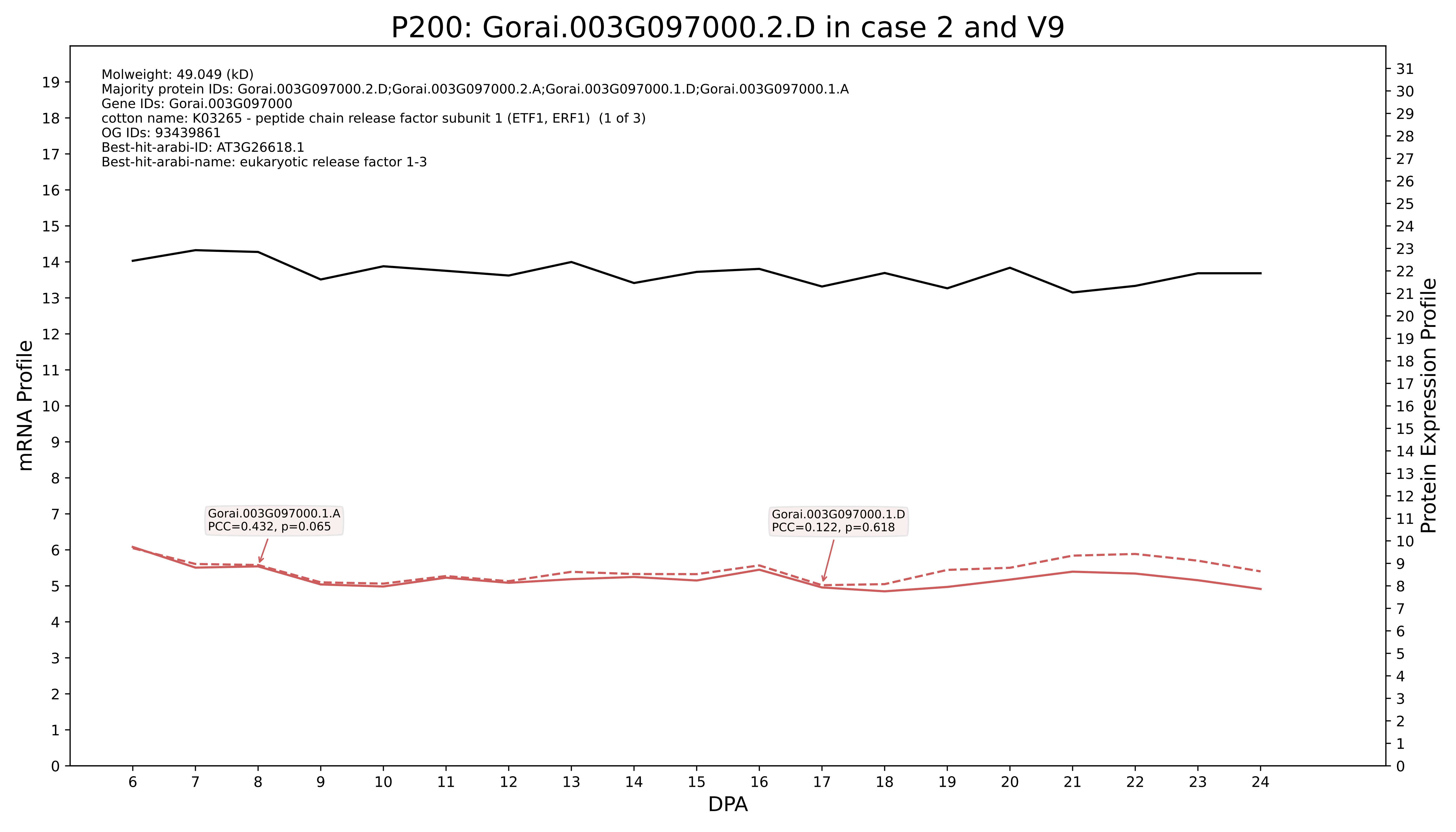
P200: Gorai.003G097000.2.D in case 2 and V9
Molweight: 49.049 (kD)
Majority protein IDs: Gorai.003G097000.2.D;Gorai.003G097000.2.A;Gorai.003G097000.1.D;Gorai.003G097000.1.A
Gene IDs: Gorai.003G097000
cotton name: K03265 - peptide chain release factor subunit 1 (ETF1, ERF1) (1 of 3)
OG IDs: 93439861
Best-hit-arabi-ID: AT3G26618.1
Best-hit-arabi-name: eukaryotic release factor 1-3
Gorai.003G097000.1.A
PCC=0.432, p=0.065
Gorai.003G097000.1.D
PCC=0.122, p=0.618
mRNA Profile
Protein Expression Profile
DPA

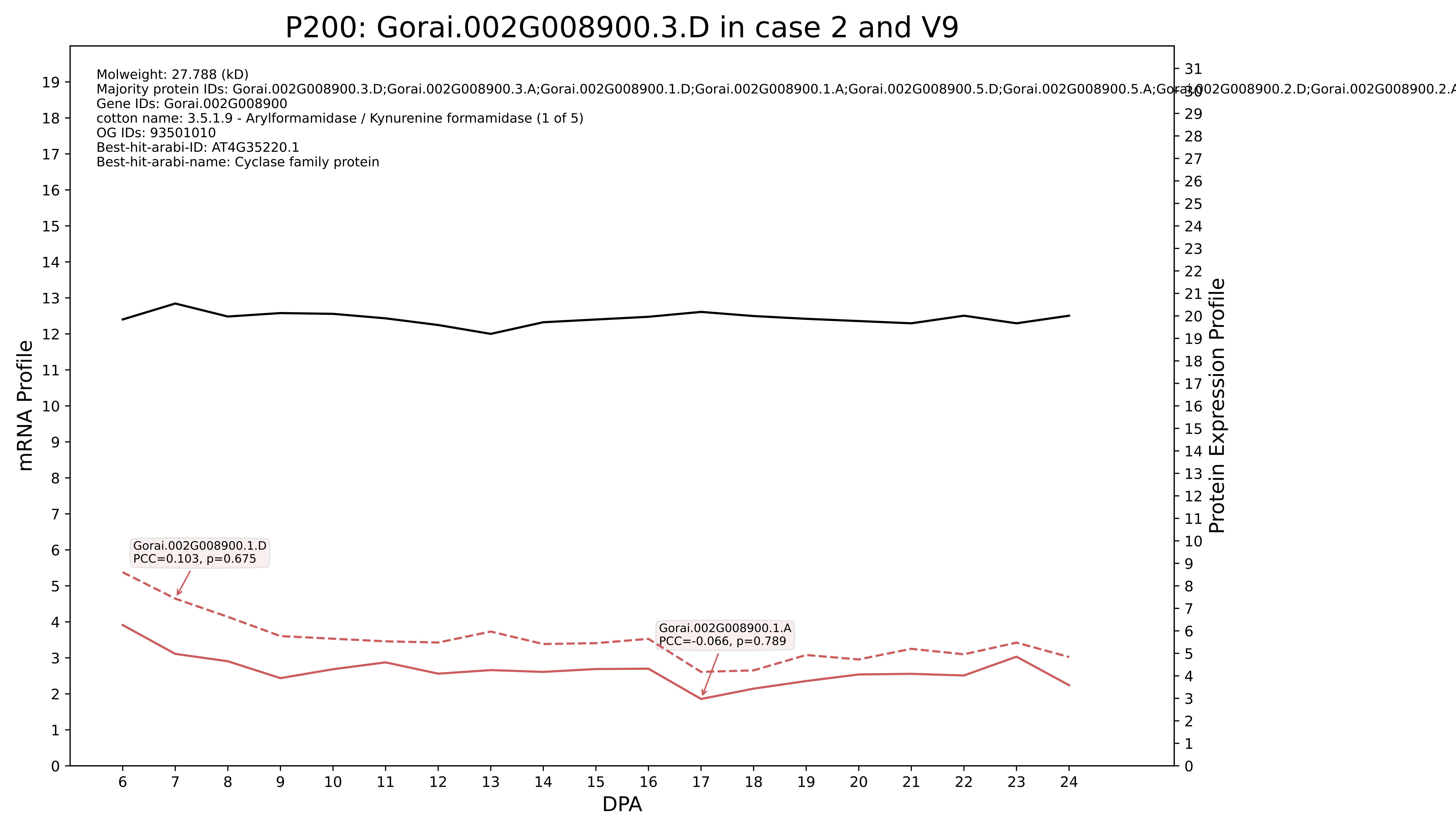
P200: Gorai.002G008900.3.D in case 2 and V9
Molweight: 27.788 (kD)
Majority protein IDs: Gorai.002G008900.3.D;Gorai.002G008900.3.A;Gorai.002G008900.1.D;Gorai.002G008900.1.A;Gorai.002G008900.5.D;Gorai.002G008900.5.A;Gorai.002G008900.2.D;Gorai.002G008900.2.A
Gene IDs: Gorai.002G008900
cotton name: 3.5.1.9 - Arylformamidase / Kynurenine formamidase (1 of 5)
OG IDs: 93501010
Best-hit-arabi-ID: AT4G35220.1
Best-hit-arabi-name: Cyclase family protein
Gorai.002G008900.1.D
PCC=0.103, p=0.675
Gorai.002G008900.1.A
PCC=-0.066, p=0.789
mRNA Profile
Protein Expression Profile
DPA

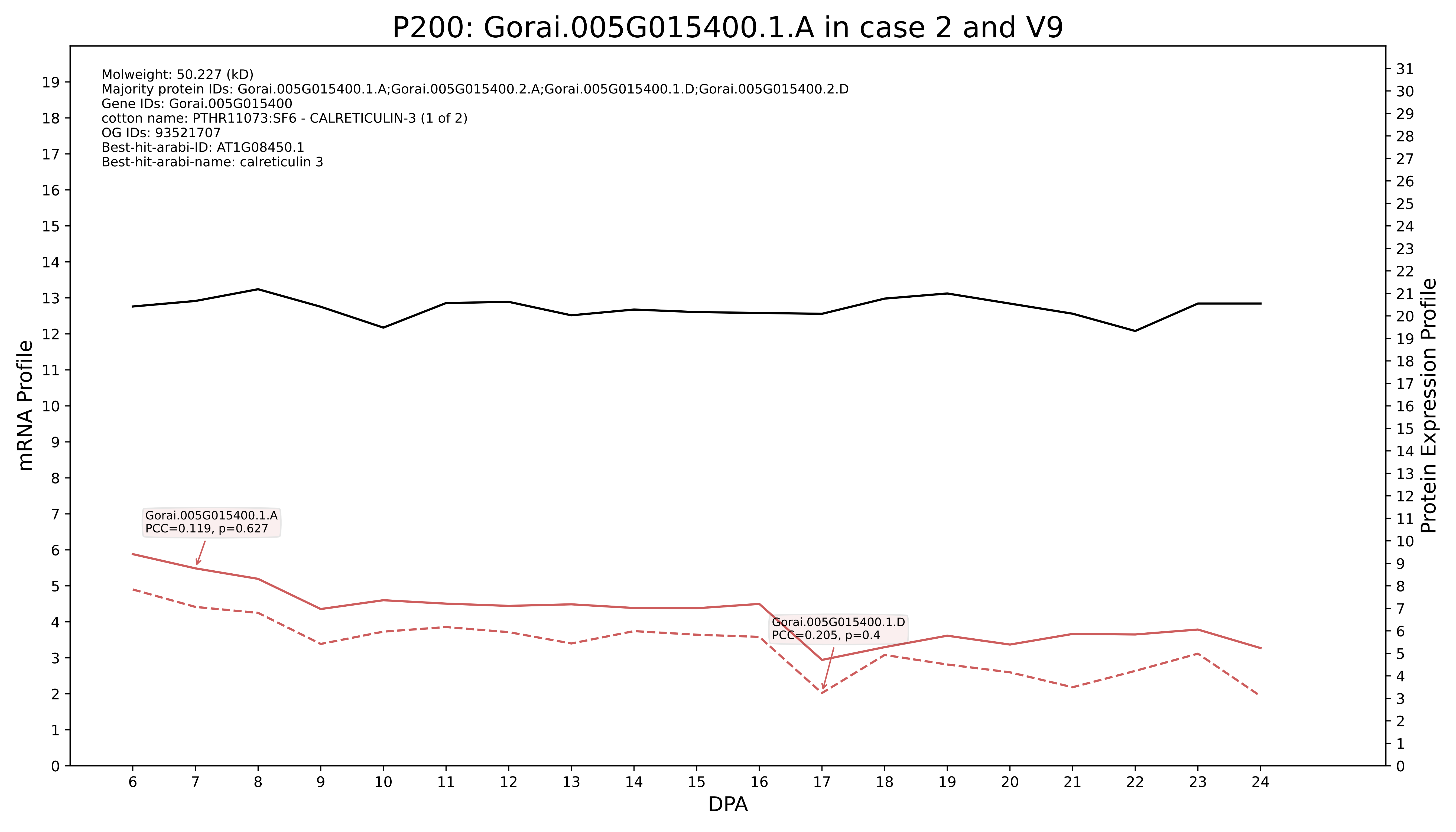

P200: Gorai.005G015400.1.A in case 2 and V9
Molweight: 50.227 (kD)
Majority protein IDs: Gorai.005G015400.1.A;Gorai.005G015400.2.A;Gorai.005G015400.1.D;Gorai.005G015400.2.D
Gene IDs: Gorai.005G015400
cotton name: PTHR11073:SF6 - CALRETICULIN-3 (1 of 2)
OG IDs: 93521707
Best-hit-arabi-ID: AT1G08450.1
Best-hit-arabi-name: calreticulin 3
Gorai.005G015400.1.A
PCC=0.119, p=0.627
Gorai.005G015400.1.D
PCC=0.205, p=0.4
mRNA Profile
Protein Expression Profile
DPA

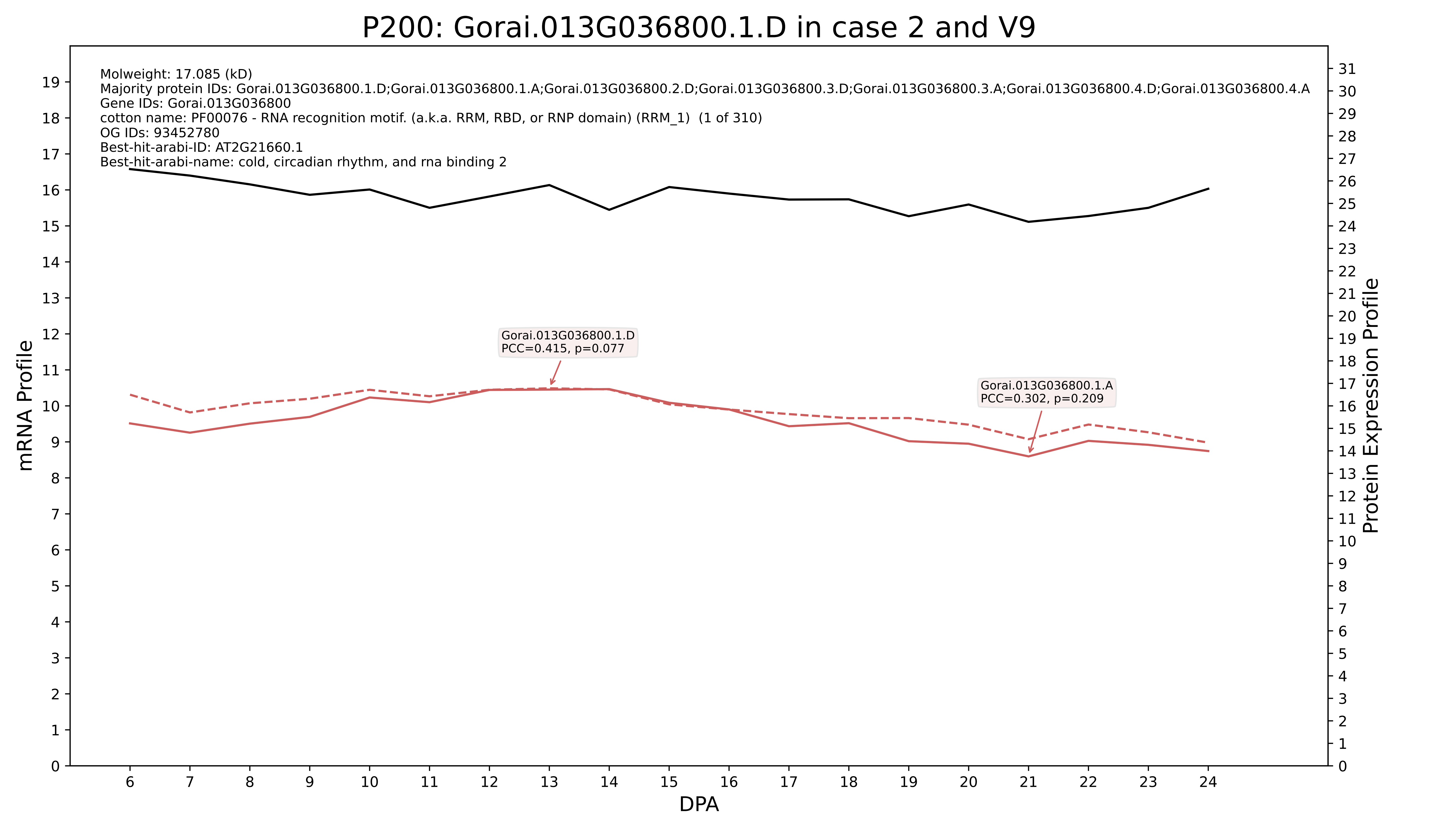
P200: Gorai.013G036800.1.D in case 2 and V9
Molweight: 17.085 (kD)
Majority protein IDs: Gorai.013G036800.1.D;Gorai.013G036800.1.A;Gorai.013G036800.2.D;Gorai.013G036800.3.D;Gorai.013G036800.3.A;Gorai.013G036800.4.D;Gorai.013G036800.4.A
Gene IDs: Gorai.013G036800
cotton name: PF00076 - RNA recognition motif. (a.k.a. RRM, RBD, or RNP domain) (RRM_1) (1 of 310)
OG IDs: 93452780
Best-hit-arabi-ID: AT2G21660.1
Best-hit-arabi-name: cold, circadian rhythm, and rna binding 2
Gorai.013G036800.1.D
PCC=0.415, p=0.077
Gorai.013G036800.1.A
PCC=0.302, p=0.209
mRNA Profile
Protein Expression Profile
DPA

P200: Gorai.005G184600.1.D in case 2 and V9

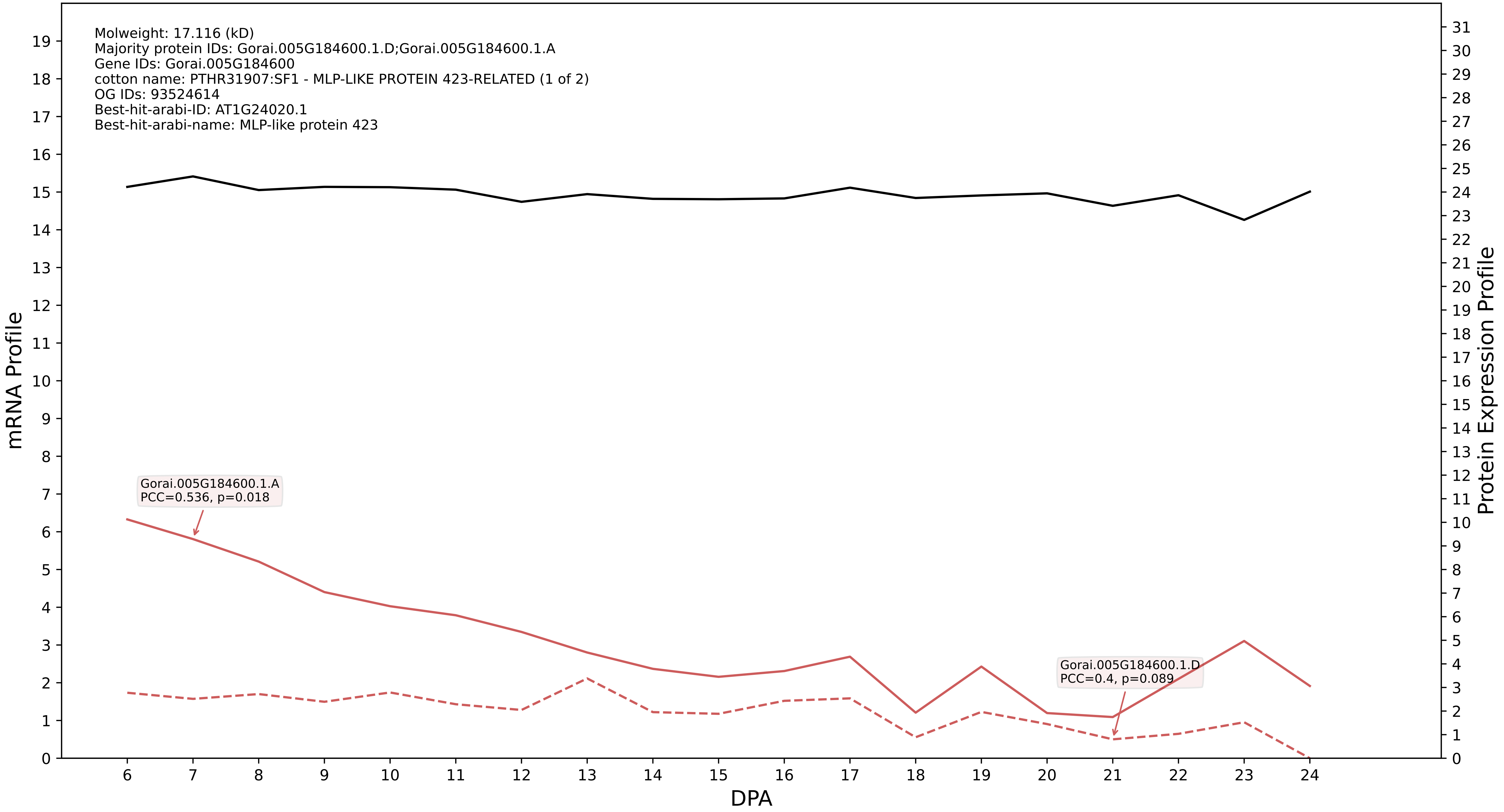

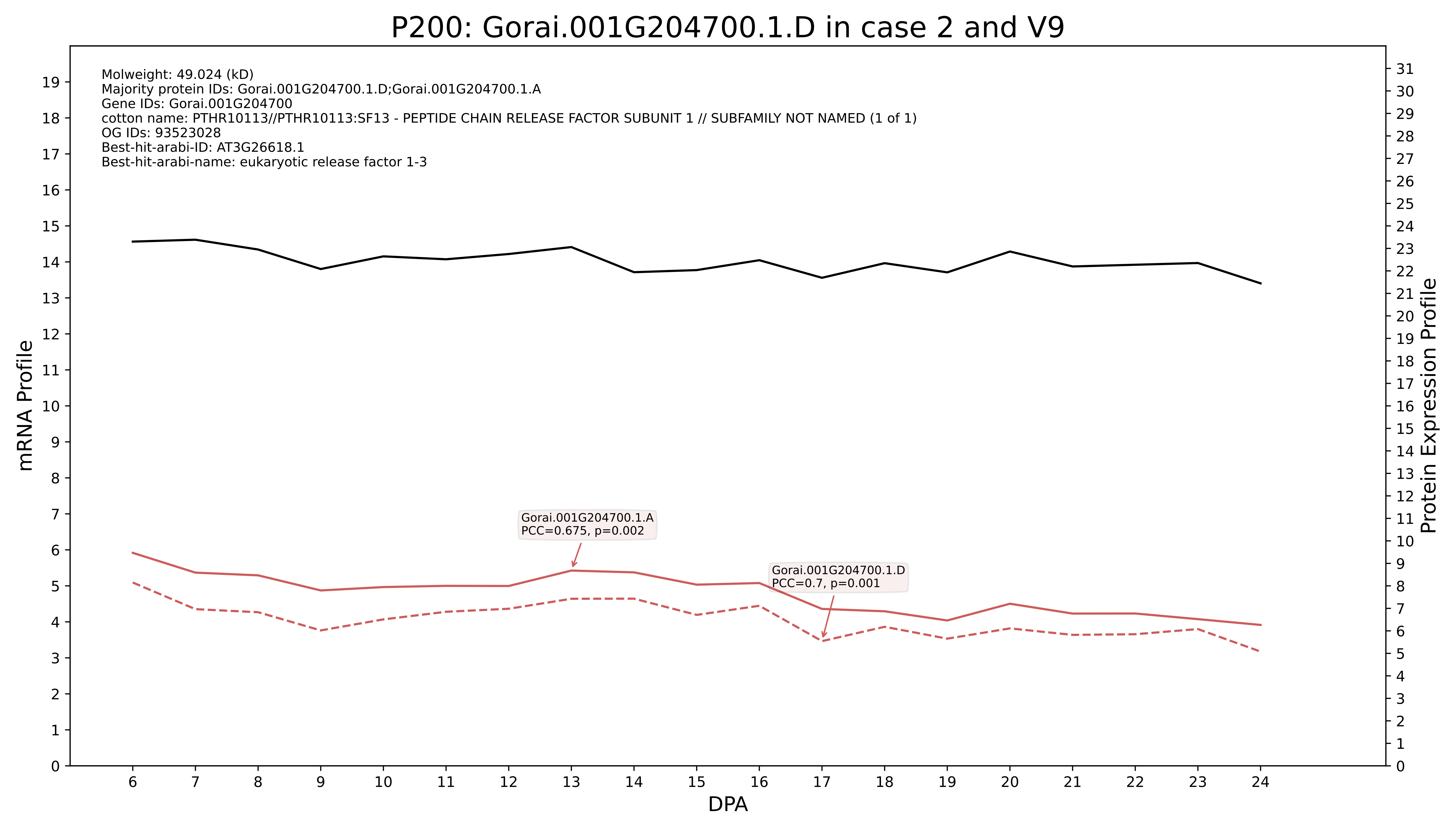
P200: Gorai.001G204700.1.D in case 2 and V9
Molweight: 49.024 (kD)
Majority protein IDs: Gorai.001G204700.1.D;Gorai.001G204700.1.A
Gene IDs: Gorai.001G204700
cotton name: PTHR10113//PTHR10113:SF13 - PEPTIDE CHAIN RELEASE FACTOR SUBUNIT 1 // SUBFAMILY NOT NAMED (1 of 1)
OG IDs: 93523028
Best-hit-arabi-ID: AT3G26618.1
Best-hit-arabi-name: eukaryotic release factor 1-3
Gorai.001G204700.1.A
PCC=0.675, p=0.002
Gorai.001G204700.1.D
PCC=0.7, p=0.001
mRNA Profile
Protein Expression Profile
DPA

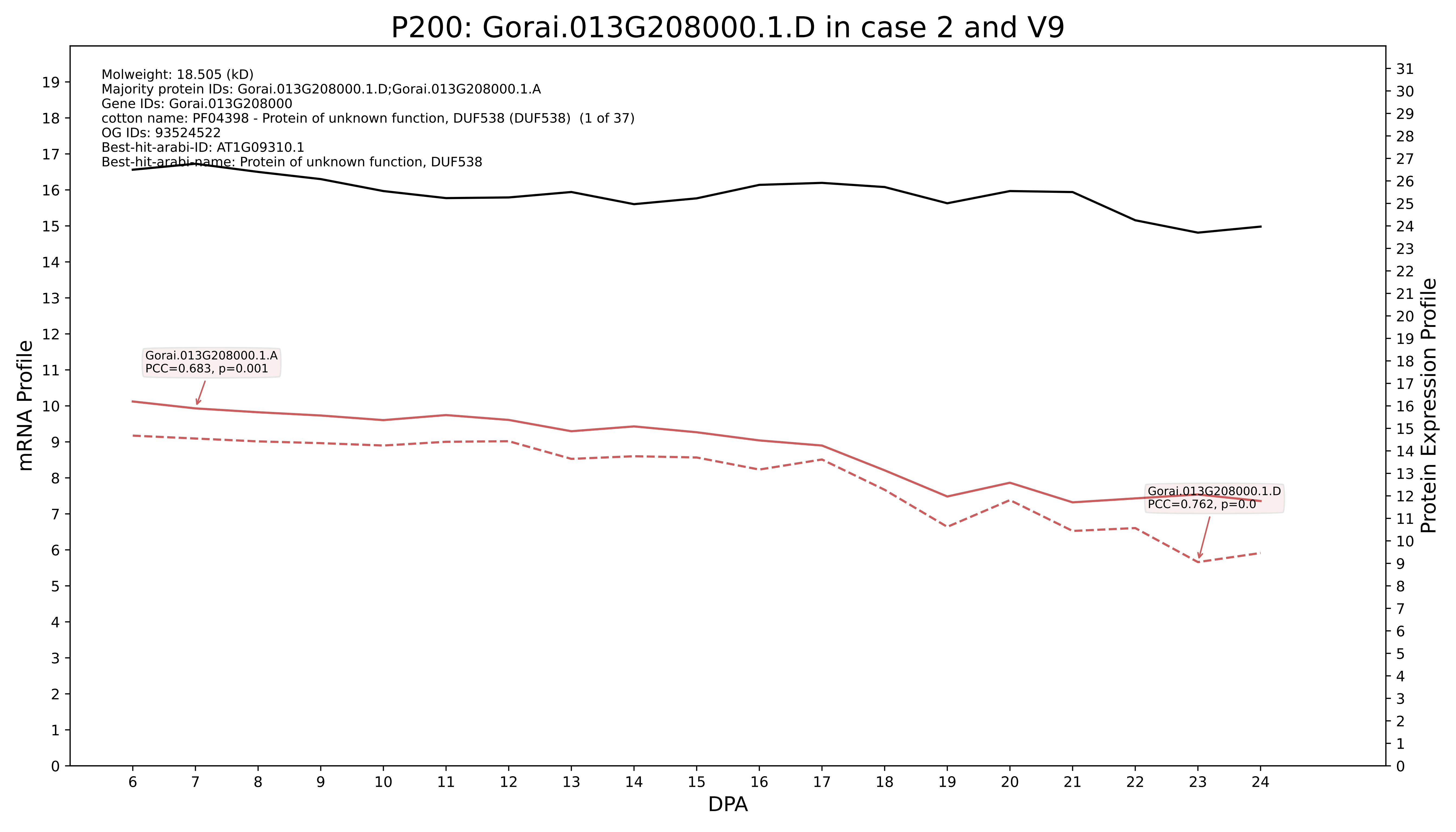
P200: Gorai.013G208000.1.D in case 2 and V9
Molweight: 18.505 (kD)
Majority protein IDs: Gorai.013G208000.1.D;Gorai.013G208000.1.A
Gene IDs: Gorai.013G208000
cotton name: PF04398 - Protein of unknown function, DUF538 (DUF538) (1 of 37)
OG IDs: 93524522
Best-hit-arabi-ID: AT1G09310.1
Best-hit-arabi-name: Protein of unknown function, DUF538
Gorai.013G208000.1.A
PCC=0.683, p=0.001
Gorai.013G208000.1.D
PCC=0.762, p=0.0
mRNA Profile
Protein Expression Profile
DPA

P200: Gorai.009G334400.1.A in case 2 and V9

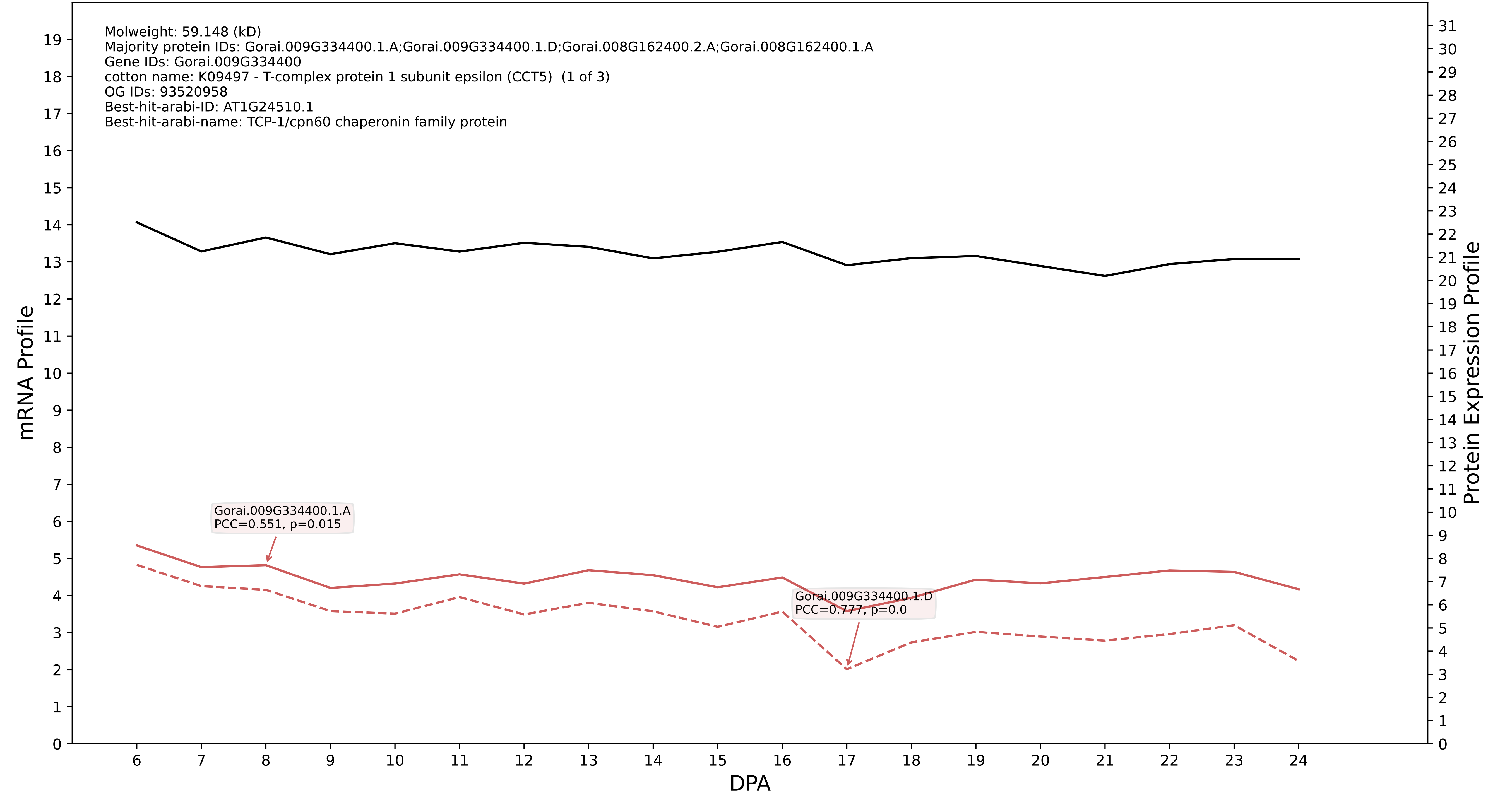

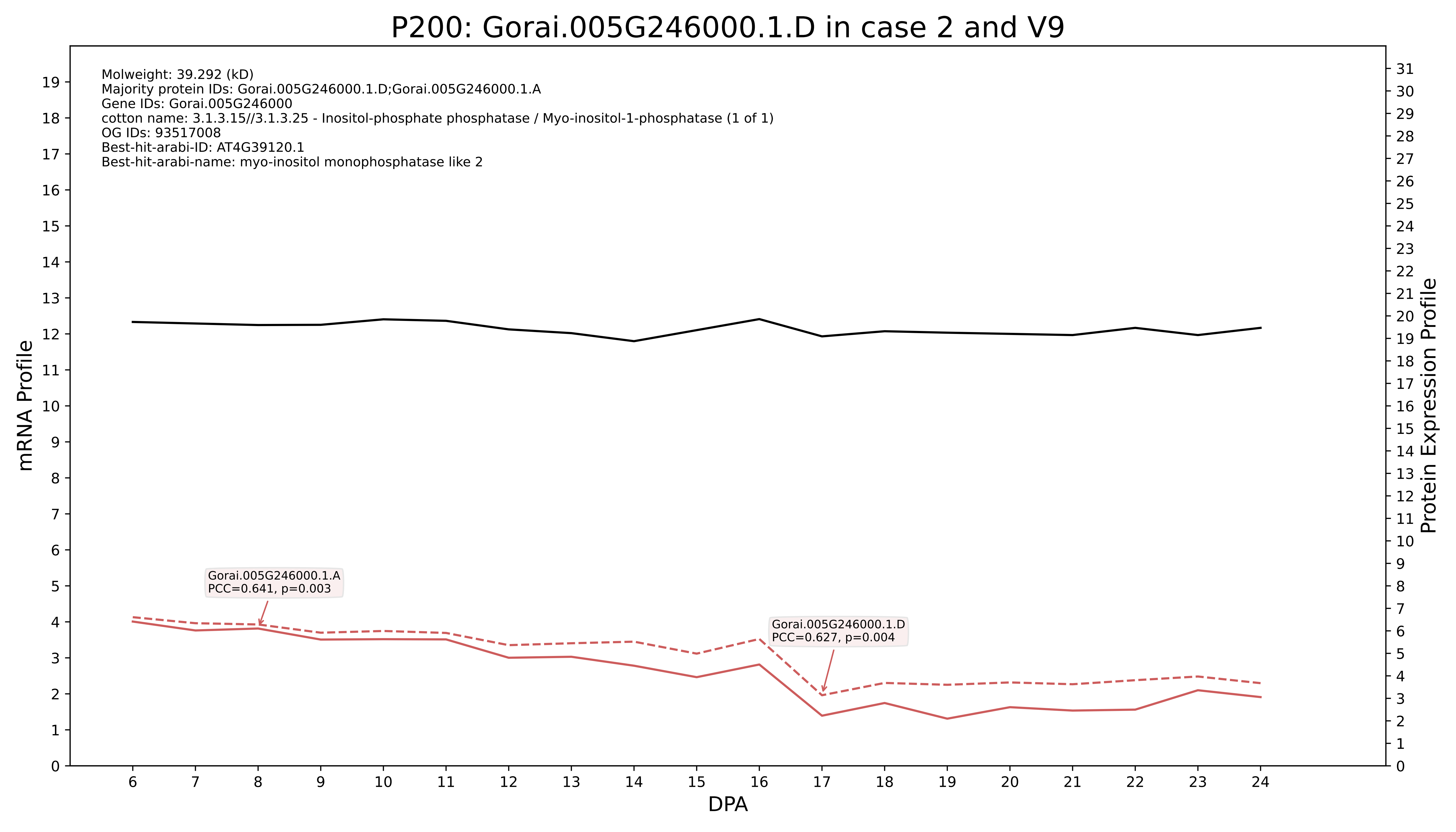

P200: Gorai.005G246000.1.D in case 2 and V9
Molweight: 39.292 (kD)
Majority protein IDs: Gorai.005G246000.1.D;Gorai.005G246000.1.A
Gene IDs: Gorai.005G246000
cotton name: 3.1.3.15//3.1.3.25 - Inositol-phosphate phosphatase / Myo-inositol-1-phosphatase (1 of 1)
OG IDs: 93517008
Best-hit-arabi-ID: AT4G39120.1
Best-hit-arabi-name: myo-inositol monophosphatase like 2
Gorai.005G246000.1.A
PCC=0.641, p=0.003
Gorai.005G246000.1.D
PCC=0.627, p=0.004
mRNA Profile
Protein Expression Profile
DPA

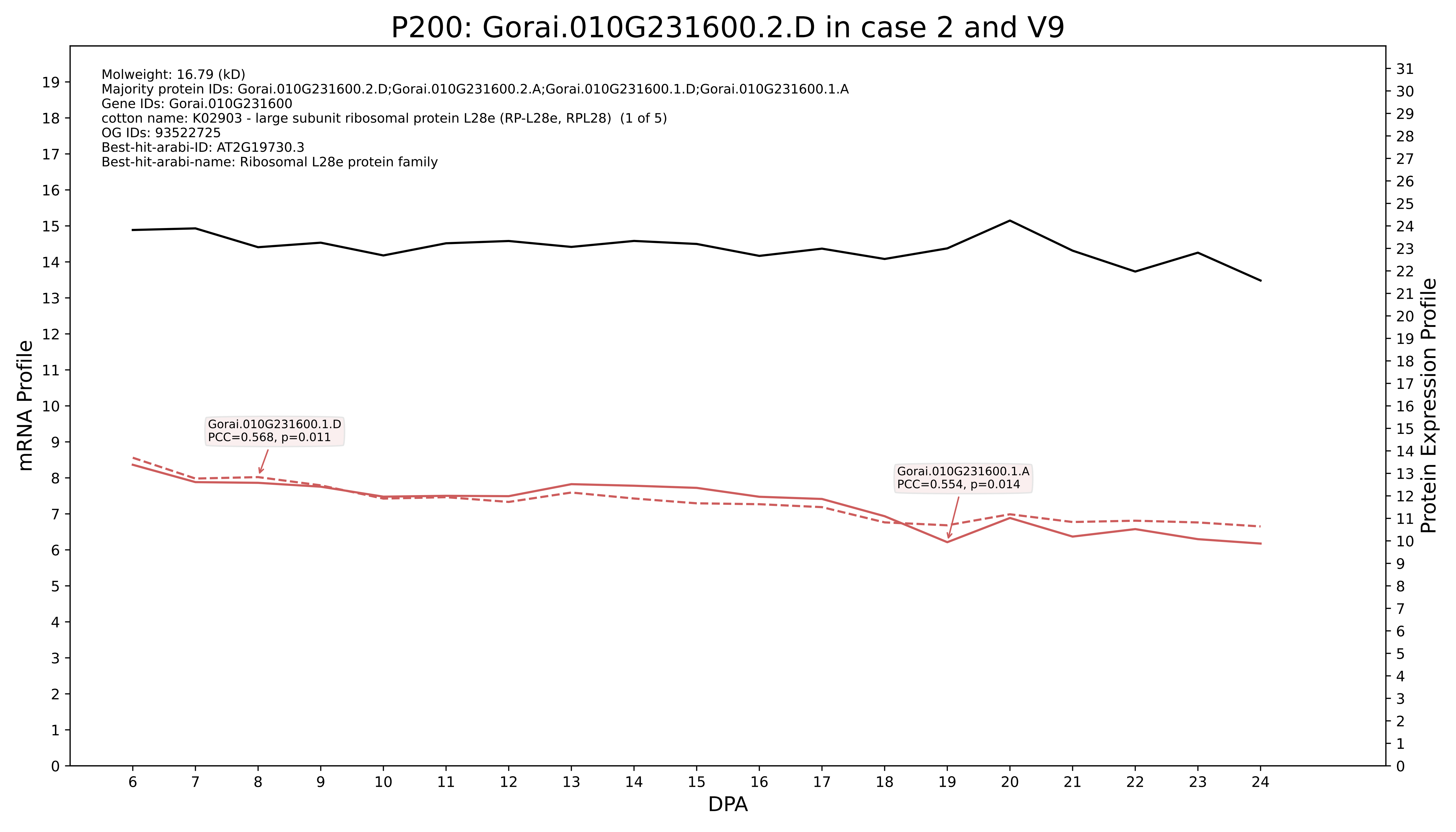
P200: Gorai.010G231600.2.D in case 2 and V9
Molweight: 16.79 (kD)
Majority protein IDs: Gorai.010G231600.2.D;Gorai.010G231600.2.A;Gorai.010G231600.1.D;Gorai.010G231600.1.A
Gene IDs: Gorai.010G231600
cotton name: K02903 - large subunit ribosomal protein L28e (RP-L28e, RPL28) (1 of 5)
OG IDs: 93522725
Best-hit-arabi-ID: AT2G19730.3
Best-hit-arabi-name: Ribosomal L28e protein family
Gorai.010G231600.1.D
PCC=0.568, p=0.011
Gorai.010G231600.1.A
PCC=0.554, p=0.014
mRNA Profile
Protein Expression Profile
DPA
0
1
2
3
4
5
6
7
8
9
10
11
12
13
14
15
16
17
18
19
20
21
22
23
24
25
26
27
28
29
30
31

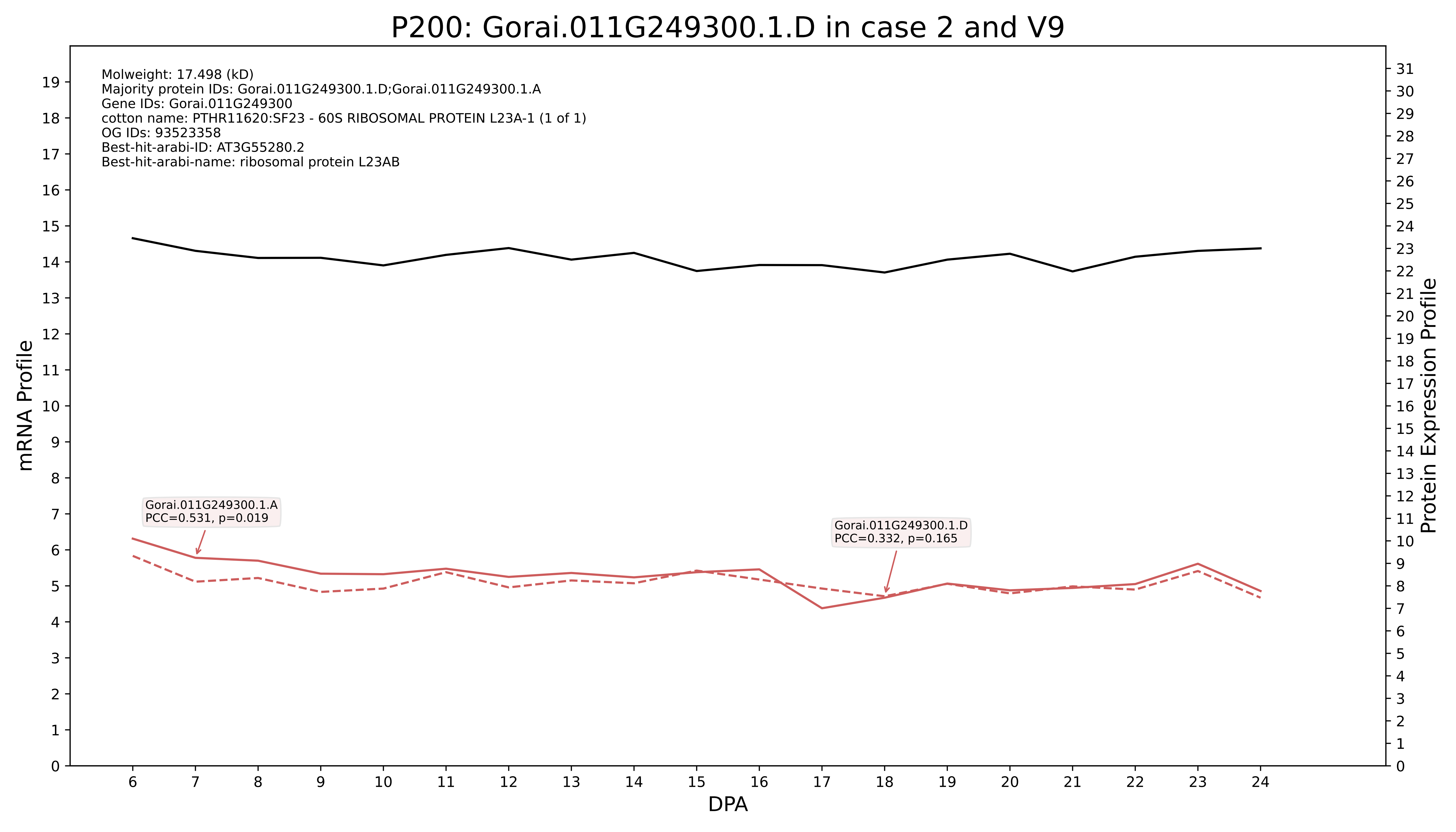
P200: Gorai.011G249300.1.D in case 2 and V9
Molweight: 17.498 (kD)
Majority protein IDs: Gorai.011G249300.1.D;Gorai.011G249300.1.A
Gene IDs: Gorai.011G249300
cotton name: PTHR11620:SF23 - 60S RIBOSOMAL PROTEIN L23A-1 (1 of 1)
OG IDs: 93523358
Best-hit-arabi-ID: AT3G55280.2
Best-hit-arabi-name: ribosomal protein L23AB
Gorai.011G249300.1.A
PCC=0.531, p=0.019
Gorai.011G249300.1.D
PCC=0.332, p=0.165
mRNA Profile
Protein Expression Profile
DPA

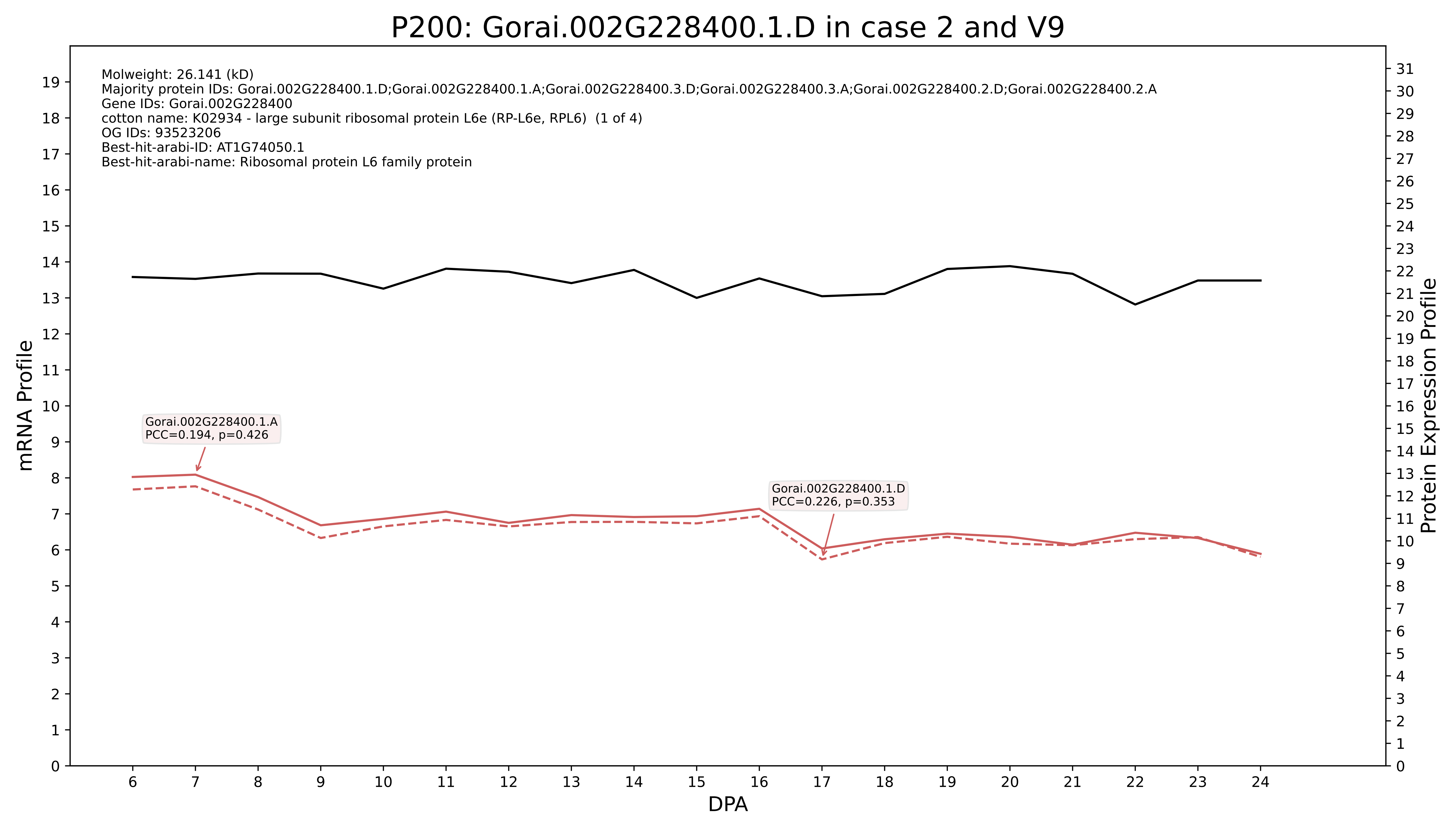

P200: Gorai.002G228400.1.D in case 2 and V9
Molweight: 26.141 (kD)
Majority protein IDs: Gorai.002G228400.1.D;Gorai.002G228400.1.A;Gorai.002G228400.3.D;Gorai.002G228400.3.A;Gorai.002G228400.2.D;Gorai.002G228400.2.A
Gene IDs: Gorai.002G228400
cotton name: K02934 - large subunit ribosomal protein L6e (RP-L6e, RPL6) (1 of 4)
OG IDs: 93523206
Best-hit-arabi-ID: AT1G74050.1
Best-hit-arabi-name: Ribosomal protein L6 family protein
Gorai.002G228400.1.A
PCC=0.194, p=0.426
Gorai.002G228400.1.D
PCC=0.226, p=0.353
mRNA Profile
Protein Expression Profile
DPA

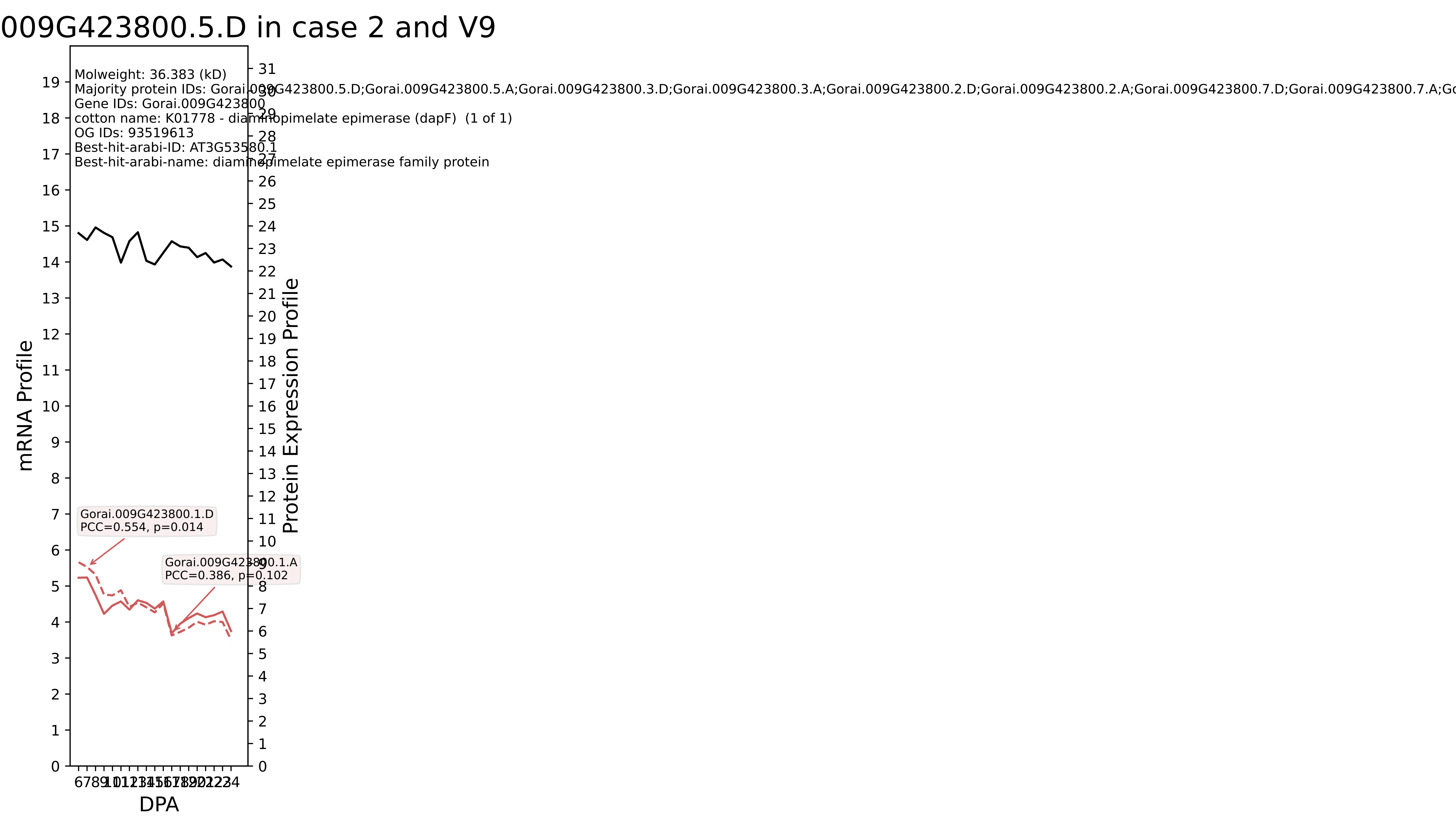
009G423800.5.D in case 2 and V9
Molweight: 36.383 (kD)
Majority protein IDs: Gorai.009G423800.5.D;Gorai.009G423800.5.A;Gorai.009G423800.3.D;Gorai.009G423800.3.A;Gorai.009G423800.2.D;Gorai.009G423800.2.A;Gorai.009G423800.7.D;Gorai.009G423800.7.A;Go
Gene IDs: Gorai.009G423800
cotton name: K01778 - diaminopimelate epimerase (dapF) (1 of 1)
OG IDs: 93519613
Best-hit-arabi-ID: AT3G53580.1
Best-hit-arabi-name: diaminopimelate epimerase family protein
Gorai.009G423800.1.D
PCC=0.554, p=0.014
Gorai.009G423800.1.A
PCC=0.386, p=0.102
mRNA Profile
Protein Expression Profile
DPA

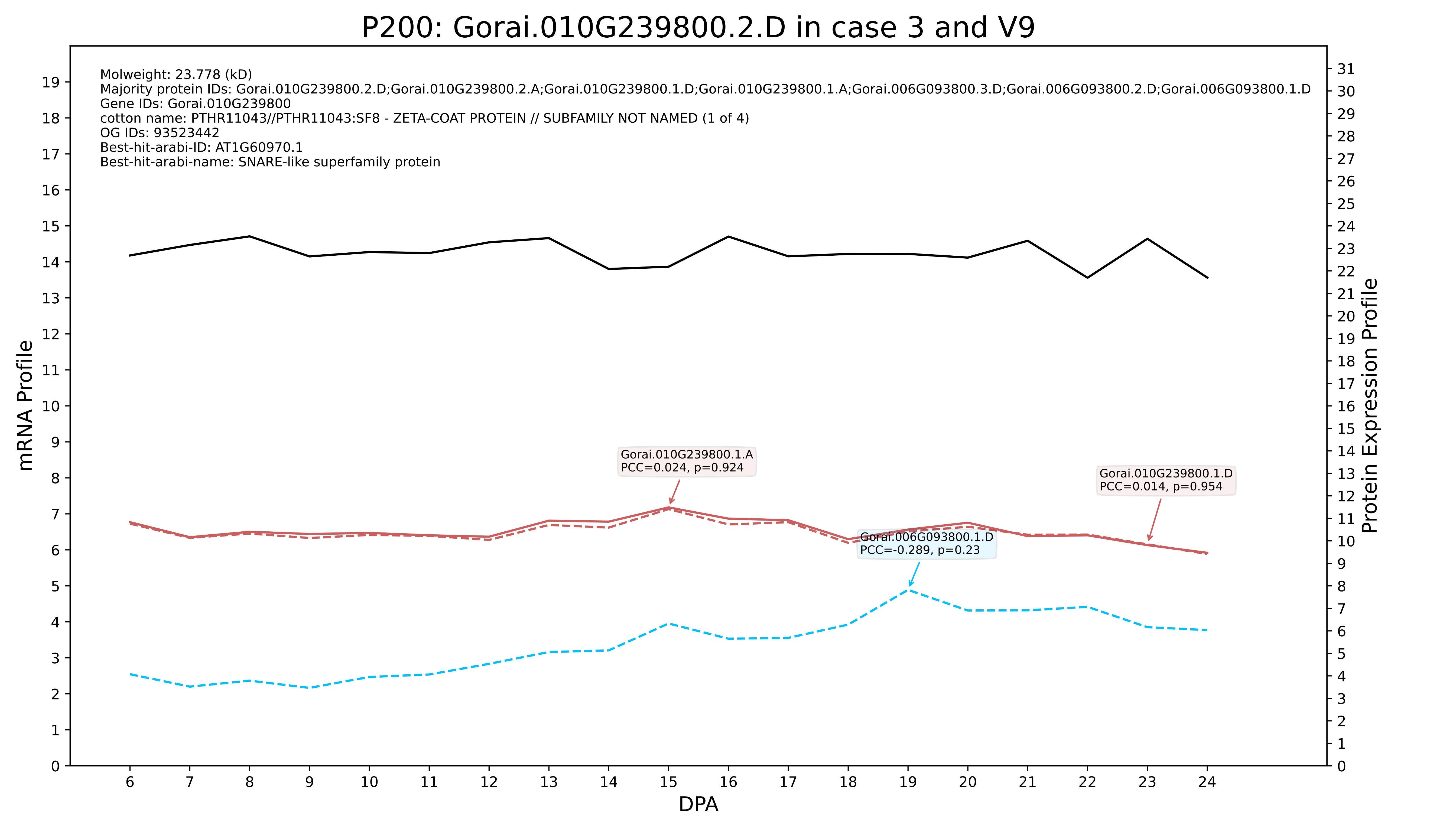
P200: Gorai.010G239800.2.D in case 3 and V9
Molweight: 23.778 (kD)
Majority protein IDs: Gorai.010G239800.2.D;Gorai.010G239800.2.A;Gorai.010G239800.1.D;Gorai.010G239800.1.A;Gorai.006G093800.3.D;Gorai.006G093800.2.D;Gorai.006G093800.1.D
Gene IDs: Gorai.010G239800
cotton name: PTHR11043//PTHR11043:SF8 - ZETA-COAT PROTEIN // SUBFAMILY NOT NAMED (1 of 4)
OG IDs: 93523442
Best-hit-arabi-ID: AT1G60970.1
Best-hit-arabi-name: SNARE-like superfamily protein
Gorai.010G239800.1.A
PCC=0.024, p=0.924
Gorai.010G239800.1.D
PCC=0.014, p=0.954
Gorai.006G093800.1.D
PCC=-0.289, p=0.23
mRNA Profile
Protein Expression Profile
DPA

200: Gorai.010G017700.2.D in case 3 and V9

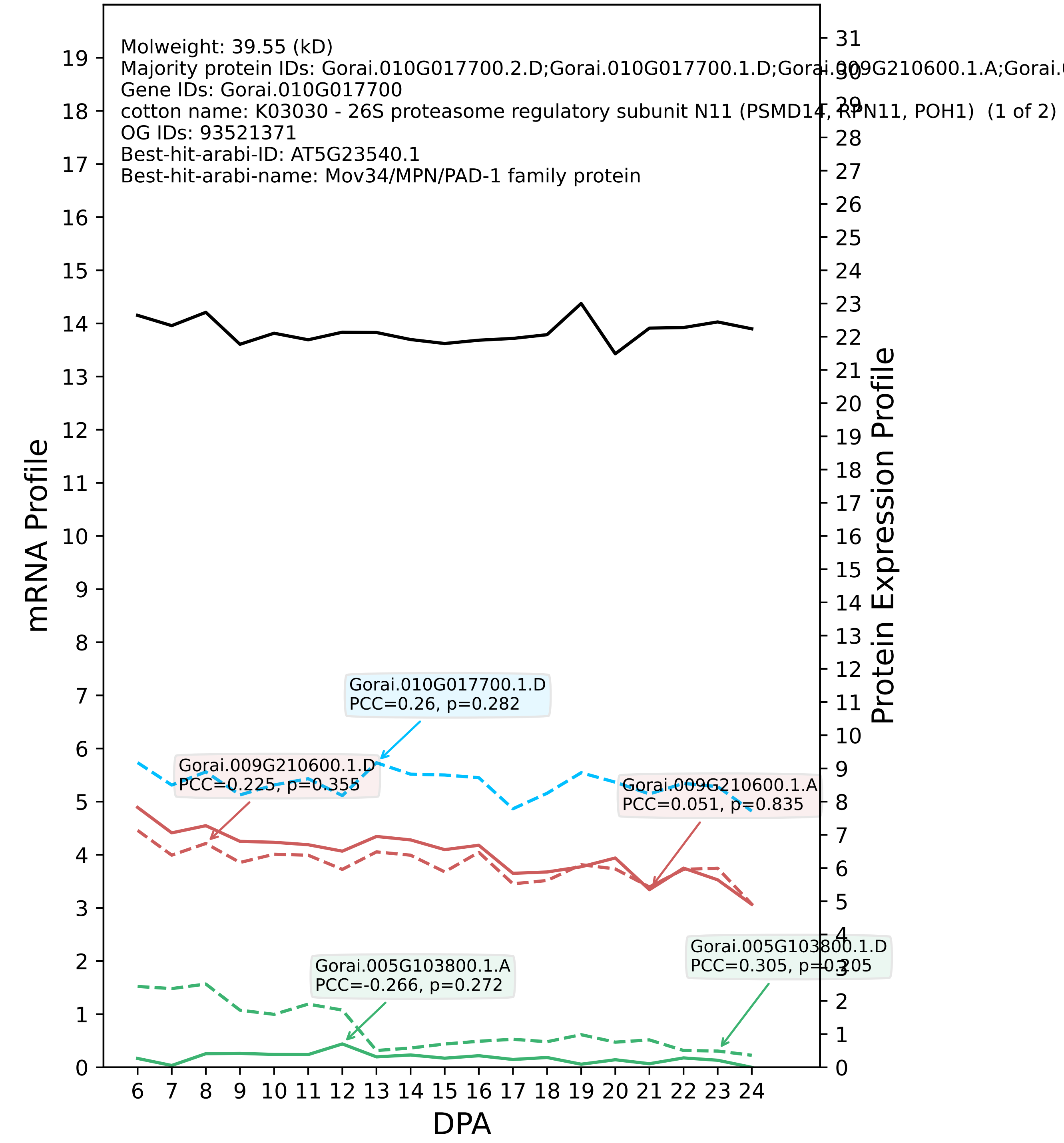

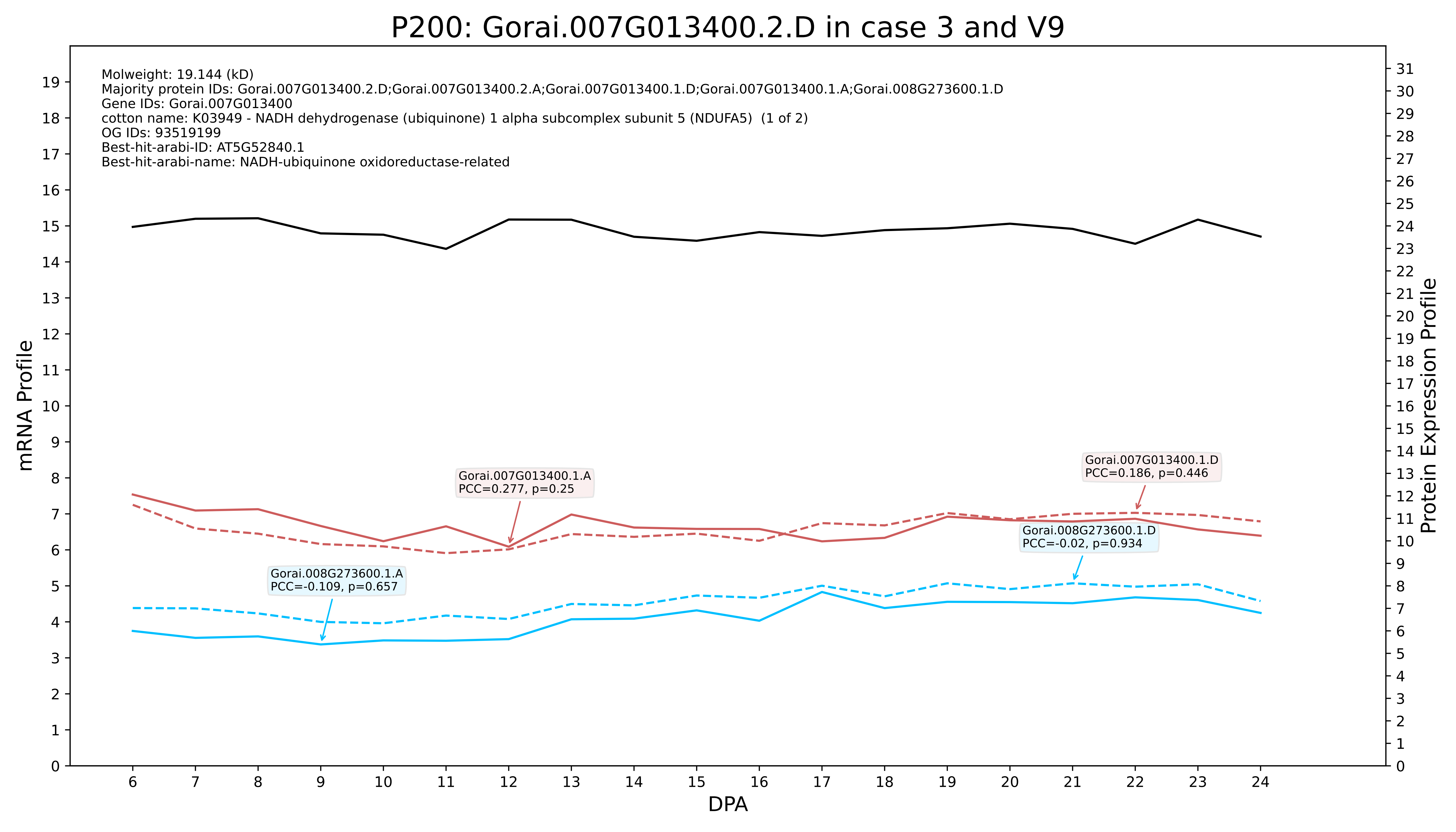
P200: Gorai.007G013400.2.D in case 3 and V9
Molweight: 19.144 (kD)
Majority protein IDs: Gorai.007G013400.2.D;Gorai.007G013400.2.A;Gorai.007G013400.1.D;Gorai.007G013400.1.A;Gorai.008G273600.1.D
Gene IDs: Gorai.007G013400
cotton name: K03949 - NADH dehydrogenase (ubiquinone) 1 alpha subcomplex subunit 5 (NDUFA5) (1 of 2)
OG IDs: 93519199
Best-hit-arabi-ID: AT5G52840.1
Best-hit-arabi-name: NADH-ubiquinone oxidoreductase-related
Gorai.007G013400.1.A
PCC=0.277, p=0.25
Gorai.007G013400.1.D
PCC=0.186, p=0.446
Gorai.008G273600.1.D
PCC=-0.02, p=0.934
Gorai.008G273600.1.A
PCC=-0.109, p=0.657
mRNA Profile
Protein Expression Profile
DPA

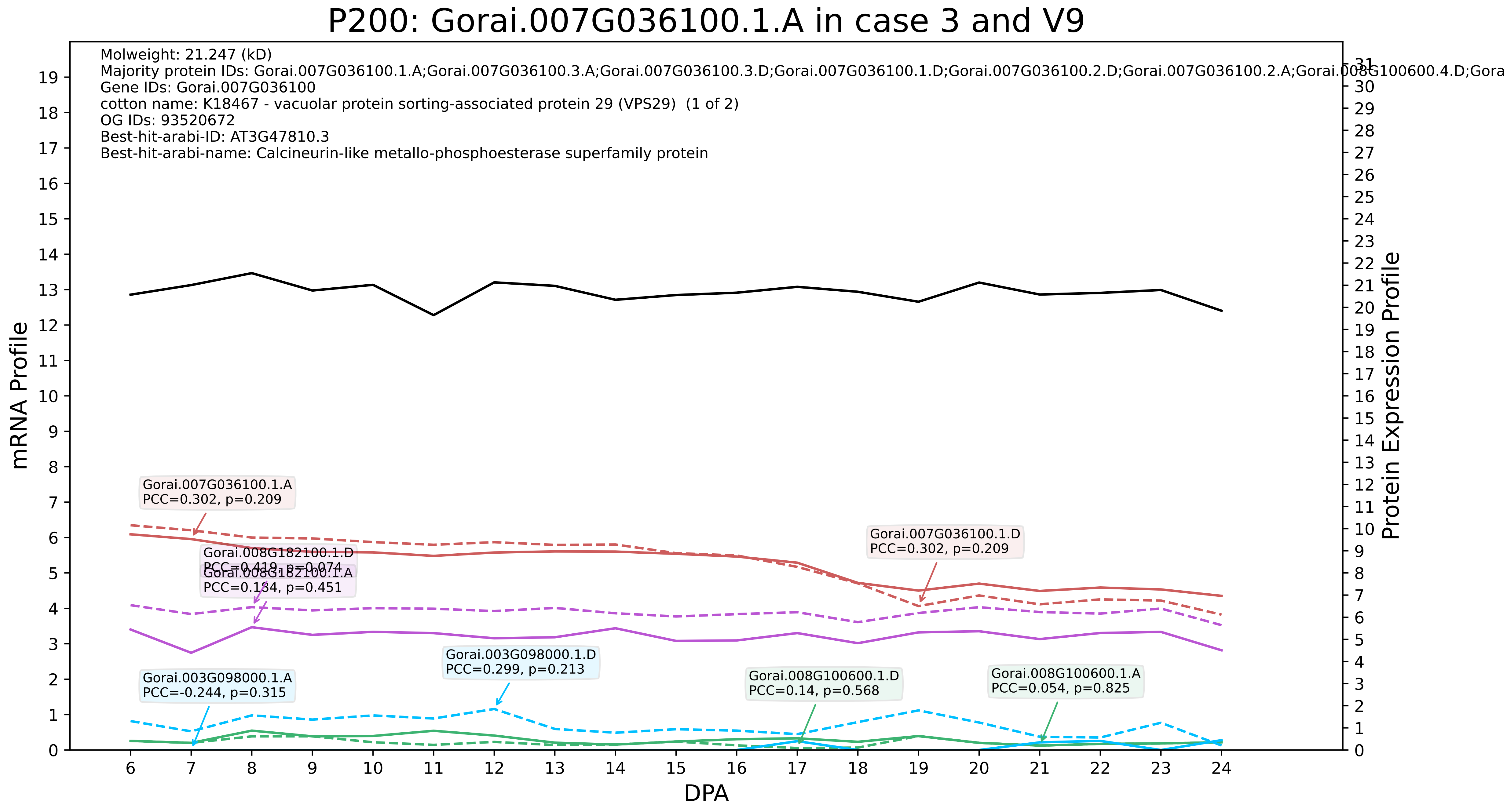
P200: Gorai.007G036100.1.A in case 3 and V9
Molweight: 21.247 (kD)
Majority protein IDs: Gorai.007G036100.1.A;Gorai.007G036100.3.A;Gorai.007G036100.3.D;Gorai.007G036100.1.D;Gorai.007G036100.2.D;Gorai.007G036100.2.A;Gorai.008G100600.4.D;Gorai
Gene IDs: Gorai.007G036100
cotton name: K18467 - vacuolar protein sorting-associated protein 29 (VPS29) (1 of 2)
OG IDs: 93520672
Best-hit-arabi-ID: AT3G47810.3
Best-hit-arabi-name: Calcineurin-like metallo-phosphoesterase superfamily protein
Gorai.007G036100.1.A
PCC=0.302, p=0.209
Gorai.007G036100.1.D
PCC=0.302, p=0.209
Gorai.008G182100.1.D
PCC=0.419, p=0.074
Gorai.008G182100.1.A
PCC=0.184, p=0.451
Gorai.003G098000.1.D
PCC=0.299, p=0.213
Gorai.003G098000.1.A
PCC=-0.244, p=0.315
Gorai.008G100600.1.D
PCC=0.14, p=0.568
Gorai.008G100600.1.A
PCC=0.054, p=0.825
mRNA Profile
Protein Expression Profile
DPA

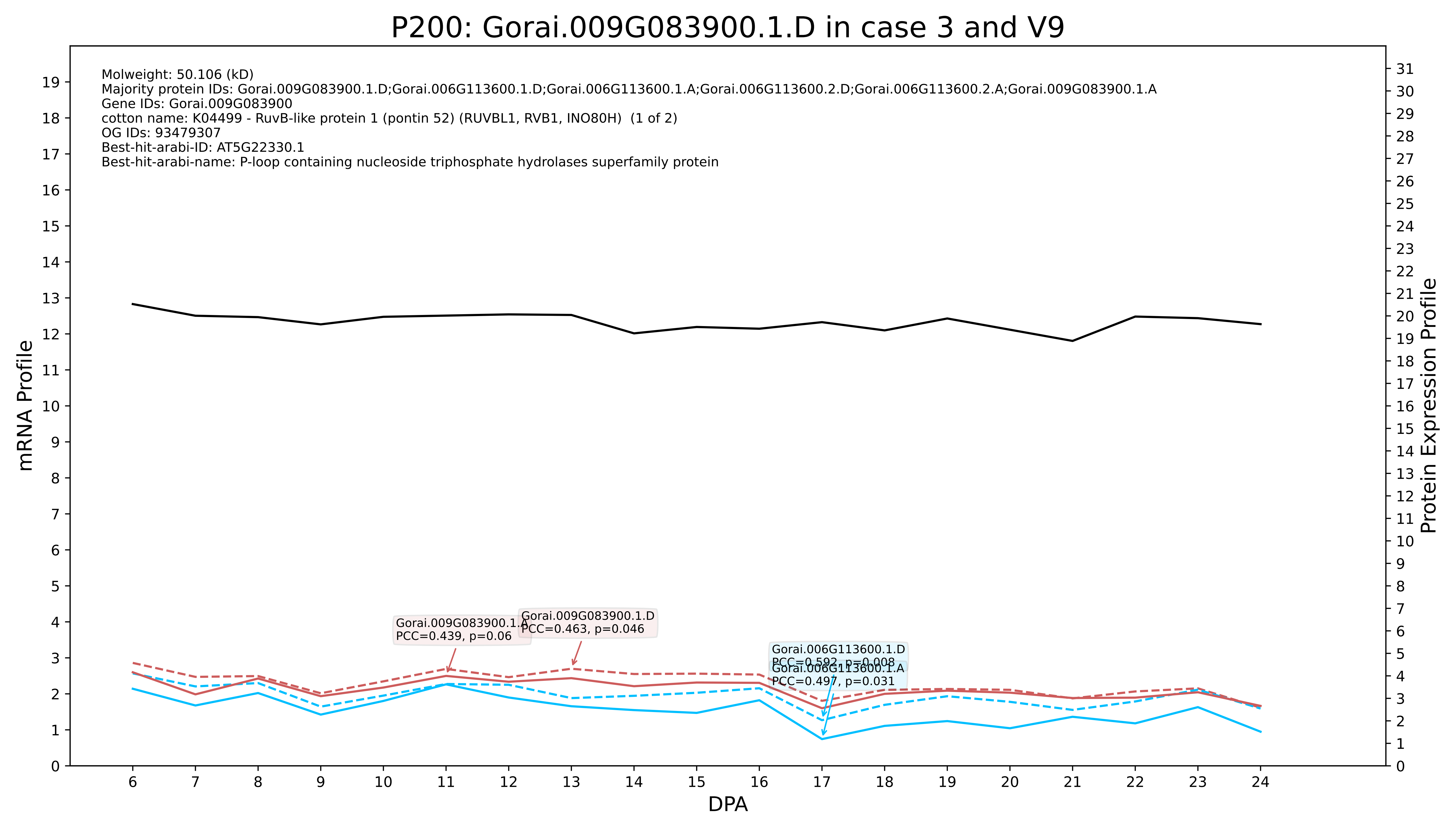
P200: Gorai.009G083900.1.D in case 3 and V9
Molweight: 50.106 (kD)
Majority protein IDs: Gorai.009G083900.1.D;Gorai.006G113600.1.D;Gorai.006G113600.1.A;Gorai.006G113600.2.D;Gorai.006G113600.2.A;Gorai.009G083900.1.A
Gene IDs: Gorai.009G083900
cotton name: K04499 - RuvB-like protein 1 (pontin 52) (RUVBL1, RVB1, INO80H) (1 of 2)
OG IDs: 93479307
Best-hit-arabi-ID: AT5G22330.1
Best-hit-arabi-name: P-loop containing nucleoside triphosphate hydrolases superfamily protein
Gorai.009G083900.1.A
PCC=0.439, p=0.06
Gorai.009G083900.1.D
PCC=0.463, p=0.046
Gorai.006G113600.1.D
PCC=0.592, p=0.008
Gorai.006G113600.1.A
PCC=0.497, p=0.031
mRNA Profile
Protein Expression Profile
DPA

P200: Gorai.006G246500.1.A in case 3 and V9

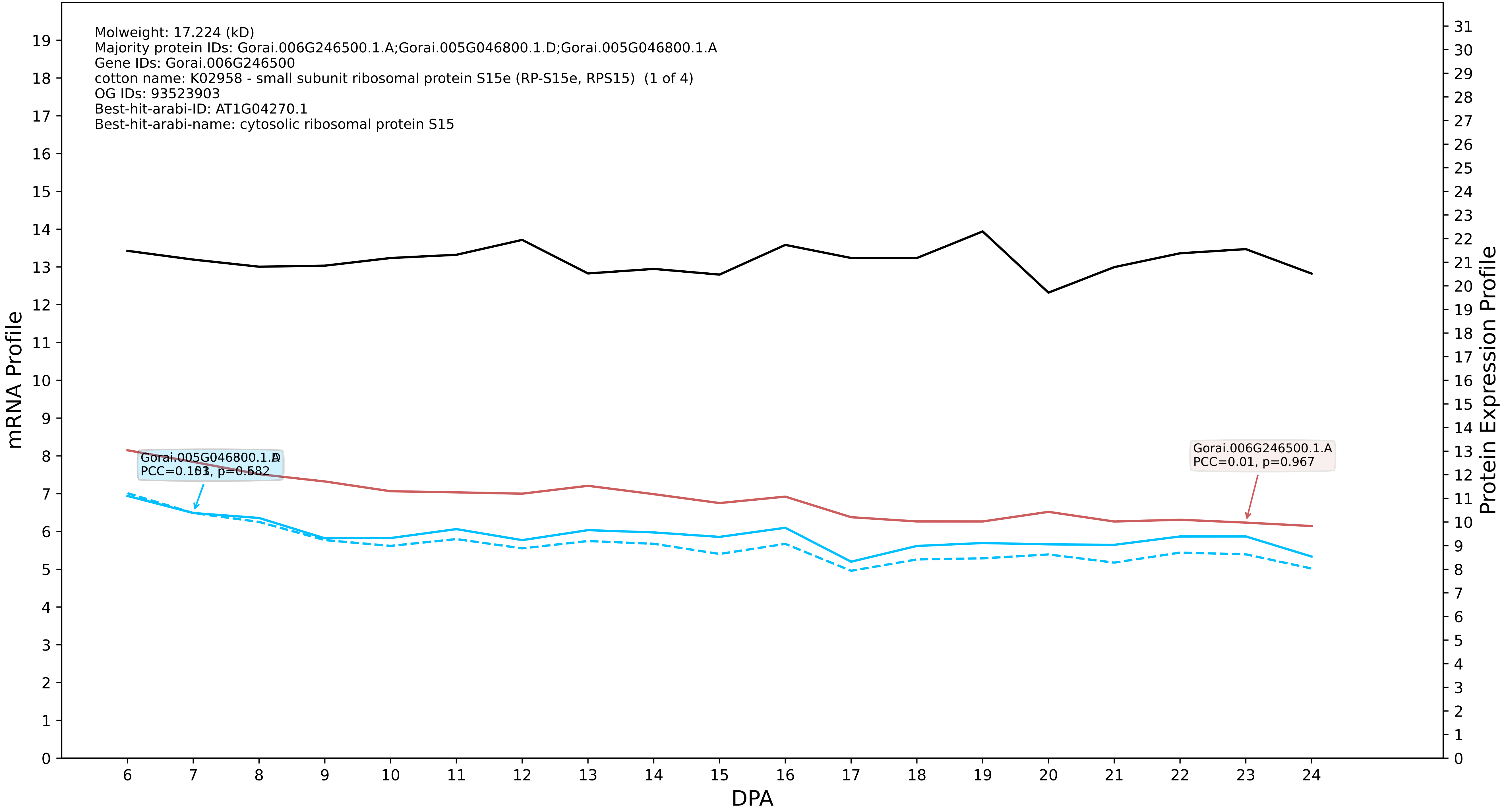

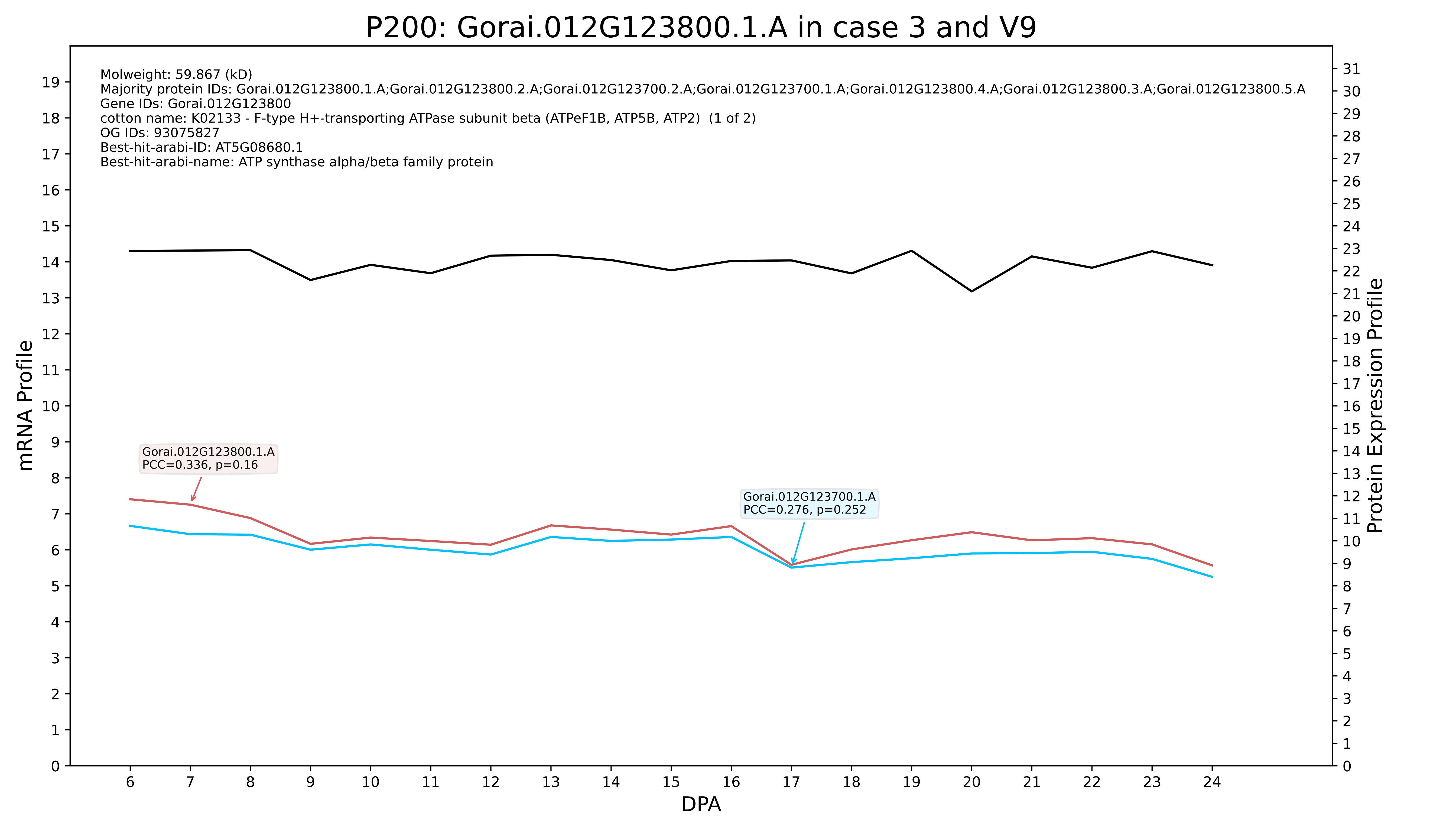

P200: Gorai.012G123800.1.A in case 3 and V9
Molweight: 59.867 (kD)
Majority protein IDs: Gorai.012G123800.1.A;Gorai.012G123800.2.A;Gorai.012G123700.2.A;Gorai.012G123700.1.A;Gorai.012G123800.4.A;Gorai.012G123800.3.A;Gorai.012G123800.5.A
Gene IDs: Gorai.012G123800
cotton name: K02133 - F-type H+-transporting ATPase subunit beta (ATPeF1B, ATP5B, ATP2) (1 of 2)
OG IDs: 93075827
Best-hit-arabi-ID: AT5G08680.1
Best-hit-arabi-name: ATP synthase alpha/beta family protein
Gorai.012G123800.1.A
PCC=0.336, p=0.16
Gorai.012G123700.1.A
PCC=0.276, p=0.252
mRNA Profile
Protein Expression Profile
DPA

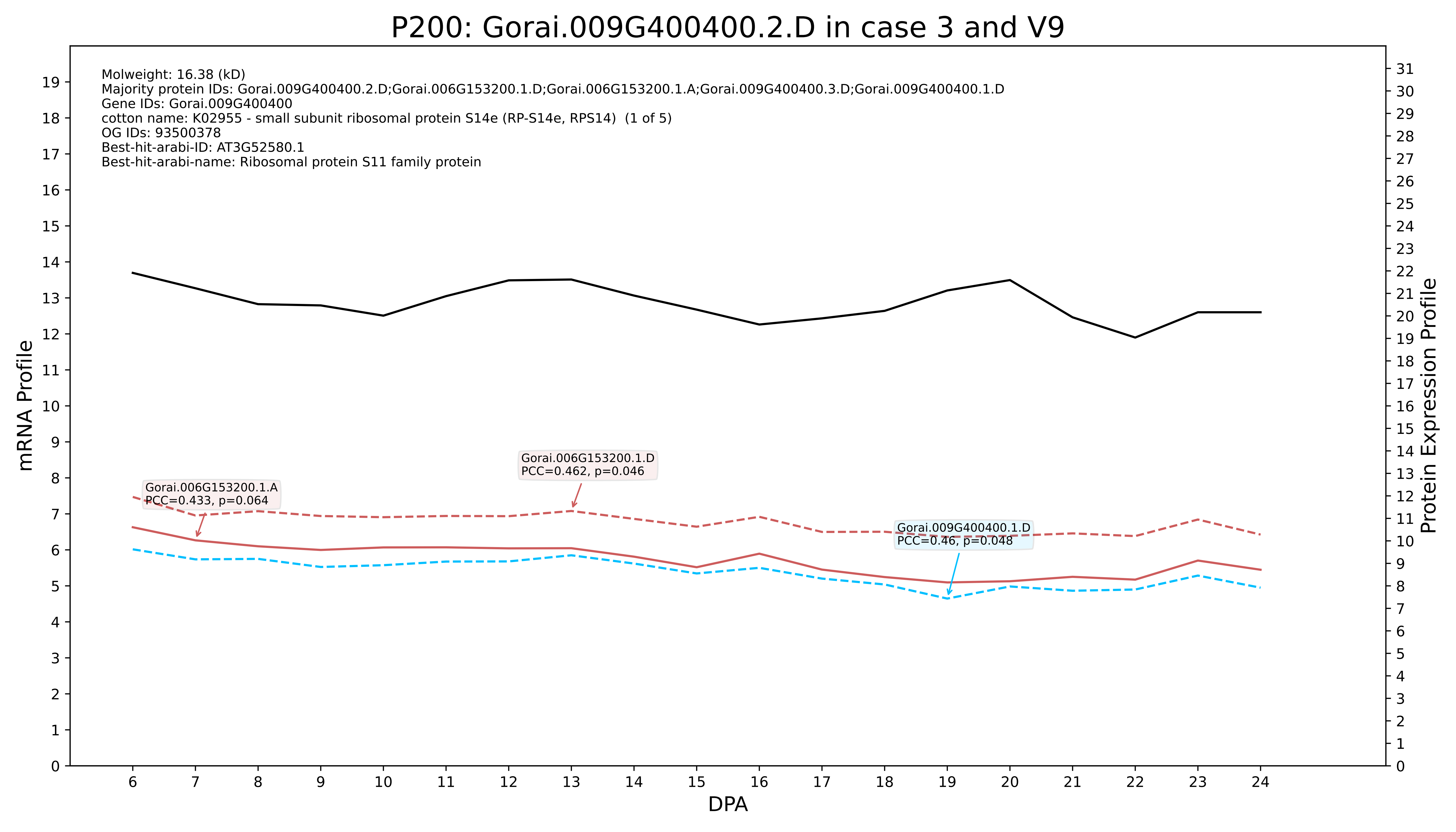

P200: Gorai.009G400400.2.D in case 3 and V9
Molweight: 16.38 (kD)
Majority protein IDs: Gorai.009G400400.2.D;Gorai.006G153200.1.D;Gorai.006G153200.1.A;Gorai.009G400400.3.D;Gorai.009G400400.1.D
Gene IDs: Gorai.009G400400
cotton name: K02955 - small subunit ribosomal protein S14e (RP-S14e, RPS14) (1 of 5)
OG IDs: 93500378
Best-hit-arabi-ID: AT3G52580.1
Best-hit-arabi-name: Ribosomal protein S11 family protein
Gorai.006G153200.1.A
PCC=0.433, p=0.064
Gorai.006G153200.1.D
PCC=0.462, p=0.046
Gorai.009G400400.1.D
PCC=0.46, p=0.048
mRNA Profile
Protein Expression Profile
DPA

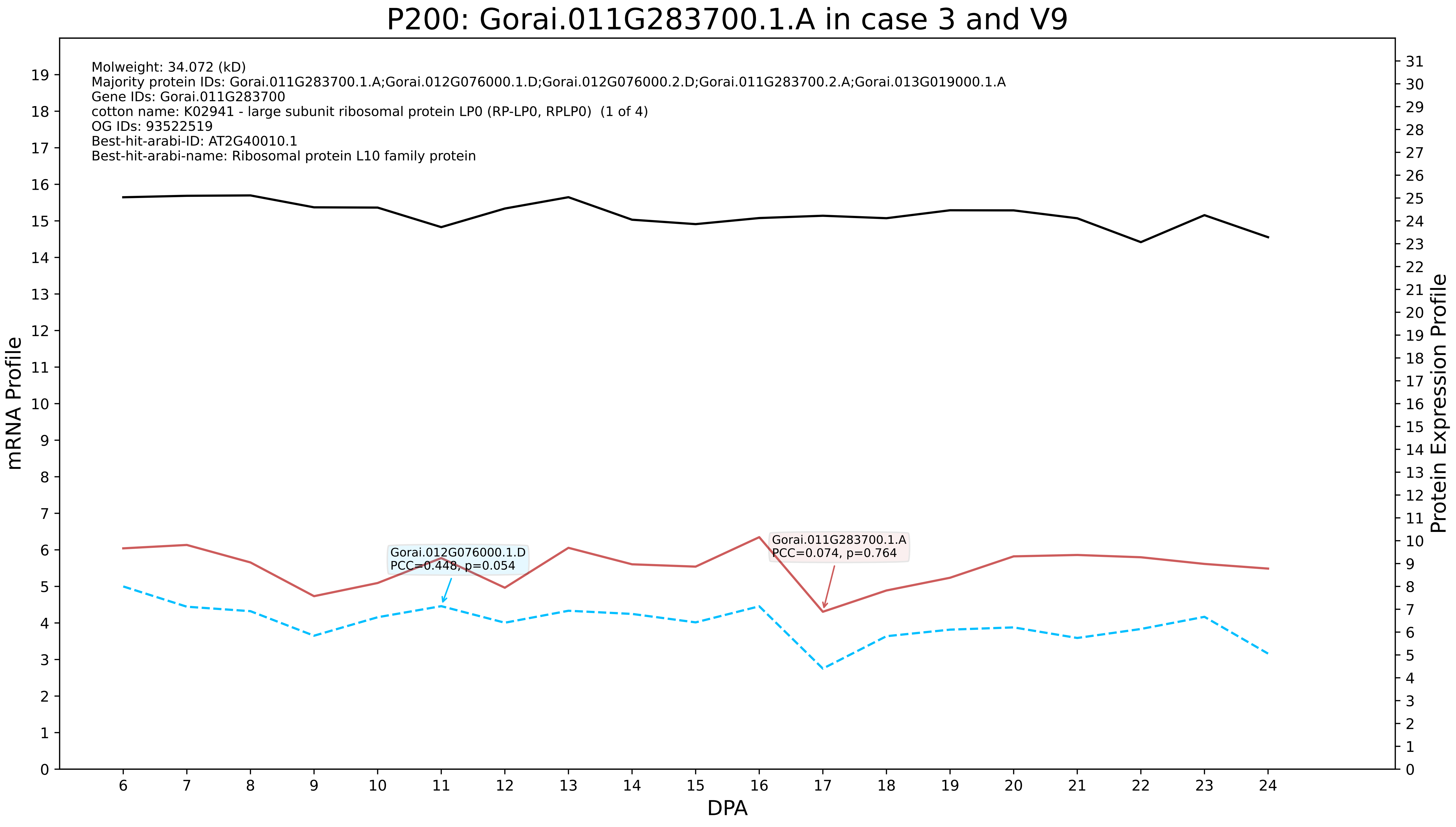
P200: Gorai.011G283700.1.A in case 3 and V9
Molweight: 34.072 (kD)
Majority protein IDs: Gorai.011G283700.1.A;Gorai.012G076000.1.D;Gorai.012G076000.2.D;Gorai.011G283700.2.A;Gorai.013G019000.1.A
Gene IDs: Gorai.011G283700
cotton name: K02941 - large subunit ribosomal protein LP0 (RP-LP0, RPLP0) (1 of 4)
OG IDs: 93522519
Best-hit-arabi-ID: AT2G40010.1
Best-hit-arabi-name: Ribosomal protein L10 family protein
Gorai.012G076000.1.D
PCC=0.448, p=0.054
Gorai.011G283700.1.A
PCC=0.074, p=0.764
mRNA Profile
Protein Expression Profile
DPA
0
1
2
3
4
5
6
7
8
9
10
11
12
13
14
15
16
17
18
19
20
21
22
23
24
25
26
27
28
29
30
31

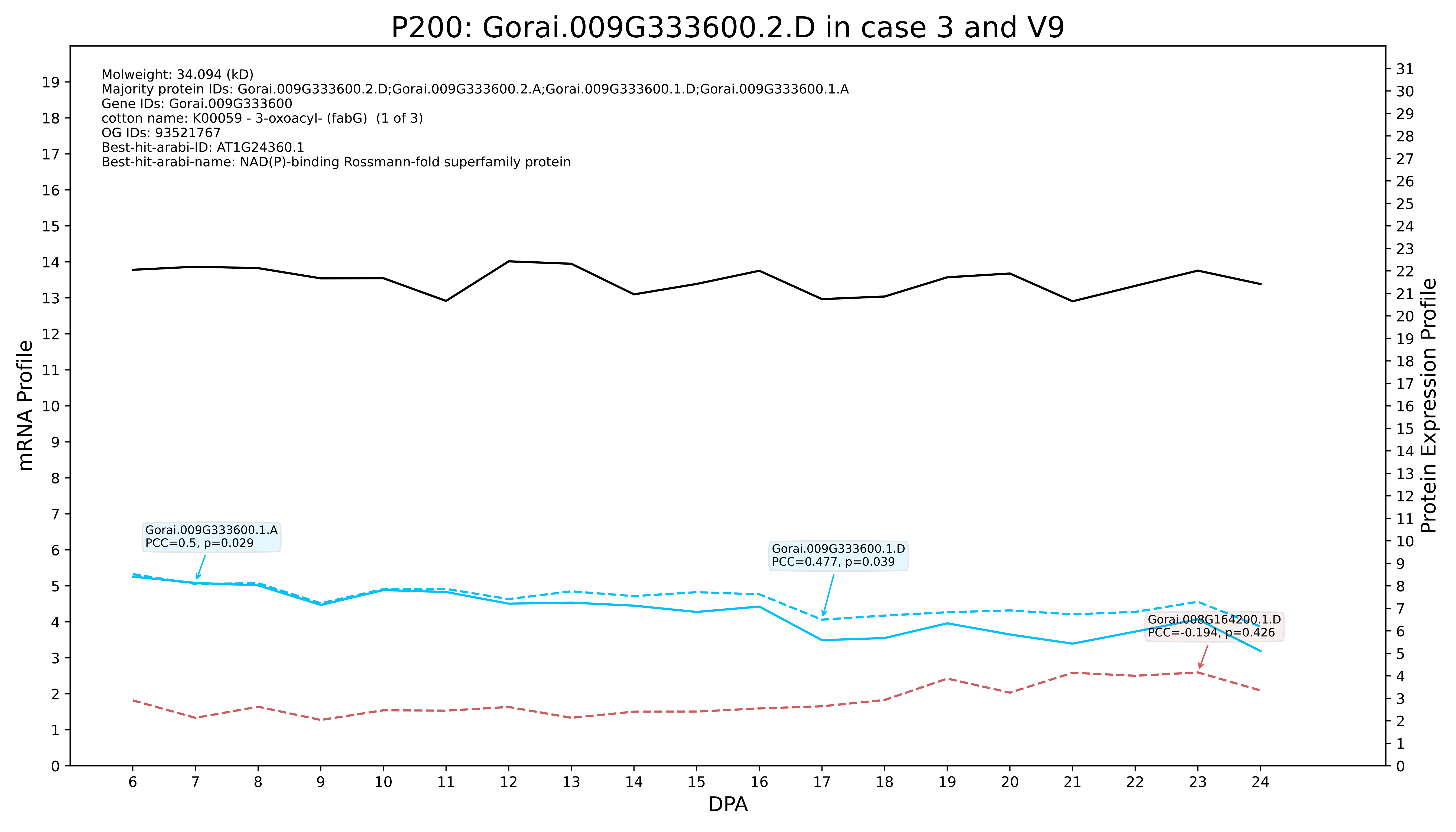
P200: Gorai.009G333600.2.D in case 3 and V9
Molweight: 34.094 (kD)
Majority protein IDs: Gorai.009G333600.2.D;Gorai.009G333600.2.A;Gorai.009G333600.1.D;Gorai.009G333600.1.A
Gene IDs: Gorai.009G333600
cotton name: K00059 - 3-oxoacyl- (fabG) (1 of 3)
OG IDs: 93521767
Best-hit-arabi-ID: AT1G24360.1
Best-hit-arabi-name: NAD(P)-binding Rossmann-fold superfamily protein
Gorai.009G333600.1.A
PCC=0.5, p=0.029
Gorai.009G333600.1.D
PCC=0.477, p=0.039
Gorai.008G164200.1.D
PCC=-0.194, p=0.426
mRNA Profile
Protein Expression Profile
DPA
0
1
2
3
4
5
6
7
8
9
10
11
12
13
14
15
16
17
18
19
20
21
22
23
24
25
26
27
28
29
30
31

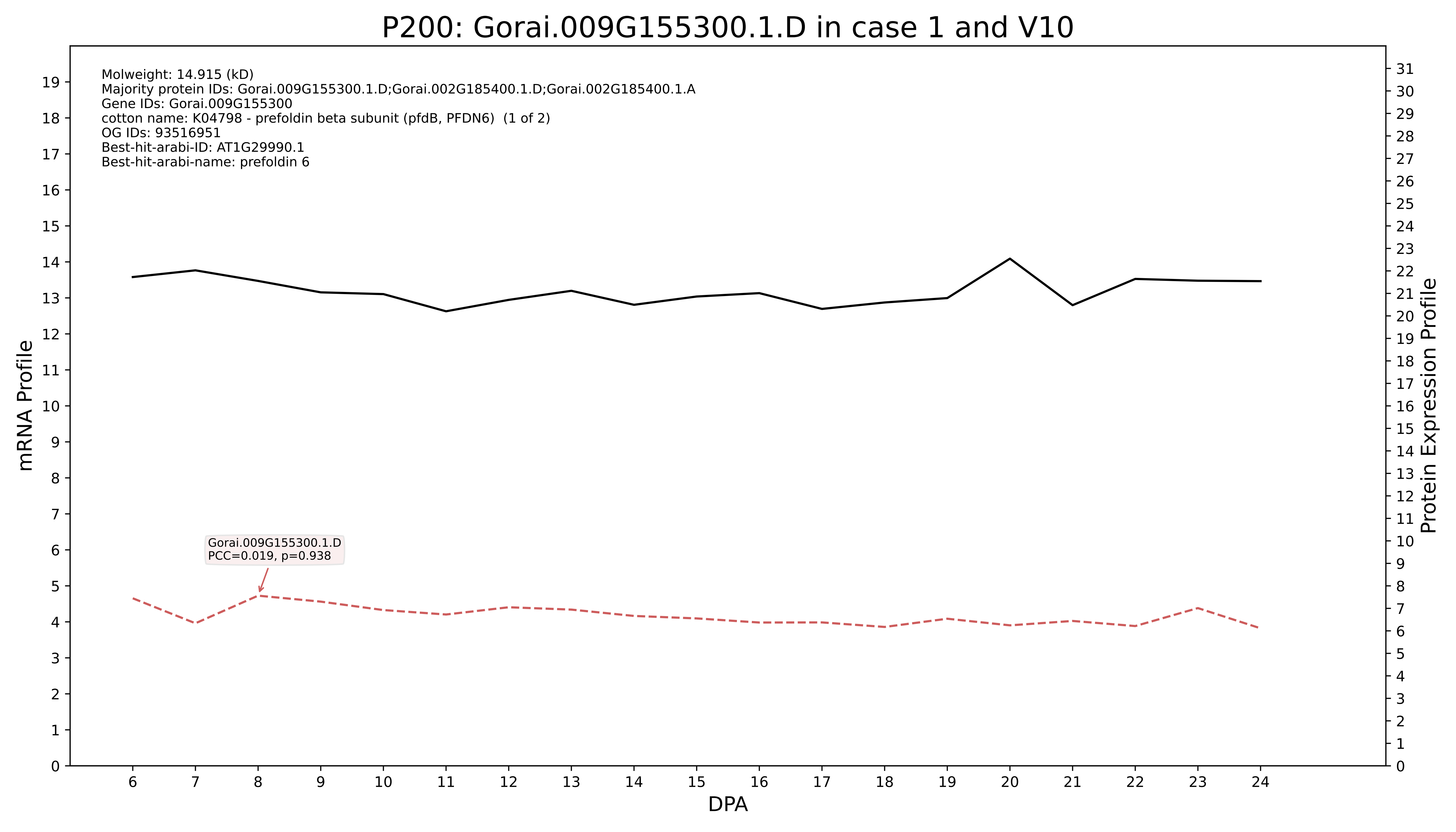

P200: Gorai.009G155300.1.D in case 1 and V10
Molweight: 14.915 (kD)
Majority protein IDs: Gorai.009G155300.1.D;Gorai.002G185400.1.D;Gorai.002G185400.1.A
Gene IDs: Gorai.009G155300
cotton name: K04798 - prefoldin beta subunit (pfdB, PFDN6) (1 of 2)
OG IDs: 93516951
Best-hit-arabi-ID: AT1G29990.1
Best-hit-arabi-name: prefoldin 6
Gorai.009G155300.1.D
PCC=0.019, p=0.938
mRNA Profile
Protein Expression Profile
DPA

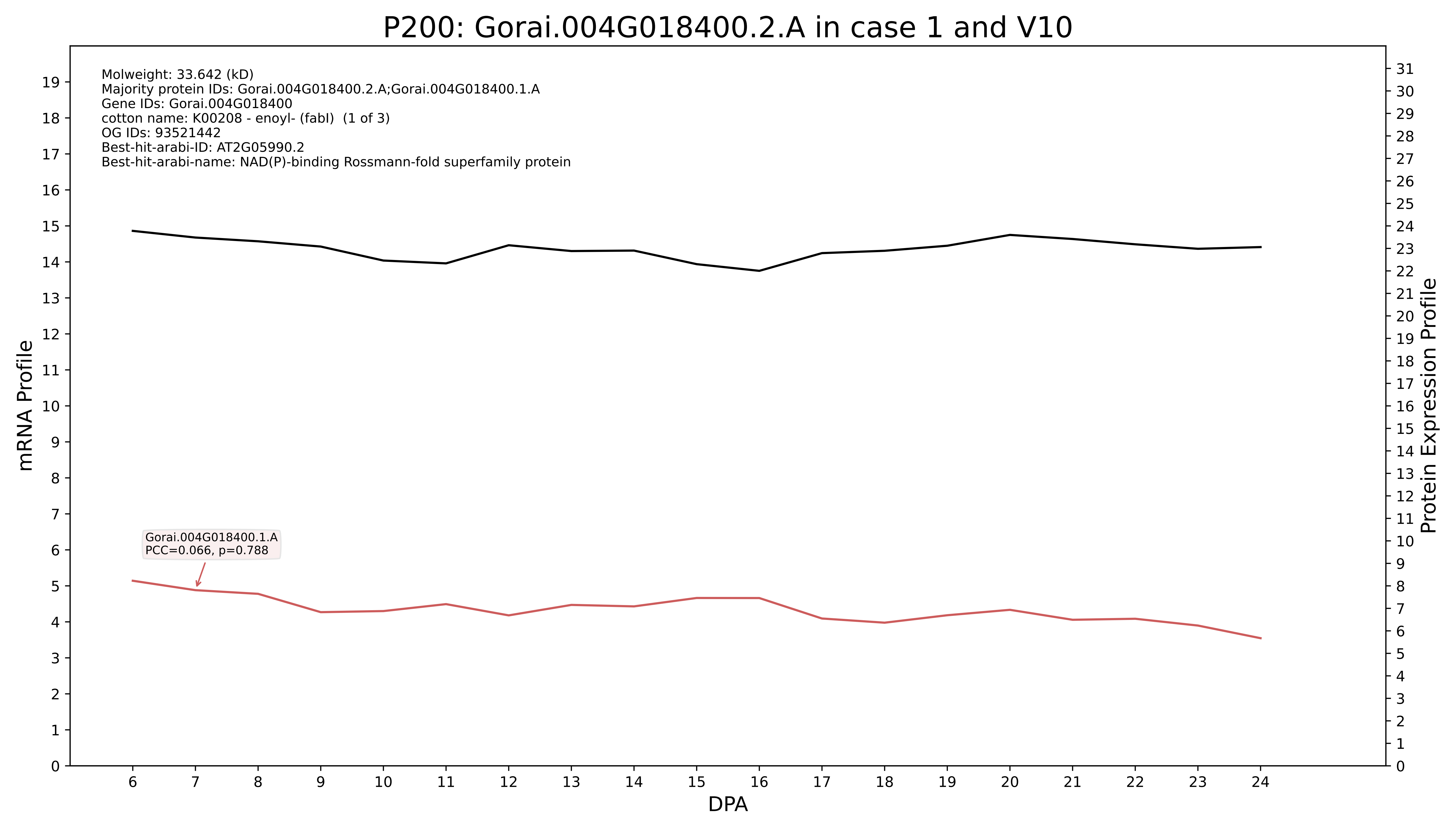
P200: Gorai.004G018400.2.A in case 1 and V10
Molweight: 33.642 (kD)
Majority protein IDs: Gorai.004G018400.2.A;Gorai.004G018400.1.A
Gene IDs: Gorai.004G018400
cotton name: K00208 - enoyl- (fabI) (1 of 3)
OG IDs: 93521442
Best-hit-arabi-ID: AT2G05990.2
Best-hit-arabi-name: NAD(P)-binding Rossmann-fold superfamily protein
Gorai.004G018400.1.A
PCC=0.066, p=0.788
mRNA Profile
Protein Expression Profile
DPA
0
1
2
3
4
5
6
7
8
9
10
11
12
13
14
15
16
17
18
19
20
21
22
23
24
25
26
27
28
29
30
31

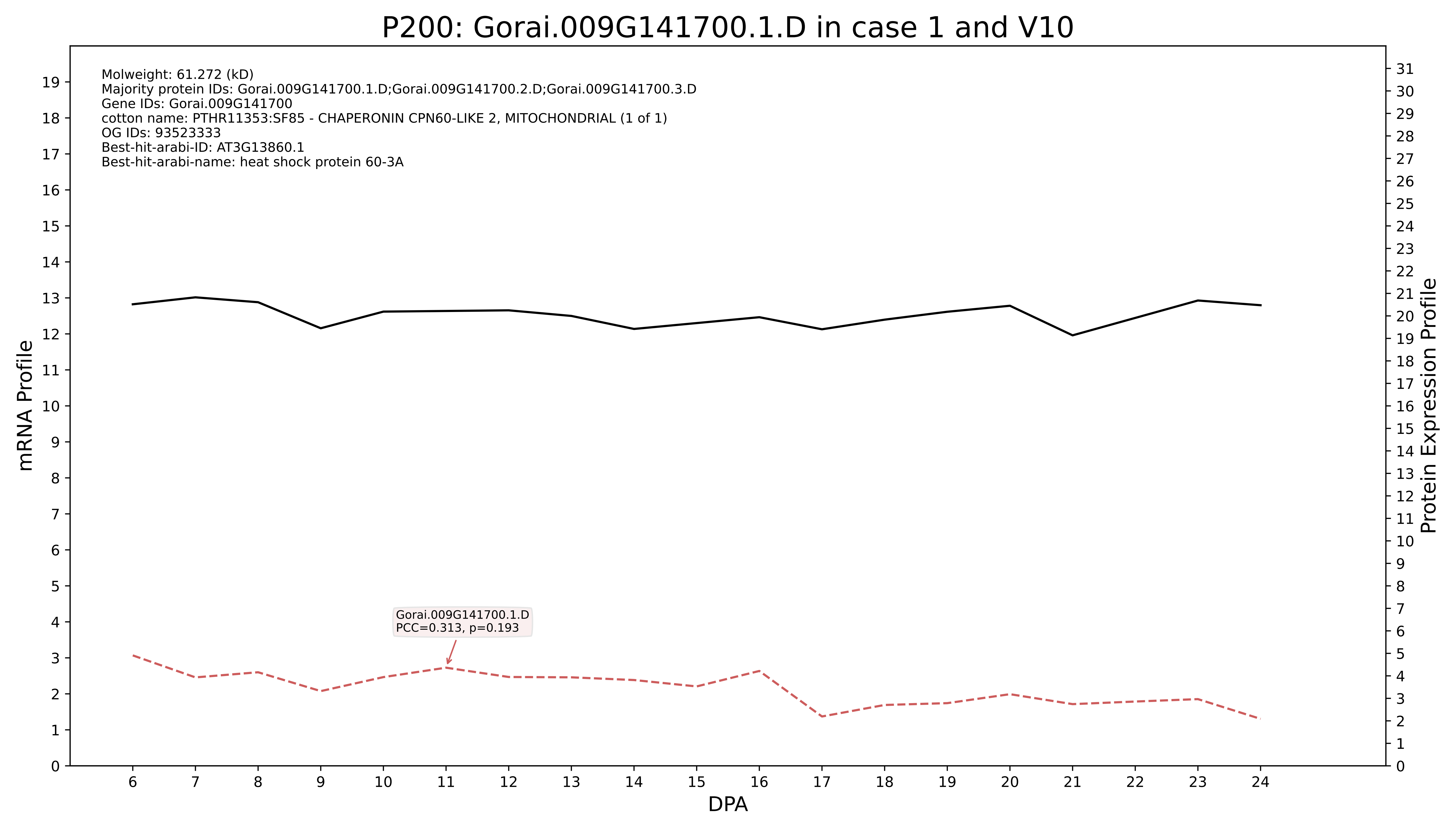
P200: Gorai.009G141700.1.D in case 1 and V10
Molweight: 61.272 (kD)
Majority protein IDs: Gorai.009G141700.1.D;Gorai.009G141700.2.D;Gorai.009G141700.3.D
Gene IDs: Gorai.009G141700
cotton name: PTHR11353:SF85 - CHAPERONIN CPN60-LIKE 2, MITOCHONDRIAL (1 of 1)
OG IDs: 93523333
Best-hit-arabi-ID: AT3G13860.1
Best-hit-arabi-name: heat shock protein 60-3A
Gorai.009G141700.1.D
PCC=0.313, p=0.193
mRNA Profile
Protein Expression Profile
DPA

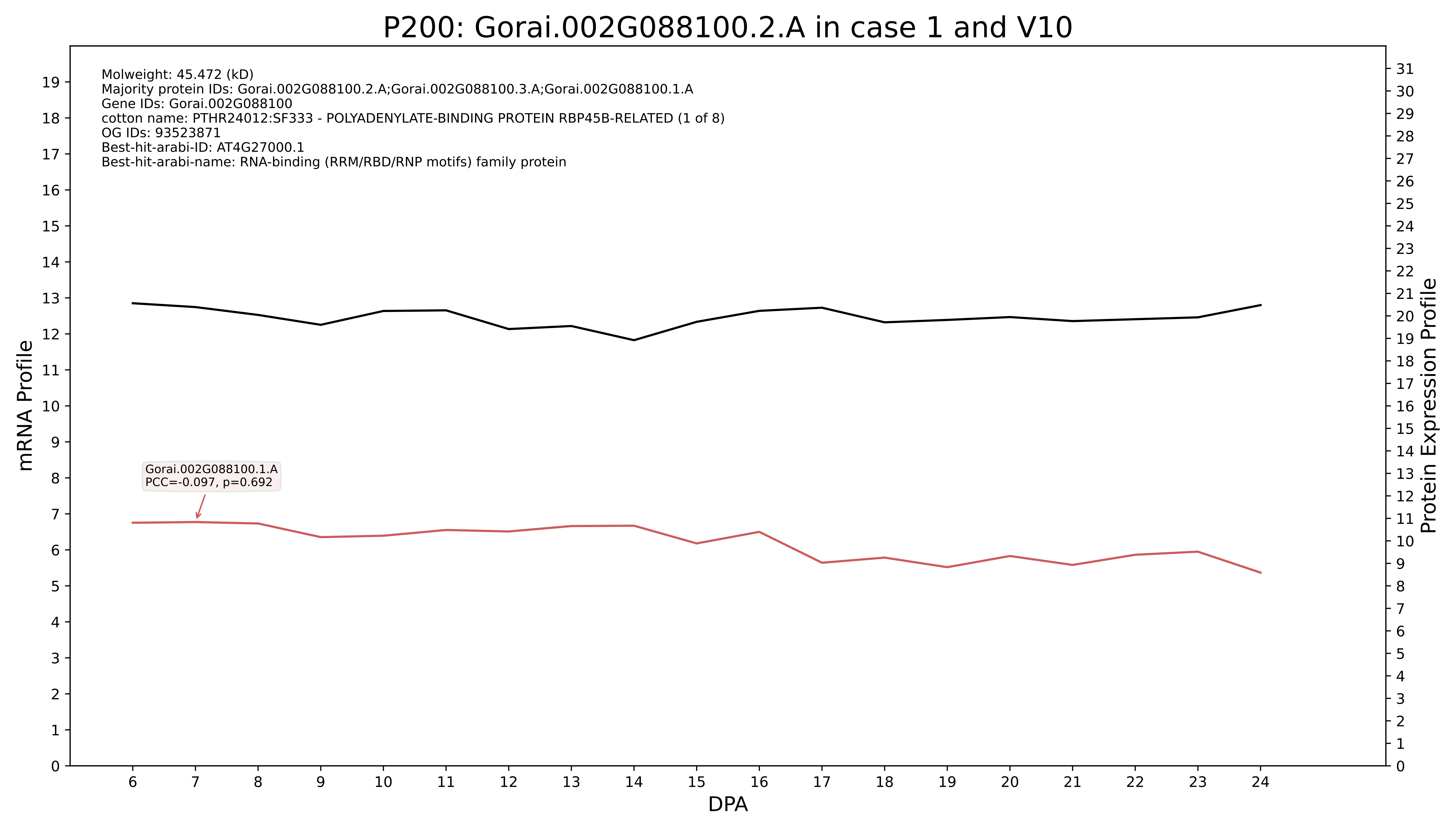
P200: Gorai.002G088100.2.A in case 1 and V10
Molweight: 45.472 (kD)
Majority protein IDs: Gorai.002G088100.2.A;Gorai.002G088100.3.A;Gorai.002G088100.1.A
Gene IDs: Gorai.002G088100
cotton name: PTHR24012:SF333 - POLYADENYLATE-BINDING PROTEIN RBP45B-RELATED (1 of 8)
OG IDs: 93523871
Best-hit-arabi-ID: AT4G27000.1
Best-hit-arabi-name: RNA-binding (RRM/RBD/RNP motifs) family protein
Gorai.002G088100.1.A
PCC=-0.097, p=0.692
mRNA Profile
Protein Expression Profile
DPA

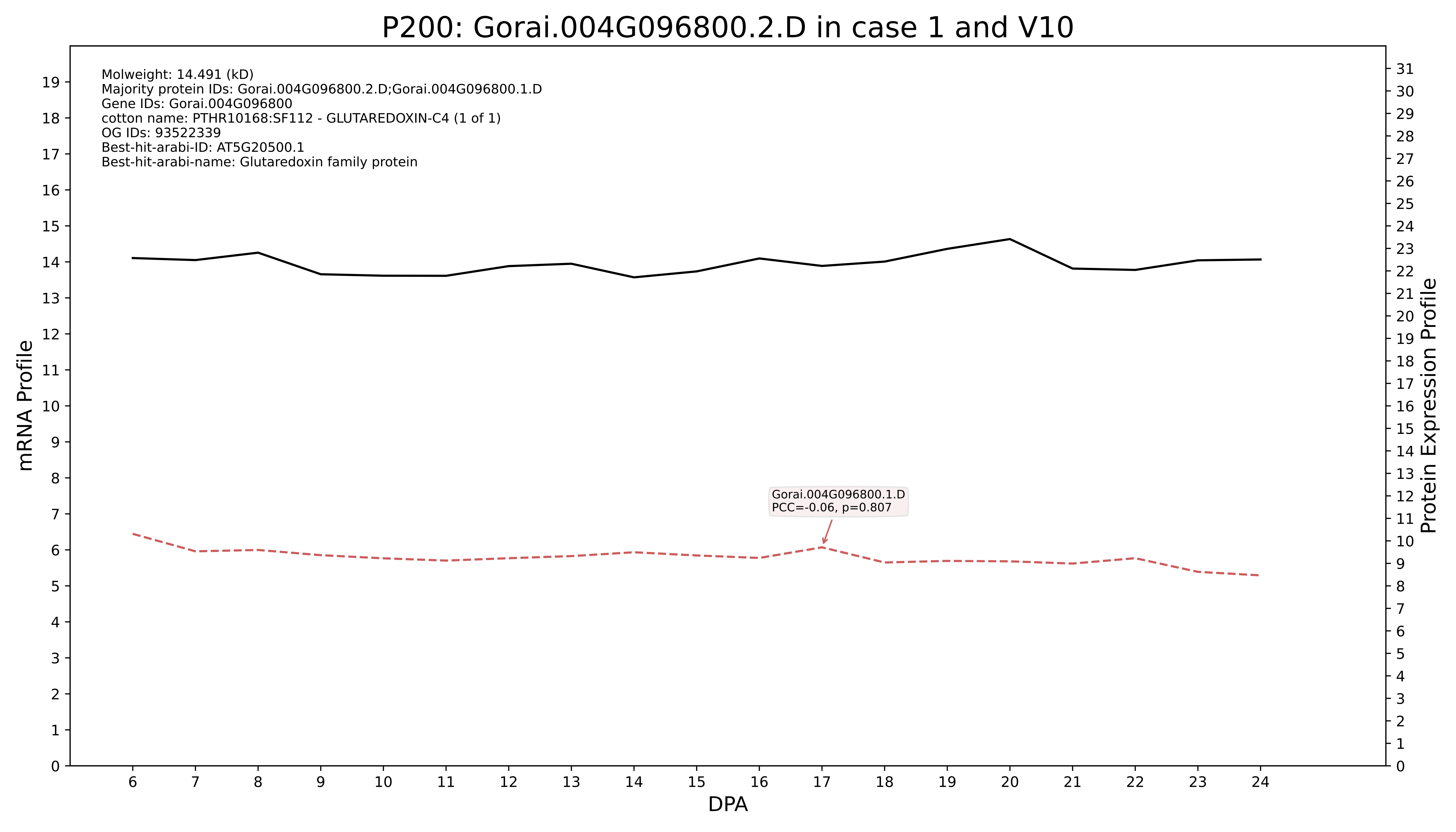
P200: Gorai.004G096800.2.D in case 1 and V10
Molweight: 14.491 (kD)
Majority protein IDs: Gorai.004G096800.2.D;Gorai.004G096800.1.D
Gene IDs: Gorai.004G096800
cotton name: PTHR10168:SF112 - GLUTAREDOXIN-C4 (1 of 1)
OG IDs: 93522339
Best-hit-arabi-ID: AT5G20500.1
Best-hit-arabi-name: Glutaredoxin family protein
Gorai.004G096800.1.D
PCC=-0.06, p=0.807
mRNA Profile
Protein Expression Profile
DPA
0
1
2
3
4
5
6
7
8
9
10
11
12
13
14
15
16
17
18
19
20
21
22
23
24
25
26
27
28
29
30
31

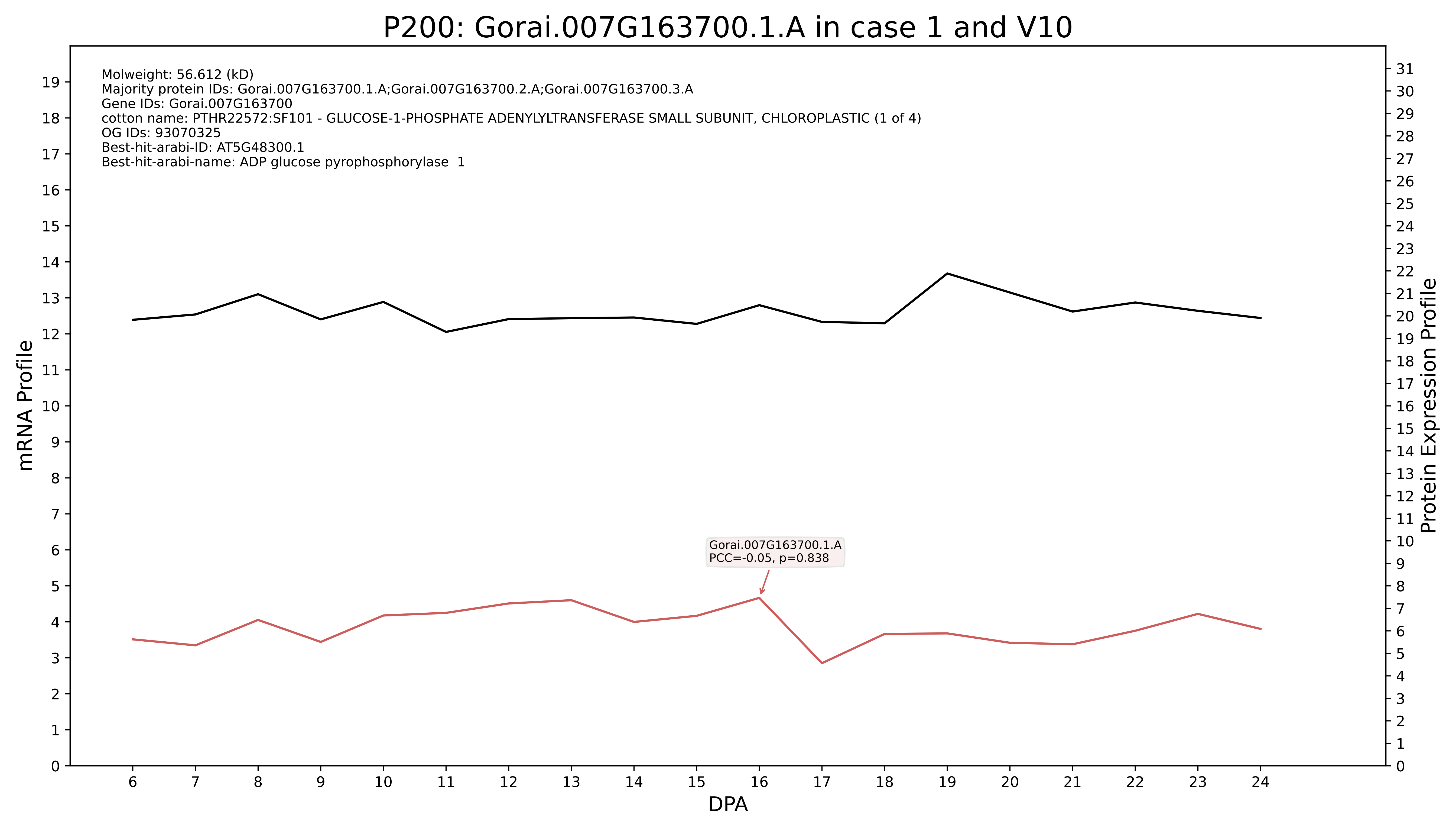
P200: Gorai.007G163700.1.A in case 1 and V10
Molweight: 56.612 (kD)
Majority protein IDs: Gorai.007G163700.1.A;Gorai.007G163700.2.A;Gorai.007G163700.3.A
Gene IDs: Gorai.007G163700
cotton name: PTHR22572:SF101 - GLUCOSE-1-PHOSPHATE ADENYLYLTRANSFERASE SMALL SUBUNIT, CHLOROPLASTIC (1 of 4)
OG IDs: 93070325
Best-hit-arabi-ID: AT5G48300.1
Best-hit-arabi-name: ADP glucose pyrophosphorylase 1
Gorai.007G163700.1.A
PCC=-0.05, p=0.838
mRNA Profile
Protein Expression Profile
DPA
0
1
2
3
4
5
6
7
8
9
10
11
12
13
14
15
16
17
18
19
20
21
22
23
24
25
26
27
28
29
30
31

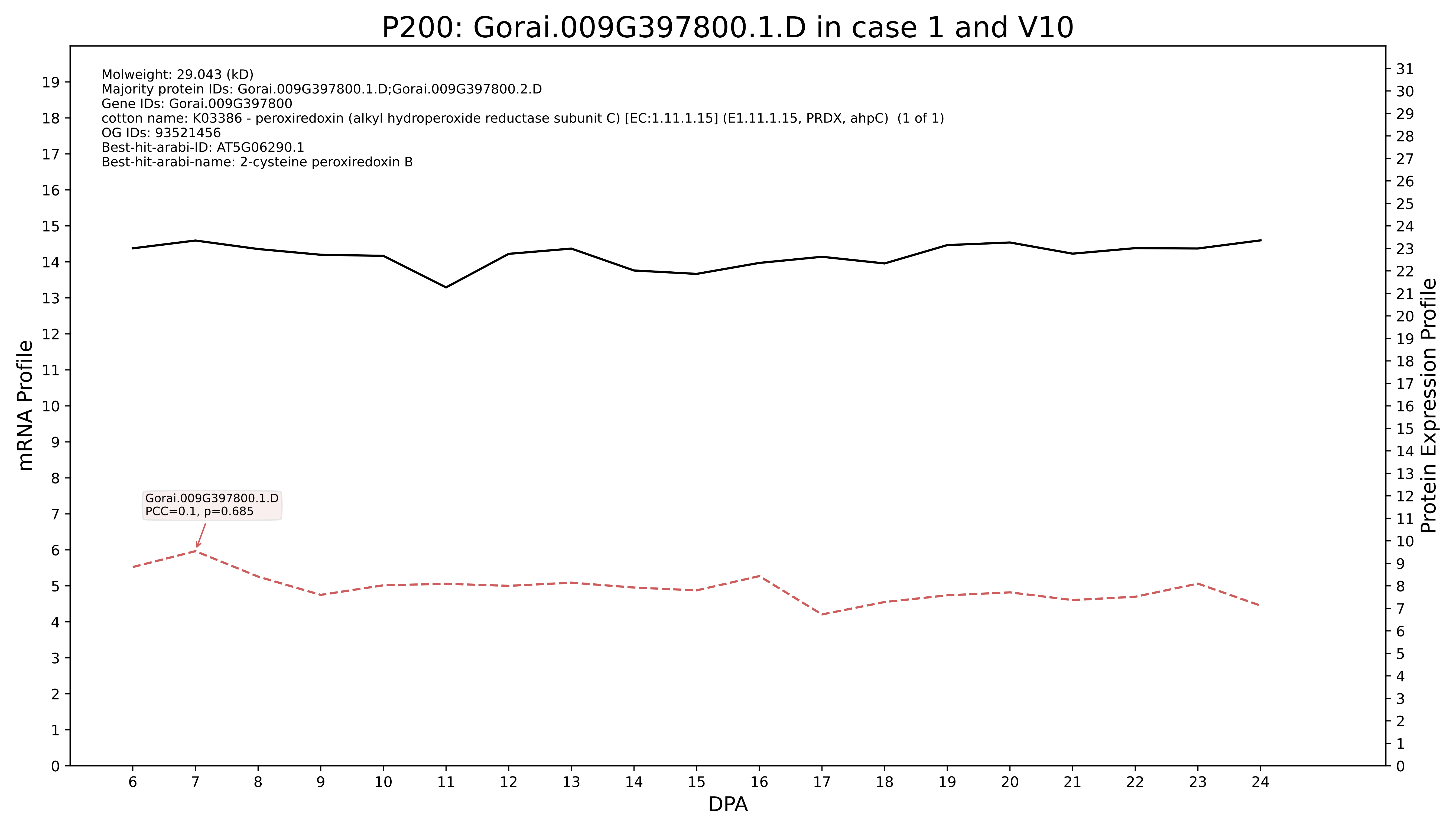
P200: Gorai.009G397800.1.D in case 1 and V10
Molweight: 29.043 (kD)
Majority protein IDs: Gorai.009G397800.1.D;Gorai.009G397800.2.D
Gene IDs: Gorai.009G397800
cotton name: K03386 - peroxiredoxin (alkyl hydroperoxide reductase subunit C) [EC:1.11.1.15] (E1.11.1.15, PRDX, ahpC) (1 of 1)
OG IDs: 93521456
Best-hit-arabi-ID: AT5G06290.1
Best-hit-arabi-name: 2-cysteine peroxiredoxin B
Gorai.009G397800.1.D
PCC=0.1, p=0.685
mRNA Profile
Protein Expression Profile
DPA

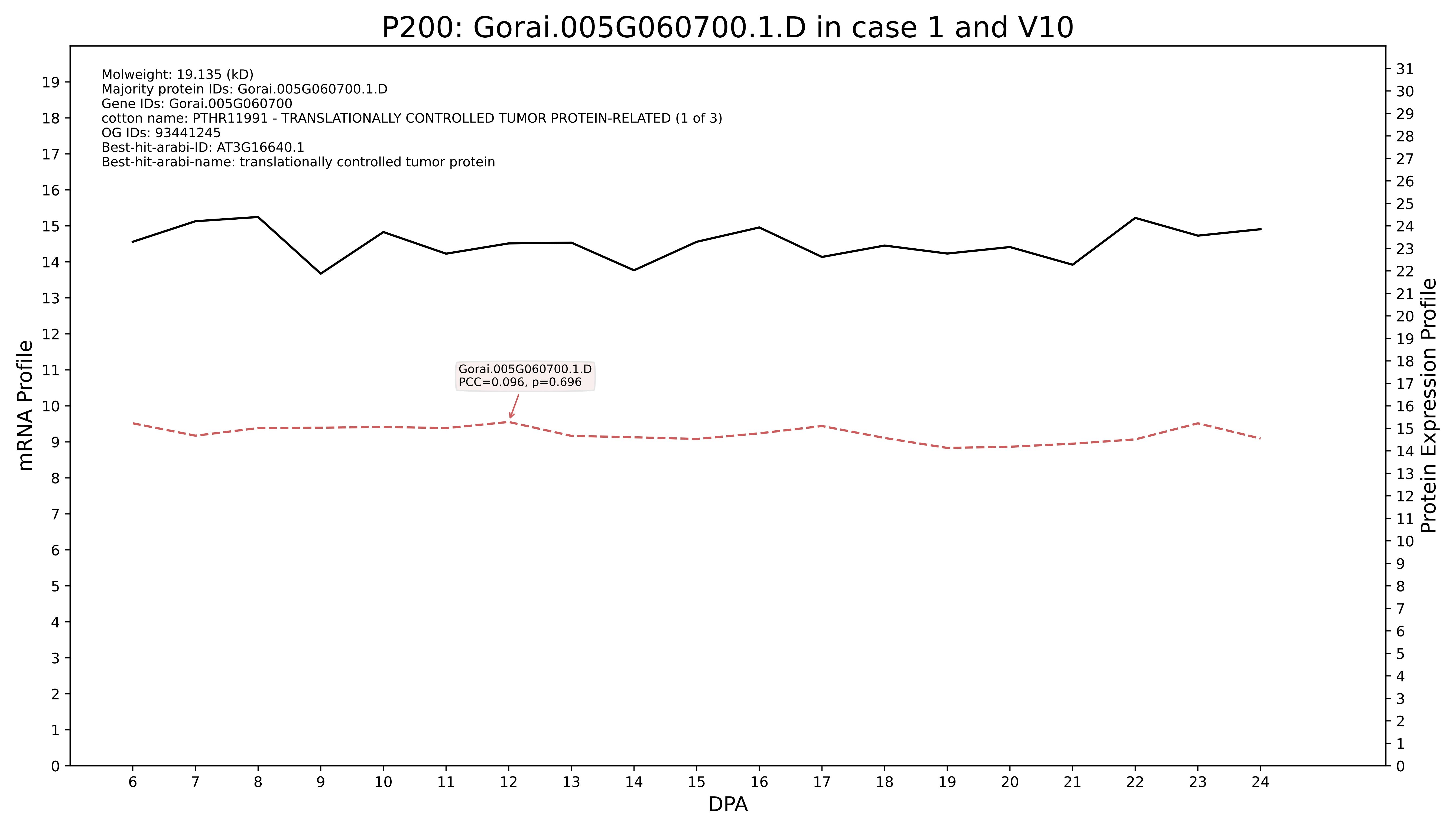

P200: Gorai.005G060700.1.D in case 1 and V10
Molweight: 19.135 (kD)
Majority protein IDs: Gorai.005G060700.1.D
Gene IDs: Gorai.005G060700
cotton name: PTHR11991 - TRANSLATIONALLY CONTROLLED TUMOR PROTEIN-RELATED (1 of 3)
OG IDs: 93441245
Best-hit-arabi-ID: AT3G16640.1
Best-hit-arabi-name: translationally controlled tumor protein
Gorai.005G060700.1.D
PCC=0.096, p=0.696
mRNA Profile
Protein Expression Profile
DPA

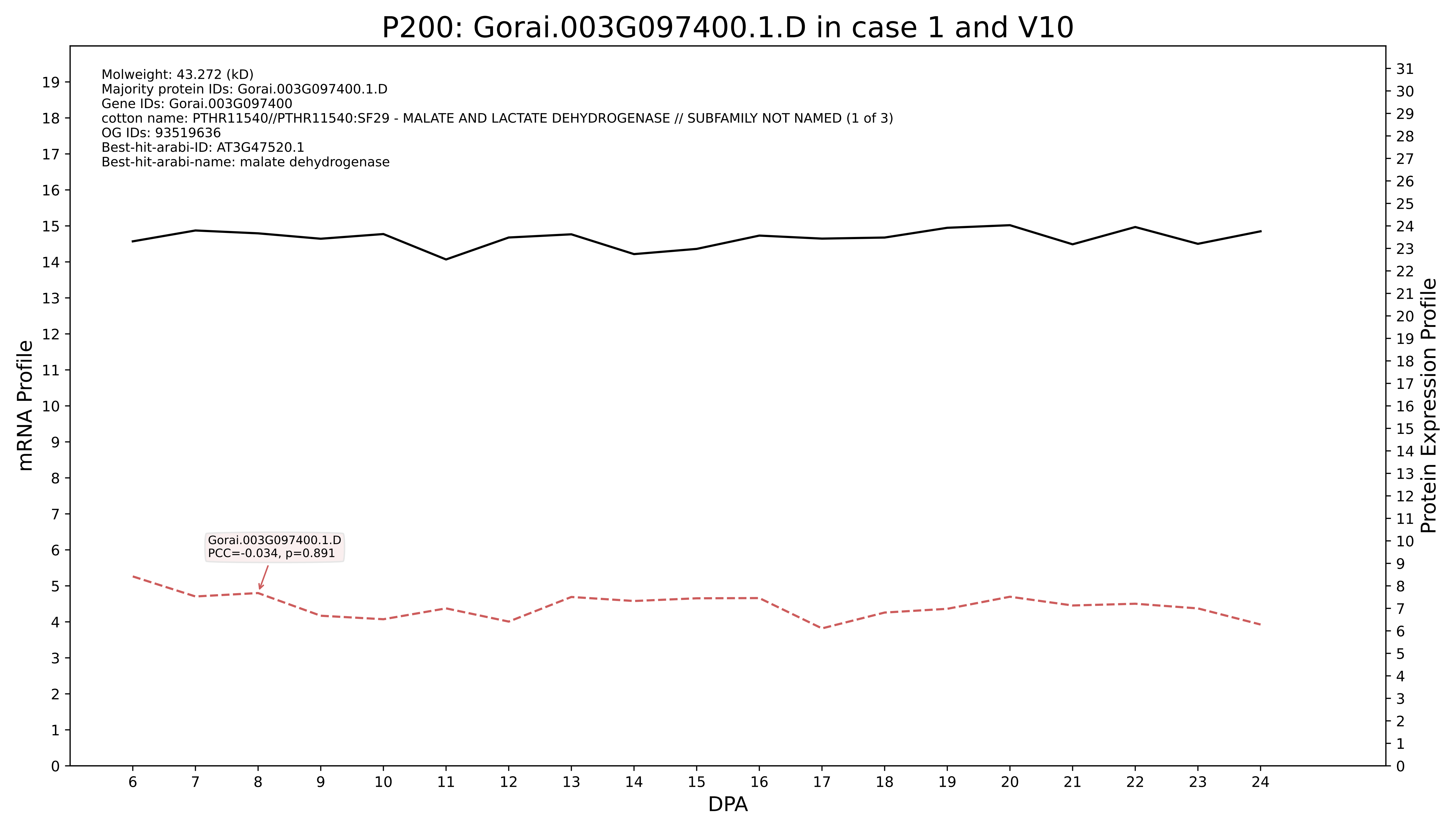

P200: Gorai.003G097400.1.D in case 1 and V10
Molweight: 43.272 (kD)
Majority protein IDs: Gorai.003G097400.1.D
Gene IDs: Gorai.003G097400
cotton name: PTHR11540//PTHR11540:SF29 - MALATE AND LACTATE DEHYDROGENASE // SUBFAMILY NOT NAMED (1 of 3)
OG IDs: 93519636
Best-hit-arabi-ID: AT3G47520.1
Best-hit-arabi-name: malate dehydrogenase
Gorai.003G097400.1.D
PCC=-0.034, p=0.891
mRNA Profile
Protein Expression Profile
DPA

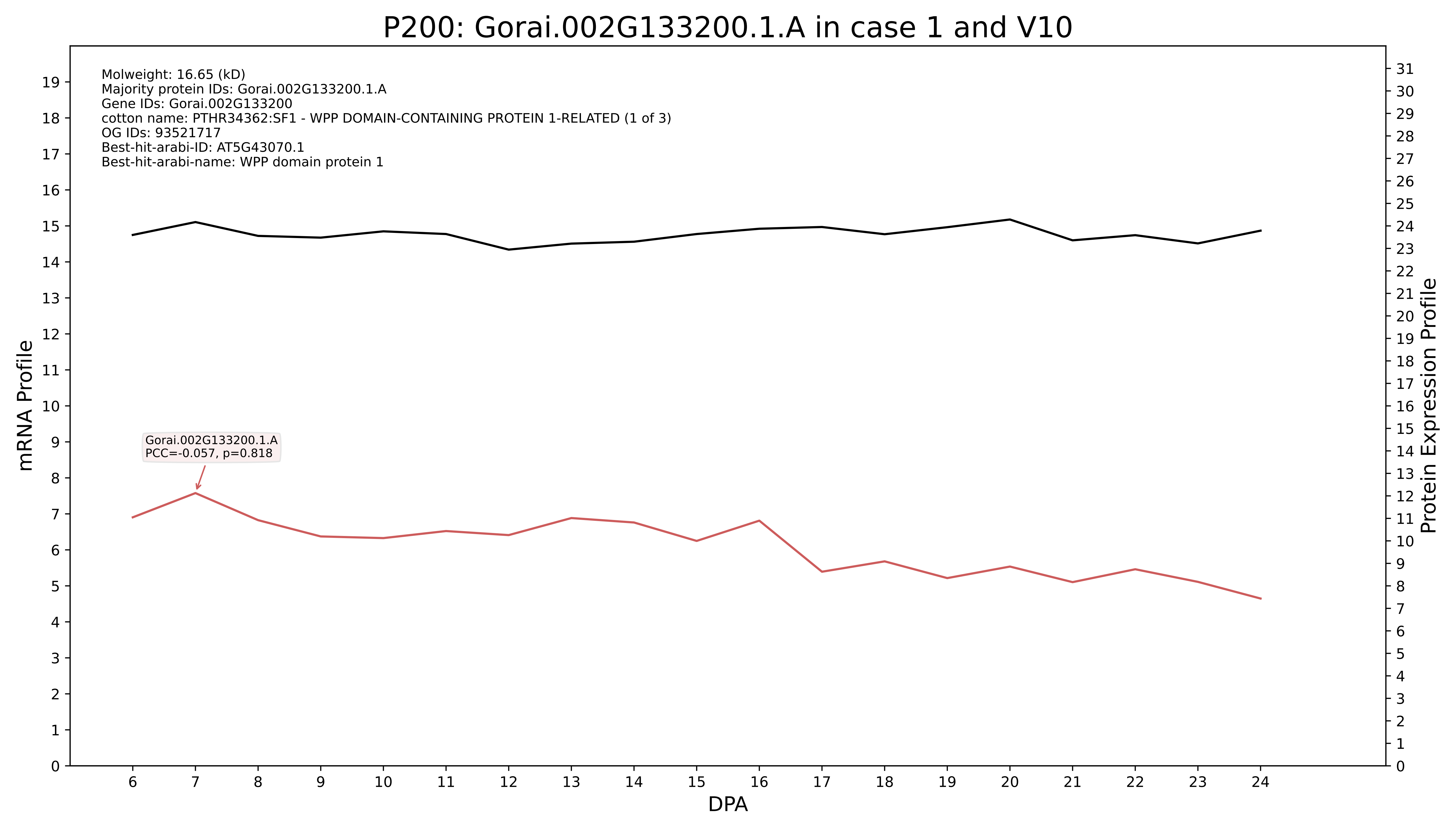

P200: Gorai.002G133200.1.A in case 1 and V10
Molweight: 16.65 (kD)
Majority protein IDs: Gorai.002G133200.1.A
Gene IDs: Gorai.002G133200
cotton name: PTHR34362:SF1 - WPP DOMAIN-CONTAINING PROTEIN 1-RELATED (1 of 3)
OG IDs: 93521717
Best-hit-arabi-ID: AT5G43070.1
Best-hit-arabi-name: WPP domain protein 1
Gorai.002G133200.1.A
PCC=-0.057, p=0.818
mRNA Profile
Protein Expression Profile
DPA

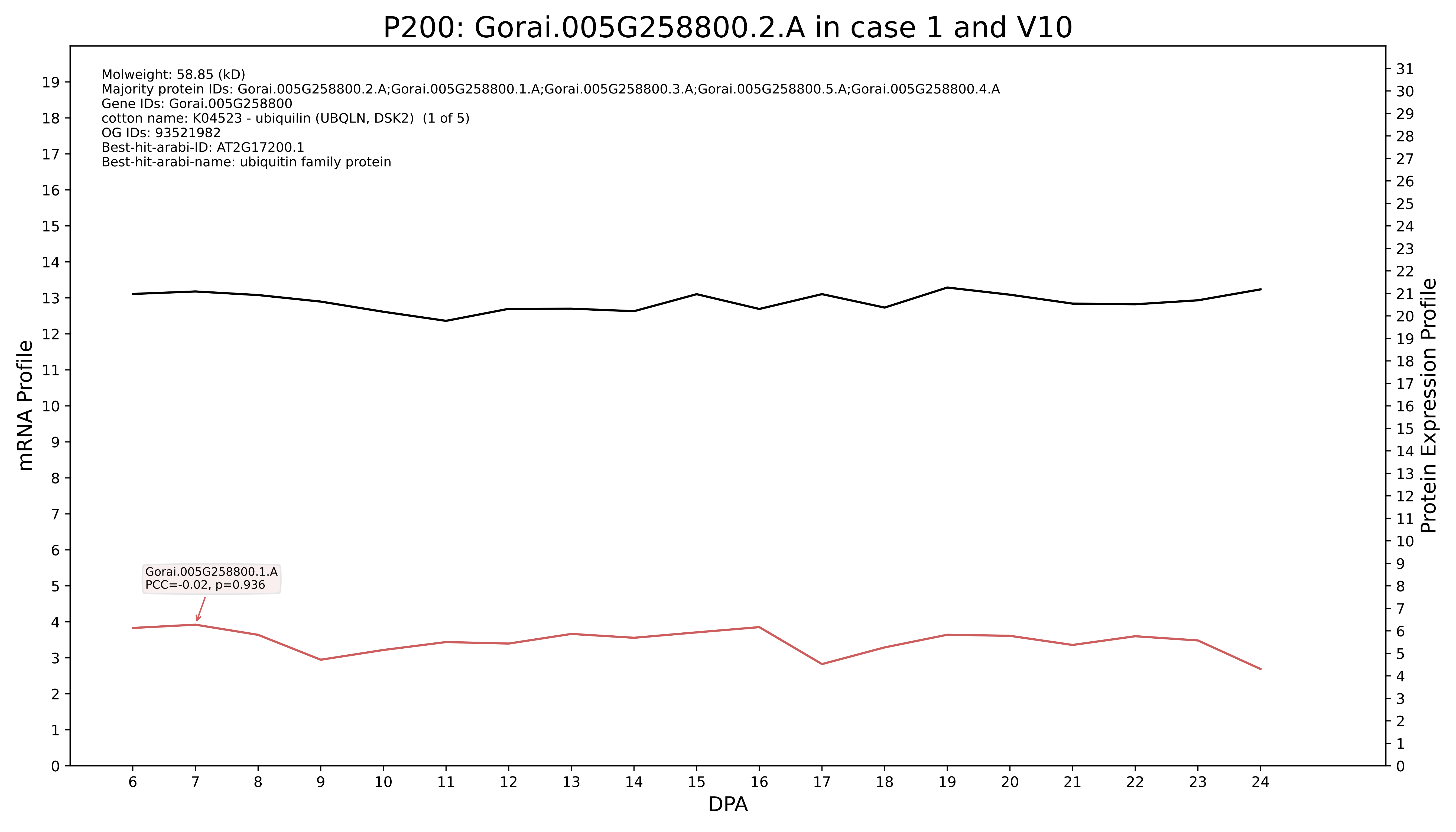
P200: Gorai.005G258800.2.A in case 1 and V10
Molweight: 58.85 (kD)
Majority protein IDs: Gorai.005G258800.2.A;Gorai.005G258800.1.A;Gorai.005G258800.3.A;Gorai.005G258800.5.A;Gorai.005G258800.4.A
Gene IDs: Gorai.005G258800
cotton name: K04523 - ubiquilin (UBQLN, DSK2) (1 of 5)
OG IDs: 93521982
Best-hit-arabi-ID: AT2G17200.1
Best-hit-arabi-name: ubiquitin family protein
Gorai.005G258800.1.A
PCC=-0.02, p=0.936
mRNA Profile
Protein Expression Profile
DPA

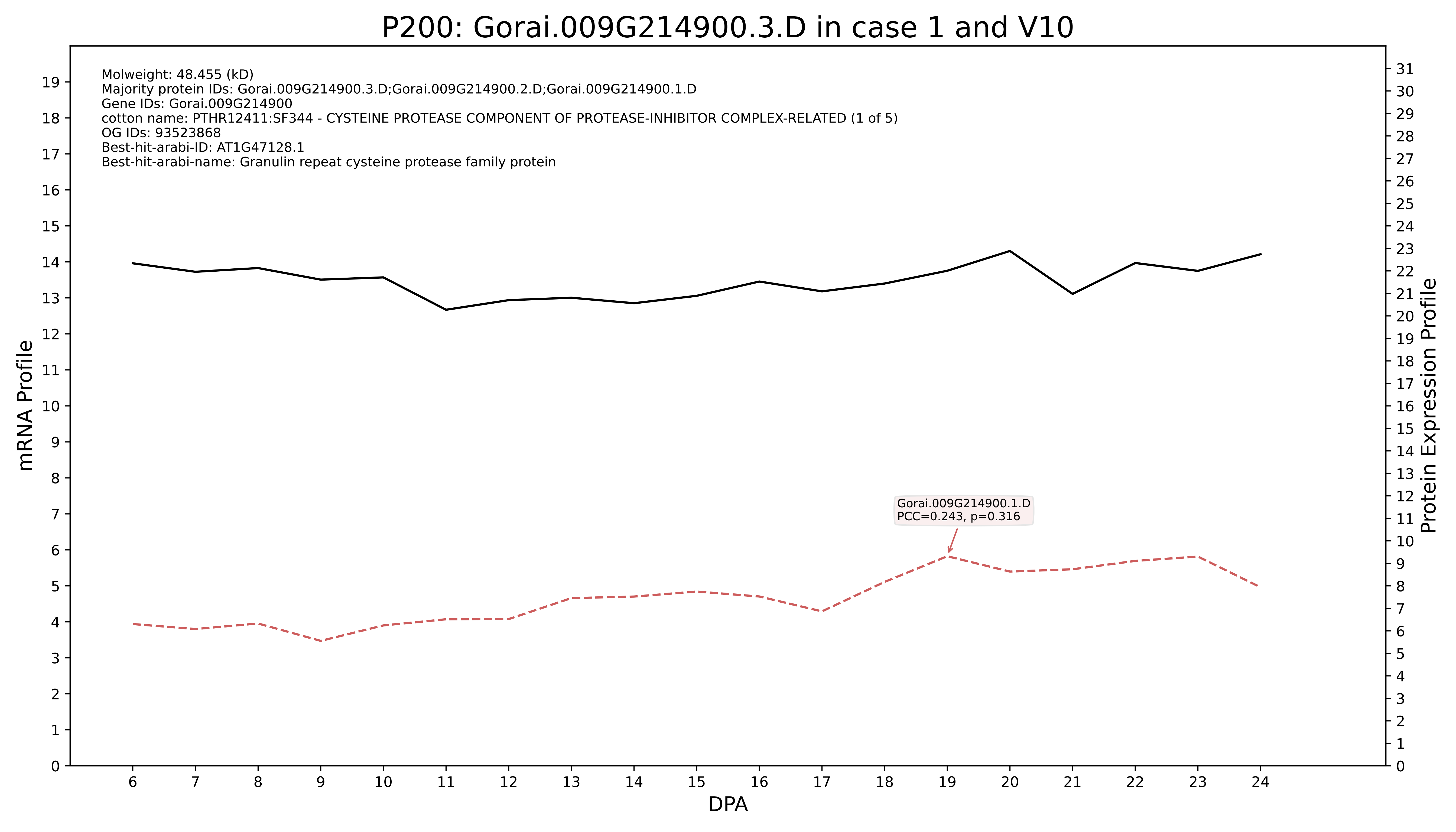

P200: Gorai.009G214900.3.D in case 1 and V10
Molweight: 48.455 (kD)
Majority protein IDs: Gorai.009G214900.3.D;Gorai.009G214900.2.D;Gorai.009G214900.1.D
Gene IDs: Gorai.009G214900
cotton name: PTHR12411:SF344 - CYSTEINE PROTEASE COMPONENT OF PROTEASE-INHIBITOR COMPLEX-RELATED (1 of 5)
OG IDs: 93523868
Best-hit-arabi-ID: AT1G47128.1
Best-hit-arabi-name: Granulin repeat cysteine protease family protein
Gorai.009G214900.1.D
PCC=0.243, p=0.316
mRNA Profile
Protein Expression Profile
DPA

P200: Gorai.010G042000.1.A in case 1 and V10

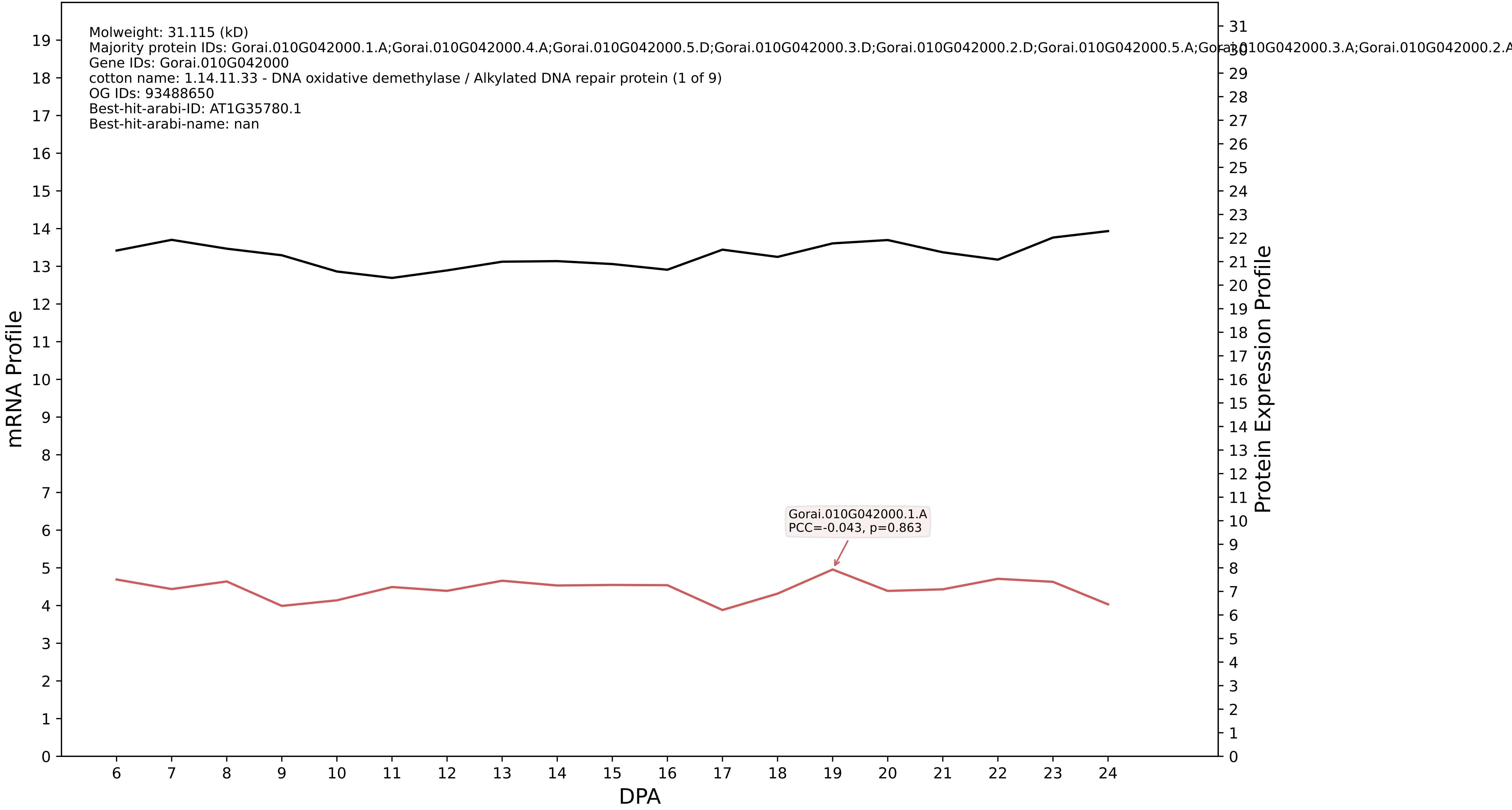

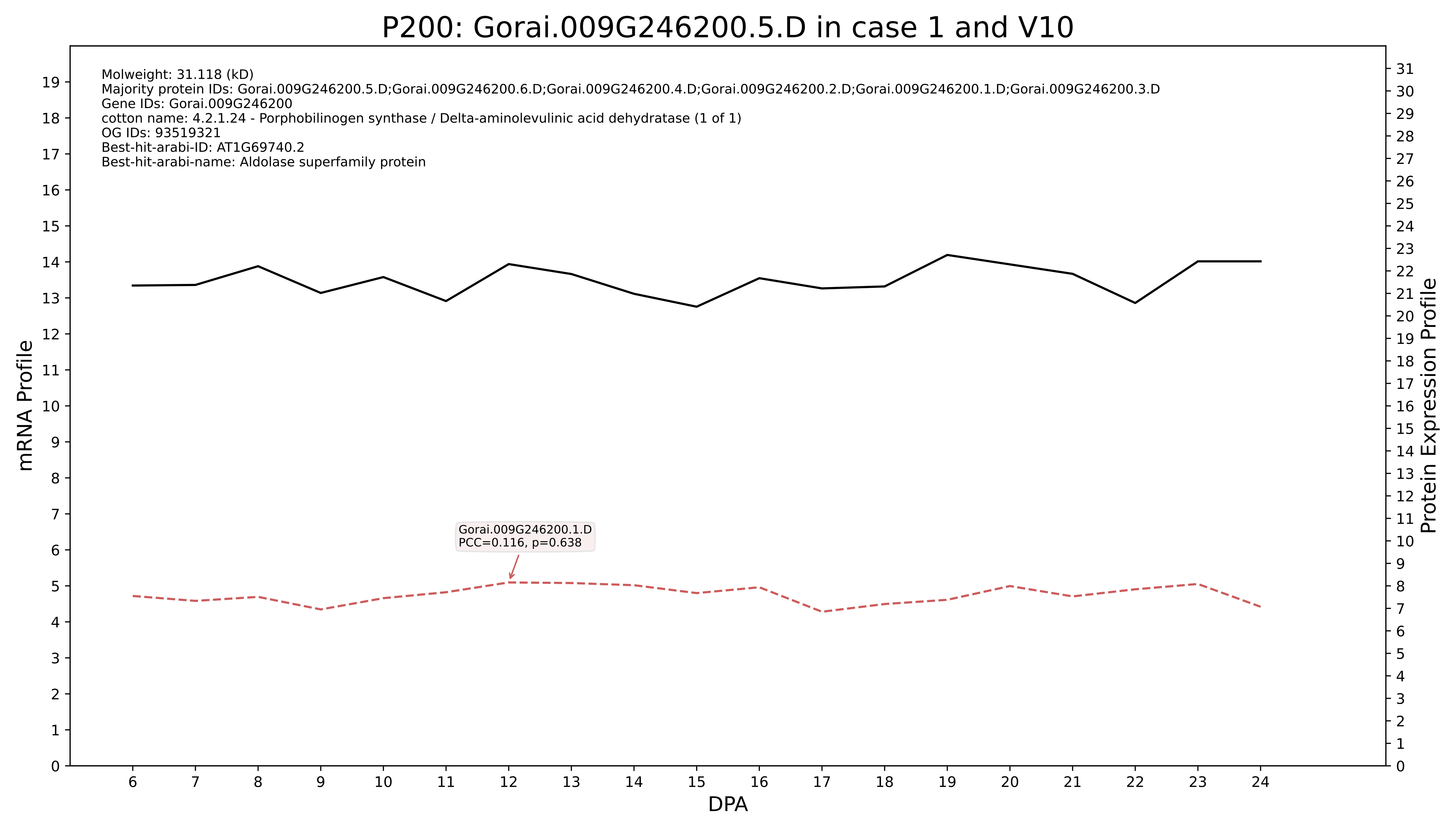
P200: Gorai.009G246200.5.D in case 1 and V10
Molweight: 31.118 (kD)
Majority protein IDs: Gorai.009G246200.5.D;Gorai.009G246200.6.D;Gorai.009G246200.4.D;Gorai.009G246200.2.D;Gorai.009G246200.1.D;Gorai.009G246200.3.D
Gene IDs: Gorai.009G246200
cotton name: 4.2.1.24 - Porphobilinogen synthase / Delta-aminolevulinic acid dehydratase (1 of 1)
OG IDs: 93519321
Best-hit-arabi-ID: AT1G69740.2
Best-hit-arabi-name: Aldolase superfamily protein
Gorai.009G246200.1.D
PCC=0.116, p=0.638
mRNA Profile
Protein Expression Profile
DPA

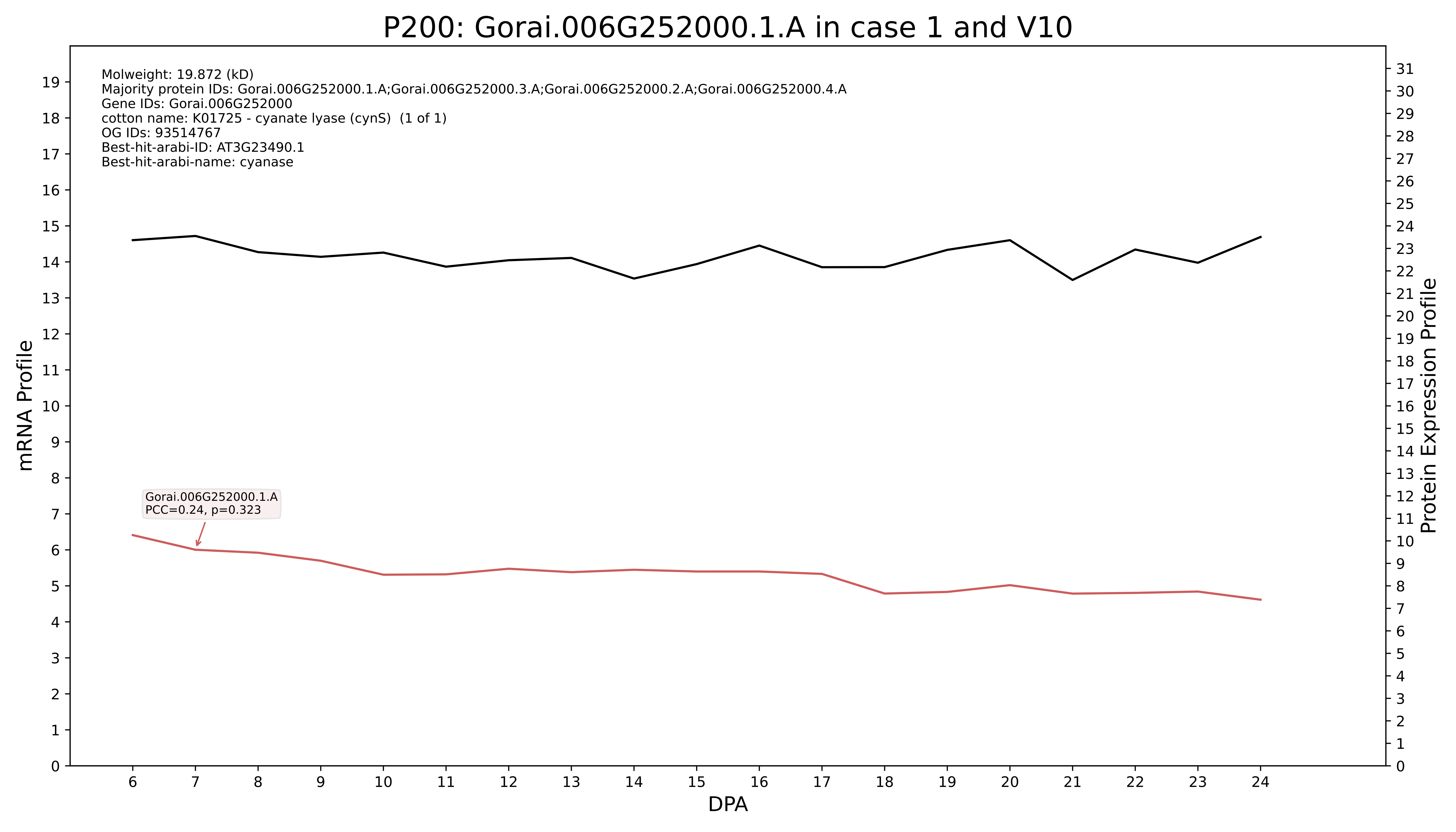

P200: Gorai.006G252000.1.A in case 1 and V10
Molweight: 19.872 (kD)
Majority protein IDs: Gorai.006G252000.1.A;Gorai.006G252000.3.A;Gorai.006G252000.2.A;Gorai.006G252000.4.A
Gene IDs: Gorai.006G252000
cotton name: K01725 - cyanate lyase (cynS) (1 of 1)
OG IDs: 93514767
Best-hit-arabi-ID: AT3G23490.1
Best-hit-arabi-name: cyanase
Gorai.006G252000.1.A
PCC=0.24, p=0.323
mRNA Profile
Protein Expression Profile
DPA

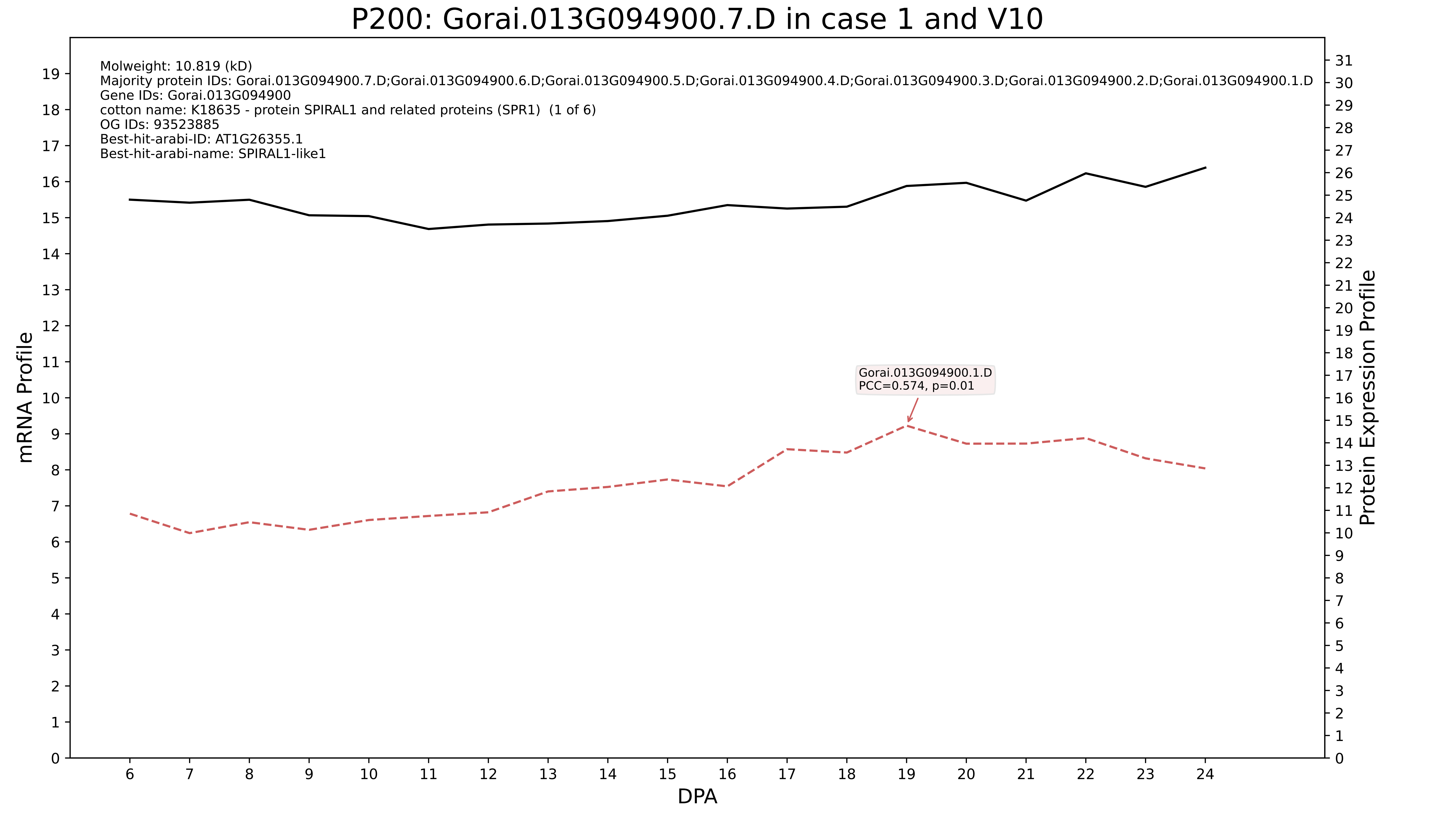

P200: Gorai.013G094900.7.D in case 1 and V10
Molweight: 10.819 (kD)
Majority protein IDs: Gorai.013G094900.7.D;Gorai.013G094900.6.D;Gorai.013G094900.5.D;Gorai.013G094900.4.D;Gorai.013G094900.3.D;Gorai.013G094900.2.D;Gorai.013G094900.1.D
Gene IDs: Gorai.013G094900
cotton name: K18635 - protein SPIRAL1 and related proteins (SPR1) (1 of 6)
OG IDs: 93523885
Best-hit-arabi-ID: AT1G26355.1
Best-hit-arabi-name: SPIRAL1-like1
Gorai.013G094900.1.D
PCC=0.574, p=0.01
mRNA Profile
Protein Expression Profile
DPA

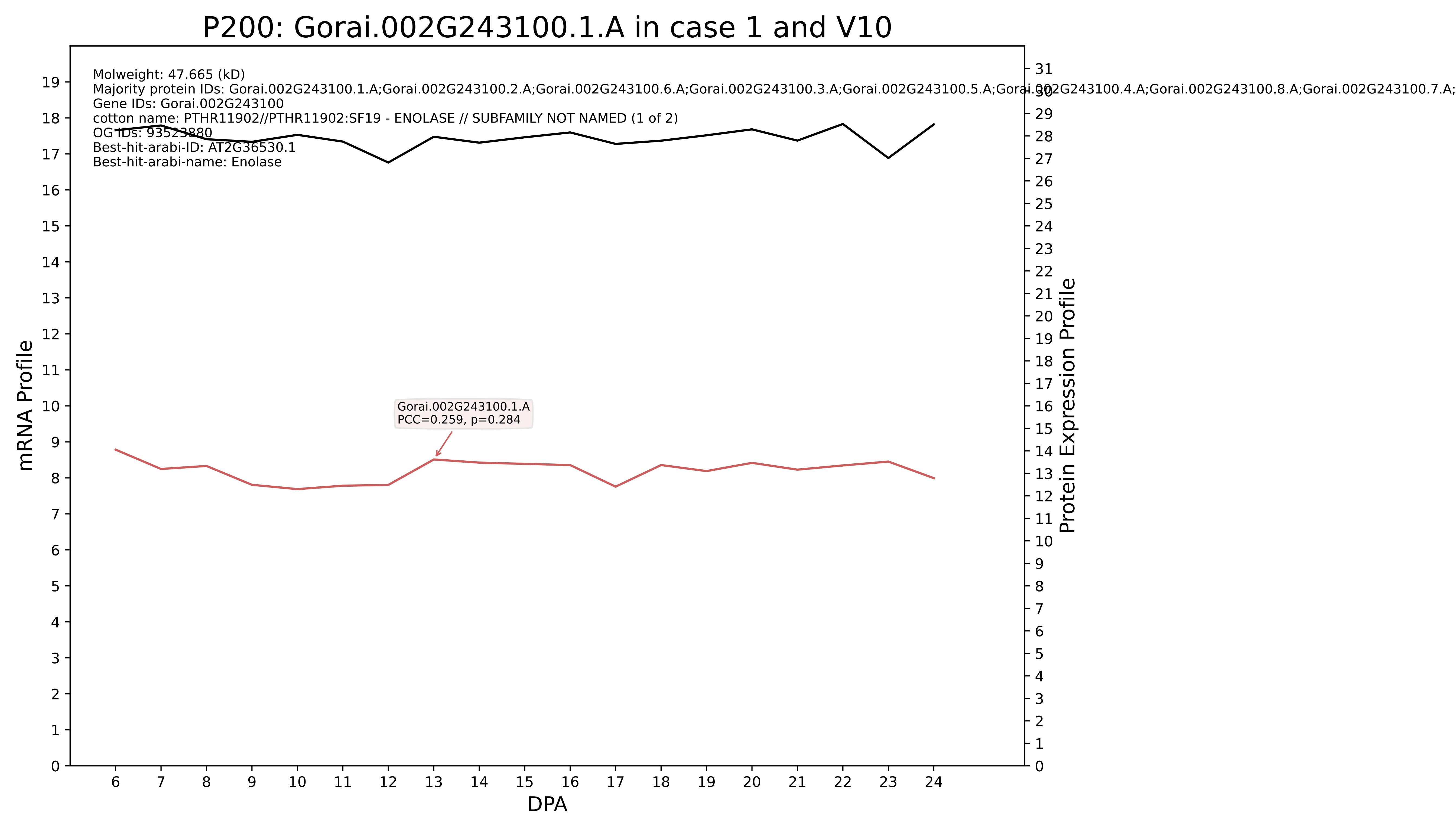
P200: Gorai.002G243100.1.A in case 1 and V10
Molweight: 47.665 (kD)
Majority protein IDs: Gorai.002G243100.1.A;Gorai.002G243100.2.A;Gorai.002G243100.6.A;Gorai.002G243100.3.A;Gorai.002G243100.5.A;Gorai.002G243100.4.A;Gorai.002G243100.8.A;Gorai.002G243100.7.A;
Gene IDs: Gorai.002G243100
cotton name: PTHR11902//PTHR11902:SF19 - ENOLASE // SUBFAMILY NOT NAMED (1 of 2)
OG IDs: 93523880
Best-hit-arabi-ID: AT2G36530.1
Best-hit-arabi-name: Enolase
Gorai.002G243100.1.A
PCC=0.259, p=0.284
mRNA Profile
Protein Expression Profile
DPA

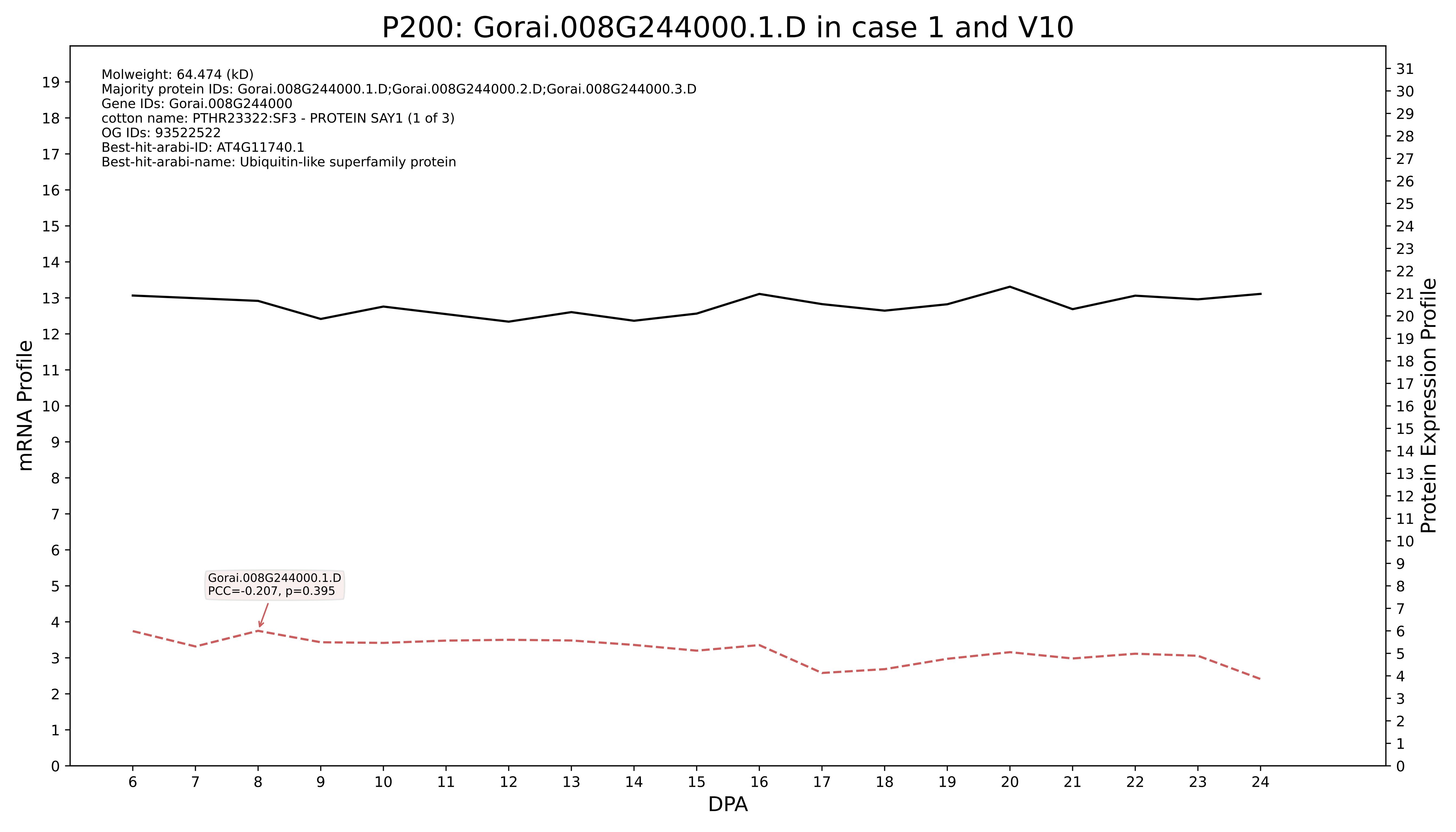

P200: Gorai.008G244000.1.D in case 1 and V10
Molweight: 64.474 (kD)
Majority protein IDs: Gorai.008G244000.1.D;Gorai.008G244000.2.D;Gorai.008G244000.3.D
Gene IDs: Gorai.008G244000
cotton name: PTHR23322:SF3 - PROTEIN SAY1 (1 of 3)
OG IDs: 93522522
Best-hit-arabi-ID: AT4G11740.1
Best-hit-arabi-name: Ubiquitin-like superfamily protein
Gorai.008G244000.1.D
PCC=-0.207, p=0.395
mRNA Profile
Protein Expression Profile
DPA

P200: Gorai.011G233300.6.A in case 1 and V10

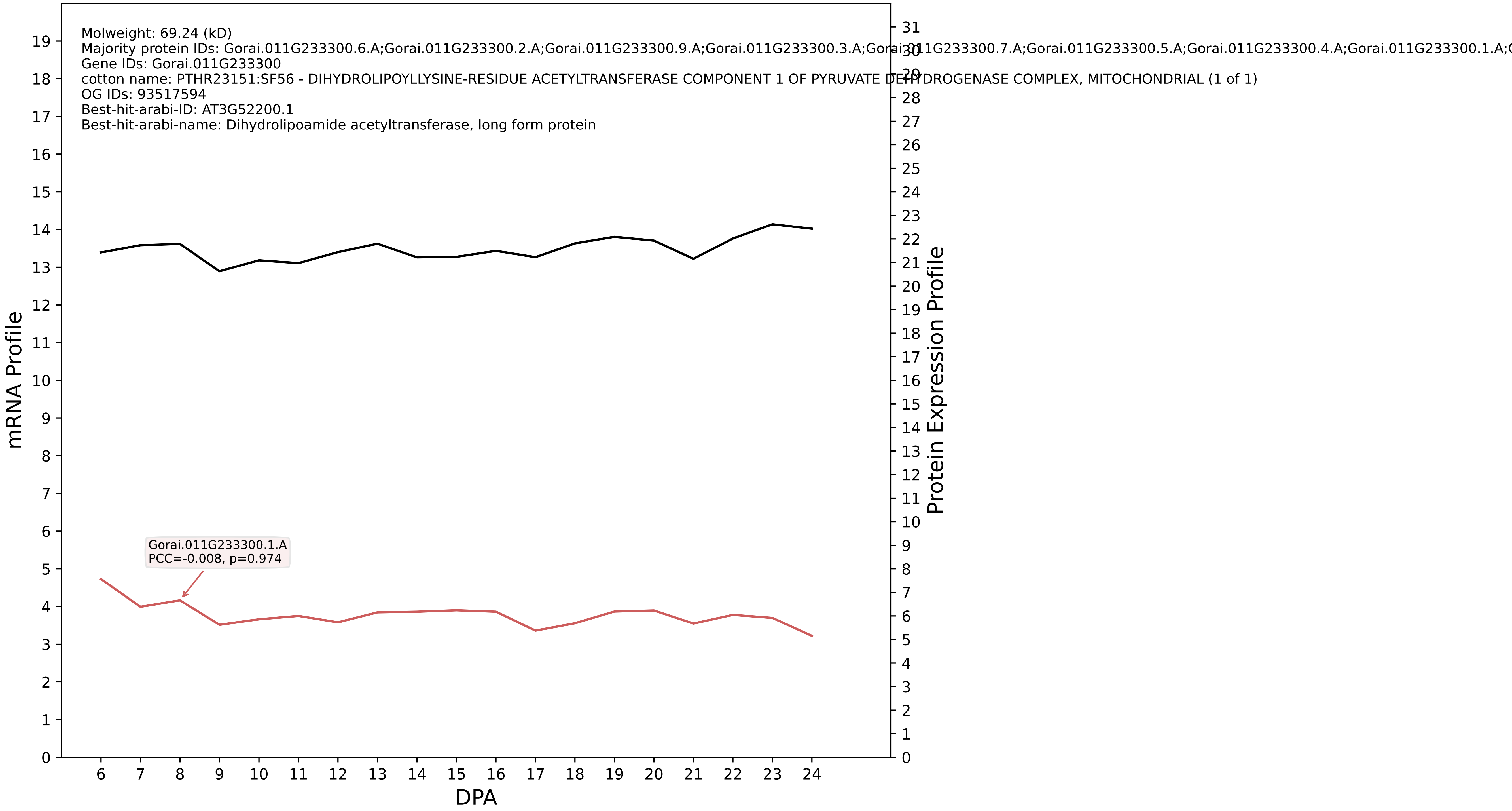

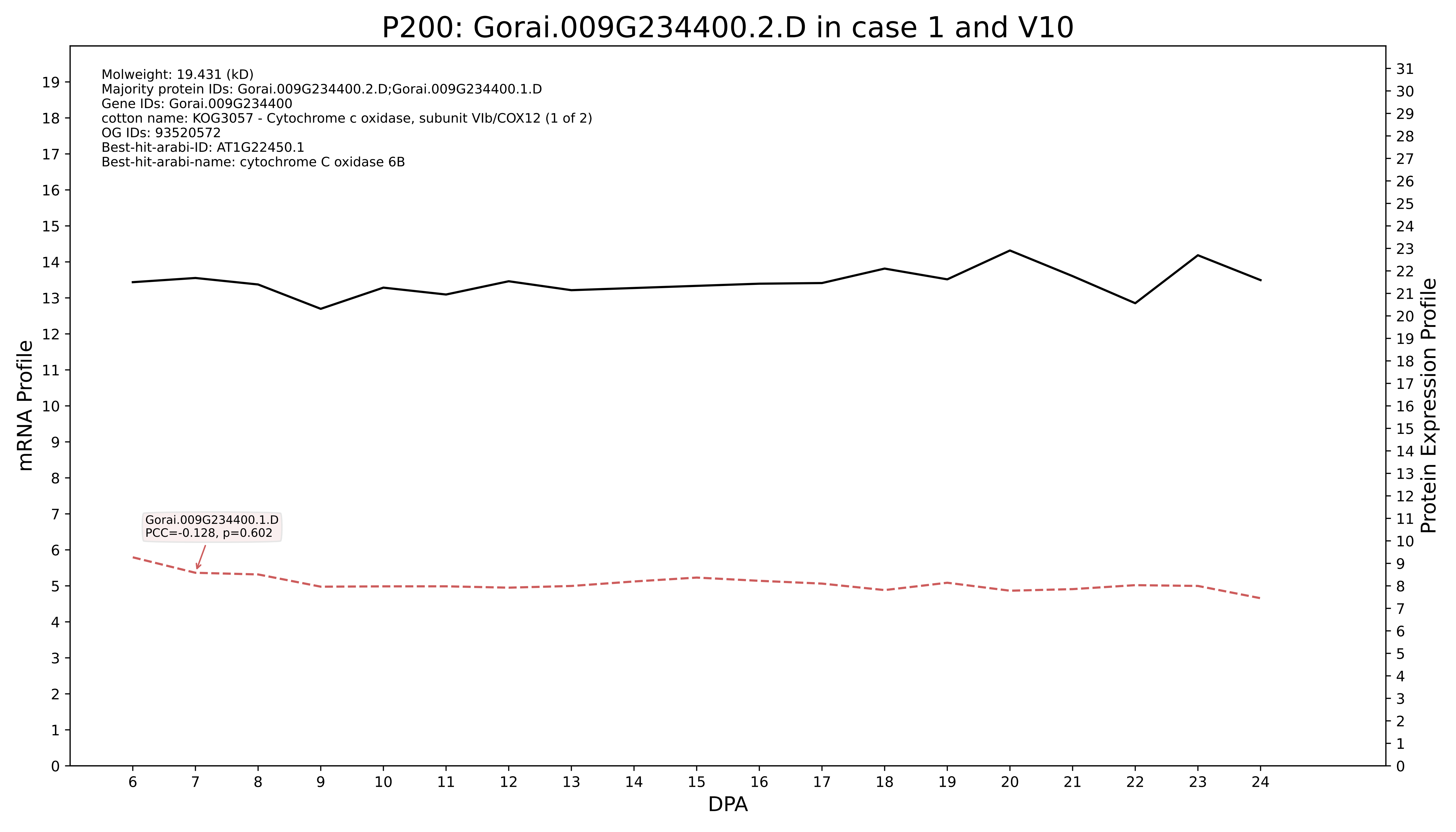
P200: Gorai.009G234400.2.D in case 1 and V10
Molweight: 19.431 (kD)
Majority protein IDs: Gorai.009G234400.2.D;Gorai.009G234400.1.D
Gene IDs: Gorai.009G234400
cotton name: KOG3057 - Cytochrome c oxidase, subunit VIb/COX12 (1 of 2)
OG IDs: 93520572
Best-hit-arabi-ID: AT1G22450.1
Best-hit-arabi-name: cytochrome C oxidase 6B
Gorai.009G234400.1.D
PCC=-0.128, p=0.602
mRNA Profile
Protein Expression Profile
DPA

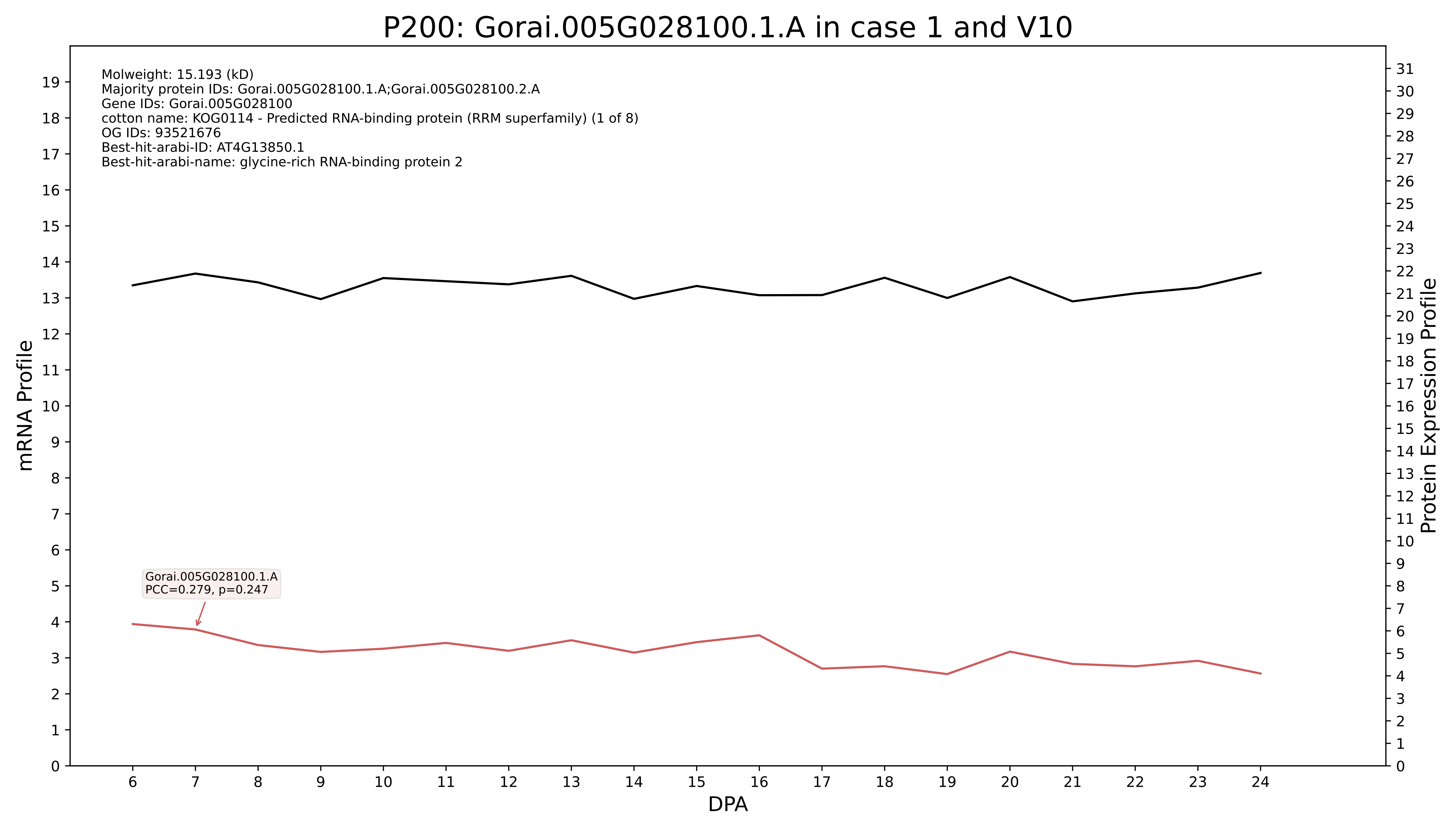
P200: Gorai.005G028100.1.A in case 1 and V10
Molweight: 15.193 (kD)
Majority protein IDs: Gorai.005G028100.1.A;Gorai.005G028100.2.A
Gene IDs: Gorai.005G028100
cotton name: KOG0114 - Predicted RNA-binding protein (RRM superfamily) (1 of 8)
OG IDs: 93521676
Best-hit-arabi-ID: AT4G13850.1
Best-hit-arabi-name: glycine-rich RNA-binding protein 2
Gorai.005G028100.1.A
PCC=0.279, p=0.247
mRNA Profile
Protein Expression Profile
DPA

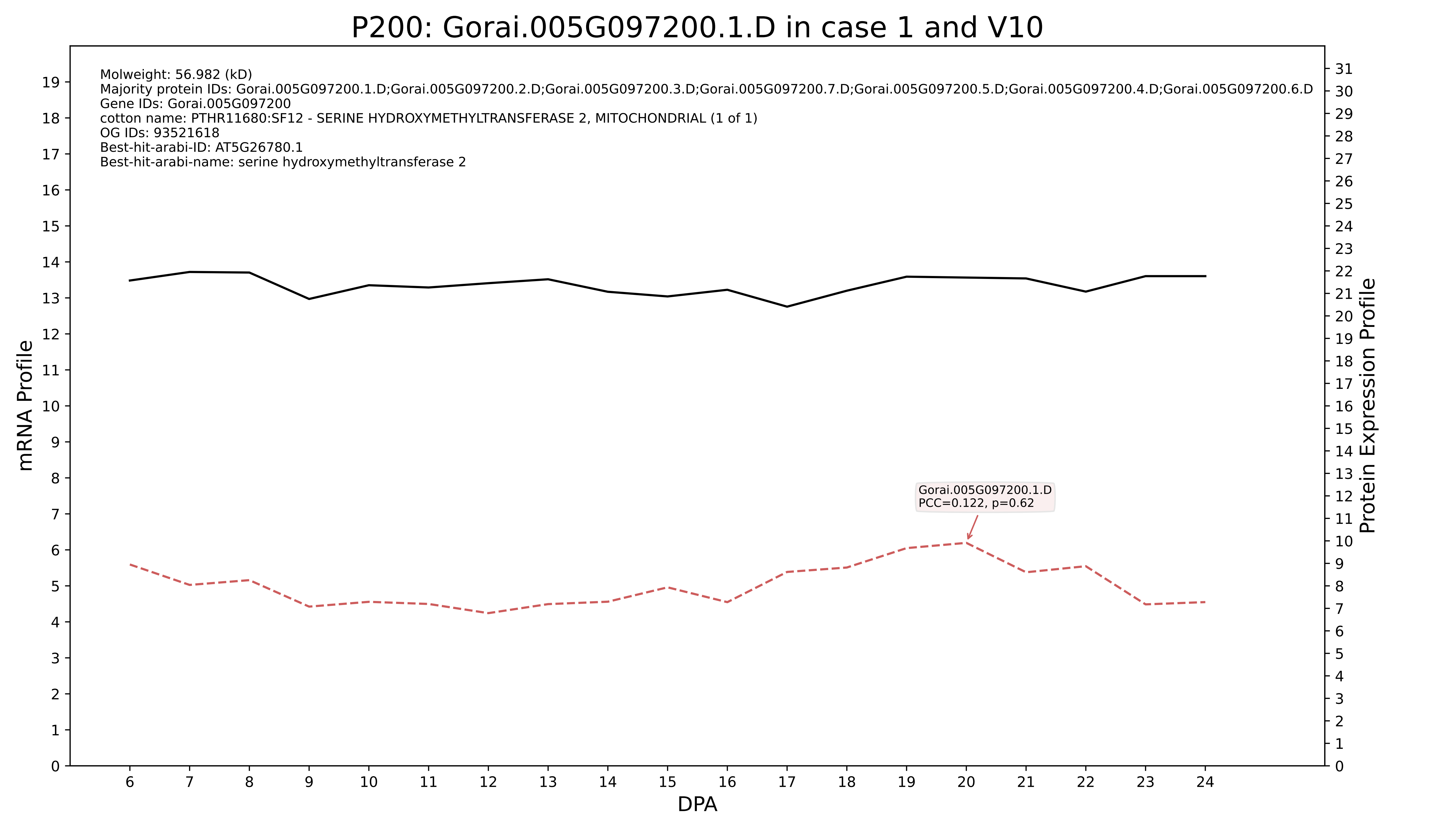
P200: Gorai.005G097200.1.D in case 1 and V10
Molweight: 56.982 (kD)
Majority protein IDs: Gorai.005G097200.1.D;Gorai.005G097200.2.D;Gorai.005G097200.3.D;Gorai.005G097200.7.D;Gorai.005G097200.5.D;Gorai.005G097200.4.D;Gorai.005G097200.6.D
Gene IDs: Gorai.005G097200
cotton name: PTHR11680:SF12 - SERINE HYDROXYMETHYLTRANSFERASE 2, MITOCHONDRIAL (1 of 1)
OG IDs: 93521618
Best-hit-arabi-ID: AT5G26780.1
Best-hit-arabi-name: serine hydroxymethyltransferase 2
Gorai.005G097200.1.D
PCC=0.122, p=0.62
mRNA Profile
Protein Expression Profile
DPA

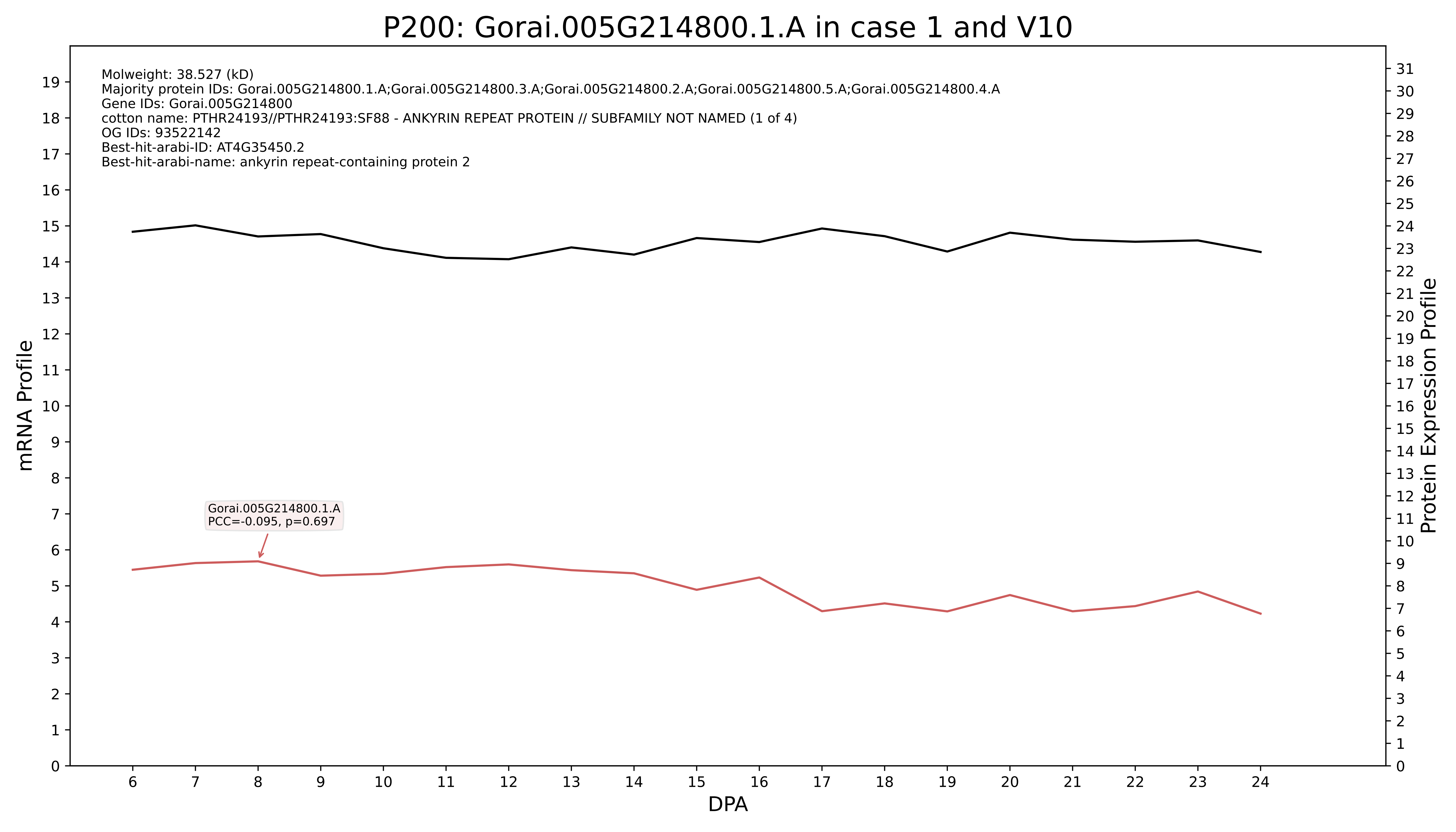
P200: Gorai.005G214800.1.A in case 1 and V10
Molweight: 38.527 (kD)
Majority protein IDs: Gorai.005G214800.1.A;Gorai.005G214800.3.A;Gorai.005G214800.2.A;Gorai.005G214800.5.A;Gorai.005G214800.4.A
Gene IDs: Gorai.005G214800
cotton name: PTHR24193//PTHR24193:SF88 - ANKYRIN REPEAT PROTEIN // SUBFAMILY NOT NAMED (1 of 4)
OG IDs: 93522142
Best-hit-arabi-ID: AT4G35450.2
Best-hit-arabi-name: ankyrin repeat-containing protein 2
Gorai.005G214800.1.A
PCC=-0.095, p=0.697
mRNA Profile
Protein Expression Profile
DPA

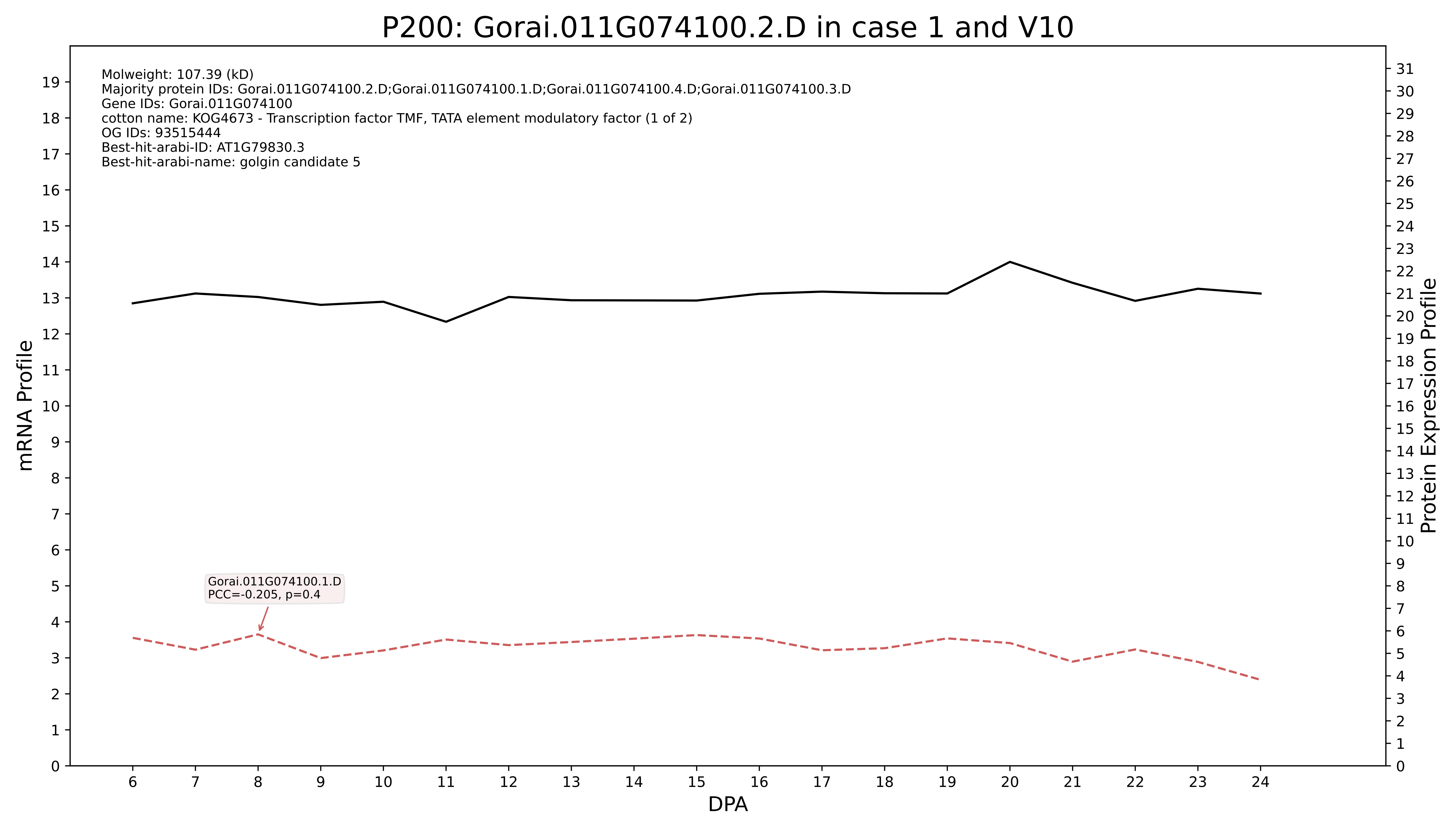
P200: Gorai.011G074100.2.D in case 1 and V10
Molweight: 107.39 (kD)
Majority protein IDs: Gorai.011G074100.2.D;Gorai.011G074100.1.D;Gorai.011G074100.4.D;Gorai.011G074100.3.D
Gene IDs: Gorai.011G074100
cotton name: KOG4673 - Transcription factor TMF, TATA element modulatory factor (1 of 2)
OG IDs: 93515444
Best-hit-arabi-ID: AT1G79830.3
Best-hit-arabi-name: golgin candidate 5
Gorai.011G074100.1.D
PCC=-0.205, p=0.4
mRNA Profile
Protein Expression Profile
DPA

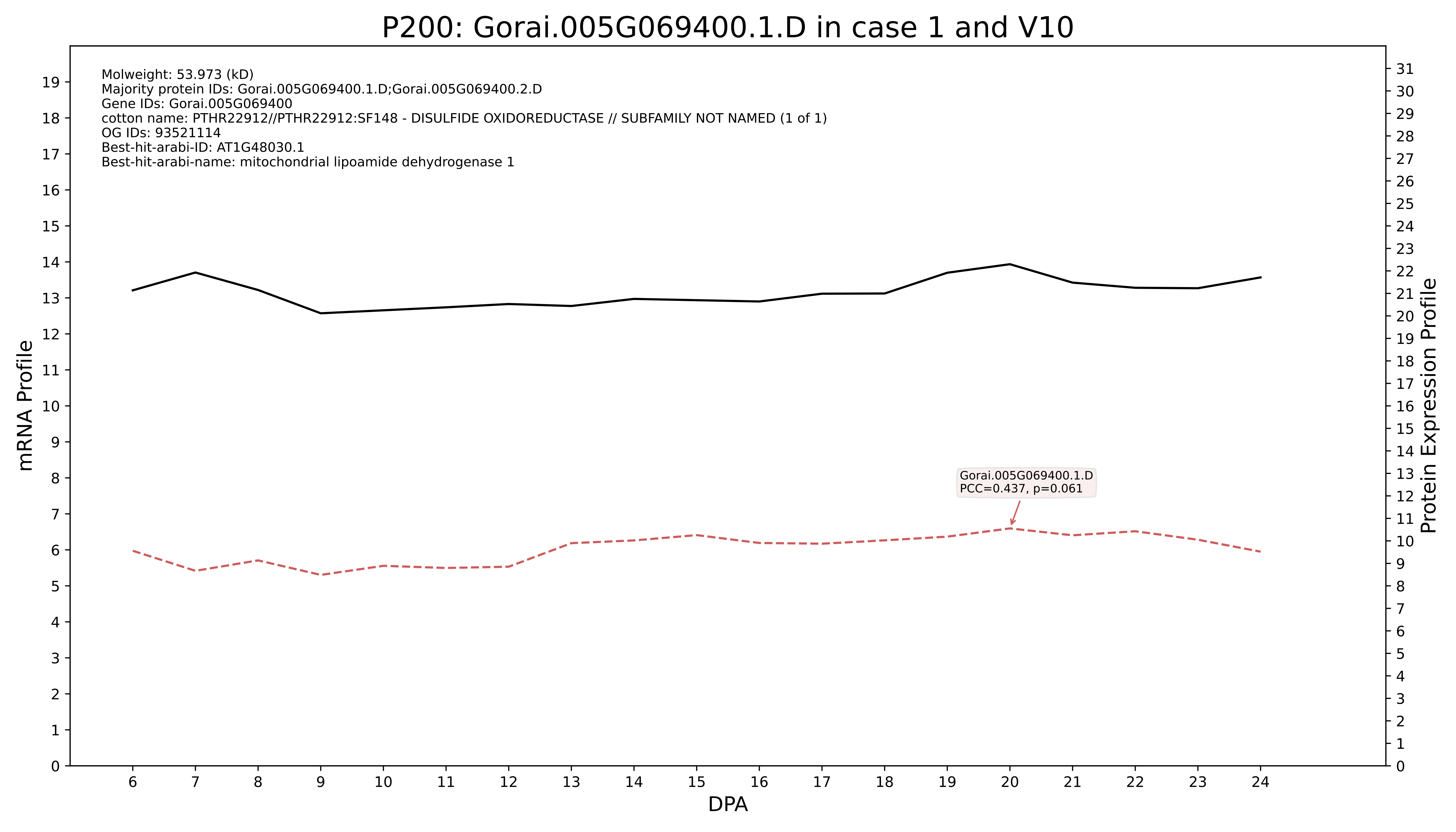
P200: Gorai.005G069400.1.D in case 1 and V10
Molweight: 53.973 (kD)
Majority protein IDs: Gorai.005G069400.1.D;Gorai.005G069400.2.D
Gene IDs: Gorai.005G069400
cotton name: PTHR22912//PTHR22912:SF148 - DISULFIDE OXIDOREDUCTASE // SUBFAMILY NOT NAMED (1 of 1)
OG IDs: 93521114
Best-hit-arabi-ID: AT1G48030.1
Best-hit-arabi-name: mitochondrial lipoamide dehydrogenase 1
Gorai.005G069400.1.D
PCC=0.437, p=0.061
mRNA Profile
Protein Expression Profile
DPA
0
1
2
3
4
5
6
7
8
9
10
11
12
13
14
15
16
17
18
19
20
21
22
23
24
25
26
27
28
29
30
31

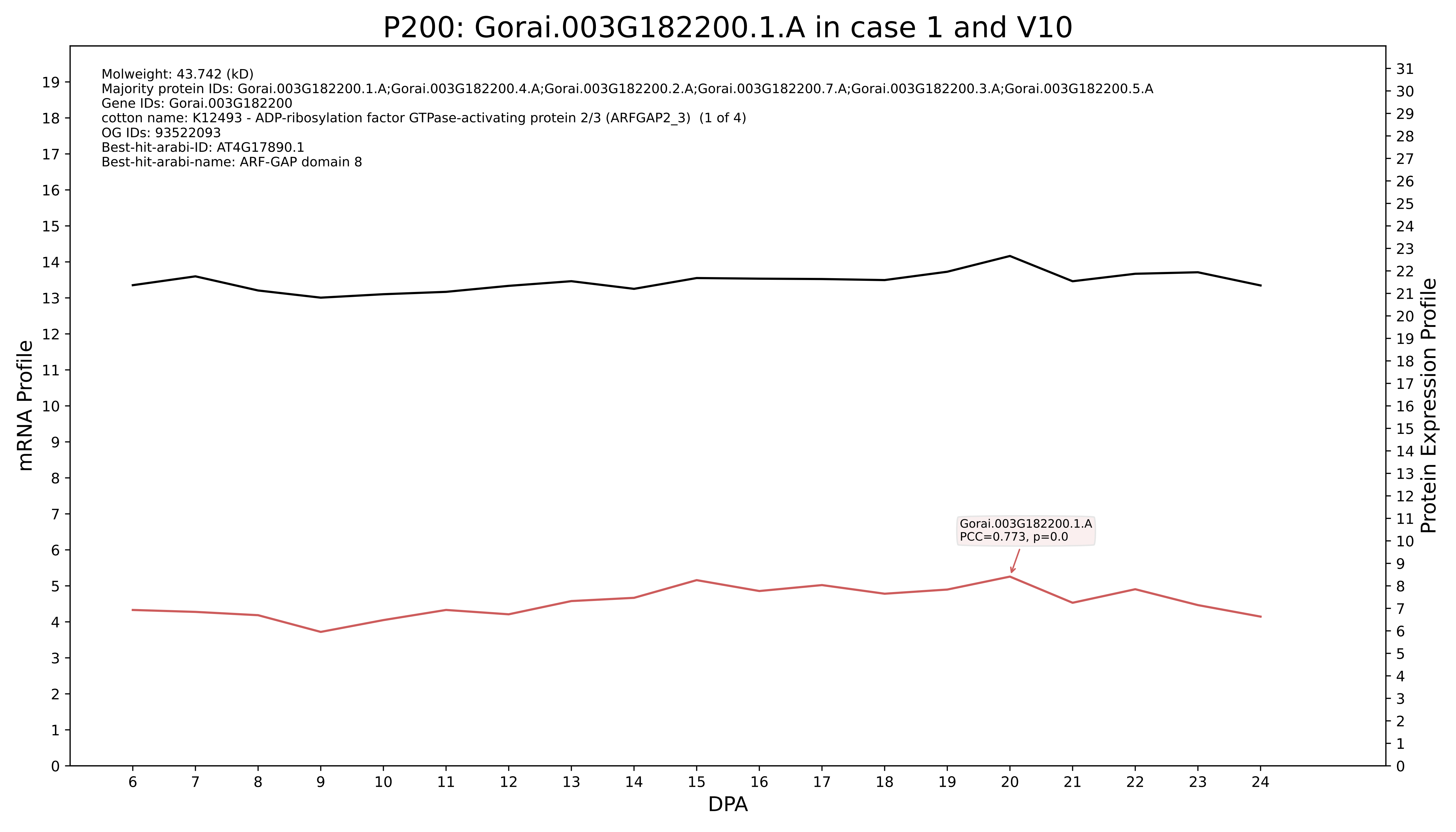

P200: Gorai.003G182200.1.A in case 1 and V10
Molweight: 43.742 (kD)
Majority protein IDs: Gorai.003G182200.1.A;Gorai.003G182200.4.A;Gorai.003G182200.2.A;Gorai.003G182200.7.A;Gorai.003G182200.3.A;Gorai.003G182200.5.A
Gene IDs: Gorai.003G182200
cotton name: K12493 - ADP-ribosylation factor GTPase-activating protein 2/3 (ARFGAP2_3) (1 of 4)
OG IDs: 93522093
Best-hit-arabi-ID: AT4G17890.1
Best-hit-arabi-name: ARF-GAP domain 8
Gorai.003G182200.1.A
PCC=0.773, p=0.0
mRNA Profile
Protein Expression Profile
DPA

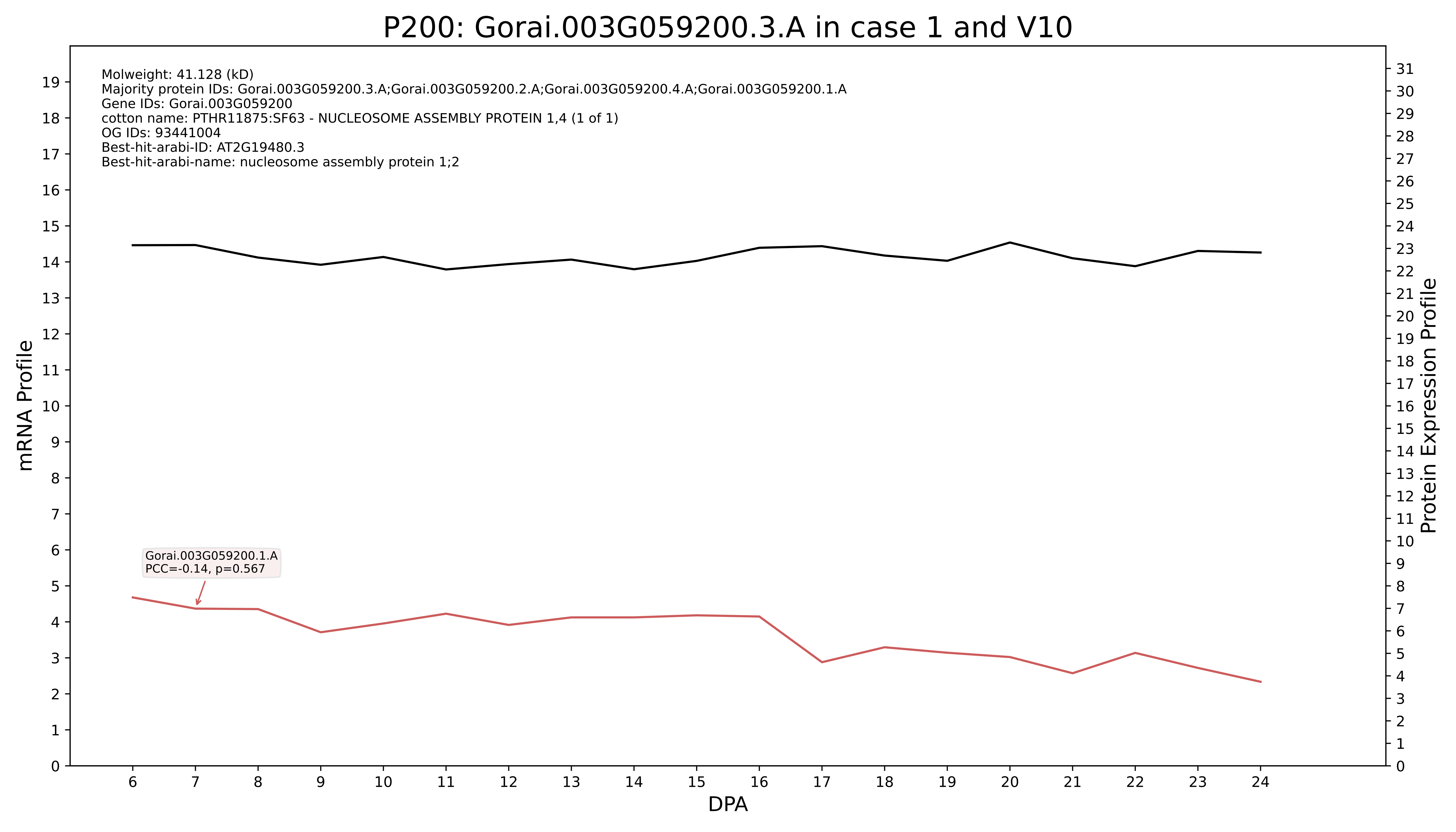
P200: Gorai.003G059200.3.A in case 1 and V10
Molweight: 41.128 (kD)
Majority protein IDs: Gorai.003G059200.3.A;Gorai.003G059200.2.A;Gorai.003G059200.4.A;Gorai.003G059200.1.A
Gene IDs: Gorai.003G059200
cotton name: PTHR11875:SF63 - NUCLEOSOME ASSEMBLY PROTEIN 1,4 (1 of 1)
OG IDs: 93441004
Best-hit-arabi-ID: AT2G19480.3
Best-hit-arabi-name: nucleosome assembly protein 1;2
Gorai.003G059200.1.A
PCC=-0.14, p=0.567
mRNA Profile
Protein Expression Profile
DPA
0
1
2
3
4
5
6
7
8
9
10
11
12
13
14
15
16
17
18
19
20
21
22
23
24
25
26
27
28
29
30
31

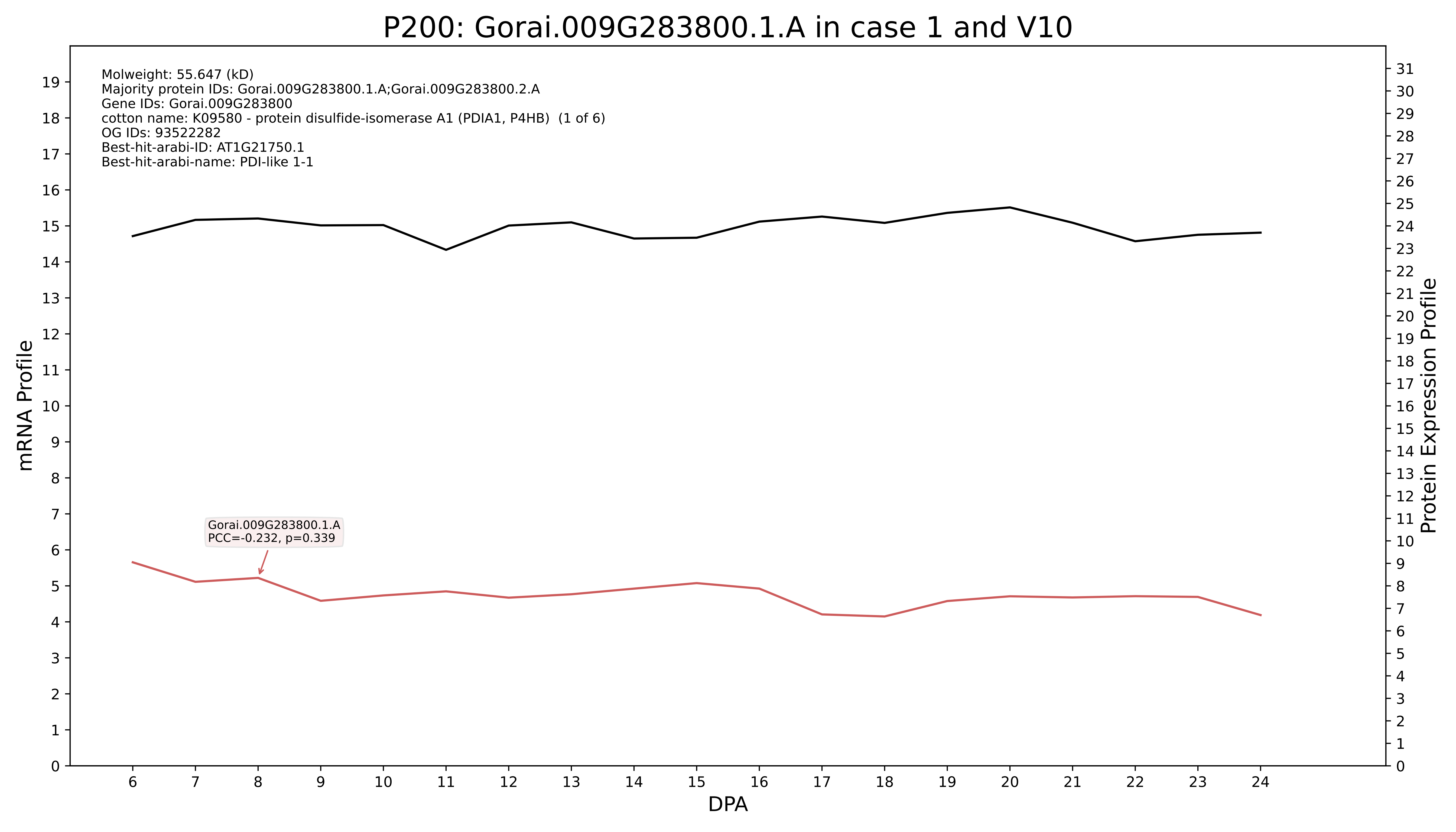
P200: Gorai.009G283800.1.A in case 1 and V10
Molweight: 55.647 (kD)
Majority protein IDs: Gorai.009G283800.1.A;Gorai.009G283800.2.A
Gene IDs: Gorai.009G283800
cotton name: K09580 - protein disulfide-isomerase A1 (PDIA1, P4HB) (1 of 6)
OG IDs: 93522282
Best-hit-arabi-ID: AT1G21750.1
Best-hit-arabi-name: PDI-like 1-1
Gorai.009G283800.1.A
PCC=-0.232, p=0.339
mRNA Profile
Protein Expression Profile
DPA

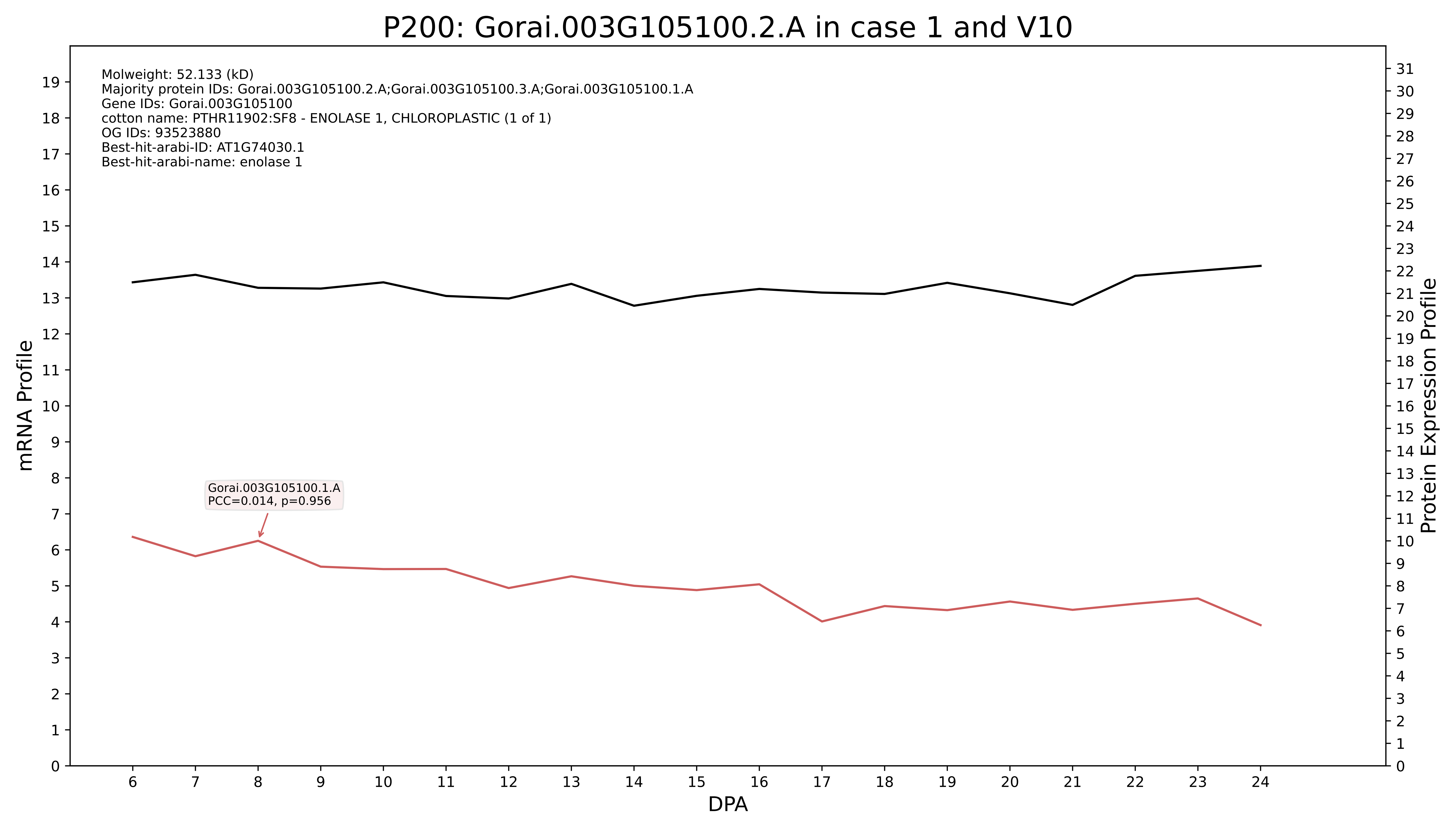
P200: Gorai.003G105100.2.A in case 1 and V10
Molweight: 52.133 (kD)
Majority protein IDs: Gorai.003G105100.2.A;Gorai.003G105100.3.A;Gorai.003G105100.1.A
Gene IDs: Gorai.003G105100
cotton name: PTHR11902:SF8 - ENOLASE 1, CHLOROPLASTIC (1 of 1)
OG IDs: 93523880
Best-hit-arabi-ID: AT1G74030.1
Best-hit-arabi-name: enolase 1
Gorai.003G105100.1.A
PCC=0.014, p=0.956
mRNA Profile
Protein Expression Profile
DPA
0
1
2
3
4
5
6
7
8
9
10
11
12
13
14
15
16
17
18
19
20
21
22
23
24
25
26
27
28
29
30
31

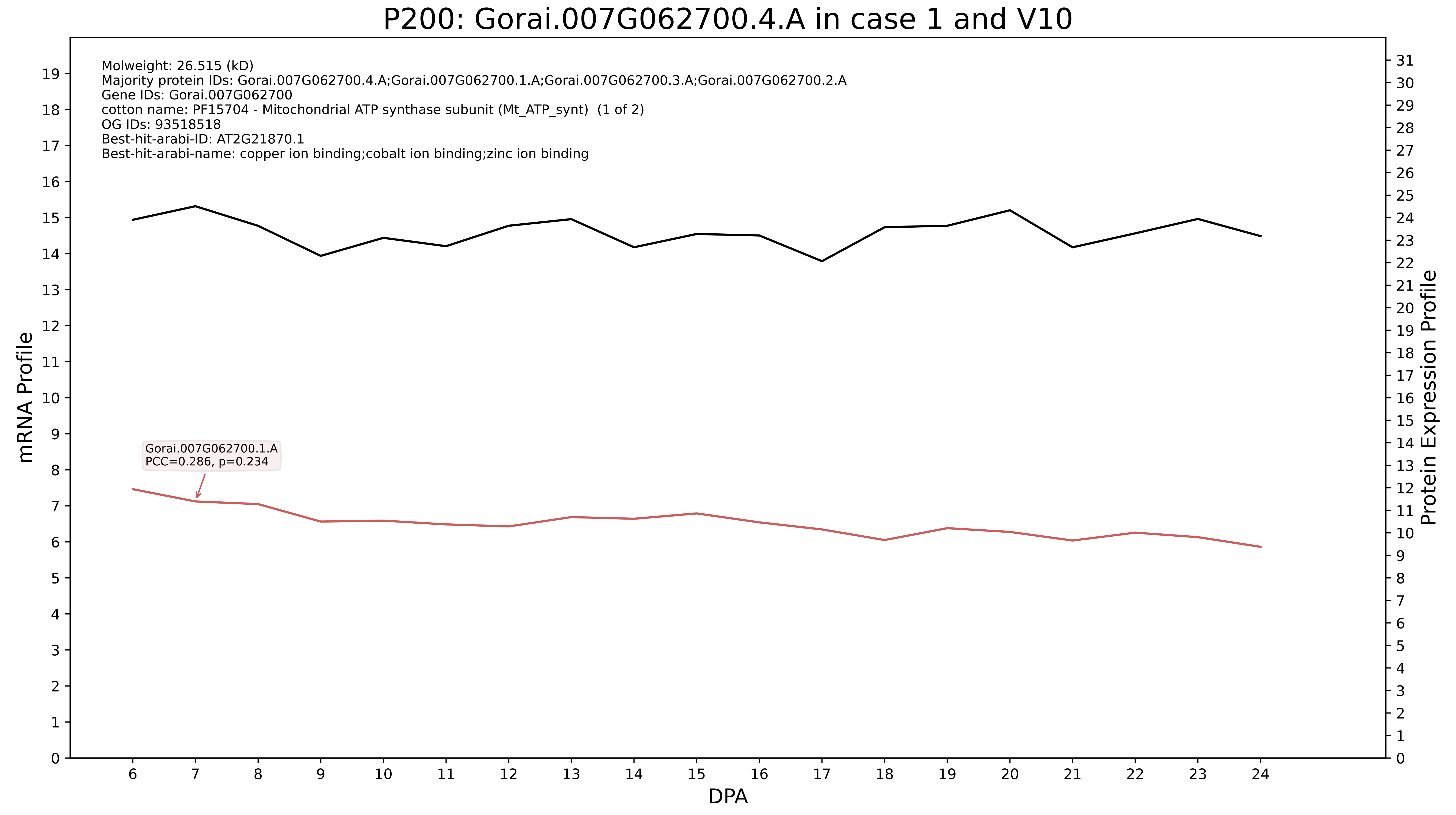

P200: Gorai.007G062700.4.A in case 1 and V10
Molweight: 26.515 (kD)
Majority protein IDs: Gorai.007G062700.4.A;Gorai.007G062700.1.A;Gorai.007G062700.3.A;Gorai.007G062700.2.A
Gene IDs: Gorai.007G062700
cotton name: PF15704 - Mitochondrial ATP synthase subunit (Mt_ATP_synt) (1 of 2)
OG IDs: 93518518
Best-hit-arabi-ID: AT2G21870.1
Best-hit-arabi-name: copper ion binding;cobalt ion binding;zinc ion binding
Gorai.007G062700.1.A
PCC=0.286, p=0.234
mRNA Profile
Protein Expression Profile
DPA

P200: Gorai.005G062400.4.D in case 1 and V10

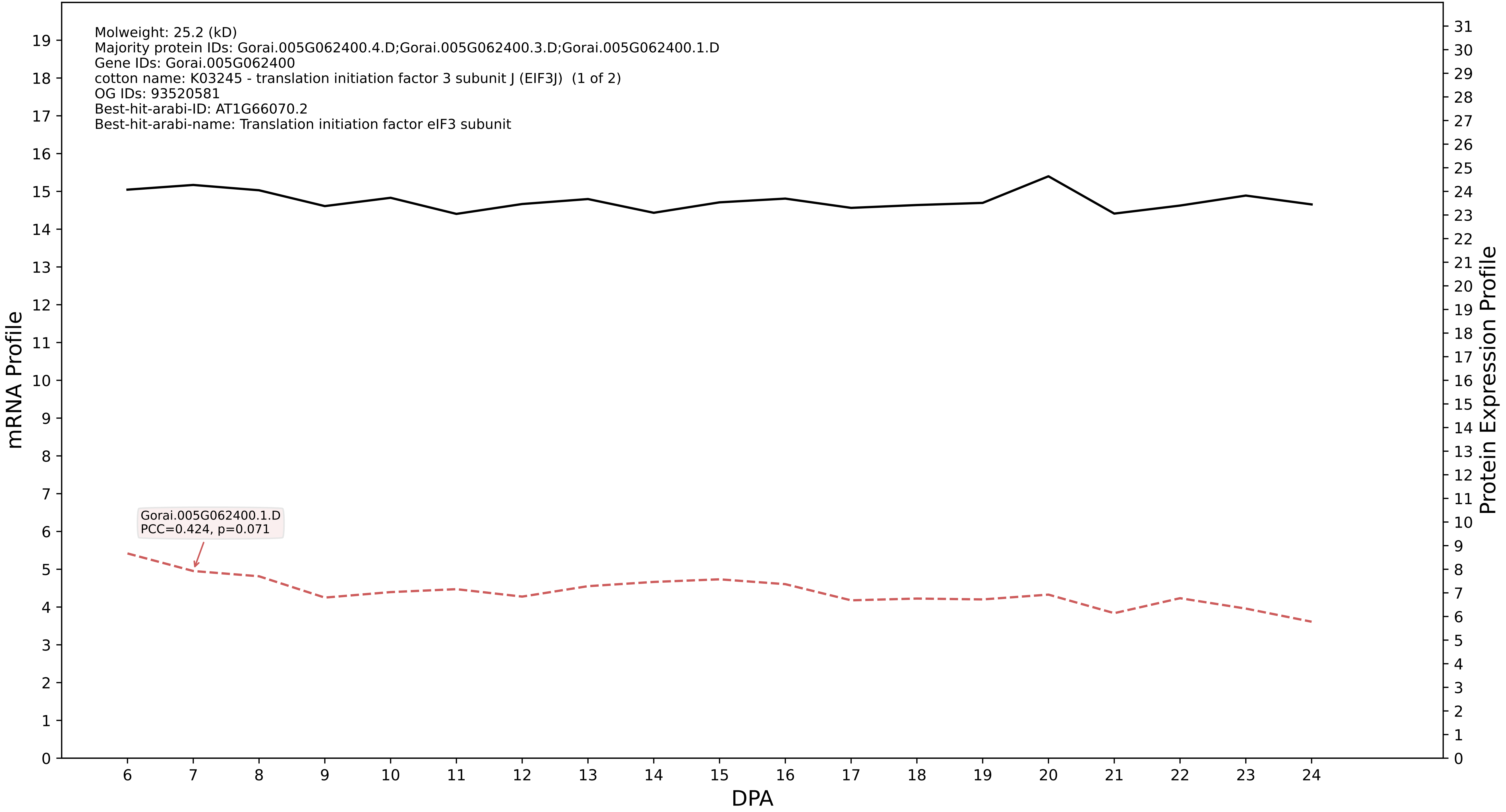

P200: Gorai.002G077600.3.D in case 1 and V10

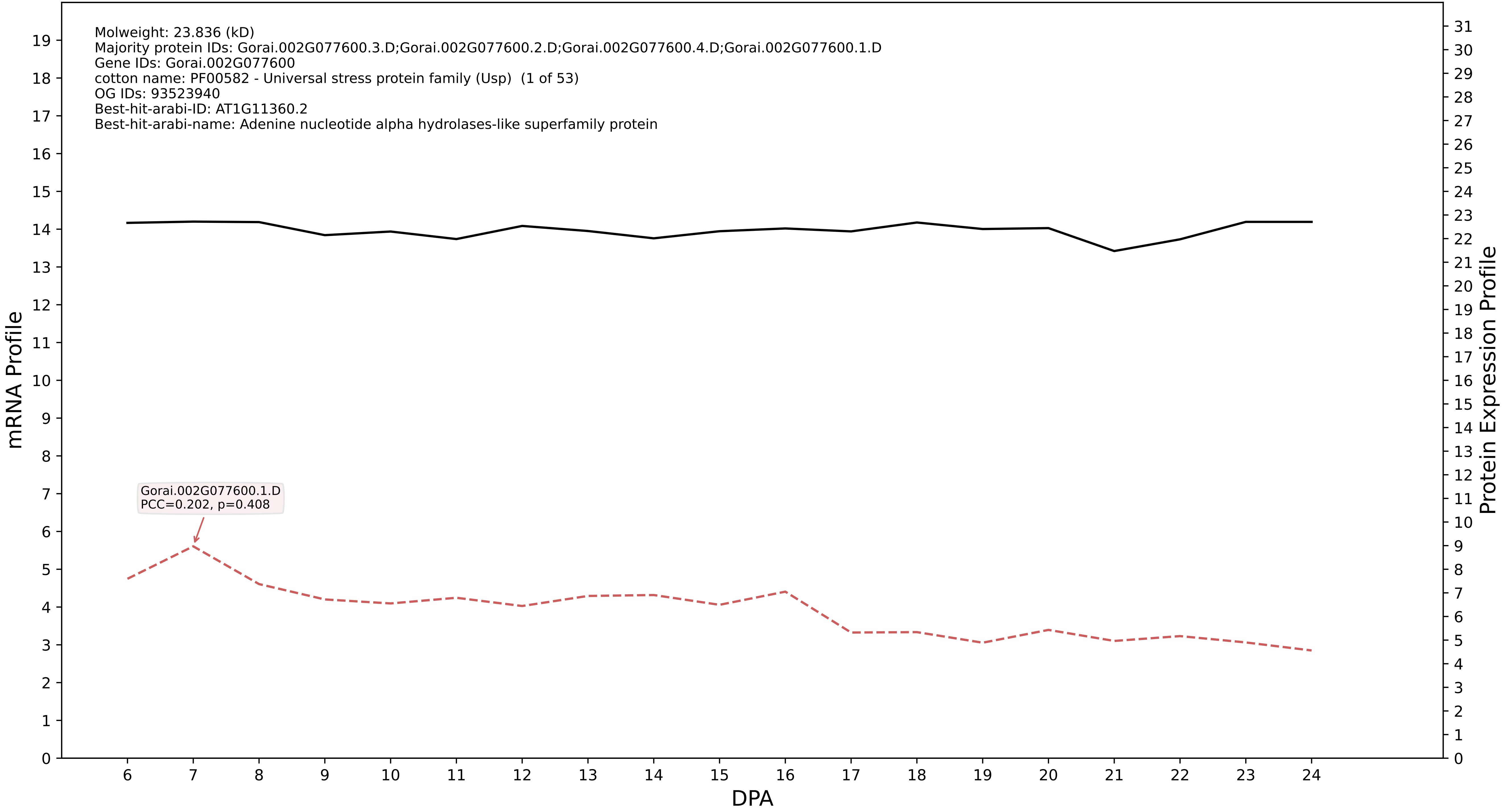

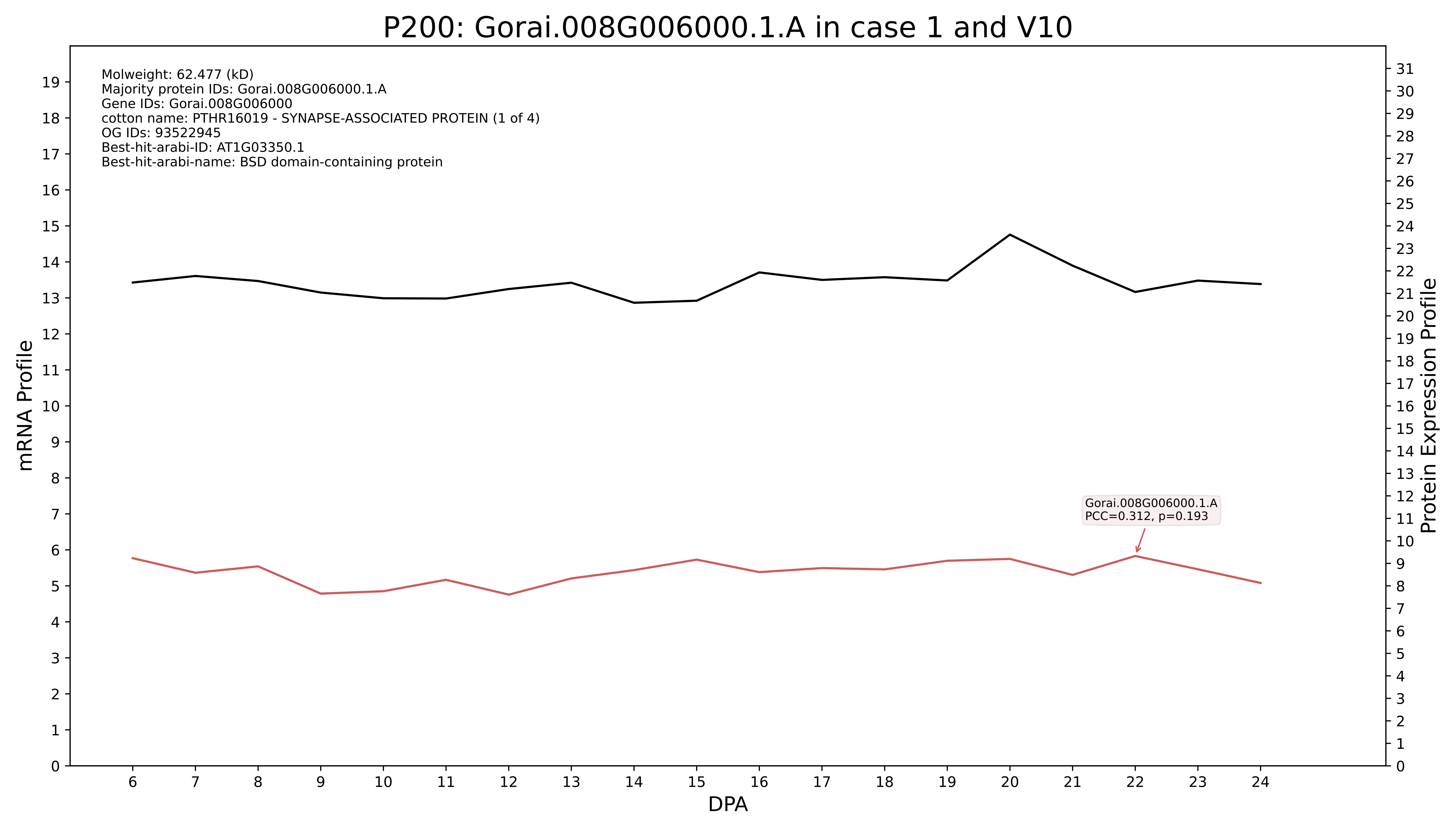
P200: Gorai.008G006000.1.A in case 1 and V10
Molweight: 62.477 (kD)
Majority protein IDs: Gorai.008G006000.1.A
Gene IDs: Gorai.008G006000
cotton name: PTHR16019 - SYNAPSE-ASSOCIATED PROTEIN (1 of 4)
OG IDs: 93522945
Best-hit-arabi-ID: AT1G03350.1
Best-hit-arabi-name: BSD domain-containing protein
Gorai.008G006000.1.A
PCC=0.312, p=0.193
mRNA Profile
Protein Expression Profile
DPA

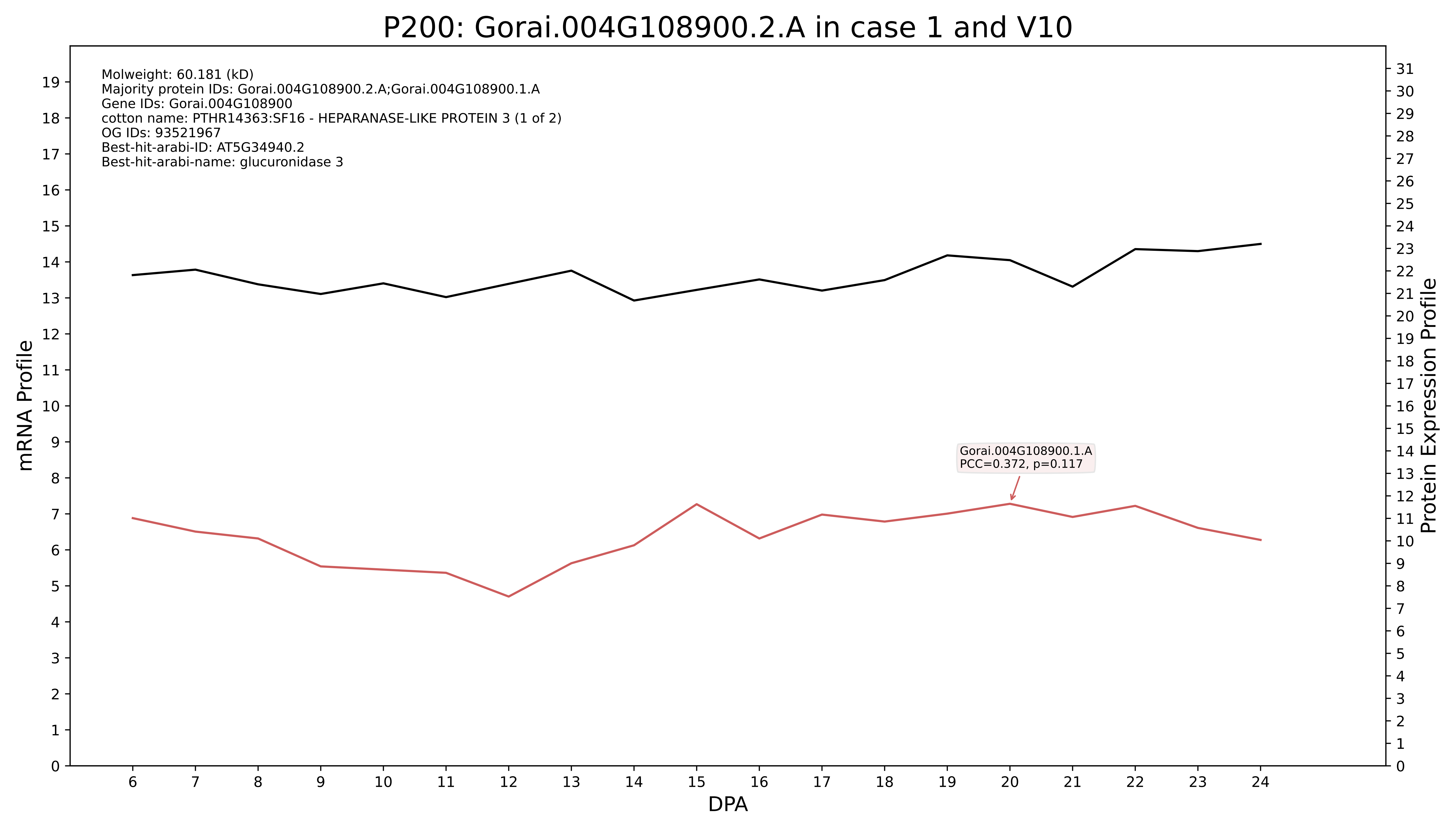
P200: Gorai.004G108900.2.A in case 1 and V10
Molweight: 60.181 (kD)
Majority protein IDs: Gorai.004G108900.2.A;Gorai.004G108900.1.A
Gene IDs: Gorai.004G108900
cotton name: PTHR14363:SF16 - HEPARANASE-LIKE PROTEIN 3 (1 of 2)
OG IDs: 93521967
Best-hit-arabi-ID: AT5G34940.2
Best-hit-arabi-name: glucuronidase 3
Gorai.004G108900.1.A
PCC=0.372, p=0.117
mRNA Profile
Protein Expression Profile
DPA

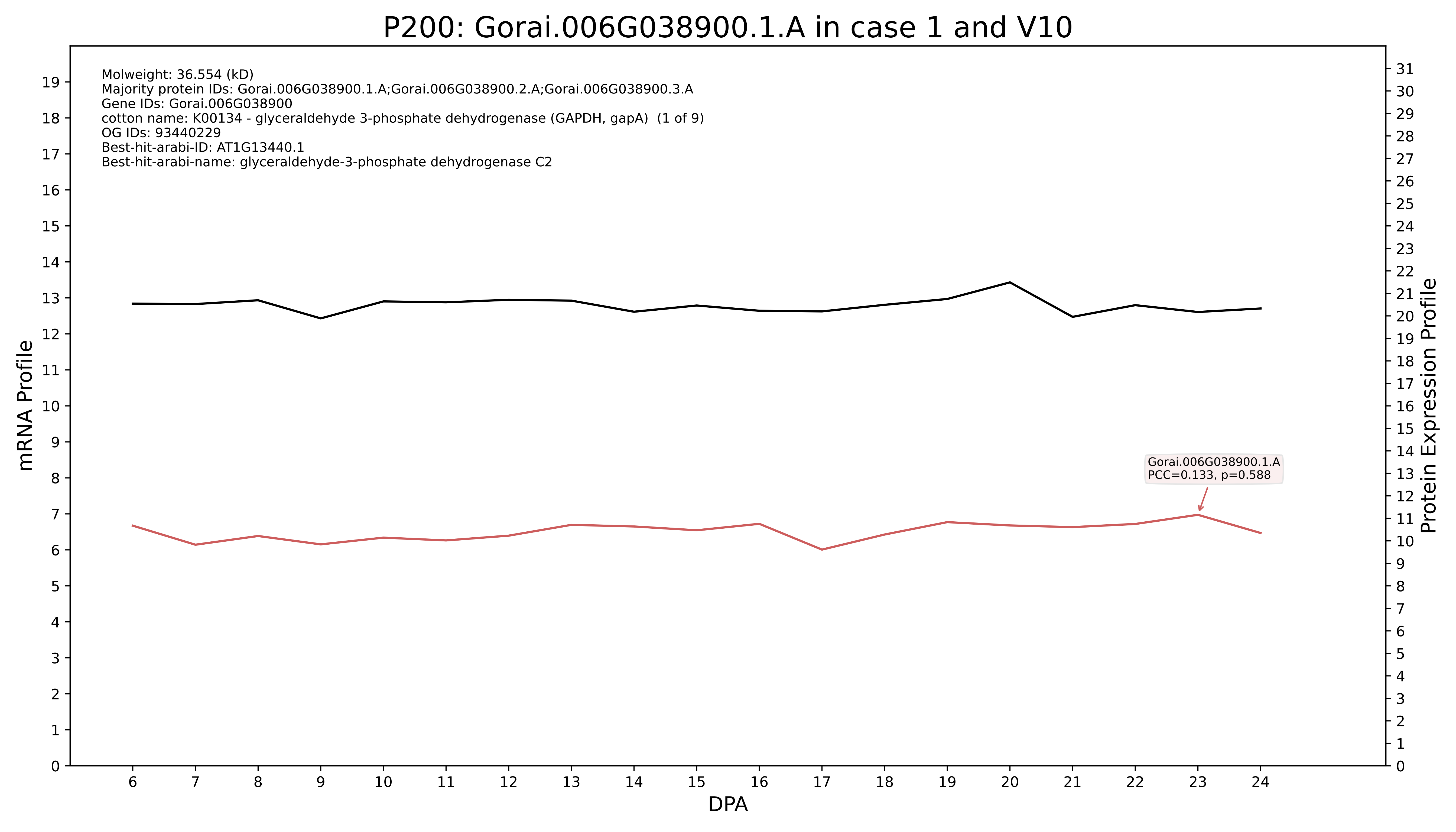
P200: Gorai.006G038900.1.A in case 1 and V10
Molweight: 36.554 (kD)
Majority protein IDs: Gorai.006G038900.1.A;Gorai.006G038900.2.A;Gorai.006G038900.3.A
Gene IDs: Gorai.006G038900
cotton name: K00134 - glyceraldehyde 3-phosphate dehydrogenase (GAPDH, gapA) (1 of 9)
OG IDs: 93440229
Best-hit-arabi-ID: AT1G13440.1
Best-hit-arabi-name: glyceraldehyde-3-phosphate dehydrogenase C2
Gorai.006G038900.1.A
PCC=0.133, p=0.588
mRNA Profile
Protein Expression Profile
DPA

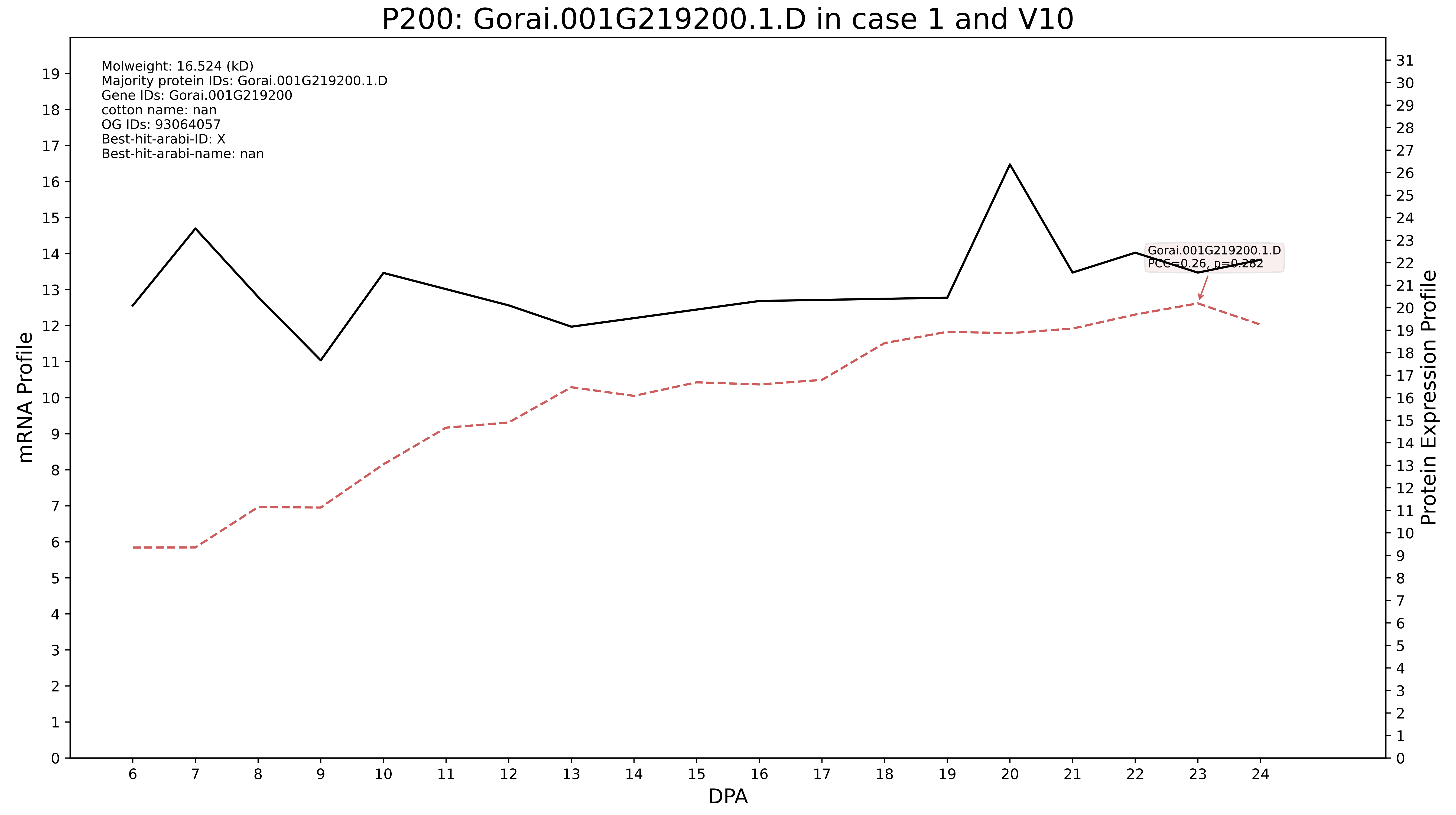

P200: Gorai.001G219200.1.D in case 1 and V10
Molweight: 16.524 (kD)
Majority protein IDs: Gorai.001G219200.1.D
Gene IDs: Gorai.001G219200
cotton name: nan
OG IDs: 93064057
Best-hit-arabi-ID: X
Best-hit-arabi-name: nan
Gorai.001G219200.1.D
PCC=0.26, p=0.282
mRNA Profile
Protein Expression Profile
DPA

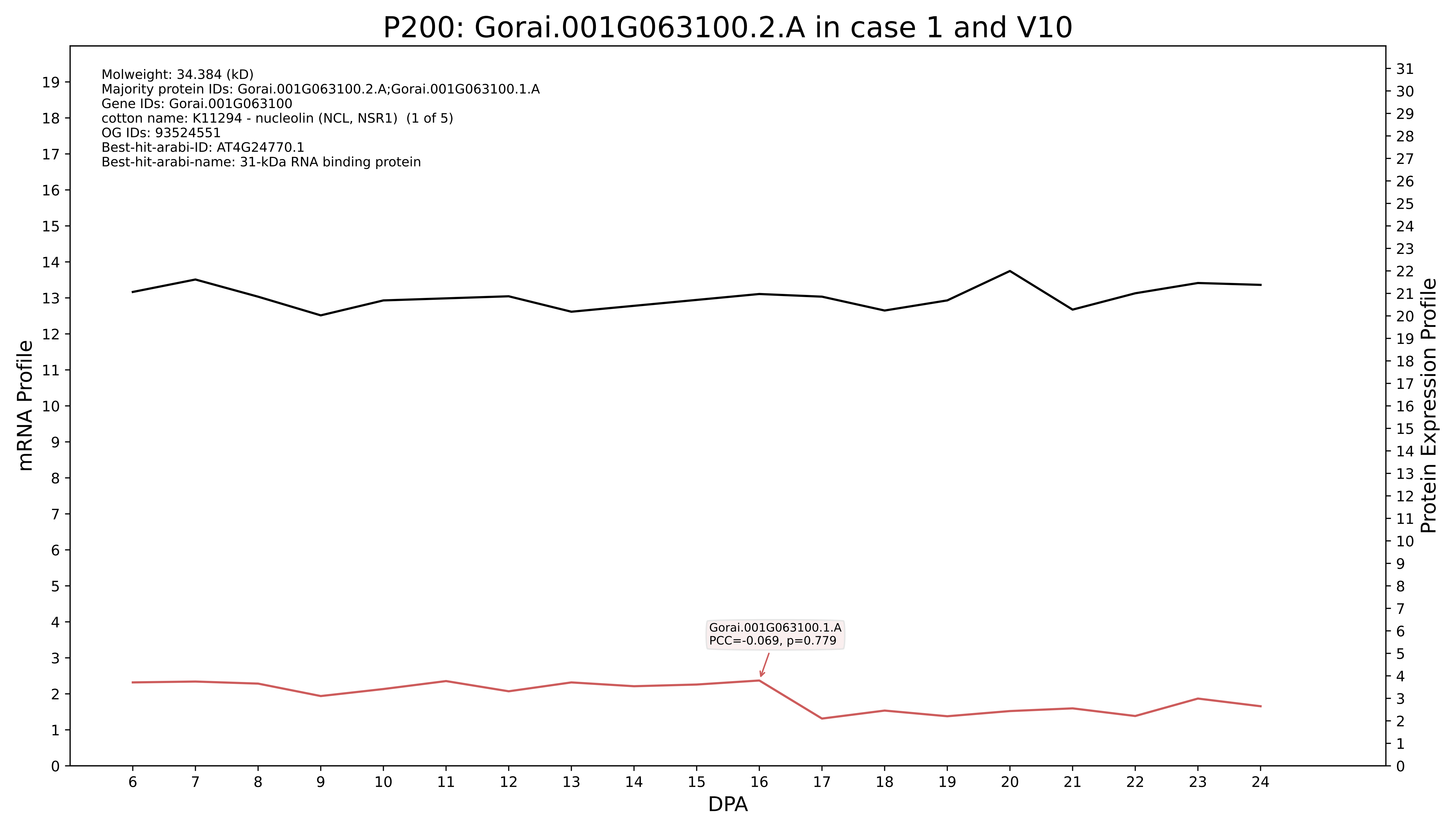
P200: Gorai.001G063100.2.A in case 1 and V10
Molweight: 34.384 (kD)
Majority protein IDs: Gorai.001G063100.2.A;Gorai.001G063100.1.A
Gene IDs: Gorai.001G063100
cotton name: K11294 - nucleolin (NCL, NSR1) (1 of 5)
OG IDs: 93524551
Best-hit-arabi-ID: AT4G24770.1
Best-hit-arabi-name: 31-kDa RNA binding protein
Gorai.001G063100.1.A
PCC=-0.069, p=0.779
mRNA Profile
Protein Expression Profile
DPA

P200: Gorai.002G133800.2.D in case 1 and V10

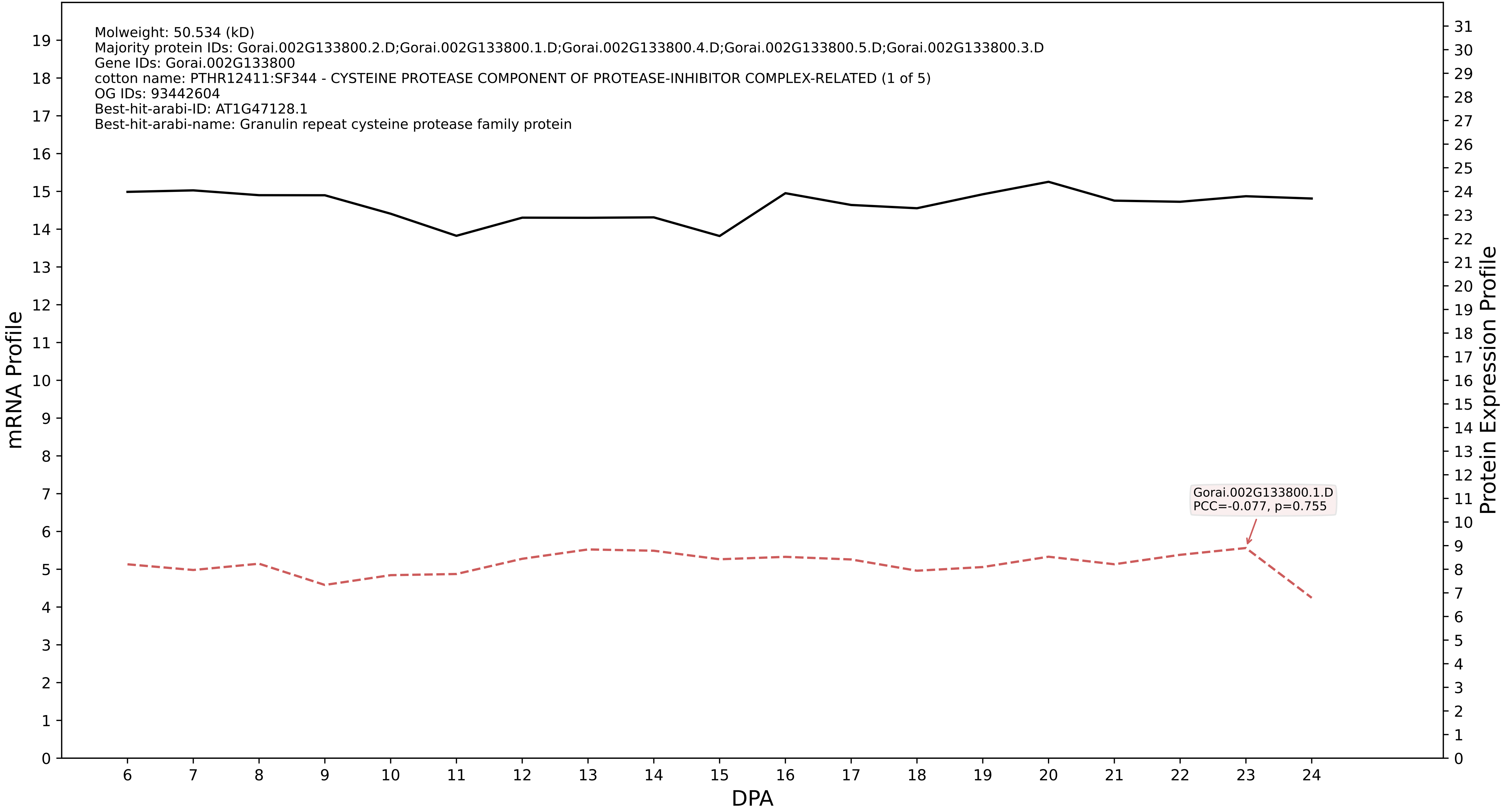

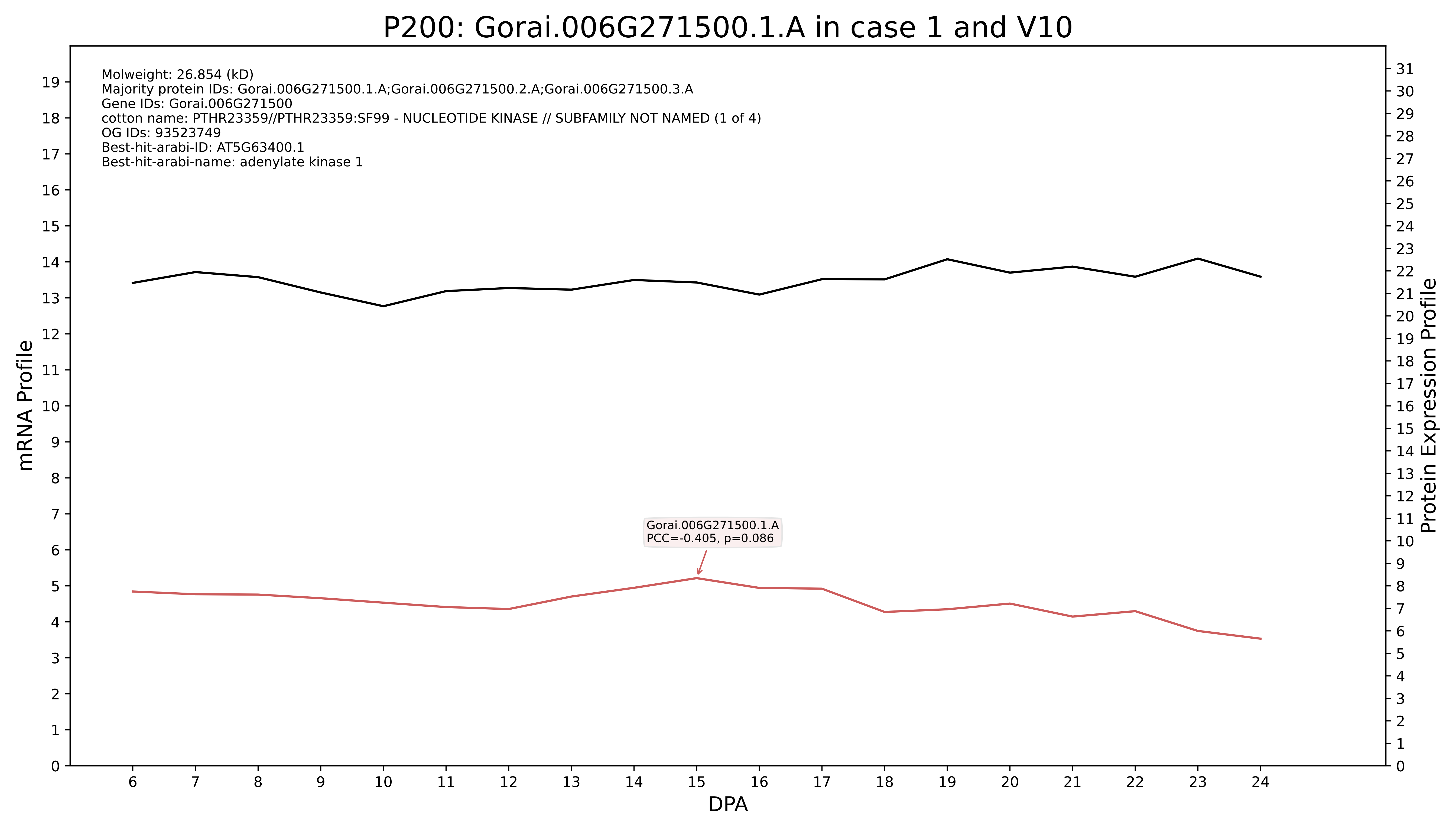
P200: Gorai.006G271500.1.A in case 1 and V10
Molweight: 26.854 (kD)
Majority protein IDs: Gorai.006G271500.1.A;Gorai.006G271500.2.A;Gorai.006G271500.3.A
Gene IDs: Gorai.006G271500
cotton name: PTHR23359//PTHR23359:SF99 - NUCLEOTIDE KINASE // SUBFAMILY NOT NAMED (1 of 4)
OG IDs: 93523749
Best-hit-arabi-ID: AT5G63400.1
Best-hit-arabi-name: adenylate kinase 1
Gorai.006G271500.1.A
PCC=-0.405, p=0.086
mRNA Profile
Protein Expression Profile
DPA

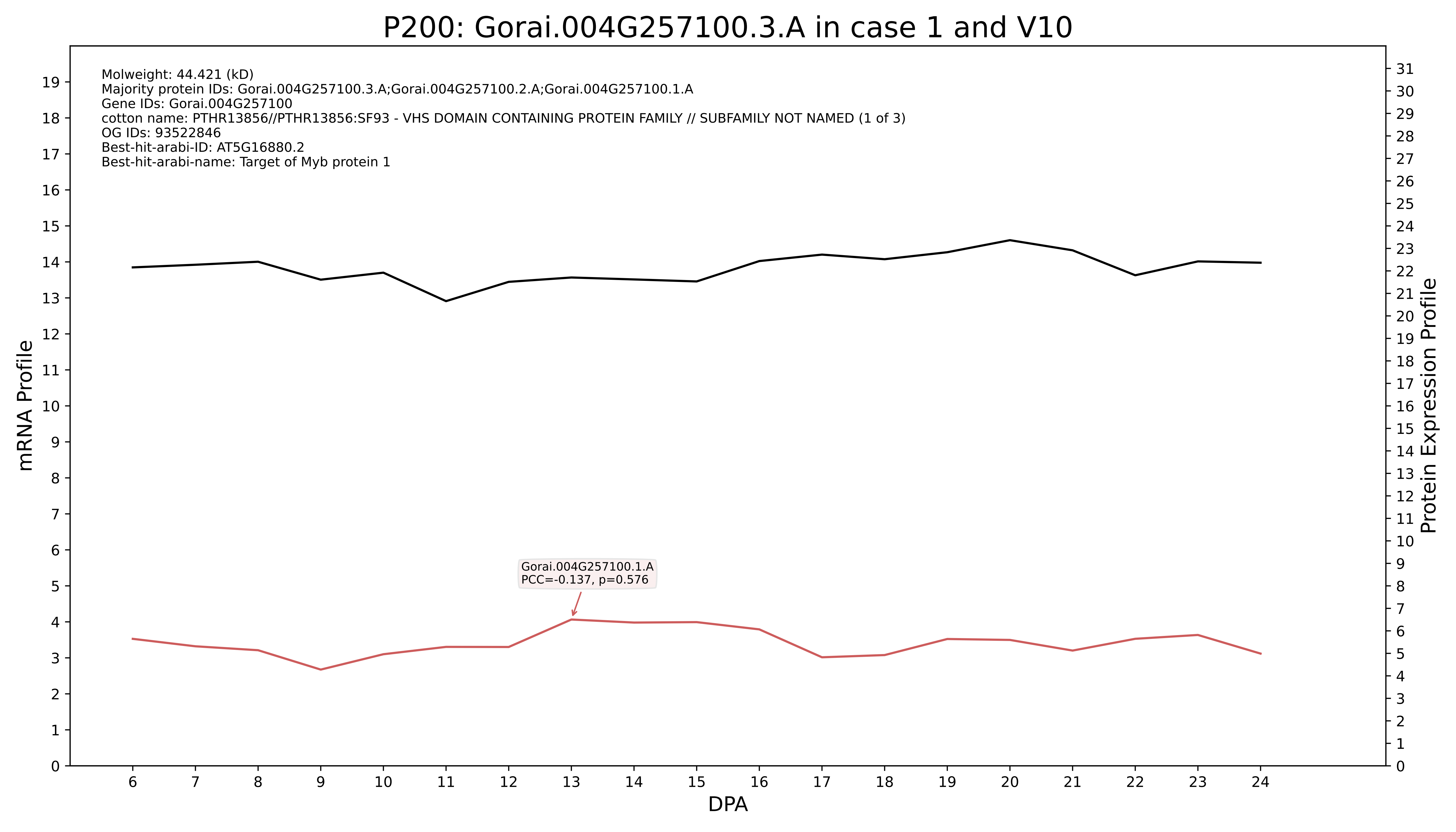
P200: Gorai.004G257100.3.A in case 1 and V10
Molweight: 44.421 (kD)
Majority protein IDs: Gorai.004G257100.3.A;Gorai.004G257100.2.A;Gorai.004G257100.1.A
Gene IDs: Gorai.004G257100
cotton name: PTHR13856//PTHR13856:SF93 - VHS DOMAIN CONTAINING PROTEIN FAMILY // SUBFAMILY NOT NAMED (1 of 3)
OG IDs: 93522846
Best-hit-arabi-ID: AT5G16880.2
Best-hit-arabi-name: Target of Myb protein 1
Gorai.004G257100.1.A
PCC=-0.137, p=0.576
mRNA Profile
Protein Expression Profile
DPA

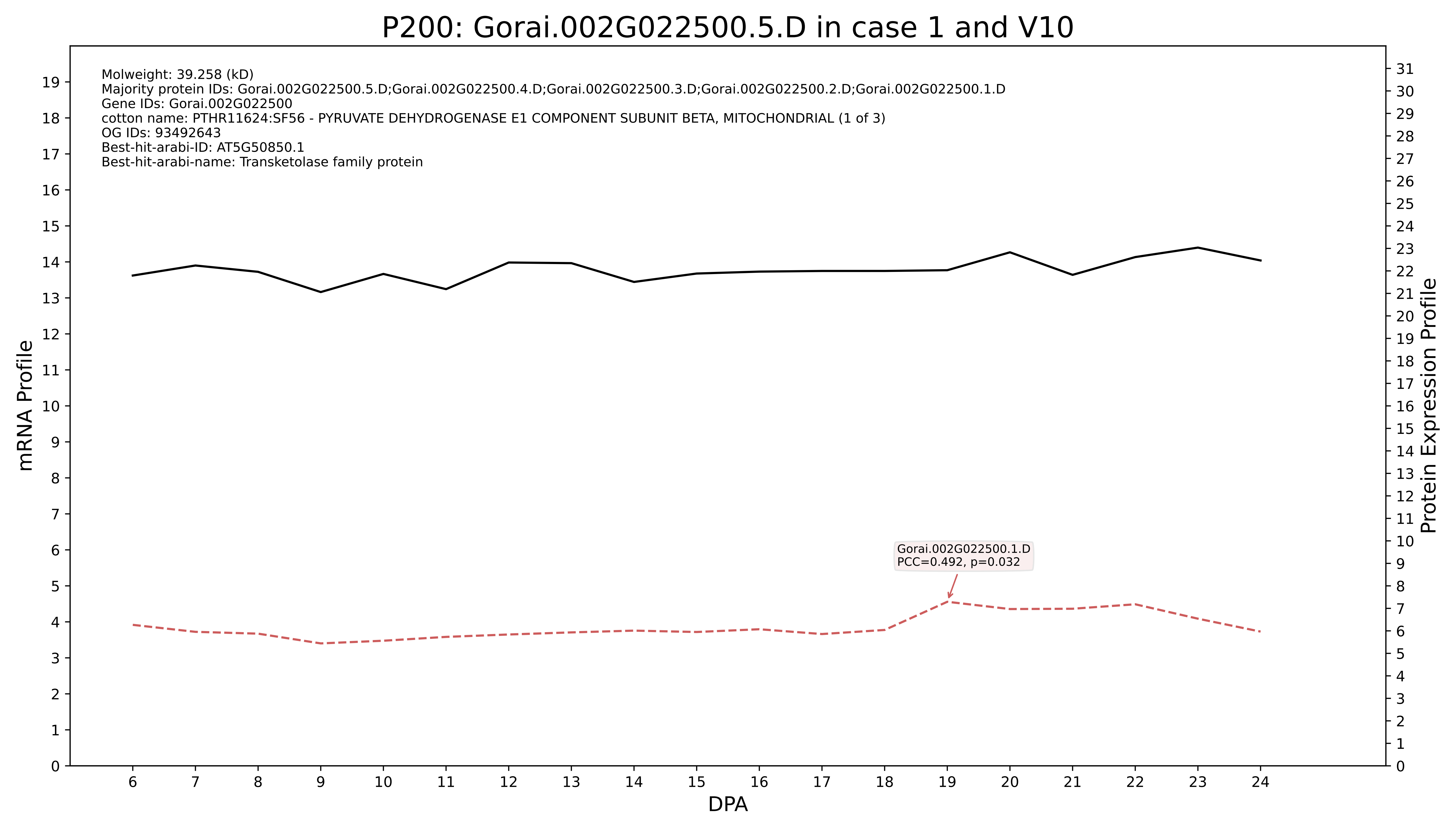
P200: Gorai.002G022500.5.D in case 1 and V10
Molweight: 39.258 (kD)
Majority protein IDs: Gorai.002G022500.5.D;Gorai.002G022500.4.D;Gorai.002G022500.3.D;Gorai.002G022500.2.D;Gorai.002G022500.1.D
Gene IDs: Gorai.002G022500
cotton name: PTHR11624:SF56 - PYRUVATE DEHYDROGENASE E1 COMPONENT SUBUNIT BETA, MITOCHONDRIAL (1 of 3)
OG IDs: 93492643
Best-hit-arabi-ID: AT5G50850.1
Best-hit-arabi-name: Transketolase family protein
Gorai.002G022500.1.D
PCC=0.492, p=0.032
mRNA Profile
Protein Expression Profile
DPA

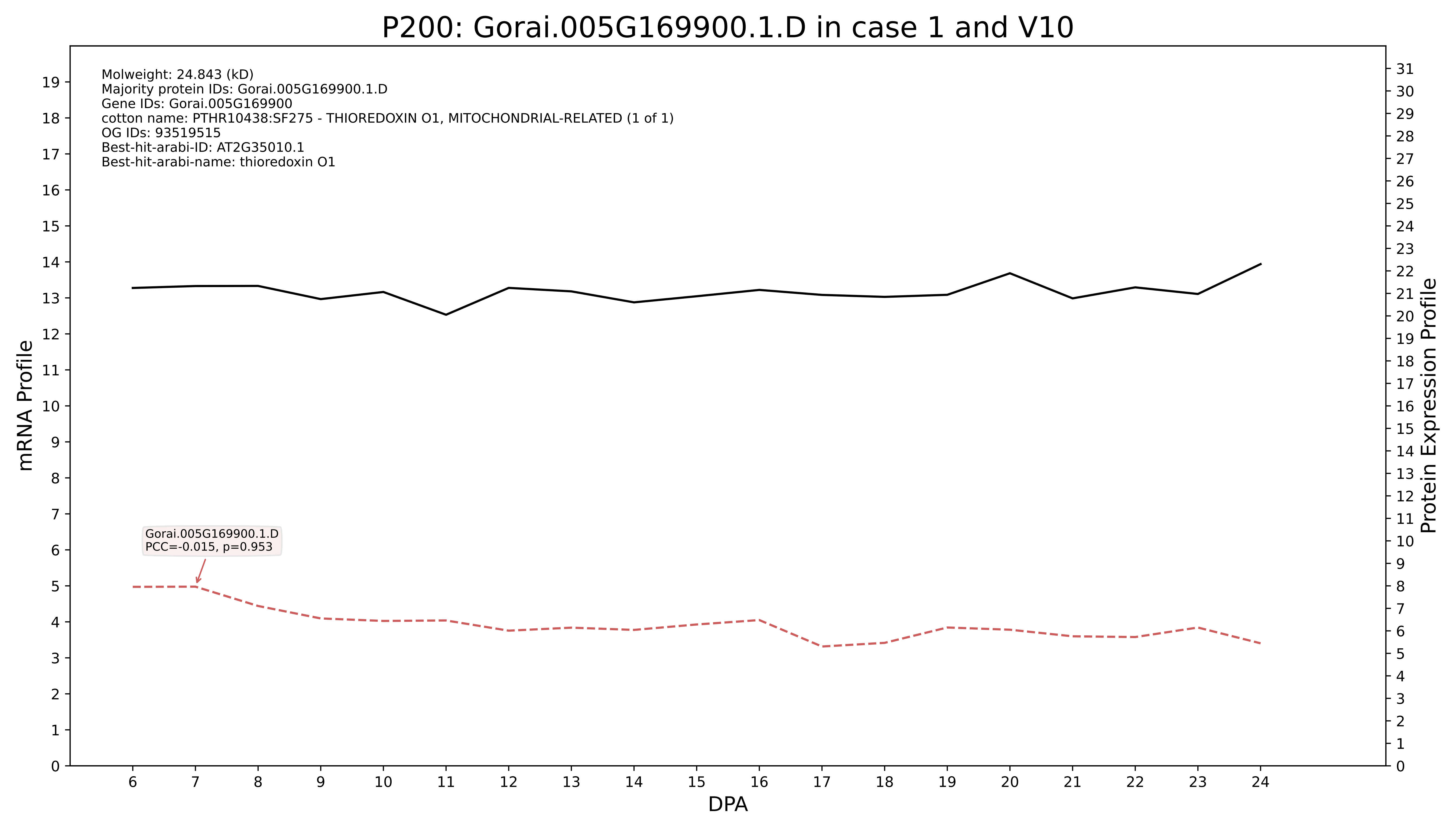
P200: Gorai.005G169900.1.D in case 1 and V10
Molweight: 24.843 (kD)
Majority protein IDs: Gorai.005G169900.1.D
Gene IDs: Gorai.005G169900
cotton name: PTHR10438:SF275 - THIOREDOXIN O1, MITOCHONDRIAL-RELATED (1 of 1)
OG IDs: 93519515
Best-hit-arabi-ID: AT2G35010.1
Best-hit-arabi-name: thioredoxin O1
Gorai.005G169900.1.D
PCC=-0.015, p=0.953
mRNA Profile
Protein Expression Profile
DPA

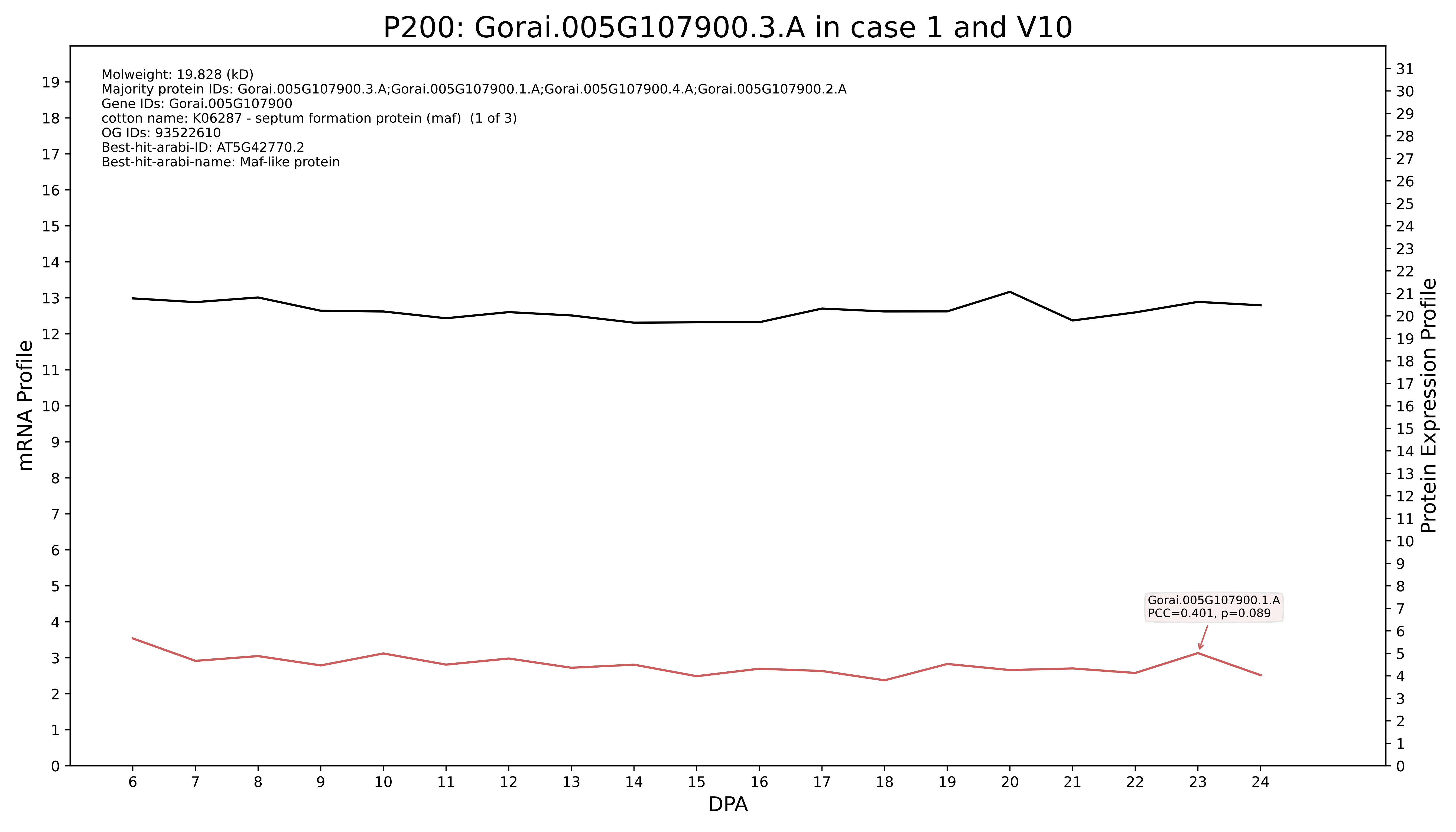
P200: Gorai.005G107900.3.A in case 1 and V10
Molweight: 19.828 (kD)
Majority protein IDs: Gorai.005G107900.3.A;Gorai.005G107900.1.A;Gorai.005G107900.4.A;Gorai.005G107900.2.A
Gene IDs: Gorai.005G107900
cotton name: K06287 - septum formation protein (maf) (1 of 3)
OG IDs: 93522610
Best-hit-arabi-ID: AT5G42770.2
Best-hit-arabi-name: Maf-like protein
Gorai.005G107900.1.A
PCC=0.401, p=0.089
mRNA Profile
Protein Expression Profile
DPA

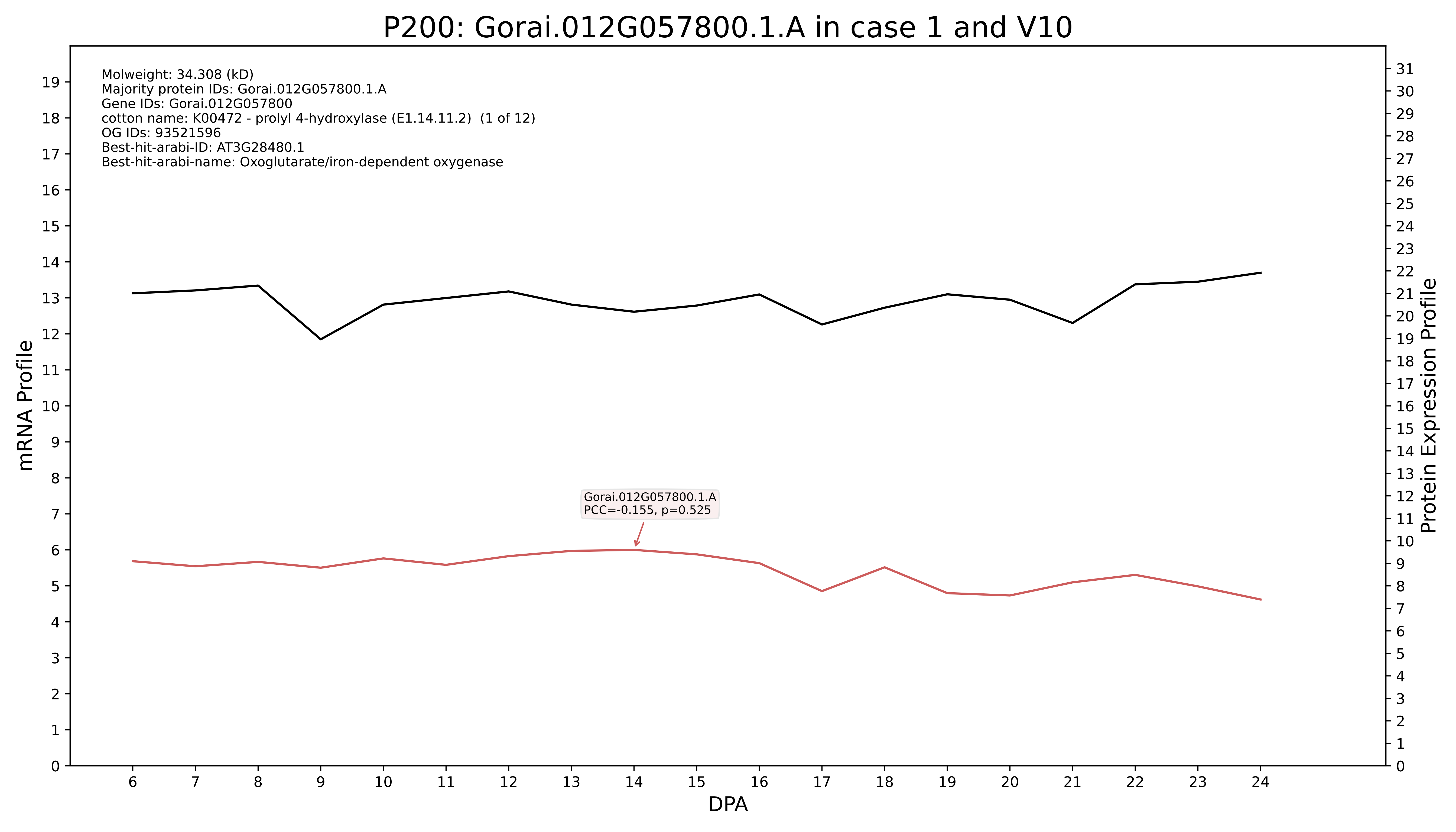

P200: Gorai.012G057800.1.A in case 1 and V10
Molweight: 34.308 (kD)
Majority protein IDs: Gorai.012G057800.1.A
Gene IDs: Gorai.012G057800
cotton name: K00472 - prolyl 4-hydroxylase (E1.14.11.2) (1 of 12)
OG IDs: 93521596
Best-hit-arabi-ID: AT3G28480.1
Best-hit-arabi-name: Oxoglutarate/iron-dependent oxygenase
Gorai.012G057800.1.A
PCC=-0.155, p=0.525
mRNA Profile
Protein Expression Profile
DPA

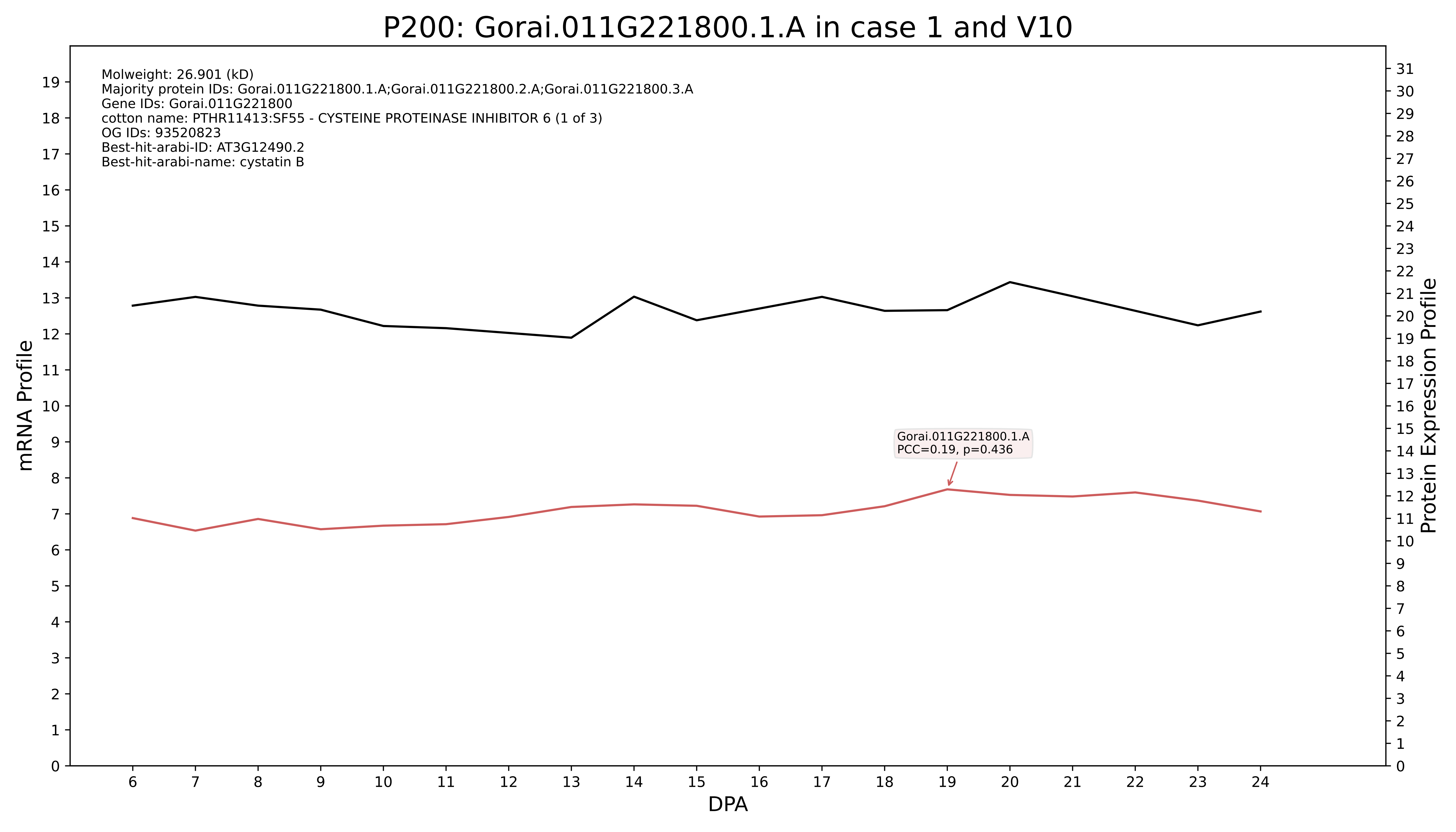
P200: Gorai.011G221800.1.A in case 1 and V10
Molweight: 26.901 (kD)
Majority protein IDs: Gorai.011G221800.1.A;Gorai.011G221800.2.A;Gorai.011G221800.3.A
Gene IDs: Gorai.011G221800
cotton name: PTHR11413:SF55 - CYSTEINE PROTEINASE INHIBITOR 6 (1 of 3)
OG IDs: 93520823
Best-hit-arabi-ID: AT3G12490.2
Best-hit-arabi-name: cystatin B
Gorai.011G221800.1.A
PCC=0.19, p=0.436
mRNA Profile
Protein Expression Profile
DPA

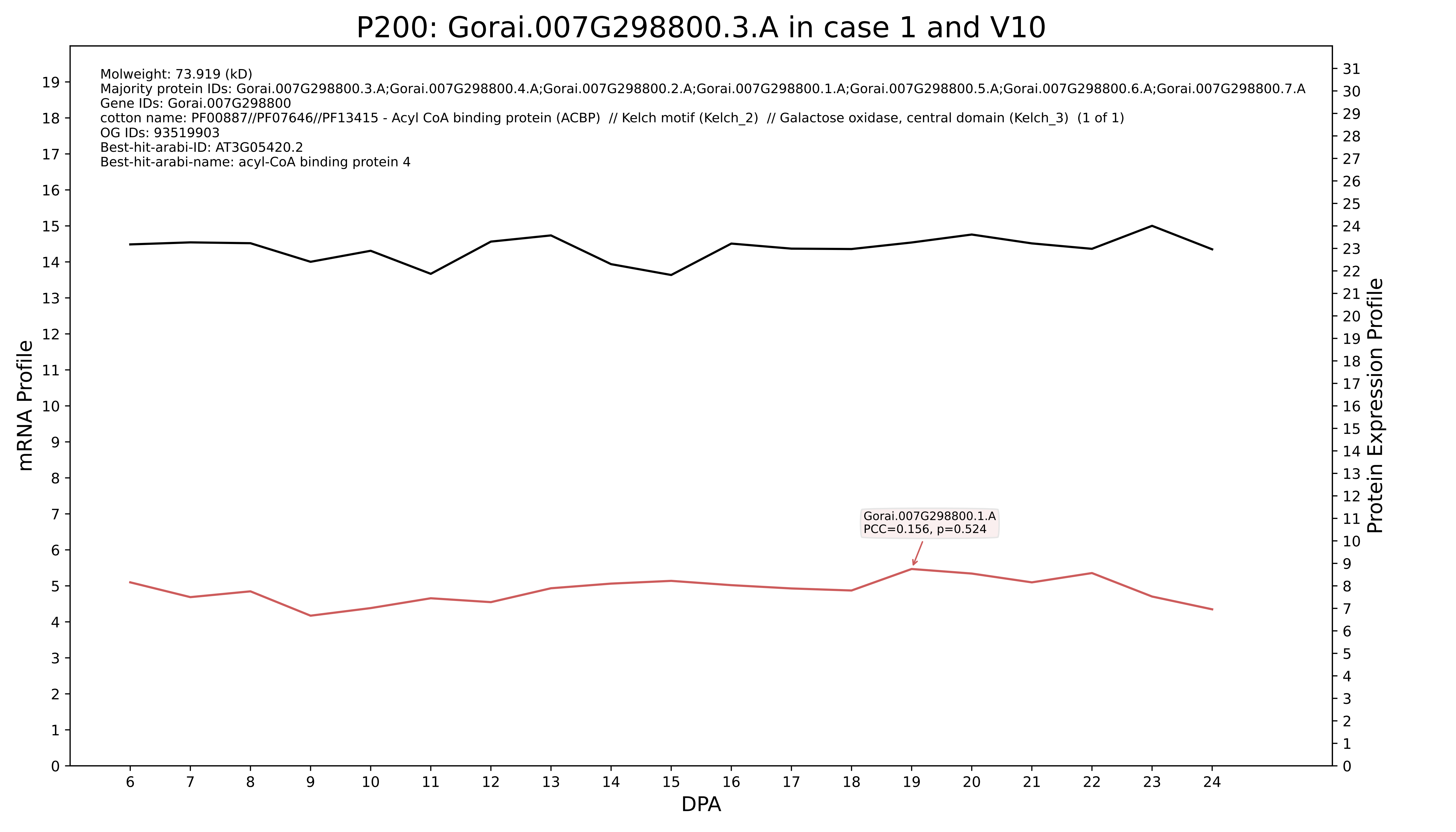
P200: Gorai.007G298800.3.A in case 1 and V10
Molweight: 73.919 (kD)
Majority protein IDs: Gorai.007G298800.3.A;Gorai.007G298800.4.A;Gorai.007G298800.2.A;Gorai.007G298800.1.A;Gorai.007G298800.5.A;Gorai.007G298800.6.A;Gorai.007G298800.7.A
Gene IDs: Gorai.007G298800
cotton name: PF00887//PF07646//PF13415 - Acyl CoA binding protein (ACBP) // Kelch motif (Kelch_2) // Galactose oxidase, central domain (Kelch_3) (1 of 1)
OG IDs: 93519903
Best-hit-arabi-ID: AT3G05420.2
Best-hit-arabi-name: acyl-CoA binding protein 4
Gorai.007G298800.1.A
PCC=0.156, p=0.524
mRNA Profile
Protein Expression Profile
DPA
0
1
2
3
4
5
6
7
8
9
10
11
12
13
14
15
16
17
18
19
20
21
22
23
24
25
26
27
28
29
30
31

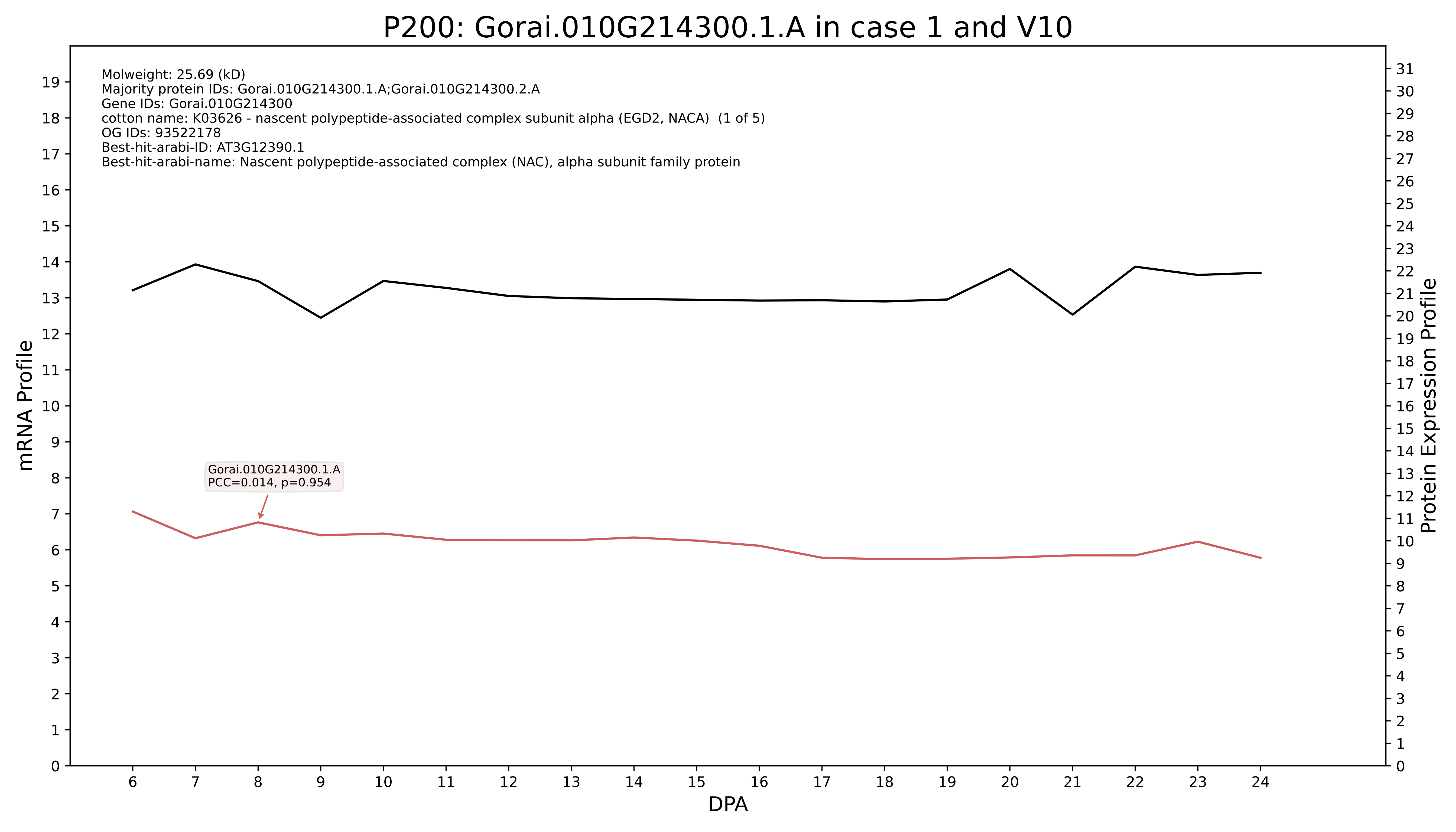
P200: Gorai.010G214300.1.A in case 1 and V10
Molweight: 25.69 (kD)
Majority protein IDs: Gorai.010G214300.1.A;Gorai.010G214300.2.A
Gene IDs: Gorai.010G214300
cotton name: K03626 - nascent polypeptide-associated complex subunit alpha (EGD2, NACA) (1 of 5)
OG IDs: 93522178
Best-hit-arabi-ID: AT3G12390.1
Best-hit-arabi-name: Nascent polypeptide-associated complex (NAC), alpha subunit family protein
Gorai.010G214300.1.A
PCC=0.014, p=0.954
mRNA Profile
Protein Expression Profile
DPA

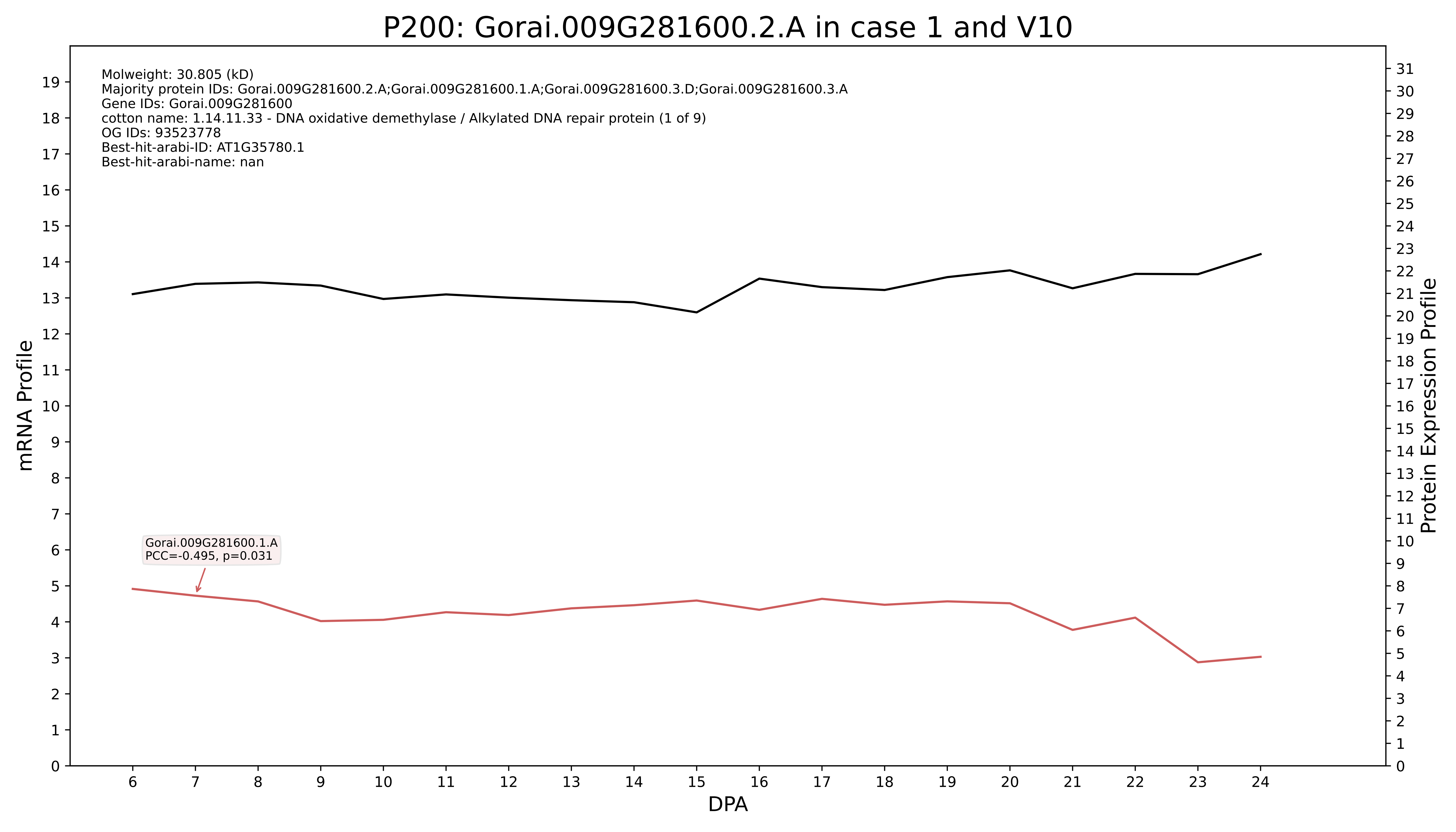

P200: Gorai.009G281600.2.A in case 1 and V10
Molweight: 30.805 (kD)
Majority protein IDs: Gorai.009G281600.2.A;Gorai.009G281600.1.A;Gorai.009G281600.3.D;Gorai.009G281600.3.A
Gene IDs: Gorai.009G281600
cotton name: 1.14.11.33 - DNA oxidative demethylase / Alkylated DNA repair protein (1 of 9)
OG IDs: 93523778
Best-hit-arabi-ID: AT1G35780.1
Best-hit-arabi-name: nan
Gorai.009G281600.1.A
PCC=-0.495, p=0.031
mRNA Profile
Protein Expression Profile
DPA

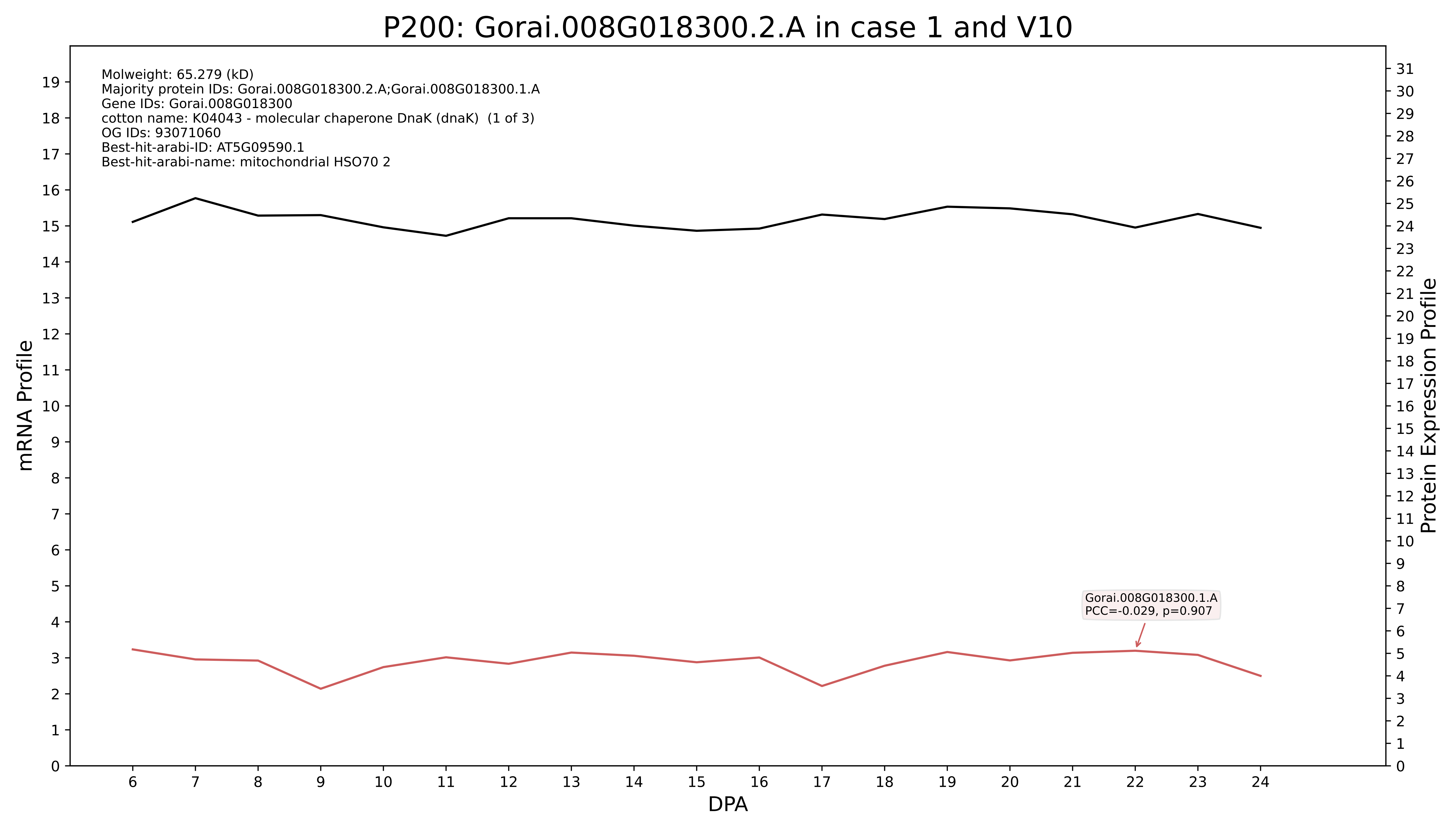

P200: Gorai.008G018300.2.A in case 1 and V10
Molweight: 65.279 (kD)
Majority protein IDs: Gorai.008G018300.2.A;Gorai.008G018300.1.A
Gene IDs: Gorai.008G018300
cotton name: K04043 - molecular chaperone DnaK (dnaK) (1 of 3)
OG IDs: 93071060
Best-hit-arabi-ID: AT5G09590.1
Best-hit-arabi-name: mitochondrial HSO70 2
Gorai.008G018300.1.A
PCC=-0.029, p=0.907
mRNA Profile
Protein Expression Profile
DPA

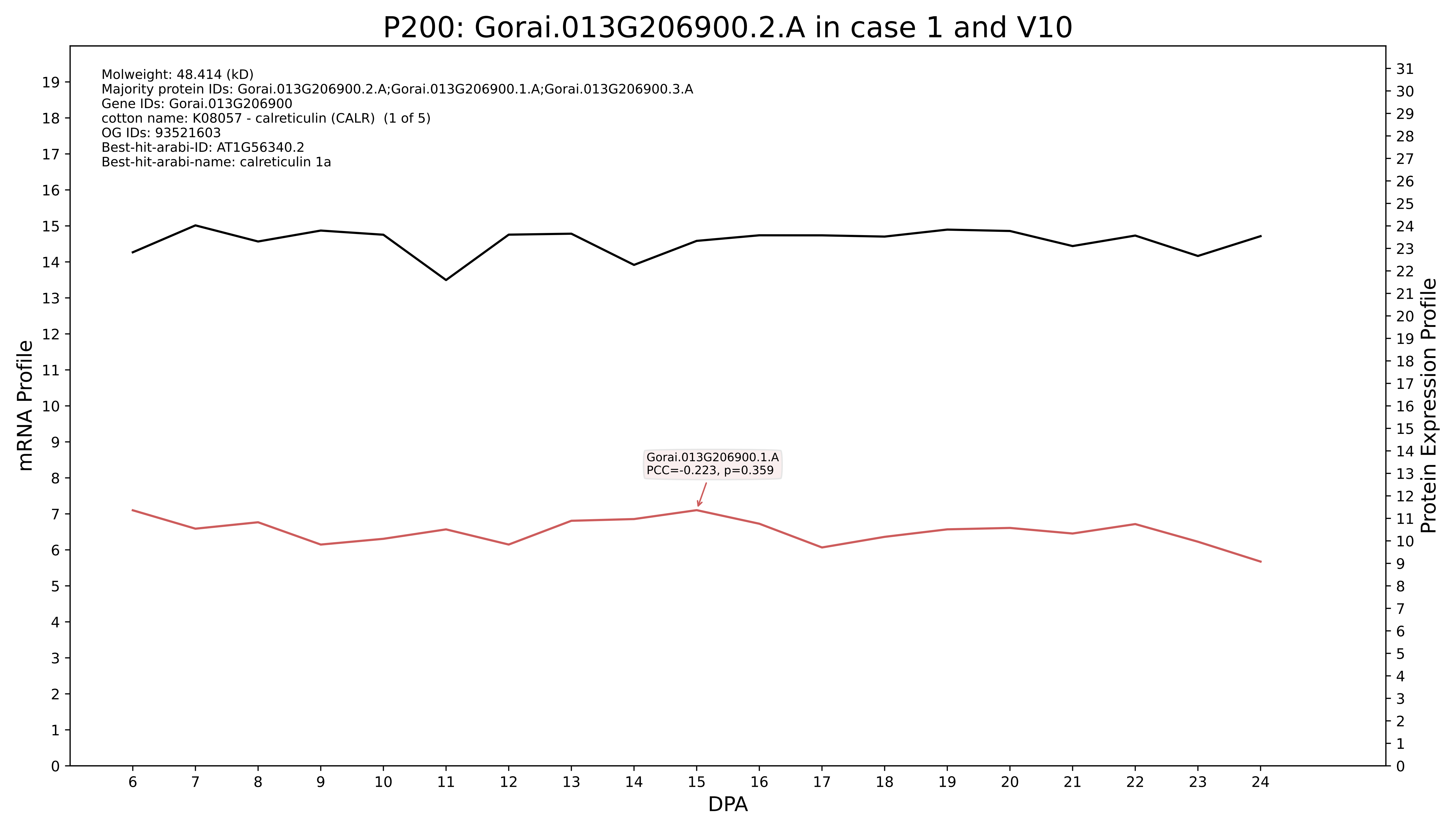
P200: Gorai.013G206900.2.A in case 1 and V10
Molweight: 48.414 (kD)
Majority protein IDs: Gorai.013G206900.2.A;Gorai.013G206900.1.A;Gorai.013G206900.3.A
Gene IDs: Gorai.013G206900
cotton name: K08057 - calreticulin (CALR) (1 of 5)
OG IDs: 93521603
Best-hit-arabi-ID: AT1G56340.2
Best-hit-arabi-name: calreticulin 1a
Gorai.013G206900.1.A
PCC=-0.223, p=0.359
mRNA Profile
Protein Expression Profile
DPA

P200: Gorai.013G149500.3.A in case 1 and V10

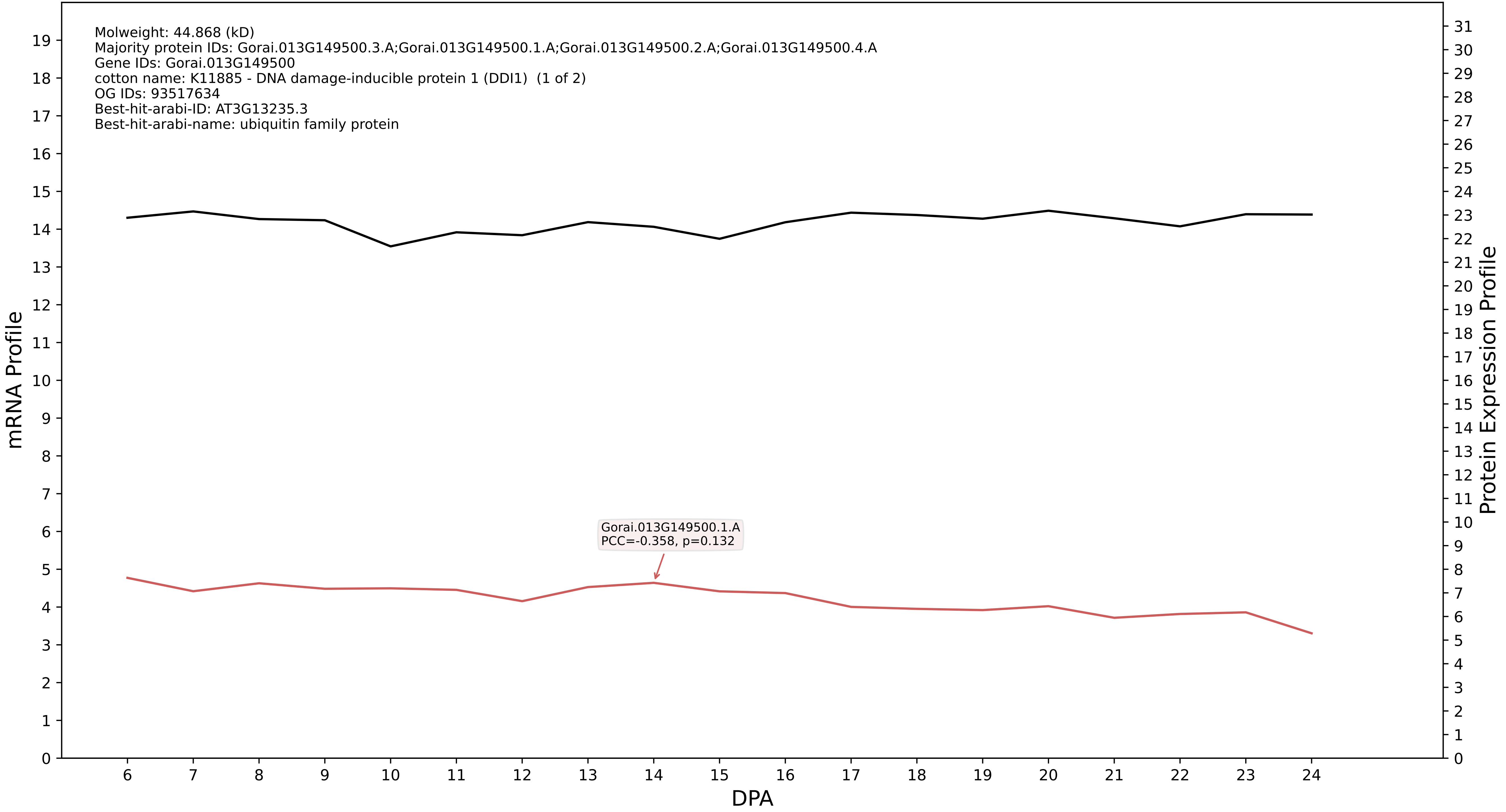

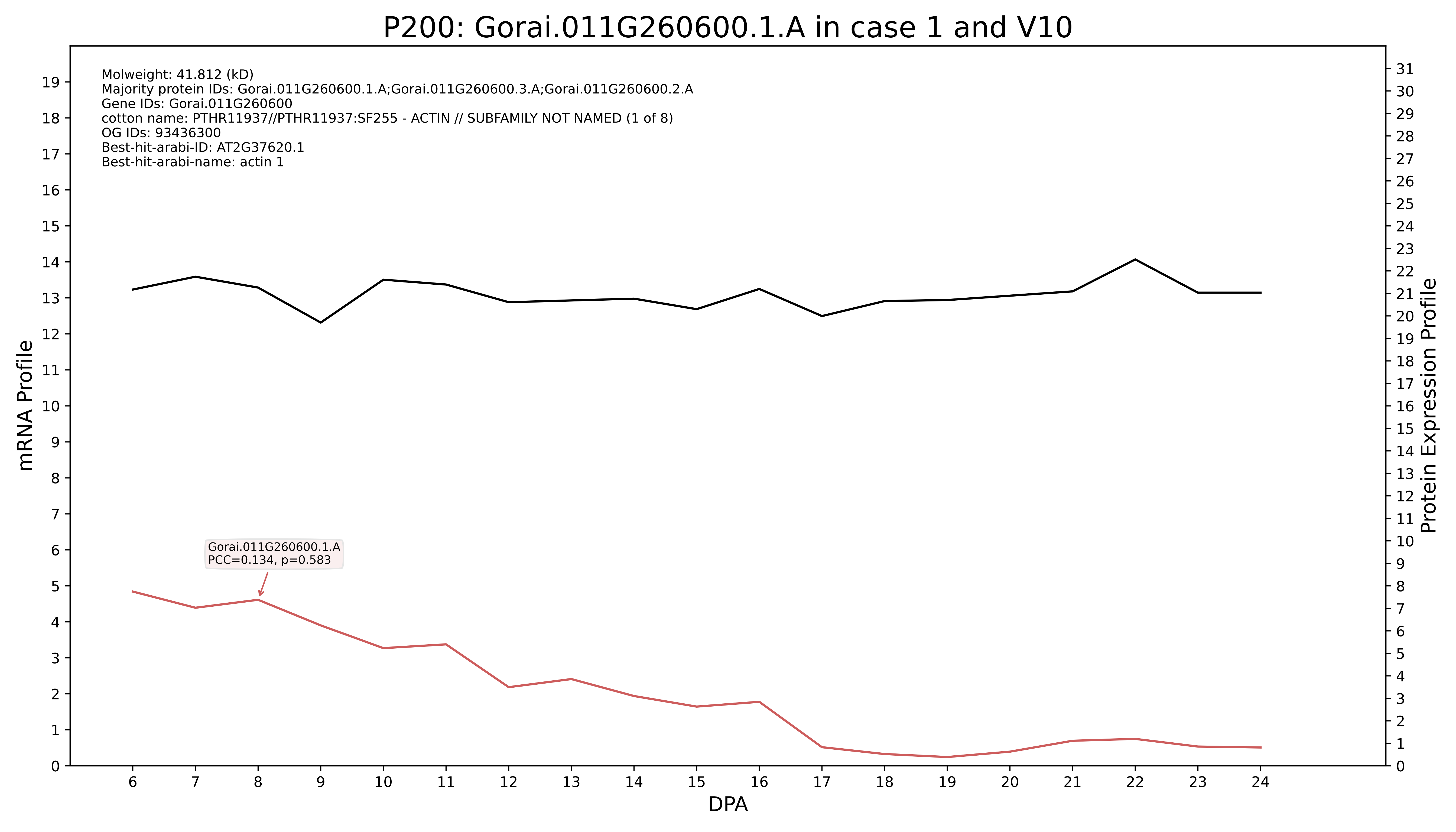
P200: Gorai.011G260600.1.A in case 1 and V10
Molweight: 41.812 (kD)
Majority protein IDs: Gorai.011G260600.1.A;Gorai.011G260600.3.A;Gorai.011G260600.2.A
Gene IDs: Gorai.011G260600
cotton name: PTHR11937//PTHR11937:SF255 - ACTIN // SUBFAMILY NOT NAMED (1 of 8)
OG IDs: 93436300
Best-hit-arabi-ID: AT2G37620.1
Best-hit-arabi-name: actin 1
Gorai.011G260600.1.A
PCC=0.134, p=0.583
mRNA Profile
Protein Expression Profile
DPA

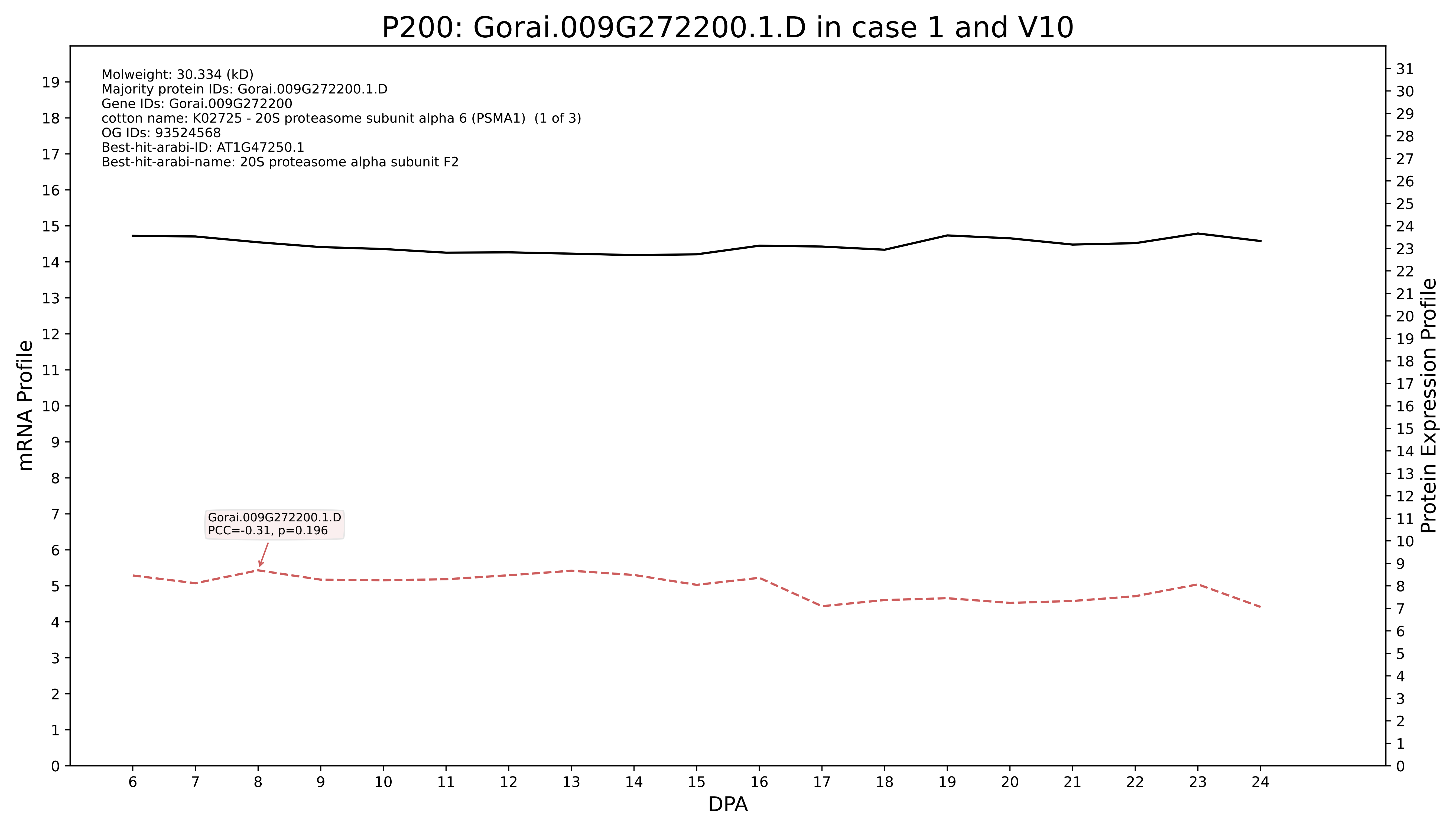
P200: Gorai.009G272200.1.D in case 1 and V10
Molweight: 30.334 (kD)
Majority protein IDs: Gorai.009G272200.1.D
Gene IDs: Gorai.009G272200
cotton name: K02725 - 20S proteasome subunit alpha 6 (PSMA1) (1 of 3)
OG IDs: 93524568
Best-hit-arabi-ID: AT1G47250.1
Best-hit-arabi-name: 20S proteasome alpha subunit F2
Gorai.009G272200.1.D
PCC=-0.31, p=0.196
mRNA Profile
Protein Expression Profile
DPA

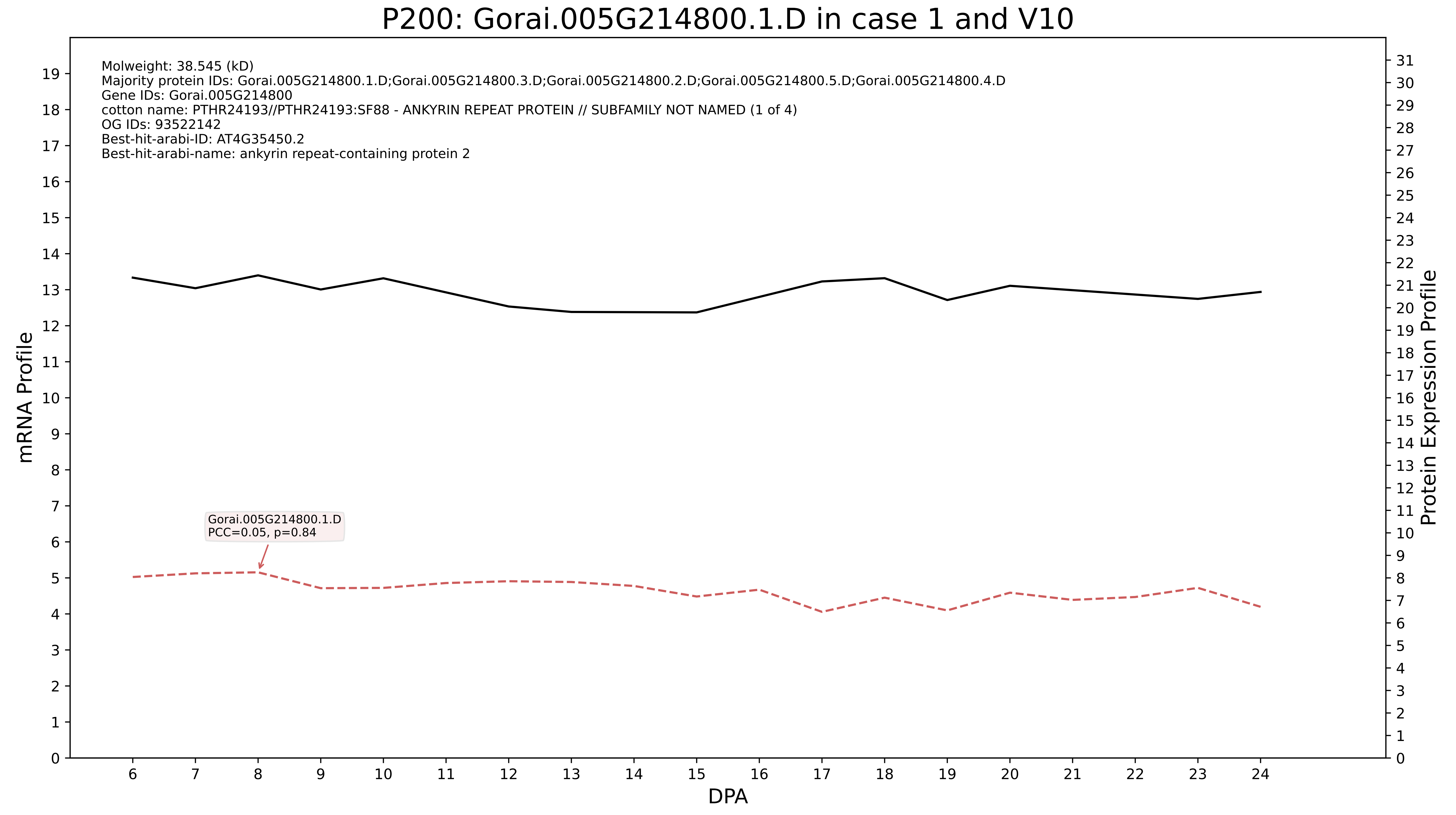

P200: Gorai.005G214800.1.D in case 1 and V10
Molweight: 38.545 (kD)
Majority protein IDs: Gorai.005G214800.1.D;Gorai.005G214800.3.D;Gorai.005G214800.2.D;Gorai.005G214800.5.D;Gorai.005G214800.4.D
Gene IDs: Gorai.005G214800
cotton name: PTHR24193//PTHR24193:SF88 - ANKYRIN REPEAT PROTEIN // SUBFAMILY NOT NAMED (1 of 4)
OG IDs: 93522142
Best-hit-arabi-ID: AT4G35450.2
Best-hit-arabi-name: ankyrin repeat-containing protein 2
Gorai.005G214800.1.D
PCC=0.05, p=0.84
mRNA Profile
Protein Expression Profile
DPA

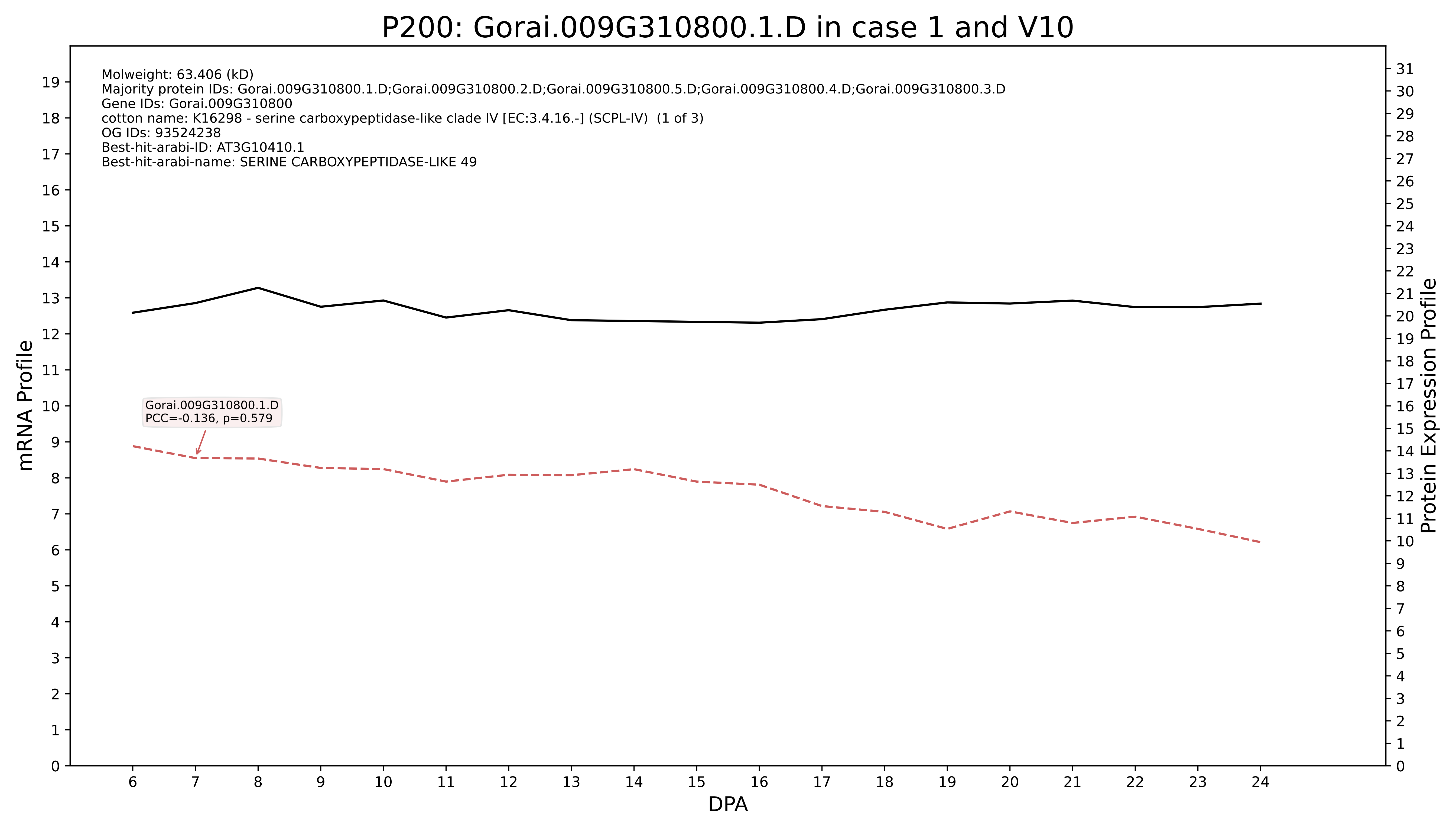
P200: Gorai.009G310800.1.D in case 1 and V10
Molweight: 63.406 (kD)
Majority protein IDs: Gorai.009G310800.1.D;Gorai.009G310800.2.D;Gorai.009G310800.5.D;Gorai.009G310800.4.D;Gorai.009G310800.3.D
Gene IDs: Gorai.009G310800
cotton name: K16298 - serine carboxypeptidase-like clade IV [EC:3.4.16.-] (SCPL-IV) (1 of 3)
OG IDs: 93524238
Best-hit-arabi-ID: AT3G10410.1
Best-hit-arabi-name: SERINE CARBOXYPEPTIDASE-LIKE 49
Gorai.009G310800.1.D
PCC=-0.136, p=0.579
mRNA Profile
Protein Expression Profile
DPA

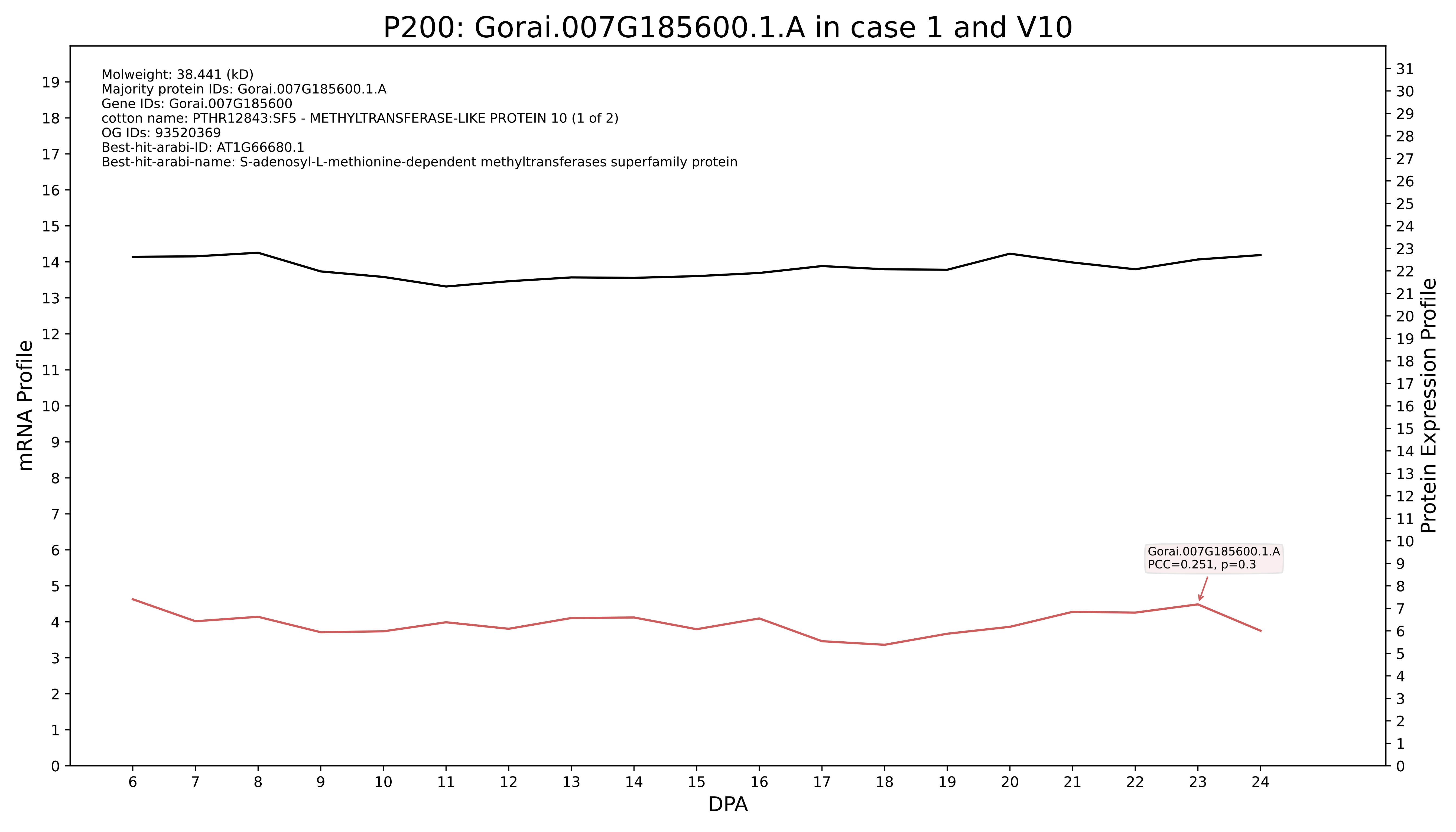
P200: Gorai.007G185600.1.A in case 1 and V10
Molweight: 38.441 (kD)
Majority protein IDs: Gorai.007G185600.1.A
Gene IDs: Gorai.007G185600
cotton name: PTHR12843:SF5 - METHYLTRANSFERASE-LIKE PROTEIN 10 (1 of 2)
OG IDs: 93520369
Best-hit-arabi-ID: AT1G66680.1
Best-hit-arabi-name: S-adenosyl-L-methionine-dependent methyltransferases superfamily protein
Gorai.007G185600.1.A
PCC=0.251, p=0.3
mRNA Profile
Protein Expression Profile
DPA

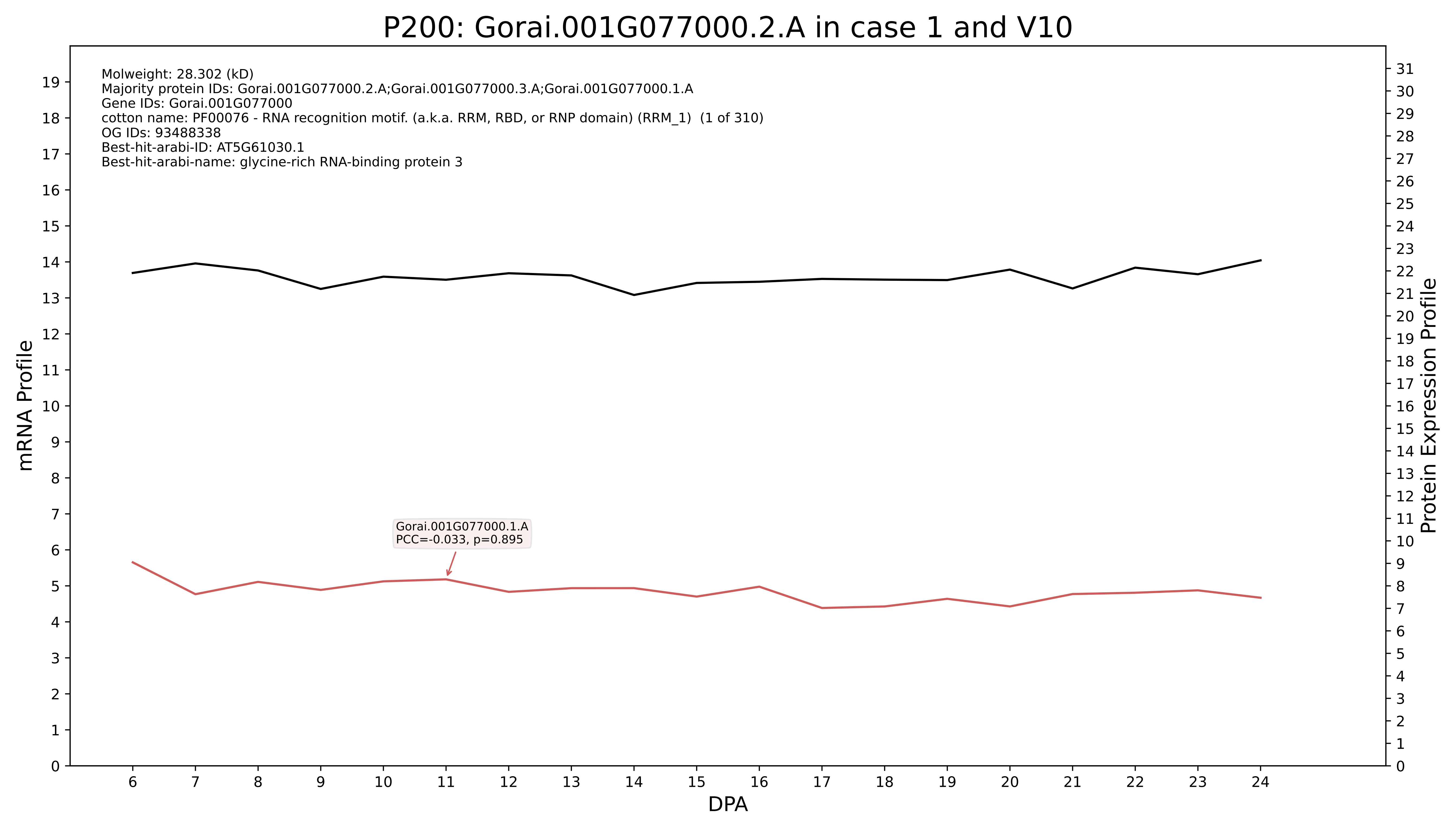
P200: Gorai.001G077000.2.A in case 1 and V10
Molweight: 28.302 (kD)
Majority protein IDs: Gorai.001G077000.2.A;Gorai.001G077000.3.A;Gorai.001G077000.1.A
Gene IDs: Gorai.001G077000
cotton name: PF00076 - RNA recognition motif. (a.k.a. RRM, RBD, or RNP domain) (RRM_1) (1 of 310)
OG IDs: 93488338
Best-hit-arabi-ID: AT5G61030.1
Best-hit-arabi-name: glycine-rich RNA-binding protein 3
Gorai.001G077000.1.A
PCC=-0.033, p=0.895
mRNA Profile
Protein Expression Profile
DPA

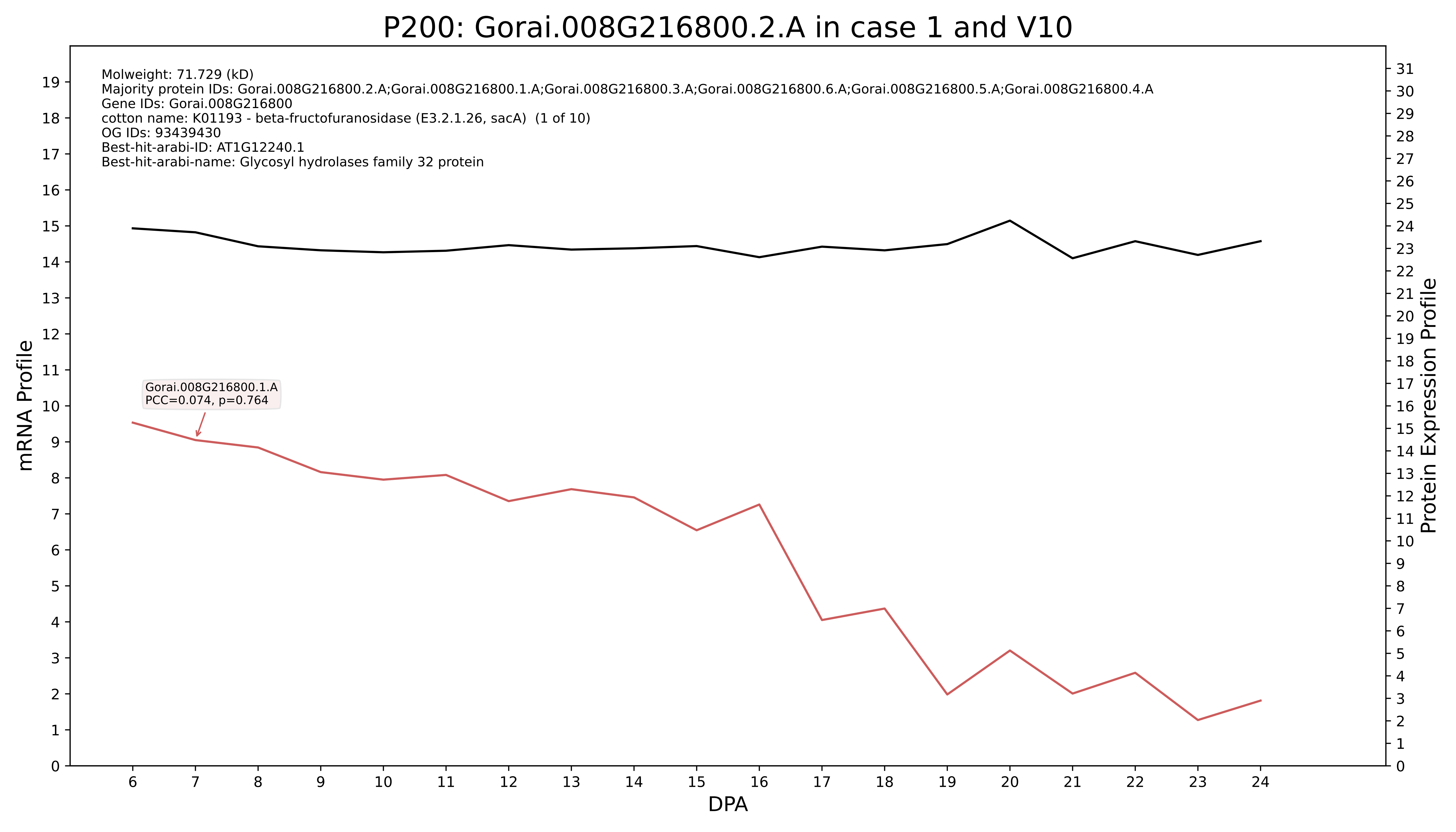
P200: Gorai.008G216800.2.A in case 1 and V10
Molweight: 71.729 (kD)
Majority protein IDs: Gorai.008G216800.2.A;Gorai.008G216800.1.A;Gorai.008G216800.3.A;Gorai.008G216800.6.A;Gorai.008G216800.5.A;Gorai.008G216800.4.A
Gene IDs: Gorai.008G216800
cotton name: K01193 - beta-fructofuranosidase (E3.2.1.26, sacA) (1 of 10)
OG IDs: 93439430
Best-hit-arabi-ID: AT1G12240.1
Best-hit-arabi-name: Glycosyl hydrolases family 32 protein
Gorai.008G216800.1.A
PCC=0.074, p=0.764
mRNA Profile
Protein Expression Profile
DPA

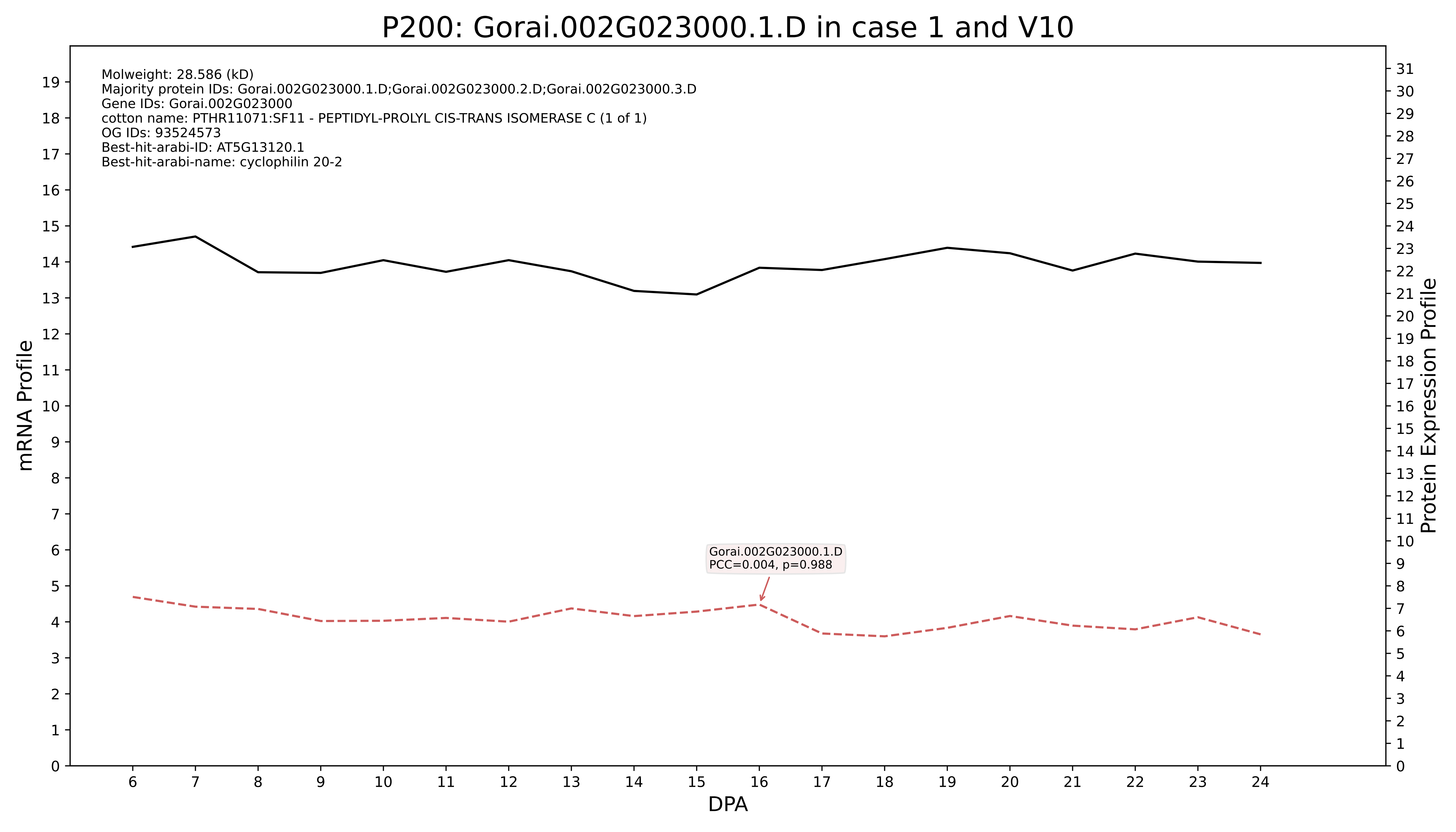
P200: Gorai.002G023000.1.D in case 1 and V10
Molweight: 28.586 (kD)
Majority protein IDs: Gorai.002G023000.1.D;Gorai.002G023000.2.D;Gorai.002G023000.3.D
Gene IDs: Gorai.002G023000
cotton name: PTHR11071:SF11 - PEPTIDYL-PROLYL CIS-TRANS ISOMERASE C (1 of 1)
OG IDs: 93524573
Best-hit-arabi-ID: AT5G13120.1
Best-hit-arabi-name: cyclophilin 20-2
Gorai.002G023000.1.D
PCC=0.004, p=0.988
mRNA Profile
Protein Expression Profile
DPA

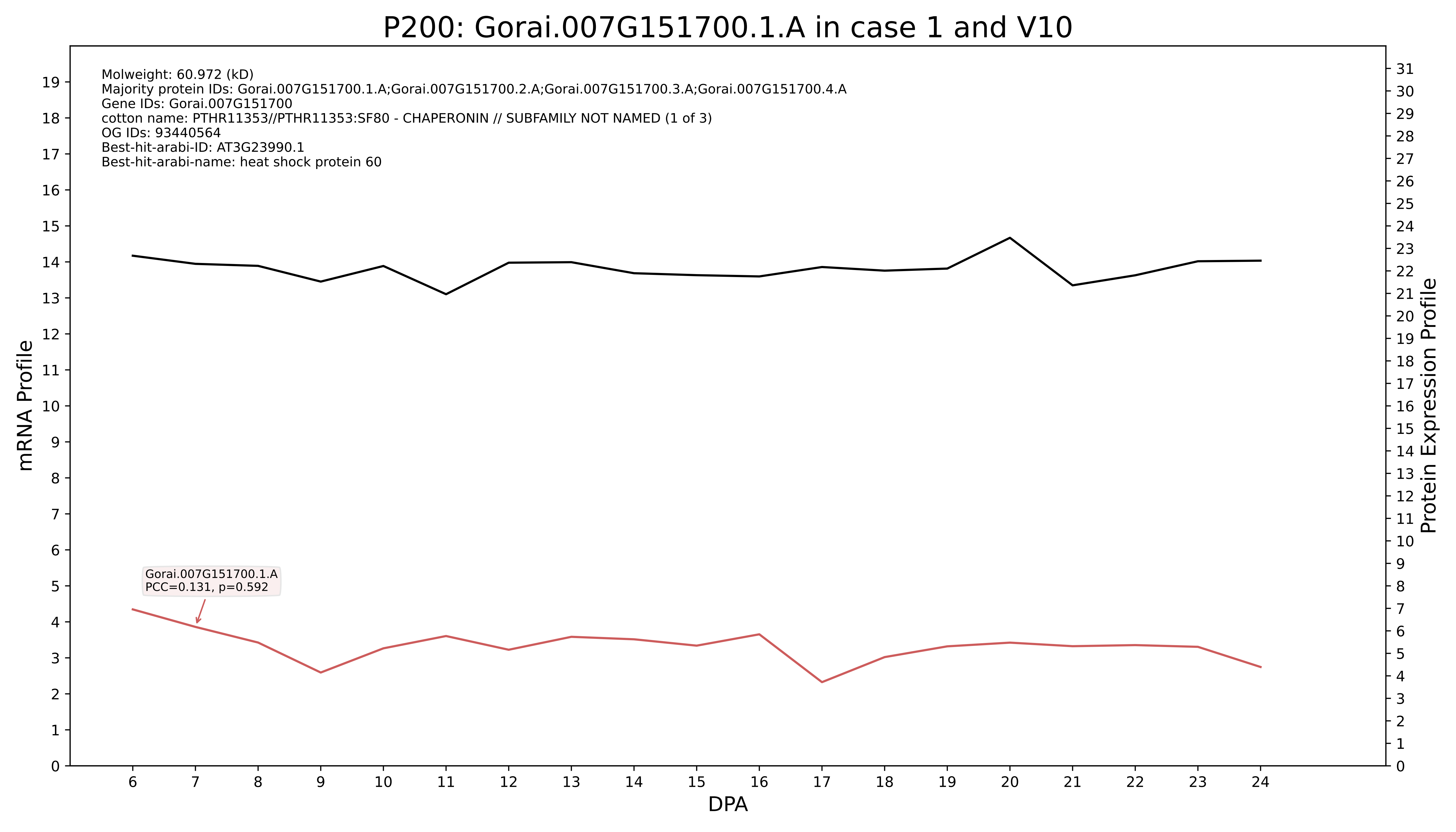
P200: Gorai.007G151700.1.A in case 1 and V10
Molweight: 60.972 (kD)
Majority protein IDs: Gorai.007G151700.1.A;Gorai.007G151700.2.A;Gorai.007G151700.3.A;Gorai.007G151700.4.A
Gene IDs: Gorai.007G151700
cotton name: PTHR11353//PTHR11353:SF80 - CHAPERONIN // SUBFAMILY NOT NAMED (1 of 3)
OG IDs: 93440564
Best-hit-arabi-ID: AT3G23990.1
Best-hit-arabi-name: heat shock protein 60
Gorai.007G151700.1.A
PCC=0.131, p=0.592
mRNA Profile
Protein Expression Profile
DPA

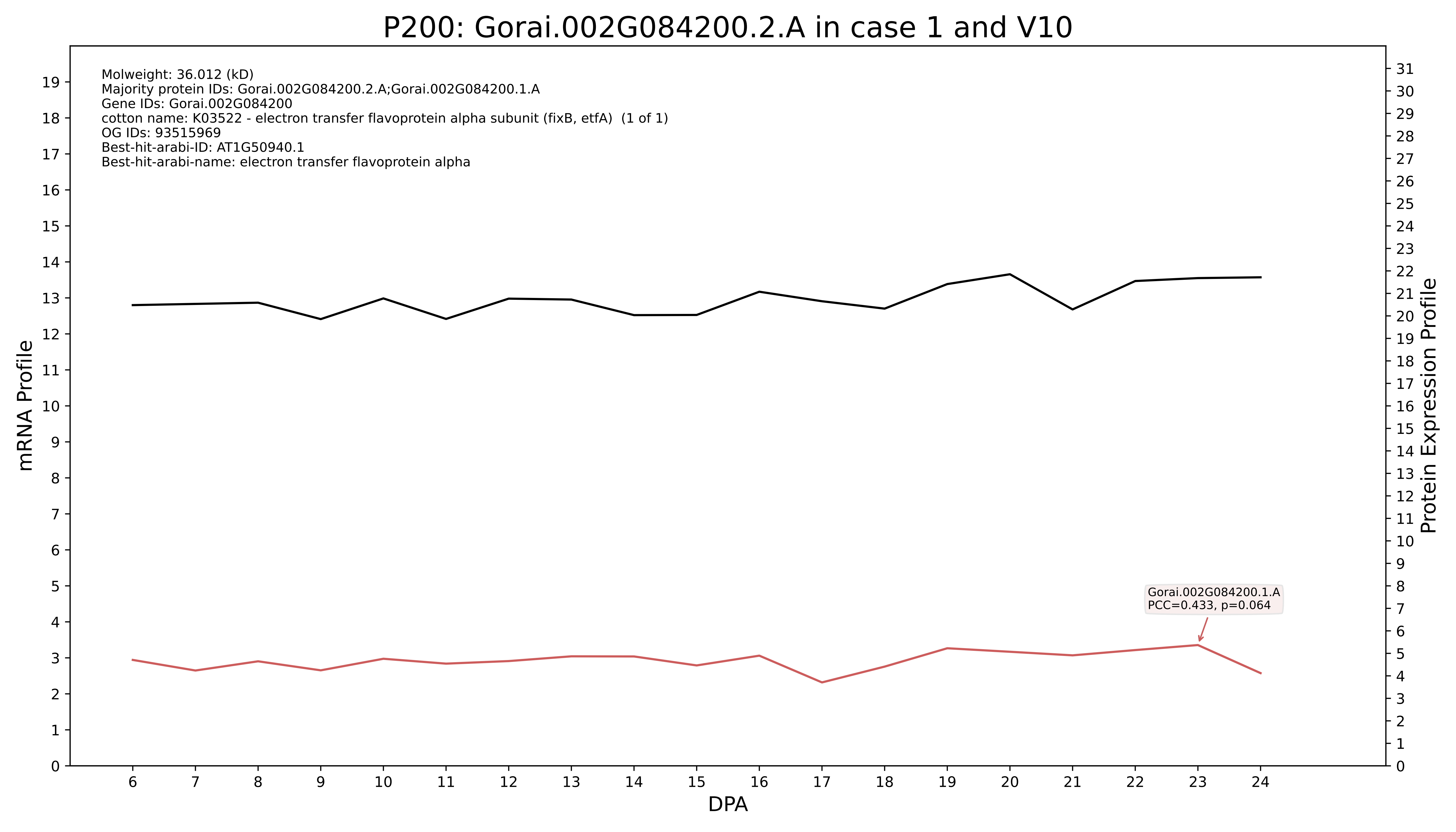
P200: Gorai.002G084200.2.A in case 1 and V10
Molweight: 36.012 (kD)
Majority protein IDs: Gorai.002G084200.2.A;Gorai.002G084200.1.A
Gene IDs: Gorai.002G084200
cotton name: K03522 - electron transfer flavoprotein alpha subunit (fixB, etfA) (1 of 1)
OG IDs: 93515969
Best-hit-arabi-ID: AT1G50940.1
Best-hit-arabi-name: electron transfer flavoprotein alpha
Gorai.002G084200.1.A
PCC=0.433, p=0.064
mRNA Profile
Protein Expression Profile
DPA

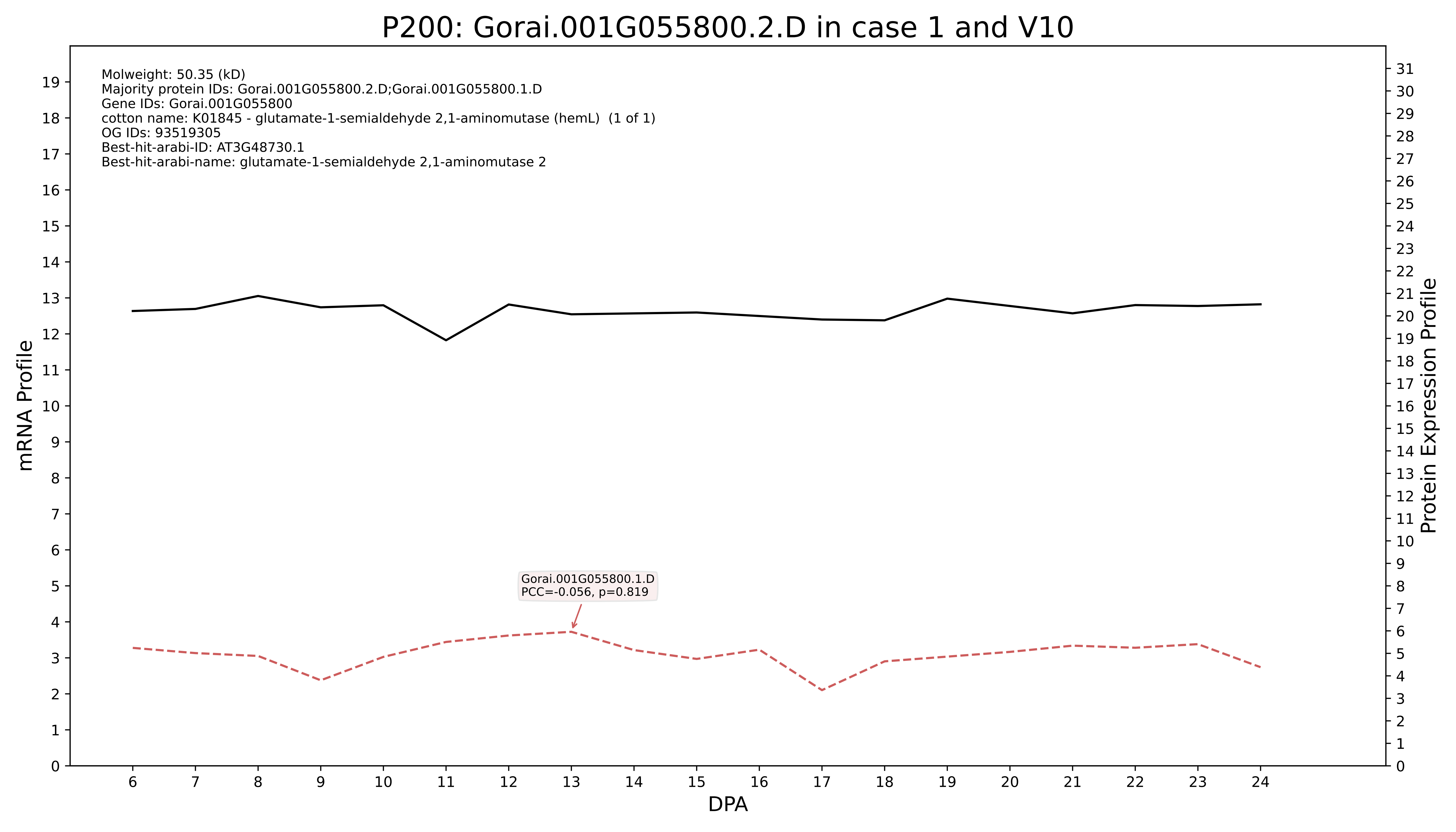
P200: Gorai.001G055800.2.D in case 1 and V10
Molweight: 50.35 (kD)
Majority protein IDs: Gorai.001G055800.2.D;Gorai.001G055800.1.D
Gene IDs: Gorai.001G055800
cotton name: K01845 - glutamate-1-semialdehyde 2,1-aminomutase (hemL) (1 of 1)
OG IDs: 93519305
Best-hit-arabi-ID: AT3G48730.1
Best-hit-arabi-name: glutamate-1-semialdehyde 2,1-aminomutase 2
Gorai.001G055800.1.D
PCC=-0.056, p=0.819
mRNA Profile
Protein Expression Profile
DPA

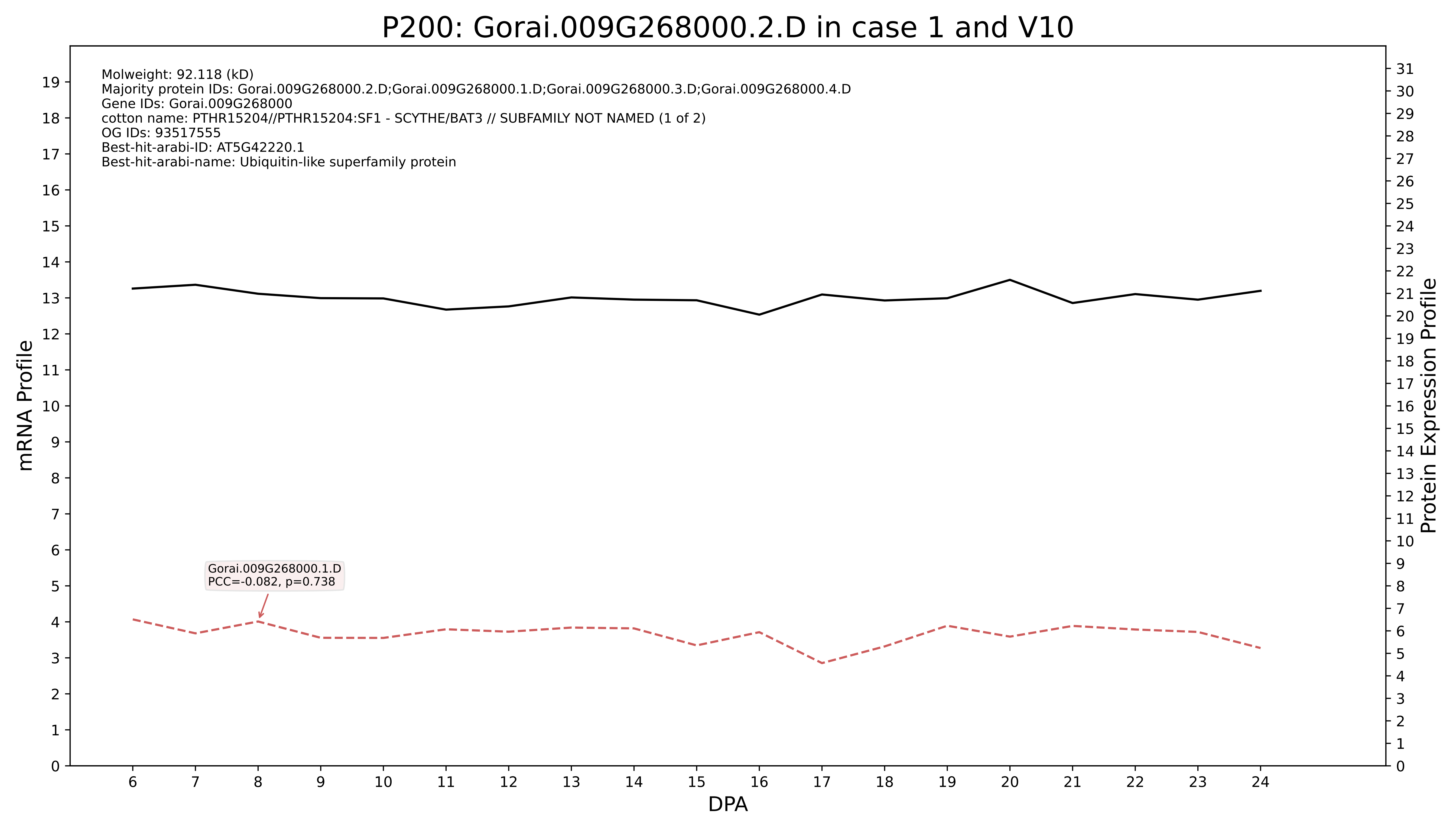
P200: Gorai.009G268000.2.D in case 1 and V10
Molweight: 92.118 (kD)
Majority protein IDs: Gorai.009G268000.2.D;Gorai.009G268000.1.D;Gorai.009G268000.3.D;Gorai.009G268000.4.D
Gene IDs: Gorai.009G268000
cotton name: PTHR15204//PTHR15204:SF1 - SCYTHE/BAT3 // SUBFAMILY NOT NAMED (1 of 2)
OG IDs: 93517555
Best-hit-arabi-ID: AT5G42220.1
Best-hit-arabi-name: Ubiquitin-like superfamily protein
Gorai.009G268000.1.D
PCC=-0.082, p=0.738
mRNA Profile
Protein Expression Profile
DPA
6
7
8
9
10
11
12
13
14
15
16
17
18
19
20
21
22
23
24

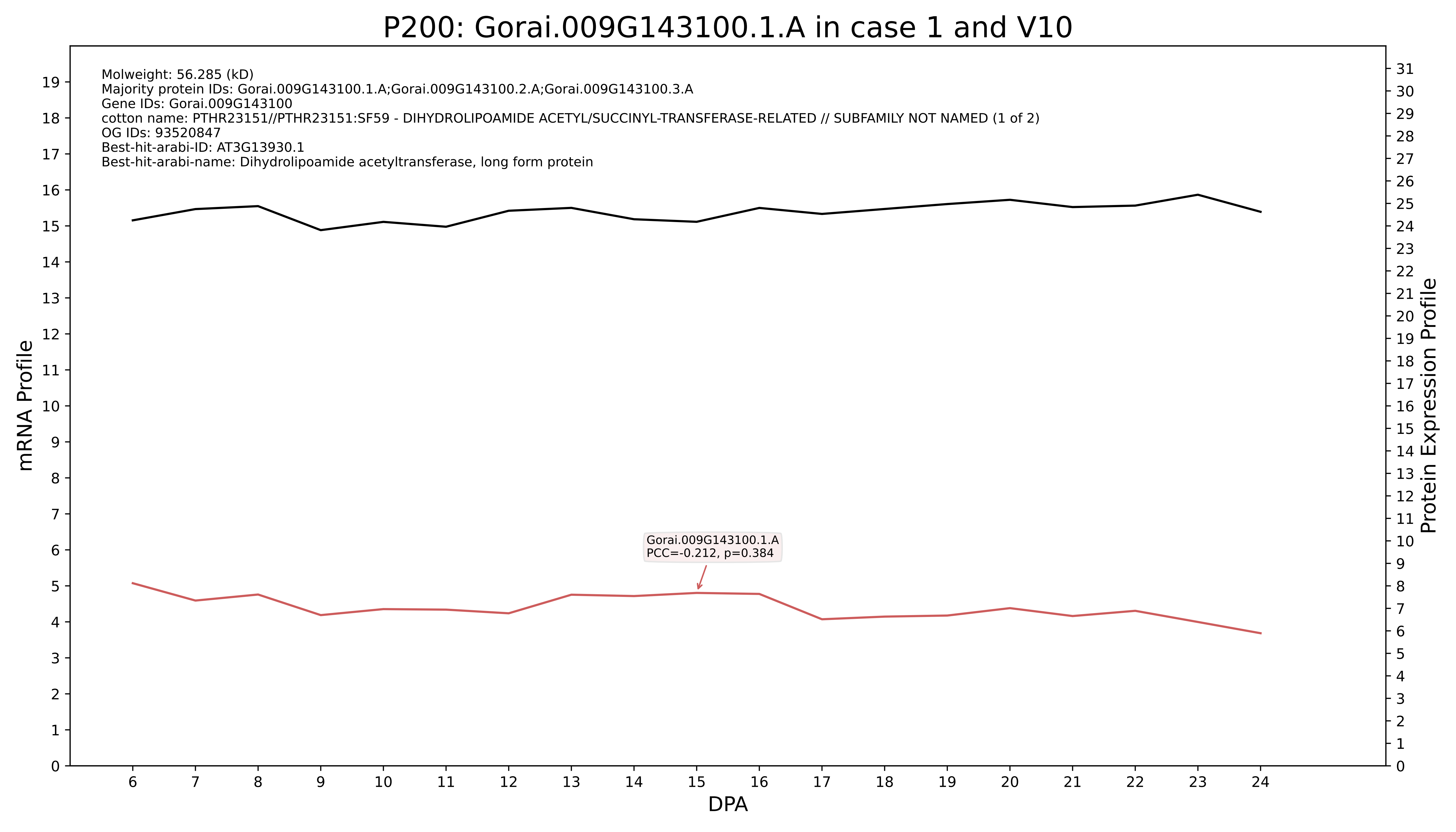
P200: Gorai.009G143100.1.A in case 1 and V10
Molweight: 56.285 (kD)
Majority protein IDs: Gorai.009G143100.1.A;Gorai.009G143100.2.A;Gorai.009G143100.3.A
Gene IDs: Gorai.009G143100
cotton name: PTHR23151//PTHR23151:SF59 - DIHYDROLIPOAMIDE ACETYL/SUCCINYL-TRANSFERASE-RELATED // SUBFAMILY NOT NAMED (1 of 2)
OG IDs: 93520847
Best-hit-arabi-ID: AT3G13930.1
Best-hit-arabi-name: Dihydrolipoamide acetyltransferase, long form protein
Gorai.009G143100.1.A
PCC=-0.212, p=0.384
mRNA Profile
Protein Expression Profile
DPA

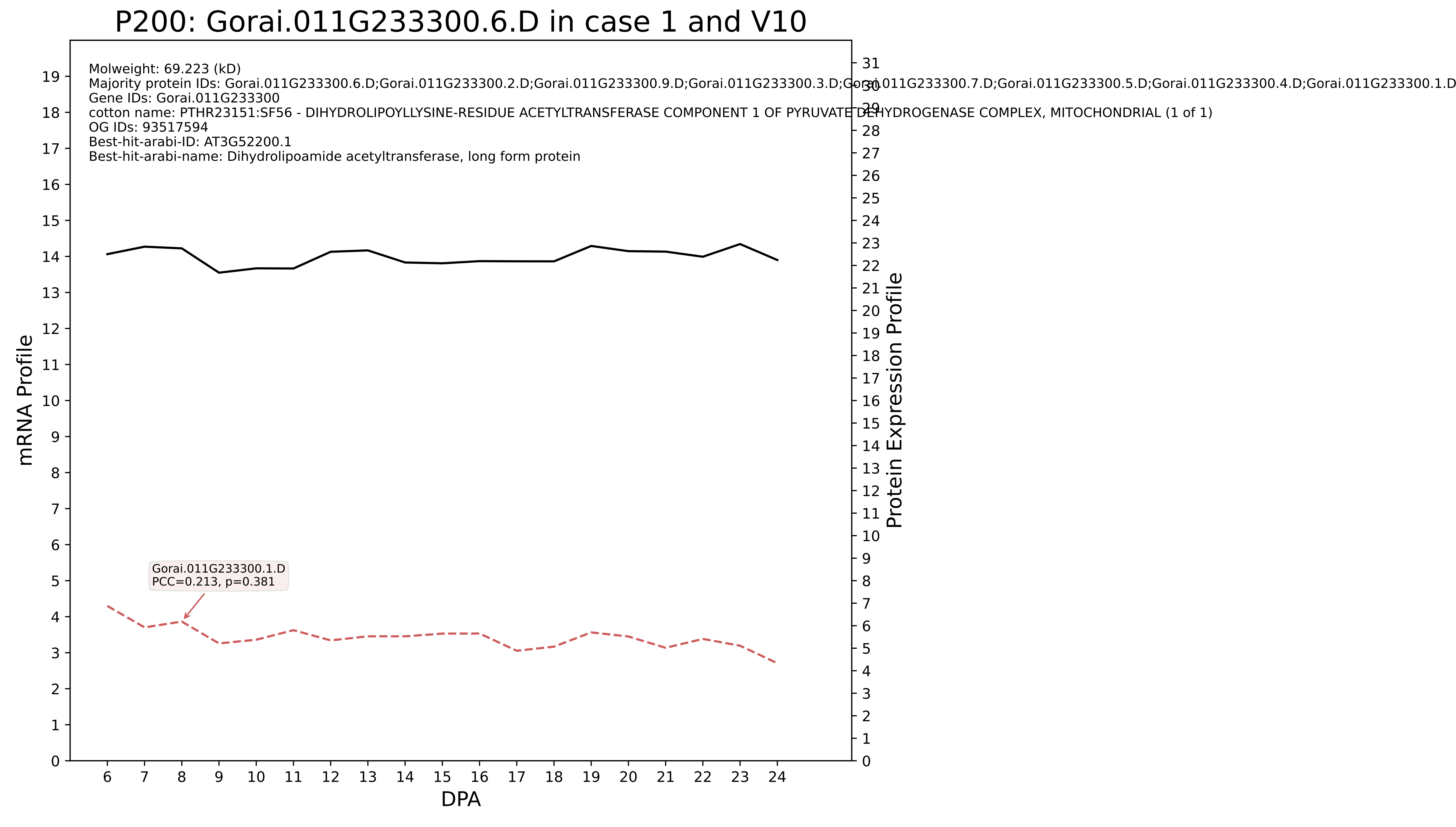
P200: Gorai.011G233300.6.D in case 1 and V10
Molweight: 69.223 (kD)
Majority protein IDs: Gorai.011G233300.6.D;Gorai.011G233300.2.D;Gorai.011G233300.9.D;Gorai.011G233300.3.D;Gorai.011G233300.7.D;Gorai.011G233300.5.D;Gorai.011G233300.4.D;Gorai.011G233300.1.D
Gene IDs: Gorai.011G233300
cotton name: PTHR23151:SF56 - DIHYDROLIPOYLLYSINE-RESIDUE ACETYLTRANSFERASE COMPONENT 1 OF PYRUVATE DEHYDROGENASE COMPLEX, MITOCHONDRIAL (1 of 1)
OG IDs: 93517594
Best-hit-arabi-ID: AT3G52200.1
Best-hit-arabi-name: Dihydrolipoamide acetyltransferase, long form protein
Gorai.011G233300.1.D
PCC=0.213, p=0.381
mRNA Profile
Protein Expression Profile
DPA

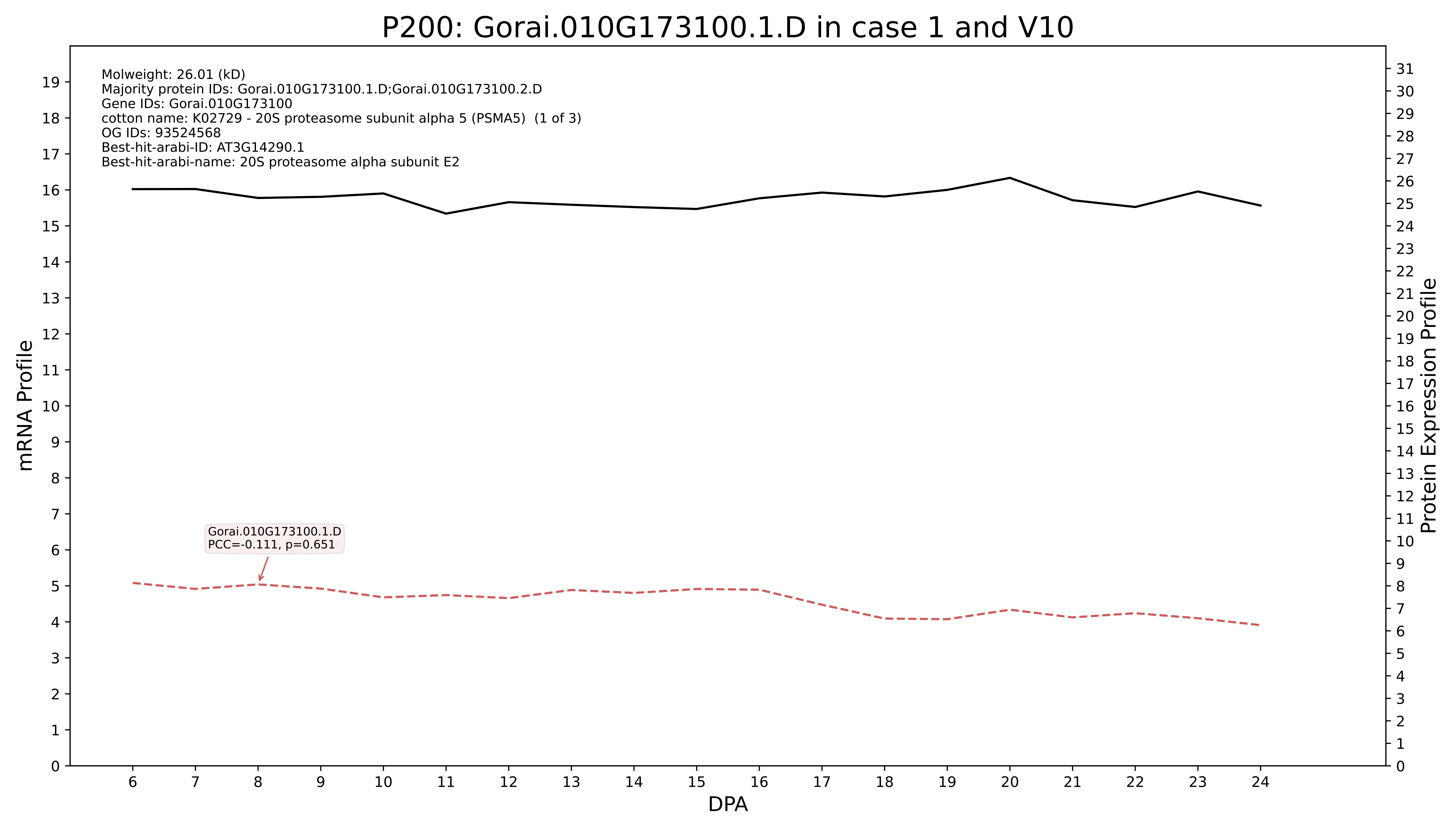
P200: Gorai.010G173100.1.D in case 1 and V10
Molweight: 26.01 (kD)
Majority protein IDs: Gorai.010G173100.1.D;Gorai.010G173100.2.D
Gene IDs: Gorai.010G173100
cotton name: K02729 - 20S proteasome subunit alpha 5 (PSMA5) (1 of 3)
OG IDs: 93524568
Best-hit-arabi-ID: AT3G14290.1
Best-hit-arabi-name: 20S proteasome alpha subunit E2
Gorai.010G173100.1.D
PCC=-0.111, p=0.651
mRNA Profile
Protein Expression Profile
DPA

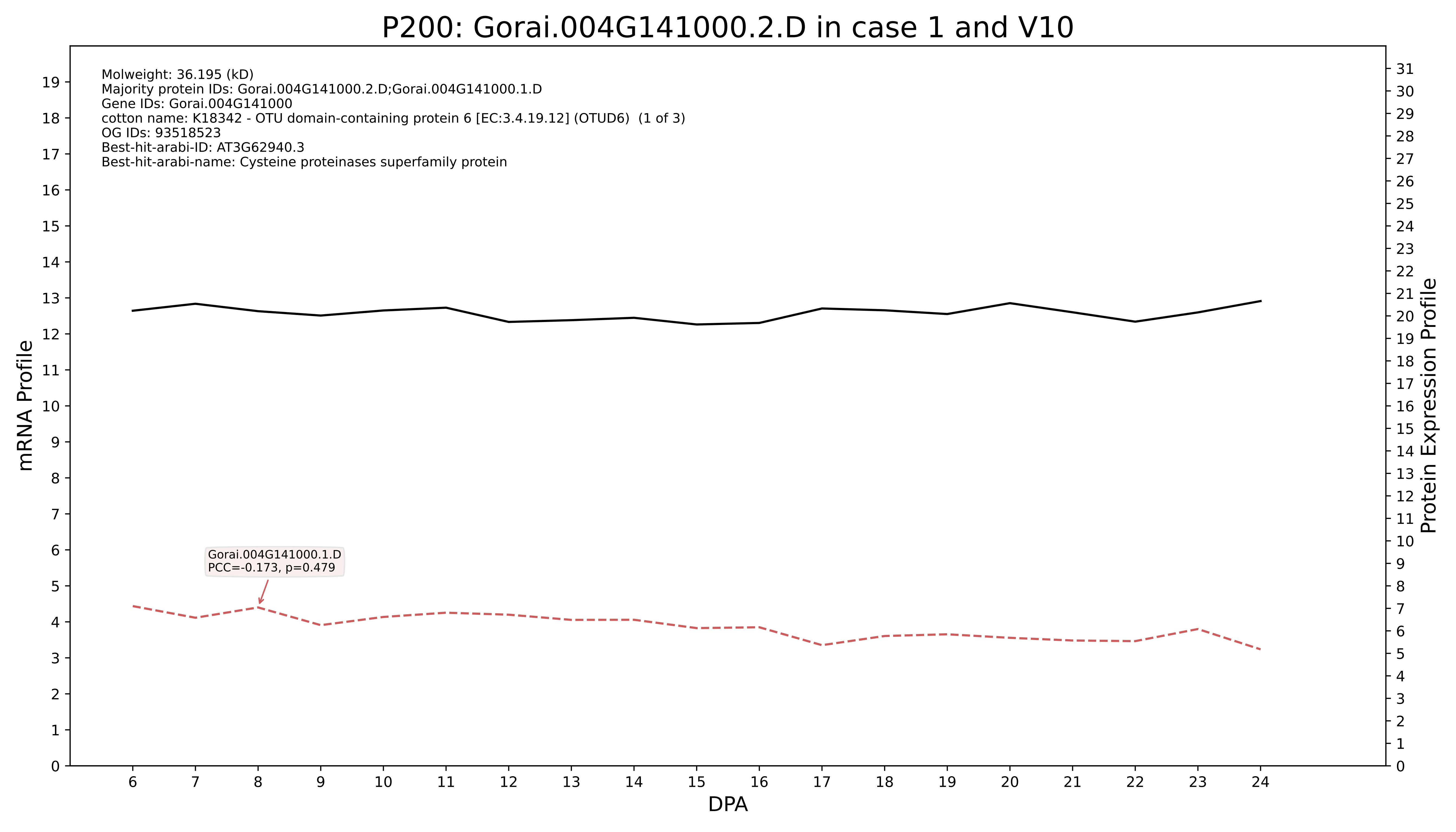
P200: Gorai.004G141000.2.D in case 1 and V10
Molweight: 36.195 (kD)
Majority protein IDs: Gorai.004G141000.2.D;Gorai.004G141000.1.D
Gene IDs: Gorai.004G141000
cotton name: K18342 - OTU domain-containing protein 6 [EC:3.4.19.12] (OTUD6) (1 of 3)
OG IDs: 93518523
Best-hit-arabi-ID: AT3G62940.3
Best-hit-arabi-name: Cysteine proteinases superfamily protein
Gorai.004G141000.1.D
PCC=-0.173, p=0.479
mRNA Profile
Protein Expression Profile
DPA

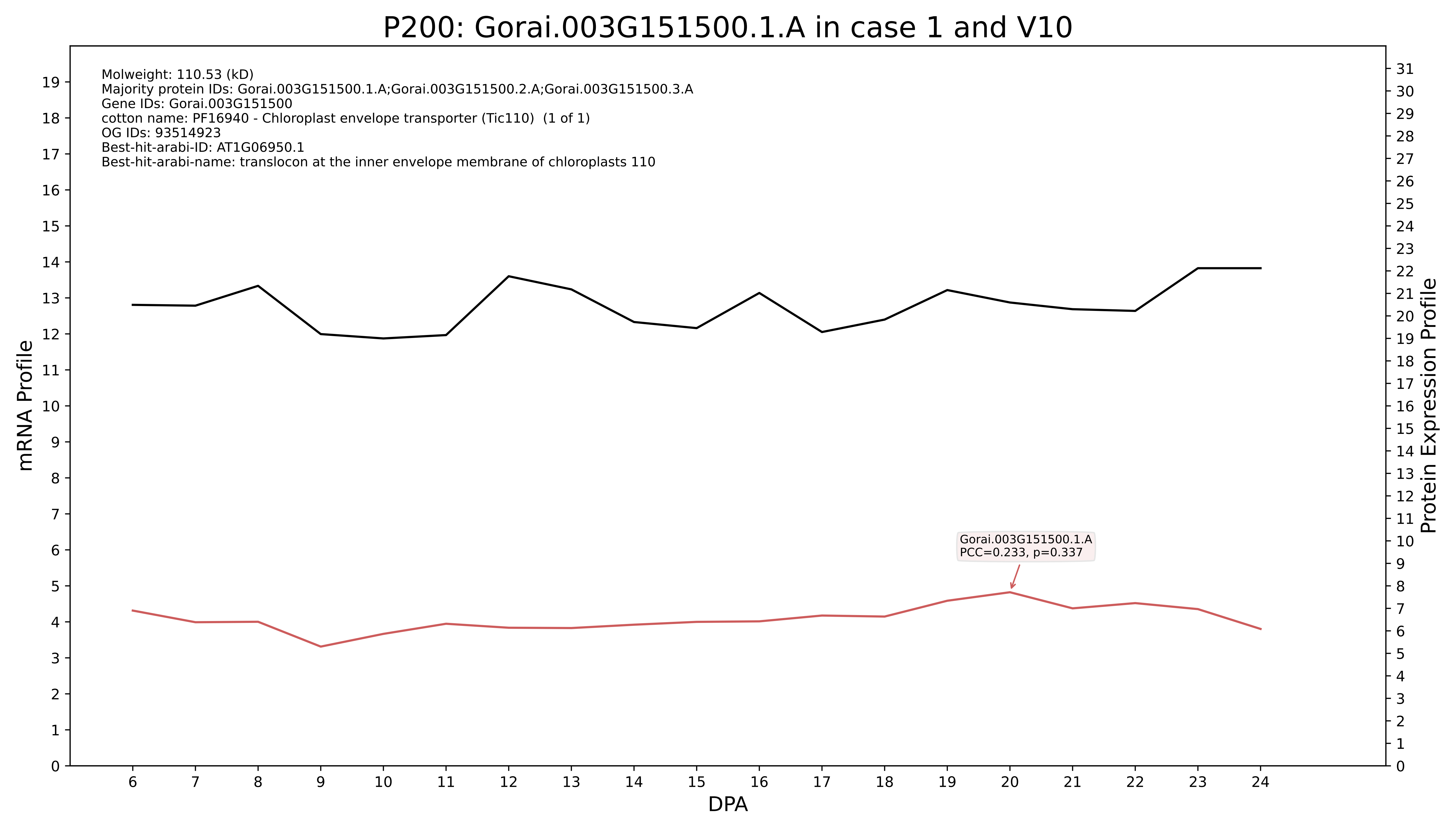
P200: Gorai.003G151500.1.A in case 1 and V10
Molweight: 110.53 (kD)
Majority protein IDs: Gorai.003G151500.1.A;Gorai.003G151500.2.A;Gorai.003G151500.3.A
Gene IDs: Gorai.003G151500
cotton name: PF16940 - Chloroplast envelope transporter (Tic110) (1 of 1)
OG IDs: 93514923
Best-hit-arabi-ID: AT1G06950.1
Best-hit-arabi-name: translocon at the inner envelope membrane of chloroplasts 110
Gorai.003G151500.1.A
PCC=0.233, p=0.337
mRNA Profile
Protein Expression Profile
DPA

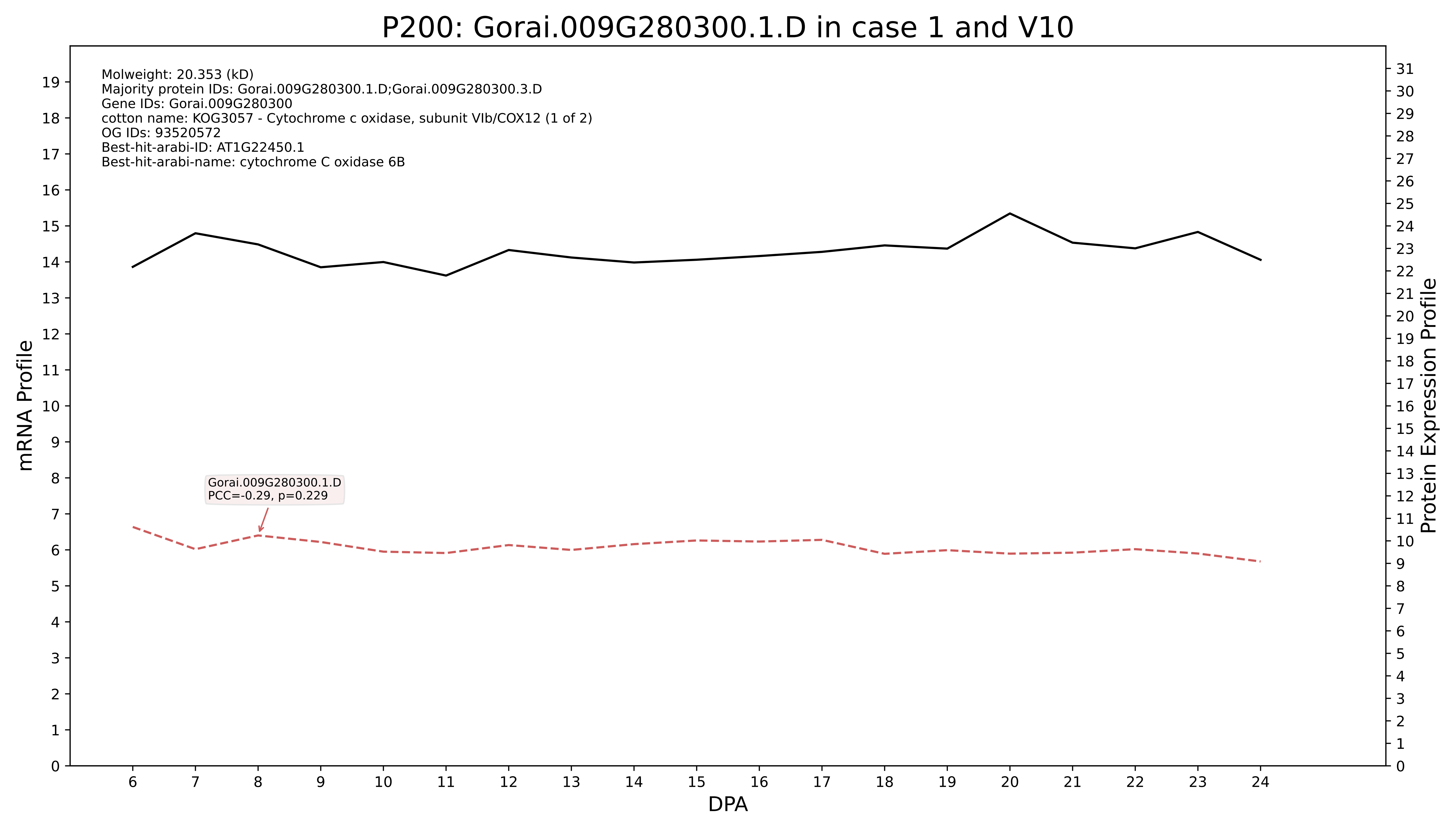

P200: Gorai.009G280300.1.D in case 1 and V10
Molweight: 20.353 (kD)
Majority protein IDs: Gorai.009G280300.1.D;Gorai.009G280300.3.D
Gene IDs: Gorai.009G280300
cotton name: KOG3057 - Cytochrome c oxidase, subunit VIb/COX12 (1 of 2)
OG IDs: 93520572
Best-hit-arabi-ID: AT1G22450.1
Best-hit-arabi-name: cytochrome C oxidase 6B
Gorai.009G280300.1.D
PCC=-0.29, p=0.229
mRNA Profile
Protein Expression Profile
DPA

P200: Gorai.011G222600.1.A in case 1 and V10

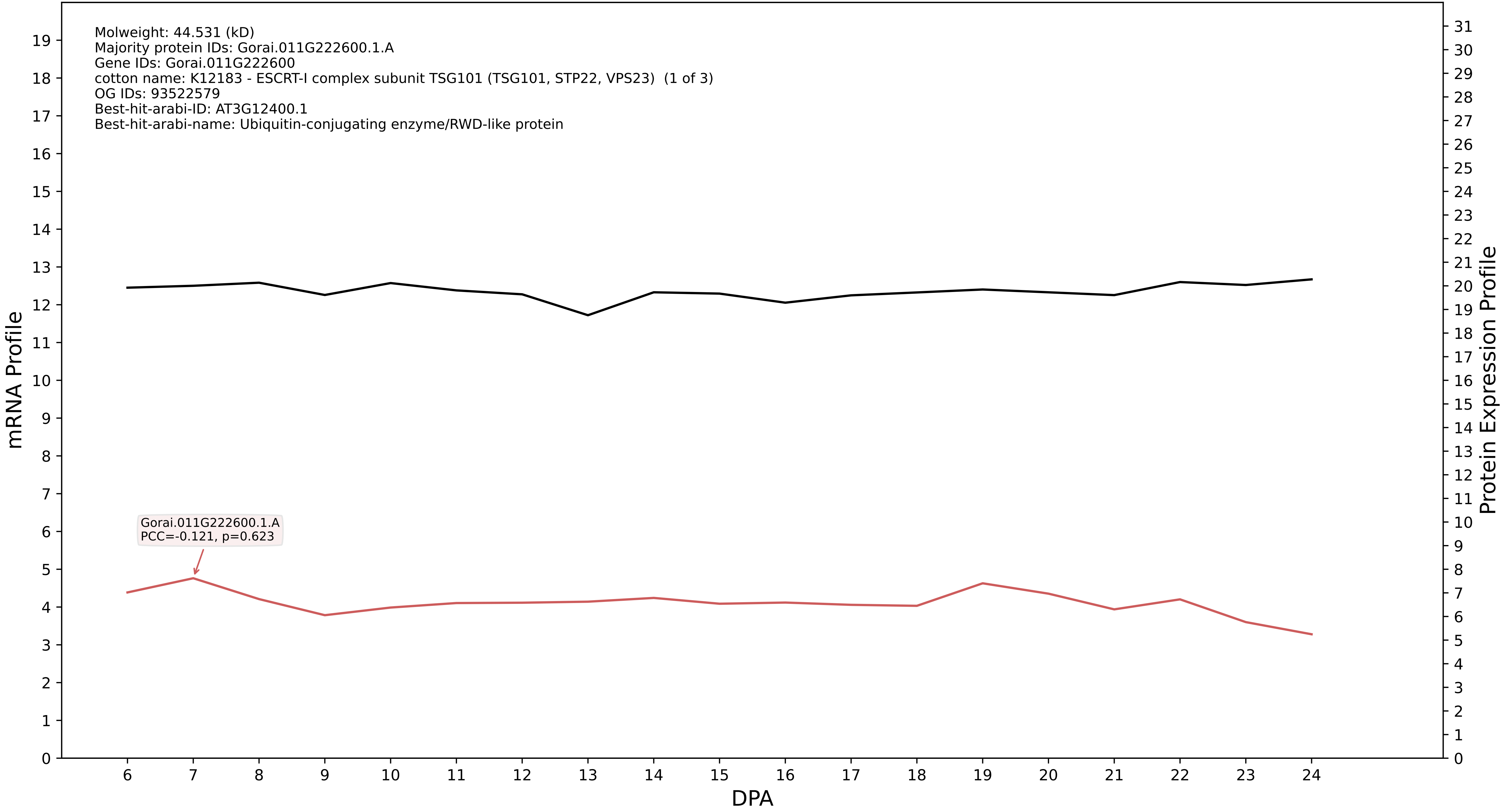

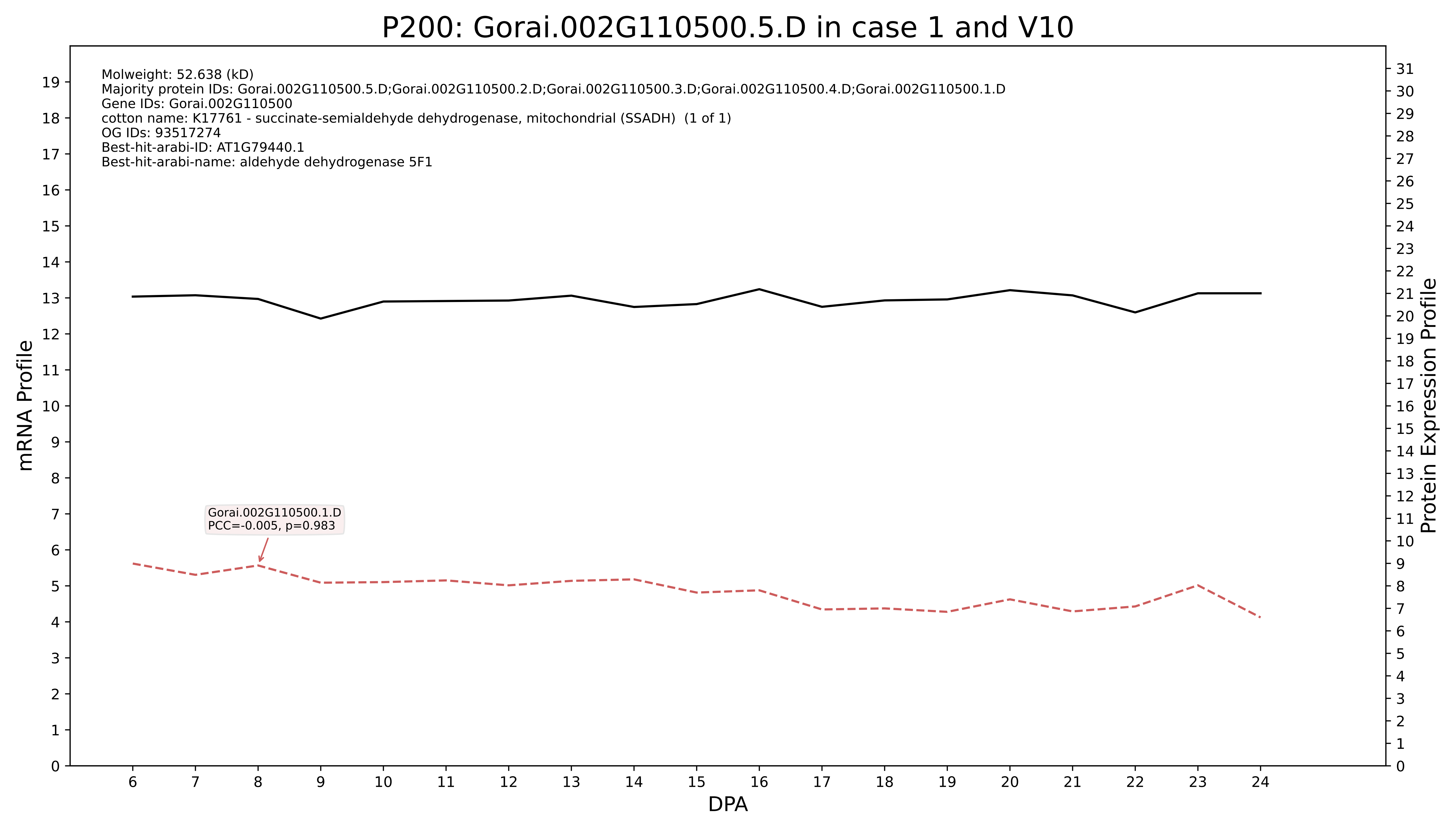

P200: Gorai.002G110500.5.D in case 1 and V10
Molweight: 52.638 (kD)
Majority protein IDs: Gorai.002G110500.5.D;Gorai.002G110500.2.D;Gorai.002G110500.3.D;Gorai.002G110500.4.D;Gorai.002G110500.1.D
Gene IDs: Gorai.002G110500
cotton name: K17761 - succinate-semialdehyde dehydrogenase, mitochondrial (SSADH) (1 of 1)
OG IDs: 93517274
Best-hit-arabi-ID: AT1G79440.1
Best-hit-arabi-name: aldehyde dehydrogenase 5F1
Gorai.002G110500.1.D
PCC=-0.005, p=0.983
mRNA Profile
Protein Expression Profile
DPA

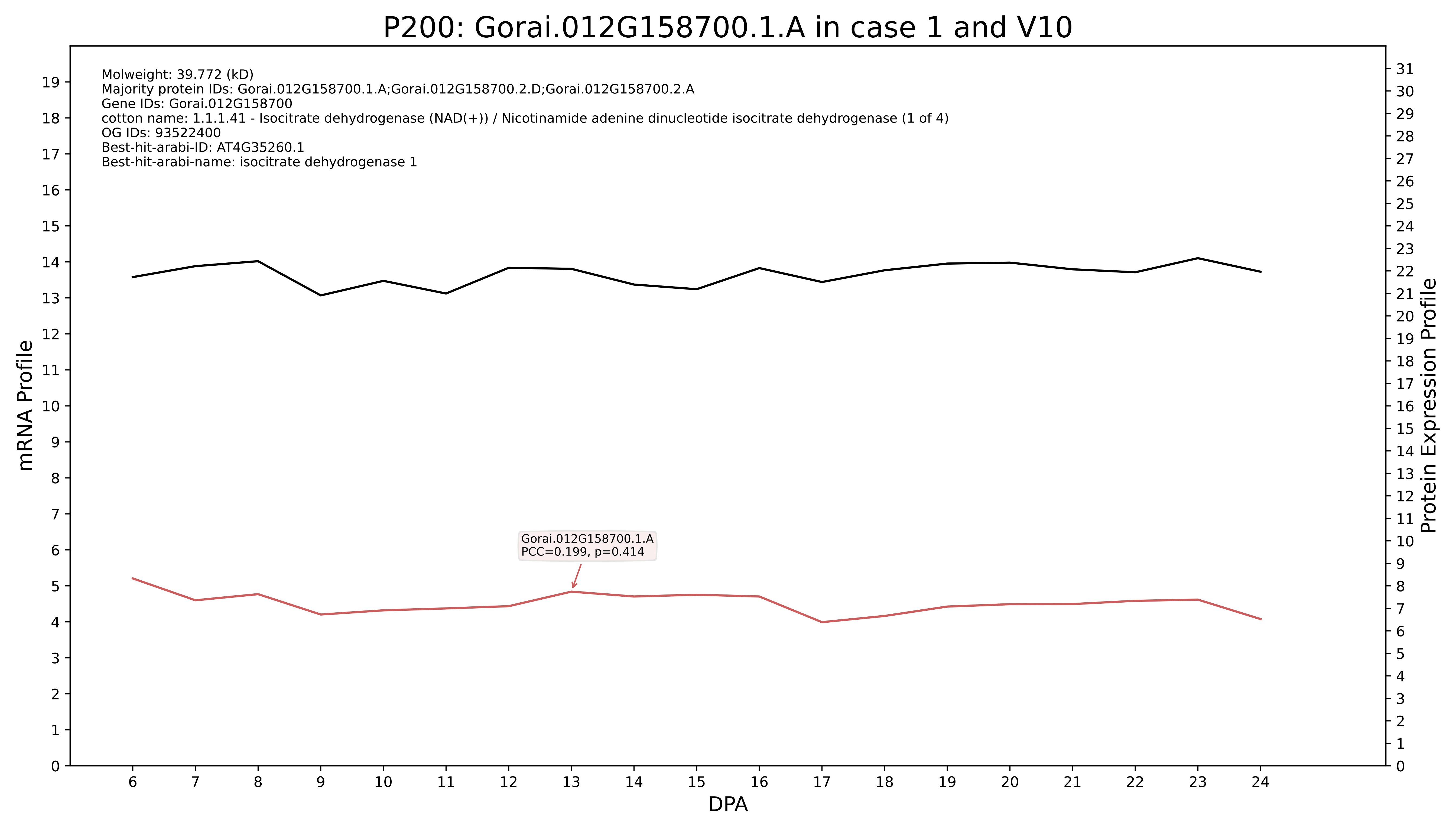
P200: Gorai.012G158700.1.A in case 1 and V10
Molweight: 39.772 (kD)
Majority protein IDs: Gorai.012G158700.1.A;Gorai.012G158700.2.D;Gorai.012G158700.2.A
Gene IDs: Gorai.012G158700
cotton name: 1.1.1.41 - Isocitrate dehydrogenase (NAD(+)) / Nicotinamide adenine dinucleotide isocitrate dehydrogenase (1 of 4)
OG IDs: 93522400
Best-hit-arabi-ID: AT4G35260.1
Best-hit-arabi-name: isocitrate dehydrogenase 1
Gorai.012G158700.1.A
PCC=0.199, p=0.414
mRNA Profile
Protein Expression Profile
DPA

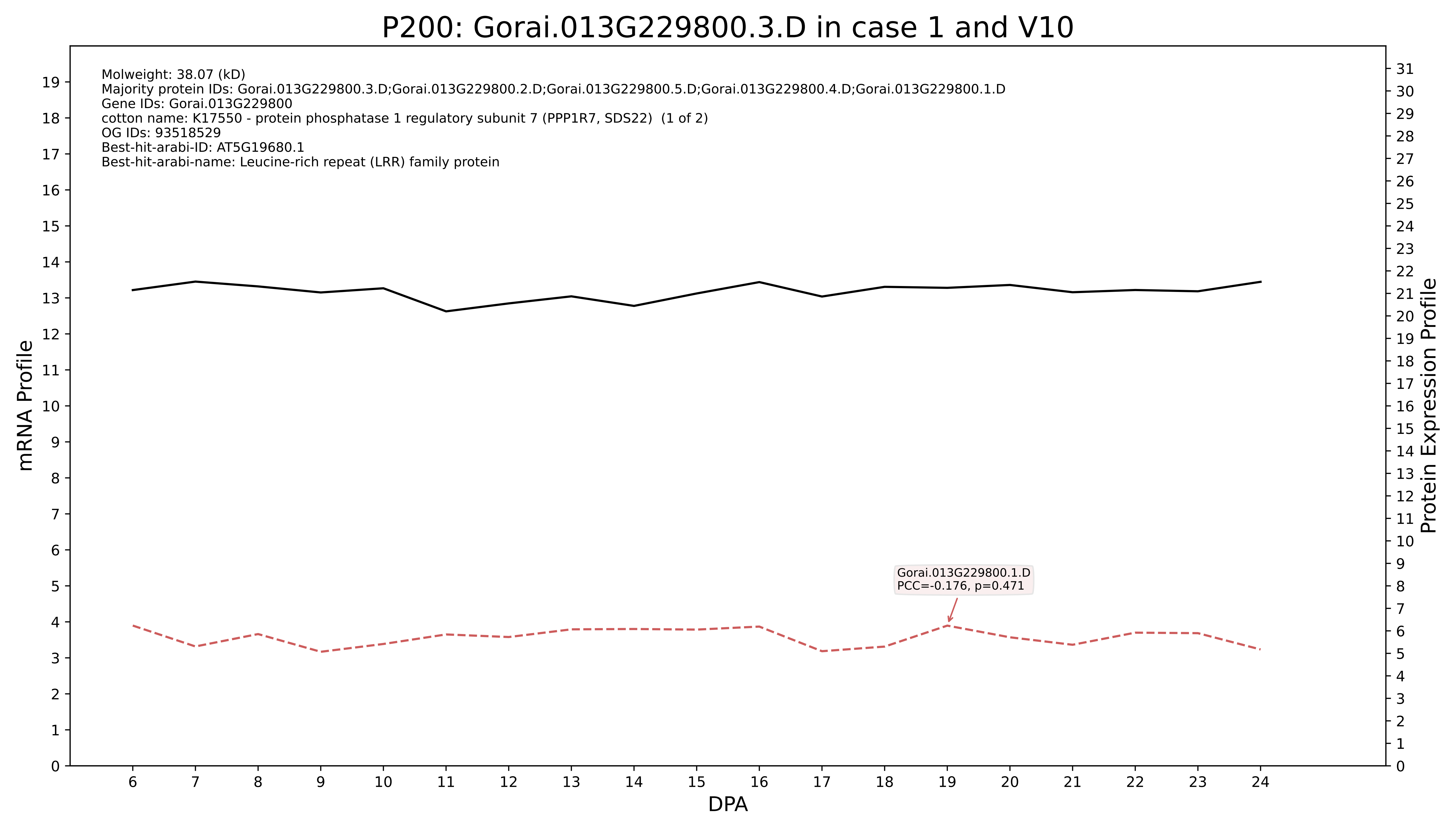
P200: Gorai.013G229800.3.D in case 1 and V10
Molweight: 38.07 (kD)
Majority protein IDs: Gorai.013G229800.3.D;Gorai.013G229800.2.D;Gorai.013G229800.5.D;Gorai.013G229800.4.D;Gorai.013G229800.1.D
Gene IDs: Gorai.013G229800
cotton name: K17550 - protein phosphatase 1 regulatory subunit 7 (PPP1R7, SDS22) (1 of 2)
OG IDs: 93518529
Best-hit-arabi-ID: AT5G19680.1
Best-hit-arabi-name: Leucine-rich repeat (LRR) family protein
Gorai.013G229800.1.D
PCC=-0.176, p=0.471
mRNA Profile
Protein Expression Profile
DPA

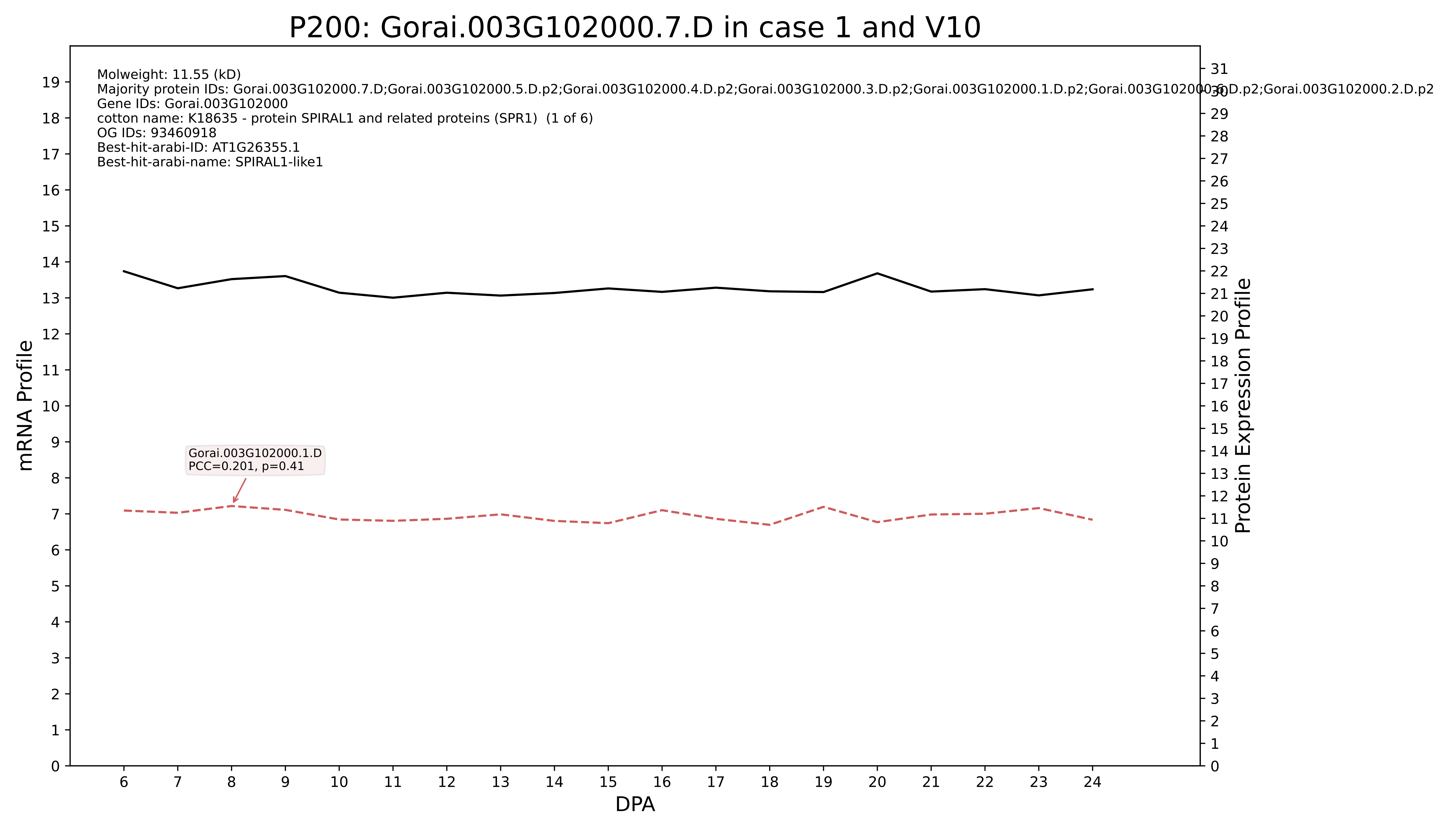
P200: Gorai.003G102000.7.D in case 1 and V10
Molweight: 11.55 (kD)
Majority protein IDs: Gorai.003G102000.7.D;Gorai.003G102000.5.D.p2;Gorai.003G102000.4.D.p2;Gorai.003G102000.3.D.p2;Gorai.003G102000.1.D.p2;Gorai.003G102000.6.D.p2;Gorai.003G102000.2.D.p2
Gene IDs: Gorai.003G102000
cotton name: K18635 - protein SPIRAL1 and related proteins (SPR1) (1 of 6)
OG IDs: 93460918
Best-hit-arabi-ID: AT1G26355.1
Best-hit-arabi-name: SPIRAL1-like1
Gorai.003G102000.1.D
PCC=0.201, p=0.41
mRNA Profile
Protein Expression Profile
DPA

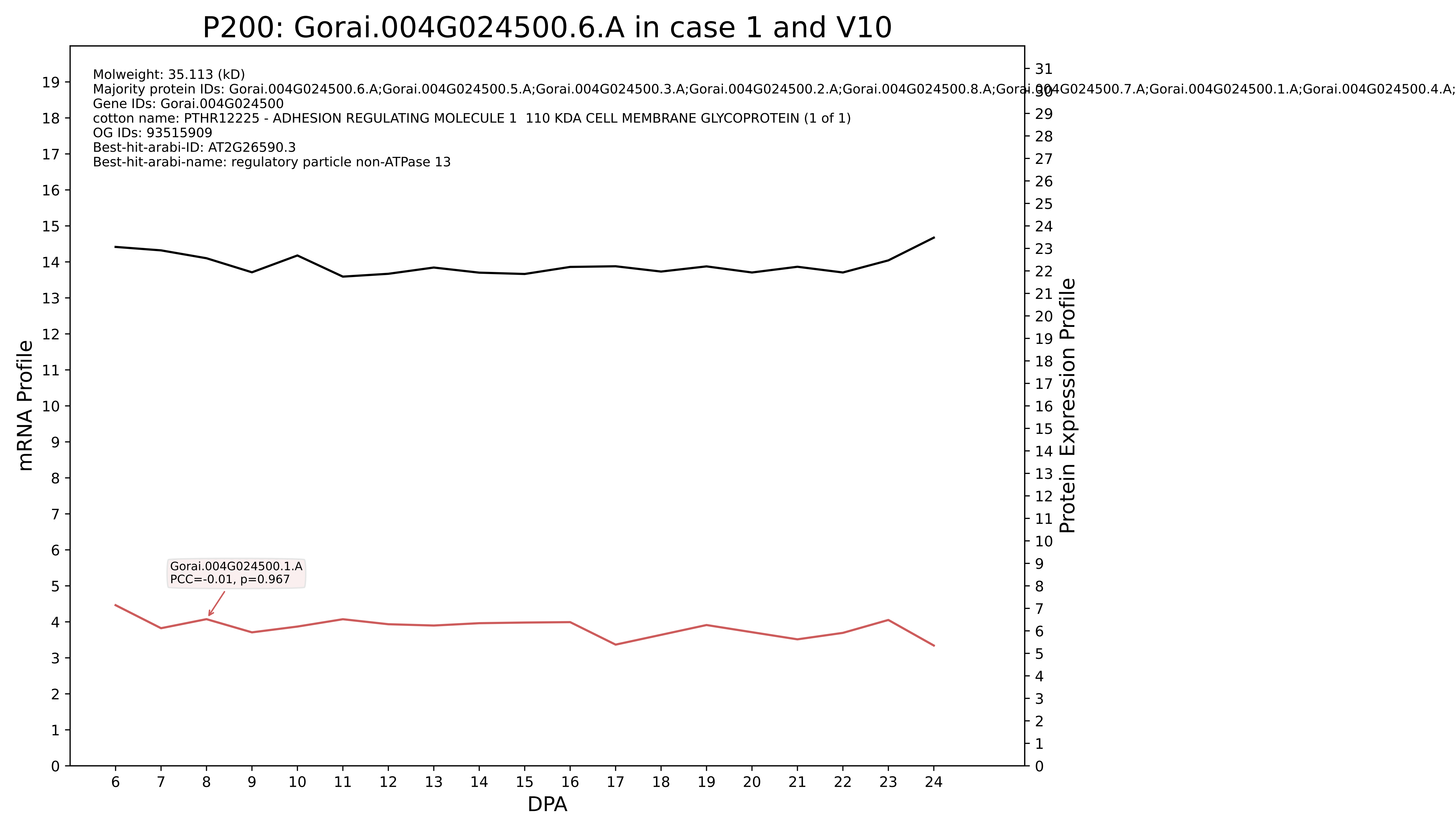

P200: Gorai.004G024500.6.A in case 1 and V10
Molweight: 35.113 (kD)
Majority protein IDs: Gorai.004G024500.6.A;Gorai.004G024500.5.A;Gorai.004G024500.3.A;Gorai.004G024500.2.A;Gorai.004G024500.8.A;Gorai.004G024500.7.A;Gorai.004G024500.1.A;Gorai.004G024500.4.A;
Gene IDs: Gorai.004G024500
cotton name: PTHR12225 - ADHESION REGULATING MOLECULE 1 110 KDA CELL MEMBRANE GLYCOPROTEIN (1 of 1)
OG IDs: 93515909
Best-hit-arabi-ID: AT2G26590.3
Best-hit-arabi-name: regulatory particle non-ATPase 13
Gorai.004G024500.1.A
PCC=-0.01, p=0.967
mRNA Profile
Protein Expression Profile
DPA

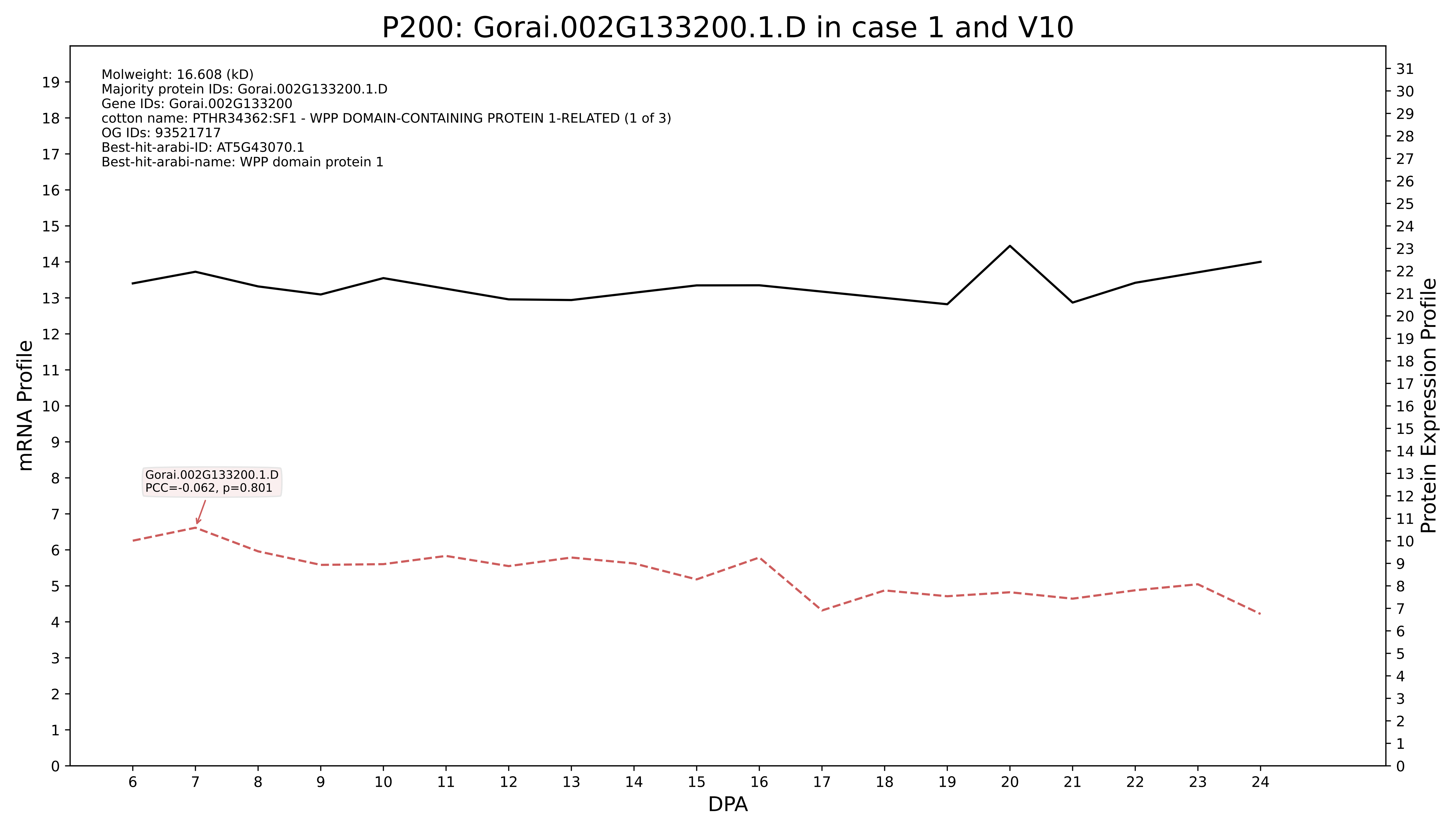

P200: Gorai.002G133200.1.D in case 1 and V10
Molweight: 16.608 (kD)
Majority protein IDs: Gorai.002G133200.1.D
Gene IDs: Gorai.002G133200
cotton name: PTHR34362:SF1 - WPP DOMAIN-CONTAINING PROTEIN 1-RELATED (1 of 3)
OG IDs: 93521717
Best-hit-arabi-ID: AT5G43070.1
Best-hit-arabi-name: WPP domain protein 1
Gorai.002G133200.1.D
PCC=-0.062, p=0.801
mRNA Profile
Protein Expression Profile
DPA

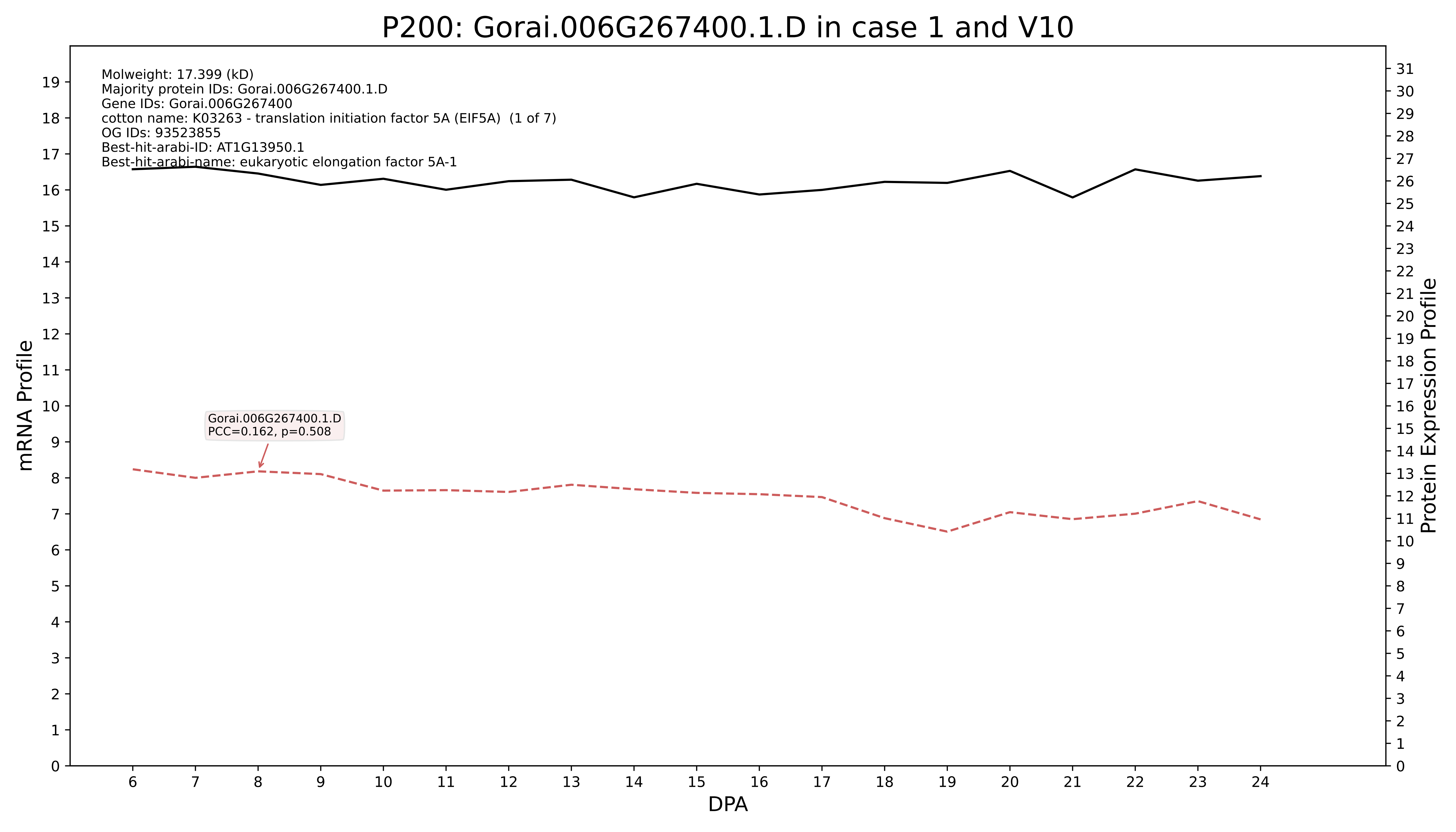
P200: Gorai.006G267400.1.D in case 1 and V10
Molweight: 17.399 (kD)
Majority protein IDs: Gorai.006G267400.1.D
Gene IDs: Gorai.006G267400
cotton name: K03263 - translation initiation factor 5A (EIF5A) (1 of 7)
OG IDs: 93523855
Best-hit-arabi-ID: AT1G13950.1
Best-hit-arabi-name: eukaryotic elongation factor 5A-1
Gorai.006G267400.1.D
PCC=0.162, p=0.508
mRNA Profile
Protein Expression Profile
DPA

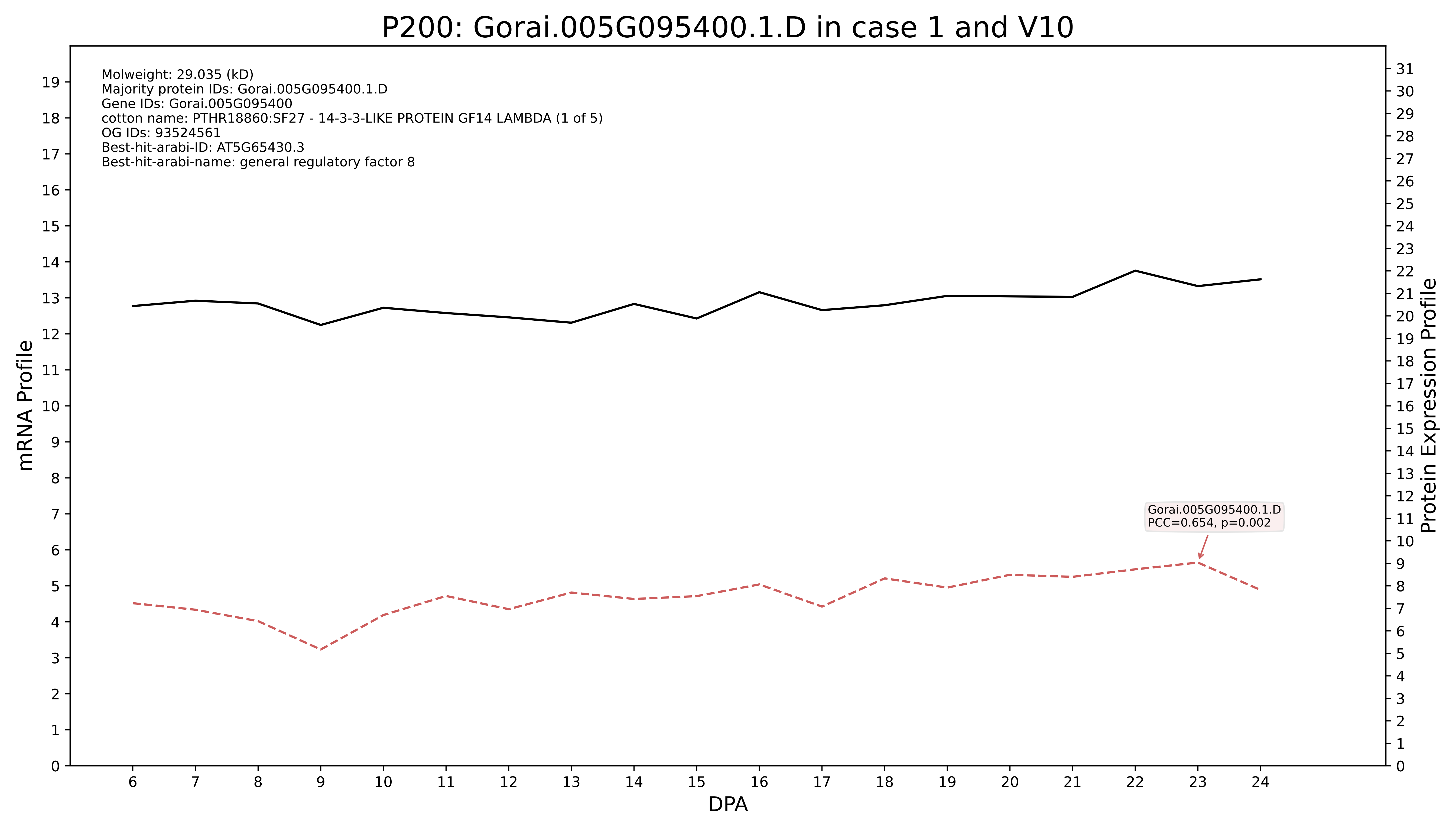
P200: Gorai.005G095400.1.D in case 1 and V10
Molweight: 29.035 (kD)
Majority protein IDs: Gorai.005G095400.1.D
Gene IDs: Gorai.005G095400
cotton name: PTHR18860:SF27 - 14-3-3-LIKE PROTEIN GF14 LAMBDA (1 of 5)
OG IDs: 93524561
Best-hit-arabi-ID: AT5G65430.3
Best-hit-arabi-name: general regulatory factor 8
Gorai.005G095400.1.D
PCC=0.654, p=0.002
mRNA Profile
Protein Expression Profile
DPA

P200: Gorai.010G256800.1.A in case 1 and V10

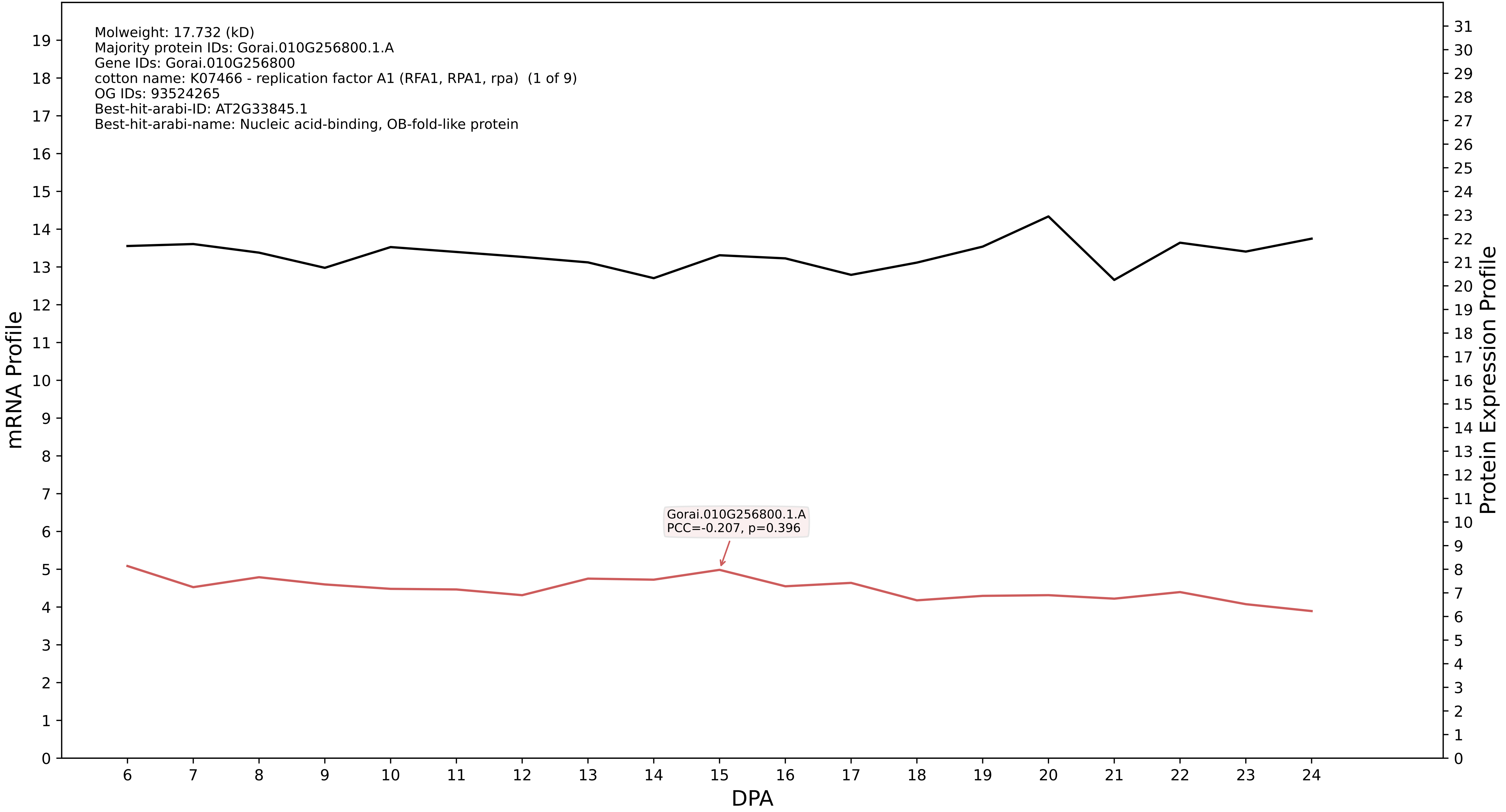

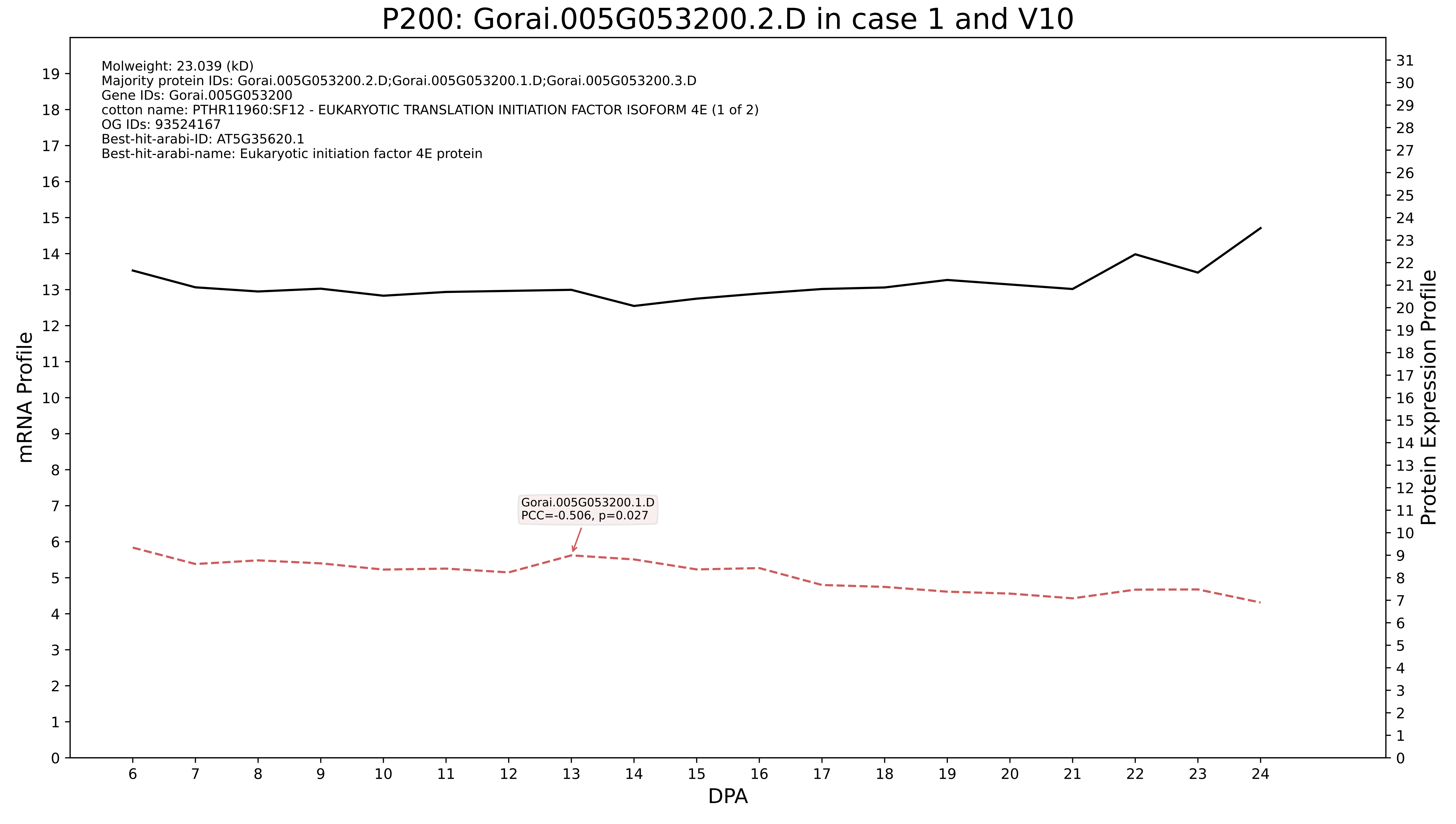

P200: Gorai.005G053200.2.D in case 1 and V10
Molweight: 23.039 (kD)
Majority protein IDs: Gorai.005G053200.2.D;Gorai.005G053200.1.D;Gorai.005G053200.3.D
Gene IDs: Gorai.005G053200
cotton name: PTHR11960:SF12 - EUKARYOTIC TRANSLATION INITIATION FACTOR ISOFORM 4E (1 of 2)
OG IDs: 93524167
Best-hit-arabi-ID: AT5G35620.1
Best-hit-arabi-name: Eukaryotic initiation factor 4E protein
Gorai.005G053200.1.D
PCC=-0.506, p=0.027
mRNA Profile
Protein Expression Profile
DPA

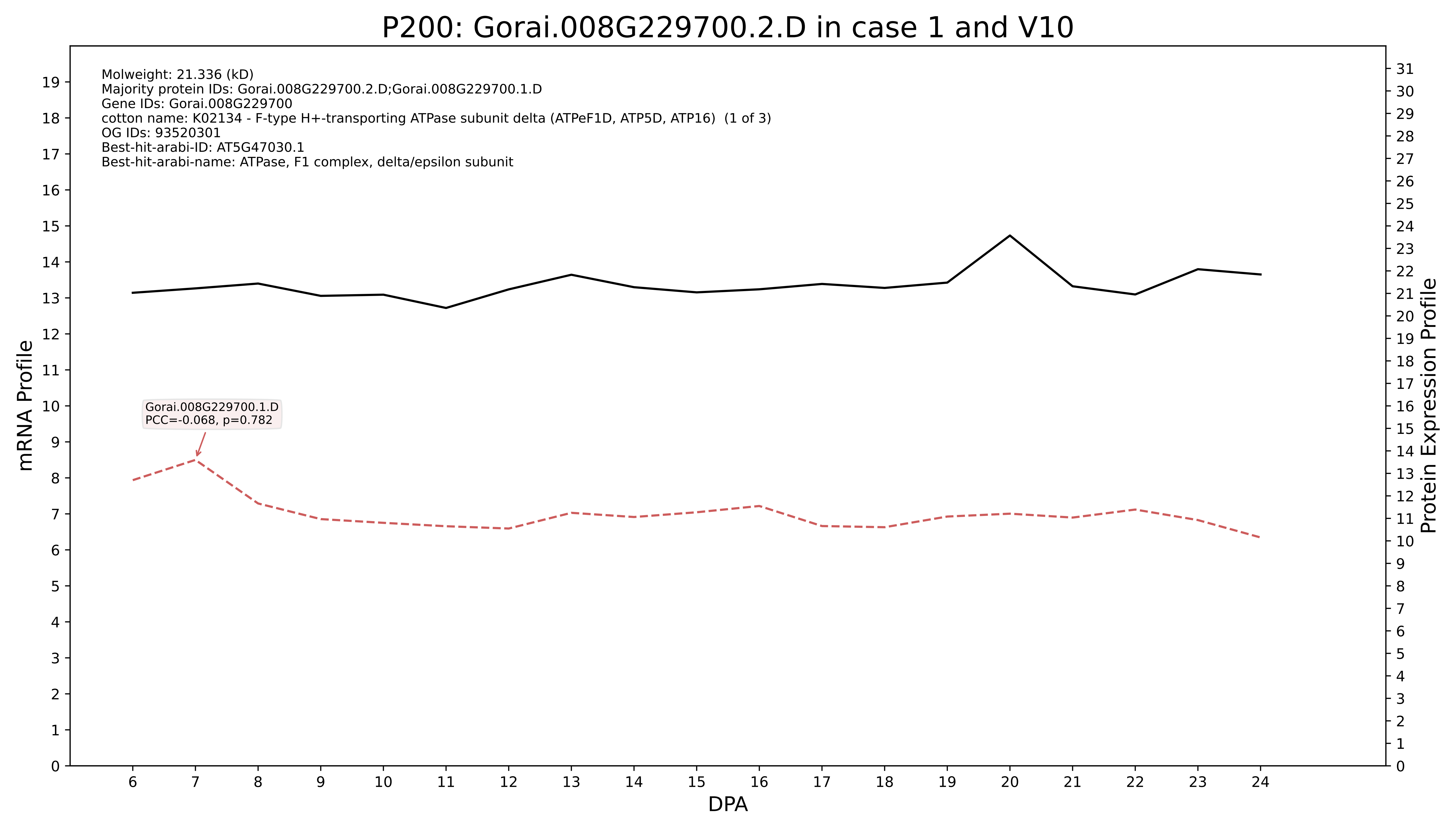

P200: Gorai.008G229700.2.D in case 1 and V10
Molweight: 21.336 (kD)
Majority protein IDs: Gorai.008G229700.2.D;Gorai.008G229700.1.D
Gene IDs: Gorai.008G229700
cotton name: K02134 - F-type H+-transporting ATPase subunit delta (ATPeF1D, ATP5D, ATP16) (1 of 3)
OG IDs: 93520301
Best-hit-arabi-ID: AT5G47030.1
Best-hit-arabi-name: ATPase, F1 complex, delta/epsilon subunit
Gorai.008G229700.1.D
PCC=-0.068, p=0.782
mRNA Profile
Protein Expression Profile
DPA

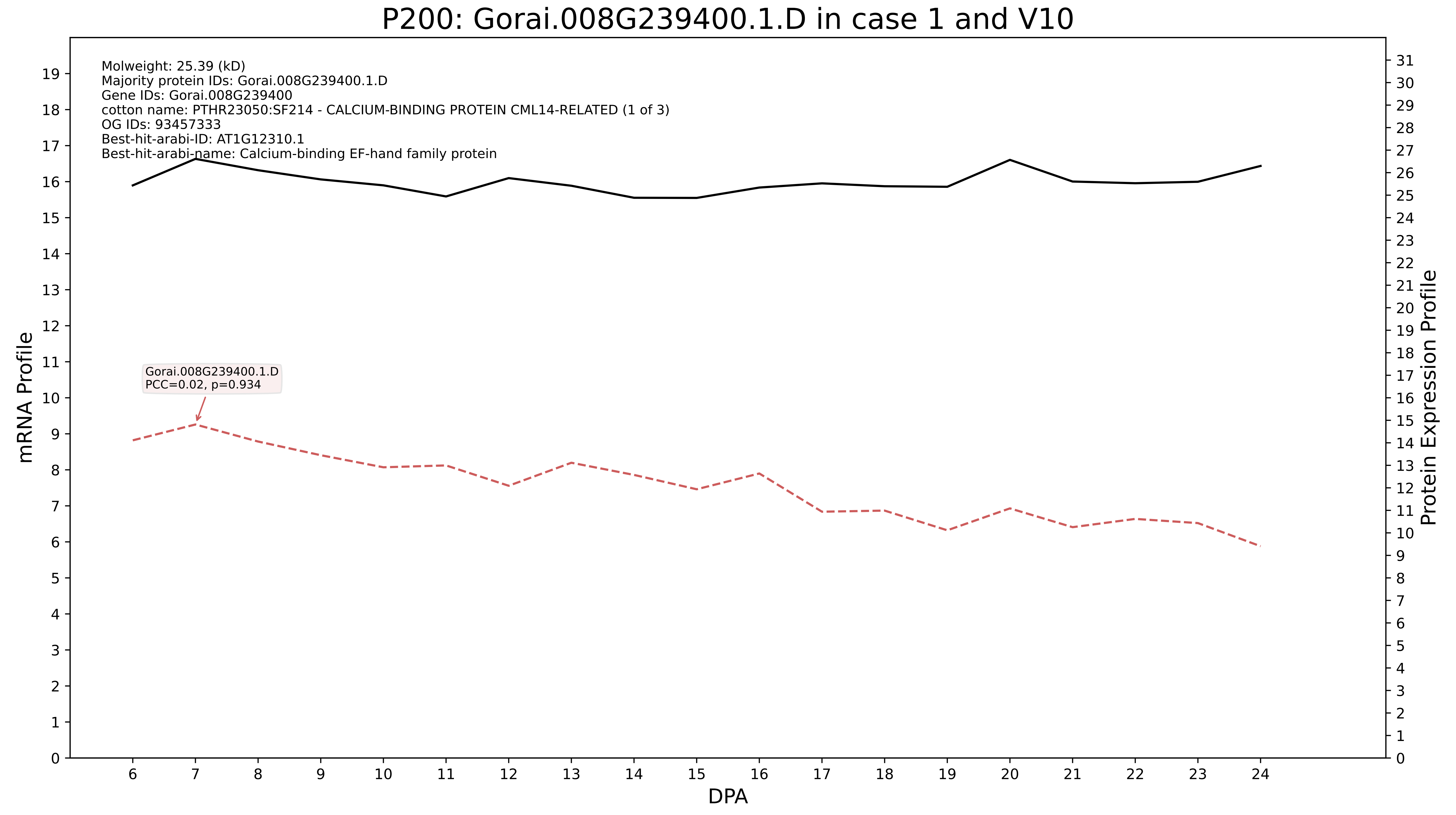

P200: Gorai.008G239400.1.D in case 1 and V10
Molweight: 25.39 (kD)
Majority protein IDs: Gorai.008G239400.1.D
Gene IDs: Gorai.008G239400
cotton name: PTHR23050:SF214 - CALCIUM-BINDING PROTEIN CML14-RELATED (1 of 3)
OG IDs: 93457333
Best-hit-arabi-ID: AT1G12310.1
Best-hit-arabi-name: Calcium-binding EF-hand family protein
Gorai.008G239400.1.D
PCC=0.02, p=0.934
mRNA Profile
Protein Expression Profile
DPA

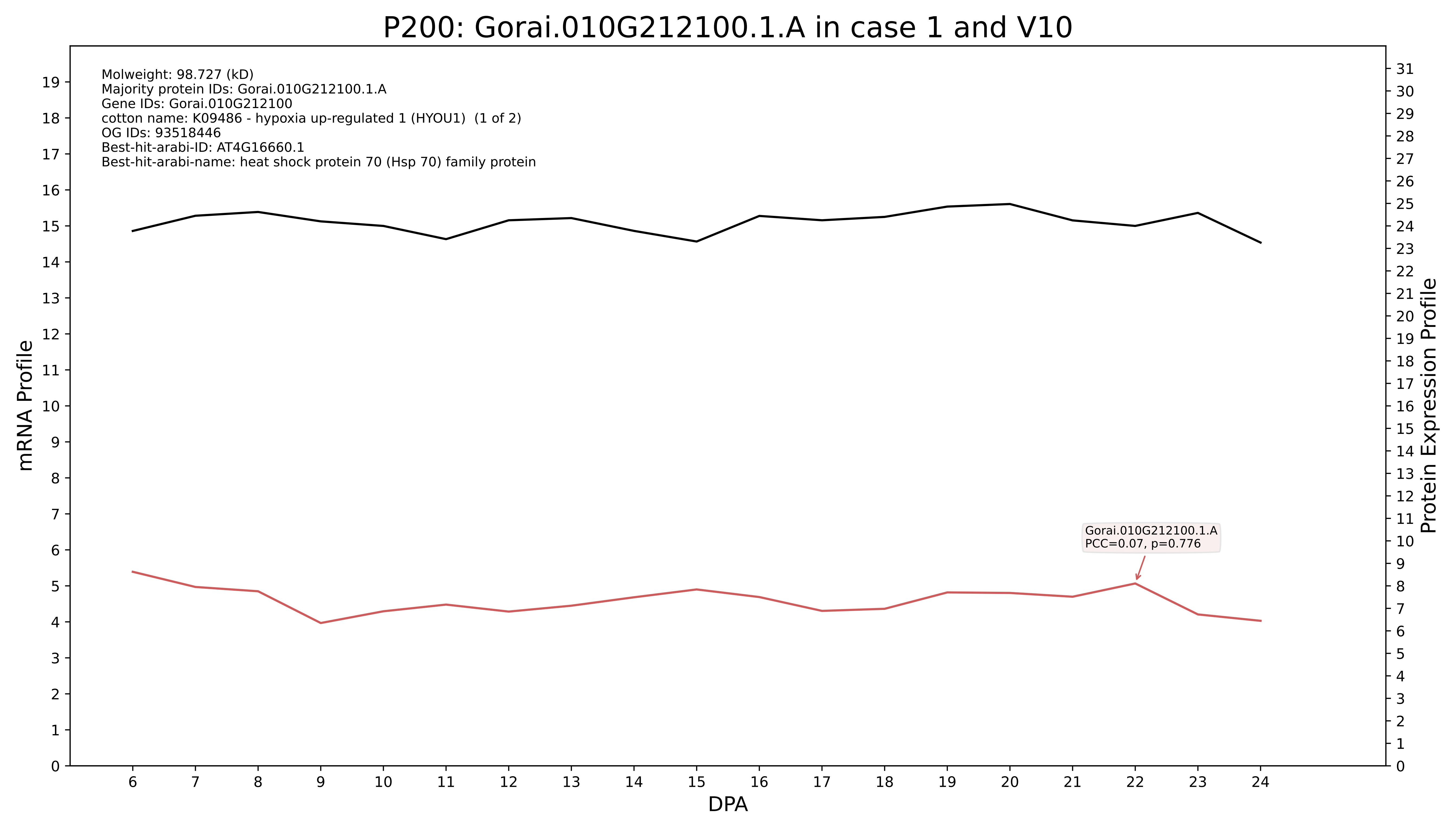
P200: Gorai.010G212100.1.A in case 1 and V10
Molweight: 98.727 (kD)
Majority protein IDs: Gorai.010G212100.1.A
Gene IDs: Gorai.010G212100
cotton name: K09486 - hypoxia up-regulated 1 (HYOU1) (1 of 2)
OG IDs: 93518446
Best-hit-arabi-ID: AT4G16660.1
Best-hit-arabi-name: heat shock protein 70 (Hsp 70) family protein
Gorai.010G212100.1.A
PCC=0.07, p=0.776
mRNA Profile
Protein Expression Profile
DPA

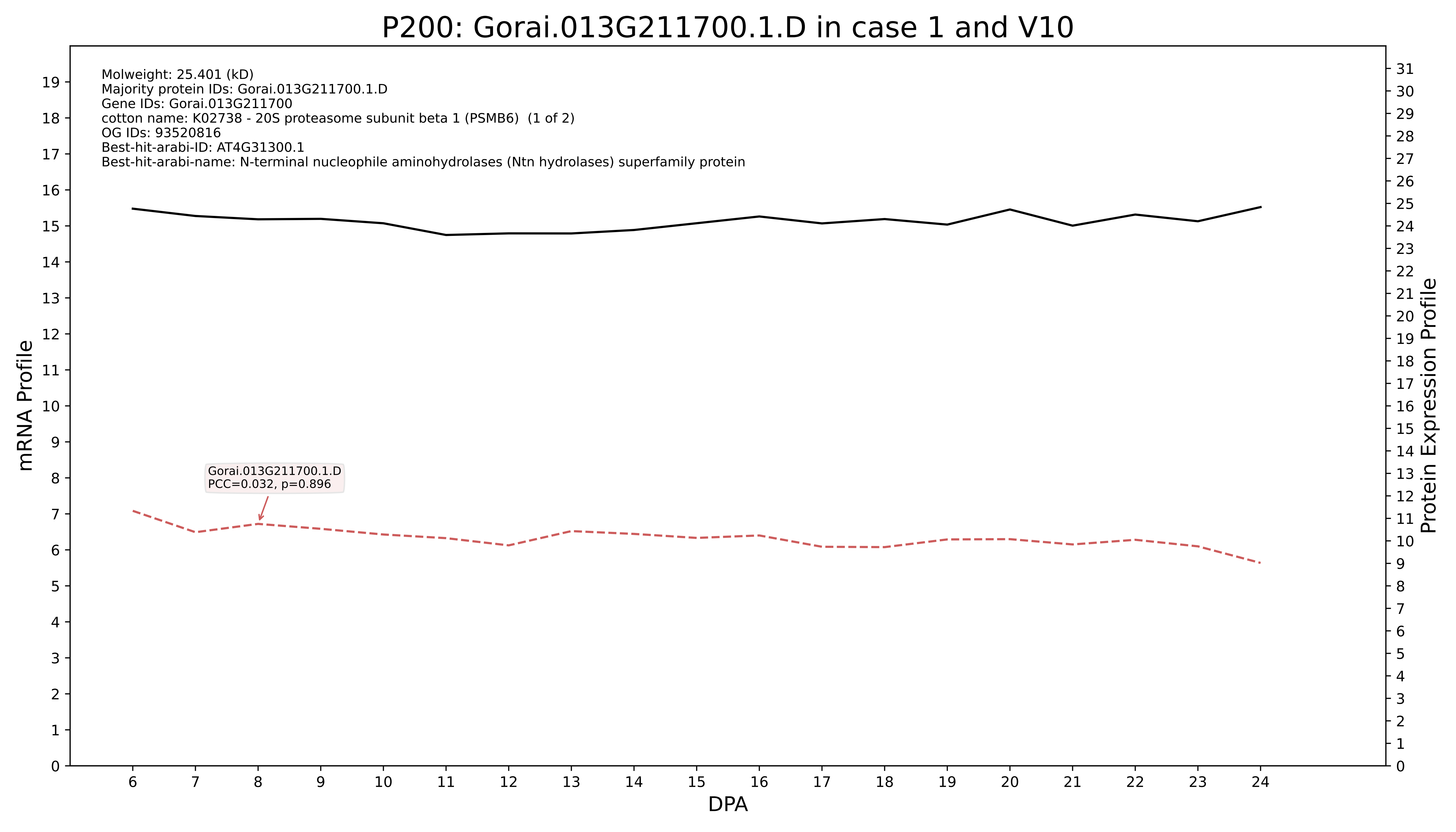
P200: Gorai.013G211700.1.D in case 1 and V10
Molweight: 25.401 (kD)
Majority protein IDs: Gorai.013G211700.1.D
Gene IDs: Gorai.013G211700
cotton name: K02738 - 20S proteasome subunit beta 1 (PSMB6) (1 of 2)
OG IDs: 93520816
Best-hit-arabi-ID: AT4G31300.1
Best-hit-arabi-name: N-terminal nucleophile aminohydrolases (Ntn hydrolases) superfamily protein
Gorai.013G211700.1.D
PCC=0.032, p=0.896
mRNA Profile
Protein Expression Profile
DPA

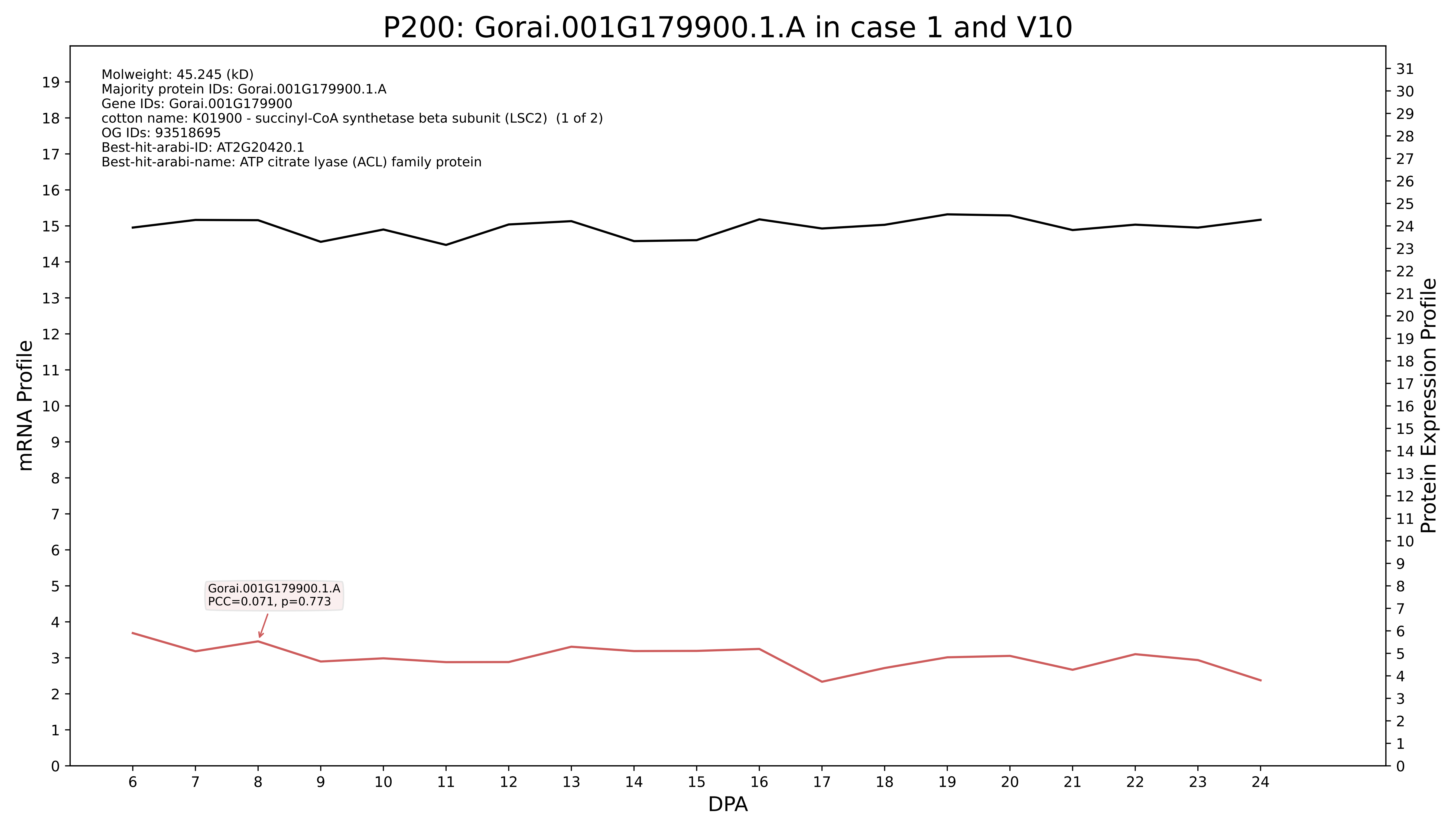
P200: Gorai.001G179900.1.A in case 1 and V10
Molweight: 45.245 (kD)
Majority protein IDs: Gorai.001G179900.1.A
Gene IDs: Gorai.001G179900
cotton name: K01900 - succinyl-CoA synthetase beta subunit (LSC2) (1 of 2)
OG IDs: 93518695
Best-hit-arabi-ID: AT2G20420.1
Best-hit-arabi-name: ATP citrate lyase (ACL) family protein
Gorai.001G179900.1.A
PCC=0.071, p=0.773
mRNA Profile
Protein Expression Profile
DPA

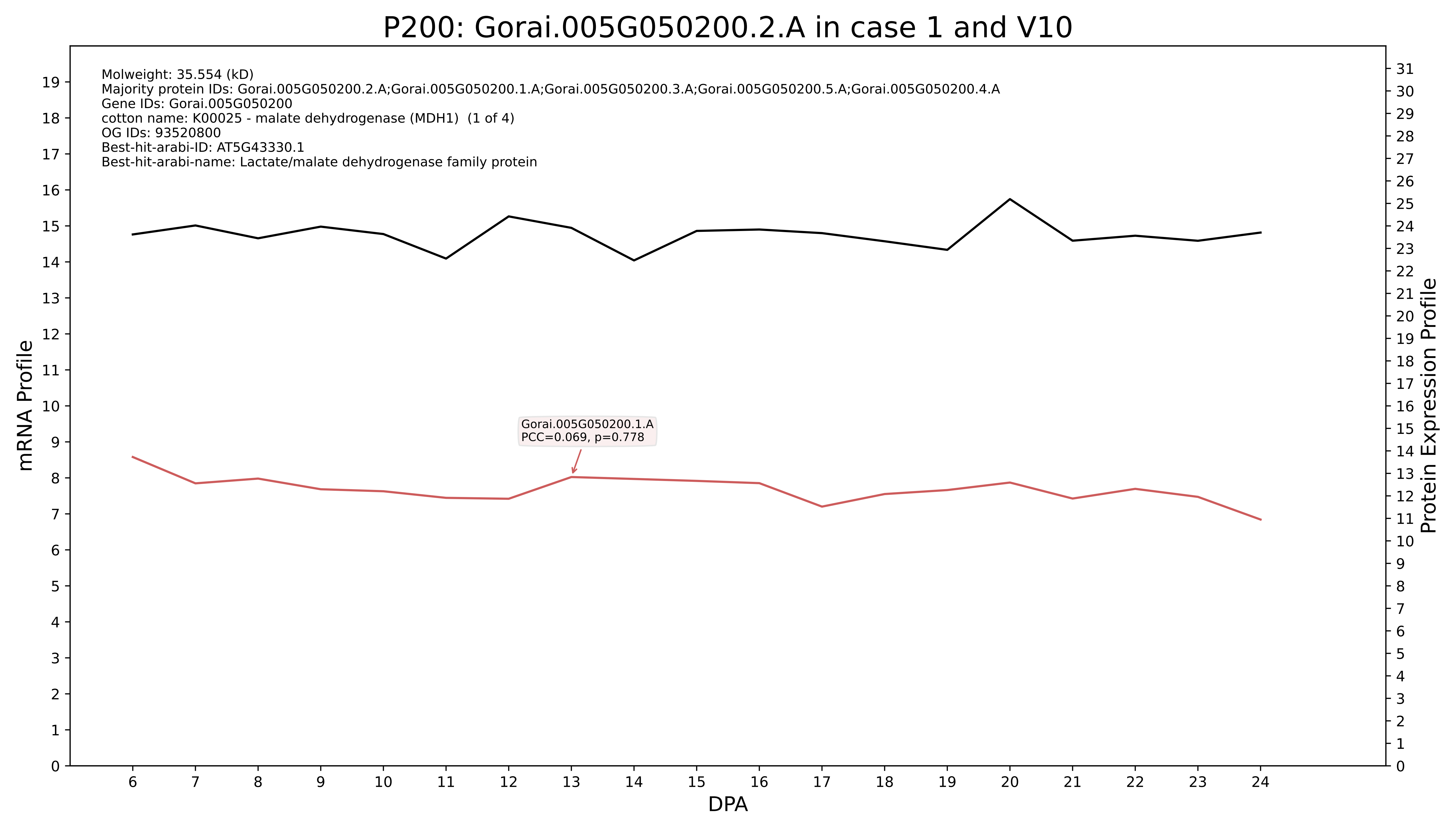

P200: Gorai.005G050200.2.A in case 1 and V10
Molweight: 35.554 (kD)
Majority protein IDs: Gorai.005G050200.2.A;Gorai.005G050200.1.A;Gorai.005G050200.3.A;Gorai.005G050200.5.A;Gorai.005G050200.4.A
Gene IDs: Gorai.005G050200
cotton name: K00025 - malate dehydrogenase (MDH1) (1 of 4)
OG IDs: 93520800
Best-hit-arabi-ID: AT5G43330.1
Best-hit-arabi-name: Lactate/malate dehydrogenase family protein
Gorai.005G050200.1.A
PCC=0.069, p=0.778
mRNA Profile
Protein Expression Profile
DPA

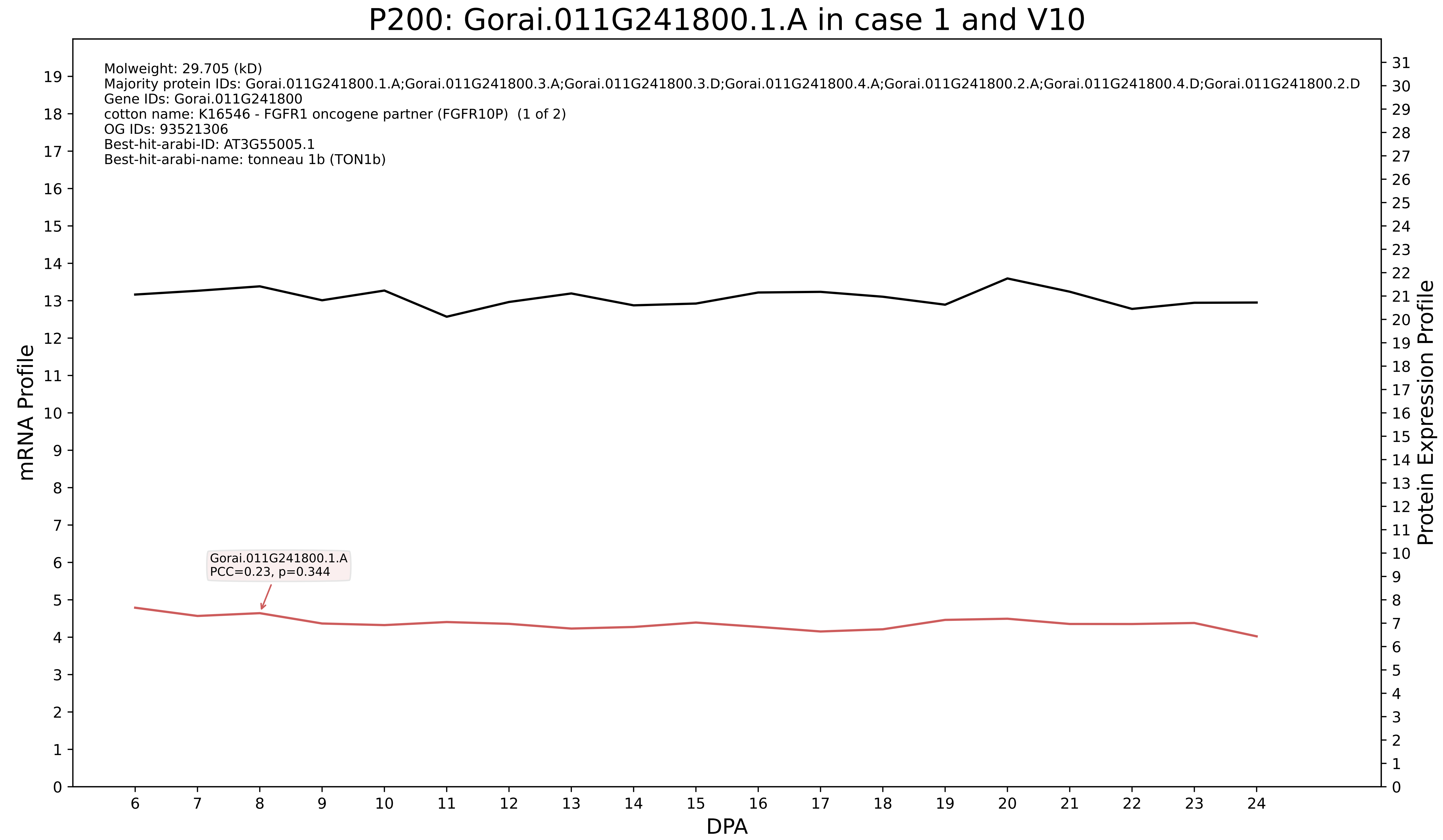

P200: Gorai.011G241800.1.A in case 1 and V10
Molweight: 29.705 (kD)
Majority protein IDs: Gorai.011G241800.1.A;Gorai.011G241800.3.A;Gorai.011G241800.3.D;Gorai.011G241800.4.A;Gorai.011G241800.2.A;Gorai.011G241800.4.D;Gorai.011G241800.2.D
Gene IDs: Gorai.011G241800
cotton name: K16546 - FGFR1 oncogene partner (FGFR10P) (1 of 2)
OG IDs: 93521306
Best-hit-arabi-ID: AT3G55005.1
Best-hit-arabi-name: tonneau 1b (TON1b)
Gorai.011G241800.1.A
PCC=0.23, p=0.344
mRNA Profile
Protein Expression Profile
DPA

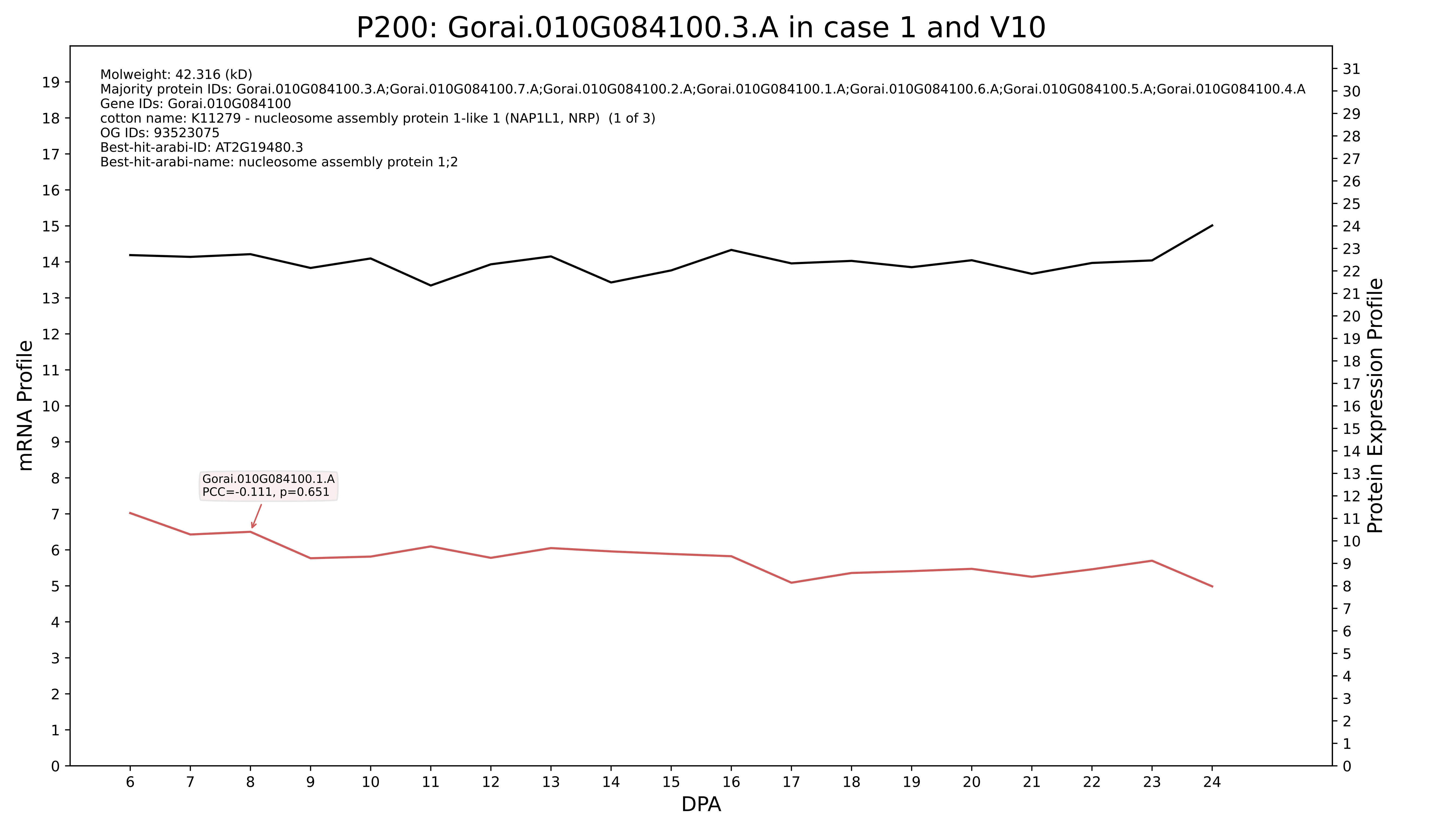

P200: Gorai.010G084100.3.A in case 1 and V10
Molweight: 42.316 (kD)
Majority protein IDs: Gorai.010G084100.3.A;Gorai.010G084100.7.A;Gorai.010G084100.2.A;Gorai.010G084100.1.A;Gorai.010G084100.6.A;Gorai.010G084100.5.A;Gorai.010G084100.4.A
Gene IDs: Gorai.010G084100
cotton name: K11279 - nucleosome assembly protein 1-like 1 (NAP1L1, NRP) (1 of 3)
OG IDs: 93523075
Best-hit-arabi-ID: AT2G19480.3
Best-hit-arabi-name: nucleosome assembly protein 1;2
Gorai.010G084100.1.A
PCC=-0.111, p=0.651
mRNA Profile
Protein Expression Profile
DPA

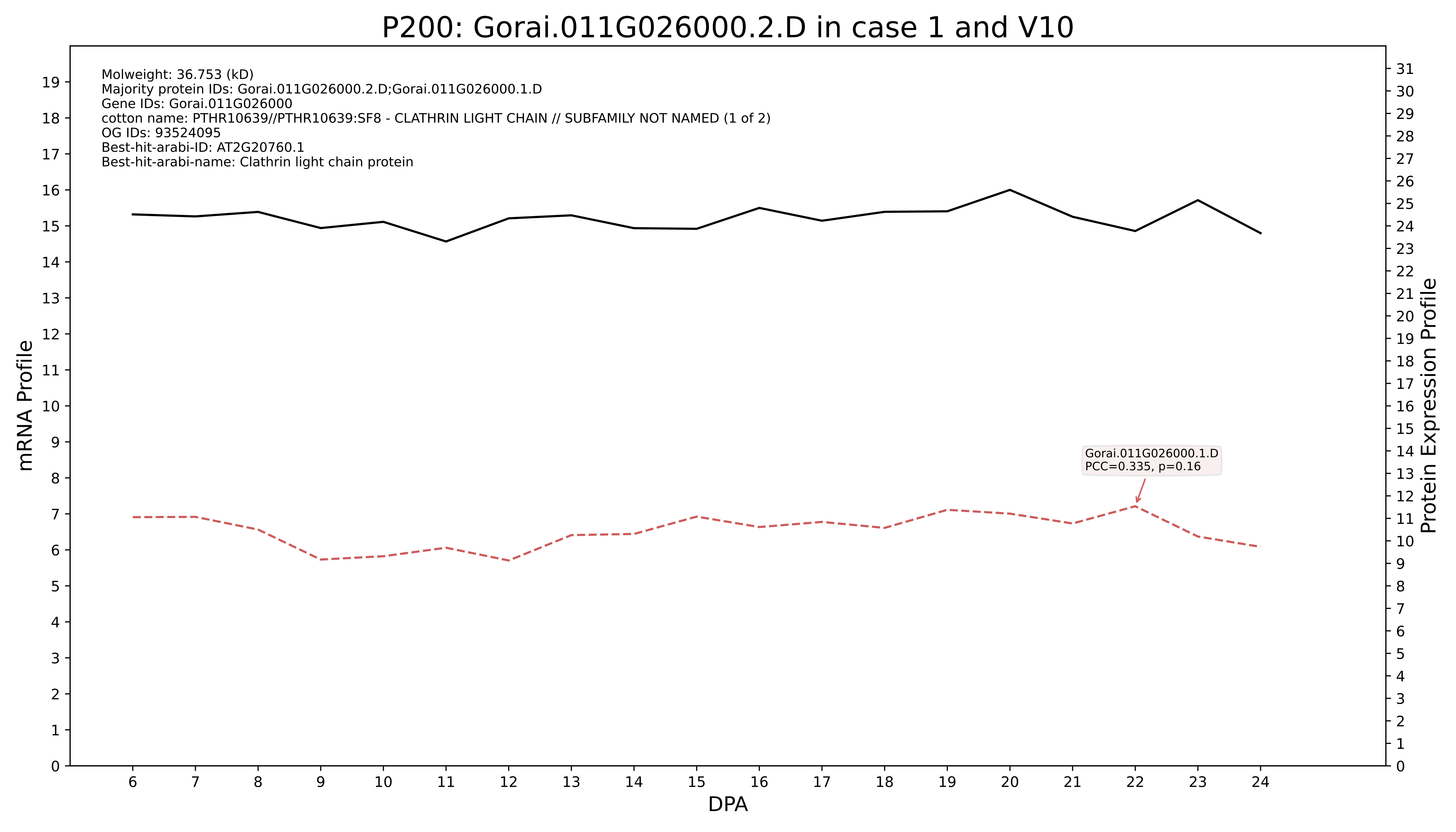

P200: Gorai.011G026000.2.D in case 1 and V10
Molweight: 36.753 (kD)
Majority protein IDs: Gorai.011G026000.2.D;Gorai.011G026000.1.D
Gene IDs: Gorai.011G026000
cotton name: PTHR10639//PTHR10639:SF8 - CLATHRIN LIGHT CHAIN // SUBFAMILY NOT NAMED (1 of 2)
OG IDs: 93524095
Best-hit-arabi-ID: AT2G20760.1
Best-hit-arabi-name: Clathrin light chain protein
Gorai.011G026000.1.D
PCC=0.335, p=0.16
mRNA Profile
Protein Expression Profile
DPA

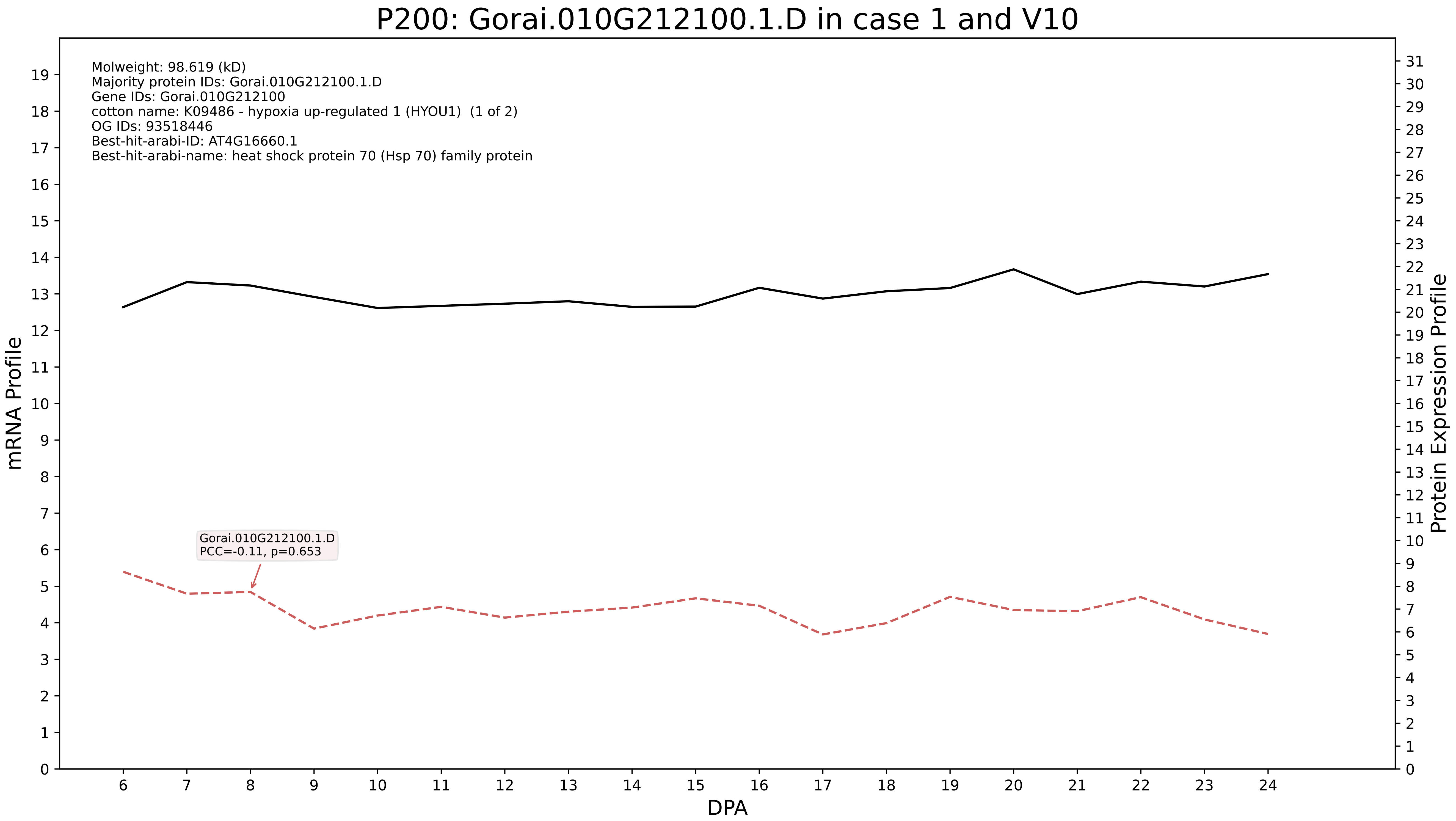
P200: Gorai.010G212100.1.D in case 1 and V10
Molweight: 98.619 (kD)
Majority protein IDs: Gorai.010G212100.1.D
Gene IDs: Gorai.010G212100
cotton name: K09486 - hypoxia up-regulated 1 (HYOU1) (1 of 2)
OG IDs: 93518446
Best-hit-arabi-ID: AT4G16660.1
Best-hit-arabi-name: heat shock protein 70 (Hsp 70) family protein
Gorai.010G212100.1.D
PCC=-0.11, p=0.653
mRNA Profile
Protein Expression Profile
DPA
0
1
2
3
4
5
6
7
8
9
10
11
12
13
14
15
16
17
18
19
20
21
22
23
24
25
26
27
28
29
30
31

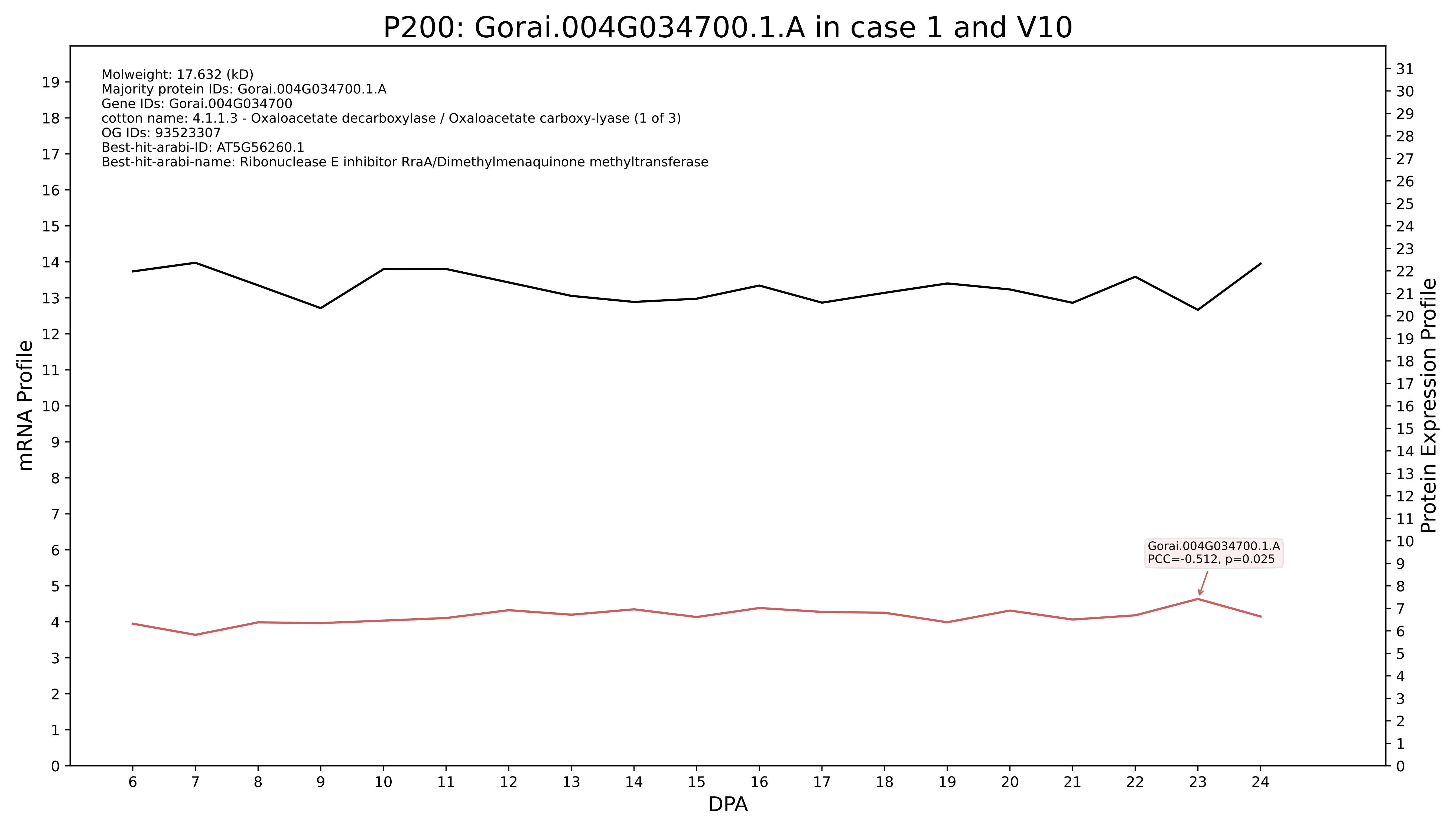
P200: Gorai.004G034700.1.A in case 1 and V10
Molweight: 17.632 (kD)
Majority protein IDs: Gorai.004G034700.1.A
Gene IDs: Gorai.004G034700
cotton name: 4.1.1.3 - Oxaloacetate decarboxylase / Oxaloacetate carboxy-lyase (1 of 3)
OG IDs: 93523307
Best-hit-arabi-ID: AT5G56260.1
Best-hit-arabi-name: Ribonuclease E inhibitor RraA/Dimethylmenaquinone methyltransferase
Gorai.004G034700.1.A
PCC=-0.512, p=0.025
mRNA Profile
Protein Expression Profile
DPA
0
1
2
3
4
5
6
7
8
9
10
11
12
13
14
15
16
17
18
19
20
21
22
23
24
25
26
27
28
29
30
31

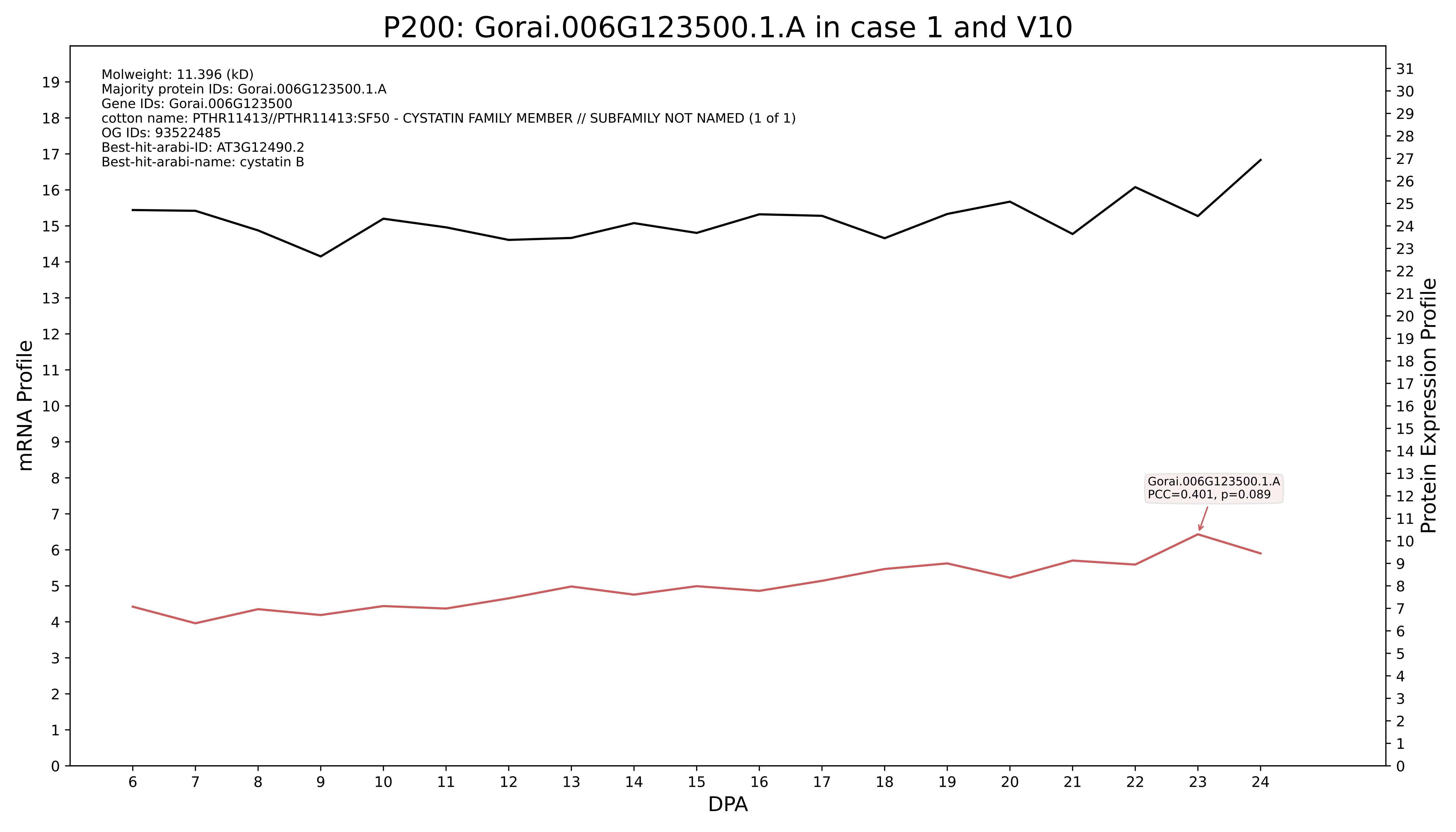

P200: Gorai.006G123500.1.A in case 1 and V10
Molweight: 11.396 (kD)
Majority protein IDs: Gorai.006G123500.1.A
Gene IDs: Gorai.006G123500
cotton name: PTHR11413//PTHR11413:SF50 - CYSTATIN FAMILY MEMBER // SUBFAMILY NOT NAMED (1 of 1)
OG IDs: 93522485
Best-hit-arabi-ID: AT3G12490.2
Best-hit-arabi-name: cystatin B
Gorai.006G123500.1.A
PCC=0.401, p=0.089
mRNA Profile
Protein Expression Profile
DPA

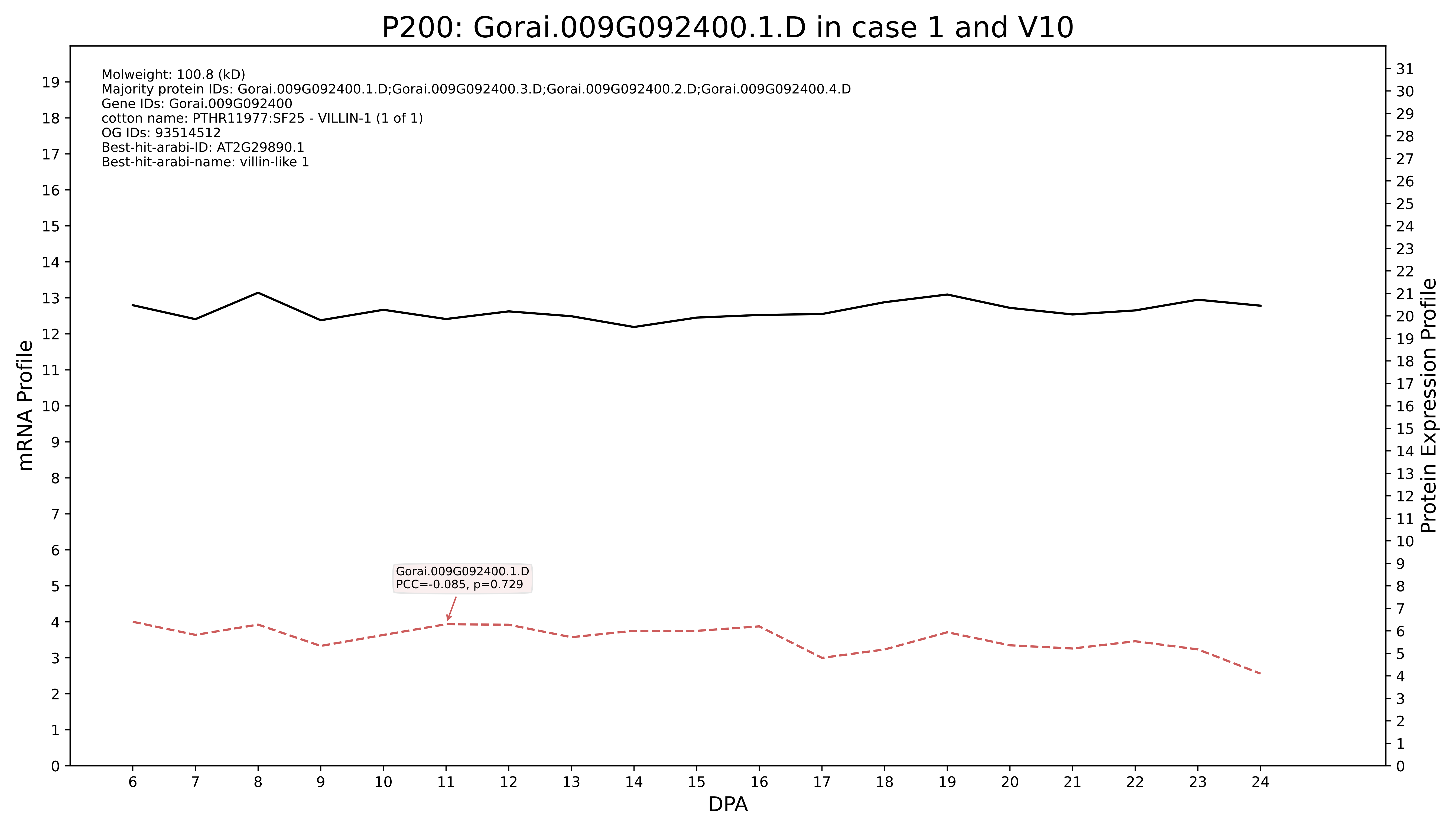

P200: Gorai.009G092400.1.D in case 1 and V10
Molweight: 100.8 (kD)
Majority protein IDs: Gorai.009G092400.1.D;Gorai.009G092400.3.D;Gorai.009G092400.2.D;Gorai.009G092400.4.D
Gene IDs: Gorai.009G092400
cotton name: PTHR11977:SF25 - VILLIN-1 (1 of 1)
OG IDs: 93514512
Best-hit-arabi-ID: AT2G29890.1
Best-hit-arabi-name: villin-like 1
Gorai.009G092400.1.D
PCC=-0.085, p=0.729
mRNA Profile
Protein Expression Profile
DPA

P200: Gorai.005G219700.1.D in case 1 and V10

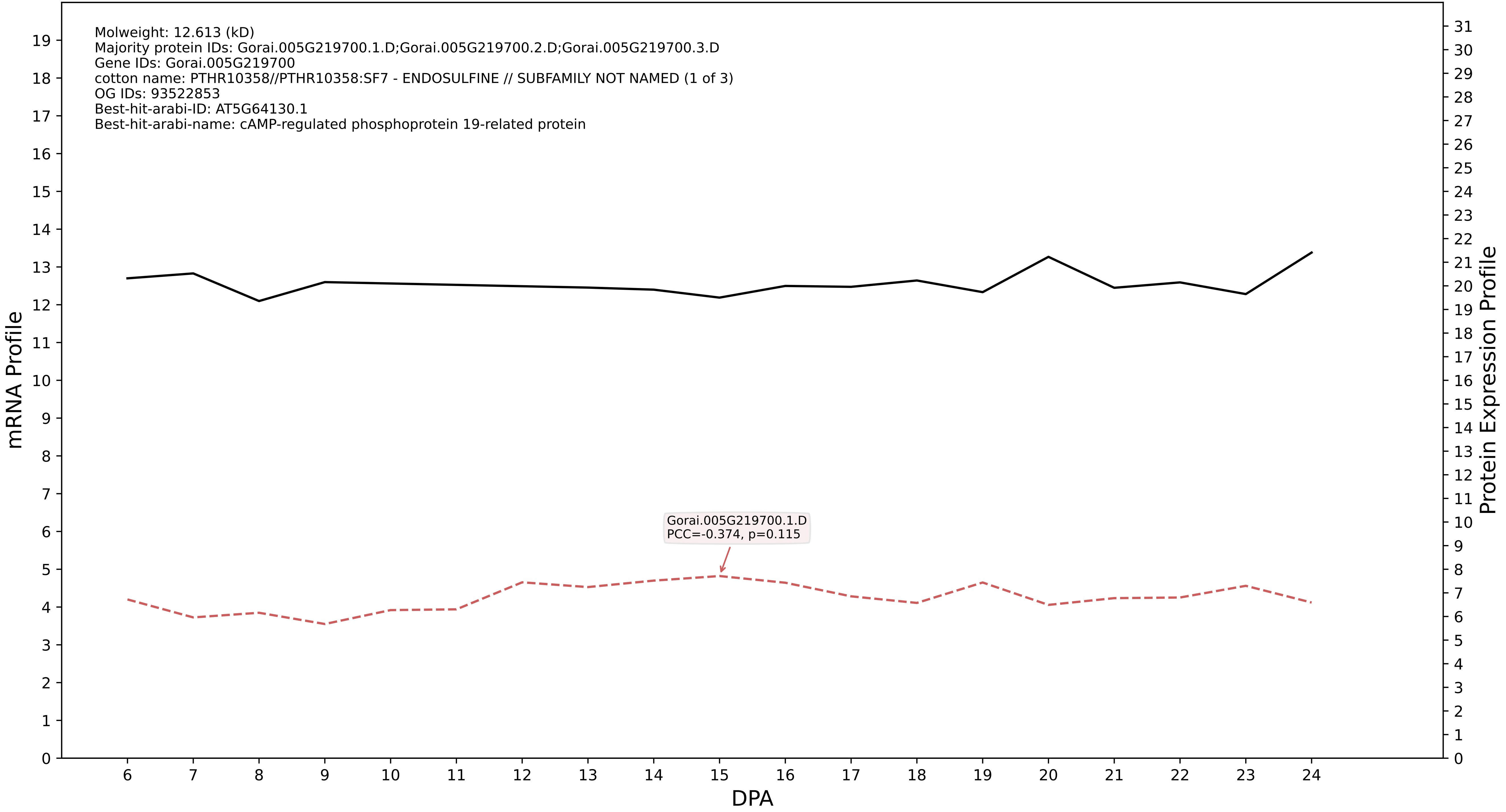

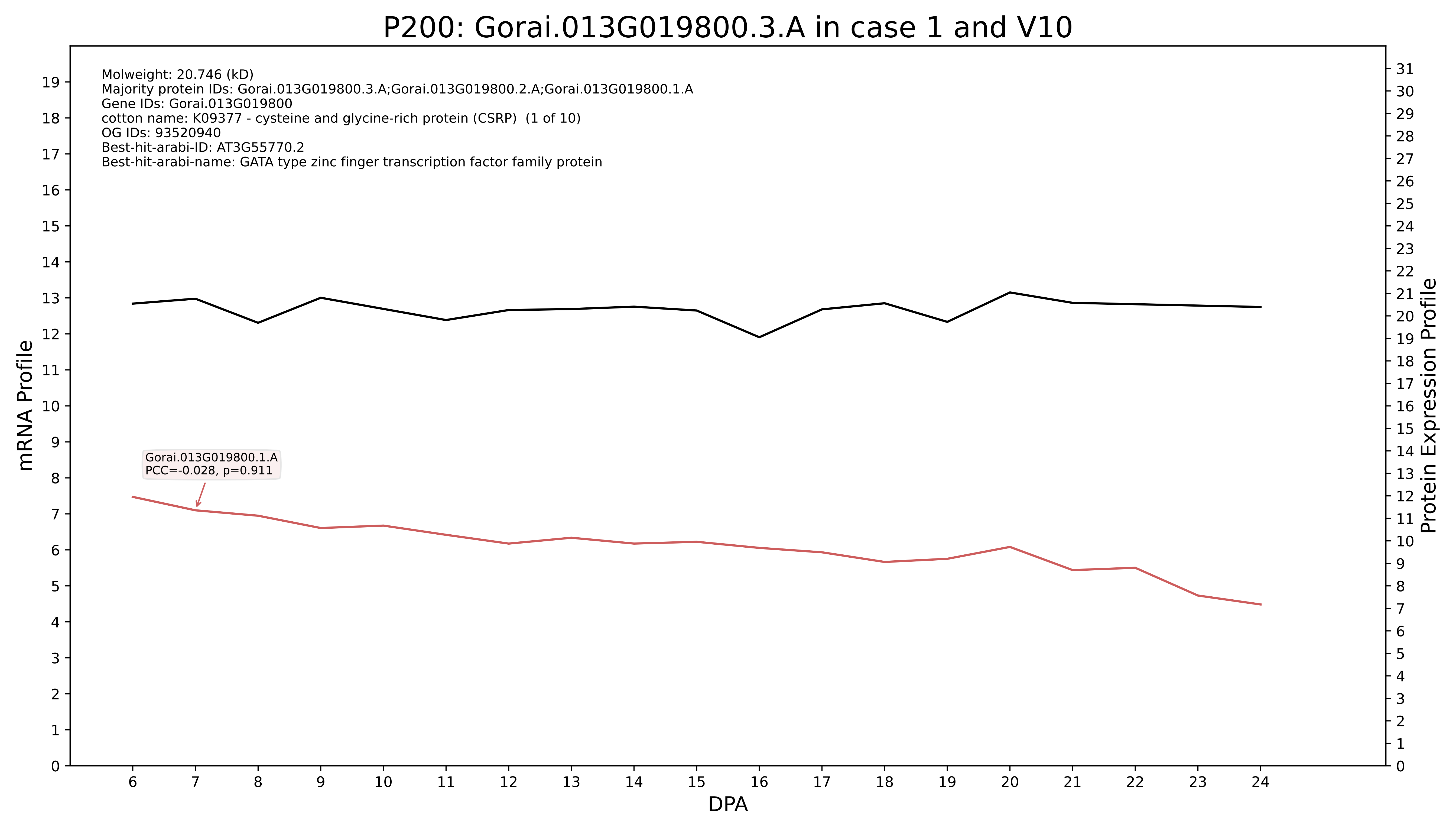
P200: Gorai.013G019800.3.A in case 1 and V10
Molweight: 20.746 (kD)
Majority protein IDs: Gorai.013G019800.3.A;Gorai.013G019800.2.A;Gorai.013G019800.1.A
Gene IDs: Gorai.013G019800
cotton name: K09377 - cysteine and glycine-rich protein (CSRP) (1 of 10)
OG IDs: 93520940
Best-hit-arabi-ID: AT3G55770.2
Best-hit-arabi-name: GATA type zinc finger transcription factor family protein
Gorai.013G019800.1.A
PCC=-0.028, p=0.911
mRNA Profile
Protein Expression Profile
DPA

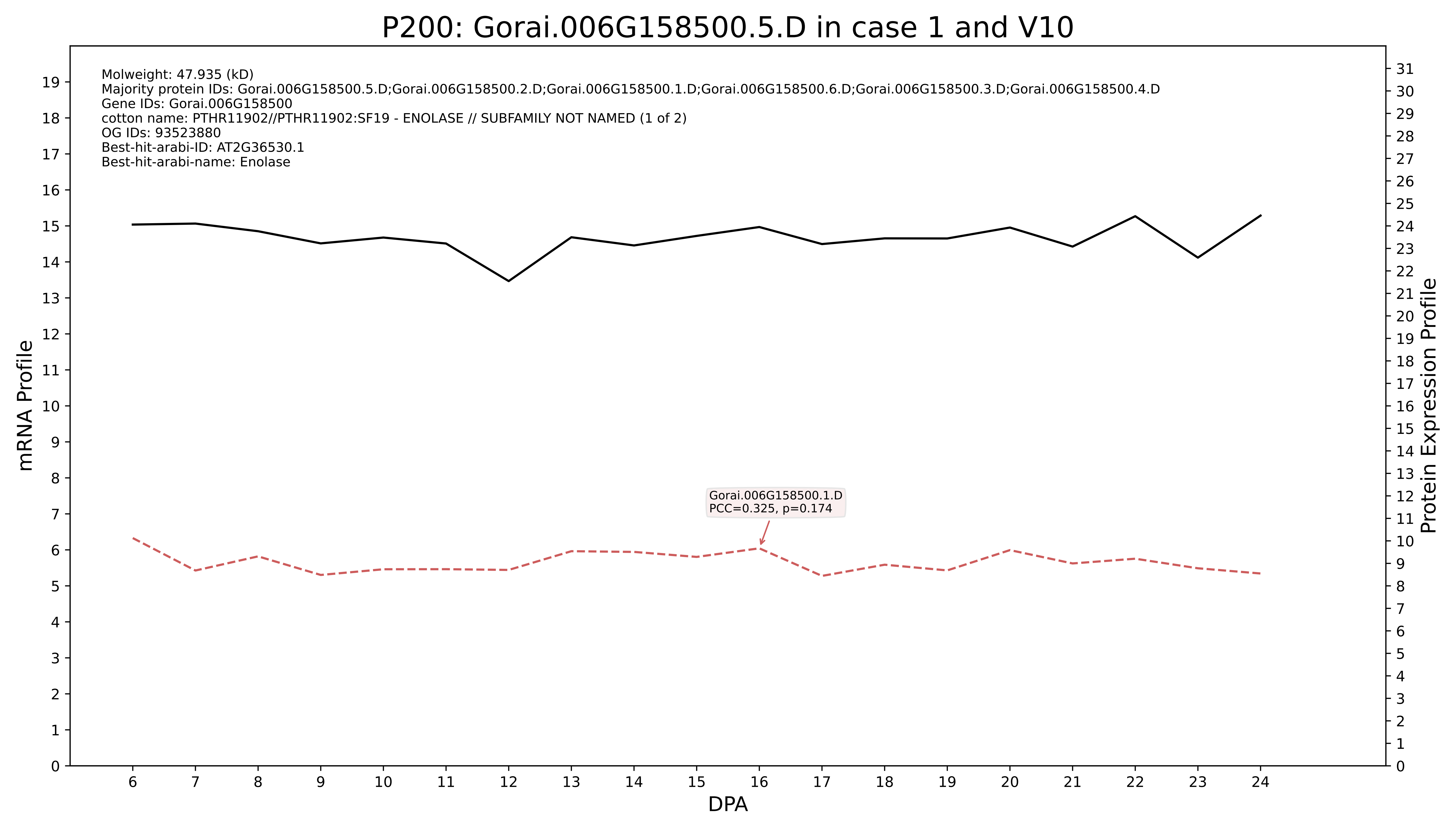

P200: Gorai.006G158500.5.D in case 1 and V10
Molweight: 47.935 (kD)
Majority protein IDs: Gorai.006G158500.5.D;Gorai.006G158500.2.D;Gorai.006G158500.1.D;Gorai.006G158500.6.D;Gorai.006G158500.3.D;Gorai.006G158500.4.D
Gene IDs: Gorai.006G158500
cotton name: PTHR11902//PTHR11902:SF19 - ENOLASE // SUBFAMILY NOT NAMED (1 of 2)
OG IDs: 93523880
Best-hit-arabi-ID: AT2G36530.1
Best-hit-arabi-name: Enolase
Gorai.006G158500.1.D
PCC=0.325, p=0.174
mRNA Profile
Protein Expression Profile
DPA

P200: Gorai.001G074600.1.A in case 1 and V10

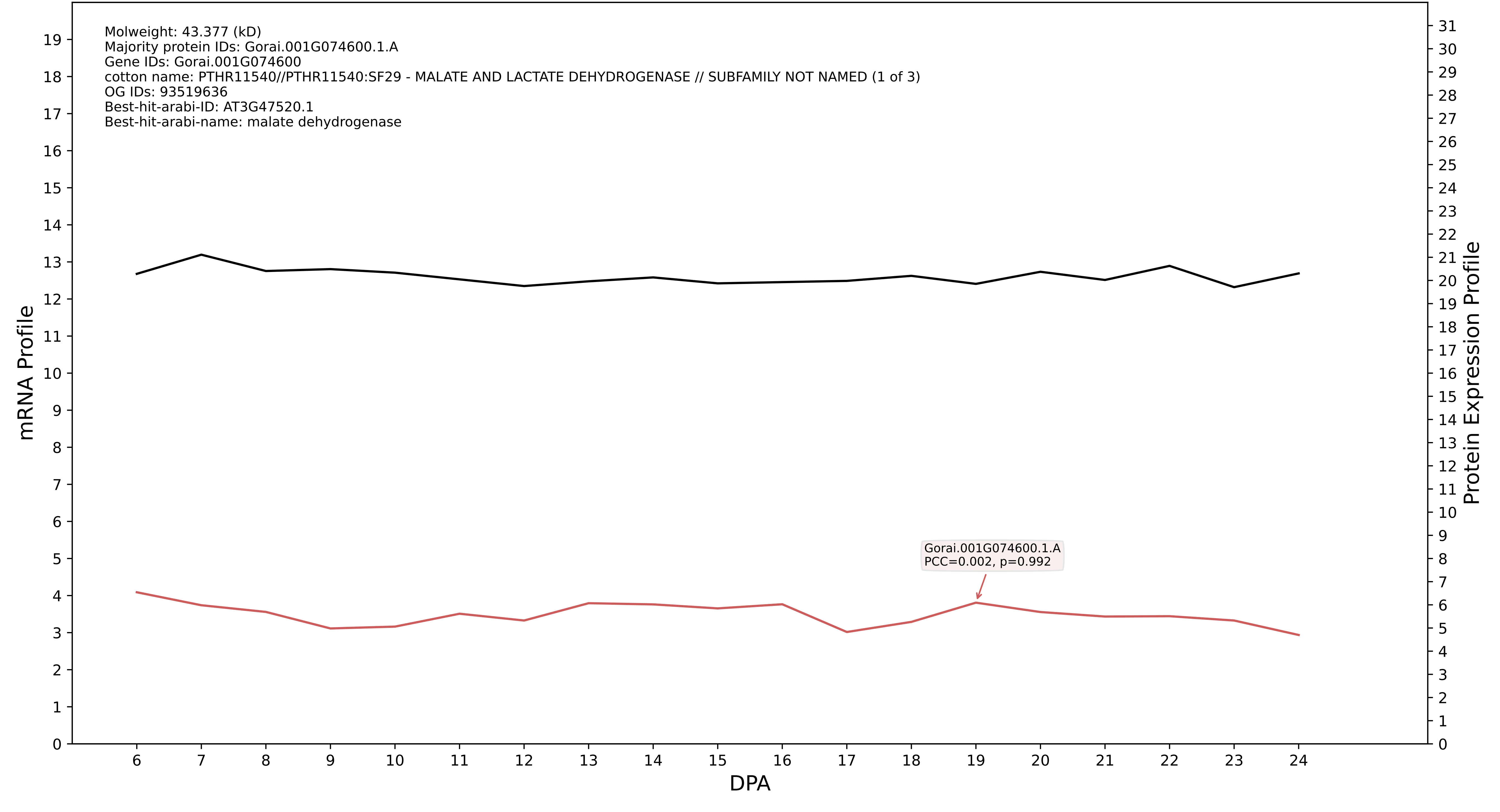

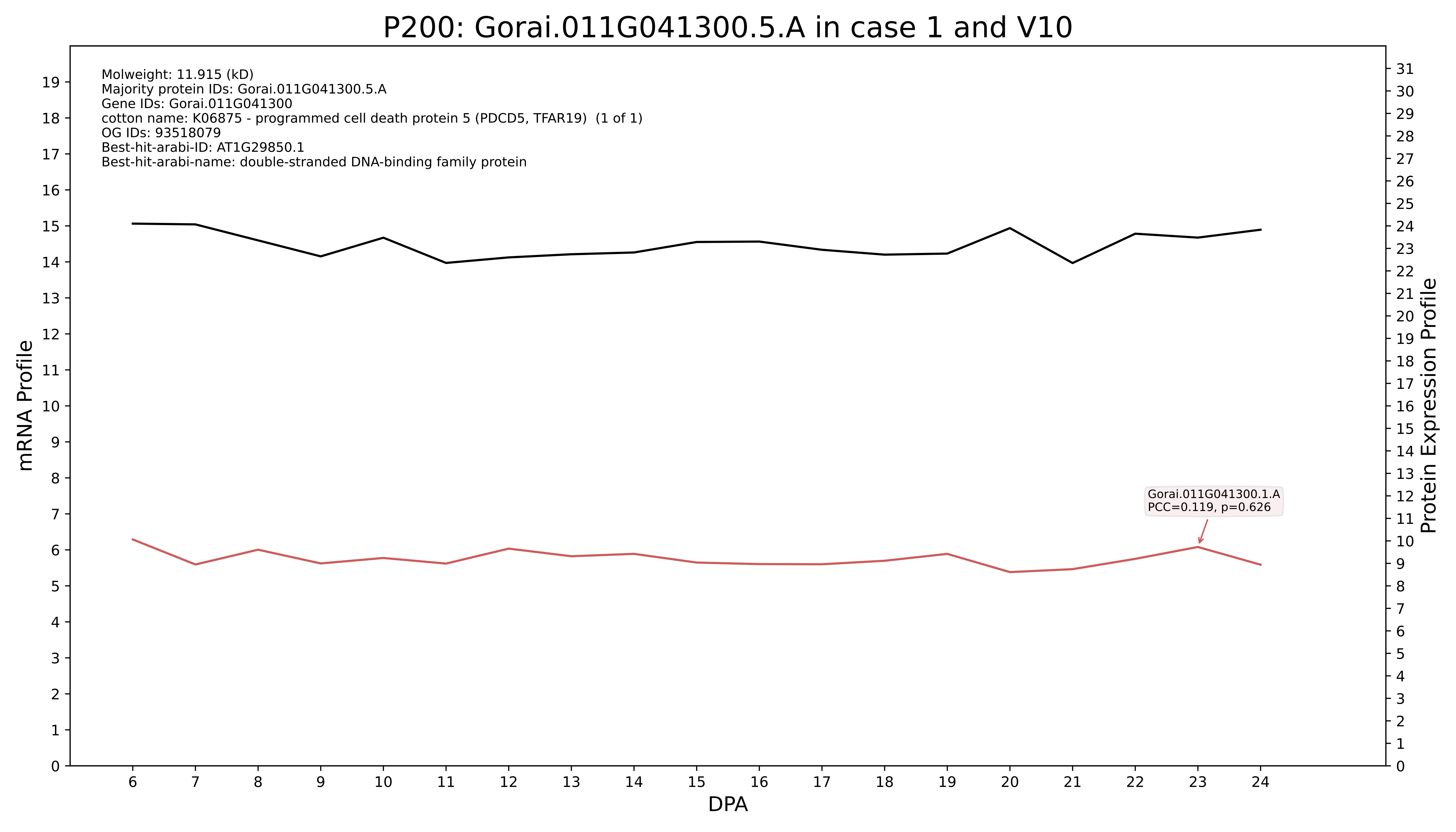
P200: Gorai.011G041300.5.A in case 1 and V10
Molweight: 11.915 (kD)
Majority protein IDs: Gorai.011G041300.5.A
Gene IDs: Gorai.011G041300
cotton name: K06875 - programmed cell death protein 5 (PDCD5, TFAR19) (1 of 1)
OG IDs: 93518079
Best-hit-arabi-ID: AT1G29850.1
Best-hit-arabi-name: double-stranded DNA-binding family protein
Gorai.011G041300.1.A
PCC=0.119, p=0.626
mRNA Profile
Protein Expression Profile
DPA

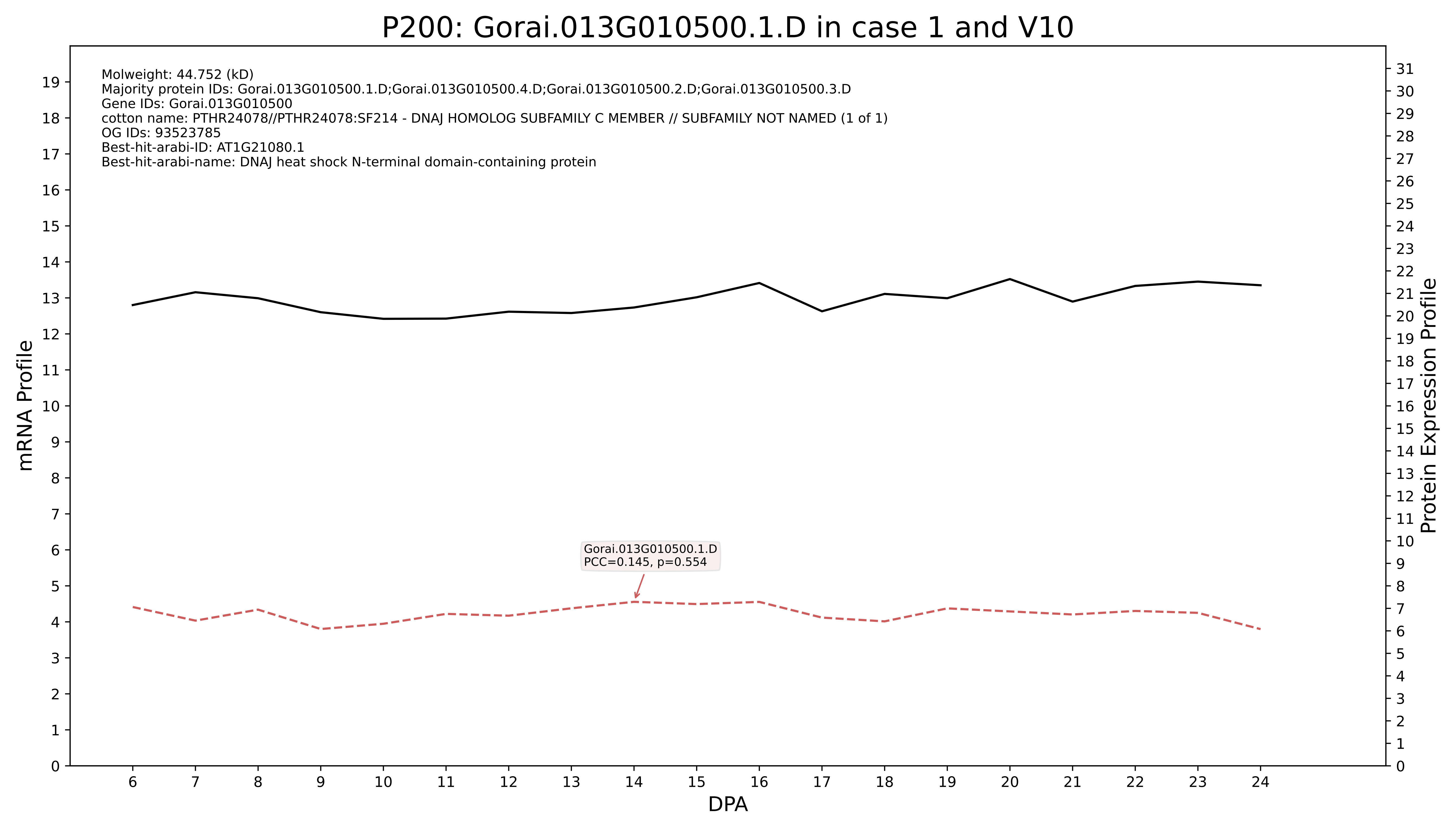
P200: Gorai.013G010500.1.D in case 1 and V10
Molweight: 44.752 (kD)
Majority protein IDs: Gorai.013G010500.1.D;Gorai.013G010500.4.D;Gorai.013G010500.2.D;Gorai.013G010500.3.D
Gene IDs: Gorai.013G010500
cotton name: PTHR24078//PTHR24078:SF214 - DNAJ HOMOLOG SUBFAMILY C MEMBER // SUBFAMILY NOT NAMED (1 of 1)
OG IDs: 93523785
Best-hit-arabi-ID: AT1G21080.1
Best-hit-arabi-name: DNAJ heat shock N-terminal domain-containing protein
Gorai.013G010500.1.D
PCC=0.145, p=0.554
mRNA Profile
Protein Expression Profile
DPA

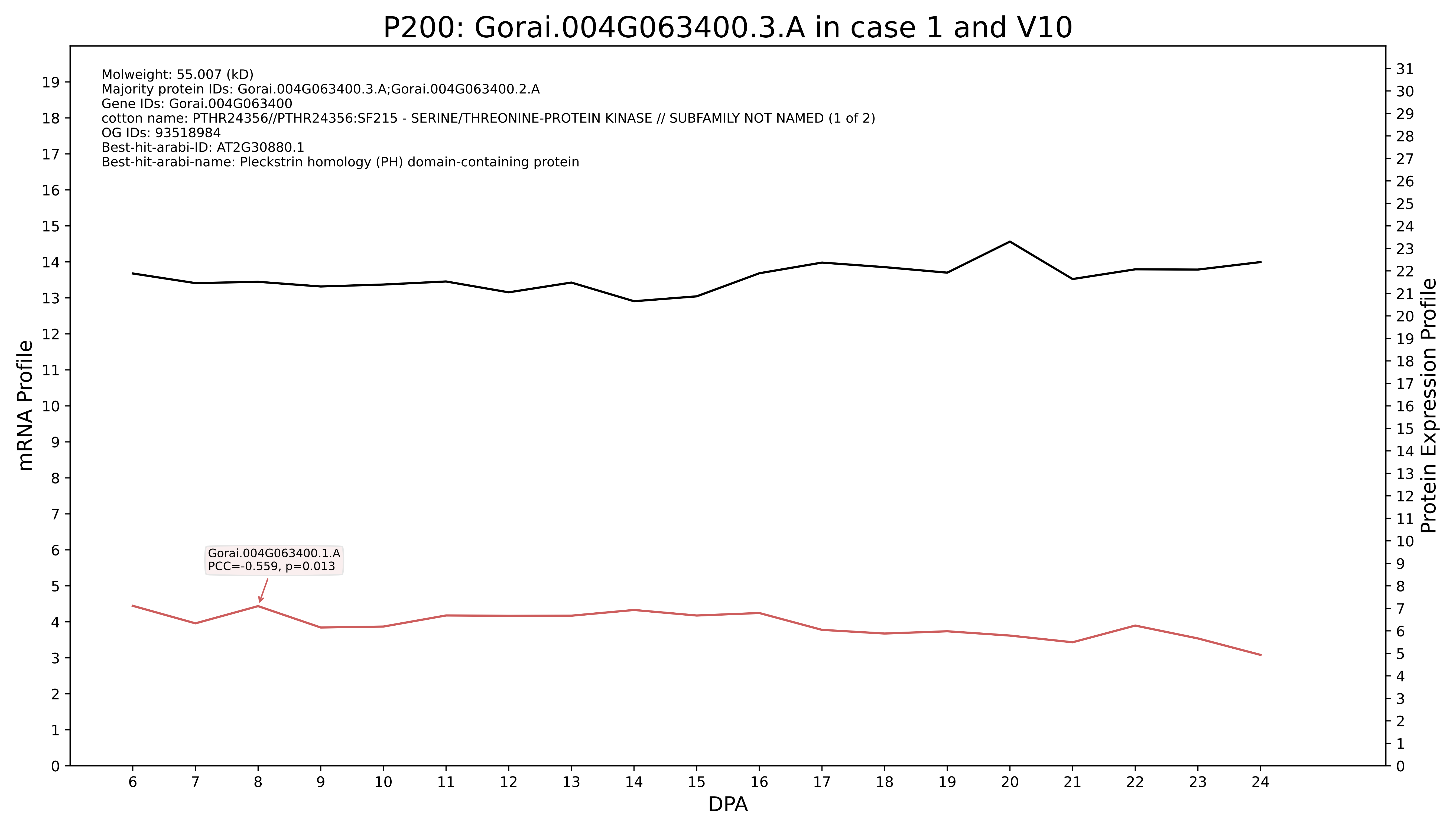
P200: Gorai.004G063400.3.A in case 1 and V10
Molweight: 55.007 (kD)
Majority protein IDs: Gorai.004G063400.3.A;Gorai.004G063400.2.A
Gene IDs: Gorai.004G063400
cotton name: PTHR24356//PTHR24356:SF215 - SERINE/THREONINE-PROTEIN KINASE // SUBFAMILY NOT NAMED (1 of 2)
OG IDs: 93518984
Best-hit-arabi-ID: AT2G30880.1
Best-hit-arabi-name: Pleckstrin homology (PH) domain-containing protein
Gorai.004G063400.1.A
PCC=-0.559, p=0.013
mRNA Profile
Protein Expression Profile
DPA

P200: Gorai.013G120900.1.A in case 1 and V10

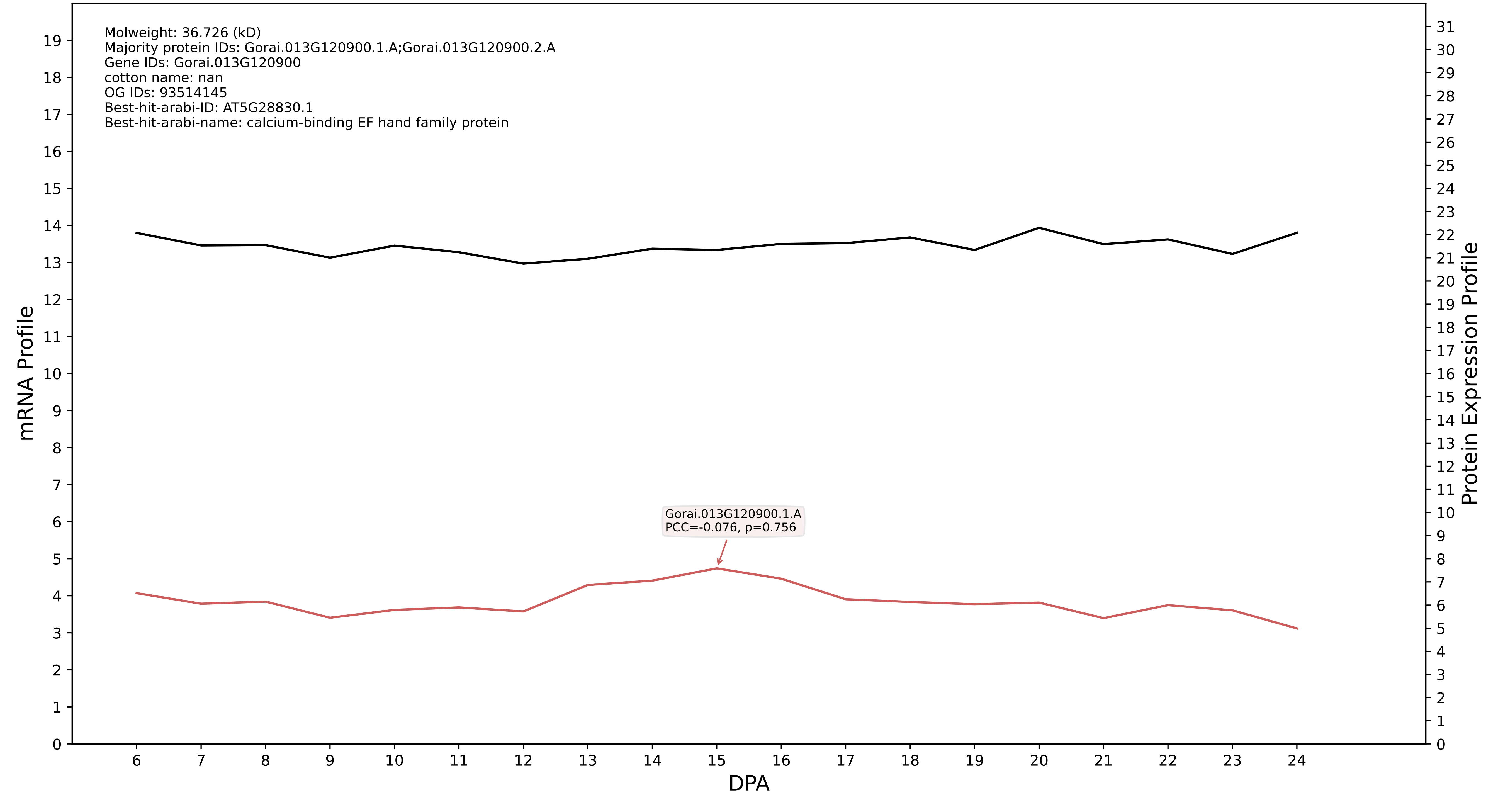

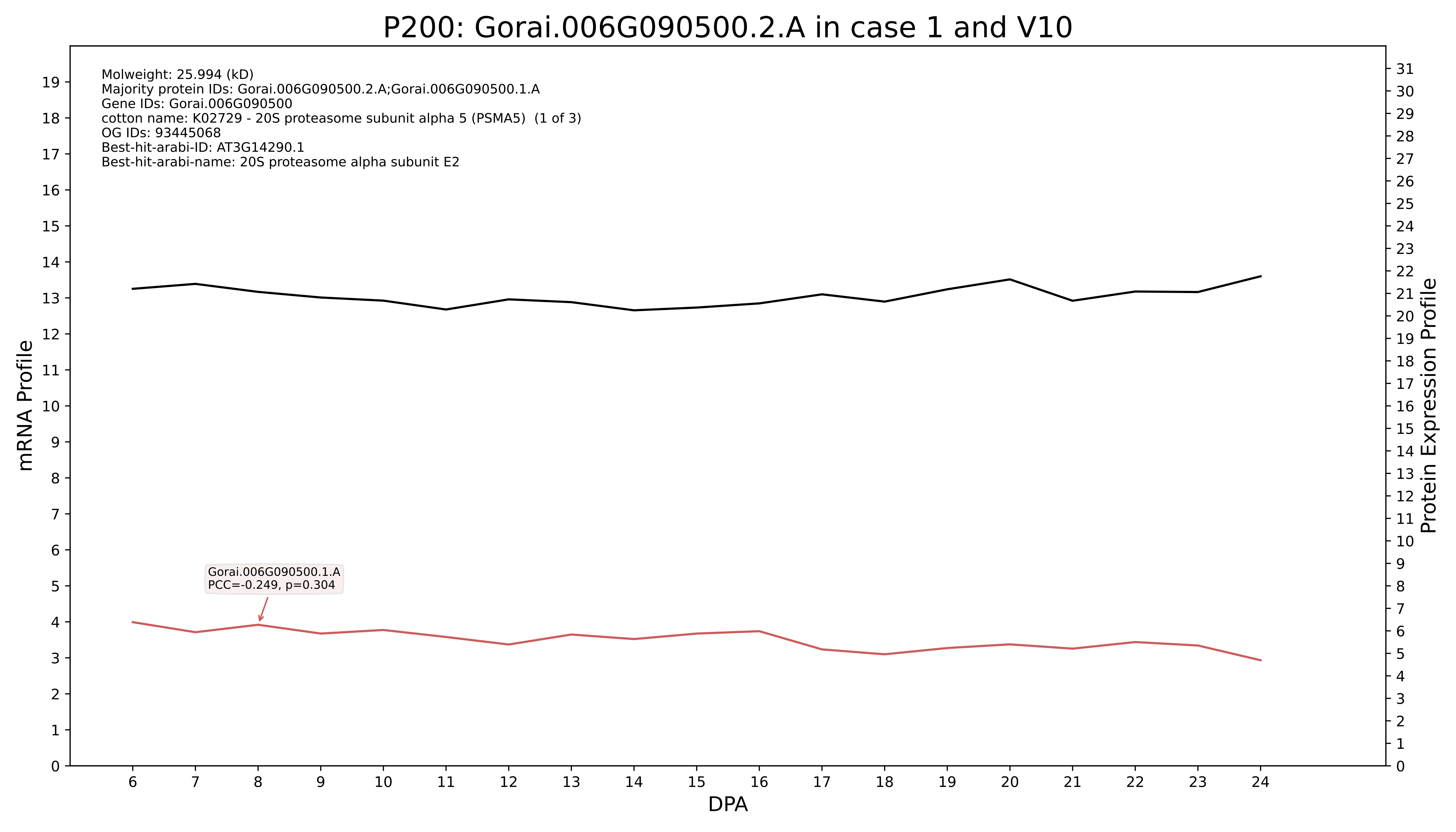
P200: Gorai.006G090500.2.A in case 1 and V10
Molweight: 25.994 (kD)
Majority protein IDs: Gorai.006G090500.2.A;Gorai.006G090500.1.A
Gene IDs: Gorai.006G090500
cotton name: K02729 - 20S proteasome subunit alpha 5 (PSMA5) (1 of 3)
OG IDs: 93445068
Best-hit-arabi-ID: AT3G14290.1
Best-hit-arabi-name: 20S proteasome alpha subunit E2
Gorai.006G090500.1.A
PCC=-0.249, p=0.304
mRNA Profile
Protein Expression Profile
DPA

P200: Gorai.009G418200.2.A in case 1 and V10

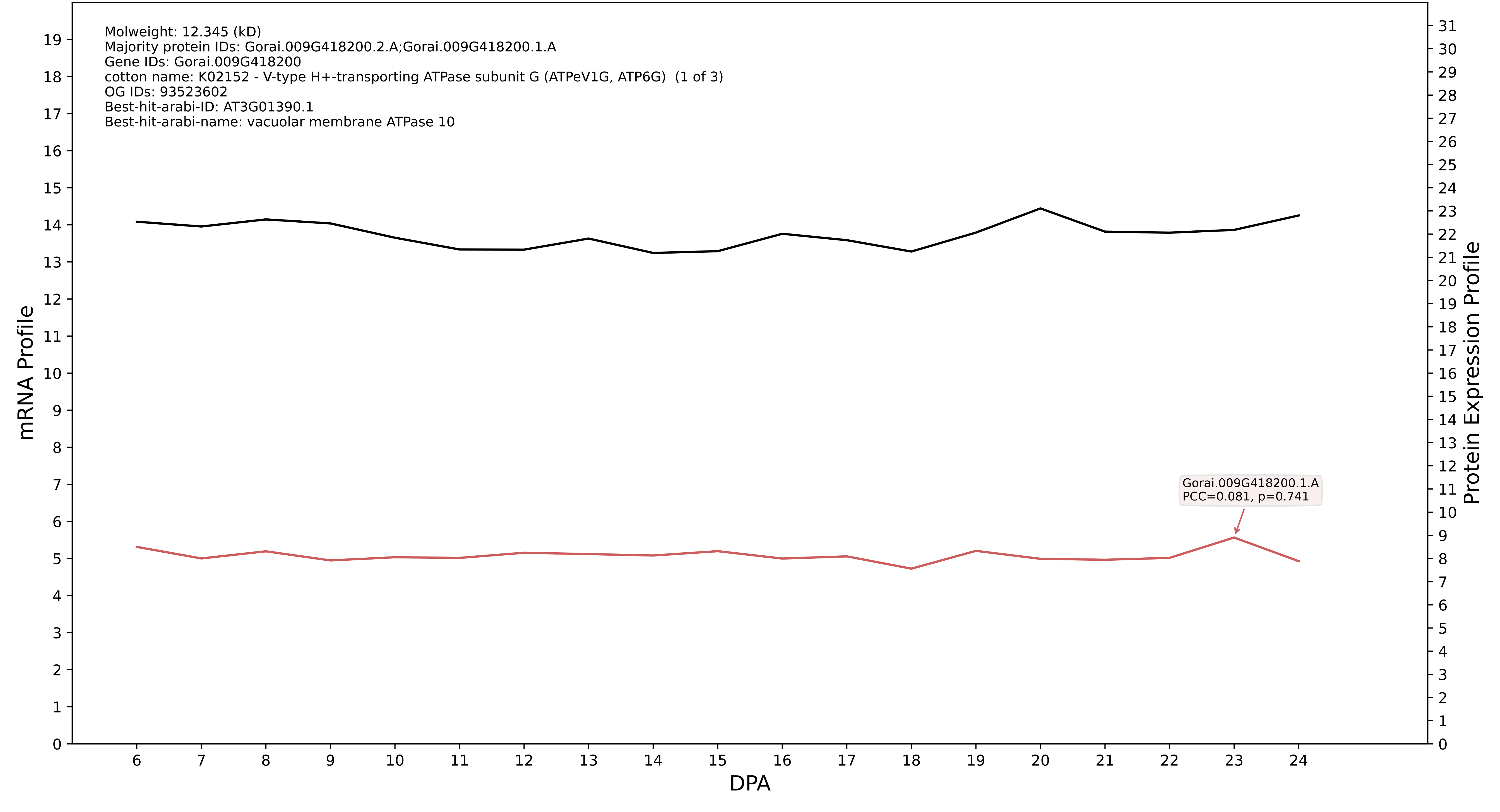

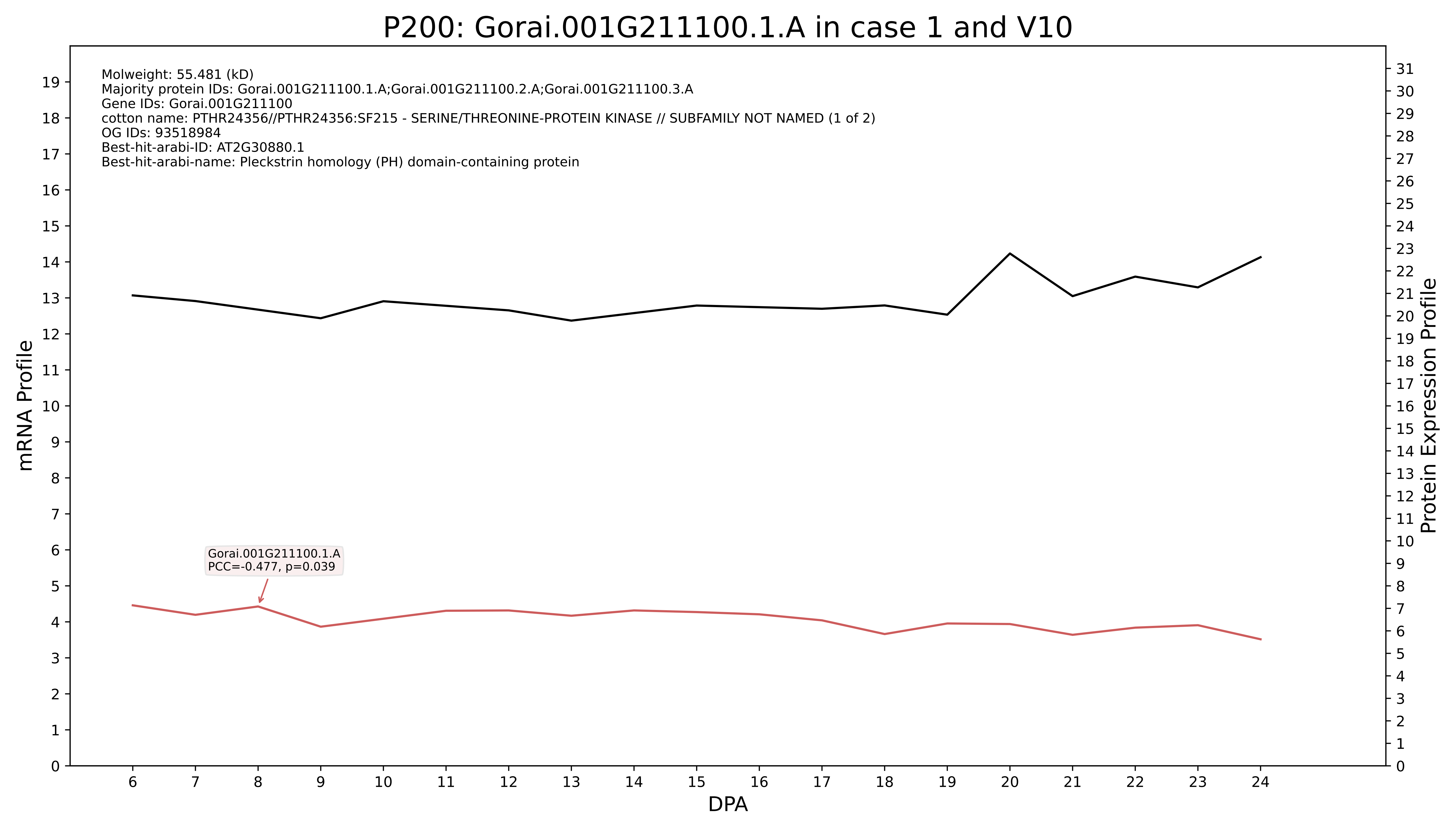
P200: Gorai.001G211100.1.A in case 1 and V10
Molweight: 55.481 (kD)
Majority protein IDs: Gorai.001G211100.1.A;Gorai.001G211100.2.A;Gorai.001G211100.3.A
Gene IDs: Gorai.001G211100
cotton name: PTHR24356//PTHR24356:SF215 - SERINE/THREONINE-PROTEIN KINASE // SUBFAMILY NOT NAMED (1 of 2)
OG IDs: 93518984
Best-hit-arabi-ID: AT2G30880.1
Best-hit-arabi-name: Pleckstrin homology (PH) domain-containing protein
Gorai.001G211100.1.A
PCC=-0.477, p=0.039
mRNA Profile
Protein Expression Profile
DPA

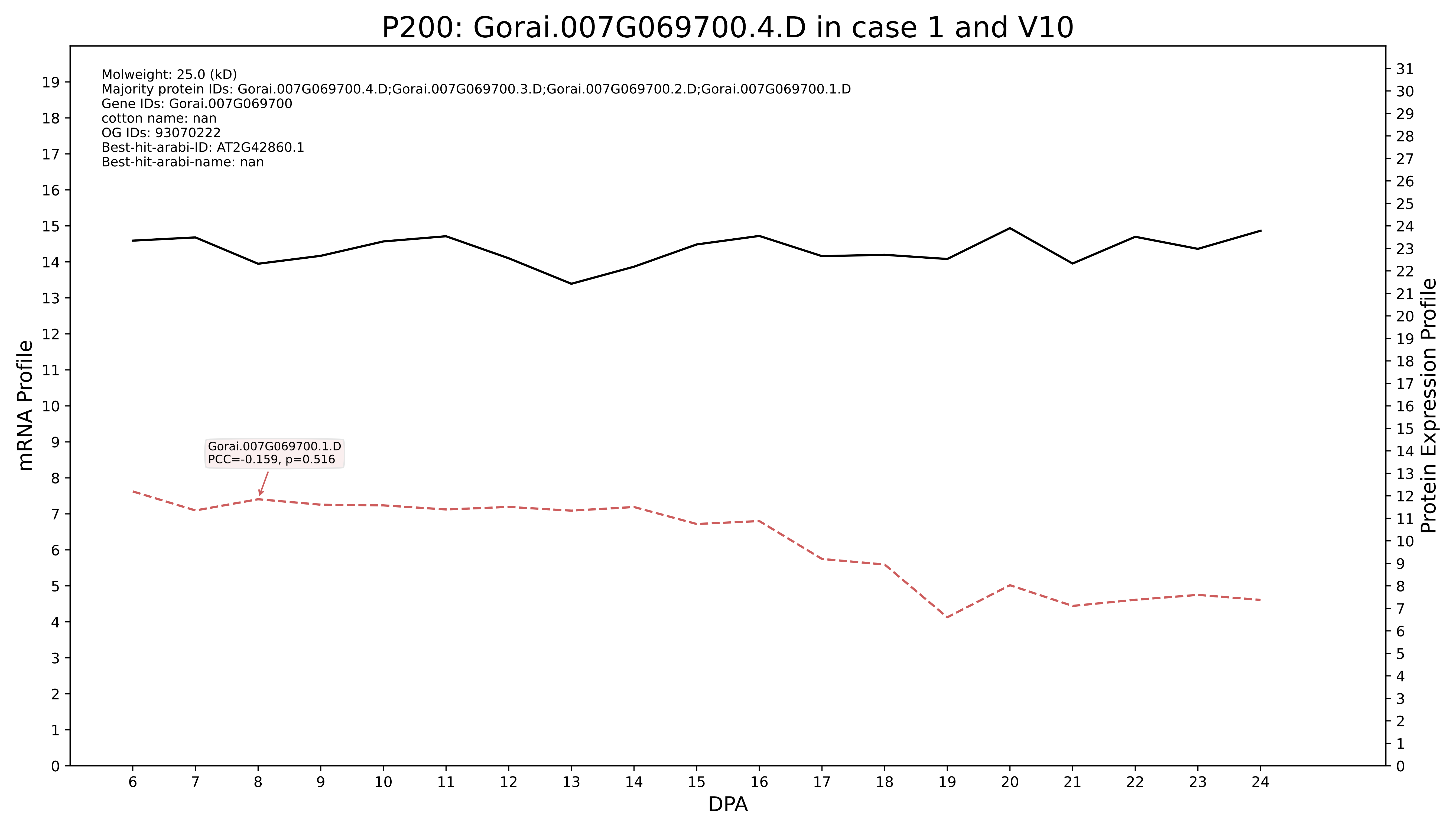

P200: Gorai.007G069700.4.D in case 1 and V10
Molweight: 25.0 (kD)
Majority protein IDs: Gorai.007G069700.4.D;Gorai.007G069700.3.D;Gorai.007G069700.2.D;Gorai.007G069700.1.D
Gene IDs: Gorai.007G069700
cotton name: nan
OG IDs: 93070222
Best-hit-arabi-ID: AT2G42860.1
Best-hit-arabi-name: nan
Gorai.007G069700.1.D
PCC=-0.159, p=0.516
mRNA Profile
Protein Expression Profile
DPA

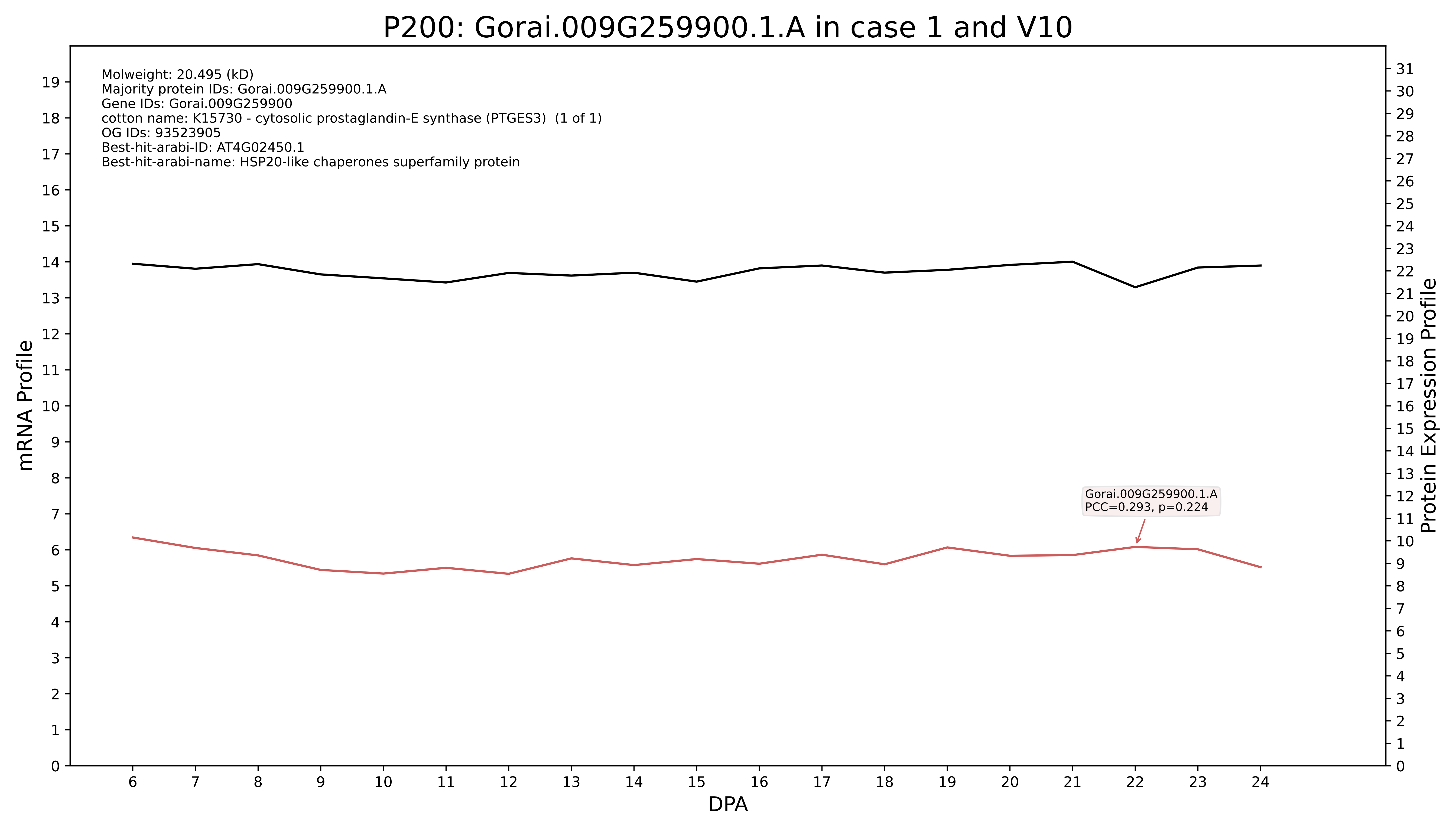
P200: Gorai.009G259900.1.A in case 1 and V10
Molweight: 20.495 (kD)
Majority protein IDs: Gorai.009G259900.1.A
Gene IDs: Gorai.009G259900
cotton name: K15730 - cytosolic prostaglandin-E synthase (PTGES3) (1 of 1)
OG IDs: 93523905
Best-hit-arabi-ID: AT4G02450.1
Best-hit-arabi-name: HSP20-like chaperones superfamily protein
Gorai.009G259900.1.A
PCC=0.293, p=0.224
mRNA Profile
Protein Expression Profile
DPA
0
1
2
3
4
5
6
7
8
9
10
11
12
13
14
15
16
17
18
19
20
21
22
23
24
25
26
27
28
29
30
31

P200: Gorai.002G063300.1.A in case 1 and V10

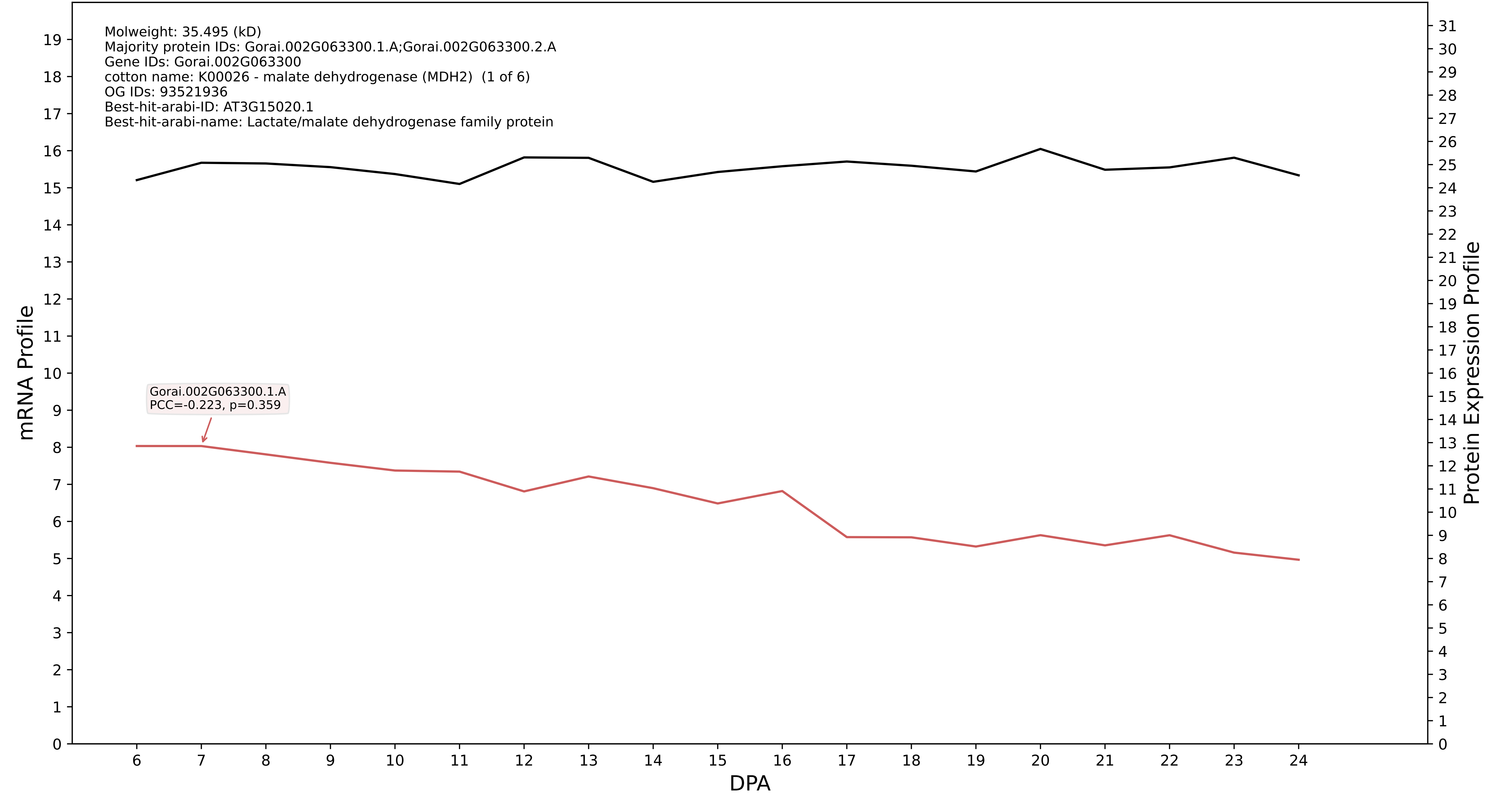

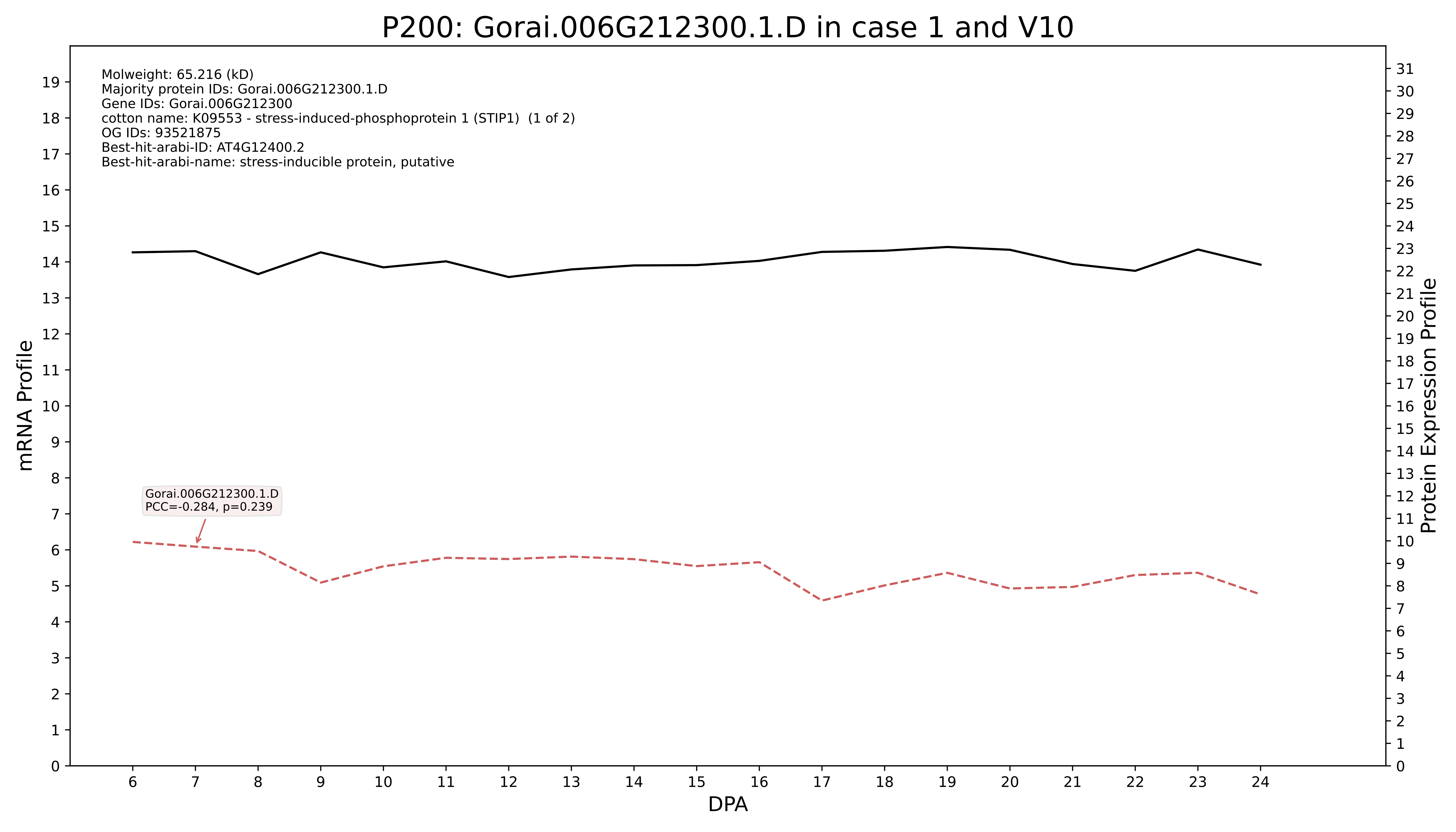
P200: Gorai.006G212300.1.D in case 1 and V10
Molweight: 65.216 (kD)
Majority protein IDs: Gorai.006G212300.1.D
Gene IDs: Gorai.006G212300
cotton name: K09553 - stress-induced-phosphoprotein 1 (STIP1) (1 of 2)
OG IDs: 93521875
Best-hit-arabi-ID: AT4G12400.2
Best-hit-arabi-name: stress-inducible protein, putative
Gorai.006G212300.1.D
PCC=-0.284, p=0.239
mRNA Profile
Protein Expression Profile
DPA

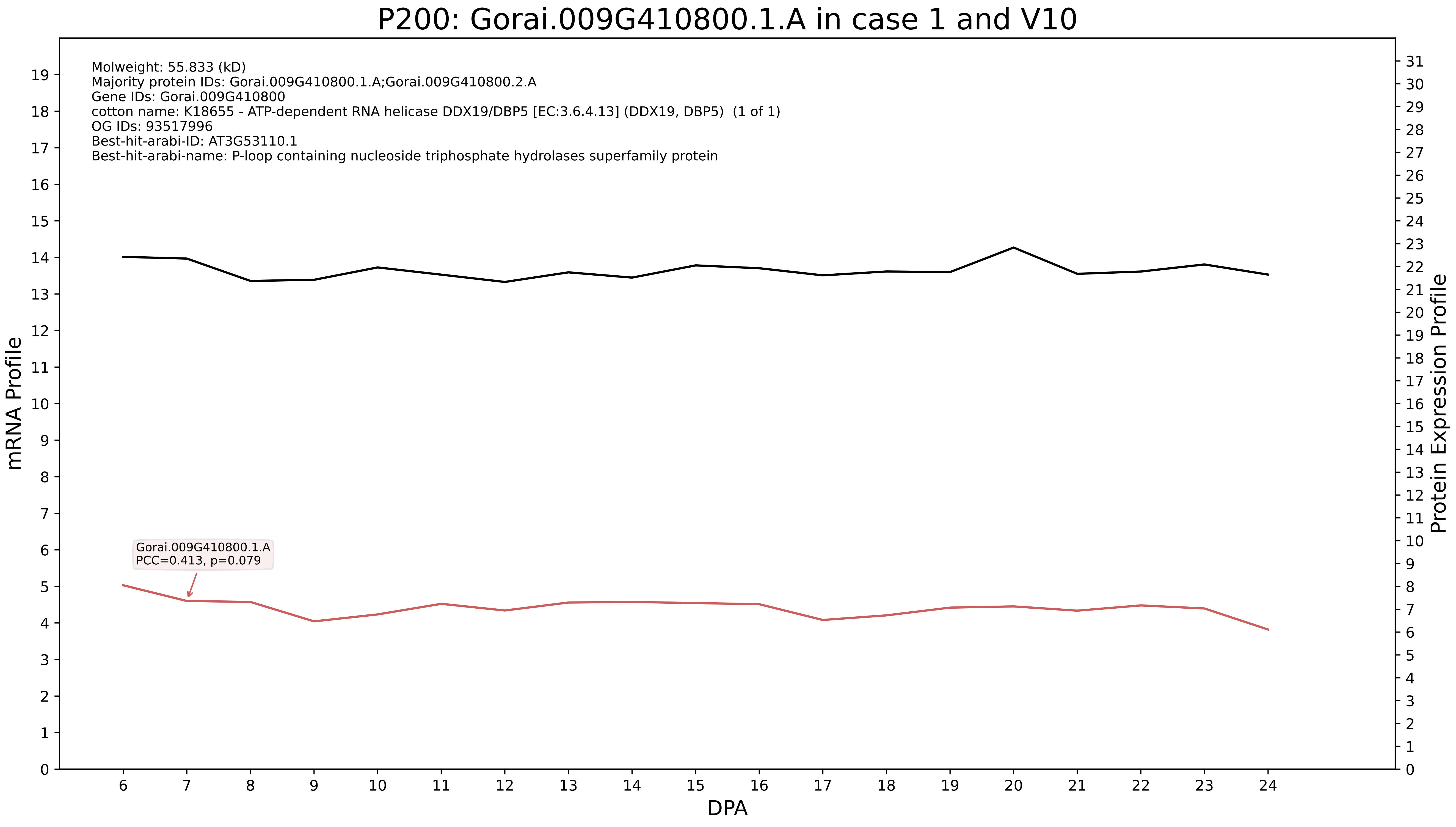
P200: Gorai.009G410800.1.A in case 1 and V10
Molweight: 55.833 (kD)
Majority protein IDs: Gorai.009G410800.1.A;Gorai.009G410800.2.A
Gene IDs: Gorai.009G410800
cotton name: K18655 - ATP-dependent RNA helicase DDX19/DBP5 [EC:3.6.4.13] (DDX19, DBP5) (1 of 1)
OG IDs: 93517996
Best-hit-arabi-ID: AT3G53110.1
Best-hit-arabi-name: P-loop containing nucleoside triphosphate hydrolases superfamily protein
Gorai.009G410800.1.A
PCC=0.413, p=0.079
mRNA Profile
Protein Expression Profile
DPA
0
1
2
3
4
5
6
7
8
9
10
11
12
13
14
15
16
17
18
19
20
21
22
23
24
25
26
27
28
29
30
31

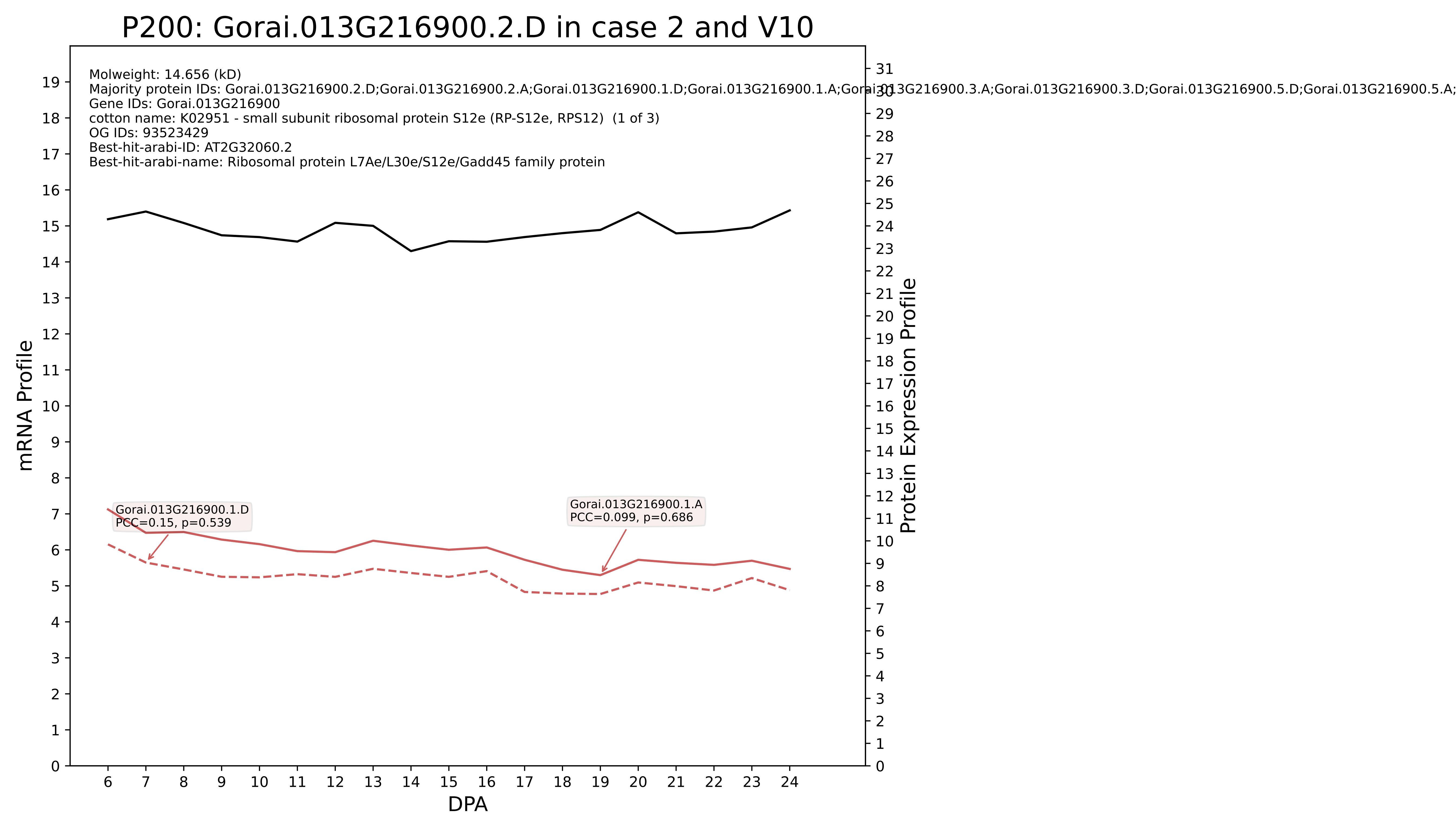
P200: Gorai.013G216900.2.D in case 2 and V10
Molweight: 14.656 (kD)
Majority protein IDs: Gorai.013G216900.2.D;Gorai.013G216900.2.A;Gorai.013G216900.1.D;Gorai.013G216900.1.A;Gorai.013G216900.3.A;Gorai.013G216900.3.D;Gorai.013G216900.5.D;Gorai.013G216900.5.A;
Gene IDs: Gorai.013G216900
cotton name: K02951 - small subunit ribosomal protein S12e (RP-S12e, RPS12) (1 of 3)
OG IDs: 93523429
Best-hit-arabi-ID: AT2G32060.2
Best-hit-arabi-name: Ribosomal protein L7Ae/L30e/S12e/Gadd45 family protein
Gorai.013G216900.1.D
PCC=0.15, p=0.539
Gorai.013G216900.1.A
PCC=0.099, p=0.686
mRNA Profile
Protein Expression Profile
DPA

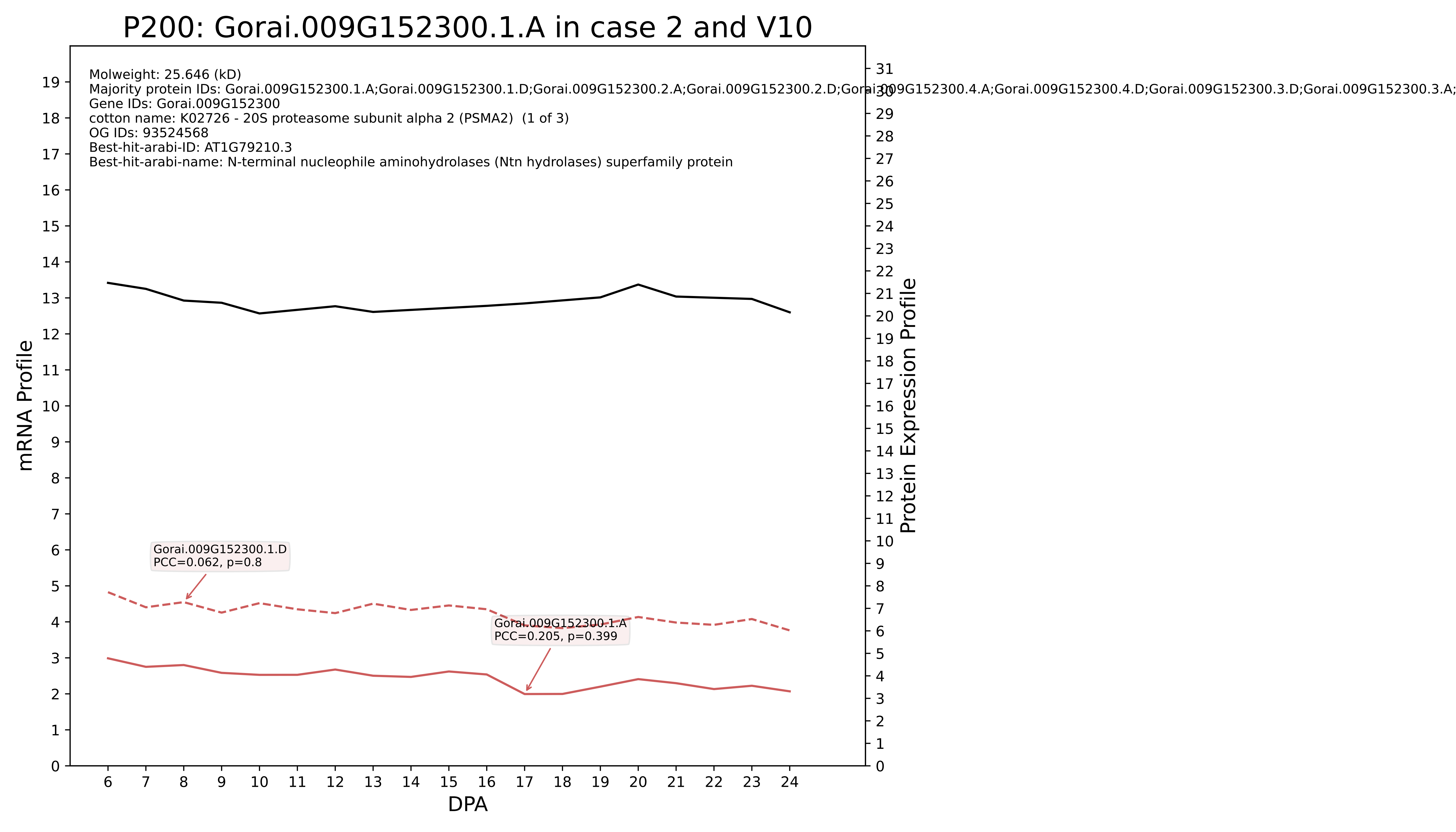
P200: Gorai.009G152300.1.A in case 2 and V10
Molweight: 25.646 (kD)
Majority protein IDs: Gorai.009G152300.1.A;Gorai.009G152300.1.D;Gorai.009G152300.2.A;Gorai.009G152300.2.D;Gorai.009G152300.4.A;Gorai.009G152300.4.D;Gorai.009G152300.3.D;Gorai.009G152300.3.A;
Gene IDs: Gorai.009G152300
cotton name: K02726 - 20S proteasome subunit alpha 2 (PSMA2) (1 of 3)
OG IDs: 93524568
Best-hit-arabi-ID: AT1G79210.3
Best-hit-arabi-name: N-terminal nucleophile aminohydrolases (Ntn hydrolases) superfamily protein
Gorai.009G152300.1.D
PCC=0.062, p=0.8
Gorai.009G152300.1.A
PCC=0.205, p=0.399
mRNA Profile
Protein Expression Profile
DPA

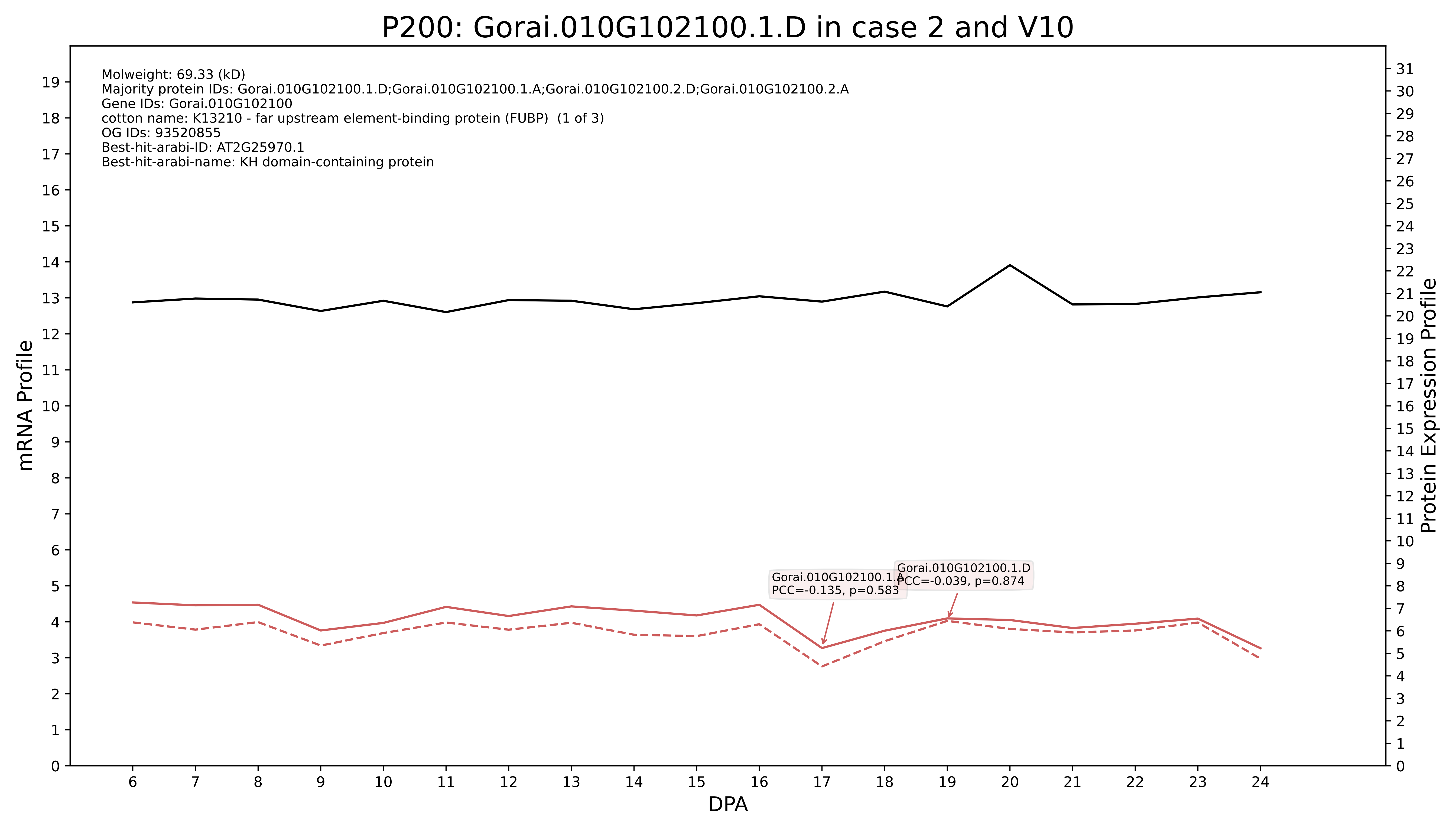
P200: Gorai.010G102100.1.D in case 2 and V10
Molweight: 69.33 (kD)
Majority protein IDs: Gorai.010G102100.1.D;Gorai.010G102100.1.A;Gorai.010G102100.2.D;Gorai.010G102100.2.A
Gene IDs: Gorai.010G102100
cotton name: K13210 - far upstream element-binding protein (FUBP) (1 of 3)
OG IDs: 93520855
Best-hit-arabi-ID: AT2G25970.1
Best-hit-arabi-name: KH domain-containing protein
Gorai.010G102100.1.A
PCC=-0.135, p=0.583
Gorai.010G102100.1.D
PCC=-0.039, p=0.874
mRNA Profile
Protein Expression Profile
DPA

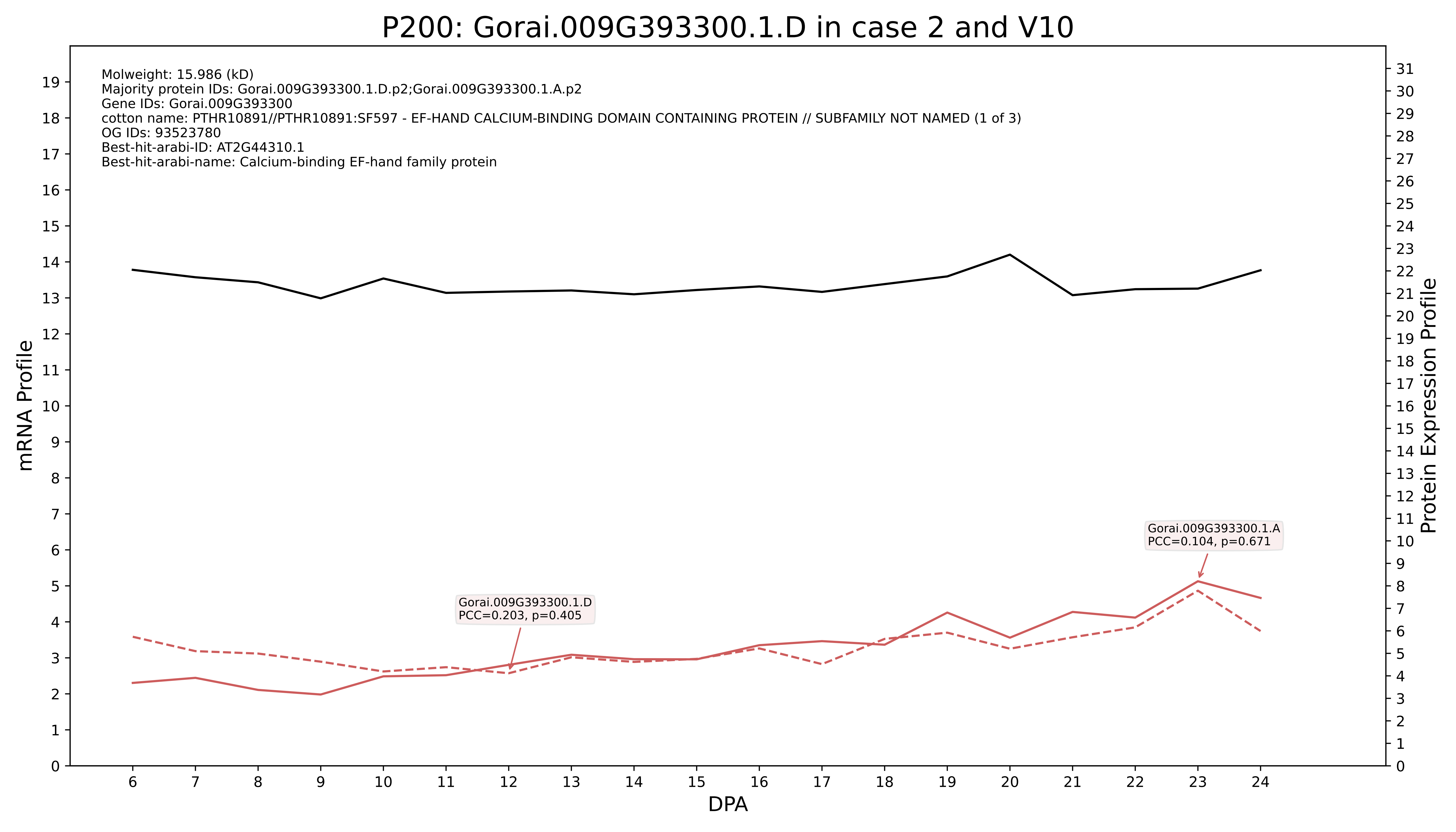

P200: Gorai.009G393300.1.D in case 2 and V10
Molweight: 15.986 (kD)
Majority protein IDs: Gorai.009G393300.1.D.p2;Gorai.009G393300.1.A.p2
Gene IDs: Gorai.009G393300
cotton name: PTHR10891//PTHR10891:SF597 - EF-HAND CALCIUM-BINDING DOMAIN CONTAINING PROTEIN // SUBFAMILY NOT NAMED (1 of 3)
OG IDs: 93523780
Best-hit-arabi-ID: AT2G44310.1
Best-hit-arabi-name: Calcium-binding EF-hand family protein
Gorai.009G393300.1.A
PCC=0.104, p=0.671
Gorai.009G393300.1.D
PCC=0.203, p=0.405
mRNA Profile
Protein Expression Profile
DPA

00: Gorai.012G025200.6.D in case 2 and V10

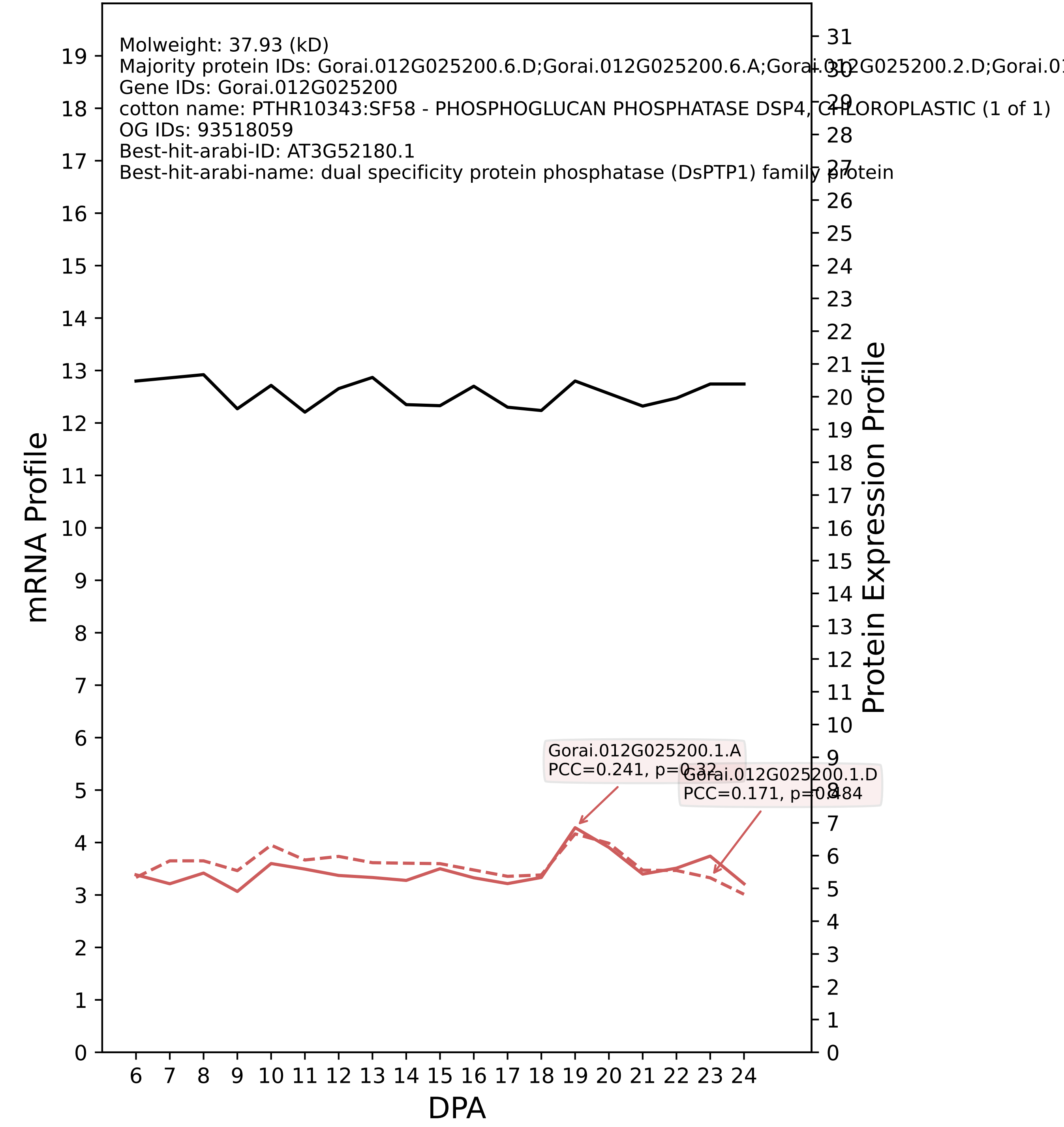

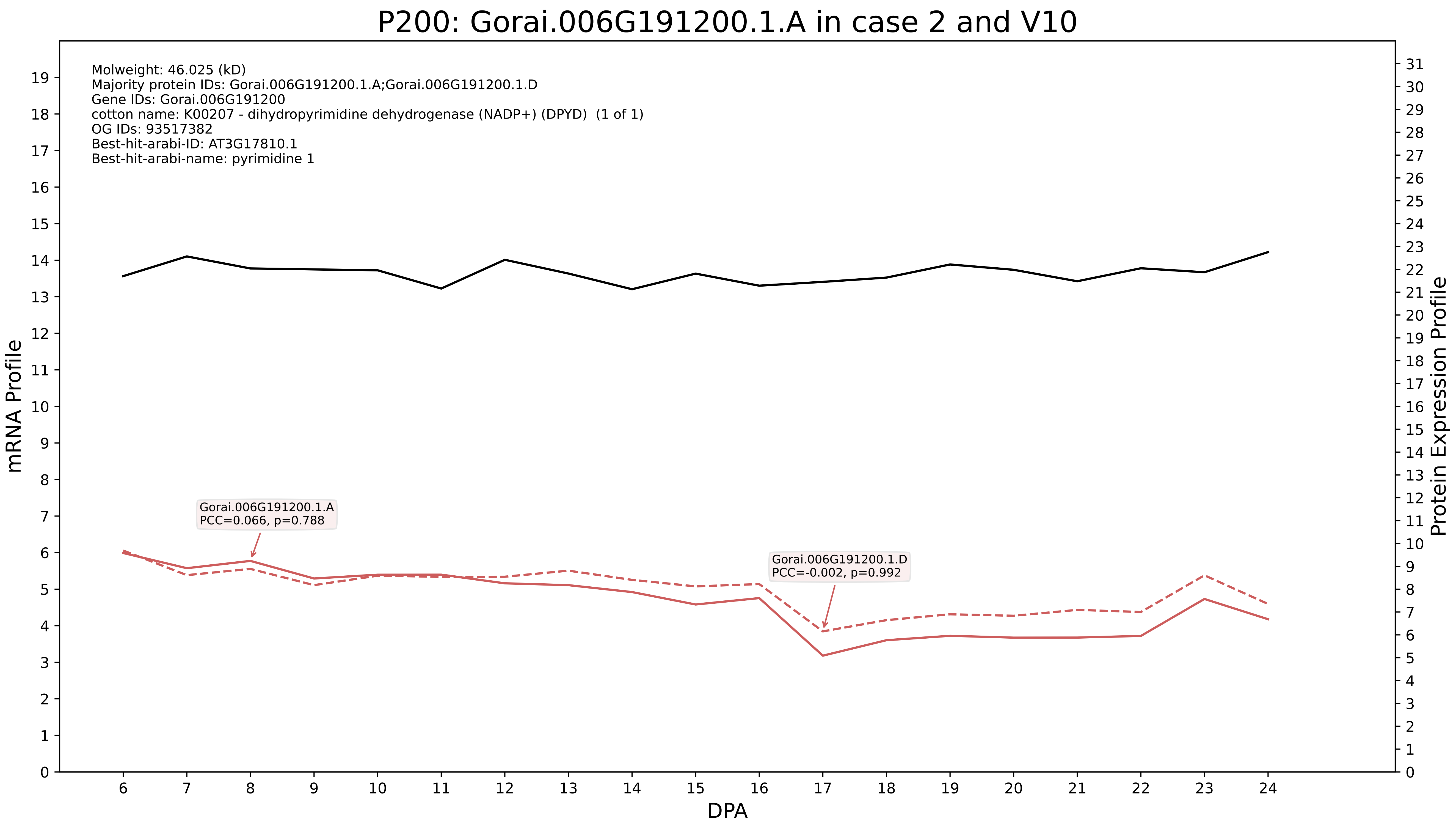
P200: Gorai.006G191200.1.A in case 2 and V10
Molweight: 46.025 (kD)
Majority protein IDs: Gorai.006G191200.1.A;Gorai.006G191200.1.D
Gene IDs: Gorai.006G191200
cotton name: K00207 - dihydropyrimidine dehydrogenase (NADP+) (DPYD) (1 of 1)
OG IDs: 93517382
Best-hit-arabi-ID: AT3G17810.1
Best-hit-arabi-name: pyrimidine 1
Gorai.006G191200.1.A
PCC=0.066, p=0.788
Gorai.006G191200.1.D
PCC=-0.002, p=0.992
mRNA Profile
Protein Expression Profile
DPA

P200: Gorai.005G195600.1.A in case 2 and V10

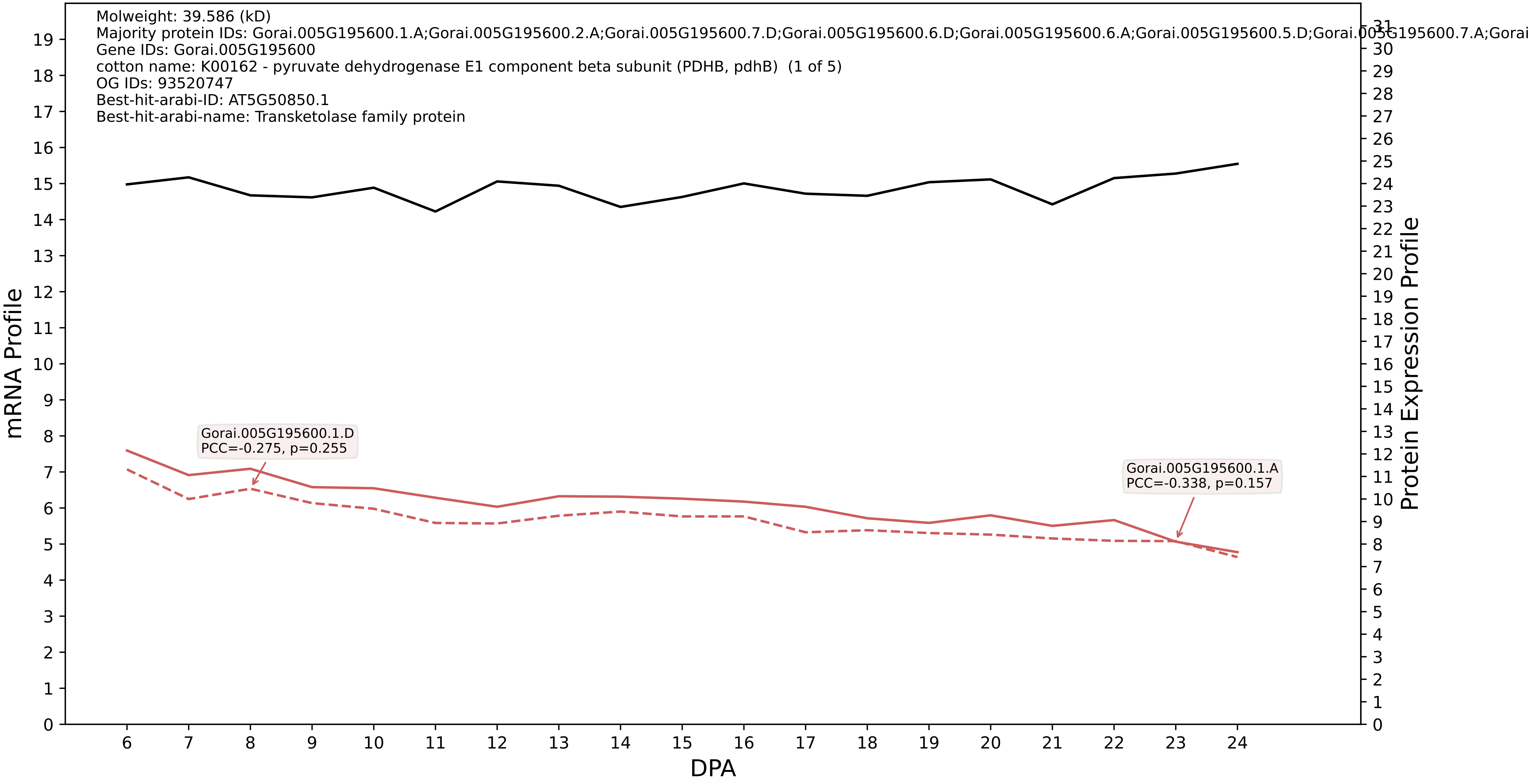

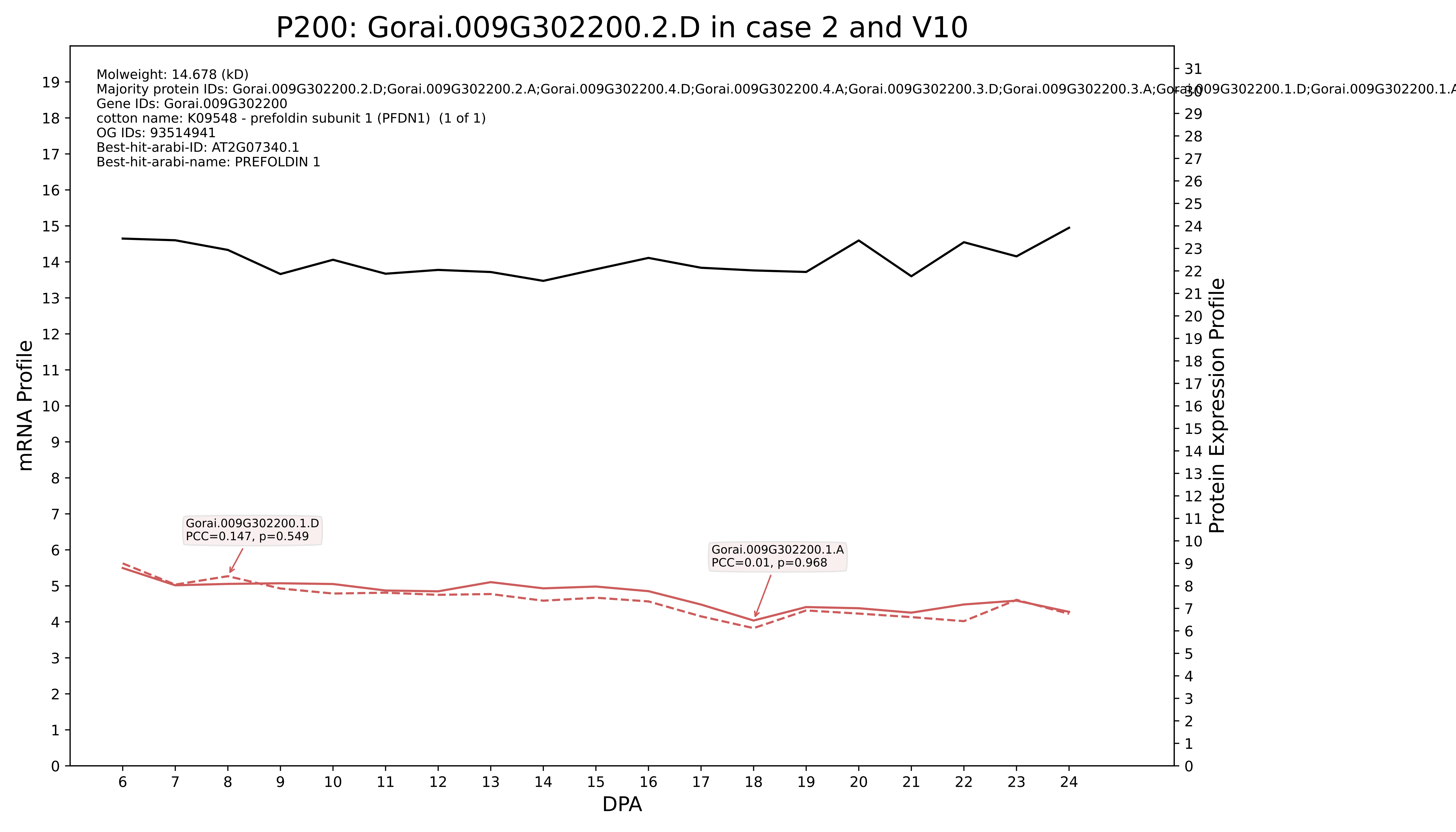
P200: Gorai.009G302200.2.D in case 2 and V10
Molweight: 14.678 (kD)
Majority protein IDs: Gorai.009G302200.2.D;Gorai.009G302200.2.A;Gorai.009G302200.4.D;Gorai.009G302200.4.A;Gorai.009G302200.3.D;Gorai.009G302200.3.A;Gorai.009G302200.1.D;Gorai.009G302200.1.A
Gene IDs: Gorai.009G302200
cotton name: K09548 - prefoldin subunit 1 (PFDN1) (1 of 1)
OG IDs: 93514941
Best-hit-arabi-ID: AT2G07340.1
Best-hit-arabi-name: PREFOLDIN 1
Gorai.009G302200.1.D
PCC=0.147, p=0.549
Gorai.009G302200.1.A
PCC=0.01, p=0.968
mRNA Profile
Protein Expression Profile
DPA

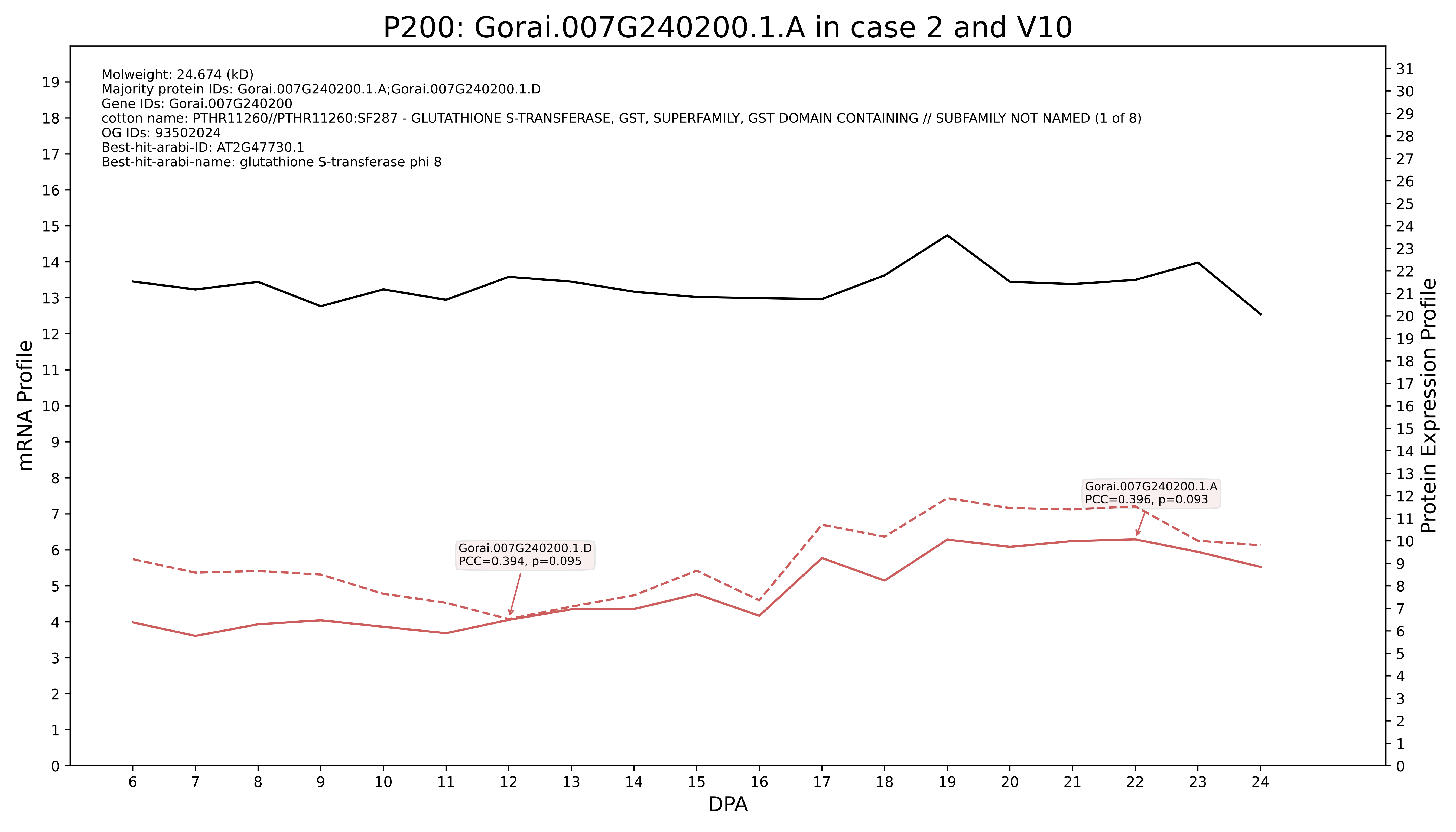
P200: Gorai.007G240200.1.A in case 2 and V10
Molweight: 24.674 (kD)
Majority protein IDs: Gorai.007G240200.1.A;Gorai.007G240200.1.D
Gene IDs: Gorai.007G240200
cotton name: PTHR11260//PTHR11260:SF287 - GLUTATHIONE S-TRANSFERASE, GST, SUPERFAMILY, GST DOMAIN CONTAINING // SUBFAMILY NOT NAMED (1 of 8)
OG IDs: 93502024
Best-hit-arabi-ID: AT2G47730.1
Best-hit-arabi-name: glutathione S-transferase phi 8
Gorai.007G240200.1.D
PCC=0.394, p=0.095
Gorai.007G240200.1.A
PCC=0.396, p=0.093
mRNA Profile
Protein Expression Profile
DPA
0
1
2
3
4
5
6
7
8
9
10
11
12
13
14
15
16
17
18
19
20
21
22
23
24
25
26
27
28
29
30
31

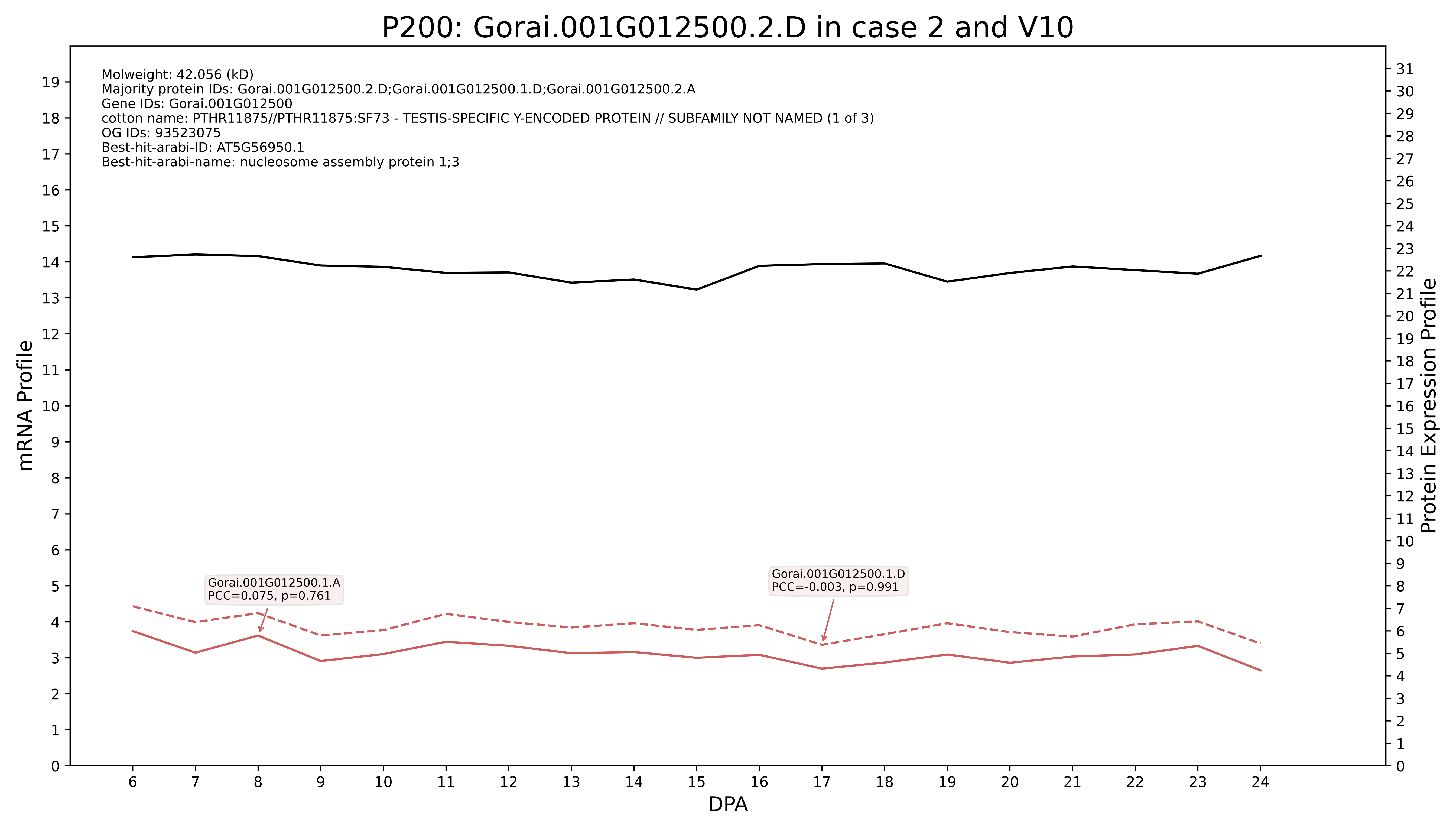
P200: Gorai.001G012500.2.D in case 2 and V10
Molweight: 42.056 (kD)
Majority protein IDs: Gorai.001G012500.2.D;Gorai.001G012500.1.D;Gorai.001G012500.2.A
Gene IDs: Gorai.001G012500
cotton name: PTHR11875//PTHR11875:SF73 - TESTIS-SPECIFIC Y-ENCODED PROTEIN // SUBFAMILY NOT NAMED (1 of 3)
OG IDs: 93523075
Best-hit-arabi-ID: AT5G56950.1
Best-hit-arabi-name: nucleosome assembly protein 1;3
Gorai.001G012500.1.A
PCC=0.075, p=0.761
Gorai.001G012500.1.D
PCC=-0.003, p=0.991
mRNA Profile
Protein Expression Profile
DPA

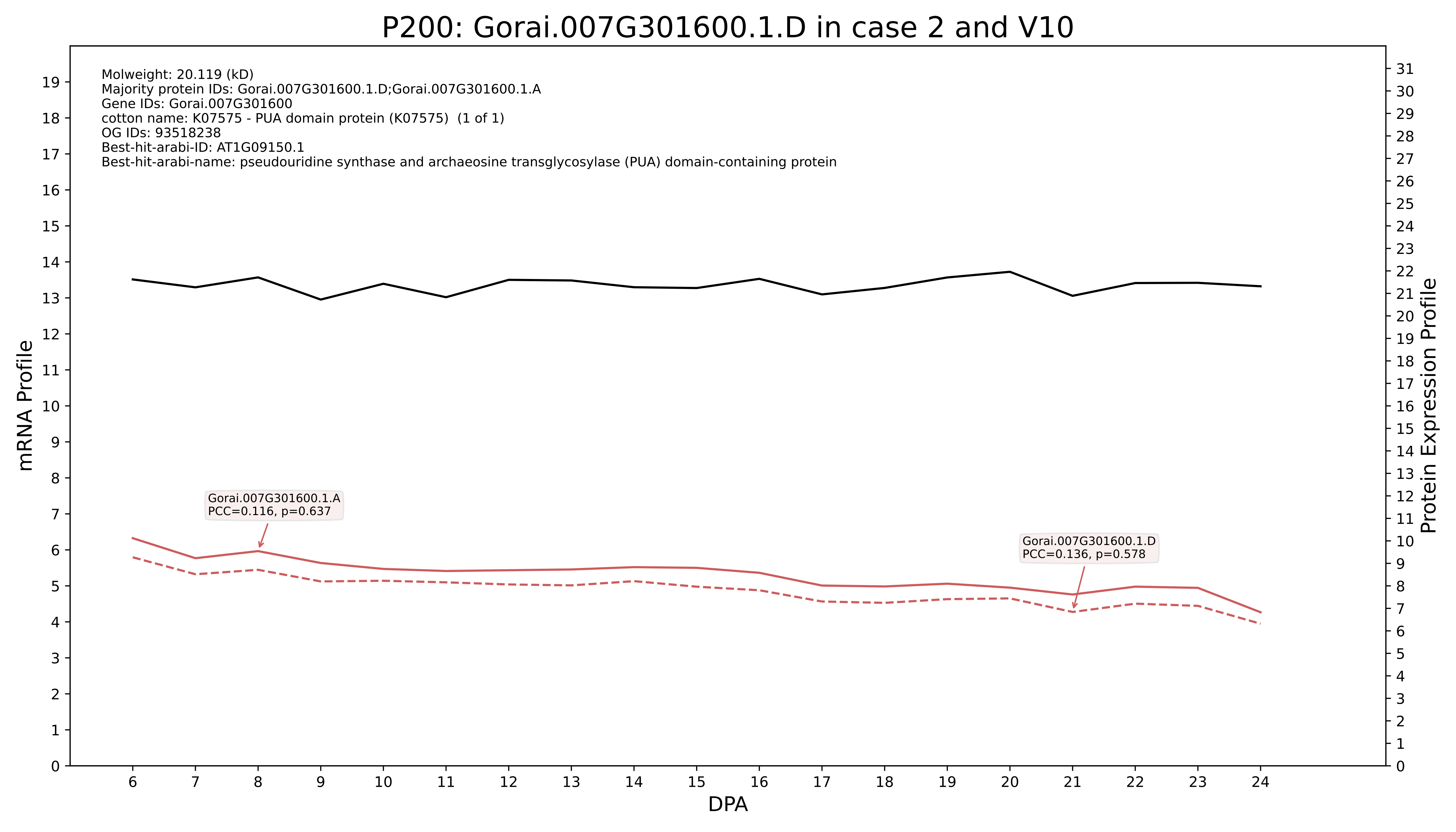
P200: Gorai.007G301600.1.D in case 2 and V10
Molweight: 20.119 (kD)
Majority protein IDs: Gorai.007G301600.1.D;Gorai.007G301600.1.A
Gene IDs: Gorai.007G301600
cotton name: K07575 - PUA domain protein (K07575) (1 of 1)
OG IDs: 93518238
Best-hit-arabi-ID: AT1G09150.1
Best-hit-arabi-name: pseudouridine synthase and archaeosine transglycosylase (PUA) domain-containing protein
Gorai.007G301600.1.A
PCC=0.116, p=0.637
Gorai.007G301600.1.D
PCC=0.136, p=0.578
mRNA Profile
Protein Expression Profile
DPA

00: Gorai.005G071800.4.D in case 2 and V10

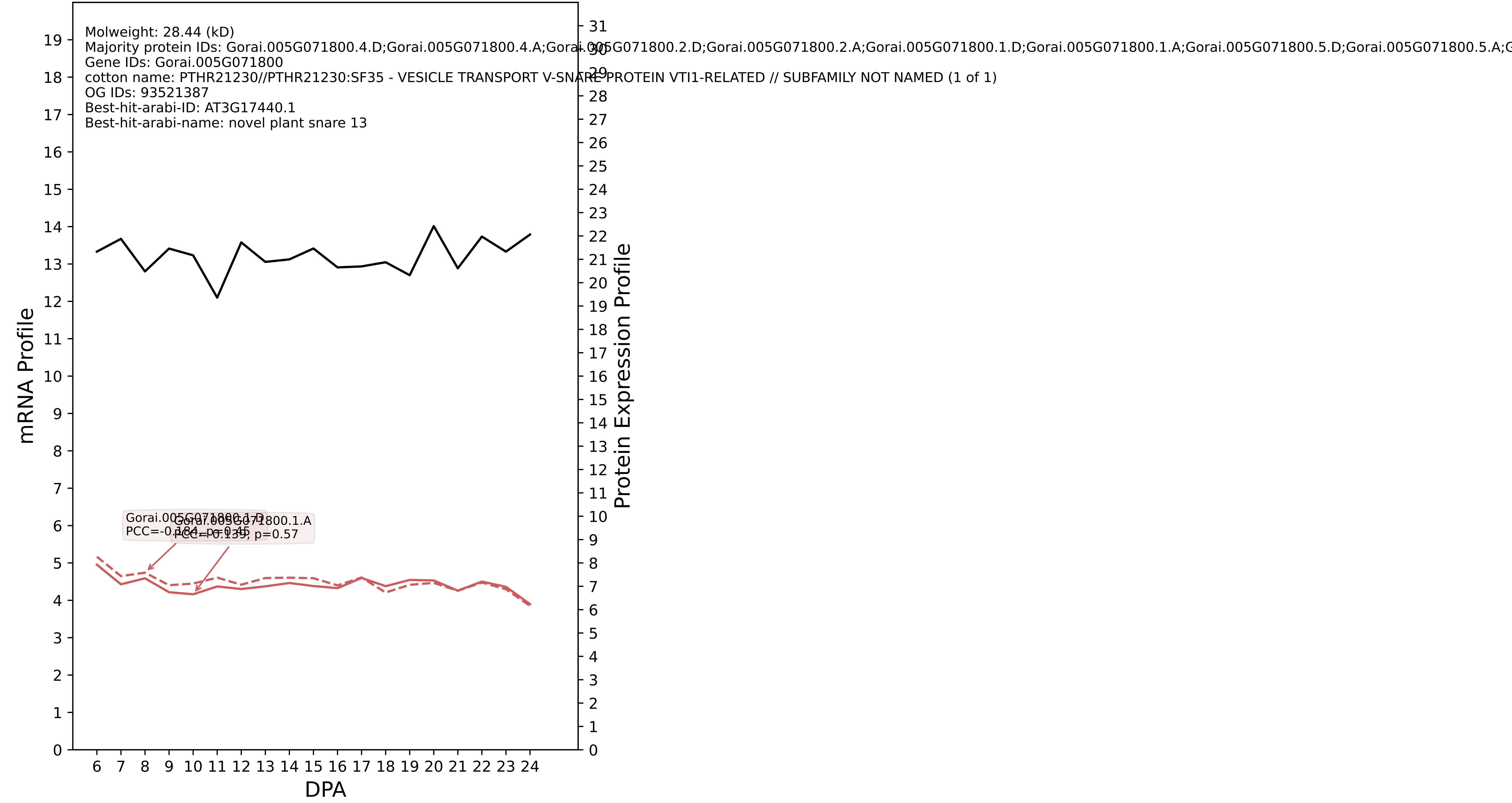

P200: Gorai.004G100400.1.D in case 2 and V10

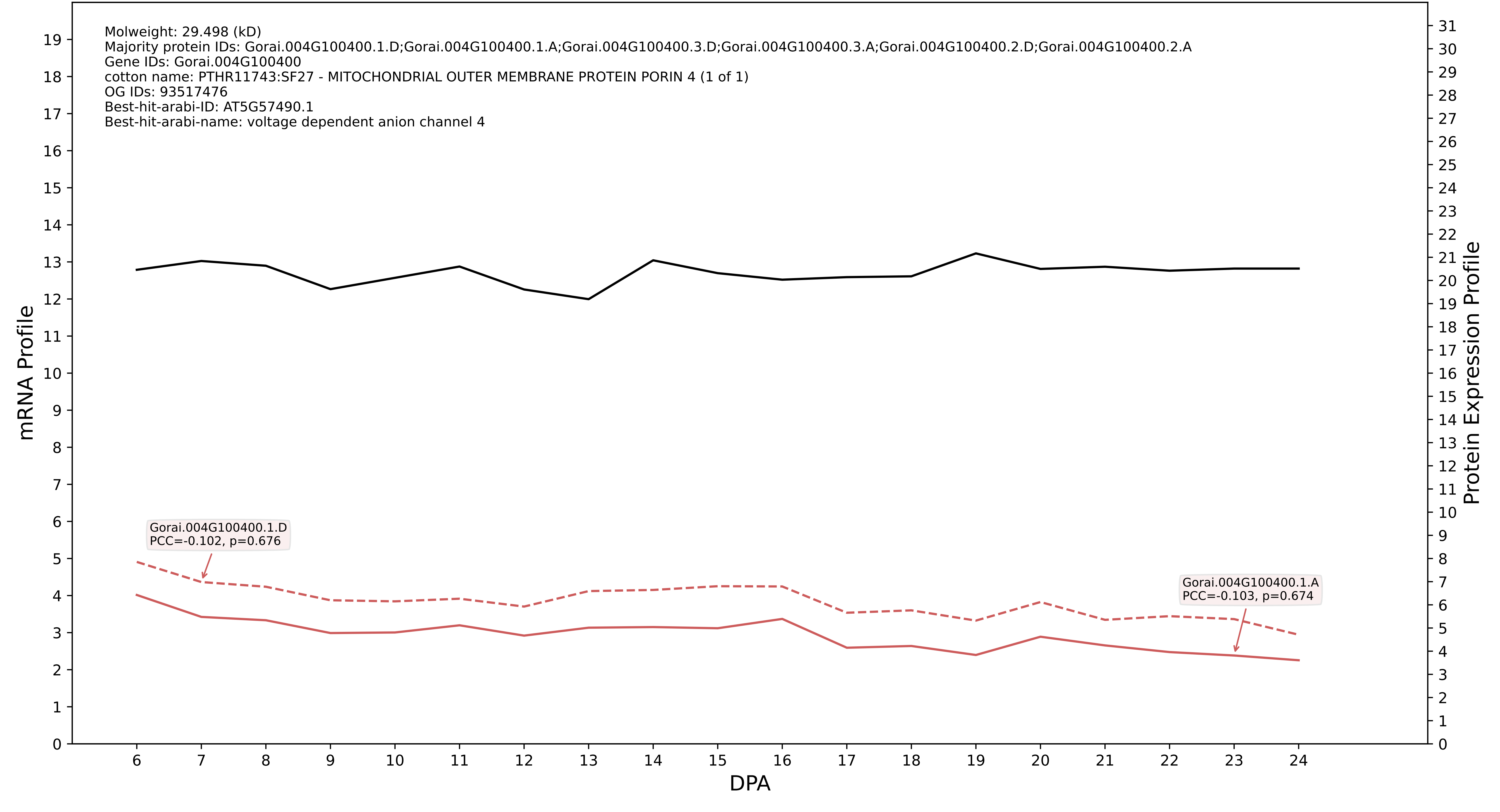

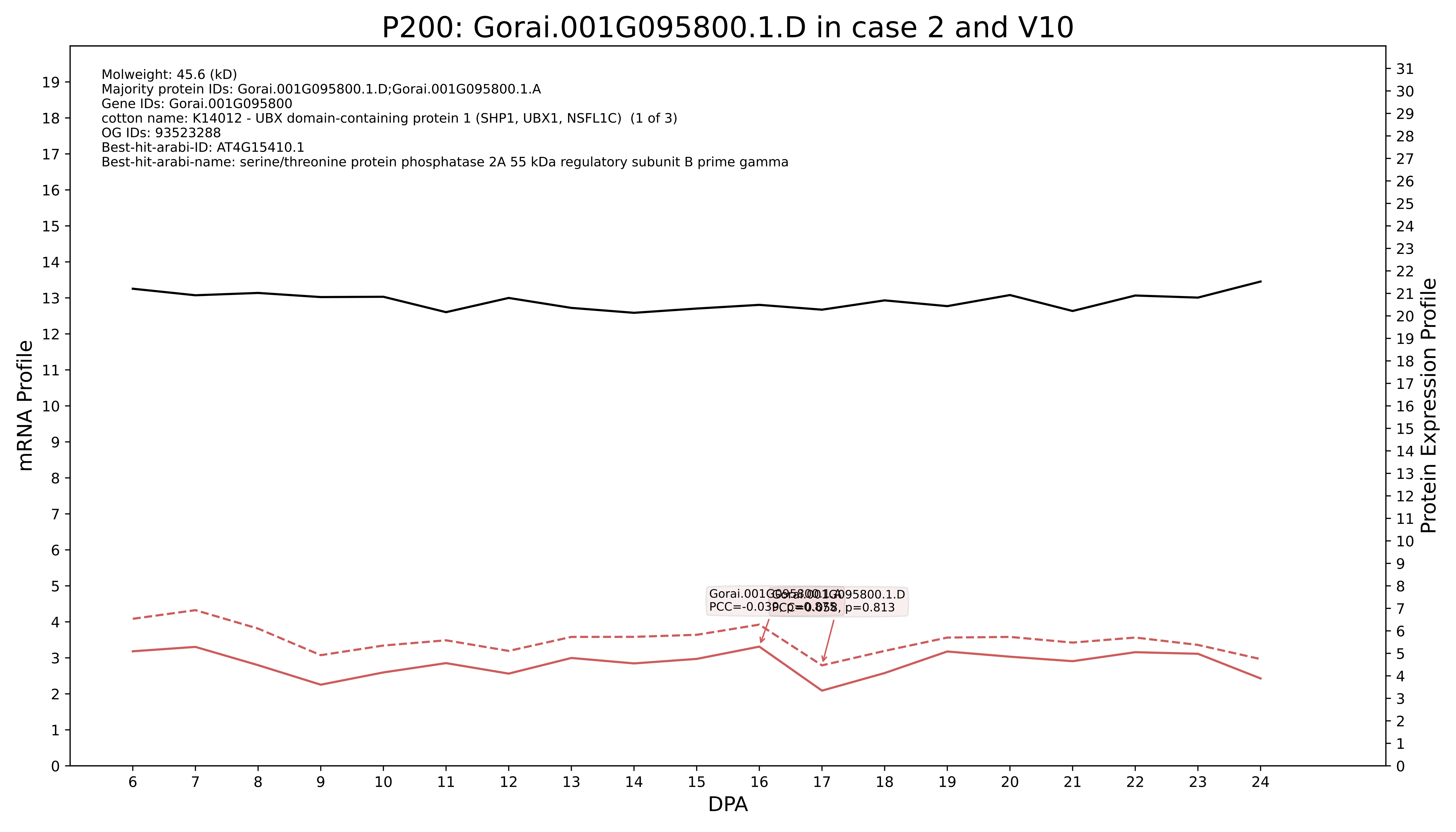

P200: Gorai.001G095800.1.D in case 2 and V10
Molweight: 45.6 (kD)
Majority protein IDs: Gorai.001G095800.1.D;Gorai.001G095800.1.A
Gene IDs: Gorai.001G095800
cotton name: K14012 - UBX domain-containing protein 1 (SHP1, UBX1, NSFL1C) (1 of 3)
OG IDs: 93523288
Best-hit-arabi-ID: AT4G15410.1
Best-hit-arabi-name: serine/threonine protein phosphatase 2A 55 kDa regulatory subunit B prime gamma
p=0.813
mRNA Profile
Protein Expression Profile
DPA
0
1
2
3
4
5
6
7
8
9
10
11
12
13
14
15
16
17
18
19
20
21
22
23
24
25
26
27
28
29
30
31

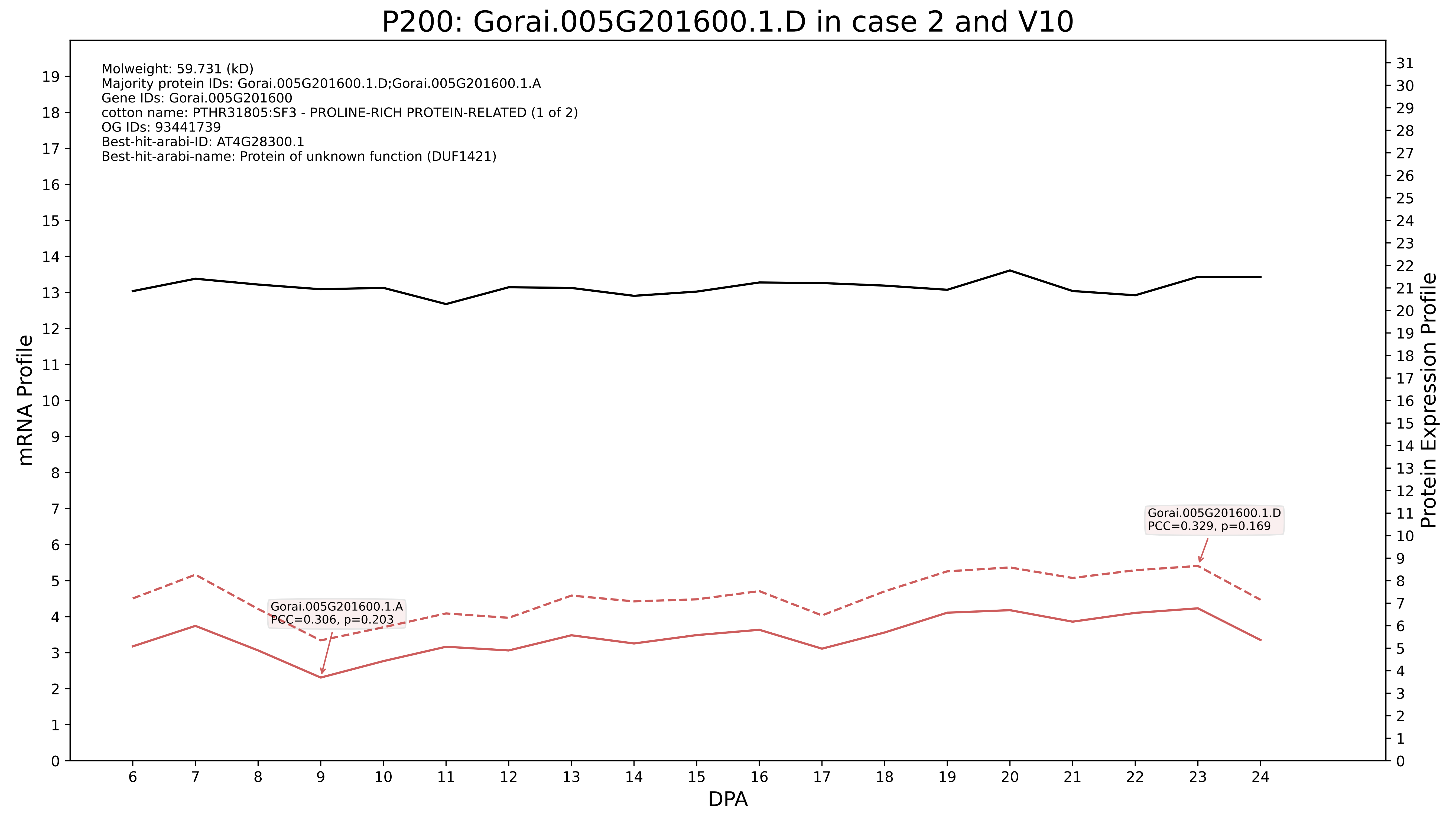
P200: Gorai.005G201600.1.D in case 2 and V10
Molweight: 59.731 (kD)
Majority protein IDs: Gorai.005G201600.1.D;Gorai.005G201600.1.A
Gene IDs: Gorai.005G201600
cotton name: PTHR31805:SF3 - PROLINE-RICH PROTEIN-RELATED (1 of 2)
OG IDs: 93441739
Best-hit-arabi-ID: AT4G28300.1
Best-hit-arabi-name: Protein of unknown function (DUF1421)
Gorai.005G201600.1.D
PCC=0.329, p=0.169
Gorai.005G201600.1.A
PCC=0.306, p=0.203
mRNA Profile
Protein Expression Profile
DPA

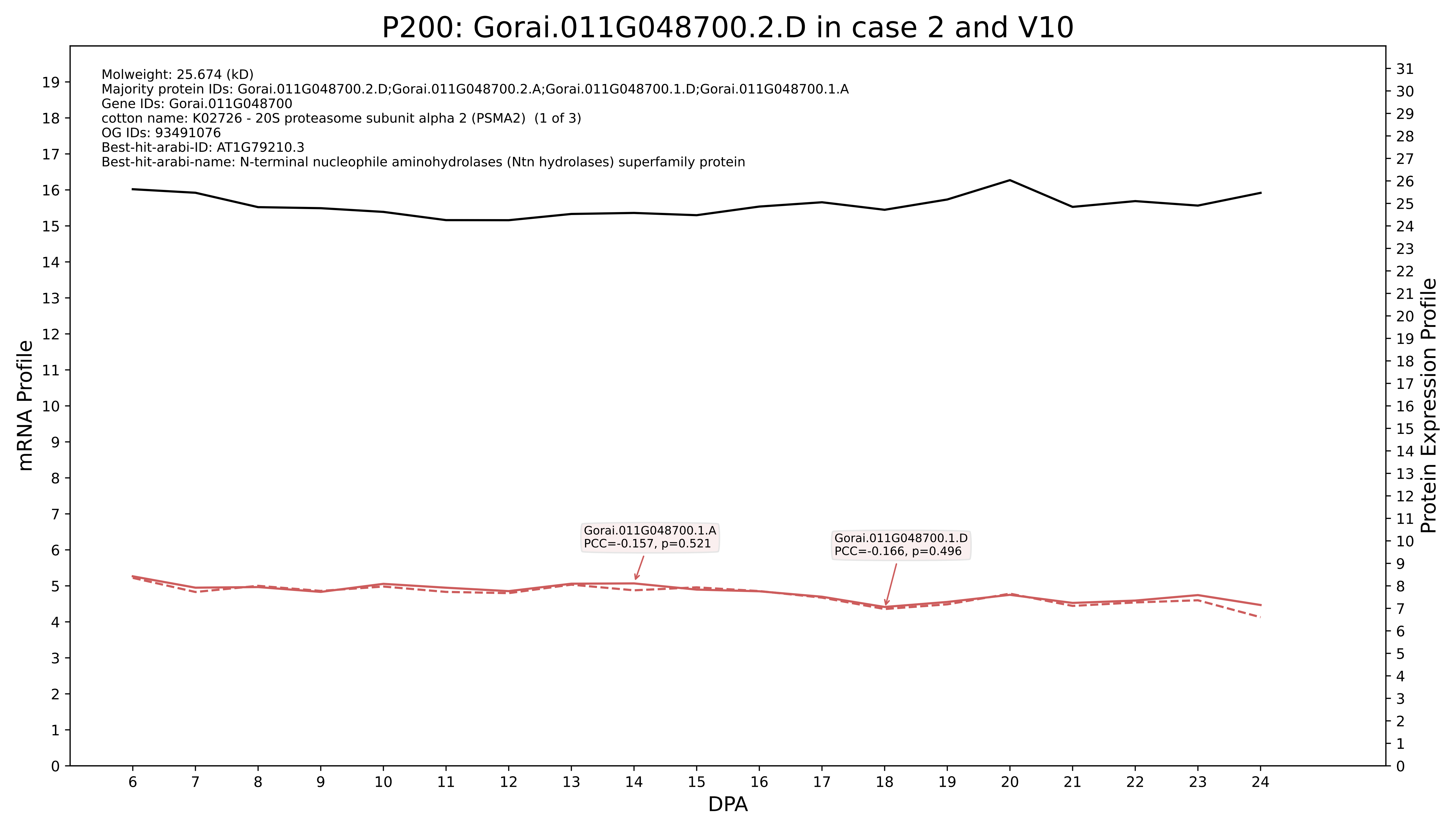
P200: Gorai.011G048700.2.D in case 2 and V10
Molweight: 25.674 (kD)
Majority protein IDs: Gorai.011G048700.2.D;Gorai.011G048700.2.A;Gorai.011G048700.1.D;Gorai.011G048700.1.A
Gene IDs: Gorai.011G048700
cotton name: K02726 - 20S proteasome subunit alpha 2 (PSMA2) (1 of 3)
OG IDs: 93491076
Best-hit-arabi-ID: AT1G79210.3
Best-hit-arabi-name: N-terminal nucleophile aminohydrolases (Ntn hydrolases) superfamily protein
Gorai.011G048700.1.A
PCC=-0.157, p=0.521
Gorai.011G048700.1.D
PCC=-0.166, p=0.496
mRNA Profile
Protein Expression Profile
DPA

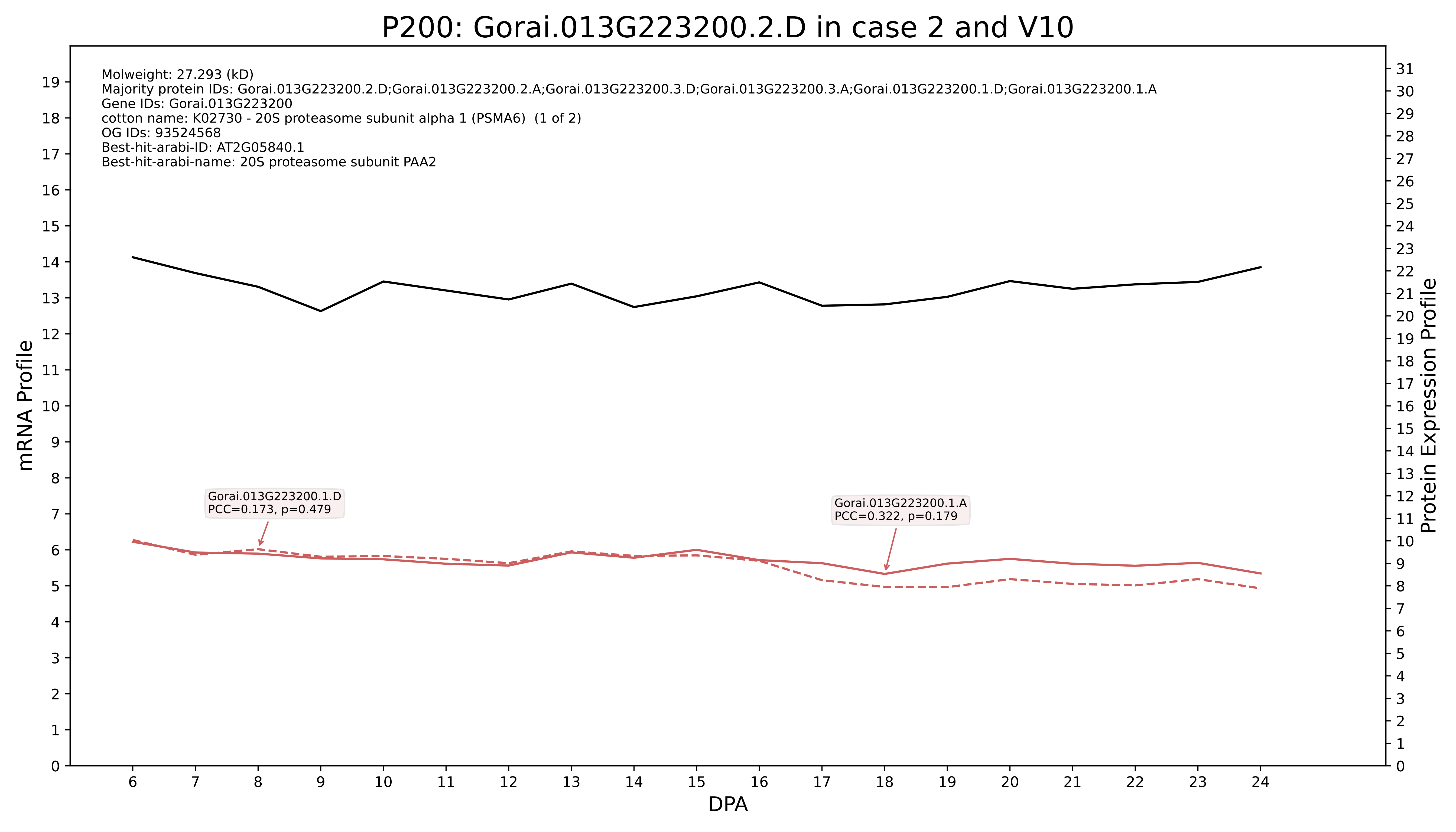
P200: Gorai.013G223200.2.D in case 2 and V10
Molweight: 27.293 (kD)
Majority protein IDs: Gorai.013G223200.2.D;Gorai.013G223200.2.A;Gorai.013G223200.3.D;Gorai.013G223200.3.A;Gorai.013G223200.1.D;Gorai.013G223200.1.A
Gene IDs: Gorai.013G223200
cotton name: K02730 - 20S proteasome subunit alpha 1 (PSMA6) (1 of 2)
OG IDs: 93524568
Best-hit-arabi-ID: AT2G05840.1
Best-hit-arabi-name: 20S proteasome subunit PAA2
Gorai.013G223200.1.D
PCC=0.173, p=0.479
Gorai.013G223200.1.A
PCC=0.322, p=0.179
mRNA Profile
Protein Expression Profile
DPA

P200: Gorai.009G367300.1.D in case 2 and V10

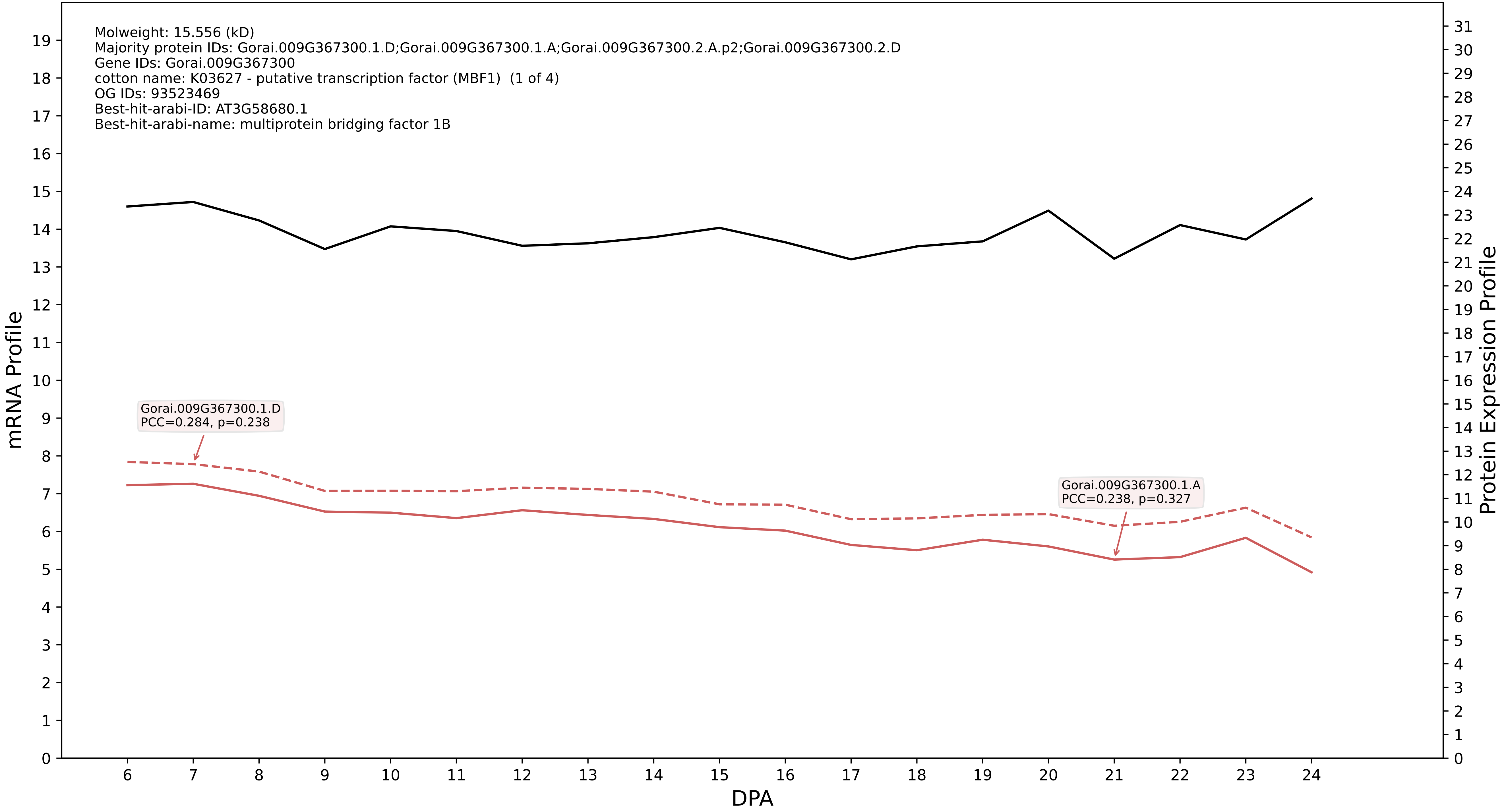

P200: Gorai.011G105500.1.D in case 2 and V10

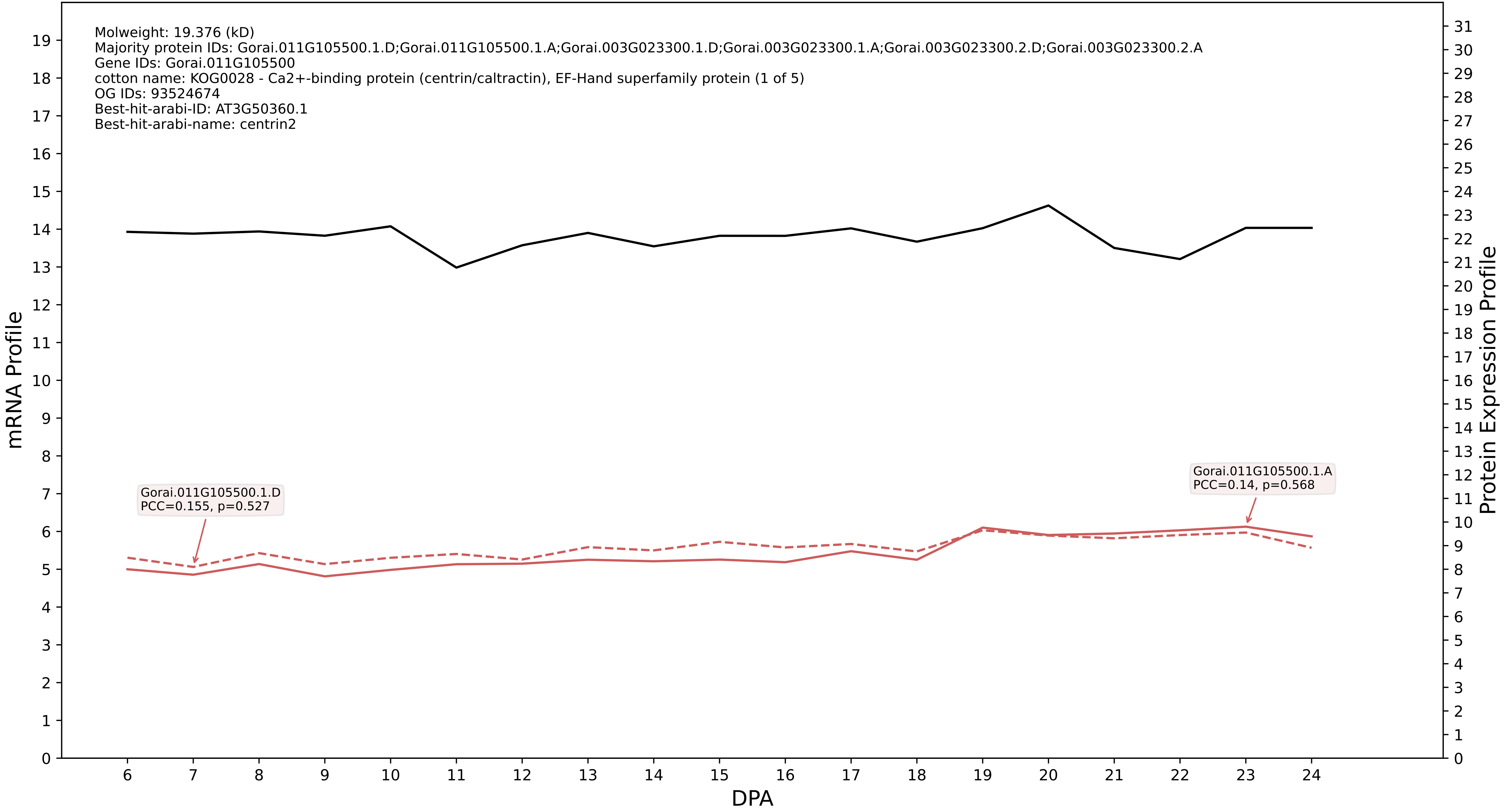

P200: Gorai.006G040100.1.D in case 2 and V10

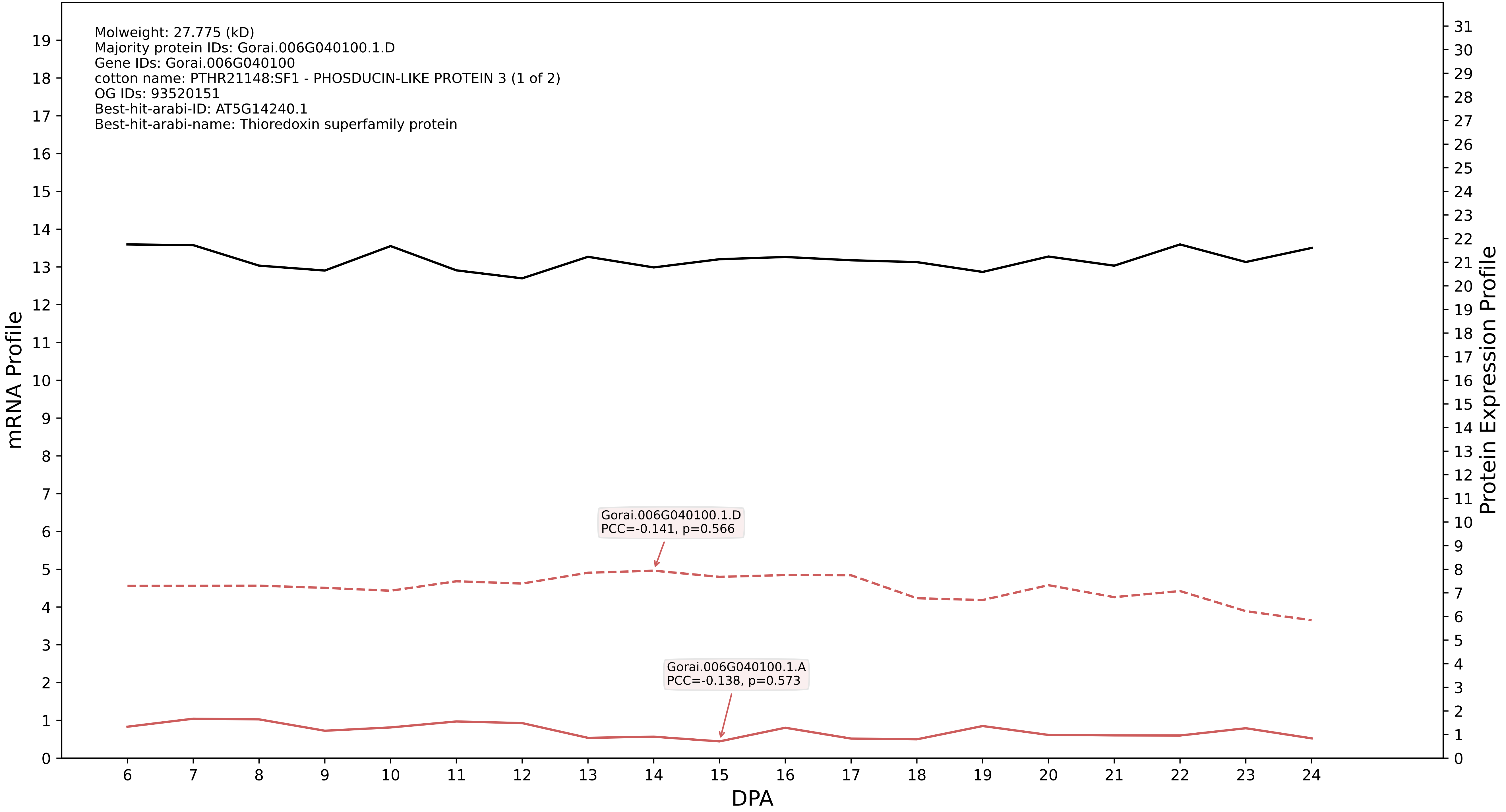

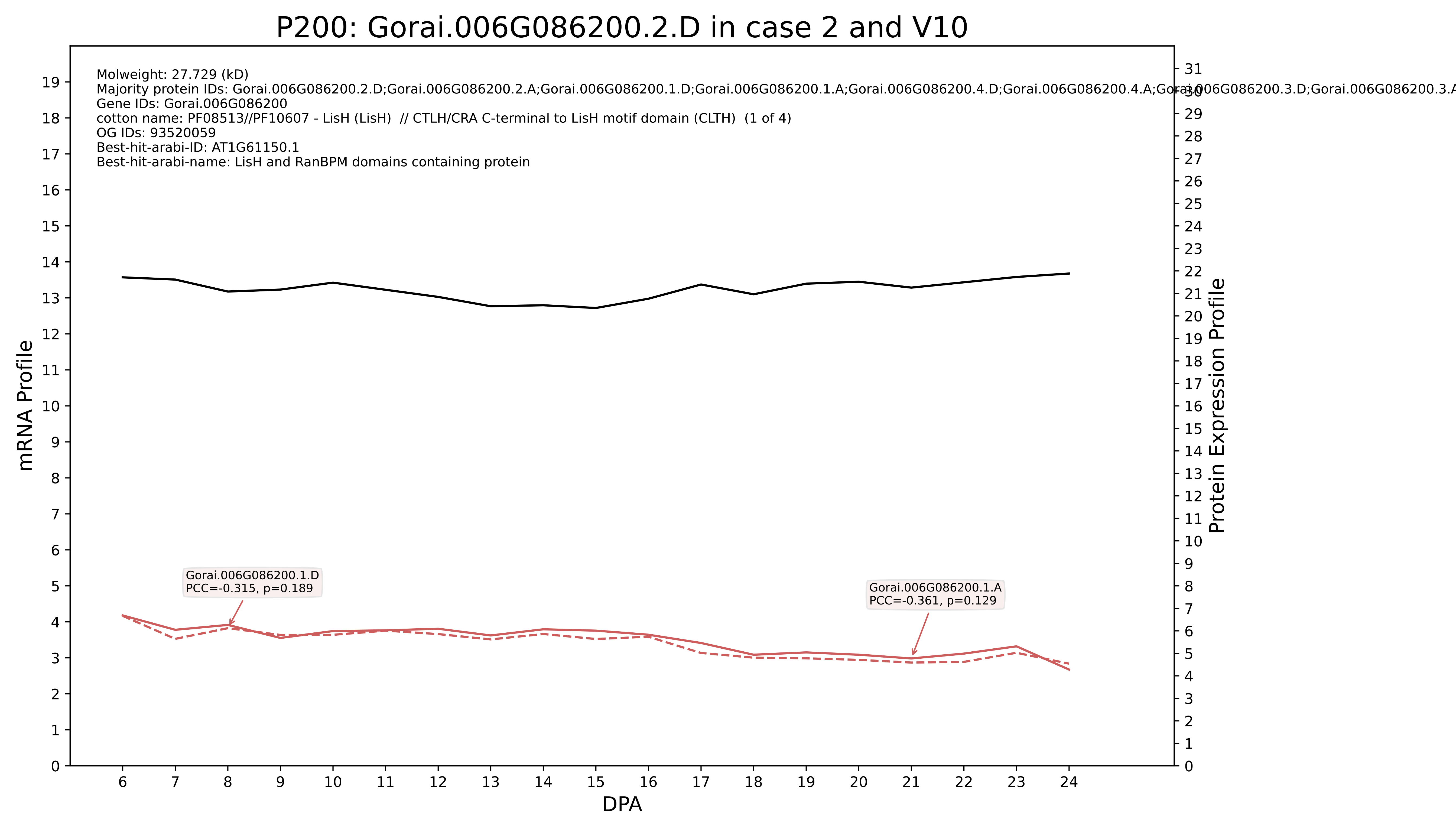

P200: Gorai.006G086200.2.D in case 2 and V10
Molweight: 27.729 (kD)
Majority protein IDs: Gorai.006G086200.2.D;Gorai.006G086200.2.A;Gorai.006G086200.1.D;Gorai.006G086200.1.A;Gorai.006G086200.4.D;Gorai.006G086200.4.A;Gorai.006G086200.3.D;Gorai.006G086200.3.A
Gene IDs: Gorai.006G086200
cotton name: PF08513//PF10607 - LisH (LisH) // CTLH/CRA C-terminal to LisH motif domain (CLTH) (1 of 4)
OG IDs: 93520059
Best-hit-arabi-ID: AT1G61150.1
Best-hit-arabi-name: LisH and RanBPM domains containing protein
Gorai.006G086200.1.D
PCC=-0.315, p=0.189
Gorai.006G086200.1.A
PCC=-0.361, p=0.129
mRNA Profile
Protein Expression Profile
DPA

P200: Gorai.004G058800.1.D in case 2 and V10

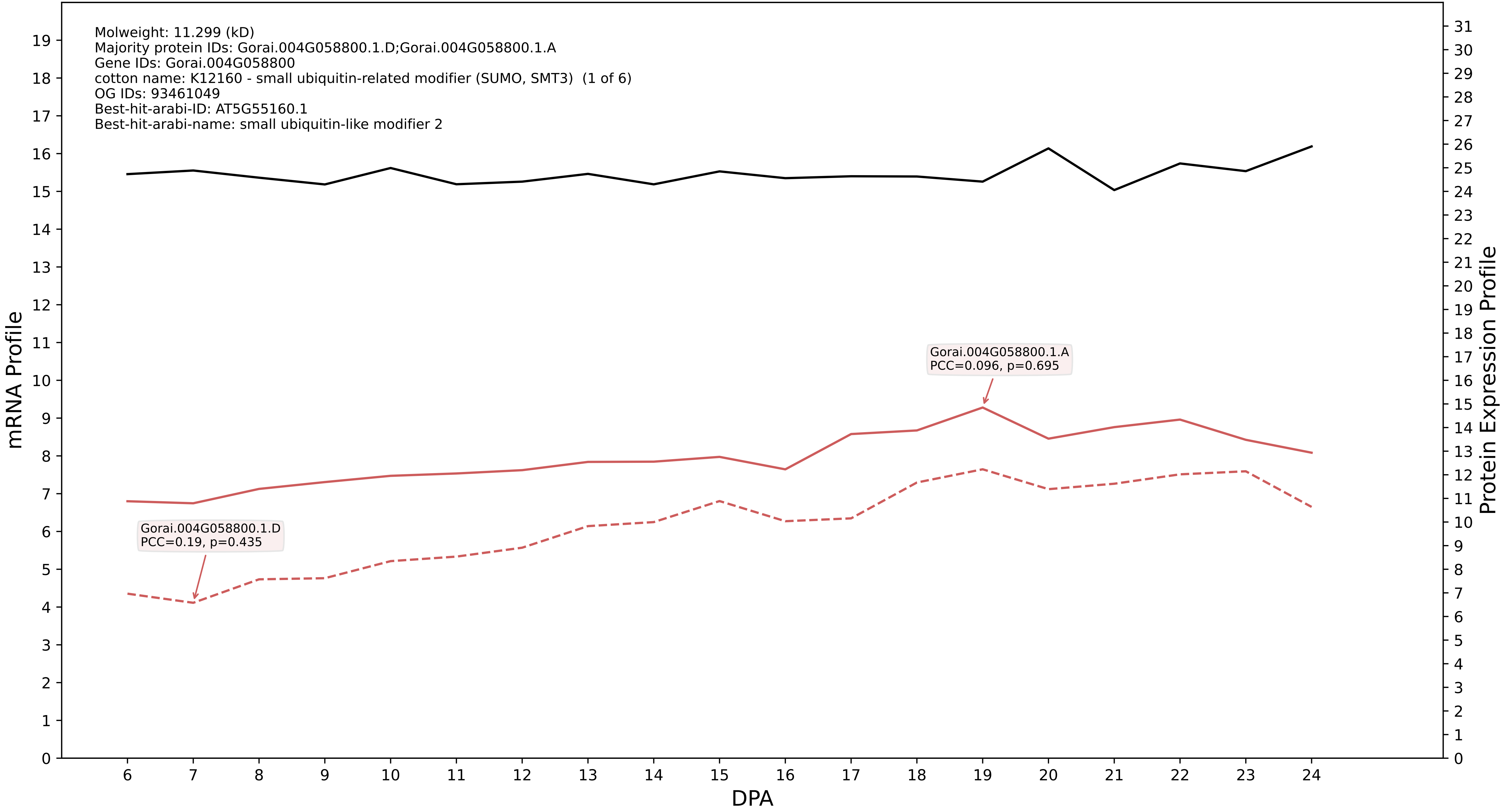

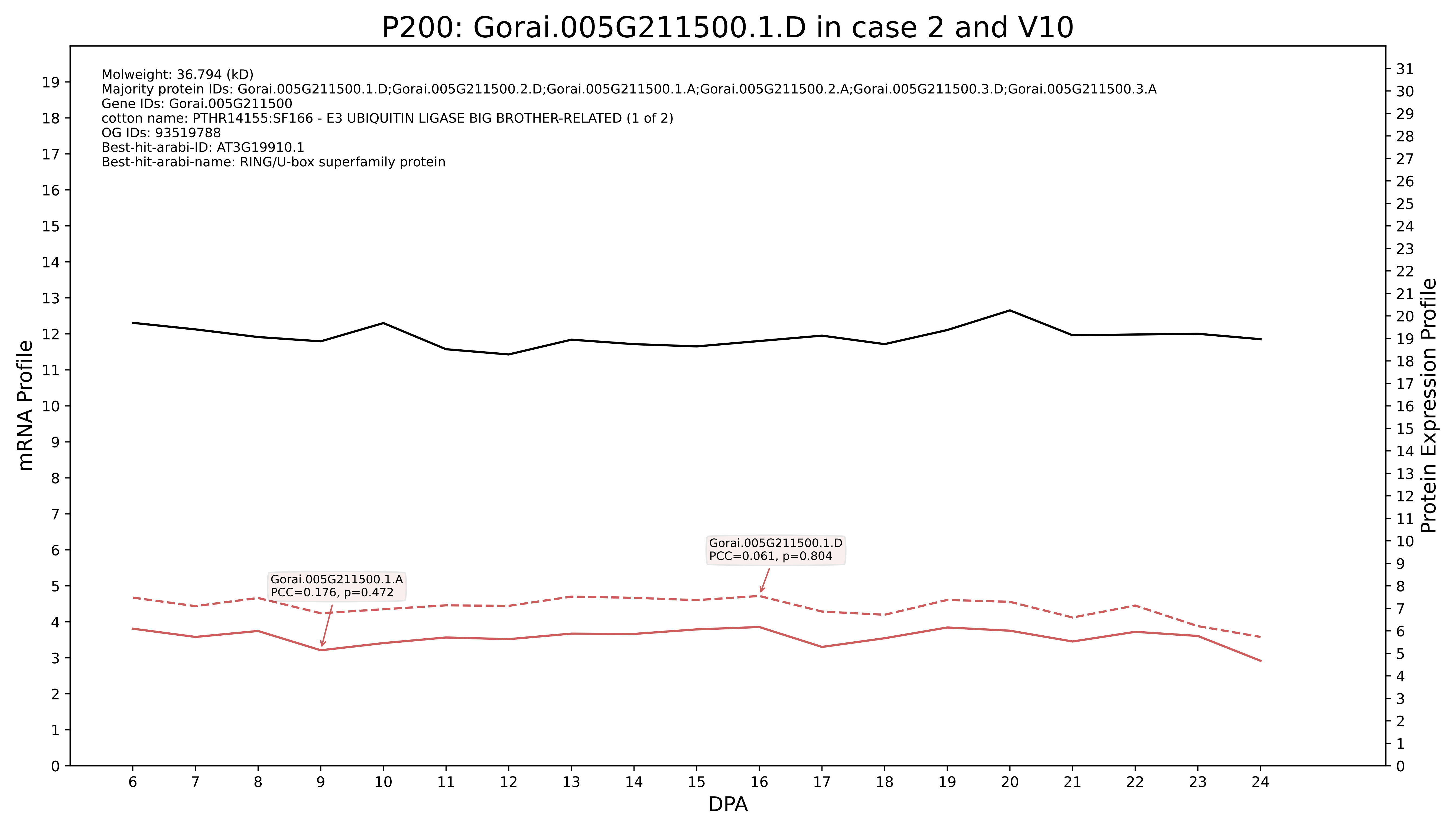
P200: Gorai.005G211500.1.D in case 2 and V10
Molweight: 36.794 (kD)
Majority protein IDs: Gorai.005G211500.1.D;Gorai.005G211500.2.D;Gorai.005G211500.1.A;Gorai.005G211500.2.A;Gorai.005G211500.3.D;Gorai.005G211500.3.A
Gene IDs: Gorai.005G211500
cotton name: PTHR14155:SF166 - E3 UBIQUITIN LIGASE BIG BROTHER-RELATED (1 of 2)
OG IDs: 93519788
Best-hit-arabi-ID: AT3G19910.1
Best-hit-arabi-name: RING/U-box superfamily protein
Gorai.005G211500.1.A
PCC=0.176, p=0.472
Gorai.005G211500.1.D
PCC=0.061, p=0.804
mRNA Profile
Protein Expression Profile
DPA

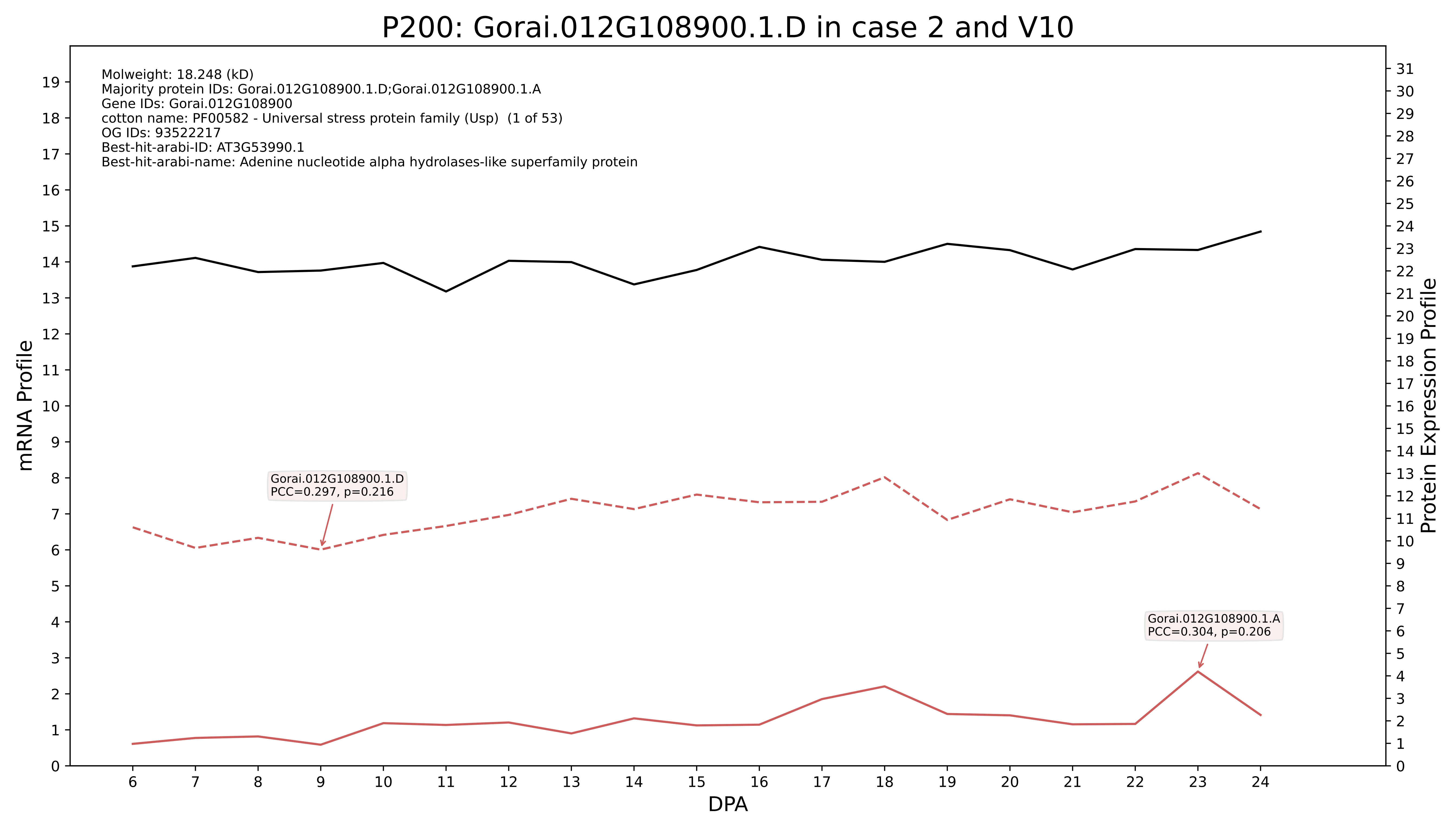
P200: Gorai.012G108900.1.D in case 2 and V10
Molweight: 18.248 (kD)
Majority protein IDs: Gorai.012G108900.1.D;Gorai.012G108900.1.A
Gene IDs: Gorai.012G108900
cotton name: PF00582 - Universal stress protein family (Usp) (1 of 53)
OG IDs: 93522217
Best-hit-arabi-ID: AT3G53990.1
Best-hit-arabi-name: Adenine nucleotide alpha hydrolases-like superfamily protein
Gorai.012G108900.1.D
PCC=0.297, p=0.216
Gorai.012G108900.1.A
PCC=0.304, p=0.206
mRNA Profile
Protein Expression Profile
DPA

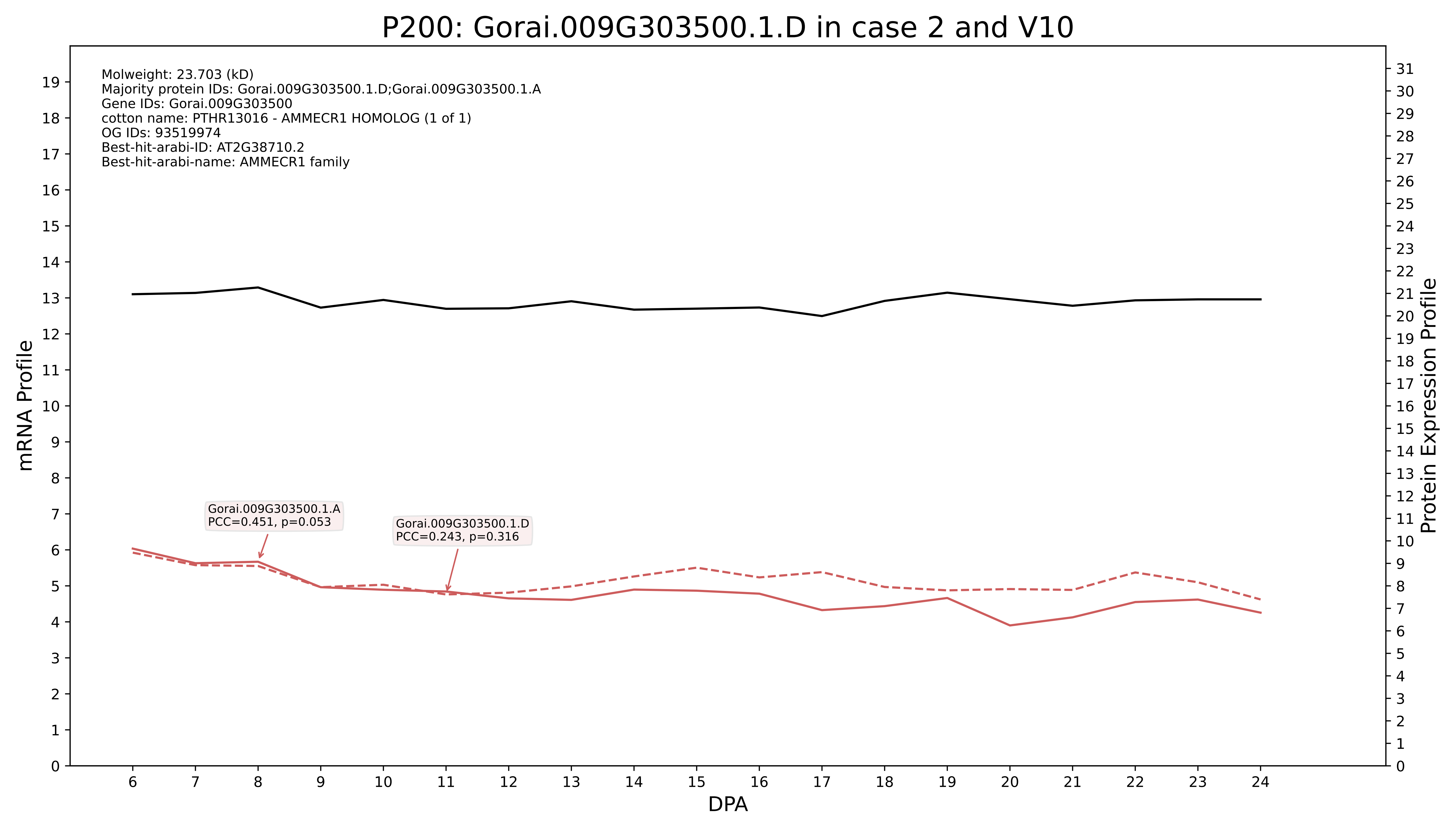

P200: Gorai.009G303500.1.D in case 2 and V10
Molweight: 23.703 (kD)
Majority protein IDs: Gorai.009G303500.1.D;Gorai.009G303500.1.A
Gene IDs: Gorai.009G303500
cotton name: PTHR13016 - AMMECR1 HOMOLOG (1 of 1)
OG IDs: 93519974
Best-hit-arabi-ID: AT2G38710.2
Best-hit-arabi-name: AMMECR1 family
Gorai.009G303500.1.A
PCC=0.451, p=0.053
Gorai.009G303500.1.D
PCC=0.243, p=0.316
mRNA Profile
Protein Expression Profile
DPA

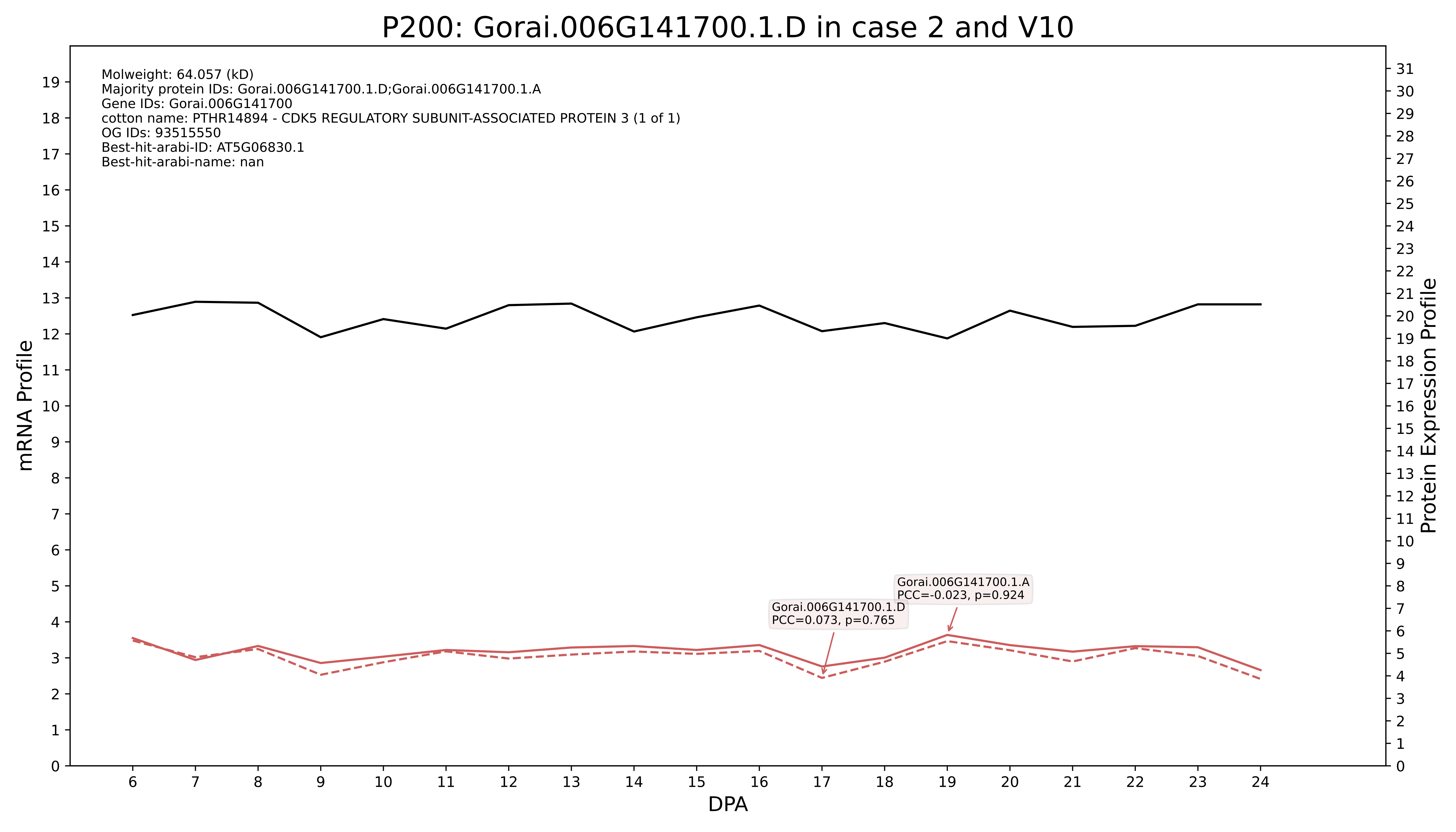
P200: Gorai.006G141700.1.D in case 2 and V10
Molweight: 64.057 (kD)
Majority protein IDs: Gorai.006G141700.1.D;Gorai.006G141700.1.A
Gene IDs: Gorai.006G141700
cotton name: PTHR14894 - CDK5 REGULATORY SUBUNIT-ASSOCIATED PROTEIN 3 (1 of 1)
OG IDs: 93515550
Best-hit-arabi-ID: AT5G06830.1
Best-hit-arabi-name: nan
Gorai.006G141700.1.A
PCC=-0.023, p=0.924
Gorai.006G141700.1.D
PCC=0.073, p=0.765
mRNA Profile
Protein Expression Profile
DPA

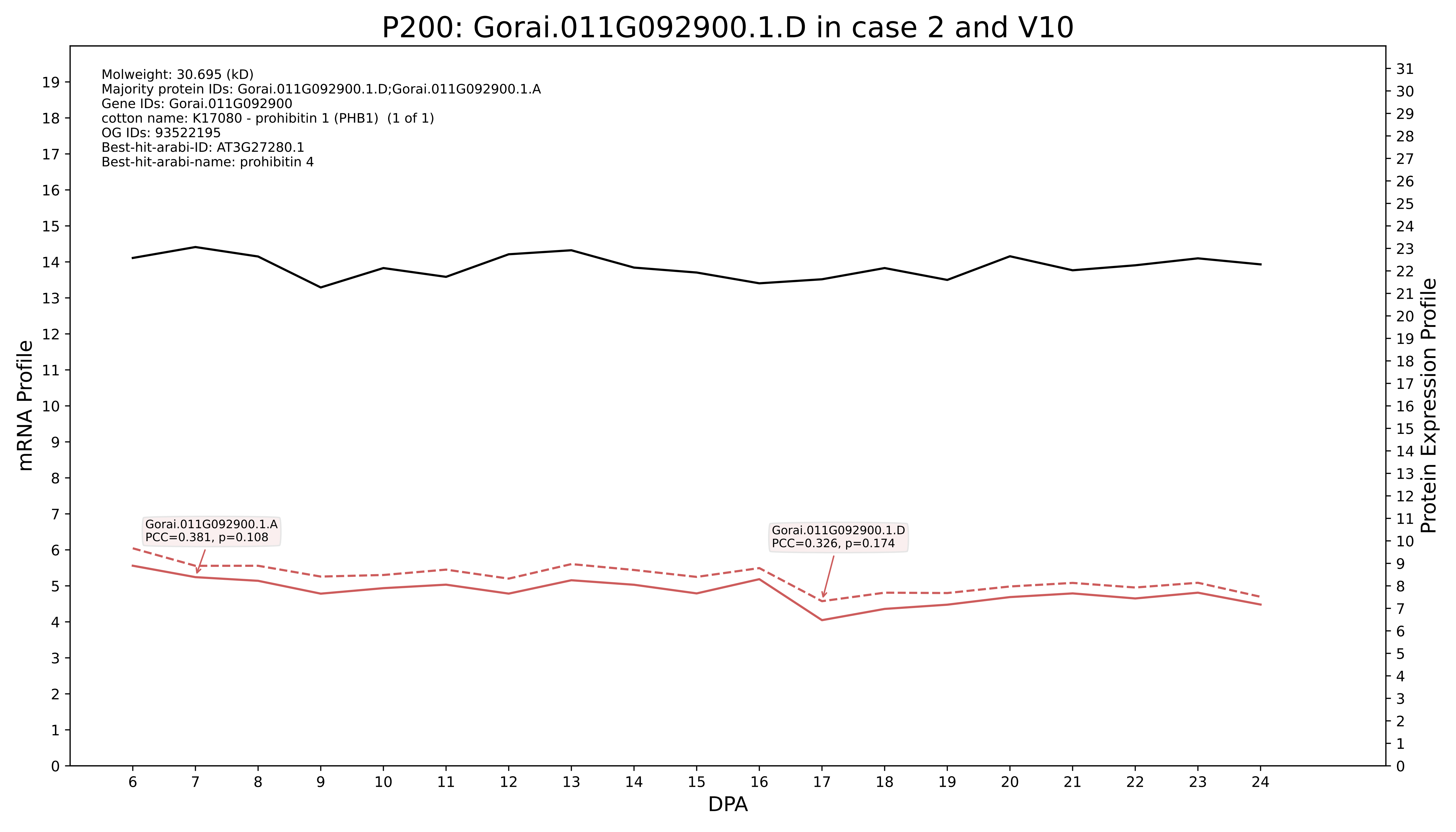
P200: Gorai.011G092900.1.D in case 2 and V10
Molweight: 30.695 (kD)
Majority protein IDs: Gorai.011G092900.1.D;Gorai.011G092900.1.A
Gene IDs: Gorai.011G092900
cotton name: K17080 - prohibitin 1 (PHB1) (1 of 1)
OG IDs: 93522195
Best-hit-arabi-ID: AT3G27280.1
Best-hit-arabi-name: prohibitin 4
Gorai.011G092900.1.A
PCC=0.381, p=0.108
Gorai.011G092900.1.D
PCC=0.326, p=0.174
mRNA Profile
Protein Expression Profile
DPA

P200: Gorai.007G068200.1.D in case 2 and V10

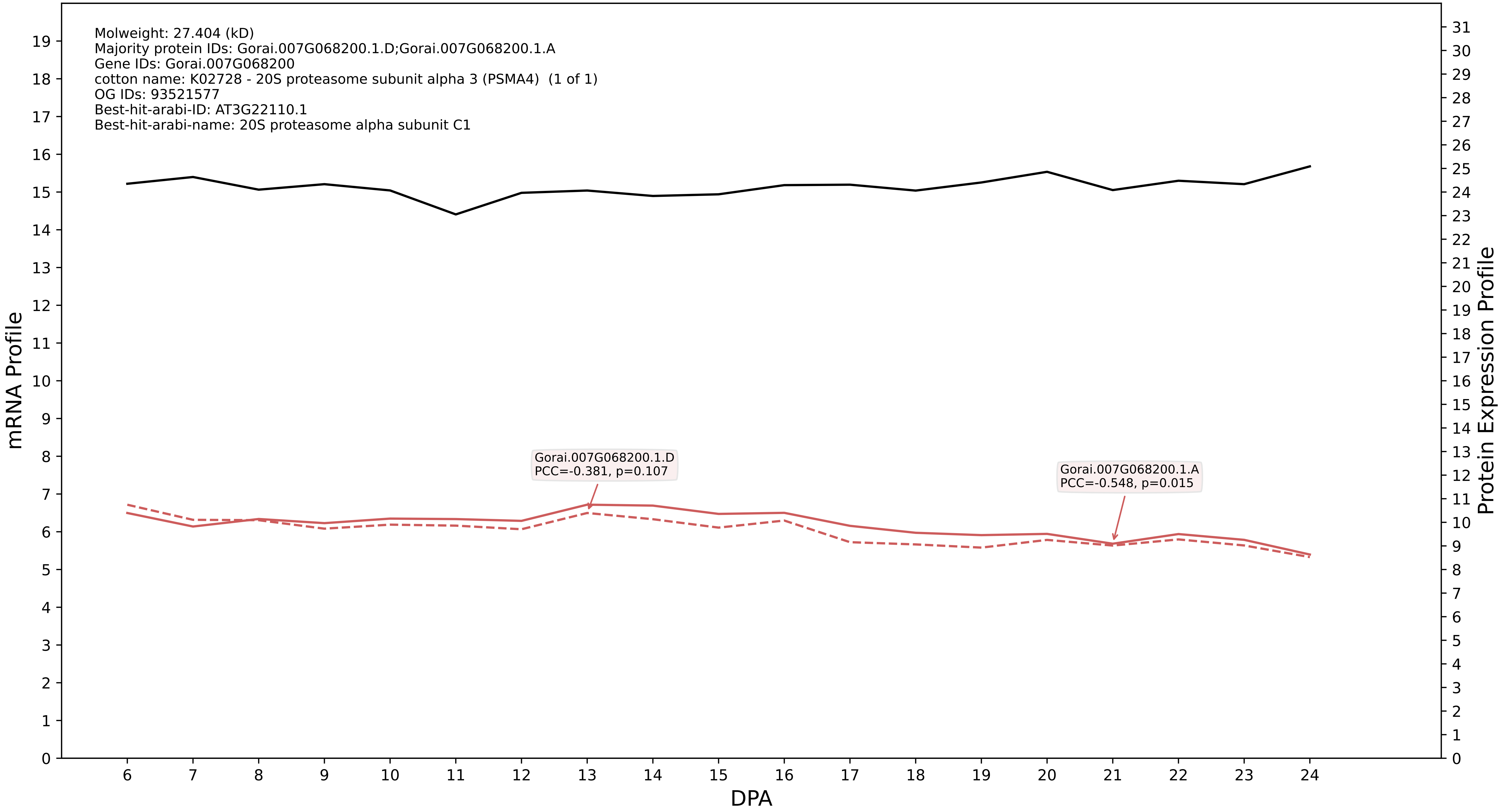

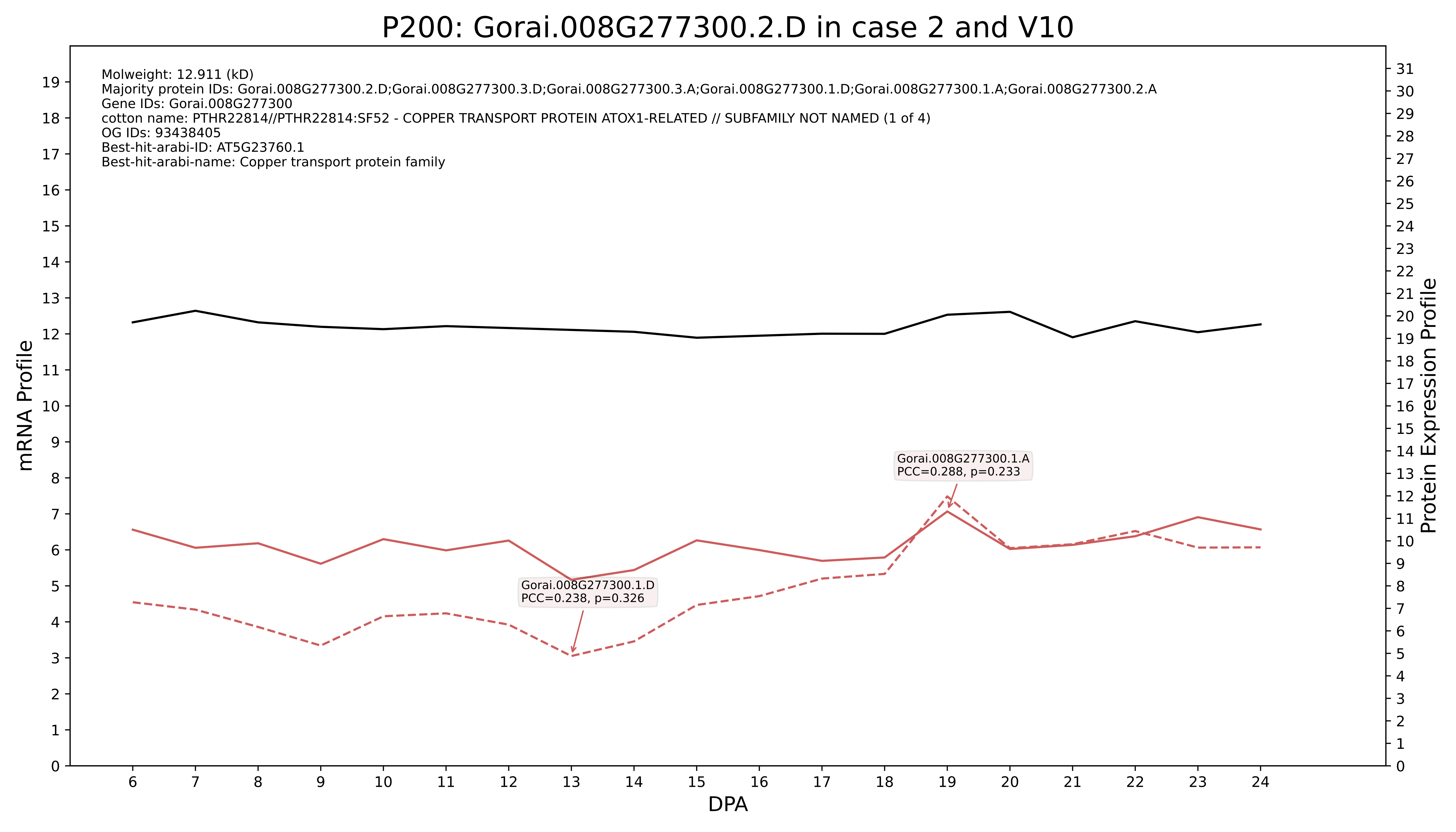
P200: Gorai.008G277300.2.D in case 2 and V10
Molweight: 12.911 (kD)
Majority protein IDs: Gorai.008G277300.2.D;Gorai.008G277300.3.D;Gorai.008G277300.3.A;Gorai.008G277300.1.D;Gorai.008G277300.1.A;Gorai.008G277300.2.A
Gene IDs: Gorai.008G277300
cotton name: PTHR22814//PTHR22814:SF52 - COPPER TRANSPORT PROTEIN ATOX1-RELATED // SUBFAMILY NOT NAMED (1 of 4)
OG IDs: 93438405
Best-hit-arabi-ID: AT5G23760.1
Best-hit-arabi-name: Copper transport protein family
Gorai.008G277300.1.A
PCC=0.288, p=0.233
Gorai.008G277300.1.D
PCC=0.238, p=0.326
mRNA Profile
Protein Expression Profile
DPA

P200: Gorai.004G143300.1.D in case 2 and V10

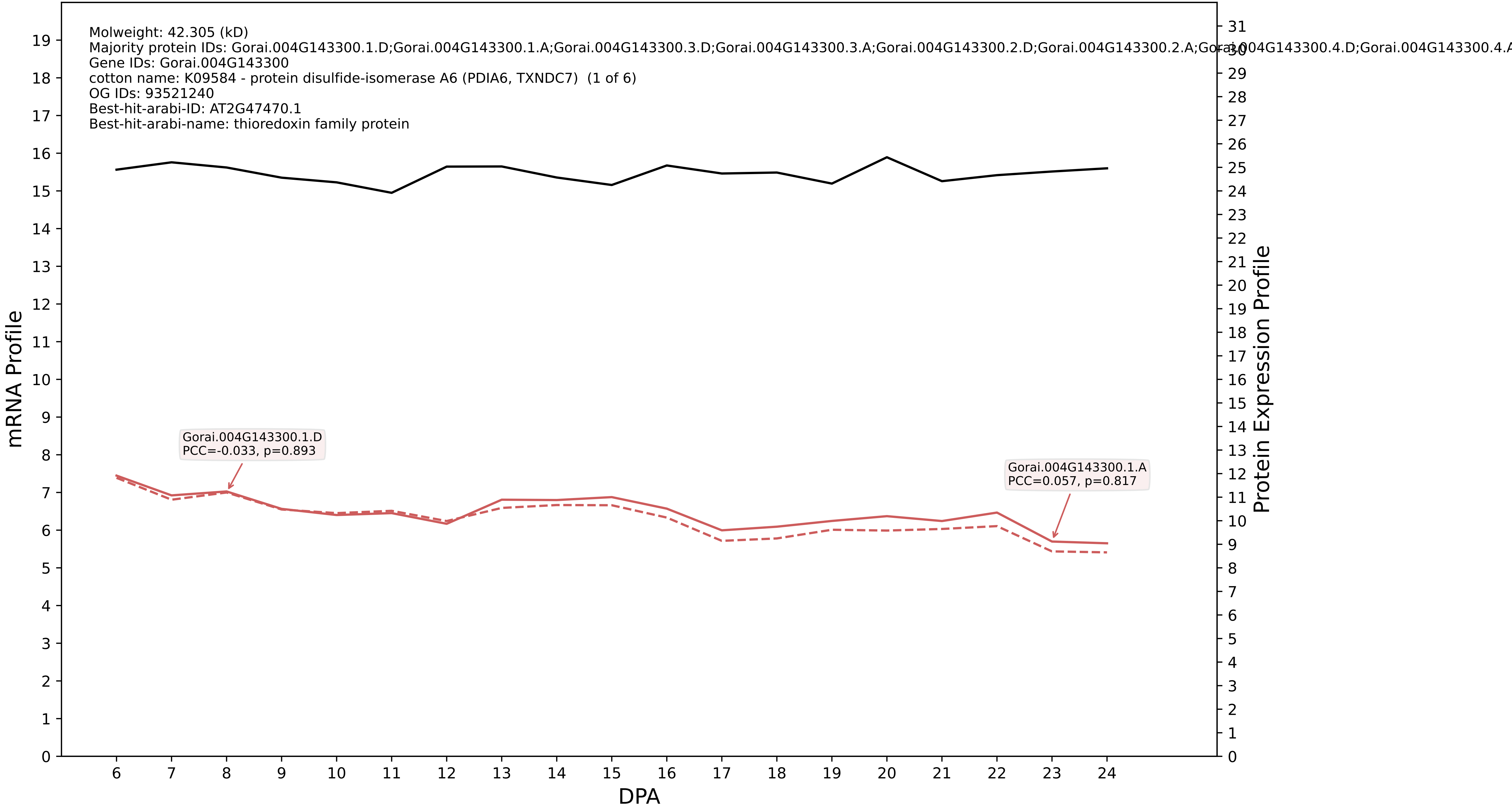

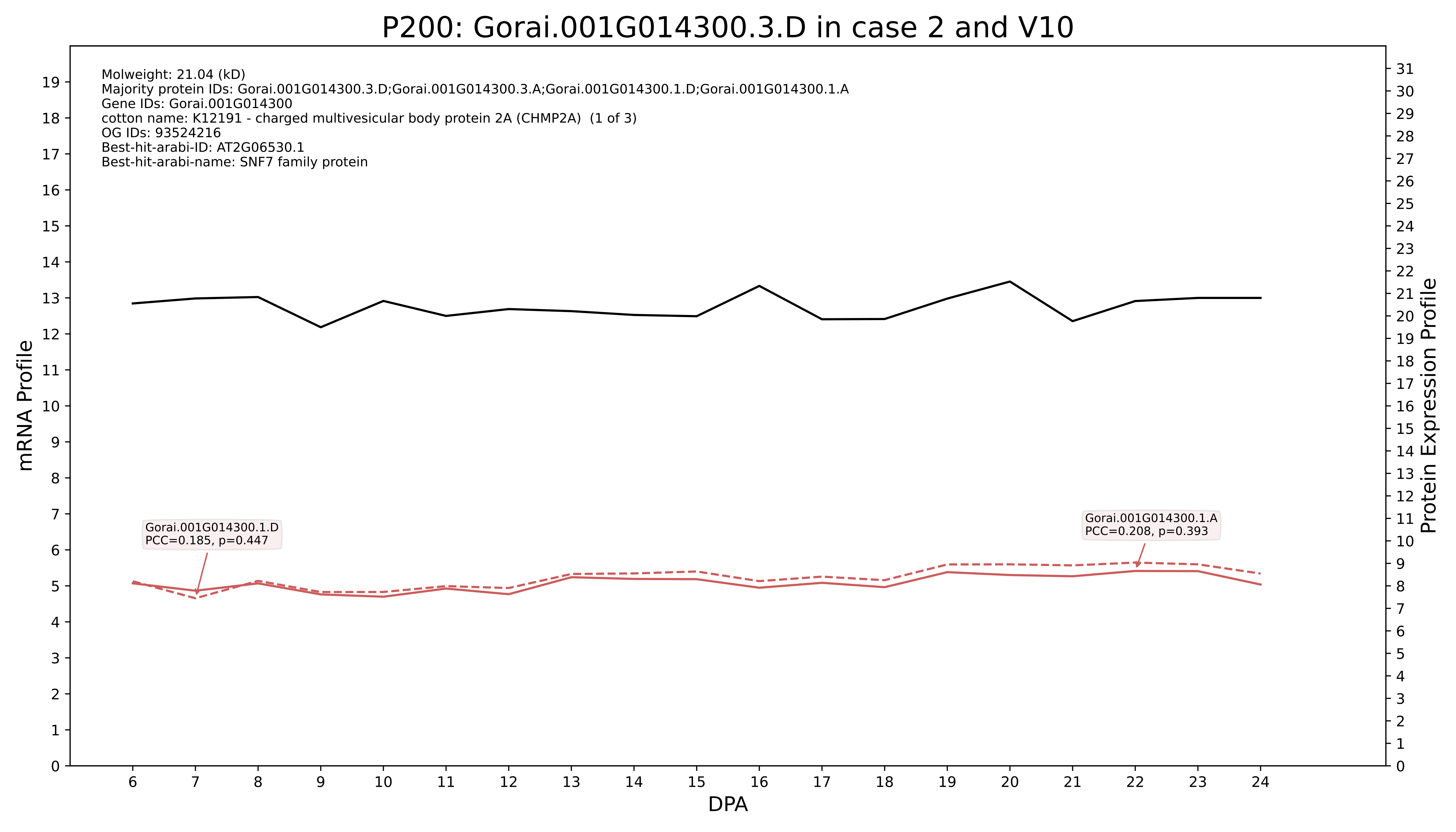

P200: Gorai.001G014300.3.D in case 2 and V10
Molweight: 21.04 (kD)
Majority protein IDs: Gorai.001G014300.3.D;Gorai.001G014300.3.A;Gorai.001G014300.1.D;Gorai.001G014300.1.A
Gene IDs: Gorai.001G014300
cotton name: K12191 - charged multivesicular body protein 2A (CHMP2A) (1 of 3)
OG IDs: 93524216
Best-hit-arabi-ID: AT2G06530.1
Best-hit-arabi-name: SNF7 family protein
Gorai.001G014300.1.D
PCC=0.185, p=0.447
Gorai.001G014300.1.A
PCC=0.208, p=0.393
mRNA Profile
Protein Expression Profile
DPA

P200: Gorai.010G120600.1.D in case 2 and V10

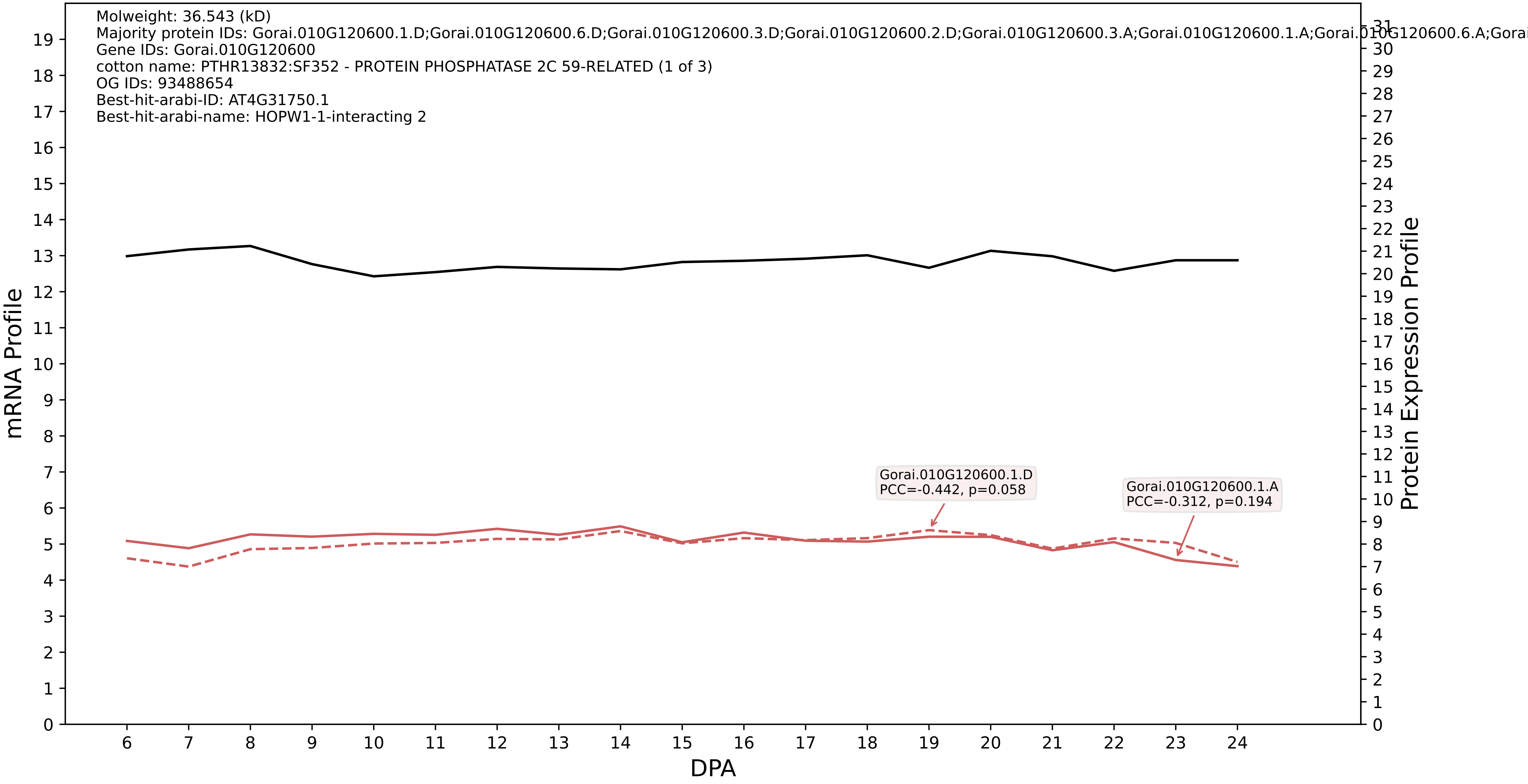

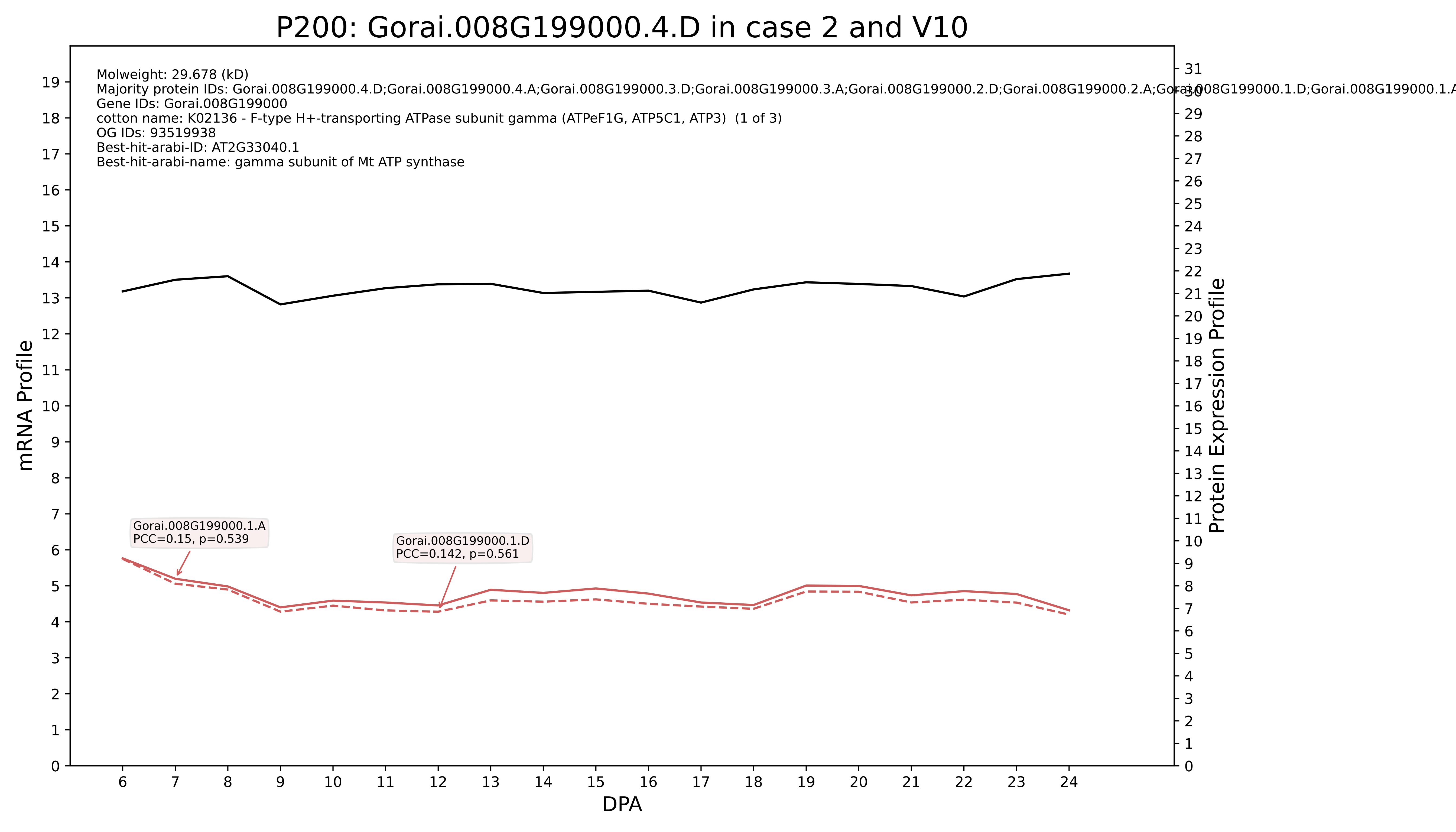
P200: Gorai.008G199000.4.D in case 2 and V10
Molweight: 29.678 (kD)
Majority protein IDs: Gorai.008G199000.4.D;Gorai.008G199000.4.A;Gorai.008G199000.3.D;Gorai.008G199000.3.A;Gorai.008G199000.2.D;Gorai.008G199000.2.A;Gorai.008G199000.1.D;Gorai.008G199000.1.A
Gene IDs: Gorai.008G199000
cotton name: K02136 - F-type H+-transporting ATPase subunit gamma (ATPeF1G, ATP5C1, ATP3) (1 of 3)
OG IDs: 93519938
Best-hit-arabi-ID: AT2G33040.1
Best-hit-arabi-name: gamma subunit of Mt ATP synthase
Gorai.008G199000.1.A
PCC=0.15, p=0.539
Gorai.008G199000.1.D
PCC=0.142, p=0.561
mRNA Profile
Protein Expression Profile
DPA

P200: Gorai.011G245600.2.D in case 2 and V10

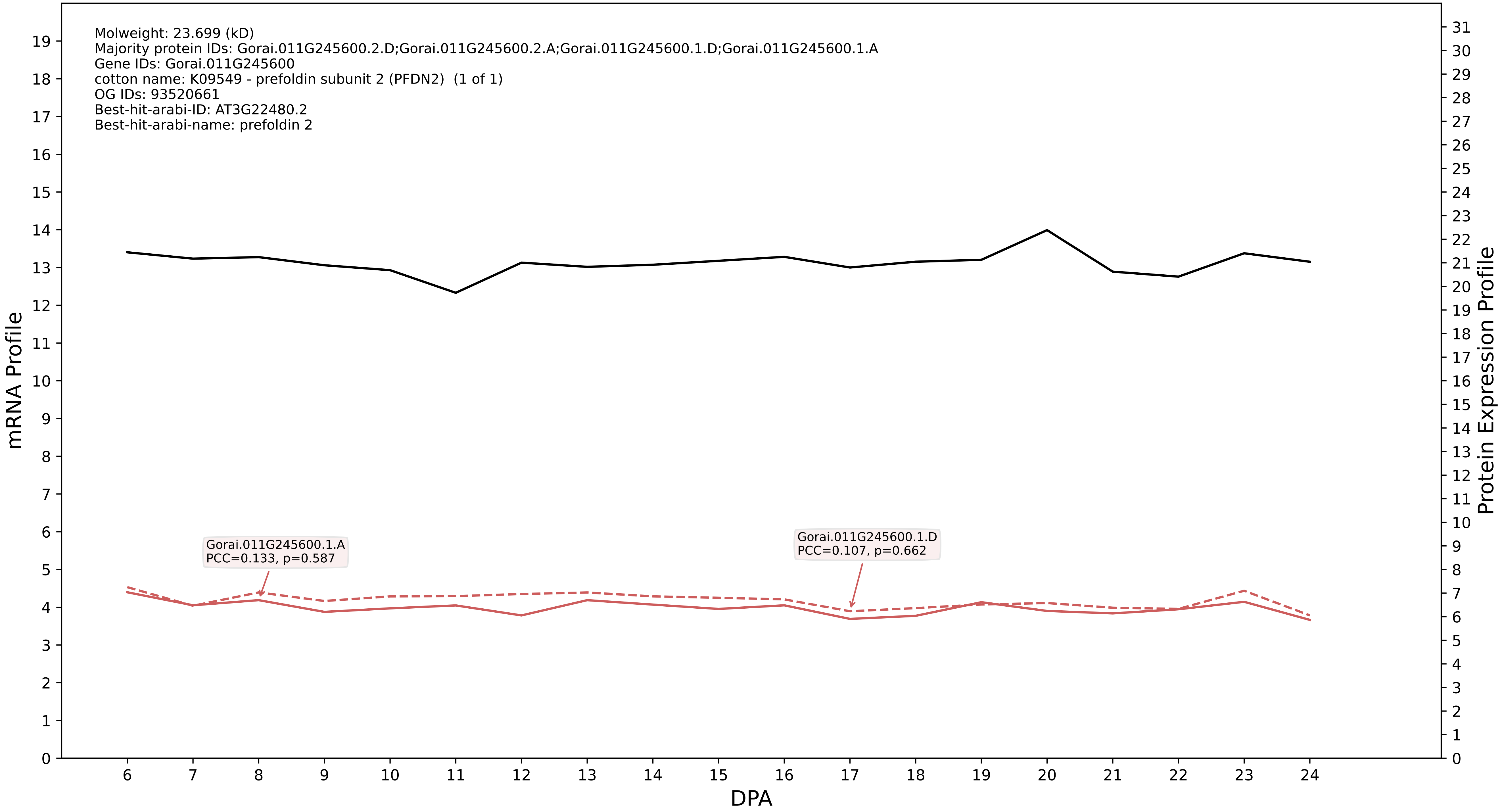

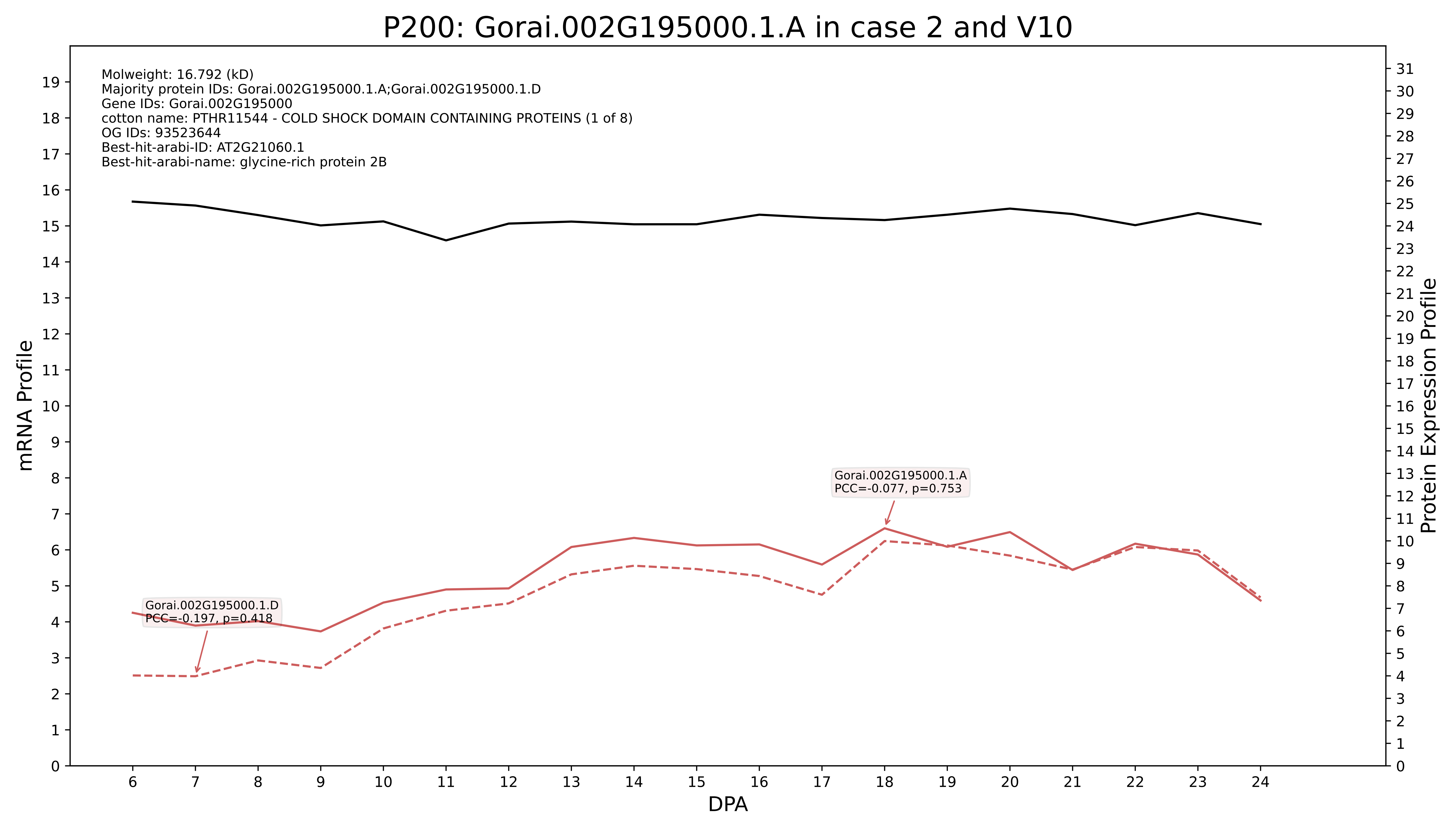
P200: Gorai.002G195000.1.A in case 2 and V10
Molweight: 16.792 (kD)
Majority protein IDs: Gorai.002G195000.1.A;Gorai.002G195000.1.D
Gene IDs: Gorai.002G195000
cotton name: PTHR11544 - COLD SHOCK DOMAIN CONTAINING PROTEINS (1 of 8)
OG IDs: 93523644
Best-hit-arabi-ID: AT2G21060.1
Best-hit-arabi-name: glycine-rich protein 2B
Gorai.002G195000.1.A
PCC=-0.077, p=0.753
Gorai.002G195000.1.D
PCC=-0.197, p=0.418
mRNA Profile
Protein Expression Profile
DPA

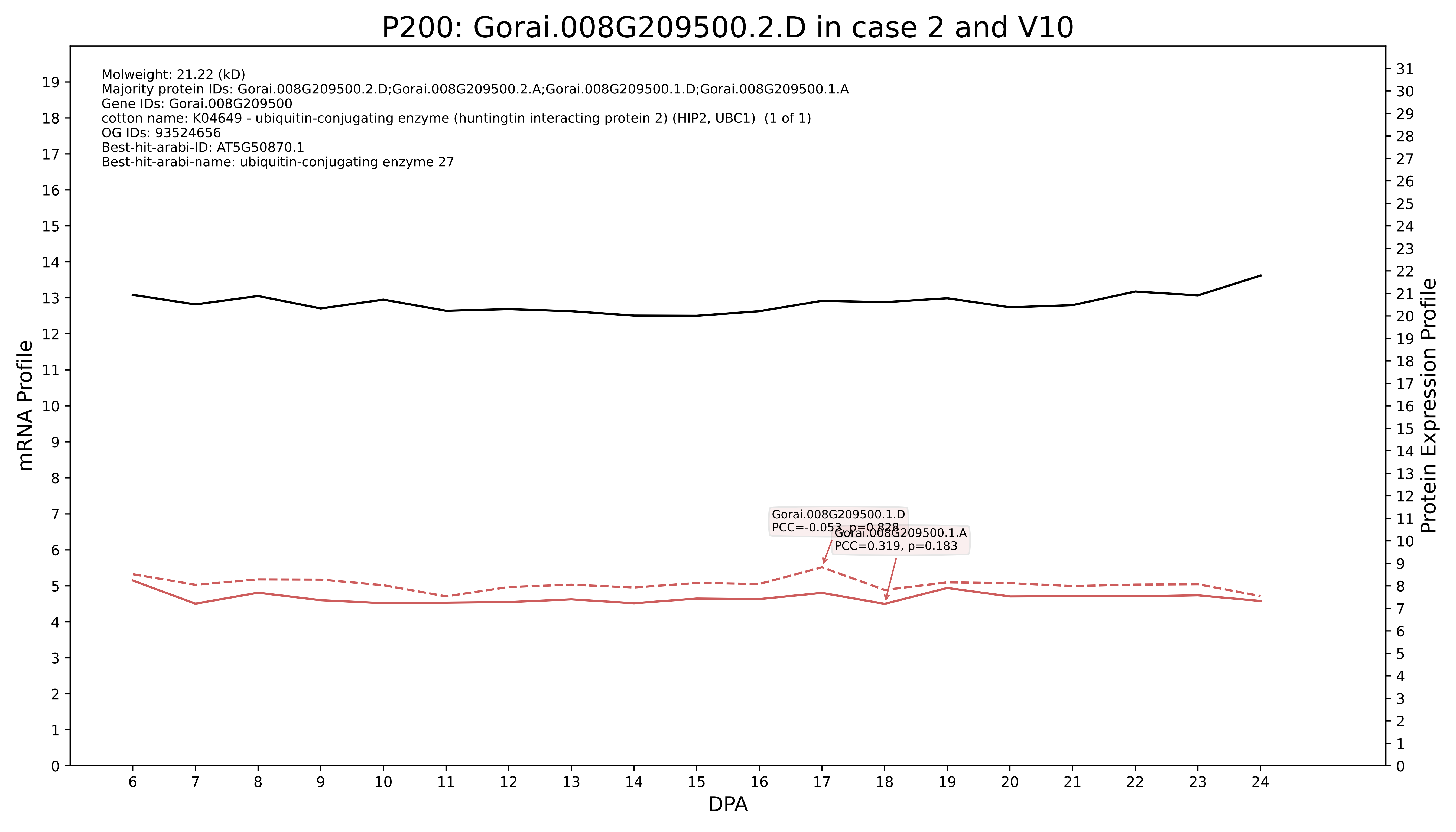
P200: Gorai.008G209500.2.D in case 2 and V10
Molweight: 21.22 (kD)
Majority protein IDs: Gorai.008G209500.2.D;Gorai.008G209500.2.A;Gorai.008G209500.1.D;Gorai.008G209500.1.A
Gene IDs: Gorai.008G209500
cotton name: K04649 - ubiquitin-conjugating enzyme (huntingtin interacting protein 2) (HIP2, UBC1) (1 of 1)
OG IDs: 93524656
Best-hit-arabi-ID: AT5G50870.1
Best-hit-arabi-name: ubiquitin-conjugating enzyme 27
Gorai.008G209500.1.D
PCC=-0.053, p=0.828
Gorai.008G209500.1.A
PCC=0.319, p=0.183
mRNA Profile
Protein Expression Profile
DPA
0
1
2
3
4
5
6
7
8
9
10
11
12
13
14
15
16
17
18
19
20
21
22
23
24
25
26
27
28
29
30
31

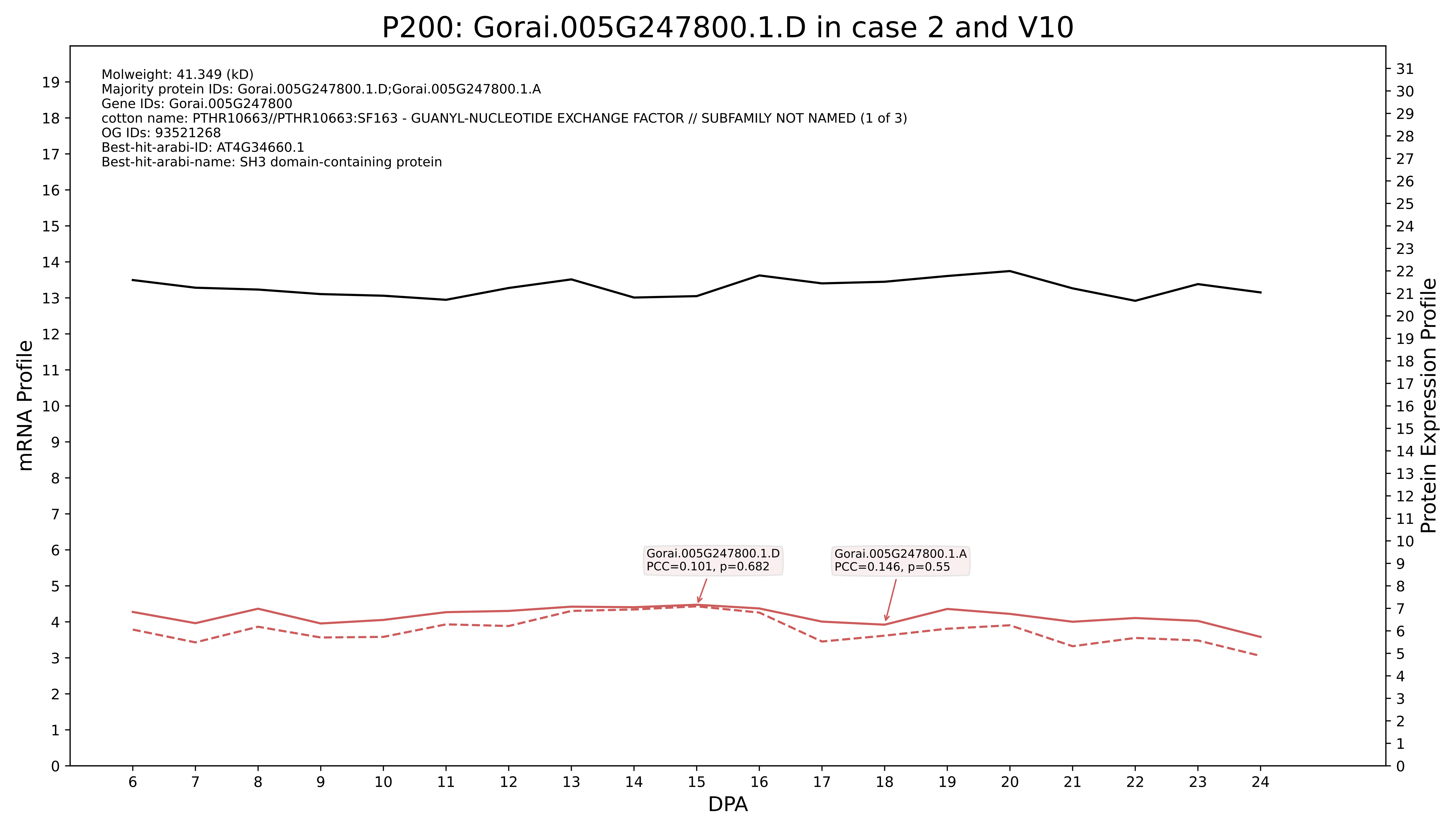
P200: Gorai.005G247800.1.D in case 2 and V10
Molweight: 41.349 (kD)
Majority protein IDs: Gorai.005G247800.1.D;Gorai.005G247800.1.A
Gene IDs: Gorai.005G247800
cotton name: PTHR10663//PTHR10663:SF163 - GUANYL-NUCLEOTIDE EXCHANGE FACTOR // SUBFAMILY NOT NAMED (1 of 3)
OG IDs: 93521268
Best-hit-arabi-ID: AT4G34660.1
Best-hit-arabi-name: SH3 domain-containing protein
Gorai.005G247800.1.D
PCC=0.101, p=0.682
Gorai.005G247800.1.A
PCC=0.146, p=0.55
mRNA Profile
Protein Expression Profile
DPA

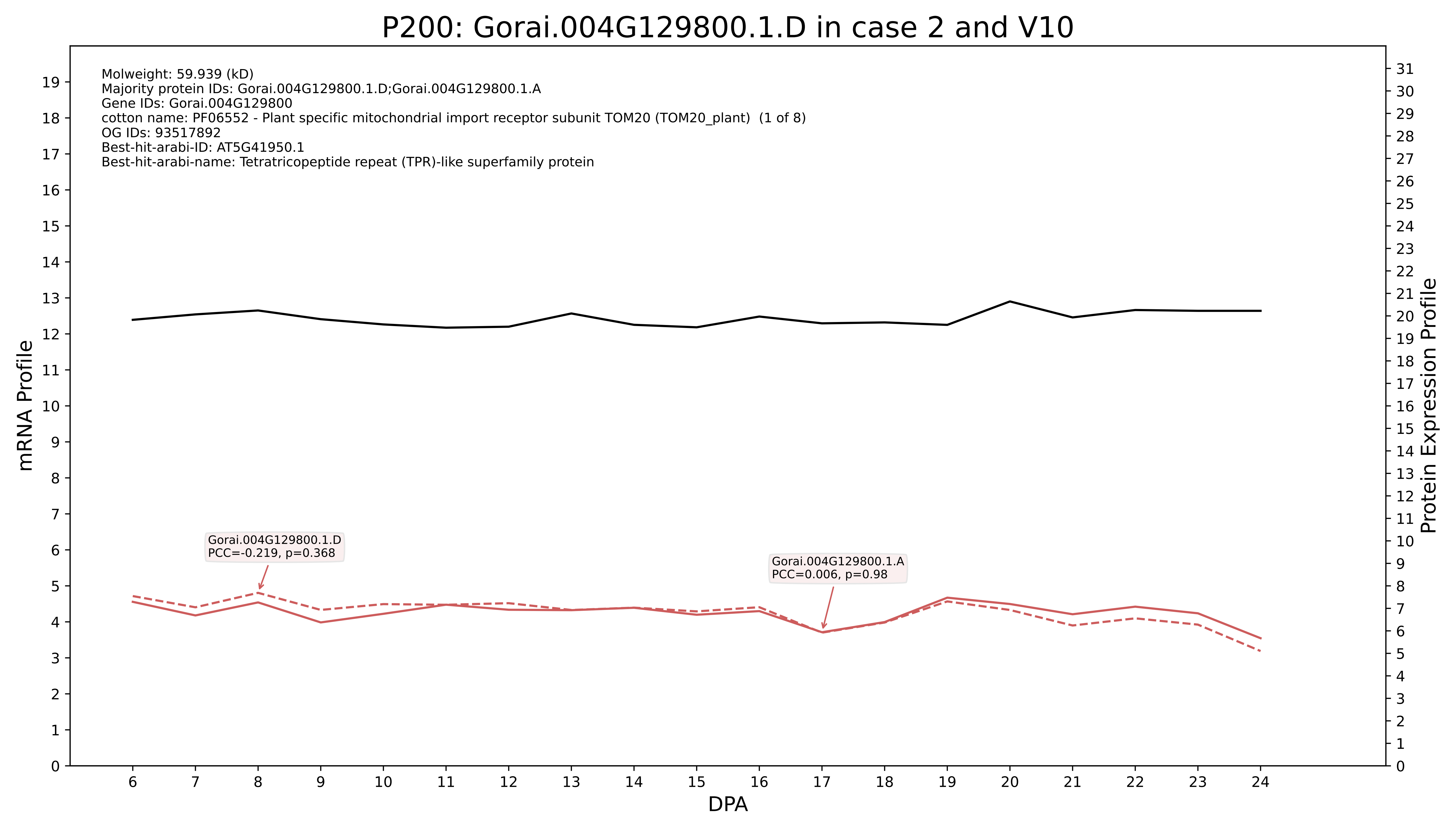

P200: Gorai.004G129800.1.D in case 2 and V10
Molweight: 59.939 (kD)
Majority protein IDs: Gorai.004G129800.1.D;Gorai.004G129800.1.A
Gene IDs: Gorai.004G129800
cotton name: PF06552 - Plant specific mitochondrial import receptor subunit TOM20 (TOM20_plant) (1 of 8)
OG IDs: 93517892
Best-hit-arabi-ID: AT5G41950.1
Best-hit-arabi-name: Tetratricopeptide repeat (TPR)-like superfamily protein
Gorai.004G129800.1.D
PCC=-0.219, p=0.368
Gorai.004G129800.1.A
PCC=0.006, p=0.98
mRNA Profile
Protein Expression Profile
DPA

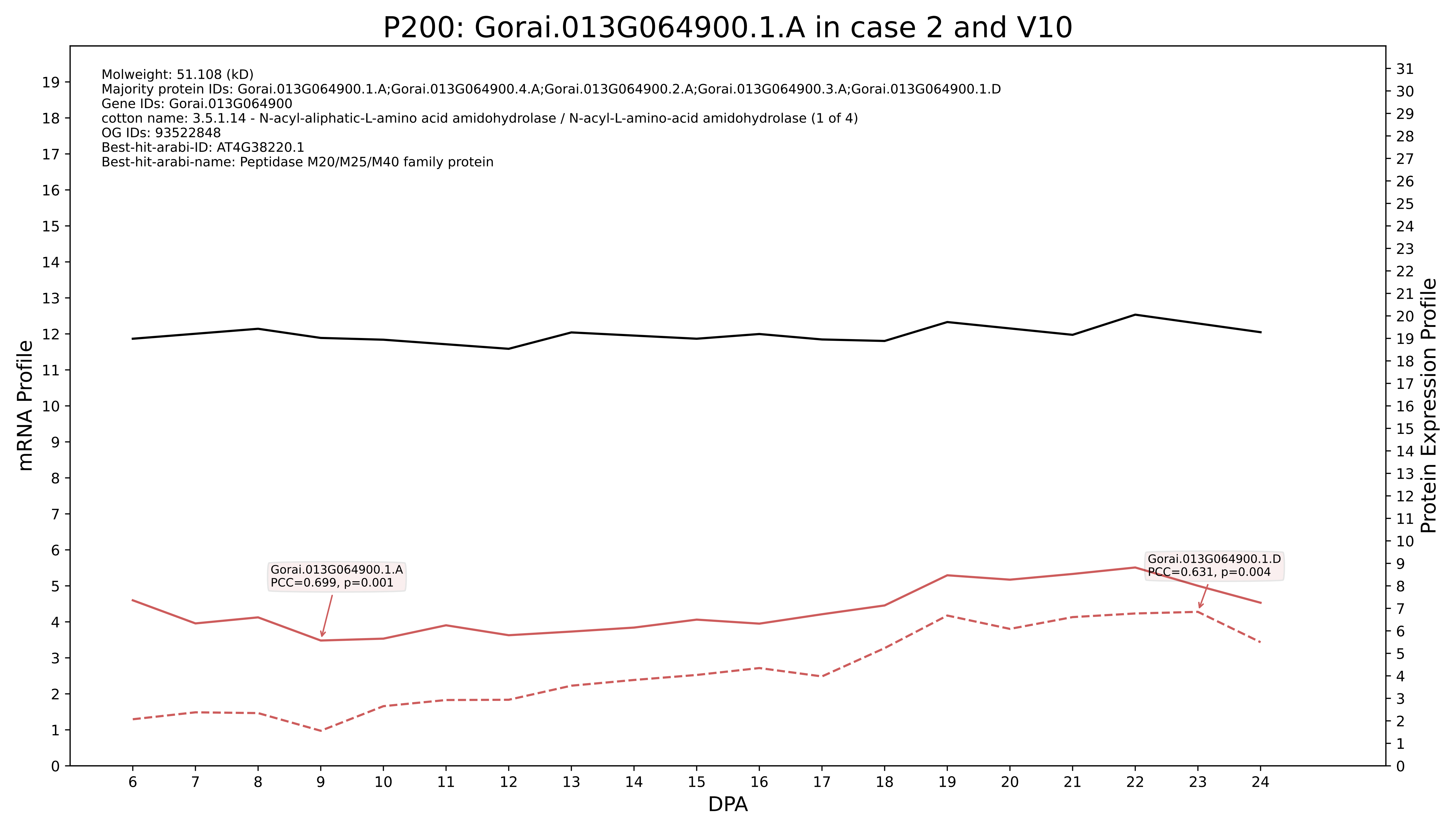
P200: Gorai.013G064900.1.A in case 2 and V10
Molweight: 51.108 (kD)
Majority protein IDs: Gorai.013G064900.1.A;Gorai.013G064900.4.A;Gorai.013G064900.2.A;Gorai.013G064900.3.A;Gorai.013G064900.1.D
Gene IDs: Gorai.013G064900
cotton name: 3.5.1.14 - N-acyl-aliphatic-L-amino acid amidohydrolase / N-acyl-L-amino-acid amidohydrolase (1 of 4)
OG IDs: 93522848
Best-hit-arabi-ID: AT4G38220.1
Best-hit-arabi-name: Peptidase M20/M25/M40 family protein
Gorai.013G064900.1.A
PCC=0.699, p=0.001
Gorai.013G064900.1.D
PCC=0.631, p=0.004
mRNA Profile
Protein Expression Profile
DPA

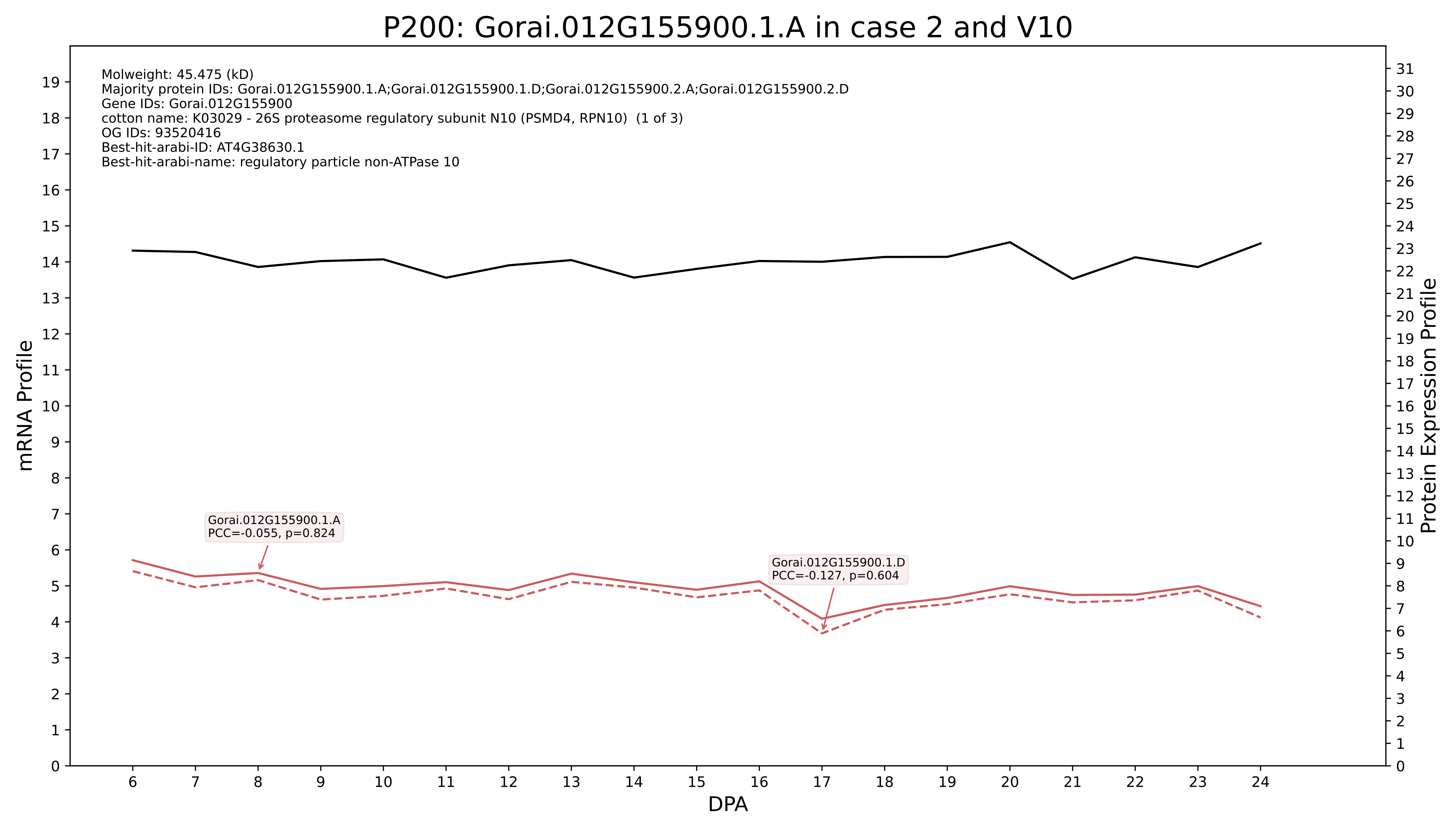
P200: Gorai.012G155900.1.A in case 2 and V10
Molweight: 45.475 (kD)
Majority protein IDs: Gorai.012G155900.1.A;Gorai.012G155900.1.D;Gorai.012G155900.2.A;Gorai.012G155900.2.D
Gene IDs: Gorai.012G155900
cotton name: K03029 - 26S proteasome regulatory subunit N10 (PSMD4, RPN10) (1 of 3)
OG IDs: 93520416
Best-hit-arabi-ID: AT4G38630.1
Best-hit-arabi-name: regulatory particle non-ATPase 10
Gorai.012G155900.1.A
PCC=-0.055, p=0.824
Gorai.012G155900.1.D
PCC=-0.127, p=0.604
mRNA Profile
Protein Expression Profile
DPA

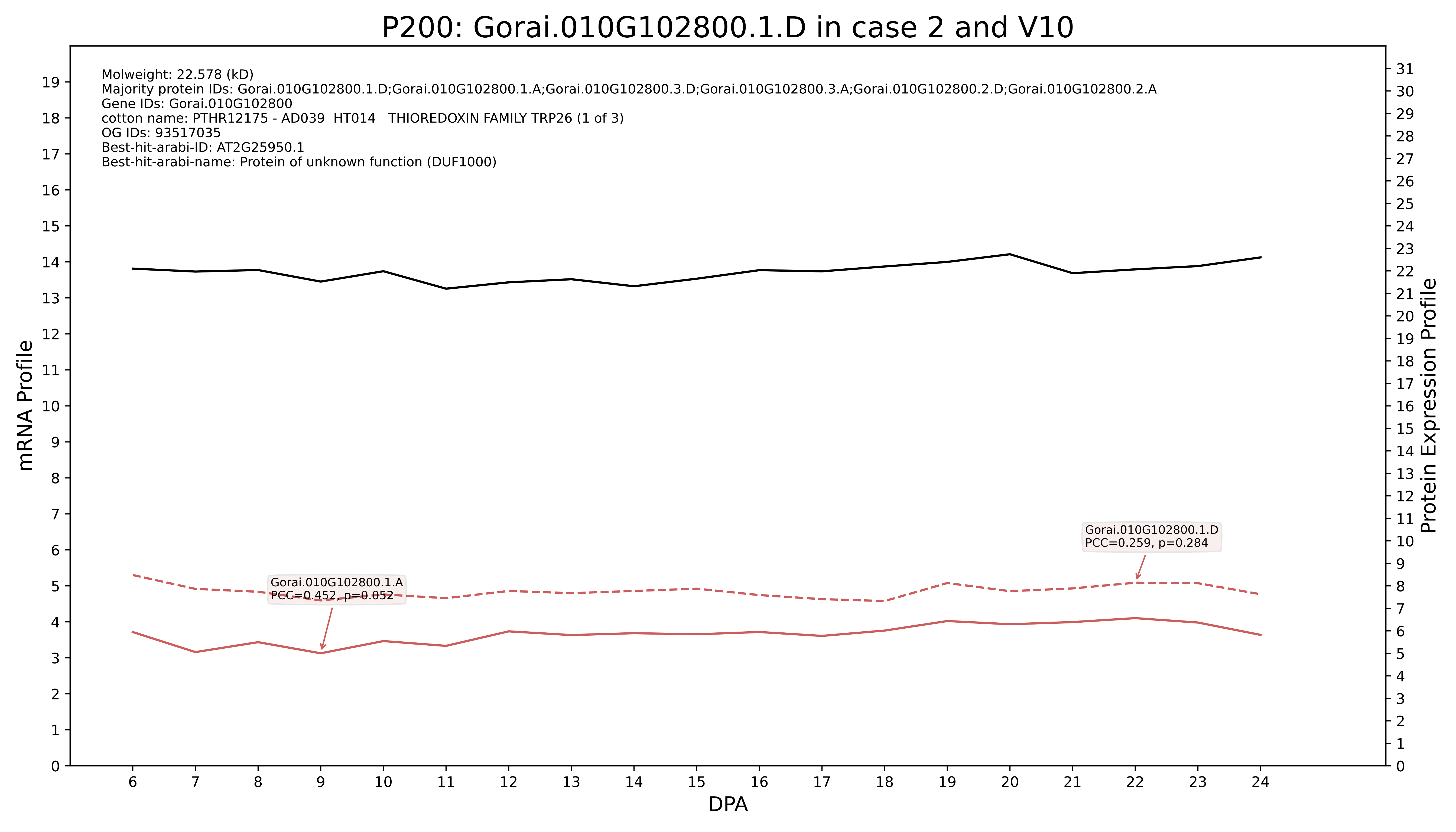

P200: Gorai.010G102800.1.D in case 2 and V10
Molweight: 22.578 (kD)
Majority protein IDs: Gorai.010G102800.1.D;Gorai.010G102800.1.A;Gorai.010G102800.3.D;Gorai.010G102800.3.A;Gorai.010G102800.2.D;Gorai.010G102800.2.A
Gene IDs: Gorai.010G102800
cotton name: PTHR12175 - AD039 HT014 THIOREDOXIN FAMILY TRP26 (1 of 3)
OG IDs: 93517035
Best-hit-arabi-ID: AT2G25950.1
Best-hit-arabi-name: Protein of unknown function (DUF1000)
Gorai.010G102800.1.A
PCC=0.452, p=0.052
Gorai.010G102800.1.D
PCC=0.259, p=0.284
mRNA Profile
Protein Expression Profile
DPA

P200: Gorai.006G064700.1.A in case 3 and V10

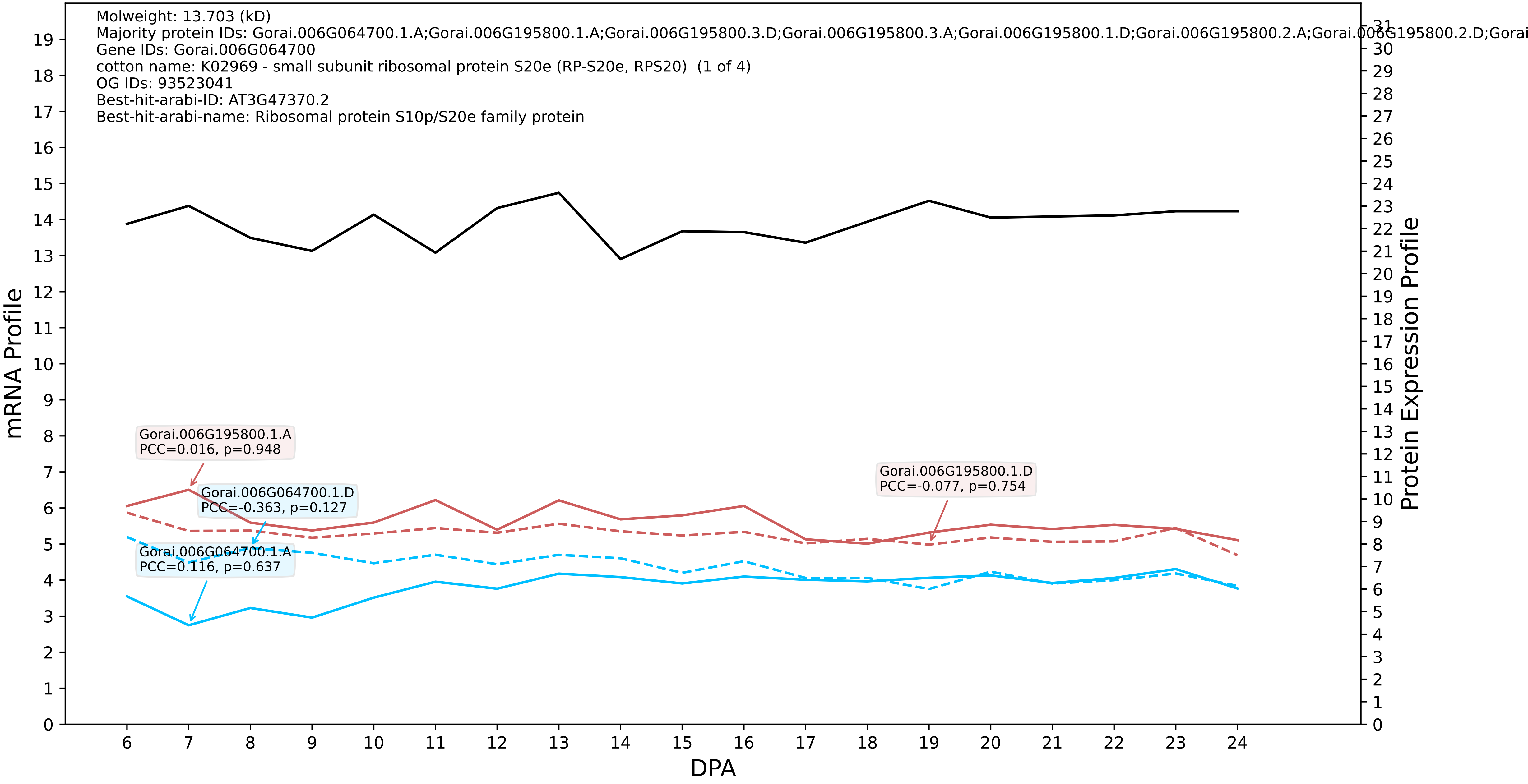

00: Gorai.013G163400.1.D in case 3 and V10

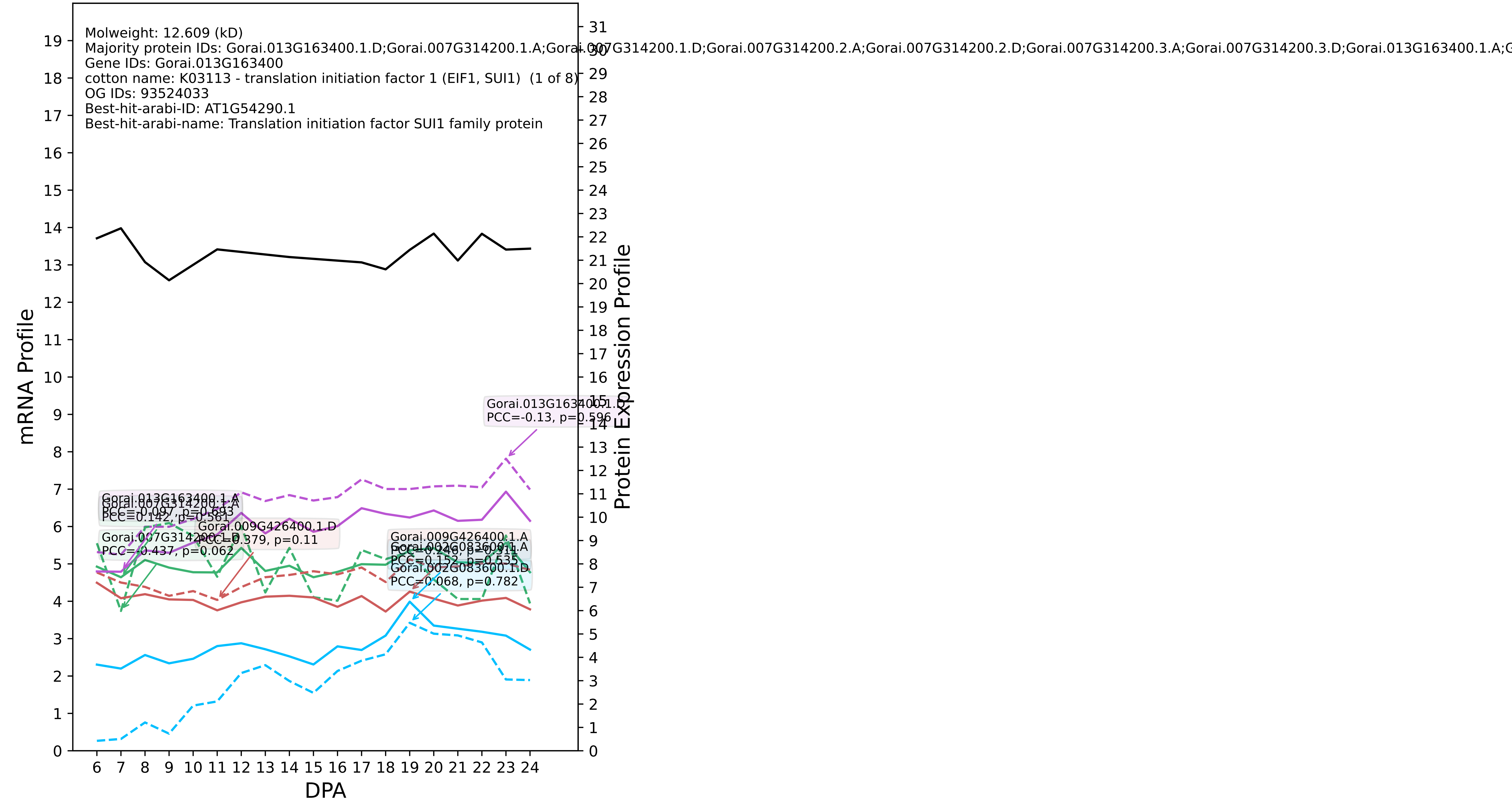

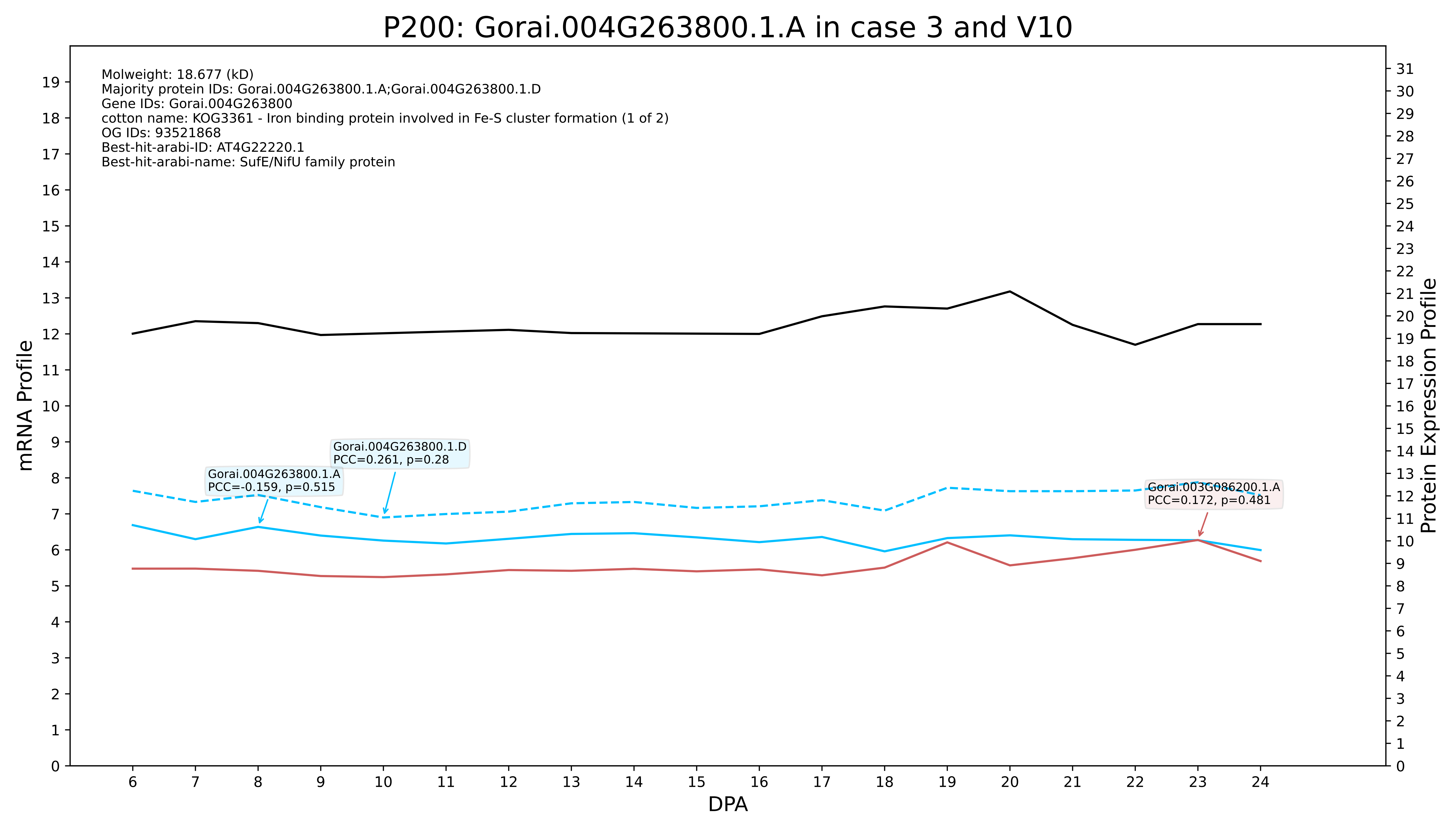
P200: Gorai.004G263800.1.A in case 3 and V10
Molweight: 18.677 (kD)
Majority protein IDs: Gorai.004G263800.1.A;Gorai.004G263800.1.D
Gene IDs: Gorai.004G263800
cotton name: KOG3361 - Iron binding protein involved in Fe-S cluster formation (1 of 2)
OG IDs: 93521868
Best-hit-arabi-ID: AT4G22220.1
Best-hit-arabi-name: SufE/NifU family protein
Gorai.004G263800.1.D
PCC=0.261, p=0.28
Gorai.004G263800.1.A
PCC=-0.159, p=0.515
Gorai.003G086200.1.A
PCC=0.172, p=0.481
mRNA Profile
Protein Expression Profile
DPA

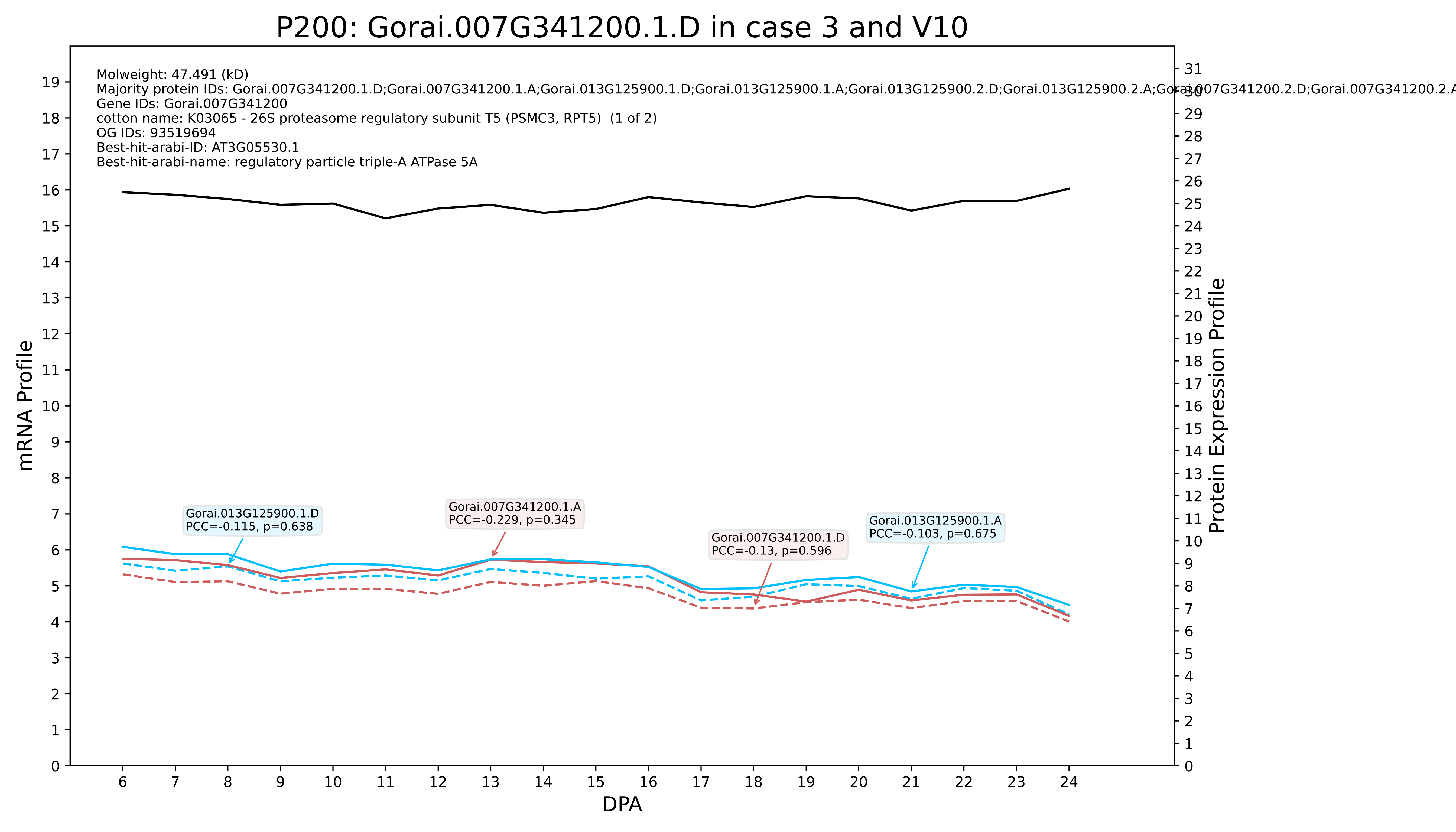
P200: Gorai.007G341200.1.D in case 3 and V10
Molweight: 47.491 (kD)
Majority protein IDs: Gorai.007G341200.1.D;Gorai.007G341200.1.A;Gorai.013G125900.1.D;Gorai.013G125900.1.A;Gorai.013G125900.2.D;Gorai.013G125900.2.A;Gorai.007G341200.2.D;Gorai.007G341200.2.A
Gene IDs: Gorai.007G341200
cotton name: K03065 - 26S proteasome regulatory subunit T5 (PSMC3, RPT5) (1 of 2)
OG IDs: 93519694
Best-hit-arabi-ID: AT3G05530.1
Best-hit-arabi-name: regulatory particle triple-A ATPase 5A
Gorai.013G125900.1.D
PCC=-0.115, p=0.638
Gorai.007G341200.1.A
PCC=-0.229, p=0.345
Gorai.007G341200.1.D
PCC=-0.13, p=0.596
Gorai.013G125900.1.A
PCC=-0.103, p=0.675
mRNA Profile
Protein Expression Profile
DPA

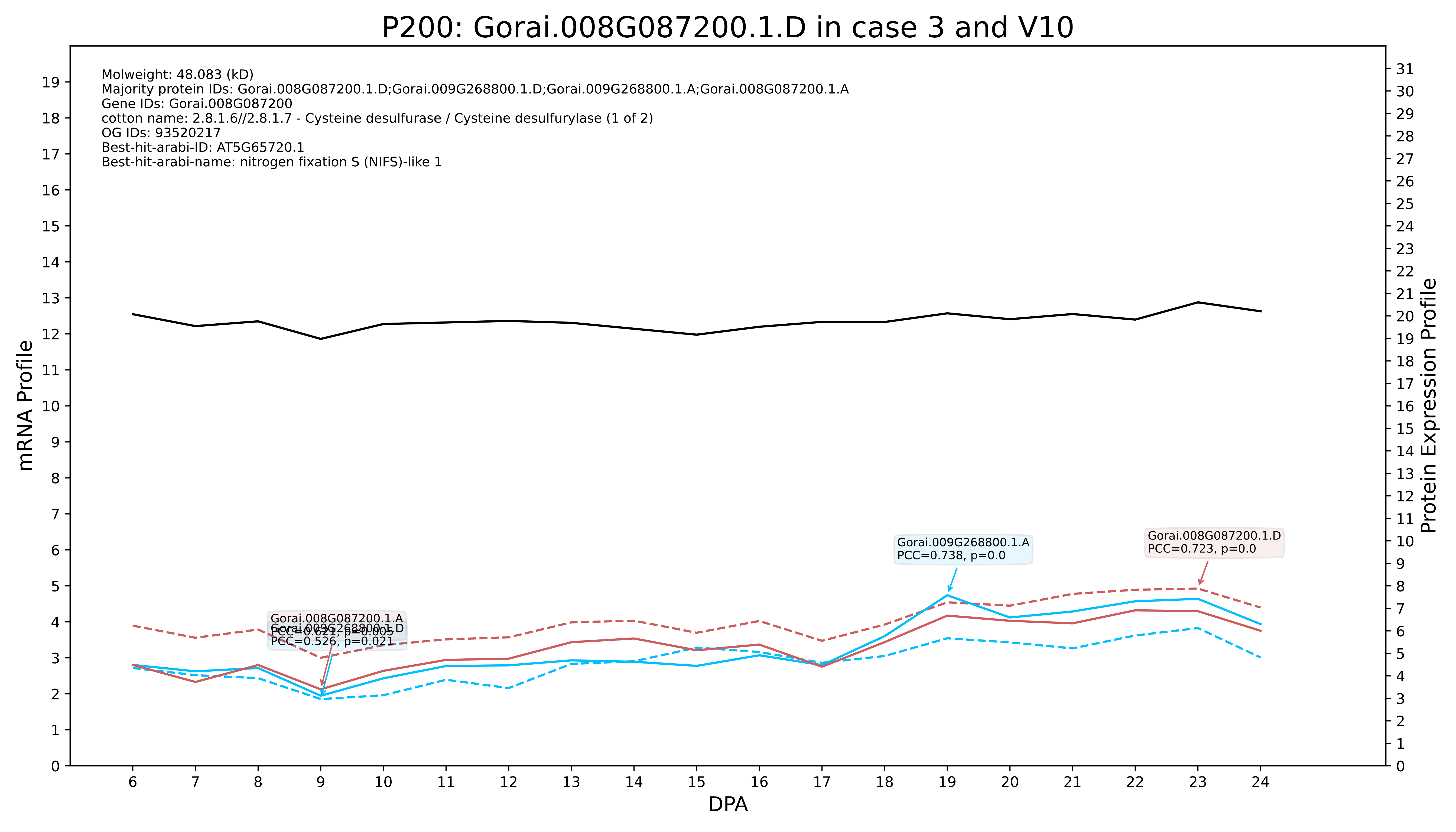
P200: Gorai.008G087200.1.D in case 3 and V10
Molweight: 48.083 (kD)
Majority protein IDs: Gorai.008G087200.1.D;Gorai.009G268800.1.D;Gorai.009G268800.1.A;Gorai.008G087200.1.A
Gene IDs: Gorai.008G087200
cotton name: 2.8.1.6//2.8.1.7 - Cysteine desulfurase / Cysteine desulfurylase (1 of 2)
OG IDs: 93520217
Best-hit-arabi-ID: AT5G65720.1
Best-hit-arabi-name: nitrogen fixation S (NIFS)-like 1
Gorai.008G087200.1.A
Gorai.009G268800.1.D
PCC=0.526, p=0.021
Gorai.009G268800.1.A
PCC=0.738, p=0.0
Gorai.008G087200.1.D
PCC=0.723, p=0.0
mRNA Profile
Protein Expression Profile
DPA

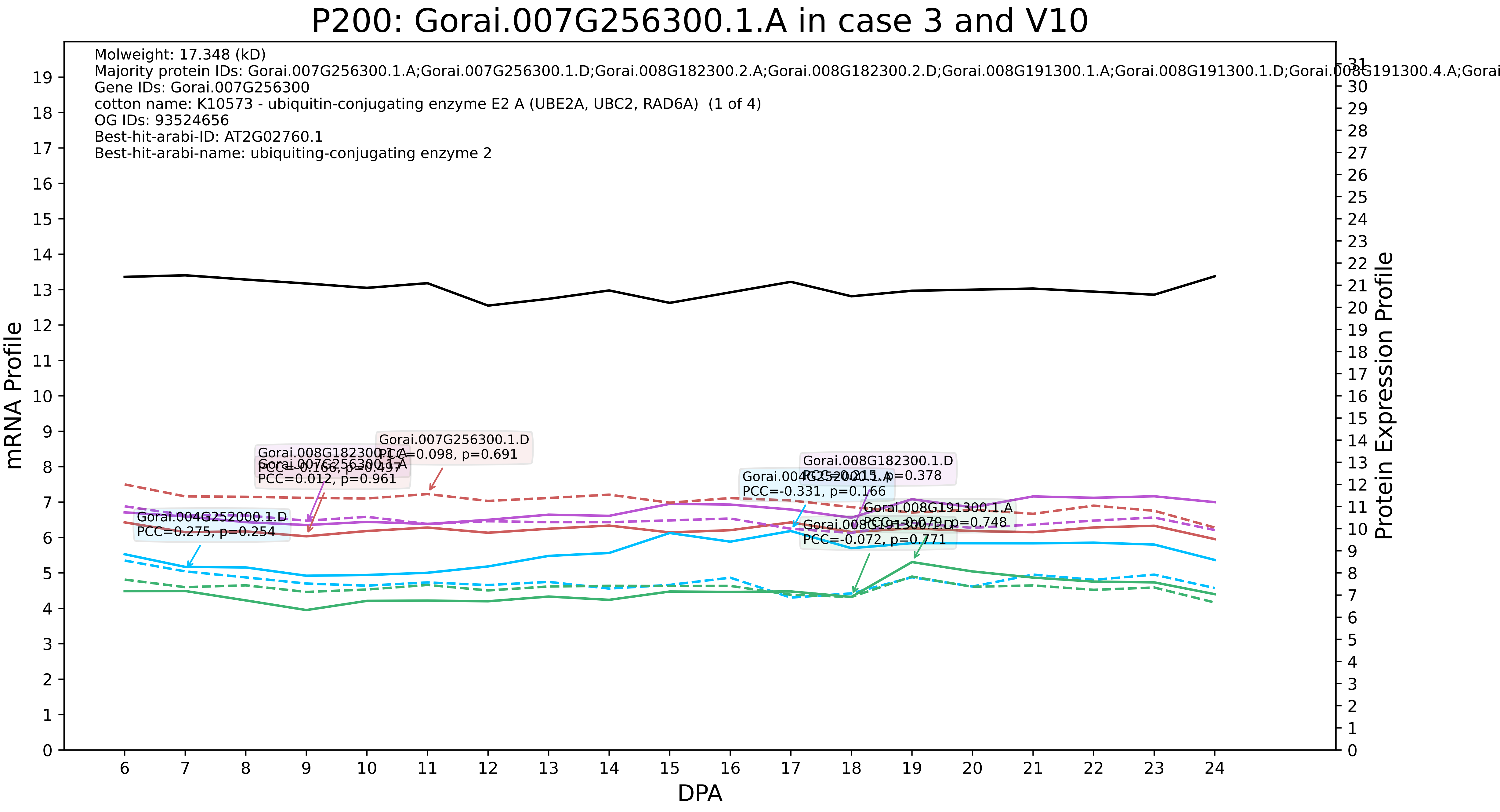

P200: Gorai.007G256300.1.A in case 3 and V10
Molweight: 17.348 (kD)
Majority protein IDs: Gorai.007G256300.1.A;Gorai.007G256300.1.D;Gorai.008G182300.2.A;Gorai.008G182300.2.D;Gorai.008G191300.1.A;Gorai.008G191300.1.D;Gorai.008G191300.4.A;Gorai
Gene IDs: Gorai.007G256300
cotton name: K10573 - ubiquitin-conjugating enzyme E2 A (UBE2A, UBC2, RAD6A) (1 of 4)
OG IDs: 93524656
Best-hit-arabi-ID: AT2G02760.1
Best-hit-arabi-name: ubiquiting-conjugating enzyme 2
mRNA Profile
Protein Expression Profile
DPA
Gorai.007G256300.1.D
PCC=0.098, p=0.691
Gorai.008G182300.1.D
PCC=0.215, p=0.378
Gorai.004G252000.1.D
PCC=0.275, p=0.254
Gorai.008G191300.1.A
PCC=0.079, p=0.748
PCC=-0.331, p=0.166
PCC=-0.072, p=0.771
PCC=0.012, p=0.961

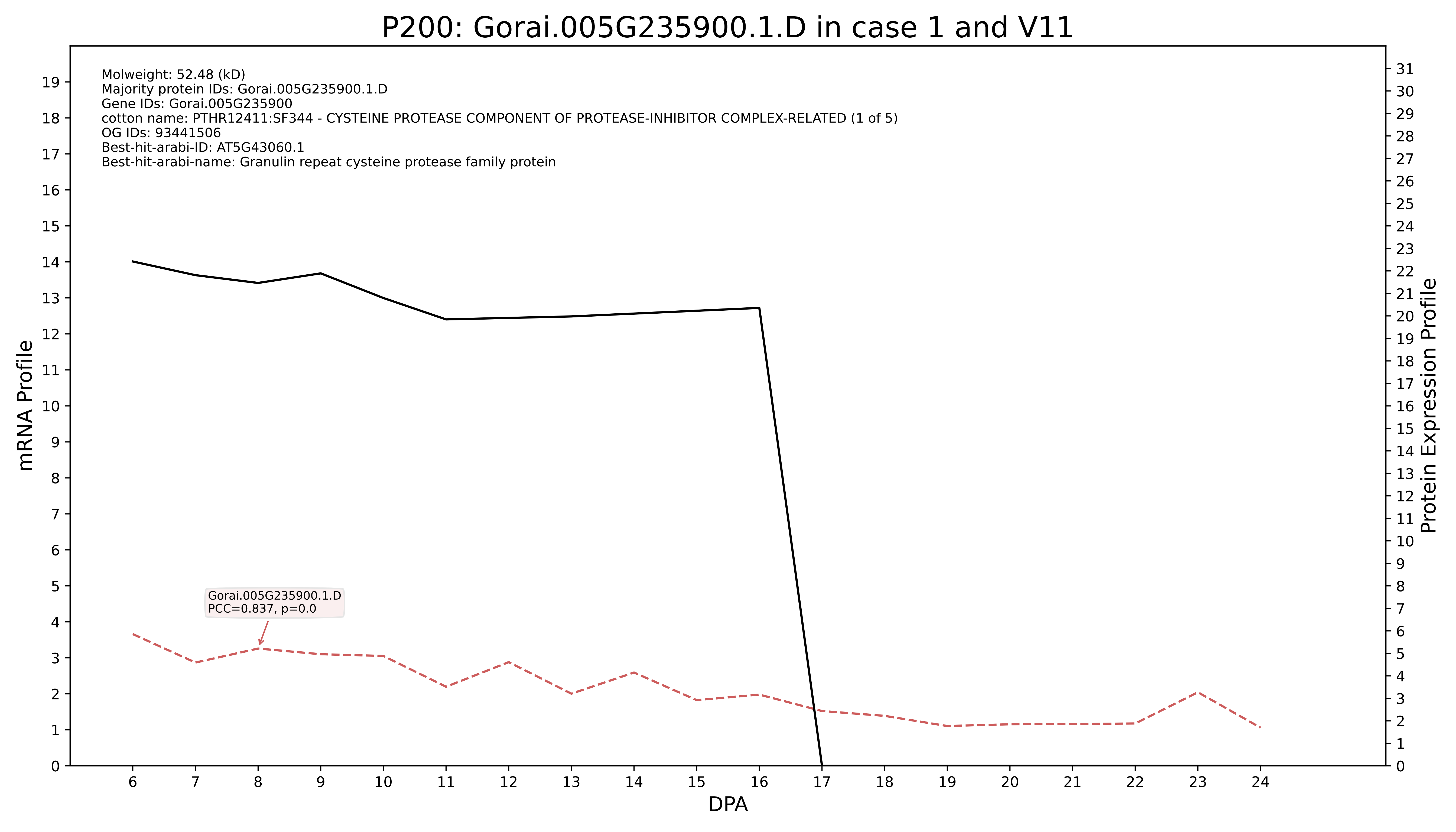

P200: Gorai.005G235900.1.D in case 1 and V11
Molweight: 52.48 (kD)
Majority protein IDs: Gorai.005G235900.1.D
Gene IDs: Gorai.005G235900
cotton name: PTHR12411:SF344 - CYSTEINE PROTEASE COMPONENT OF PROTEASE-INHIBITOR COMPLEX-RELATED (1 of 5)
OG IDs: 93441506
Best-hit-arabi-ID: AT5G43060.1
Best-hit-arabi-name: Granulin repeat cysteine protease family protein
Gorai.005G235900.1.D
PCC=0.837, p=0.0
mRNA Profile
Protein Expression Profile
DPA

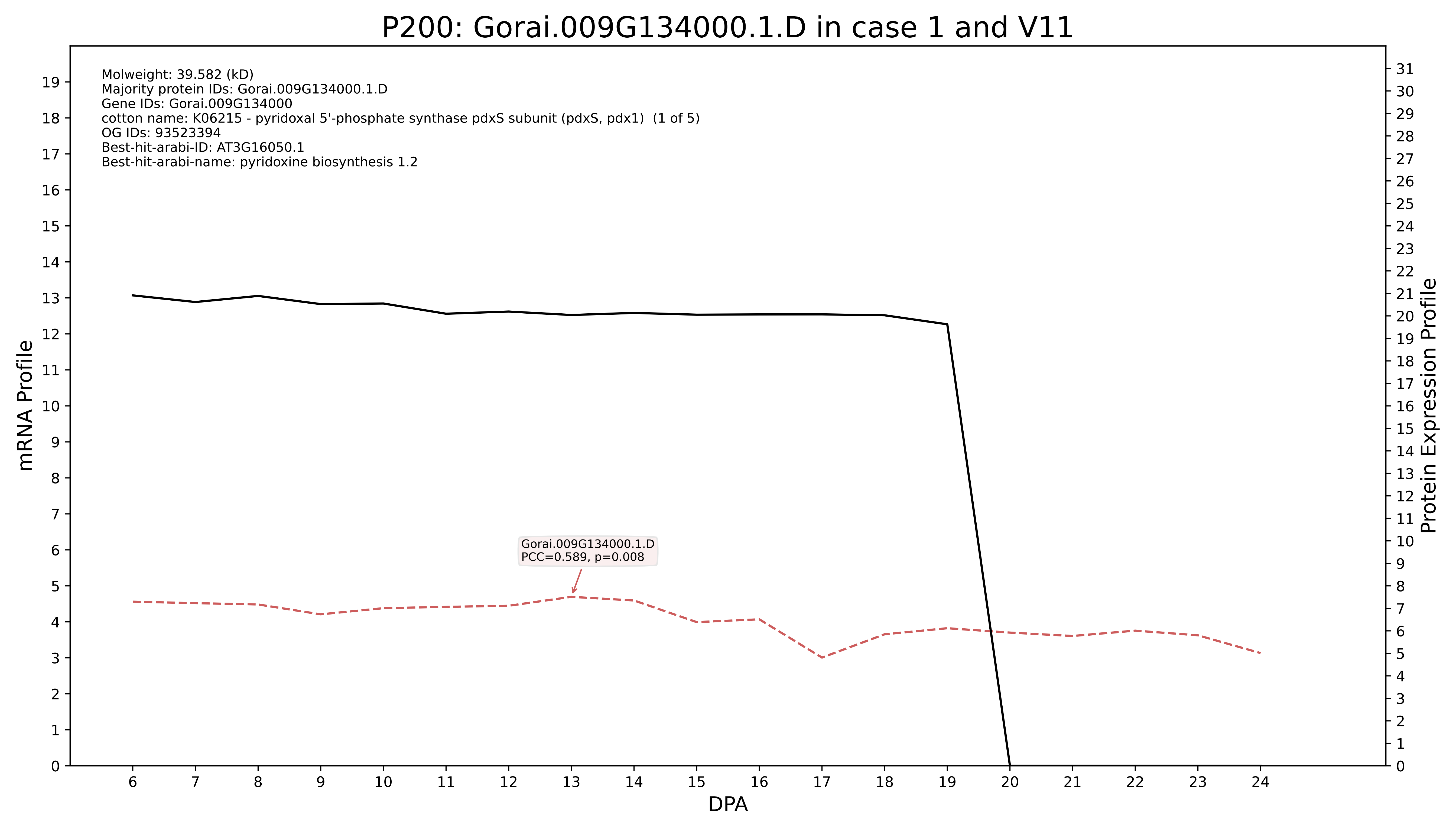
P200: Gorai.009G134000.1.D in case 1 and V11
Molweight: 39.582 (kD)
Majority protein IDs: Gorai.009G134000.1.D
Gene IDs: Gorai.009G134000
cotton name: K06215 - pyridoxal 5'-phosphate synthase pdxS subunit (pdxS, pdx1) (1 of 5)
OG IDs: 93523394
Best-hit-arabi-ID: AT3G16050.1
Best-hit-arabi-name: pyridoxine biosynthesis 1.2
Gorai.009G134000.1.D
PCC=0.589, p=0.008
mRNA Profile
Protein Expression Profile
DPA
0
1
2
3
4
5
6
7
8
9
10
11
12
13
14
15
16
17
18
19
20
21
22
23
24
25
26
27
28
29
30
31

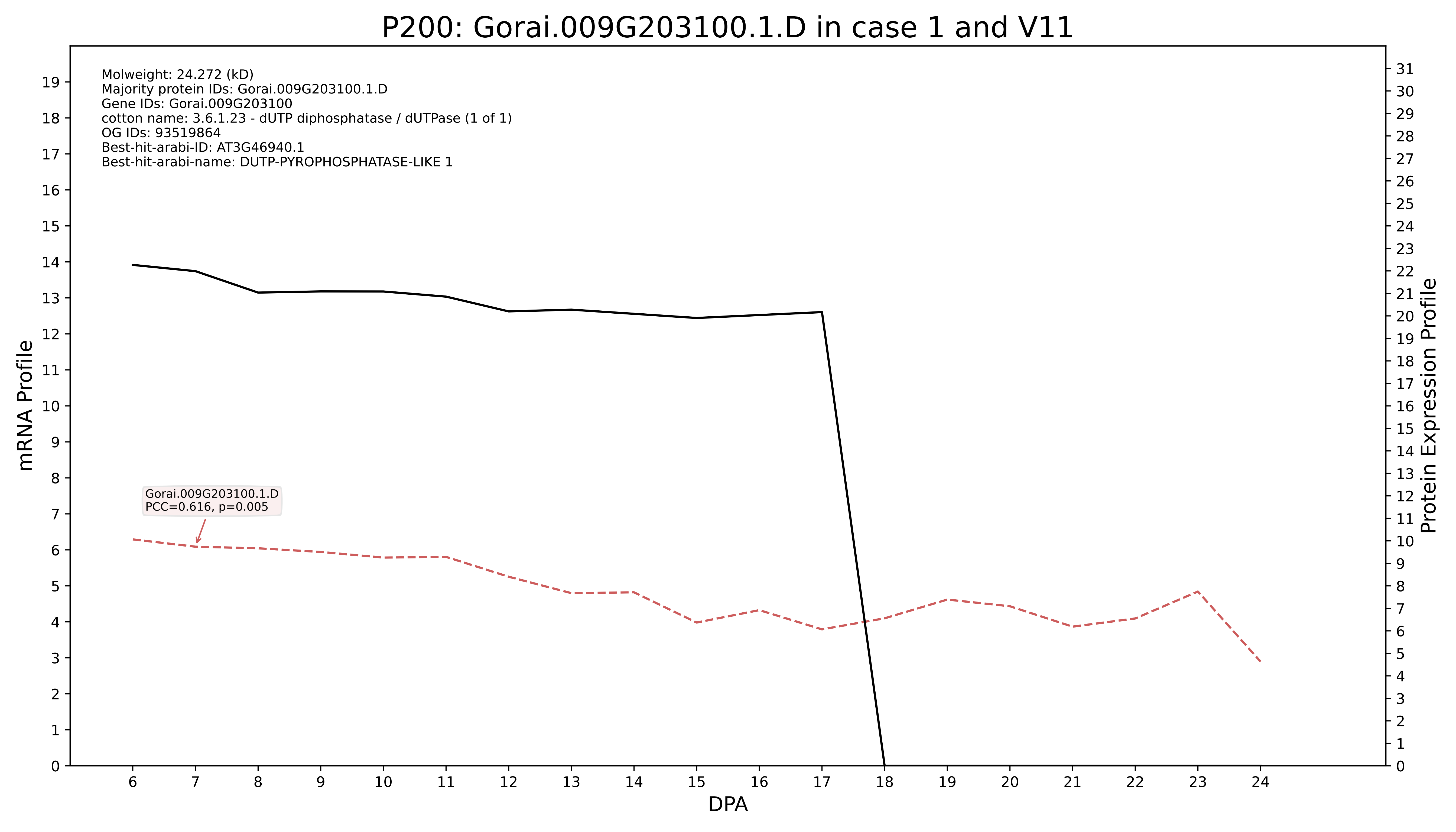

P200: Gorai.009G203100.1.D in case 1 and V11
Molweight: 24.272 (kD)
Majority protein IDs: Gorai.009G203100.1.D
Gene IDs: Gorai.009G203100
cotton name: 3.6.1.23 - dUTP diphosphatase / dUTPase (1 of 1)
OG IDs: 93519864
Best-hit-arabi-ID: AT3G46940.1
Best-hit-arabi-name: DUTP-PYROPHOSPHATASE-LIKE 1
Gorai.009G203100.1.D
PCC=0.616, p=0.005
mRNA Profile
Protein Expression Profile
DPA

P200: Gorai.001G121200.1.A in case 1 and V11

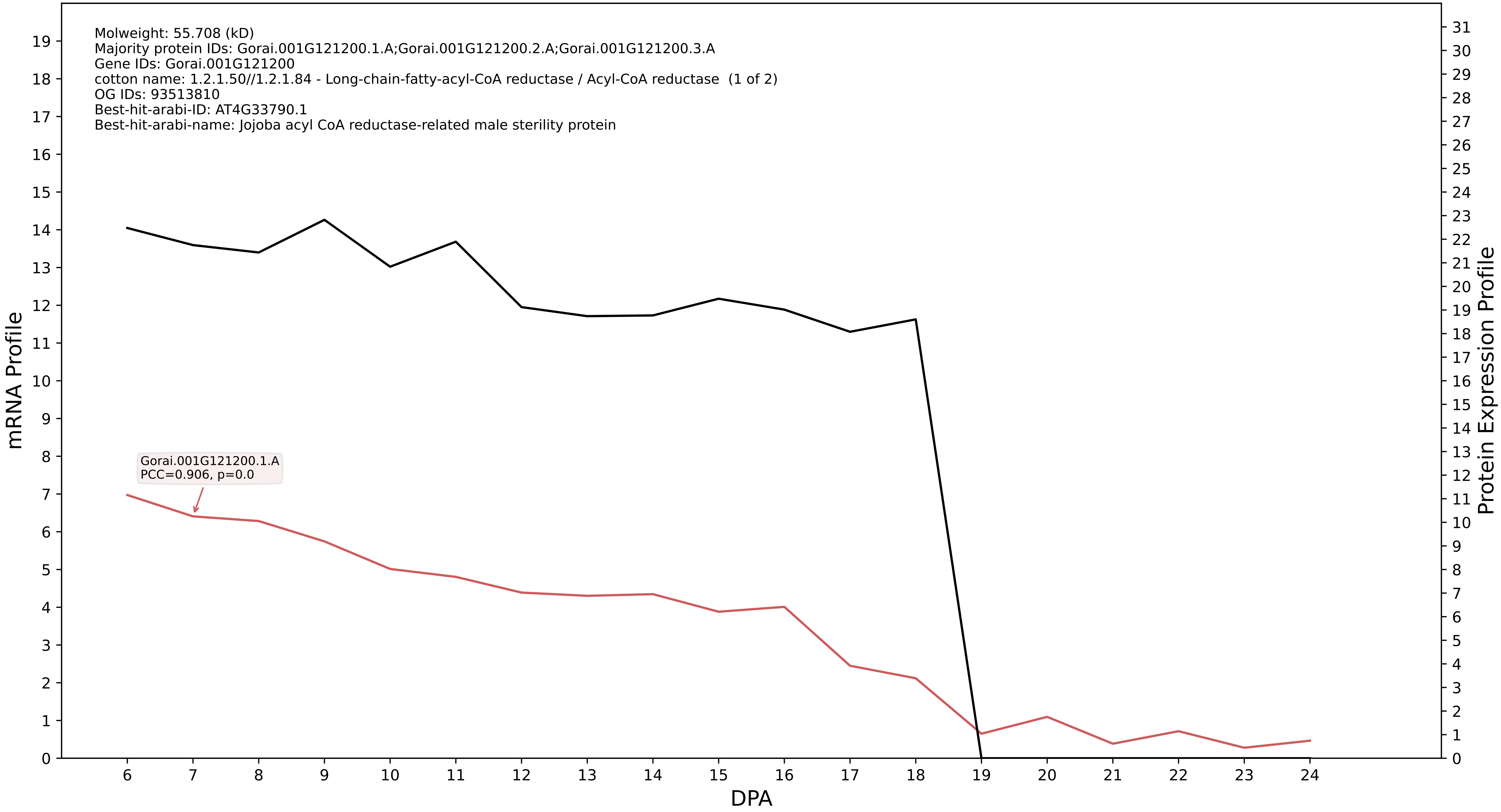

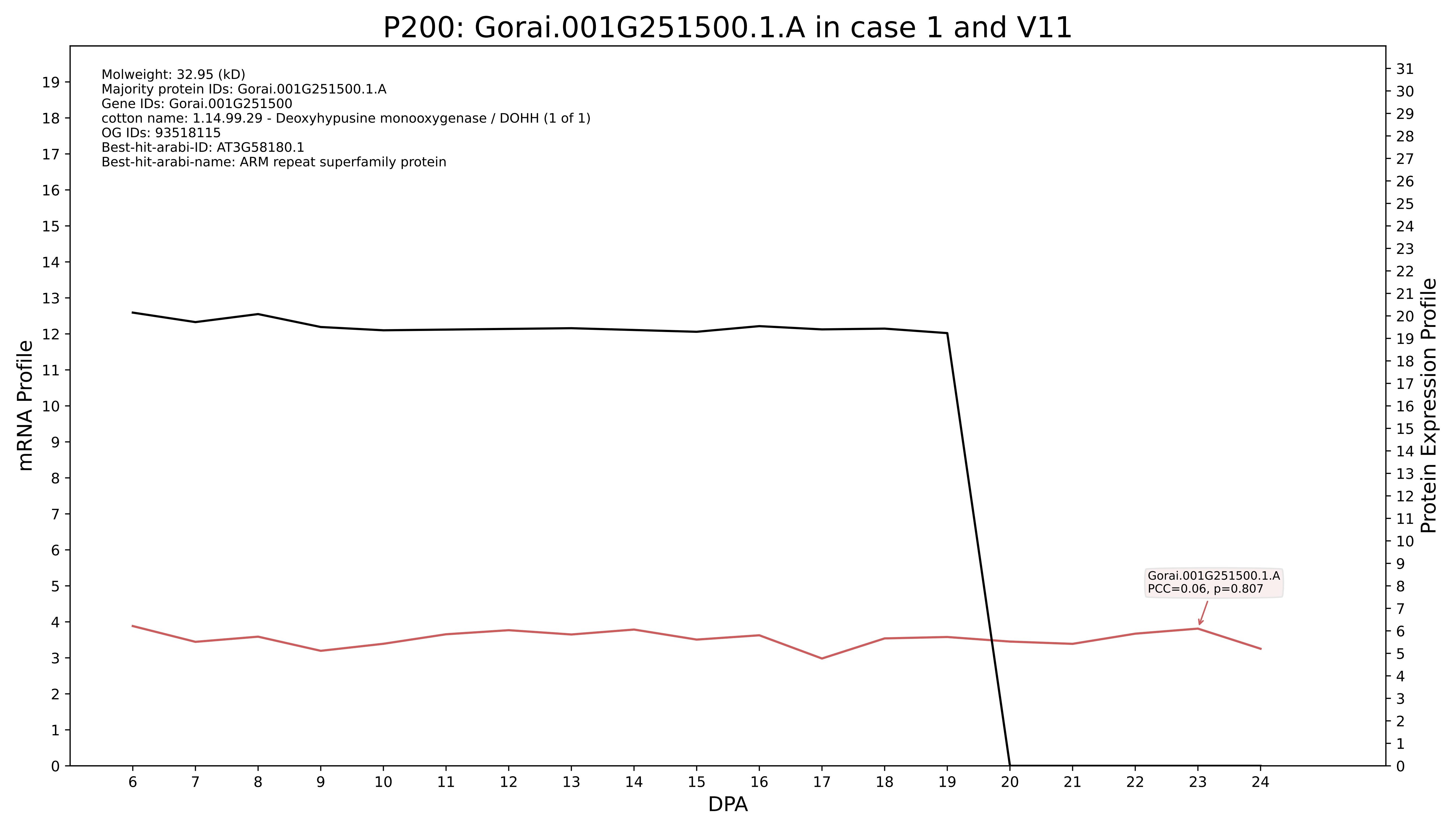

P200: Gorai.001G251500.1.A in case 1 and V11
Molweight: 32.95 (kD)
Majority protein IDs: Gorai.001G251500.1.A
Gene IDs: Gorai.001G251500
cotton name: 1.14.99.29 - Deoxyhypusine monooxygenase / DOHH (1 of 1)
OG IDs: 93518115
Best-hit-arabi-ID: AT3G58180.1
Best-hit-arabi-name: ARM repeat superfamily protein
Gorai.001G251500.1.A
PCC=0.06, p=0.807
mRNA Profile
Protein Expression Profile
DPA

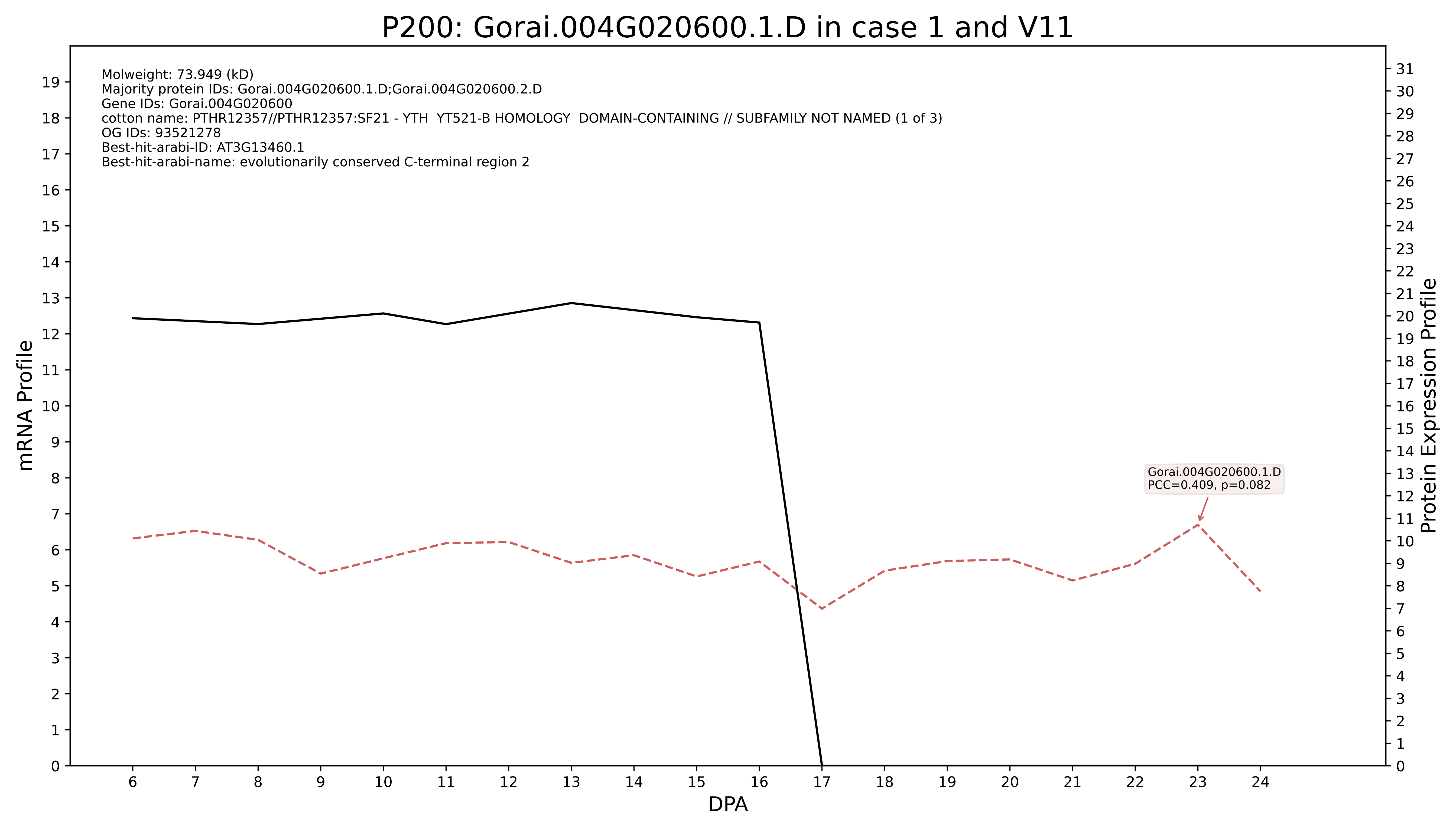
P200: Gorai.004G020600.1.D in case 1 and V11
Molweight: 73.949 (kD)
Majority protein IDs: Gorai.004G020600.1.D;Gorai.004G020600.2.D
Gene IDs: Gorai.004G020600
cotton name: PTHR12357//PTHR12357:SF21 - YTH YT521-B HOMOLOGY DOMAIN-CONTAINING // SUBFAMILY NOT NAMED (1 of 3)
OG IDs: 93521278
Best-hit-arabi-ID: AT3G13460.1
Best-hit-arabi-name: evolutionarily conserved C-terminal region 2
Gorai.004G020600.1.D
PCC=0.409, p=0.082
mRNA Profile
Protein Expression Profile
DPA
0
1
2
3
4
5
6
7
8
9
10
11
12
13
14
15
16
17
18
19
20
21
22
23
24
25
26
27
28
29
30
31

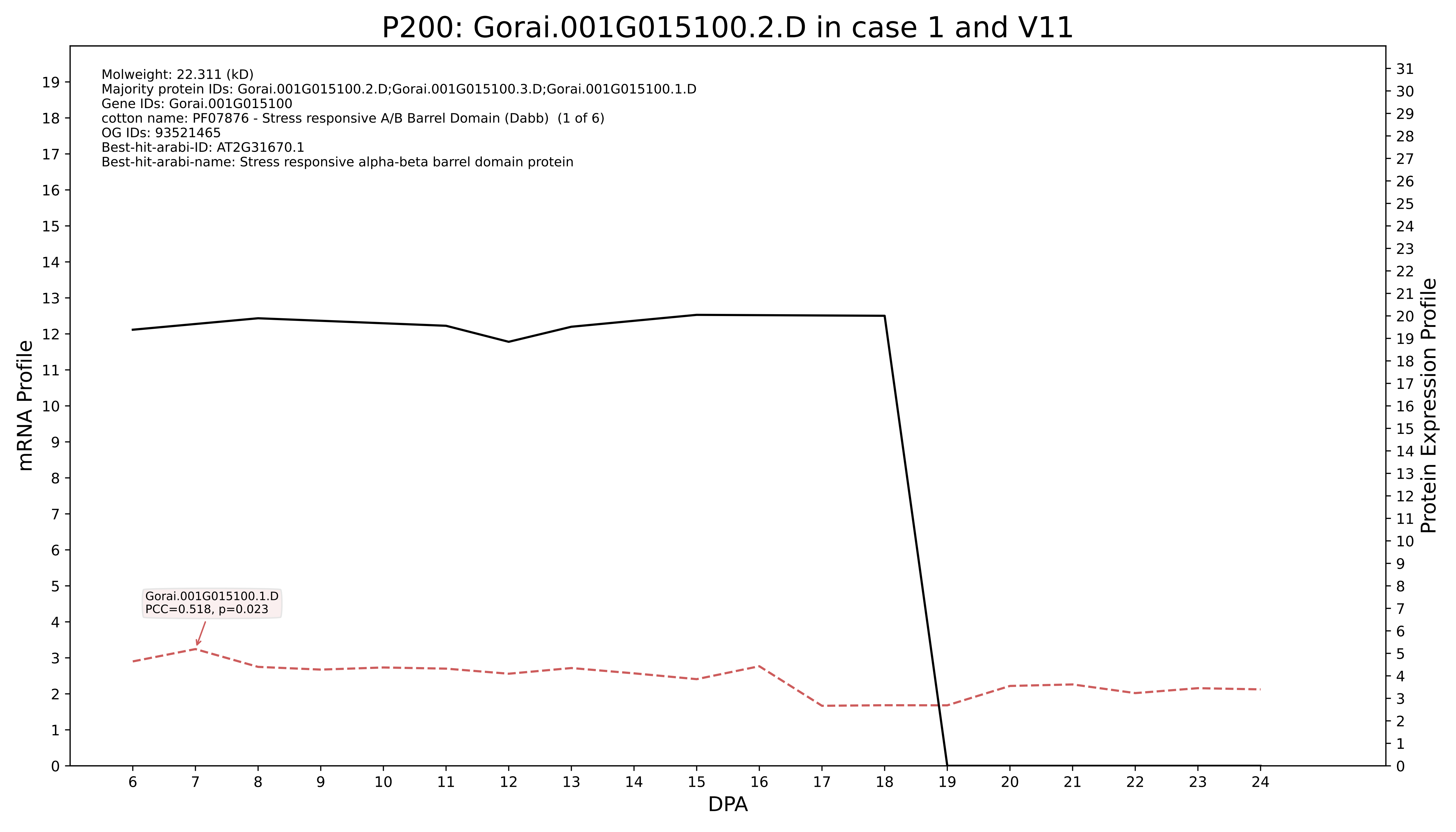

P200: Gorai.001G015100.2.D in case 1 and V11
Molweight: 22.311 (kD)
Majority protein IDs: Gorai.001G015100.2.D;Gorai.001G015100.3.D;Gorai.001G015100.1.D
Gene IDs: Gorai.001G015100
cotton name: PF07876 - Stress responsive A/B Barrel Domain (Dabb) (1 of 6)
OG IDs: 93521465
Best-hit-arabi-ID: AT2G31670.1
Best-hit-arabi-name: Stress responsive alpha-beta barrel domain protein
Gorai.001G015100.1.D
PCC=0.518, p=0.023
mRNA Profile
Protein Expression Profile
DPA

P200: Gorai.011G130700.3.A in case 1 and V11

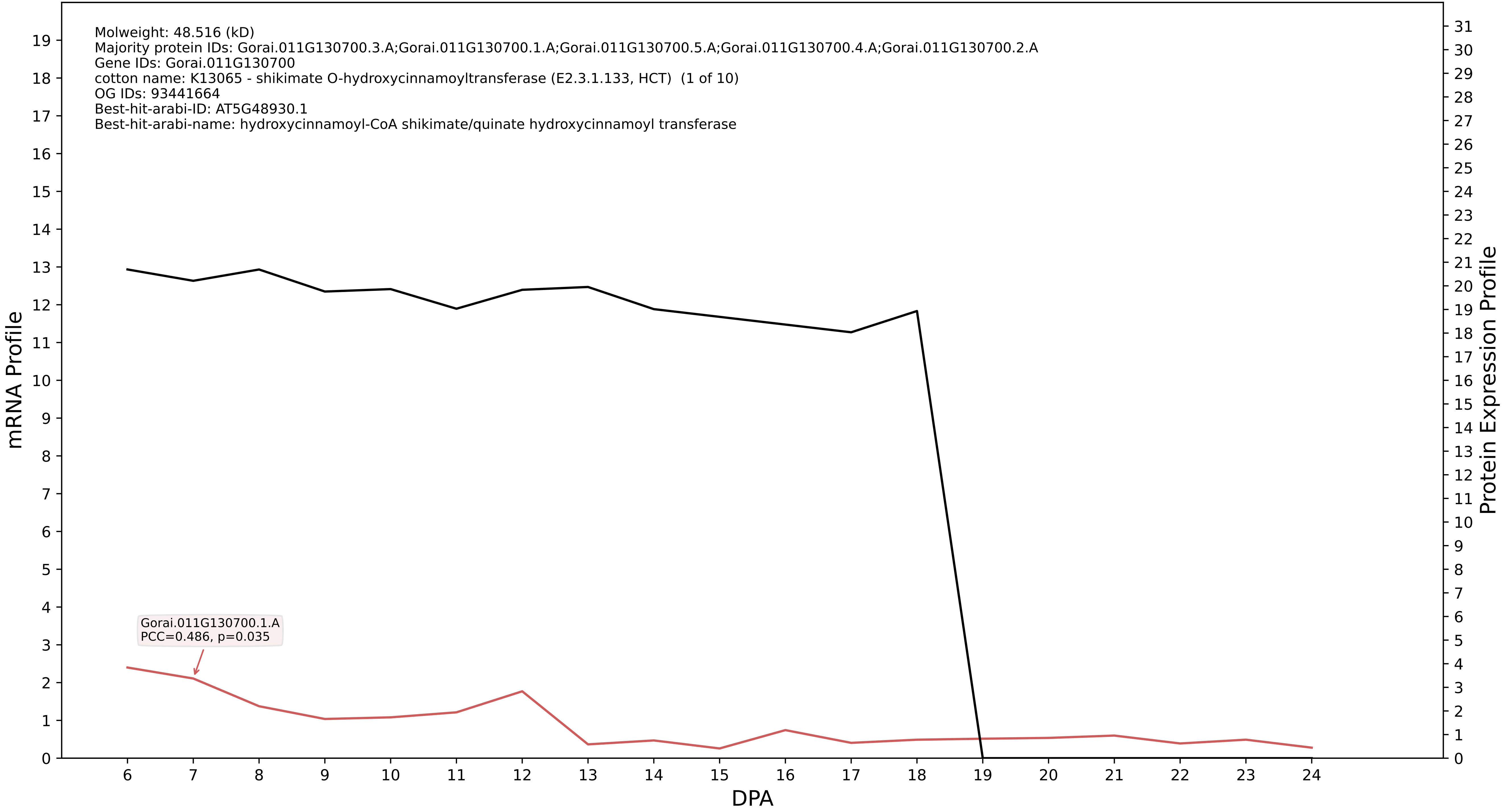

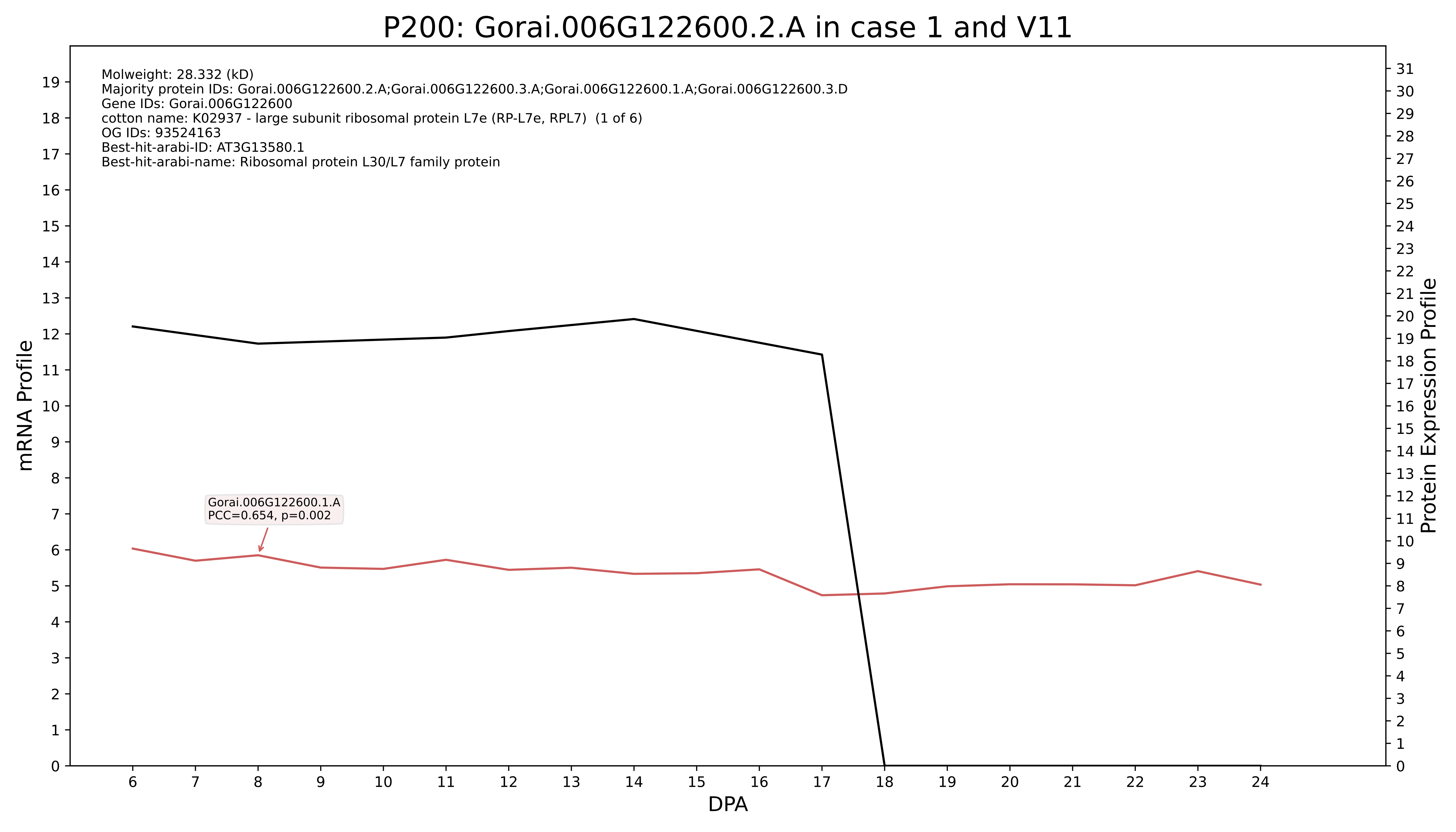
P200: Gorai.006G122600.2.A in case 1 and V11
Molweight: 28.332 (kD)
Majority protein IDs: Gorai.006G122600.2.A;Gorai.006G122600.3.A;Gorai.006G122600.1.A;Gorai.006G122600.3.D
Gene IDs: Gorai.006G122600
cotton name: K02937 - large subunit ribosomal protein L7e (RP-L7e, RPL7) (1 of 6)
OG IDs: 93524163
Best-hit-arabi-ID: AT3G13580.1
Best-hit-arabi-name: Ribosomal protein L30/L7 family protein
Gorai.006G122600.1.A
PCC=0.654, p=0.002
mRNA Profile
Protein Expression Profile
DPA
0 1 2 3 4 5 6 7 8 9 10 11 12 13 14 15 16 17 18 19
0 1 2 3 4 5 6 7 8 9 10 11 12 13 14 15 16 17 18 19 20 21 22 23 24 25 26 27 28 29 30 31
6 7 8 9 10 11 12 13 14 15 16 17 18 19 20 21 22 23 24

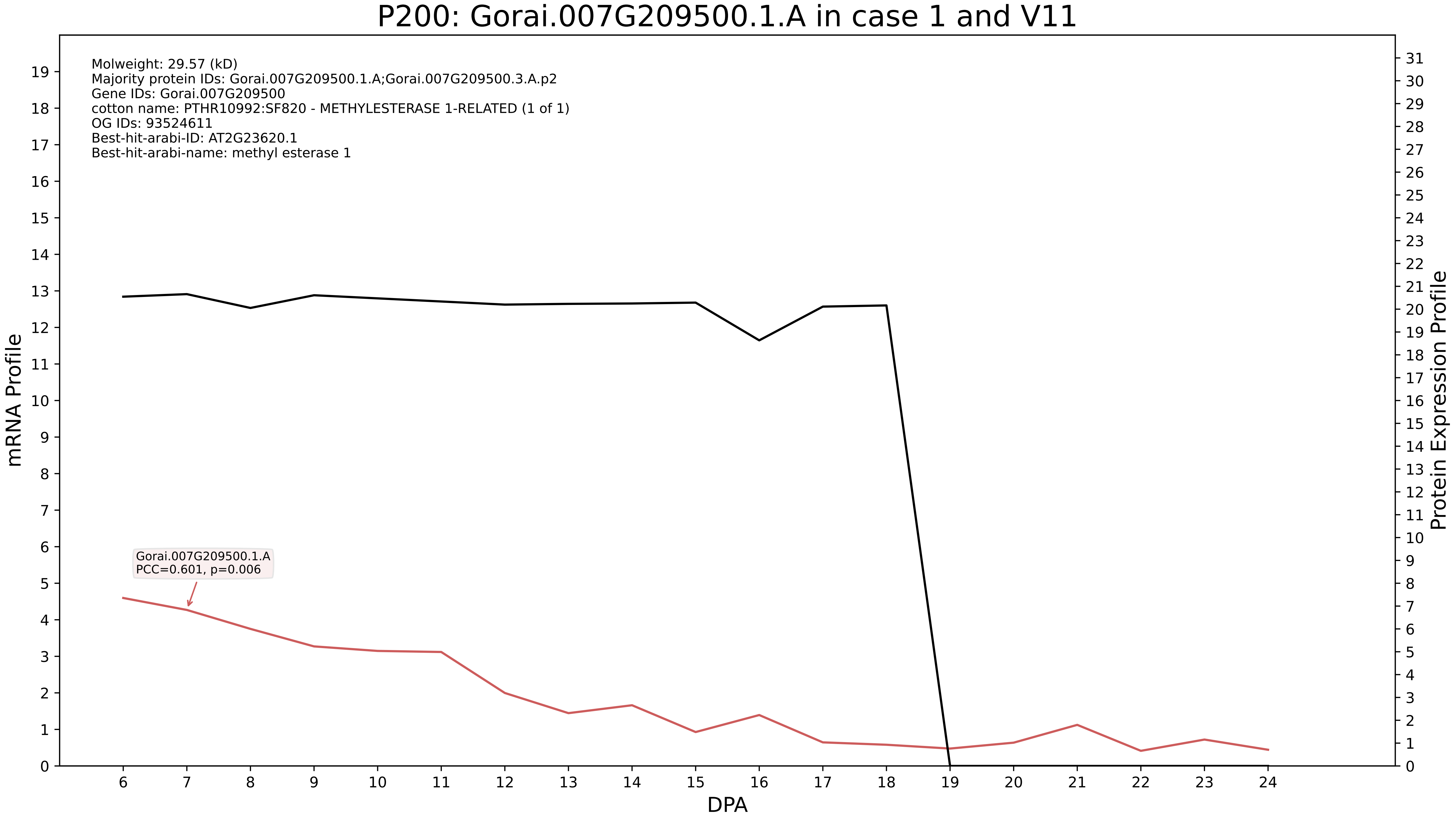
P200: Gorai.007G209500.1.A in case 1 and V11
Molweight: 29.57 (kD)
Majority protein IDs: Gorai.007G209500.1.A;Gorai.007G209500.3.A.p2
Gene IDs: Gorai.007G209500
cotton name: PTHR10992:SF820 - METHYLESTERASE 1-RELATED (1 of 1)
OG IDs: 93524611
Best-hit-arabi-ID: AT2G23620.1
Best-hit-arabi-name: methyl esterase 1
Gorai.007G209500.1.A
PCC=0.601, p=0.006
mRNA Profile
Protein Expression Profile
DPA
0
1
2
3
4
5
6
7
8
9
10
11
12
13
14
15
16
17
18
19
20
21
22
23
24
25
26
27
28
29
30
31

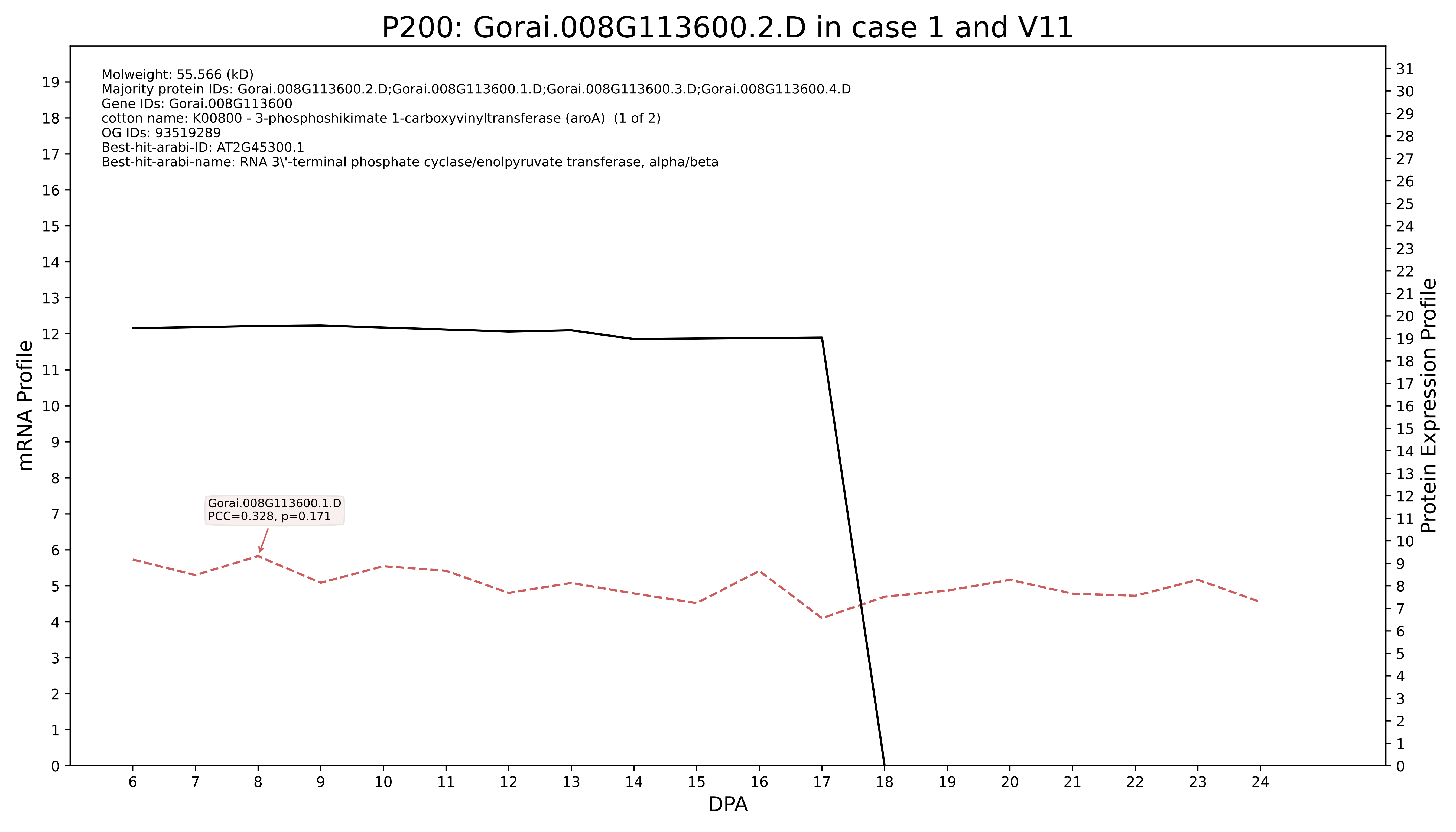
P200: Gorai.008G113600.2.D in case 1 and V11
Molweight: 55.566 (kD)
Majority protein IDs: Gorai.008G113600.2.D;Gorai.008G113600.1.D;Gorai.008G113600.3.D;Gorai.008G113600.4.D
Gene IDs: Gorai.008G113600
cotton name: K00800 - 3-phosphoshikimate 1-carboxyvinyltransferase (aroA) (1 of 2)
OG IDs: 93519289
Best-hit-arabi-ID: AT2G45300.1
Best-hit-arabi-name: RNA 3\'-terminal phosphate cyclase/enolpyruvate transferase, alpha/beta
Gorai.008G113600.1.D
PCC=0.328, p=0.171
mRNA Profile
Protein Expression Profile
DPA

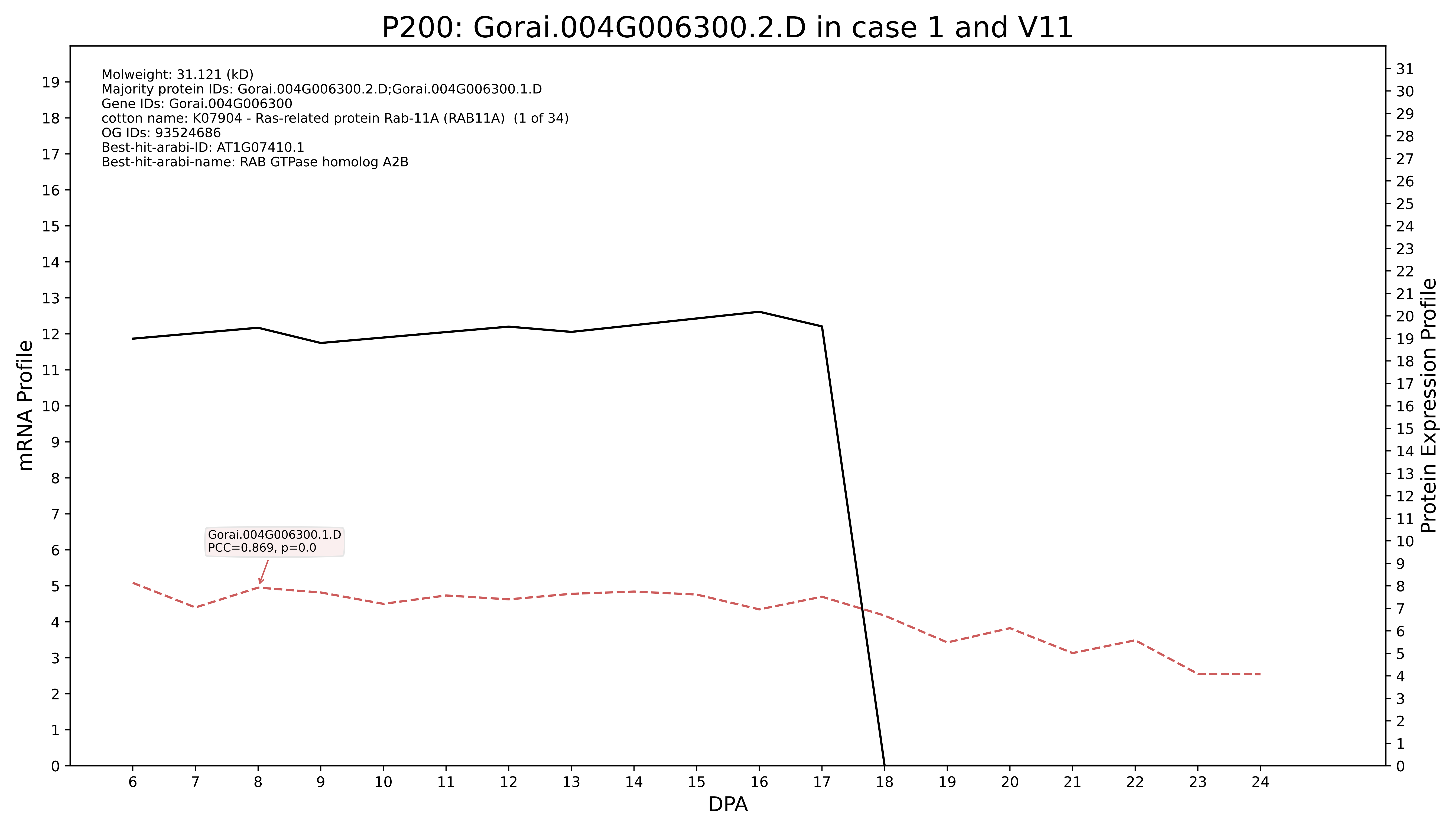
P200: Gorai.004G006300.2.D in case 1 and V11
Molweight: 31.121 (kD)
Majority protein IDs: Gorai.004G006300.2.D;Gorai.004G006300.1.D
Gene IDs: Gorai.004G006300
cotton name: K07904 - Ras-related protein Rab-11A (RAB11A) (1 of 34)
OG IDs: 93524686
Best-hit-arabi-ID: AT1G07410.1
Best-hit-arabi-name: RAB GTPase homolog A2B
Gorai.004G006300.1.D
PCC=0.869, p=0.0
mRNA Profile
Protein Expression Profile
DPA

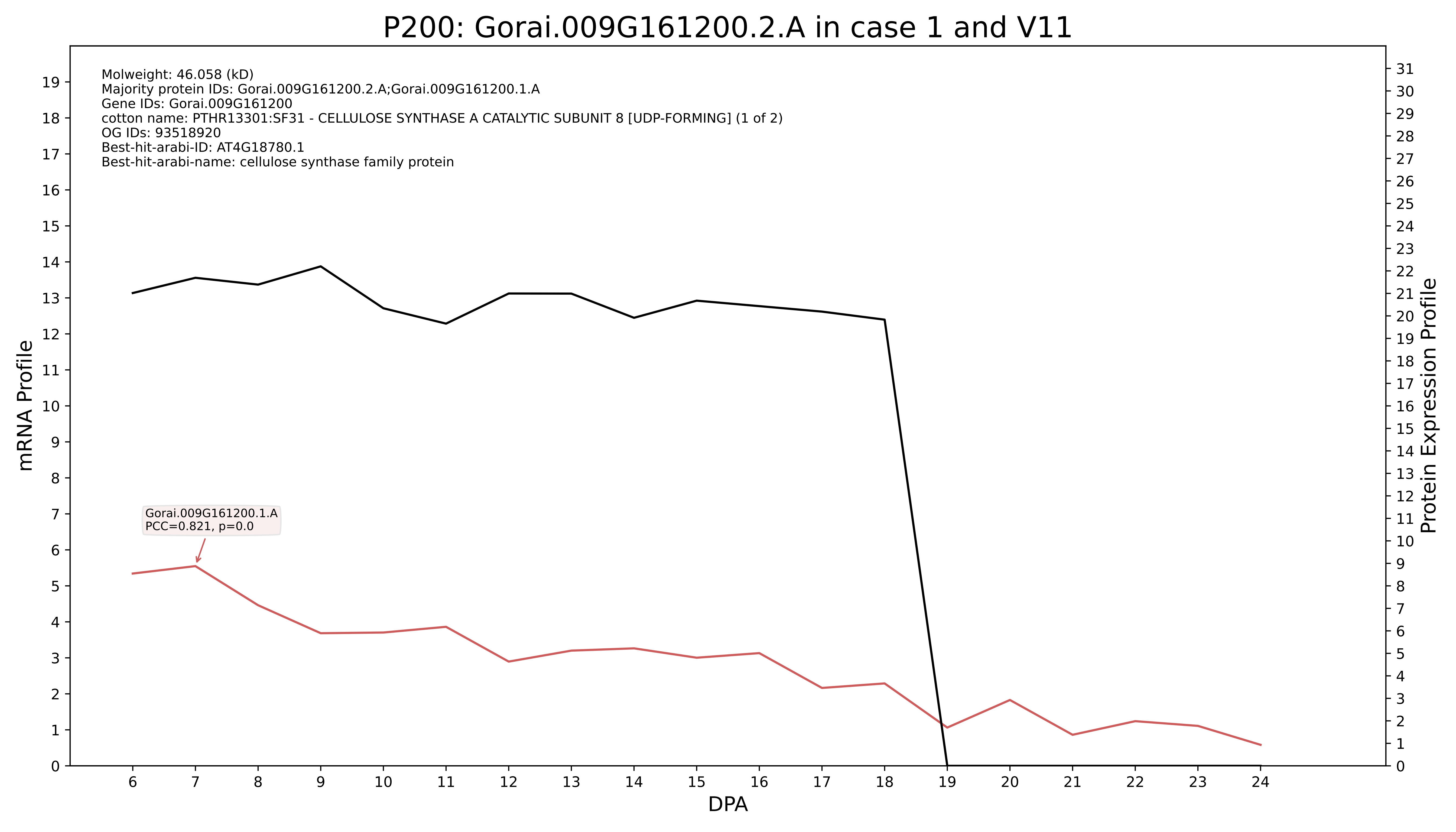

P200: Gorai.009G161200.2.A in case 1 and V11
Molweight: 46.058 (kD)
Majority protein IDs: Gorai.009G161200.2.A;Gorai.009G161200.1.A
Gene IDs: Gorai.009G161200
cotton name: PTHR13301:SF31 - CELLULOSE SYNTHASE A CATALYTIC SUBUNIT 8 [UDP-FORMING] (1 of 2)
OG IDs: 93518920
Best-hit-arabi-ID: AT4G18780.1
Best-hit-arabi-name: cellulose synthase family protein
Gorai.009G161200.1.A
PCC=0.821, p=0.0
mRNA Profile
Protein Expression Profile
DPA

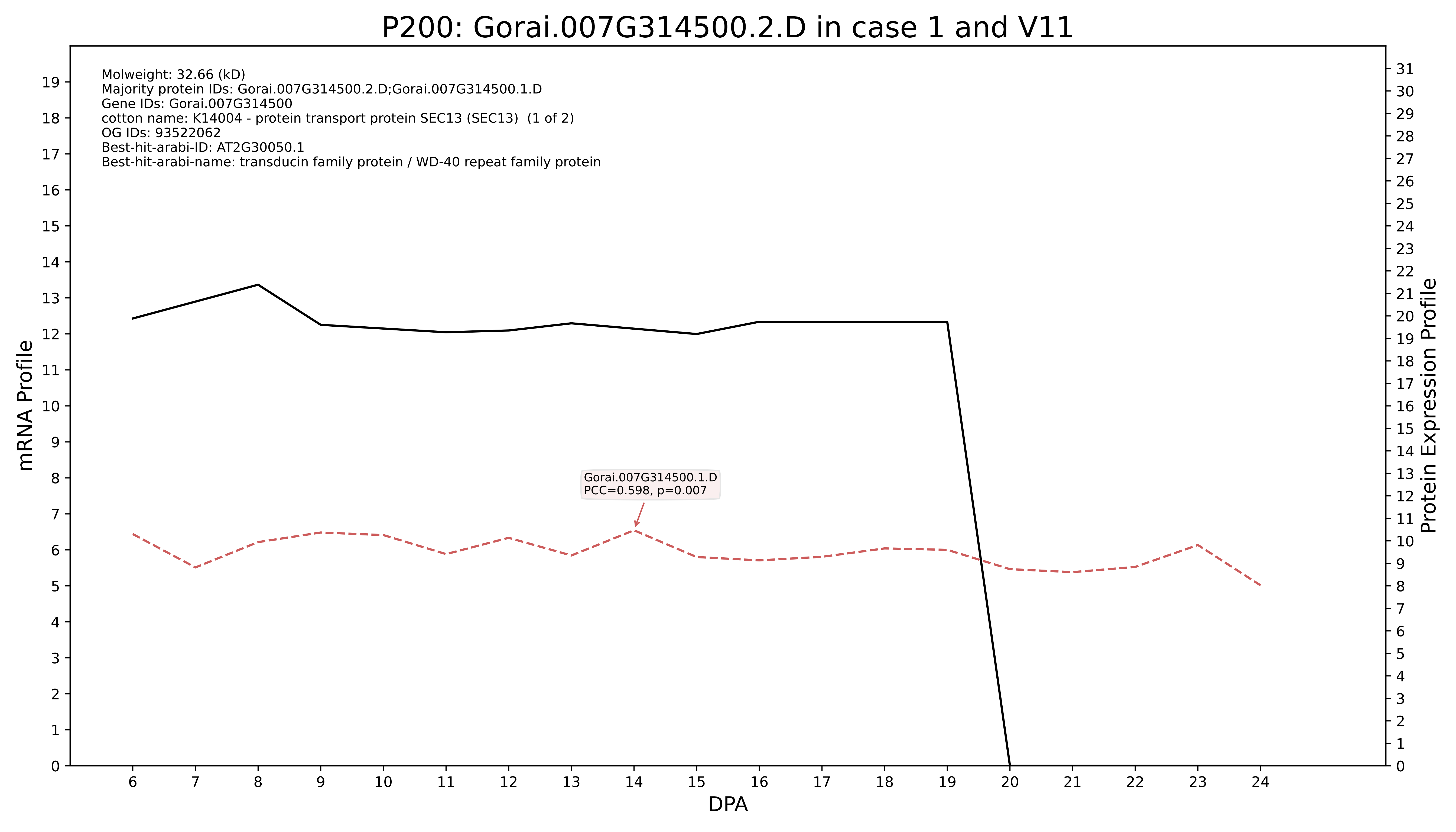
P200: Gorai.007G314500.2.D in case 1 and V11
Molweight: 32.66 (kD)
Majority protein IDs: Gorai.007G314500.2.D;Gorai.007G314500.1.D
Gene IDs: Gorai.007G314500
cotton name: K14004 - protein transport protein SEC13 (SEC13) (1 of 2)
OG IDs: 93522062
Best-hit-arabi-ID: AT2G30050.1
Best-hit-arabi-name: transducin family protein / WD-40 repeat family protein
Gorai.007G314500.1.D
PCC=0.598, p=0.007
mRNA Profile
Protein Expression Profile
DPA

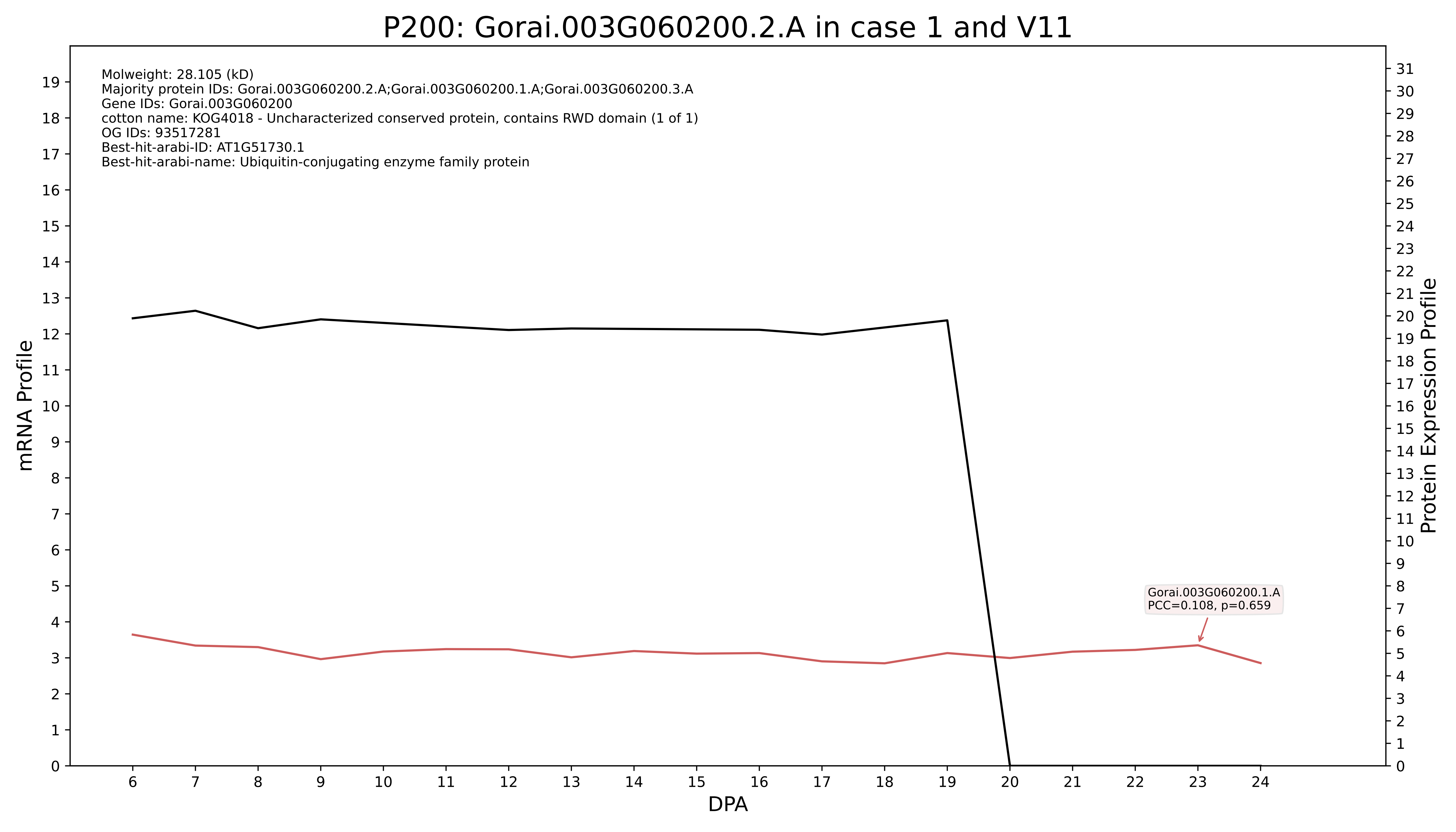

P200: Gorai.003G060200.2.A in case 1 and V11
Molweight: 28.105 (kD)
Majority protein IDs: Gorai.003G060200.2.A;Gorai.003G060200.1.A;Gorai.003G060200.3.A
Gene IDs: Gorai.003G060200
cotton name: KOG4018 - Uncharacterized conserved protein, contains RWD domain (1 of 1)
OG IDs: 93517281
Best-hit-arabi-ID: AT1G51730.1
Best-hit-arabi-name: Ubiquitin-conjugating enzyme family protein
Gorai.003G060200.1.A
PCC=0.108, p=0.659
mRNA Profile
Protein Expression Profile
DPA

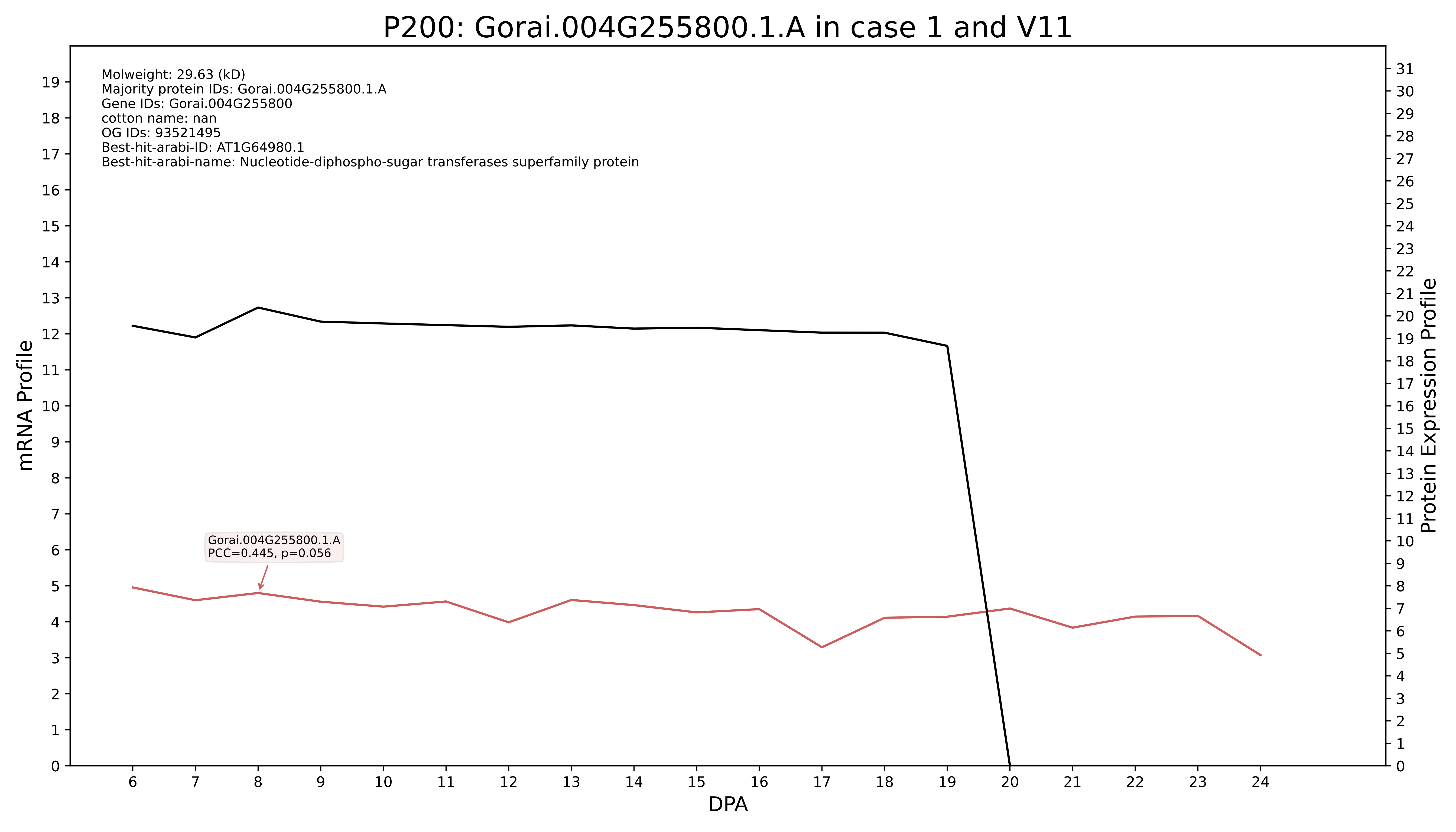
P200: Gorai.004G255800.1.A in case 1 and V11
Molweight: 29.63 (kD)
Majority protein IDs: Gorai.004G255800.1.A
Gene IDs: Gorai.004G255800
cotton name: nan
OG IDs: 93521495
Best-hit-arabi-ID: AT1G64980.1
Best-hit-arabi-name: Nucleotide-diphospho-sugar transferases superfamily protein
Gorai.004G255800.1.A
PCC=0.445, p=0.056
mRNA Profile
Protein Expression Profile
DPA

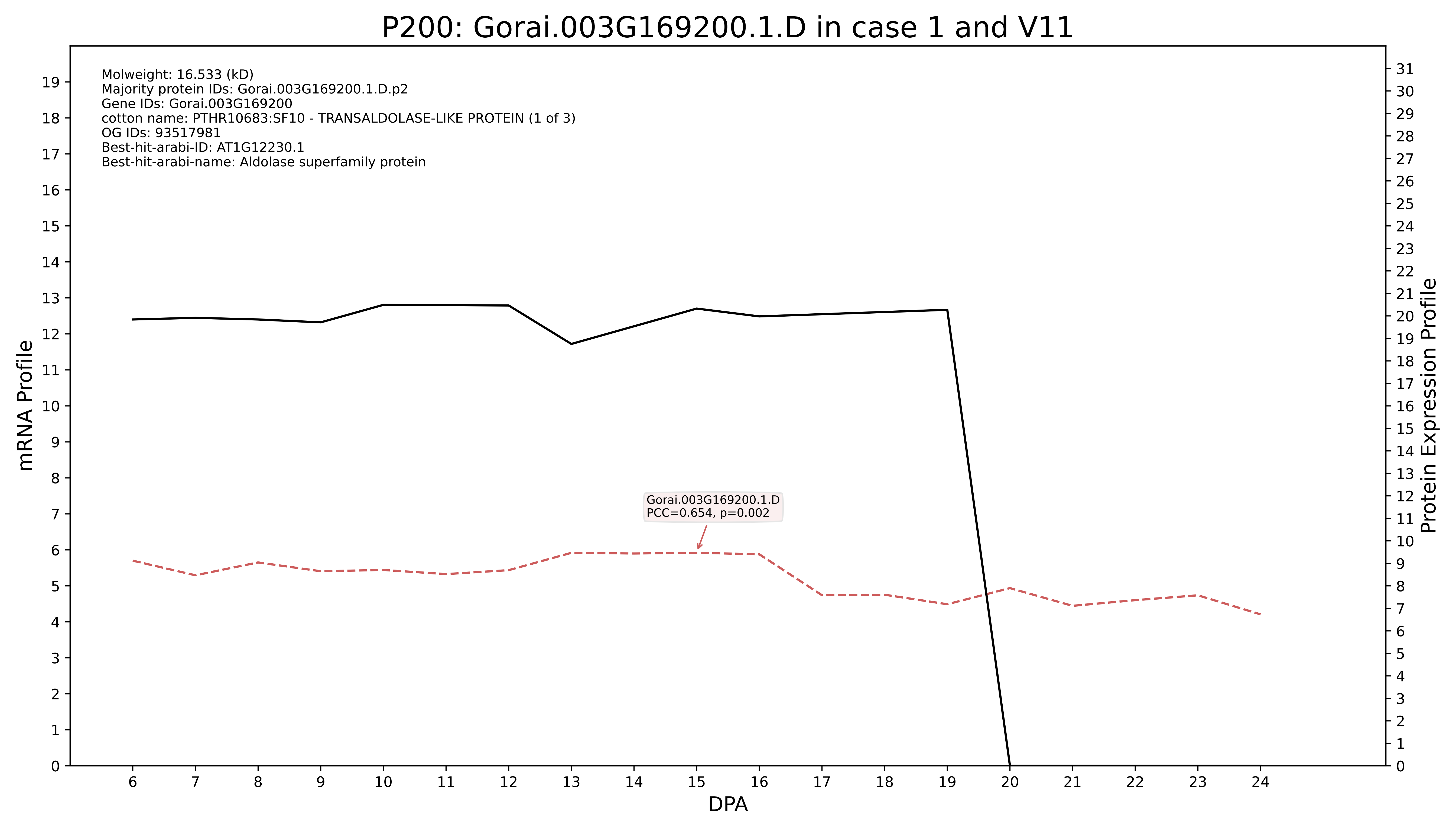

P200: Gorai.003G169200.1.D in case 1 and V11
Molweight: 16.533 (kD)
Majority protein IDs: Gorai.003G169200.1.D.p2
Gene IDs: Gorai.003G169200
cotton name: PTHR10683:SF10 - TRANSALDOLASE-LIKE PROTEIN (1 of 3)
OG IDs: 93517981
Best-hit-arabi-ID: AT1G12230.1
Best-hit-arabi-name: Aldolase superfamily protein
Gorai.003G169200.1.D
PCC=0.654, p=0.002
mRNA Profile
Protein Expression Profile
DPA

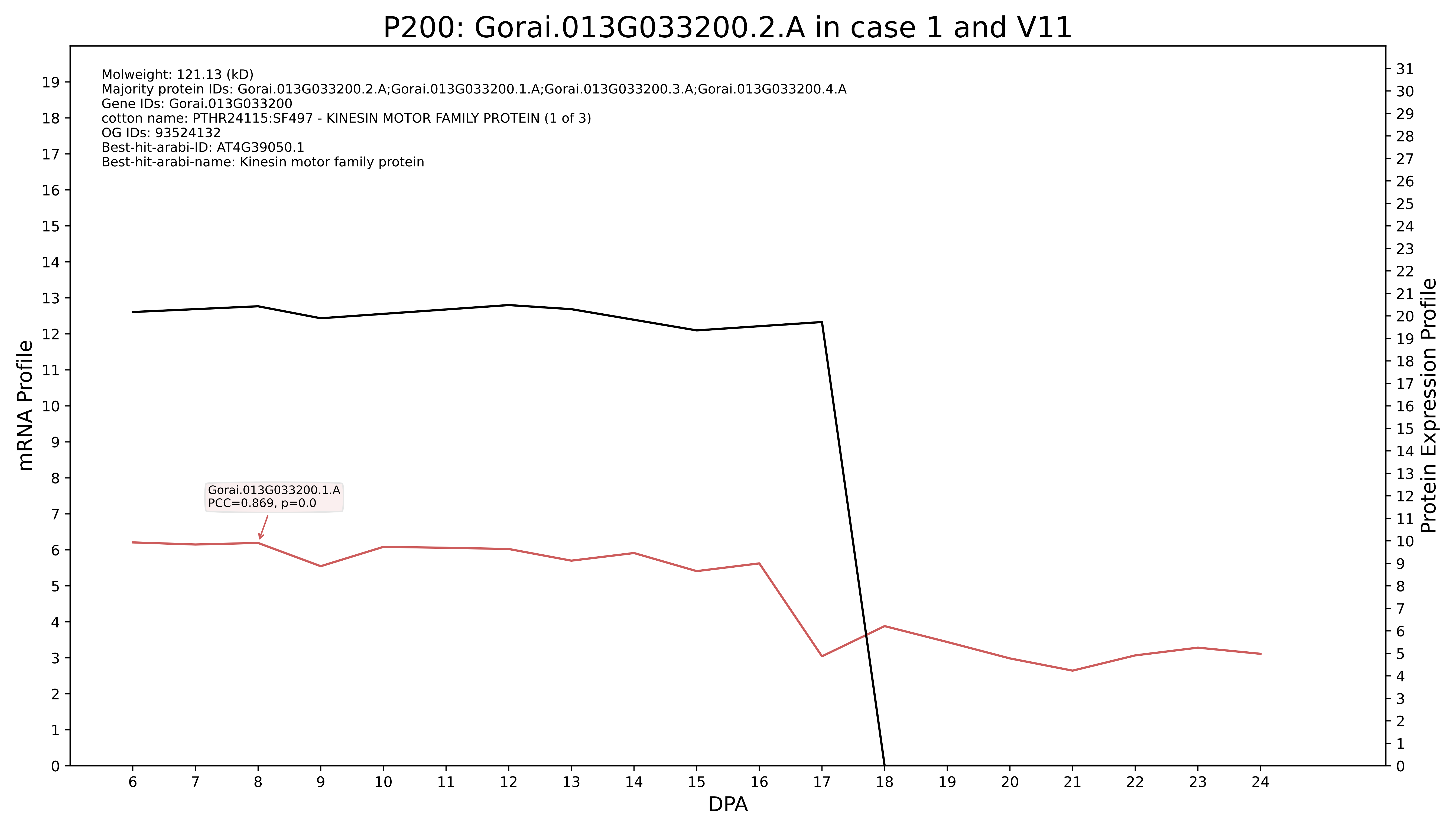

P200: Gorai.013G033200.2.A in case 1 and V11
Molweight: 121.13 (kD)
Majority protein IDs: Gorai.013G033200.2.A;Gorai.013G033200.1.A;Gorai.013G033200.3.A;Gorai.013G033200.4.A
Gene IDs: Gorai.013G033200
cotton name: PTHR24115:SF497 - KINESIN MOTOR FAMILY PROTEIN (1 of 3)
OG IDs: 93524132
Best-hit-arabi-ID: AT4G39050.1
Best-hit-arabi-name: Kinesin motor family protein
Gorai.013G033200.1.A
PCC=0.869, p=0.0
mRNA Profile
Protein Expression Profile
DPA

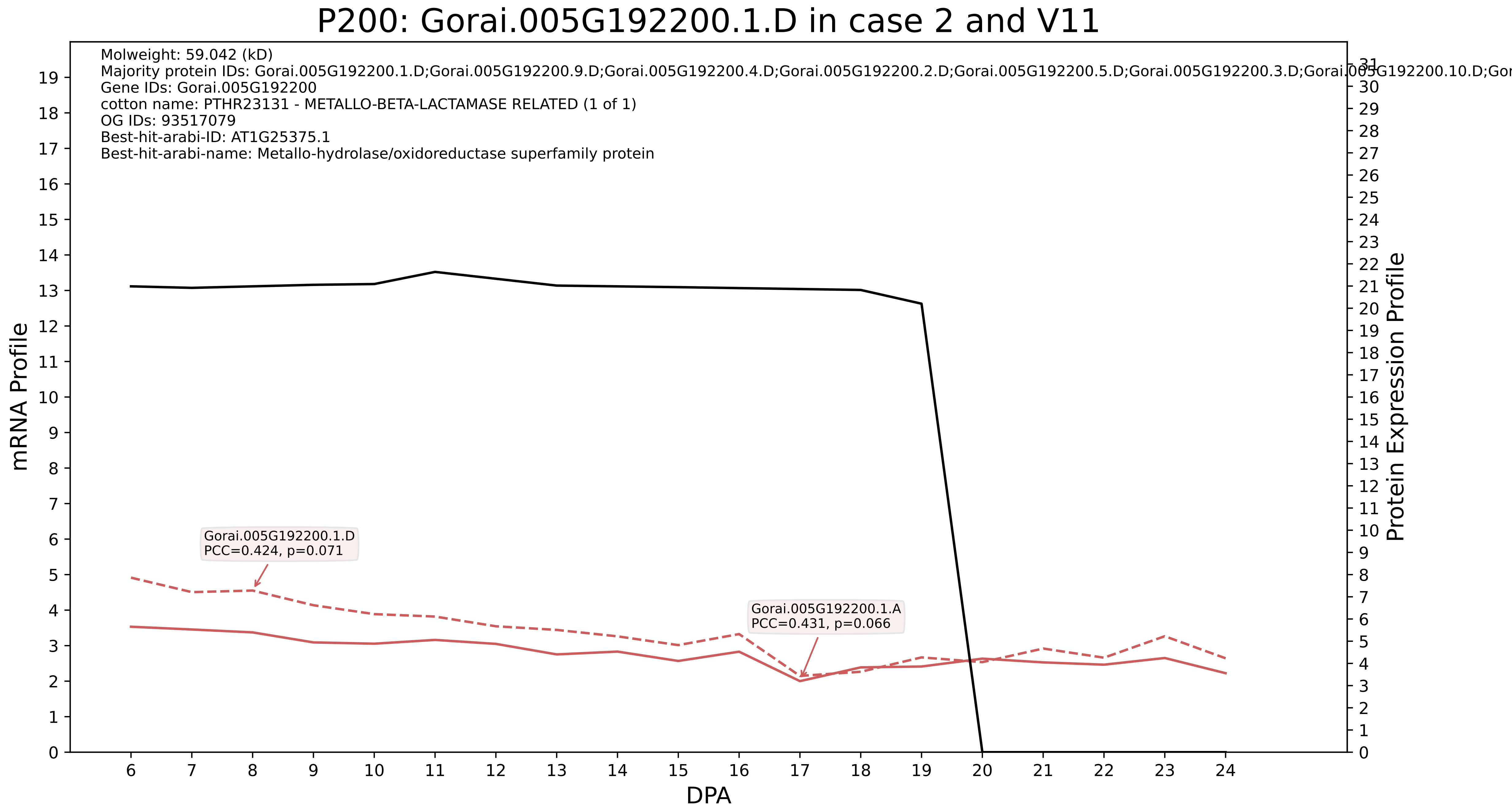

P200: Gorai.005G192200.1.D in case 2 and V11
Molweight: 59.042 (kD)
Majority protein IDs: Gorai.005G192200.1.D;Gorai.005G192200.9.D;Gorai.005G192200.4.D;Gorai.005G192200.2.D;Gorai.005G192200.5.D;Gorai.005G192200.3.D;Gorai.005G192200.10.D;Gor
Gene IDs: Gorai.005G192200
cotton name: PTHR23131 - METALLO-BETA-LACTAMASE RELATED (1 of 1)
OG IDs: 93517079
Best-hit-arabi-ID: AT1G25375.1
Best-hit-arabi-name: Metallo-hydrolase/oxidoreductase superfamily protein
Gorai.005G192200.1.D
PCC=0.424, p=0.071
Gorai.005G192200.1.A
PCC=0.431, p=0.066
mRNA Profile
Protein Expression Profile
DPA

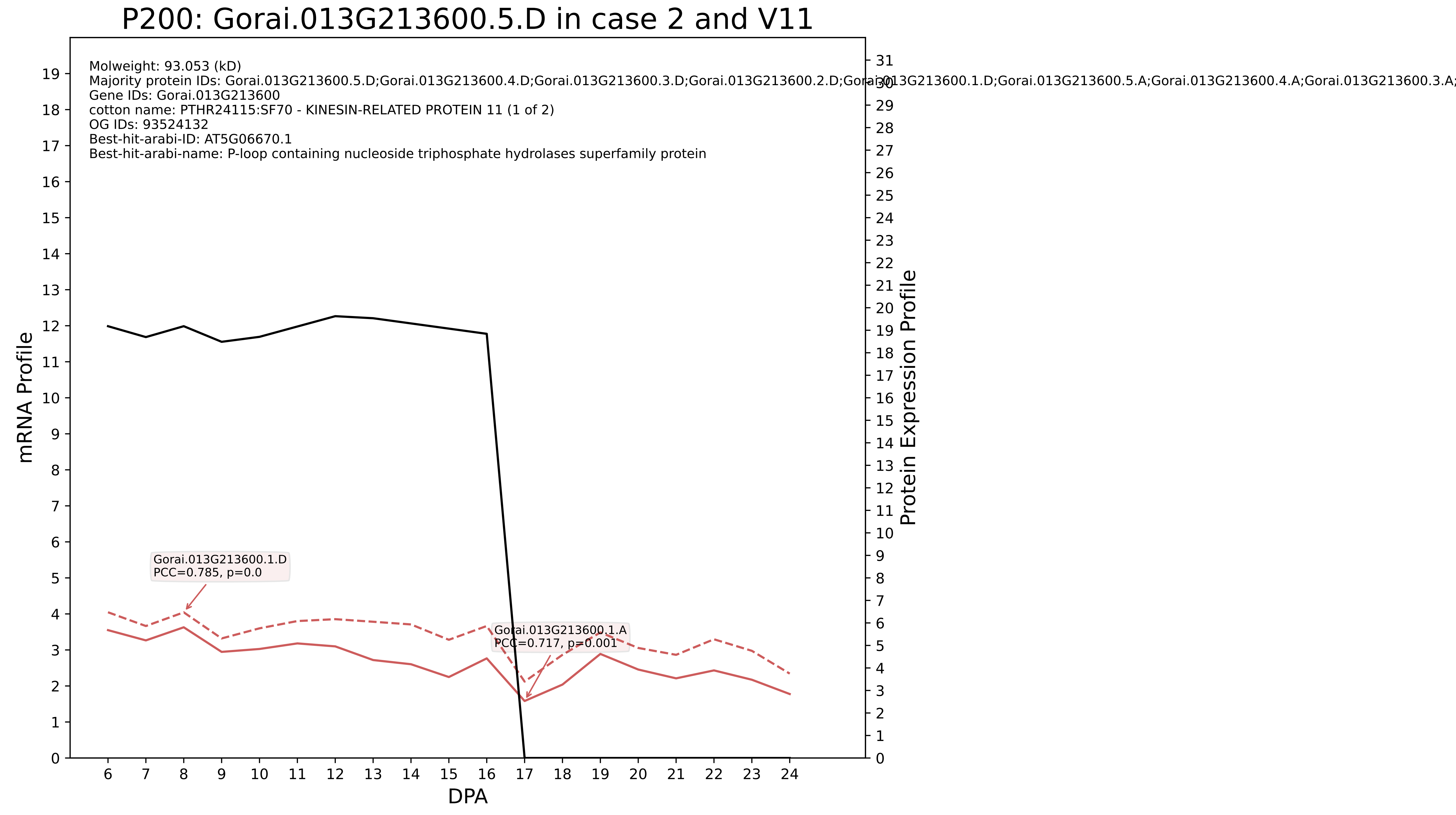
P200: Gorai.013G213600.5.D in case 2 and V11
Molweight: 93.053 (kD)
Majority protein IDs: Gorai.013G213600.5.D;Gorai.013G213600.4.D;Gorai.013G213600.3.D;Gorai.013G213600.2.D;Gorai.013G213600.1.D;Gorai.013G213600.5.A;Gorai.013G213600.4.A;Gorai.013G213600.3.A;
Gene IDs: Gorai.013G213600
cotton name: PTHR24115:SF70 - KINESIN-RELATED PROTEIN 11 (1 of 2)
OG IDs: 93524132
Best-hit-arabi-ID: AT5G06670.1
Best-hit-arabi-name: P-loop containing nucleoside triphosphate hydrolases superfamily protein
Gorai.013G213600.1.D
PCC=0.785, p=0.0
Gorai.013G213600.1.A
PCC=0.717, p=0.001
mRNA Profile
Protein Expression Profile
DPA

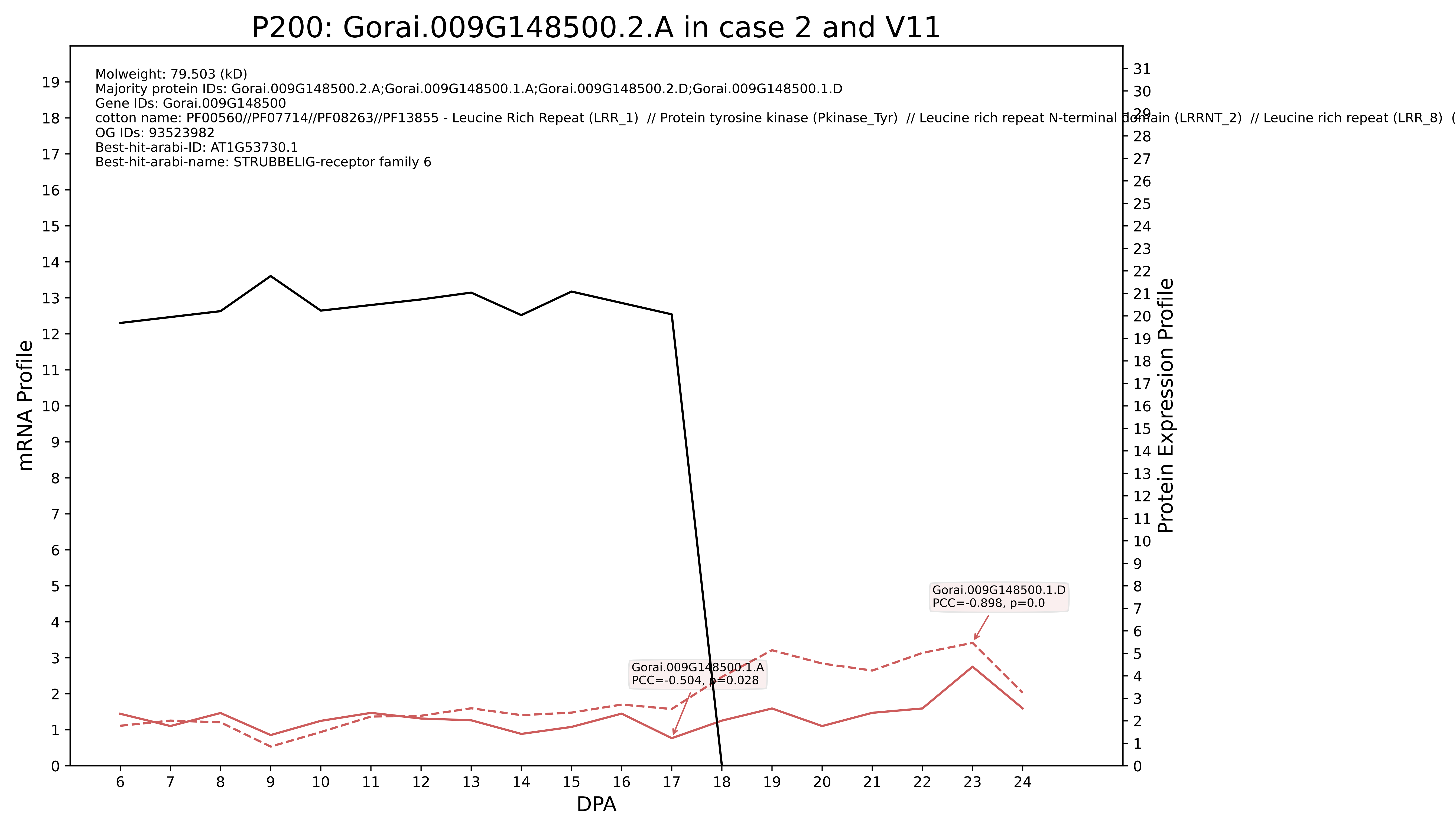

P200: Gorai.009G148500.2.A in case 2 and V11
Molweight: 79.503 (kD)
Majority protein IDs: Gorai.009G148500.2.A;Gorai.009G148500.1.A;Gorai.009G148500.2.D;Gorai.009G148500.1.D
Gene IDs: Gorai.009G148500
cotton name: PF00560//PF07714//PF08263//PF13855 - Leucine Rich Repeat (LRR_1) // Protein tyrosine kinase (Pkinase_Tyr) // Leucine rich repeat N-terminal domain (LRRNT_2) // Leucine rich repeat (LRR_8) (
OG IDs: 93523982
Best-hit-arabi-ID: AT1G53730.1
Best-hit-arabi-name: STRUBBELIG-receptor family 6
Gorai.009G148500.1.D
PCC=-0.898, p=0.0
Gorai.009G148500.1.A
PCC=-0.504, p=0.028
mRNA Profile
Protein Expression Profile
DPA

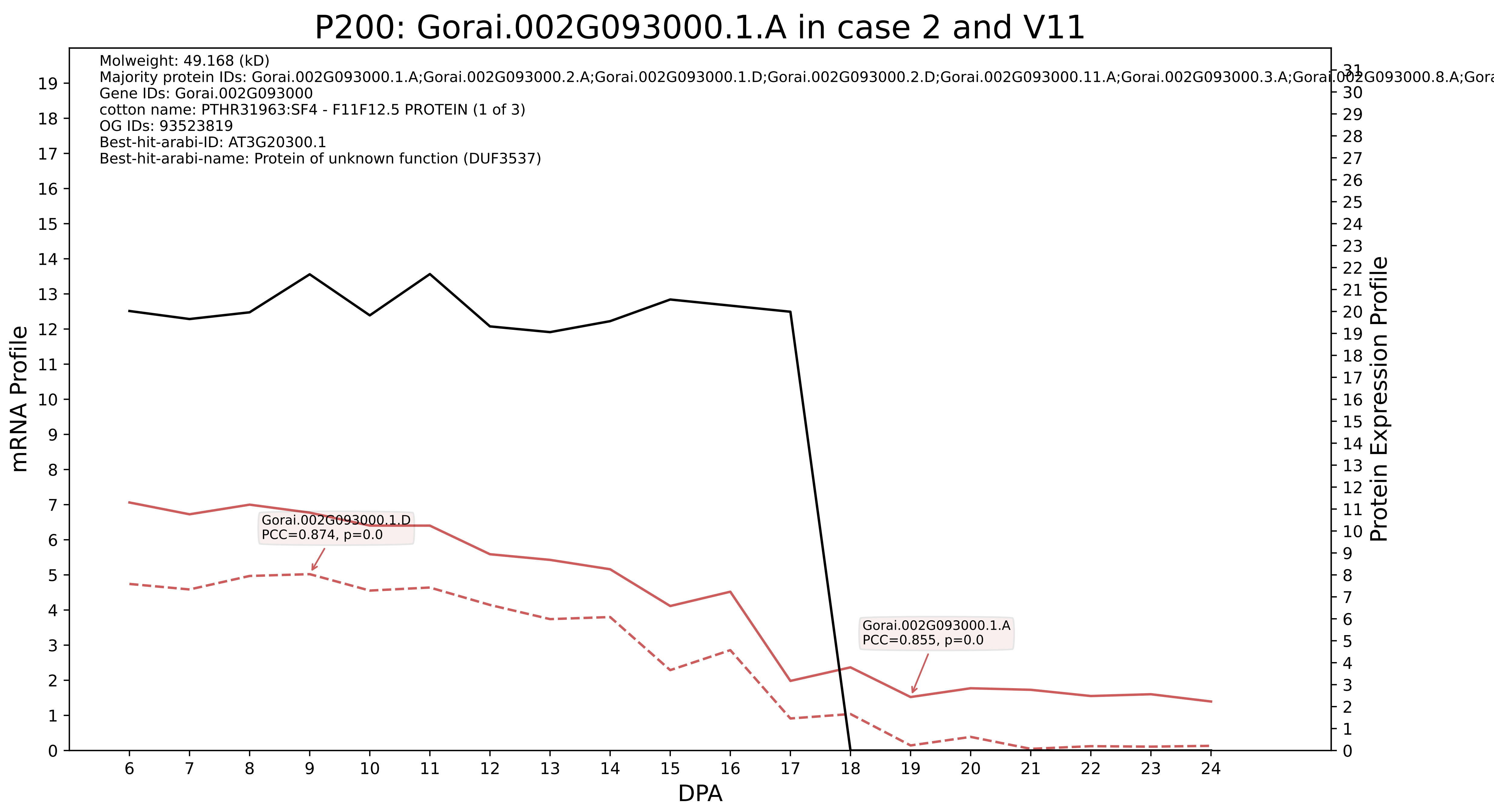

P200: Gorai.002G093000.1.A in case 2 and V11
Molweight: 49.168 (kD)
Majority protein IDs: Gorai.002G093000.1.A;Gorai.002G093000.2.A;Gorai.002G093000.1.D;Gorai.002G093000.2.D;Gorai.002G093000.11.A;Gorai.002G093000.3.A;Gorai.002G093000.8.A;Gora
Gene IDs: Gorai.002G093000
cotton name: PTHR31963:SF4 - F11F12.5 PROTEIN (1 of 3)
OG IDs: 93523819
Best-hit-arabi-ID: AT3G20300.1
Best-hit-arabi-name: Protein of unknown function (DUF3537)
Gorai.002G093000.1.D
PCC=0.874, p=0.0
Gorai.002G093000.1.A
PCC=0.855, p=0.0
mRNA Profile
Protein Expression Profile
DPA

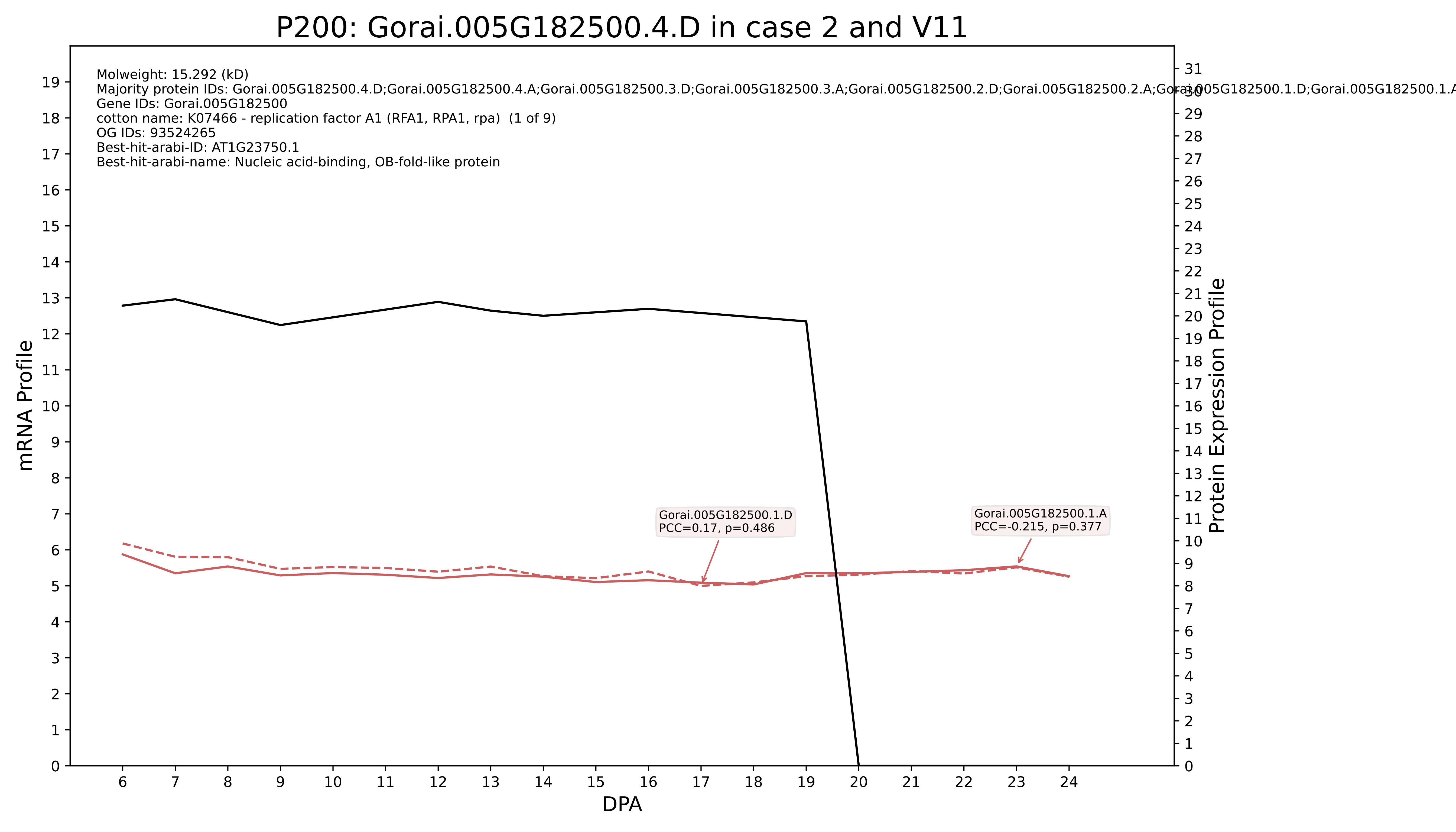
P200: Gorai.005G182500.4.D in case 2 and V11
Molweight: 15.292 (kD)
Majority protein IDs: Gorai.005G182500.4.D;Gorai.005G182500.4.A;Gorai.005G182500.3.D;Gorai.005G182500.3.A;Gorai.005G182500.2.D;Gorai.005G182500.2.A;Gorai.005G182500.1.D;Gorai.005G182500.1.A
Gene IDs: Gorai.005G182500
cotton name: K07466 - replication factor A1 (RFA1, RPA1, rpa) (1 of 9)
OG IDs: 93524265
Best-hit-arabi-ID: AT1G23750.1
Best-hit-arabi-name: Nucleic acid-binding, OB-fold-like protein
Gorai.005G182500.1.D
PCC=0.17, p=0.486
Gorai.005G182500.1.A
PCC=-0.215, p=0.377
mRNA Profile
Protein Expression Profile
DPA

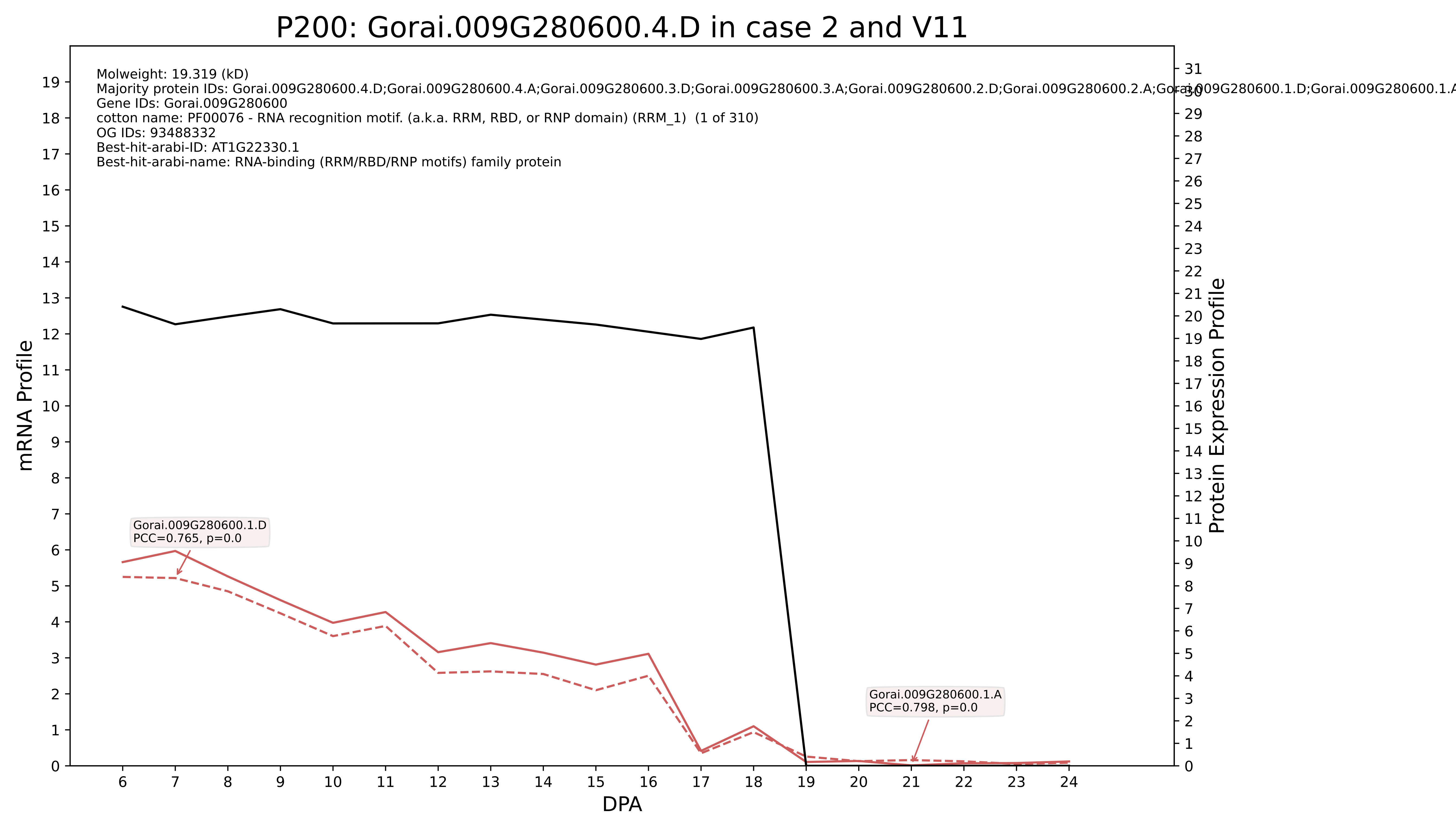
P200: Gorai.009G280600.4.D in case 2 and V11
Molweight: 19.319 (kD)
Majority protein IDs: Gorai.009G280600.4.D;Gorai.009G280600.4.A;Gorai.009G280600.3.D;Gorai.009G280600.3.A;Gorai.009G280600.2.D;Gorai.009G280600.2.A;Gorai.009G280600.1.D;Gorai.009G280600.1.A
Gene IDs: Gorai.009G280600
cotton name: PF00076 - RNA recognition motif. (a.k.a. RRM, RBD, or RNP domain) (RRM_1) (1 of 310)
OG IDs: 93488332
Best-hit-arabi-ID: AT1G22330.1
Best-hit-arabi-name: RNA-binding (RRM/RBD/RNP motifs) family protein
Gorai.009G280600.1.D
PCC=0.765, p=0.0
Gorai.009G280600.1.A
PCC=0.798, p=0.0
mRNA Profile
Protein Expression Profile
DPA

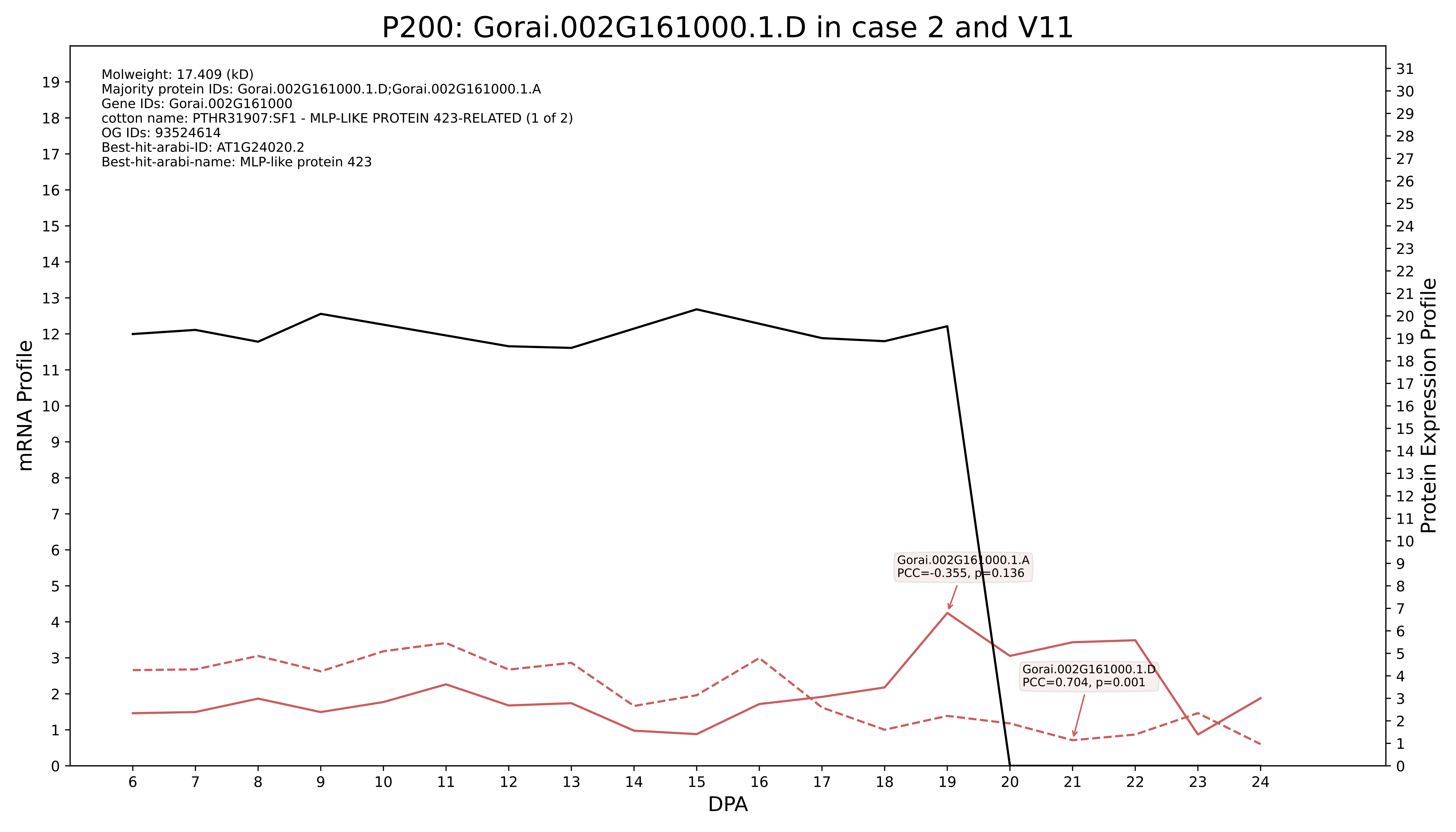
P200: Gorai.002G161000.1.D in case 2 and V11
Molweight: 17.409 (kD)
Majority protein IDs: Gorai.002G161000.1.D;Gorai.002G161000.1.A
Gene IDs: Gorai.002G161000
cotton name: PTHR31907:SF1 - MLP-LIKE PROTEIN 423-RELATED (1 of 2)
OG IDs: 93524614
Best-hit-arabi-ID: AT1G24020.2
Best-hit-arabi-name: MLP-like protein 423
Gorai.002G161000.1.A
PCC=-0.355, p=0.136
Gorai.002G161000.1.D
PCC=0.704, p=0.001
mRNA Profile
Protein Expression Profile
DPA
0
1
2
3
4
5
6
7
8
9
10
11
12
13
14
15
16
17
18
19
20
21
22
23
24
25
26
27
28
29
30
31

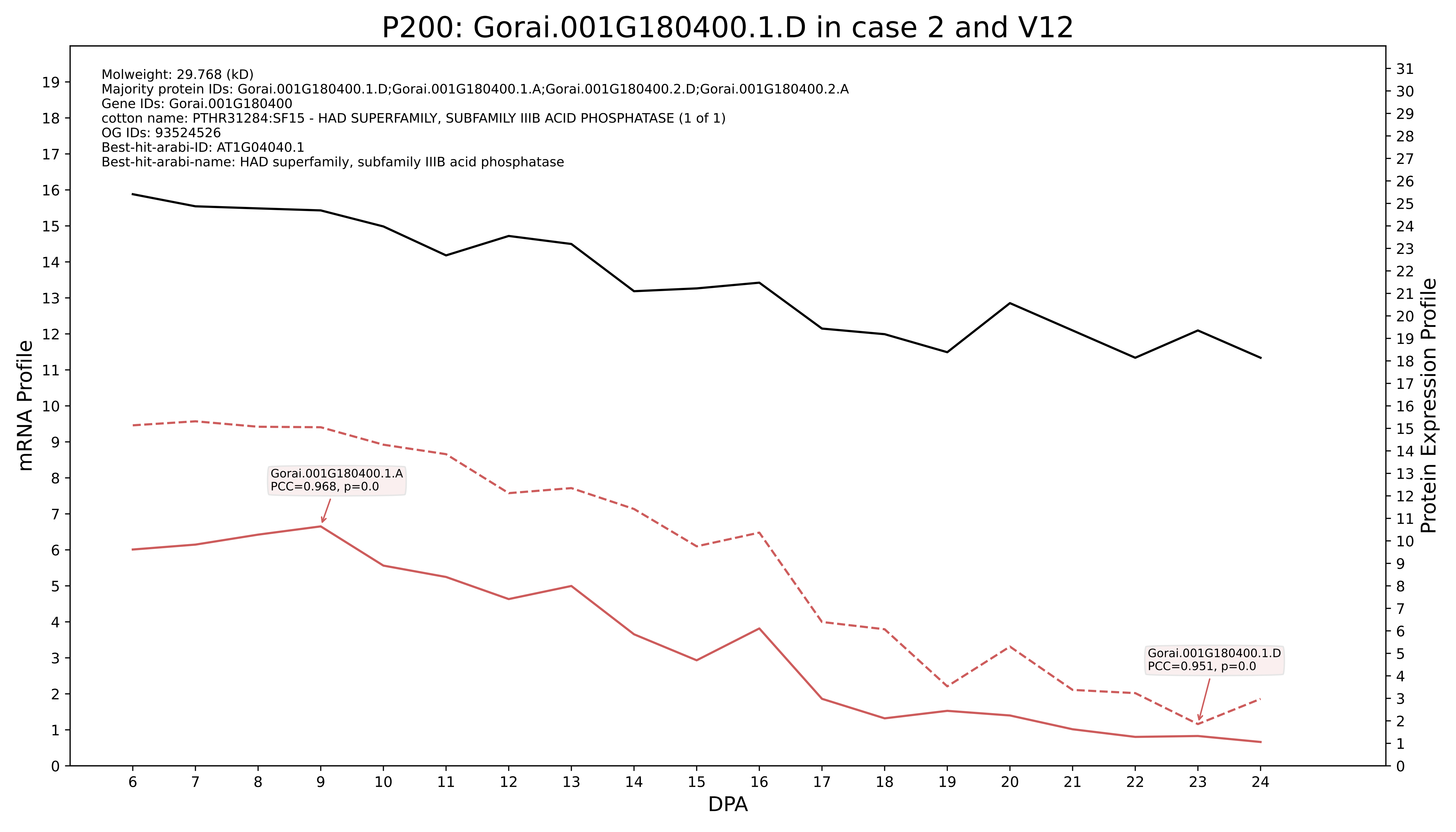
P200: Gorai.001G180400.1.D in case 2 and V12
Molweight: 29.768 (kD)
Majority protein IDs: Gorai.001G180400.1.D;Gorai.001G180400.1.A;Gorai.001G180400.2.D;Gorai.001G180400.2.A
Gene IDs: Gorai.001G180400
cotton name: PTHR31284:SF15 - HAD SUPERFAMILY, SUBFAMILY IIIB ACID PHOSPHATASE (1 of 1)
OG IDs: 93524526
Best-hit-arabi-ID: AT1G04040.1
Best-hit-arabi-name: HAD superfamily, subfamily IIIB acid phosphatase
Gorai.001G180400.1.A
PCC=0.968, p=0.0
Gorai.001G180400.1.D
PCC=0.951, p=0.0
mRNA Profile
Protein Expression Profile
DPA
0 1 2 3 4 5 6 7 8 9 10 11 12 13 14 15 16 17 18 19
0 1 2 3 4 5 6 7 8 9 10 11 12 13 14 15 16 17 18 19 20 21 22 23 24 25 26 27 28 29 30 31
6 7 8 9 10 11 12 13 14 15 16 17 18 19 20 21 22 23 24

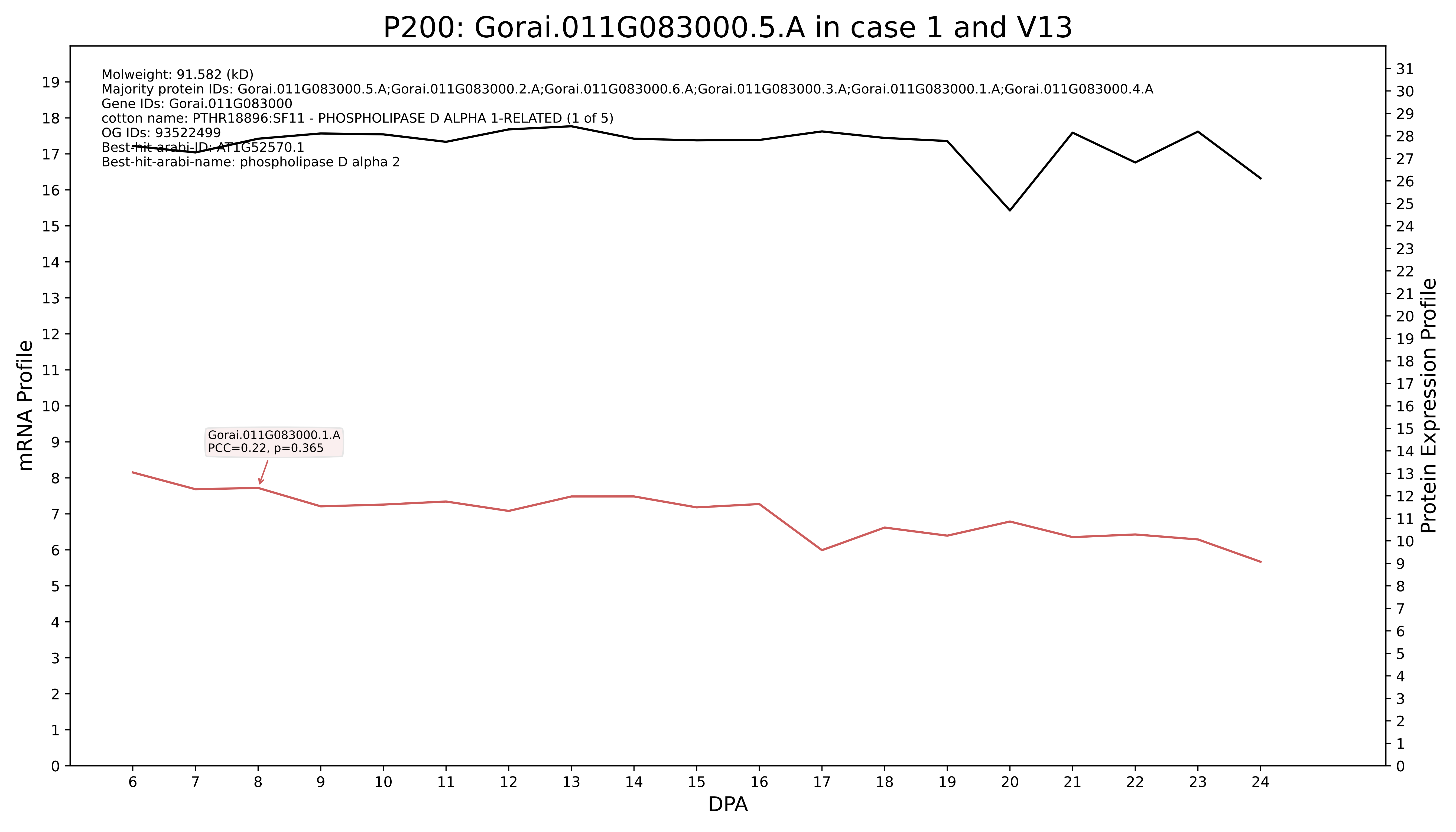
P200: Gorai.011G083000.5.A in case 1 and V13
Molweight: 91.582 (kD)
Majority protein IDs: Gorai.011G083000.5.A;Gorai.011G083000.2.A;Gorai.011G083000.6.A;Gorai.011G083000.3.A;Gorai.011G083000.1.A;Gorai.011G083000.4.A
Gene IDs: Gorai.011G083000
cotton name: PTHR18896:SF11 - PHOSPHOLIPASE D ALPHA 1-RELATED (1 of 5)
OG IDs: 93522499
Best-hit-arabi-ID: AT1G52570.1
Best-hit-arabi-name: phospholipase D alpha 2
Gorai.011G083000.1.A
PCC=0.22, p=0.365
mRNA Profile
Protein Expression Profile
DPA

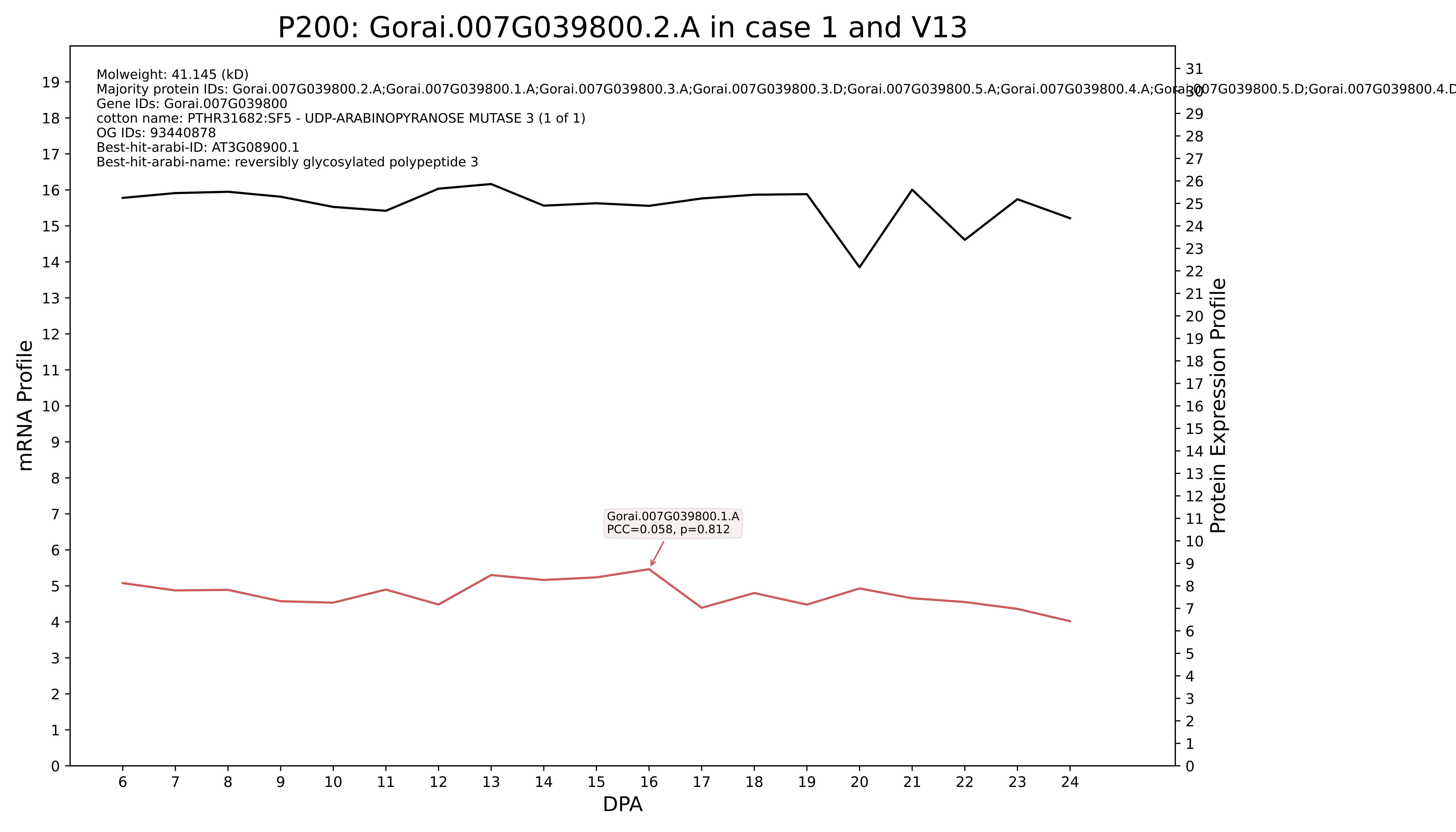

P200: Gorai.007G039800.2.A in case 1 and V13
Molweight: 41.145 (kD)
Majority protein IDs: Gorai.007G039800.2.A;Gorai.007G039800.1.A;Gorai.007G039800.3.A;Gorai.007G039800.3.D;Gorai.007G039800.5.A;Gorai.007G039800.4.A;Gorai.007G039800.5.D;Gorai.007G039800.4.D
Gene IDs: Gorai.007G039800
cotton name: PTHR31682:SF5 - UDP-ARABINOPYRANOSE MUTASE 3 (1 of 1)
OG IDs: 93440878
Best-hit-arabi-ID: AT3G08900.1
Best-hit-arabi-name: reversibly glycosylated polypeptide 3
Gorai.007G039800.1.A
PCC=0.058, p=0.812
mRNA Profile
Protein Expression Profile
DPA

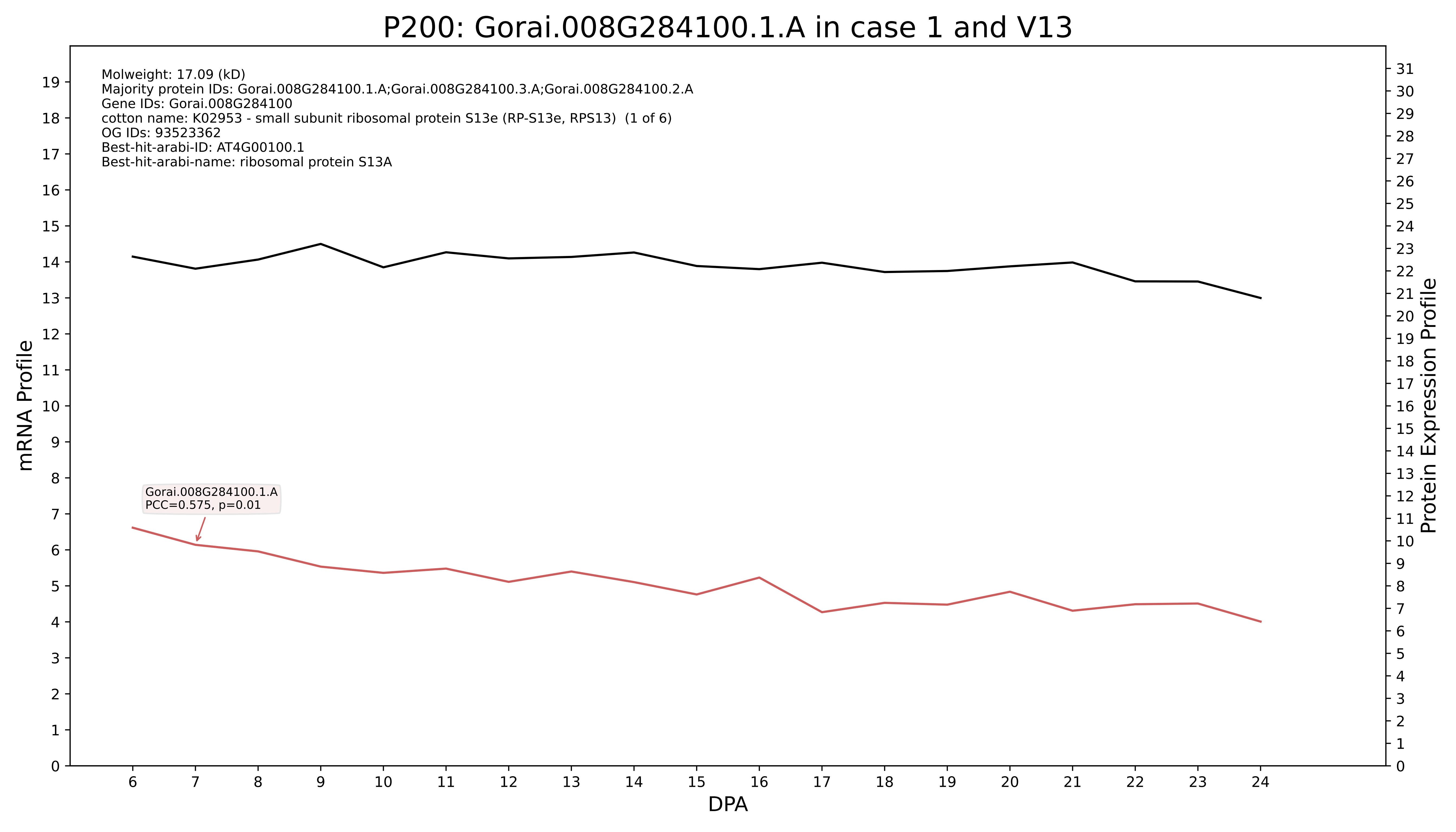
P200: Gorai.008G284100.1.A in case 1 and V13
Molweight: 17.09 (kD)
Majority protein IDs: Gorai.008G284100.1.A;Gorai.008G284100.3.A;Gorai.008G284100.2.A
Gene IDs: Gorai.008G284100
cotton name: K02953 - small subunit ribosomal protein S13e (RP-S13e, RPS13) (1 of 6)
OG IDs: 93523362
Best-hit-arabi-ID: AT4G00100.1
Best-hit-arabi-name: ribosomal protein S13A
Gorai.008G284100.1.A
PCC=0.575, p=0.01
mRNA Profile
Protein Expression Profile
DPA
0
1
2
3
4
5
6
7
8
9
10
11
12
13
14
15
16
17
18
19
20
21
22
23
24
25
26
27
28
29
30
31

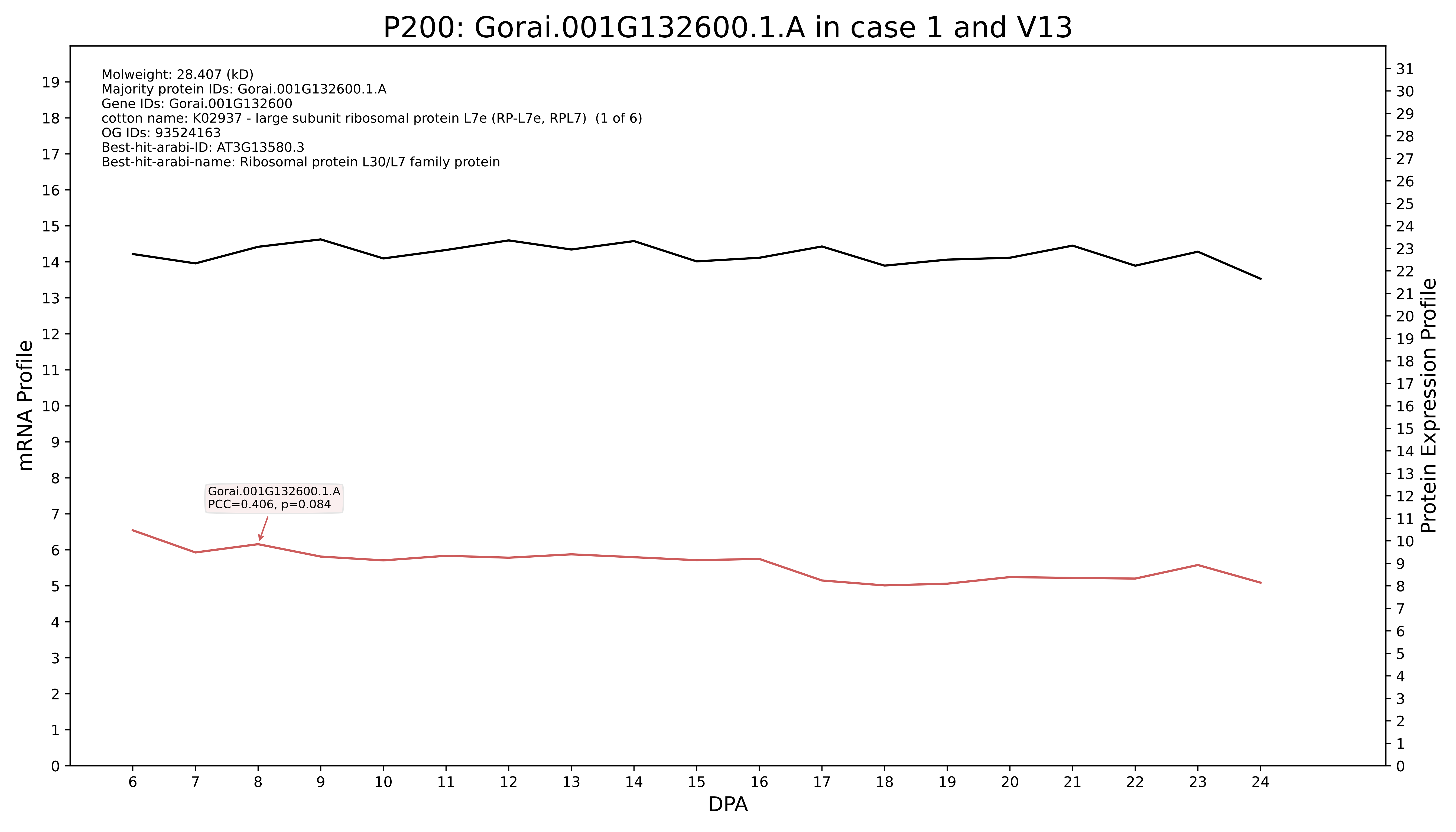
P200: Gorai.001G132600.1.A in case 1 and V13
Molweight: 28.407 (kD)
Majority protein IDs: Gorai.001G132600.1.A
Gene IDs: Gorai.001G132600
cotton name: K02937 - large subunit ribosomal protein L7e (RP-L7e, RPL7) (1 of 6)
OG IDs: 93524163
Best-hit-arabi-ID: AT3G13580.3
Best-hit-arabi-name: Ribosomal protein L30/L7 family protein
Gorai.001G132600.1.A
PCC=0.406, p=0.084
mRNA Profile
Protein Expression Profile
DPA

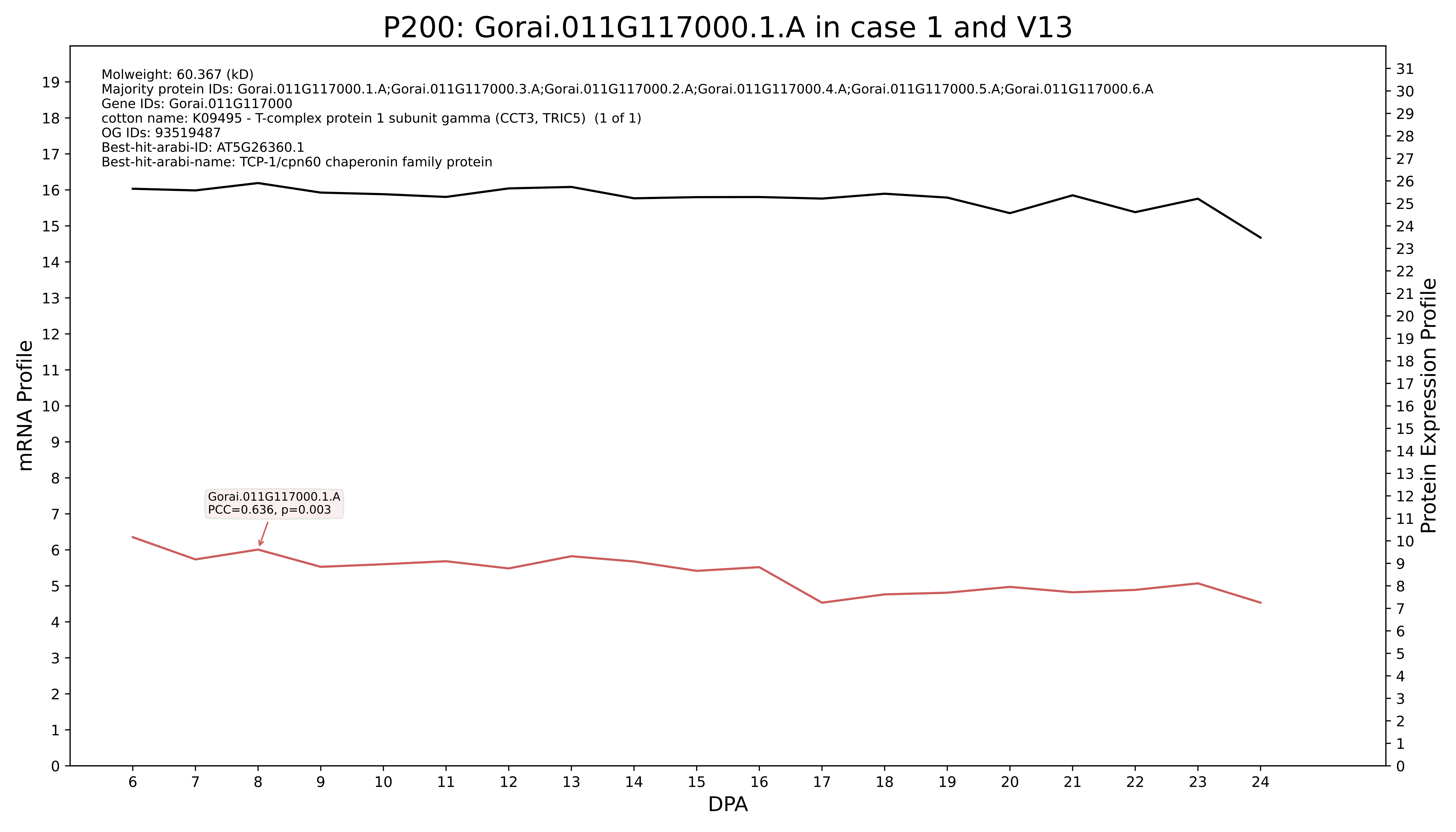
P200: Gorai.011G117000.1.A in case 1 and V13
Molweight: 60.367 (kD)
Majority protein IDs: Gorai.011G117000.1.A;Gorai.011G117000.3.A;Gorai.011G117000.2.A;Gorai.011G117000.4.A;Gorai.011G117000.5.A;Gorai.011G117000.6.A
Gene IDs: Gorai.011G117000
cotton name: K09495 - T-complex protein 1 subunit gamma (CCT3, TRIC5) (1 of 1)
OG IDs: 93519487
Best-hit-arabi-ID: AT5G26360.1
Best-hit-arabi-name: TCP-1/cpn60 chaperonin family protein
Gorai.011G117000.1.A
PCC=0.636, p=0.003
mRNA Profile
Protein Expression Profile
DPA

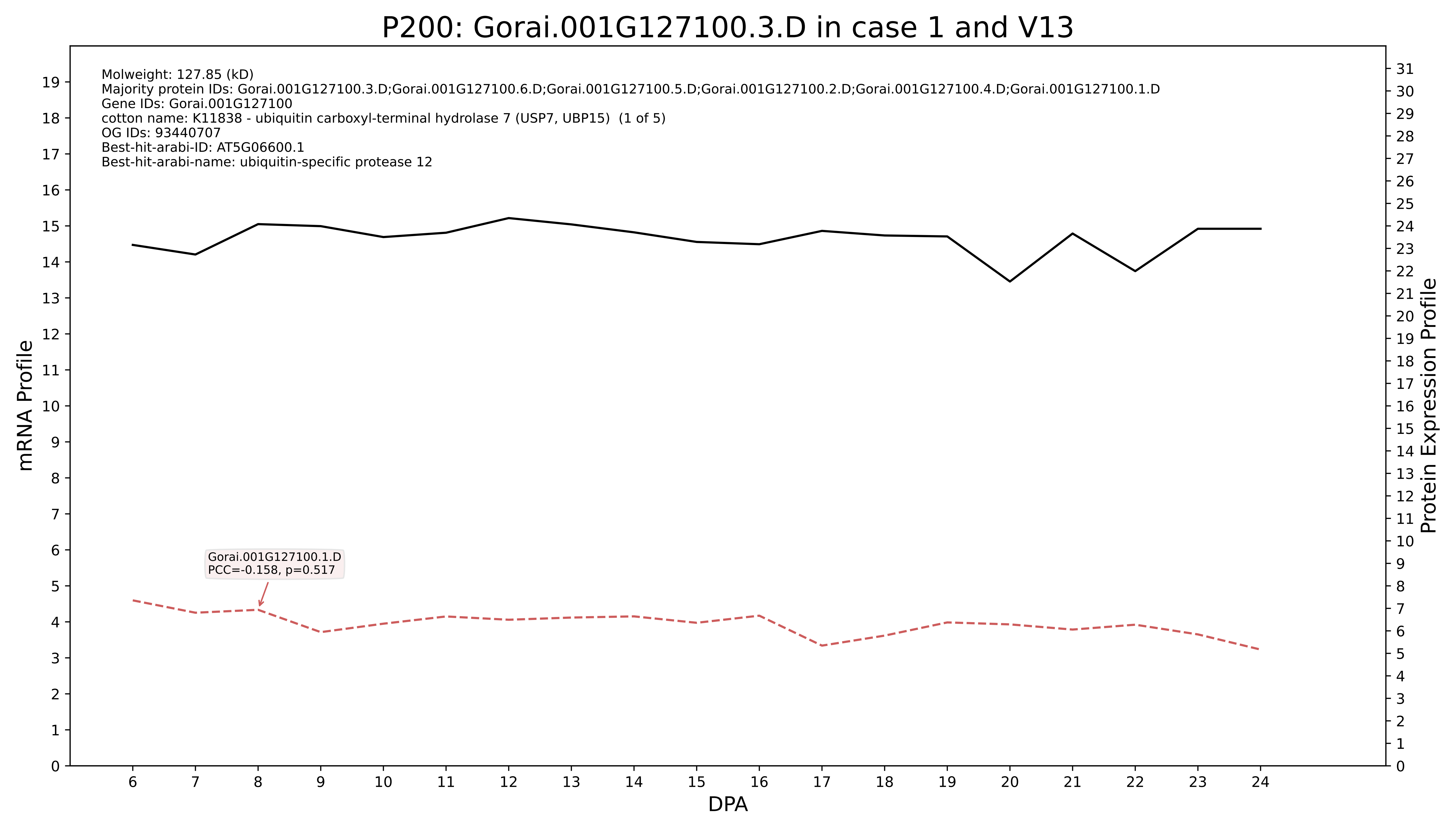
P200: Gorai.001G127100.3.D in case 1 and V13
Molweight: 127.85 (kD)
Majority protein IDs: Gorai.001G127100.3.D;Gorai.001G127100.6.D;Gorai.001G127100.5.D;Gorai.001G127100.2.D;Gorai.001G127100.4.D;Gorai.001G127100.1.D
Gene IDs: Gorai.001G127100
cotton name: K11838 - ubiquitin carboxyl-terminal hydrolase 7 (USP7, UBP15) (1 of 5)
OG IDs: 93440707
Best-hit-arabi-ID: AT5G06600.1
Best-hit-arabi-name: ubiquitin-specific protease 12
Gorai.001G127100.1.D
PCC=-0.158, p=0.517
mRNA Profile
Protein Expression Profile
DPA

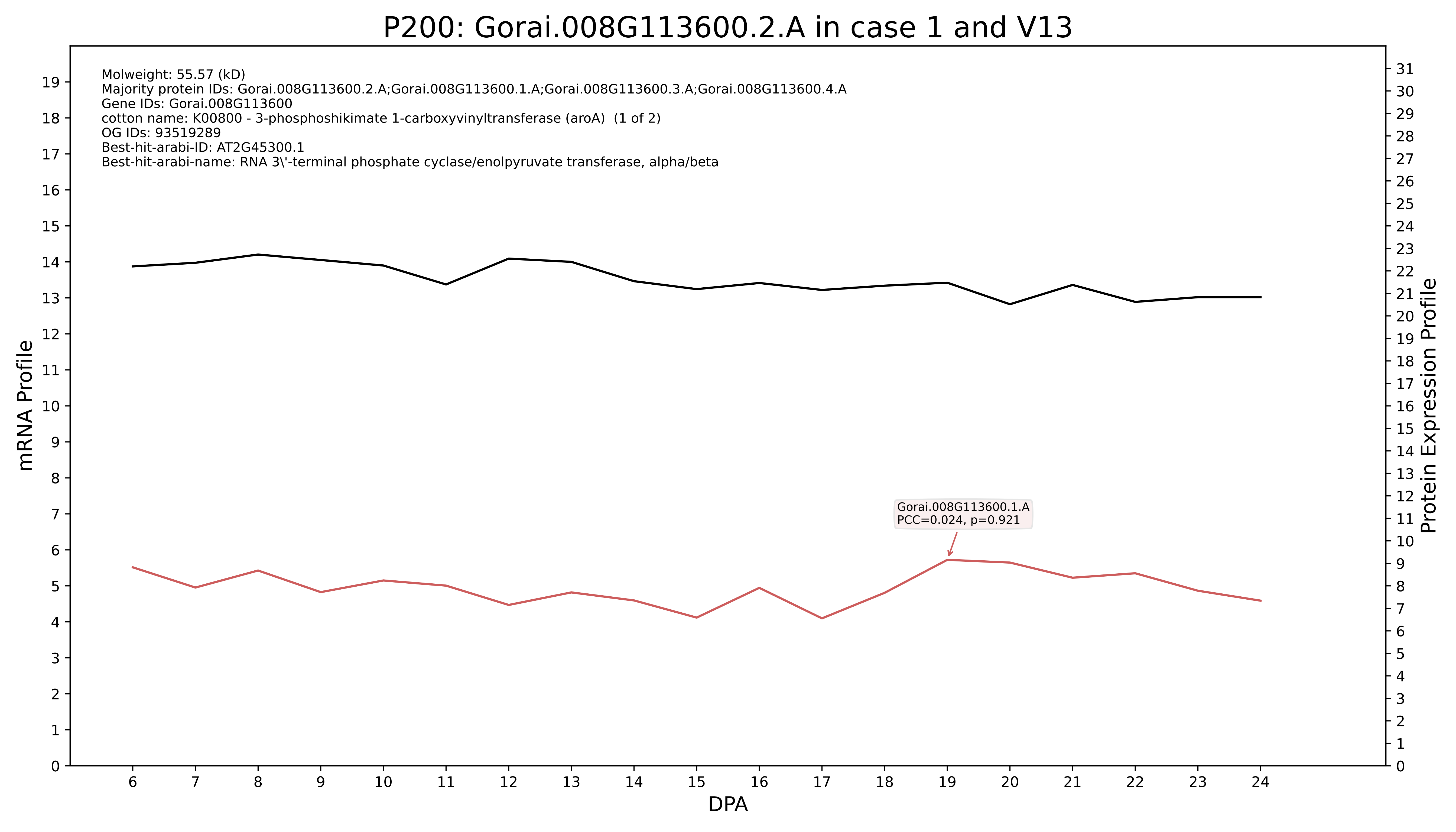
P200: Gorai.008G113600.2.A in case 1 and V13
Molweight: 55.57 (kD)
Majority protein IDs: Gorai.008G113600.2.A;Gorai.008G113600.1.A;Gorai.008G113600.3.A;Gorai.008G113600.4.A
Gene IDs: Gorai.008G113600
cotton name: K00800 - 3-phosphoshikimate 1-carboxyvinyltransferase (aroA) (1 of 2)
OG IDs: 93519289
Best-hit-arabi-ID: AT2G45300.1
Best-hit-arabi-name: RNA 3\'-terminal phosphate cyclase/enolpyruvate transferase, alpha/beta
Gorai.008G113600.1.A
PCC=0.024, p=0.921
mRNA Profile
Protein Expression Profile
DPA

P200: Gorai.005G125000.1.D in case 1 and V13

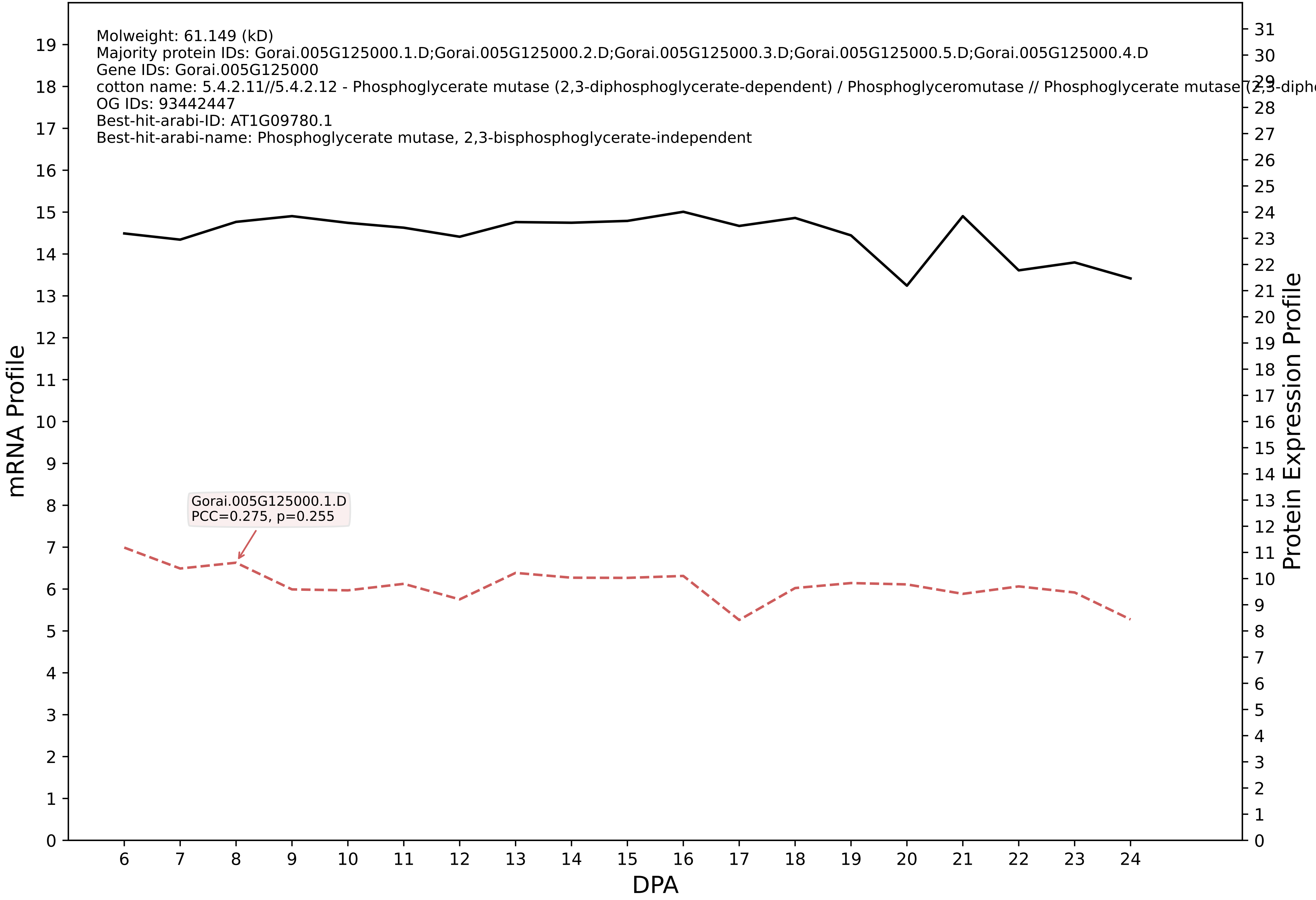

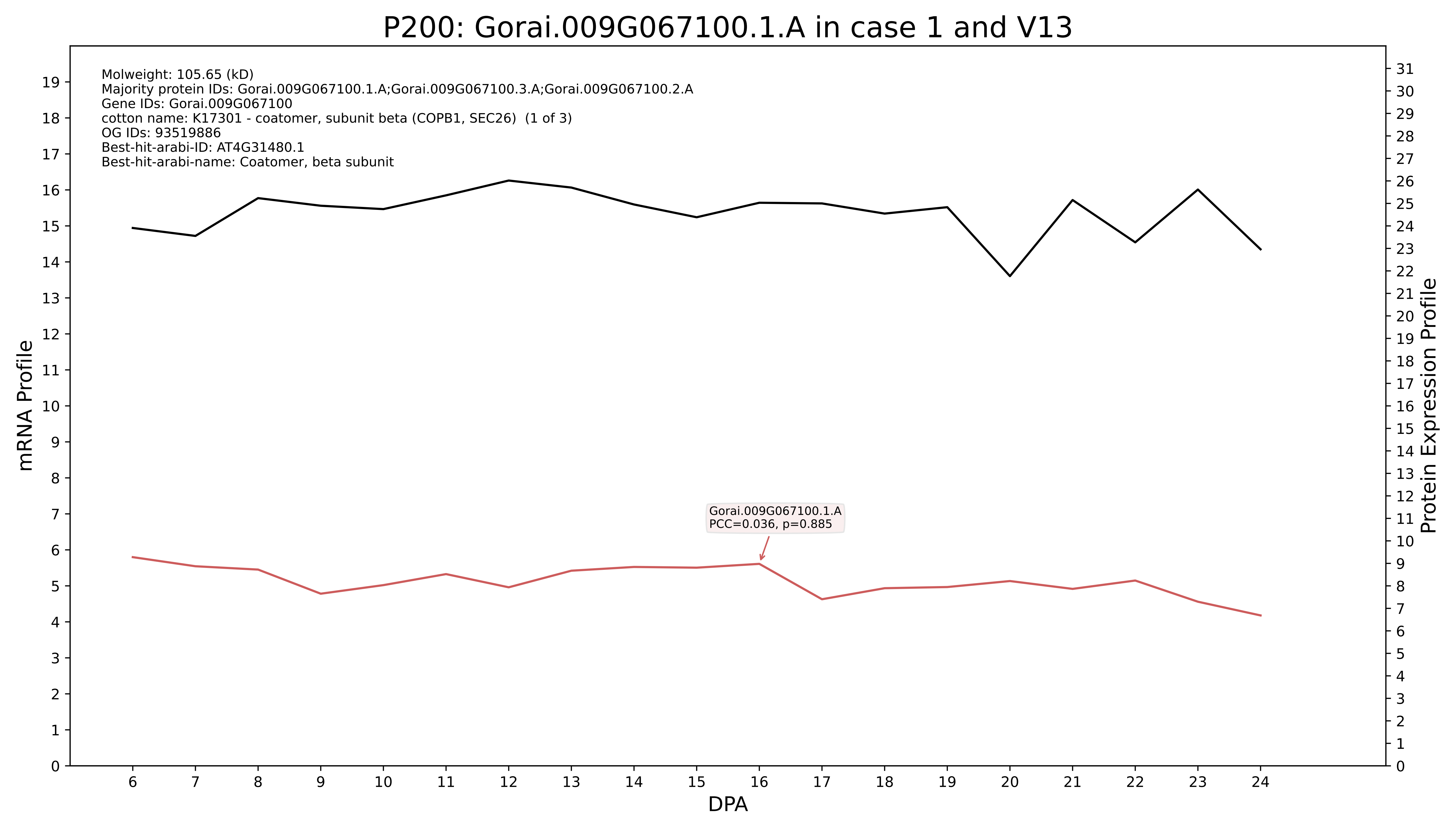
P200: Gorai.009G067100.1.A in case 1 and V13
Molweight: 105.65 (kD)
Majority protein IDs: Gorai.009G067100.1.A;Gorai.009G067100.3.A;Gorai.009G067100.2.A
Gene IDs: Gorai.009G067100
cotton name: K17301 - coatomer, subunit beta (COPB1, SEC26) (1 of 3)
OG IDs: 93519886
Best-hit-arabi-ID: AT4G31480.1
Best-hit-arabi-name: Coatomer, beta subunit
Gorai.009G067100.1.A
PCC=0.036, p=0.885
mRNA Profile
Protein Expression Profile
DPA

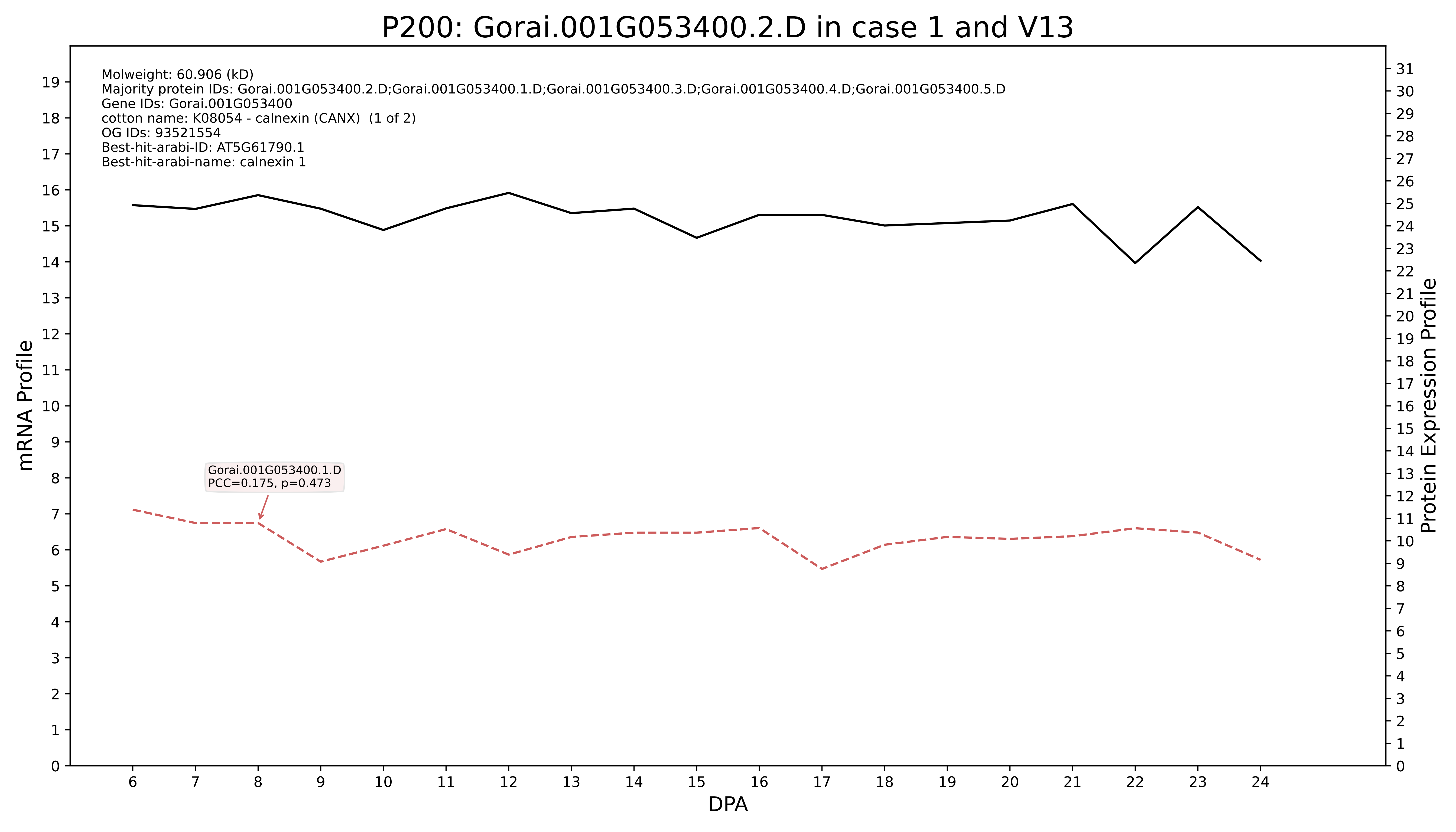

P200: Gorai.001G053400.2.D in case 1 and V13
Molweight: 60.906 (kD)
Majority protein IDs: Gorai.001G053400.2.D;Gorai.001G053400.1.D;Gorai.001G053400.3.D;Gorai.001G053400.4.D;Gorai.001G053400.5.D
Gene IDs: Gorai.001G053400
cotton name: K08054 - calnexin (CANX) (1 of 2)
OG IDs: 93521554
Best-hit-arabi-ID: AT5G61790.1
Best-hit-arabi-name: calnexin 1
Gorai.001G053400.1.D
PCC=0.175, p=0.473
mRNA Profile
Protein Expression Profile
DPA

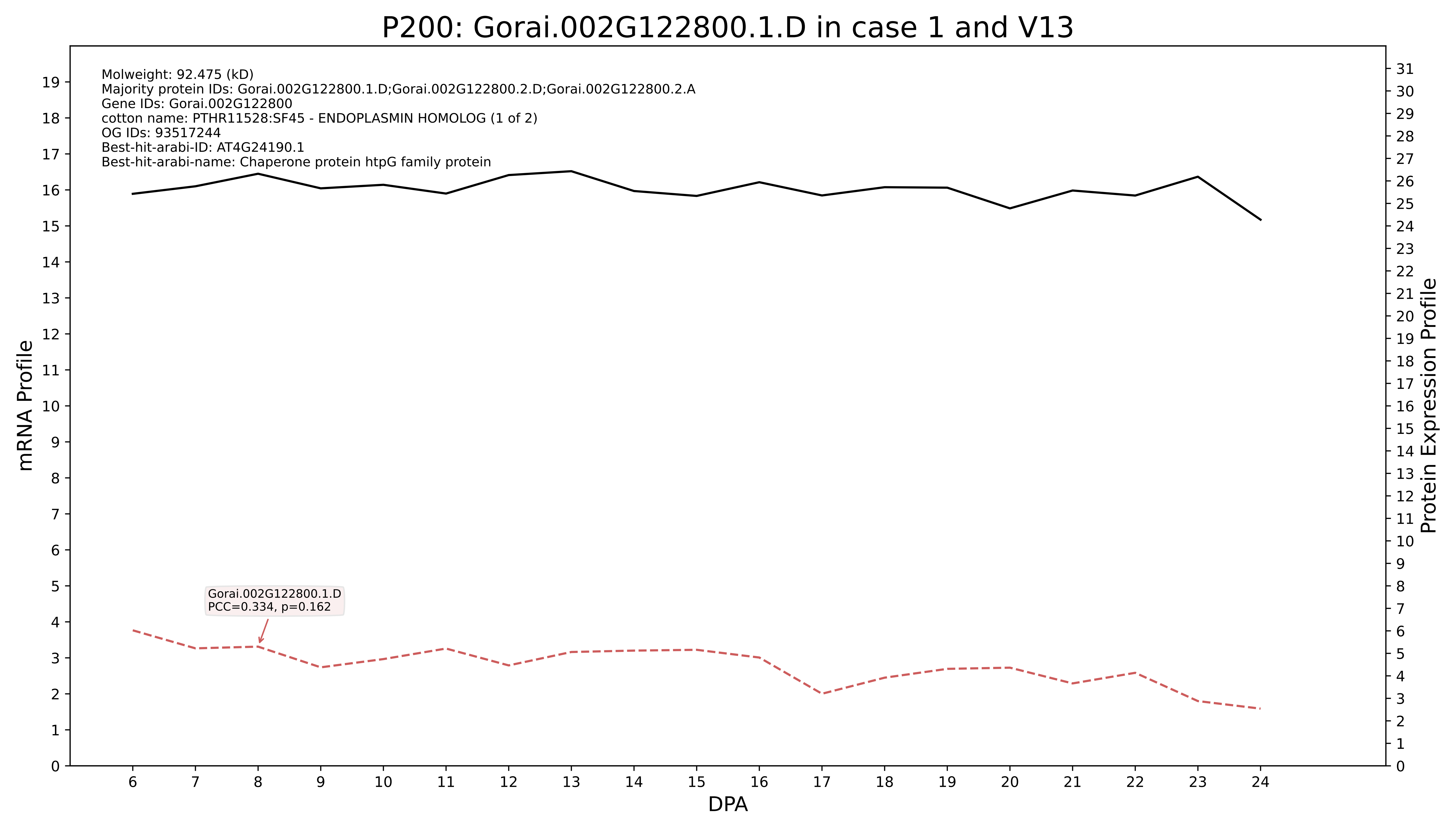
P200: Gorai.002G122800.1.D in case 1 and V13
Molweight: 92.475 (kD)
Majority protein IDs: Gorai.002G122800.1.D;Gorai.002G122800.2.D;Gorai.002G122800.2.A
Gene IDs: Gorai.002G122800
cotton name: PTHR11528:SF45 - ENDOPLASMIN HOMOLOG (1 of 2)
OG IDs: 93517244
Best-hit-arabi-ID: AT4G24190.1
Best-hit-arabi-name: Chaperone protein htpG family protein
Gorai.002G122800.1.D
PCC=0.334, p=0.162
mRNA Profile
Protein Expression Profile
DPA

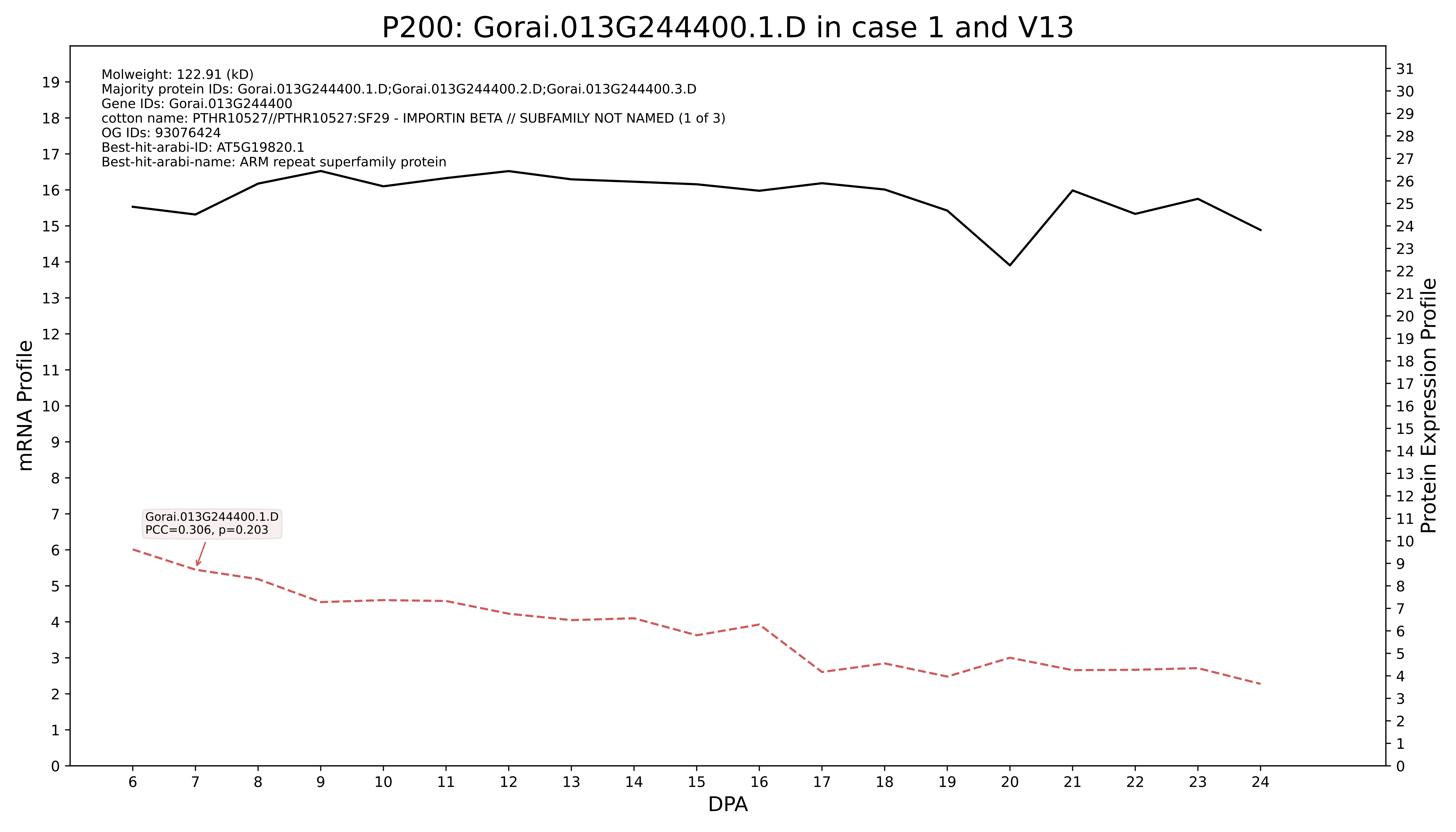
P200: Gorai.013G244400.1.D in case 1 and V13
Molweight: 122.91 (kD)
Majority protein IDs: Gorai.013G244400.1.D;Gorai.013G244400.2.D;Gorai.013G244400.3.D
Gene IDs: Gorai.013G244400
cotton name: PTHR10527//PTHR10527:SF29 - IMPORTIN BETA // SUBFAMILY NOT NAMED (1 of 3)
OG IDs: 93076424
Best-hit-arabi-ID: AT5G19820.1
Best-hit-arabi-name: ARM repeat superfamily protein
Gorai.013G244400.1.D
PCC=0.306, p=0.203
mRNA Profile
Protein Expression Profile
DPA

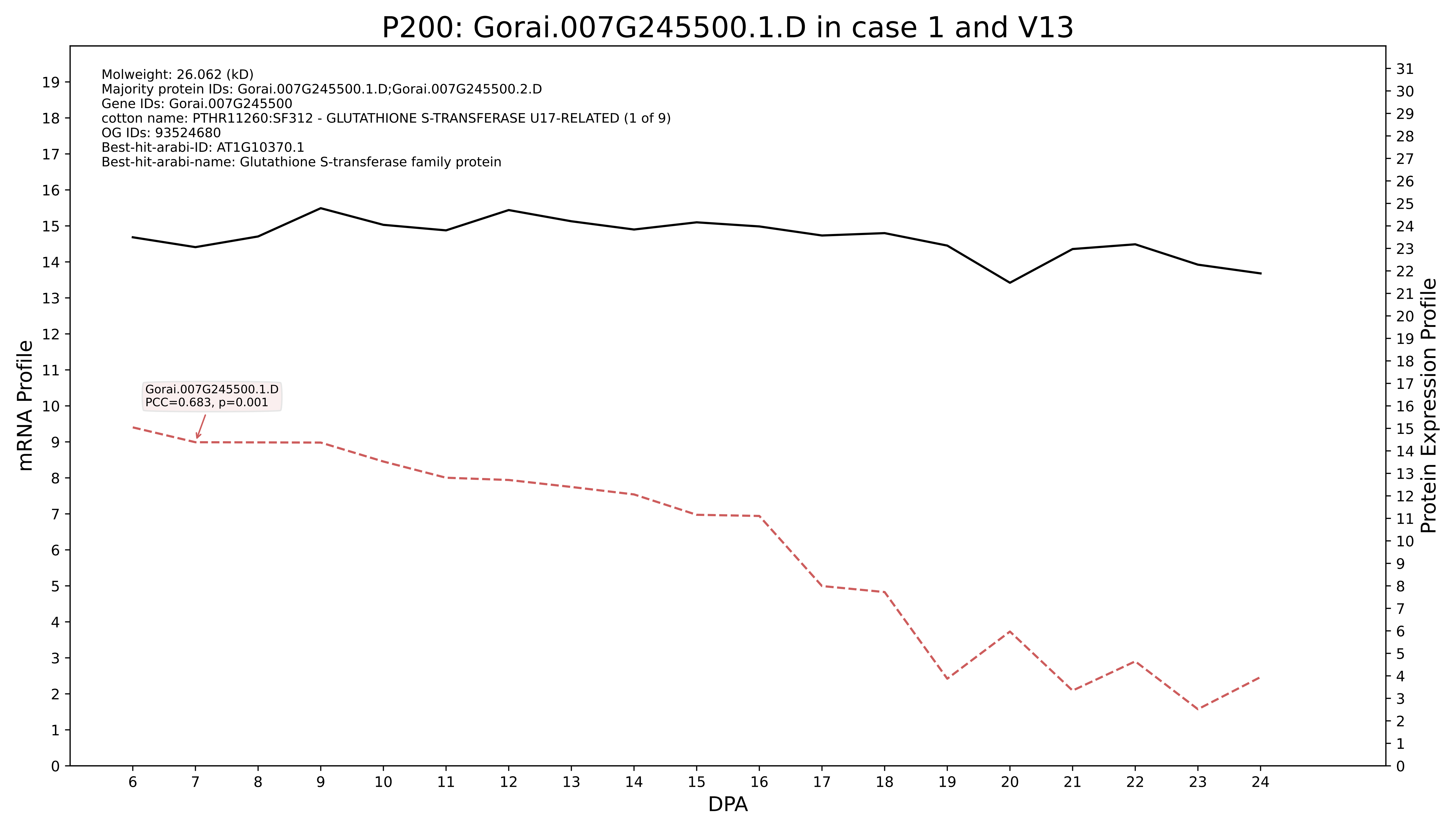
P200: Gorai.007G245500.1.D in case 1 and V13
Molweight: 26.062 (kD)
Majority protein IDs: Gorai.007G245500.1.D;Gorai.007G245500.2.D
Gene IDs: Gorai.007G245500
cotton name: PTHR11260:SF312 - GLUTATHIONE S-TRANSFERASE U17-RELATED (1 of 9)
OG IDs: 93524680
Best-hit-arabi-ID: AT1G10370.1
Best-hit-arabi-name: Glutathione S-transferase family protein
Gorai.007G245500.1.D
PCC=0.683, p=0.001
mRNA Profile
Protein Expression Profile
DPA
0 1 2 3 4 5 6 7 8 9 10 11 12 13 14 15 16 17 18 19
0 1 2 3 4 5 6 7 8 9 10 11 12 13 14 15 16 17 18 19 20 21 22 23 24 25 26 27 28 29 30 31
6 7 8 9 10 11 12 13 14 15 16 17 18 19 20 21 22 23 24

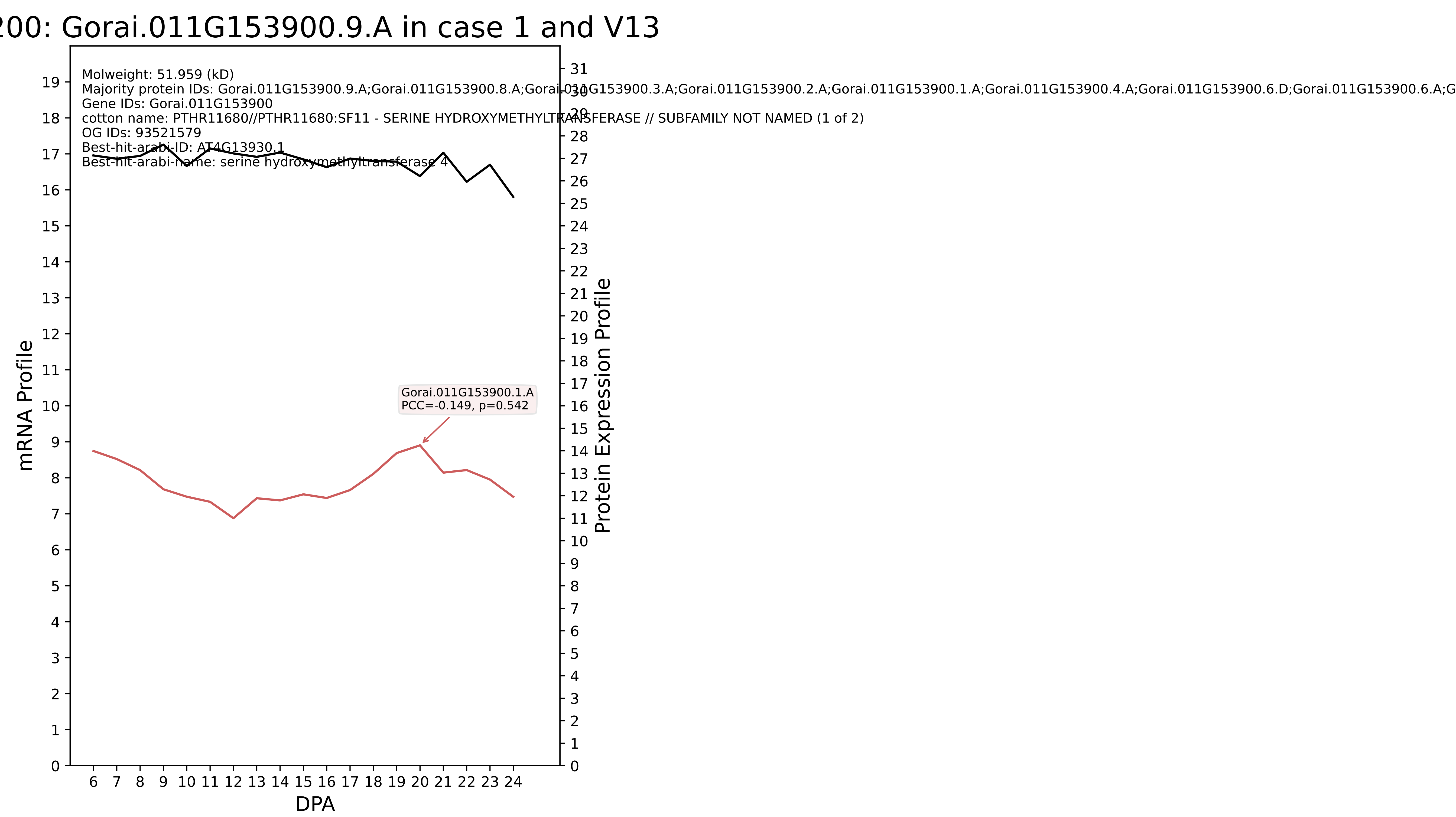
200: Gorai.011G153900.9.A in case 1 and V13
Molweight: 51.959 (kD)
Majority protein IDs: Gorai.011G153900.9.A;Gorai.011G153900.8.A;Gorai.011G153900.3.A;Gorai.011G153900.2.A;Gorai.011G153900.1.A;Gorai.011G153900.4.A;Gorai.011G153900.6.D;Gorai.011G153900.6.A;G
Gene IDs: Gorai.011G153900
cotton name: PTHR11680//PTHR11680:SF11 - SERINE HYDROXYMETHYLTRANSFERASE // SUBFAMILY NOT NAMED (1 of 2)
OG IDs: 93521579
Best-hit-arabi-ID: AT4G13930.1
Best-hit-arabi-name: serine hydroxymethyltransferase 4
Gorai.011G153900.1.A
PCC=-0.149, p=0.542
mRNA Profile
Protein Expression Profile
DPA

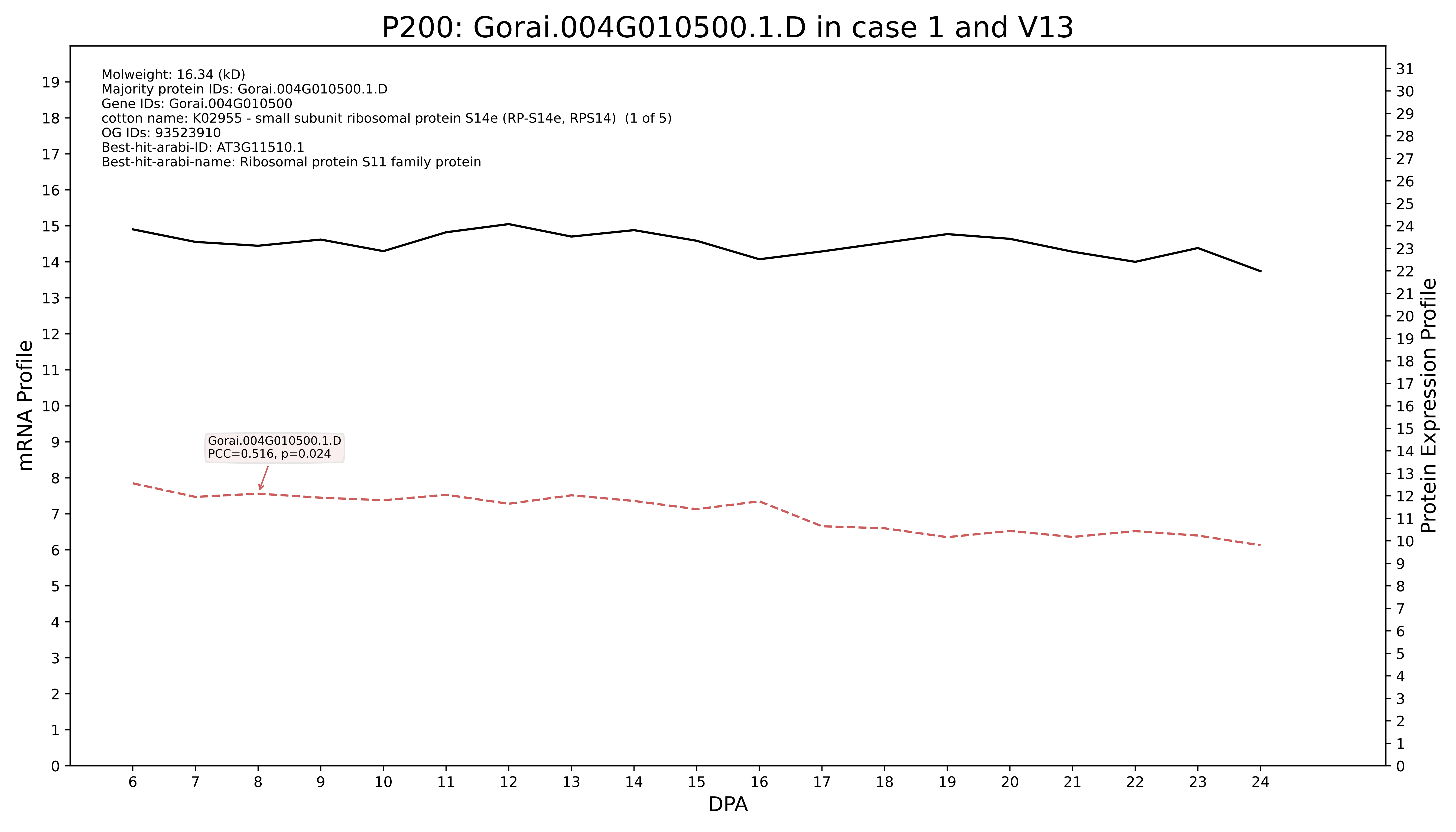
P200: Gorai.004G010500.1.D in case 1 and V13
Molweight: 16.34 (kD)
Majority protein IDs: Gorai.004G010500.1.D
Gene IDs: Gorai.004G010500
cotton name: K02955 - small subunit ribosomal protein S14e (RP-S14e, RPS14) (1 of 5)
OG IDs: 93523910
Best-hit-arabi-ID: AT3G11510.1
Best-hit-arabi-name: Ribosomal protein S11 family protein
Gorai.004G010500.1.D
PCC=0.516, p=0.024
mRNA Profile
Protein Expression Profile
DPA

P200: Gorai.006G212500.5.A in case 1 and V13

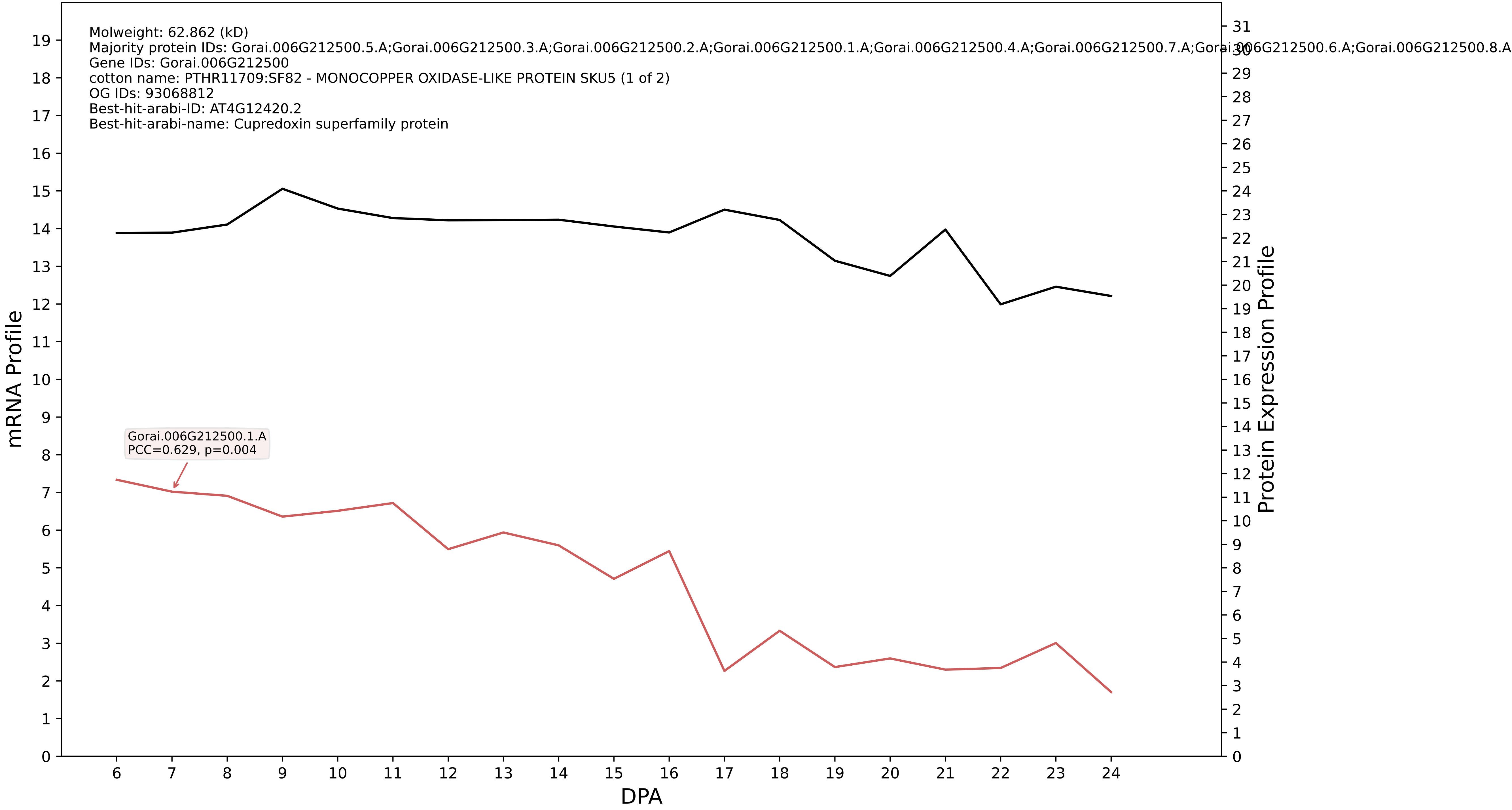

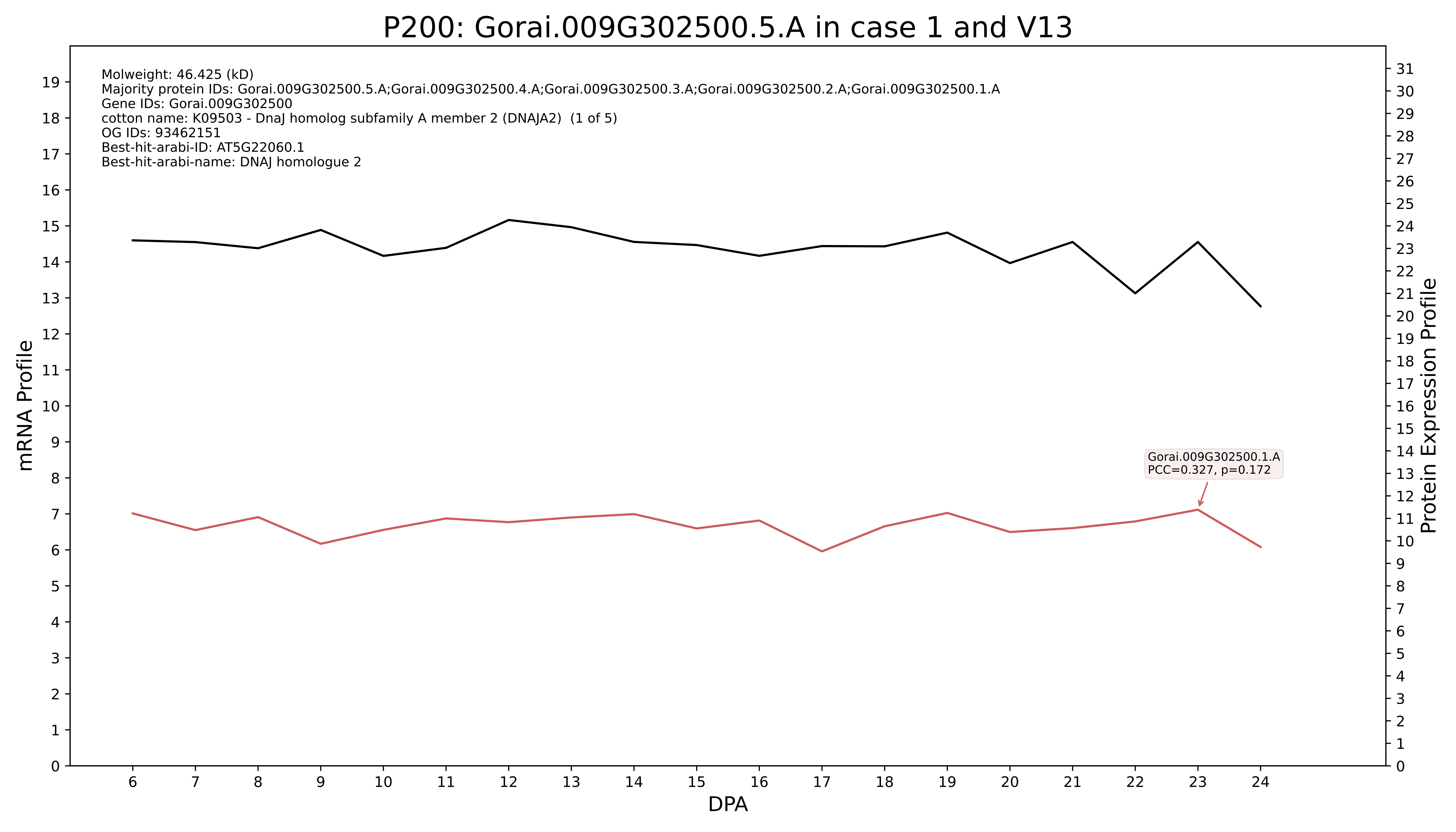
P200: Gorai.009G302500.5.A in case 1 and V13
Molweight: 46.425 (kD)
Majority protein IDs: Gorai.009G302500.5.A;Gorai.009G302500.4.A;Gorai.009G302500.3.A;Gorai.009G302500.2.A;Gorai.009G302500.1.A
Gene IDs: Gorai.009G302500
cotton name: K09503 - DnaJ homolog subfamily A member 2 (DNAJA2) (1 of 5)
OG IDs: 93462151
Best-hit-arabi-ID: AT5G22060.1
Best-hit-arabi-name: DNAJ homologue 2
Gorai.009G302500.1.A
PCC=0.327, p=0.172
mRNA Profile
Protein Expression Profile
DPA

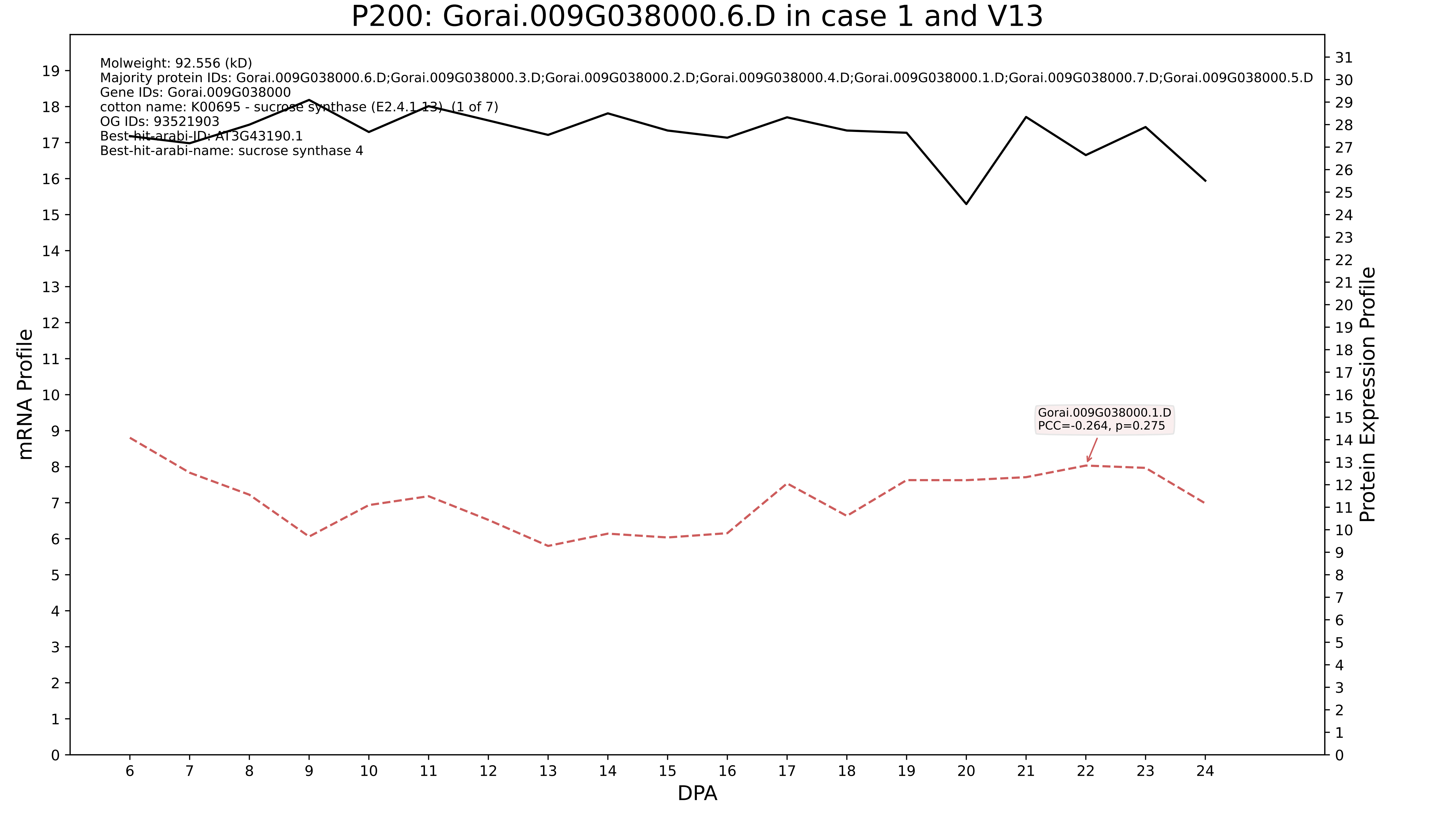
P200: Gorai.009G038000.6.D in case 1 and V13
Molweight: 92.556 (kD)
Majority protein IDs: Gorai.009G038000.6.D;Gorai.009G038000.3.D;Gorai.009G038000.2.D;Gorai.009G038000.4.D;Gorai.009G038000.1.D;Gorai.009G038000.7.D;Gorai.009G038000.5.D
Gene IDs: Gorai.009G038000
cotton name: K00695 - sucrose synthase (E2.4.1.13) (1 of 7)
OG IDs: 93521903
Best-hit-arabi-ID: AT3G43190.1
Best-hit-arabi-name: sucrose synthase 4
Gorai.009G038000.1.D
PCC=-0.264, p=0.275
mRNA Profile
Protein Expression Profile
DPA

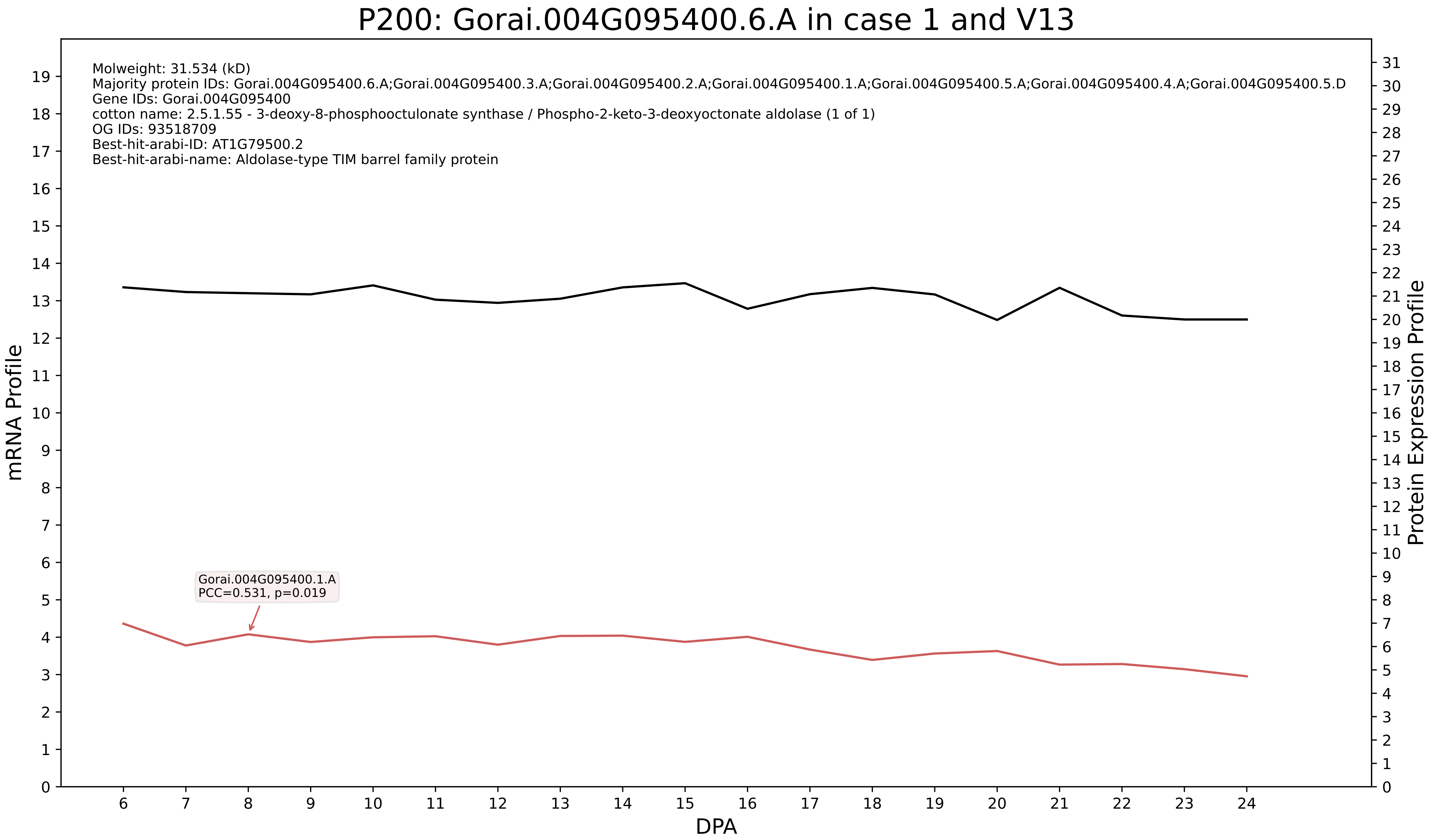
P200: Gorai.004G095400.6.A in case 1 and V13
Molweight: 31.534 (kD)
Majority protein IDs: Gorai.004G095400.6.A;Gorai.004G095400.3.A;Gorai.004G095400.2.A;Gorai.004G095400.1.A;Gorai.004G095400.5.A;Gorai.004G095400.4.A;Gorai.004G095400.5.D
Gene IDs: Gorai.004G095400
cotton name: 2.5.1.55 - 3-deoxy-8-phosphooctulonate synthase / Phospho-2-keto-3-deoxyoctonate aldolase (1 of 1)
OG IDs: 93518709
Best-hit-arabi-ID: AT1G79500.2
Best-hit-arabi-name: Aldolase-type TIM barrel family protein
Gorai.004G095400.1.A
PCC=0.531, p=0.019
mRNA Profile
Protein Expression Profile
DPA

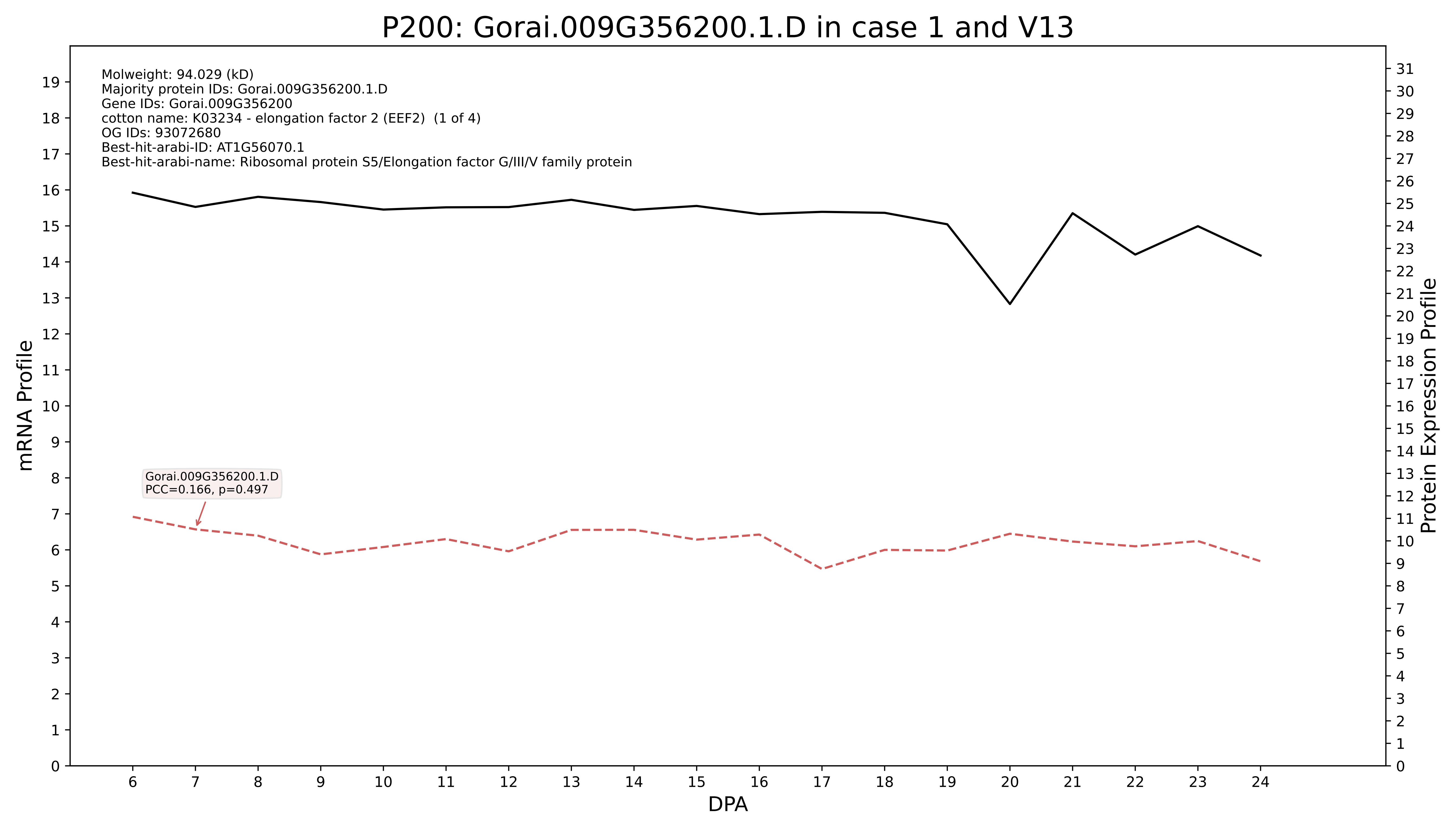

P200: Gorai.009G356200.1.D in case 1 and V13
Molweight: 94.029 (kD)
Majority protein IDs: Gorai.009G356200.1.D
Gene IDs: Gorai.009G356200
cotton name: K03234 - elongation factor 2 (EEF2) (1 of 4)
OG IDs: 93072680
Best-hit-arabi-ID: AT1G56070.1
Best-hit-arabi-name: Ribosomal protein S5/Elongation factor G/III/V family protein
Gorai.009G356200.1.D
PCC=0.166, p=0.497
mRNA Profile
Protein Expression Profile
DPA

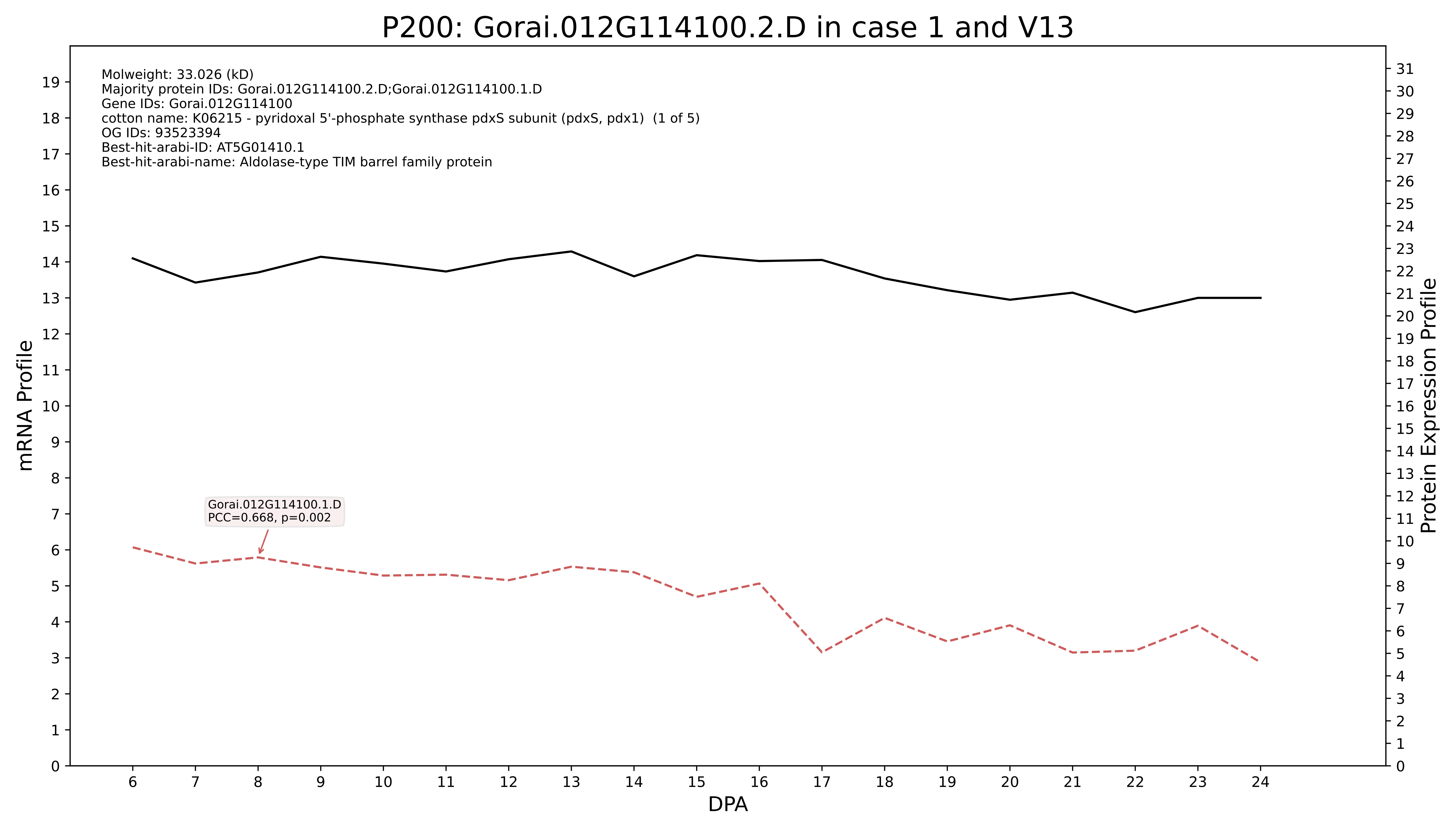

P200: Gorai.012G114100.2.D in case 1 and V13
Molweight: 33.026 (kD)
Majority protein IDs: Gorai.012G114100.2.D;Gorai.012G114100.1.D
Gene IDs: Gorai.012G114100
cotton name: K06215 - pyridoxal 5'-phosphate synthase pdxS subunit (pdxS, pdx1) (1 of 5)
OG IDs: 93523394
Best-hit-arabi-ID: AT5G01410.1
Best-hit-arabi-name: Aldolase-type TIM barrel family protein
Gorai.012G114100.1.D
PCC=0.668, p=0.002
mRNA Profile
Protein Expression Profile
DPA
0
1
2
3
4
5
6
7
8
9
10
11
12
13
14
15
16
17
18
19
20
21
22
23
24
25
26
27
28
29
30
31

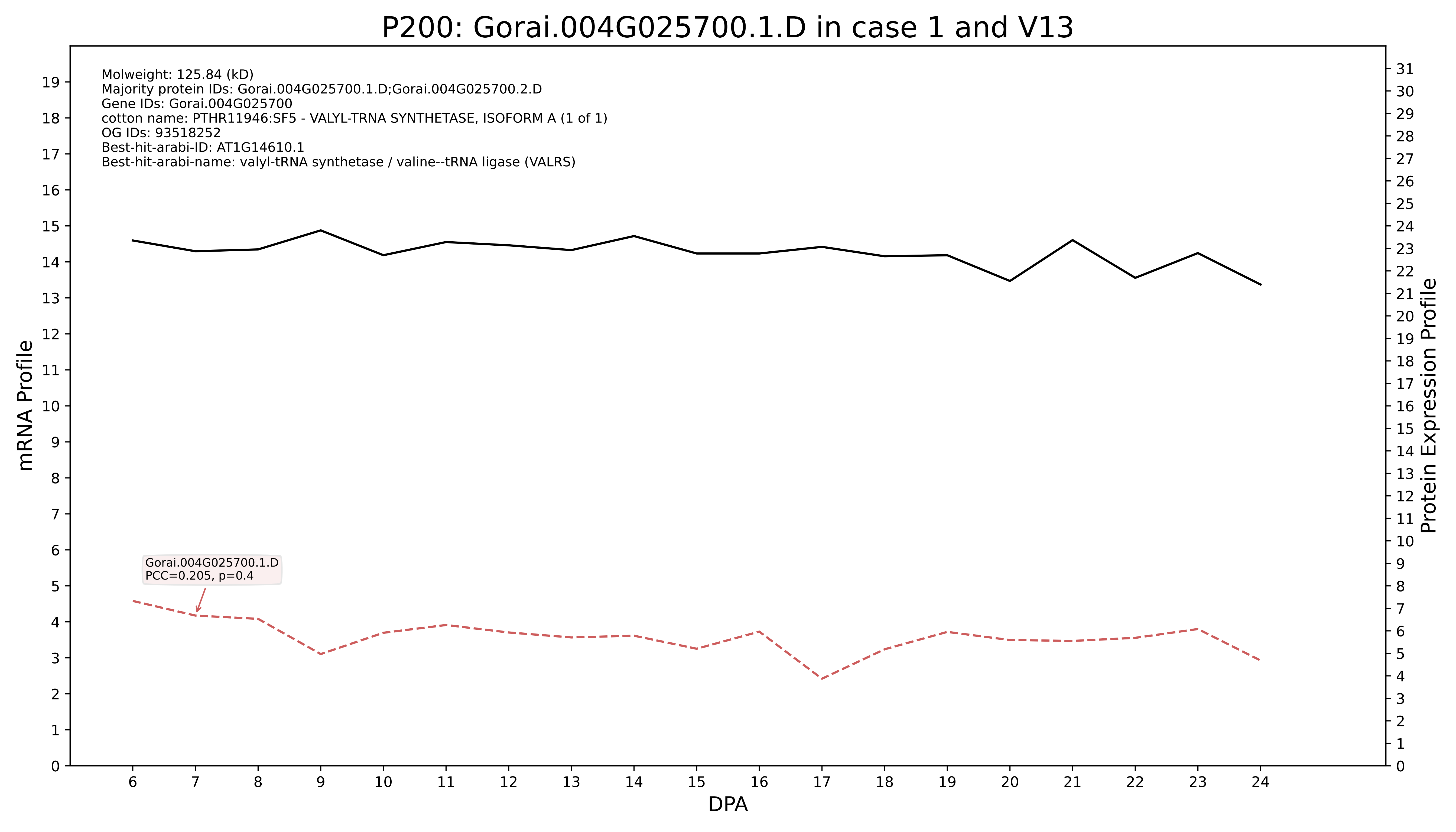
P200: Gorai.004G025700.1.D in case 1 and V13
Molweight: 125.84 (kD)
Majority protein IDs: Gorai.004G025700.1.D;Gorai.004G025700.2.D
Gene IDs: Gorai.004G025700
cotton name: PTHR11946:SF5 - VALYL-TRNA SYNTHETASE, ISOFORM A (1 of 1)
OG IDs: 93518252
Best-hit-arabi-ID: AT1G14610.1
Best-hit-arabi-name: valyl-tRNA synthetase / valine--tRNA ligase (VALRS)
Gorai.004G025700.1.D
PCC=0.205, p=0.4
mRNA Profile
Protein Expression Profile
DPA

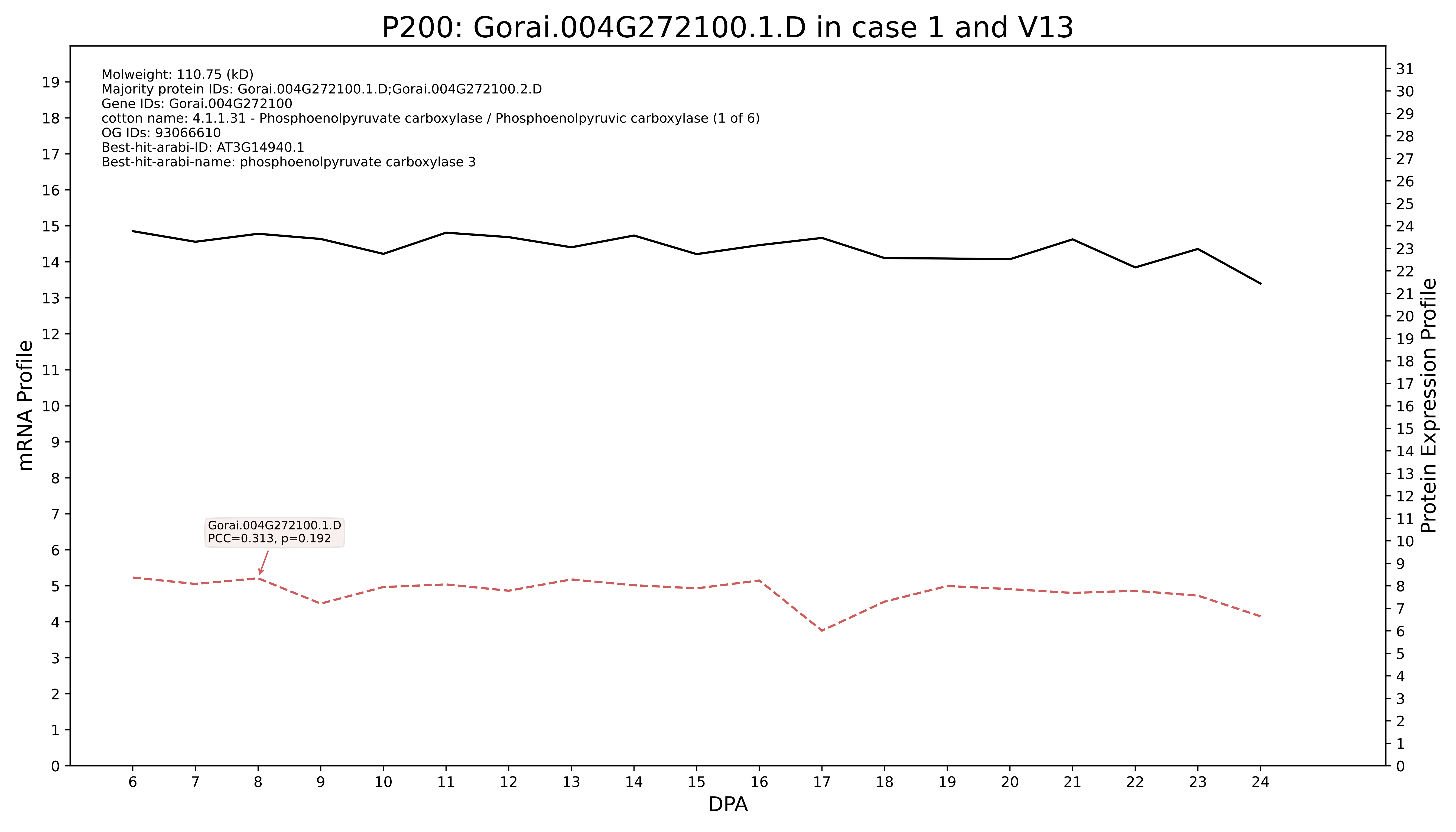
P200: Gorai.004G272100.1.D in case 1 and V13
Molweight: 110.75 (kD)
Majority protein IDs: Gorai.004G272100.1.D;Gorai.004G272100.2.D
Gene IDs: Gorai.004G272100
cotton name: 4.1.1.31 - Phosphoenolpyruvate carboxylase / Phosphoenolpyruvic carboxylase (1 of 6)
OG IDs: 93066610
Best-hit-arabi-ID: AT3G14940.1
Best-hit-arabi-name: phosphoenolpyruvate carboxylase 3
Gorai.004G272100.1.D
PCC=0.313, p=0.192
mRNA Profile
Protein Expression Profile
DPA

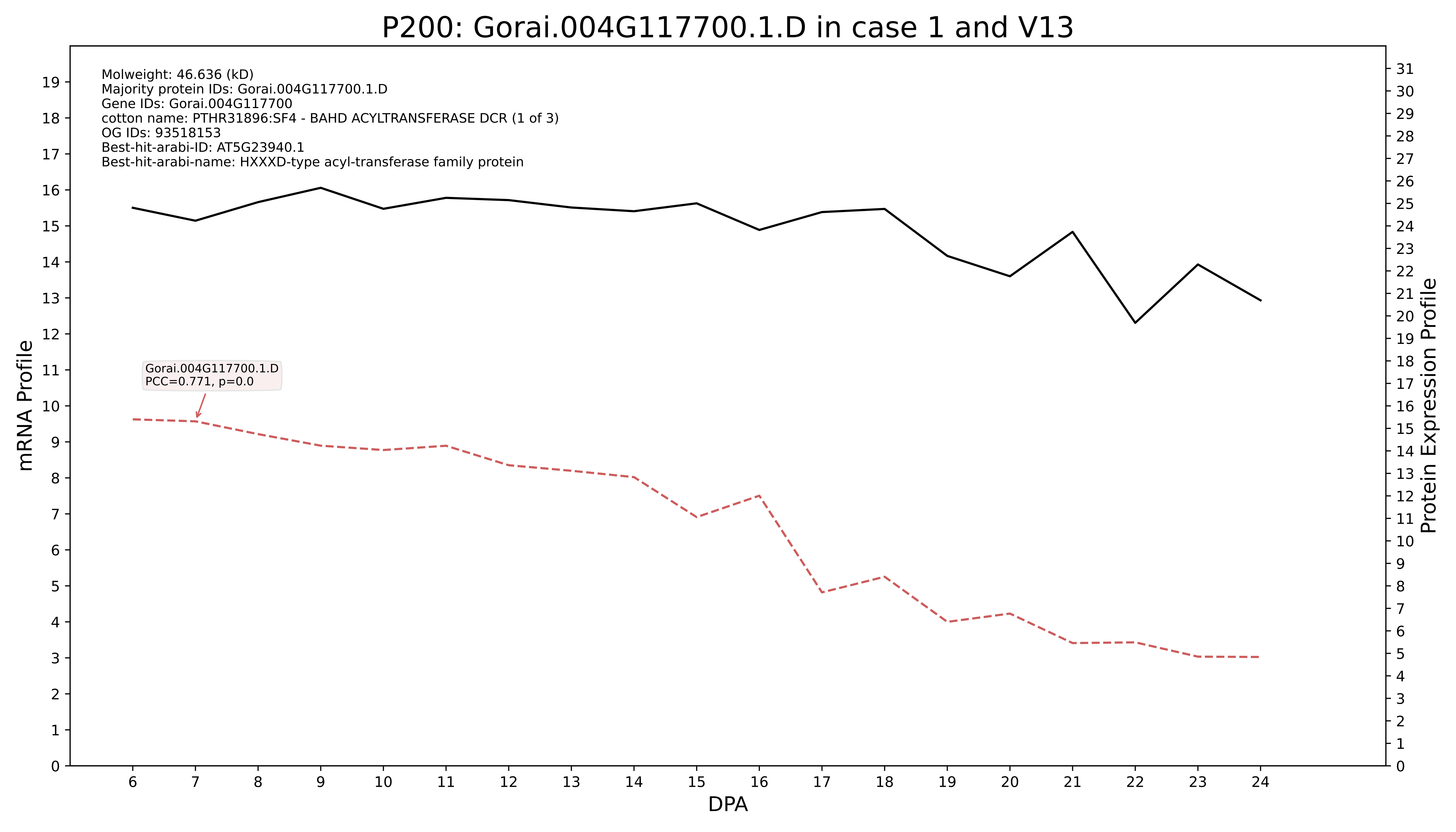

P200: Gorai.004G117700.1.D in case 1 and V13
Molweight: 46.636 (kD)
Majority protein IDs: Gorai.004G117700.1.D
Gene IDs: Gorai.004G117700
cotton name: PTHR31896:SF4 - BAHD ACYLTRANSFERASE DCR (1 of 3)
OG IDs: 93518153
Best-hit-arabi-ID: AT5G23940.1
Best-hit-arabi-name: HXXXD-type acyl-transferase family protein
Gorai.004G117700.1.D
PCC=0.771, p=0.0
mRNA Profile
Protein Expression Profile
DPA

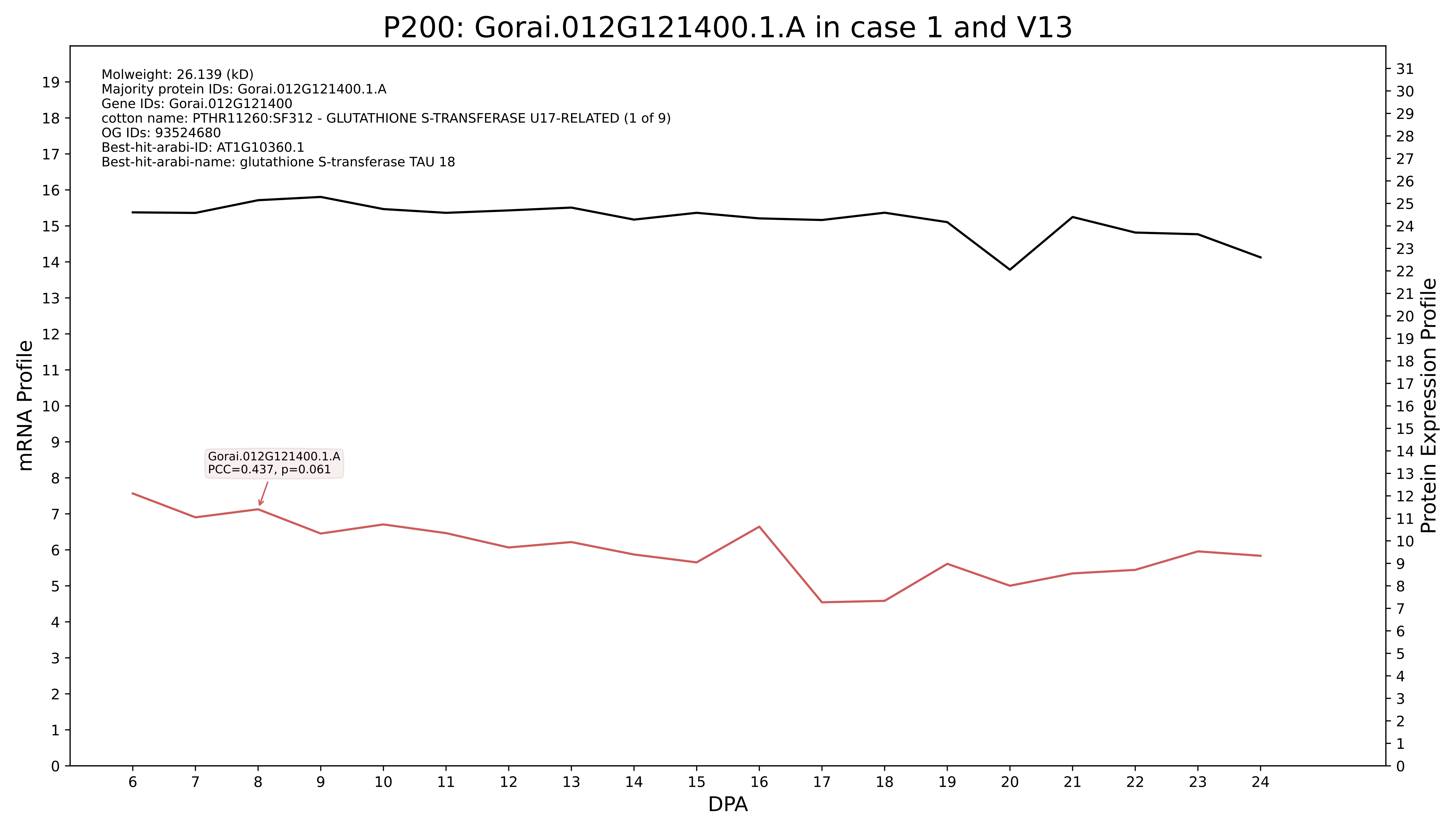
P200: Gorai.012G121400.1.A in case 1 and V13
Molweight: 26.139 (kD)
Majority protein IDs: Gorai.012G121400.1.A
Gene IDs: Gorai.012G121400
cotton name: PTHR11260:SF312 - GLUTATHIONE S-TRANSFERASE U17-RELATED (1 of 9)
OG IDs: 93524680
Best-hit-arabi-ID: AT1G10360.1
Best-hit-arabi-name: glutathione S-transferase TAU 18
Gorai.012G121400.1.A
PCC=0.437, p=0.061
mRNA Profile
Protein Expression Profile
DPA
0 1 2 3 4 5 6 7 8 9 10 11 12 13 14 15 16 17 18 19
0 1 2 3 4 5 6 7 8 9 10 11 12 13 14 15 16 17 18 19 20 21 22 23 24 25 26 27 28 29 30 31
6 7 8 9 10 11 12 13 14 15 16 17 18 19 20 21 22 23 24

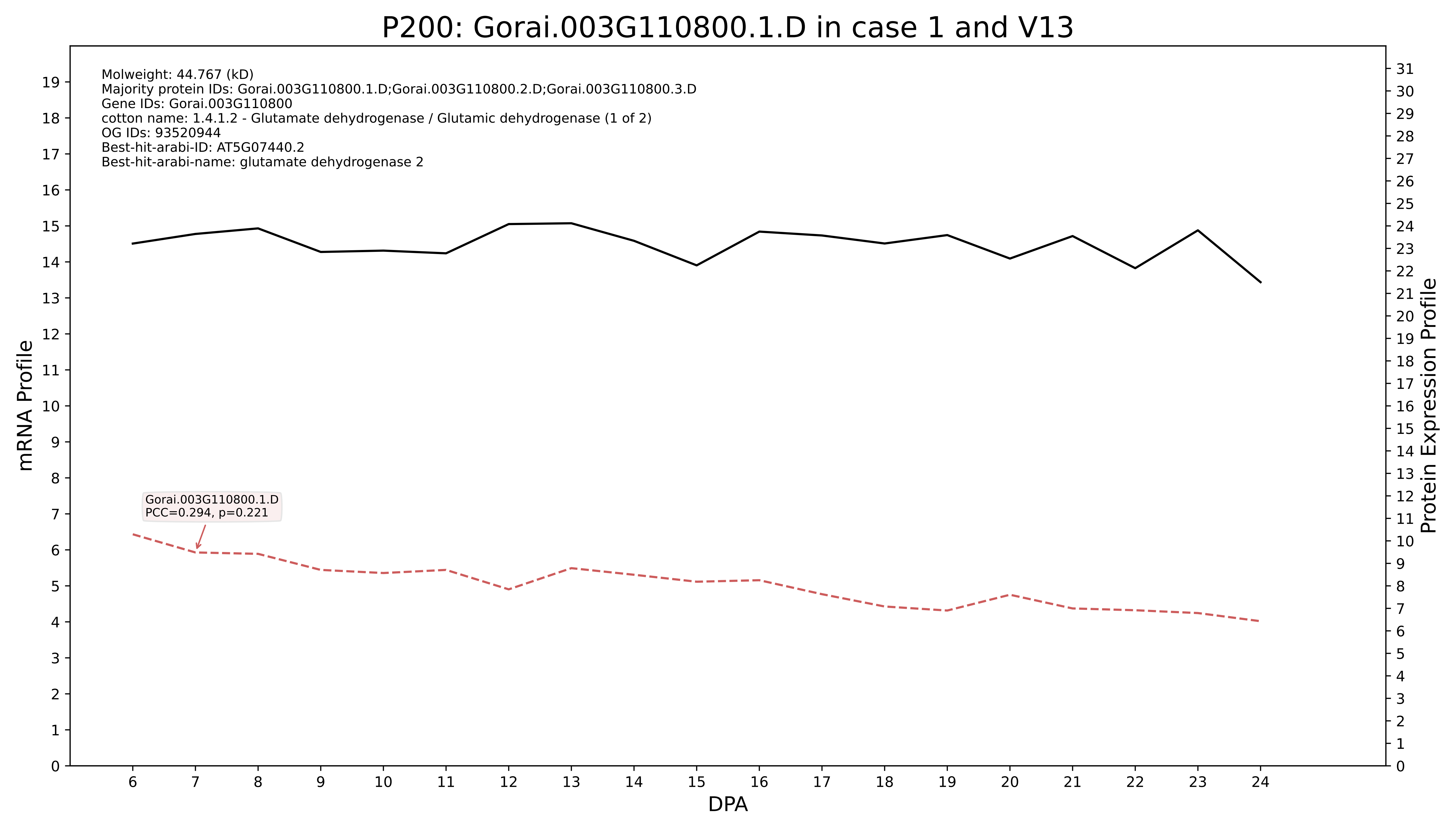
P200: Gorai.003G110800.1.D in case 1 and V13
Molweight: 44.767 (kD)
Majority protein IDs: Gorai.003G110800.1.D;Gorai.003G110800.2.D;Gorai.003G110800.3.D
Gene IDs: Gorai.003G110800
cotton name: 1.4.1.2 - Glutamate dehydrogenase / Glutamic dehydrogenase (1 of 2)
OG IDs: 93520944
Best-hit-arabi-ID: AT5G07440.2
Best-hit-arabi-name: glutamate dehydrogenase 2
Gorai.003G110800.1.D
PCC=0.294, p=0.221
mRNA Profile
Protein Expression Profile
DPA

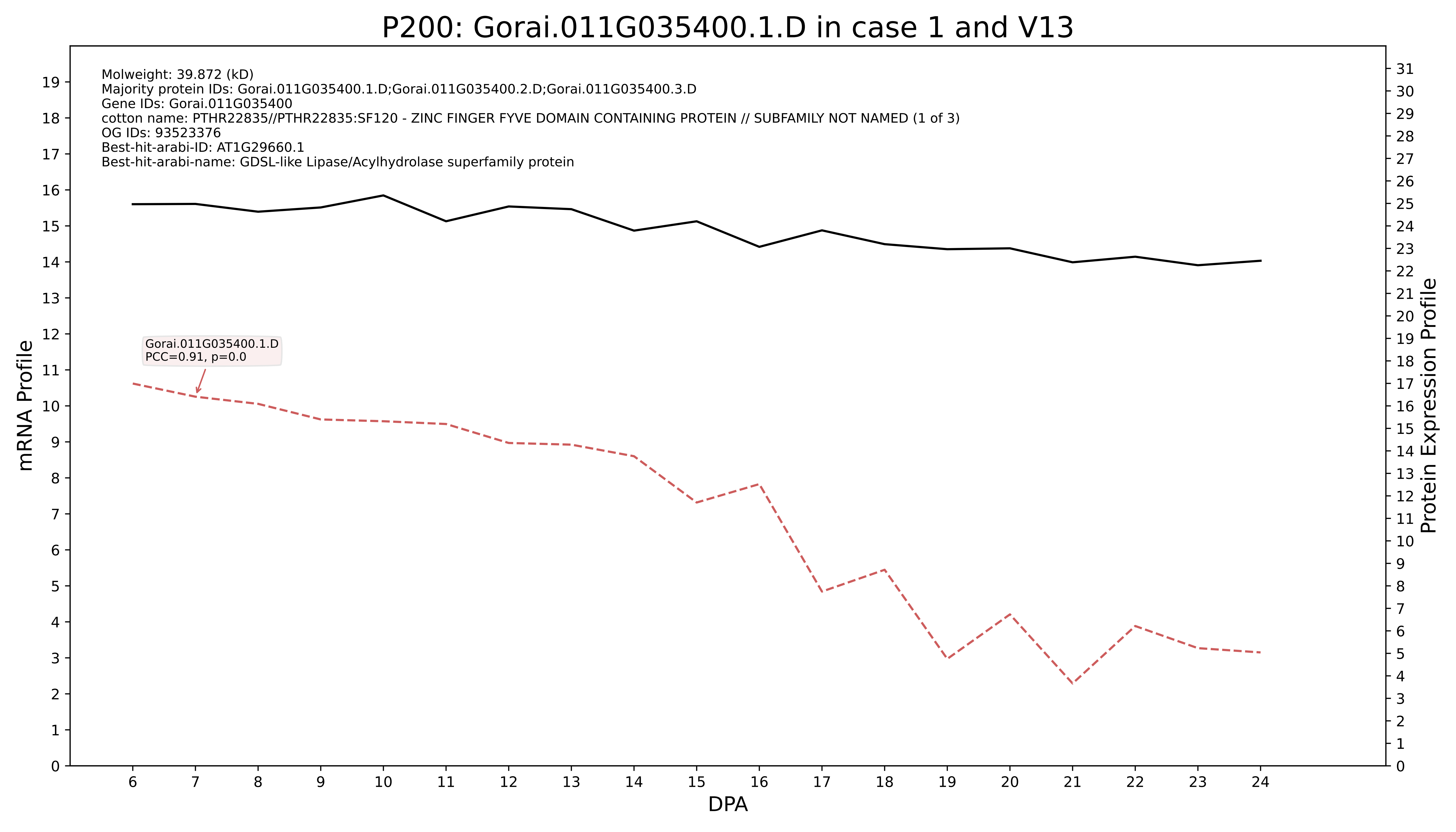

P200: Gorai.011G035400.1.D in case 1 and V13
Molweight: 39.872 (kD)
Majority protein IDs: Gorai.011G035400.1.D;Gorai.011G035400.2.D;Gorai.011G035400.3.D
Gene IDs: Gorai.011G035400
cotton name: PTHR22835//PTHR22835:SF120 - ZINC FINGER FYVE DOMAIN CONTAINING PROTEIN // SUBFAMILY NOT NAMED (1 of 3)
OG IDs: 93523376
Best-hit-arabi-ID: AT1G29660.1
Best-hit-arabi-name: GDSL-like Lipase/Acylhydrolase superfamily protein
Gorai.011G035400.1.D
PCC=0.91, p=0.0
mRNA Profile
Protein Expression Profile
DPA

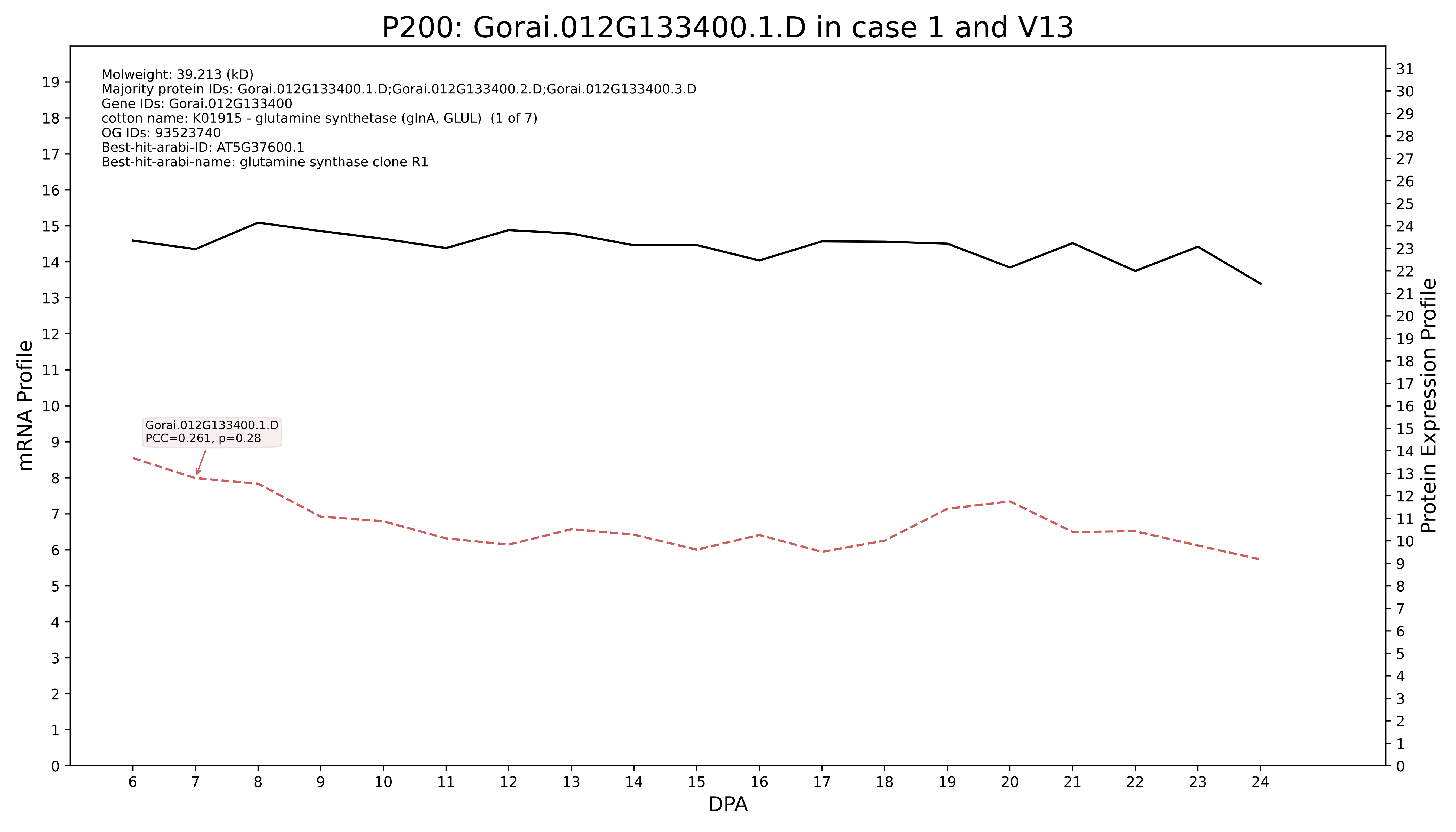
P200: Gorai.012G133400.1.D in case 1 and V13
Molweight: 39.213 (kD)
Majority protein IDs: Gorai.012G133400.1.D;Gorai.012G133400.2.D;Gorai.012G133400.3.D
Gene IDs: Gorai.012G133400
cotton name: K01915 - glutamine synthetase (glnA, GLUL) (1 of 7)
OG IDs: 93523740
Best-hit-arabi-ID: AT5G37600.1
Best-hit-arabi-name: glutamine synthase clone R1
Gorai.012G133400.1.D
PCC=0.261, p=0.28
mRNA Profile
Protein Expression Profile
DPA
0
1
2
3
4
5
6
7
8
9
10
11
12
13
14
15
16
17
18
19
20
21
22
23
24
25
26
27
28
29
30
31

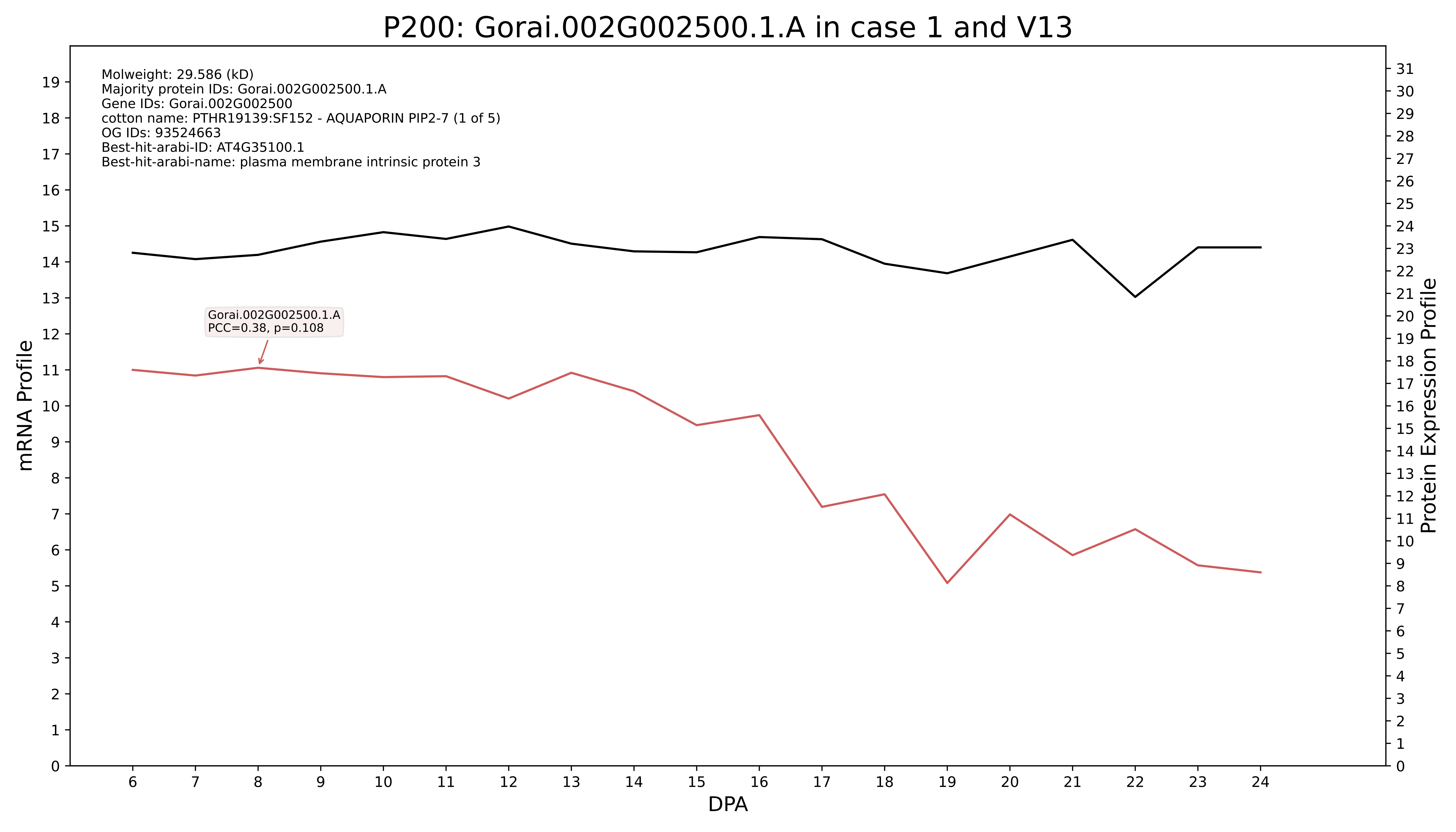
P200: Gorai.002G002500.1.A in case 1 and V13
Molweight: 29.586 (kD)
Majority protein IDs: Gorai.002G002500.1.A
Gene IDs: Gorai.002G002500
cotton name: PTHR19139:SF152 - AQUAPORIN PIP2-7 (1 of 5)
OG IDs: 93524663
Best-hit-arabi-ID: AT4G35100.1
Best-hit-arabi-name: plasma membrane intrinsic protein 3
Gorai.002G002500.1.A
PCC=0.38, p=0.108
mRNA Profile
Protein Expression Profile
DPA

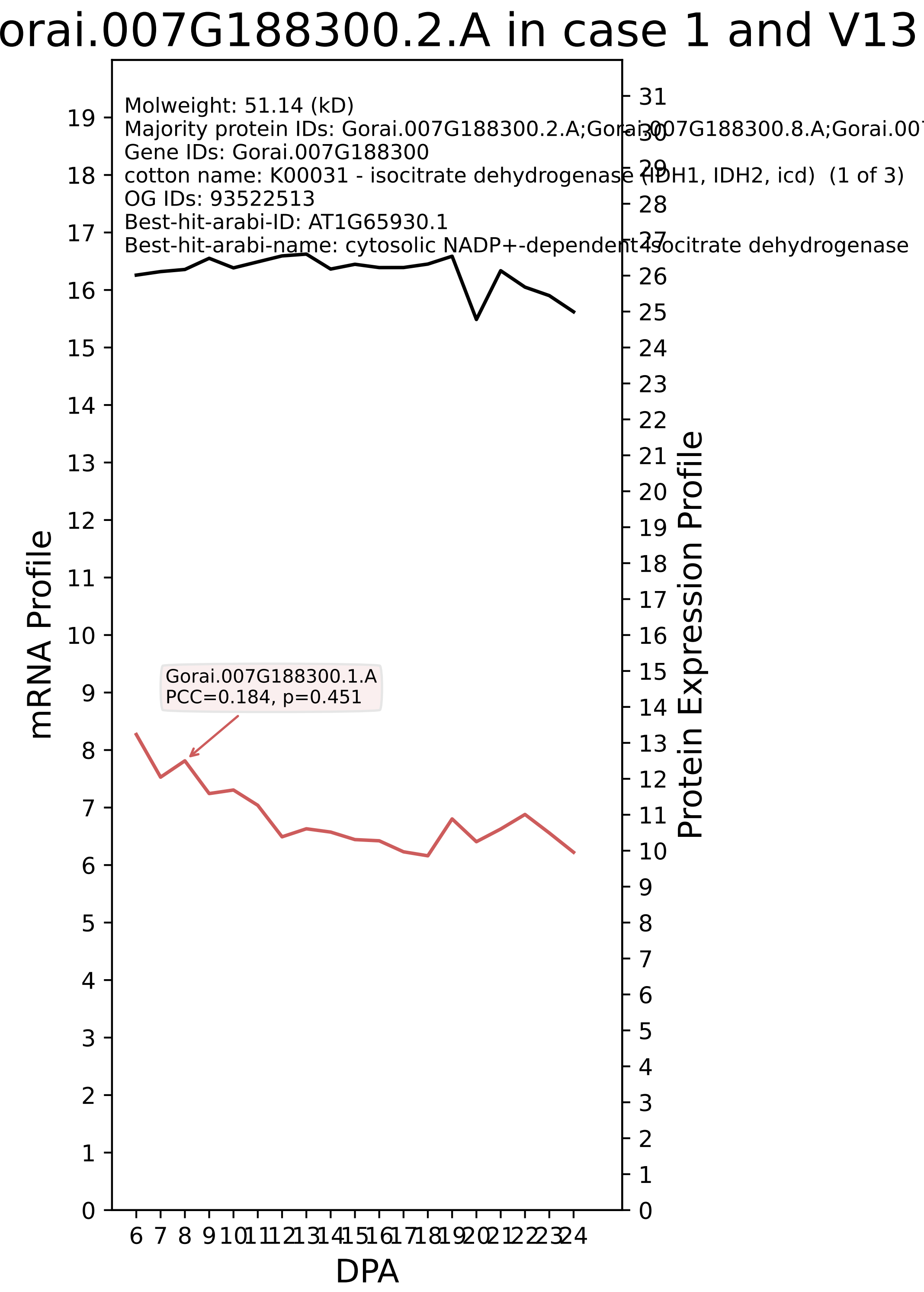
orai.007G188300.2.A in case 1 and V13
Molweight: 51.14 (kD)
Majority protein IDs: Gorai.007G188300.2.A;Gorai.007G188300.8.A;Gorai.007G188300.1.A;Gorai.007G188300.3.A;Gorai.007G188300.4.A;Gorai.007G188300.5.A;Gorai.007G188300.6.A;Gorai.007G188300.7.A;Go
Gene IDs: Gorai.007G188300
cotton name: K00031 - isocitrate dehydrogenase (IDH1, IDH2, icd) (1 of 3)
OG IDs: 93522513
Best-hit-arabi-ID: AT1G65930.1
Best-hit-arabi-name: cytosolic NADP+-dependent isocitrate dehydrogenase
Gorai.007G188300.1.A
PCC=0.184, p=0.451
mRNA Profile
Protein Expression Profile
DPA

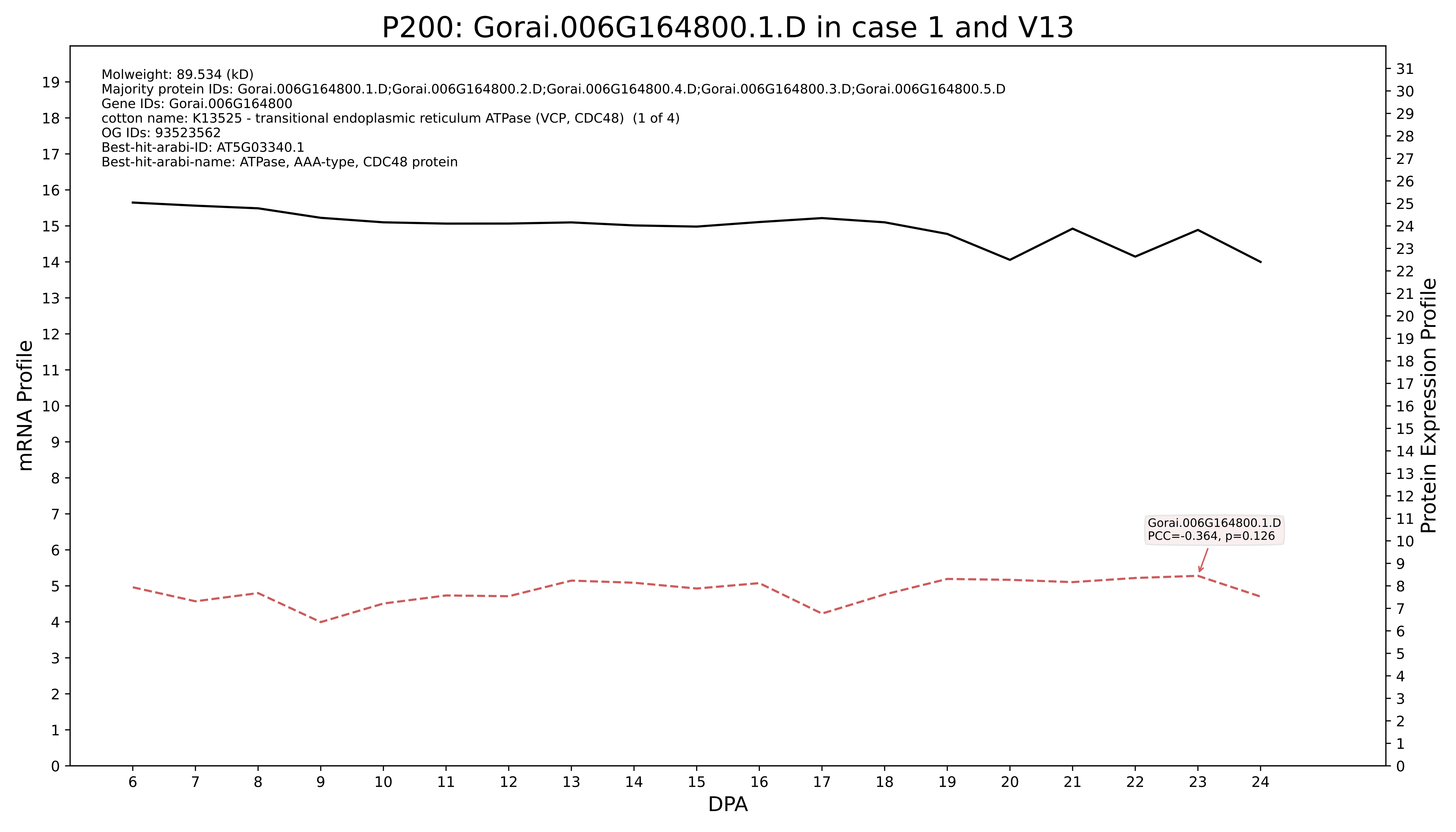

P200: Gorai.006G164800.1.D in case 1 and V13
Molweight: 89.534 (kD)
Majority protein IDs: Gorai.006G164800.1.D;Gorai.006G164800.2.D;Gorai.006G164800.4.D;Gorai.006G164800.3.D;Gorai.006G164800.5.D
Gene IDs: Gorai.006G164800
cotton name: K13525 - transitional endoplasmic reticulum ATPase (VCP, CDC48) (1 of 4)
OG IDs: 93523562
Best-hit-arabi-ID: AT5G03340.1
Best-hit-arabi-name: ATPase, AAA-type, CDC48 protein
Gorai.006G164800.1.D
PCC=-0.364, p=0.126
mRNA Profile
Protein Expression Profile
DPA

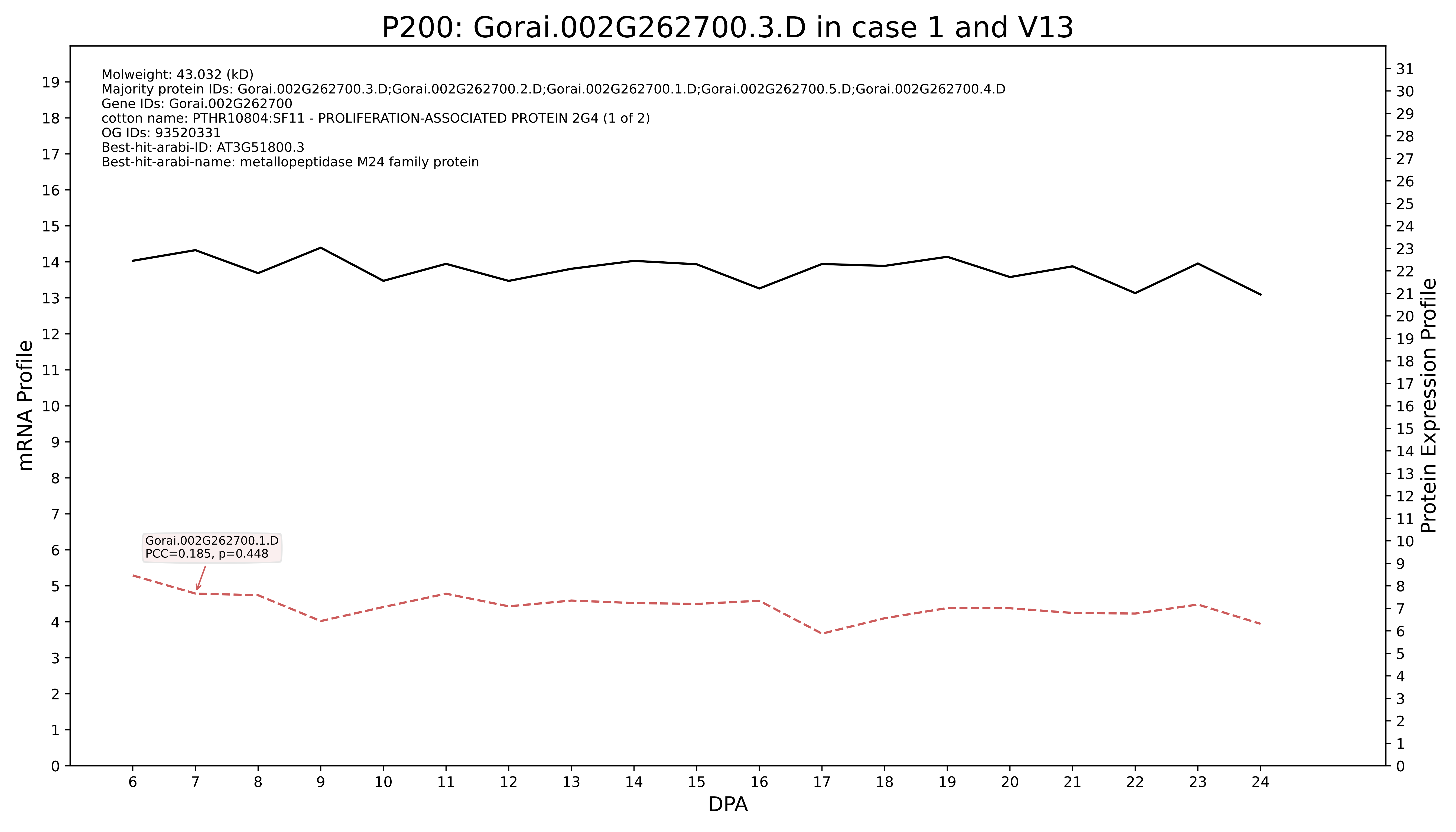
P200: Gorai.002G262700.3.D in case 1 and V13
Molweight: 43.032 (kD)
Majority protein IDs: Gorai.002G262700.3.D;Gorai.002G262700.2.D;Gorai.002G262700.1.D;Gorai.002G262700.5.D;Gorai.002G262700.4.D
Gene IDs: Gorai.002G262700
cotton name: PTHR10804:SF11 - PROLIFERATION-ASSOCIATED PROTEIN 2G4 (1 of 2)
OG IDs: 93520331
Best-hit-arabi-ID: AT3G51800.3
Best-hit-arabi-name: metallopeptidase M24 family protein
Gorai.002G262700.1.D
PCC=0.185, p=0.448
mRNA Profile
Protein Expression Profile
DPA

P200: Gorai.005G210000.1.A in case 1 and V13

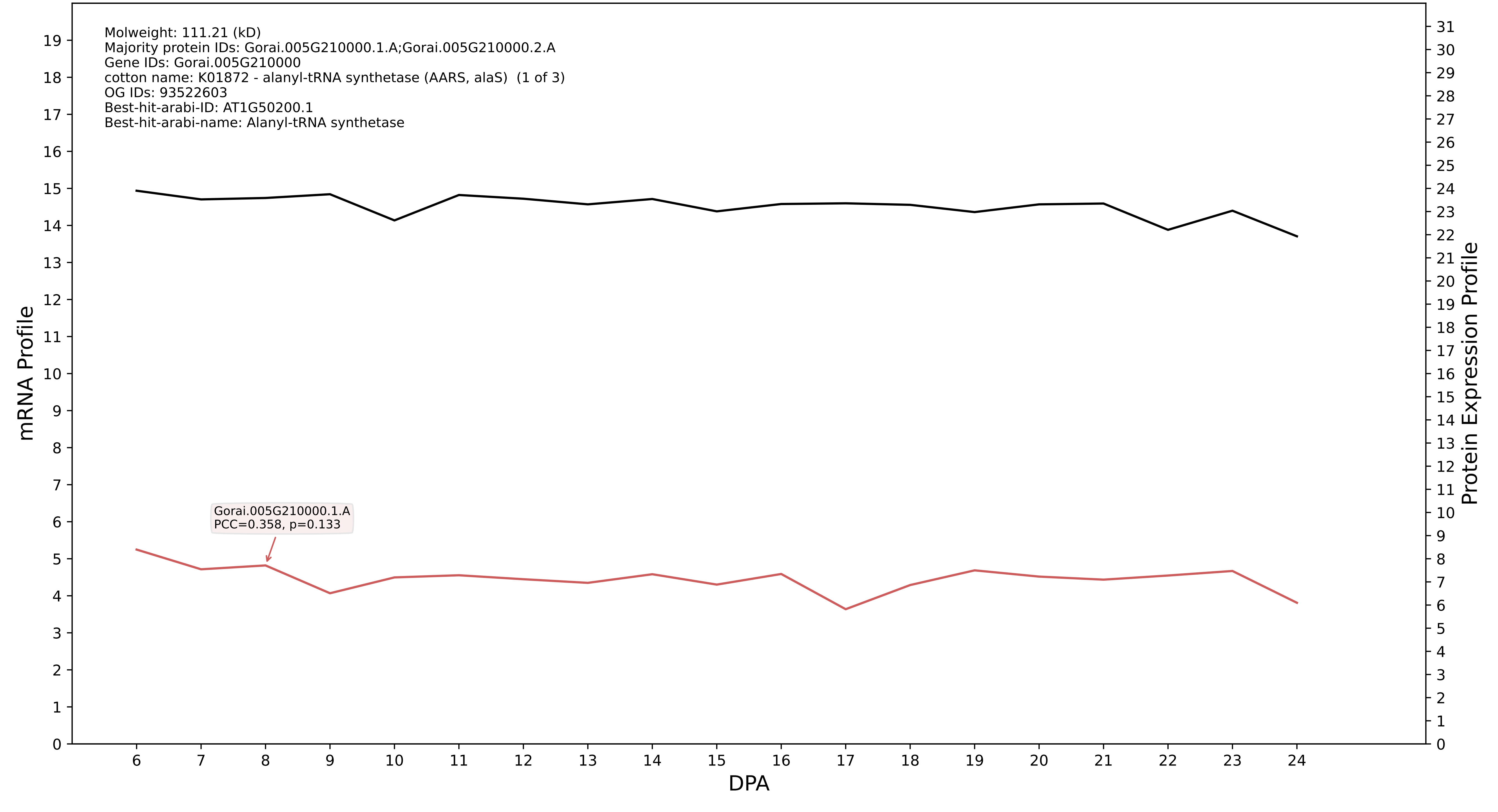

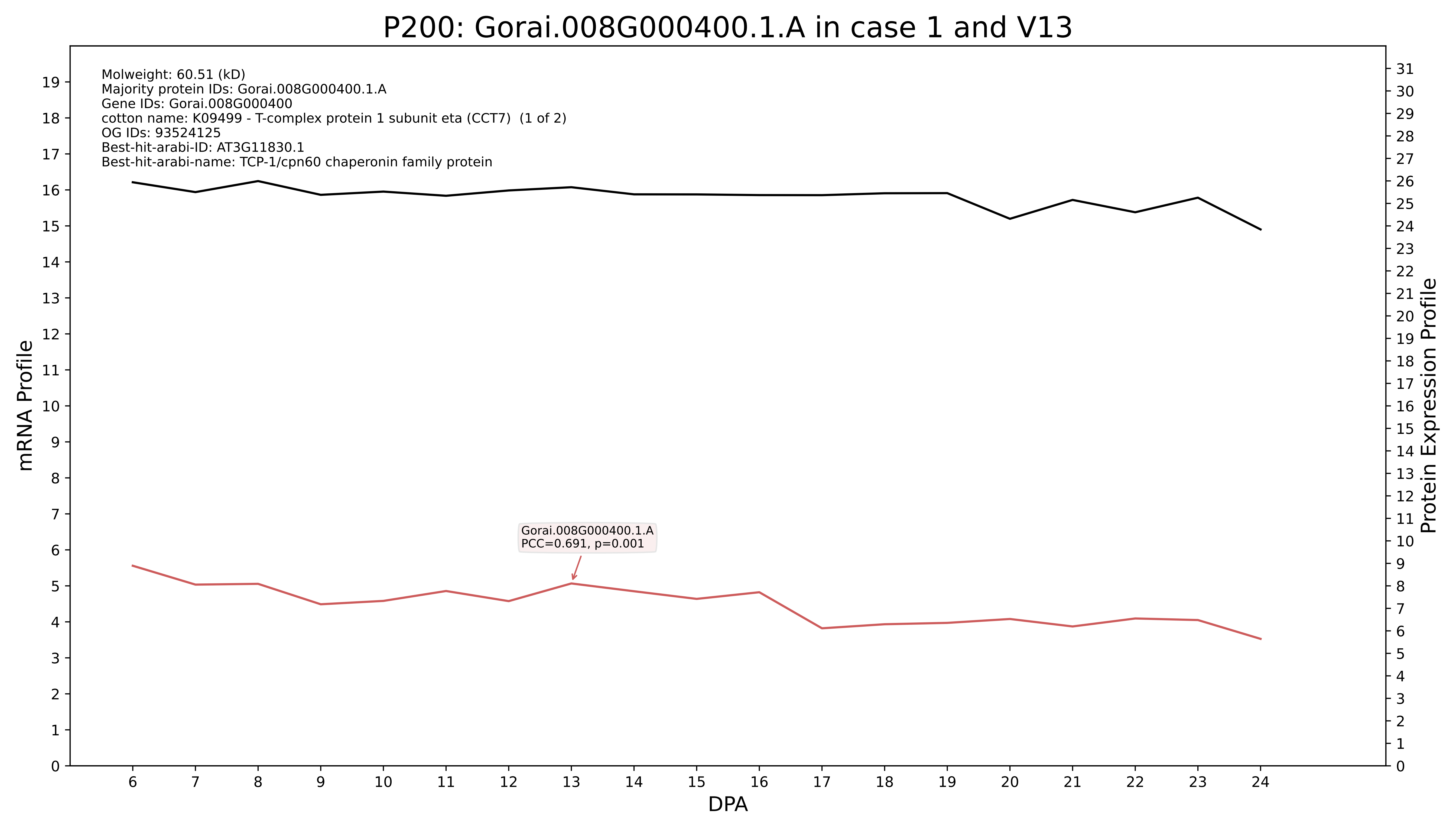

P200: Gorai.008G000400.1.A in case 1 and V13
Molweight: 60.51 (kD)
Majority protein IDs: Gorai.008G000400.1.A
Gene IDs: Gorai.008G000400
cotton name: K09499 - T-complex protein 1 subunit eta (CCT7) (1 of 2)
OG IDs: 93524125
Best-hit-arabi-ID: AT3G11830.1
Best-hit-arabi-name: TCP-1/cpn60 chaperonin family protein
Gorai.008G000400.1.A
PCC=0.691, p=0.001
mRNA Profile
Protein Expression Profile
DPA

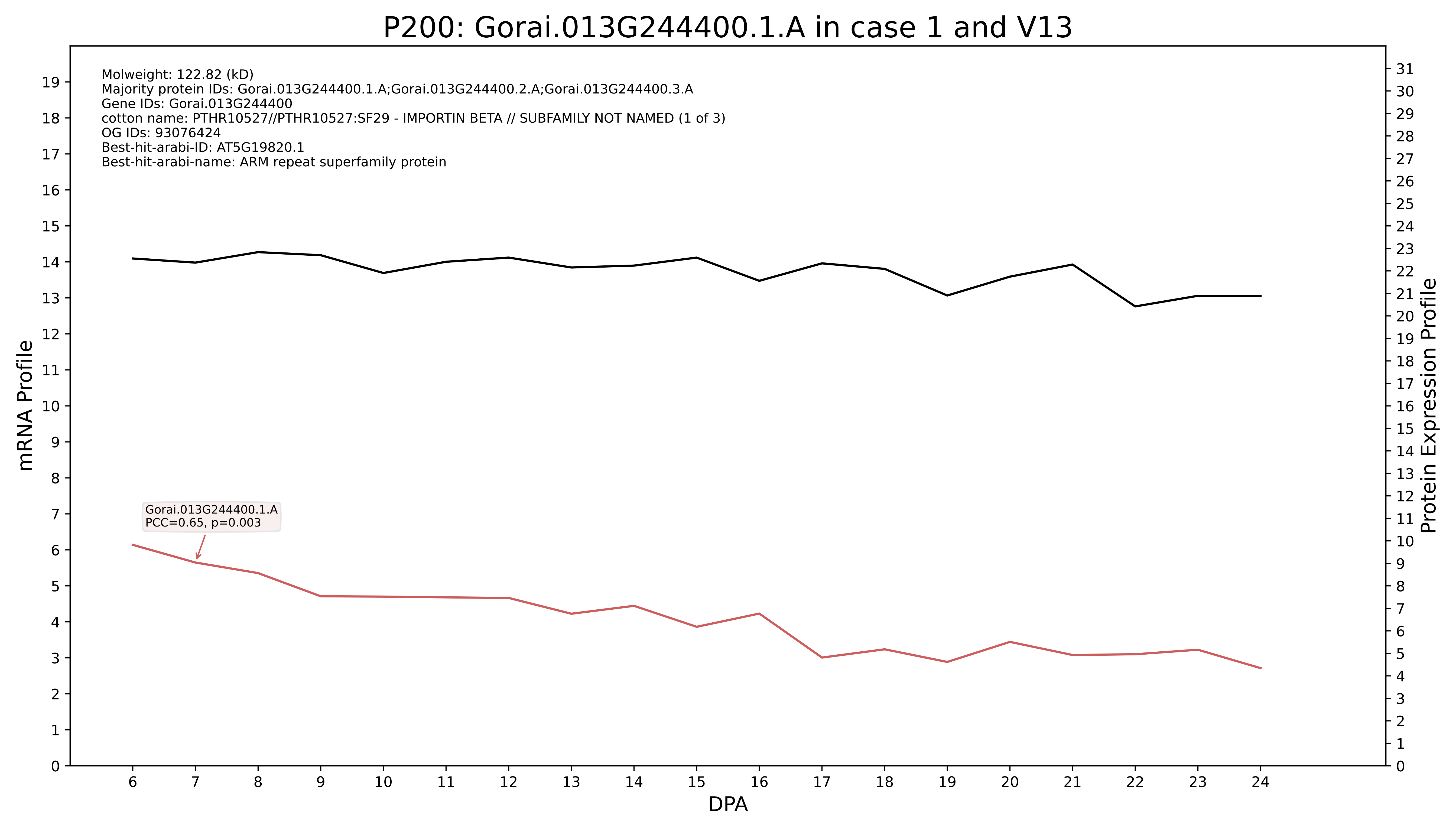
P200: Gorai.013G244400.1.A in case 1 and V13
Molweight: 122.82 (kD)
Majority protein IDs: Gorai.013G244400.1.A;Gorai.013G244400.2.A;Gorai.013G244400.3.A
Gene IDs: Gorai.013G244400
cotton name: PTHR10527//PTHR10527:SF29 - IMPORTIN BETA // SUBFAMILY NOT NAMED (1 of 3)
OG IDs: 93076424
Best-hit-arabi-ID: AT5G19820.1
Best-hit-arabi-name: ARM repeat superfamily protein
Gorai.013G244400.1.A
PCC=0.65, p=0.003
mRNA Profile
Protein Expression Profile
DPA

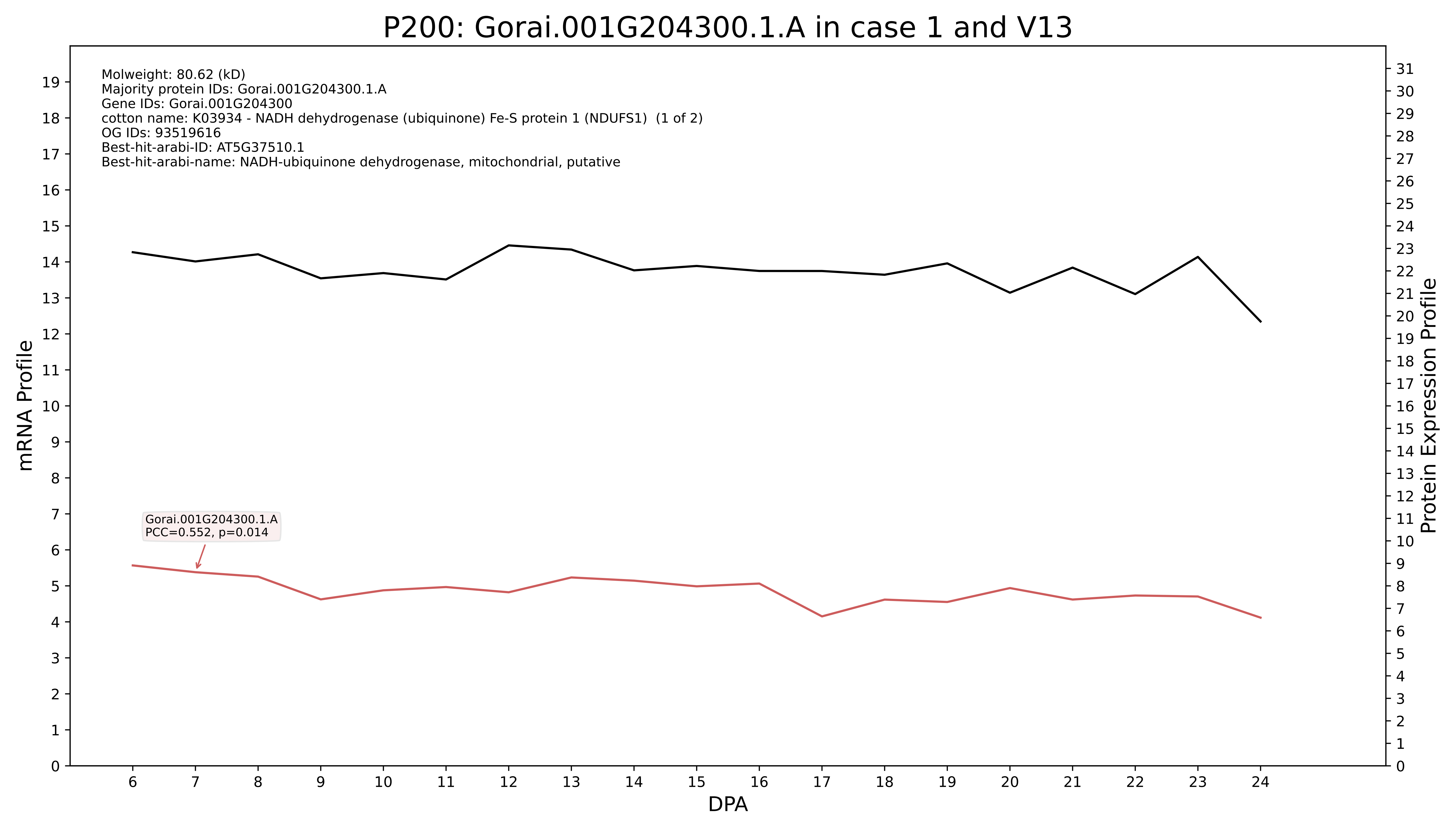
P200: Gorai.001G204300.1.A in case 1 and V13
Molweight: 80.62 (kD)
Majority protein IDs: Gorai.001G204300.1.A
Gene IDs: Gorai.001G204300
cotton name: K03934 - NADH dehydrogenase (ubiquinone) Fe-S protein 1 (NDUFS1) (1 of 2)
OG IDs: 93519616
Best-hit-arabi-ID: AT5G37510.1
Best-hit-arabi-name: NADH-ubiquinone dehydrogenase, mitochondrial, putative
Gorai.001G204300.1.A
PCC=0.552, p=0.014
mRNA Profile
Protein Expression Profile
DPA
0
1
2
3
4
5
6
7
8
9
10
11
12
13
14
15
16
17
18
19
20
21
22
23
24
25
26
27
28
29
30
31

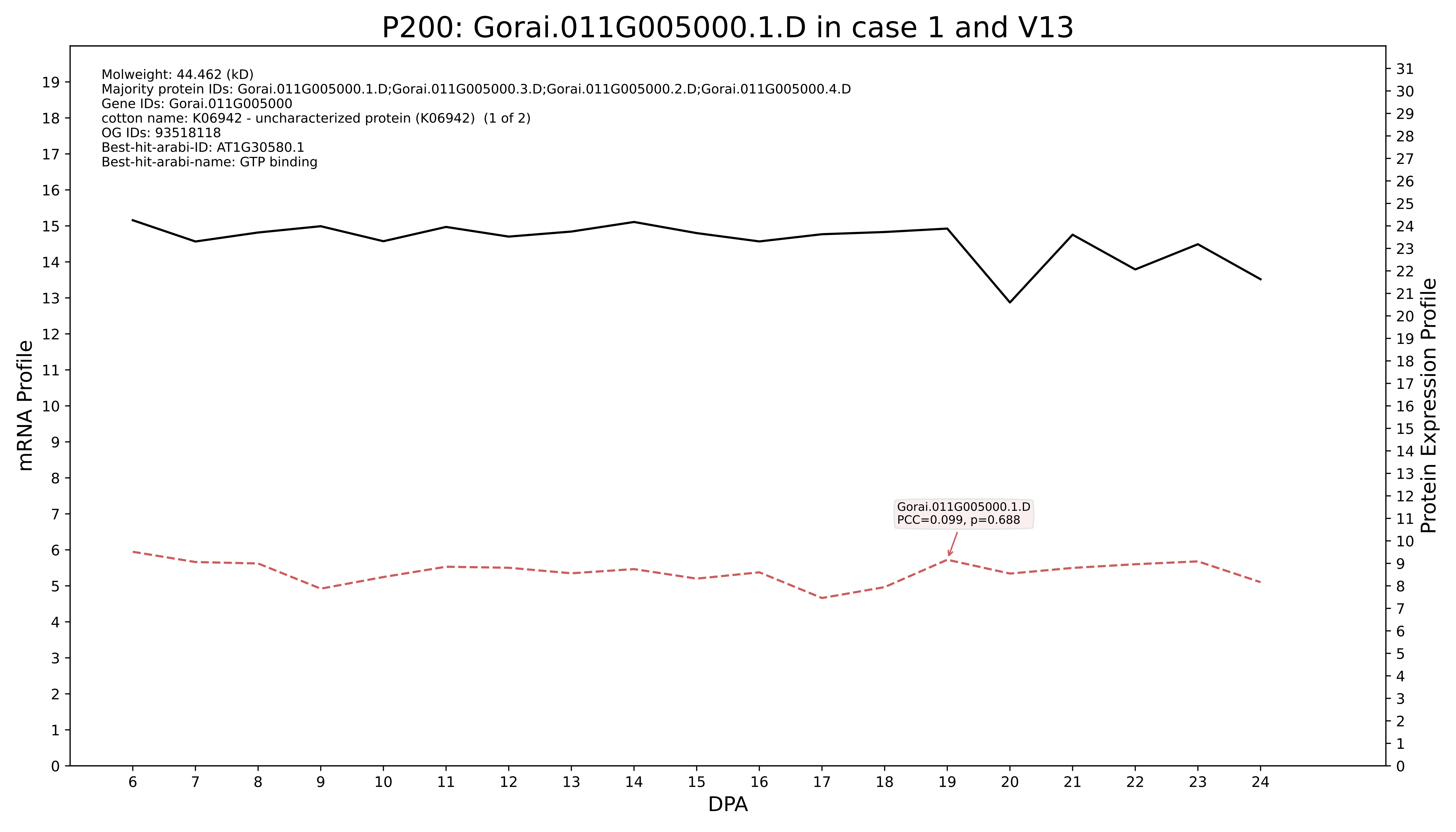
P200: Gorai.011G005000.1.D in case 1 and V13
Molweight: 44.462 (kD)
Majority protein IDs: Gorai.011G005000.1.D;Gorai.011G005000.3.D;Gorai.011G005000.2.D;Gorai.011G005000.4.D
Gene IDs: Gorai.011G005000
cotton name: K06942 - uncharacterized protein (K06942) (1 of 2)
OG IDs: 93518118
Best-hit-arabi-ID: AT1G30580.1
Best-hit-arabi-name: GTP binding
Gorai.011G005000.1.D
PCC=0.099, p=0.688
mRNA Profile
Protein Expression Profile
DPA

P200: Gorai.013G270300.1.D in case 1 and V13

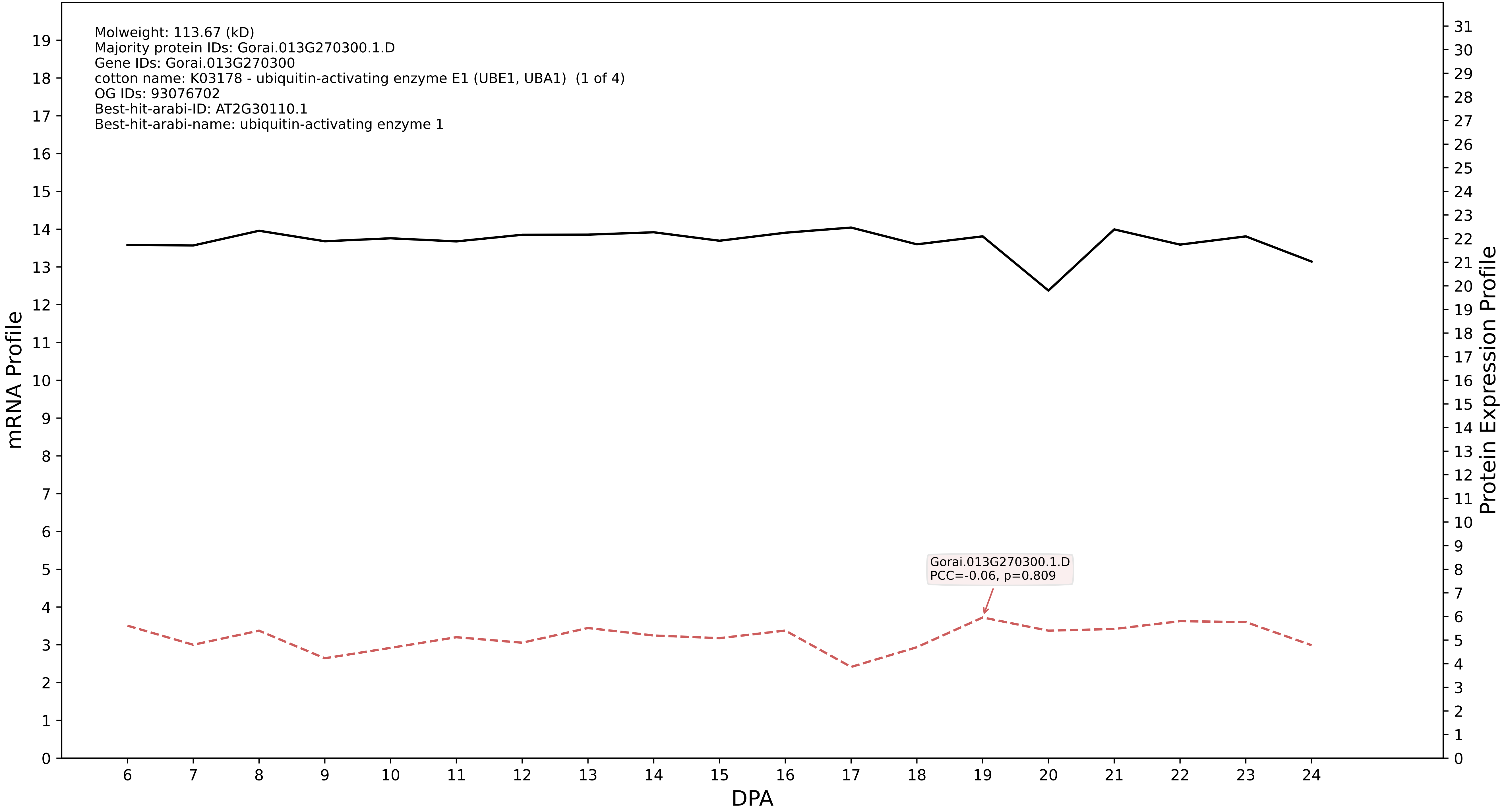

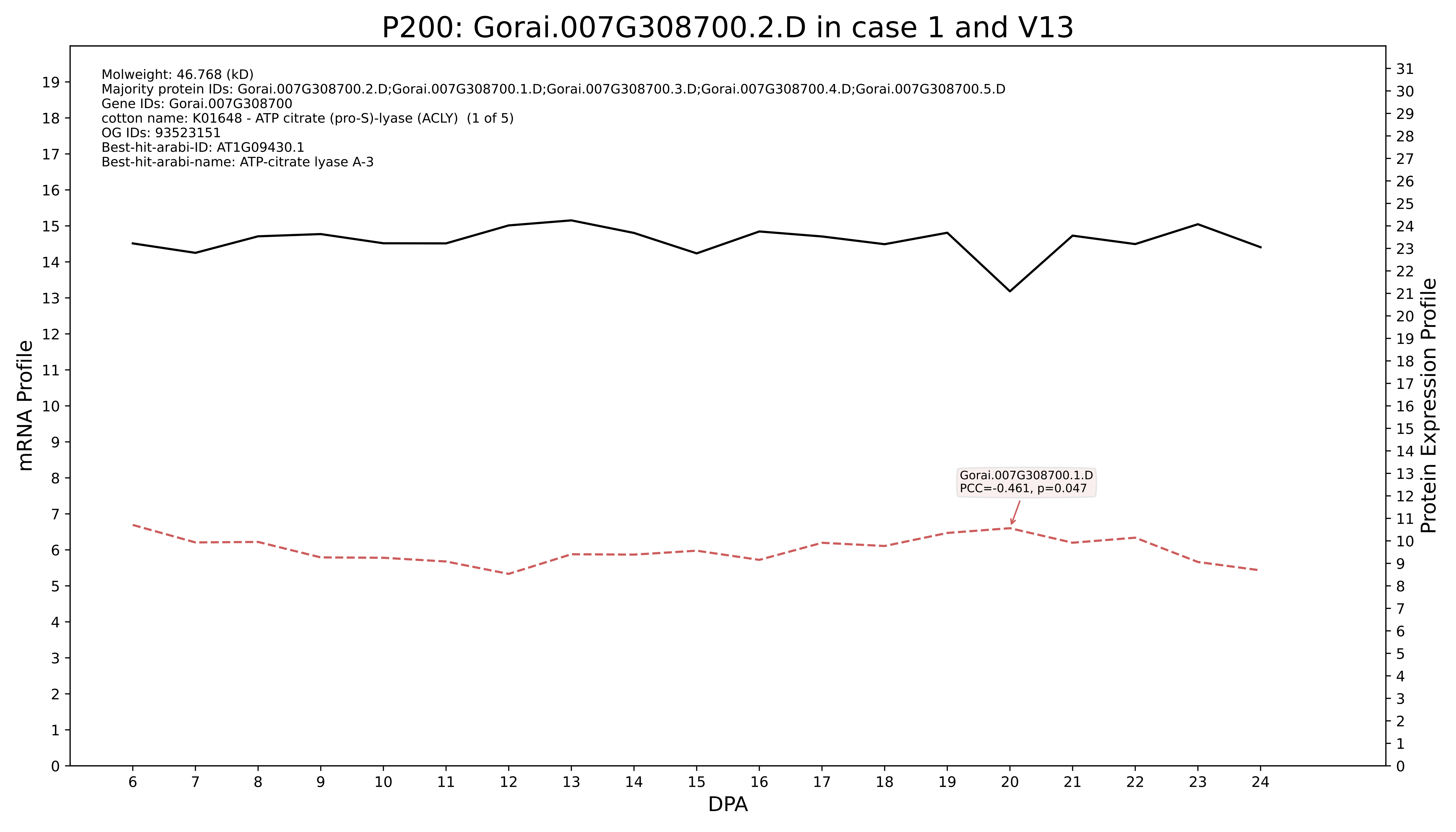
P200: Gorai.007G308700.2.D in case 1 and V13
Molweight: 46.768 (kD)
Majority protein IDs: Gorai.007G308700.2.D;Gorai.007G308700.1.D;Gorai.007G308700.3.D;Gorai.007G308700.4.D;Gorai.007G308700.5.D
Gene IDs: Gorai.007G308700
cotton name: K01648 - ATP citrate (pro-S)-lyase (ACLY) (1 of 5)
OG IDs: 93523151
Best-hit-arabi-ID: AT1G09430.1
Best-hit-arabi-name: ATP-citrate lyase A-3
Gorai.007G308700.1.D
PCC=-0.461, p=0.047
mRNA Profile
Protein Expression Profile
DPA

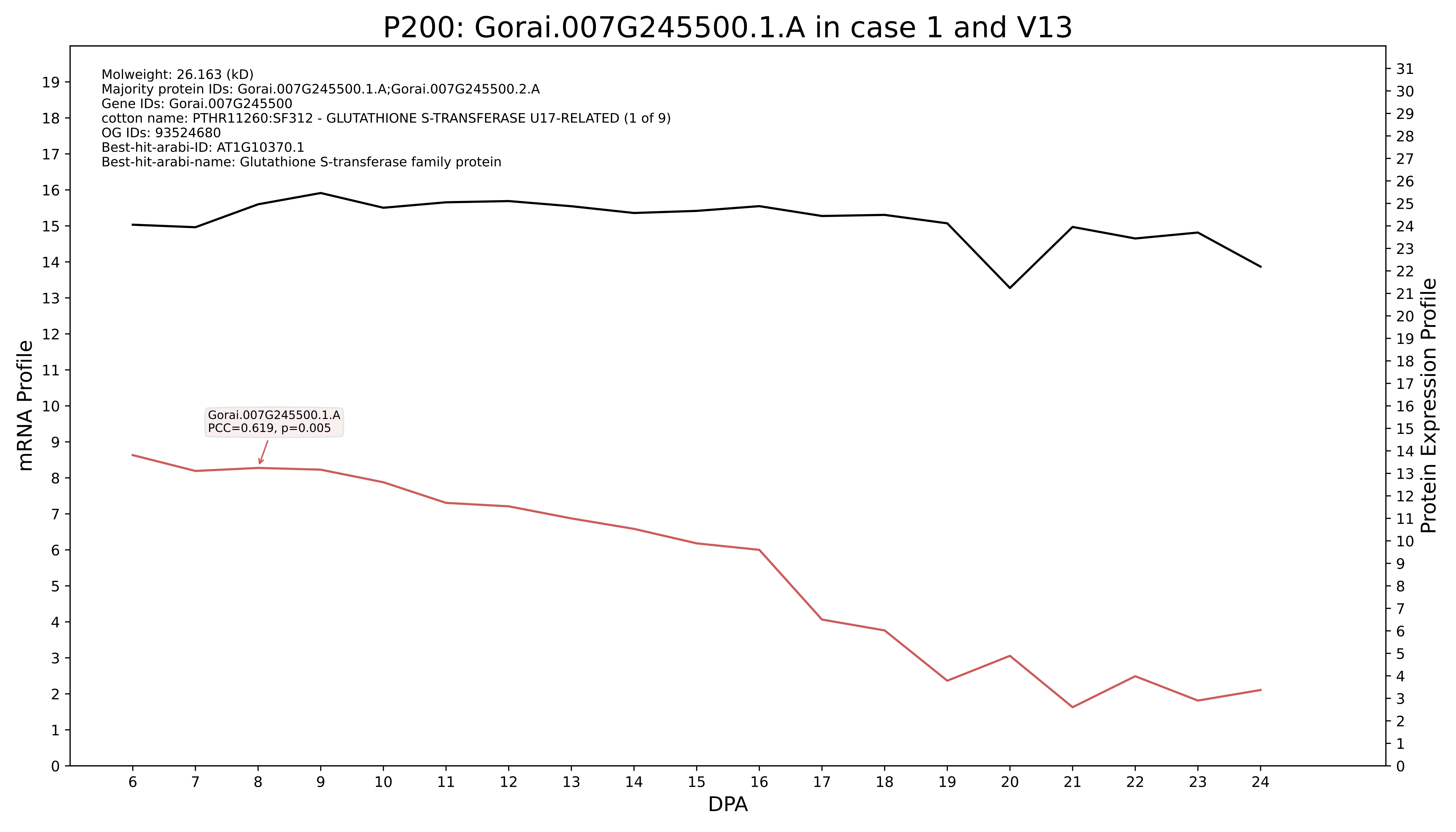

P200: Gorai.007G245500.1.A in case 1 and V13
Molweight: 26.163 (kD)
Majority protein IDs: Gorai.007G245500.1.A;Gorai.007G245500.2.A
Gene IDs: Gorai.007G245500
cotton name: PTHR11260:SF312 - GLUTATHIONE S-TRANSFERASE U17-RELATED (1 of 9)
OG IDs: 93524680
Best-hit-arabi-ID: AT1G10370.1
Best-hit-arabi-name: Glutathione S-transferase family protein
Gorai.007G245500.1.A
PCC=0.619, p=0.005
mRNA Profile
Protein Expression Profile
DPA

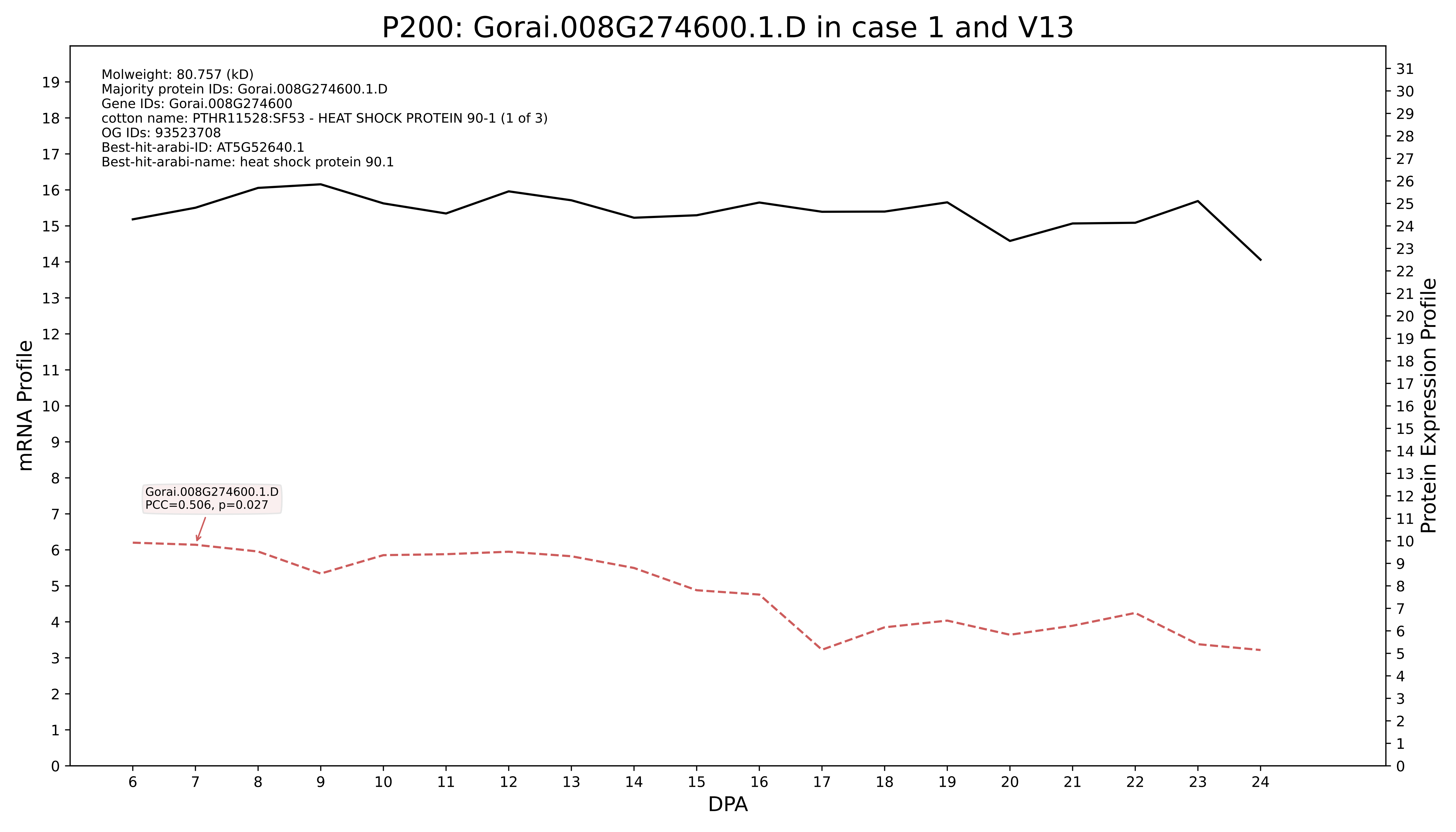
P200: Gorai.008G274600.1.D in case 1 and V13
Molweight: 80.757 (kD)
Majority protein IDs: Gorai.008G274600.1.D
Gene IDs: Gorai.008G274600
cotton name: PTHR11528:SF53 - HEAT SHOCK PROTEIN 90-1 (1 of 3)
OG IDs: 93523708
Best-hit-arabi-ID: AT5G52640.1
Best-hit-arabi-name: heat shock protein 90.1
Gorai.008G274600.1.D
PCC=0.506, p=0.027
mRNA Profile
Protein Expression Profile
DPA

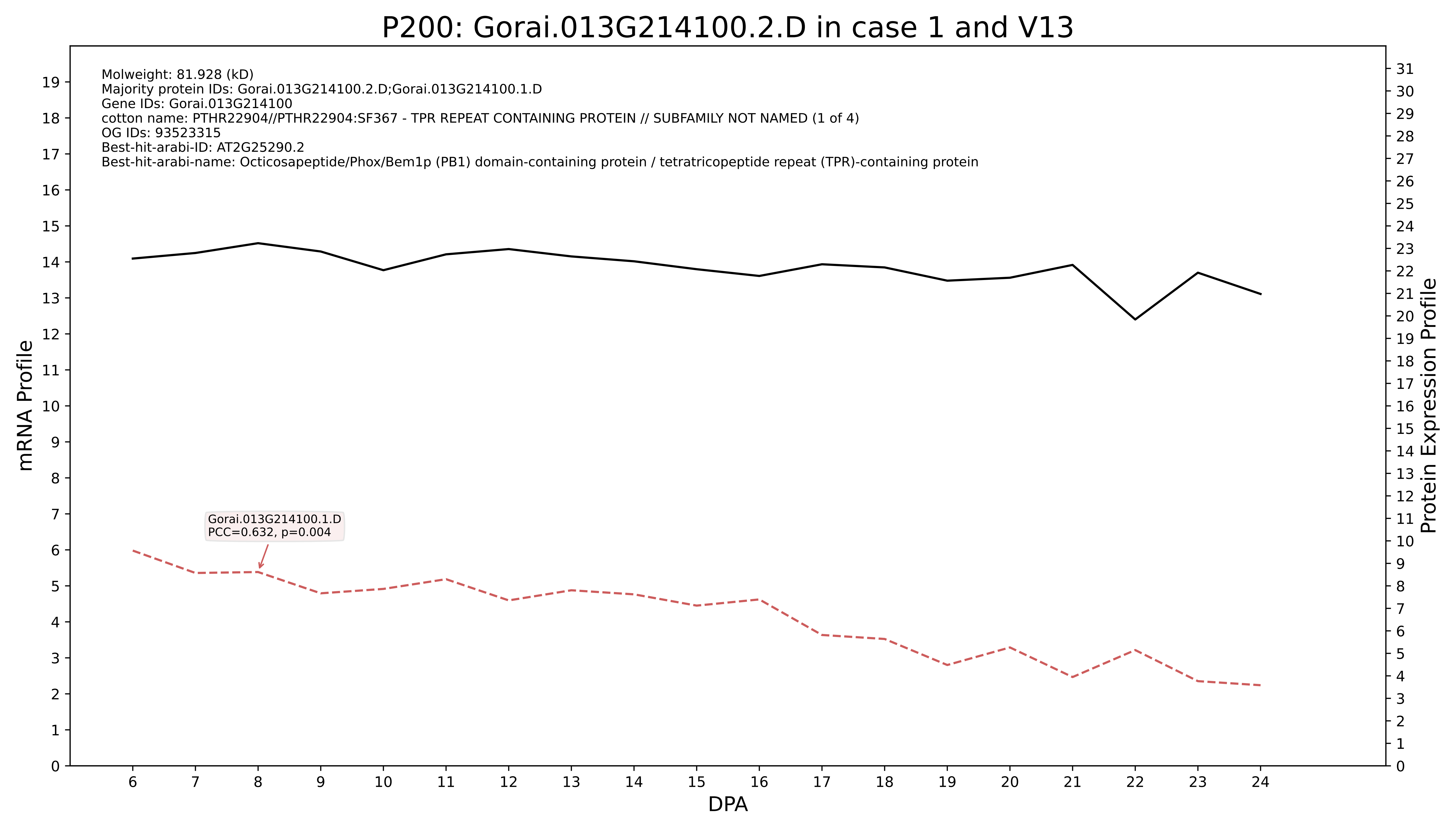
P200: Gorai.013G214100.2.D in case 1 and V13
Molweight: 81.928 (kD)
Majority protein IDs: Gorai.013G214100.2.D;Gorai.013G214100.1.D
Gene IDs: Gorai.013G214100
cotton name: PTHR22904//PTHR22904:SF367 - TPR REPEAT CONTAINING PROTEIN // SUBFAMILY NOT NAMED (1 of 4)
OG IDs: 93523315
Best-hit-arabi-ID: AT2G25290.2
Best-hit-arabi-name: Octicosapeptide/Phox/Bem1p (PB1) domain-containing protein / tetratricopeptide repeat (TPR)-containing protein
Gorai.013G214100.1.D
PCC=0.632, p=0.004
mRNA Profile
Protein Expression Profile
DPA

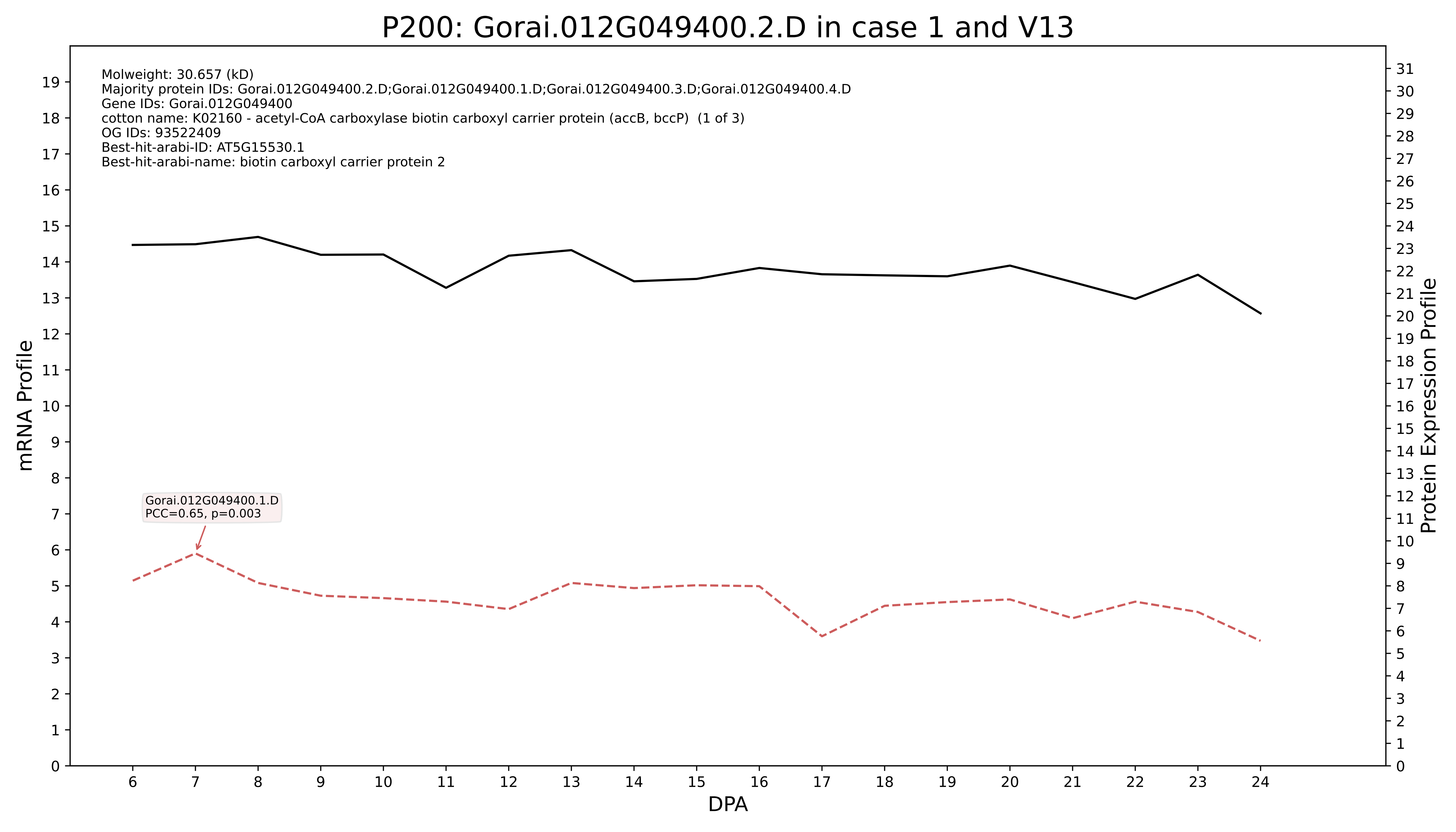

P200: Gorai.012G049400.2.D in case 1 and V13
Molweight: 30.657 (kD)
Majority protein IDs: Gorai.012G049400.2.D;Gorai.012G049400.1.D;Gorai.012G049400.3.D;Gorai.012G049400.4.D
Gene IDs: Gorai.012G049400
cotton name: K02160 - acetyl-CoA carboxylase biotin carboxyl carrier protein (accB, bccP) (1 of 3)
OG IDs: 93522409
Best-hit-arabi-ID: AT5G15530.1
Best-hit-arabi-name: biotin carboxyl carrier protein 2
Gorai.012G049400.1.D
PCC=0.65, p=0.003
mRNA Profile
Protein Expression Profile
DPA

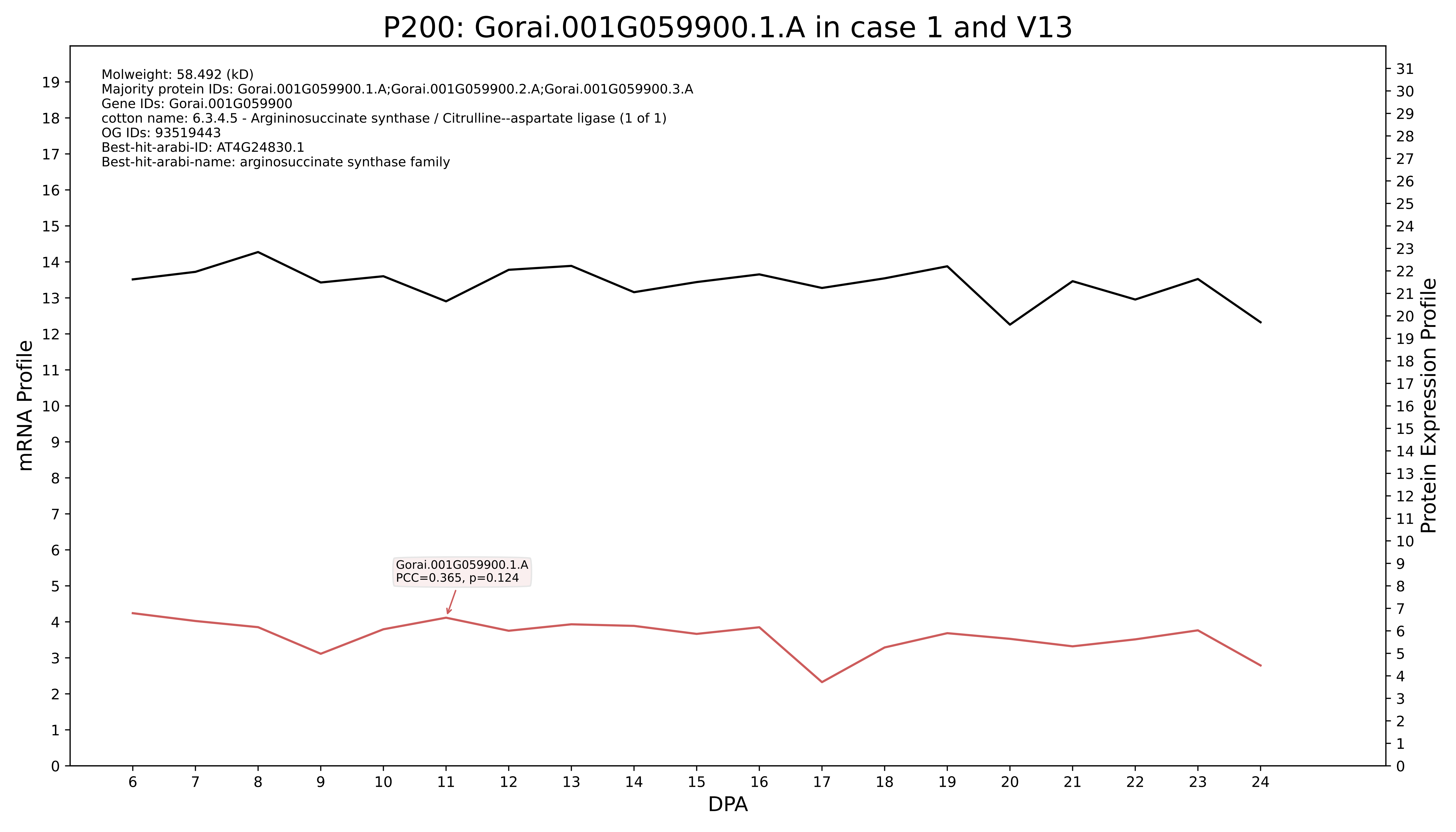

P200: Gorai.001G059900.1.A in case 1 and V13
Molweight: 58.492 (kD)
Majority protein IDs: Gorai.001G059900.1.A;Gorai.001G059900.2.A;Gorai.001G059900.3.A
Gene IDs: Gorai.001G059900
cotton name: 6.3.4.5 - Argininosuccinate synthase / Citrulline--aspartate ligase (1 of 1)
OG IDs: 93519443
Best-hit-arabi-ID: AT4G24830.1
Best-hit-arabi-name: arginosuccinate synthase family
Gorai.001G059900.1.A
PCC=0.365, p=0.124
mRNA Profile
Protein Expression Profile
DPA

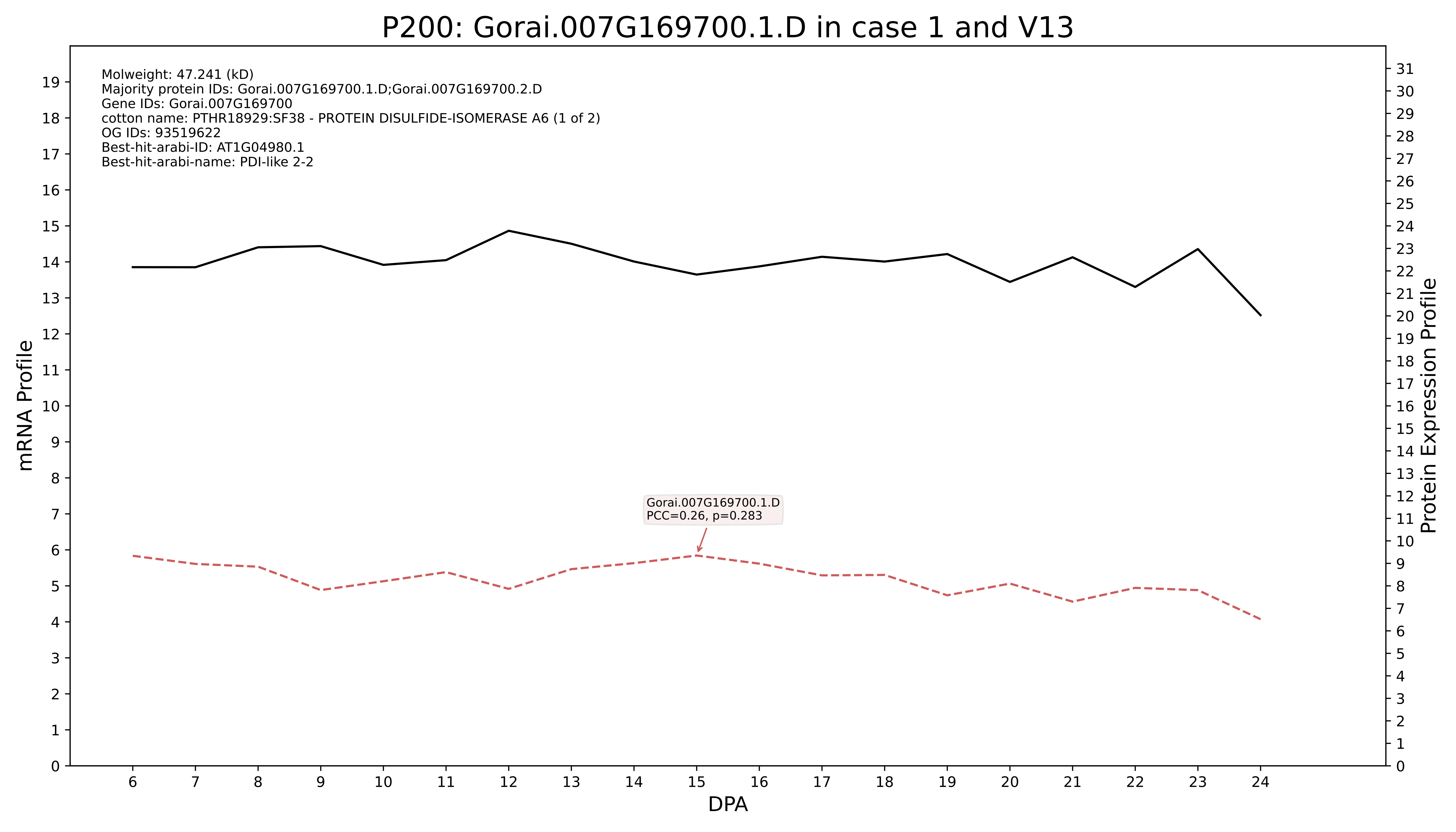

P200: Gorai.007G169700.1.D in case 1 and V13
Molweight: 47.241 (kD)
Majority protein IDs: Gorai.007G169700.1.D;Gorai.007G169700.2.D
Gene IDs: Gorai.007G169700
cotton name: PTHR18929:SF38 - PROTEIN DISULFIDE-ISOMERASE A6 (1 of 2)
OG IDs: 93519622
Best-hit-arabi-ID: AT1G04980.1
Best-hit-arabi-name: PDI-like 2-2
Gorai.007G169700.1.D
PCC=0.26, p=0.283
mRNA Profile
Protein Expression Profile
DPA

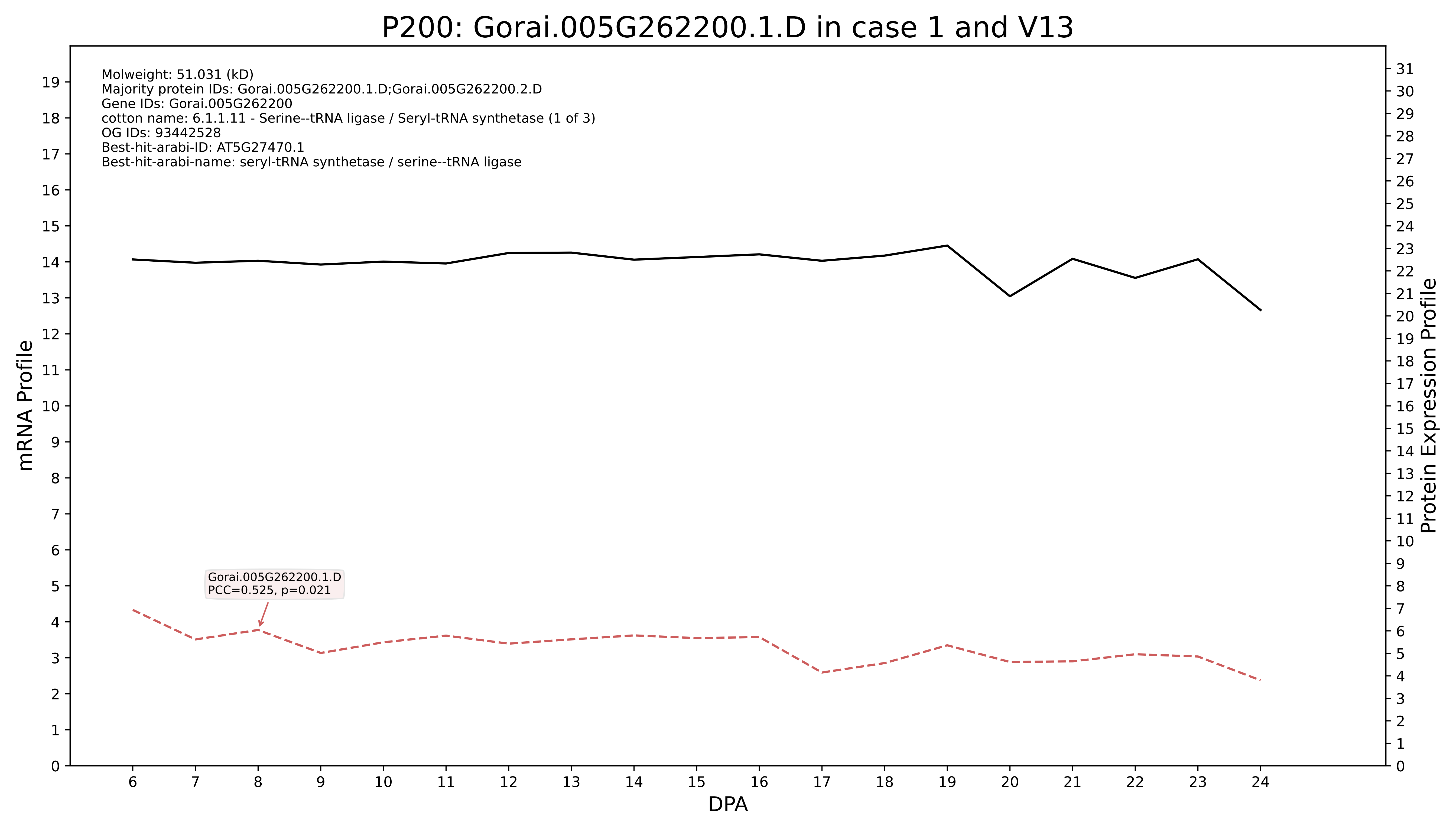
P200: Gorai.005G262200.1.D in case 1 and V13
Molweight: 51.031 (kD)
Majority protein IDs: Gorai.005G262200.1.D;Gorai.005G262200.2.D
Gene IDs: Gorai.005G262200
cotton name: 6.1.1.11 - Serine--tRNA ligase / Seryl-tRNA synthetase (1 of 3)
OG IDs: 93442528
Best-hit-arabi-ID: AT5G27470.1
Best-hit-arabi-name: seryl-tRNA synthetase / serine--tRNA ligase
Gorai.005G262200.1.D
PCC=0.525, p=0.021
mRNA Profile
Protein Expression Profile
DPA

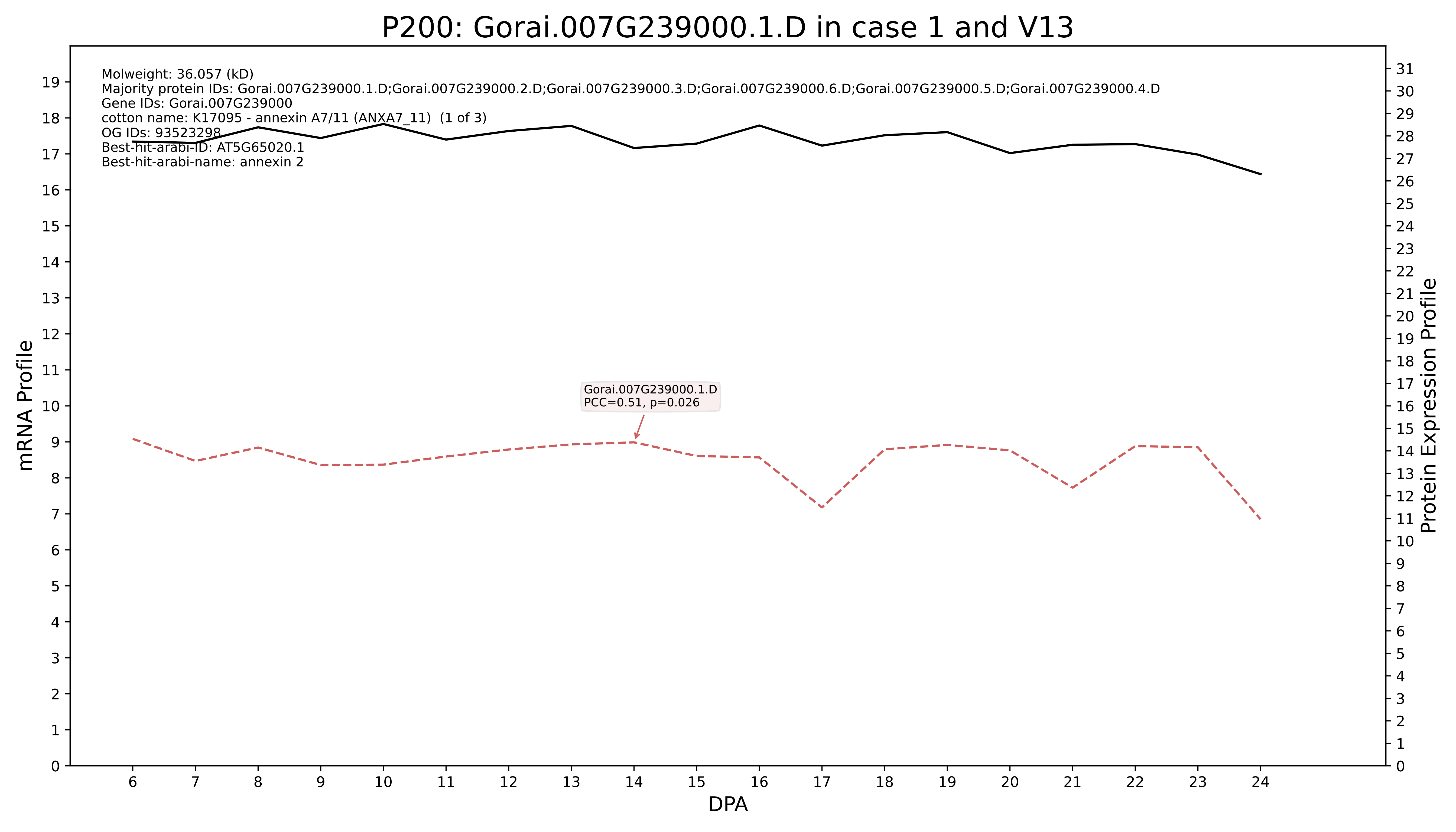
P200: Gorai.007G239000.1.D in case 1 and V13
Molweight: 36.057 (kD)
Majority protein IDs: Gorai.007G239000.1.D;Gorai.007G239000.2.D;Gorai.007G239000.3.D;Gorai.007G239000.6.D;Gorai.007G239000.5.D;Gorai.007G239000.4.D
Gene IDs: Gorai.007G239000
cotton name: K17095 - annexin A7/11 (ANXA7_11) (1 of 3)
OG IDs: 93523298
Best-hit-arabi-ID: AT5G65020.1
Best-hit-arabi-name: annexin 2
Gorai.007G239000.1.D
PCC=0.51, p=0.026
mRNA Profile
Protein Expression Profile
DPA
0
1
2
3
4
5
6
7
8
9
10
11
12
13
14
15
16
17
18
19
20
21
22
23
24
25
26
27
28
29
30
31

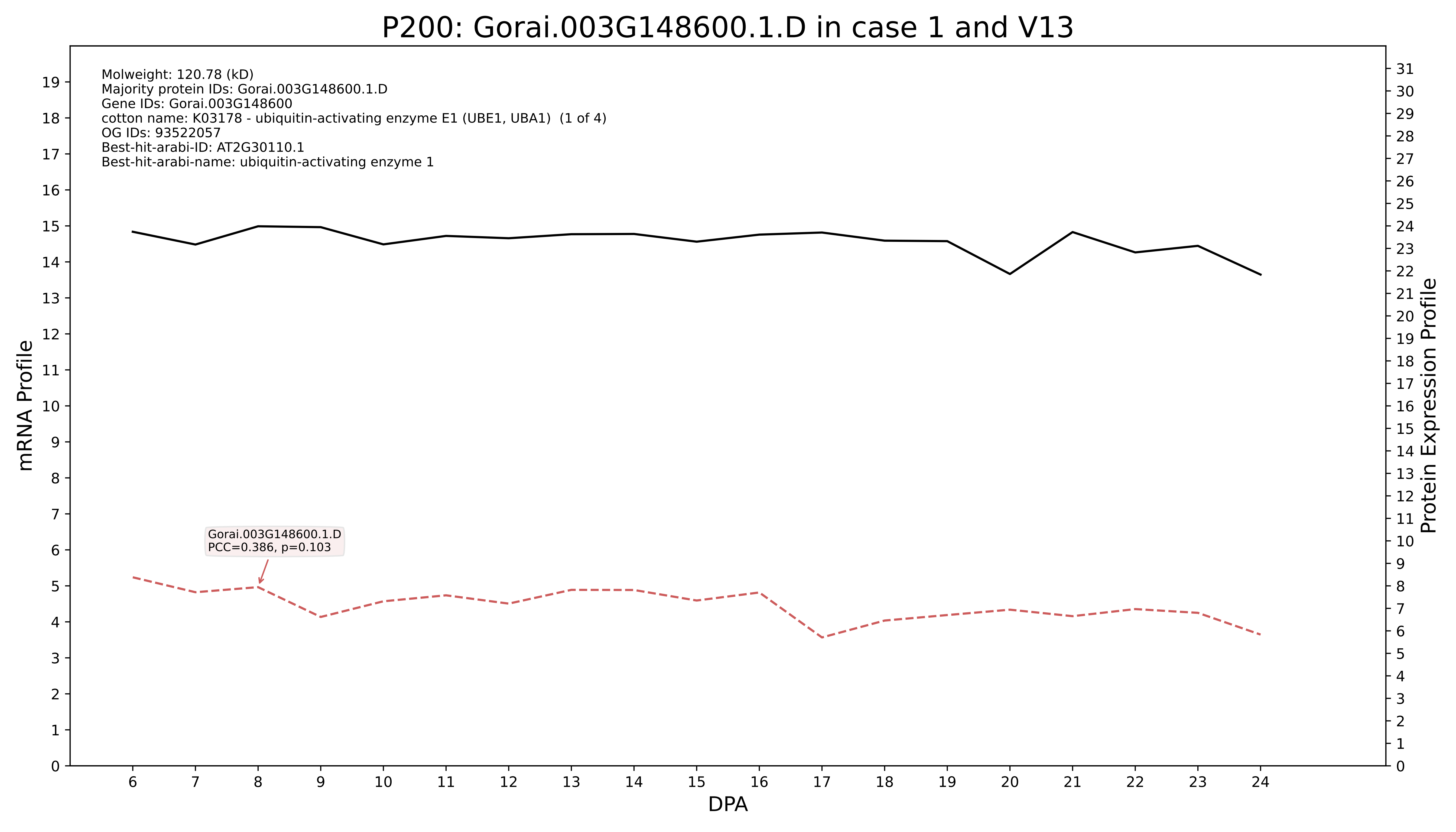

P200: Gorai.003G148600.1.D in case 1 and V13
Molweight: 120.78 (kD)
Majority protein IDs: Gorai.003G148600.1.D
Gene IDs: Gorai.003G148600
cotton name: K03178 - ubiquitin-activating enzyme E1 (UBE1, UBA1) (1 of 4)
OG IDs: 93522057
Best-hit-arabi-ID: AT2G30110.1
Best-hit-arabi-name: ubiquitin-activating enzyme 1
Gorai.003G148600.1.D
PCC=0.386, p=0.103
mRNA Profile
Protein Expression Profile
DPA

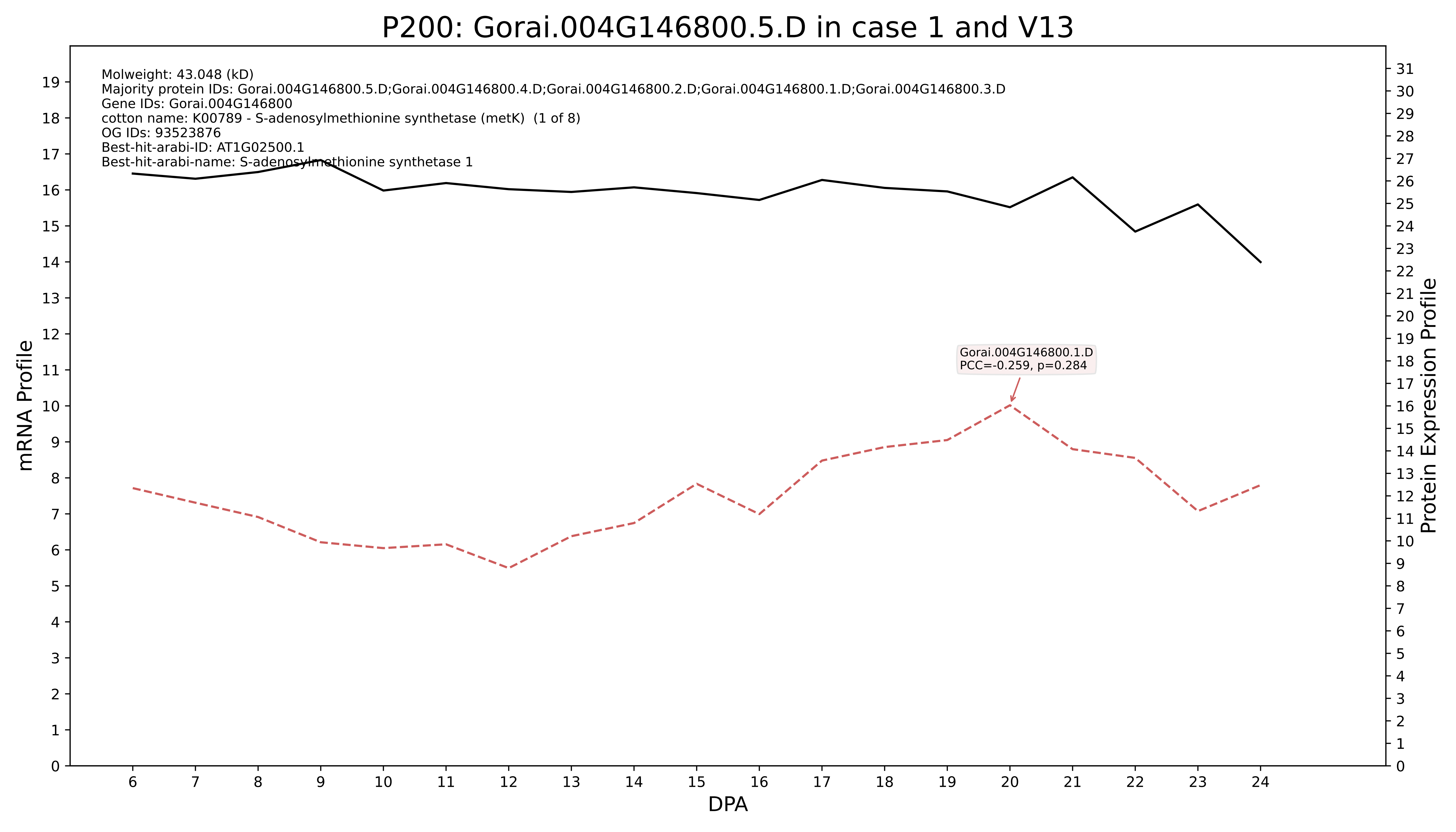

P200: Gorai.004G146800.5.D in case 1 and V13
Molweight: 43.048 (kD)
Majority protein IDs: Gorai.004G146800.5.D;Gorai.004G146800.4.D;Gorai.004G146800.2.D;Gorai.004G146800.1.D;Gorai.004G146800.3.D
Gene IDs: Gorai.004G146800
cotton name: K00789 - S-adenosylmethionine synthetase (metK) (1 of 8)
OG IDs: 93523876
Best-hit-arabi-ID: AT1G02500.1
Best-hit-arabi-name: S-adenosylmethionine synthetase 1
Gorai.004G146800.1.D
PCC=-0.259, p=0.284
mRNA Profile
Protein Expression Profile
DPA

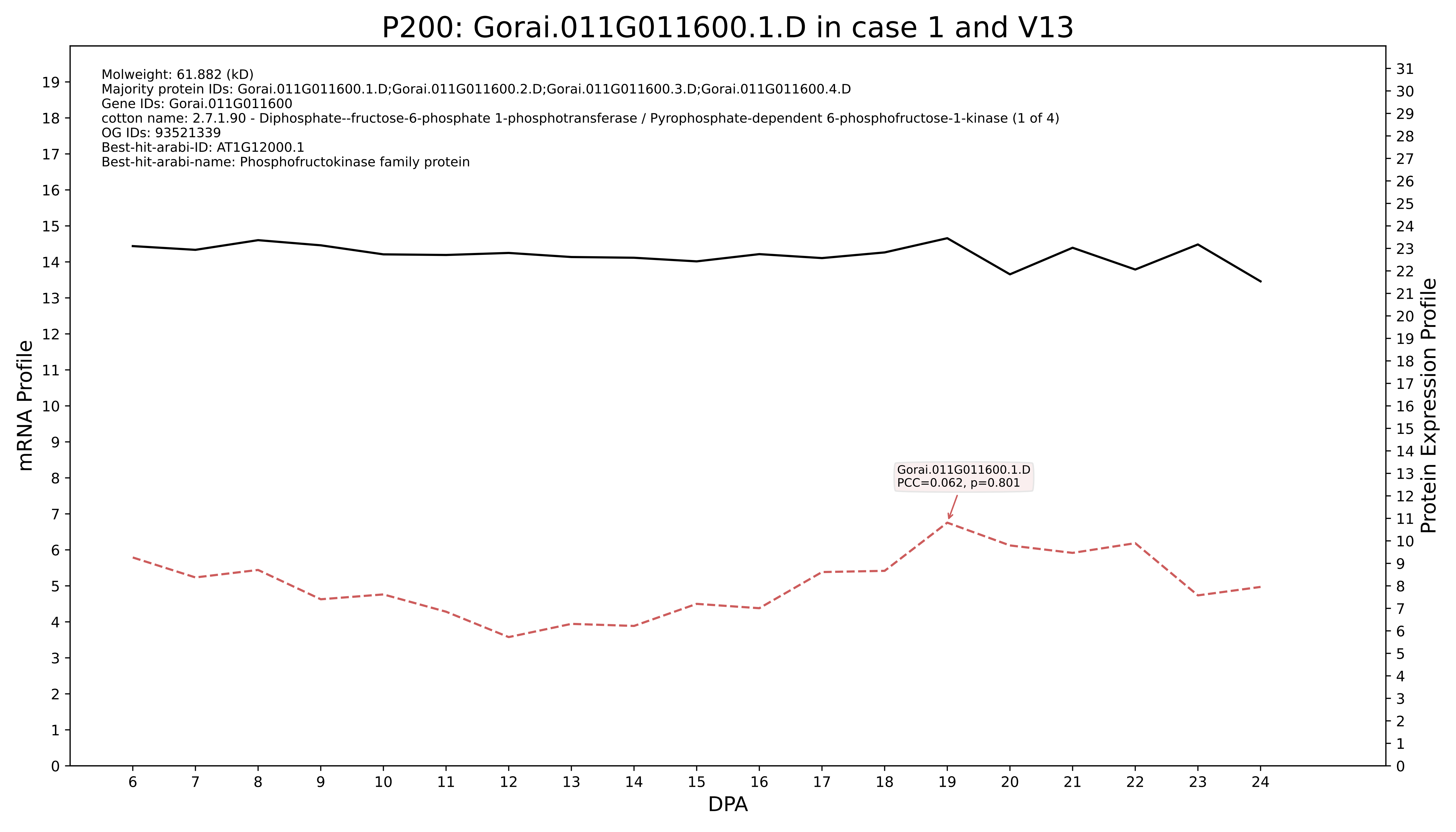
P200: Gorai.011G011600.1.D in case 1 and V13
Molweight: 61.882 (kD)
Majority protein IDs: Gorai.011G011600.1.D;Gorai.011G011600.2.D;Gorai.011G011600.3.D;Gorai.011G011600.4.D
Gene IDs: Gorai.011G011600
cotton name: 2.7.1.90 - Diphosphate--fructose-6-phosphate 1-phosphotransferase / Pyrophosphate-dependent 6-phosphofructose-1-kinase (1 of 4)
OG IDs: 93521339
Best-hit-arabi-ID: AT1G12000.1
Best-hit-arabi-name: Phosphofructokinase family protein
Gorai.011G011600.1.D
PCC=0.062, p=0.801
mRNA Profile
Protein Expression Profile
DPA

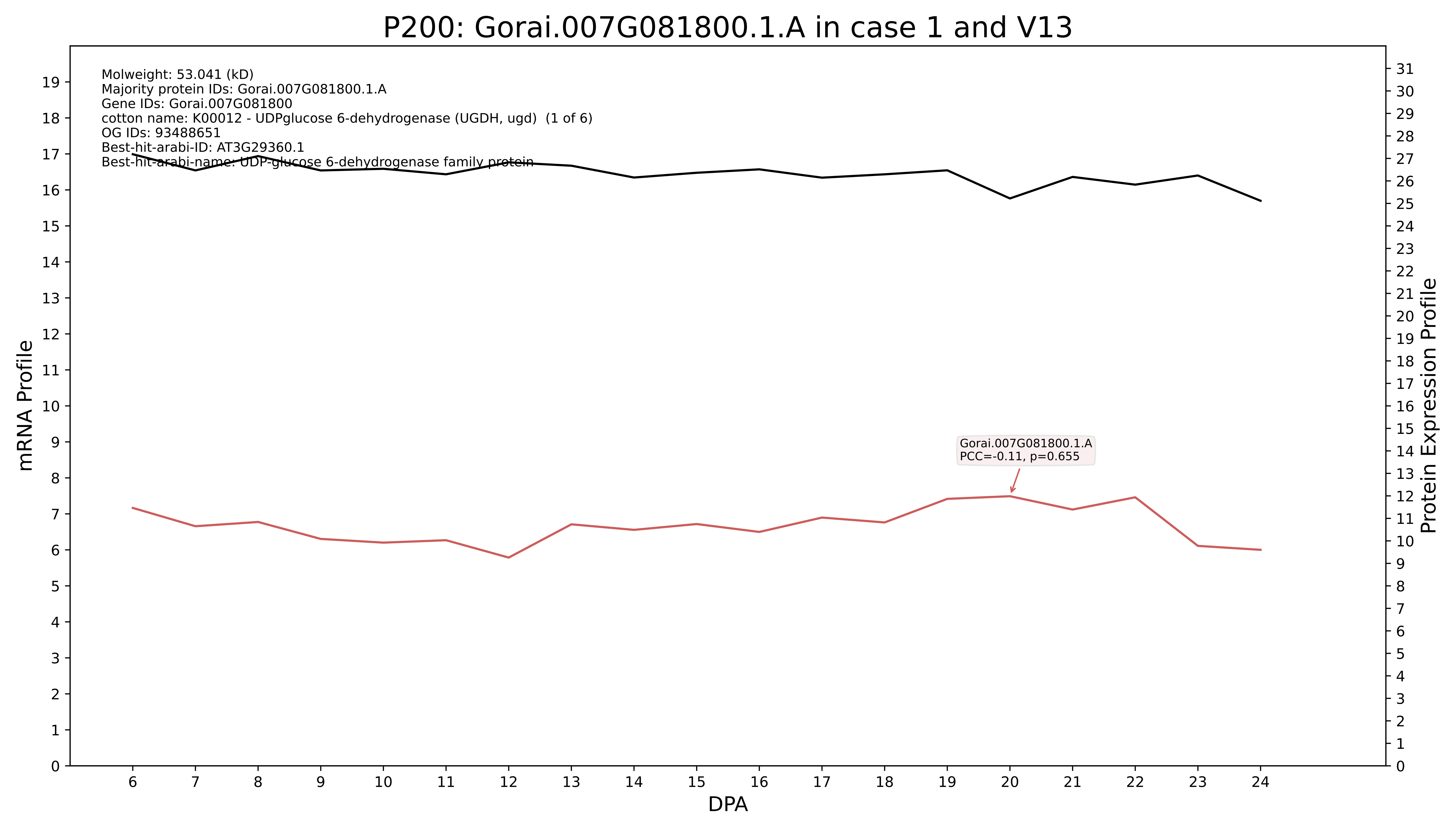

P200: Gorai.007G081800.1.A in case 1 and V13
Molweight: 53.041 (kD)
Majority protein IDs: Gorai.007G081800.1.A
Gene IDs: Gorai.007G081800
cotton name: K00012 - UDPglucose 6-dehydrogenase (UGDH, ugd) (1 of 6)
OG IDs: 93488651
Best-hit-arabi-ID: AT3G29360.1
Best-hit-arabi-name: UDP-glucose 6-dehydrogenase family protein
Gorai.007G081800.1.A
PCC=-0.11, p=0.655
mRNA Profile
Protein Expression Profile
DPA

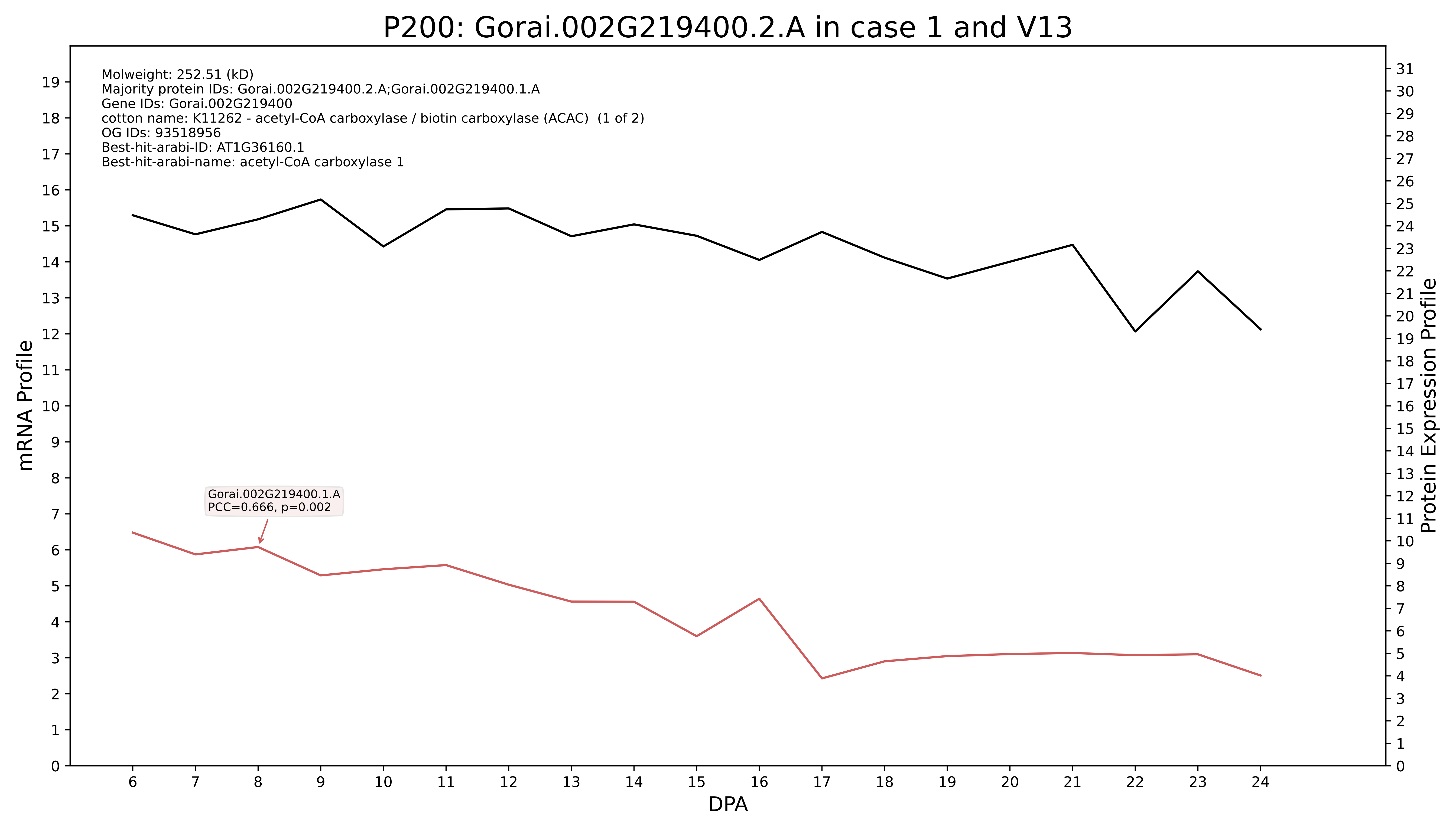
P200: Gorai.002G219400.2.A in case 1 and V13
Molweight: 252.51 (kD)
Majority protein IDs: Gorai.002G219400.2.A;Gorai.002G219400.1.A
Gene IDs: Gorai.002G219400
cotton name: K11262 - acetyl-CoA carboxylase / biotin carboxylase (ACAC) (1 of 2)
OG IDs: 93518956
Best-hit-arabi-ID: AT1G36160.1
Best-hit-arabi-name: acetyl-CoA carboxylase 1
Gorai.002G219400.1.A
PCC=0.666, p=0.002
mRNA Profile
Protein Expression Profile
DPA
0
1
2
3
4
5
6
7
8
9
10
11
12
13
14
15
16
17
18
19
20
21
22
23
24
25
26
27
28
29
30
31

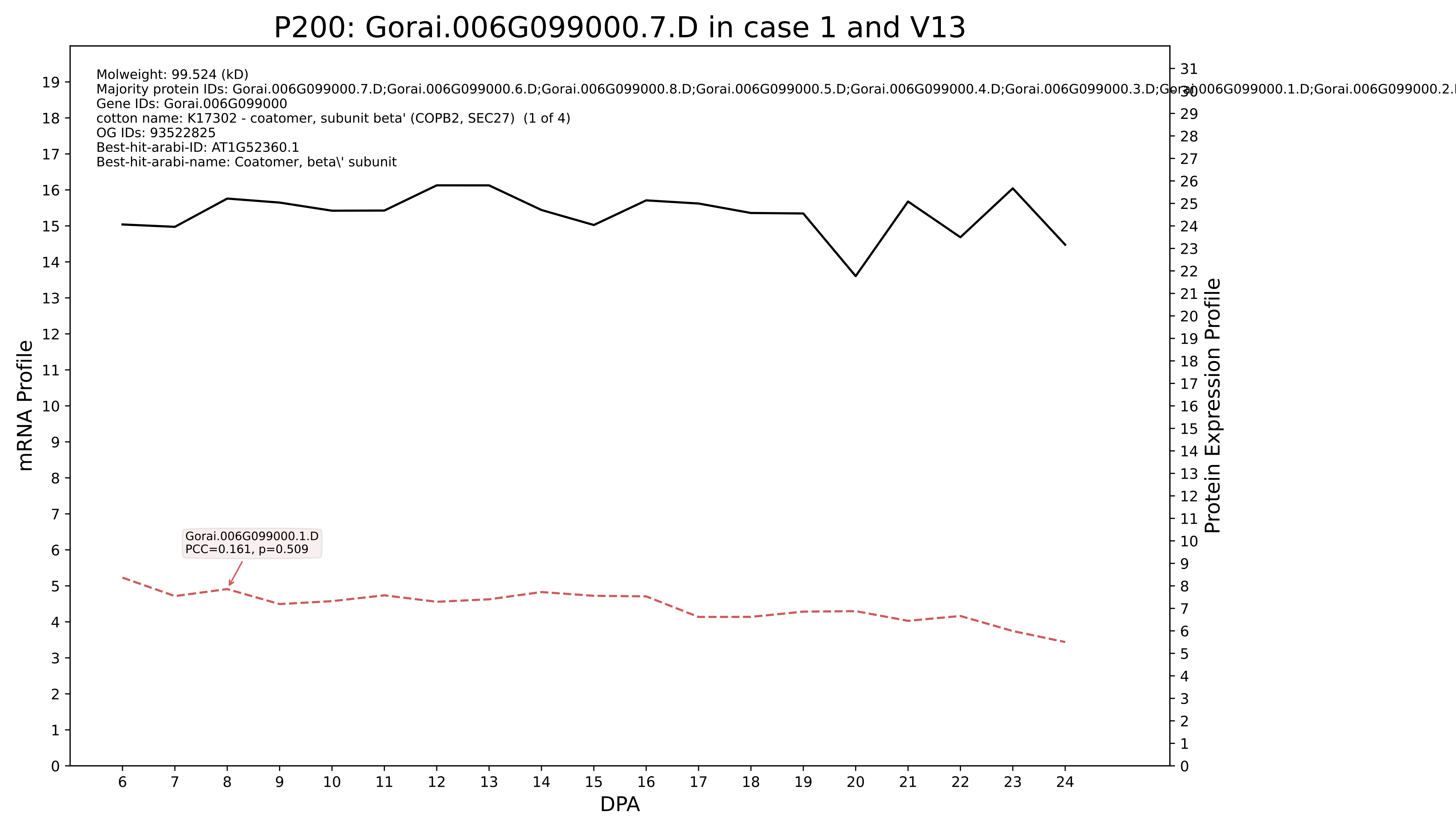
P200: Gorai.006G099000.7.D in case 1 and V13
Molweight: 99.524 (kD)
Majority protein IDs: Gorai.006G099000.7.D;Gorai.006G099000.6.D;Gorai.006G099000.8.D;Gorai.006G099000.5.D;Gorai.006G099000.4.D;Gorai.006G099000.3.D;Gorai.006G099000.1.D;Gorai.006G099000.2.
Gene IDs: Gorai.006G099000
cotton name: K17302 - coatomer, subunit beta' (COPB2, SEC27) (1 of 4)
OG IDs: 93522825
Best-hit-arabi-ID: AT1G52360.1
Best-hit-arabi-name: Coatomer, beta\' subunit
Gorai.006G099000.1.D
PCC=0.161, p=0.509
mRNA Profile
Protein Expression Profile
DPA

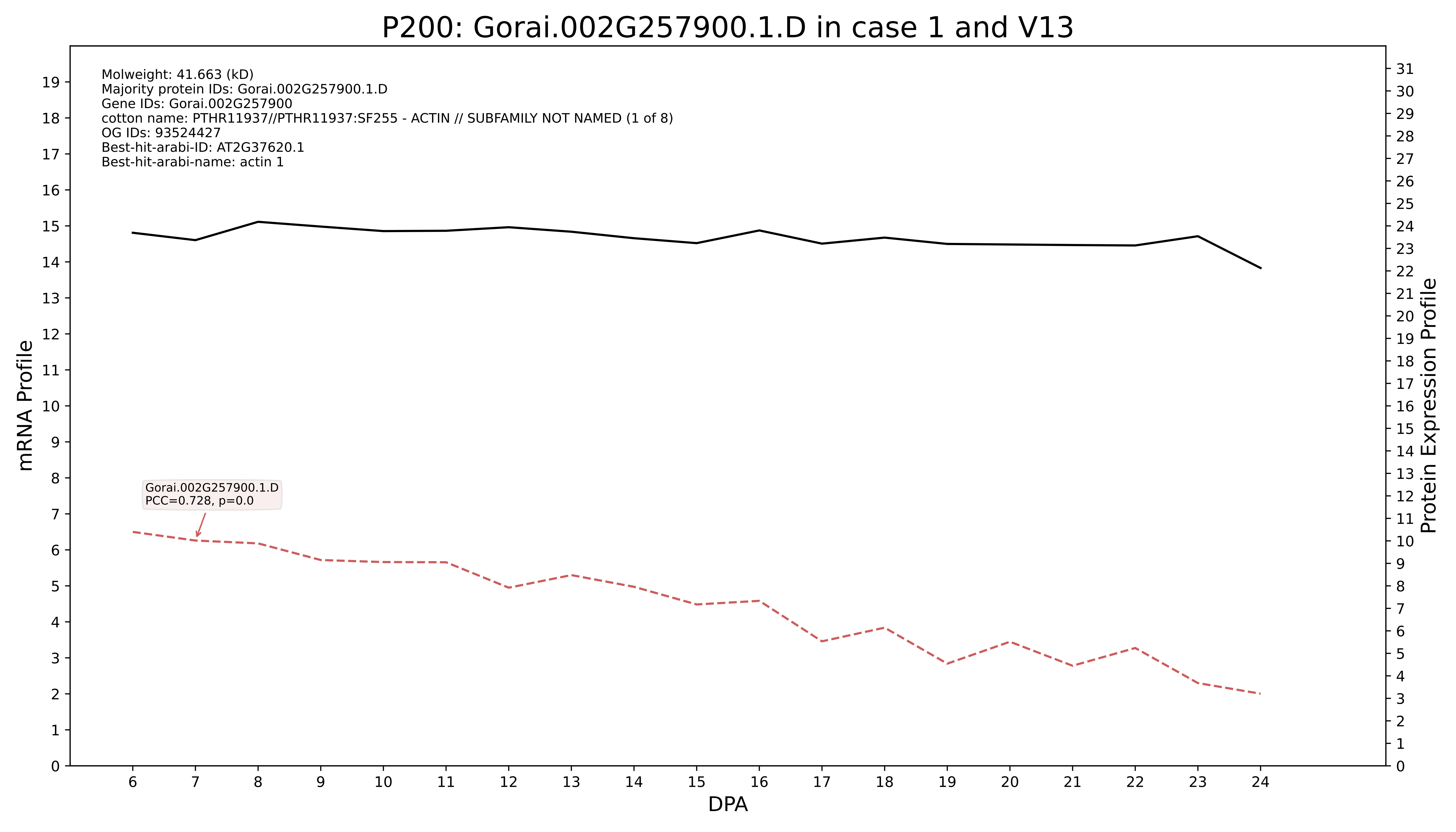
P200: Gorai.002G257900.1.D in case 1 and V13
Molweight: 41.663 (kD)
Majority protein IDs: Gorai.002G257900.1.D
Gene IDs: Gorai.002G257900
cotton name: PTHR11937//PTHR11937:SF255 - ACTIN // SUBFAMILY NOT NAMED (1 of 8)
OG IDs: 93524427
Best-hit-arabi-ID: AT2G37620.1
Best-hit-arabi-name: actin 1
Gorai.002G257900.1.D
PCC=0.728, p=0.0
mRNA Profile
Protein Expression Profile
DPA

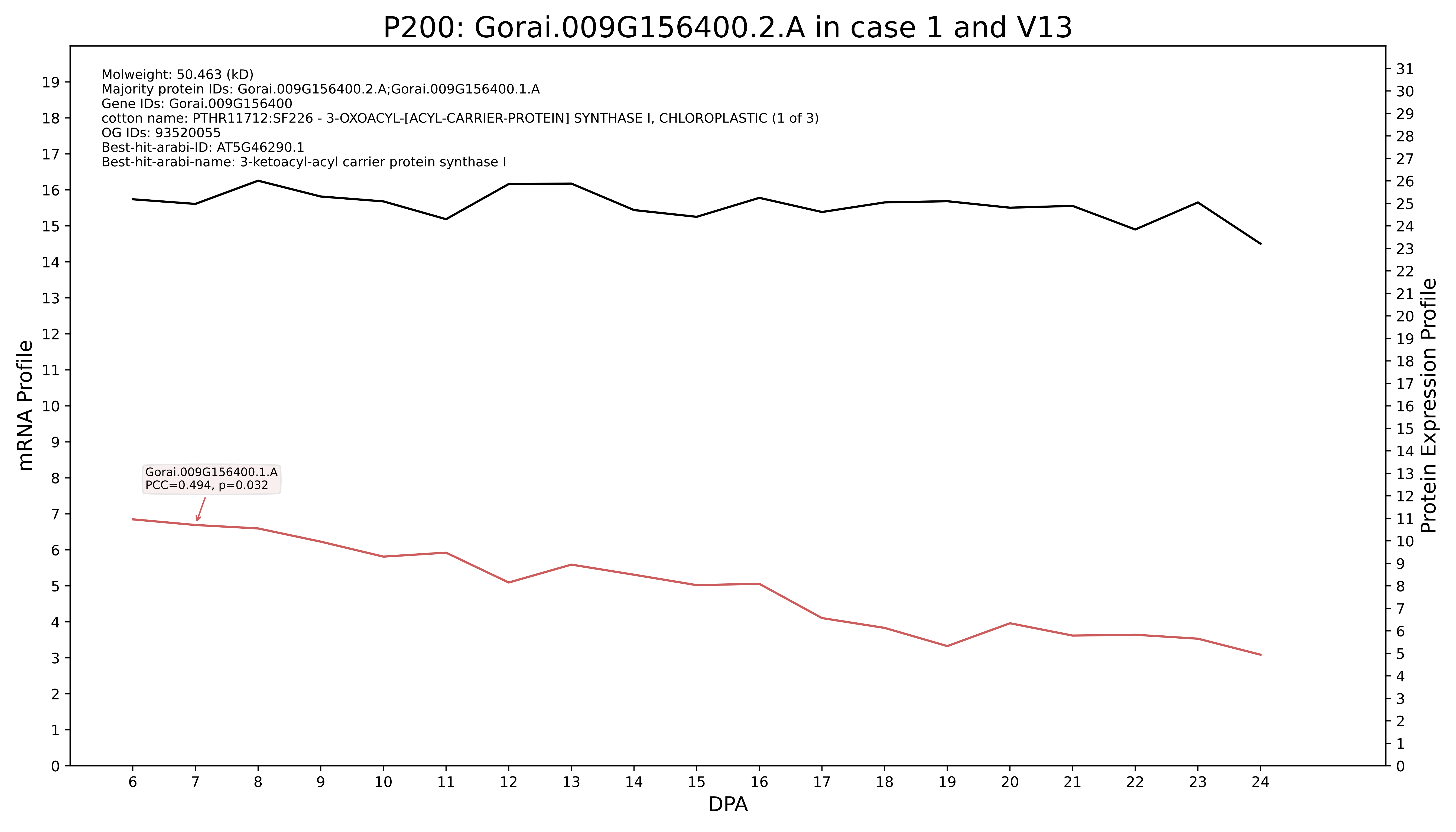
P200: Gorai.009G156400.2.A in case 1 and V13
Molweight: 50.463 (kD)
Majority protein IDs: Gorai.009G156400.2.A;Gorai.009G156400.1.A
Gene IDs: Gorai.009G156400
cotton name: PTHR11712:SF226 - 3-OXOACYL-[ACYL-CARRIER-PROTEIN] SYNTHASE I, CHLOROPLASTIC (1 of 3)
OG IDs: 93520055
Best-hit-arabi-ID: AT5G46290.1
Best-hit-arabi-name: 3-ketoacyl-acyl carrier protein synthase I
Gorai.009G156400.1.A
PCC=0.494, p=0.032
mRNA Profile
Protein Expression Profile
DPA

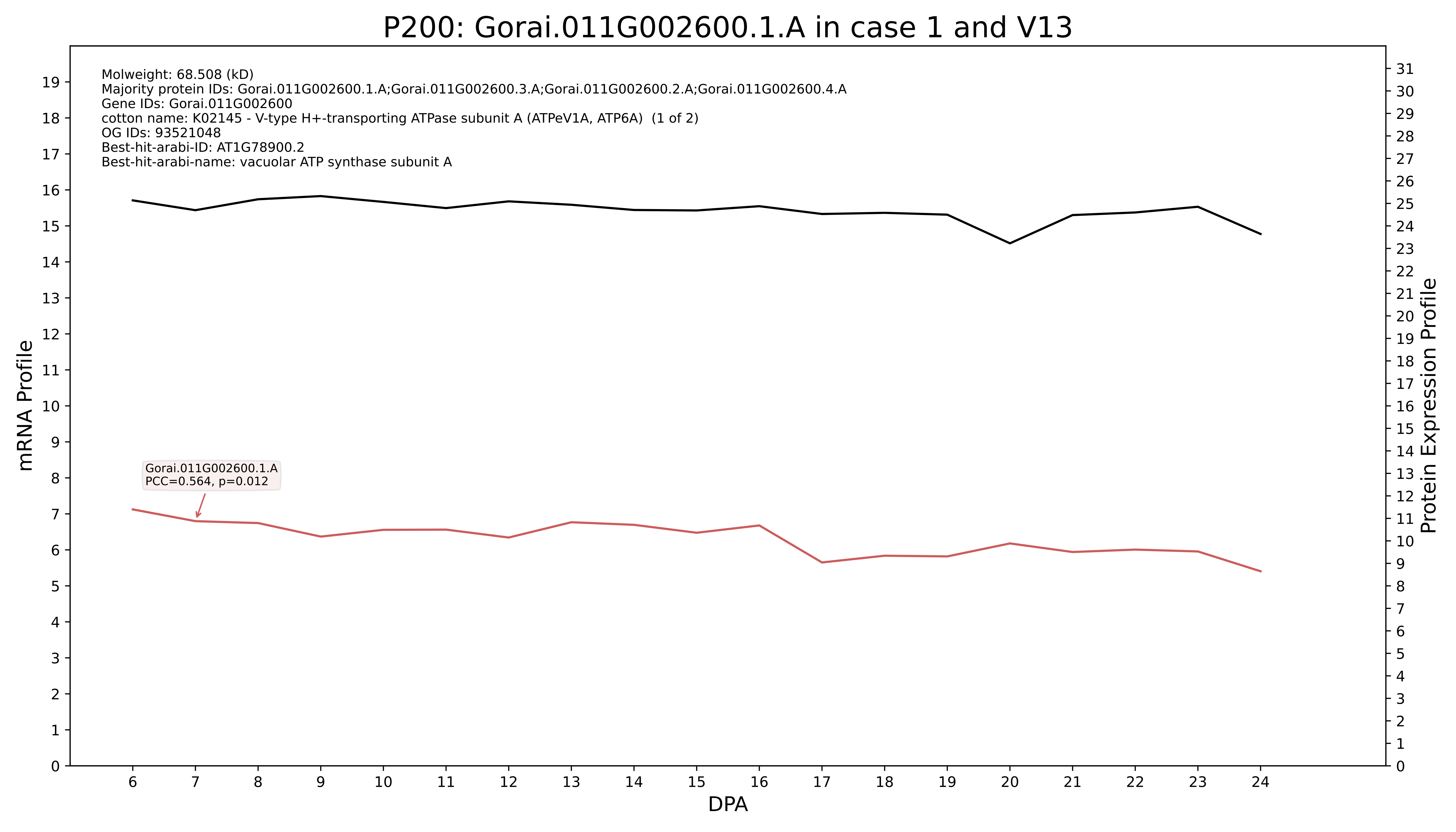

P200: Gorai.011G002600.1.A in case 1 and V13
Molweight: 68.508 (kD)
Majority protein IDs: Gorai.011G002600.1.A;Gorai.011G002600.3.A;Gorai.011G002600.2.A;Gorai.011G002600.4.A
Gene IDs: Gorai.011G002600
cotton name: K02145 - V-type H+-transporting ATPase subunit A (ATPeV1A, ATP6A) (1 of 2)
OG IDs: 93521048
Best-hit-arabi-ID: AT1G78900.2
Best-hit-arabi-name: vacuolar ATP synthase subunit A
Gorai.011G002600.1.A
PCC=0.564, p=0.012
mRNA Profile
Protein Expression Profile
DPA

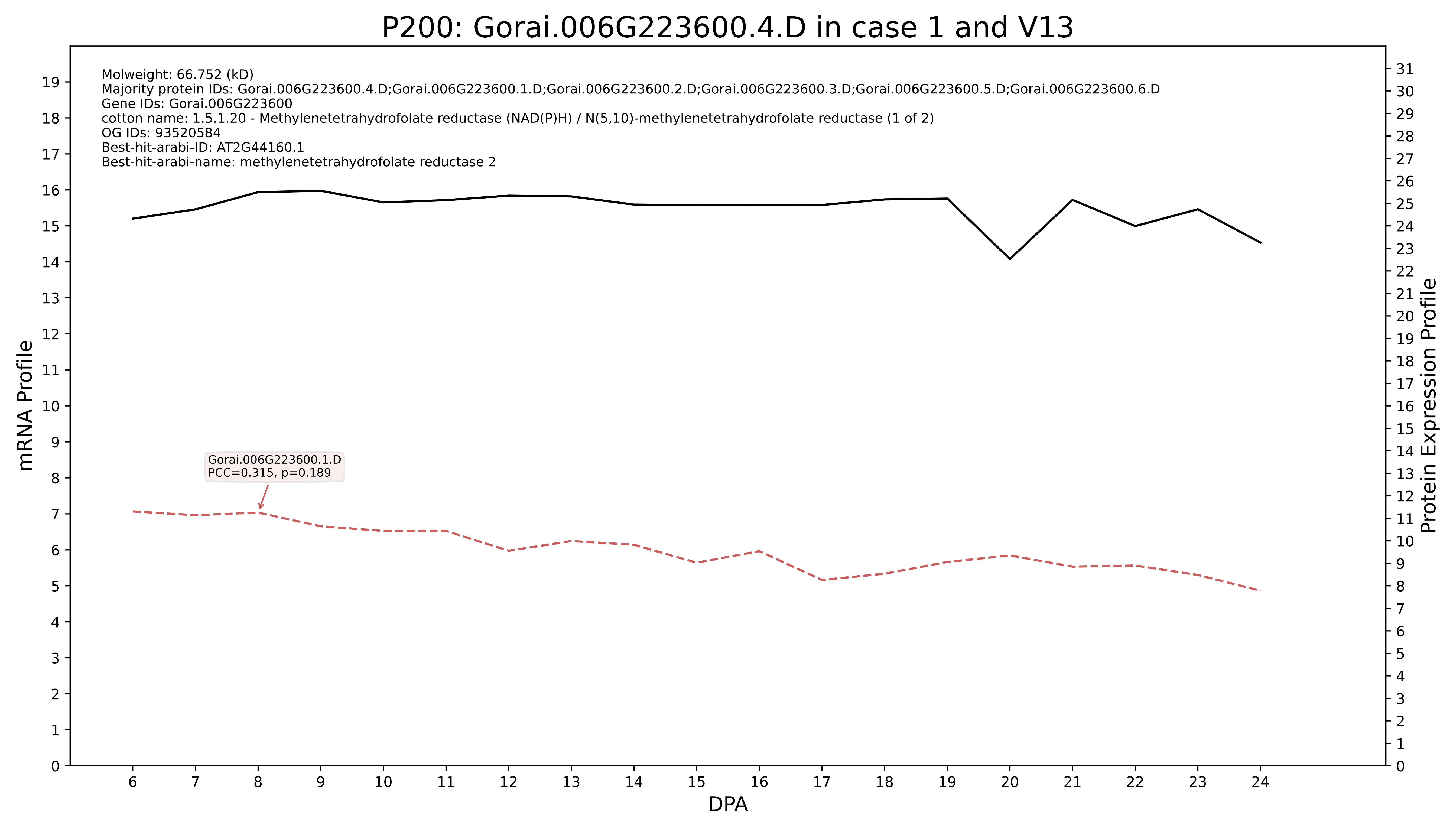
P200: Gorai.006G223600.4.D in case 1 and V13
Molweight: 66.752 (kD)
Majority protein IDs: Gorai.006G223600.4.D;Gorai.006G223600.1.D;Gorai.006G223600.2.D;Gorai.006G223600.3.D;Gorai.006G223600.5.D;Gorai.006G223600.6.D
Gene IDs: Gorai.006G223600
cotton name: 1.5.1.20 - Methylenetetrahydrofolate reductase (NAD(P)H) / N(5,10)-methylenetetrahydrofolate reductase (1 of 2)
OG IDs: 93520584
Best-hit-arabi-ID: AT2G44160.1
Best-hit-arabi-name: methylenetetrahydrofolate reductase 2
Gorai.006G223600.1.D
PCC=0.315, p=0.189
mRNA Profile
Protein Expression Profile
DPA

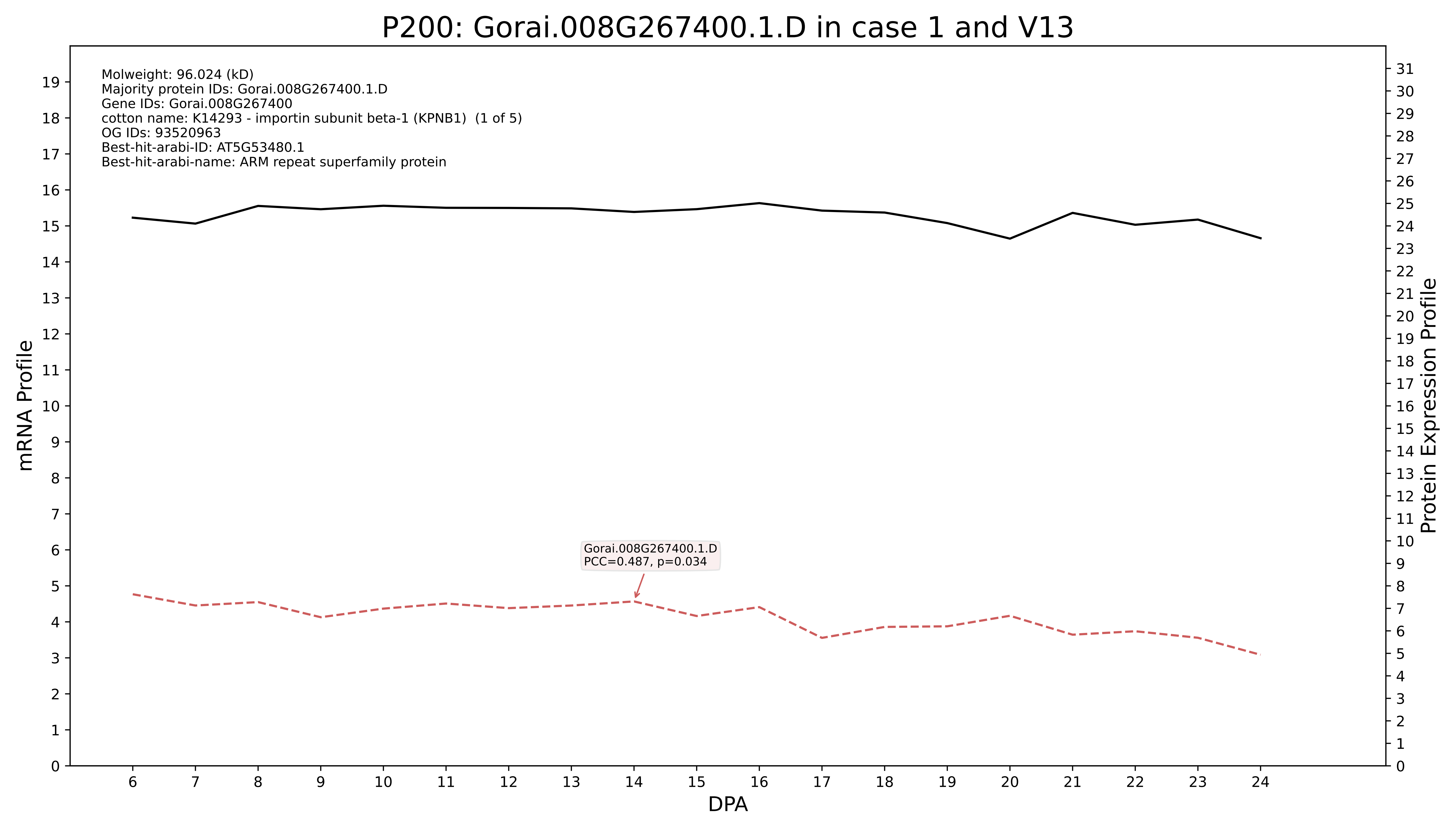

P200: Gorai.008G267400.1.D in case 1 and V13
Molweight: 96.024 (kD)
Majority protein IDs: Gorai.008G267400.1.D
Gene IDs: Gorai.008G267400
cotton name: K14293 - importin subunit beta-1 (KPNB1) (1 of 5)
OG IDs: 93520963
Best-hit-arabi-ID: AT5G53480.1
Best-hit-arabi-name: ARM repeat superfamily protein
Gorai.008G267400.1.D
PCC=0.487, p=0.034
mRNA Profile
Protein Expression Profile
DPA

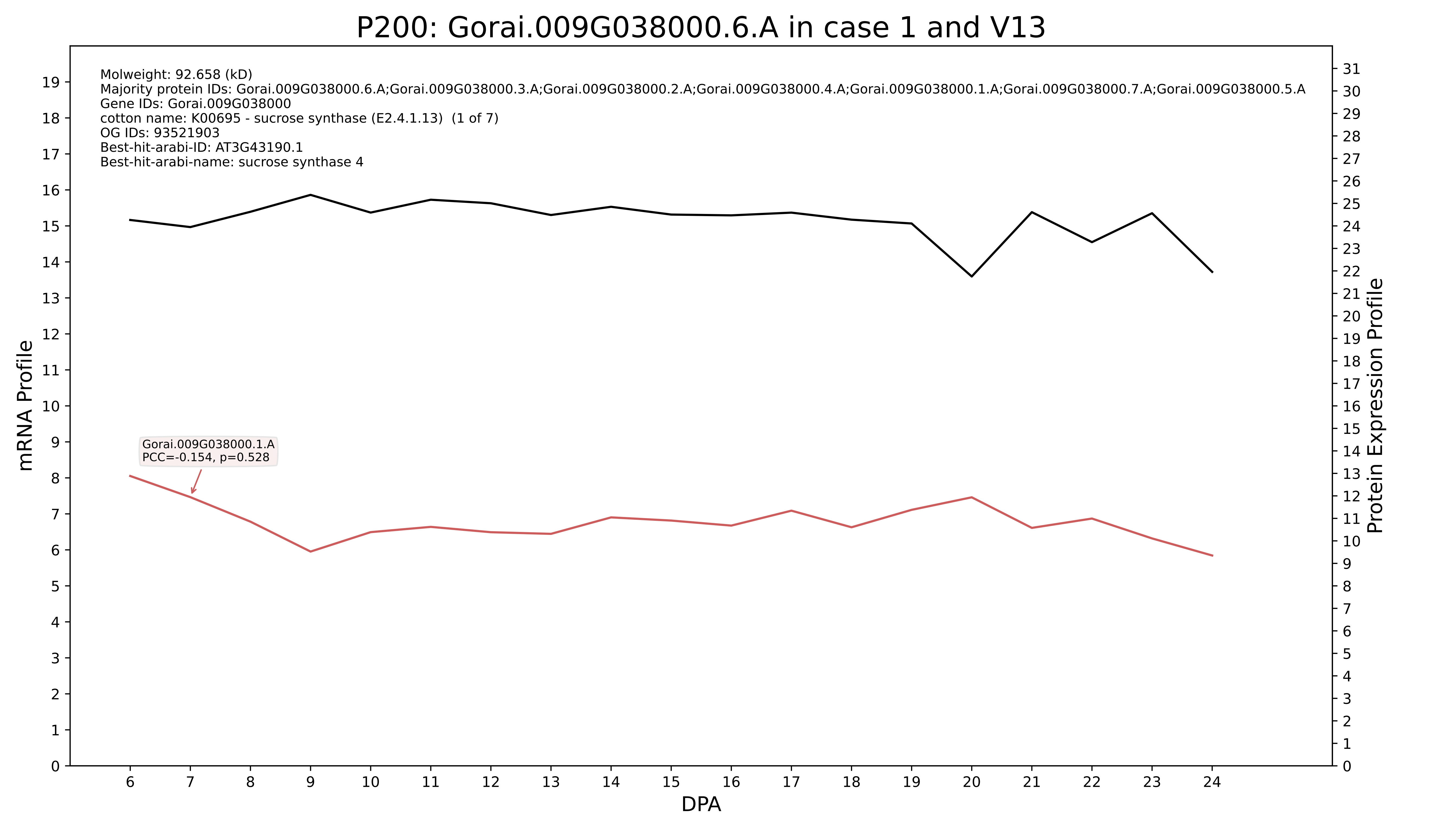

P200: Gorai.009G038000.6.A in case 1 and V13
Molweight: 92.658 (kD)
Majority protein IDs: Gorai.009G038000.6.A;Gorai.009G038000.3.A;Gorai.009G038000.2.A;Gorai.009G038000.4.A;Gorai.009G038000.1.A;Gorai.009G038000.7.A;Gorai.009G038000.5.A
Gene IDs: Gorai.009G038000
cotton name: K00695 - sucrose synthase (E2.4.1.13) (1 of 7)
OG IDs: 93521903
Best-hit-arabi-ID: AT3G43190.1
Best-hit-arabi-name: sucrose synthase 4
Gorai.009G038000.1.A
PCC=-0.154, p=0.528
mRNA Profile
Protein Expression Profile
DPA

P200: Gorai.012G136300.1.D in case 1 and V13

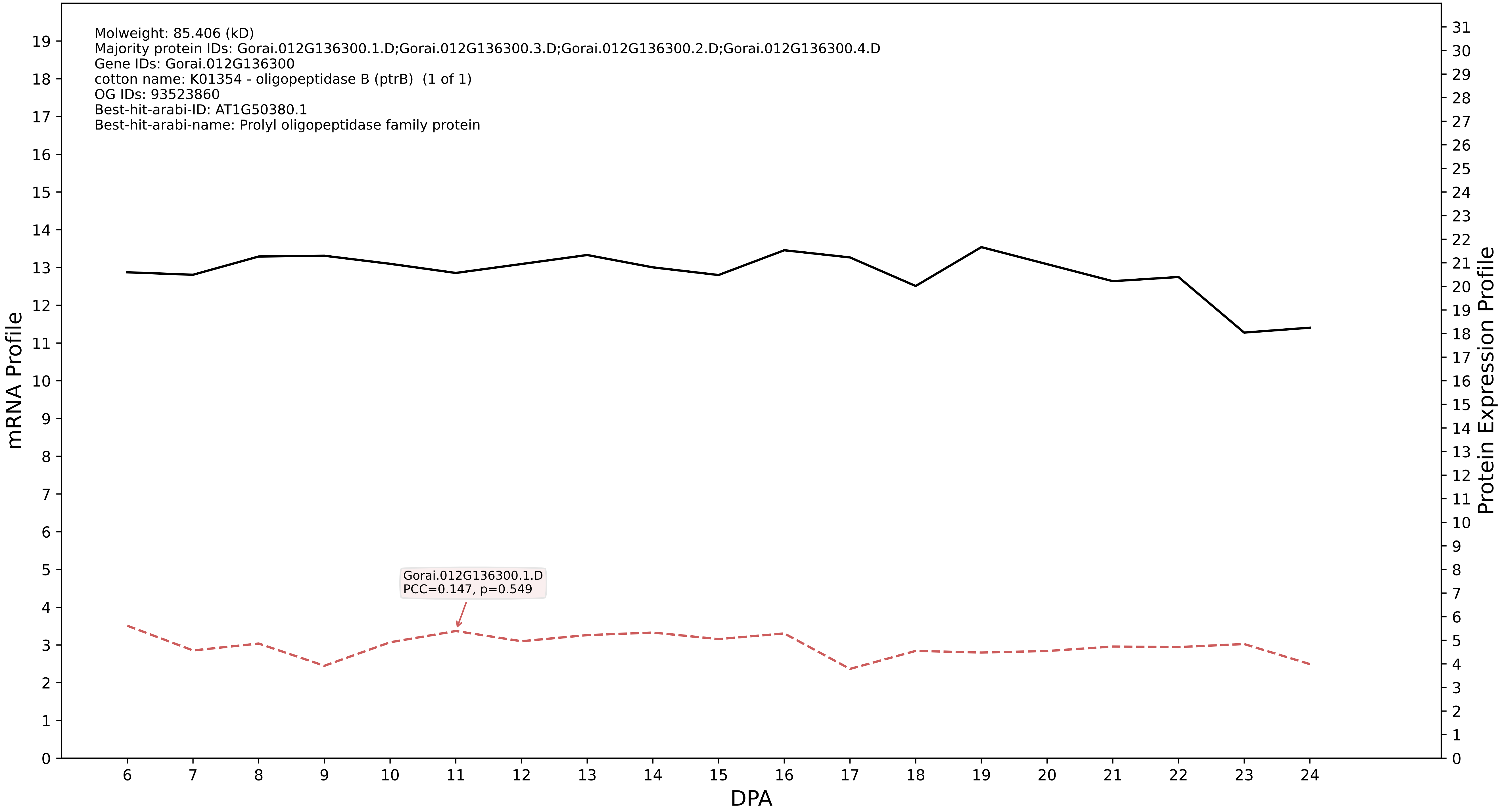

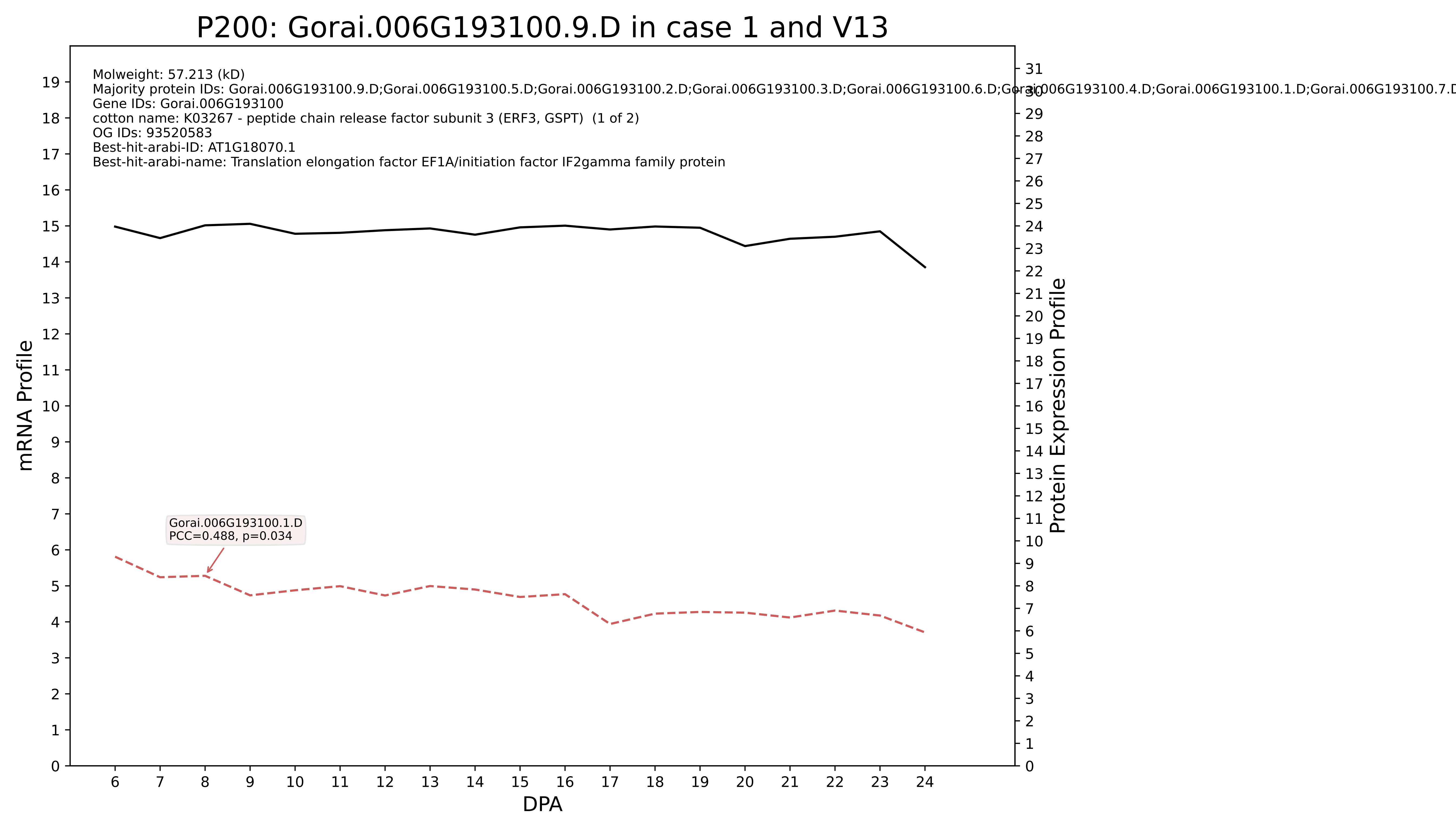
P200: Gorai.006G193100.9.D in case 1 and V13
Molweight: 57.213 (kD)
Majority protein IDs: Gorai.006G193100.9.D;Gorai.006G193100.5.D;Gorai.006G193100.2.D;Gorai.006G193100.3.D;Gorai.006G193100.6.D;Gorai.006G193100.4.D;Gorai.006G193100.1.D;Gorai.006G193100.7.D
Gene IDs: Gorai.006G193100
cotton name: K03267 - peptide chain release factor subunit 3 (ERF3, GSPT) (1 of 2)
OG IDs: 93520583
Best-hit-arabi-ID: AT1G18070.1
Best-hit-arabi-name: Translation elongation factor EF1A/initiation factor IF2gamma family protein
Gorai.006G193100.1.D
PCC=0.488, p=0.034
mRNA Profile
Protein Expression Profile
DPA

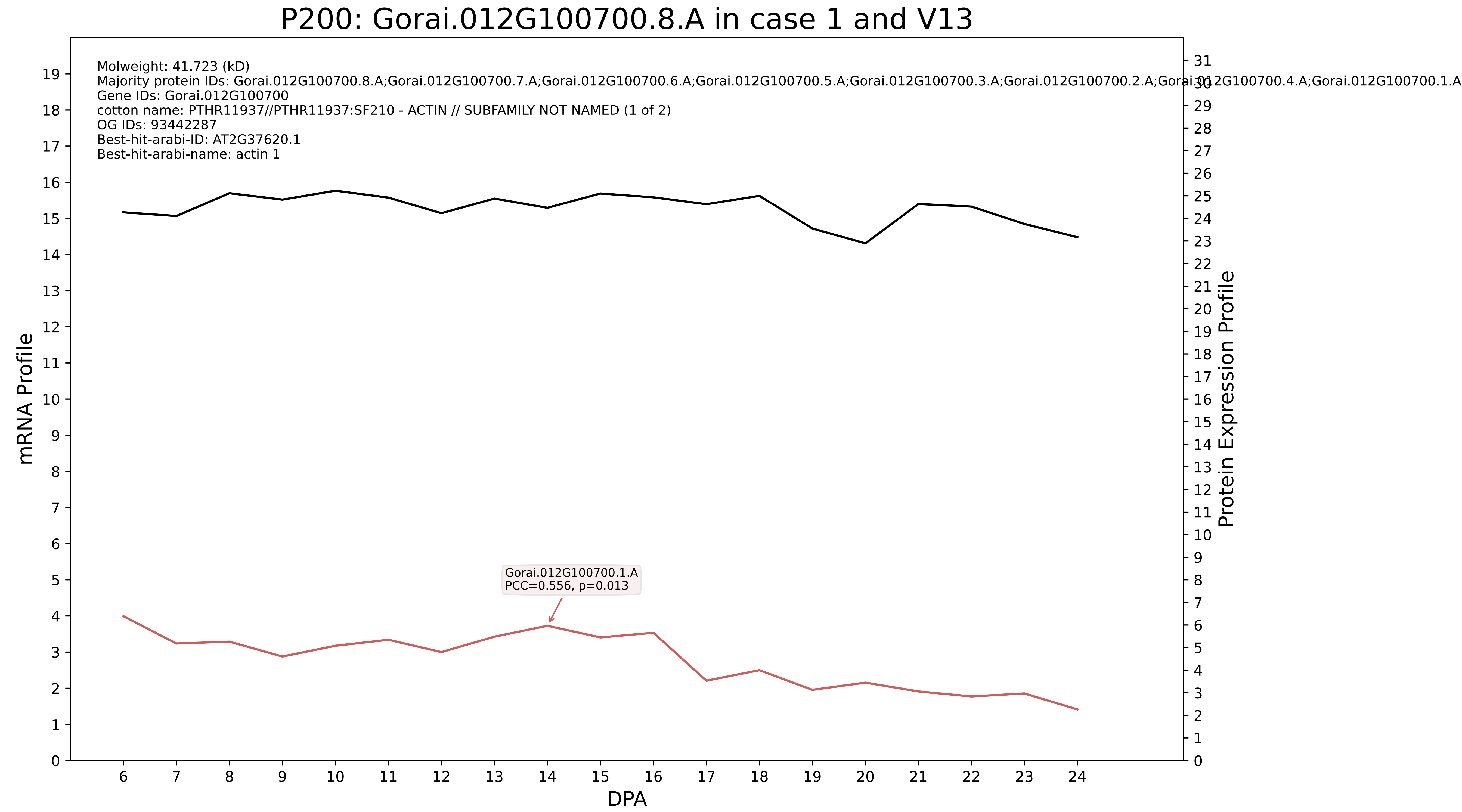

P200: Gorai.012G100700.8.A in case 1 and V13
Molweight: 41.723 (kD)
Majority protein IDs: Gorai.012G100700.8.A;Gorai.012G100700.7.A;Gorai.012G100700.6.A;Gorai.012G100700.5.A;Gorai.012G100700.3.A;Gorai.012G100700.2.A;Gorai.012G100700.4.A;Gorai.012G100700.1.A
Gene IDs: Gorai.012G100700
cotton name: PTHR11937//PTHR11937:SF210 - ACTIN // SUBFAMILY NOT NAMED (1 of 2)
OG IDs: 93442287
Best-hit-arabi-ID: AT2G37620.1
Best-hit-arabi-name: actin 1
Gorai.012G100700.1.A
PCC=0.556, p=0.013
mRNA Profile
Protein Expression Profile
DPA

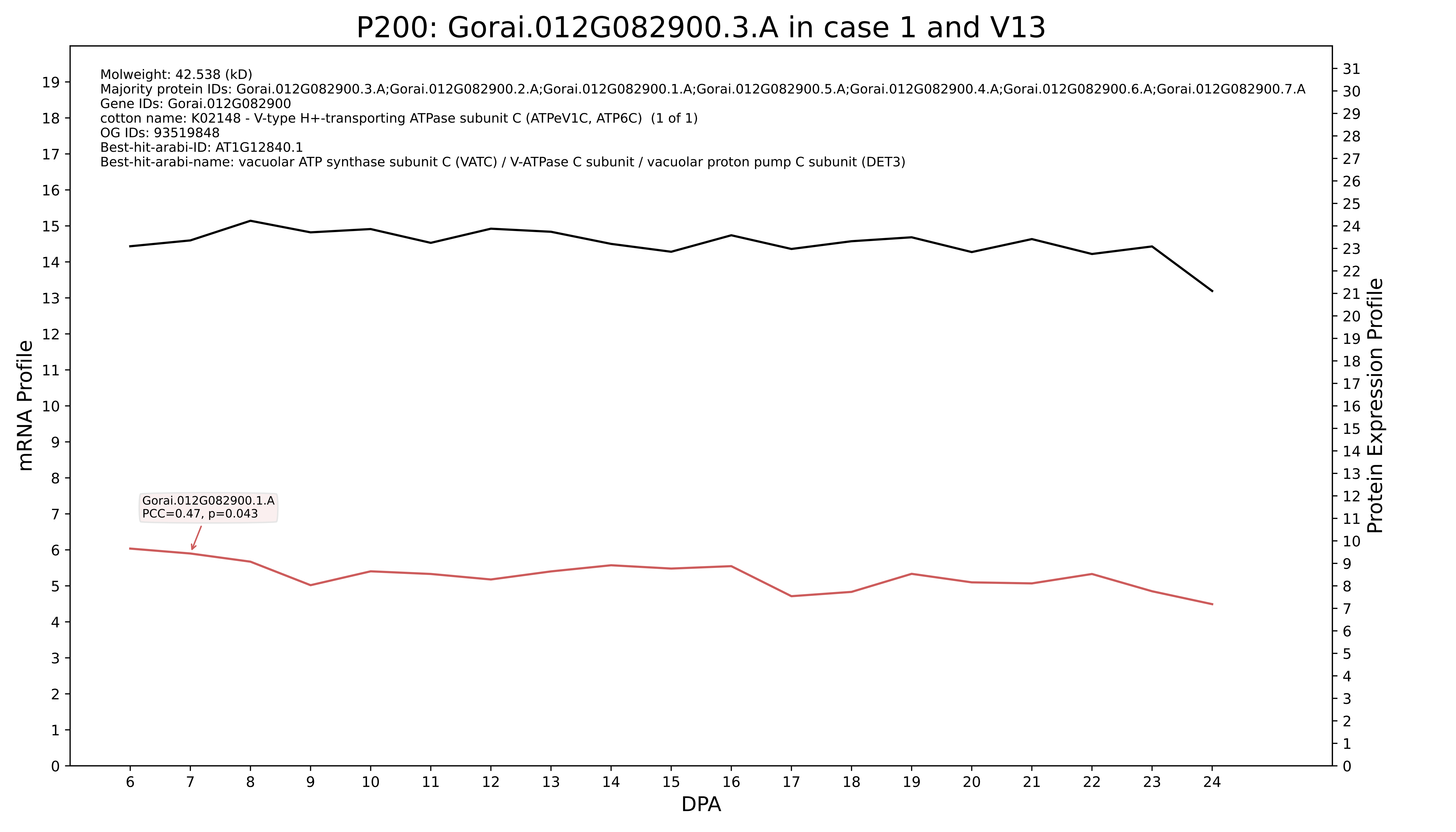

P200: Gorai.012G082900.3.A in case 1 and V13
Molweight: 42.538 (kD)
Majority protein IDs: Gorai.012G082900.3.A;Gorai.012G082900.2.A;Gorai.012G082900.1.A;Gorai.012G082900.5.A;Gorai.012G082900.4.A;Gorai.012G082900.6.A;Gorai.012G082900.7.A
Gene IDs: Gorai.012G082900
cotton name: K02148 - V-type H+-transporting ATPase subunit C (ATPeV1C, ATP6C) (1 of 1)
OG IDs: 93519848
Best-hit-arabi-ID: AT1G12840.1
Best-hit-arabi-name: vacuolar ATP synthase subunit C (VATC) / V-ATPase C subunit / vacuolar proton pump C subunit (DET3)
Gorai.012G082900.1.A
PCC=0.47, p=0.043
mRNA Profile
Protein Expression Profile
DPA

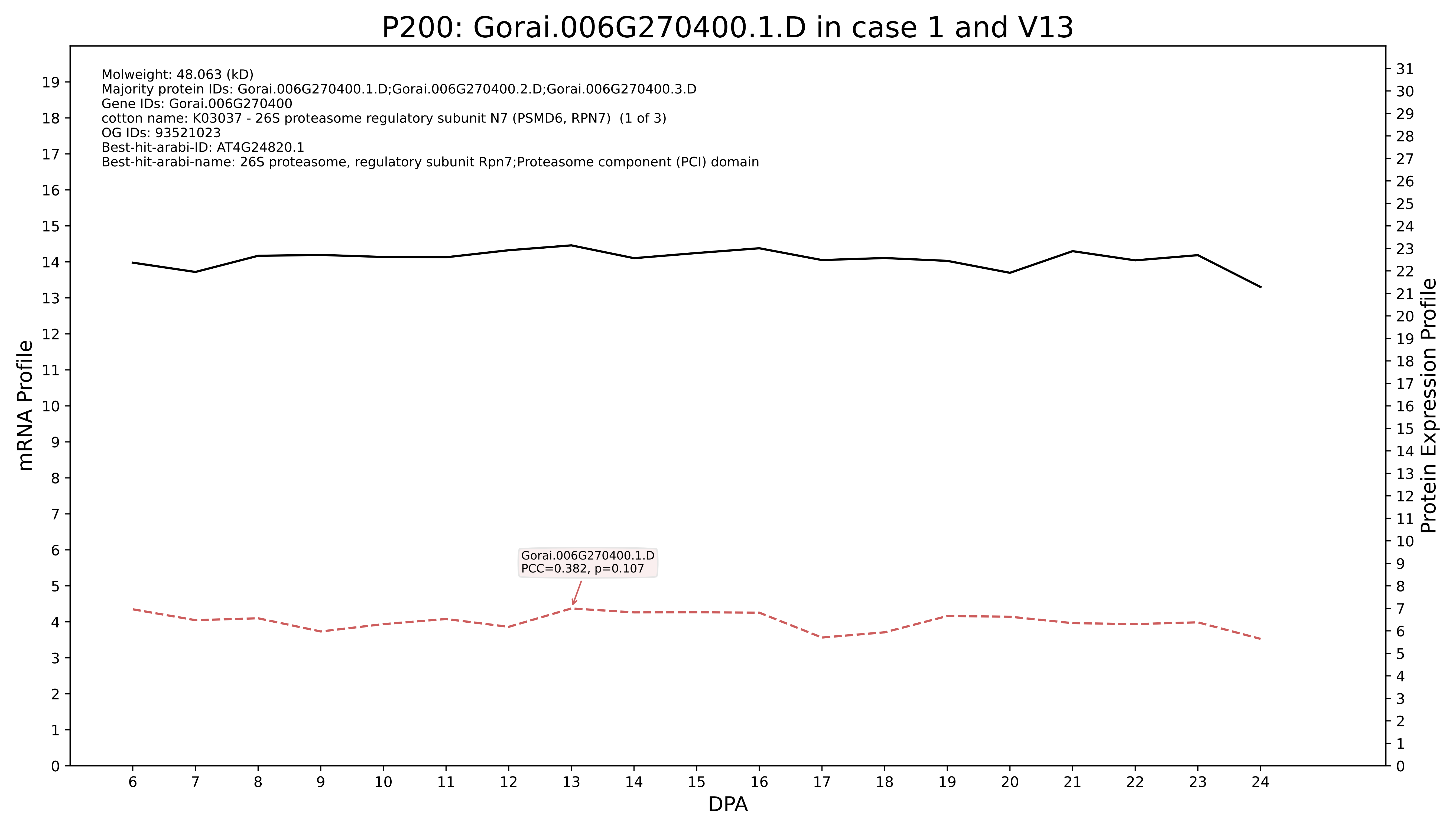
P200: Gorai.006G270400.1.D in case 1 and V13
Molweight: 48.063 (kD)
Majority protein IDs: Gorai.006G270400.1.D;Gorai.006G270400.2.D;Gorai.006G270400.3.D
Gene IDs: Gorai.006G270400
cotton name: K03037 - 26S proteasome regulatory subunit N7 (PSMD6, RPN7) (1 of 3)
OG IDs: 93521023
Best-hit-arabi-ID: AT4G24820.1
Best-hit-arabi-name: 26S proteasome, regulatory subunit Rpn7;Proteasome component (PCI) domain
Gorai.006G270400.1.D
PCC=0.382, p=0.107
mRNA Profile
Protein Expression Profile
DPA

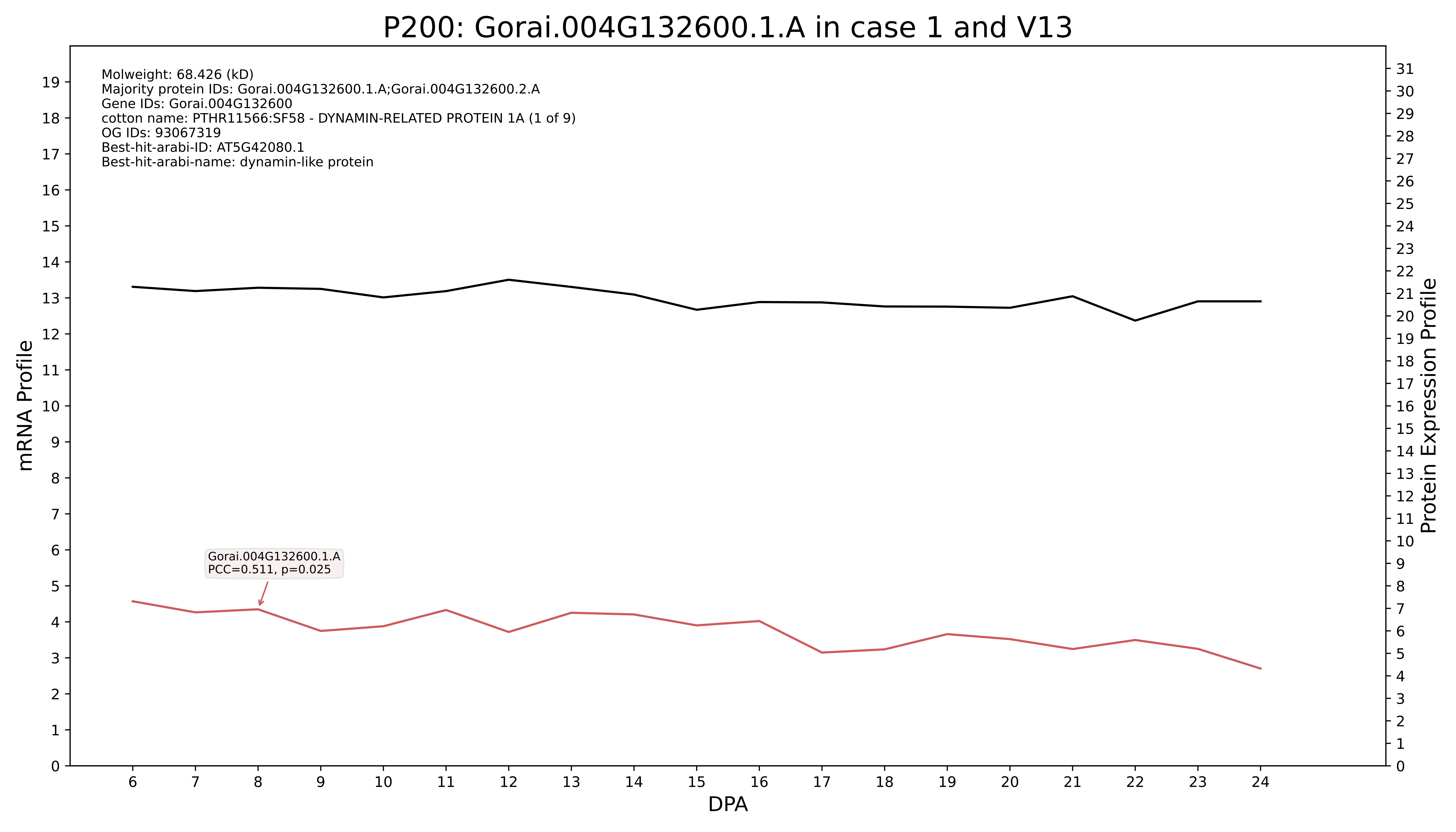

P200: Gorai.004G132600.1.A in case 1 and V13
Molweight: 68.426 (kD)
Majority protein IDs: Gorai.004G132600.1.A;Gorai.004G132600.2.A
Gene IDs: Gorai.004G132600
cotton name: PTHR11566:SF58 - DYNAMIN-RELATED PROTEIN 1A (1 of 9)
OG IDs: 93067319
Best-hit-arabi-ID: AT5G42080.1
Best-hit-arabi-name: dynamin-like protein
Gorai.004G132600.1.A
PCC=0.511, p=0.025
mRNA Profile
Protein Expression Profile
DPA

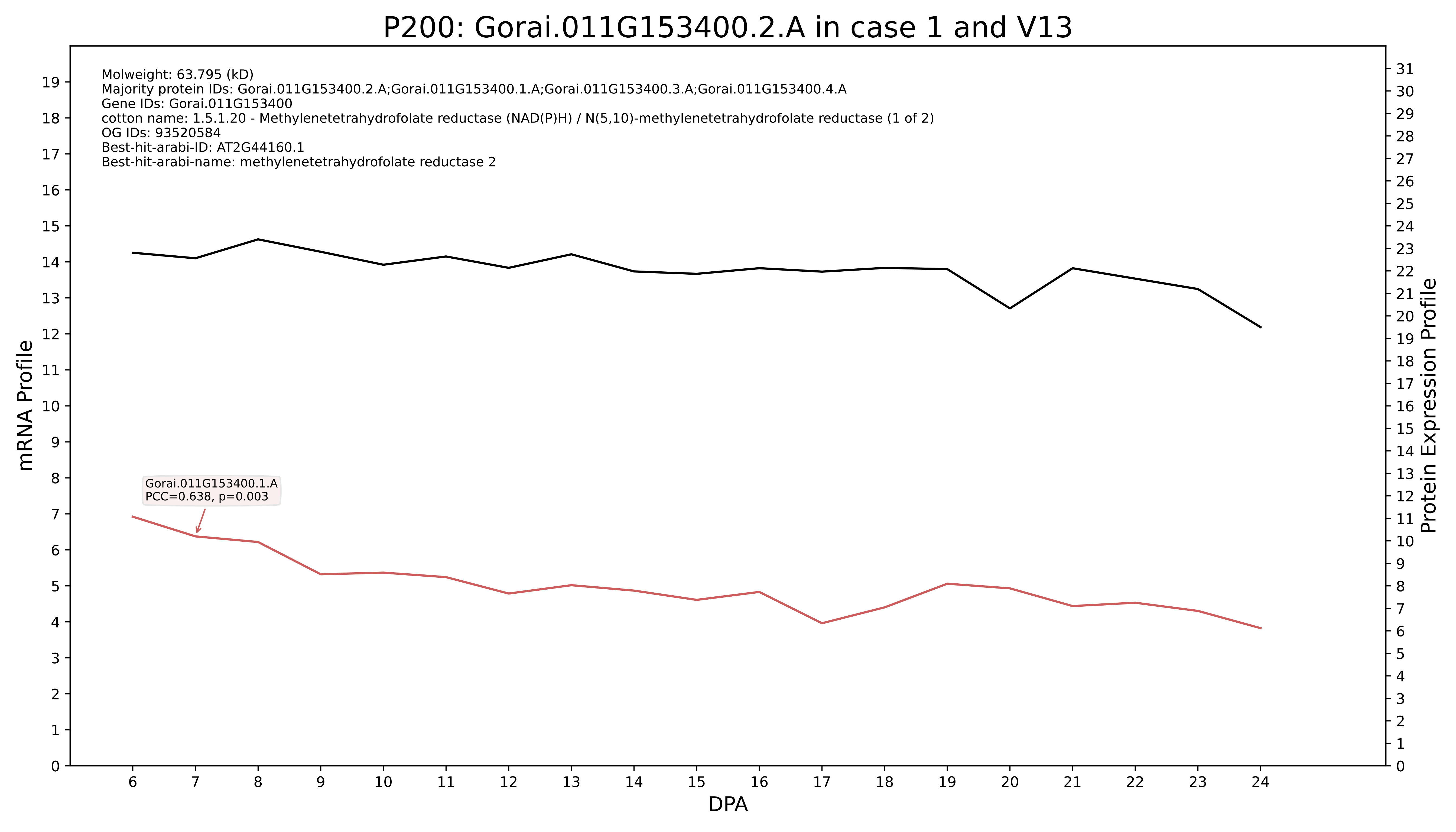

P200: Gorai.011G153400.2.A in case 1 and V13
Molweight: 63.795 (kD)
Majority protein IDs: Gorai.011G153400.2.A;Gorai.011G153400.1.A;Gorai.011G153400.3.A;Gorai.011G153400.4.A
Gene IDs: Gorai.011G153400
cotton name: 1.5.1.20 - Methylenetetrahydrofolate reductase (NAD(P)H) / N(5,10)-methylenetetrahydrofolate reductase (1 of 2)
OG IDs: 93520584
Best-hit-arabi-ID: AT2G44160.1
Best-hit-arabi-name: methylenetetrahydrofolate reductase 2
Gorai.011G153400.1.A
PCC=0.638, p=0.003
mRNA Profile
Protein Expression Profile
DPA

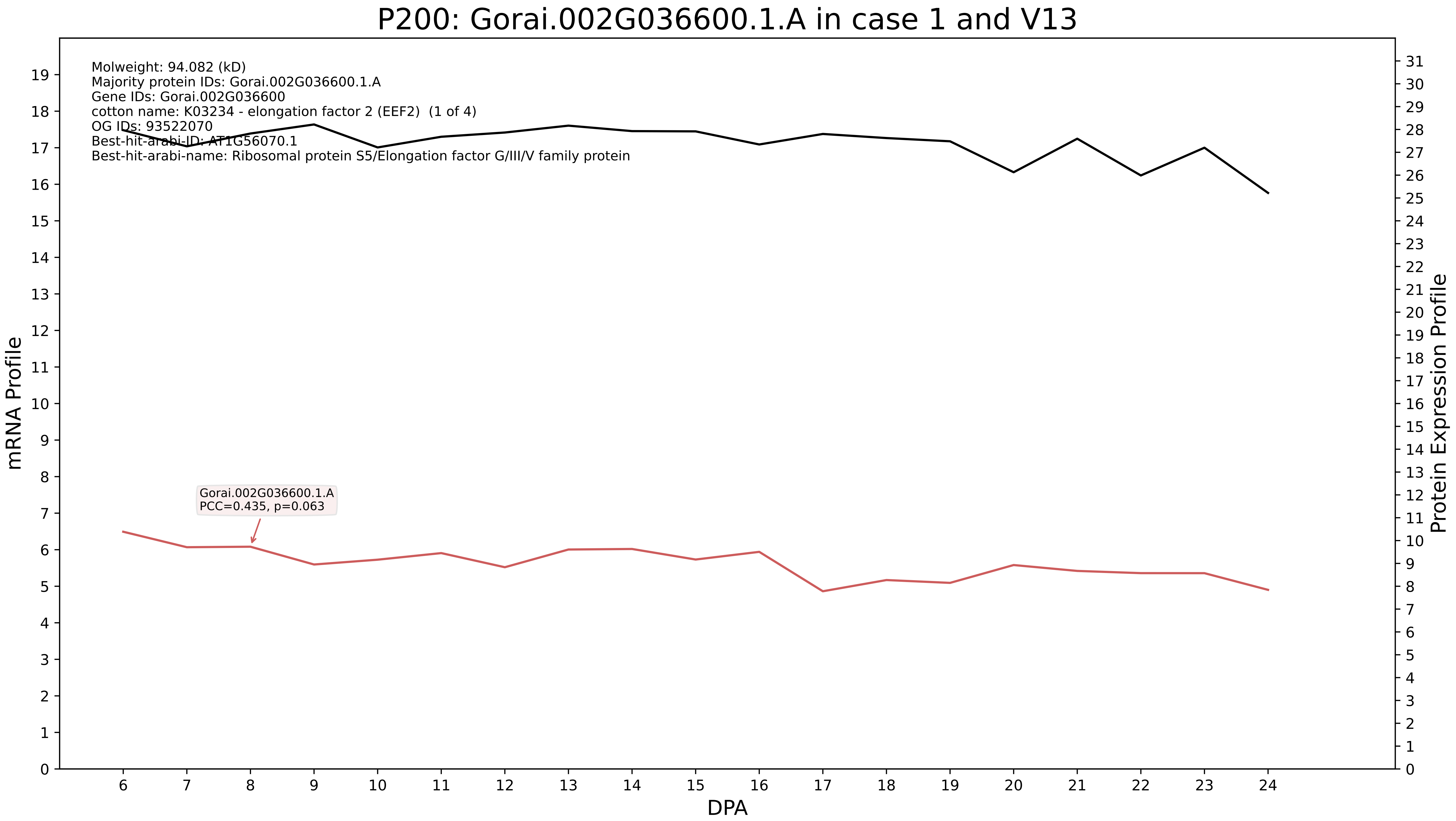
P200: Gorai.002G036600.1.A in case 1 and V13
Molweight: 94.082 (kD)
Majority protein IDs: Gorai.002G036600.1.A
Gene IDs: Gorai.002G036600
cotton name: K03234 - elongation factor 2 (EEF2) (1 of 4)
OG IDs: 93522070
Best-hit-arabi-ID: AT1G56070.1
Best-hit-arabi-name: Ribosomal protein S5/Elongation factor G/III/V family protein
Gorai.002G036600.1.A
PCC=0.435, p=0.063
mRNA Profile
Protein Expression Profile
DPA
0
1
2
3
4
5
6
7
8
9
10
11
12
13
14
15
16
17
18
19
20
21
22
23
24
25
26
27
28
29
30
31

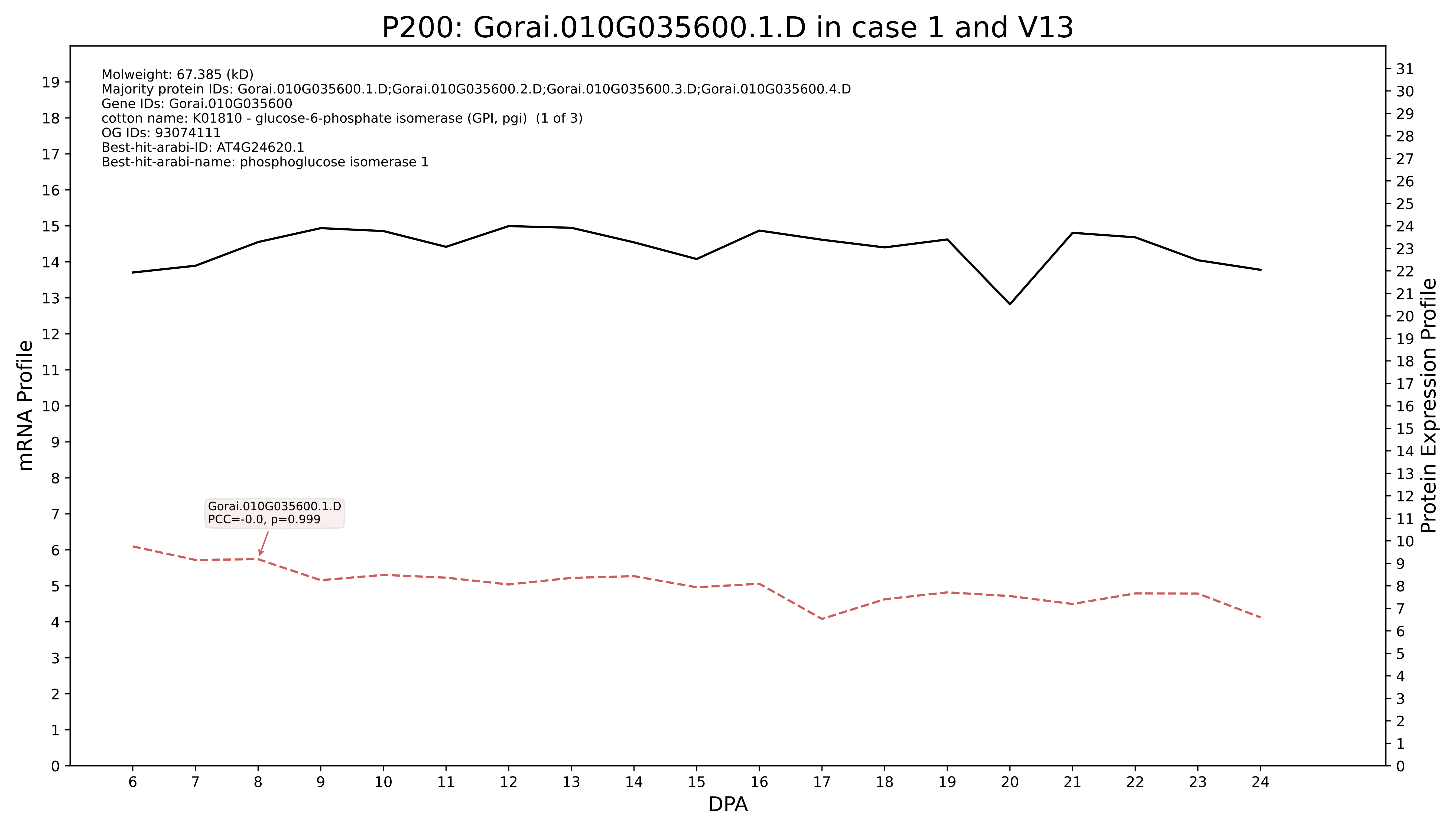
P200: Gorai.010G035600.1.D in case 1 and V13
Molweight: 67.385 (kD)
Majority protein IDs: Gorai.010G035600.1.D;Gorai.010G035600.2.D;Gorai.010G035600.3.D;Gorai.010G035600.4.D
Gene IDs: Gorai.010G035600
cotton name: K01810 - glucose-6-phosphate isomerase (GPI, pgi) (1 of 3)
OG IDs: 93074111
Best-hit-arabi-ID: AT4G24620.1
Best-hit-arabi-name: phosphoglucose isomerase 1
Gorai.010G035600.1.D
PCC=-0.0, p=0.999
mRNA Profile
Protein Expression Profile
DPA

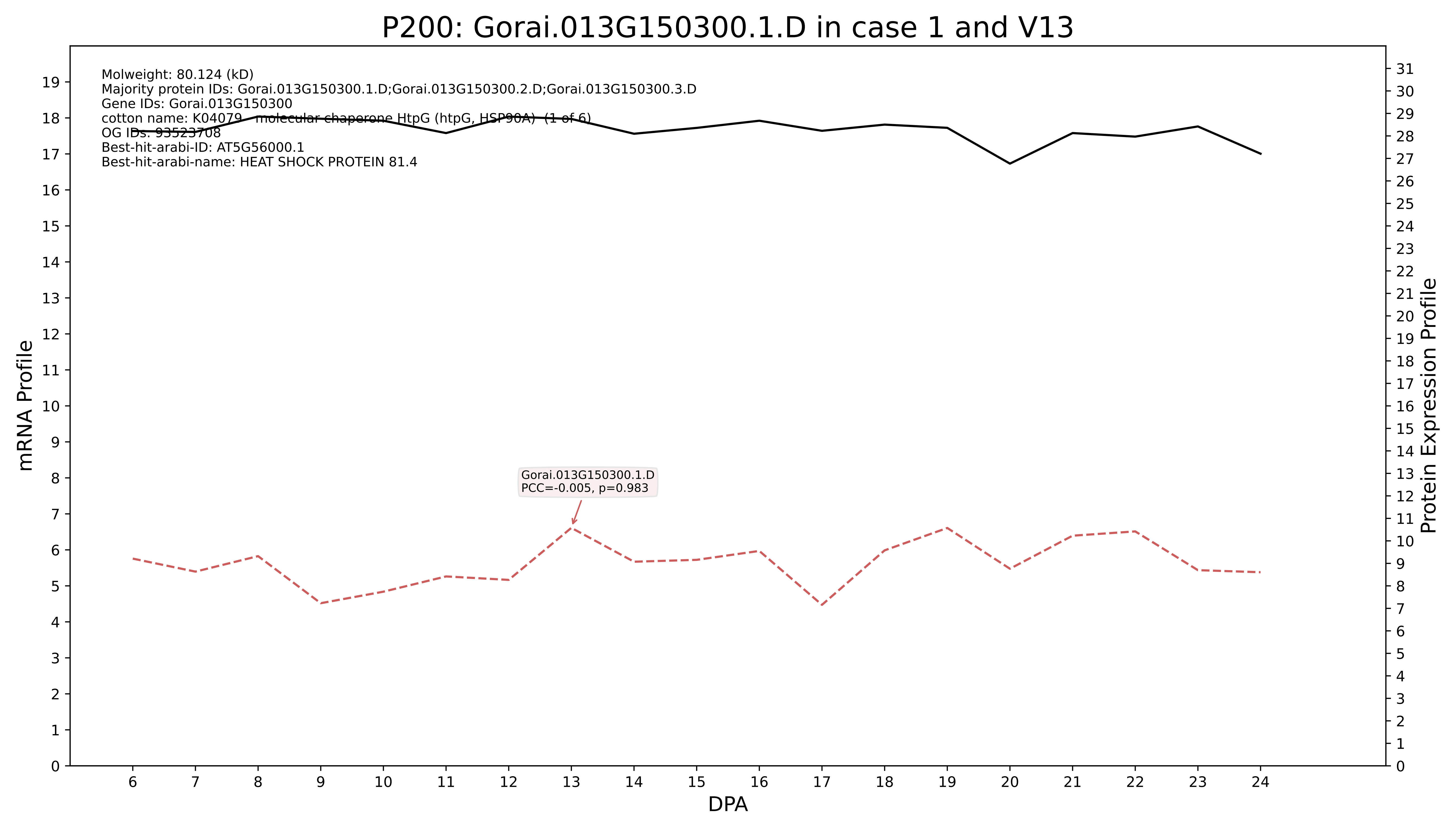

P200: Gorai.013G150300.1.D in case 1 and V13
Molweight: 80.124 (kD)
Majority protein IDs: Gorai.013G150300.1.D;Gorai.013G150300.2.D;Gorai.013G150300.3.D
Gene IDs: Gorai.013G150300
cotton name: K04079 molecular chaperone HtpG (htpG, HSP90A) (1 of 6)
OG IDs: 93523708
Best-hit-arabi-ID: AT5G56000.1
Best-hit-arabi-name: HEAT SHOCK PROTEIN 81.4
Gorai.013G150300.1.D
PCC=-0.005, p=0.983
mRNA Profile
Protein Expression Profile
DPA

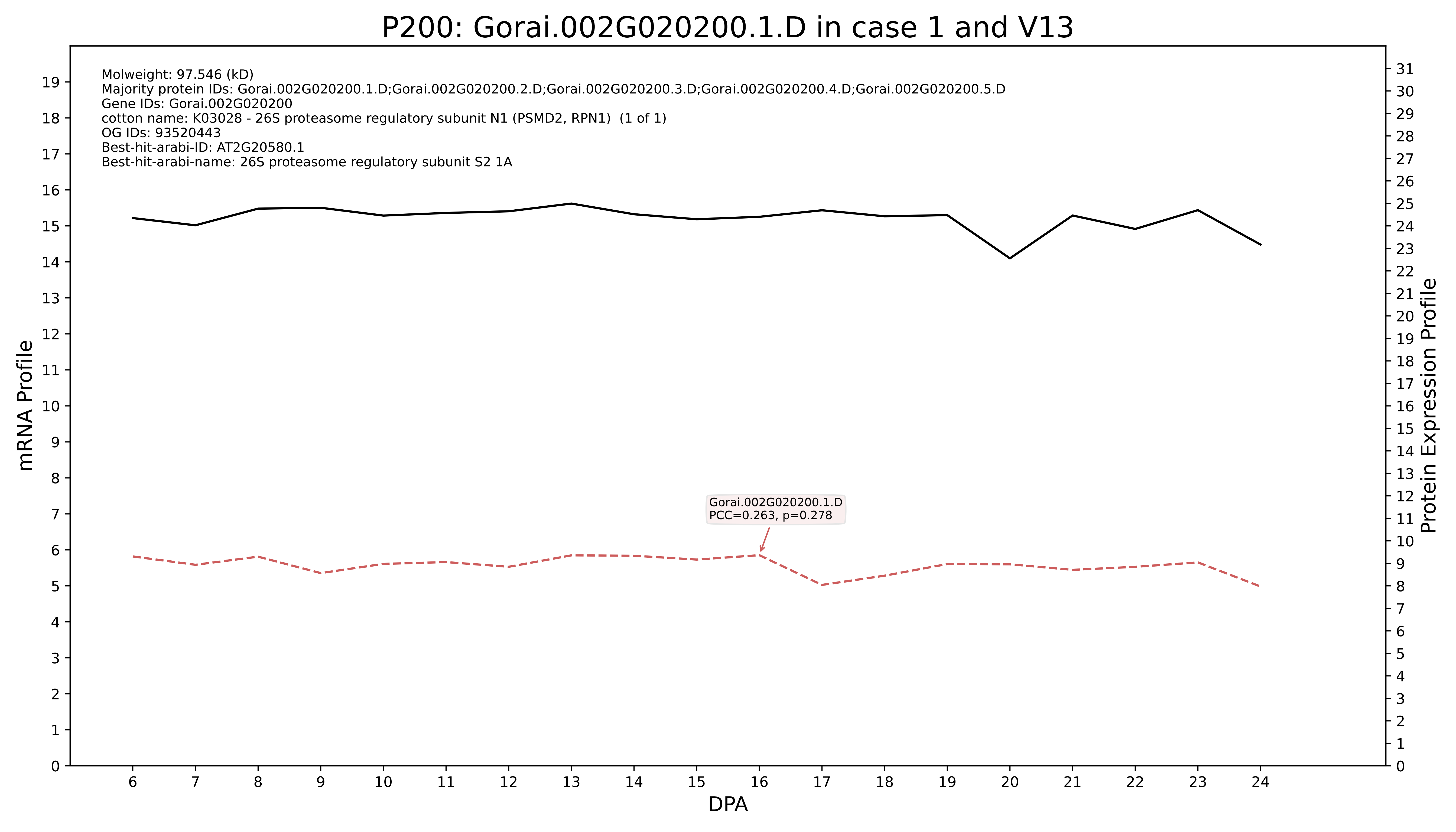

P200: Gorai.002G020200.1.D in case 1 and V13
Molweight: 97.546 (kD)
Majority protein IDs: Gorai.002G020200.1.D;Gorai.002G020200.2.D;Gorai.002G020200.3.D;Gorai.002G020200.4.D;Gorai.002G020200.5.D
Gene IDs: Gorai.002G020200
cotton name: K03028 - 26S proteasome regulatory subunit N1 (PSMD2, RPN1) (1 of 1)
OG IDs: 93520443
Best-hit-arabi-ID: AT2G20580.1
Best-hit-arabi-name: 26S proteasome regulatory subunit S2 1A
Gorai.002G020200.1.D
PCC=0.263, p=0.278
mRNA Profile
Protein Expression Profile
DPA

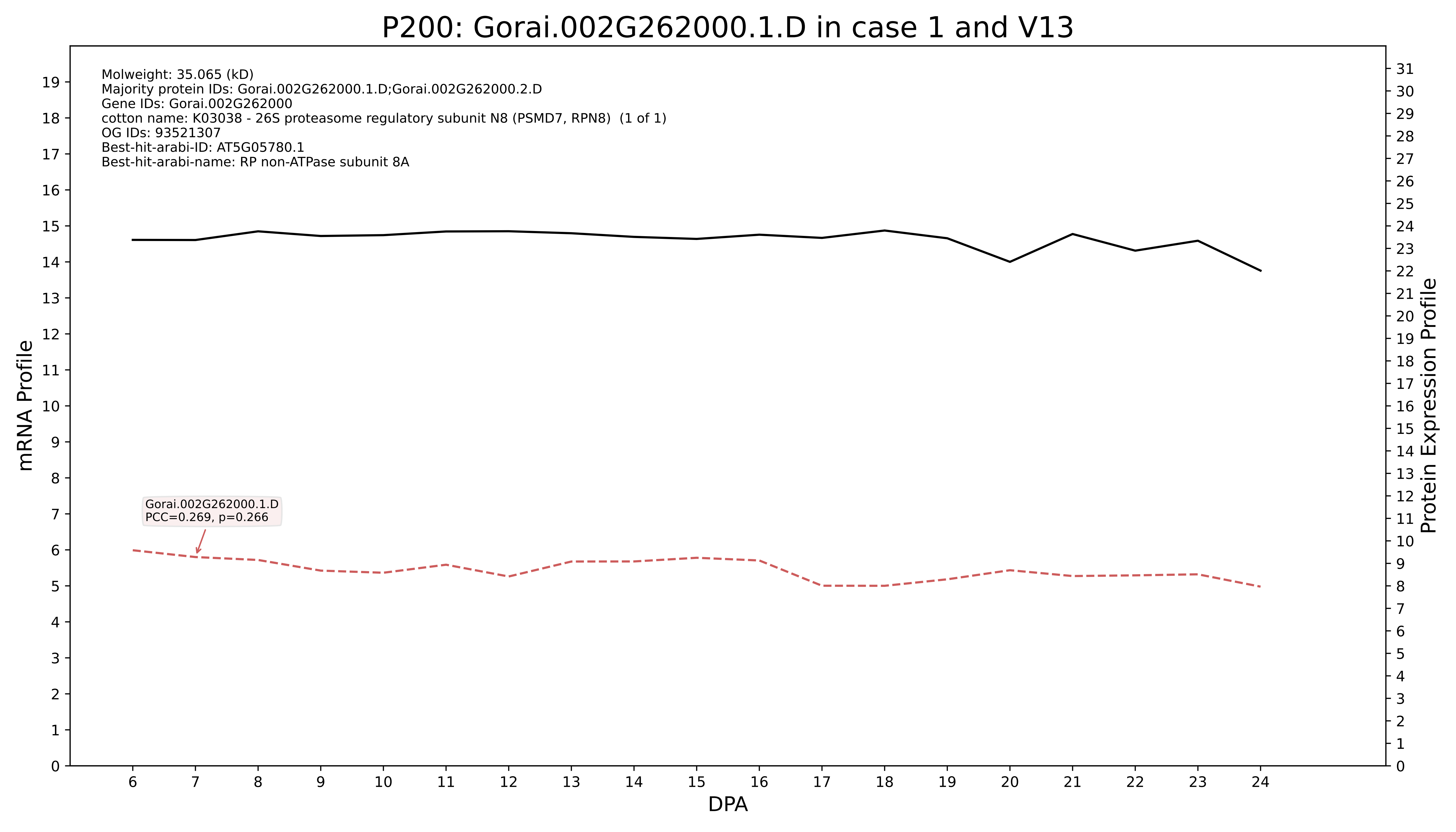

P200: Gorai.002G262000.1.D in case 1 and V13
Molweight: 35.065 (kD)
Majority protein IDs: Gorai.002G262000.1.D;Gorai.002G262000.2.D
Gene IDs: Gorai.002G262000
cotton name: K03038 - 26S proteasome regulatory subunit N8 (PSMD7, RPN8) (1 of 1)
OG IDs: 93521307
Best-hit-arabi-ID: AT5G05780.1
Best-hit-arabi-name: RP non-ATPase subunit 8A
Gorai.002G262000.1.D
PCC=0.269, p=0.266
mRNA Profile
Protein Expression Profile
DPA

P200: Gorai.004G250800.1.D in case 1 and V13

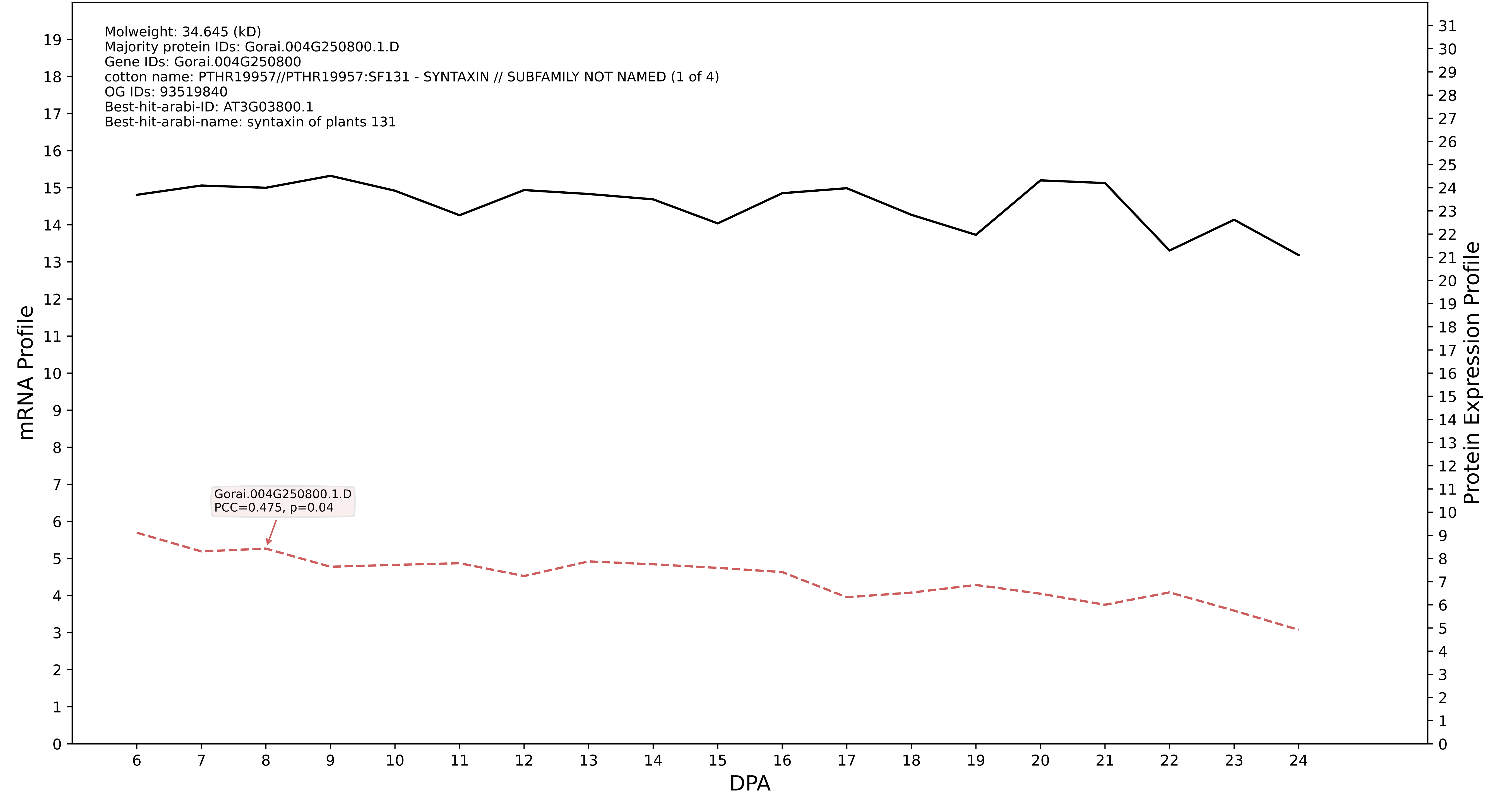

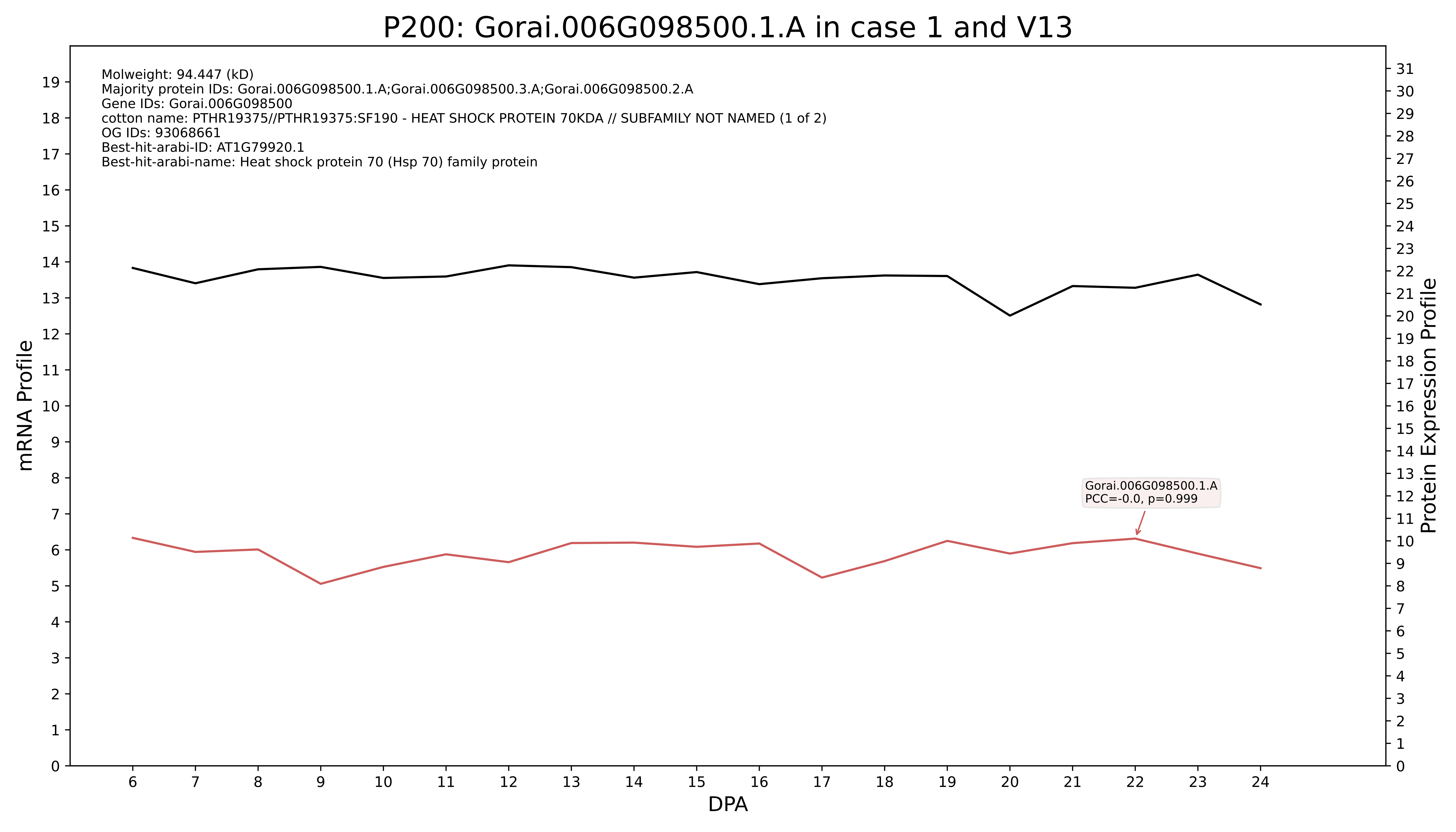

P200: Gorai.006G098500.1.A in case 1 and V13
Molweight: 94.447 (kD)
Majority protein IDs: Gorai.006G098500.1.A;Gorai.006G098500.3.A;Gorai.006G098500.2.A
Gene IDs: Gorai.006G098500
cotton name: PTHR19375//PTHR19375:SF190 - HEAT SHOCK PROTEIN 70KDA // SUBFAMILY NOT NAMED (1 of 2)
OG IDs: 93068661
Best-hit-arabi-ID: AT1G79920.1
Best-hit-arabi-name: Heat shock protein 70 (Hsp 70) family protein
Gorai.006G098500.1.A
PCC=-0.0, p=0.999
mRNA Profile
Protein Expression Profile
DPA

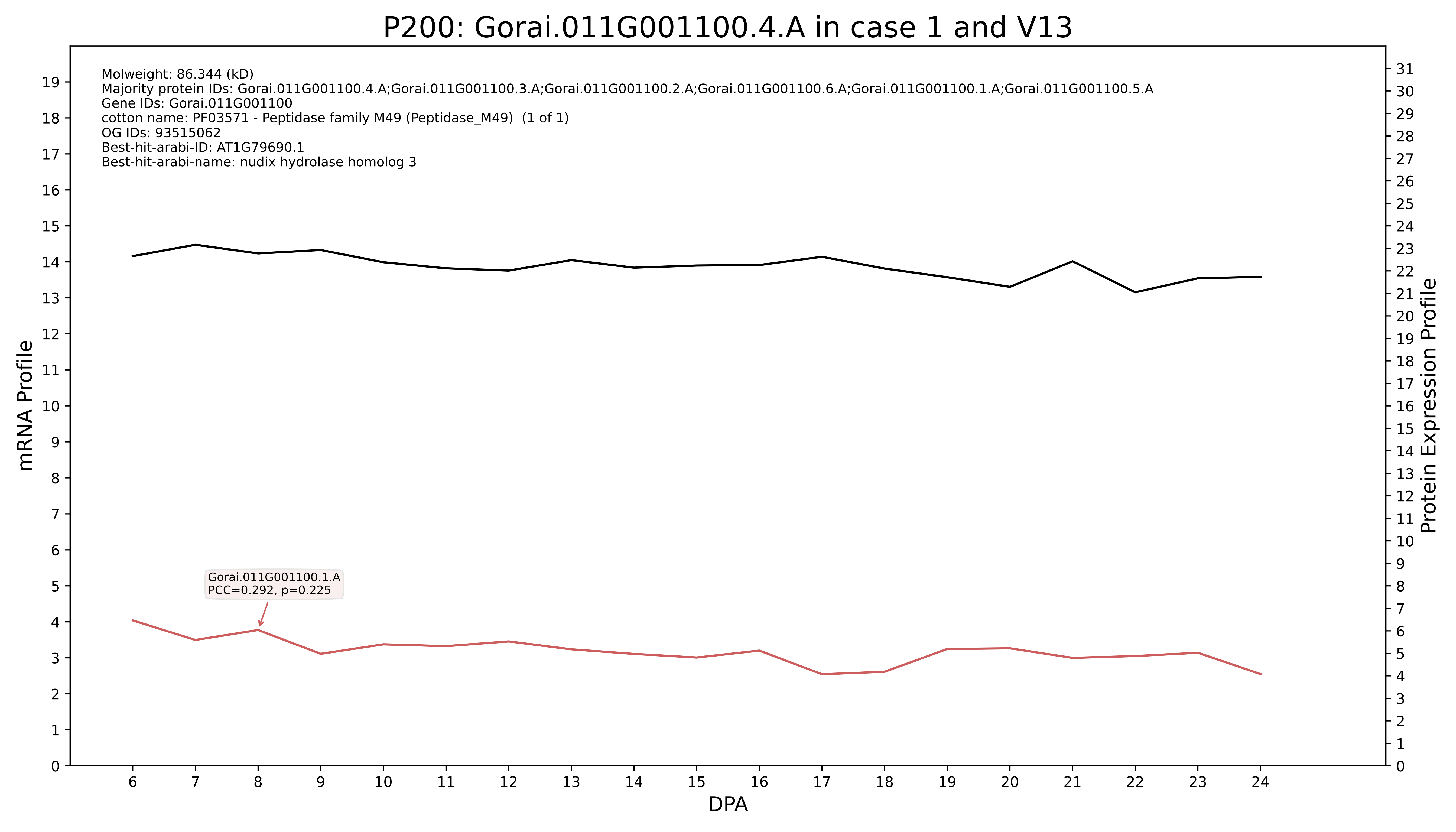
P200: Gorai.011G001100.4.A in case 1 and V13
Molweight: 86.344 (kD)
Majority protein IDs: Gorai.011G001100.4.A;Gorai.011G001100.3.A;Gorai.011G001100.2.A;Gorai.011G001100.6.A;Gorai.011G001100.1.A;Gorai.011G001100.5.A
Gene IDs: Gorai.011G001100
cotton name: PF03571 - Peptidase family M49 (Peptidase_M49) (1 of 1)
OG IDs: 93515062
Best-hit-arabi-ID: AT1G79690.1
Best-hit-arabi-name: nudix hydrolase homolog 3
Gorai.011G001100.1.A
PCC=0.292, p=0.225
mRNA Profile
Protein Expression Profile
DPA
0
1
2
3
4
5
6
7
8
9
10
11
12
13
14
15
16
17
18
19
20
21
22
23
24
25
26
27
28
29
30
31

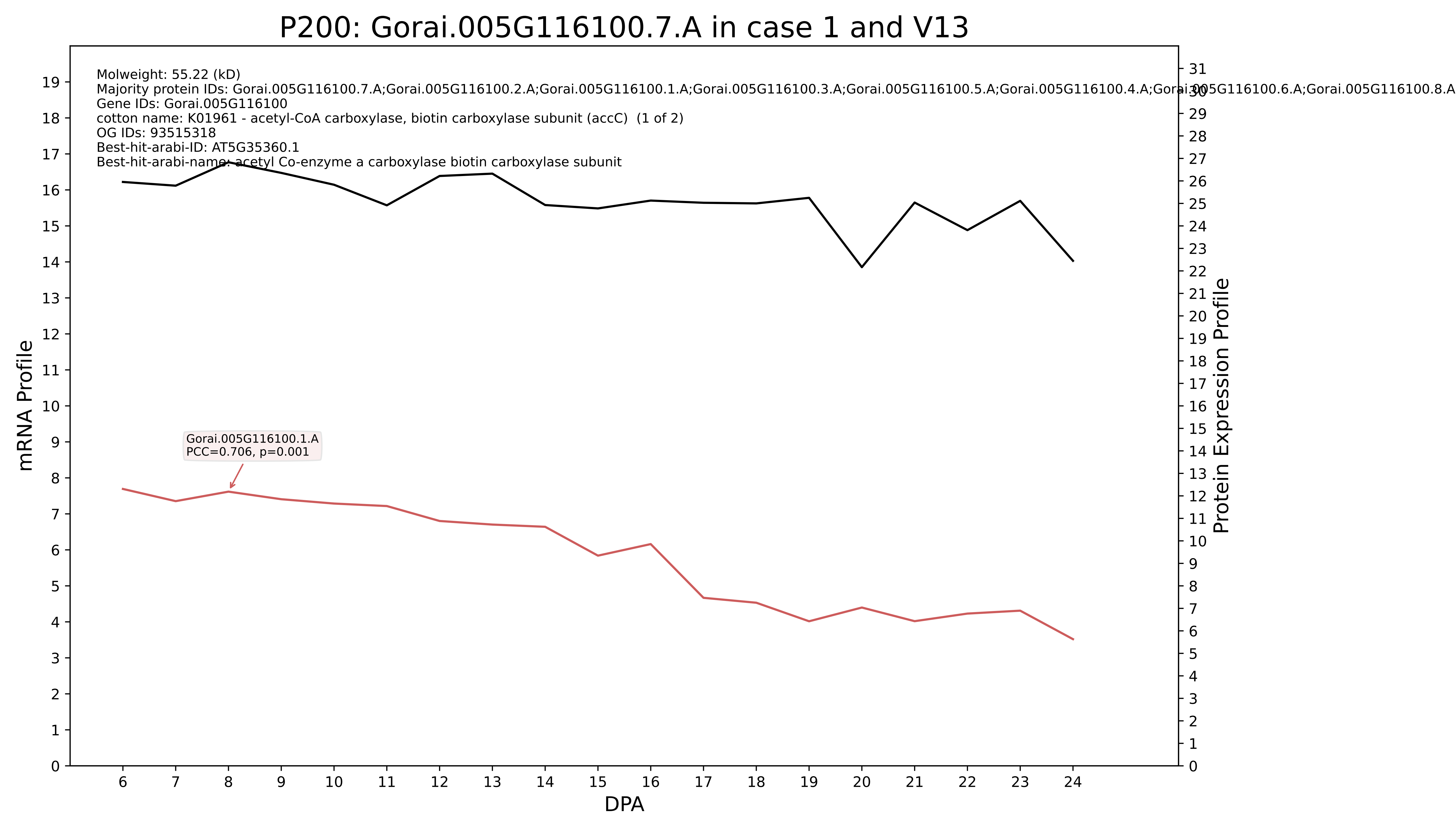
P200: Gorai.005G116100.7.A in case 1 and V13
Molweight: 55.22 (kD)
Majority protein IDs: Gorai.005G116100.7.A;Gorai.005G116100.2.A;Gorai.005G116100.1.A;Gorai.005G116100.3.A;Gorai.005G116100.5.A;Gorai.005G116100.4.A;Gorai.005G116100.6.A;Gorai.005G116100.8.A
Gene IDs: Gorai.005G116100
cotton name: K01961 - acetyl-CoA carboxylase, biotin carboxylase subunit (accC) (1 of 2)
OG IDs: 93515318
Best-hit-arabi-ID: AT5G35360.1
Best-hit-arabi-name: acetyl Co-enzyme a carboxylase biotin carboxylase subunit
Gorai.005G116100.1.A
PCC=0.706, p=0.001
mRNA Profile
Protein Expression Profile
DPA
0
1
2
3
4
5
6
7
8
9
10
11
12
13
14
15
16
17
18
19
20
21
22
23
24
25
26
27
28
29
30
31

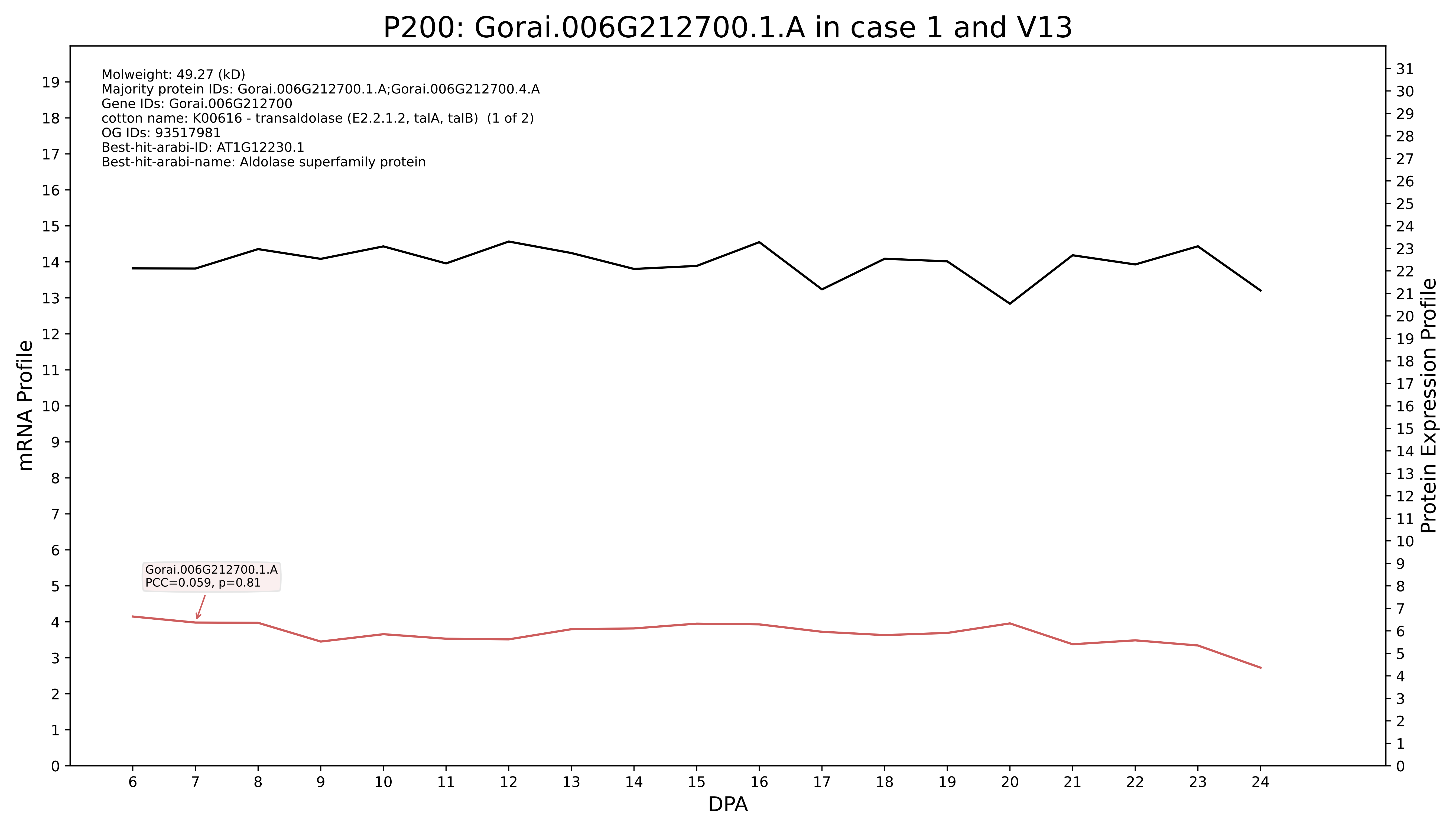

P200: Gorai.006G212700.1.A in case 1 and V13
Molweight: 49.27 (kD)
Majority protein IDs: Gorai.006G212700.1.A;Gorai.006G212700.4.A
Gene IDs: Gorai.006G212700
cotton name: K00616 - transaldolase (E2.2.1.2, talA, talB) (1 of 2)
OG IDs: 93517981
Best-hit-arabi-ID: AT1G12230.1
Best-hit-arabi-name: Aldolase superfamily protein
Gorai.006G212700.1.A
PCC=0.059, p=0.81
mRNA Profile
Protein Expression Profile
DPA

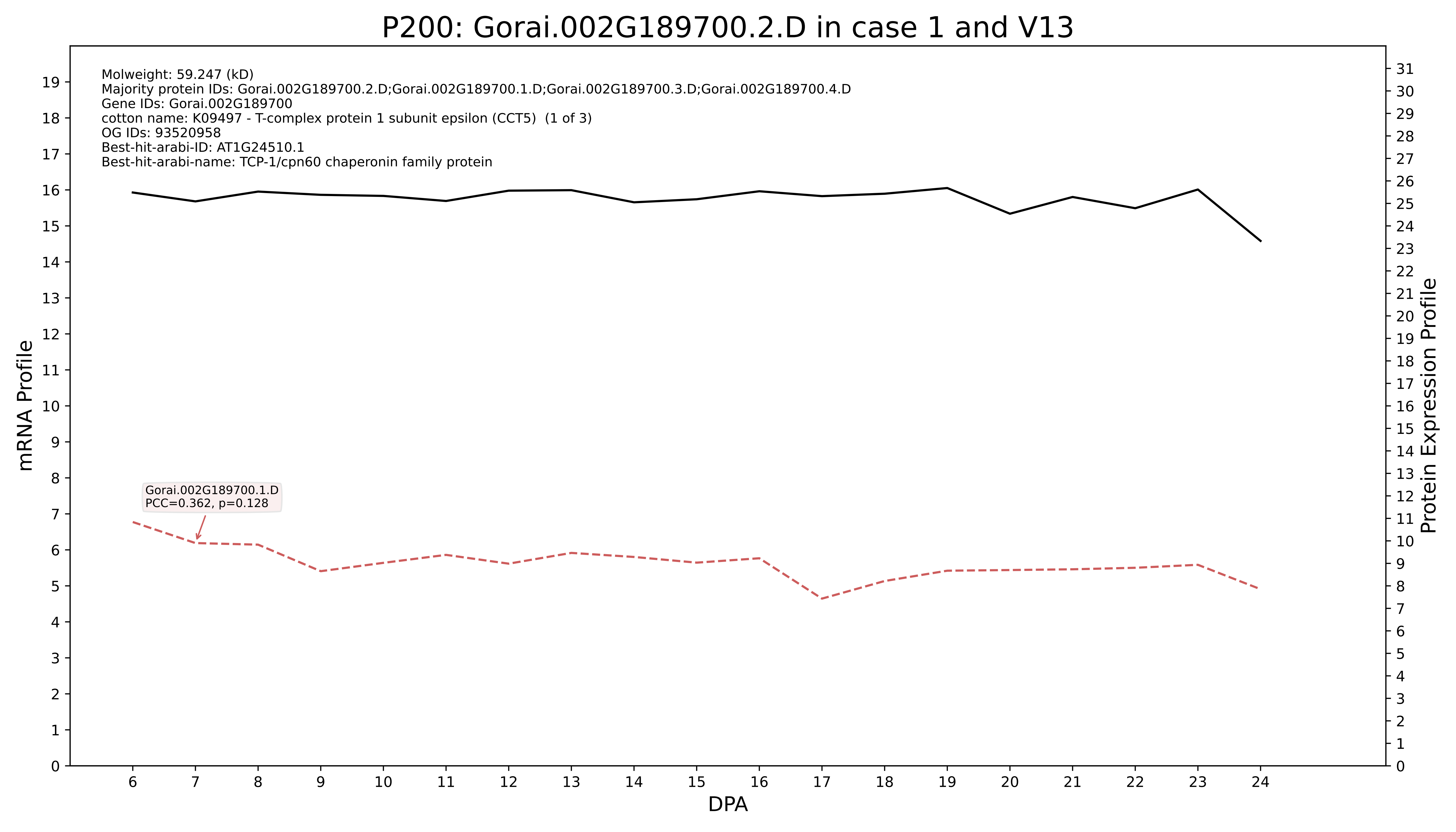
P200: Gorai.002G189700.2.D in case 1 and V13
Molweight: 59.247 (kD)
Majority protein IDs: Gorai.002G189700.2.D;Gorai.002G189700.1.D;Gorai.002G189700.3.D;Gorai.002G189700.4.D
Gene IDs: Gorai.002G189700
cotton name: K09497 - T-complex protein 1 subunit epsilon (CCT5) (1 of 3)
OG IDs: 93520958
Best-hit-arabi-ID: AT1G24510.1
Best-hit-arabi-name: TCP-1/cpn60 chaperonin family protein
Gorai.002G189700.1.D
PCC=0.362, p=0.128
mRNA Profile
Protein Expression Profile
DPA

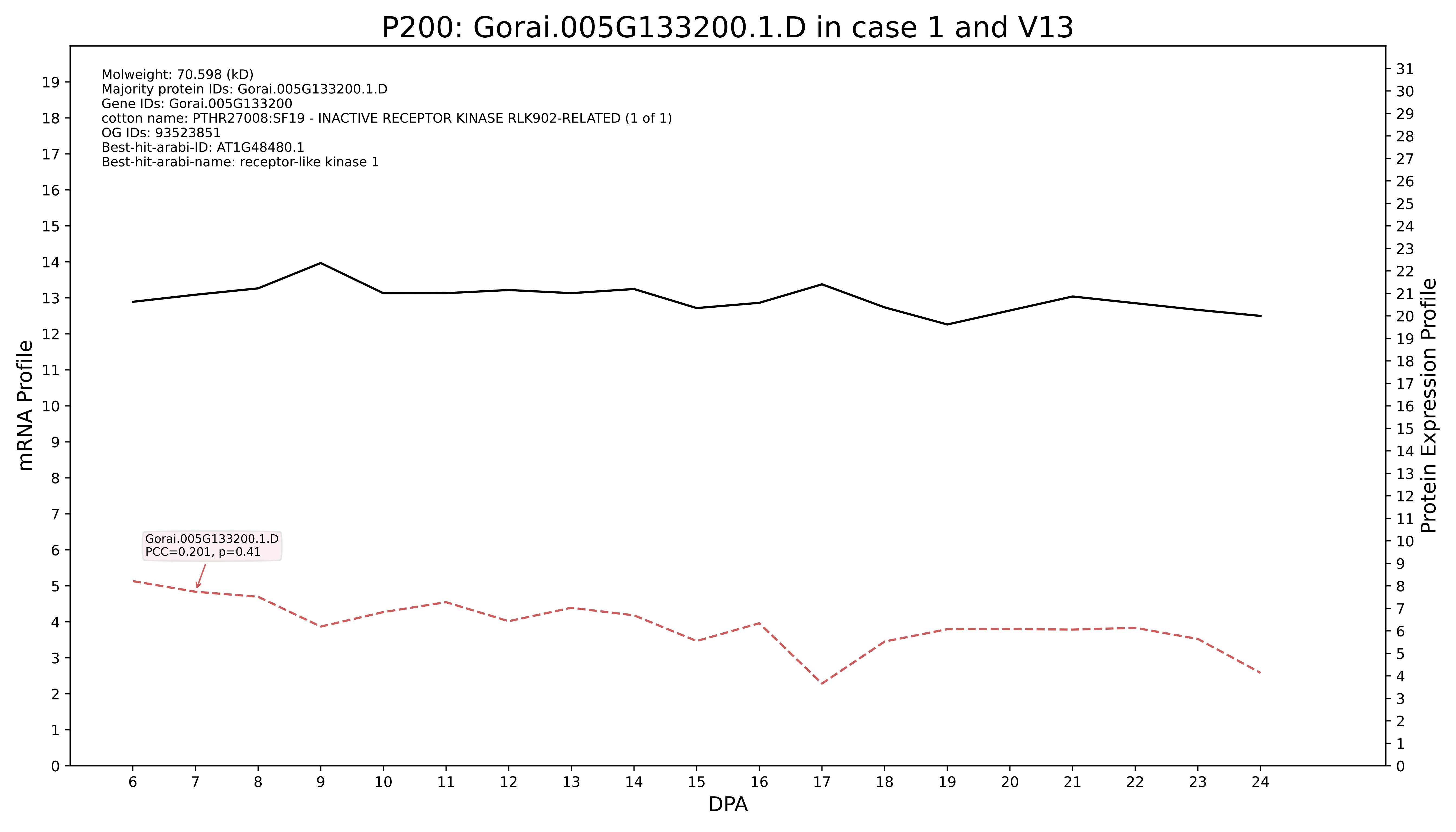
P200: Gorai.005G133200.1.D in case 1 and V13
Molweight: 70.598 (kD)
Majority protein IDs: Gorai.005G133200.1.D
Gene IDs: Gorai.005G133200
cotton name: PTHR27008:SF19 - INACTIVE RECEPTOR KINASE RLK902-RELATED (1 of 1)
OG IDs: 93523851
Best-hit-arabi-ID: AT1G48480.1
Best-hit-arabi-name: receptor-like kinase 1
Gorai.005G133200.1.D
PCC=0.201, p=0.41
mRNA Profile
Protein Expression Profile
DPA

P200: Gorai.009G175500.3.A in case 1 and V13

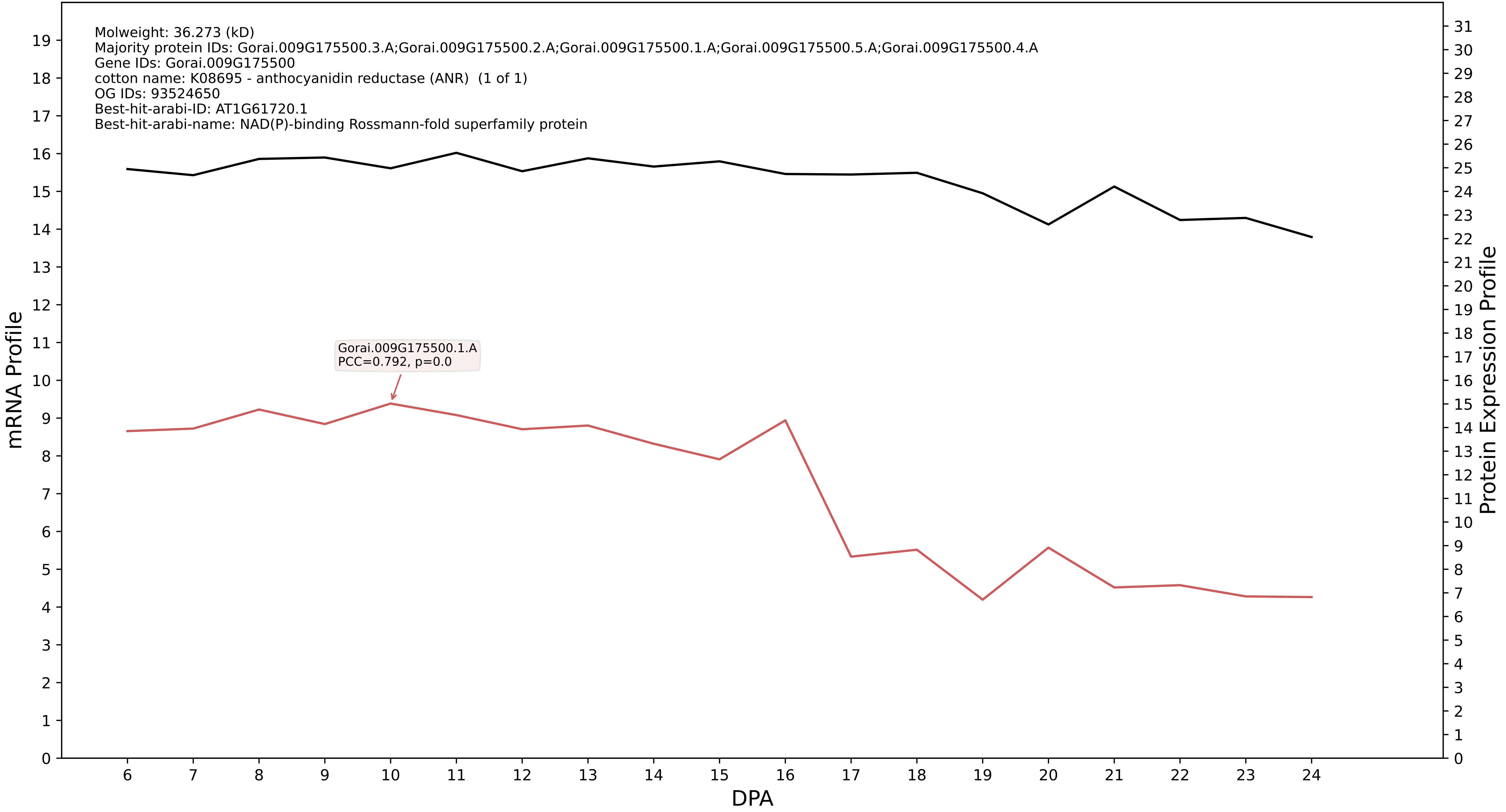

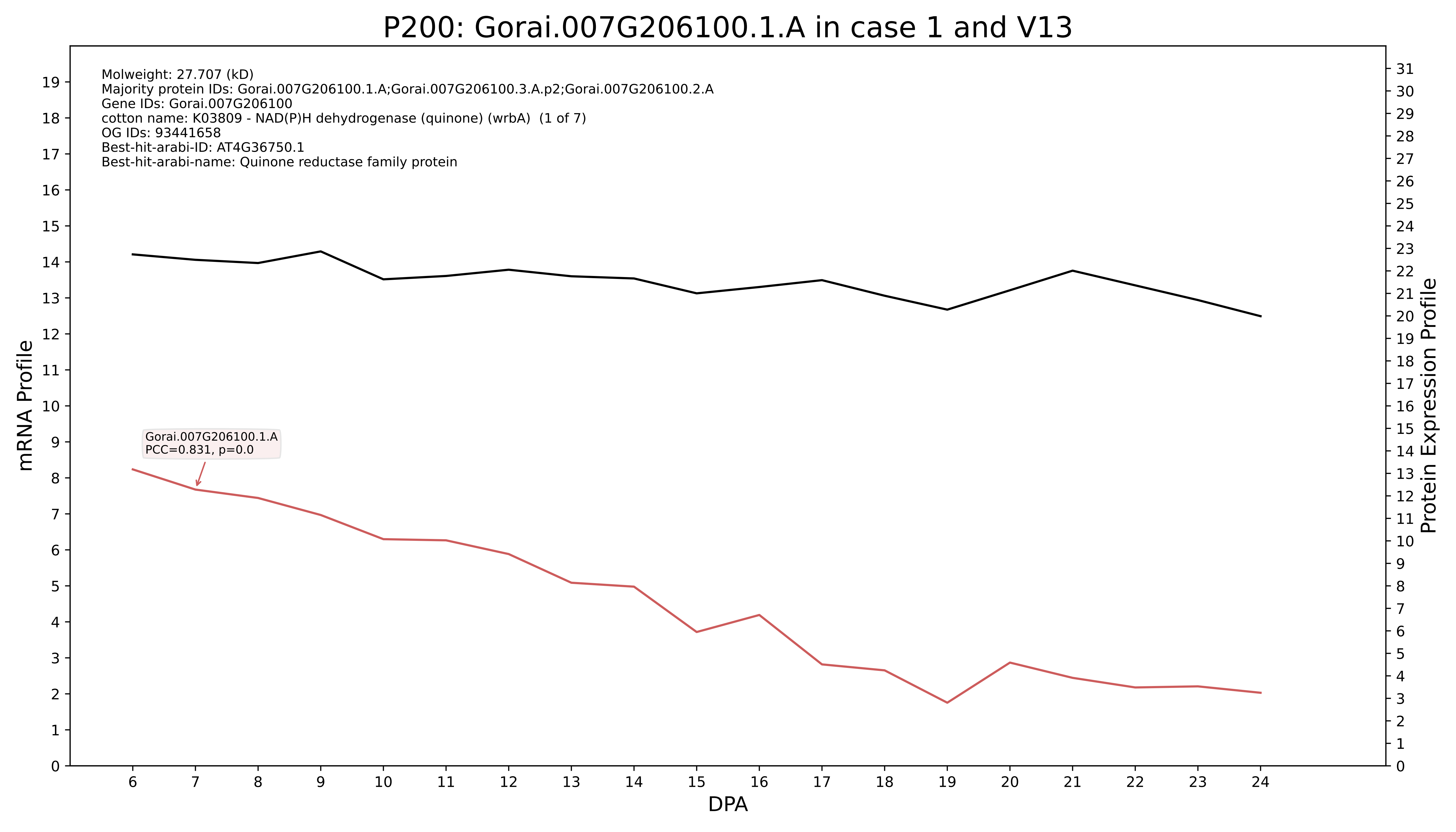
P200: Gorai.007G206100.1.A in case 1 and V13
Molweight: 27.707 (kD)
Majority protein IDs: Gorai.007G206100.1.A;Gorai.007G206100.3.A.p2;Gorai.007G206100.2.A
Gene IDs: Gorai.007G206100
cotton name: K03809 - NAD(P)H dehydrogenase (quinone) (wrbA) (1 of 7)
OG IDs: 93441658
Best-hit-arabi-ID: AT4G36750.1
Best-hit-arabi-name: Quinone reductase family protein
Gorai.007G206100.1.A
PCC=0.831, p=0.0
mRNA Profile
Protein Expression Profile
DPA
0
1
2
3
4
5
6
7
8
9
10
11
12
13
14
15
16
17
18
19
20
21
22
23
24
25
26
27
28
29
30
31

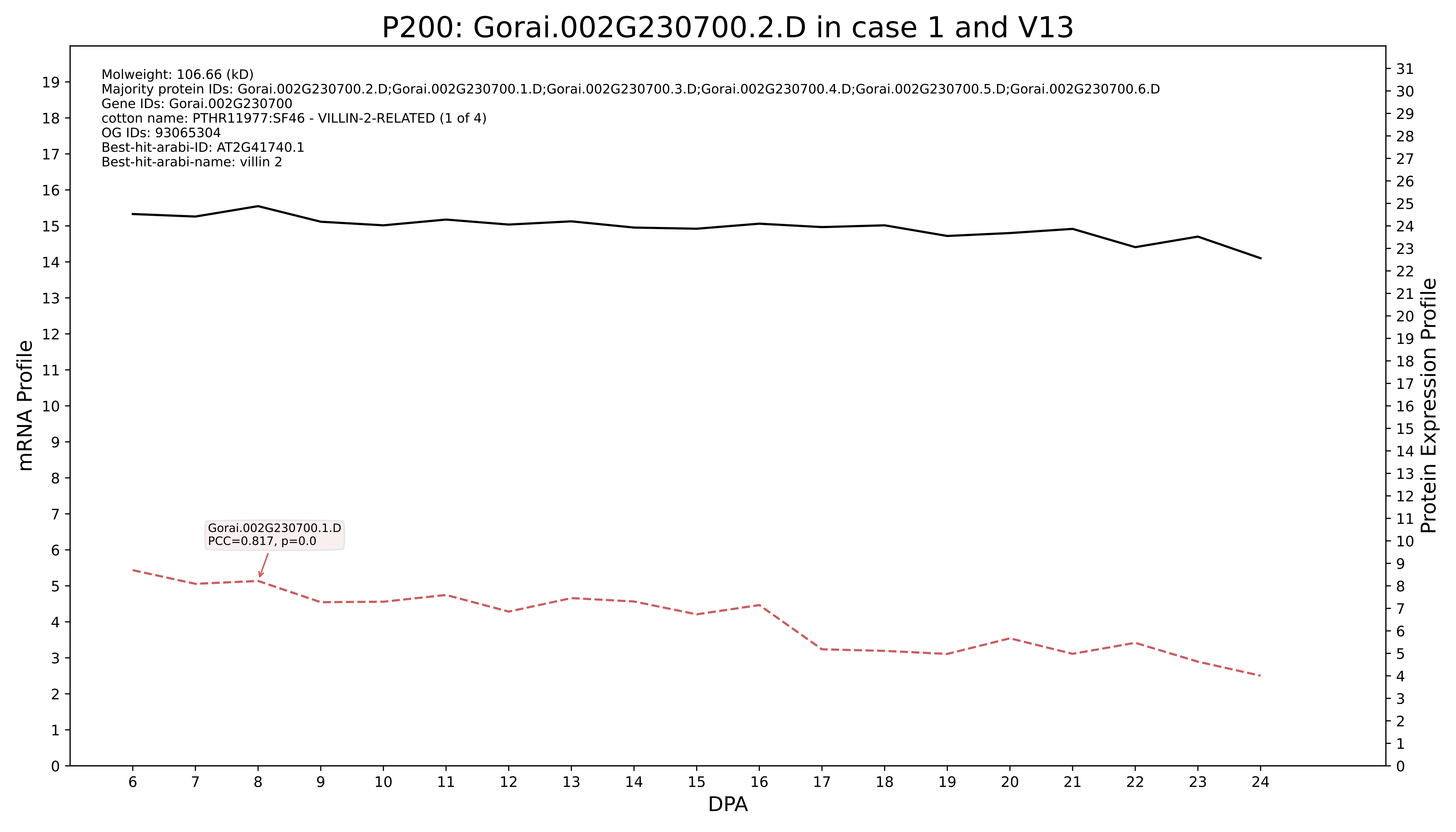

P200: Gorai.002G230700.2.D in case 1 and V13
Molweight: 106.66 (kD)
Majority protein IDs: Gorai.002G230700.2.D;Gorai.002G230700.1.D;Gorai.002G230700.3.D;Gorai.002G230700.4.D;Gorai.002G230700.5.D;Gorai.002G230700.6.D
Gene IDs: Gorai.002G230700
cotton name: PTHR11977:SF46 - VILLIN-2-RELATED (1 of 4)
OG IDs: 93065304
Best-hit-arabi-ID: AT2G41740.1
Best-hit-arabi-name: villin 2
Gorai.002G230700.1.D
PCC=0.817, p=0.0
mRNA Profile
Protein Expression Profile
DPA

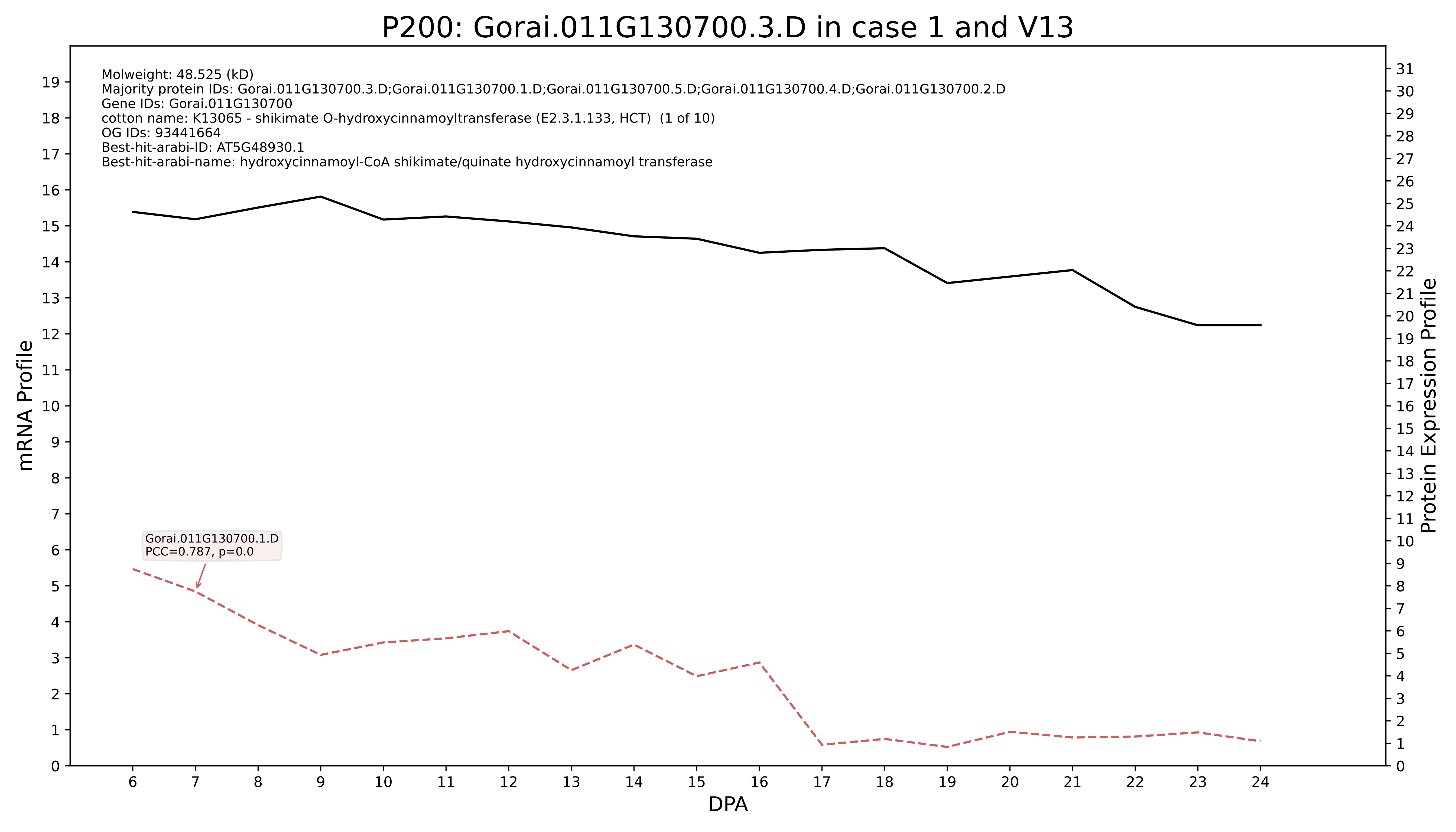

P200: Gorai.011G130700.3.D in case 1 and V13
Molweight: 48.525 (kD)
Majority protein IDs: Gorai.011G130700.3.D;Gorai.011G130700.1.D;Gorai.011G130700.5.D;Gorai.011G130700.4.D;Gorai.011G130700.2.D
Gene IDs: Gorai.011G130700
cotton name: K13065 - shikimate O-hydroxycinnamoyltransferase (E2.3.1.133, HCT) (1 of 10)
OG IDs: 93441664
Best-hit-arabi-ID: AT5G48930.1
Best-hit-arabi-name: hydroxycinnamoyl-CoA shikimate/quinate hydroxycinnamoyl transferase
Gorai.011G130700.1.D
PCC=0.787, p=0.0
mRNA Profile
Protein Expression Profile
DPA

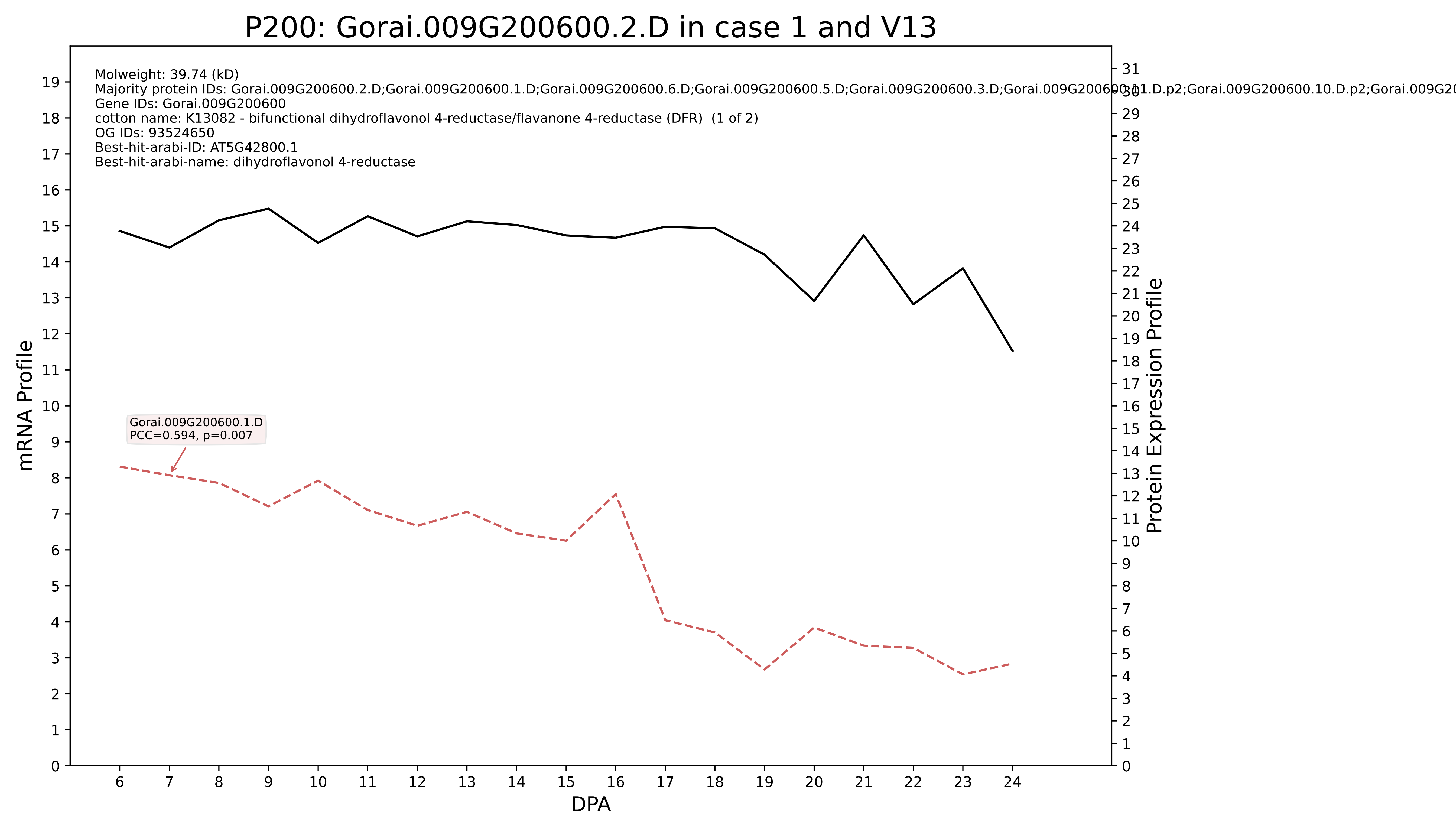
P200: Gorai.009G200600.2.D in case 1 and V13
Molweight: 39.74 (kD)
Majority protein IDs: Gorai.009G200600.2.D;Gorai.009G200600.1.D;Gorai.009G200600.6.D;Gorai.009G200600.5.D;Gorai.009G200600.3.D;Gorai.009G200600.11.D.p2;Gorai.009G200600.10.D.p2;Gorai.009G20
Gene IDs: Gorai.009G200600
cotton name: K13082 - bifunctional dihydroflavonol 4-reductase/flavanone 4-reductase (DFR) (1 of 2)
OG IDs: 93524650
Best-hit-arabi-ID: AT5G42800.1
Best-hit-arabi-name: dihydroflavonol 4-reductase
Gorai.009G200600.1.D
PCC=0.594, p=0.007
mRNA Profile
Protein Expression Profile
DPA

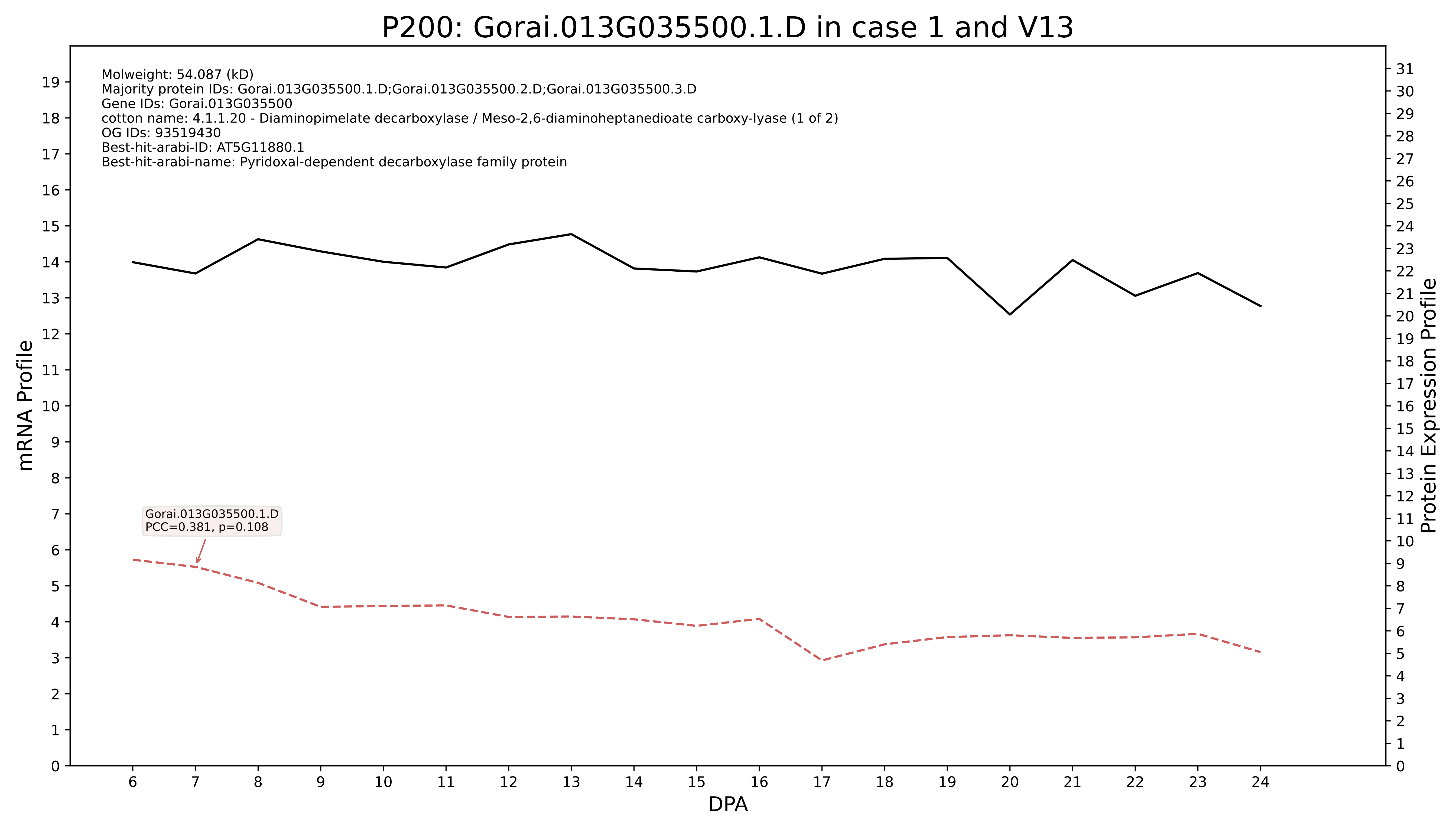

P200: Gorai.013G035500.1.D in case 1 and V13
Molweight: 54.087 (kD)
Majority protein IDs: Gorai.013G035500.1.D;Gorai.013G035500.2.D;Gorai.013G035500.3.D
Gene IDs: Gorai.013G035500
cotton name: 4.1.1.20 - Diaminopimelate decarboxylase / Meso-2,6-diaminoheptanedioate carboxy-lyase (1 of 2)
OG IDs: 93519430
Best-hit-arabi-ID: AT5G11880.1
Best-hit-arabi-name: Pyridoxal-dependent decarboxylase family protein
Gorai.013G035500.1.D
PCC=0.381, p=0.108
mRNA Profile
Protein Expression Profile
DPA

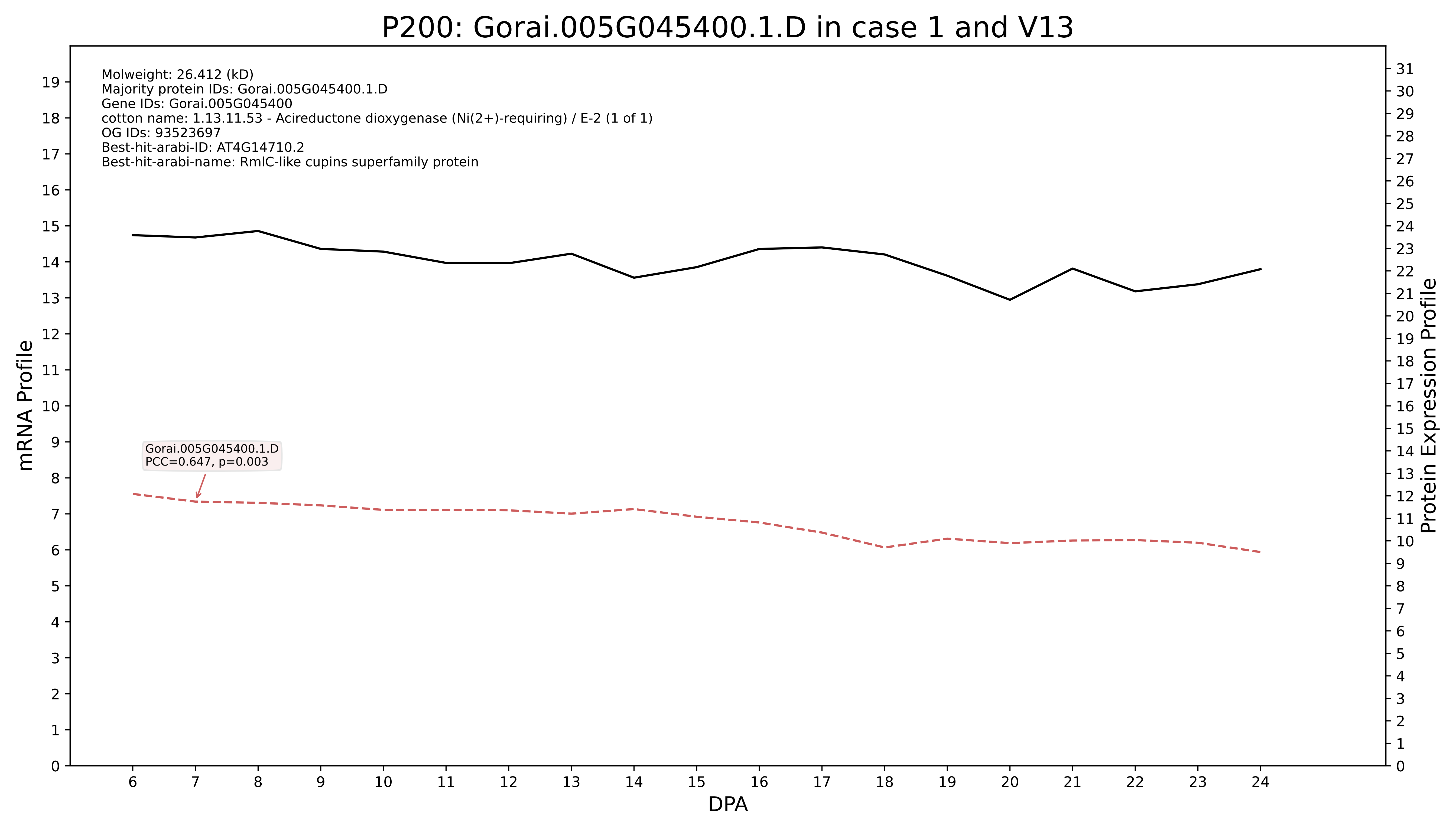

P200: Gorai.005G045400.1.D in case 1 and V13
Molweight: 26.412 (kD)
Majority protein IDs: Gorai.005G045400.1.D
Gene IDs: Gorai.005G045400
cotton name: 1.13.11.53 - Acireductone dioxygenase (Ni(2+)-requiring) / E-2 (1 of 1)
OG IDs: 93523697
Best-hit-arabi-ID: AT4G14710.2
Best-hit-arabi-name: RmlC-like cupins superfamily protein
Gorai.005G045400.1.D
PCC=0.647, p=0.003
mRNA Profile
Protein Expression Profile
DPA

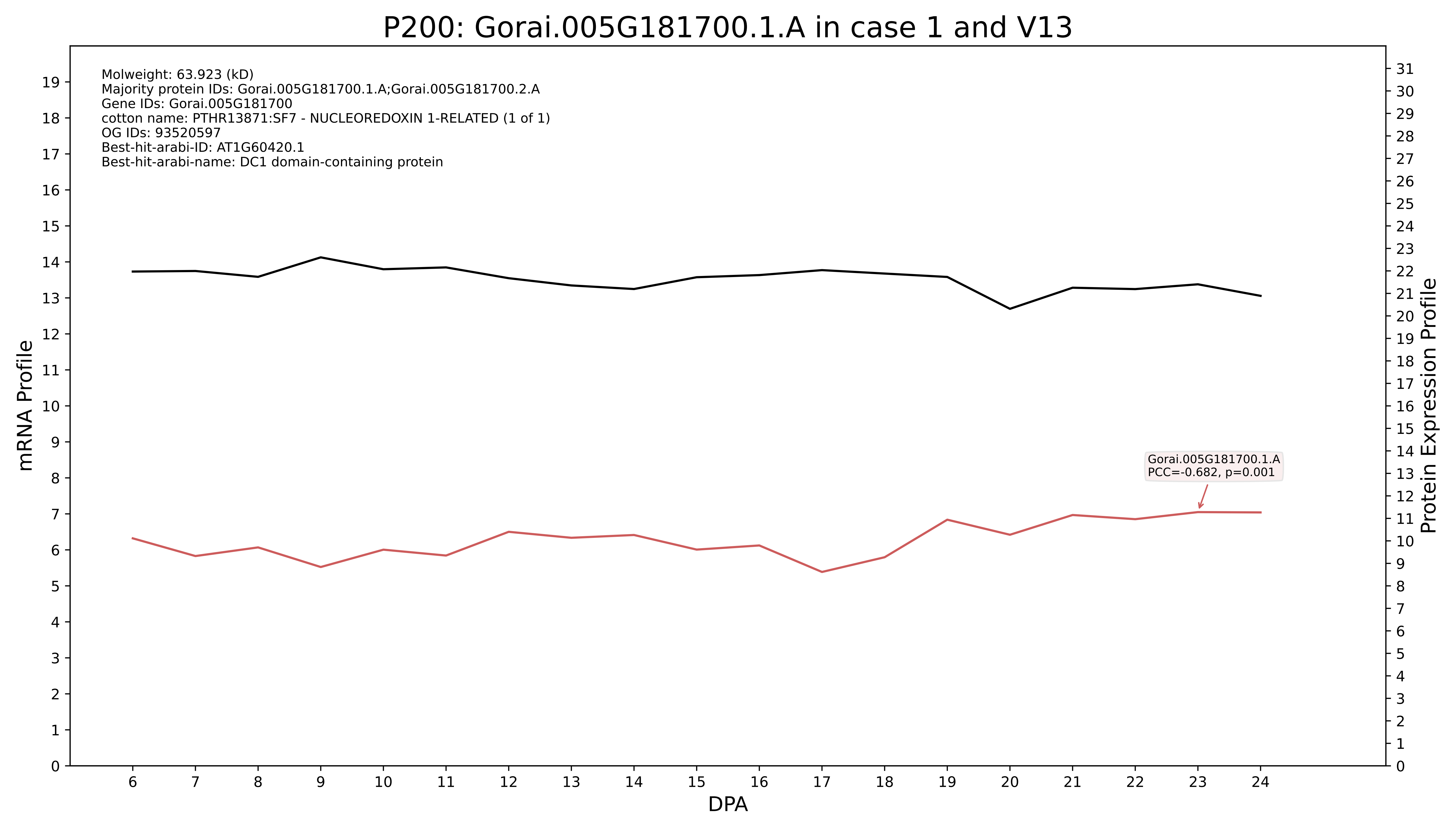
P200: Gorai.005G181700.1.A in case 1 and V13
Molweight: 63.923 (kD)
Majority protein IDs: Gorai.005G181700.1.A;Gorai.005G181700.2.A
Gene IDs: Gorai.005G181700
cotton name: PTHR13871:SF7 - NUCLEOREDOXIN 1-RELATED (1 of 1)
OG IDs: 93520597
Best-hit-arabi-ID: AT1G60420.1
Best-hit-arabi-name: DC1 domain-containing protein
Gorai.005G181700.1.A
PCC=-0.682, p=0.001
mRNA Profile
Protein Expression Profile
DPA

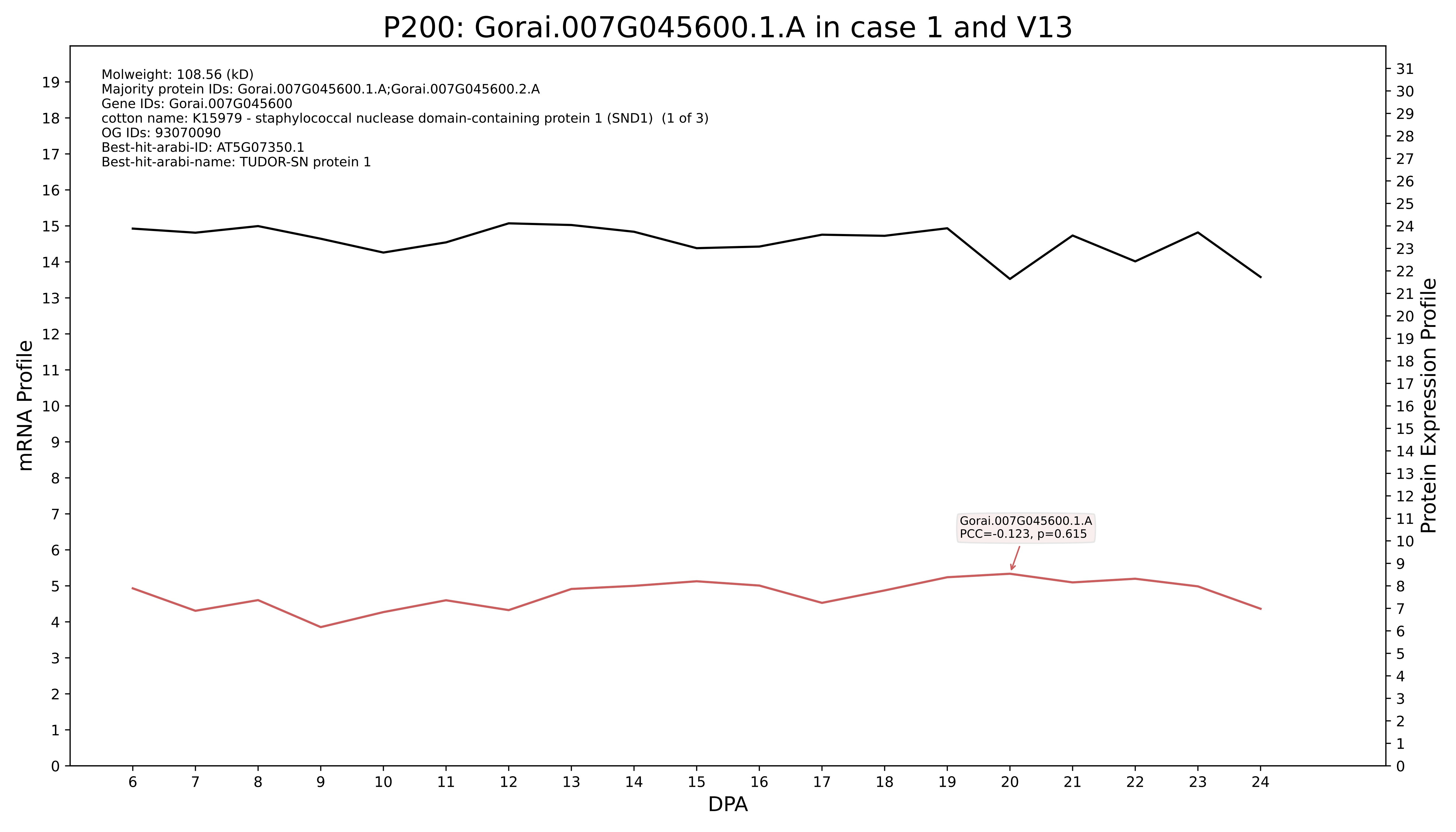
P200: Gorai.007G045600.1.A in case 1 and V13
Molweight: 108.56 (kD)
Majority protein IDs: Gorai.007G045600.1.A;Gorai.007G045600.2.A
Gene IDs: Gorai.007G045600
cotton name: K15979 - staphylococcal nuclease domain-containing protein 1 (SND1) (1 of 3)
OG IDs: 93070090
Best-hit-arabi-ID: AT5G07350.1
Best-hit-arabi-name: TUDOR-SN protein 1
Gorai.007G045600.1.A
PCC=-0.123, p=0.615
mRNA Profile
Protein Expression Profile
DPA

P200: Gorai.006G076400.1.D in case 1 and V13

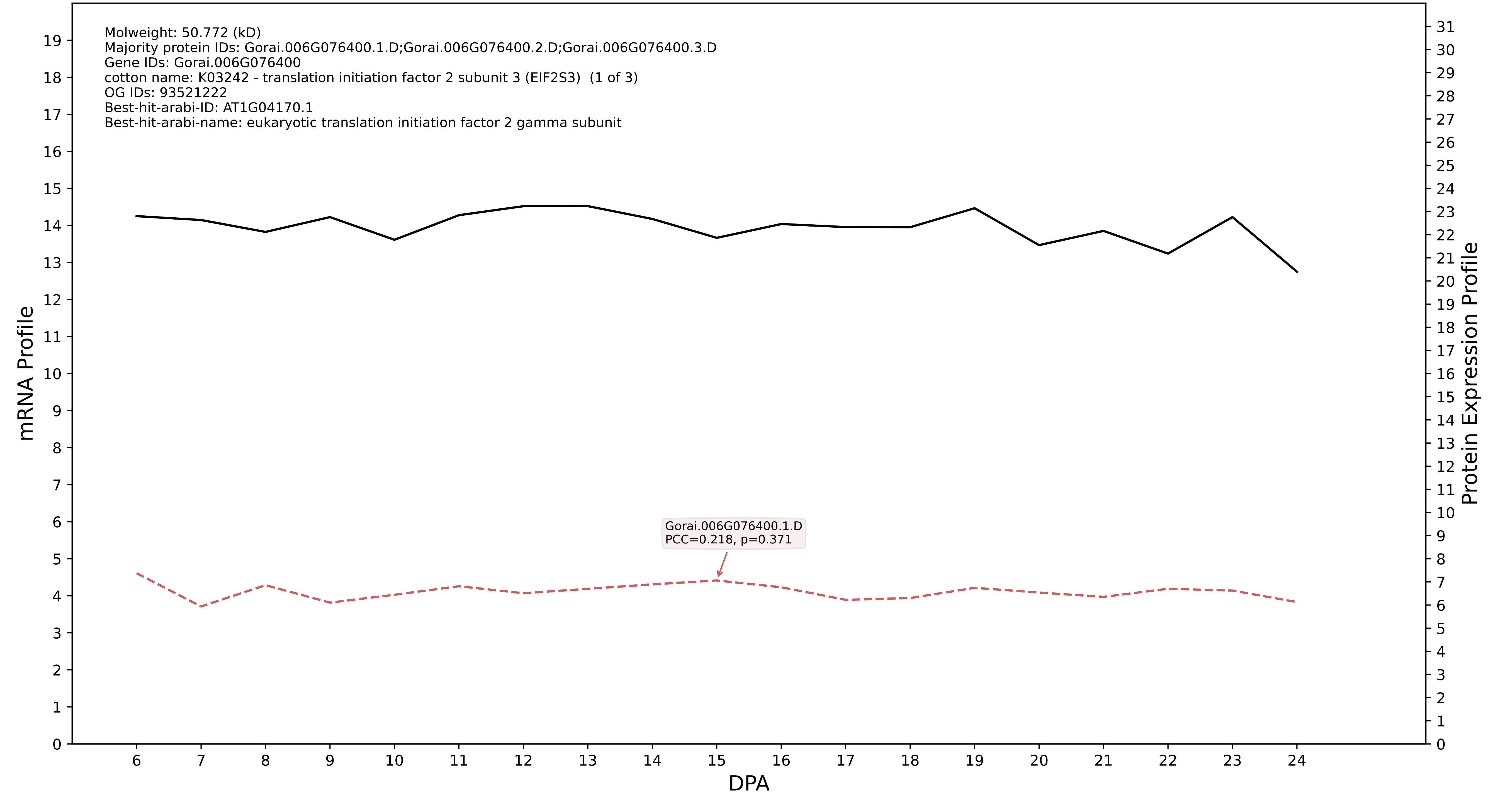

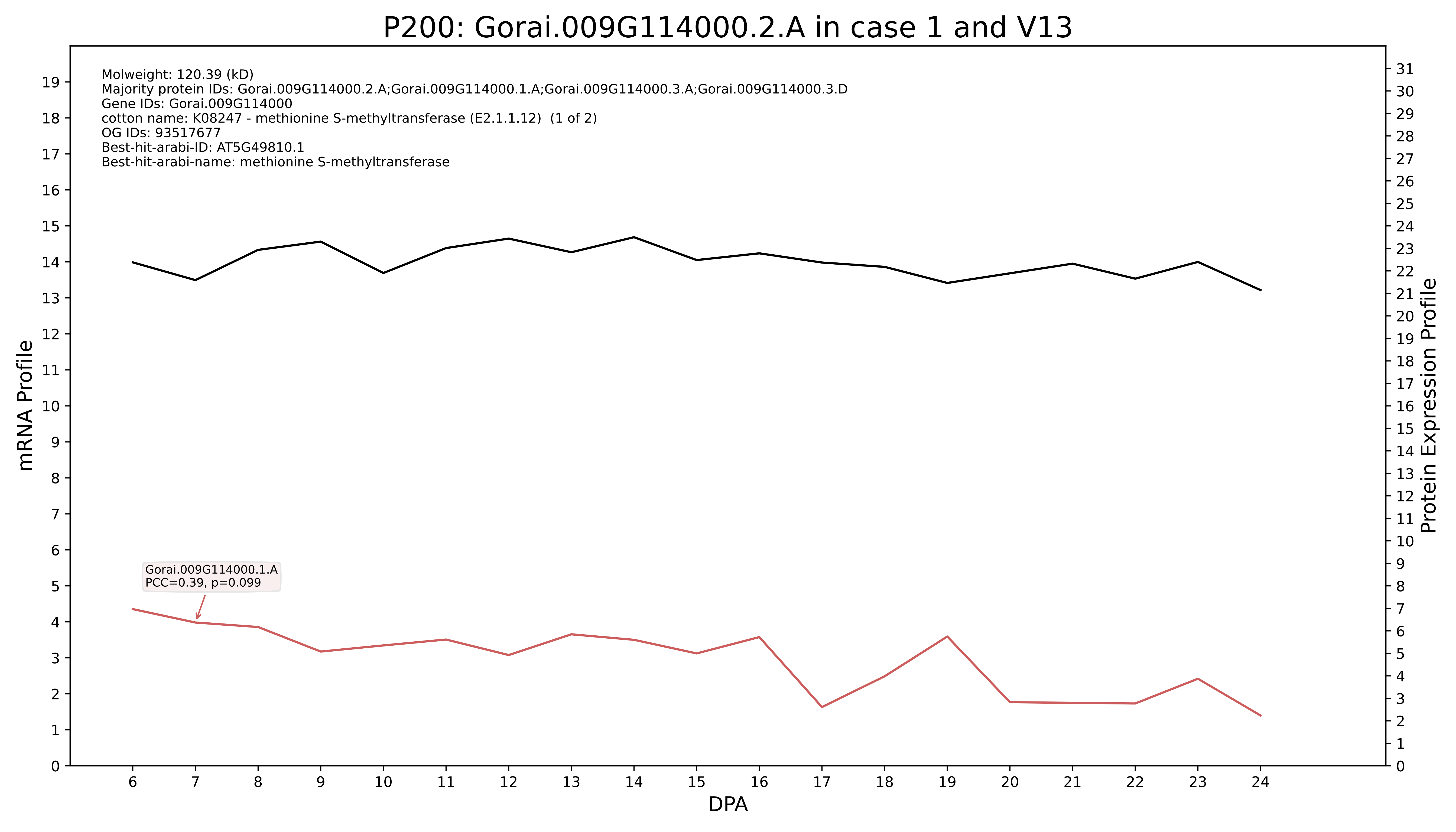

P200: Gorai.009G114000.2.A in case 1 and V13
Molweight: 120.39 (kD)
Majority protein IDs: Gorai.009G114000.2.A;Gorai.009G114000.1.A;Gorai.009G114000.3.A;Gorai.009G114000.3.D
Gene IDs: Gorai.009G114000
cotton name: K08247 - methionine S-methyltransferase (E2.1.1.12) (1 of 2)
OG IDs: 93517677
Best-hit-arabi-ID: AT5G49810.1
Best-hit-arabi-name: methionine S-methyltransferase
Gorai.009G114000.1.A
PCC=0.39, p=0.099
mRNA Profile
Protein Expression Profile
DPA

P200: Gorai.010G103900.1.D in case 1 and V13

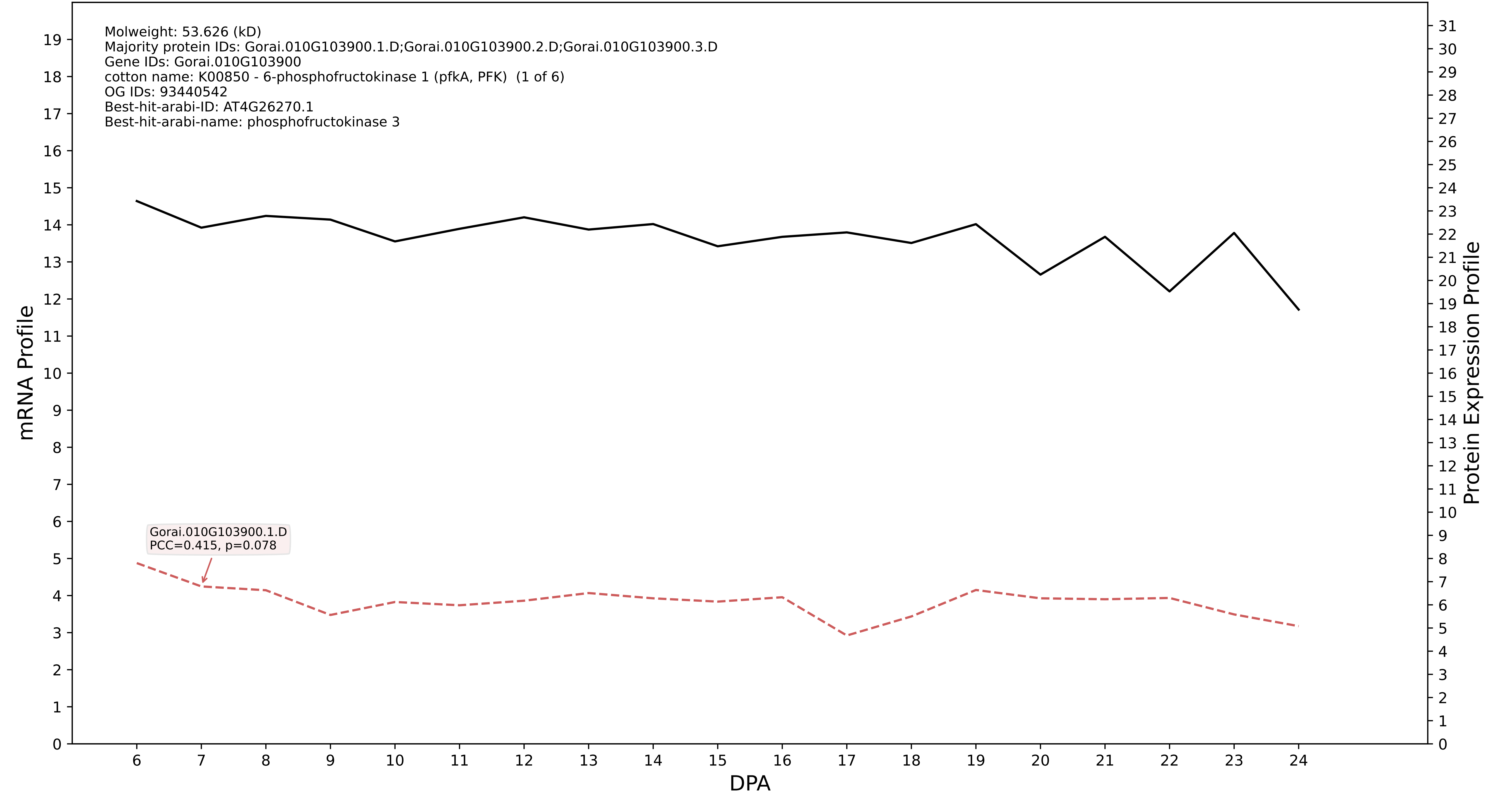

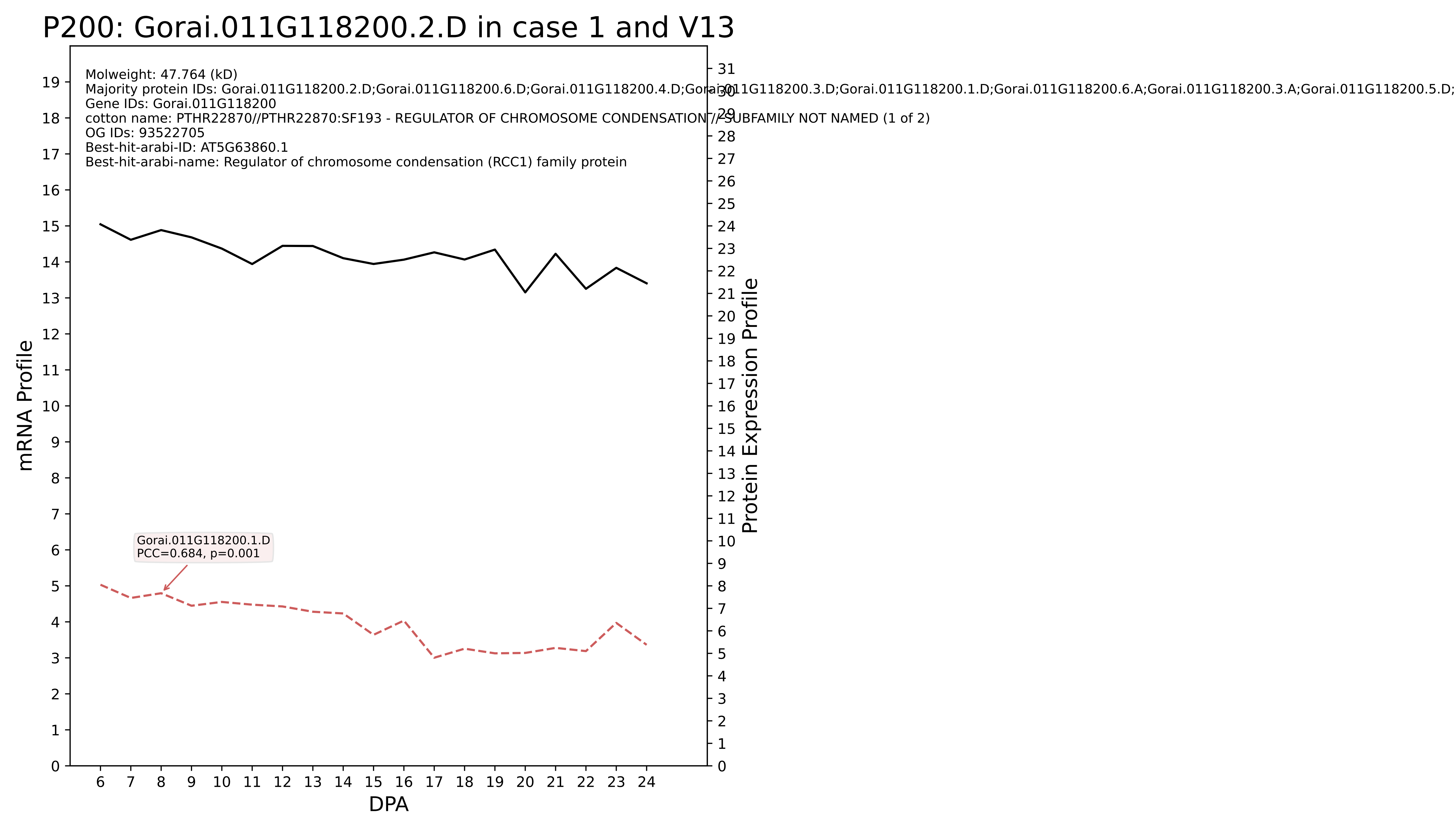

P200: Gorai.011G118200.2.D in case 1 and V13
Molweight: 47.764 (kD)
Majority protein IDs: Gorai.011G118200.2.D;Gorai.011G118200.6.D;Gorai.011G118200.4.D;Gorai.011G118200.3.D;Gorai.011G118200.1.D;Gorai.011G118200.6.A;Gorai.011G118200.3.A;Gorai.011G118200.5.D;
Gene IDs: Gorai.011G118200
cotton name: PTHR22870//PTHR22870:SF193 - REGULATOR OF CHROMOSOME CONDENSATION // SUBFAMILY NOT NAMED (1 of 2)
OG IDs: 93522705
Best-hit-arabi-ID: AT5G63860.1
Best-hit-arabi-name: Regulator of chromosome condensation (RCC1) family protein
Gorai.011G118200.1.D
PCC=0.684, p=0.001
mRNA Profile
Protein Expression Profile
DPA

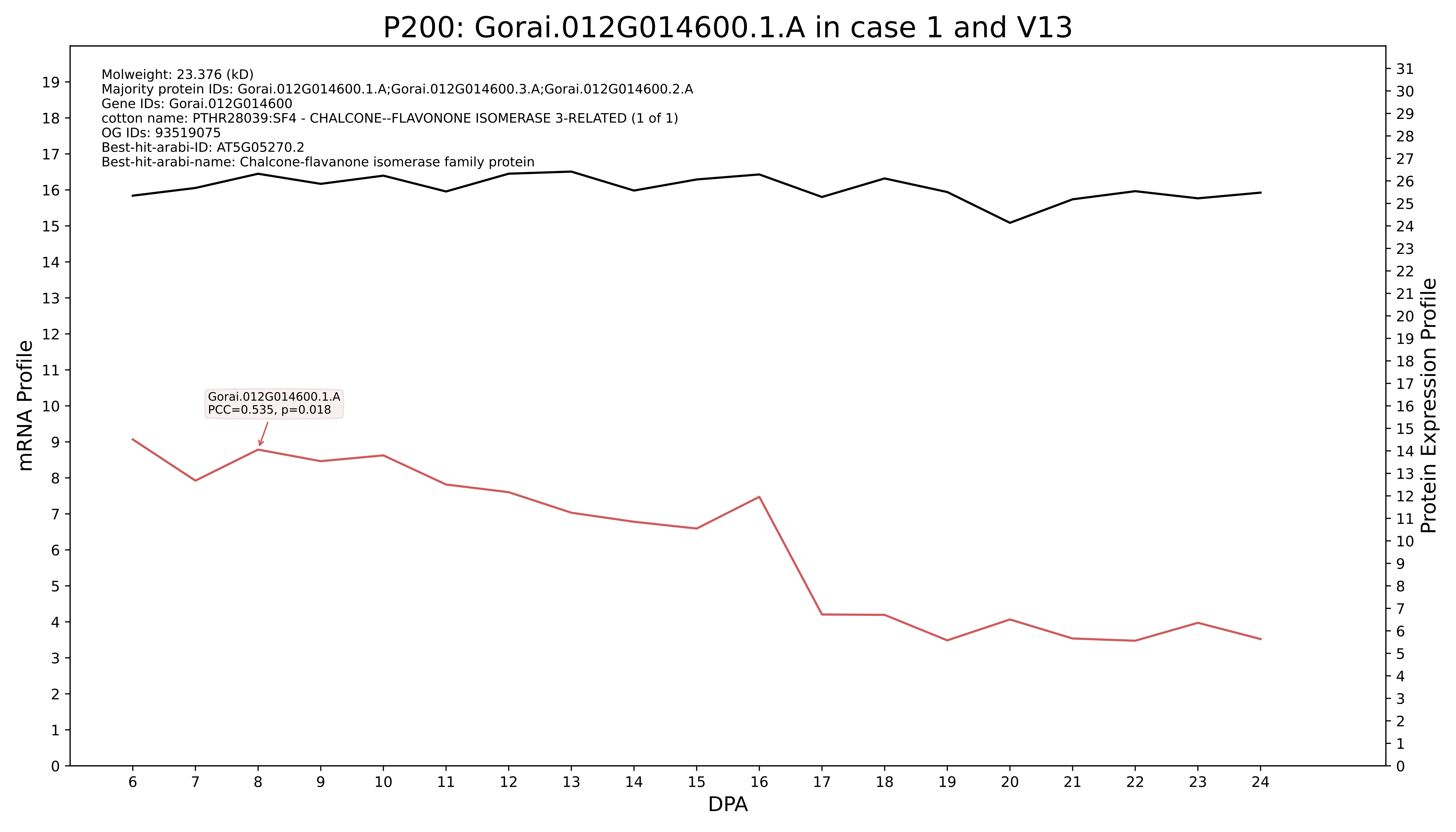
P200: Gorai.012G014600.1.A in case 1 and V13
Molweight: 23.376 (kD)
Majority protein IDs: Gorai.012G014600.1.A;Gorai.012G014600.3.A;Gorai.012G014600.2.A
Gene IDs: Gorai.012G014600
cotton name: PTHR28039:SF4 - CHALCONE--FLAVONONE ISOMERASE 3-RELATED (1 of 1)
OG IDs: 93519075
Best-hit-arabi-ID: AT5G05270.2
Best-hit-arabi-name: Chalcone-flavanone isomerase family protein
Gorai.012G014600.1.A
PCC=0.535, p=0.018
mRNA Profile
Protein Expression Profile
DPA

P200: Gorai.007G355800.1.A in case 1 and V13

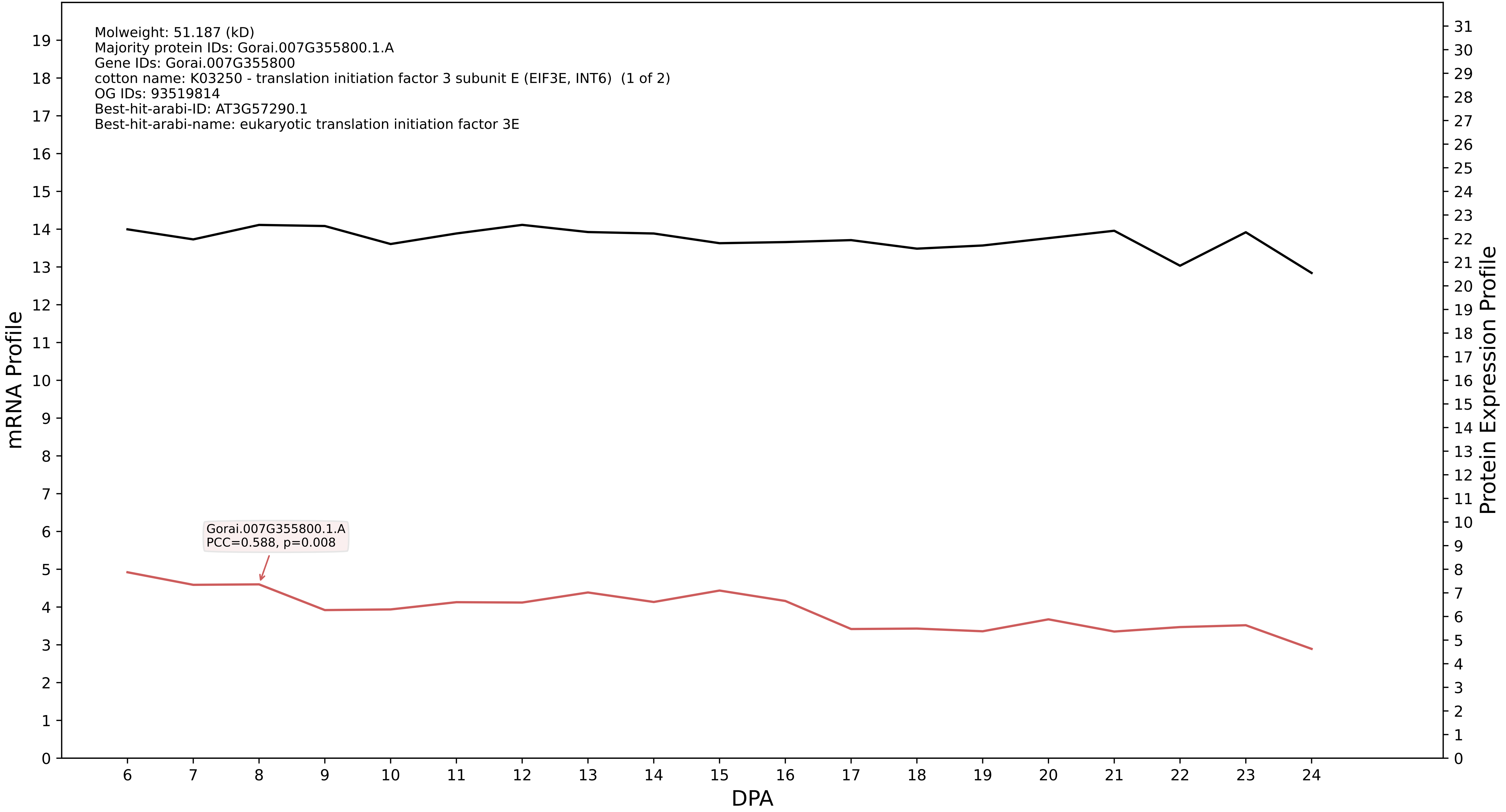

P200: Gorai.006G207300.2.D in case 1 and V13

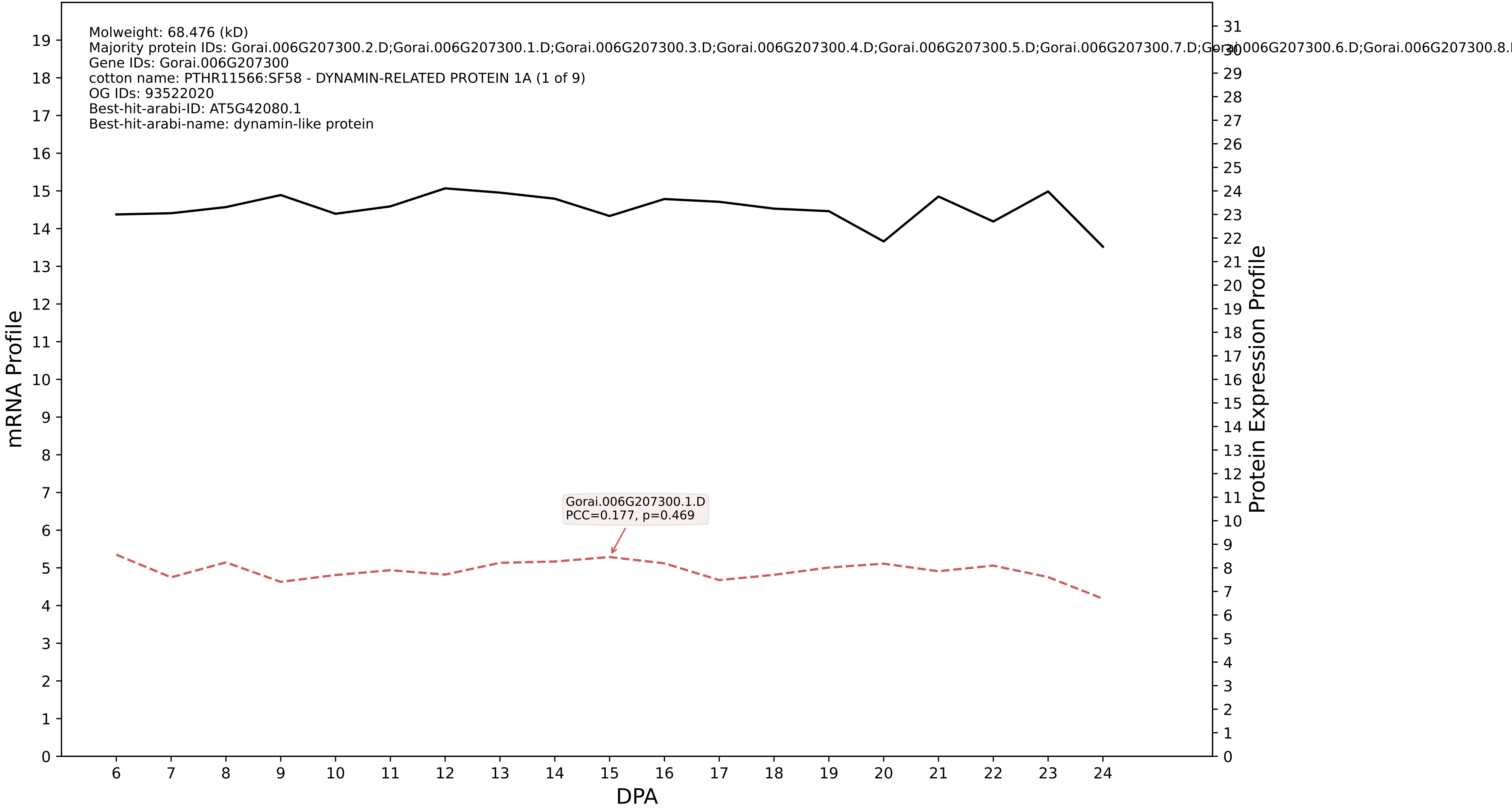

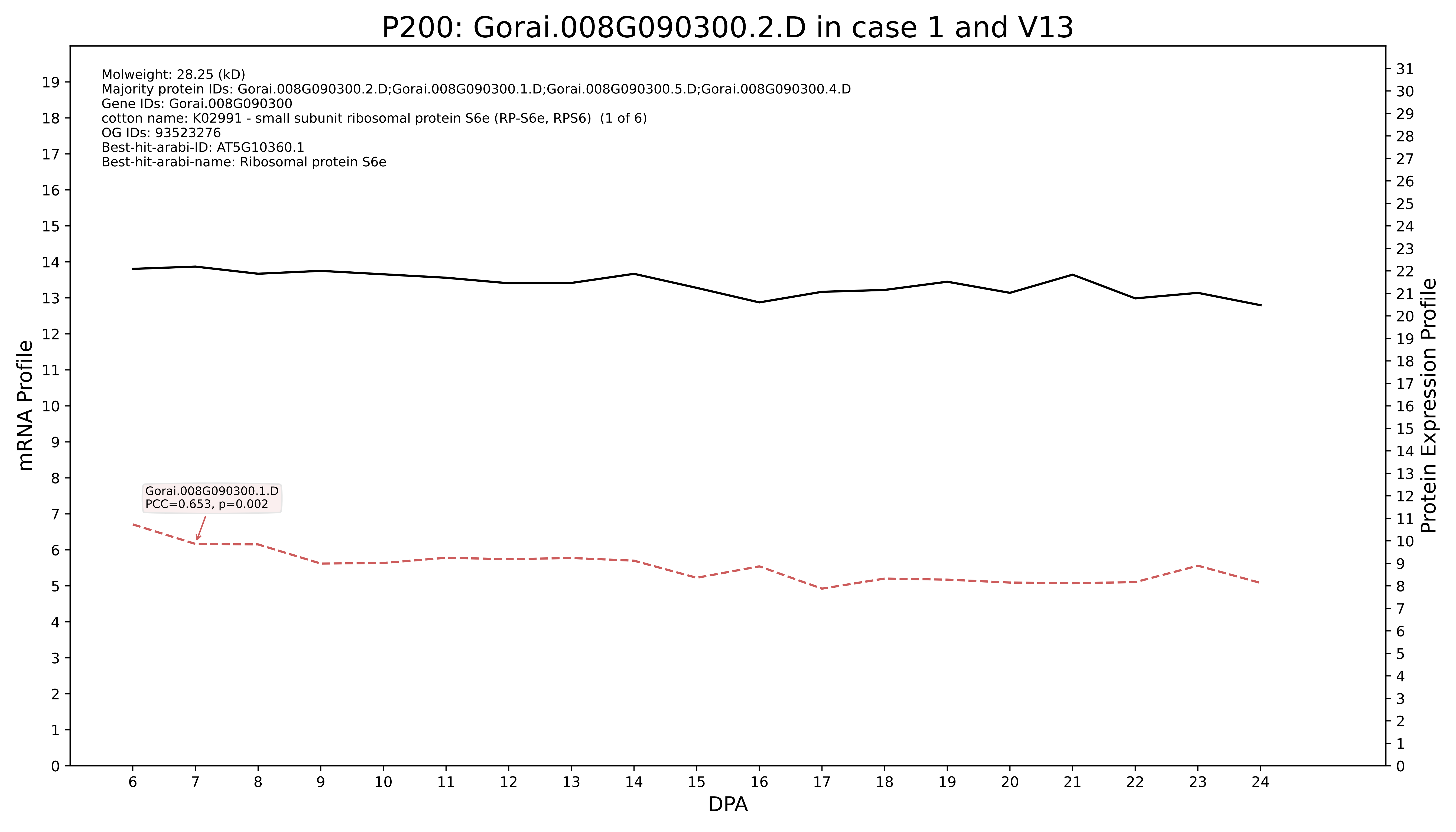
P200: Gorai.008G090300.2.D in case 1 and V13
Molweight: 28.25 (kD)
Majority protein IDs: Gorai.008G090300.2.D;Gorai.008G090300.1.D;Gorai.008G090300.5.D;Gorai.008G090300.4.D
Gene IDs: Gorai.008G090300
cotton name: K02991 - small subunit ribosomal protein S6e (RP-S6e, RPS6) (1 of 6)
OG IDs: 93523276
Best-hit-arabi-ID: AT5G10360.1
Best-hit-arabi-name: Ribosomal protein S6e
Gorai.008G090300.1.D
PCC=0.653, p=0.002
mRNA Profile
Protein Expression Profile
DPA

P200: Gorai.007G011200.1.A in case 1 and V13

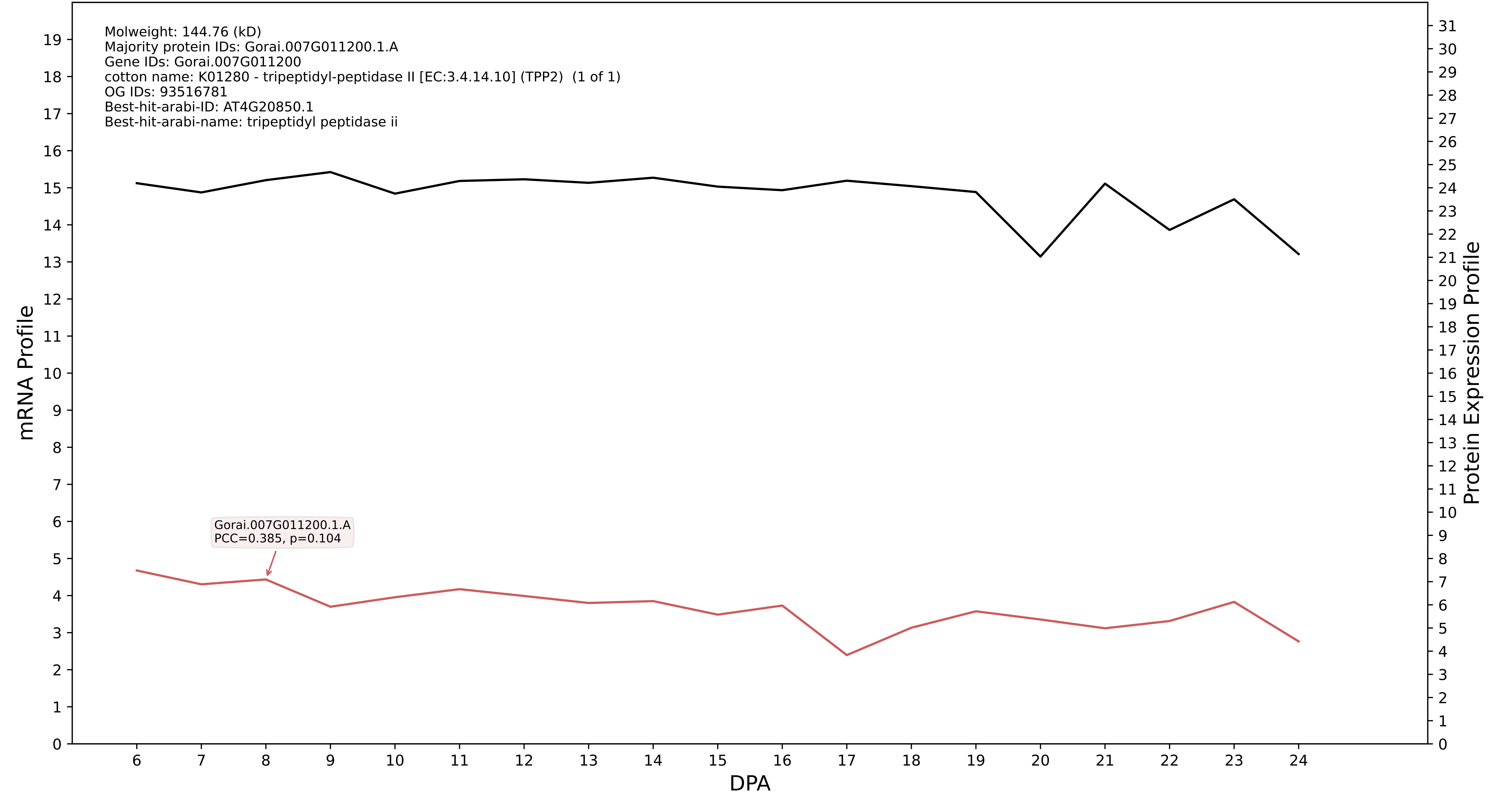

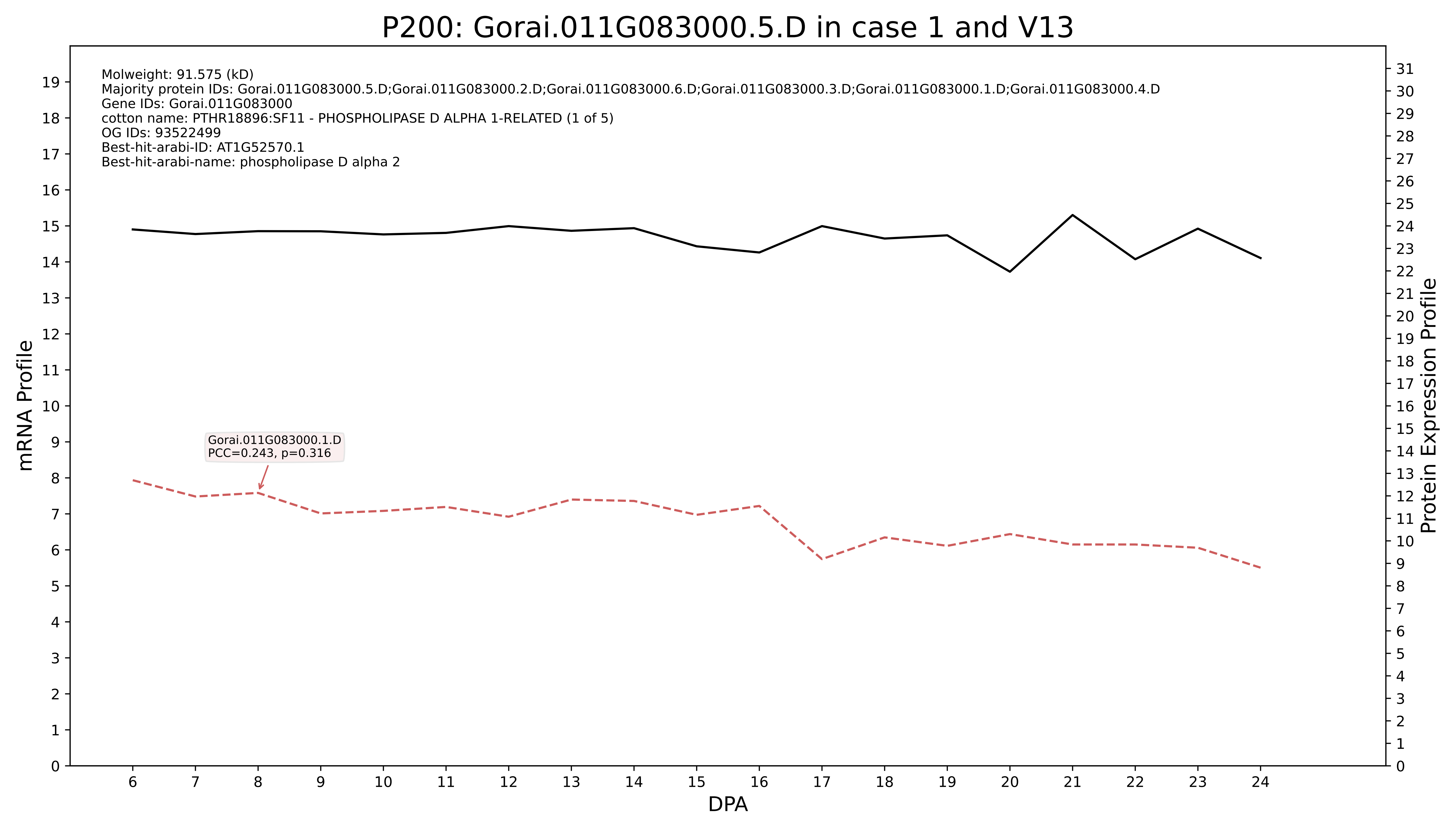

P200: Gorai.011G083000.5.D in case 1 and V13
Molweight: 91.575 (kD)
Majority protein IDs: Gorai.011G083000.5.D;Gorai.011G083000.2.D;Gorai.011G083000.6.D;Gorai.011G083000.3.D;Gorai.011G083000.1.D;Gorai.011G083000.4.D
Gene IDs: Gorai.011G083000
cotton name: PTHR18896:SF11 - PHOSPHOLIPASE D ALPHA 1-RELATED (1 of 5)
OG IDs: 93522499
Best-hit-arabi-ID: AT1G52570.1
Best-hit-arabi-name: phospholipase D alpha 2
Gorai.011G083000.1.D
PCC=0.243, p=0.316
mRNA Profile
Protein Expression Profile
DPA

P200: Gorai.007G373300.1.A in case 1 and V13

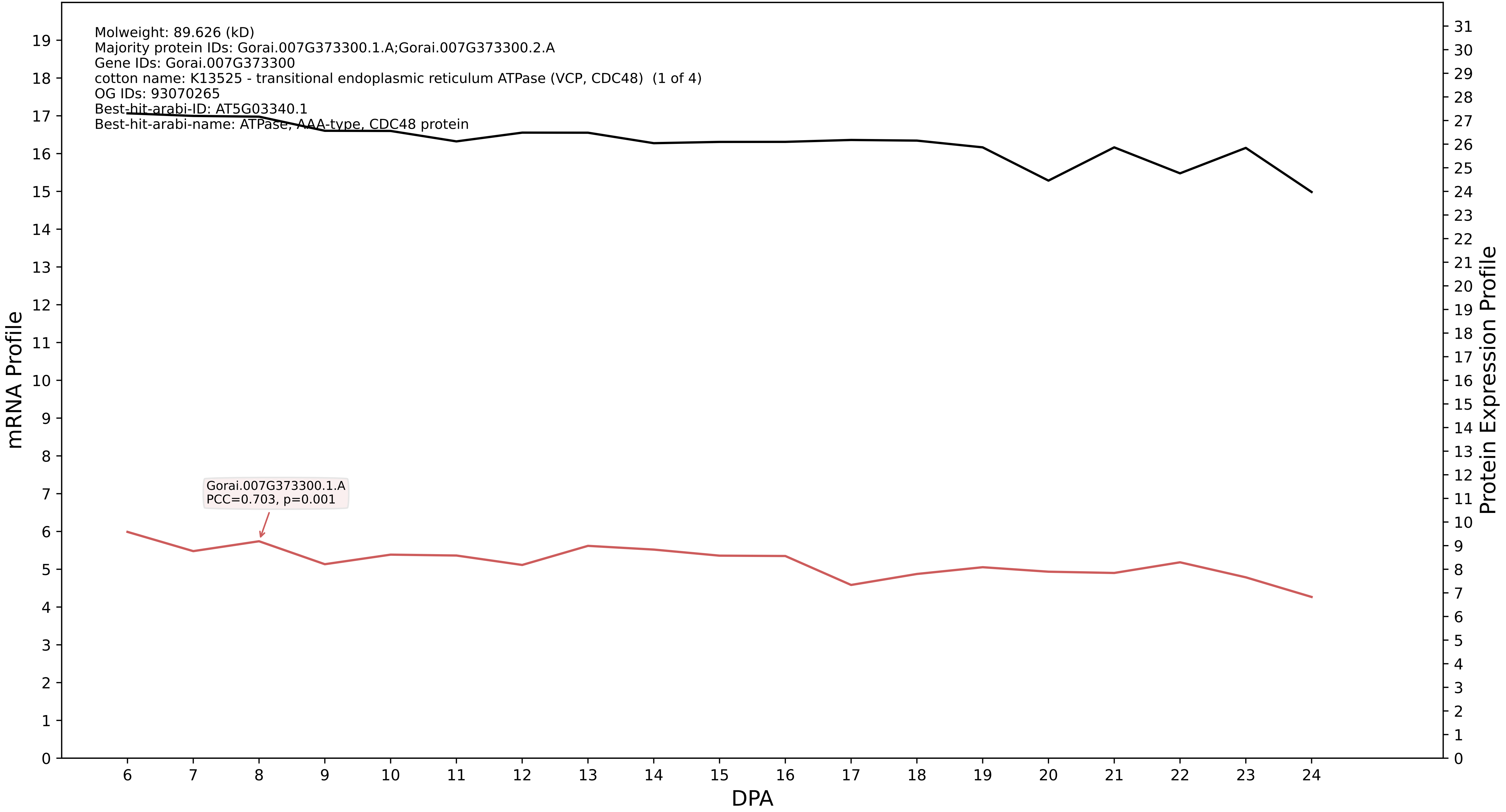

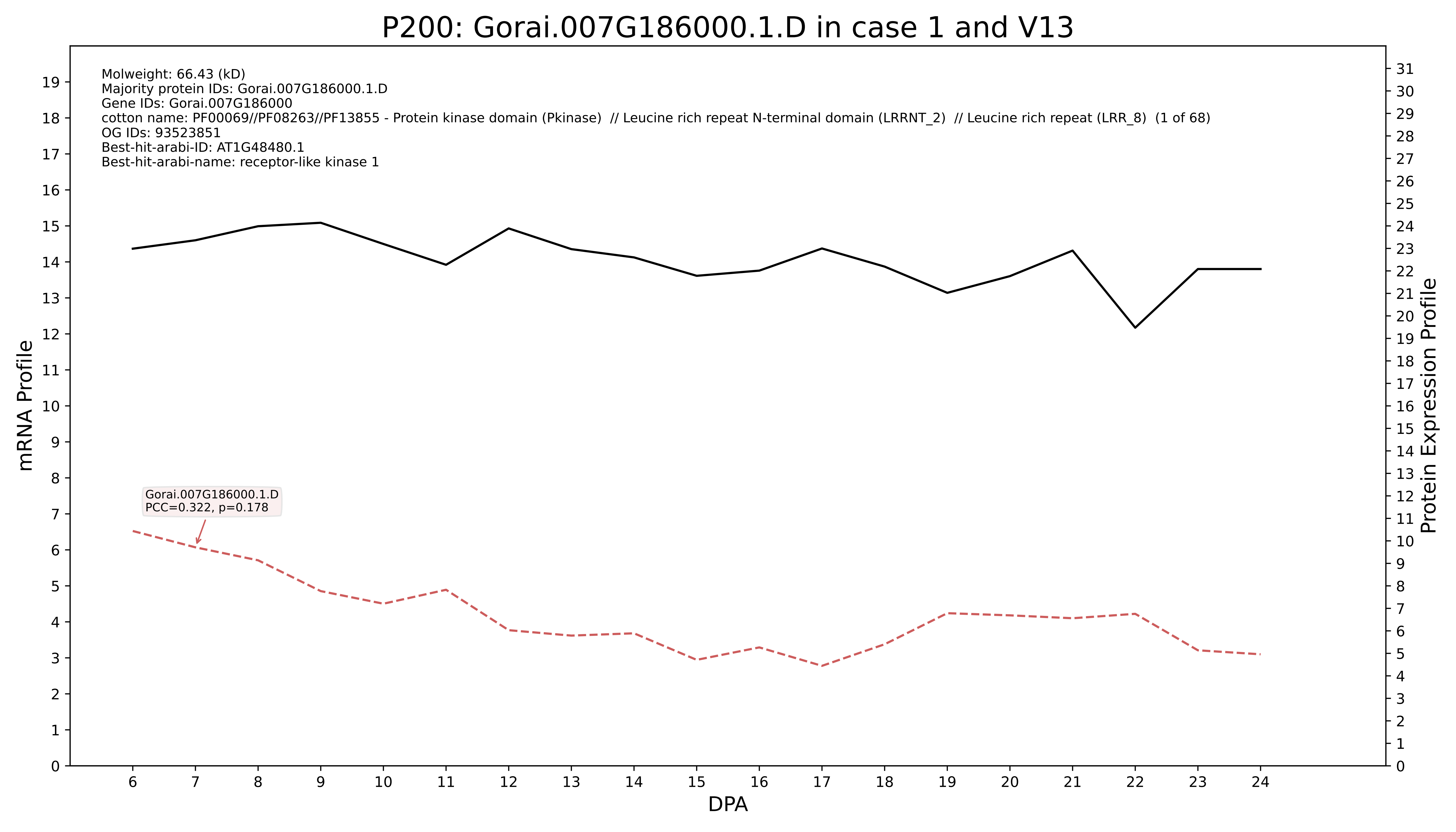

P200: Gorai.007G186000.1.D in case 1 and V13
Molweight: 66.43 (kD)
Majority protein IDs: Gorai.007G186000.1.D
Gene IDs: Gorai.007G186000
cotton name: PF00069//PF08263//PF13855 - Protein kinase domain (Pkinase) // Leucine rich repeat N-terminal domain (LRRNT_2) // Leucine rich repeat (LRR_8) (1 of 68)
OG IDs: 93523851
Best-hit-arabi-ID: AT1G48480.1
Best-hit-arabi-name: receptor-like kinase 1
Gorai.007G186000.1.D
PCC=0.322, p=0.178
mRNA Profile
Protein Expression Profile
DPA

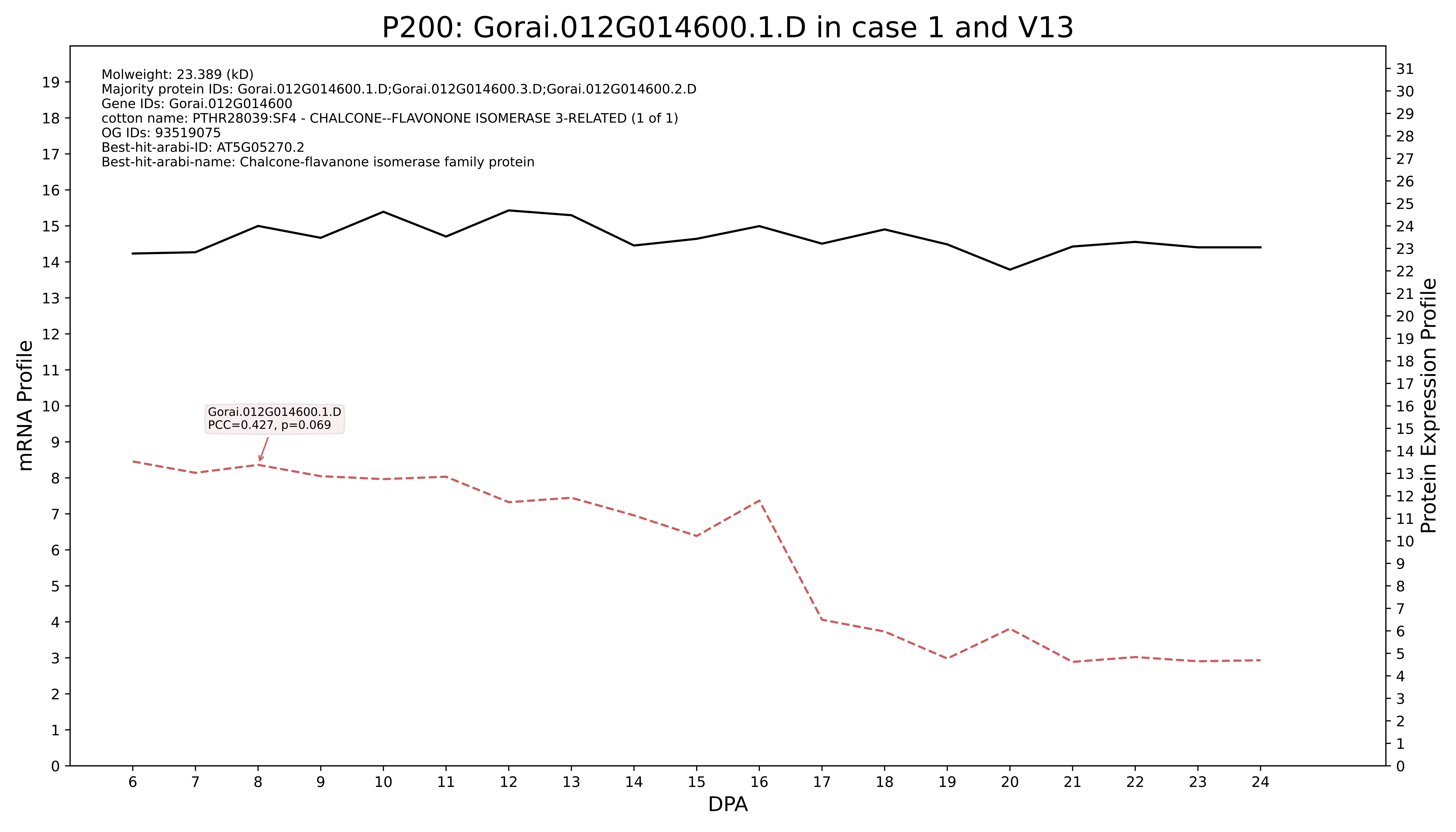
P200: Gorai.012G014600.1.D in case 1 and V13
Molweight: 23.389 (kD)
Majority protein IDs: Gorai.012G014600.1.D;Gorai.012G014600.3.D;Gorai.012G014600.2.D
Gene IDs: Gorai.012G014600
cotton name: PTHR28039:SF4 - CHALCONE--FLAVONONE ISOMERASE 3-RELATED (1 of 1)
OG IDs: 93519075
Best-hit-arabi-ID: AT5G05270.2
Best-hit-arabi-name: Chalcone-flavanone isomerase family protein
Gorai.012G014600.1.D
PCC=0.427, p=0.069
mRNA Profile
Protein Expression Profile
DPA

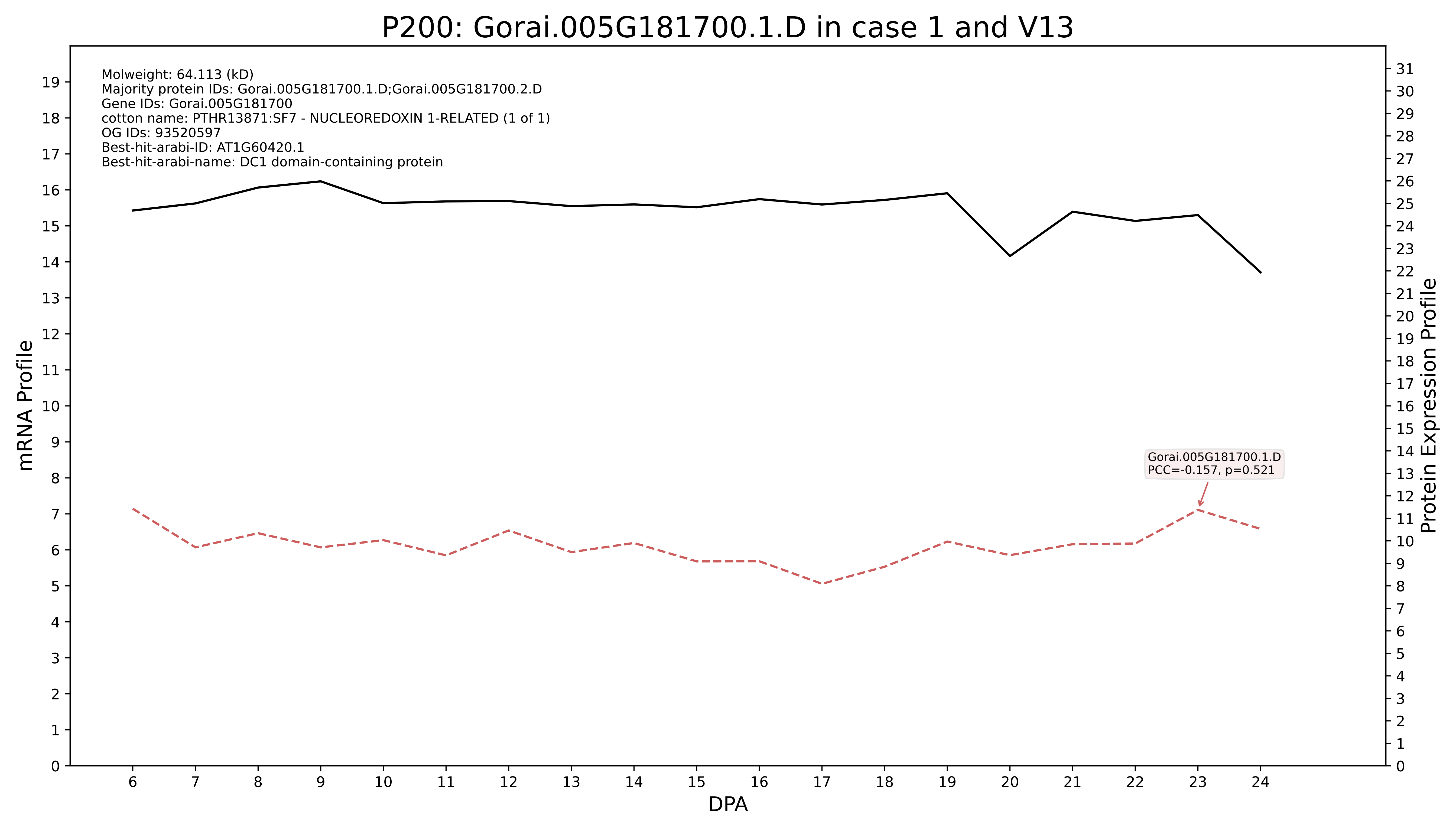
P200: Gorai.005G181700.1.D in case 1 and V13
Molweight: 64.113 (kD)
Majority protein IDs: Gorai.005G181700.1.D;Gorai.005G181700.2.D
Gene IDs: Gorai.005G181700
cotton name: PTHR13871:SF7 - NUCLEOREDOXIN 1-RELATED (1 of 1)
OG IDs: 93520597
Best-hit-arabi-ID: AT1G60420.1
Best-hit-arabi-name: DC1 domain-containing protein
Gorai.005G181700.1.D
PCC=-0.157, p=0.521
mRNA Profile
Protein Expression Profile
DPA

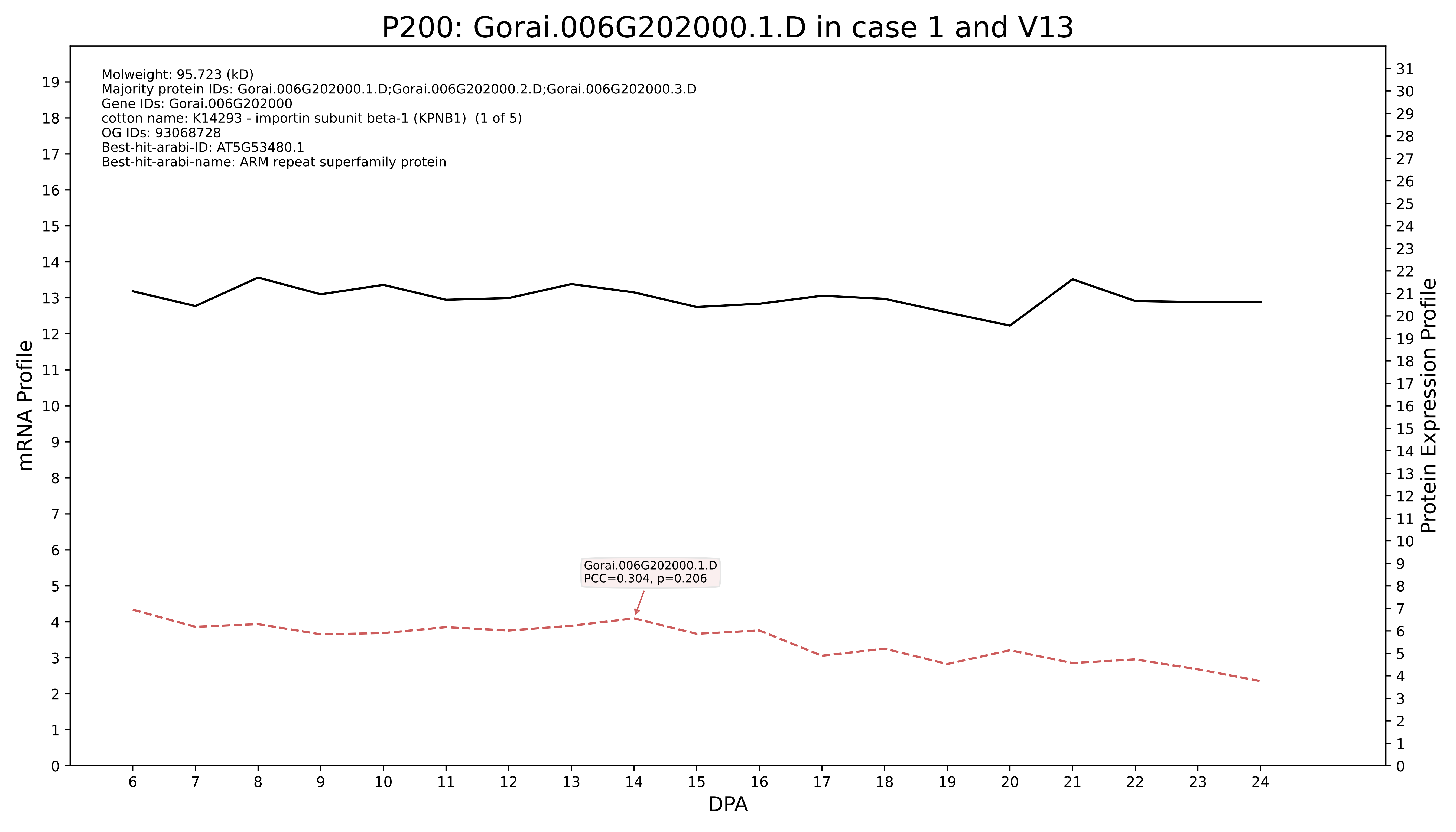
P200: Gorai.006G202000.1.D in case 1 and V13
Molweight: 95.723 (kD)
Majority protein IDs: Gorai.006G202000.1.D;Gorai.006G202000.2.D;Gorai.006G202000.3.D
Gene IDs: Gorai.006G202000
cotton name: K14293 - importin subunit beta-1 (KPNB1) (1 of 5)
OG IDs: 93068728
Best-hit-arabi-ID: AT5G53480.1
Best-hit-arabi-name: ARM repeat superfamily protein
Gorai.006G202000.1.D
PCC=0.304, p=0.206
mRNA Profile
Protein Expression Profile
DPA

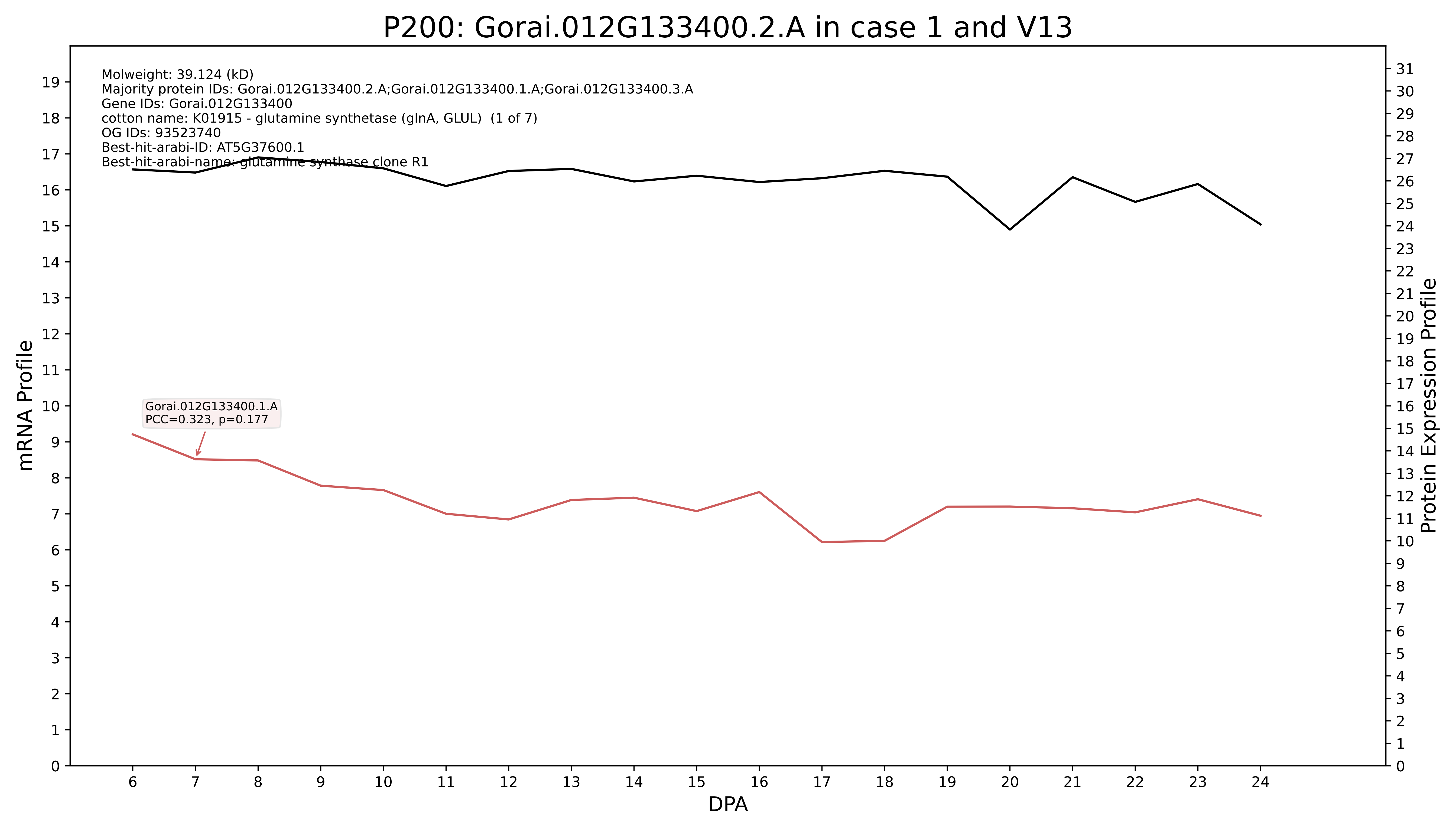

P200: Gorai.012G133400.2.A in case 1 and V13
Molweight: 39.124 (kD)
Majority protein IDs: Gorai.012G133400.2.A;Gorai.012G133400.1.A;Gorai.012G133400.3.A
Gene IDs: Gorai.012G133400
cotton name: K01915 - glutamine synthetase (glnA, GLUL) (1 of 7)
OG IDs: 93523740
Best-hit-arabi-ID: AT5G37600.1
Best-hit-arabi-name: glutamine synthase clone R1
Gorai.012G133400.1.A
PCC=0.323, p=0.177
mRNA Profile
Protein Expression Profile
DPA

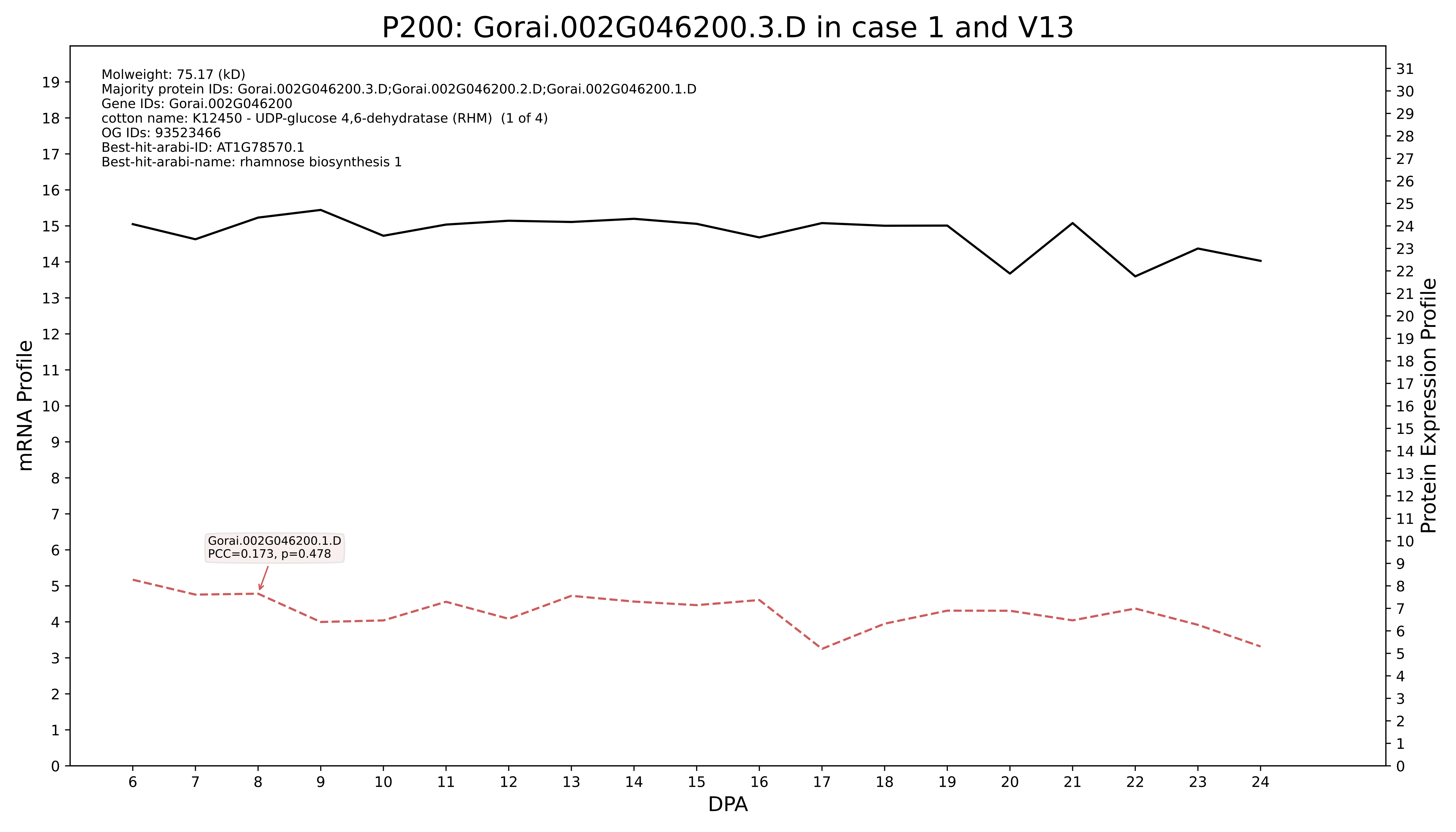
P200: Gorai.002G046200.3.D in case 1 and V13
Molweight: 75.17 (kD)
Majority protein IDs: Gorai.002G046200.3.D;Gorai.002G046200.2.D;Gorai.002G046200.1.D
Gene IDs: Gorai.002G046200
cotton name: K12450 - UDP-glucose 4,6-dehydratase (RHM) (1 of 4)
OG IDs: 93523466
Best-hit-arabi-ID: AT1G78570.1
Best-hit-arabi-name: rhamnose biosynthesis 1
Gorai.002G046200.1.D
PCC=0.173, p=0.478
mRNA Profile
Protein Expression Profile
DPA

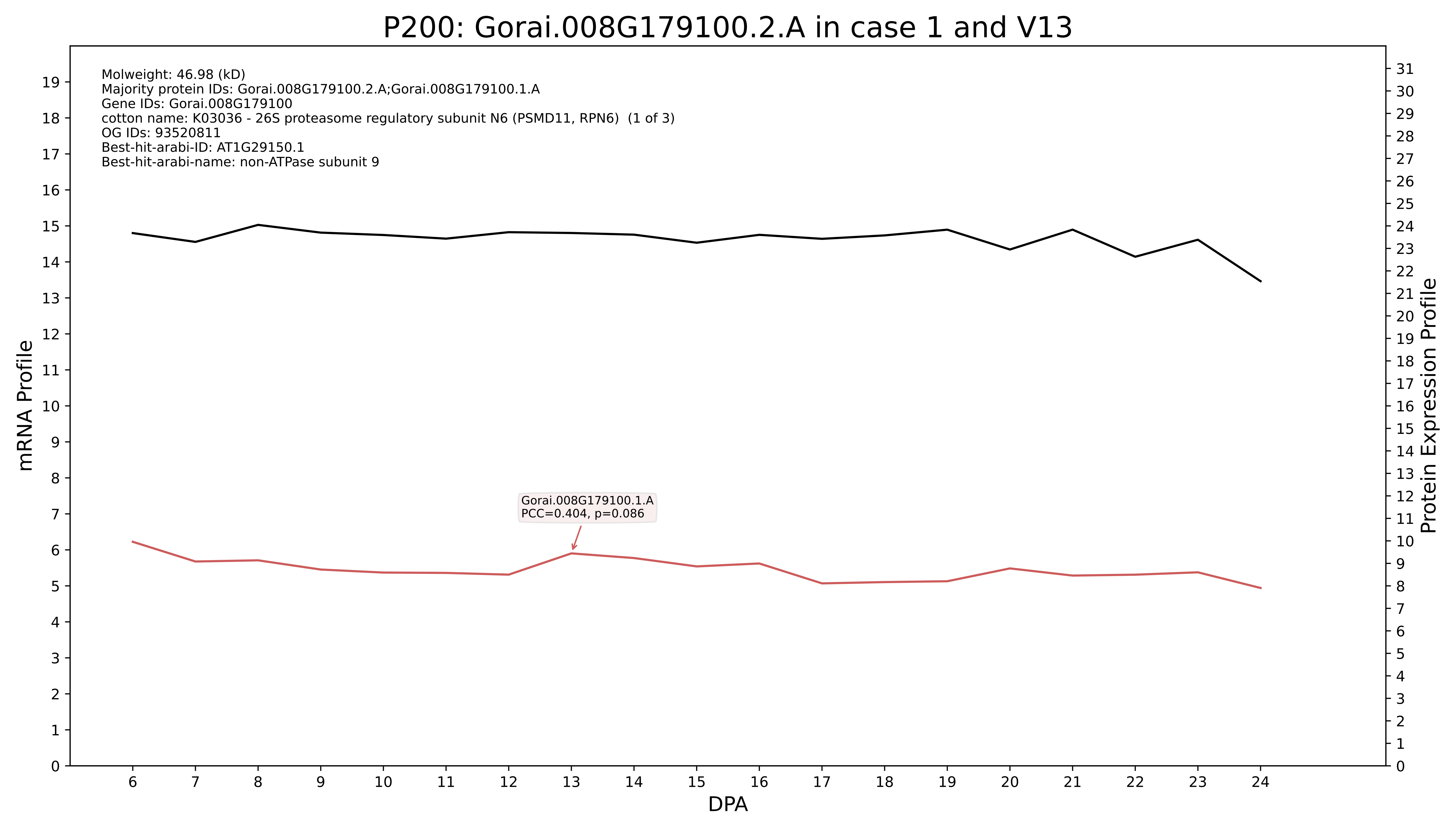
P200: Gorai.008G179100.2.A in case 1 and V13
Molweight: 46.98 (kD)
Majority protein IDs: Gorai.008G179100.2.A;Gorai.008G179100.1.A
Gene IDs: Gorai.008G179100
cotton name: K03036 - 26S proteasome regulatory subunit N6 (PSMD11, RPN6) (1 of 3)
OG IDs: 93520811
Best-hit-arabi-ID: AT1G29150.1
Best-hit-arabi-name: non-ATPase subunit 9
Gorai.008G179100.1.A
PCC=0.404, p=0.086
mRNA Profile
Protein Expression Profile
DPA

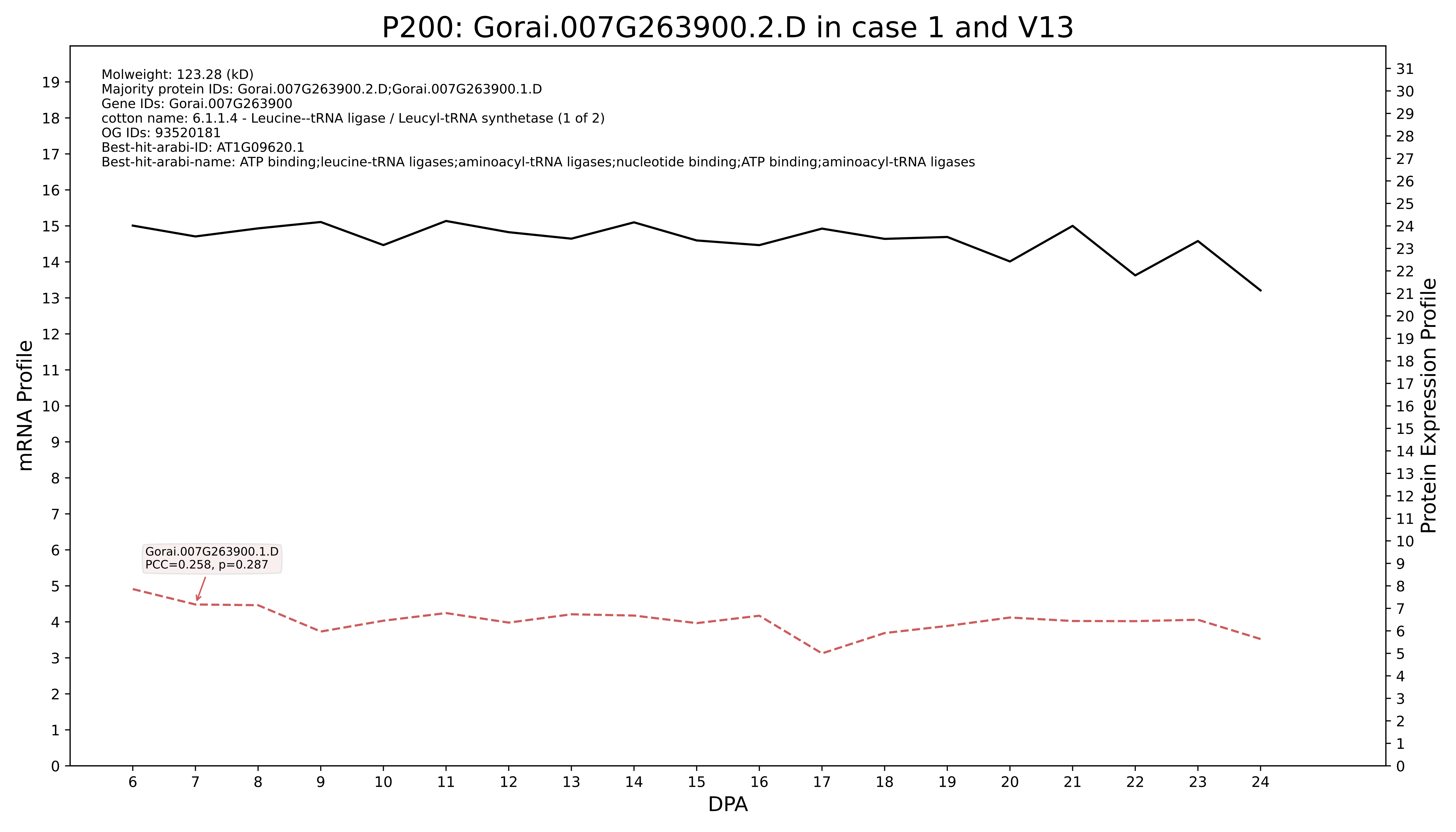
P200: Gorai.007G263900.2.D in case 1 and V13
Molweight: 123.28 (kD)
Majority protein IDs: Gorai.007G263900.2.D;Gorai.007G263900.1.D
Gene IDs: Gorai.007G263900
cotton name: 6.1.1.4 - Leucine--tRNA ligase / Leucyl-tRNA synthetase (1 of 2)
OG IDs: 93520181
Best-hit-arabi-ID: AT1G09620.1
Best-hit-arabi-name: ATP binding;leucine-tRNA ligases;aminoacyl-tRNA ligases;nucleotide binding;ATP binding;aminoacyl-tRNA ligases
Gorai.007G263900.1.D
PCC=0.258, p=0.287
mRNA Profile
Protein Expression Profile
DPA

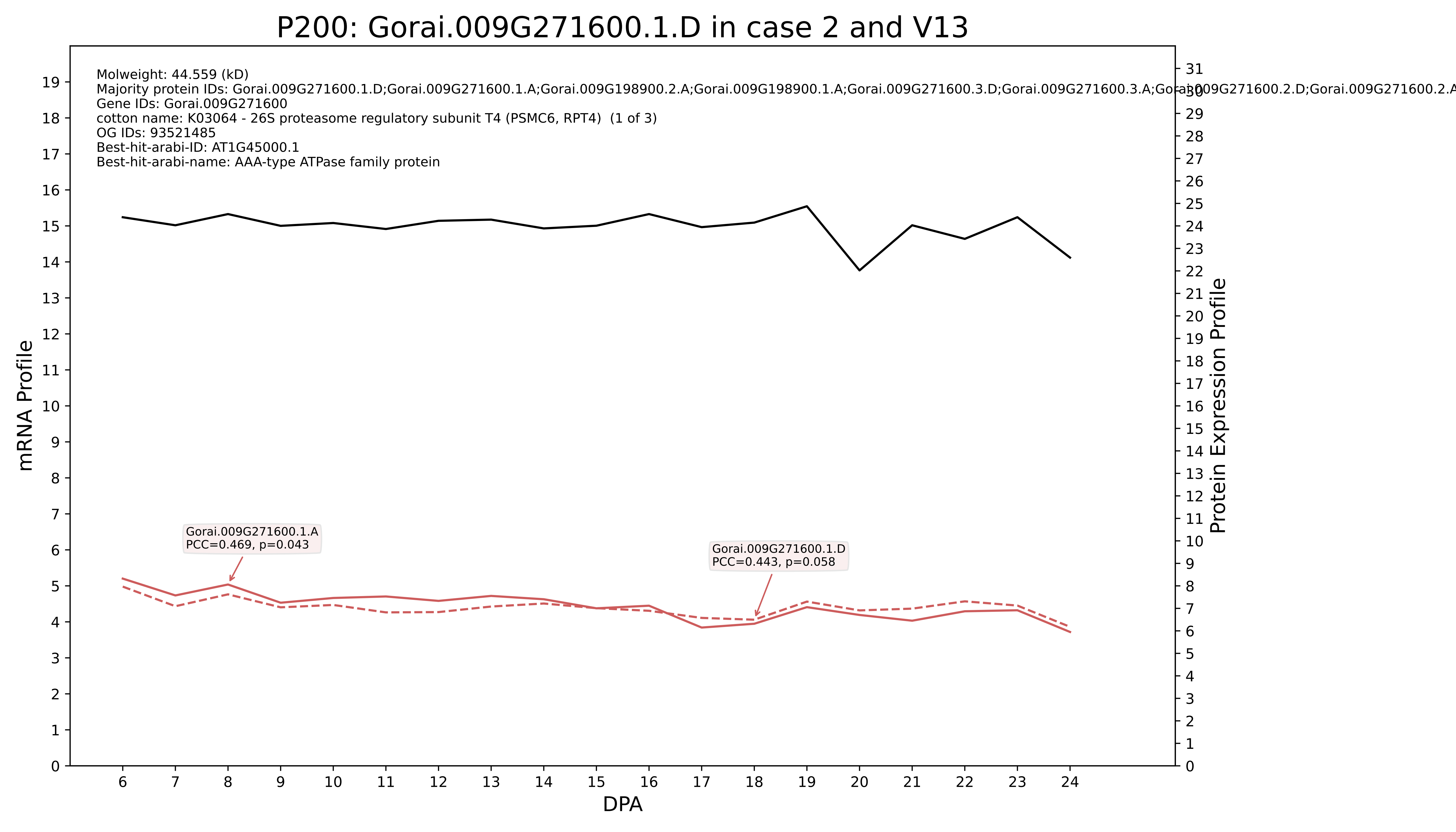
P200: Gorai.009G271600.1.D in case 2 and V13
Molweight: 44.559 (kD)
Majority protein IDs: Gorai.009G271600.1.D;Gorai.009G271600.1.A;Gorai.009G198900.2.A;Gorai.009G198900.1.A;Gorai.009G271600.3.D;Gorai.009G271600.3.A;Gorai.009G271600.2.D;Gorai.009G271600.2.A
Gene IDs: Gorai.009G271600
cotton name: K03064 - 26S proteasome regulatory subunit T4 (PSMC6, RPT4) (1 of 3)
OG IDs: 93521485
Best-hit-arabi-ID: AT1G45000.1
Best-hit-arabi-name: AAA-type ATPase family protein
Gorai.009G271600.1.A
PCC=0.469, p=0.043
Gorai.009G271600.1.D
PCC=0.443, p=0.058
mRNA Profile
Protein Expression Profile
DPA

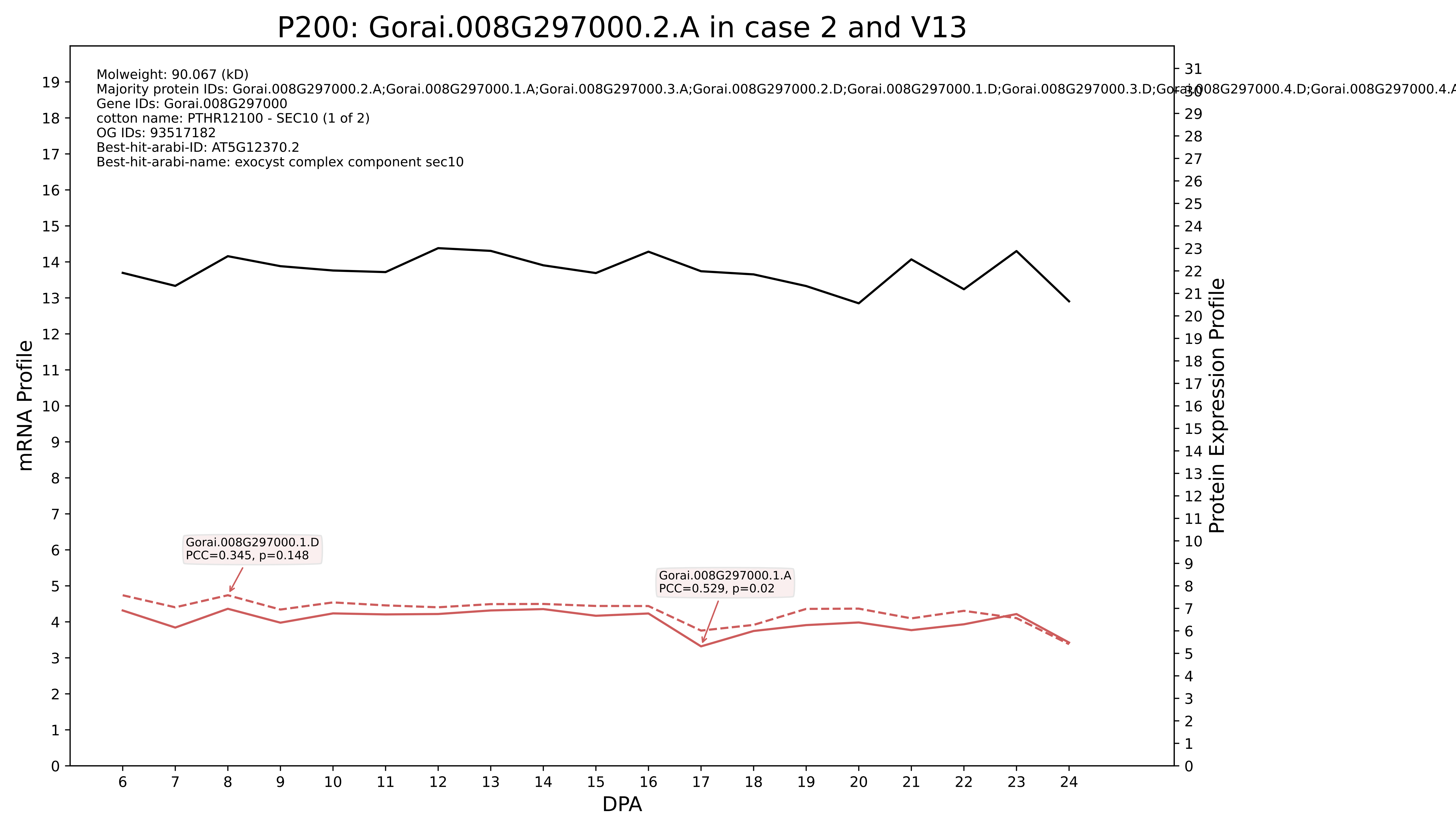

P200: Gorai.008G297000.2.A in case 2 and V13
Molweight: 90.067 (kD)
Majority protein IDs: Gorai.008G297000.2.A;Gorai.008G297000.1.A;Gorai.008G297000.3.A;Gorai.008G297000.2.D;Gorai.008G297000.1.D;Gorai.008G297000.3.D;Gorai.008G297000.4.D;Gorai.008G297000.4.A
Gene IDs: Gorai.008G297000
cotton name: PTHR12100 - SEC10 (1 of 2)
OG IDs: 93517182
Best-hit-arabi-ID: AT5G12370.2
Best-hit-arabi-name: exocyst complex component sec10
Gorai.008G297000.1.D
PCC=0.345, p=0.148
Gorai.008G297000.1.A
PCC=0.529, p=0.02
mRNA Profile
Protein Expression Profile
DPA

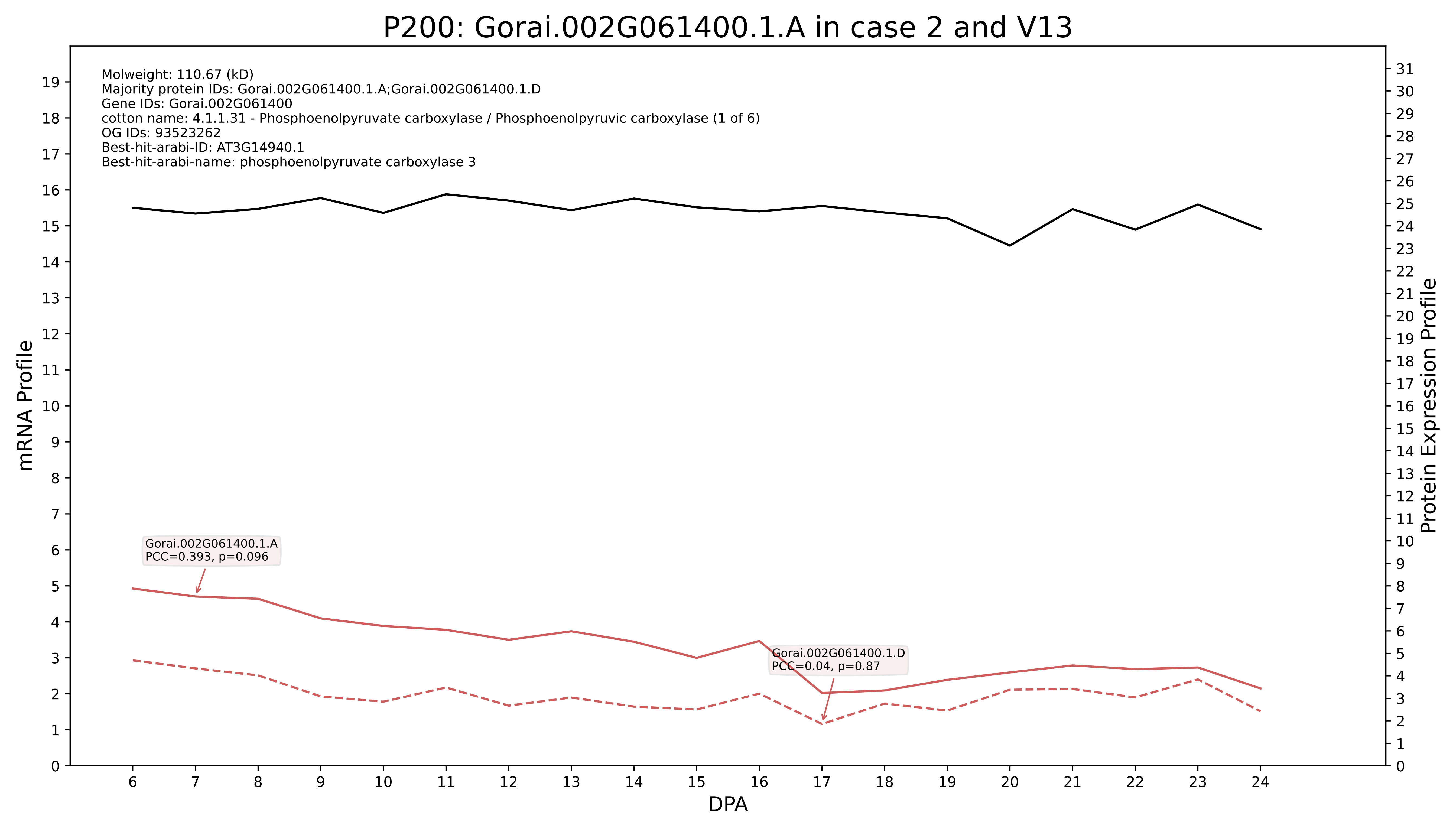
P200: Gorai.002G061400.1.A in case 2 and V13
Molweight: 110.67 (kD)
Majority protein IDs: Gorai.002G061400.1.A;Gorai.002G061400.1.D
Gene IDs: Gorai.002G061400
cotton name: 4.1.1.31 - Phosphoenolpyruvate carboxylase / Phosphoenolpyruvic carboxylase (1 of 6)
OG IDs: 93523262
Best-hit-arabi-ID: AT3G14940.1
Best-hit-arabi-name: phosphoenolpyruvate carboxylase 3
Gorai.002G061400.1.A
PCC=0.393, p=0.096
Gorai.002G061400.1.D
PCC=0.04, p=0.87
mRNA Profile
Protein Expression Profile
DPA

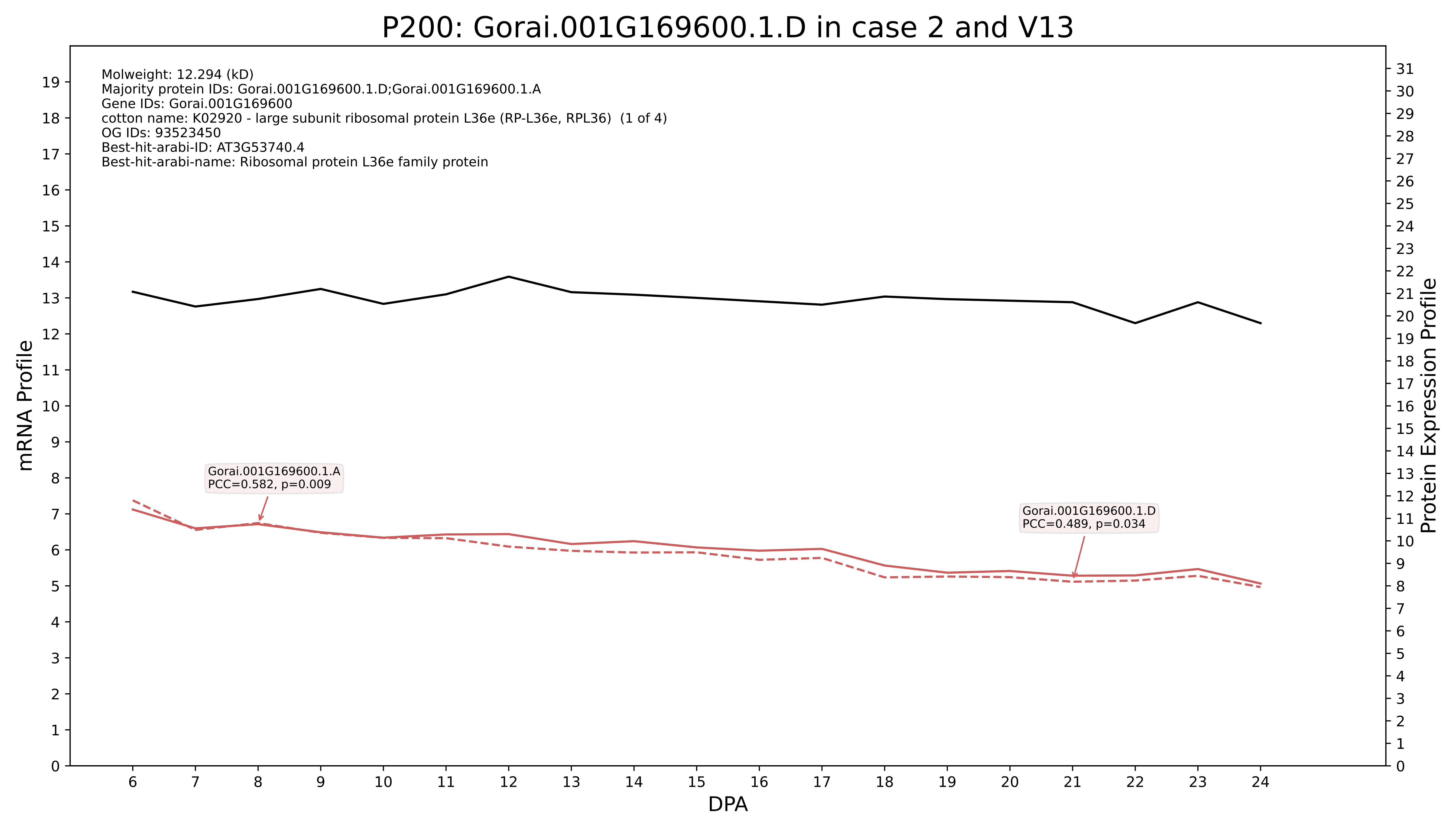
P200: Gorai.001G169600.1.D in case 2 and V13
Molweight: 12.294 (kD)
Majority protein IDs: Gorai.001G169600.1.D;Gorai.001G169600.1.A
Gene IDs: Gorai.001G169600
cotton name: K02920 - large subunit ribosomal protein L36e (RP-L36e, RPL36) (1 of 4)
OG IDs: 93523450
Best-hit-arabi-ID: AT3G53740.4
Best-hit-arabi-name: Ribosomal protein L36e family protein
Gorai.001G169600.1.A
PCC=0.582, p=0.009
Gorai.001G169600.1.D
PCC=0.489, p=0.034
mRNA Profile
Protein Expression Profile
DPA

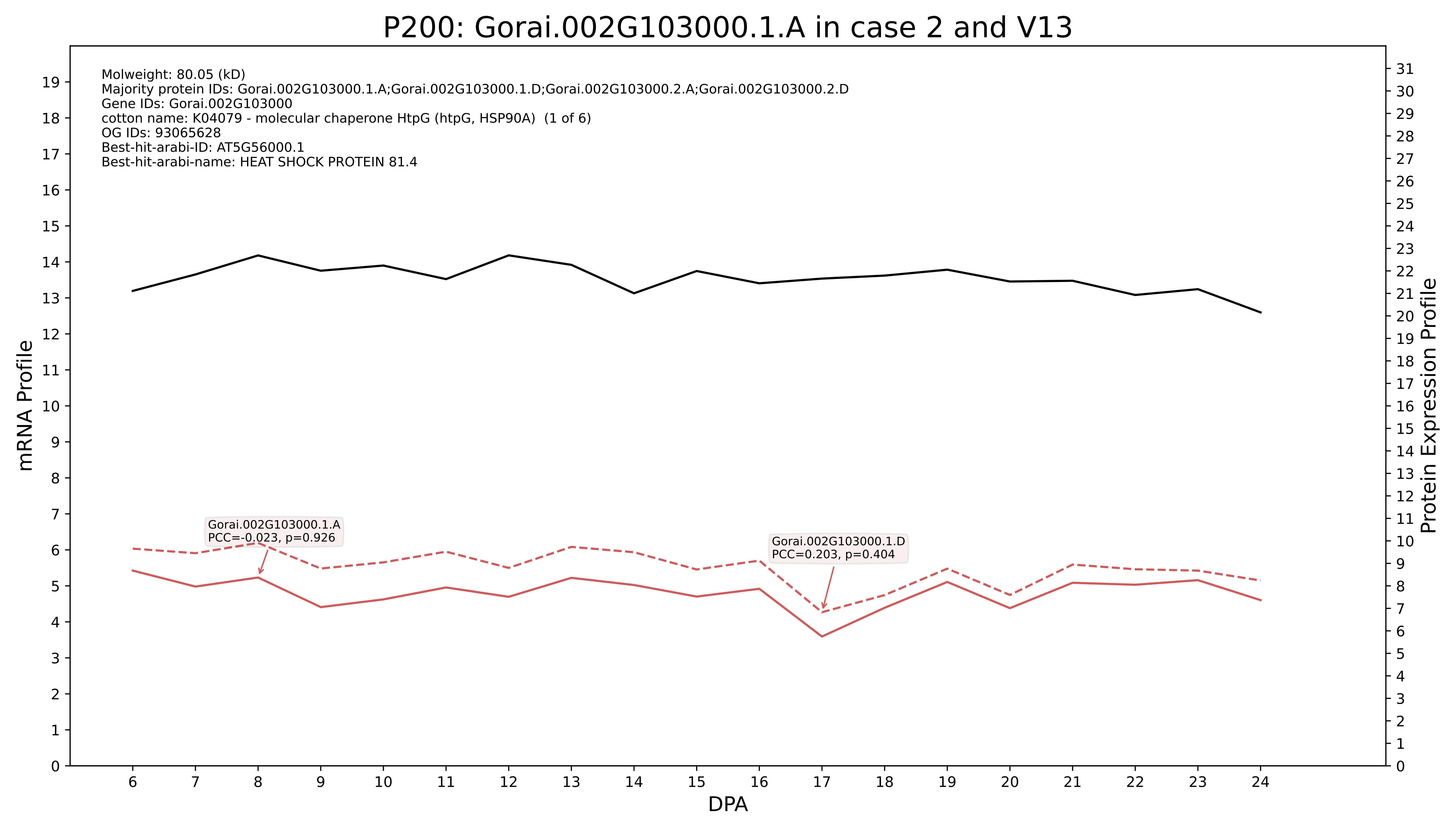
P200: Gorai.002G103000.1.A in case 2 and V13
Molweight: 80.05 (kD)
Majority protein IDs: Gorai.002G103000.1.A;Gorai.002G103000.1.D;Gorai.002G103000.2.A;Gorai.002G103000.2.D
Gene IDs: Gorai.002G103000
cotton name: K04079 - molecular chaperone HtpG (htpG, HSP90A) (1 of 6)
OG IDs: 93065628
Best-hit-arabi-ID: AT5G56000.1
Best-hit-arabi-name: HEAT SHOCK PROTEIN 81.4
Gorai.002G103000.1.A
PCC=-0.023, p=0.926
Gorai.002G103000.1.D
PCC=0.203, p=0.404
mRNA Profile
Protein Expression Profile
DPA

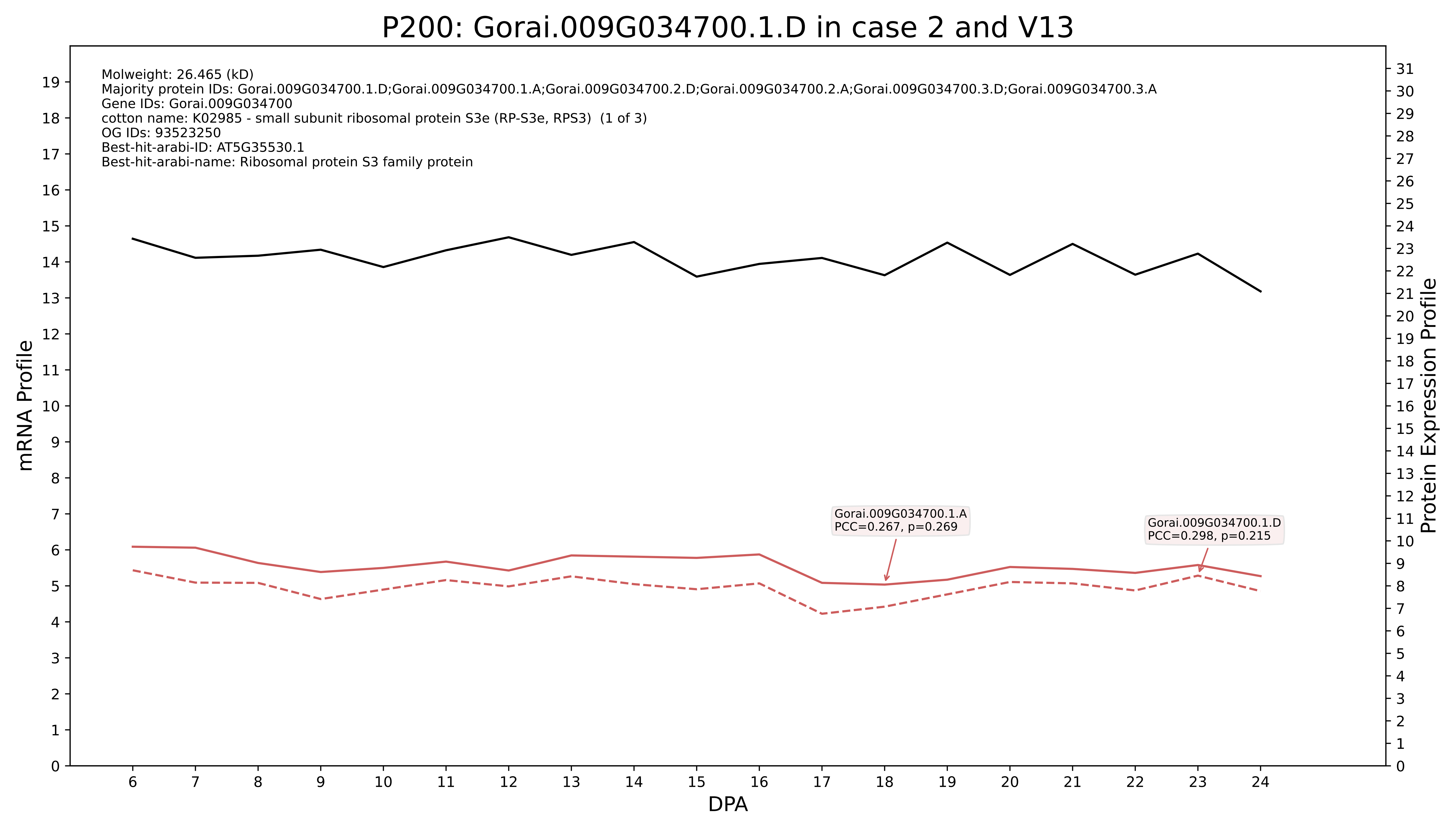
P200: Gorai.009G034700.1.D in case 2 and V13
Molweight: 26.465 (kD)
Majority protein IDs: Gorai.009G034700.1.D;Gorai.009G034700.1.A;Gorai.009G034700.2.D;Gorai.009G034700.2.A;Gorai.009G034700.3.D;Gorai.009G034700.3.A
Gene IDs: Gorai.009G034700
cotton name: K02985 - small subunit ribosomal protein S3e (RP-S3e, RPS3) (1 of 3)
OG IDs: 93523250
Best-hit-arabi-ID: AT5G35530.1
Best-hit-arabi-name: Ribosomal protein S3 family protein
Gorai.009G034700.1.A
PCC=0.267, p=0.269
Gorai.009G034700.1.D
PCC=0.298, p=0.215
mRNA Profile
Protein Expression Profile
DPA
0 1 2 3 4 5 6 7 8 9 10 11 12 13 14 15 16 17 18 19
0 1 2 3 4 5 6 7 8 9 10 11 12 13 14 15 16 17 18 19 20 21 22 23 24 25 26 27 28 29 30 31
6 7 8 9 10 11 12 13 14 15 16 17 18 19 20 21 22 23 24

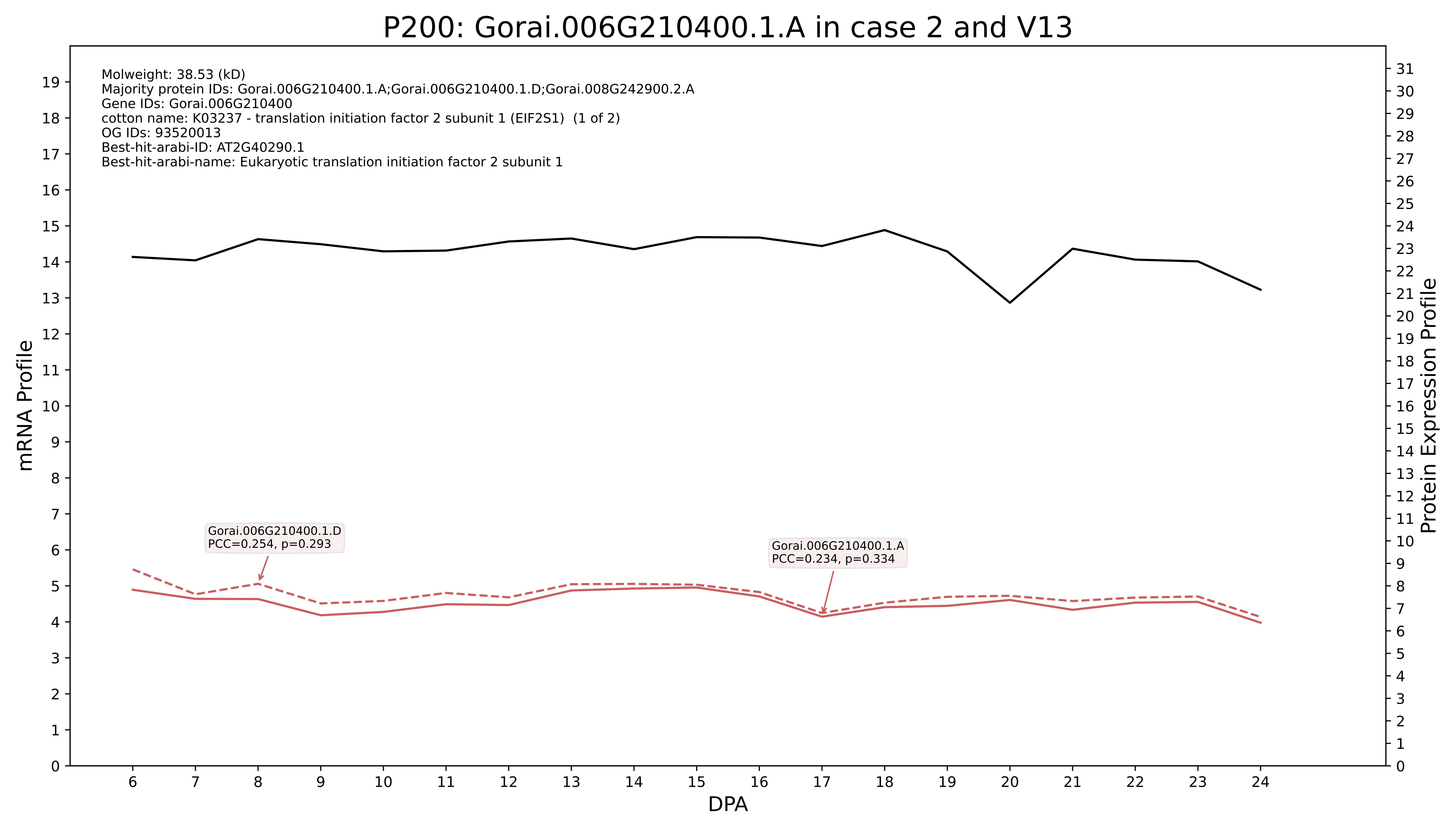

P200: Gorai.006G210400.1.A in case 2 and V13
Molweight: 38.53 (kD)
Majority protein IDs: Gorai.006G210400.1.A;Gorai.006G210400.1.D;Gorai.008G242900.2.A
Gene IDs: Gorai.006G210400
cotton name: K03237 - translation initiation factor 2 subunit 1 (EIF2S1) (1 of 2)
OG IDs: 93520013
Best-hit-arabi-ID: AT2G40290.1
Best-hit-arabi-name: Eukaryotic translation initiation factor 2 subunit 1
Gorai.006G210400.1.D
PCC=0.254, p=0.293
Gorai.006G210400.1.A
PCC=0.234, p=0.334
mRNA Profile
Protein Expression Profile
DPA

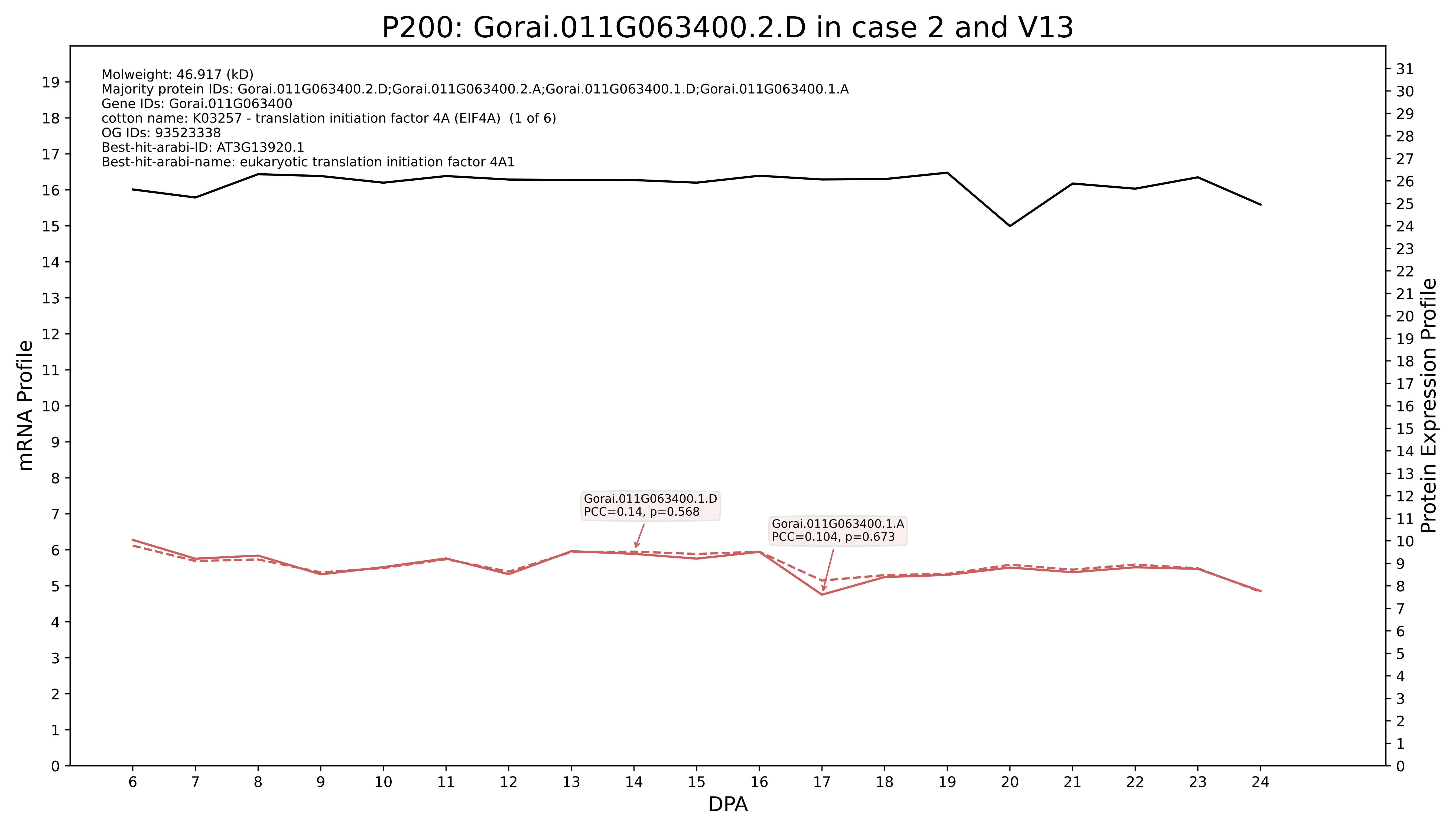
P200: Gorai.011G063400.2.D in case 2 and V13
Molweight: 46.917 (kD)
Majority protein IDs: Gorai.011G063400.2.D;Gorai.011G063400.2.A;Gorai.011G063400.1.D;Gorai.011G063400.1.A
Gene IDs: Gorai.011G063400
cotton name: K03257 - translation initiation factor 4A (EIF4A) (1 of 6)
OG IDs: 93523338
Best-hit-arabi-ID: AT3G13920.1
Best-hit-arabi-name: eukaryotic translation initiation factor 4A1
Gorai.011G063400.1.D
PCC=0.14, p=0.568
Gorai.011G063400.1.A
PCC=0.104, p=0.673
mRNA Profile
Protein Expression Profile
DPA

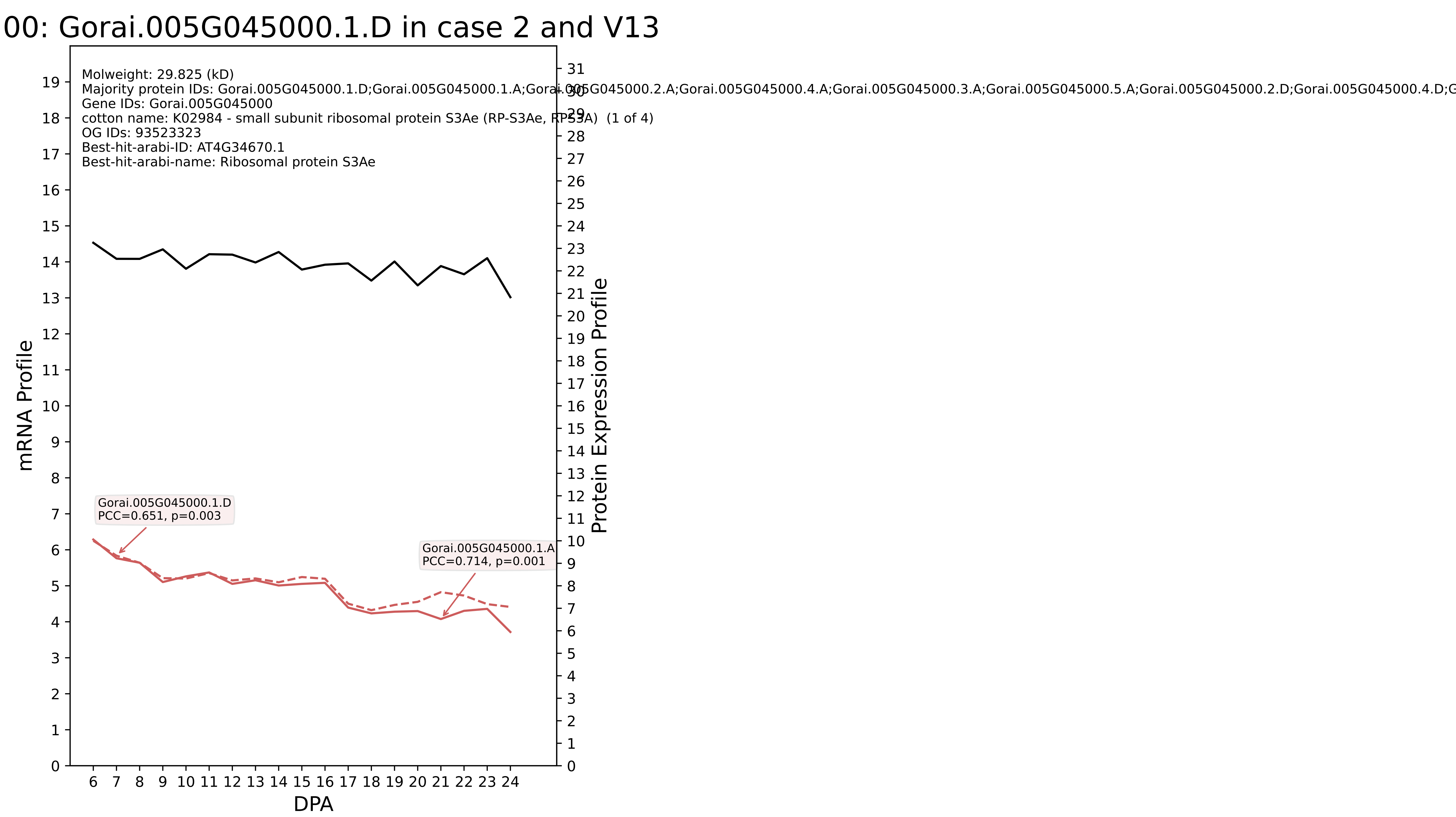
00: Gorai.005G045000.1.D in case 2 and V13
Molweight: 29.825 (kD)
Majority protein IDs: Gorai.005G045000.1.D;Gorai.005G045000.1.A;Gorai.005G045000.2.A;Gorai.005G045000.4.A;Gorai.005G045000.3.A;Gorai.005G045000.5.A;Gorai.005G045000.2.D;Gorai.005G045000.4.D;G
Gene IDs: Gorai.005G045000
cotton name: K02984 - small subunit ribosomal protein S3Ae (RP-S3Ae, RPS3A) (1 of 4)
OG IDs: 93523323
Best-hit-arabi-ID: AT4G34670.1
Best-hit-arabi-name: Ribosomal protein S3Ae
Gorai.005G045000.1.D
PCC=0.651, p=0.003
Gorai.005G045000.1.A
PCC=0.714, p=0.001
mRNA Profile
Protein Expression Profile
DPA

00: Gorai.012G070300.5.D in case 2 and V13

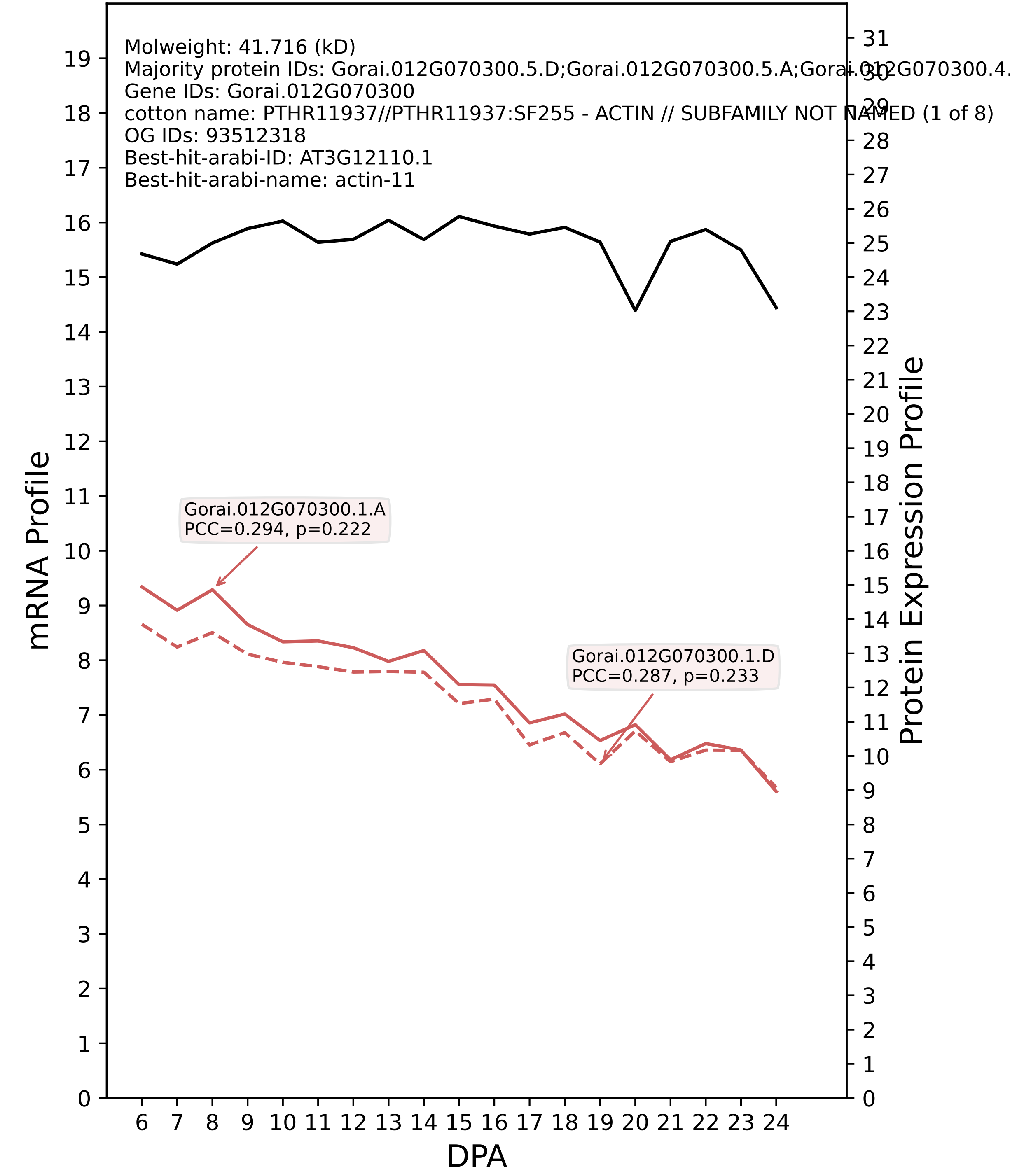

00: Gorai.011G000800.3.D in case 2 and V13

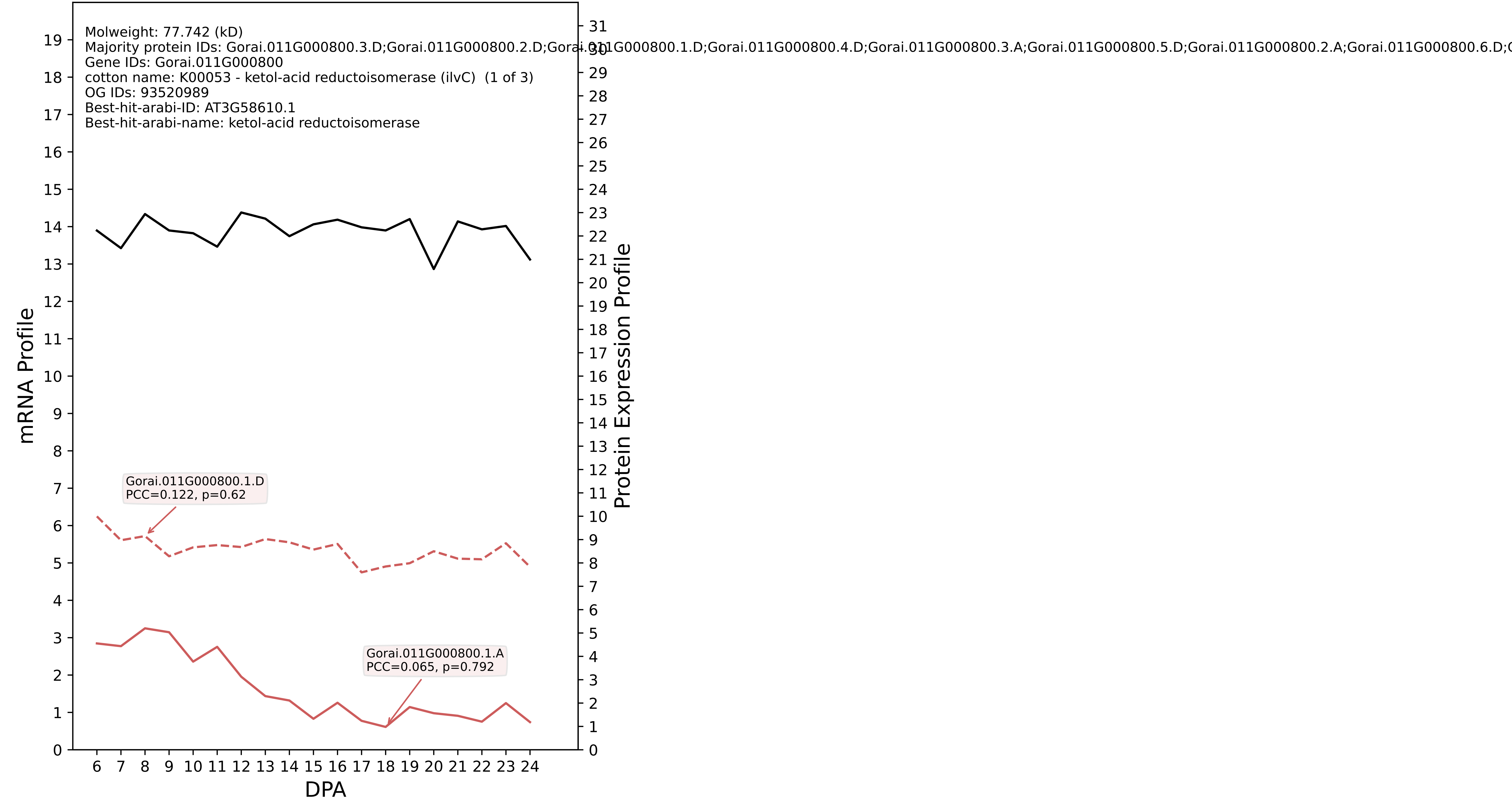

P200: Gorai.009G285900.1.D in case 2 and V13

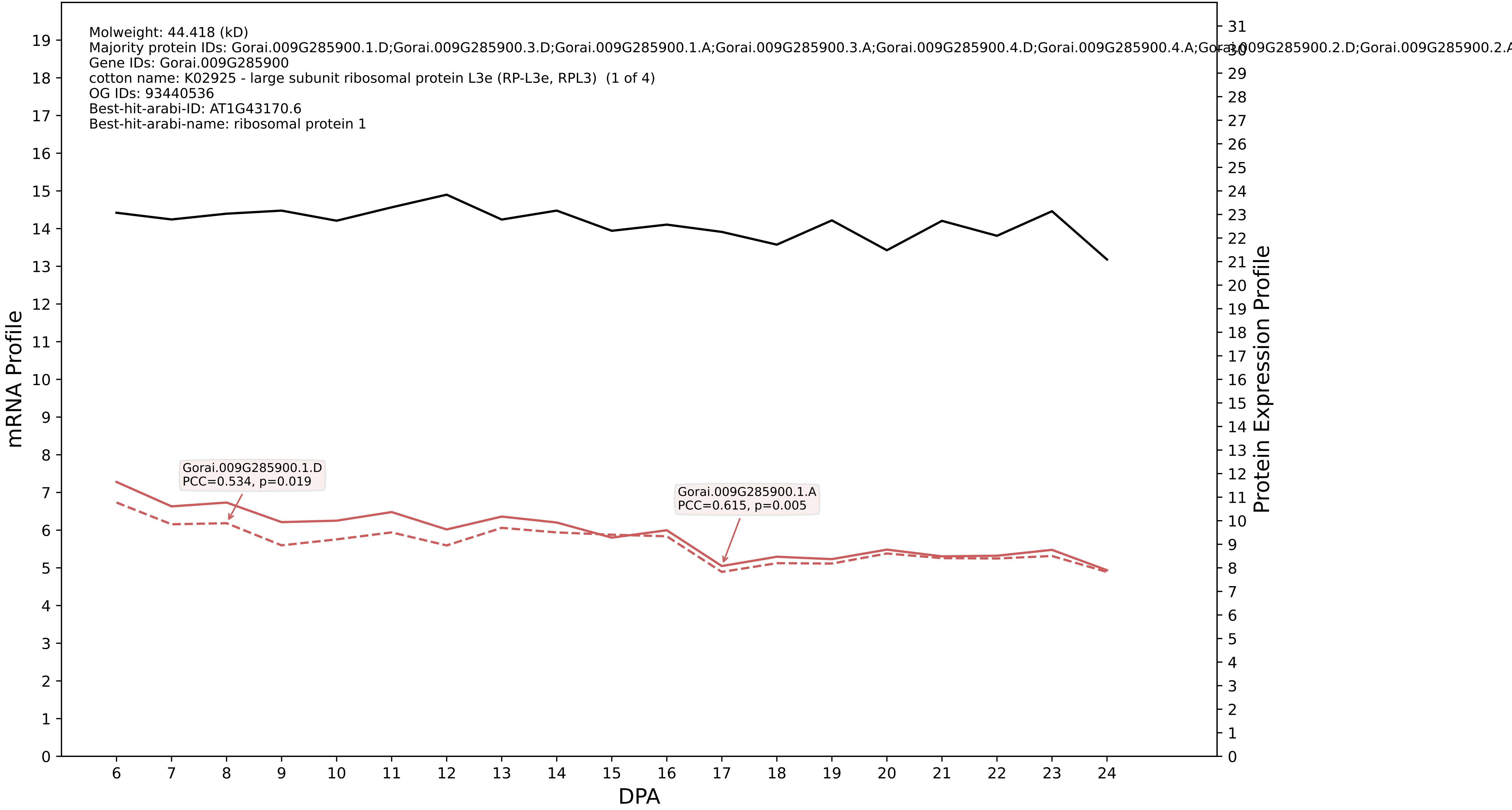

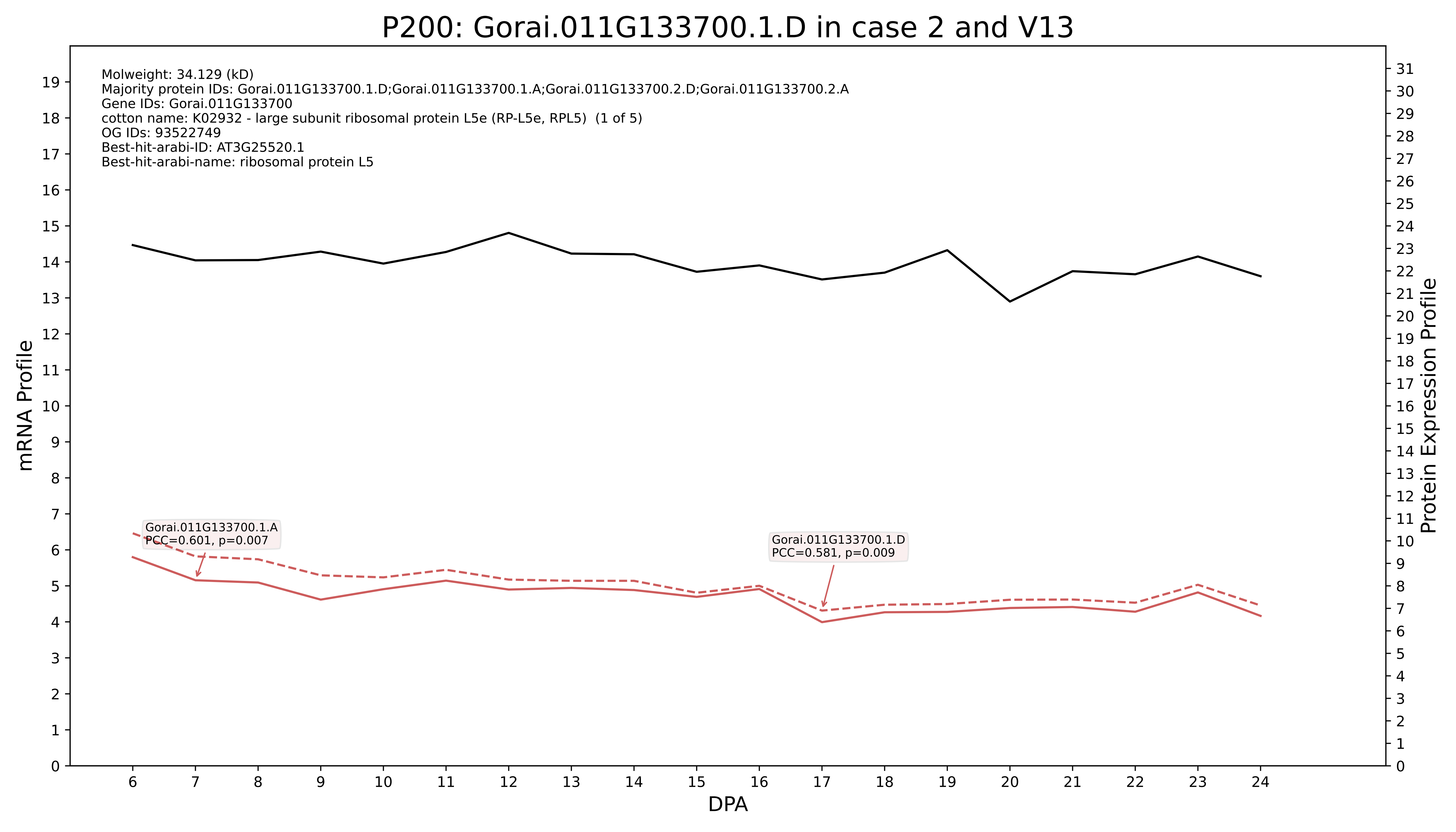
P200: Gorai.011G133700.1.D in case 2 and V13
Molweight: 34.129 (kD)
Majority protein IDs: Gorai.011G133700.1.D;Gorai.011G133700.1.A;Gorai.011G133700.2.D;Gorai.011G133700.2.A
Gene IDs: Gorai.011G133700
cotton name: K02932 - large subunit ribosomal protein L5e (RP-L5e, RPL5) (1 of 5)
OG IDs: 93522749
Best-hit-arabi-ID: AT3G25520.1
Best-hit-arabi-name: ribosomal protein L5
Gorai.011G133700.1.A
PCC=0.601, p=0.007
Gorai.011G133700.1.D
PCC=0.581, p=0.009
mRNA Profile
Protein Expression Profile
DPA
0
1
2
3
4
5
6
7
8
9
10
11
12
13
14
15
16
17
18
19
20
21
22
23
24
25
26
27
28
29
30
31

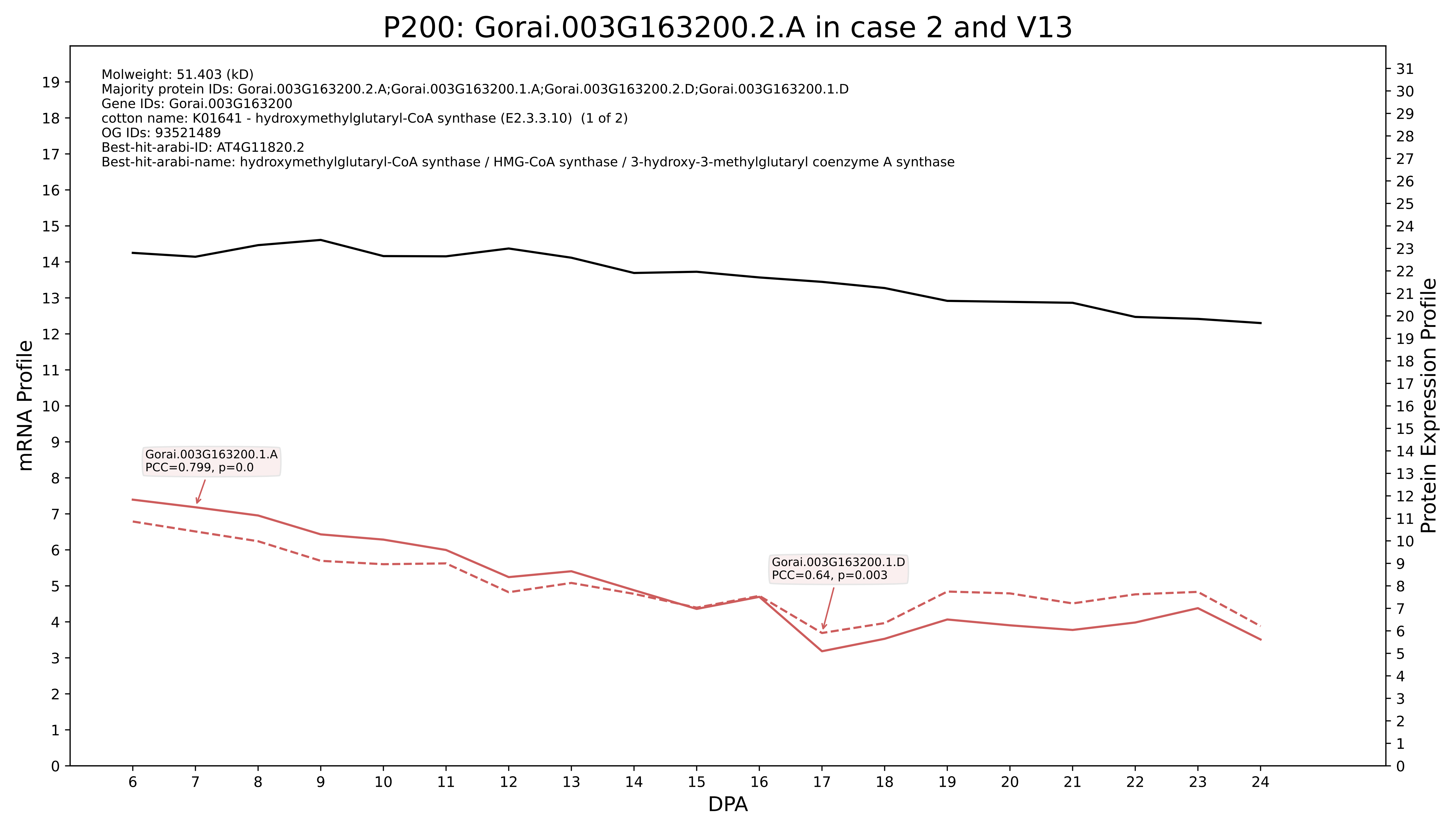

P200: Gorai.003G163200.2.A in case 2 and V13
Molweight: 51.403 (kD)
Majority protein IDs: Gorai.003G163200.2.A;Gorai.003G163200.1.A;Gorai.003G163200.2.D;Gorai.003G163200.1.D
Gene IDs: Gorai.003G163200
cotton name: K01641 - hydroxymethylglutaryl-CoA synthase (E2.3.3.10) (1 of 2)
OG IDs: 93521489
Best-hit-arabi-ID: AT4G11820.2
Best-hit-arabi-name: hydroxymethylglutaryl-CoA synthase / HMG-CoA synthase / 3-hydroxy-3-methylglutaryl coenzyme A synthase
Gorai.003G163200.1.A
PCC=0.799, p=0.0
Gorai.003G163200.1.D
PCC=0.64, p=0.003
mRNA Profile
Protein Expression Profile
DPA

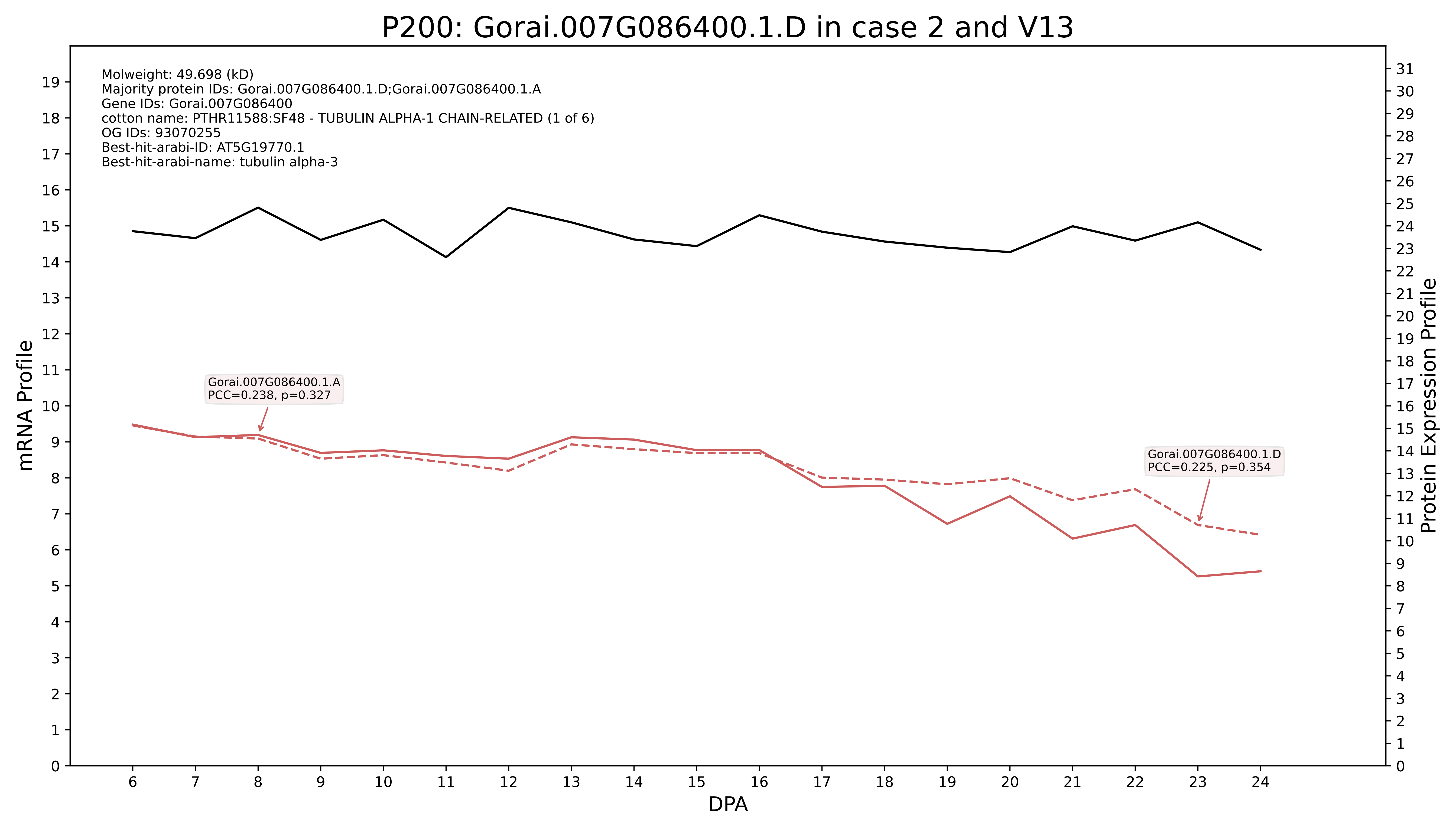
P200: Gorai.007G086400.1.D in case 2 and V13
Molweight: 49.698 (kD)
Majority protein IDs: Gorai.007G086400.1.D;Gorai.007G086400.1.A
Gene IDs: Gorai.007G086400
cotton name: PTHR11588:SF48 - TUBULIN ALPHA-1 CHAIN-RELATED (1 of 6)
OG IDs: 93070255
Best-hit-arabi-ID: AT5G19770.1
Best-hit-arabi-name: tubulin alpha-3
Gorai.007G086400.1.A
PCC=0.238, p=0.327
Gorai.007G086400.1.D
PCC=0.225, p=0.354
mRNA Profile
Protein Expression Profile
DPA

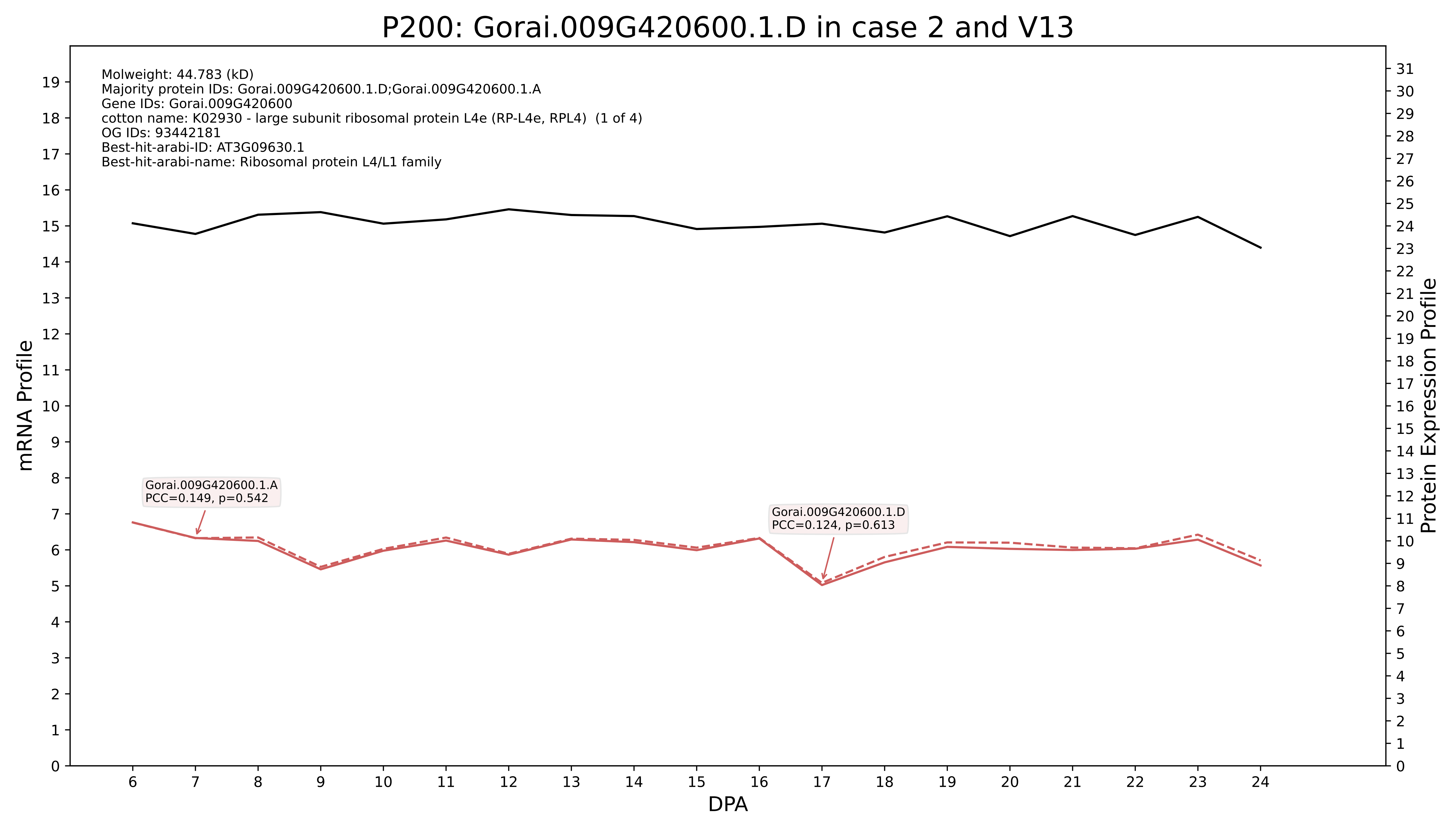
P200: Gorai.009G420600.1.D in case 2 and V13
Molweight: 44.783 (kD)
Majority protein IDs: Gorai.009G420600.1.D;Gorai.009G420600.1.A
Gene IDs: Gorai.009G420600
cotton name: K02930 - large subunit ribosomal protein L4e (RP-L4e, RPL4) (1 of 4)
OG IDs: 93442181
Best-hit-arabi-ID: AT3G09630.1
Best-hit-arabi-name: Ribosomal protein L4/L1 family
Gorai.009G420600.1.A
PCC=0.149, p=0.542
Gorai.009G420600.1.D
PCC=0.124, p=0.613
mRNA Profile
Protein Expression Profile
DPA

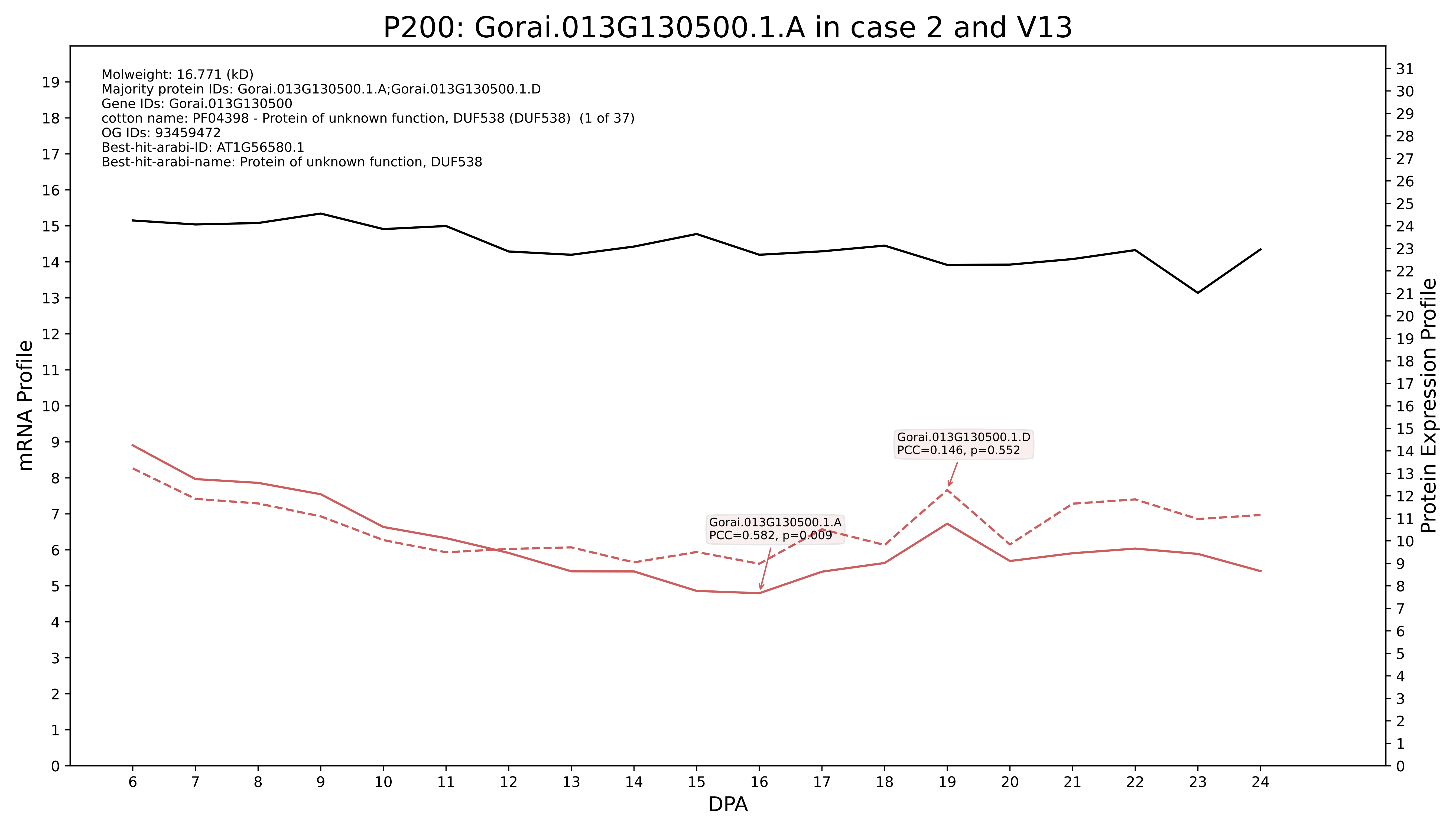

P200: Gorai.013G130500.1.A in case 2 and V13
Molweight: 16.771 (kD)
Majority protein IDs: Gorai.013G130500.1.A;Gorai.013G130500.1.D
Gene IDs: Gorai.013G130500
cotton name: PF04398 - Protein of unknown function, DUF538 (DUF538) (1 of 37)
OG IDs: 93459472
Best-hit-arabi-ID: AT1G56580.1
Best-hit-arabi-name: Protein of unknown function, DUF538
Gorai.013G130500.1.D
PCC=0.146, p=0.552
Gorai.013G130500.1.A
PCC=0.582, p=0.009
mRNA Profile
Protein Expression Profile
DPA

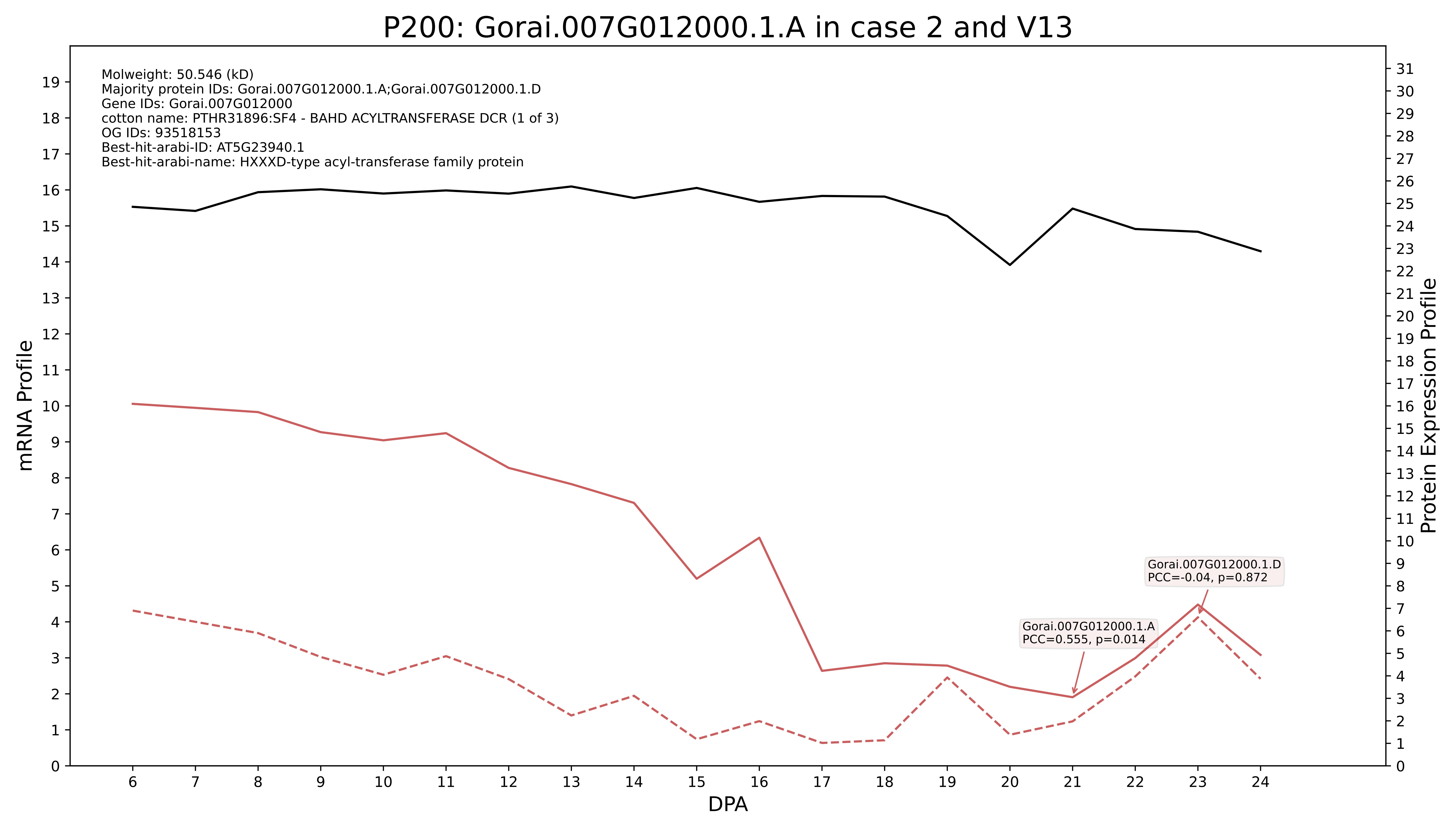
P200: Gorai.007G012000.1.A in case 2 and V13
Molweight: 50.546 (kD)
Majority protein IDs: Gorai.007G012000.1.A;Gorai.007G012000.1.D
Gene IDs: Gorai.007G012000
cotton name: PTHR31896:SF4 - BAHD ACYLTRANSFERASE DCR (1 of 3)
OG IDs: 93518153
Best-hit-arabi-ID: AT5G23940.1
Best-hit-arabi-name: HXXXD-type acyl-transferase family protein
Gorai.007G012000.1.D
PCC=-0.04, p=0.872
Gorai.007G012000.1.A
PCC=0.555, p=0.014
mRNA Profile
Protein Expression Profile
DPA

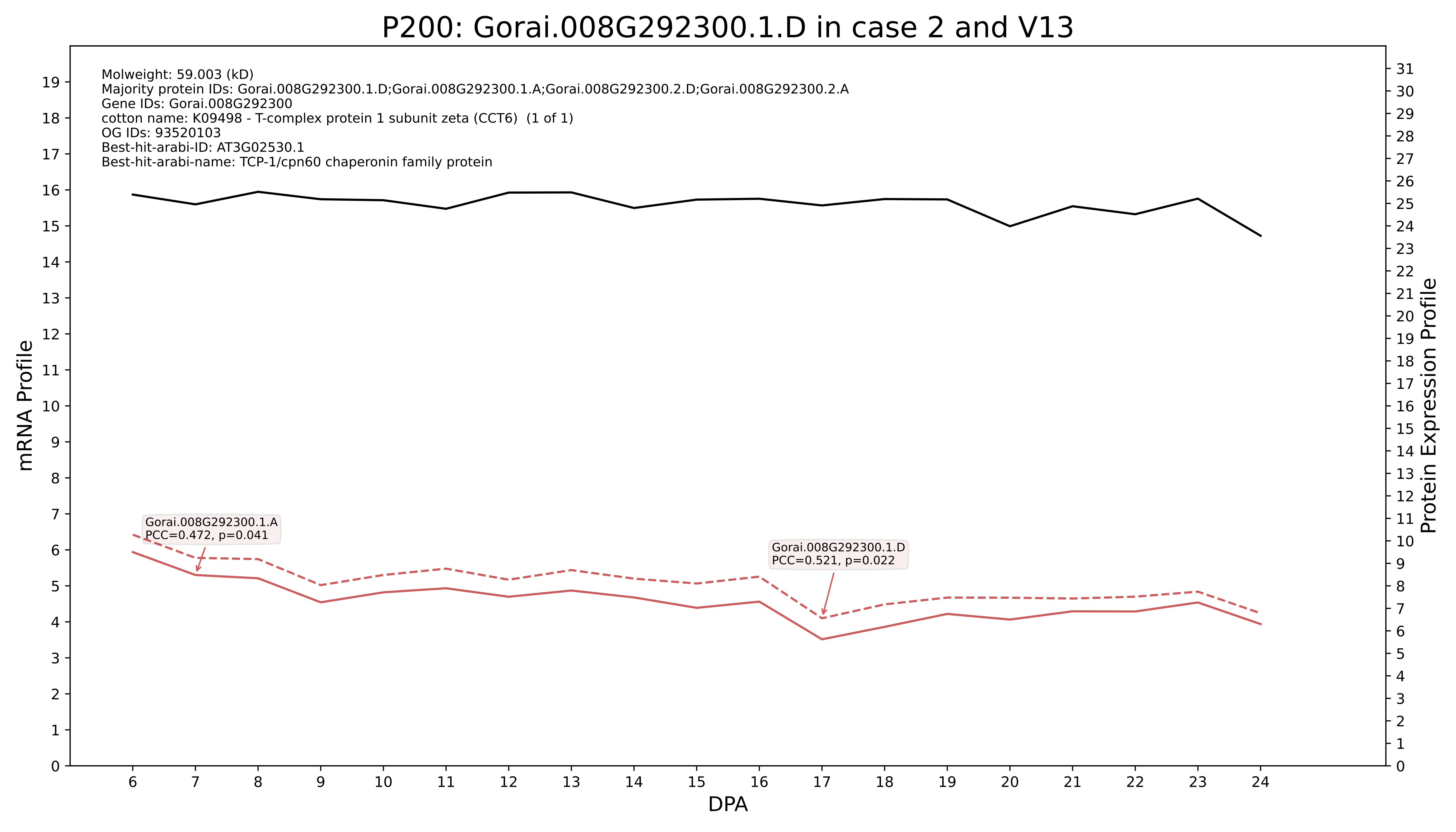
P200: Gorai.008G292300.1.D in case 2 and V13
Molweight: 59.003 (kD)
Majority protein IDs: Gorai.008G292300.1.D;Gorai.008G292300.1.A;Gorai.008G292300.2.D;Gorai.008G292300.2.A
Gene IDs: Gorai.008G292300
cotton name: K09498 - T-complex protein 1 subunit zeta (CCT6) (1 of 1)
OG IDs: 93520103
Best-hit-arabi-ID: AT3G02530.1
Best-hit-arabi-name: TCP-1/cpn60 chaperonin family protein
Gorai.008G292300.1.A
PCC=0.472, p=0.041
Gorai.008G292300.1.D
PCC=0.521, p=0.022
mRNA Profile
Protein Expression Profile
DPA
0
1
2
3
4
5
6
7
8
9
10
11
12
13
14
15
16
17
18
19
20
21
22
23
24
25
26
27
28
29
30
31

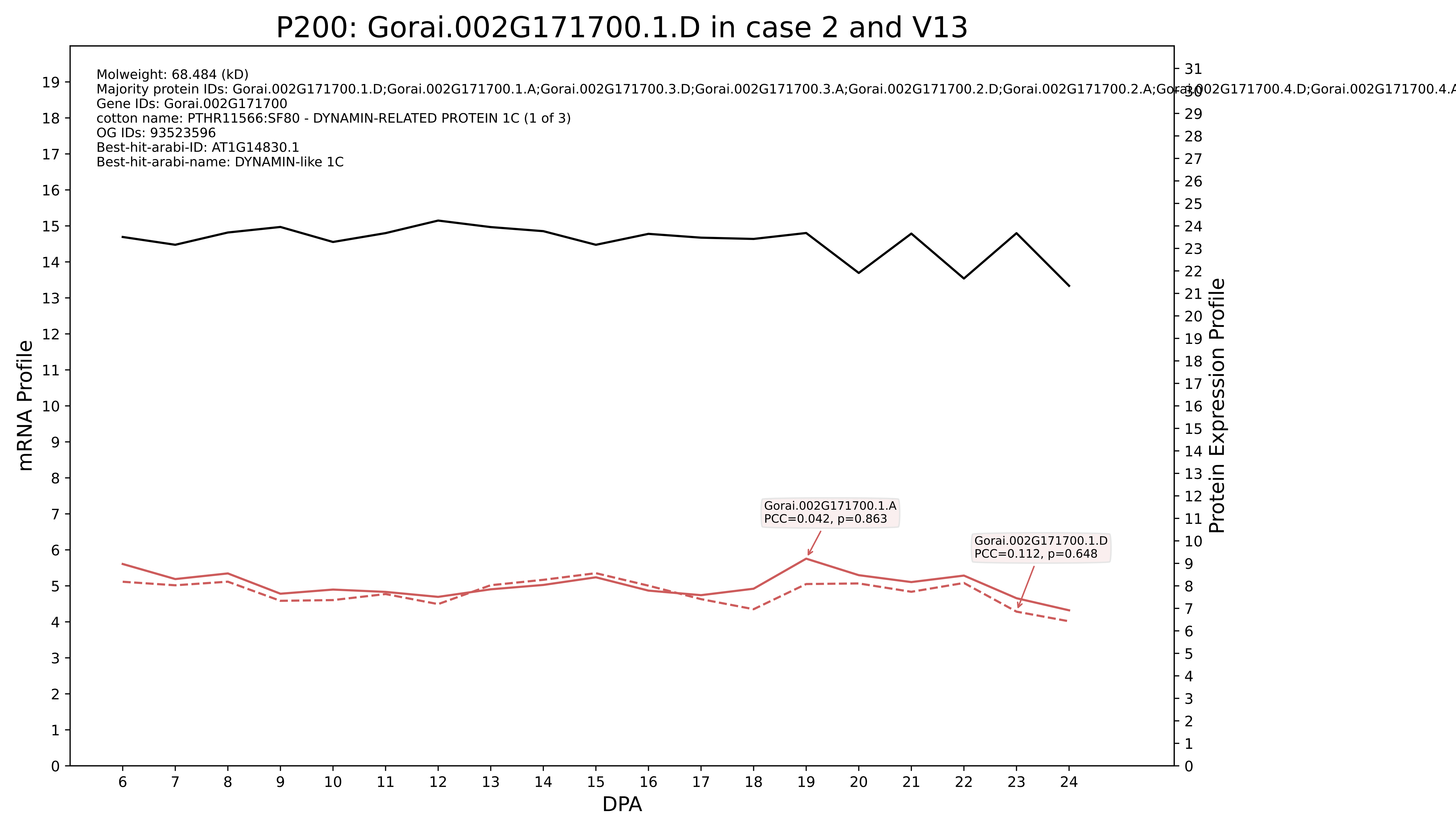
P200: Gorai.002G171700.1.D in case 2 and V13
Molweight: 68.484 (kD)
Majority protein IDs: Gorai.002G171700.1.D;Gorai.002G171700.1.A;Gorai.002G171700.3.D;Gorai.002G171700.3.A;Gorai.002G171700.2.D;Gorai.002G171700.2.A;Gorai.002G171700.4.D;Gorai.002G171700.4.A
Gene IDs: Gorai.002G171700
cotton name: PTHR11566:SF80 - DYNAMIN-RELATED PROTEIN 1C (1 of 3)
OG IDs: 93523596
Best-hit-arabi-ID: AT1G14830.1
Best-hit-arabi-name: DYNAMIN-like 1C
Gorai.002G171700.1.A
PCC=0.042, p=0.863
Gorai.002G171700.1.D
PCC=0.112, p=0.648
mRNA Profile
Protein Expression Profile
DPA

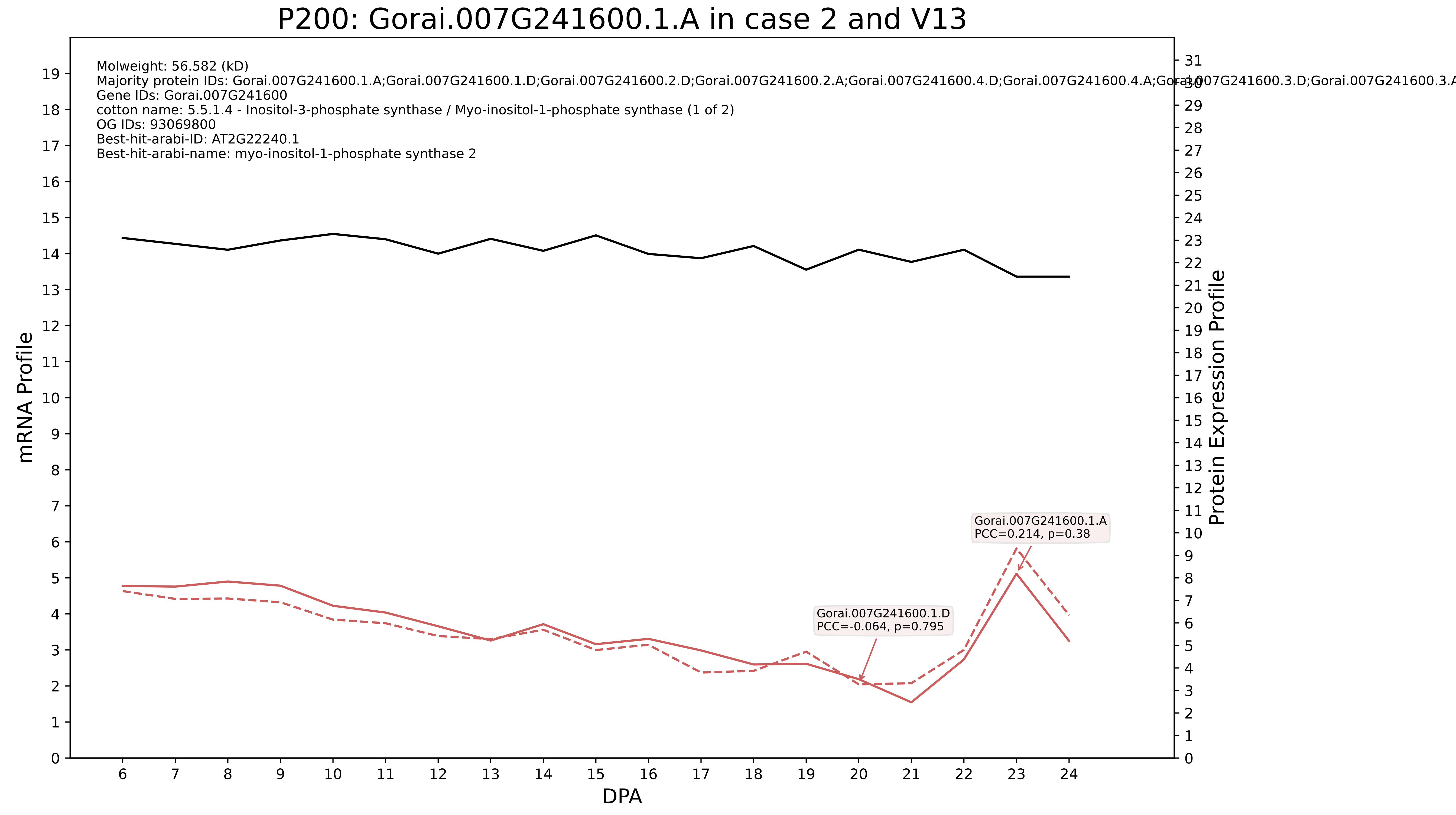
P200: Gorai.007G241600.1.A in case 2 and V13
Molweight: 56.582 (kD)
Majority protein IDs: Gorai.007G241600.1.A;Gorai.007G241600.1.D;Gorai.007G241600.2.D;Gorai.007G241600.2.A;Gorai.007G241600.4.D;Gorai.007G241600.4.A;Gorai.007G241600.3.D;Gorai.007G241600.3.A
Gene IDs: Gorai.007G241600
cotton name: 5.5.1.4 - Inositol-3-phosphate synthase / Myo-inositol-1-phosphate synthase (1 of 2)
OG IDs: 93069800
Best-hit-arabi-ID: AT2G22240.1
Best-hit-arabi-name: myo-inositol-1-phosphate synthase 2
Gorai.007G241600.1.A
PCC=0.214, p=0.38
Gorai.007G241600.1.D
PCC=-0.064, p=0.795
mRNA Profile
Protein Expression Profile
DPA

P200: Gorai.001G128200.1.D in case 2 and V13

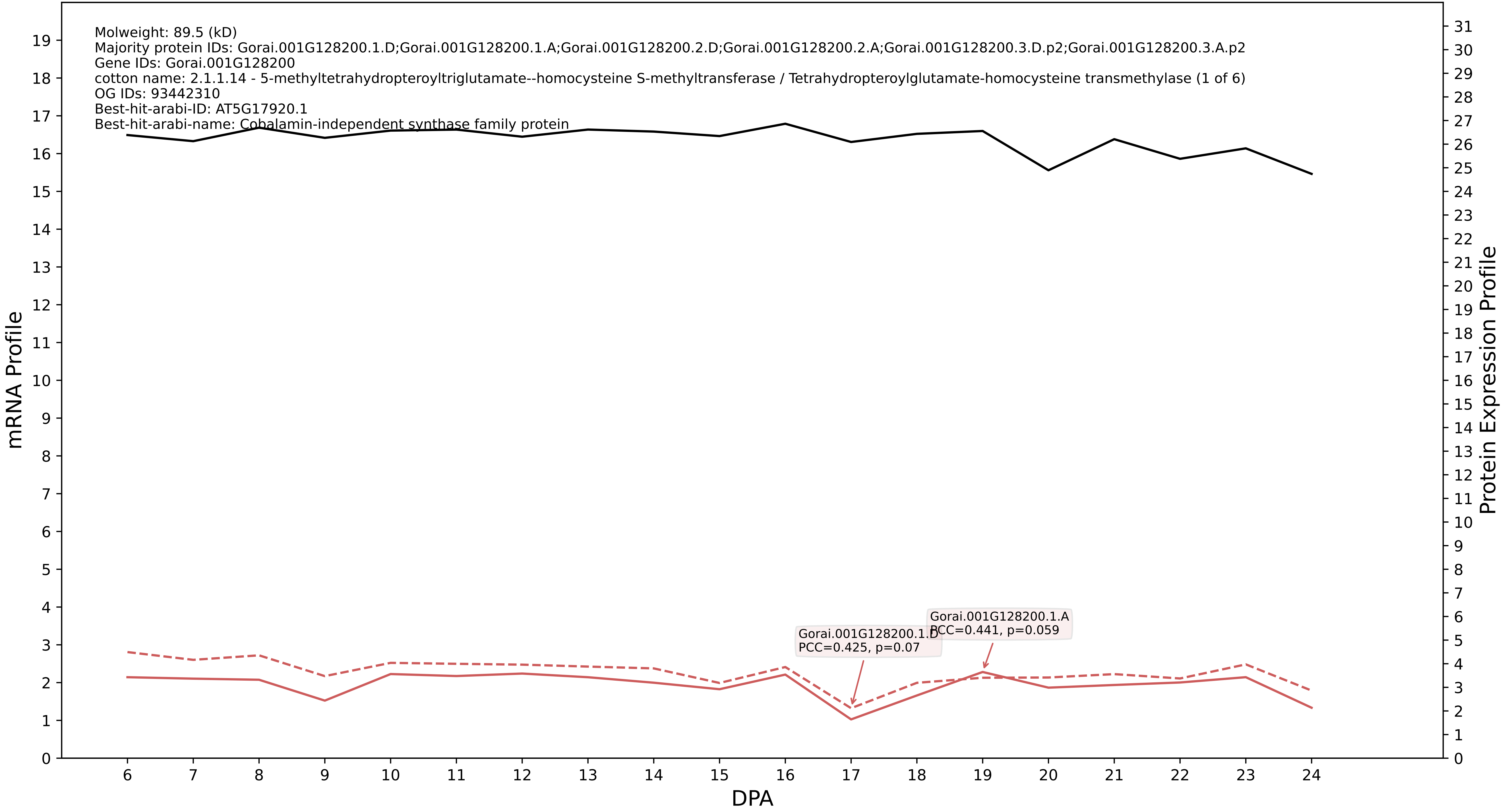

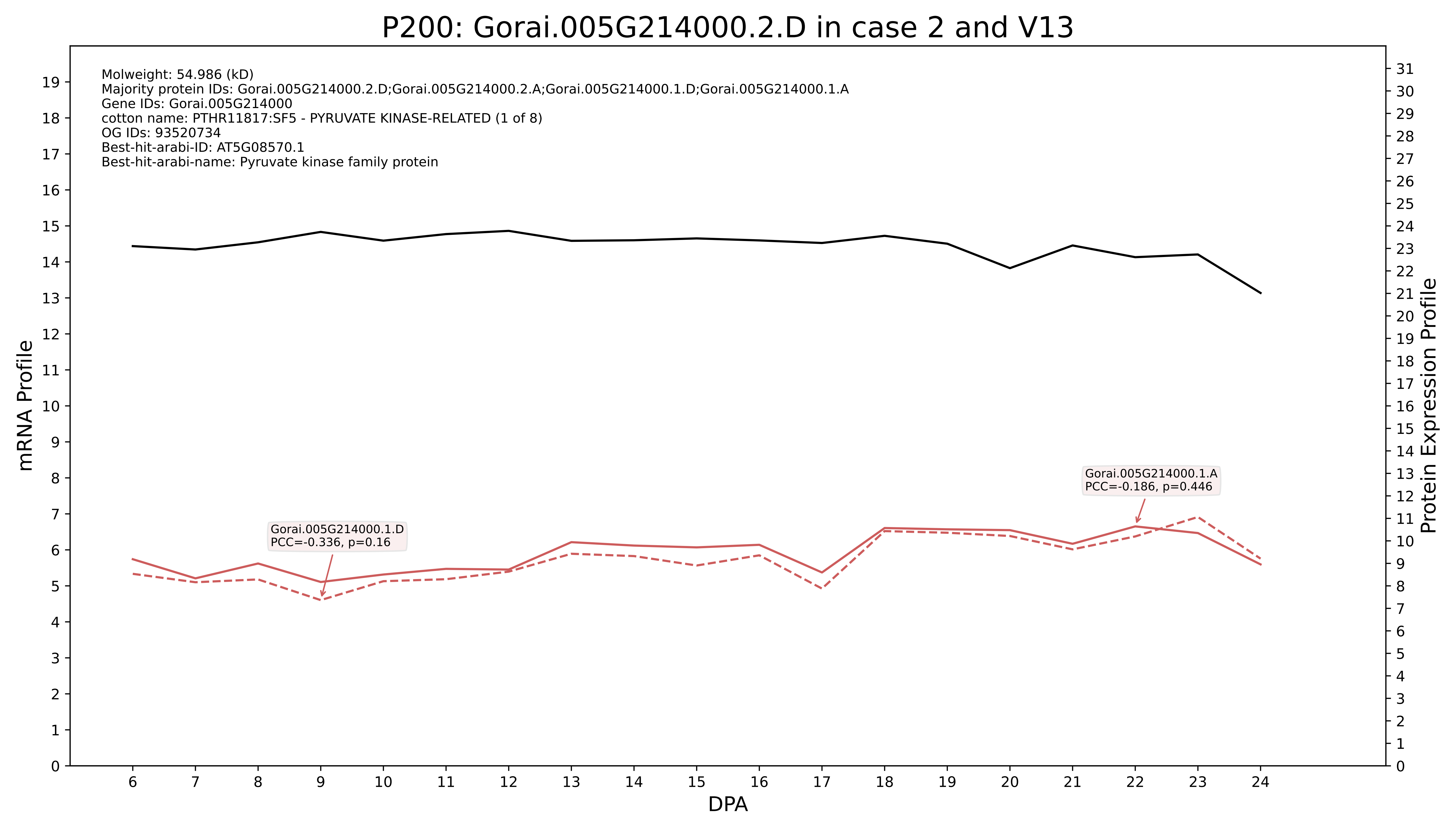

P200: Gorai.005G214000.2.D in case 2 and V13
Molweight: 54.986 (kD)
Majority protein IDs: Gorai.005G214000.2.D;Gorai.005G214000.2.A;Gorai.005G214000.1.D;Gorai.005G214000.1.A
Gene IDs: Gorai.005G214000
cotton name: PTHR11817:SF5 - PYRUVATE KINASE-RELATED (1 of 8)
OG IDs: 93520734
Best-hit-arabi-ID: AT5G08570.1
Best-hit-arabi-name: Pyruvate kinase family protein
Gorai.005G214000.1.D
PCC=-0.336, p=0.16
Gorai.005G214000.1.A
PCC=-0.186, p=0.446
mRNA Profile
Protein Expression Profile
DPA

012G055000.1.D in case 3 and V13

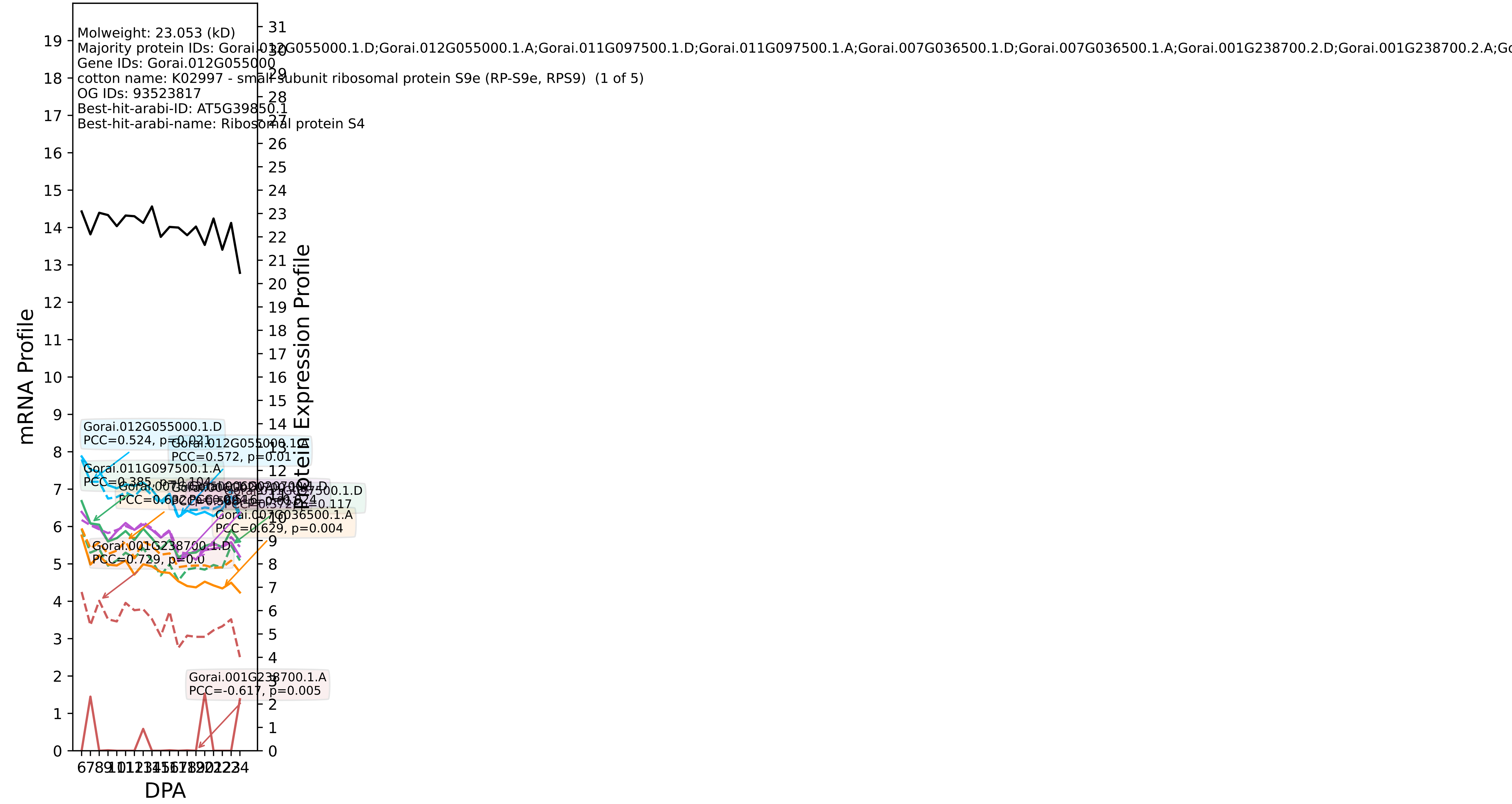

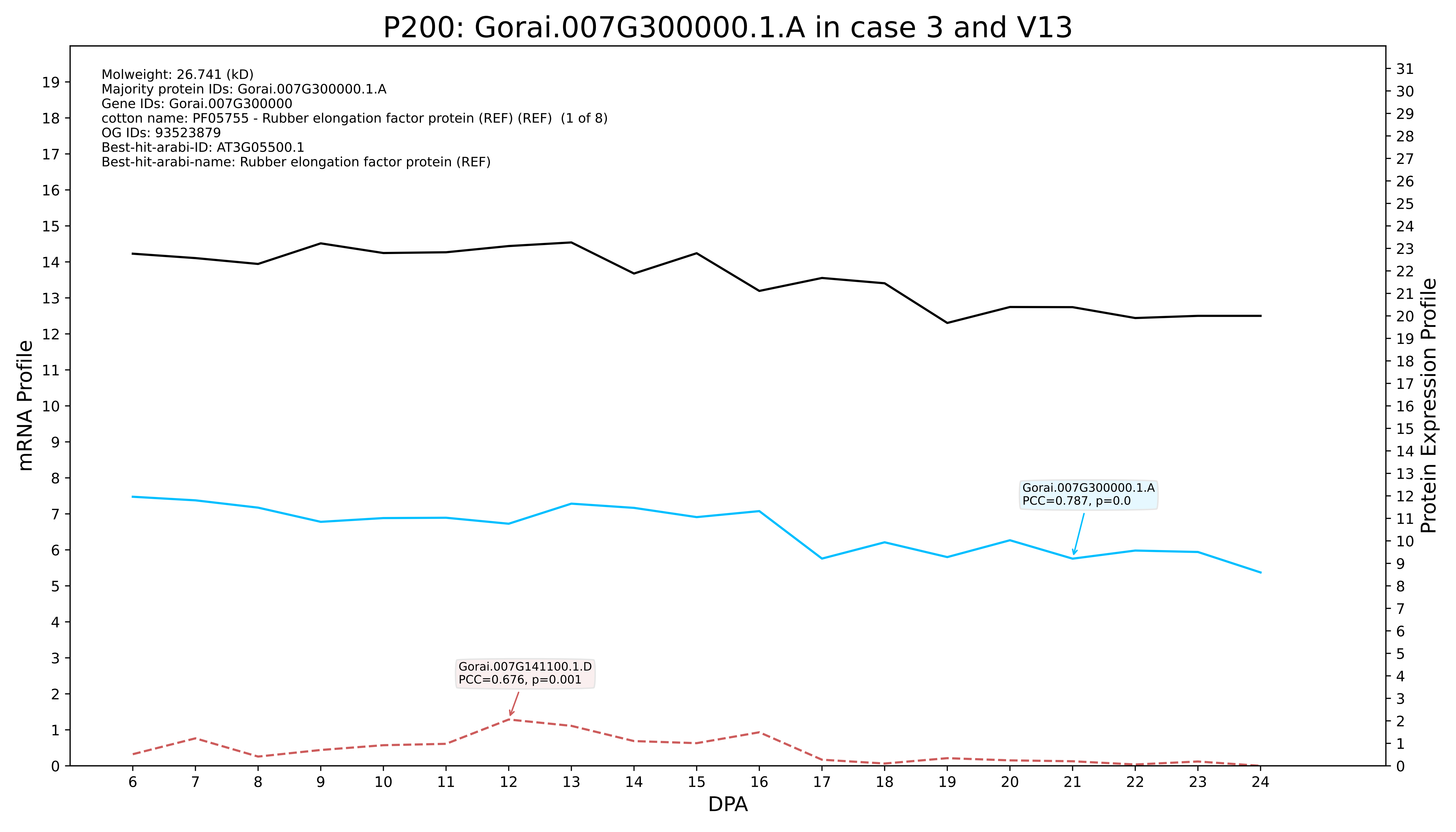
P200: Gorai.007G300000.1.A in case 3 and V13
Molweight: 26.741 (kD)
Majority protein IDs: Gorai.007G300000.1.A
Gene IDs: Gorai.007G300000
cotton name: PF05755 - Rubber elongation factor protein (REF) (REF) (1 of 8)
OG IDs: 93523879
Best-hit-arabi-ID: AT3G05500.1
Best-hit-arabi-name: Rubber elongation factor protein (REF)
Gorai.007G300000.1.A
PCC=0.787, p=0.0
Gorai.007G141100.1.D
PCC=0.676, p=0.001
mRNA Profile
Protein Expression Profile
DPA

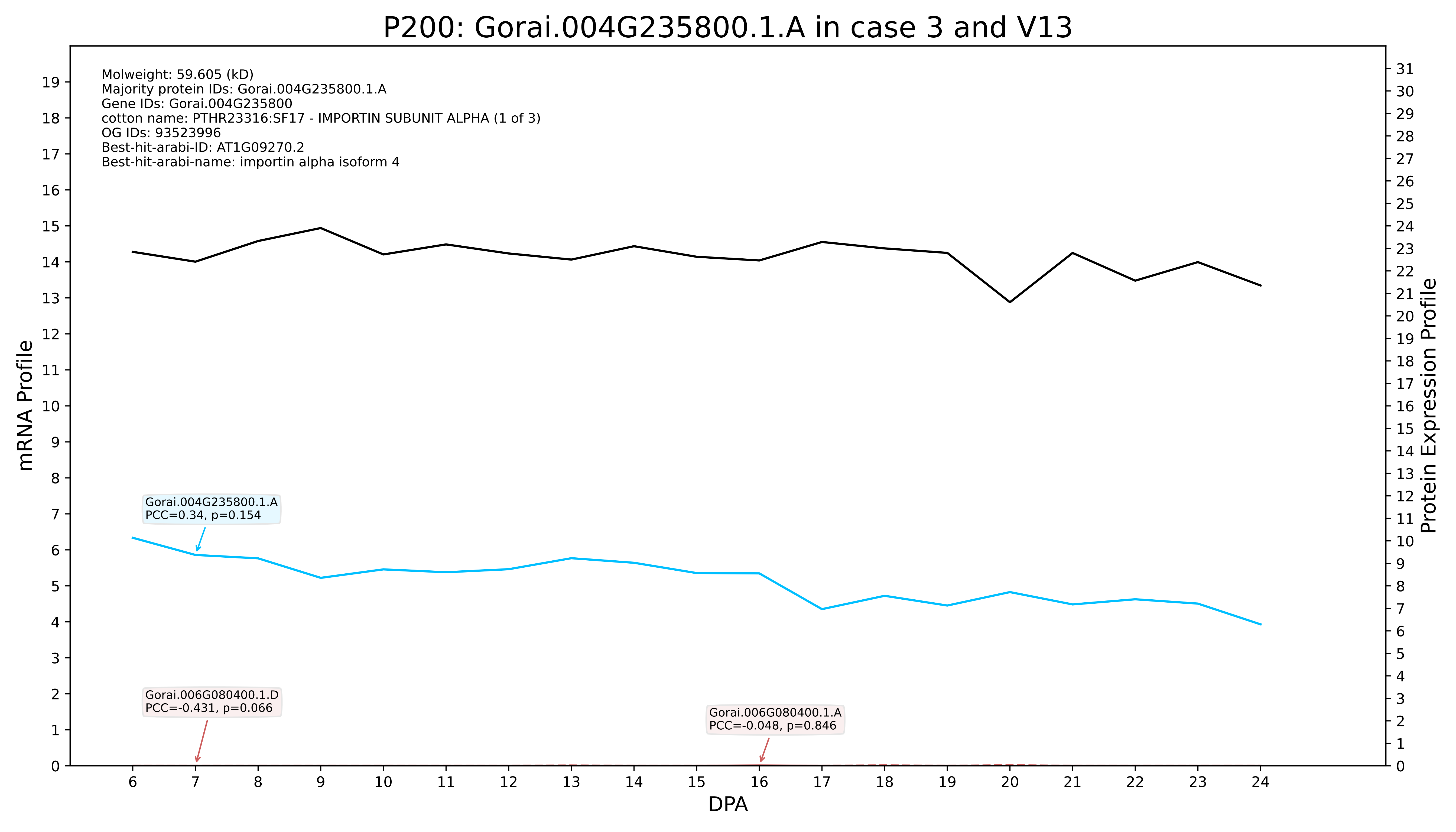
P200: Gorai.004G235800.1.A in case 3 and V13
Molweight: 59.605 (kD)
Majority protein IDs: Gorai.004G235800.1.A
Gene IDs: Gorai.004G235800
cotton name: PTHR23316:SF17 - IMPORTIN SUBUNIT ALPHA (1 of 3)
OG IDs: 93523996
Best-hit-arabi-ID: AT1G09270.2
Best-hit-arabi-name: importin alpha isoform 4
Gorai.004G235800.1.A
PCC=0.34, p=0.154
Gorai.006G080400.1.D
PCC=-0.431, p=0.066
Gorai.006G080400.1.A
PCC=-0.048, p=0.846
mRNA Profile
Protein Expression Profile
DPA

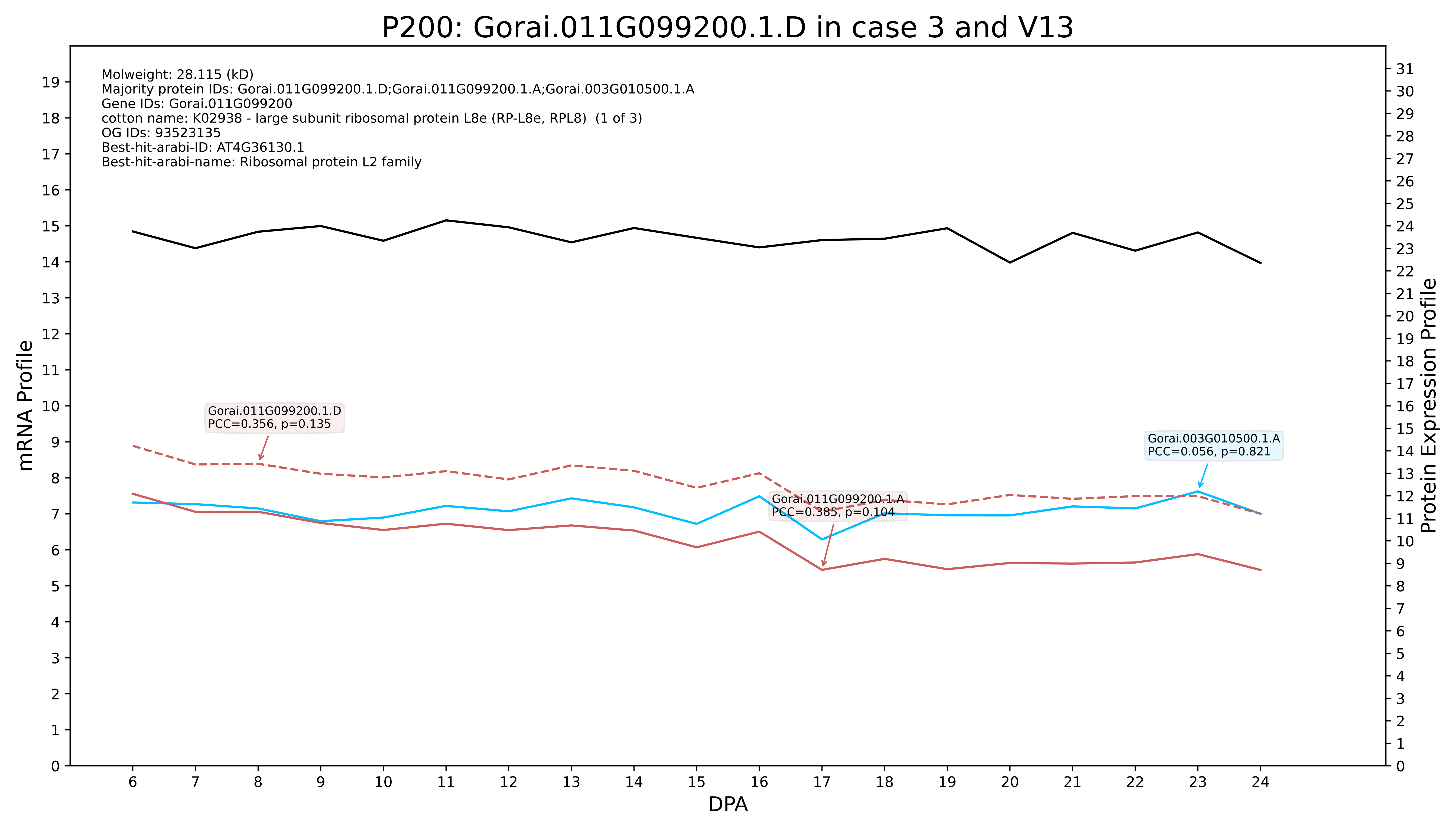
P200: Gorai.011G099200.1.D in case 3 and V13
Molweight: 28.115 (kD)
Majority protein IDs: Gorai.011G099200.1.D;Gorai.011G099200.1.A;Gorai.003G010500.1.A
Gene IDs: Gorai.011G099200
cotton name: K02938 - large subunit ribosomal protein L8e (RP-L8e, RPL8) (1 of 3)
OG IDs: 93523135
Best-hit-arabi-ID: AT4G36130.1
Best-hit-arabi-name: Ribosomal protein L2 family
Gorai.011G099200.1.D
PCC=0.356, p=0.135
Gorai.011G099200.1.A
PCC=0.385, p=0.104
Gorai.003G010500.1.A
PCC=0.056, p=0.821
mRNA Profile
Protein Expression Profile
DPA

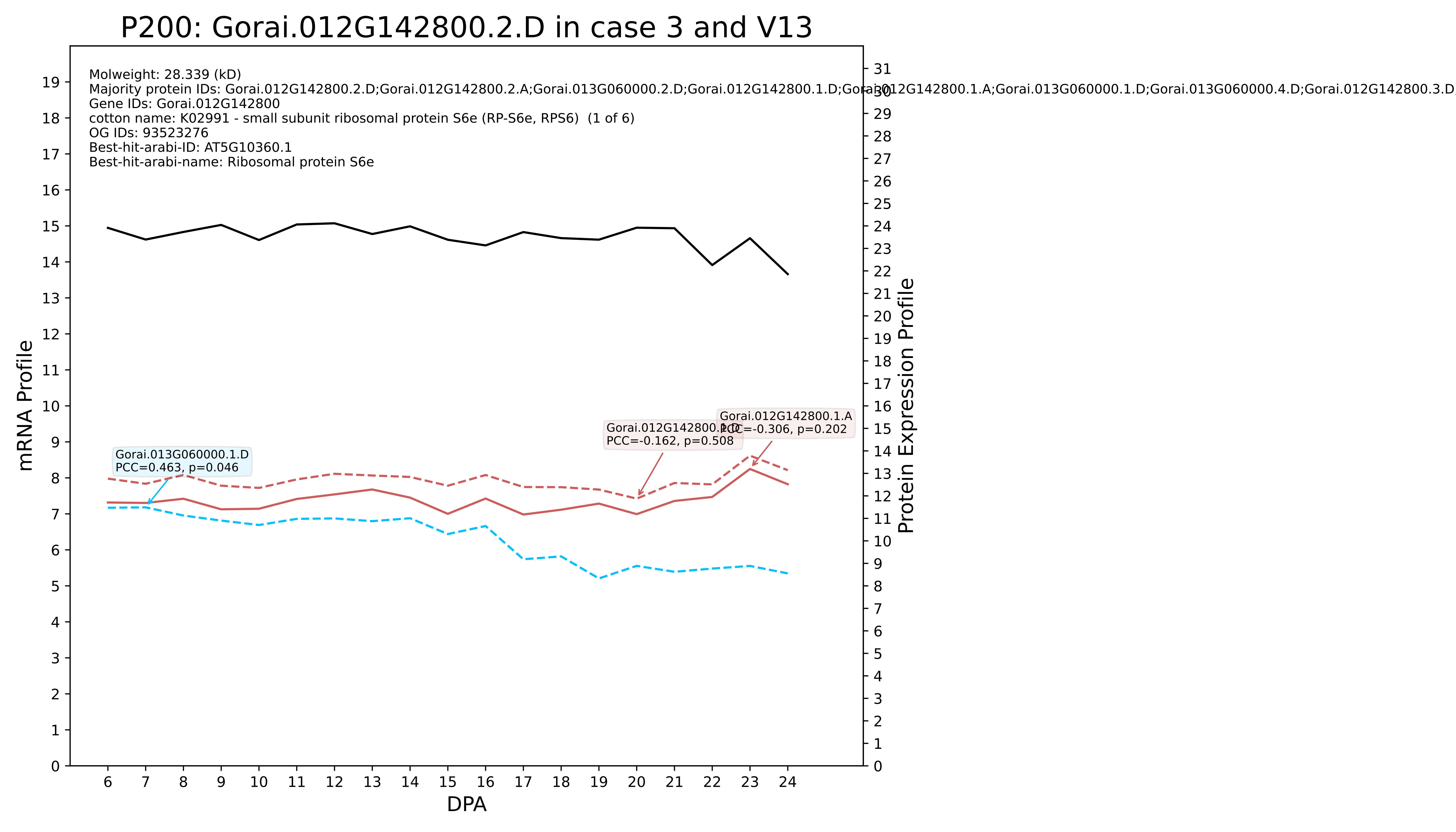
P200: Gorai.012G142800.2.D in case 3 and V13
Molweight: 28.339 (kD)
Majority protein IDs: Gorai.012G142800.2.D;Gorai.012G142800.2.A;Gorai.013G060000.2.D;Gorai.012G142800.1.D;Gorai.012G142800.1.A;Gorai.013G060000.1.D;Gorai.013G060000.4.D;Gorai.012G142800.3.D
Gene IDs: Gorai.012G142800
cotton name: K02991 - small subunit ribosomal protein S6e (RP-S6e, RPS6) (1 of 6)
OG IDs: 93523276
Best-hit-arabi-ID: AT5G10360.1
Best-hit-arabi-name: Ribosomal protein S6e
Gorai.013G060000.1.D
PCC=0.463, p=0.046
Gorai.012G142800.1.D
PCC=-0.162, p=0.508
Gorai.012G142800.1.A
PCC=-0.306, p=0.202
mRNA Profile
Protein Expression Profile
DPA

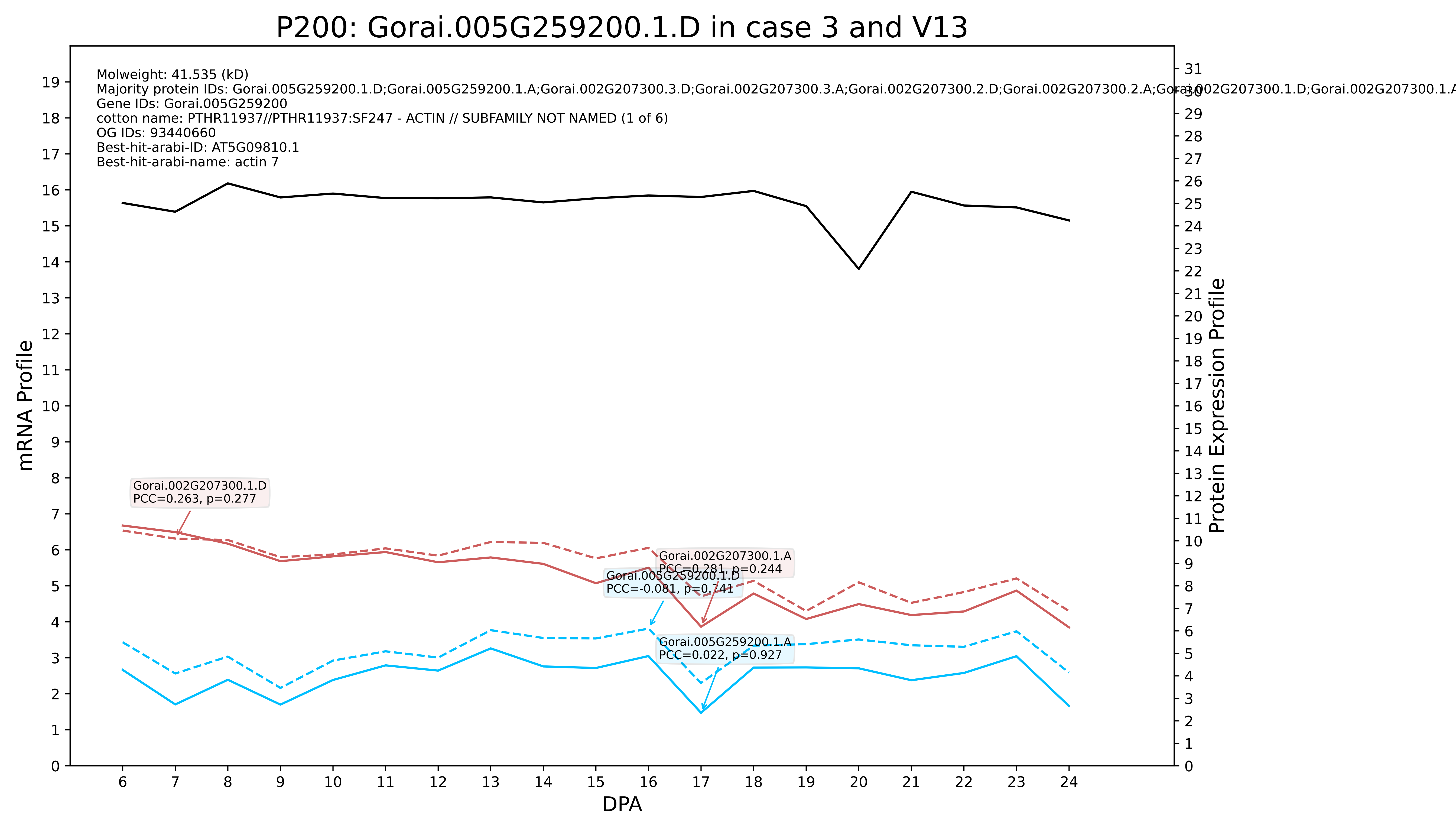

P200: Gorai.005G259200.1.D in case 3 and V13
Molweight: 41.535 (kD)
Majority protein IDs: Gorai.005G259200.1.D;Gorai.005G259200.1.A;Gorai.002G207300.3.D;Gorai.002G207300.3.A;Gorai.002G207300.2.D;Gorai.002G207300.2.A;Gorai.002G207300.1.D;Gorai.002G207300.1.A
Gene IDs: Gorai.005G259200
cotton name: PTHR11937//PTHR11937:SF247 - ACTIN // SUBFAMILY NOT NAMED (1 of 6)
OG IDs: 93440660
Best-hit-arabi-ID: AT5G09810.1
Best-hit-arabi-name: actin 7
Gorai.002G207300.1.D
PCC=0.263, p=0.277
Gorai.002G207300.1.A
PCC=0.281, p=0.244
Gorai.005G259200.1.D
PCC=-0.081, p=0.741
Gorai.005G259200.1.A
PCC=0.022, p=0.927
mRNA Profile
Protein Expression Profile
DPA

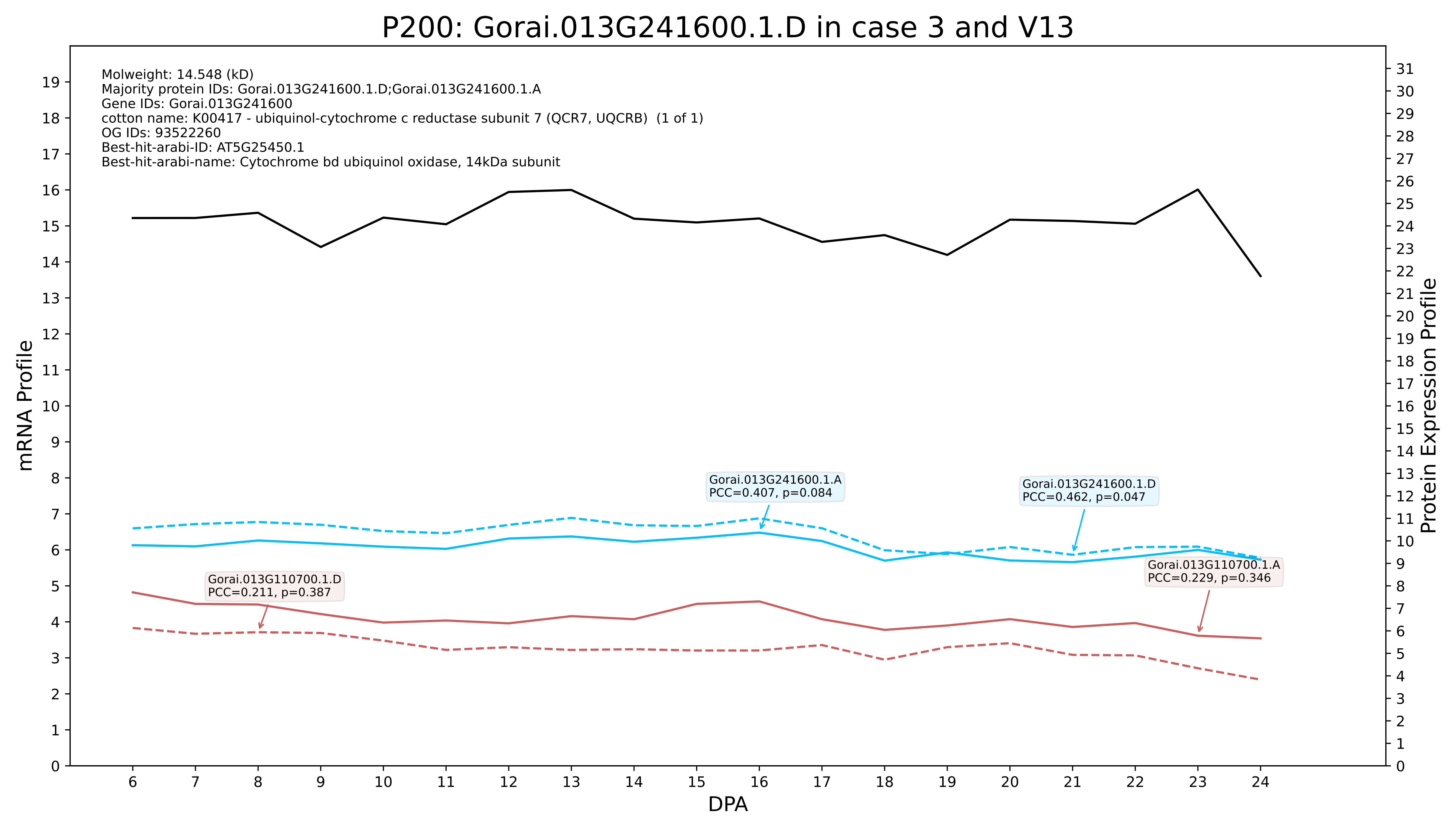

P200: Gorai.013G241600.1.D in case 3 and V13
Molweight: 14.548 (kD)
Majority protein IDs: Gorai.013G241600.1.D;Gorai.013G241600.1.A
Gene IDs: Gorai.013G241600
cotton name: K00417 - ubiquinol-cytochrome c reductase subunit 7 (QCR7, UQCRB) (1 of 1)
OG IDs: 93522260
Best-hit-arabi-ID: AT5G25450.1
Best-hit-arabi-name: Cytochrome bd ubiquinol oxidase, 14kDa subunit
Gorai.013G241600.1.A
PCC=0.407, p=0.084
Gorai.013G241600.1.D
PCC=0.462, p=0.047
Gorai.013G110700.1.D
PCC=0.211, p=0.387
Gorai.013G110700.1.A
PCC=0.229, p=0.346
mRNA Profile
Protein Expression Profile
DPA

P200: Gorai.006G144300.1.D in case 3 and V13

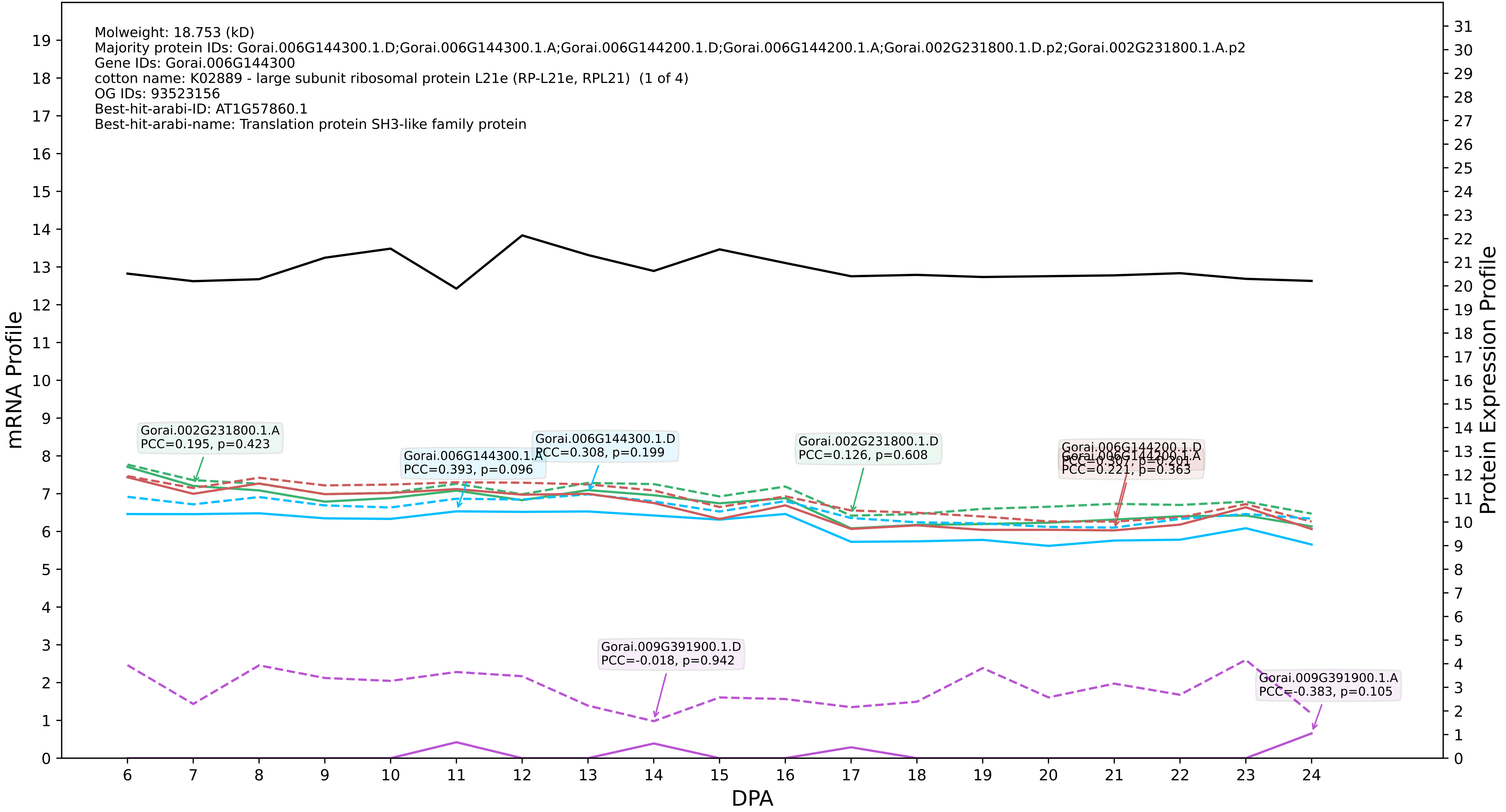

P200: Gorai.010G236400.1.D in case 3 and V13

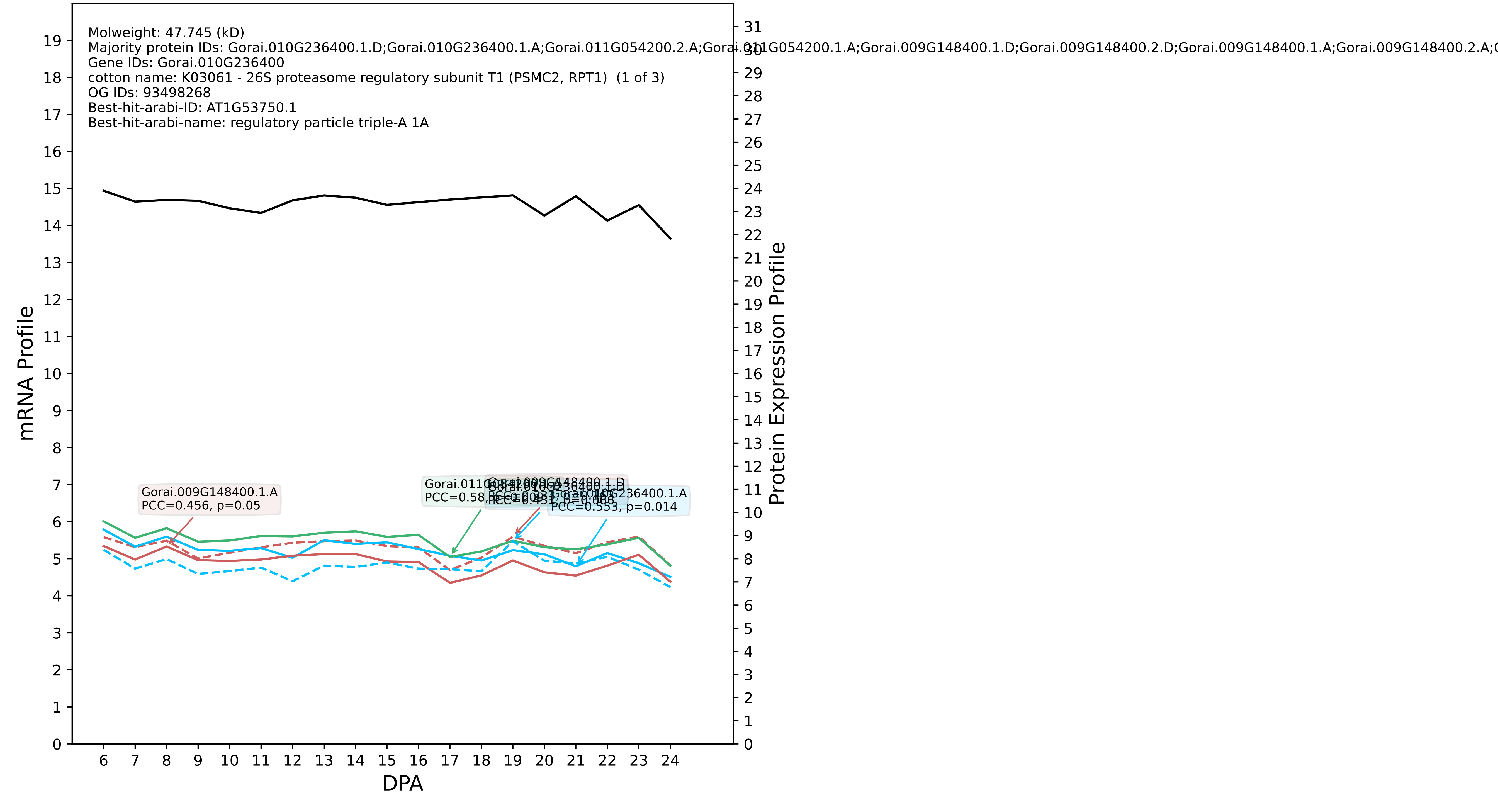

P200: Gorai.012G174700.6.D in case 1 and V14

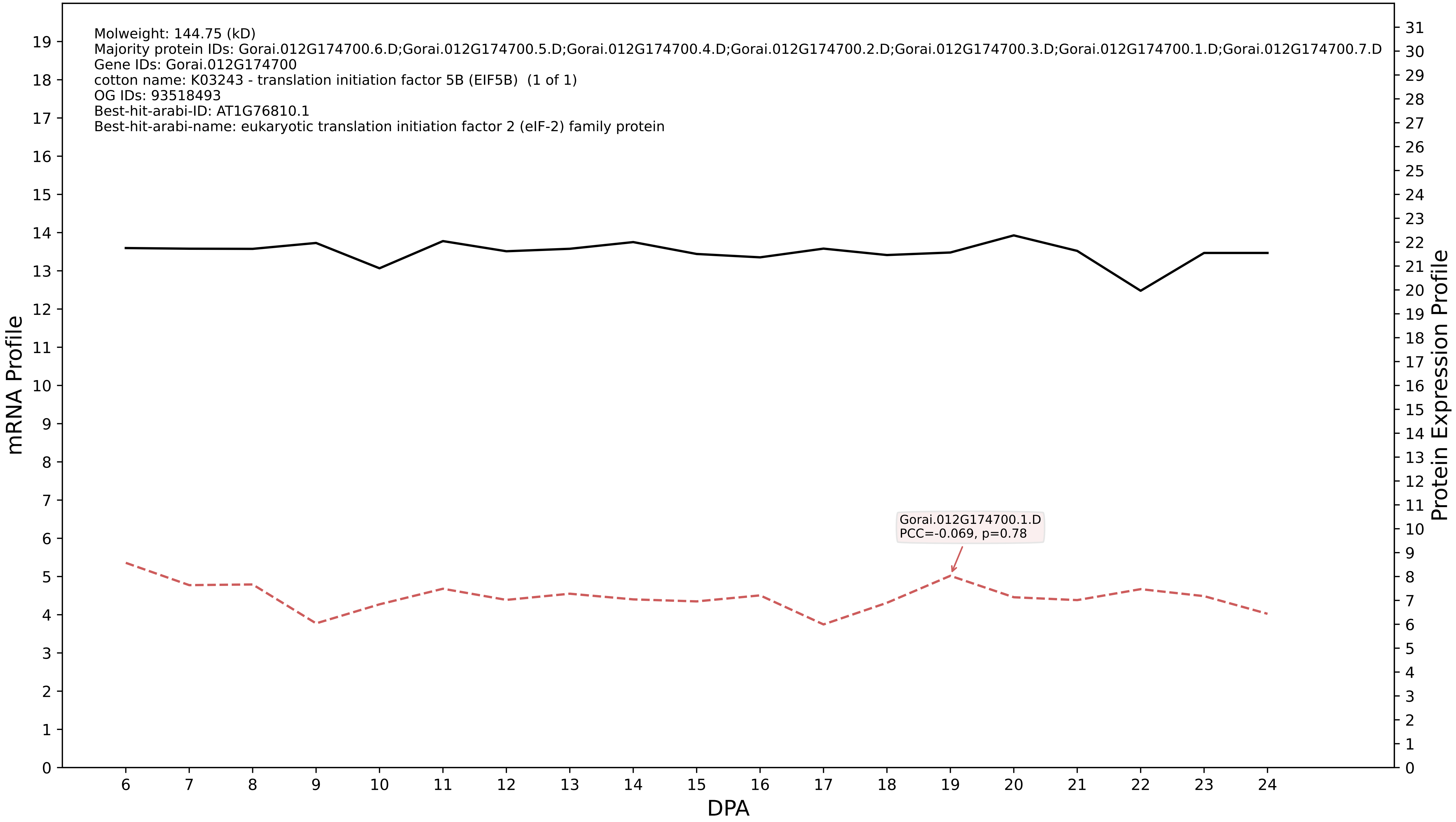

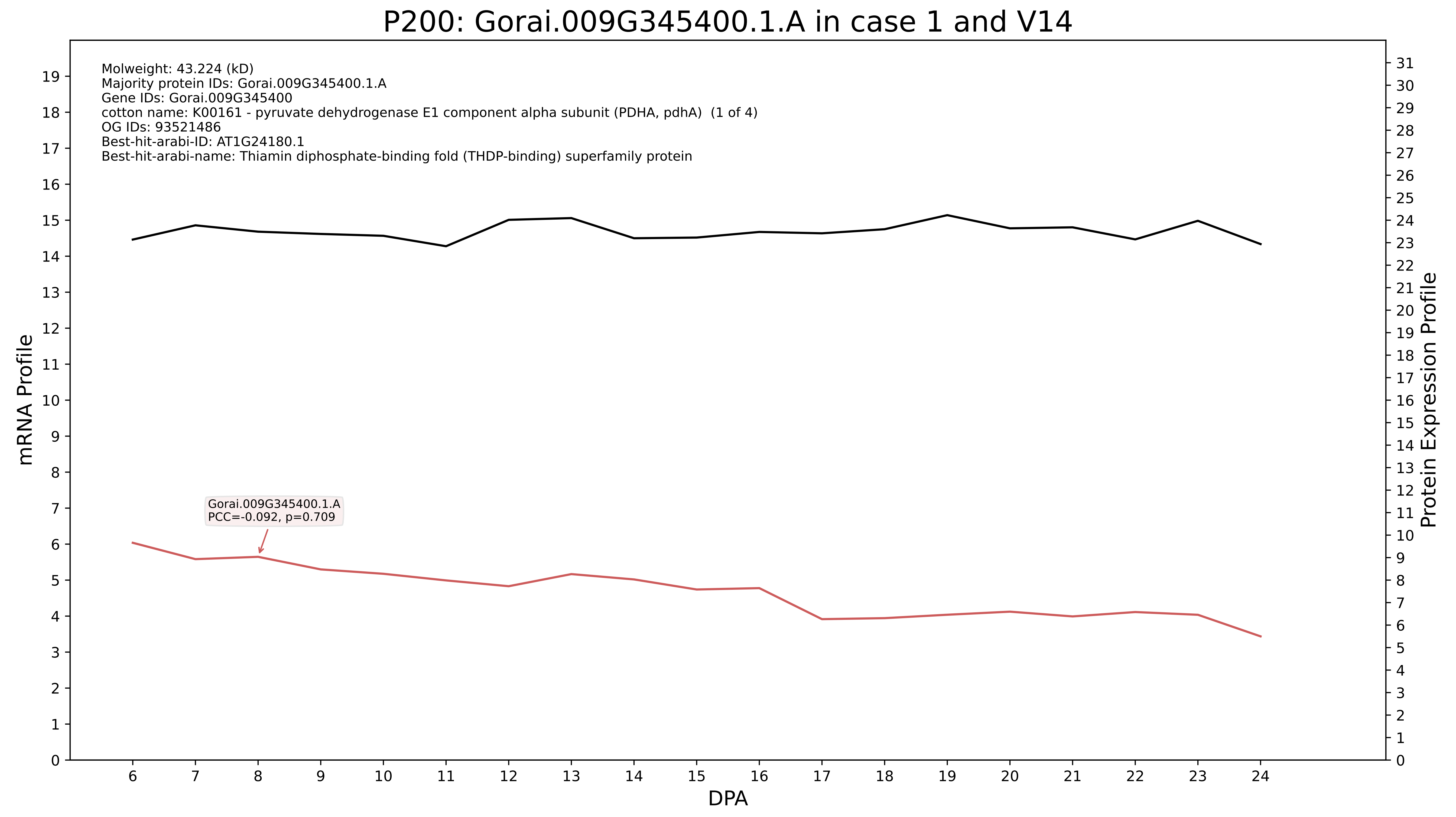
P200: Gorai.009G345400.1.A in case 1 and V14
Molweight: 43.224 (kD)
Majority protein IDs: Gorai.009G345400.1.A
Gene IDs: Gorai.009G345400
cotton name: K00161 - pyruvate dehydrogenase E1 component alpha subunit (PDHA, pdhA) (1 of 4)
OG IDs: 93521486
Best-hit-arabi-ID: AT1G24180.1
Best-hit-arabi-name: Thiamin diphosphate-binding fold (THDP-binding) superfamily protein
Gorai.009G345400.1.A
PCC=-0.092, p=0.709
mRNA Profile
Protein Expression Profile
DPA

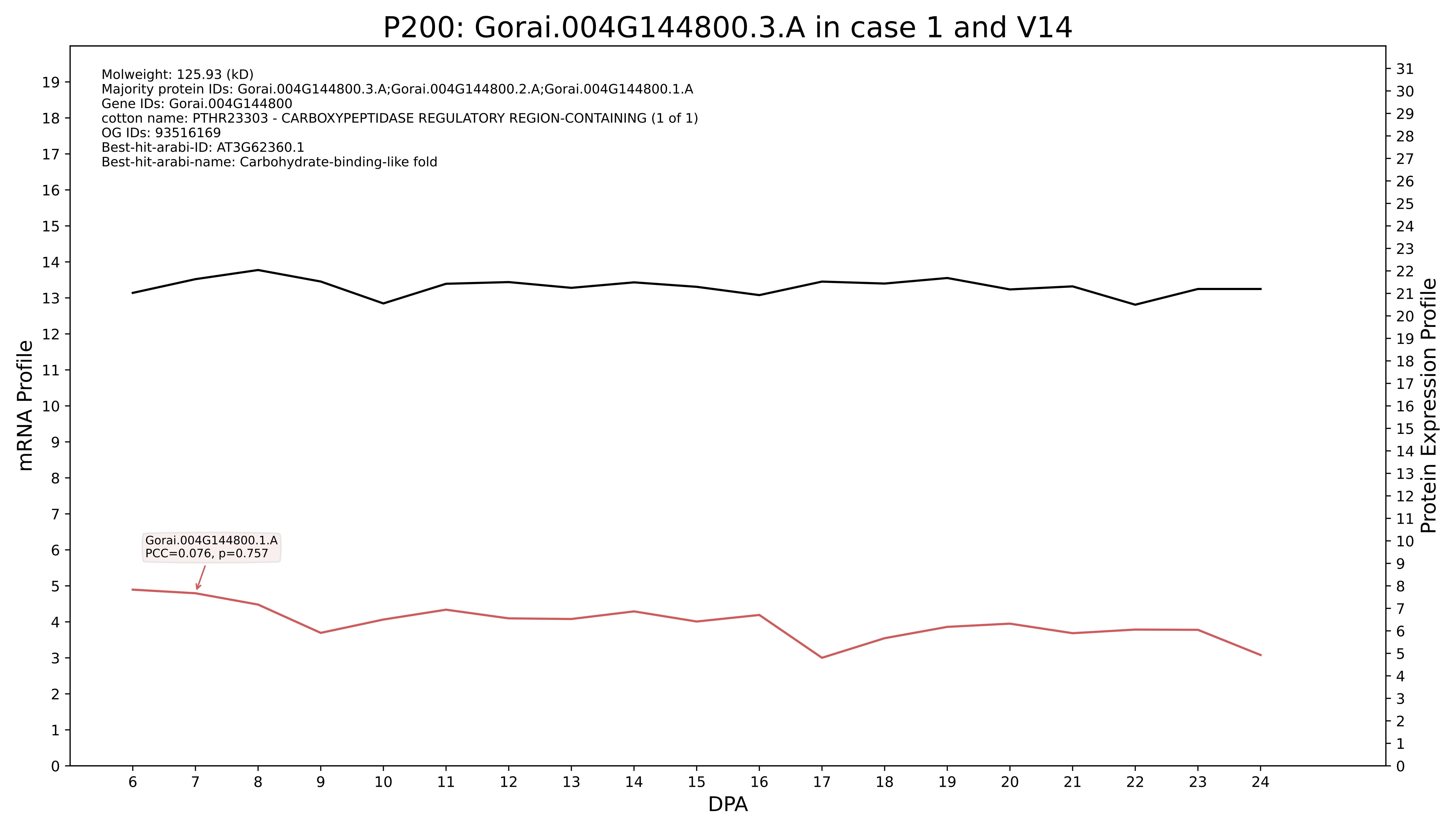
P200: Gorai.004G144800.3.A in case 1 and V14
Molweight: 125.93 (kD)
Majority protein IDs: Gorai.004G144800.3.A;Gorai.004G144800.2.A;Gorai.004G144800.1.A
Gene IDs: Gorai.004G144800
cotton name: PTHR23303 - CARBOXYPEPTIDASE REGULATORY REGION-CONTAINING (1 of 1)
OG IDs: 93516169
Best-hit-arabi-ID: AT3G62360.1
Best-hit-arabi-name: Carbohydrate-binding-like fold
Gorai.004G144800.1.A
PCC=0.076, p=0.757
mRNA Profile
Protein Expression Profile
DPA

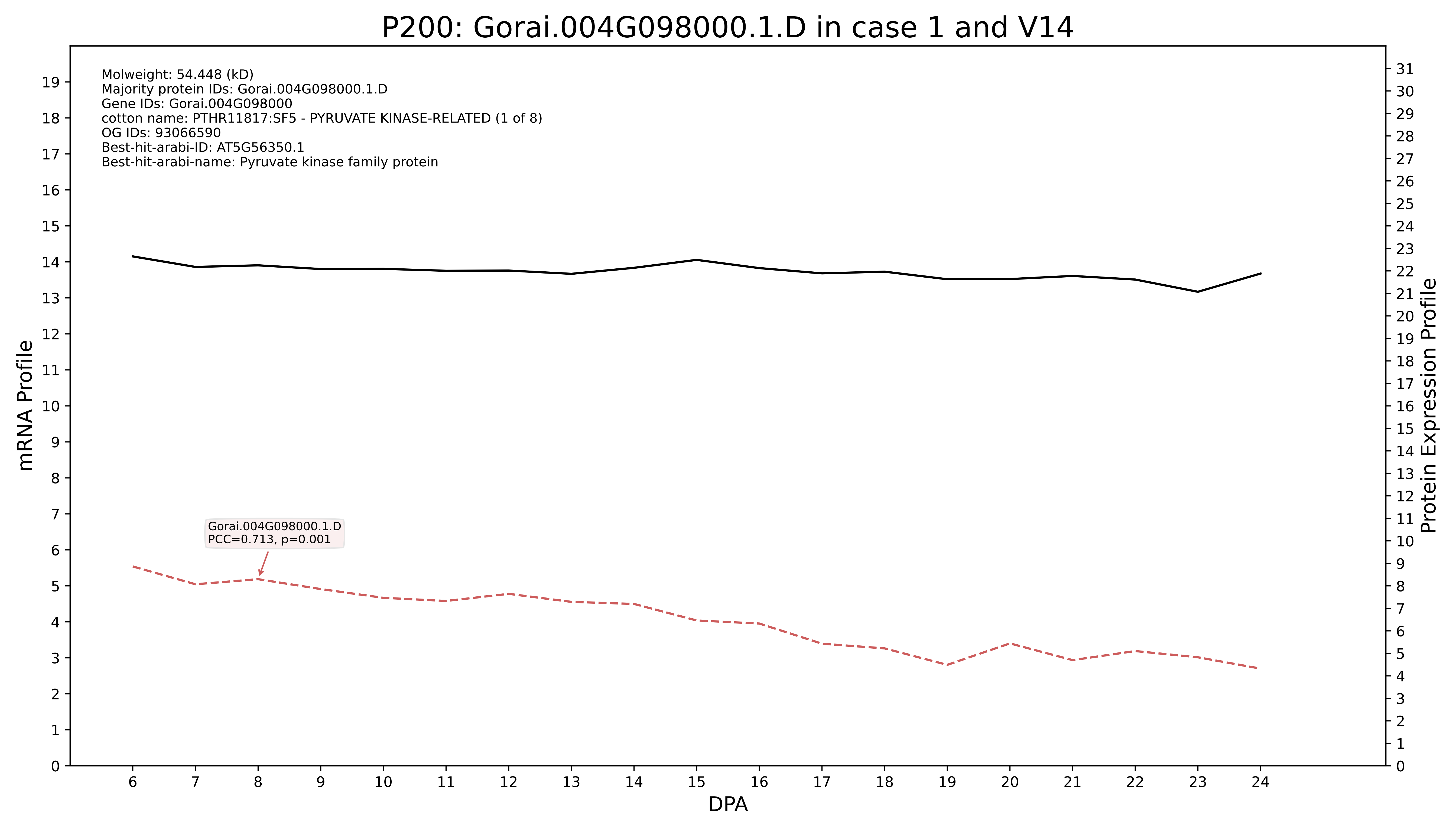
P200: Gorai.004G098000.1.D in case 1 and V14
Molweight: 54.448 (kD)
Majority protein IDs: Gorai.004G098000.1.D
Gene IDs: Gorai.004G098000
cotton name: PTHR11817:SF5 - PYRUVATE KINASE-RELATED (1 of 8)
OG IDs: 93066590
Best-hit-arabi-ID: AT5G56350.1
Best-hit-arabi-name: Pyruvate kinase family protein
Gorai.004G098000.1.D
PCC=0.713, p=0.001
mRNA Profile
Protein Expression Profile
DPA

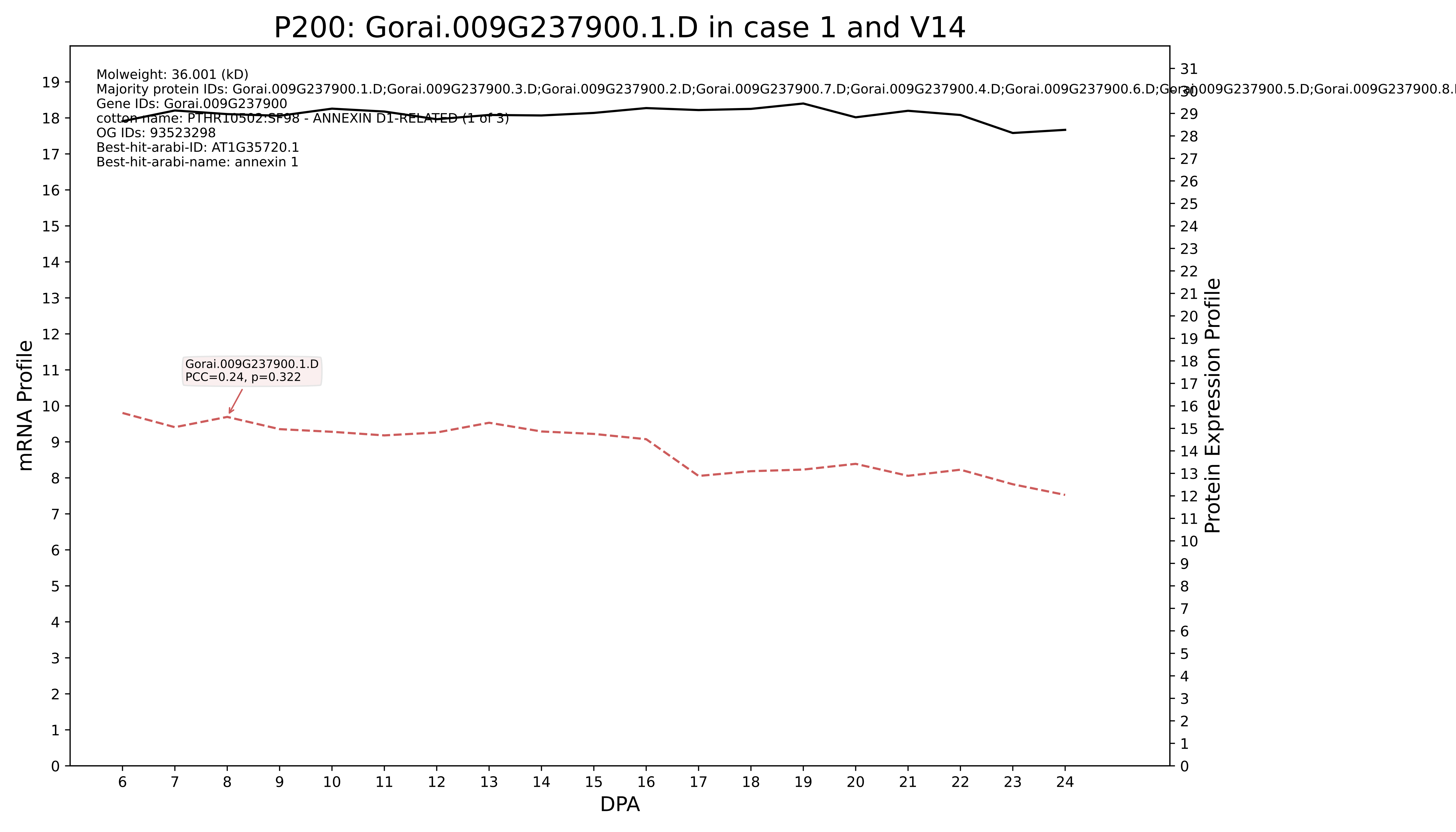
P200: Gorai.009G237900.1.D in case 1 and V14
Molweight: 36.001 (kD)
Majority protein IDs: Gorai.009G237900.1.D;Gorai.009G237900.3.D;Gorai.009G237900.2.D;Gorai.009G237900.7.D;Gorai.009G237900.4.D;Gorai.009G237900.6.D;Gorai.009G237900.5.D;Gorai.009G237900.8.D
Gene IDs: Gorai.009G237900
cotton name: PTHR10502:SF98 - ANNEXIN D1-RELATED (1 of 3)
OG IDs: 93523298
Best-hit-arabi-ID: AT1G35720.1
Best-hit-arabi-name: annexin 1
Gorai.009G237900.1.D
PCC=0.24, p=0.322
mRNA Profile
Protein Expression Profile
DPA

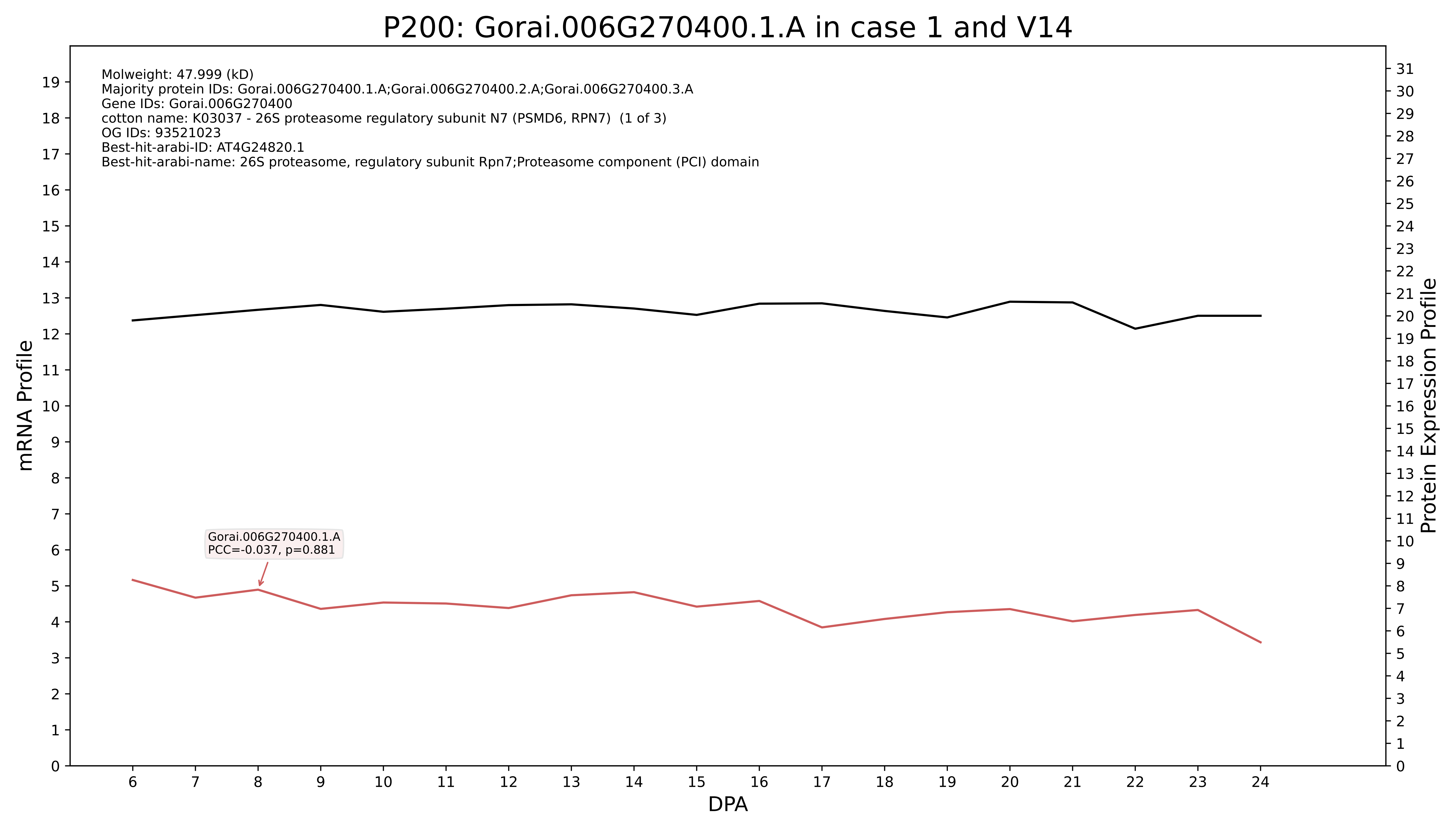

P200: Gorai.006G270400.1.A in case 1 and V14
Molweight: 47.999 (kD)
Majority protein IDs: Gorai.006G270400.1.A;Gorai.006G270400.2.A;Gorai.006G270400.3.A
Gene IDs: Gorai.006G270400
cotton name: K03037 - 26S proteasome regulatory subunit N7 (PSMD6, RPN7) (1 of 3)
OG IDs: 93521023
Best-hit-arabi-ID: AT4G24820.1
Best-hit-arabi-name: 26S proteasome, regulatory subunit Rpn7;Proteasome component (PCI) domain
Gorai.006G270400.1.A
PCC=-0.037, p=0.881
mRNA Profile
Protein Expression Profile
DPA

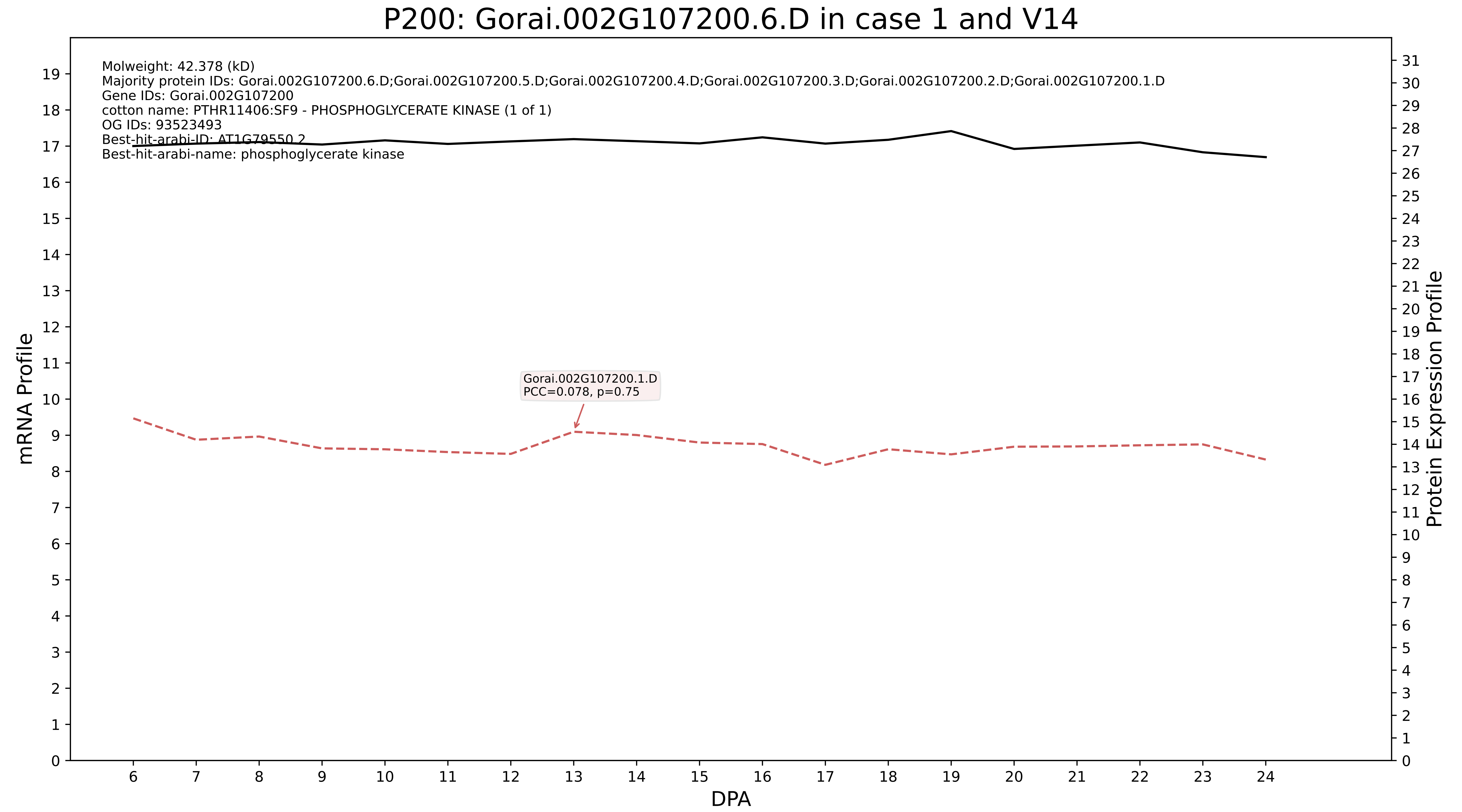

P200: Gorai.002G107200.6.D in case 1 and V14
Molweight: 42.378 (kD)
Majority protein IDs: Gorai.002G107200.6.D;Gorai.002G107200.5.D;Gorai.002G107200.4.D;Gorai.002G107200.3.D;Gorai.002G107200.2.D;Gorai.002G107200.1.D
Gene IDs: Gorai.002G107200
cotton name: PTHR11406:SF9 - PHOSPHOGLYCERATE KINASE (1 of 1)
OG IDs: 93523493
Best-hit-arabi-ID: AT1G79550.2
Best-hit-arabi-name: phosphoglycerate kinase
Gorai.002G107200.1.D
PCC=0.078, p=0.75
mRNA Profile
Protein Expression Profile
DPA

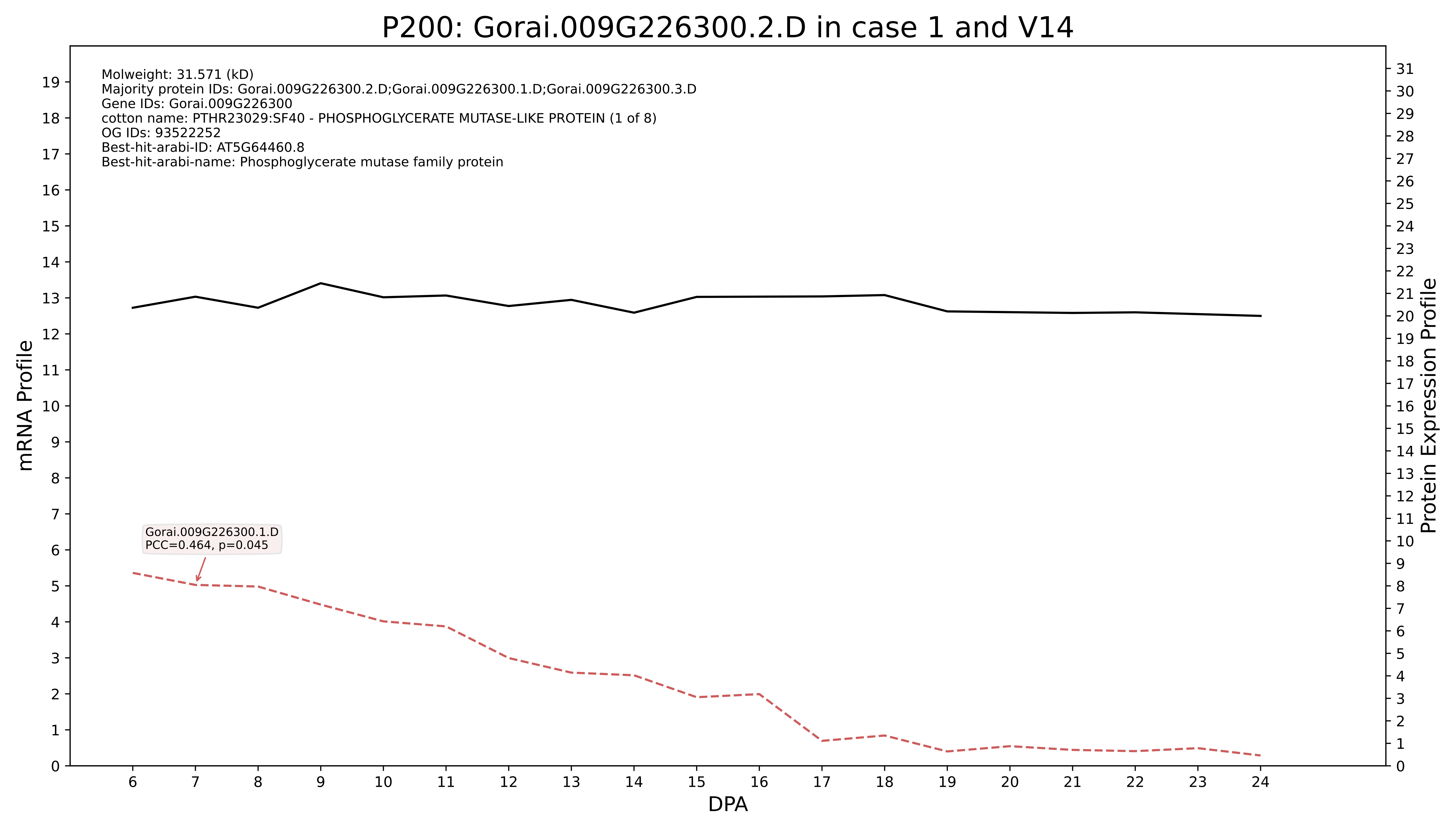
P200: Gorai.009G226300.2.D in case 1 and V14
Molweight: 31.571 (kD)
Majority protein IDs: Gorai.009G226300.2.D;Gorai.009G226300.1.D;Gorai.009G226300.3.D
Gene IDs: Gorai.009G226300
cotton name: PTHR23029:SF40 - PHOSPHOGLYCERATE MUTASE-LIKE PROTEIN (1 of 8)
OG IDs: 93522252
Best-hit-arabi-ID: AT5G64460.8
Best-hit-arabi-name: Phosphoglycerate mutase family protein
Gorai.009G226300.1.D
PCC=0.464, p=0.045
mRNA Profile
Protein Expression Profile
DPA

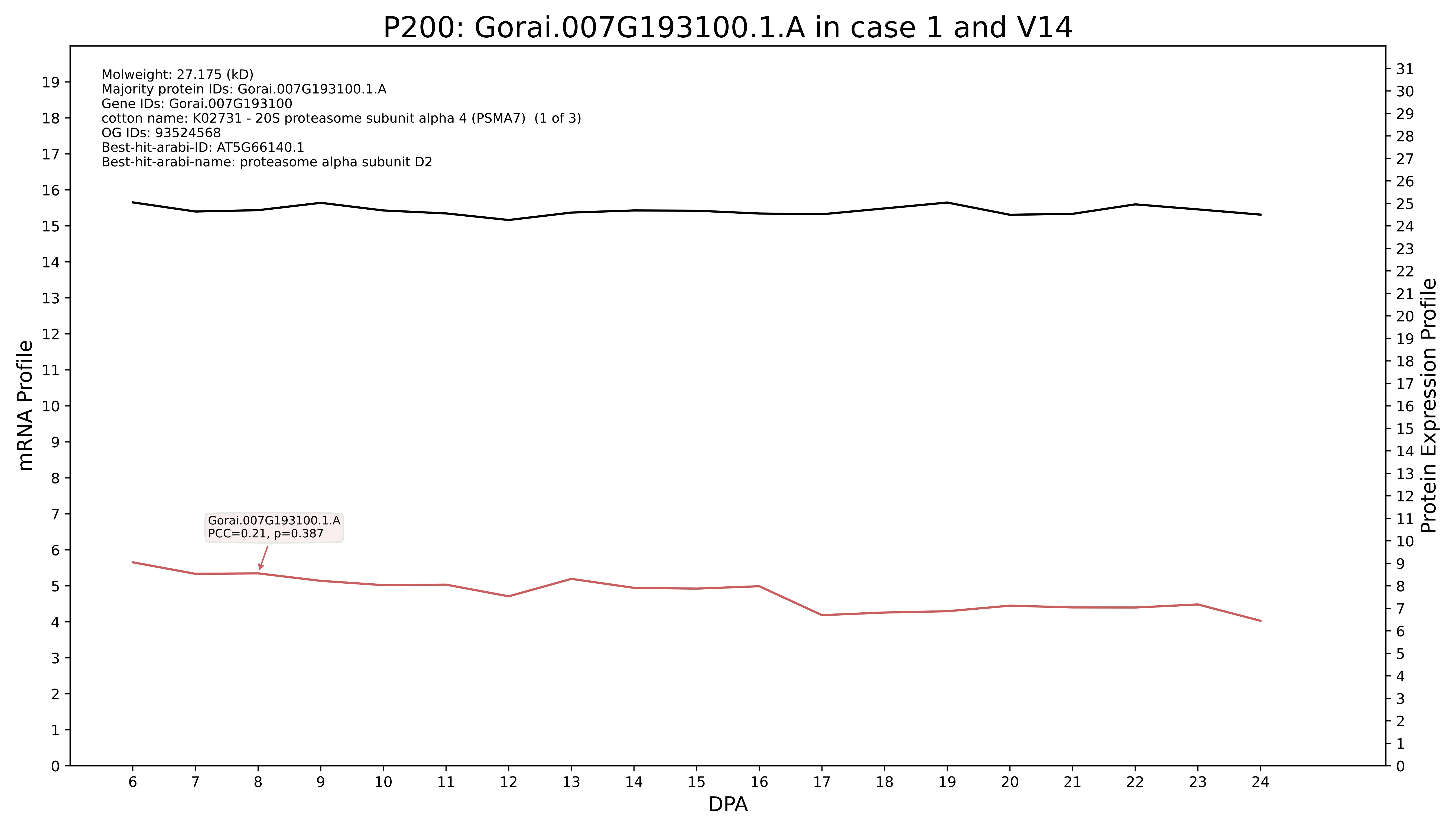
P200: Gorai.007G193100.1.A in case 1 and V14
Molweight: 27.175 (kD)
Majority protein IDs: Gorai.007G193100.1.A
Gene IDs: Gorai.007G193100
cotton name: K02731 - 20S proteasome subunit alpha 4 (PSMA7) (1 of 3)
OG IDs: 93524568
Best-hit-arabi-ID: AT5G66140.1
Best-hit-arabi-name: proteasome alpha subunit D2
Gorai.007G193100.1.A
PCC=0.21, p=0.387
mRNA Profile
Protein Expression Profile
DPA

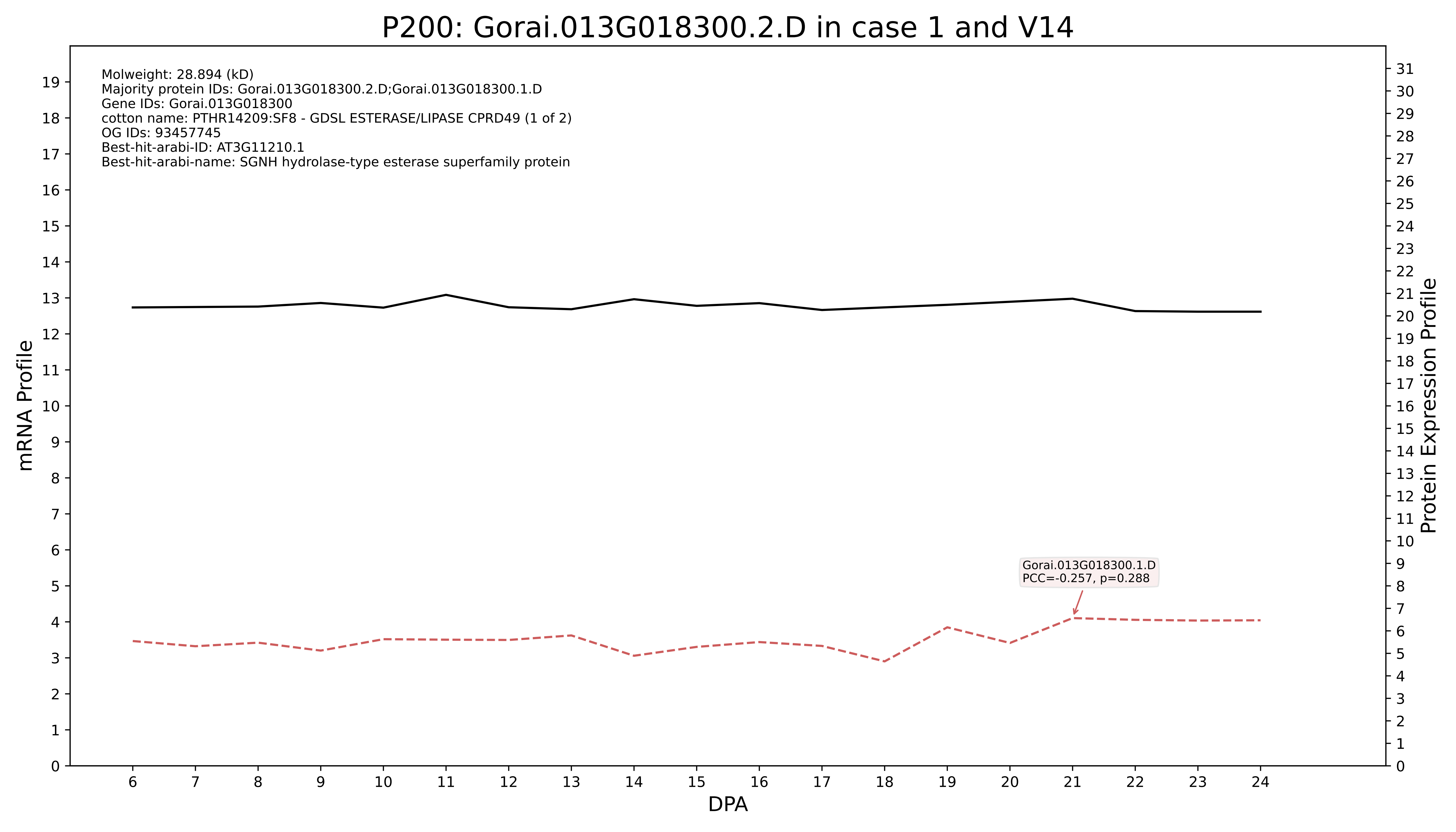
P200: Gorai.013G018300.2.D in case 1 and V14
Molweight: 28.894 (kD)
Majority protein IDs: Gorai.013G018300.2.D;Gorai.013G018300.1.D
Gene IDs: Gorai.013G018300
cotton name: PTHR14209:SF8 - GDSL ESTERASE/LIPASE CPRD49 (1 of 2)
OG IDs: 93457745
Best-hit-arabi-ID: AT3G11210.1
Best-hit-arabi-name: SGNH hydrolase-type esterase superfamily protein
Gorai.013G018300.1.D
PCC=-0.257, p=0.288
mRNA Profile
Protein Expression Profile
DPA

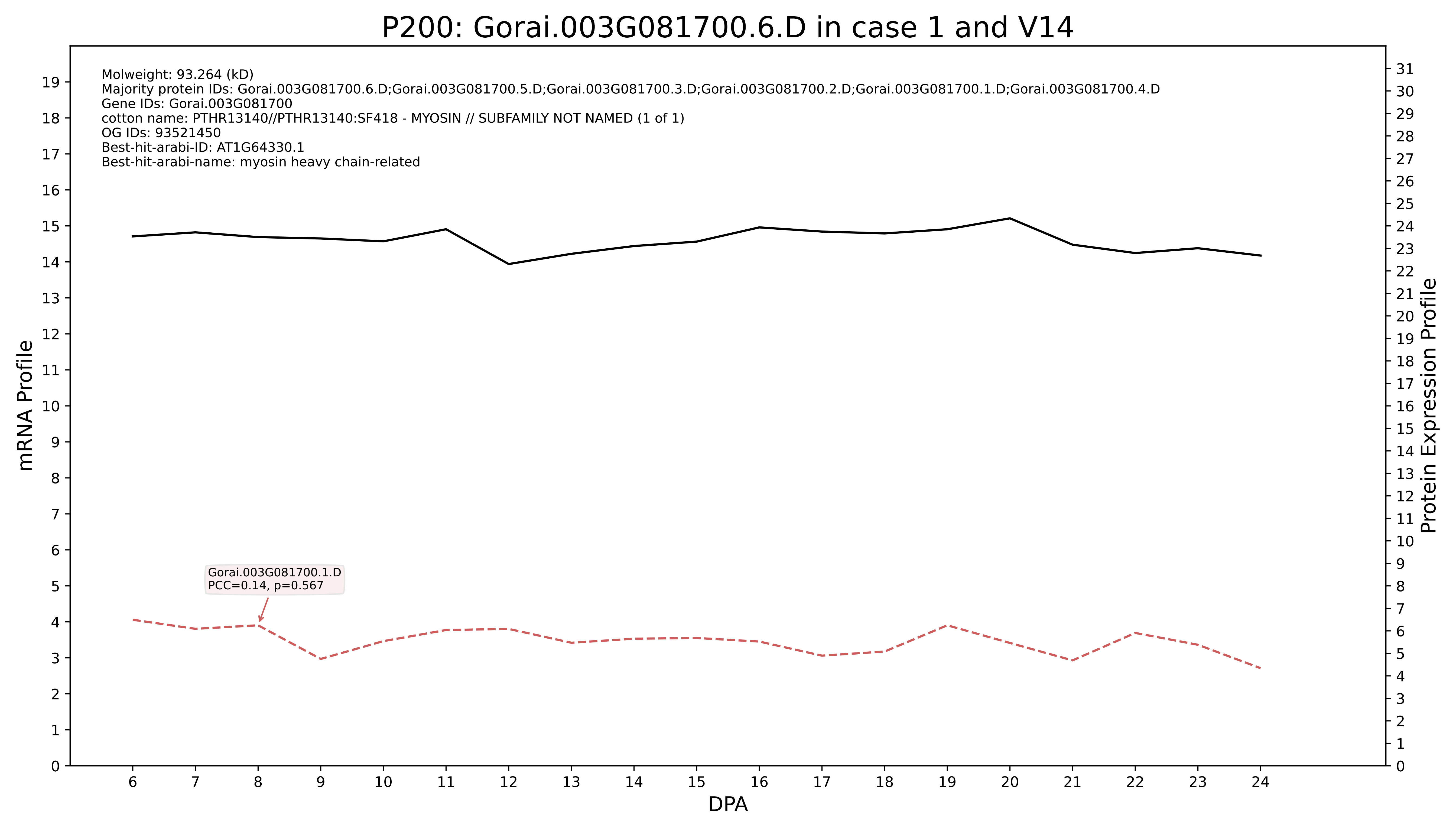
P200: Gorai.003G081700.6.D in case 1 and V14
Molweight: 93.264 (kD)
Majority protein IDs: Gorai.003G081700.6.D;Gorai.003G081700.5.D;Gorai.003G081700.3.D;Gorai.003G081700.2.D;Gorai.003G081700.1.D;Gorai.003G081700.4.D
Gene IDs: Gorai.003G081700
cotton name: PTHR13140//PTHR13140:SF418 - MYOSIN // SUBFAMILY NOT NAMED (1 of 1)
OG IDs: 93521450
Best-hit-arabi-ID: AT1G64330.1
Best-hit-arabi-name: myosin heavy chain-related
Gorai.003G081700.1.D
PCC=0.14, p=0.567
mRNA Profile
Protein Expression Profile
DPA

P200: Gorai.009G047500.8.A in case 1 and V14

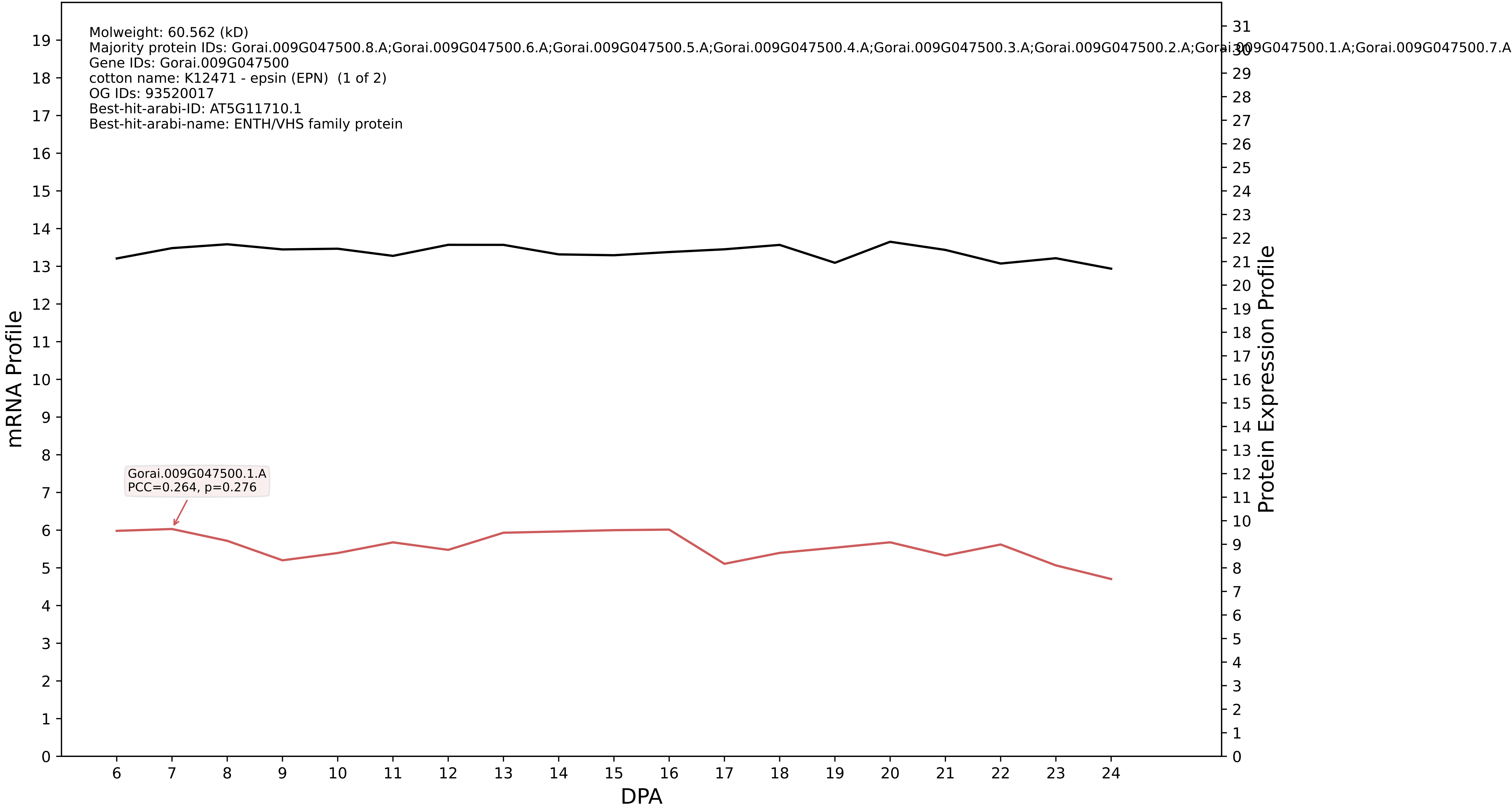

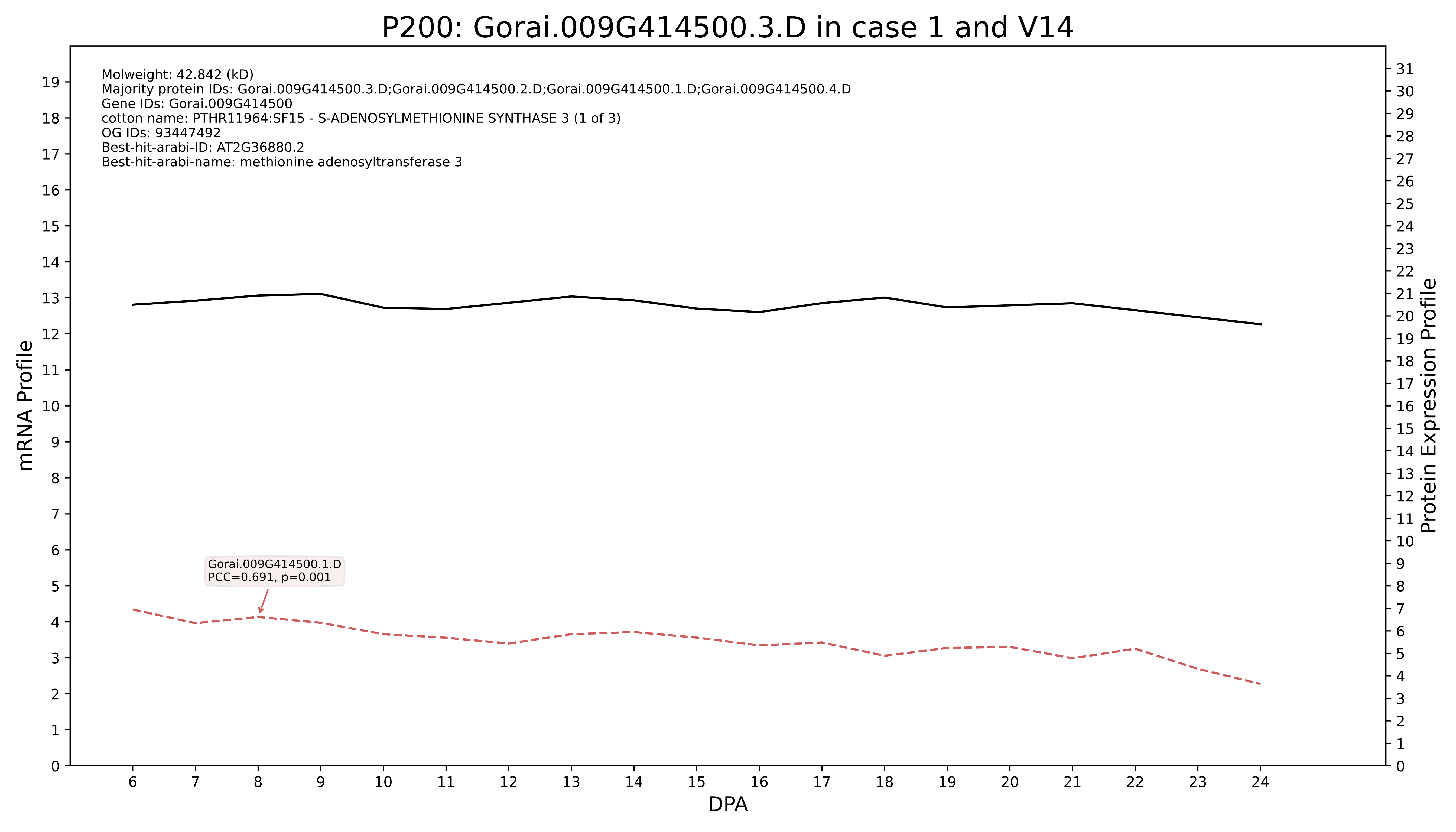

P200: Gorai.009G414500.3.D in case 1 and V14
Molweight: 42.842 (kD)
Majority protein IDs: Gorai.009G414500.3.D;Gorai.009G414500.2.D;Gorai.009G414500.1.D;Gorai.009G414500.4.D
Gene IDs: Gorai.009G414500
cotton name: PTHR11964:SF15 - S-ADENOSYLMETHIONINE SYNTHASE 3 (1 of 3)
OG IDs: 93447492
Best-hit-arabi-ID: AT2G36880.2
Best-hit-arabi-name: methionine adenosyltransferase 3
Gorai.009G414500.1.D
PCC=0.691, p=0.001
mRNA Profile
Protein Expression Profile
DPA

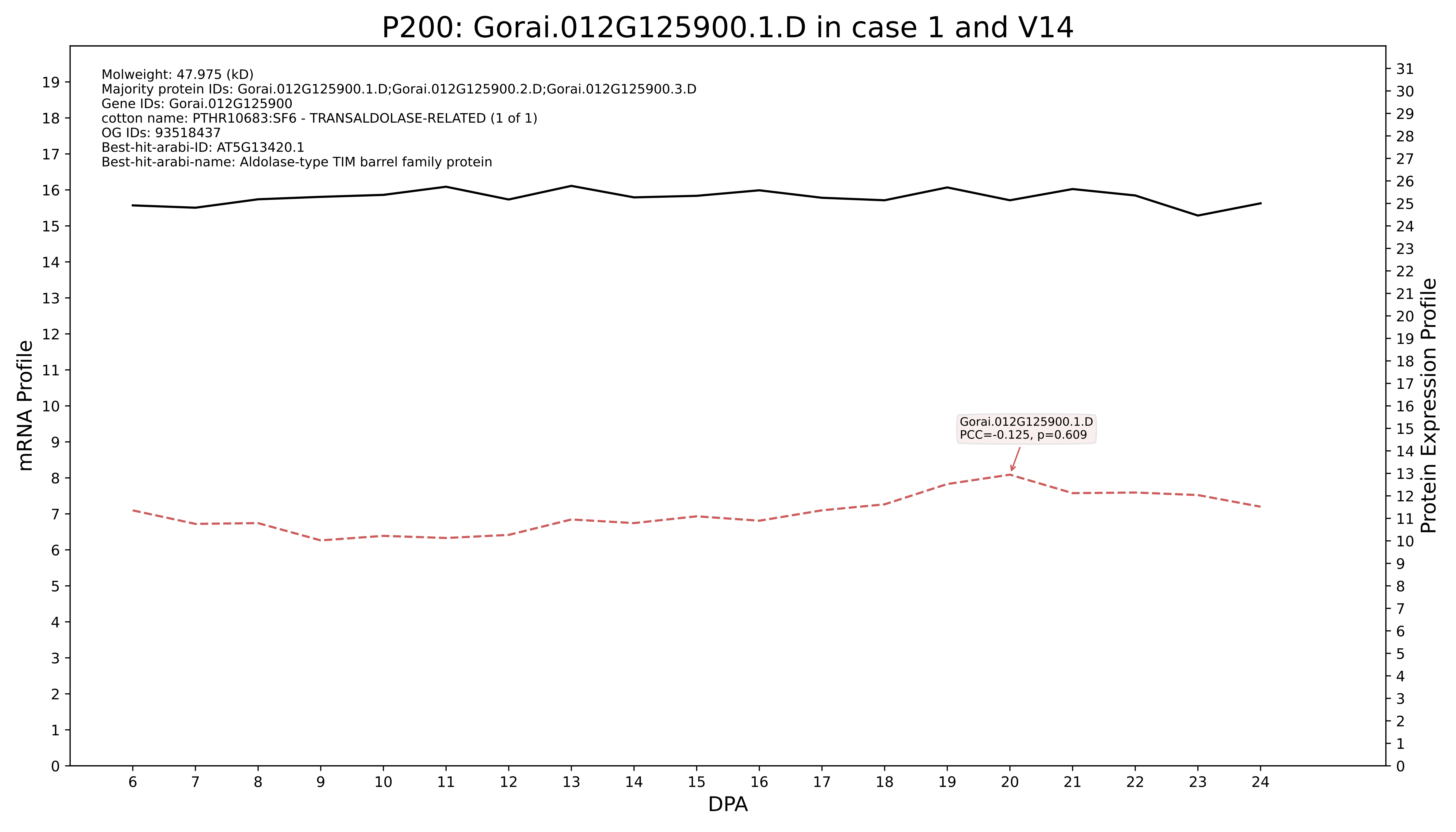
P200: Gorai.012G125900.1.D in case 1 and V14
Molweight: 47.975 (kD)
Majority protein IDs: Gorai.012G125900.1.D;Gorai.012G125900.2.D;Gorai.012G125900.3.D
Gene IDs: Gorai.012G125900
cotton name: PTHR10683:SF6 - TRANSALDOLASE-RELATED (1 of 1)
OG IDs: 93518437
Best-hit-arabi-ID: AT5G13420.1
Best-hit-arabi-name: Aldolase-type TIM barrel family protein
Gorai.012G125900.1.D
PCC=-0.125, p=0.609
mRNA Profile
Protein Expression Profile
DPA

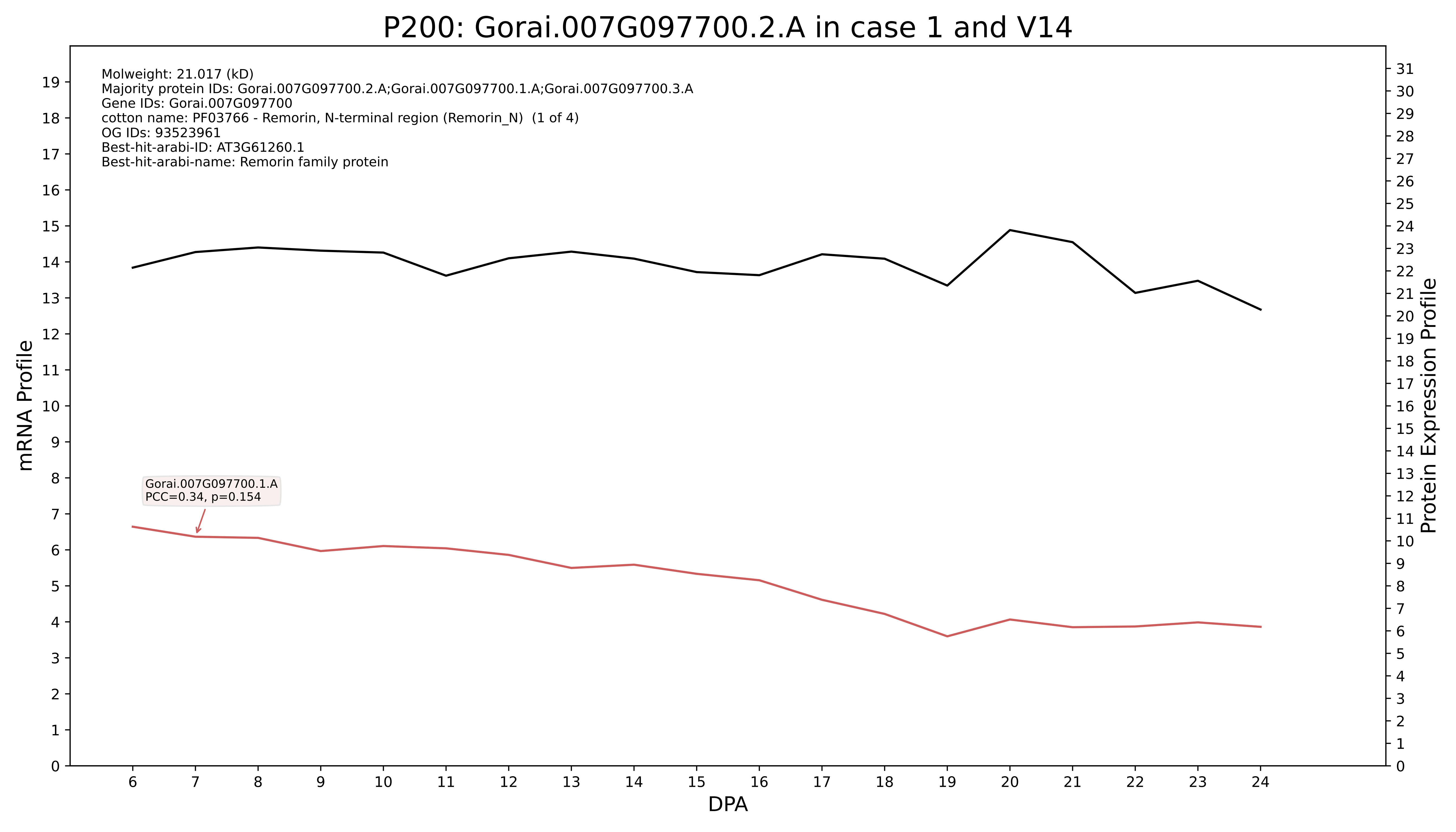
P200: Gorai.007G097700.2.A in case 1 and V14
Molweight: 21.017 (kD)
Majority protein IDs: Gorai.007G097700.2.A;Gorai.007G097700.1.A;Gorai.007G097700.3.A
Gene IDs: Gorai.007G097700
cotton name: PF03766 - Remorin, N-terminal region (Remorin_N) (1 of 4)
OG IDs: 93523961
Best-hit-arabi-ID: AT3G61260.1
Best-hit-arabi-name: Remorin family protein
Gorai.007G097700.1.A
PCC=0.34, p=0.154
mRNA Profile
Protein Expression Profile
DPA

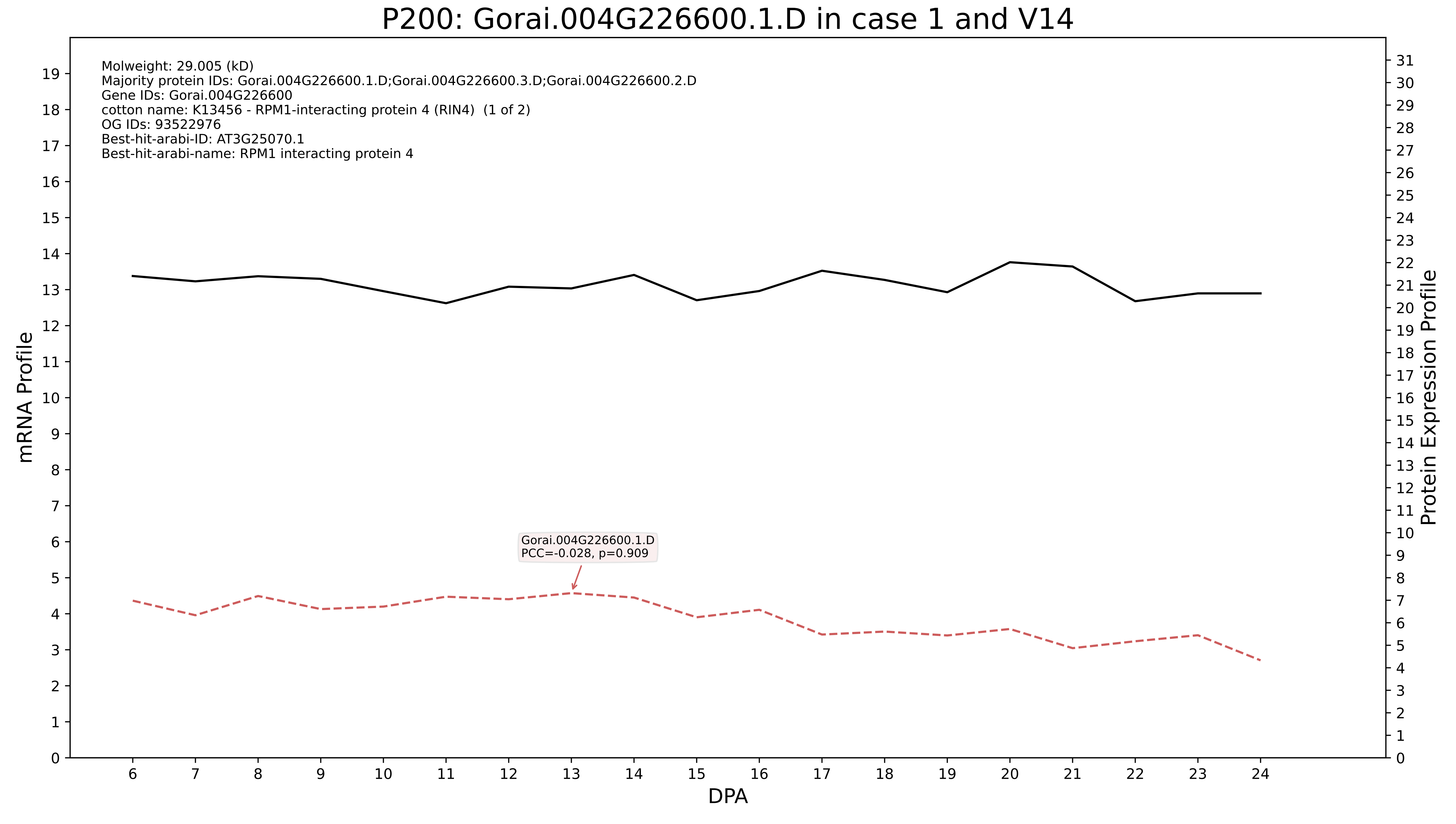
P200: Gorai.004G226600.1.D in case 1 and V14
Molweight: 29.005 (kD)
Majority protein IDs: Gorai.004G226600.1.D;Gorai.004G226600.3.D;Gorai.004G226600.2.D
Gene IDs: Gorai.004G226600
cotton name: K13456 - RPM1-interacting protein 4 (RIN4) (1 of 2)
OG IDs: 93522976
Best-hit-arabi-ID: AT3G25070.1
Best-hit-arabi-name: RPM1 interacting protein 4
Gorai.004G226600.1.D
PCC=-0.028, p=0.909
mRNA Profile
Protein Expression Profile
DPA

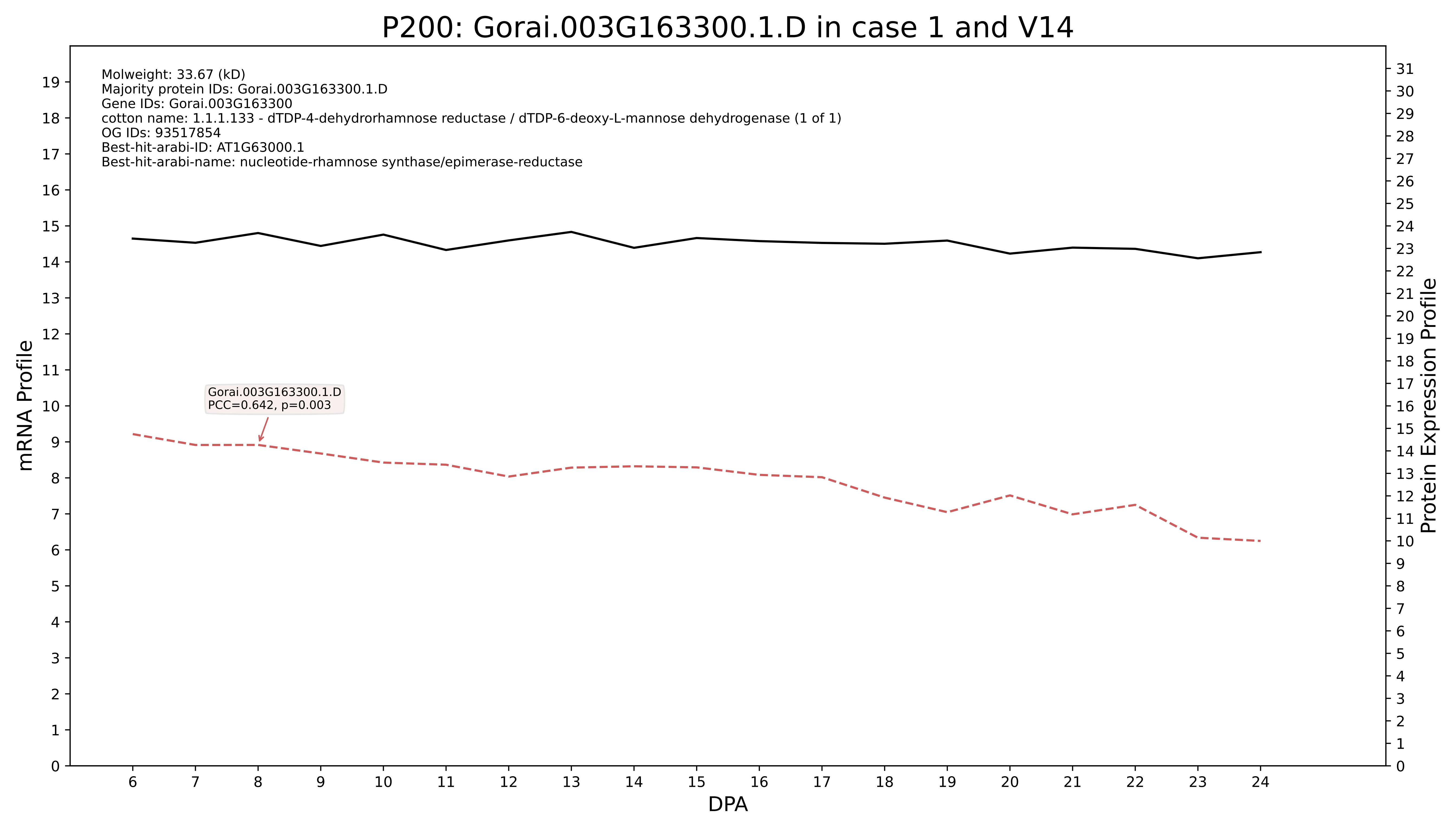
P200: Gorai.003G163300.1.D in case 1 and V14
Molweight: 33.67 (kD)
Majority protein IDs: Gorai.003G163300.1.D
Gene IDs: Gorai.003G163300
cotton name: 1.1.1.133 - dTDP-4-dehydrorhamnose reductase / dTDP-6-deoxy-L-mannose dehydrogenase (1 of 1)
OG IDs: 93517854
Best-hit-arabi-ID: AT1G63000.1
Best-hit-arabi-name: nucleotide-rhamnose synthase/epimerase-reductase
Gorai.003G163300.1.D
PCC=0.642, p=0.003
mRNA Profile
Protein Expression Profile
DPA

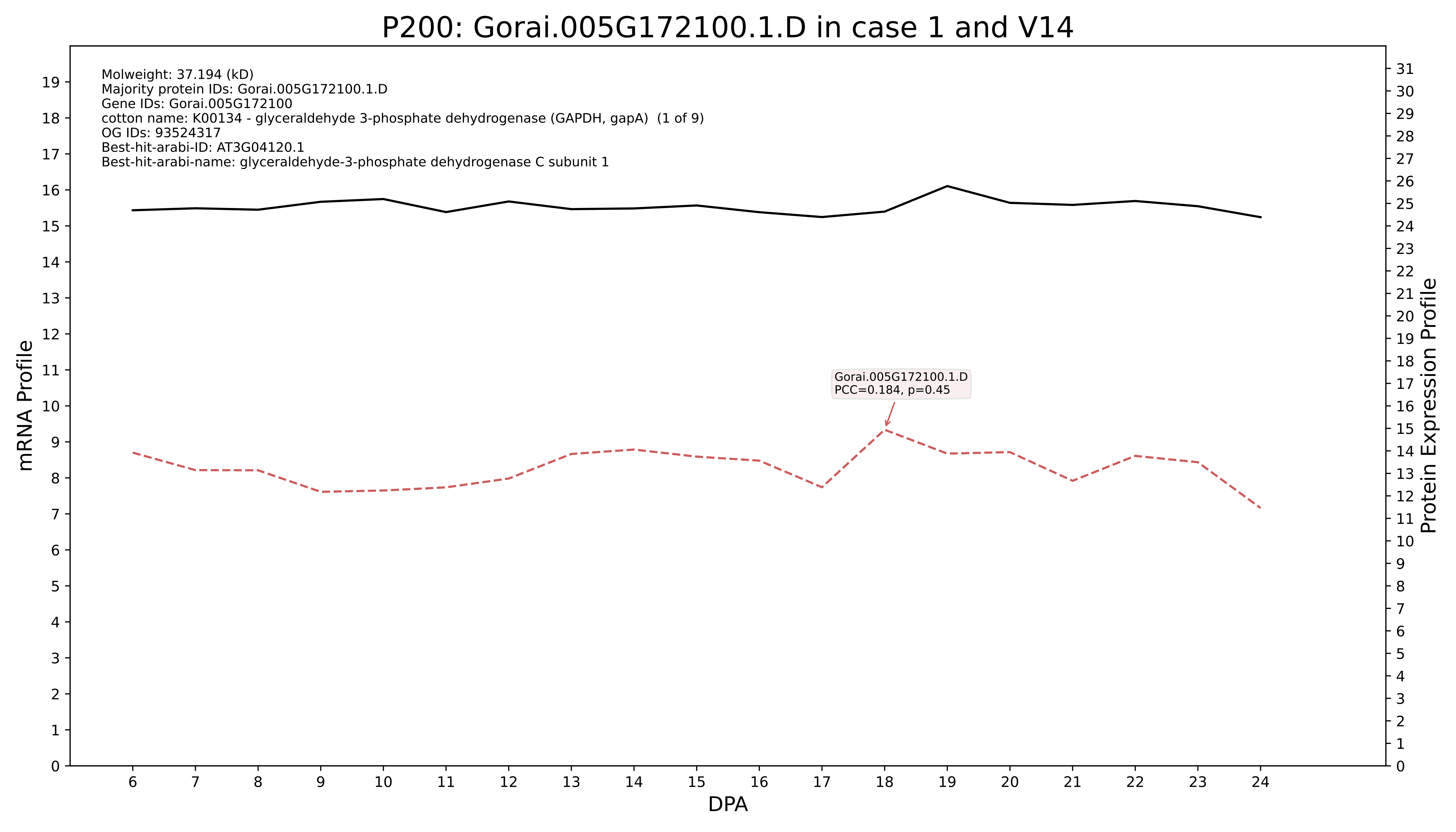
P200: Gorai.005G172100.1.D in case 1 and V14
Molweight: 37.194 (kD)
Majority protein IDs: Gorai.005G172100.1.D
Gene IDs: Gorai.005G172100
cotton name: K00134 - glyceraldehyde 3-phosphate dehydrogenase (GAPDH, gapA) (1 of 9)
OG IDs: 93524317
Best-hit-arabi-ID: AT3G04120.1
Best-hit-arabi-name: glyceraldehyde-3-phosphate dehydrogenase C subunit 1
Gorai.005G172100.1.D
PCC=0.184, p=0.45
mRNA Profile
Protein Expression Profile
DPA

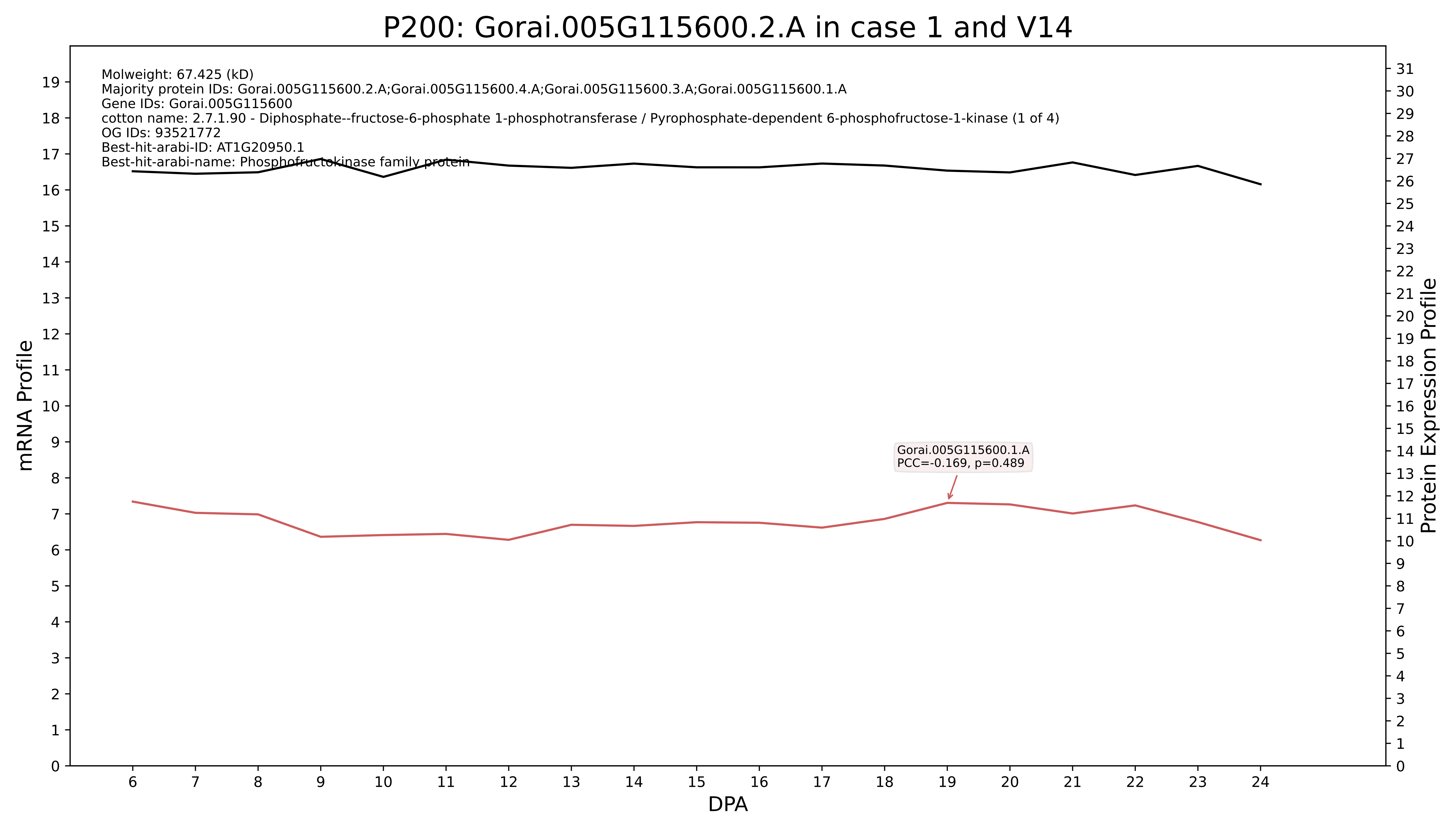

P200: Gorai.005G115600.2.A in case 1 and V14
Molweight: 67.425 (kD)
Majority protein IDs: Gorai.005G115600.2.A;Gorai.005G115600.4.A;Gorai.005G115600.3.A;Gorai.005G115600.1.A
Gene IDs: Gorai.005G115600
cotton name: 2.7.1.90 - Diphosphate--fructose-6-phosphate 1-phosphotransferase / Pyrophosphate-dependent 6-phosphofructose-1-kinase (1 of 4)
OG IDs: 93521772
Best-hit-arabi-ID: AT1G20950.1
Best-hit-arabi-name: Phosphofructokinase family protein
Gorai.005G115600.1.A
PCC=-0.169, p=0.489
mRNA Profile
Protein Expression Profile
DPA

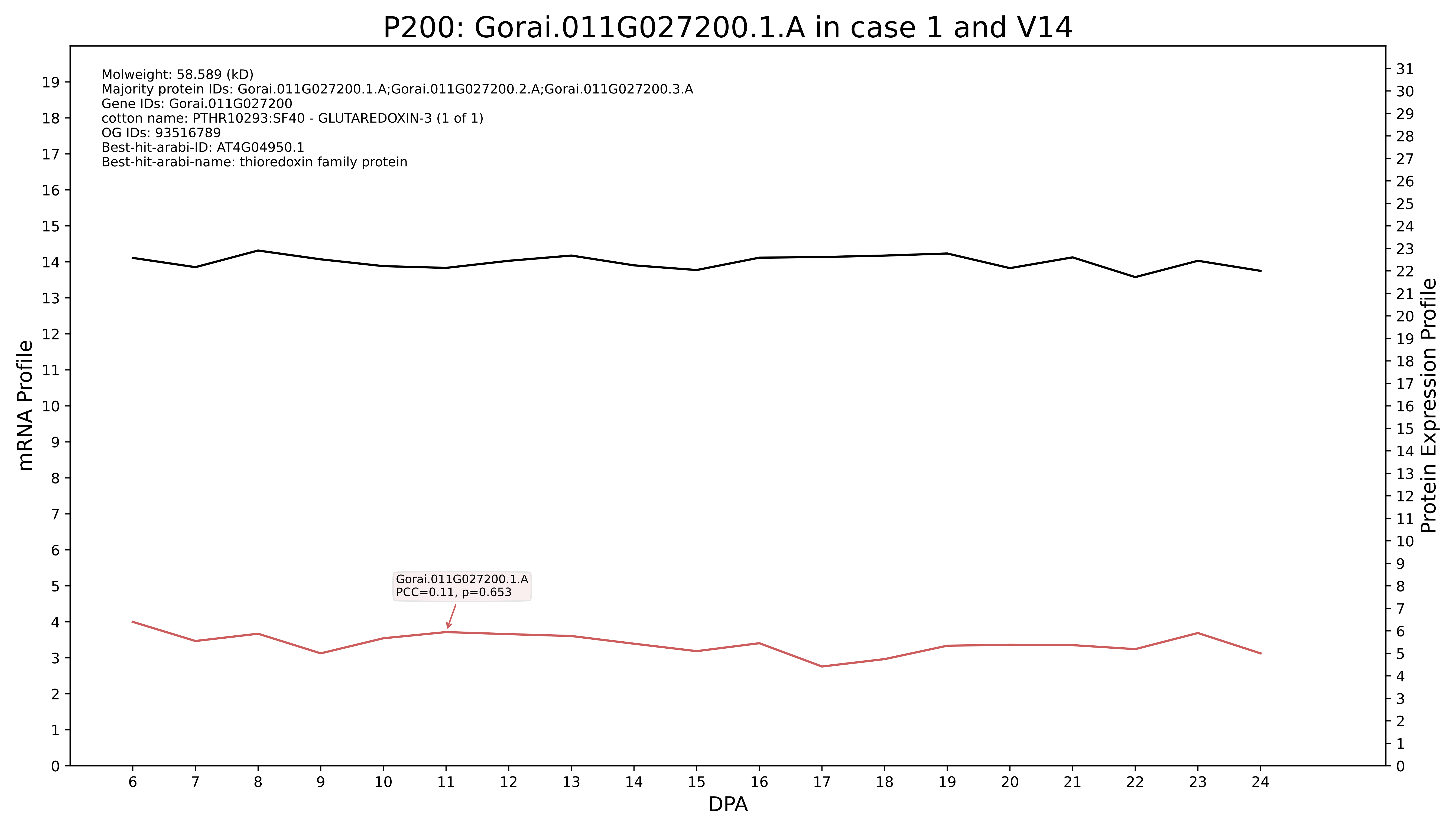
P200: Gorai.011G027200.1.A in case 1 and V14
Molweight: 58.589 (kD)
Majority protein IDs: Gorai.011G027200.1.A;Gorai.011G027200.2.A;Gorai.011G027200.3.A
Gene IDs: Gorai.011G027200
cotton name: PTHR10293:SF40 - GLUTAREDOXIN-3 (1 of 1)
OG IDs: 93516789
Best-hit-arabi-ID: AT4G04950.1
Best-hit-arabi-name: thioredoxin family protein
Gorai.011G027200.1.A
PCC=0.11, p=0.653
mRNA Profile
Protein Expression Profile
DPA

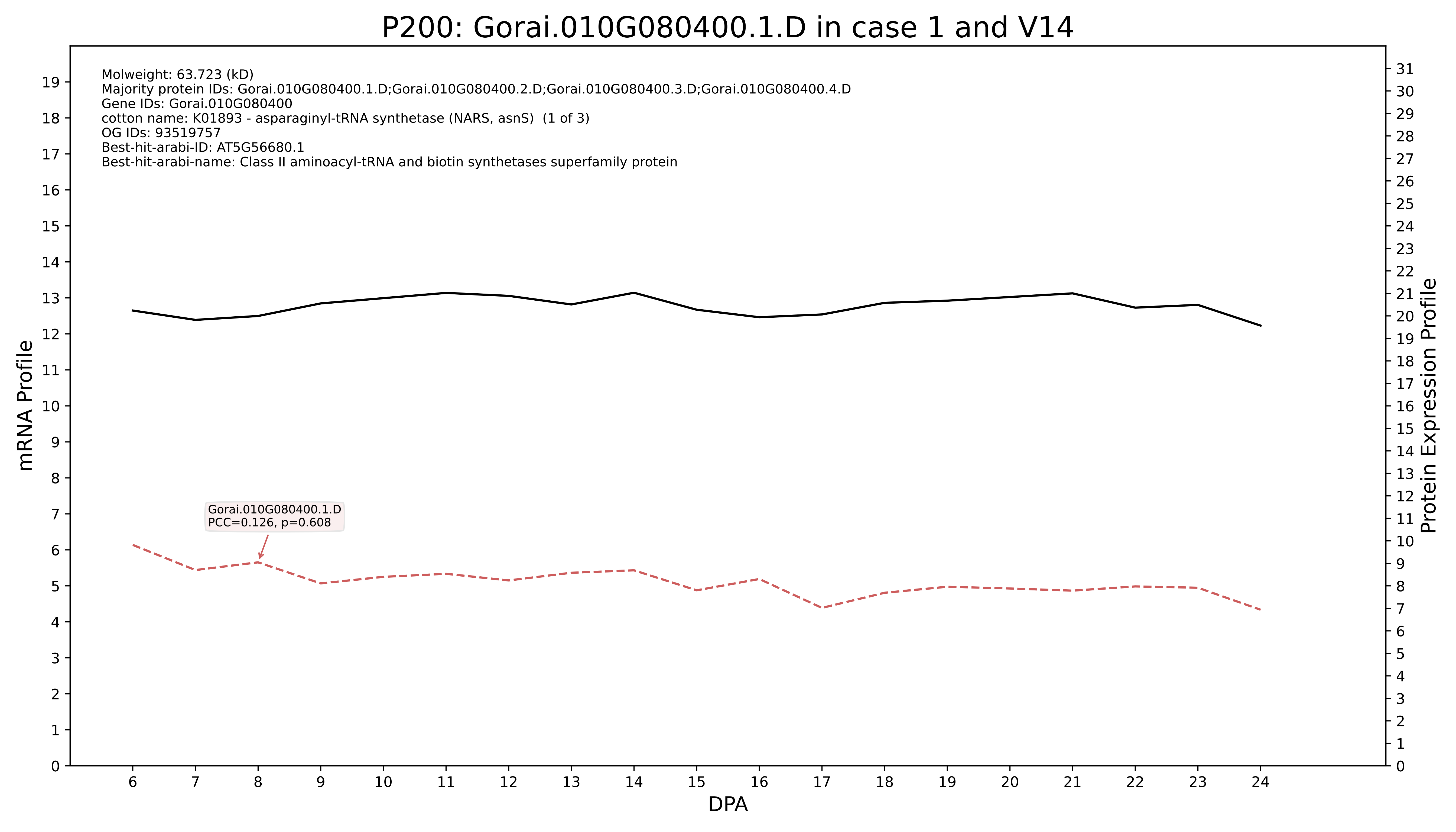

P200: Gorai.010G080400.1.D in case 1 and V14
Molweight: 63.723 (kD)
Majority protein IDs: Gorai.010G080400.1.D;Gorai.010G080400.2.D;Gorai.010G080400.3.D;Gorai.010G080400.4.D
Gene IDs: Gorai.010G080400
cotton name: K01893 - asparaginyl-tRNA synthetase (NARS, asnS) (1 of 3)
OG IDs: 93519757
Best-hit-arabi-ID: AT5G56680.1
Best-hit-arabi-name: Class II aminoacyl-tRNA and biotin synthetases superfamily protein
Gorai.010G080400.1.D
PCC=0.126, p=0.608
mRNA Profile
Protein Expression Profile
DPA

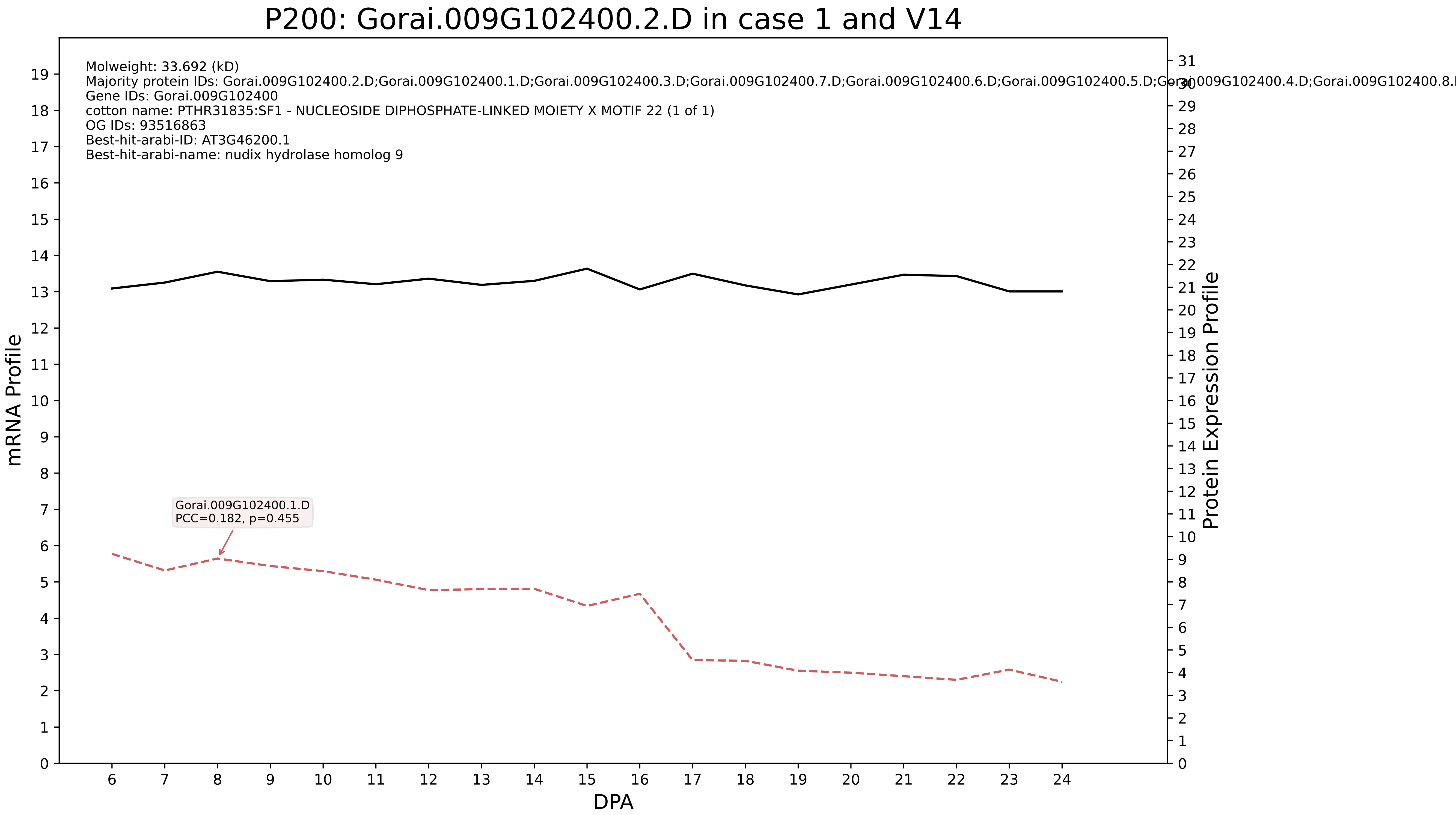
P200: Gorai.009G102400.2.D in case 1 and V14
Molweight: 33.692 (kD)
Majority protein IDs: Gorai.009G102400.2.D;Gorai.009G102400.1.D;Gorai.009G102400.3.D;Gorai.009G102400.7.D;Gorai.009G102400.6.D;Gorai.009G102400.5.D;Gorai.009G102400.4.D;Gorai.009G102400.8.D
Gene IDs: Gorai.009G102400
cotton name: PTHR31835:SF1 - NUCLEOSIDE DIPHOSPHATE-LINKED MOIETY X MOTIF 22 (1 of 1)
OG IDs: 93516863
Best-hit-arabi-ID: AT3G46200.1
Best-hit-arabi-name: nudix hydrolase homolog 9
Gorai.009G102400.1.D
PCC=0.182, p=0.455
mRNA Profile
Protein Expression Profile
DPA

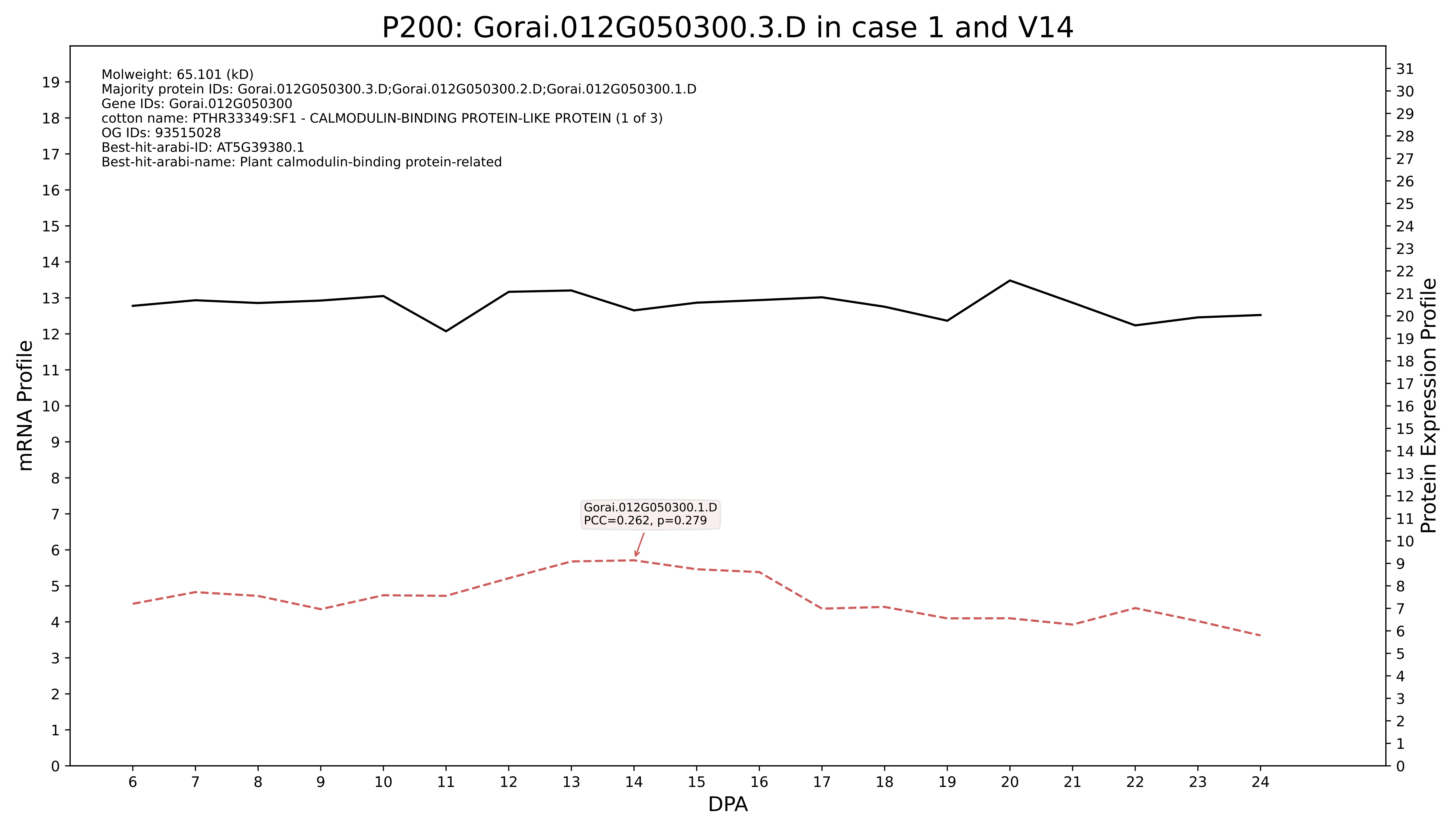
P200: Gorai.012G050300.3.D in case 1 and V14
Molweight: 65.101 (kD)
Majority protein IDs: Gorai.012G050300.3.D;Gorai.012G050300.2.D;Gorai.012G050300.1.D
Gene IDs: Gorai.012G050300
cotton name: PTHR33349:SF1 - CALMODULIN-BINDING PROTEIN-LIKE PROTEIN (1 of 3)
OG IDs: 93515028
Best-hit-arabi-ID: AT5G39380.1
Best-hit-arabi-name: Plant calmodulin-binding protein-related
Gorai.012G050300.1.D
PCC=0.262, p=0.279
mRNA Profile
Protein Expression Profile
DPA
0
1
2
3
4
5
6
7
8
9
10
11
12
13
14
15
16
17
18
19
20
21
22
23
24
25
26
27
28
29
30
31

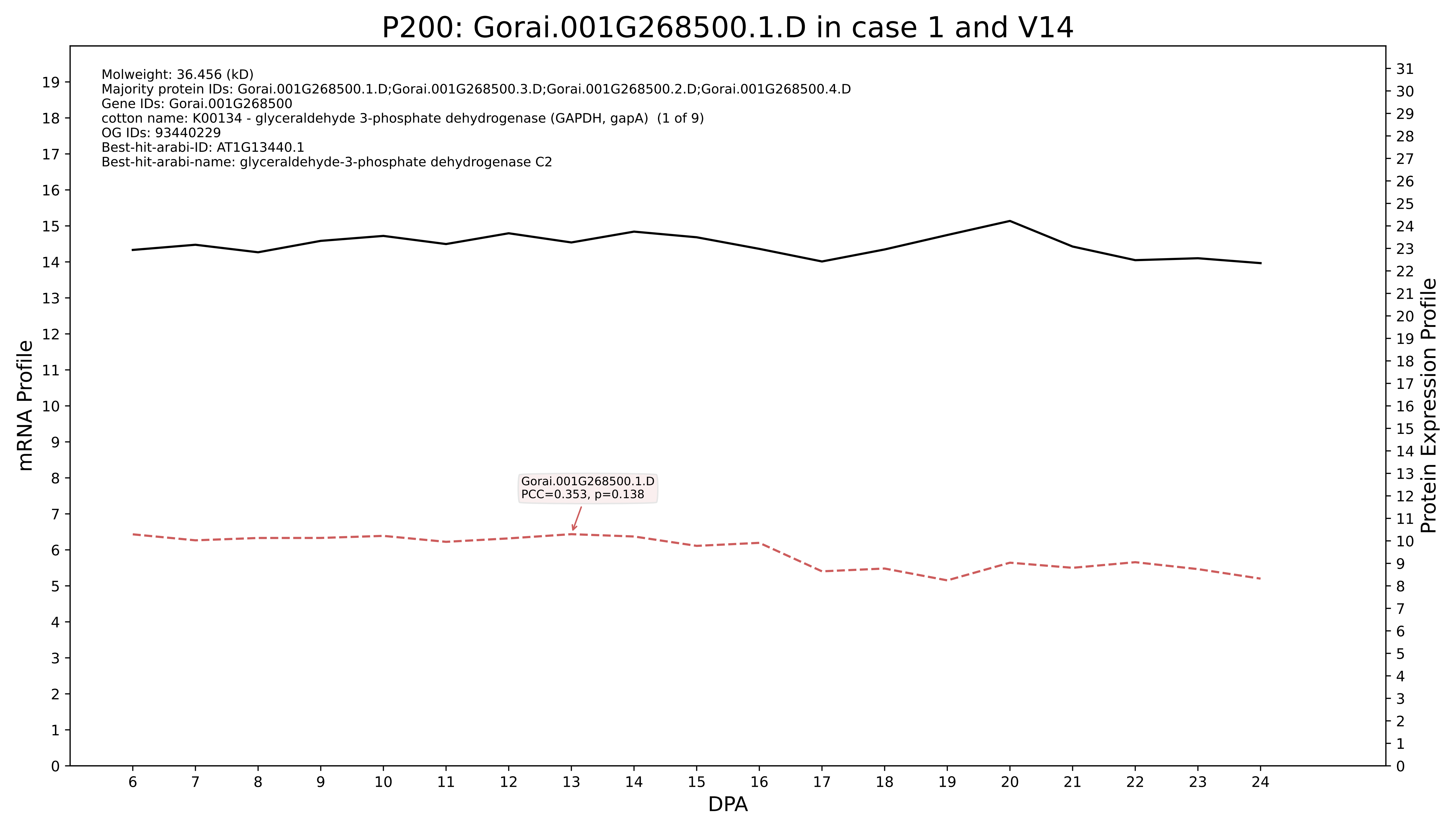
P200: Gorai.001G268500.1.D in case 1 and V14
Molweight: 36.456 (kD)
Majority protein IDs: Gorai.001G268500.1.D;Gorai.001G268500.3.D;Gorai.001G268500.2.D;Gorai.001G268500.4.D
Gene IDs: Gorai.001G268500
cotton name: K00134 - glyceraldehyde 3-phosphate dehydrogenase (GAPDH, gapA) (1 of 9)
OG IDs: 93440229
Best-hit-arabi-ID: AT1G13440.1
Best-hit-arabi-name: glyceraldehyde-3-phosphate dehydrogenase C2
Gorai.001G268500.1.D
PCC=0.353, p=0.138
mRNA Profile
Protein Expression Profile
DPA

P200: Gorai.010G072900.1.A in case 1 and V14

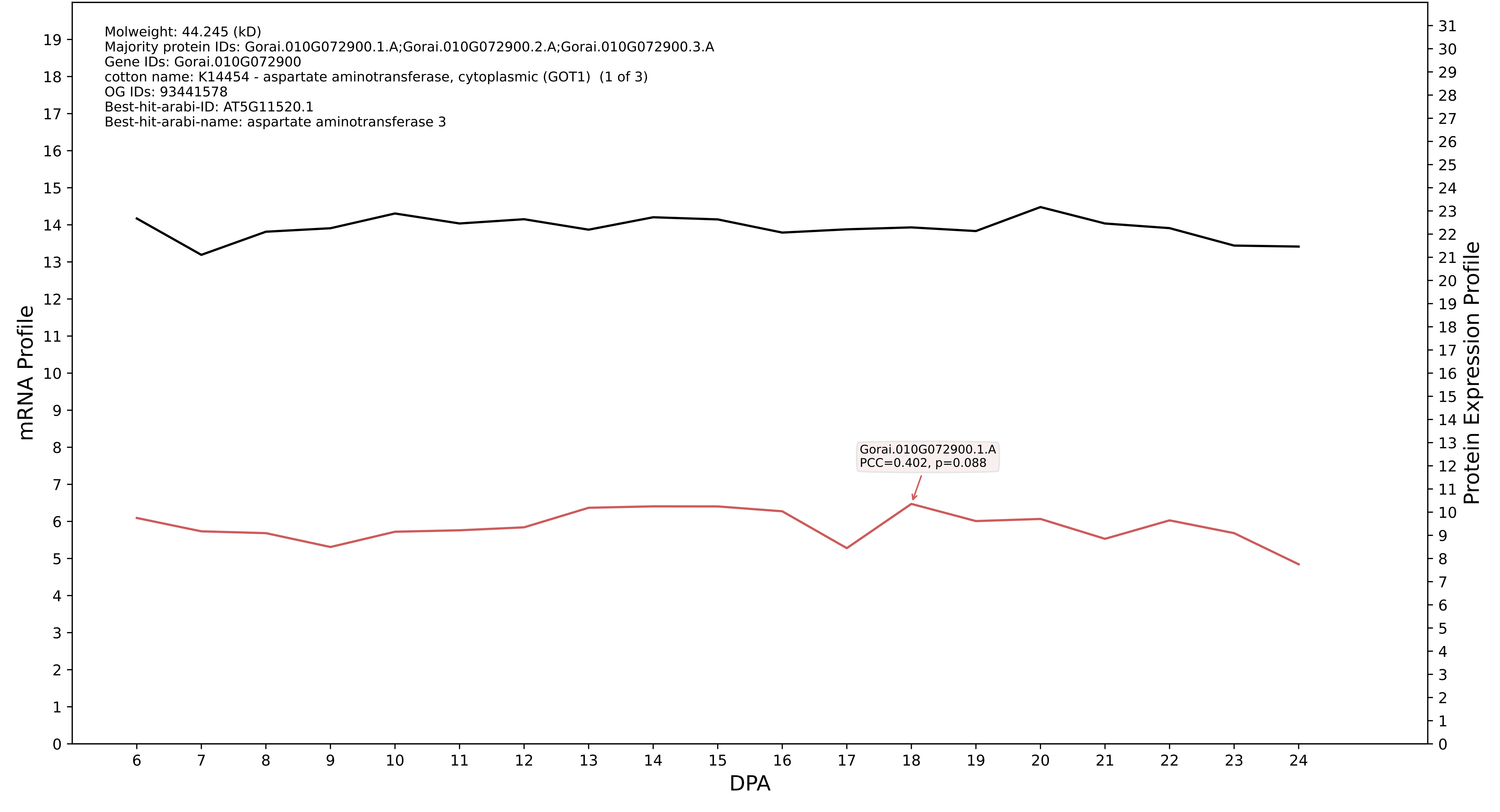

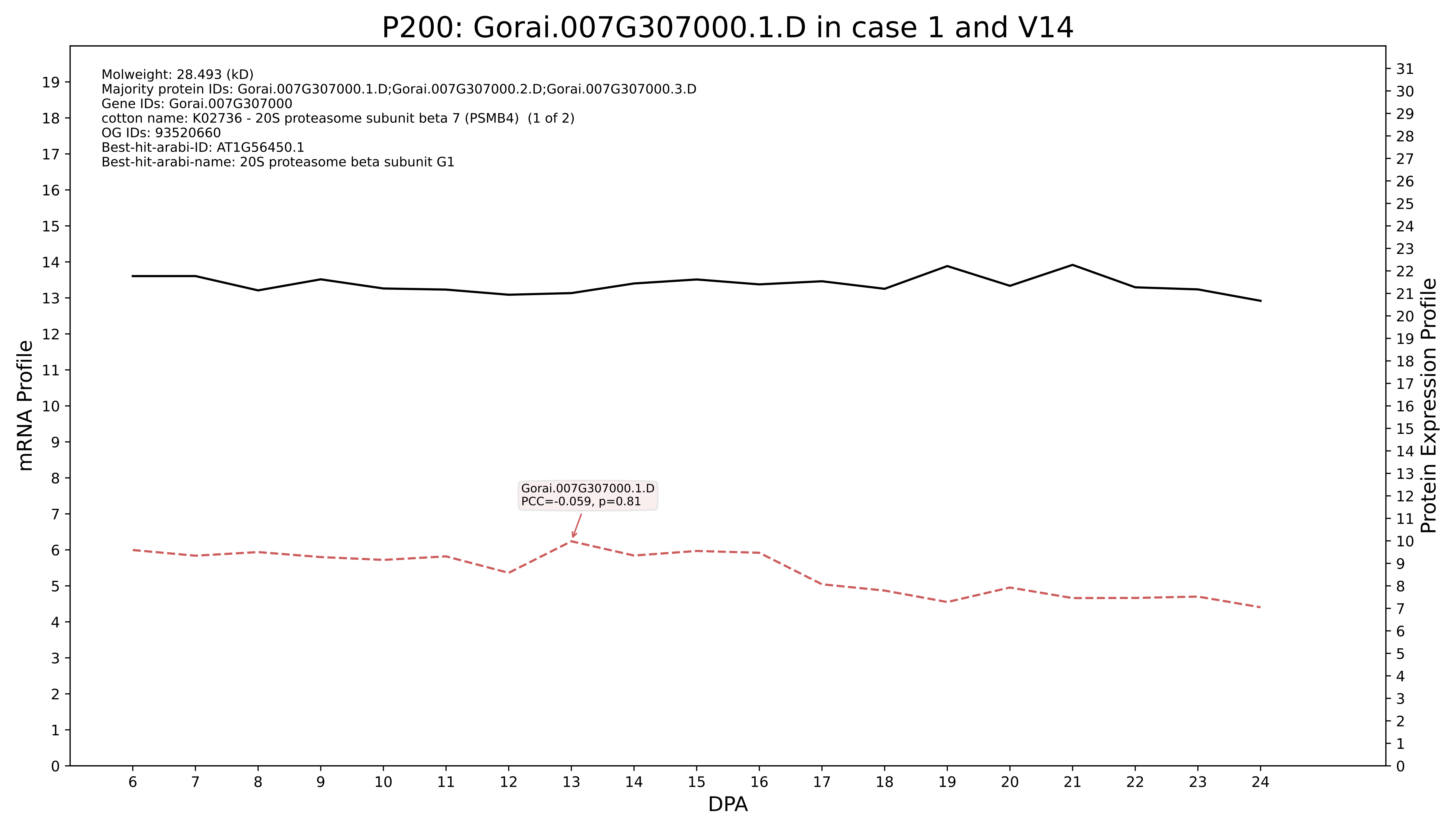
P200: Gorai.007G307000.1.D in case 1 and V14
Molweight: 28.493 (kD)
Majority protein IDs: Gorai.007G307000.1.D;Gorai.007G307000.2.D;Gorai.007G307000.3.D
Gene IDs: Gorai.007G307000
cotton name: K02736 - 20S proteasome subunit beta 7 (PSMB4) (1 of 2)
OG IDs: 93520660
Best-hit-arabi-ID: AT1G56450.1
Best-hit-arabi-name: 20S proteasome beta subunit G1
Gorai.007G307000.1.D
PCC=-0.059, p=0.81
mRNA Profile
Protein Expression Profile
DPA

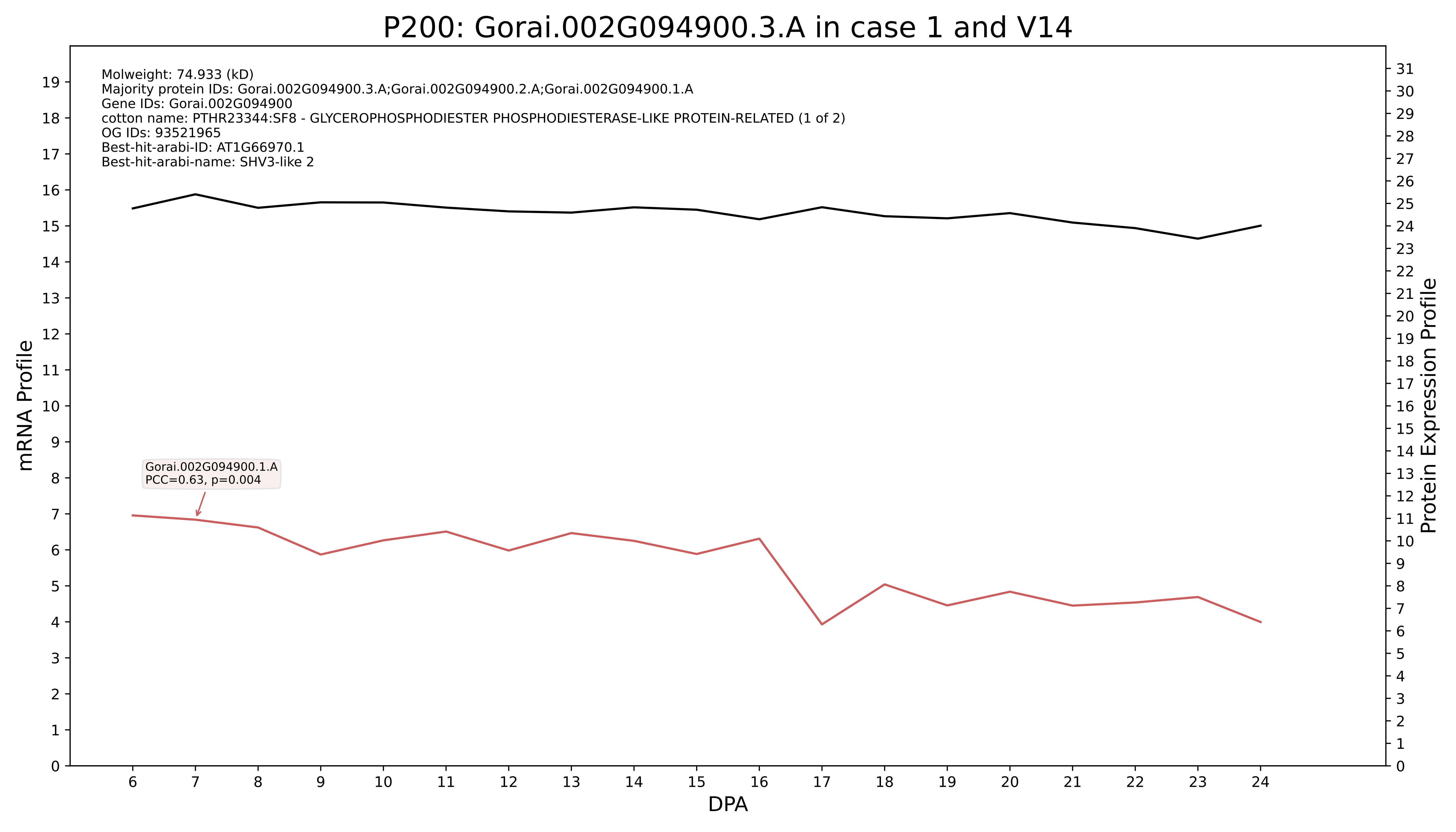
P200: Gorai.002G094900.3.A in case 1 and V14
Molweight: 74.933 (kD)
Majority protein IDs: Gorai.002G094900.3.A;Gorai.002G094900.2.A;Gorai.002G094900.1.A
Gene IDs: Gorai.002G094900
cotton name: PTHR23344:SF8 - GLYCEROPHOSPHODIESTER PHOSPHODIESTERASE-LIKE PROTEIN-RELATED (1 of 2)
OG IDs: 93521965
Best-hit-arabi-ID: AT1G66970.1
Best-hit-arabi-name: SHV3-like 2
Gorai.002G094900.1.A
PCC=0.63, p=0.004
mRNA Profile
Protein Expression Profile
DPA

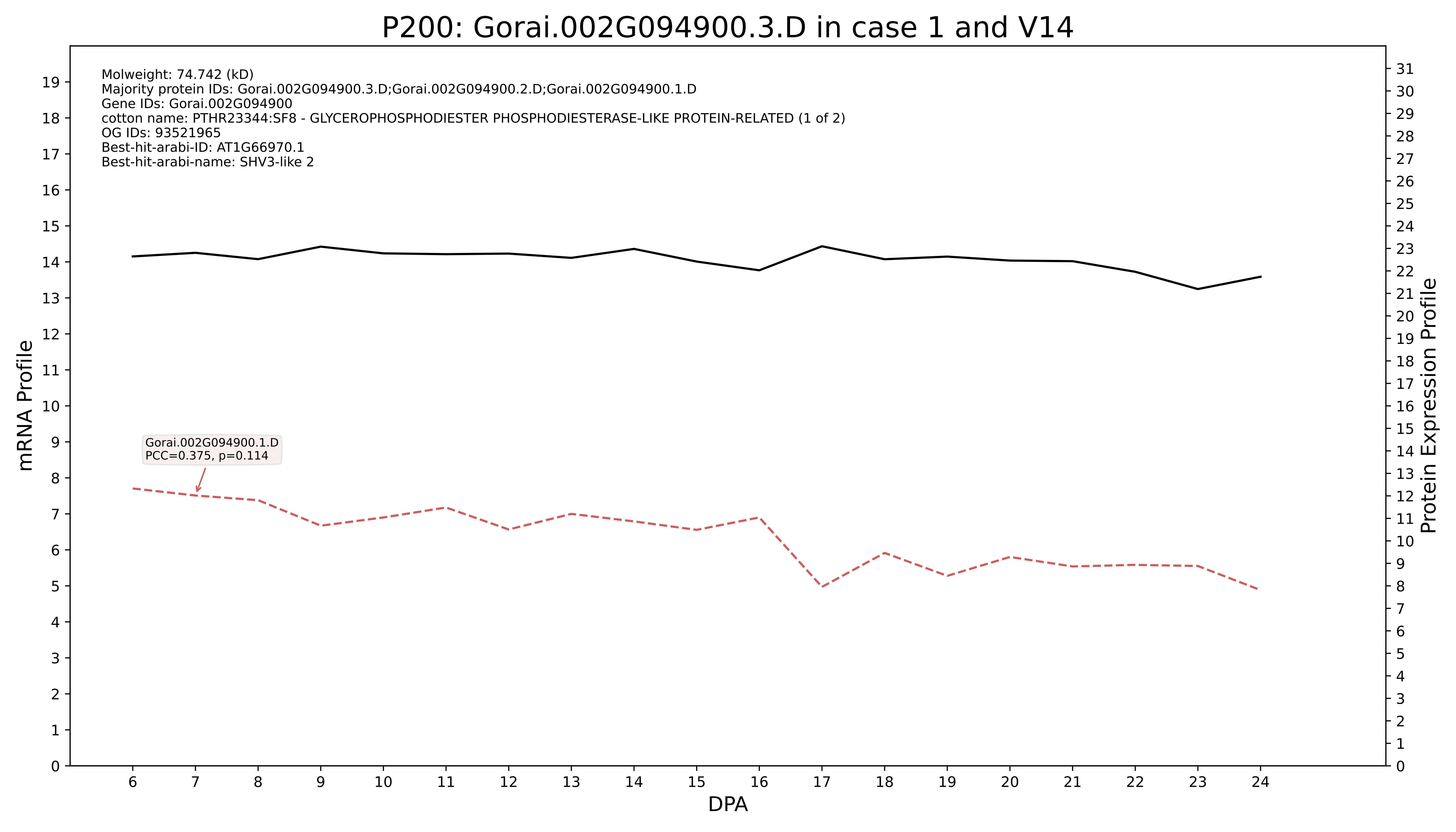
P200: Gorai.002G094900.3.D in case 1 and V14
Molweight: 74.742 (kD)
Majority protein IDs: Gorai.002G094900.3.D;Gorai.002G094900.2.D;Gorai.002G094900.1.D
Gene IDs: Gorai.002G094900
cotton name: PTHR23344:SF8 - GLYCEROPHOSPHODIESTER PHOSPHODIESTERASE-LIKE PROTEIN-RELATED (1 of 2)
OG IDs: 93521965
Best-hit-arabi-ID: AT1G66970.1
Best-hit-arabi-name: SHV3-like 2
Gorai.002G094900.1.D
PCC=0.375, p=0.114
mRNA Profile
Protein Expression Profile
DPA
0
1
2
3
4
5
6
7
8
9
10
11
12
13
14
15
16
17
18
19
20
21
22
23
24
25
26
27
28
29
30
31

P200: Gorai.001G071200.2.D in case 1 and V14

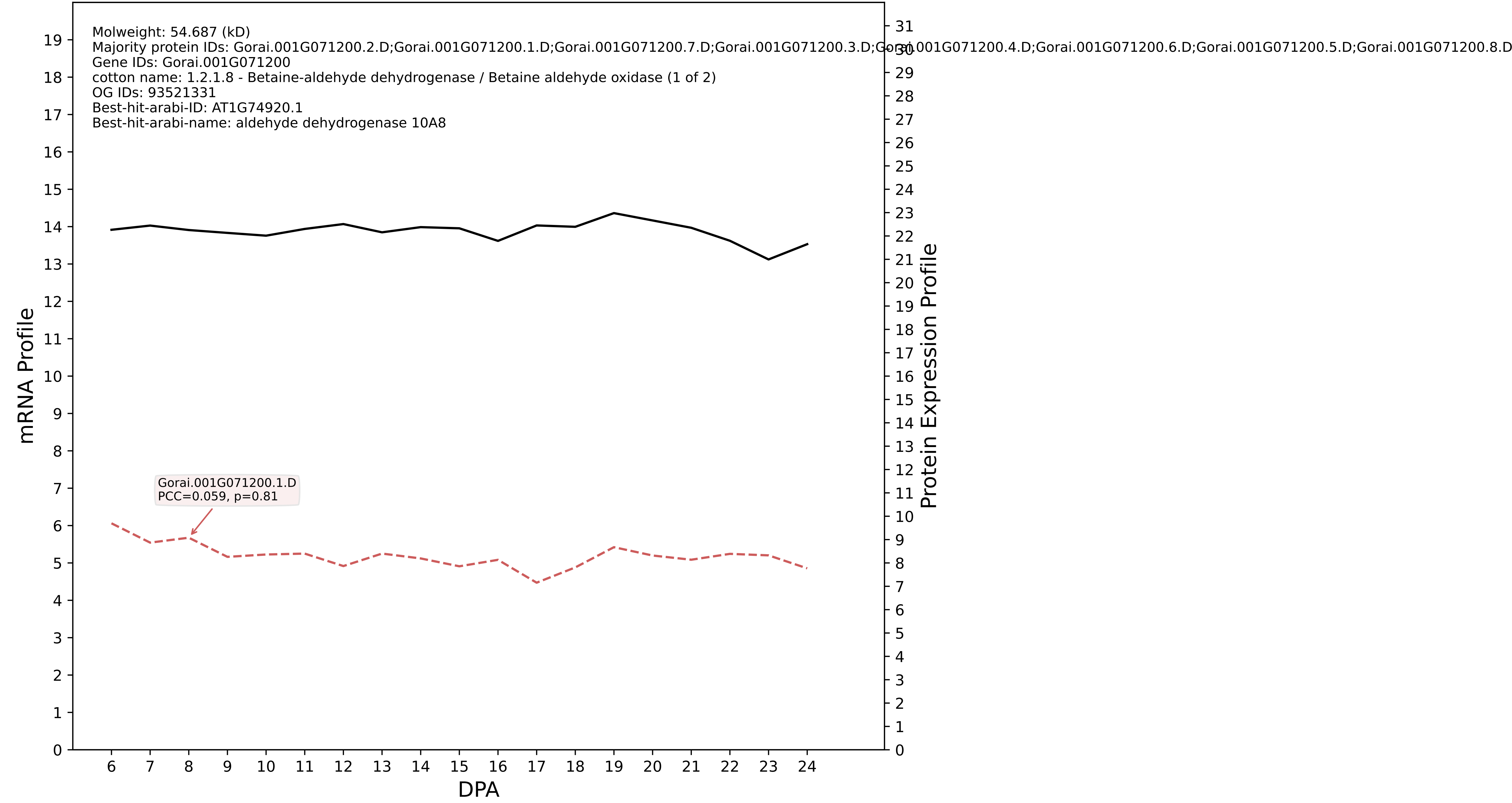

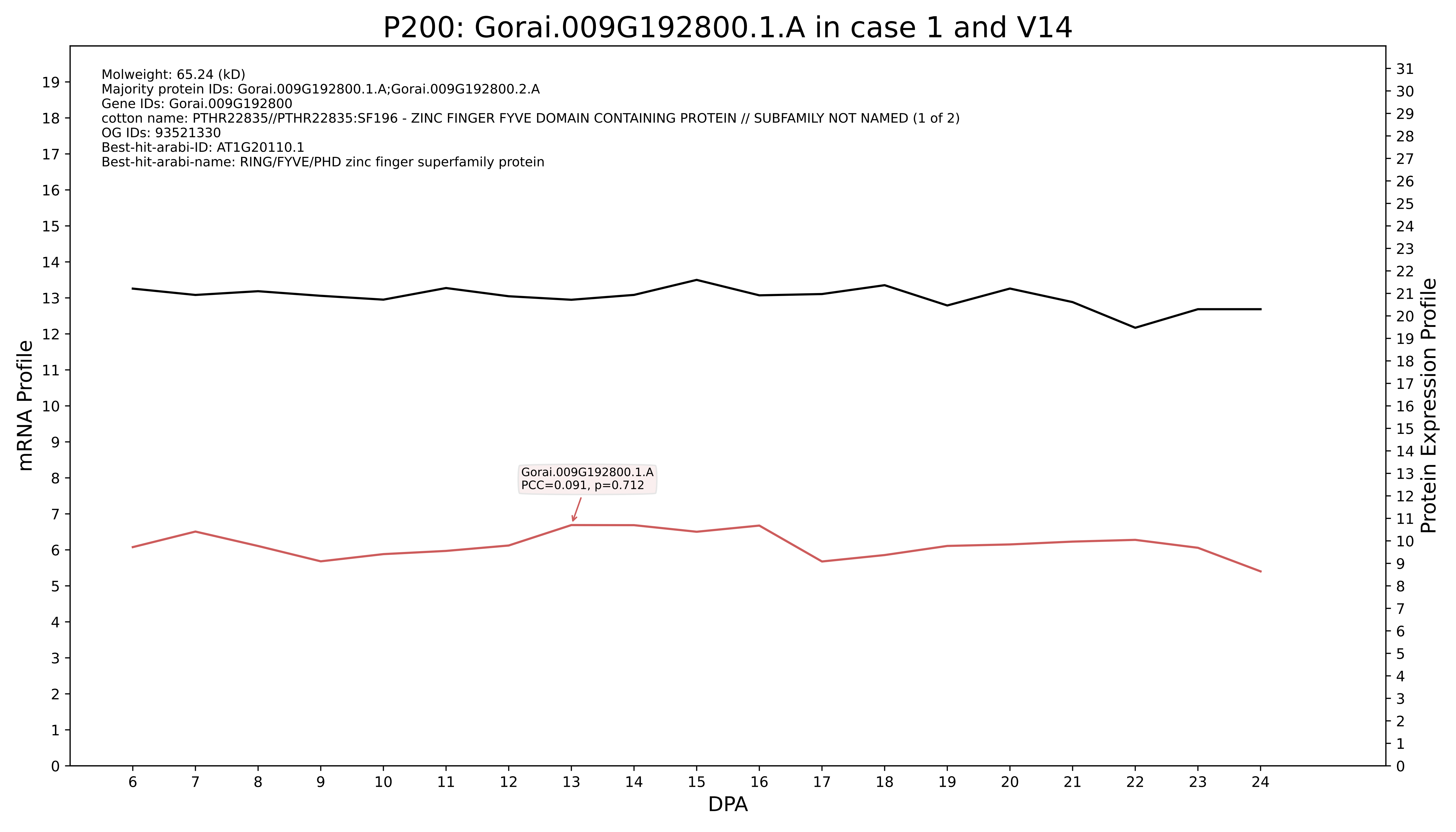

P200: Gorai.009G192800.1.A in case 1 and V14
Molweight: 65.24 (kD)
Majority protein IDs: Gorai.009G192800.1.A;Gorai.009G192800.2.A
Gene IDs: Gorai.009G192800
cotton name: PTHR22835//PTHR22835:SF196 - ZINC FINGER FYVE DOMAIN CONTAINING PROTEIN // SUBFAMILY NOT NAMED (1 of 2)
OG IDs: 93521330
Best-hit-arabi-ID: AT1G20110.1
Best-hit-arabi-name: RING/FYVE/PHD zinc finger superfamily protein
Gorai.009G192800.1.A
PCC=0.091, p=0.712
mRNA Profile
Protein Expression Profile
DPA

P200: Gorai.002G147300.4.A in case 1 and V14

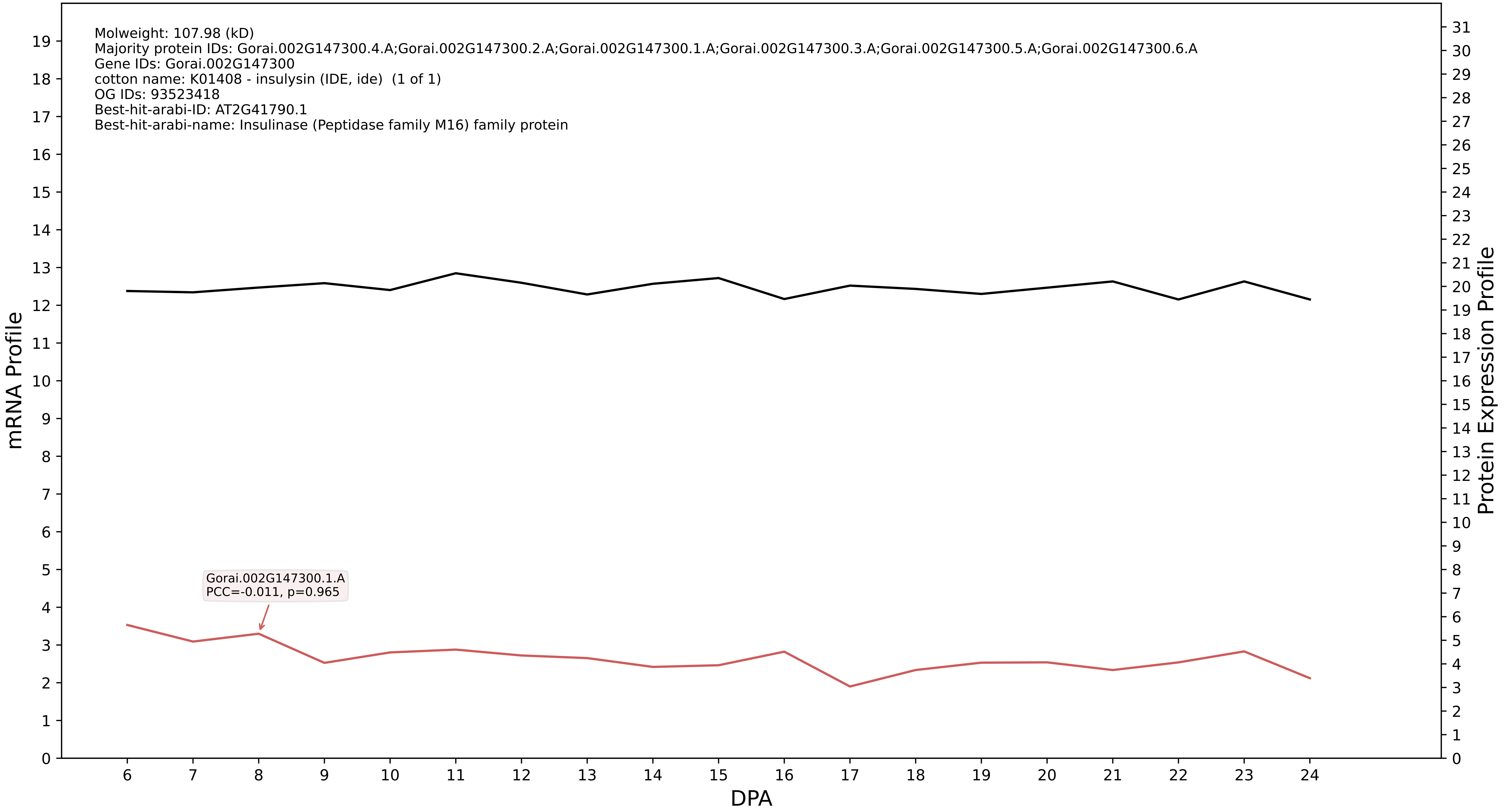

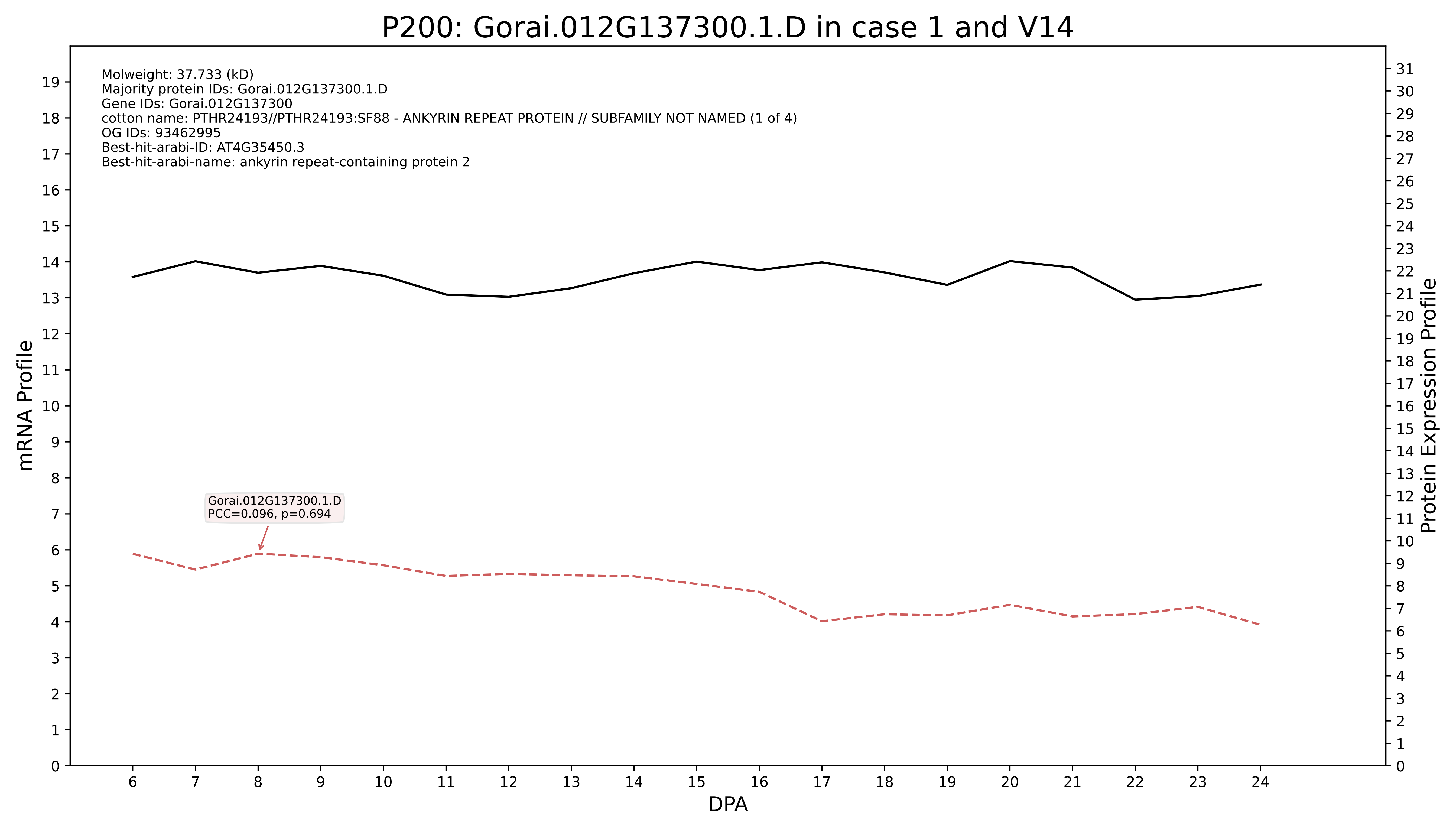
P200: Gorai.012G137300.1.D in case 1 and V14
Molweight: 37.733 (kD)
Majority protein IDs: Gorai.012G137300.1.D
Gene IDs: Gorai.012G137300
cotton name: PTHR24193//PTHR24193:SF88 - ANKYRIN REPEAT PROTEIN // SUBFAMILY NOT NAMED (1 of 4)
OG IDs: 93462995
Best-hit-arabi-ID: AT4G35450.3
Best-hit-arabi-name: ankyrin repeat-containing protein 2
Gorai.012G137300.1.D
PCC=0.096, p=0.694
mRNA Profile
Protein Expression Profile
DPA

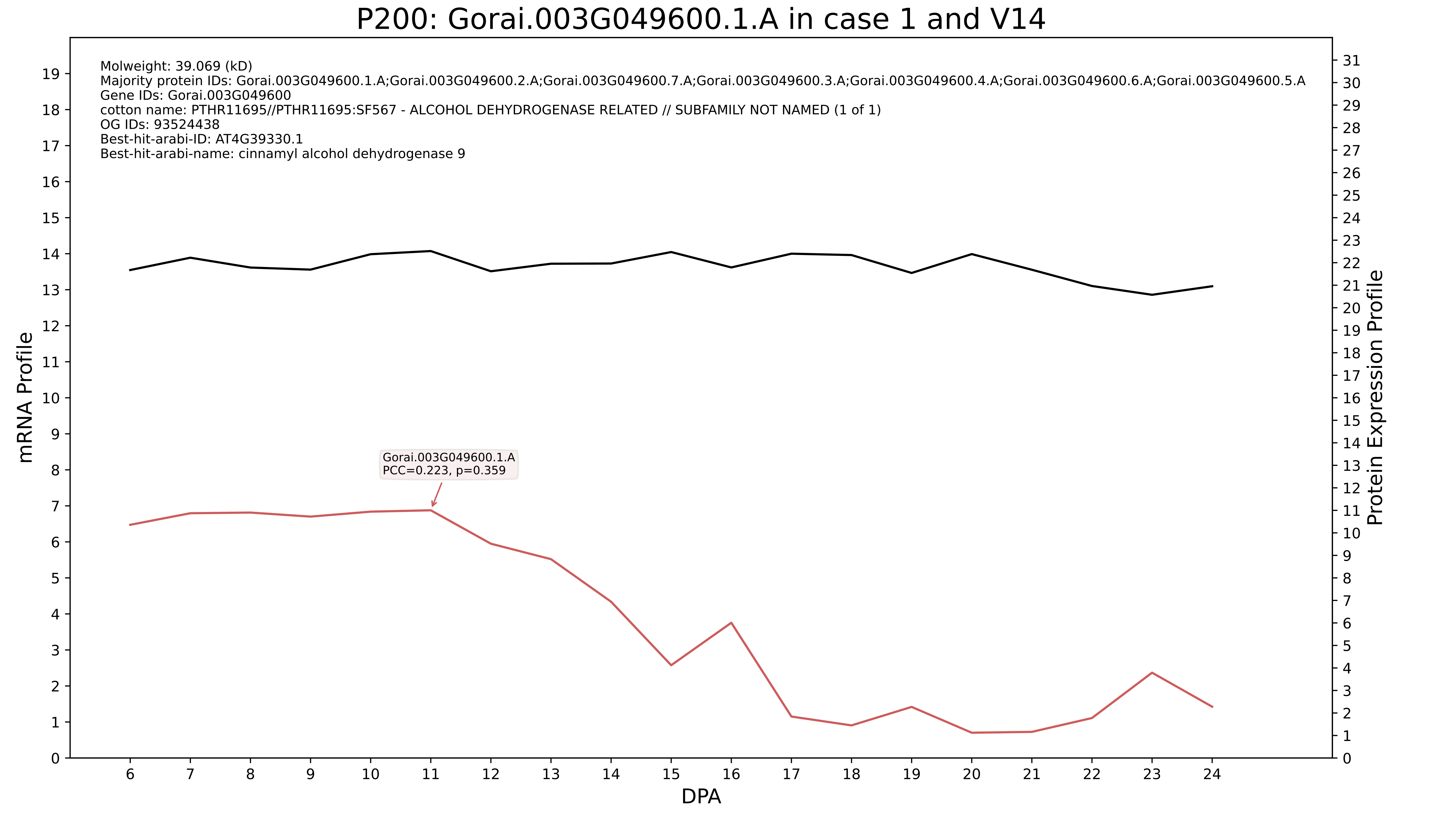

P200: Gorai.003G049600.1.A in case 1 and V14
Molweight: 39.069 (kD)
Majority protein IDs: Gorai.003G049600.1.A;Gorai.003G049600.2.A;Gorai.003G049600.7.A;Gorai.003G049600.3.A;Gorai.003G049600.4.A;Gorai.003G049600.6.A;Gorai.003G049600.5.A
Gene IDs: Gorai.003G049600
cotton name: PTHR11695//PTHR11695:SF567 - ALCOHOL DEHYDROGENASE RELATED // SUBFAMILY NOT NAMED (1 of 1)
OG IDs: 93524438
Best-hit-arabi-ID: AT4G39330.1
Best-hit-arabi-name: cinnamyl alcohol dehydrogenase 9
Gorai.003G049600.1.A
PCC=0.223, p=0.359
mRNA Profile
Protein Expression Profile
DPA

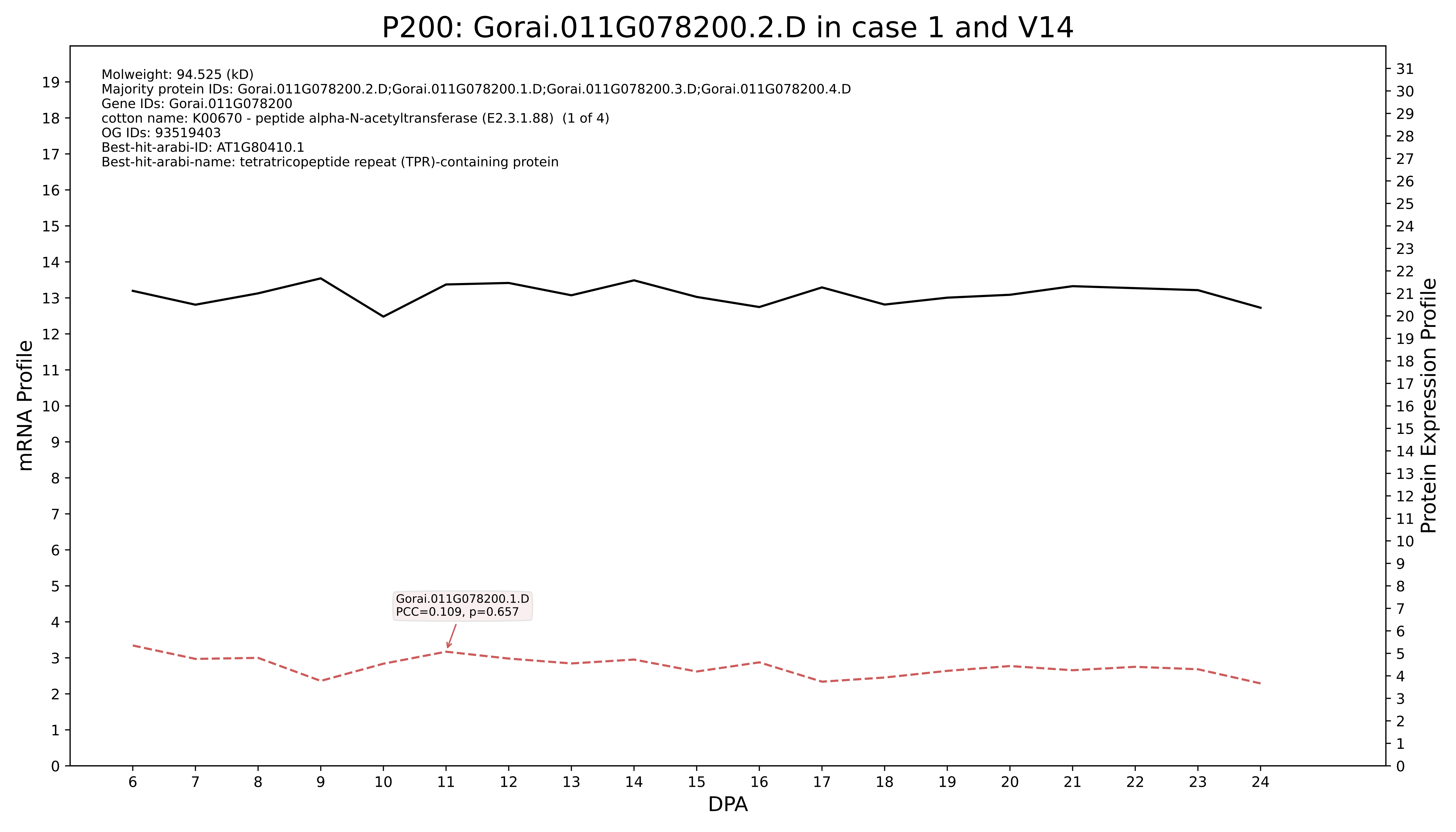

P200: Gorai.011G078200.2.D in case 1 and V14
Molweight: 94.525 (kD)
Majority protein IDs: Gorai.011G078200.2.D;Gorai.011G078200.1.D;Gorai.011G078200.3.D;Gorai.011G078200.4.D
Gene IDs: Gorai.011G078200
cotton name: K00670 - peptide alpha-N-acetyltransferase (E2.3.1.88) (1 of 4)
OG IDs: 93519403
Best-hit-arabi-ID: AT1G80410.1
Best-hit-arabi-name: tetratricopeptide repeat (TPR)-containing protein
Gorai.011G078200.1.D
PCC=0.109, p=0.657
mRNA Profile
Protein Expression Profile
DPA

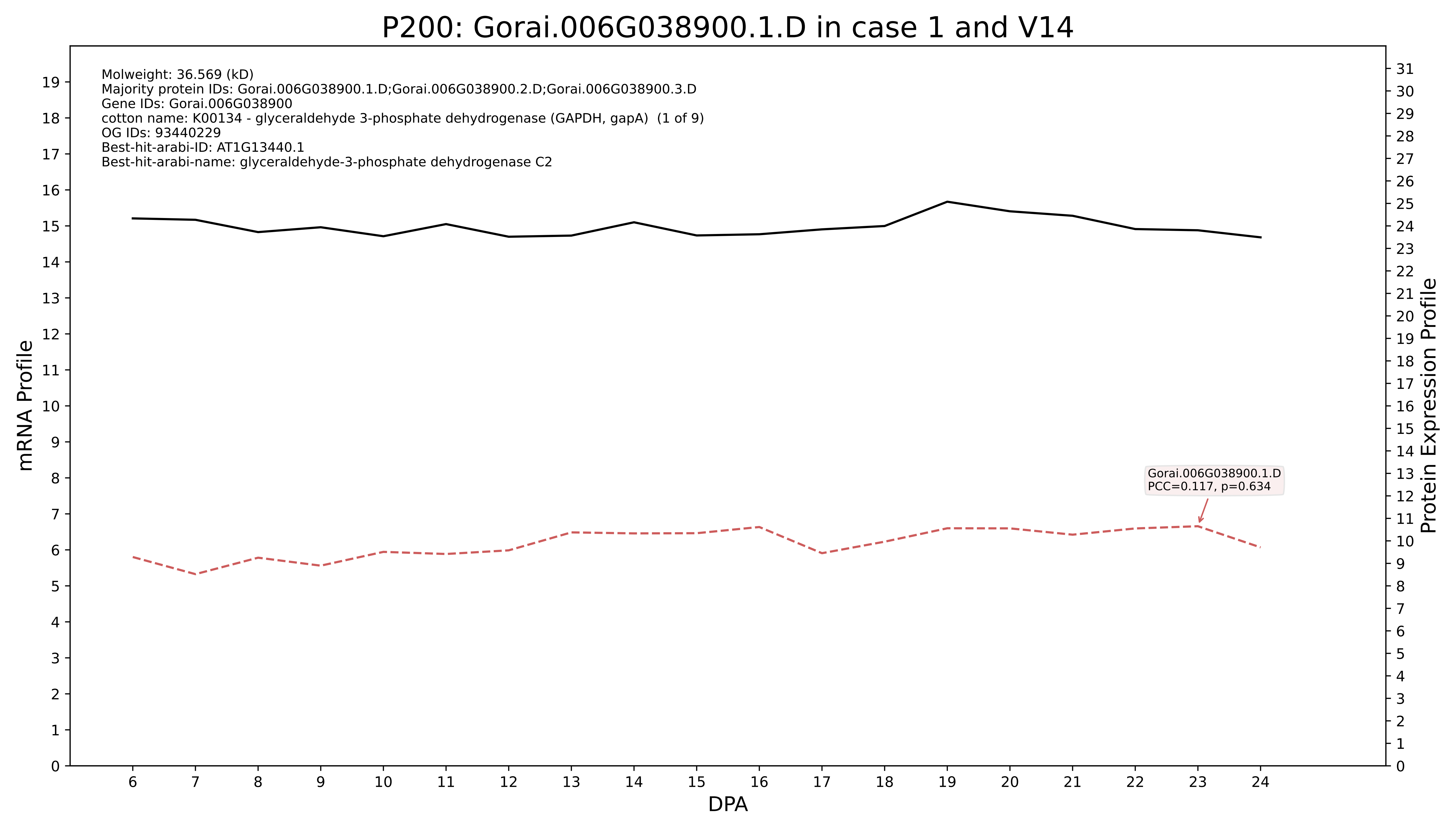
P200: Gorai.006G038900.1.D in case 1 and V14
Molweight: 36.569 (kD)
Majority protein IDs: Gorai.006G038900.1.D;Gorai.006G038900.2.D;Gorai.006G038900.3.D
Gene IDs: Gorai.006G038900
cotton name: K00134 - glyceraldehyde 3-phosphate dehydrogenase (GAPDH, gapA) (1 of 9)
OG IDs: 93440229
Best-hit-arabi-ID: AT1G13440.1
Best-hit-arabi-name: glyceraldehyde-3-phosphate dehydrogenase C2
Gorai.006G038900.1.D
PCC=0.117, p=0.634
mRNA Profile
Protein Expression Profile
DPA

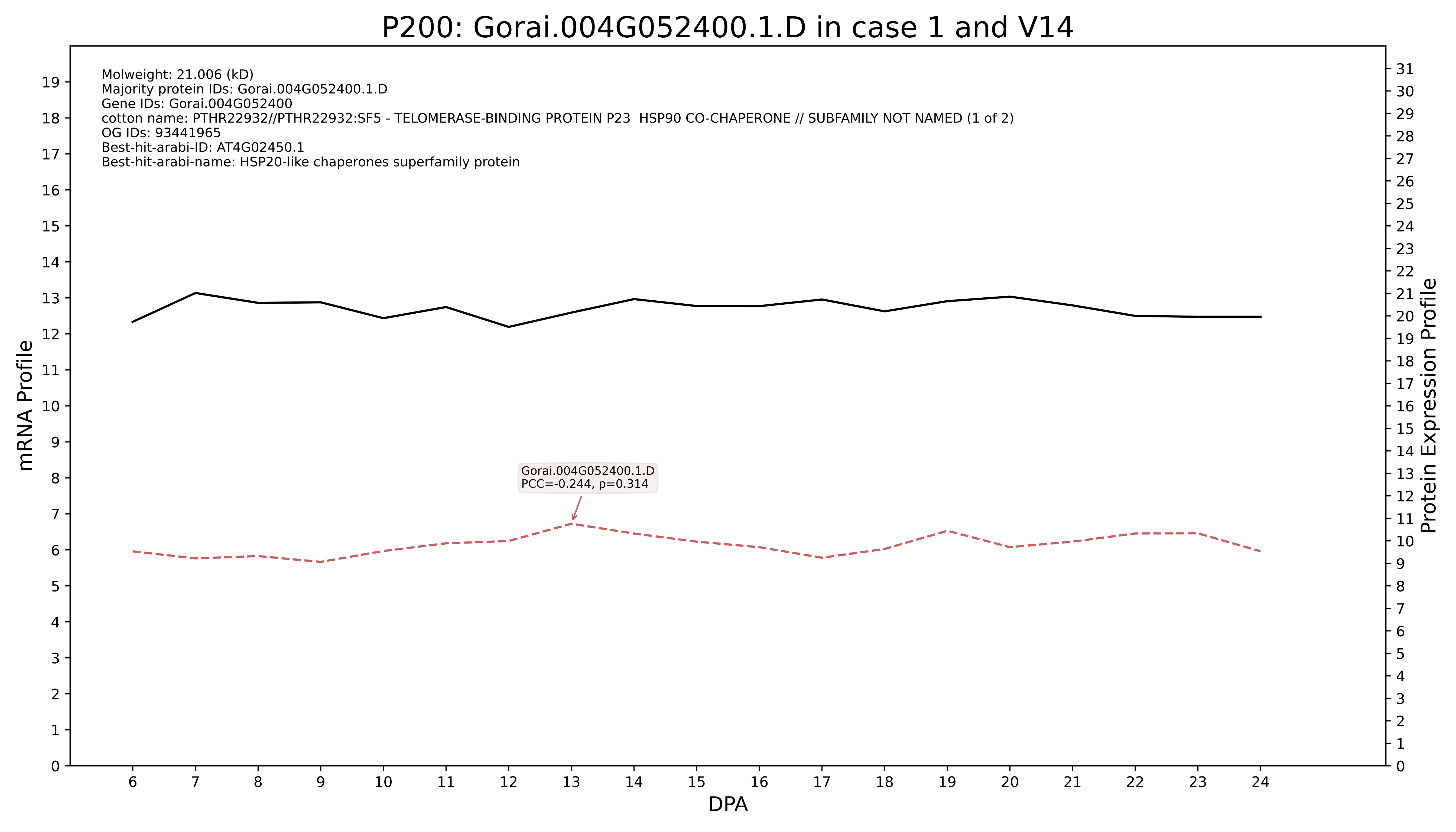

P200: Gorai.004G052400.1.D in case 1 and V14
Molweight: 21.006 (kD)
Majority protein IDs: Gorai.004G052400.1.D
Gene IDs: Gorai.004G052400
cotton name: PTHR22932//PTHR22932:SF5 - TELOMERASE-BINDING PROTEIN P23 HSP90 CO-CHAPERONE // SUBFAMILY NOT NAMED (1 of 2)
OG IDs: 93441965
Best-hit-arabi-ID: AT4G02450.1
Best-hit-arabi-name: HSP20-like chaperones superfamily protein
Gorai.004G052400.1.D
PCC=-0.244, p=0.314
mRNA Profile
Protein Expression Profile
DPA

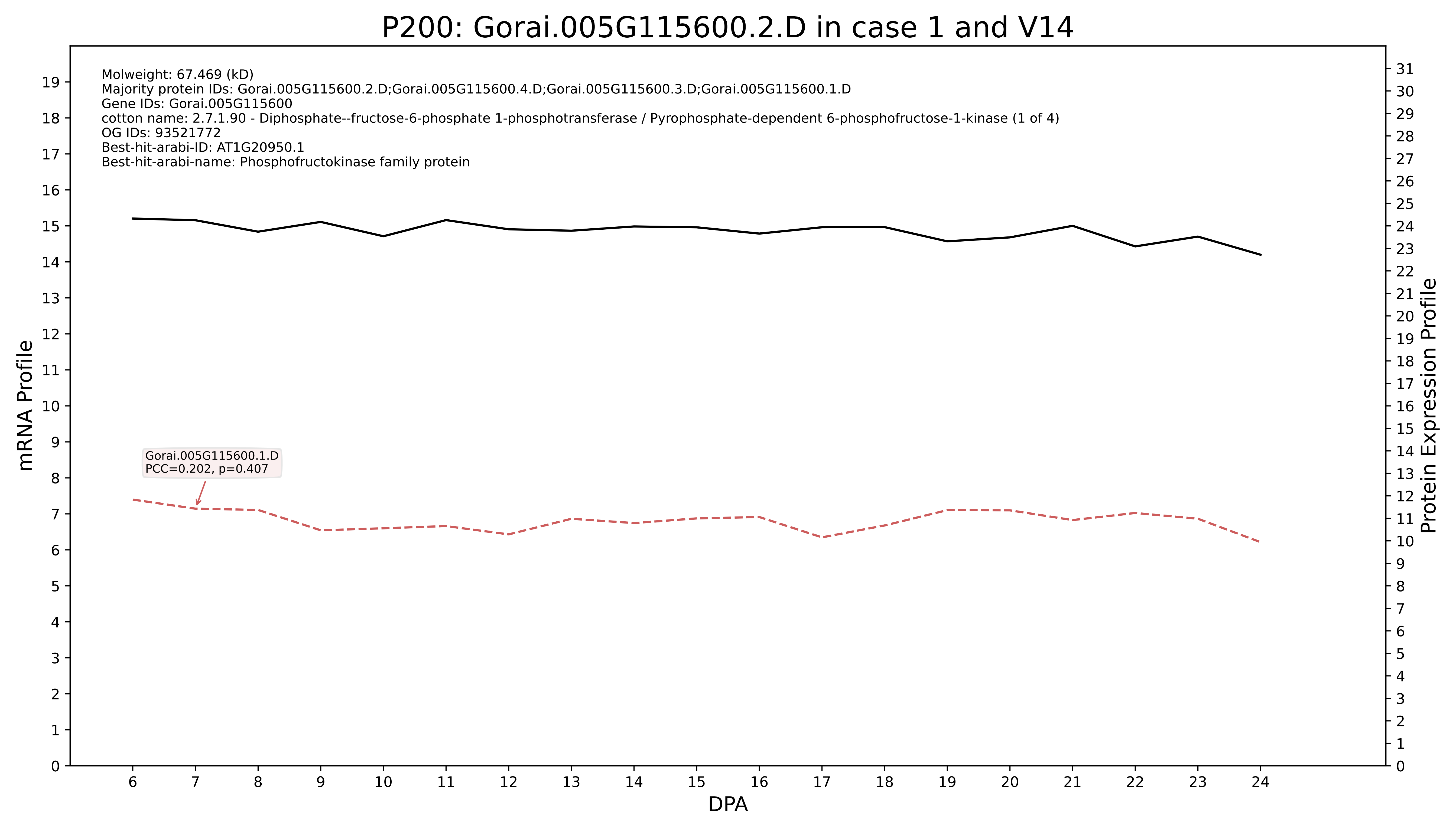
P200: Gorai.005G115600.2.D in case 1 and V14
Molweight: 67.469 (kD)
Majority protein IDs: Gorai.005G115600.2.D;Gorai.005G115600.4.D;Gorai.005G115600.3.D;Gorai.005G115600.1.D
Gene IDs: Gorai.005G115600
cotton name: 2.7.1.90 - Diphosphate--fructose-6-phosphate 1-phosphotransferase / Pyrophosphate-dependent 6-phosphofructose-1-kinase (1 of 4)
OG IDs: 93521772
Best-hit-arabi-ID: AT1G20950.1
Best-hit-arabi-name: Phosphofructokinase family protein
Gorai.005G115600.1.D
PCC=0.202, p=0.407
mRNA Profile
Protein Expression Profile
DPA

P200: Gorai.013G146600.1.A in case 1 and V14

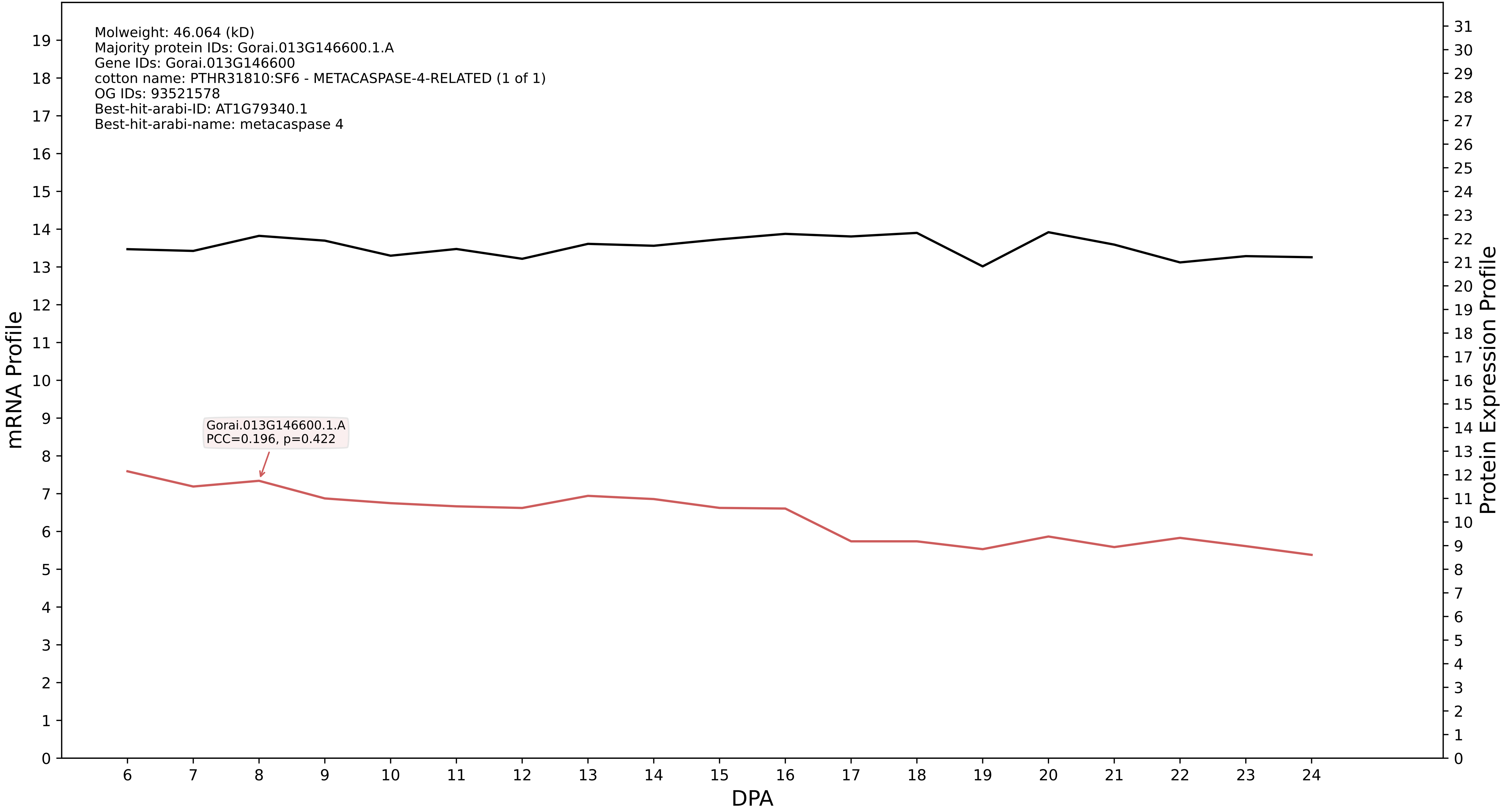

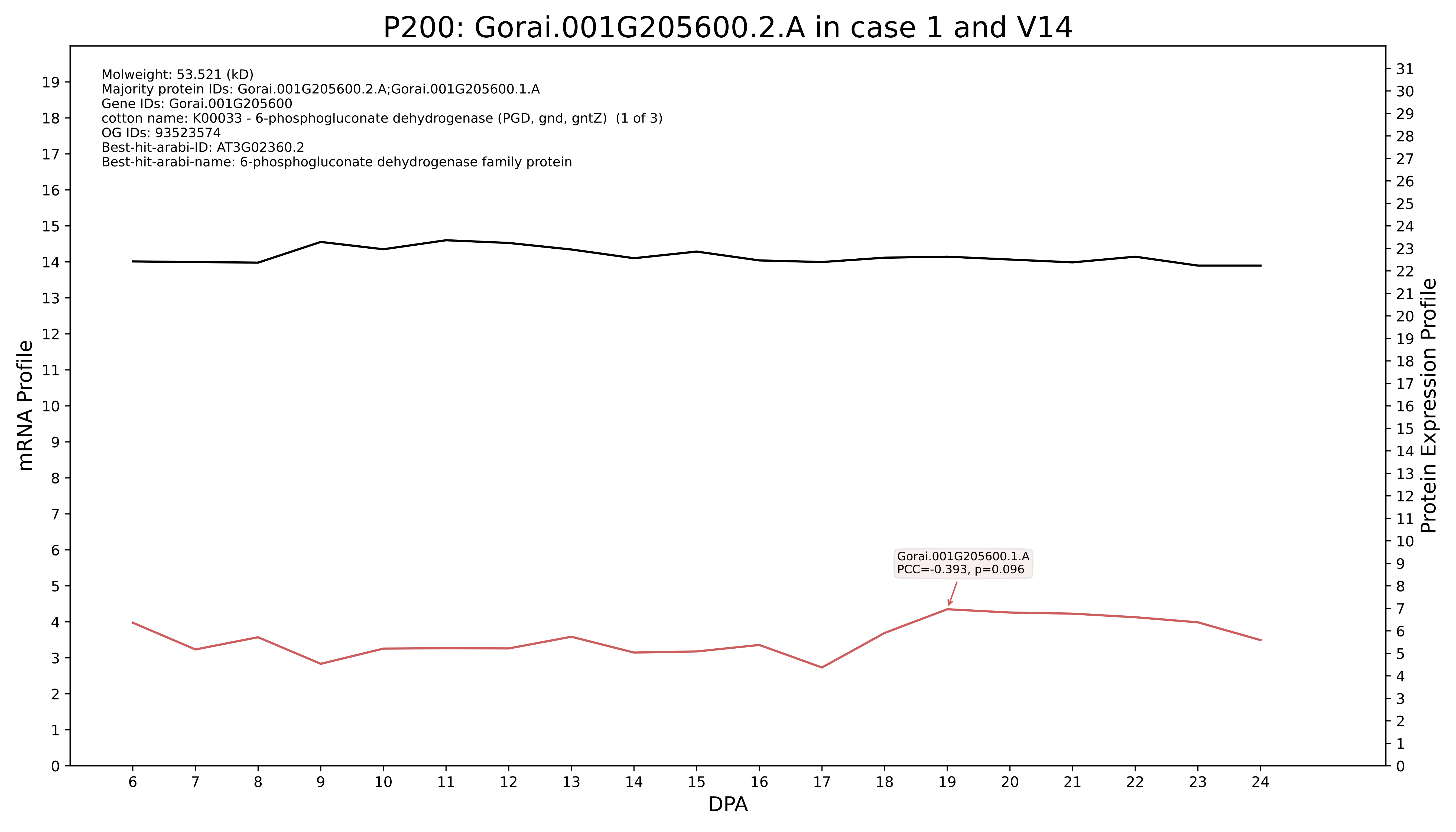
P200: Gorai.001G205600.2.A in case 1 and V14
Molweight: 53.521 (kD)
Majority protein IDs: Gorai.001G205600.2.A;Gorai.001G205600.1.A
Gene IDs: Gorai.001G205600
cotton name: K00033 - 6-phosphogluconate dehydrogenase (PGD, gnd, gntZ) (1 of 3)
OG IDs: 93523574
Best-hit-arabi-ID: AT3G02360.2
Best-hit-arabi-name: 6-phosphogluconate dehydrogenase family protein
Gorai.001G205600.1.A
PCC=-0.393, p=0.096
mRNA Profile
Protein Expression Profile
DPA
0
1
2
3
4
5
6
7
8
9
10
11
12
13
14
15
16
17
18
19
20
21
22
23
24
25
26
27
28
29
30
31

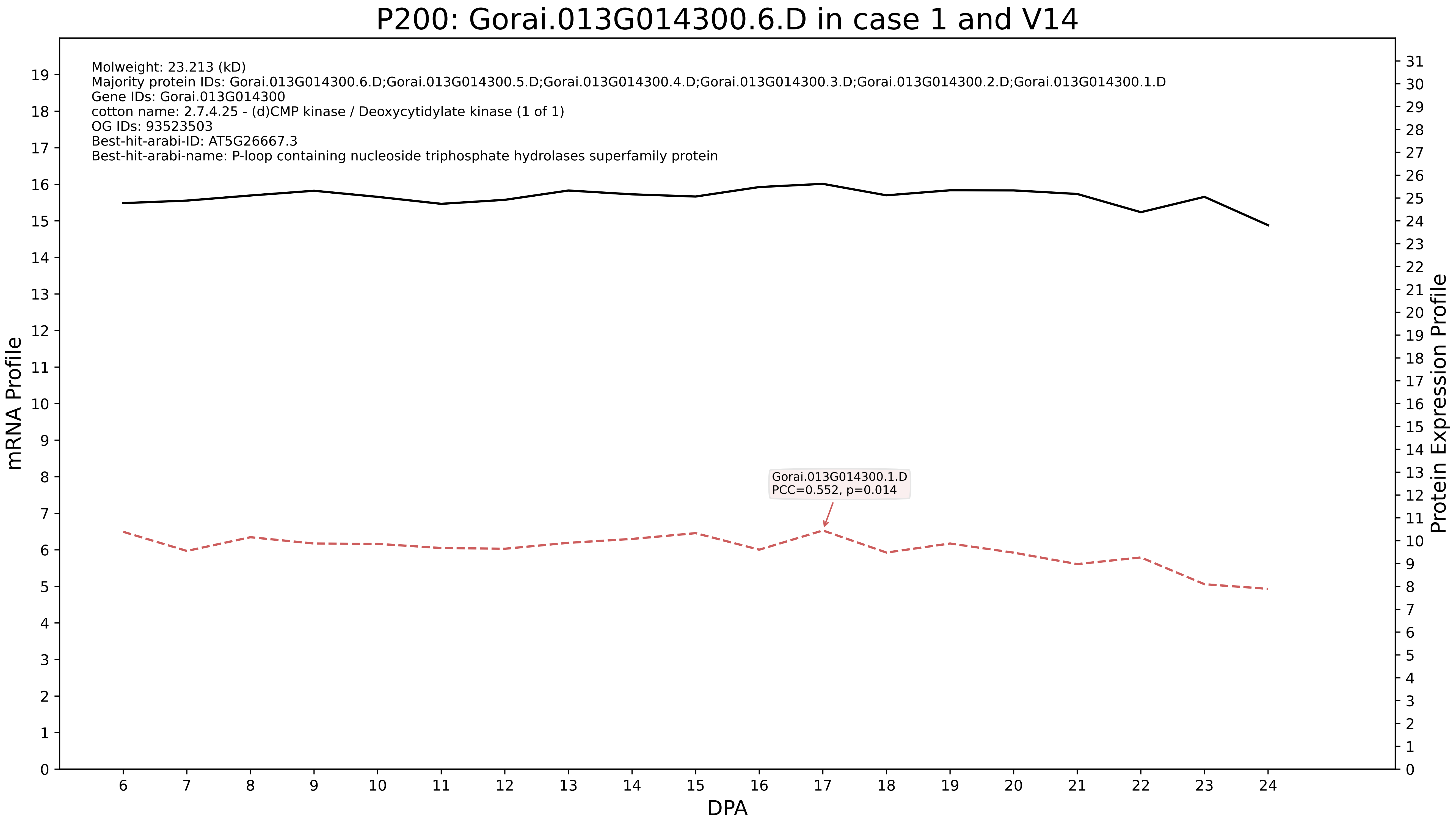
P200: Gorai.013G014300.6.D in case 1 and V14
Molweight: 23.213 (kD)
Majority protein IDs: Gorai.013G014300.6.D;Gorai.013G014300.5.D;Gorai.013G014300.4.D;Gorai.013G014300.3.D;Gorai.013G014300.2.D;Gorai.013G014300.1.D
Gene IDs: Gorai.013G014300
cotton name: 2.7.4.25 - (d)CMP kinase / Deoxycytidylate kinase (1 of 1)
OG IDs: 93523503
Best-hit-arabi-ID: AT5G26667.3
Best-hit-arabi-name: P-loop containing nucleoside triphosphate hydrolases superfamily protein
Gorai.013G014300.1.D
PCC=0.552, p=0.014
mRNA Profile
Protein Expression Profile
DPA

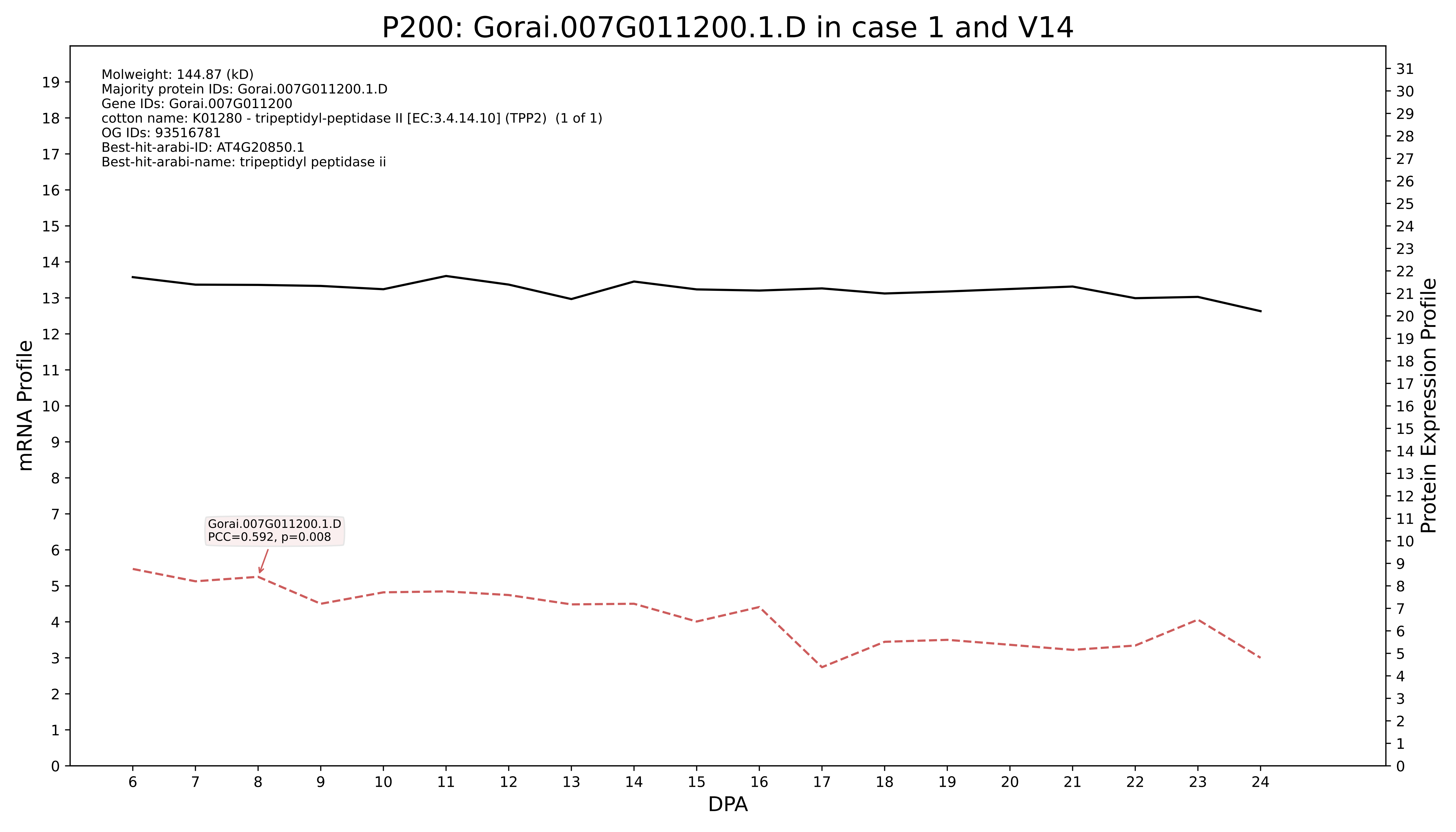
P200: Gorai.007G011200.1.D in case 1 and V14
Molweight: 144.87 (kD)
Majority protein IDs: Gorai.007G011200.1.D
Gene IDs: Gorai.007G011200
cotton name: K01280 - tripeptidyl-peptidase II [EC:3.4.14.10] (TPP2) (1 of 1)
OG IDs: 93516781
Best-hit-arabi-ID: AT4G20850.1
Best-hit-arabi-name: tripeptidyl peptidase ii
Gorai.007G011200.1.D
PCC=0.592, p=0.008
mRNA Profile
Protein Expression Profile
DPA

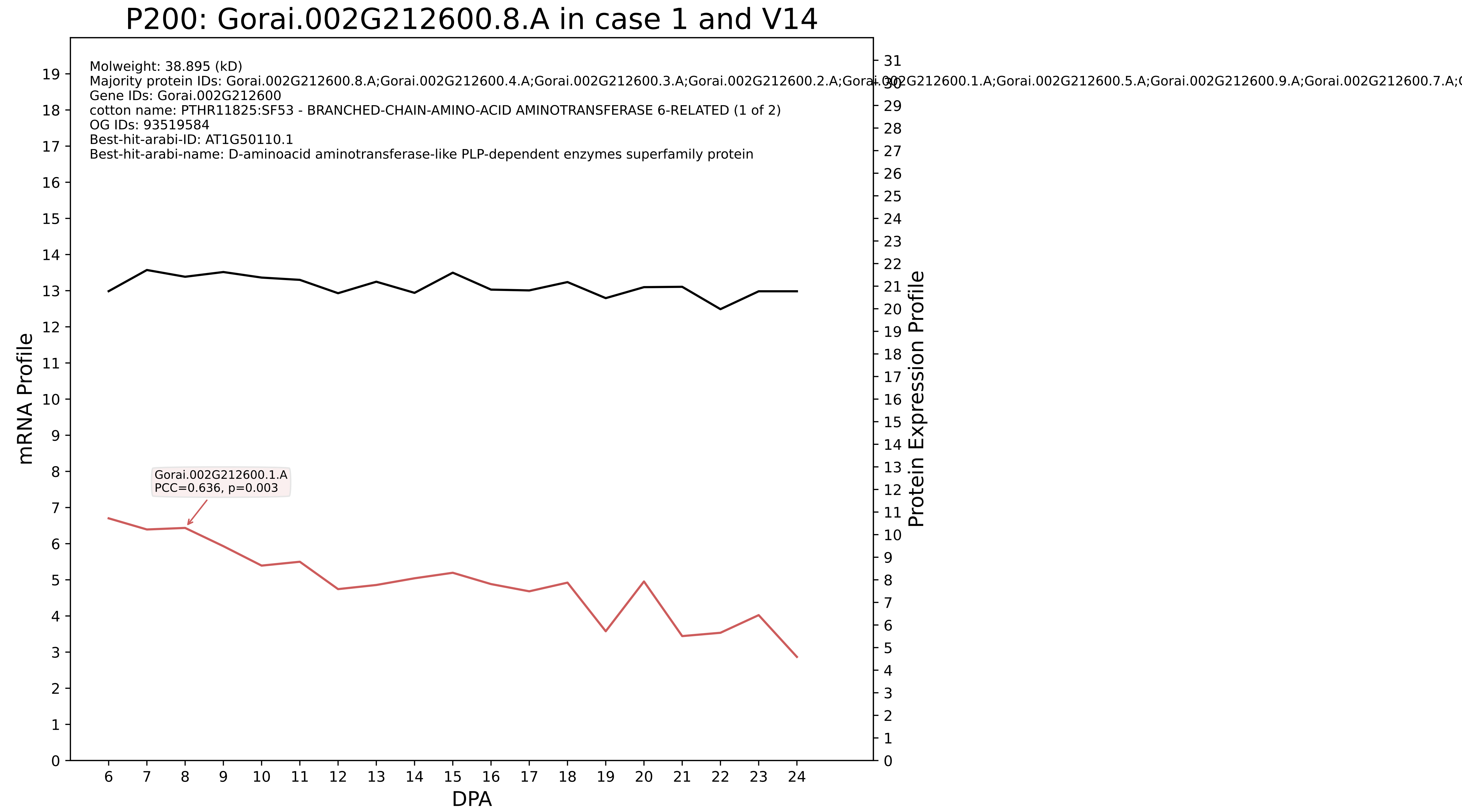

P200: Gorai.002G212600.8.A in case 1 and V14
Molweight: 38.895 (kD)
Majority protein IDs: Gorai.002G212600.8.A;Gorai.002G212600.4.A;Gorai.002G212600.3.A;Gorai.002G212600.2.A;Gorai.002G212600.1.A;Gorai.002G212600.5.A;Gorai.002G212600.9.A;Gorai.002G212600.7.A;G
Gene IDs: Gorai.002G212600
cotton name: PTHR11825:SF53 - BRANCHED-CHAIN-AMINO-ACID AMINOTRANSFERASE 6-RELATED (1 of 2)
OG IDs: 93519584
Best-hit-arabi-ID: AT1G50110.1
Best-hit-arabi-name: D-aminoacid aminotransferase-like PLP-dependent enzymes superfamily protein
Gorai.002G212600.1.A
PCC=0.636, p=0.003
mRNA Profile
Protein Expression Profile
DPA

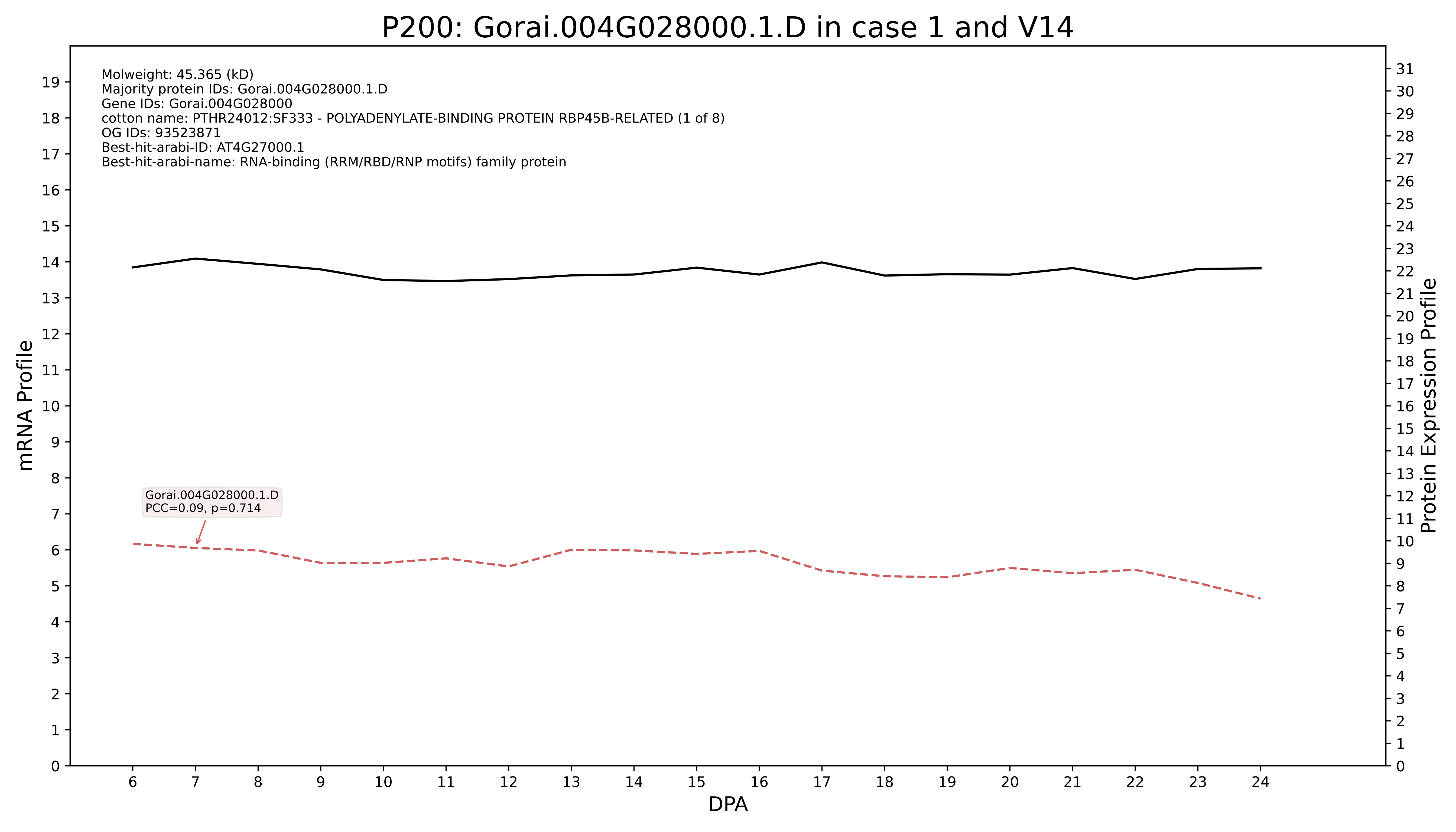
P200: Gorai.004G028000.1.D in case 1 and V14
Molweight: 45.365 (kD)
Majority protein IDs: Gorai.004G028000.1.D
Gene IDs: Gorai.004G028000
cotton name: PTHR24012:SF333 - POLYADENYLATE-BINDING PROTEIN RBP45B-RELATED (1 of 8)
OG IDs: 93523871
Best-hit-arabi-ID: AT4G27000.1
Best-hit-arabi-name: RNA-binding (RRM/RBD/RNP motifs) family protein
Gorai.004G028000.1.D
PCC=0.09, p=0.714
mRNA Profile
Protein Expression Profile
DPA

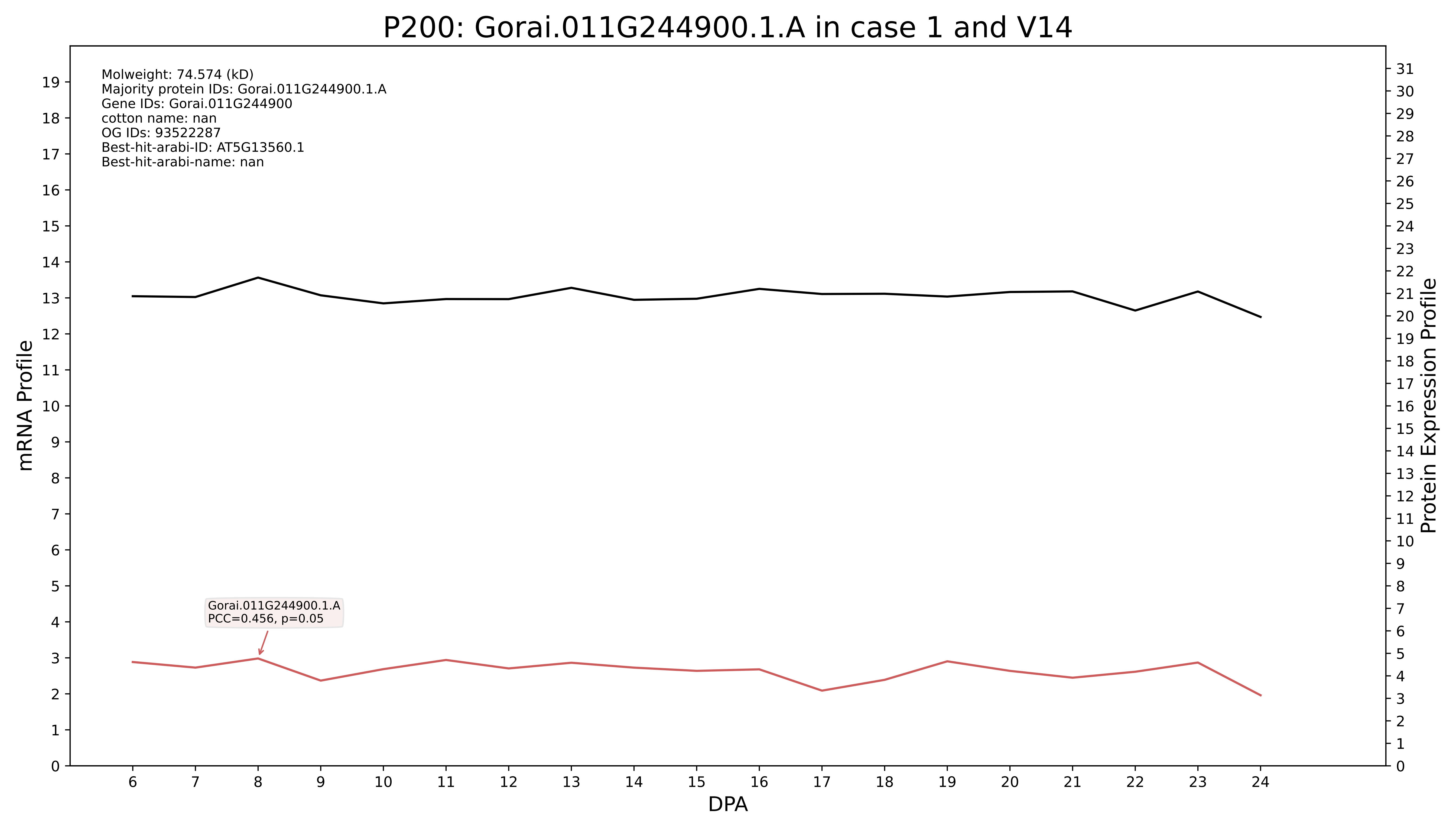
P200: Gorai.011G244900.1.A in case 1 and V14
Molweight: 74.574 (kD)
Majority protein IDs: Gorai.011G244900.1.A
Gene IDs: Gorai.011G244900
cotton name: nan
OG IDs: 93522287
Best-hit-arabi-ID: AT5G13560.1
Best-hit-arabi-name: nan
Gorai.011G244900.1.A
PCC=0.456, p=0.05
mRNA Profile
Protein Expression Profile
DPA

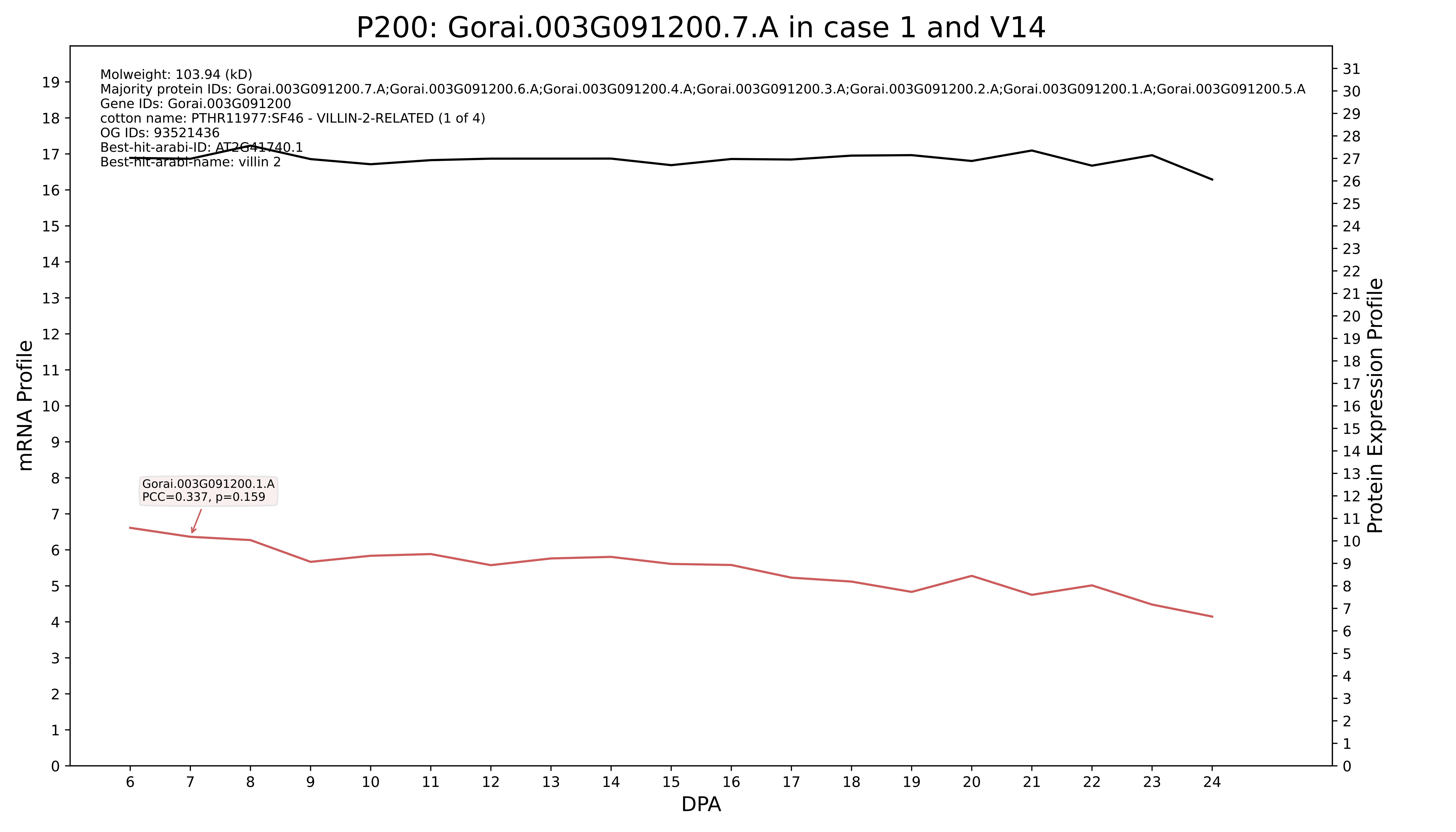
P200: Gorai.003G091200.7.A in case 1 and V14
Molweight: 103.94 (kD)
Majority protein IDs: Gorai.003G091200.7.A;Gorai.003G091200.6.A;Gorai.003G091200.4.A;Gorai.003G091200.3.A;Gorai.003G091200.2.A;Gorai.003G091200.1.A;Gorai.003G091200.5.A
Gene IDs: Gorai.003G091200
cotton name: PTHR11977:SF46 - VILLIN-2-RELATED (1 of 4)
OG IDs: 93521436
Best-hit-arabi-ID: AT2G41740.1
Best-hit-arabi-name: villin 2
Gorai.003G091200.1.A
PCC=0.337, p=0.159
mRNA Profile
Protein Expression Profile
DPA

P200: Gorai.010G098500.4.D in case 1 and V14

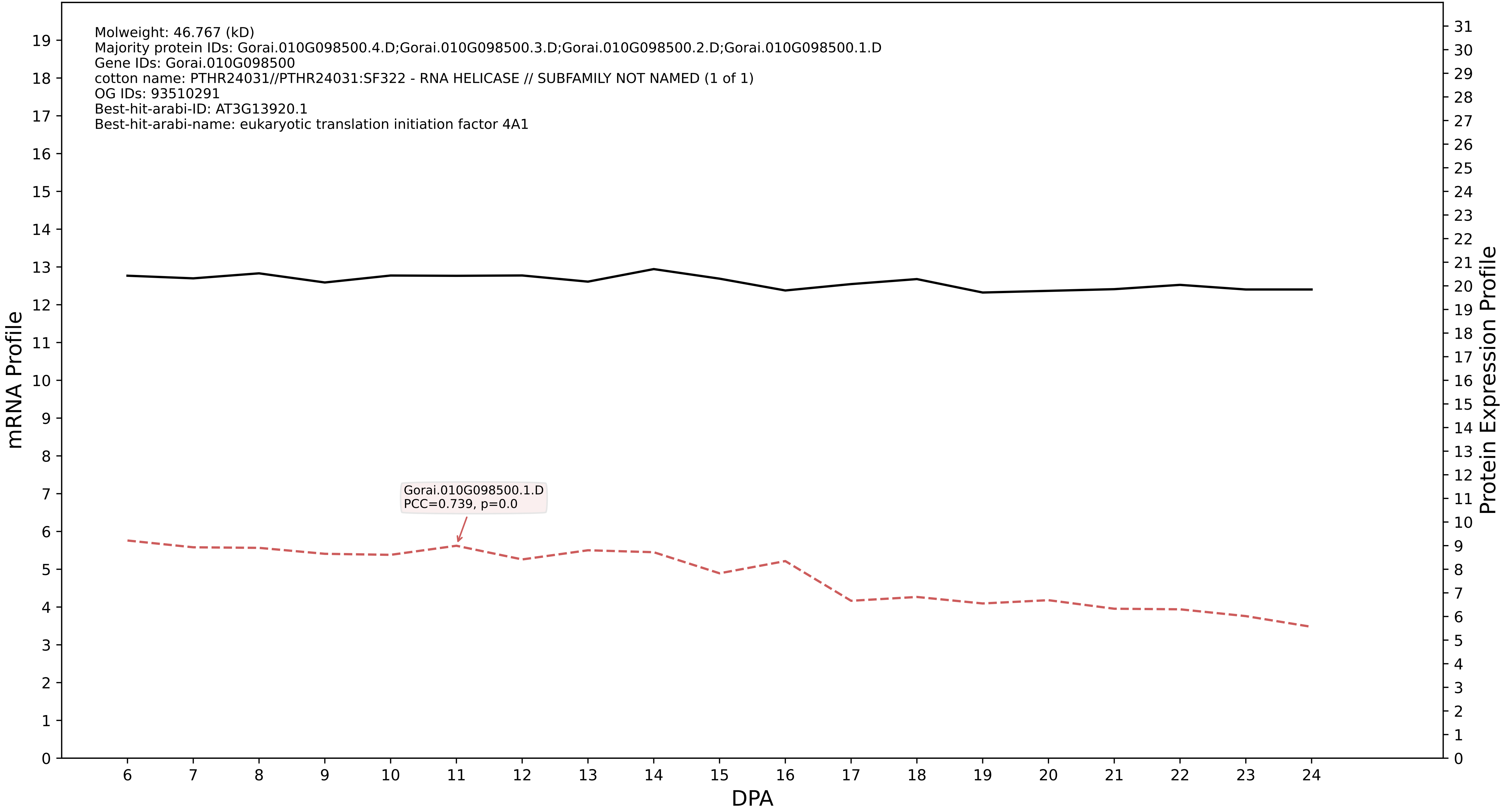

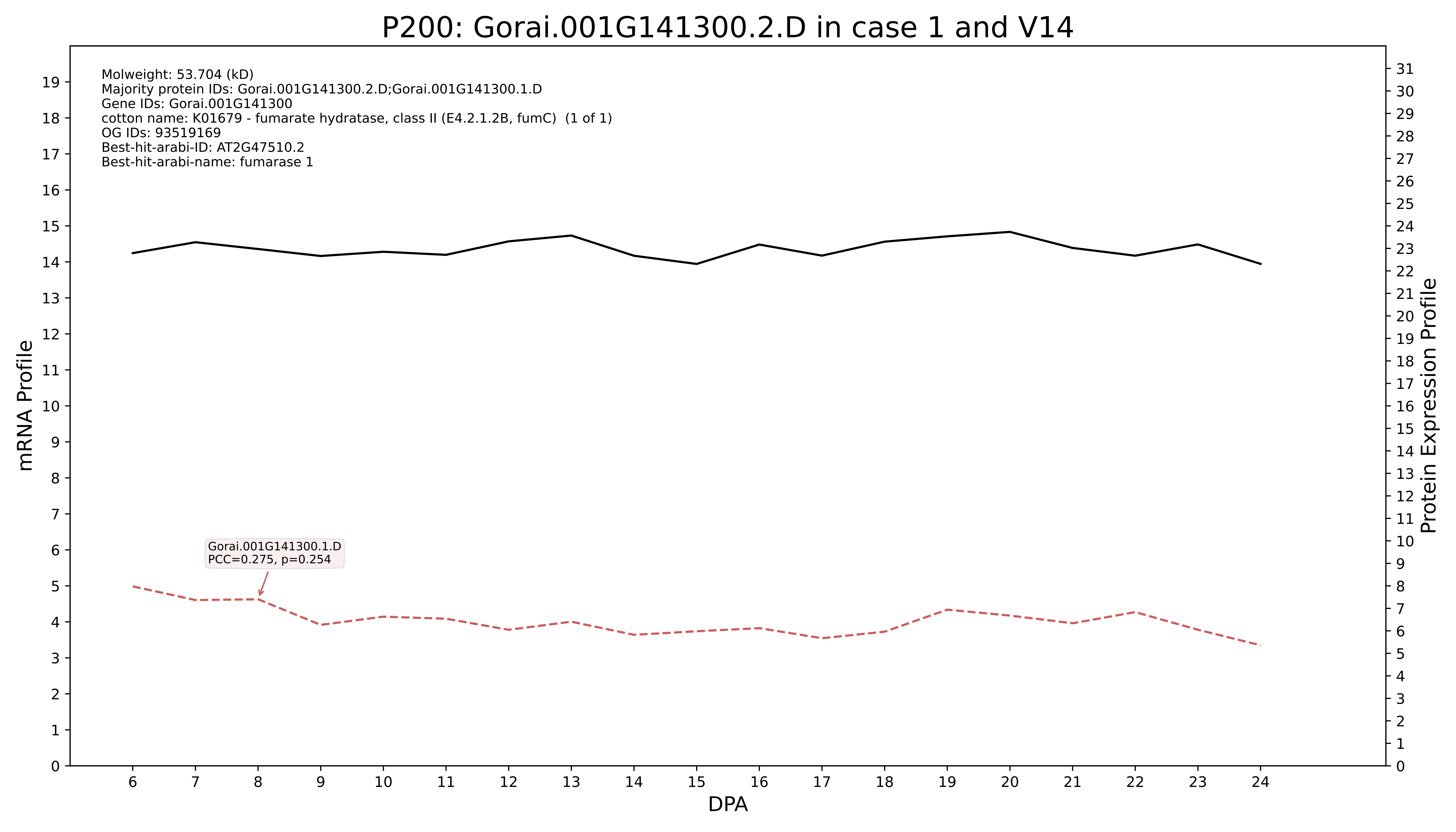
P200: Gorai.001G141300.2.D in case 1 and V14
Molweight: 53.704 (kD)
Majority protein IDs: Gorai.001G141300.2.D;Gorai.001G141300.1.D
Gene IDs: Gorai.001G141300
cotton name: K01679 - fumarate hydratase, class II (E4.2.1.2B, fumC) (1 of 1)
OG IDs: 93519169
Best-hit-arabi-ID: AT2G47510.2
Best-hit-arabi-name: fumarase 1
Gorai.001G141300.1.D
PCC=0.275, p=0.254
mRNA Profile
Protein Expression Profile
DPA
0
1
2
3
4
5
6
7
8
9
10
11
12
13
14
15
16
17
18
19
20
21
22
23
24
25
26
27
28
29
30
31

P200: Gorai.008G010500.3.A in case 1 and V14

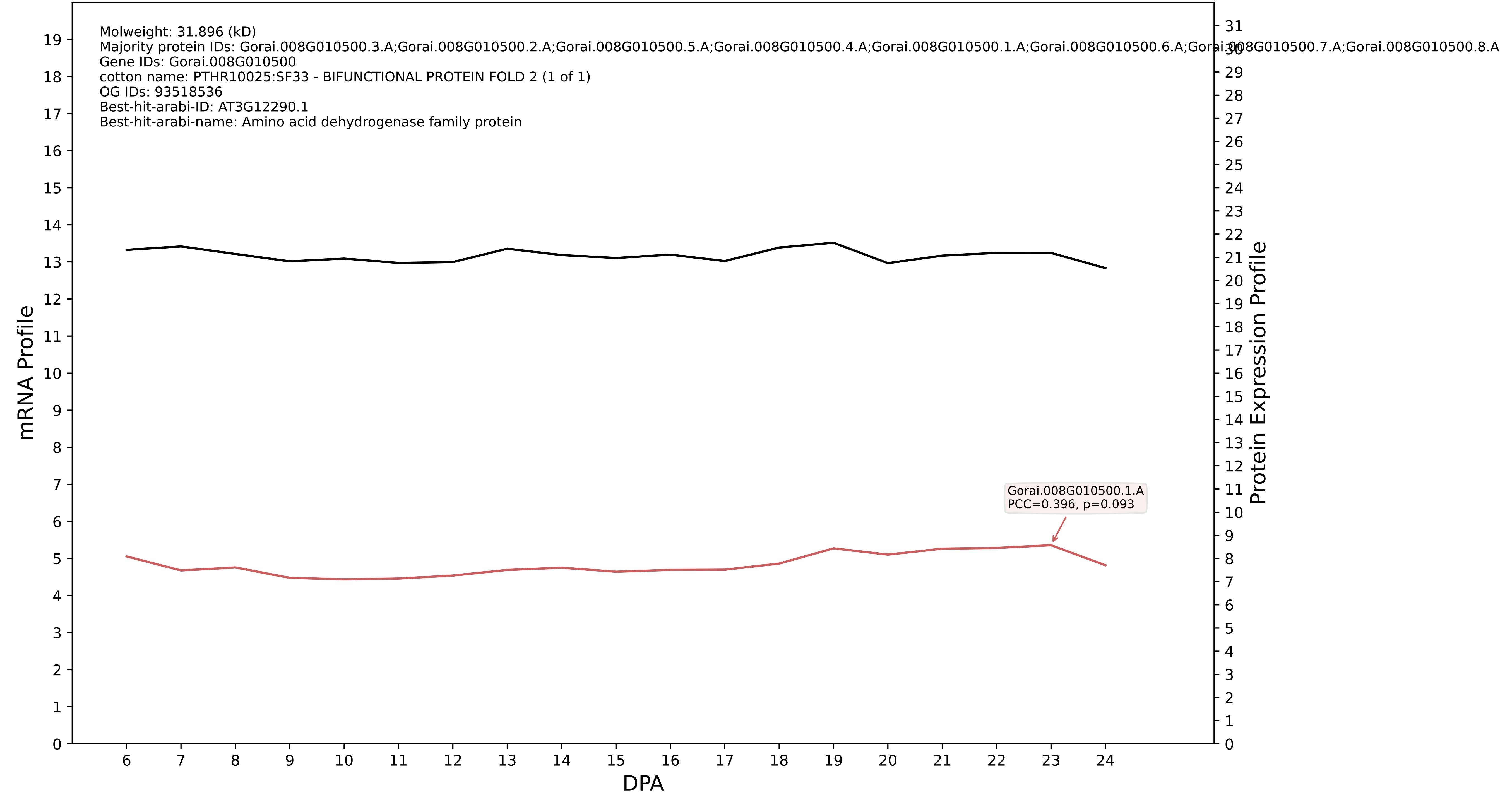

P200: Gorai.010G184300.1.D in case 1 and V14

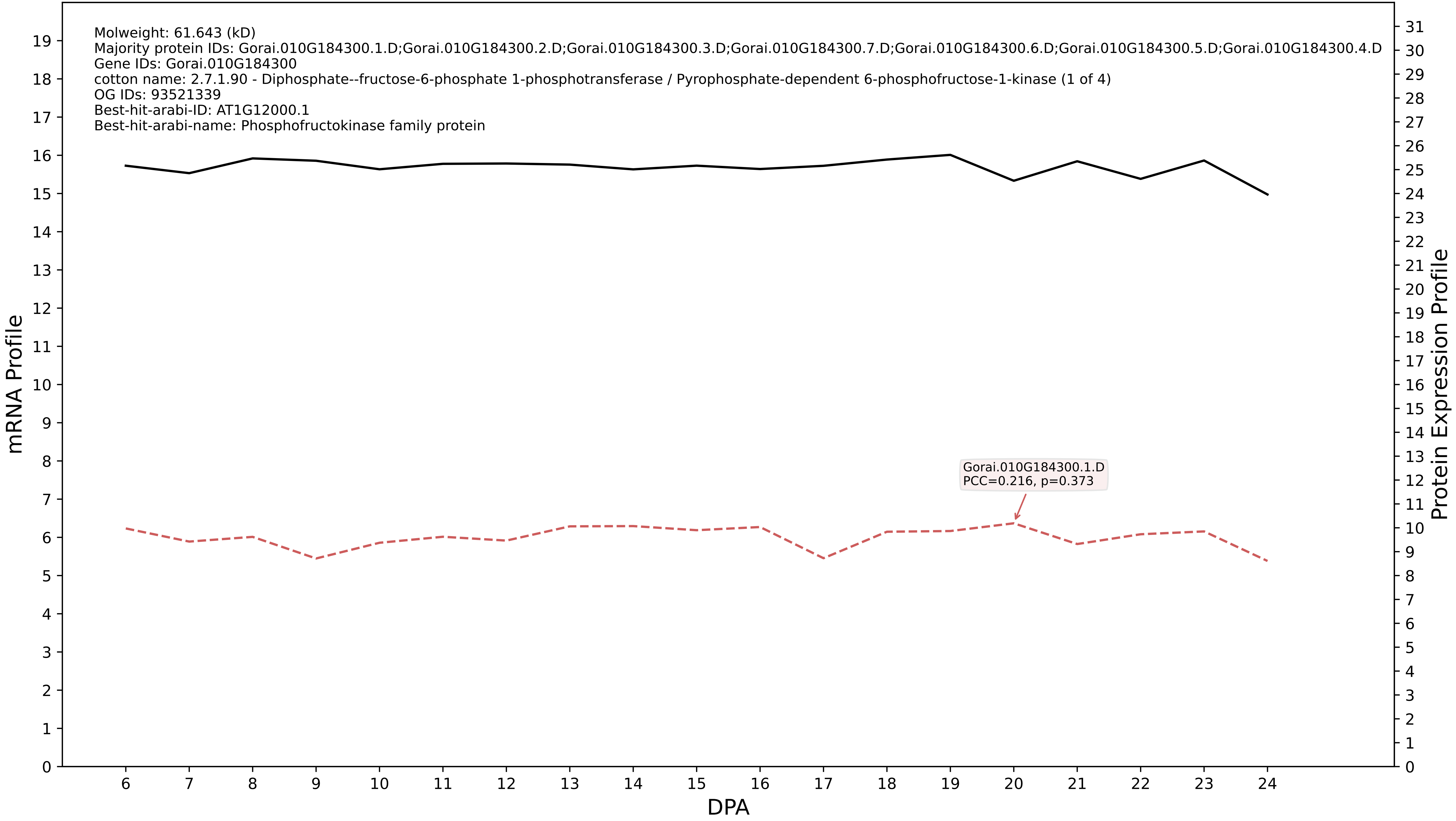

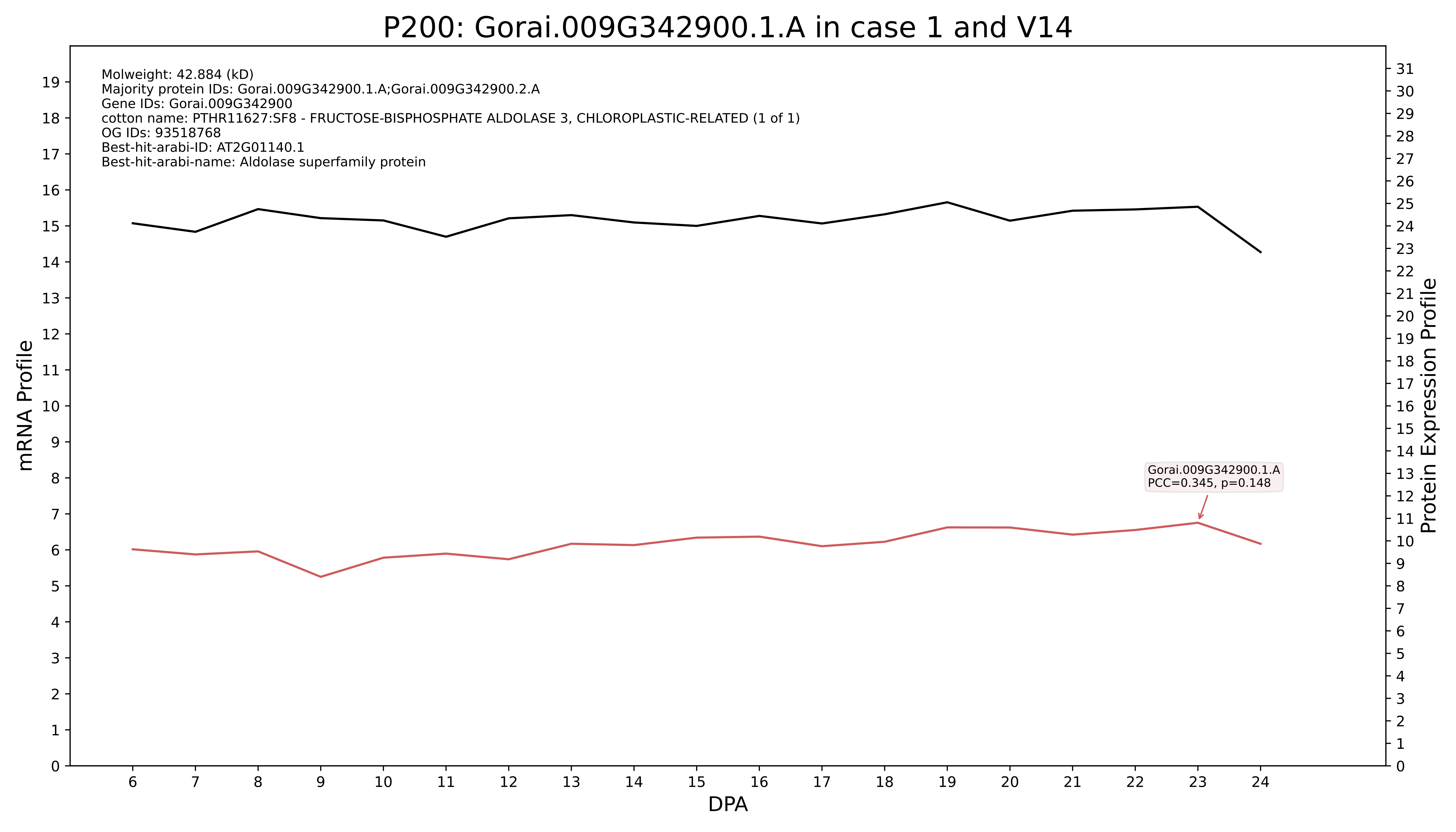
P200: Gorai.009G342900.1.A in case 1 and V14
Molweight: 42.884 (kD)
Majority protein IDs: Gorai.009G342900.1.A;Gorai.009G342900.2.A
Gene IDs: Gorai.009G342900
cotton name: PTHR11627:SF8 - FRUCTOSE-BISPHOSPHATE ALDOLASE 3, CHLOROPLASTIC-RELATED (1 of 1)
OG IDs: 93518768
Best-hit-arabi-ID: AT2G01140.1
Best-hit-arabi-name: Aldolase superfamily protein
Gorai.009G342900.1.A
PCC=0.345, p=0.148
mRNA Profile
Protein Expression Profile
DPA

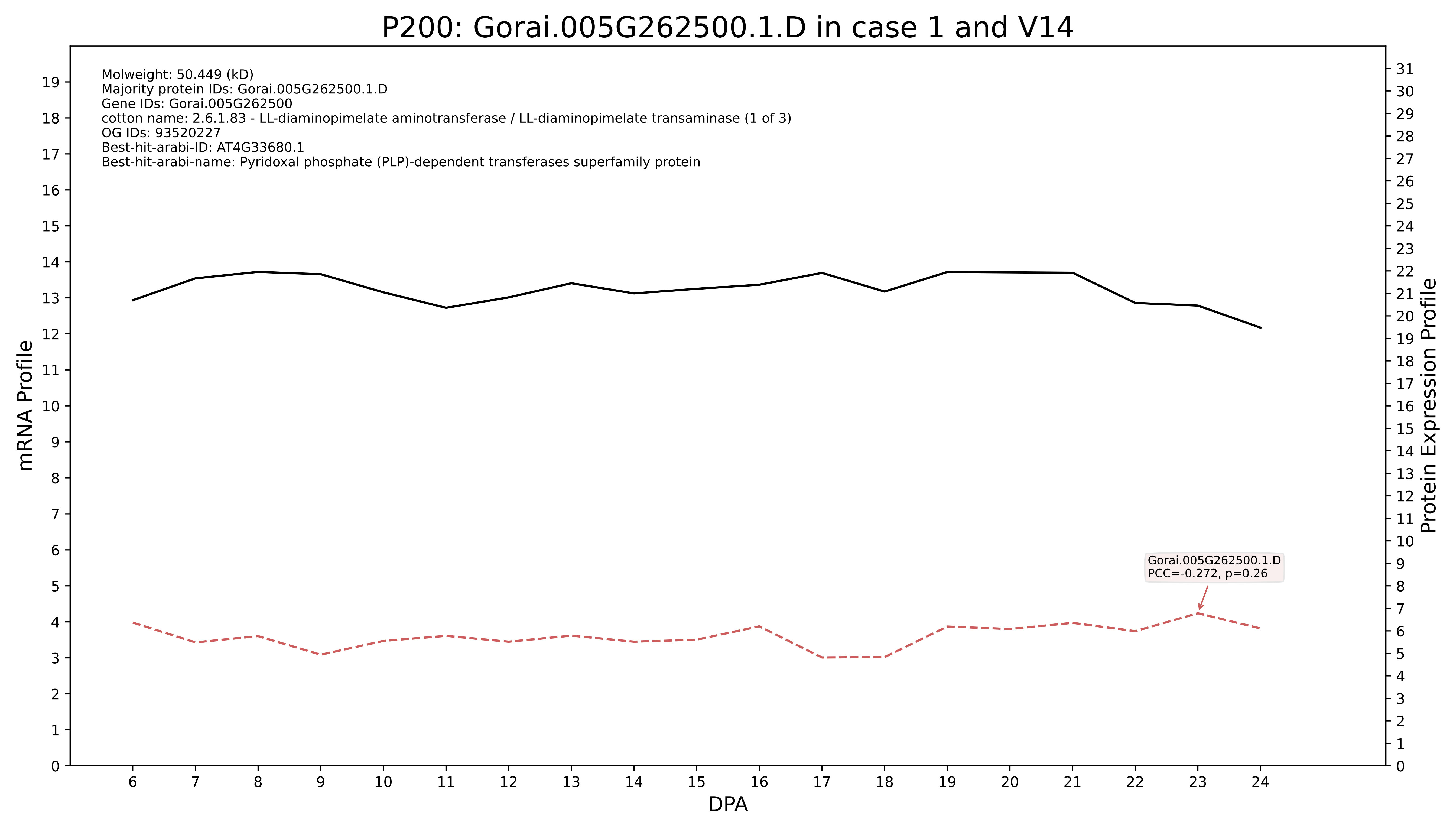
P200: Gorai.005G262500.1.D in case 1 and V14
Molweight: 50.449 (kD)
Majority protein IDs: Gorai.005G262500.1.D
Gene IDs: Gorai.005G262500
cotton name: 2.6.1.83 - LL-diaminopimelate aminotransferase / LL-diaminopimelate transaminase (1 of 3)
OG IDs: 93520227
Best-hit-arabi-ID: AT4G33680.1
Best-hit-arabi-name: Pyridoxal phosphate (PLP)-dependent transferases superfamily protein
Gorai.005G262500.1.D
PCC=-0.272, p=0.26
mRNA Profile
Protein Expression Profile
DPA

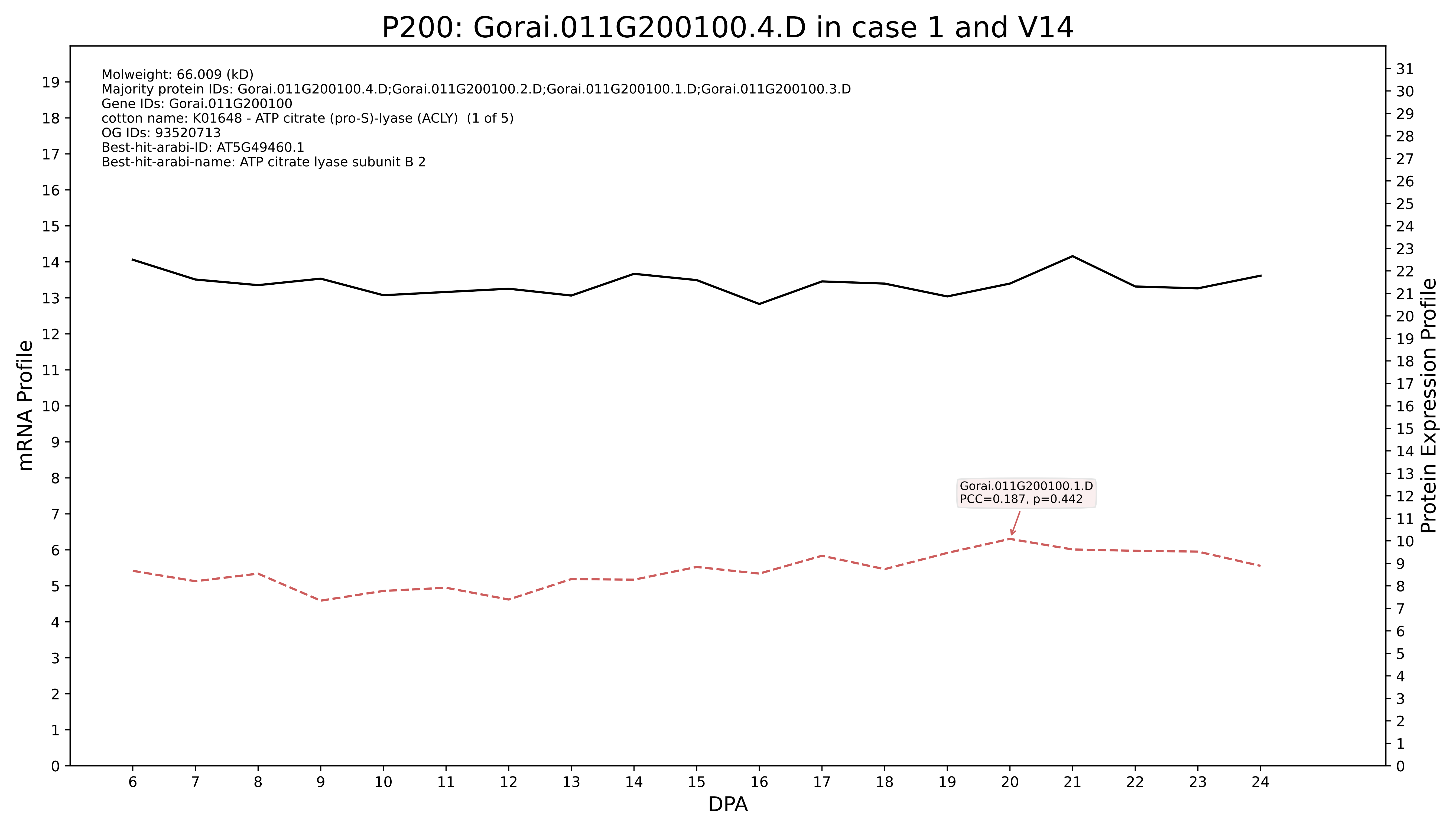
P200: Gorai.011G200100.4.D in case 1 and V14
Molweight: 66.009 (kD)
Majority protein IDs: Gorai.011G200100.4.D;Gorai.011G200100.2.D;Gorai.011G200100.1.D;Gorai.011G200100.3.D
Gene IDs: Gorai.011G200100
cotton name: K01648 - ATP citrate (pro-S)-lyase (ACLY) (1 of 5)
OG IDs: 93520713
Best-hit-arabi-ID: AT5G49460.1
Best-hit-arabi-name: ATP citrate lyase subunit B 2
Gorai.011G200100.1.D
PCC=0.187, p=0.442
mRNA Profile
Protein Expression Profile
DPA

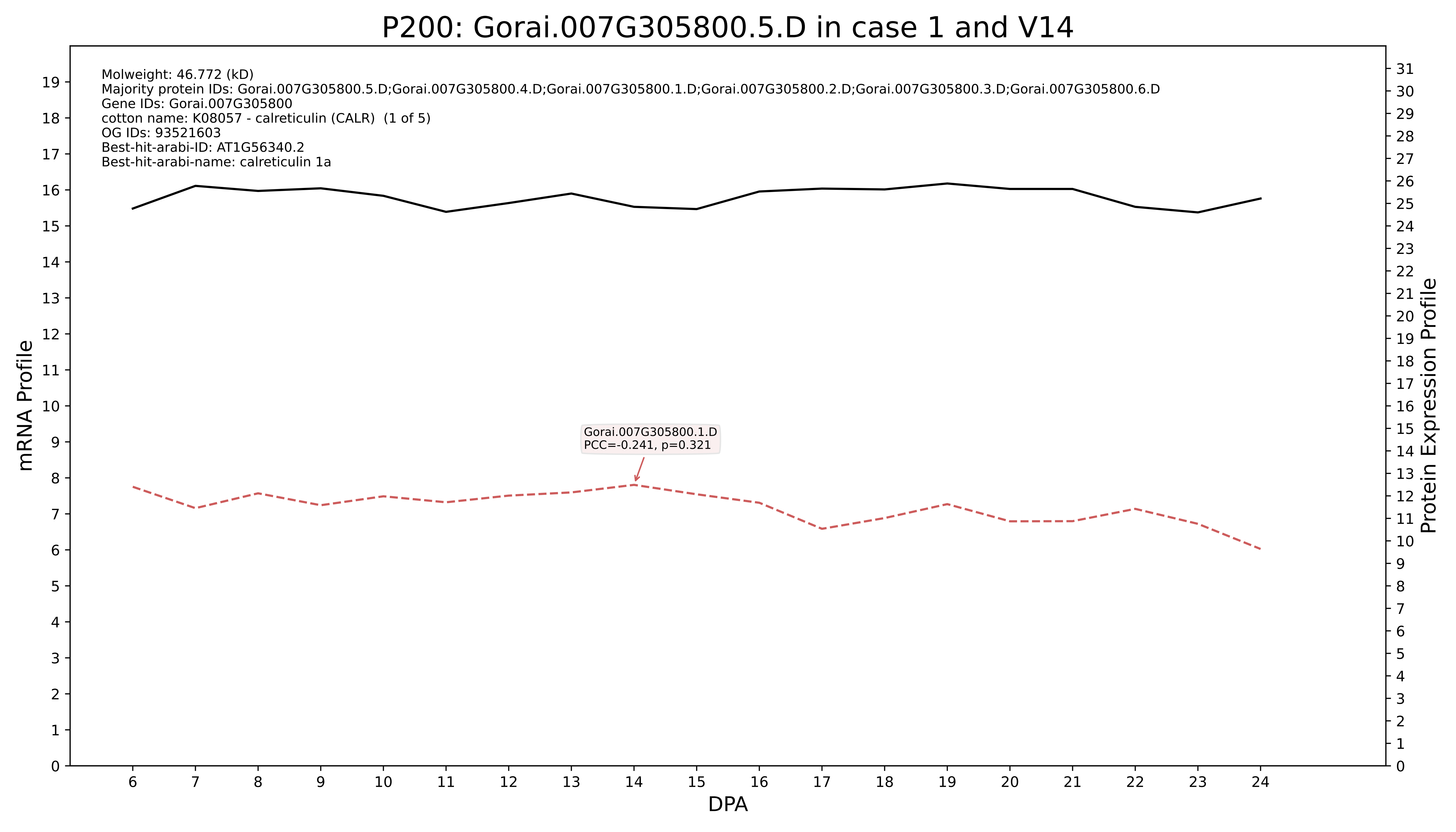
P200: Gorai.007G305800.5.D in case 1 and V14
Molweight: 46.772 (kD)
Majority protein IDs: Gorai.007G305800.5.D;Gorai.007G305800.4.D;Gorai.007G305800.1.D;Gorai.007G305800.2.D;Gorai.007G305800.3.D;Gorai.007G305800.6.D
Gene IDs: Gorai.007G305800
cotton name: K08057 - calreticulin (CALR) (1 of 5)
OG IDs: 93521603
Best-hit-arabi-ID: AT1G56340.2
Best-hit-arabi-name: calreticulin 1a
Gorai.007G305800.1.D
PCC=-0.241, p=0.321
mRNA Profile
Protein Expression Profile
DPA

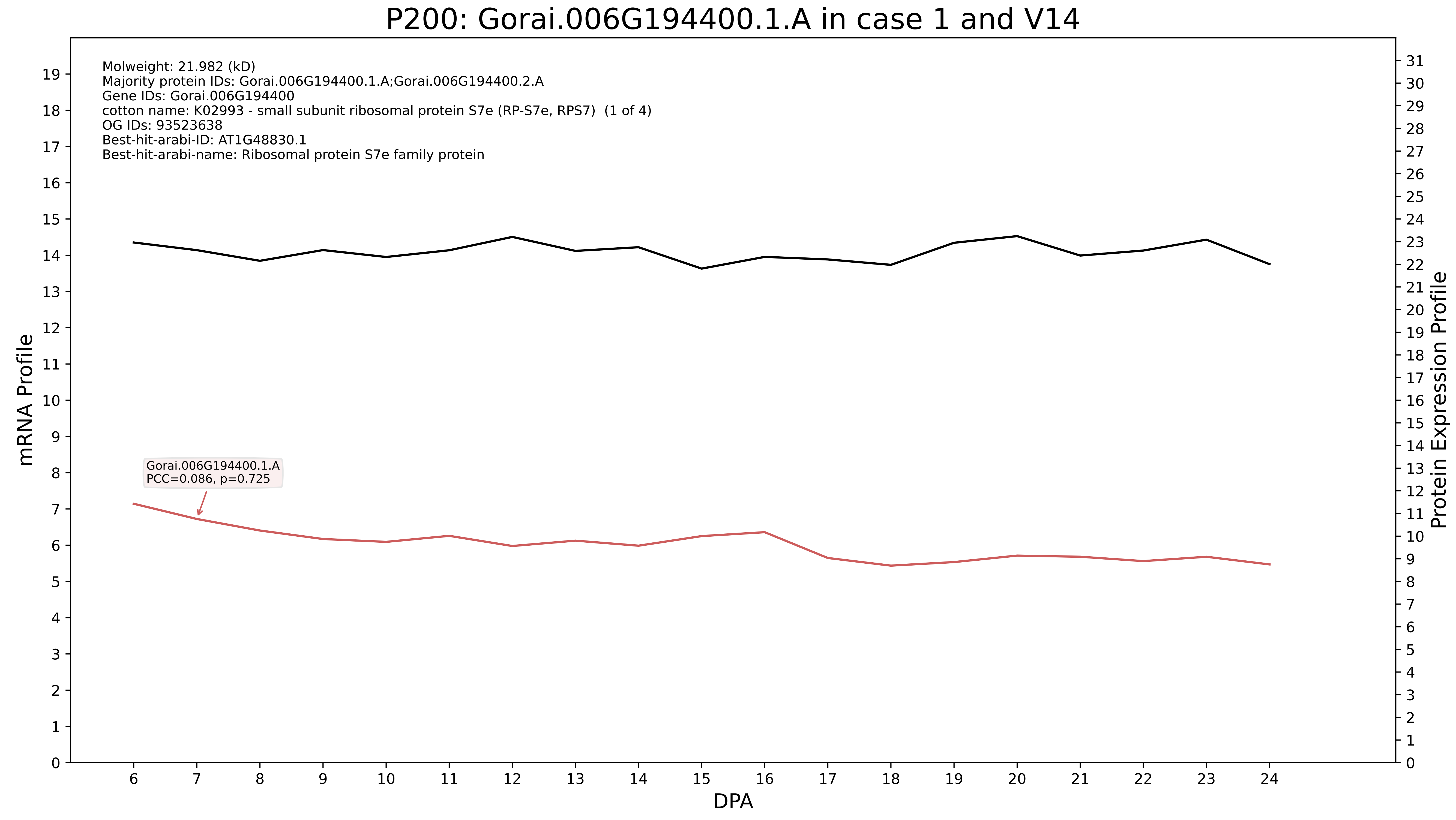
P200: Gorai.006G194400.1.A in case 1 and V14
Molweight: 21.982 (kD)
Majority protein IDs: Gorai.006G194400.1.A;Gorai.006G194400.2.A
Gene IDs: Gorai.006G194400
cotton name: K02993 - small subunit ribosomal protein S7e (RP-S7e, RPS7) (1 of 4)
OG IDs: 93523638
Best-hit-arabi-ID: AT1G48830.1
Best-hit-arabi-name: Ribosomal protein S7e family protein
Gorai.006G194400.1.A
PCC=0.086, p=0.725
mRNA Profile
Protein Expression Profile
DPA

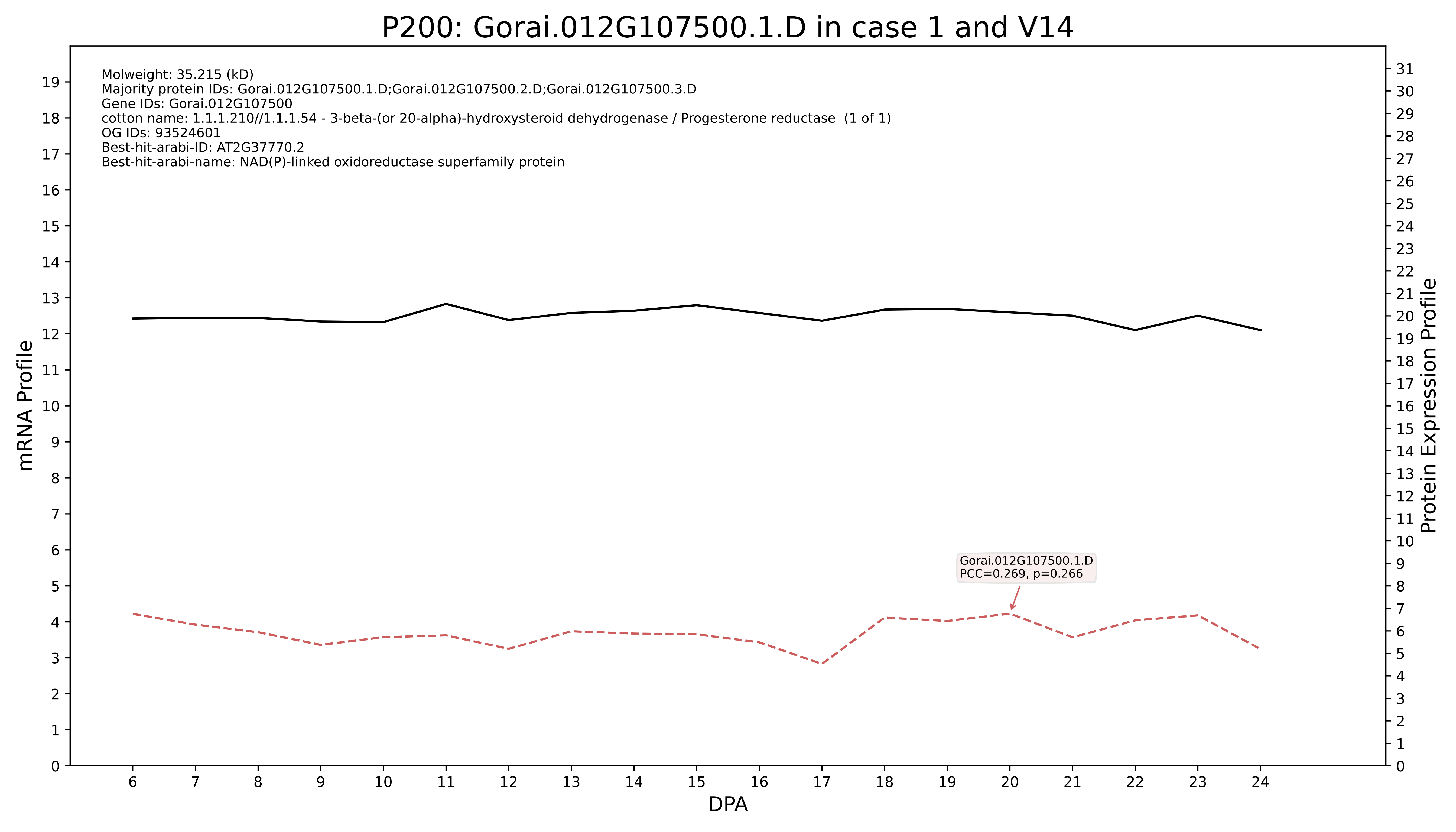
P200: Gorai.012G107500.1.D in case 1 and V14
Molweight: 35.215 (kD)
Majority protein IDs: Gorai.012G107500.1.D;Gorai.012G107500.2.D;Gorai.012G107500.3.D
Gene IDs: Gorai.012G107500
cotton name: 1.1.1.210//1.1.1.54 - 3-beta-(or 20-alpha)-hydroxysteroid dehydrogenase / Progesterone reductase (1 of 1)
OG IDs: 93524601
Best-hit-arabi-ID: AT2G37770.2
Best-hit-arabi-name: NAD(P)-linked oxidoreductase superfamily protein
Gorai.012G107500.1.D
PCC=0.269, p=0.266
mRNA Profile
Protein Expression Profile
DPA
6
7
8
9
10
11
12
13
14
15
16
17
18
19
20
21
22
23
24

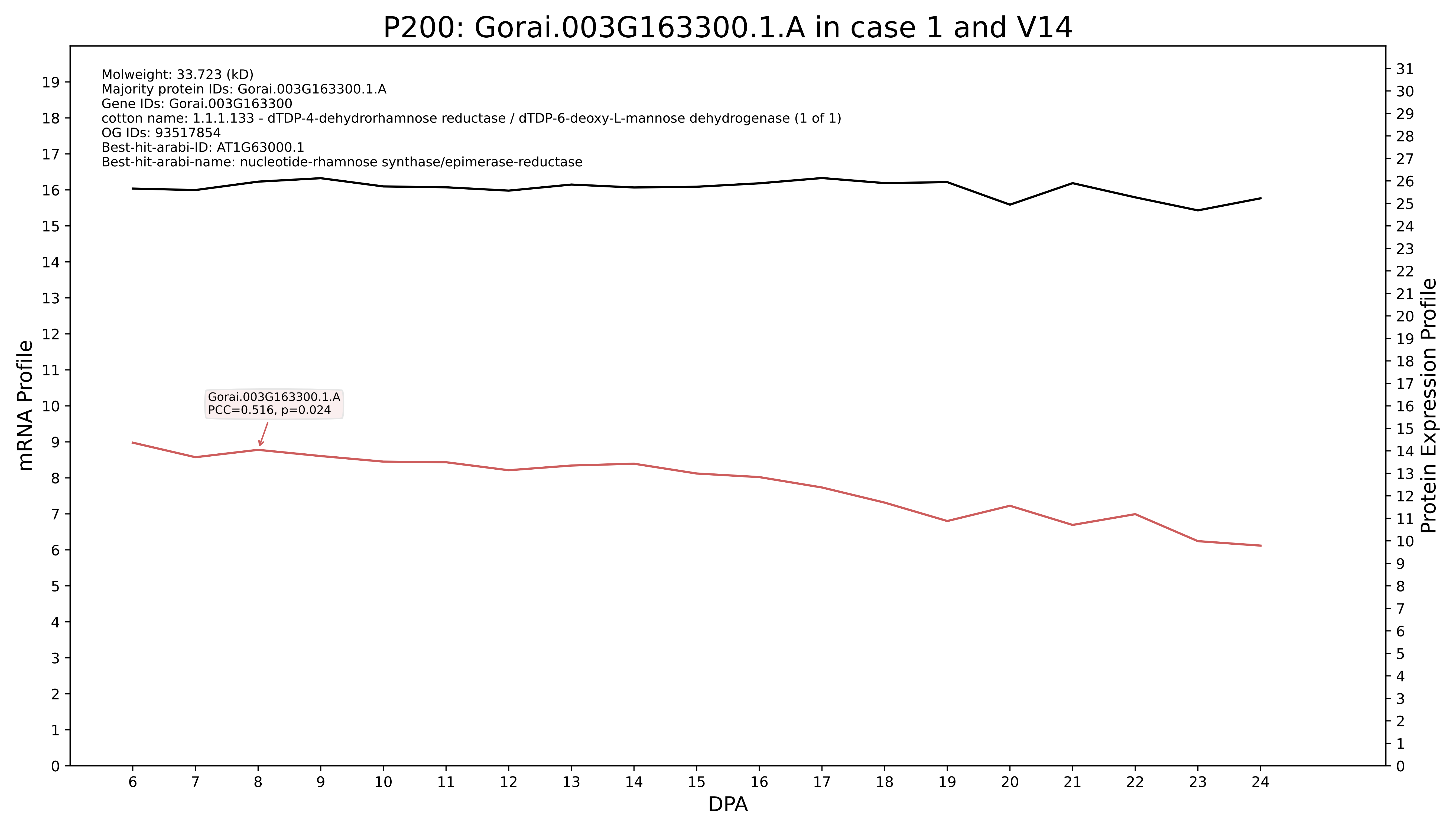
P200: Gorai.003G163300.1.A in case 1 and V14
Molweight: 33.723 (kD)
Majority protein IDs: Gorai.003G163300.1.A
Gene IDs: Gorai.003G163300
cotton name: 1.1.1.133 - dTDP-4-dehydrorhamnose reductase / dTDP-6-deoxy-L-mannose dehydrogenase (1 of 1)
OG IDs: 93517854
Best-hit-arabi-ID: AT1G63000.1
Best-hit-arabi-name: nucleotide-rhamnose synthase/epimerase-reductase
Gorai.003G163300.1.A
PCC=0.516, p=0.024
mRNA Profile
Protein Expression Profile
DPA

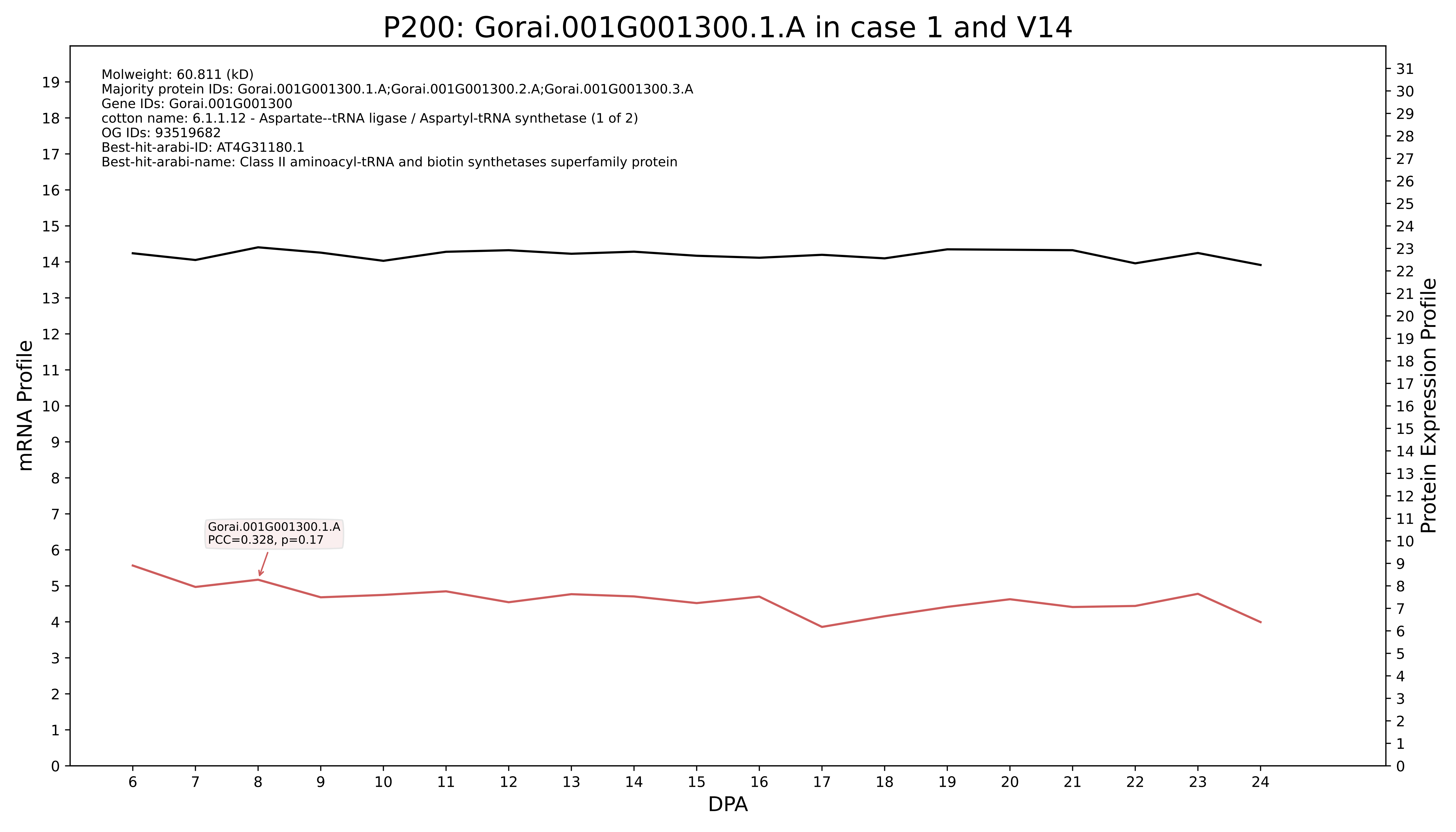
P200: Gorai.001G001300.1.A in case 1 and V14
Molweight: 60.811 (kD)
Majority protein IDs: Gorai.001G001300.1.A;Gorai.001G001300.2.A;Gorai.001G001300.3.A
Gene IDs: Gorai.001G001300
cotton name: 6.1.1.12 - Aspartate--tRNA ligase / Aspartyl-tRNA synthetase (1 of 2)
OG IDs: 93519682
Best-hit-arabi-ID: AT4G31180.1
Best-hit-arabi-name: Class II aminoacyl-tRNA and biotin synthetases superfamily protein
Gorai.001G001300.1.A
PCC=0.328, p=0.17
mRNA Profile
Protein Expression Profile
DPA

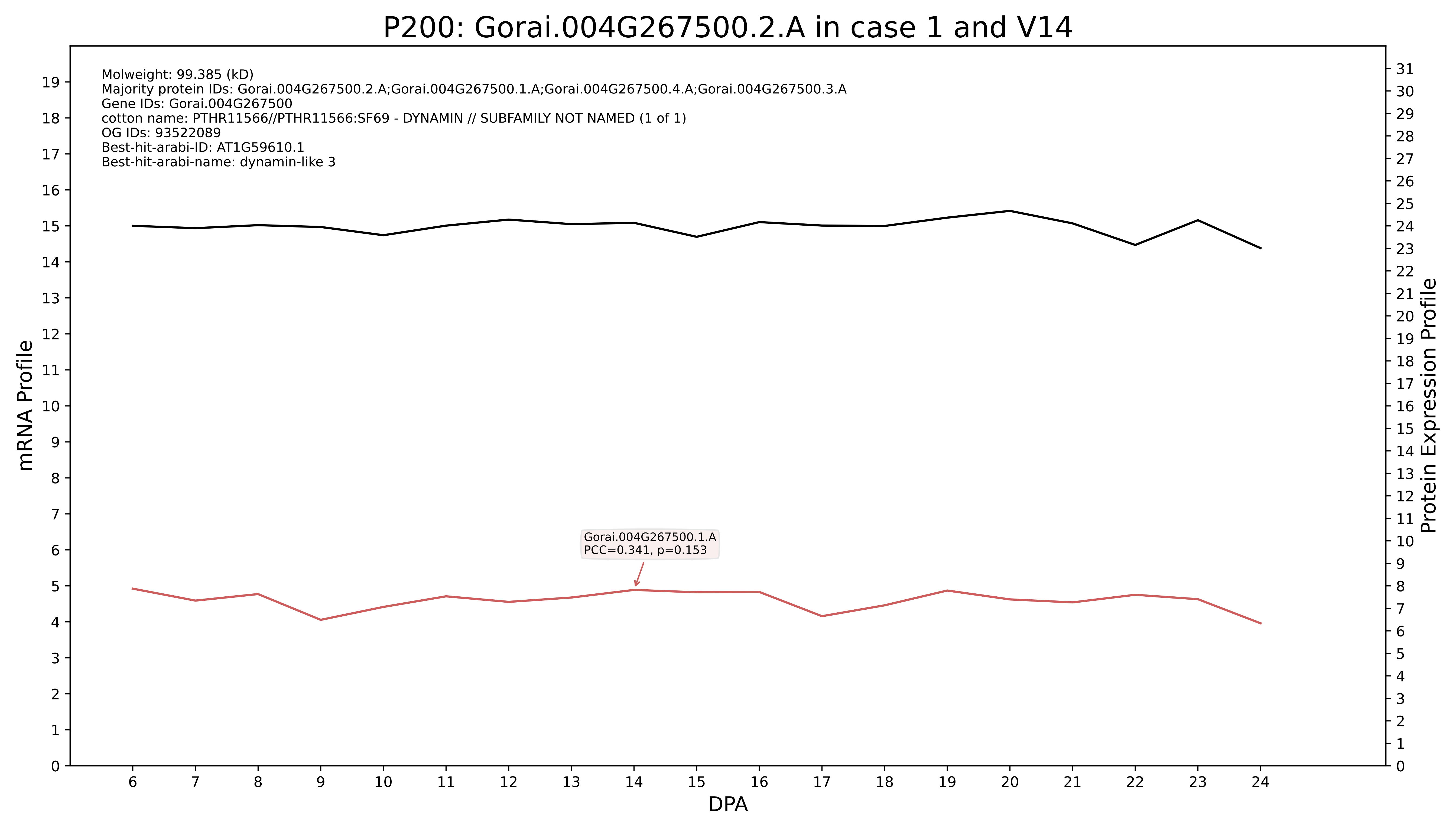

P200: Gorai.004G267500.2.A in case 1 and V14
Molweight: 99.385 (kD)
Majority protein IDs: Gorai.004G267500.2.A;Gorai.004G267500.1.A;Gorai.004G267500.4.A;Gorai.004G267500.3.A
Gene IDs: Gorai.004G267500
cotton name: PTHR11566//PTHR11566:SF69 - DYNAMIN // SUBFAMILY NOT NAMED (1 of 1)
OG IDs: 93522089
Best-hit-arabi-ID: AT1G59610.1
Best-hit-arabi-name: dynamin-like 3
Gorai.004G267500.1.A
PCC=0.341, p=0.153
mRNA Profile
Protein Expression Profile
DPA

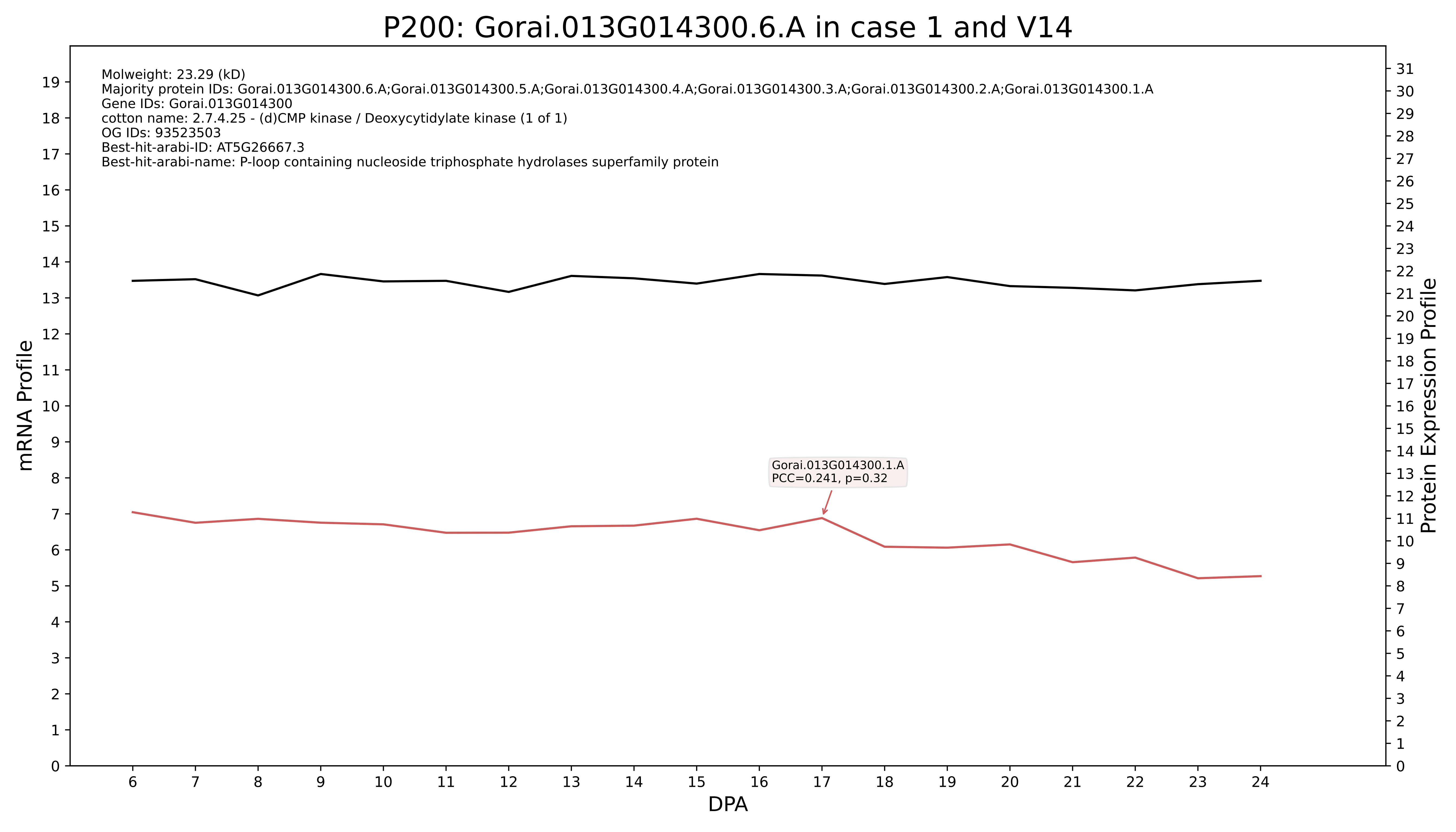
P200: Gorai.013G014300.6.A in case 1 and V14
Molweight: 23.29 (kD)
Majority protein IDs: Gorai.013G014300.6.A;Gorai.013G014300.5.A;Gorai.013G014300.4.A;Gorai.013G014300.3.A;Gorai.013G014300.2.A;Gorai.013G014300.1.A
Gene IDs: Gorai.013G014300
cotton name: 2.7.4.25 - (d)CMP kinase / Deoxycytidylate kinase (1 of 1)
OG IDs: 93523503
Best-hit-arabi-ID: AT5G26667.3
Best-hit-arabi-name: P-loop containing nucleoside triphosphate hydrolases superfamily protein
Gorai.013G014300.1.A
PCC=0.241, p=0.32
mRNA Profile
Protein Expression Profile
DPA

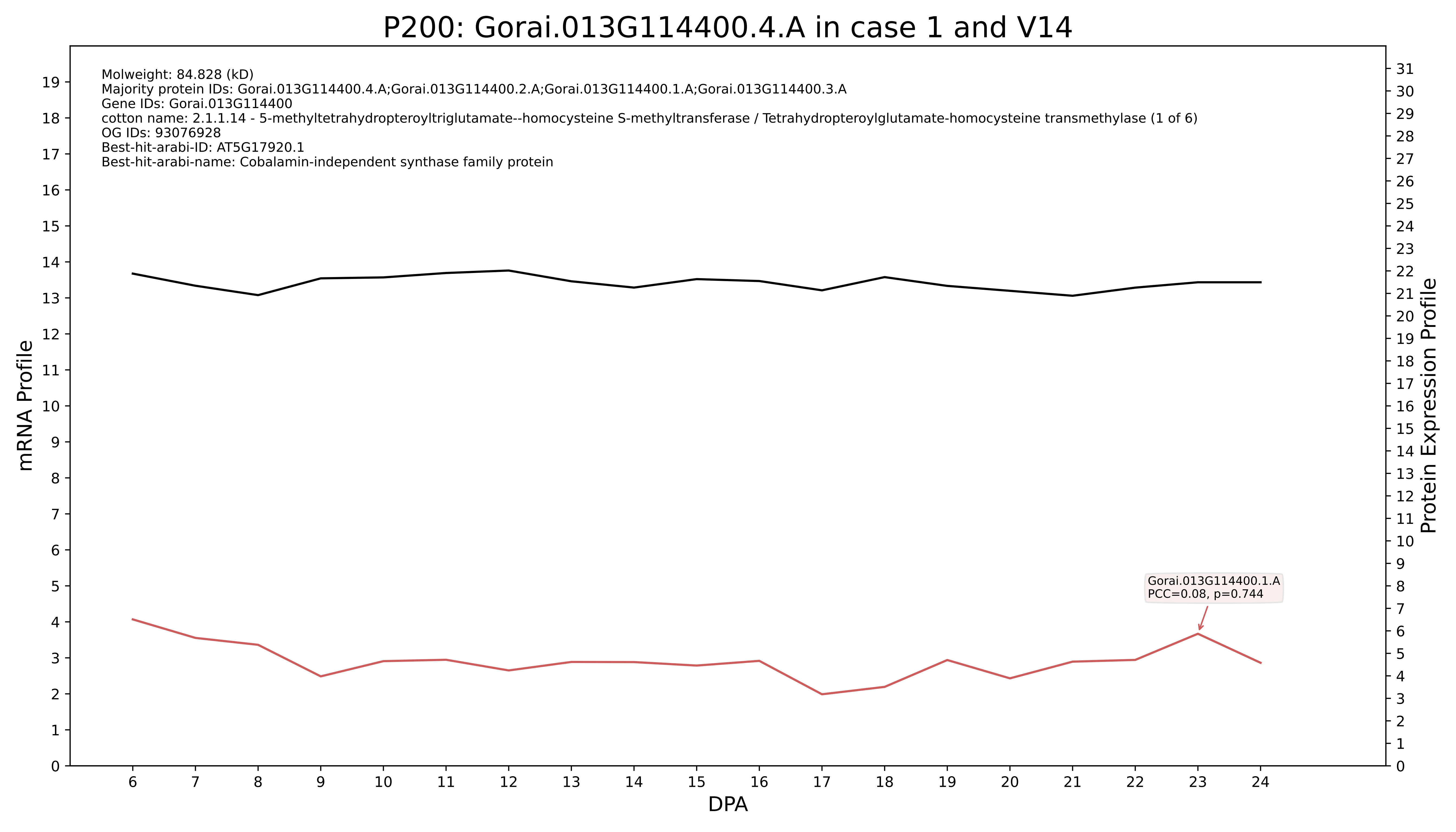

P200: Gorai.013G114400.4.A in case 1 and V14
Molweight: 84.828 (kD)
Majority protein IDs: Gorai.013G114400.4.A;Gorai.013G114400.2.A;Gorai.013G114400.1.A;Gorai.013G114400.3.A
Gene IDs: Gorai.013G114400
cotton name: 2.1.1.14 - 5-methyltetrahydropteroyltriglutamate--homocysteine S-methyltransferase / Tetrahydropteroylglutamate-homocysteine transmethylase (1 of 6)
OG IDs: 93076928
Best-hit-arabi-ID: AT5G17920.1
Best-hit-arabi-name: Cobalamin-independent synthase family protein
Gorai.013G114400.1.A
PCC=0.08, p=0.744
mRNA Profile
Protein Expression Profile
DPA

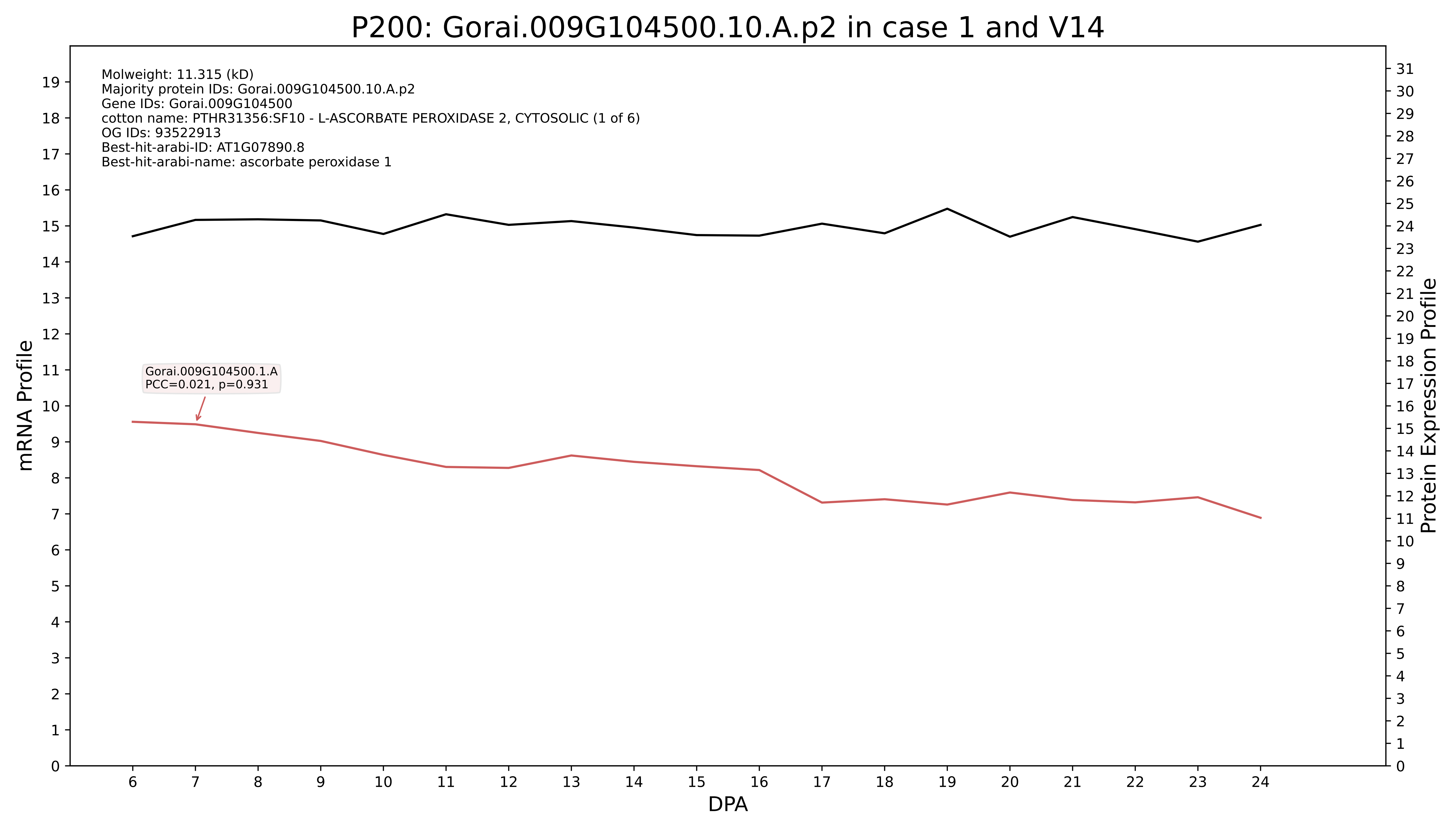
P200: Gorai.009G104500.10.A.p2 in case 1 and V14
Molweight: 11.315 (kD)
Majority protein IDs: Gorai.009G104500.10.A.p2
Gene IDs: Gorai.009G104500
cotton name: PTHR31356:SF10 - L-ASCORBATE PEROXIDASE 2, CYTOSOLIC (1 of 6)
OG IDs: 93522913
Best-hit-arabi-ID: AT1G07890.8
Best-hit-arabi-name: ascorbate peroxidase 1
Gorai.009G104500.1.A
PCC=0.021, p=0.931
mRNA Profile
Protein Expression Profile
DPA

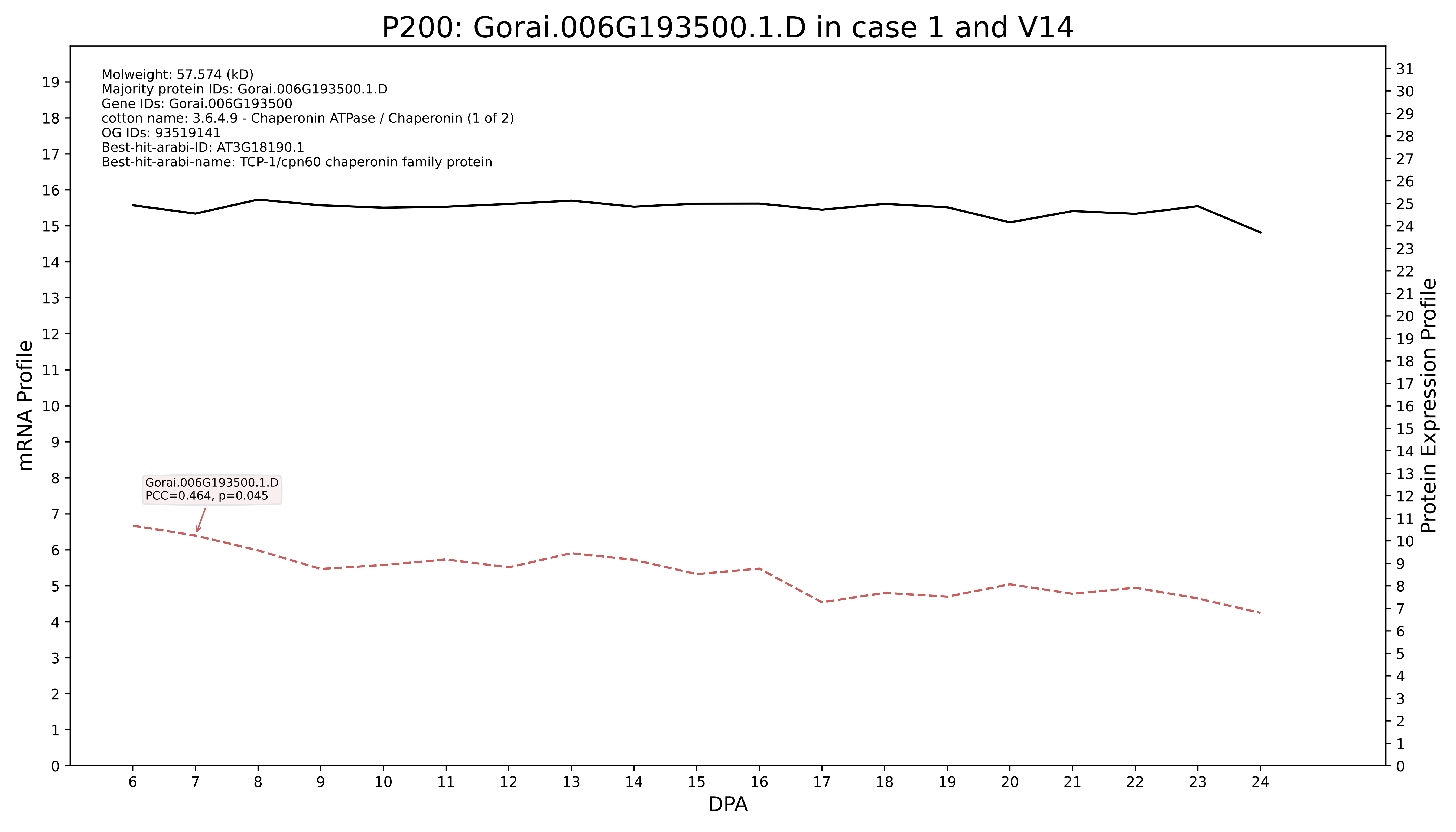

P200: Gorai.006G193500.1.D in case 1 and V14
Molweight: 57.574 (kD)
Majority protein IDs: Gorai.006G193500.1.D
Gene IDs: Gorai.006G193500
cotton name: 3.6.4.9 - Chaperonin ATPase / Chaperonin (1 of 2)
OG IDs: 93519141
Best-hit-arabi-ID: AT3G18190.1
Best-hit-arabi-name: TCP-1/cpn60 chaperonin family protein
Gorai.006G193500.1.D
PCC=0.464, p=0.045
mRNA Profile
Protein Expression Profile
DPA

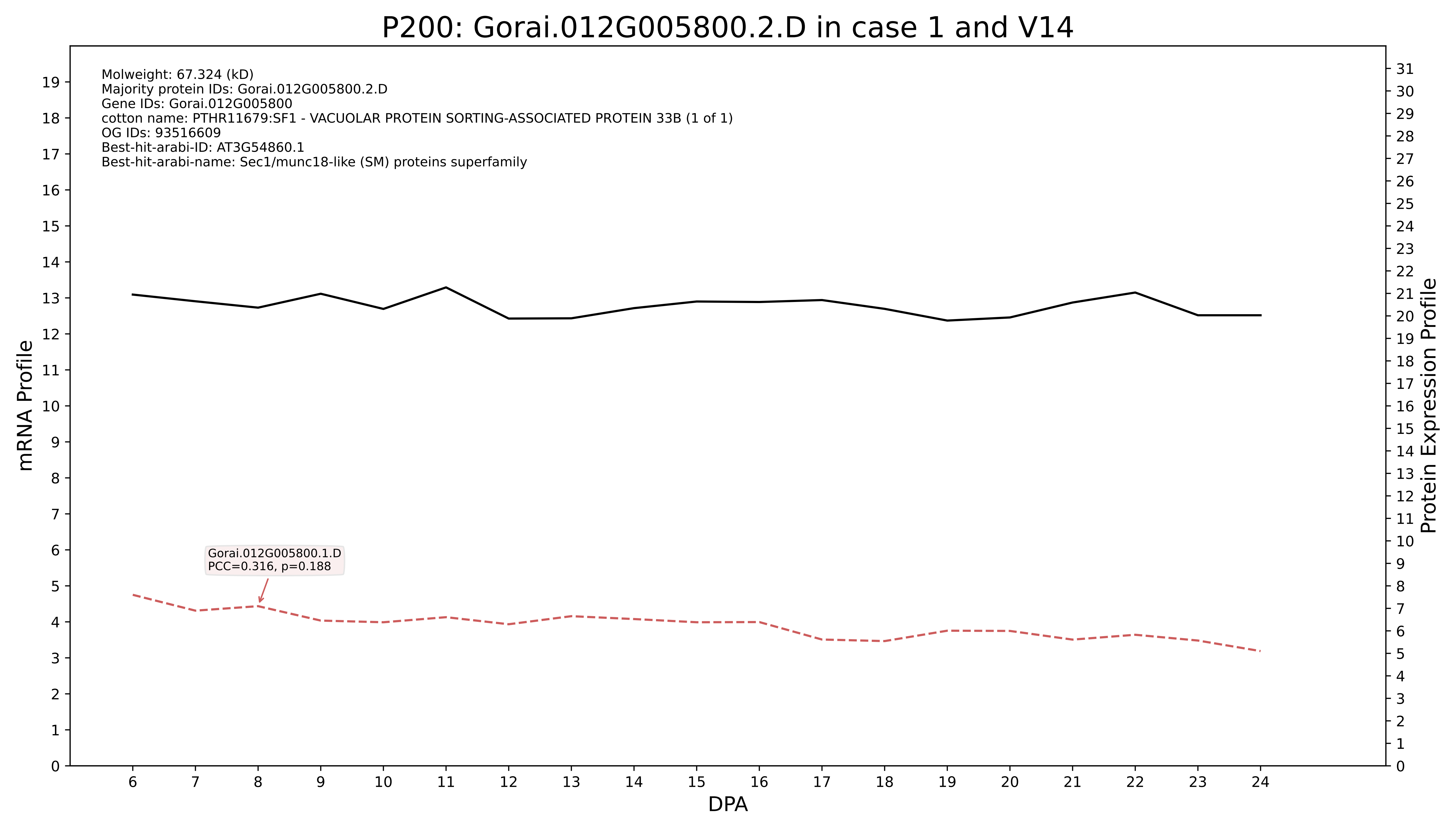

P200: Gorai.012G005800.2.D in case 1 and V14
Molweight: 67.324 (kD)
Majority protein IDs: Gorai.012G005800.2.D
Gene IDs: Gorai.012G005800
cotton name: PTHR11679:SF1 - VACUOLAR PROTEIN SORTING-ASSOCIATED PROTEIN 33B (1 of 1)
OG IDs: 93516609
Best-hit-arabi-ID: AT3G54860.1
Best-hit-arabi-name: Sec1/munc18-like (SM) proteins superfamily
Gorai.012G005800.1.D
PCC=0.316, p=0.188
mRNA Profile
Protein Expression Profile
DPA

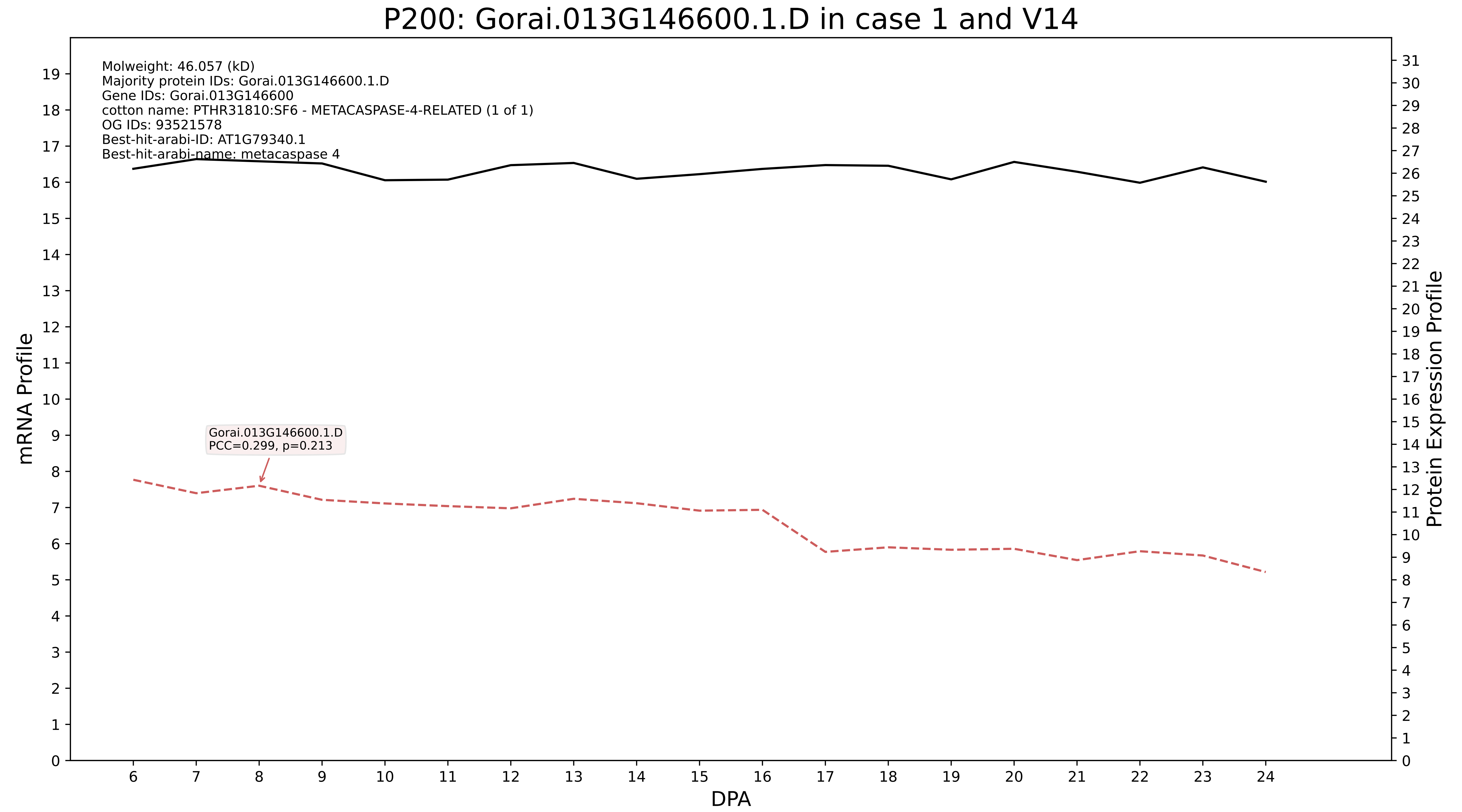

P200: Gorai.013G146600.1.D in case 1 and V14
Molweight: 46.057 (kD)
Majority protein IDs: Gorai.013G146600.1.D
Gene IDs: Gorai.013G146600
cotton name: PTHR31810:SF6 - METACASPASE-4-RELATED (1 of 1)
OG IDs: 93521578
Best-hit-arabi-ID: AT1G79340.1
Best-hit-arabi-name: metacaspase 4
Gorai.013G146600.1.D
PCC=0.299, p=0.213
mRNA Profile
Protein Expression Profile
DPA

P200: Gorai.006G130500.3.A in case 1 and V14

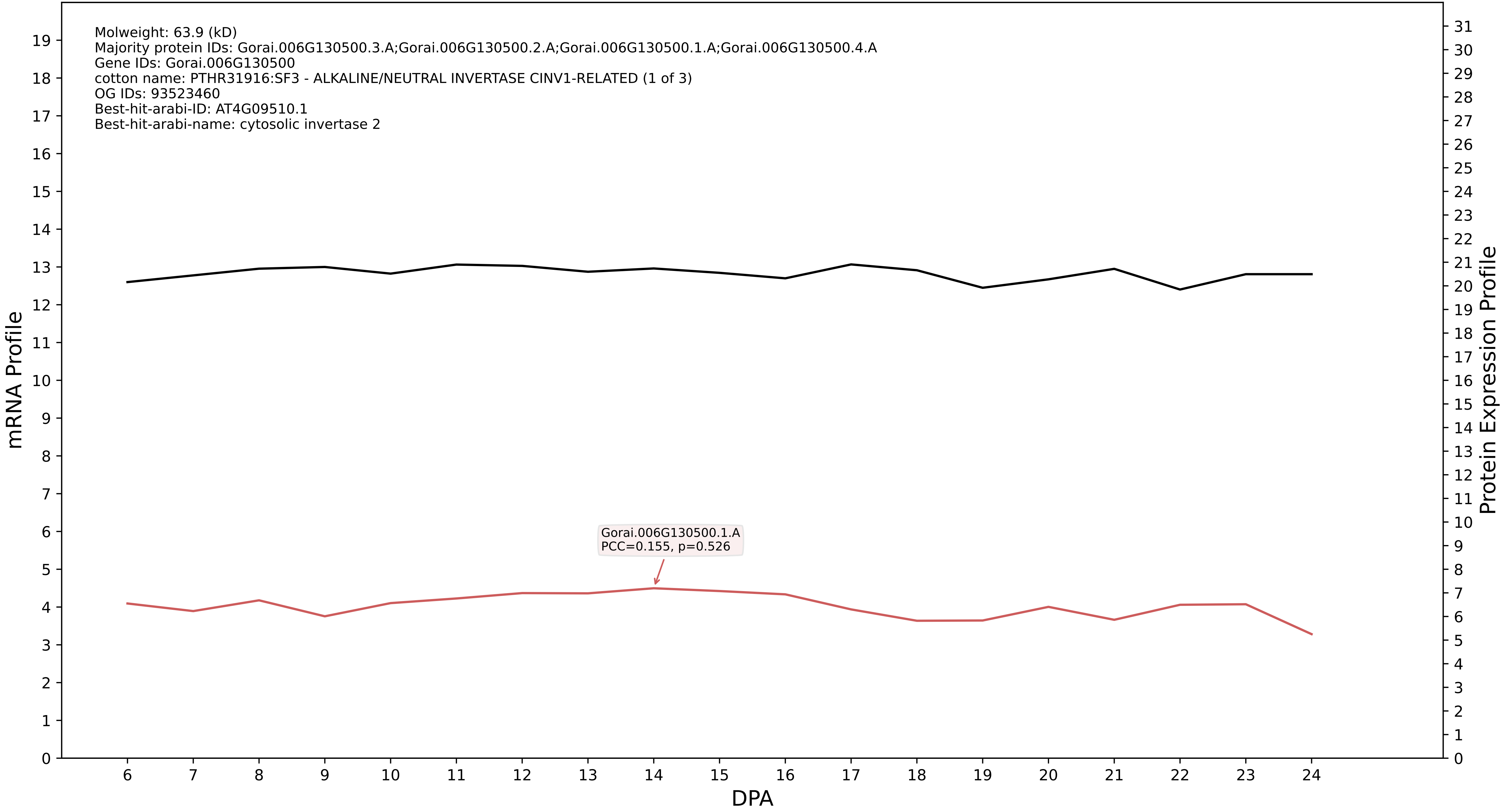

P200: Gorai.001G133500.1.D in case 1 and V14

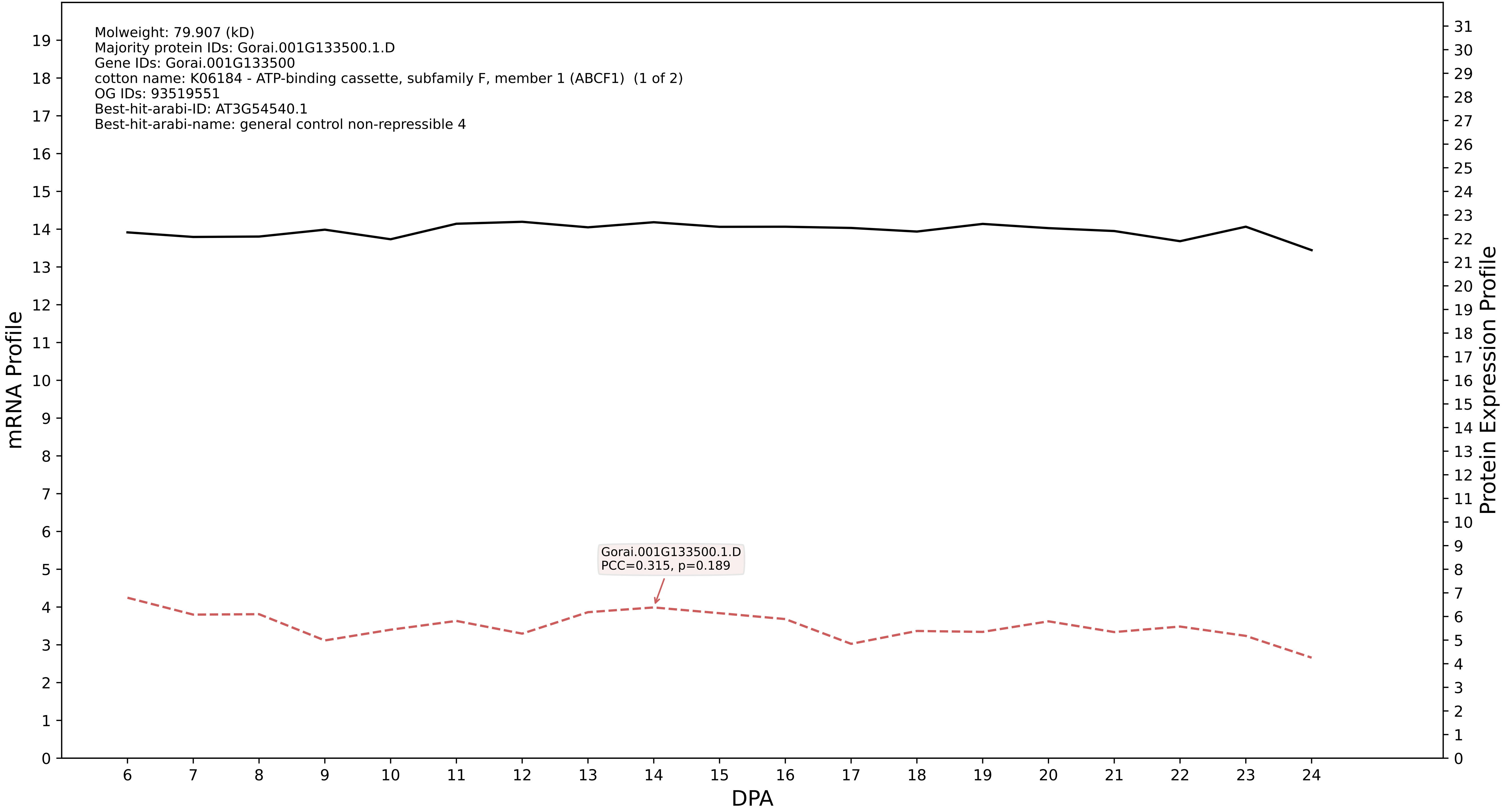

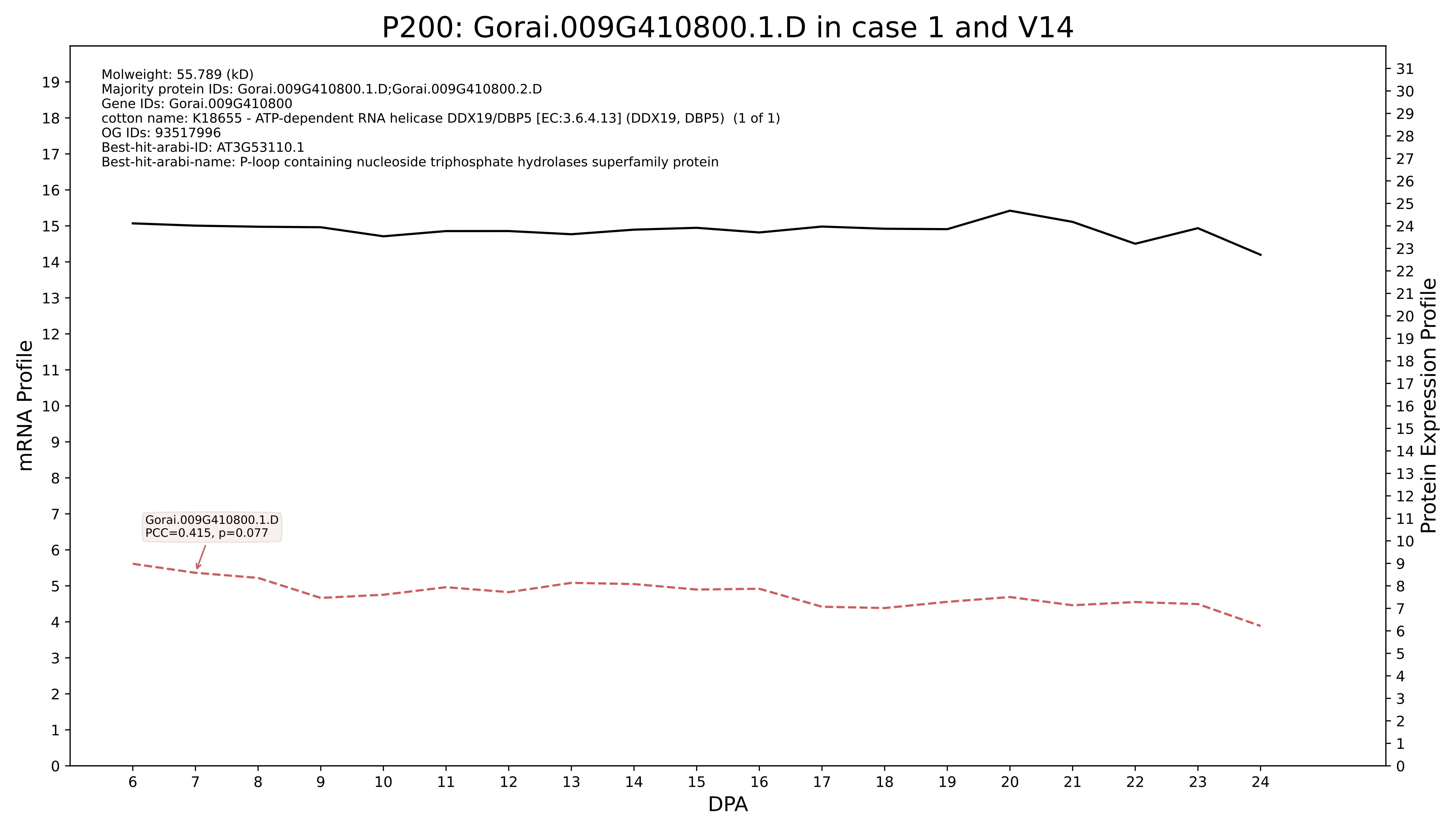

P200: Gorai.009G410800.1.D in case 1 and V14
Molweight: 55.789 (kD)
Majority protein IDs: Gorai.009G410800.1.D;Gorai.009G410800.2.D
Gene IDs: Gorai.009G410800
cotton name: K18655 - ATP-dependent RNA helicase DDX19/DBP5 [EC:3.6.4.13] (DDX19, DBP5) (1 of 1)
OG IDs: 93517996
Best-hit-arabi-ID: AT3G53110.1
Best-hit-arabi-name: P-loop containing nucleoside triphosphate hydrolases superfamily protein
Gorai.009G410800.1.D
PCC=0.415, p=0.077
mRNA Profile
Protein Expression Profile
DPA
0
1
2
3
4
5
6
7
8
9
10
11
12
13
14
15
16
17
18
19
20
21
22
23
24
25
26
27
28
29
30
31

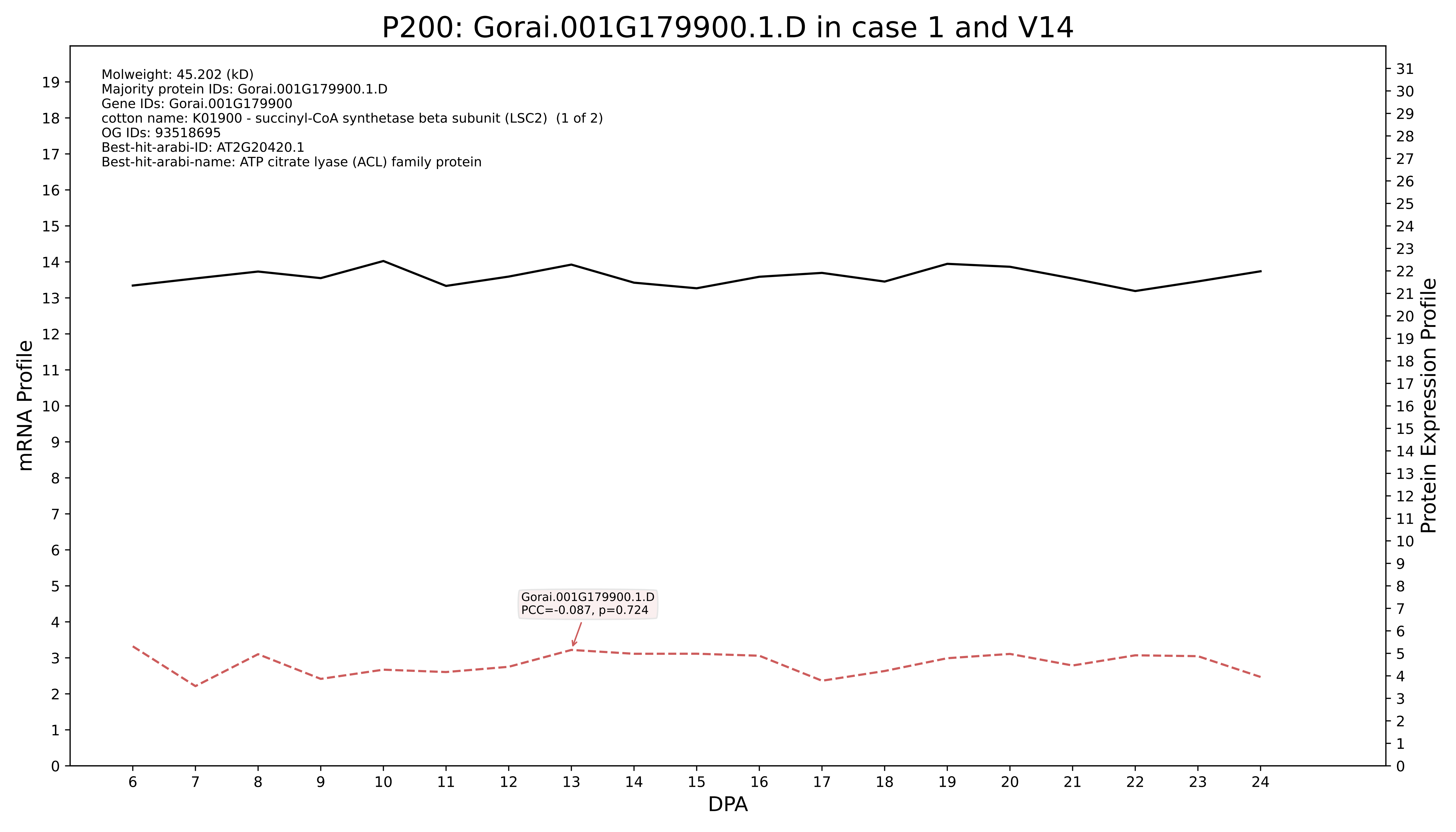
P200: Gorai.001G179900.1.D in case 1 and V14
Molweight: 45.202 (kD)
Majority protein IDs: Gorai.001G179900.1.D
Gene IDs: Gorai.001G179900
cotton name: K01900 - succinyl-CoA synthetase beta subunit (LSC2) (1 of 2)
OG IDs: 93518695
Best-hit-arabi-ID: AT2G20420.1
Best-hit-arabi-name: ATP citrate lyase (ACL) family protein
Gorai.001G179900.1.D
PCC=-0.087, p=0.724
mRNA Profile
Protein Expression Profile
DPA

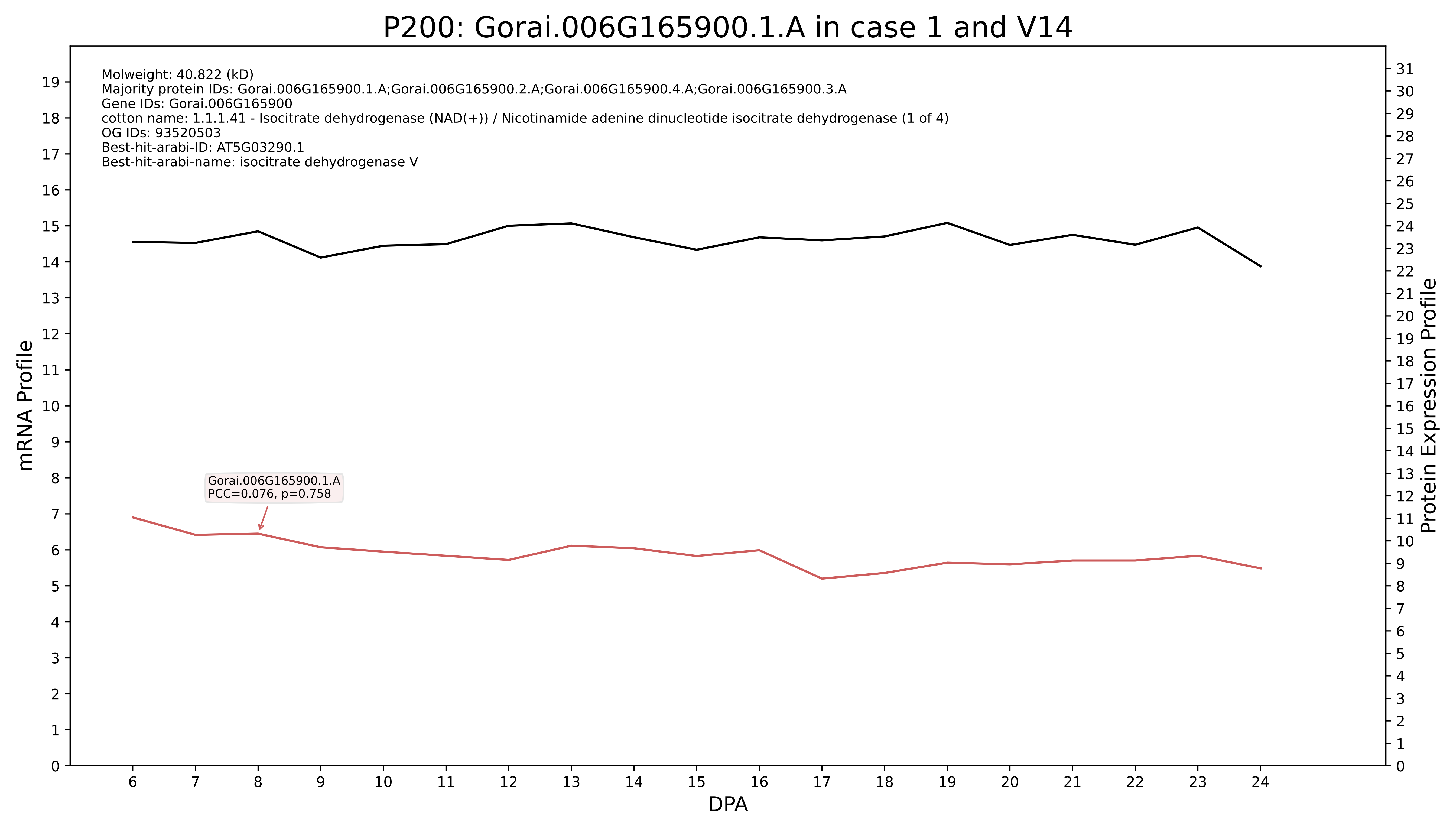

P200: Gorai.006G165900.1.A in case 1 and V14
Molweight: 40.822 (kD)
Majority protein IDs: Gorai.006G165900.1.A;Gorai.006G165900.2.A;Gorai.006G165900.4.A;Gorai.006G165900.3.A
Gene IDs: Gorai.006G165900
cotton name: 1.1.1.41 - Isocitrate dehydrogenase (NAD(+)) / Nicotinamide adenine dinucleotide isocitrate dehydrogenase (1 of 4)
OG IDs: 93520503
Best-hit-arabi-ID: AT5G03290.1
Best-hit-arabi-name: isocitrate dehydrogenase V
Gorai.006G165900.1.A
PCC=0.076, p=0.758
mRNA Profile
Protein Expression Profile
DPA

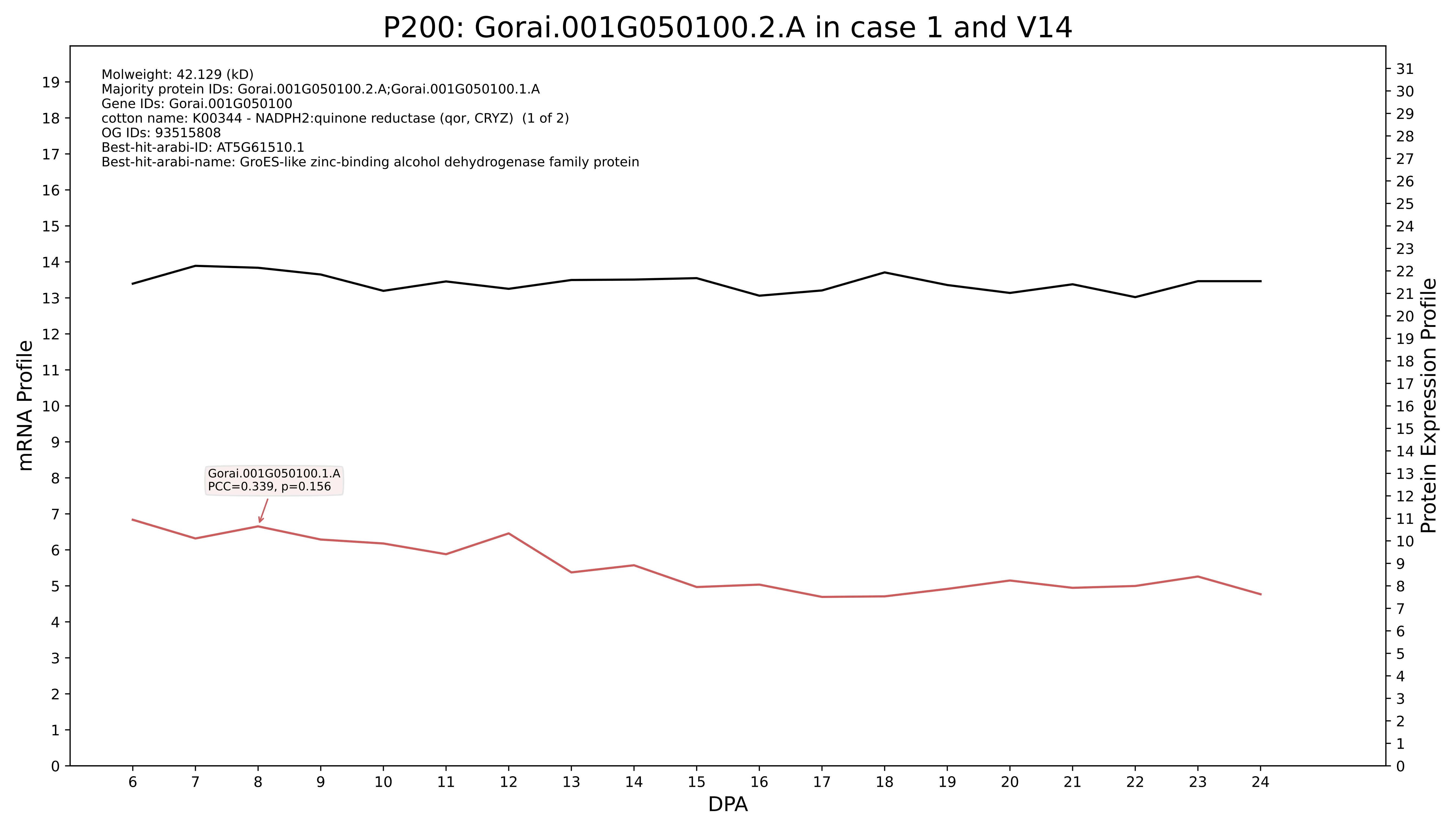
P200: Gorai.001G050100.2.A in case 1 and V14
Molweight: 42.129 (kD)
Majority protein IDs: Gorai.001G050100.2.A;Gorai.001G050100.1.A
Gene IDs: Gorai.001G050100
cotton name: K00344 - NADPH2:quinone reductase (qor, CRYZ) (1 of 2)
OG IDs: 93515808
Best-hit-arabi-ID: AT5G61510.1
Best-hit-arabi-name: GroES-like zinc-binding alcohol dehydrogenase family protein
Gorai.001G050100.1.A
PCC=0.339, p=0.156
mRNA Profile
Protein Expression Profile
DPA

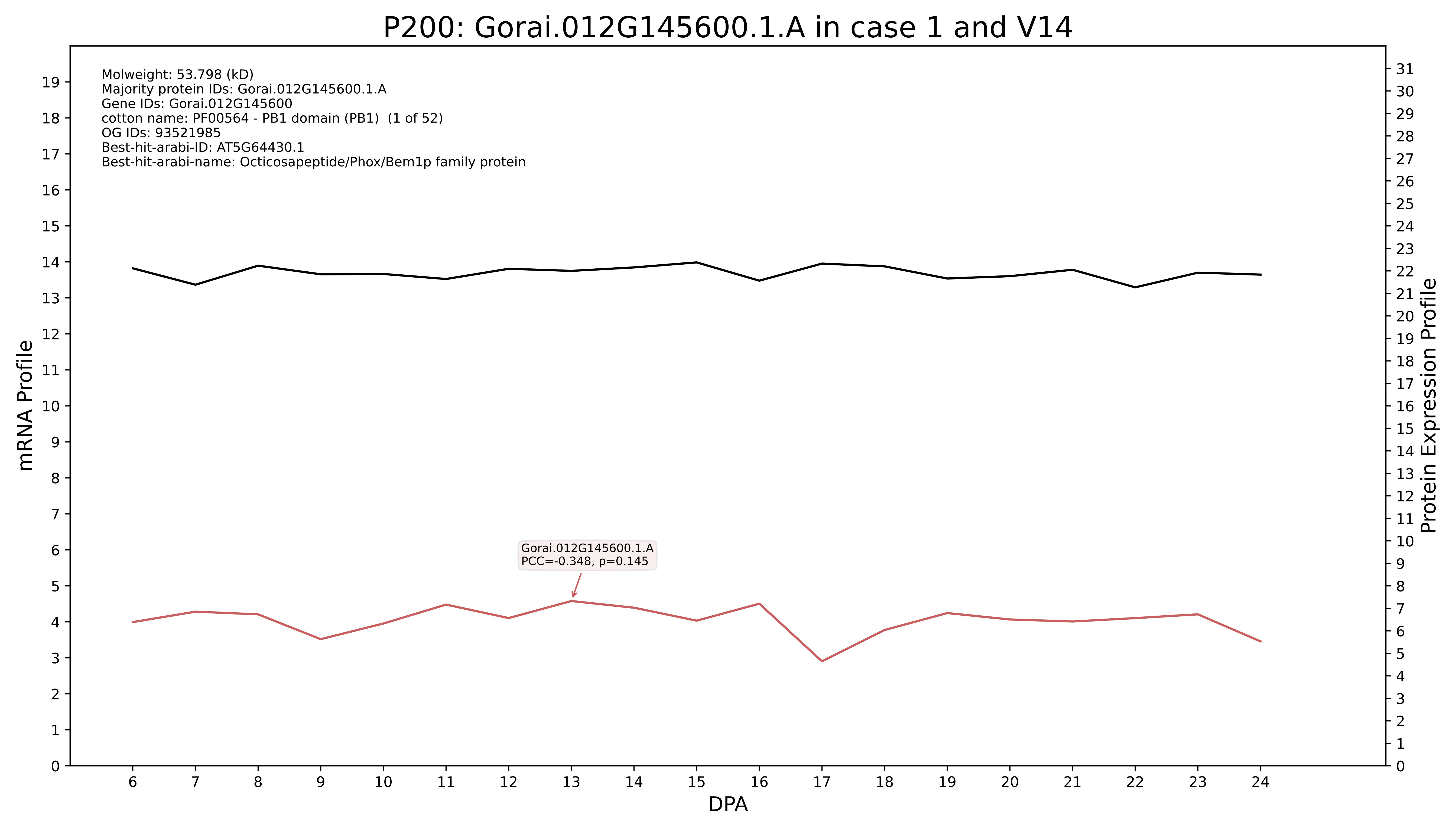
P200: Gorai.012G145600.1.A in case 1 and V14
Molweight: 53.798 (kD)
Majority protein IDs: Gorai.012G145600.1.A
Gene IDs: Gorai.012G145600
cotton name: PF00564 - PB1 domain (PB1) (1 of 52)
OG IDs: 93521985
Best-hit-arabi-ID: AT5G64430.1
Best-hit-arabi-name: Octicosapeptide/Phox/Bem1p family protein
Gorai.012G145600.1.A
PCC=-0.348, p=0.145
mRNA Profile
Protein Expression Profile
DPA

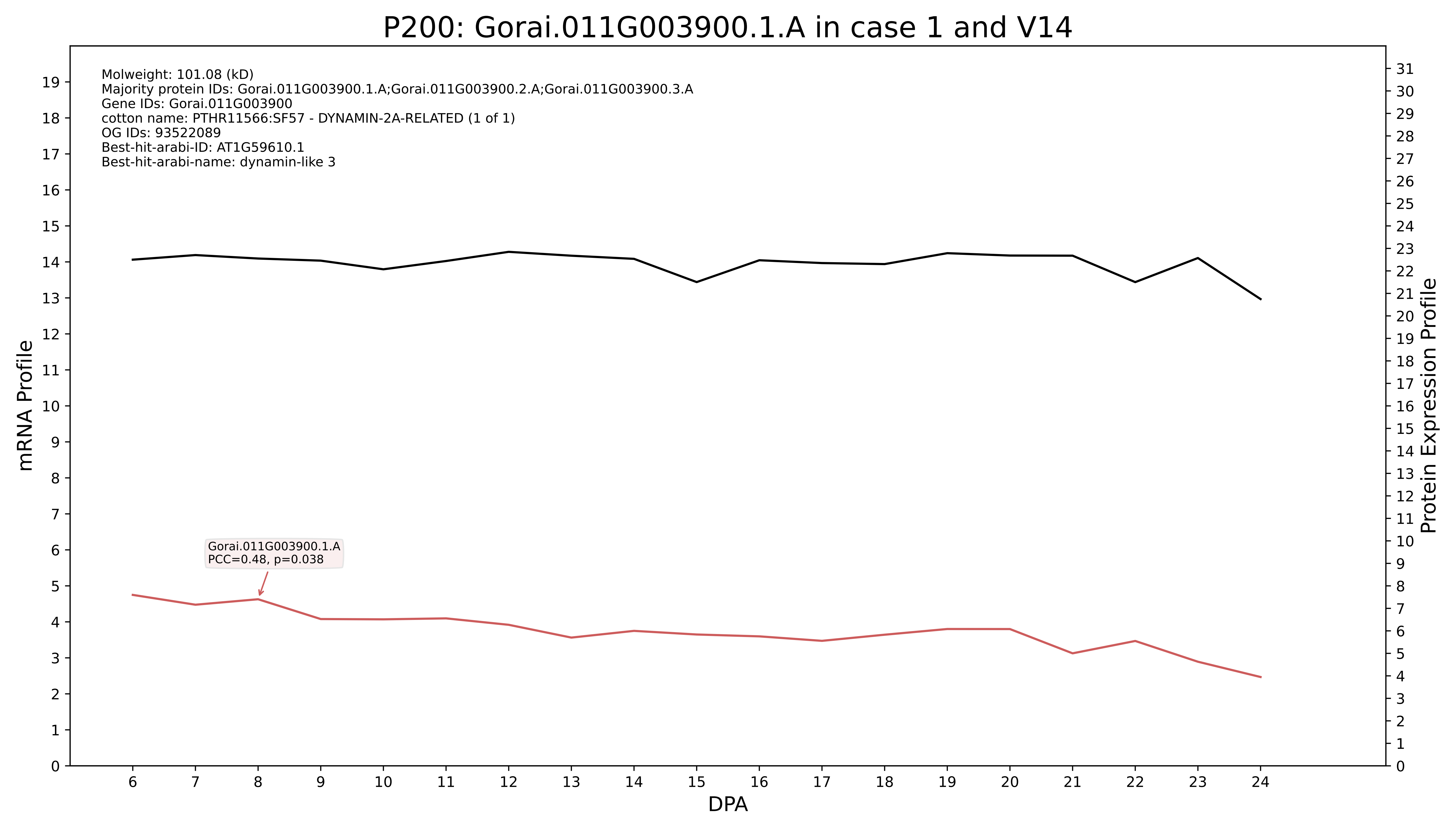
P200: Gorai.011G003900.1.A in case 1 and V14
Molweight: 101.08 (kD)
Majority protein IDs: Gorai.011G003900.1.A;Gorai.011G003900.2.A;Gorai.011G003900.3.A
Gene IDs: Gorai.011G003900
cotton name: PTHR11566:SF57 - DYNAMIN-2A-RELATED (1 of 1)
OG IDs: 93522089
Best-hit-arabi-ID: AT1G59610.1
Best-hit-arabi-name: dynamin-like 3
Gorai.011G003900.1.A
PCC=0.48, p=0.038
mRNA Profile
Protein Expression Profile
DPA

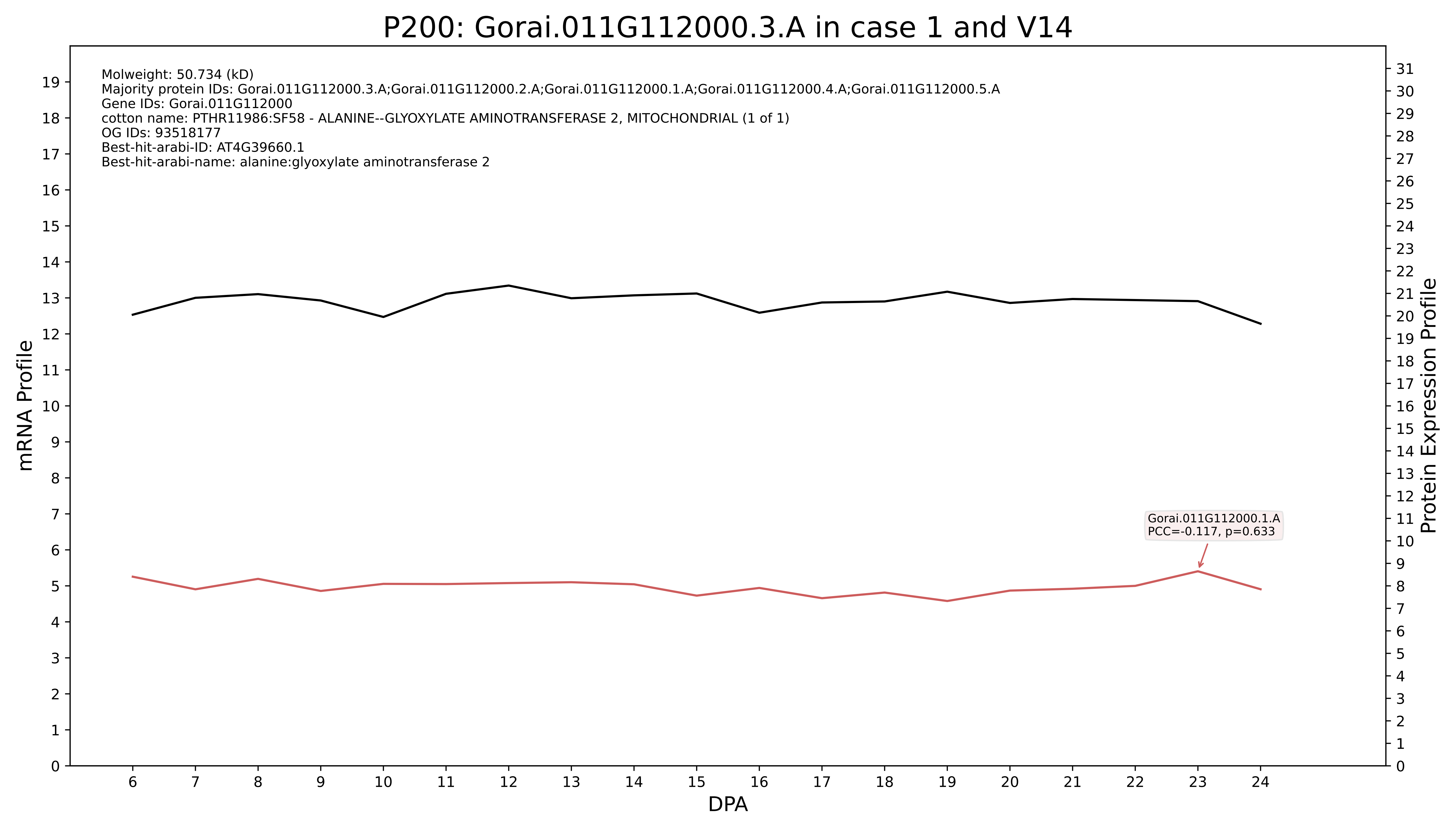

P200: Gorai.011G112000.3.A in case 1 and V14
Molweight: 50.734 (kD)
Majority protein IDs: Gorai.011G112000.3.A;Gorai.011G112000.2.A;Gorai.011G112000.1.A;Gorai.011G112000.4.A;Gorai.011G112000.5.A
Gene IDs: Gorai.011G112000
cotton name: PTHR11986:SF58 - ALANINE--GLYOXYLATE AMINOTRANSFERASE 2, MITOCHONDRIAL (1 of 1)
OG IDs: 93518177
Best-hit-arabi-ID: AT4G39660.1
Best-hit-arabi-name: alanine:glyoxylate aminotransferase 2
Gorai.011G112000.1.A
PCC=-0.117, p=0.633
mRNA Profile
Protein Expression Profile
DPA

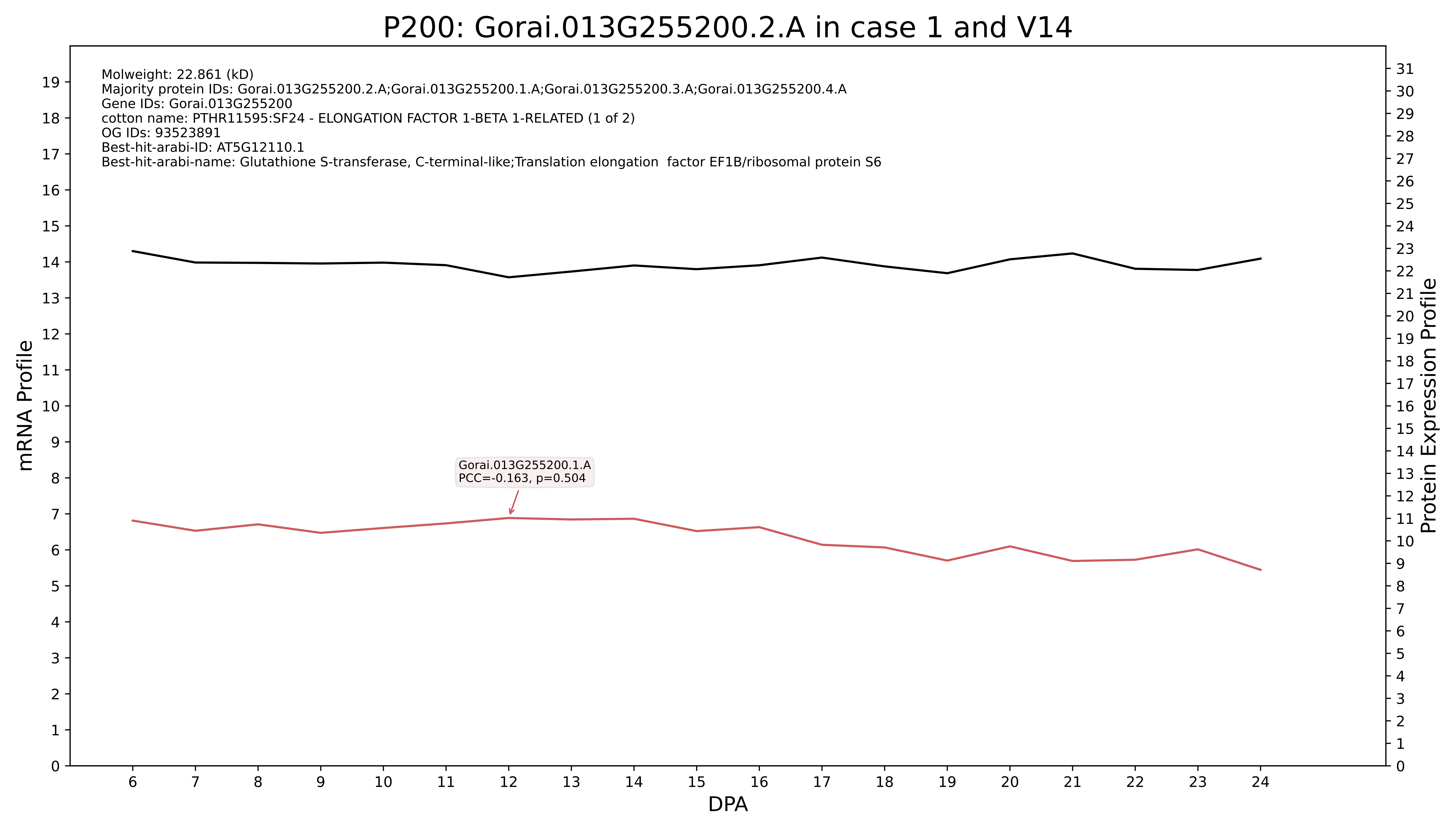

P200: Gorai.013G255200.2.A in case 1 and V14
Molweight: 22.861 (kD)
Majority protein IDs: Gorai.013G255200.2.A;Gorai.013G255200.1.A;Gorai.013G255200.3.A;Gorai.013G255200.4.A
Gene IDs: Gorai.013G255200
cotton name: PTHR11595:SF24 - ELONGATION FACTOR 1-BETA 1-RELATED (1 of 2)
OG IDs: 93523891
Best-hit-arabi-ID: AT5G12110.1
Best-hit-arabi-name: Glutathione S-transferase, C-terminal-like;Translation elongation factor EF1B/ribosomal protein S6
Gorai.013G255200.1.A
PCC=-0.163, p=0.504
mRNA Profile
Protein Expression Profile
DPA

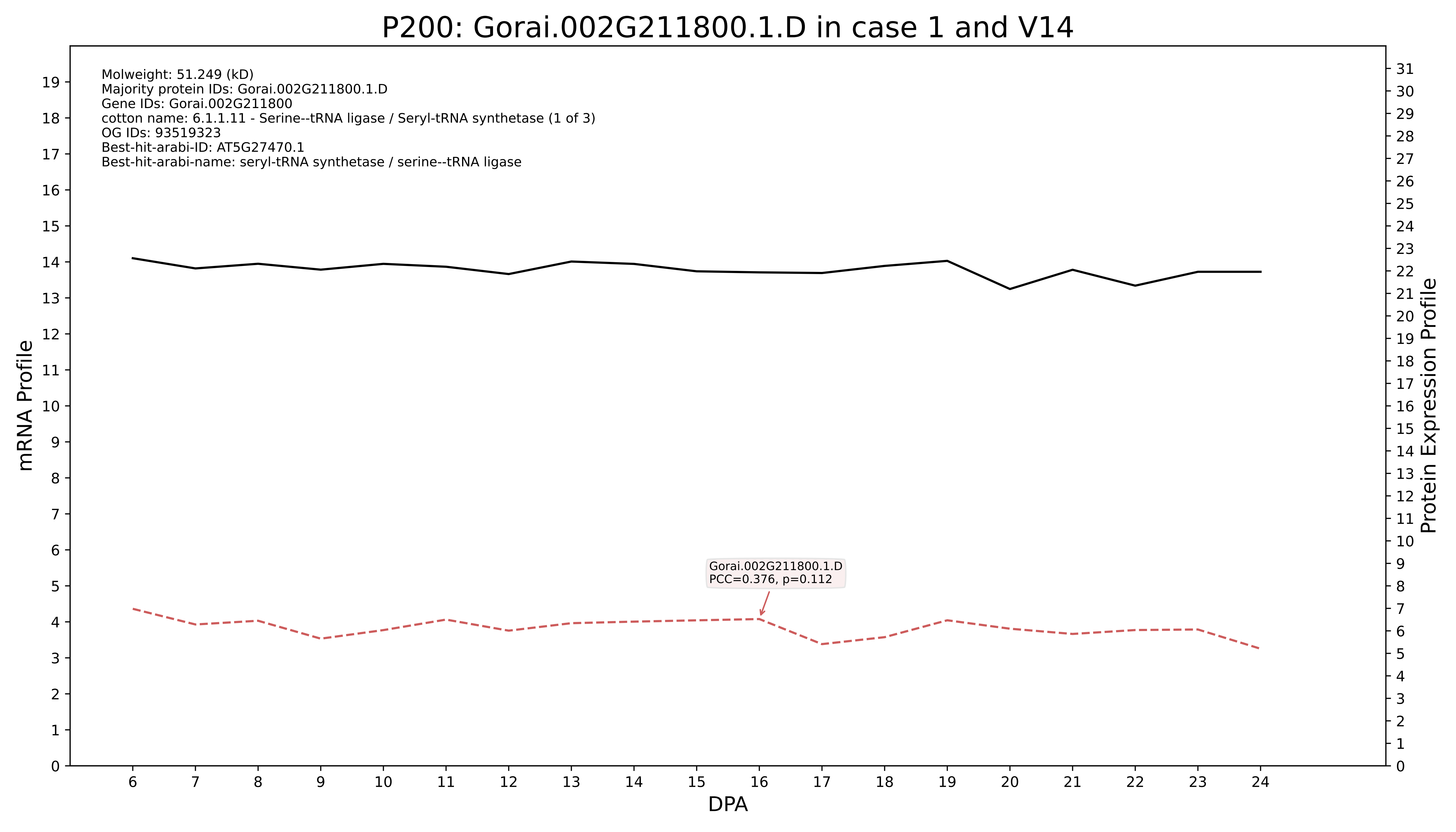
P200: Gorai.002G211800.1.D in case 1 and V14
Molweight: 51.249 (kD)
Majority protein IDs: Gorai.002G211800.1.D
Gene IDs: Gorai.002G211800
cotton name: 6.1.1.11 - Serine--tRNA ligase / Seryl-tRNA synthetase (1 of 3)
OG IDs: 93519323
Best-hit-arabi-ID: AT5G27470.1
Best-hit-arabi-name: seryl-tRNA synthetase / serine--tRNA ligase
Gorai.002G211800.1.D
PCC=0.376, p=0.112
mRNA Profile
Protein Expression Profile
DPA

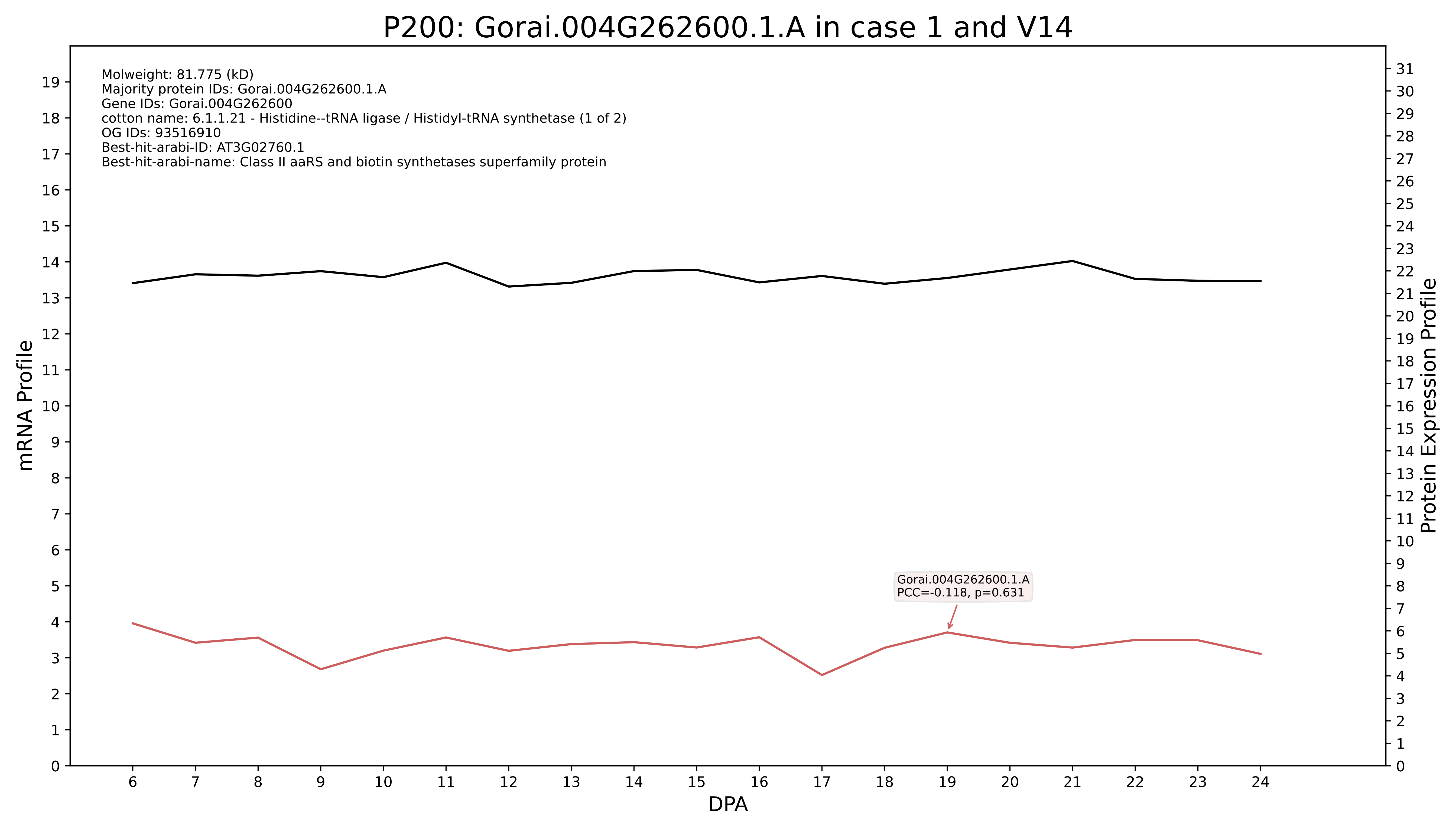
P200: Gorai.004G262600.1.A in case 1 and V14
Molweight: 81.775 (kD)
Majority protein IDs: Gorai.004G262600.1.A
Gene IDs: Gorai.004G262600
cotton name: 6.1.1.21 - Histidine--tRNA ligase / Histidyl-tRNA synthetase (1 of 2)
OG IDs: 93516910
Best-hit-arabi-ID: AT3G02760.1
Best-hit-arabi-name: Class II aaRS and biotin synthetases superfamily protein
Gorai.004G262600.1.A
PCC=-0.118, p=0.631
mRNA Profile
Protein Expression Profile
DPA
0
1
2
3
4
5
6
7
8
9
10
11
12
13
14
15
16
17
18
19
20
21
22
23
24
25
26
27
28
29
30
31

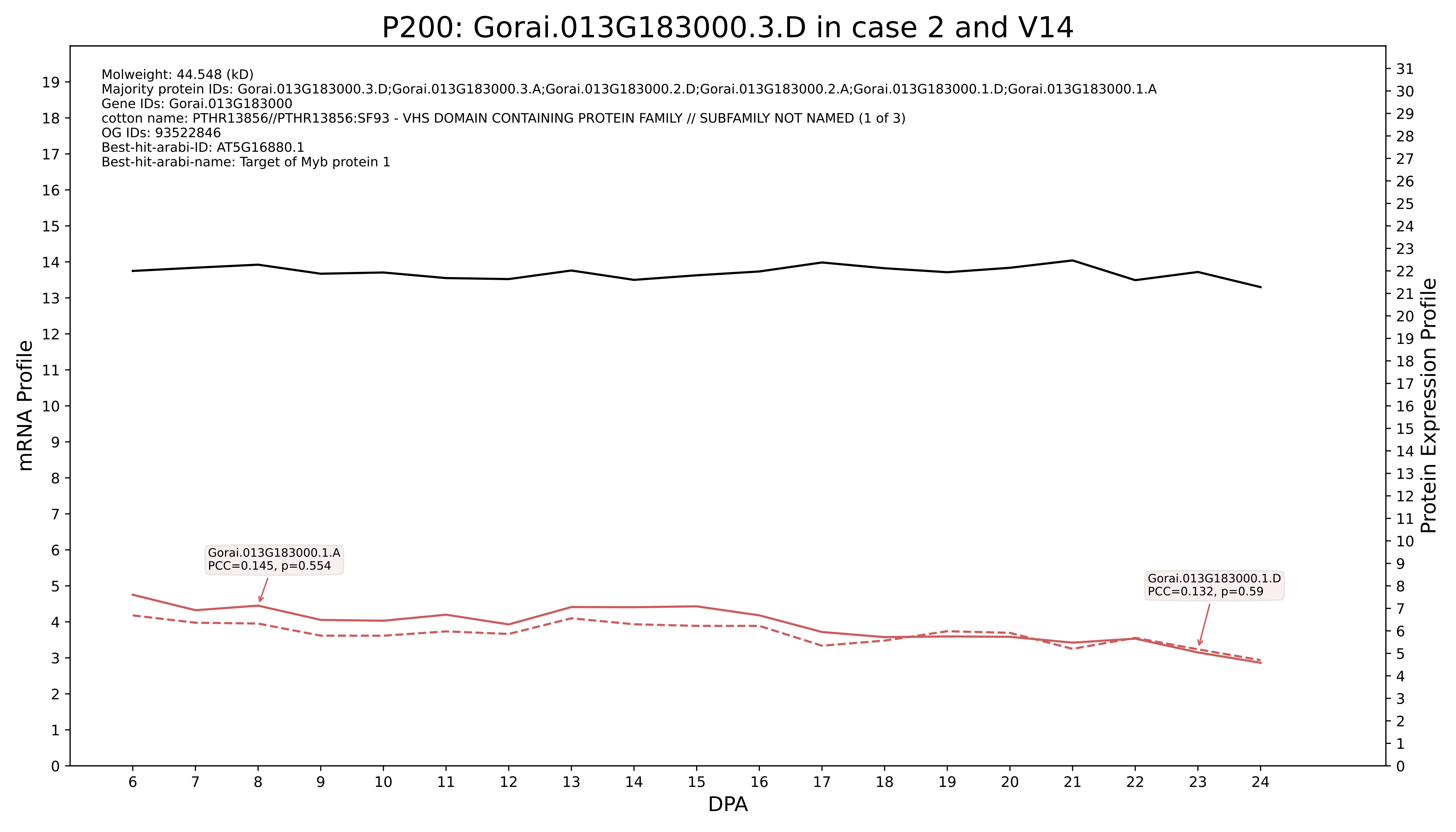
P200: Gorai.013G183000.3.D in case 2 and V14
Molweight: 44.548 (kD)
Majority protein IDs: Gorai.013G183000.3.D;Gorai.013G183000.3.A;Gorai.013G183000.2.D;Gorai.013G183000.2.A;Gorai.013G183000.1.D;Gorai.013G183000.1.A
Gene IDs: Gorai.013G183000
cotton name: PTHR13856//PTHR13856:SF93 - VHS DOMAIN CONTAINING PROTEIN FAMILY // SUBFAMILY NOT NAMED (1 of 3)
OG IDs: 93522846
Best-hit-arabi-ID: AT5G16880.1
Best-hit-arabi-name: Target of Myb protein 1
Gorai.013G183000.1.A
PCC=0.145, p=0.554
Gorai.013G183000.1.D
PCC=0.132, p=0.59
mRNA Profile
Protein Expression Profile
DPA

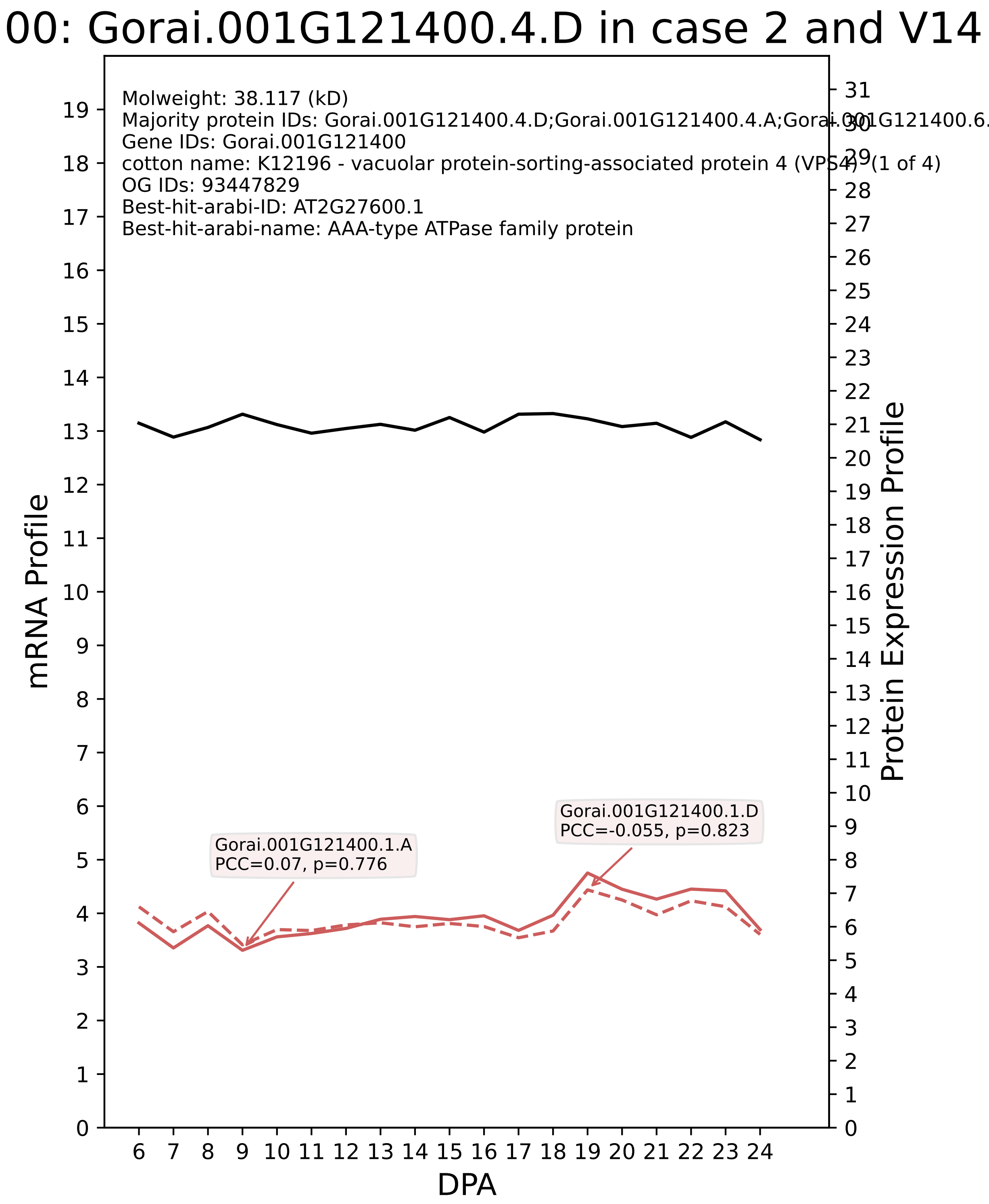
00: Gorai.001G121400.4.D in case 2 and V14
Molweight: 38.117 (kD)
Majority protein IDs: Gorai.001G121400.4.D;Gorai.001G121400.4.A;Gorai.001G121400.6.D;Gorai.001G121400.6.A;Gorai.001G121400.3.D;Gorai.001G121400.3.A;Gorai.001G121400.1.D;Gorai.001G121400.1.A;G
Gene IDs: Gorai.001G121400
cotton name: K12196 - vacuolar protein-sorting-associated protein 4 (VPS4) (1 of 4)
OG IDs: 93447829
Best-hit-arabi-ID: AT2G27600.1
Best-hit-arabi-name: AAA-type ATPase family protein
Gorai.001G121400.1.A
PCC=0.07, p=0.776
Gorai.001G121400.1.D
PCC=-0.055, p=0.823
mRNA Profile
Protein Expression Profile
DPA

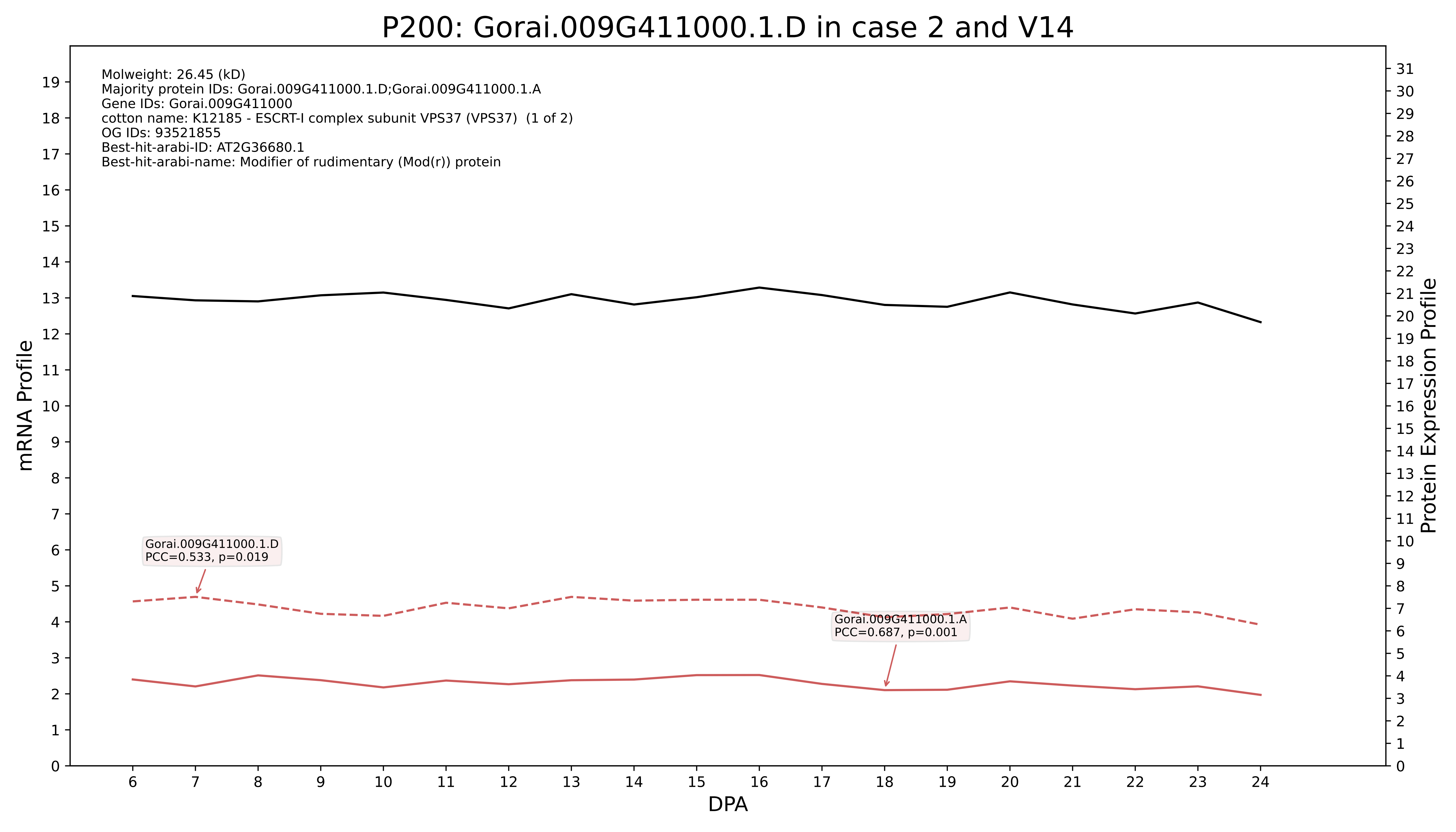

P200: Gorai.009G411000.1.D in case 2 and V14
Molweight: 26.45 (kD)
Majority protein IDs: Gorai.009G411000.1.D;Gorai.009G411000.1.A
Gene IDs: Gorai.009G411000
cotton name: K12185 - ESCRT-I complex subunit VPS37 (VPS37) (1 of 2)
OG IDs: 93521855
Best-hit-arabi-ID: AT2G36680.1
Best-hit-arabi-name: Modifier of rudimentary (Mod(r)) protein
Gorai.009G411000.1.D
PCC=0.533, p=0.019
Gorai.009G411000.1.A
PCC=0.687, p=0.001
mRNA Profile
Protein Expression Profile
DPA

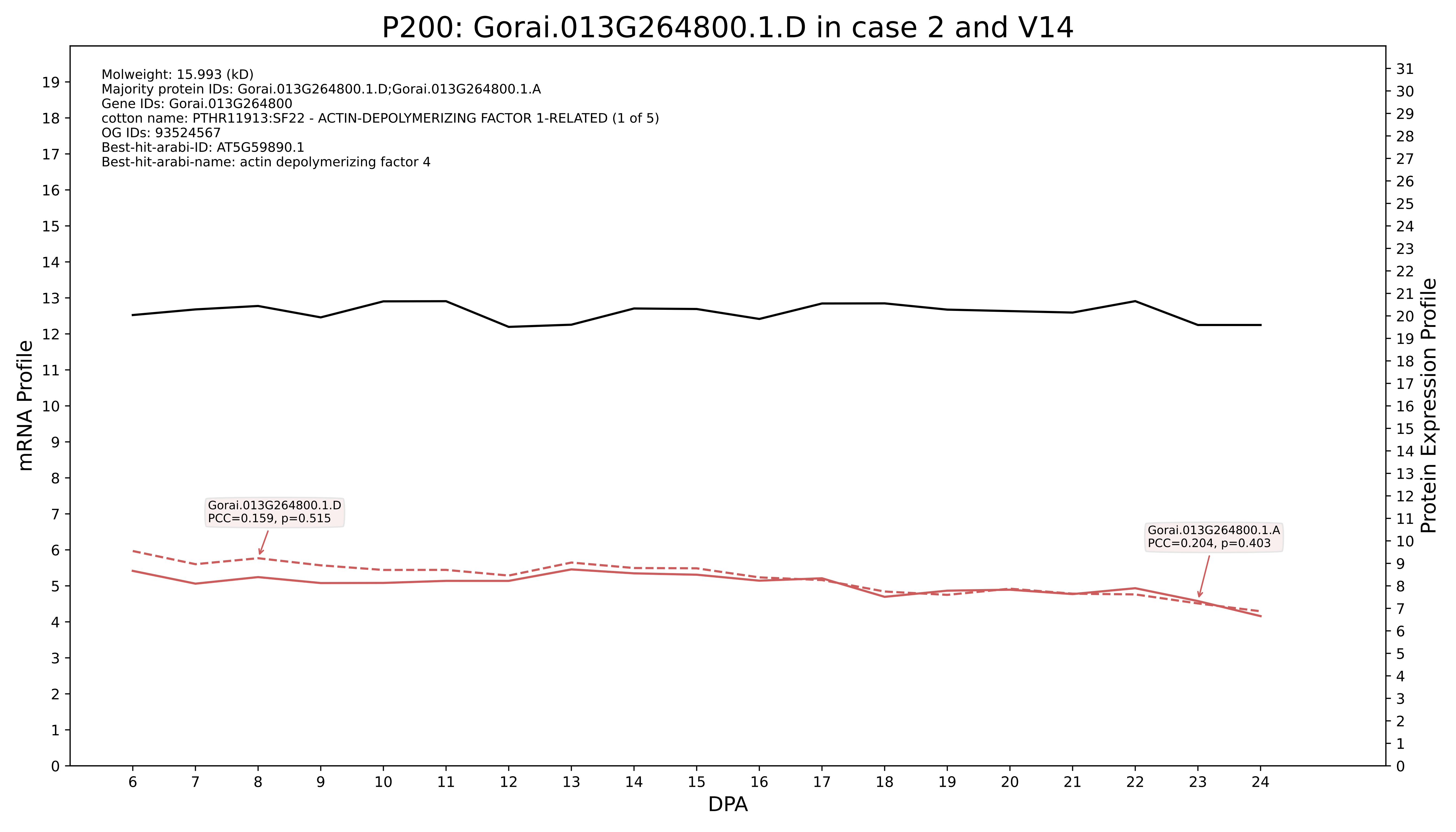
P200: Gorai.013G264800.1.D in case 2 and V14
Molweight: 15.993 (kD)
Majority protein IDs: Gorai.013G264800.1.D;Gorai.013G264800.1.A
Gene IDs: Gorai.013G264800
cotton name: PTHR11913:SF22 - ACTIN-DEPOLYMERIZING FACTOR 1-RELATED (1 of 5)
OG IDs: 93524567
Best-hit-arabi-ID: AT5G59890.1
Best-hit-arabi-name: actin depolymerizing factor 4
Gorai.013G264800.1.D
PCC=0.159, p=0.515
Gorai.013G264800.1.A
PCC=0.204, p=0.403
mRNA Profile
Protein Expression Profile
DPA

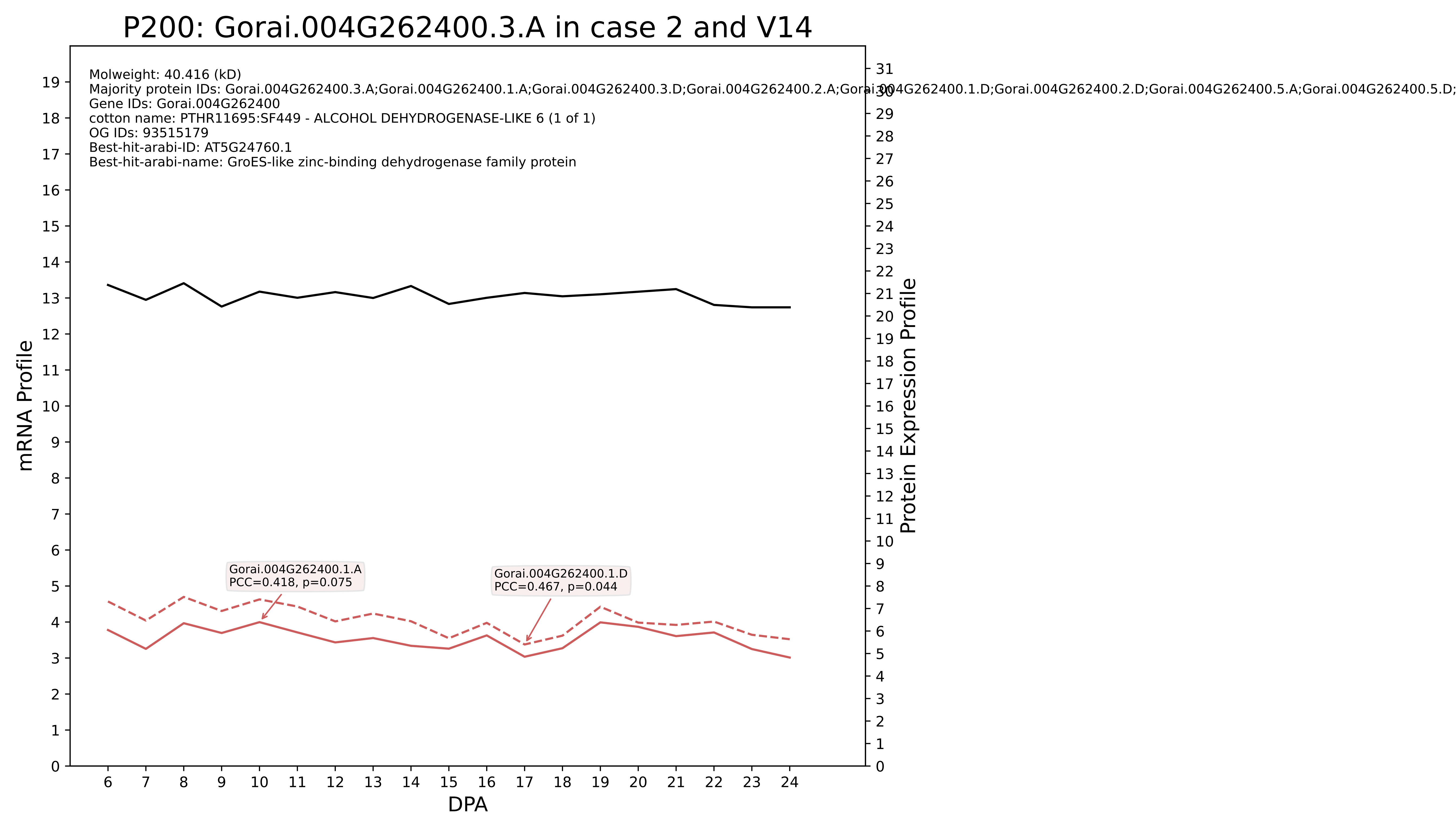
P200: Gorai.004G262400.3.A in case 2 and V14
Molweight: 40.416 (kD)
Majority protein IDs: Gorai.004G262400.3.A;Gorai.004G262400.1.A;Gorai.004G262400.3.D;Gorai.004G262400.2.A;Gorai.004G262400.1.D;Gorai.004G262400.2.D;Gorai.004G262400.5.A;Gorai.004G262400.5.D;
Gene IDs: Gorai.004G262400
cotton name: PTHR11695:SF449 - ALCOHOL DEHYDROGENASE-LIKE 6 (1 of 1)
OG IDs: 93515179
Best-hit-arabi-ID: AT5G24760.1
Best-hit-arabi-name: GroES-like zinc-binding dehydrogenase family protein
Gorai.004G262400.1.A
PCC=0.418, p=0.075
Gorai.004G262400.1.D
PCC=0.467, p=0.044
mRNA Profile
Protein Expression Profile
DPA

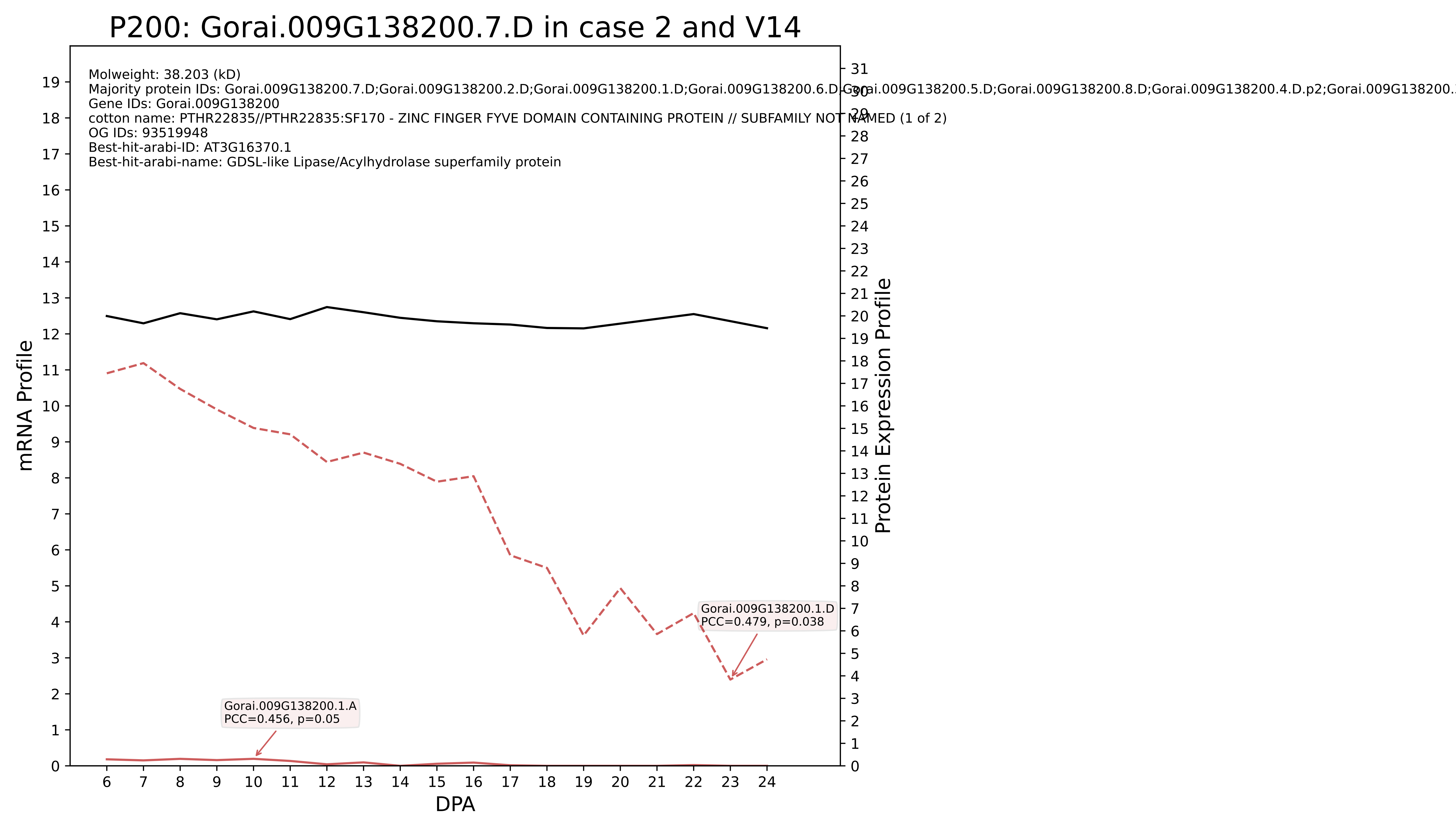
P200: Gorai.009G138200.7.D in case 2 and V14
Molweight: 38.203 (kD)
Majority protein IDs: Gorai.009G138200.7.D;Gorai.009G138200.2.D;Gorai.009G138200.1.D;Gorai.009G138200.6.D;Gorai.009G138200.5.D;Gorai.009G138200.8.D;Gorai.009G138200.4.D.p2;Gorai.009G138200.
Gene IDs: Gorai.009G138200
cotton name: PTHR22835//PTHR22835:SF170 - ZINC FINGER FYVE DOMAIN CONTAINING PROTEIN // SUBFAMILY NOT NAMED (1 of 2)
OG IDs: 93519948
Best-hit-arabi-ID: AT3G16370.1
Best-hit-arabi-name: GDSL-like Lipase/Acylhydrolase superfamily protein
Gorai.009G138200.1.D
PCC=0.479, p=0.038
Gorai.009G138200.1.A
PCC=0.456, p=0.05
mRNA Profile
Protein Expression Profile
DPA

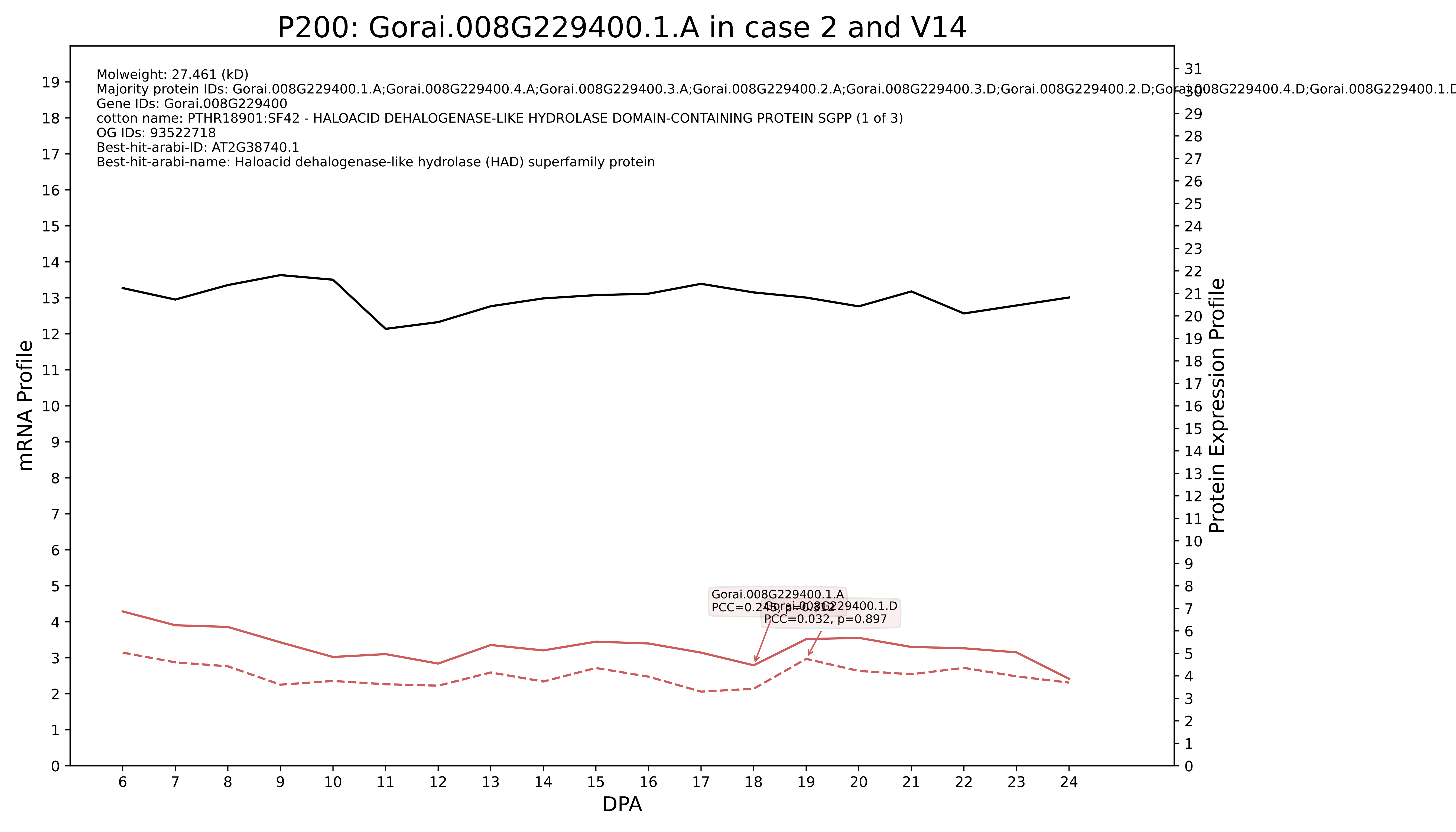
P200: Gorai.008G229400.1.A in case 2 and V14
Molweight: 27.461 (kD)
Majority protein IDs: Gorai.008G229400.1.A;Gorai.008G229400.4.A;Gorai.008G229400.3.A;Gorai.008G229400.2.A;Gorai.008G229400.3.D;Gorai.008G229400.2.D;Gorai.008G229400.4.D;Gorai.008G229400.1.D
Gene IDs: Gorai.008G229400
cotton name: PTHR18901:SF42 - HALOACID DEHALOGENASE-LIKE HYDROLASE DOMAIN-CONTAINING PROTEIN SGPP (1 of 3)
OG IDs: 93522718
Best-hit-arabi-ID: AT2G38740.1
Best-hit-arabi-name: Haloacid dehalogenase-like hydrolase (HAD) superfamily protein
Gorai.008G229400.1.A
PCC=0.243, p=0.312
Gorai.008G229400.1.D
PCC=0.032, p=0.897
mRNA Profile
Protein Expression Profile
DPA

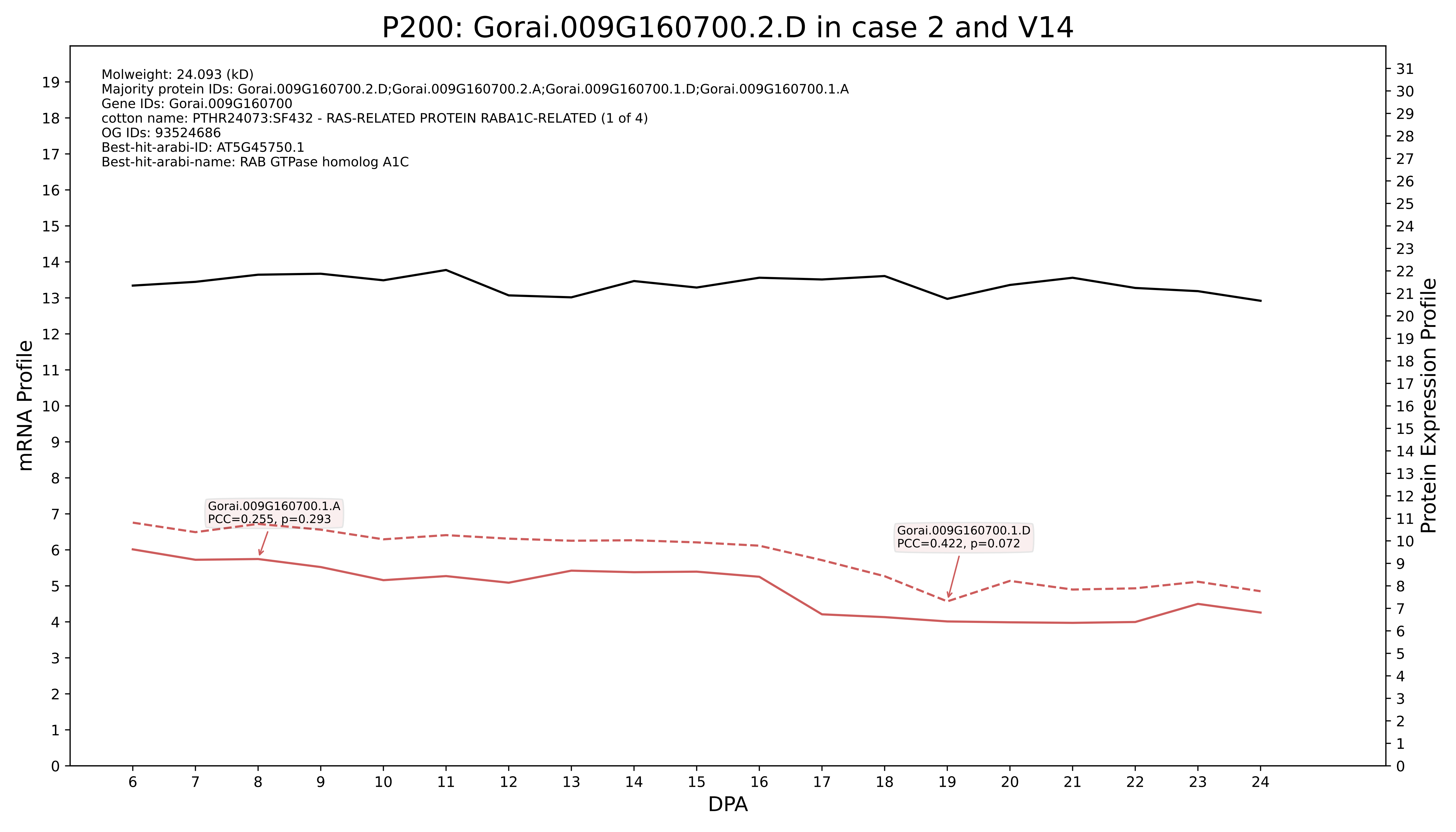

P200: Gorai.009G160700.2.D in case 2 and V14
Molweight: 24.093 (kD)
Majority protein IDs: Gorai.009G160700.2.D;Gorai.009G160700.2.A;Gorai.009G160700.1.D;Gorai.009G160700.1.A
Gene IDs: Gorai.009G160700
cotton name: PTHR24073:SF432 - RAS-RELATED PROTEIN RABA1C-RELATED (1 of 4)
OG IDs: 93524686
Best-hit-arabi-ID: AT5G45750.1
Best-hit-arabi-name: RAB GTPase homolog A1C
Gorai.009G160700.1.A
PCC=0.255, p=0.293
Gorai.009G160700.1.D
PCC=0.422, p=0.072
mRNA Profile
Protein Expression Profile
DPA

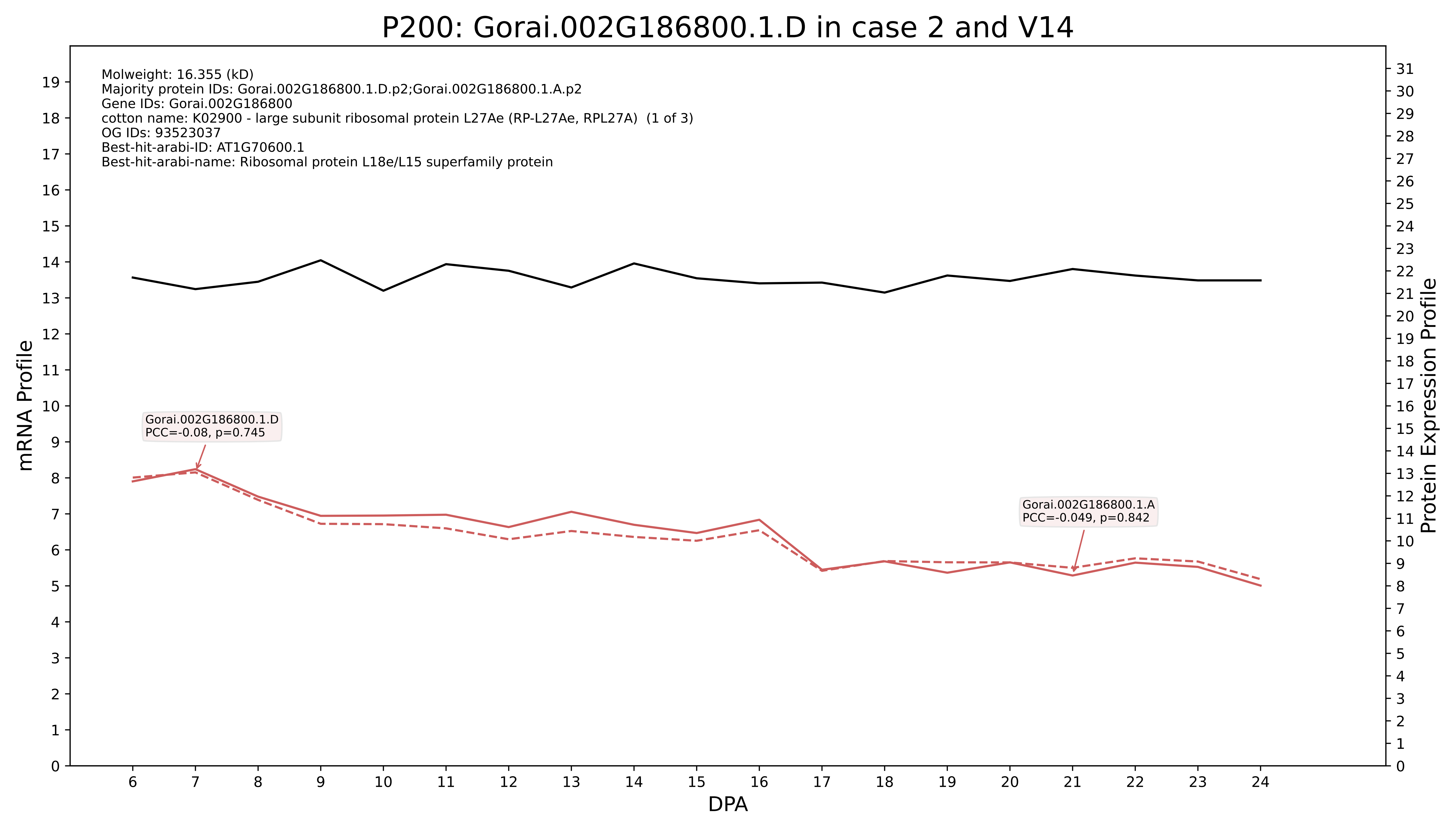
P200: Gorai.002G186800.1.D in case 2 and V14
Molweight: 16.355 (kD)
Majority protein IDs: Gorai.002G186800.1.D.p2;Gorai.002G186800.1.A.p2
Gene IDs: Gorai.002G186800
cotton name: K02900 - large subunit ribosomal protein L27Ae (RP-L27Ae, RPL27A) (1 of 3)
OG IDs: 93523037
Best-hit-arabi-ID: AT1G70600.1
Best-hit-arabi-name: Ribosomal protein L18e/L15 superfamily protein
Gorai.002G186800.1.D
PCC=-0.08, p=0.745
Gorai.002G186800.1.A
PCC=-0.049, p=0.842
mRNA Profile
Protein Expression Profile
DPA

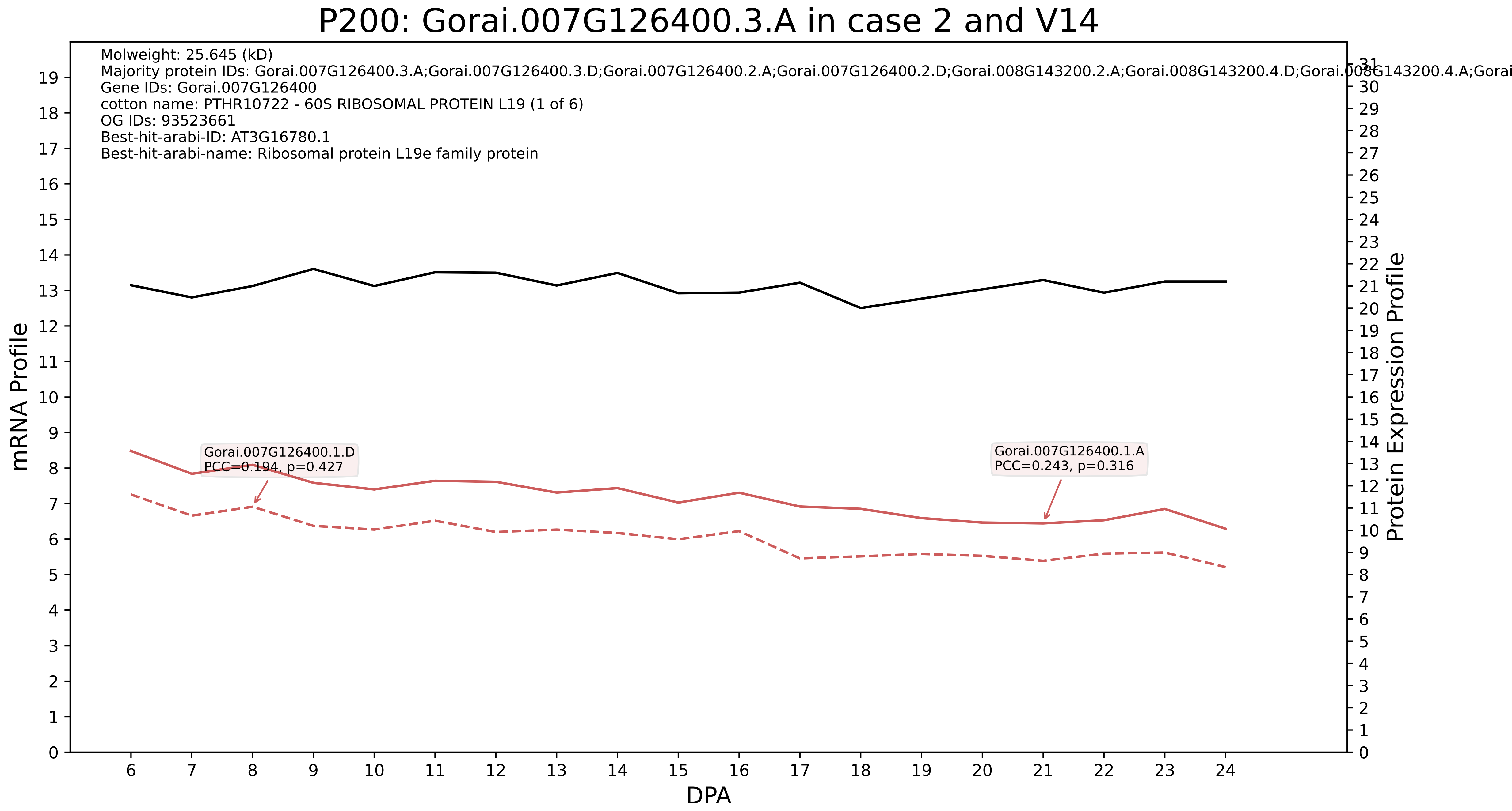

P200: Gorai.007G126400.3.A in case 2 and V14
Molweight: 25.645 (kD)
Majority protein IDs: Gorai.007G126400.3.A;Gorai.007G126400.3.D;Gorai.007G126400.2.A;Gorai.007G126400.2.D;Gorai.008G143200.2.A;Gorai.008G143200.4.D;Gorai.008G143200.4.A;Gorai
Gene IDs: Gorai.007G126400
cotton name: PTHR10722 - 60S RIBOSOMAL PROTEIN L19 (1 of 6)
OG IDs: 93523661
Best-hit-arabi-ID: AT3G16780.1
Best-hit-arabi-name: Ribosomal protein L19e family protein
Gorai.007G126400.1.D
PCC=0.194, p=0.427
Gorai.007G126400.1.A
PCC=0.243, p=0.316
mRNA Profile
Protein Expression Profile
DPA

P200: Gorai.009G200700.1.D in case 2 and V14

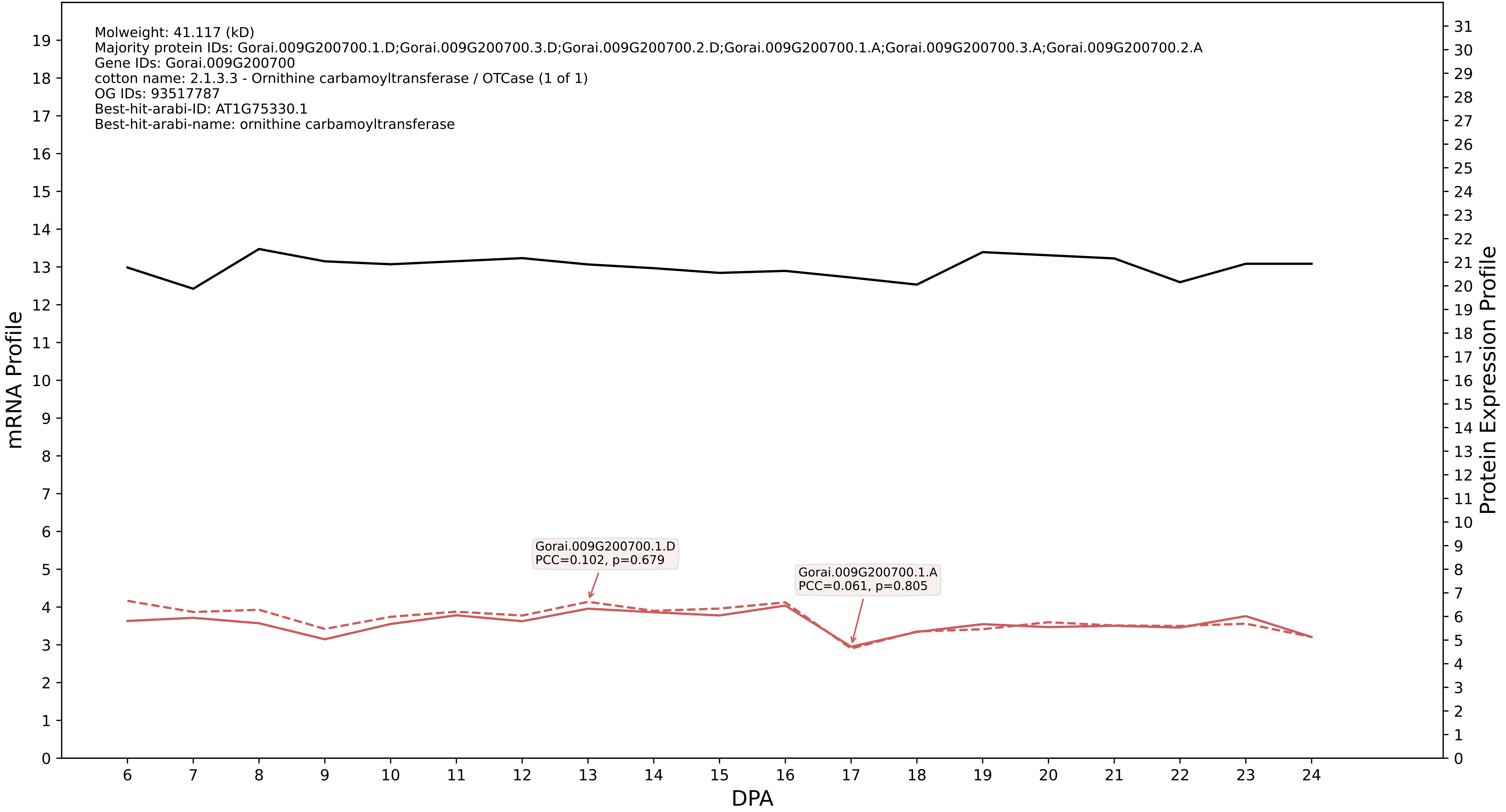

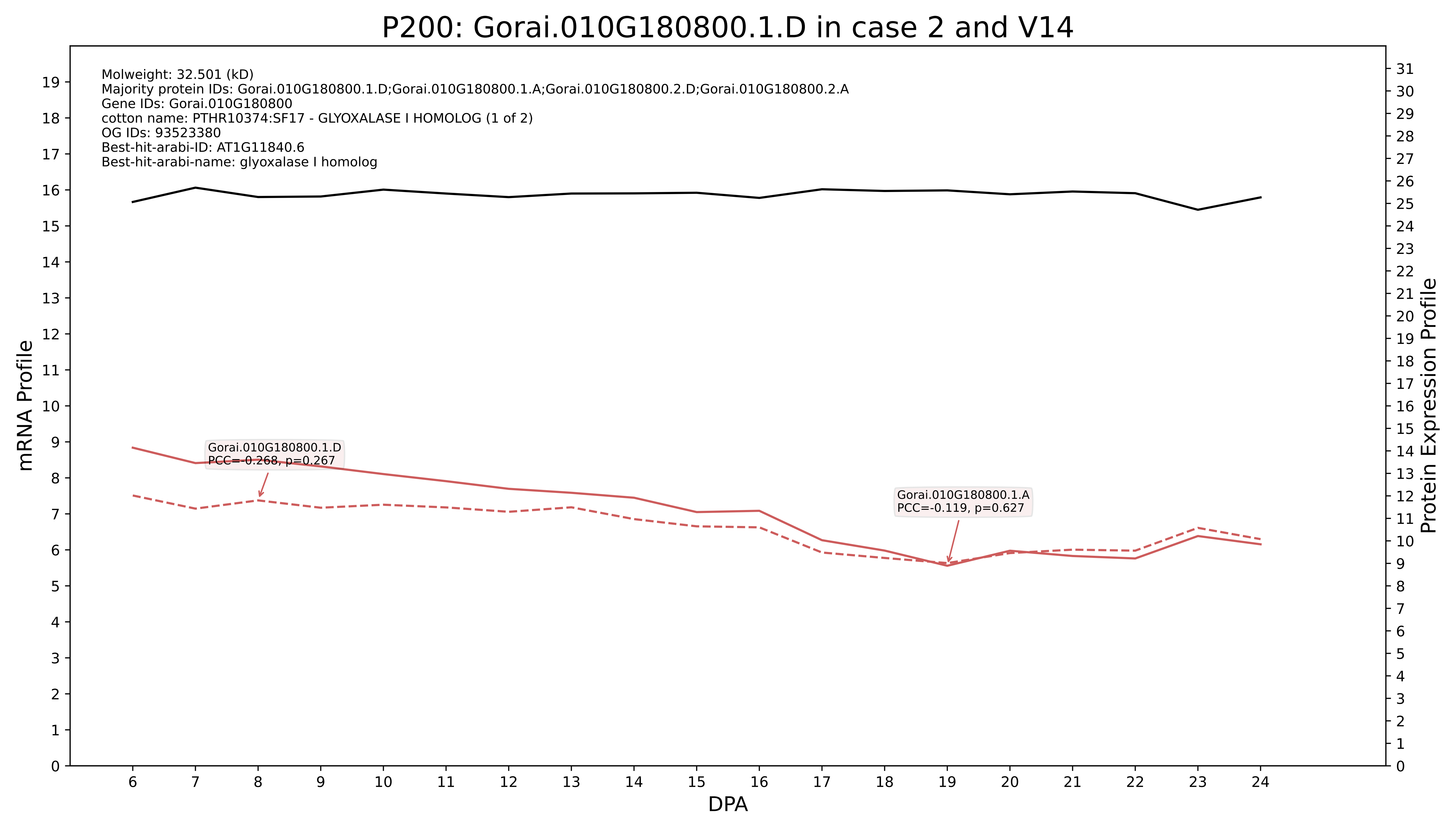

P200: Gorai.010G180800.1.D in case 2 and V14
Molweight: 32.501 (kD)
Majority protein IDs: Gorai.010G180800.1.D;Gorai.010G180800.1.A;Gorai.010G180800.2.D;Gorai.010G180800.2.A
Gene IDs: Gorai.010G180800
cotton name: PTHR10374:SF17 - GLYOXALASE I HOMOLOG (1 of 2)
OG IDs: 93523380
Best-hit-arabi-ID: AT1G11840.6
Best-hit-arabi-name: glyoxalase I homolog
Gorai.010G180800.1.D
PCC=-0.268, p=0.267
Gorai.010G180800.1.A
PCC=-0.119, p=0.627
mRNA Profile
Protein Expression Profile
DPA

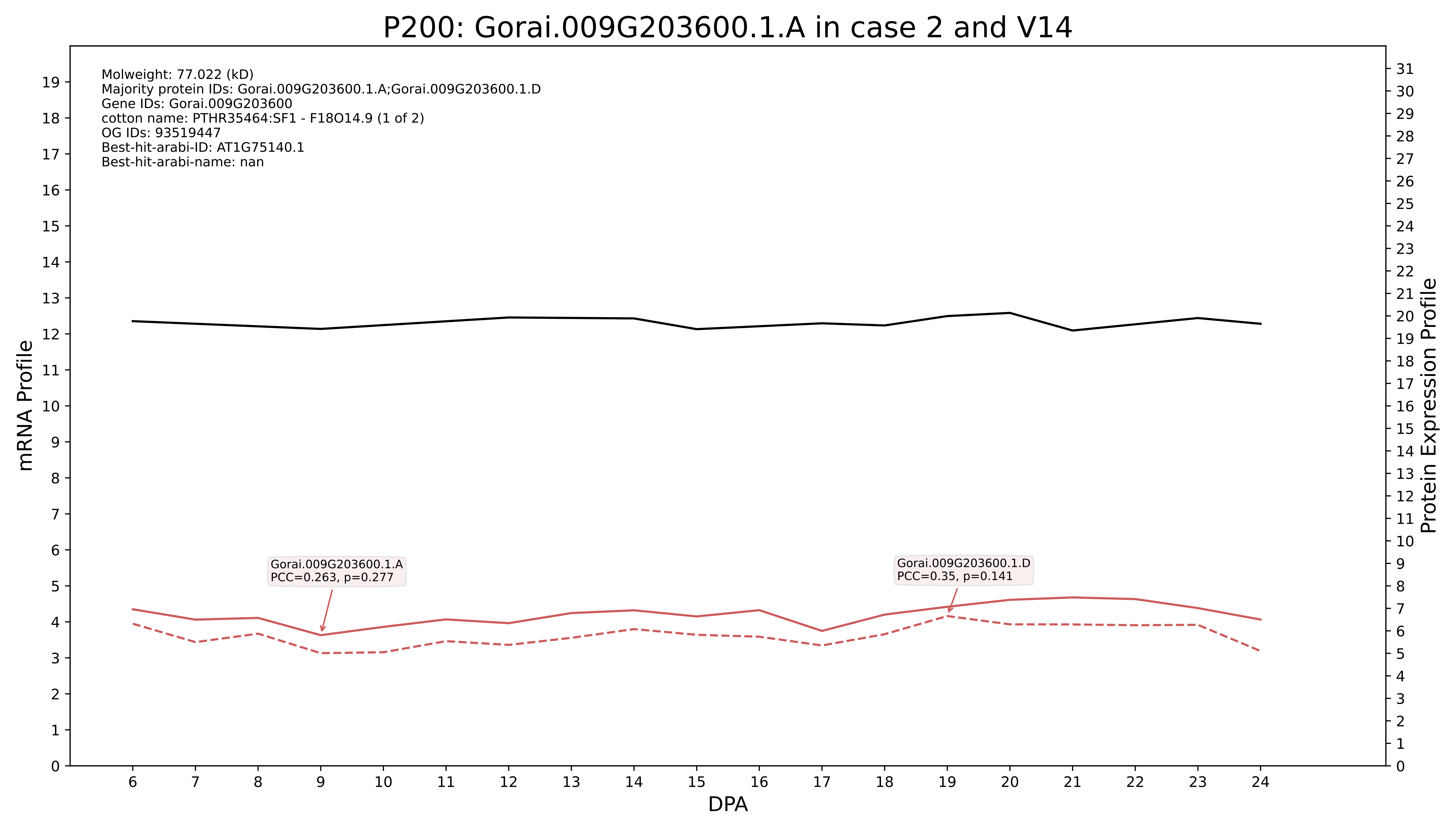
P200: Gorai.009G203600.1.A in case 2 and V14
Molweight: 77.022 (kD)
Majority protein IDs: Gorai.009G203600.1.A;Gorai.009G203600.1.D
Gene IDs: Gorai.009G203600
cotton name: PTHR35464:SF1 - F18O14.9 (1 of 2)
OG IDs: 93519447
Best-hit-arabi-ID: AT1G75140.1
Best-hit-arabi-name: nan
Gorai.009G203600.1.A
PCC=0.263, p=0.277
Gorai.009G203600.1.D
PCC=0.35, p=0.141
mRNA Profile
Protein Expression Profile
DPA

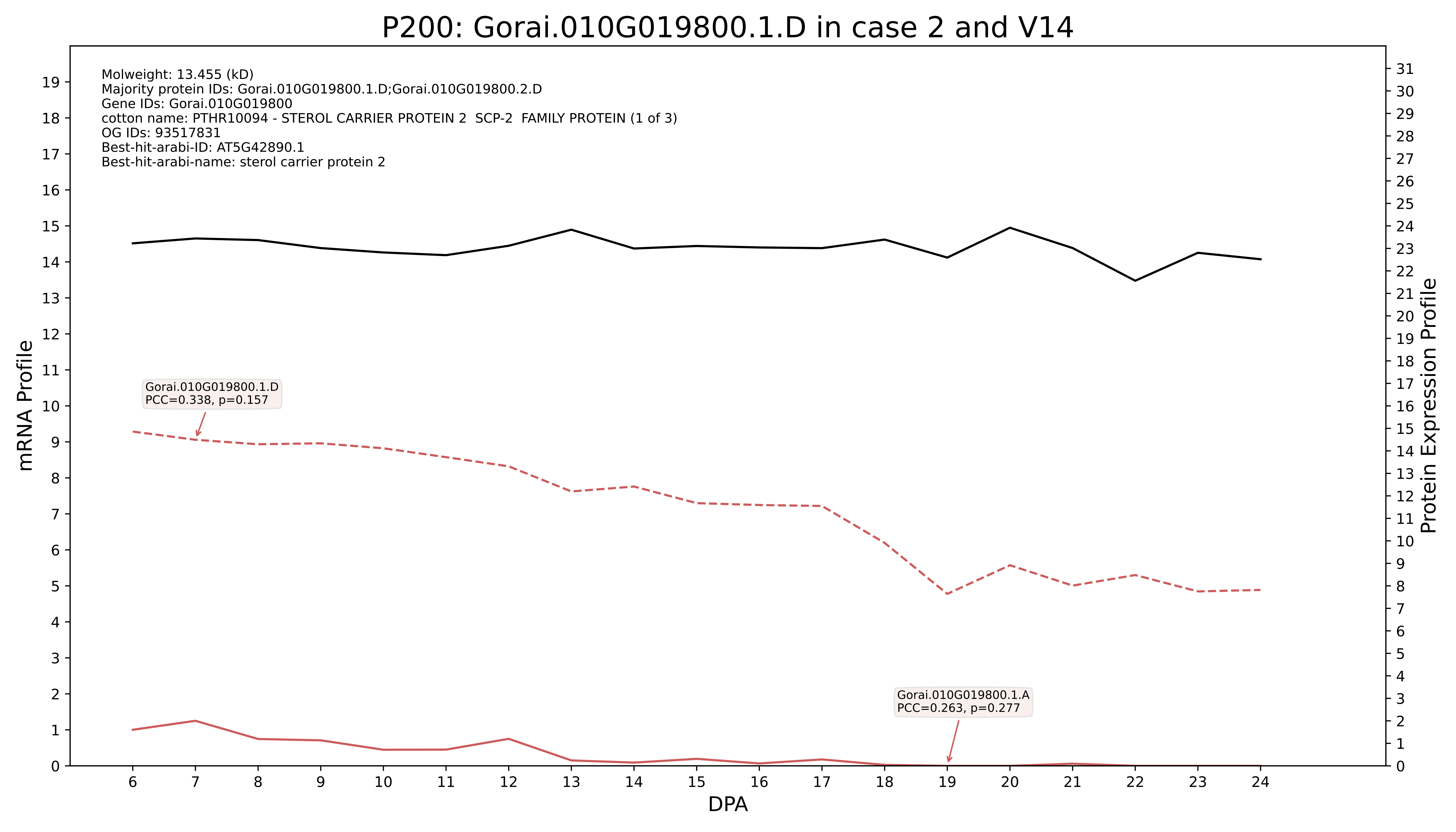
P200: Gorai.010G019800.1.D in case 2 and V14
Molweight: 13.455 (kD)
Majority protein IDs: Gorai.010G019800.1.D;Gorai.010G019800.2.D
Gene IDs: Gorai.010G019800
cotton name: PTHR10094 - STEROL CARRIER PROTEIN 2 SCP-2 FAMILY PROTEIN (1 of 3)
OG IDs: 93517831
Best-hit-arabi-ID: AT5G42890.1
Best-hit-arabi-name: sterol carrier protein 2
Gorai.010G019800.1.D
PCC=0.338, p=0.157
Gorai.010G019800.1.A
PCC=0.263, p=0.277
mRNA Profile
Protein Expression Profile
DPA

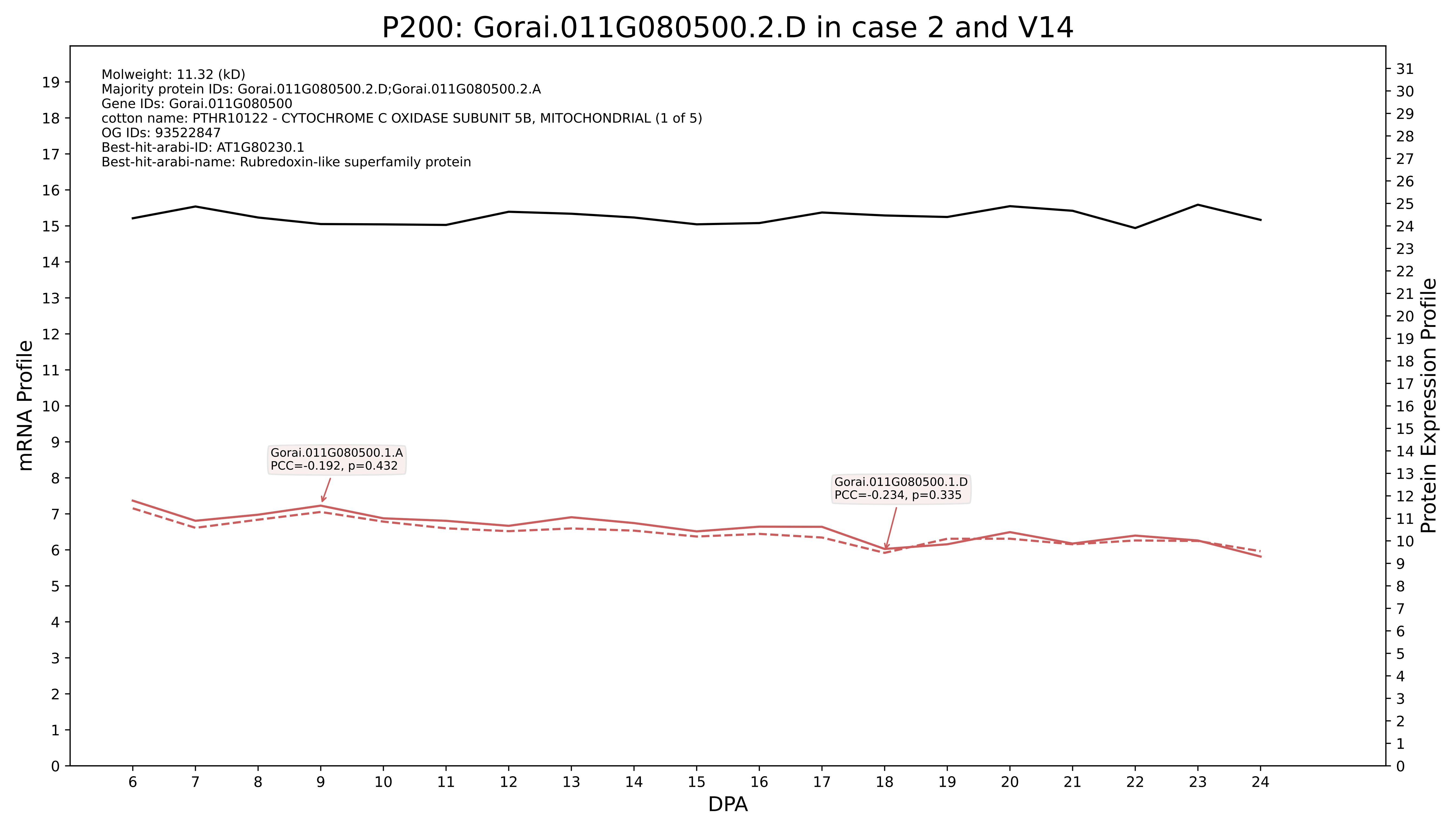

P200: Gorai.011G080500.2.D in case 2 and V14
Molweight: 11.32 (kD)
Majority protein IDs: Gorai.011G080500.2.D;Gorai.011G080500.2.A
Gene IDs: Gorai.011G080500
cotton name: PTHR10122 - CYTOCHROME C OXIDASE SUBUNIT 5B, MITOCHONDRIAL (1 of 5)
OG IDs: 93522847
Best-hit-arabi-ID: AT1G80230.1
Best-hit-arabi-name: Rubredoxin-like superfamily protein
Gorai.011G080500.1.A
PCC=-0.192, p=0.432
Gorai.011G080500.1.D
PCC=-0.234, p=0.335
mRNA Profile
Protein Expression Profile
DPA

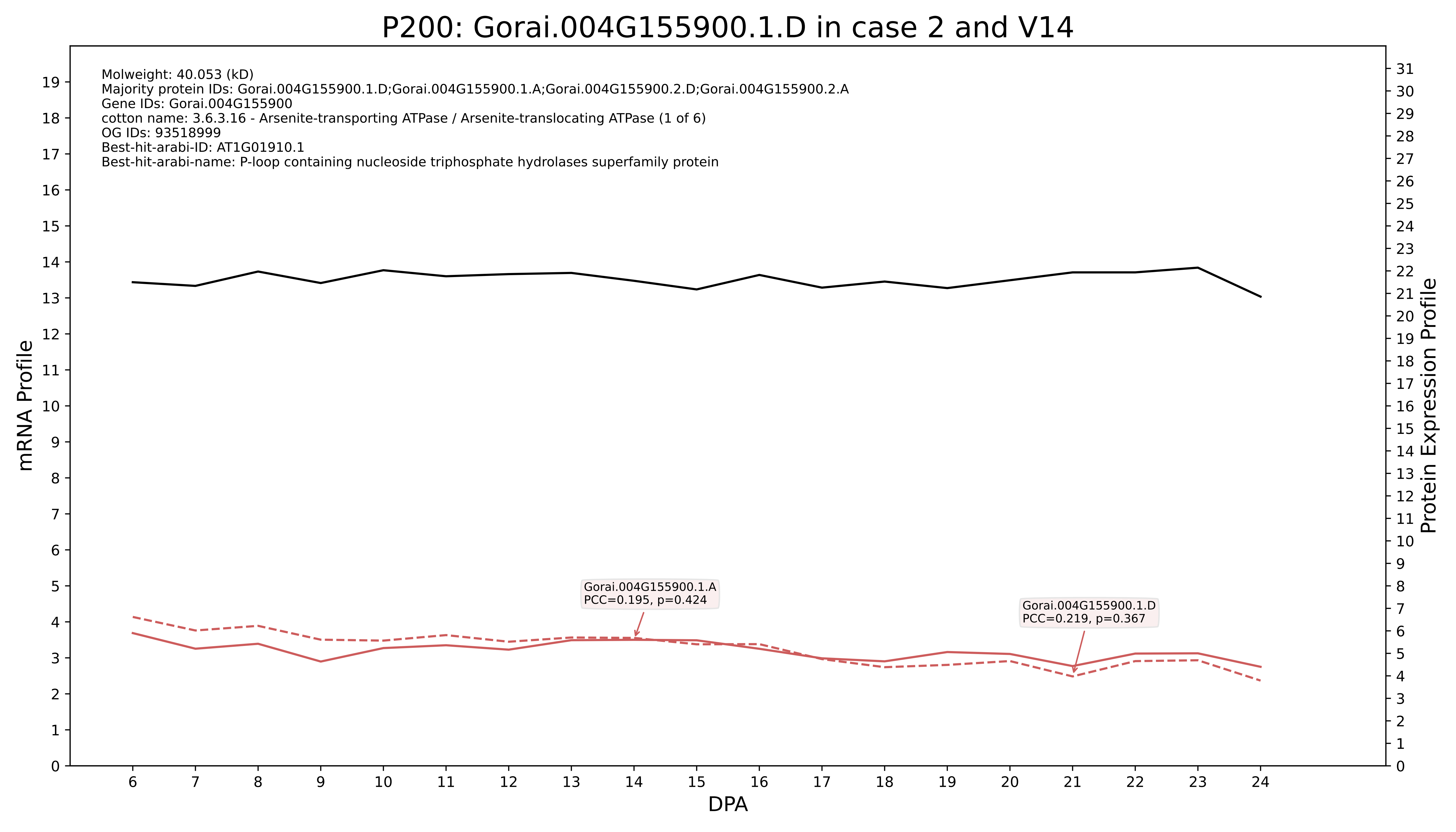
P200: Gorai.004G155900.1.D in case 2 and V14
Molweight: 40.053 (kD)
Majority protein IDs: Gorai.004G155900.1.D;Gorai.004G155900.1.A;Gorai.004G155900.2.D;Gorai.004G155900.2.A
Gene IDs: Gorai.004G155900
cotton name: 3.6.3.16 - Arsenite-transporting ATPase / Arsenite-translocating ATPase (1 of 6)
OG IDs: 93518999
Best-hit-arabi-ID: AT1G01910.1
Best-hit-arabi-name: P-loop containing nucleoside triphosphate hydrolases superfamily protein
Gorai.004G155900.1.A
PCC=0.195, p=0.424
Gorai.004G155900.1.D
PCC=0.219, p=0.367
mRNA Profile
Protein Expression Profile
DPA
0
1
2
3
4
5
6
7
8
9
10
11
12
13
14
15
16
17
18
19
0
1
2
3
4
5
6
7
8
9
10
11
12
13
14
15
16
17
18
19
20
21
22
23
24
25
26
27
28
29
30
31
6
7
8
9
10
11
12
13
14
15
16
17
18
19
20
21
22
23
24

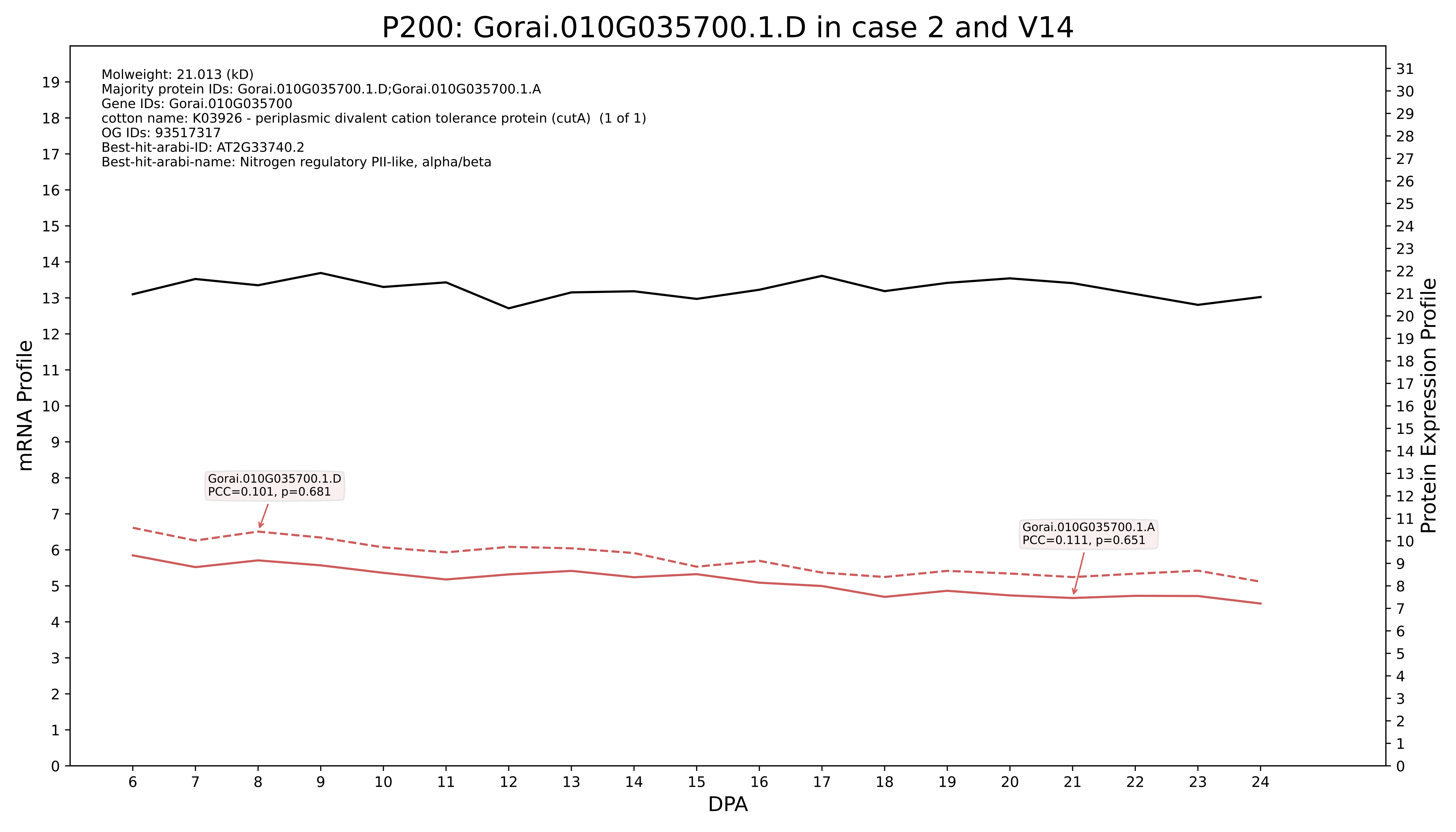
P200: Gorai.010G035700.1.D in case 2 and V14
Molweight: 21.013 (kD)
Majority protein IDs: Gorai.010G035700.1.D;Gorai.010G035700.1.A
Gene IDs: Gorai.010G035700
cotton name: K03926 - periplasmic divalent cation tolerance protein (cutA) (1 of 1)
OG IDs: 93517317
Best-hit-arabi-ID: AT2G33740.2
Best-hit-arabi-name: Nitrogen regulatory PII-like, alpha/beta
Gorai.010G035700.1.D
PCC=0.101, p=0.681
Gorai.010G035700.1.A
PCC=0.111, p=0.651
mRNA Profile
Protein Expression Profile
DPA
0
1
2
3
4
5
6
7
8
9
10
11
12
13
14
15
16
17
18
19
20
21
22
23
24
25
26
27
28
29
30
31

P200: Gorai.013G012400.1.A in case 2 and V14

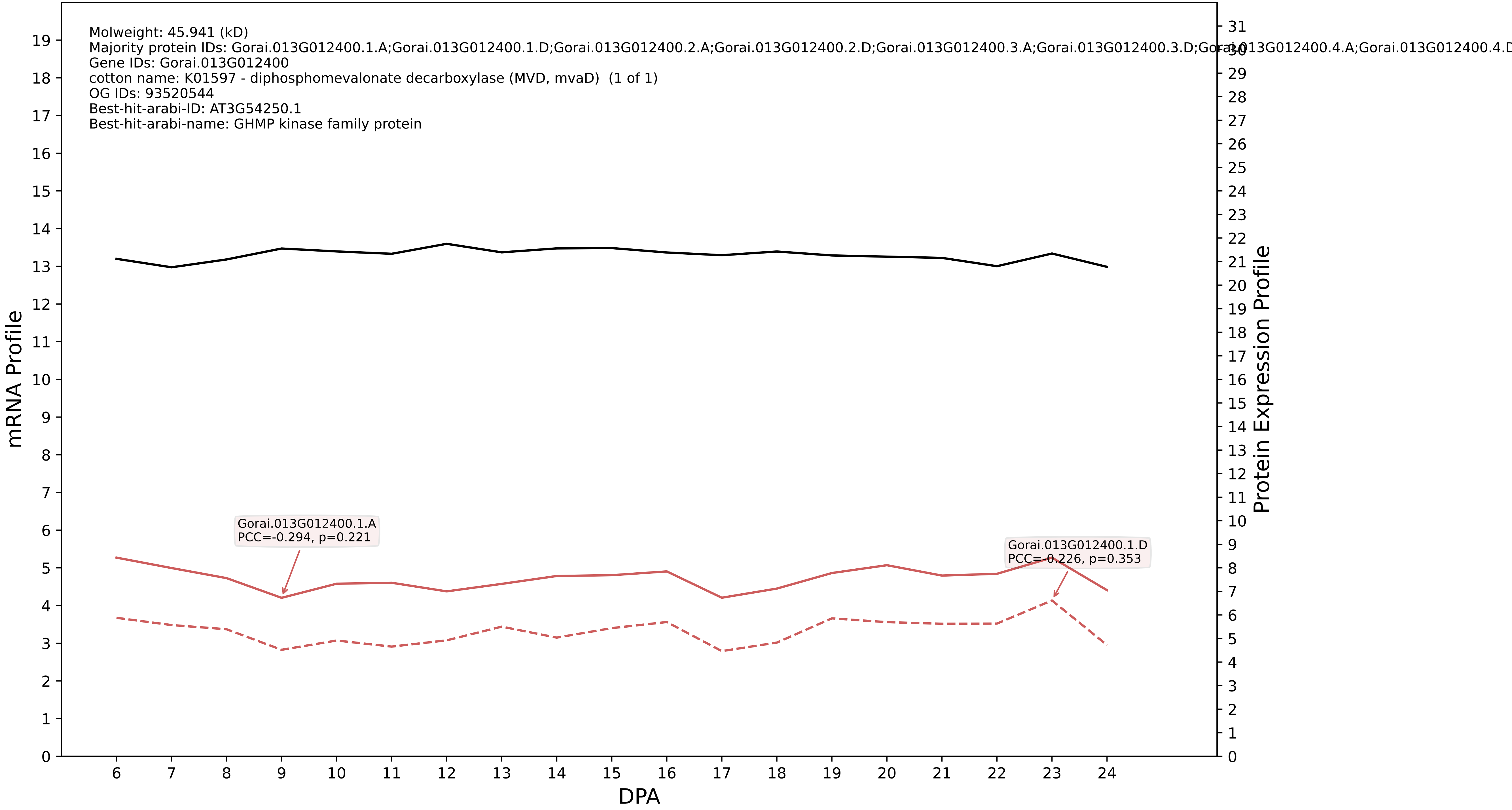

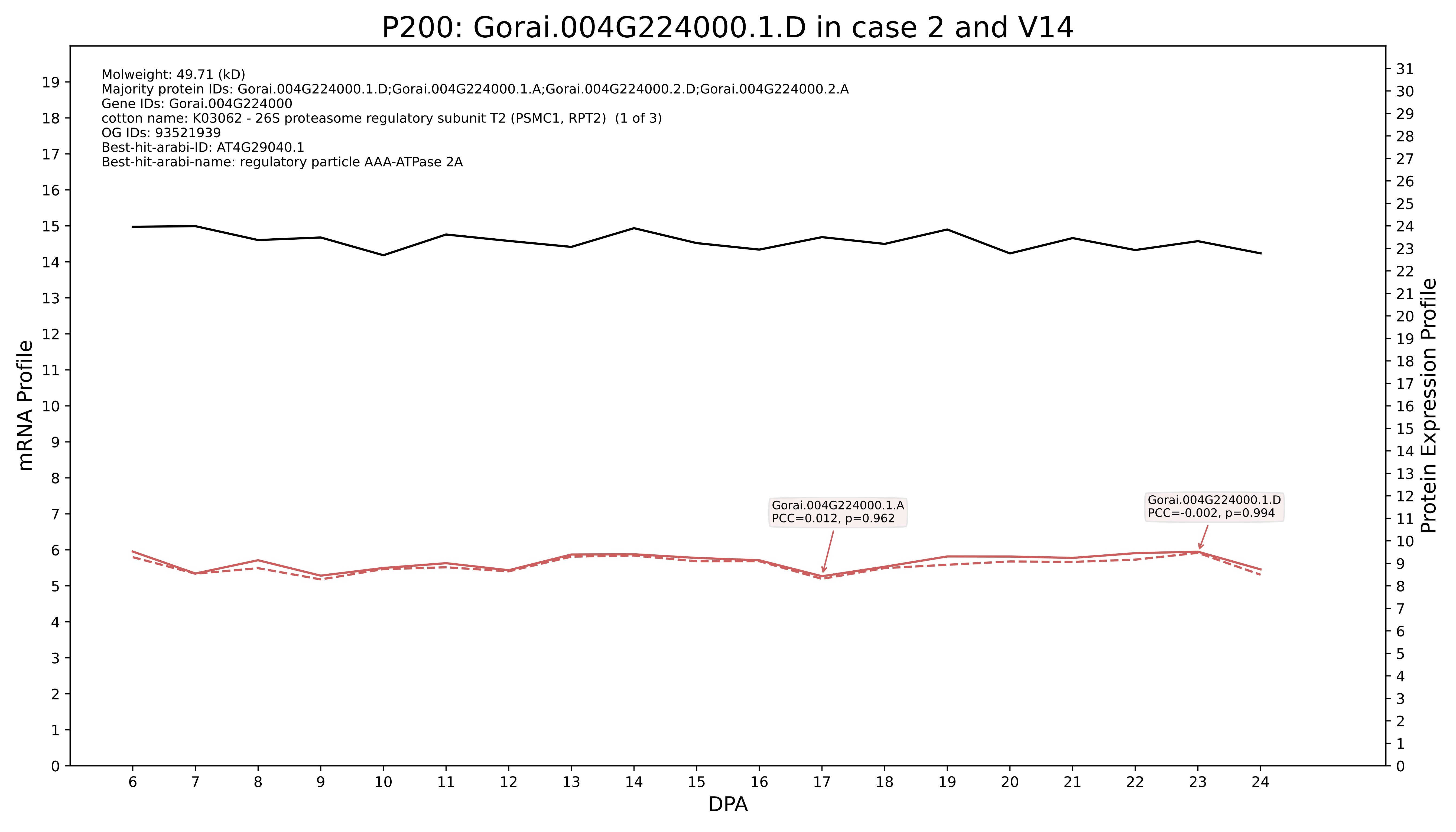
P200: Gorai.004G224000.1.D in case 2 and V14
Molweight: 49.71 (kD)
Majority protein IDs: Gorai.004G224000.1.D;Gorai.004G224000.1.A;Gorai.004G224000.2.D;Gorai.004G224000.2.A
Gene IDs: Gorai.004G224000
cotton name: K03062 - 26S proteasome regulatory subunit T2 (PSMC1, RPT2) (1 of 3)
OG IDs: 93521939
Best-hit-arabi-ID: AT4G29040.1
Best-hit-arabi-name: regulatory particle AAA-ATPase 2A
Gorai.004G224000.1.A
PCC=0.012, p=0.962
Gorai.004G224000.1.D
PCC=-0.002, p=0.994
mRNA Profile
Protein Expression Profile
DPA

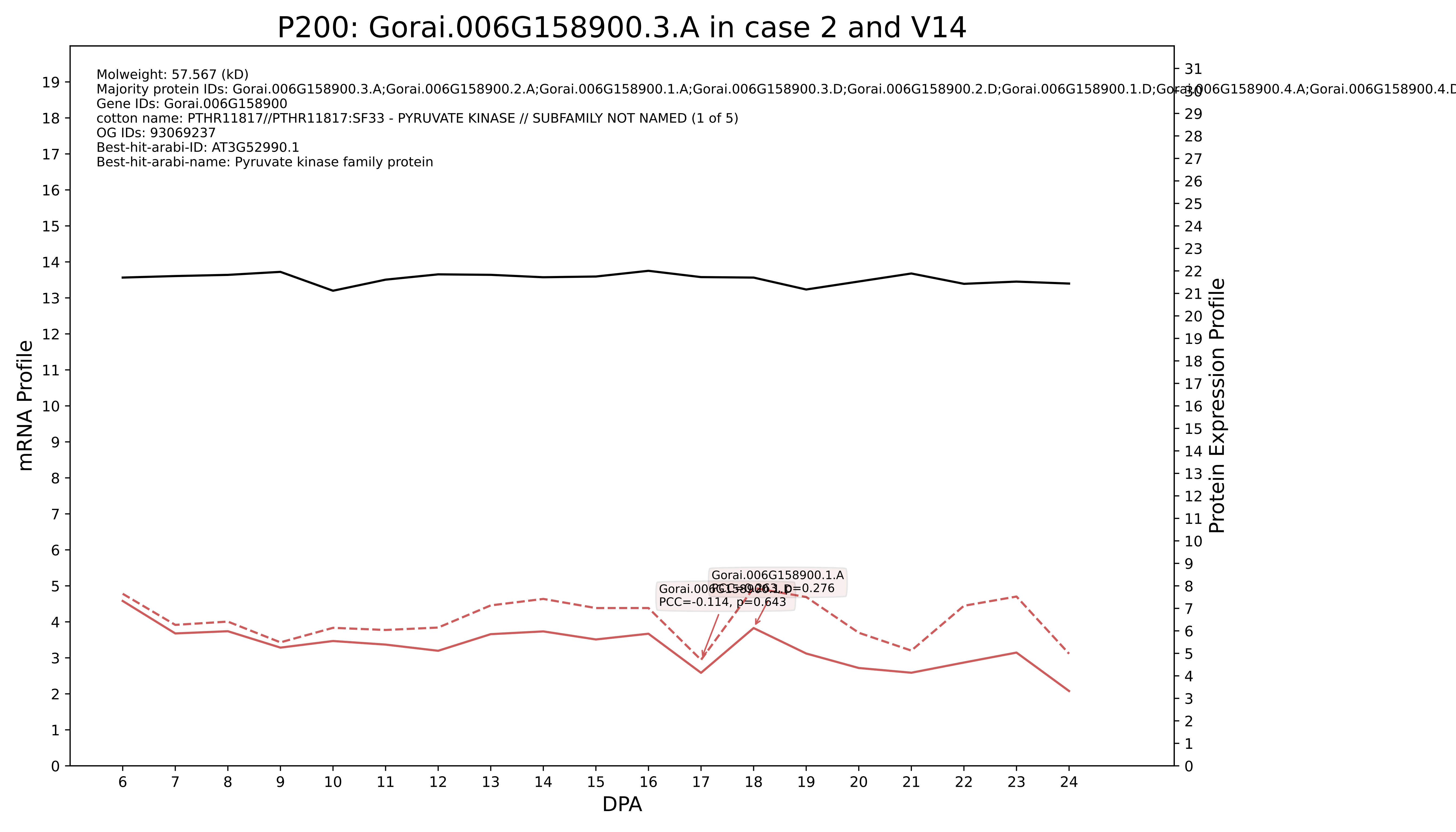
P200: Gorai.006G158900.3.A in case 2 and V14
Molweight: 57.567 (kD)
Majority protein IDs: Gorai.006G158900.3.A;Gorai.006G158900.2.A;Gorai.006G158900.1.A;Gorai.006G158900.3.D;Gorai.006G158900.2.D;Gorai.006G158900.1.D;Gorai.006G158900.4.A;Gorai.006G158900.4.D
Gene IDs: Gorai.006G158900
cotton name: PTHR11817//PTHR11817:SF33 - PYRUVATE KINASE // SUBFAMILY NOT NAMED (1 of 5)
OG IDs: 93069237
Best-hit-arabi-ID: AT3G52990.1
Best-hit-arabi-name: Pyruvate kinase family protein
Gorai.006G158900.1.A
PCC=0.263, p=0.276
PCC=-0.114, p=0.643
mRNA Profile
Protein Expression Profile
DPA

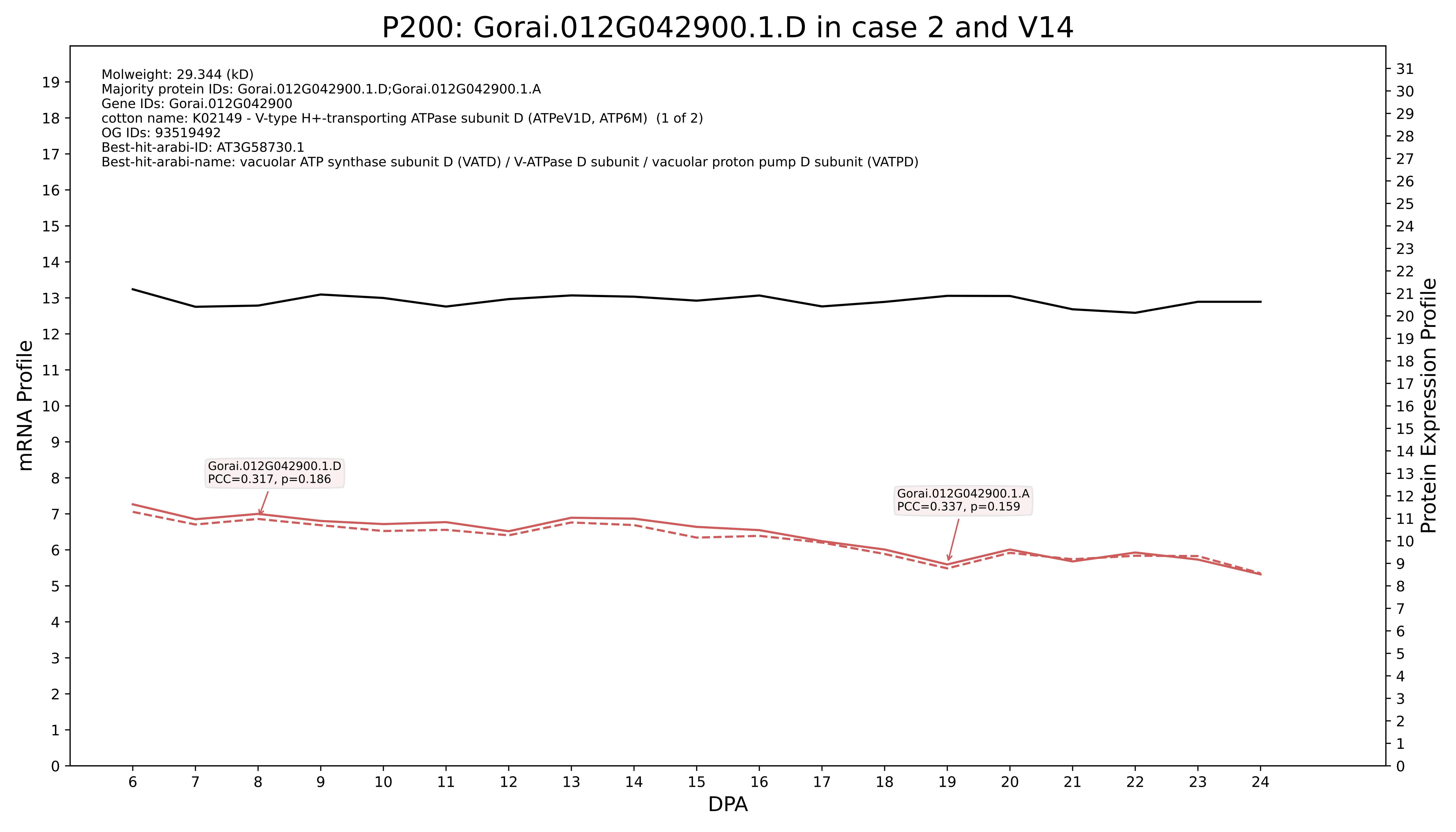
P200: Gorai.012G042900.1.D in case 2 and V14
Molweight: 29.344 (kD)
Majority protein IDs: Gorai.012G042900.1.D;Gorai.012G042900.1.A
Gene IDs: Gorai.012G042900
cotton name: K02149 - V-type H+-transporting ATPase subunit D (ATPeV1D, ATP6M) (1 of 2)
OG IDs: 93519492
Best-hit-arabi-ID: AT3G58730.1
Best-hit-arabi-name: vacuolar ATP synthase subunit D (VATD) / V-ATPase D subunit / vacuolar proton pump D subunit (VATPD)
Gorai.012G042900.1.D
PCC=0.317, p=0.186
Gorai.012G042900.1.A
PCC=0.337, p=0.159
mRNA Profile
Protein Expression Profile
DPA

P200: Gorai.002G258500.1.D in case 2 and V14

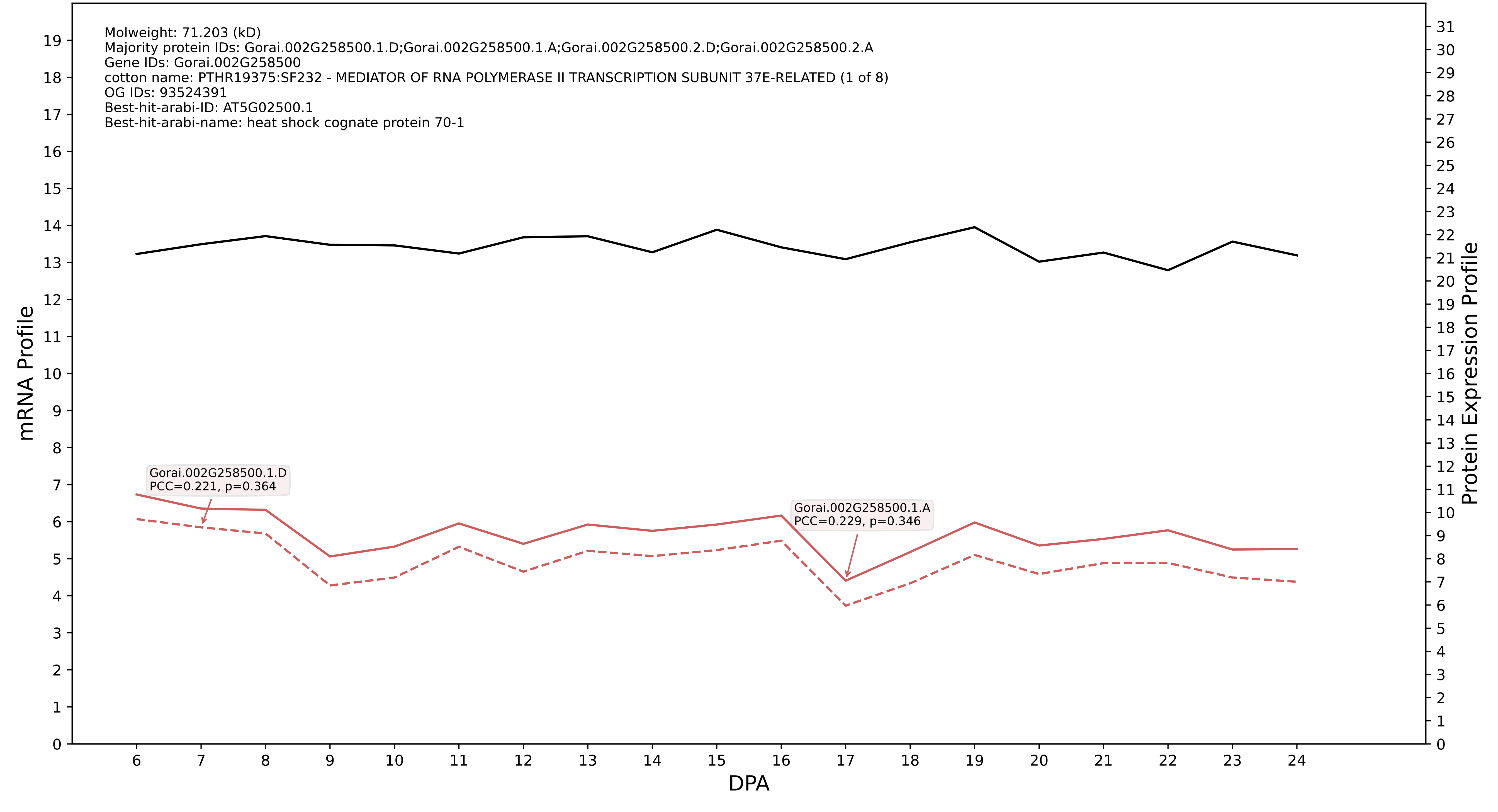

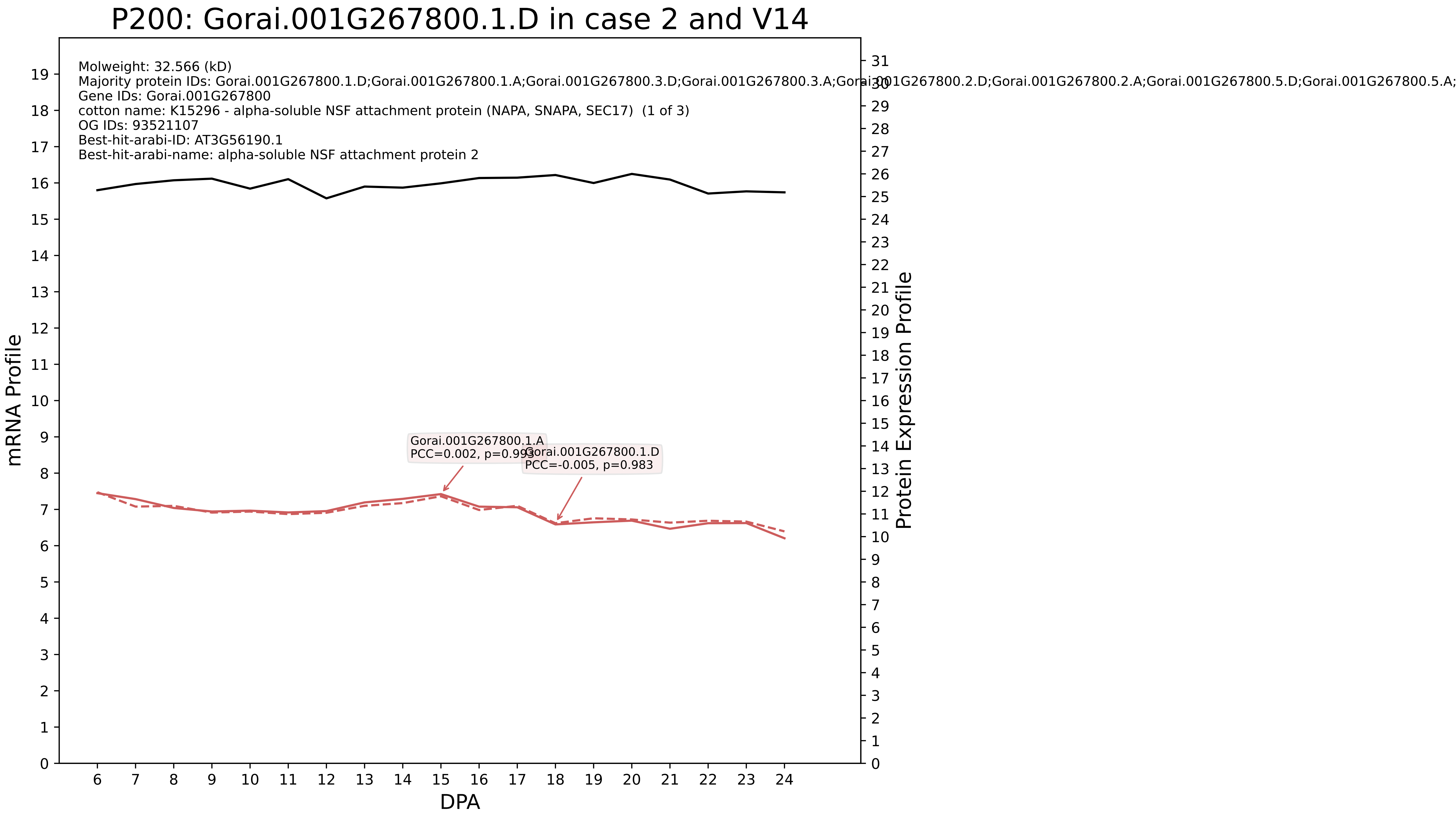
P200: Gorai.001G267800.1.D in case 2 and V14
Molweight: 32.566 (kD)
Majority protein IDs: Gorai.001G267800.1.D;Gorai.001G267800.1.A;Gorai.001G267800.3.D;Gorai.001G267800.3.A;Gorai.001G267800.2.D;Gorai.001G267800.2.A;Gorai.001G267800.5.D;Gorai.001G267800.5.A;
Gene IDs: Gorai.001G267800
cotton name: K15296 - alpha-soluble NSF attachment protein (NAPA, SNAPA, SEC17) (1 of 3)
OG IDs: 93521107
Best-hit-arabi-ID: AT3G56190.1
Best-hit-arabi-name: alpha-soluble NSF attachment protein 2
Gorai.001G267800.1.A
PCC=0.002, p=0.993
Gorai.001G267800.1.D
PCC=-0.005, p=0.983
mRNA Profile
Protein Expression Profile
DPA

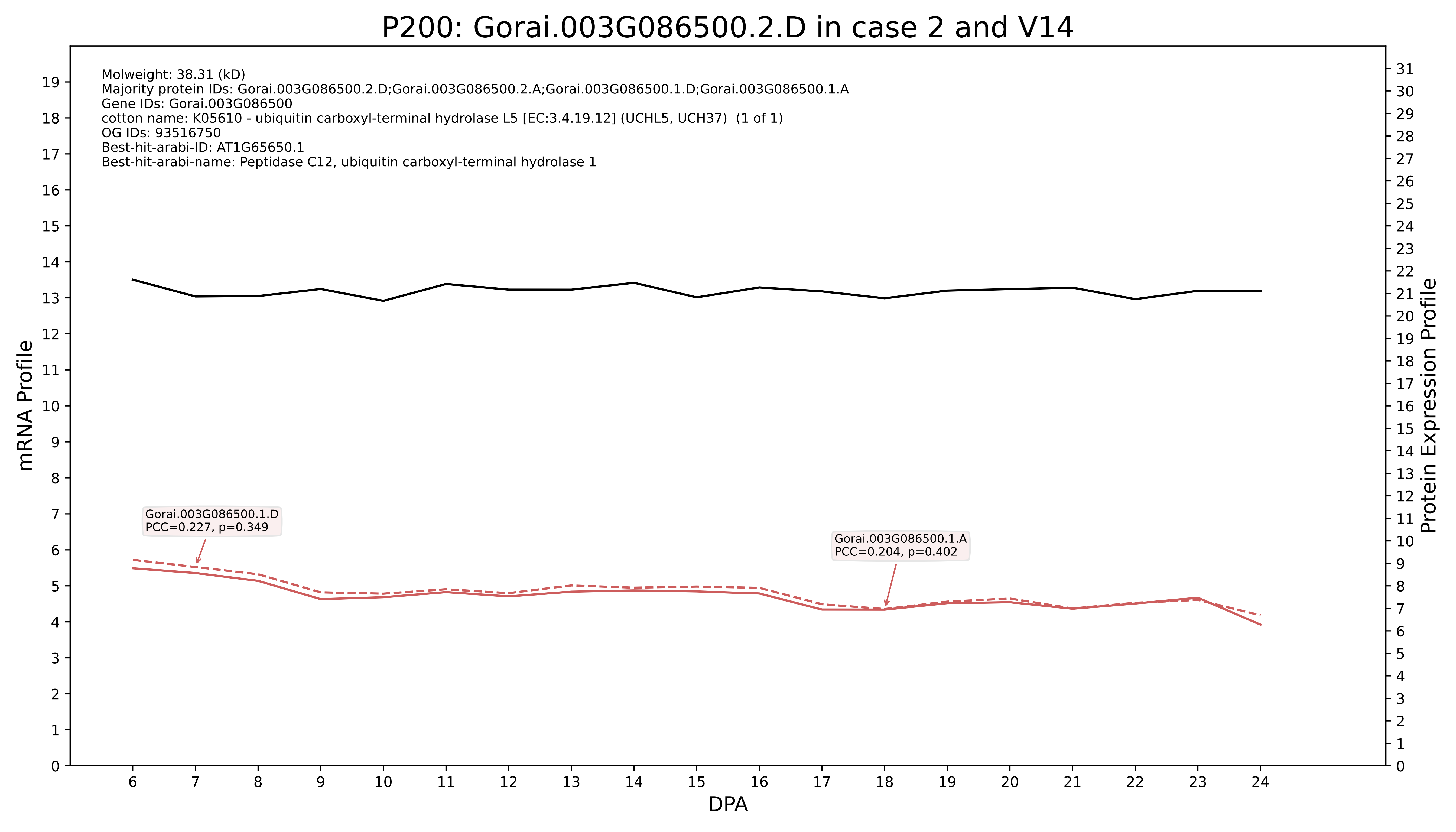
P200: Gorai.003G086500.2.D in case 2 and V14
Molweight: 38.31 (kD)
Majority protein IDs: Gorai.003G086500.2.D;Gorai.003G086500.2.A;Gorai.003G086500.1.D;Gorai.003G086500.1.A
Gene IDs: Gorai.003G086500
cotton name: K05610 - ubiquitin carboxyl-terminal hydrolase L5 [EC:3.4.19.12] (UCHL5, UCH37) (1 of 1)
OG IDs: 93516750
Best-hit-arabi-ID: AT1G65650.1
Best-hit-arabi-name: Peptidase C12, ubiquitin carboxyl-terminal hydrolase 1
Gorai.003G086500.1.D
PCC=0.227, p=0.349
Gorai.003G086500.1.A
PCC=0.204, p=0.402
mRNA Profile
Protein Expression Profile
DPA

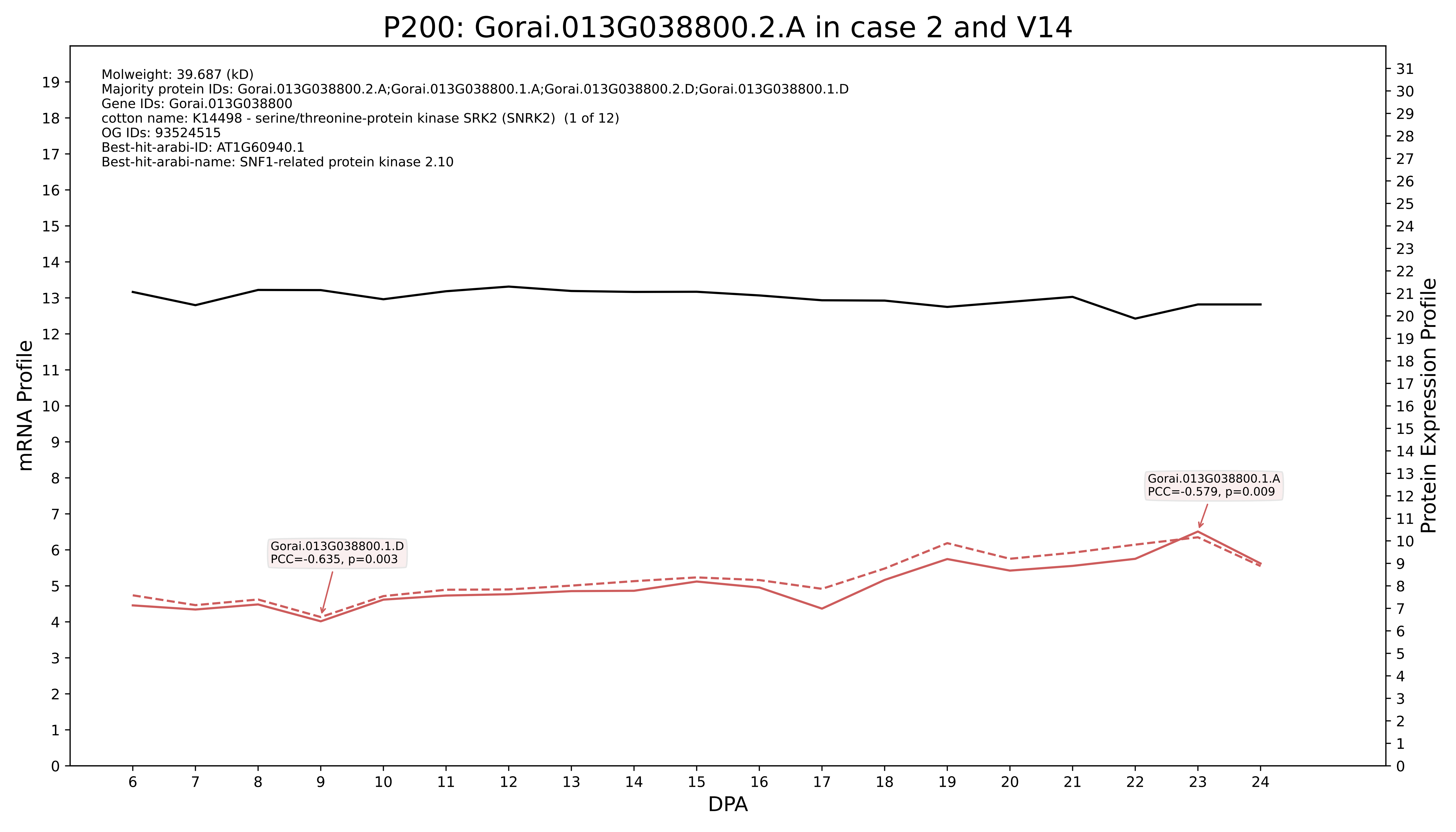
P200: Gorai.013G038800.2.A in case 2 and V14
Molweight: 39.687 (kD)
Majority protein IDs: Gorai.013G038800.2.A;Gorai.013G038800.1.A;Gorai.013G038800.2.D;Gorai.013G038800.1.D
Gene IDs: Gorai.013G038800
cotton name: K14498 - serine/threonine-protein kinase SRK2 (SNRK2) (1 of 12)
OG IDs: 93524515
Best-hit-arabi-ID: AT1G60940.1
Best-hit-arabi-name: SNF1-related protein kinase 2.10
Gorai.013G038800.1.D
PCC=-0.635, p=0.003
Gorai.013G038800.1.A
PCC=-0.579, p=0.009
mRNA Profile
Protein Expression Profile
DPA

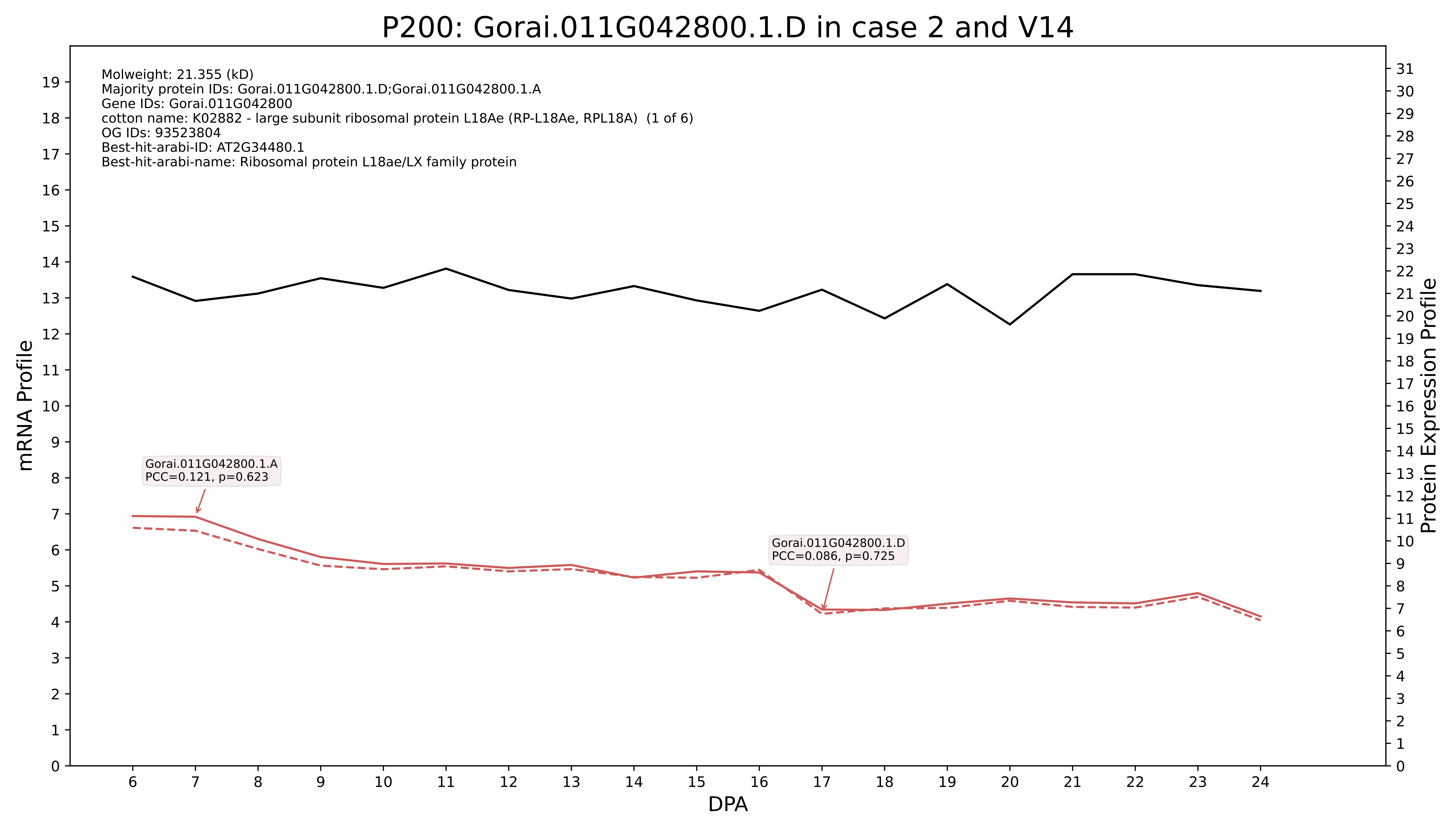
P200: Gorai.011G042800.1.D in case 2 and V14
Molweight: 21.355 (kD)
Majority protein IDs: Gorai.011G042800.1.D;Gorai.011G042800.1.A
Gene IDs: Gorai.011G042800
cotton name: K02882 - large subunit ribosomal protein L18Ae (RP-L18Ae, RPL18A) (1 of 6)
OG IDs: 93523804
Best-hit-arabi-ID: AT2G34480.1
Best-hit-arabi-name: Ribosomal protein L18ae/LX family protein
Gorai.011G042800.1.A
PCC=0.121, p=0.623
Gorai.011G042800.1.D
PCC=0.086, p=0.725
mRNA Profile
Protein Expression Profile
DPA

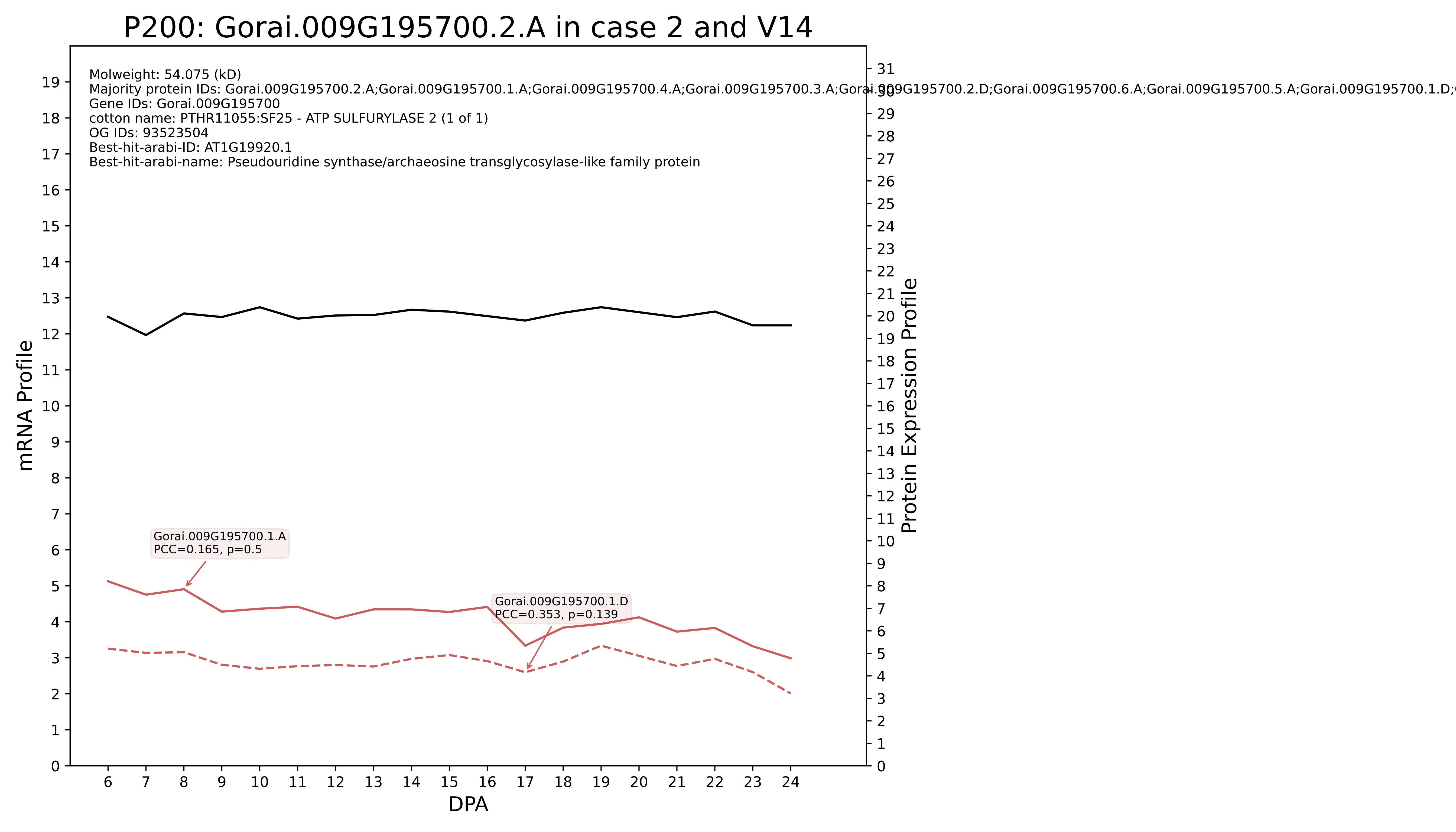
P200: Gorai.009G195700.2.A in case 2 and V14
Molweight: 54.075 (kD)
Majority protein IDs: Gorai.009G195700.2.A;Gorai.009G195700.1.A;Gorai.009G195700.4.A;Gorai.009G195700.3.A;Gorai.009G195700.2.D;Gorai.009G195700.6.A;Gorai.009G195700.5.A;Gorai.009G195700.1.D;
Gene IDs: Gorai.009G195700
cotton name: PTHR11055:SF25 - ATP SULFURYLASE 2 (1 of 1)
OG IDs: 93523504
Best-hit-arabi-ID: AT1G19920.1
Best-hit-arabi-name: Pseudouridine synthase/archaeosine transglycosylase-like family protein
Gorai.009G195700.1.A
PCC=0.165, p=0.5
Gorai.009G195700.1.D
PCC=0.353, p=0.139
mRNA Profile
Protein Expression Profile
DPA

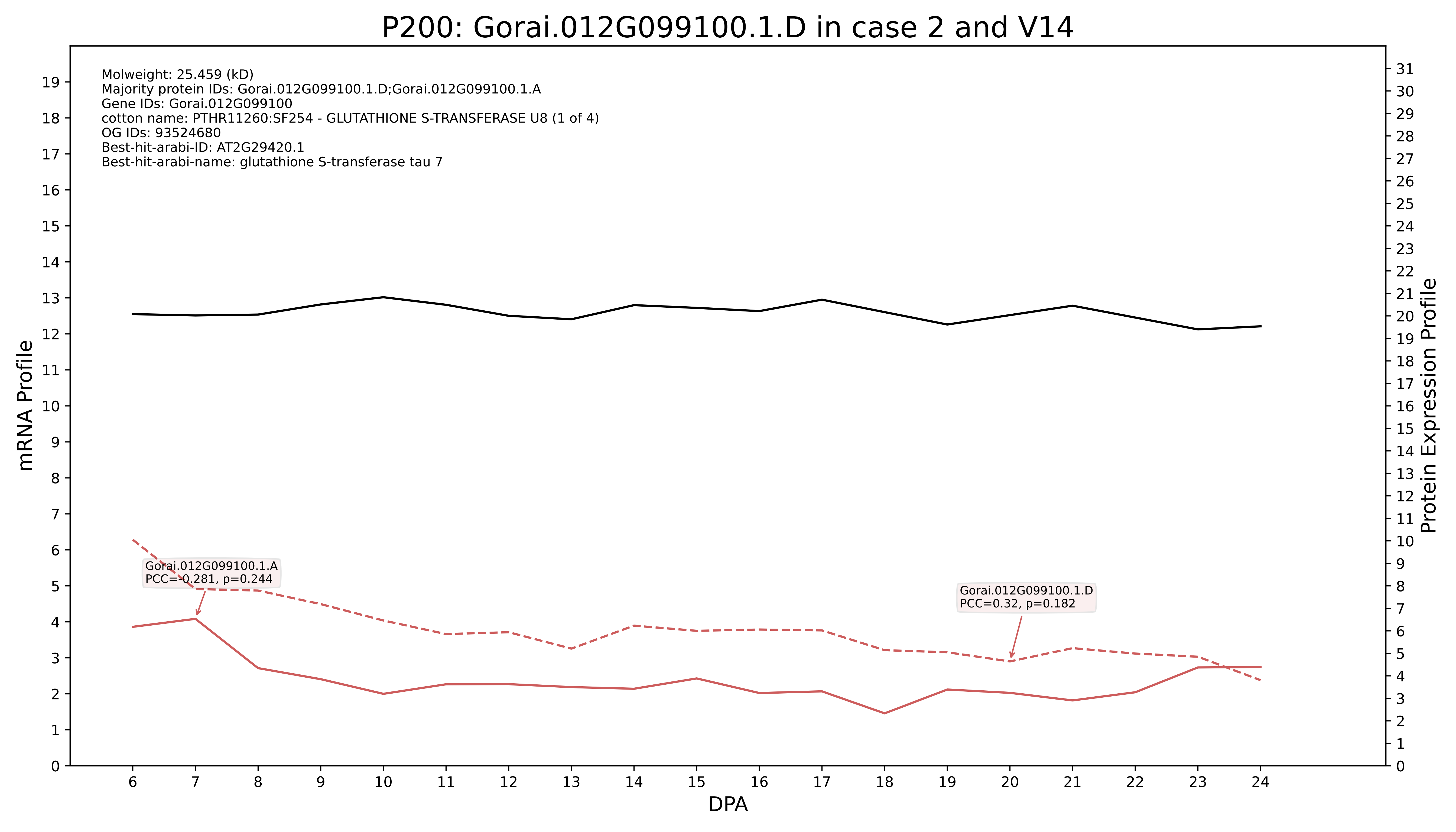
P200: Gorai.012G099100.1.D in case 2 and V14
Molweight: 25.459 (kD)
Majority protein IDs: Gorai.012G099100.1.D;Gorai.012G099100.1.A
Gene IDs: Gorai.012G099100
cotton name: PTHR11260:SF254 - GLUTATHIONE S-TRANSFERASE U8 (1 of 4)
OG IDs: 93524680
Best-hit-arabi-ID: AT2G29420.1
Best-hit-arabi-name: glutathione S-transferase tau 7
Gorai.012G099100.1.A
PCC=-0.281, p=0.244
Gorai.012G099100.1.D
PCC=0.32, p=0.182
mRNA Profile
Protein Expression Profile
DPA
0
1
2
3
4
5
6
7
8
9
10
11
12
13
14
15
16
17
18
19
20
21
22
23
24
25
26
27
28
29
30
31

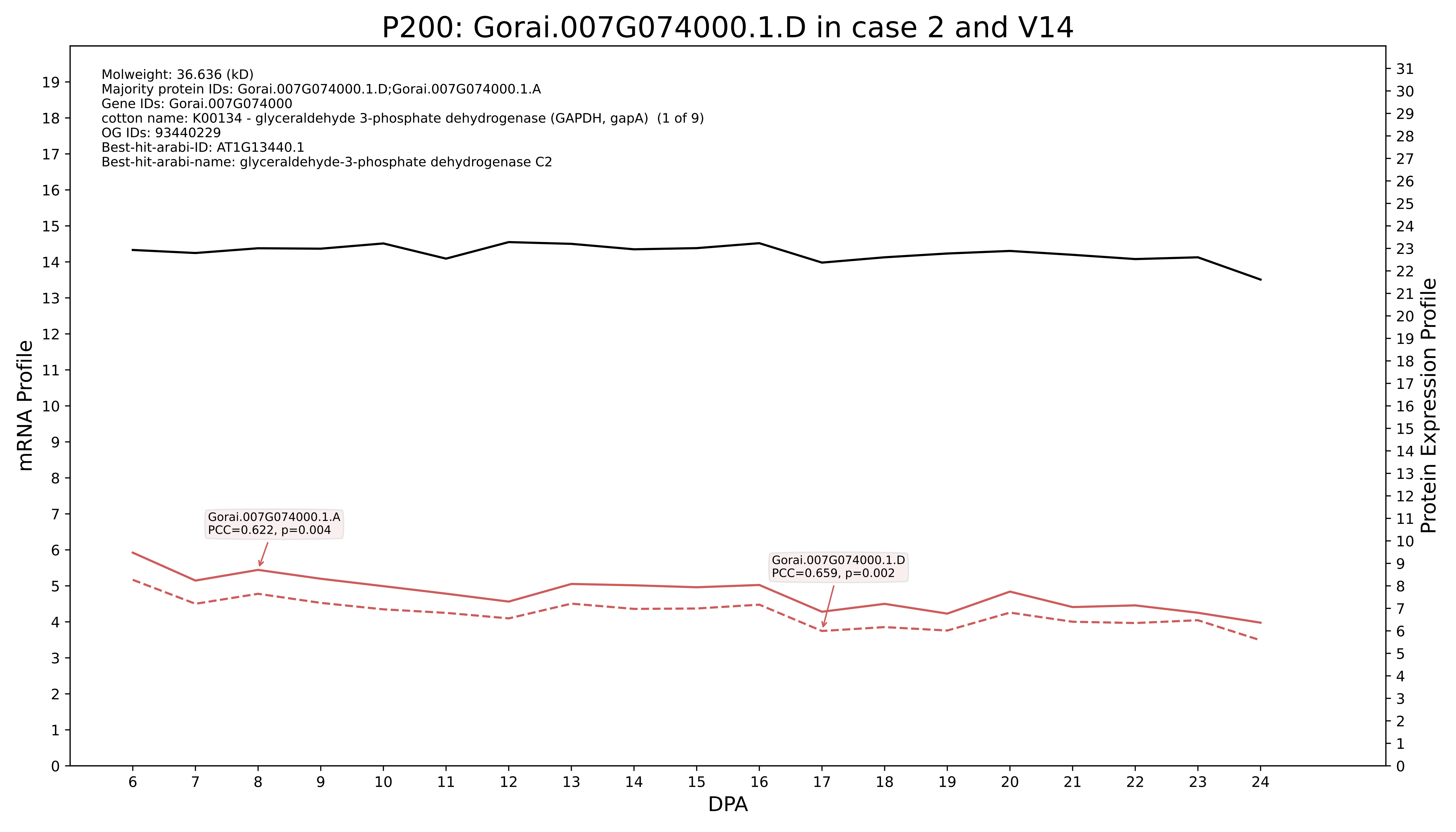
P200: Gorai.007G074000.1.D in case 2 and V14
Molweight: 36.636 (kD)
Majority protein IDs: Gorai.007G074000.1.D;Gorai.007G074000.1.A
Gene IDs: Gorai.007G074000
cotton name: K00134 - glyceraldehyde 3-phosphate dehydrogenase (GAPDH, gapA) (1 of 9)
OG IDs: 93440229
Best-hit-arabi-ID: AT1G13440.1
Best-hit-arabi-name: glyceraldehyde-3-phosphate dehydrogenase C2
Gorai.007G074000.1.A
PCC=0.622, p=0.004
Gorai.007G074000.1.D
PCC=0.659, p=0.002
mRNA Profile
Protein Expression Profile
DPA
0
1
2
3
4
5
6
7
8
9
10
11
12
13
14
15
16
17
18
19
20
21
22
23
24
25
26
27
28
29
30
31

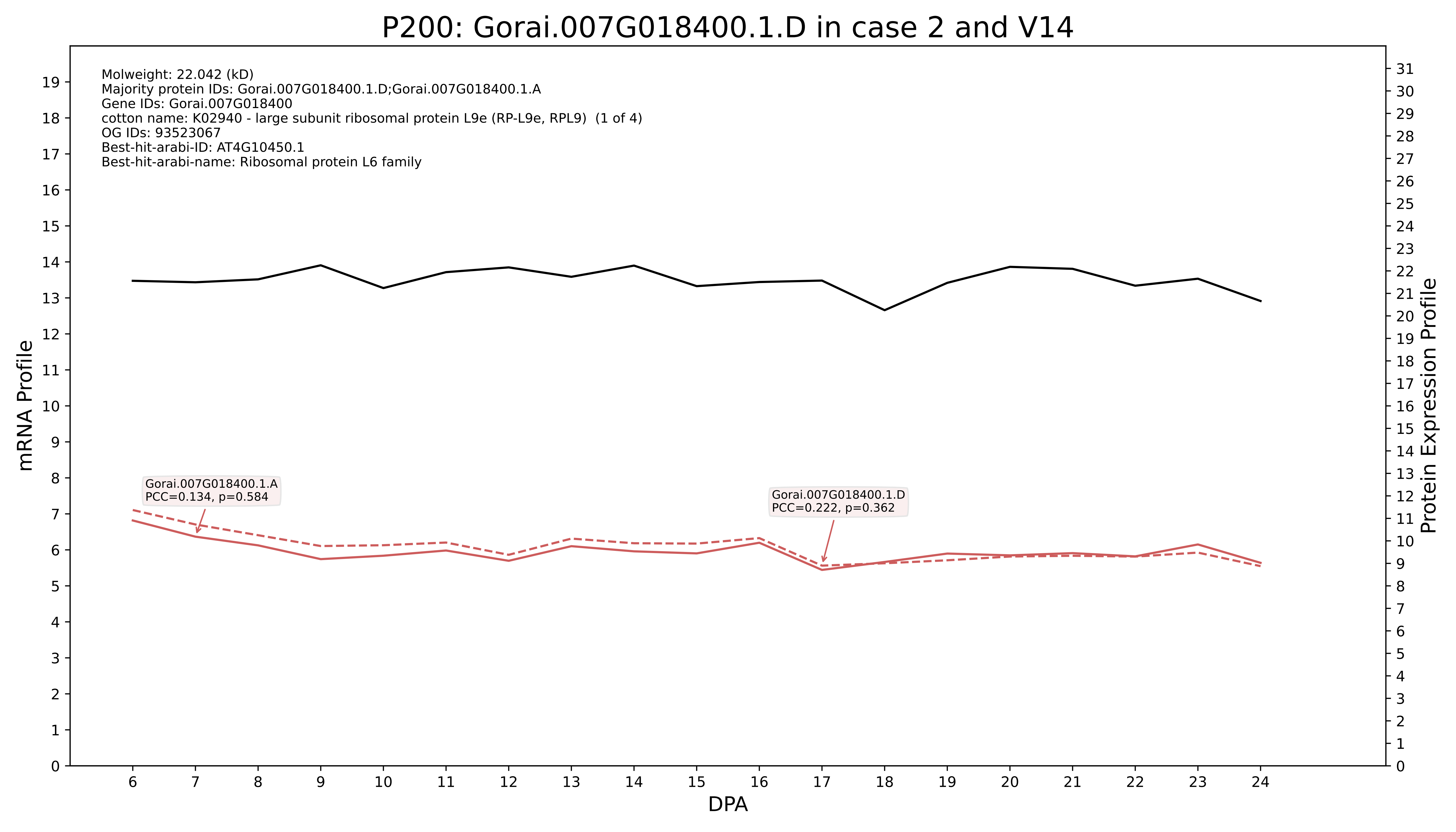
P200: Gorai.007G018400.1.D in case 2 and V14
Molweight: 22.042 (kD)
Majority protein IDs: Gorai.007G018400.1.D;Gorai.007G018400.1.A
Gene IDs: Gorai.007G018400
cotton name: K02940 - large subunit ribosomal protein L9e (RP-L9e, RPL9) (1 of 4)
OG IDs: 93523067
Best-hit-arabi-ID: AT4G10450.1
Best-hit-arabi-name: Ribosomal protein L6 family
Gorai.007G018400.1.A
PCC=0.134, p=0.584
Gorai.007G018400.1.D
PCC=0.222, p=0.362
mRNA Profile
Protein Expression Profile
DPA

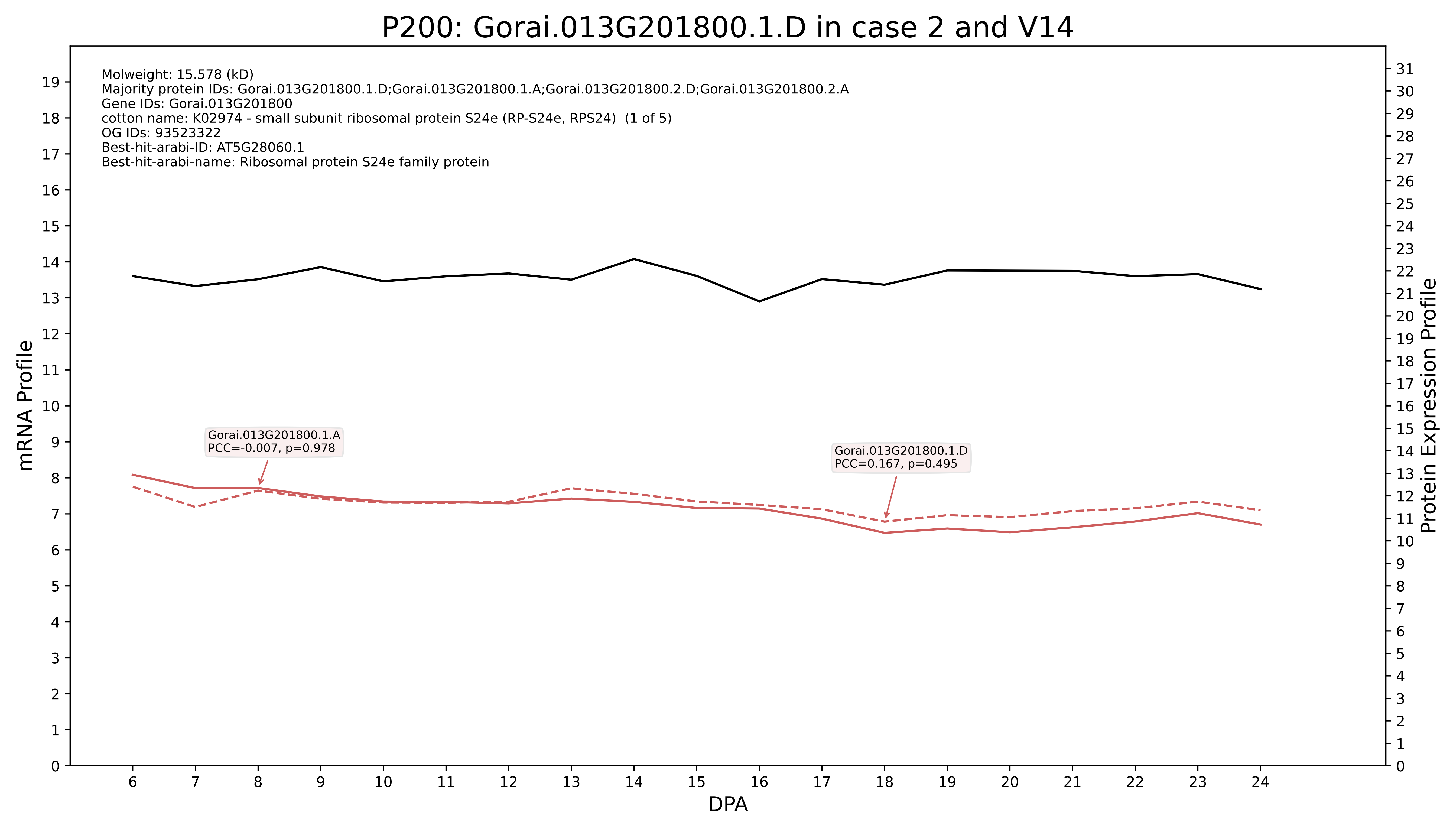
P200: Gorai.013G201800.1.D in case 2 and V14
Molweight: 15.578 (kD)
Majority protein IDs: Gorai.013G201800.1.D;Gorai.013G201800.1.A;Gorai.013G201800.2.D;Gorai.013G201800.2.A
Gene IDs: Gorai.013G201800
cotton name: K02974 - small subunit ribosomal protein S24e (RP-S24e, RPS24) (1 of 5)
OG IDs: 93523322
Best-hit-arabi-ID: AT5G28060.1
Best-hit-arabi-name: Ribosomal protein S24e family protein
Gorai.013G201800.1.A
PCC=-0.007, p=0.978
Gorai.013G201800.1.D
PCC=0.167, p=0.495
mRNA Profile
Protein Expression Profile
DPA

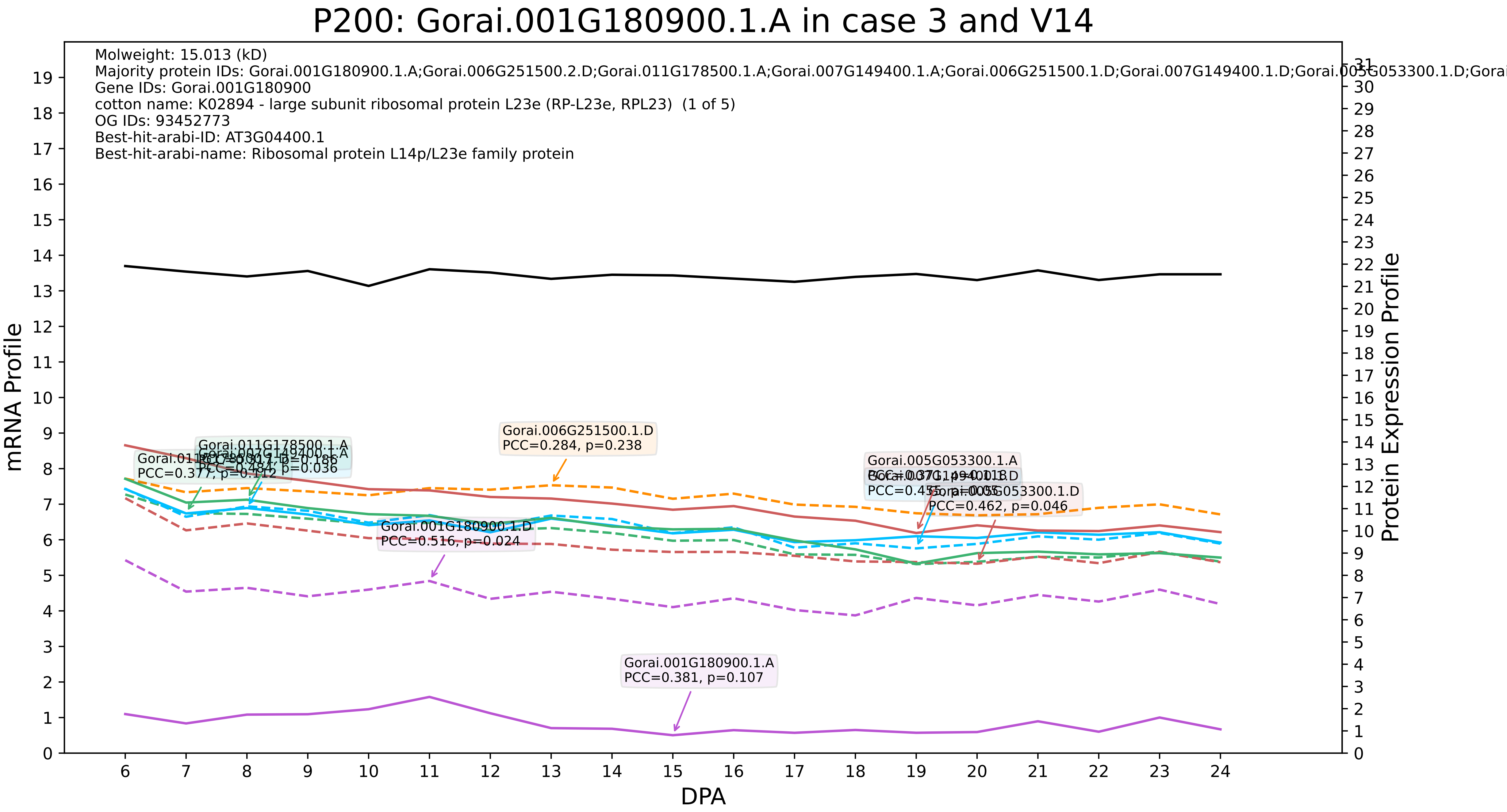
P200: Gorai.001G180900.1.A in case 3 and V14
Molweight: 15.013 (kD)
Majority protein IDs: Gorai.001G180900.1.A;Gorai.006G251500.2.D;Gorai.011G178500.1.A;Gorai.007G149400.1.A;Gorai.006G251500.1.D;Gorai.007G149400.1.D;Gorai.005G053300.1.D;Gorai
Gene IDs: Gorai.001G180900
cotton name: K02894 - large subunit ribosomal protein L23e (RP-L23e, RPL23) (1 of 5)
OG IDs: 93452773
Best-hit-arabi-ID: AT3G04400.1
Best-hit-arabi-name: Ribosomal protein L14p/L23e family protein
mRNA Profile
Protein Expression Profile
DPA
Gorai.006G251500.1.D
PCC=0.284, p=0.238
Gorai.011G178500.1.A
PCC=0.377, p=0.112
Gorai.005G053300.1.A
Gorai.005G053300.1.D
PCC=0.462, p=0.046
Gorai.001G180900.1.D
PCC=0.516, p=0.024
Gorai.001G180900.1.A
PCC=0.381, p=0.107

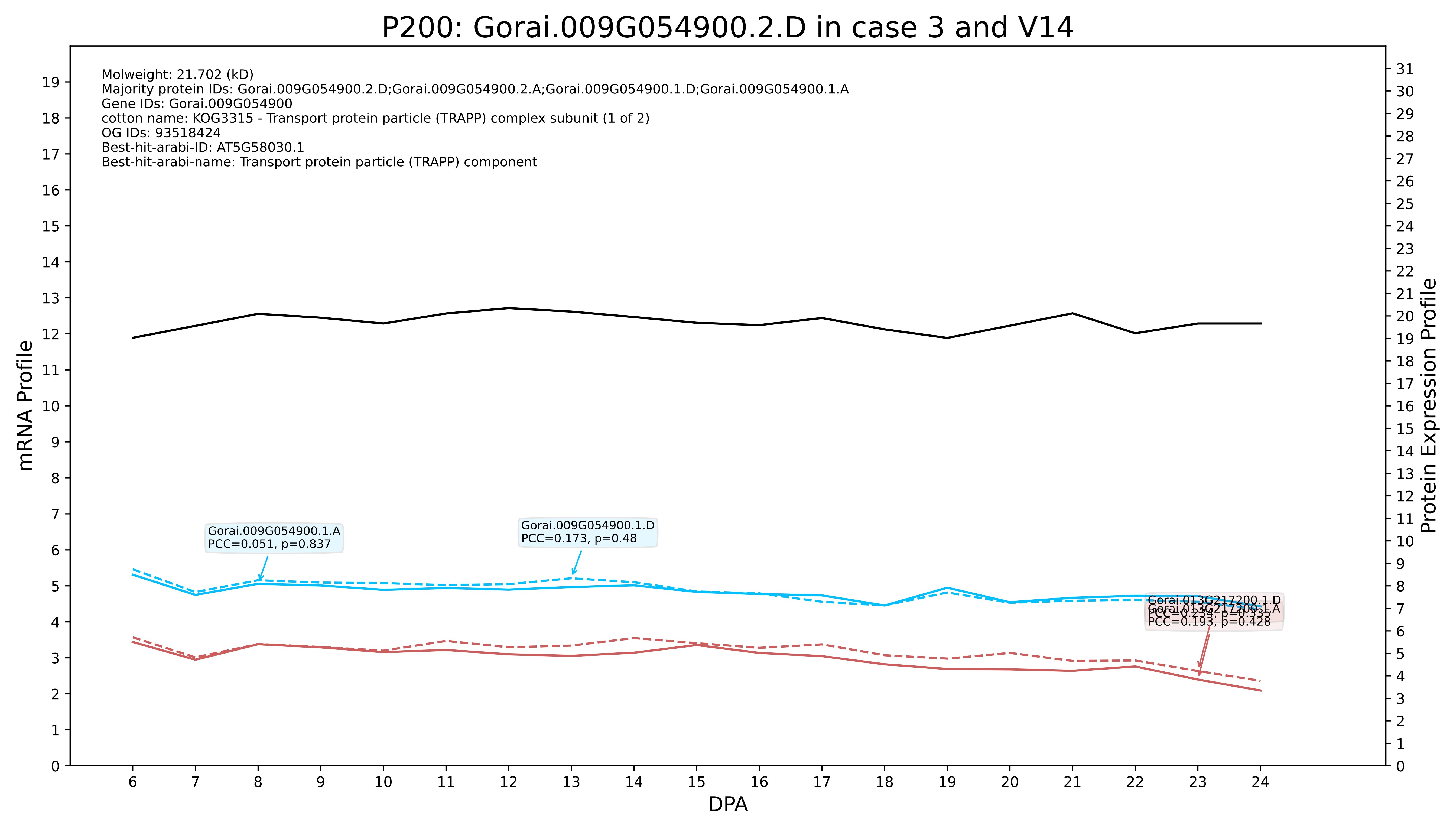
P200: Gorai.009G054900.2.D in case 3 and V14
Molweight: 21.702 (kD)
Majority protein IDs: Gorai.009G054900.2.D;Gorai.009G054900.2.A;Gorai.009G054900.1.D;Gorai.009G054900.1.A
Gene IDs: Gorai.009G054900
cotton name: KOG3315 - Transport protein particle (TRAPP) complex subunit (1 of 2)
OG IDs: 93518424
Best-hit-arabi-ID: AT5G58030.1
Best-hit-arabi-name: Transport protein particle (TRAPP) component
Gorai.009G054900.1.A
PCC=0.051, p=0.837
Gorai.009G054900.1.D
PCC=0.173, p=0.48
Gorai.013G217200.1.D
PCC=0.234, p=0.335
Gorai.013G217200.1.A
PCC=0.193, p=0.428
mRNA Profile
Protein Expression Profile
DPA
0
1
2
3
4
5
6
7
8
9
10
11
12
13
14
15
16
17
18
19
20
21
22
23
24
25
26
27
28
29
30
31

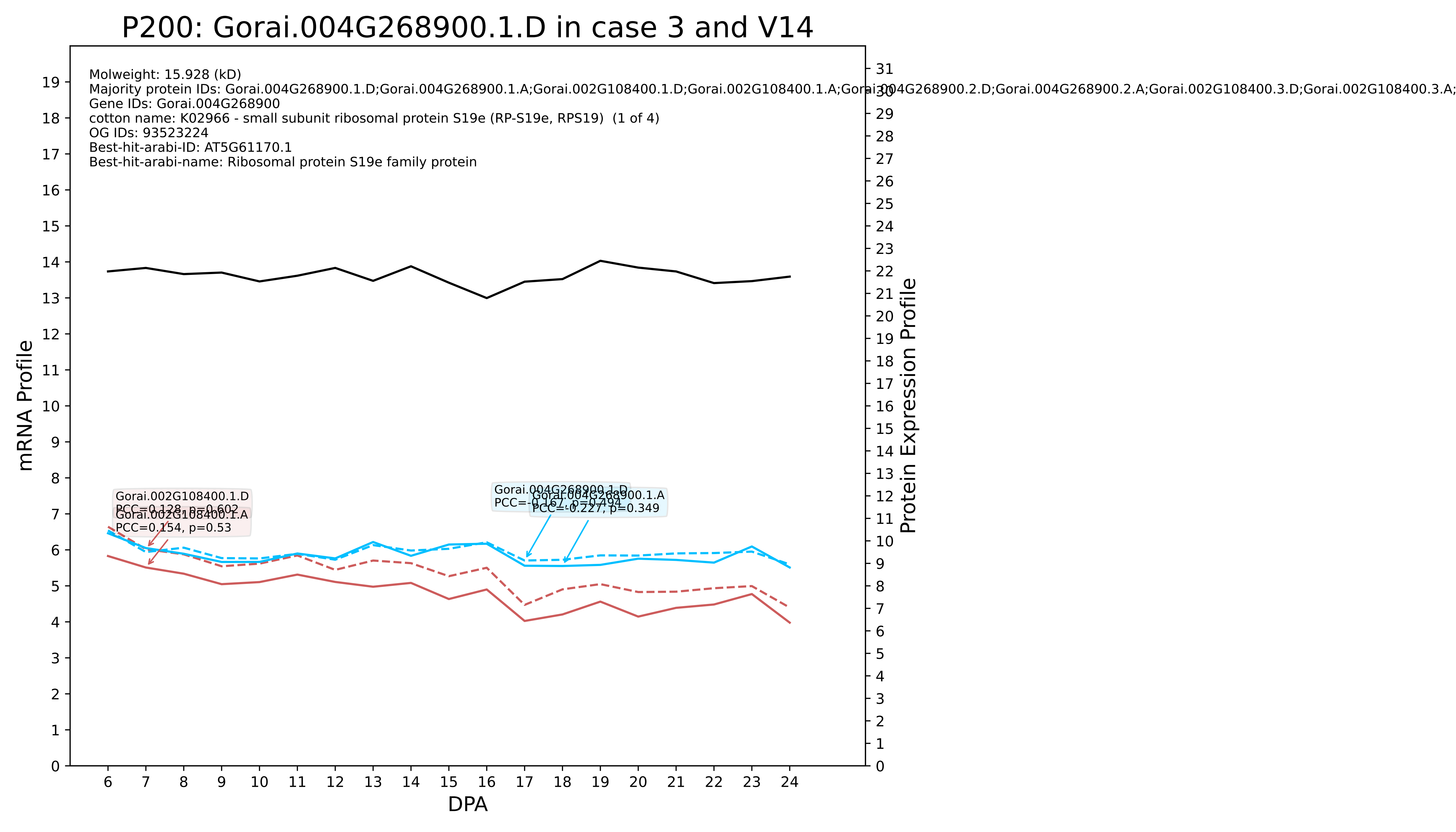
P200: Gorai.004G268900.1.D in case 3 and V14
Molweight: 15.928 (kD)
Majority protein IDs: Gorai.004G268900.1.D;Gorai.004G268900.1.A;Gorai.002G108400.1.D;Gorai.002G108400.1.A;Gorai.004G268900.2.D;Gorai.004G268900.2.A;Gorai.002G108400.3.D;Gorai.002G108400.3.A;
Gene IDs: Gorai.004G268900
cotton name: K02966 - small subunit ribosomal protein S19e (RP-S19e, RPS19) (1 of 4)
OG IDs: 93523224
Best-hit-arabi-ID: AT5G61170.1
Best-hit-arabi-name: Ribosomal protein S19e family protein
Gorai.002G108400.1.D
PCC=0.128, p=0.602
Gorai.002G108400.1.A
PCC=0.154, p=0.53
Gorai.004G268900.1.D
PCC=-0.167, p=0.494
Gorai.004G268900.1.A
PCC=-0.227, p=0.349
mRNA Profile
Protein Expression Profile
DPA

P200: Gorai.009G230200.2.D in case 3 and V14

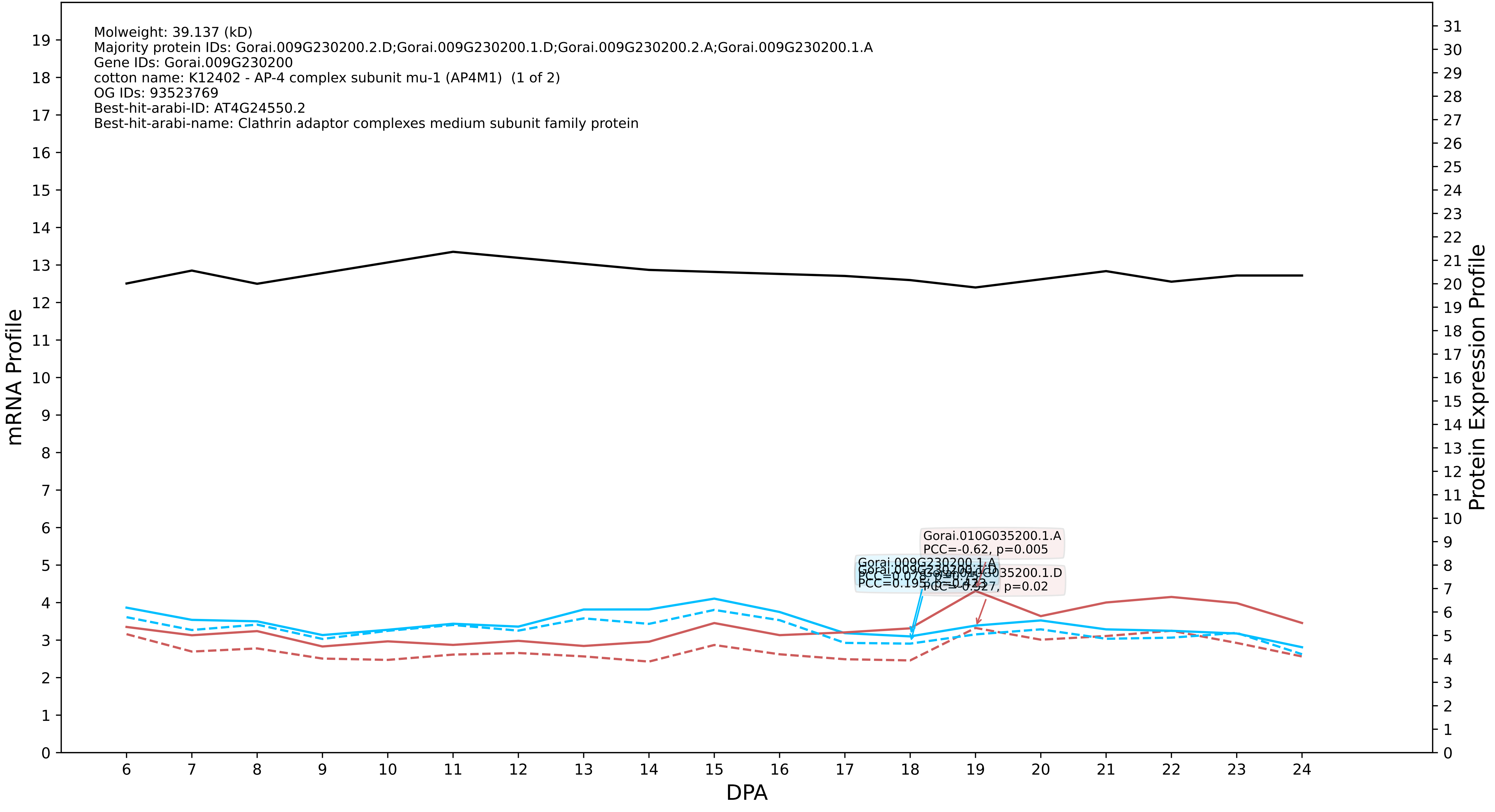

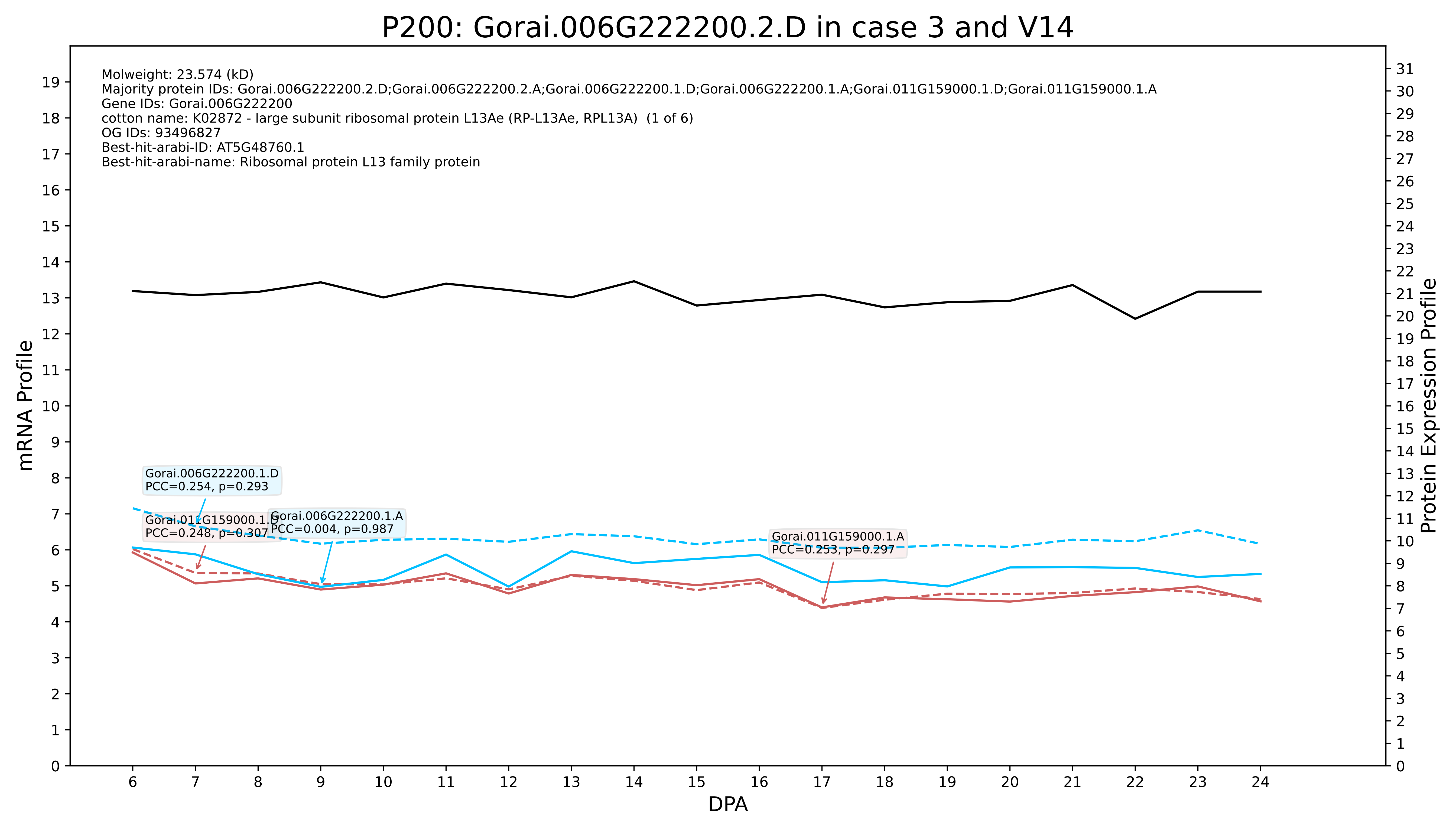
P200: Gorai.006G222200.2.D in case 3 and V14
Molweight: 23.574 (kD)
Majority protein IDs: Gorai.006G222200.2.D;Gorai.006G222200.2.A;Gorai.006G222200.1.D;Gorai.006G222200.1.A;Gorai.011G159000.1.D;Gorai.011G159000.1.A
Gene IDs: Gorai.006G222200
cotton name: K02872 - large subunit ribosomal protein L13Ae (RP-L13Ae, RPL13A) (1 of 6)
OG IDs: 93496827
Best-hit-arabi-ID: AT5G48760.1
Best-hit-arabi-name: Ribosomal protein L13 family protein
Gorai.006G222200.1.D
PCC=0.254, p=0.293
Gorai.011G159000.1.D
PCC=0.248, p=0.307
Gorai.006G222200.1.A
PCC=0.004, p=0.987
Gorai.011G159000.1.A
PCC=0.253, p=0.297
mRNA Profile
Protein Expression Profile
DPA

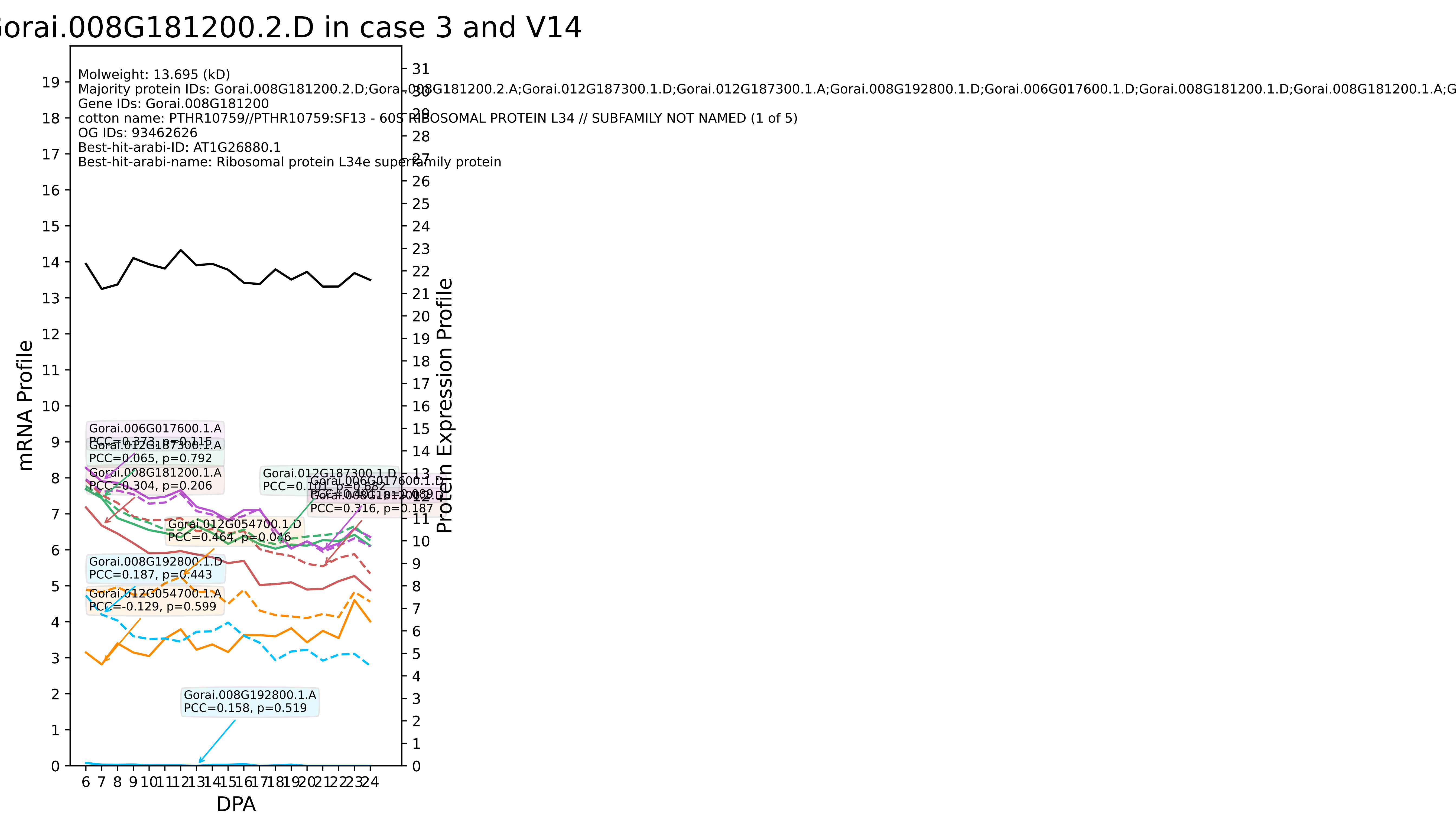
Gorai.008G181200.2.D in case 3 and V14
Molweight: 13.695 (kD)
Majority protein IDs: Gorai.008G181200.2.D;Gorai.008G181200.2.A;Gorai.012G187300.1.D;Gorai.012G187300.1.A;Gorai.008G192800.1.D;Gorai.006G017600.1.D;Gorai.008G181200.1.D;Gorai.008G181200.1.A;G
Gene IDs: Gorai.008G181200
cotton name: PTHR10759//PTHR10759:SF13 - 60S RIBOSOMAL PROTEIN L34 // SUBFAMILY NOT NAMED (1 of 5)
OG IDs: 93462626
Best-hit-arabi-ID: AT1G26880.1
Best-hit-arabi-name: Ribosomal protein L34e superfamily protein
Gorai.006G017600.1.A
Gorai.012G187300.1.A
PCC=0.065, p=0.792
Gorai.008G181200.1.A
PCC=0.304, p=0.206
Gorai.012G187300.1.D
PCC=0.101, p=0.682
PCC=0.316, p=0.187
Gorai.012G054700.1.D
PCC=0.464, p=0.046
Gorai.008G192800.1.D
PCC=0.187, p=0.443
Gorai.012G054700.1.A
PCC=-0.129, p=0.599
Gorai.008G192800.1.A
PCC=0.158, p=0.519
mRNA Profile
Protein Expression Profile
DPA

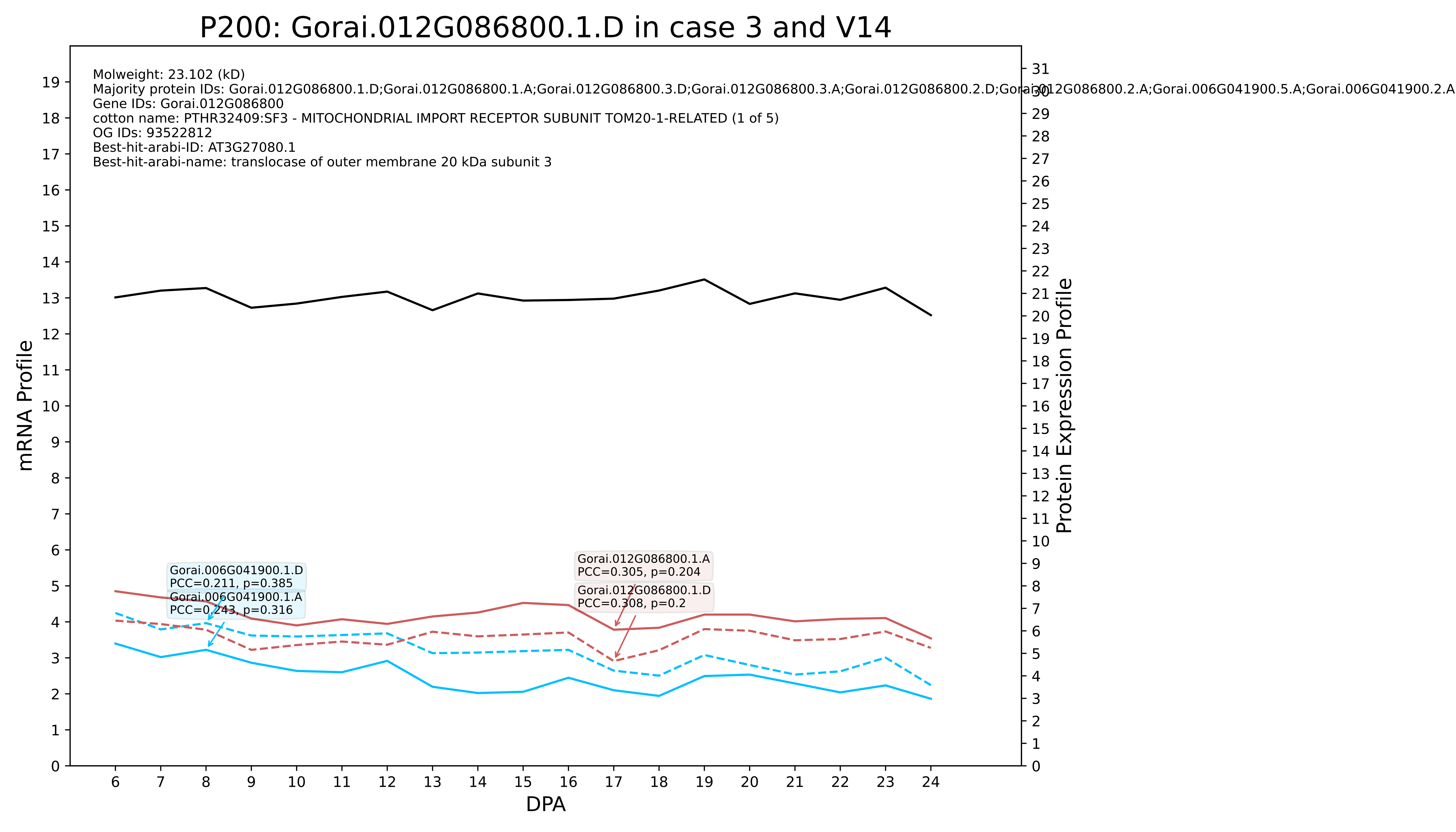
P200: Gorai.012G086800.1.D in case 3 and V14
Molweight: 23.102 (kD)
Majority protein IDs: Gorai.012G086800.1.D;Gorai.012G086800.1.A;Gorai.012G086800.3.D;Gorai.012G086800.3.A;Gorai.012G086800.2.D;Gorai.012G086800.2.A;Gorai.006G041900.5.A;Gorai.006G041900.2.A
Gene IDs: Gorai.012G086800
cotton name: PTHR32409:SF3 - MITOCHONDRIAL IMPORT RECEPTOR SUBUNIT TOM20-1-RELATED (1 of 5)
OG IDs: 93522812
Best-hit-arabi-ID: AT3G27080.1
Best-hit-arabi-name: translocase of outer membrane 20 kDa subunit 3
Gorai.006G041900.1.D
PCC=0.211, p=0.385
Gorai.006G041900.1.A
PCC=0.243, p=0.316
Gorai.012G086800.1.A
PCC=0.305, p=0.204
Gorai.012G086800.1.D
PCC=0.308, p=0.2
mRNA Profile
Protein Expression Profile
DPA

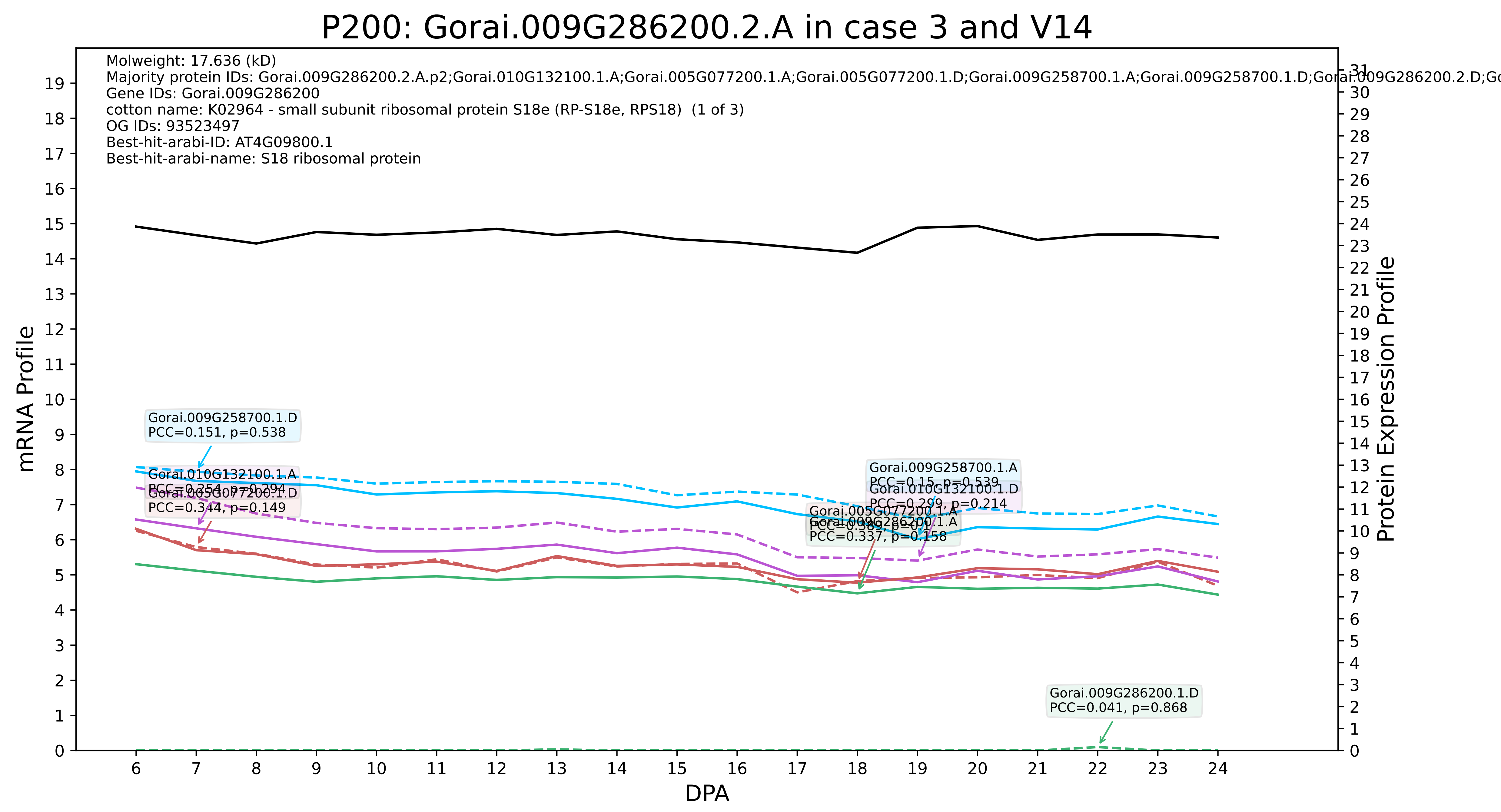
P200: Gorai.009G286200.2.A in case 3 and V14
Molweight: 17.636 (kD)
Majority protein IDs: Gorai.009G286200.2.A.p2;Gorai.010G132100.1.A;Gorai.005G077200.1.A;Gorai.005G077200.1.D;Gorai.009G258700.1.A;Gorai.009G258700.1.D;Gorai.009G286200.2.D;Go
Gene IDs: Gorai.009G286200
cotton name: K02964 - small subunit ribosomal protein S18e (RP-S18e, RPS18) (1 of 3)
OG IDs: 93523497
Best-hit-arabi-ID: AT4G09800.1
Best-hit-arabi-name: S18 ribosomal protein
Gorai.009G258700.1.D
PCC=0.151, p=0.538
Gorai.010G132100.1.A
PCC=0.254, p=0.294
Gorai.005G077200.1.D
PCC=0.344, p=0.149
Gorai.009G258700.1.A
PCC=0.15, p=0.539
Gorai.010G132100.1.D
PCC=0.299, p=0.214
Gorai.005G077200.1.A
Gorai.009G286200.1.A
PCC=0.337, p=0.158
Gorai.009G286200.1.D
PCC=0.041, p=0.868
mRNA Profile
Protein Expression Profile
DPA

P200: Gorai.007G188300.8.D in case 3 and V14

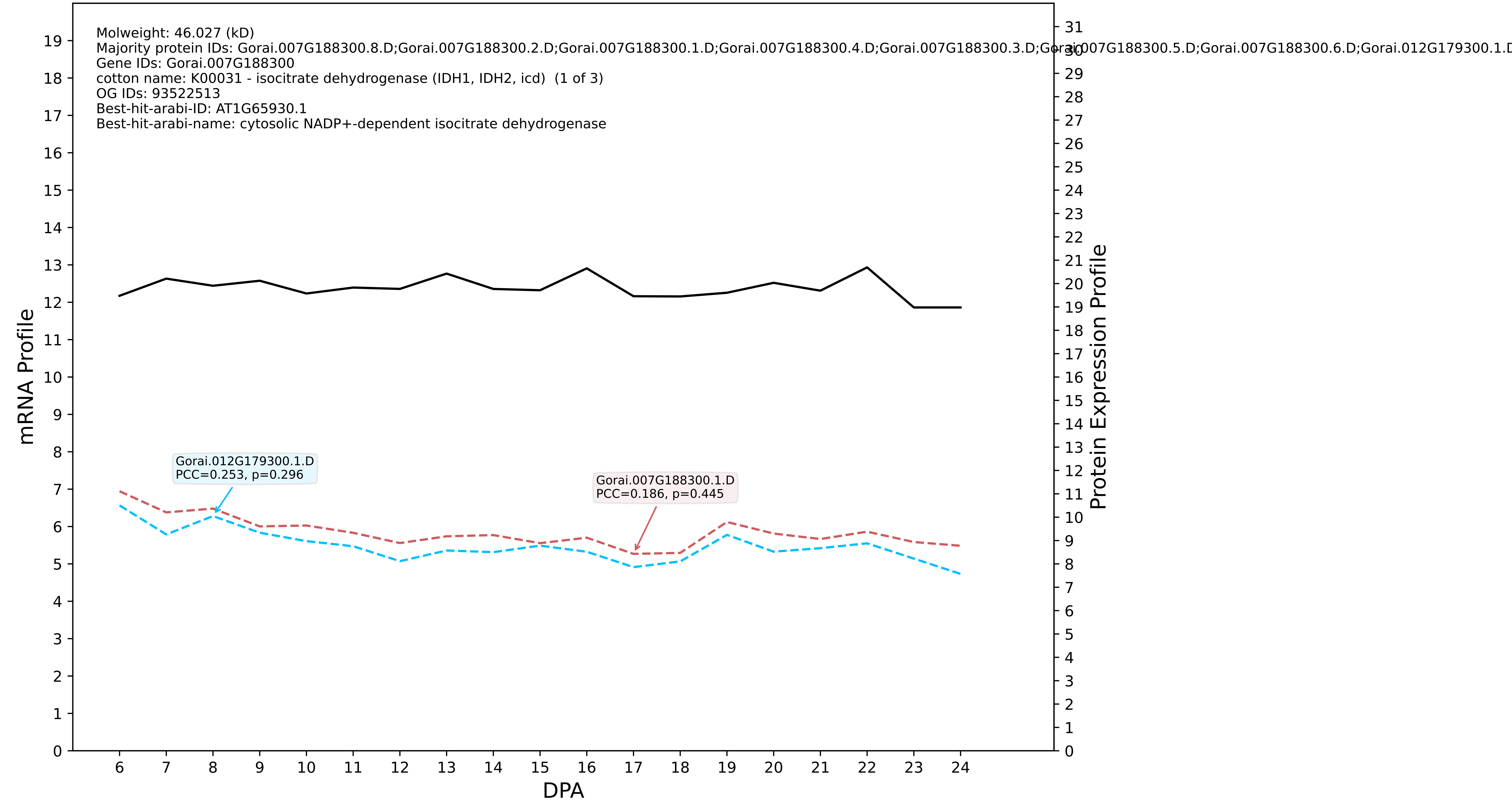

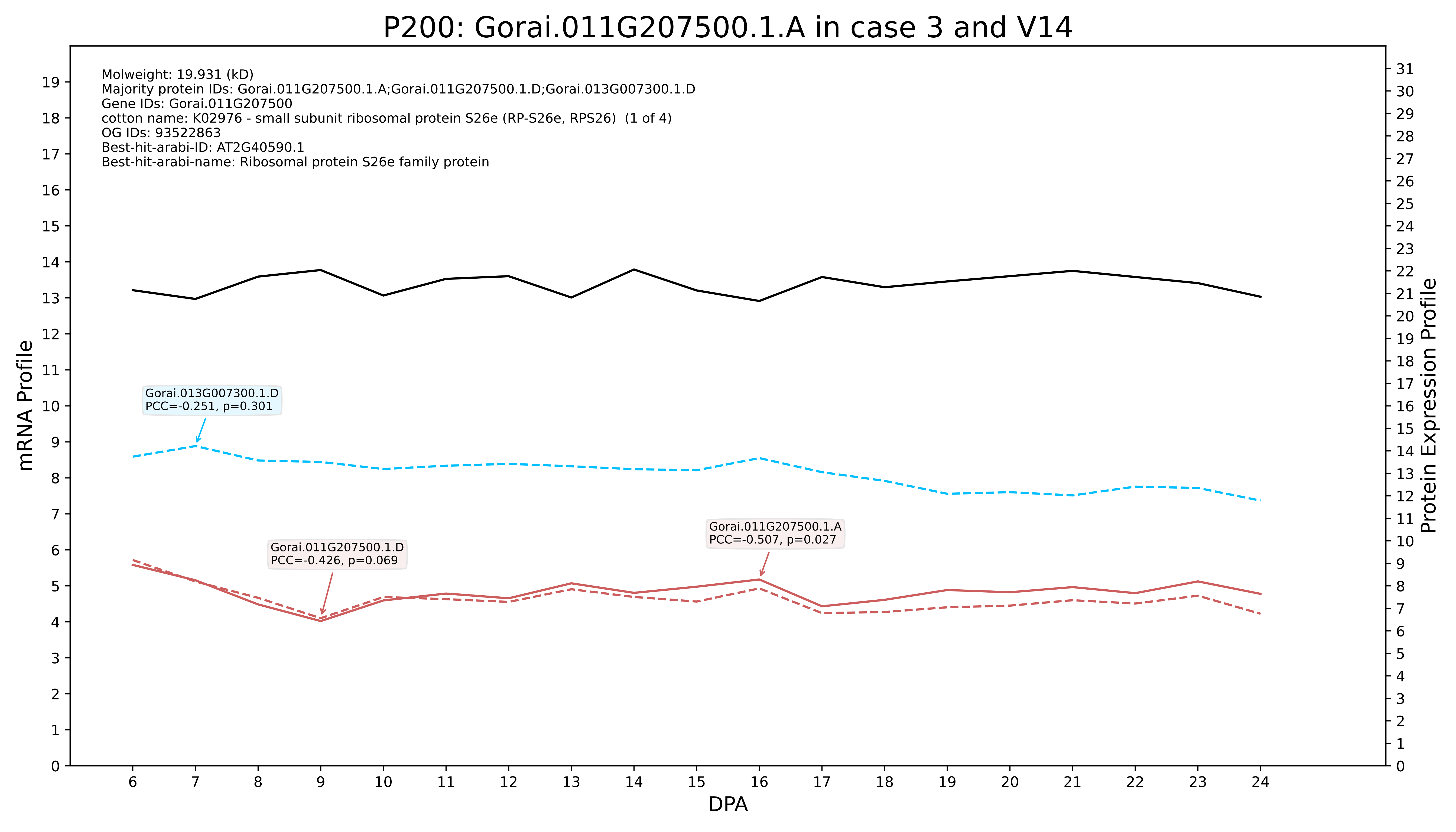
P200: Gorai.011G207500.1.A in case 3 and V14
Molweight: 19.931 (kD)
Majority protein IDs: Gorai.011G207500.1.A;Gorai.011G207500.1.D;Gorai.013G007300.1.D
Gene IDs: Gorai.011G207500
cotton name: K02976 - small subunit ribosomal protein S26e (RP-S26e, RPS26) (1 of 4)
OG IDs: 93522863
Best-hit-arabi-ID: AT2G40590.1
Best-hit-arabi-name: Ribosomal protein S26e family protein
Gorai.013G007300.1.D
PCC=-0.251, p=0.301
Gorai.011G207500.1.D
PCC=-0.426, p=0.069
Gorai.011G207500.1.A
PCC=-0.507, p=0.027
mRNA Profile
Protein Expression Profile
DPA

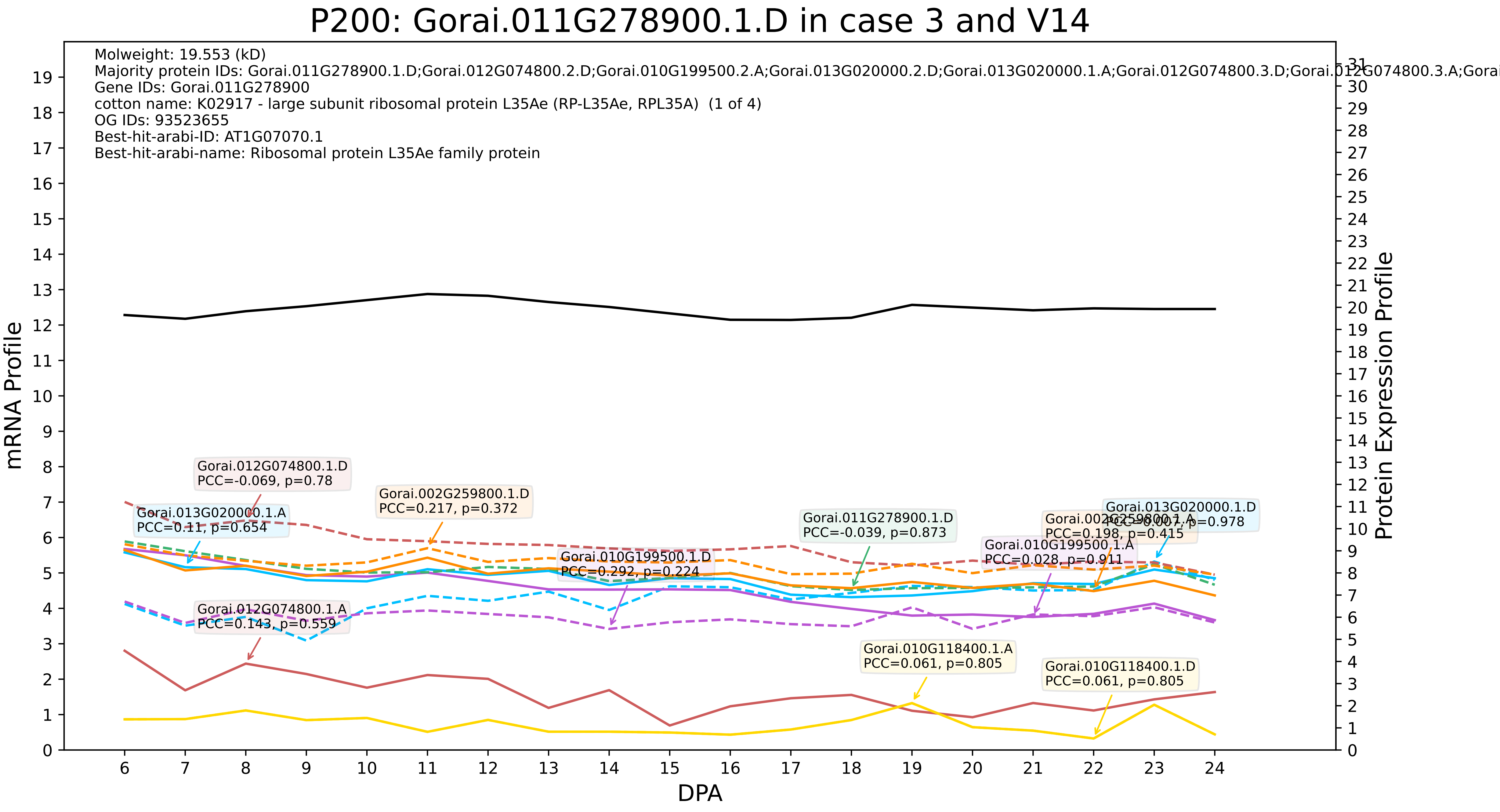
P200: Gorai.011G278900.1.D in case 3 and V14
Molweight: 19.553 (kD)
Majority protein IDs: Gorai.011G278900.1.D;Gorai.012G074800.2.D;Gorai.010G199500.2.A;Gorai.013G020000.2.D;Gorai.013G020000.1.A;Gorai.012G074800.3.D;Gorai.012G074800.3.A;Gorai
Gene IDs: Gorai.011G278900
cotton name: K02917 - large subunit ribosomal protein L35Ae (RP-L35Ae, RPL35A) (1 of 4)
OG IDs: 93523655
Best-hit-arabi-ID: AT1G07070.1
Best-hit-arabi-name: Ribosomal protein L35Ae family protein
Gorai.012G074800.1.D
PCC=-0.069, p=0.78
Gorai.013G020000.1.A
PCC=0.11, p=0.654
Gorai.002G259800.1.D
PCC=0.217, p=0.372
Gorai.011G278900.1.D
PCC=-0.039, p=0.873
Gorai.013G020000.1.D
PCC=0.007, p=0.978
Gorai.002G259800.1.A
PCC=0.198, p=0.415
Gorai.010G199500.1.A
PCC=0.028, p=0.911
Gorai.010G199500.1.D
PCC=0.292, p=0.224
Gorai.012G074800.1.A
PCC=0.143, p=0.559
Gorai.010G118400.1.A
PCC=0.061, p=0.805
Gorai.010G118400.1.D
PCC=0.061, p=0.805
mRNA Profile
Protein Expression Profile
DPA

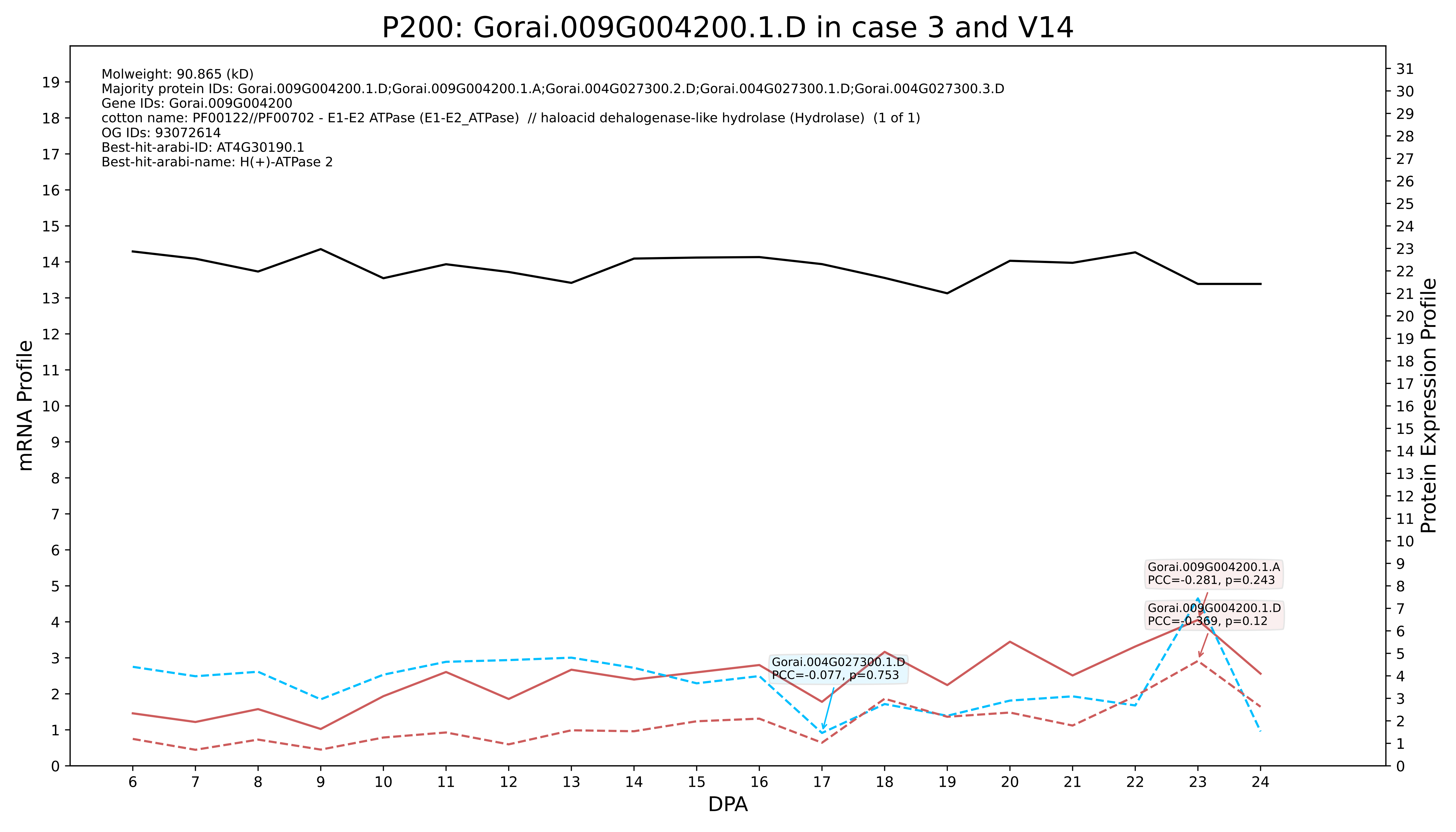
P200: Gorai.009G004200.1.D in case 3 and V14
Molweight: 90.865 (kD)
Majority protein IDs: Gorai.009G004200.1.D;Gorai.009G004200.1.A;Gorai.004G027300.2.D;Gorai.004G027300.1.D;Gorai.004G027300.3.D
Gene IDs: Gorai.009G004200
cotton name: PF00122//PF00702 - E1-E2 ATPase (E1-E2_ATPase) // haloacid dehalogenase-like hydrolase (Hydrolase) (1 of 1)
OG IDs: 93072614
Best-hit-arabi-ID: AT4G30190.1
Best-hit-arabi-name: H(+)-ATPase 2
Gorai.009G004200.1.A
PCC=-0.281, p=0.243
Gorai.009G004200.1.D
PCC=-0.369, p=0.12
Gorai.004G027300.1.D
PCC=-0.077, p=0.753
mRNA Profile
Protein Expression Profile
DPA

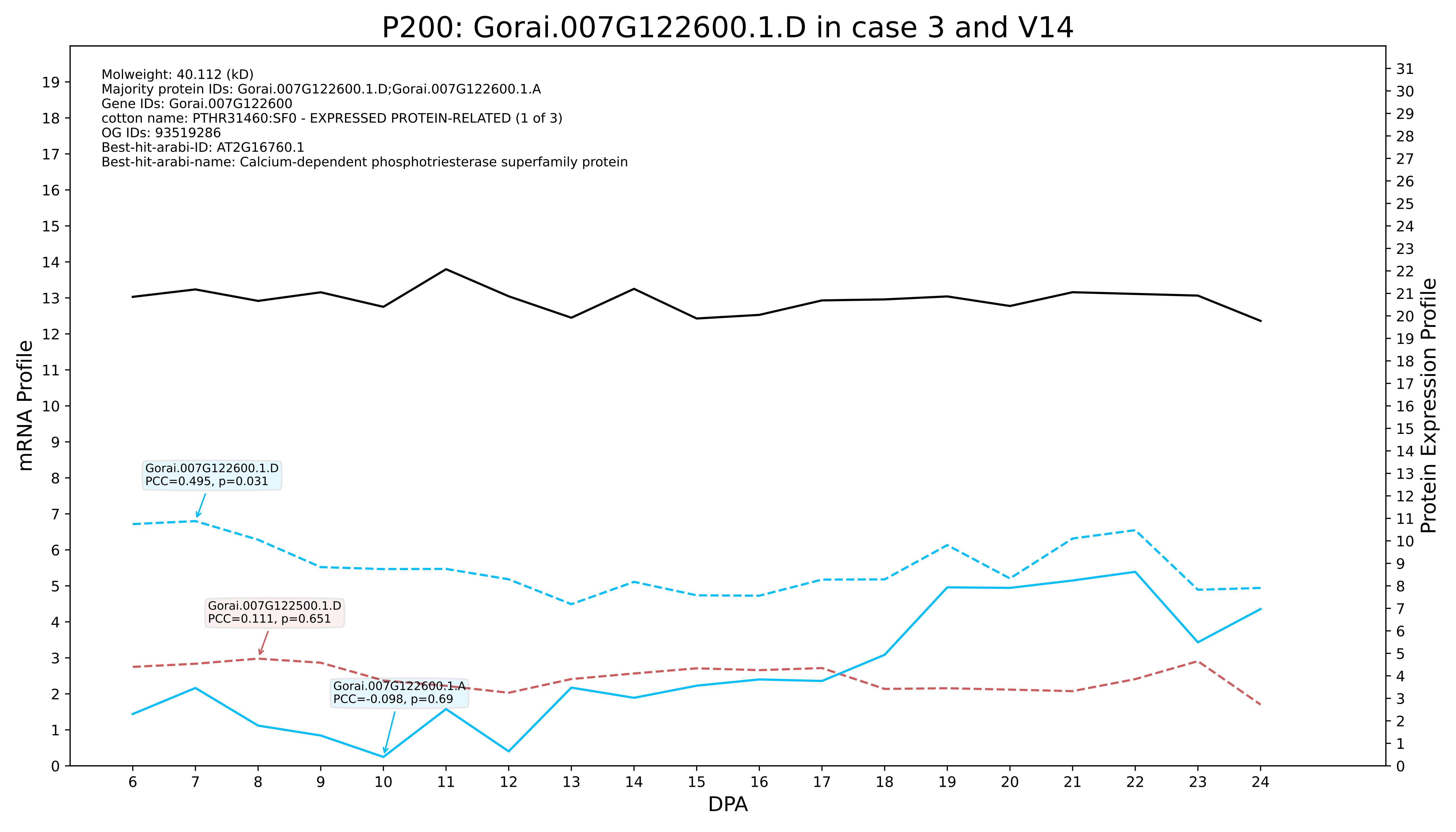
P200: Gorai.007G122600.1.D in case 3 and V14
Molweight: 40.112 (kD)
Majority protein IDs: Gorai.007G122600.1.D;Gorai.007G122600.1.A
Gene IDs: Gorai.007G122600
cotton name: PTHR31460:SF0 - EXPRESSED PROTEIN-RELATED (1 of 3)
OG IDs: 93519286
Best-hit-arabi-ID: AT2G16760.1
Best-hit-arabi-name: Calcium-dependent phosphotriesterase superfamily protein
Gorai.007G122600.1.D
PCC=0.495, p=0.031
Gorai.007G122500.1.D
PCC=0.111, p=0.651
Gorai.007G122600.1.A
PCC=-0.098, p=0.69
mRNA Profile
Protein Expression Profile
DPA

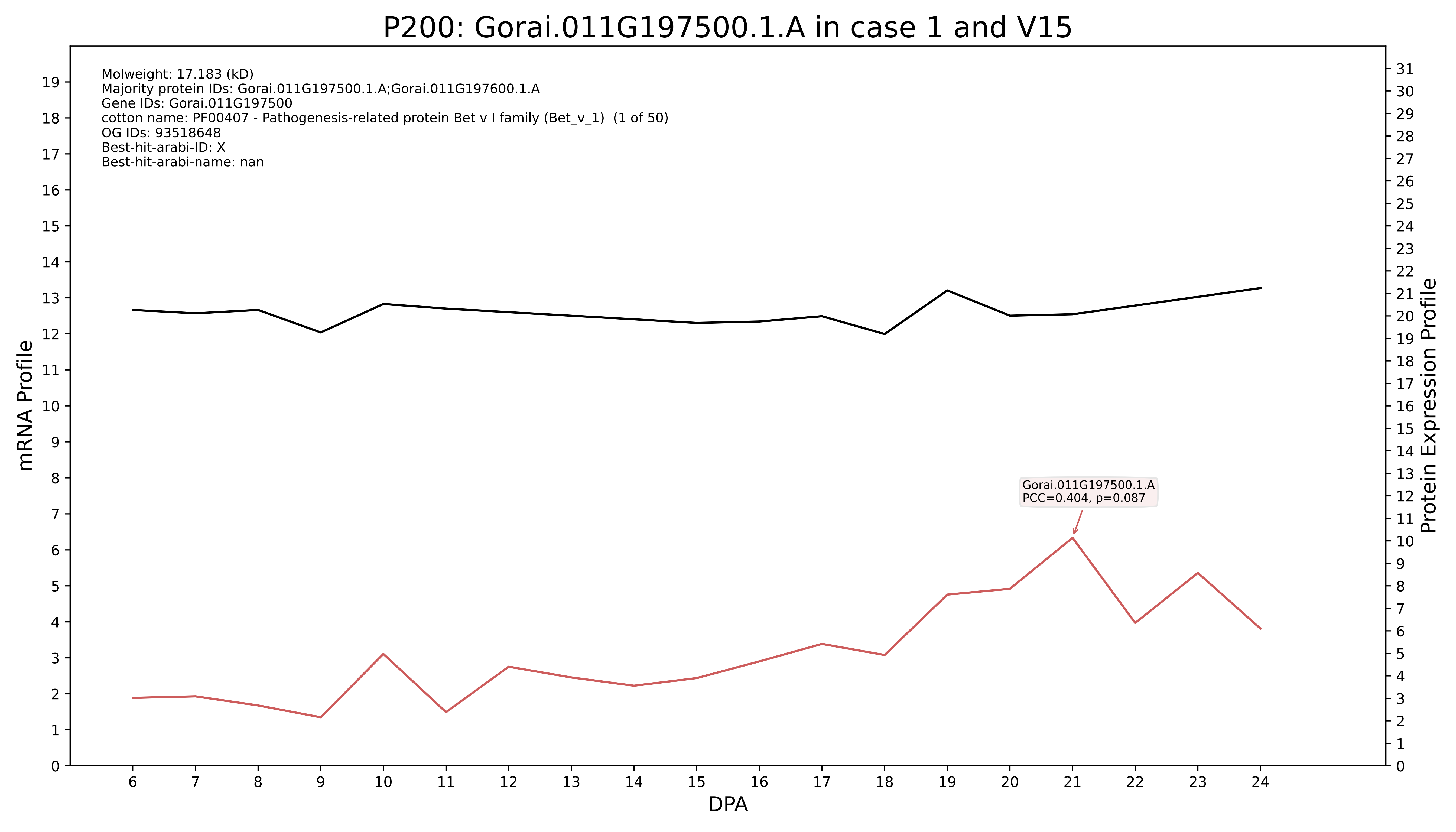
P200: Gorai.011G197500.1.A in case 1 and V15
Molweight: 17.183 (kD)
Majority protein IDs: Gorai.011G197500.1.A;Gorai.011G197600.1.A
Gene IDs: Gorai.011G197500
cotton name: PF00407 - Pathogenesis-related protein Bet v I family (Bet_v_1) (1 of 50)
OG IDs: 93518648
Best-hit-arabi-ID: X
Best-hit-arabi-name: nan
Gorai.011G197500.1.A
PCC=0.404, p=0.087
mRNA Profile
Protein Expression Profile
DPA

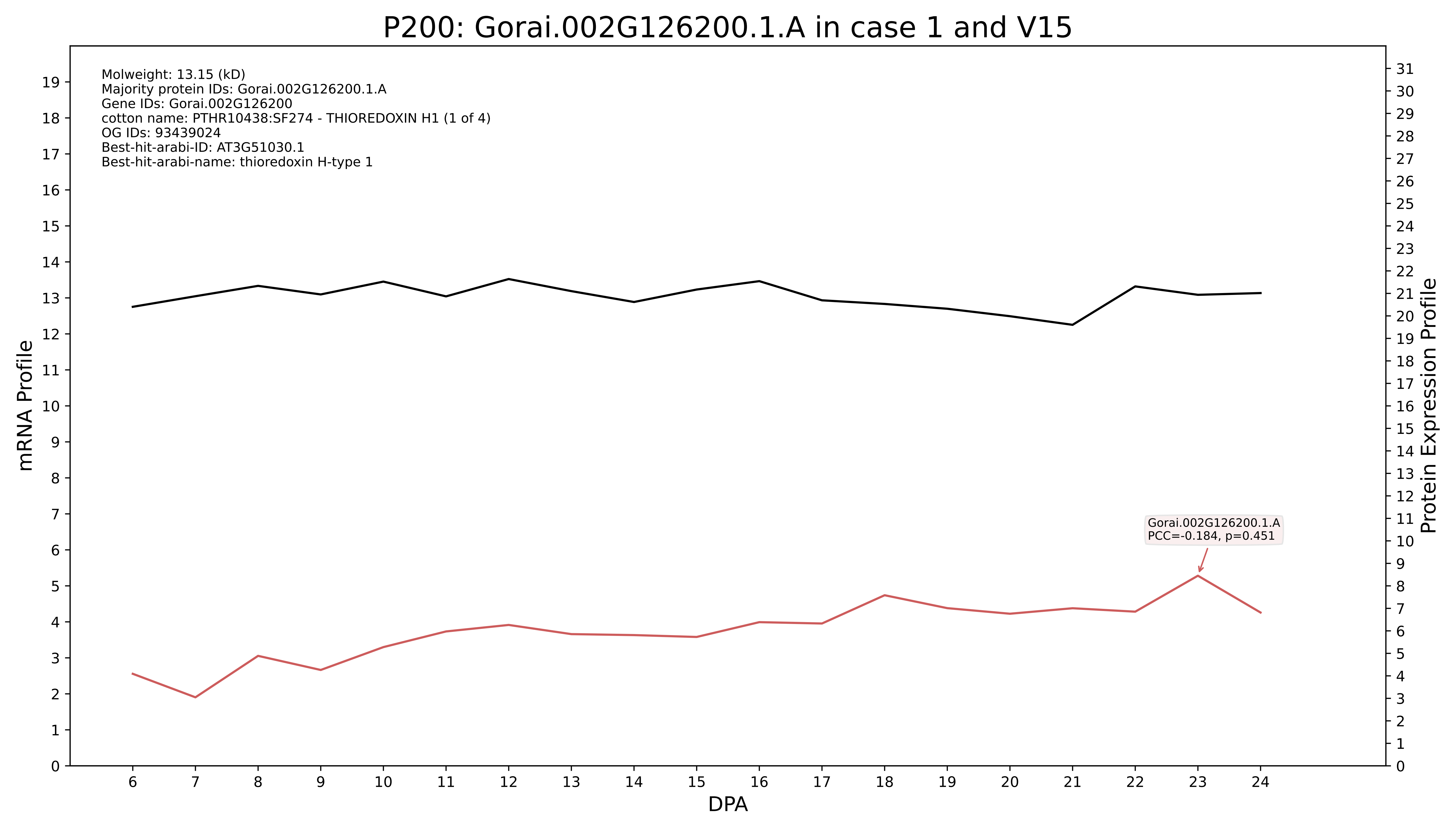
P200: Gorai.002G126200.1.A in case 1 and V15
Molweight: 13.15 (kD)
Majority protein IDs: Gorai.002G126200.1.A
Gene IDs: Gorai.002G126200
cotton name: PTHR10438:SF274 - THIOREDOXIN H1 (1 of 4)
OG IDs: 93439024
Best-hit-arabi-ID: AT3G51030.1
Best-hit-arabi-name: thioredoxin H-type 1
Gorai.002G126200.1.A
PCC=-0.184, p=0.451
mRNA Profile
Protein Expression Profile
DPA
0
1
2
3
4
5
6
7
8
9
10
11
12
13
14
15
16
17
18
19
20
21
22
23
24
25
26
27
28
29
30
31

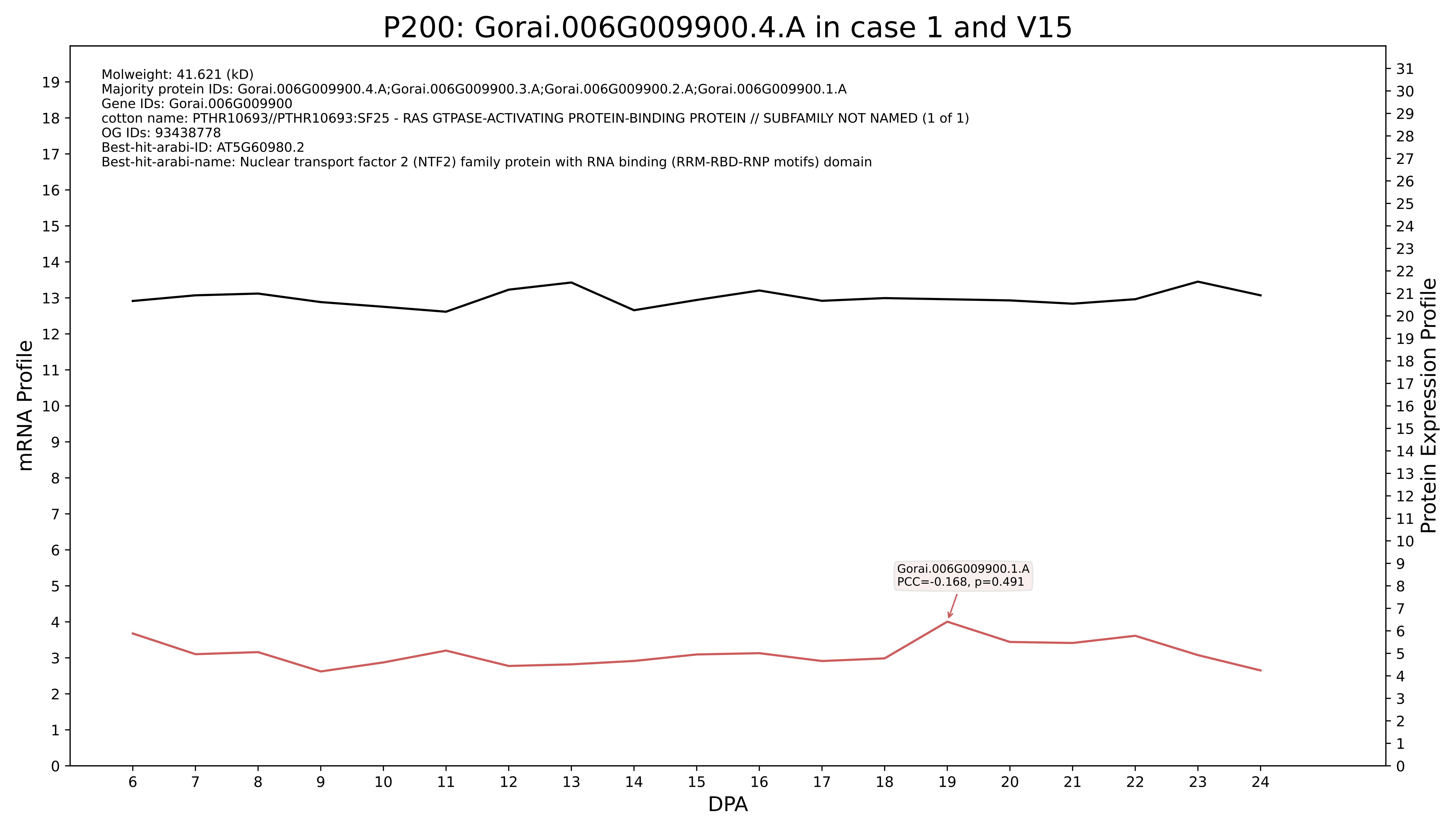
P200: Gorai.006G009900.4.A in case 1 and V15
Molweight: 41.621 (kD)
Majority protein IDs: Gorai.006G009900.4.A;Gorai.006G009900.3.A;Gorai.006G009900.2.A;Gorai.006G009900.1.A
Gene IDs: Gorai.006G009900
cotton name: PTHR10693//PTHR10693:SF25 - RAS GTPASE-ACTIVATING PROTEIN-BINDING PROTEIN // SUBFAMILY NOT NAMED (1 of 1)
OG IDs: 93438778
Best-hit-arabi-ID: AT5G60980.2
Best-hit-arabi-name: Nuclear transport factor 2 (NTF2) family protein with RNA binding (RRM-RBD-RNP motifs) domain
Gorai.006G009900.1.A
PCC=-0.168, p=0.491
mRNA Profile
Protein Expression Profile
DPA

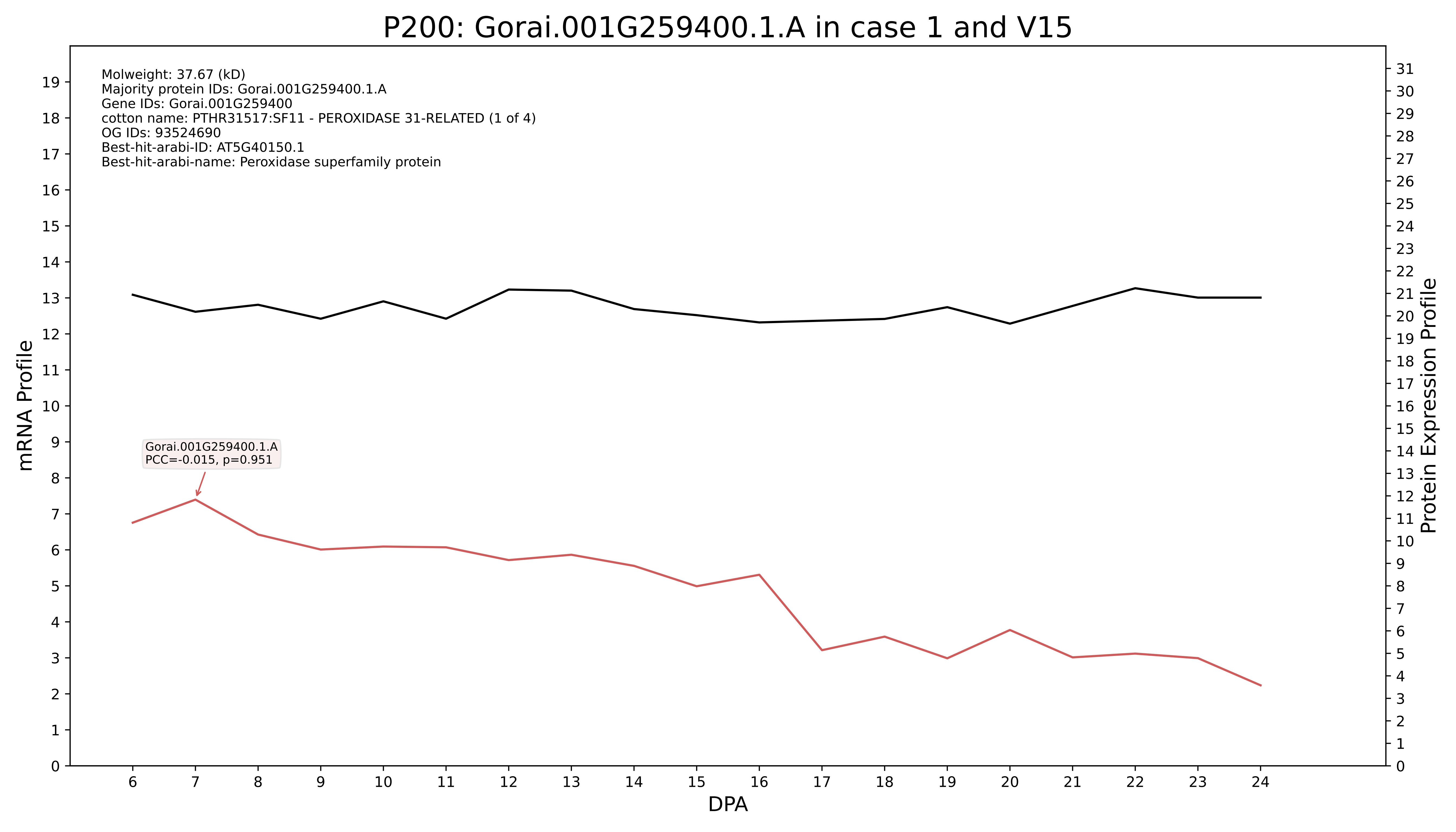
P200: Gorai.001G259400.1.A in case 1 and V15
Molweight: 37.67 (kD)
Majority protein IDs: Gorai.001G259400.1.A
Gene IDs: Gorai.001G259400
cotton name: PTHR31517:SF11 - PEROXIDASE 31-RELATED (1 of 4)
OG IDs: 93524690
Best-hit-arabi-ID: AT5G40150.1
Best-hit-arabi-name: Peroxidase superfamily protein
Gorai.001G259400.1.A
PCC=-0.015, p=0.951
mRNA Profile
Protein Expression Profile
DPA

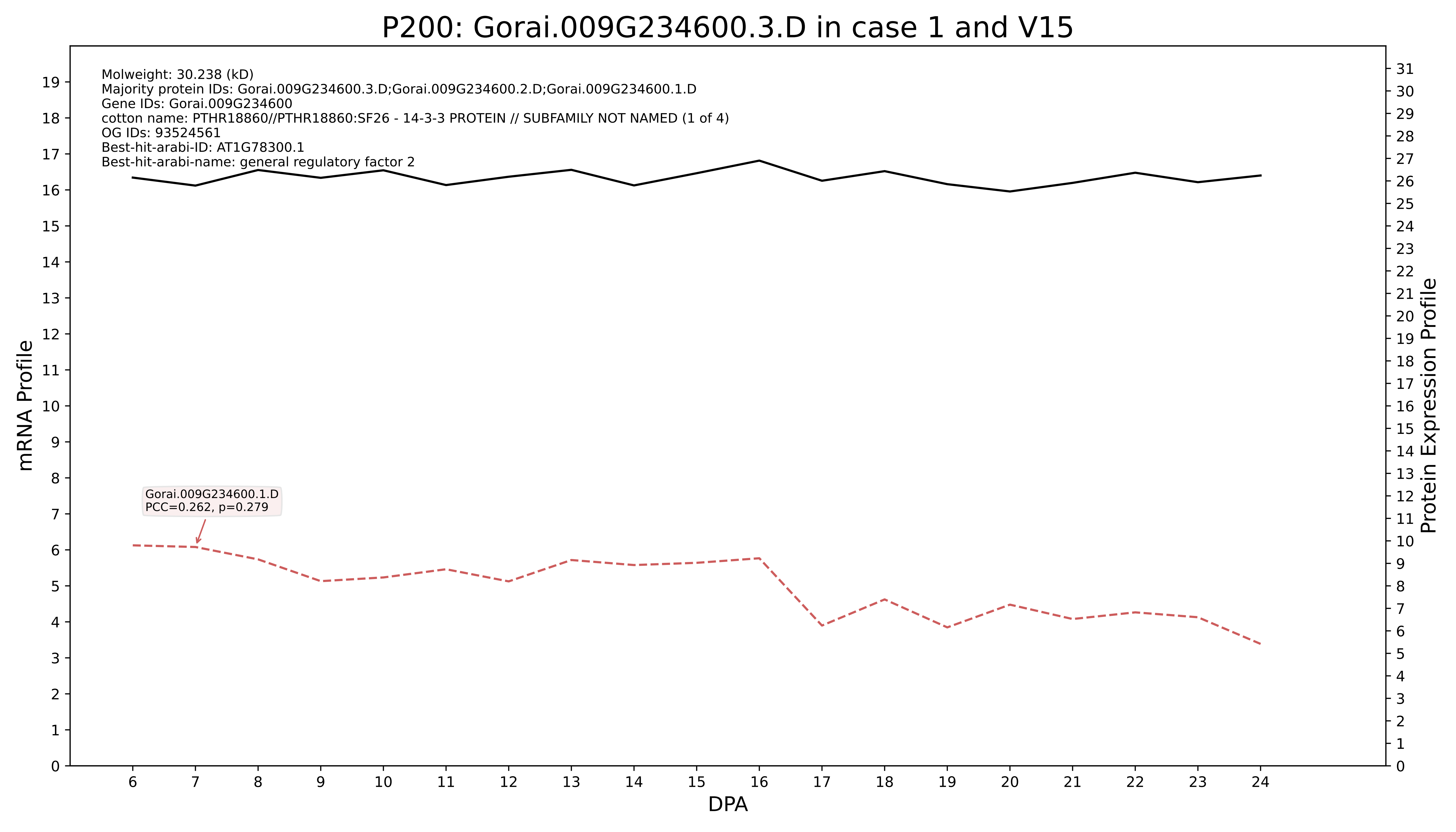
P200: Gorai.009G234600.3.D in case 1 and V15
Molweight: 30.238 (kD)
Majority protein IDs: Gorai.009G234600.3.D;Gorai.009G234600.2.D;Gorai.009G234600.1.D
Gene IDs: Gorai.009G234600
cotton name: PTHR18860//PTHR18860:SF26 - 14-3-3 PROTEIN // SUBFAMILY NOT NAMED (1 of 4)
OG IDs: 93524561
Best-hit-arabi-ID: AT1G78300.1
Best-hit-arabi-name: general regulatory factor 2
Gorai.009G234600.1.D
PCC=0.262, p=0.279
mRNA Profile
Protein Expression Profile
DPA

P200: Gorai.001G198400.3.A in case 1 and V15

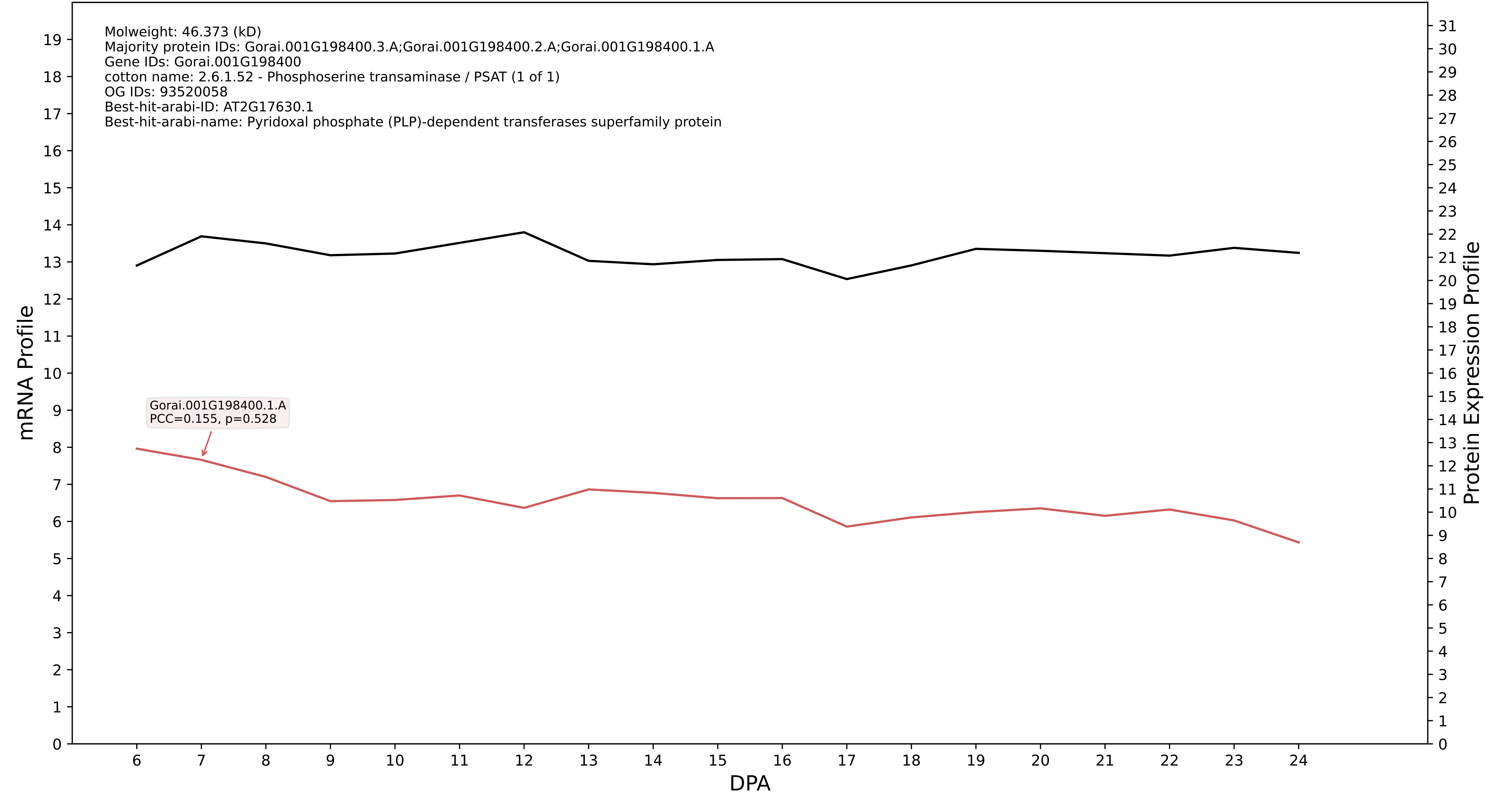

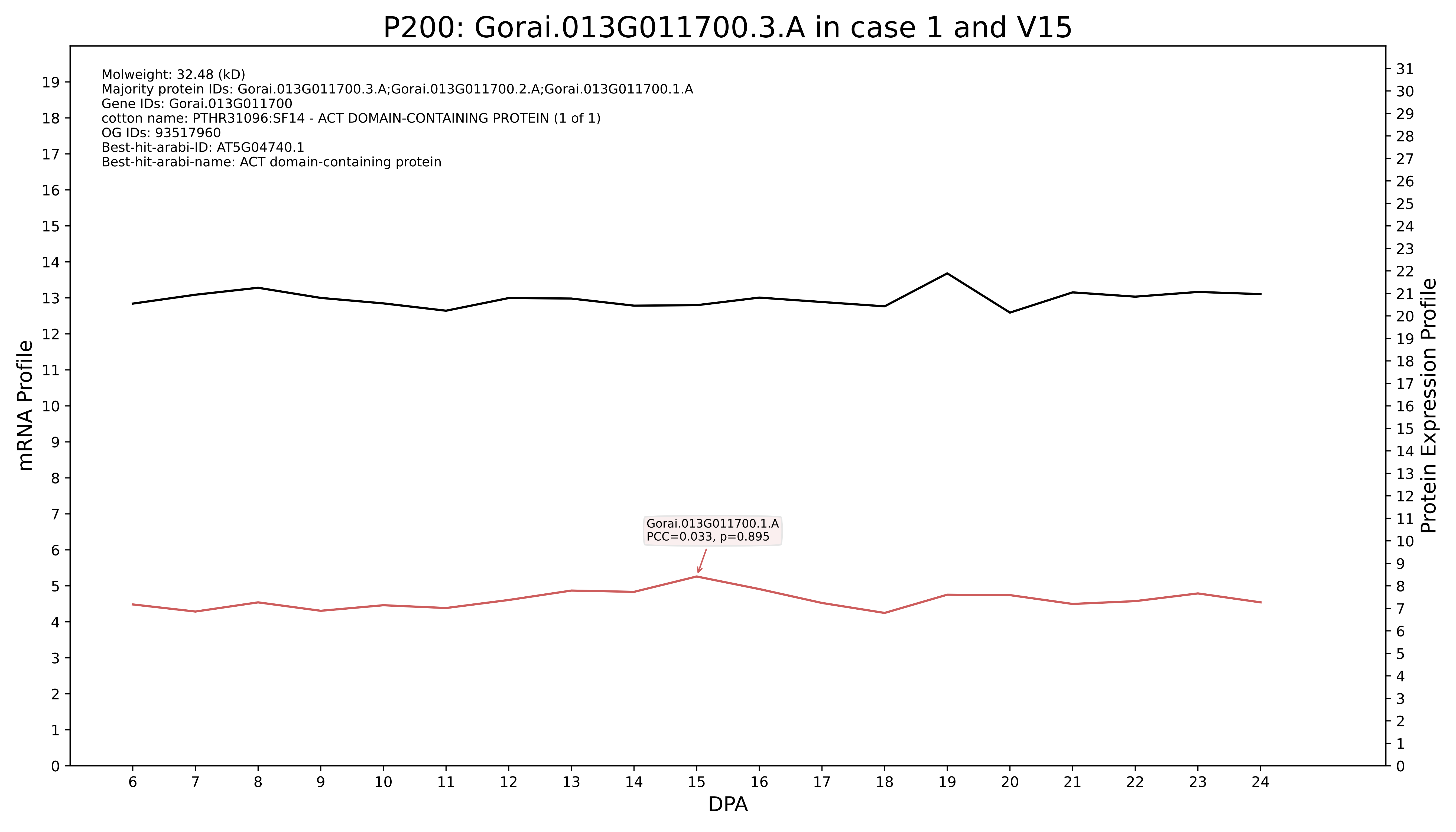
P200: Gorai.013G011700.3.A in case 1 and V15
Molweight: 32.48 (kD)
Majority protein IDs: Gorai.013G011700.3.A;Gorai.013G011700.2.A;Gorai.013G011700.1.A
Gene IDs: Gorai.013G011700
cotton name: PTHR31096:SF14 - ACT DOMAIN-CONTAINING PROTEIN (1 of 1)
OG IDs: 93517960
Best-hit-arabi-ID: AT5G04740.1
Best-hit-arabi-name: ACT domain-containing protein
Gorai.013G011700.1.A
PCC=0.033, p=0.895
mRNA Profile
Protein Expression Profile
DPA
0
1
2
3
4
5
6
7
8
9
10
11
12
13
14
15
16
17
18
19
20
21
22
23
24
25
26
27
28
29
30
31

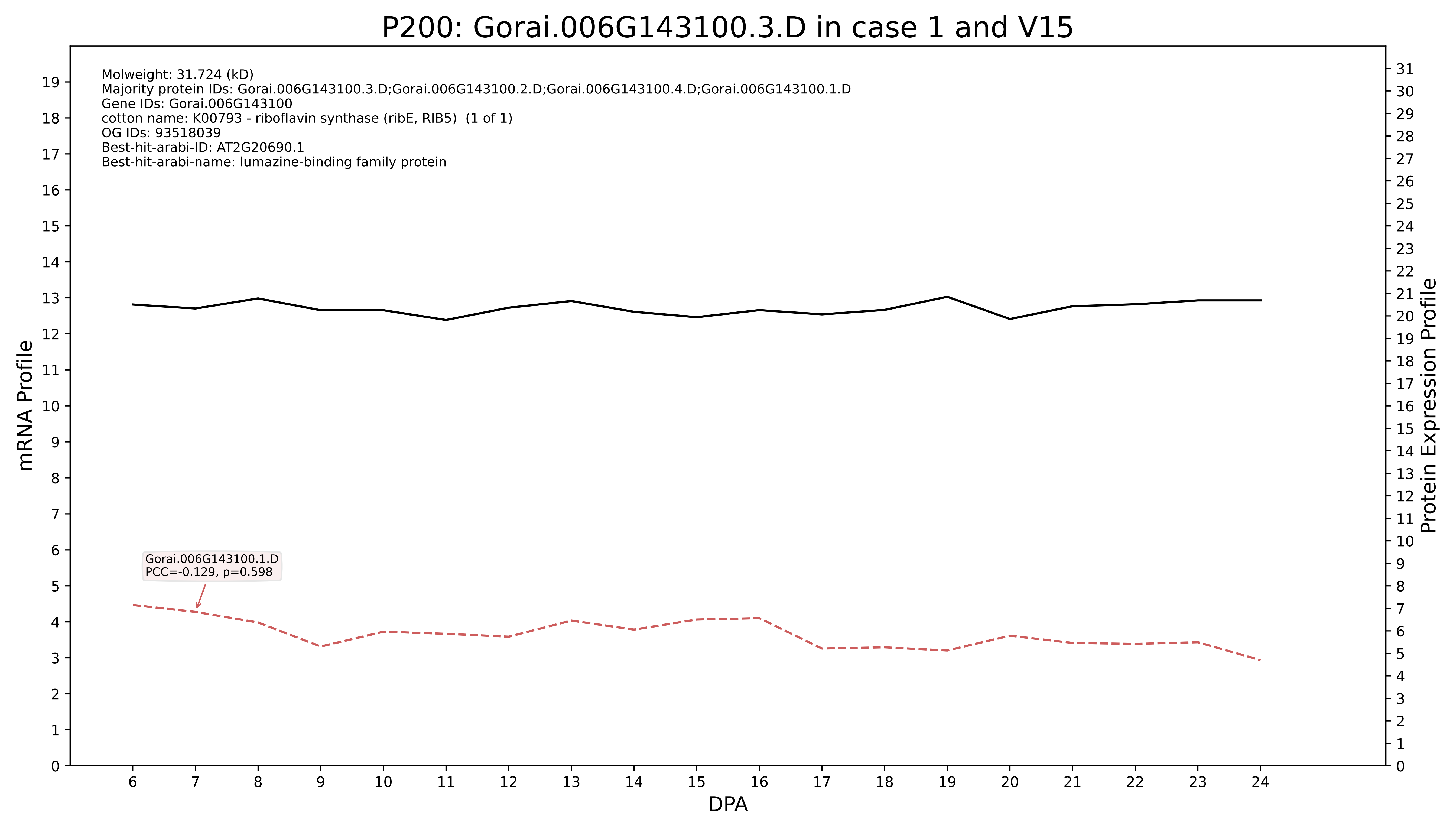
P200: Gorai.006G143100.3.D in case 1 and V15
Molweight: 31.724 (kD)
Majority protein IDs: Gorai.006G143100.3.D;Gorai.006G143100.2.D;Gorai.006G143100.4.D;Gorai.006G143100.1.D
Gene IDs: Gorai.006G143100
cotton name: K00793 - riboflavin synthase (ribE, RIB5) (1 of 1)
OG IDs: 93518039
Best-hit-arabi-ID: AT2G20690.1
Best-hit-arabi-name: lumazine-binding family protein
Gorai.006G143100.1.D
PCC=-0.129, p=0.598
mRNA Profile
Protein Expression Profile
DPA
0
1
2
3
4
5
6
7
8
9
10
11
12
13
14
15
16
17
18
19
20
21
22
23
24
25
26
27
28
29
30
31

P200: Gorai.008G097100.4.A in case 1 and V15

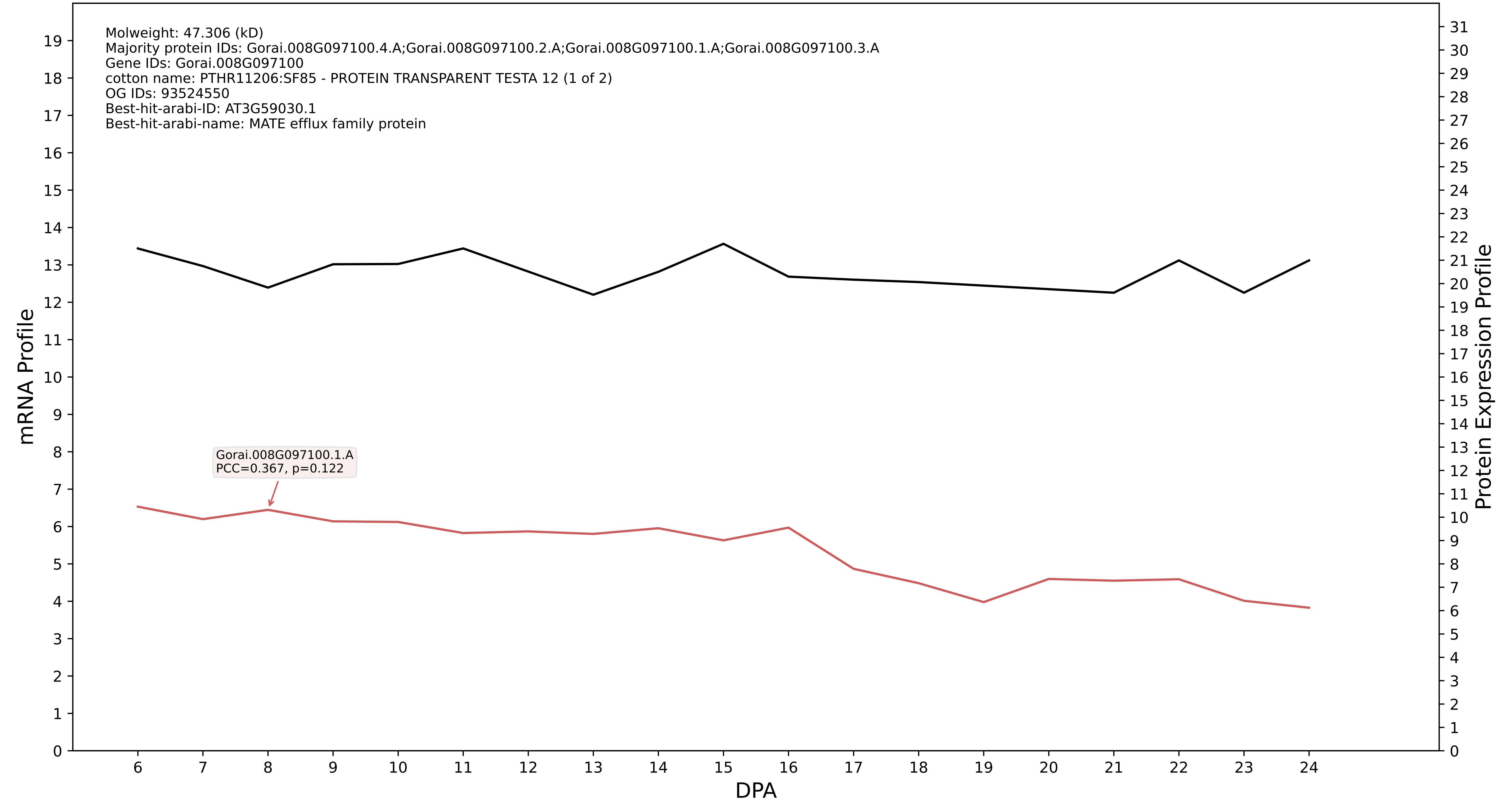

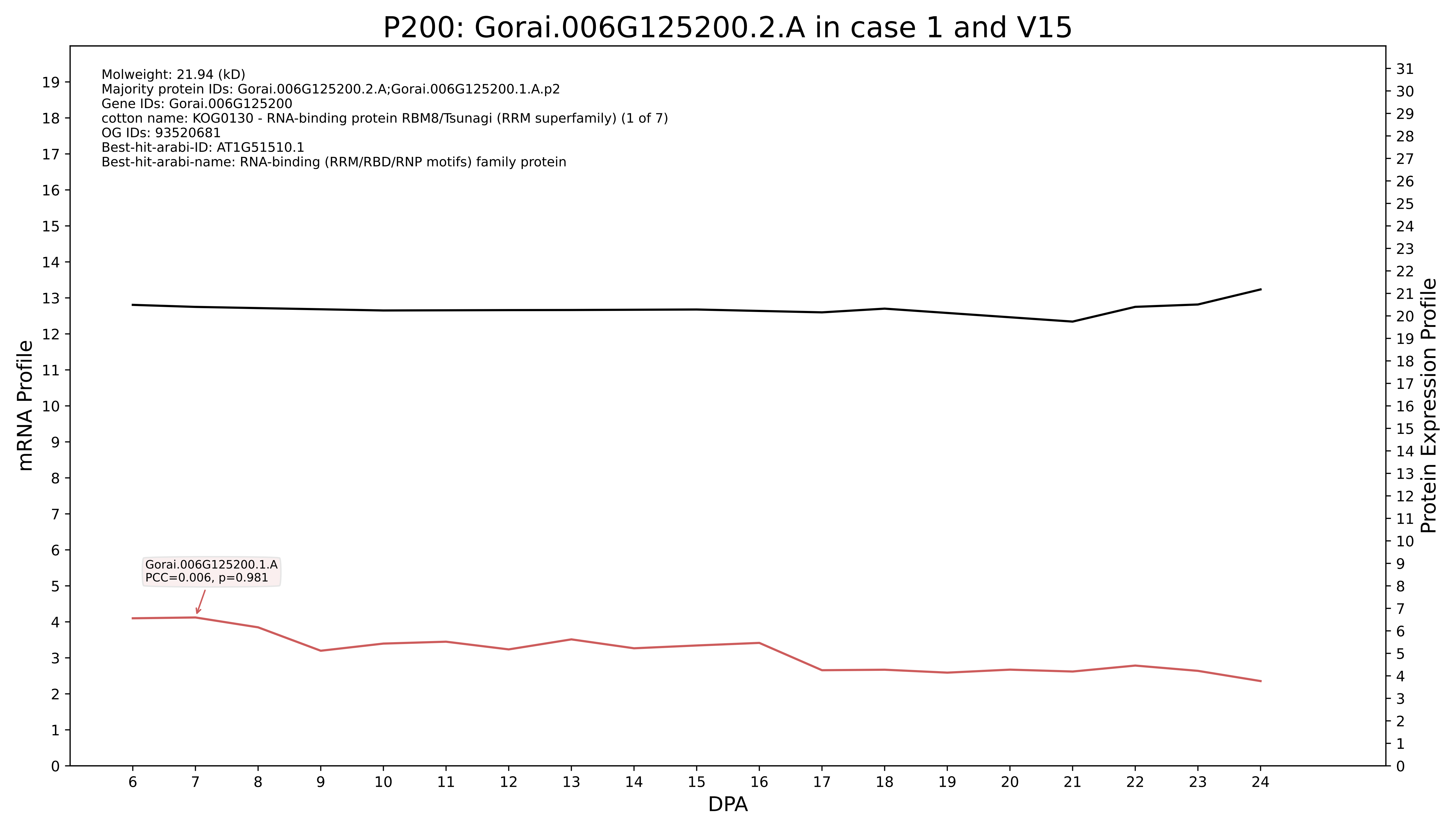

P200: Gorai.006G125200.2.A in case 1 and V15
Molweight: 21.94 (kD)
Majority protein IDs: Gorai.006G125200.2.A;Gorai.006G125200.1.A.p2
Gene IDs: Gorai.006G125200
cotton name: KOG0130 - RNA-binding protein RBM8/Tsunagi (RRM superfamily) (1 of 7)
OG IDs: 93520681
Best-hit-arabi-ID: AT1G51510.1
Best-hit-arabi-name: RNA-binding (RRM/RBD/RNP motifs) family protein
Gorai.006G125200.1.A
PCC=0.006, p=0.981
mRNA Profile
Protein Expression Profile
DPA

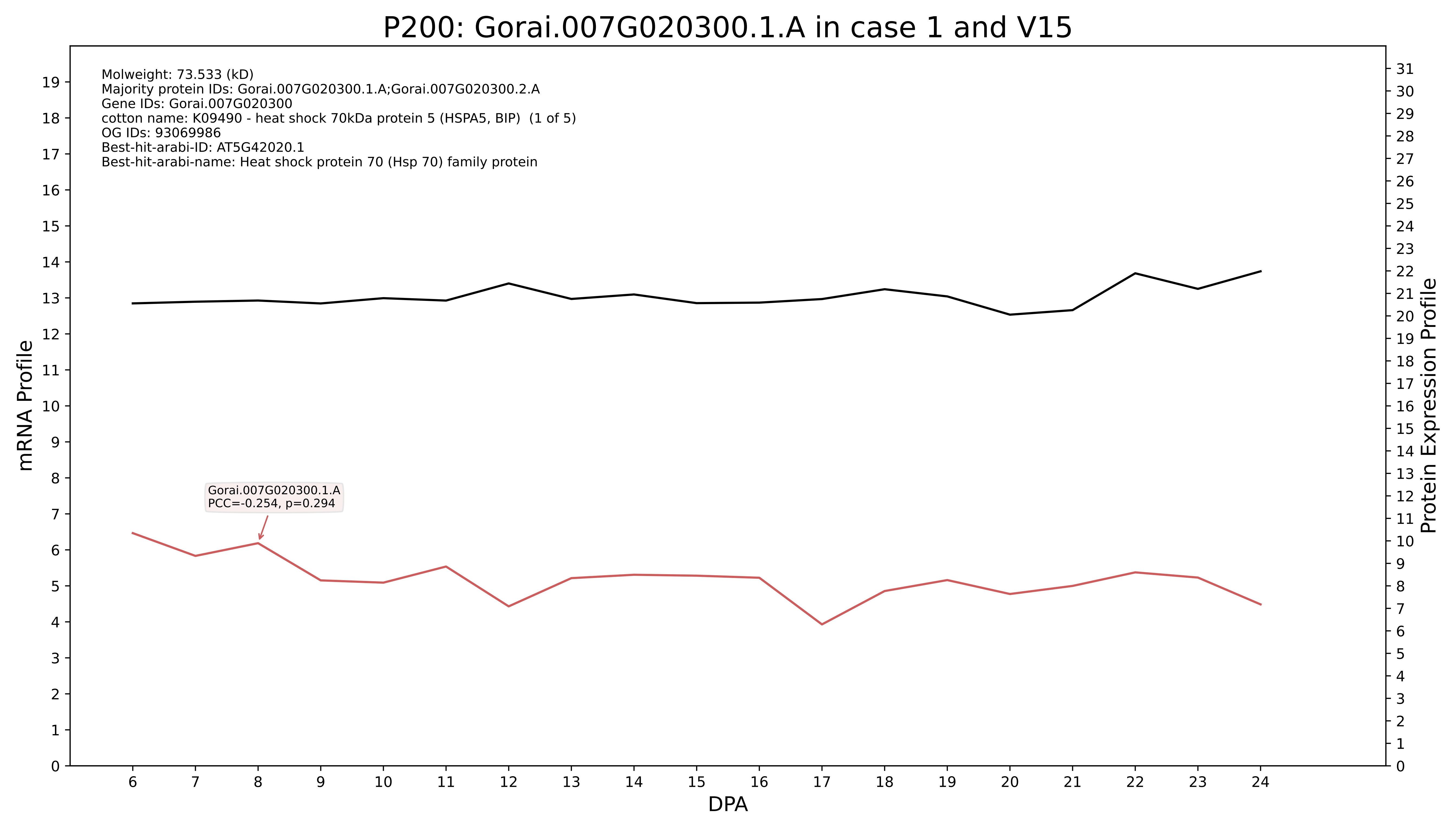
P200: Gorai.007G020300.1.A in case 1 and V15
Molweight: 73.533 (kD)
Majority protein IDs: Gorai.007G020300.1.A;Gorai.007G020300.2.A
Gene IDs: Gorai.007G020300
cotton name: K09490 - heat shock 70kDa protein 5 (HSPA5, BIP) (1 of 5)
OG IDs: 93069986
Best-hit-arabi-ID: AT5G42020.1
Best-hit-arabi-name: Heat shock protein 70 (Hsp 70) family protein
Gorai.007G020300.1.A
PCC=-0.254, p=0.294
mRNA Profile
Protein Expression Profile
DPA

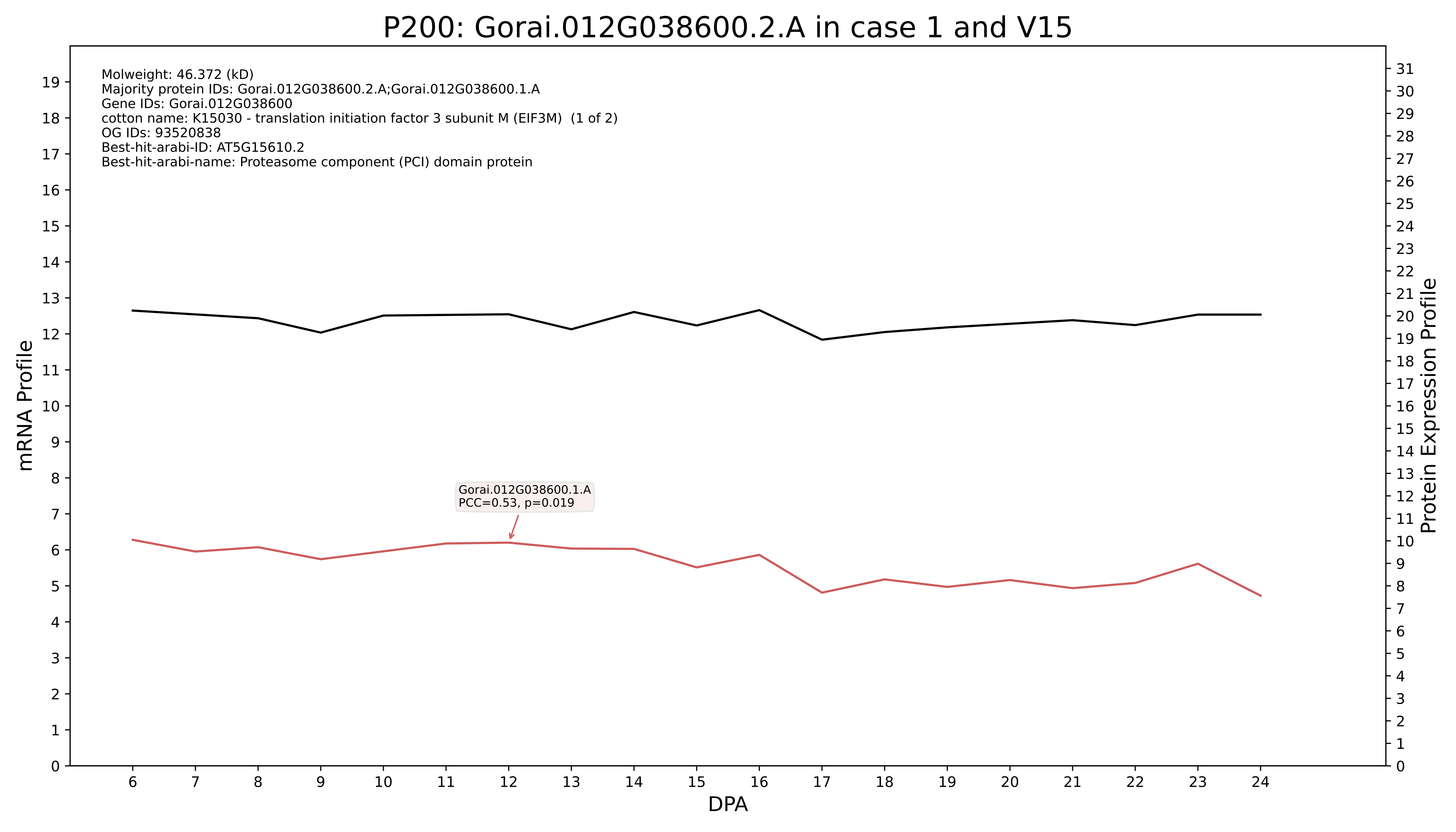
P200: Gorai.012G038600.2.A in case 1 and V15
Molweight: 46.372 (kD)
Majority protein IDs: Gorai.012G038600.2.A;Gorai.012G038600.1.A
Gene IDs: Gorai.012G038600
cotton name: K15030 - translation initiation factor 3 subunit M (EIF3M) (1 of 2)
OG IDs: 93520838
Best-hit-arabi-ID: AT5G15610.2
Best-hit-arabi-name: Proteasome component (PCI) domain protein
Gorai.012G038600.1.A
PCC=0.53, p=0.019
mRNA Profile
Protein Expression Profile
DPA

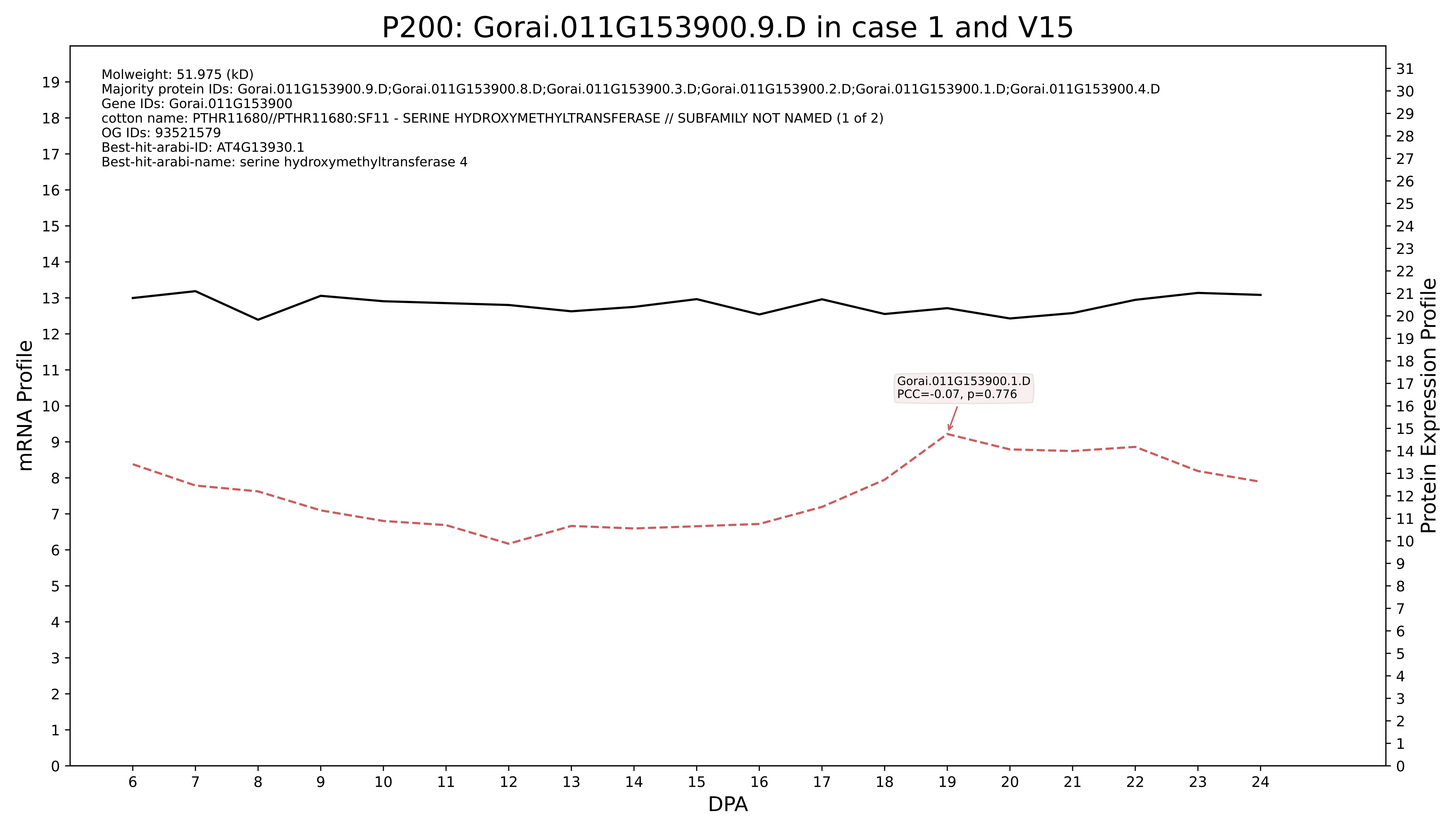
P200: Gorai.011G153900.9.D in case 1 and V15
Molweight: 51.975 (kD)
Majority protein IDs: Gorai.011G153900.9.D;Gorai.011G153900.8.D;Gorai.011G153900.3.D;Gorai.011G153900.2.D;Gorai.011G153900.1.D;Gorai.011G153900.4.D
Gene IDs: Gorai.011G153900
cotton name: PTHR11680//PTHR11680:SF11 - SERINE HYDROXYMETHYLTRANSFERASE // SUBFAMILY NOT NAMED (1 of 2)
OG IDs: 93521579
Best-hit-arabi-ID: AT4G13930.1
Best-hit-arabi-name: serine hydroxymethyltransferase 4
Gorai.011G153900.1.D
PCC=-0.07, p=0.776
mRNA Profile
Protein Expression Profile
DPA

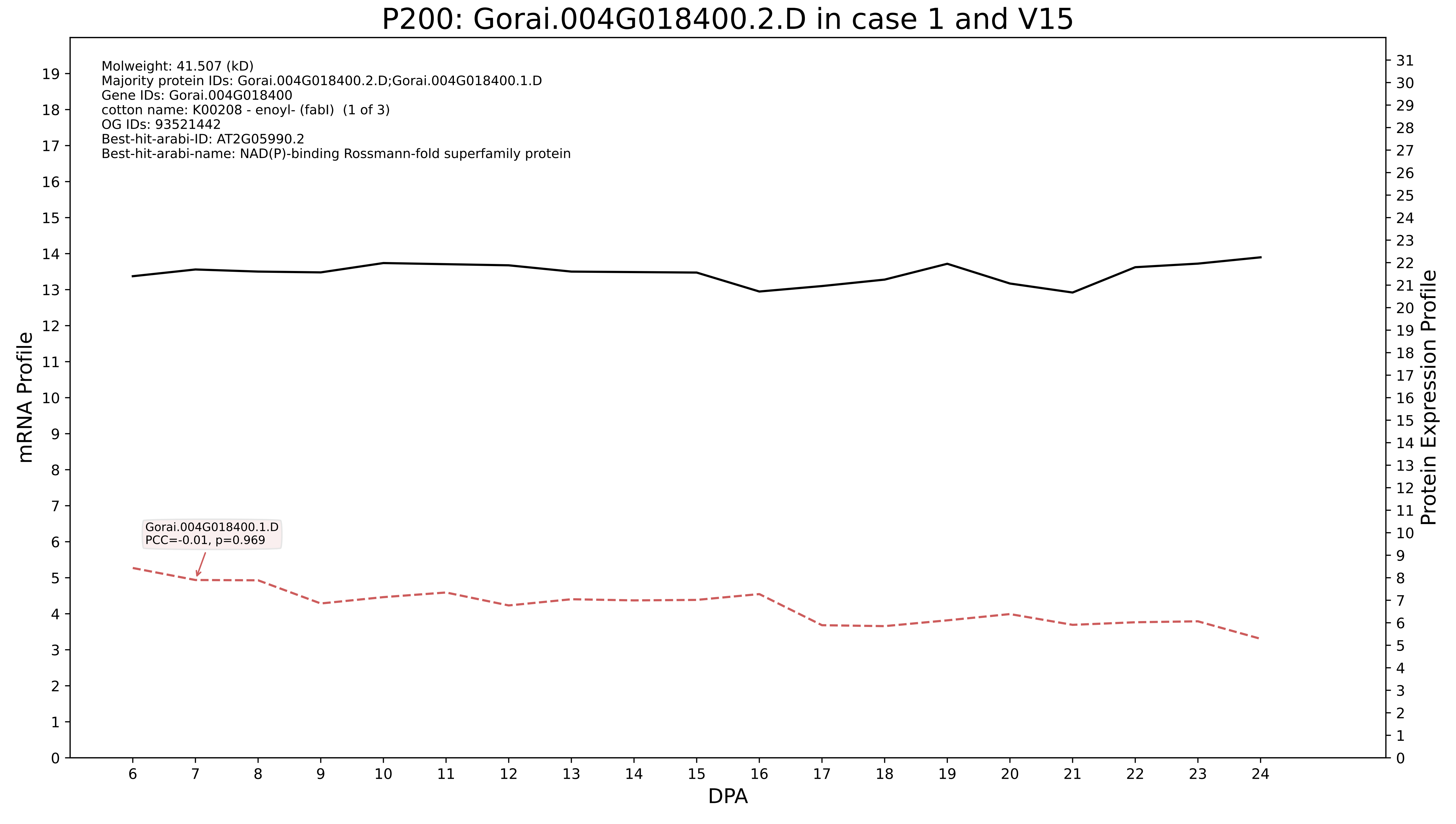
P200: Gorai.004G018400.2.D in case 1 and V15
Molweight: 41.507 (kD)
Majority protein IDs: Gorai.004G018400.2.D;Gorai.004G018400.1.D
Gene IDs: Gorai.004G018400
cotton name: K00208 - enoyl- (fabI) (1 of 3)
OG IDs: 93521442
Best-hit-arabi-ID: AT2G05990.2
Best-hit-arabi-name: NAD(P)-binding Rossmann-fold superfamily protein
Gorai.004G018400.1.D
PCC=-0.01, p=0.969
mRNA Profile
Protein Expression Profile
DPA

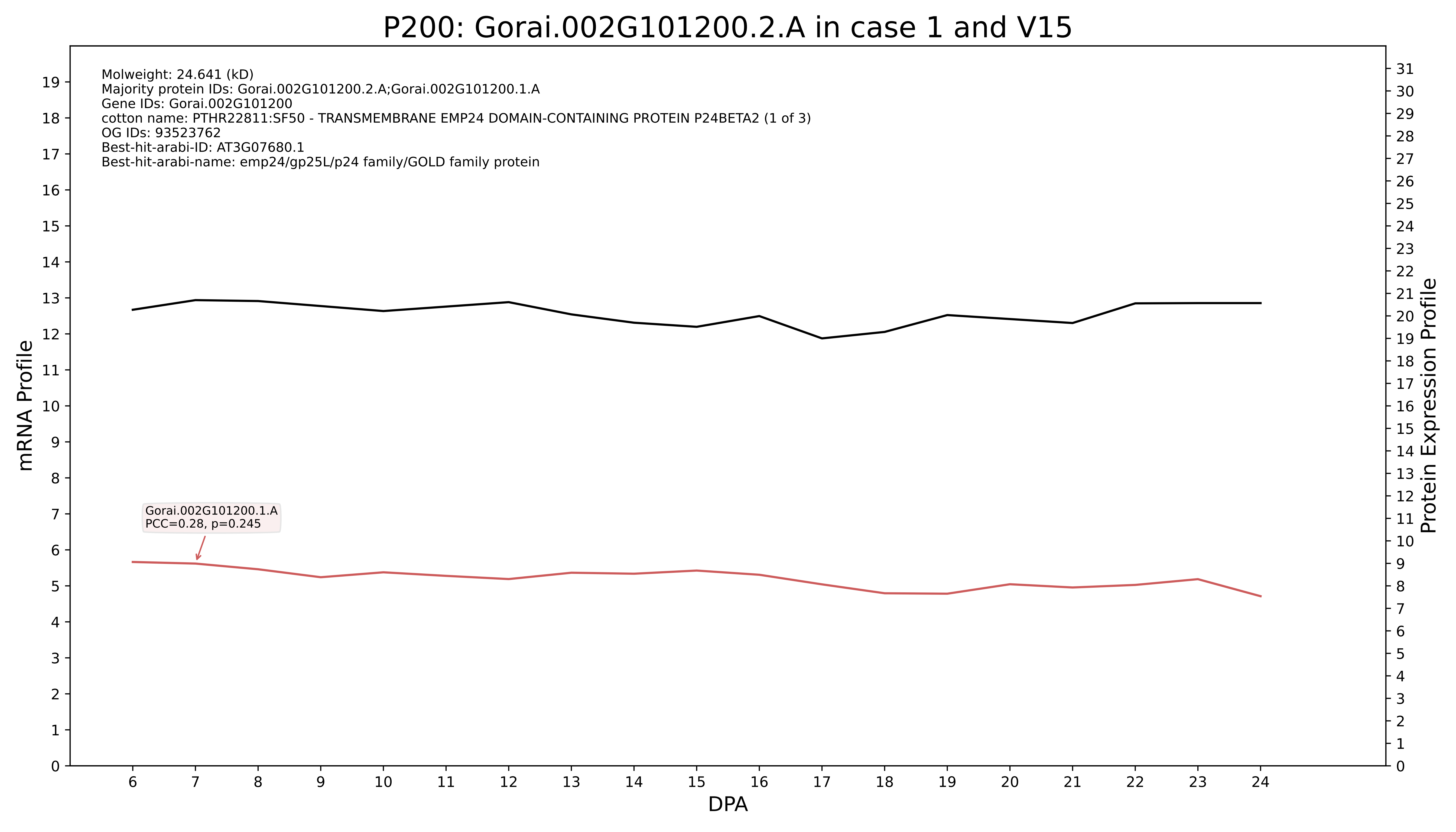
P200: Gorai.002G101200.2.A in case 1 and V15
Molweight: 24.641 (kD)
Majority protein IDs: Gorai.002G101200.2.A;Gorai.002G101200.1.A
Gene IDs: Gorai.002G101200
cotton name: PTHR22811:SF50 - TRANSMEMBRANE EMP24 DOMAIN-CONTAINING PROTEIN P24BETA2 (1 of 3)
OG IDs: 93523762
Best-hit-arabi-ID: AT3G07680.1
Best-hit-arabi-name: emp24/gp25L/p24 family/GOLD family protein
Gorai.002G101200.1.A
PCC=0.28, p=0.245
mRNA Profile
Protein Expression Profile
DPA

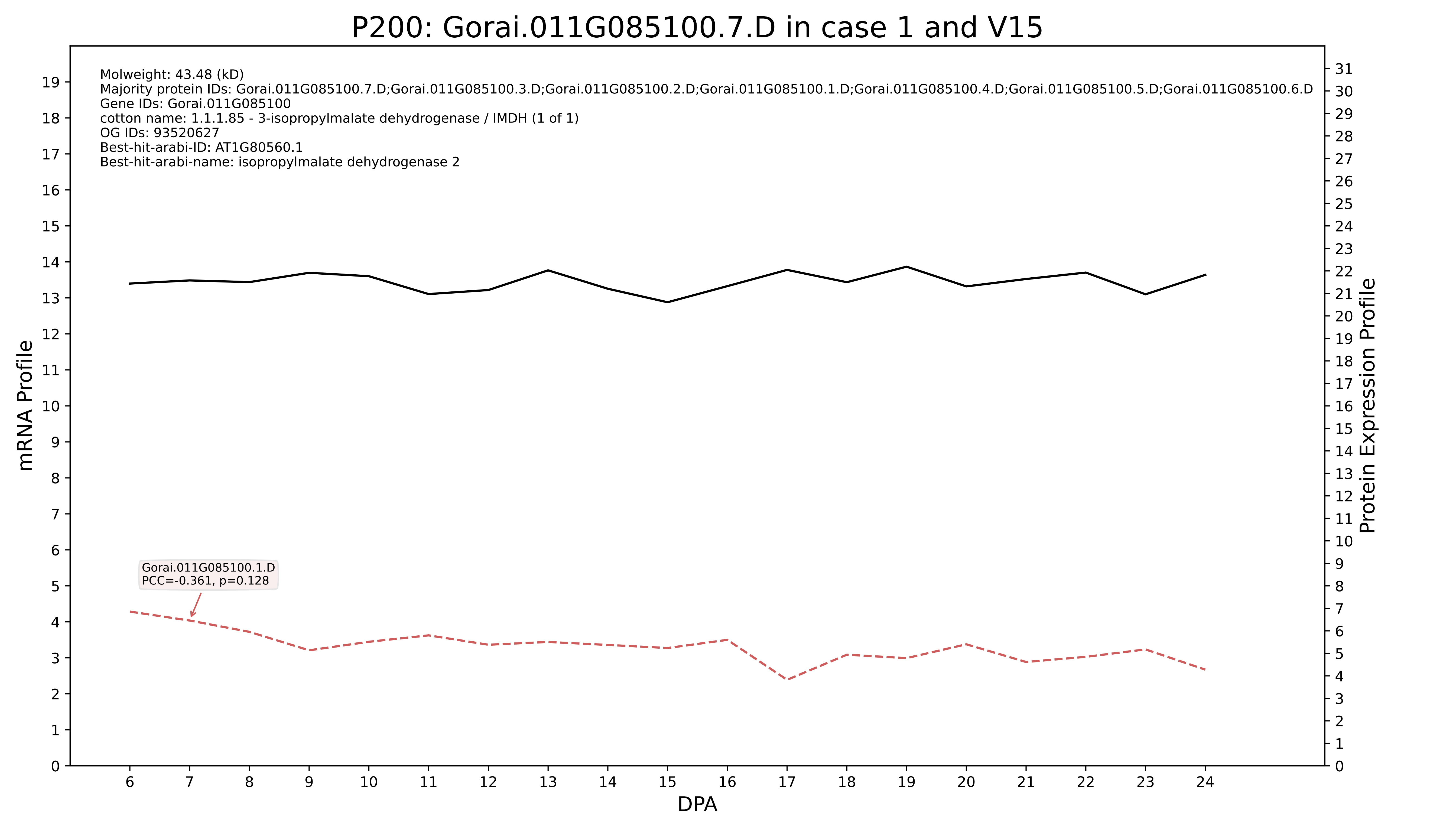

P200: Gorai.011G085100.7.D in case 1 and V15
Molweight: 43.48 (kD)
Majority protein IDs: Gorai.011G085100.7.D;Gorai.011G085100.3.D;Gorai.011G085100.2.D;Gorai.011G085100.1.D;Gorai.011G085100.4.D;Gorai.011G085100.5.D;Gorai.011G085100.6.D
Gene IDs: Gorai.011G085100
cotton name: 1.1.1.85 - 3-isopropylmalate dehydrogenase / IMDH (1 of 1)
OG IDs: 93520627
Best-hit-arabi-ID: AT1G80560.1
Best-hit-arabi-name: isopropylmalate dehydrogenase 2
Gorai.011G085100.1.D
PCC=-0.361, p=0.128
mRNA Profile
Protein Expression Profile
DPA

P200: Gorai.004G024500.6.D in case 1 and V15

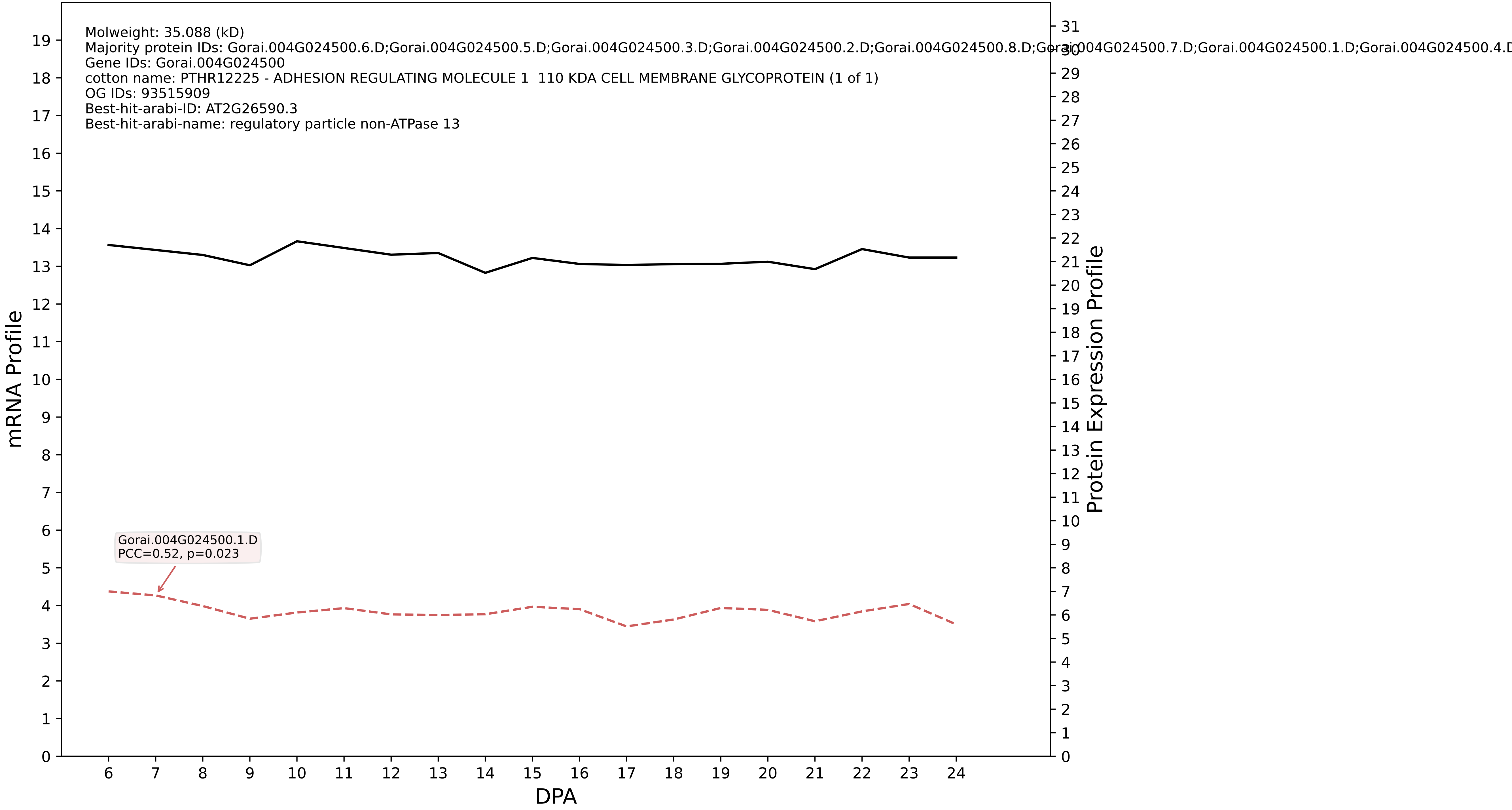

P200: Gorai.006G243700.1.A in case 1 and V15

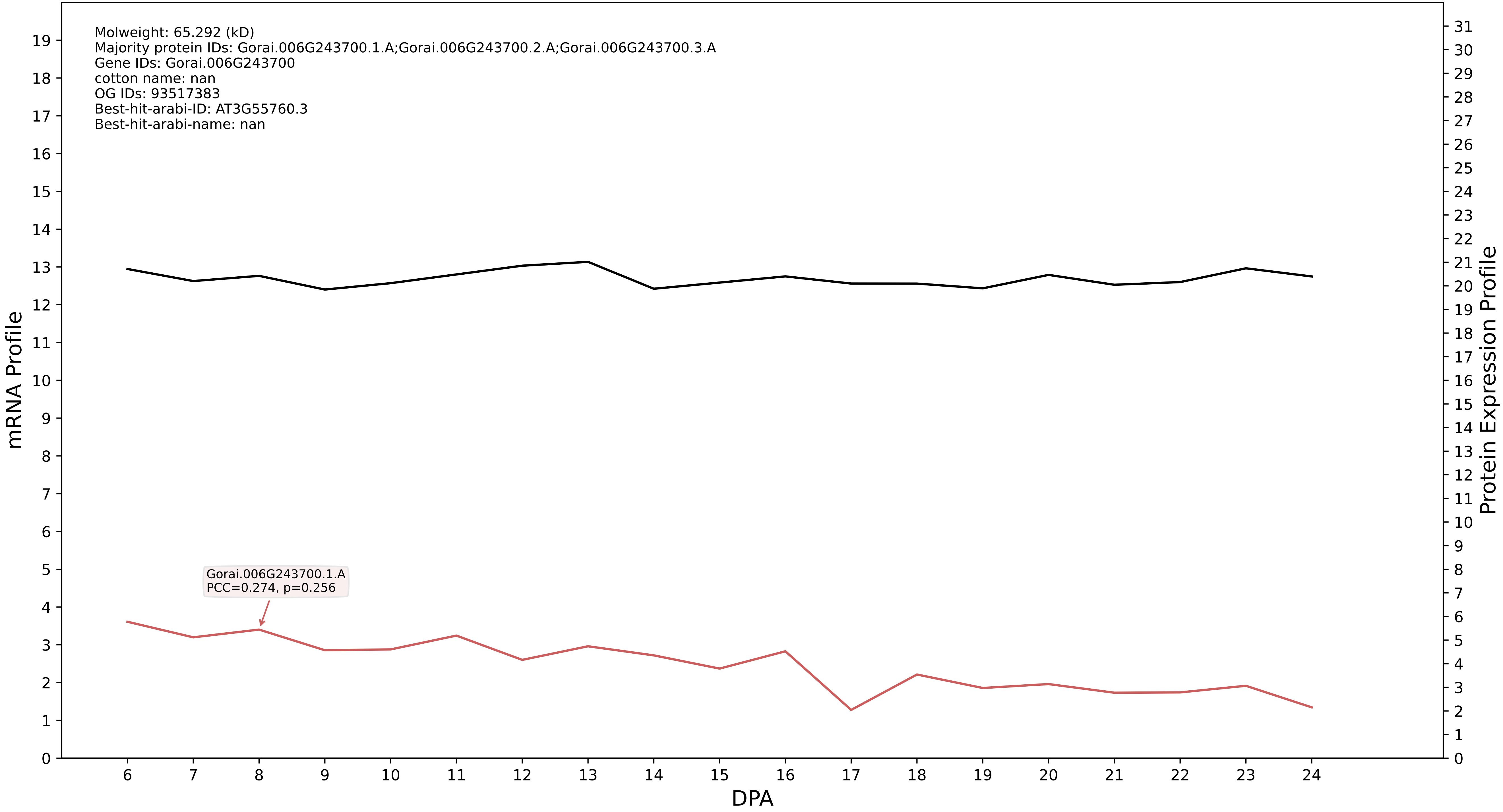

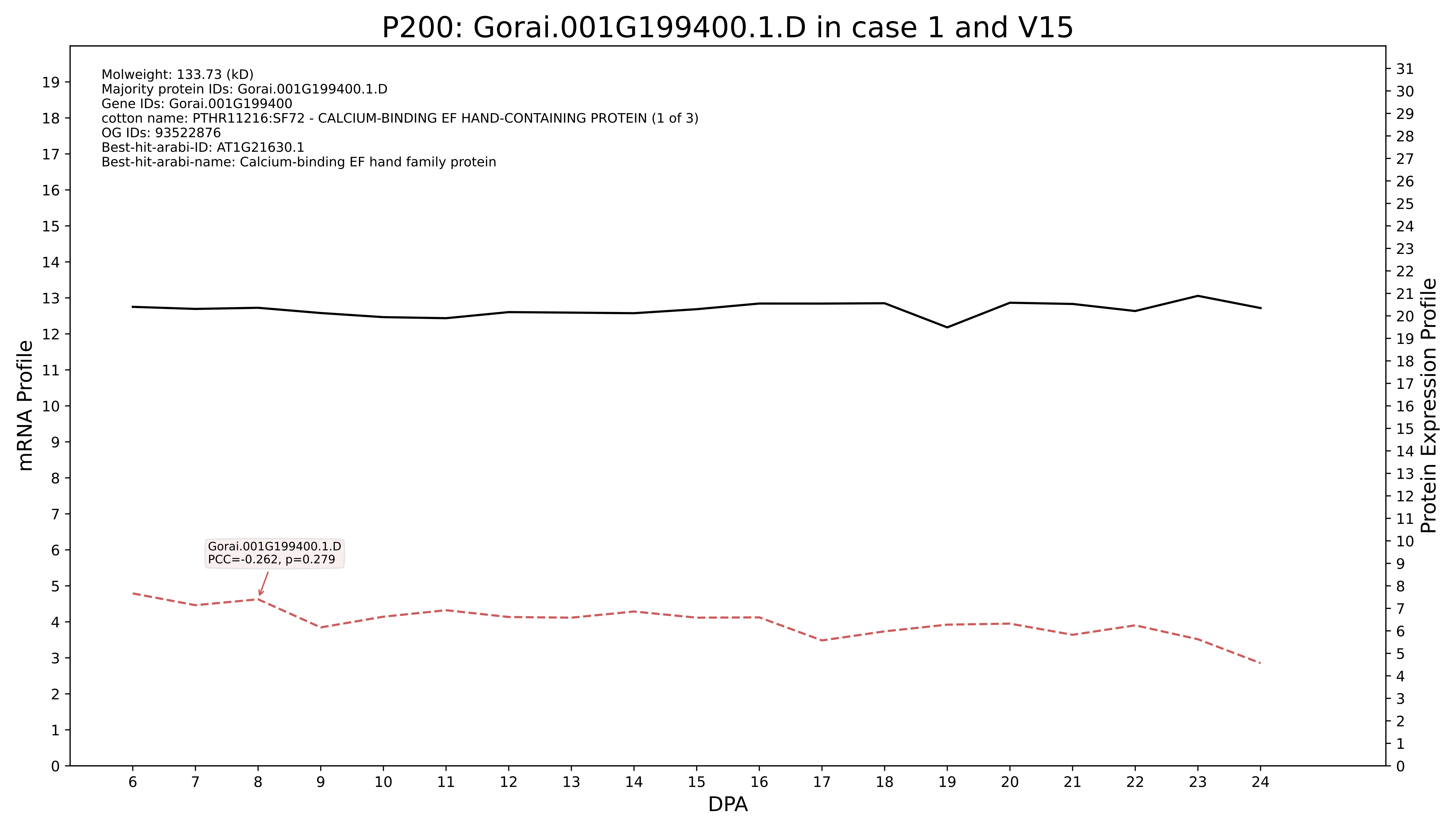
P200: Gorai.001G199400.1.D in case 1 and V15
Molweight: 133.73 (kD)
Majority protein IDs: Gorai.001G199400.1.D
Gene IDs: Gorai.001G199400
cotton name: PTHR11216:SF72 - CALCIUM-BINDING EF HAND-CONTAINING PROTEIN (1 of 3)
OG IDs: 93522876
Best-hit-arabi-ID: AT1G21630.1
Best-hit-arabi-name: Calcium-binding EF hand family protein
Gorai.001G199400.1.D
PCC=-0.262, p=0.279
mRNA Profile
Protein Expression Profile
DPA
0
1
2
3
4
5
6
7
8
9
10
11
12
13
14
15
16
17
18
19
20
21
22
23
24
25
26
27
28
29
30
31

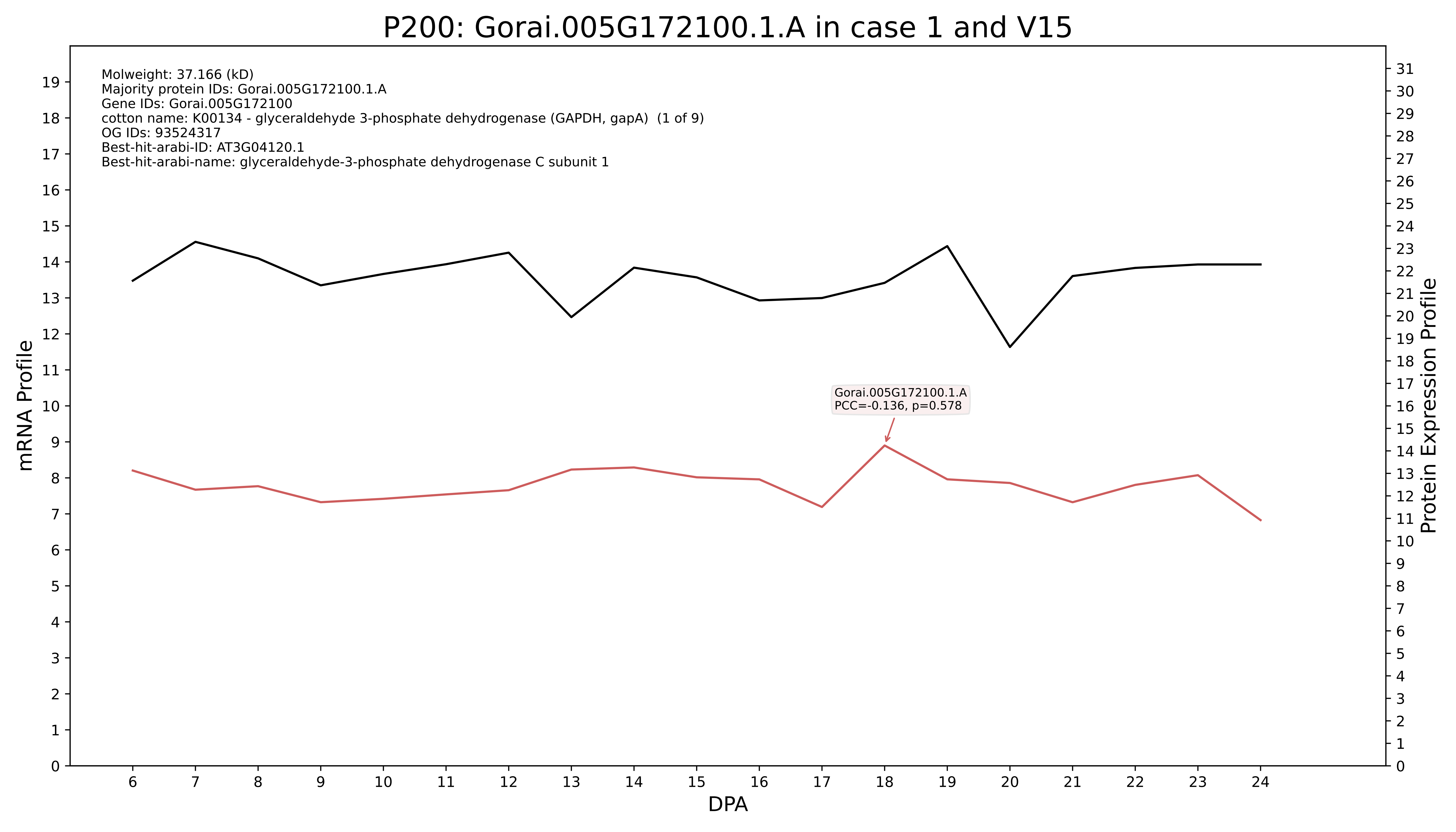
P200: Gorai.005G172100.1.A in case 1 and V15
Molweight: 37.166 (kD)
Majority protein IDs: Gorai.005G172100.1.A
Gene IDs: Gorai.005G172100
cotton name: K00134 - glyceraldehyde 3-phosphate dehydrogenase (GAPDH, gapA) (1 of 9)
OG IDs: 93524317
Best-hit-arabi-ID: AT3G04120.1
Best-hit-arabi-name: glyceraldehyde-3-phosphate dehydrogenase C subunit 1
Gorai.005G172100.1.A
PCC=-0.136, p=0.578
mRNA Profile
Protein Expression Profile
DPA

P200: Gorai.005G216700.1.D in case 1 and V15

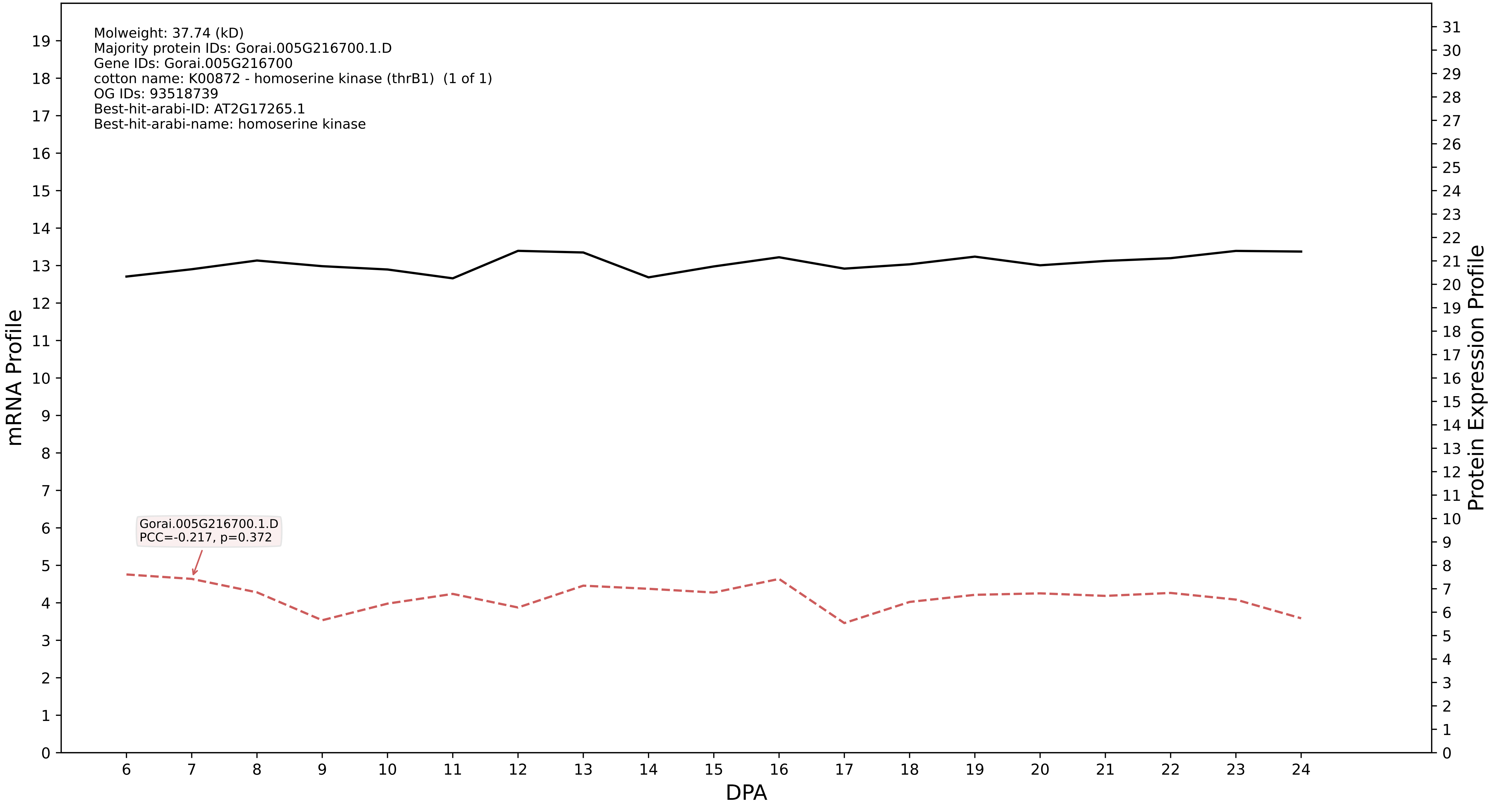

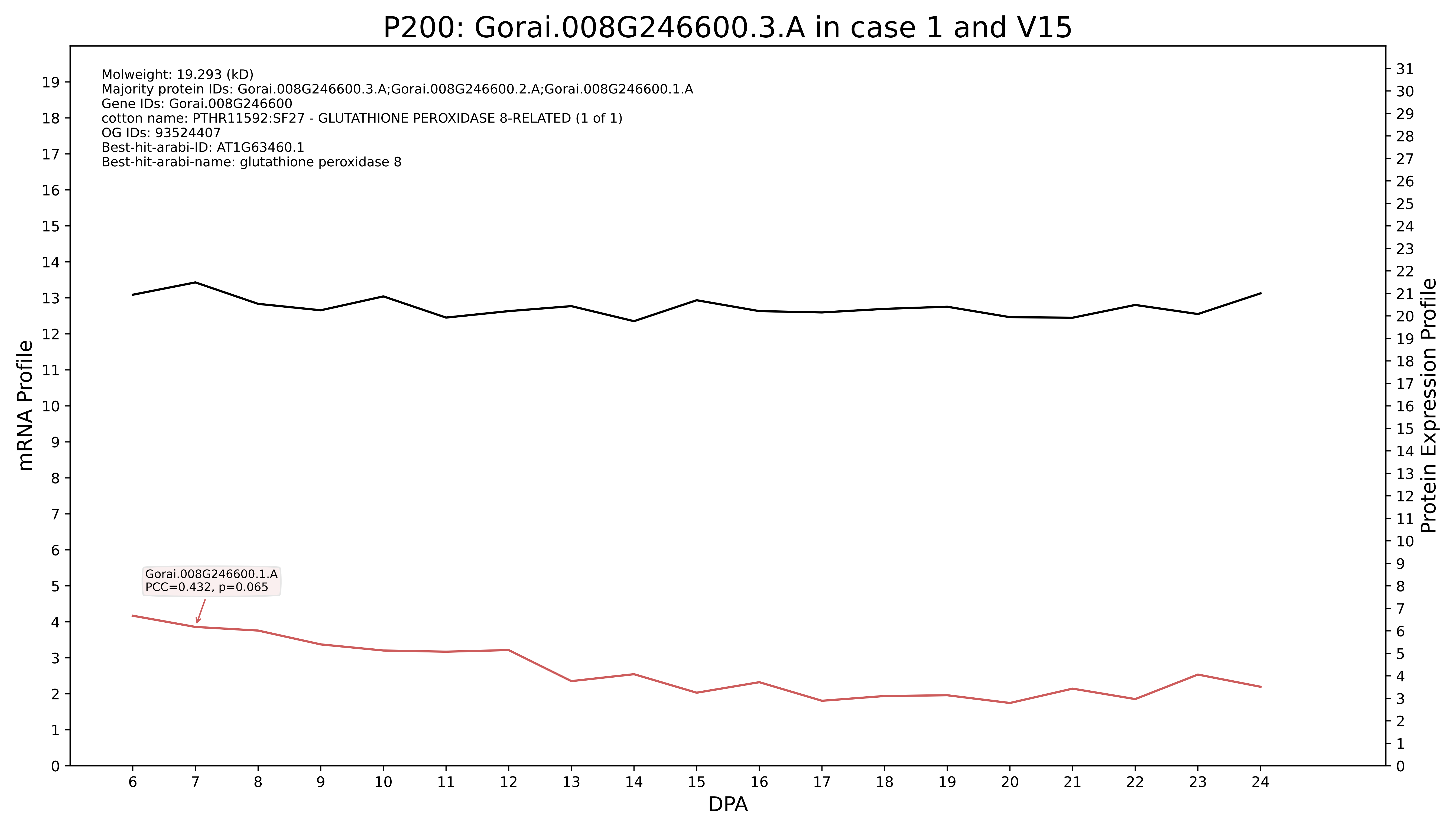

P200: Gorai.008G246600.3.A in case 1 and V15
Molweight: 19.293 (kD)
Majority protein IDs: Gorai.008G246600.3.A;Gorai.008G246600.2.A;Gorai.008G246600.1.A
Gene IDs: Gorai.008G246600
cotton name: PTHR11592:SF27 - GLUTATHIONE PEROXIDASE 8-RELATED (1 of 1)
OG IDs: 93524407
Best-hit-arabi-ID: AT1G63460.1
Best-hit-arabi-name: glutathione peroxidase 8
Gorai.008G246600.1.A
PCC=0.432, p=0.065
mRNA Profile
Protein Expression Profile
DPA

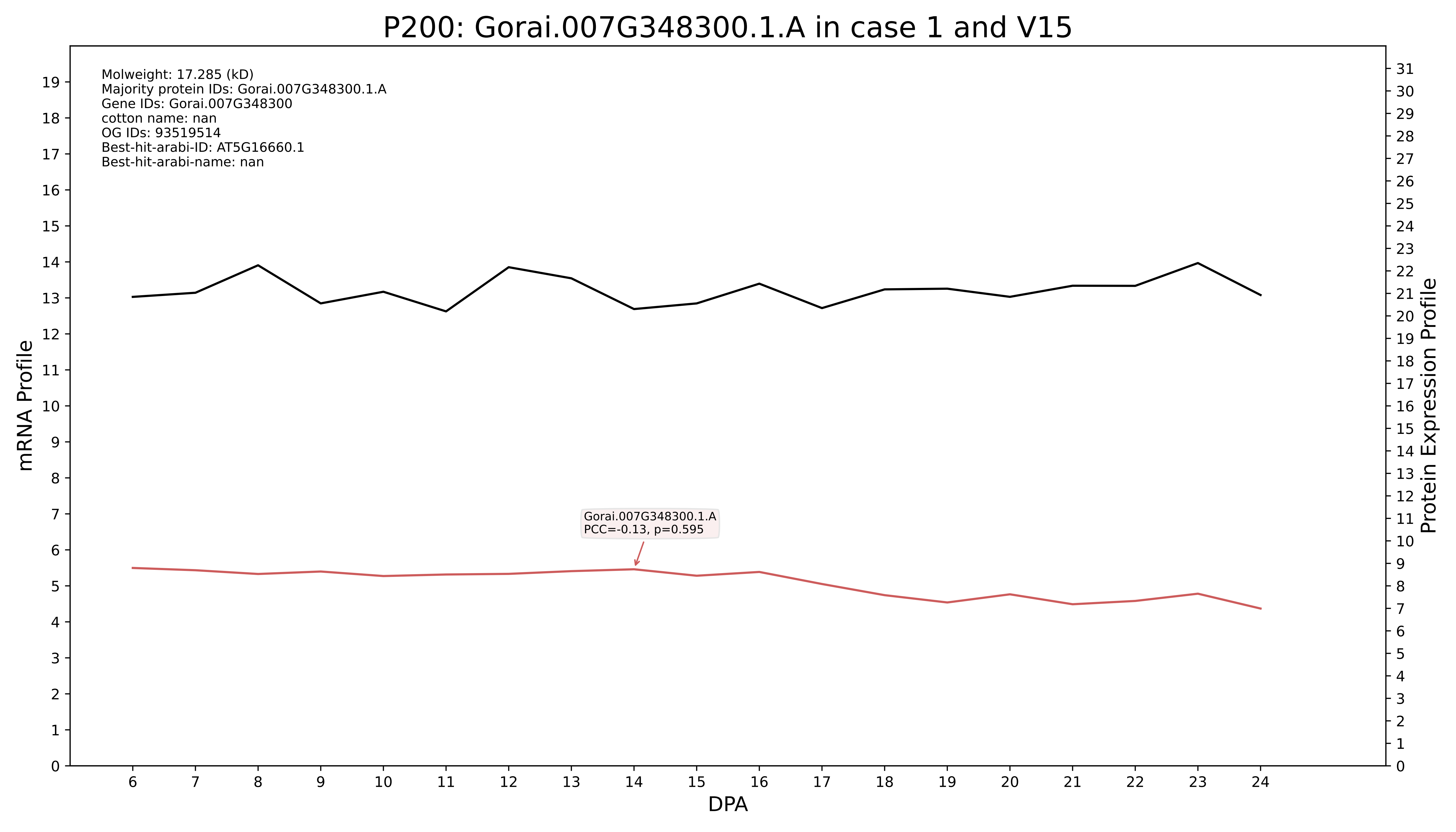
P200: Gorai.007G348300.1.A in case 1 and V15
Molweight: 17.285 (kD)
Majority protein IDs: Gorai.007G348300.1.A
Gene IDs: Gorai.007G348300
cotton name: nan
OG IDs: 93519514
Best-hit-arabi-ID: AT5G16660.1
Best-hit-arabi-name: nan
Gorai.007G348300.1.A
PCC=-0.13, p=0.595
mRNA Profile
Protein Expression Profile
DPA
0
1
2
3
4
5
6
7
8
9
10
11
12
13
14
15
16
17
18
19
20
21
22
23
24
25
26
27
28
29
30
31

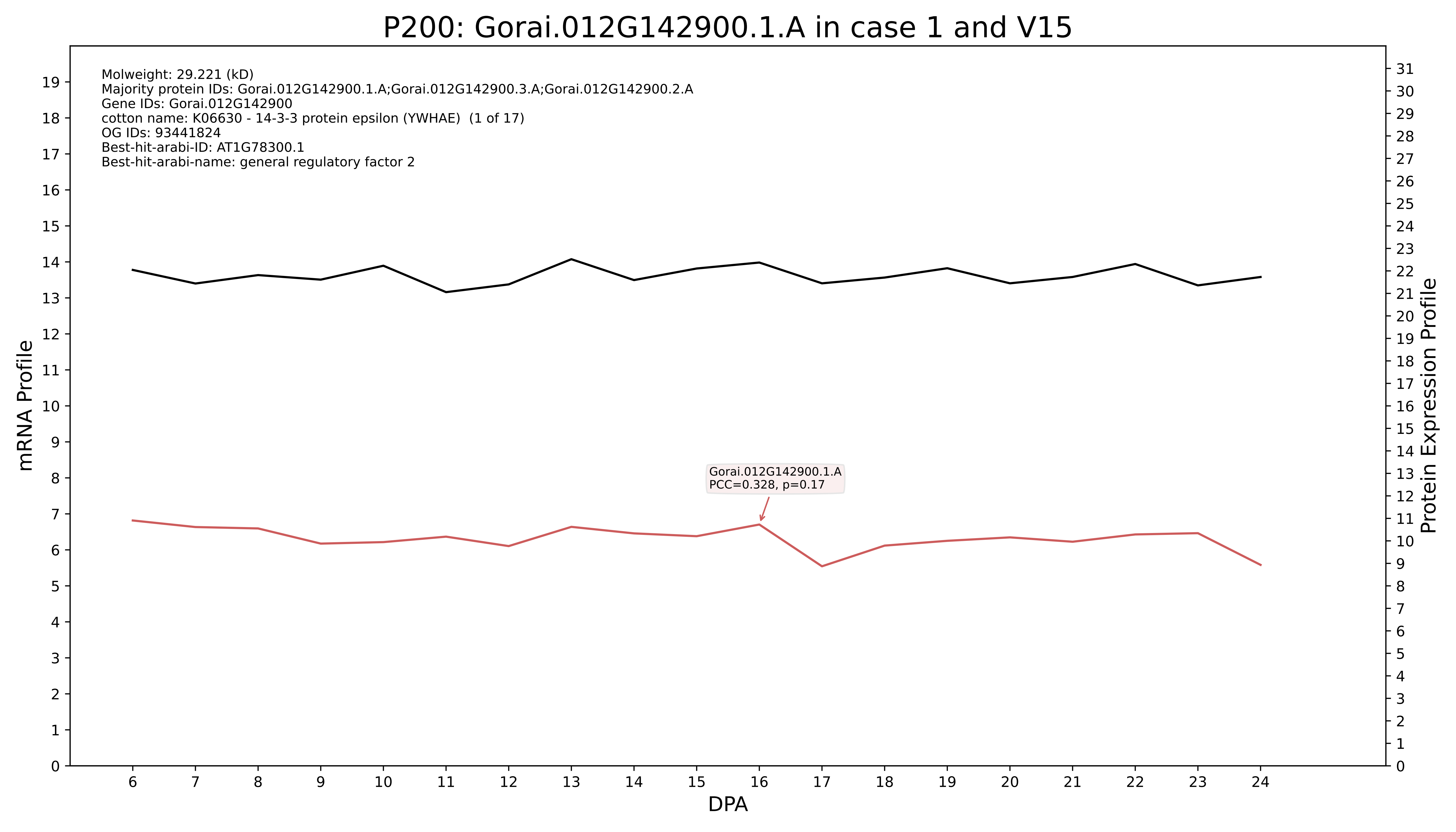

P200: Gorai.012G142900.1.A in case 1 and V15
Molweight: 29.221 (kD)
Majority protein IDs: Gorai.012G142900.1.A;Gorai.012G142900.3.A;Gorai.012G142900.2.A
Gene IDs: Gorai.012G142900
cotton name: K06630 - 14-3-3 protein epsilon (YWHAE) (1 of 17)
OG IDs: 93441824
Best-hit-arabi-ID: AT1G78300.1
Best-hit-arabi-name: general regulatory factor 2
Gorai.012G142900.1.A
PCC=0.328, p=0.17
mRNA Profile
Protein Expression Profile
DPA

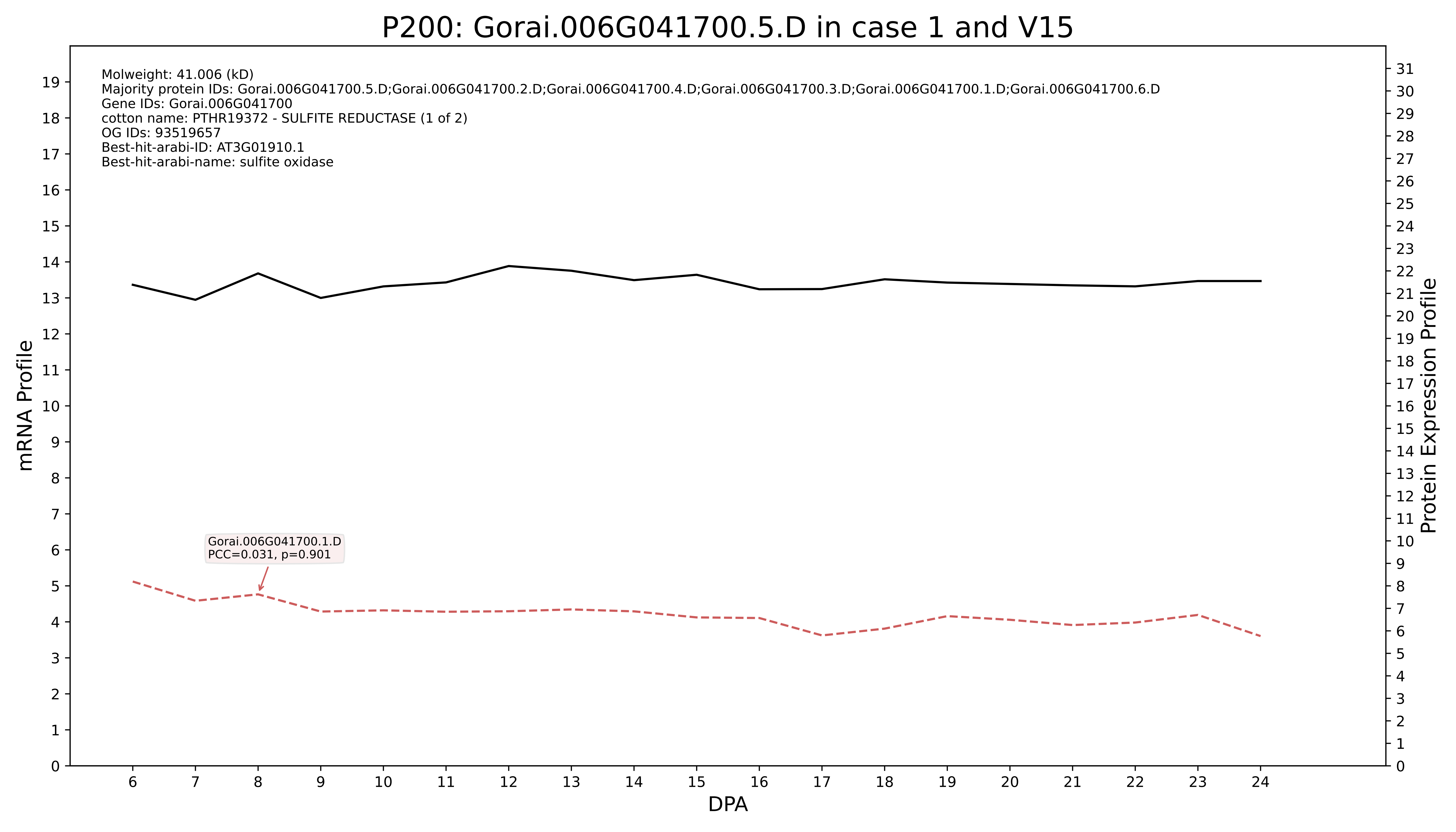
P200: Gorai.006G041700.5.D in case 1 and V15
Molweight: 41.006 (kD)
Majority protein IDs: Gorai.006G041700.5.D;Gorai.006G041700.2.D;Gorai.006G041700.4.D;Gorai.006G041700.3.D;Gorai.006G041700.1.D;Gorai.006G041700.6.D
Gene IDs: Gorai.006G041700
cotton name: PTHR19372 - SULFITE REDUCTASE (1 of 2)
OG IDs: 93519657
Best-hit-arabi-ID: AT3G01910.1
Best-hit-arabi-name: sulfite oxidase
Gorai.006G041700.1.D
PCC=0.031, p=0.901
mRNA Profile
Protein Expression Profile
DPA
0
1
2
3
4
5
6
7
8
9
10
11
12
13
14
15
16
17
18
19
20
21
22
23
24
25
26
27
28
29
30
31

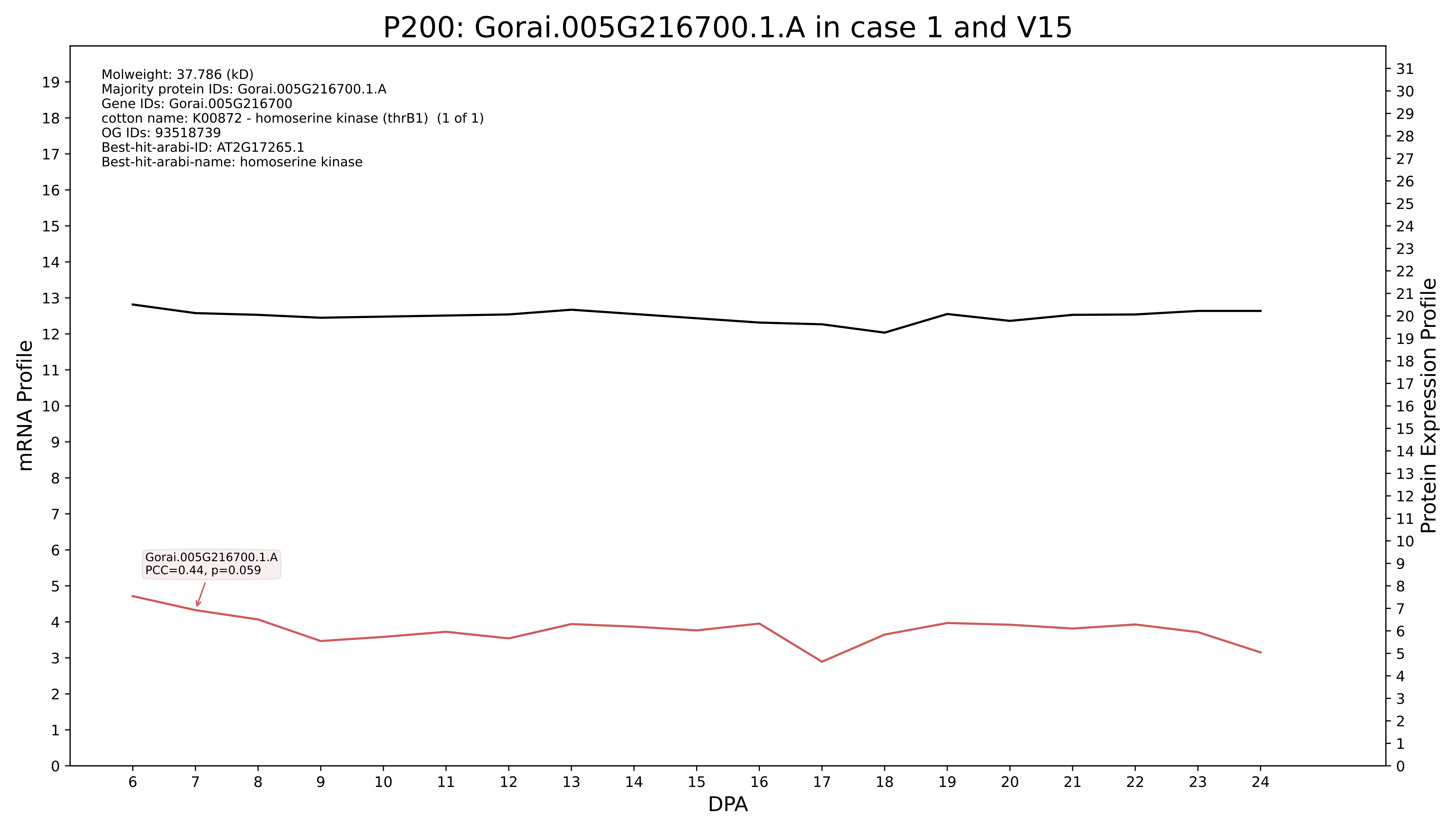

P200: Gorai.005G216700.1.A in case 1 and V15
Molweight: 37.786 (kD)
Majority protein IDs: Gorai.005G216700.1.A
Gene IDs: Gorai.005G216700
cotton name: K00872 - homoserine kinase (thrB1) (1 of 1)
OG IDs: 93518739
Best-hit-arabi-ID: AT2G17265.1
Best-hit-arabi-name: homoserine kinase
Gorai.005G216700.1.A
PCC=0.44, p=0.059
mRNA Profile
Protein Expression Profile
DPA

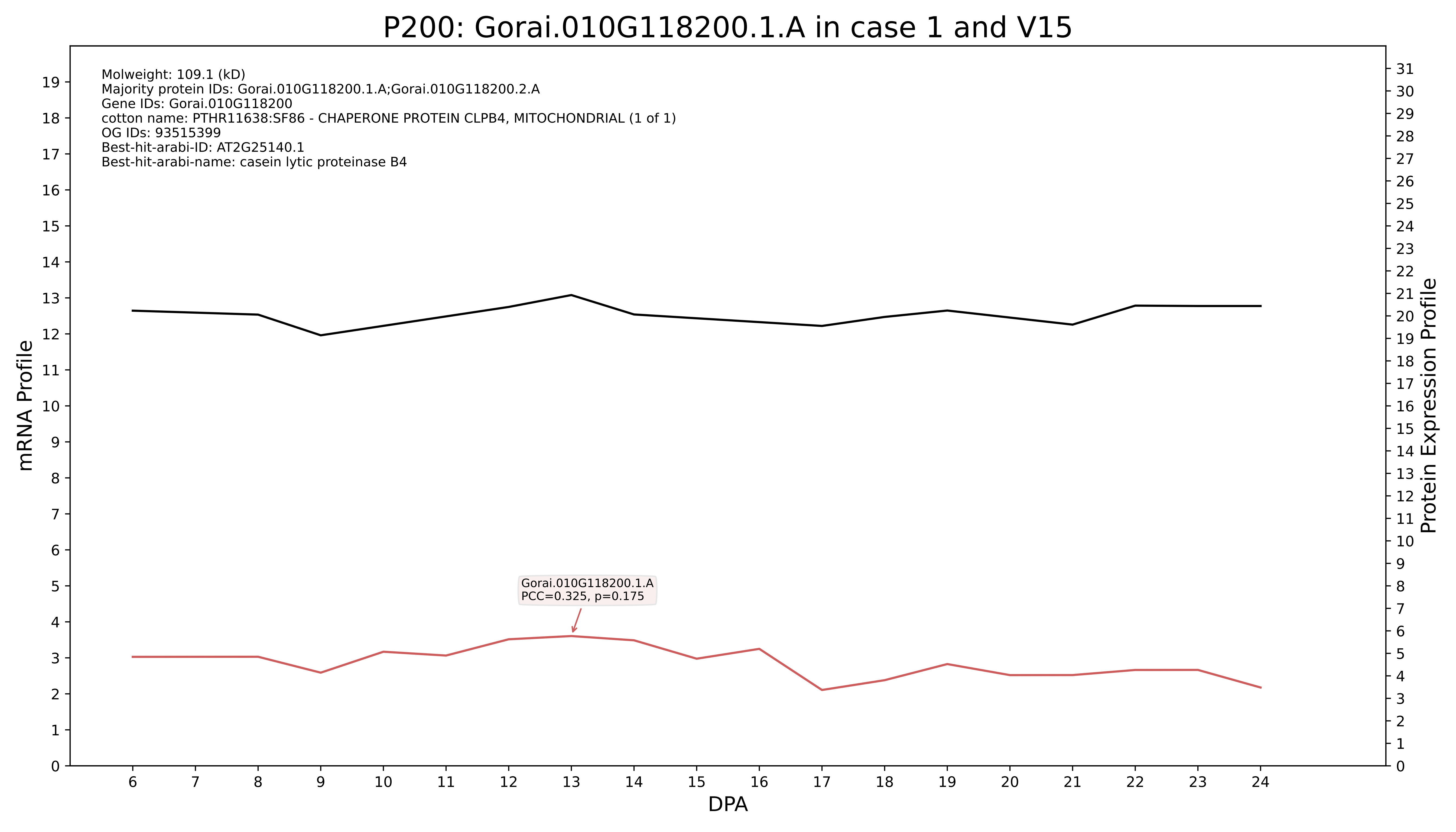
P200: Gorai.010G118200.1.A in case 1 and V15
Molweight: 109.1 (kD)
Majority protein IDs: Gorai.010G118200.1.A;Gorai.010G118200.2.A
Gene IDs: Gorai.010G118200
cotton name: PTHR11638:SF86 - CHAPERONE PROTEIN CLPB4, MITOCHONDRIAL (1 of 1)
OG IDs: 93515399
Best-hit-arabi-ID: AT2G25140.1
Best-hit-arabi-name: casein lytic proteinase B4
Gorai.010G118200.1.A
PCC=0.325, p=0.175
mRNA Profile
Protein Expression Profile
DPA

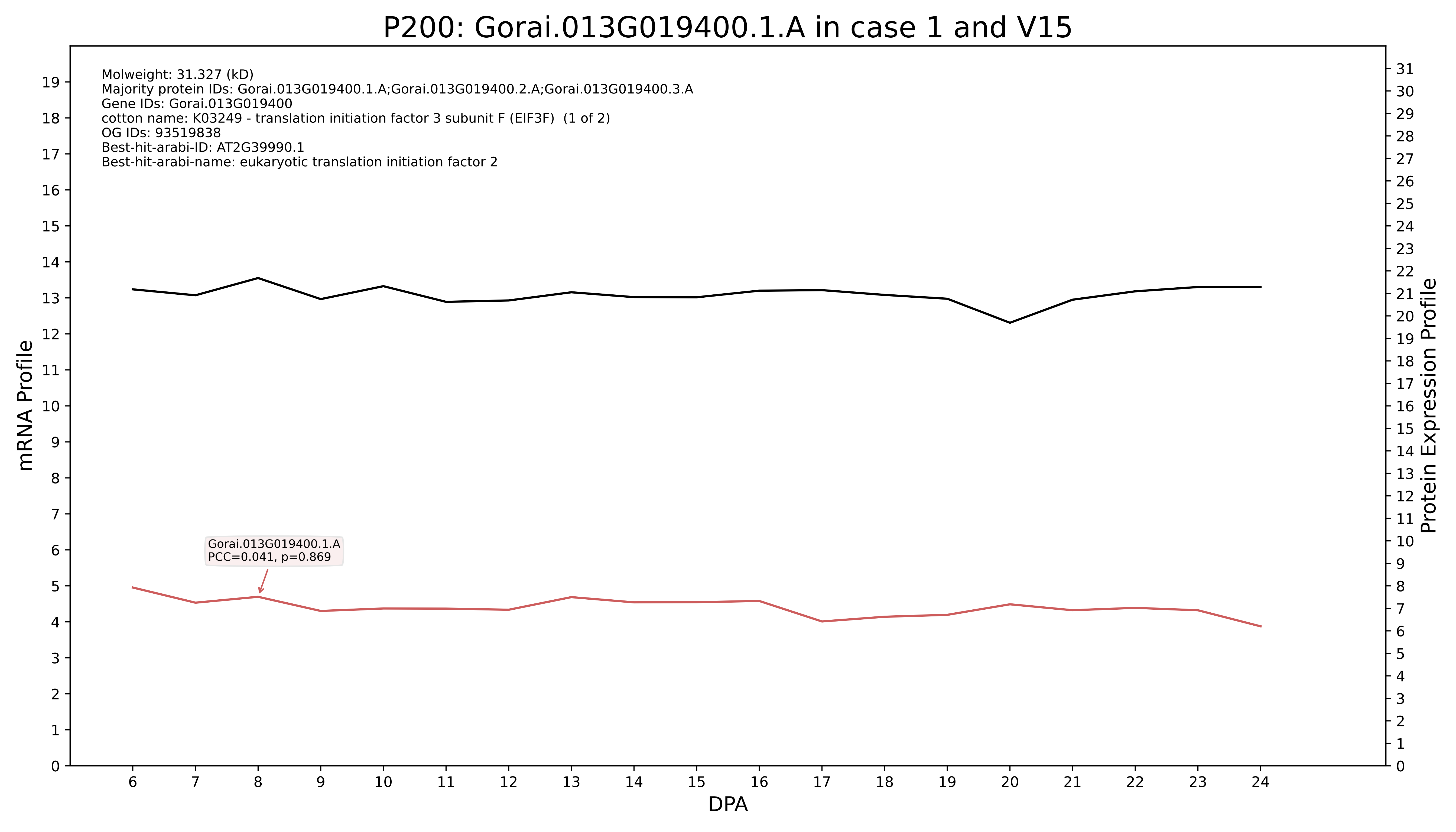
P200: Gorai.013G019400.1.A in case 1 and V15
Molweight: 31.327 (kD)
Majority protein IDs: Gorai.013G019400.1.A;Gorai.013G019400.2.A;Gorai.013G019400.3.A
Gene IDs: Gorai.013G019400
cotton name: K03249 - translation initiation factor 3 subunit F (EIF3F) (1 of 2)
OG IDs: 93519838
Best-hit-arabi-ID: AT2G39990.1
Best-hit-arabi-name: eukaryotic translation initiation factor 2
Gorai.013G019400.1.A
PCC=0.041, p=0.869
mRNA Profile
Protein Expression Profile
DPA

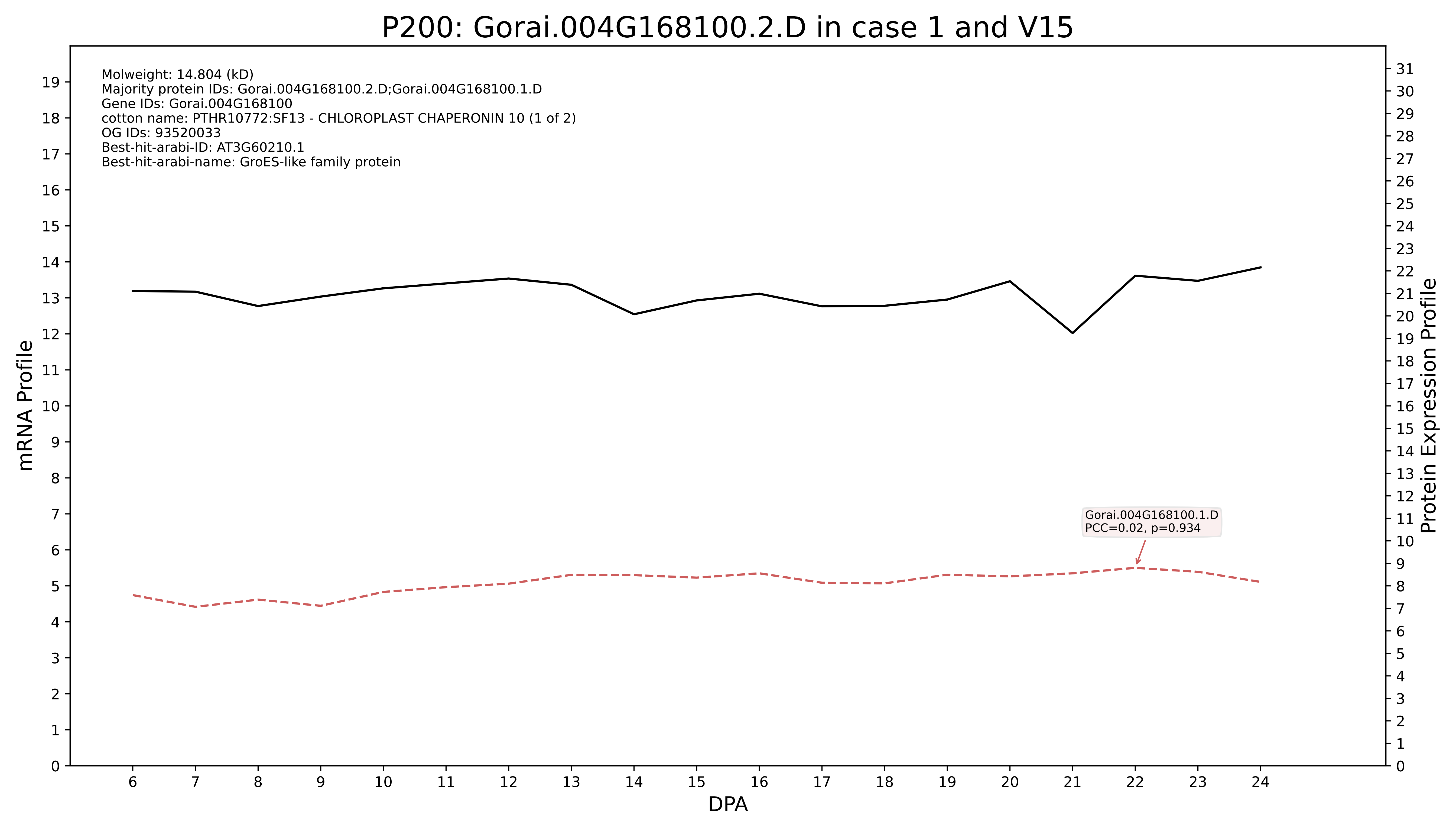
P200: Gorai.004G168100.2.D in case 1 and V15
Molweight: 14.804 (kD)
Majority protein IDs: Gorai.004G168100.2.D;Gorai.004G168100.1.D
Gene IDs: Gorai.004G168100
cotton name: PTHR10772:SF13 - CHLOROPLAST CHAPERONIN 10 (1 of 2)
OG IDs: 93520033
Best-hit-arabi-ID: AT3G60210.1
Best-hit-arabi-name: GroES-like family protein
Gorai.004G168100.1.D
PCC=0.02, p=0.934
mRNA Profile
Protein Expression Profile
DPA

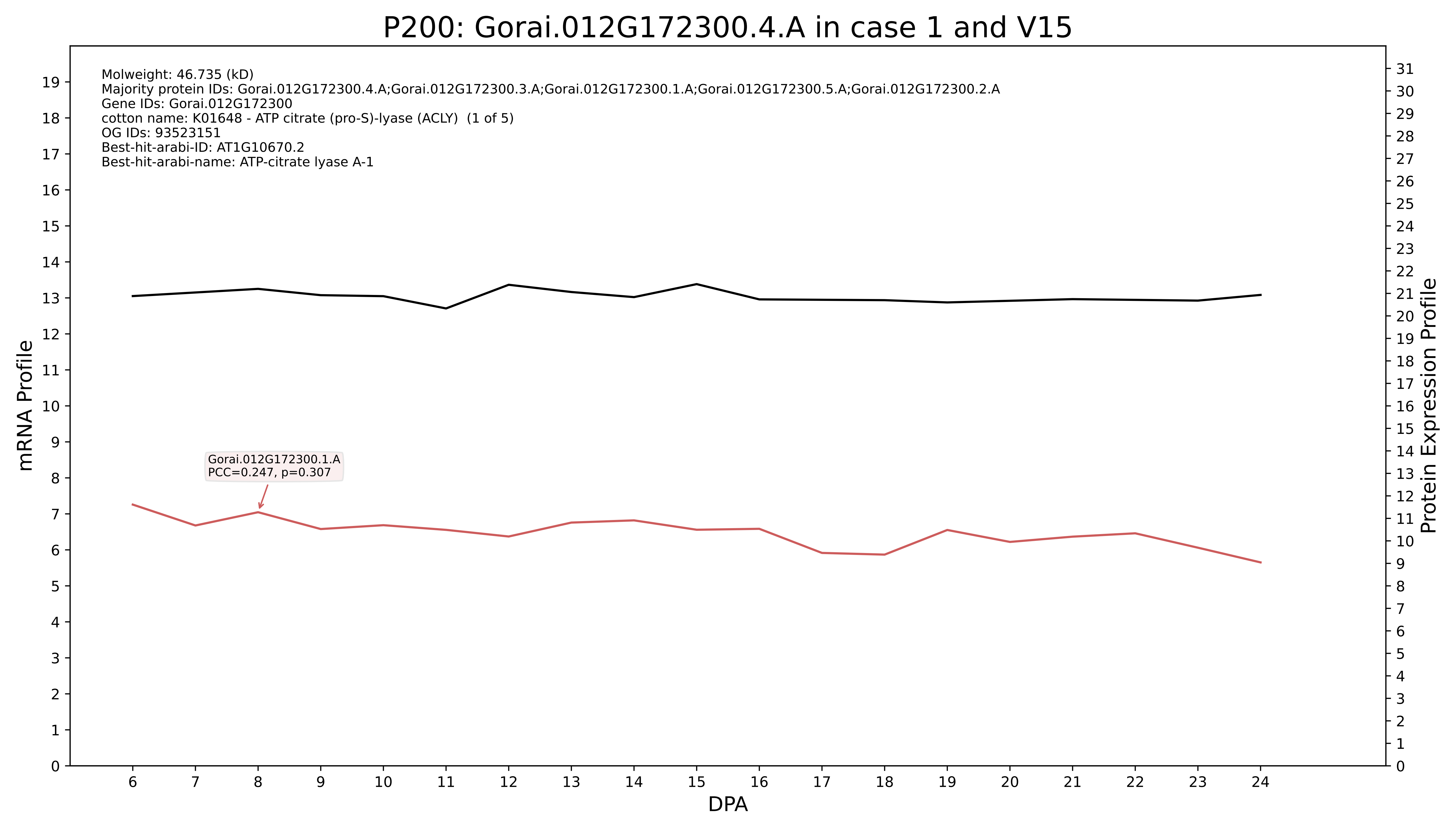
P200: Gorai.012G172300.4.A in case 1 and V15
Molweight: 46.735 (kD)
Majority protein IDs: Gorai.012G172300.4.A;Gorai.012G172300.3.A;Gorai.012G172300.1.A;Gorai.012G172300.5.A;Gorai.012G172300.2.A
Gene IDs: Gorai.012G172300
cotton name: K01648 - ATP citrate (pro-S)-lyase (ACLY) (1 of 5)
OG IDs: 93523151
Best-hit-arabi-ID: AT1G10670.2
Best-hit-arabi-name: ATP-citrate lyase A-1
Gorai.012G172300.1.A
PCC=0.247, p=0.307
mRNA Profile
Protein Expression Profile
DPA

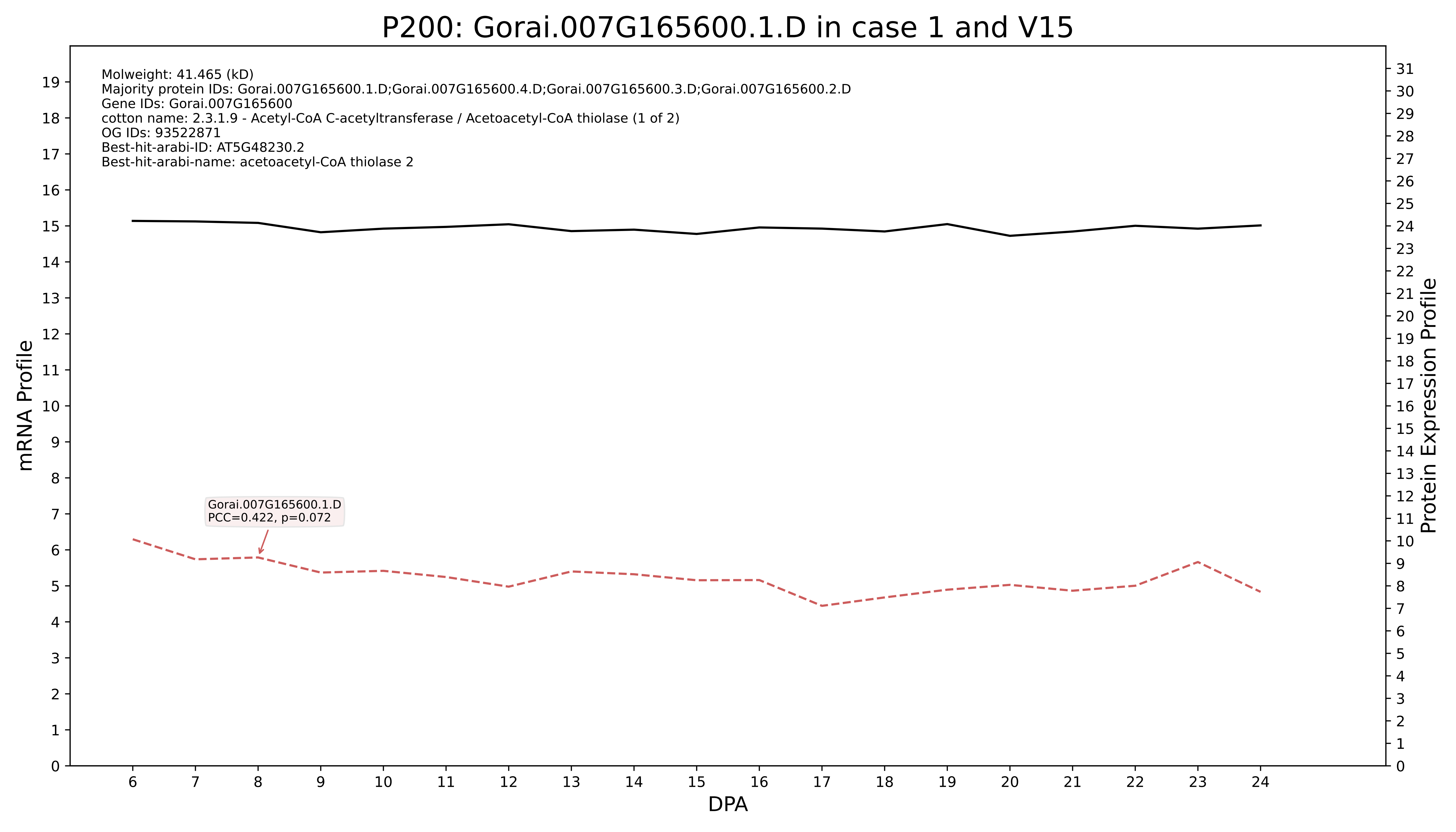

P200: Gorai.007G165600.1.D in case 1 and V15
Molweight: 41.465 (kD)
Majority protein IDs: Gorai.007G165600.1.D;Gorai.007G165600.4.D;Gorai.007G165600.3.D;Gorai.007G165600.2.D
Gene IDs: Gorai.007G165600
cotton name: 2.3.1.9 - Acetyl-CoA C-acetyltransferase / Acetoacetyl-CoA thiolase (1 of 2)
OG IDs: 93522871
Best-hit-arabi-ID: AT5G48230.2
Best-hit-arabi-name: acetoacetyl-CoA thiolase 2
Gorai.007G165600.1.D
PCC=0.422, p=0.072
mRNA Profile
Protein Expression Profile
DPA

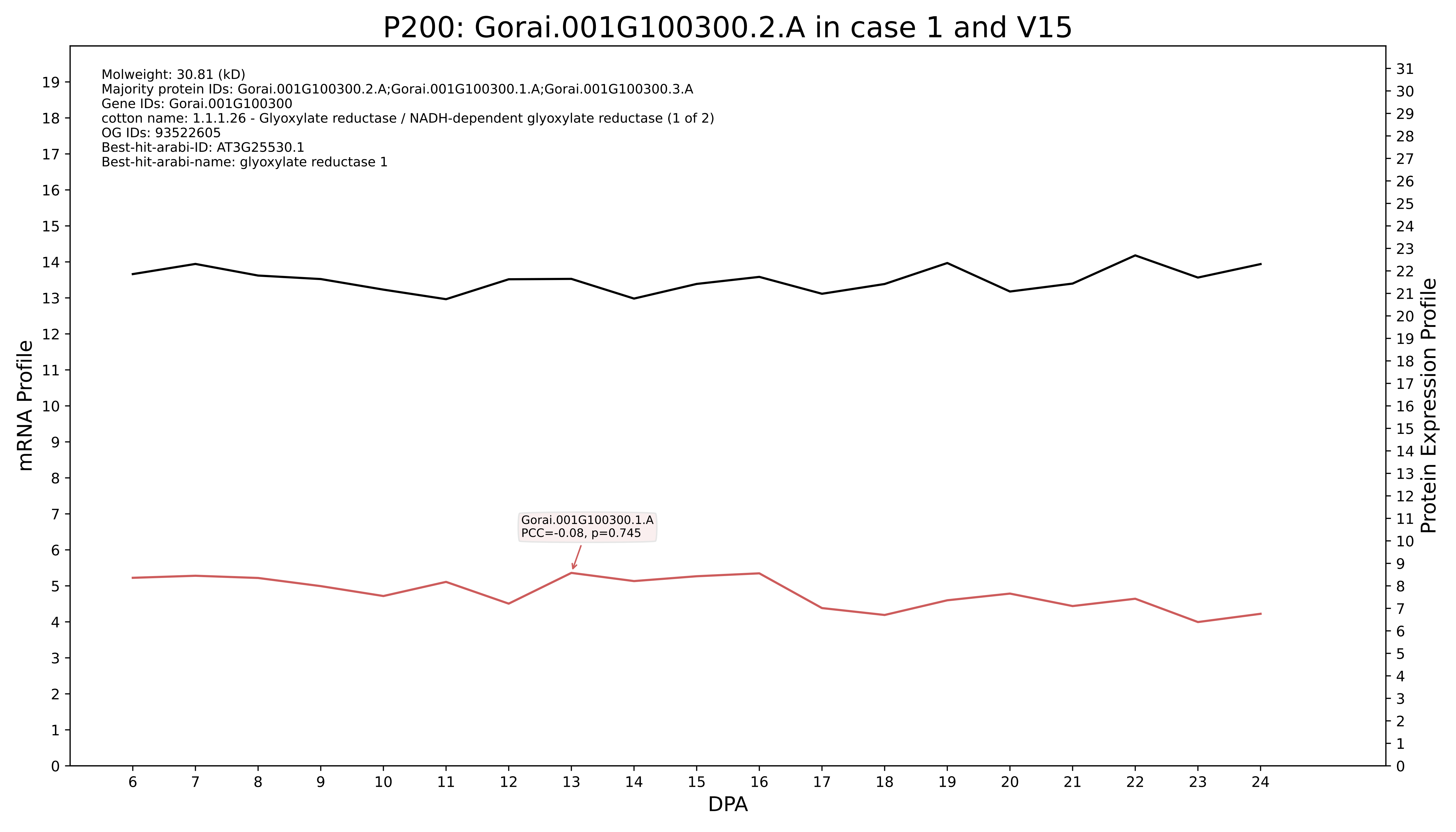

P200: Gorai.001G100300.2.A in case 1 and V15
Molweight: 30.81 (kD)
Majority protein IDs: Gorai.001G100300.2.A;Gorai.001G100300.1.A;Gorai.001G100300.3.A
Gene IDs: Gorai.001G100300
cotton name: 1.1.1.26 - Glyoxylate reductase / NADH-dependent glyoxylate reductase (1 of 2)
OG IDs: 93522605
Best-hit-arabi-ID: AT3G25530.1
Best-hit-arabi-name: glyoxylate reductase 1
Gorai.001G100300.1.A
PCC=-0.08, p=0.745
mRNA Profile
Protein Expression Profile
DPA

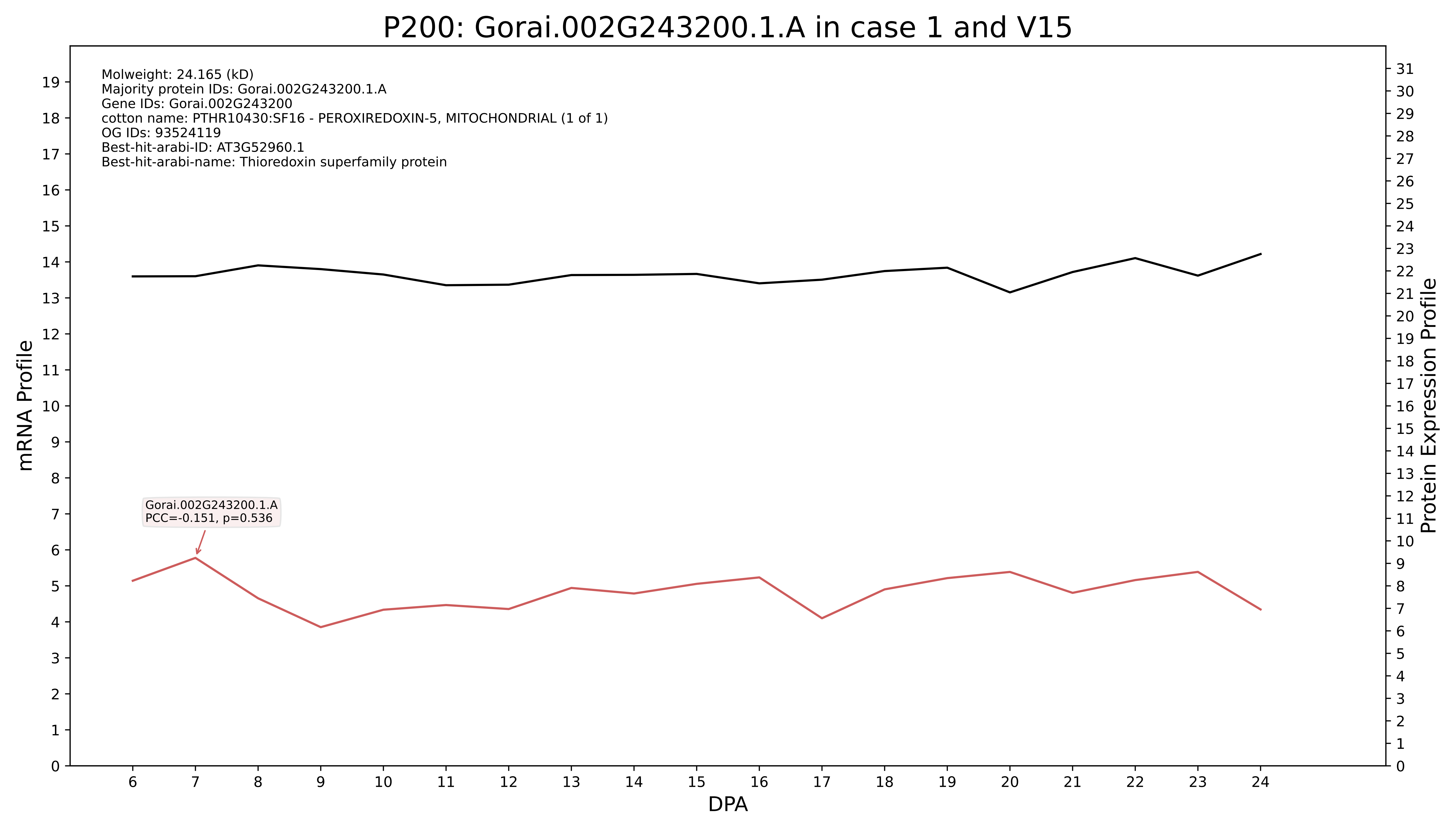
P200: Gorai.002G243200.1.A in case 1 and V15
Molweight: 24.165 (kD)
Majority protein IDs: Gorai.002G243200.1.A
Gene IDs: Gorai.002G243200
cotton name: PTHR10430:SF16 - PEROXIREDOXIN-5, MITOCHONDRIAL (1 of 1)
OG IDs: 93524119
Best-hit-arabi-ID: AT3G52960.1
Best-hit-arabi-name: Thioredoxin superfamily protein
Gorai.002G243200.1.A
PCC=-0.151, p=0.536
mRNA Profile
Protein Expression Profile
DPA

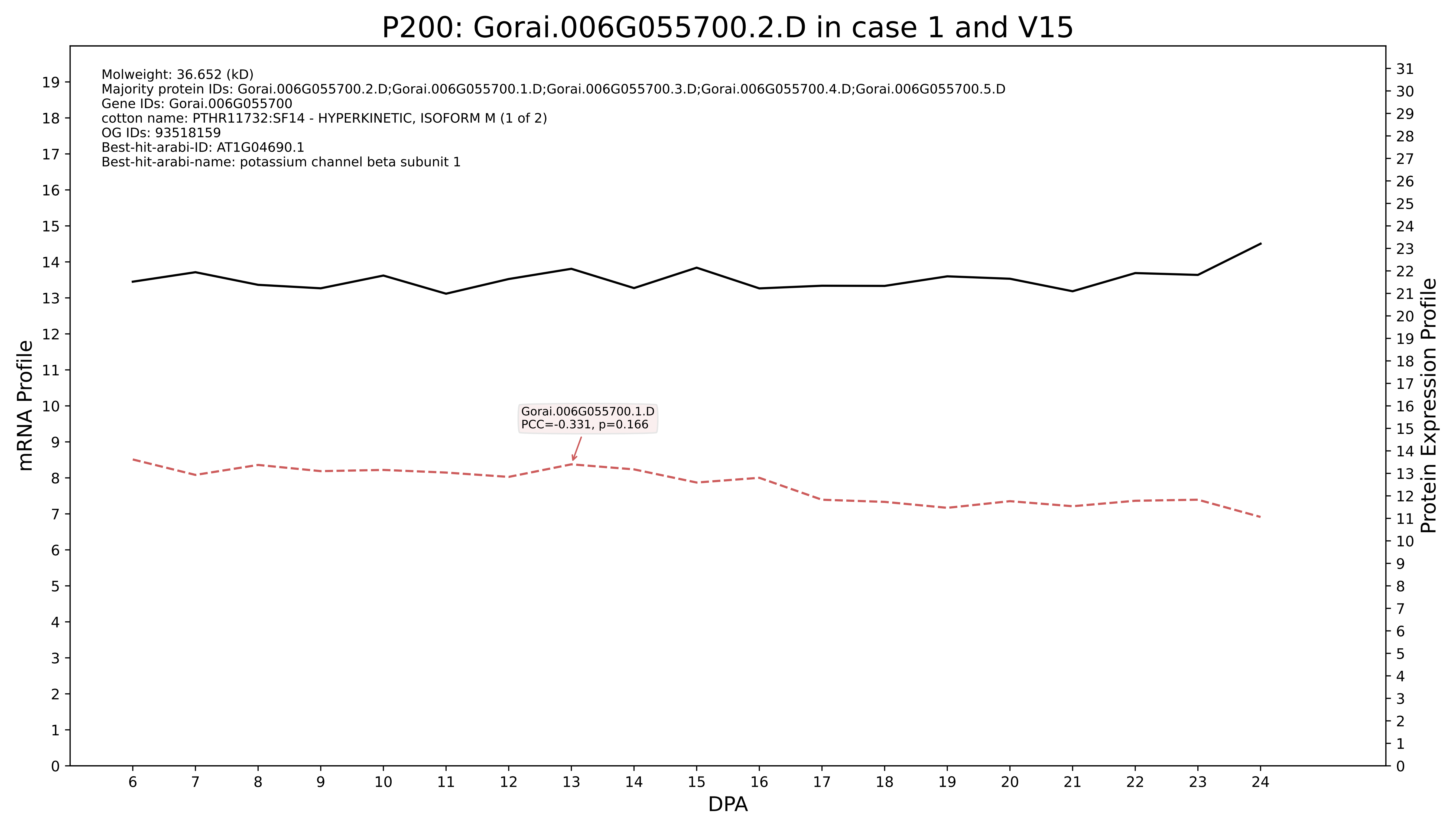
P200: Gorai.006G055700.2.D in case 1 and V15
Molweight: 36.652 (kD)
Majority protein IDs: Gorai.006G055700.2.D;Gorai.006G055700.1.D;Gorai.006G055700.3.D;Gorai.006G055700.4.D;Gorai.006G055700.5.D
Gene IDs: Gorai.006G055700
cotton name: PTHR11732:SF14 - HYPERKINETIC, ISOFORM M (1 of 2)
OG IDs: 93518159
Best-hit-arabi-ID: AT1G04690.1
Best-hit-arabi-name: potassium channel beta subunit 1
Gorai.006G055700.1.D
PCC=-0.331, p=0.166
mRNA Profile
Protein Expression Profile
DPA

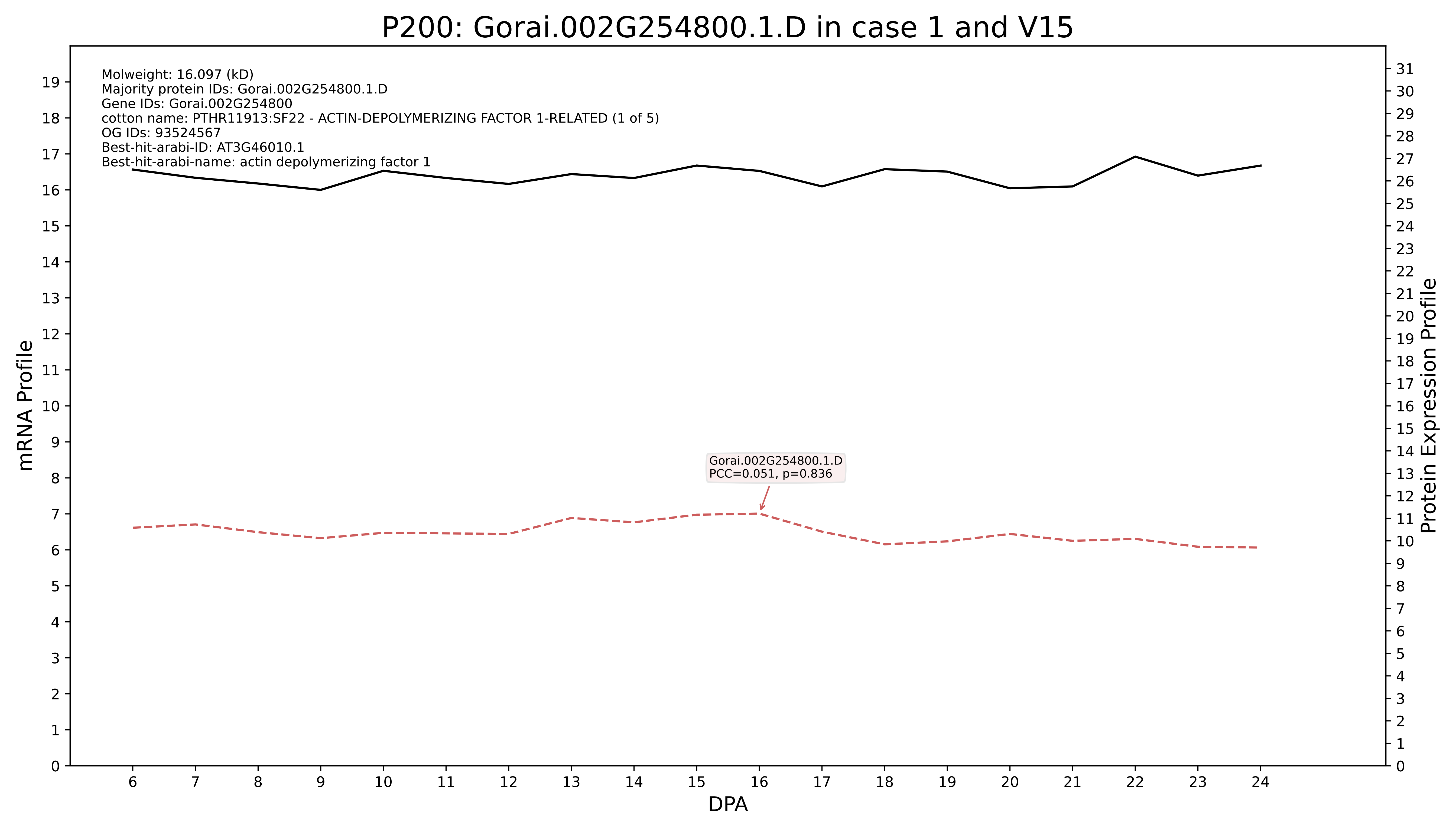
P200: Gorai.002G254800.1.D in case 1 and V15
Molweight: 16.097 (kD)
Majority protein IDs: Gorai.002G254800.1.D
Gene IDs: Gorai.002G254800
cotton name: PTHR11913:SF22 - ACTIN-DEPOLYMERIZING FACTOR 1-RELATED (1 of 5)
OG IDs: 93524567
Best-hit-arabi-ID: AT3G46010.1
Best-hit-arabi-name: actin depolymerizing factor 1
Gorai.002G254800.1.D
PCC=0.051, p=0.836
mRNA Profile
Protein Expression Profile
DPA

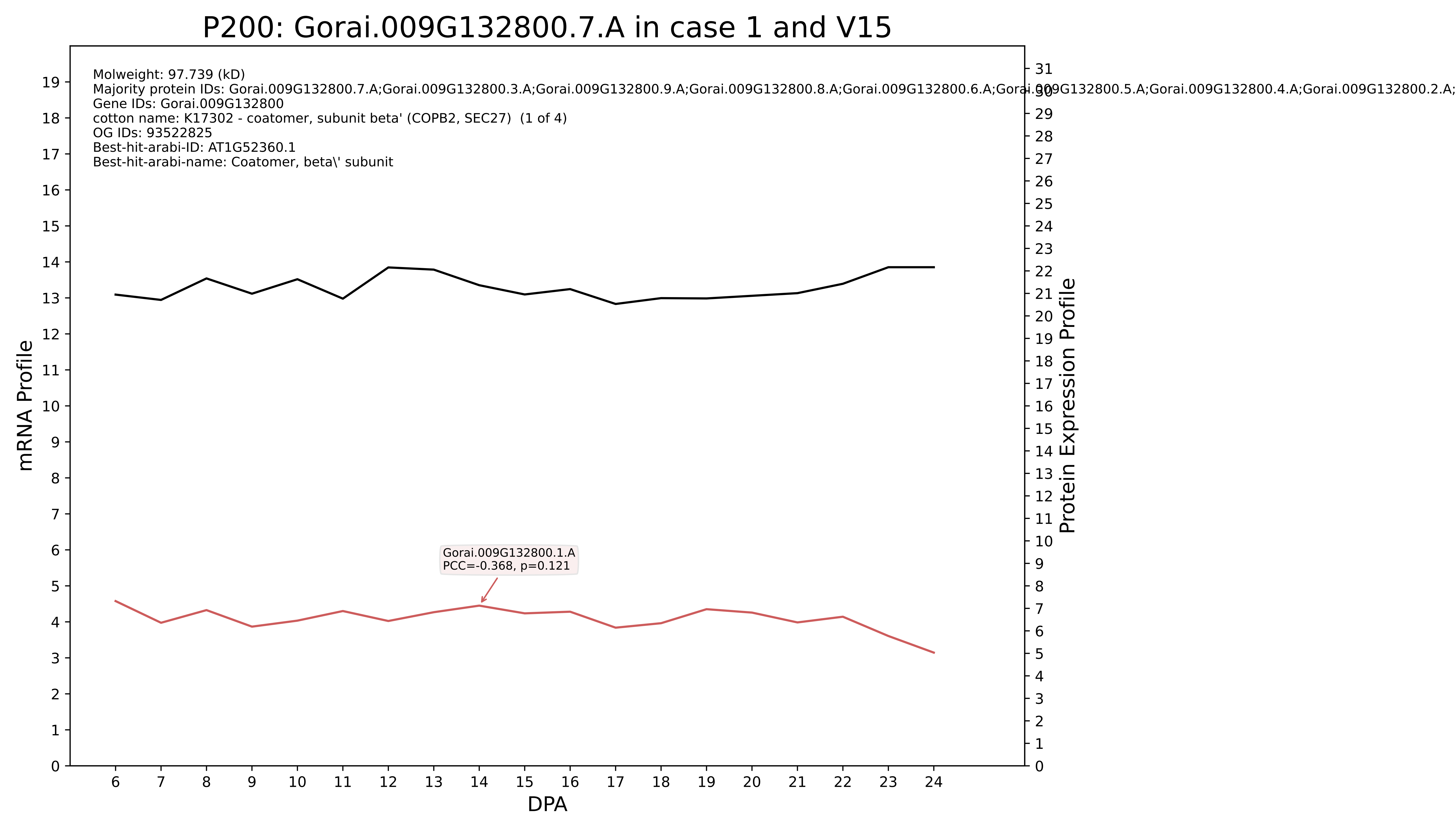

P200: Gorai.009G132800.7.A in case 1 and V15
Molweight: 97.739 (kD)
Majority protein IDs: Gorai.009G132800.7.A;Gorai.009G132800.3.A;Gorai.009G132800.9.A;Gorai.009G132800.8.A;Gorai.009G132800.6.A;Gorai.009G132800.5.A;Gorai.009G132800.4.A;Gorai.009G132800.2.A;
Gene IDs: Gorai.009G132800
cotton name: K17302 - coatomer, subunit beta' (COPB2, SEC27) (1 of 4)
OG IDs: 93522825
Best-hit-arabi-ID: AT1G52360.1
Best-hit-arabi-name: Coatomer, beta\' subunit
Gorai.009G132800.1.A
PCC=-0.368, p=0.121
mRNA Profile
Protein Expression Profile
DPA

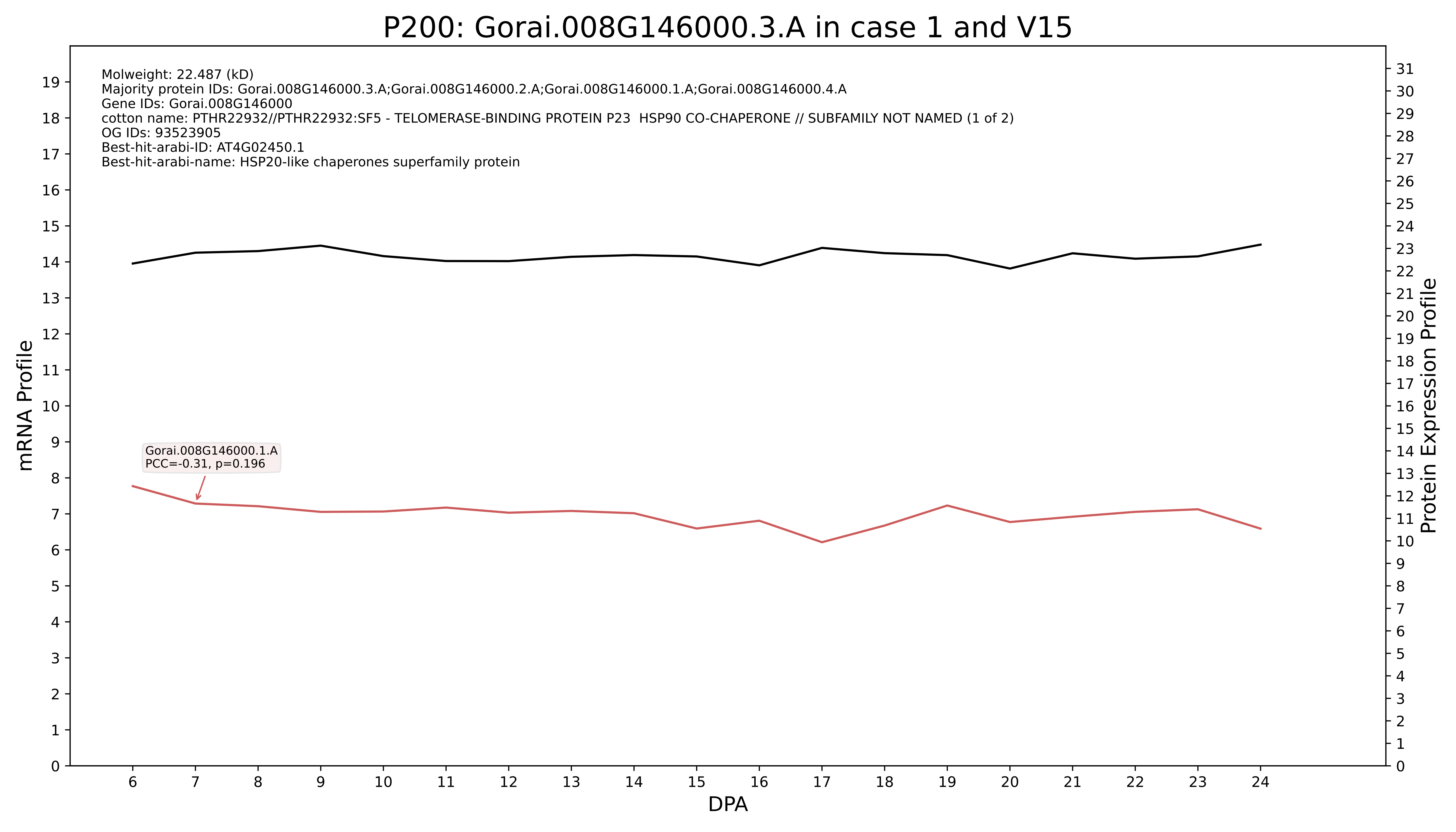
P200: Gorai.008G146000.3.A in case 1 and V15
Molweight: 22.487 (kD)
Majority protein IDs: Gorai.008G146000.3.A;Gorai.008G146000.2.A;Gorai.008G146000.1.A;Gorai.008G146000.4.A
Gene IDs: Gorai.008G146000
cotton name: PTHR22932//PTHR22932:SF5 - TELOMERASE-BINDING PROTEIN P23 HSP90 CO-CHAPERONE // SUBFAMILY NOT NAMED (1 of 2)
OG IDs: 93523905
Best-hit-arabi-ID: AT4G02450.1
Best-hit-arabi-name: HSP20-like chaperones superfamily protein
Gorai.008G146000.1.A
PCC=-0.31, p=0.196
mRNA Profile
Protein Expression Profile
DPA

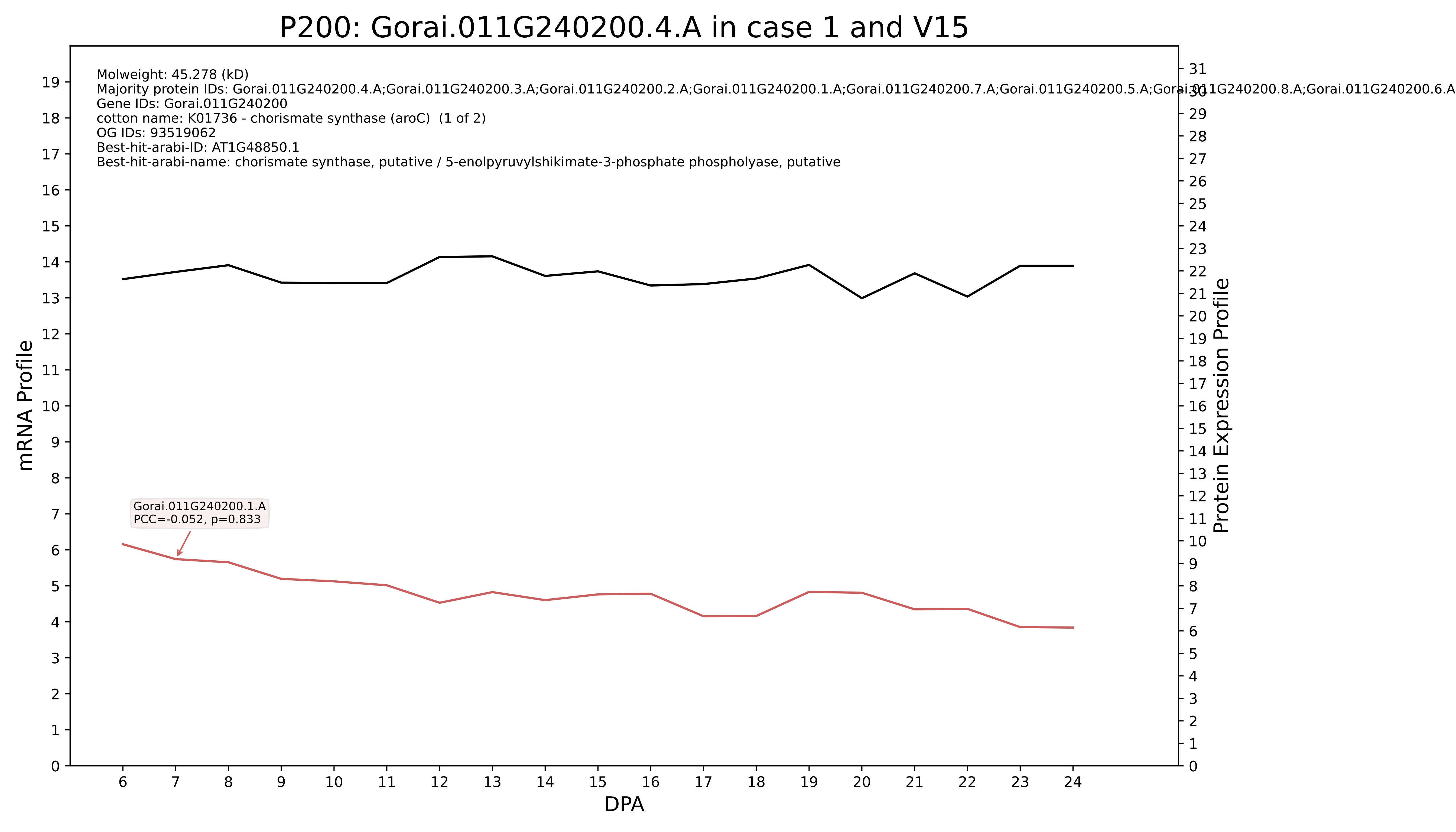

P200: Gorai.011G240200.4.A in case 1 and V15
Molweight: 45.278 (kD)
Majority protein IDs: Gorai.011G240200.4.A;Gorai.011G240200.3.A;Gorai.011G240200.2.A;Gorai.011G240200.1.A;Gorai.011G240200.7.A;Gorai.011G240200.5.A;Gorai.011G240200.8.A;Gorai.011G240200.6.A
Gene IDs: Gorai.011G240200
cotton name: K01736 - chorismate synthase (aroC) (1 of 2)
OG IDs: 93519062
Best-hit-arabi-ID: AT1G48850.1
Best-hit-arabi-name: chorismate synthase, putative / 5-enolpyruvylshikimate-3-phosphate phospholyase, putative
Gorai.011G240200.1.A
PCC=-0.052, p=0.833
mRNA Profile
Protein Expression Profile
DPA

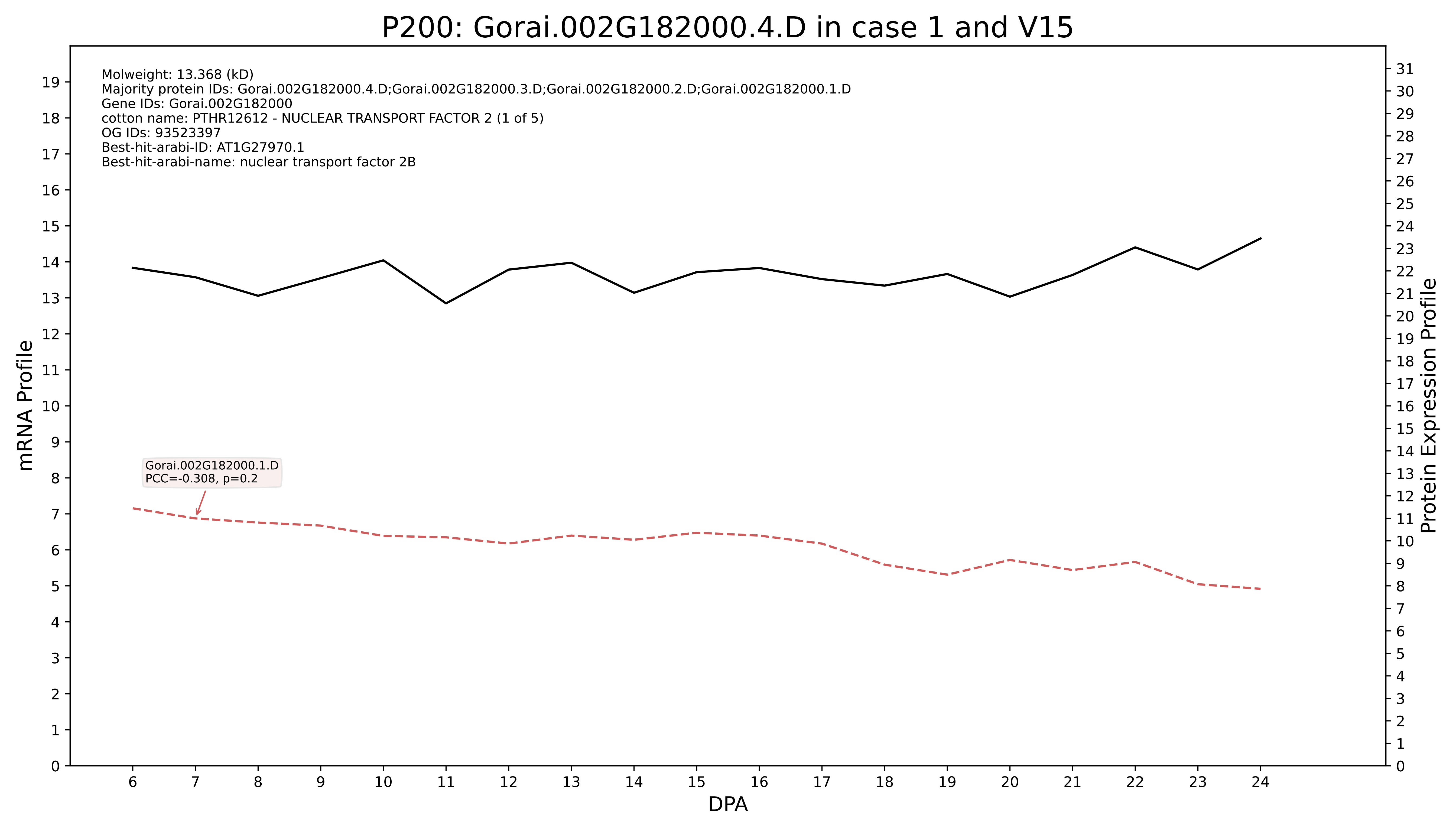
P200: Gorai.002G182000.4.D in case 1 and V15
Molweight: 13.368 (kD)
Majority protein IDs: Gorai.002G182000.4.D;Gorai.002G182000.3.D;Gorai.002G182000.2.D;Gorai.002G182000.1.D
Gene IDs: Gorai.002G182000
cotton name: PTHR12612 - NUCLEAR TRANSPORT FACTOR 2 (1 of 5)
OG IDs: 93523397
Best-hit-arabi-ID: AT1G27970.1
Best-hit-arabi-name: nuclear transport factor 2B
Gorai.002G182000.1.D
PCC=-0.308, p=0.2
mRNA Profile
Protein Expression Profile
DPA

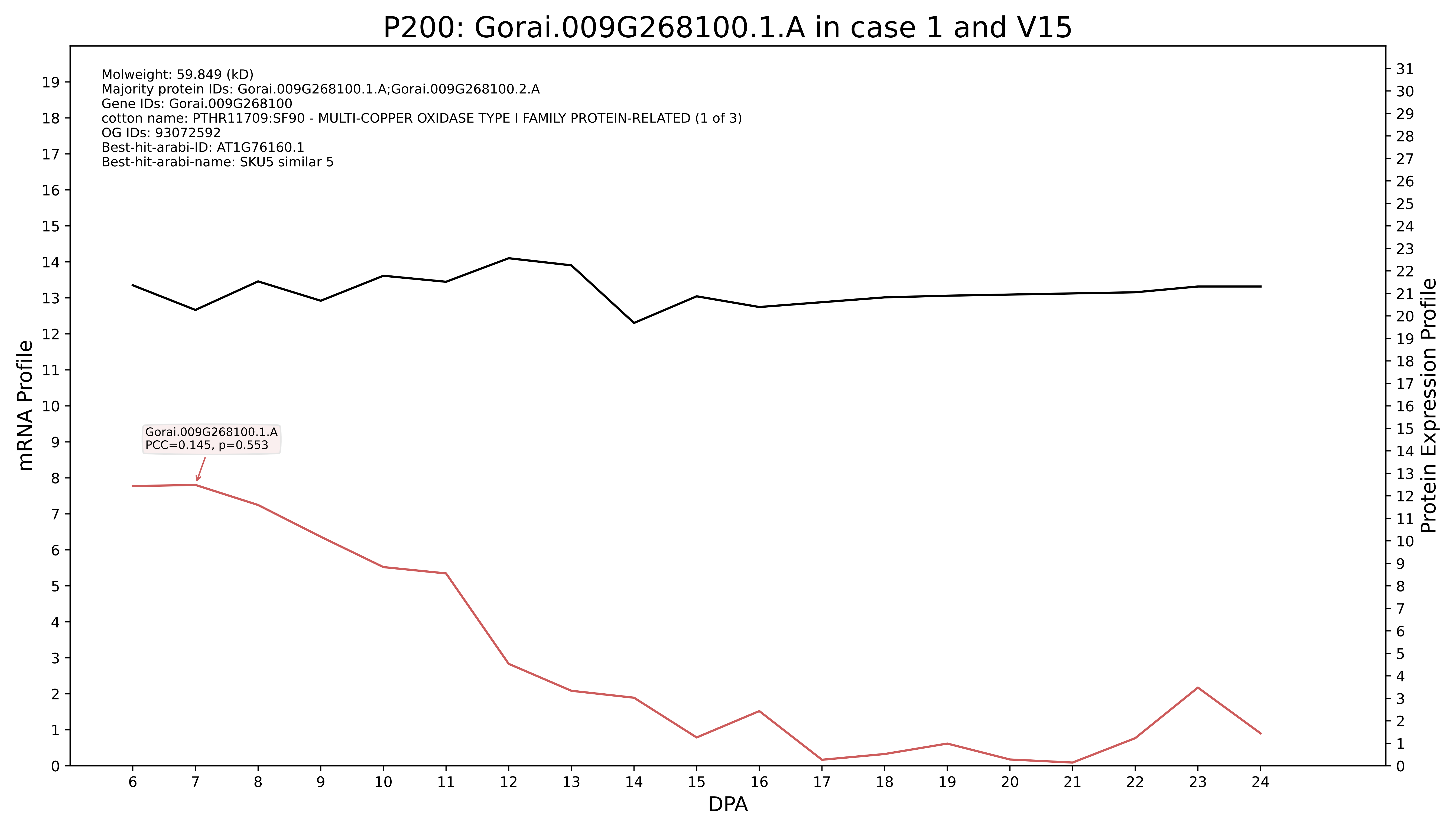
P200: Gorai.009G268100.1.A in case 1 and V15
Molweight: 59.849 (kD)
Majority protein IDs: Gorai.009G268100.1.A;Gorai.009G268100.2.A
Gene IDs: Gorai.009G268100
cotton name: PTHR11709:SF90 - MULTI-COPPER OXIDASE TYPE I FAMILY PROTEIN-RELATED (1 of 3)
OG IDs: 93072592
Best-hit-arabi-ID: AT1G76160.1
Best-hit-arabi-name: SKU5 similar 5
Gorai.009G268100.1.A
PCC=0.145, p=0.553
mRNA Profile
Protein Expression Profile
DPA

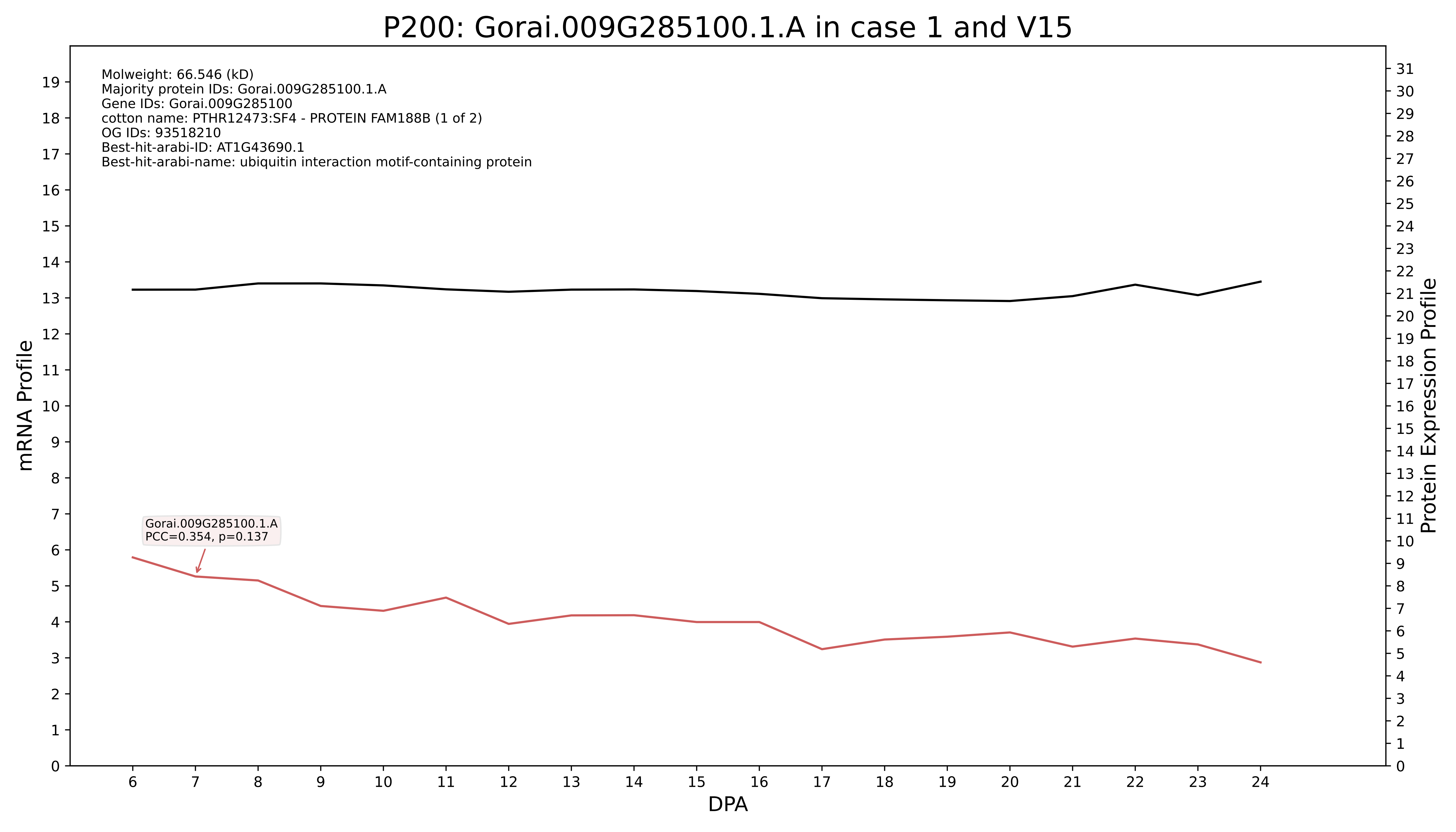

P200: Gorai.009G285100.1.A in case 1 and V15
Molweight: 66.546 (kD)
Majority protein IDs: Gorai.009G285100.1.A
Gene IDs: Gorai.009G285100
cotton name: PTHR12473:SF4 - PROTEIN FAM188B (1 of 2)
OG IDs: 93518210
Best-hit-arabi-ID: AT1G43690.1
Best-hit-arabi-name: ubiquitin interaction motif-containing protein
Gorai.009G285100.1.A
PCC=0.354, p=0.137
mRNA Profile
Protein Expression Profile
DPA

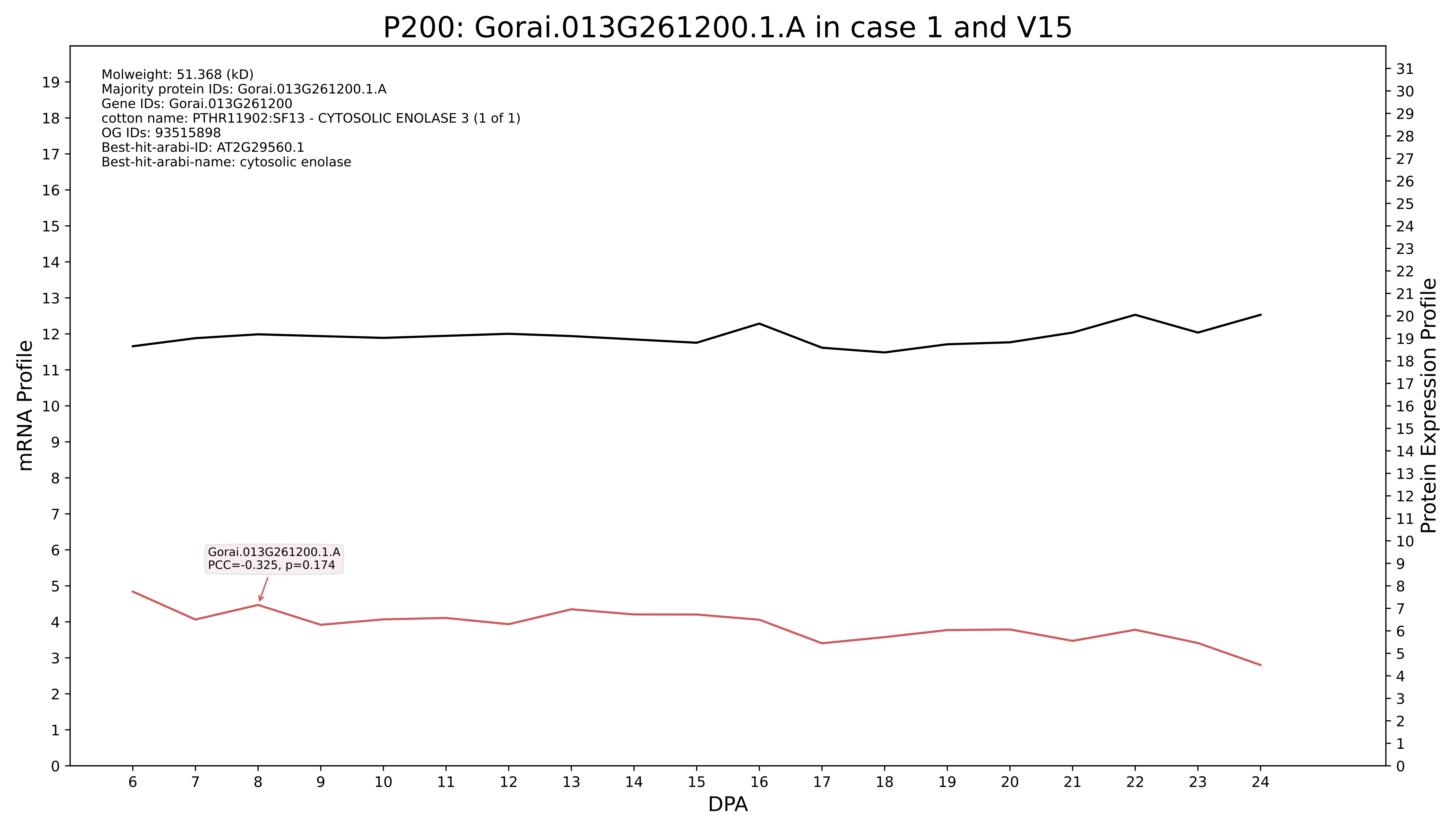
P200: Gorai.013G261200.1.A in case 1 and V15
Molweight: 51.368 (kD)
Majority protein IDs: Gorai.013G261200.1.A
Gene IDs: Gorai.013G261200
cotton name: PTHR11902:SF13 - CYTOSOLIC ENOLASE 3 (1 of 1)
OG IDs: 93515898
Best-hit-arabi-ID: AT2G29560.1
Best-hit-arabi-name: cytosolic enolase
Gorai.013G261200.1.A
PCC=-0.325, p=0.174
mRNA Profile
Protein Expression Profile
DPA
0
1
2
3
4
5
6
7
8
9
10
11
12
13
14
15
16
17
18
19
20
21
22
23
24
25
26
27
28
29
30
31

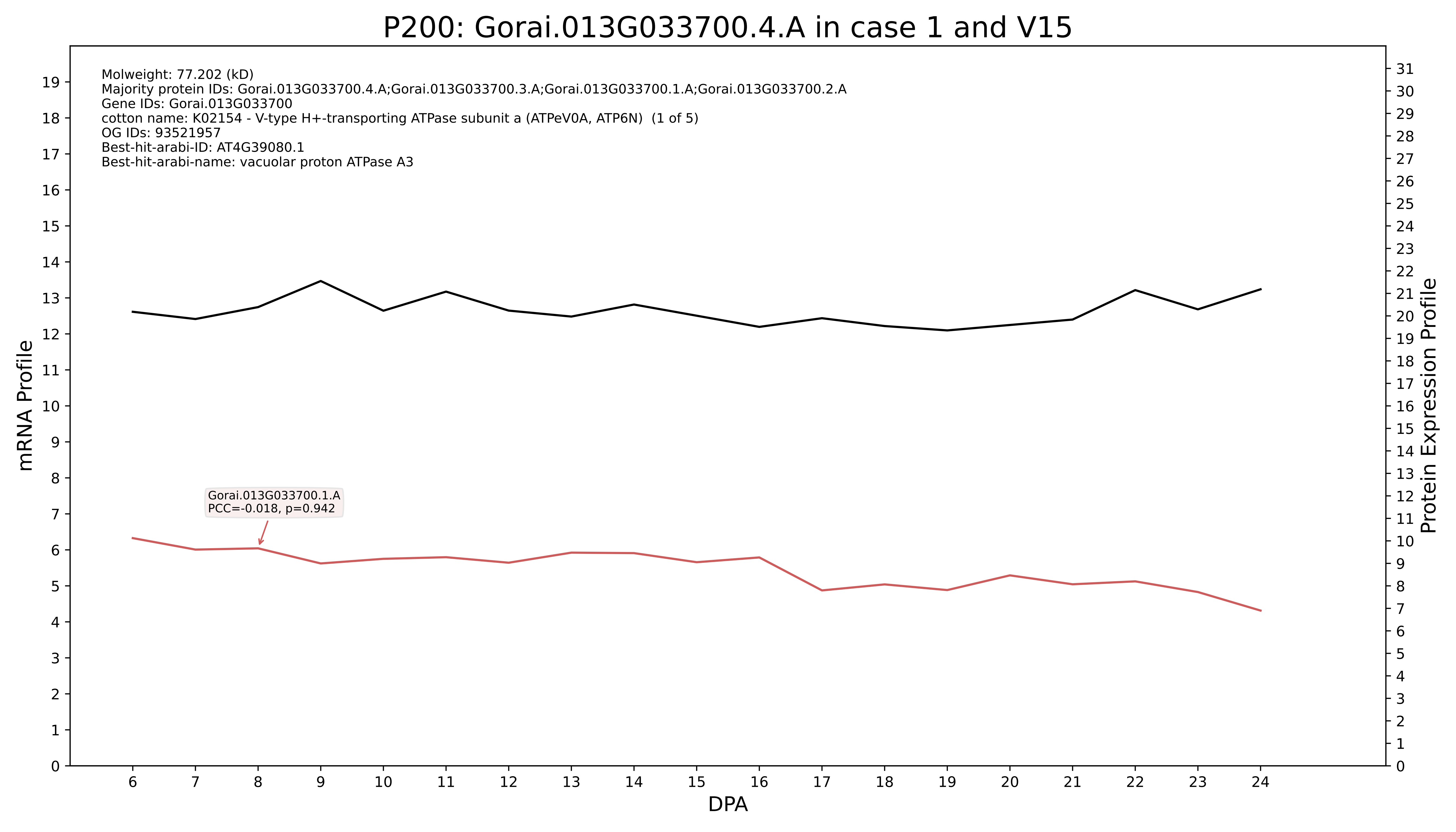
P200: Gorai.013G033700.4.A in case 1 and V15
Molweight: 77.202 (kD)
Majority protein IDs: Gorai.013G033700.4.A;Gorai.013G033700.3.A;Gorai.013G033700.1.A;Gorai.013G033700.2.A
Gene IDs: Gorai.013G033700
cotton name: K02154 - V-type H+-transporting ATPase subunit a (ATPeV0A, ATP6N) (1 of 5)
OG IDs: 93521957
Best-hit-arabi-ID: AT4G39080.1
Best-hit-arabi-name: vacuolar proton ATPase A3
Gorai.013G033700.1.A
PCC=-0.018, p=0.942
mRNA Profile
Protein Expression Profile
DPA
6
7
8
9
10
11
12
13
14
15
16
17
18
19
20
21
22
23
24

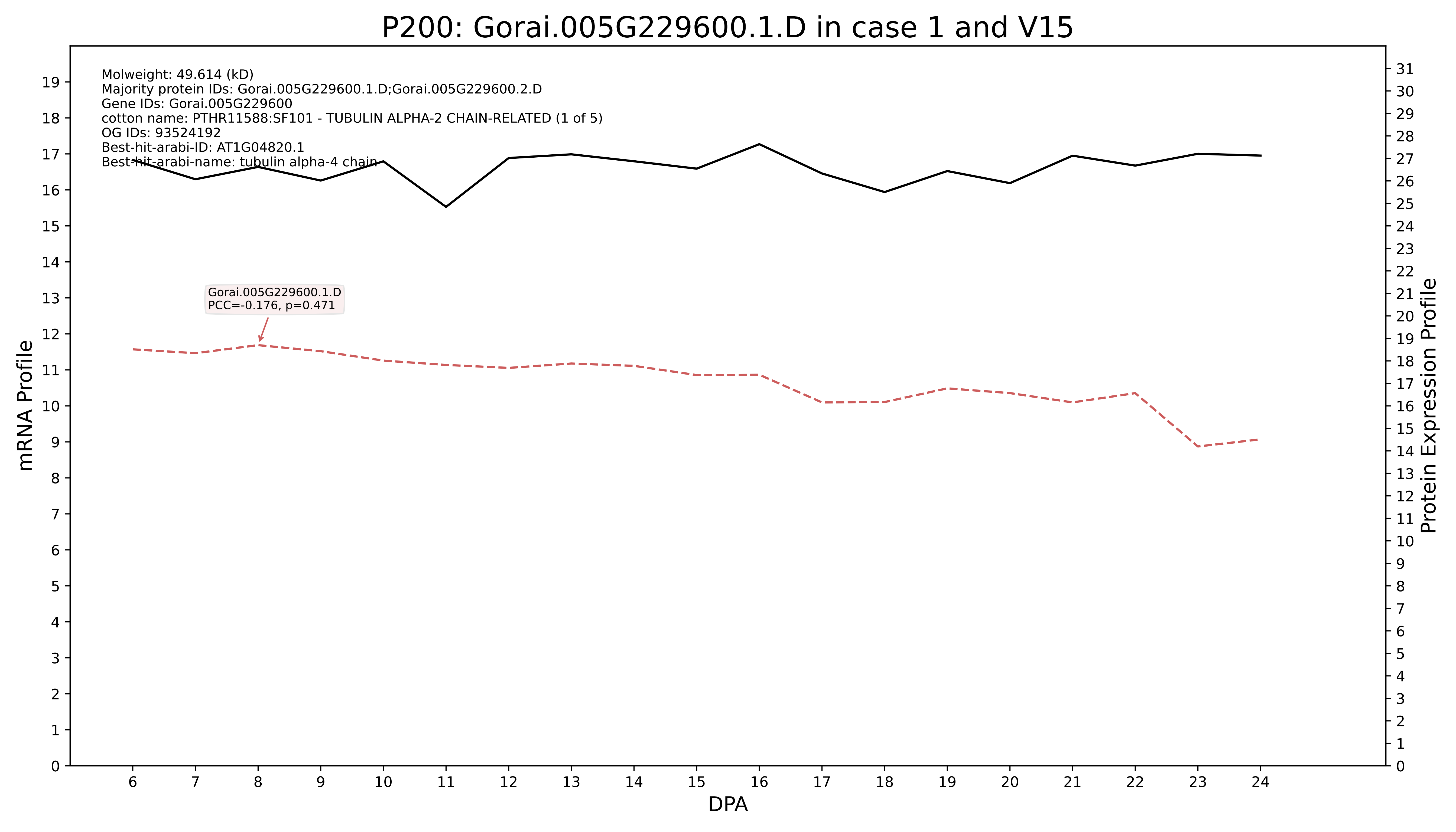

P200: Gorai.005G229600.1.D in case 1 and V15
Molweight: 49.614 (kD)
Majority protein IDs: Gorai.005G229600.1.D;Gorai.005G229600.2.D
Gene IDs: Gorai.005G229600
cotton name: PTHR11588:SF101 - TUBULIN ALPHA-2 CHAIN-RELATED (1 of 5)
OG IDs: 93524192
Best-hit-arabi-ID: AT1G04820.1
Best-hit-arabi-name: tubulin alpha-4 chain
Gorai.005G229600.1.D
PCC=-0.176, p=0.471
mRNA Profile
Protein Expression Profile
DPA

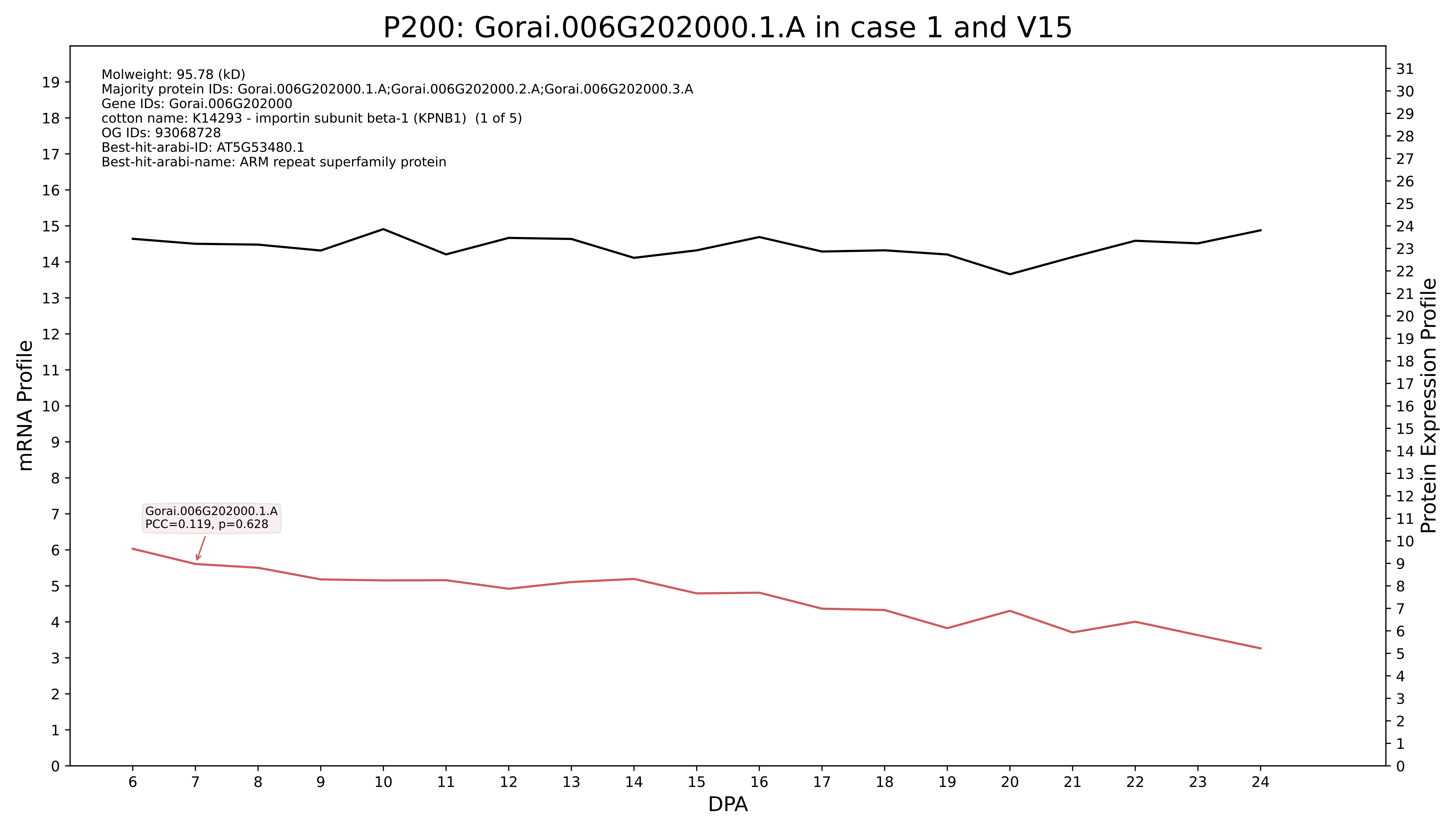
P200: Gorai.006G202000.1.A in case 1 and V15
Molweight: 95.78 (kD)
Majority protein IDs: Gorai.006G202000.1.A;Gorai.006G202000.2.A;Gorai.006G202000.3.A
Gene IDs: Gorai.006G202000
cotton name: K14293 - importin subunit beta-1 (KPNB1) (1 of 5)
OG IDs: 93068728
Best-hit-arabi-ID: AT5G53480.1
Best-hit-arabi-name: ARM repeat superfamily protein
Gorai.006G202000.1.A
PCC=0.119, p=0.628
mRNA Profile
Protein Expression Profile
DPA

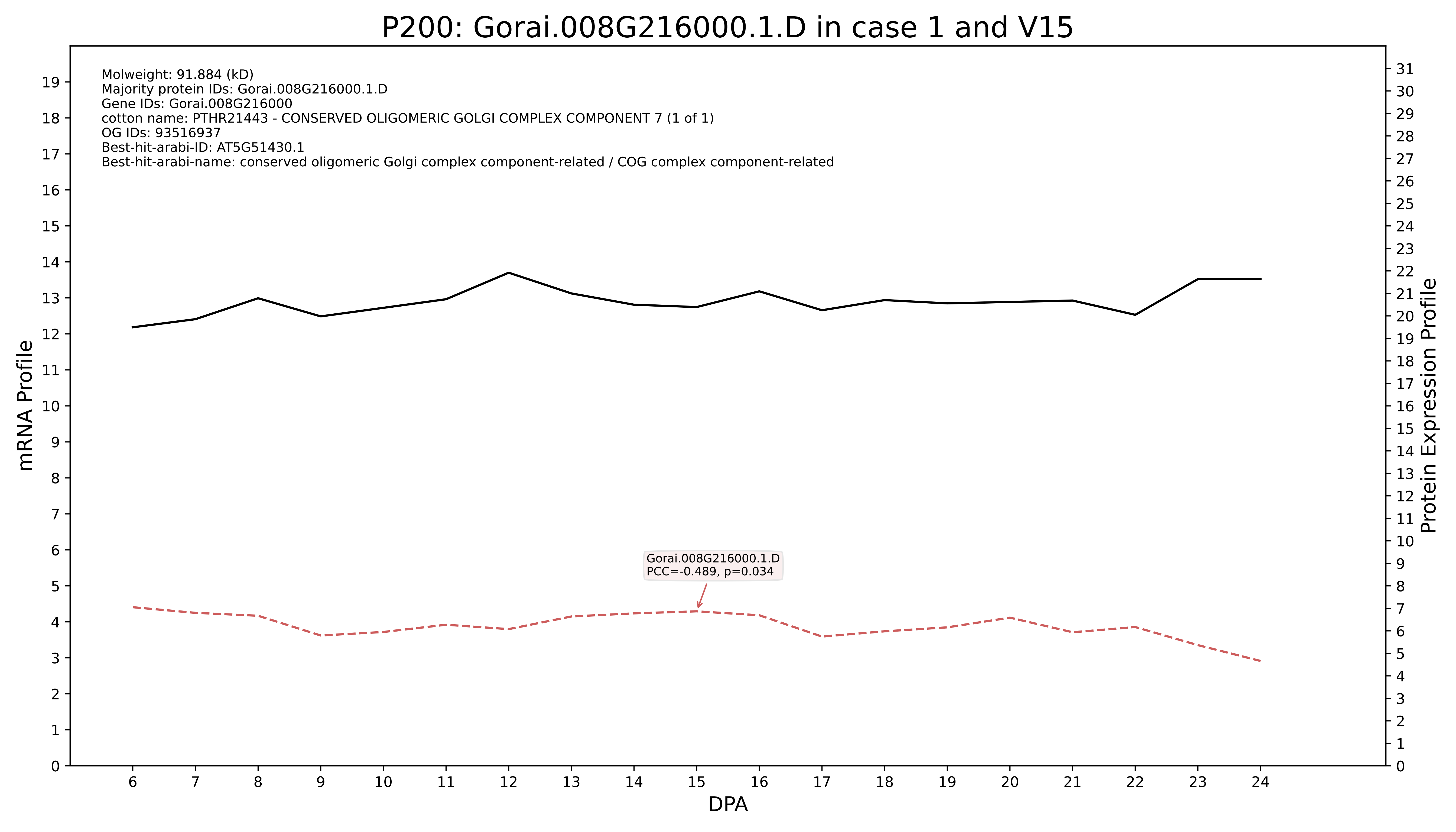
P200: Gorai.008G216000.1.D in case 1 and V15
Molweight: 91.884 (kD)
Majority protein IDs: Gorai.008G216000.1.D
Gene IDs: Gorai.008G216000
cotton name: PTHR21443 - CONSERVED OLIGOMERIC GOLGI COMPLEX COMPONENT 7 (1 of 1)
OG IDs: 93516937
Best-hit-arabi-ID: AT5G51430.1
Best-hit-arabi-name: conserved oligomeric Golgi complex component-related / COG complex component-related
Gorai.008G216000.1.D
PCC=-0.489, p=0.034
mRNA Profile
Protein Expression Profile
DPA

rai.005G130000.2.D in case 1 and V15

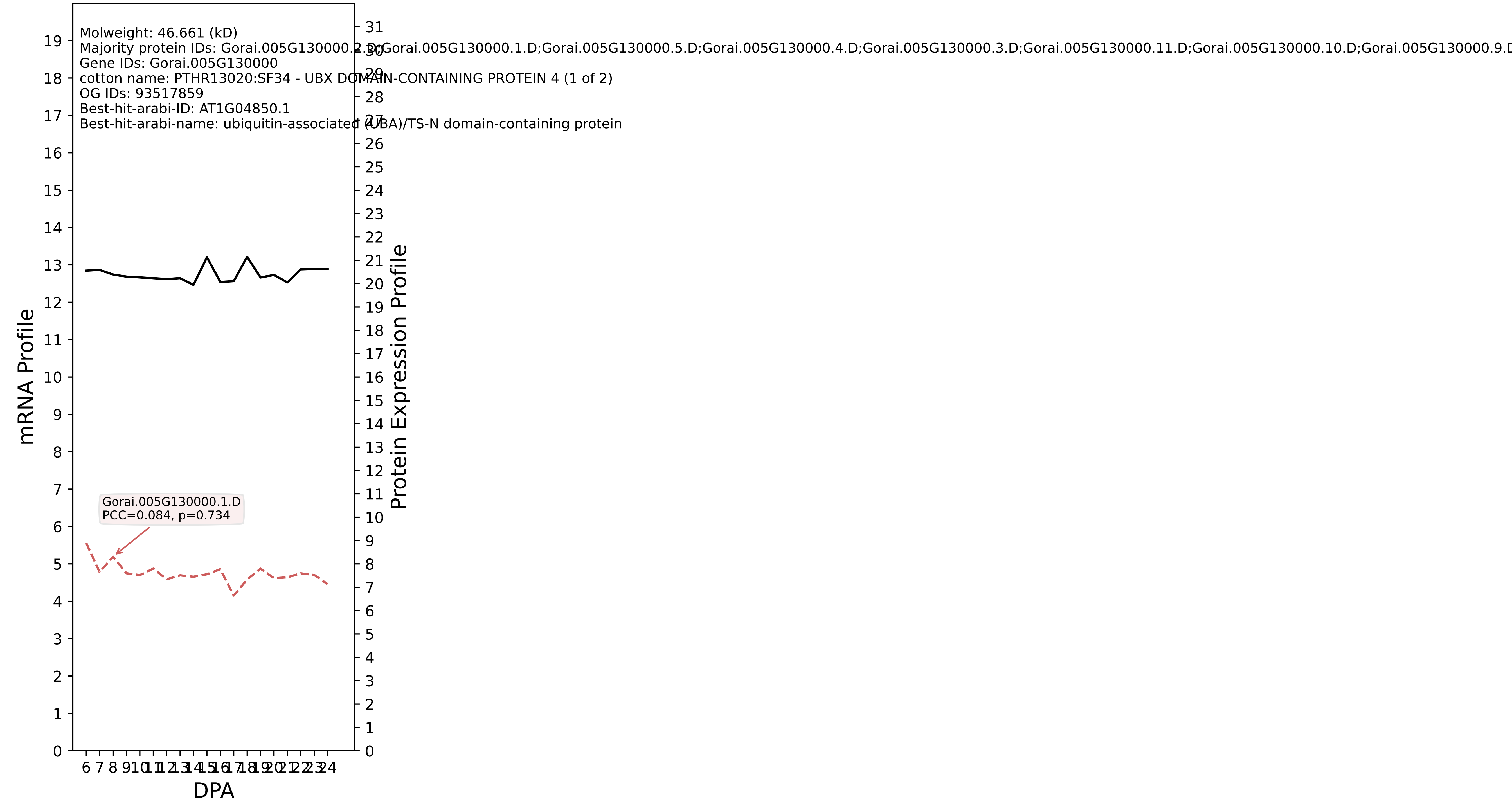

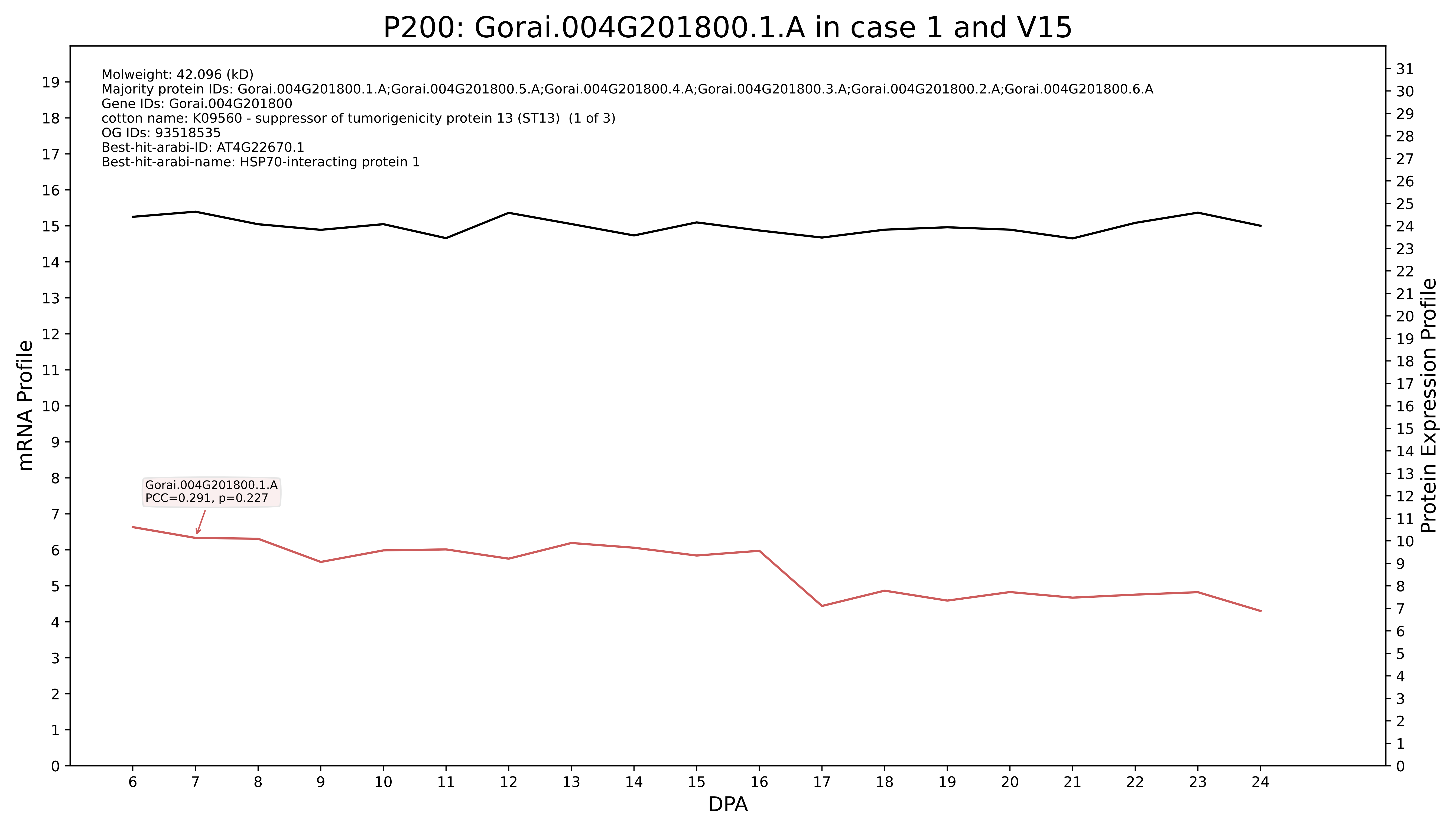

P200: Gorai.004G201800.1.A in case 1 and V15
Molweight: 42.096 (kD)
Majority protein IDs: Gorai.004G201800.1.A;Gorai.004G201800.5.A;Gorai.004G201800.4.A;Gorai.004G201800.3.A;Gorai.004G201800.2.A;Gorai.004G201800.6.A
Gene IDs: Gorai.004G201800
cotton name: K09560 - suppressor of tumorigenicity protein 13 (ST13) (1 of 3)
OG IDs: 93518535
Best-hit-arabi-ID: AT4G22670.1
Best-hit-arabi-name: HSP70-interacting protein 1
Gorai.004G201800.1.A
PCC=0.291, p=0.227
mRNA Profile
Protein Expression Profile
DPA

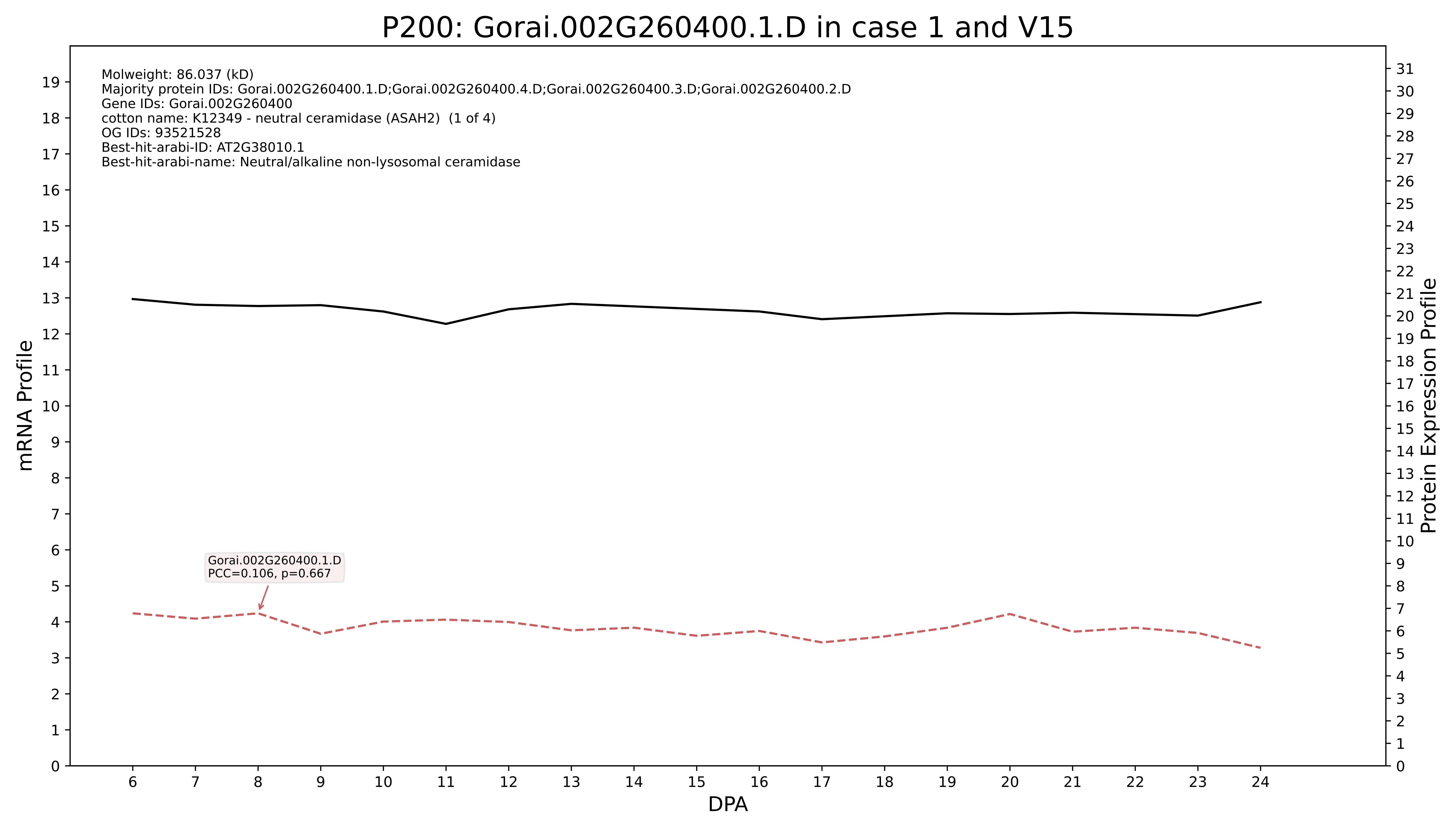
P200: Gorai.002G260400.1.D in case 1 and V15
Molweight: 86.037 (kD)
Majority protein IDs: Gorai.002G260400.1.D;Gorai.002G260400.4.D;Gorai.002G260400.3.D;Gorai.002G260400.2.D
Gene IDs: Gorai.002G260400
cotton name: K12349 - neutral ceramidase (ASAH2) (1 of 4)
OG IDs: 93521528
Best-hit-arabi-ID: AT2G38010.1
Best-hit-arabi-name: Neutral/alkaline non-lysosomal ceramidase
Gorai.002G260400.1.D
PCC=0.106, p=0.667
mRNA Profile
Protein Expression Profile
DPA

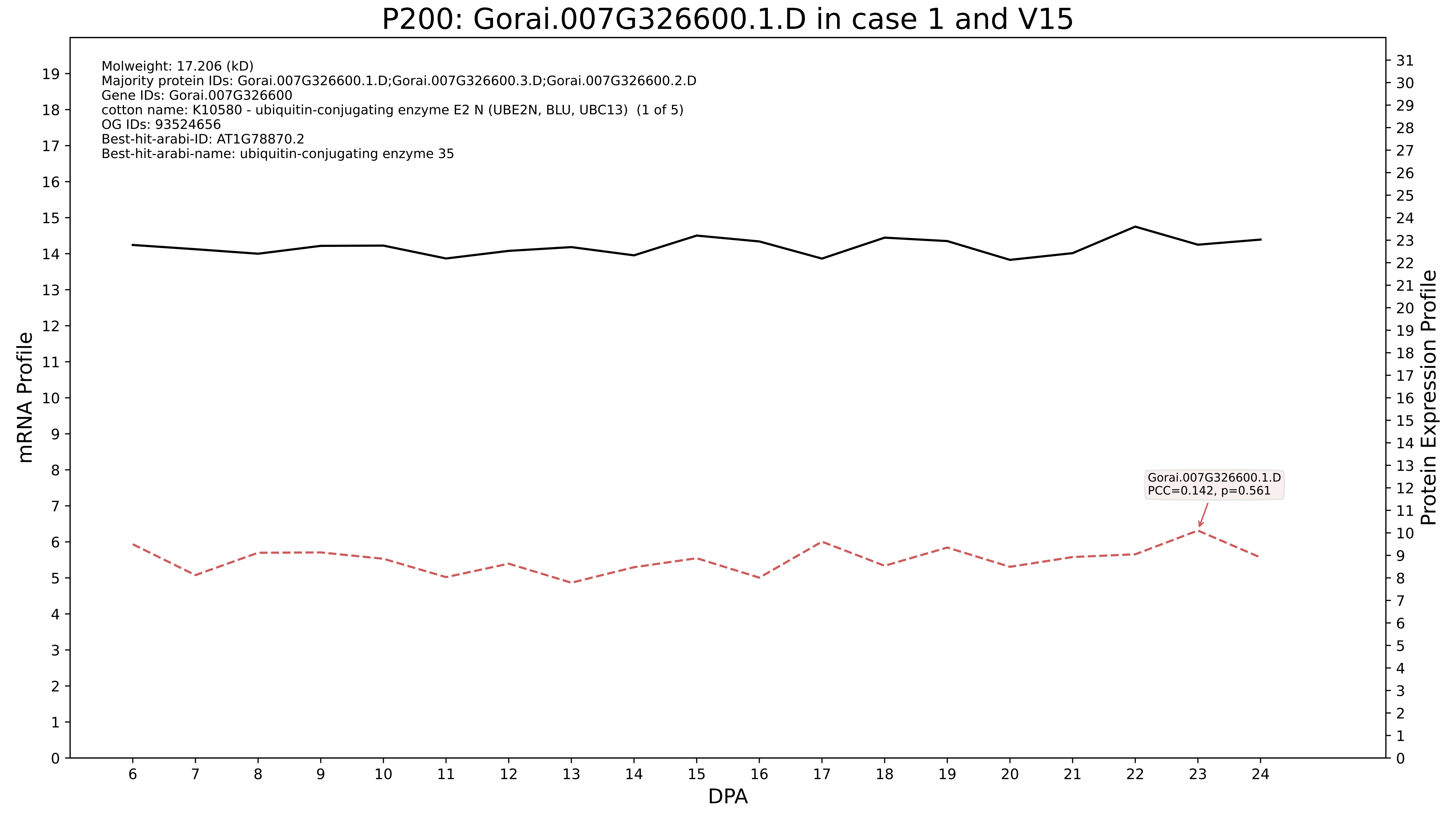

P200: Gorai.007G326600.1.D in case 1 and V15
Molweight: 17.206 (kD)
Majority protein IDs: Gorai.007G326600.1.D;Gorai.007G326600.3.D;Gorai.007G326600.2.D
Gene IDs: Gorai.007G326600
cotton name: K10580 - ubiquitin-conjugating enzyme E2 N (UBE2N, BLU, UBC13) (1 of 5)
OG IDs: 93524656
Best-hit-arabi-ID: AT1G78870.2
Best-hit-arabi-name: ubiquitin-conjugating enzyme 35
Gorai.007G326600.1.D
PCC=0.142, p=0.561
mRNA Profile
Protein Expression Profile
DPA

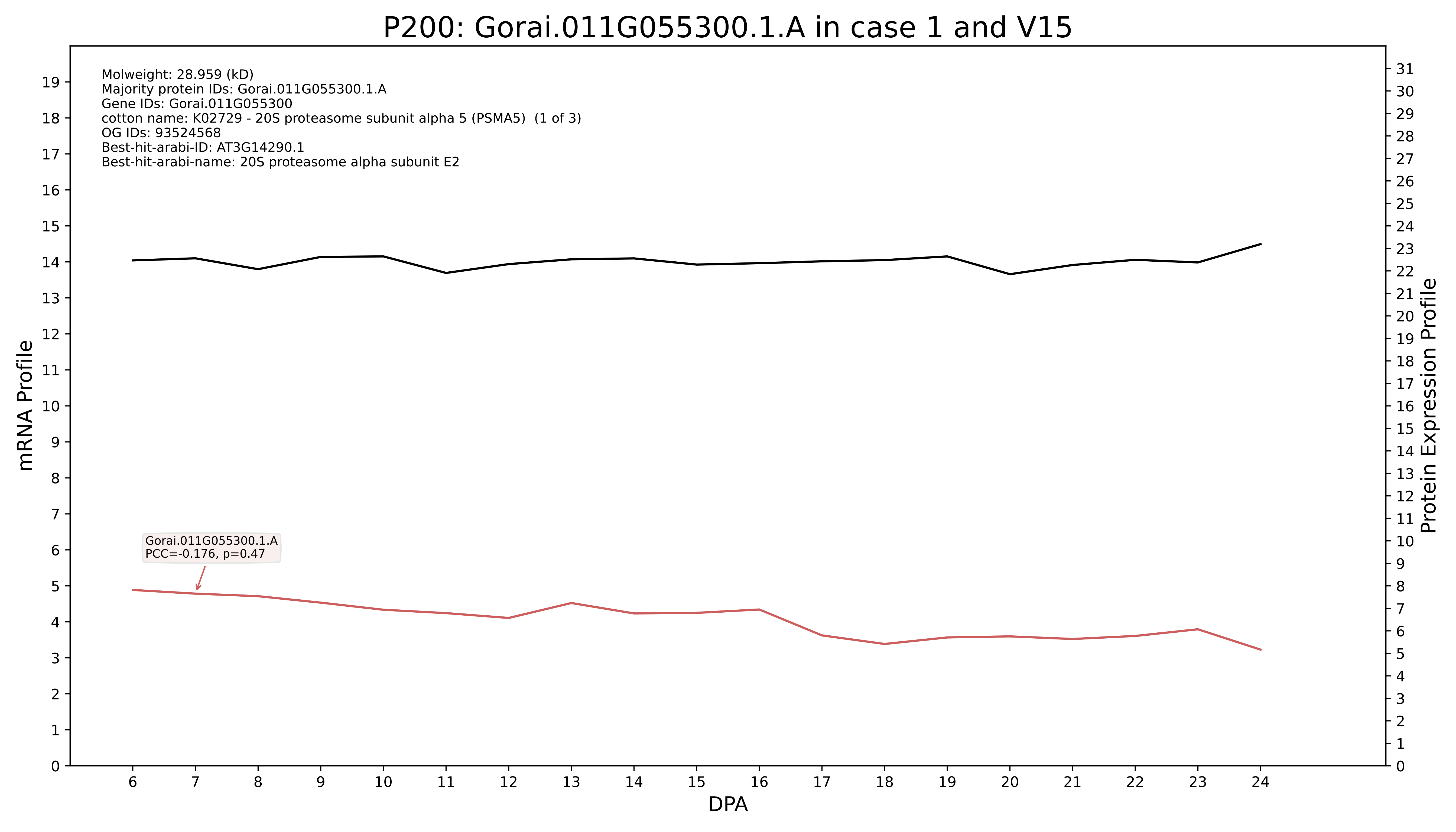
P200: Gorai.011G055300.1.A in case 1 and V15
Molweight: 28.959 (kD)
Majority protein IDs: Gorai.011G055300.1.A
Gene IDs: Gorai.011G055300
cotton name: K02729 - 20S proteasome subunit alpha 5 (PSMA5) (1 of 3)
OG IDs: 93524568
Best-hit-arabi-ID: AT3G14290.1
Best-hit-arabi-name: 20S proteasome alpha subunit E2
Gorai.011G055300.1.A
PCC=-0.176, p=0.47
mRNA Profile
Protein Expression Profile
DPA

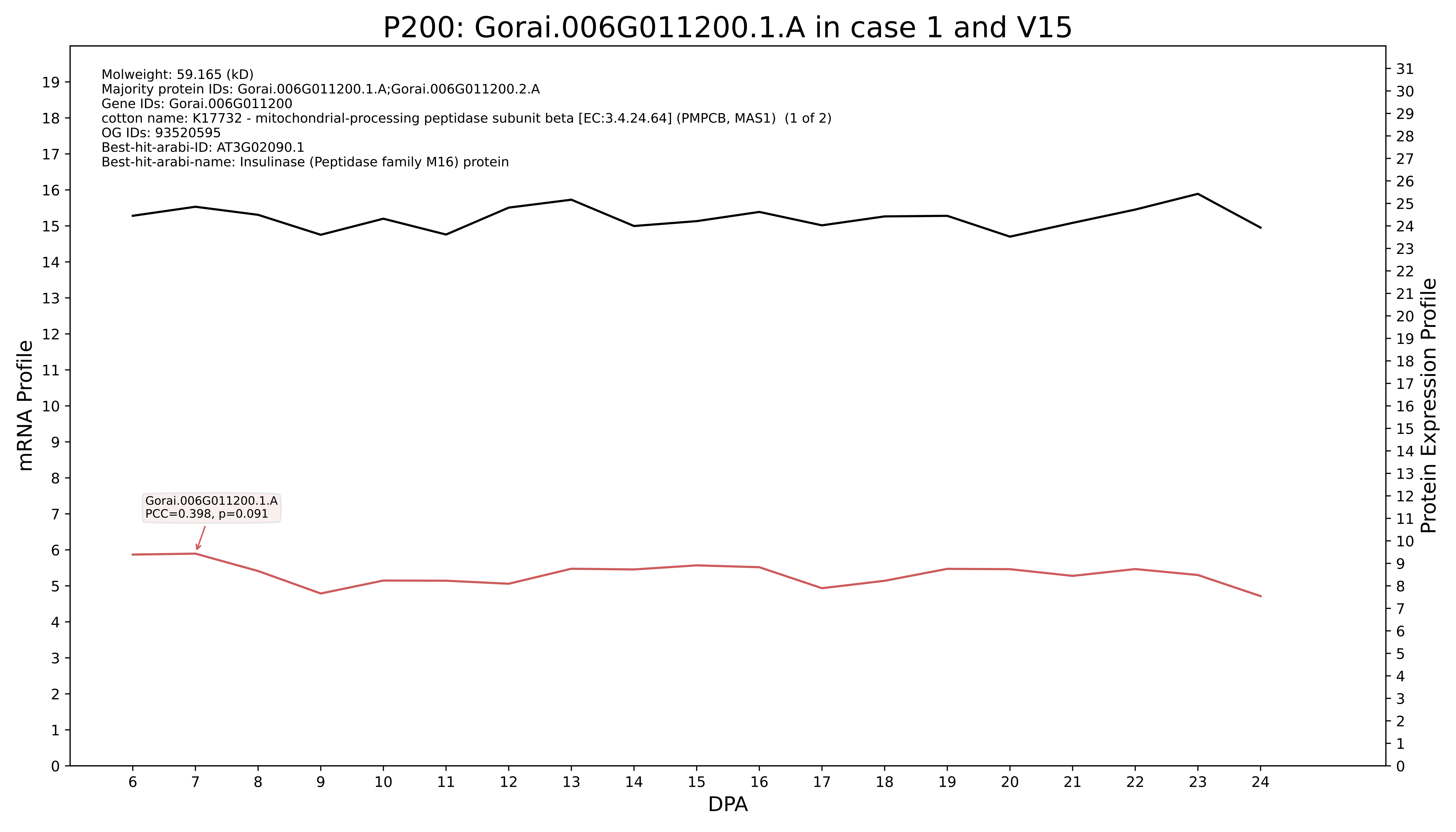
P200: Gorai.006G011200.1.A in case 1 and V15
Molweight: 59.165 (kD)
Majority protein IDs: Gorai.006G011200.1.A;Gorai.006G011200.2.A
Gene IDs: Gorai.006G011200
cotton name: K17732 - mitochondrial-processing peptidase subunit beta [EC:3.4.24.64] (PMPCB, MAS1) (1 of 2)
OG IDs: 93520595
Best-hit-arabi-ID: AT3G02090.1
Best-hit-arabi-name: Insulinase (Peptidase family M16) protein
Gorai.006G011200.1.A
PCC=0.398, p=0.091
mRNA Profile
Protein Expression Profile
DPA

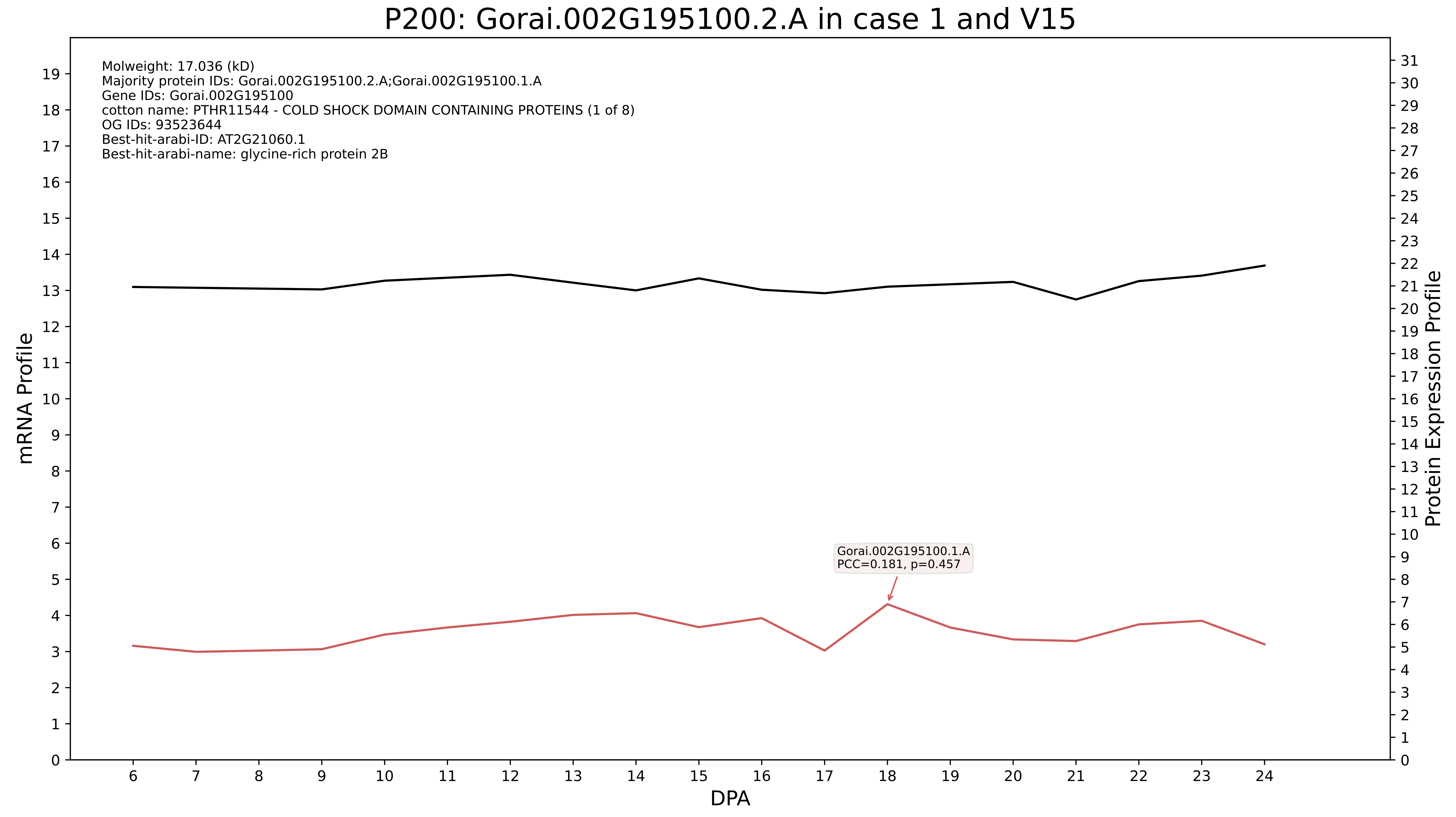
P200: Gorai.002G195100.2.A in case 1 and V15
Molweight: 17.036 (kD)
Majority protein IDs: Gorai.002G195100.2.A;Gorai.002G195100.1.A
Gene IDs: Gorai.002G195100
cotton name: PTHR11544 - COLD SHOCK DOMAIN CONTAINING PROTEINS (1 of 8)
OG IDs: 93523644
Best-hit-arabi-ID: AT2G21060.1
Best-hit-arabi-name: glycine-rich protein 2B
Gorai.002G195100.1.A
PCC=0.181, p=0.457
mRNA Profile
Protein Expression Profile
DPA
0
1
2
3
4
5
6
7
8
9
10
11
12
13
14
15
16
17
18
19
20
21
22
23
24
25
26
27
28
29
30
31

P200: Gorai.013G270300.1.A in case 1 and V15

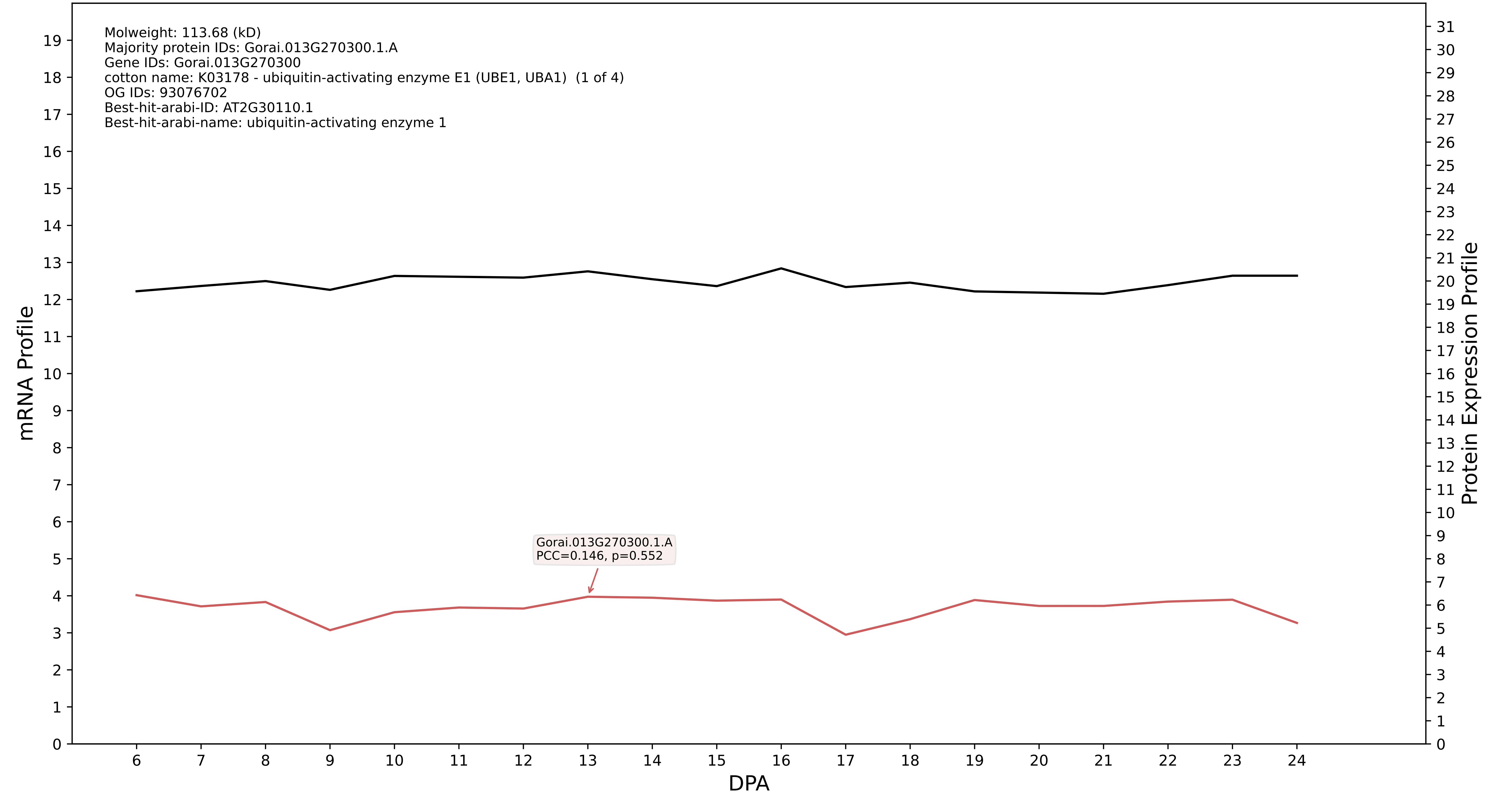

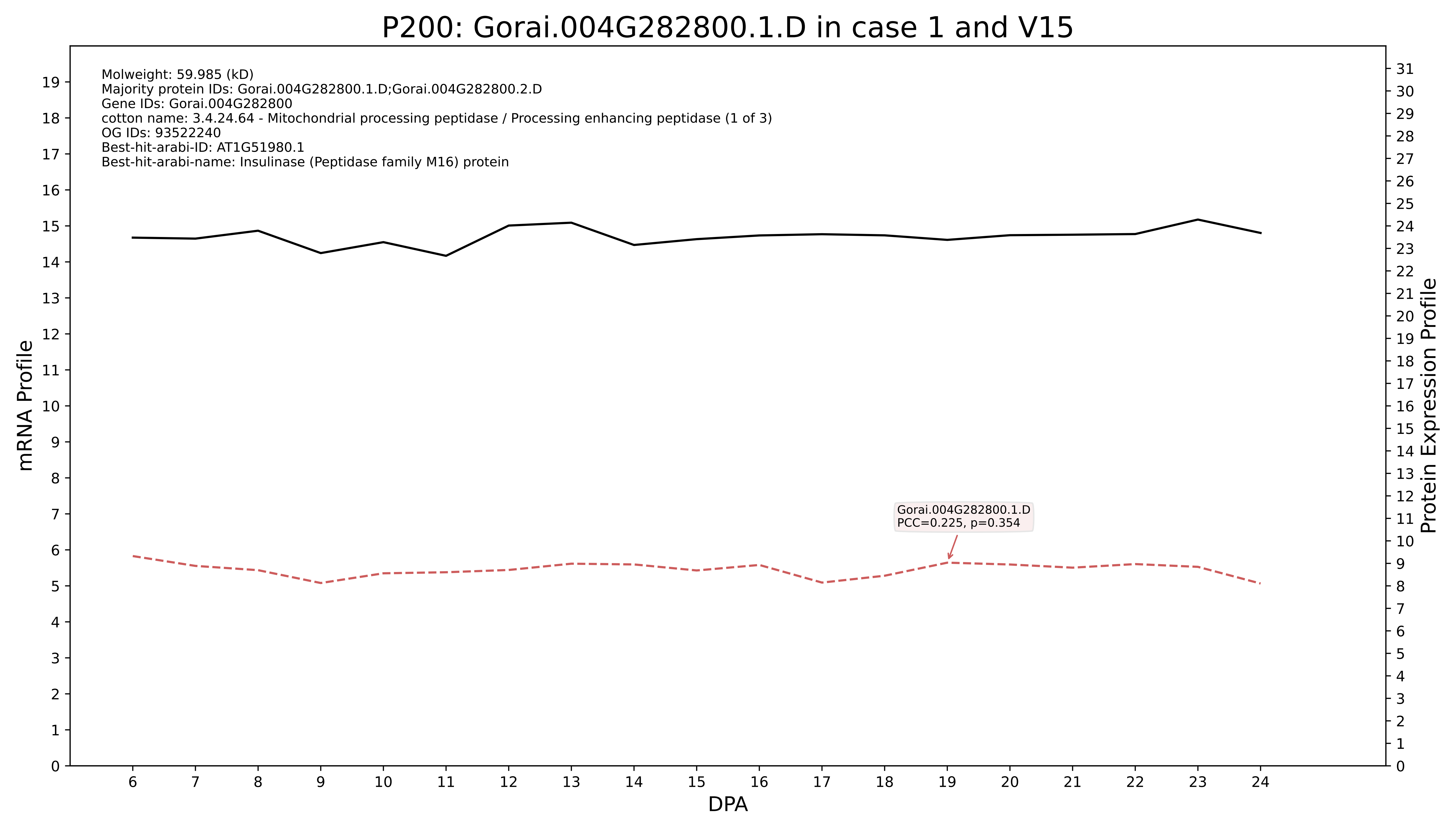
P200: Gorai.004G282800.1.D in case 1 and V15
Molweight: 59.985 (kD)
Majority protein IDs: Gorai.004G282800.1.D;Gorai.004G282800.2.D
Gene IDs: Gorai.004G282800
cotton name: 3.4.24.64 - Mitochondrial processing peptidase / Processing enhancing peptidase (1 of 3)
OG IDs: 93522240
Best-hit-arabi-ID: AT1G51980.1
Best-hit-arabi-name: Insulinase (Peptidase family M16) protein
Gorai.004G282800.1.D
PCC=0.225, p=0.354
mRNA Profile
Protein Expression Profile
DPA

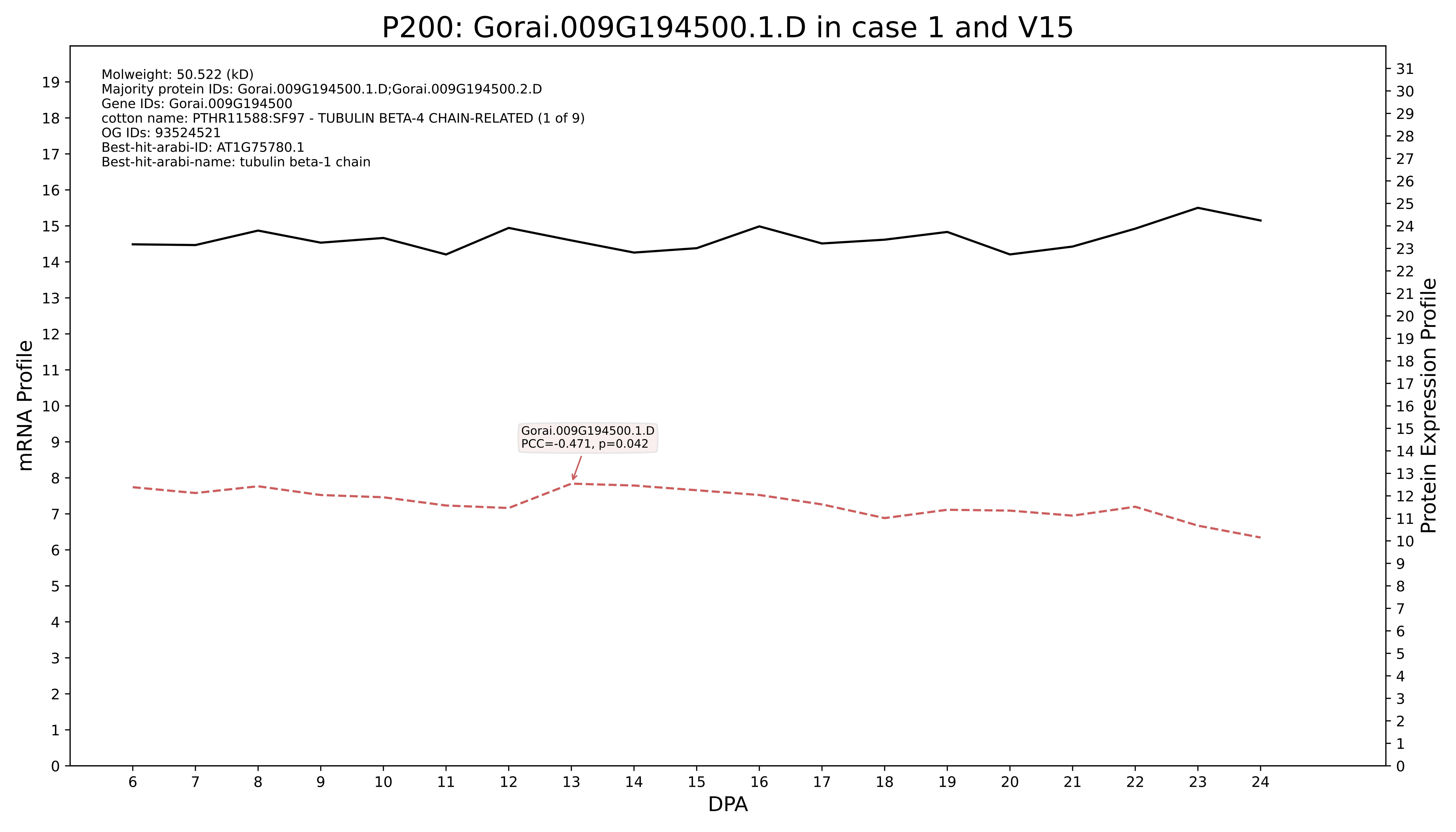
P200: Gorai.009G194500.1.D in case 1 and V15
Molweight: 50.522 (kD)
Majority protein IDs: Gorai.009G194500.1.D;Gorai.009G194500.2.D
Gene IDs: Gorai.009G194500
cotton name: PTHR11588:SF97 - TUBULIN BETA-4 CHAIN-RELATED (1 of 9)
OG IDs: 93524521
Best-hit-arabi-ID: AT1G75780.1
Best-hit-arabi-name: tubulin beta-1 chain
Gorai.009G194500.1.D
PCC=-0.471, p=0.042
mRNA Profile
Protein Expression Profile
DPA

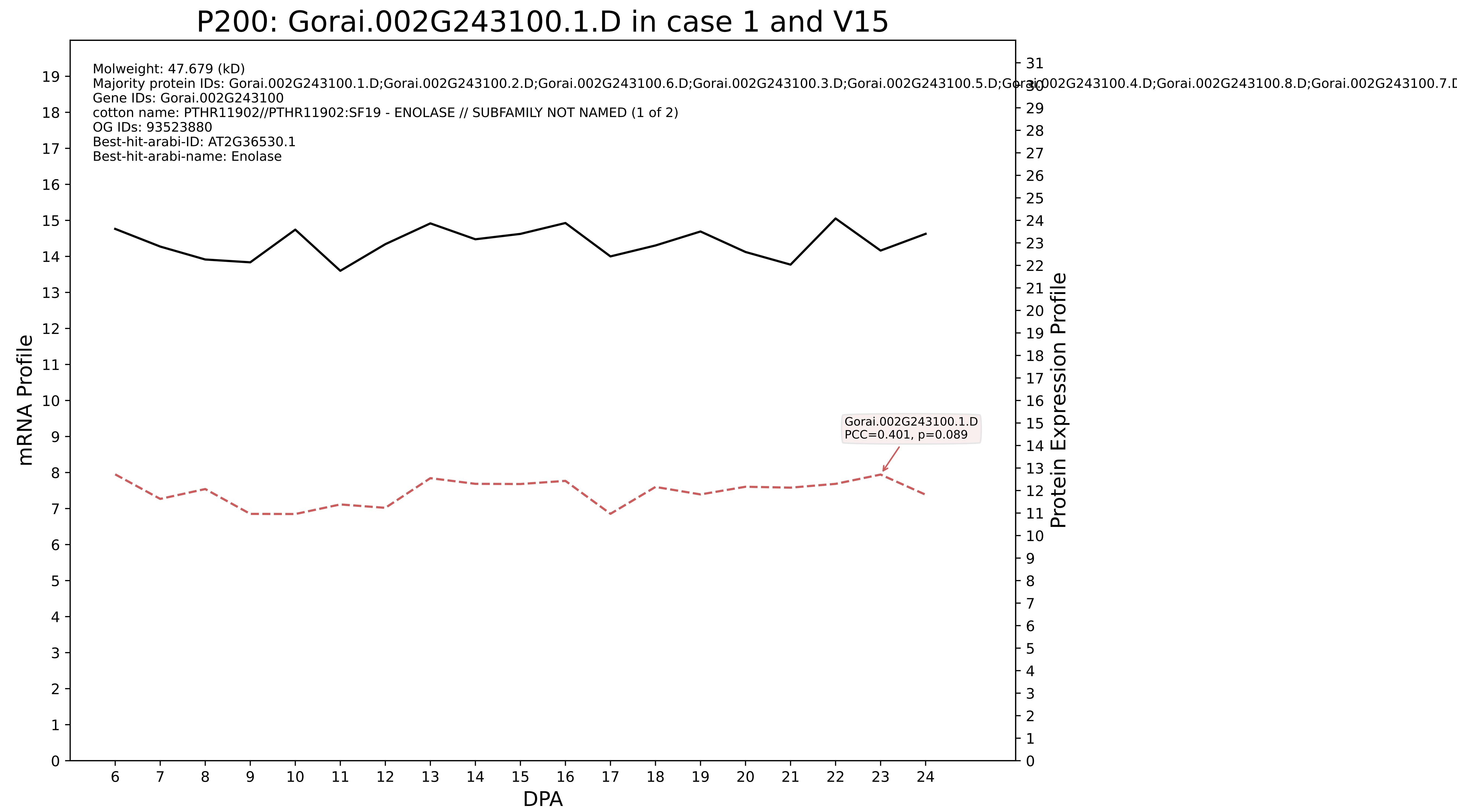
P200: Gorai.002G243100.1.D in case 1 and V15
Molweight: 47.679 (kD)
Majority protein IDs: Gorai.002G243100.1.D;Gorai.002G243100.2.D;Gorai.002G243100.6.D;Gorai.002G243100.3.D;Gorai.002G243100.5.D;Gorai.002G243100.4.D;Gorai.002G243100.8.D;Gorai.002G243100.7.D
Gene IDs: Gorai.002G243100
cotton name: PTHR11902//PTHR11902:SF19 - ENOLASE // SUBFAMILY NOT NAMED (1 of 2)
OG IDs: 93523880
Best-hit-arabi-ID: AT2G36530.1
Best-hit-arabi-name: Enolase
Gorai.002G243100.1.D
PCC=0.401, p=0.089
mRNA Profile
Protein Expression Profile
DPA

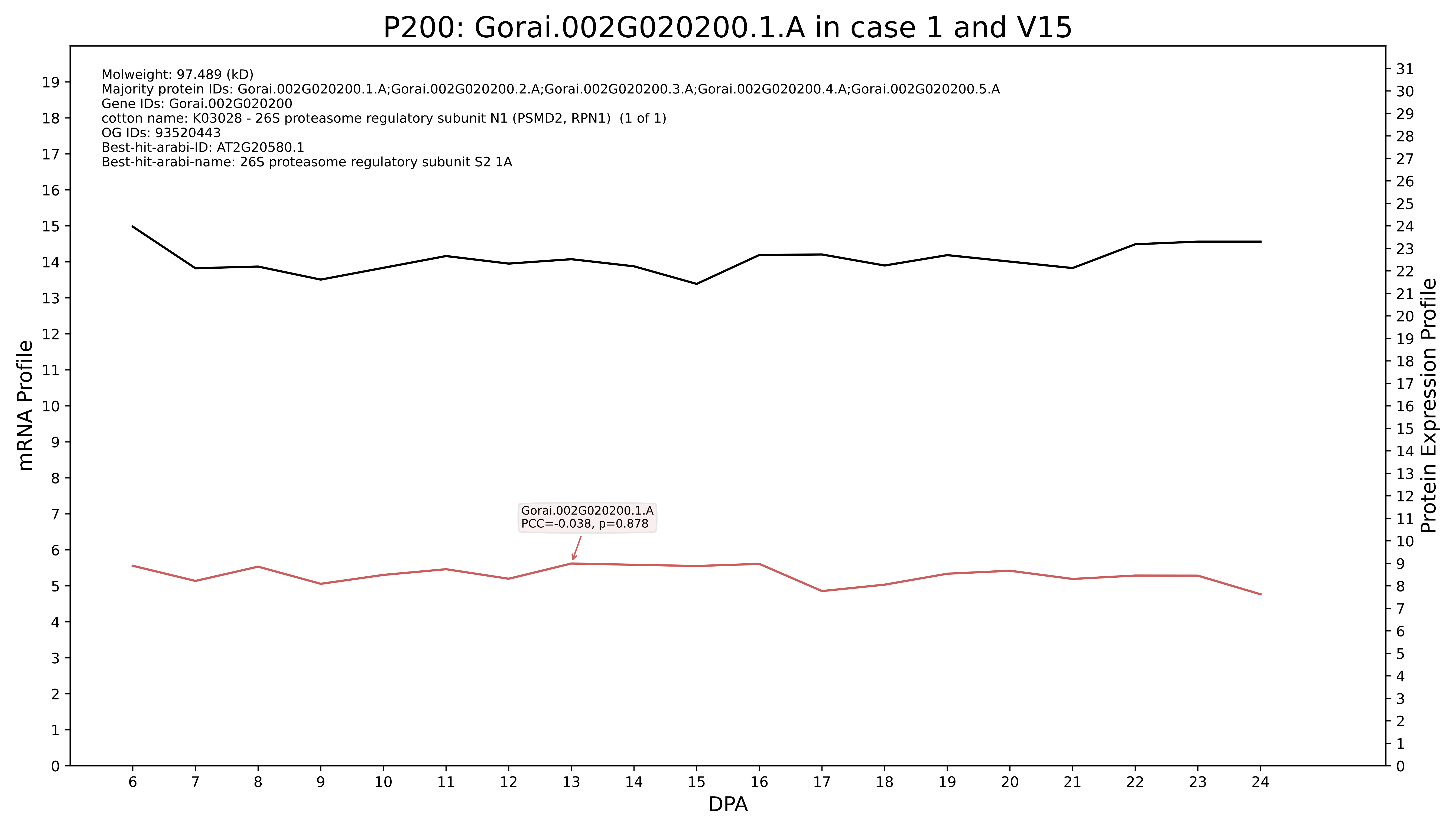

P200: Gorai.002G020200.1.A in case 1 and V15
Molweight: 97.489 (kD)
Majority protein IDs: Gorai.002G020200.1.A;Gorai.002G020200.2.A;Gorai.002G020200.3.A;Gorai.002G020200.4.A;Gorai.002G020200.5.A
Gene IDs: Gorai.002G020200
cotton name: K03028 - 26S proteasome regulatory subunit N1 (PSMD2, RPN1) (1 of 1)
OG IDs: 93520443
Best-hit-arabi-ID: AT2G20580.1
Best-hit-arabi-name: 26S proteasome regulatory subunit S2 1A
Gorai.002G020200.1.A
PCC=-0.038, p=0.878
mRNA Profile
Protein Expression Profile
DPA

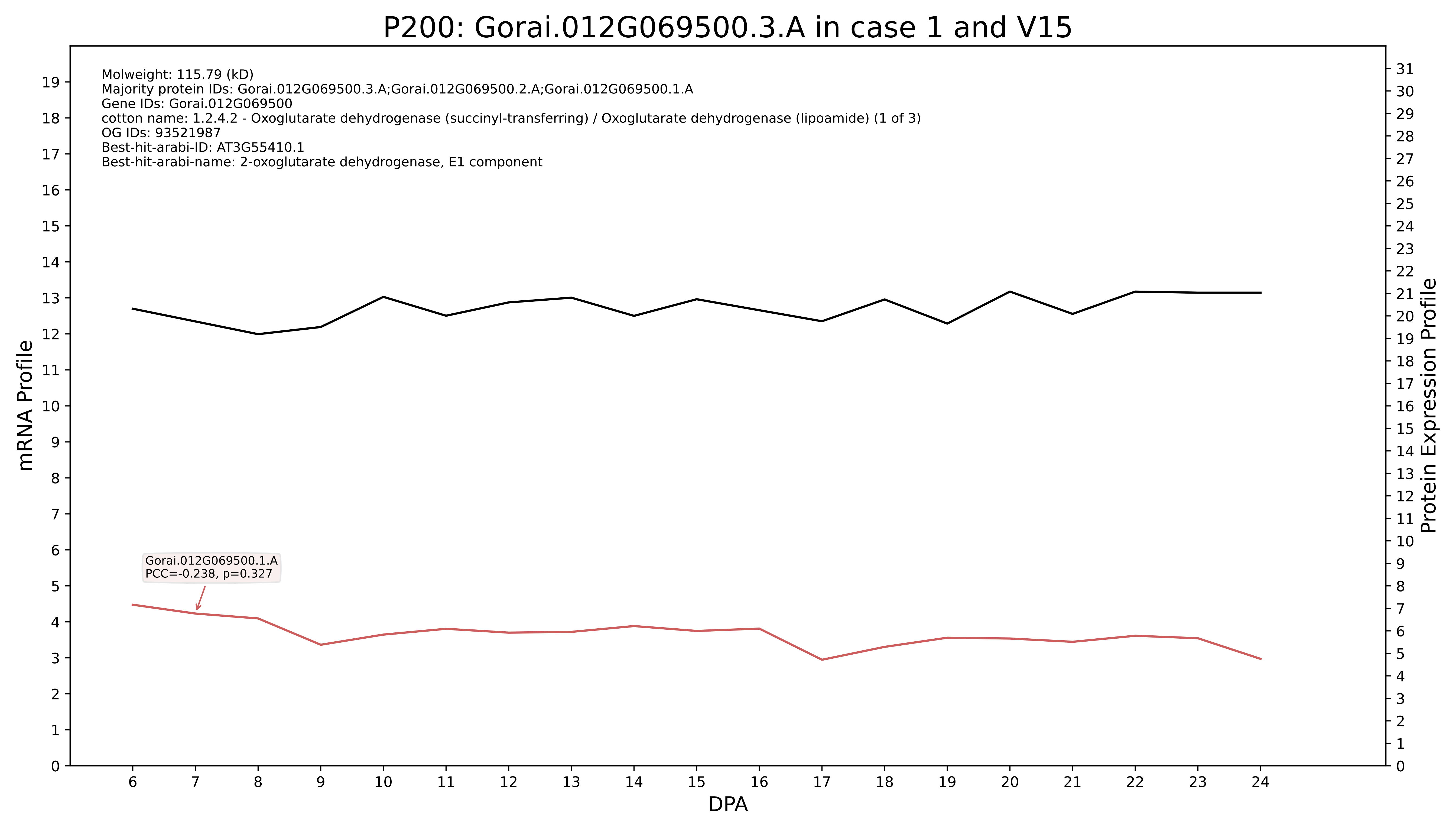
P200: Gorai.012G069500.3.A in case 1 and V15
Molweight: 115.79 (kD)
Majority protein IDs: Gorai.012G069500.3.A;Gorai.012G069500.2.A;Gorai.012G069500.1.A
Gene IDs: Gorai.012G069500
cotton name: 1.2.4.2 - Oxoglutarate dehydrogenase (succinyl-transferring) / Oxoglutarate dehydrogenase (lipoamide) (1 of 3)
OG IDs: 93521987
Best-hit-arabi-ID: AT3G55410.1
Best-hit-arabi-name: 2-oxoglutarate dehydrogenase, E1 component
Gorai.012G069500.1.A
PCC=-0.238, p=0.327
mRNA Profile
Protein Expression Profile
DPA

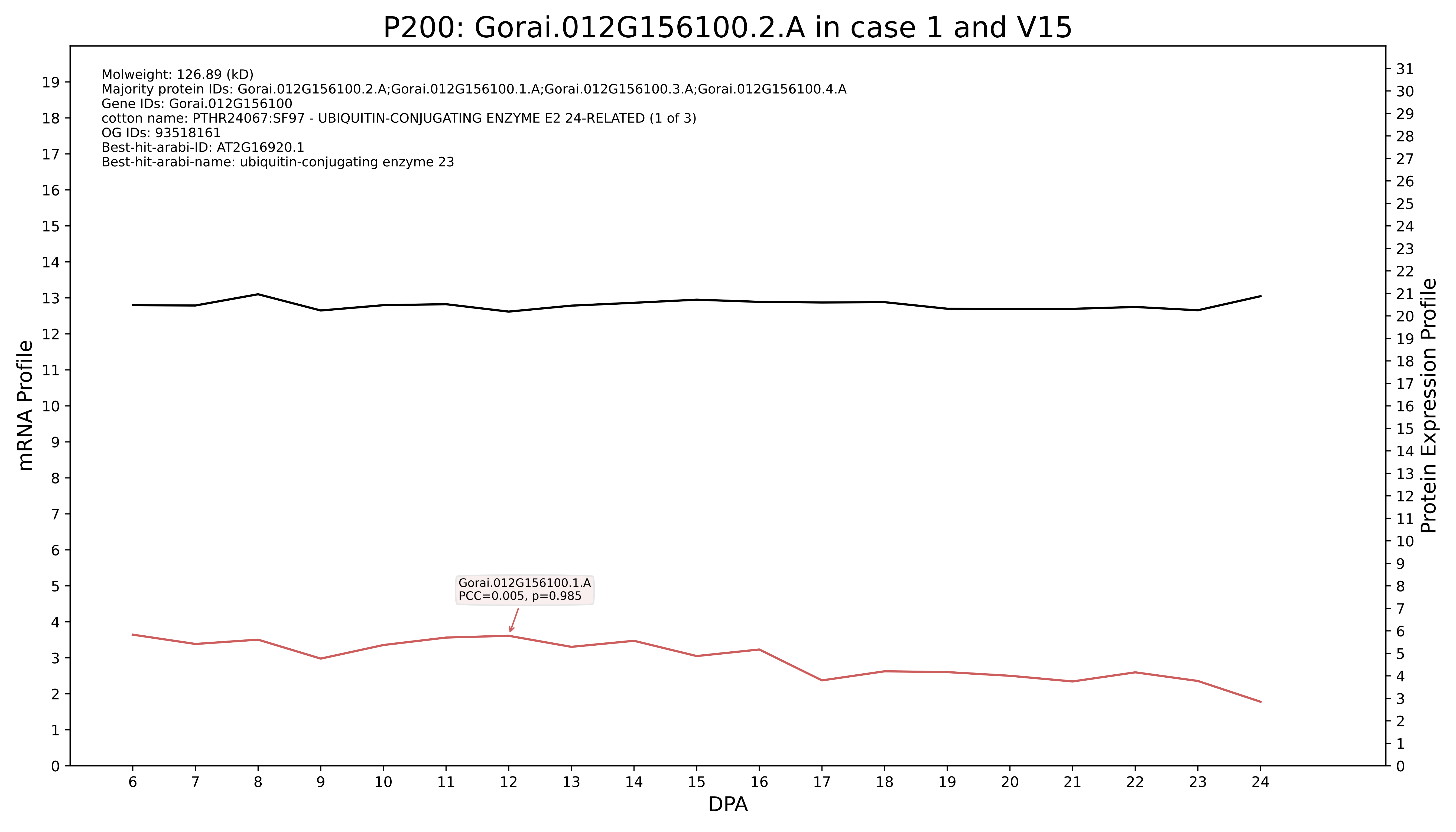
P200: Gorai.012G156100.2.A in case 1 and V15
Molweight: 126.89 (kD)
Majority protein IDs: Gorai.012G156100.2.A;Gorai.012G156100.1.A;Gorai.012G156100.3.A;Gorai.012G156100.4.A
Gene IDs: Gorai.012G156100
cotton name: PTHR24067:SF97 - UBIQUITIN-CONJUGATING ENZYME E2 24-RELATED (1 of 3)
OG IDs: 93518161
Best-hit-arabi-ID: AT2G16920.1
Best-hit-arabi-name: ubiquitin-conjugating enzyme 23
Gorai.012G156100.1.A
PCC=0.005, p=0.985
mRNA Profile
Protein Expression Profile
DPA

P200: Gorai.001G197200.7.D in case 1 and V15

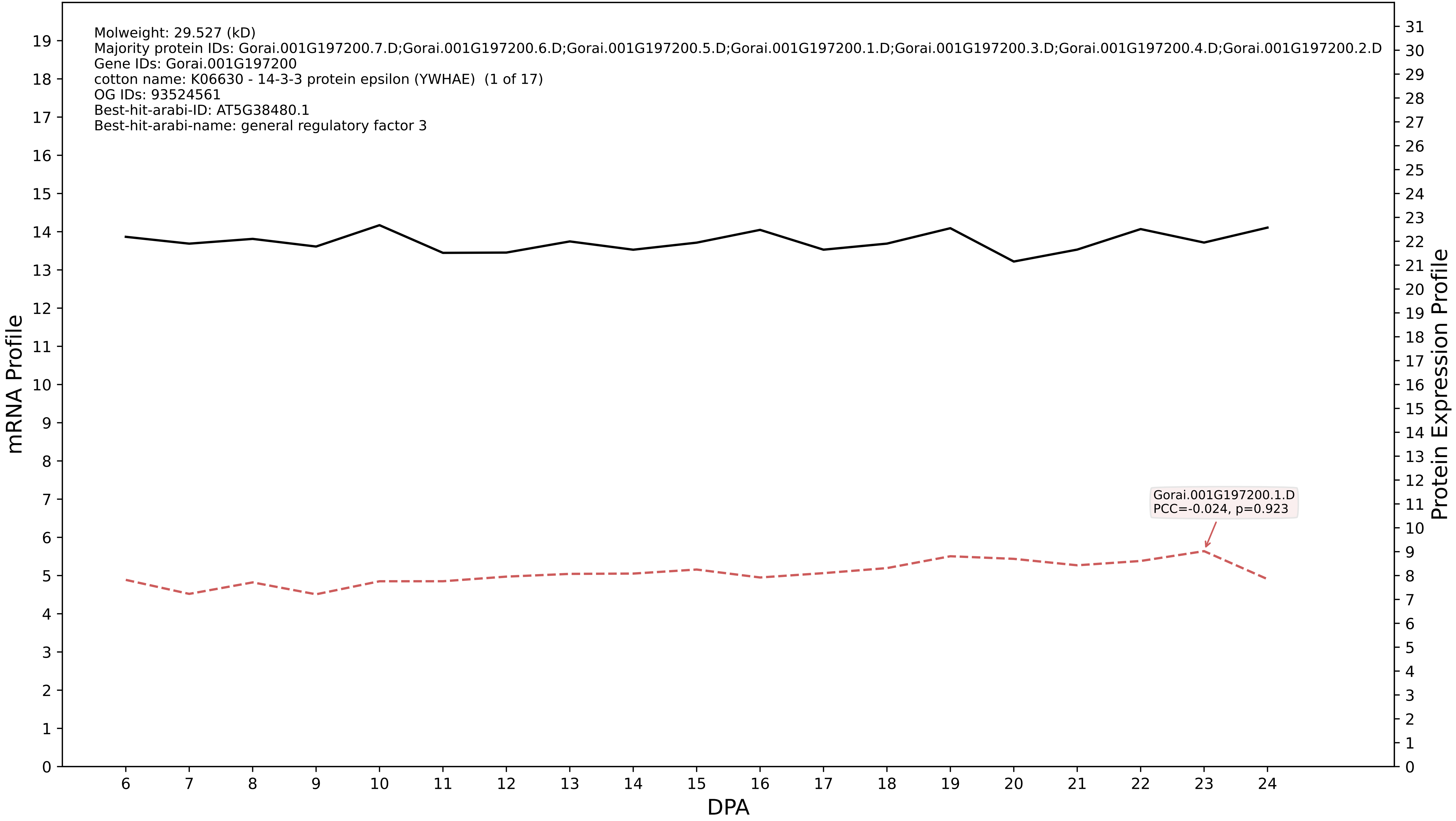

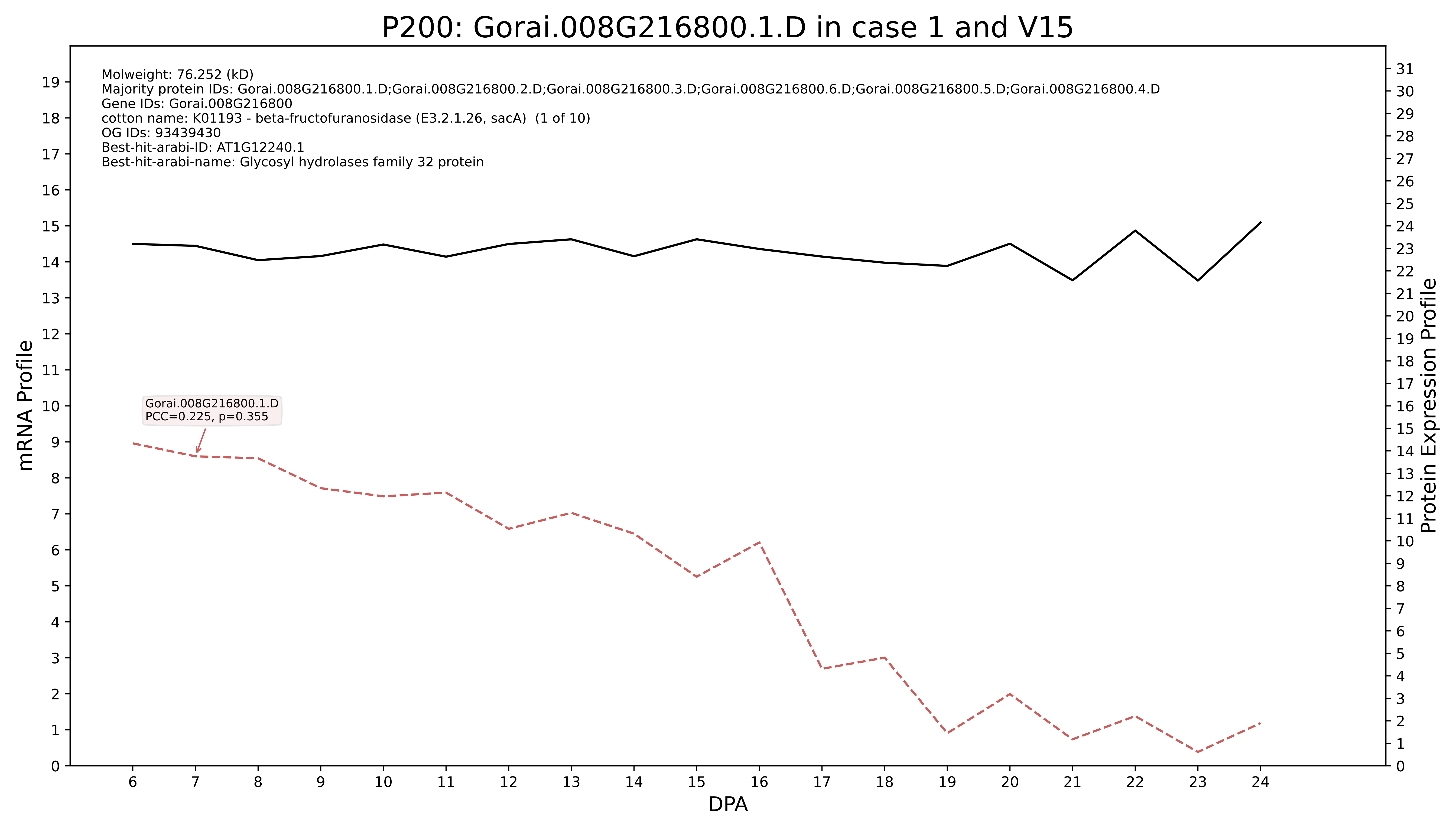
P200: Gorai.008G216800.1.D in case 1 and V15
Molweight: 76.252 (kD)
Majority protein IDs: Gorai.008G216800.1.D;Gorai.008G216800.2.D;Gorai.008G216800.3.D;Gorai.008G216800.6.D;Gorai.008G216800.5.D;Gorai.008G216800.4.D
Gene IDs: Gorai.008G216800
cotton name: K01193 - beta-fructofuranosidase (E3.2.1.26, sacA) (1 of 10)
OG IDs: 93439430
Best-hit-arabi-ID: AT1G12240.1
Best-hit-arabi-name: Glycosyl hydrolases family 32 protein
Gorai.008G216800.1.D
PCC=0.225, p=0.355
mRNA Profile
Protein Expression Profile
DPA

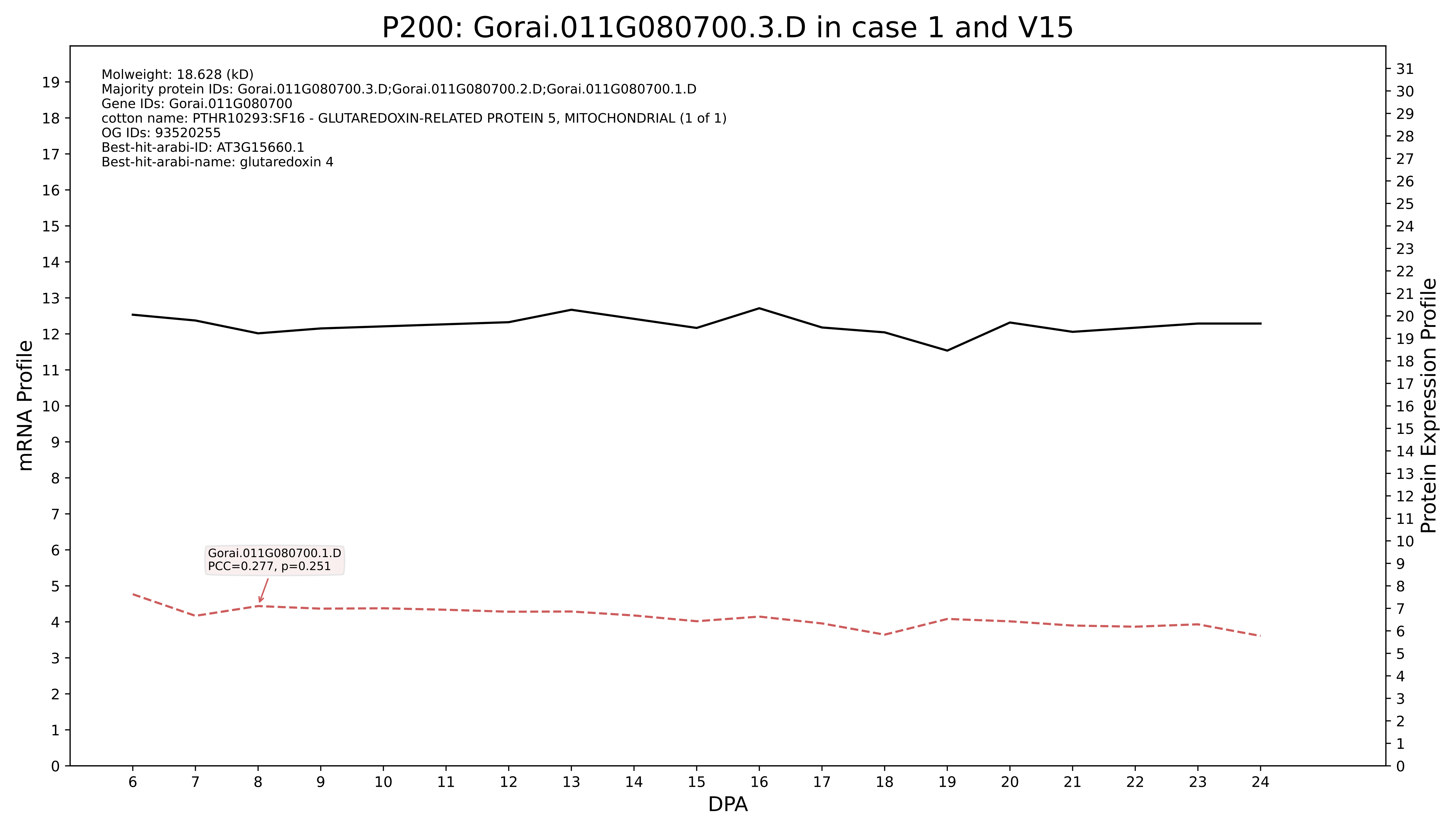
P200: Gorai.011G080700.3.D in case 1 and V15
Molweight: 18.628 (kD)
Majority protein IDs: Gorai.011G080700.3.D;Gorai.011G080700.2.D;Gorai.011G080700.1.D
Gene IDs: Gorai.011G080700
cotton name: PTHR10293:SF16 - GLUTAREDOXIN-RELATED PROTEIN 5, MITOCHONDRIAL (1 of 1)
OG IDs: 93520255
Best-hit-arabi-ID: AT3G15660.1
Best-hit-arabi-name: glutaredoxin 4
Gorai.011G080700.1.D
PCC=0.277, p=0.251
mRNA Profile
Protein Expression Profile
DPA

P200: Gorai.009G000700.1.A in case 1 and V15

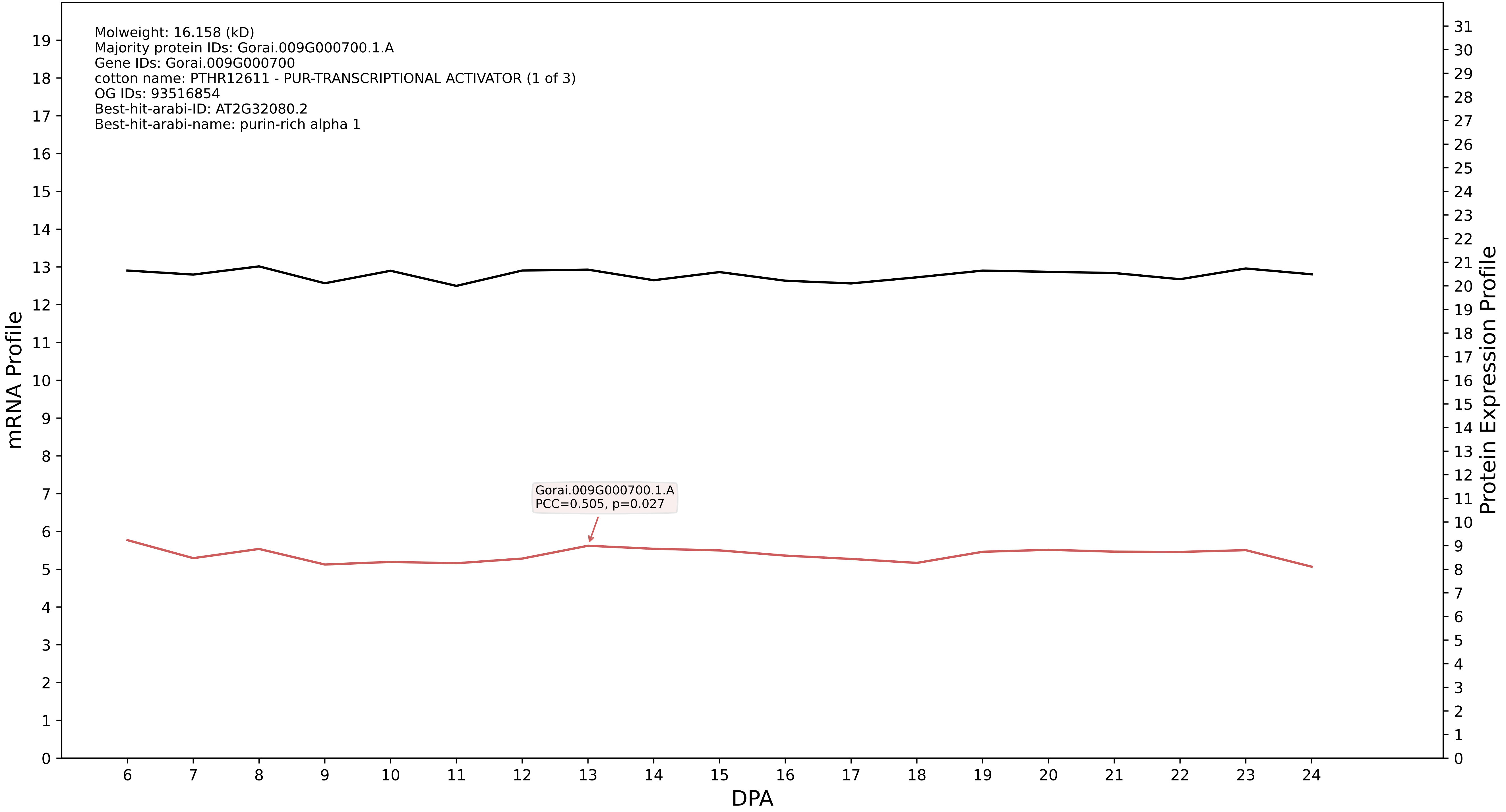

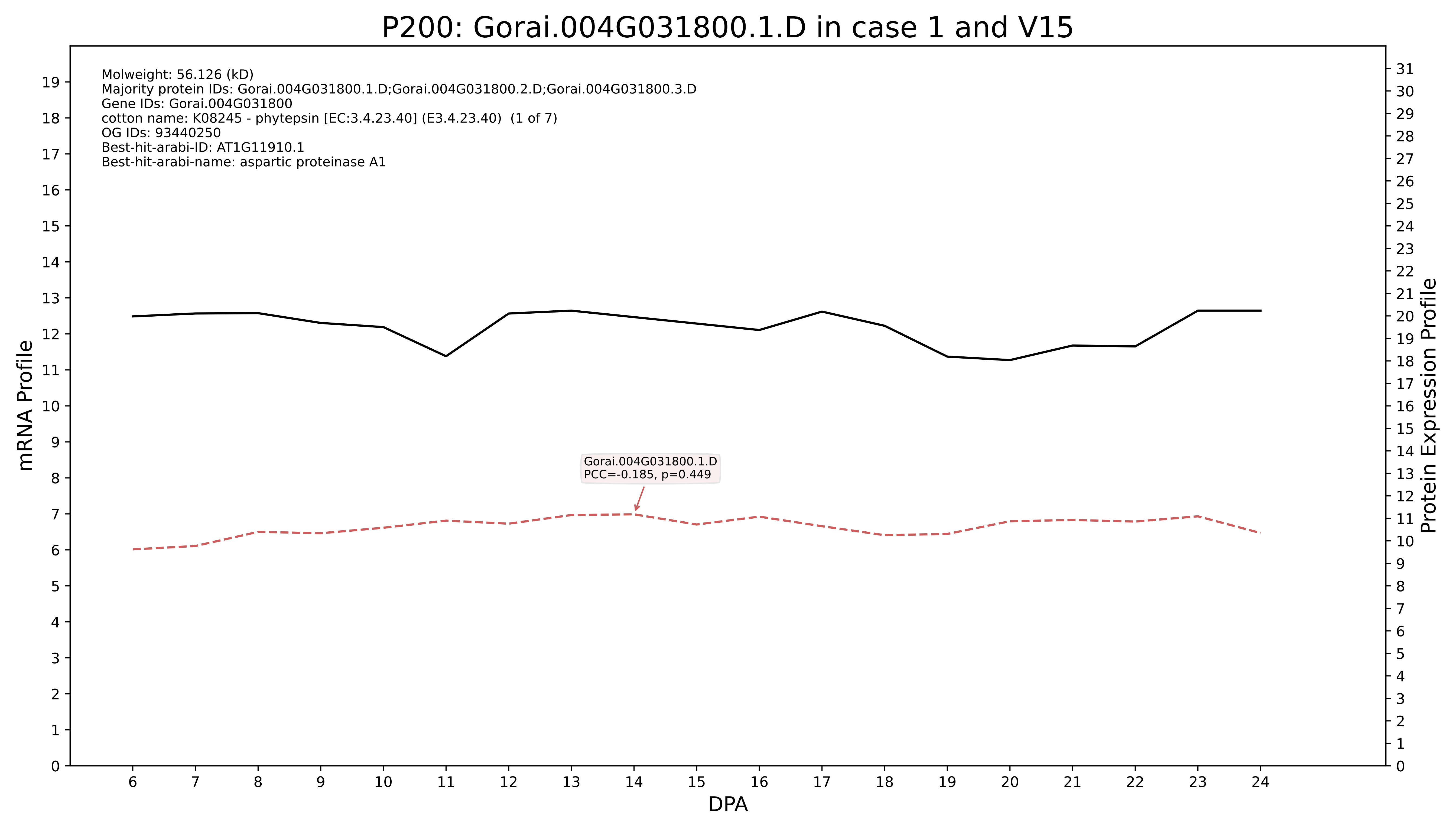
P200: Gorai.004G031800.1.D in case 1 and V15
Molweight: 56.126 (kD)
Majority protein IDs: Gorai.004G031800.1.D;Gorai.004G031800.2.D;Gorai.004G031800.3.D
Gene IDs: Gorai.004G031800
cotton name: K08245 - phytepsin [EC:3.4.23.40] (E3.4.23.40) (1 of 7)
OG IDs: 93440250
Best-hit-arabi-ID: AT1G11910.1
Best-hit-arabi-name: aspartic proteinase A1
Gorai.004G031800.1.D
PCC=-0.185, p=0.449
mRNA Profile
Protein Expression Profile
DPA

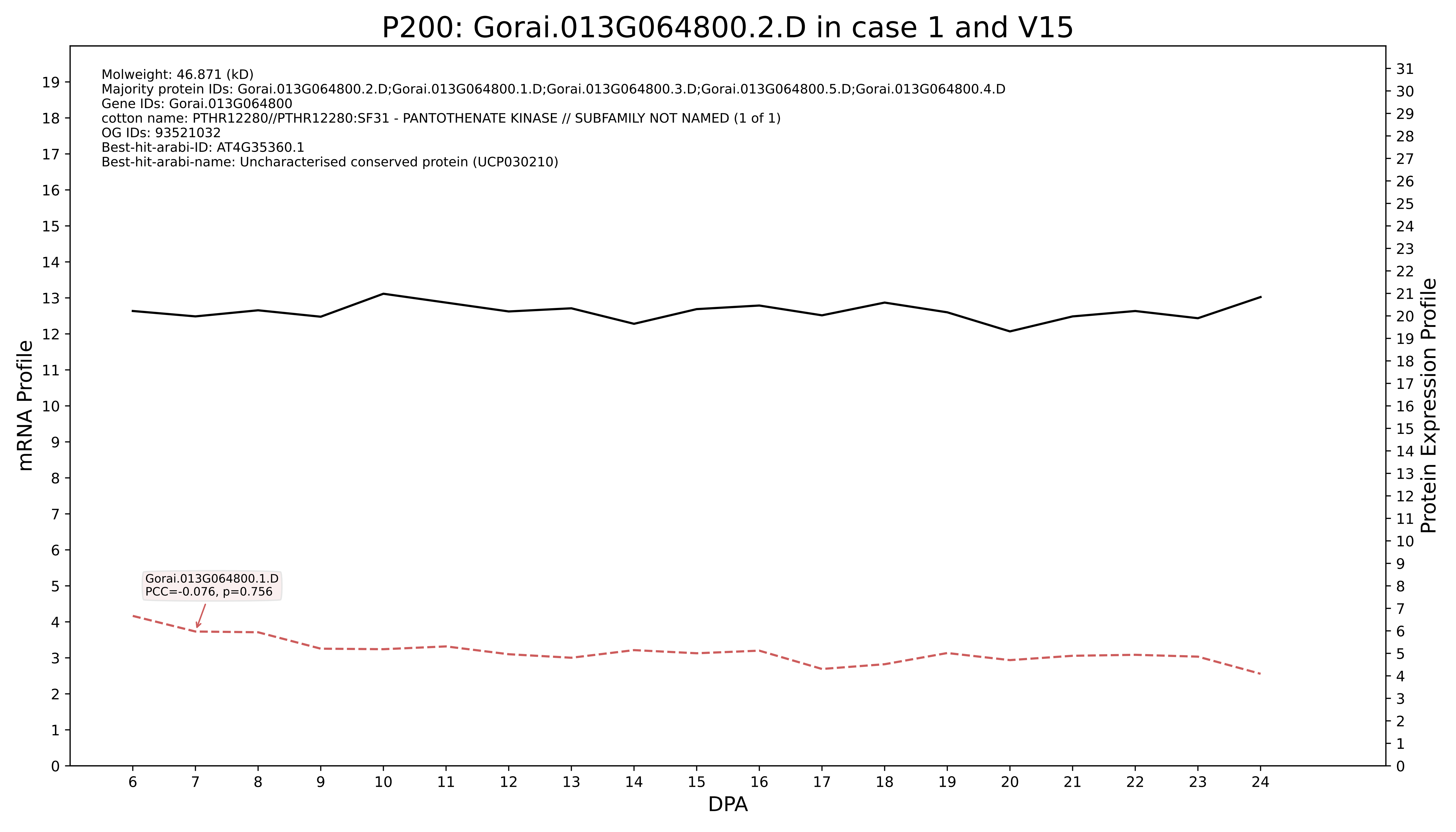

P200: Gorai.013G064800.2.D in case 1 and V15
Molweight: 46.871 (kD)
Majority protein IDs: Gorai.013G064800.2.D;Gorai.013G064800.1.D;Gorai.013G064800.3.D;Gorai.013G064800.5.D;Gorai.013G064800.4.D
Gene IDs: Gorai.013G064800
cotton name: PTHR12280//PTHR12280:SF31 - PANTOTHENATE KINASE // SUBFAMILY NOT NAMED (1 of 1)
OG IDs: 93521032
Best-hit-arabi-ID: AT4G35360.1
Best-hit-arabi-name: Uncharacterised conserved protein (UCP030210)
Gorai.013G064800.1.D
PCC=-0.076, p=0.756
mRNA Profile
Protein Expression Profile
DPA

P200: Gorai.003G107800.3.A in case 1 and V15

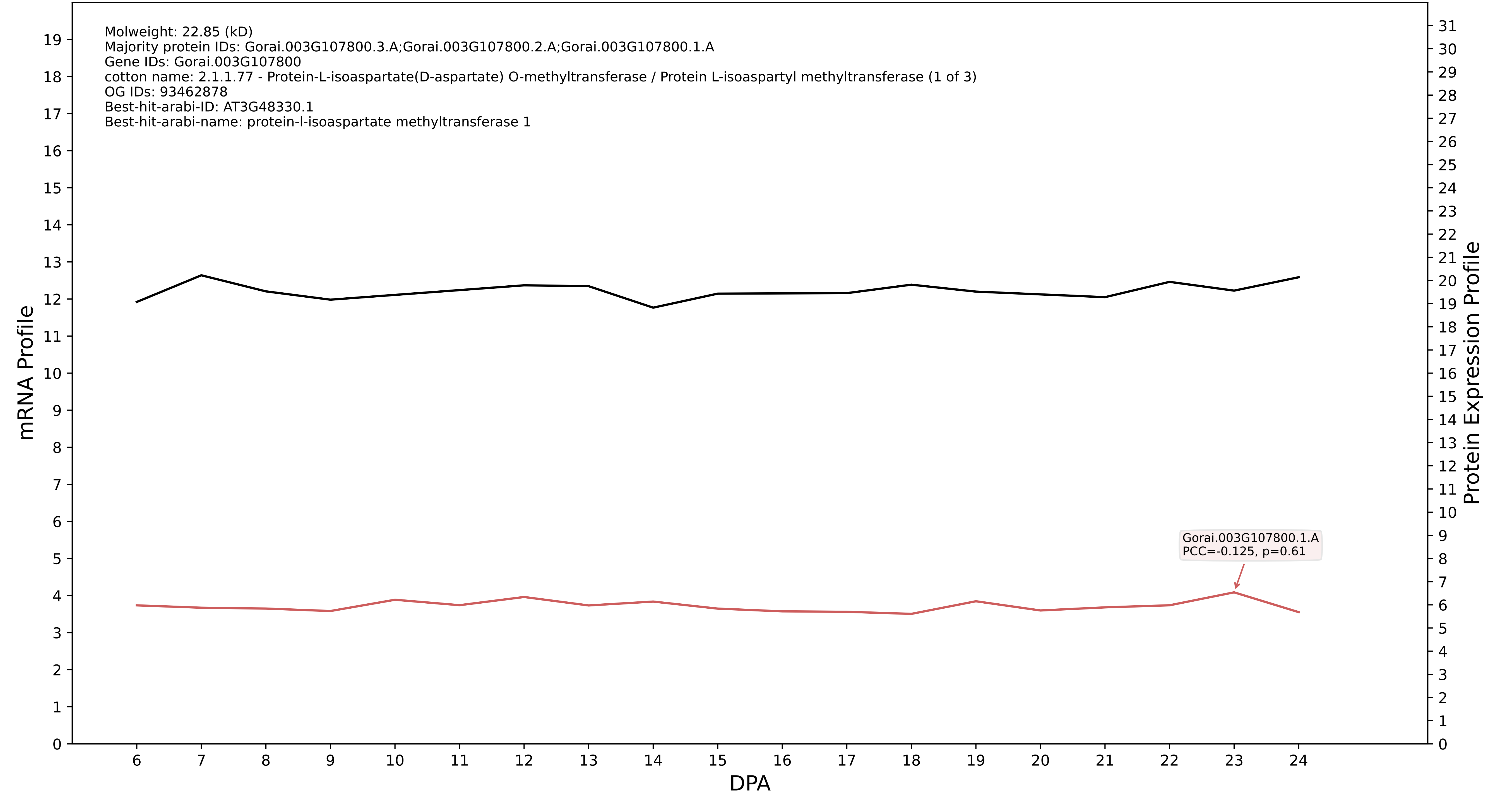

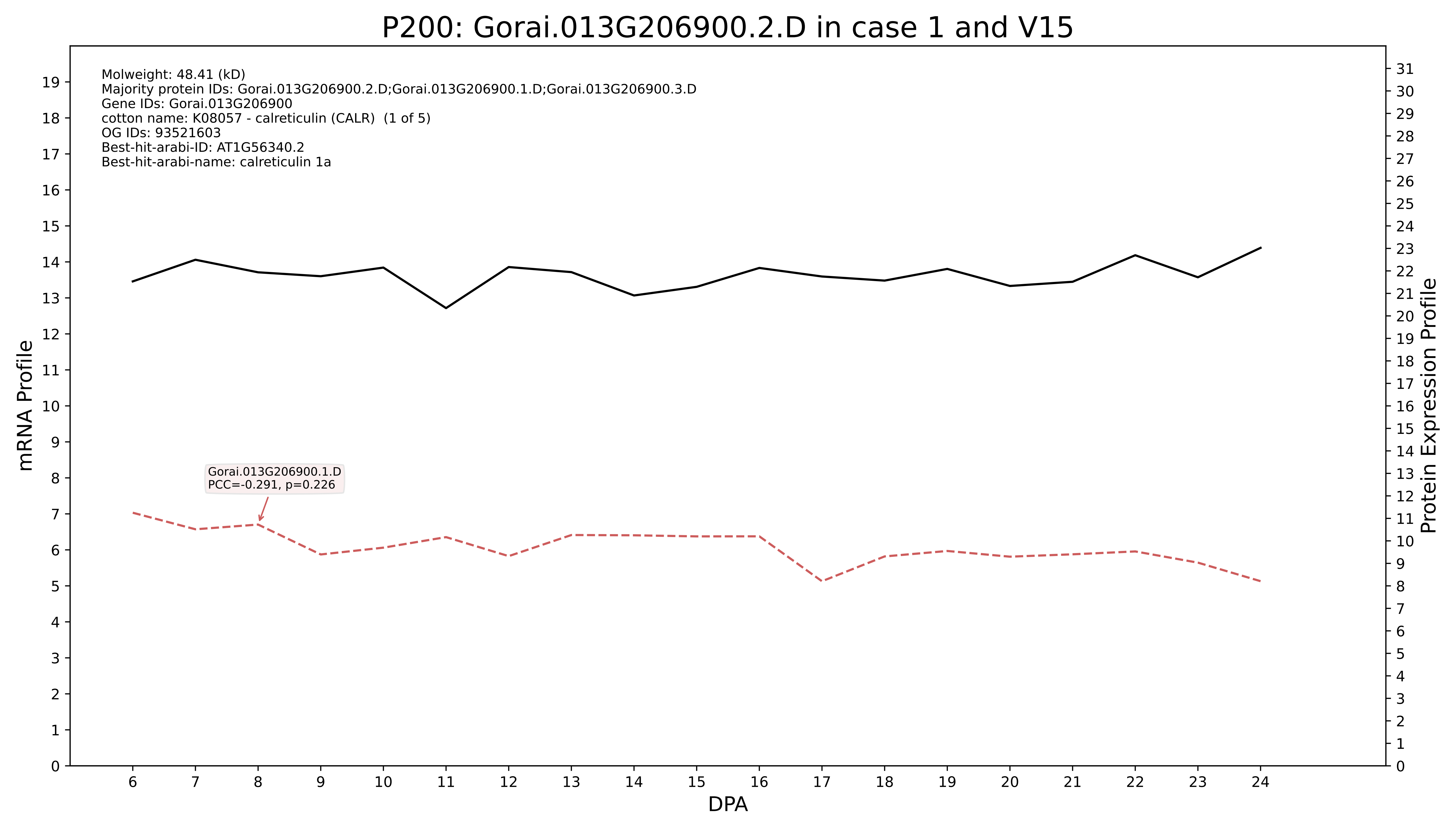
P200: Gorai.013G206900.2.D in case 1 and V15
Molweight: 48.41 (kD)
Majority protein IDs: Gorai.013G206900.2.D;Gorai.013G206900.1.D;Gorai.013G206900.3.D
Gene IDs: Gorai.013G206900
cotton name: K08057 - calreticulin (CALR) (1 of 5)
OG IDs: 93521603
Best-hit-arabi-ID: AT1G56340.2
Best-hit-arabi-name: calreticulin 1a
Gorai.013G206900.1.D
PCC=-0.291, p=0.226
mRNA Profile
Protein Expression Profile
DPA

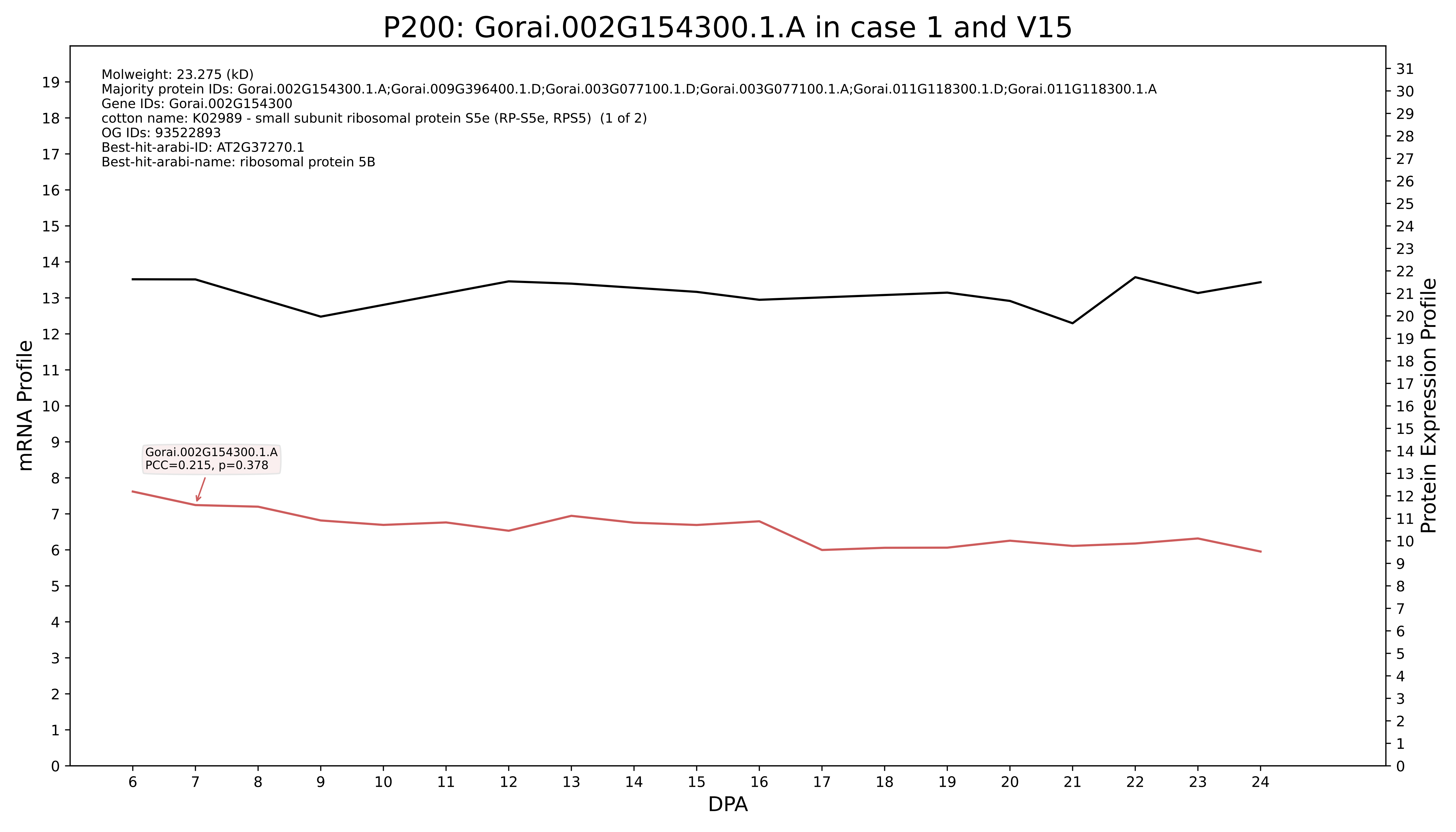
P200: Gorai.002G154300.1.A in case 1 and V15
Molweight: 23.275 (kD)
Majority protein IDs: Gorai.002G154300.1.A;Gorai.009G396400.1.D;Gorai.003G077100.1.D;Gorai.003G077100.1.A;Gorai.011G118300.1.D;Gorai.011G118300.1.A
Gene IDs: Gorai.002G154300
cotton name: K02989 - small subunit ribosomal protein S5e (RP-S5e, RPS5) (1 of 2)
OG IDs: 93522893
Best-hit-arabi-ID: AT2G37270.1
Best-hit-arabi-name: ribosomal protein 5B
Gorai.002G154300.1.A
PCC=0.215, p=0.378
mRNA Profile
Protein Expression Profile
DPA

P200: Gorai.005G226100.1.D in case 1 and V15

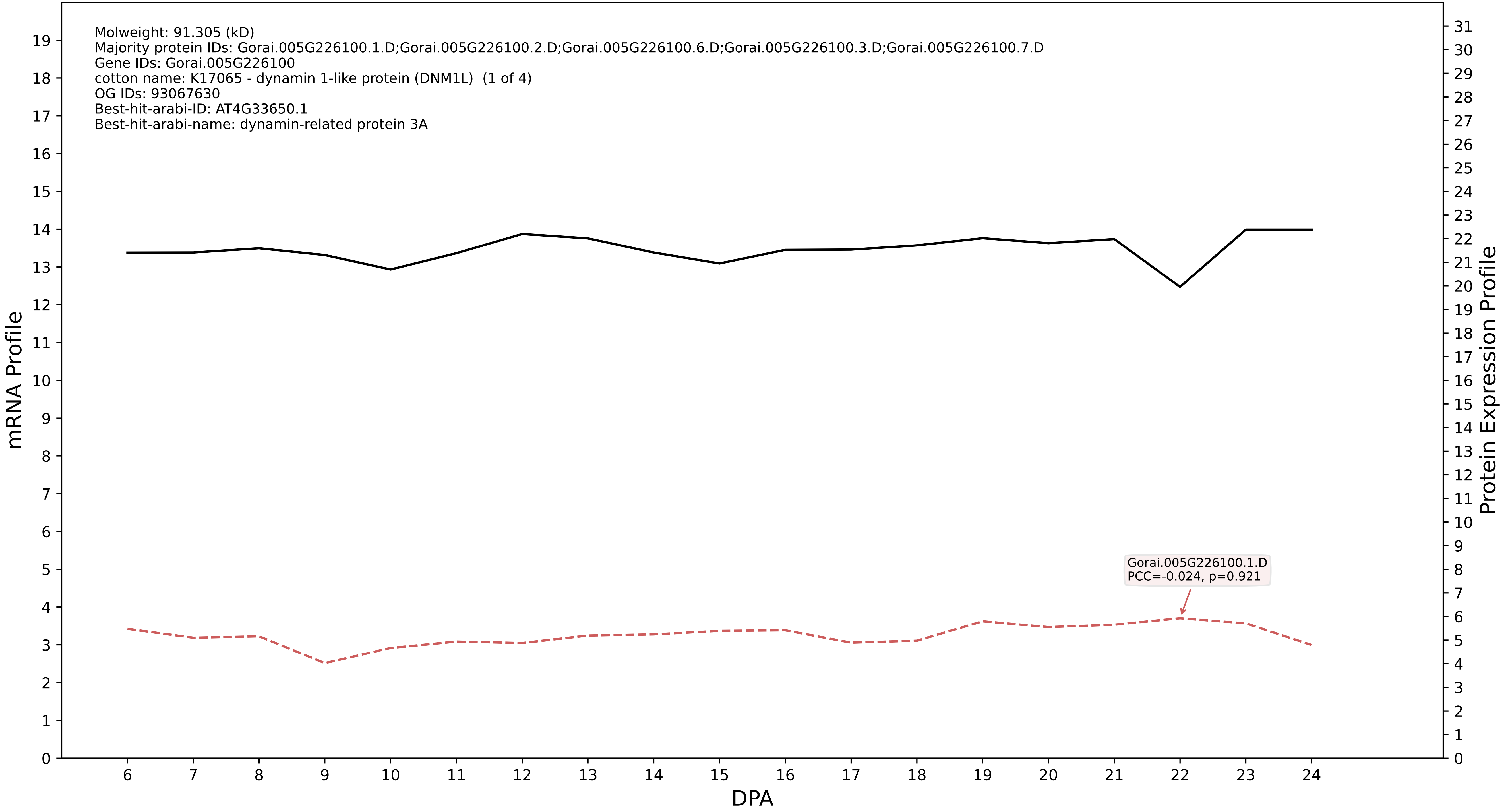

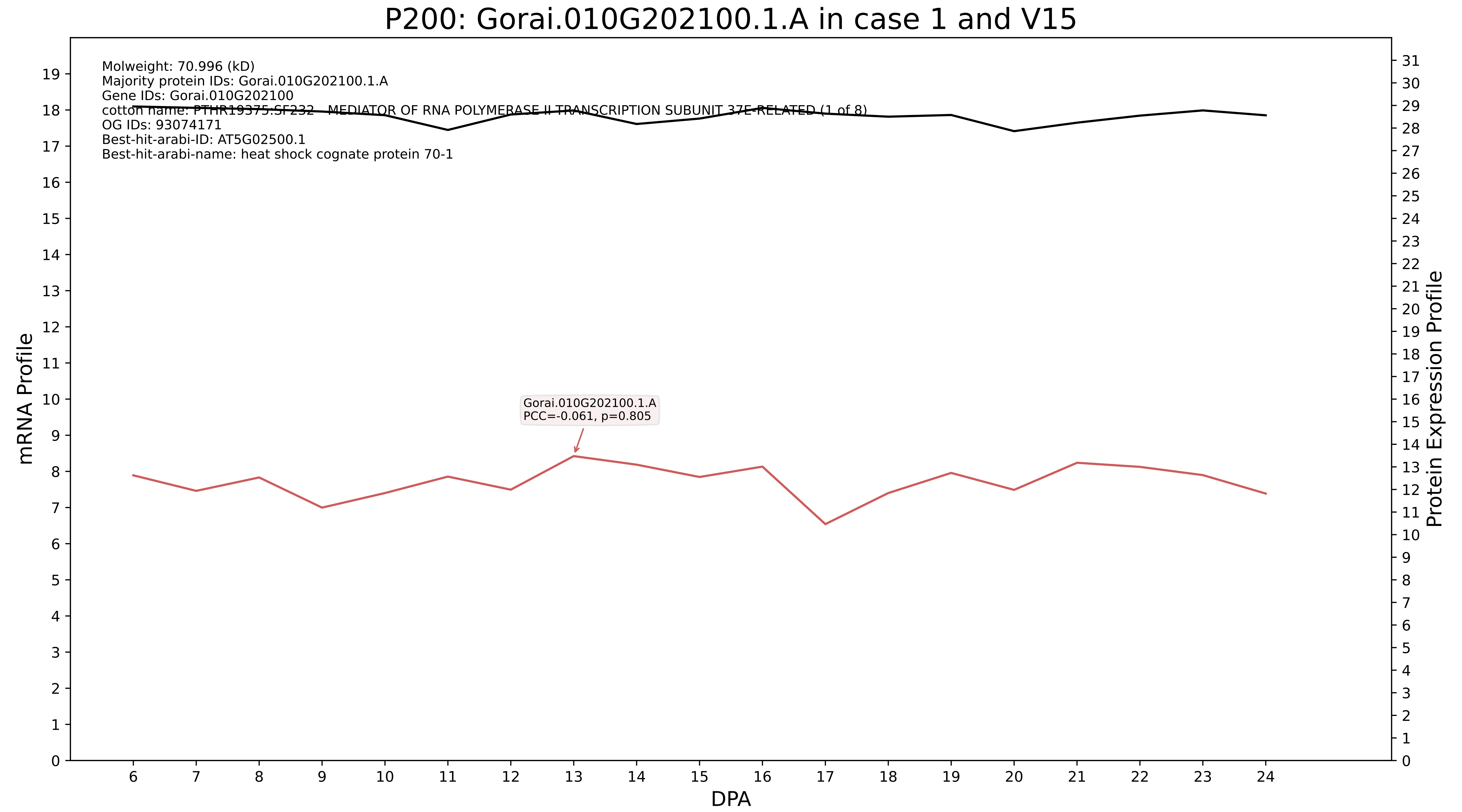
P200: Gorai.010G202100.1.A in case 1 and V15
Molweight: 70.996 (kD)
Majority protein IDs: Gorai.010G202100.1.A
Gene IDs: Gorai.010G202100
cotton name: PTHR19375:SF232 - MEDIATOR OF RNA POLYMERASE II TRANSCRIPTION SUBUNIT 37E-RELATED (1 of 8)
OG IDs: 93074171
Best-hit-arabi-ID: AT5G02500.1
Best-hit-arabi-name: heat shock cognate protein 70-1
Gorai.010G202100.1.A
PCC=-0.061, p=0.805
mRNA Profile
Protein Expression Profile
DPA

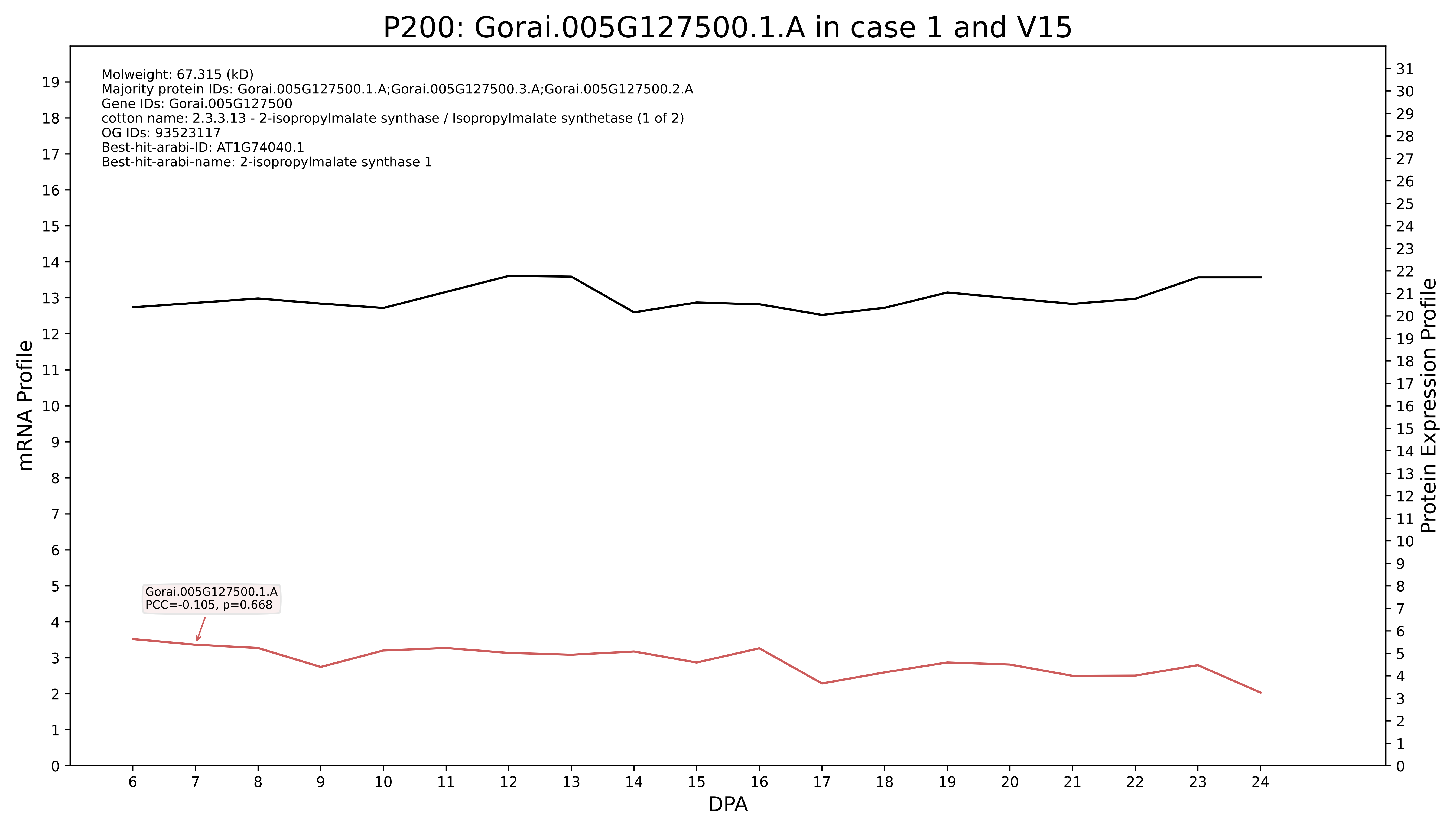

P200: Gorai.005G127500.1.A in case 1 and V15
Molweight: 67.315 (kD)
Majority protein IDs: Gorai.005G127500.1.A;Gorai.005G127500.3.A;Gorai.005G127500.2.A
Gene IDs: Gorai.005G127500
cotton name: 2.3.3.13 - 2-isopropylmalate synthase / Isopropylmalate synthetase (1 of 2)
OG IDs: 93523117
Best-hit-arabi-ID: AT1G74040.1
Best-hit-arabi-name: 2-isopropylmalate synthase 1
Gorai.005G127500.1.A
PCC=-0.105, p=0.668
mRNA Profile
Protein Expression Profile
DPA

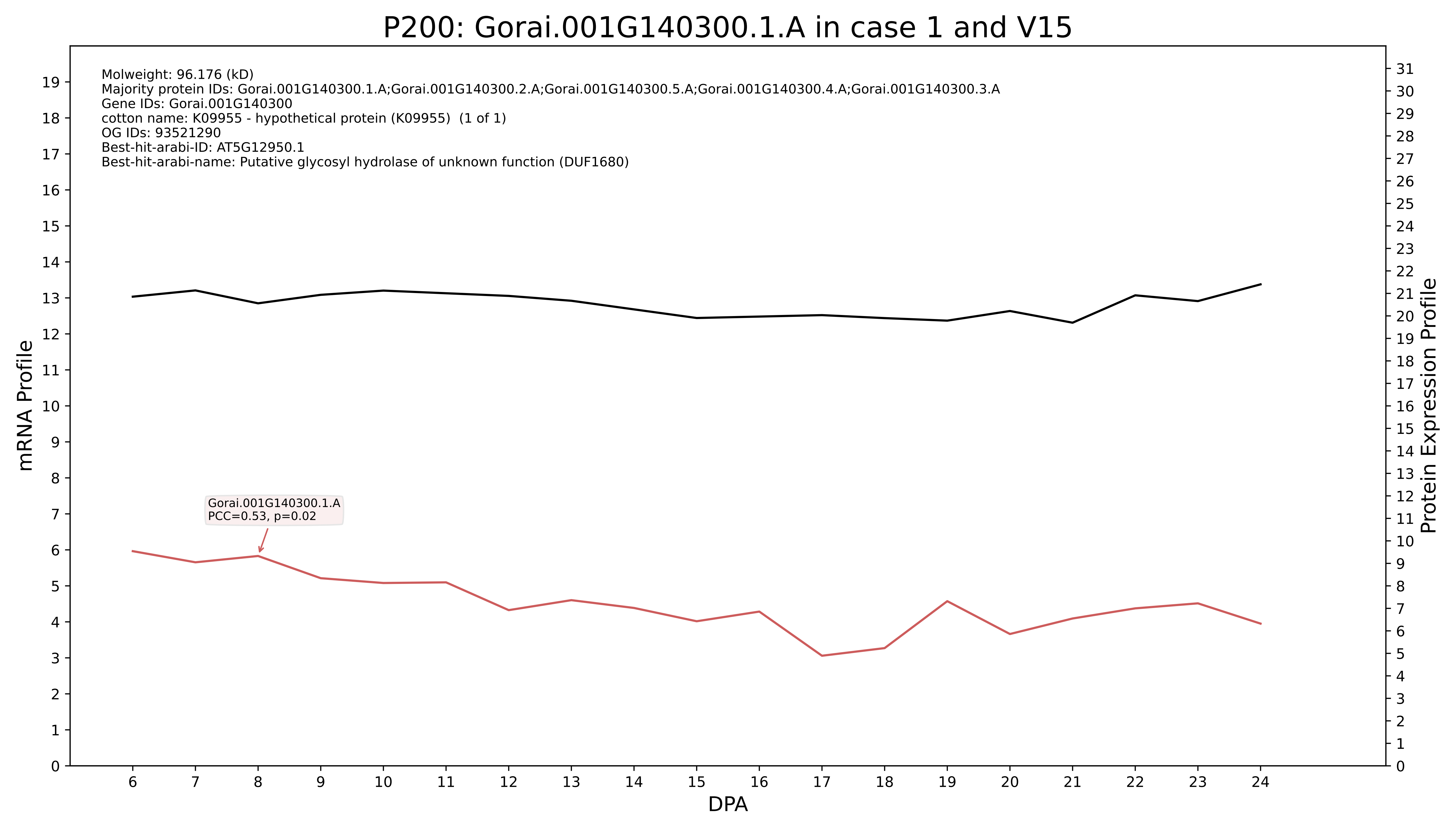
P200: Gorai.001G140300.1.A in case 1 and V15
Molweight: 96.176 (kD)
Majority protein IDs: Gorai.001G140300.1.A;Gorai.001G140300.2.A;Gorai.001G140300.5.A;Gorai.001G140300.4.A;Gorai.001G140300.3.A
Gene IDs: Gorai.001G140300
cotton name: K09955 - hypothetical protein (K09955) (1 of 1)
OG IDs: 93521290
Best-hit-arabi-ID: AT5G12950.1
Best-hit-arabi-name: Putative glycosyl hydrolase of unknown function (DUF1680)
Gorai.001G140300.1.A
PCC=0.53, p=0.02
mRNA Profile
Protein Expression Profile
DPA
0
1
2
3
4
5
6
7
8
9
10
11
12
13
14
15
16
17
18
19
20
21
22
23
24
25
26
27
28
29
30
31

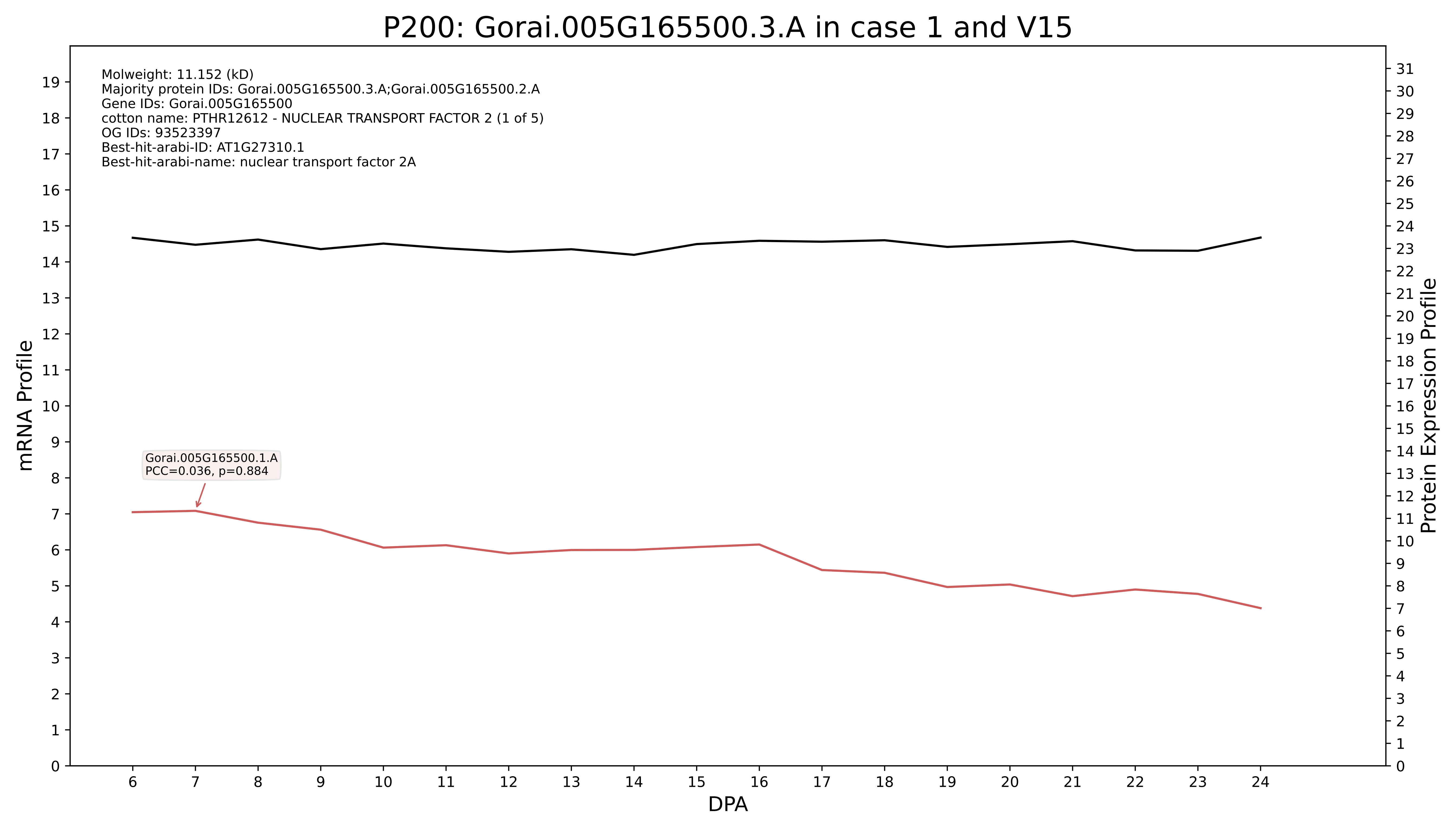

P200: Gorai.005G165500.3.A in case 1 and V15
Molweight: 11.152 (kD)
Majority protein IDs: Gorai.005G165500.3.A;Gorai.005G165500.2.A
Gene IDs: Gorai.005G165500
cotton name: PTHR12612 - NUCLEAR TRANSPORT FACTOR 2 (1 of 5)
OG IDs: 93523397
Best-hit-arabi-ID: AT1G27310.1
Best-hit-arabi-name: nuclear transport factor 2A
Gorai.005G165500.1.A
PCC=0.036, p=0.884
mRNA Profile
Protein Expression Profile
DPA

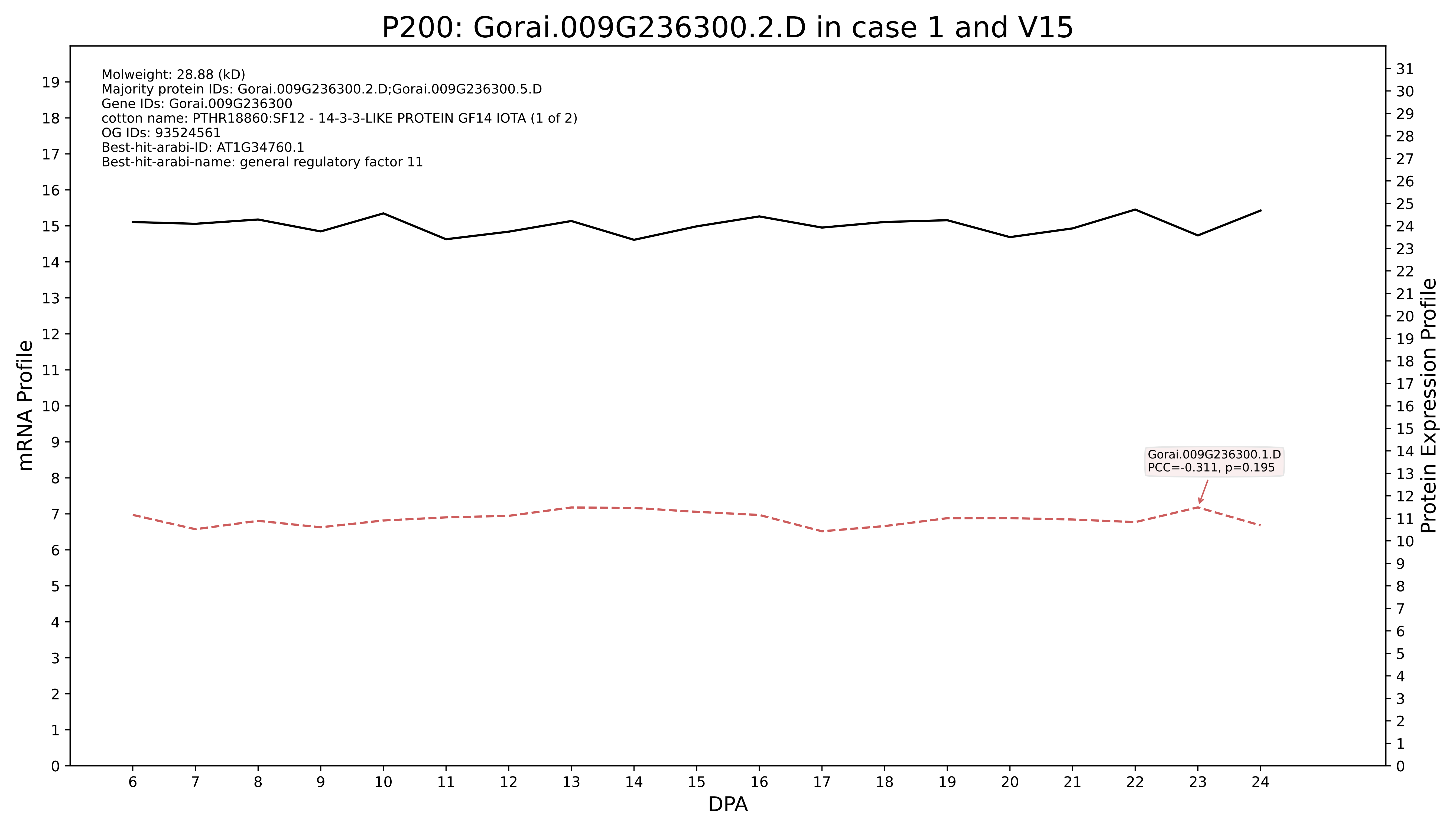

P200: Gorai.009G236300.2.D in case 1 and V15
Molweight: 28.88 (kD)
Majority protein IDs: Gorai.009G236300.2.D;Gorai.009G236300.5.D
Gene IDs: Gorai.009G236300
cotton name: PTHR18860:SF12 - 14-3-3-LIKE PROTEIN GF14 IOTA (1 of 2)
OG IDs: 93524561
Best-hit-arabi-ID: AT1G34760.1
Best-hit-arabi-name: general regulatory factor 11
Gorai.009G236300.1.D
PCC=-0.311, p=0.195
mRNA Profile
Protein Expression Profile
DPA

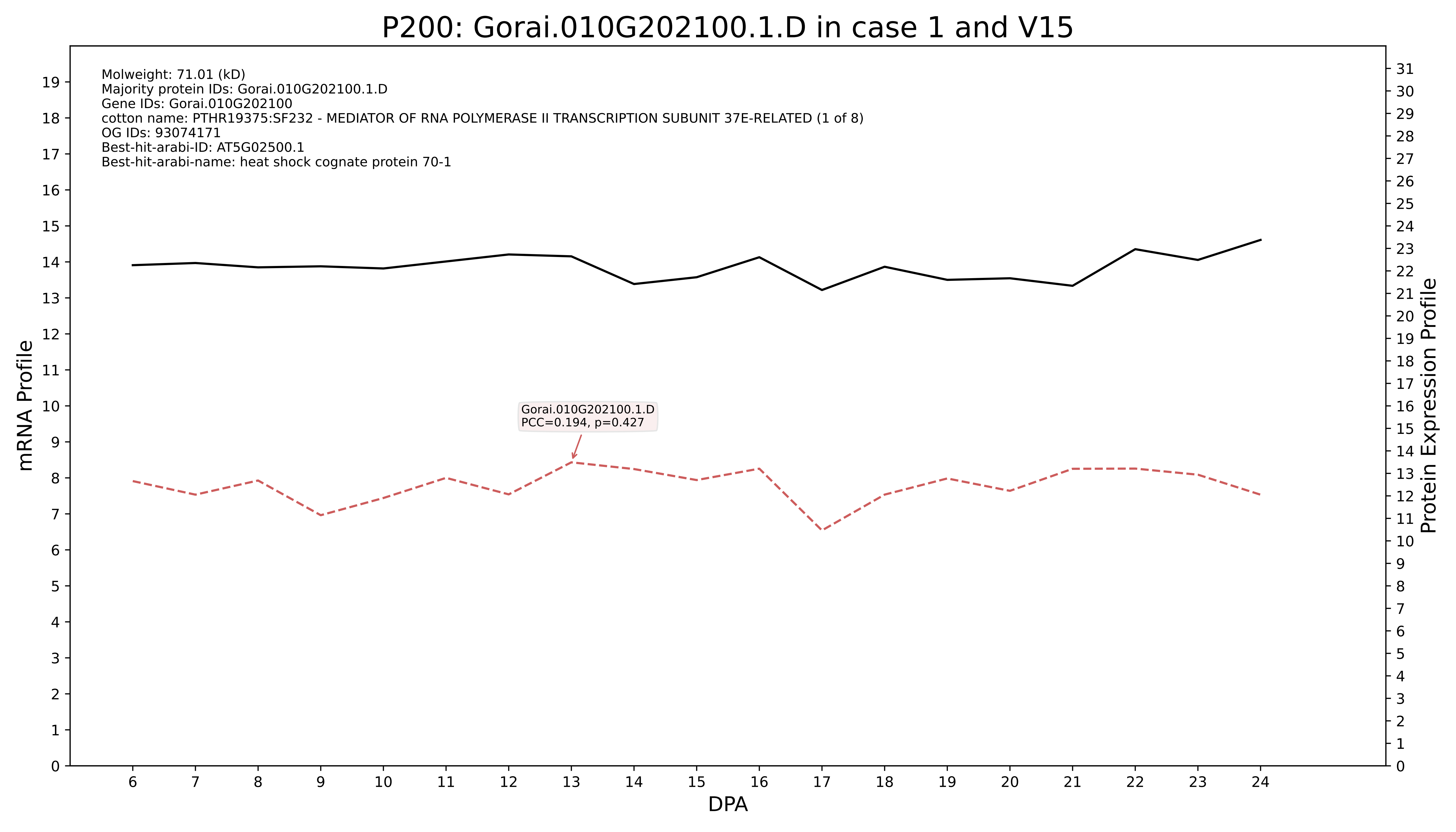

P200: Gorai.010G202100.1.D in case 1 and V15
Molweight: 71.01 (kD)
Majority protein IDs: Gorai.010G202100.1.D
Gene IDs: Gorai.010G202100
cotton name: PTHR19375:SF232 - MEDIATOR OF RNA POLYMERASE II TRANSCRIPTION SUBUNIT 37E-RELATED (1 of 8)
OG IDs: 93074171
Best-hit-arabi-ID: AT5G02500.1
Best-hit-arabi-name: heat shock cognate protein 70-1
Gorai.010G202100.1.D
PCC=0.194, p=0.427
mRNA Profile
Protein Expression Profile
DPA

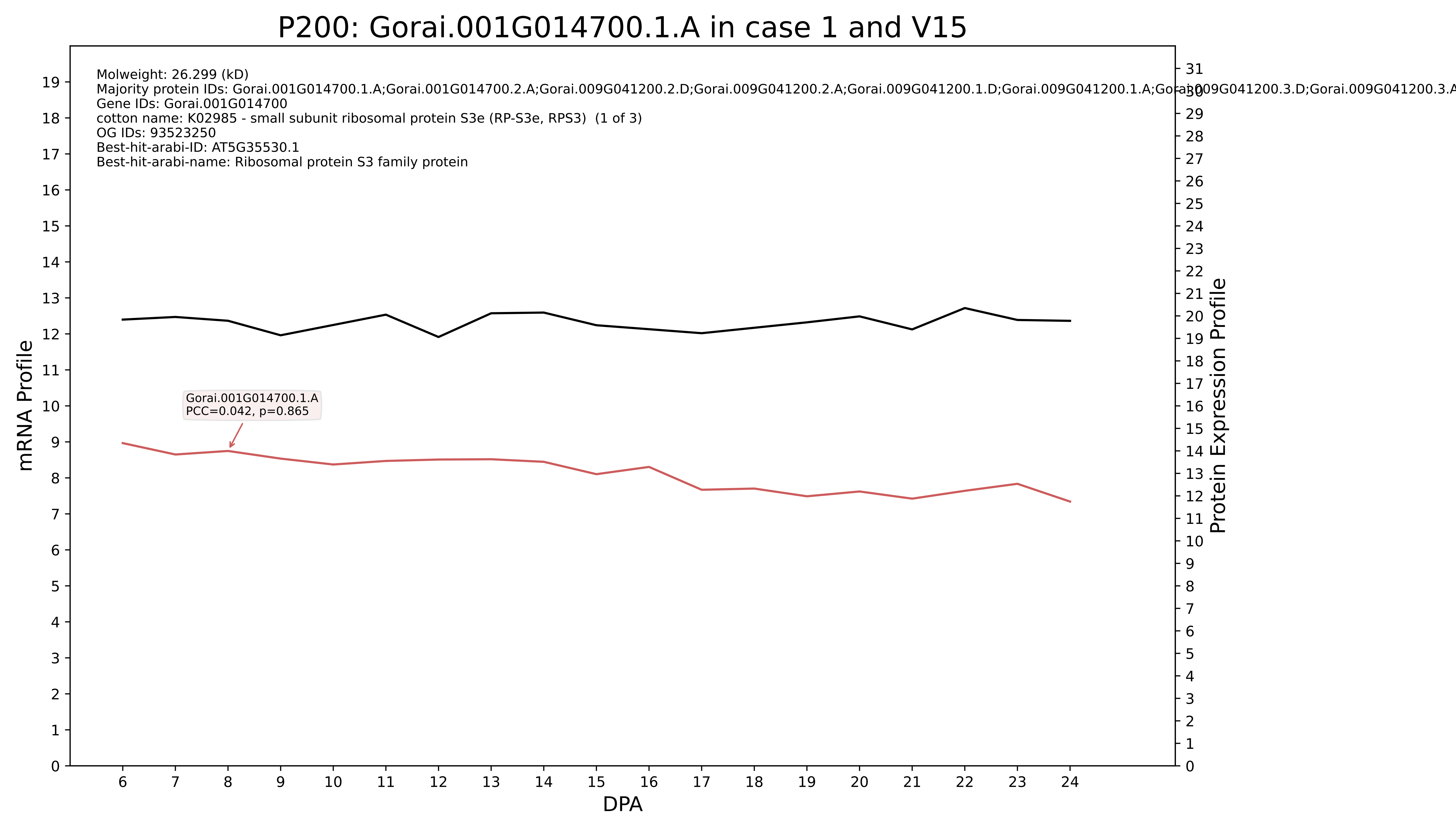

P200: Gorai.001G014700.1.A in case 1 and V15
Molweight: 26.299 (kD)
Majority protein IDs: Gorai.001G014700.1.A;Gorai.001G014700.2.A;Gorai.009G041200.2.D;Gorai.009G041200.2.A;Gorai.009G041200.1.D;Gorai.009G041200.1.A;Gorai.009G041200.3.D;Gorai.009G041200.3.A
Gene IDs: Gorai.001G014700
cotton name: K02985 - small subunit ribosomal protein S3e (RP-S3e, RPS3) (1 of 3)
OG IDs: 93523250
Best-hit-arabi-ID: AT5G35530.1
Best-hit-arabi-name: Ribosomal protein S3 family protein
Gorai.001G014700.1.A
PCC=0.042, p=0.865
mRNA Profile
Protein Expression Profile
DPA

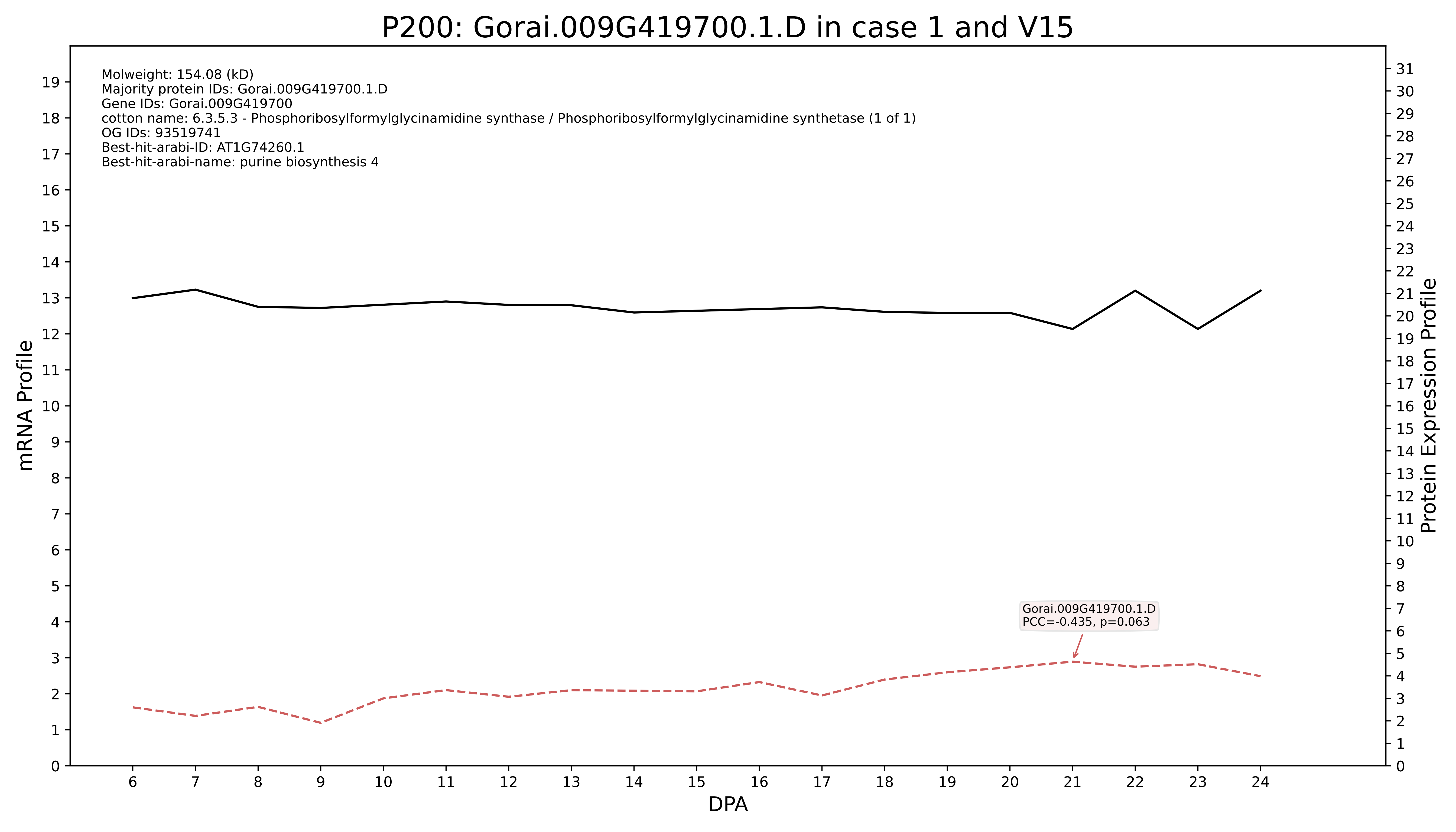
P200: Gorai.009G419700.1.D in case 1 and V15
Molweight: 154.08 (kD)
Majority protein IDs: Gorai.009G419700.1.D
Gene IDs: Gorai.009G419700
cotton name: 6.3.5.3 - Phosphoribosylformylglycinamidine synthase / Phosphoribosylformylglycinamidine synthetase (1 of 1)
OG IDs: 93519741
Best-hit-arabi-ID: AT1G74260.1
Best-hit-arabi-name: purine biosynthesis 4
Gorai.009G419700.1.D
PCC=-0.435, p=0.063
mRNA Profile
Protein Expression Profile
DPA

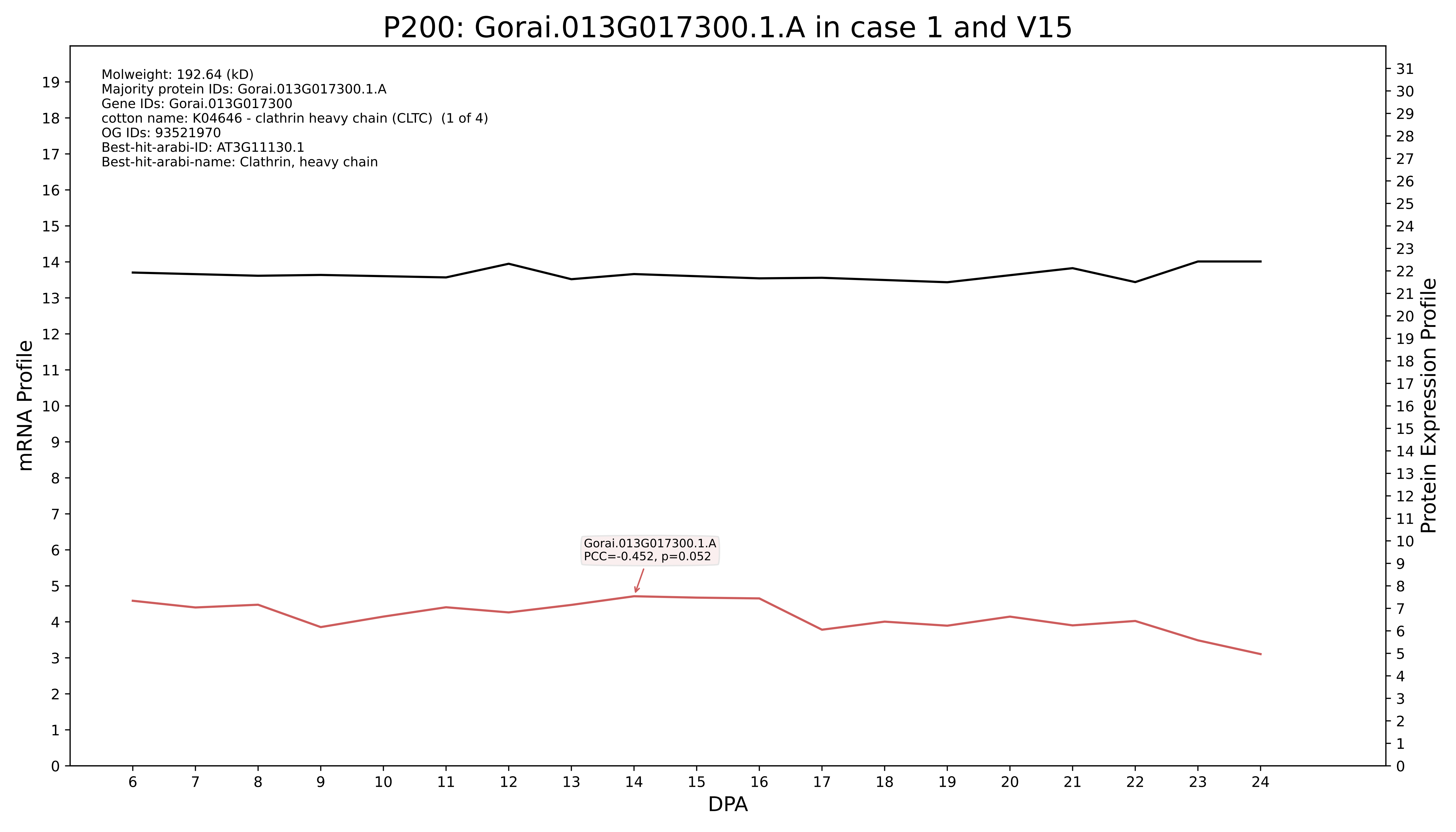

P200: Gorai.013G017300.1.A in case 1 and V15
Molweight: 192.64 (kD)
Majority protein IDs: Gorai.013G017300.1.A
Gene IDs: Gorai.013G017300
cotton name: K04646 - clathrin heavy chain (CLTC) (1 of 4)
OG IDs: 93521970
Best-hit-arabi-ID: AT3G11130.1
Best-hit-arabi-name: Clathrin, heavy chain
Gorai.013G017300.1.A
PCC=-0.452, p=0.052
mRNA Profile
Protein Expression Profile
DPA

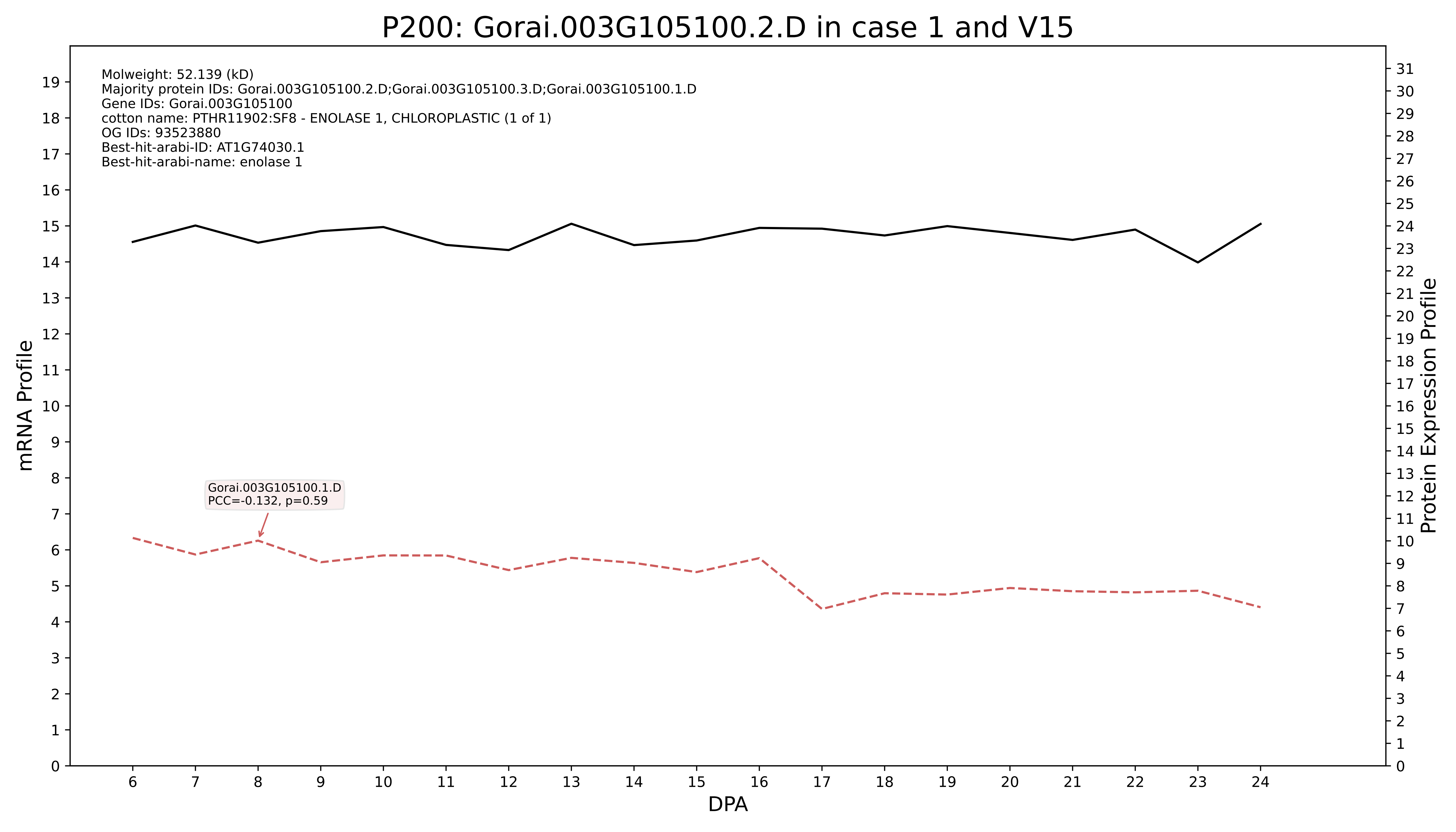
P200: Gorai.003G105100.2.D in case 1 and V15
Molweight: 52.139 (kD)
Majority protein IDs: Gorai.003G105100.2.D;Gorai.003G105100.3.D;Gorai.003G105100.1.D
Gene IDs: Gorai.003G105100
cotton name: PTHR11902:SF8 - ENOLASE 1, CHLOROPLASTIC (1 of 1)
OG IDs: 93523880
Best-hit-arabi-ID: AT1G74030.1
Best-hit-arabi-name: enolase 1
Gorai.003G105100.1.D
PCC=-0.132, p=0.59
mRNA Profile
Protein Expression Profile
DPA

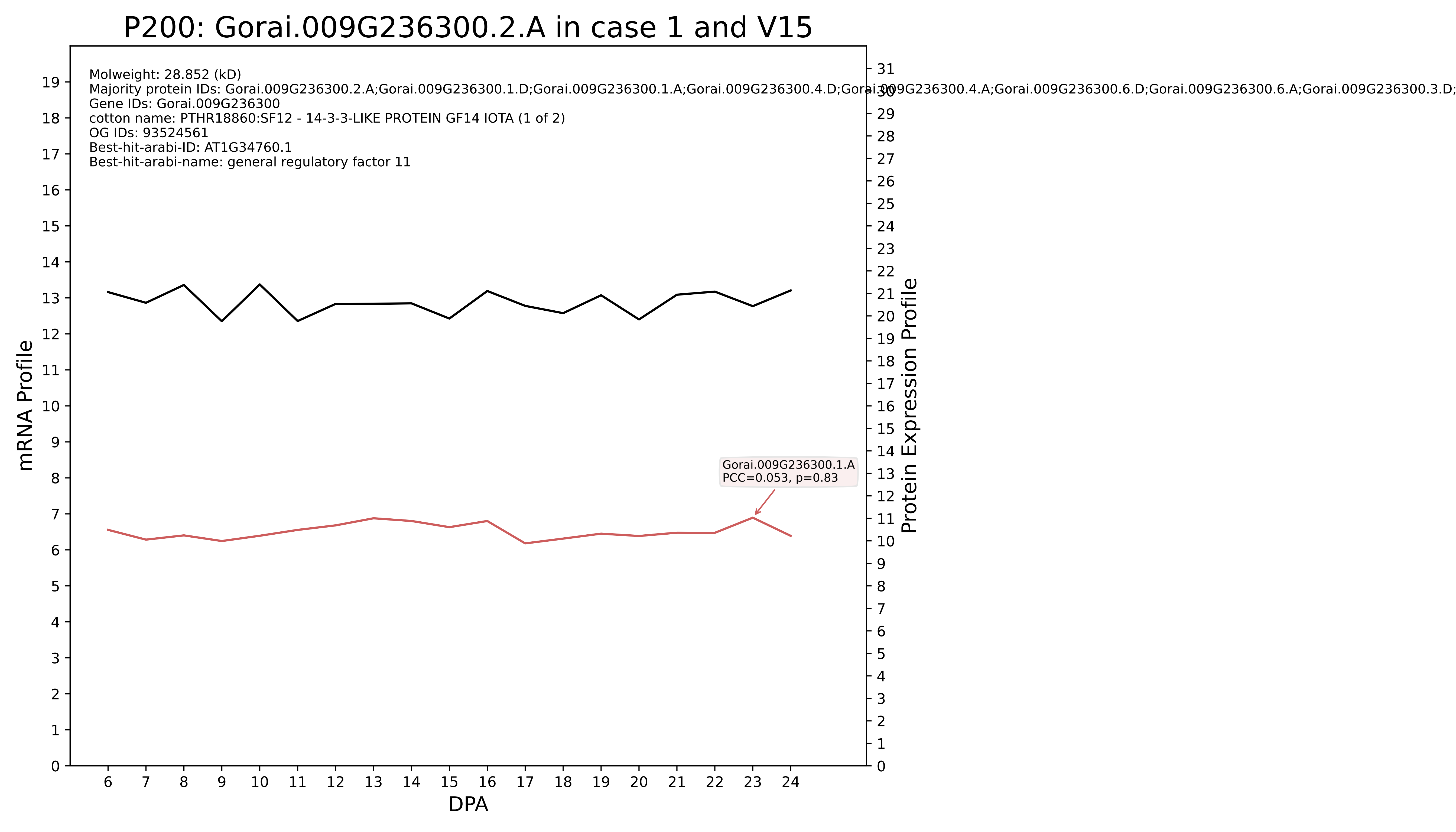
P200: Gorai.009G236300.2.A in case 1 and V15
Molweight: 28.852 (kD)
Majority protein IDs: Gorai.009G236300.2.A;Gorai.009G236300.1.D;Gorai.009G236300.1.A;Gorai.009G236300.4.D;Gorai.009G236300.4.A;Gorai.009G236300.6.D;Gorai.009G236300.6.A;Gorai.009G236300.3.D;
Gene IDs: Gorai.009G236300
cotton name: PTHR18860:SF12 - 14-3-3-LIKE PROTEIN GF14 IOTA (1 of 2)
OG IDs: 93524561
Best-hit-arabi-ID: AT1G34760.1
Best-hit-arabi-name: general regulatory factor 11
Gorai.009G236300.1.A
PCC=0.053, p=0.83
mRNA Profile
Protein Expression Profile
DPA

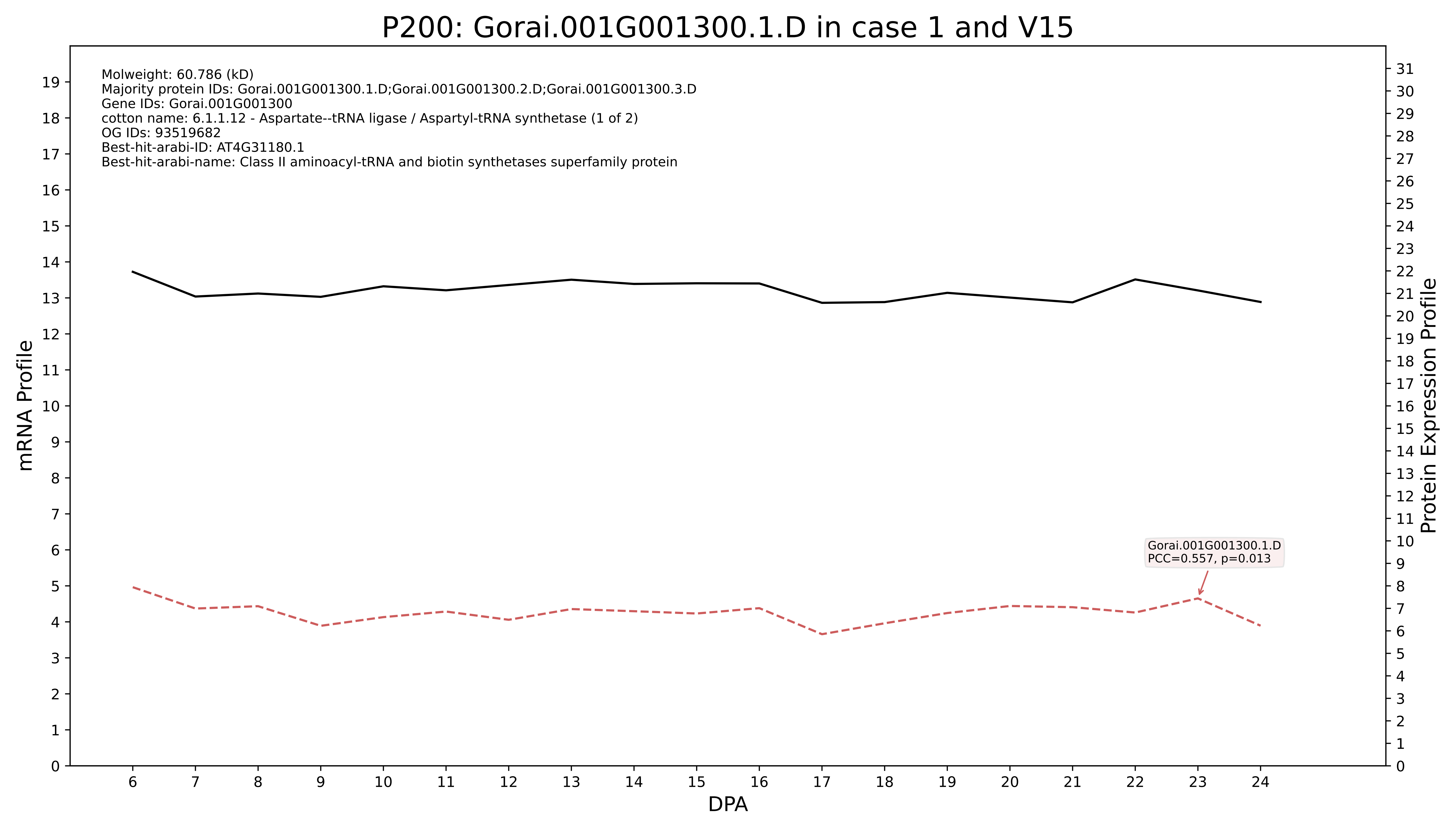
P200: Gorai.001G001300.1.D in case 1 and V15
Molweight: 60.786 (kD)
Majority protein IDs: Gorai.001G001300.1.D;Gorai.001G001300.2.D;Gorai.001G001300.3.D
Gene IDs: Gorai.001G001300
cotton name: 6.1.1.12 - Aspartate--tRNA ligase / Aspartyl-tRNA synthetase (1 of 2)
OG IDs: 93519682
Best-hit-arabi-ID: AT4G31180.1
Best-hit-arabi-name: Class II aminoacyl-tRNA and biotin synthetases superfamily protein
Gorai.001G001300.1.D
PCC=0.557, p=0.013
mRNA Profile
Protein Expression Profile
DPA

P200: Gorai.013G046900.2.D in case 1 and V15

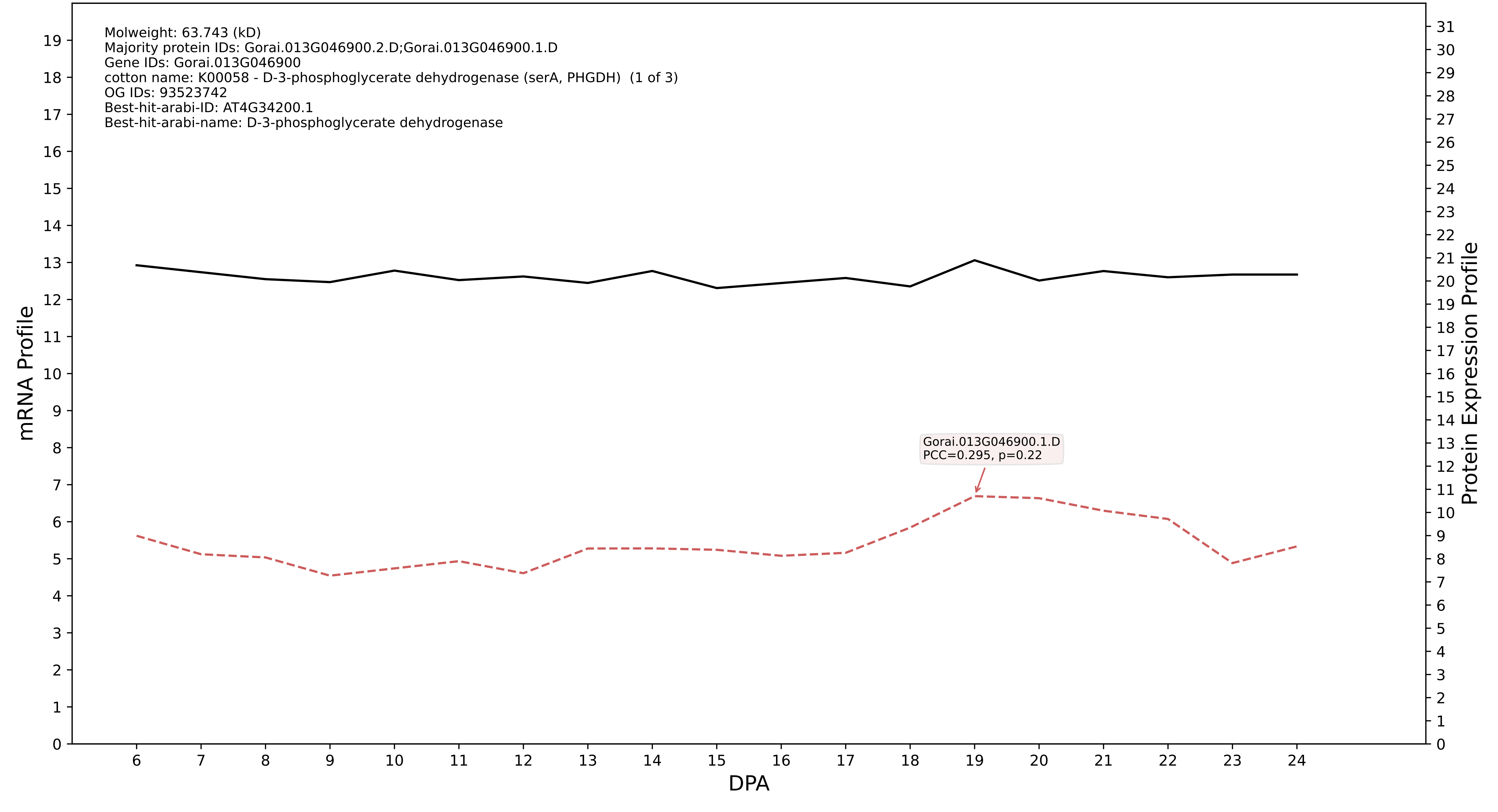

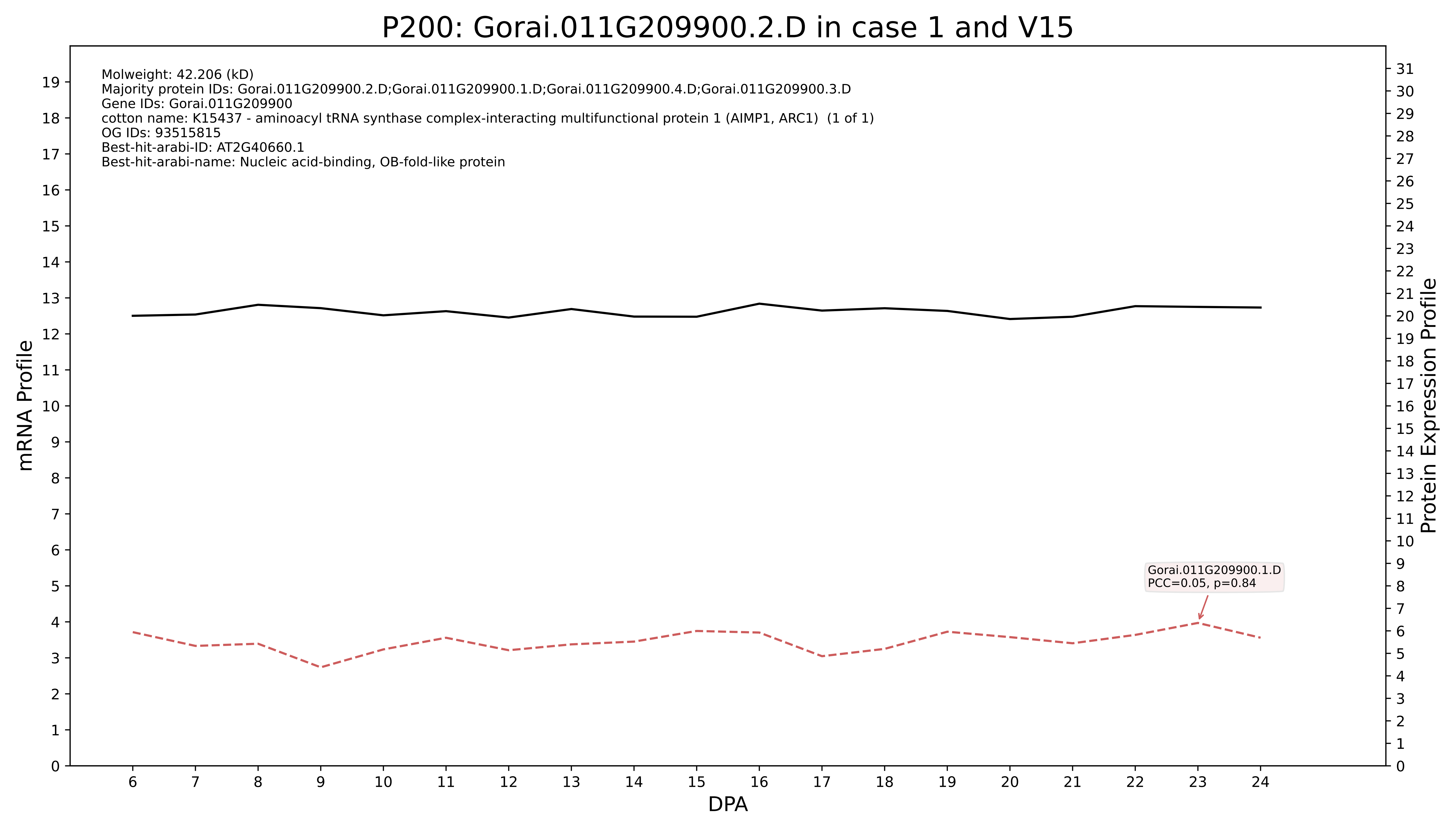

P200: Gorai.011G209900.2.D in case 1 and V15
Molweight: 42.206 (kD)
Majority protein IDs: Gorai.011G209900.2.D;Gorai.011G209900.1.D;Gorai.011G209900.4.D;Gorai.011G209900.3.D
Gene IDs: Gorai.011G209900
cotton name: K15437 - aminoacyl tRNA synthase complex-interacting multifunctional protein 1 (AIMP1, ARC1) (1 of 1)
OG IDs: 93515815
Best-hit-arabi-ID: AT2G40660.1
Best-hit-arabi-name: Nucleic acid-binding, OB-fold-like protein
Gorai.011G209900.1.D
PCC=0.05, p=0.84
mRNA Profile
Protein Expression Profile
DPA

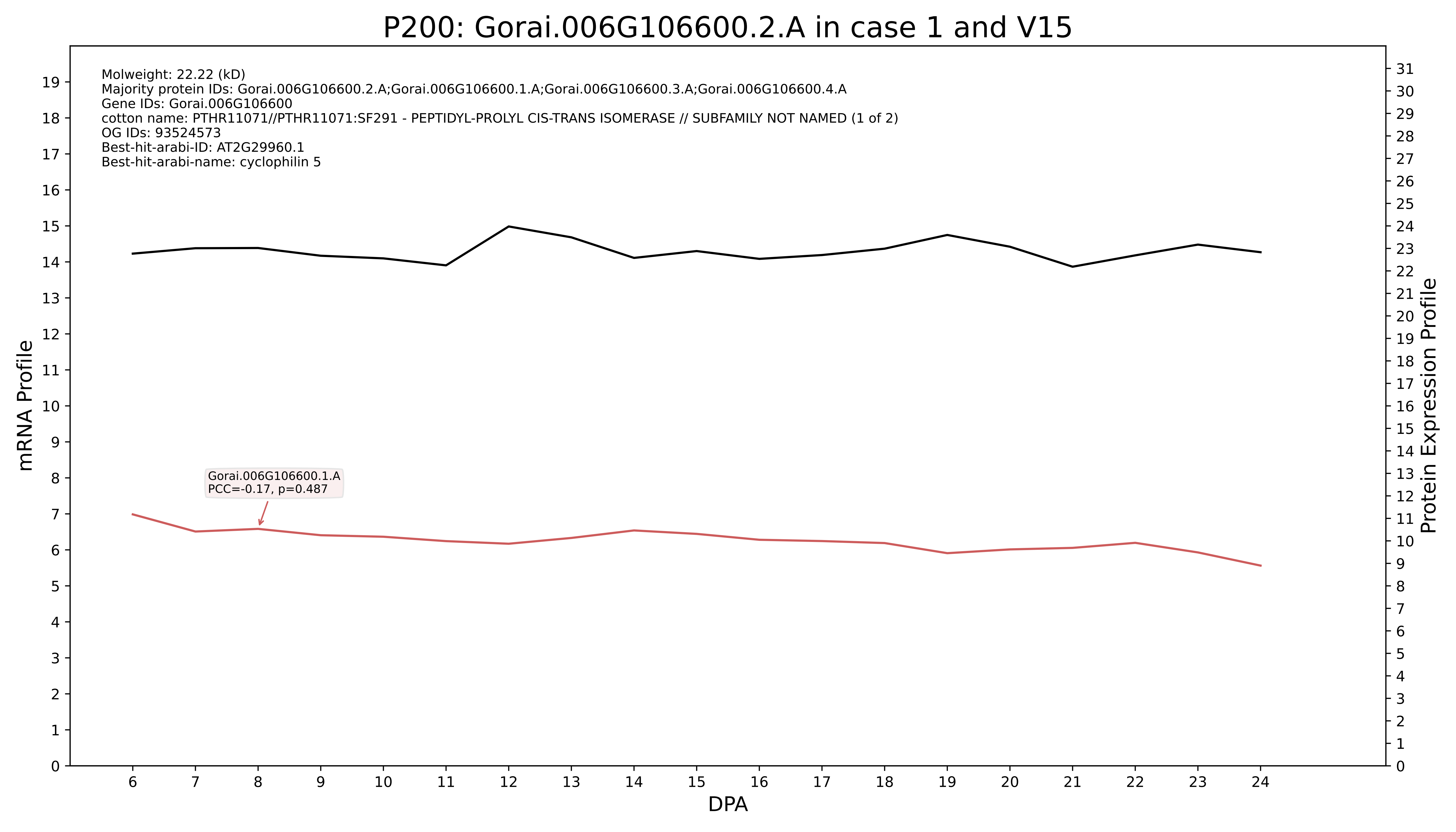
P200: Gorai.006G106600.2.A in case 1 and V15
Molweight: 22.22 (kD)
Majority protein IDs: Gorai.006G106600.2.A;Gorai.006G106600.1.A;Gorai.006G106600.3.A;Gorai.006G106600.4.A
Gene IDs: Gorai.006G106600
cotton name: PTHR11071//PTHR11071:SF291 - PEPTIDYL-PROLYL CIS-TRANS ISOMERASE // SUBFAMILY NOT NAMED (1 of 2)
OG IDs: 93524573
Best-hit-arabi-ID: AT2G29960.1
Best-hit-arabi-name: cyclophilin 5
Gorai.006G106600.1.A
PCC=-0.17, p=0.487
mRNA Profile
Protein Expression Profile
DPA

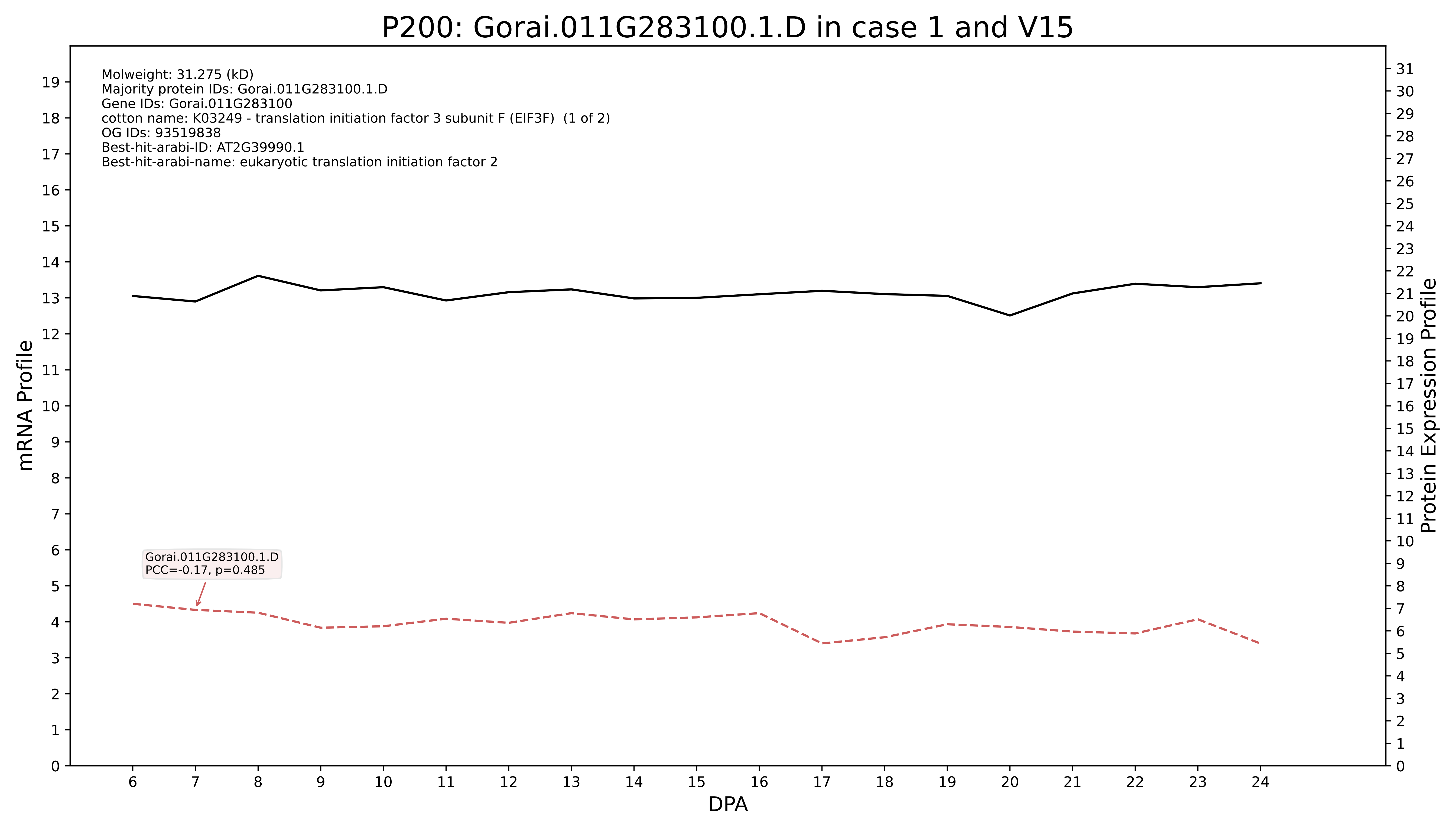
P200: Gorai.011G283100.1.D in case 1 and V15
Molweight: 31.275 (kD)
Majority protein IDs: Gorai.011G283100.1.D
Gene IDs: Gorai.011G283100
cotton name: K03249 - translation initiation factor 3 subunit F (EIF3F) (1 of 2)
OG IDs: 93519838
Best-hit-arabi-ID: AT2G39990.1
Best-hit-arabi-name: eukaryotic translation initiation factor 2
Gorai.011G283100.1.D
PCC=-0.17, p=0.485
mRNA Profile
Protein Expression Profile
DPA

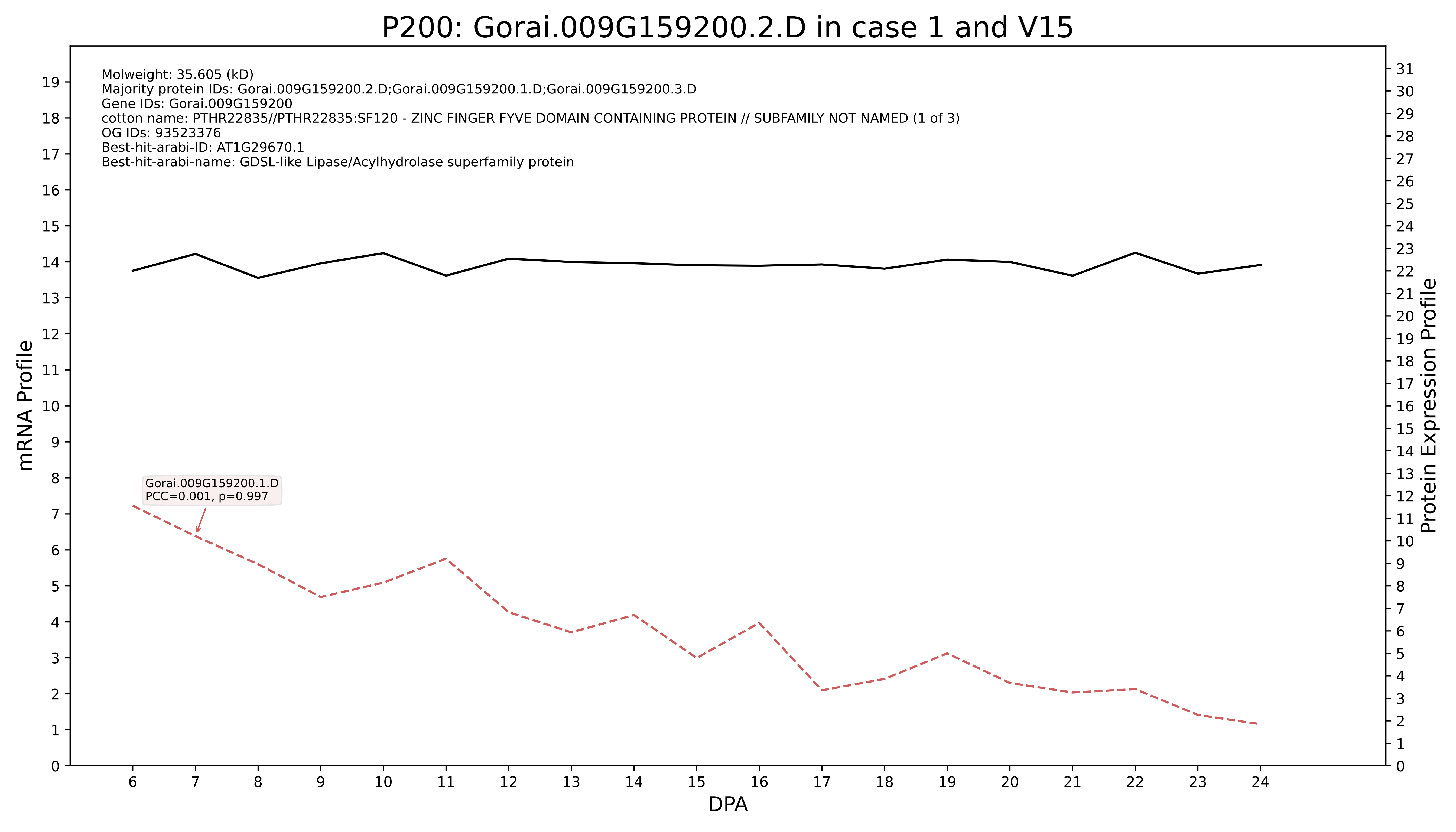
P200: Gorai.009G159200.2.D in case 1 and V15
Molweight: 35.605 (kD)
Majority protein IDs: Gorai.009G159200.2.D;Gorai.009G159200.1.D;Gorai.009G159200.3.D
Gene IDs: Gorai.009G159200
cotton name: PTHR22835//PTHR22835:SF120 - ZINC FINGER FYVE DOMAIN CONTAINING PROTEIN // SUBFAMILY NOT NAMED (1 of 3)
OG IDs: 93523376
Best-hit-arabi-ID: AT1G29670.1
Best-hit-arabi-name: GDSL-like Lipase/Acylhydrolase superfamily protein
Gorai.009G159200.1.D
PCC=0.001, p=0.997
mRNA Profile
Protein Expression Profile
DPA

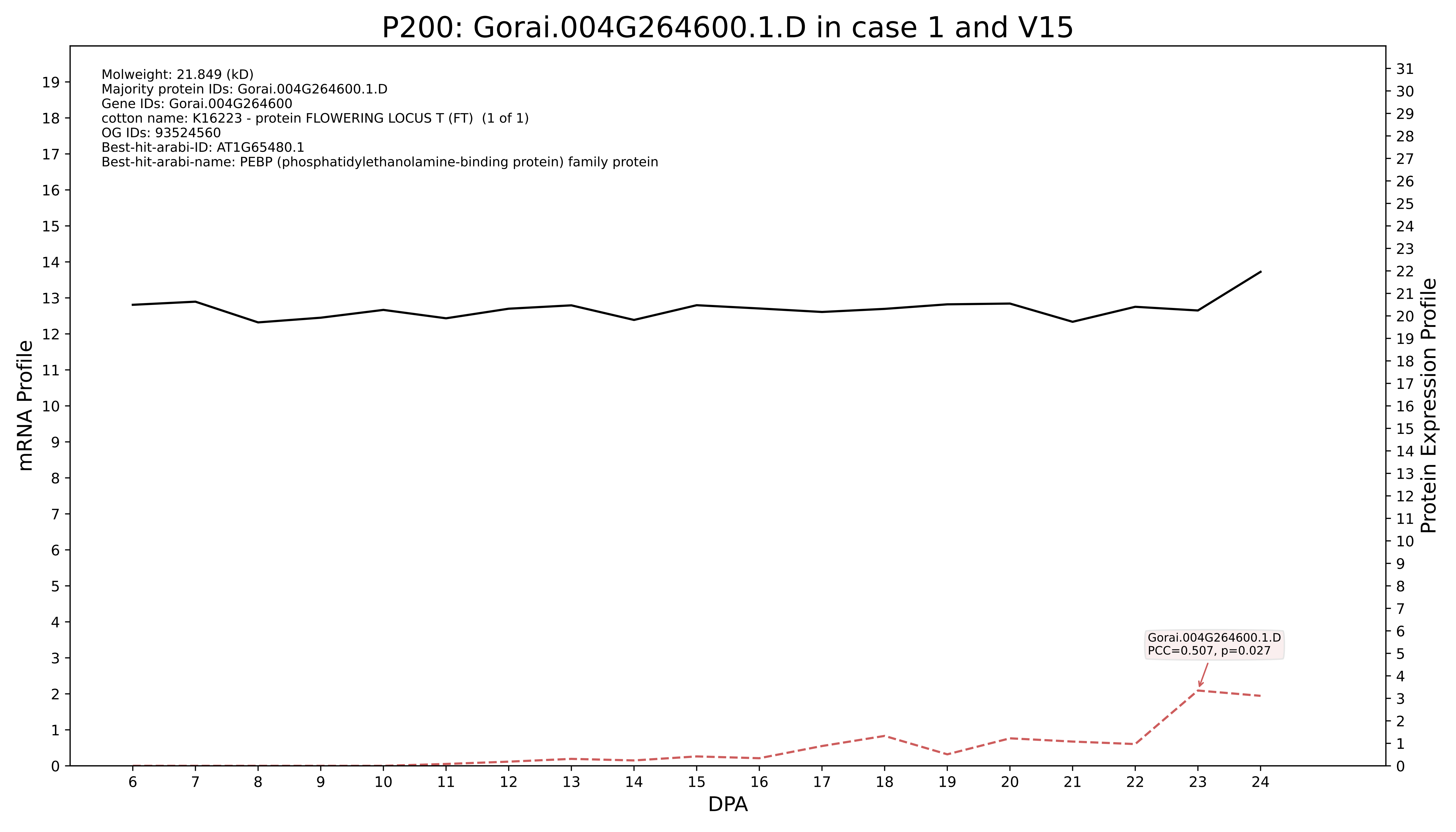
P200: Gorai.004G264600.1.D in case 1 and V15
Molweight: 21.849 (kD)
Majority protein IDs: Gorai.004G264600.1.D
Gene IDs: Gorai.004G264600
cotton name: K16223 - protein FLOWERING LOCUS T (FT) (1 of 1)
OG IDs: 93524560
Best-hit-arabi-ID: AT1G65480.1
Best-hit-arabi-name: PEBP (phosphatidylethanolamine-binding protein) family protein
Gorai.004G264600.1.D
PCC=0.507, p=0.027
mRNA Profile
Protein Expression Profile
DPA

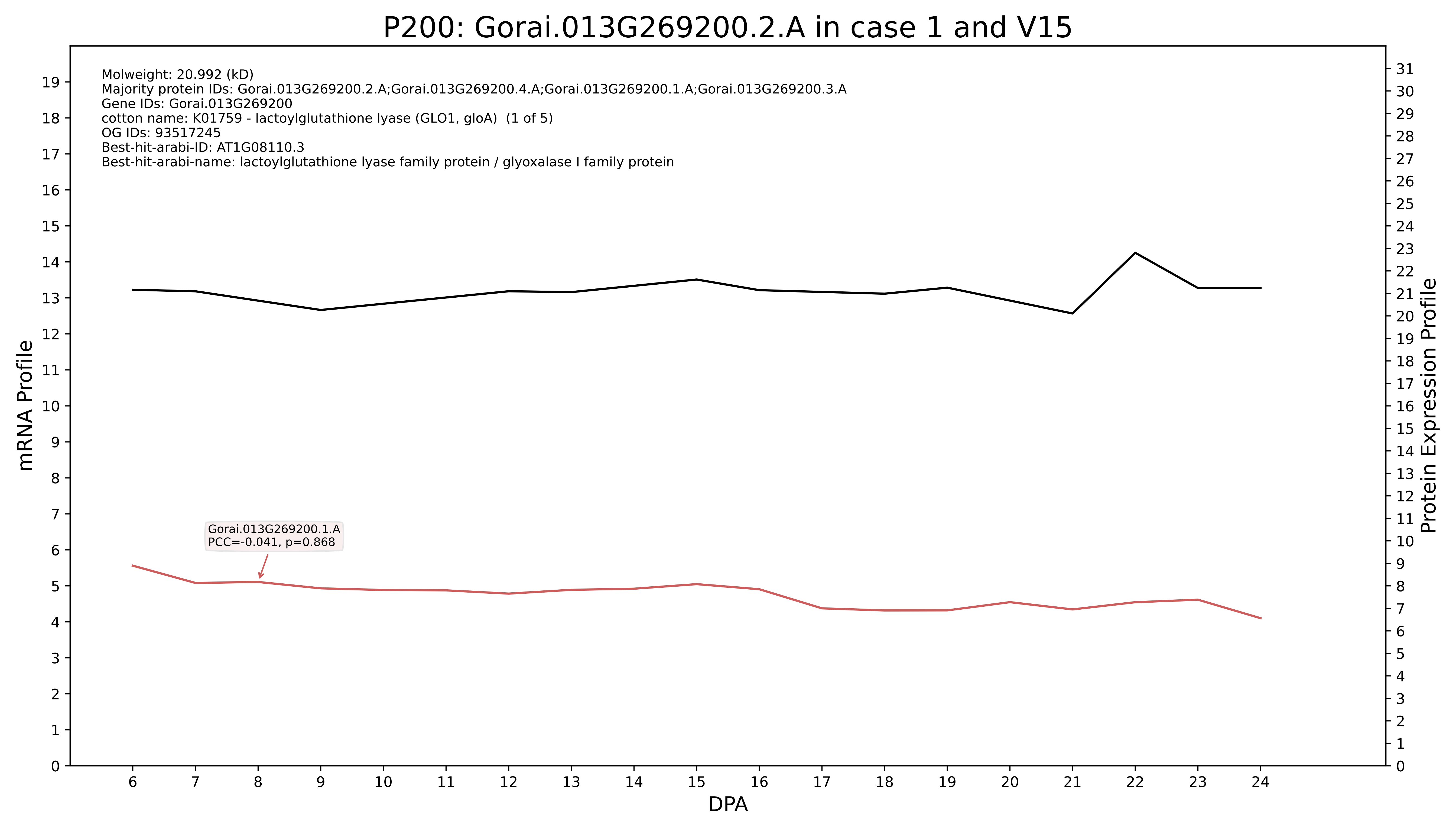
P200: Gorai.013G269200.2.A in case 1 and V15
Molweight: 20.992 (kD)
Majority protein IDs: Gorai.013G269200.2.A;Gorai.013G269200.4.A;Gorai.013G269200.1.A;Gorai.013G269200.3.A
Gene IDs: Gorai.013G269200
cotton name: K01759 - lactoylglutathione lyase (GLO1, gloA) (1 of 5)
OG IDs: 93517245
Best-hit-arabi-ID: AT1G08110.3
Best-hit-arabi-name: lactoylglutathione lyase family protein / glyoxalase I family protein
Gorai.013G269200.1.A
PCC=-0.041, p=0.868
mRNA Profile
Protein Expression Profile
DPA

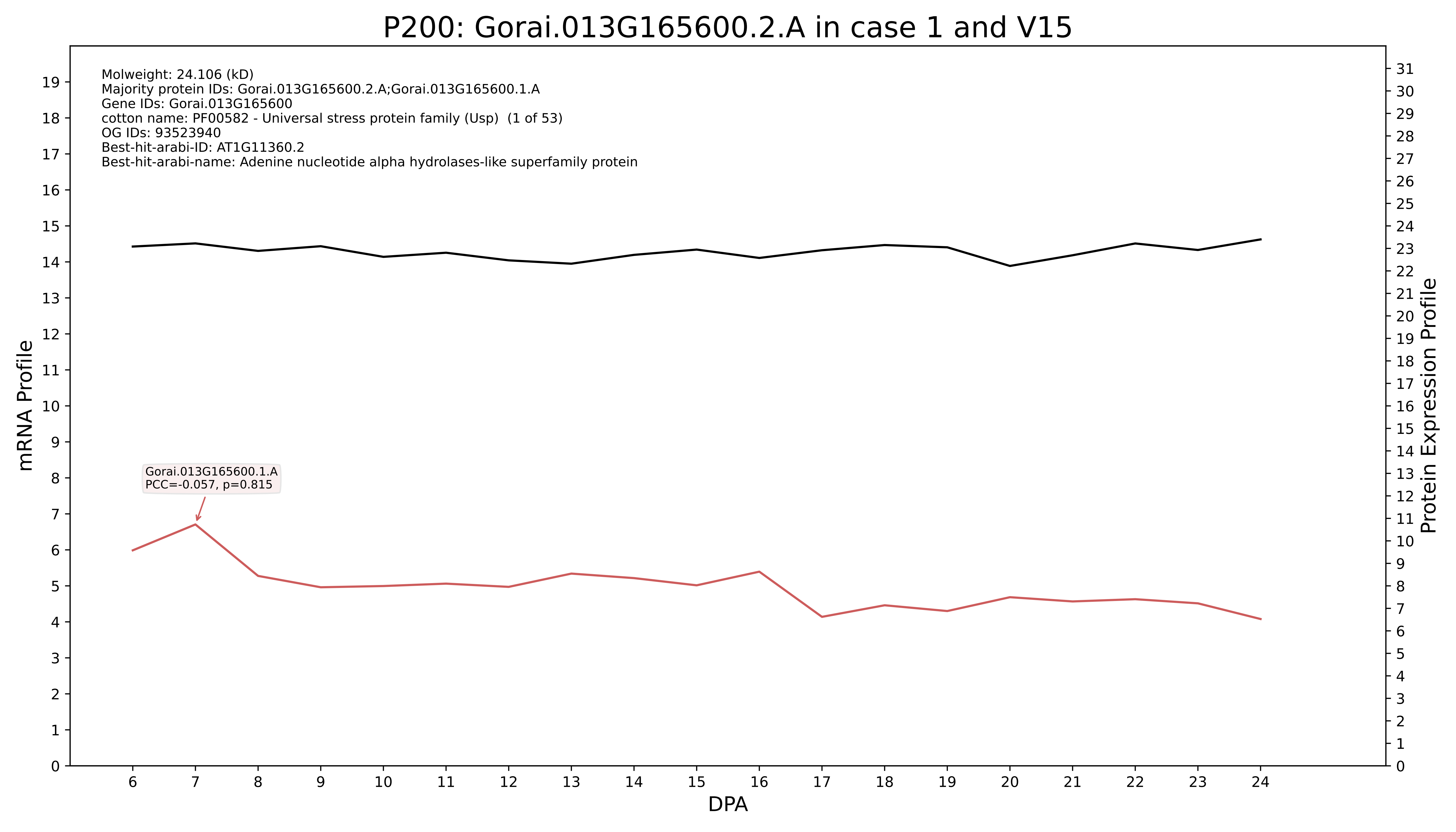
P200: Gorai.013G165600.2.A in case 1 and V15
Molweight: 24.106 (kD)
Majority protein IDs: Gorai.013G165600.2.A;Gorai.013G165600.1.A
Gene IDs: Gorai.013G165600
cotton name: PF00582 - Universal stress protein family (Usp) (1 of 53)
OG IDs: 93523940
Best-hit-arabi-ID: AT1G11360.2
Best-hit-arabi-name: Adenine nucleotide alpha hydrolases-like superfamily protein
Gorai.013G165600.1.A
PCC=-0.057, p=0.815
mRNA Profile
Protein Expression Profile
DPA

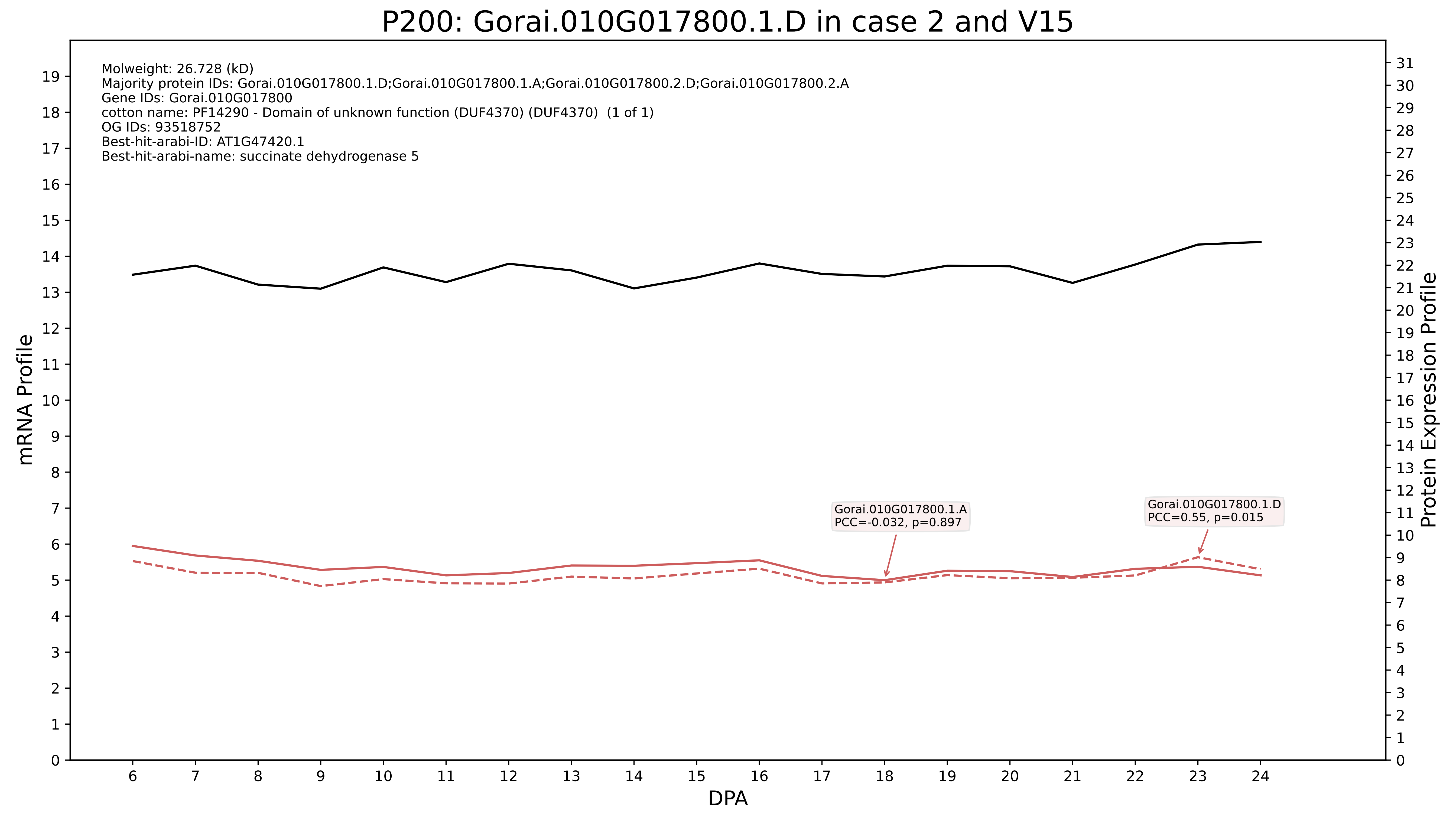
P200: Gorai.010G017800.1.D in case 2 and V15
Molweight: 26.728 (kD)
Majority protein IDs: Gorai.010G017800.1.D;Gorai.010G017800.1.A;Gorai.010G017800.2.D;Gorai.010G017800.2.A
Gene IDs: Gorai.010G017800
cotton name: PF14290 - Domain of unknown function (DUF4370) (DUF4370) (1 of 1)
OG IDs: 93518752
Best-hit-arabi-ID: AT1G47420.1
Best-hit-arabi-name: succinate dehydrogenase 5
Gorai.010G017800.1.A
PCC=-0.032, p=0.897
Gorai.010G017800.1.D
PCC=0.55, p=0.015
mRNA Profile
Protein Expression Profile
DPA

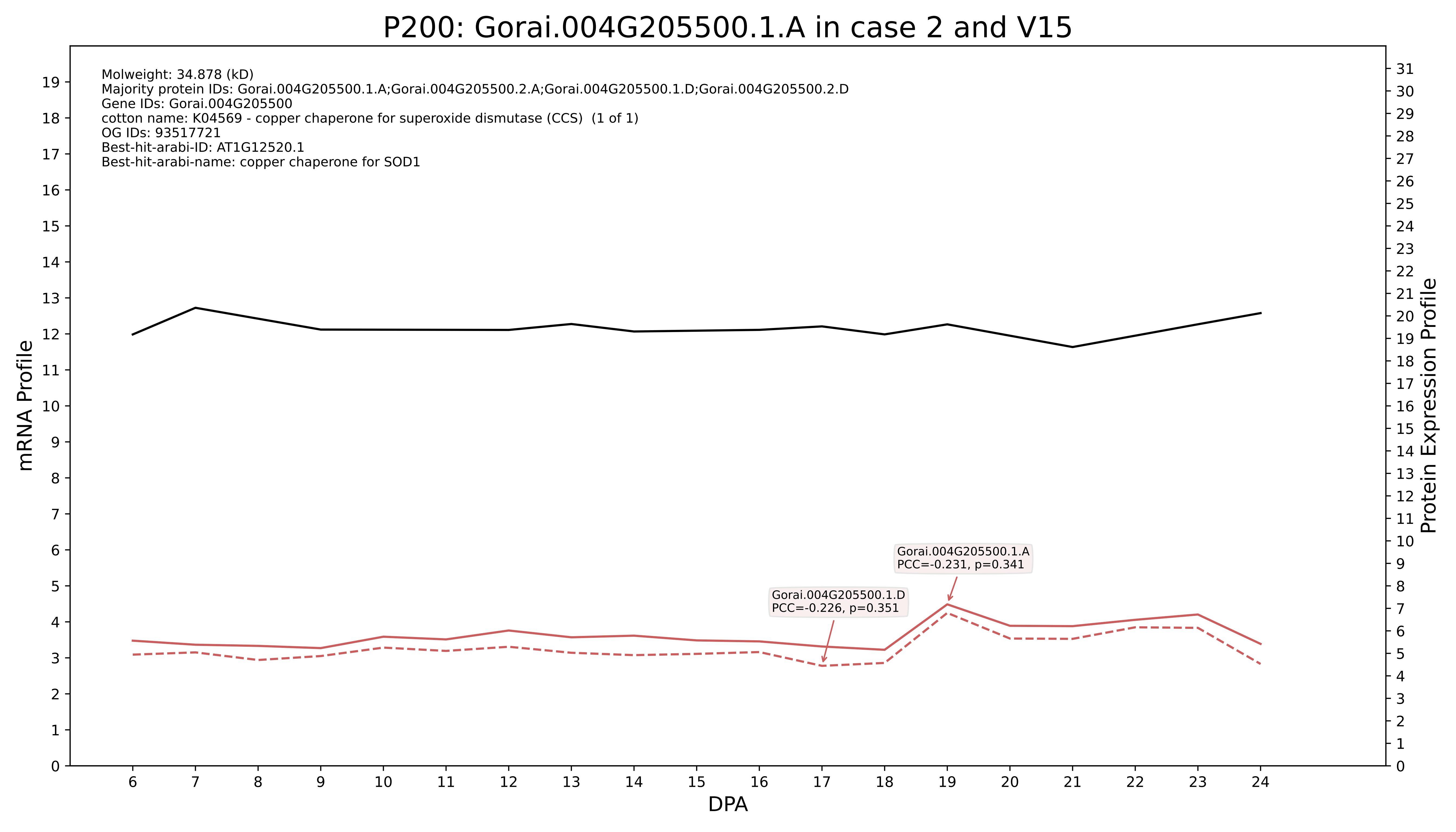
P200: Gorai.004G205500.1.A in case 2 and V15
Molweight: 34.878 (kD)
Majority protein IDs: Gorai.004G205500.1.A;Gorai.004G205500.2.A;Gorai.004G205500.1.D;Gorai.004G205500.2.D
Gene IDs: Gorai.004G205500
cotton name: K04569 - copper chaperone for superoxide dismutase (CCS) (1 of 1)
OG IDs: 93517721
Best-hit-arabi-ID: AT1G12520.1
Best-hit-arabi-name: copper chaperone for SOD1
Gorai.004G205500.1.A
PCC=-0.231, p=0.341
Gorai.004G205500.1.D
PCC=-0.226, p=0.351
mRNA Profile
Protein Expression Profile
DPA

P200: Gorai.002G249600.3.D in case 2 and V15

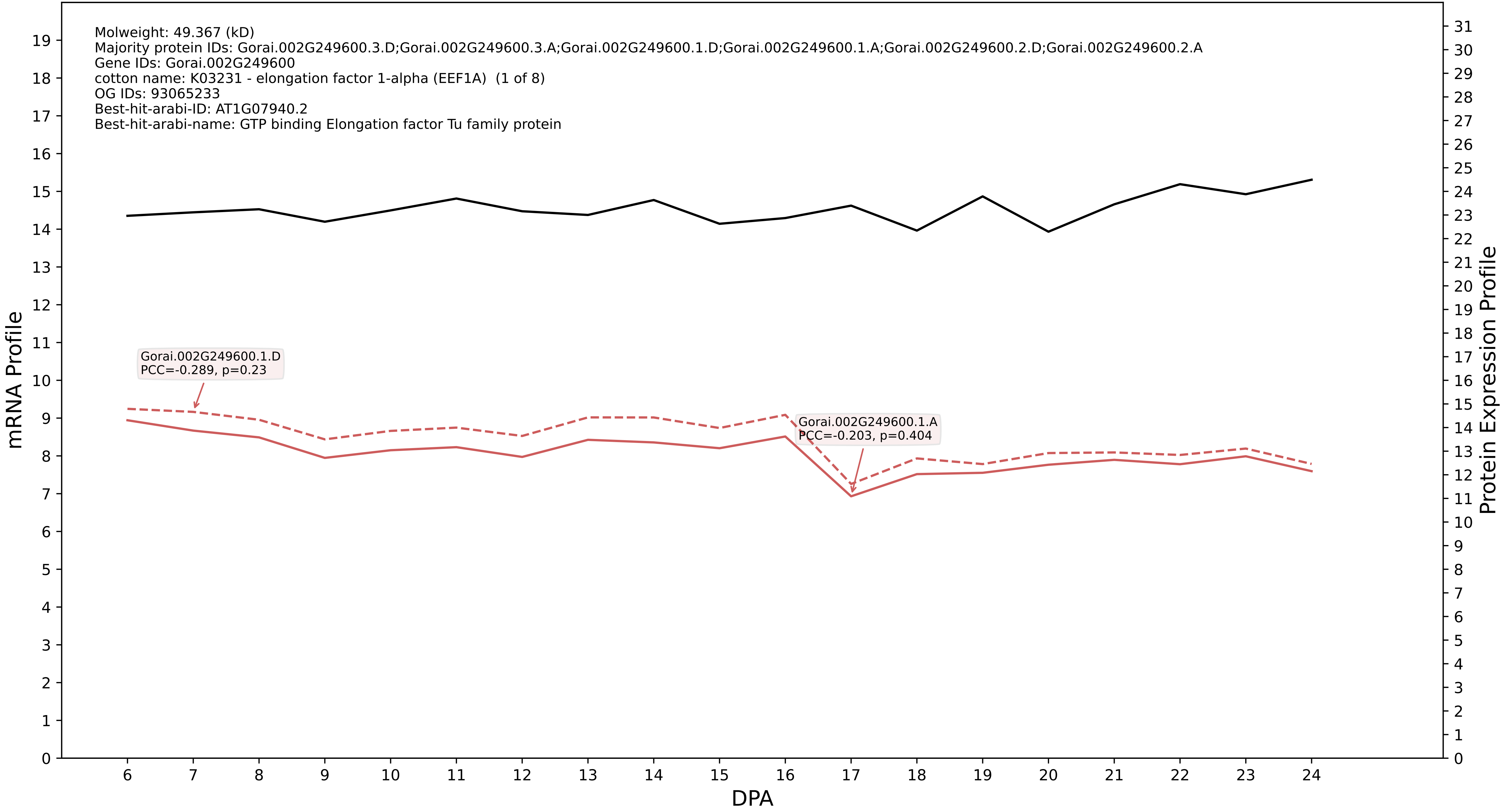

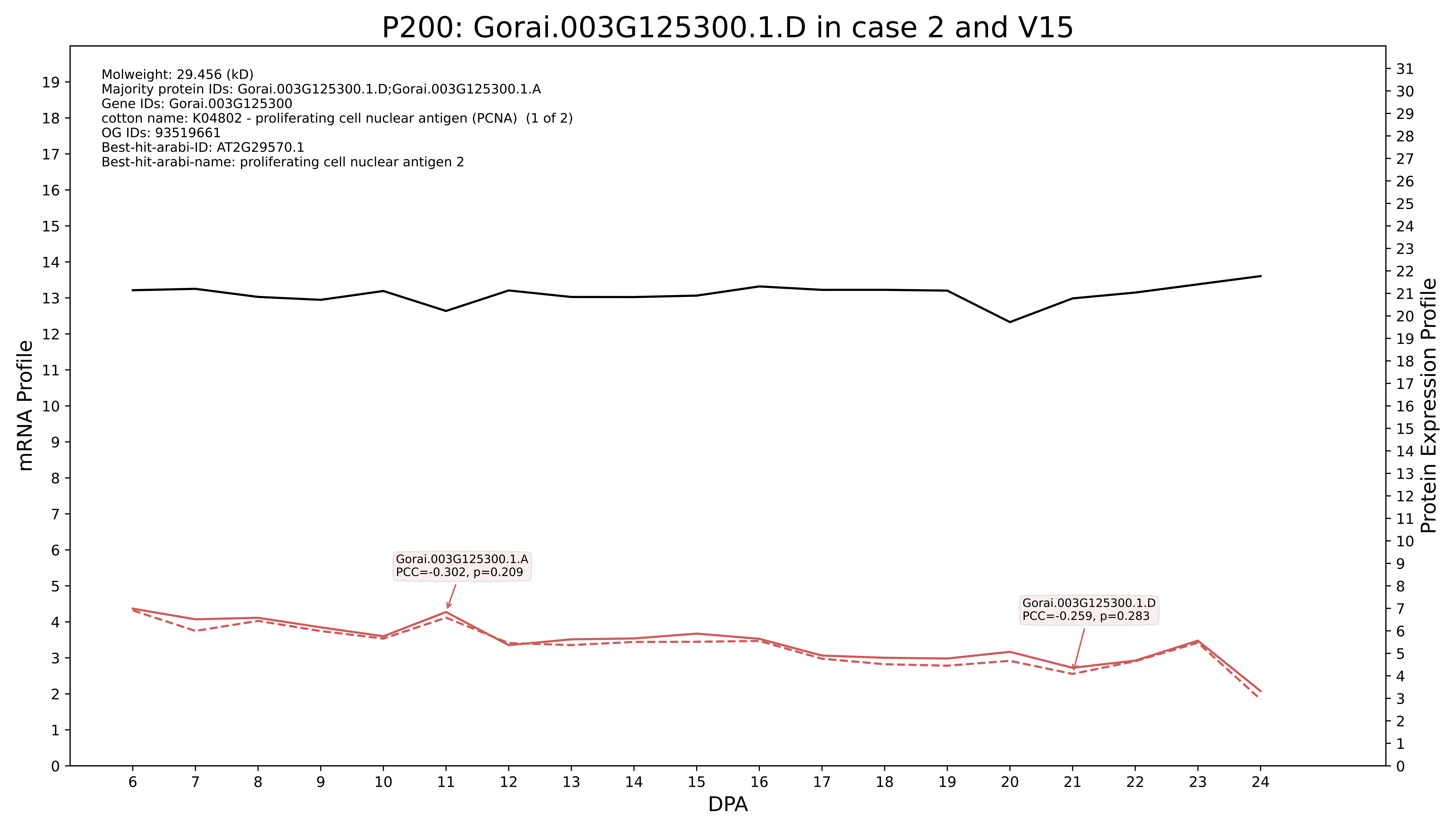
P200: Gorai.003G125300.1.D in case 2 and V15
Molweight: 29.456 (kD)
Majority protein IDs: Gorai.003G125300.1.D;Gorai.003G125300.1.A
Gene IDs: Gorai.003G125300
cotton name: K04802 - proliferating cell nuclear antigen (PCNA) (1 of 2)
OG IDs: 93519661
Best-hit-arabi-ID: AT2G29570.1
Best-hit-arabi-name: proliferating cell nuclear antigen 2
Gorai.003G125300.1.A
PCC=-0.302, p=0.209
Gorai.003G125300.1.D
PCC=-0.259, p=0.283
mRNA Profile
Protein Expression Profile
DPA

00: Gorai.001G213900.5.D in case 2 and V15

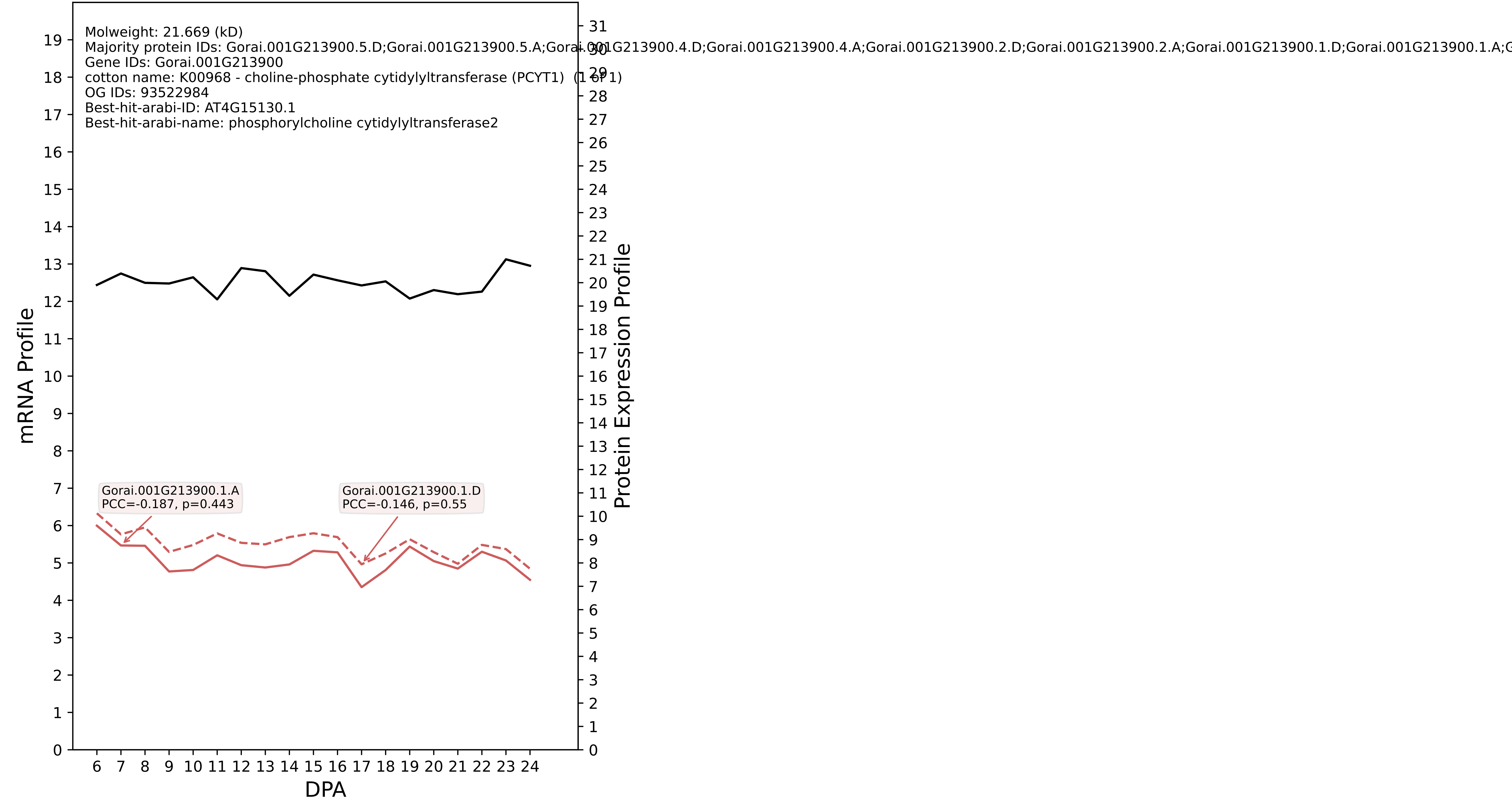

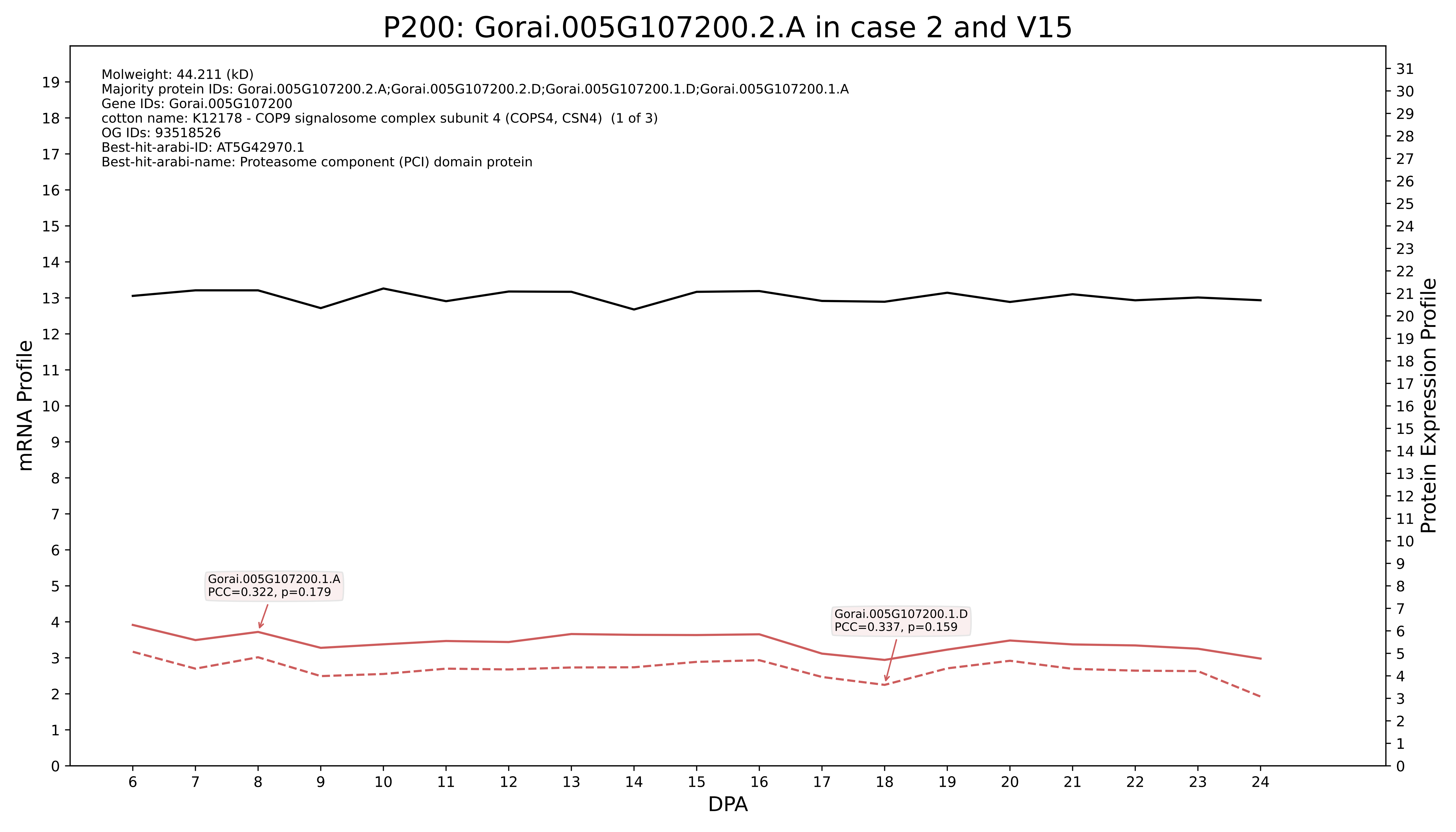

P200: Gorai.005G107200.2.A in case 2 and V15
Molweight: 44.211 (kD)
Majority protein IDs: Gorai.005G107200.2.A;Gorai.005G107200.2.D;Gorai.005G107200.1.D;Gorai.005G107200.1.A
Gene IDs: Gorai.005G107200
cotton name: K12178 - COP9 signalosome complex subunit 4 (COPS4, CSN4) (1 of 3)
OG IDs: 93518526
Best-hit-arabi-ID: AT5G42970.1
Best-hit-arabi-name: Proteasome component (PCI) domain protein
Gorai.005G107200.1.A
PCC=0.322, p=0.179
Gorai.005G107200.1.D
PCC=0.337, p=0.159
mRNA Profile
Protein Expression Profile
DPA

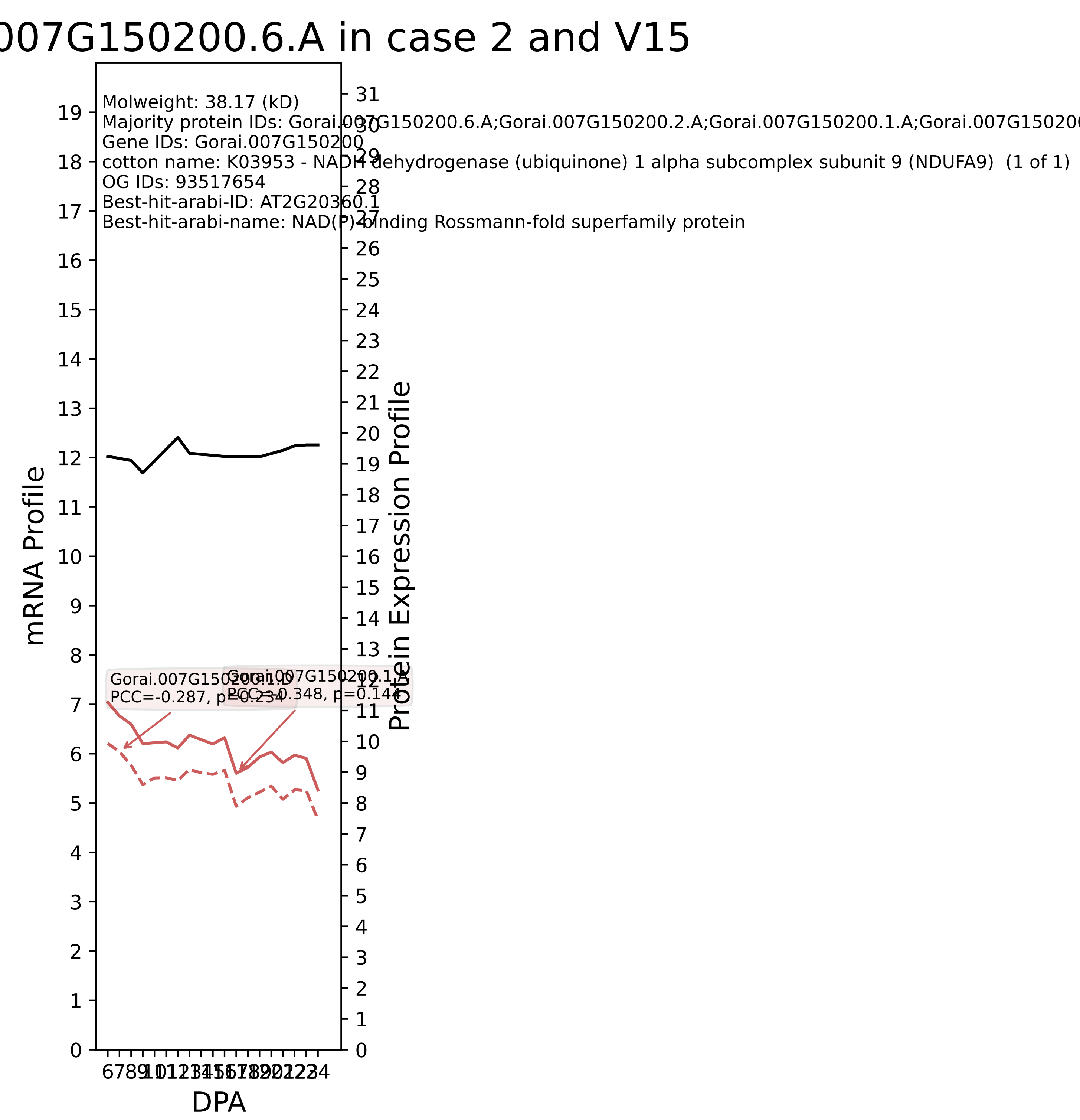

007G150200.6.A in case 2 and V15
Molweight: 38.17 (kD)
Majority protein IDs: Gorai.007G150200.6.A;Gorai.007G150200.2.A;Gorai.007G150200.1.A;Gorai.007G150200.7.A;Gorai.007G150200.6.D;Gorai.007G150200.5.A;Gorai.007G150200.2.D;Gorai.007G150200.1.D;Go
Gene IDs: Gorai.007G150200
cotton name: K03953 - NADH dehydrogenase (ubiquinone) 1 alpha subcomplex subunit 9 (NDUFA9) (1 of 1)
OG IDs: 93517654
Best-hit-arabi-ID: AT2G20360.1
Best-hit-arabi-name: NAD(P)-binding Rossmann-fold superfamily protein
Gorai.007G150200.1.D
PCC=-0.287, p=0.234
Gorai.007G150200.1.A
PCC=-0.348, p=0.144
mRNA Profile
Protein Expression Profile
DPA

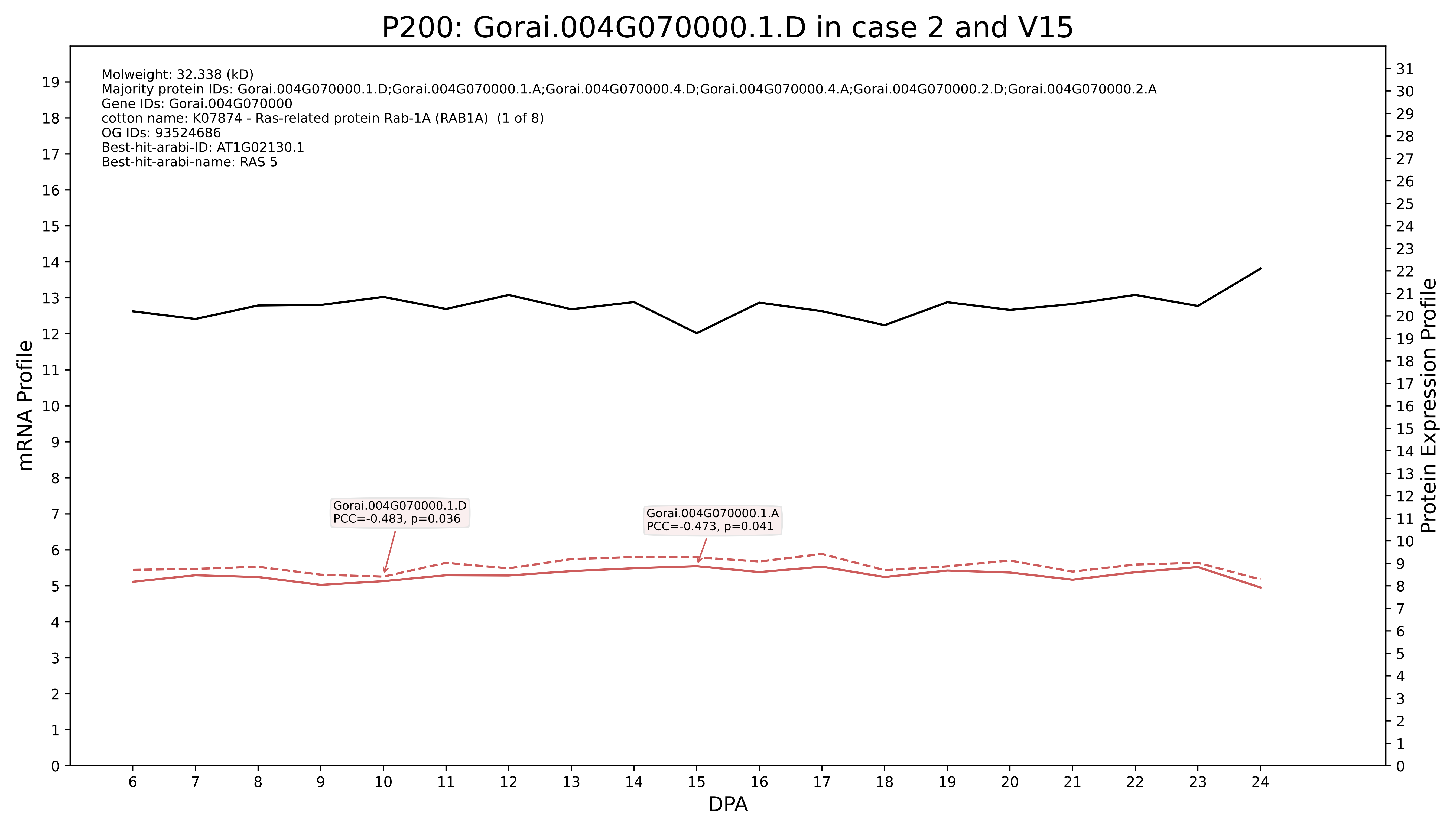
P200: Gorai.004G070000.1.D in case 2 and V15
Molweight: 32.338 (kD)
Majority protein IDs: Gorai.004G070000.1.D;Gorai.004G070000.1.A;Gorai.004G070000.4.D;Gorai.004G070000.4.A;Gorai.004G070000.2.D;Gorai.004G070000.2.A
Gene IDs: Gorai.004G070000
cotton name: K07874 - Ras-related protein Rab-1A (RAB1A) (1 of 8)
OG IDs: 93524686
Best-hit-arabi-ID: AT1G02130.1
Best-hit-arabi-name: RAS 5
Gorai.004G070000.1.D
PCC=-0.483, p=0.036
Gorai.004G070000.1.A
PCC=-0.473, p=0.041
mRNA Profile
Protein Expression Profile
DPA

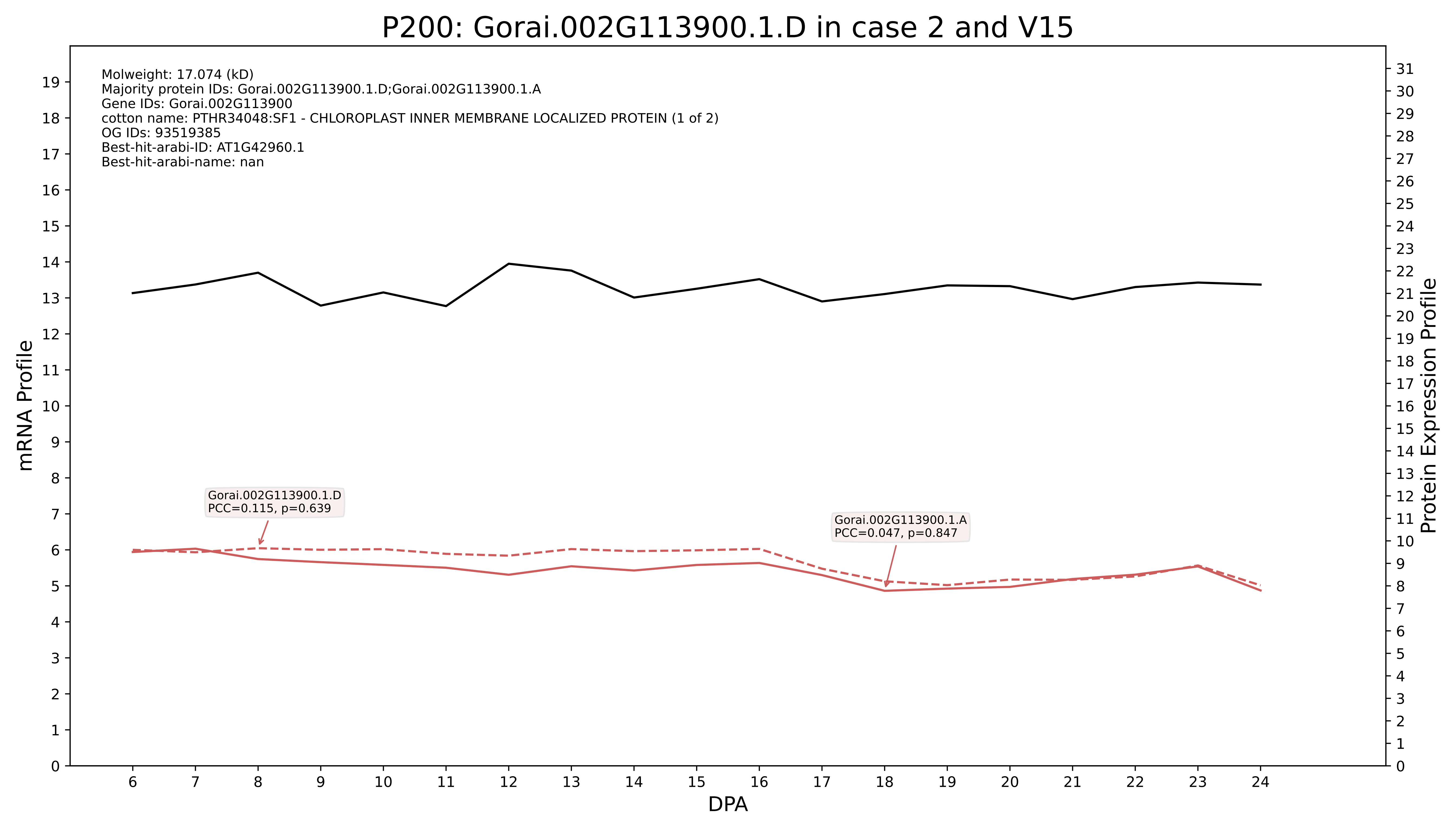
P200: Gorai.002G113900.1.D in case 2 and V15
Molweight: 17.074 (kD)
Majority protein IDs: Gorai.002G113900.1.D;Gorai.002G113900.1.A
Gene IDs: Gorai.002G113900
cotton name: PTHR34048:SF1 - CHLOROPLAST INNER MEMBRANE LOCALIZED PROTEIN (1 of 2)
OG IDs: 93519385
Best-hit-arabi-ID: AT1G42960.1
Best-hit-arabi-name: nan
Gorai.002G113900.1.D
PCC=0.115, p=0.639
Gorai.002G113900.1.A
PCC=0.047, p=0.847
mRNA Profile
Protein Expression Profile
DPA

009G005400.1.D in case 2 and V15

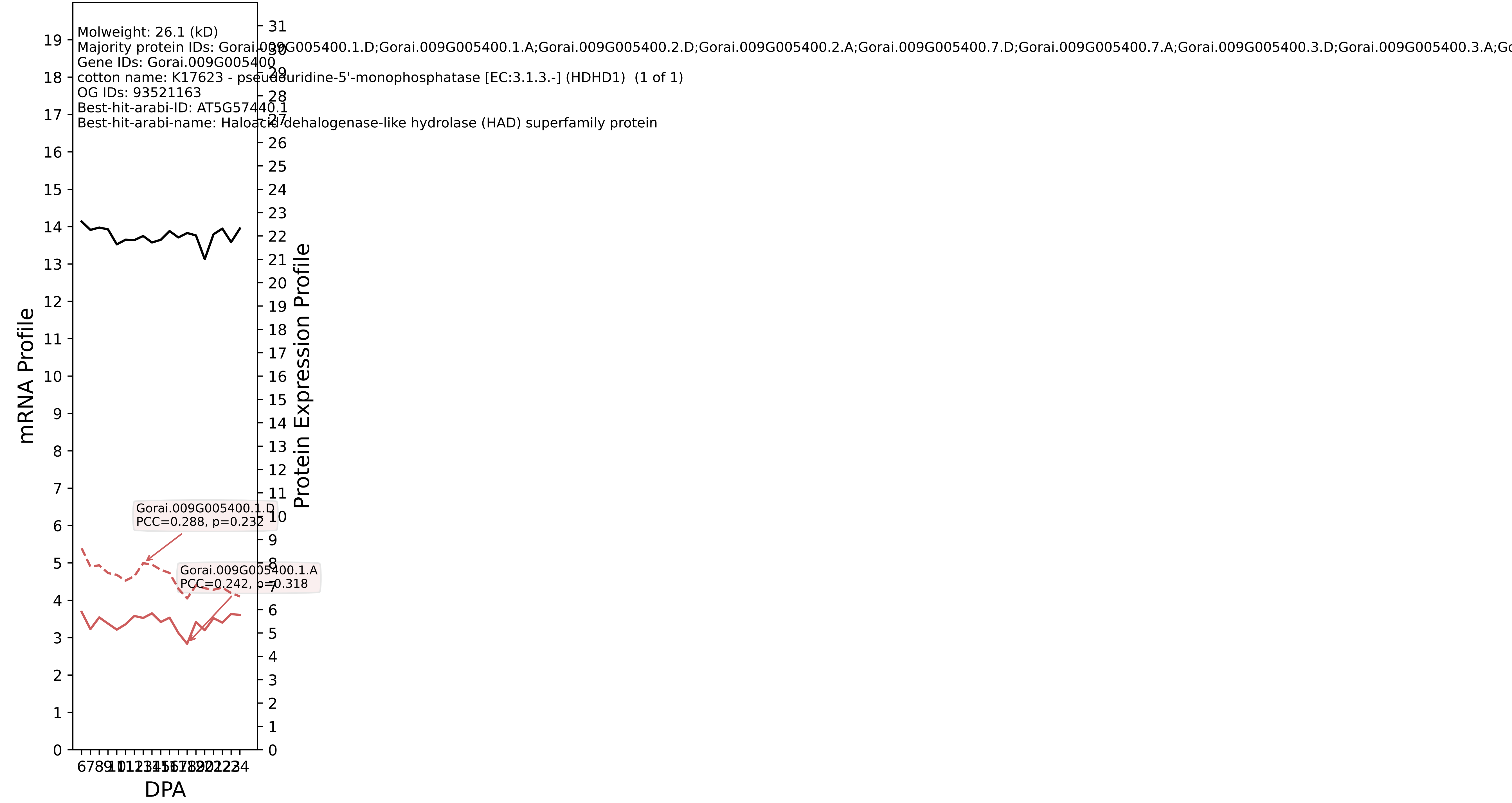

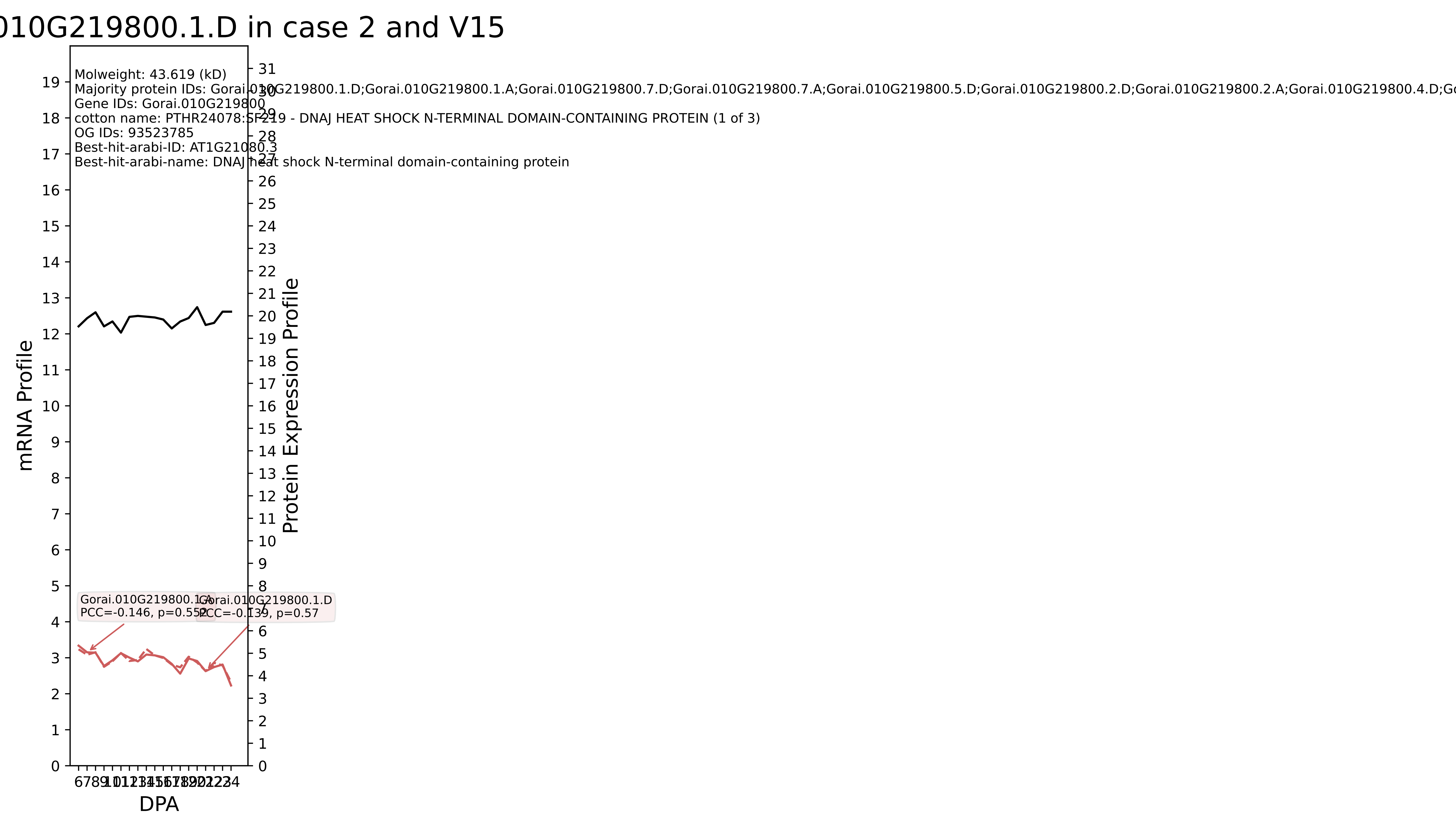
010G219800.1.D in case 2 and V15
Molweight: 43.619 (kD)
Majority protein IDs: Gorai.010G219800.1.D;Gorai.010G219800.1.A;Gorai.010G219800.7.D;Gorai.010G219800.7.A;Gorai.010G219800.5.D;Gorai.010G219800.2.D;Gorai.010G219800.2.A;Gorai.010G219800.4.D;Go
Gene IDs: Gorai.010G219800
cotton name: PTHR24078:SF219 - DNAJ HEAT SHOCK N-TERMINAL DOMAIN-CONTAINING PROTEIN (1 of 3)
OG IDs: 93523785
Best-hit-arabi-ID: AT1G21080.3
Best-hit-arabi-name: DNAJ heat shock N-terminal domain-containing protein
Gorai.010G219800.1.A
PCC=-0.146, p=0.552
Gorai.010G219800.1.D
PCC=-0.139, p=0.57
mRNA Profile
Protein Expression Profile
DPA

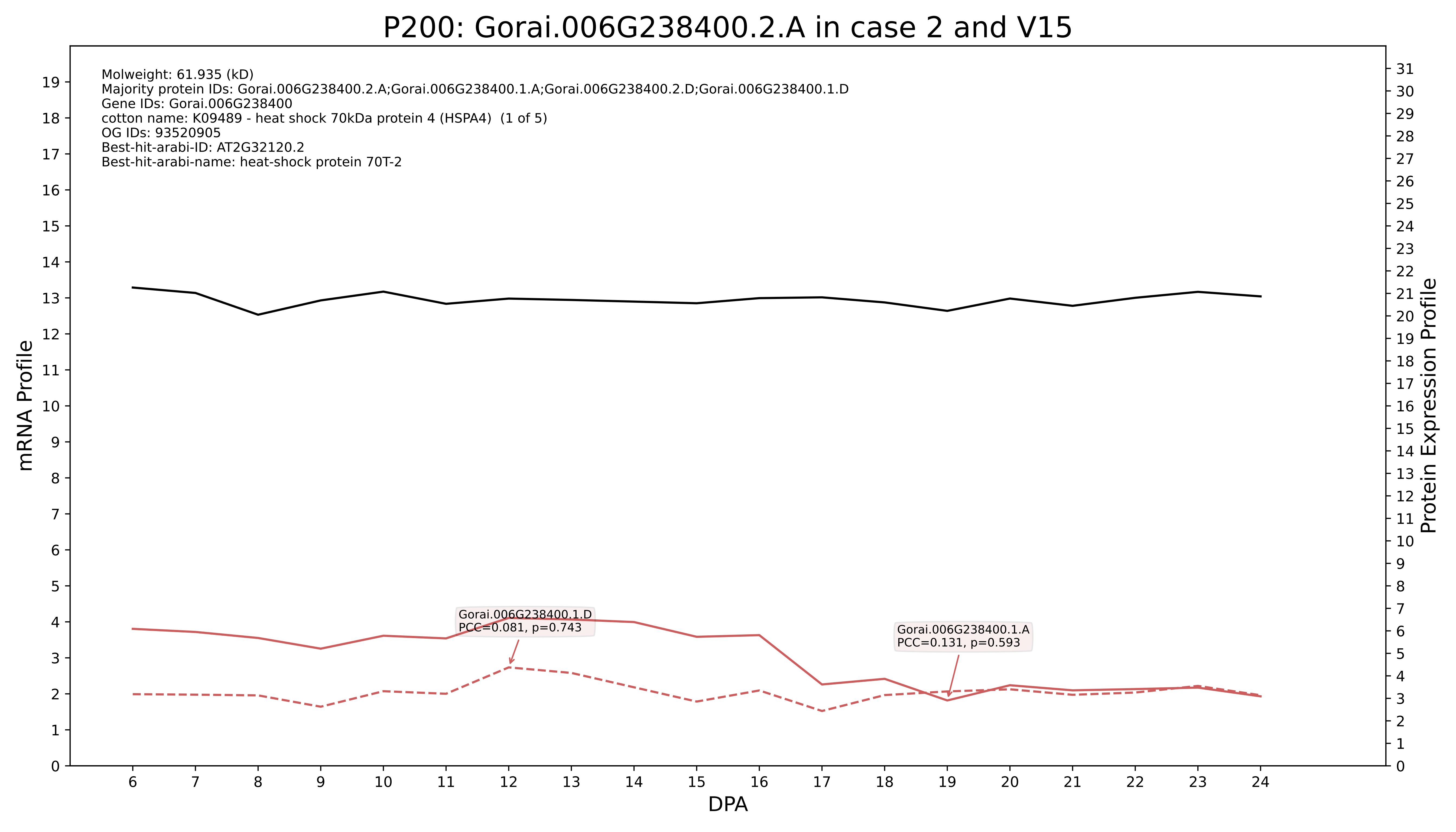
P200: Gorai.006G238400.2.A in case 2 and V15
Molweight: 61.935 (kD)
Majority protein IDs: Gorai.006G238400.2.A;Gorai.006G238400.1.A;Gorai.006G238400.2.D;Gorai.006G238400.1.D
Gene IDs: Gorai.006G238400
cotton name: K09489 - heat shock 70kDa protein 4 (HSPA4) (1 of 5)
OG IDs: 93520905
Best-hit-arabi-ID: AT2G32120.2
Best-hit-arabi-name: heat-shock protein 70T-2
Gorai.006G238400.1.D
PCC=0.081, p=0.743
Gorai.006G238400.1.A
PCC=0.131, p=0.593
mRNA Profile
Protein Expression Profile
DPA
0
1
2
3
4
5
6
7
8
9
10
11
12
13
14
15
16
17
18
19
20
21
22
23
24
25
26
27
28
29
30
31

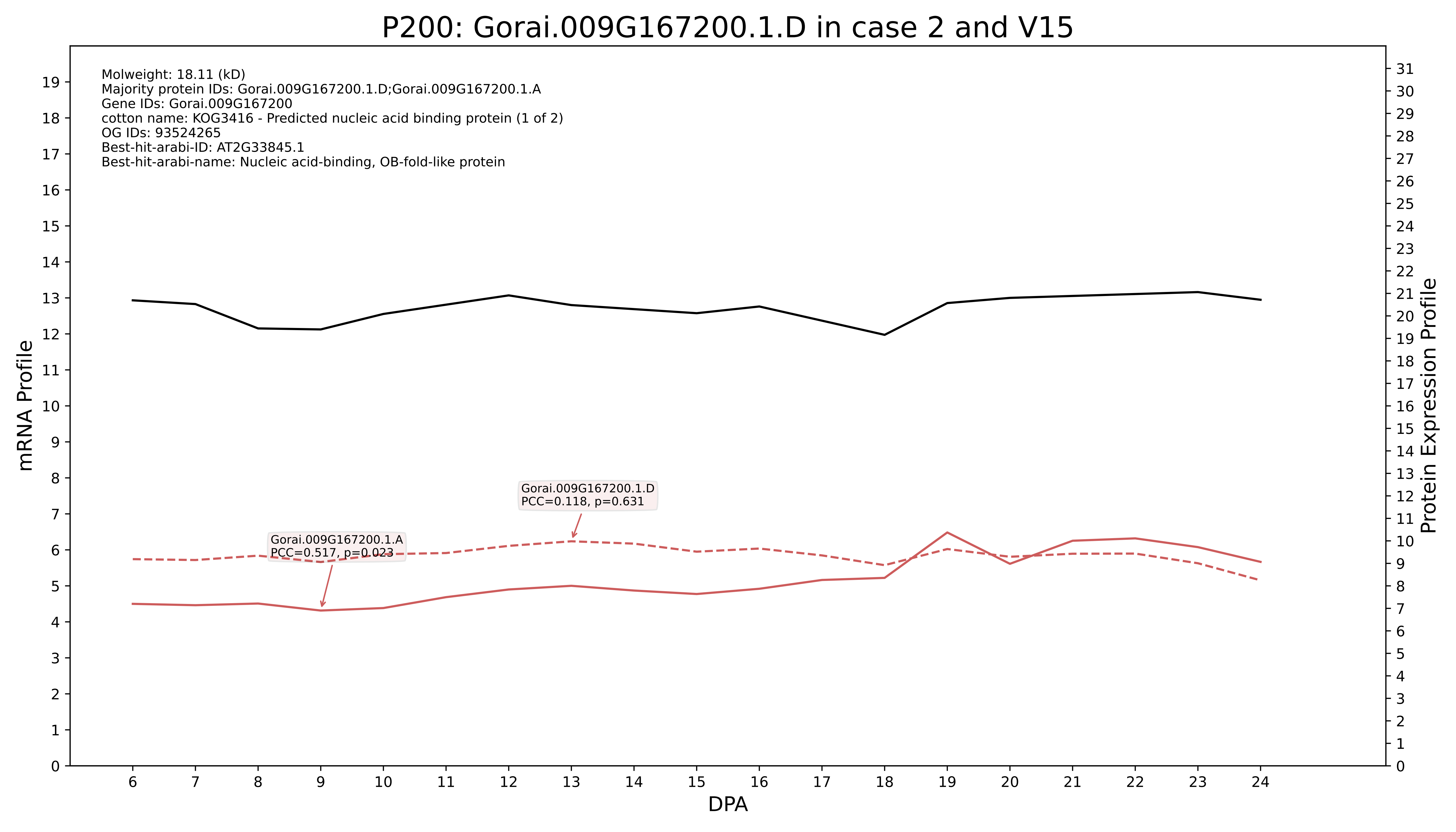

P200: Gorai.009G167200.1.D in case 2 and V15
Molweight: 18.11 (kD)
Majority protein IDs: Gorai.009G167200.1.D;Gorai.009G167200.1.A
Gene IDs: Gorai.009G167200
cotton name: KOG3416 - Predicted nucleic acid binding protein (1 of 2)
OG IDs: 93524265
Best-hit-arabi-ID: AT2G33845.1
Best-hit-arabi-name: Nucleic acid-binding, OB-fold-like protein
Gorai.009G167200.1.D
PCC=0.118, p=0.631
Gorai.009G167200.1.A
PCC=0.517, p=0.023
mRNA Profile
Protein Expression Profile
DPA

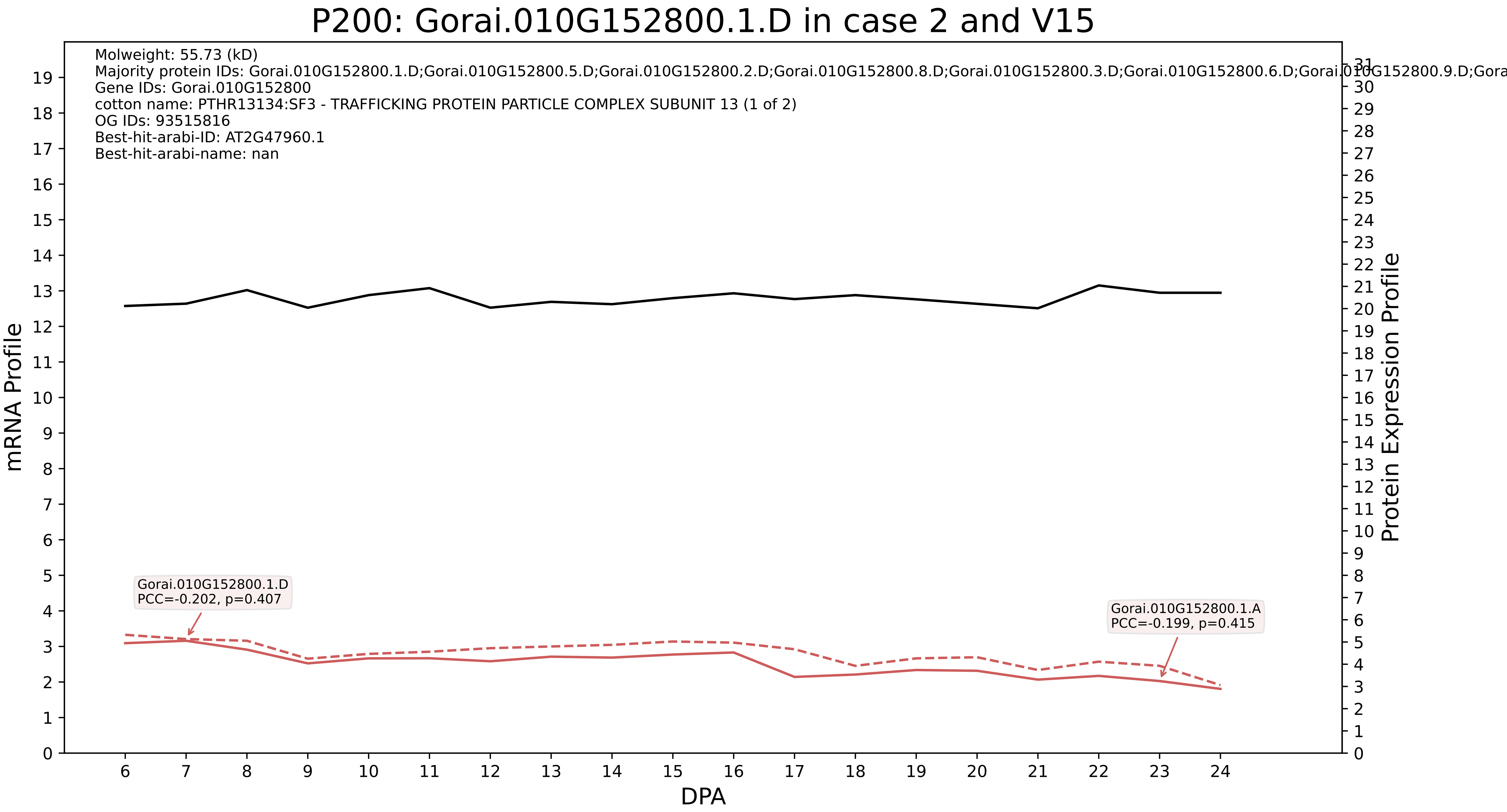

P200: Gorai.010G152800.1.D in case 2 and V15
Molweight: 55.73 (kD)
Majority protein IDs: Gorai.010G152800.1.D;Gorai.010G152800.5.D;Gorai.010G152800.2.D;Gorai.010G152800.8.D;Gorai.010G152800.3.D;Gorai.010G152800.6.D;Gorai.010G152800.9.D;Gora
Gene IDs: Gorai.010G152800
cotton name: PTHR13134:SF3 - TRAFFICKING PROTEIN PARTICLE COMPLEX SUBUNIT 13 (1 of 2)
OG IDs: 93515816
Best-hit-arabi-ID: AT2G47960.1
Best-hit-arabi-name: nan
Gorai.010G152800.1.D
PCC=-0.202, p=0.407
Gorai.010G152800.1.A
PCC=-0.199, p=0.415
mRNA Profile
Protein Expression Profile
DPA

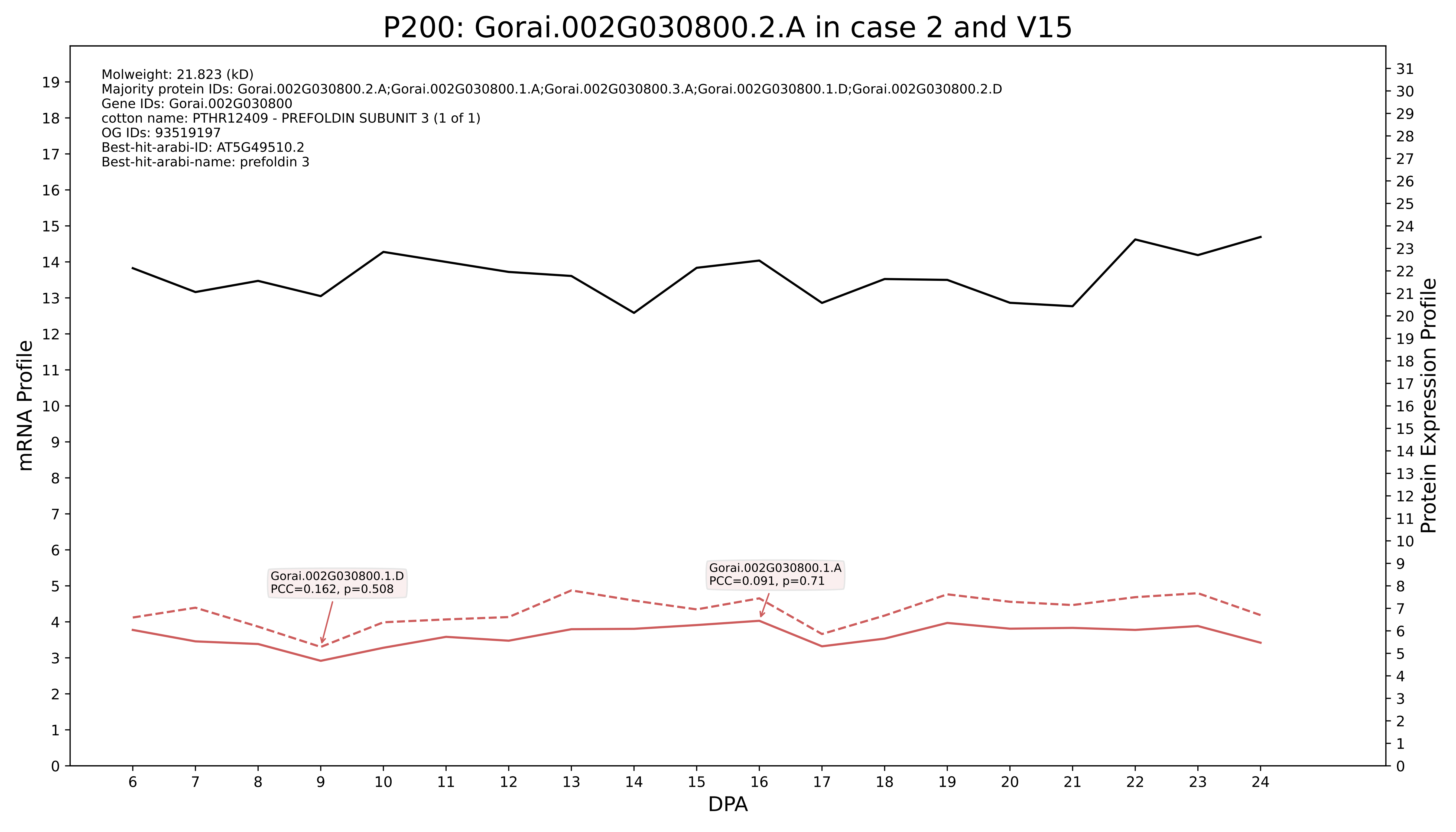

P200: Gorai.002G030800.2.A in case 2 and V15
Molweight: 21.823 (kD)
Majority protein IDs: Gorai.002G030800.2.A;Gorai.002G030800.1.A;Gorai.002G030800.3.A;Gorai.002G030800.1.D;Gorai.002G030800.2.D
Gene IDs: Gorai.002G030800
cotton name: PTHR12409 - PREFOLDIN SUBUNIT 3 (1 of 1)
OG IDs: 93519197
Best-hit-arabi-ID: AT5G49510.2
Best-hit-arabi-name: prefoldin 3
Gorai.002G030800.1.D
PCC=0.162, p=0.508
Gorai.002G030800.1.A
PCC=0.091, p=0.71
mRNA Profile
Protein Expression Profile
DPA

00: Gorai.003G069800.6.D in case 2 and V15

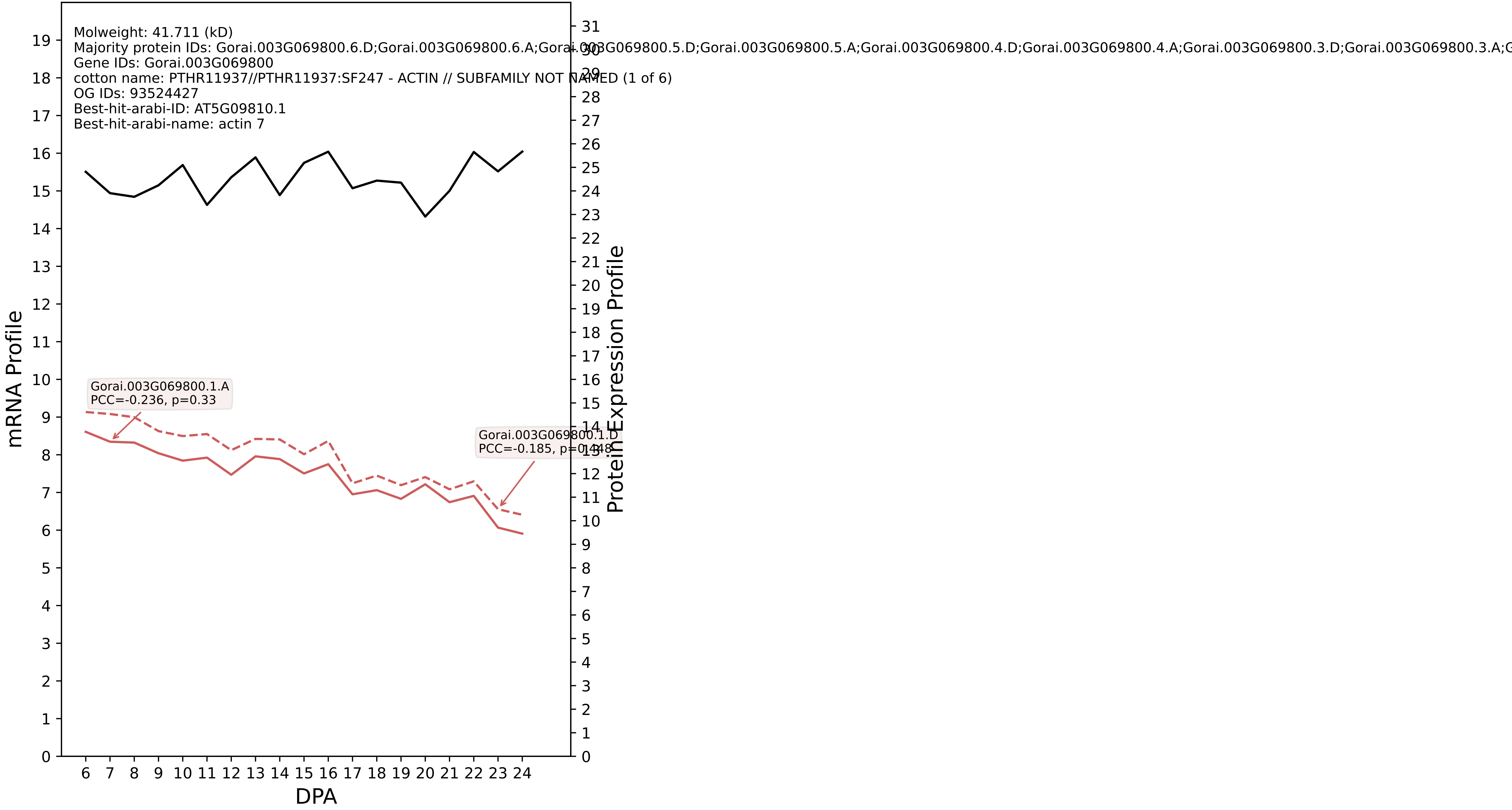

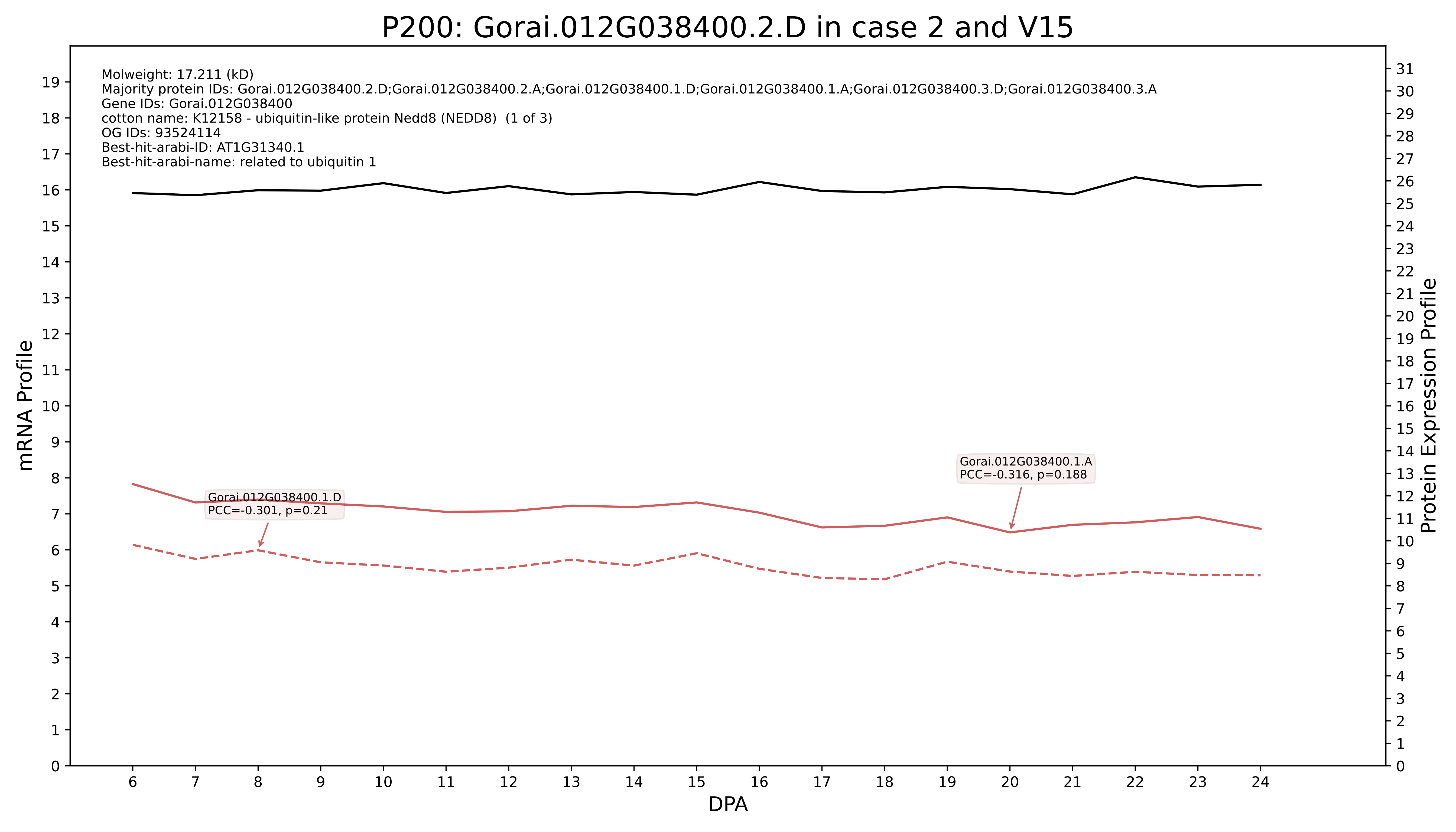
P200: Gorai.012G038400.2.D in case 2 and V15
Molweight: 17.211 (kD)
Majority protein IDs: Gorai.012G038400.2.D;Gorai.012G038400.2.A;Gorai.012G038400.1.D;Gorai.012G038400.1.A;Gorai.012G038400.3.D;Gorai.012G038400.3.A
Gene IDs: Gorai.012G038400
cotton name: K12158 - ubiquitin-like protein Nedd8 (NEDD8) (1 of 3)
OG IDs: 93524114
Best-hit-arabi-ID: AT1G31340.1
Best-hit-arabi-name: related to ubiquitin 1
Gorai.012G038400.1.D
PCC=-0.301, p=0.21
Gorai.012G038400.1.A
PCC=-0.316, p=0.188
mRNA Profile
Protein Expression Profile
DPA

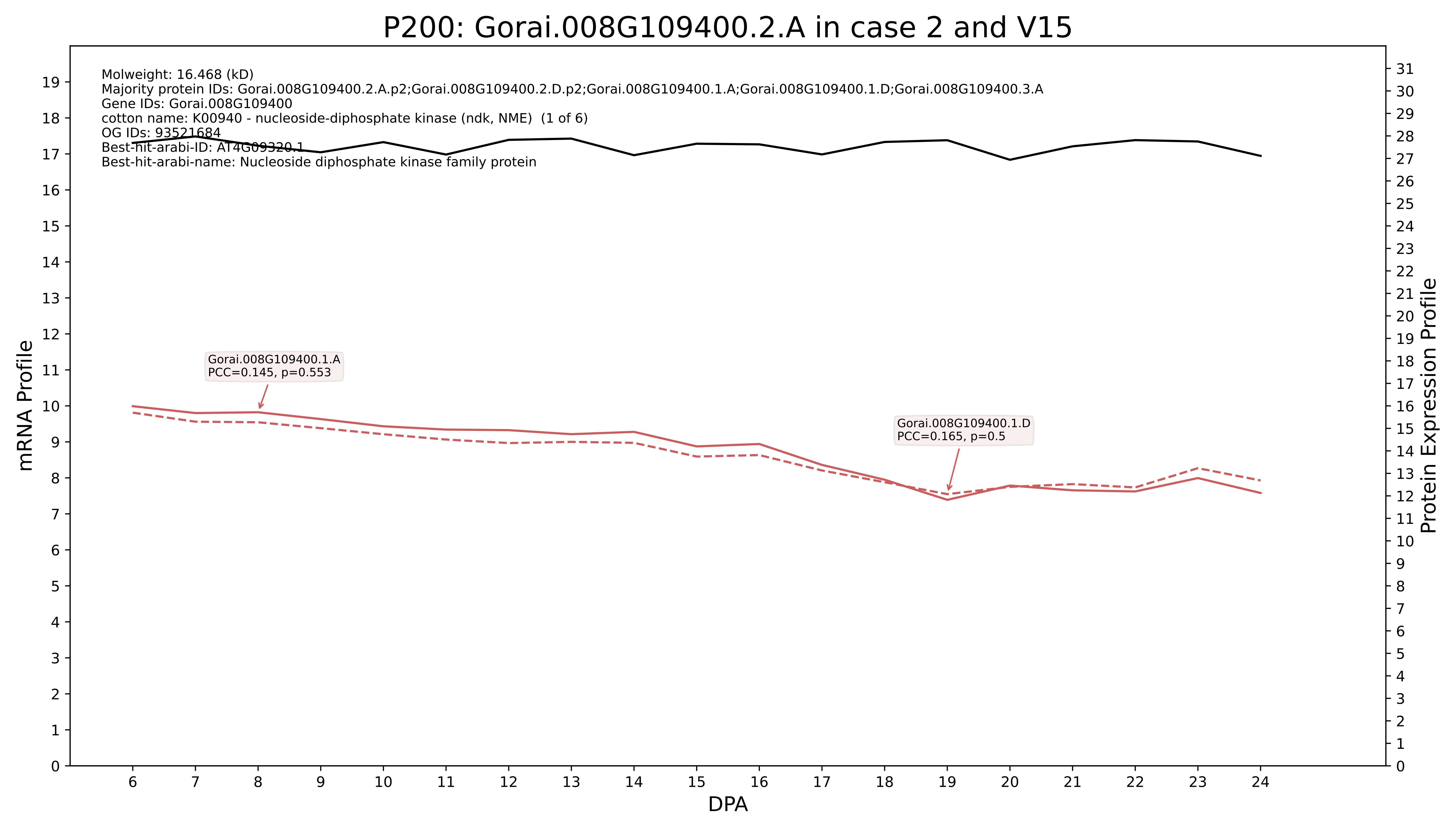
P200: Gorai.008G109400.2.A in case 2 and V15
Molweight: 16.468 (kD)
Majority protein IDs: Gorai.008G109400.2.A.p2;Gorai.008G109400.2.D.p2;Gorai.008G109400.1.A;Gorai.008G109400.1.D;Gorai.008G109400.3.A
Gene IDs: Gorai.008G109400
cotton name: K00940 - nucleoside-diphosphate kinase (ndk, NME) (1 of 6)
OG IDs: 93521684
Best-hit-arabi-ID: AT4G09320.1
Best-hit-arabi-name: Nucleoside diphosphate kinase family protein
Gorai.008G109400.1.A
PCC=0.145, p=0.553
Gorai.008G109400.1.D
PCC=0.165, p=0.5
mRNA Profile
Protein Expression Profile
DPA

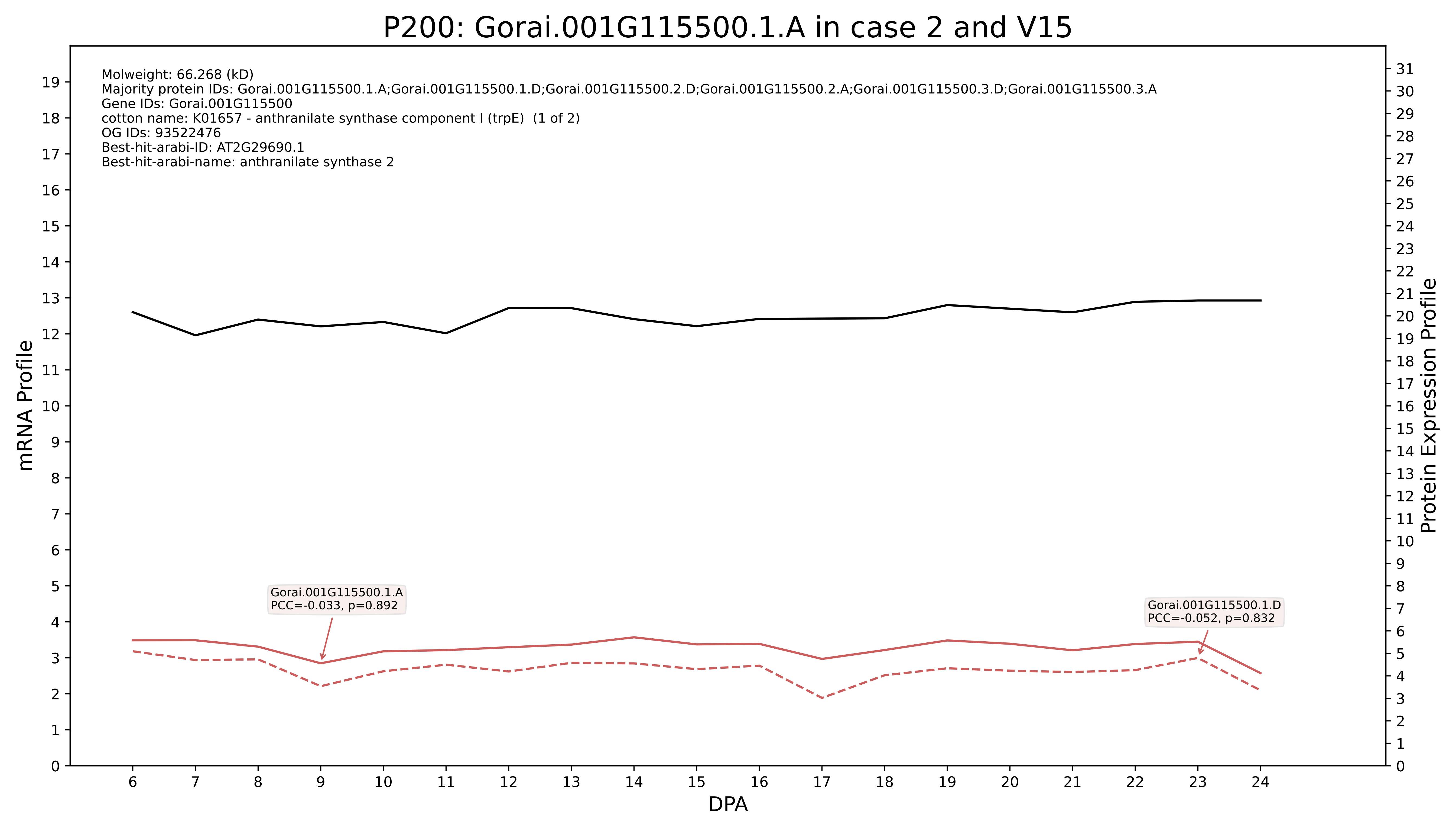
P200: Gorai.001G115500.1.A in case 2 and V15
Molweight: 66.268 (kD)
Majority protein IDs: Gorai.001G115500.1.A;Gorai.001G115500.1.D;Gorai.001G115500.2.D;Gorai.001G115500.2.A;Gorai.001G115500.3.D;Gorai.001G115500.3.A
Gene IDs: Gorai.001G115500
cotton name: K01657 - anthranilate synthase component I (trpE) (1 of 2)
OG IDs: 93522476
Best-hit-arabi-ID: AT2G29690.1
Best-hit-arabi-name: anthranilate synthase 2
Gorai.001G115500.1.A
PCC=-0.033, p=0.892
Gorai.001G115500.1.D
PCC=-0.052, p=0.832
mRNA Profile
Protein Expression Profile
DPA

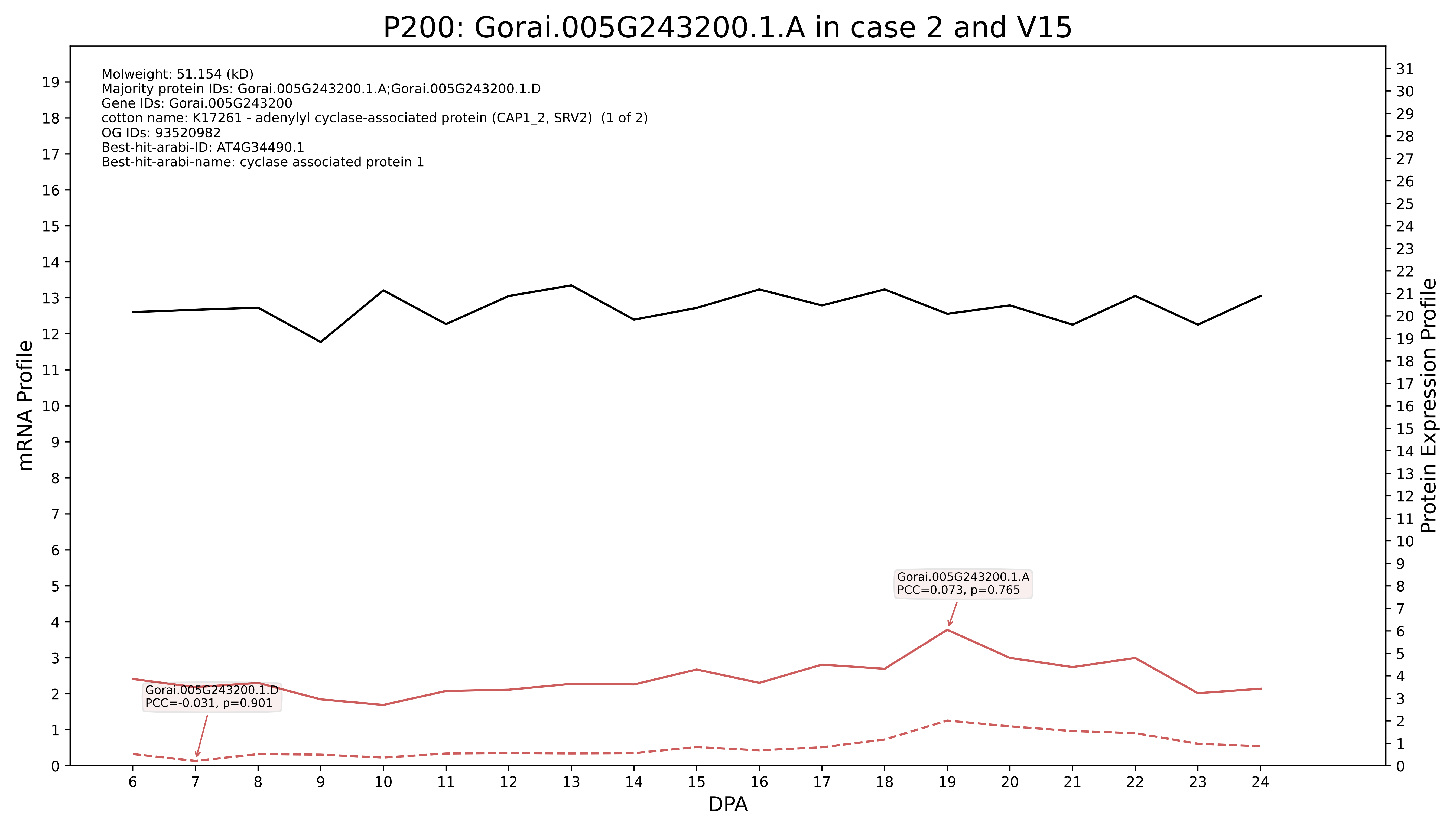

P200: Gorai.005G243200.1.A in case 2 and V15
Molweight: 51.154 (kD)
Majority protein IDs: Gorai.005G243200.1.A;Gorai.005G243200.1.D
Gene IDs: Gorai.005G243200
cotton name: K17261 - adenylyl cyclase-associated protein (CAP1_2, SRV2) (1 of 2)
OG IDs: 93520982
Best-hit-arabi-ID: AT4G34490.1
Best-hit-arabi-name: cyclase associated protein 1
Gorai.005G243200.1.A
PCC=0.073, p=0.765
Gorai.005G243200.1.D
PCC=-0.031, p=0.901
mRNA Profile
Protein Expression Profile
DPA

P200: Gorai.003G177600.1.D in case 2 and V15

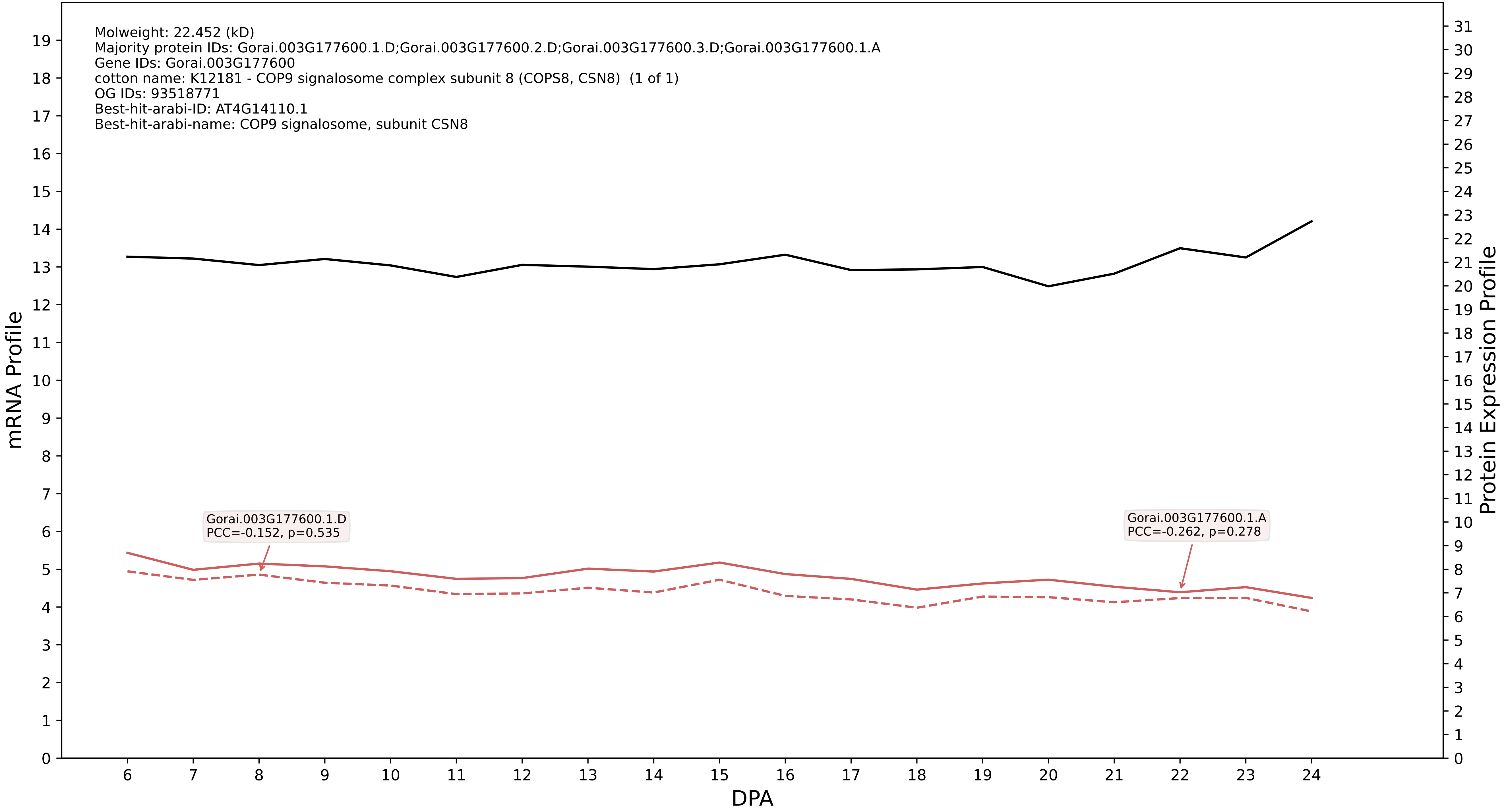

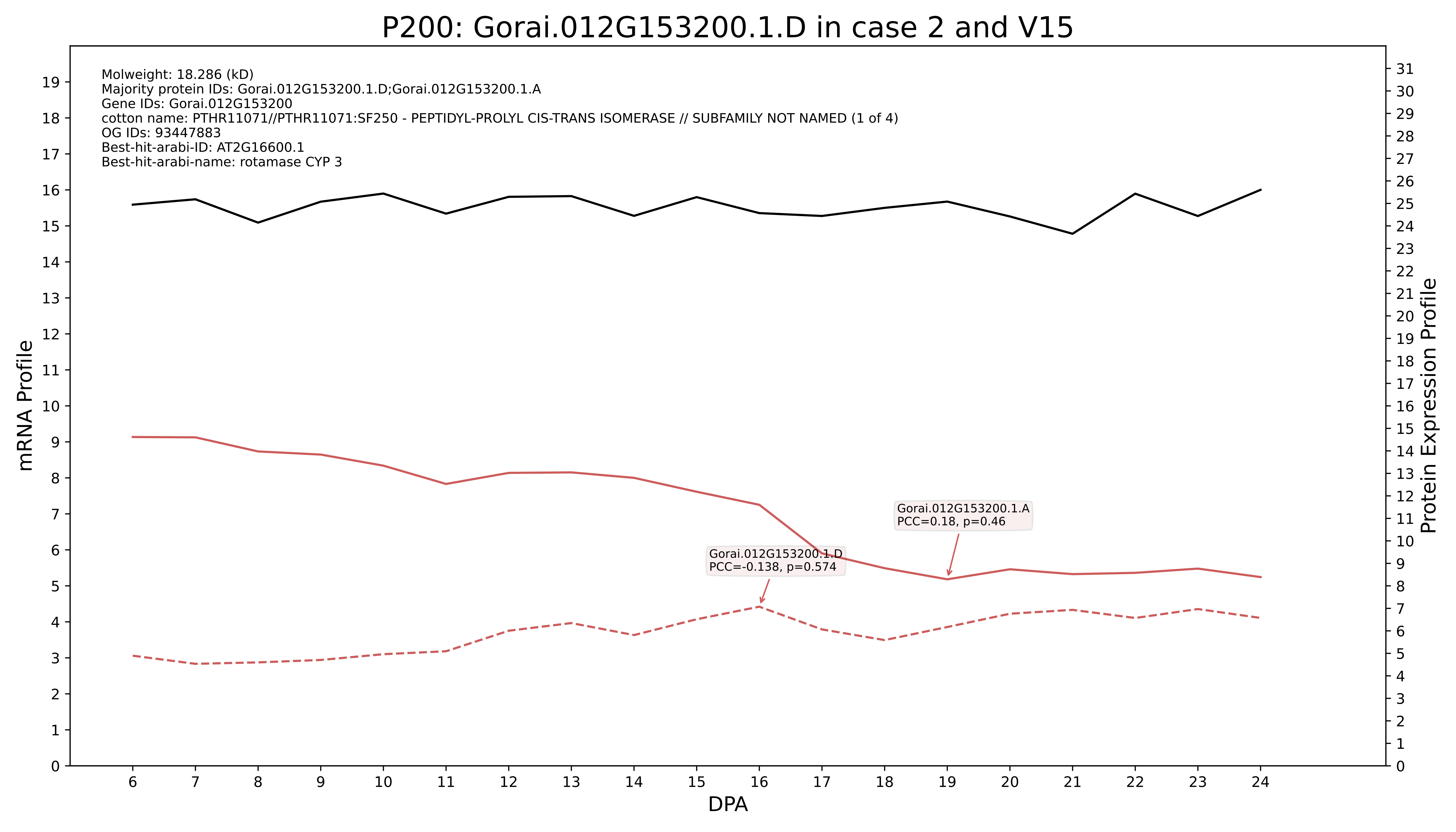
P200: Gorai.012G153200.1.D in case 2 and V15
Molweight: 18.286 (kD)
Majority protein IDs: Gorai.012G153200.1.D;Gorai.012G153200.1.A
Gene IDs: Gorai.012G153200
cotton name: PTHR11071//PTHR11071:SF250 - PEPTIDYL-PROLYL CIS-TRANS ISOMERASE // SUBFAMILY NOT NAMED (1 of 4)
OG IDs: 93447883
Best-hit-arabi-ID: AT2G16600.1
Best-hit-arabi-name: rotamase CYP 3
Gorai.012G153200.1.A
PCC=0.18, p=0.46
Gorai.012G153200.1.D
PCC=-0.138, p=0.574
mRNA Profile
Protein Expression Profile
DPA

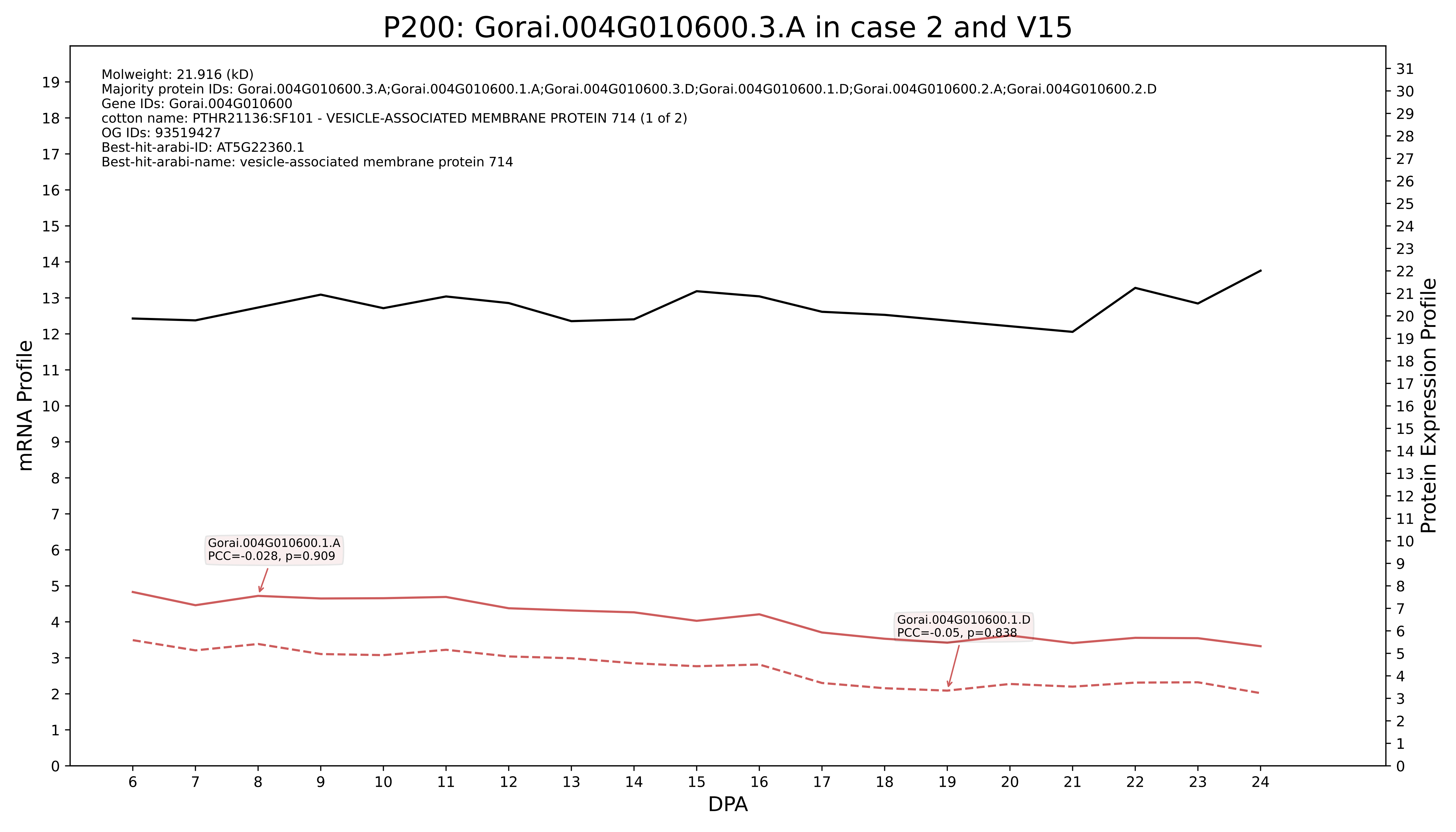

P200: Gorai.004G010600.3.A in case 2 and V15
Molweight: 21.916 (kD)
Majority protein IDs: Gorai.004G010600.3.A;Gorai.004G010600.1.A;Gorai.004G010600.3.D;Gorai.004G010600.1.D;Gorai.004G010600.2.A;Gorai.004G010600.2.D
Gene IDs: Gorai.004G010600
cotton name: PTHR21136:SF101 - VESICLE-ASSOCIATED MEMBRANE PROTEIN 714 (1 of 2)
OG IDs: 93519427
Best-hit-arabi-ID: AT5G22360.1
Best-hit-arabi-name: vesicle-associated membrane protein 714
Gorai.004G010600.1.A
PCC=-0.028, p=0.909
Gorai.004G010600.1.D
PCC=-0.05, p=0.838
mRNA Profile
Protein Expression Profile
DPA

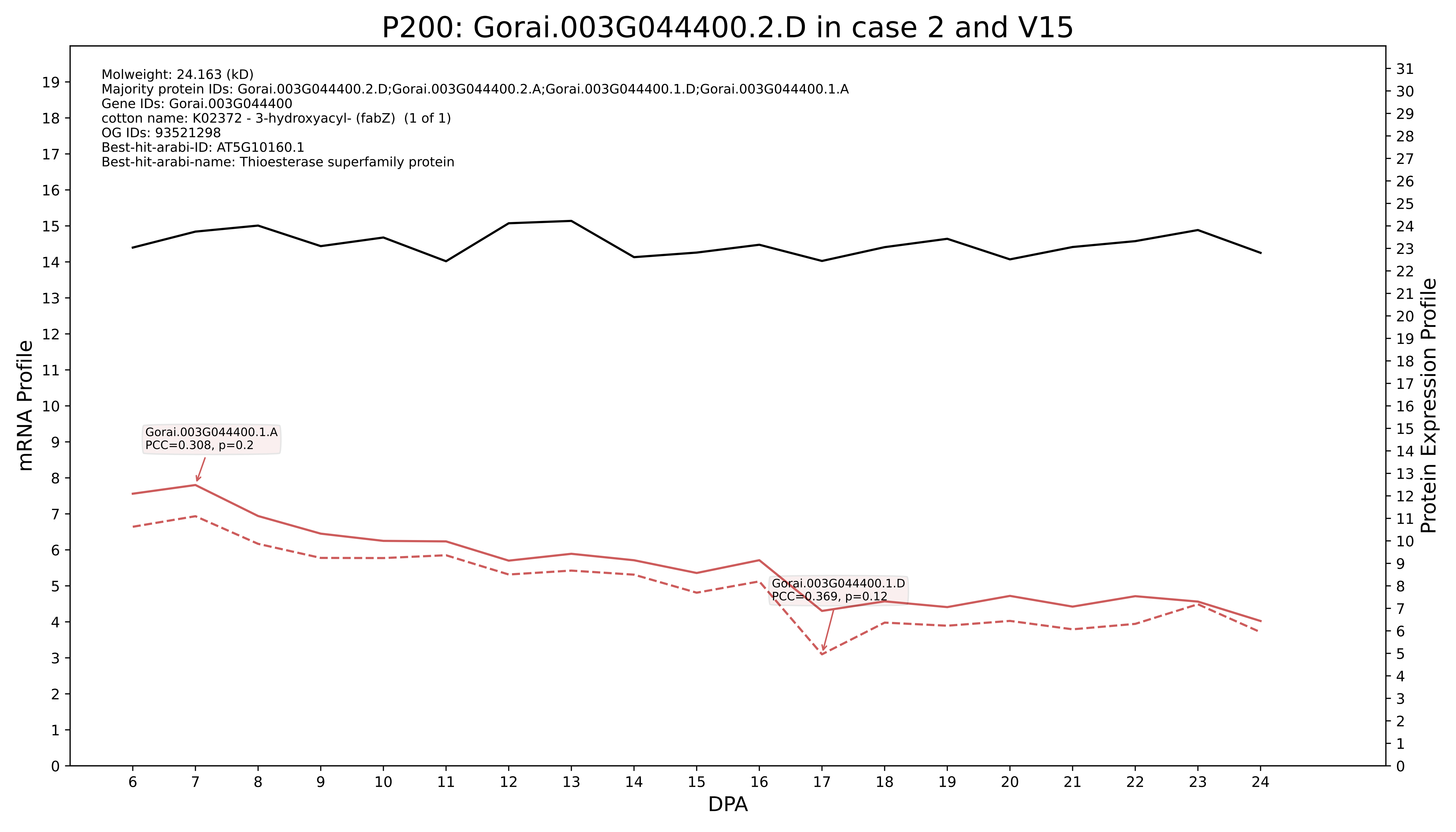

P200: Gorai.003G044400.2.D in case 2 and V15
Molweight: 24.163 (kD)
Majority protein IDs: Gorai.003G044400.2.D;Gorai.003G044400.2.A;Gorai.003G044400.1.D;Gorai.003G044400.1.A
Gene IDs: Gorai.003G044400
cotton name: K02372 - 3-hydroxyacyl- (fabZ) (1 of 1)
OG IDs: 93521298
Best-hit-arabi-ID: AT5G10160.1
Best-hit-arabi-name: Thioesterase superfamily protein
Gorai.003G044400.1.A
PCC=0.308, p=0.2
Gorai.003G044400.1.D
PCC=0.369, p=0.12
mRNA Profile
Protein Expression Profile
DPA

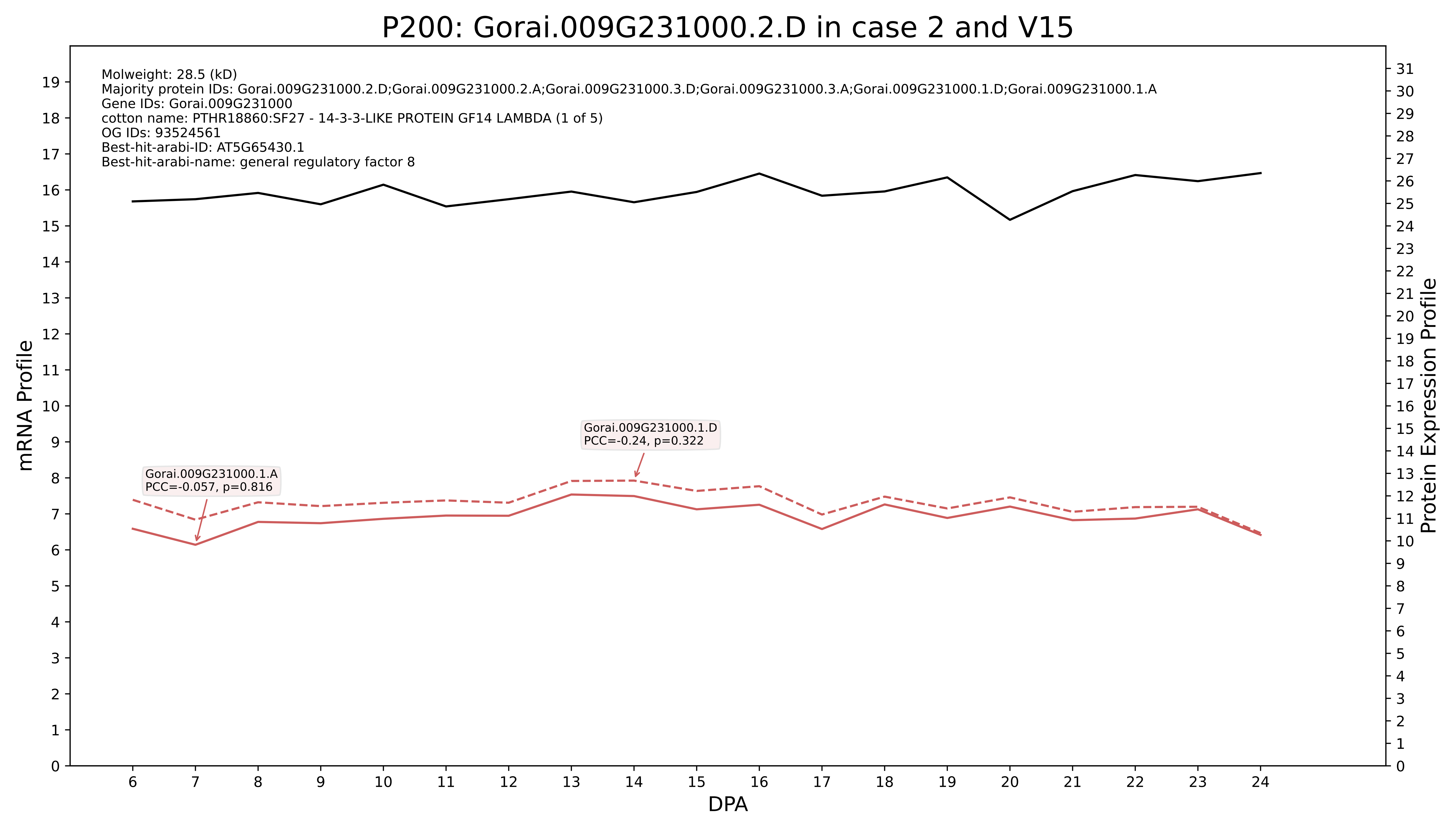
P200: Gorai.009G231000.2.D in case 2 and V15
Molweight: 28.5 (kD)
Majority protein IDs: Gorai.009G231000.2.D;Gorai.009G231000.2.A;Gorai.009G231000.3.D;Gorai.009G231000.3.A;Gorai.009G231000.1.D;Gorai.009G231000.1.A
Gene IDs: Gorai.009G231000
cotton name: PTHR18860:SF27 - 14-3-3-LIKE PROTEIN GF14 LAMBDA (1 of 5)
OG IDs: 93524561
Best-hit-arabi-ID: AT5G65430.1
Best-hit-arabi-name: general regulatory factor 8
Gorai.009G231000.1.D
PCC=-0.24, p=0.322
Gorai.009G231000.1.A
PCC=-0.057, p=0.816
mRNA Profile
Protein Expression Profile
DPA

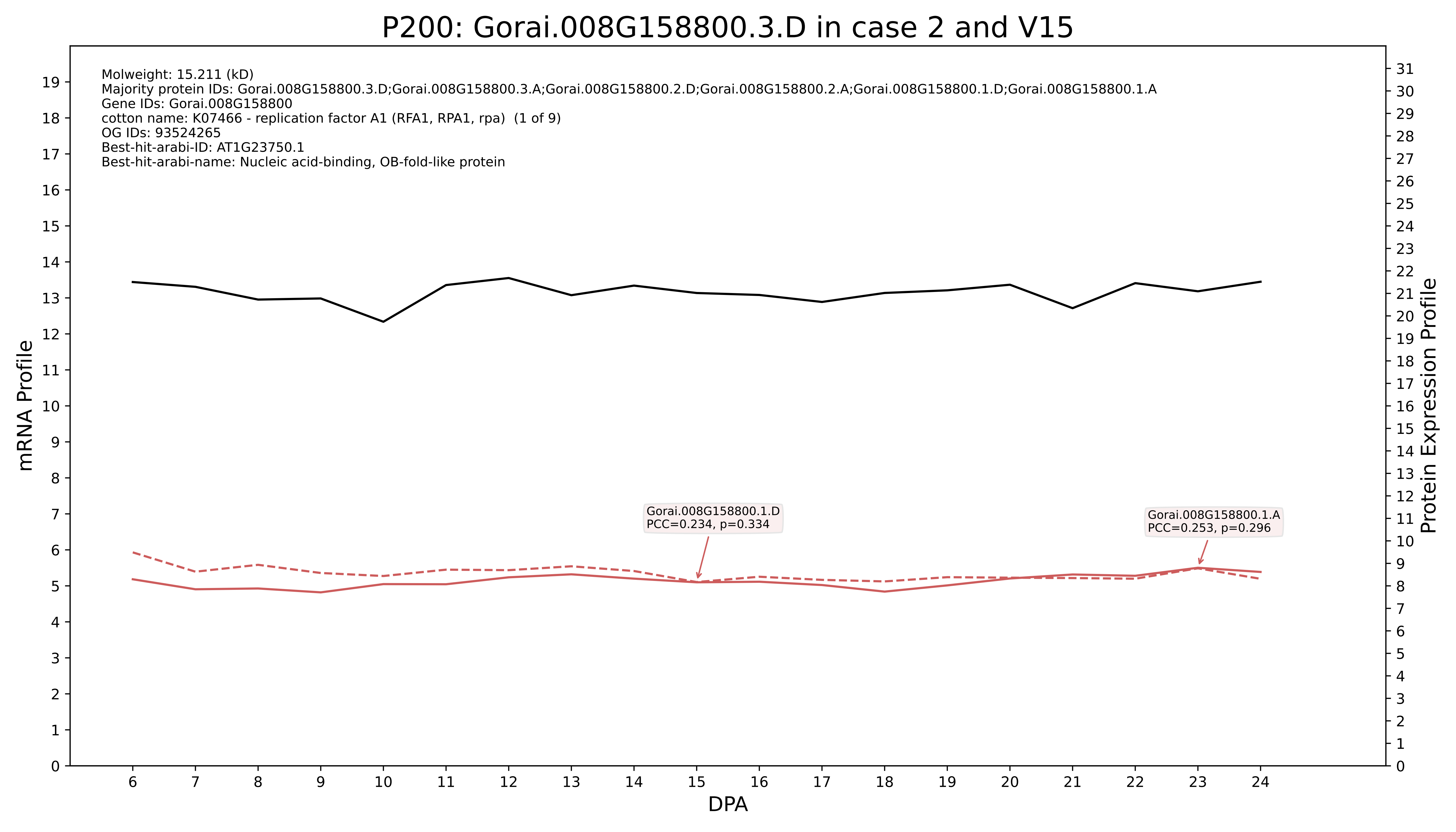

P200: Gorai.008G158800.3.D in case 2 and V15
Molweight: 15.211 (kD)
Majority protein IDs: Gorai.008G158800.3.D;Gorai.008G158800.3.A;Gorai.008G158800.2.D;Gorai.008G158800.2.A;Gorai.008G158800.1.D;Gorai.008G158800.1.A
Gene IDs: Gorai.008G158800
cotton name: K07466 - replication factor A1 (RFA1, RPA1, rpa) (1 of 9)
OG IDs: 93524265
Best-hit-arabi-ID: AT1G23750.1
Best-hit-arabi-name: Nucleic acid-binding, OB-fold-like protein
Gorai.008G158800.1.D
PCC=0.234, p=0.334
Gorai.008G158800.1.A
PCC=0.253, p=0.296
mRNA Profile
Protein Expression Profile
DPA

00: Gorai.005G242800.2.D in case 2 and V15

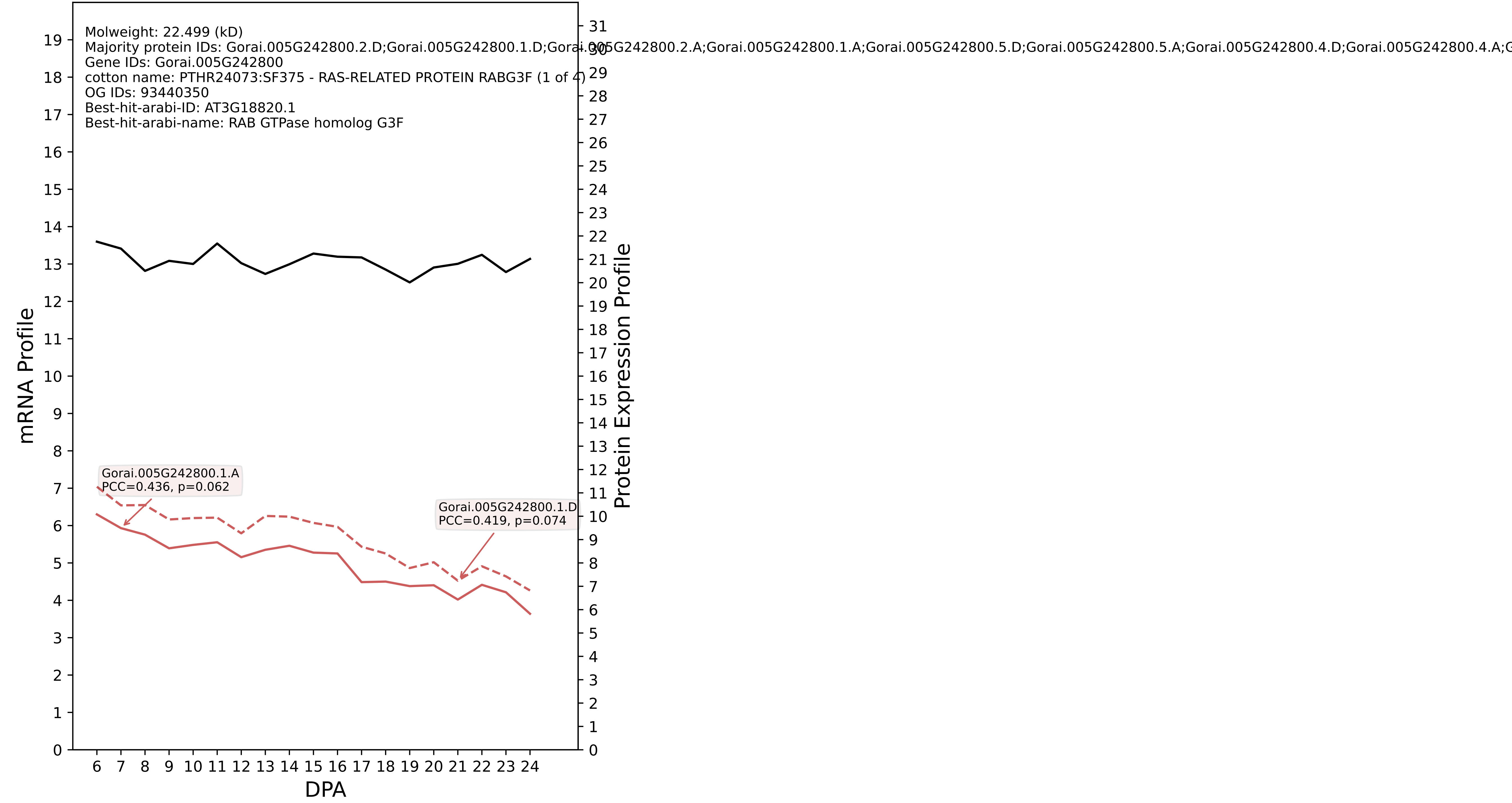

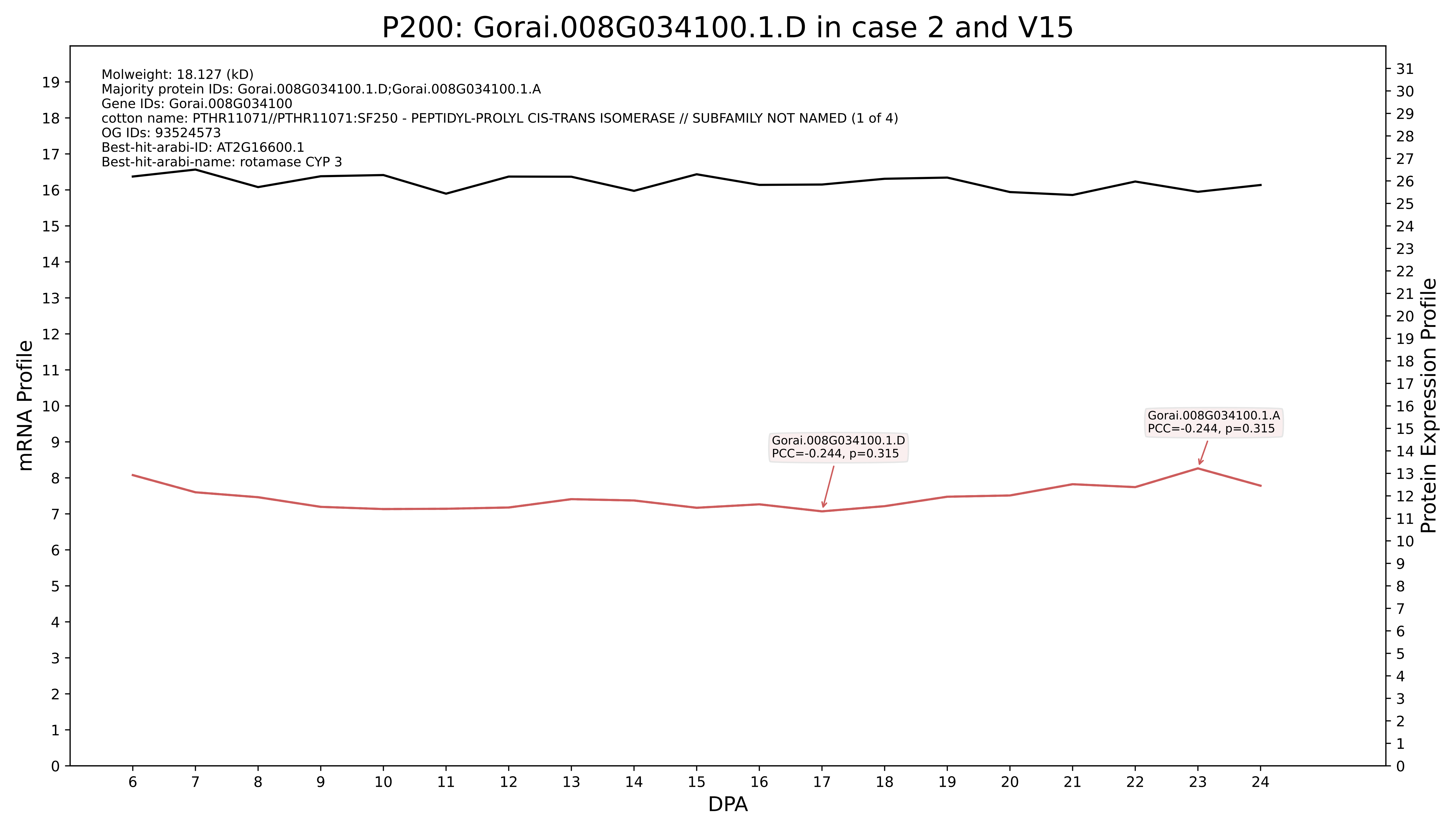

P200: Gorai.008G034100.1.D in case 2 and V15
Molweight: 18.127 (kD)
Majority protein IDs: Gorai.008G034100.1.D;Gorai.008G034100.1.A
Gene IDs: Gorai.008G034100
cotton name: PTHR11071//PTHR11071:SF250 - PEPTIDYL-PROLYL CIS-TRANS ISOMERASE // SUBFAMILY NOT NAMED (1 of 4)
OG IDs: 93524573
Best-hit-arabi-ID: AT2G16600.1
Best-hit-arabi-name: rotamase CYP 3
Gorai.008G034100.1.D
PCC=-0.244, p=0.315
Gorai.008G034100.1.A
PCC=-0.244, p=0.315
mRNA Profile
Protein Expression Profile
DPA
0
1
2
3
4
5
6
7
8
9
10
11
12
13
14
15
16
17
18
19
20
21
22
23
24
25
26
27
28
29
30
31

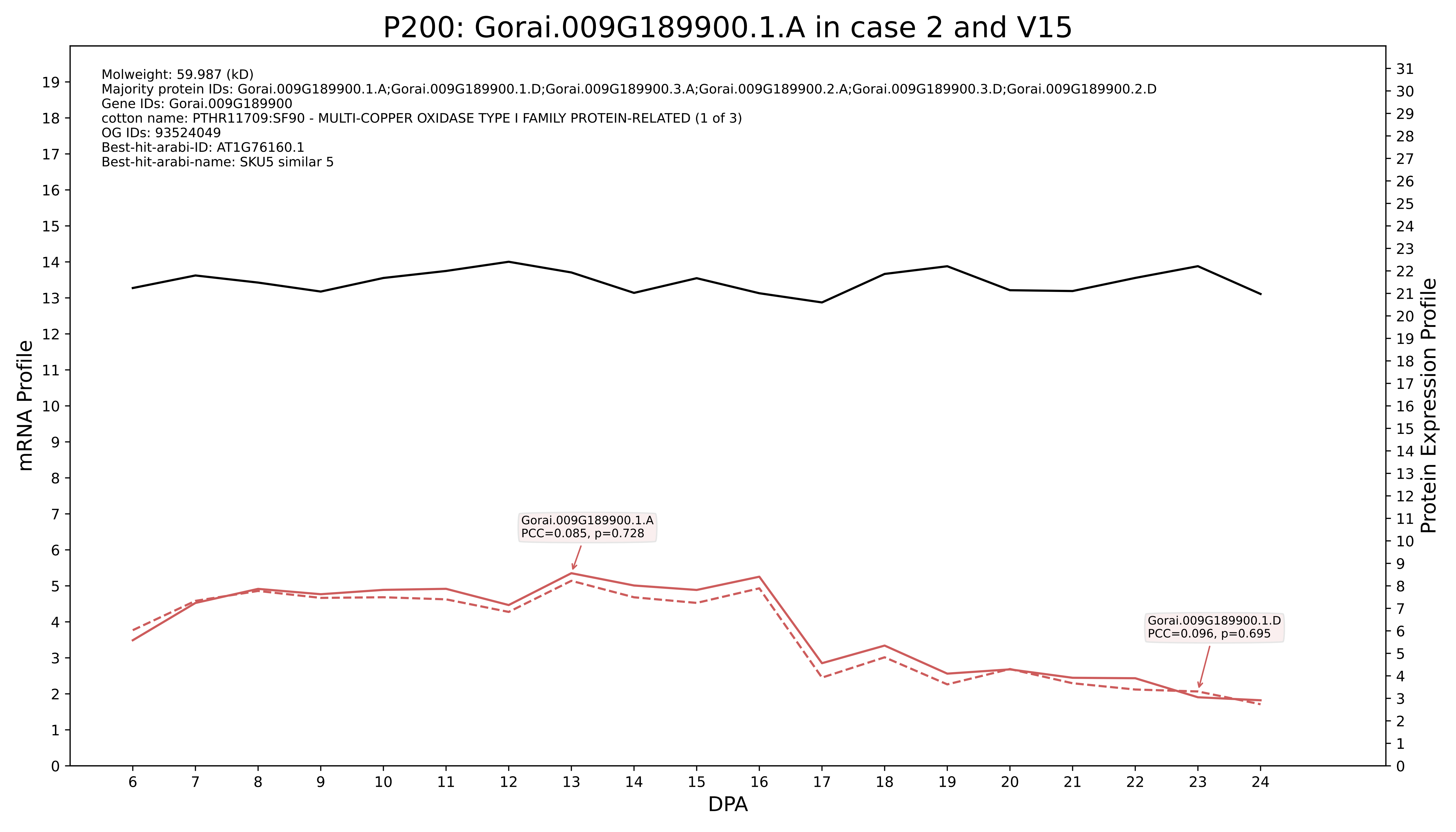
P200: Gorai.009G189900.1.A in case 2 and V15
Molweight: 59.987 (kD)
Majority protein IDs: Gorai.009G189900.1.A;Gorai.009G189900.1.D;Gorai.009G189900.3.A;Gorai.009G189900.2.A;Gorai.009G189900.3.D;Gorai.009G189900.2.D
Gene IDs: Gorai.009G189900
cotton name: PTHR11709:SF90 - MULTI-COPPER OXIDASE TYPE I FAMILY PROTEIN-RELATED (1 of 3)
OG IDs: 93524049
Best-hit-arabi-ID: AT1G76160.1
Best-hit-arabi-name: SKU5 similar 5
Gorai.009G189900.1.A
PCC=0.085, p=0.728
Gorai.009G189900.1.D
PCC=0.096, p=0.695
mRNA Profile
Protein Expression Profile
DPA

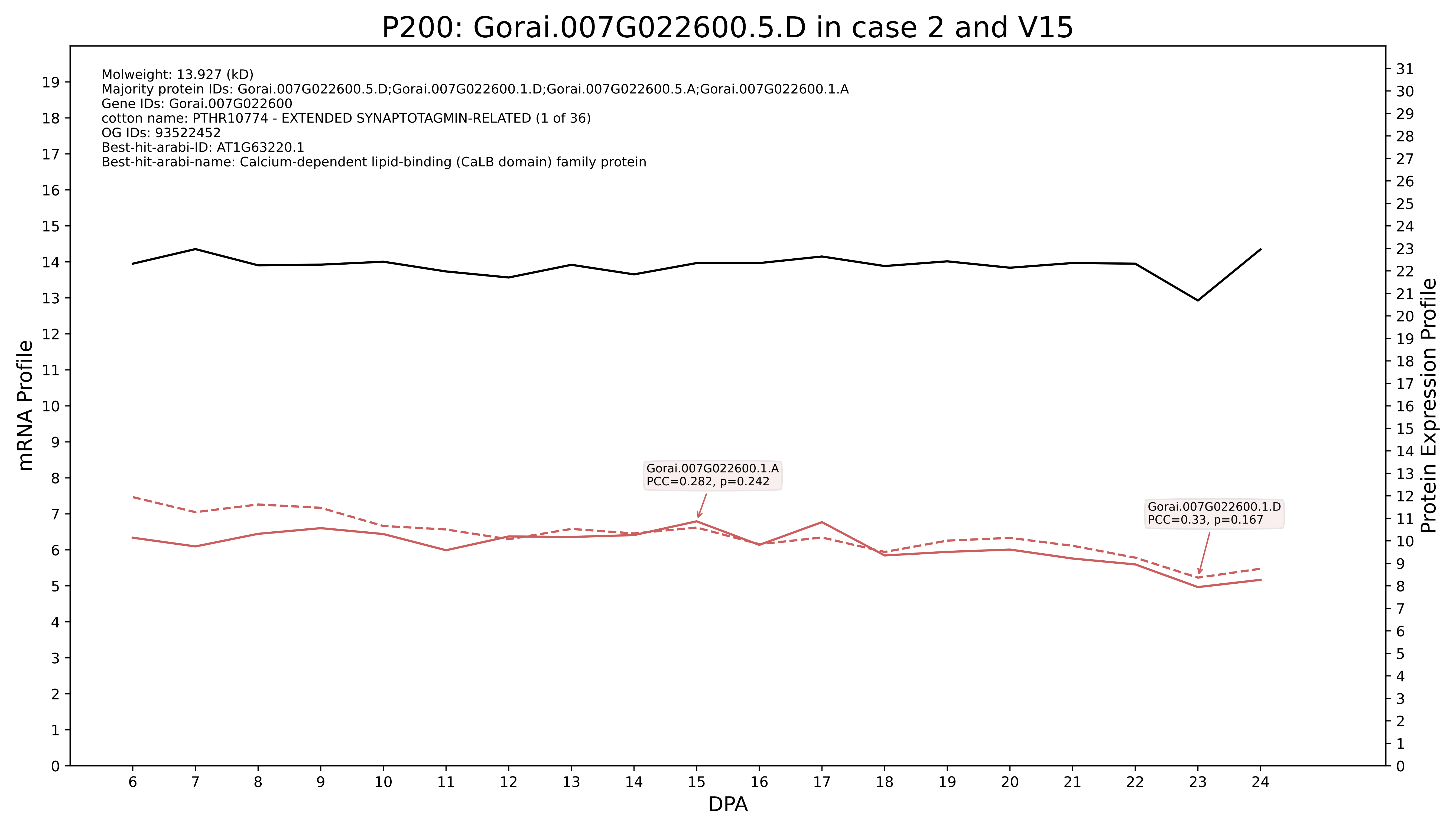

P200: Gorai.007G022600.5.D in case 2 and V15
Molweight: 13.927 (kD)
Majority protein IDs: Gorai.007G022600.5.D;Gorai.007G022600.1.D;Gorai.007G022600.5.A;Gorai.007G022600.1.A
Gene IDs: Gorai.007G022600
cotton name: PTHR10774 - EXTENDED SYNAPTOTAGMIN-RELATED (1 of 36)
OG IDs: 93522452
Best-hit-arabi-ID: AT1G63220.1
Best-hit-arabi-name: Calcium-dependent lipid-binding (CaLB domain) family protein
Gorai.007G022600.1.A
PCC=0.282, p=0.242
Gorai.007G022600.1.D
PCC=0.33, p=0.167
mRNA Profile
Protein Expression Profile
DPA

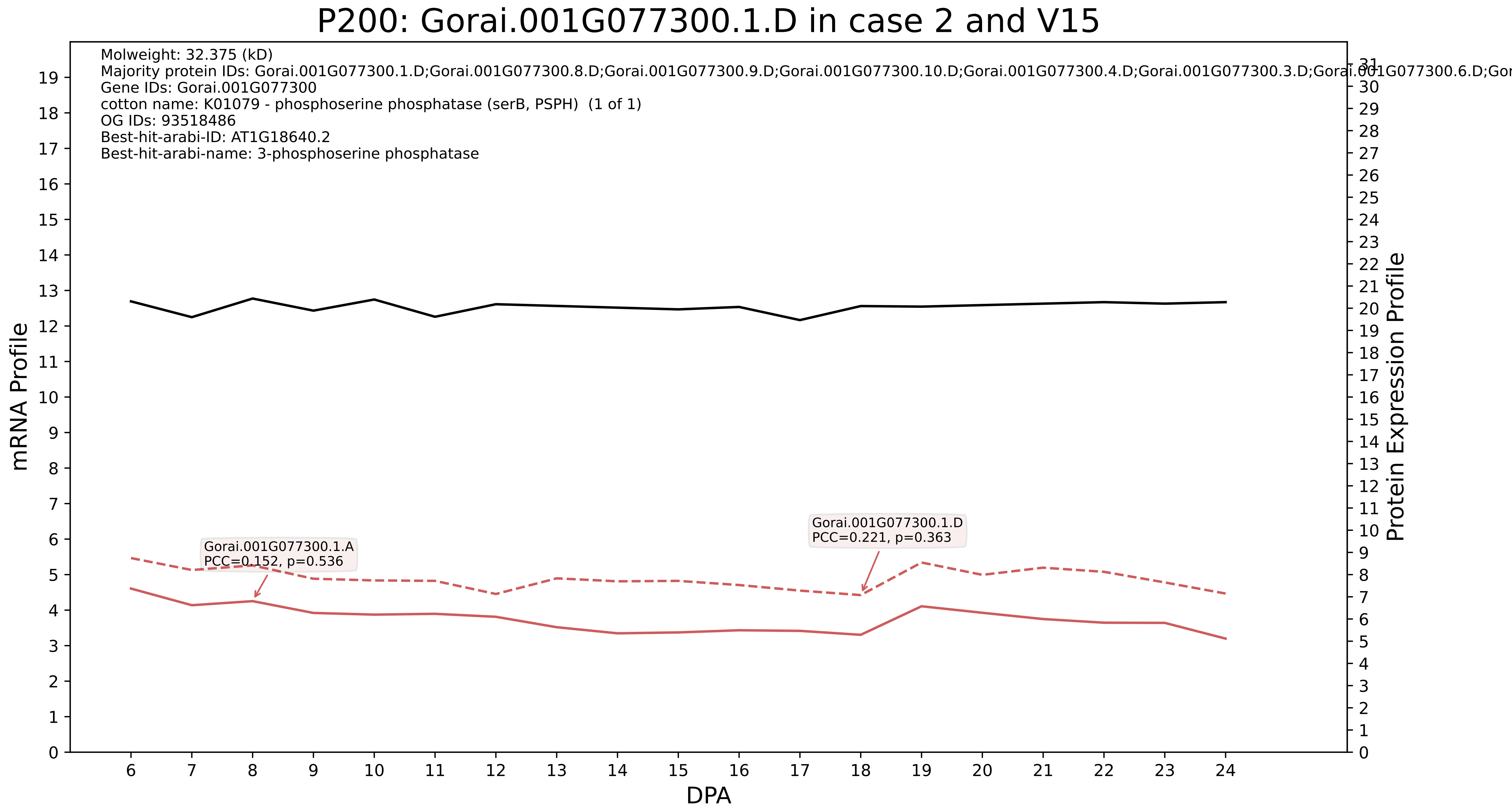
P200: Gorai.001G077300.1.D in case 2 and V15
Molweight: 32.375 (kD)
Majority protein IDs: Gorai.001G077300.1.D;Gorai.001G077300.8.D;Gorai.001G077300.9.D;Gorai.001G077300.10.D;Gorai.001G077300.4.D;Gorai.001G077300.3.D;Gorai.001G077300.6.D;Gor
Gene IDs: Gorai.001G077300
cotton name: K01079 - phosphoserine phosphatase (serB, PSPH) (1 of 1)
OG IDs: 93518486
Best-hit-arabi-ID: AT1G18640.2
Best-hit-arabi-name: 3-phosphoserine phosphatase
Gorai.001G077300.1.A
PCC=0.152, p=0.536
Gorai.001G077300.1.D
PCC=0.221, p=0.363
mRNA Profile
Protein Expression Profile
DPA

P200: Gorai.003G154600.3.D in case 2 and V15

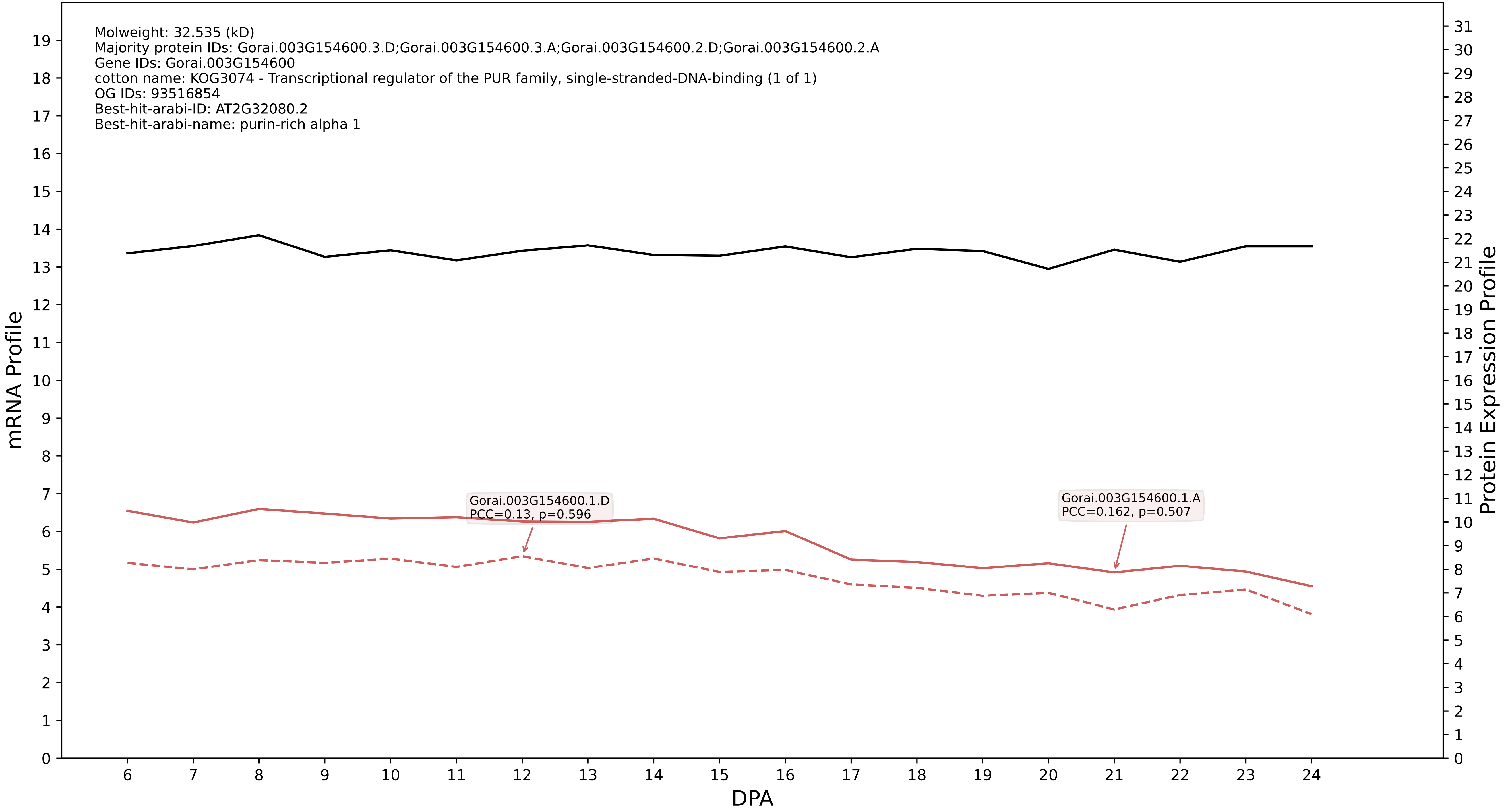

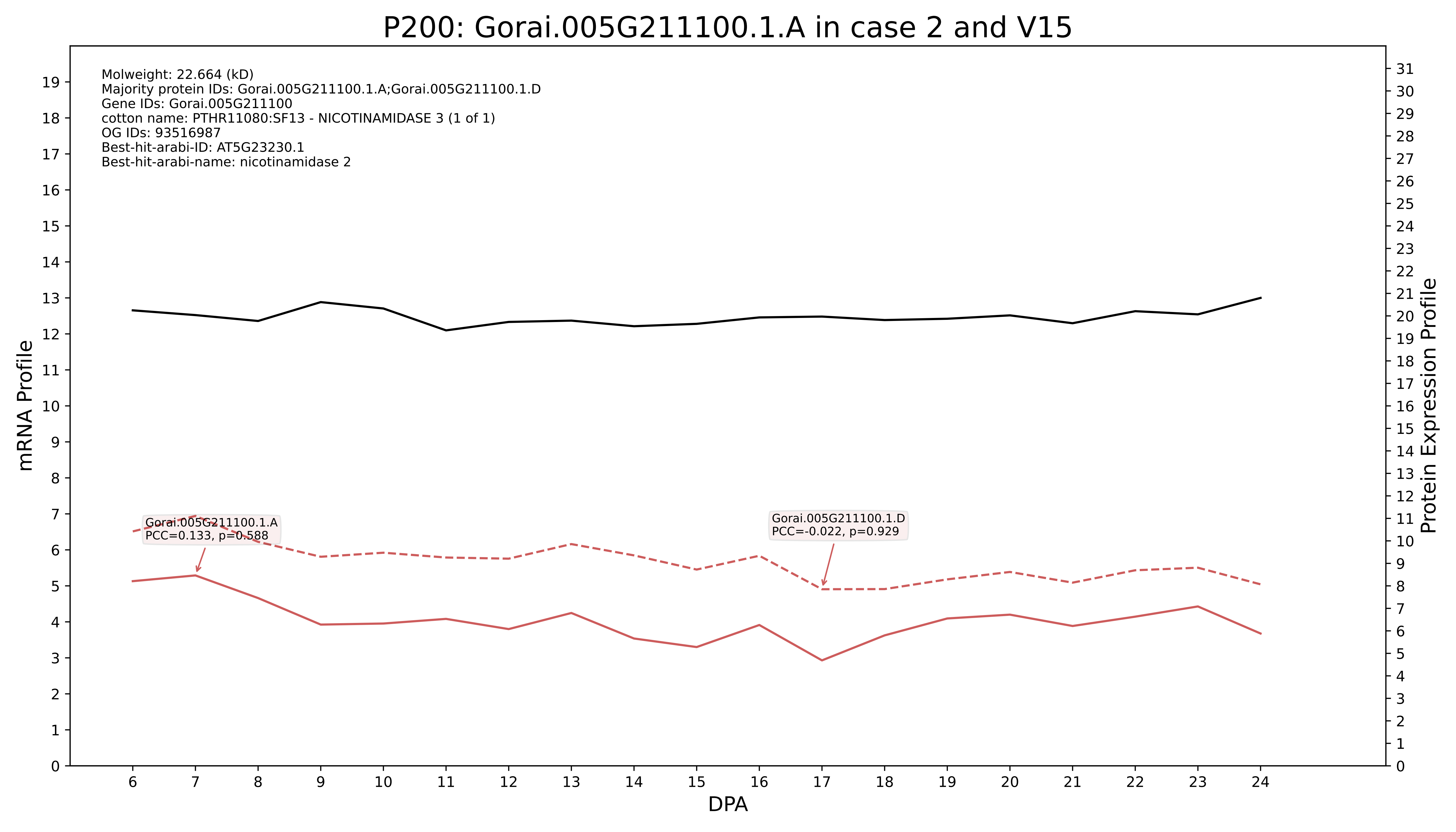

P200: Gorai.005G211100.1.A in case 2 and V15
Molweight: 22.664 (kD)
Majority protein IDs: Gorai.005G211100.1.A;Gorai.005G211100.1.D
Gene IDs: Gorai.005G211100
cotton name: PTHR11080:SF13 - NICOTINAMIDASE 3 (1 of 1)
OG IDs: 93516987
Best-hit-arabi-ID: AT5G23230.1
Best-hit-arabi-name: nicotinamidase 2
Gorai.005G211100.1.A
PCC=0.133, p=0.588
Gorai.005G211100.1.D
PCC=-0.022, p=0.929
mRNA Profile
Protein Expression Profile
DPA

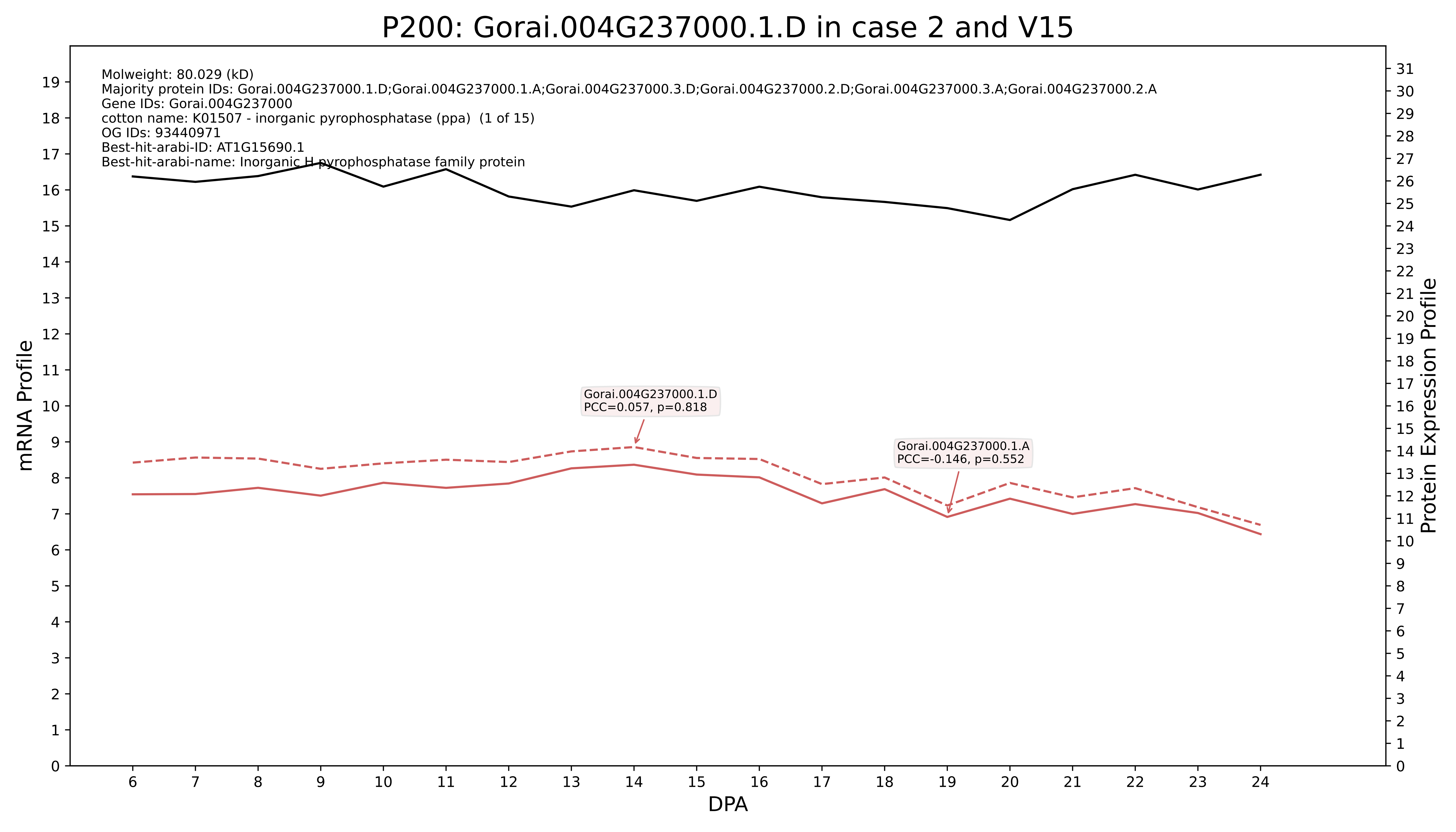
P200: Gorai.004G237000.1.D in case 2 and V15
Molweight: 80.029 (kD)
Majority protein IDs: Gorai.004G237000.1.D;Gorai.004G237000.1.A;Gorai.004G237000.3.D;Gorai.004G237000.2.D;Gorai.004G237000.3.A;Gorai.004G237000.2.A
Gene IDs: Gorai.004G237000
cotton name: K01507 - inorganic pyrophosphatase (ppa) (1 of 15)
OG IDs: 93440971
Best-hit-arabi-ID: AT1G15690.1
Best-hit-arabi-name: Inorganic H pyrophosphatase family protein
Gorai.004G237000.1.D
PCC=0.057, p=0.818
Gorai.004G237000.1.A
PCC=-0.146, p=0.552
mRNA Profile
Protein Expression Profile
DPA

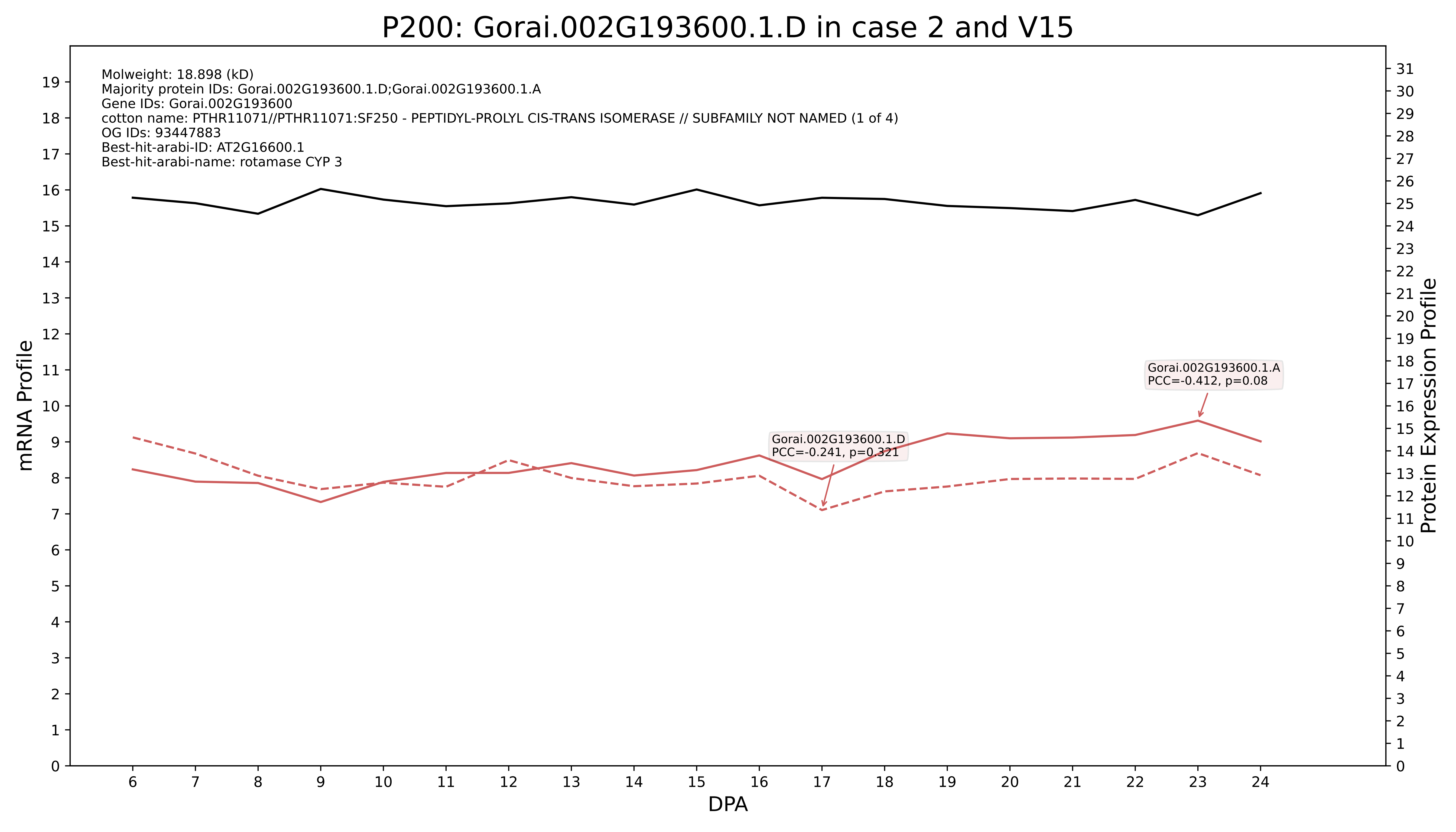

P200: Gorai.002G193600.1.D in case 2 and V15
Molweight: 18.898 (kD)
Majority protein IDs: Gorai.002G193600.1.D;Gorai.002G193600.1.A
Gene IDs: Gorai.002G193600
cotton name: PTHR11071//PTHR11071:SF250 - PEPTIDYL-PROLYL CIS-TRANS ISOMERASE // SUBFAMILY NOT NAMED (1 of 4)
OG IDs: 93447883
Best-hit-arabi-ID: AT2G16600.1
Best-hit-arabi-name: rotamase CYP 3
Gorai.002G193600.1.A
PCC=-0.412, p=0.08
Gorai.002G193600.1.D
PCC=-0.241, p=0.321
mRNA Profile
Protein Expression Profile
DPA

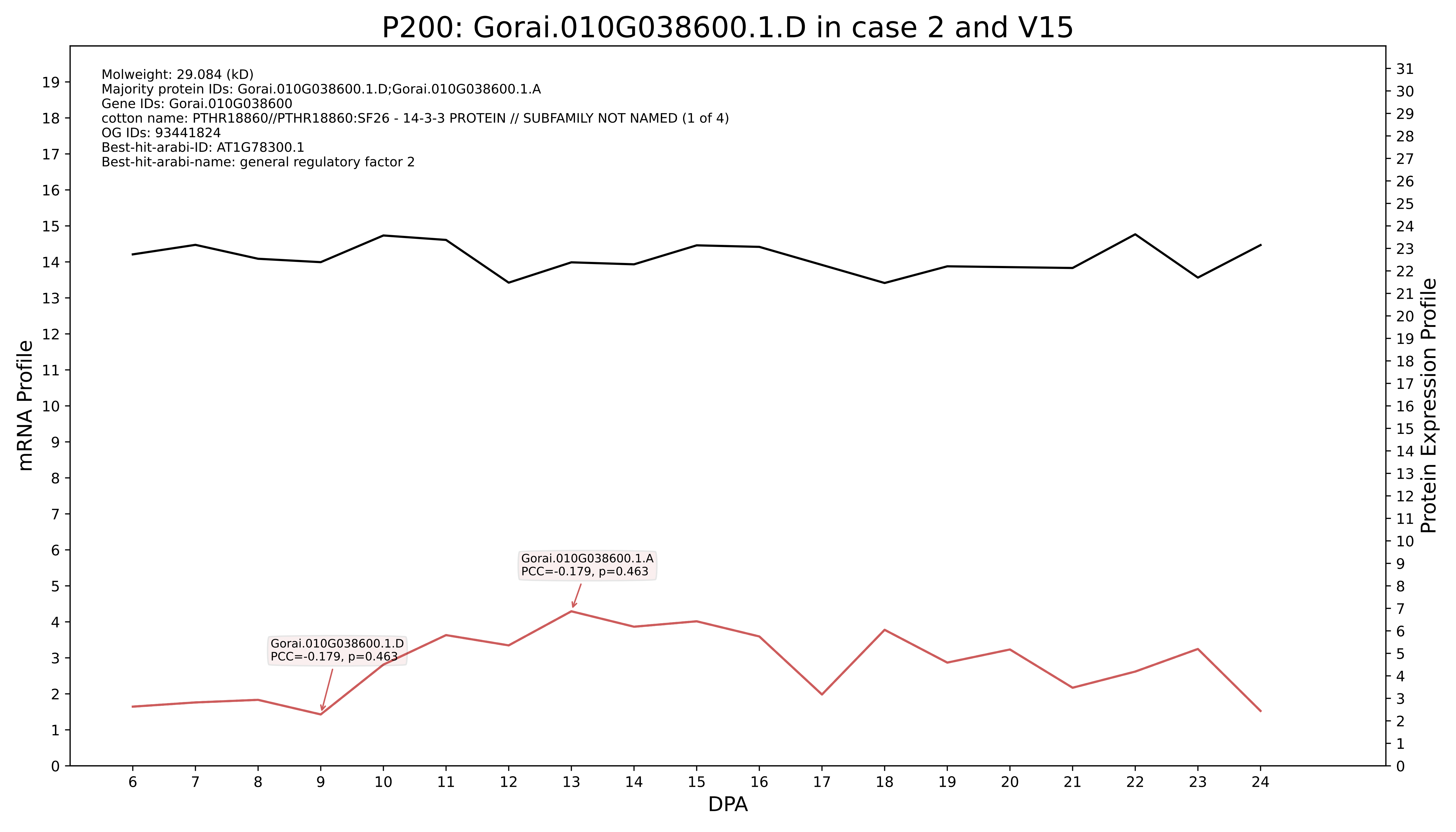
P200: Gorai.010G038600.1.D in case 2 and V15
Molweight: 29.084 (kD)
Majority protein IDs: Gorai.010G038600.1.D;Gorai.010G038600.1.A
Gene IDs: Gorai.010G038600
cotton name: PTHR18860//PTHR18860:SF26 - 14-3-3 PROTEIN // SUBFAMILY NOT NAMED (1 of 4)
OG IDs: 93441824
Best-hit-arabi-ID: AT1G78300.1
Best-hit-arabi-name: general regulatory factor 2
Gorai.010G038600.1.A
PCC=-0.179, p=0.463
Gorai.010G038600.1.D
PCC=-0.179, p=0.463
mRNA Profile
Protein Expression Profile
DPA

P200: Gorai.009G292700.2.D in case 2 and V15

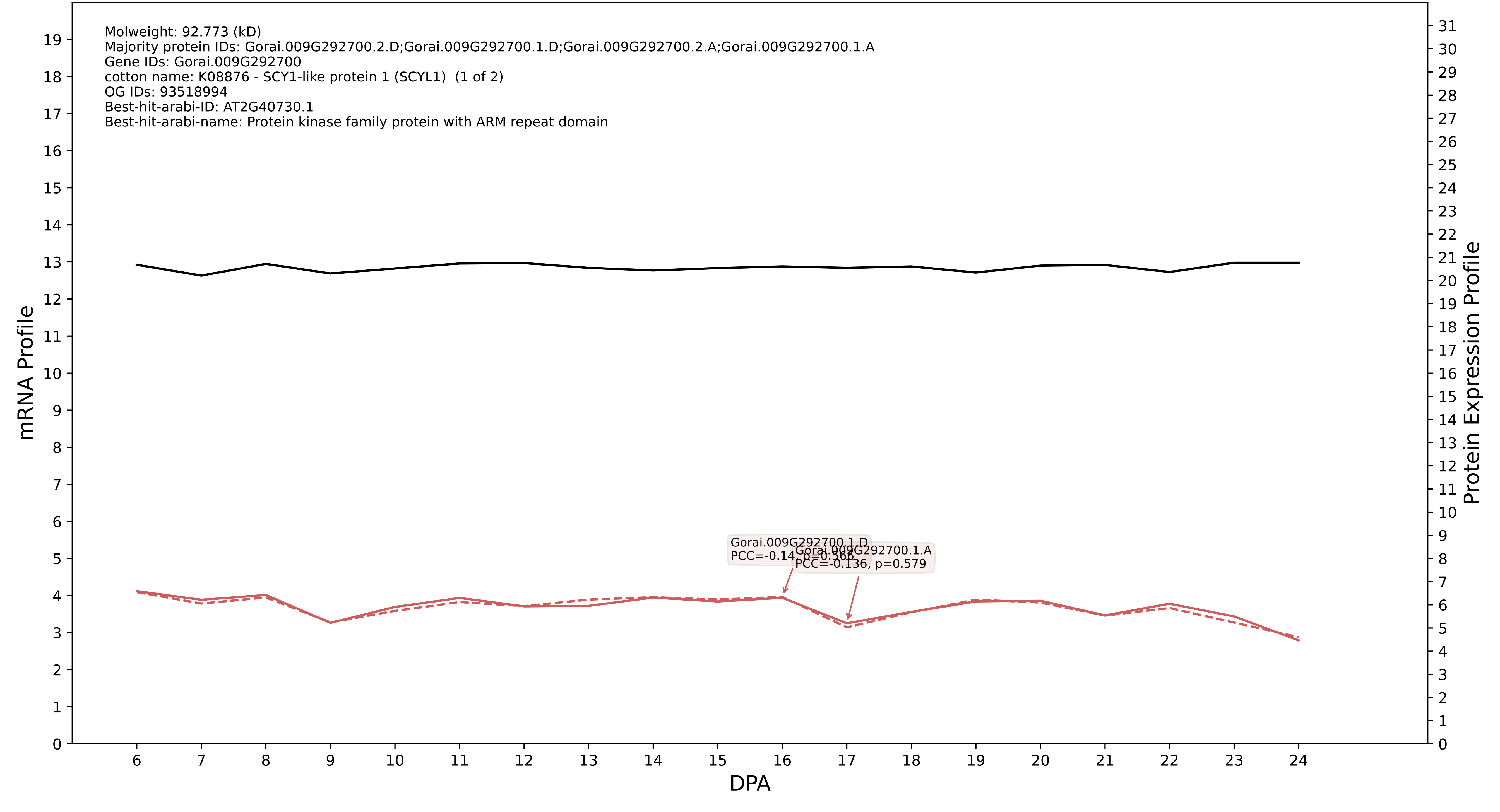

P200: Gorai.006G227100.1.D in case 2 and V15

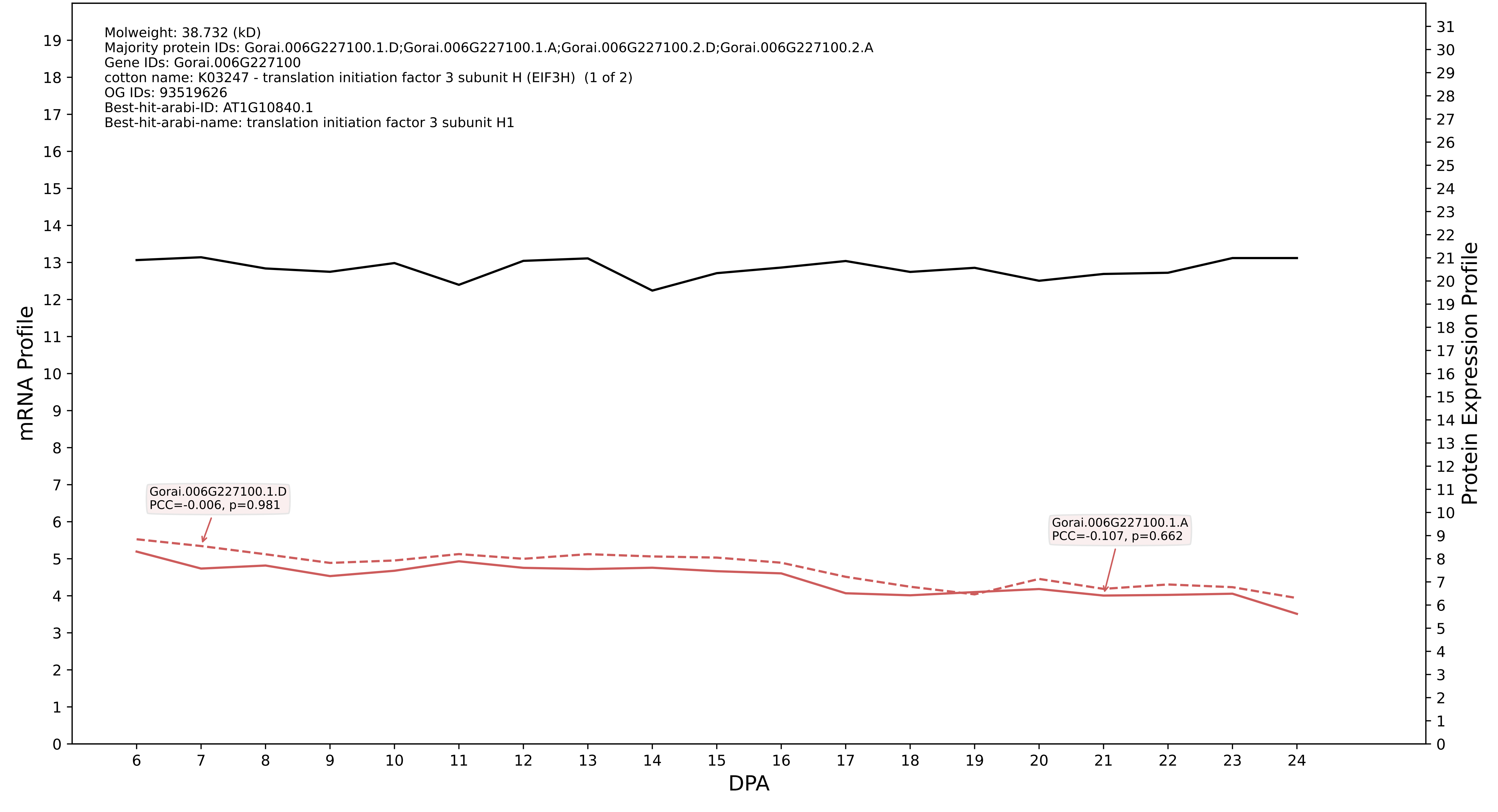

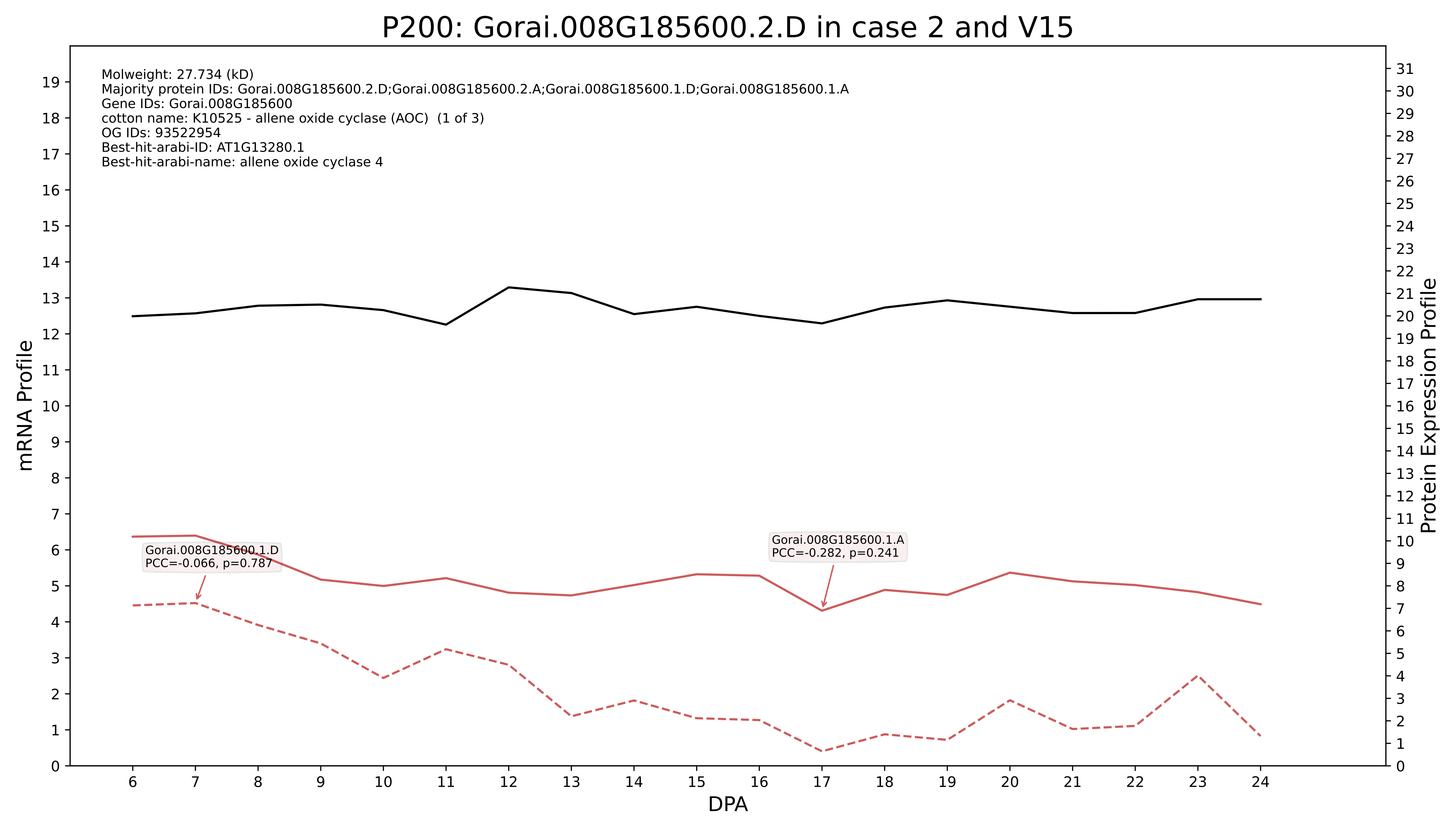
P200: Gorai.008G185600.2.D in case 2 and V15
Molweight: 27.734 (kD)
Majority protein IDs: Gorai.008G185600.2.D;Gorai.008G185600.2.A;Gorai.008G185600.1.D;Gorai.008G185600.1.A
Gene IDs: Gorai.008G185600
cotton name: K10525 - allene oxide cyclase (AOC) (1 of 3)
OG IDs: 93522954
Best-hit-arabi-ID: AT1G13280.1
Best-hit-arabi-name: allene oxide cyclase 4
Gorai.008G185600.1.D
PCC=-0.066, p=0.787
Gorai.008G185600.1.A
PCC=-0.282, p=0.241
mRNA Profile
Protein Expression Profile
DPA

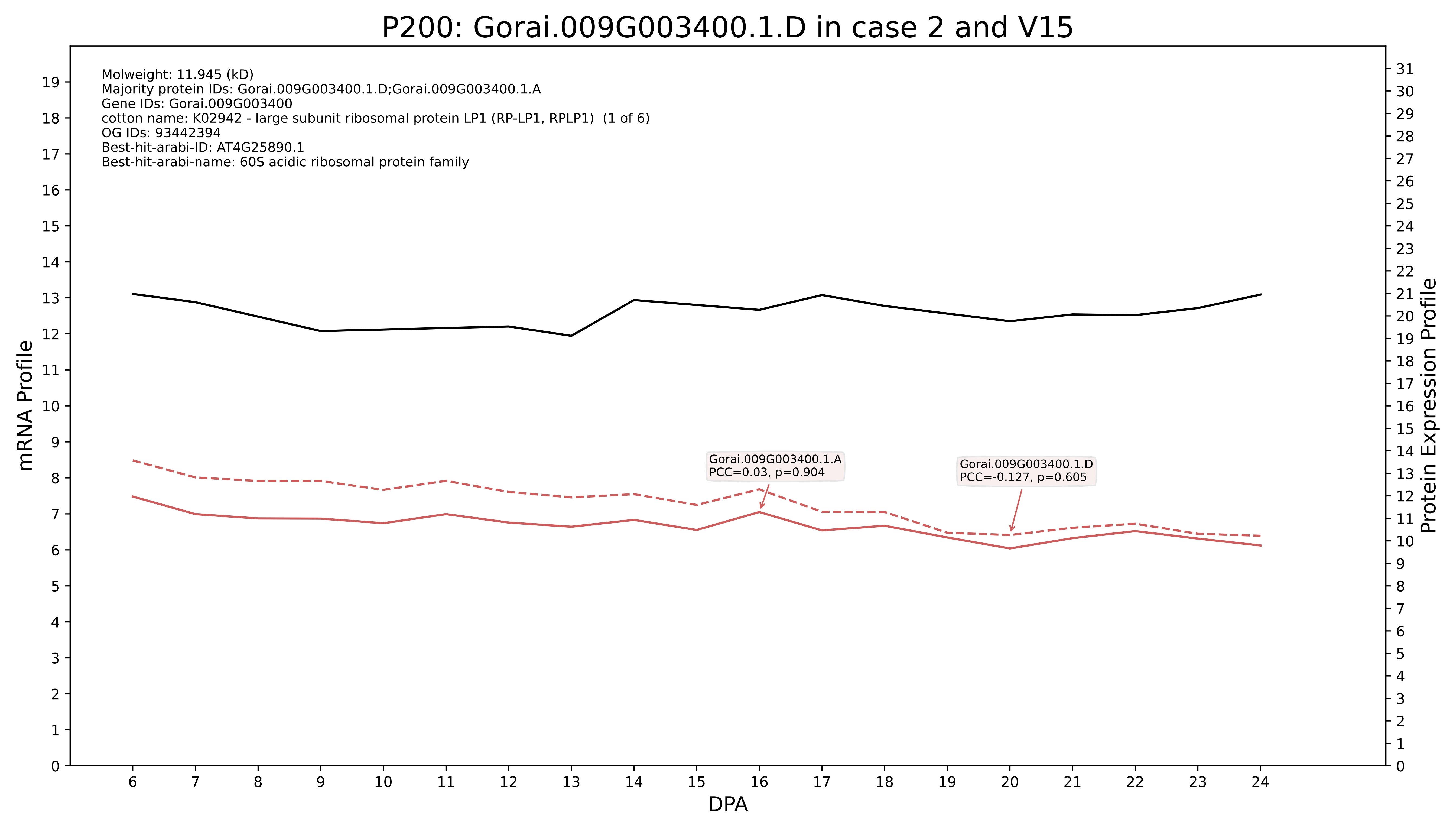
P200: Gorai.009G003400.1.D in case 2 and V15
Molweight: 11.945 (kD)
Majority protein IDs: Gorai.009G003400.1.D;Gorai.009G003400.1.A
Gene IDs: Gorai.009G003400
cotton name: K02942 - large subunit ribosomal protein LP1 (RP-LP1, RPLP1) (1 of 6)
OG IDs: 93442394
Best-hit-arabi-ID: AT4G25890.1
Best-hit-arabi-name: 60S acidic ribosomal protein family
Gorai.009G003400.1.A
PCC=0.03, p=0.904
Gorai.009G003400.1.D
PCC=-0.127, p=0.605
mRNA Profile
Protein Expression Profile
DPA

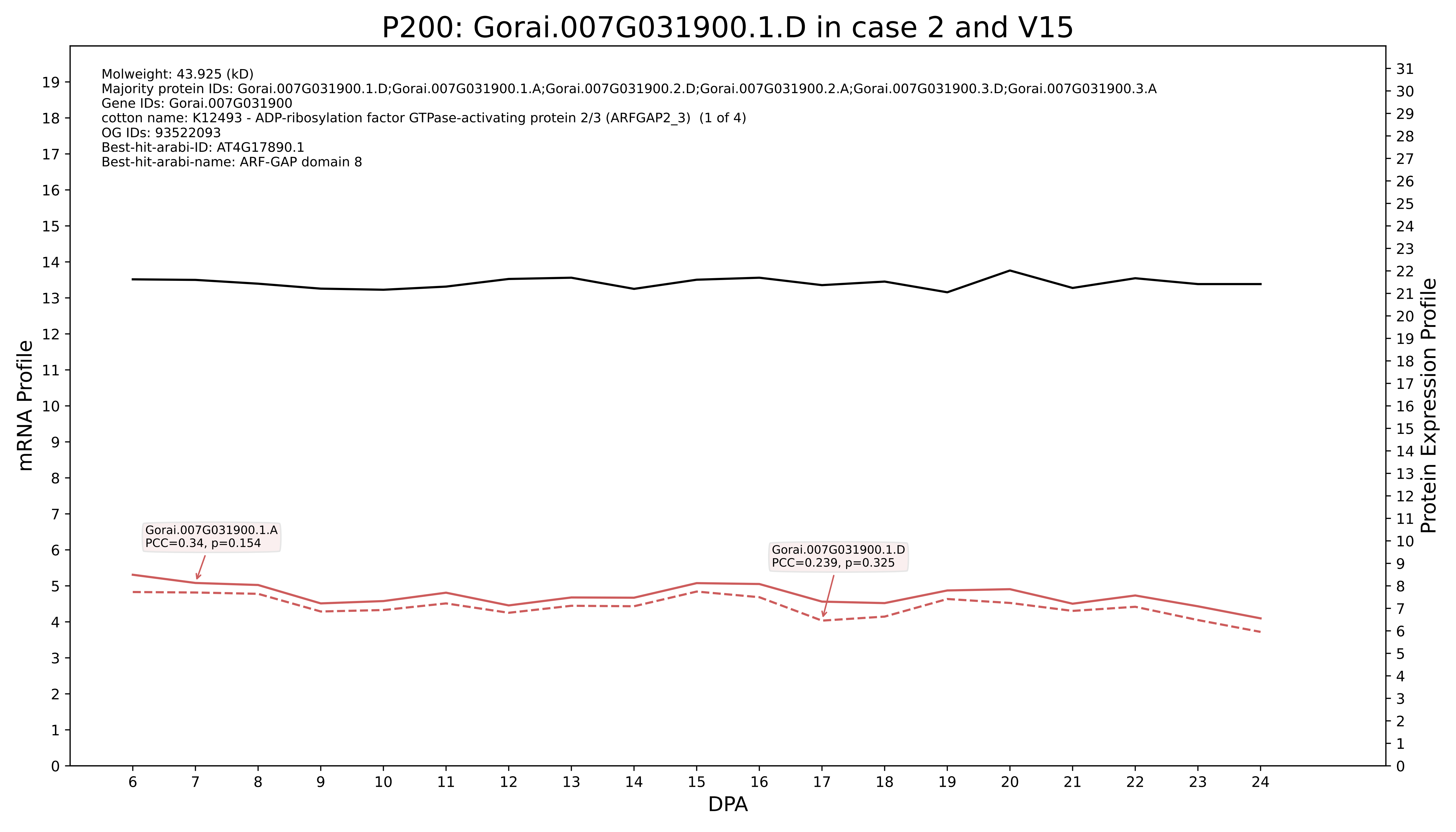
P200: Gorai.007G031900.1.D in case 2 and V15
Molweight: 43.925 (kD)
Majority protein IDs: Gorai.007G031900.1.D;Gorai.007G031900.1.A;Gorai.007G031900.2.D;Gorai.007G031900.2.A;Gorai.007G031900.3.D;Gorai.007G031900.3.A
Gene IDs: Gorai.007G031900
cotton name: K12493 - ADP-ribosylation factor GTPase-activating protein 2/3 (ARFGAP2_3) (1 of 4)
OG IDs: 93522093
Best-hit-arabi-ID: AT4G17890.1
Best-hit-arabi-name: ARF-GAP domain 8
Gorai.007G031900.1.A
PCC=0.34, p=0.154
Gorai.007G031900.1.D
PCC=0.239, p=0.325
mRNA Profile
Protein Expression Profile
DPA
0
1
2
3
4
5
6
7
8
9
10
11
12
13
14
15
16
17
18
19
20
21
22
23
24
25
26
27
28
29
30
31

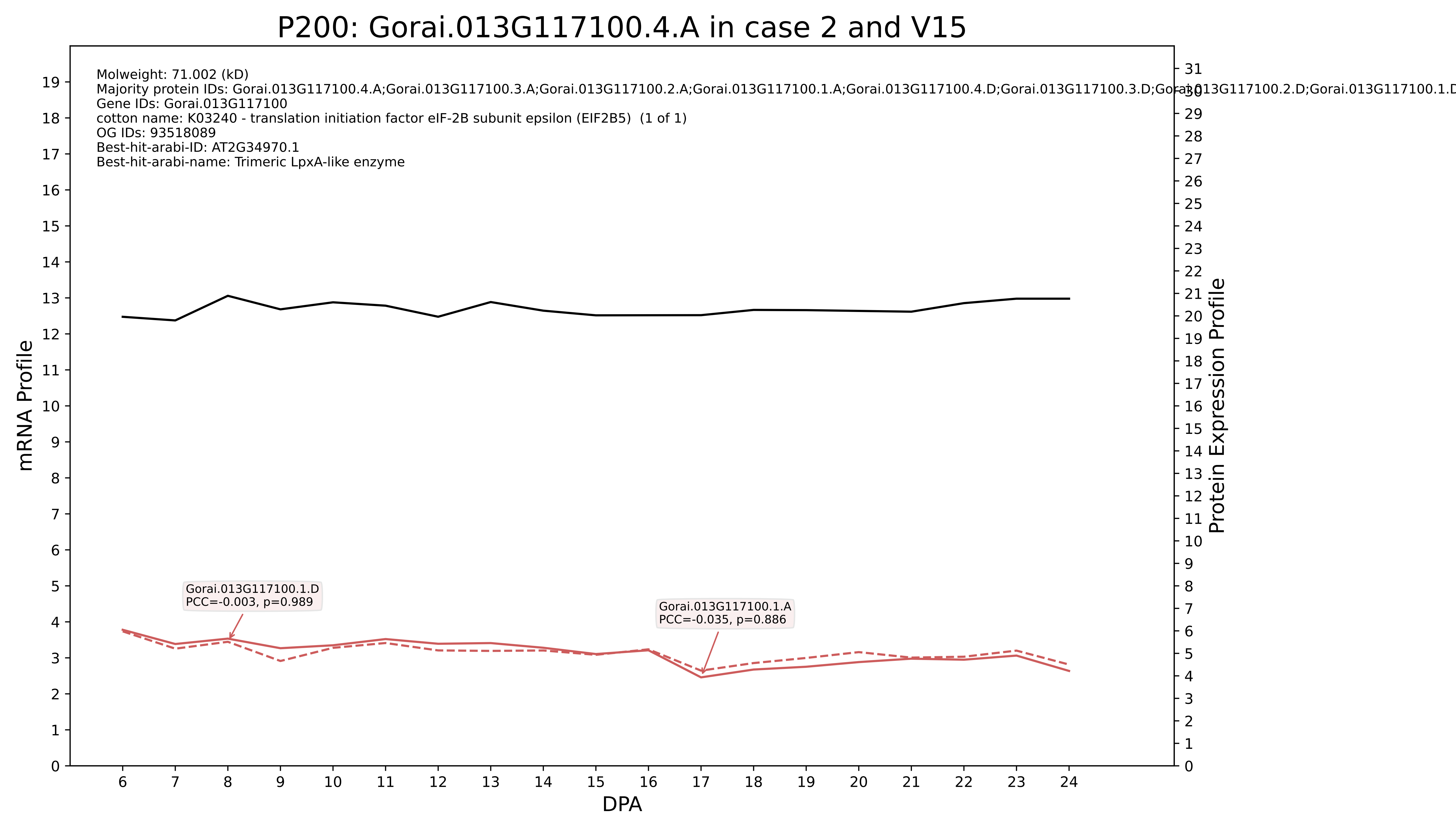

P200: Gorai.013G117100.4.A in case 2 and V15
Molweight: 71.002 (kD)
Majority protein IDs: Gorai.013G117100.4.A;Gorai.013G117100.3.A;Gorai.013G117100.2.A;Gorai.013G117100.1.A;Gorai.013G117100.4.D;Gorai.013G117100.3.D;Gorai.013G117100.2.D;Gorai.013G117100.1.D
Gene IDs: Gorai.013G117100
cotton name: K03240 - translation initiation factor eIF-2B subunit epsilon (EIF2B5) (1 of 1)
OG IDs: 93518089
Best-hit-arabi-ID: AT2G34970.1
Best-hit-arabi-name: Trimeric LpxA-like enzyme
Gorai.013G117100.1.D
PCC=-0.003, p=0.989
Gorai.013G117100.1.A
PCC=-0.035, p=0.886
mRNA Profile
Protein Expression Profile
DPA

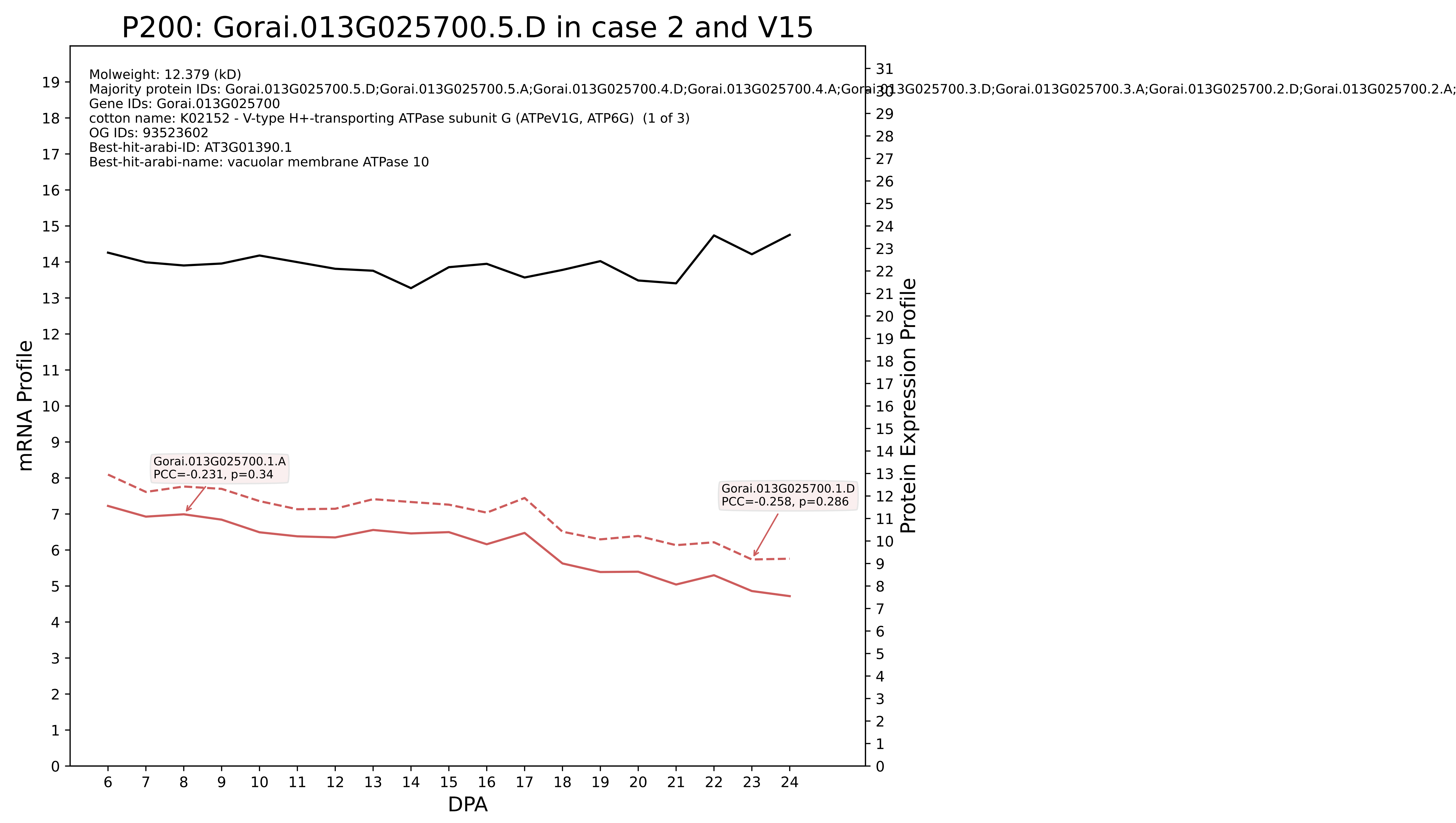
P200: Gorai.013G025700.5.D in case 2 and V15
Molweight: 12.379 (kD)
Majority protein IDs: Gorai.013G025700.5.D;Gorai.013G025700.5.A;Gorai.013G025700.4.D;Gorai.013G025700.4.A;Gorai.013G025700.3.D;Gorai.013G025700.3.A;Gorai.013G025700.2.D;Gorai.013G025700.2.A;
Gene IDs: Gorai.013G025700
cotton name: K02152 - V-type H+-transporting ATPase subunit G (ATPeV1G, ATP6G) (1 of 3)
OG IDs: 93523602
Best-hit-arabi-ID: AT3G01390.1
Best-hit-arabi-name: vacuolar membrane ATPase 10
Gorai.013G025700.1.A
PCC=-0.231, p=0.34
Gorai.013G025700.1.D
PCC=-0.258, p=0.286
mRNA Profile
Protein Expression Profile
DPA

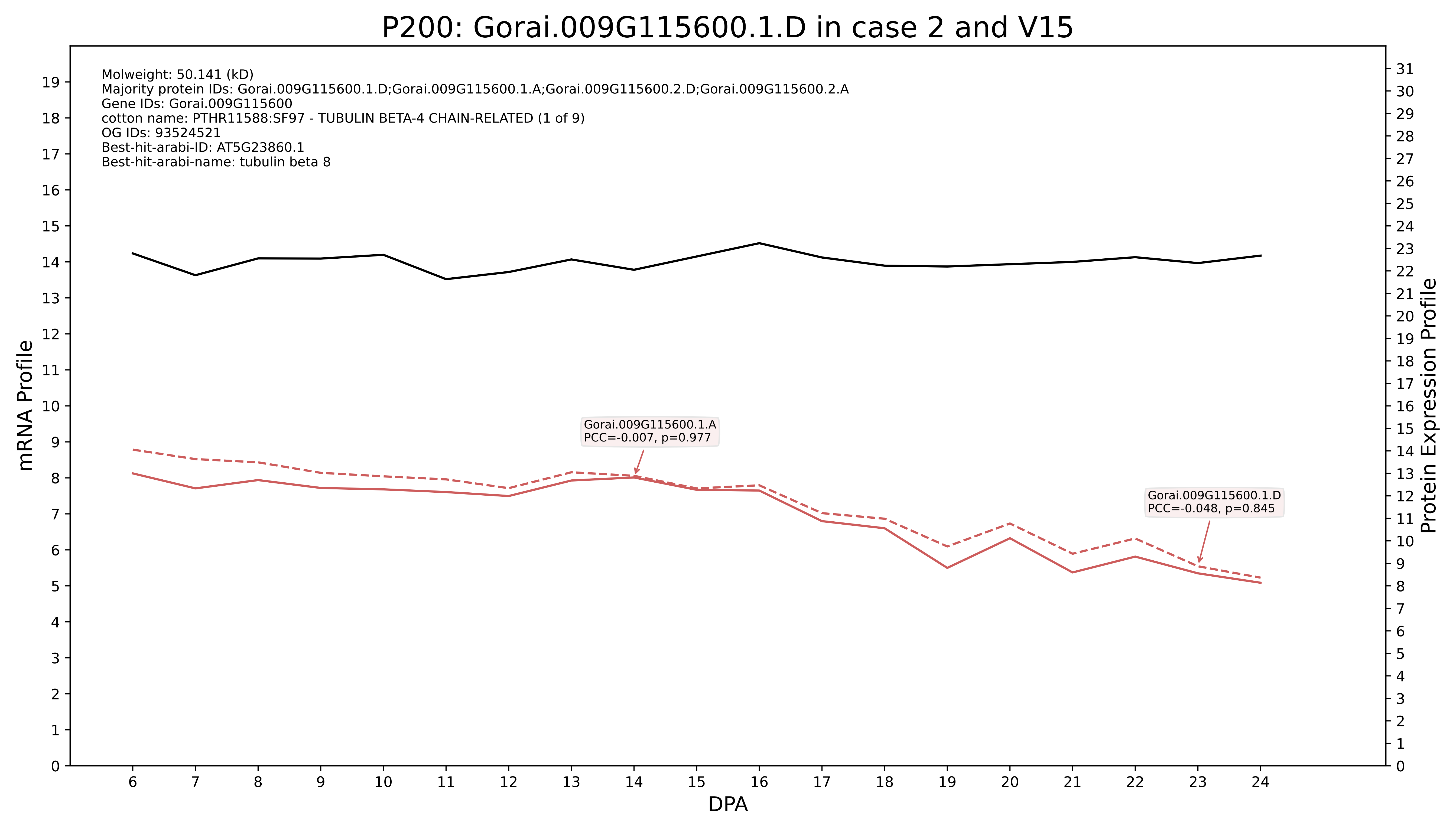
P200: Gorai.009G115600.1.D in case 2 and V15
Molweight: 50.141 (kD)
Majority protein IDs: Gorai.009G115600.1.D;Gorai.009G115600.1.A;Gorai.009G115600.2.D;Gorai.009G115600.2.A
Gene IDs: Gorai.009G115600
cotton name: PTHR11588:SF97 - TUBULIN BETA-4 CHAIN-RELATED (1 of 9)
OG IDs: 93524521
Best-hit-arabi-ID: AT5G23860.1
Best-hit-arabi-name: tubulin beta 8
Gorai.009G115600.1.A
PCC=-0.007, p=0.977
Gorai.009G115600.1.D
PCC=-0.048, p=0.845
mRNA Profile
Protein Expression Profile
DPA

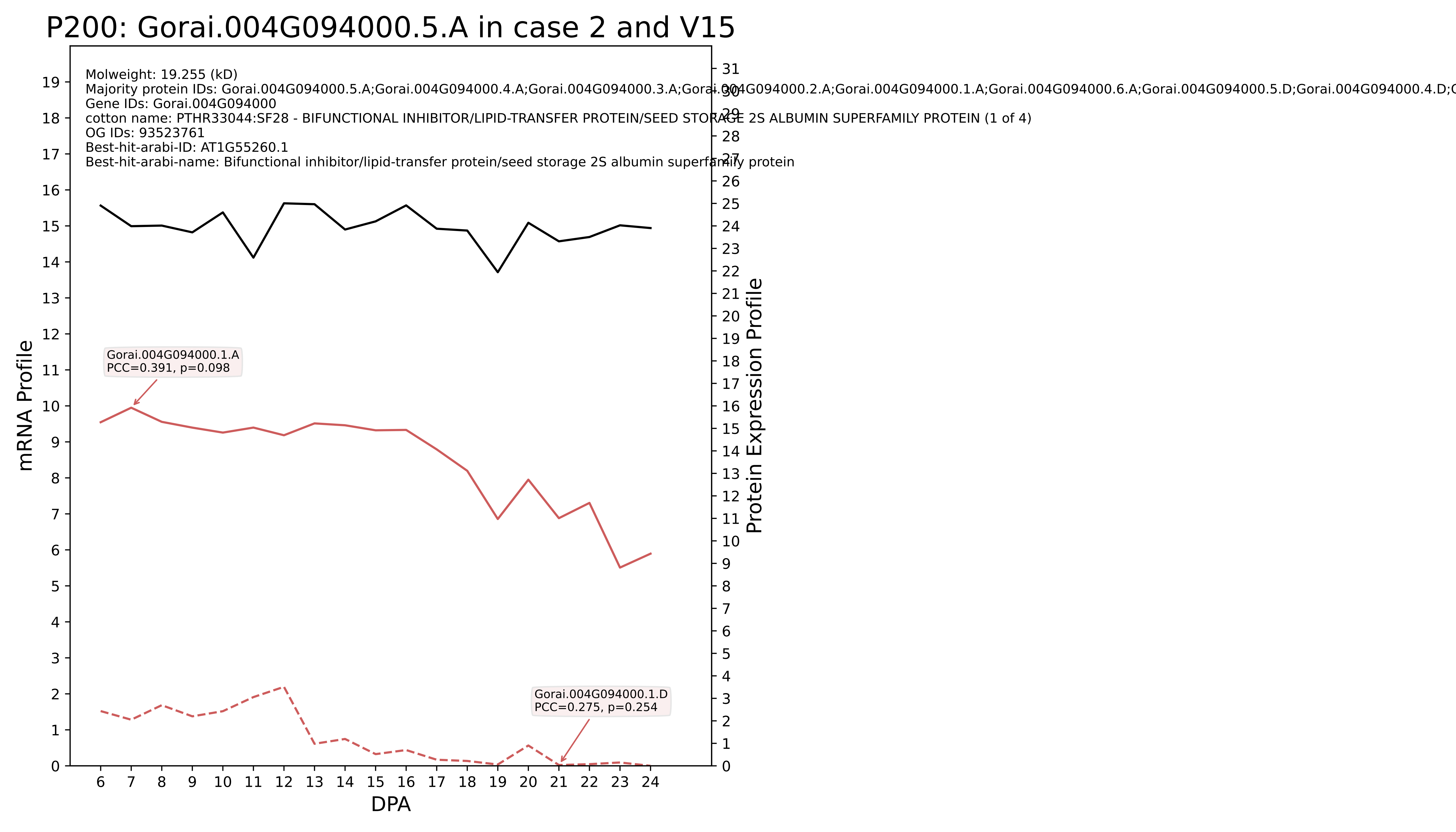
P200: Gorai.004G094000.5.A in case 2 and V15
Molweight: 19.255 (kD)
Majority protein IDs: Gorai.004G094000.5.A;Gorai.004G094000.4.A;Gorai.004G094000.3.A;Gorai.004G094000.2.A;Gorai.004G094000.1.A;Gorai.004G094000.6.A;Gorai.004G094000.5.D;Gorai.004G094000.4.D;G
Gene IDs: Gorai.004G094000
cotton name: PTHR33044:SF28 - BIFUNCTIONAL INHIBITOR/LIPID-TRANSFER PROTEIN/SEED STORAGE 2S ALBUMIN SUPERFAMILY PROTEIN (1 of 4)
OG IDs: 93523761
Best-hit-arabi-ID: AT1G55260.1
Best-hit-arabi-name: Bifunctional inhibitor/lipid-transfer protein/seed storage 2S albumin superfamily protein
Gorai.004G094000.1.A
PCC=0.391, p=0.098
Gorai.004G094000.1.D
PCC=0.275, p=0.254
mRNA Profile
Protein Expression Profile
DPA
0
1
2
3
4
5
6
7
8
9
10
11
12
13
14
15
16
17
18
19
20
21
22
23
24
25
26
27
28
29
30
31

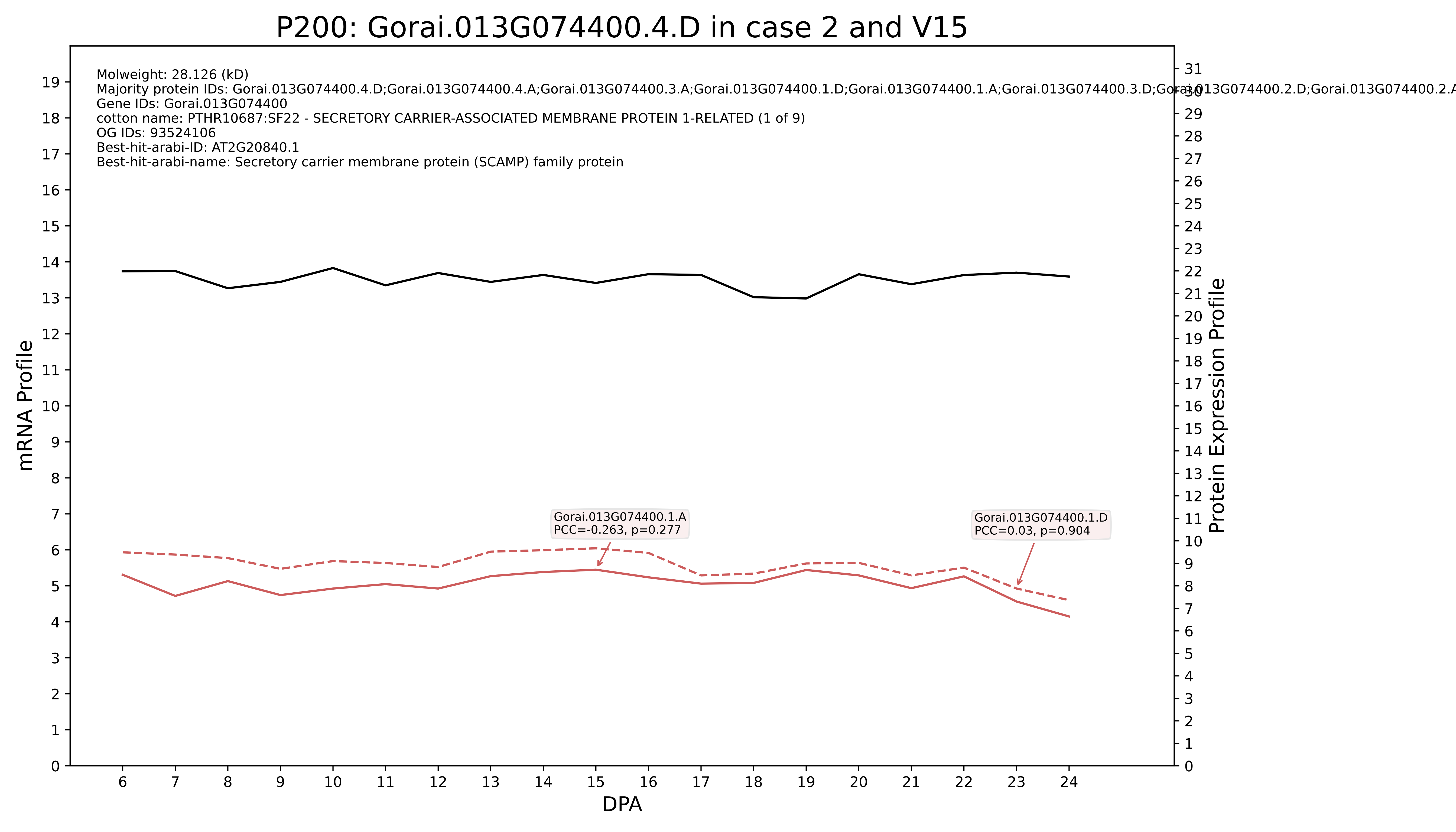
P200: Gorai.013G074400.4.D in case 2 and V15
Molweight: 28.126 (kD)
Majority protein IDs: Gorai.013G074400.4.D;Gorai.013G074400.4.A;Gorai.013G074400.3.A;Gorai.013G074400.1.D;Gorai.013G074400.1.A;Gorai.013G074400.3.D;Gorai.013G074400.2.D;Gorai.013G074400.2.A
Gene IDs: Gorai.013G074400
cotton name: PTHR10687:SF22 - SECRETORY CARRIER-ASSOCIATED MEMBRANE PROTEIN 1-RELATED (1 of 9)
OG IDs: 93524106
Best-hit-arabi-ID: AT2G20840.1
Best-hit-arabi-name: Secretory carrier membrane protein (SCAMP) family protein
Gorai.013G074400.1.A
PCC=-0.263, p=0.277
Gorai.013G074400.1.D
PCC=0.03, p=0.904
mRNA Profile
Protein Expression Profile
DPA

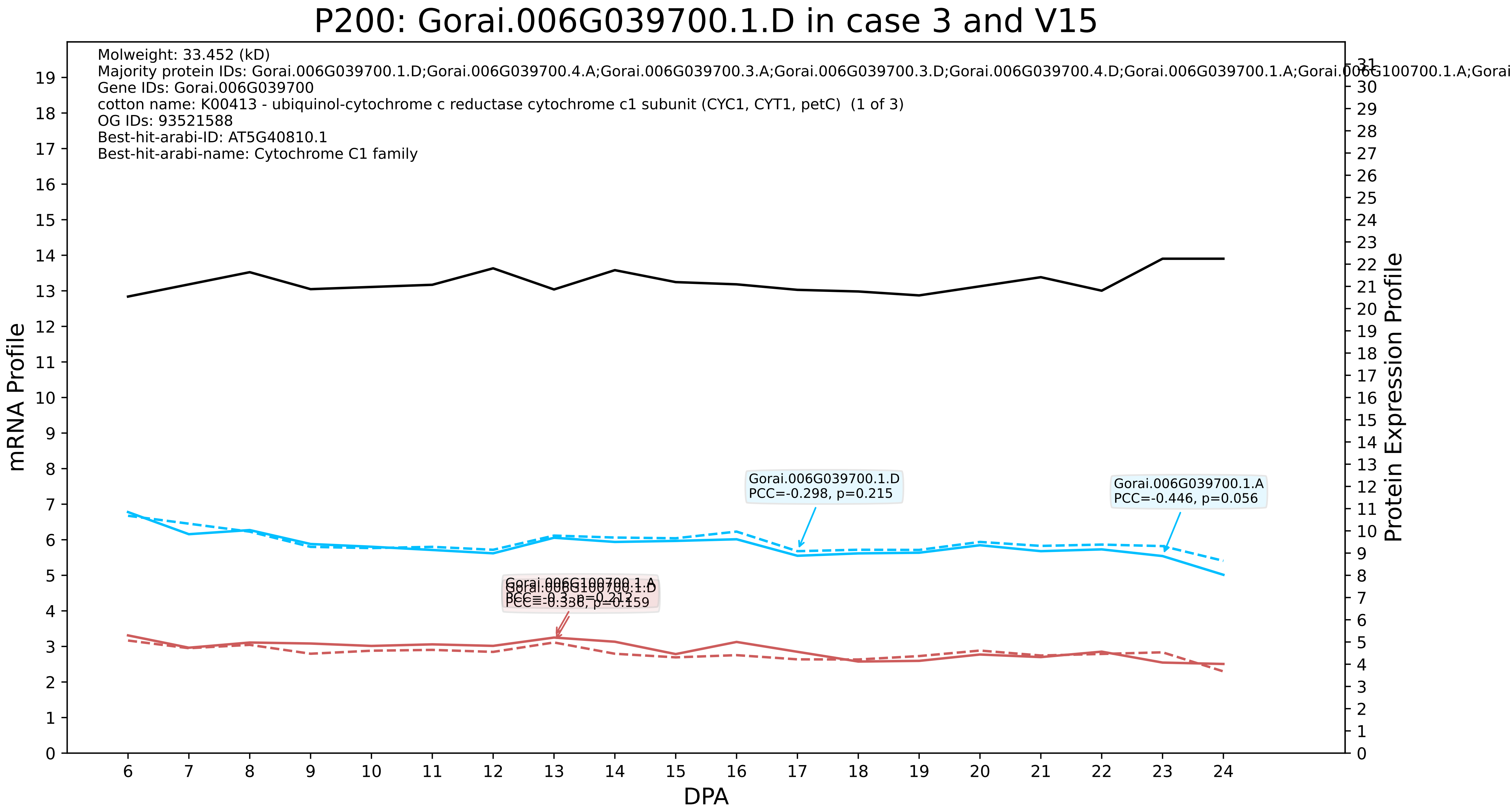

P200: Gorai.006G039700.1.D in case 3 and V15
Molweight: 33.452 (kD)
Majority protein IDs: Gorai.006G039700.1.D;Gorai.006G039700.4.A;Gorai.006G039700.3.A;Gorai.006G039700.3.D;Gorai.006G039700.4.D;Gorai.006G039700.1.A;Gorai.006G100700.1.A;Gorai
Gene IDs: Gorai.006G039700
cotton name: K00413 - ubiquinol-cytochrome c reductase cytochrome c1 subunit (CYC1, CYT1, petC) (1 of 3)
OG IDs: 93521588
Best-hit-arabi-ID: AT5G40810.1
Best-hit-arabi-name: Cytochrome C1 family
Gorai.006G039700.1.D
PCC=-0.298, p=0.215
Gorai.006G039700.1.A
PCC=-0.446, p=0.056
mRNA Profile
Protein Expression Profile
DPA

P200: Gorai.004G218600.2.D in case 3 and V15

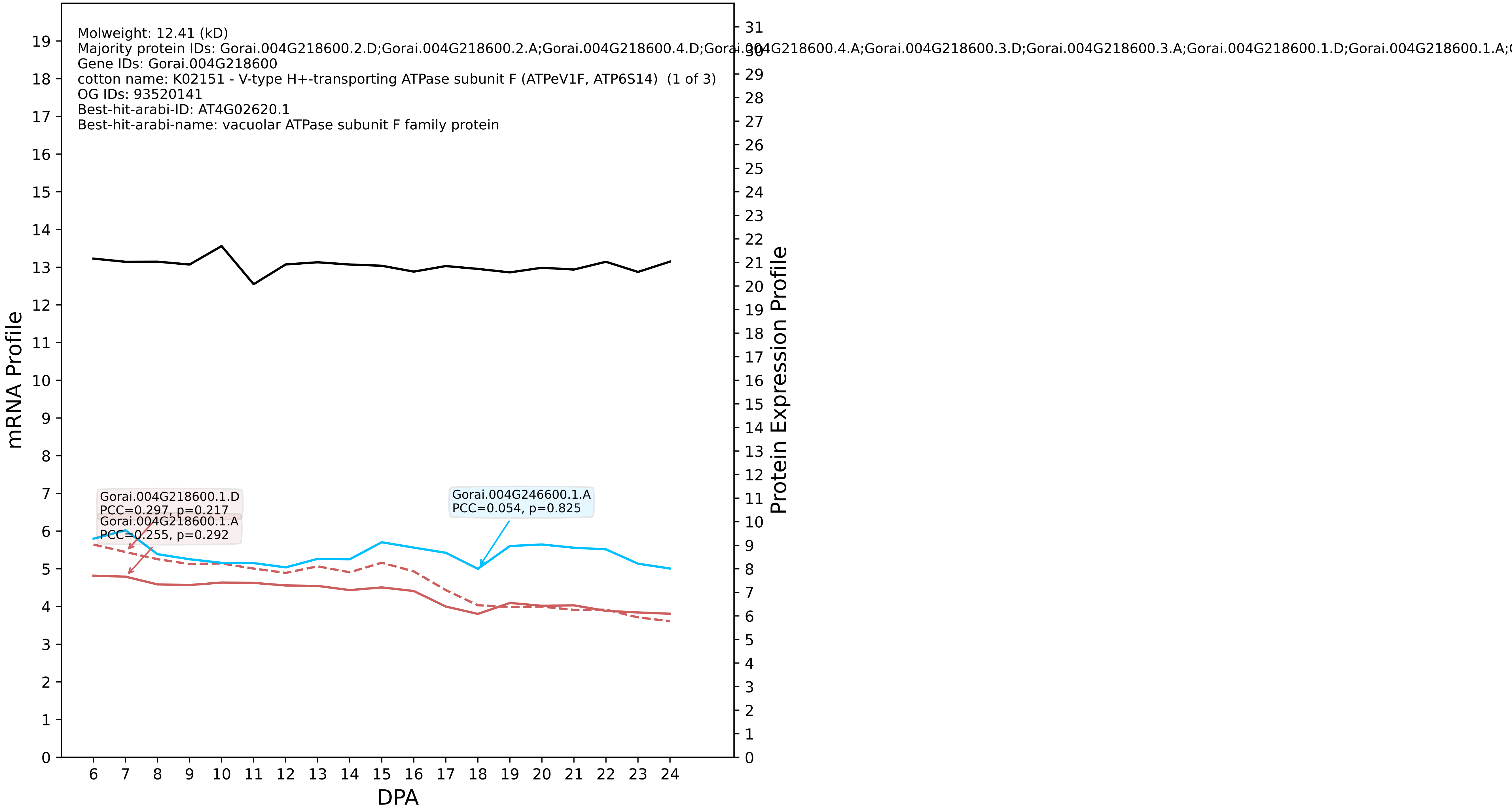

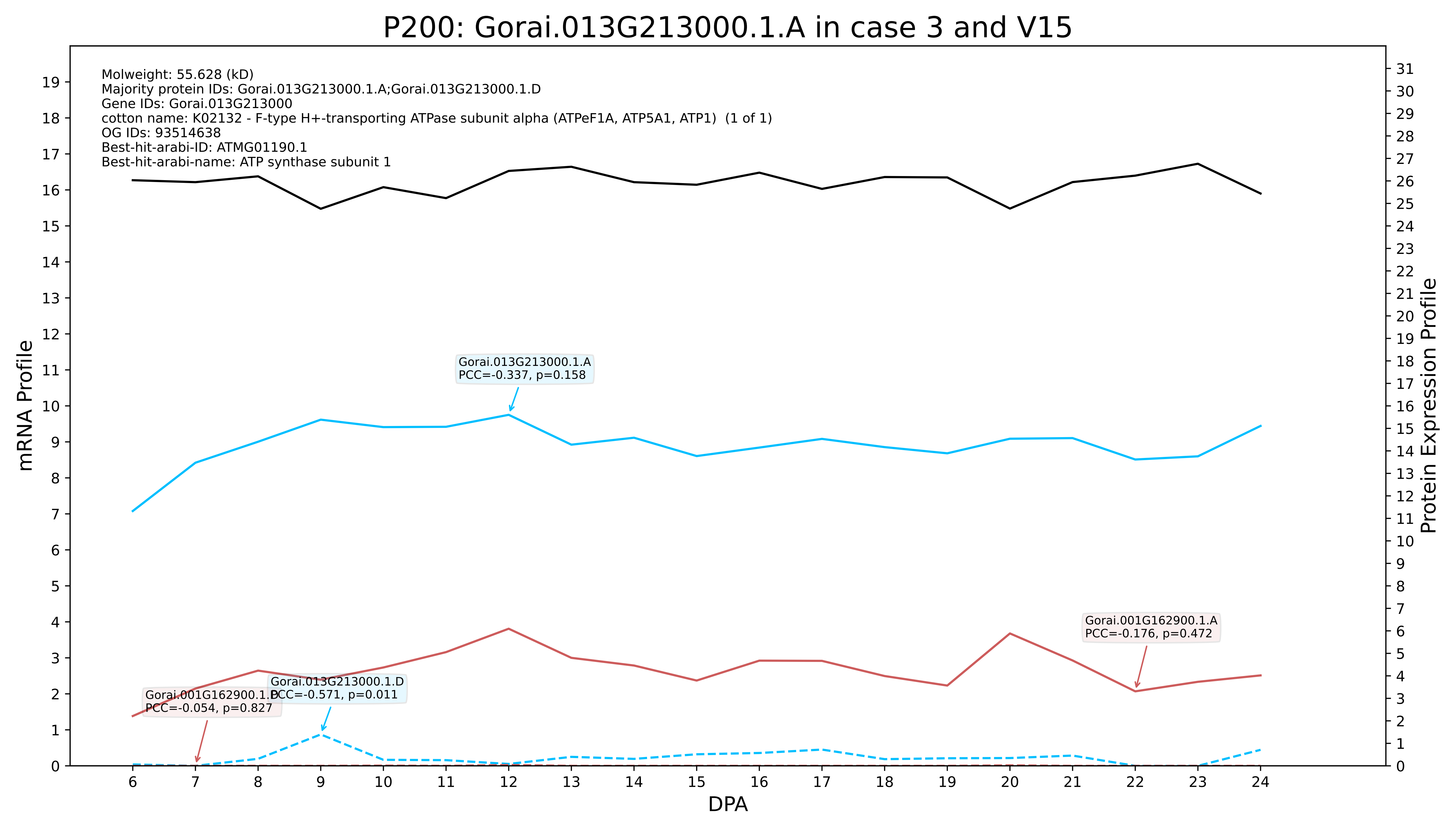
P200: Gorai.013G213000.1.A in case 3 and V15
Molweight: 55.628 (kD)
Majority protein IDs: Gorai.013G213000.1.A;Gorai.013G213000.1.D
Gene IDs: Gorai.013G213000
cotton name: K02132 - F-type H+-transporting ATPase subunit alpha (ATPeF1A, ATP5A1, ATP1) (1 of 1)
OG IDs: 93514638
Best-hit-arabi-ID: ATMG01190.1
Best-hit-arabi-name: ATP synthase subunit 1
Gorai.013G213000.1.A
PCC=-0.337, p=0.158
Gorai.001G162900.1.A
PCC=-0.176, p=0.472
Gorai.013G213000.1.D
PCC=-0.571, p=0.011
Gorai.001G162900.1.D
PCC=-0.054, p=0.827
mRNA Profile
Protein Expression Profile
DPA

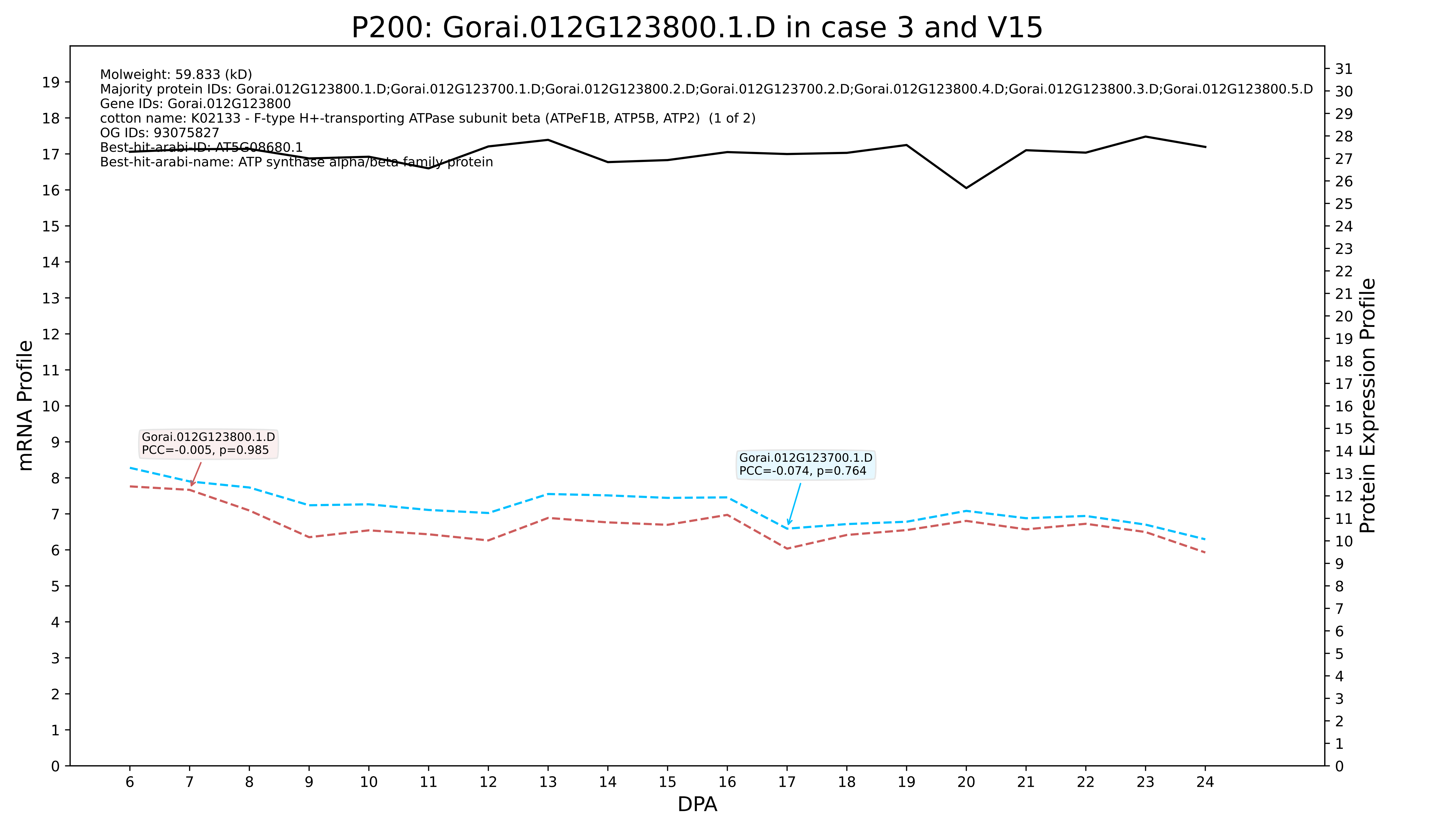
P200: Gorai.012G123800.1.D in case 3 and V15
Molweight: 59.833 (kD)
Majority protein IDs: Gorai.012G123800.1.D;Gorai.012G123700.1.D;Gorai.012G123800.2.D;Gorai.012G123700.2.D;Gorai.012G123800.4.D;Gorai.012G123800.3.D;Gorai.012G123800.5.D
Gene IDs: Gorai.012G123800
cotton name: K02133 - F-type H+-transporting ATPase subunit beta (ATPeF1B, ATP5B, ATP2) (1 of 2)
OG IDs: 93075827
Best-hit-arabi-ID: AT5G08680.1
Best-hit-arabi-name: ATP synthase alpha/beta family protein
Gorai.012G123800.1.D
PCC=-0.005, p=0.985
Gorai.012G123700.1.D
PCC=-0.074, p=0.764
mRNA Profile
Protein Expression Profile
DPA

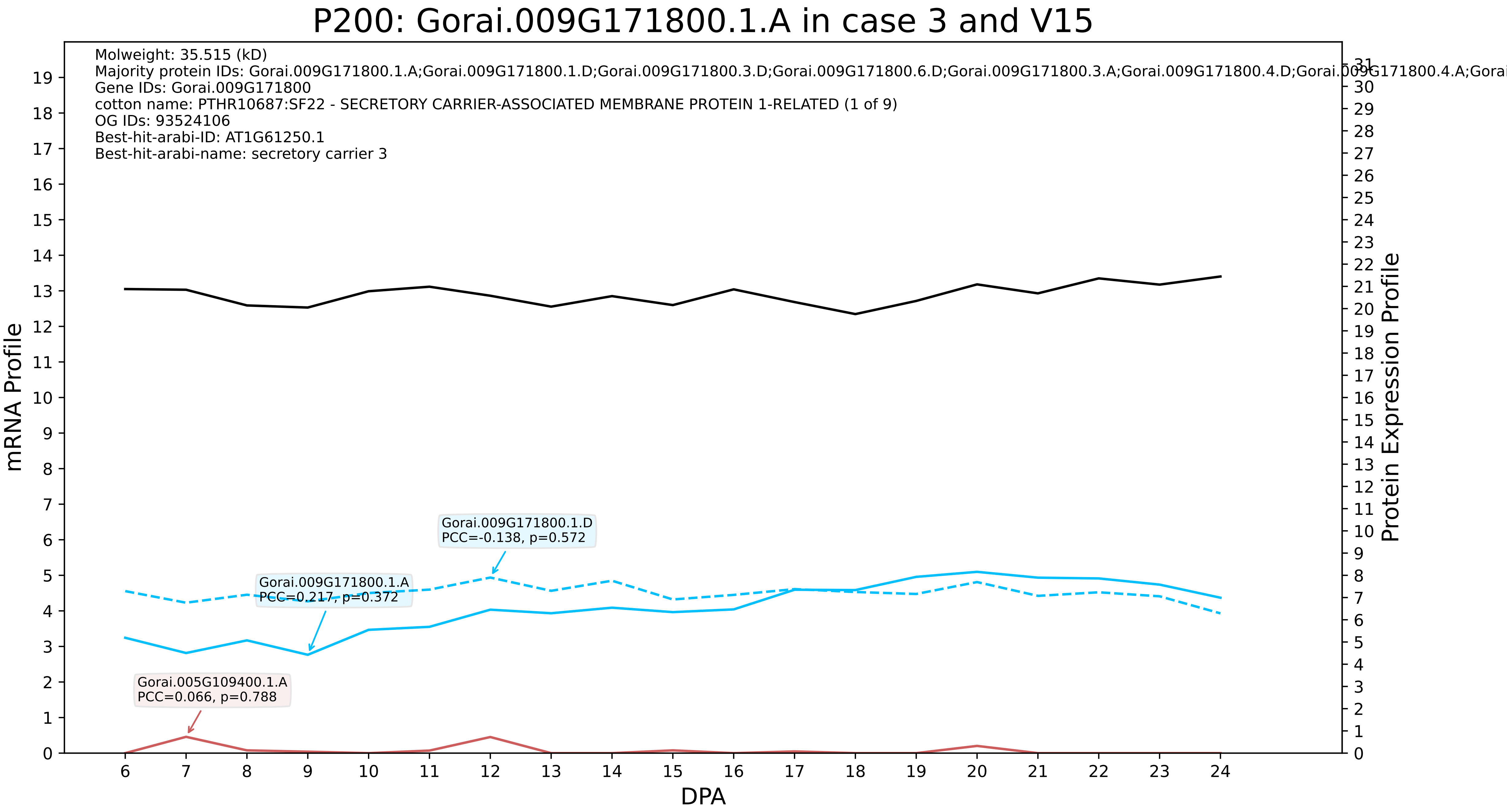
P200: Gorai.009G171800.1.A in case 3 and V15
Molweight: 35.515 (kD)
Majority protein IDs: Gorai.009G171800.1.A;Gorai.009G171800.1.D;Gorai.009G171800.3.D;Gorai.009G171800.6.D;Gorai.009G171800.3.A;Gorai.009G171800.4.D;Gorai.009G171800.4.A;Gorai
Gene IDs: Gorai.009G171800
cotton name: PTHR10687:SF22 - SECRETORY CARRIER-ASSOCIATED MEMBRANE PROTEIN 1-RELATED (1 of 9)
OG IDs: 93524106
Best-hit-arabi-ID: AT1G61250.1
Best-hit-arabi-name: secretory carrier 3
Gorai.009G171800.1.D
PCC=-0.138, p=0.572
Gorai.009G171800.1.A
PCC=0.217, p=0.372
Gorai.005G109400.1.A
PCC=0.066, p=0.788
mRNA Profile
Protein Expression Profile
DPA

P200: Gorai.009G057600.1.A in case 3 and V15

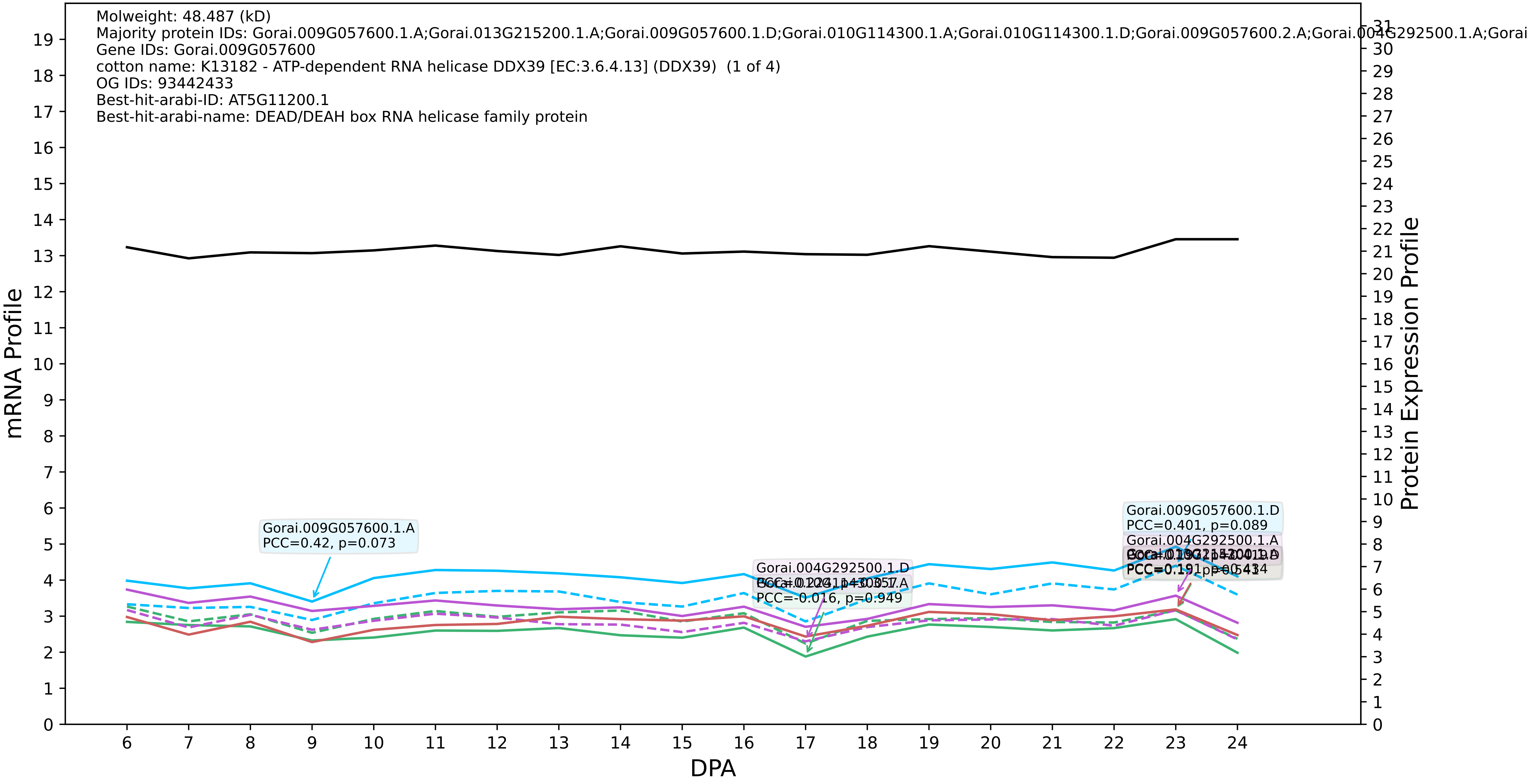

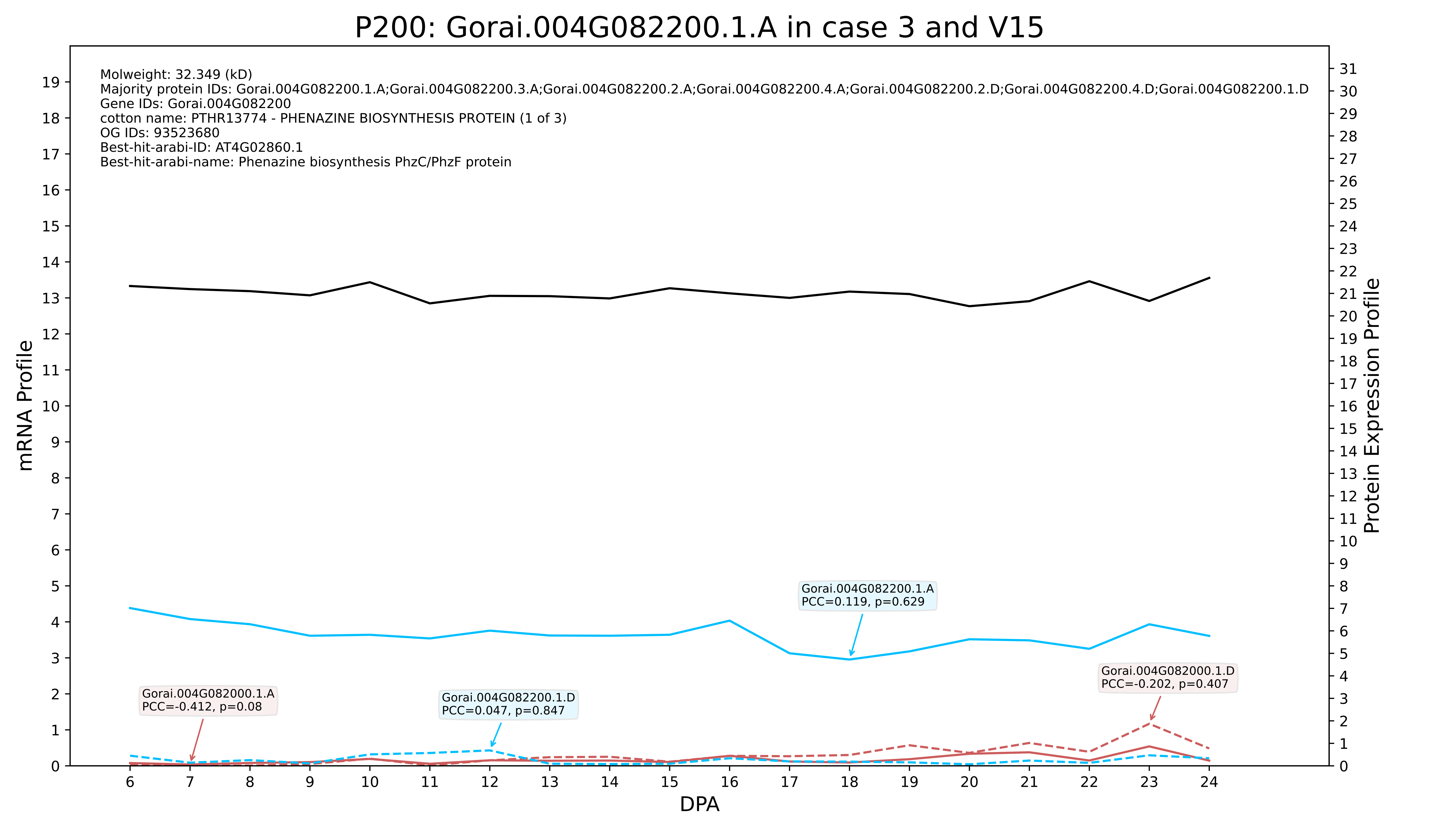
P200: Gorai.004G082200.1.A in case 3 and V15
Molweight: 32.349 (kD)
Majority protein IDs: Gorai.004G082200.1.A;Gorai.004G082200.3.A;Gorai.004G082200.2.A;Gorai.004G082200.4.A;Gorai.004G082200.2.D;Gorai.004G082200.4.D;Gorai.004G082200.1.D
Gene IDs: Gorai.004G082200
cotton name: PTHR13774 - PHENAZINE BIOSYNTHESIS PROTEIN (1 of 3)
OG IDs: 93523680
Best-hit-arabi-ID: AT4G02860.1
Best-hit-arabi-name: Phenazine biosynthesis PhzC/PhzF protein
Gorai.004G082200.1.A
PCC=0.119, p=0.629
Gorai.004G082000.1.D
PCC=-0.202, p=0.407
Gorai.004G082000.1.A
PCC=-0.412, p=0.08
Gorai.004G082200.1.D
PCC=0.047, p=0.847
mRNA Profile
Protein Expression Profile
DPA

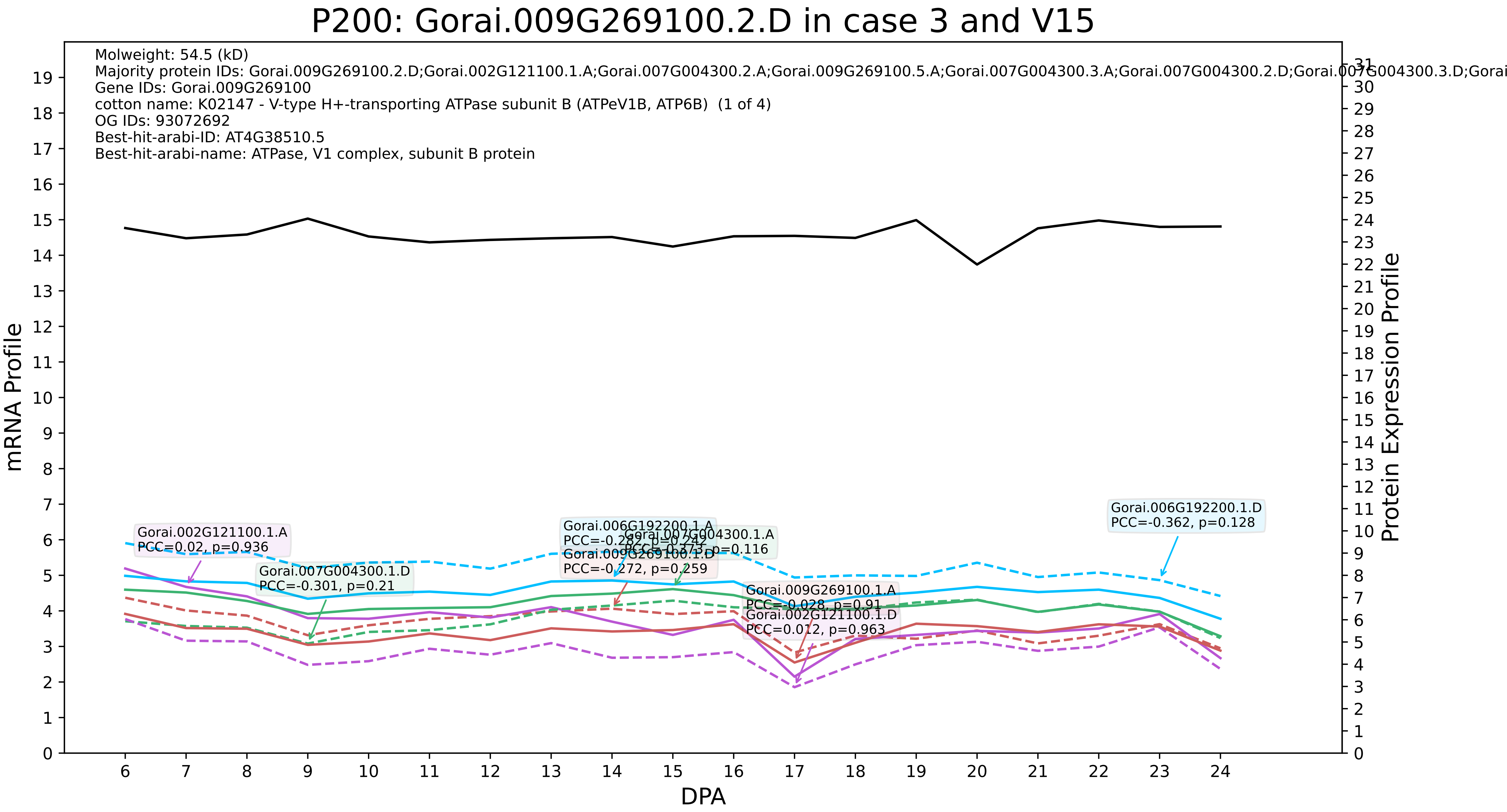
P200: Gorai.009G269100.2.D in case 3 and V15
Molweight: 54.5 (kD)
Majority protein IDs: Gorai.009G269100.2.D;Gorai.002G121100.1.A;Gorai.007G004300.2.A;Gorai.009G269100.5.A;Gorai.007G004300.3.A;Gorai.007G004300.2.D;Gorai.007G004300.3.D;Gorai
Gene IDs: Gorai.009G269100
cotton name: K02147 - V-type H+-transporting ATPase subunit B (ATPeV1B, ATP6B) (1 of 4)
OG IDs: 93072692
Best-hit-arabi-ID: AT4G38510.5
Best-hit-arabi-name: ATPase, V1 complex, subunit B protein
Gorai.002G121100.1.A
PCC=0.02, p=0.936
Gorai.007G004300.1.D
PCC=-0.301, p=0.21
Gorai.006G192200.1.A
PCC=-0.282, p=0.242
Gorai.007G004300.1.A
PCC=-0.373, p=0.116
Gorai.009G269100.1.D
PCC=-0.272, p=0.259
Gorai.009G269100.1.A
PCC=-0.028, p=0.91
Gorai.002G121100.1.D
PCC=0.012, p=0.963
Gorai.006G192200.1.D
PCC=-0.362, p=0.128
mRNA Profile
Protein Expression Profile
DPA

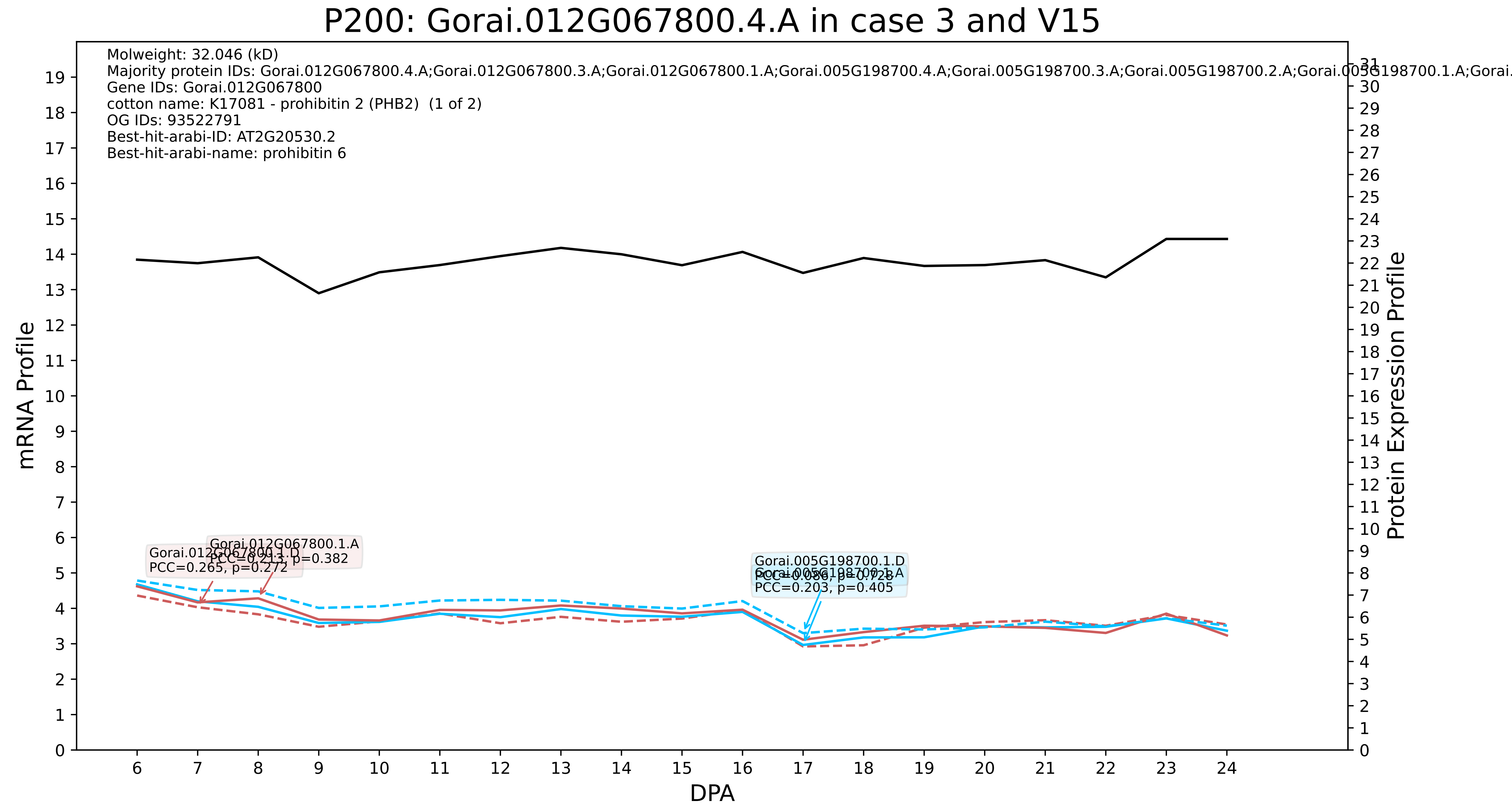

P200: Gorai.012G067800.4.A in case 3 and V15
Molweight: 32.046 (kD)
Majority protein IDs: Gorai.012G067800.4.A;Gorai.012G067800.3.A;Gorai.012G067800.1.A;Gorai.005G198700.4.A;Gorai.005G198700.3.A;Gorai.005G198700.2.A;Gorai.005G198700.1.A;Gorai.
Gene IDs: Gorai.012G067800
cotton name: K17081 - prohibitin 2 (PHB2) (1 of 2)
OG IDs: 93522791
Best-hit-arabi-ID: AT2G20530.2
Best-hit-arabi-name: prohibitin 6
Gorai.012G067800.1.D
PCC=0.265, p=0.272
Gorai.012G067800.1.A
PCC=0.213, p=0.382
Gorai.005G198700.1.D
PCC=0.086, p=0.728
Gorai.005G198700.1.A
PCC=0.203, p=0.405
mRNA Profile
Protein Expression Profile
DPA

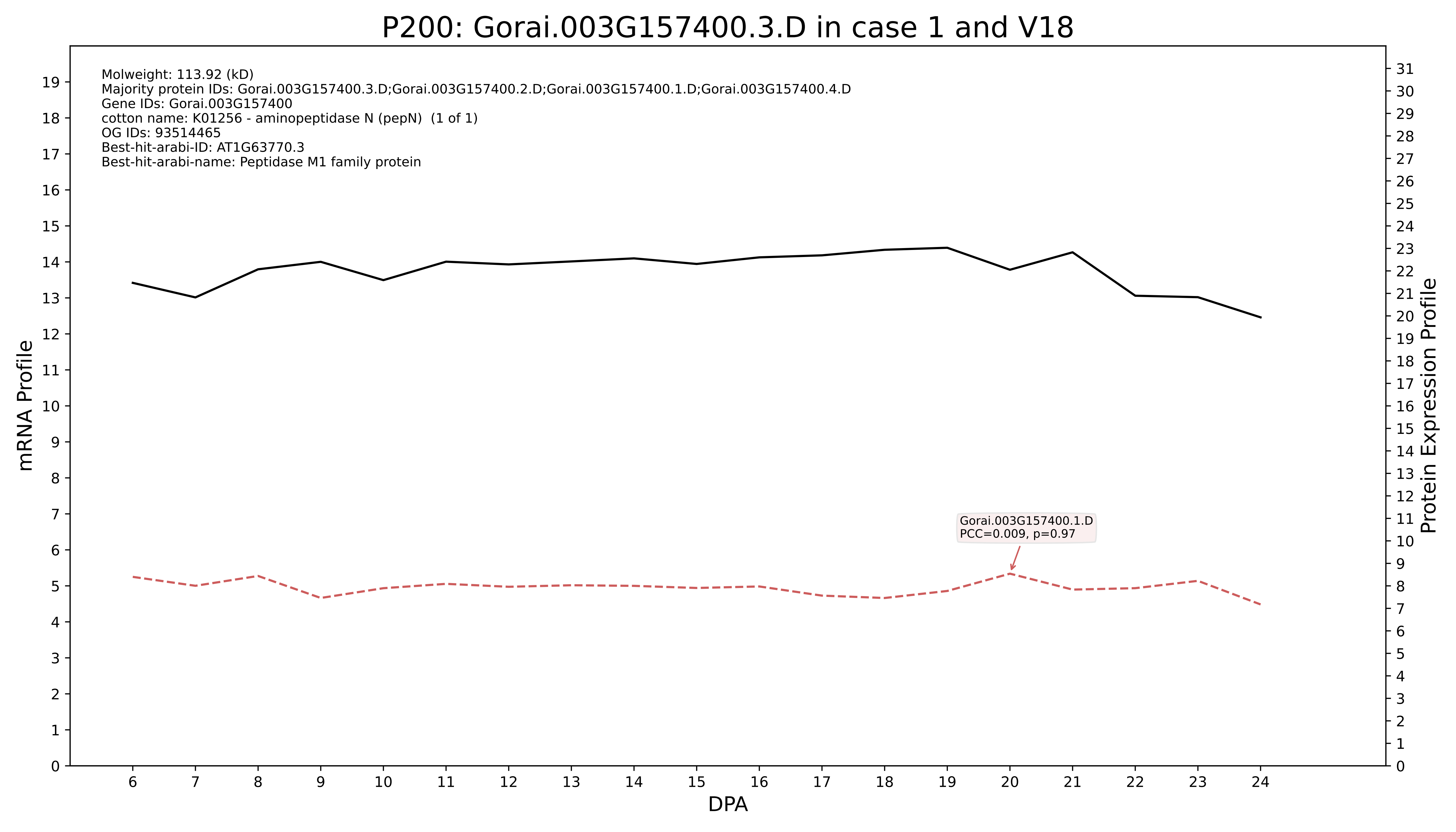

P200: Gorai.003G157400.3.D in case 1 and V18
Molweight: 113.92 (kD)
Majority protein IDs: Gorai.003G157400.3.D;Gorai.003G157400.2.D;Gorai.003G157400.1.D;Gorai.003G157400.4.D
Gene IDs: Gorai.003G157400
cotton name: K01256 - aminopeptidase N (pepN) (1 of 1)
OG IDs: 93514465
Best-hit-arabi-ID: AT1G63770.3
Best-hit-arabi-name: Peptidase M1 family protein
Gorai.003G157400.1.D
PCC=0.009, p=0.97
mRNA Profile
Protein Expression Profile
DPA

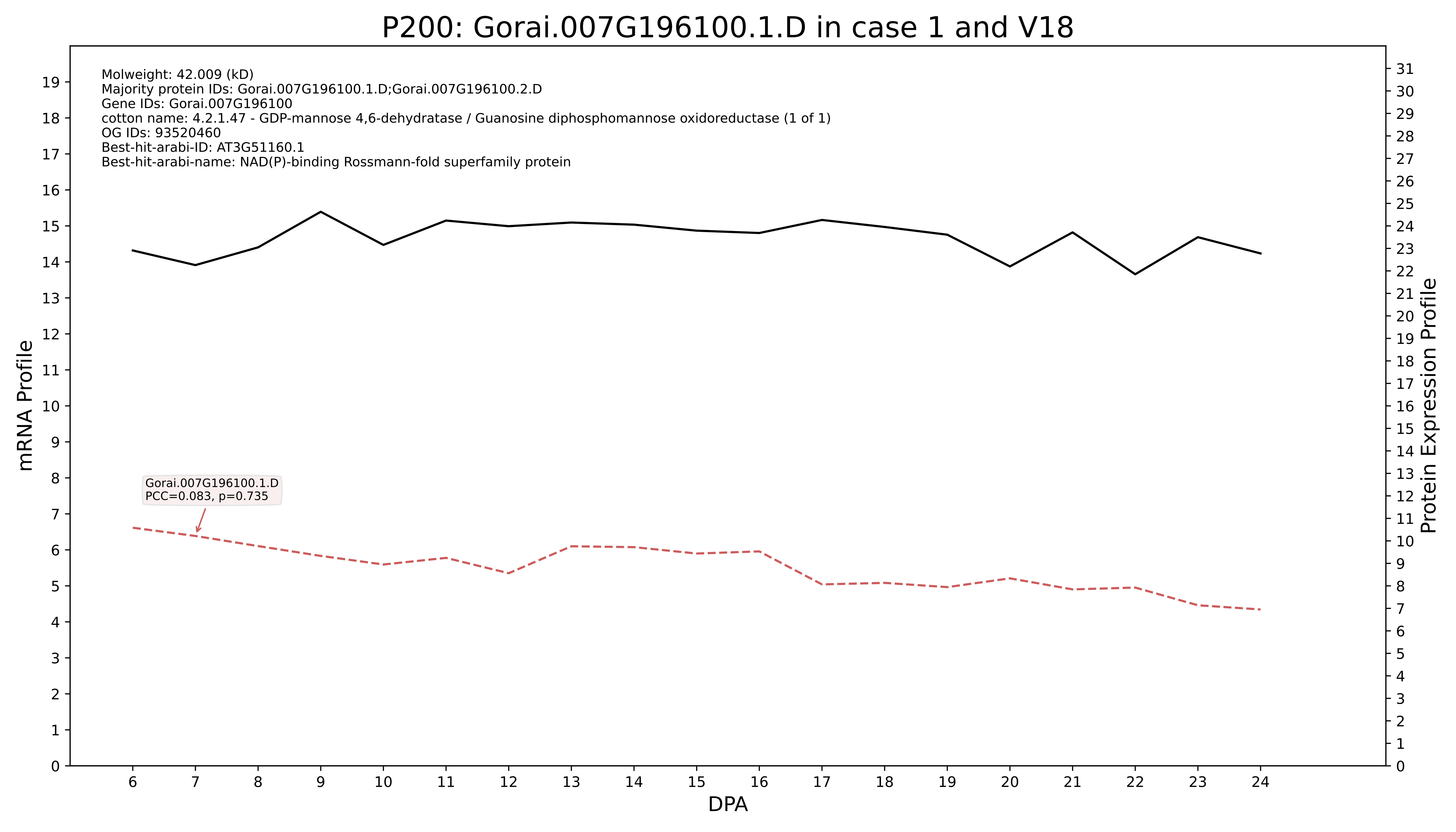
P200: Gorai.007G196100.1.D in case 1 and V18
Molweight: 42.009 (kD)
Majority protein IDs: Gorai.007G196100.1.D;Gorai.007G196100.2.D
Gene IDs: Gorai.007G196100
cotton name: 4.2.1.47 - GDP-mannose 4,6-dehydratase / Guanosine diphosphomannose oxidoreductase (1 of 1)
OG IDs: 93520460
Best-hit-arabi-ID: AT3G51160.1
Best-hit-arabi-name: NAD(P)-binding Rossmann-fold superfamily protein
Gorai.007G196100.1.D
PCC=0.083, p=0.735
mRNA Profile
Protein Expression Profile
DPA

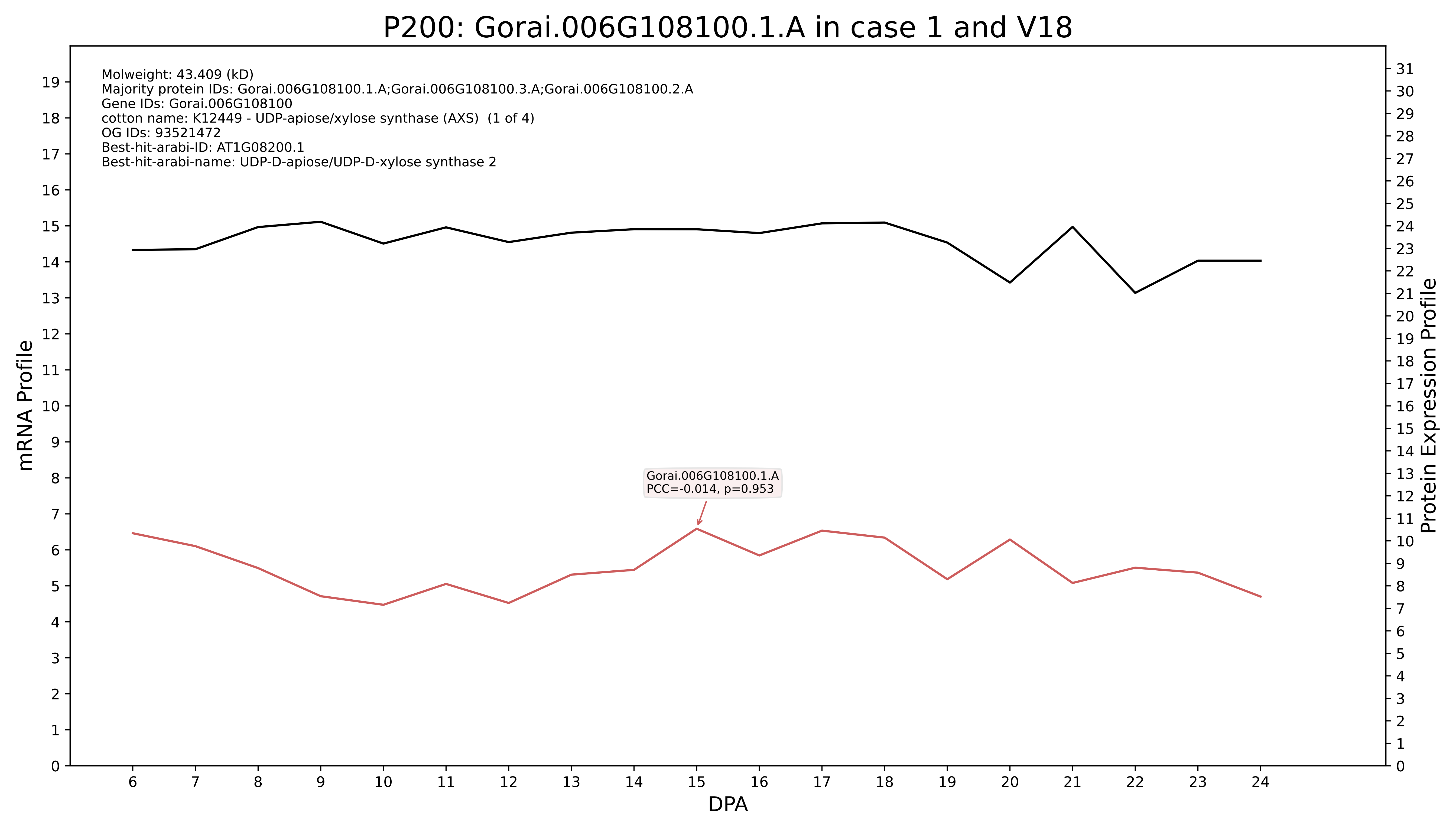
P200: Gorai.006G108100.1.A in case 1 and V18
Molweight: 43.409 (kD)
Majority protein IDs: Gorai.006G108100.1.A;Gorai.006G108100.3.A;Gorai.006G108100.2.A
Gene IDs: Gorai.006G108100
cotton name: K12449 - UDP-apiose/xylose synthase (AXS) (1 of 4)
OG IDs: 93521472
Best-hit-arabi-ID: AT1G08200.1
Best-hit-arabi-name: UDP-D-apiose/UDP-D-xylose synthase 2
Gorai.006G108100.1.A
PCC=-0.014, p=0.953
mRNA Profile
Protein Expression Profile
DPA

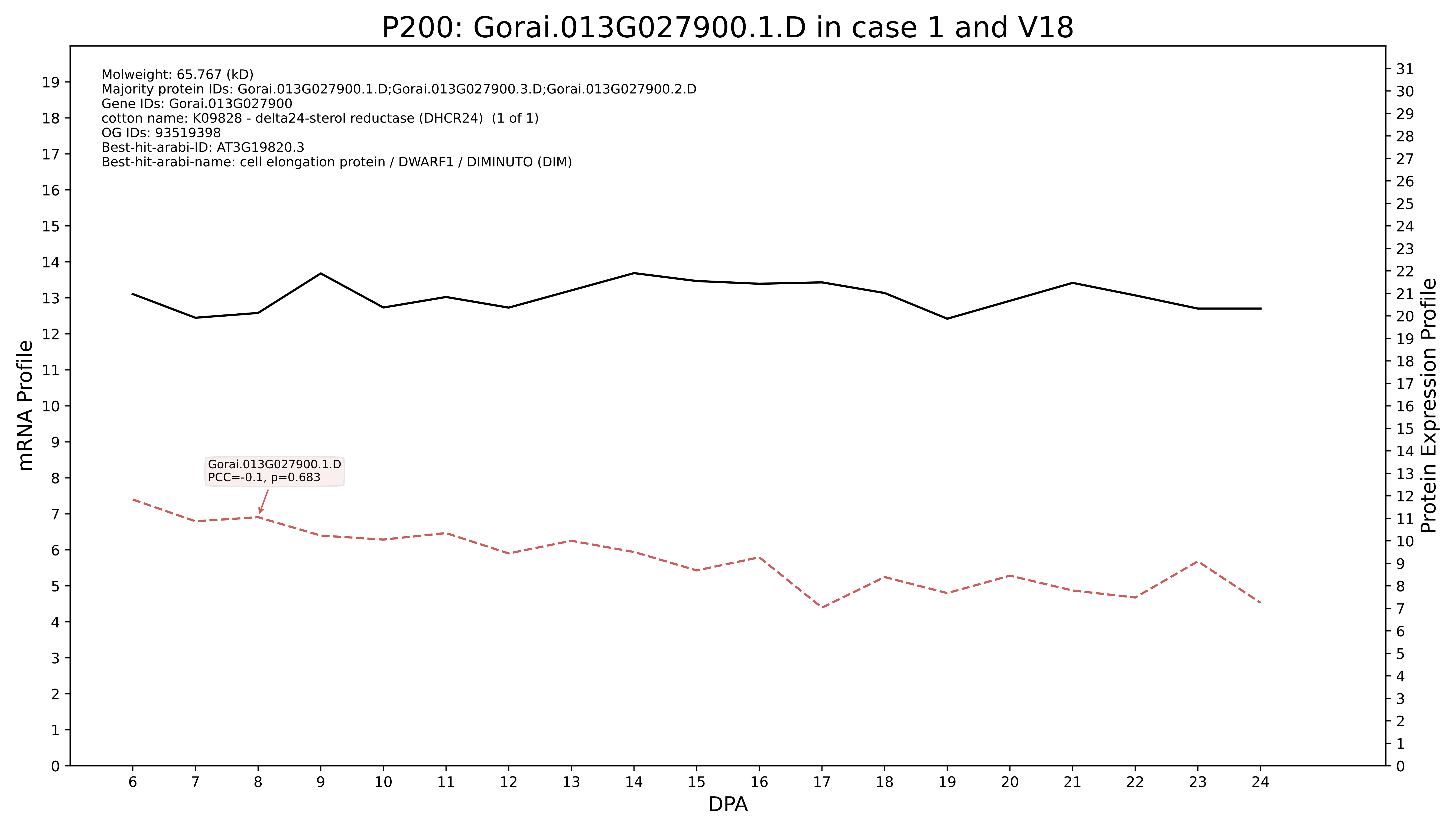
P200: Gorai.013G027900.1.D in case 1 and V18
Molweight: 65.767 (kD)
Majority protein IDs: Gorai.013G027900.1.D;Gorai.013G027900.3.D;Gorai.013G027900.2.D
Gene IDs: Gorai.013G027900
cotton name: K09828 - delta24-sterol reductase (DHCR24) (1 of 1)
OG IDs: 93519398
Best-hit-arabi-ID: AT3G19820.3
Best-hit-arabi-name: cell elongation protein / DWARF1 / DIMINUTO (DIM)
Gorai.013G027900.1.D
PCC=-0.1, p=0.683
mRNA Profile
Protein Expression Profile
DPA

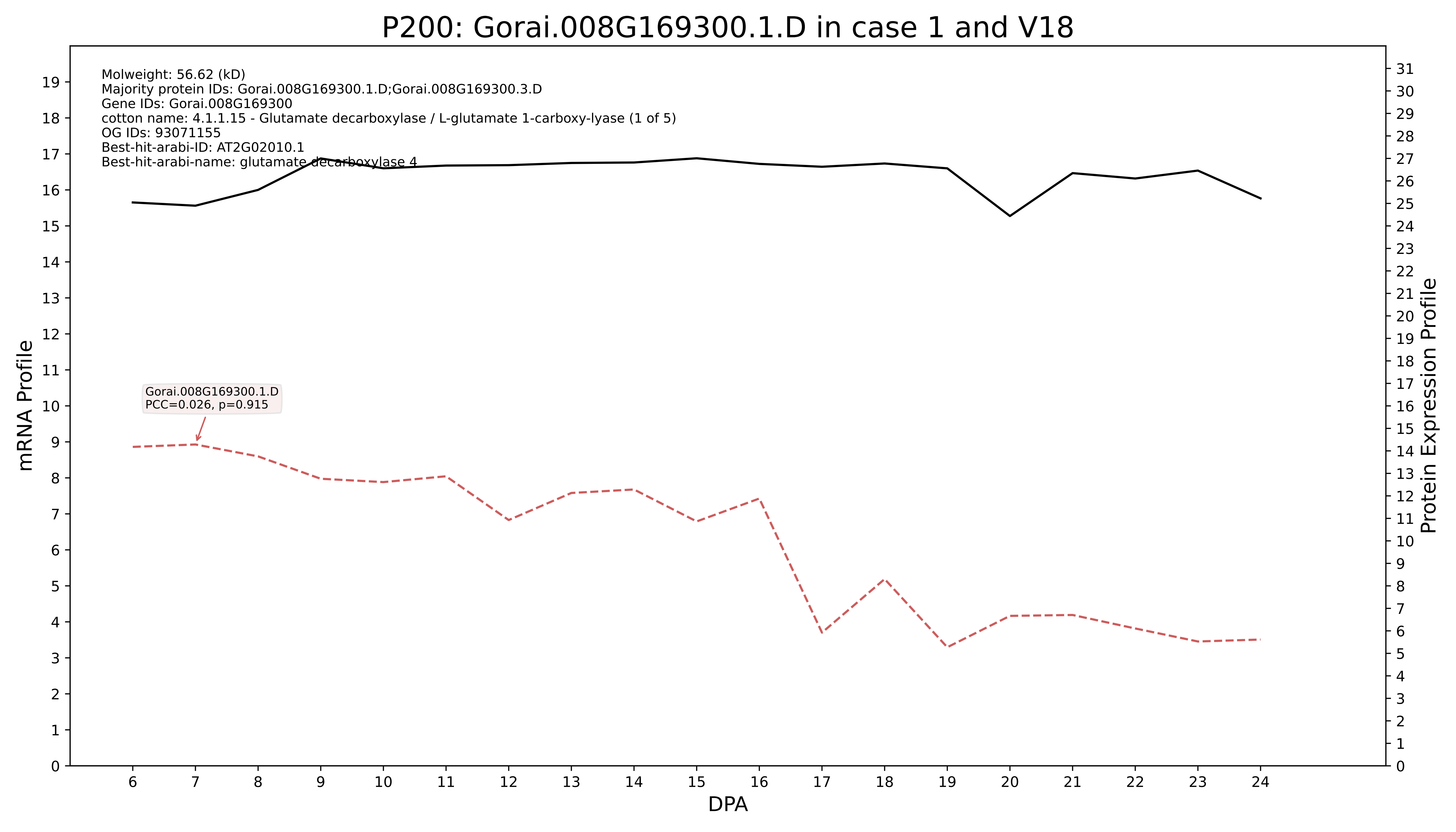

P200: Gorai.008G169300.1.D in case 1 and V18
Molweight: 56.62 (kD)
Majority protein IDs: Gorai.008G169300.1.D;Gorai.008G169300.3.D
Gene IDs: Gorai.008G169300
cotton name: 4.1.1.15 - Glutamate decarboxylase / L-glutamate 1-carboxy-lyase (1 of 5)
OG IDs: 93071155
Best-hit-arabi-ID: AT2G02010.1
Best-hit-arabi-name: glutamate decarboxylase 4
Gorai.008G169300.1.D
PCC=0.026, p=0.915
mRNA Profile
Protein Expression Profile
DPA
0 1 2 3 4 5 6 7 8 9 10 11 12 13 14 15 16 17 18 19
0 1 2 3 4 5 6 7 8 9 10 11 12 13 14 15 16 17 18 19 20 21 22 23 24 25 26 27 28 29 30 31
6 7 8 9 10 11 12 13 14 15 16 17 18 19 20 21 22 23 24

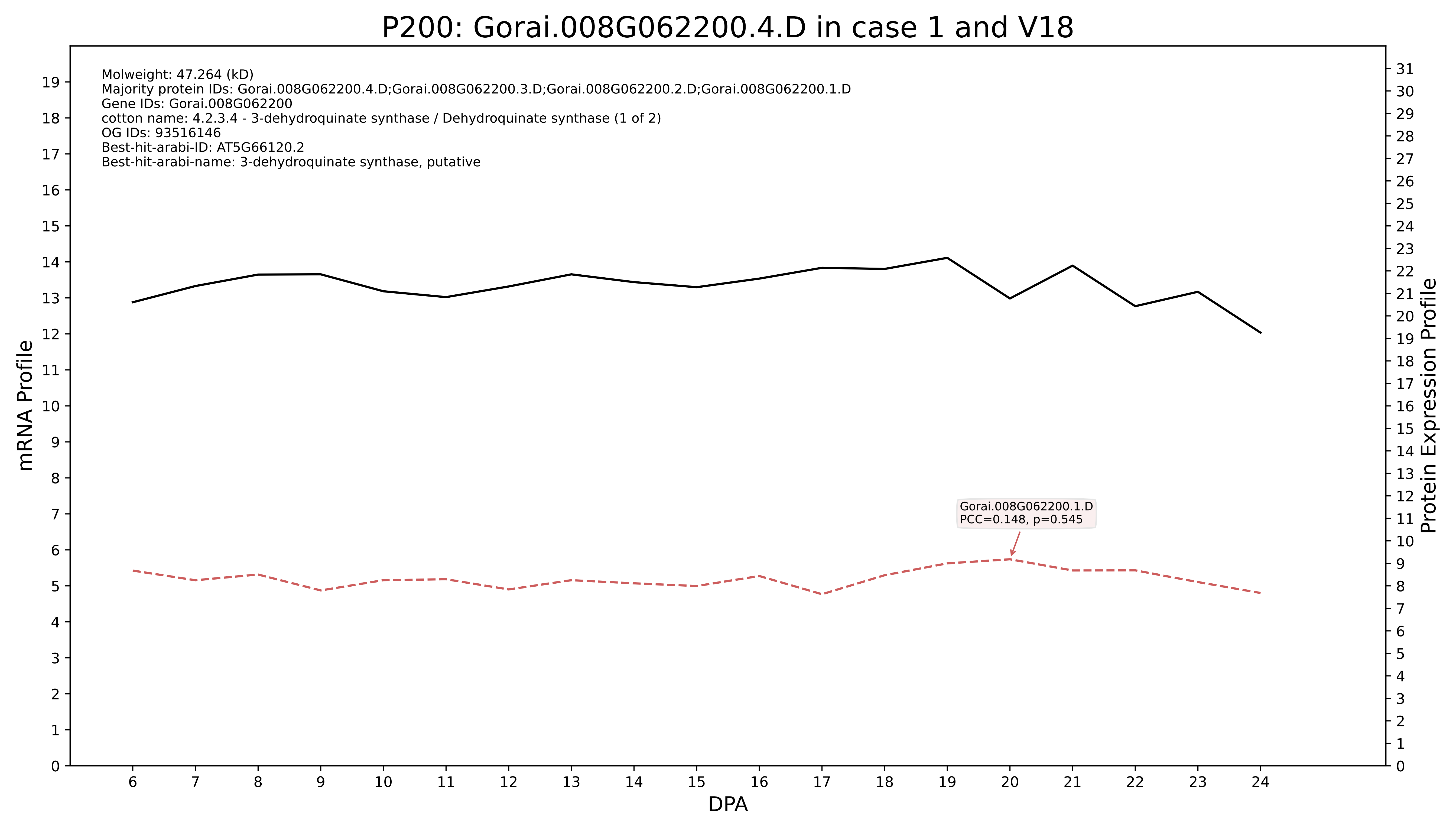
P200: Gorai.008G062200.4.D in case 1 and V18
Molweight: 47.264 (kD)
Majority protein IDs: Gorai.008G062200.4.D;Gorai.008G062200.3.D;Gorai.008G062200.2.D;Gorai.008G062200.1.D
Gene IDs: Gorai.008G062200
cotton name: 4.2.3.4 - 3-dehydroquinate synthase / Dehydroquinate synthase (1 of 2)
OG IDs: 93516146
Best-hit-arabi-ID: AT5G66120.2
Best-hit-arabi-name: 3-dehydroquinate synthase, putative
Gorai.008G062200.1.D
PCC=0.148, p=0.545
mRNA Profile
Protein Expression Profile
DPA
0
1
2
3
4
5
6
7
8
9
10
11
12
13
14
15
16
17
18
19
20
21
22
23
24
25
26
27
28
29
30
31

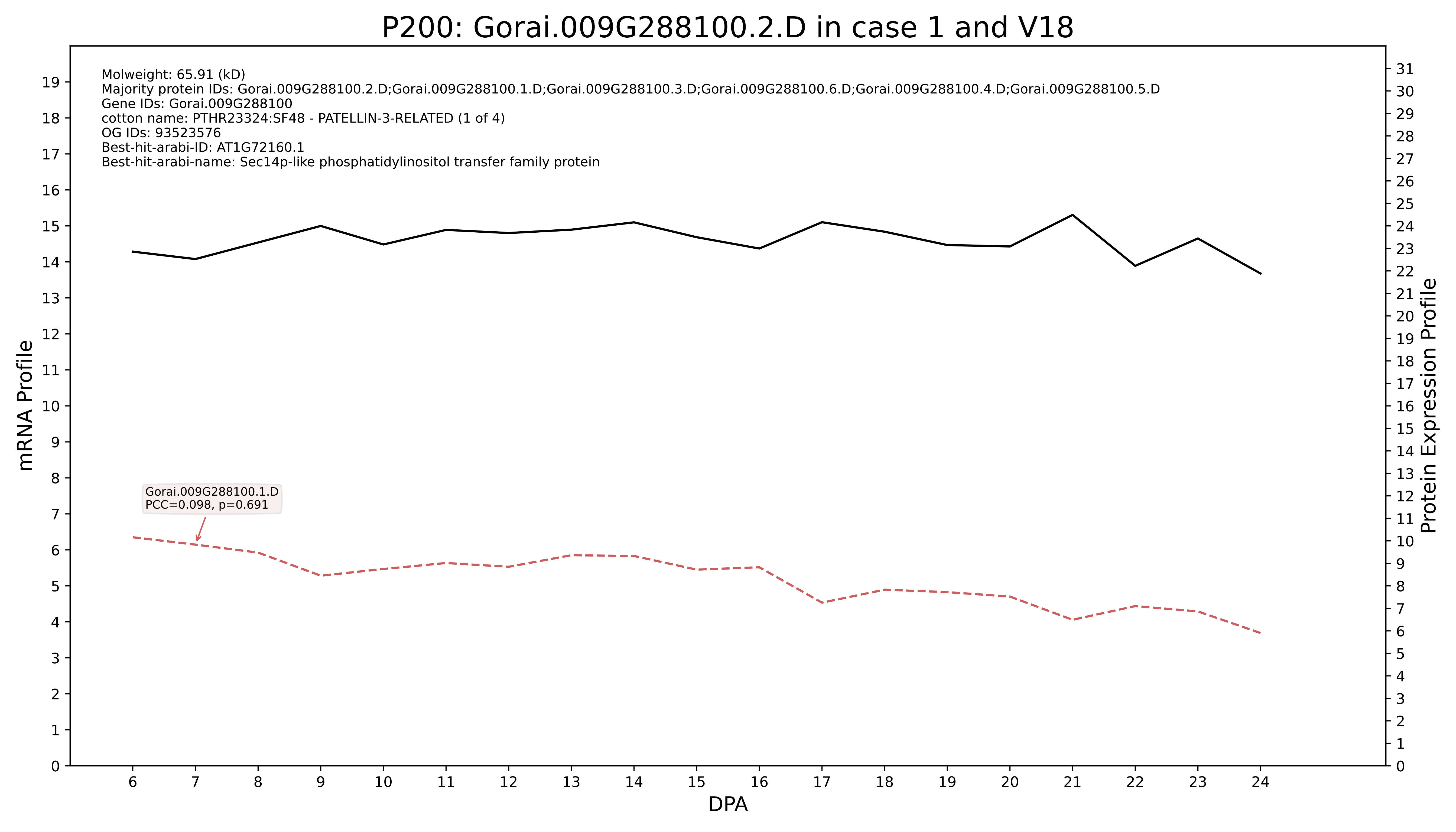

P200: Gorai.009G288100.2.D in case 1 and V18
Molweight: 65.91 (kD)
Majority protein IDs: Gorai.009G288100.2.D;Gorai.009G288100.1.D;Gorai.009G288100.3.D;Gorai.009G288100.6.D;Gorai.009G288100.4.D;Gorai.009G288100.5.D
Gene IDs: Gorai.009G288100
cotton name: PTHR23324:SF48 - PATELLIN-3-RELATED (1 of 4)
OG IDs: 93523576
Best-hit-arabi-ID: AT1G72160.1
Best-hit-arabi-name: Sec14p-like phosphatidylinositol transfer family protein
Gorai.009G288100.1.D
PCC=0.098, p=0.691
mRNA Profile
Protein Expression Profile
DPA

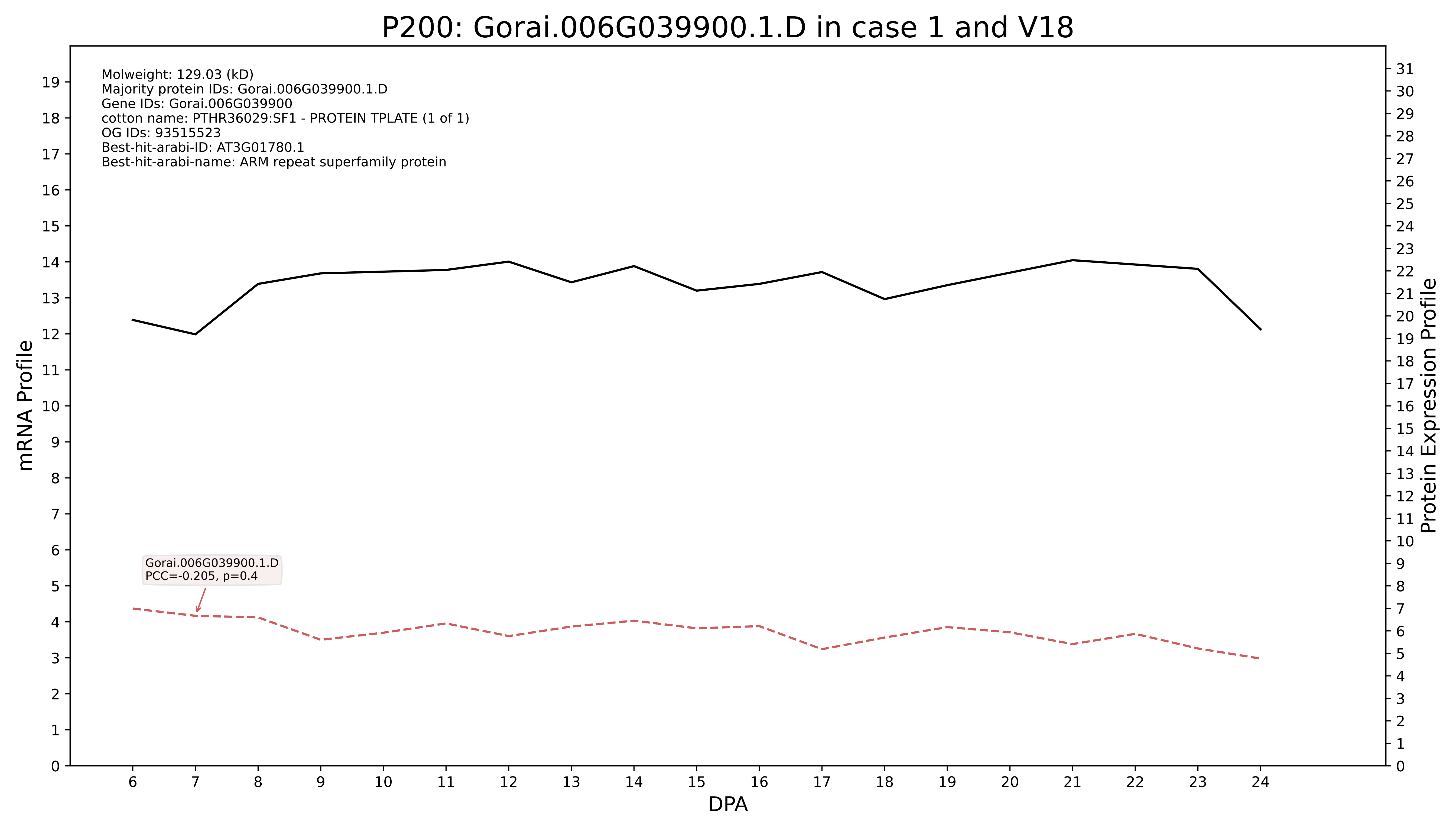

P200: Gorai.006G039900.1.D in case 1 and V18
Molweight: 129.03 (kD)
Majority protein IDs: Gorai.006G039900.1.D
Gene IDs: Gorai.006G039900
cotton name: PTHR36029:SF1 - PROTEIN TPLATE (1 of 1)
OG IDs: 93515523
Best-hit-arabi-ID: AT3G01780.1
Best-hit-arabi-name: ARM repeat superfamily protein
Gorai.006G039900.1.D
PCC=-0.205, p=0.4
mRNA Profile
Protein Expression Profile
DPA

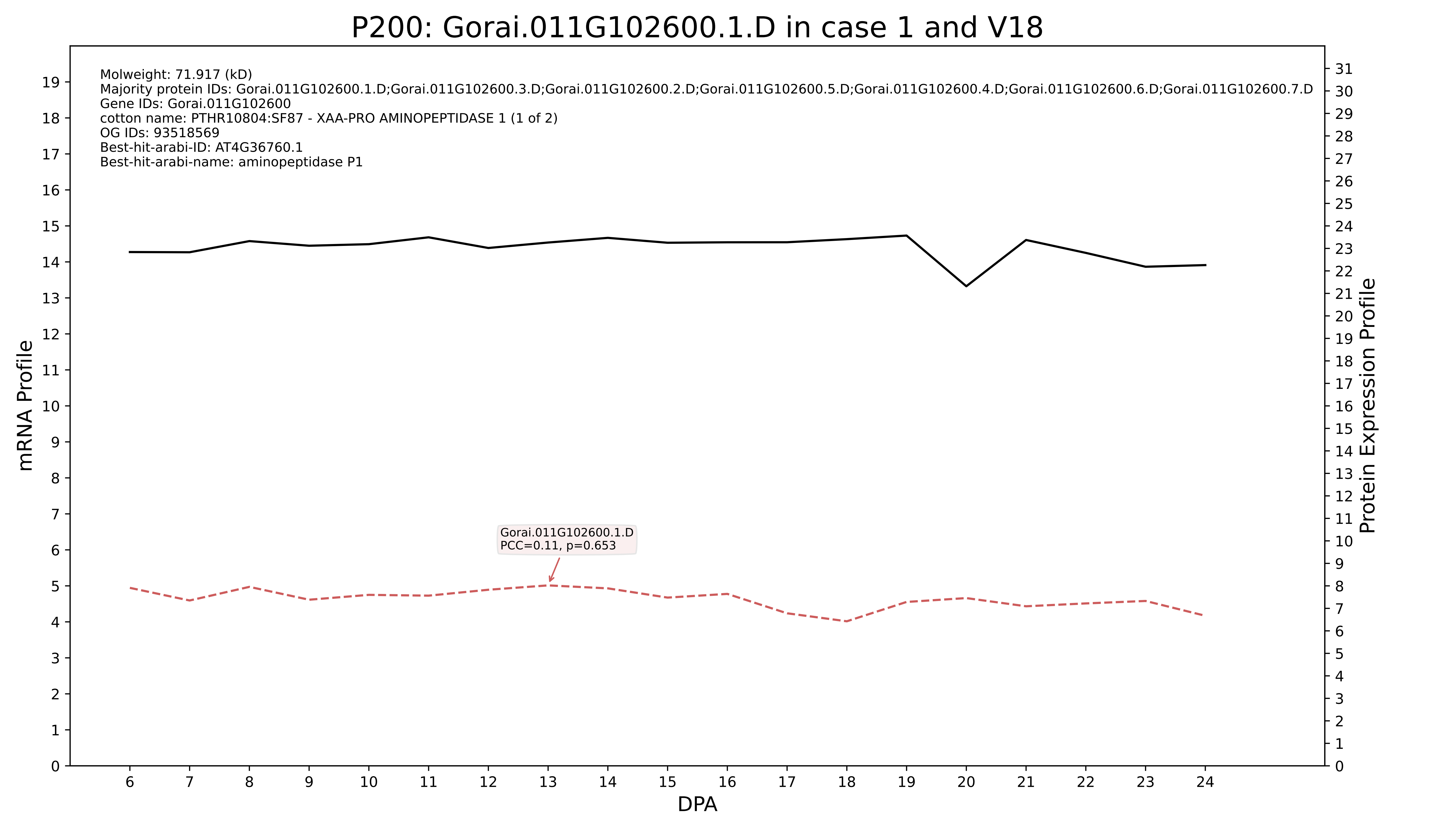
P200: Gorai.011G102600.1.D in case 1 and V18
Molweight: 71.917 (kD)
Majority protein IDs: Gorai.011G102600.1.D;Gorai.011G102600.3.D;Gorai.011G102600.2.D;Gorai.011G102600.5.D;Gorai.011G102600.4.D;Gorai.011G102600.6.D;Gorai.011G102600.7.D
Gene IDs: Gorai.011G102600
cotton name: PTHR10804:SF87 - XAA-PRO AMINOPEPTIDASE 1 (1 of 2)
OG IDs: 93518569
Best-hit-arabi-ID: AT4G36760.1
Best-hit-arabi-name: aminopeptidase P1
Gorai.011G102600.1.D
PCC=0.11, p=0.653
mRNA Profile
Protein Expression Profile
DPA

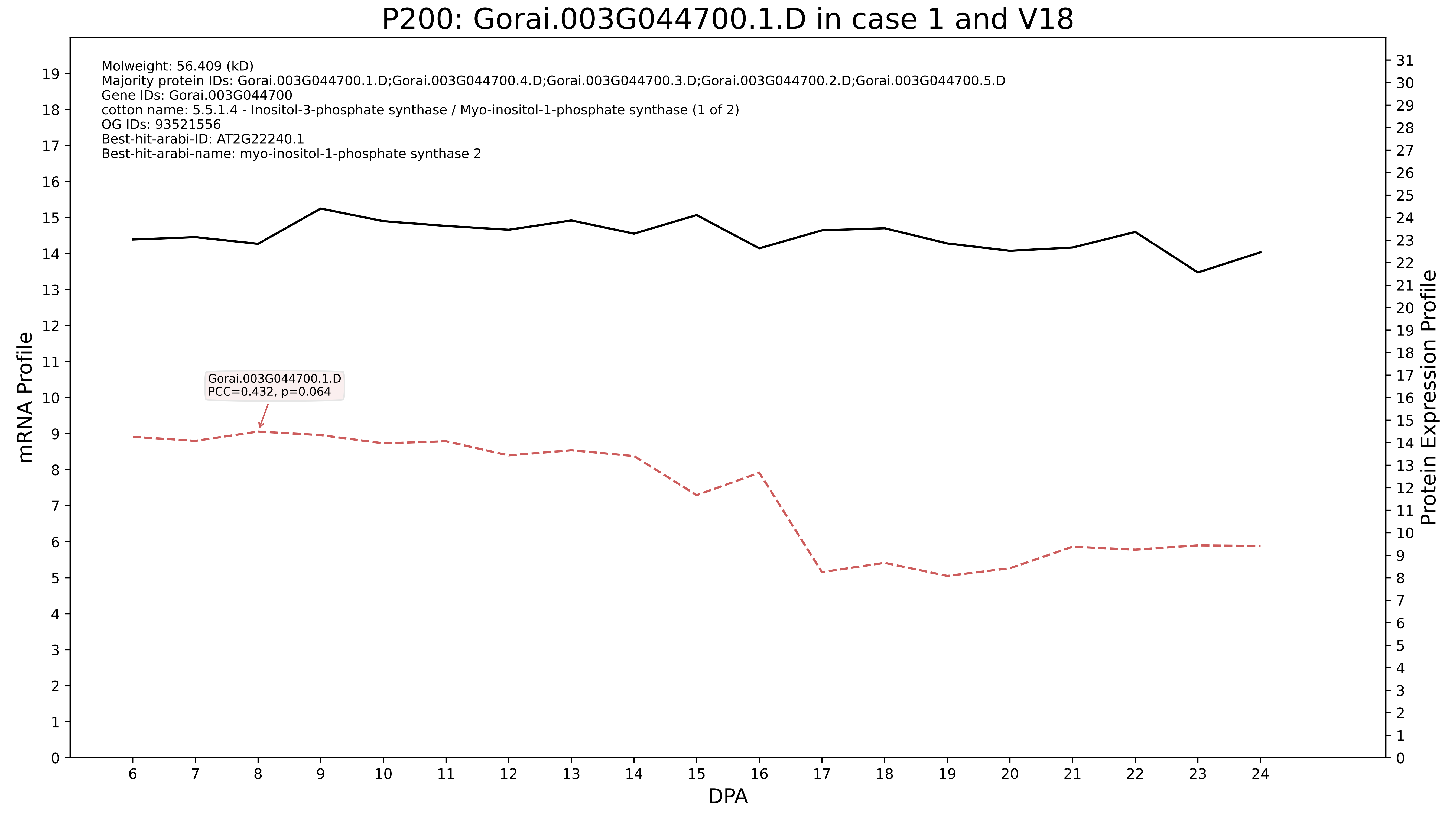
P200: Gorai.003G044700.1.D in case 1 and V18
Molweight: 56.409 (kD)
Majority protein IDs: Gorai.003G044700.1.D;Gorai.003G044700.4.D;Gorai.003G044700.3.D;Gorai.003G044700.2.D;Gorai.003G044700.5.D
Gene IDs: Gorai.003G044700
cotton name: 5.5.1.4 - Inositol-3-phosphate synthase / Myo-inositol-1-phosphate synthase (1 of 2)
OG IDs: 93521556
Best-hit-arabi-ID: AT2G22240.1
Best-hit-arabi-name: myo-inositol-1-phosphate synthase 2
Gorai.003G044700.1.D
PCC=0.432, p=0.064
mRNA Profile
Protein Expression Profile
DPA

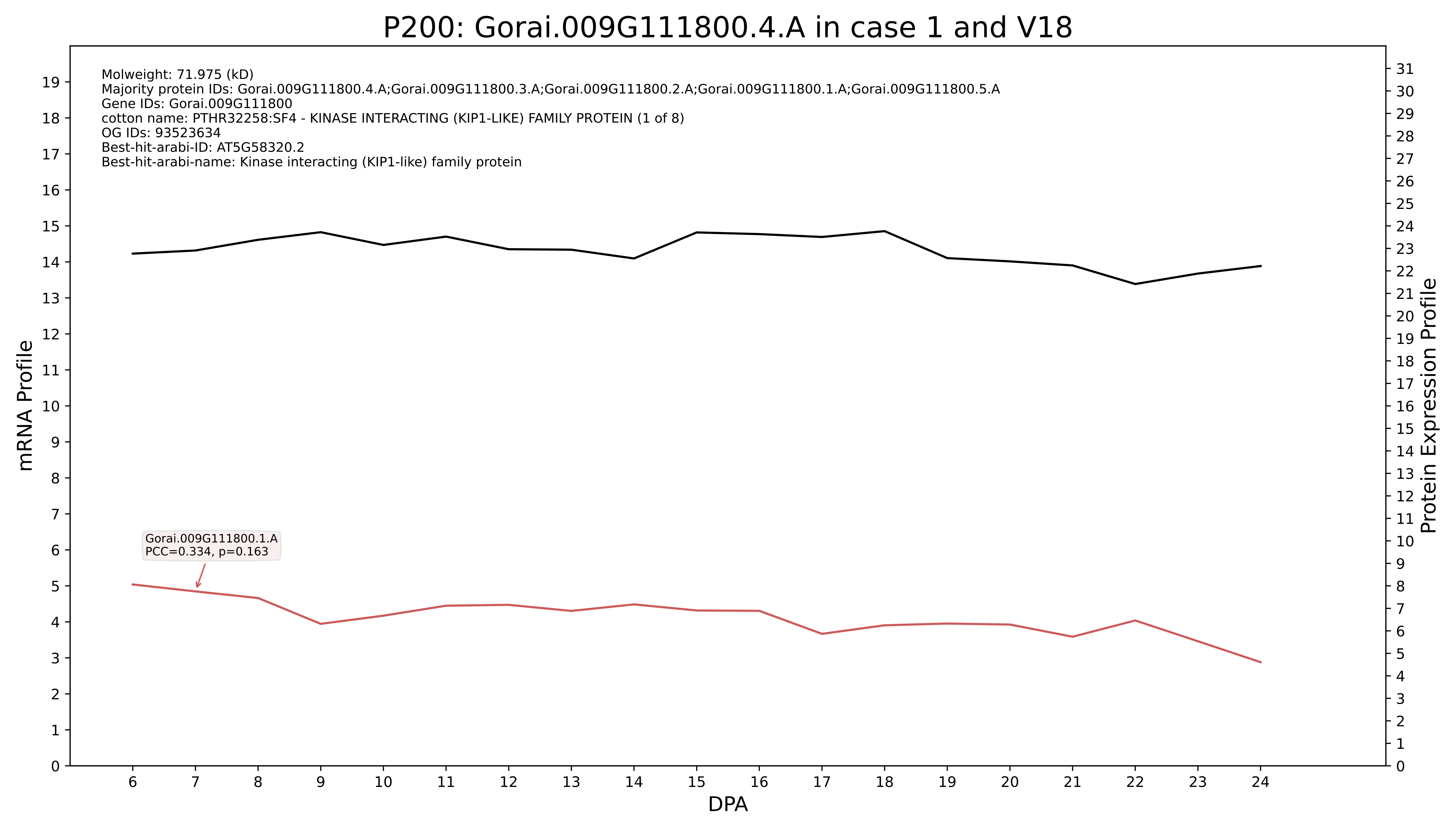
P200: Gorai.009G111800.4.A in case 1 and V18
Molweight: 71.975 (kD)
Majority protein IDs: Gorai.009G111800.4.A;Gorai.009G111800.3.A;Gorai.009G111800.2.A;Gorai.009G111800.1.A;Gorai.009G111800.5.A
Gene IDs: Gorai.009G111800
cotton name: PTHR32258:SF4 - KINASE INTERACTING (KIP1-LIKE) FAMILY PROTEIN (1 of 8)
OG IDs: 93523634
Best-hit-arabi-ID: AT5G58320.2
Best-hit-arabi-name: Kinase interacting (KIP1-like) family protein
Gorai.009G111800.1.A
PCC=0.334, p=0.163
mRNA Profile
Protein Expression Profile
DPA

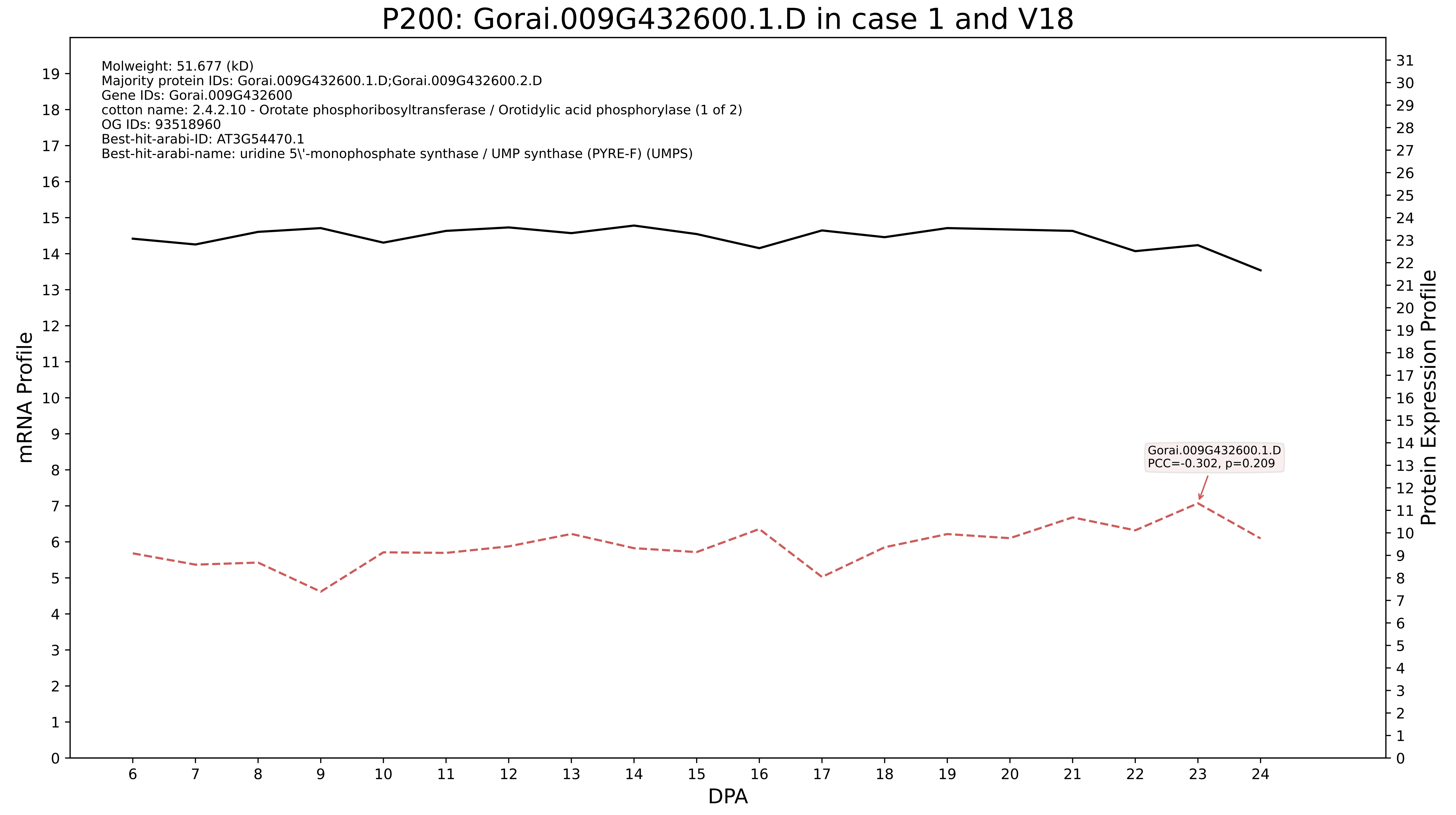
P200: Gorai.009G432600.1.D in case 1 and V18
Molweight: 51.677 (kD)
Majority protein IDs: Gorai.009G432600.1.D;Gorai.009G432600.2.D
Gene IDs: Gorai.009G432600
cotton name: 2.4.2.10 - Orotate phosphoribosyltransferase / Orotidylic acid phosphorylase (1 of 2)
OG IDs: 93518960
Best-hit-arabi-ID: AT3G54470.1
Best-hit-arabi-name: uridine 5\'-monophosphate synthase / UMP synthase (PYRE-F) (UMPS)
Gorai.009G432600.1.D
PCC=-0.302, p=0.209
mRNA Profile
Protein Expression Profile
DPA
0
1
2
3
4
5
6
7
8
9
10
11
12
13
14
15
16
17
18
19
20
21
22
23
24
25
26
27
28
29
30
31

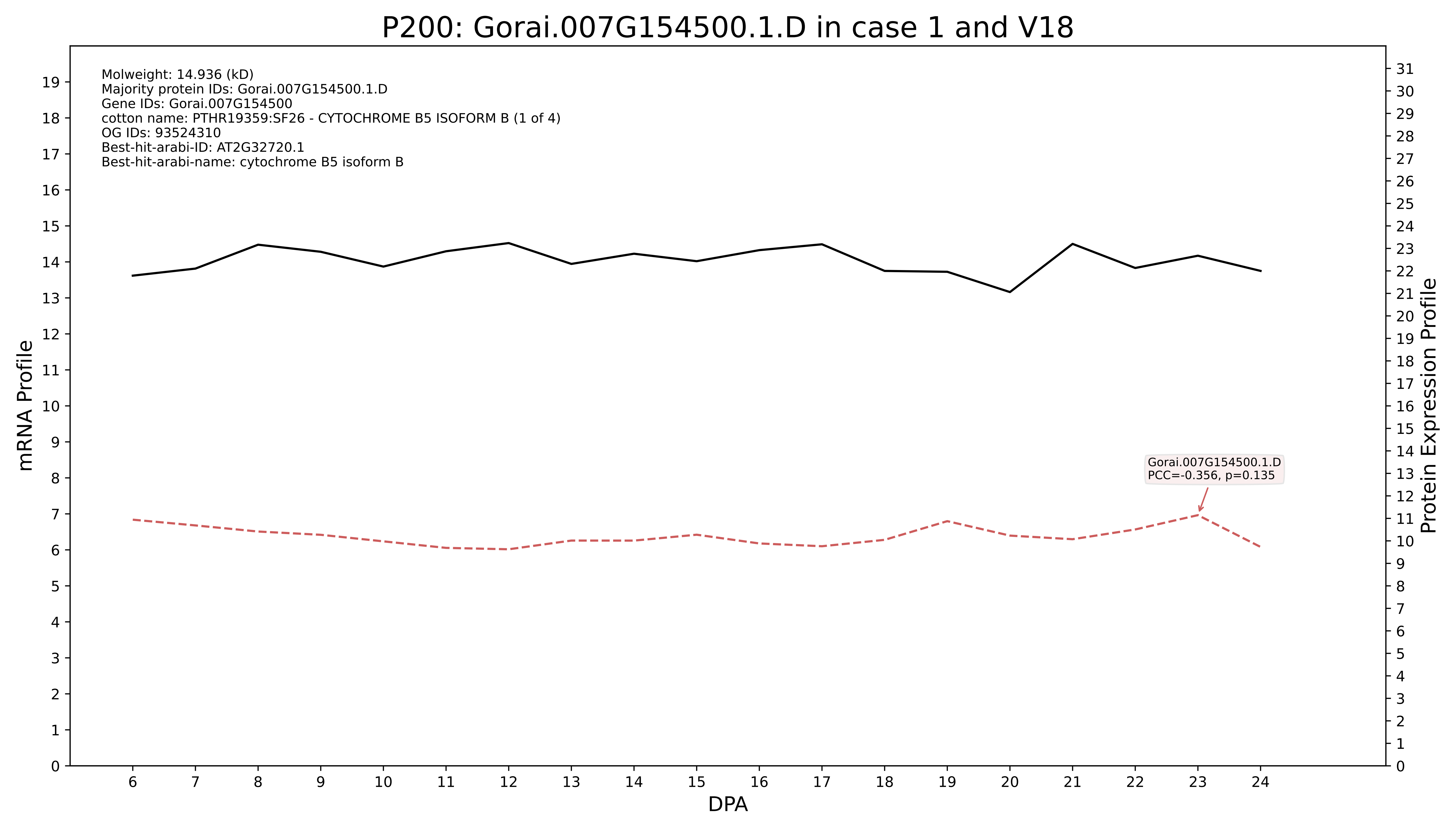

P200: Gorai.007G154500.1.D in case 1 and V18
Molweight: 14.936 (kD)
Majority protein IDs: Gorai.007G154500.1.D
Gene IDs: Gorai.007G154500
cotton name: PTHR19359:SF26 - CYTOCHROME B5 ISOFORM B (1 of 4)
OG IDs: 93524310
Best-hit-arabi-ID: AT2G32720.1
Best-hit-arabi-name: cytochrome B5 isoform B
Gorai.007G154500.1.D
PCC=-0.356, p=0.135
mRNA Profile
Protein Expression Profile
DPA

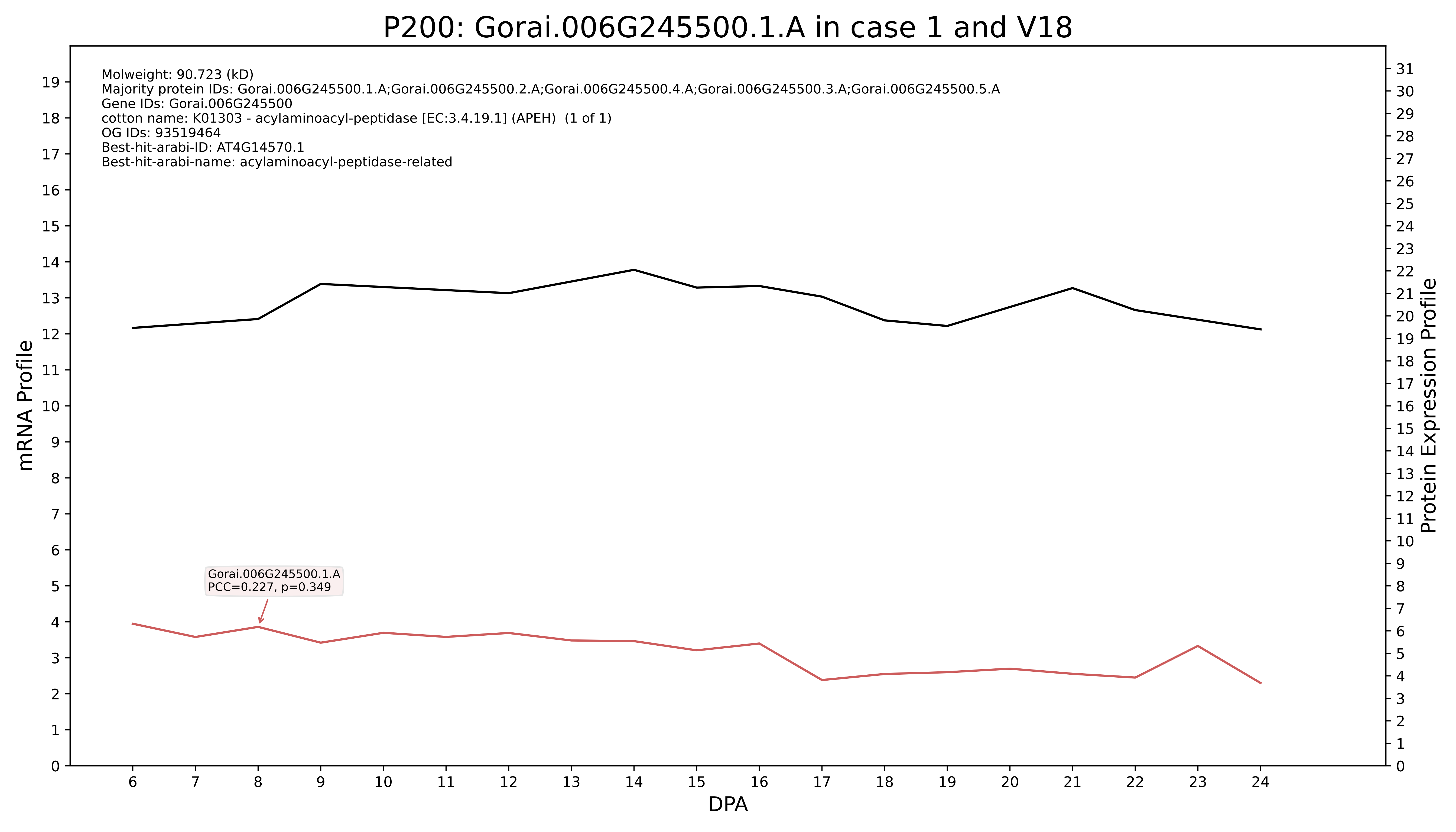
P200: Gorai.006G245500.1.A in case 1 and V18
Molweight: 90.723 (kD)
Majority protein IDs: Gorai.006G245500.1.A;Gorai.006G245500.2.A;Gorai.006G245500.4.A;Gorai.006G245500.3.A;Gorai.006G245500.5.A
Gene IDs: Gorai.006G245500
cotton name: K01303 - acylaminoacyl-peptidase [EC:3.4.19.1] (APEH) (1 of 1)
OG IDs: 93519464
Best-hit-arabi-ID: AT4G14570.1
Best-hit-arabi-name: acylaminoacyl-peptidase-related
Gorai.006G245500.1.A
PCC=0.227, p=0.349
mRNA Profile
Protein Expression Profile
DPA

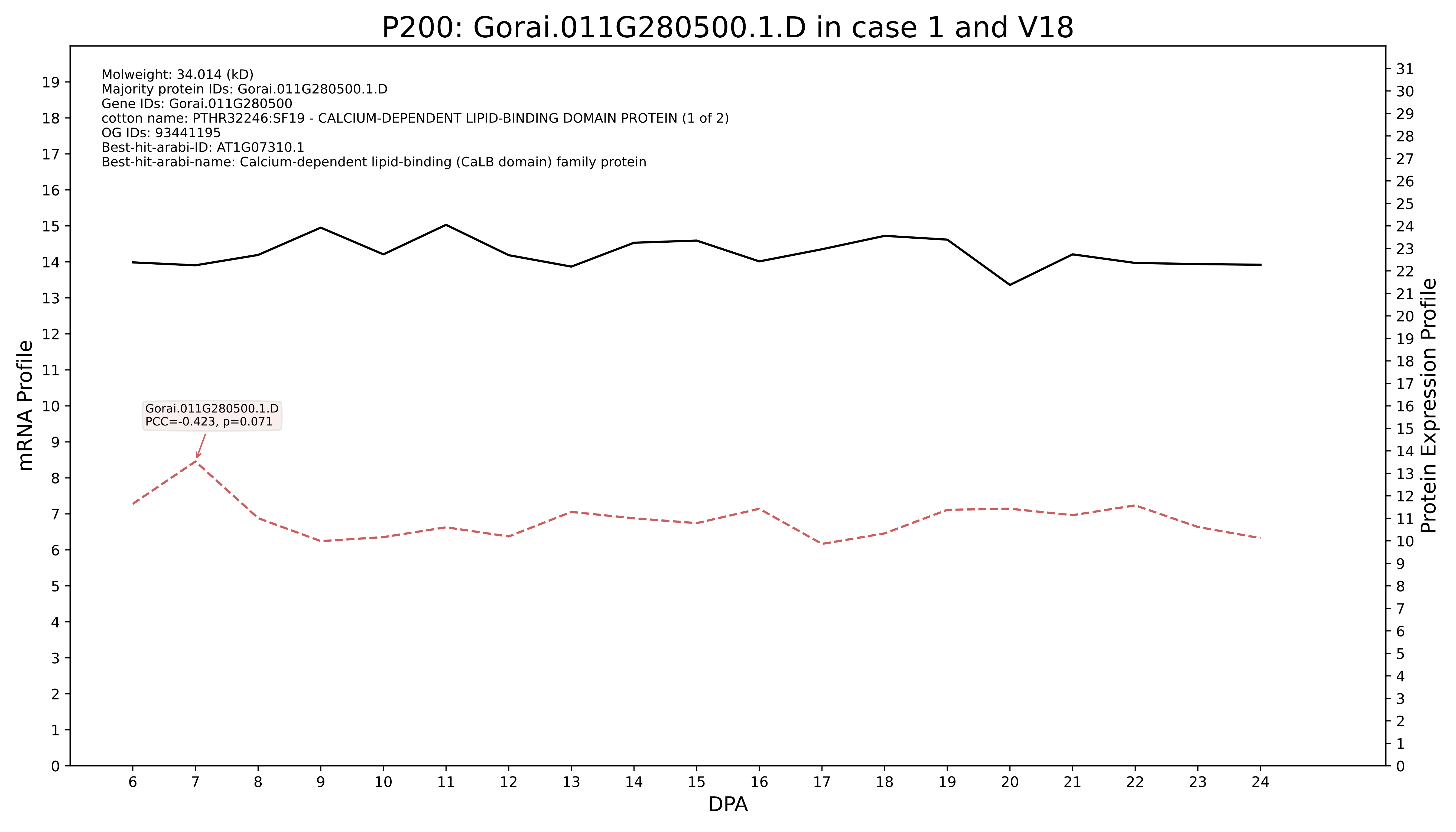
P200: Gorai.011G280500.1.D in case 1 and V18
Molweight: 34.014 (kD)
Majority protein IDs: Gorai.011G280500.1.D
Gene IDs: Gorai.011G280500
cotton name: PTHR32246:SF19 - CALCIUM-DEPENDENT LIPID-BINDING DOMAIN PROTEIN (1 of 2)
OG IDs: 93441195
Best-hit-arabi-ID: AT1G07310.1
Best-hit-arabi-name: Calcium-dependent lipid-binding (CaLB domain) family protein
Gorai.011G280500.1.D
PCC=-0.423, p=0.071
mRNA Profile
Protein Expression Profile
DPA
0
1
2
3
4
5
6
7
8
9
10
11
12
13
14
15
16
17
18
19
20
21
22
23
24
25
26
27
28
29
30
31

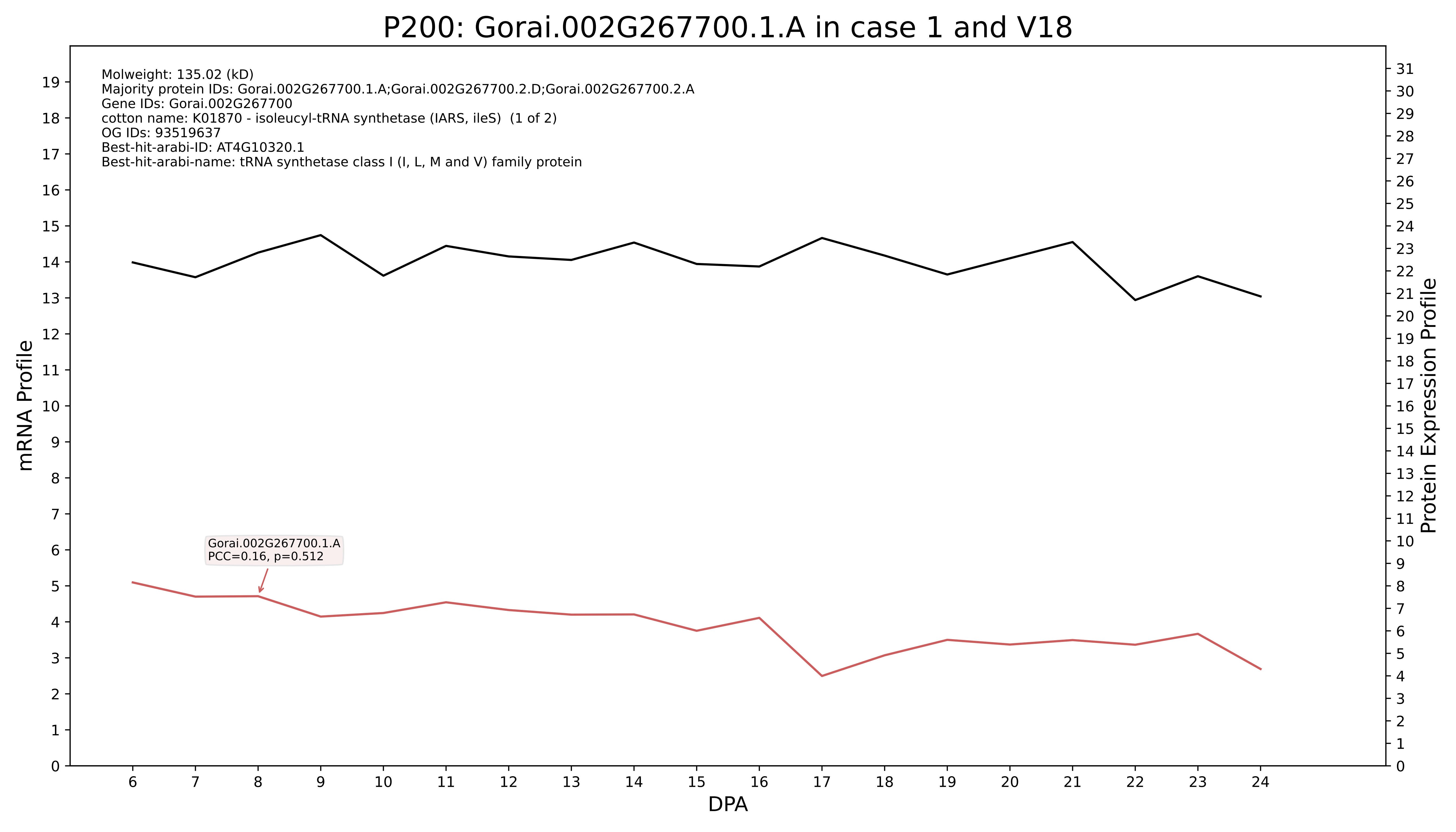
P200: Gorai.002G267700.1.A in case 1 and V18
Molweight: 135.02 (kD)
Majority protein IDs: Gorai.002G267700.1.A;Gorai.002G267700.2.D;Gorai.002G267700.2.A
Gene IDs: Gorai.002G267700
cotton name: K01870 - isoleucyl-tRNA synthetase (IARS, ileS) (1 of 2)
OG IDs: 93519637
Best-hit-arabi-ID: AT4G10320.1
Best-hit-arabi-name: tRNA synthetase class I (I, L, M and V) family protein
Gorai.002G267700.1.A
PCC=0.16, p=0.512
mRNA Profile
Protein Expression Profile
DPA
0
1
2
3
4
5
6
7
8
9
10
11
12
13
14
15
16
17
18
19
20
21
22
23
24
25
26
27
28
29
30
31

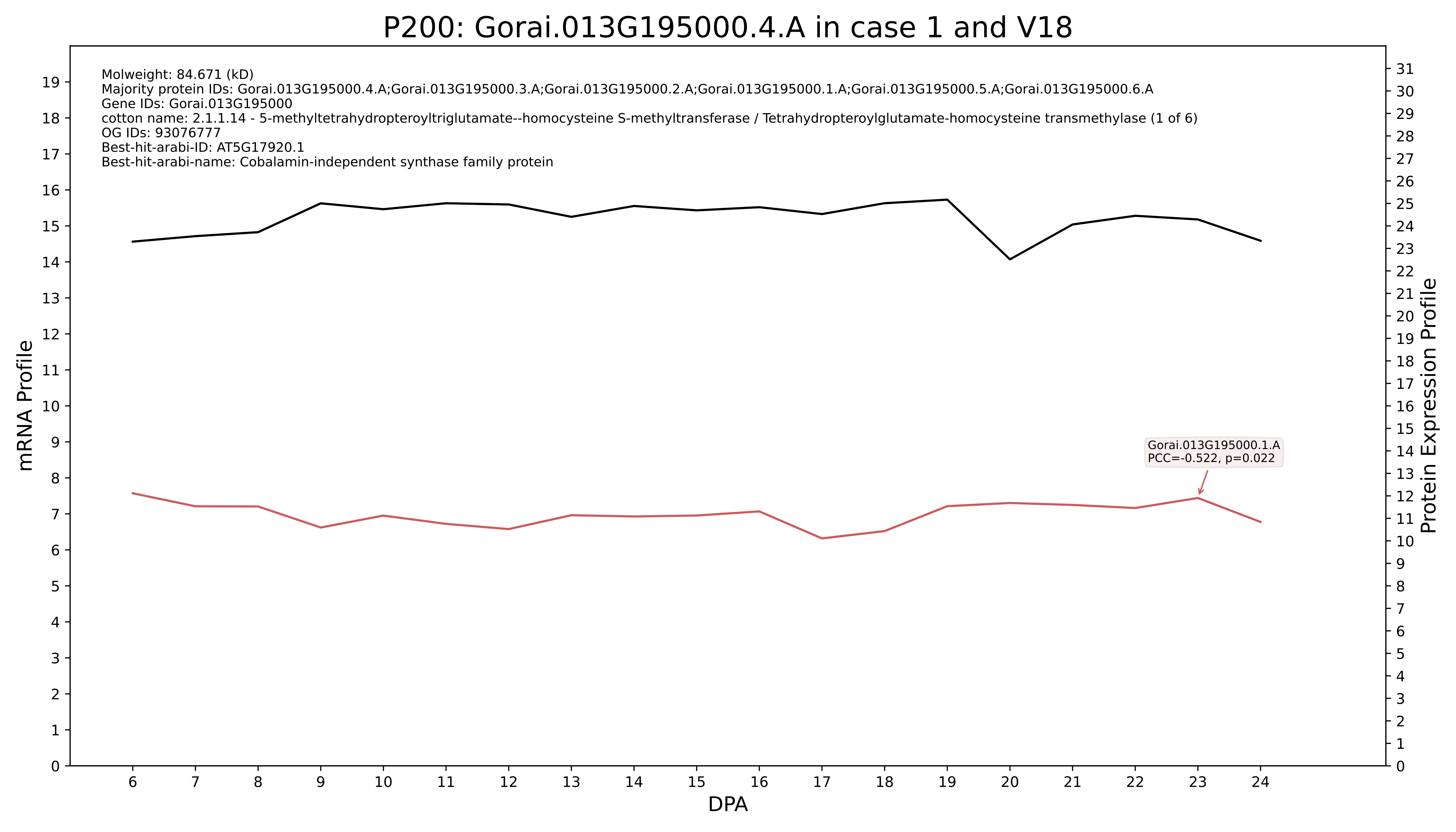
P200: Gorai.013G195000.4.A in case 1 and V18
Molweight: 84.671 (kD)
Majority protein IDs: Gorai.013G195000.4.A;Gorai.013G195000.3.A;Gorai.013G195000.2.A;Gorai.013G195000.1.A;Gorai.013G195000.5.A;Gorai.013G195000.6.A
Gene IDs: Gorai.013G195000
cotton name: 2.1.1.14 - 5-methyltetrahydropteroyltriglutamate--homocysteine S-methyltransferase / Tetrahydropteroylglutamate-homocysteine transmethylase (1 of 6)
OG IDs: 93076777
Best-hit-arabi-ID: AT5G17920.1
Best-hit-arabi-name: Cobalamin-independent synthase family protein
Gorai.013G195000.1.A
PCC=-0.522, p=0.022
mRNA Profile
Protein Expression Profile
DPA

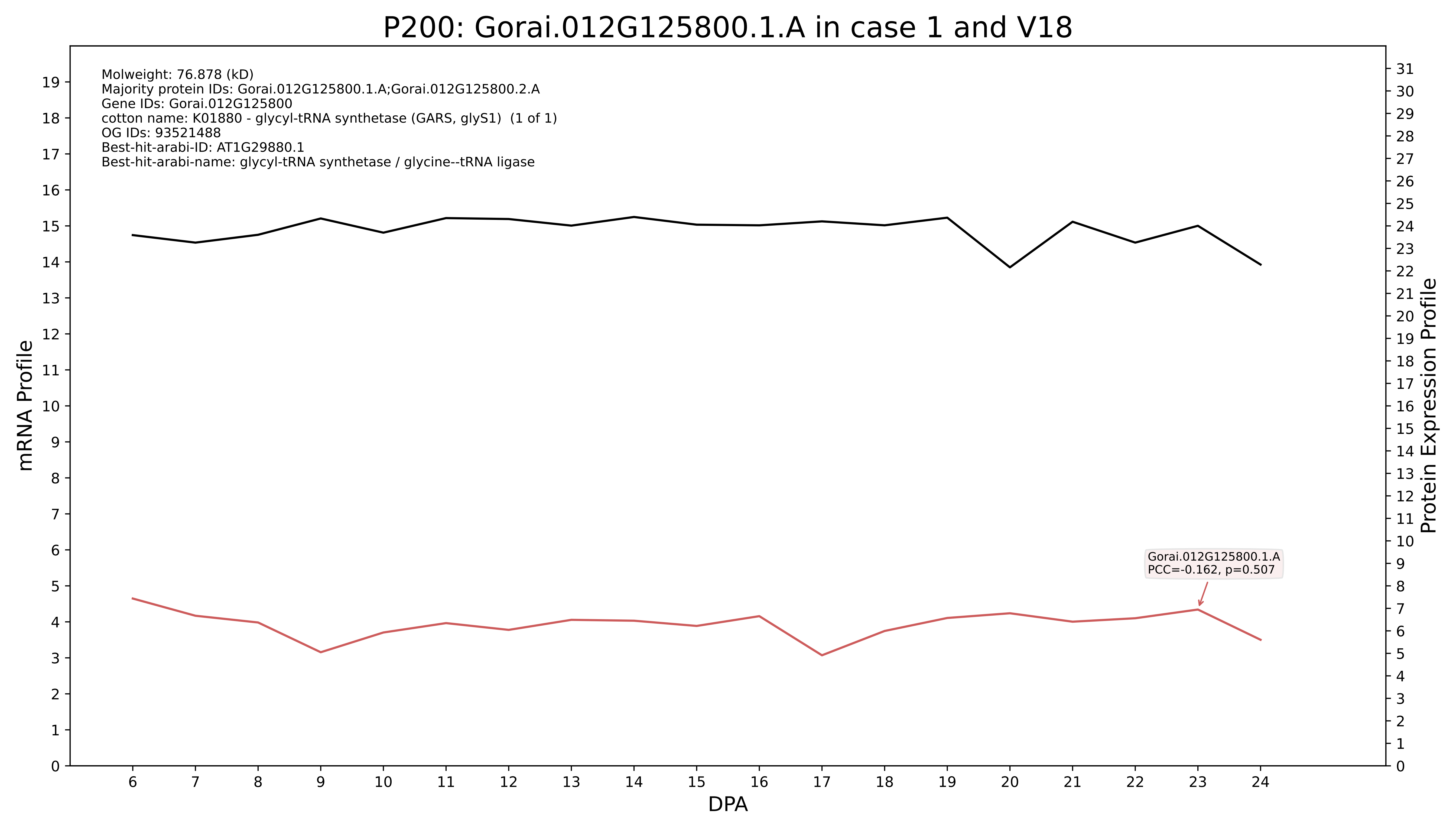
P200: Gorai.012G125800.1.A in case 1 and V18
Molweight: 76.878 (kD)
Majority protein IDs: Gorai.012G125800.1.A;Gorai.012G125800.2.A
Gene IDs: Gorai.012G125800
cotton name: K01880 - glycyl-tRNA synthetase (GARS, glyS1) (1 of 1)
OG IDs: 93521488
Best-hit-arabi-ID: AT1G29880.1
Best-hit-arabi-name: glycyl-tRNA synthetase / glycine--tRNA ligase
Gorai.012G125800.1.A
PCC=-0.162, p=0.507
mRNA Profile
Protein Expression Profile
DPA

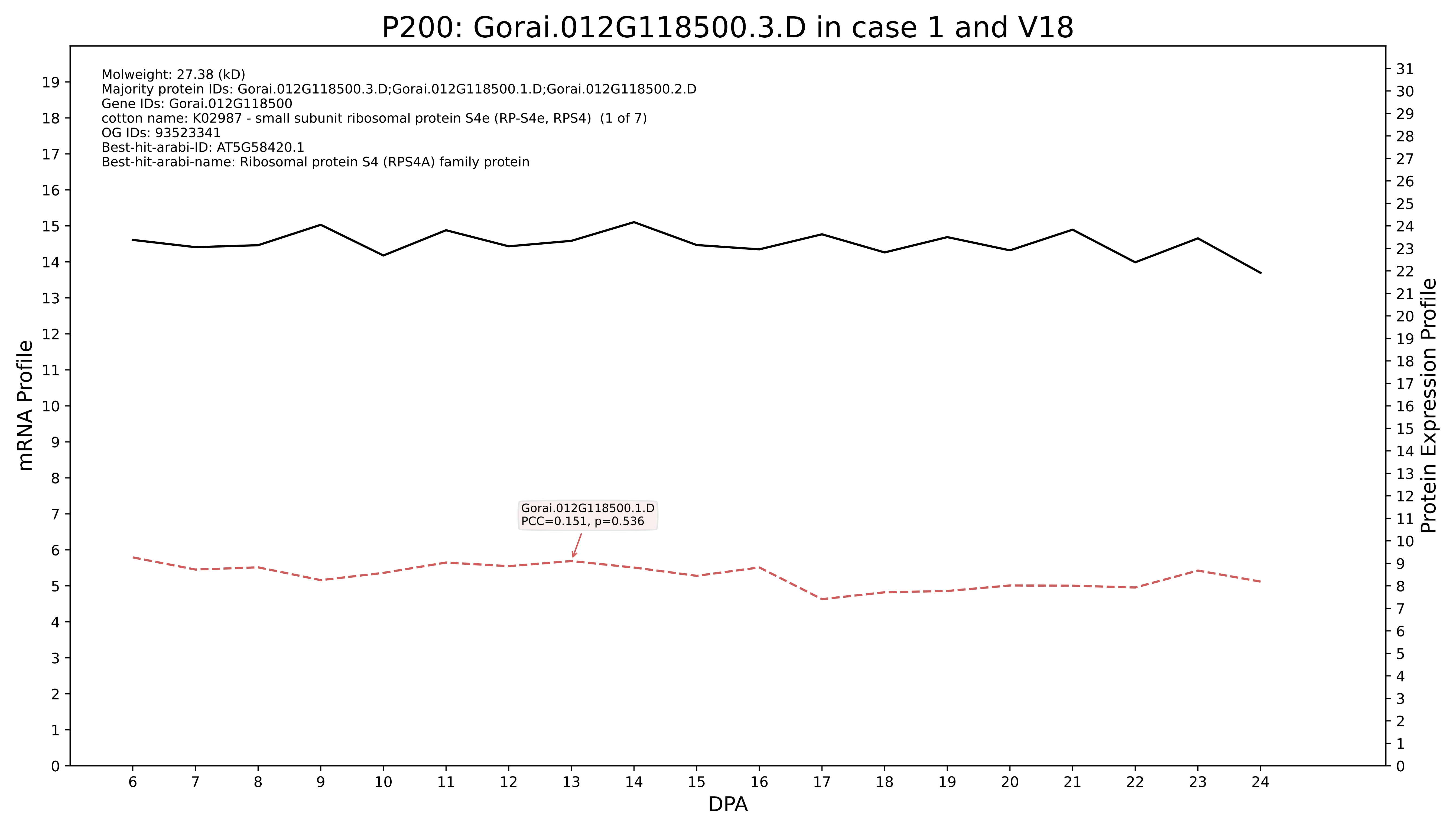
P200: Gorai.012G118500.3.D in case 1 and V18
Molweight: 27.38 (kD)
Majority protein IDs: Gorai.012G118500.3.D;Gorai.012G118500.1.D;Gorai.012G118500.2.D
Gene IDs: Gorai.012G118500
cotton name: K02987 - small subunit ribosomal protein S4e (RP-S4e, RPS4) (1 of 7)
OG IDs: 93523341
Best-hit-arabi-ID: AT5G58420.1
Best-hit-arabi-name: Ribosomal protein S4 (RPS4A) family protein
Gorai.012G118500.1.D
PCC=0.151, p=0.536
mRNA Profile
Protein Expression Profile
DPA
0
1
2
3
4
5
6
7
8
9
10
11
12
13
14
15
16
17
18
19
20
21
22
23
24
25
26
27
28
29
30
31

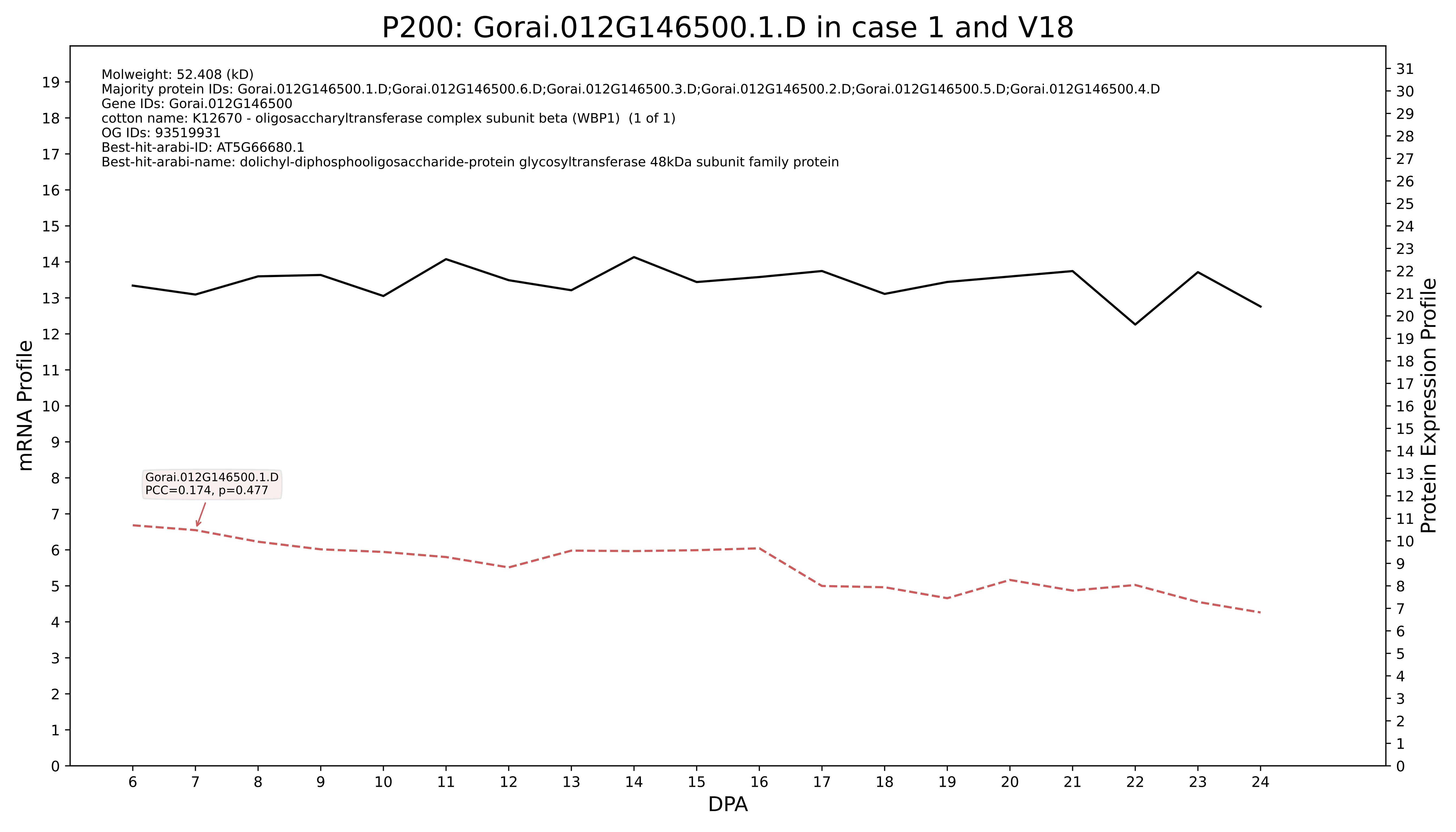
P200: Gorai.012G146500.1.D in case 1 and V18
Molweight: 52.408 (kD)
Majority protein IDs: Gorai.012G146500.1.D;Gorai.012G146500.6.D;Gorai.012G146500.3.D;Gorai.012G146500.2.D;Gorai.012G146500.5.D;Gorai.012G146500.4.D
Gene IDs: Gorai.012G146500
cotton name: K12670 - oligosaccharyltransferase complex subunit beta (WBP1) (1 of 1)
OG IDs: 93519931
Best-hit-arabi-ID: AT5G66680.1
Best-hit-arabi-name: dolichyl-diphosphooligosaccharide-protein glycosyltransferase 48kDa subunit family protein
Gorai.012G146500.1.D
PCC=0.174, p=0.477
mRNA Profile
Protein Expression Profile
DPA

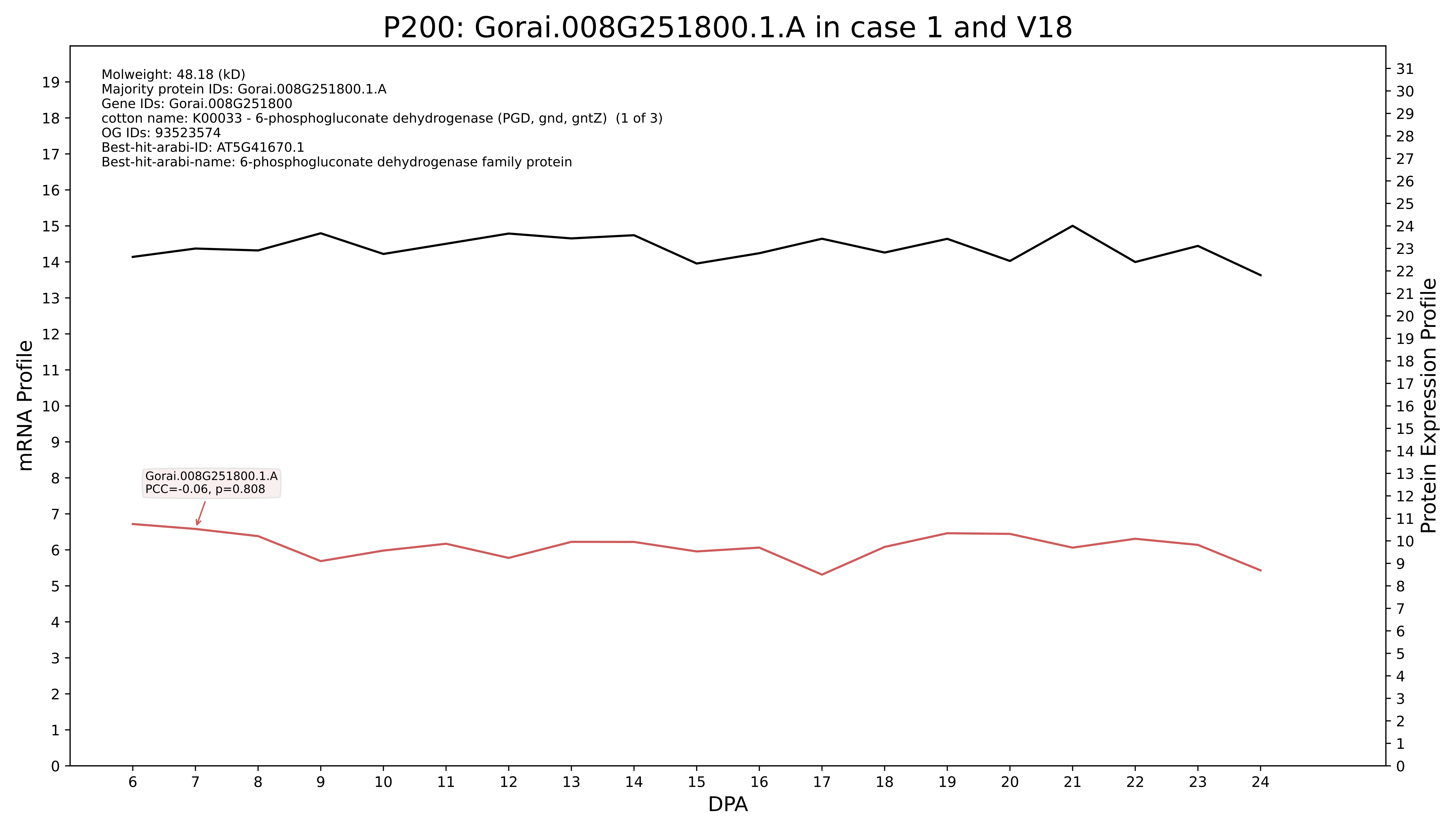
P200: Gorai.008G251800.1.A in case 1 and V18
Molweight: 48.18 (kD)
Majority protein IDs: Gorai.008G251800.1.A
Gene IDs: Gorai.008G251800
cotton name: K00033 - 6-phosphogluconate dehydrogenase (PGD, gnd, gntZ) (1 of 3)
OG IDs: 93523574
Best-hit-arabi-ID: AT5G41670.1
Best-hit-arabi-name: 6-phosphogluconate dehydrogenase family protein
Gorai.008G251800.1.A
PCC=-0.06, p=0.808
mRNA Profile
Protein Expression Profile
DPA
0
1
2
3
4
5
6
7
8
9
10
11
12
13
14
15
16
17
18
19
20
21
22
23
24
25
26
27
28
29
30
31

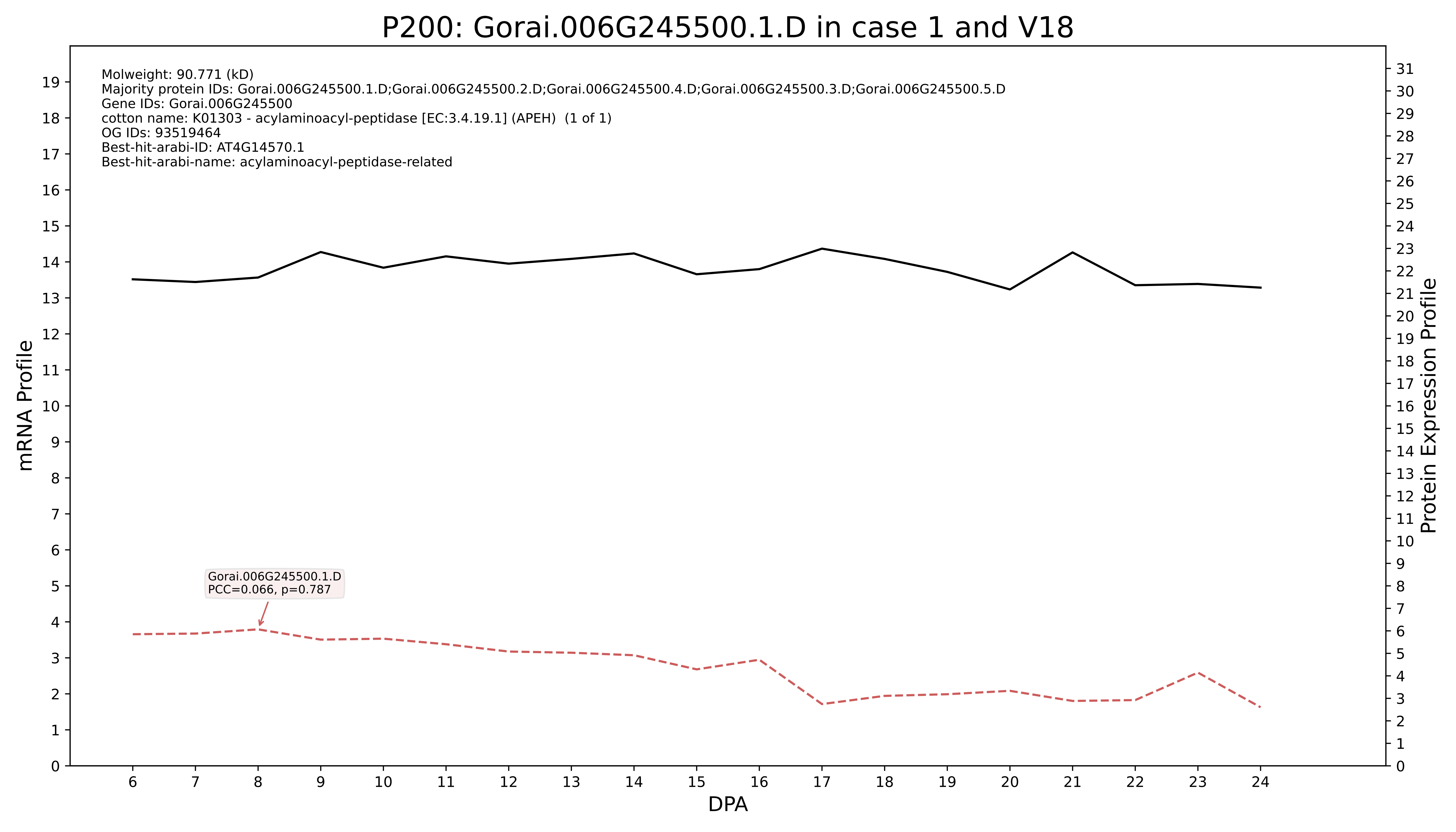
P200: Gorai.006G245500.1.D in case 1 and V18
Molweight: 90.771 (kD)
Majority protein IDs: Gorai.006G245500.1.D;Gorai.006G245500.2.D;Gorai.006G245500.4.D;Gorai.006G245500.3.D;Gorai.006G245500.5.D
Gene IDs: Gorai.006G245500
cotton name: K01303 - acylaminoacyl-peptidase [EC:3.4.19.1] (APEH) (1 of 1)
OG IDs: 93519464
Best-hit-arabi-ID: AT4G14570.1
Best-hit-arabi-name: acylaminoacyl-peptidase-related
Gorai.006G245500.1.D
PCC=0.066, p=0.787
mRNA Profile
Protein Expression Profile
DPA

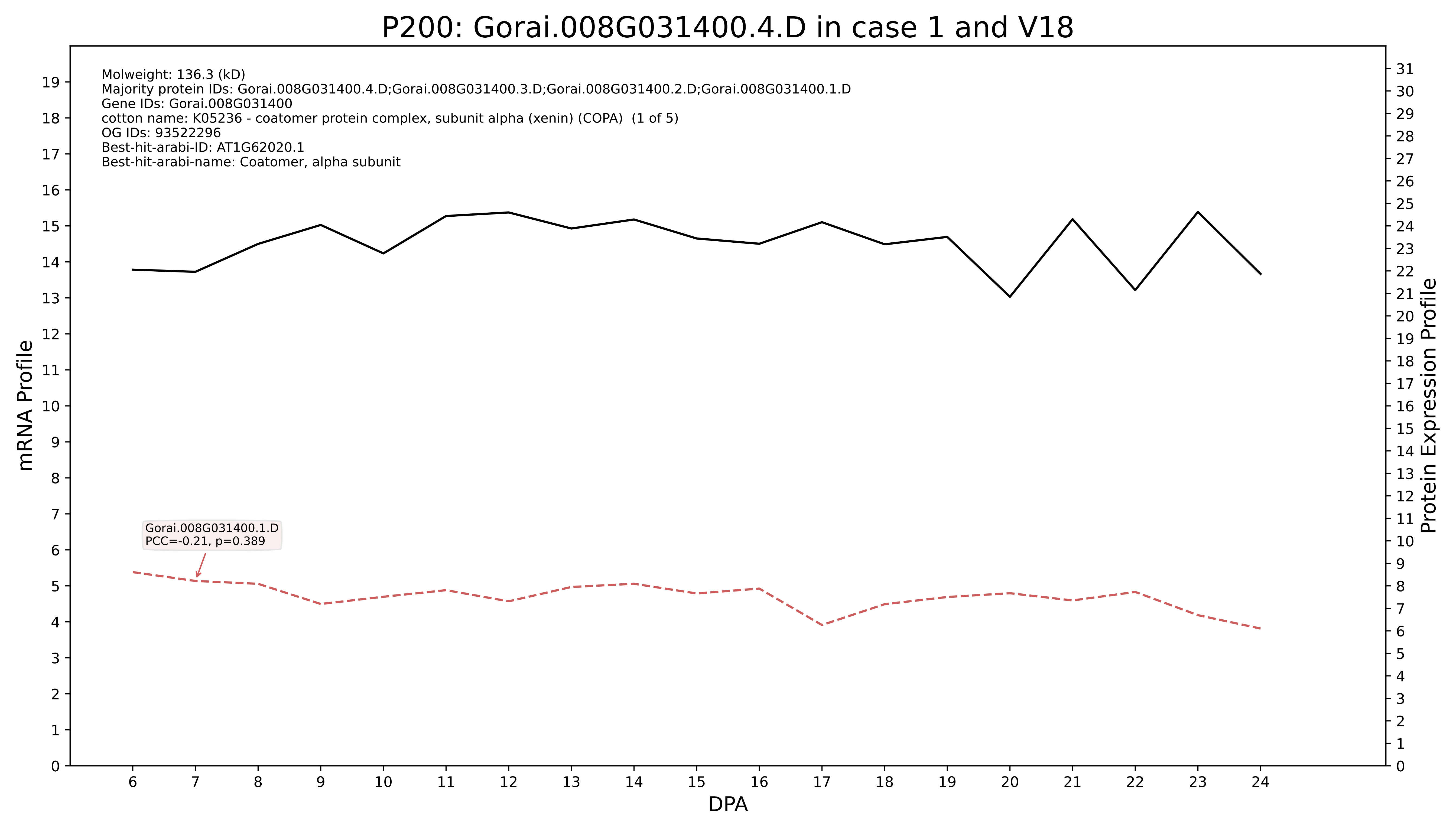
P200: Gorai.008G031400.4.D in case 1 and V18
Molweight: 136.3 (kD)
Majority protein IDs: Gorai.008G031400.4.D;Gorai.008G031400.3.D;Gorai.008G031400.2.D;Gorai.008G031400.1.D
Gene IDs: Gorai.008G031400
cotton name: K05236 - coatomer protein complex, subunit alpha (xenin) (COPA) (1 of 5)
OG IDs: 93522296
Best-hit-arabi-ID: AT1G62020.1
Best-hit-arabi-name: Coatomer, alpha subunit
Gorai.008G031400.1.D
PCC=-0.21, p=0.389
mRNA Profile
Protein Expression Profile
DPA

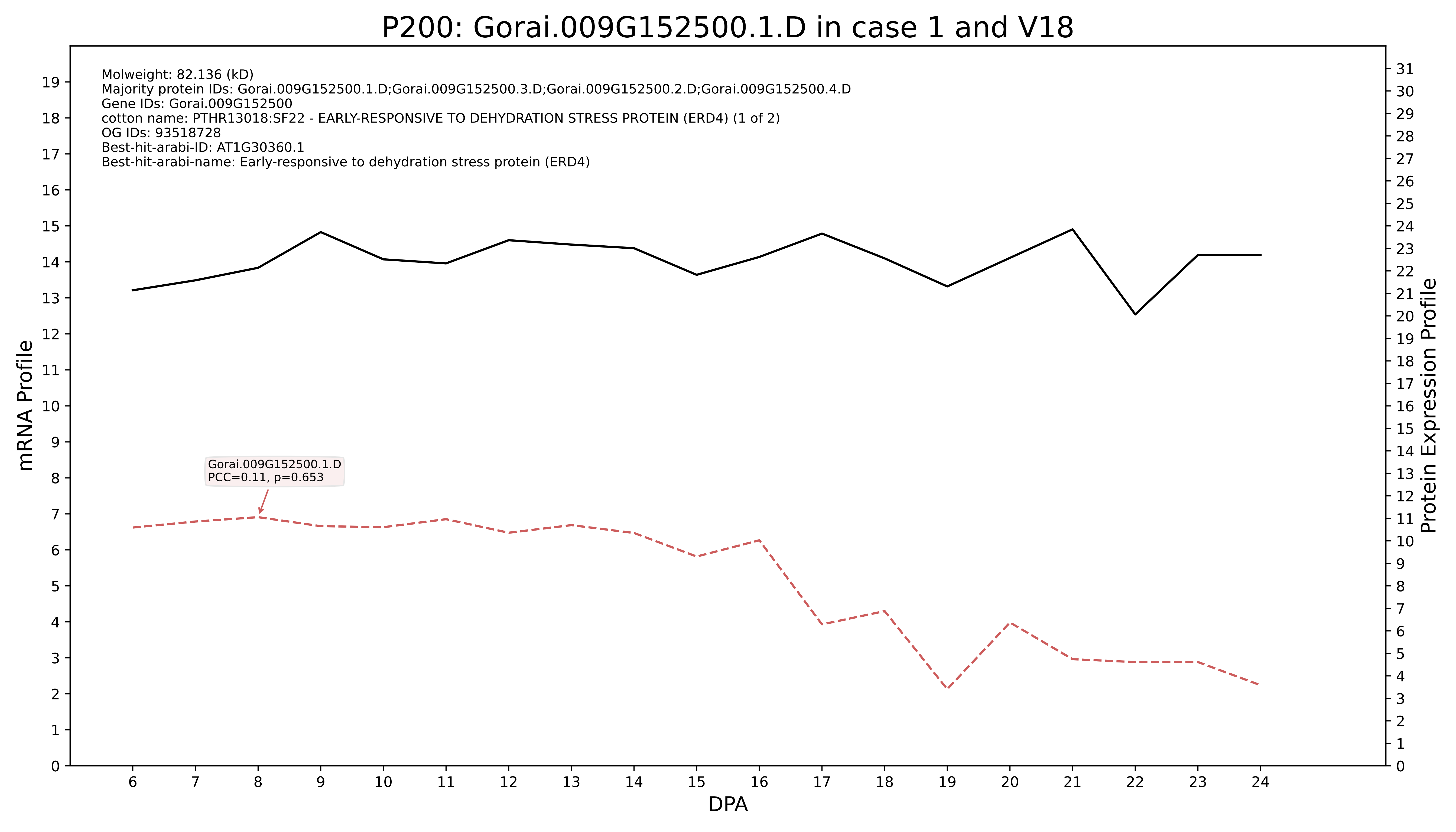
P200: Gorai.009G152500.1.D in case 1 and V18
Molweight: 82.136 (kD)
Majority protein IDs: Gorai.009G152500.1.D;Gorai.009G152500.3.D;Gorai.009G152500.2.D;Gorai.009G152500.4.D
Gene IDs: Gorai.009G152500
cotton name: PTHR13018:SF22 - EARLY-RESPONSIVE TO DEHYDRATION STRESS PROTEIN (ERD4) (1 of 2)
OG IDs: 93518728
Best-hit-arabi-ID: AT1G30360.1
Best-hit-arabi-name: Early-responsive to dehydration stress protein (ERD4)
Gorai.009G152500.1.D
PCC=0.11, p=0.653
mRNA Profile
Protein Expression Profile
DPA

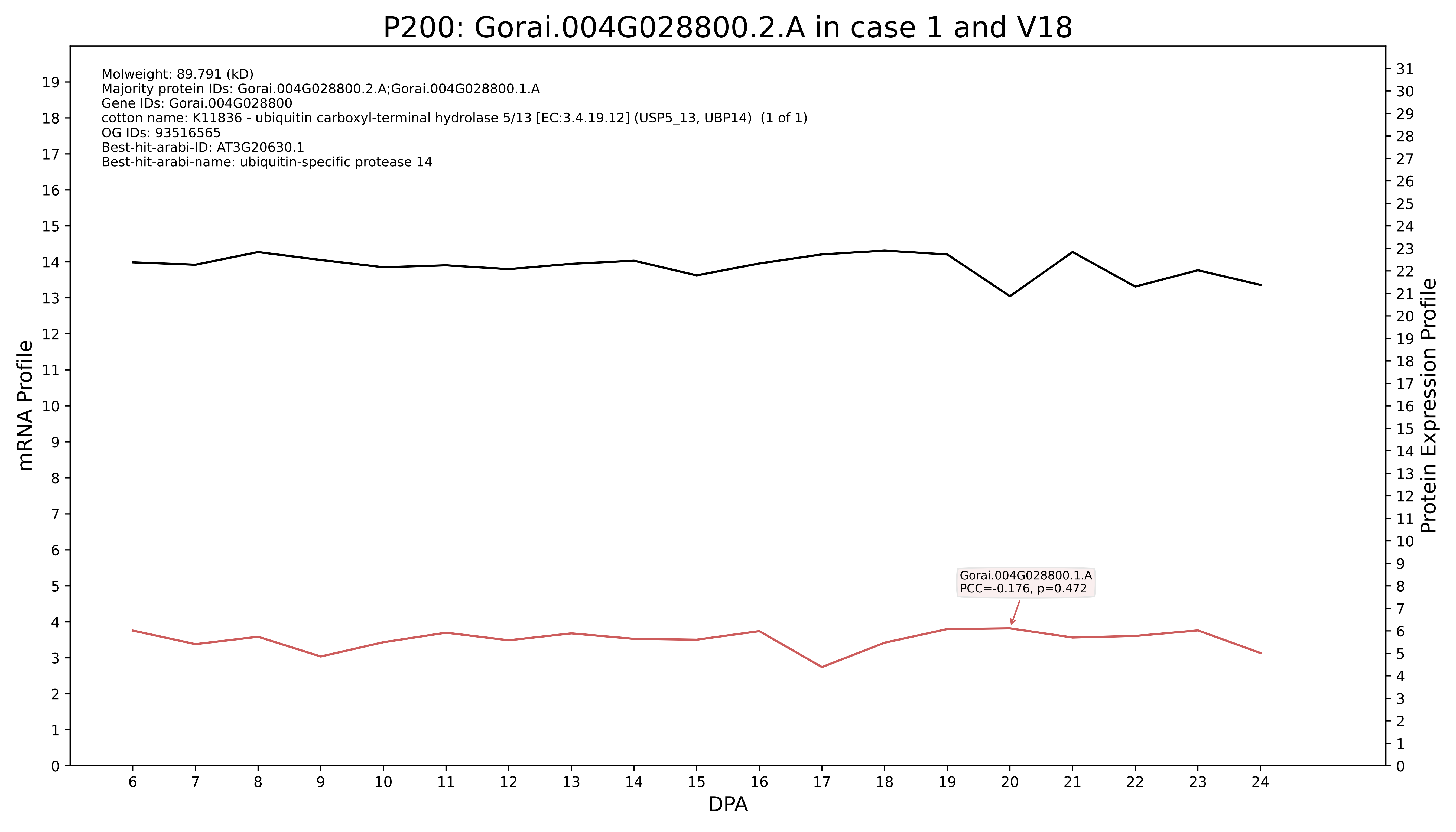
P200: Gorai.004G028800.2.A in case 1 and V18
Molweight: 89.791 (kD)
Majority protein IDs: Gorai.004G028800.2.A;Gorai.004G028800.1.A
Gene IDs: Gorai.004G028800
cotton name: K11836 - ubiquitin carboxyl-terminal hydrolase 5/13 [EC:3.4.19.12] (USP5_13, UBP14) (1 of 1)
OG IDs: 93516565
Best-hit-arabi-ID: AT3G20630.1
Best-hit-arabi-name: ubiquitin-specific protease 14
Gorai.004G028800.1.A
PCC=-0.176, p=0.472
mRNA Profile
Protein Expression Profile
DPA

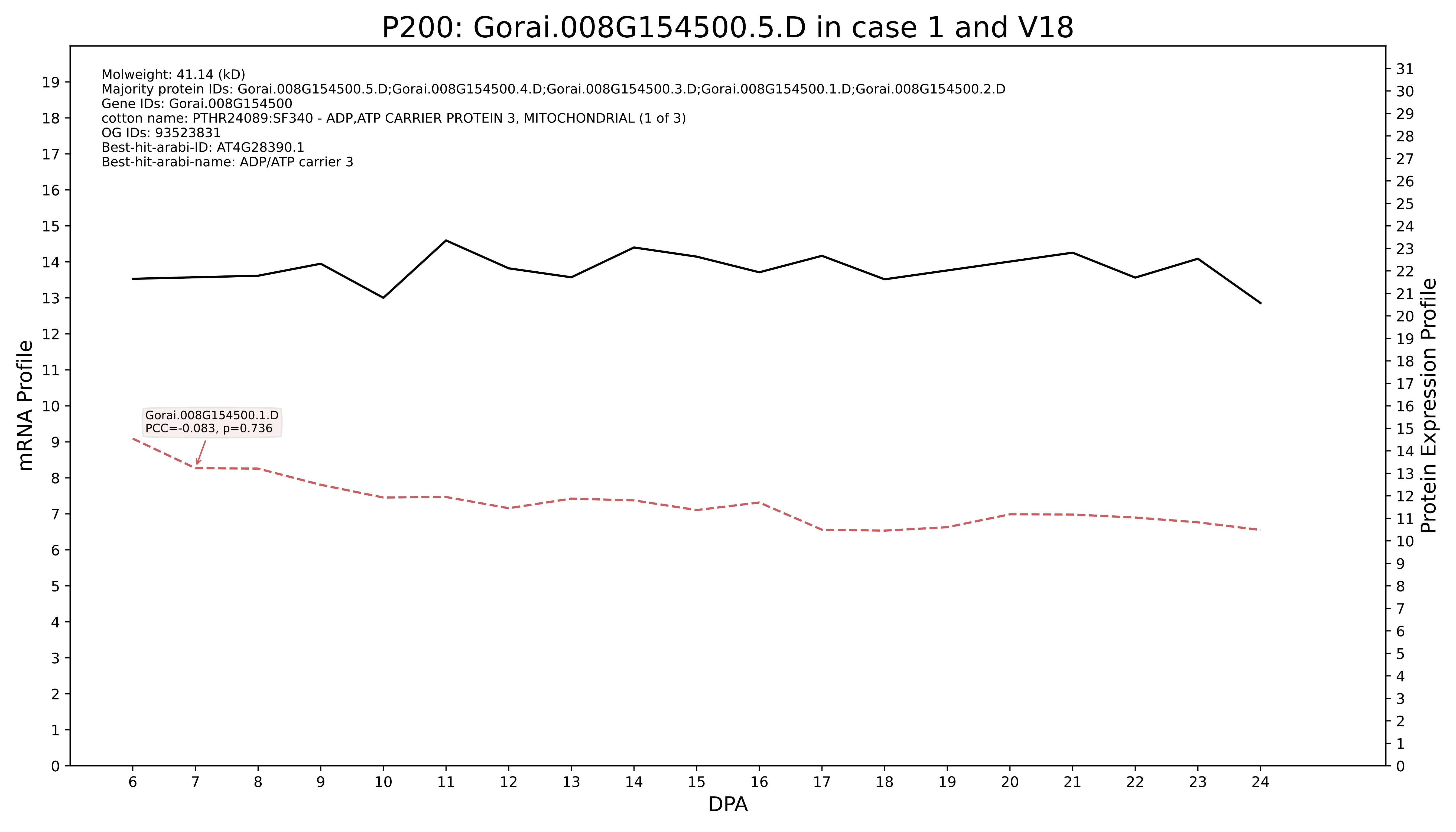

P200: Gorai.008G154500.5.D in case 1 and V18
Molweight: 41.14 (kD)
Majority protein IDs: Gorai.008G154500.5.D;Gorai.008G154500.4.D;Gorai.008G154500.3.D;Gorai.008G154500.1.D;Gorai.008G154500.2.D
Gene IDs: Gorai.008G154500
cotton name: PTHR24089:SF340 - ADP,ATP CARRIER PROTEIN 3, MITOCHONDRIAL (1 of 3)
OG IDs: 93523831
Best-hit-arabi-ID: AT4G28390.1
Best-hit-arabi-name: ADP/ATP carrier 3
Gorai.008G154500.1.D
PCC=-0.083, p=0.736
mRNA Profile
Protein Expression Profile
DPA

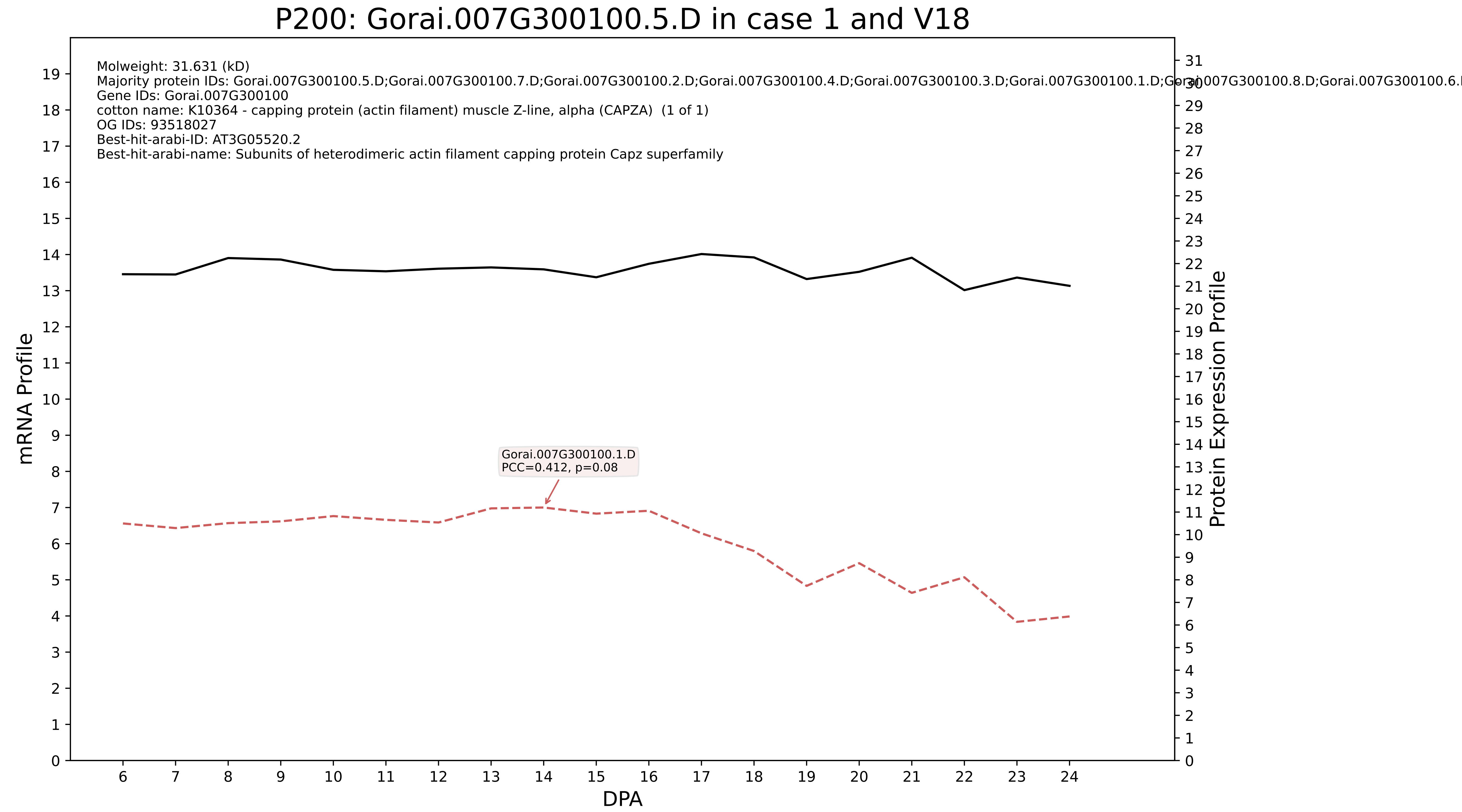

P200: Gorai.007G300100.5.D in case 1 and V18
Molweight: 31.631 (kD)
Majority protein IDs: Gorai.007G300100.5.D;Gorai.007G300100.7.D;Gorai.007G300100.2.D;Gorai.007G300100.4.D;Gorai.007G300100.3.D;Gorai.007G300100.1.D;Gorai.007G300100.8.D;Gorai.007G300100.6.
Gene IDs: Gorai.007G300100
cotton name: K10364 - capping protein (actin filament) muscle Z-line, alpha (CAPZA) (1 of 1)
OG IDs: 93518027
Best-hit-arabi-ID: AT3G05520.2
Best-hit-arabi-name: Subunits of heterodimeric actin filament capping protein Capz superfamily
Gorai.007G300100.1.D
PCC=0.412, p=0.08
mRNA Profile
Protein Expression Profile
DPA

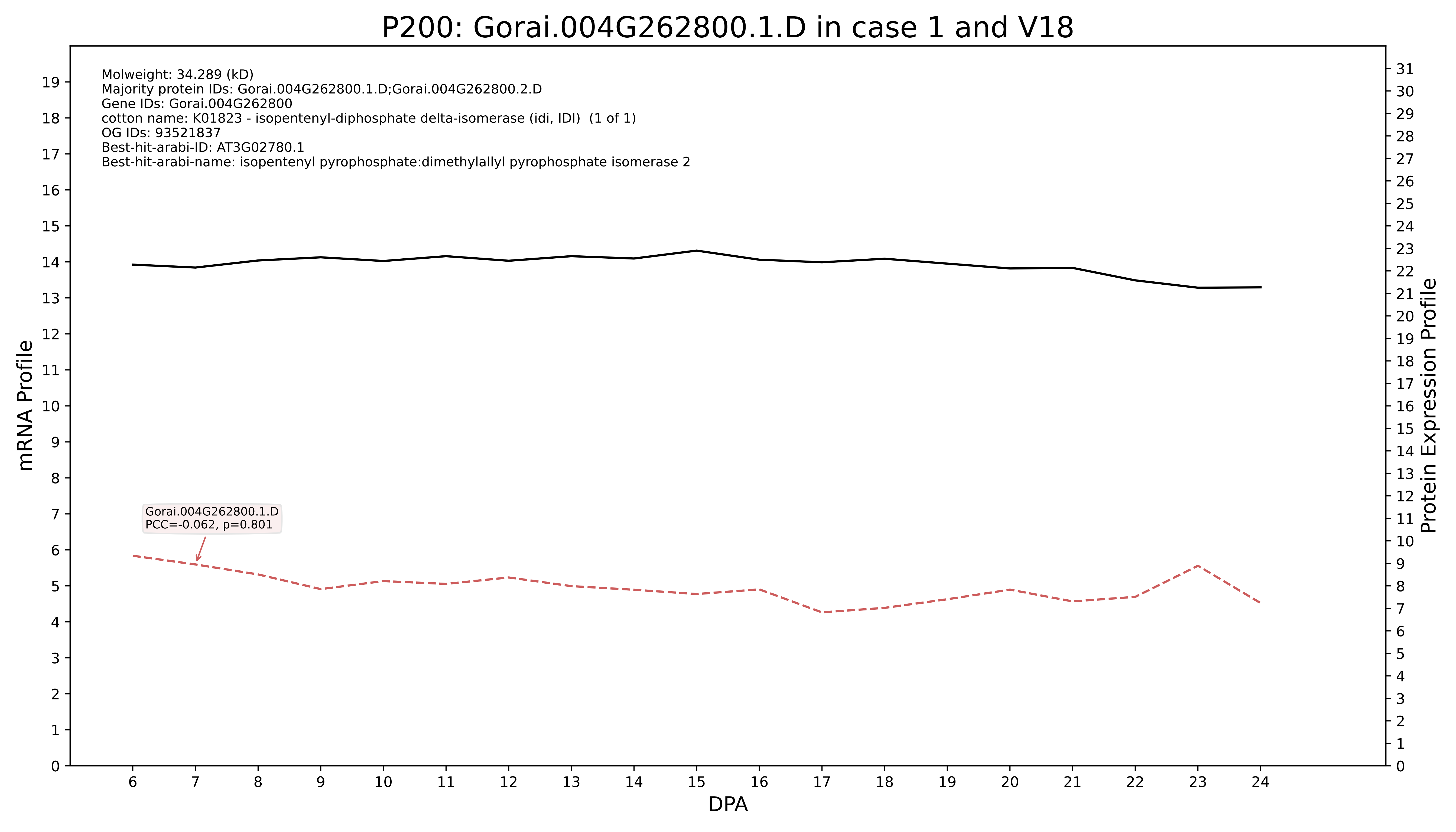
P200: Gorai.004G262800.1.D in case 1 and V18
Molweight: 34.289 (kD)
Majority protein IDs: Gorai.004G262800.1.D;Gorai.004G262800.2.D
Gene IDs: Gorai.004G262800
cotton name: K01823 - isopentenyl-diphosphate delta-isomerase (idi, IDI) (1 of 1)
OG IDs: 93521837
Best-hit-arabi-ID: AT3G02780.1
Best-hit-arabi-name: isopentenyl pyrophosphate:dimethylallyl pyrophosphate isomerase 2
Gorai.004G262800.1.D
PCC=-0.062, p=0.801
mRNA Profile
Protein Expression Profile
DPA

P200: Gorai.001G154000.3.A in case 1 and V18

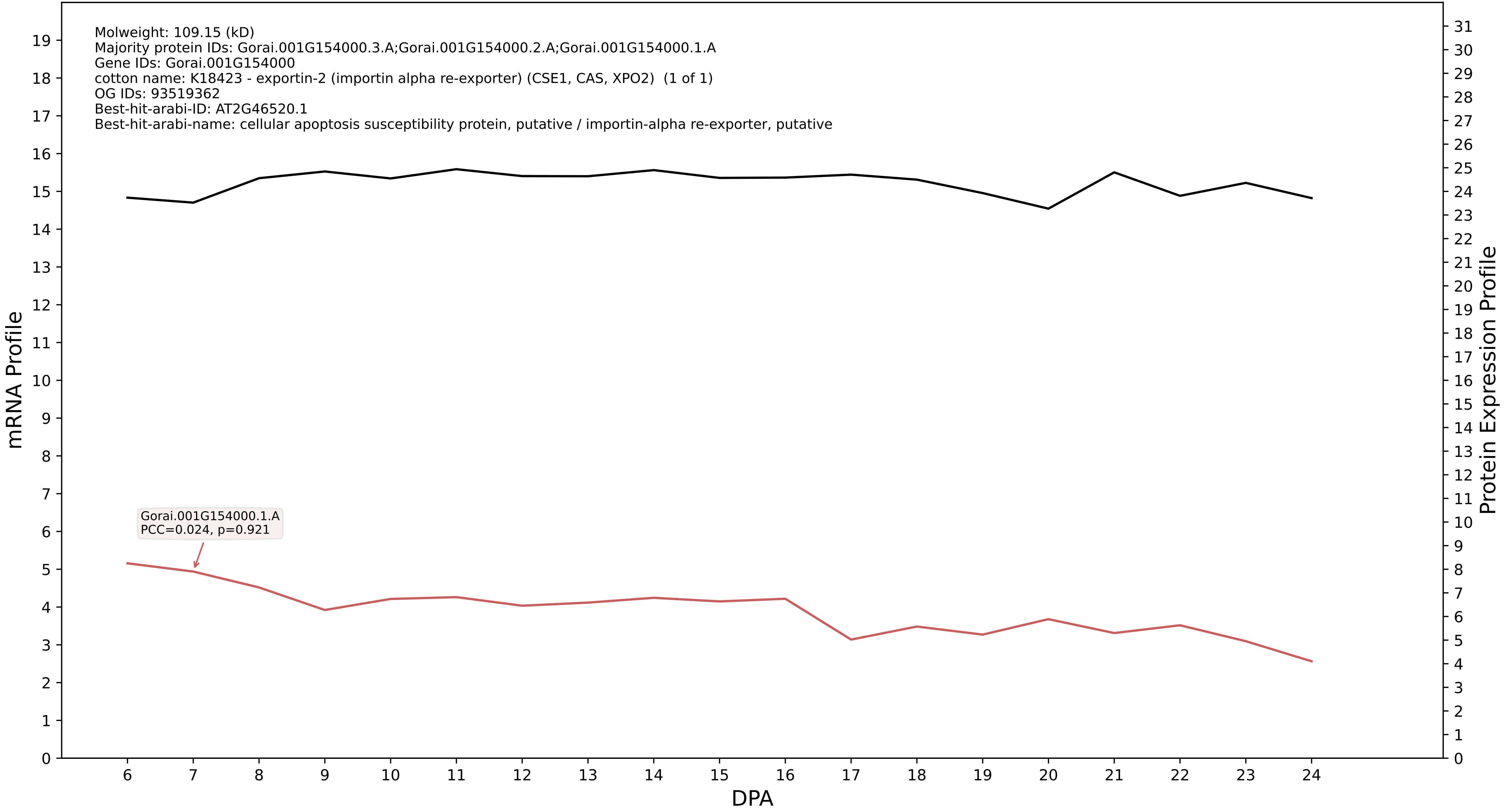

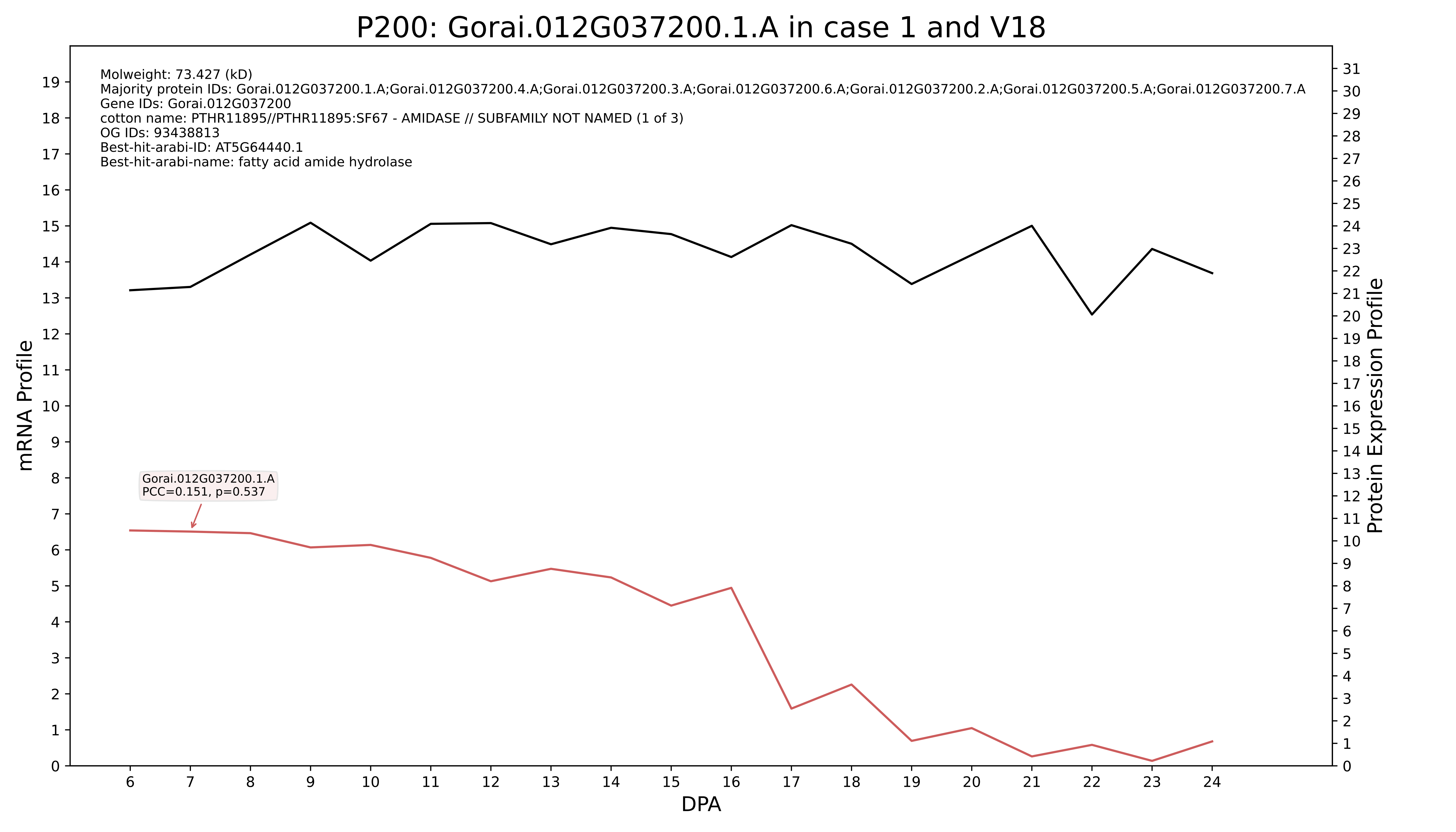
P200: Gorai.012G037200.1.A in case 1 and V18
Molweight: 73.427 (kD)
Majority protein IDs: Gorai.012G037200.1.A;Gorai.012G037200.4.A;Gorai.012G037200.3.A;Gorai.012G037200.6.A;Gorai.012G037200.2.A;Gorai.012G037200.5.A;Gorai.012G037200.7.A
Gene IDs: Gorai.012G037200
cotton name: PTHR11895//PTHR11895:SF67 - AMIDASE // SUBFAMILY NOT NAMED (1 of 3)
OG IDs: 93438813
Best-hit-arabi-ID: AT5G64440.1
Best-hit-arabi-name: fatty acid amide hydrolase
Gorai.012G037200.1.A
PCC=0.151, p=0.537
mRNA Profile
Protein Expression Profile
DPA

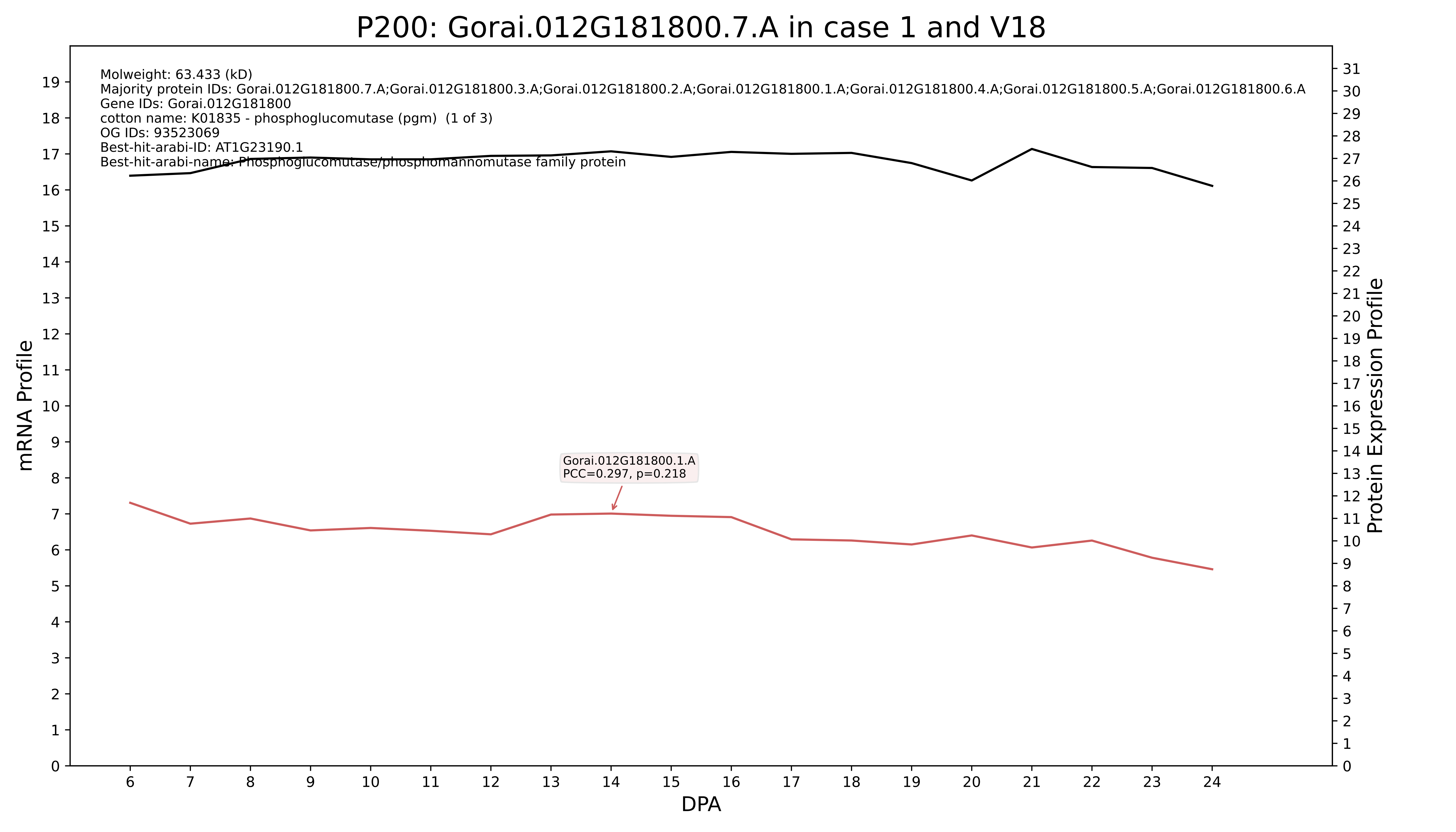

P200: Gorai.012G181800.7.A in case 1 and V18
Molweight: 63.433 (kD)
Majority protein IDs: Gorai.012G181800.7.A;Gorai.012G181800.3.A;Gorai.012G181800.2.A;Gorai.012G181800.1.A;Gorai.012G181800.4.A;Gorai.012G181800.5.A;Gorai.012G181800.6.A
Gene IDs: Gorai.012G181800
cotton name: K01835 - phosphoglucomutase (pgm) (1 of 3)
OG IDs: 93523069
Best-hit-arabi-ID: AT1G23190.1
Best-hit-arabi-name: Phosphoglucomutase/phosphomannomutase family protein
Gorai.012G181800.1.A
PCC=0.297, p=0.218
mRNA Profile
Protein Expression Profile
DPA

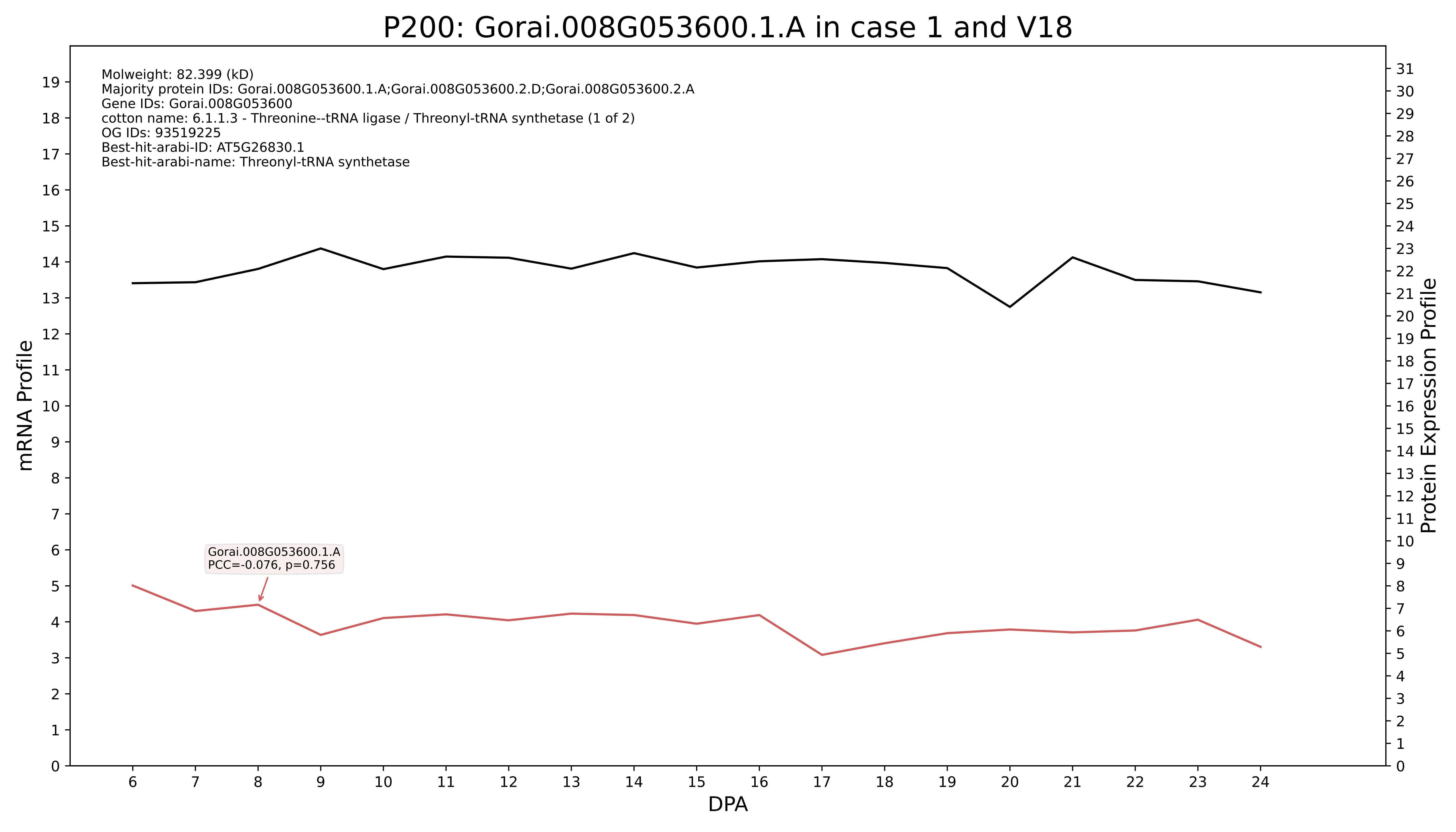

P200: Gorai.008G053600.1.A in case 1 and V18
Molweight: 82.399 (kD)
Majority protein IDs: Gorai.008G053600.1.A;Gorai.008G053600.2.D;Gorai.008G053600.2.A
Gene IDs: Gorai.008G053600
cotton name: 6.1.1.3 - Threonine--tRNA ligase / Threonyl-tRNA synthetase (1 of 2)
OG IDs: 93519225
Best-hit-arabi-ID: AT5G26830.1
Best-hit-arabi-name: Threonyl-tRNA synthetase
Gorai.008G053600.1.A
PCC=-0.076, p=0.756
mRNA Profile
Protein Expression Profile
DPA

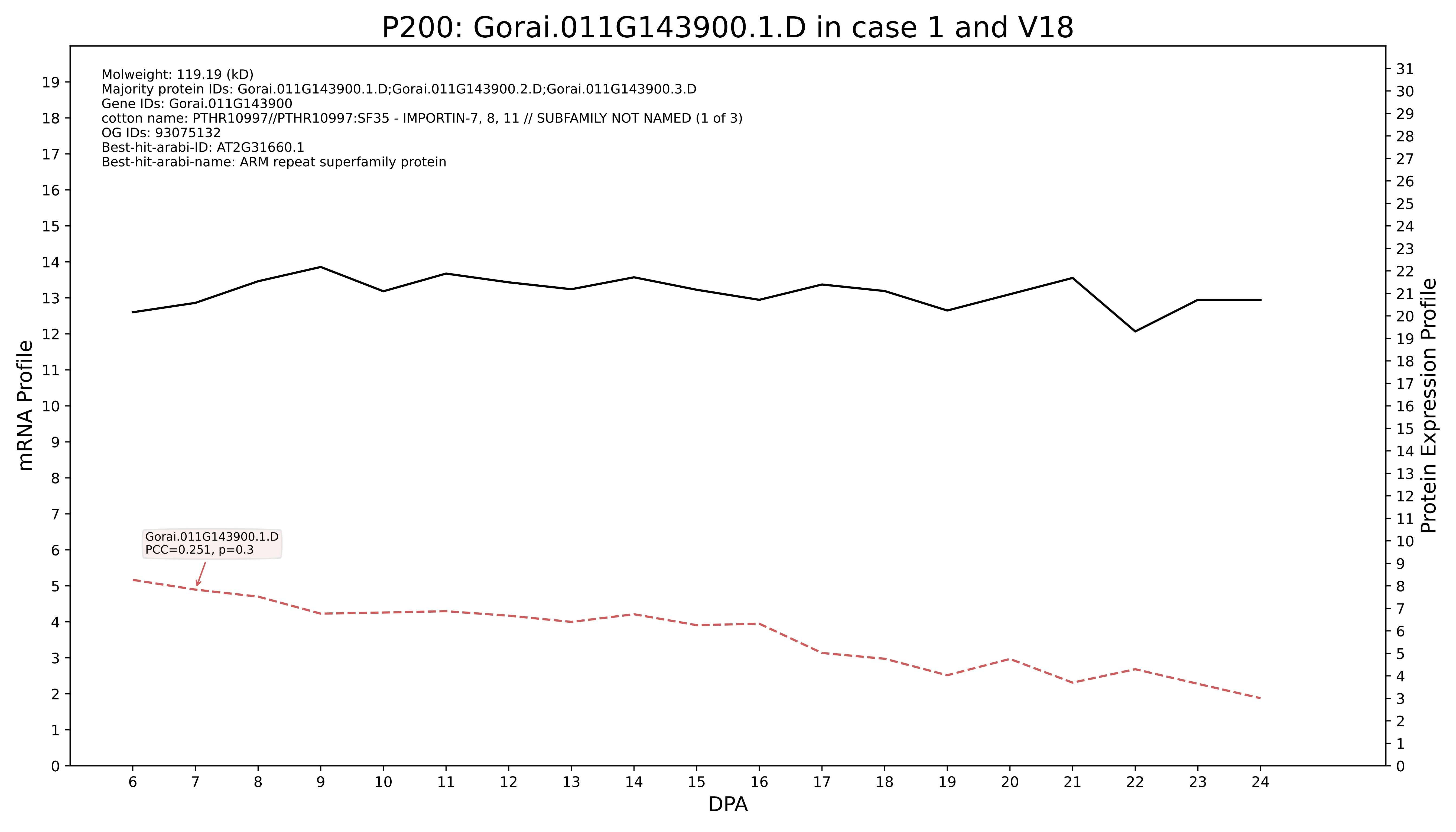

P200: Gorai.011G143900.1.D in case 1 and V18
Molweight: 119.19 (kD)
Majority protein IDs: Gorai.011G143900.1.D;Gorai.011G143900.2.D;Gorai.011G143900.3.D
Gene IDs: Gorai.011G143900
cotton name: PTHR10997//PTHR10997:SF35 - IMPORTIN-7, 8, 11 // SUBFAMILY NOT NAMED (1 of 3)
OG IDs: 93075132
Best-hit-arabi-ID: AT2G31660.1
Best-hit-arabi-name: ARM repeat superfamily protein
Gorai.011G143900.1.D
PCC=0.251, p=0.3
mRNA Profile
Protein Expression Profile
DPA

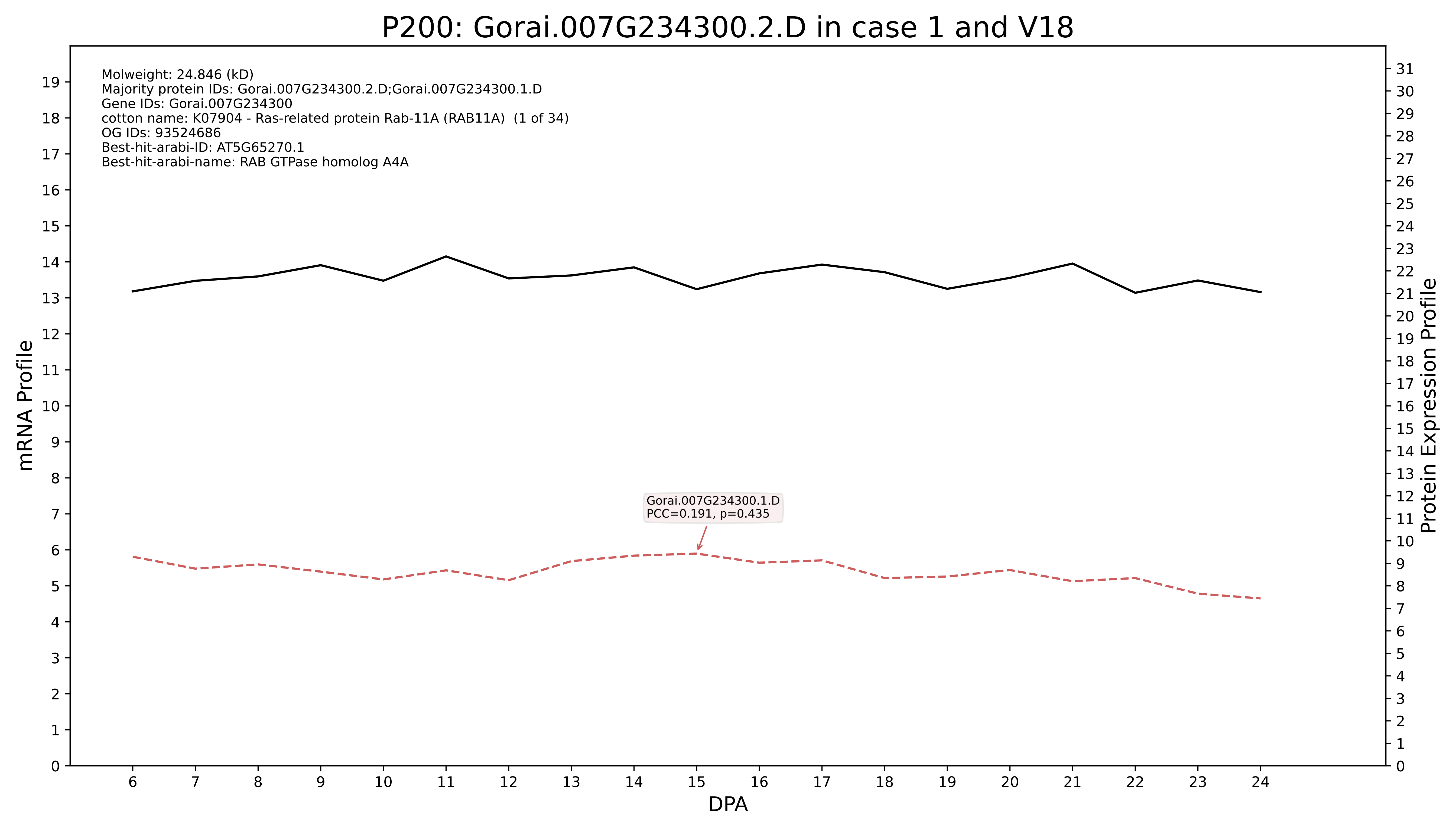

P200: Gorai.007G234300.2.D in case 1 and V18
Molweight: 24.846 (kD)
Majority protein IDs: Gorai.007G234300.2.D;Gorai.007G234300.1.D
Gene IDs: Gorai.007G234300
cotton name: K07904 - Ras-related protein Rab-11A (RAB11A) (1 of 34)
OG IDs: 93524686
Best-hit-arabi-ID: AT5G65270.1
Best-hit-arabi-name: RAB GTPase homolog A4A
Gorai.007G234300.1.D
PCC=0.191, p=0.435
mRNA Profile
Protein Expression Profile
DPA

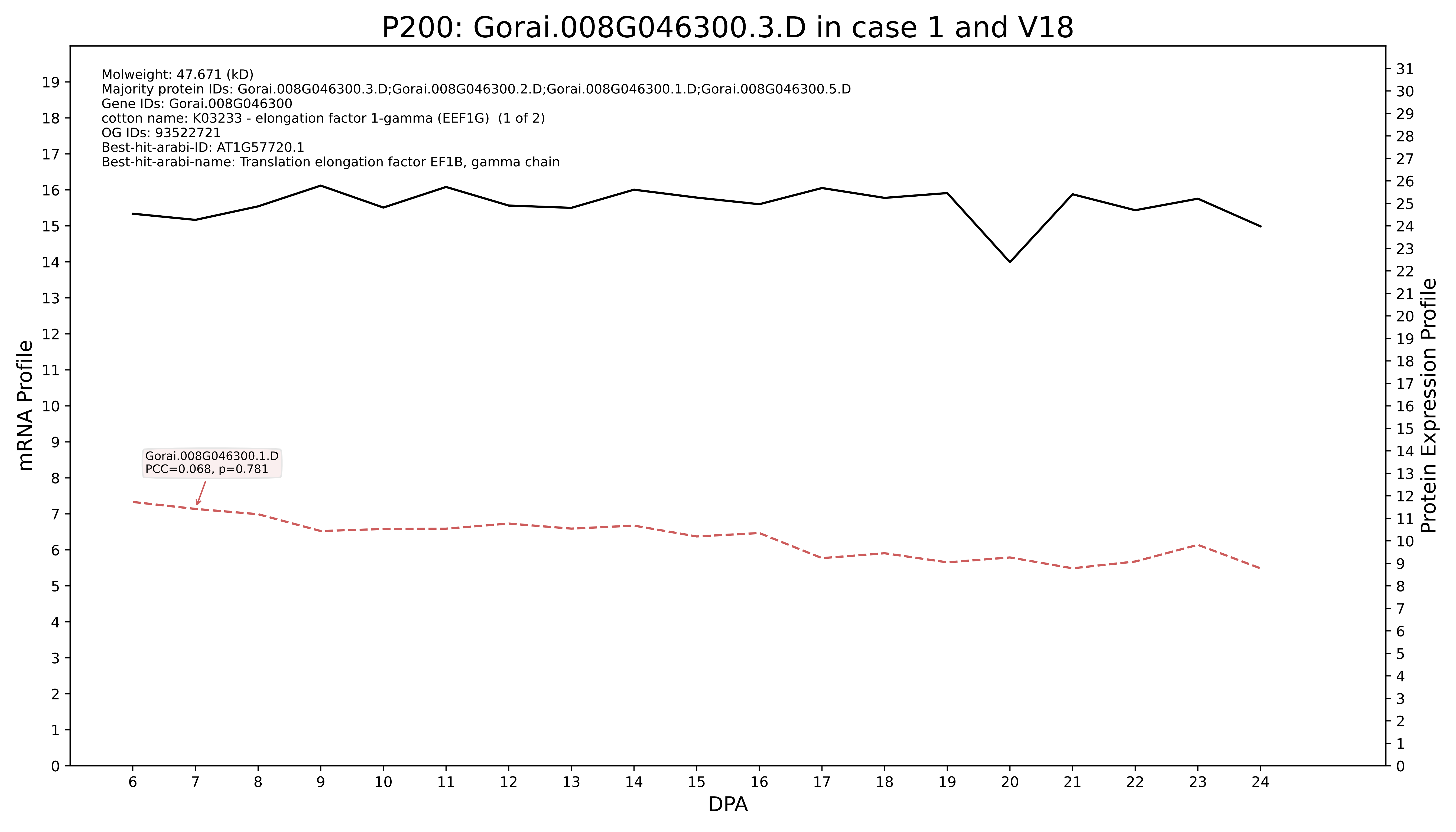

P200: Gorai.008G046300.3.D in case 1 and V18
Molweight: 47.671 (kD)
Majority protein IDs: Gorai.008G046300.3.D;Gorai.008G046300.2.D;Gorai.008G046300.1.D;Gorai.008G046300.5.D
Gene IDs: Gorai.008G046300
cotton name: K03233 - elongation factor 1-gamma (EEF1G) (1 of 2)
OG IDs: 93522721
Best-hit-arabi-ID: AT1G57720.1
Best-hit-arabi-name: Translation elongation factor EF1B, gamma chain
Gorai.008G046300.1.D
PCC=0.068, p=0.781
mRNA Profile
Protein Expression Profile
DPA

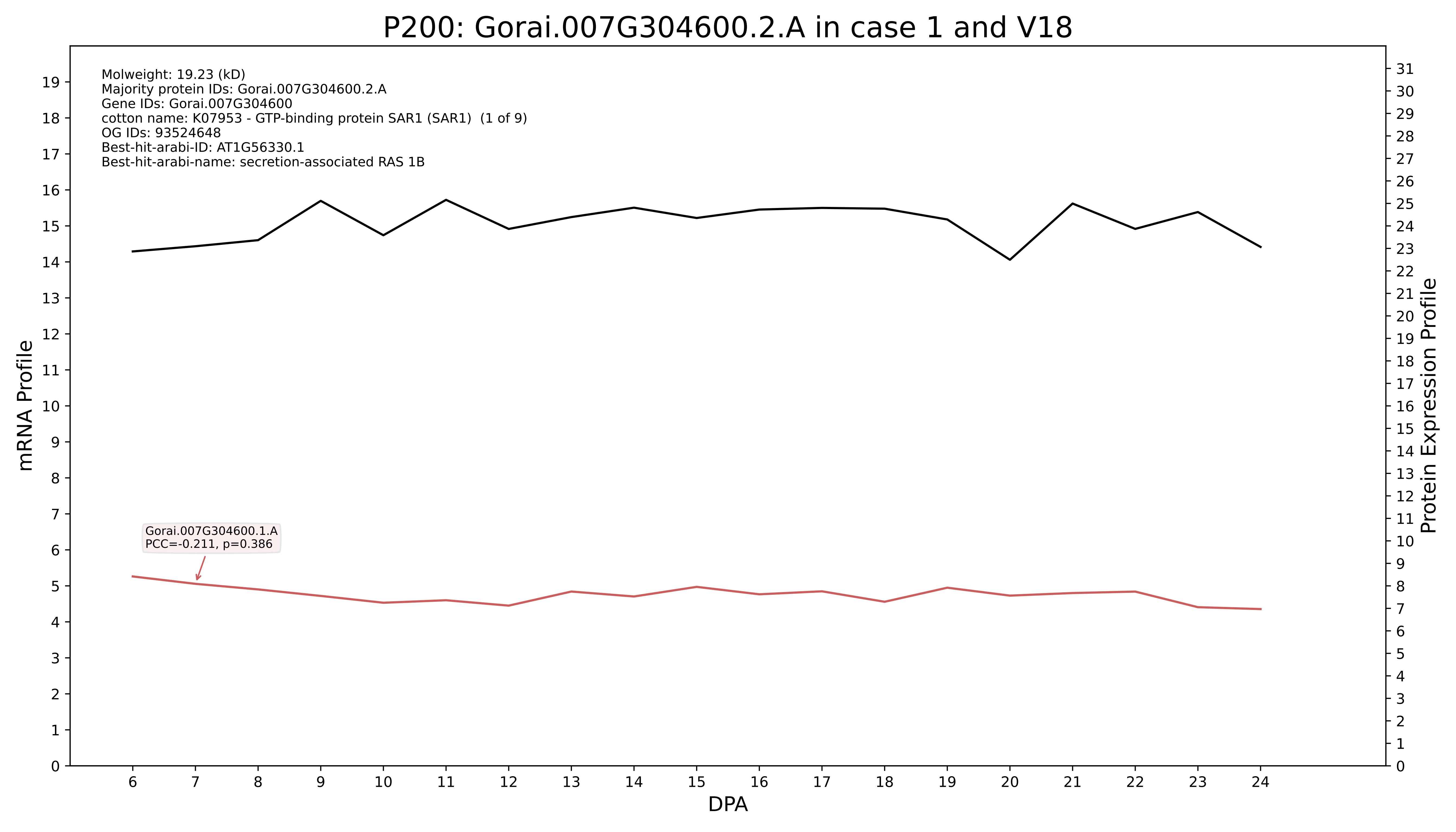
P200: Gorai.007G304600.2.A in case 1 and V18
Molweight: 19.23 (kD)
Majority protein IDs: Gorai.007G304600.2.A
Gene IDs: Gorai.007G304600
cotton name: K07953 - GTP-binding protein SAR1 (SAR1) (1 of 9)
OG IDs: 93524648
Best-hit-arabi-ID: AT1G56330.1
Best-hit-arabi-name: secretion-associated RAS 1B
Gorai.007G304600.1.A
PCC=-0.211, p=0.386
mRNA Profile
Protein Expression Profile
DPA

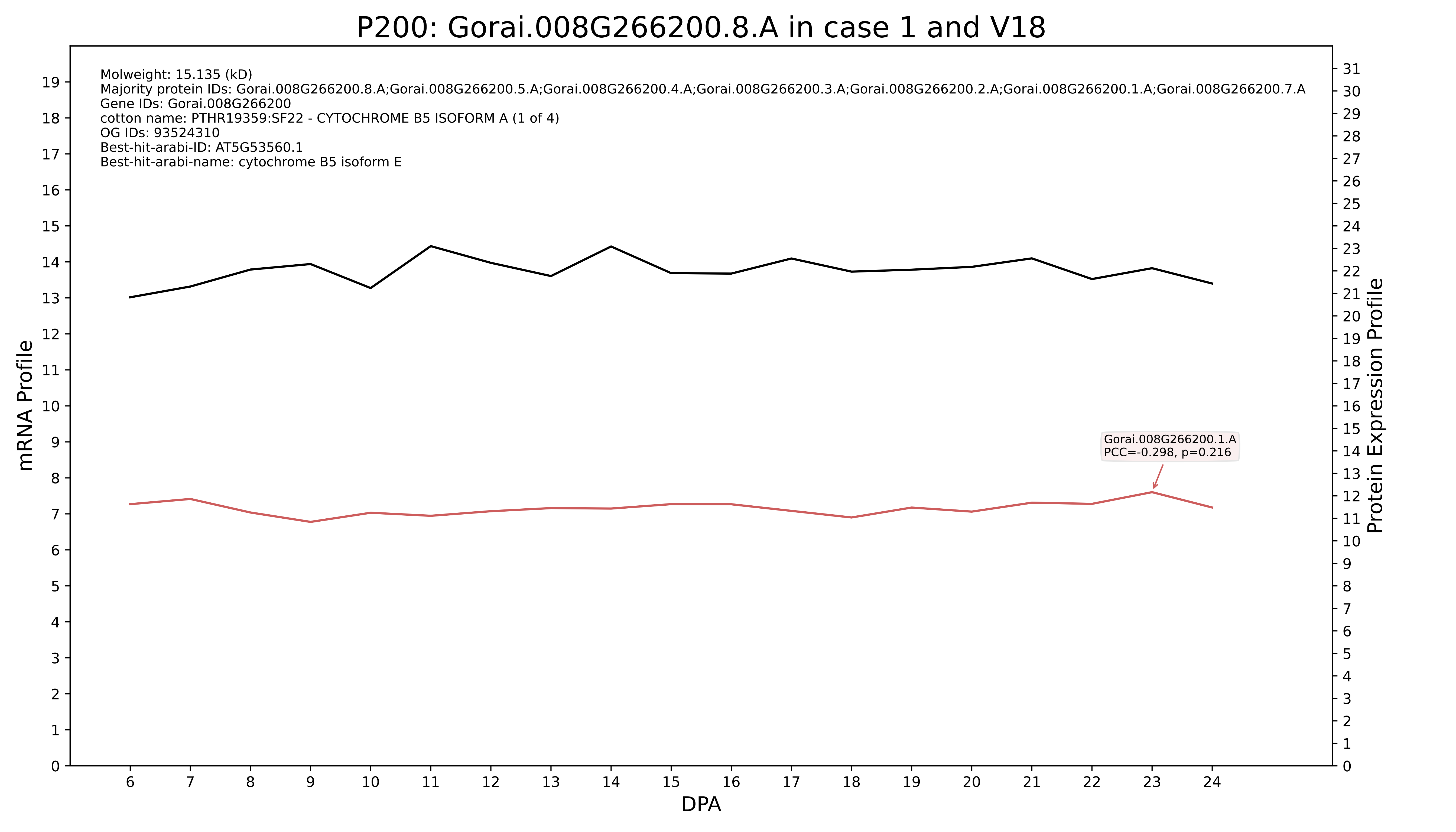

P200: Gorai.008G266200.8.A in case 1 and V18
Molweight: 15.135 (kD)
Majority protein IDs: Gorai.008G266200.8.A;Gorai.008G266200.5.A;Gorai.008G266200.4.A;Gorai.008G266200.3.A;Gorai.008G266200.2.A;Gorai.008G266200.1.A;Gorai.008G266200.7.A
Gene IDs: Gorai.008G266200
cotton name: PTHR19359:SF22 - CYTOCHROME B5 ISOFORM A (1 of 4)
OG IDs: 93524310
Best-hit-arabi-ID: AT5G53560.1
Best-hit-arabi-name: cytochrome B5 isoform E
Gorai.008G266200.1.A
PCC=-0.298, p=0.216
mRNA Profile
Protein Expression Profile
DPA

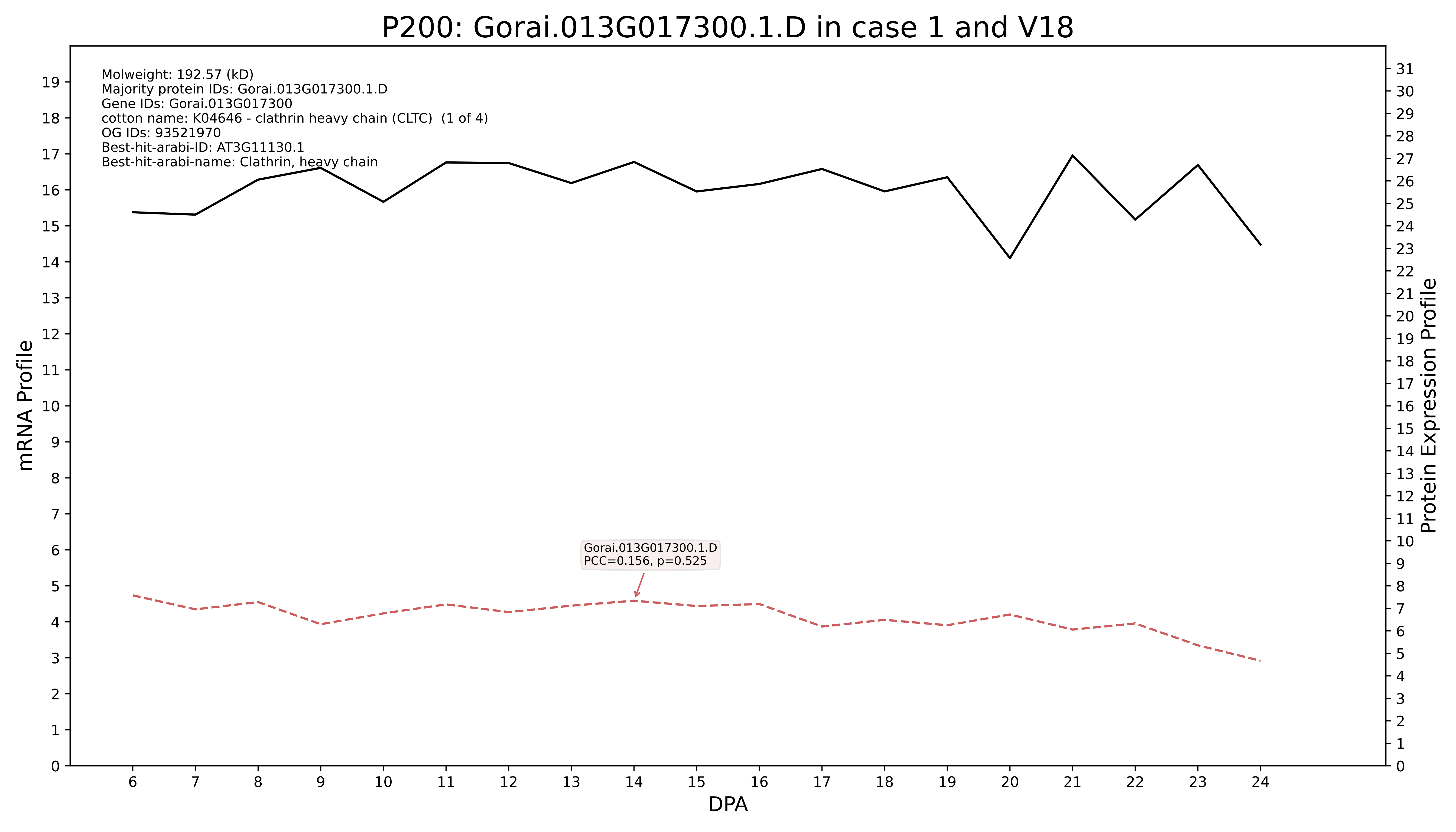
P200: Gorai.013G017300.1.D in case 1 and V18
Molweight: 192.57 (kD)
Majority protein IDs: Gorai.013G017300.1.D
Gene IDs: Gorai.013G017300
cotton name: K04646 - clathrin heavy chain (CLTC) (1 of 4)
OG IDs: 93521970
Best-hit-arabi-ID: AT3G11130.1
Best-hit-arabi-name: Clathrin, heavy chain
Gorai.013G017300.1.D
PCC=0.156, p=0.525
mRNA Profile
Protein Expression Profile
DPA
0
1
2
3
4
5
6
7
8
9
10
11
12
13
14
15
16
17
18
19
20
21
22
23
24
25
26
27
28
29
30
31

P200: Gorai.007G090400.1.D in case 1 and V18

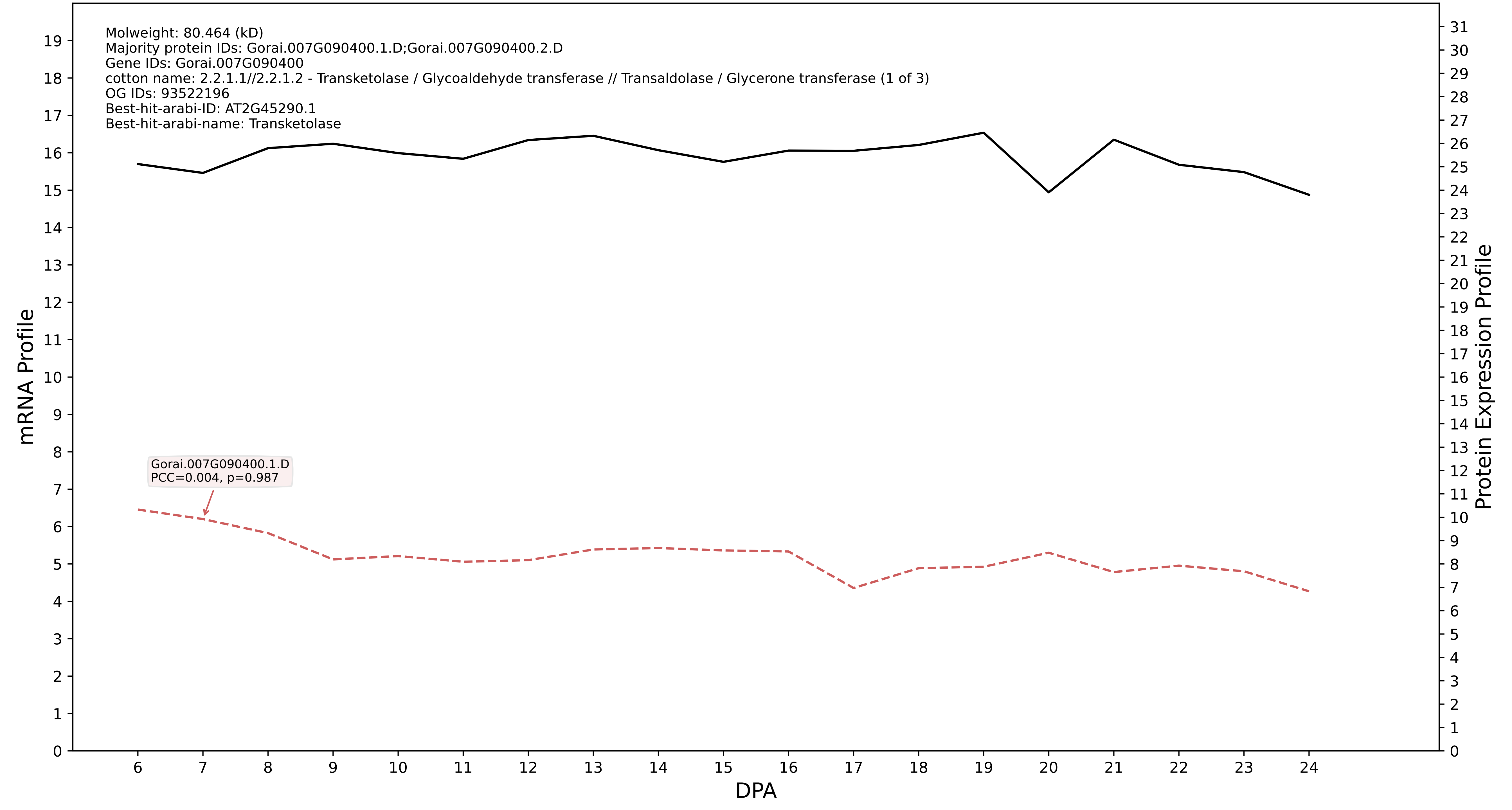

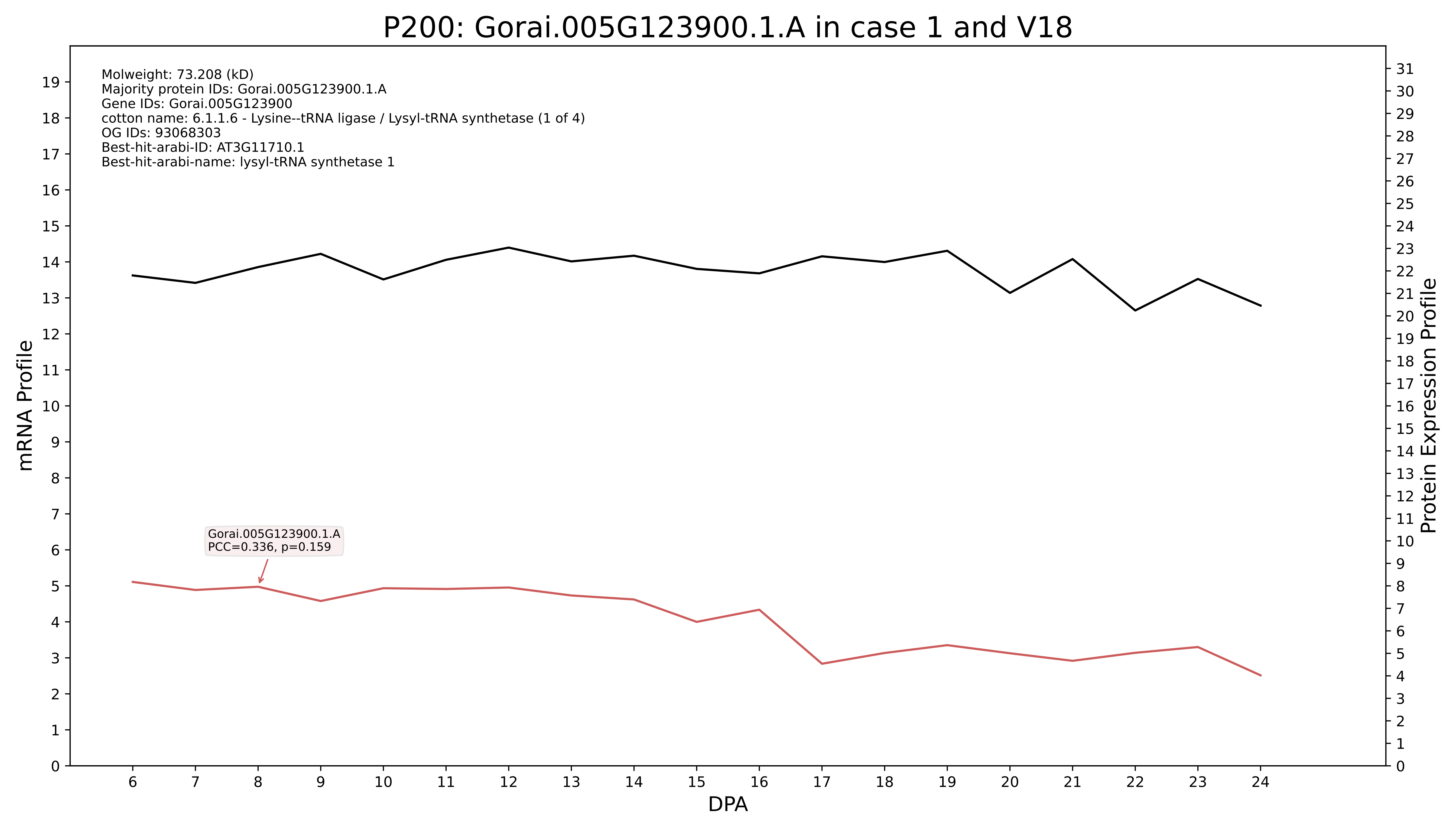
P200: Gorai.005G123900.1.A in case 1 and V18
Molweight: 73.208 (kD)
Majority protein IDs: Gorai.005G123900.1.A
Gene IDs: Gorai.005G123900
cotton name: 6.1.1.6 - Lysine--tRNA ligase / Lysyl-tRNA synthetase (1 of 4)
OG IDs: 93068303
Best-hit-arabi-ID: AT3G11710.1
Best-hit-arabi-name: lysyl-tRNA synthetase 1
Gorai.005G123900.1.A
PCC=0.336, p=0.159
mRNA Profile
Protein Expression Profile
DPA

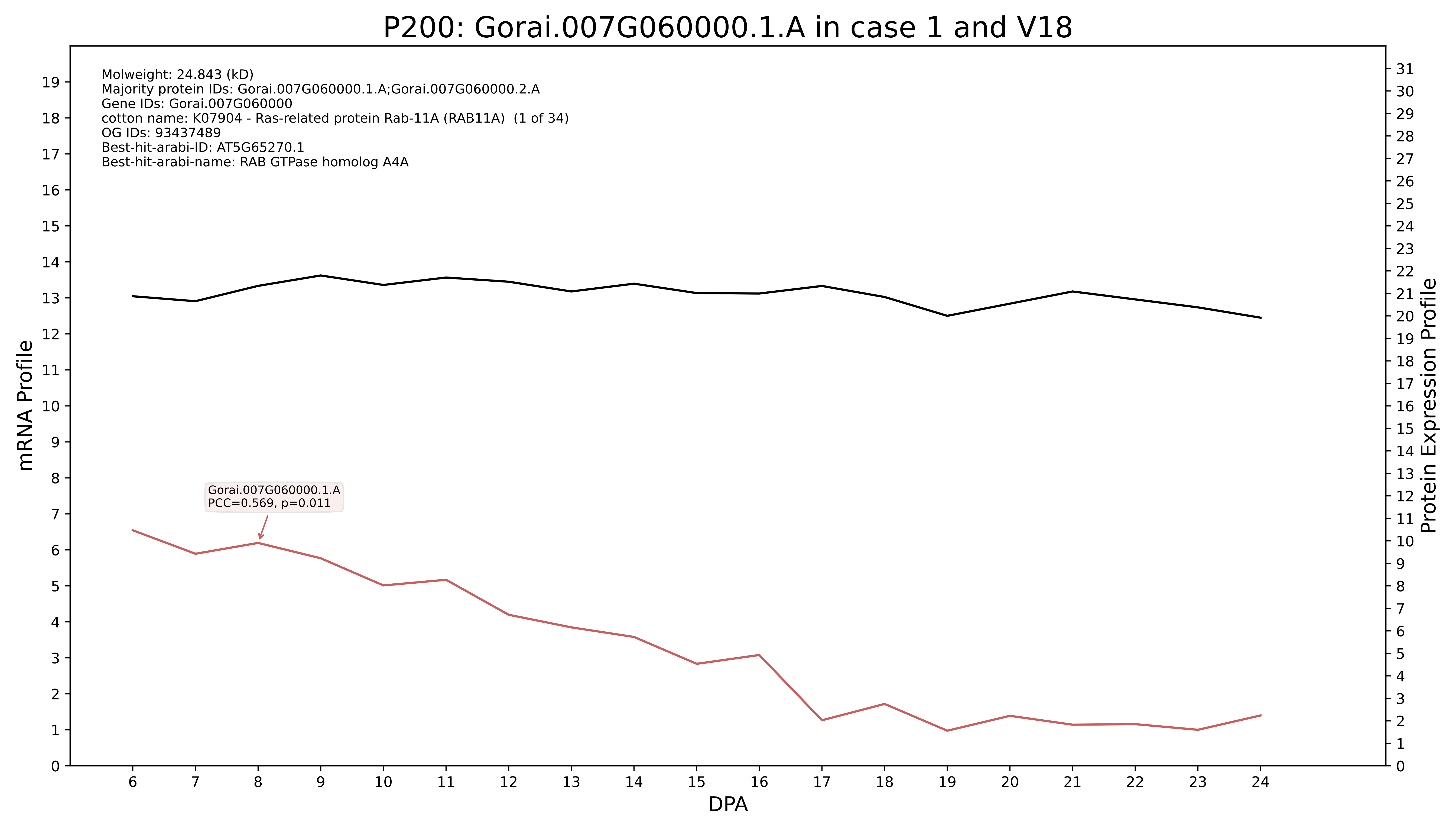
P200: Gorai.007G060000.1.A in case 1 and V18
Molweight: 24.843 (kD)
Majority protein IDs: Gorai.007G060000.1.A;Gorai.007G060000.2.A
Gene IDs: Gorai.007G060000
cotton name: K07904 - Ras-related protein Rab-11A (RAB11A) (1 of 34)
OG IDs: 93437489
Best-hit-arabi-ID: AT5G65270.1
Best-hit-arabi-name: RAB GTPase homolog A4A
Gorai.007G060000.1.A
PCC=0.569, p=0.011
mRNA Profile
Protein Expression Profile
DPA
0
1
2
3
4
5
6
7
8
9
10
11
12
13
14
15
16
17
18
19
20
21
22
23
24
25
26
27
28
29
30
31

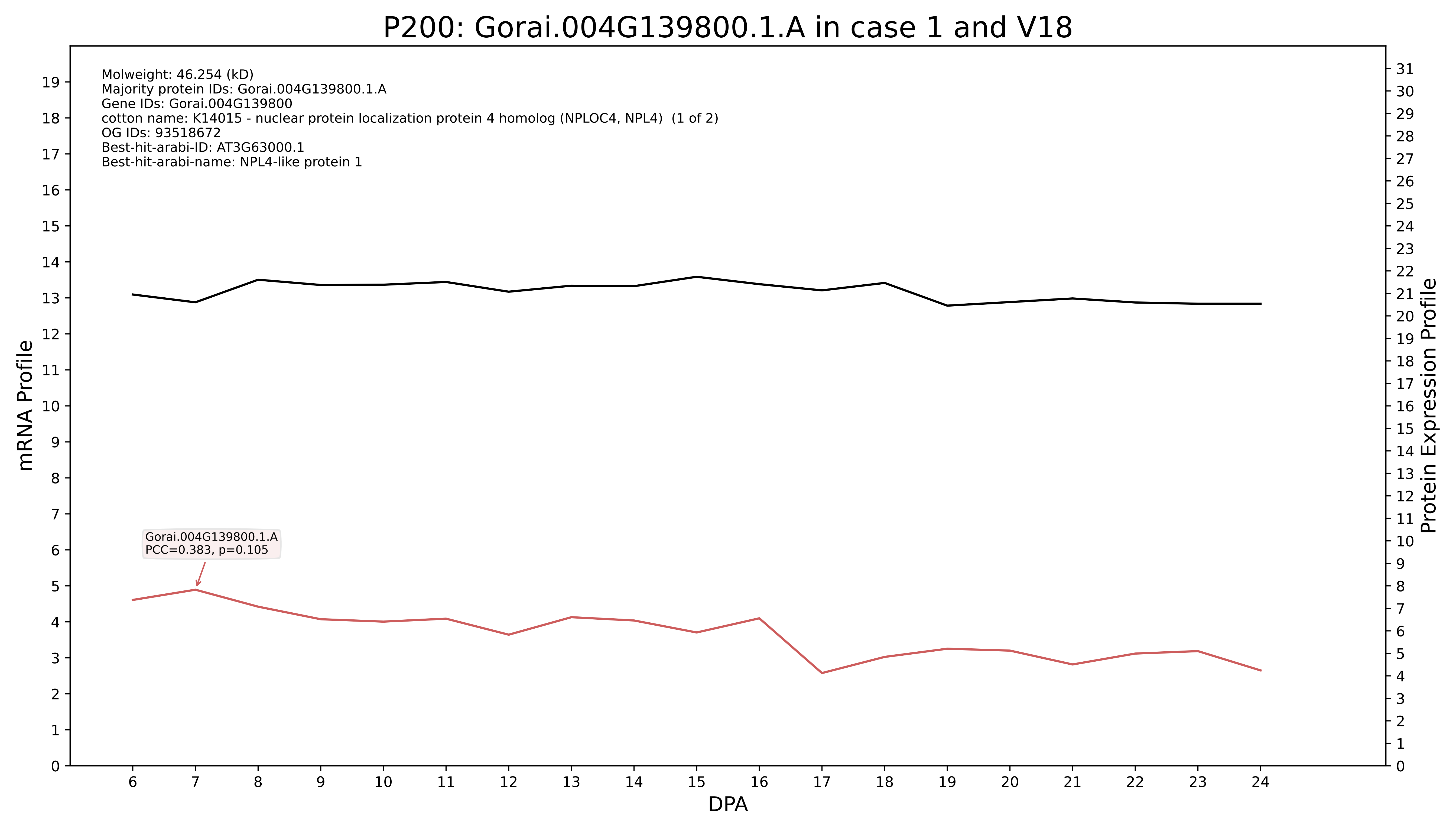
P200: Gorai.004G139800.1.A in case 1 and V18
Molweight: 46.254 (kD)
Majority protein IDs: Gorai.004G139800.1.A
Gene IDs: Gorai.004G139800
cotton name: K14015 - nuclear protein localization protein 4 homolog (NPLOC4, NPL4) (1 of 2)
OG IDs: 93518672
Best-hit-arabi-ID: AT3G63000.1
Best-hit-arabi-name: NPL4-like protein 1
Gorai.004G139800.1.A
PCC=0.383, p=0.105
mRNA Profile
Protein Expression Profile
DPA

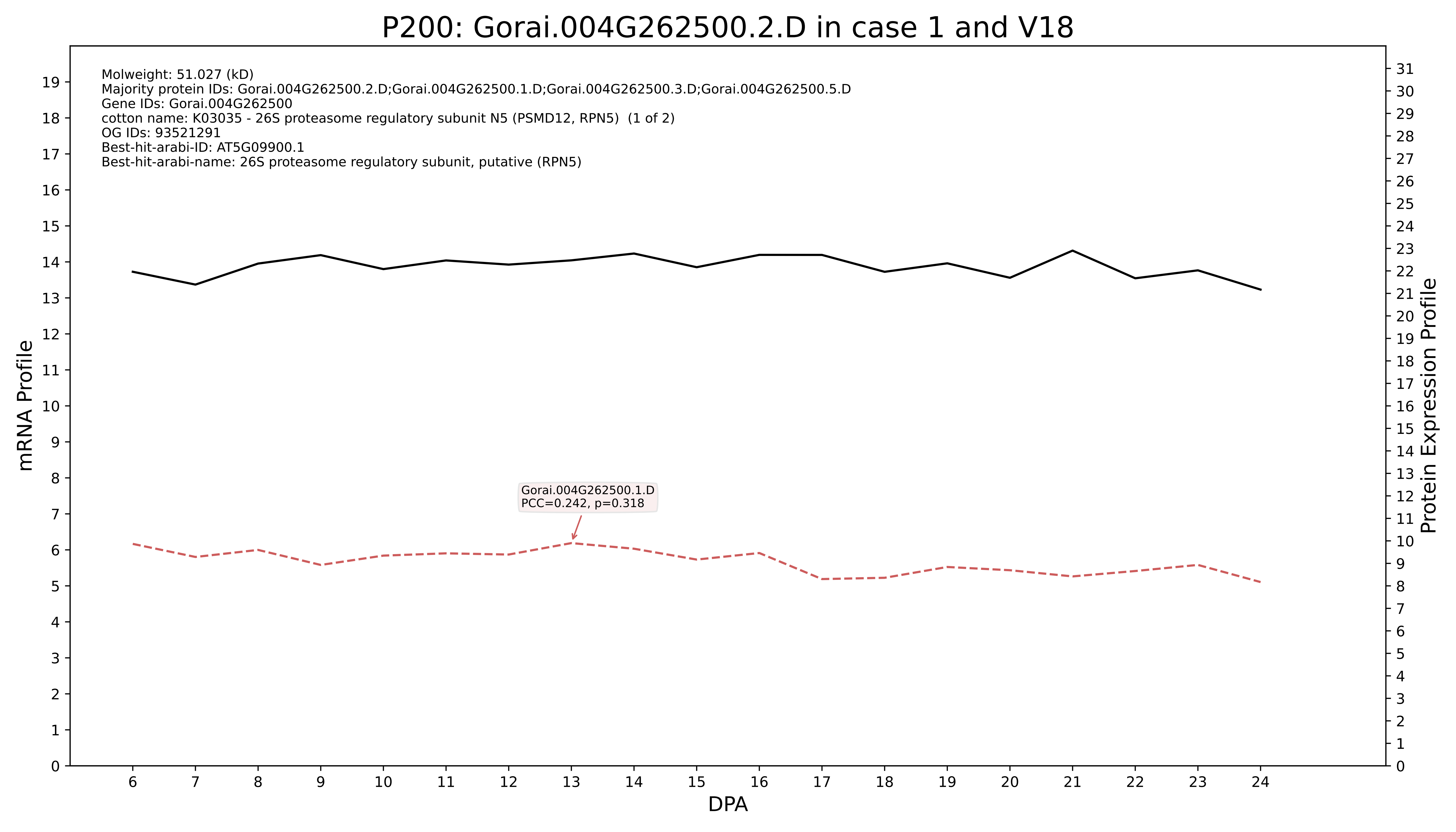
P200: Gorai.004G262500.2.D in case 1 and V18
Molweight: 51.027 (kD)
Majority protein IDs: Gorai.004G262500.2.D;Gorai.004G262500.1.D;Gorai.004G262500.3.D;Gorai.004G262500.5.D
Gene IDs: Gorai.004G262500
cotton name: K03035 - 26S proteasome regulatory subunit N5 (PSMD12, RPN5) (1 of 2)
OG IDs: 93521291
Best-hit-arabi-ID: AT5G09900.1
Best-hit-arabi-name: 26S proteasome regulatory subunit, putative (RPN5)
Gorai.004G262500.1.D
PCC=0.242, p=0.318
mRNA Profile
Protein Expression Profile
DPA

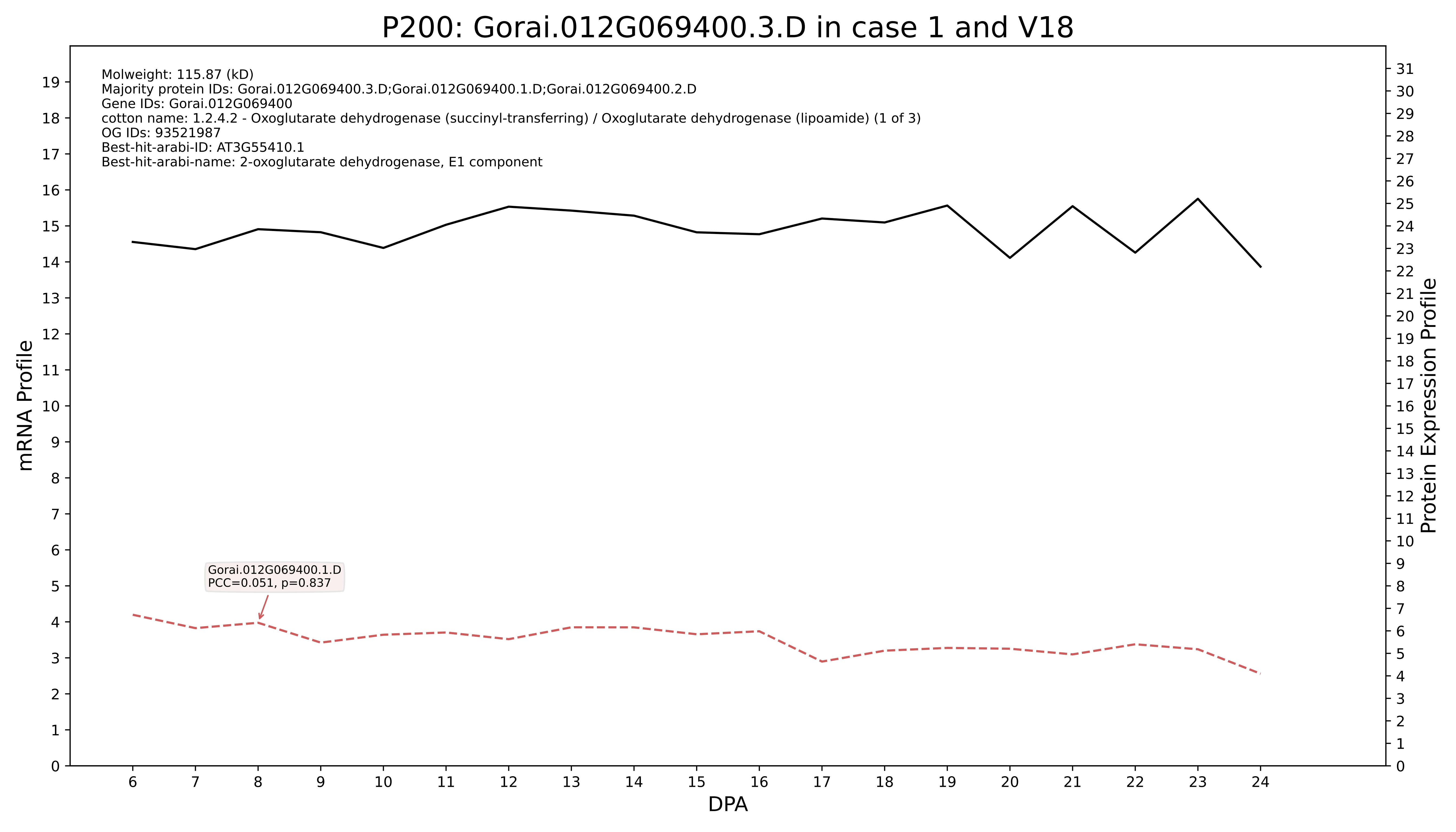
P200: Gorai.012G069400.3.D in case 1 and V18
Molweight: 115.87 (kD)
Majority protein IDs: Gorai.012G069400.3.D;Gorai.012G069400.1.D;Gorai.012G069400.2.D
Gene IDs: Gorai.012G069400
cotton name: 1.2.4.2 - Oxoglutarate dehydrogenase (succinyl-transferring) / Oxoglutarate dehydrogenase (lipoamide) (1 of 3)
OG IDs: 93521987
Best-hit-arabi-ID: AT3G55410.1
Best-hit-arabi-name: 2-oxoglutarate dehydrogenase, E1 component
Gorai.012G069400.1.D
PCC=0.051, p=0.837
mRNA Profile
Protein Expression Profile
DPA
0
1
2
3
4
5
6
7
8
9
10
11
12
13
14
15
16
17
18
19
20
21
22
23
24
25
26
27
28
29
30
31

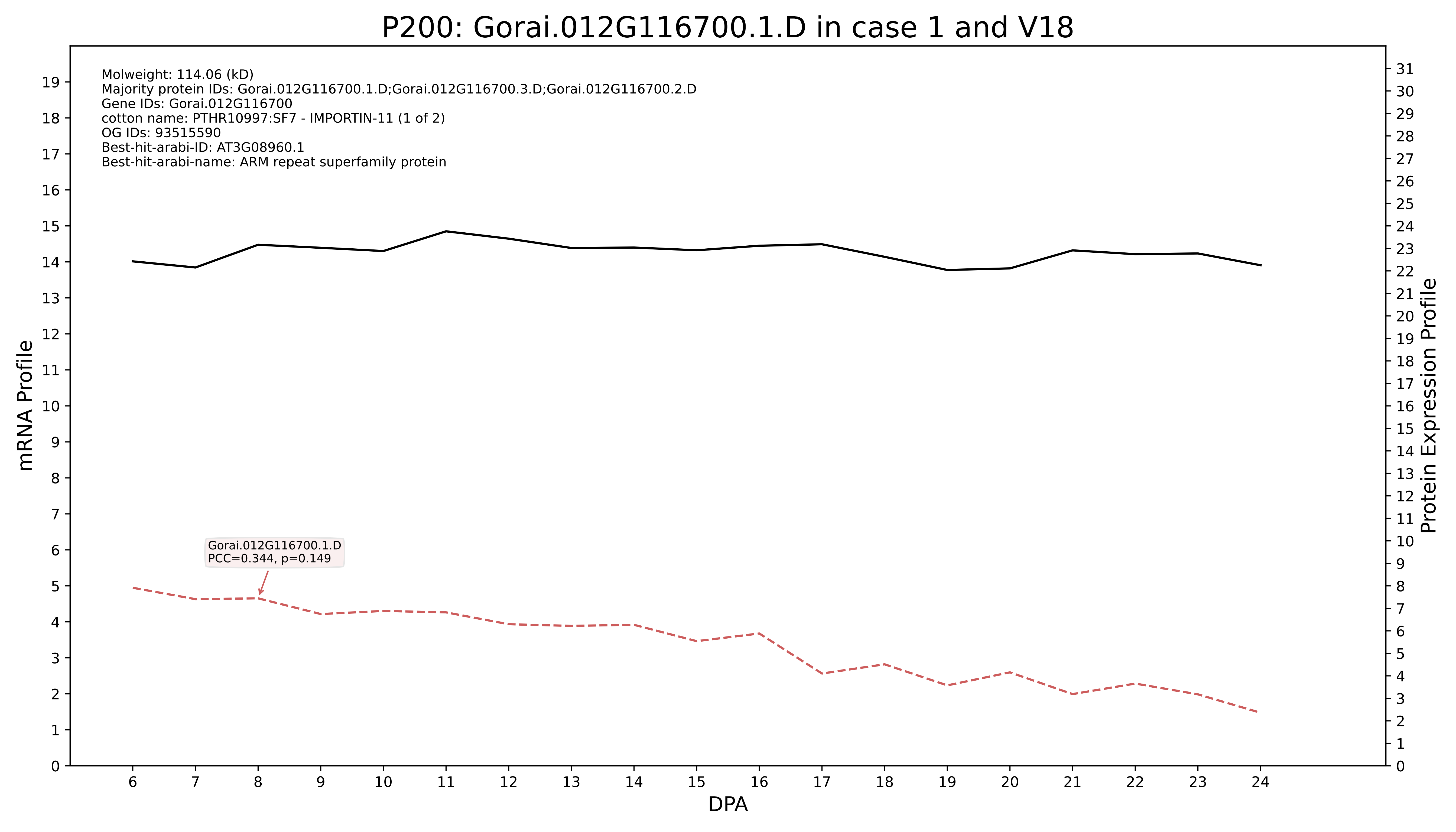
P200: Gorai.012G116700.1.D in case 1 and V18
Molweight: 114.06 (kD)
Majority protein IDs: Gorai.012G116700.1.D;Gorai.012G116700.3.D;Gorai.012G116700.2.D
Gene IDs: Gorai.012G116700
cotton name: PTHR10997:SF7 - IMPORTIN-11 (1 of 2)
OG IDs: 93515590
Best-hit-arabi-ID: AT3G08960.1
Best-hit-arabi-name: ARM repeat superfamily protein
Gorai.012G116700.1.D
PCC=0.344, p=0.149
mRNA Profile
Protein Expression Profile
DPA
0
1
2
3
4
5
6
7
8
9
10
11
12
13
14
15
16
17
18
19
20
21
22
23
24
25
26
27
28
29
30
31

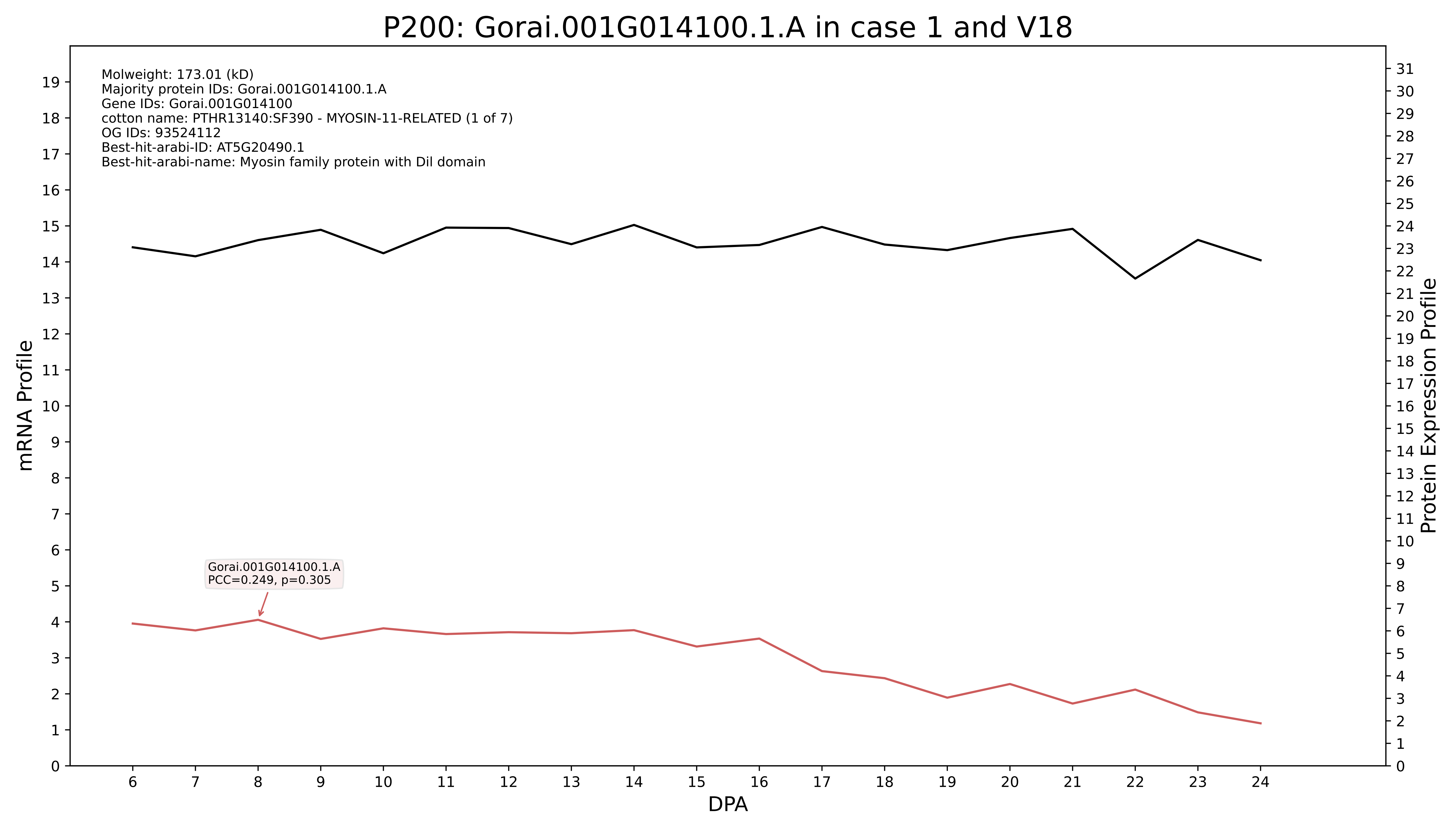
P200: Gorai.001G014100.1.A in case 1 and V18
Molweight: 173.01 (kD)
Majority protein IDs: Gorai.001G014100.1.A
Gene IDs: Gorai.001G014100
cotton name: PTHR13140:SF390 - MYOSIN-11-RELATED (1 of 7)
OG IDs: 93524112
Best-hit-arabi-ID: AT5G20490.1
Best-hit-arabi-name: Myosin family protein with Dil domain
Gorai.001G014100.1.A
PCC=0.249, p=0.305
mRNA Profile
Protein Expression Profile
DPA

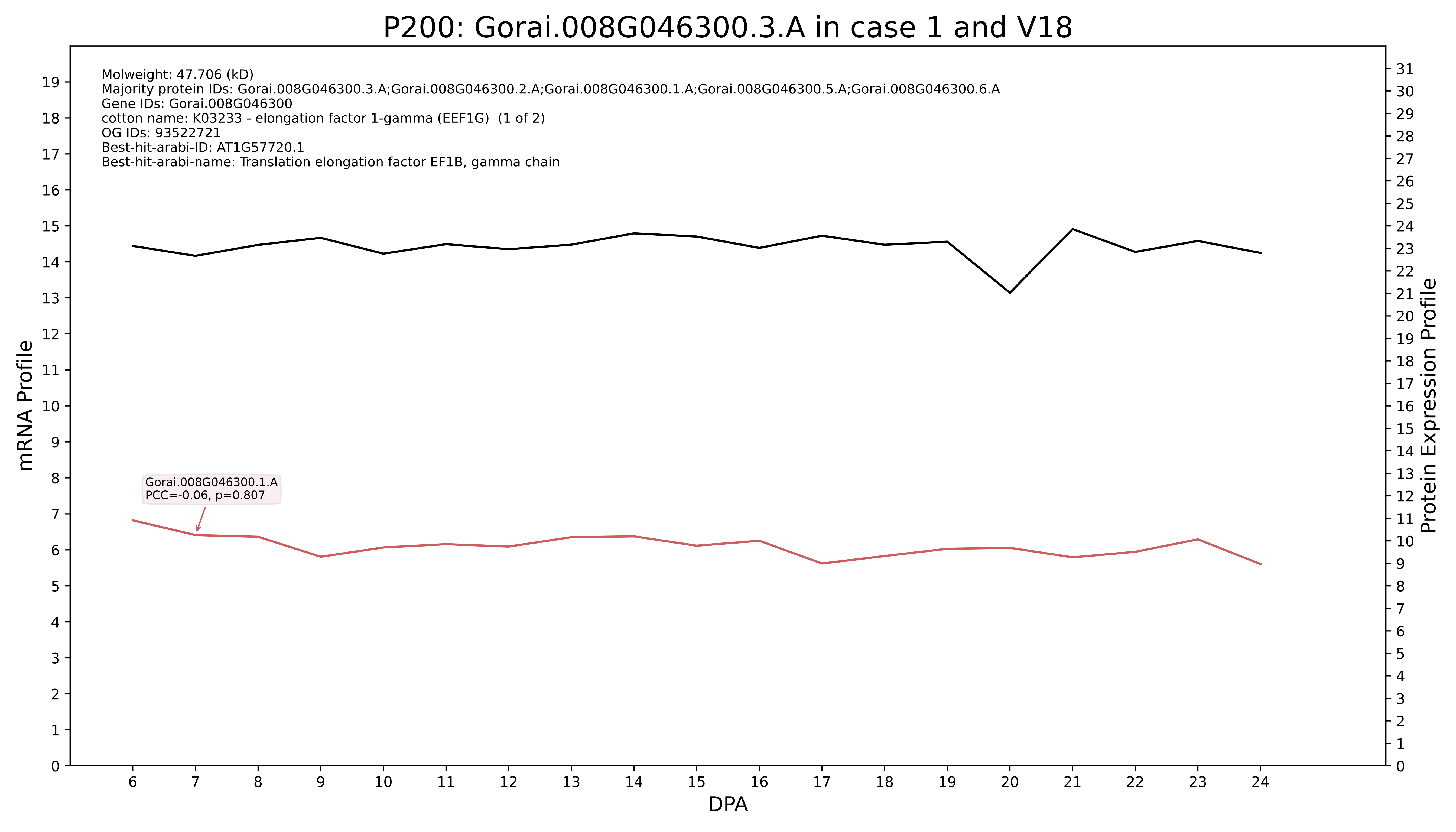
P200: Gorai.008G046300.3.A in case 1 and V18
Molweight: 47.706 (kD)
Majority protein IDs: Gorai.008G046300.3.A;Gorai.008G046300.2.A;Gorai.008G046300.1.A;Gorai.008G046300.5.A;Gorai.008G046300.6.A
Gene IDs: Gorai.008G046300
cotton name: K03233 - elongation factor 1-gamma (EEF1G) (1 of 2)
OG IDs: 93522721
Best-hit-arabi-ID: AT1G57720.1
Best-hit-arabi-name: Translation elongation factor EF1B, gamma chain
Gorai.008G046300.1.A
PCC=-0.06, p=0.807
mRNA Profile
Protein Expression Profile
DPA

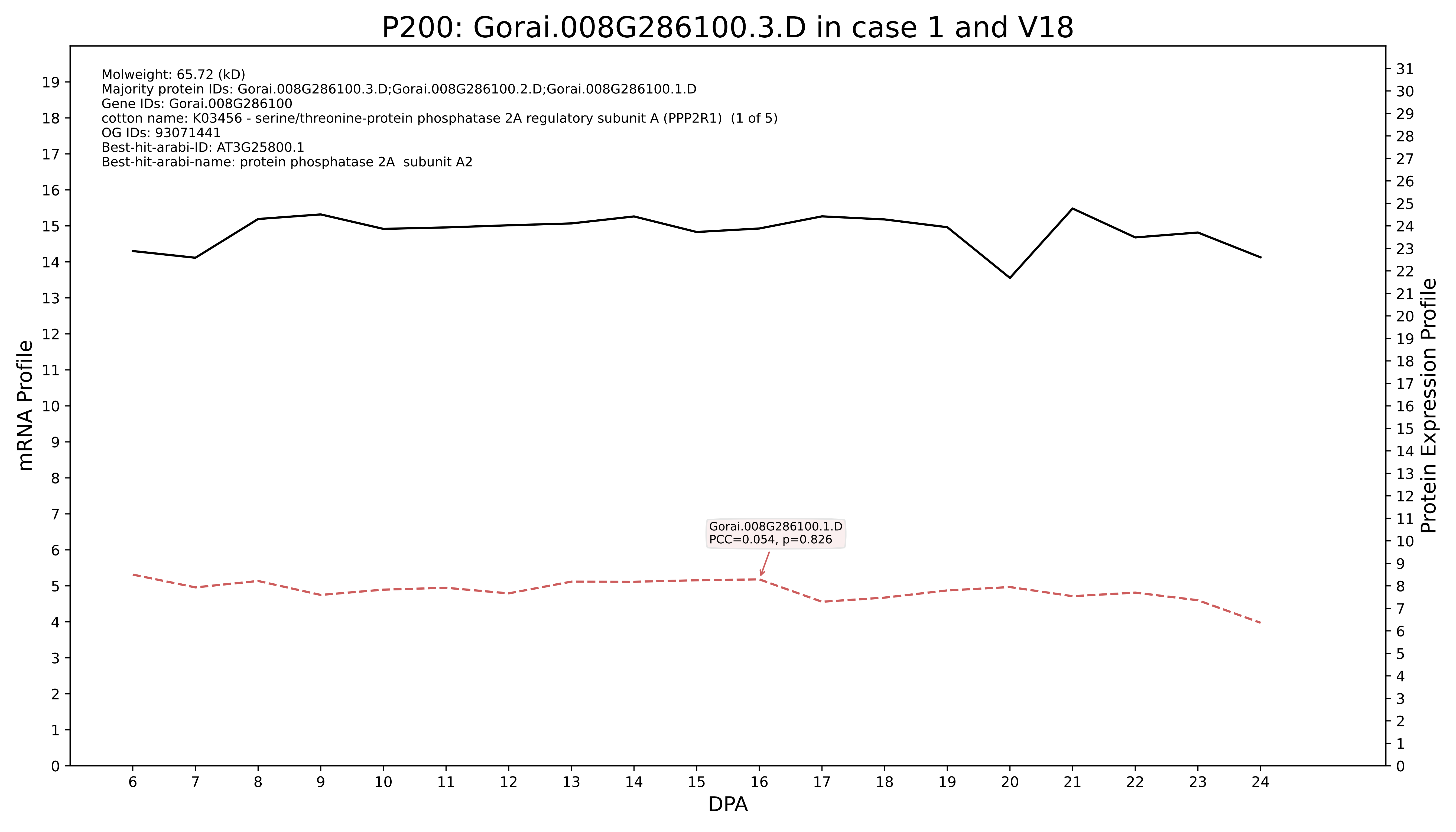
P200: Gorai.008G286100.3.D in case 1 and V18
Molweight: 65.72 (kD)
Majority protein IDs: Gorai.008G286100.3.D;Gorai.008G286100.2.D;Gorai.008G286100.1.D
Gene IDs: Gorai.008G286100
cotton name: K03456 - serine/threonine-protein phosphatase 2A regulatory subunit A (PPP2R1) (1 of 5)
OG IDs: 93071441
Best-hit-arabi-ID: AT3G25800.1
Best-hit-arabi-name: protein phosphatase 2A subunit A2
Gorai.008G286100.1.D
PCC=0.054, p=0.826
mRNA Profile
Protein Expression Profile
DPA

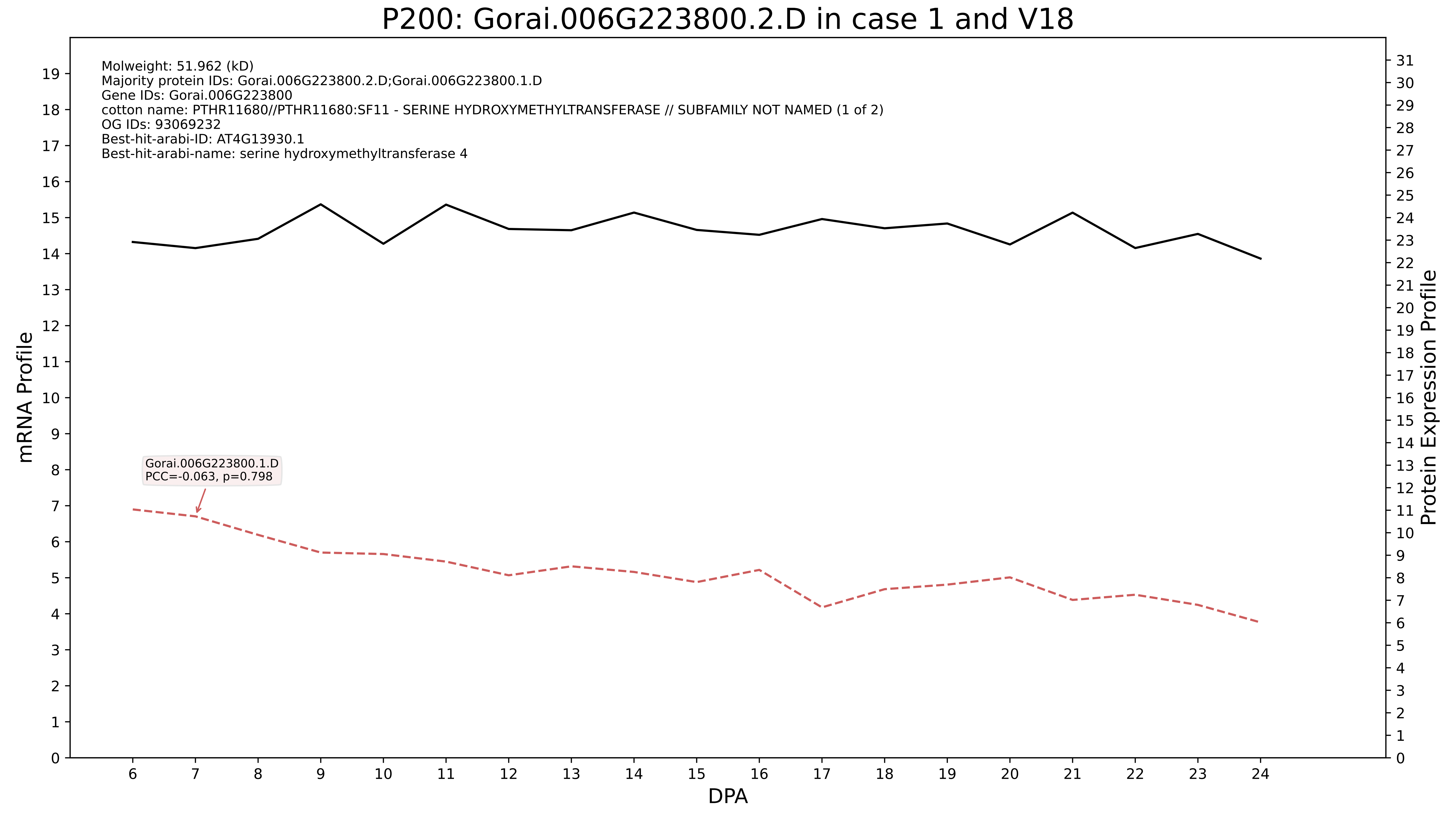
P200: Gorai.006G223800.2.D in case 1 and V18
Molweight: 51.962 (kD)
Majority protein IDs: Gorai.006G223800.2.D;Gorai.006G223800.1.D
Gene IDs: Gorai.006G223800
cotton name: PTHR11680//PTHR11680:SF11 - SERINE HYDROXYMETHYLTRANSFERASE // SUBFAMILY NOT NAMED (1 of 2)
OG IDs: 93069232
Best-hit-arabi-ID: AT4G13930.1
Best-hit-arabi-name: serine hydroxymethyltransferase 4
Gorai.006G223800.1.D
PCC=-0.063, p=0.798
mRNA Profile
Protein Expression Profile
DPA
0
1
2
3
4
5
6
7
8
9
10
11
12
13
14
15
16
17
18
19
20
21
22
23
24
25
26
27
28
29
30
31

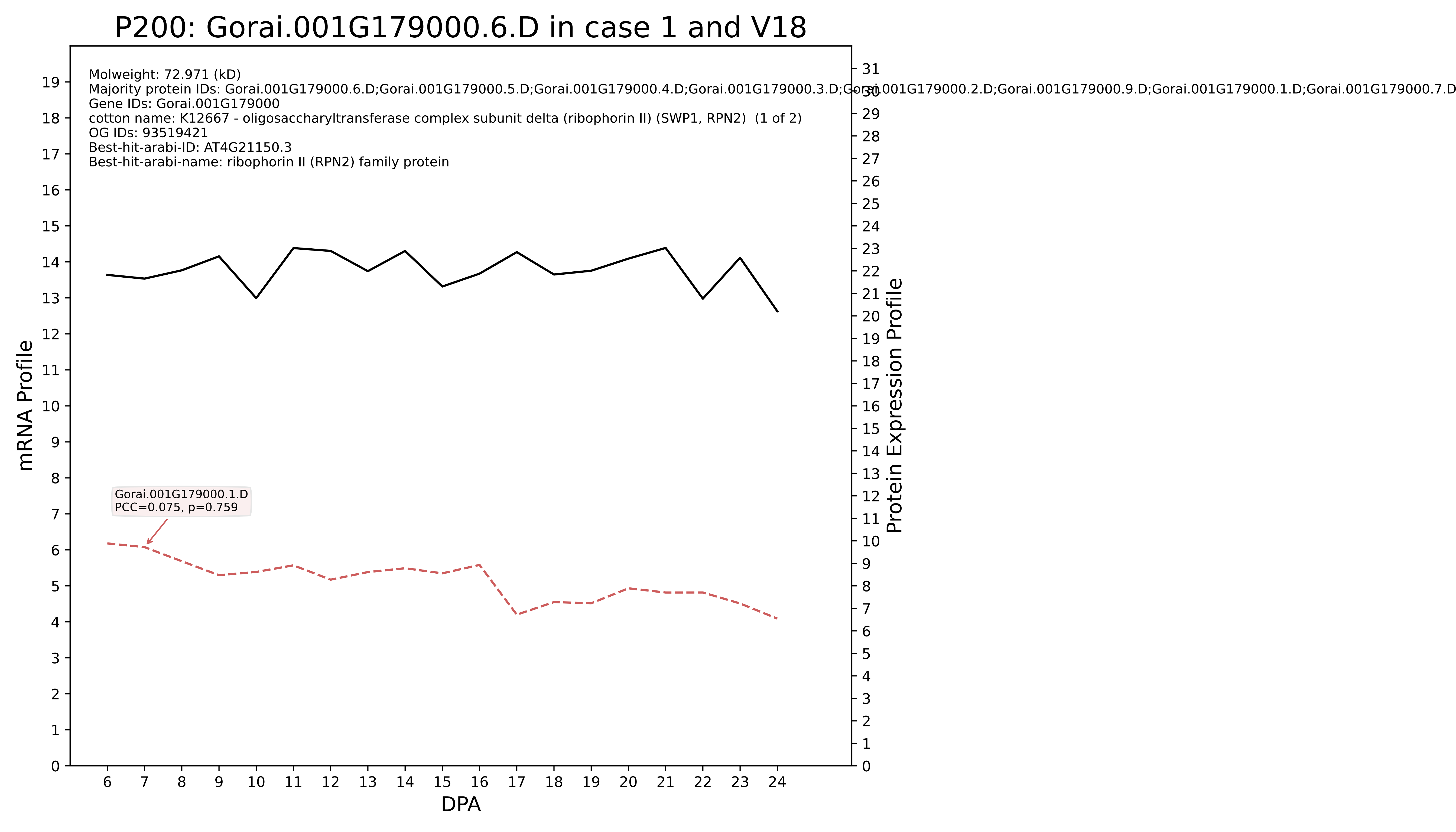
P200: Gorai.001G179000.6.D in case 1 and V18
Molweight: 72.971 (kD)
Majority protein IDs: Gorai.001G179000.6.D;Gorai.001G179000.5.D;Gorai.001G179000.4.D;Gorai.001G179000.3.D;Gorai.001G179000.2.D;Gorai.001G179000.9.D;Gorai.001G179000.1.D;Gorai.001G179000.7.D
Gene IDs: Gorai.001G179000
cotton name: K12667 - oligosaccharyltransferase complex subunit delta (ribophorin II) (SWP1, RPN2) (1 of 2)
OG IDs: 93519421
Best-hit-arabi-ID: AT4G21150.3
Best-hit-arabi-name: ribophorin II (RPN2) family protein
Gorai.001G179000.1.D
PCC=0.075, p=0.759
mRNA Profile
Protein Expression Profile
DPA

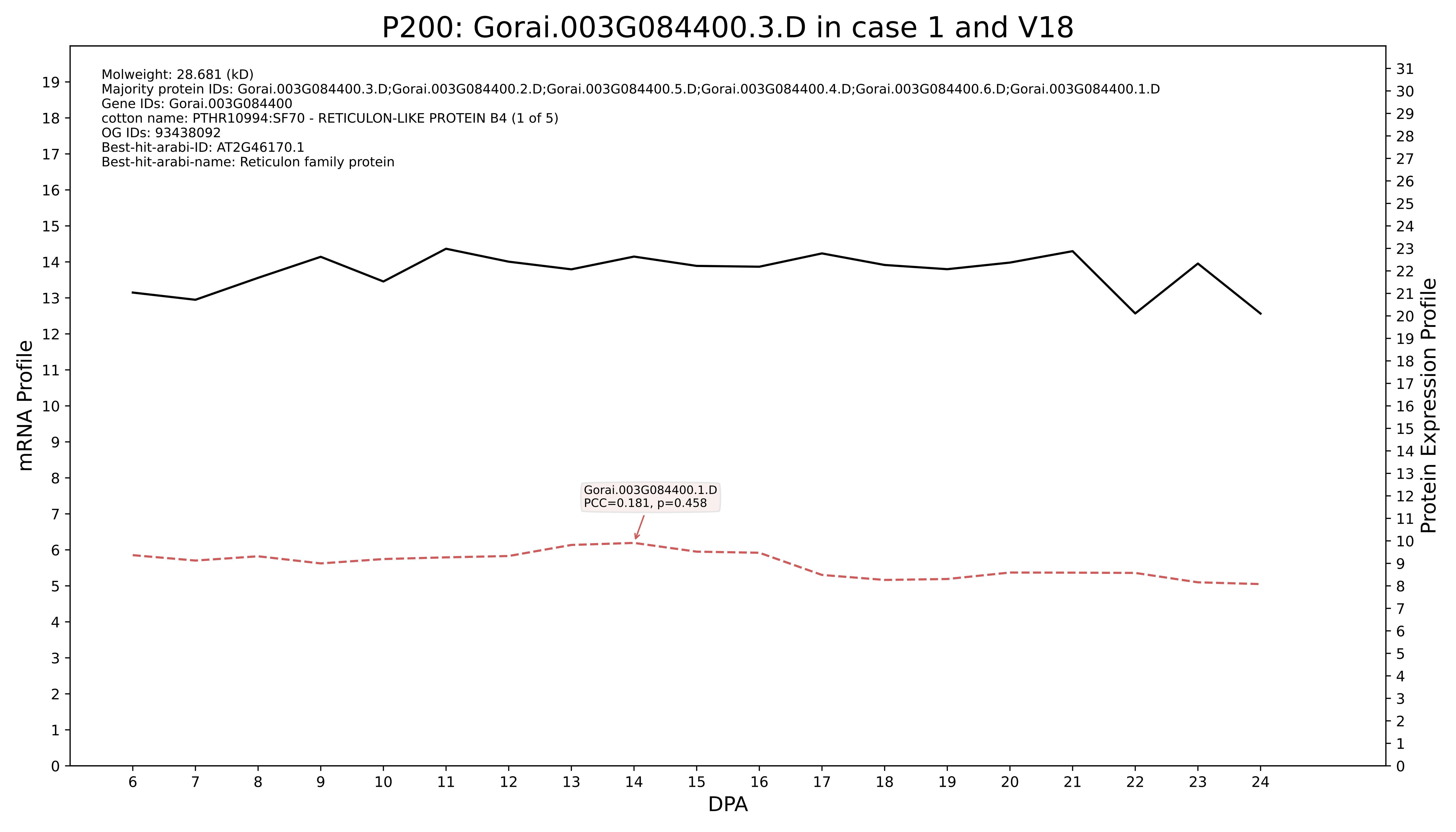
P200: Gorai.003G084400.3.D in case 1 and V18
Molweight: 28.681 (kD)
Majority protein IDs: Gorai.003G084400.3.D;Gorai.003G084400.2.D;Gorai.003G084400.5.D;Gorai.003G084400.4.D;Gorai.003G084400.6.D;Gorai.003G084400.1.D
Gene IDs: Gorai.003G084400
cotton name: PTHR10994:SF70 - RETICULON-LIKE PROTEIN B4 (1 of 5)
OG IDs: 93438092
Best-hit-arabi-ID: AT2G46170.1
Best-hit-arabi-name: Reticulon family protein
Gorai.003G084400.1.D
PCC=0.181, p=0.458
mRNA Profile
Protein Expression Profile
DPA

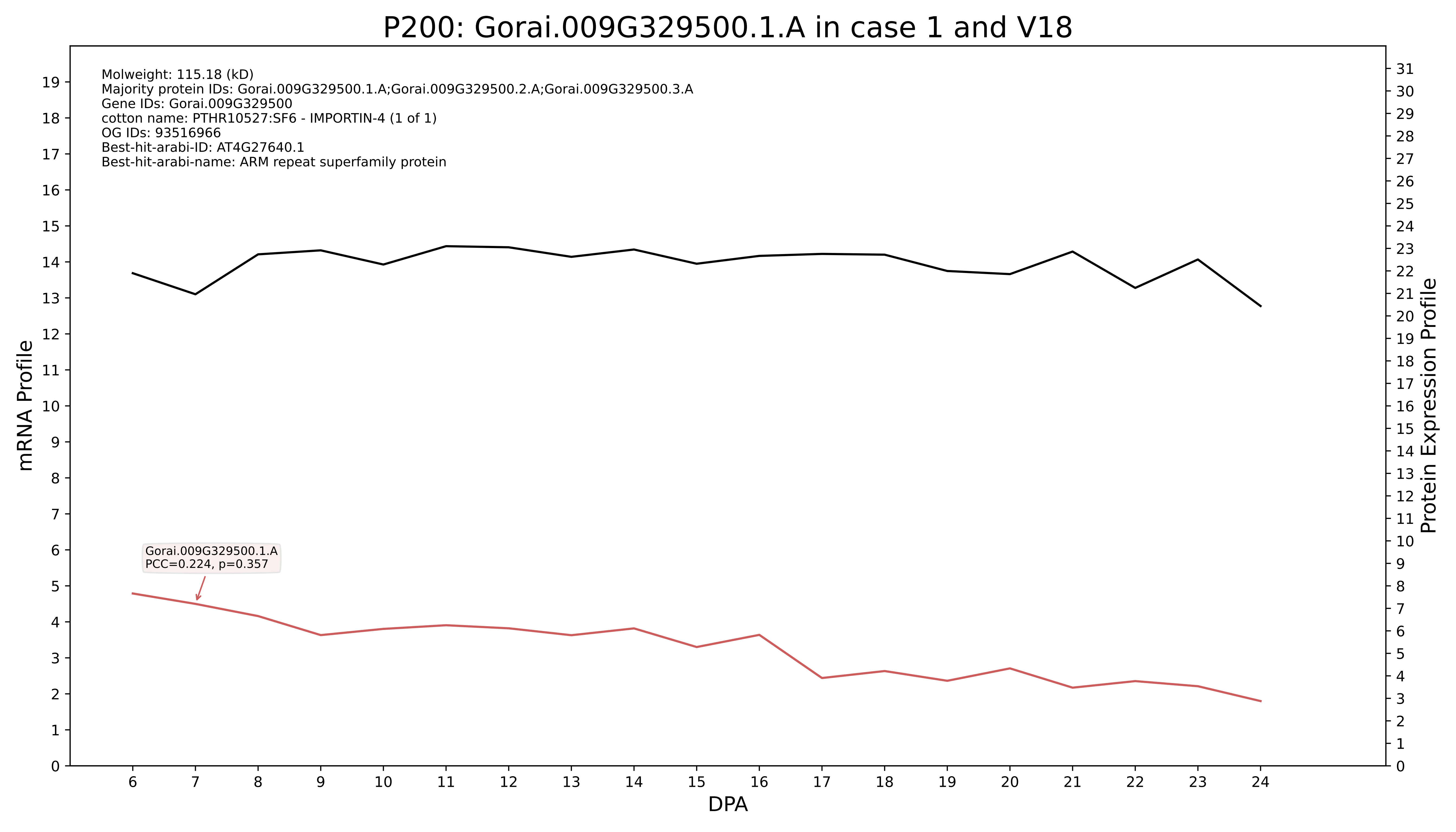
P200: Gorai.009G329500.1.A in case 1 and V18
Molweight: 115.18 (kD)
Majority protein IDs: Gorai.009G329500.1.A;Gorai.009G329500.2.A;Gorai.009G329500.3.A
Gene IDs: Gorai.009G329500
cotton name: PTHR10527:SF6 - IMPORTIN-4 (1 of 1)
OG IDs: 93516966
Best-hit-arabi-ID: AT4G27640.1
Best-hit-arabi-name: ARM repeat superfamily protein
Gorai.009G329500.1.A
PCC=0.224, p=0.357
mRNA Profile
Protein Expression Profile
DPA

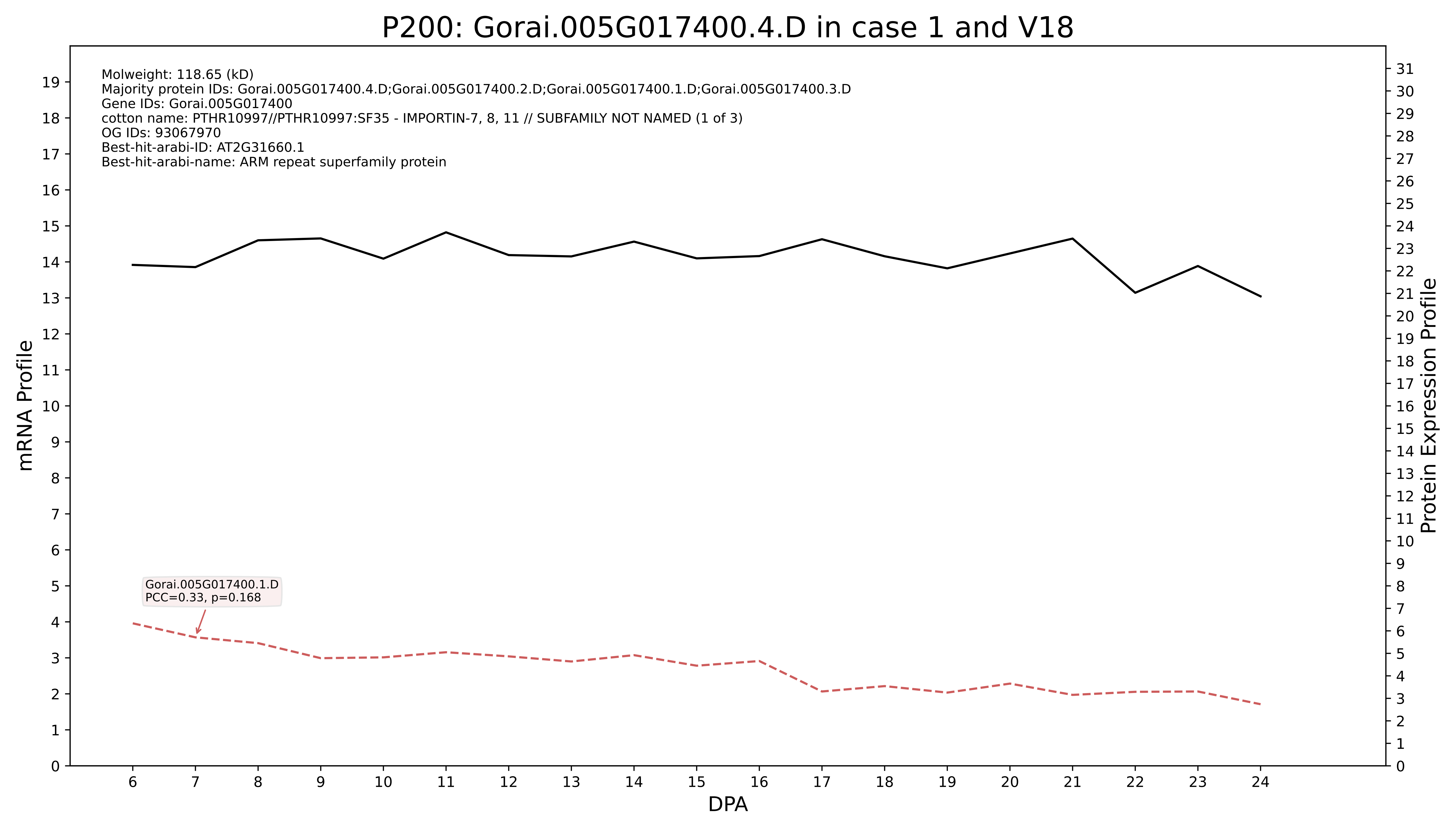
P200: Gorai.005G017400.4.D in case 1 and V18
Molweight: 118.65 (kD)
Majority protein IDs: Gorai.005G017400.4.D;Gorai.005G017400.2.D;Gorai.005G017400.1.D;Gorai.005G017400.3.D
Gene IDs: Gorai.005G017400
cotton name: PTHR10997//PTHR10997:SF35 - IMPORTIN-7, 8, 11 // SUBFAMILY NOT NAMED (1 of 3)
OG IDs: 93067970
Best-hit-arabi-ID: AT2G31660.1
Best-hit-arabi-name: ARM repeat superfamily protein
Gorai.005G017400.1.D
PCC=0.33, p=0.168
mRNA Profile
Protein Expression Profile
DPA
6
7
8
9
10
11
12
13
14
15
16
17
18
19
20
21
22
23
24

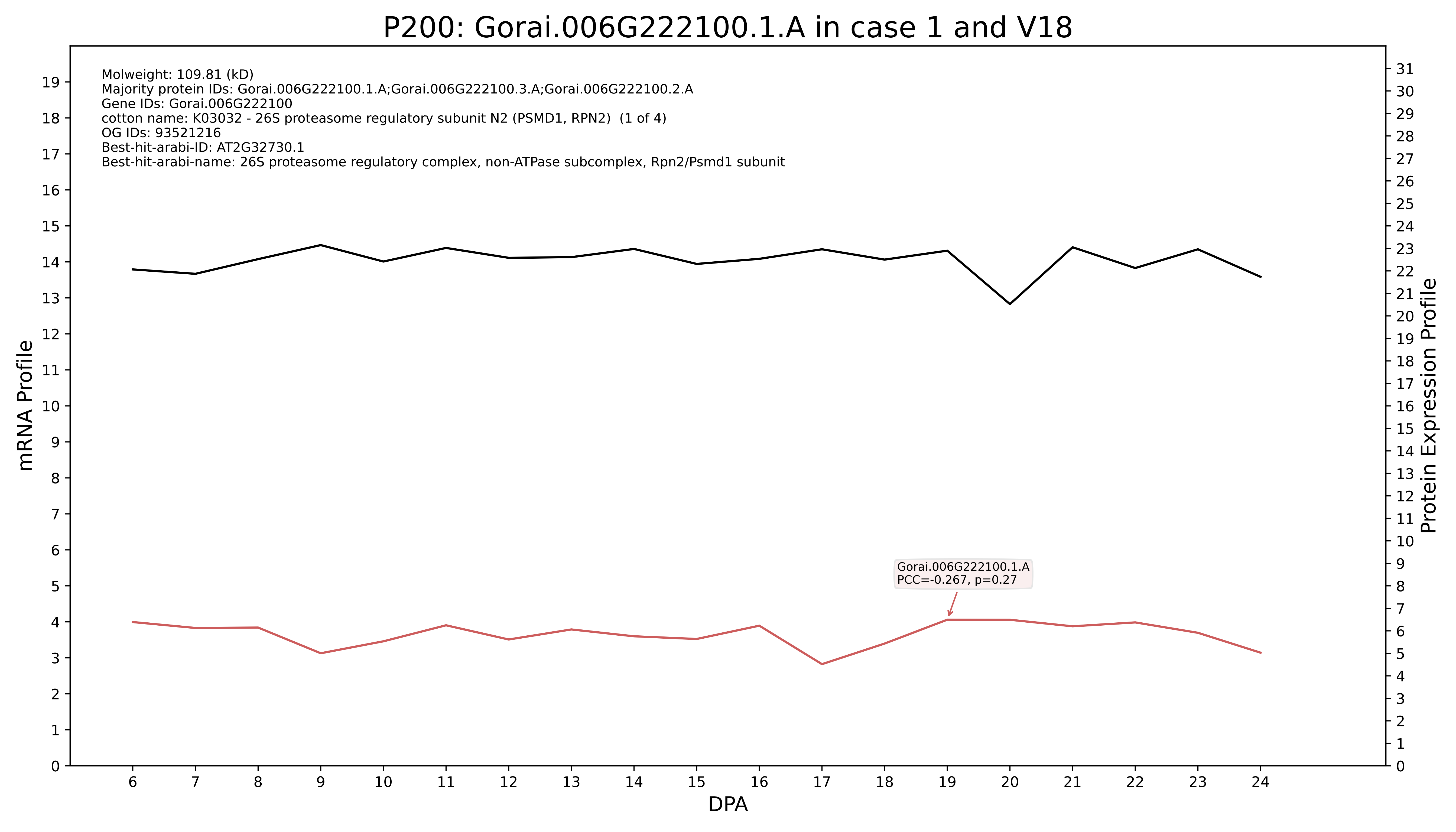
P200: Gorai.006G222100.1.A in case 1 and V18
Molweight: 109.81 (kD)
Majority protein IDs: Gorai.006G222100.1.A;Gorai.006G222100.3.A;Gorai.006G222100.2.A
Gene IDs: Gorai.006G222100
cotton name: K03032 - 26S proteasome regulatory subunit N2 (PSMD1, RPN2) (1 of 4)
OG IDs: 93521216
Best-hit-arabi-ID: AT2G32730.1
Best-hit-arabi-name: 26S proteasome regulatory complex, non-ATPase subcomplex, Rpn2/Psmd1 subunit
Gorai.006G222100.1.A
PCC=-0.267, p=0.27
mRNA Profile
Protein Expression Profile
DPA

P200: Gorai.013G116900.3.D in case 1 and V18

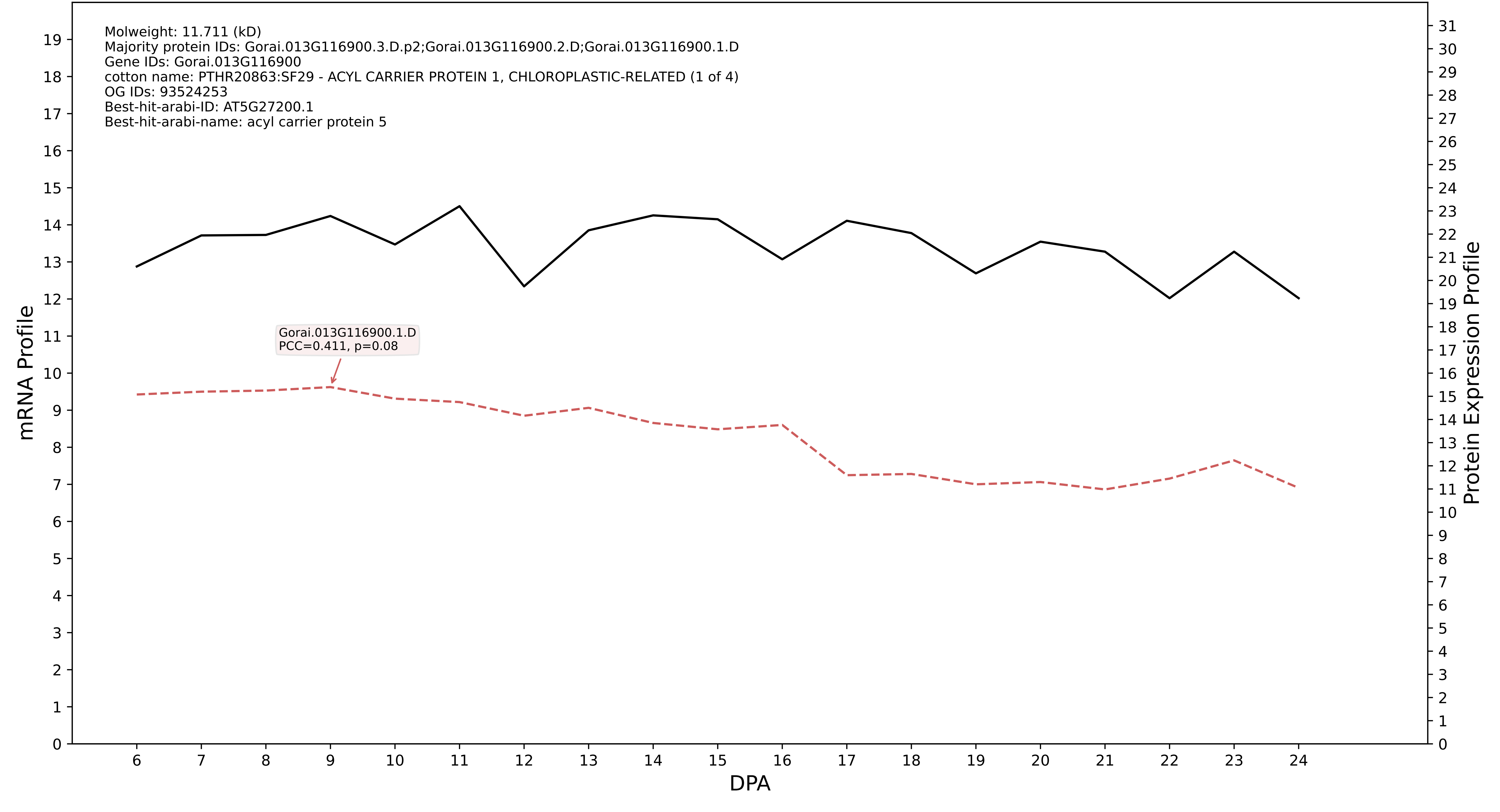

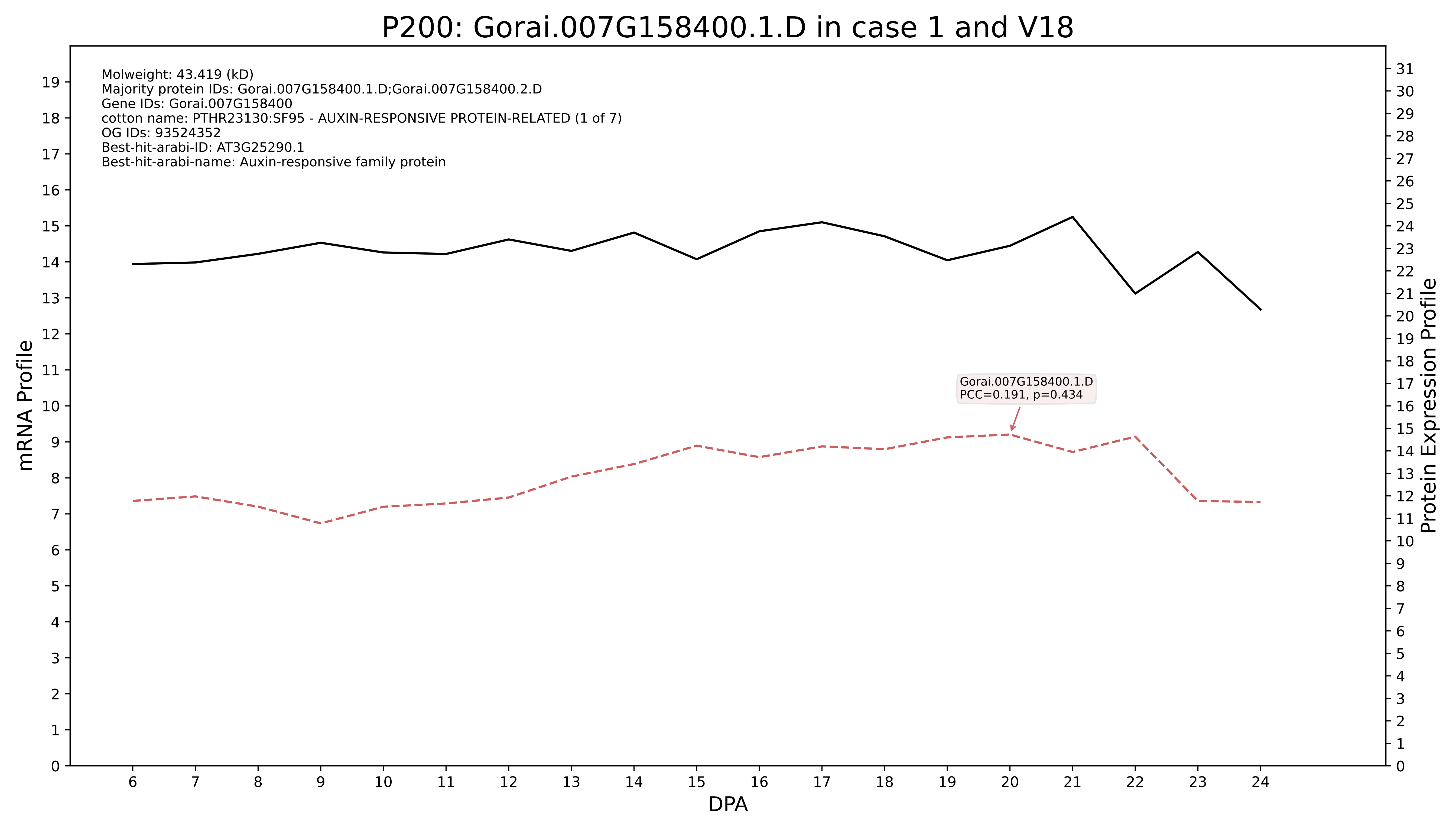
P200: Gorai.007G158400.1.D in case 1 and V18
Molweight: 43.419 (kD)
Majority protein IDs: Gorai.007G158400.1.D;Gorai.007G158400.2.D
Gene IDs: Gorai.007G158400
cotton name: PTHR23130:SF95 - AUXIN-RESPONSIVE PROTEIN-RELATED (1 of 7)
OG IDs: 93524352
Best-hit-arabi-ID: AT3G25290.1
Best-hit-arabi-name: Auxin-responsive family protein
Gorai.007G158400.1.D
PCC=0.191, p=0.434
mRNA Profile
Protein Expression Profile
DPA
0
1
2
3
4
5
6
7
8
9
10
11
12
13
14
15
16
17
18
19
20
21
22
23
24
25
26
27
28
29
30
31

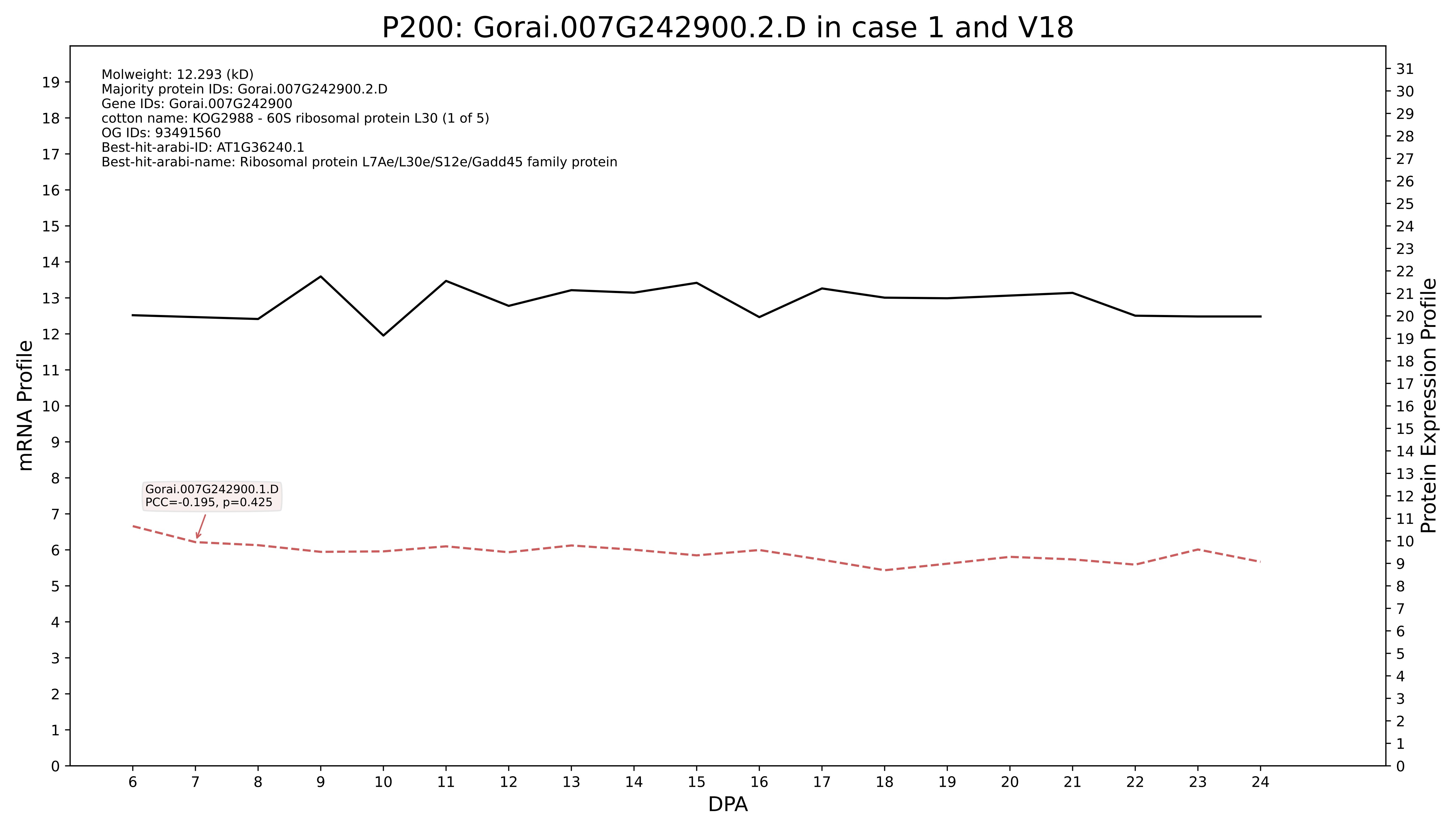
P200: Gorai.007G242900.2.D in case 1 and V18
Molweight: 12.293 (kD)
Majority protein IDs: Gorai.007G242900.2.D
Gene IDs: Gorai.007G242900
cotton name: KOG2988 - 60S ribosomal protein L30 (1 of 5)
OG IDs: 93491560
Best-hit-arabi-ID: AT1G36240.1
Best-hit-arabi-name: Ribosomal protein L7Ae/L30e/S12e/Gadd45 family protein
Gorai.007G242900.1.D
PCC=-0.195, p=0.425
mRNA Profile
Protein Expression Profile
DPA

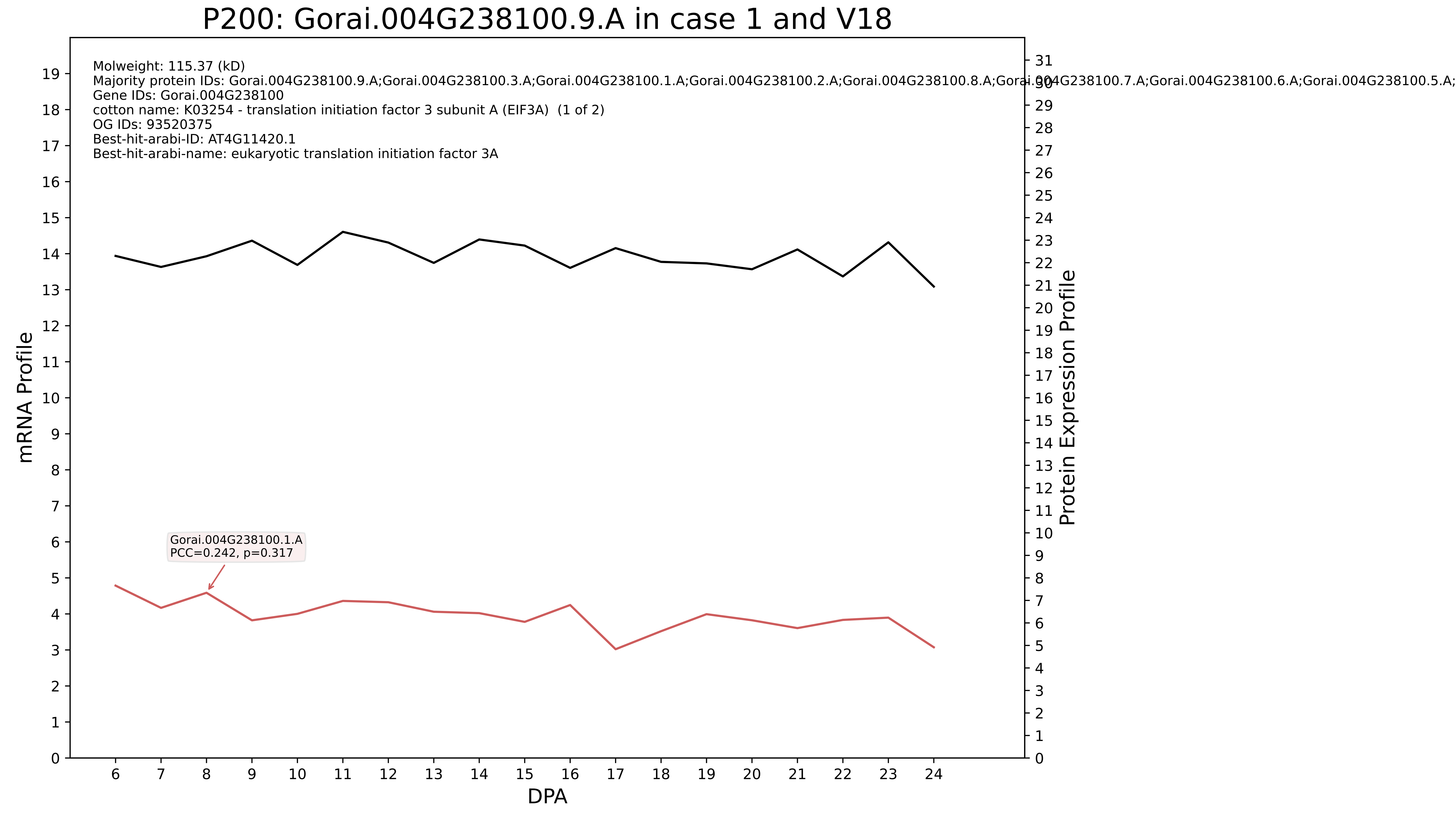

P200: Gorai.004G238100.9.A in case 1 and V18
Molweight: 115.37 (kD)
Majority protein IDs: Gorai.004G238100.9.A;Gorai.004G238100.3.A;Gorai.004G238100.1.A;Gorai.004G238100.2.A;Gorai.004G238100.8.A;Gorai.004G238100.7.A;Gorai.004G238100.6.A;Gorai.004G238100.5.A;
Gene IDs: Gorai.004G238100
cotton name: K03254 - translation initiation factor 3 subunit A (EIF3A) (1 of 2)
OG IDs: 93520375
Best-hit-arabi-ID: AT4G11420.1
Best-hit-arabi-name: eukaryotic translation initiation factor 3A
Gorai.004G238100.1.A
PCC=0.242, p=0.317
mRNA Profile
Protein Expression Profile
DPA

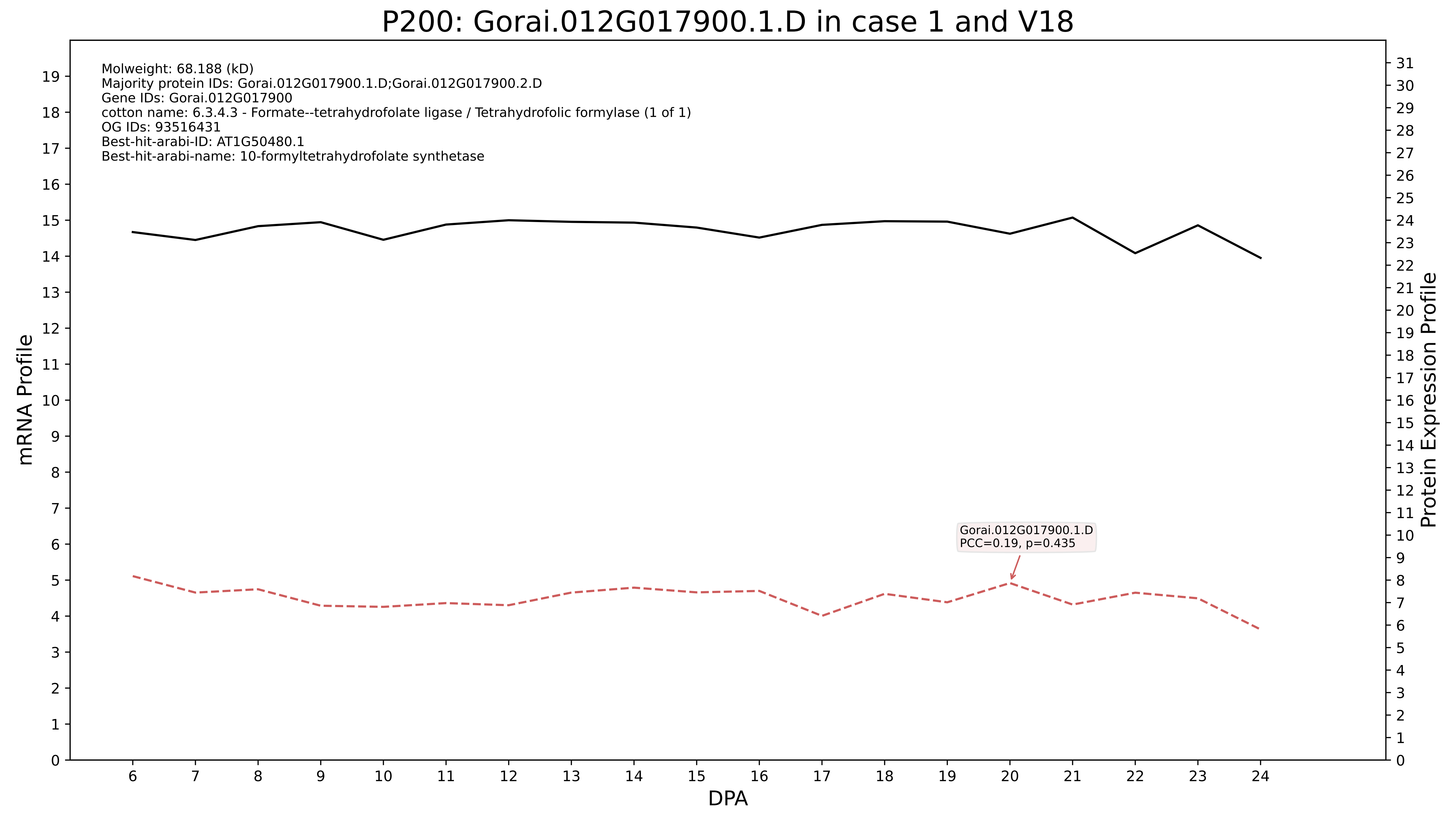
P200: Gorai.012G017900.1.D in case 1 and V18
Molweight: 68.188 (kD)
Majority protein IDs: Gorai.012G017900.1.D;Gorai.012G017900.2.D
Gene IDs: Gorai.012G017900
cotton name: 6.3.4.3 - Formate--tetrahydrofolate ligase / Tetrahydrofolic formylase (1 of 1)
OG IDs: 93516431
Best-hit-arabi-ID: AT1G50480.1
Best-hit-arabi-name: 10-formyltetrahydrofolate synthetase
Gorai.012G017900.1.D
PCC=0.19, p=0.435
mRNA Profile
Protein Expression Profile
DPA
0
1
2
3
4
5
6
7
8
9
10
11
12
13
14
15
16
17
18
19
20
21
22
23
24
25
26
27
28
29
30
31

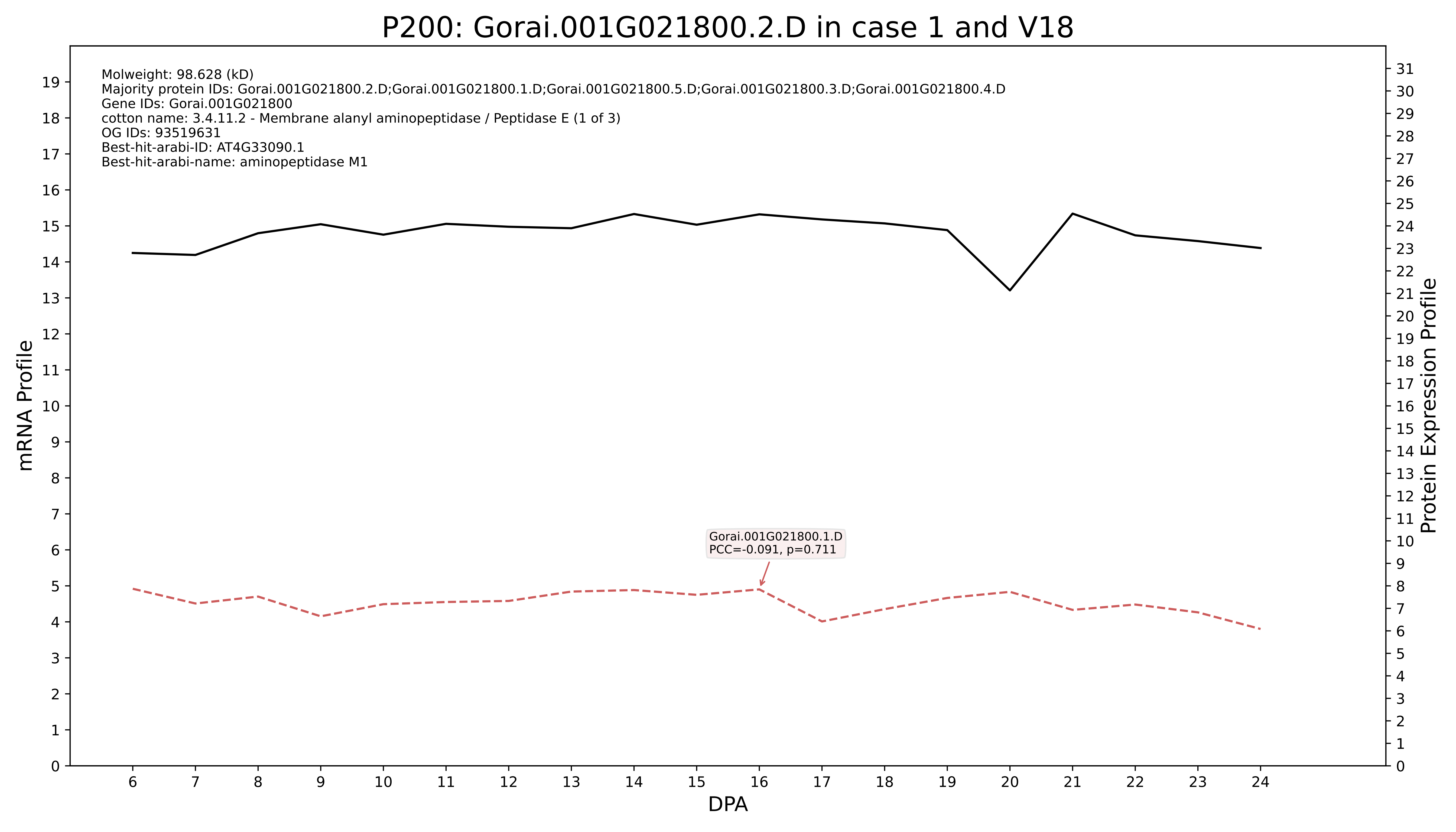

P200: Gorai.001G021800.2.D in case 1 and V18
Molweight: 98.628 (kD)
Majority protein IDs: Gorai.001G021800.2.D;Gorai.001G021800.1.D;Gorai.001G021800.5.D;Gorai.001G021800.3.D;Gorai.001G021800.4.D
Gene IDs: Gorai.001G021800
cotton name: 3.4.11.2 - Membrane alanyl aminopeptidase / Peptidase E (1 of 3)
OG IDs: 93519631
Best-hit-arabi-ID: AT4G33090.1
Best-hit-arabi-name: aminopeptidase M1
Gorai.001G021800.1.D
PCC=-0.091, p=0.711
mRNA Profile
Protein Expression Profile
DPA

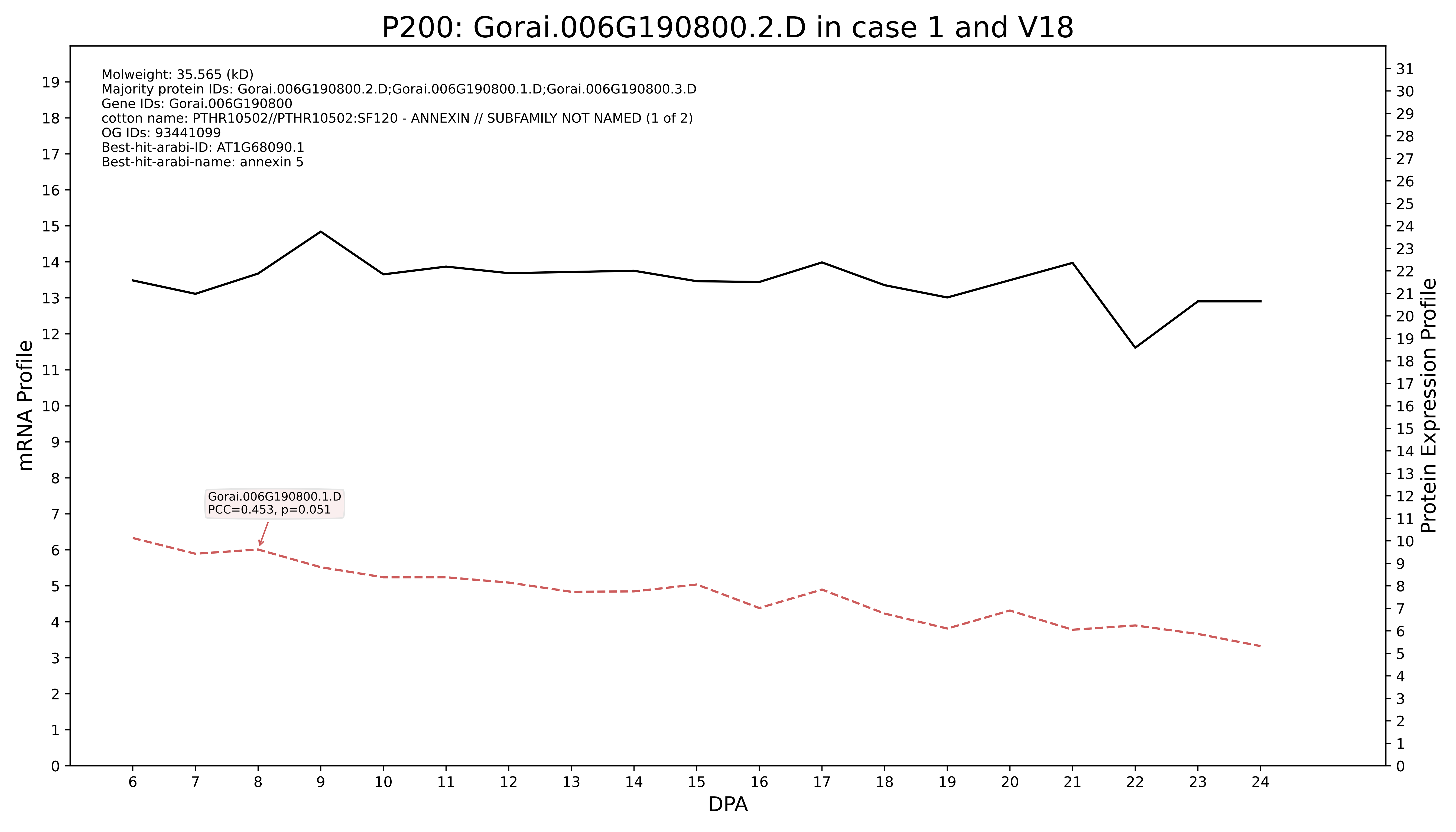

P200: Gorai.006G190800.2.D in case 1 and V18
Molweight: 35.565 (kD)
Majority protein IDs: Gorai.006G190800.2.D;Gorai.006G190800.1.D;Gorai.006G190800.3.D
Gene IDs: Gorai.006G190800
cotton name: PTHR10502//PTHR10502:SF120 - ANNEXIN // SUBFAMILY NOT NAMED (1 of 2)
OG IDs: 93441099
Best-hit-arabi-ID: AT1G68090.1
Best-hit-arabi-name: annexin 5
Gorai.006G190800.1.D
PCC=0.453, p=0.051
mRNA Profile
Protein Expression Profile
DPA

P200: Gorai.007G026300.2.D in case 1 and V18

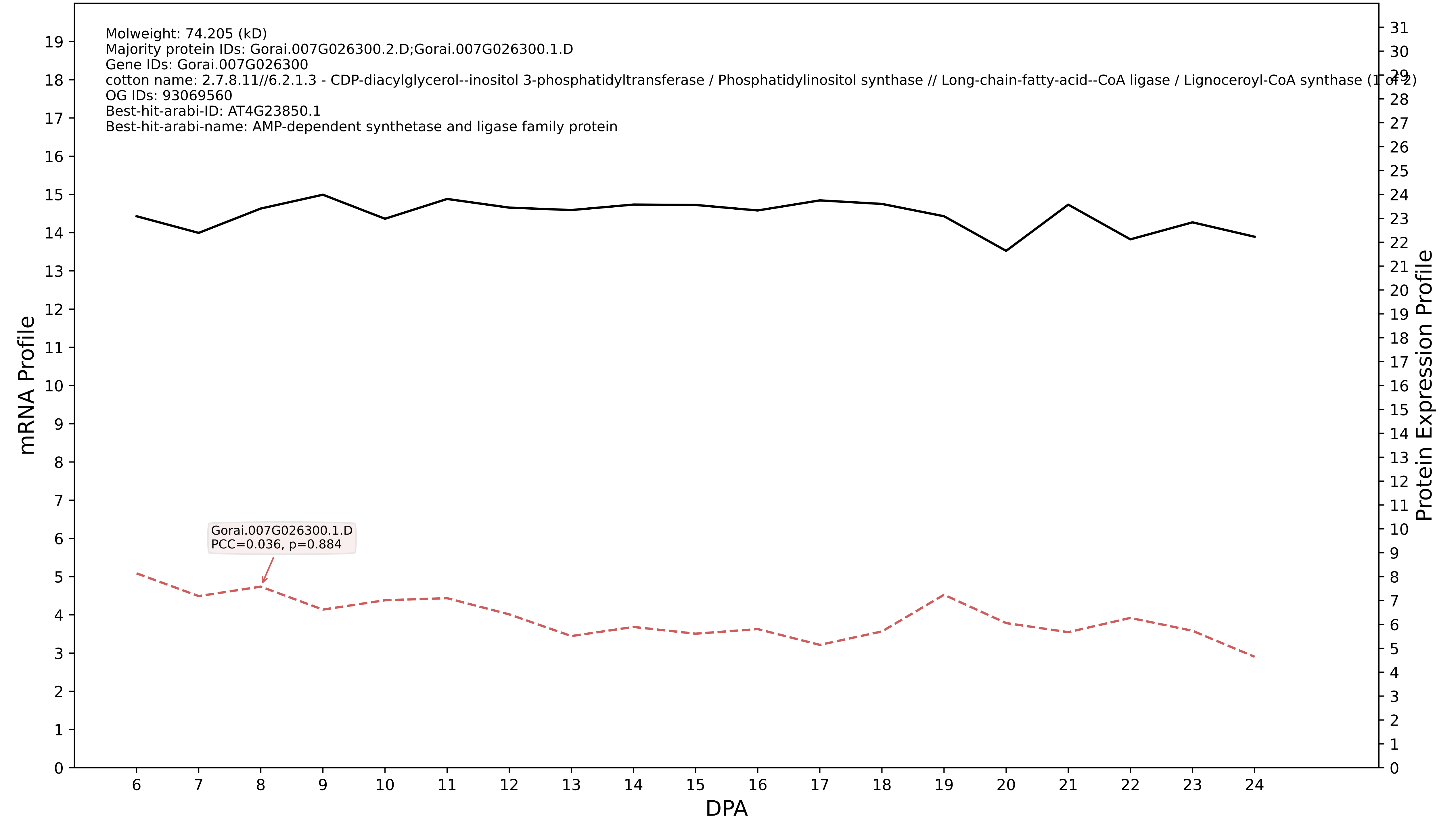

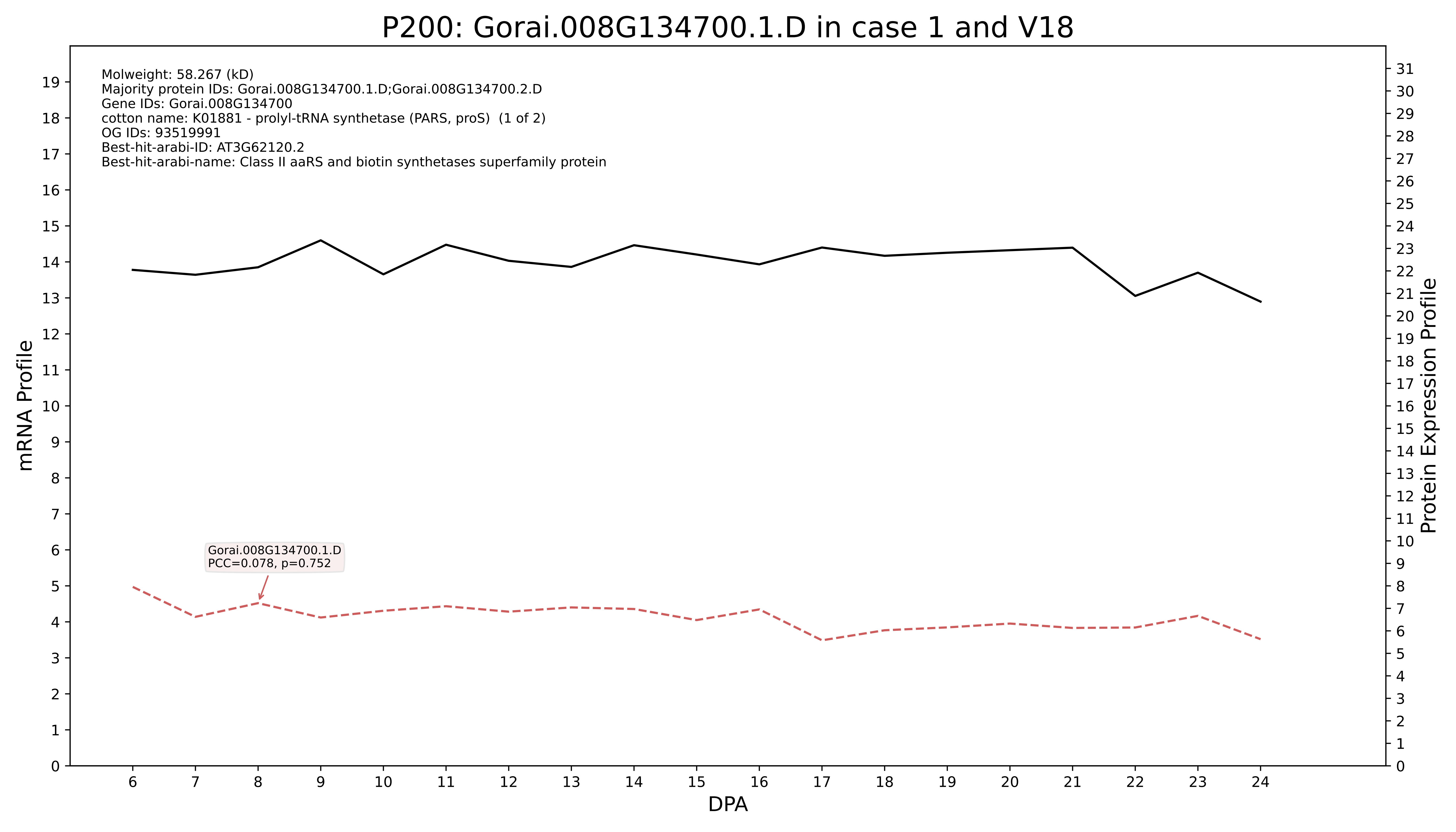
P200: Gorai.008G134700.1.D in case 1 and V18
Molweight: 58.267 (kD)
Majority protein IDs: Gorai.008G134700.1.D;Gorai.008G134700.2.D
Gene IDs: Gorai.008G134700
cotton name: K01881 - prolyl-tRNA synthetase (PARS, proS) (1 of 2)
OG IDs: 93519991
Best-hit-arabi-ID: AT3G62120.2
Best-hit-arabi-name: Class II aaRS and biotin synthetases superfamily protein
Gorai.008G134700.1.D
PCC=0.078, p=0.752
mRNA Profile
Protein Expression Profile
DPA

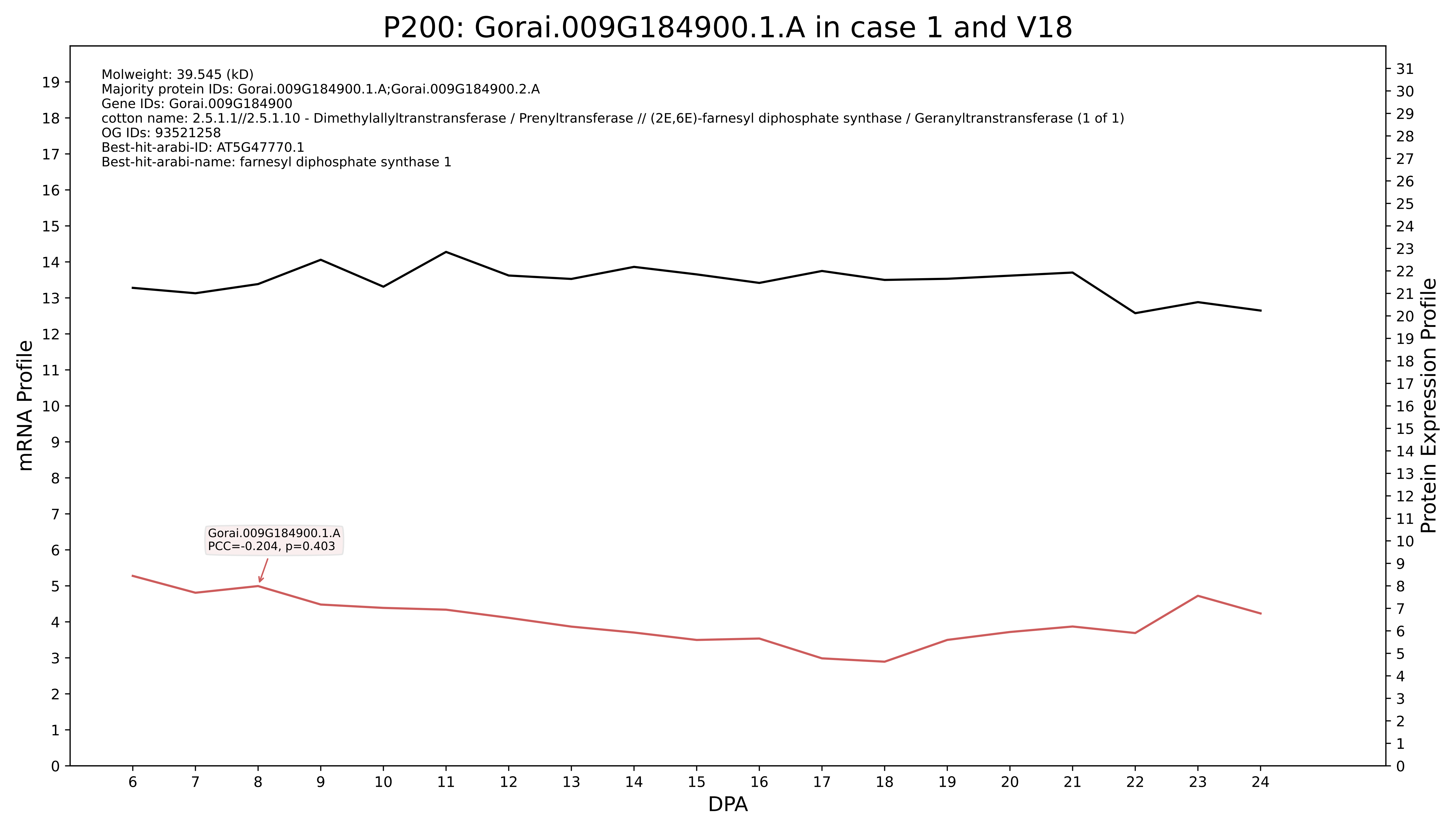
P200: Gorai.009G184900.1.A in case 1 and V18
Molweight: 39.545 (kD)
Majority protein IDs: Gorai.009G184900.1.A;Gorai.009G184900.2.A
Gene IDs: Gorai.009G184900
cotton name: 2.5.1.1//2.5.1.10 - Dimethylallyltranstransferase / Prenyltransferase // (2E,6E)-farnesyl diphosphate synthase / Geranyltranstransferase (1 of 1)
OG IDs: 93521258
Best-hit-arabi-ID: AT5G47770.1
Best-hit-arabi-name: farnesyl diphosphate synthase 1
Gorai.009G184900.1.A
PCC=-0.204, p=0.403
mRNA Profile
Protein Expression Profile
DPA
6 7 8 9 10 11 12 13 14 15 16 17 18 19 20 21 22 23 24

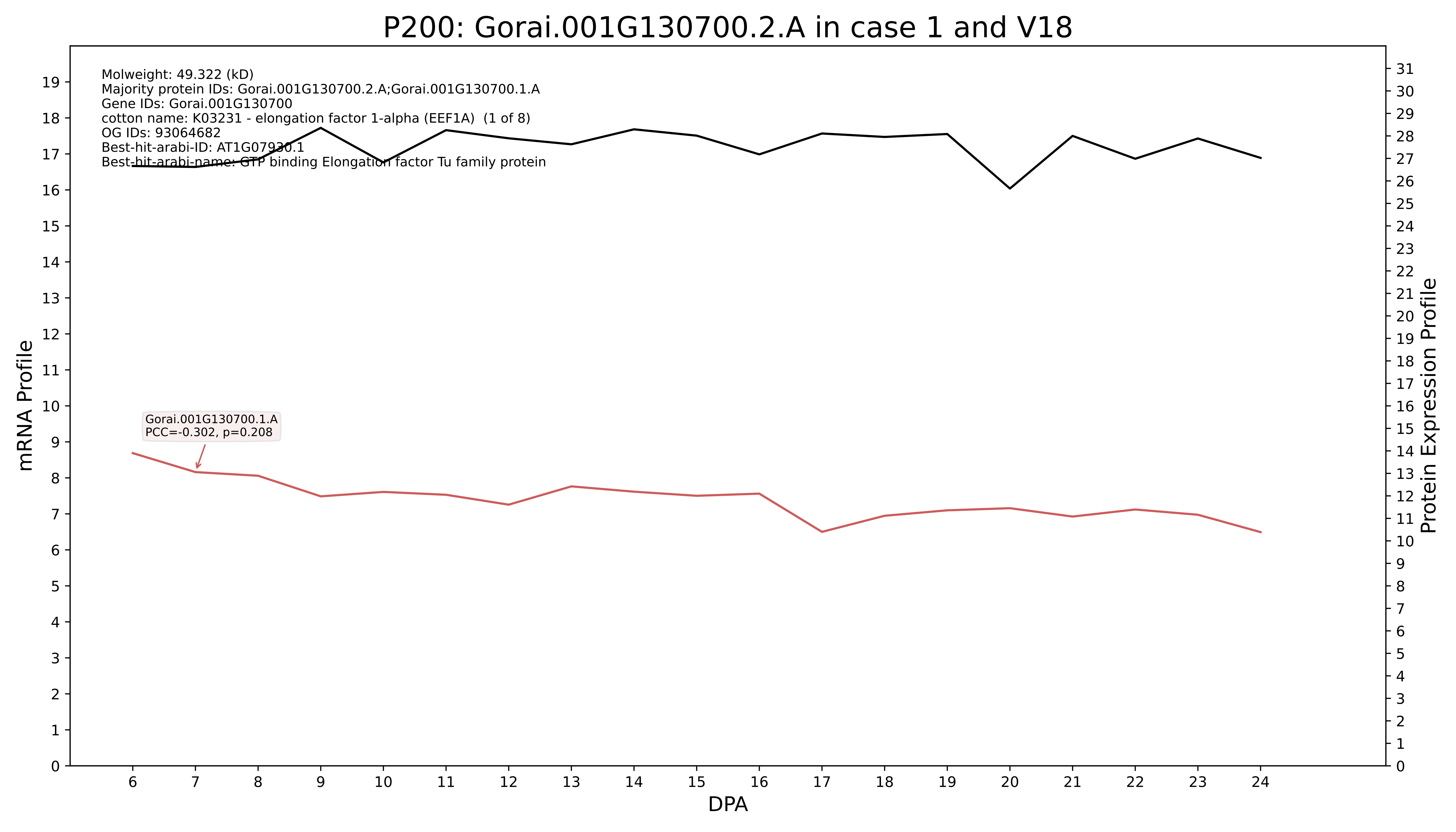
P200: Gorai.001G130700.2.A in case 1 and V18
Molweight: 49.322 (kD)
Majority protein IDs: Gorai.001G130700.2.A;Gorai.001G130700.1.A
Gene IDs: Gorai.001G130700
cotton name: K03231 - elongation factor 1-alpha (EEF1A) (1 of 8)
OG IDs: 93064682
Best-hit-arabi-ID: AT1G07930.1
Best-hit-arabi-name: GTP binding Elongation factor Tu family protein
Gorai.001G130700.1.A
PCC=-0.302, p=0.208
mRNA Profile
Protein Expression Profile
DPA

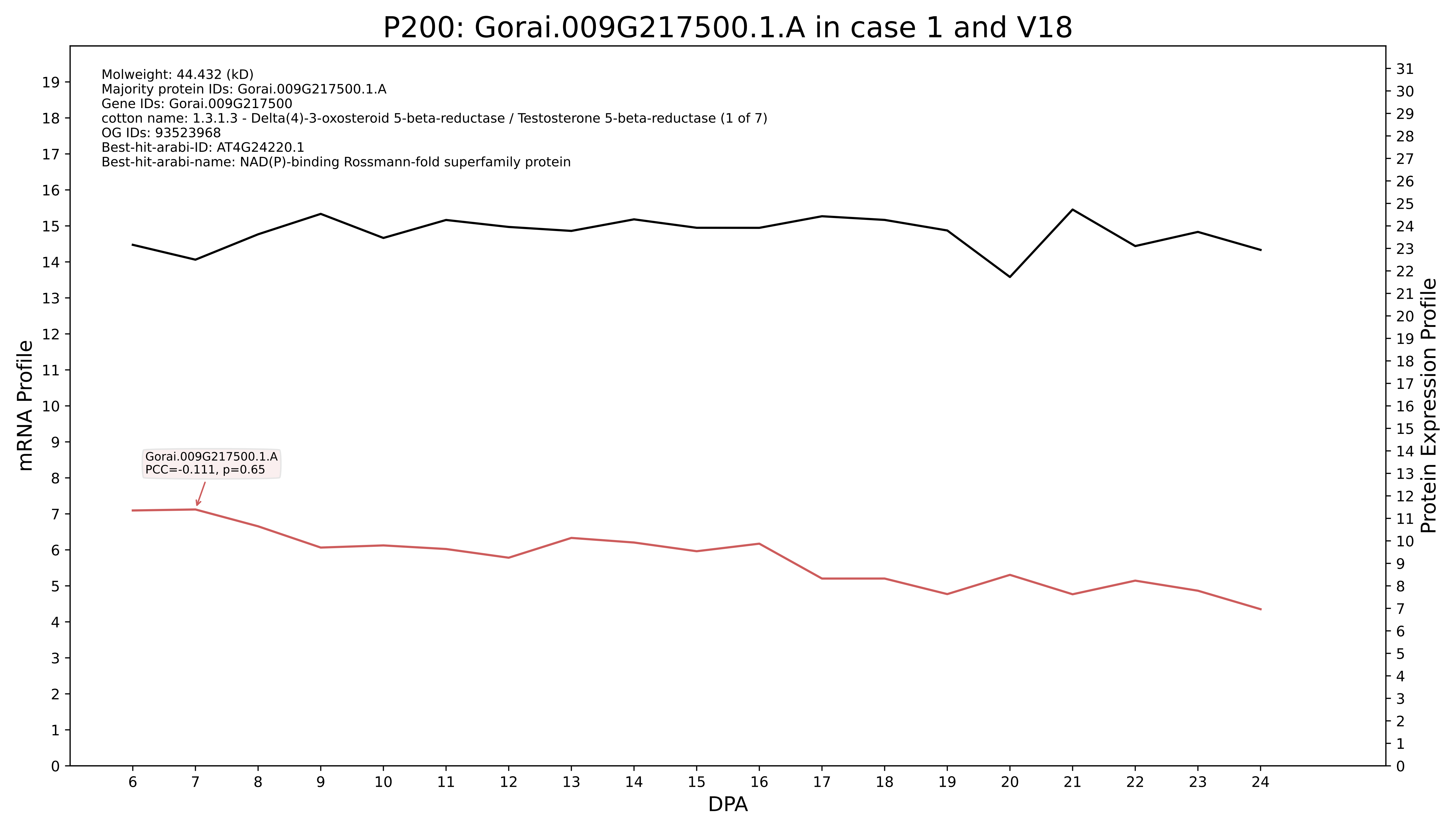
P200: Gorai.009G217500.1.A in case 1 and V18
Molweight: 44.432 (kD)
Majority protein IDs: Gorai.009G217500.1.A
Gene IDs: Gorai.009G217500
cotton name: 1.3.1.3 - Delta(4)-3-oxosteroid 5-beta-reductase / Testosterone 5-beta-reductase (1 of 7)
OG IDs: 93523968
Best-hit-arabi-ID: AT4G24220.1
Best-hit-arabi-name: NAD(P)-binding Rossmann-fold superfamily protein
Gorai.009G217500.1.A
PCC=-0.111, p=0.65
mRNA Profile
Protein Expression Profile
DPA

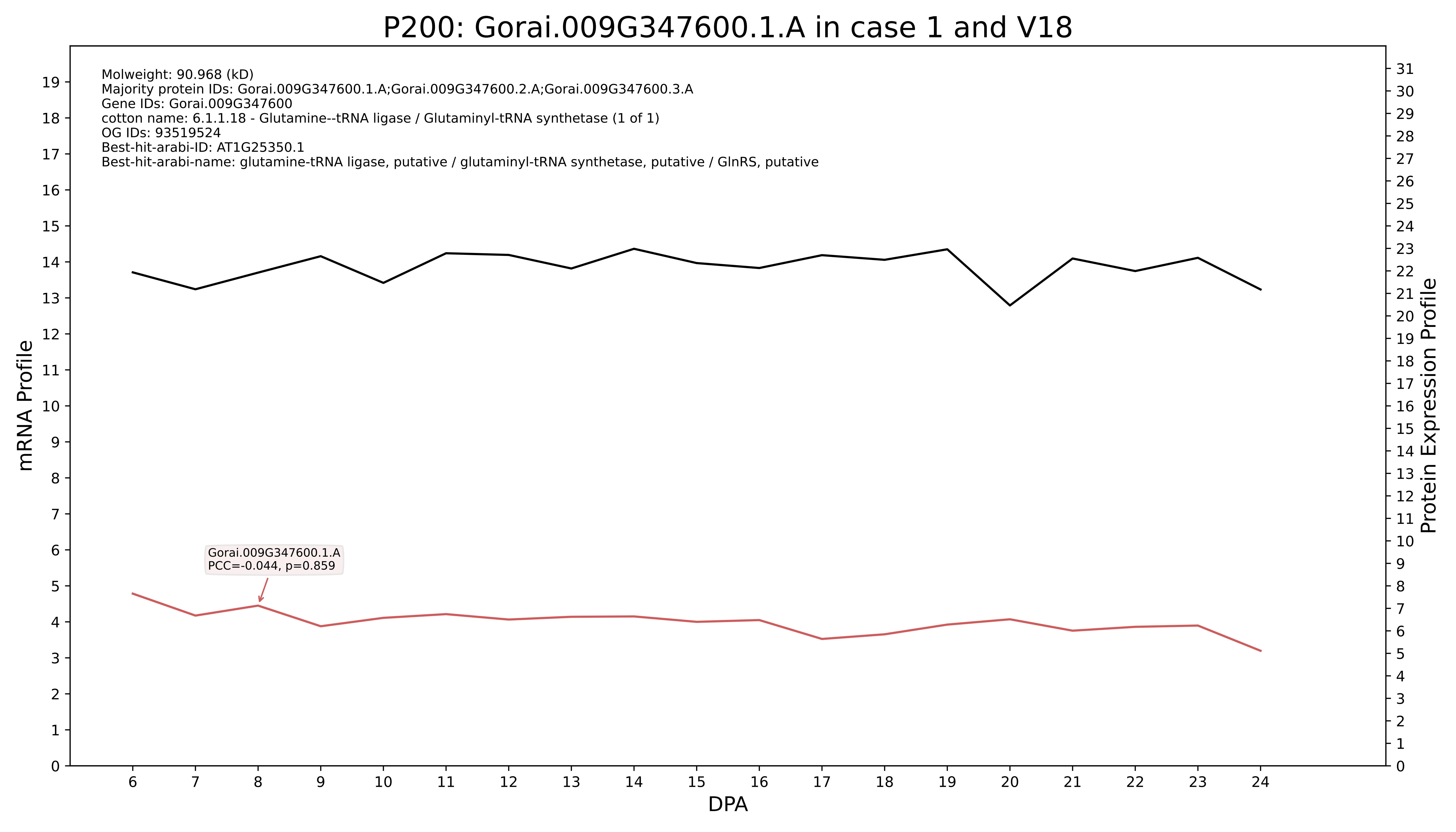
P200: Gorai.009G347600.1.A in case 1 and V18
Molweight: 90.968 (kD)
Majority protein IDs: Gorai.009G347600.1.A;Gorai.009G347600.2.A;Gorai.009G347600.3.A
Gene IDs: Gorai.009G347600
cotton name: 6.1.1.18 - Glutamine--tRNA ligase / Glutaminyl-tRNA synthetase (1 of 1)
OG IDs: 93519524
Best-hit-arabi-ID: AT1G25350.1
Best-hit-arabi-name: glutamine-tRNA ligase, putative / glutaminyl-tRNA synthetase, putative / GlnRS, putative
Gorai.009G347600.1.A
PCC=-0.044, p=0.859
mRNA Profile
Protein Expression Profile
DPA
0
1
2
3
4
5
6
7
8
9
10
11
12
13
14
15
16
17
18
19
20
21
22
23
24
25
26
27
28
29
30
31

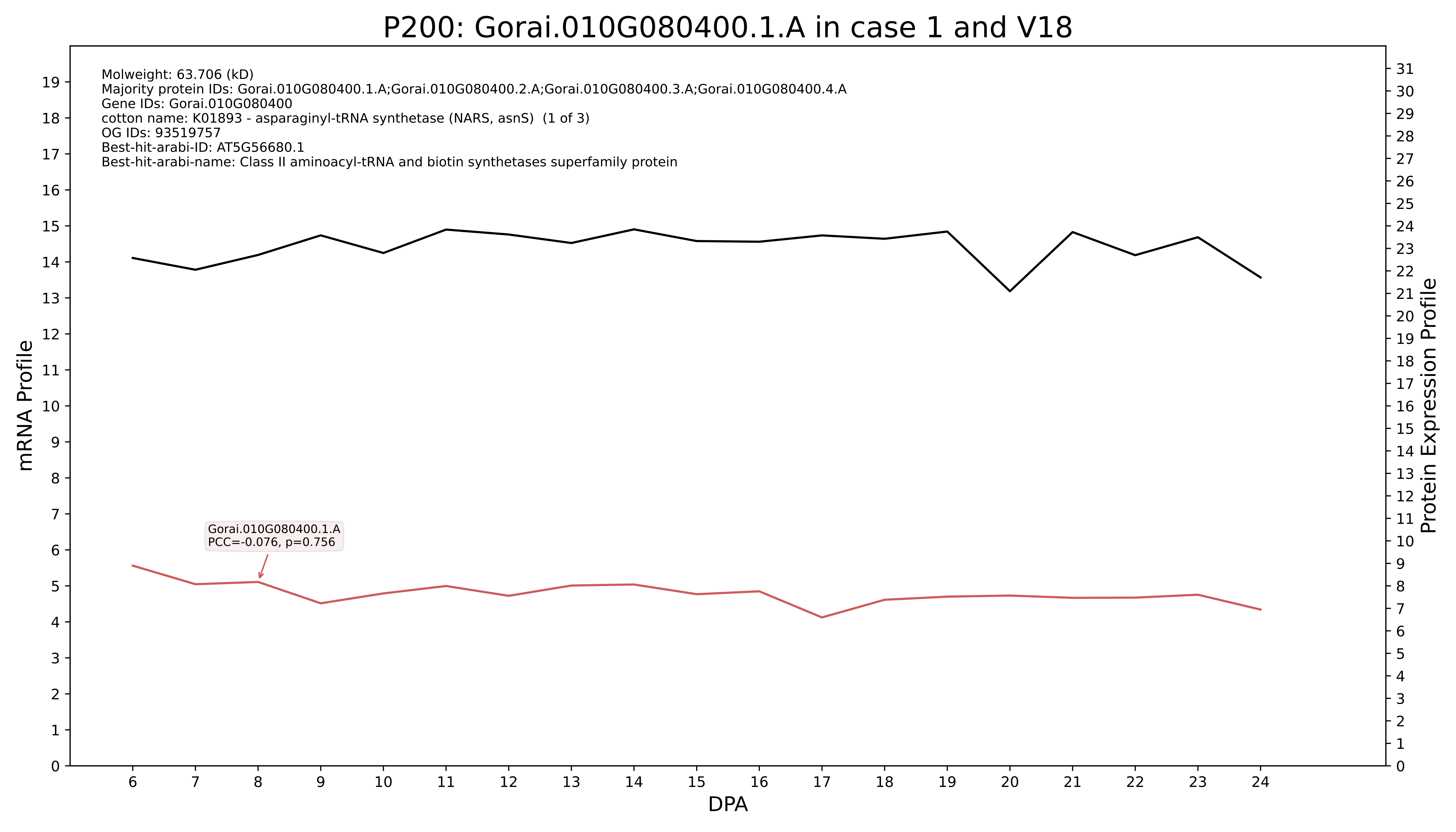
P200: Gorai.010G080400.1.A in case 1 and V18
Molweight: 63.706 (kD)
Majority protein IDs: Gorai.010G080400.1.A;Gorai.010G080400.2.A;Gorai.010G080400.3.A;Gorai.010G080400.4.A
Gene IDs: Gorai.010G080400
cotton name: K01893 - asparaginyl-tRNA synthetase (NARS, asnS) (1 of 3)
OG IDs: 93519757
Best-hit-arabi-ID: AT5G56680.1
Best-hit-arabi-name: Class II aminoacyl-tRNA and biotin synthetases superfamily protein
Gorai.010G080400.1.A
PCC=-0.076, p=0.756
mRNA Profile
Protein Expression Profile
DPA

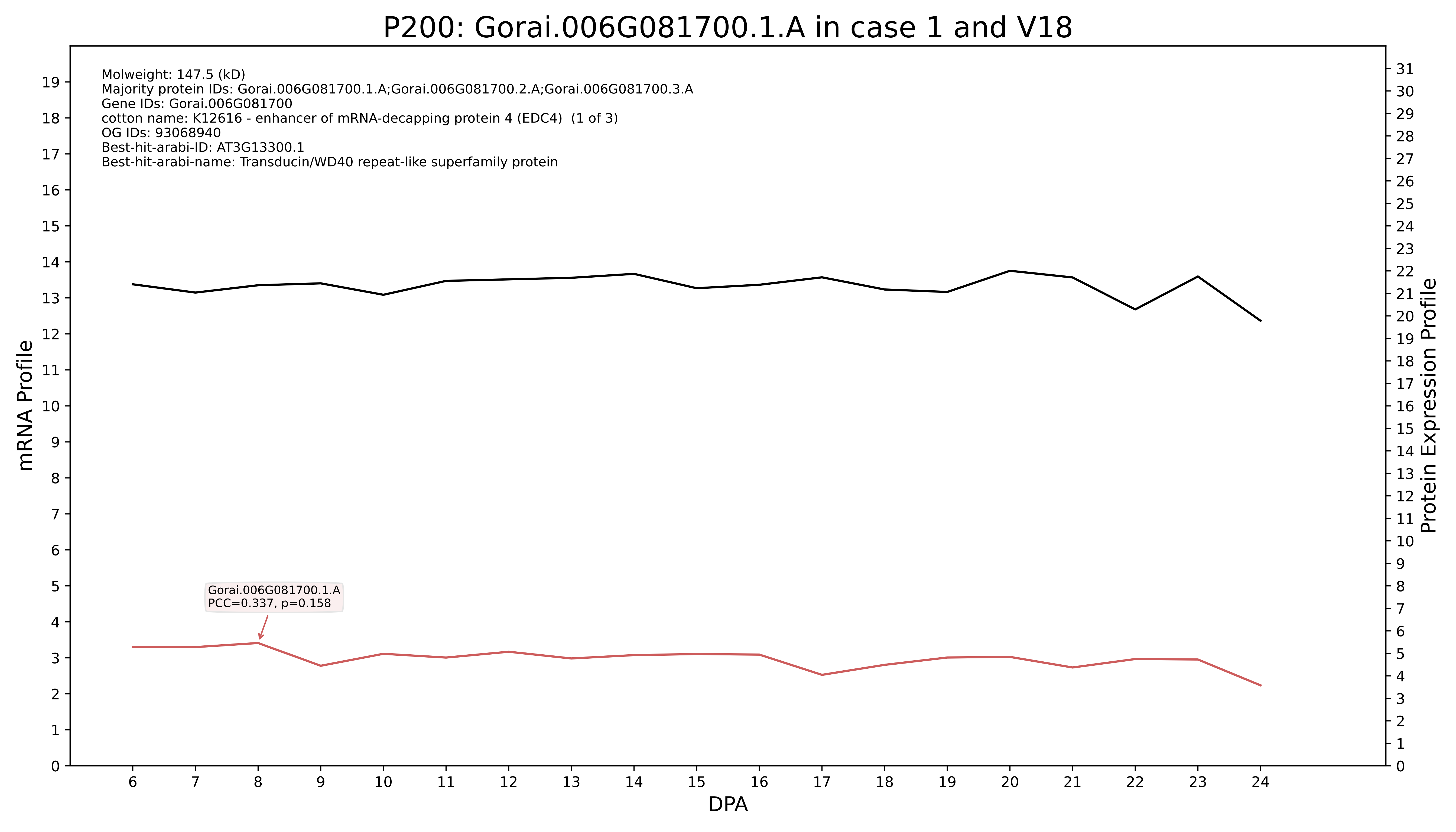
P200: Gorai.006G081700.1.A in case 1 and V18
Molweight: 147.5 (kD)
Majority protein IDs: Gorai.006G081700.1.A;Gorai.006G081700.2.A;Gorai.006G081700.3.A
Gene IDs: Gorai.006G081700
cotton name: K12616 - enhancer of mRNA-decapping protein 4 (EDC4) (1 of 3)
OG IDs: 93068940
Best-hit-arabi-ID: AT3G13300.1
Best-hit-arabi-name: Transducin/WD40 repeat-like superfamily protein
Gorai.006G081700.1.A
PCC=0.337, p=0.158
mRNA Profile
Protein Expression Profile
DPA

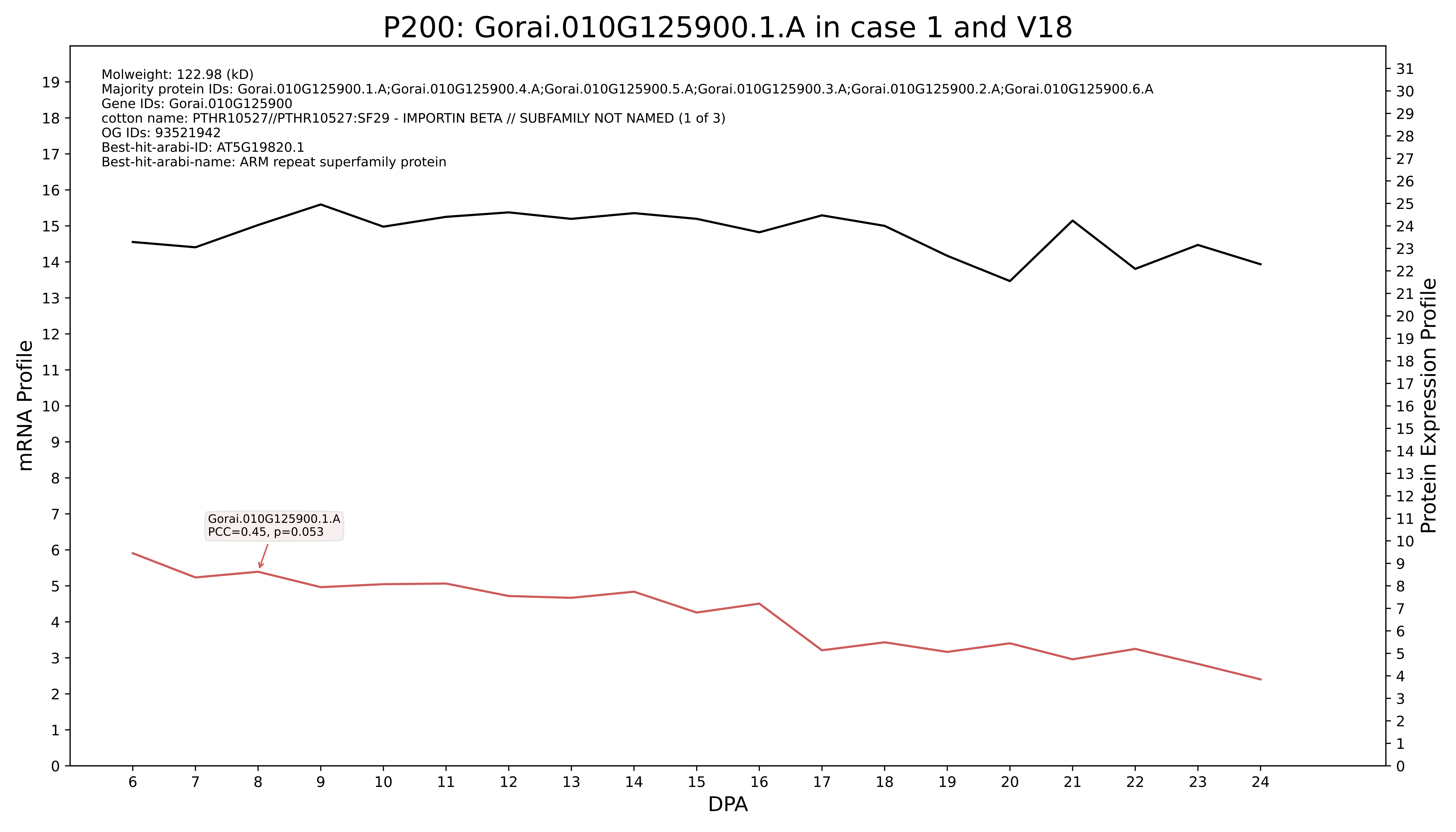
P200: Gorai.010G125900.1.A in case 1 and V18
Molweight: 122.98 (kD)
Majority protein IDs: Gorai.010G125900.1.A;Gorai.010G125900.4.A;Gorai.010G125900.5.A;Gorai.010G125900.3.A;Gorai.010G125900.2.A;Gorai.010G125900.6.A
Gene IDs: Gorai.010G125900
cotton name: PTHR10527//PTHR10527:SF29 - IMPORTIN BETA // SUBFAMILY NOT NAMED (1 of 3)
OG IDs: 93521942
Best-hit-arabi-ID: AT5G19820.1
Best-hit-arabi-name: ARM repeat superfamily protein
Gorai.010G125900.1.A
PCC=0.45, p=0.053
mRNA Profile
Protein Expression Profile
DPA

P200: Gorai.010G153600.1.A in case 1 and V18

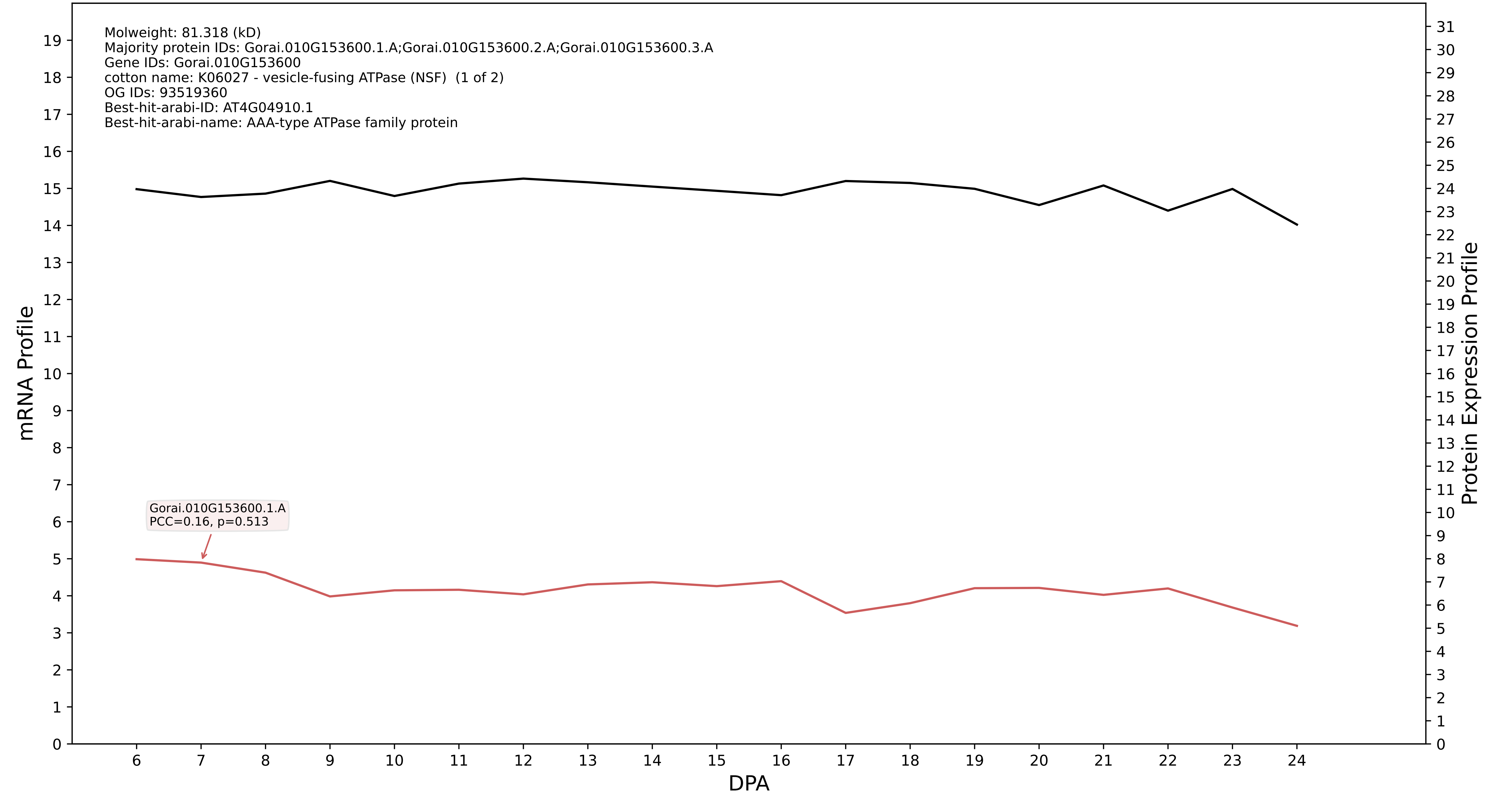

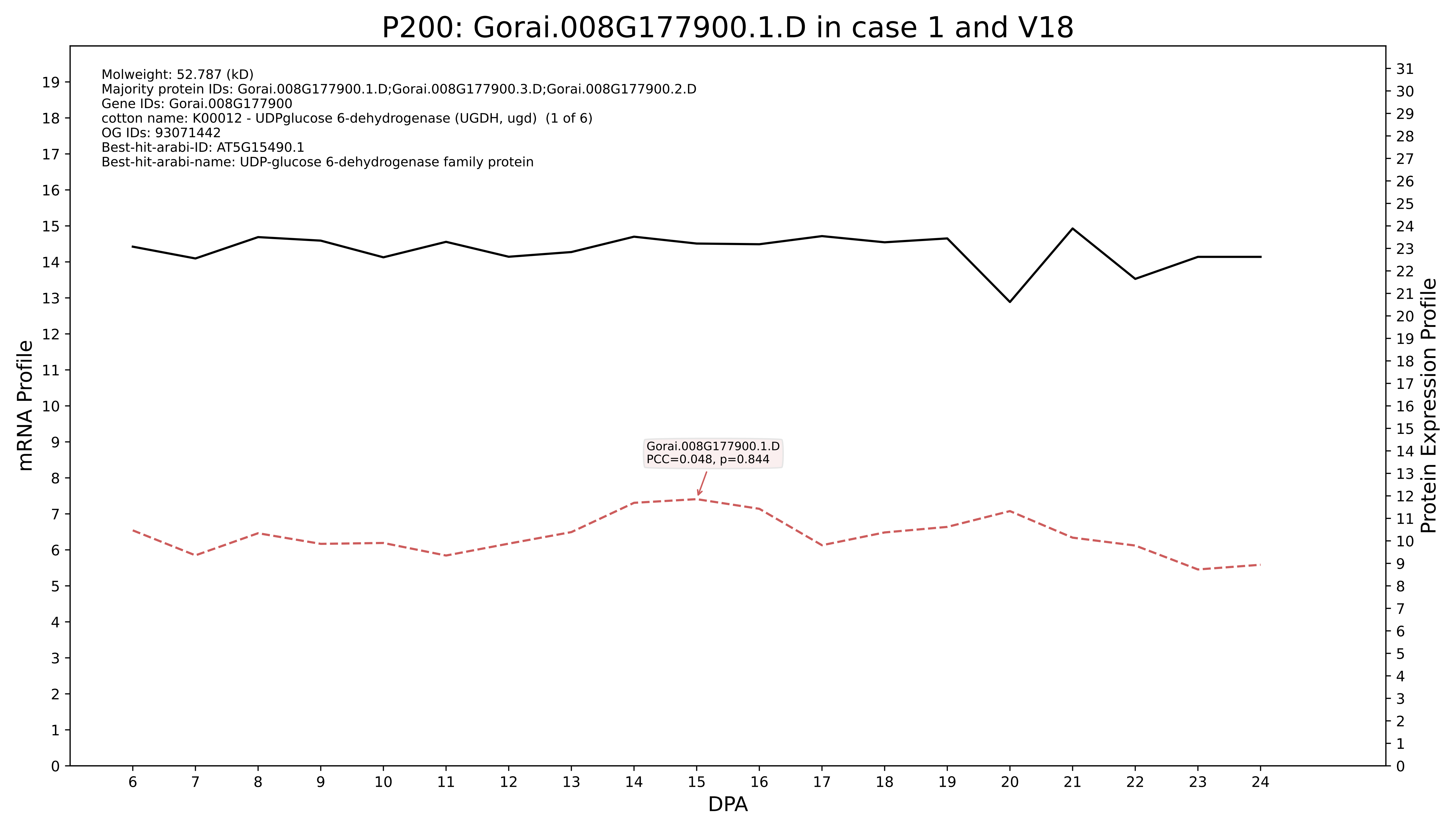
P200: Gorai.008G177900.1.D in case 1 and V18
Molweight: 52.787 (kD)
Majority protein IDs: Gorai.008G177900.1.D;Gorai.008G177900.3.D;Gorai.008G177900.2.D
Gene IDs: Gorai.008G177900
cotton name: K00012 - UDPglucose 6-dehydrogenase (UGDH, ugd) (1 of 6)
OG IDs: 93071442
Best-hit-arabi-ID: AT5G15490.1
Best-hit-arabi-name: UDP-glucose 6-dehydrogenase family protein
Gorai.008G177900.1.D
PCC=0.048, p=0.844
mRNA Profile
Protein Expression Profile
DPA

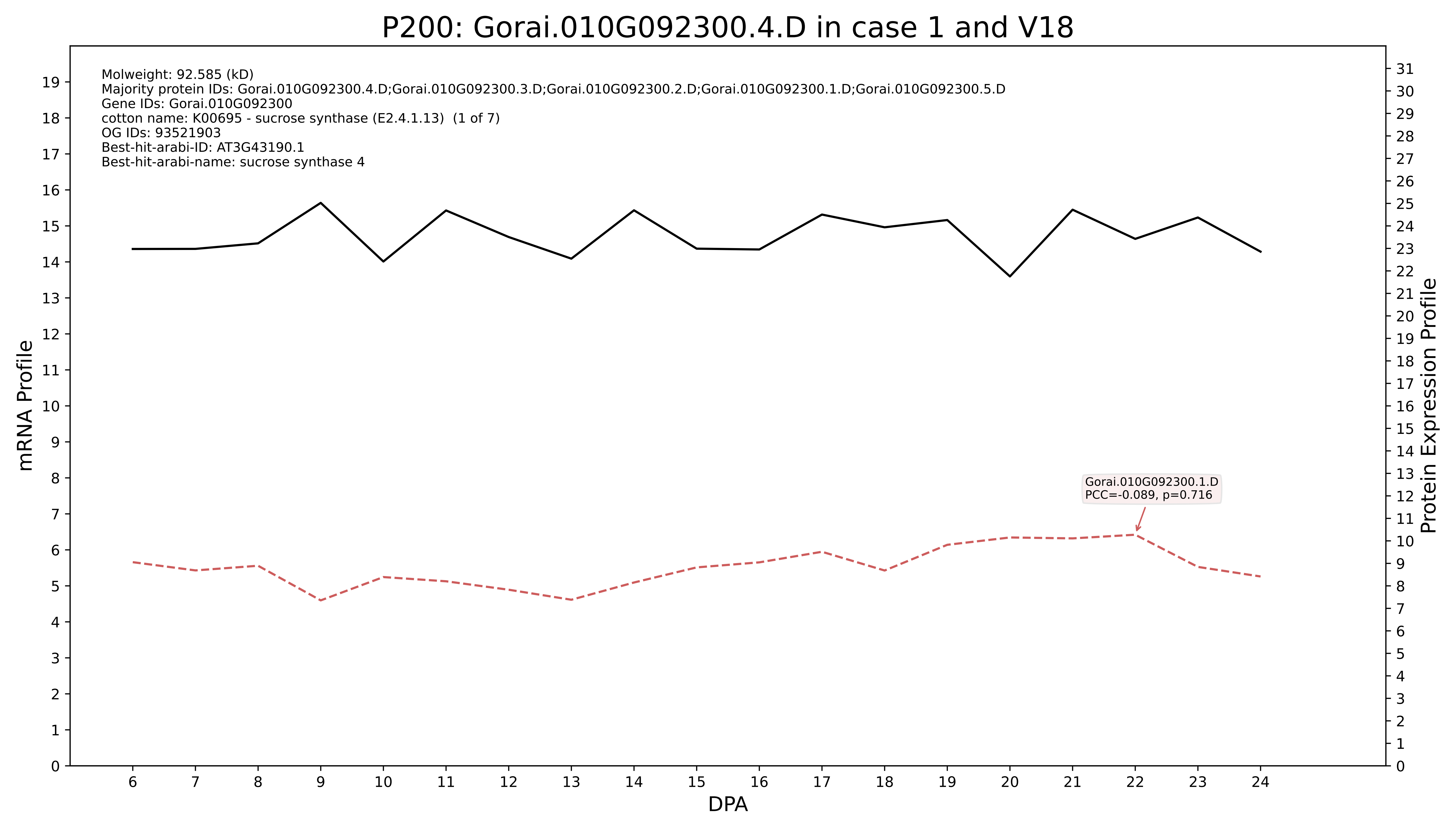
P200: Gorai.010G092300.4.D in case 1 and V18
Molweight: 92.585 (kD)
Majority protein IDs: Gorai.010G092300.4.D;Gorai.010G092300.3.D;Gorai.010G092300.2.D;Gorai.010G092300.1.D;Gorai.010G092300.5.D
Gene IDs: Gorai.010G092300
cotton name: K00695 - sucrose synthase (E2.4.1.13) (1 of 7)
OG IDs: 93521903
Best-hit-arabi-ID: AT3G43190.1
Best-hit-arabi-name: sucrose synthase 4
Gorai.010G092300.1.D
PCC=-0.089, p=0.716
mRNA Profile
Protein Expression Profile
DPA

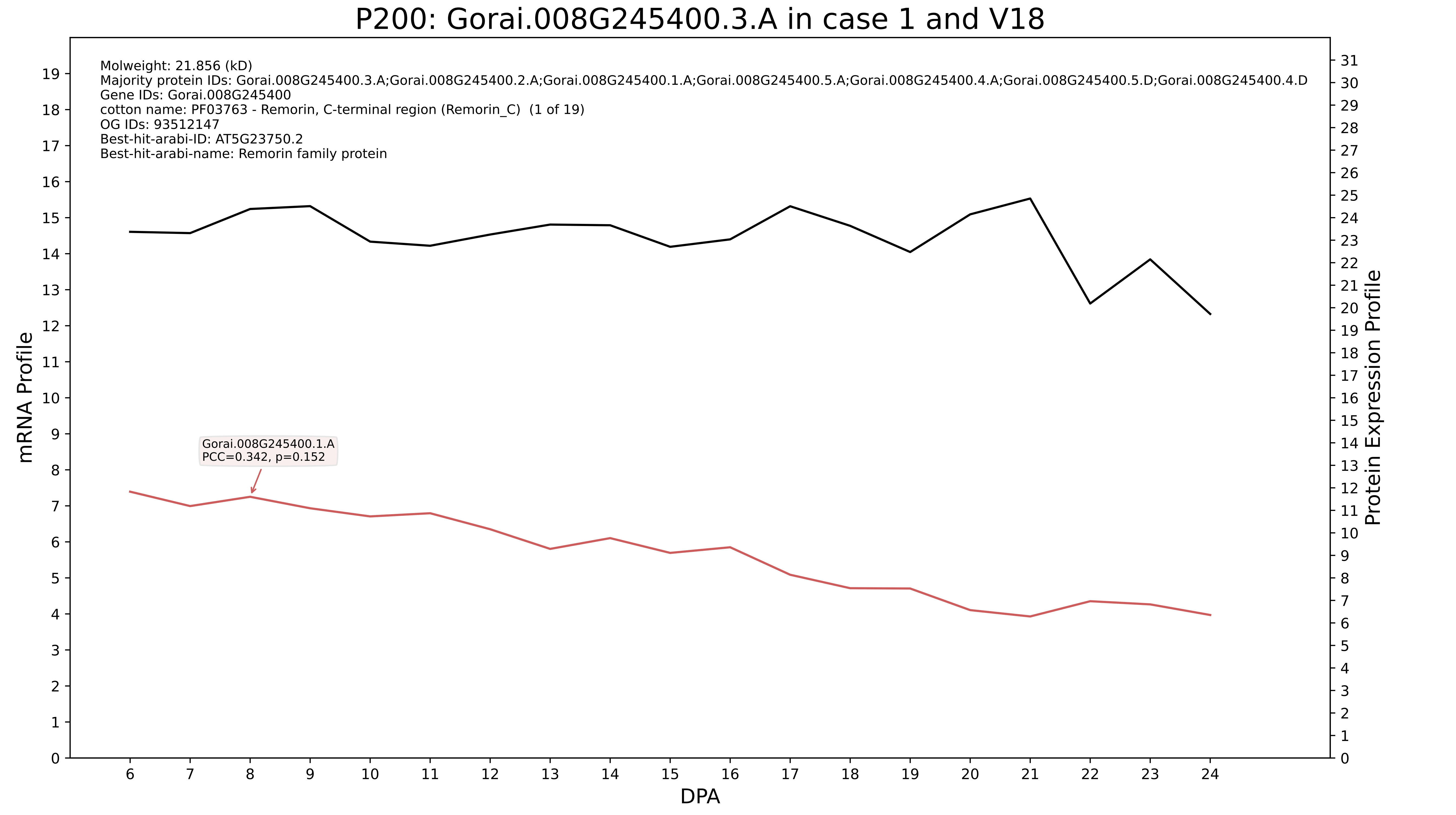
P200: Gorai.008G245400.3.A in case 1 and V18
Molweight: 21.856 (kD)
Majority protein IDs: Gorai.008G245400.3.A;Gorai.008G245400.2.A;Gorai.008G245400.1.A;Gorai.008G245400.5.A;Gorai.008G245400.4.A;Gorai.008G245400.5.D;Gorai.008G245400.4.D
Gene IDs: Gorai.008G245400
cotton name: PF03763 - Remorin, C-terminal region (Remorin_C) (1 of 19)
OG IDs: 93512147
Best-hit-arabi-ID: AT5G23750.2
Best-hit-arabi-name: Remorin family protein
Gorai.008G245400.1.A
PCC=0.342, p=0.152
mRNA Profile
Protein Expression Profile
DPA

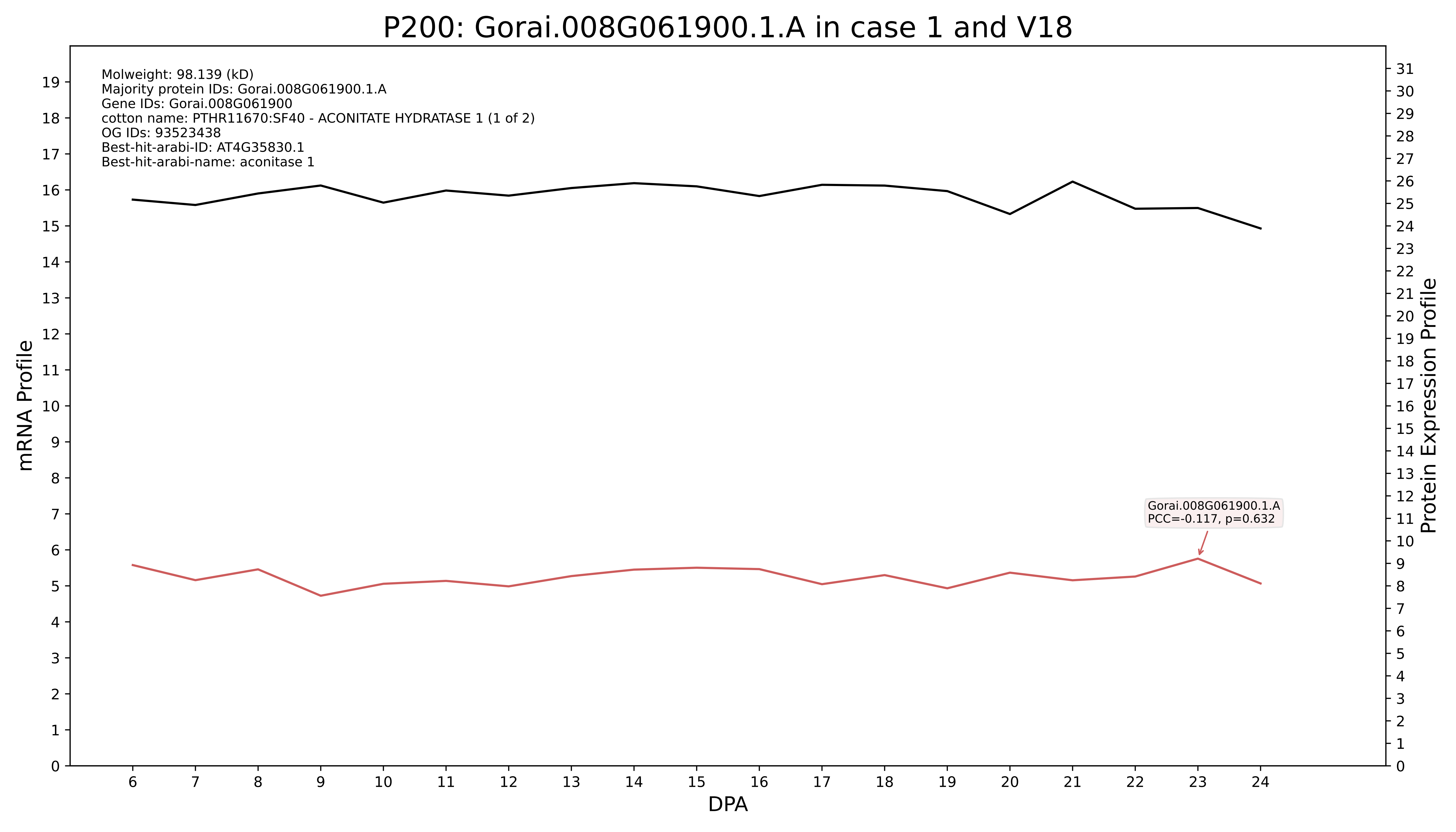

P200: Gorai.008G061900.1.A in case 1 and V18
Molweight: 98.139 (kD)
Majority protein IDs: Gorai.008G061900.1.A
Gene IDs: Gorai.008G061900
cotton name: PTHR11670:SF40 - ACONITATE HYDRATASE 1 (1 of 2)
OG IDs: 93523438
Best-hit-arabi-ID: AT4G35830.1
Best-hit-arabi-name: aconitase 1
Gorai.008G061900.1.A
PCC=-0.117, p=0.632
mRNA Profile
Protein Expression Profile
DPA

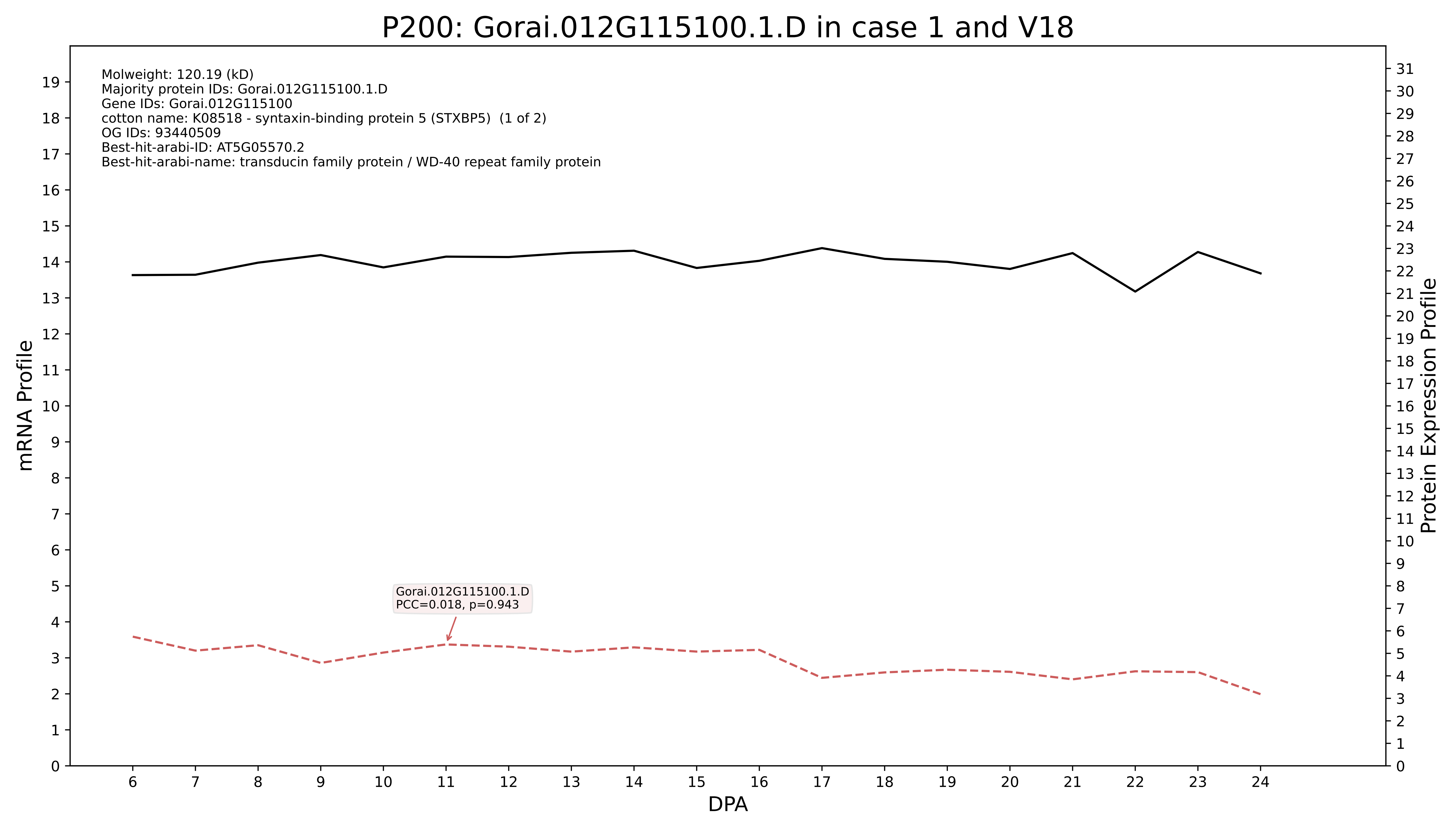

P200: Gorai.012G115100.1.D in case 1 and V18
Molweight: 120.19 (kD)
Majority protein IDs: Gorai.012G115100.1.D
Gene IDs: Gorai.012G115100
cotton name: K08518 - syntaxin-binding protein 5 (STXBP5) (1 of 2)
OG IDs: 93440509
Best-hit-arabi-ID: AT5G05570.2
Best-hit-arabi-name: transducin family protein / WD-40 repeat family protein
Gorai.012G115100.1.D
PCC=0.018, p=0.943
mRNA Profile
Protein Expression Profile
DPA

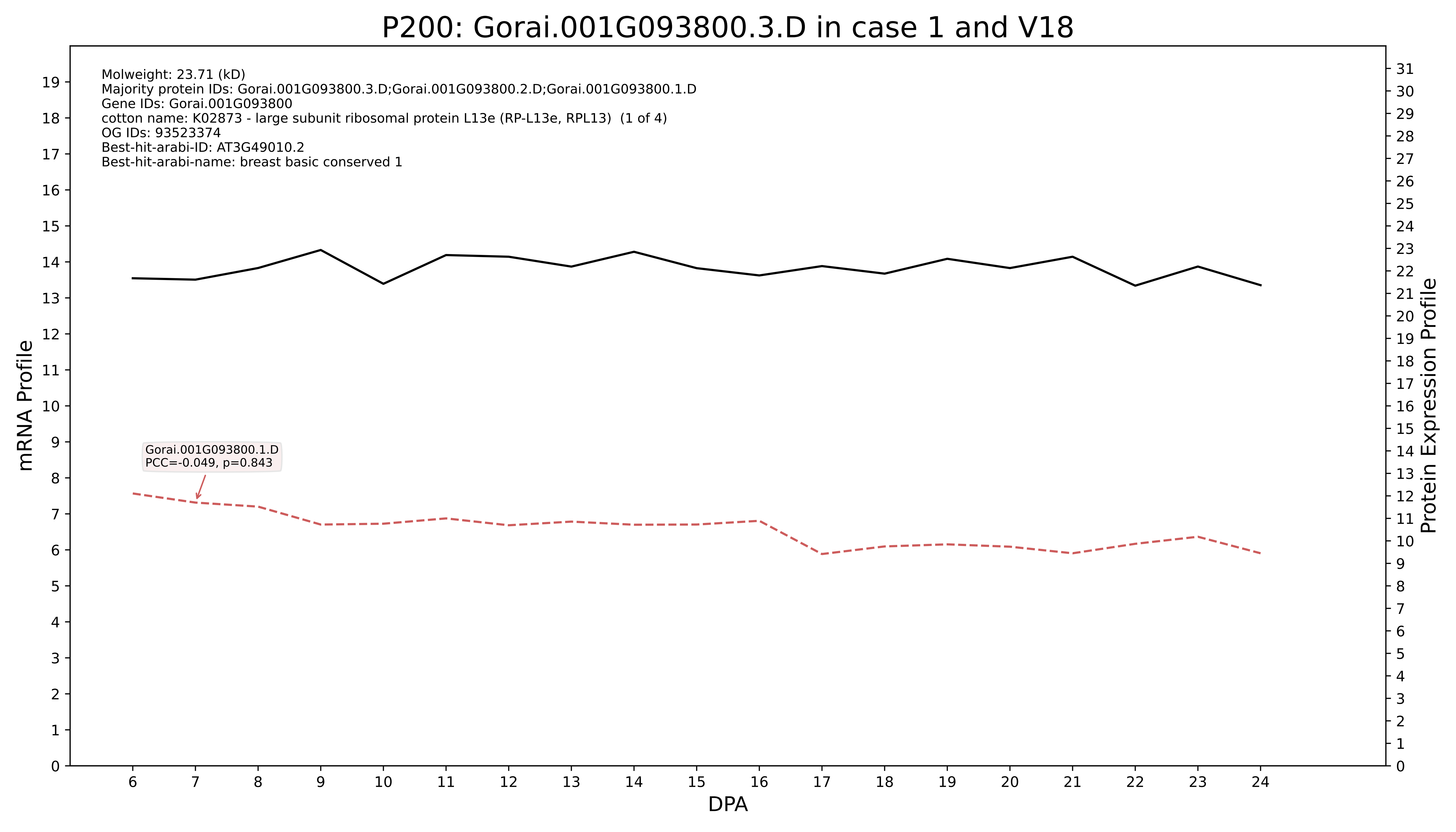
P200: Gorai.001G093800.3.D in case 1 and V18
Molweight: 23.71 (kD)
Majority protein IDs: Gorai.001G093800.3.D;Gorai.001G093800.2.D;Gorai.001G093800.1.D
Gene IDs: Gorai.001G093800
cotton name: K02873 - large subunit ribosomal protein L13e (RP-L13e, RPL13) (1 of 4)
OG IDs: 93523374
Best-hit-arabi-ID: AT3G49010.2
Best-hit-arabi-name: breast basic conserved 1
Gorai.001G093800.1.D
PCC=-0.049, p=0.843
mRNA Profile
Protein Expression Profile
DPA
6
7
8
9
10
11
12
13
14
15
16
17
18
19
20
21
22
23
24

P200: Gorai.007G004500.1.D in case 1 and V18

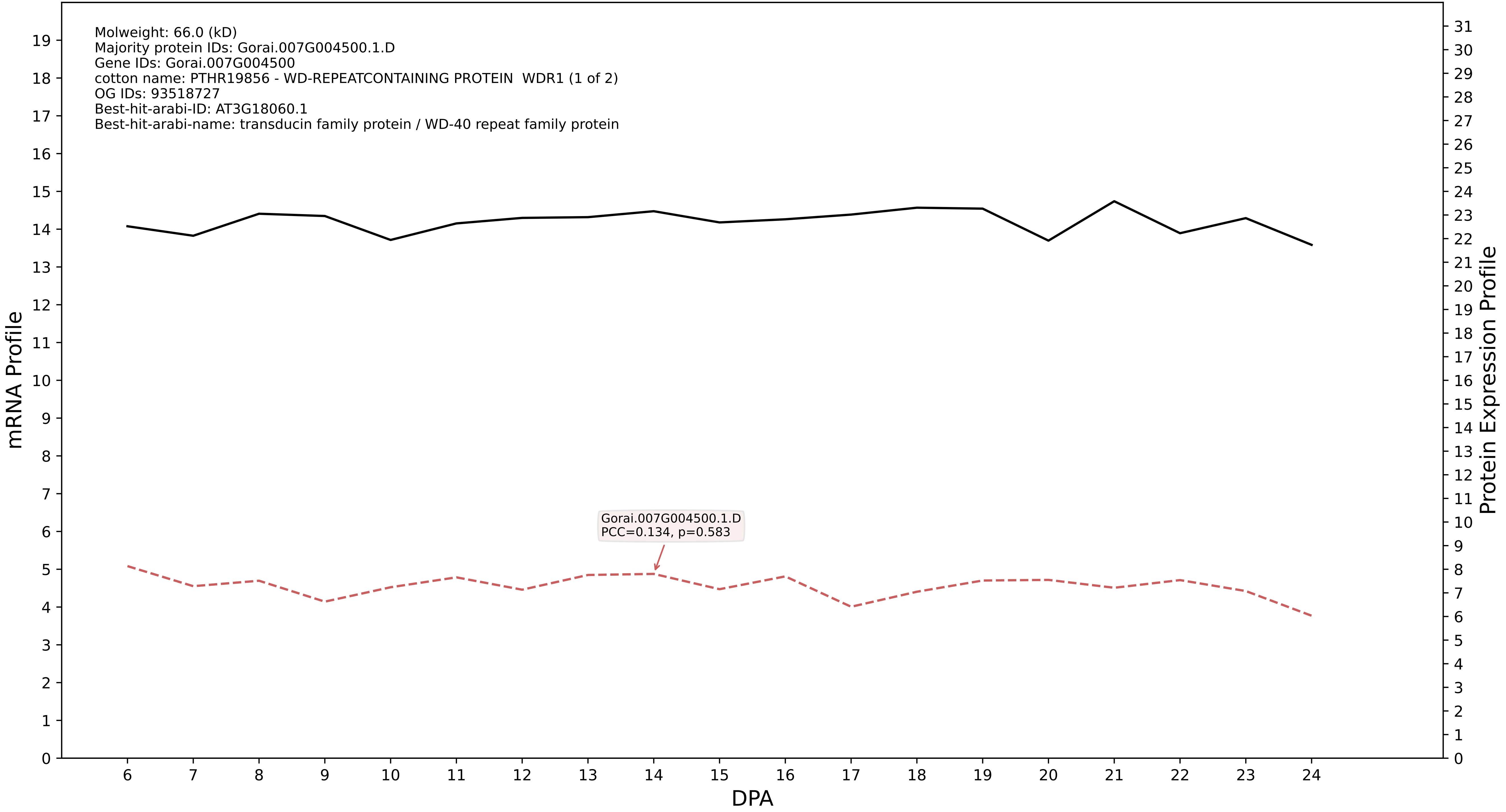

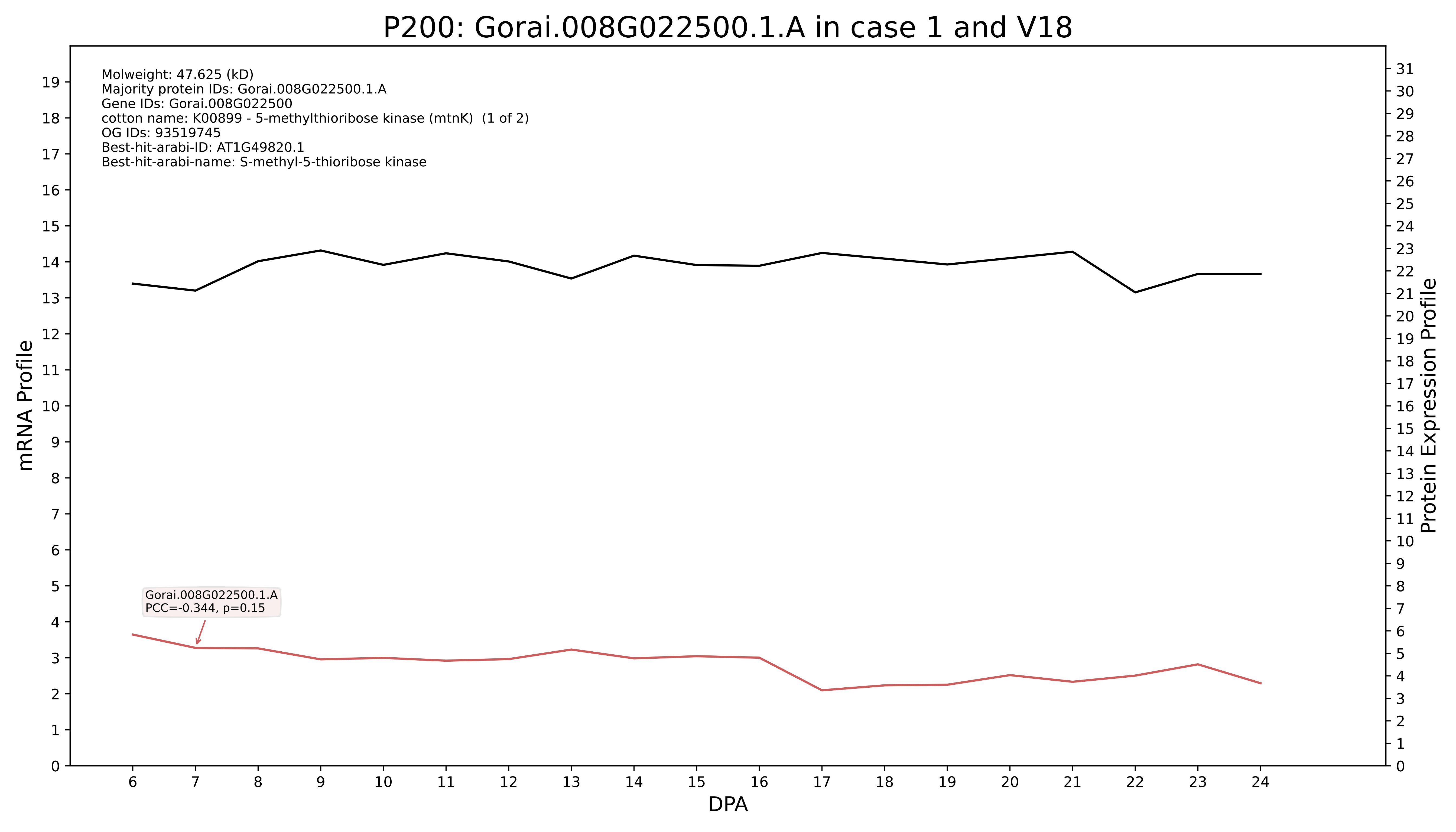

P200: Gorai.008G022500.1.A in case 1 and V18
Molweight: 47.625 (kD)
Majority protein IDs: Gorai.008G022500.1.A
Gene IDs: Gorai.008G022500
cotton name: K00899 - 5-methylthioribose kinase (mtnK) (1 of 2)
OG IDs: 93519745
Best-hit-arabi-ID: AT1G49820.1
Best-hit-arabi-name: S-methyl-5-thioribose kinase
Gorai.008G022500.1.A
PCC=-0.344, p=0.15
mRNA Profile
Protein Expression Profile
DPA

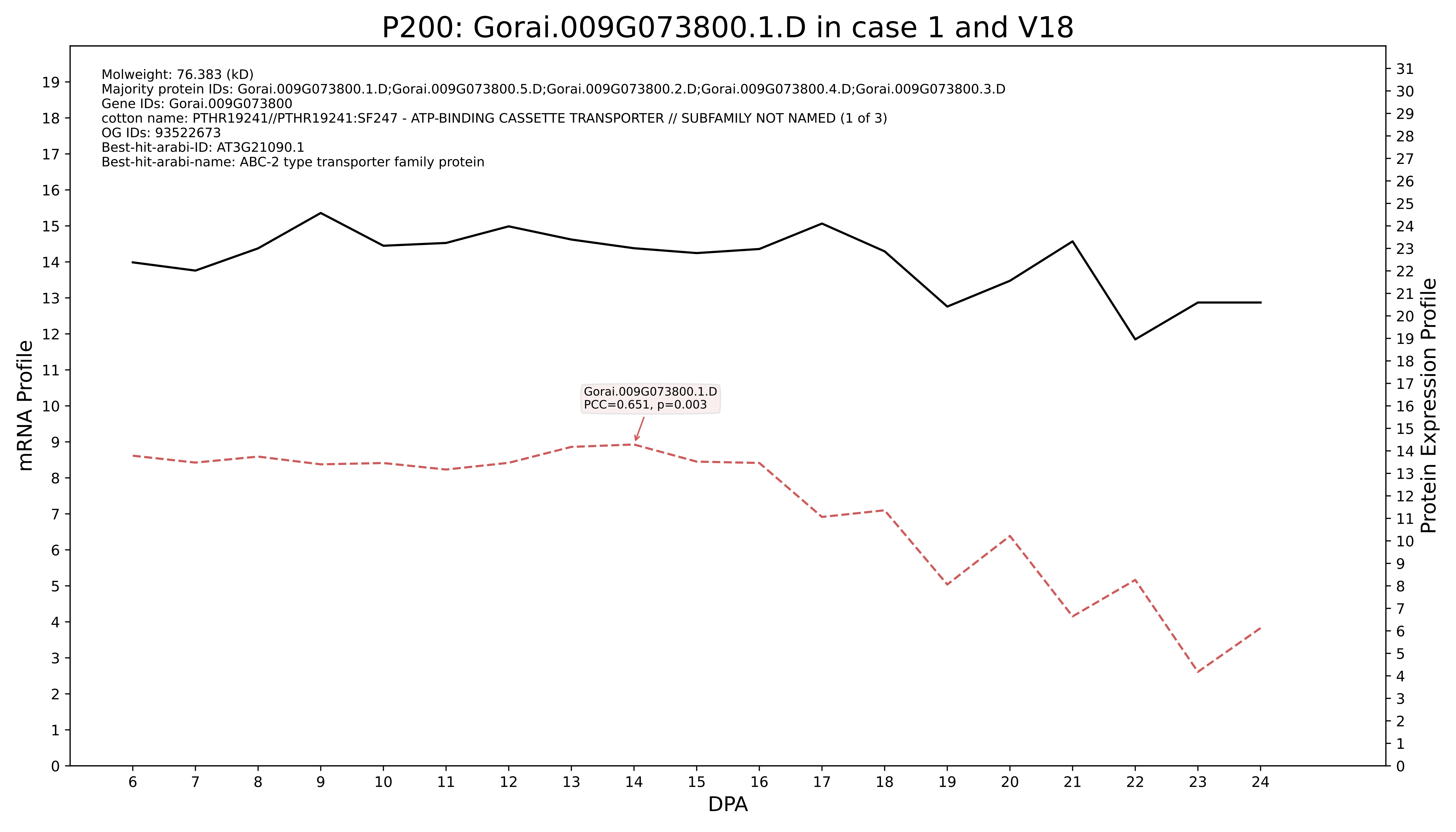
P200: Gorai.009G073800.1.D in case 1 and V18
Molweight: 76.383 (kD)
Majority protein IDs: Gorai.009G073800.1.D;Gorai.009G073800.5.D;Gorai.009G073800.2.D;Gorai.009G073800.4.D;Gorai.009G073800.3.D
Gene IDs: Gorai.009G073800
cotton name: PTHR19241//PTHR19241:SF247 - ATP-BINDING CASSETTE TRANSPORTER // SUBFAMILY NOT NAMED (1 of 3)
OG IDs: 93522673
Best-hit-arabi-ID: AT3G21090.1
Best-hit-arabi-name: ABC-2 type transporter family protein
Gorai.009G073800.1.D
PCC=0.651, p=0.003
mRNA Profile
Protein Expression Profile
DPA

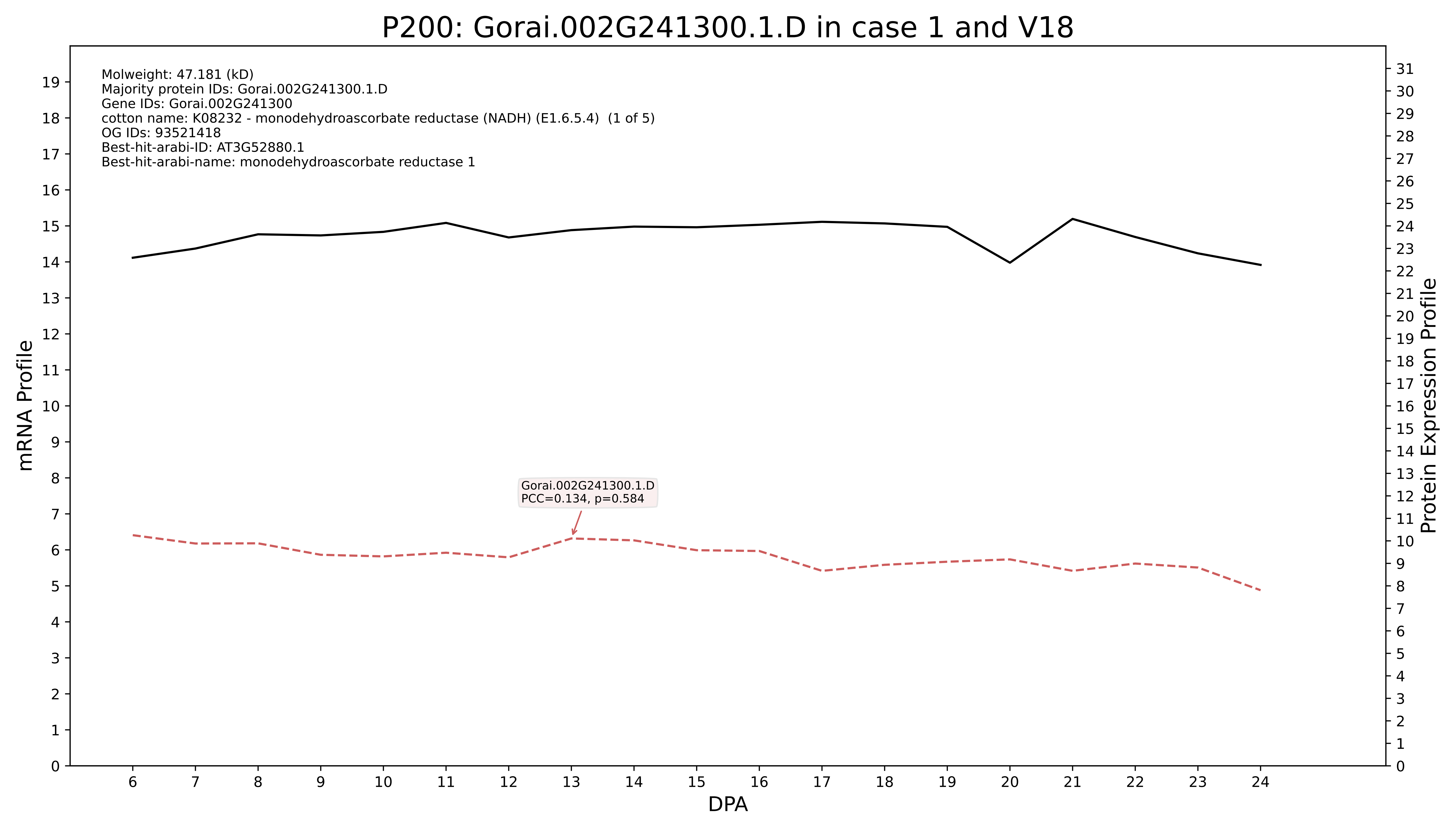
P200: Gorai.002G241300.1.D in case 1 and V18
Molweight: 47.181 (kD)
Majority protein IDs: Gorai.002G241300.1.D
Gene IDs: Gorai.002G241300
cotton name: K08232 - monodehydroascorbate reductase (NADH) (E1.6.5.4) (1 of 5)
OG IDs: 93521418
Best-hit-arabi-ID: AT3G52880.1
Best-hit-arabi-name: monodehydroascorbate reductase 1
Gorai.002G241300.1.D
PCC=0.134, p=0.584
mRNA Profile
Protein Expression Profile
DPA

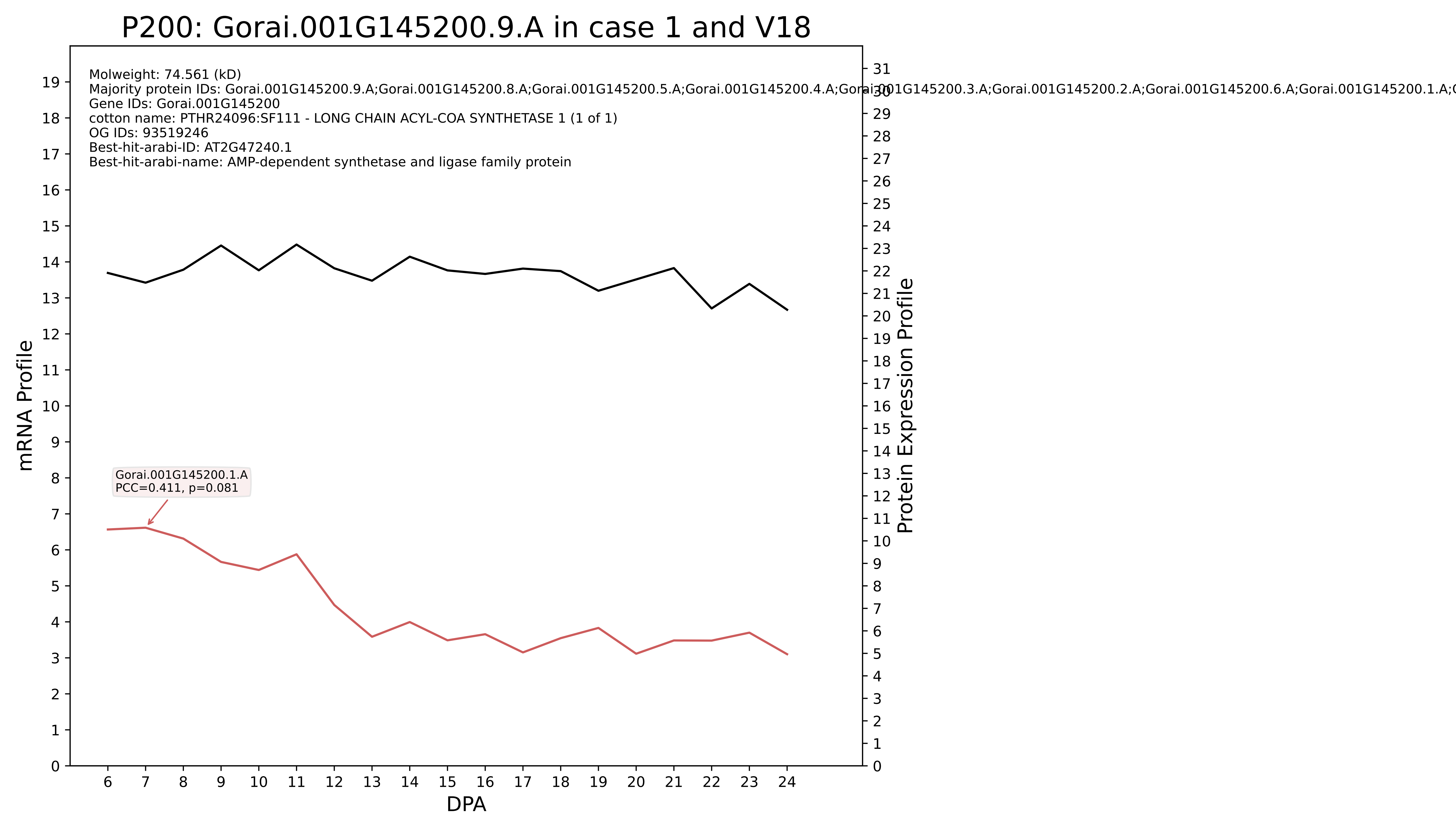
P200: Gorai.001G145200.9.A in case 1 and V18
Molweight: 74.561 (kD)
Majority protein IDs: Gorai.001G145200.9.A;Gorai.001G145200.8.A;Gorai.001G145200.5.A;Gorai.001G145200.4.A;Gorai.001G145200.3.A;Gorai.001G145200.2.A;Gorai.001G145200.6.A;Gorai.001G145200.1.A;
Gene IDs: Gorai.001G145200
cotton name: PTHR24096:SF111 - LONG CHAIN ACYL-COA SYNTHETASE 1 (1 of 1)
OG IDs: 93519246
Best-hit-arabi-ID: AT2G47240.1
Best-hit-arabi-name: AMP-dependent synthetase and ligase family protein
Gorai.001G145200.1.A
PCC=0.411, p=0.081
mRNA Profile
Protein Expression Profile
DPA

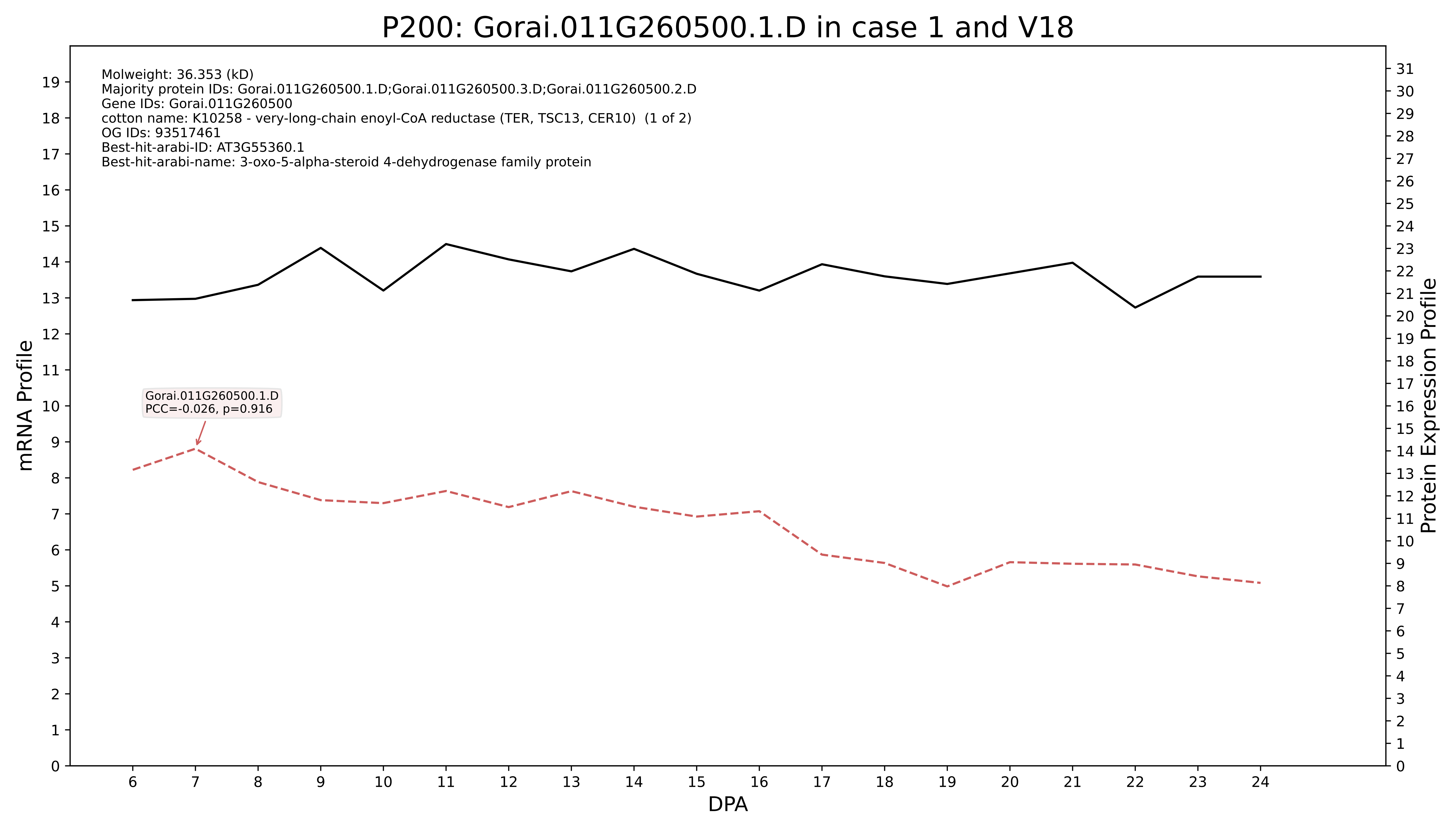

P200: Gorai.011G260500.1.D in case 1 and V18
Molweight: 36.353 (kD)
Majority protein IDs: Gorai.011G260500.1.D;Gorai.011G260500.3.D;Gorai.011G260500.2.D
Gene IDs: Gorai.011G260500
cotton name: K10258 - very-long-chain enoyl-CoA reductase (TER, TSC13, CER10) (1 of 2)
OG IDs: 93517461
Best-hit-arabi-ID: AT3G55360.1
Best-hit-arabi-name: 3-oxo-5-alpha-steroid 4-dehydrogenase family protein
Gorai.011G260500.1.D
PCC=-0.026, p=0.916
mRNA Profile
Protein Expression Profile
DPA

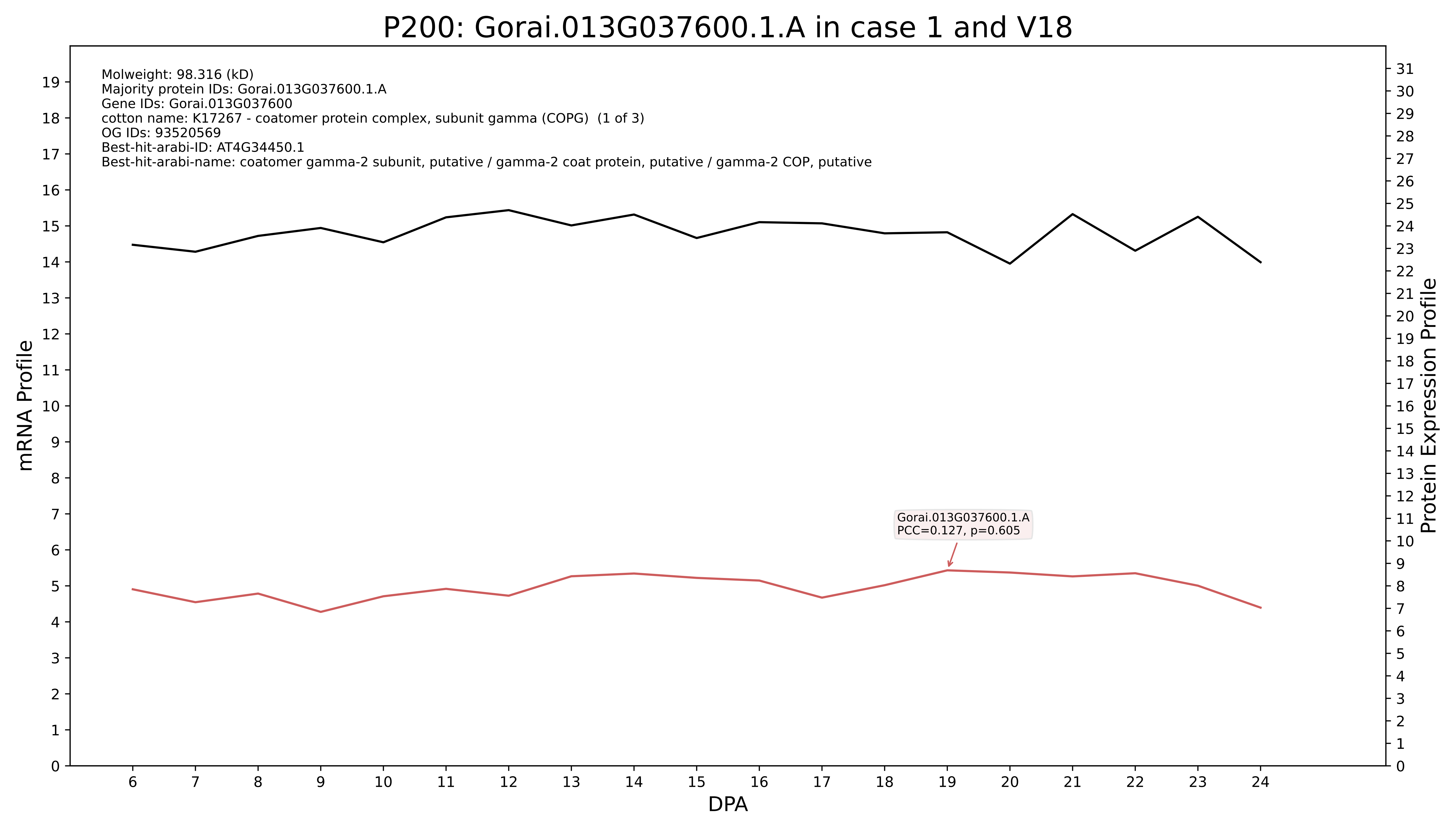
P200: Gorai.013G037600.1.A in case 1 and V18
Molweight: 98.316 (kD)
Majority protein IDs: Gorai.013G037600.1.A
Gene IDs: Gorai.013G037600
cotton name: K17267 - coatomer protein complex, subunit gamma (COPG) (1 of 3)
OG IDs: 93520569
Best-hit-arabi-ID: AT4G34450.1
Best-hit-arabi-name: coatomer gamma-2 subunit, putative / gamma-2 coat protein, putative / gamma-2 COP, putative
Gorai.013G037600.1.A
PCC=0.127, p=0.605
mRNA Profile
Protein Expression Profile
DPA

P200: Gorai.006G073400.1.A in case 1 and V18

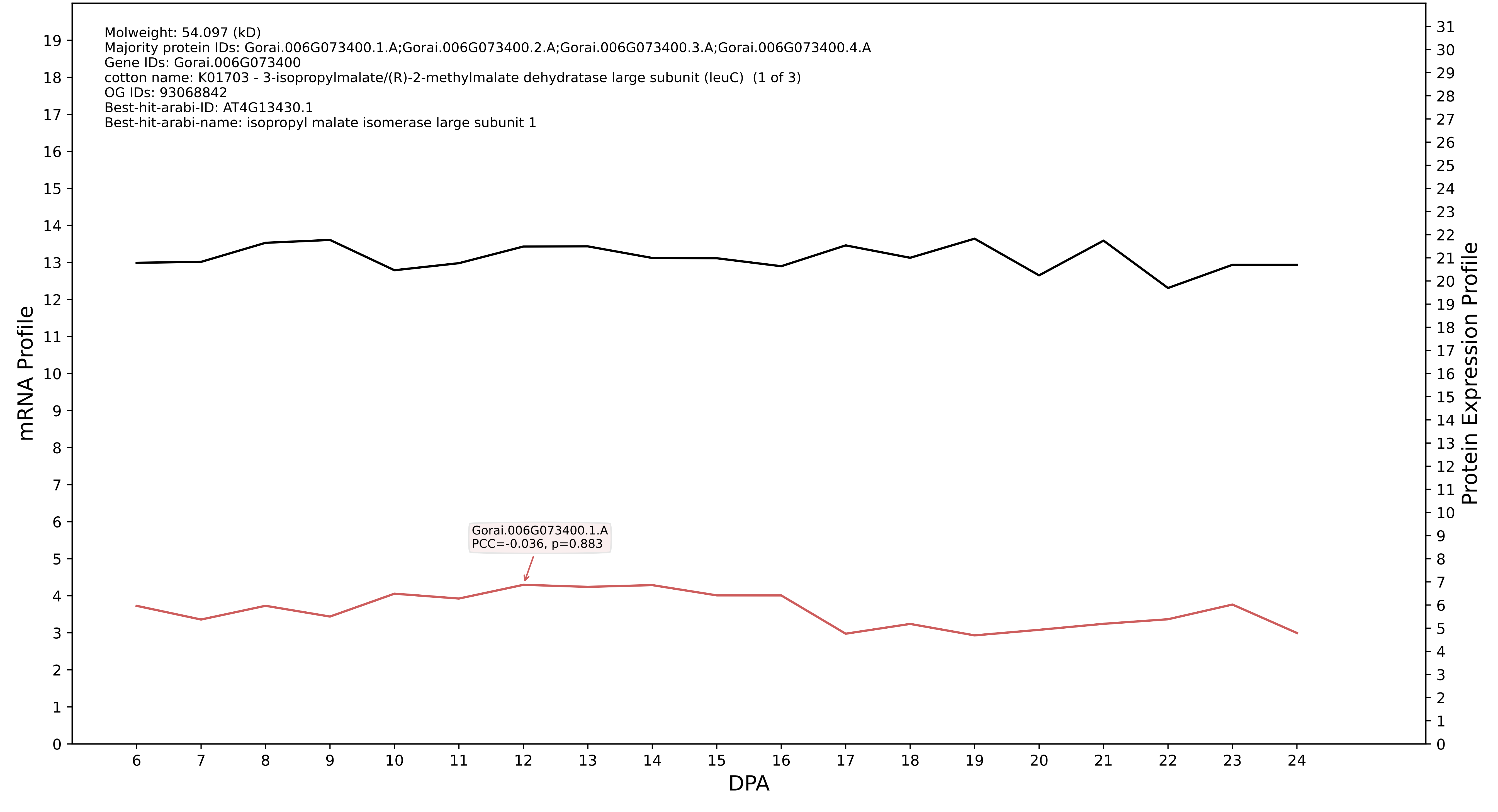

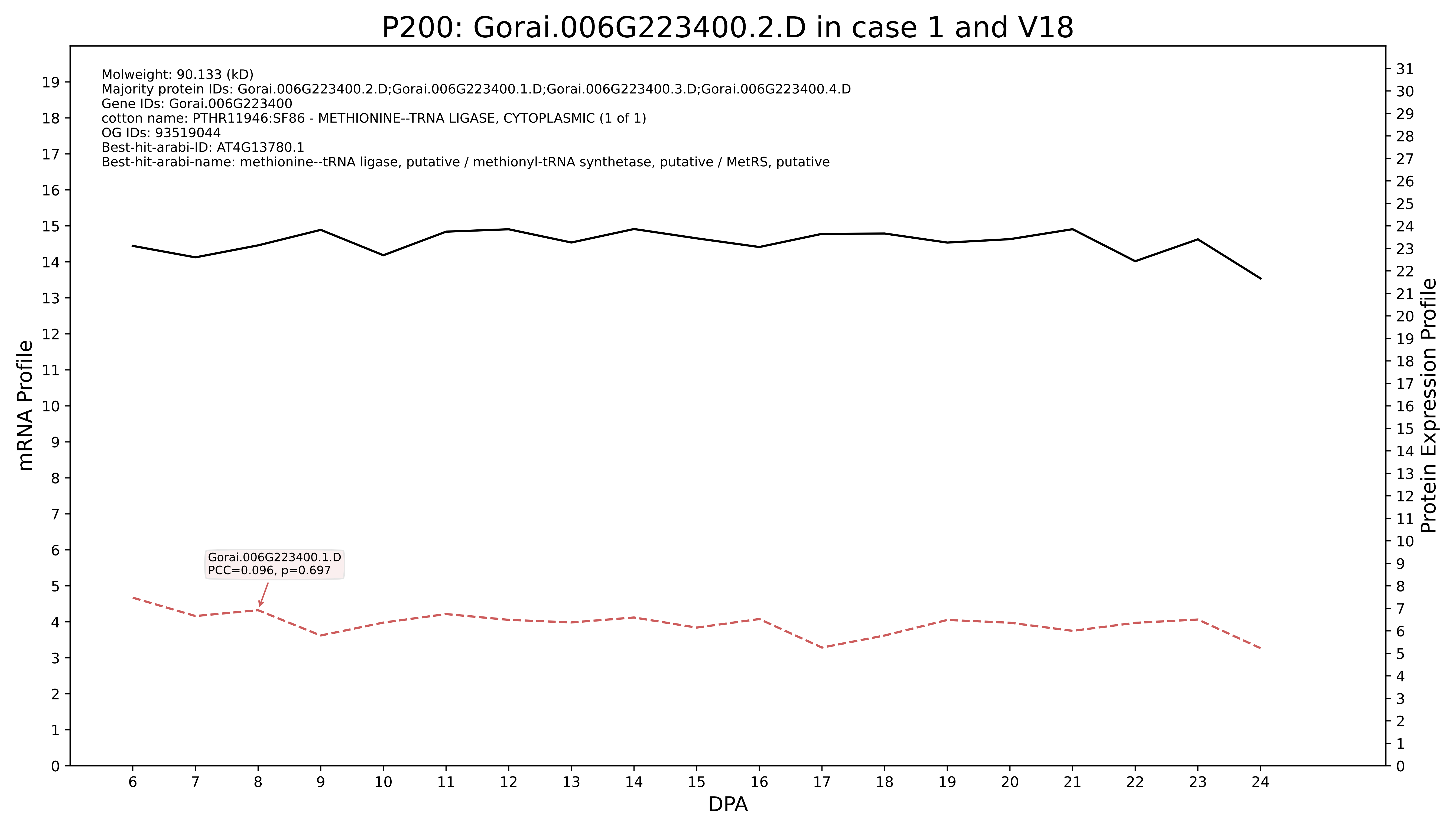

P200: Gorai.006G223400.2.D in case 1 and V18
Molweight: 90.133 (kD)
Majority protein IDs: Gorai.006G223400.2.D;Gorai.006G223400.1.D;Gorai.006G223400.3.D;Gorai.006G223400.4.D
Gene IDs: Gorai.006G223400
cotton name: PTHR11946:SF86 - METHIONINE--TRNA LIGASE, CYTOPLASMIC (1 of 1)
OG IDs: 93519044
Best-hit-arabi-ID: AT4G13780.1
Best-hit-arabi-name: methionine--tRNA ligase, putative / methionyl-tRNA synthetase, putative / MetRS, putative
Gorai.006G223400.1.D
PCC=0.096, p=0.697
mRNA Profile
Protein Expression Profile
DPA

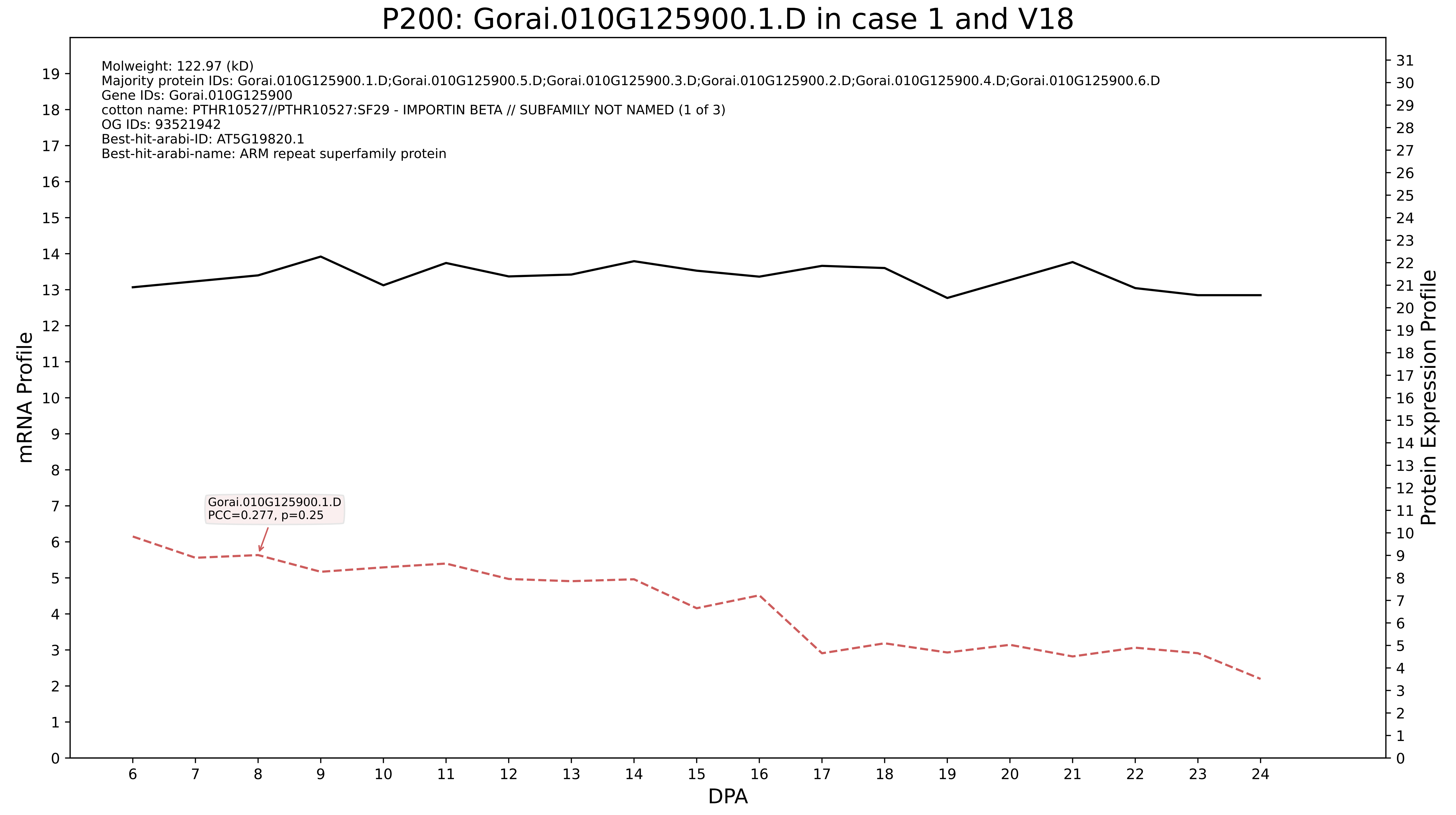

P200: Gorai.010G125900.1.D in case 1 and V18
Molweight: 122.97 (kD)
Majority protein IDs: Gorai.010G125900.1.D;Gorai.010G125900.5.D;Gorai.010G125900.3.D;Gorai.010G125900.2.D;Gorai.010G125900.4.D;Gorai.010G125900.6.D
Gene IDs: Gorai.010G125900
cotton name: PTHR10527//PTHR10527:SF29 - IMPORTIN BETA // SUBFAMILY NOT NAMED (1 of 3)
OG IDs: 93521942
Best-hit-arabi-ID: AT5G19820.1
Best-hit-arabi-name: ARM repeat superfamily protein
Gorai.010G125900.1.D
PCC=0.277, p=0.25
mRNA Profile
Protein Expression Profile
DPA

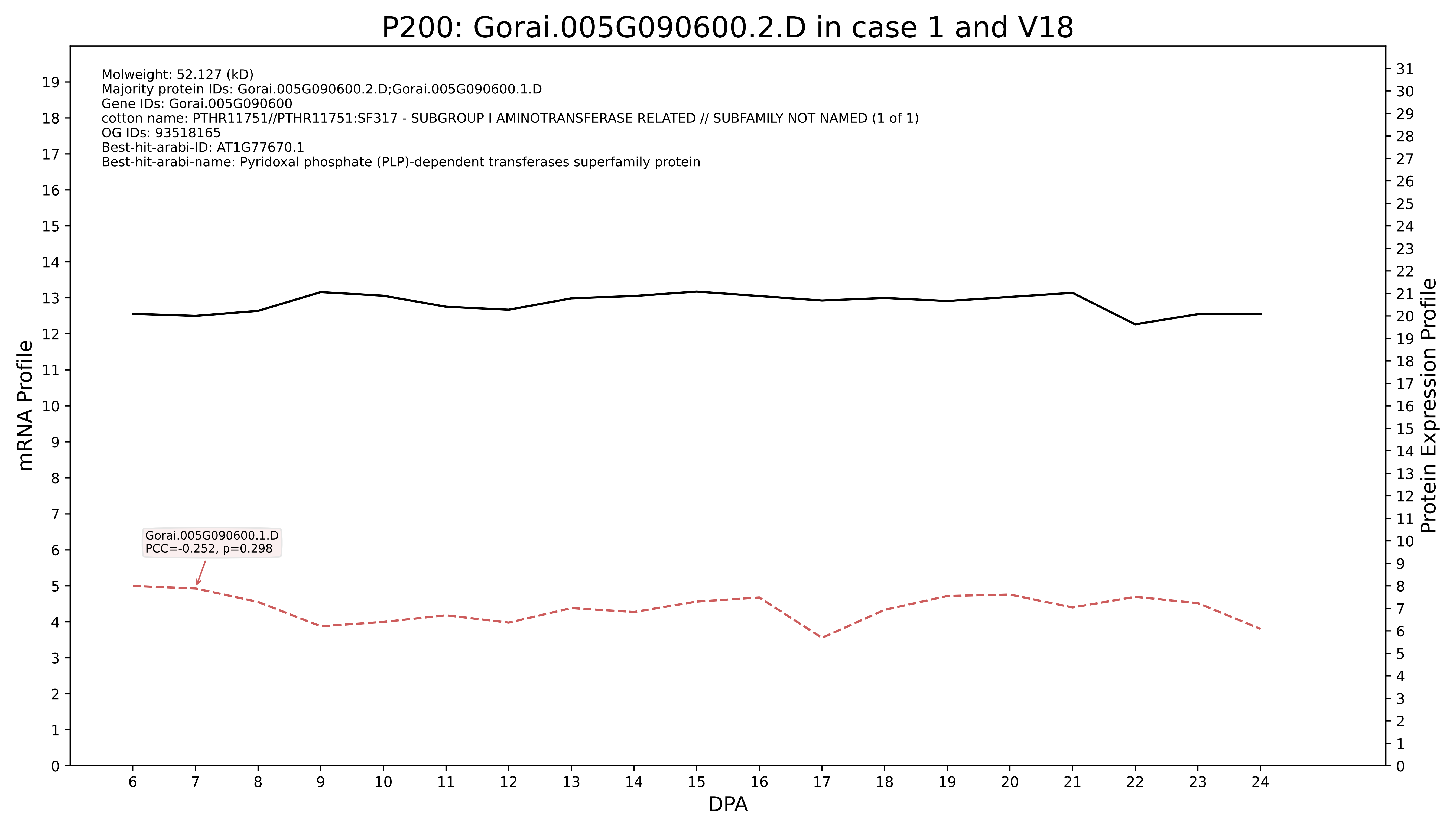
P200: Gorai.005G090600.2.D in case 1 and V18
Molweight: 52.127 (kD)
Majority protein IDs: Gorai.005G090600.2.D;Gorai.005G090600.1.D
Gene IDs: Gorai.005G090600
cotton name: PTHR11751//PTHR11751:SF317 - SUBGROUP I AMINOTRANSFERASE RELATED // SUBFAMILY NOT NAMED (1 of 1)
OG IDs: 93518165
Best-hit-arabi-ID: AT1G77670.1
Best-hit-arabi-name: Pyridoxal phosphate (PLP)-dependent transferases superfamily protein
Gorai.005G090600.1.D
PCC=-0.252, p=0.298
mRNA Profile
Protein Expression Profile
DPA

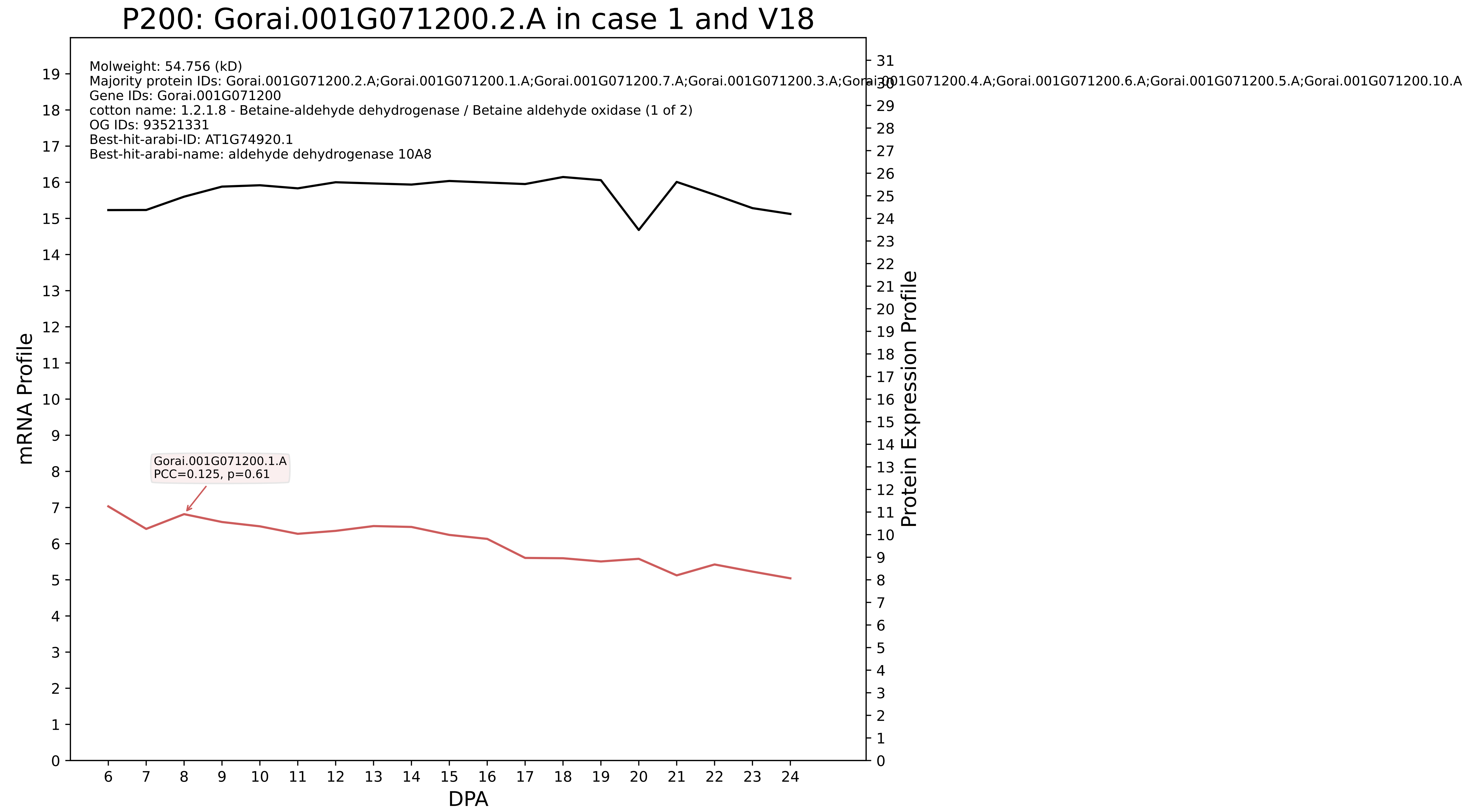

P200: Gorai.001G071200.2.A in case 1 and V18
Molweight: 54.756 (kD)
Majority protein IDs: Gorai.001G071200.2.A;Gorai.001G071200.1.A;Gorai.001G071200.7.A;Gorai.001G071200.3.A;Gorai.001G071200.4.A;Gorai.001G071200.6.A;Gorai.001G071200.5.A;Gorai.001G071200.10.A
Gene IDs: Gorai.001G071200
cotton name: 1.2.1.8 - Betaine-aldehyde dehydrogenase / Betaine aldehyde oxidase (1 of 2)
OG IDs: 93521331
Best-hit-arabi-ID: AT1G74920.1
Best-hit-arabi-name: aldehyde dehydrogenase 10A8
Gorai.001G071200.1.A
PCC=0.125, p=0.61
mRNA Profile
Protein Expression Profile
DPA

P200: Gorai.008G218700.1.D in case 1 and V18

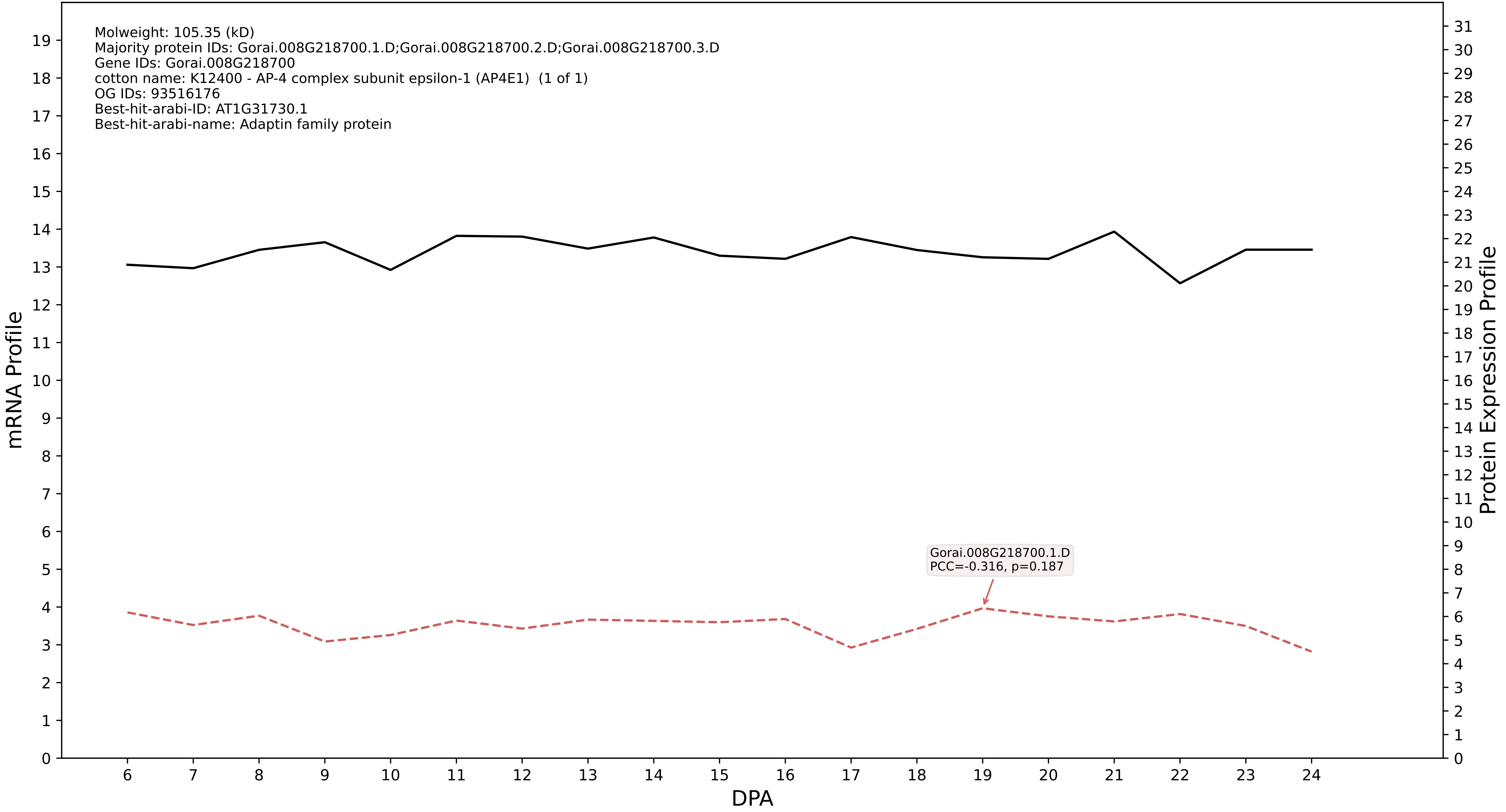

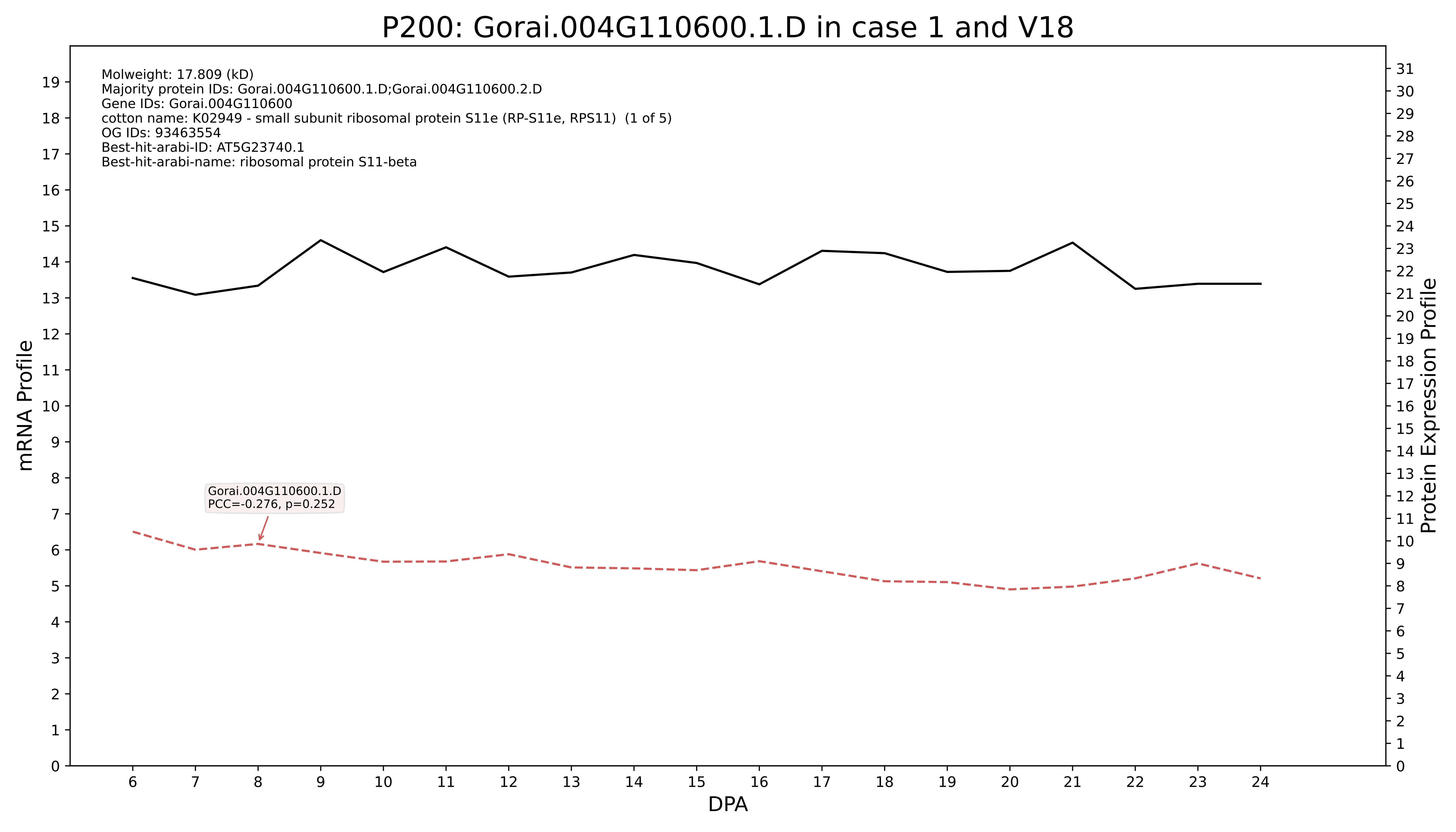
P200: Gorai.004G110600.1.D in case 1 and V18
Molweight: 17.809 (kD)
Majority protein IDs: Gorai.004G110600.1.D;Gorai.004G110600.2.D
Gene IDs: Gorai.004G110600
cotton name: K02949 - small subunit ribosomal protein S11e (RP-S11e, RPS11) (1 of 5)
OG IDs: 93463554
Best-hit-arabi-ID: AT5G23740.1
Best-hit-arabi-name: ribosomal protein S11-beta
Gorai.004G110600.1.D
PCC=-0.276, p=0.252
mRNA Profile
Protein Expression Profile
DPA
6
7
8
9
10
11
12
13
14
15
16
17
18
19
20
21
22
23
24

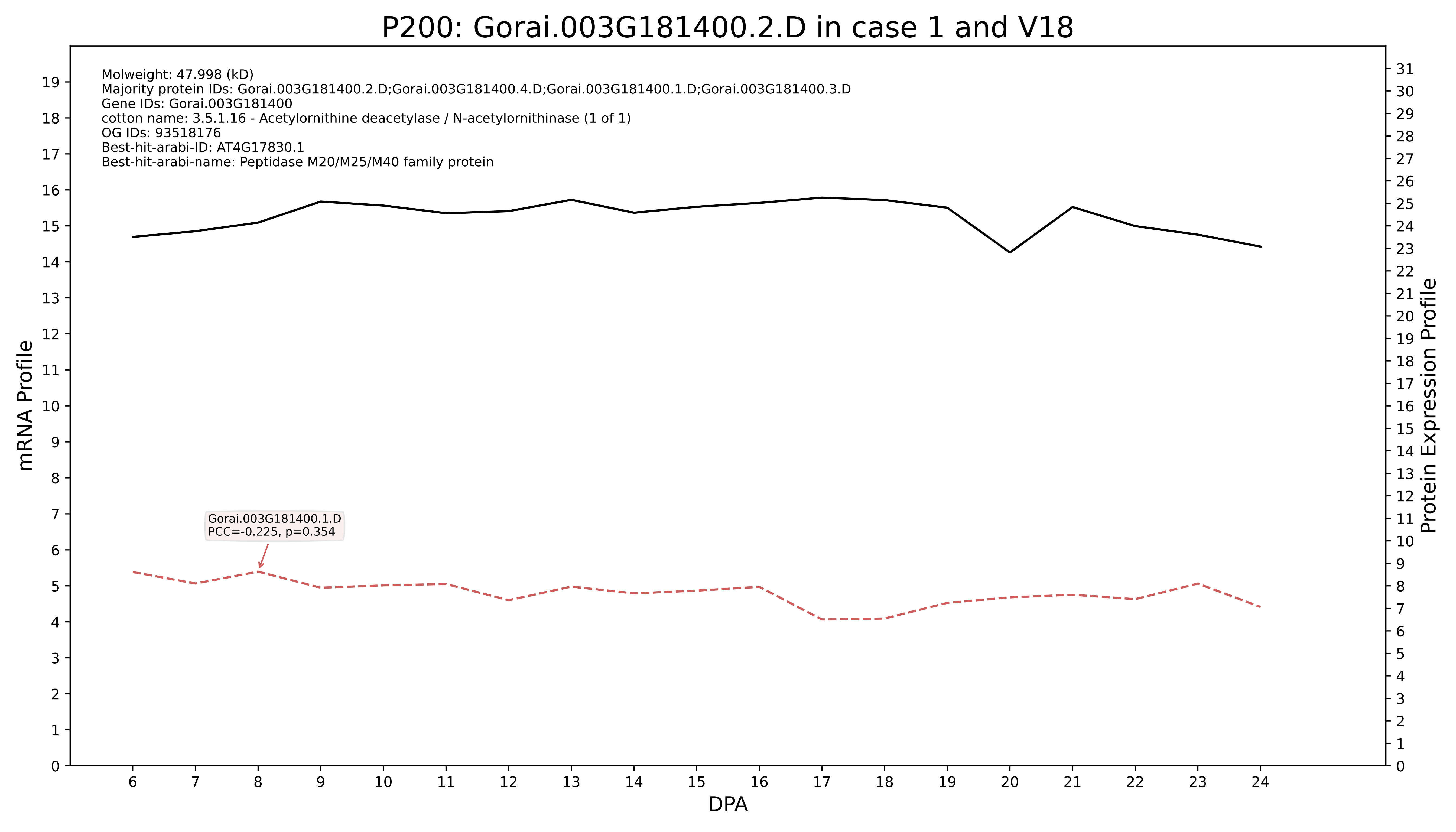

P200: Gorai.003G181400.2.D in case 1 and V18
Molweight: 47.998 (kD)
Majority protein IDs: Gorai.003G181400.2.D;Gorai.003G181400.4.D;Gorai.003G181400.1.D;Gorai.003G181400.3.D
Gene IDs: Gorai.003G181400
cotton name: 3.5.1.16 - Acetylornithine deacetylase / N-acetylornithinase (1 of 1)
OG IDs: 93518176
Best-hit-arabi-ID: AT4G17830.1
Best-hit-arabi-name: Peptidase M20/M25/M40 family protein
Gorai.003G181400.1.D
PCC=-0.225, p=0.354
mRNA Profile
Protein Expression Profile
DPA

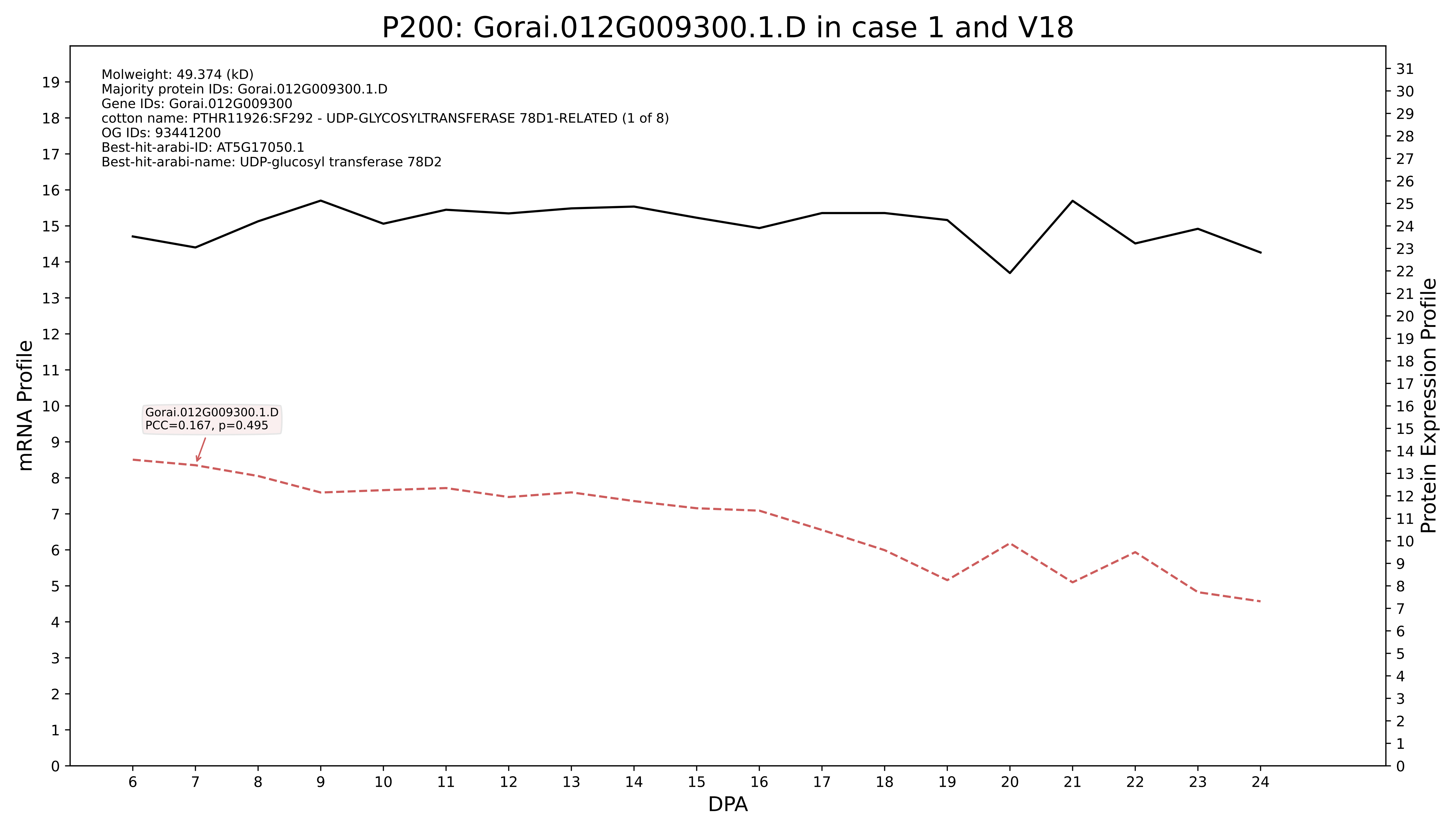
P200: Gorai.012G009300.1.D in case 1 and V18
Molweight: 49.374 (kD)
Majority protein IDs: Gorai.012G009300.1.D
Gene IDs: Gorai.012G009300
cotton name: PTHR11926:SF292 - UDP-GLYCOSYLTRANSFERASE 78D1-RELATED (1 of 8)
OG IDs: 93441200
Best-hit-arabi-ID: AT5G17050.1
Best-hit-arabi-name: UDP-glucosyl transferase 78D2
Gorai.012G009300.1.D
PCC=0.167, p=0.495
mRNA Profile
Protein Expression Profile
DPA
0 1 2 3 4 5 6 7 8 9 10 11 12 13 14 15 16 17 18 19
0 1 2 3 4 5 6 7 8 9 10 11 12 13 14 15 16 17 18 19 20 21 22 23 24 25 26 27 28 29 30 31
6 7 8 9 10 11 12 13 14 15 16 17 18 19 20 21 22 23 24

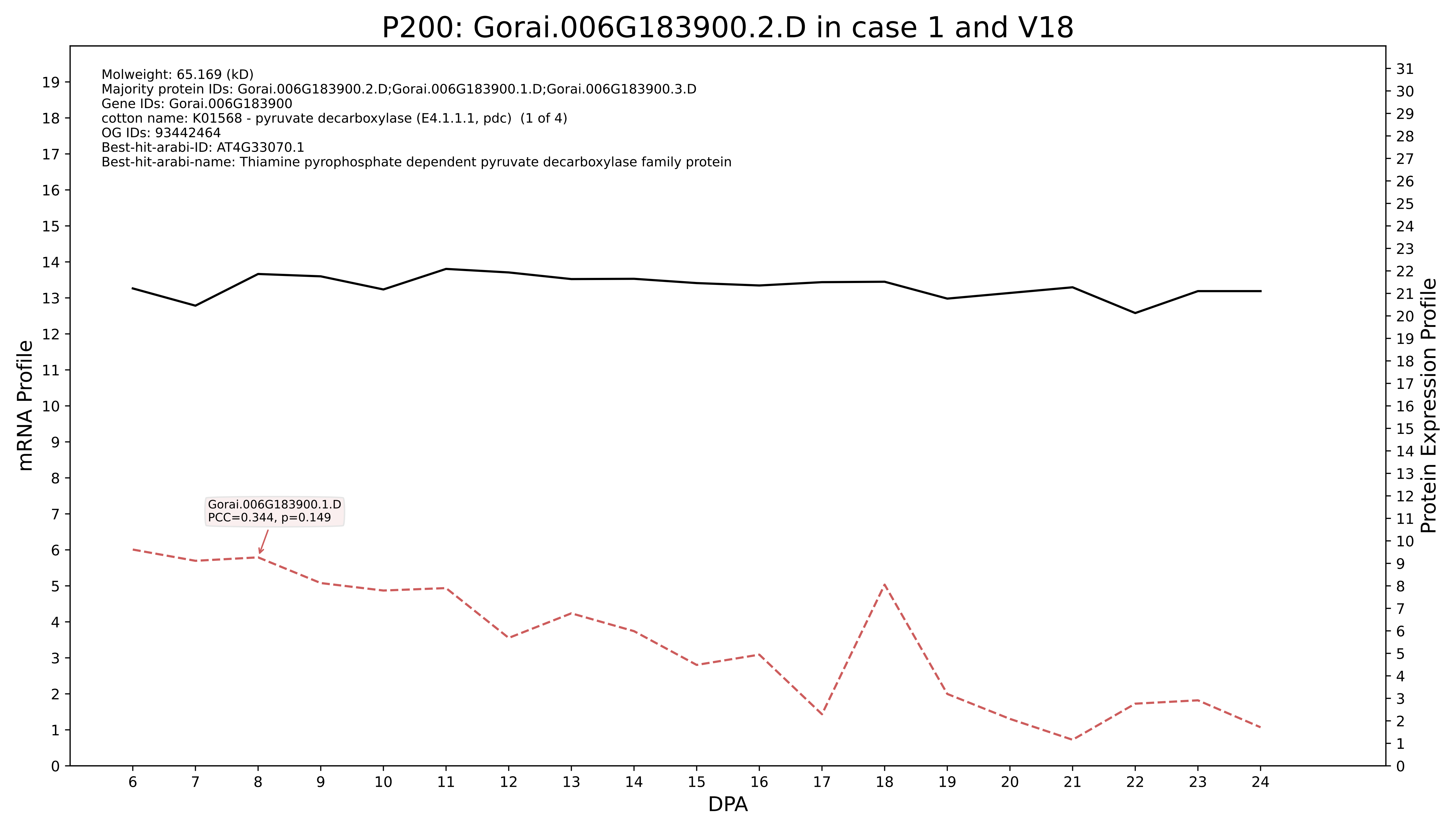
P200: Gorai.006G183900.2.D in case 1 and V18
Molweight: 65.169 (kD)
Majority protein IDs: Gorai.006G183900.2.D;Gorai.006G183900.1.D;Gorai.006G183900.3.D
Gene IDs: Gorai.006G183900
cotton name: K01568 - pyruvate decarboxylase (E4.1.1.1, pdc) (1 of 4)
OG IDs: 93442464
Best-hit-arabi-ID: AT4G33070.1
Best-hit-arabi-name: Thiamine pyrophosphate dependent pyruvate decarboxylase family protein
Gorai.006G183900.1.D
PCC=0.344, p=0.149
mRNA Profile
Protein Expression Profile
DPA

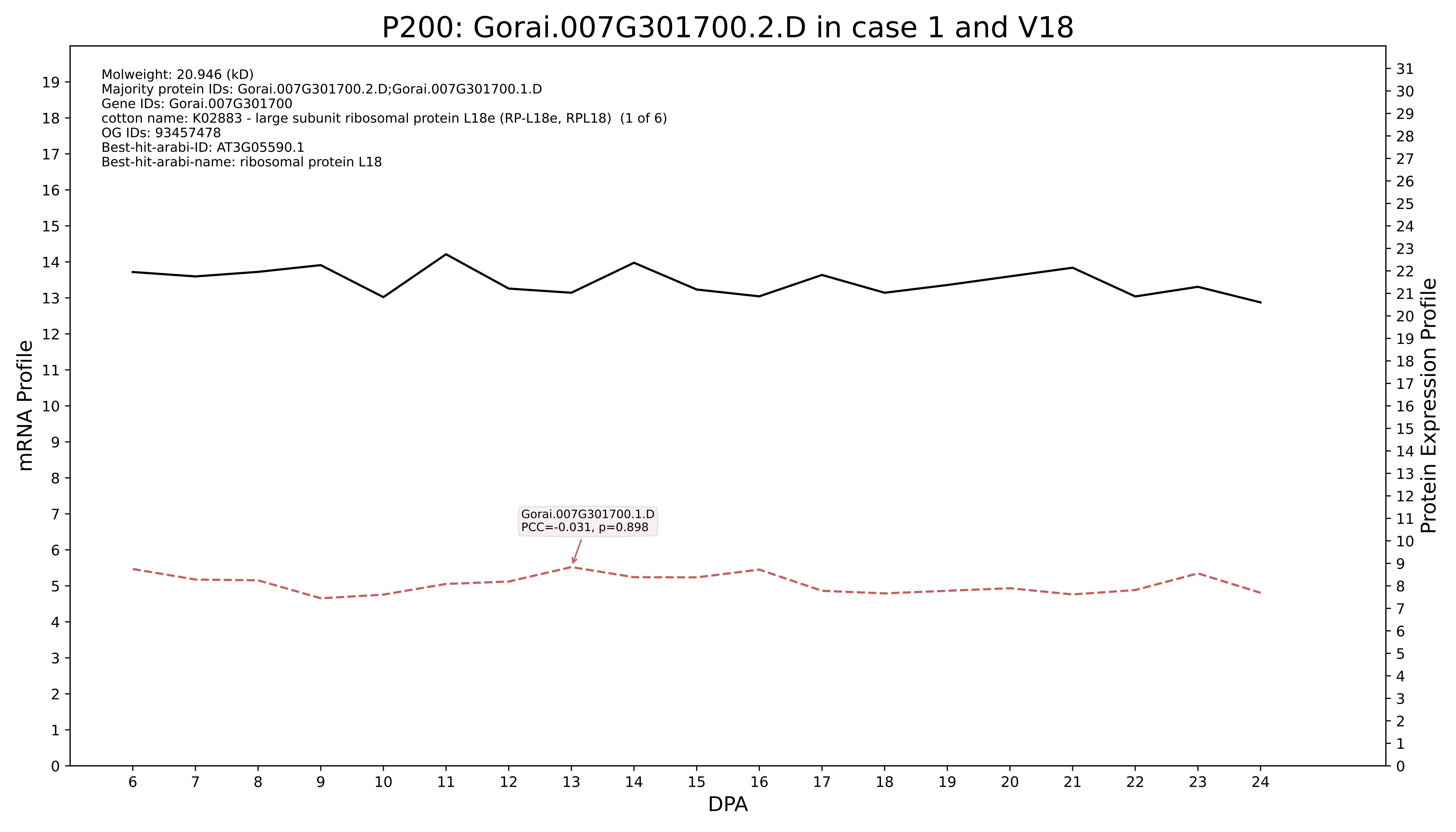

P200: Gorai.007G301700.2.D in case 1 and V18
Molweight: 20.946 (kD)
Majority protein IDs: Gorai.007G301700.2.D;Gorai.007G301700.1.D
Gene IDs: Gorai.007G301700
cotton name: K02883 - large subunit ribosomal protein L18e (RP-L18e, RPL18) (1 of 6)
OG IDs: 93457478
Best-hit-arabi-ID: AT3G05590.1
Best-hit-arabi-name: ribosomal protein L18
Gorai.007G301700.1.D
PCC=-0.031, p=0.898
mRNA Profile
Protein Expression Profile
DPA

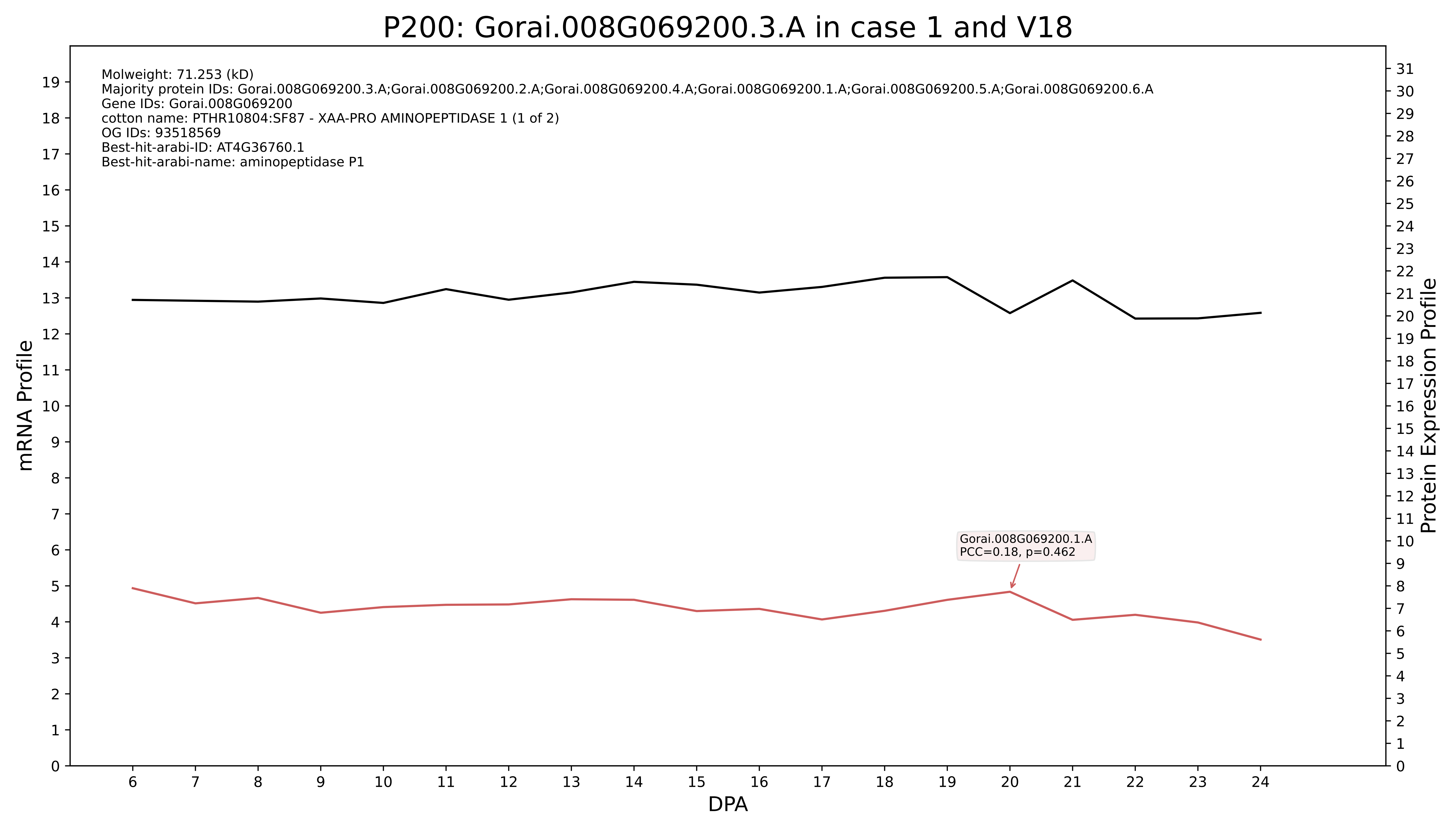
P200: Gorai.008G069200.3.A in case 1 and V18
Molweight: 71.253 (kD)
Majority protein IDs: Gorai.008G069200.3.A;Gorai.008G069200.2.A;Gorai.008G069200.4.A;Gorai.008G069200.1.A;Gorai.008G069200.5.A;Gorai.008G069200.6.A
Gene IDs: Gorai.008G069200
cotton name: PTHR10804:SF87 - XAA-PRO AMINOPEPTIDASE 1 (1 of 2)
OG IDs: 93518569
Best-hit-arabi-ID: AT4G36760.1
Best-hit-arabi-name: aminopeptidase P1
Gorai.008G069200.1.A
PCC=0.18, p=0.462
mRNA Profile
Protein Expression Profile
DPA

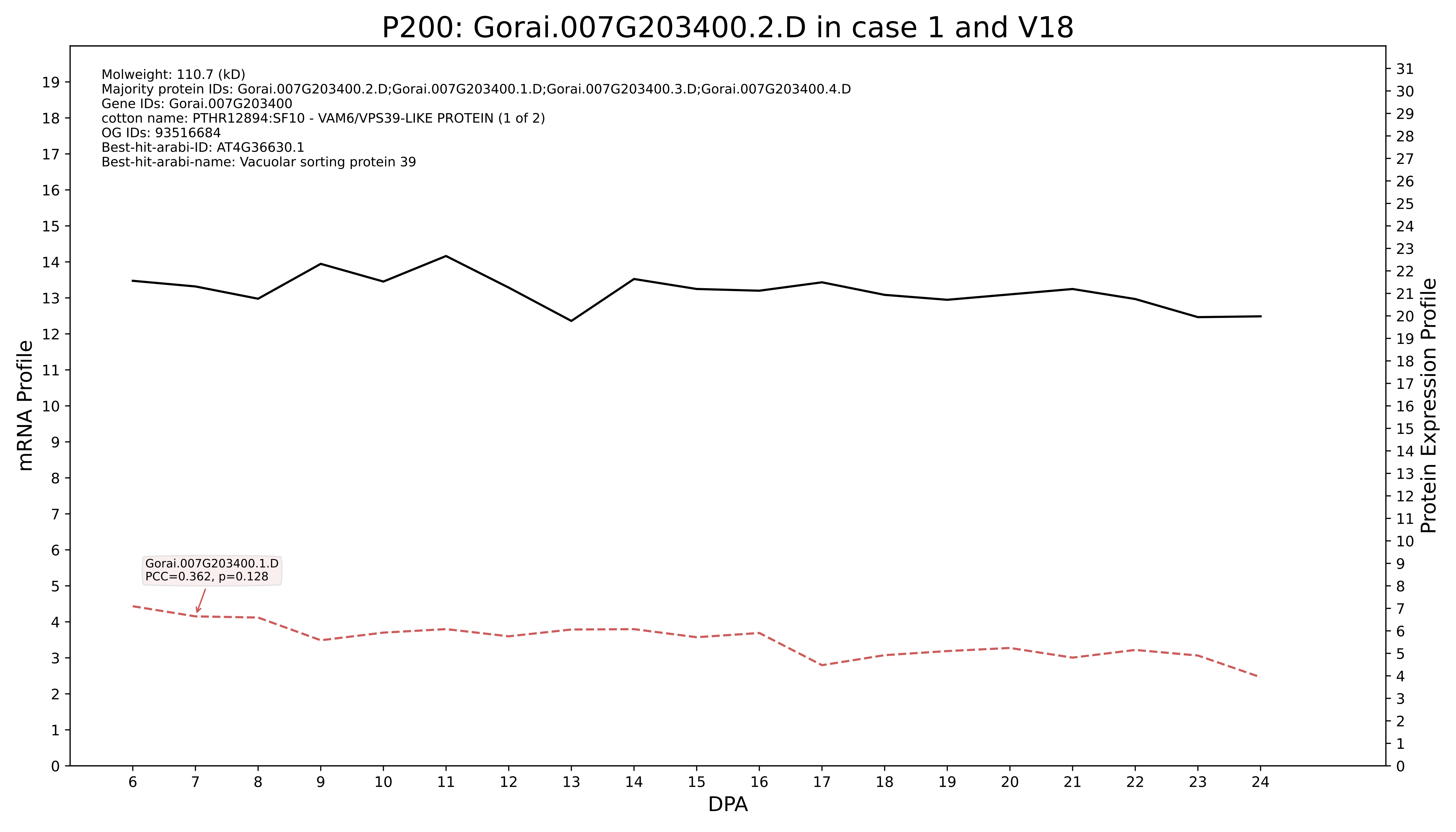

P200: Gorai.007G203400.2.D in case 1 and V18
Molweight: 110.7 (kD)
Majority protein IDs: Gorai.007G203400.2.D;Gorai.007G203400.1.D;Gorai.007G203400.3.D;Gorai.007G203400.4.D
Gene IDs: Gorai.007G203400
cotton name: PTHR12894:SF10 - VAM6/VPS39-LIKE PROTEIN (1 of 2)
OG IDs: 93516684
Best-hit-arabi-ID: AT4G36630.1
Best-hit-arabi-name: Vacuolar sorting protein 39
Gorai.007G203400.1.D
PCC=0.362, p=0.128
mRNA Profile
Protein Expression Profile
DPA

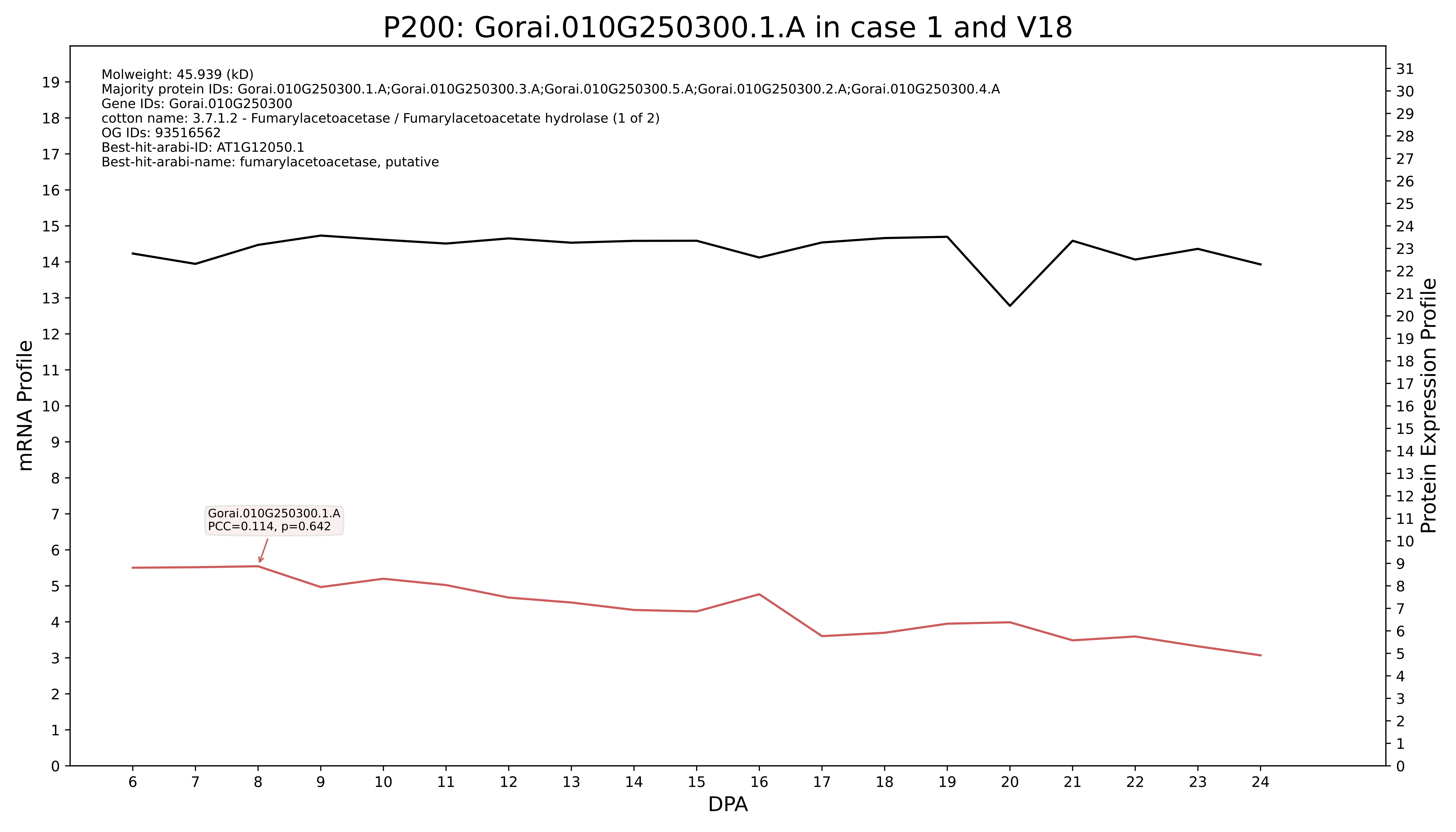
P200: Gorai.010G250300.1.A in case 1 and V18
Molweight: 45.939 (kD)
Majority protein IDs: Gorai.010G250300.1.A;Gorai.010G250300.3.A;Gorai.010G250300.5.A;Gorai.010G250300.2.A;Gorai.010G250300.4.A
Gene IDs: Gorai.010G250300
cotton name: 3.7.1.2 - Fumarylacetoacetase / Fumarylacetoacetate hydrolase (1 of 2)
OG IDs: 93516562
Best-hit-arabi-ID: AT1G12050.1
Best-hit-arabi-name: fumarylacetoacetase, putative
Gorai.010G250300.1.A
PCC=0.114, p=0.642
mRNA Profile
Protein Expression Profile
DPA

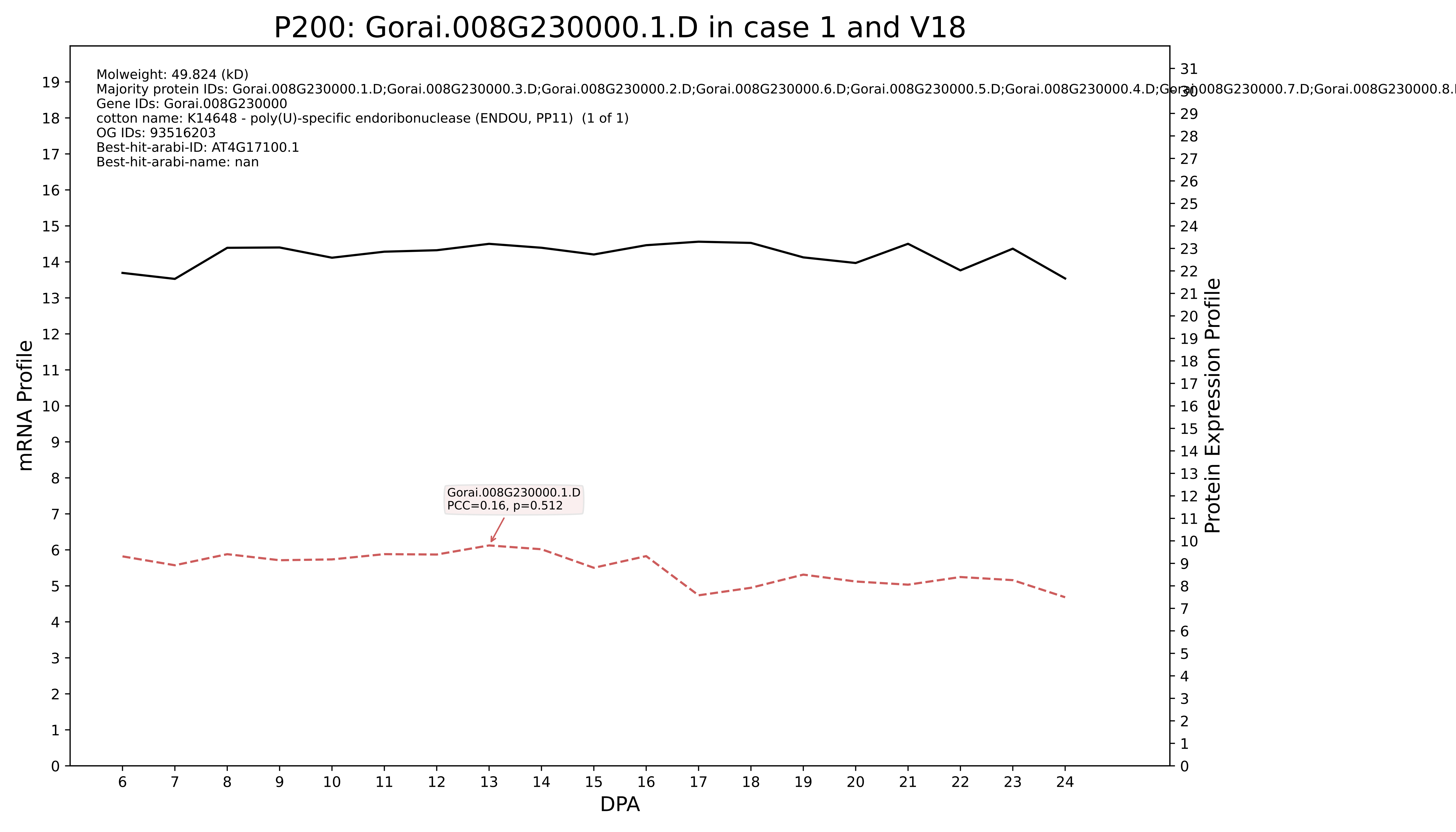

P200: Gorai.008G230000.1.D in case 1 and V18
Molweight: 49.824 (kD)
Majority protein IDs: Gorai.008G230000.1.D;Gorai.008G230000.3.D;Gorai.008G230000.2.D;Gorai.008G230000.6.D;Gorai.008G230000.5.D;Gorai.008G230000.4.D;Gorai.008G230000.7.D;Gorai.008G230000.8.D
Gene IDs: Gorai.008G230000
cotton name: K14648 - poly(U)-specific endoribonuclease (ENDOU, PP11) (1 of 1)
OG IDs: 93516203
Best-hit-arabi-ID: AT4G17100.1
Best-hit-arabi-name: nan
Gorai.008G230000.1.D
PCC=0.16, p=0.512
mRNA Profile
Protein Expression Profile
DPA

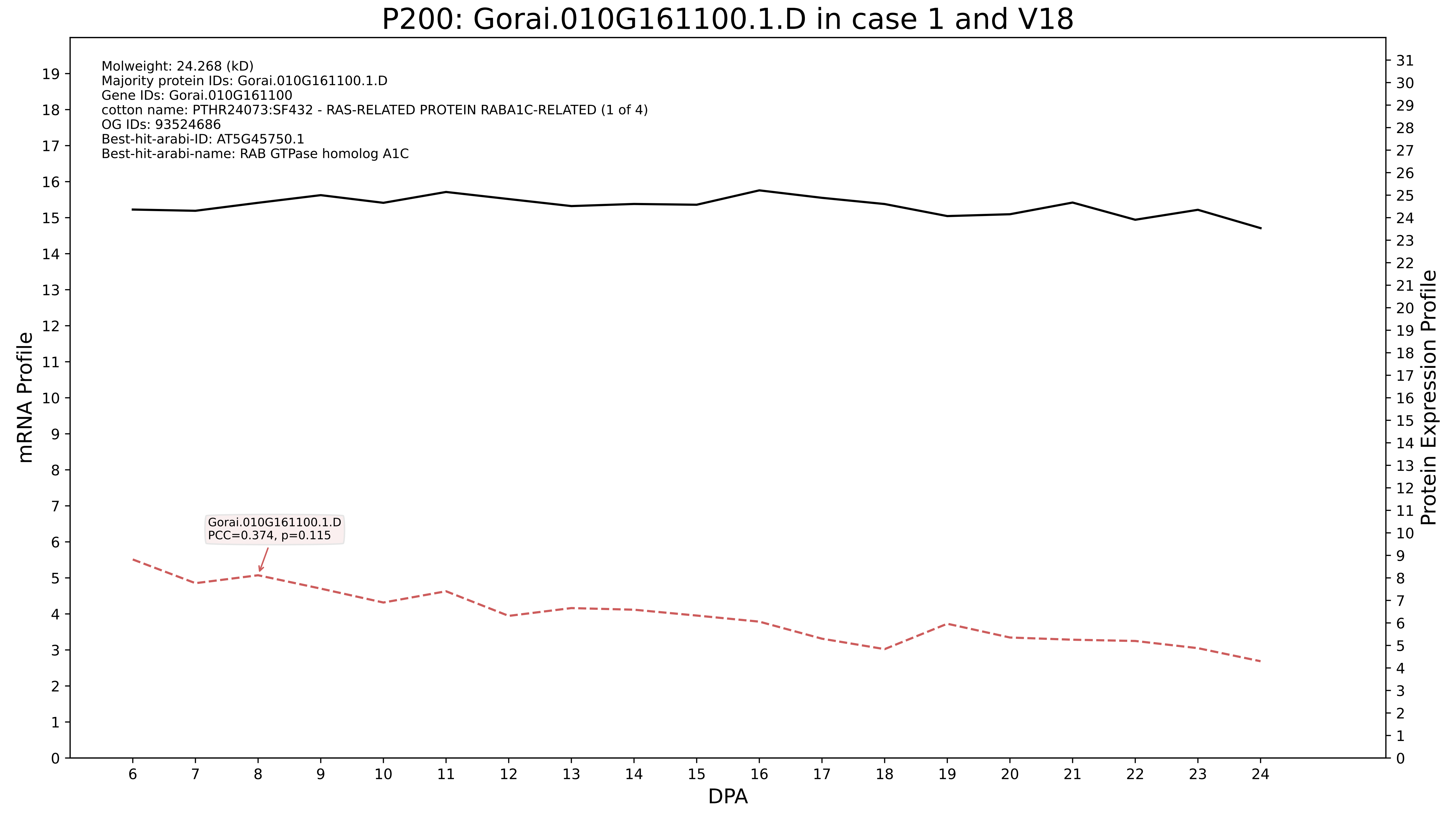
P200: Gorai.010G161100.1.D in case 1 and V18
Molweight: 24.268 (kD)
Majority protein IDs: Gorai.010G161100.1.D
Gene IDs: Gorai.010G161100
cotton name: PTHR24073:SF432 - RAS-RELATED PROTEIN RABA1C-RELATED (1 of 4)
OG IDs: 93524686
Best-hit-arabi-ID: AT5G45750.1
Best-hit-arabi-name: RAB GTPase homolog A1C
Gorai.010G161100.1.D
PCC=0.374, p=0.115
mRNA Profile
Protein Expression Profile
DPA

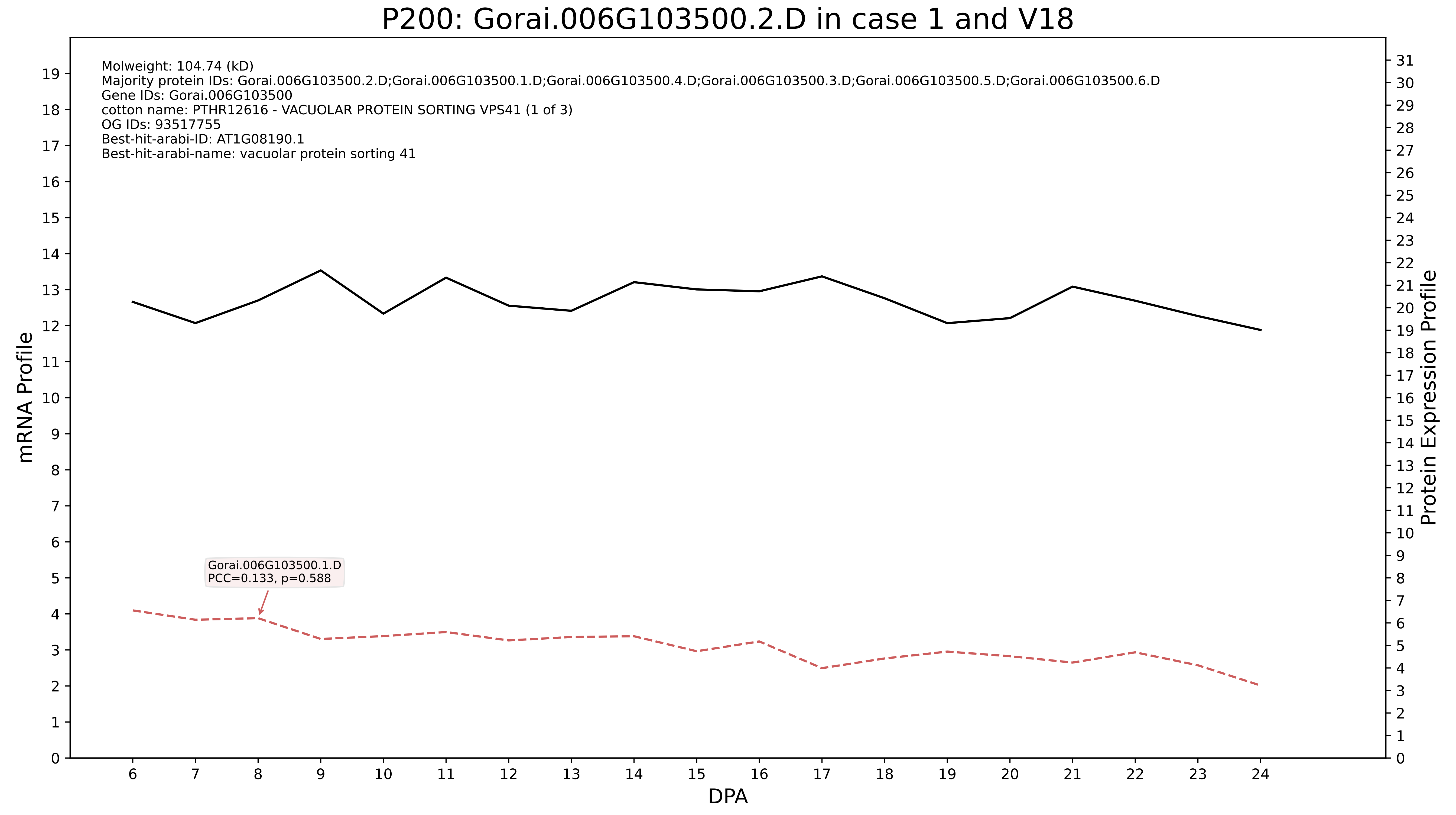
P200: Gorai.006G103500.2.D in case 1 and V18
Molweight: 104.74 (kD)
Majority protein IDs: Gorai.006G103500.2.D;Gorai.006G103500.1.D;Gorai.006G103500.4.D;Gorai.006G103500.3.D;Gorai.006G103500.5.D;Gorai.006G103500.6.D
Gene IDs: Gorai.006G103500
cotton name: PTHR12616 - VACUOLAR PROTEIN SORTING VPS41 (1 of 3)
OG IDs: 93517755
Best-hit-arabi-ID: AT1G08190.1
Best-hit-arabi-name: vacuolar protein sorting 41
Gorai.006G103500.1.D
PCC=0.133, p=0.588
mRNA Profile
Protein Expression Profile
DPA

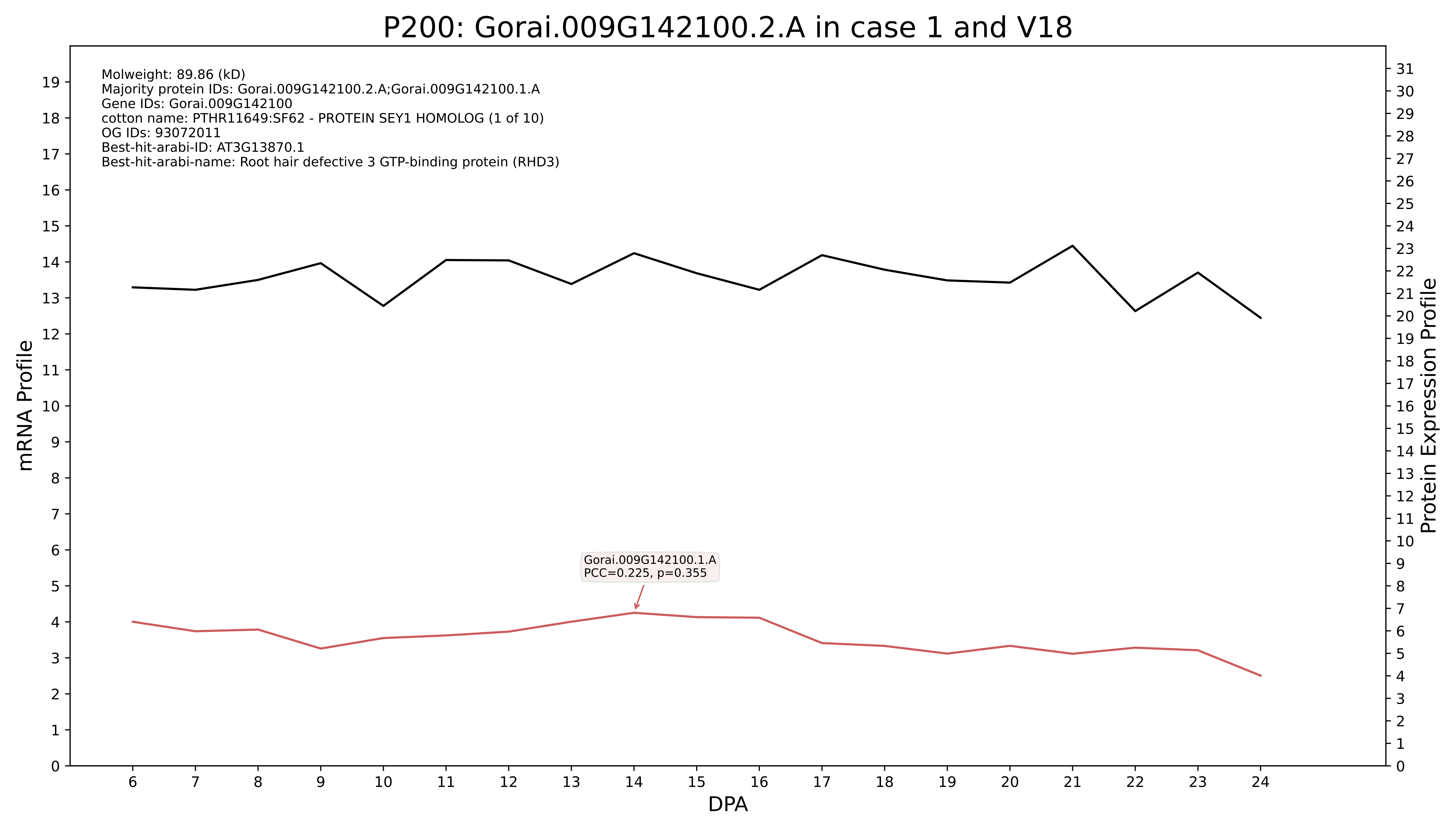
P200: Gorai.009G142100.2.A in case 1 and V18
Molweight: 89.86 (kD)
Majority protein IDs: Gorai.009G142100.2.A;Gorai.009G142100.1.A
Gene IDs: Gorai.009G142100
cotton name: PTHR11649:SF62 - PROTEIN SEY1 HOMOLOG (1 of 10)
OG IDs: 93072011
Best-hit-arabi-ID: AT3G13870.1
Best-hit-arabi-name: Root hair defective 3 GTP-binding protein (RHD3)
Gorai.009G142100.1.A
PCC=0.225, p=0.355
mRNA Profile
Protein Expression Profile
DPA

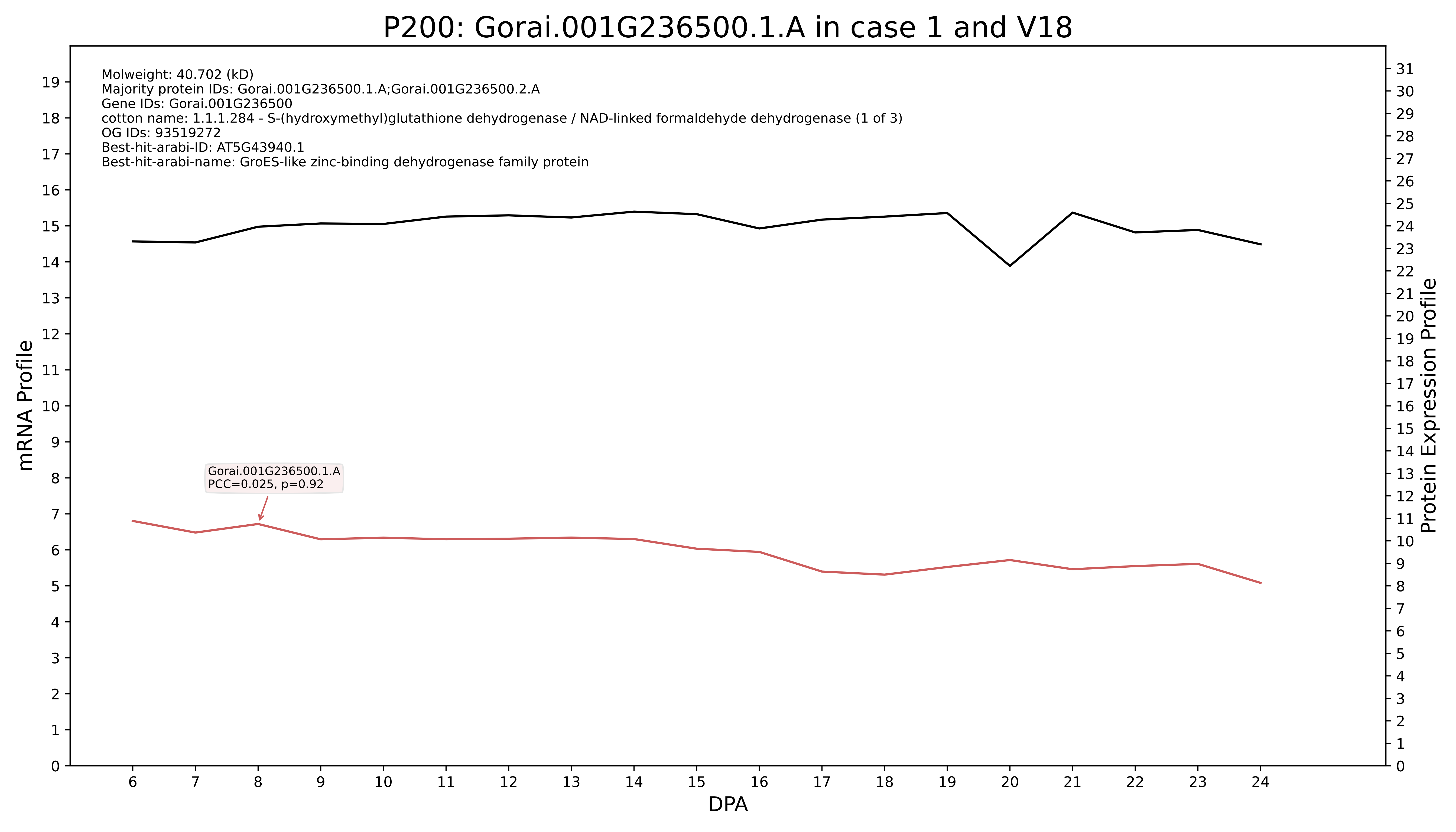
P200: Gorai.001G236500.1.A in case 1 and V18
Molweight: 40.702 (kD)
Majority protein IDs: Gorai.001G236500.1.A;Gorai.001G236500.2.A
Gene IDs: Gorai.001G236500
cotton name: 1.1.1.284 - S-(hydroxymethyl)glutathione dehydrogenase / NAD-linked formaldehyde dehydrogenase (1 of 3)
OG IDs: 93519272
Best-hit-arabi-ID: AT5G43940.1
Best-hit-arabi-name: GroES-like zinc-binding dehydrogenase family protein
Gorai.001G236500.1.A
PCC=0.025, p=0.92
mRNA Profile
Protein Expression Profile
DPA

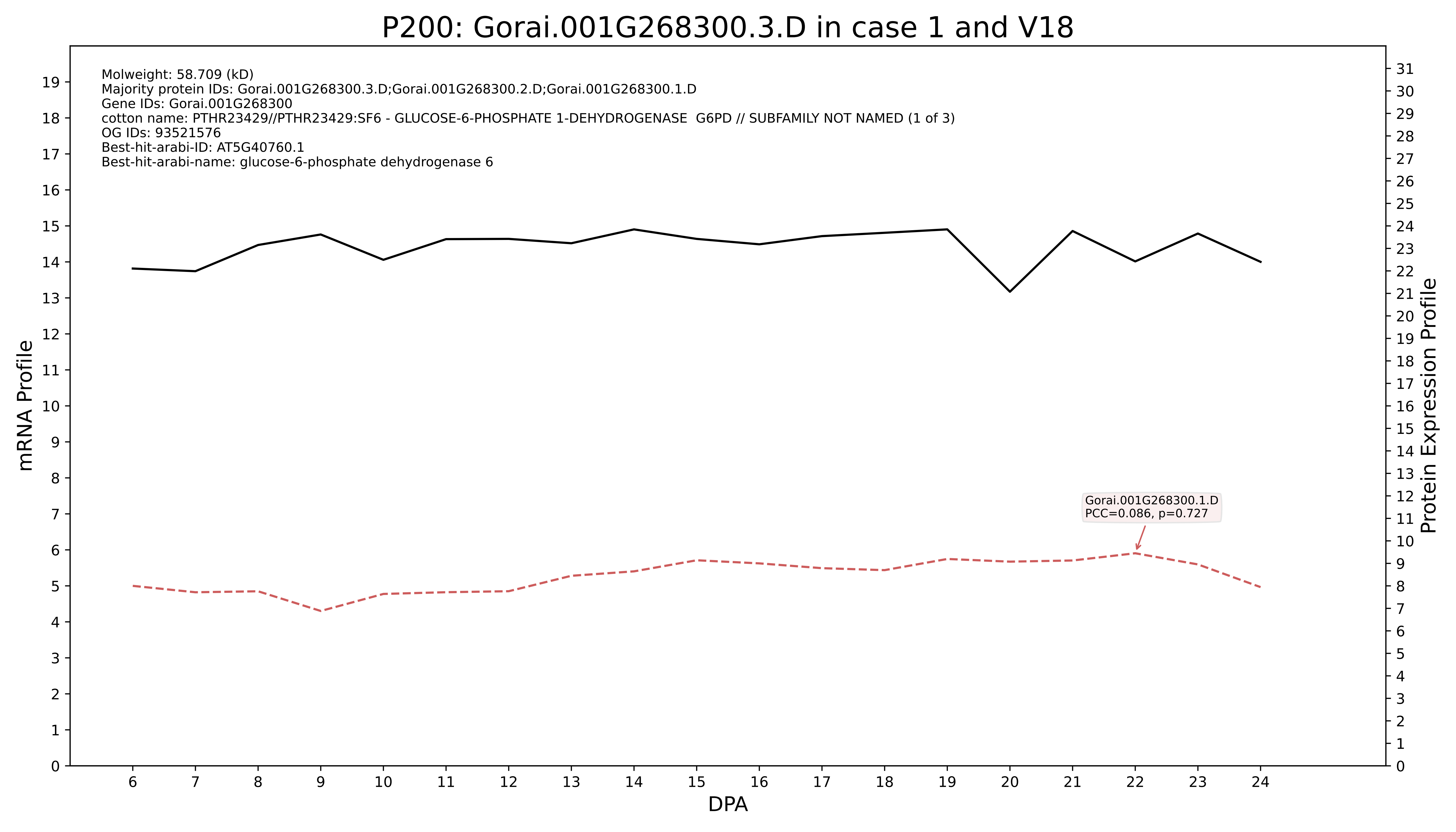

P200: Gorai.001G268300.3.D in case 1 and V18
Molweight: 58.709 (kD)
Majority protein IDs: Gorai.001G268300.3.D;Gorai.001G268300.2.D;Gorai.001G268300.1.D
Gene IDs: Gorai.001G268300
cotton name: PTHR23429//PTHR23429:SF6 - GLUCOSE-6-PHOSPHATE 1-DEHYDROGENASE G6PD // SUBFAMILY NOT NAMED (1 of 3)
OG IDs: 93521576
Best-hit-arabi-ID: AT5G40760.1
Best-hit-arabi-name: glucose-6-phosphate dehydrogenase 6
Gorai.001G268300.1.D
PCC=0.086, p=0.727
mRNA Profile
Protein Expression Profile
DPA

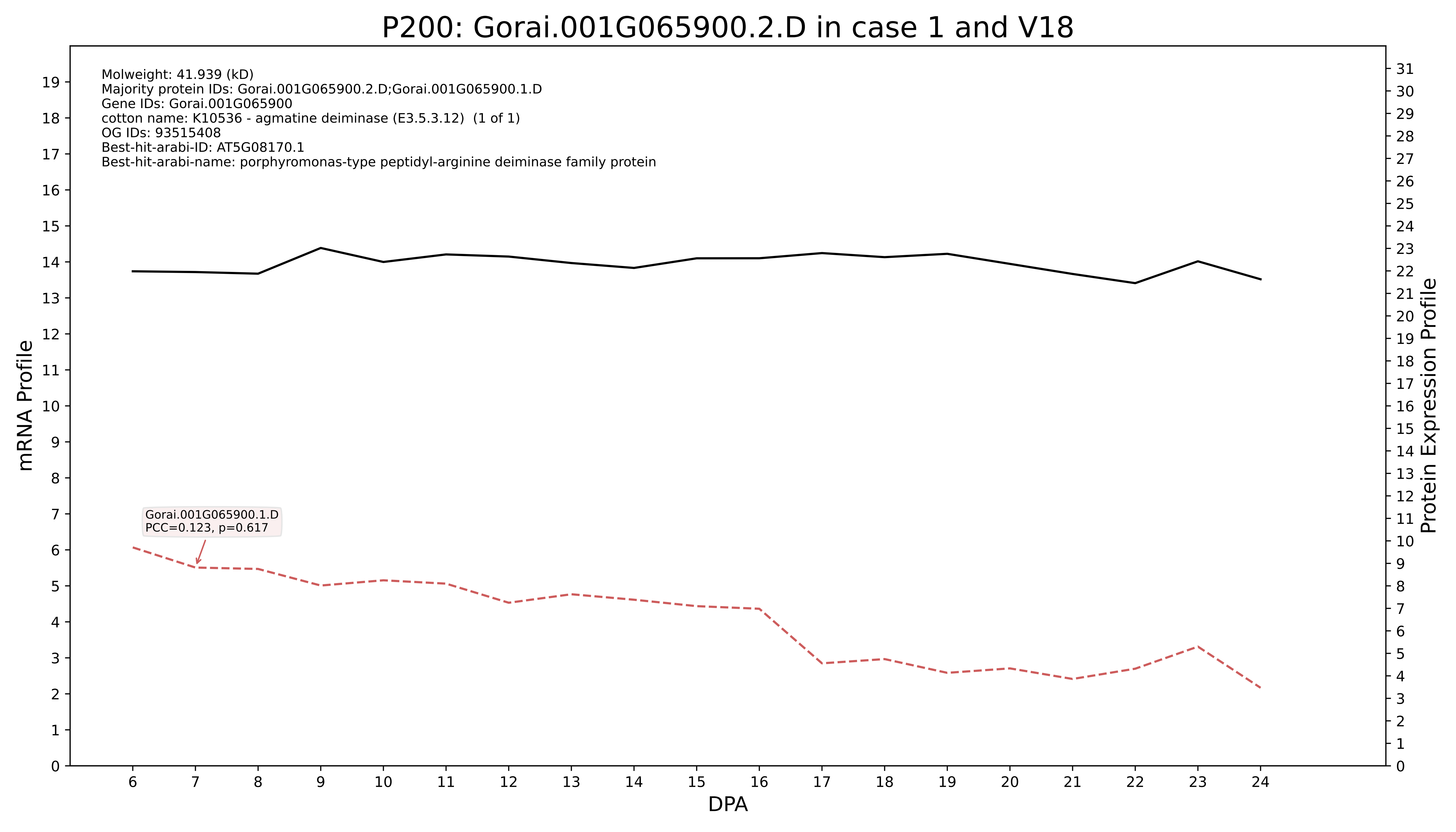

P200: Gorai.001G065900.2.D in case 1 and V18
Molweight: 41.939 (kD)
Majority protein IDs: Gorai.001G065900.2.D;Gorai.001G065900.1.D
Gene IDs: Gorai.001G065900
cotton name: K10536 - agmatine deiminase (E3.5.3.12) (1 of 1)
OG IDs: 93515408
Best-hit-arabi-ID: AT5G08170.1
Best-hit-arabi-name: porphyromonas-type peptidyl-arginine deiminase family protein
Gorai.001G065900.1.D
PCC=0.123, p=0.617
mRNA Profile
Protein Expression Profile
DPA

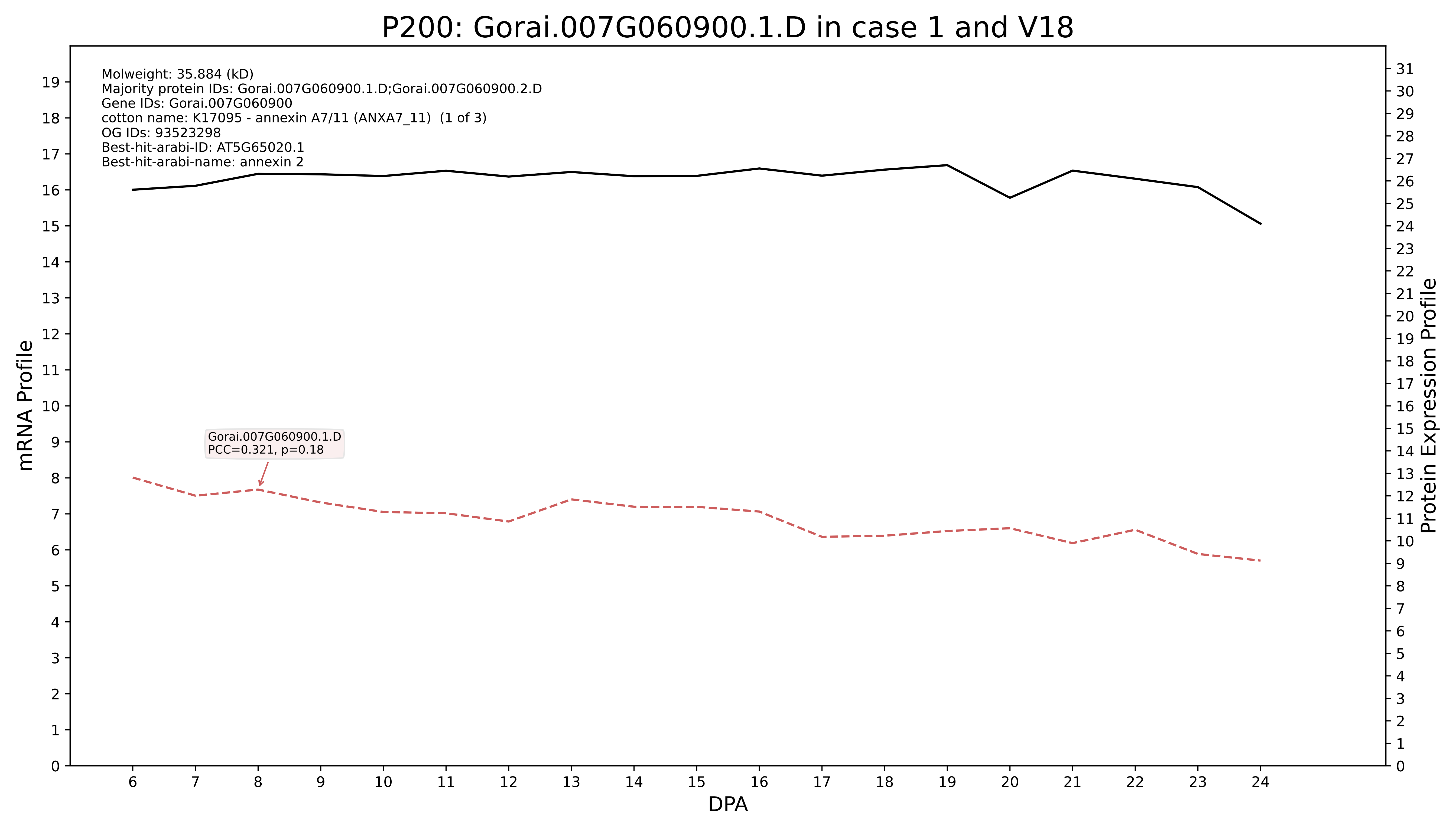

P200: Gorai.007G060900.1.D in case 1 and V18
Molweight: 35.884 (kD)
Majority protein IDs: Gorai.007G060900.1.D;Gorai.007G060900.2.D
Gene IDs: Gorai.007G060900
cotton name: K17095 - annexin A7/11 (ANXA7_11) (1 of 3)
OG IDs: 93523298
Best-hit-arabi-ID: AT5G65020.1
Best-hit-arabi-name: annexin 2
Gorai.007G060900.1.D
PCC=0.321, p=0.18
mRNA Profile
Protein Expression Profile
DPA

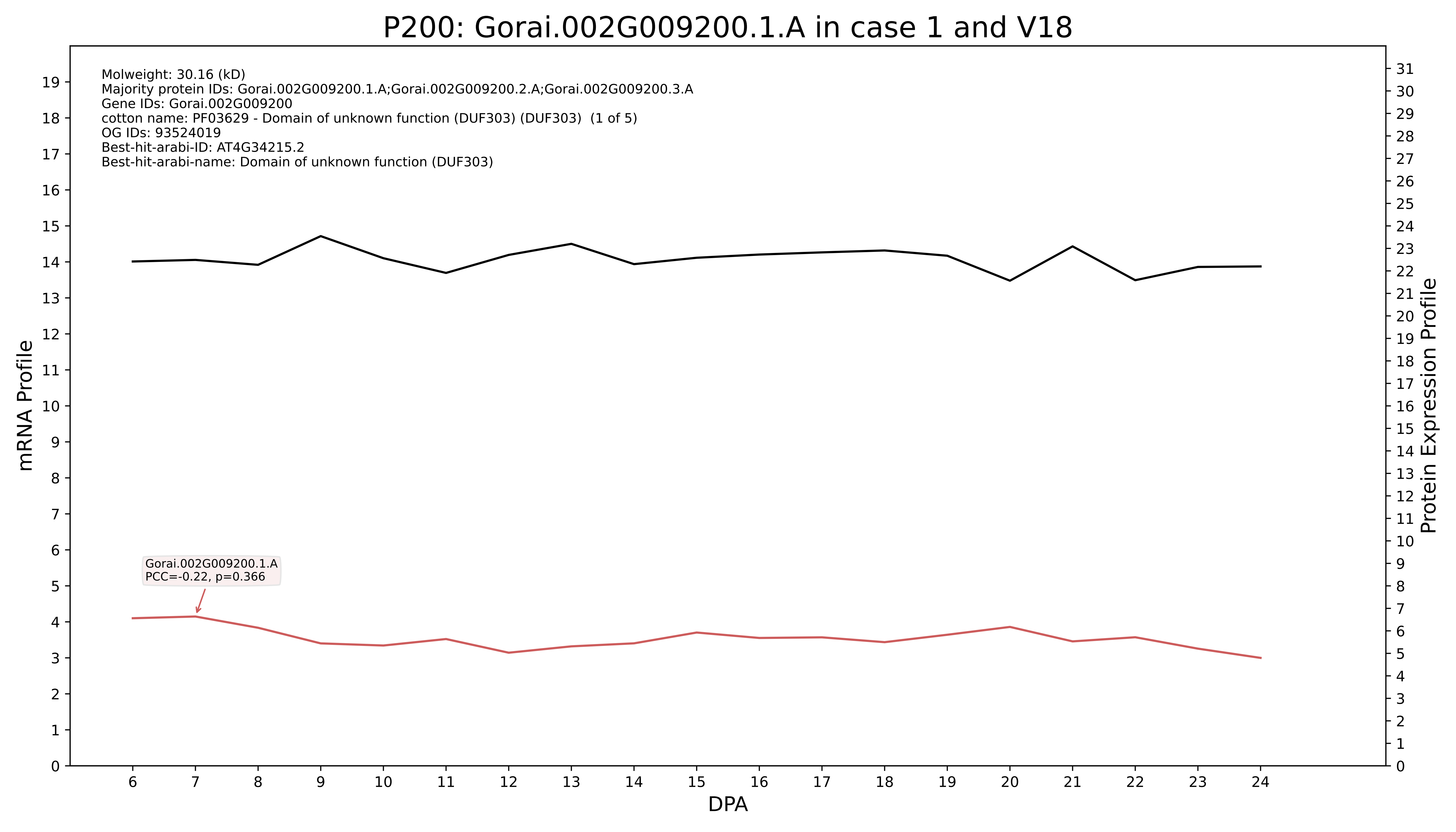
P200: Gorai.002G009200.1.A in case 1 and V18
Molweight: 30.16 (kD)
Majority protein IDs: Gorai.002G009200.1.A;Gorai.002G009200.2.A;Gorai.002G009200.3.A
Gene IDs: Gorai.002G009200
cotton name: PF03629 - Domain of unknown function (DUF303) (DUF303) (1 of 5)
OG IDs: 93524019
Best-hit-arabi-ID: AT4G34215.2
Best-hit-arabi-name: Domain of unknown function (DUF303)
Gorai.002G009200.1.A
PCC=-0.22, p=0.366
mRNA Profile
Protein Expression Profile
DPA
6
7
8
9
10
11
12
13
14
15
16
17
18
19
20
21
22
23
24
0
1
2
3
4
5
6
7
8
9
10
11
12
13
14
15
16
17
18
19
20
21
22
23
24
25
26
27
28
29
30
31

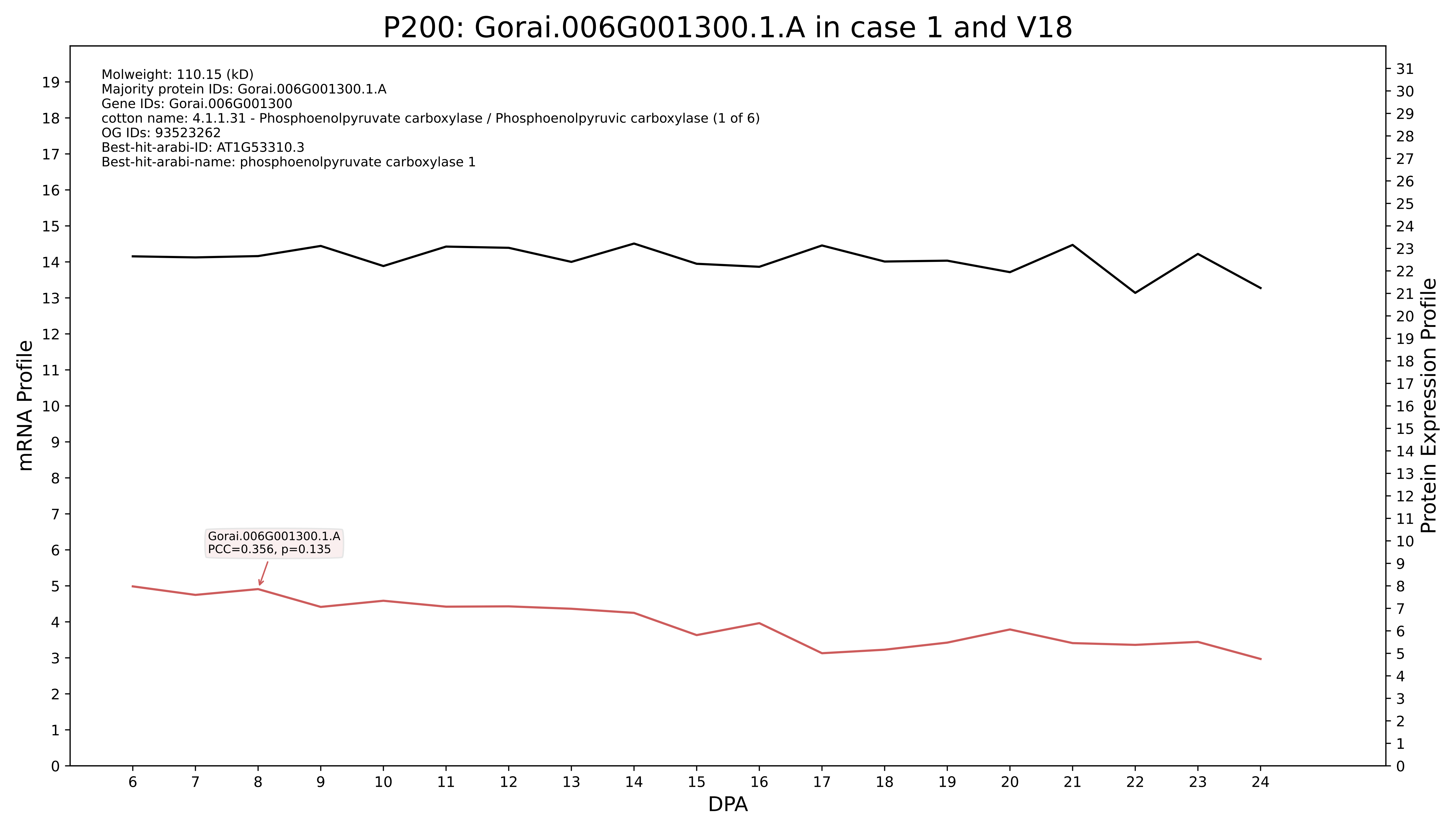
P200: Gorai.006G001300.1.A in case 1 and V18
Molweight: 110.15 (kD)
Majority protein IDs: Gorai.006G001300.1.A
Gene IDs: Gorai.006G001300
cotton name: 4.1.1.31 - Phosphoenolpyruvate carboxylase / Phosphoenolpyruvic carboxylase (1 of 6)
OG IDs: 93523262
Best-hit-arabi-ID: AT1G53310.3
Best-hit-arabi-name: phosphoenolpyruvate carboxylase 1
Gorai.006G001300.1.A
PCC=0.356, p=0.135
mRNA Profile
Protein Expression Profile
DPA

P200: Gorai.010G181500.1.D in case 1 and V18

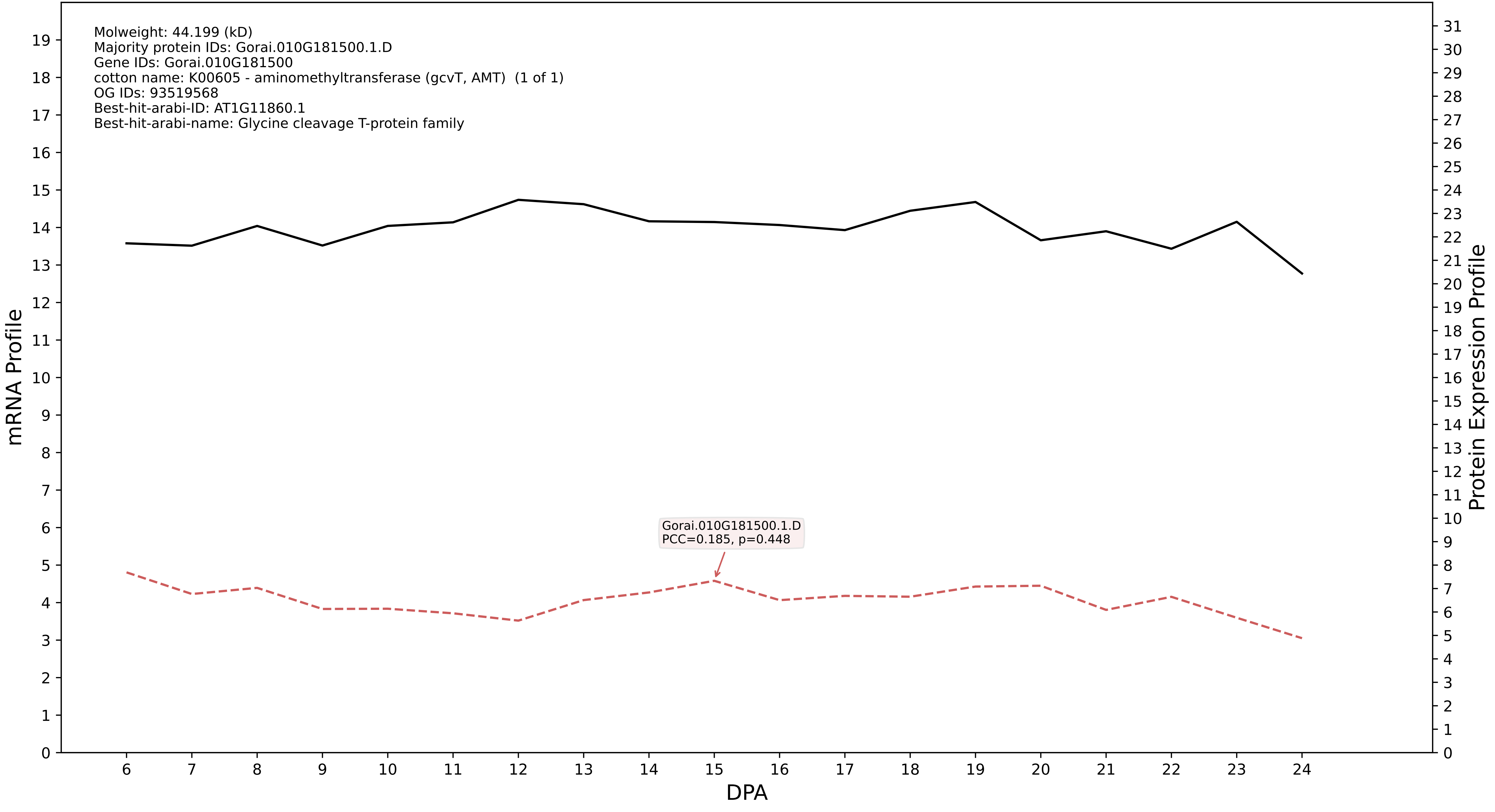

P200: Gorai.004G212800.3.D in case 2 and V18

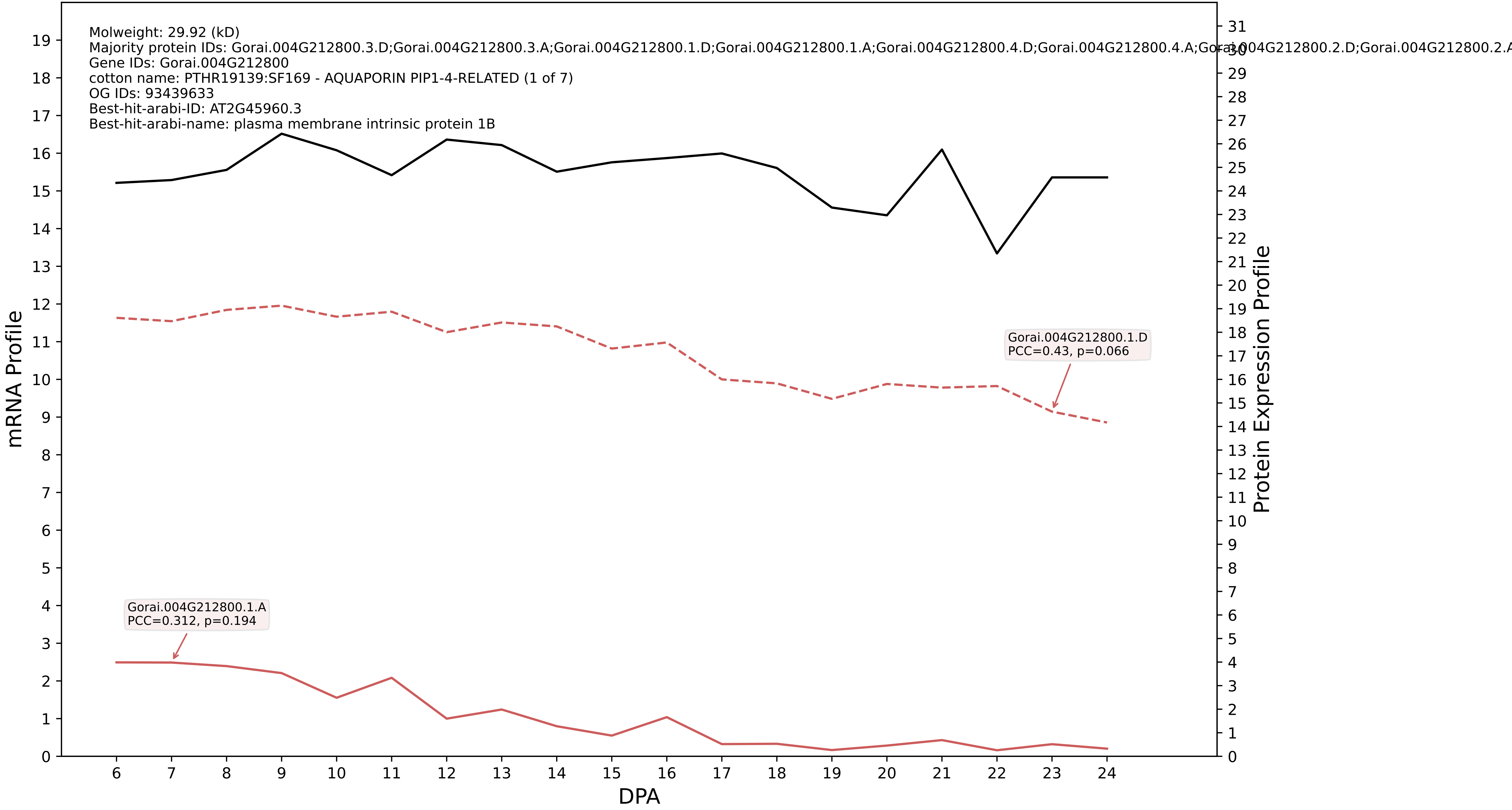

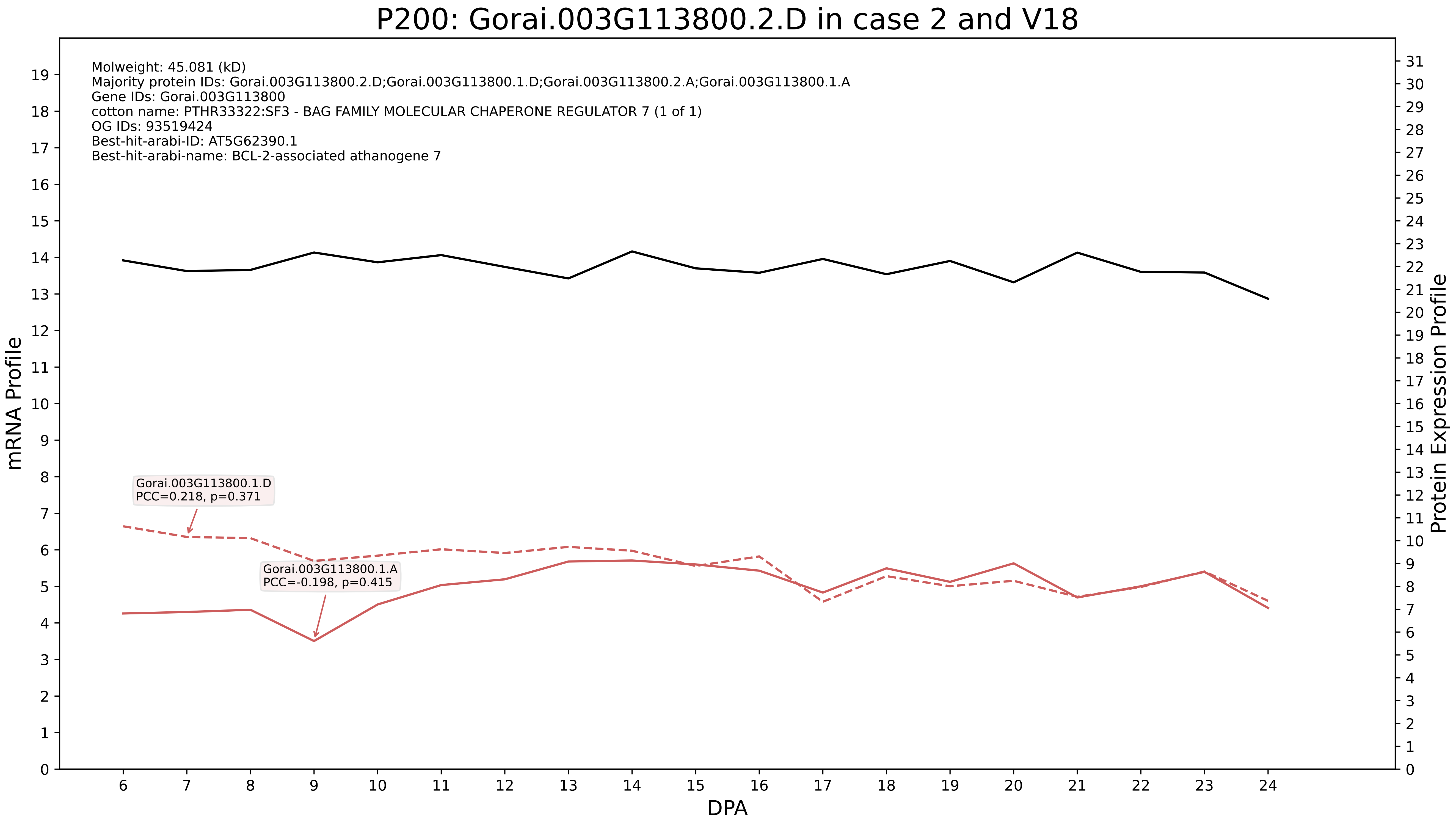
P200: Gorai.003G113800.2.D in case 2 and V18
Molweight: 45.081 (kD)
Majority protein IDs: Gorai.003G113800.2.D;Gorai.003G113800.1.D;Gorai.003G113800.2.A;Gorai.003G113800.1.A
Gene IDs: Gorai.003G113800
cotton name: PTHR33322:SF3 - BAG FAMILY MOLECULAR CHAPERONE REGULATOR 7 (1 of 1)
OG IDs: 93519424
Best-hit-arabi-ID: AT5G62390.1
Best-hit-arabi-name: BCL-2-associated athanogene 7
Gorai.003G113800.1.D
PCC=0.218, p=0.371
Gorai.003G113800.1.A
PCC=-0.198, p=0.415
mRNA Profile
Protein Expression Profile
DPA

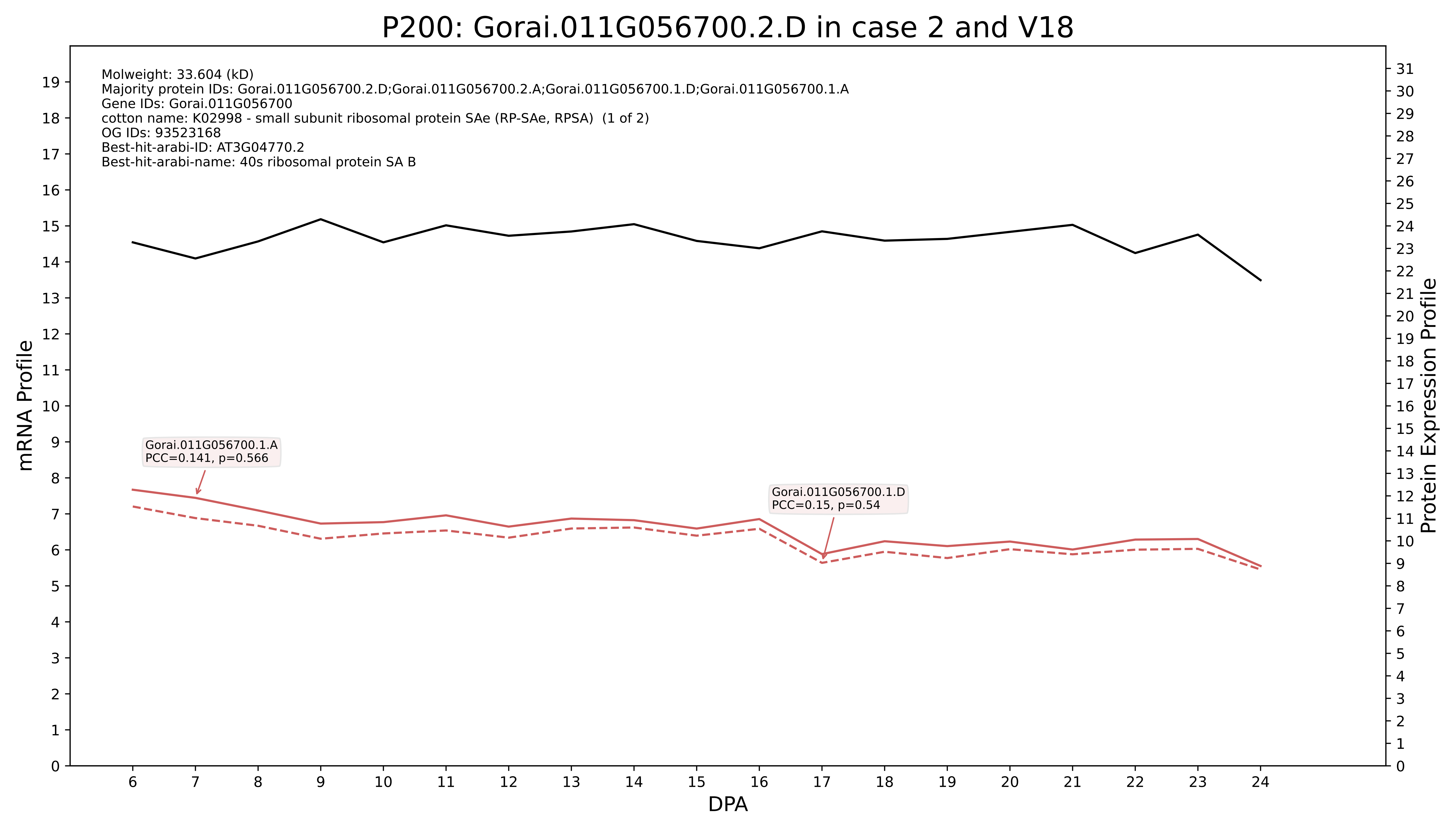
P200: Gorai.011G056700.2.D in case 2 and V18
Molweight: 33.604 (kD)
Majority protein IDs: Gorai.011G056700.2.D;Gorai.011G056700.2.A;Gorai.011G056700.1.D;Gorai.011G056700.1.A
Gene IDs: Gorai.011G056700
cotton name: K02998 - small subunit ribosomal protein SAe (RP-SAe, RPSA) (1 of 2)
OG IDs: 93523168
Best-hit-arabi-ID: AT3G04770.2
Best-hit-arabi-name: 40s ribosomal protein SA B
Gorai.011G056700.1.A
PCC=0.141, p=0.566
Gorai.011G056700.1.D
PCC=0.15, p=0.54
mRNA Profile
Protein Expression Profile
DPA

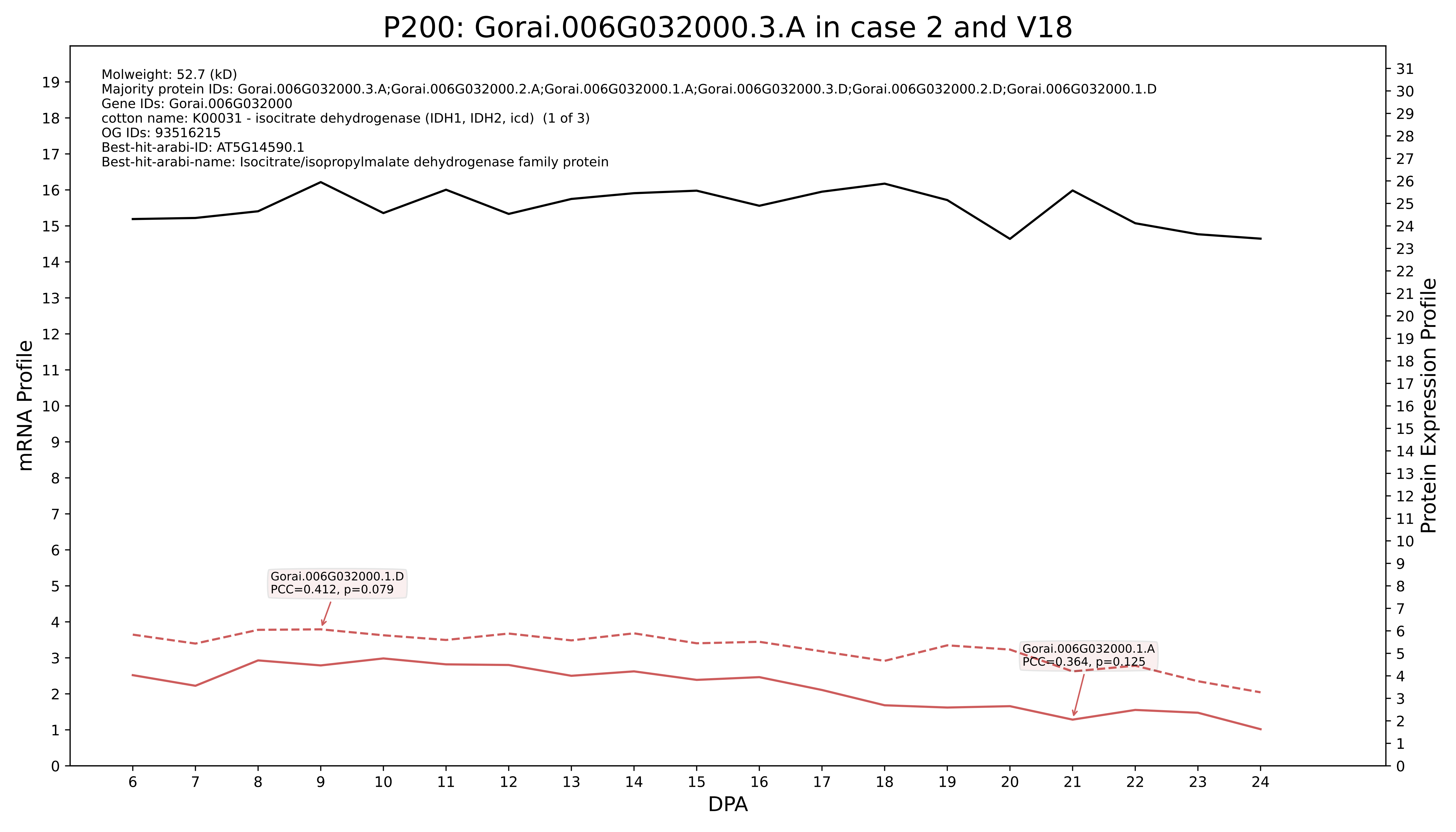

P200: Gorai.006G032000.3.A in case 2 and V18
Molweight: 52.7 (kD)
Majority protein IDs: Gorai.006G032000.3.A;Gorai.006G032000.2.A;Gorai.006G032000.1.A;Gorai.006G032000.3.D;Gorai.006G032000.2.D;Gorai.006G032000.1.D
Gene IDs: Gorai.006G032000
cotton name: K00031 - isocitrate dehydrogenase (IDH1, IDH2, icd) (1 of 3)
OG IDs: 93516215
Best-hit-arabi-ID: AT5G14590.1
Best-hit-arabi-name: Isocitrate/isopropylmalate dehydrogenase family protein
Gorai.006G032000.1.D
PCC=0.412, p=0.079
Gorai.006G032000.1.A
PCC=0.364, p=0.125
mRNA Profile
Protein Expression Profile
DPA
0 1 2 3 4 5 6 7 8 9 10 11 12 13 14 15 16 17 18 19
0 1 2 3 4 5 6 7 8 9 10 11 12 13 14 15 16 17 18 19 20 21 22 23 24 25 26 27 28 29 30 31
6 7 8 9 10 11 12 13 14 15 16 17 18 19 20 21 22 23 24

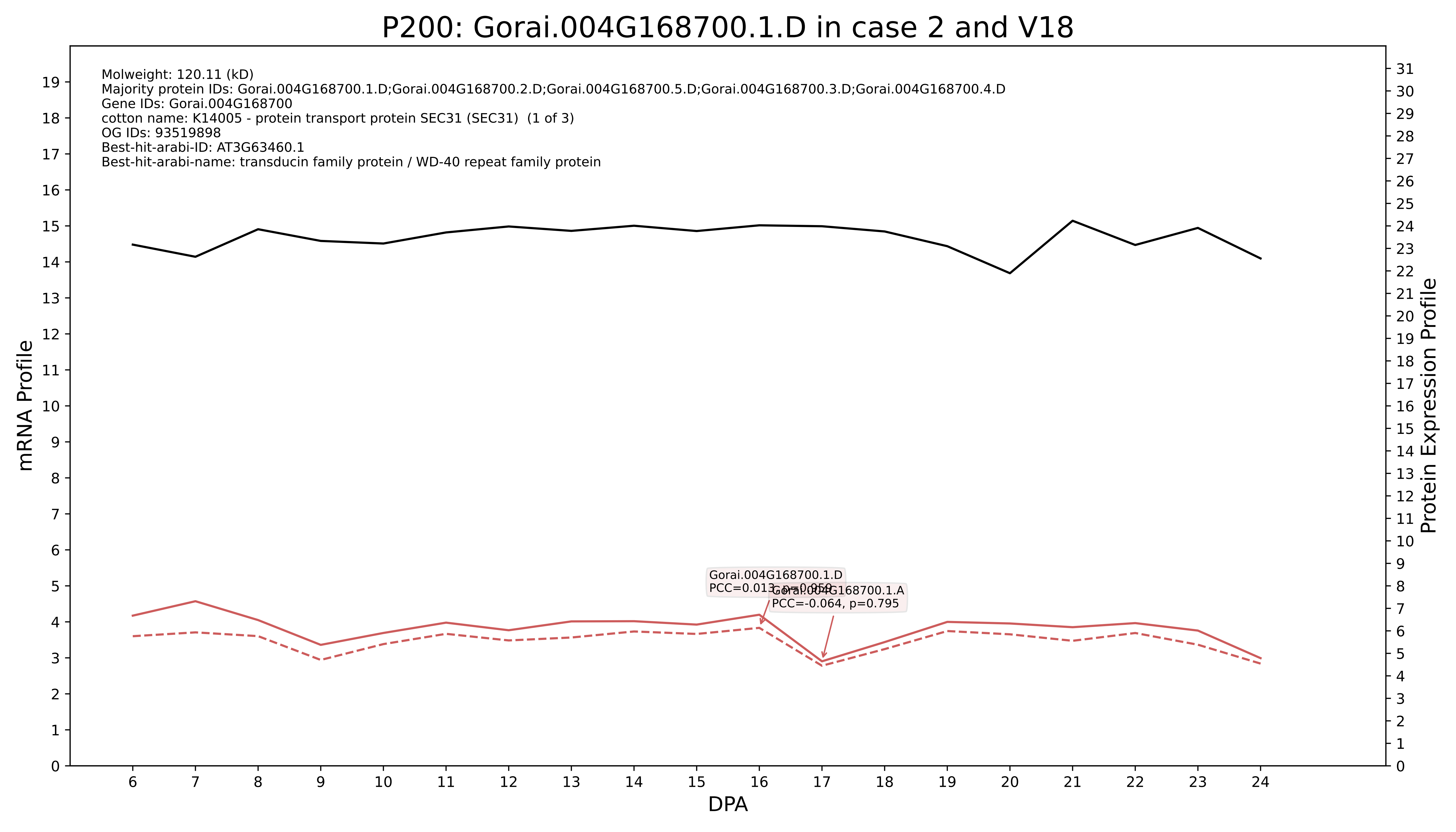

P200: Gorai.004G168700.1.D in case 2 and V18
Molweight: 120.11 (kD)
Majority protein IDs: Gorai.004G168700.1.D;Gorai.004G168700.2.D;Gorai.004G168700.5.D;Gorai.004G168700.3.D;Gorai.004G168700.4.D
Gene IDs: Gorai.004G168700
cotton name: K14005 - protein transport protein SEC31 (SEC31) (1 of 3)
OG IDs: 93519898
Best-hit-arabi-ID: AT3G63460.1
Best-hit-arabi-name: transducin family protein / WD-40 repeat family protein
Gorai.004G168700.1.D
PCC=0.013, p=0.959
Gorai.004G168700.1.A
PCC=-0.064, p=0.795
mRNA Profile
Protein Expression Profile
DPA

P200: Gorai.010G196100.3.D in case 2 and V18

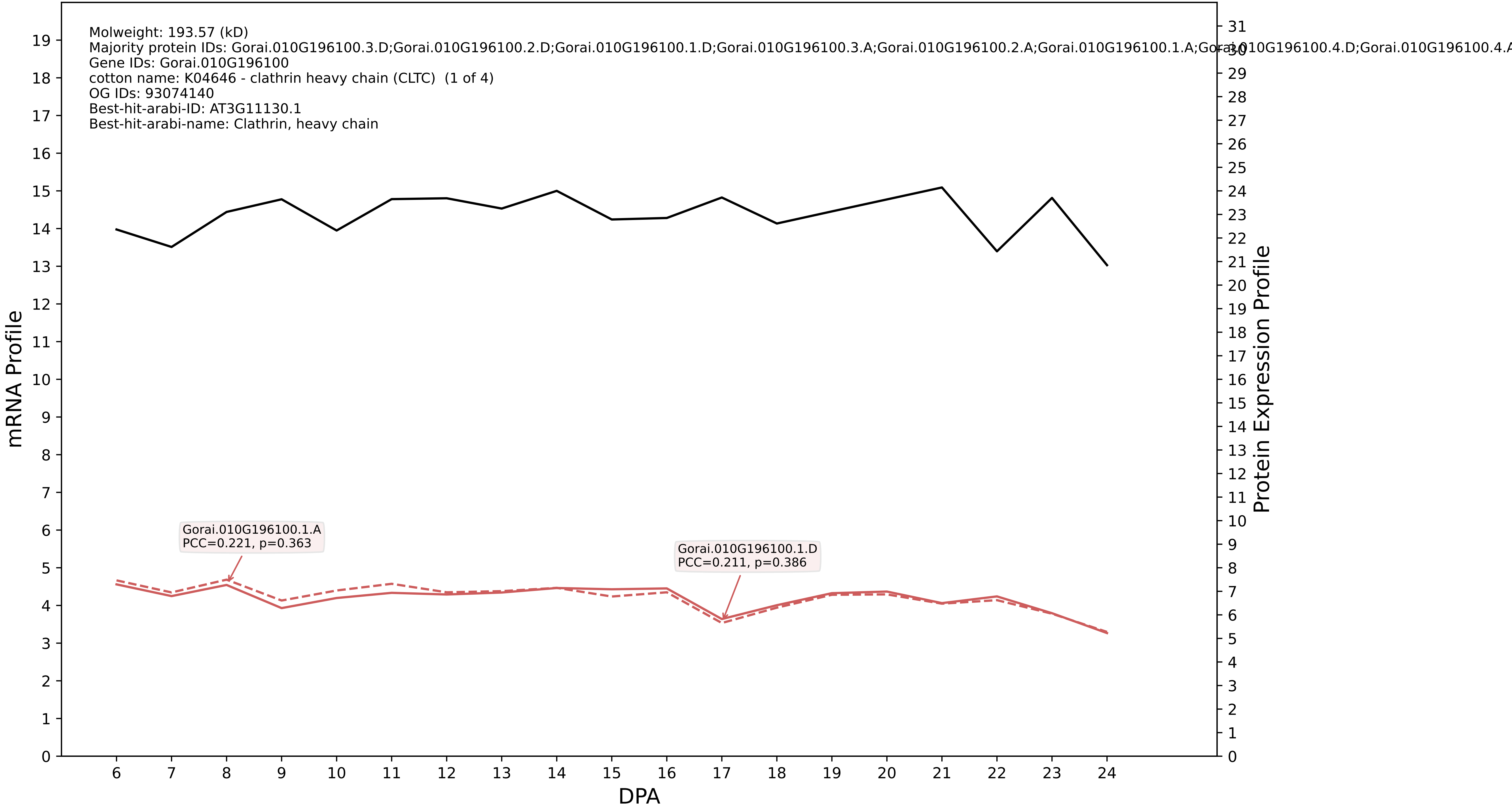

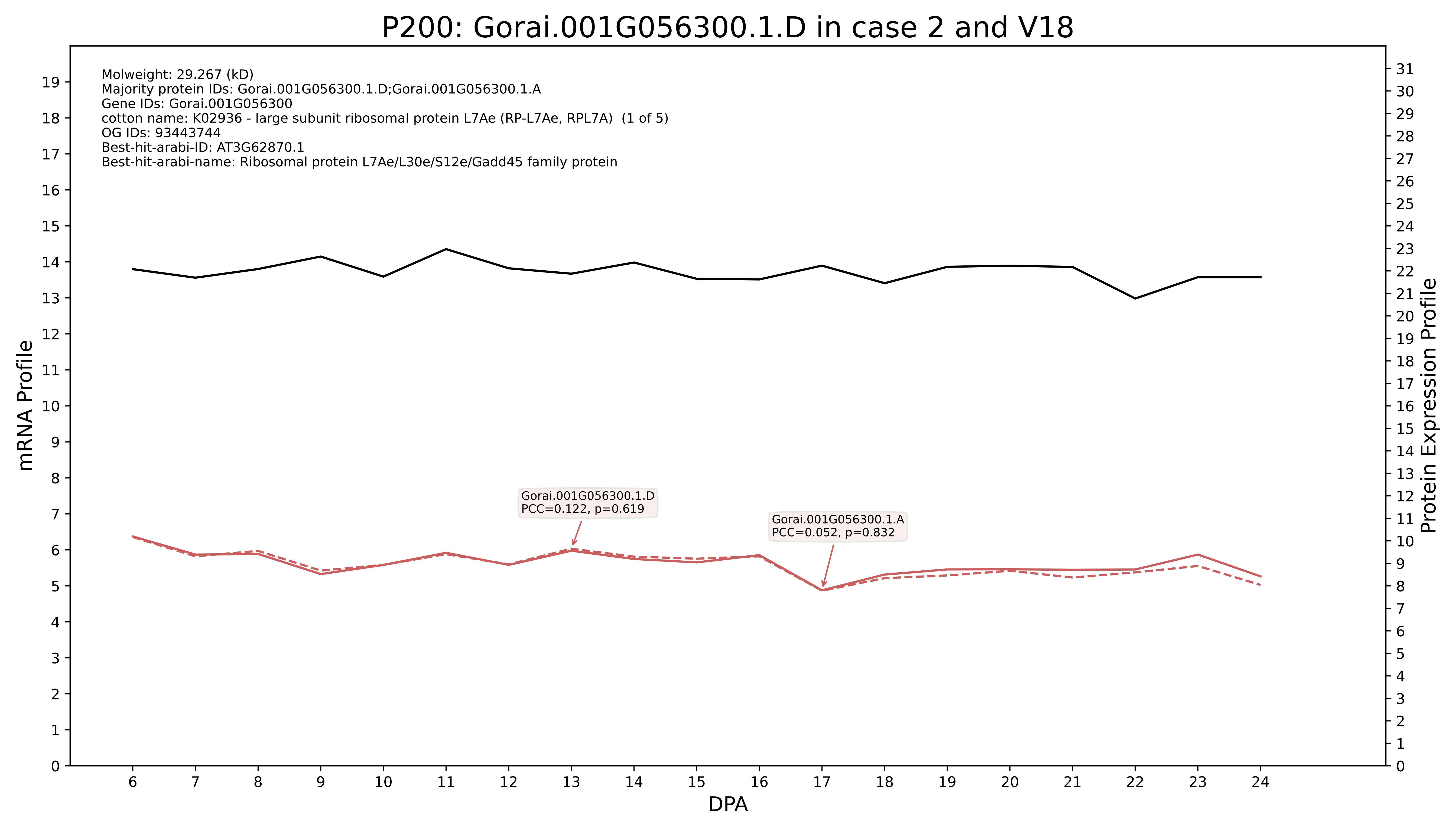
P200: Gorai.001G056300.1.D in case 2 and V18
Molweight: 29.267 (kD)
Majority protein IDs: Gorai.001G056300.1.D;Gorai.001G056300.1.A
Gene IDs: Gorai.001G056300
cotton name: K02936 - large subunit ribosomal protein L7Ae (RP-L7Ae, RPL7A) (1 of 5)
OG IDs: 93443744
Best-hit-arabi-ID: AT3G62870.1
Best-hit-arabi-name: Ribosomal protein L7Ae/L30e/S12e/Gadd45 family protein
Gorai.001G056300.1.D
PCC=0.122, p=0.619
Gorai.001G056300.1.A
PCC=0.052, p=0.832
mRNA Profile
Protein Expression Profile
DPA

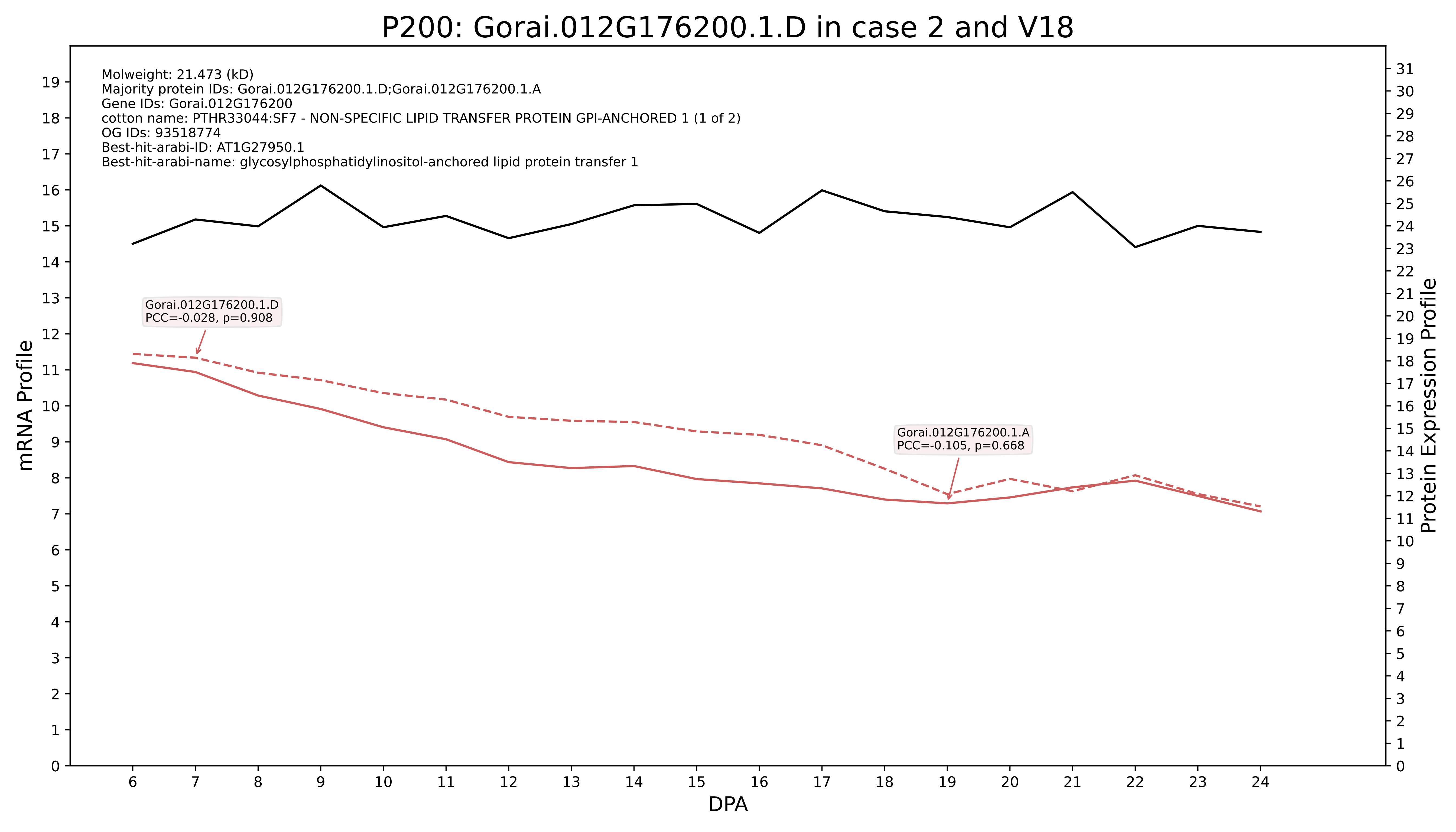

P200: Gorai.012G176200.1.D in case 2 and V18
Molweight: 21.473 (kD)
Majority protein IDs: Gorai.012G176200.1.D;Gorai.012G176200.1.A
Gene IDs: Gorai.012G176200
cotton name: PTHR33044:SF7 - NON-SPECIFIC LIPID TRANSFER PROTEIN GPI-ANCHORED 1 (1 of 2)
OG IDs: 93518774
Best-hit-arabi-ID: AT1G27950.1
Best-hit-arabi-name: glycosylphosphatidylinositol-anchored lipid protein transfer 1
Gorai.012G176200.1.D
PCC=-0.028, p=0.908
Gorai.012G176200.1.A
PCC=-0.105, p=0.668
mRNA Profile
Protein Expression Profile
DPA

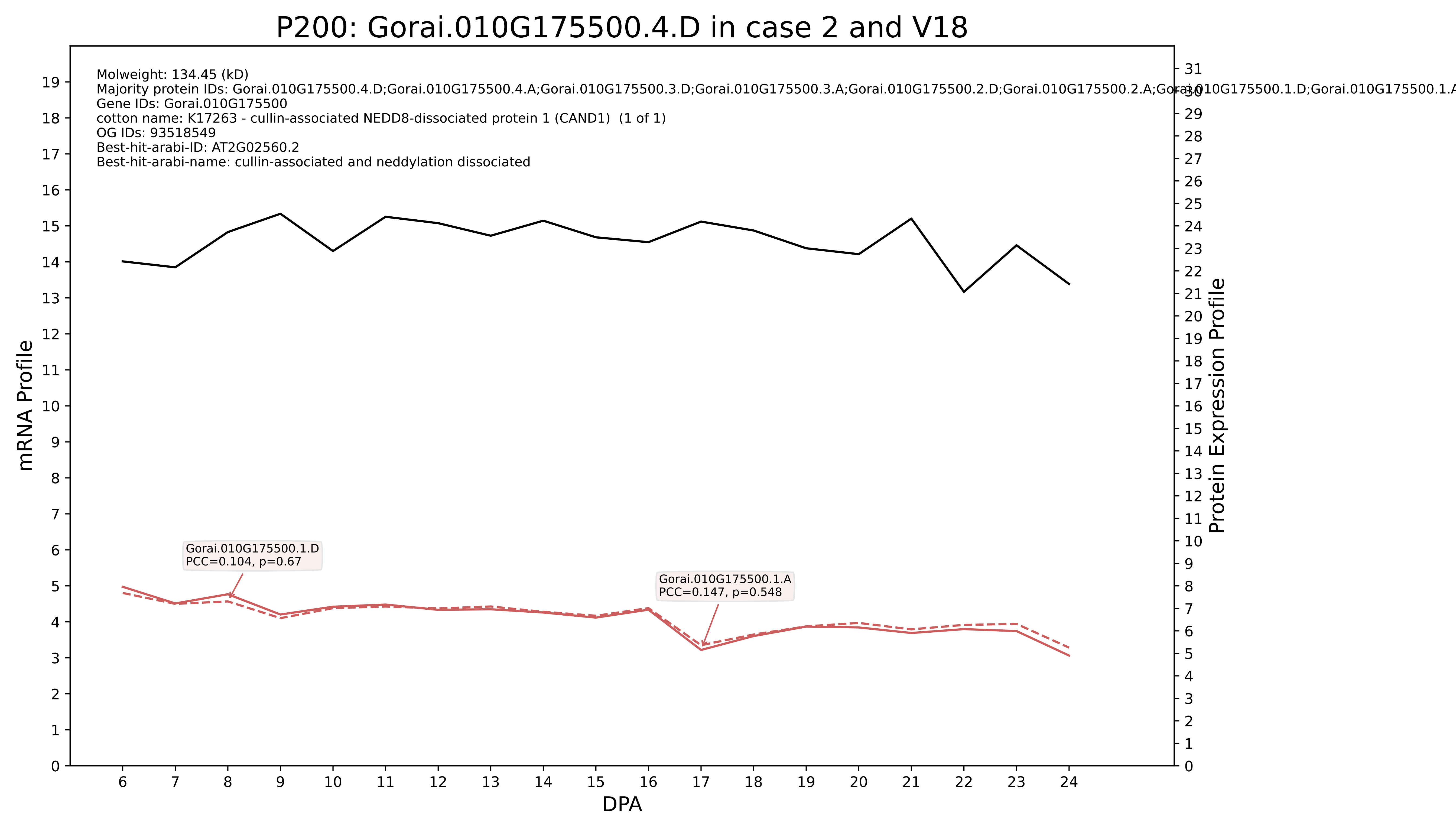

P200: Gorai.010G175500.4.D in case 2 and V18
Molweight: 134.45 (kD)
Majority protein IDs: Gorai.010G175500.4.D;Gorai.010G175500.4.A;Gorai.010G175500.3.D;Gorai.010G175500.3.A;Gorai.010G175500.2.D;Gorai.010G175500.2.A;Gorai.010G175500.1.D;Gorai.010G175500.1.A
Gene IDs: Gorai.010G175500
cotton name: K17263 - cullin-associated NEDD8-dissociated protein 1 (CAND1) (1 of 1)
OG IDs: 93518549
Best-hit-arabi-ID: AT2G02560.2
Best-hit-arabi-name: cullin-associated and neddylation dissociated
Gorai.010G175500.1.D
PCC=0.104, p=0.67
Gorai.010G175500.1.A
PCC=0.147, p=0.548
mRNA Profile
Protein Expression Profile
DPA

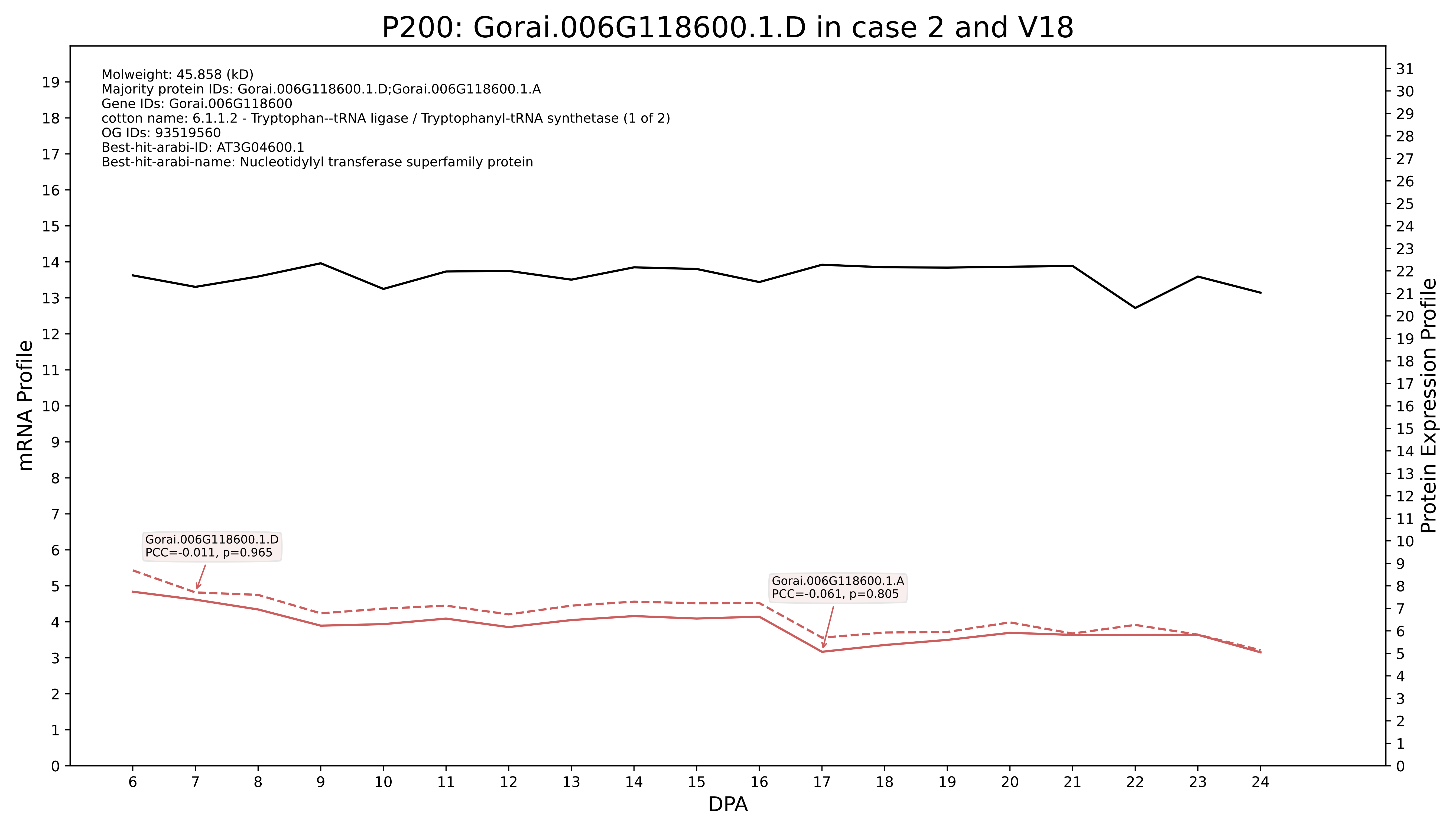
P200: Gorai.006G118600.1.D in case 2 and V18
Molweight: 45.858 (kD)
Majority protein IDs: Gorai.006G118600.1.D;Gorai.006G118600.1.A
Gene IDs: Gorai.006G118600
cotton name: 6.1.1.2 - Tryptophan--tRNA ligase / Tryptophanyl-tRNA synthetase (1 of 2)
OG IDs: 93519560
Best-hit-arabi-ID: AT3G04600.1
Best-hit-arabi-name: Nucleotidylyl transferase superfamily protein
Gorai.006G118600.1.D
PCC=-0.011, p=0.965
Gorai.006G118600.1.A
PCC=-0.061, p=0.805
mRNA Profile
Protein Expression Profile
DPA
6
7
8
9
10
11
12
13
14
15
16
17
18
19
20
21
22
23
24

200: Gorai.001G147400.6.A in case 2 and V18

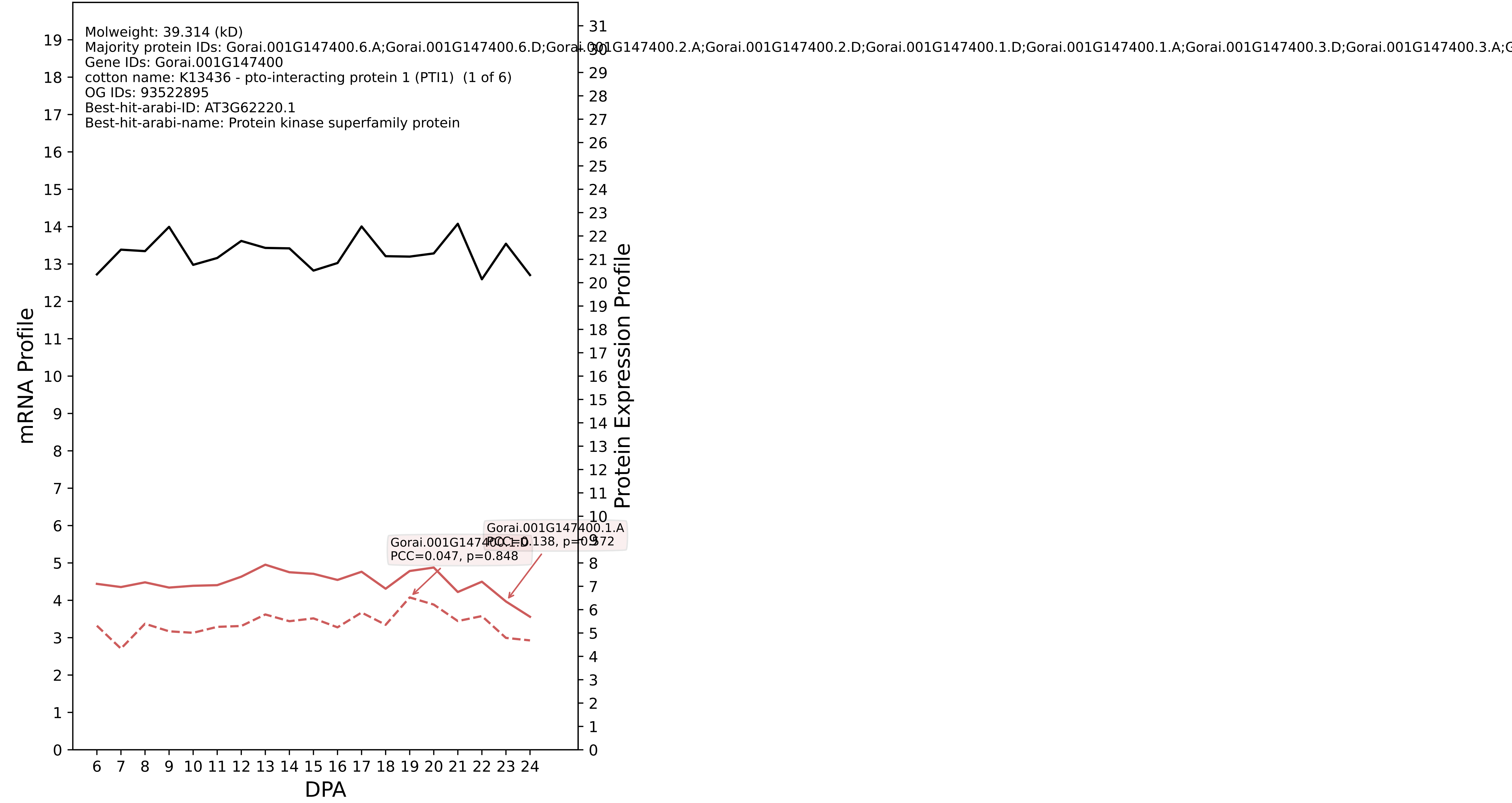

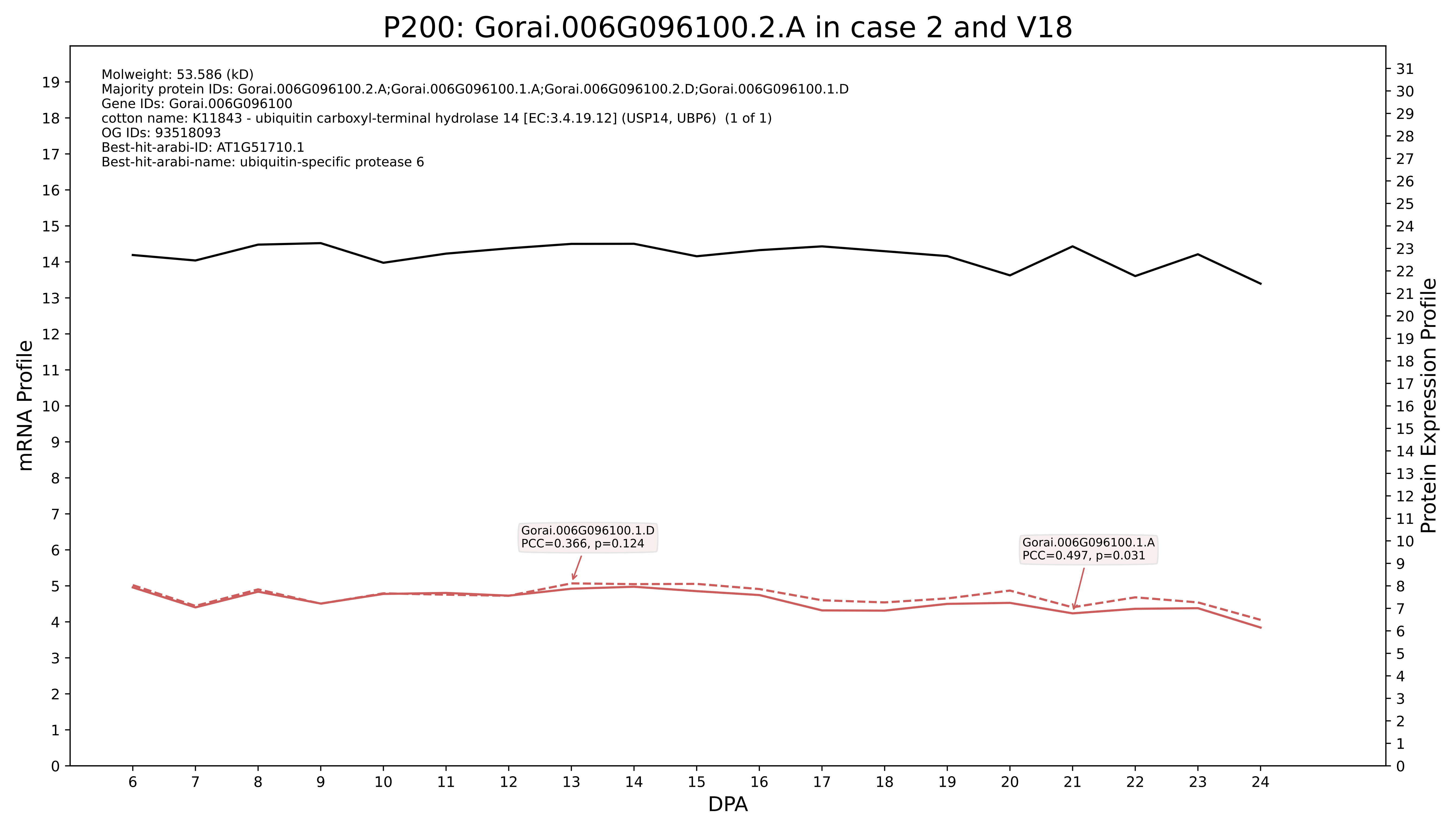
P200: Gorai.006G096100.2.A in case 2 and V18
Molweight: 53.586 (kD)
Majority protein IDs: Gorai.006G096100.2.A;Gorai.006G096100.1.A;Gorai.006G096100.2.D;Gorai.006G096100.1.D
Gene IDs: Gorai.006G096100
cotton name: K11843 - ubiquitin carboxyl-terminal hydrolase 14 [EC:3.4.19.12] (USP14, UBP6) (1 of 1)
OG IDs: 93518093
Best-hit-arabi-ID: AT1G51710.1
Best-hit-arabi-name: ubiquitin-specific protease 6
Gorai.006G096100.1.D
PCC=0.366, p=0.124
Gorai.006G096100.1.A
PCC=0.497, p=0.031
mRNA Profile
Protein Expression Profile
DPA
0
1
2
3
4
5
6
7
8
9
10
11
12
13
14
15
16
17
18
19
20
21
22
23
24
25
26
27
28
29
30
31

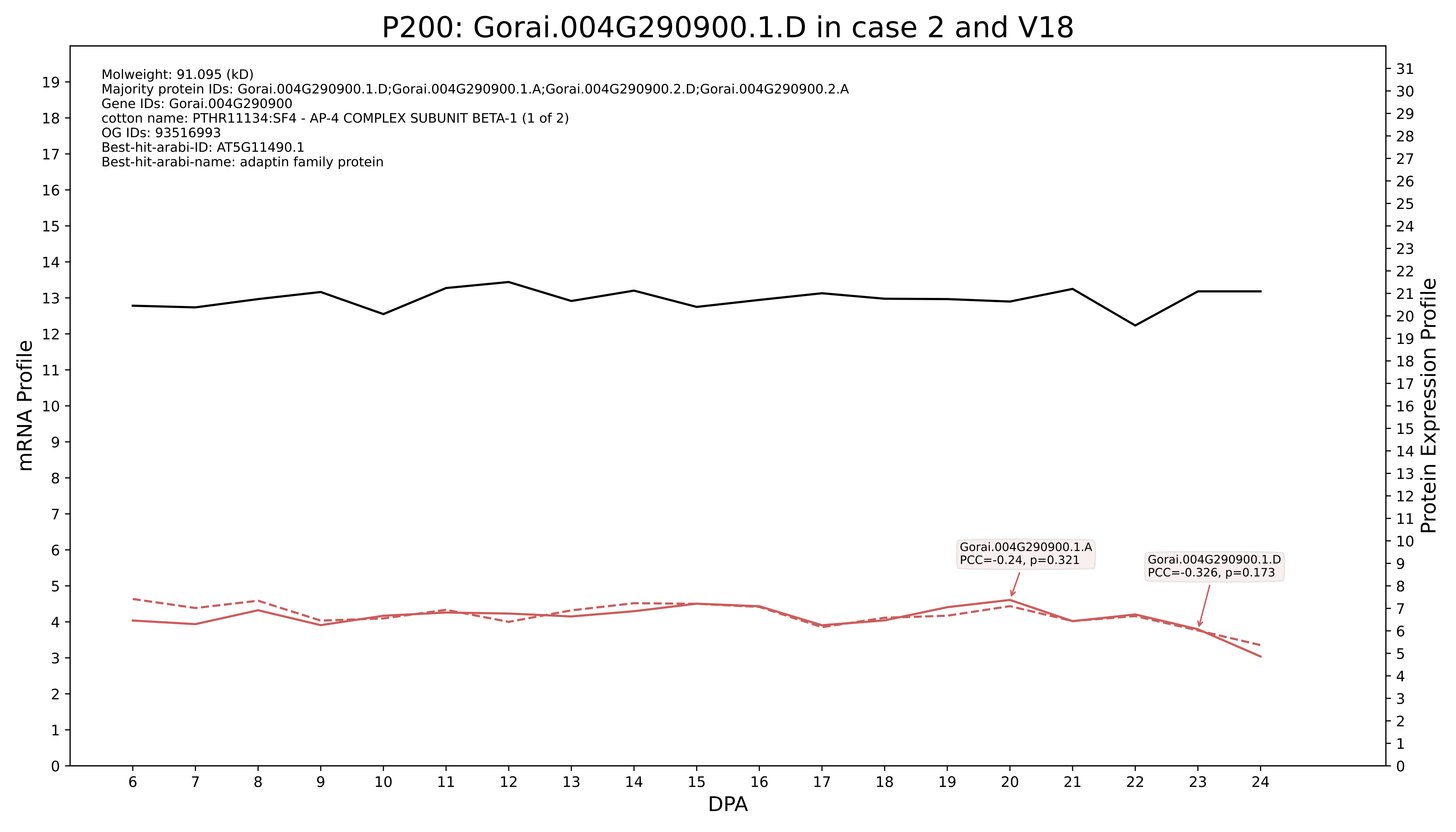

P200: Gorai.004G290900.1.D in case 2 and V18
Molweight: 91.095 (kD)
Majority protein IDs: Gorai.004G290900.1.D;Gorai.004G290900.1.A;Gorai.004G290900.2.D;Gorai.004G290900.2.A
Gene IDs: Gorai.004G290900
cotton name: PTHR11134:SF4 - AP-4 COMPLEX SUBUNIT BETA-1 (1 of 2)
OG IDs: 93516993
Best-hit-arabi-ID: AT5G11490.1
Best-hit-arabi-name: adaptin family protein
Gorai.004G290900.1.A
PCC=-0.24, p=0.321
Gorai.004G290900.1.D
PCC=-0.326, p=0.173
mRNA Profile
Protein Expression Profile
DPA

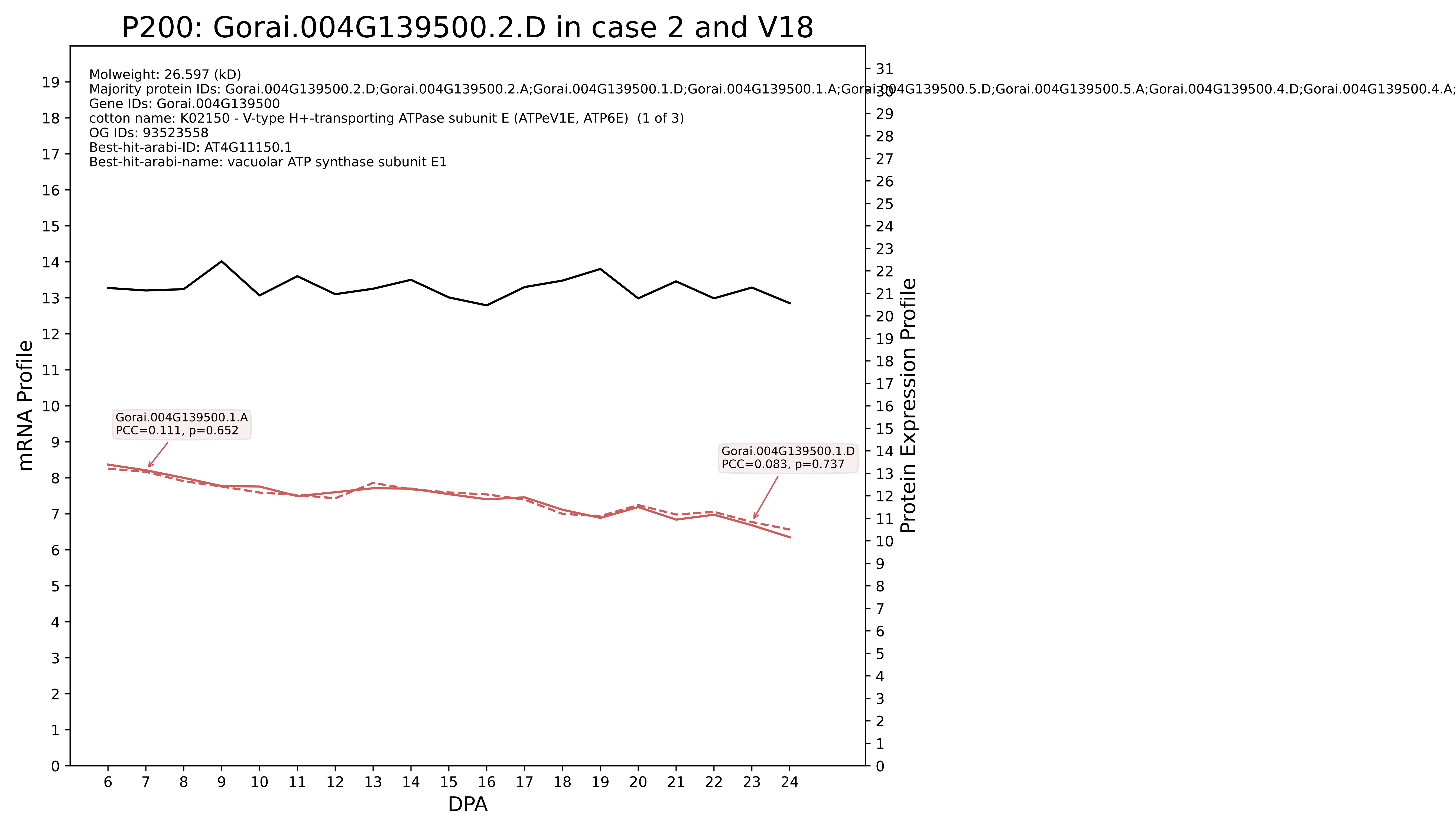

P200: Gorai.004G139500.2.D in case 2 and V18
Molweight: 26.597 (kD)
Majority protein IDs: Gorai.004G139500.2.D;Gorai.004G139500.2.A;Gorai.004G139500.1.D;Gorai.004G139500.1.A;Gorai.004G139500.5.D;Gorai.004G139500.5.A;Gorai.004G139500.4.D;Gorai.004G139500.4.A;
Gene IDs: Gorai.004G139500
cotton name: K02150 - V-type H+-transporting ATPase subunit E (ATPeV1E, ATP6E) (1 of 3)
OG IDs: 93523558
Best-hit-arabi-ID: AT4G11150.1
Best-hit-arabi-name: vacuolar ATP synthase subunit E1
Gorai.004G139500.1.A
PCC=0.111, p=0.652
Gorai.004G139500.1.D
PCC=0.083, p=0.737
mRNA Profile
Protein Expression Profile
DPA

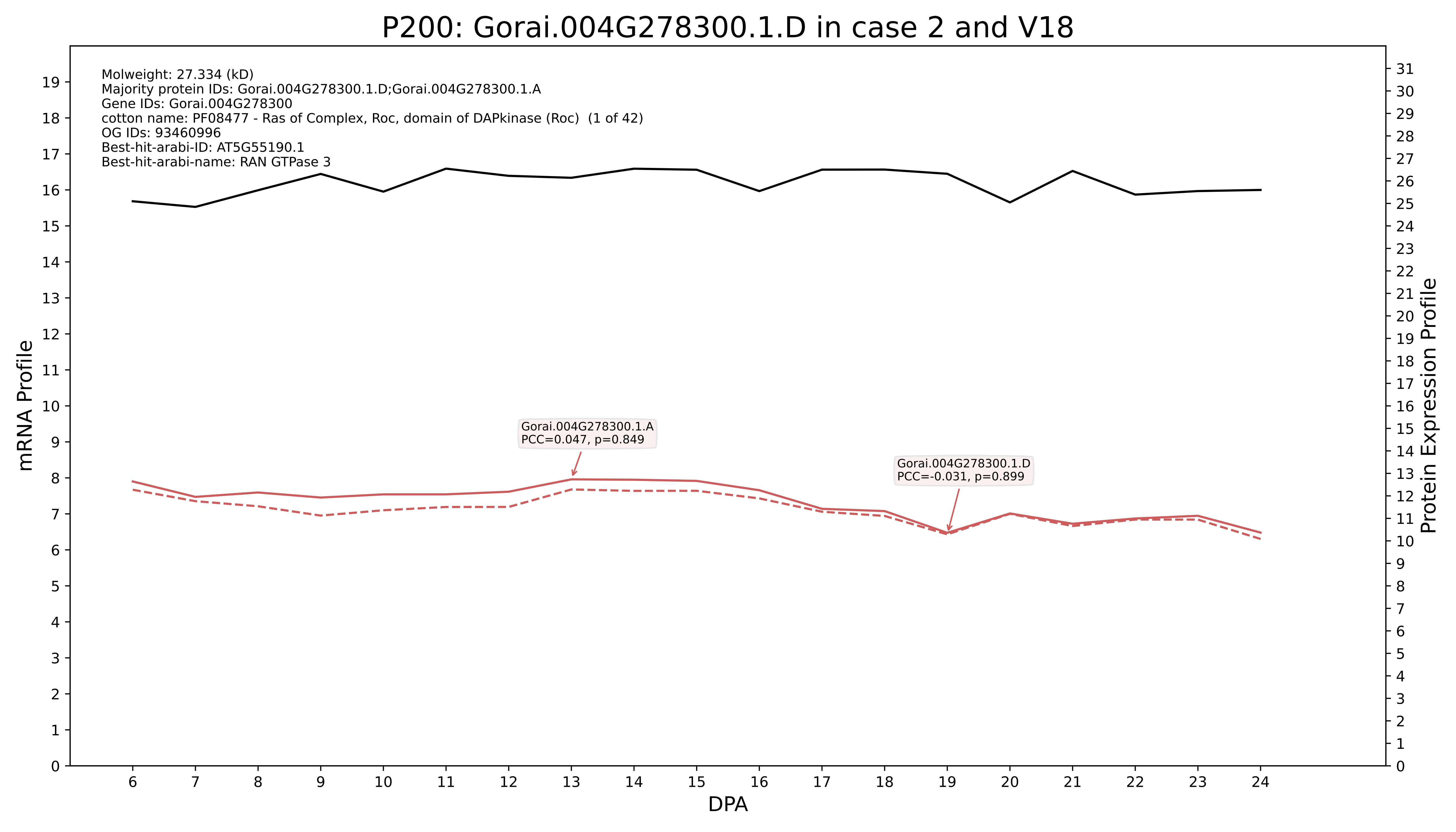
P200: Gorai.004G278300.1.D in case 2 and V18
Molweight: 27.334 (kD)
Majority protein IDs: Gorai.004G278300.1.D;Gorai.004G278300.1.A
Gene IDs: Gorai.004G278300
cotton name: PF08477 - Ras of Complex, Roc, domain of DAPkinase (Roc) (1 of 42)
OG IDs: 93460996
Best-hit-arabi-ID: AT5G55190.1
Best-hit-arabi-name: RAN GTPase 3
Gorai.004G278300.1.A
PCC=0.047, p=0.849
Gorai.004G278300.1.D
PCC=-0.031, p=0.899
mRNA Profile
Protein Expression Profile
DPA

P200: Gorai.002G200500.2.A in case 2 and V18

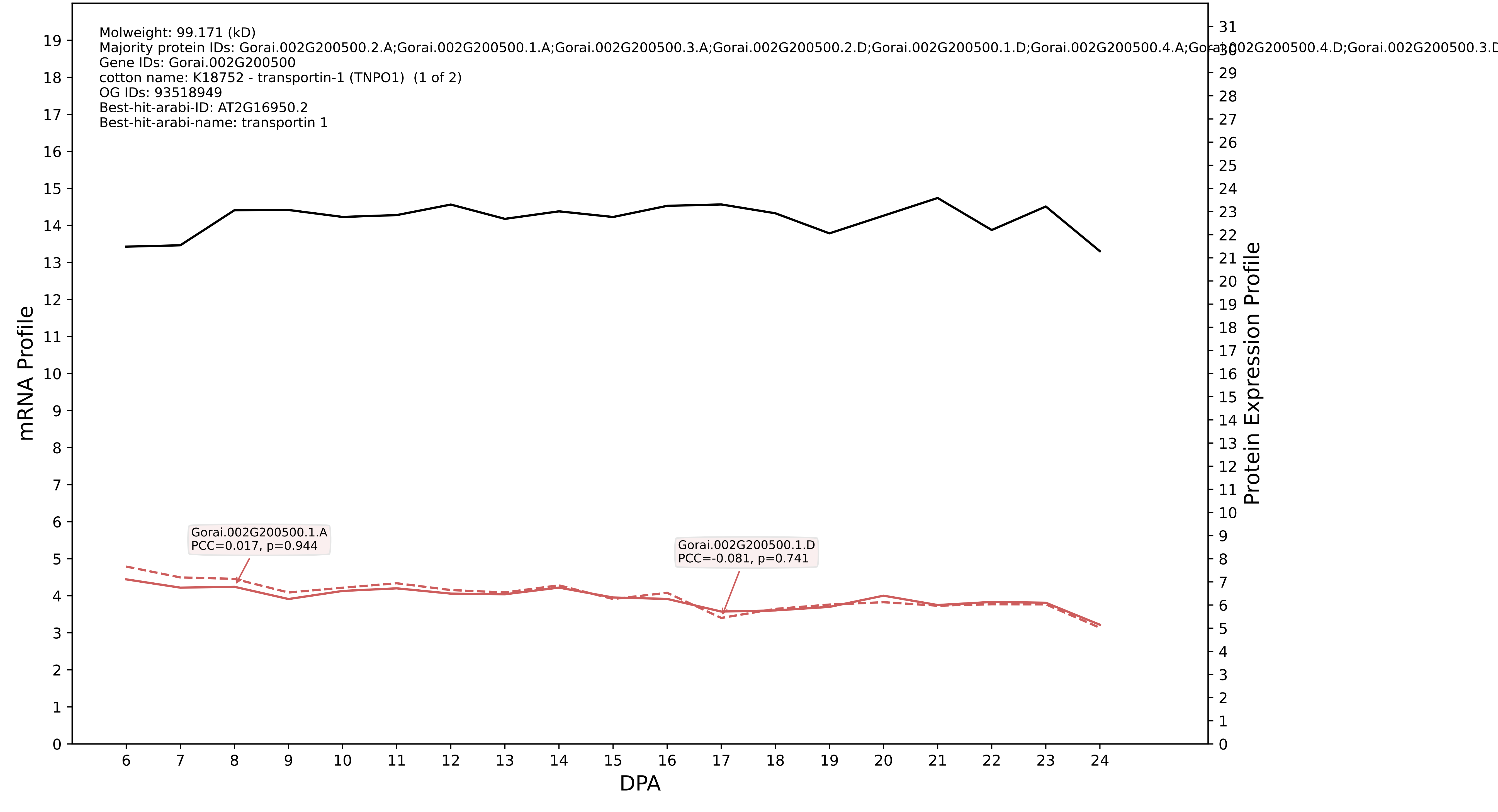

00: Gorai.005G216500.1.D in case 2 and V18

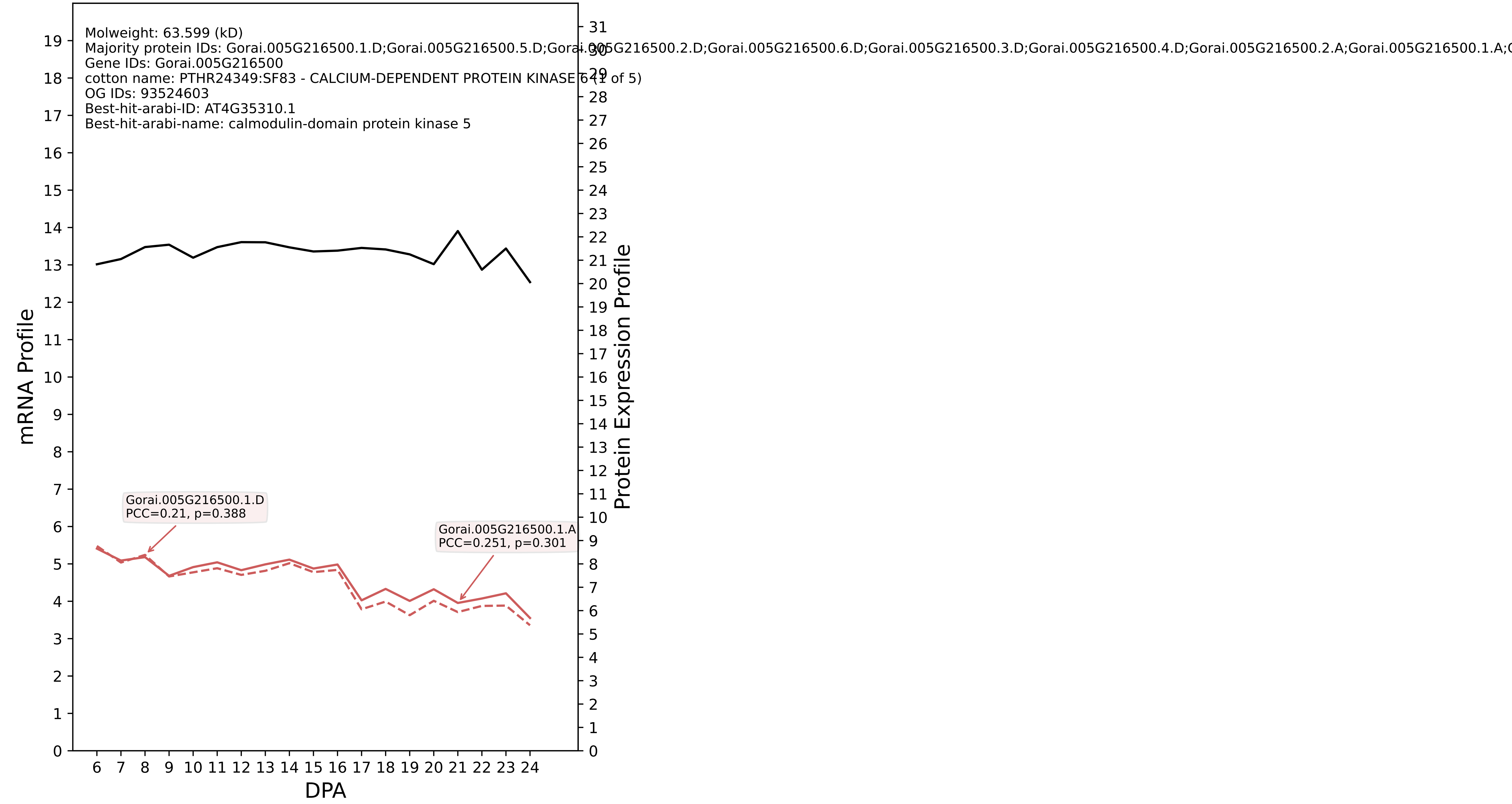

P200: Gorai.013G030300.5.D in case 2 and V18

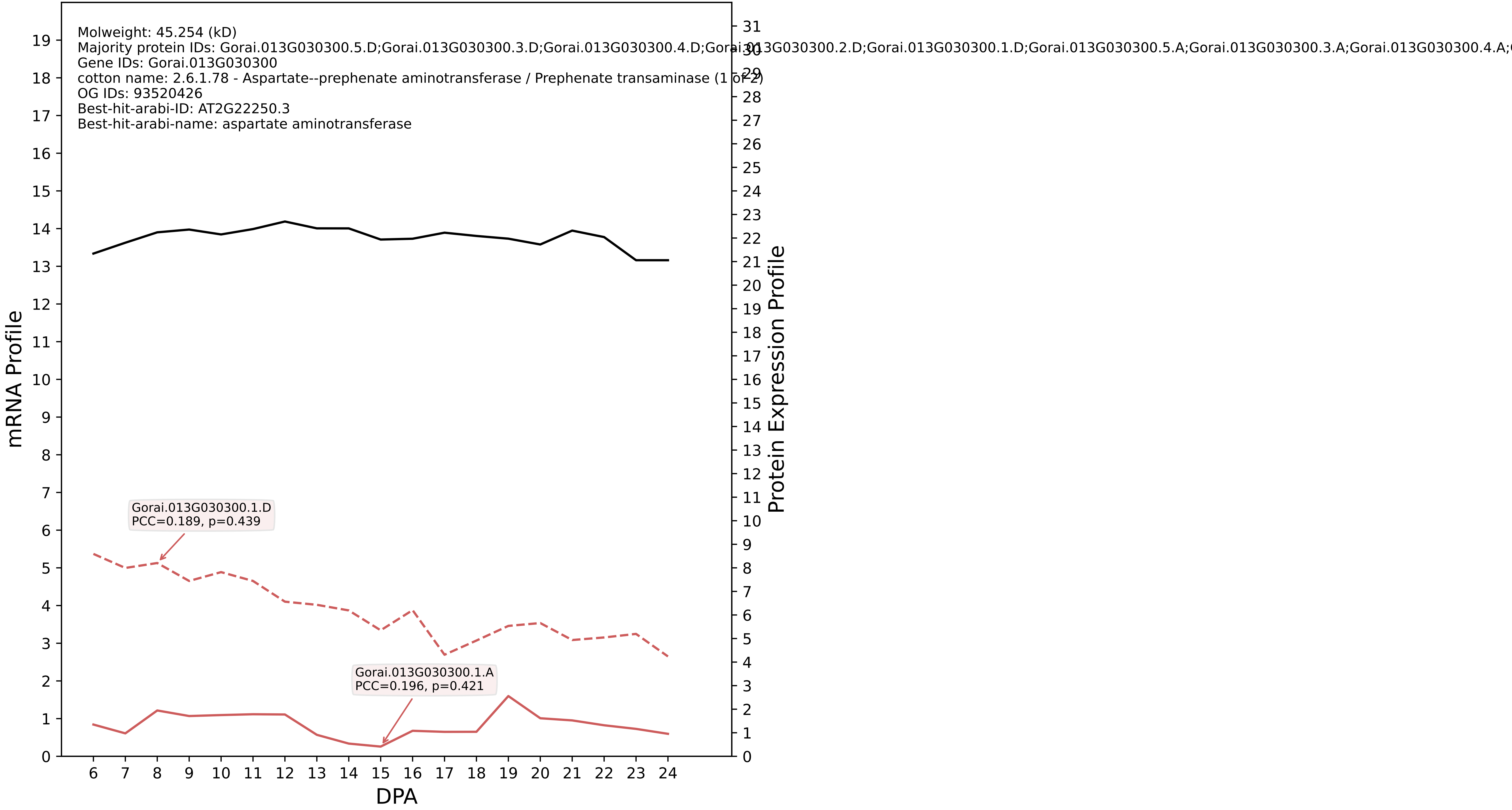

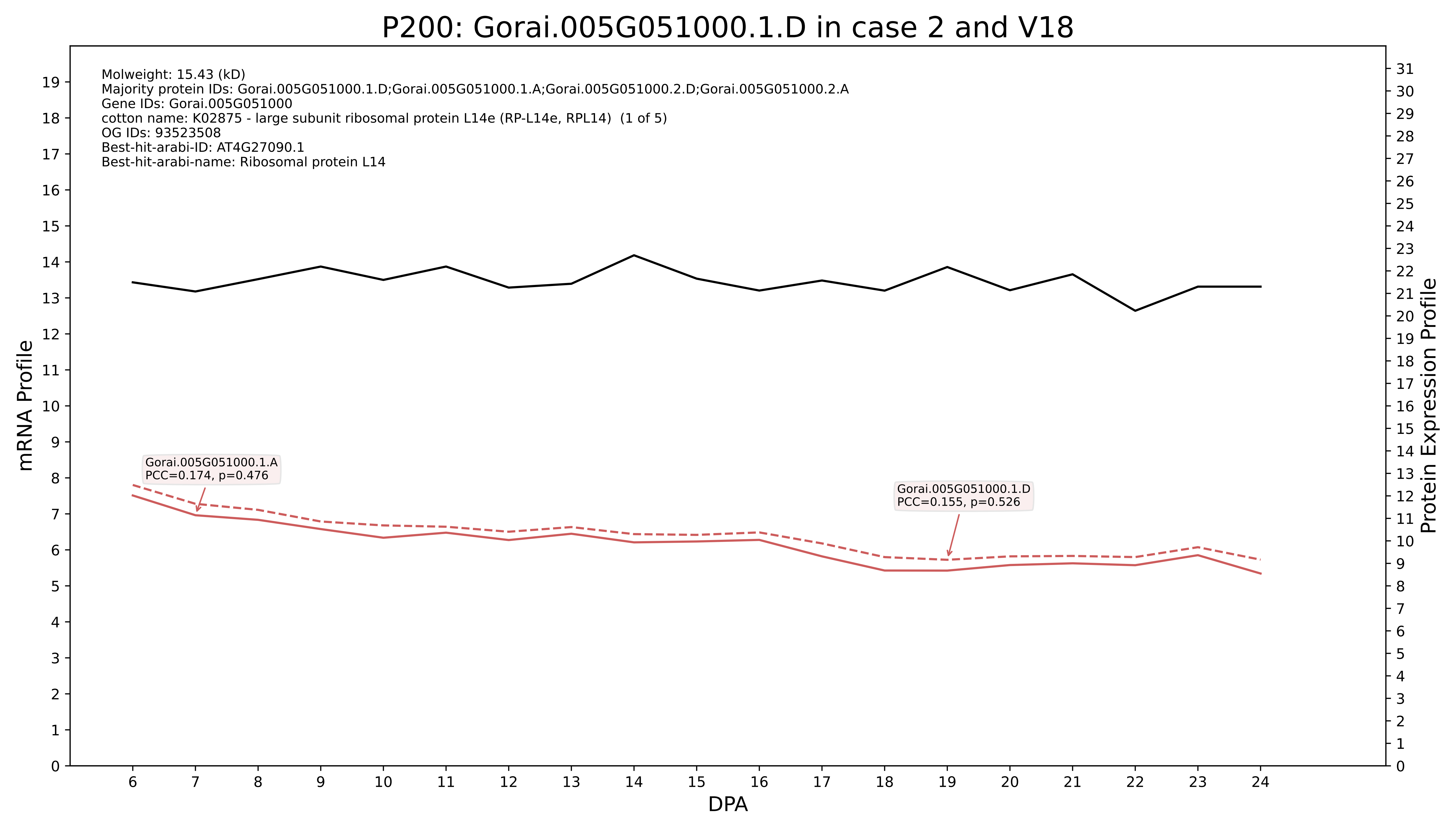
P200: Gorai.005G051000.1.D in case 2 and V18
Molweight: 15.43 (kD)
Majority protein IDs: Gorai.005G051000.1.D;Gorai.005G051000.1.A;Gorai.005G051000.2.D;Gorai.005G051000.2.A
Gene IDs: Gorai.005G051000
cotton name: K02875 - large subunit ribosomal protein L14e (RP-L14e, RPL14) (1 of 5)
OG IDs: 93523508
Best-hit-arabi-ID: AT4G27090.1
Best-hit-arabi-name: Ribosomal protein L14
Gorai.005G051000.1.A
PCC=0.174, p=0.476
Gorai.005G051000.1.D
PCC=0.155, p=0.526
mRNA Profile
Protein Expression Profile
DPA

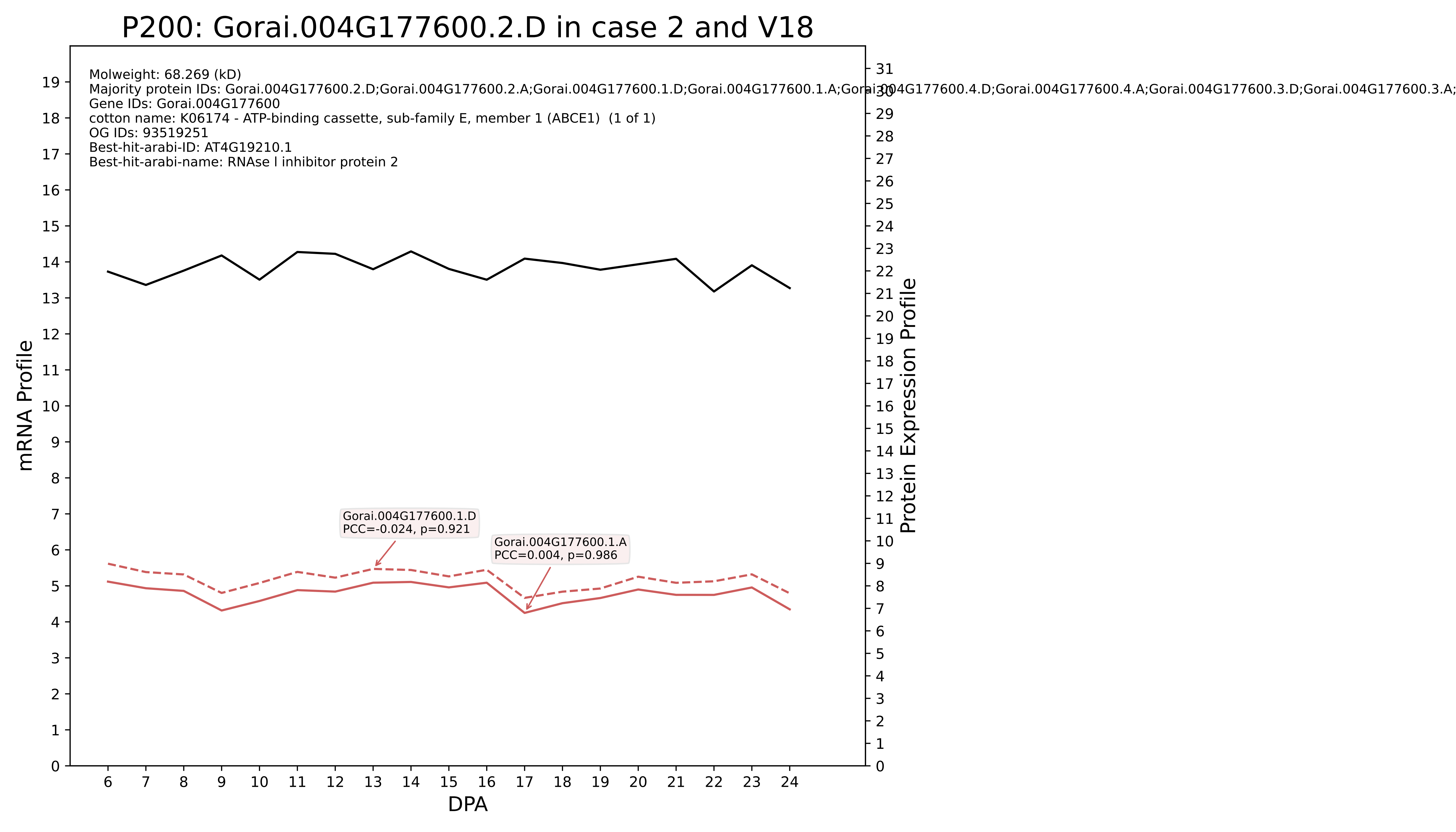

P200: Gorai.004G177600.2.D in case 2 and V18
Molweight: 68.269 (kD)
Majority protein IDs: Gorai.004G177600.2.D;Gorai.004G177600.2.A;Gorai.004G177600.1.D;Gorai.004G177600.1.A;Gorai.004G177600.4.D;Gorai.004G177600.4.A;Gorai.004G177600.3.D;Gorai.004G177600.3.A;
Gene IDs: Gorai.004G177600
cotton name: K06174 - ATP-binding cassette, sub-family E, member 1 (ABCE1) (1 of 1)
OG IDs: 93519251
Best-hit-arabi-ID: AT4G19210.1
Best-hit-arabi-name: RNAse l inhibitor protein 2
Gorai.004G177600.1.D
PCC=-0.024, p=0.921
Gorai.004G177600.1.A
PCC=0.004, p=0.986
mRNA Profile
Protein Expression Profile
DPA

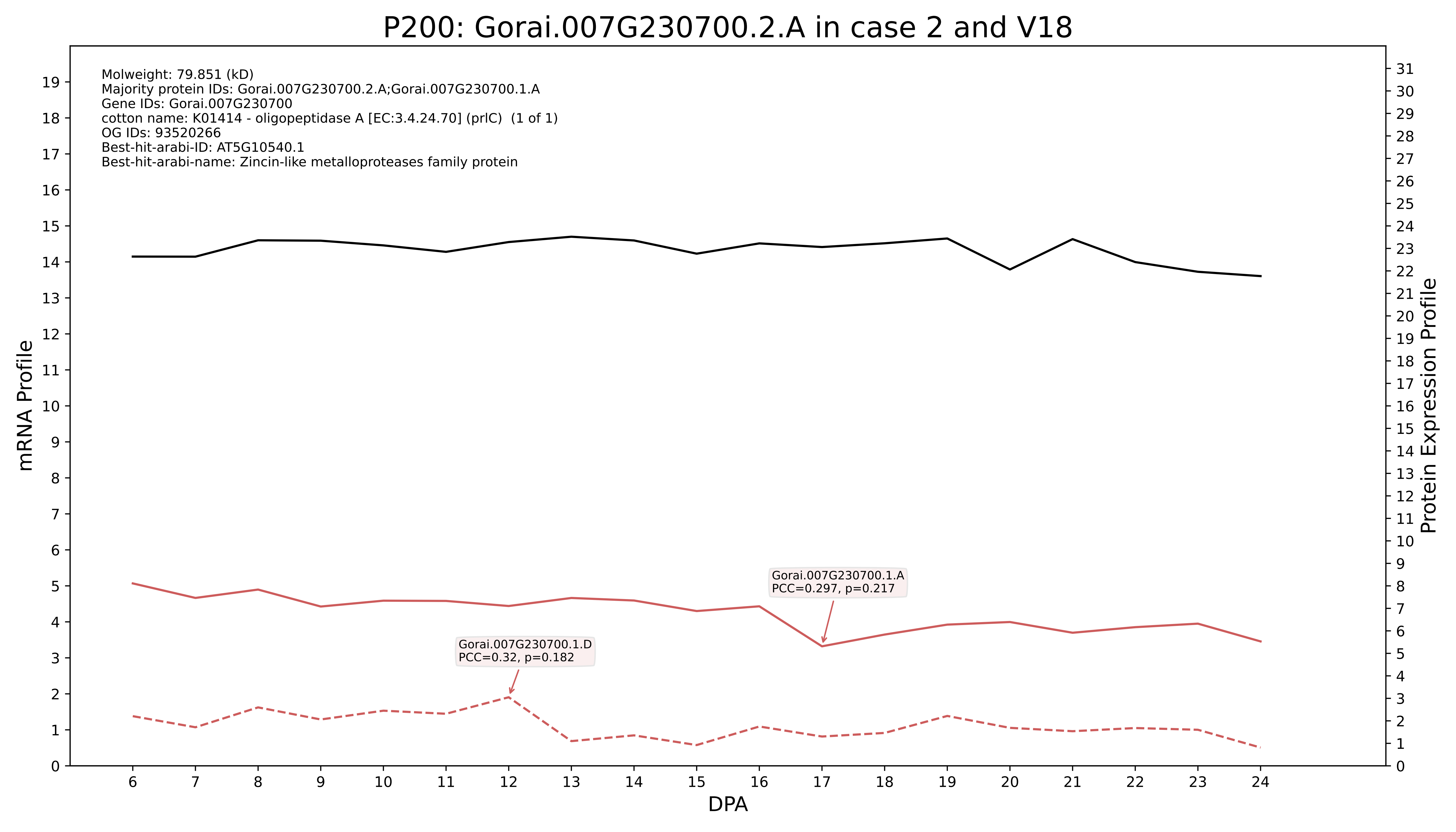
P200: Gorai.007G230700.2.A in case 2 and V18
Molweight: 79.851 (kD)
Majority protein IDs: Gorai.007G230700.2.A;Gorai.007G230700.1.A
Gene IDs: Gorai.007G230700
cotton name: K01414 - oligopeptidase A [EC:3.4.24.70] (prlC) (1 of 1)
OG IDs: 93520266
Best-hit-arabi-ID: AT5G10540.1
Best-hit-arabi-name: Zincin-like metalloproteases family protein
Gorai.007G230700.1.A
PCC=0.297, p=0.217
Gorai.007G230700.1.D
PCC=0.32, p=0.182
mRNA Profile
Protein Expression Profile
DPA
0
1
2
3
4
5
6
7
8
9
10
11
12
13
14
15
16
17
18
19
20
21
22
23
24
25
26
27
28
29
30
31

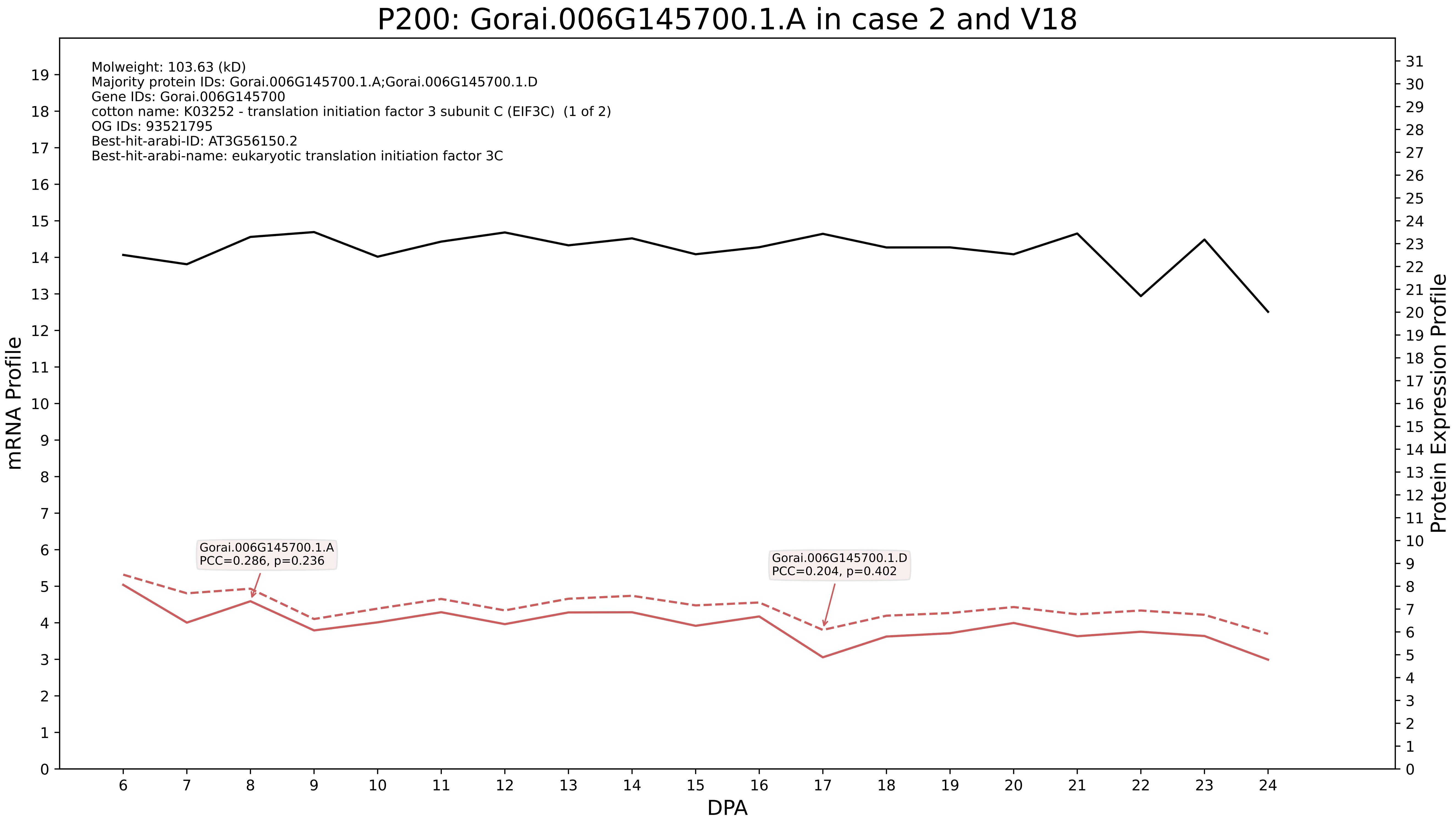
P200: Gorai.006G145700.1.A in case 2 and V18
Molweight: 103.63 (kD)
Majority protein IDs: Gorai.006G145700.1.A;Gorai.006G145700.1.D
Gene IDs: Gorai.006G145700
cotton name: K03252 - translation initiation factor 3 subunit C (EIF3C) (1 of 2)
OG IDs: 93521795
Best-hit-arabi-ID: AT3G56150.2
Best-hit-arabi-name: eukaryotic translation initiation factor 3C
Gorai.006G145700.1.A
PCC=0.286, p=0.236
Gorai.006G145700.1.D
PCC=0.204, p=0.402
mRNA Profile
Protein Expression Profile
DPA
0
1
2
3
4
5
6
7
8
9
10
11
12
13
14
15
16
17
18
19
20
21
22
23
24
25
26
27
28
29
30
31

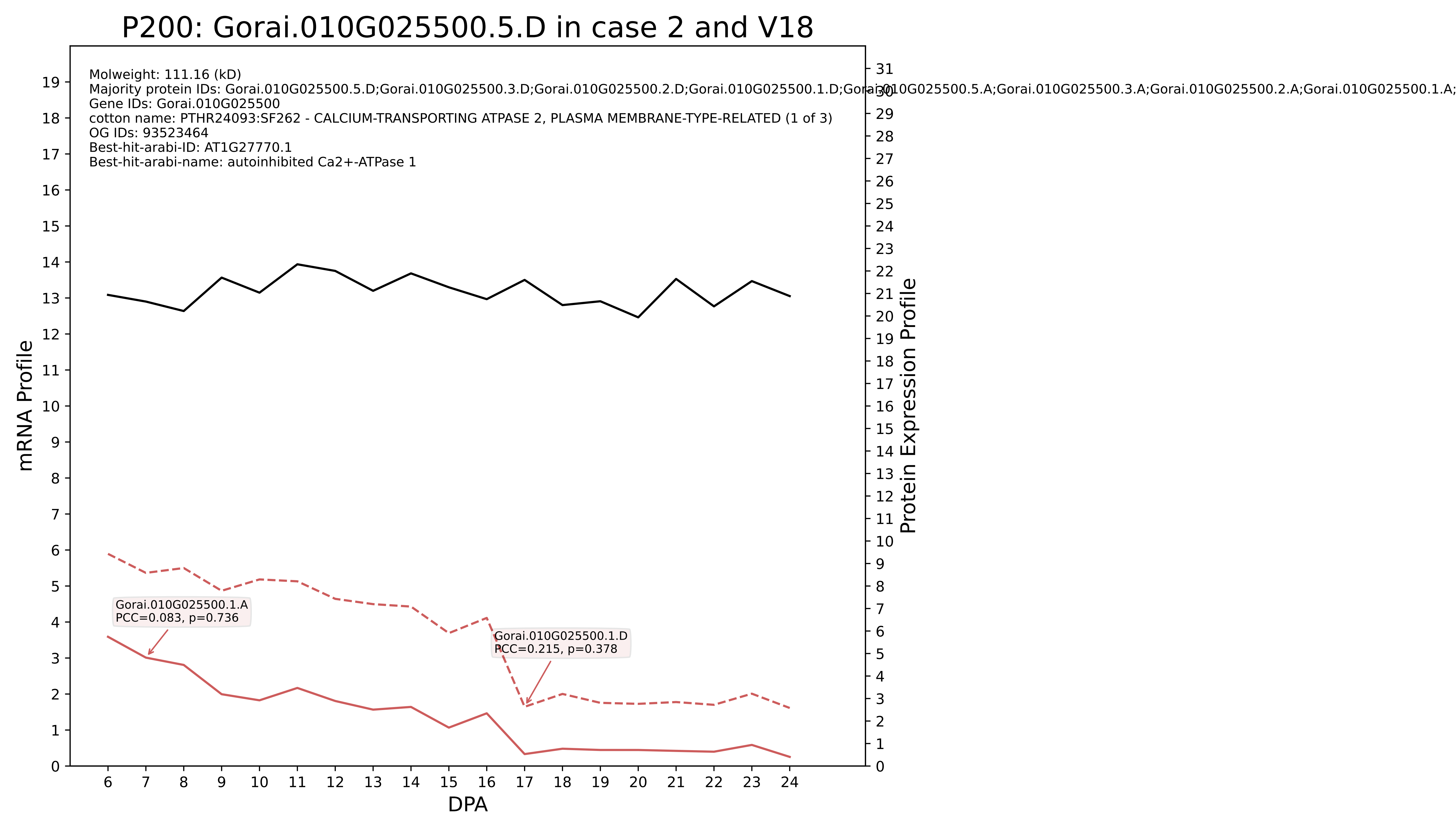
P200: Gorai.010G025500.5.D in case 2 and V18
Molweight: 111.16 (kD)
Majority protein IDs: Gorai.010G025500.5.D;Gorai.010G025500.3.D;Gorai.010G025500.2.D;Gorai.010G025500.1.D;Gorai.010G025500.5.A;Gorai.010G025500.3.A;Gorai.010G025500.2.A;Gorai.010G025500.1.A;
Gene IDs: Gorai.010G025500
cotton name: PTHR24093:SF262 - CALCIUM-TRANSPORTING ATPASE 2, PLASMA MEMBRANE-TYPE-RELATED (1 of 3)
OG IDs: 93523464
Best-hit-arabi-ID: AT1G27770.1
Best-hit-arabi-name: autoinhibited Ca2+-ATPase 1
Gorai.010G025500.1.A
PCC=0.083, p=0.736
Gorai.010G025500.1.D
PCC=0.215, p=0.378
mRNA Profile
Protein Expression Profile
DPA

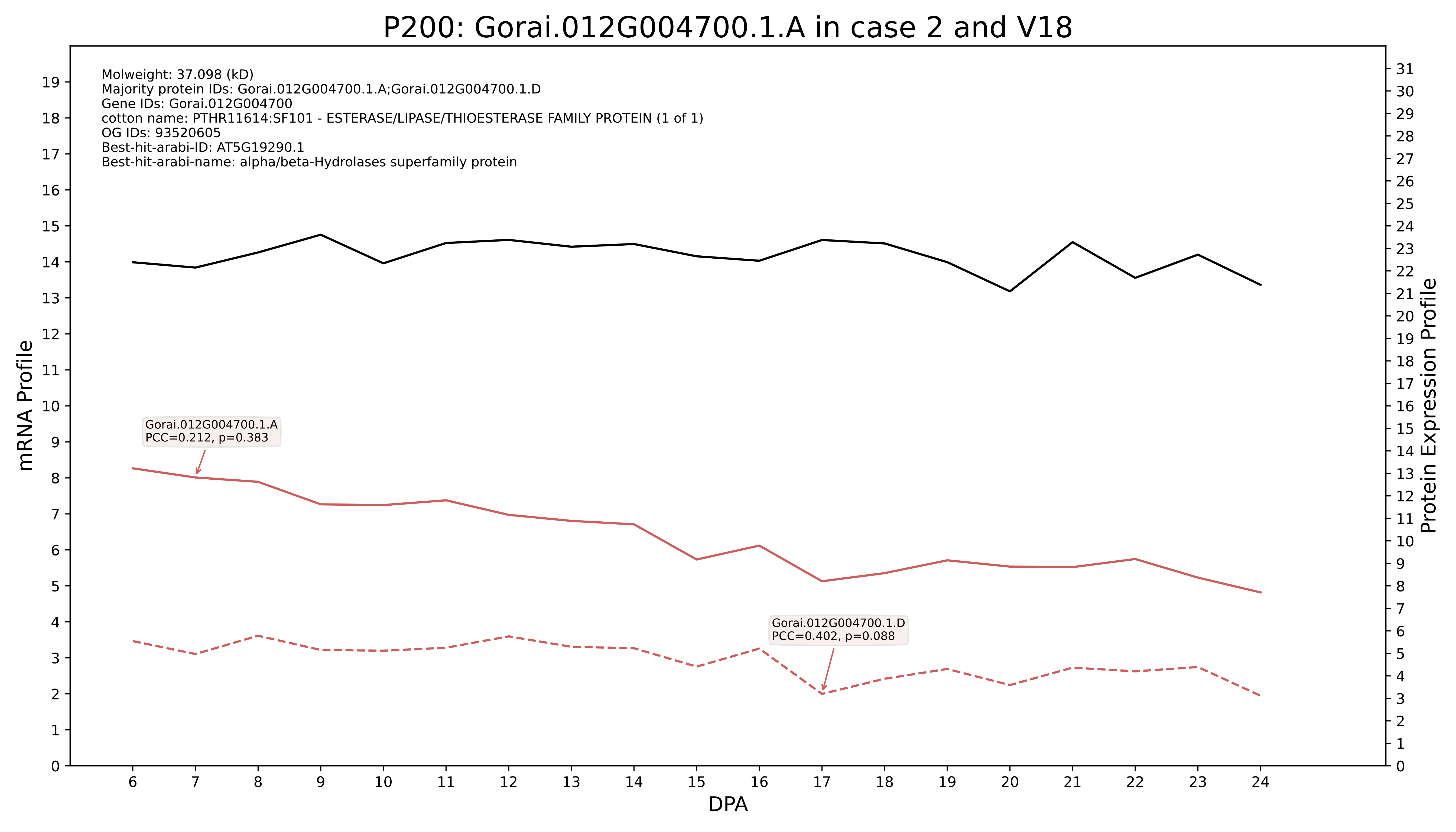

P200: Gorai.012G004700.1.A in case 2 and V18
Molweight: 37.098 (kD)
Majority protein IDs: Gorai.012G004700.1.A;Gorai.012G004700.1.D
Gene IDs: Gorai.012G004700
cotton name: PTHR11614:SF101 - ESTERASE/LIPASE/THIOESTERASE FAMILY PROTEIN (1 of 1)
OG IDs: 93520605
Best-hit-arabi-ID: AT5G19290.1
Best-hit-arabi-name: alpha/beta-Hydrolases superfamily protein
Gorai.012G004700.1.A
PCC=0.212, p=0.383
Gorai.012G004700.1.D
PCC=0.402, p=0.088
mRNA Profile
Protein Expression Profile
DPA

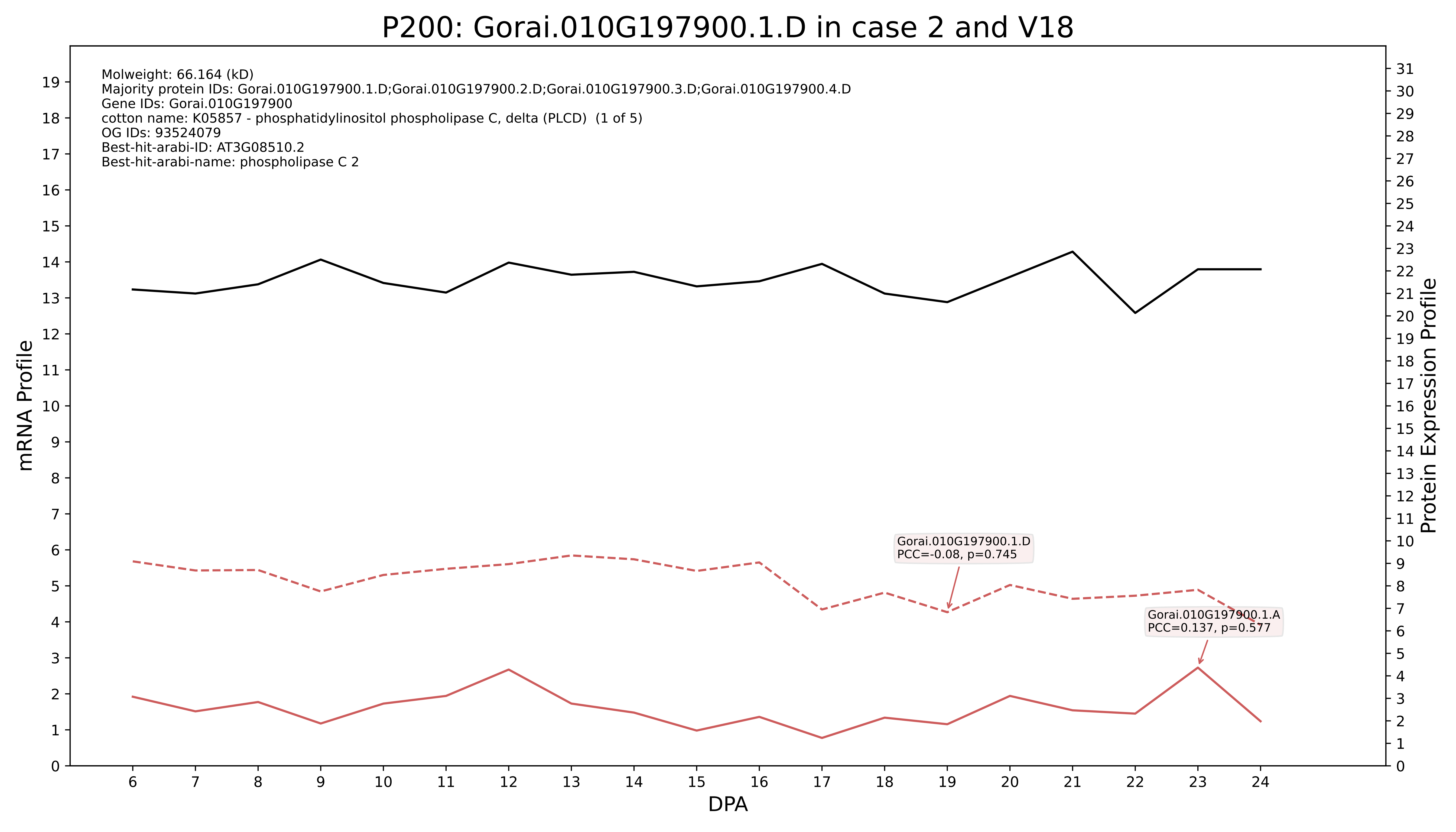

P200: Gorai.010G197900.1.D in case 2 and V18
Molweight: 66.164 (kD)
Majority protein IDs: Gorai.010G197900.1.D;Gorai.010G197900.2.D;Gorai.010G197900.3.D;Gorai.010G197900.4.D
Gene IDs: Gorai.010G197900
cotton name: K05857 - phosphatidylinositol phospholipase C, delta (PLCD) (1 of 5)
OG IDs: 93524079
Best-hit-arabi-ID: AT3G08510.2
Best-hit-arabi-name: phospholipase C 2
Gorai.010G197900.1.D
PCC=-0.08, p=0.745
Gorai.010G197900.1.A
PCC=0.137, p=0.577
mRNA Profile
Protein Expression Profile
DPA

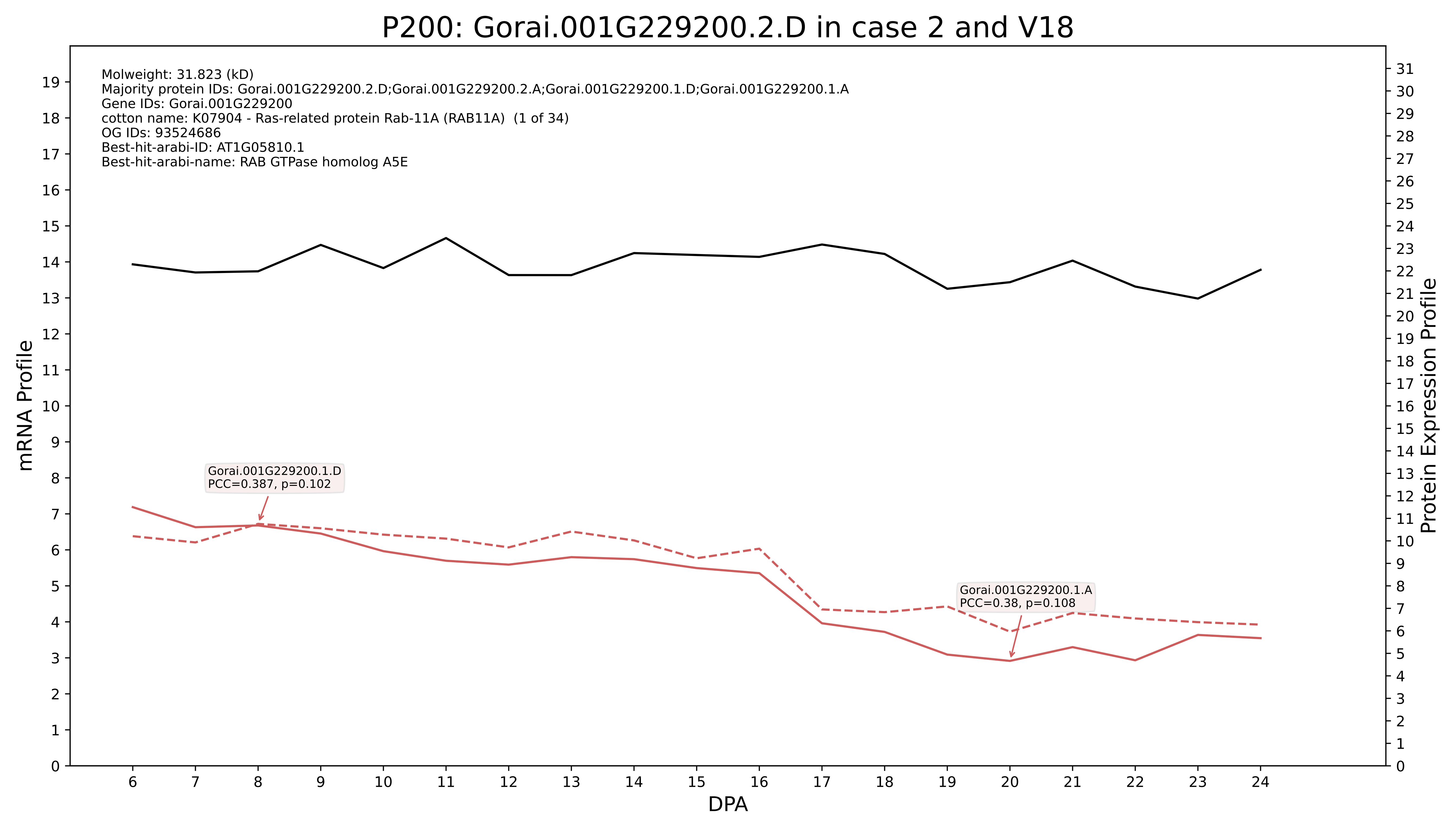

P200: Gorai.001G229200.2.D in case 2 and V18
Molweight: 31.823 (kD)
Majority protein IDs: Gorai.001G229200.2.D;Gorai.001G229200.2.A;Gorai.001G229200.1.D;Gorai.001G229200.1.A
Gene IDs: Gorai.001G229200
cotton name: K07904 - Ras-related protein Rab-11A (RAB11A) (1 of 34)
OG IDs: 93524686
Best-hit-arabi-ID: AT1G05810.1
Best-hit-arabi-name: RAB GTPase homolog A5E
Gorai.001G229200.1.D
PCC=0.387, p=0.102
Gorai.001G229200.1.A
PCC=0.38, p=0.108
mRNA Profile
Protein Expression Profile
DPA

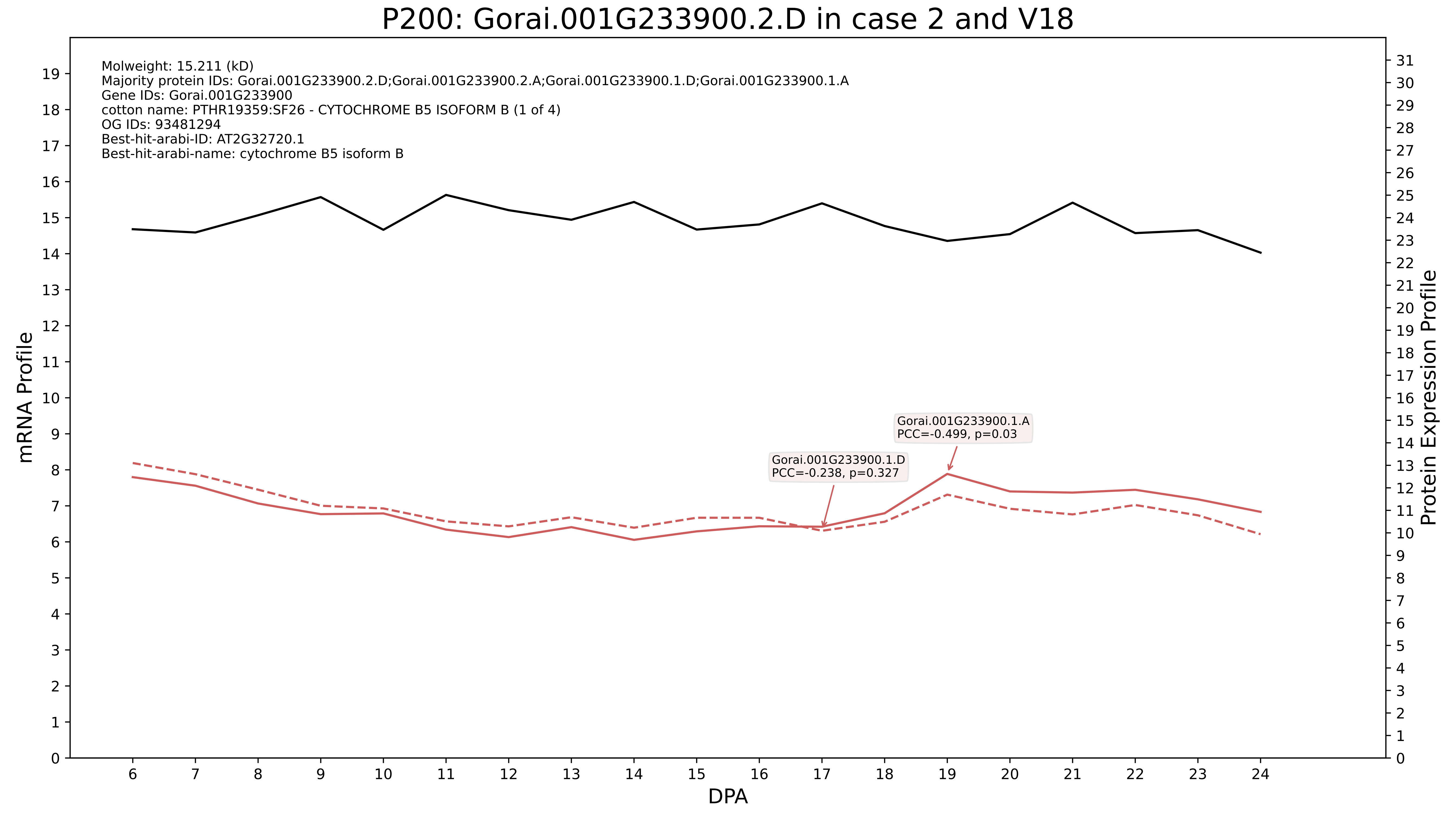
P200: Gorai.001G233900.2.D in case 2 and V18
Molweight: 15.211 (kD)
Majority protein IDs: Gorai.001G233900.2.D;Gorai.001G233900.2.A;Gorai.001G233900.1.D;Gorai.001G233900.1.A
Gene IDs: Gorai.001G233900
cotton name: PTHR19359:SF26 - CYTOCHROME B5 ISOFORM B (1 of 4)
OG IDs: 93481294
Best-hit-arabi-ID: AT2G32720.1
Best-hit-arabi-name: cytochrome B5 isoform B
Gorai.001G233900.1.A
PCC=-0.499, p=0.03
Gorai.001G233900.1.D
PCC=-0.238, p=0.327
mRNA Profile
Protein Expression Profile
DPA

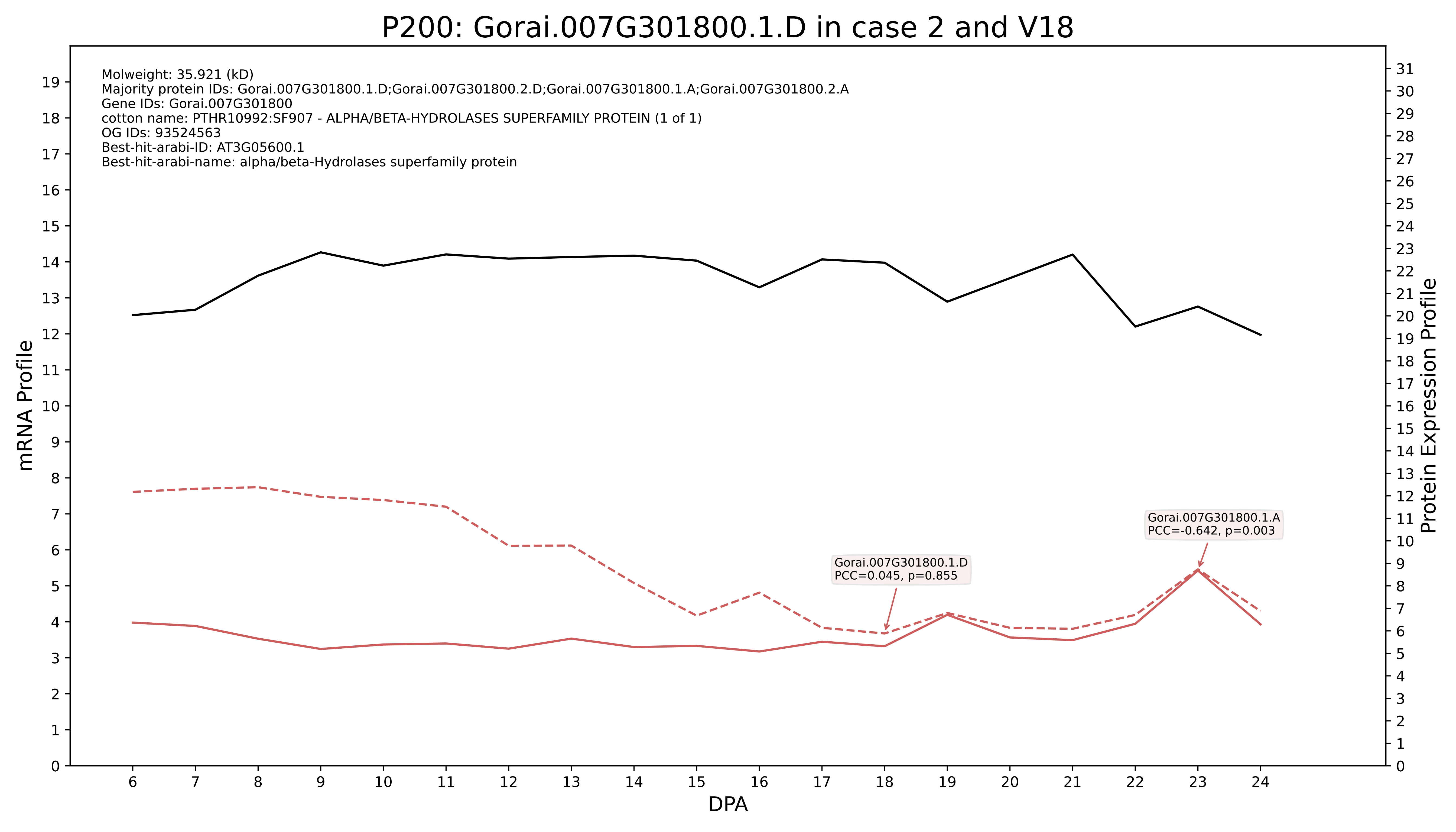

P200: Gorai.007G301800.1.D in case 2 and V18
Molweight: 35.921 (kD)
Majority protein IDs: Gorai.007G301800.1.D;Gorai.007G301800.2.D;Gorai.007G301800.1.A;Gorai.007G301800.2.A
Gene IDs: Gorai.007G301800
cotton name: PTHR10992:SF907 - ALPHA/BETA-HYDROLASES SUPERFAMILY PROTEIN (1 of 1)
OG IDs: 93524563
Best-hit-arabi-ID: AT3G05600.1
Best-hit-arabi-name: alpha/beta-Hydrolases superfamily protein
Gorai.007G301800.1.A
PCC=-0.642, p=0.003
Gorai.007G301800.1.D
PCC=0.045, p=0.855
mRNA Profile
Protein Expression Profile
DPA

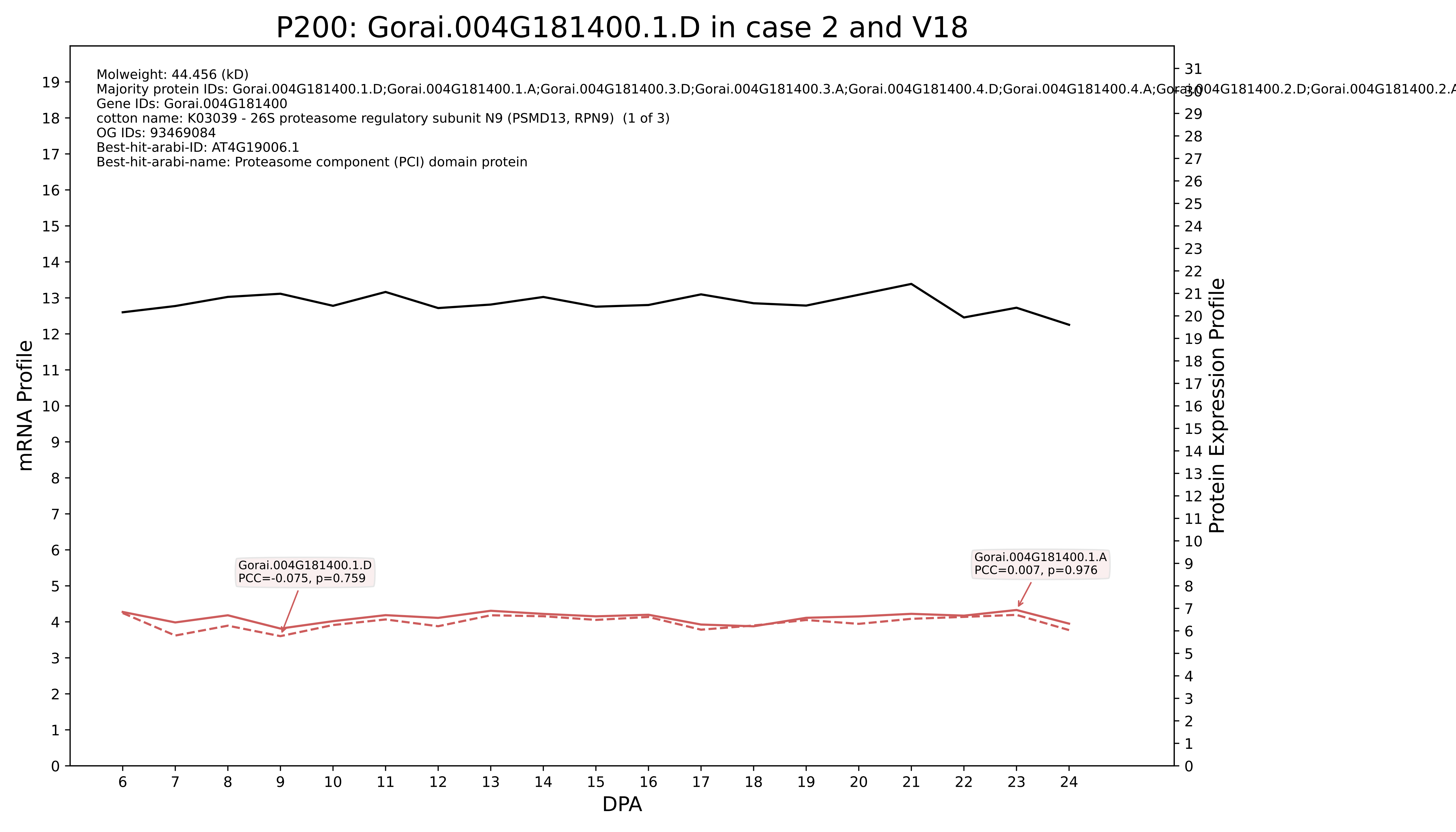

P200: Gorai.004G181400.1.D in case 2 and V18
Molweight: 44.456 (kD)
Majority protein IDs: Gorai.004G181400.1.D;Gorai.004G181400.1.A;Gorai.004G181400.3.D;Gorai.004G181400.3.A;Gorai.004G181400.4.D;Gorai.004G181400.4.A;Gorai.004G181400.2.D;Gorai.004G181400.2.A
Gene IDs: Gorai.004G181400
cotton name: K03039 - 26S proteasome regulatory subunit N9 (PSMD13, RPN9) (1 of 3)
OG IDs: 93469084
Best-hit-arabi-ID: AT4G19006.1
Best-hit-arabi-name: Proteasome component (PCI) domain protein
Gorai.004G181400.1.D
PCC=-0.075, p=0.759
Gorai.004G181400.1.A
PCC=0.007, p=0.976
mRNA Profile
Protein Expression Profile
DPA

001G000900.2.A in case 2 and V18

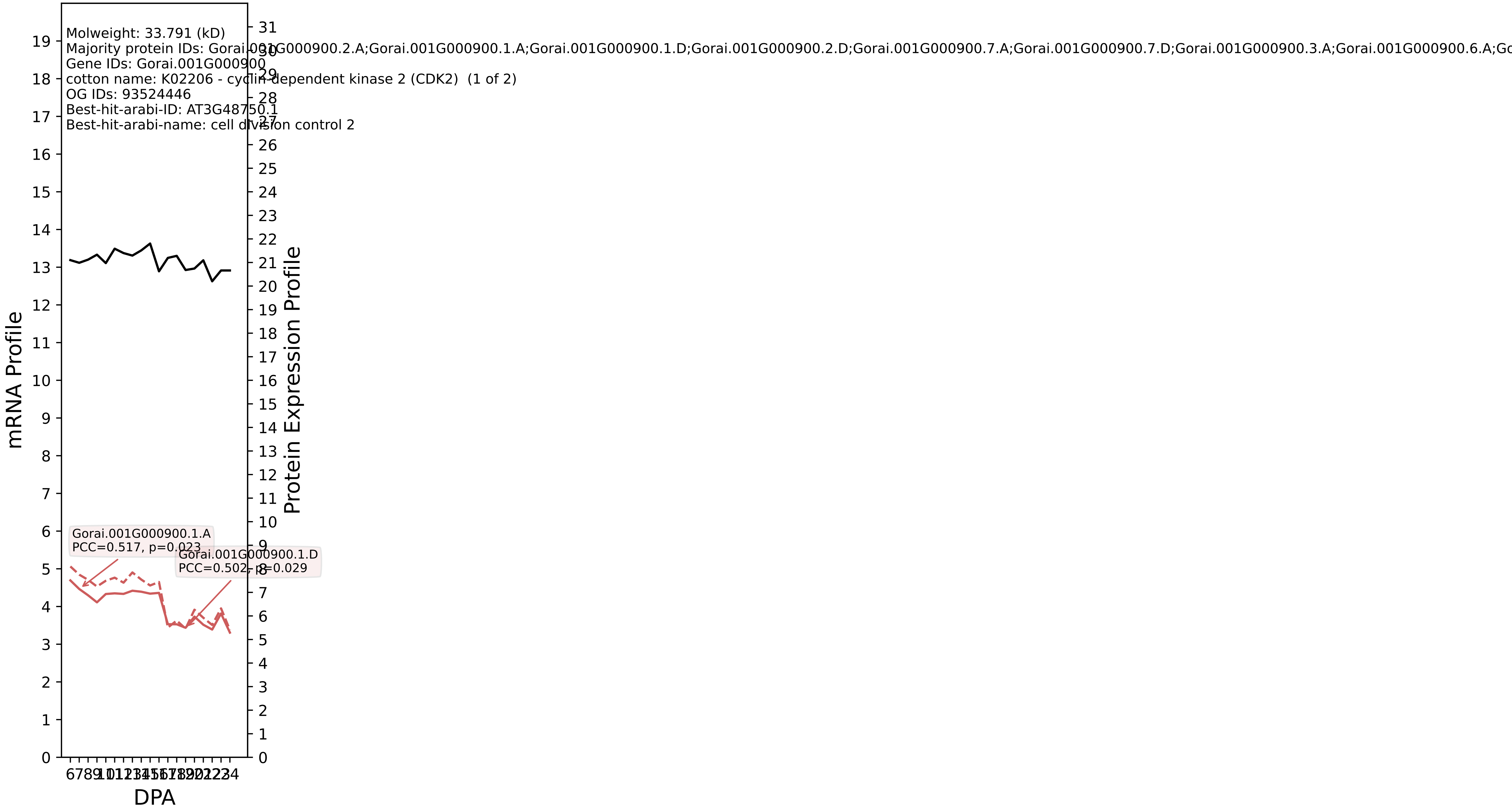

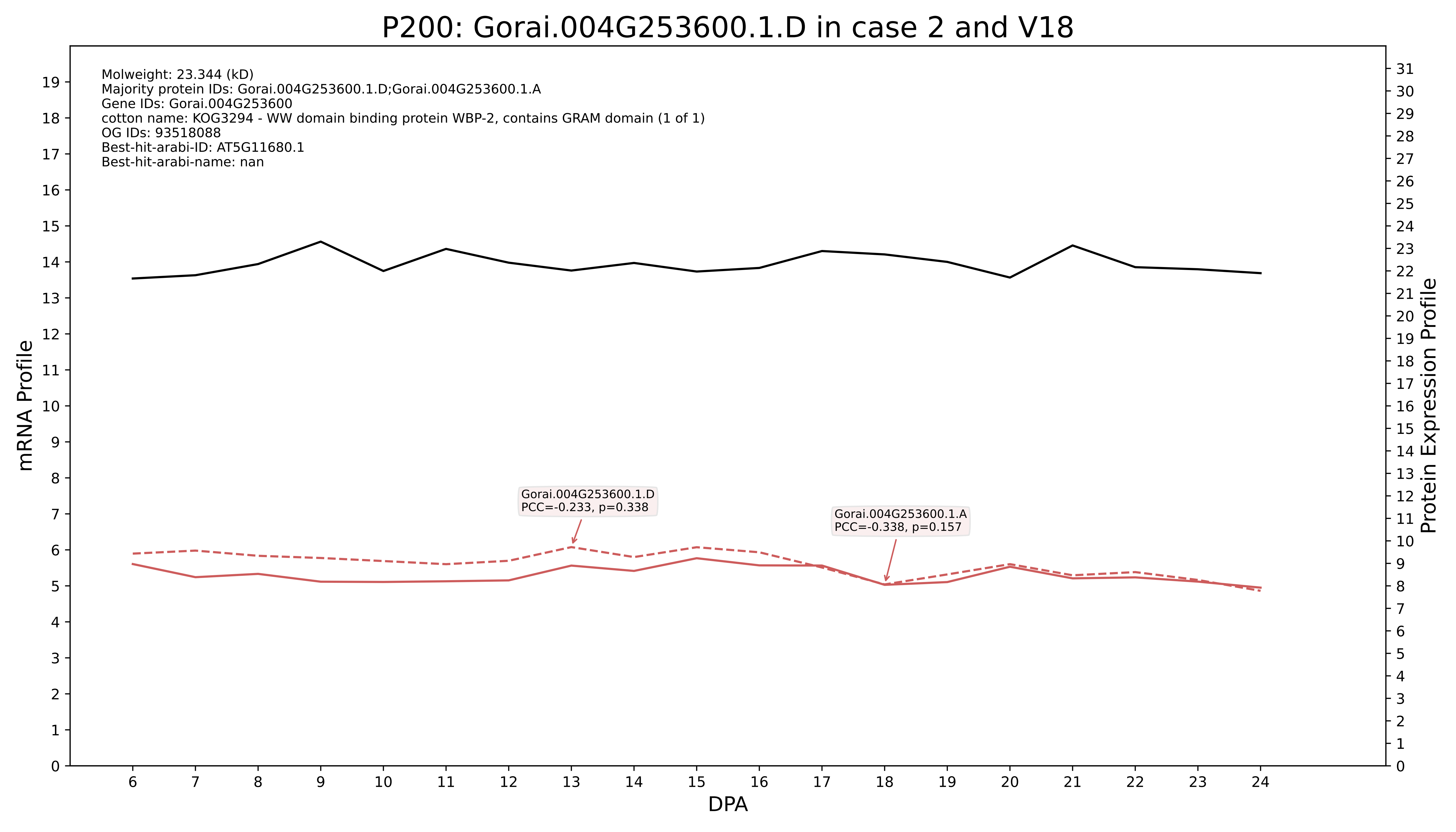

P200: Gorai.004G253600.1.D in case 2 and V18
Molweight: 23.344 (kD)
Majority protein IDs: Gorai.004G253600.1.D;Gorai.004G253600.1.A
Gene IDs: Gorai.004G253600
cotton name: KOG3294 - WW domain binding protein WBP-2, contains GRAM domain (1 of 1)
OG IDs: 93518088
Best-hit-arabi-ID: AT5G11680.1
Best-hit-arabi-name: nan
Gorai.004G253600.1.D
PCC=-0.233, p=0.338
Gorai.004G253600.1.A
PCC=-0.338, p=0.157
mRNA Profile
Protein Expression Profile
DPA

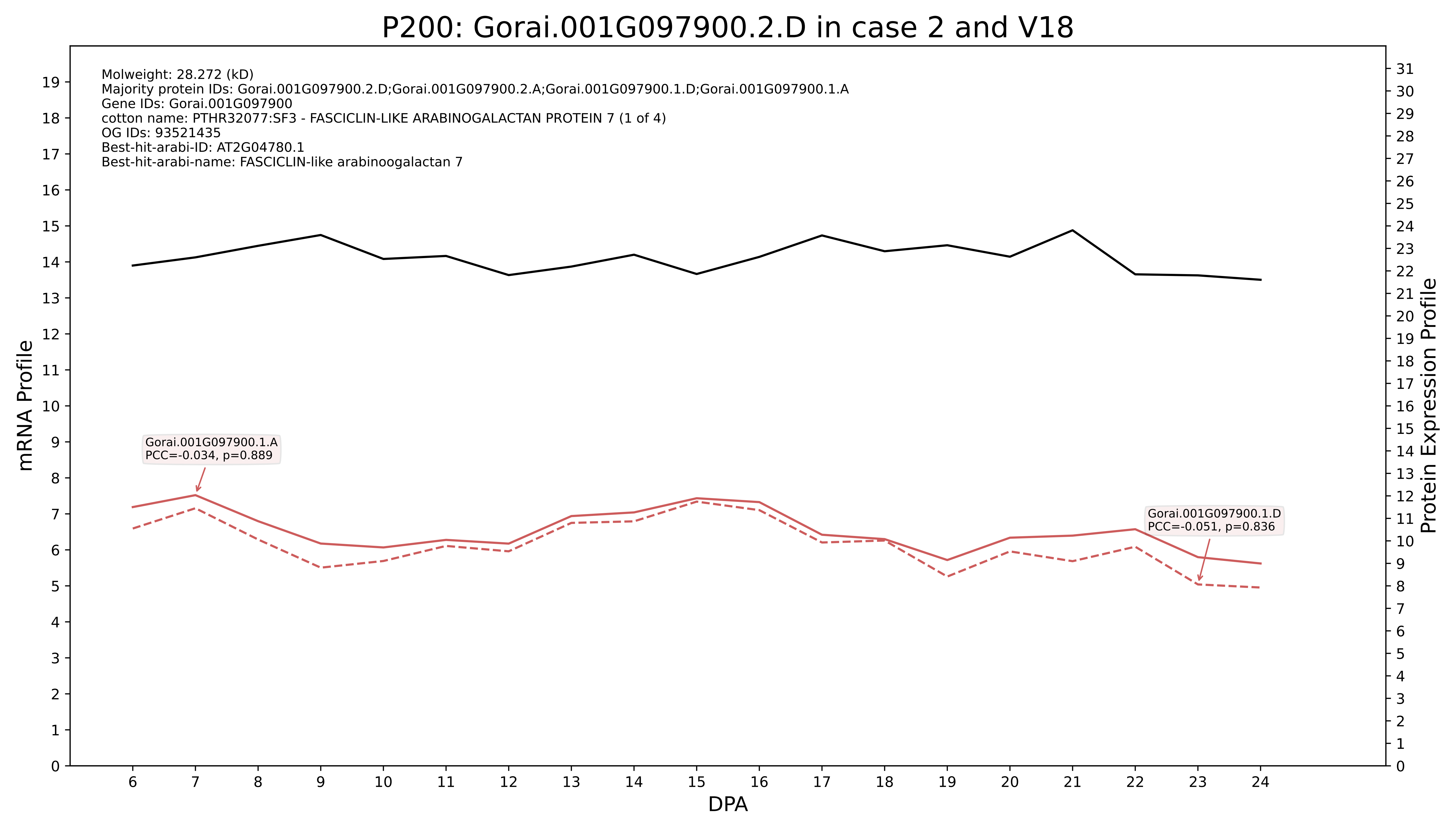

P200: Gorai.001G097900.2.D in case 2 and V18
Molweight: 28.272 (kD)
Majority protein IDs: Gorai.001G097900.2.D;Gorai.001G097900.2.A;Gorai.001G097900.1.D;Gorai.001G097900.1.A
Gene IDs: Gorai.001G097900
cotton name: PTHR32077:SF3 - FASCICLIN-LIKE ARABINOGALACTAN PROTEIN 7 (1 of 4)
OG IDs: 93521435
Best-hit-arabi-ID: AT2G04780.1
Best-hit-arabi-name: FASCICLIN-like arabinoogalactan 7
Gorai.001G097900.1.A
PCC=-0.034, p=0.889
Gorai.001G097900.1.D
PCC=-0.051, p=0.836
mRNA Profile
Protein Expression Profile
DPA

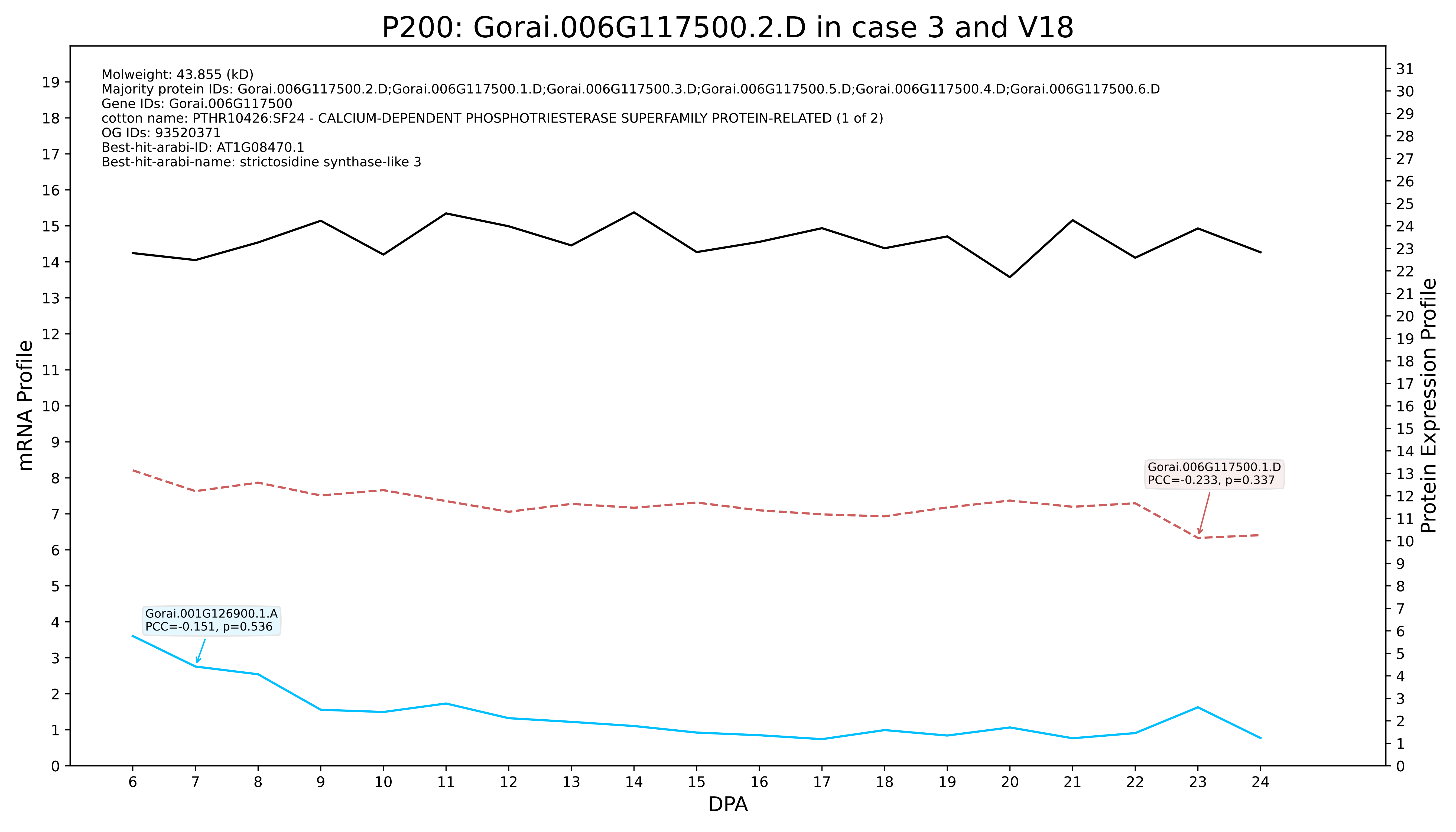
P200: Gorai.006G117500.2.D in case 3 and V18
Molweight: 43.855 (kD)
Majority protein IDs: Gorai.006G117500.2.D;Gorai.006G117500.1.D;Gorai.006G117500.3.D;Gorai.006G117500.5.D;Gorai.006G117500.4.D;Gorai.006G117500.6.D
Gene IDs: Gorai.006G117500
cotton name: PTHR10426:SF24 - CALCIUM-DEPENDENT PHOSPHOTRIESTERASE SUPERFAMILY PROTEIN-RELATED (1 of 2)
OG IDs: 93520371
Best-hit-arabi-ID: AT1G08470.1
Best-hit-arabi-name: strictosidine synthase-like 3
Gorai.006G117500.1.D
PCC=-0.233, p=0.337
Gorai.001G126900.1.A
PCC=-0.151, p=0.536
mRNA Profile
Protein Expression Profile
DPA

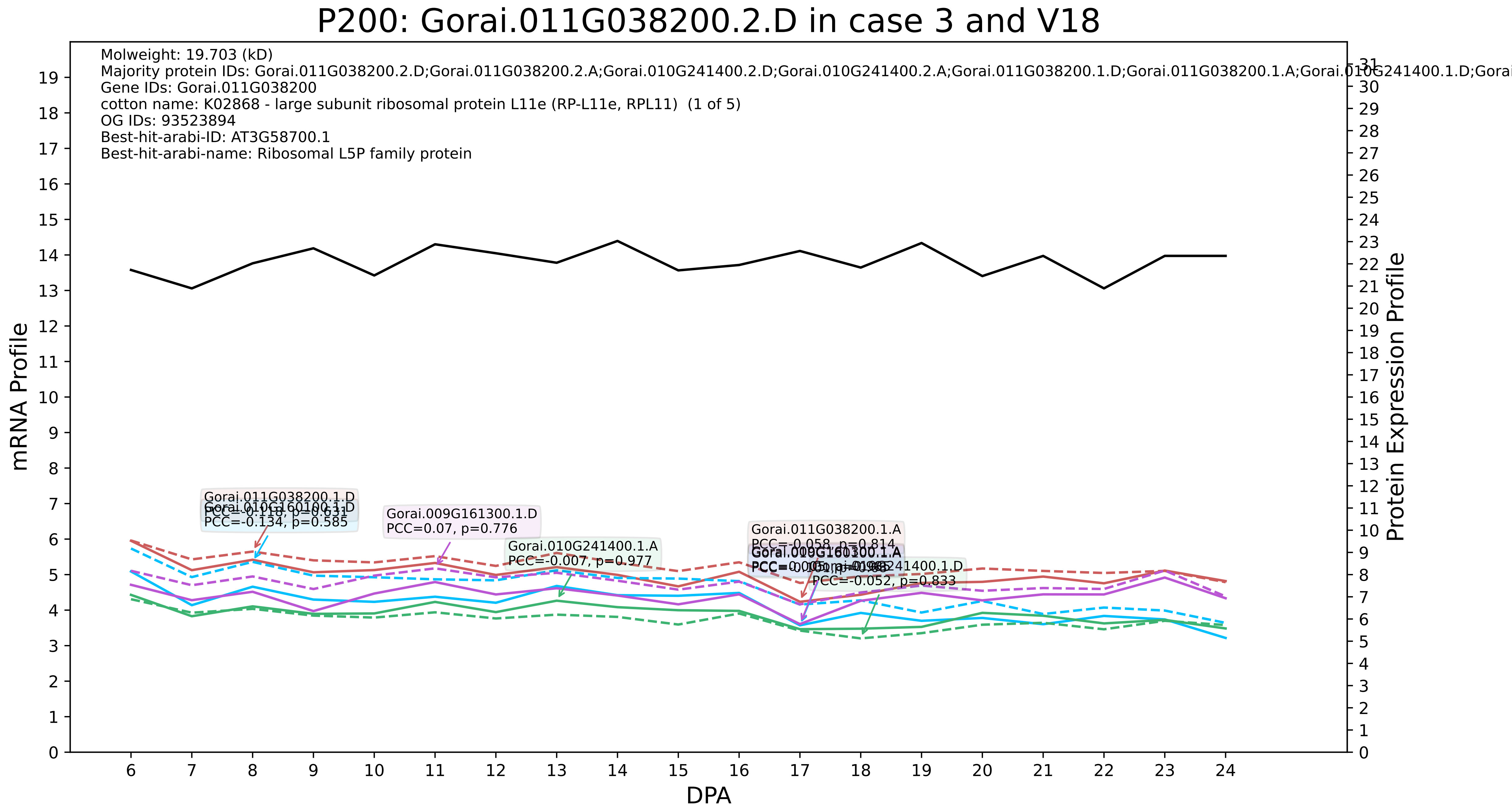
P200: Gorai.011G038200.2.D in case 3 and V18
Molweight: 19.703 (kD)
Majority protein IDs: Gorai.011G038200.2.D;Gorai.011G038200.2.A;Gorai.010G241400.2.D;Gorai.010G241400.2.A;Gorai.011G038200.1.D;Gorai.011G038200.1.A;Gorai.010G241400.1.D;Gorai
Gene IDs: Gorai.011G038200
cotton name: K02868 - large subunit ribosomal protein L11e (RP-L11e, RPL11) (1 of 5)
OG IDs: 93523894
Best-hit-arabi-ID: AT3G58700.1
Best-hit-arabi-name: Ribosomal L5P family protein
Gorai.011G038200.1.D
PCC=-0.118, p=0.631
Gorai.010G160100.1.D
PCC=-0.134, p=0.585
Gorai.009G161300.1.D
PCC=0.07, p=0.776
Gorai.010G241400.1.A
PCC=-0.007, p=0.977
Gorai.011G038200.1.A
PCC=-0.058, p=0.814
Gorai.010G241400.1.D
PCC=-0.052, p=0.833
mRNA Profile
Protein Expression Profile
DPA

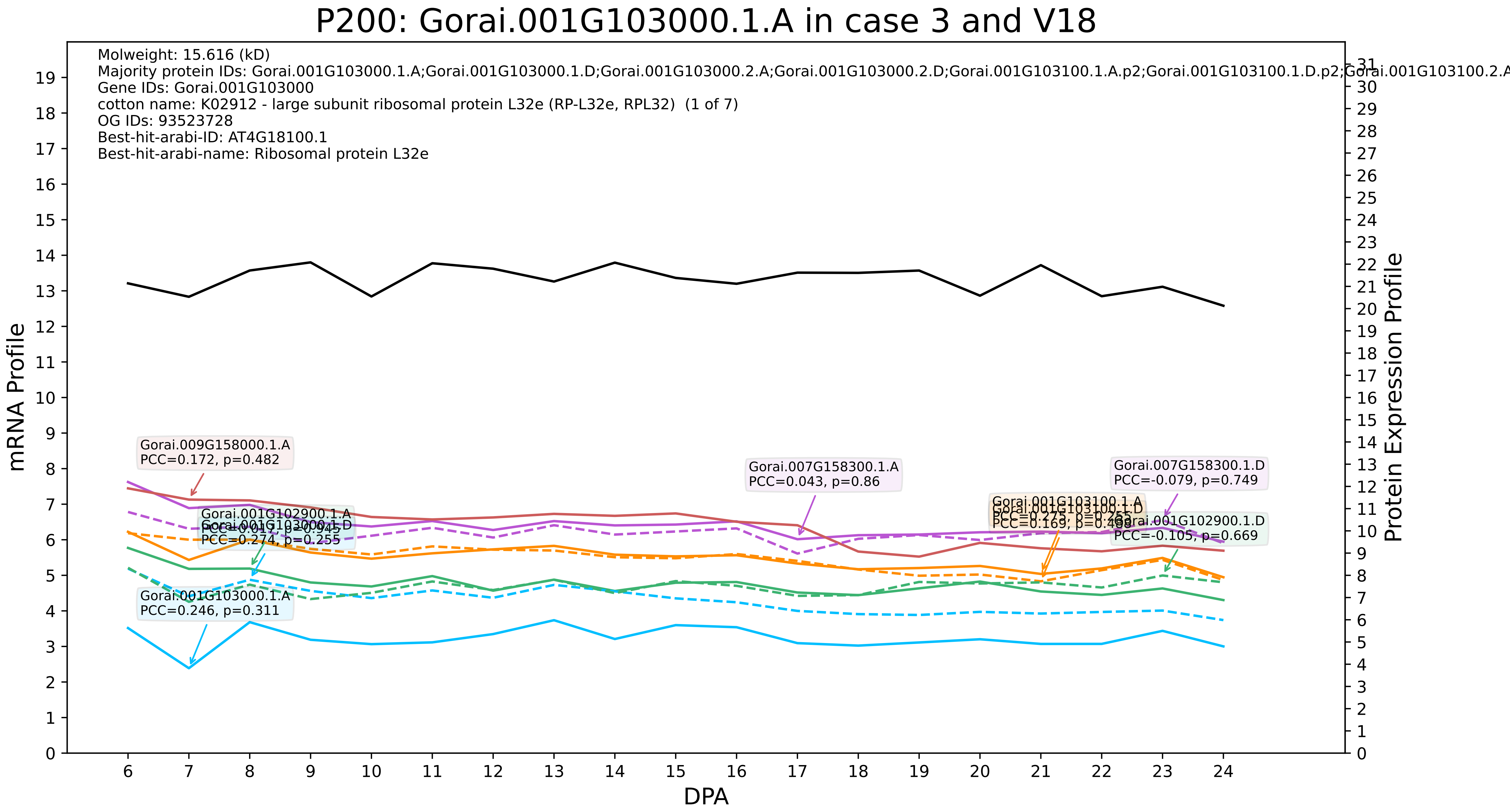

P200: Gorai.001G103000.1.A in case 3 and V18
Molweight: 15.616 (kD)
Majority protein IDs: Gorai.001G103000.1.A;Gorai.001G103000.1.D;Gorai.001G103000.2.A;Gorai.001G103000.2.D;Gorai.001G103100.1.A.p2;Gorai.001G103100.1.D.p2;Gorai.001G103100.2.A
Gene IDs: Gorai.001G103000
cotton name: K02912 - large subunit ribosomal protein L32e (RP-L32e, RPL32) (1 of 7)
OG IDs: 93523728
Best-hit-arabi-ID: AT4G18100.1
Best-hit-arabi-name: Ribosomal protein L32e
Gorai.009G158000.1.A
PCC=0.172, p=0.482
Gorai.001G102900.1.A
PCC=0.274, p=0.255
Gorai.007G158300.1.A
PCC=0.043, p=0.86
Gorai.007G158300.1.D
PCC=-0.079, p=0.749
Gorai.001G103100.1.A
Gorai.001G102900.1.D
PCC=-0.105, p=0.669
Gorai.001G103000.1.A
PCC=0.246, p=0.311
mRNA Profile
Protein Expression Profile
DPA

P200: Gorai.003G136100.4.D in case 3 and V18

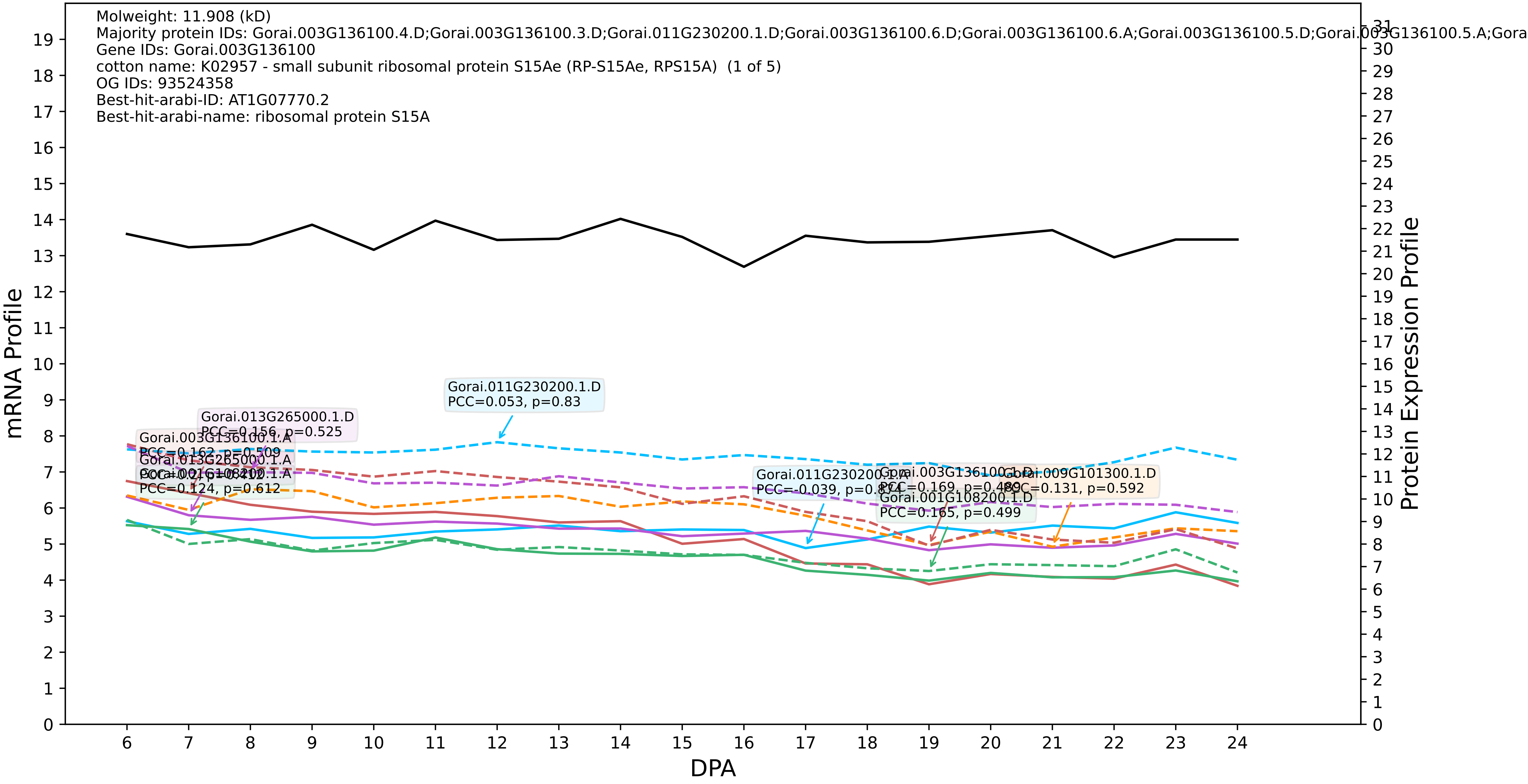

P200: Gorai.006G112000.4.D in case 3 and V18

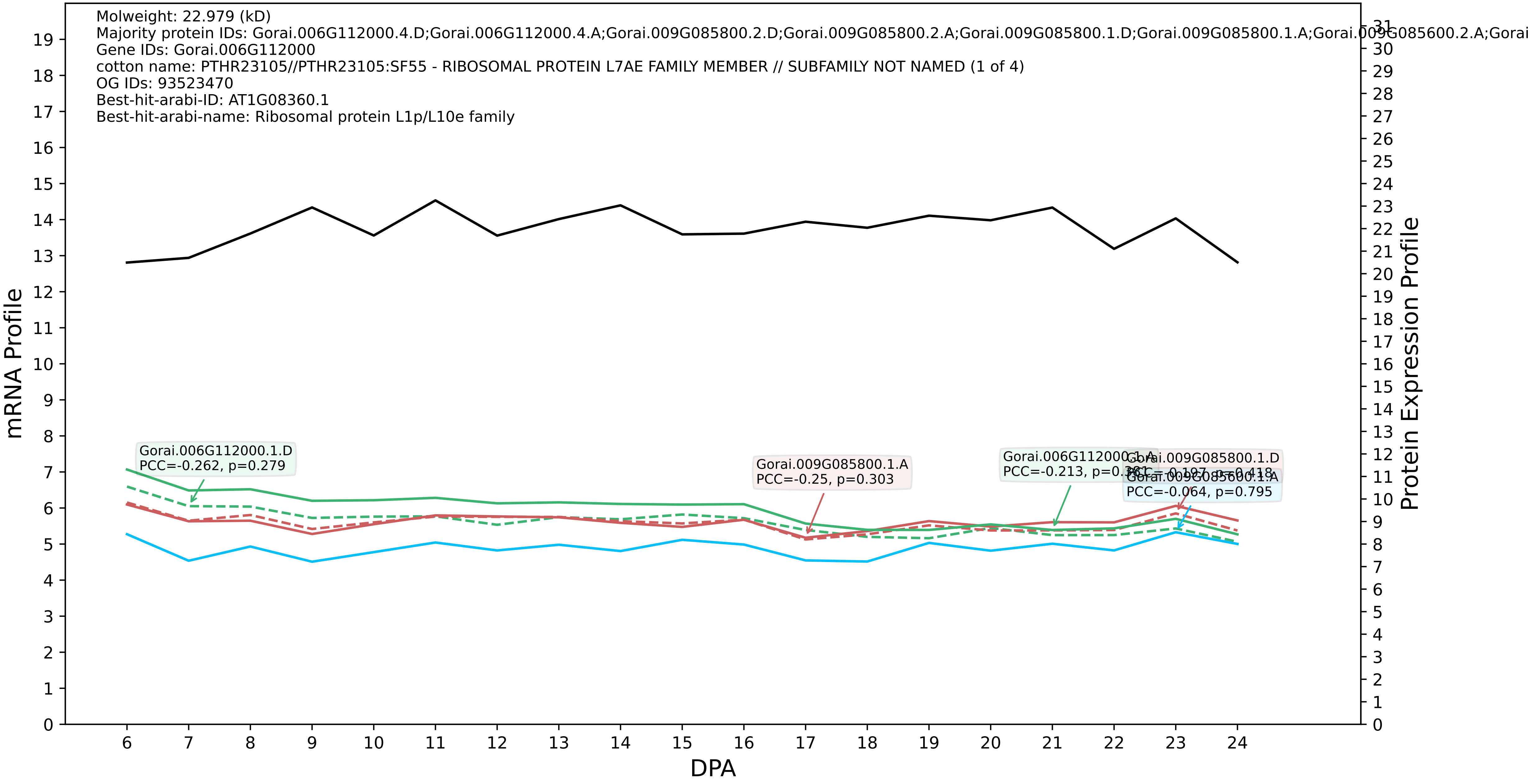

P200: Gorai.003G099200.1.D in case 3 and V18

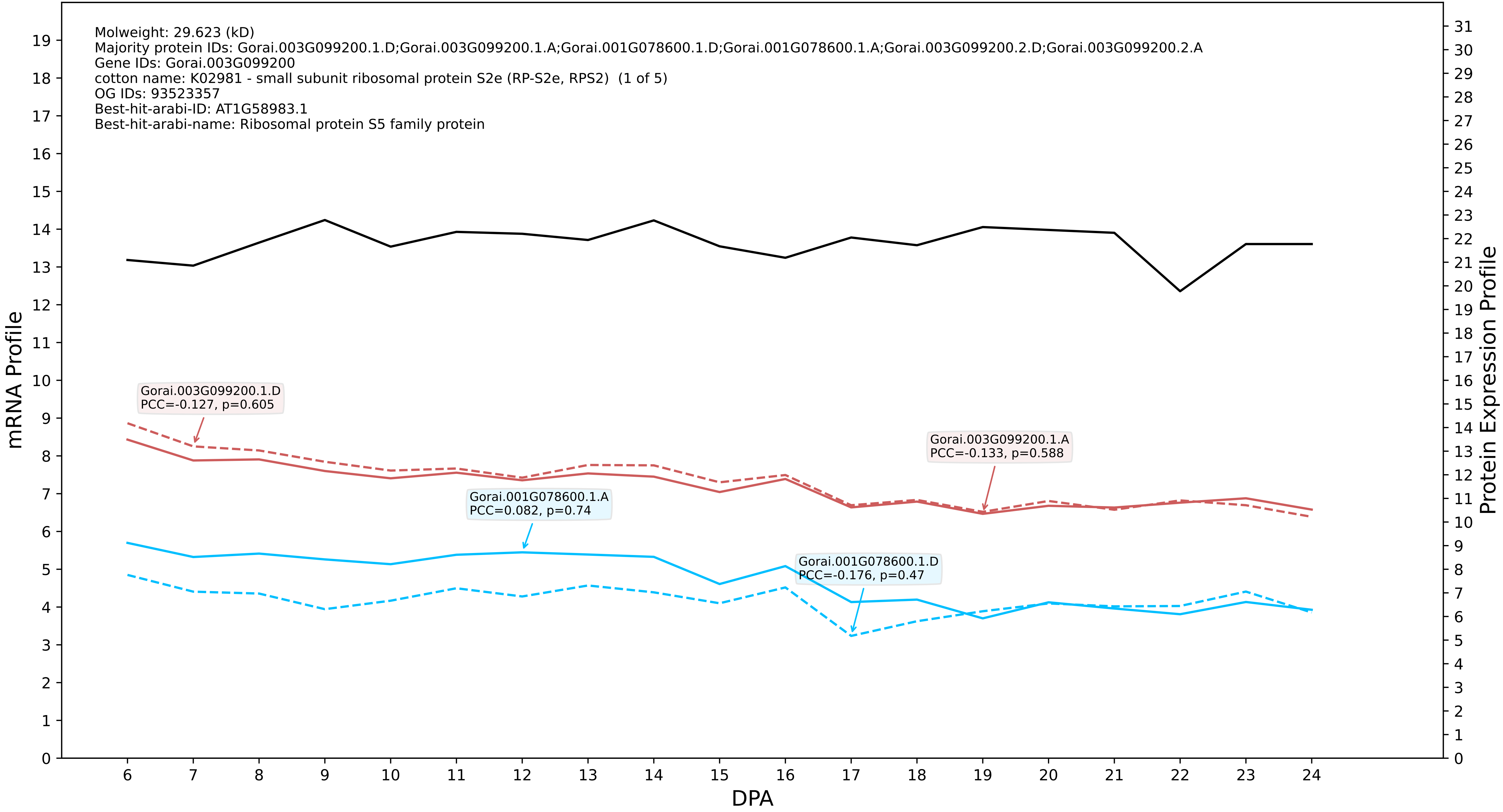

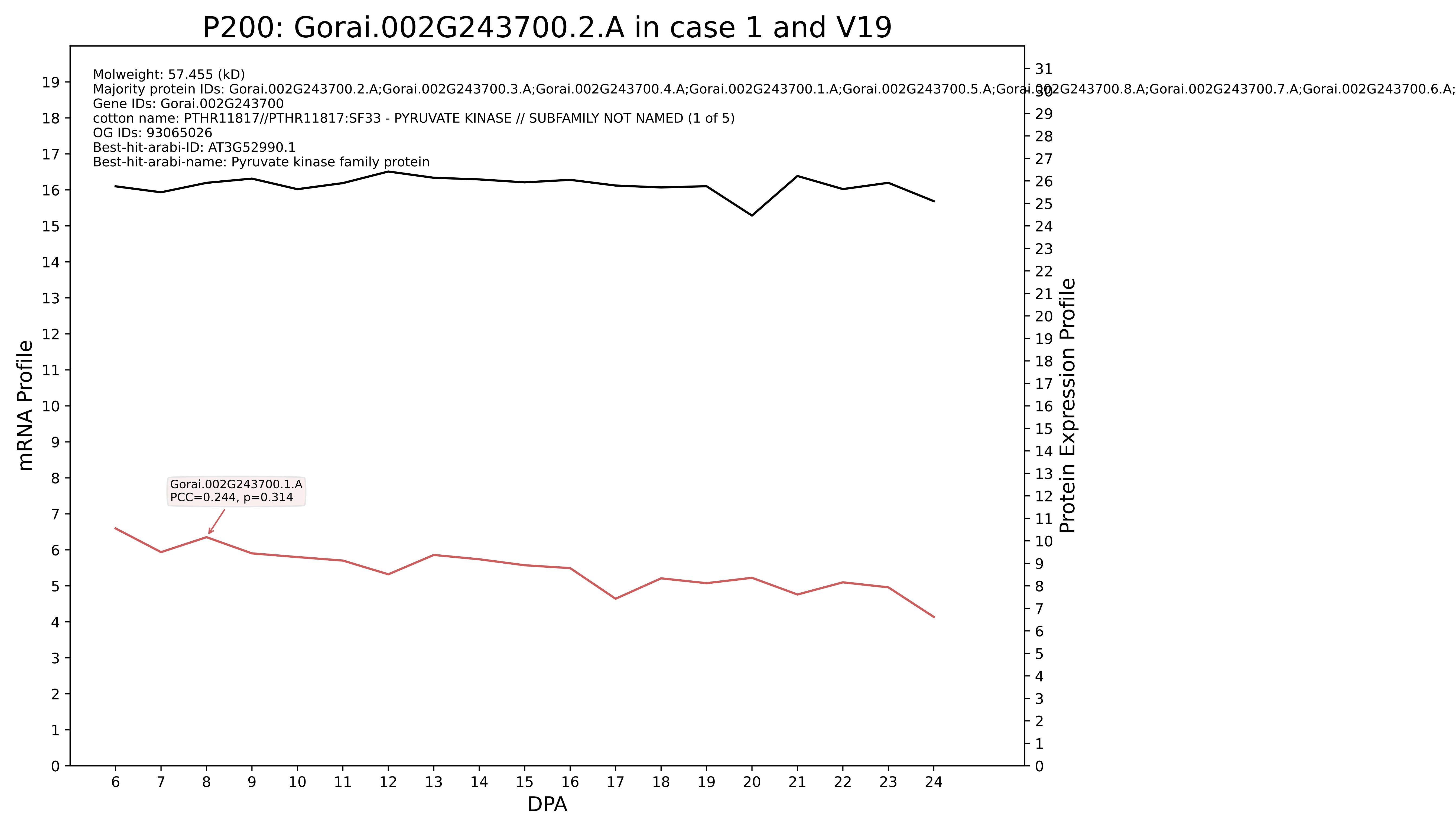
P200: Gorai.002G243700.2.A in case 1 and V19
Molweight: 57.455 (kD)
Majority protein IDs: Gorai.002G243700.2.A;Gorai.002G243700.3.A;Gorai.002G243700.4.A;Gorai.002G243700.1.A;Gorai.002G243700.5.A;Gorai.002G243700.8.A;Gorai.002G243700.7.A;Gorai.002G243700.6.A;
Gene IDs: Gorai.002G243700
cotton name: PTHR11817//PTHR11817:SF33 - PYRUVATE KINASE // SUBFAMILY NOT NAMED (1 of 5)
OG IDs: 93065026
Best-hit-arabi-ID: AT3G52990.1
Best-hit-arabi-name: Pyruvate kinase family protein
Gorai.002G243700.1.A
PCC=0.244, p=0.314
mRNA Profile
Protein Expression Profile
DPA

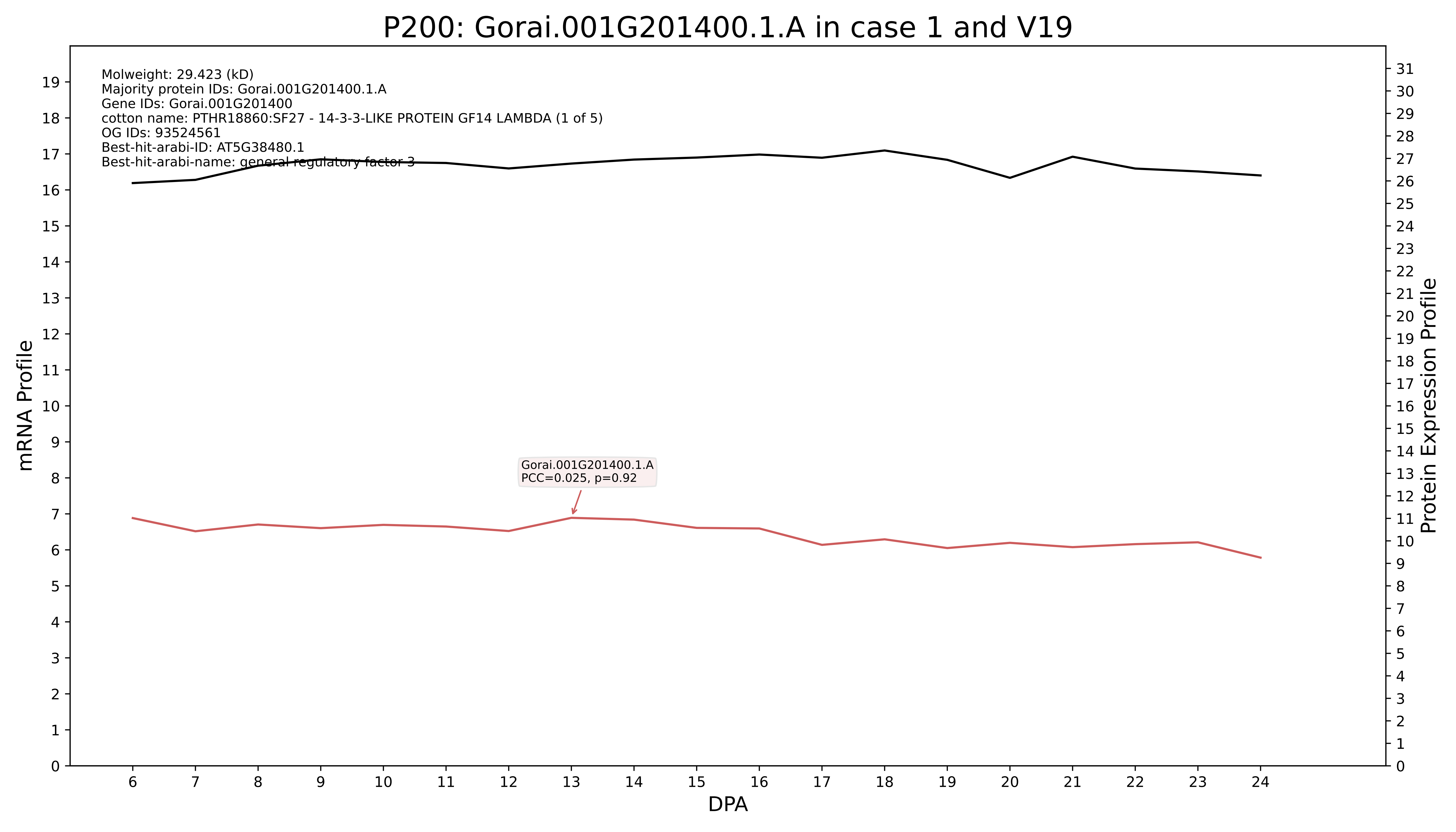
P200: Gorai.001G201400.1.A in case 1 and V19
Molweight: 29.423 (kD)
Majority protein IDs: Gorai.001G201400.1.A
Gene IDs: Gorai.001G201400
cotton name: PTHR18860:SF27 - 14-3-3-LIKE PROTEIN GF14 LAMBDA (1 of 5)
OG IDs: 93524561
Best-hit-arabi-ID: AT5G38480.1
Best-hit-arabi-name: general regulatory factor 3
Gorai.001G201400.1.A
PCC=0.025, p=0.92
mRNA Profile
Protein Expression Profile
DPA

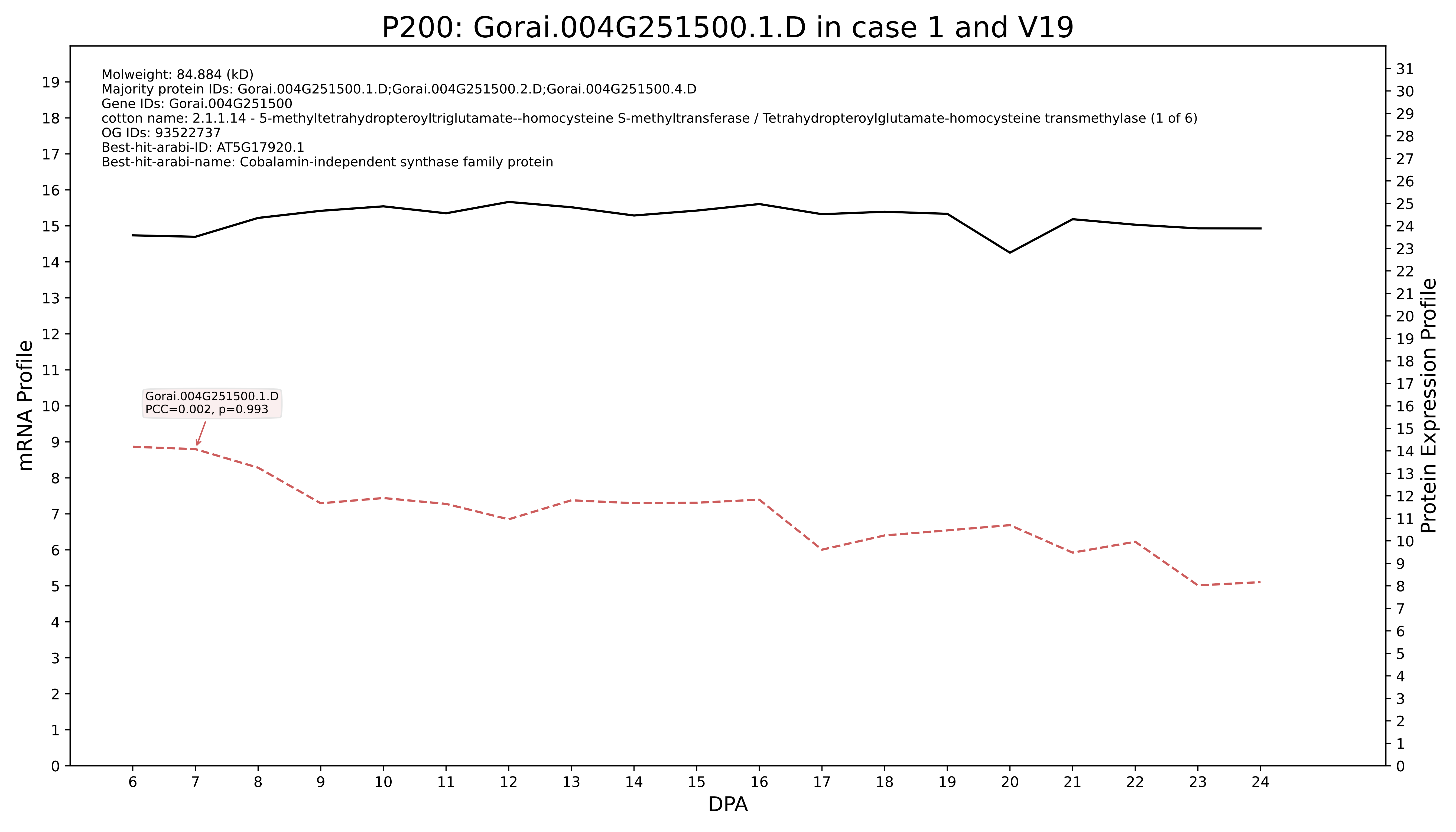
P200: Gorai.004G251500.1.D in case 1 and V19
Molweight: 84.884 (kD)
Majority protein IDs: Gorai.004G251500.1.D;Gorai.004G251500.2.D;Gorai.004G251500.4.D
Gene IDs: Gorai.004G251500
cotton name: 2.1.1.14 - 5-methyltetrahydropteroyltriglutamate--homocysteine S-methyltransferase / Tetrahydropteroylglutamate-homocysteine transmethylase (1 of 6)
OG IDs: 93522737
Best-hit-arabi-ID: AT5G17920.1
Best-hit-arabi-name: Cobalamin-independent synthase family protein
Gorai.004G251500.1.D
PCC=0.002, p=0.993
mRNA Profile
Protein Expression Profile
DPA

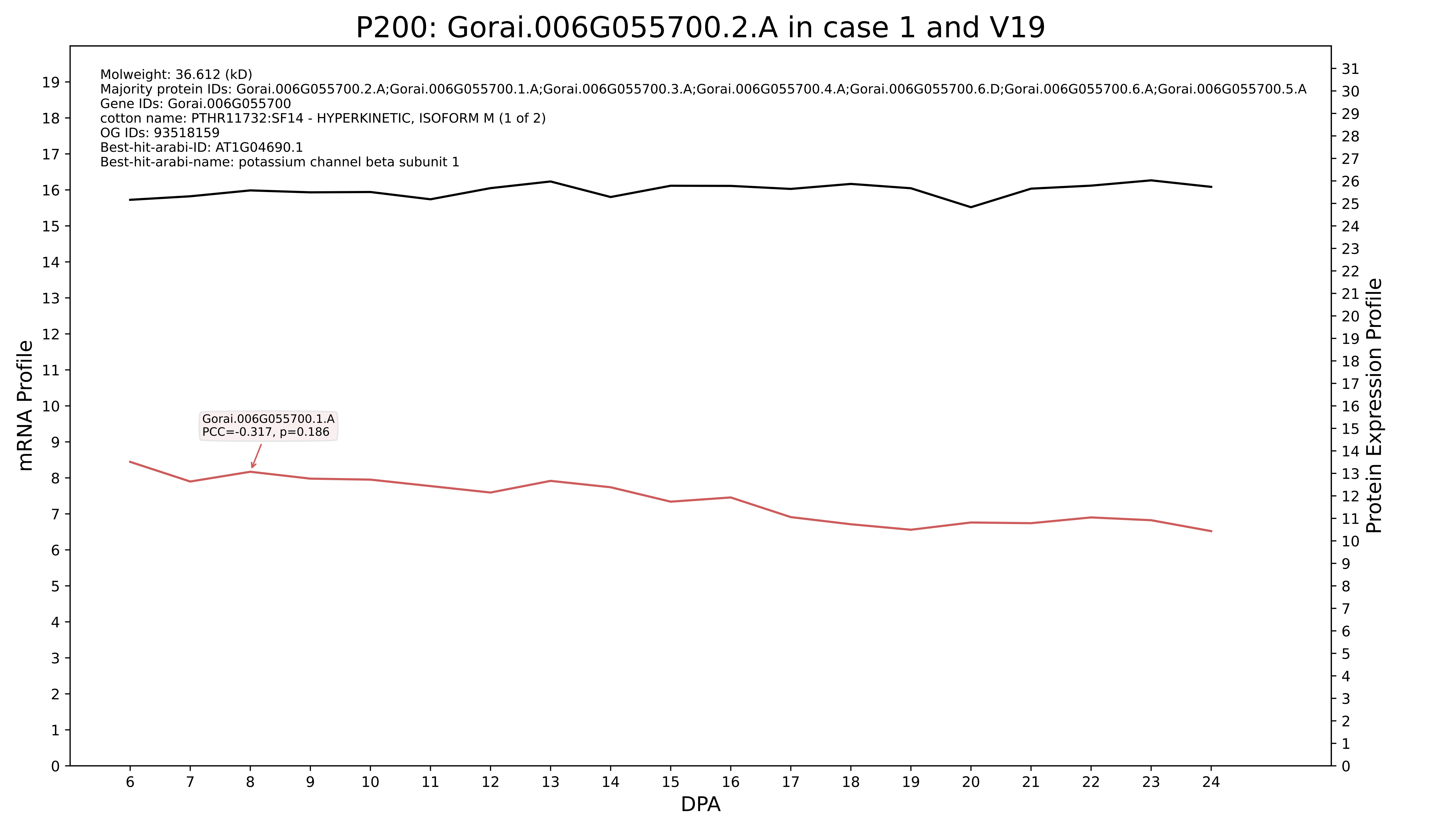
P200: Gorai.006G055700.2.A in case 1 and V19
Molweight: 36.612 (kD)
Majority protein IDs: Gorai.006G055700.2.A;Gorai.006G055700.1.A;Gorai.006G055700.3.A;Gorai.006G055700.4.A;Gorai.006G055700.6.D;Gorai.006G055700.6.A;Gorai.006G055700.5.A
Gene IDs: Gorai.006G055700
cotton name: PTHR11732:SF14 - HYPERKINETIC, ISOFORM M (1 of 2)
OG IDs: 93518159
Best-hit-arabi-ID: AT1G04690.1
Best-hit-arabi-name: potassium channel beta subunit 1
Gorai.006G055700.1.A
PCC=-0.317, p=0.186
mRNA Profile
Protein Expression Profile
DPA

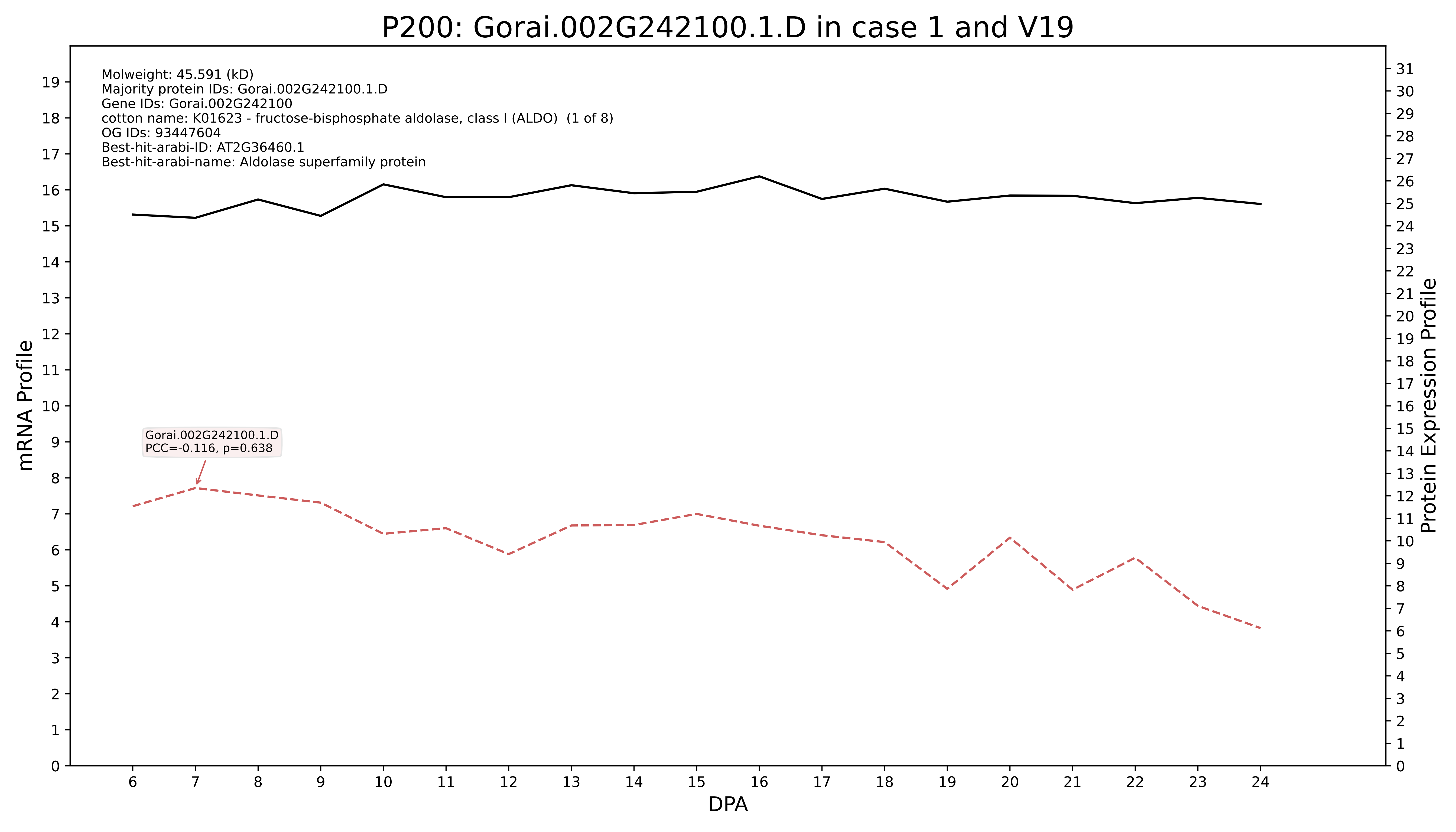

P200: Gorai.002G242100.1.D in case 1 and V19
Molweight: 45.591 (kD)
Majority protein IDs: Gorai.002G242100.1.D
Gene IDs: Gorai.002G242100
cotton name: K01623 - fructose-bisphosphate aldolase, class I (ALDO) (1 of 8)
OG IDs: 93447604
Best-hit-arabi-ID: AT2G36460.1
Best-hit-arabi-name: Aldolase superfamily protein
Gorai.002G242100.1.D
PCC=-0.116, p=0.638
mRNA Profile
Protein Expression Profile
DPA

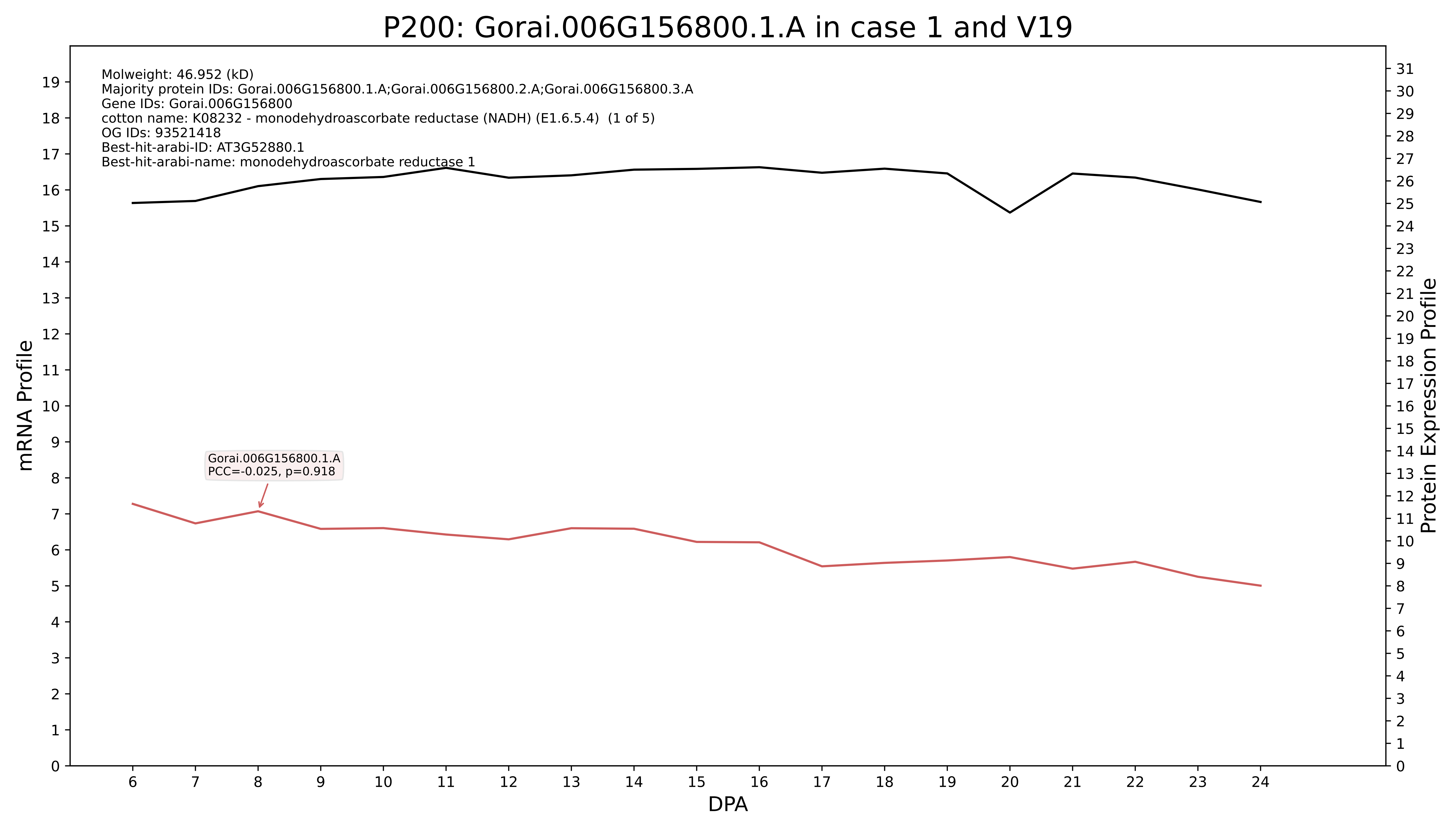
P200: Gorai.006G156800.1.A in case 1 and V19
Molweight: 46.952 (kD)
Majority protein IDs: Gorai.006G156800.1.A;Gorai.006G156800.2.A;Gorai.006G156800.3.A
Gene IDs: Gorai.006G156800
cotton name: K08232 - monodehydroascorbate reductase (NADH) (E1.6.5.4) (1 of 5)
OG IDs: 93521418
Best-hit-arabi-ID: AT3G52880.1
Best-hit-arabi-name: monodehydroascorbate reductase 1
Gorai.006G156800.1.A
PCC=-0.025, p=0.918
mRNA Profile
Protein Expression Profile
DPA

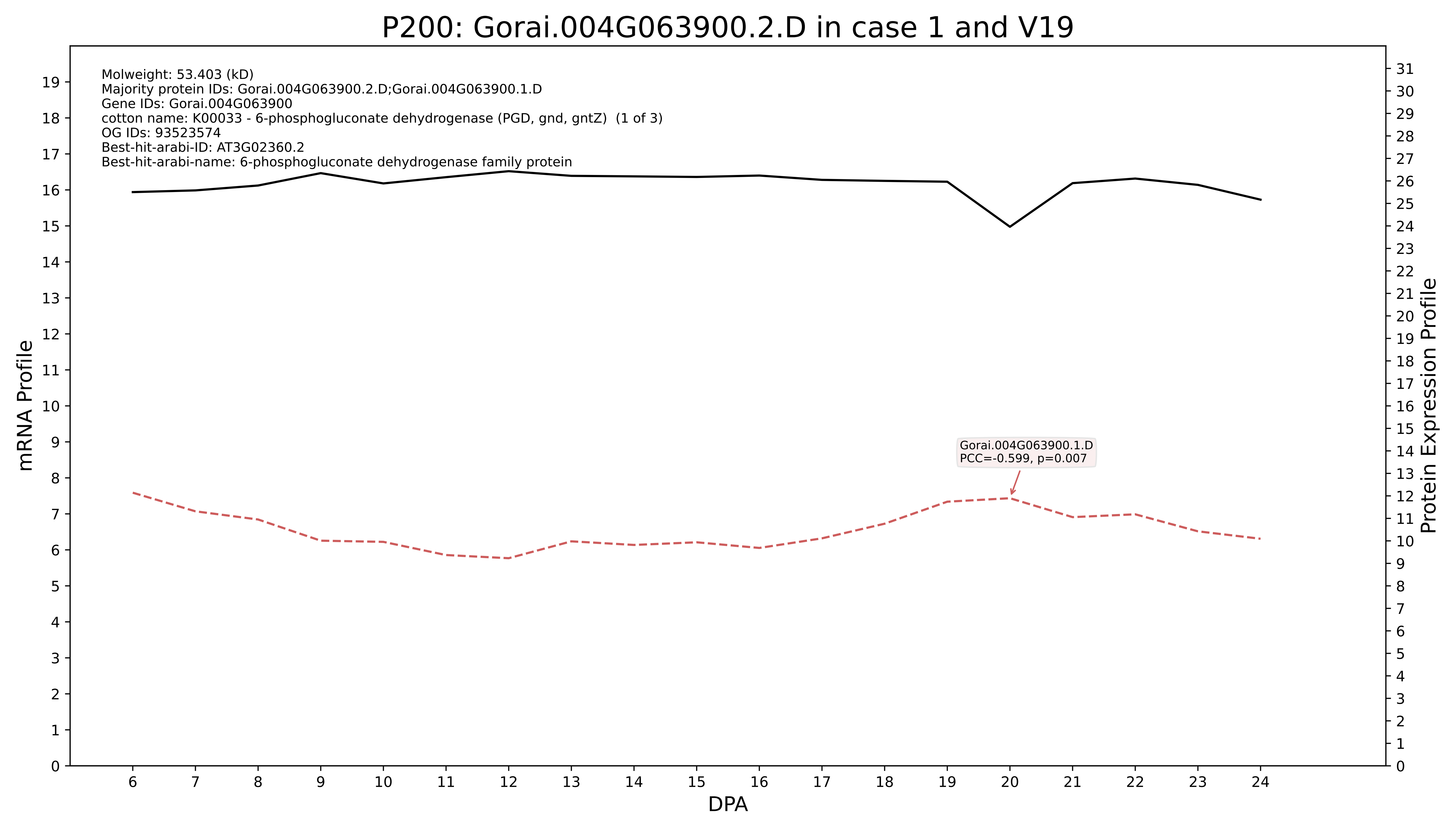
P200: Gorai.004G063900.2.D in case 1 and V19
Molweight: 53.403 (kD)
Majority protein IDs: Gorai.004G063900.2.D;Gorai.004G063900.1.D
Gene IDs: Gorai.004G063900
cotton name: K00033 - 6-phosphogluconate dehydrogenase (PGD, gnd, gntZ) (1 of 3)
OG IDs: 93523574
Best-hit-arabi-ID: AT3G02360.2
Best-hit-arabi-name: 6-phosphogluconate dehydrogenase family protein
Gorai.004G063900.1.D
PCC=-0.599, p=0.007
mRNA Profile
Protein Expression Profile
DPA
0
1
2
3
4
5
6
7
8
9
10
11
12
13
14
15
16
17
18
19
20
21
22
23
24
25
26
27
28
29
30
31

P200: Gorai.007G375600.2.D in case 1 and V19

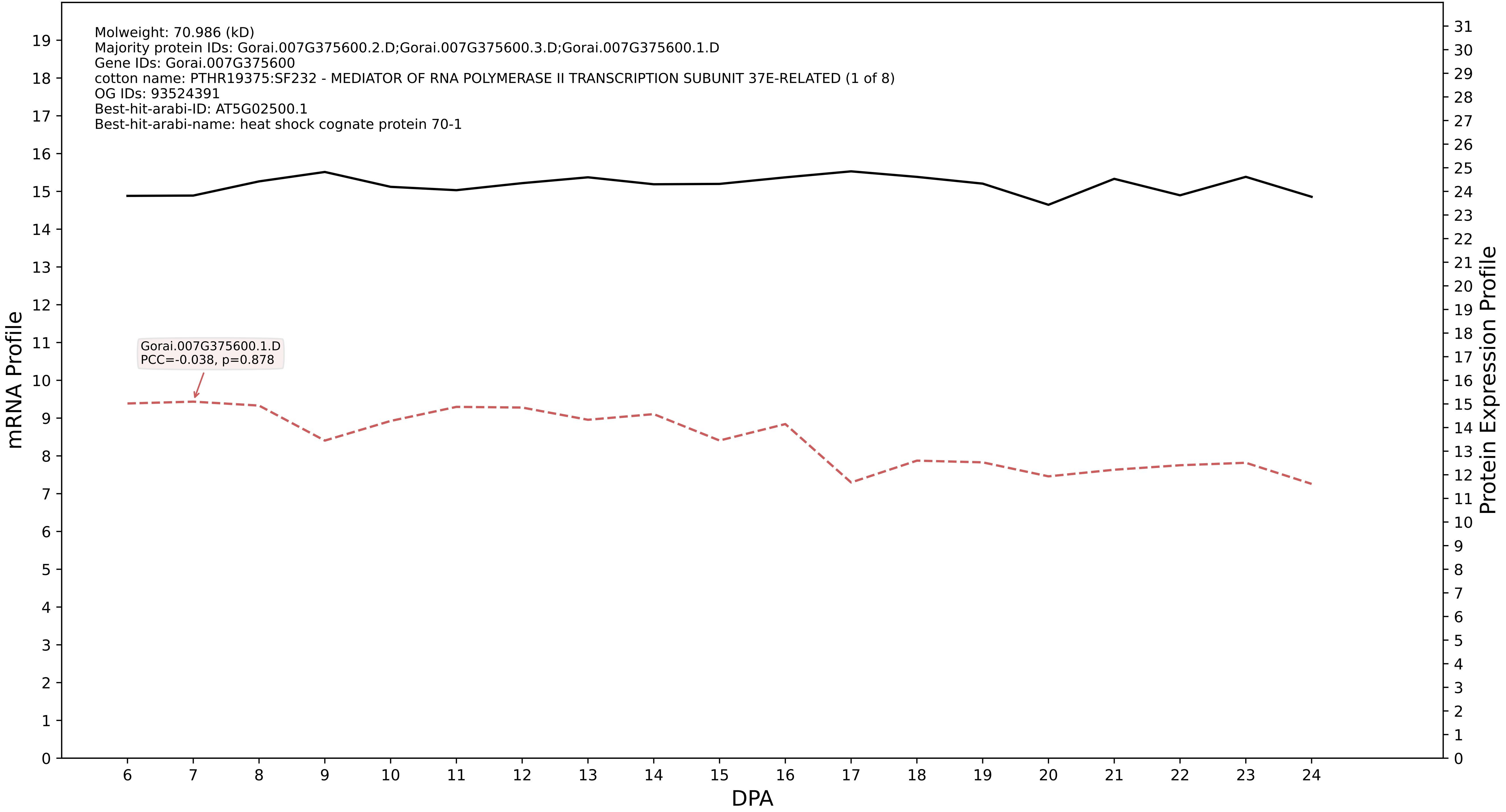

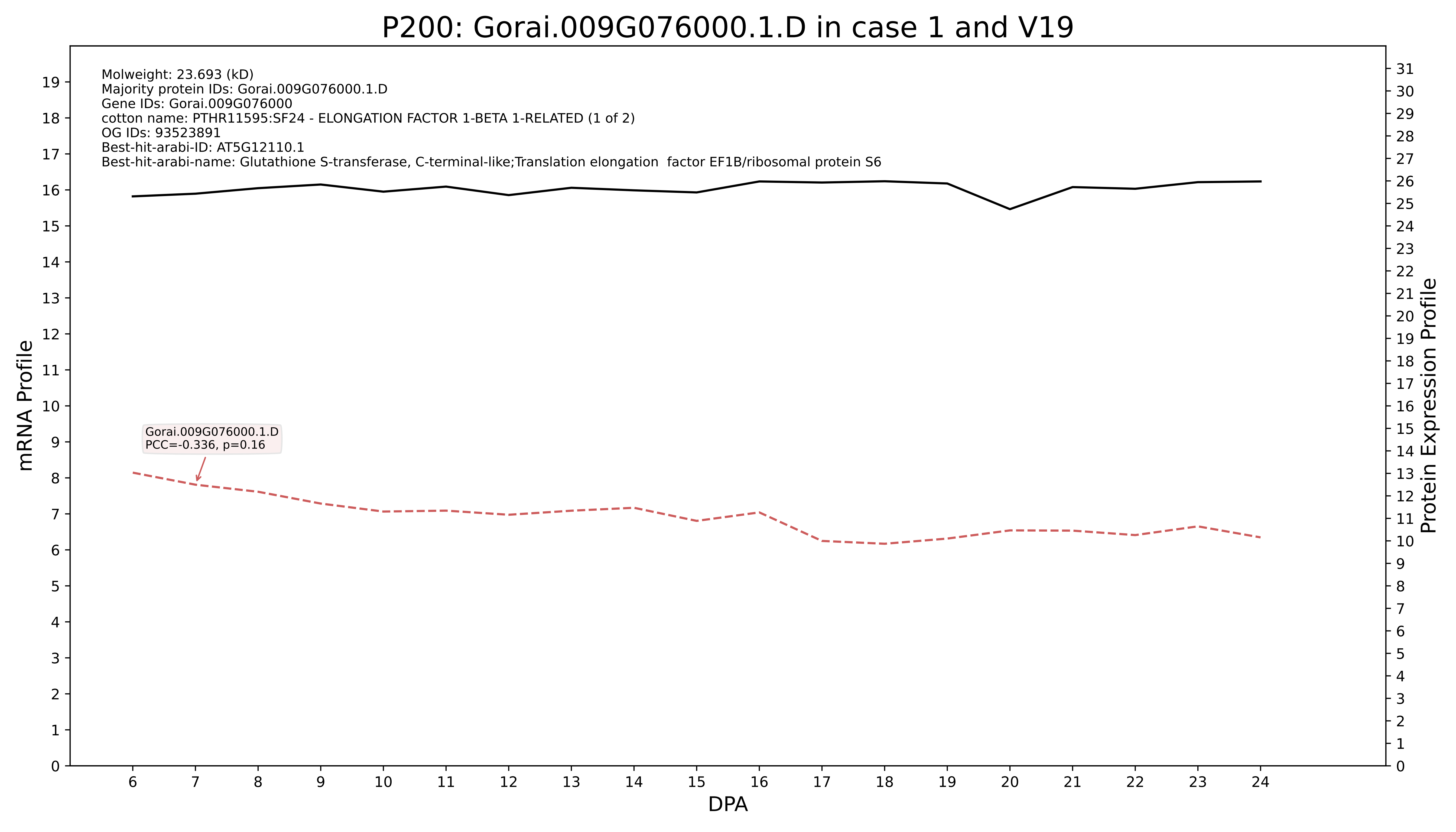

P200: Gorai.009G076000.1.D in case 1 and V19
Molweight: 23.693 (kD)
Majority protein IDs: Gorai.009G076000.1.D
Gene IDs: Gorai.009G076000
cotton name: PTHR11595:SF24 - ELONGATION FACTOR 1-BETA 1-RELATED (1 of 2)
OG IDs: 93523891
Best-hit-arabi-ID: AT5G12110.1
Best-hit-arabi-name: Glutathione S-transferase, C-terminal-like;Translation elongation factor EF1B/ribosomal protein S6
Gorai.009G076000.1.D
PCC=-0.336, p=0.16
mRNA Profile
Protein Expression Profile
DPA

P200: Gorai.001G062000.3.D in case 1 and V19

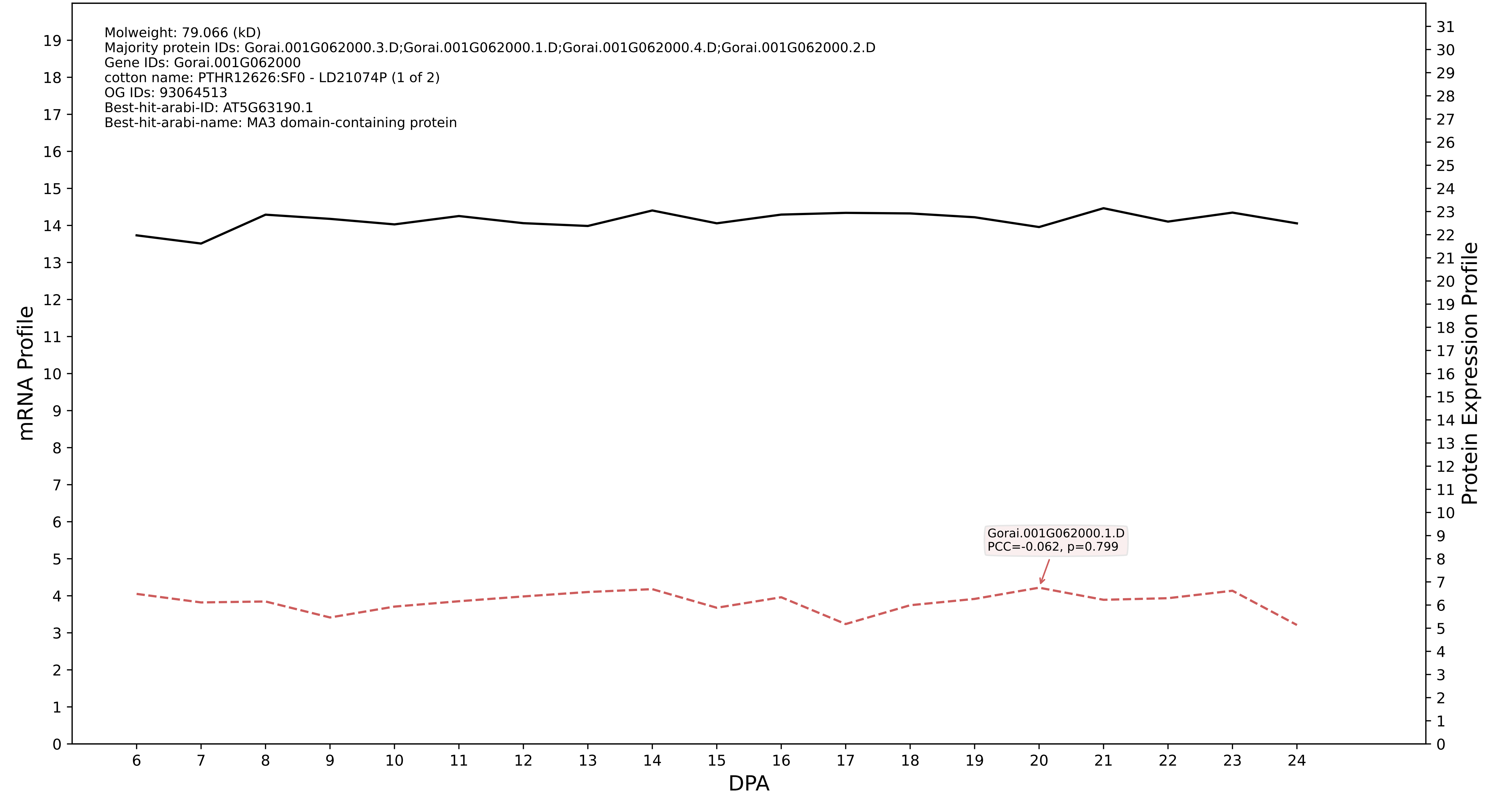

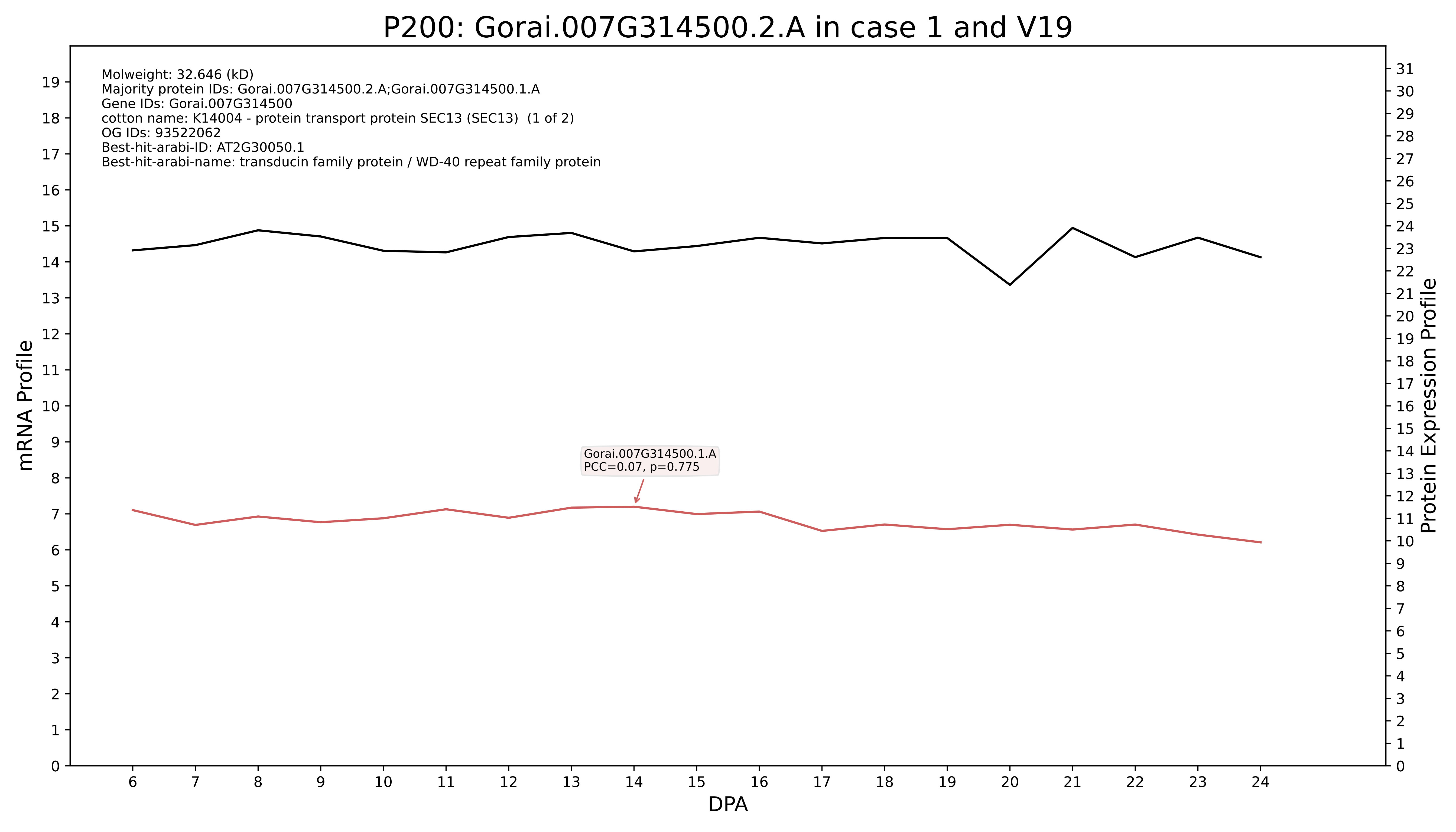

P200: Gorai.007G314500.2.A in case 1 and V19
Molweight: 32.646 (kD)
Majority protein IDs: Gorai.007G314500.2.A;Gorai.007G314500.1.A
Gene IDs: Gorai.007G314500
cotton name: K14004 - protein transport protein SEC13 (SEC13) (1 of 2)
OG IDs: 93522062
Best-hit-arabi-ID: AT2G30050.1
Best-hit-arabi-name: transducin family protein / WD-40 repeat family protein
Gorai.007G314500.1.A
PCC=0.07, p=0.775
mRNA Profile
Protein Expression Profile
DPA

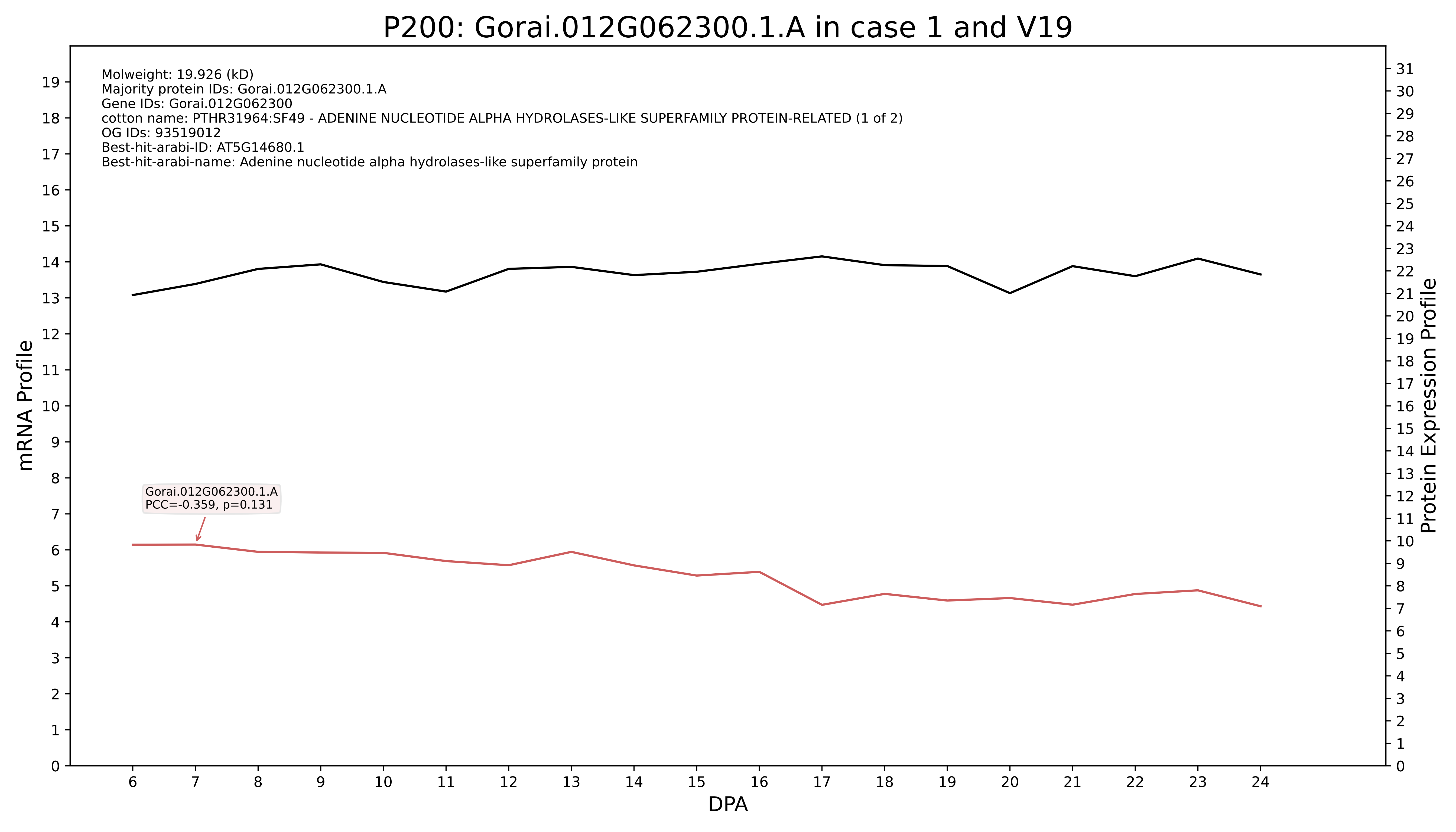
P200: Gorai.012G062300.1.A in case 1 and V19
Molweight: 19.926 (kD)
Majority protein IDs: Gorai.012G062300.1.A
Gene IDs: Gorai.012G062300
cotton name: PTHR31964:SF49 - ADENINE NUCLEOTIDE ALPHA HYDROLASES-LIKE SUPERFAMILY PROTEIN-RELATED (1 of 2)
OG IDs: 93519012
Best-hit-arabi-ID: AT5G14680.1
Best-hit-arabi-name: Adenine nucleotide alpha hydrolases-like superfamily protein
Gorai.012G062300.1.A
PCC=-0.359, p=0.131
mRNA Profile
Protein Expression Profile
DPA

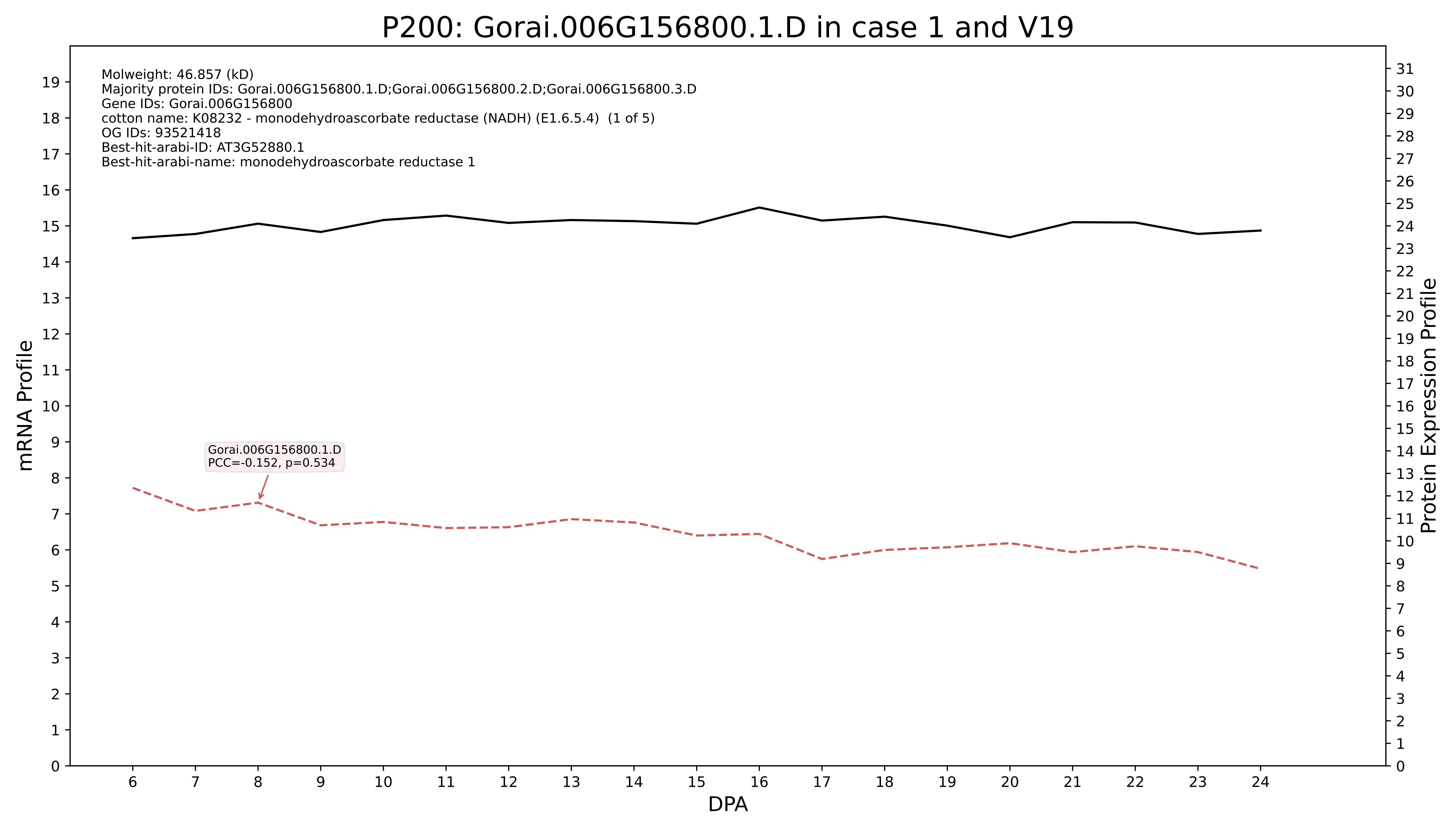
P200: Gorai.006G156800.1.D in case 1 and V19
Molweight: 46.857 (kD)
Majority protein IDs: Gorai.006G156800.1.D;Gorai.006G156800.2.D;Gorai.006G156800.3.D
Gene IDs: Gorai.006G156800
cotton name: K08232 - monodehydroascorbate reductase (NADH) (E1.6.5.4) (1 of 5)
OG IDs: 93521418
Best-hit-arabi-ID: AT3G52880.1
Best-hit-arabi-name: monodehydroascorbate reductase 1
Gorai.006G156800.1.D
PCC=-0.152, p=0.534
mRNA Profile
Protein Expression Profile
DPA

P200: Gorai.004G246300.1.A in case 1 and V19

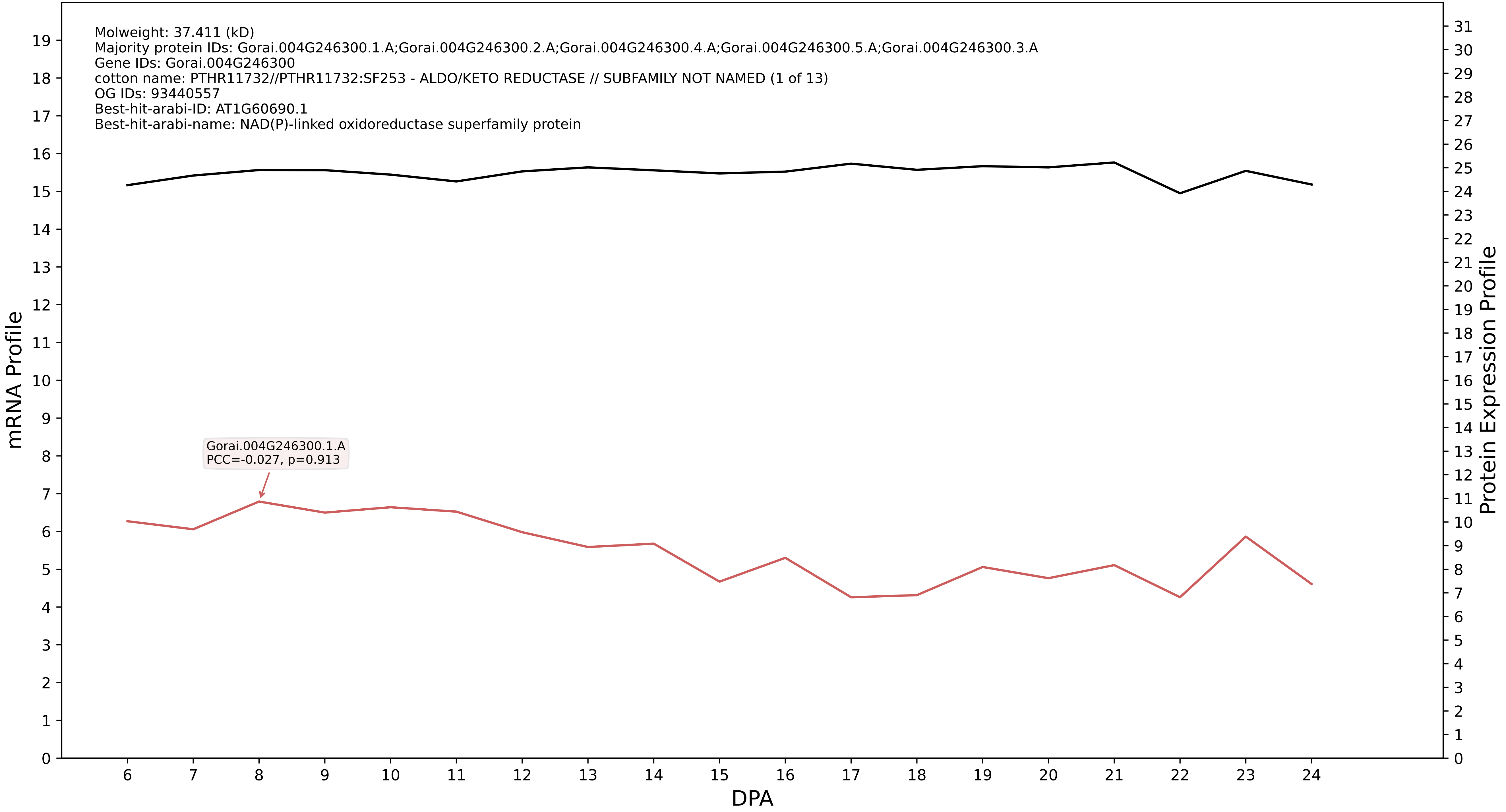

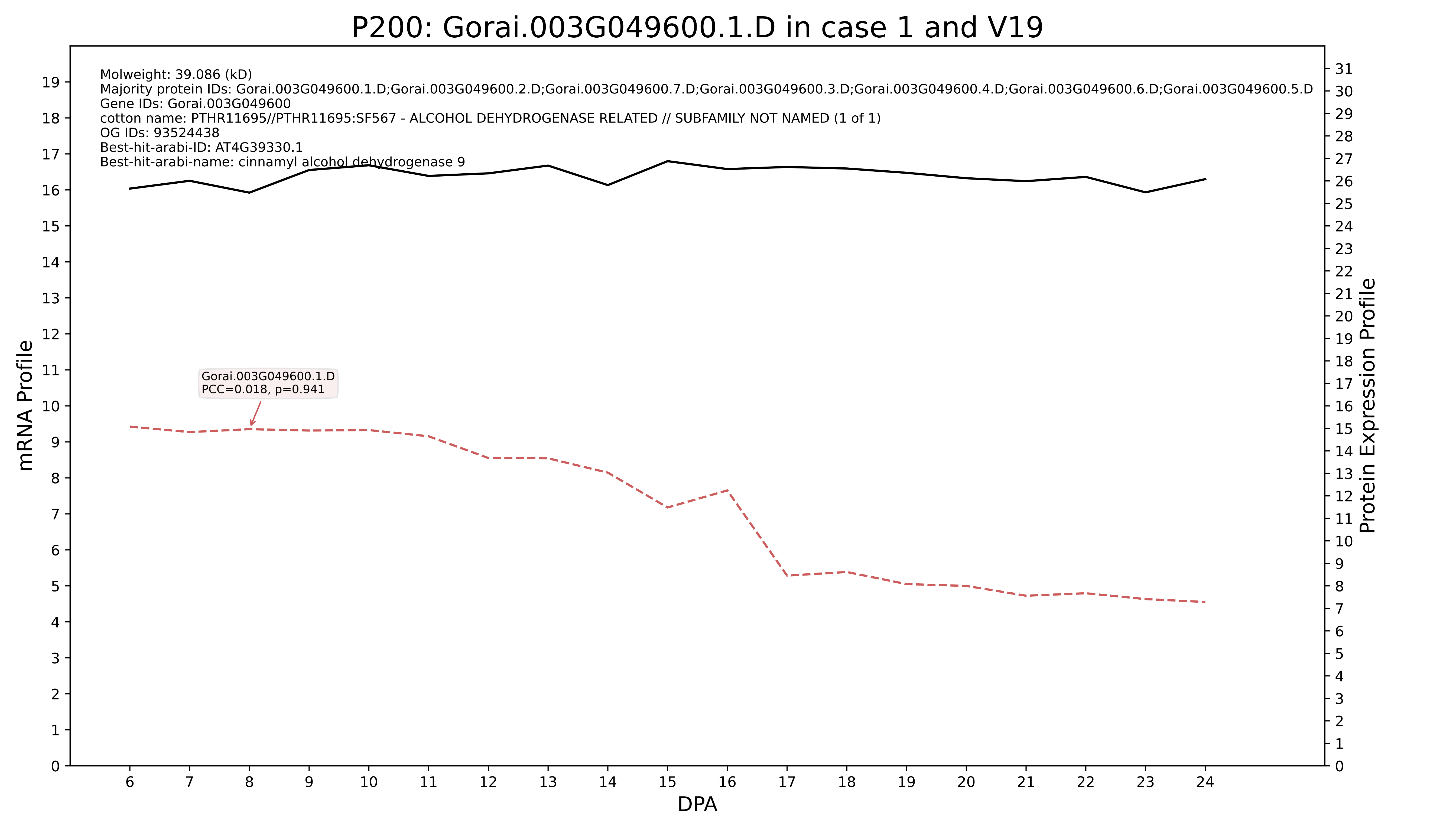
P200: Gorai.003G049600.1.D in case 1 and V19
Molweight: 39.086 (kD)
Majority protein IDs: Gorai.003G049600.1.D;Gorai.003G049600.2.D;Gorai.003G049600.7.D;Gorai.003G049600.3.D;Gorai.003G049600.4.D;Gorai.003G049600.6.D;Gorai.003G049600.5.D
Gene IDs: Gorai.003G049600
cotton name: PTHR11695//PTHR11695:SF567 - ALCOHOL DEHYDROGENASE RELATED // SUBFAMILY NOT NAMED (1 of 1)
OG IDs: 93524438
Best-hit-arabi-ID: AT4G39330.1
Best-hit-arabi-name: cinnamyl alcohol dehydrogenase 9
Gorai.003G049600.1.D
PCC=0.018, p=0.941
mRNA Profile
Protein Expression Profile
DPA

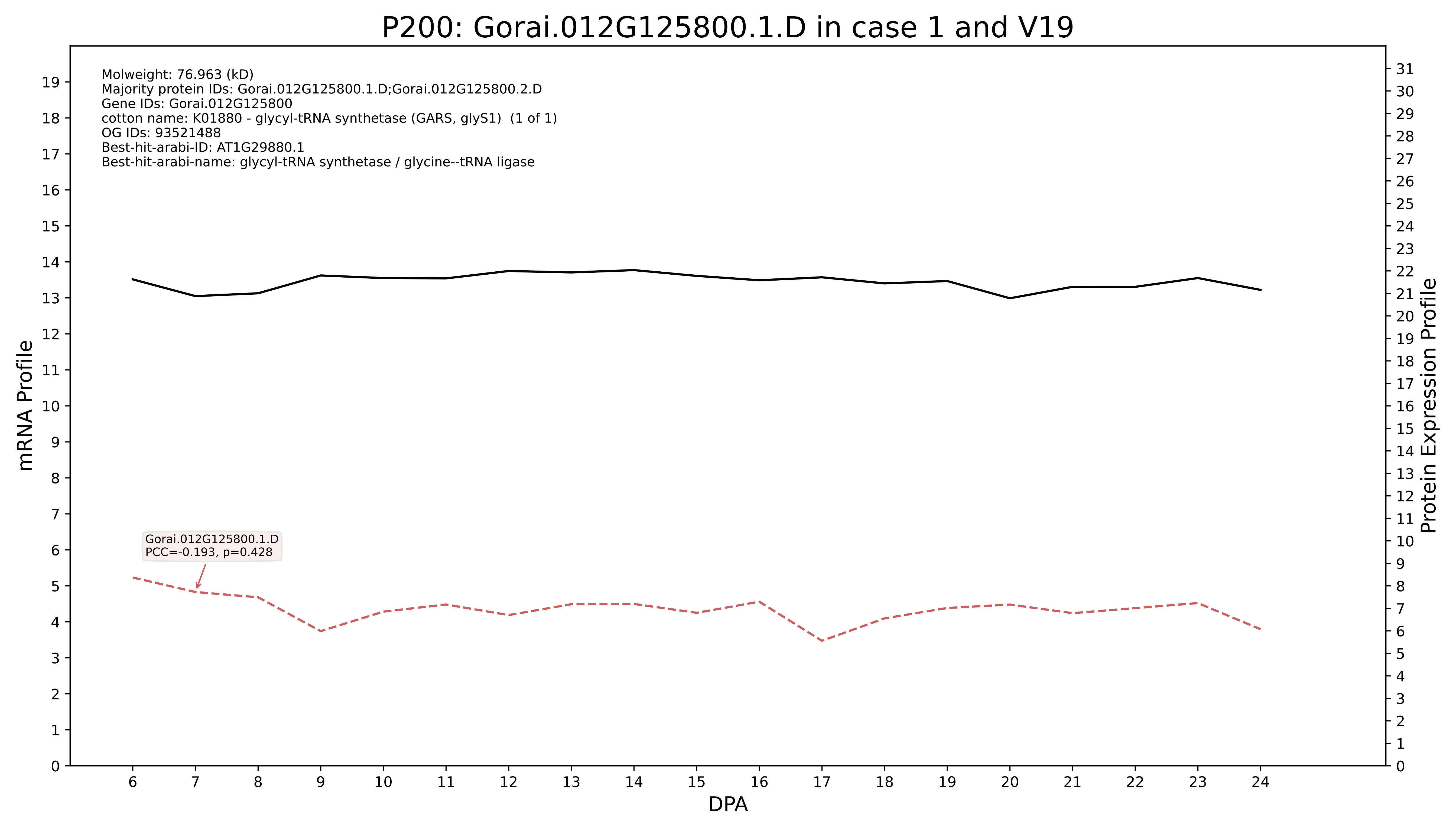
P200: Gorai.012G125800.1.D in case 1 and V19
Molweight: 76.963 (kD)
Majority protein IDs: Gorai.012G125800.1.D;Gorai.012G125800.2.D
Gene IDs: Gorai.012G125800
cotton name: K01880 - glycyl-tRNA synthetase (GARS, glyS1) (1 of 1)
OG IDs: 93521488
Best-hit-arabi-ID: AT1G29880.1
Best-hit-arabi-name: glycyl-tRNA synthetase / glycine--tRNA ligase
Gorai.012G125800.1.D
PCC=-0.193, p=0.428
mRNA Profile
Protein Expression Profile
DPA

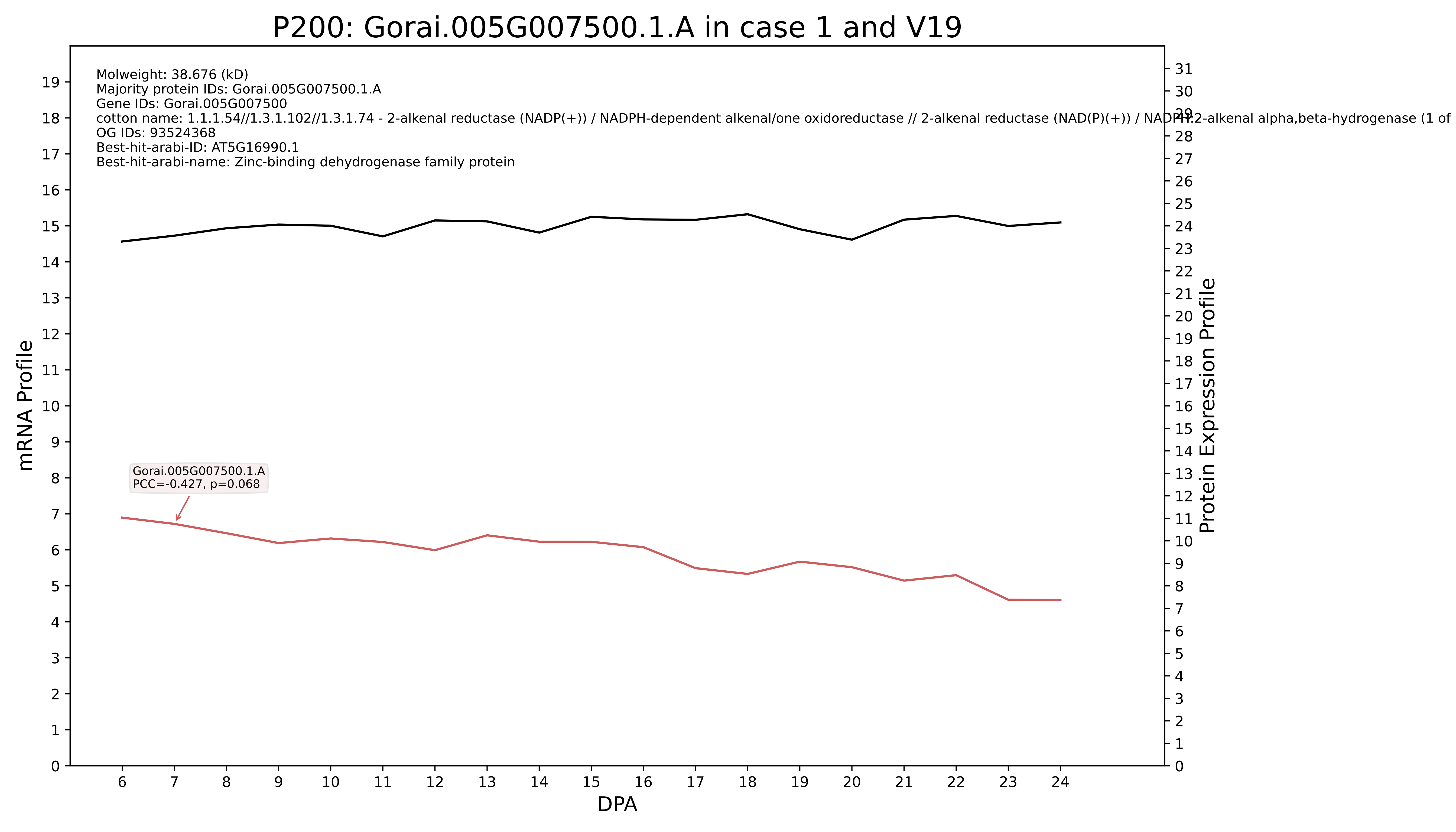

P200: Gorai.005G007500.1.A in case 1 and V19
Molweight: 38.676 (kD)
Majority protein IDs: Gorai.005G007500.1.A
Gene IDs: Gorai.005G007500
cotton name: 1.1.1.54//1.3.1.102//1.3.1.74 - 2-alkenal reductase (NADP(+)) / NADPH-dependent alkenal/one oxidoreductase // 2-alkenal reductase (NAD(P)(+)) / NADPH:2-alkenal alpha,beta-hydrogenase (1 of
OG IDs: 93524368
Best-hit-arabi-ID: AT5G16990.1
Best-hit-arabi-name: Zinc-binding dehydrogenase family protein
Gorai.005G007500.1.A
PCC=-0.427, p=0.068
mRNA Profile
Protein Expression Profile
DPA

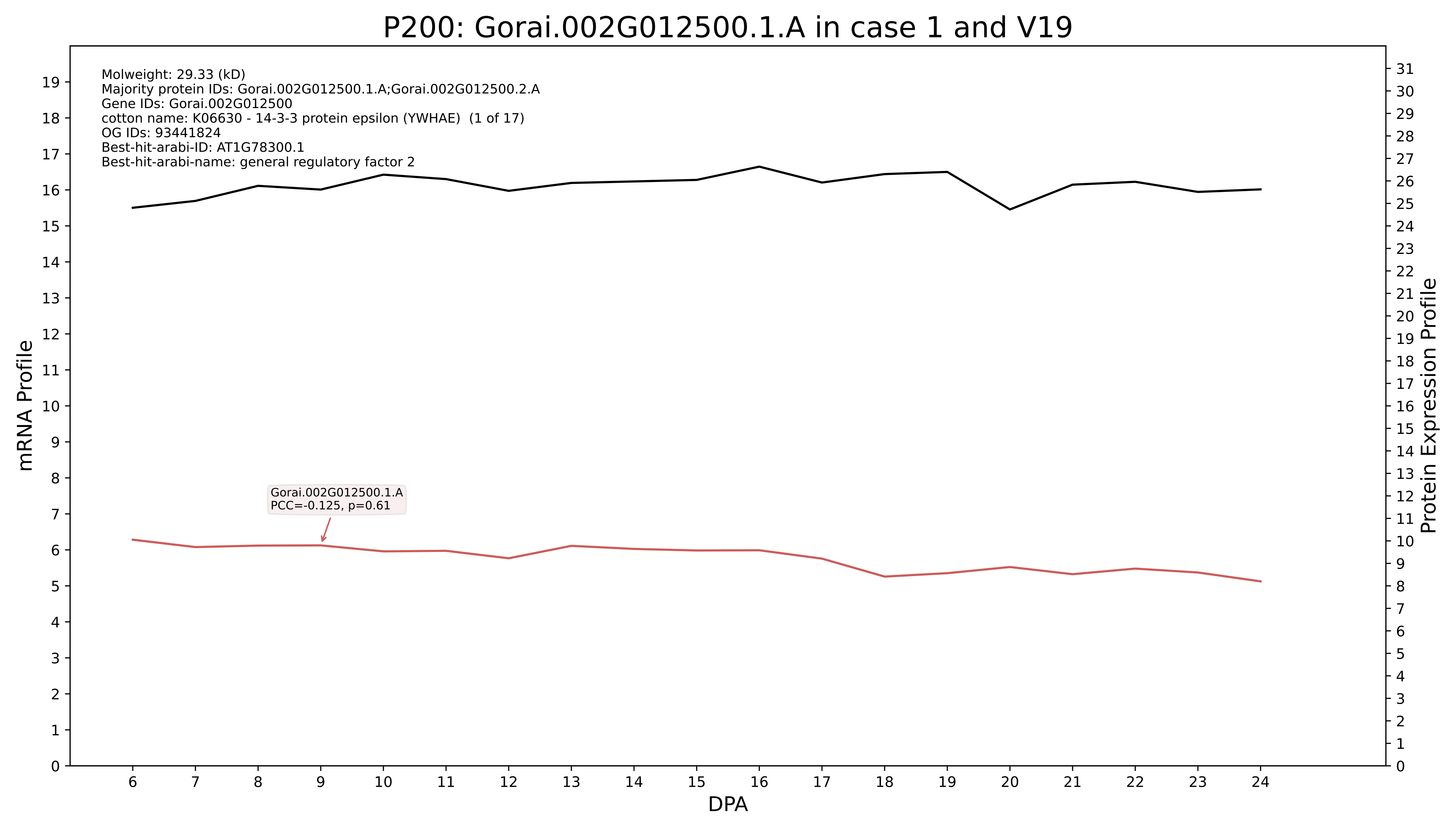
P200: Gorai.002G012500.1.A in case 1 and V19
Molweight: 29.33 (kD)
Majority protein IDs: Gorai.002G012500.1.A;Gorai.002G012500.2.A
Gene IDs: Gorai.002G012500
cotton name: K06630 - 14-3-3 protein epsilon (YWHAE) (1 of 17)
OG IDs: 93441824
Best-hit-arabi-ID: AT1G78300.1
Best-hit-arabi-name: general regulatory factor 2
Gorai.002G012500.1.A
PCC=-0.125, p=0.61
mRNA Profile
Protein Expression Profile
DPA

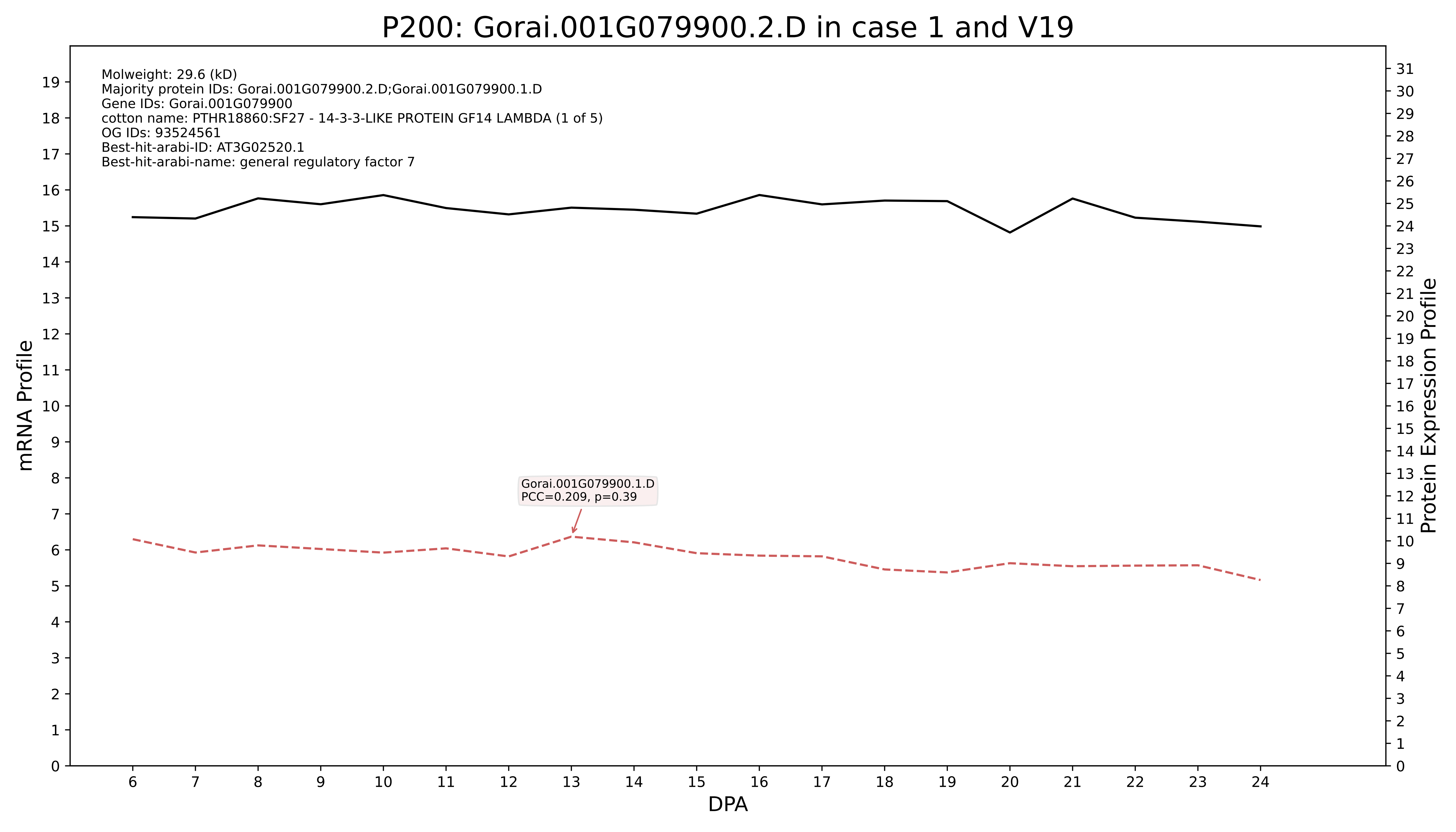
P200: Gorai.001G079900.2.D in case 1 and V19
Molweight: 29.6 (kD)
Majority protein IDs: Gorai.001G079900.2.D;Gorai.001G079900.1.D
Gene IDs: Gorai.001G079900
cotton name: PTHR18860:SF27 - 14-3-3-LIKE PROTEIN GF14 LAMBDA (1 of 5)
OG IDs: 93524561
Best-hit-arabi-ID: AT3G02520.1
Best-hit-arabi-name: general regulatory factor 7
Gorai.001G079900.1.D
PCC=0.209, p=0.39
mRNA Profile
Protein Expression Profile
DPA

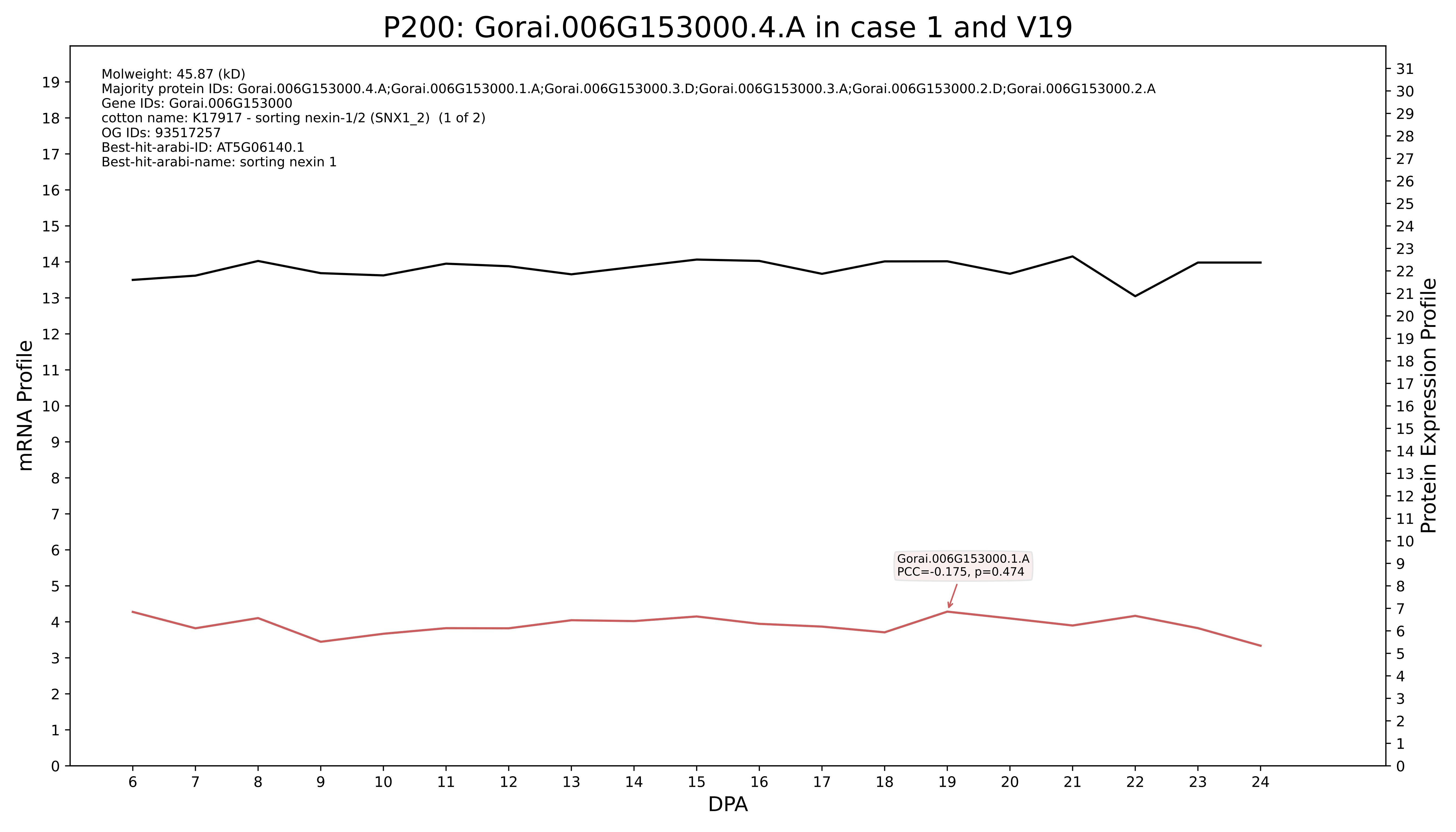
P200: Gorai.006G153000.4.A in case 1 and V19
Molweight: 45.87 (kD)
Majority protein IDs: Gorai.006G153000.4.A;Gorai.006G153000.1.A;Gorai.006G153000.3.D;Gorai.006G153000.3.A;Gorai.006G153000.2.D;Gorai.006G153000.2.A
Gene IDs: Gorai.006G153000
cotton name: K17917 - sorting nexin-1/2 (SNX1_2) (1 of 2)
OG IDs: 93517257
Best-hit-arabi-ID: AT5G06140.1
Best-hit-arabi-name: sorting nexin 1
Gorai.006G153000.1.A
PCC=-0.175, p=0.474
mRNA Profile
Protein Expression Profile
DPA

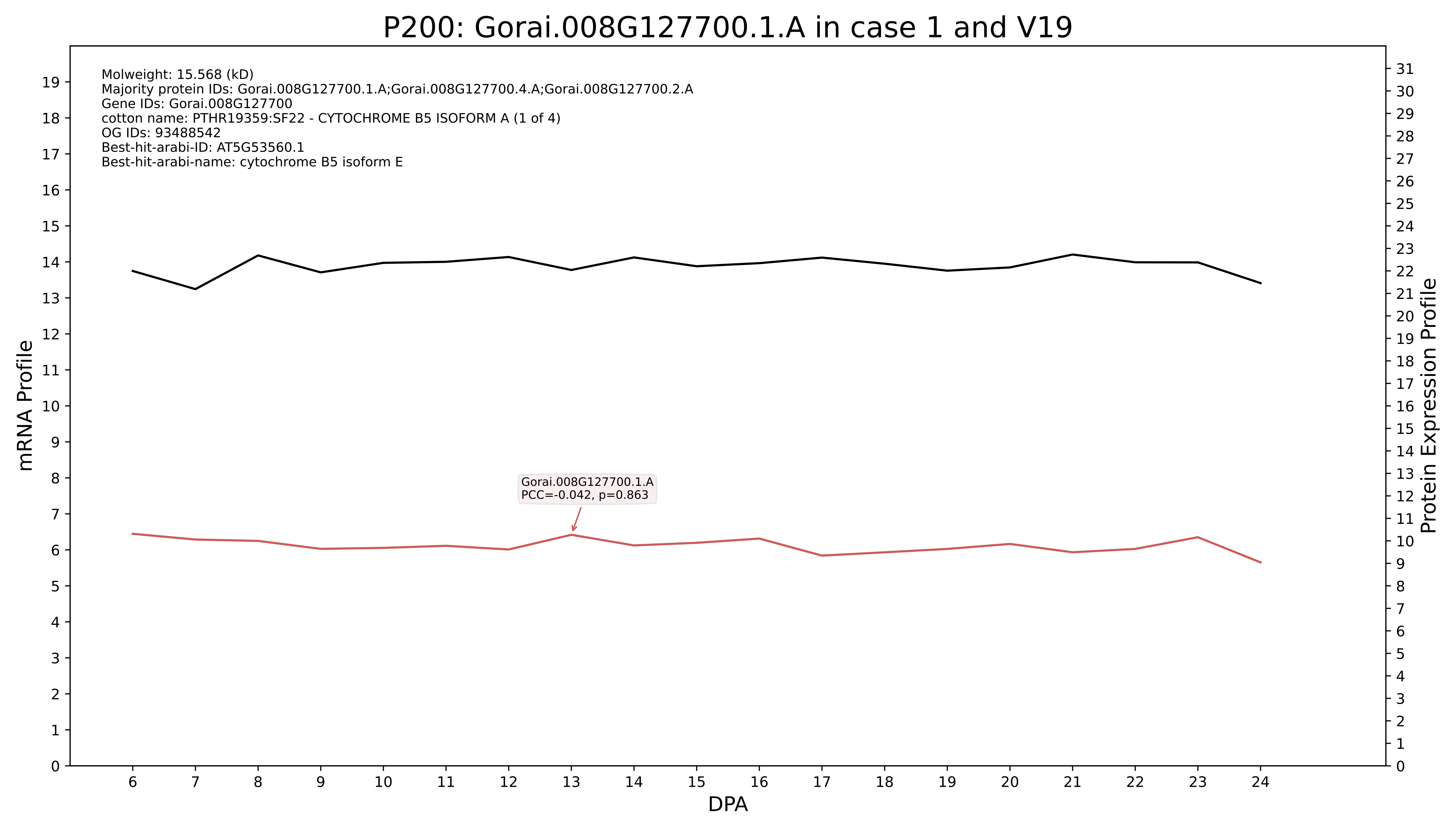

P200: Gorai.008G127700.1.A in case 1 and V19
Molweight: 15.568 (kD)
Majority protein IDs: Gorai.008G127700.1.A;Gorai.008G127700.4.A;Gorai.008G127700.2.A
Gene IDs: Gorai.008G127700
cotton name: PTHR19359:SF22 - CYTOCHROME B5 ISOFORM A (1 of 4)
OG IDs: 93488542
Best-hit-arabi-ID: AT5G53560.1
Best-hit-arabi-name: cytochrome B5 isoform E
Gorai.008G127700.1.A
PCC=-0.042, p=0.863
mRNA Profile
Protein Expression Profile
DPA

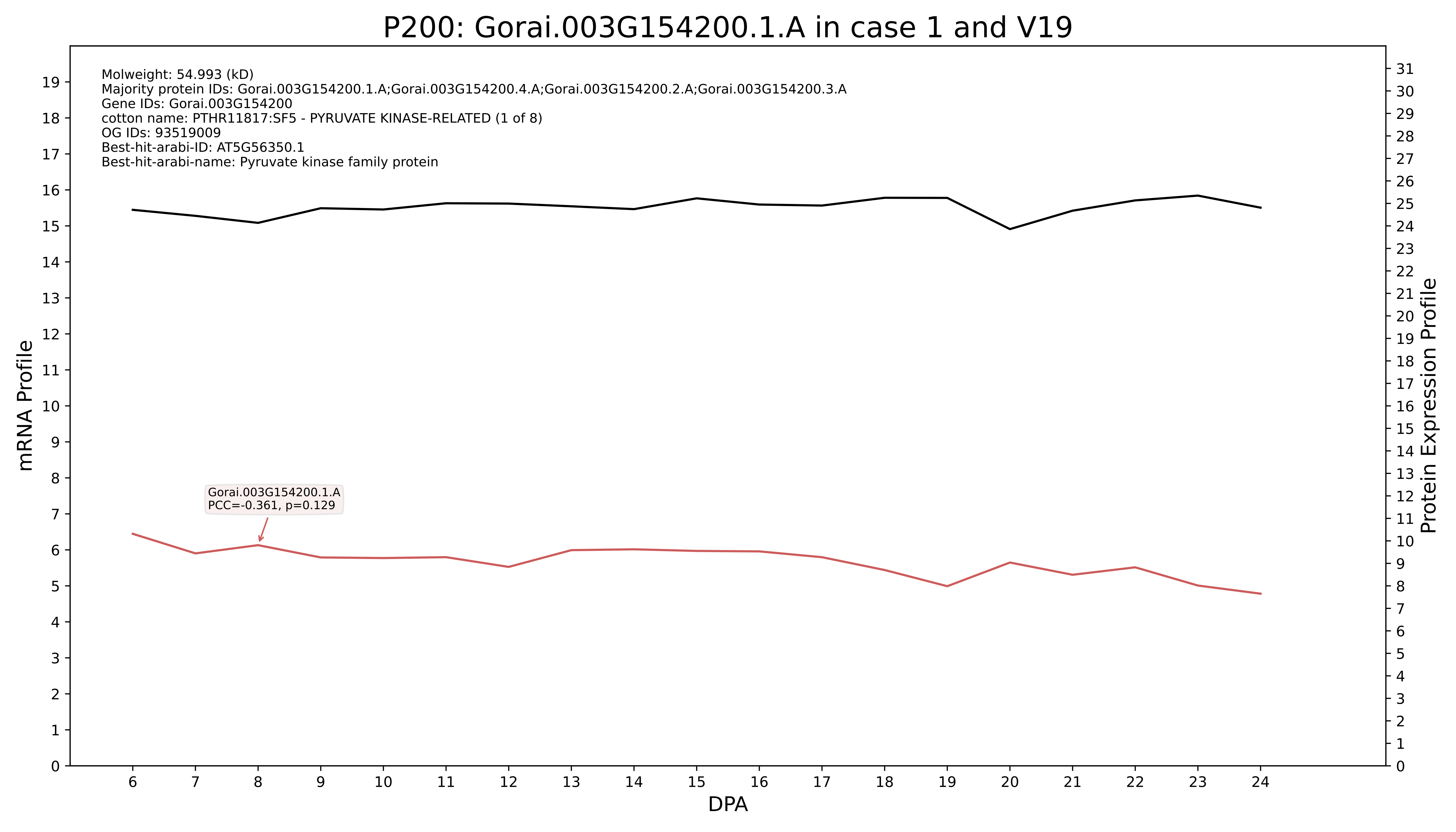

P200: Gorai.003G154200.1.A in case 1 and V19
Molweight: 54.993 (kD)
Majority protein IDs: Gorai.003G154200.1.A;Gorai.003G154200.4.A;Gorai.003G154200.2.A;Gorai.003G154200.3.A
Gene IDs: Gorai.003G154200
cotton name: PTHR11817:SF5 - PYRUVATE KINASE-RELATED (1 of 8)
OG IDs: 93519009
Best-hit-arabi-ID: AT5G56350.1
Best-hit-arabi-name: Pyruvate kinase family protein
Gorai.003G154200.1.A
PCC=-0.361, p=0.129
mRNA Profile
Protein Expression Profile
DPA

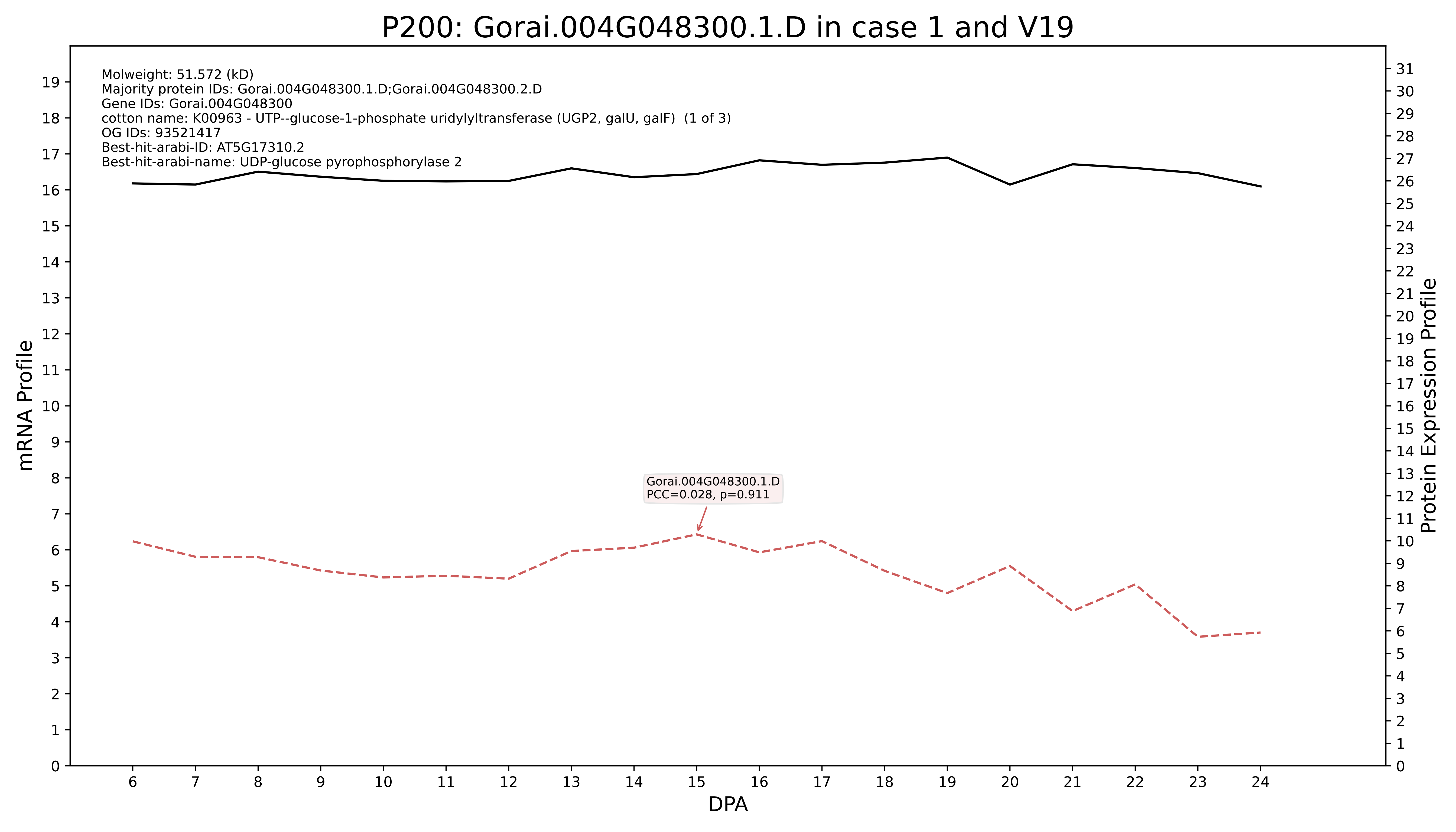

P200: Gorai.004G048300.1.D in case 1 and V19
Molweight: 51.572 (kD)
Majority protein IDs: Gorai.004G048300.1.D;Gorai.004G048300.2.D
Gene IDs: Gorai.004G048300
cotton name: K00963 - UTP--glucose-1-phosphate uridylyltransferase (UGP2, galU, galF) (1 of 3)
OG IDs: 93521417
Best-hit-arabi-ID: AT5G17310.2
Best-hit-arabi-name: UDP-glucose pyrophosphorylase 2
Gorai.004G048300.1.D
PCC=0.028, p=0.911
mRNA Profile
Protein Expression Profile
DPA

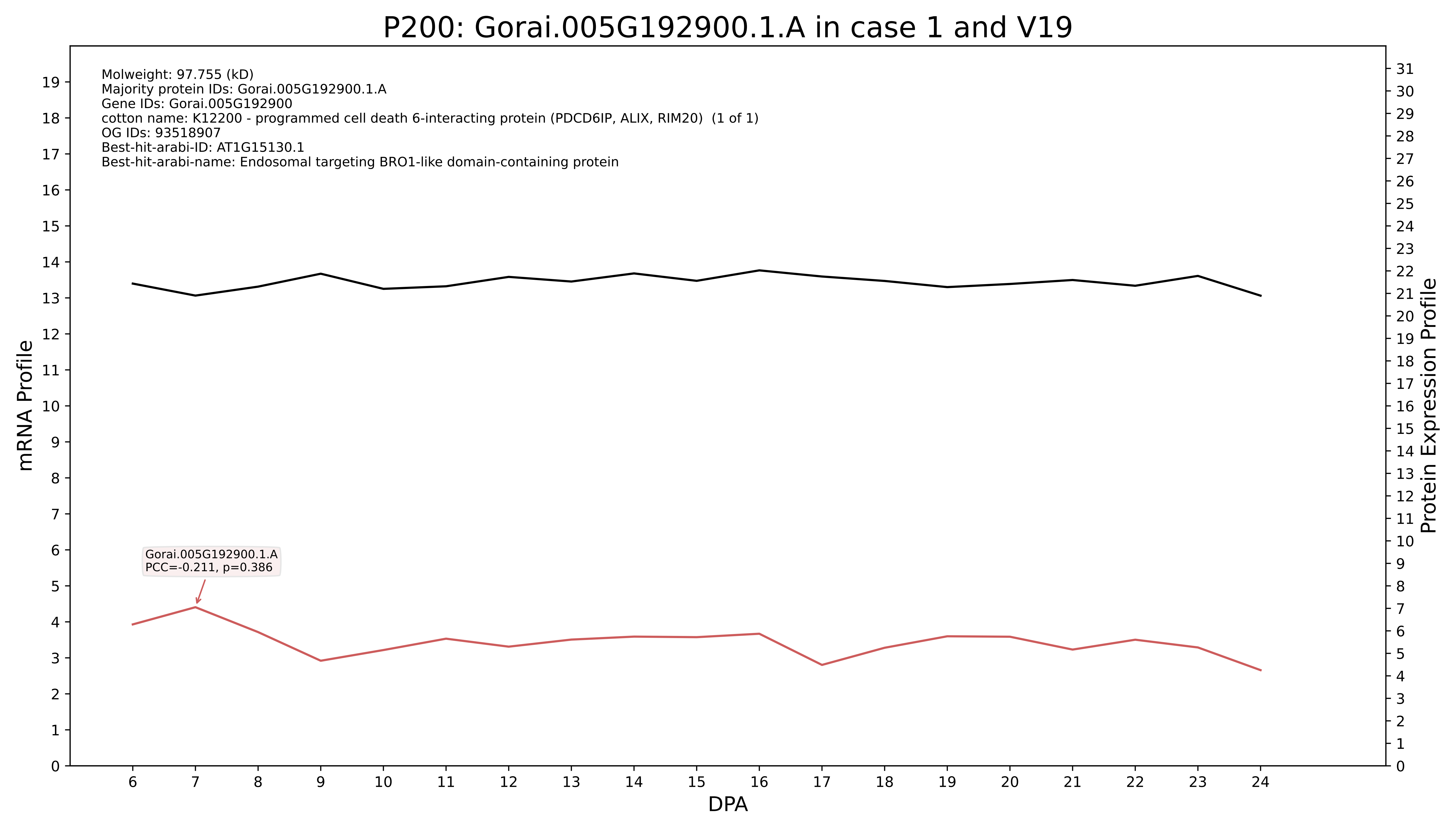

P200: Gorai.005G192900.1.A in case 1 and V19
Molweight: 97.755 (kD)
Majority protein IDs: Gorai.005G192900.1.A
Gene IDs: Gorai.005G192900
cotton name: K12200 - programmed cell death 6-interacting protein (PDCD6IP, ALIX, RIM20) (1 of 1)
OG IDs: 93518907
Best-hit-arabi-ID: AT1G15130.1
Best-hit-arabi-name: Endosomal targeting BRO1-like domain-containing protein
Gorai.005G192900.1.A
PCC=-0.211, p=0.386
mRNA Profile
Protein Expression Profile
DPA

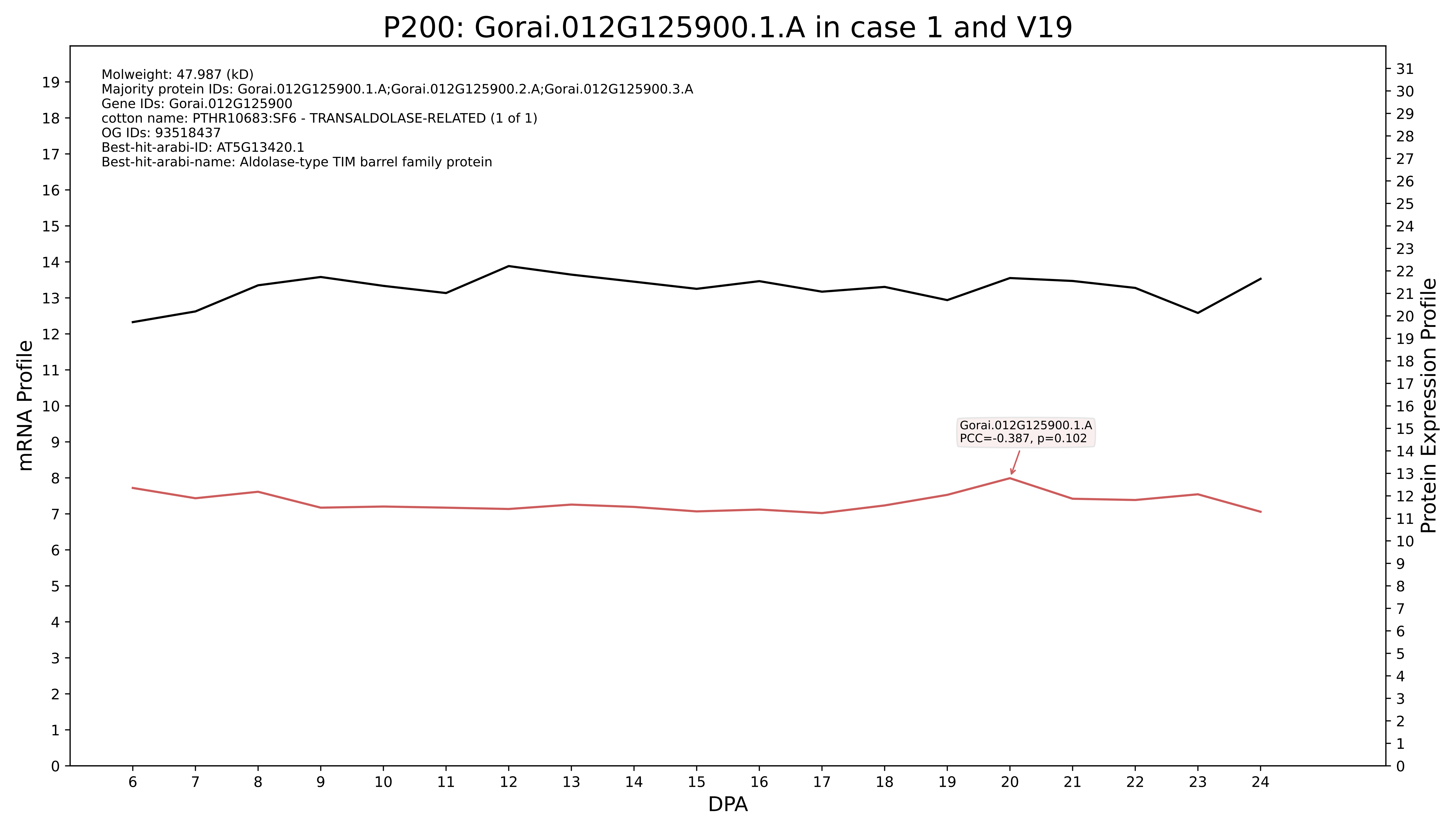

P200: Gorai.012G125900.1.A in case 1 and V19
Molweight: 47.987 (kD)
Majority protein IDs: Gorai.012G125900.1.A;Gorai.012G125900.2.A;Gorai.012G125900.3.A
Gene IDs: Gorai.012G125900
cotton name: PTHR10683:SF6 - TRANSALDOLASE-RELATED (1 of 1)
OG IDs: 93518437
Best-hit-arabi-ID: AT5G13420.1
Best-hit-arabi-name: Aldolase-type TIM barrel family protein
Gorai.012G125900.1.A
PCC=-0.387, p=0.102
mRNA Profile
Protein Expression Profile
DPA

P200: Gorai.004G260100.1.D in case 1 and V19

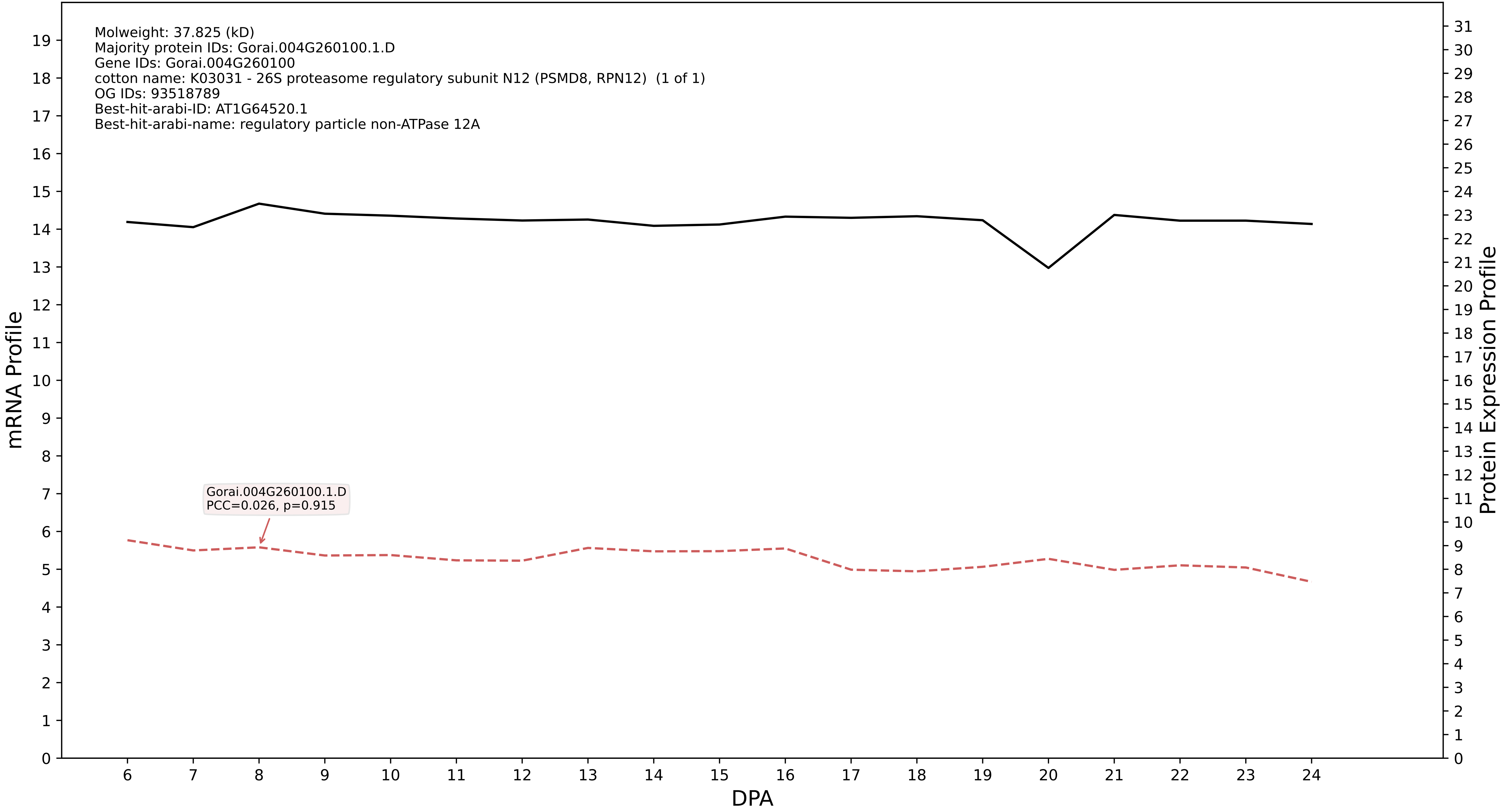

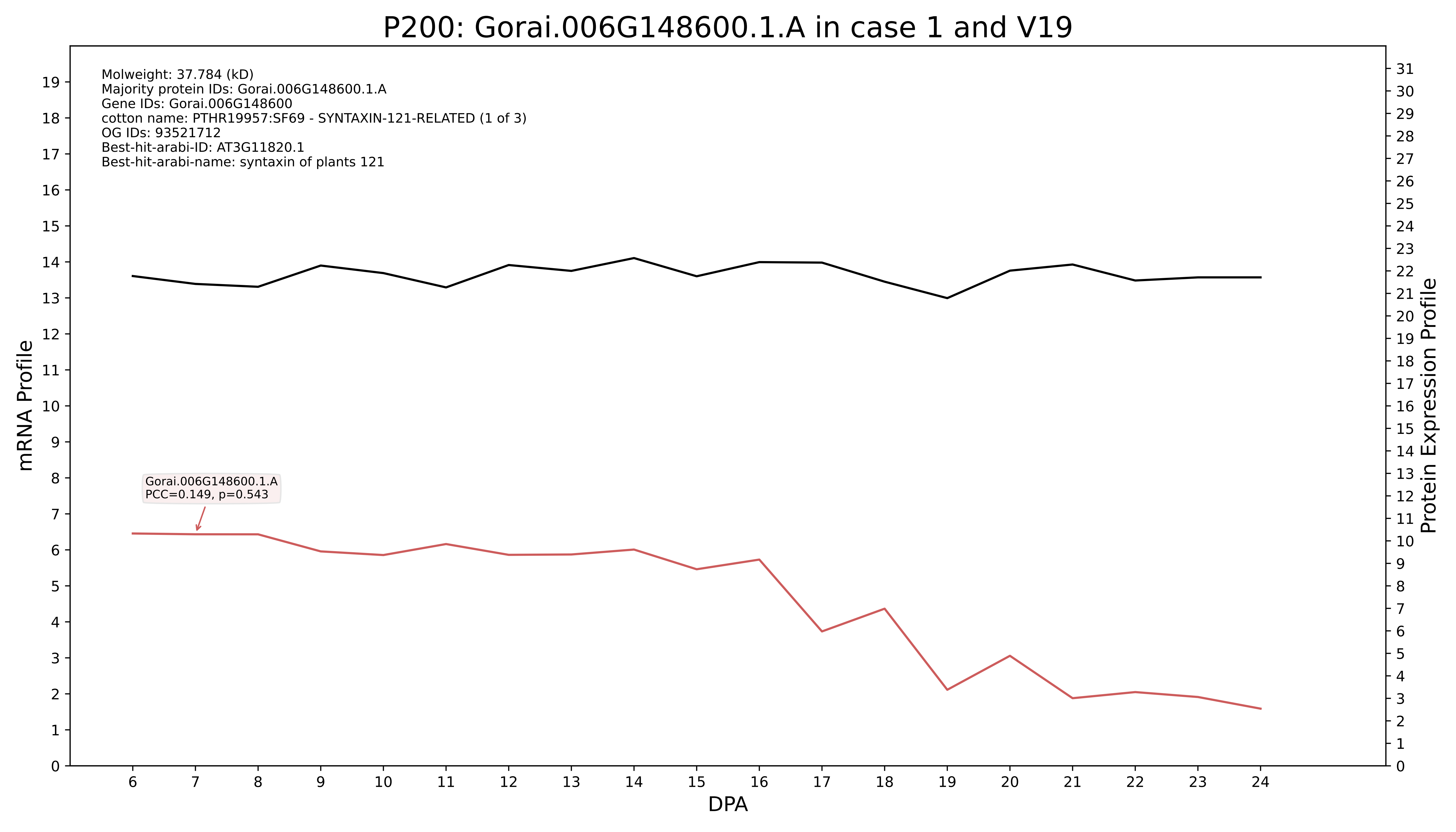
P200: Gorai.006G148600.1.A in case 1 and V19
Molweight: 37.784 (kD)
Majority protein IDs: Gorai.006G148600.1.A
Gene IDs: Gorai.006G148600
cotton name: PTHR19957:SF69 - SYNTAXIN-121-RELATED (1 of 3)
OG IDs: 93521712
Best-hit-arabi-ID: AT3G11820.1
Best-hit-arabi-name: syntaxin of plants 121
Gorai.006G148600.1.A
PCC=0.149, p=0.543
mRNA Profile
Protein Expression Profile
DPA

P200: Gorai.008G113500.1.D in case 1 and V19

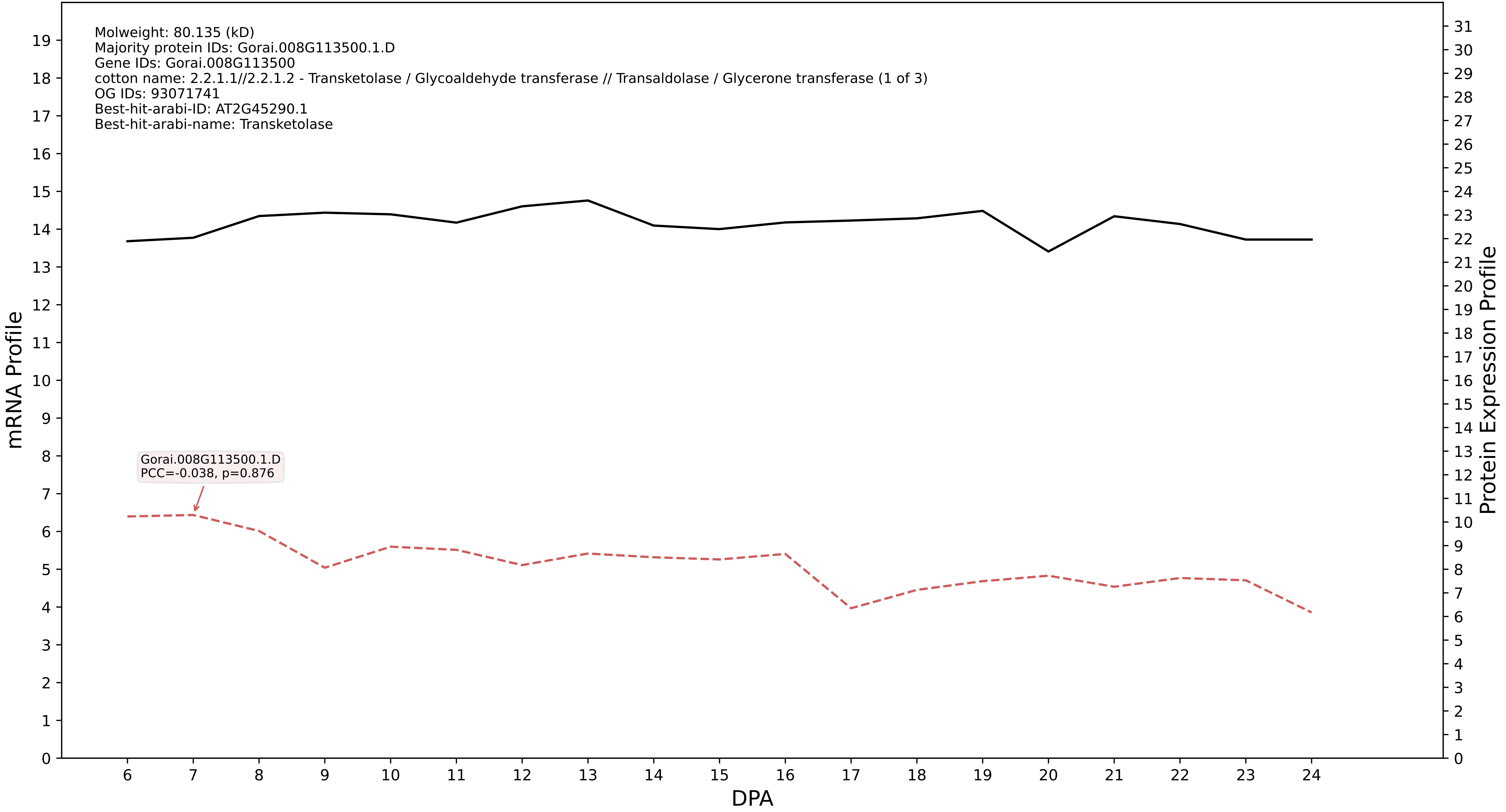

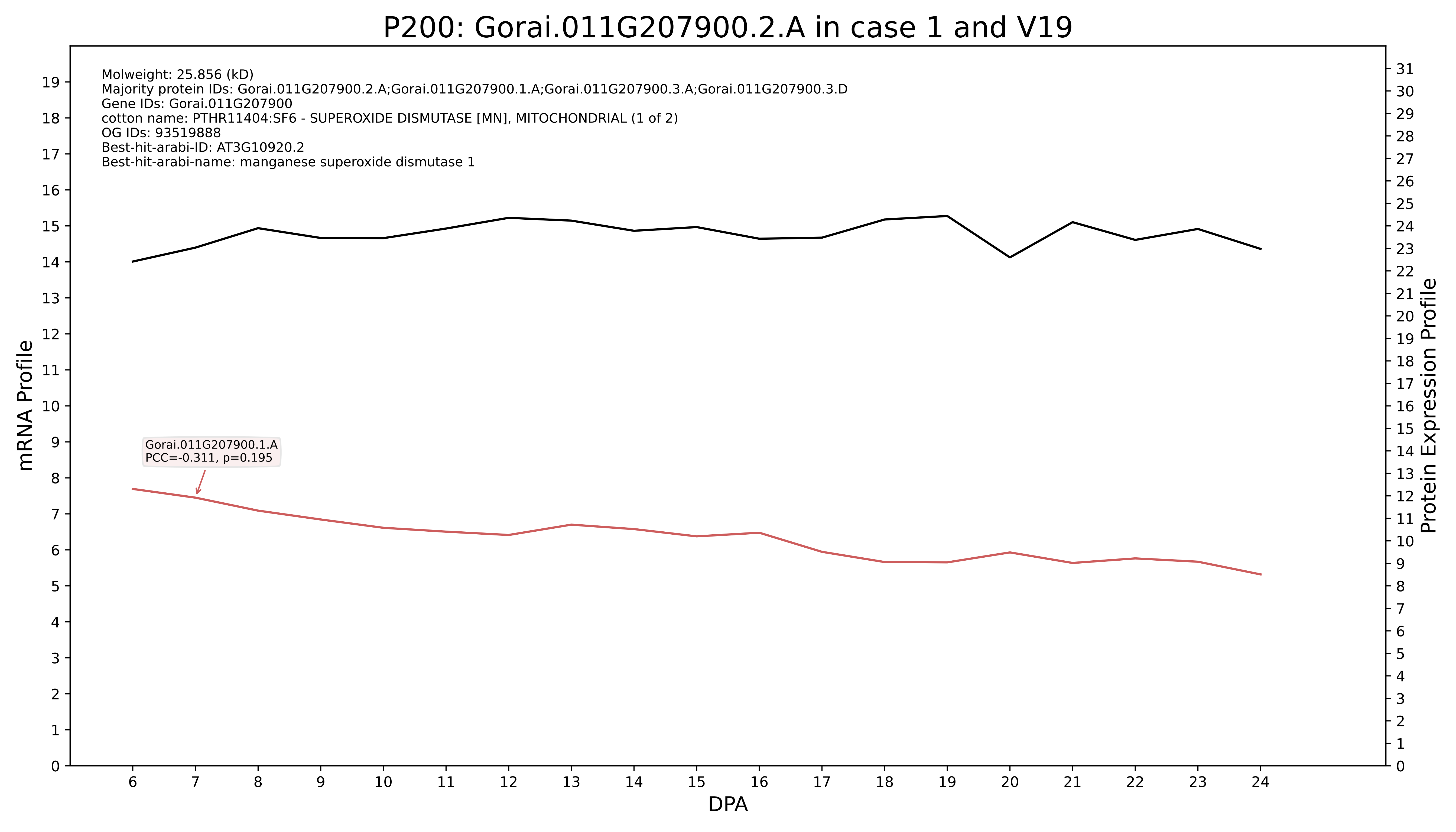
P200: Gorai.011G207900.2.A in case 1 and V19
Molweight: 25.856 (kD)
Majority protein IDs: Gorai.011G207900.2.A;Gorai.011G207900.1.A;Gorai.011G207900.3.A;Gorai.011G207900.3.D
Gene IDs: Gorai.011G207900
cotton name: PTHR11404:SF6 - SUPEROXIDE DISMUTASE [MN], MITOCHONDRIAL (1 of 2)
OG IDs: 93519888
Best-hit-arabi-ID: AT3G10920.2
Best-hit-arabi-name: manganese superoxide dismutase 1
Gorai.011G207900.1.A
PCC=-0.311, p=0.195
mRNA Profile
Protein Expression Profile
DPA
0
1
2
3
4
5
6
7
8
9
10
11
12
13
14
15
16
17
18
19
20
21
22
23
24
25
26
27
28
29
30
31

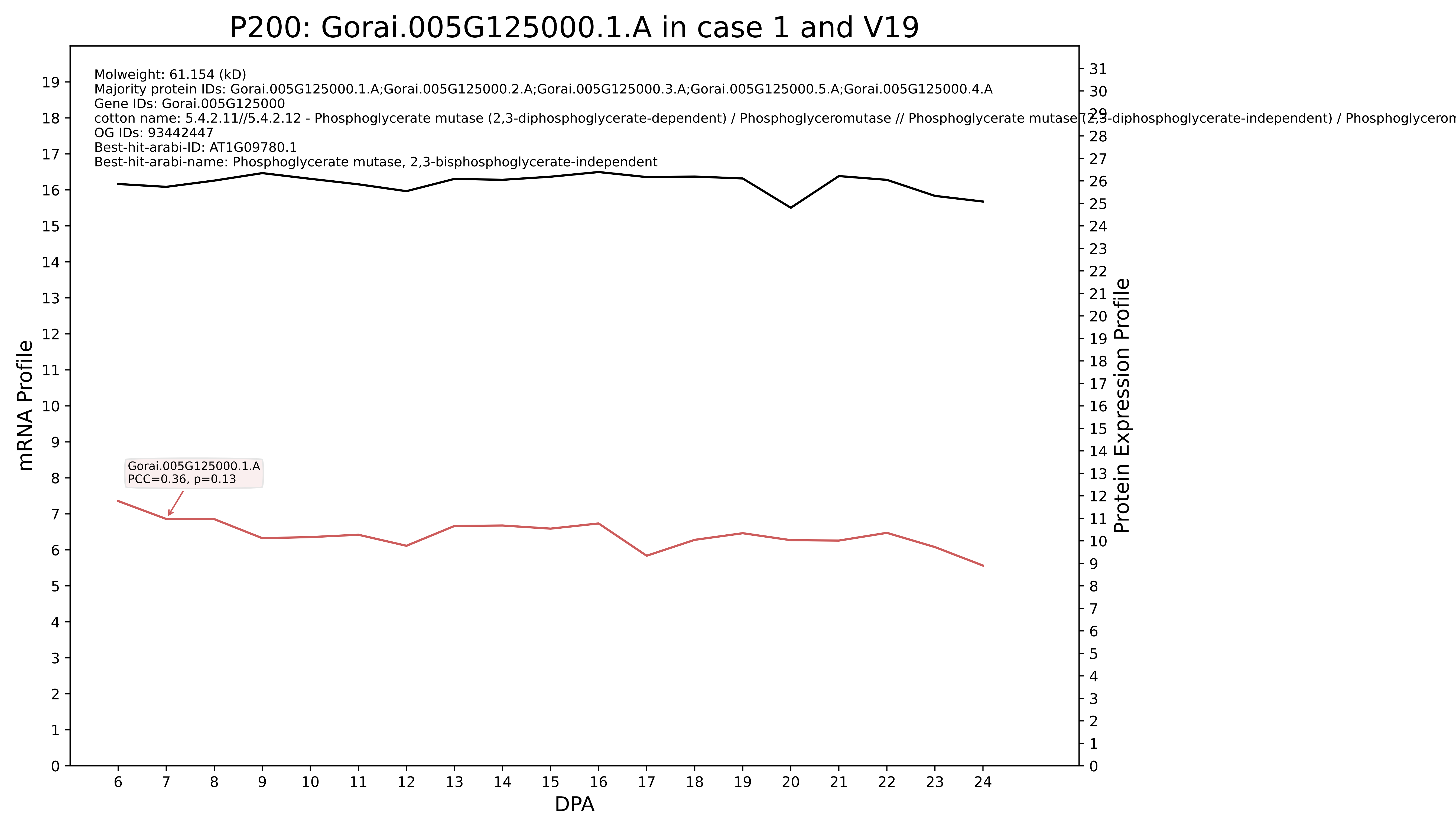

P200: Gorai.005G125000.1.A in case 1 and V19
Molweight: 61.154 (kD)
Majority protein IDs: Gorai.005G125000.1.A;Gorai.005G125000.2.A;Gorai.005G125000.3.A;Gorai.005G125000.5.A;Gorai.005G125000.4.A
Gene IDs: Gorai.005G125000
cotton name: 5.4.2.11//5.4.2.12 - Phosphoglycerate mutase (2,3-diphosphoglycerate-dependent) / Phosphoglyceromutase // Phosphoglycerate mutase (2,3-diphosphoglycerate-independent) / Phosphoglyceron
OG IDs: 93442447
Best-hit-arabi-ID: AT1G09780.1
Best-hit-arabi-name: Phosphoglycerate mutase, 2,3-bisphosphoglycerate-independent
Gorai.005G125000.1.A
PCC=0.36, p=0.13
mRNA Profile
Protein Expression Profile
DPA

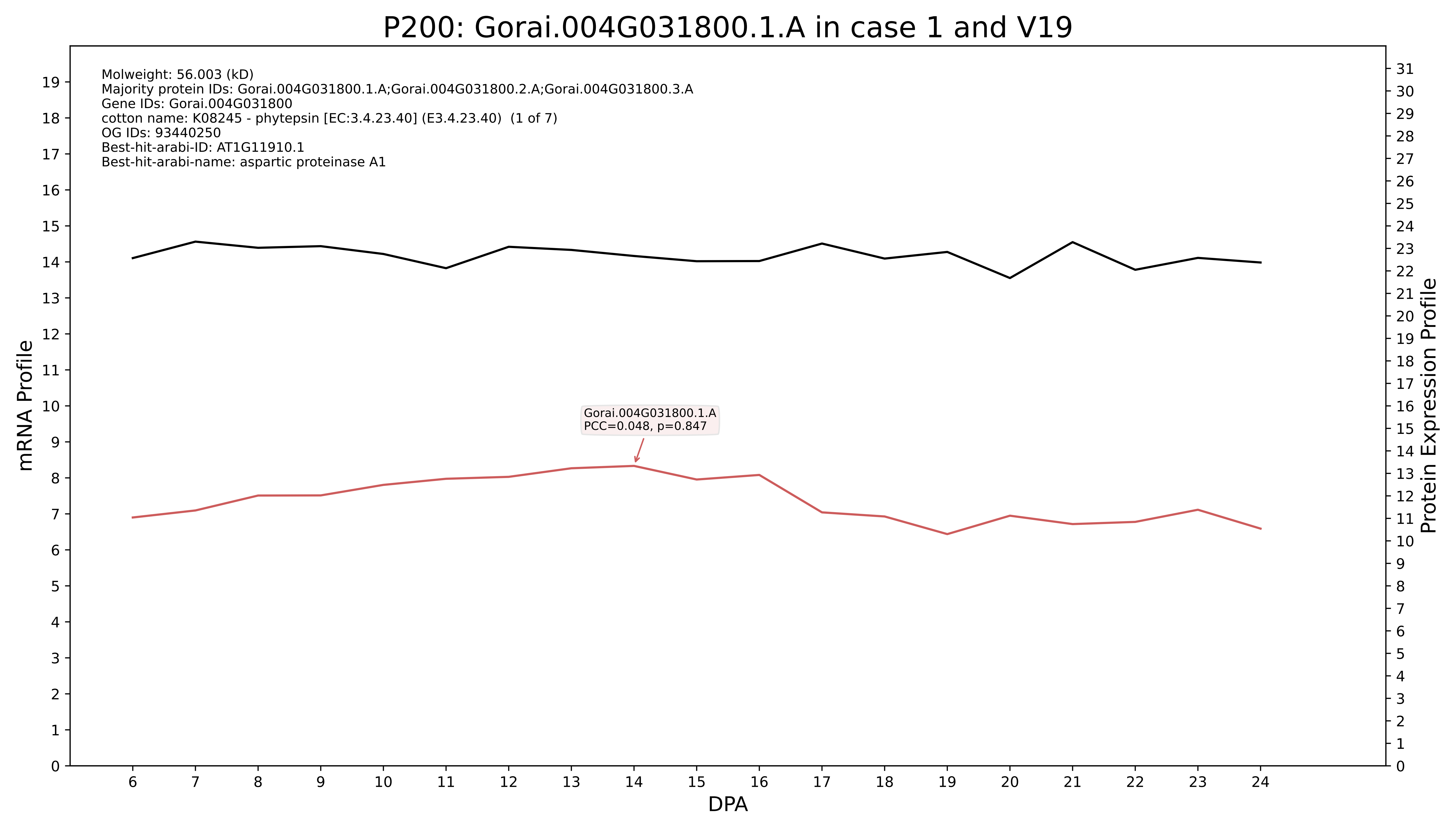
P200: Gorai.004G031800.1.A in case 1 and V19
Molweight: 56.003 (kD)
Majority protein IDs: Gorai.004G031800.1.A;Gorai.004G031800.2.A;Gorai.004G031800.3.A
Gene IDs: Gorai.004G031800
cotton name: K08245 - phytepsin [EC:3.4.23.40] (E3.4.23.40) (1 of 7)
OG IDs: 93440250
Best-hit-arabi-ID: AT1G11910.1
Best-hit-arabi-name: aspartic proteinase A1
Gorai.004G031800.1.A
PCC=0.048, p=0.847
mRNA Profile
Protein Expression Profile
DPA

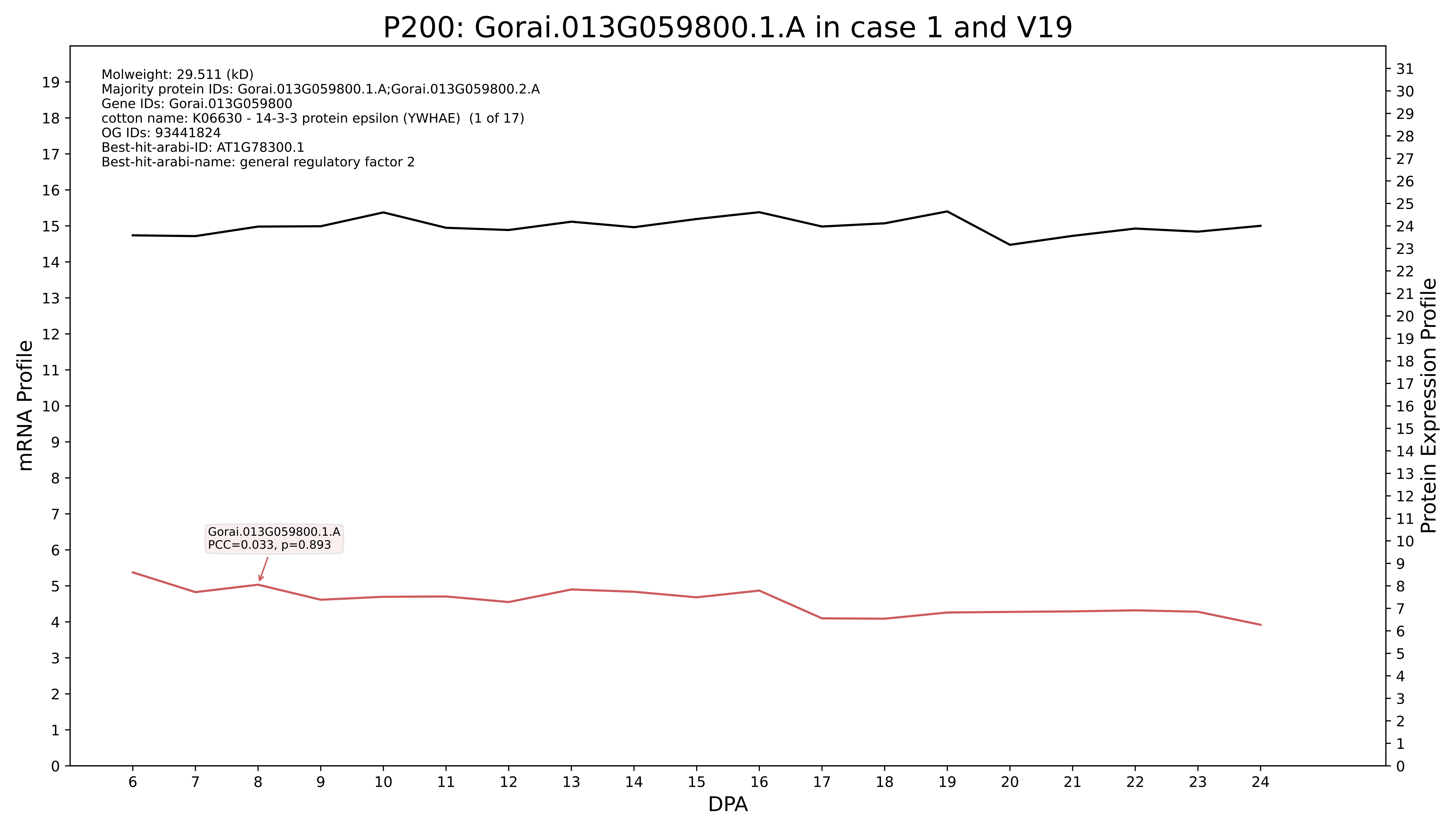
P200: Gorai.013G059800.1.A in case 1 and V19
Molweight: 29.511 (kD)
Majority protein IDs: Gorai.013G059800.1.A;Gorai.013G059800.2.A
Gene IDs: Gorai.013G059800
cotton name: K06630 - 14-3-3 protein epsilon (YWHAE) (1 of 17)
OG IDs: 93441824
Best-hit-arabi-ID: AT1G78300.1
Best-hit-arabi-name: general regulatory factor 2
Gorai.013G059800.1.A
PCC=0.033, p=0.893
mRNA Profile
Protein Expression Profile
DPA

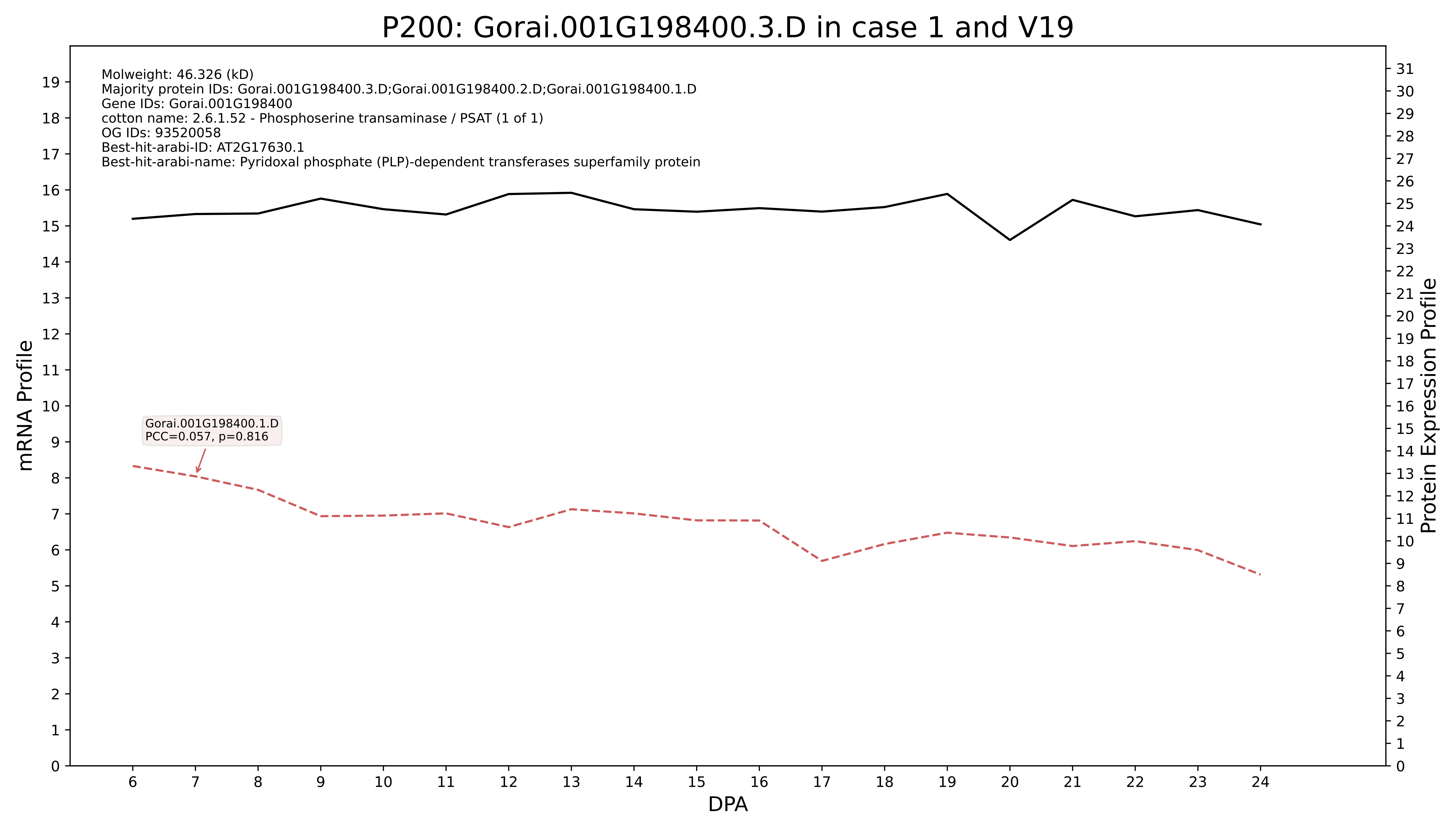
P200: Gorai.001G198400.3.D in case 1 and V19
Molweight: 46.326 (kD)
Majority protein IDs: Gorai.001G198400.3.D;Gorai.001G198400.2.D;Gorai.001G198400.1.D
Gene IDs: Gorai.001G198400
cotton name: 2.6.1.52 - Phosphoserine transaminase / PSAT (1 of 1)
OG IDs: 93520058
Best-hit-arabi-ID: AT2G17630.1
Best-hit-arabi-name: Pyridoxal phosphate (PLP)-dependent transferases superfamily protein
Gorai.001G198400.1.D
PCC=0.057, p=0.816
mRNA Profile
Protein Expression Profile
DPA

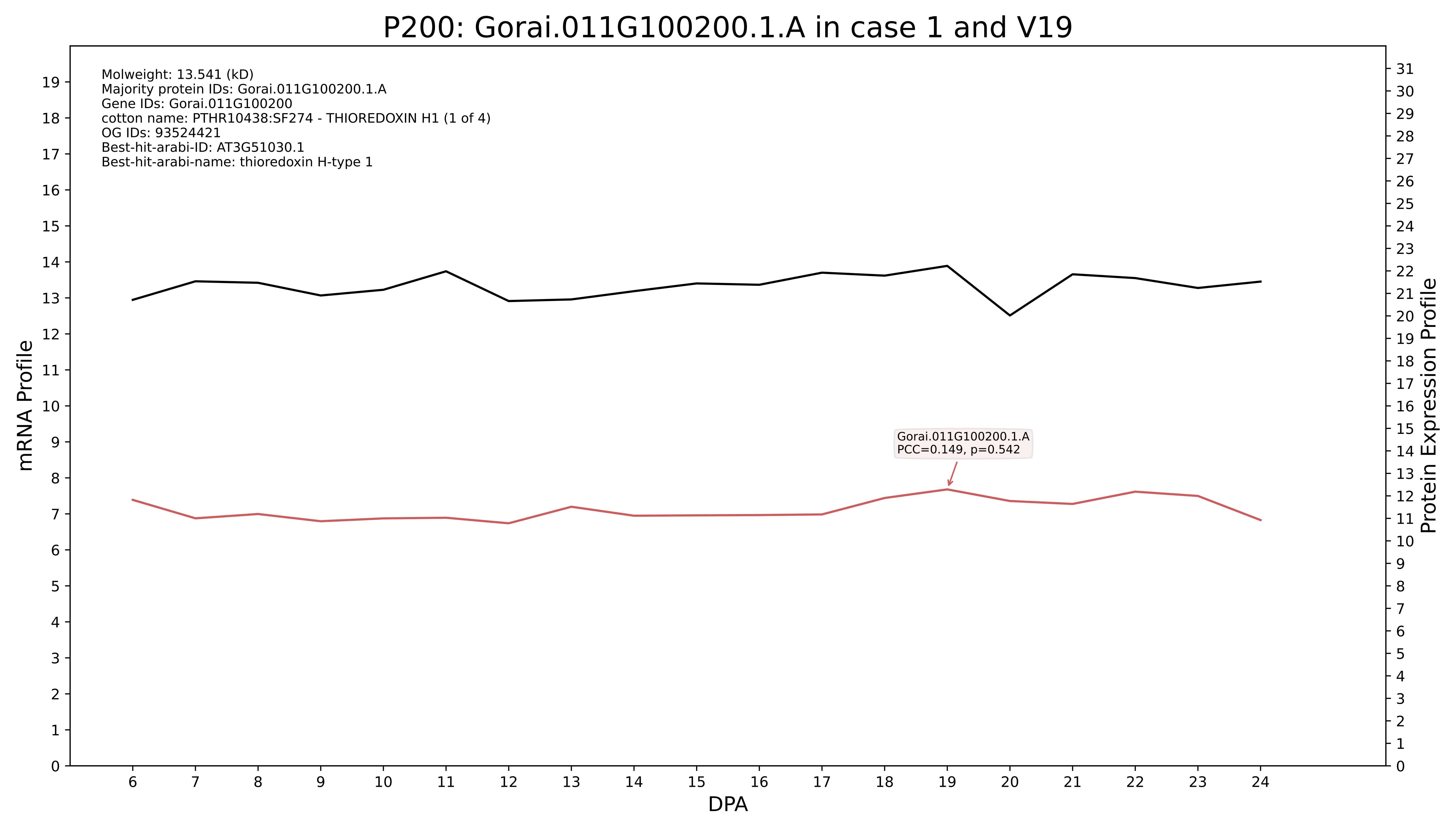

P200: Gorai.011G100200.1.A in case 1 and V19
Molweight: 13.541 (kD)
Majority protein IDs: Gorai.011G100200.1.A
Gene IDs: Gorai.011G100200
cotton name: PTHR10438:SF274 - THIOREDOXIN H1 (1 of 4)
OG IDs: 93524421
Best-hit-arabi-ID: AT3G51030.1
Best-hit-arabi-name: thioredoxin H-type 1
Gorai.011G100200.1.A
PCC=0.149, p=0.542
mRNA Profile
Protein Expression Profile
DPA

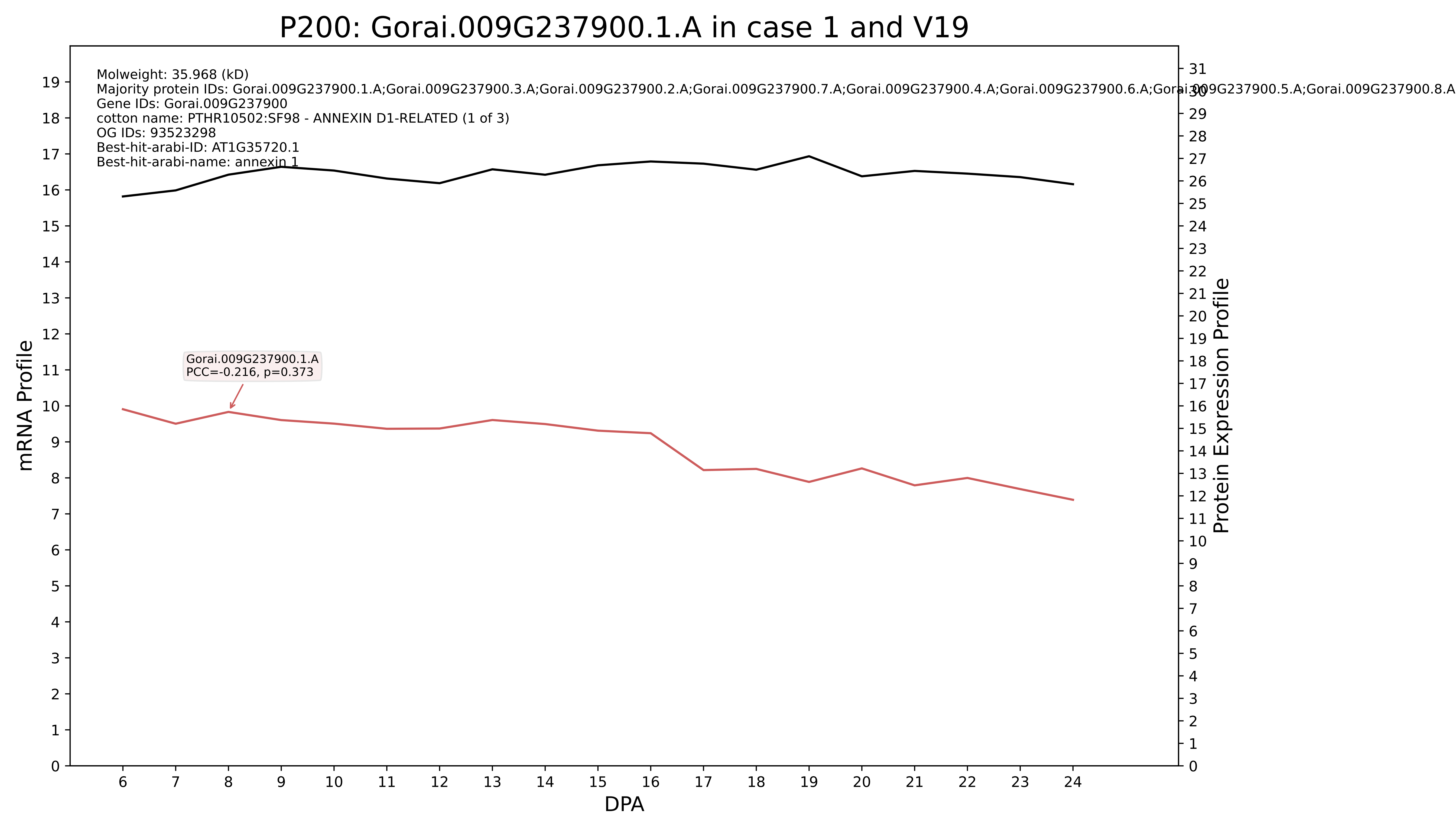

P200: Gorai.009G237900.1.A in case 1 and V19
Molweight: 35.968 (kD)
Majority protein IDs: Gorai.009G237900.1.A;Gorai.009G237900.3.A;Gorai.009G237900.2.A;Gorai.009G237900.7.A;Gorai.009G237900.4.A;Gorai.009G237900.6.A;Gorai.009G237900.5.A;Gorai.009G237900.8.A
Gene IDs: Gorai.009G237900
cotton name: PTHR10502:SF98 - ANNEXIN D1-RELATED (1 of 3)
OG IDs: 93523298
Best-hit-arabi-ID: AT1G35720.1
Best-hit-arabi-name: annexin 1
Gorai.009G237900.1.A
PCC=-0.216, p=0.373
mRNA Profile
Protein Expression Profile
DPA

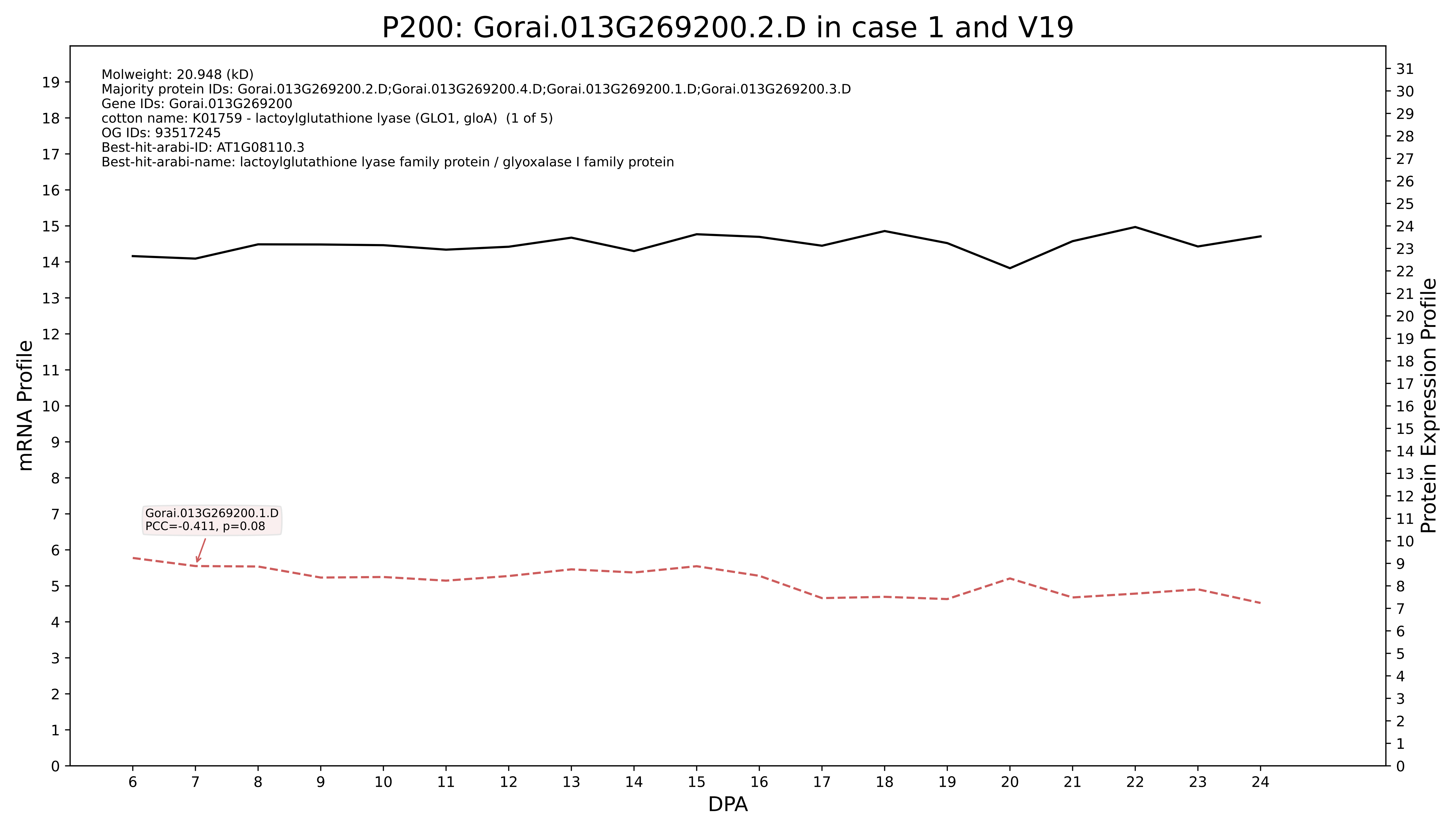

P200: Gorai.013G269200.2.D in case 1 and V19
Molweight: 20.948 (kD)
Majority protein IDs: Gorai.013G269200.2.D;Gorai.013G269200.4.D;Gorai.013G269200.1.D;Gorai.013G269200.3.D
Gene IDs: Gorai.013G269200
cotton name: K01759 - lactoylglutathione lyase (GLO1, gloA) (1 of 5)
OG IDs: 93517245
Best-hit-arabi-ID: AT1G08110.3
Best-hit-arabi-name: lactoylglutathione lyase family protein / glyoxalase I family protein
Gorai.013G269200.1.D
PCC=-0.411, p=0.08
mRNA Profile
Protein Expression Profile
DPA

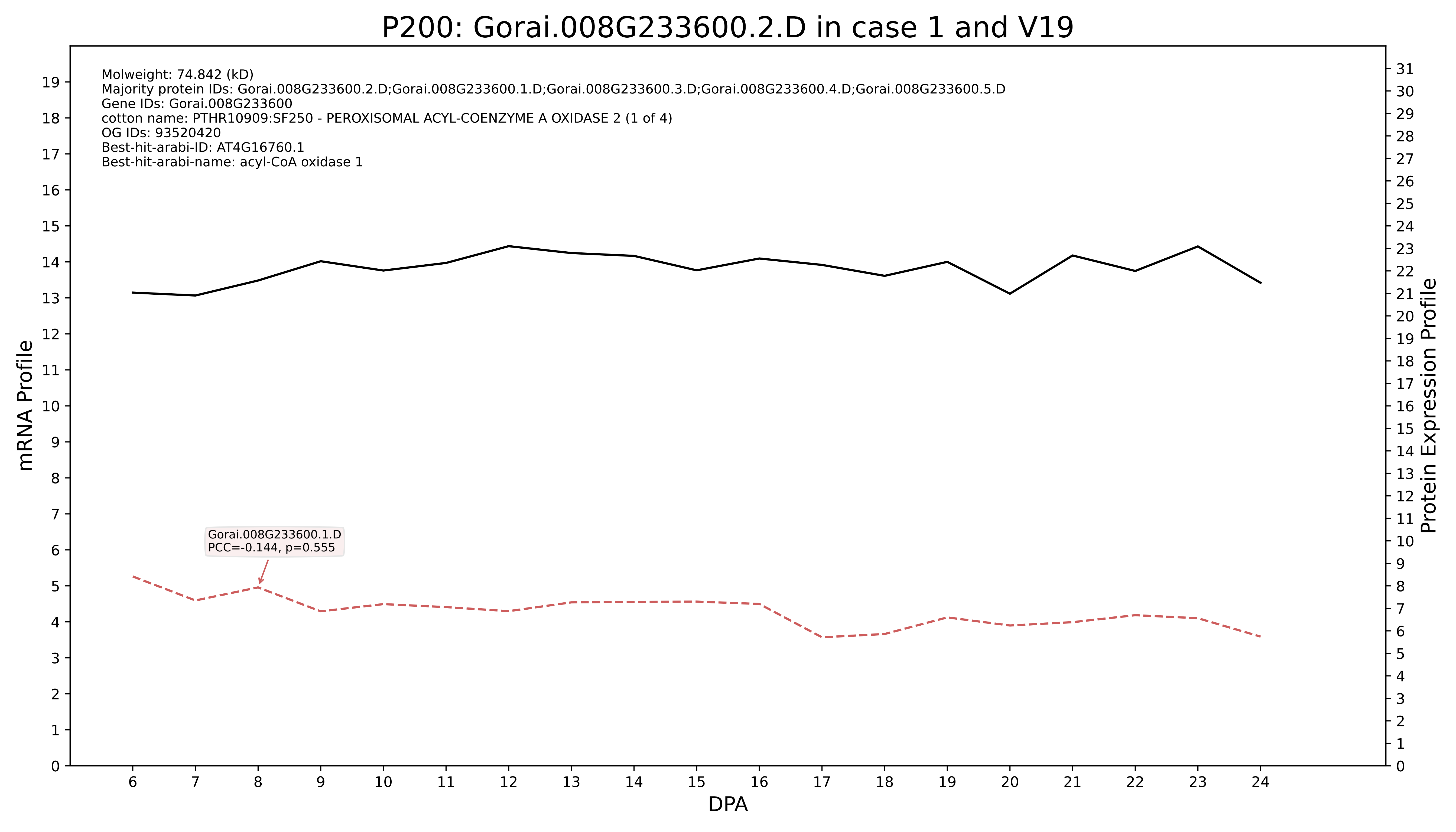
P200: Gorai.008G233600.2.D in case 1 and V19
Molweight: 74.842 (kD)
Majority protein IDs: Gorai.008G233600.2.D;Gorai.008G233600.1.D;Gorai.008G233600.3.D;Gorai.008G233600.4.D;Gorai.008G233600.5.D
Gene IDs: Gorai.008G233600
cotton name: PTHR10909:SF250 - PEROXISOMAL ACYL-COENZYME A OXIDASE 2 (1 of 4)
OG IDs: 93520420
Best-hit-arabi-ID: AT4G16760.1
Best-hit-arabi-name: acyl-CoA oxidase 1
Gorai.008G233600.1.D
PCC=-0.144, p=0.555
mRNA Profile
Protein Expression Profile
DPA

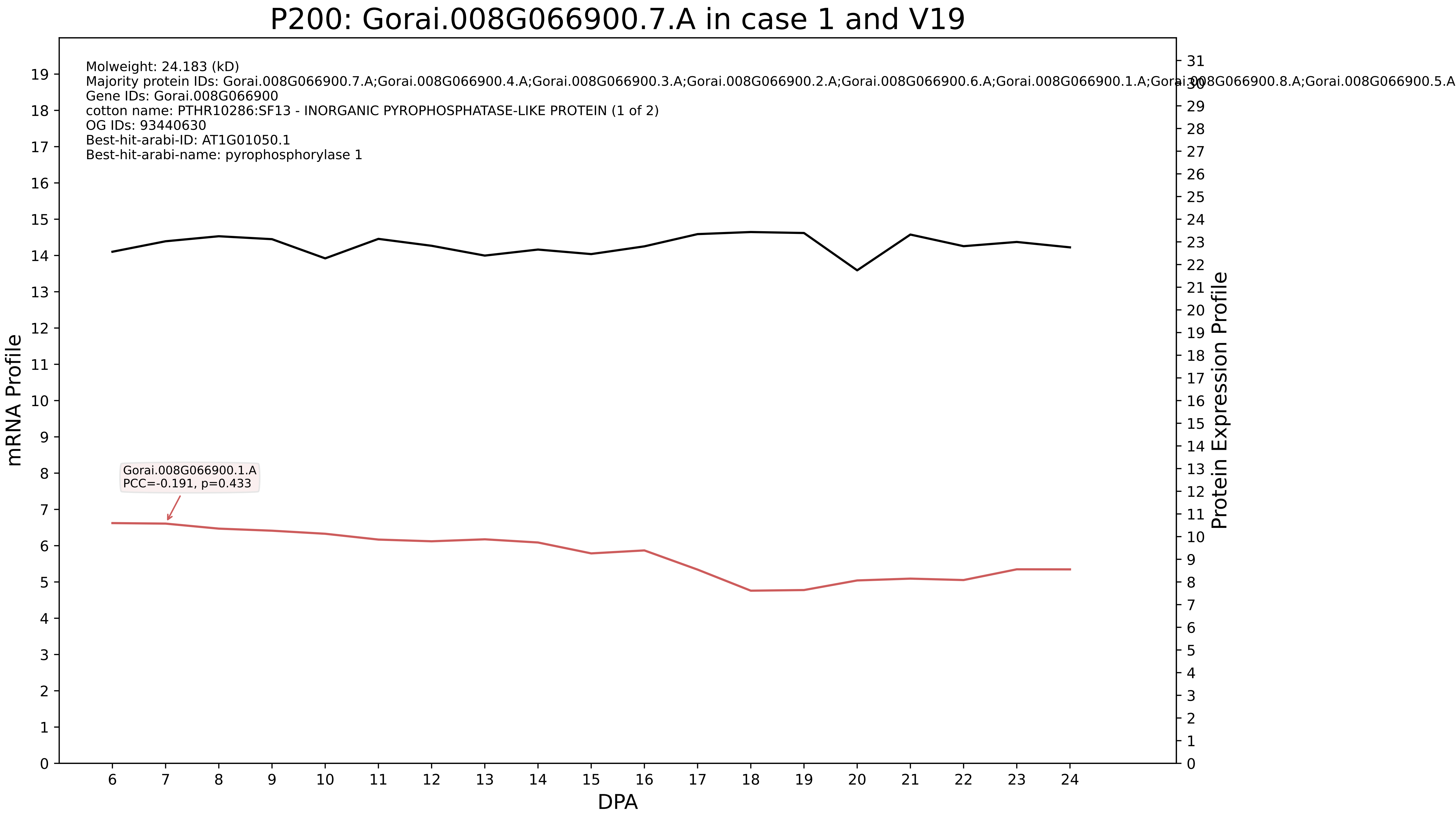
P200: Gorai.008G066900.7.A in case 1 and V19
Molweight: 24.183 (kD)
Majority protein IDs: Gorai.008G066900.7.A;Gorai.008G066900.4.A;Gorai.008G066900.3.A;Gorai.008G066900.2.A;Gorai.008G066900.6.A;Gorai.008G066900.1.A;Gorai.008G066900.8.A;Gorai.008G066900.5.A
Gene IDs: Gorai.008G066900
cotton name: PTHR10286:SF13 - INORGANIC PYROPHOSPHATASE-LIKE PROTEIN (1 of 2)
OG IDs: 93440630
Best-hit-arabi-ID: AT1G01050.1
Best-hit-arabi-name: pyrophosphorylase 1
Gorai.008G066900.1.A
PCC=-0.191, p=0.433
mRNA Profile
Protein Expression Profile
DPA
0
1
2
3
4
5
6
7
8
9
10
11
12
13
14
15
16
17
18
19
20
21
22
23
24
25
26
27
28
29
30
31

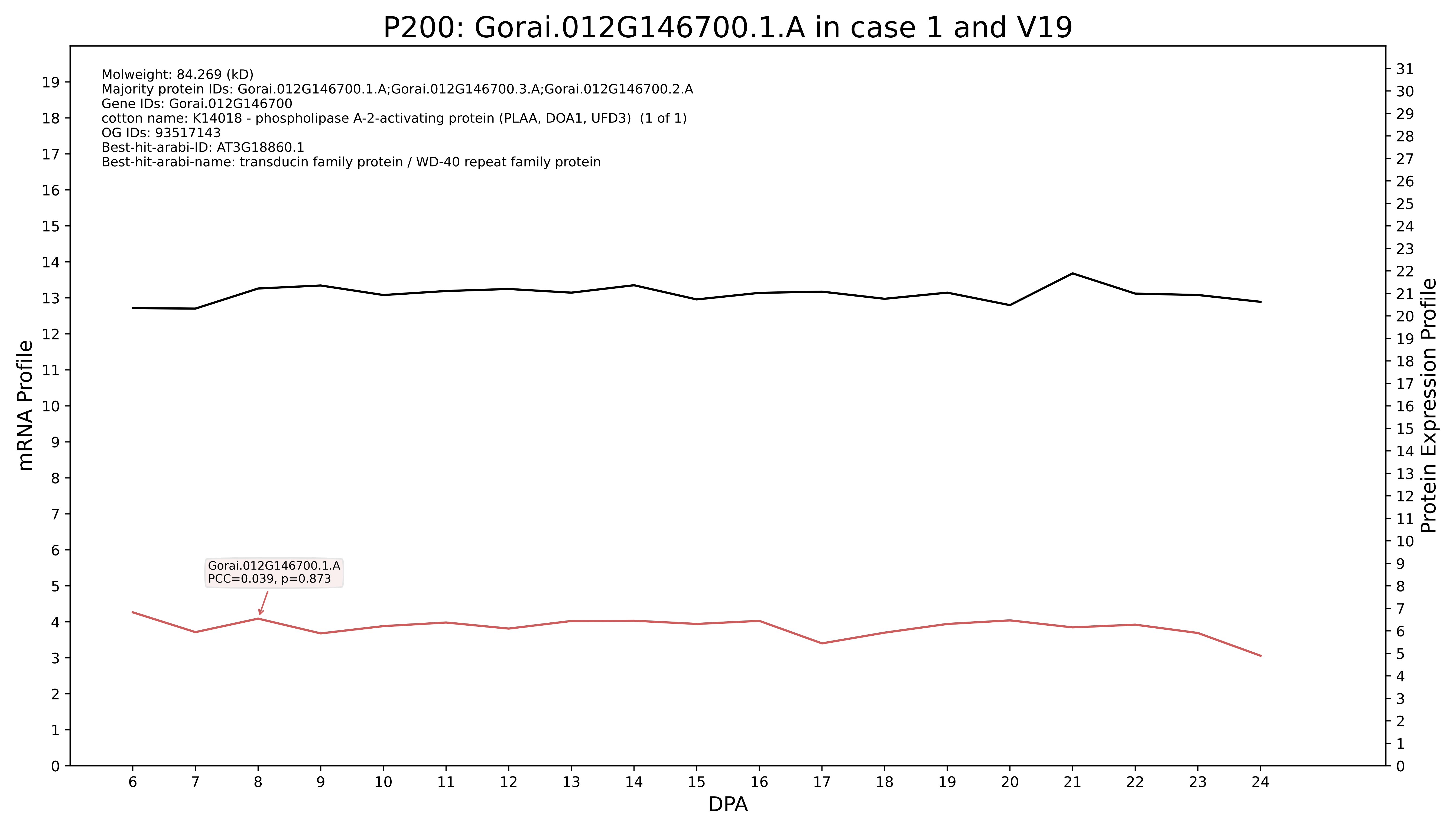
P200: Gorai.012G146700.1.A in case 1 and V19
Molweight: 84.269 (kD)
Majority protein IDs: Gorai.012G146700.1.A;Gorai.012G146700.3.A;Gorai.012G146700.2.A
Gene IDs: Gorai.012G146700
cotton name: K14018 - phospholipase A-2-activating protein (PLAA, DOA1, UFD3) (1 of 1)
OG IDs: 93517143
Best-hit-arabi-ID: AT3G18860.1
Best-hit-arabi-name: transducin family protein / WD-40 repeat family protein
Gorai.012G146700.1.A
PCC=0.039, p=0.873
mRNA Profile
Protein Expression Profile
DPA
0
1
2
3
4
5
6
7
8
9
10
11
12
13
14
15
16
17
18
19
20
21
22
23
24
25
26
27
28
29
30
31

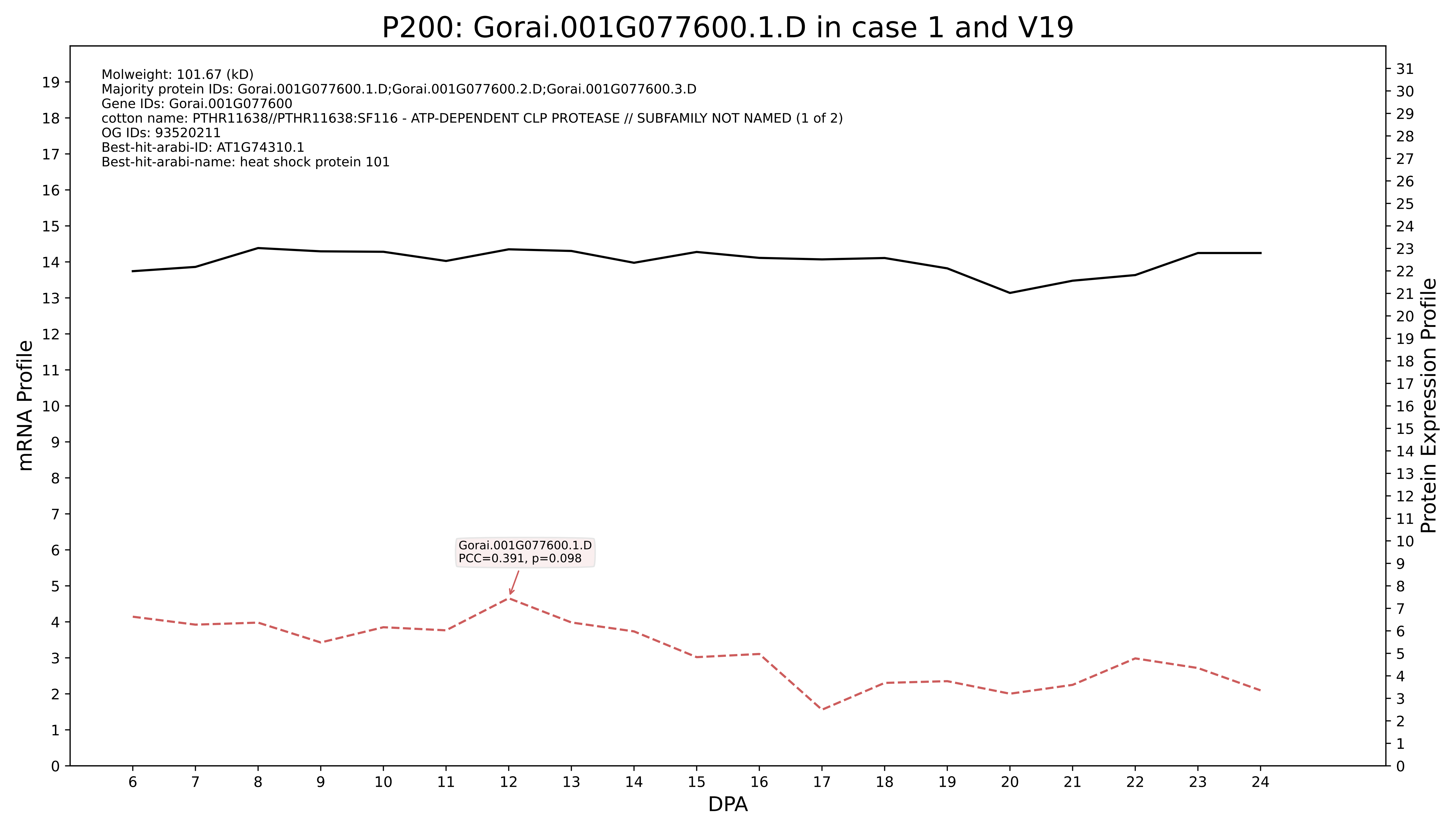

P200: Gorai.001G077600.1.D in case 1 and V19
Molweight: 101.67 (kD)
Majority protein IDs: Gorai.001G077600.1.D;Gorai.001G077600.2.D;Gorai.001G077600.3.D
Gene IDs: Gorai.001G077600
cotton name: PTHR11638//PTHR11638:SF116 - ATP-DEPENDENT CLP PROTEASE // SUBFAMILY NOT NAMED (1 of 2)
OG IDs: 93520211
Best-hit-arabi-ID: AT1G74310.1
Best-hit-arabi-name: heat shock protein 101
Gorai.001G077600.1.D
PCC=0.391, p=0.098
mRNA Profile
Protein Expression Profile
DPA

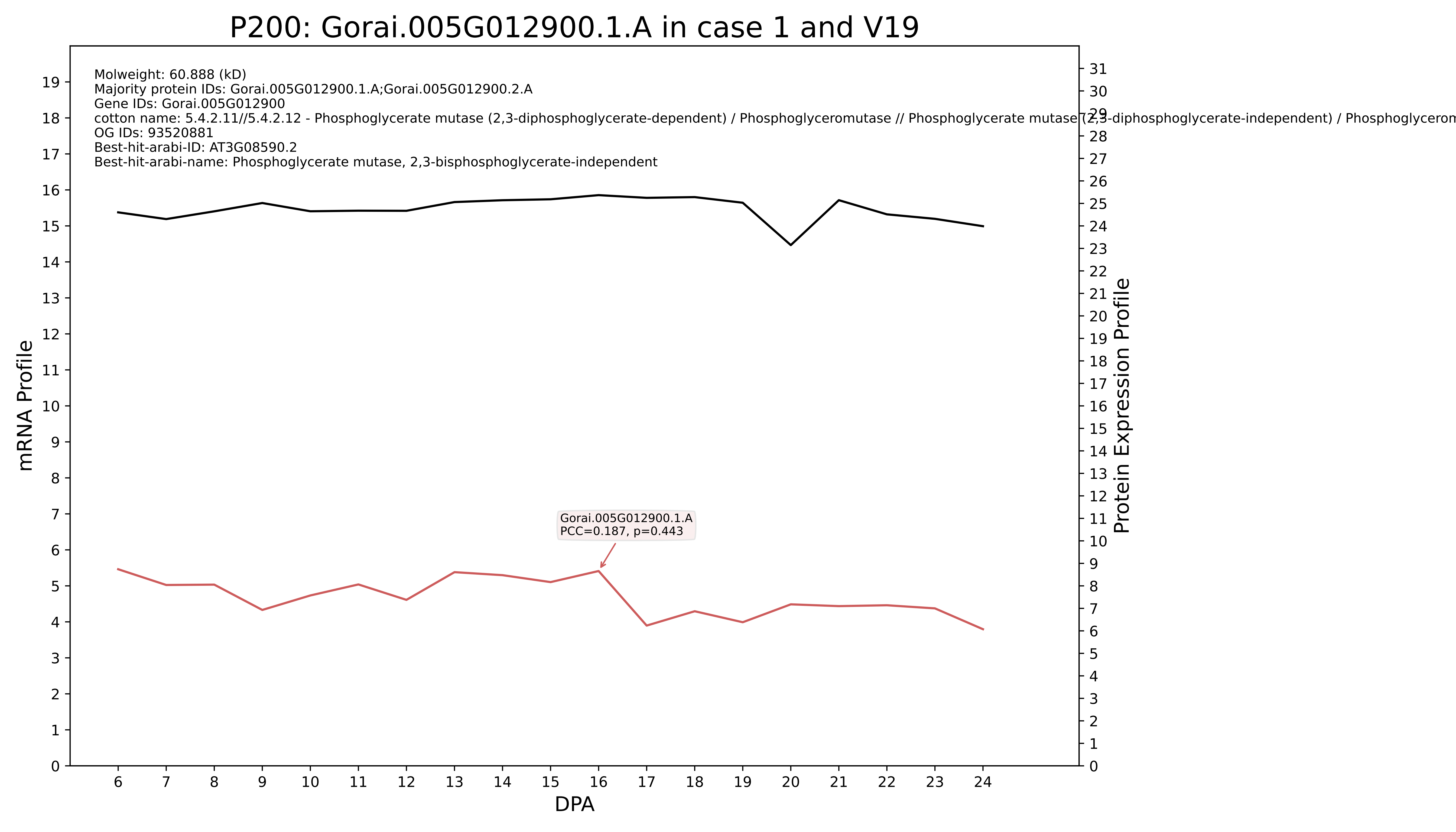

P200: Gorai.005G012900.1.A in case 1 and V19
Molweight: 60.888 (kD)
Majority protein IDs: Gorai.005G012900.1.A;Gorai.005G012900.2.A
Gene IDs: Gorai.005G012900
cotton name: 5.4.2.11//5.4.2.12 - Phosphoglycerate mutase (2,3-diphosphoglycerate-dependent) / Phosphoglyceromutase // Phosphoglycerate mutase (2,3-diphosphoglycerate-independent) / Phosphoglyceron
OG IDs: 93520881
Best-hit-arabi-ID: AT3G08590.2
Best-hit-arabi-name: Phosphoglycerate mutase, 2,3-bisphosphoglycerate-independent
Gorai.005G012900.1.A
PCC=0.187, p=0.443
mRNA Profile
Protein Expression Profile
DPA

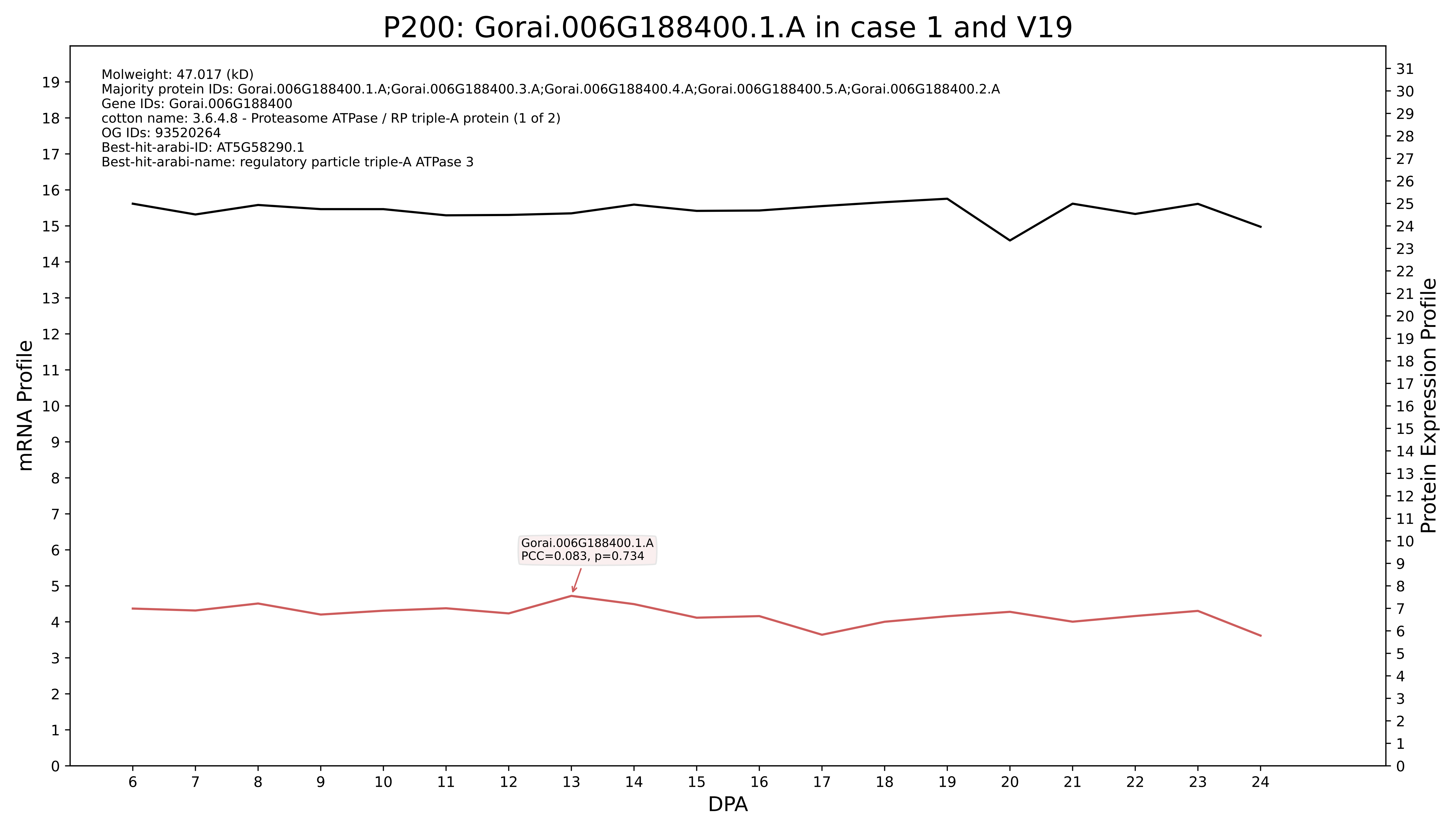
P200: Gorai.006G188400.1.A in case 1 and V19
Molweight: 47.017 (kD)
Majority protein IDs: Gorai.006G188400.1.A;Gorai.006G188400.3.A;Gorai.006G188400.4.A;Gorai.006G188400.5.A;Gorai.006G188400.2.A
Gene IDs: Gorai.006G188400
cotton name: 3.6.4.8 - Proteasome ATPase / RP triple-A protein (1 of 2)
OG IDs: 93520264
Best-hit-arabi-ID: AT5G58290.1
Best-hit-arabi-name: regulatory particle triple-A ATPase 3
Gorai.006G188400.1.A
PCC=0.083, p=0.734
mRNA Profile
Protein Expression Profile
DPA

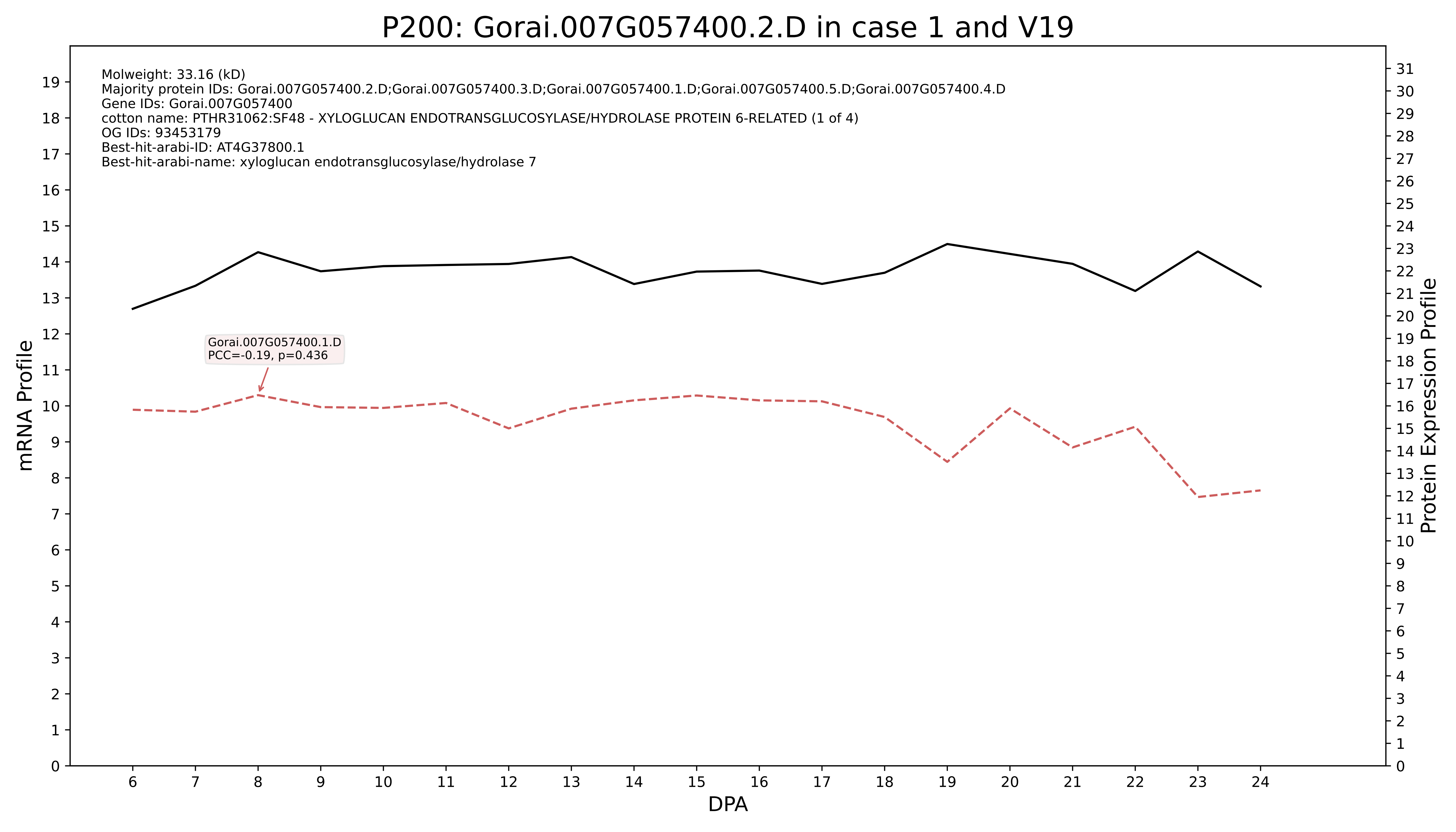

P200: Gorai.007G057400.2.D in case 1 and V19
Molweight: 33.16 (kD)
Majority protein IDs: Gorai.007G057400.2.D;Gorai.007G057400.3.D;Gorai.007G057400.1.D;Gorai.007G057400.5.D;Gorai.007G057400.4.D
Gene IDs: Gorai.007G057400
cotton name: PTHR31062:SF48 - XYLOGLUCAN ENDOTRANSGLUCOSYLASE/HYDROLASE PROTEIN 6-RELATED (1 of 4)
OG IDs: 93453179
Best-hit-arabi-ID: AT4G37800.1
Best-hit-arabi-name: xyloglucan endotransglucosylase/hydrolase 7
Gorai.007G057400.1.D
PCC=-0.19, p=0.436
mRNA Profile
Protein Expression Profile
DPA

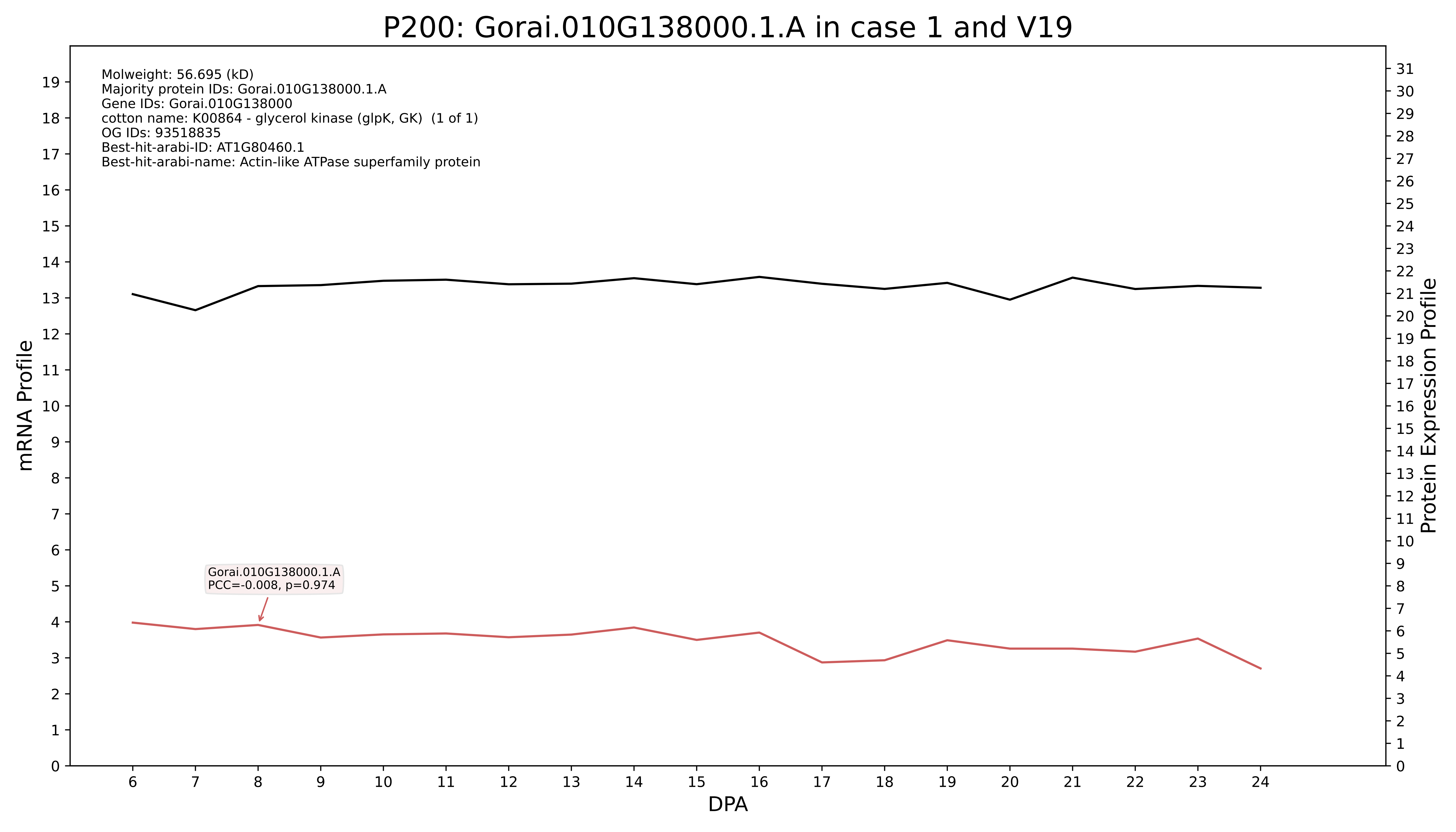
P200: Gorai.010G138000.1.A in case 1 and V19
Molweight: 56.695 (kD)
Majority protein IDs: Gorai.010G138000.1.A
Gene IDs: Gorai.010G138000
cotton name: K00864 - glycerol kinase (glpK, GK) (1 of 1)
OG IDs: 93518835
Best-hit-arabi-ID: AT1G80460.1
Best-hit-arabi-name: Actin-like ATPase superfamily protein
Gorai.010G138000.1.A
PCC=-0.008, p=0.974
mRNA Profile
Protein Expression Profile
DPA

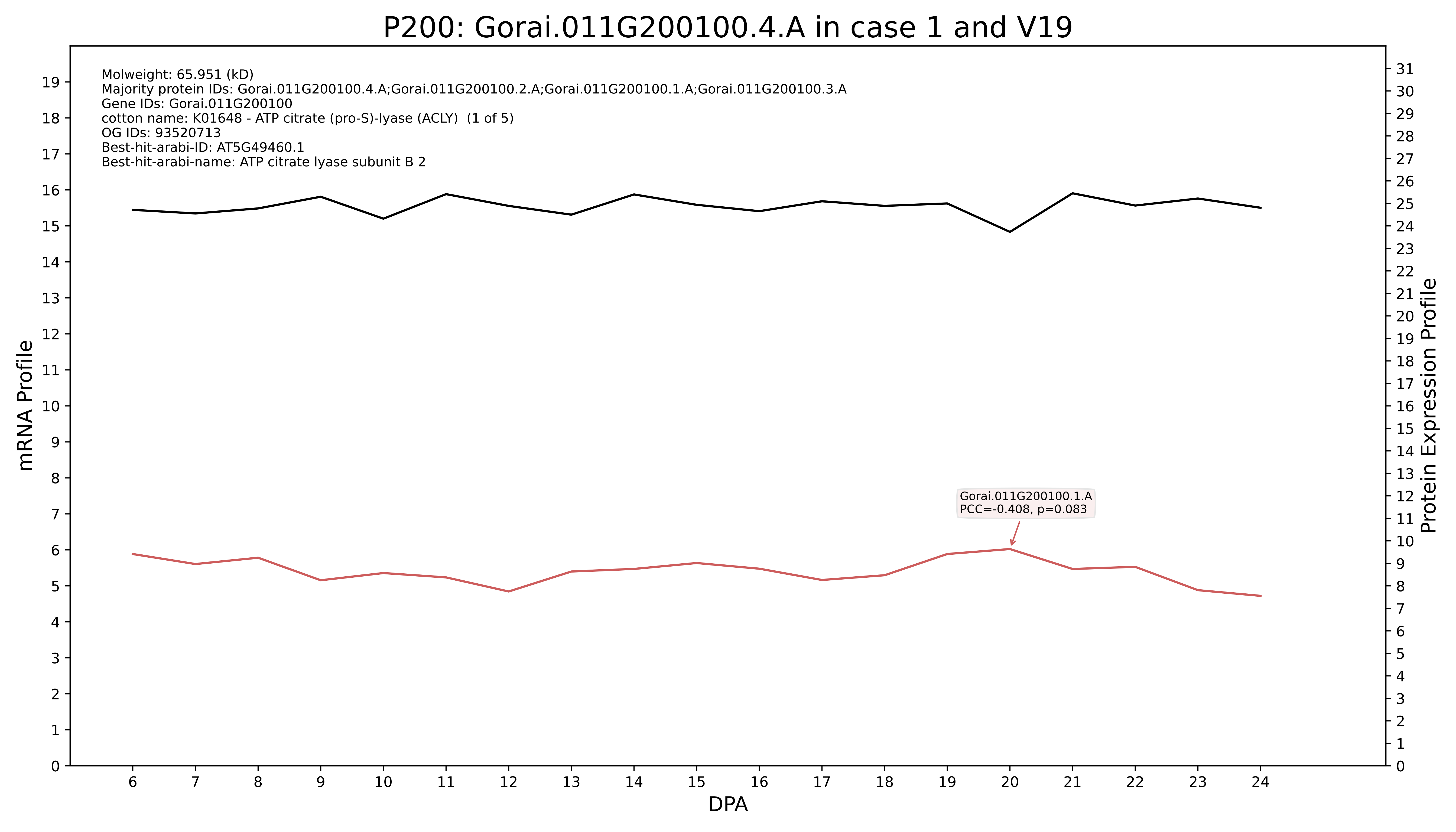
P200: Gorai.011G200100.4.A in case 1 and V19
Molweight: 65.951 (kD)
Majority protein IDs: Gorai.011G200100.4.A;Gorai.011G200100.2.A;Gorai.011G200100.1.A;Gorai.011G200100.3.A
Gene IDs: Gorai.011G200100
cotton name: K01648 - ATP citrate (pro-S)-lyase (ACLY) (1 of 5)
OG IDs: 93520713
Best-hit-arabi-ID: AT5G49460.1
Best-hit-arabi-name: ATP citrate lyase subunit B 2
Gorai.011G200100.1.A
PCC=-0.408, p=0.083
mRNA Profile
Protein Expression Profile
DPA

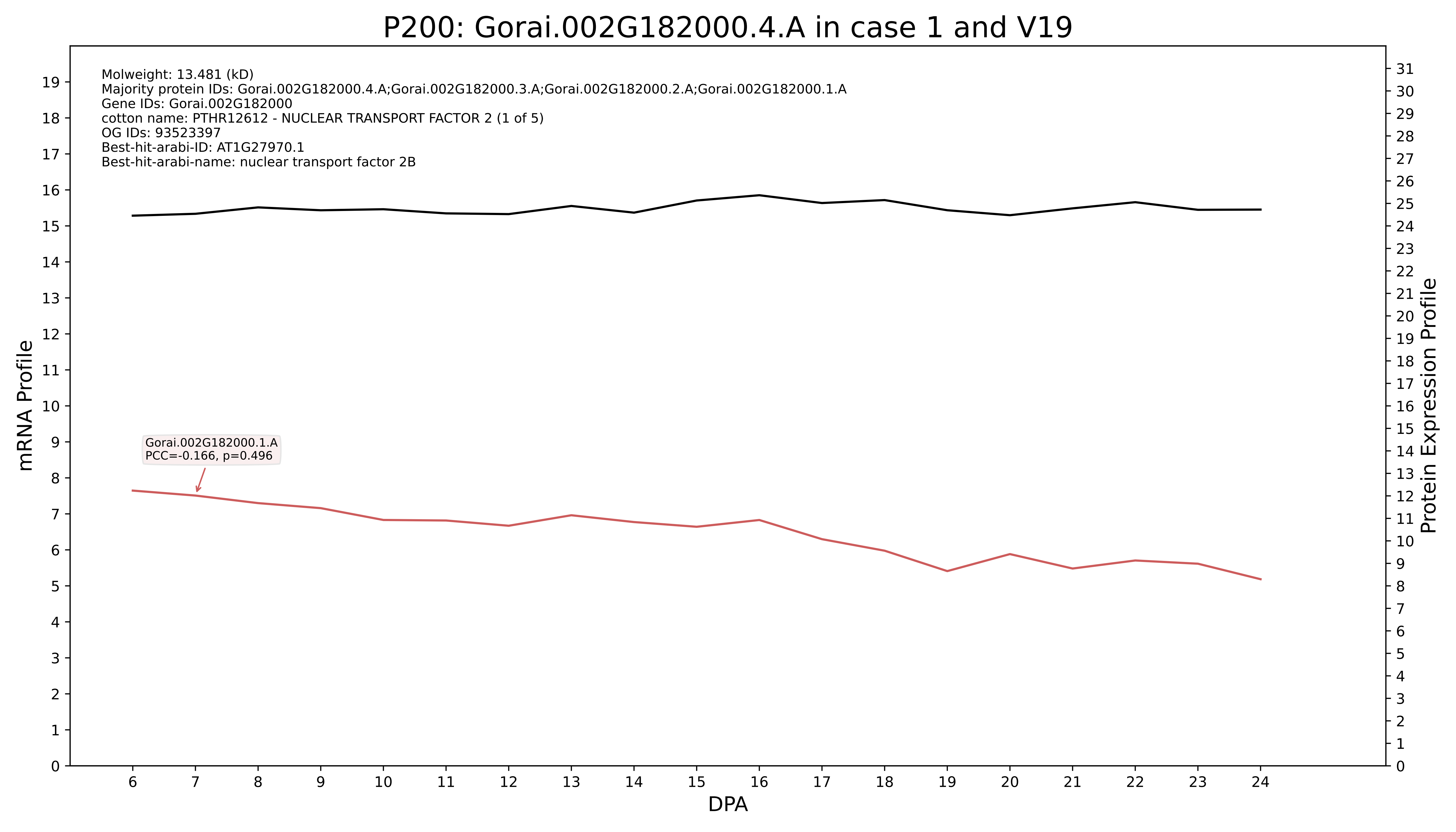
P200: Gorai.002G182000.4.A in case 1 and V19
Molweight: 13.481 (kD)
Majority protein IDs: Gorai.002G182000.4.A;Gorai.002G182000.3.A;Gorai.002G182000.2.A;Gorai.002G182000.1.A
Gene IDs: Gorai.002G182000
cotton name: PTHR12612 - NUCLEAR TRANSPORT FACTOR 2 (1 of 5)
OG IDs: 93523397
Best-hit-arabi-ID: AT1G27970.1
Best-hit-arabi-name: nuclear transport factor 2B
Gorai.002G182000.1.A
PCC=-0.166, p=0.496
mRNA Profile
Protein Expression Profile
DPA

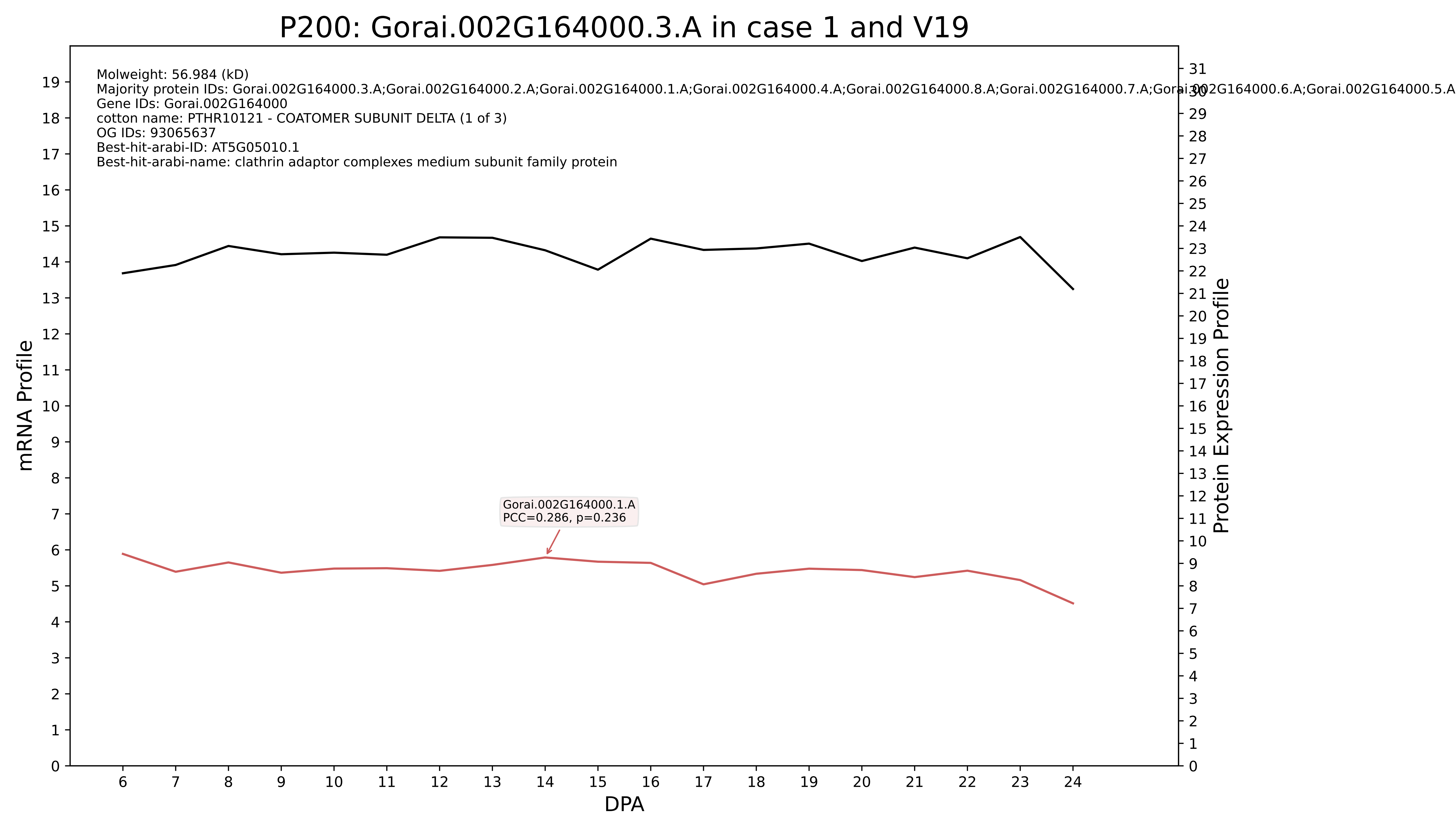
P200: Gorai.002G164000.3.A in case 1 and V19
Molweight: 56.984 (kD)
Majority protein IDs: Gorai.002G164000.3.A;Gorai.002G164000.2.A;Gorai.002G164000.1.A;Gorai.002G164000.4.A;Gorai.002G164000.8.A;Gorai.002G164000.7.A;Gorai.002G164000.6.A;Gorai.002G164000.5.A
Gene IDs: Gorai.002G164000
cotton name: PTHR10121 - COATOMER SUBUNIT DELTA (1 of 3)
OG IDs: 93065637
Best-hit-arabi-ID: AT5G05010.1
Best-hit-arabi-name: clathrin adaptor complexes medium subunit family protein
Gorai.002G164000.1.A
PCC=0.286, p=0.236
mRNA Profile
Protein Expression Profile
DPA

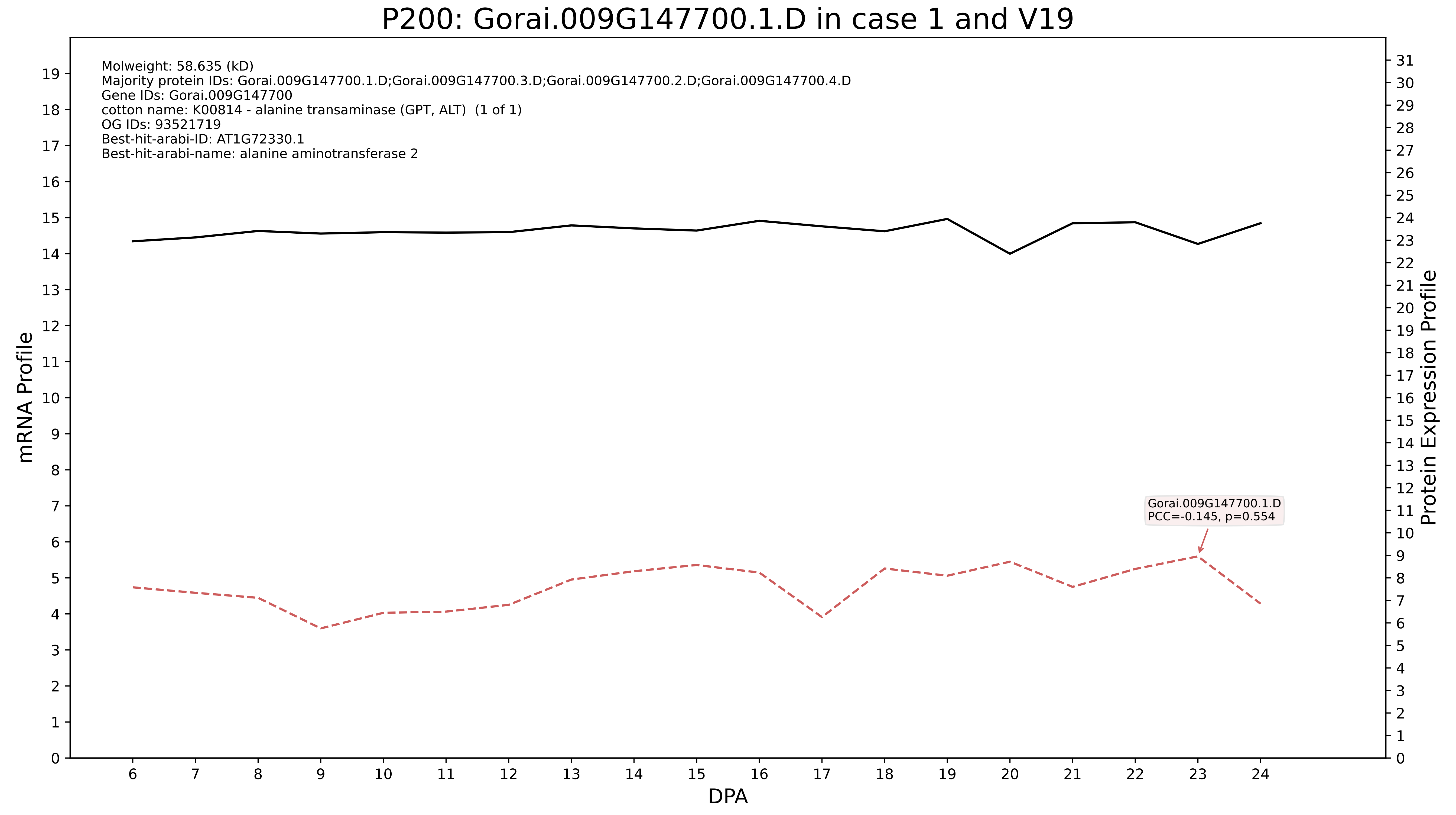
P200: Gorai.009G147700.1.D in case 1 and V19
Molweight: 58.635 (kD)
Majority protein IDs: Gorai.009G147700.1.D;Gorai.009G147700.3.D;Gorai.009G147700.2.D;Gorai.009G147700.4.D
Gene IDs: Gorai.009G147700
cotton name: K00814 - alanine transaminase (GPT, ALT) (1 of 1)
OG IDs: 93521719
Best-hit-arabi-ID: AT1G72330.1
Best-hit-arabi-name: alanine aminotransferase 2
Gorai.009G147700.1.D
PCC=-0.145, p=0.554
mRNA Profile
Protein Expression Profile
DPA

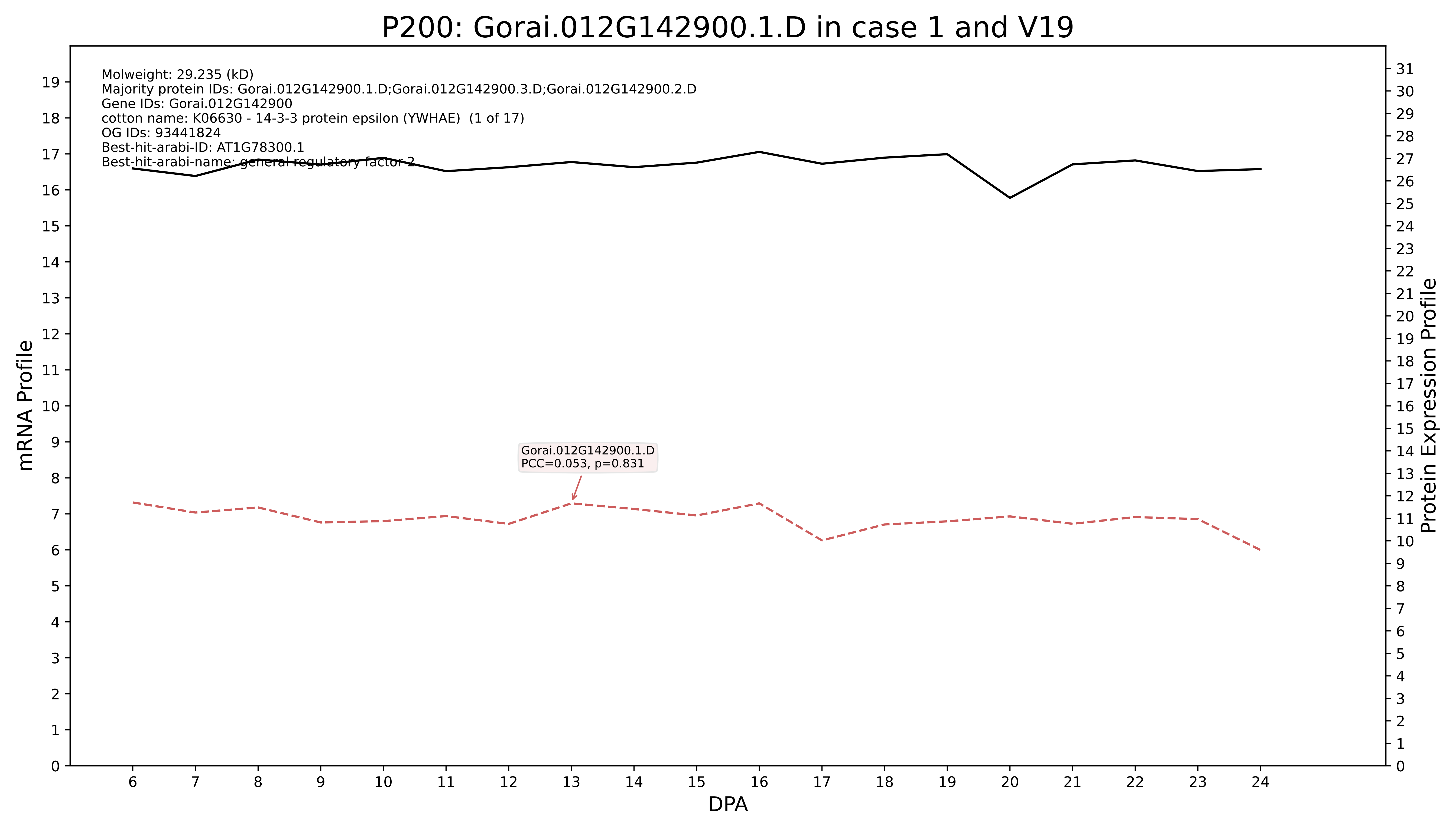
P200: Gorai.012G142900.1.D in case 1 and V19
Molweight: 29.235 (kD)
Majority protein IDs: Gorai.012G142900.1.D;Gorai.012G142900.3.D;Gorai.012G142900.2.D
Gene IDs: Gorai.012G142900
cotton name: K06630 - 14-3-3 protein epsilon (YWHAE) (1 of 17)
OG IDs: 93441824
Best-hit-arabi-ID: AT1G78300.1
Best-hit-arabi-name: general regulatory factor 2
Gorai.012G142900.1.D
PCC=0.053, p=0.831
mRNA Profile
Protein Expression Profile
DPA

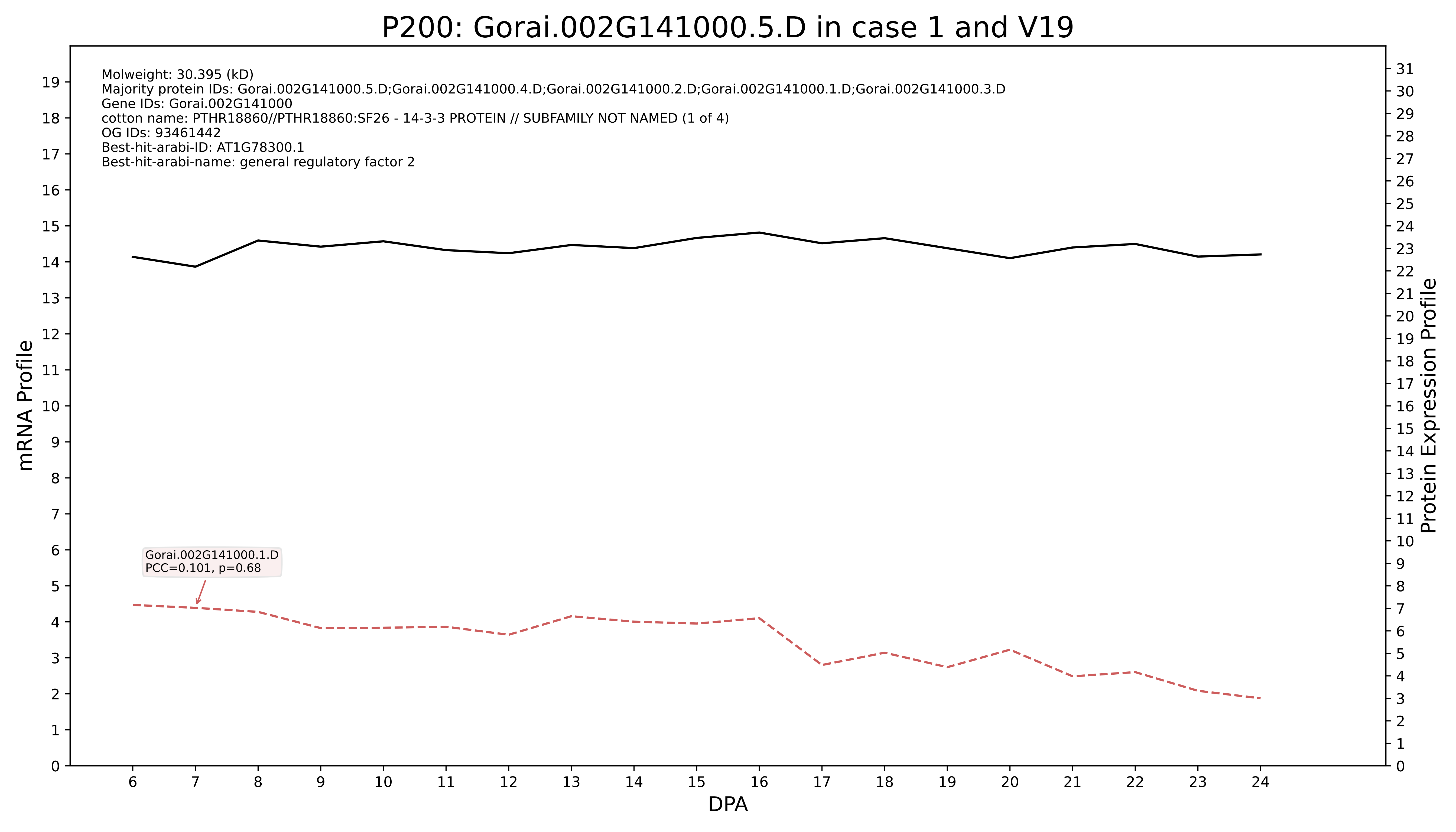
P200: Gorai.002G141000.5.D in case 1 and V19
Molweight: 30.395 (kD)
Majority protein IDs: Gorai.002G141000.5.D;Gorai.002G141000.4.D;Gorai.002G141000.2.D;Gorai.002G141000.1.D;Gorai.002G141000.3.D
Gene IDs: Gorai.002G141000
cotton name: PTHR18860//PTHR18860:SF26 - 14-3-3 PROTEIN // SUBFAMILY NOT NAMED (1 of 4)
OG IDs: 93461442
Best-hit-arabi-ID: AT1G78300.1
Best-hit-arabi-name: general regulatory factor 2
Gorai.002G141000.1.D
PCC=0.101, p=0.68
mRNA Profile
Protein Expression Profile
DPA

P200: Gorai.012G038600.2.D in case 1 and V19

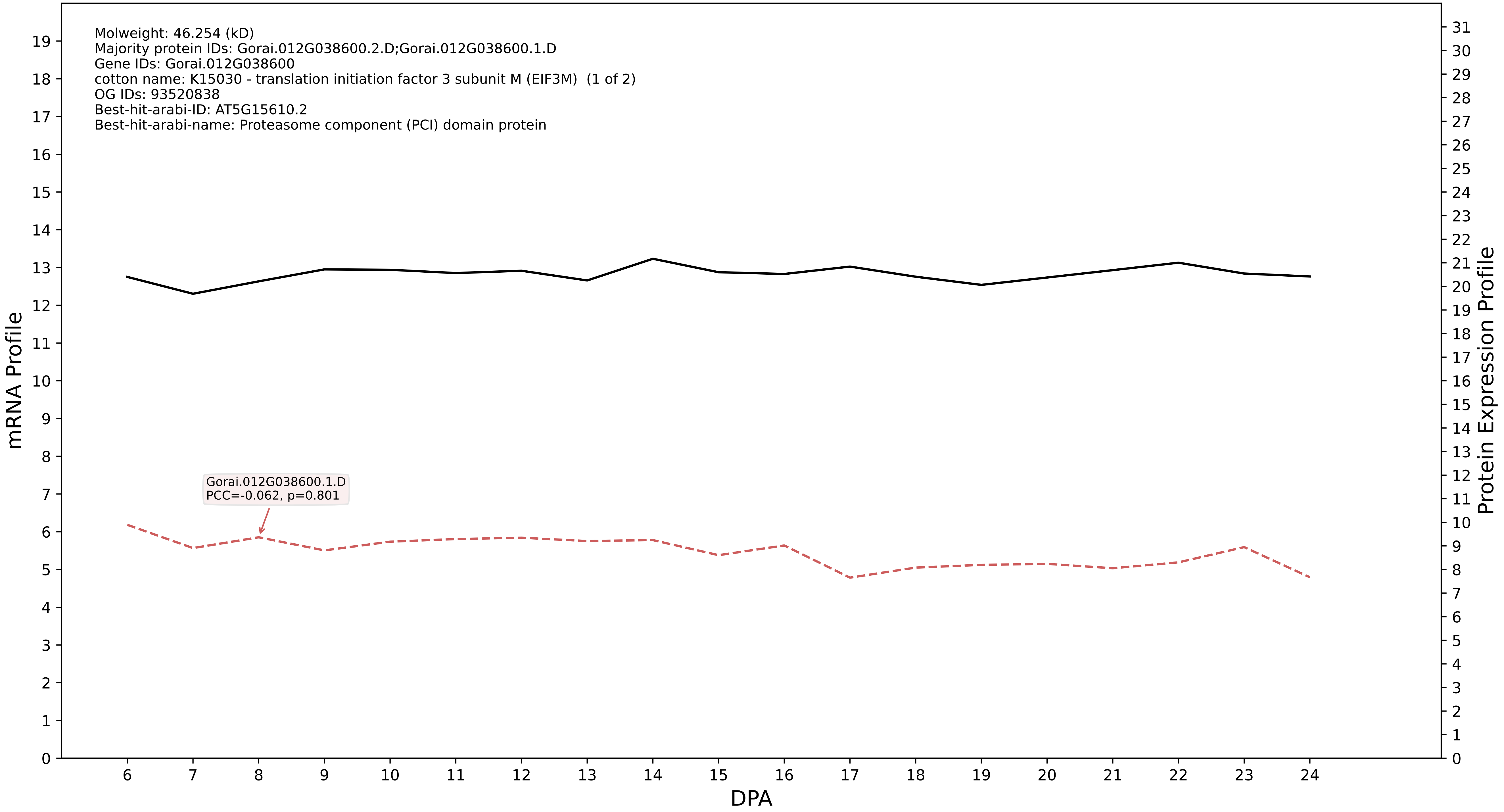

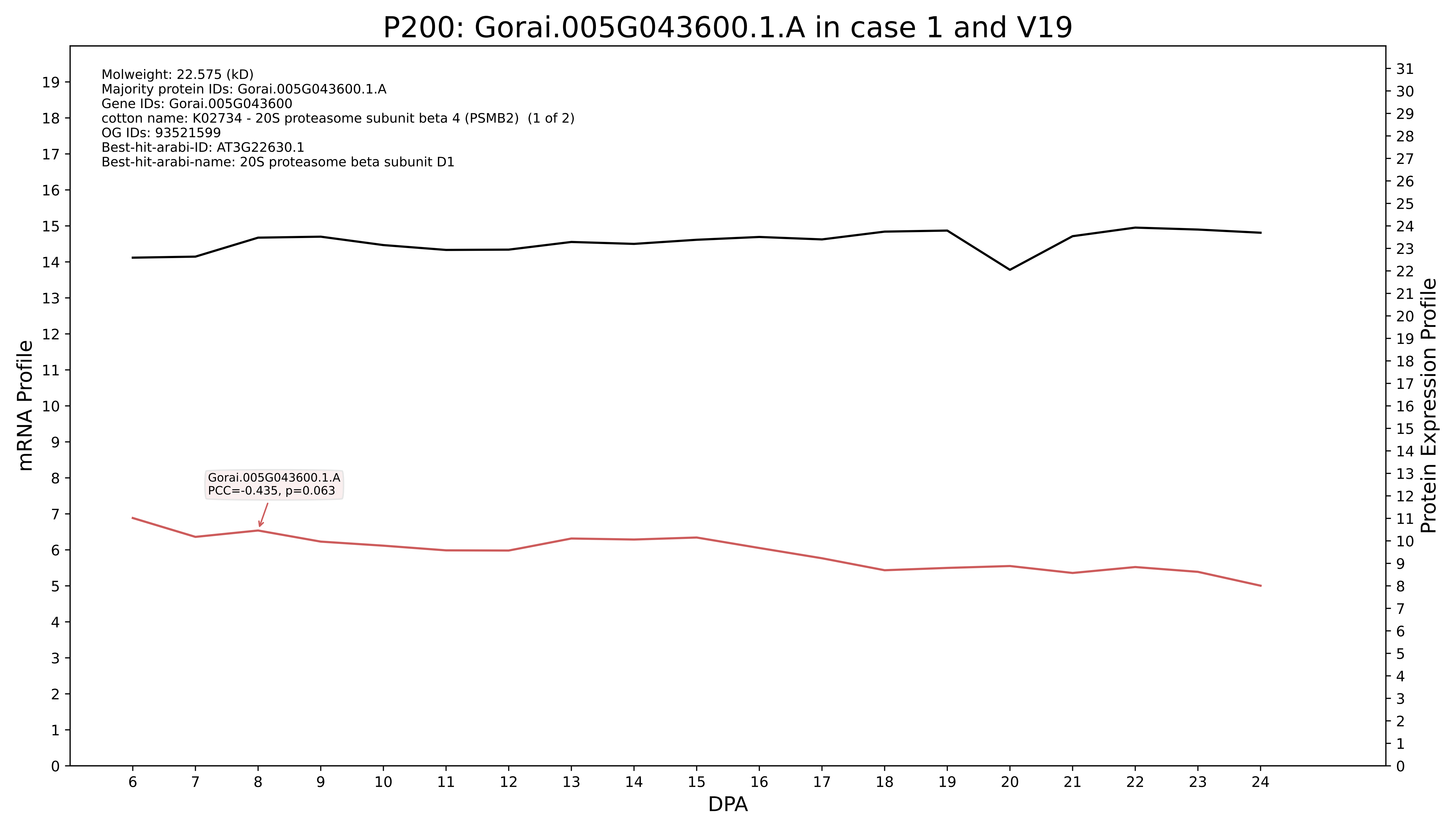
P200: Gorai.005G043600.1.A in case 1 and V19
Molweight: 22.575 (kD)
Majority protein IDs: Gorai.005G043600.1.A
Gene IDs: Gorai.005G043600
cotton name: K02734 - 20S proteasome subunit beta 4 (PSMB2) (1 of 2)
OG IDs: 93521599
Best-hit-arabi-ID: AT3G22630.1
Best-hit-arabi-name: 20S proteasome beta subunit D1
Gorai.005G043600.1.A
PCC=-0.435, p=0.063
mRNA Profile
Protein Expression Profile
DPA
0
1
2
3
4
5
6
7
8
9
10
11
12
13
14
15
16
17
18
19
20
21
22
23
24
25
26
27
28
29
30
31

P200: Gorai.004G048300.1.A in case 1 and V19

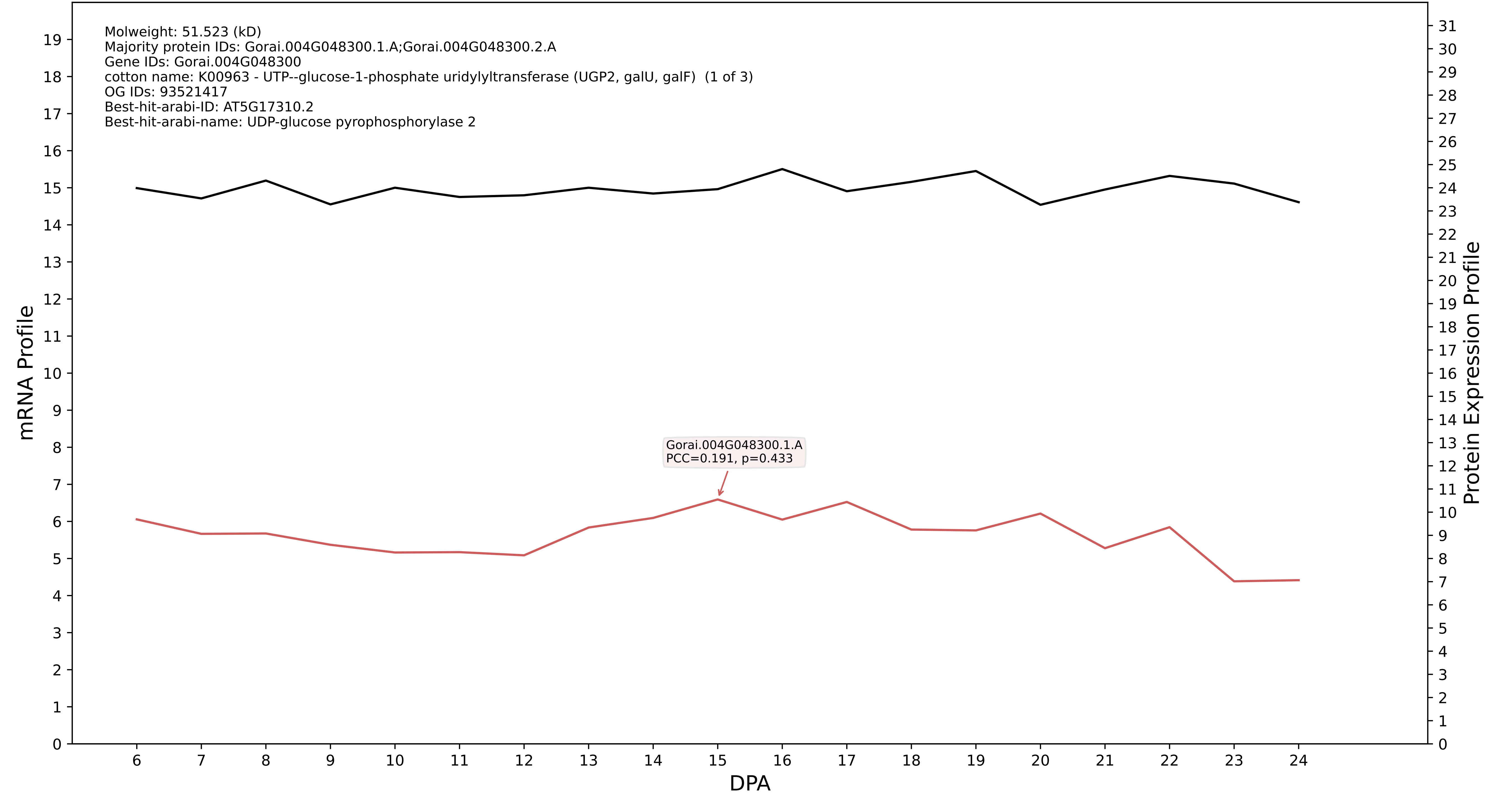

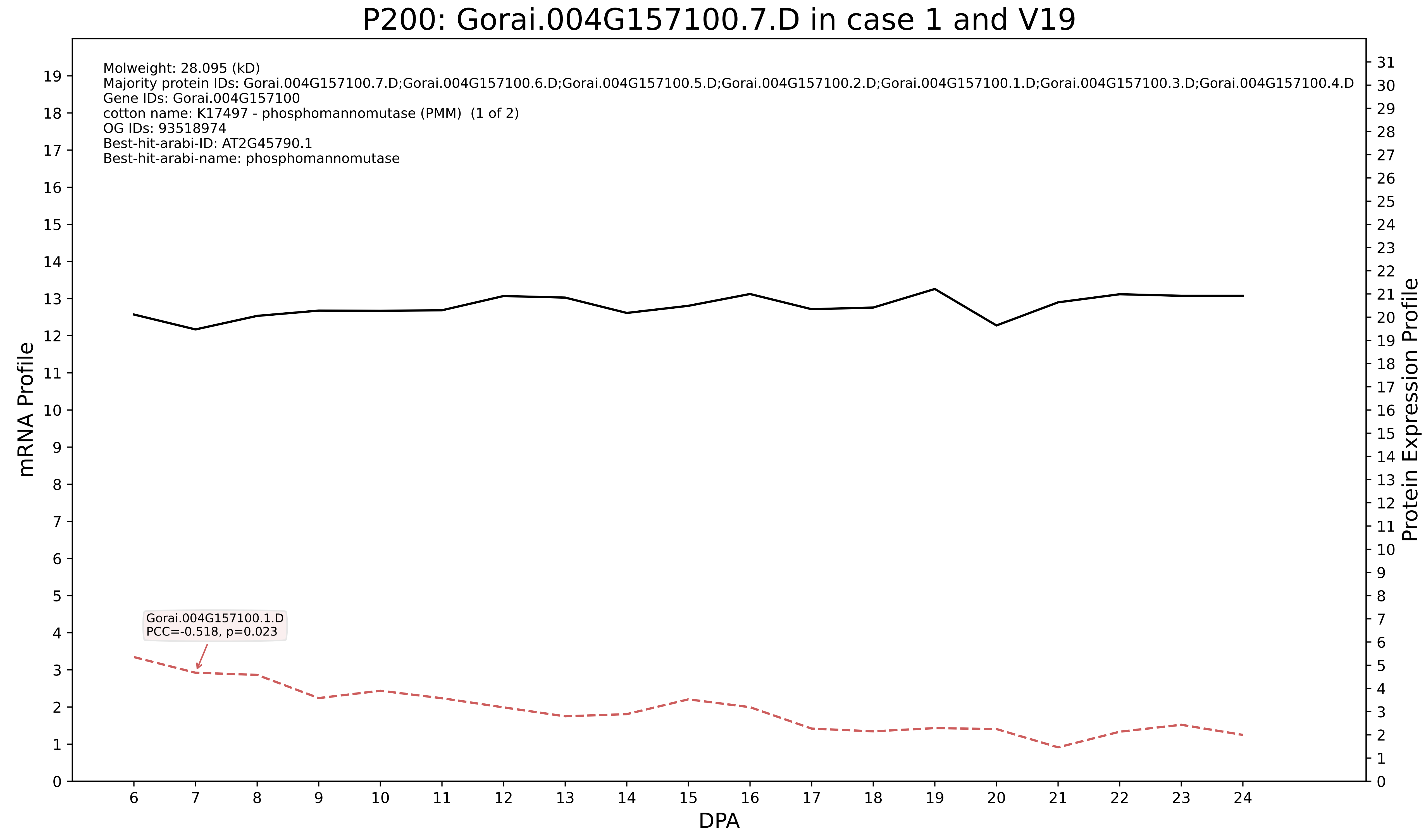

P200: Gorai.004G157100.7.D in case 1 and V19
Molweight: 28.095 (kD)
Majority protein IDs: Gorai.004G157100.7.D;Gorai.004G157100.6.D;Gorai.004G157100.5.D;Gorai.004G157100.2.D;Gorai.004G157100.1.D;Gorai.004G157100.3.D;Gorai.004G157100.4.D
Gene IDs: Gorai.004G157100
cotton name: K17497 - phosphomannomutase (PMM) (1 of 2)
OG IDs: 93518974
Best-hit-arabi-ID: AT2G45790.1
Best-hit-arabi-name: phosphomannomutase
Gorai.004G157100.1.D
PCC=-0.518, p=0.023
mRNA Profile
Protein Expression Profile
DPA

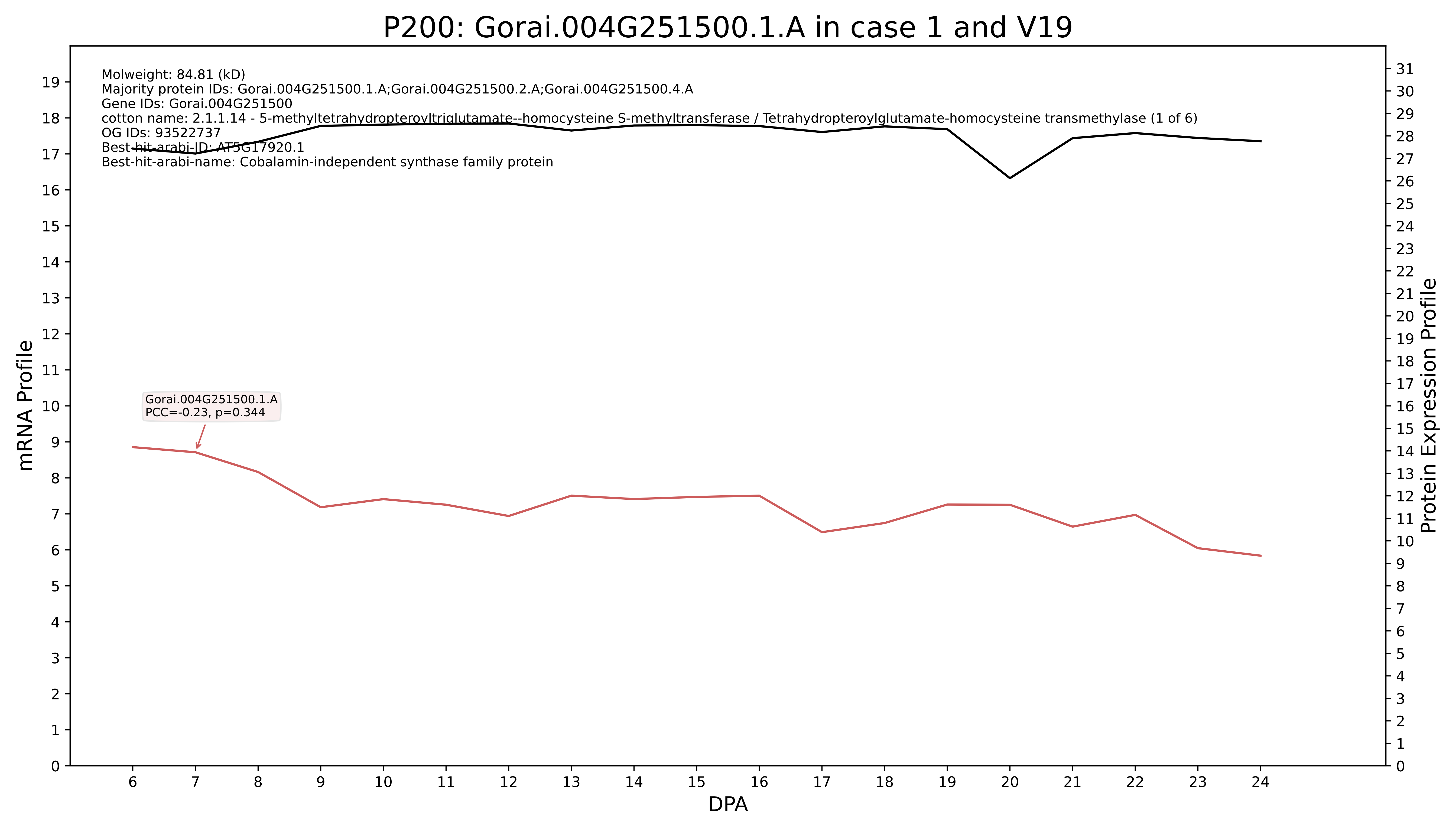
P200: Gorai.004G251500.1.A in case 1 and V19
Molweight: 84.81 (kD)
Majority protein IDs: Gorai.004G251500.1.A;Gorai.004G251500.2.A;Gorai.004G251500.4.A
Gene IDs: Gorai.004G251500
cotton name: 2.1.1.14 - 5-methyltetrahydropteroyltriglutamate--homocysteine S-methyltransferase / Tetrahydropteroylglutamate-homocysteine transmethylase (1 of 6)
OG IDs: 93522737
Best-hit-arabi-ID: AT5G17920.1
Best-hit-arabi-name: Cobalamin-independent synthase family protein
Gorai.004G251500.1.A
PCC=-0.23, p=0.344
mRNA Profile
Protein Expression Profile
DPA
0
1
2
3
4
5
6
7
8
9
10
11
12
13
14
15
16
17
18
19
20
21
22
23
24
25
26
27
28
29
30
31

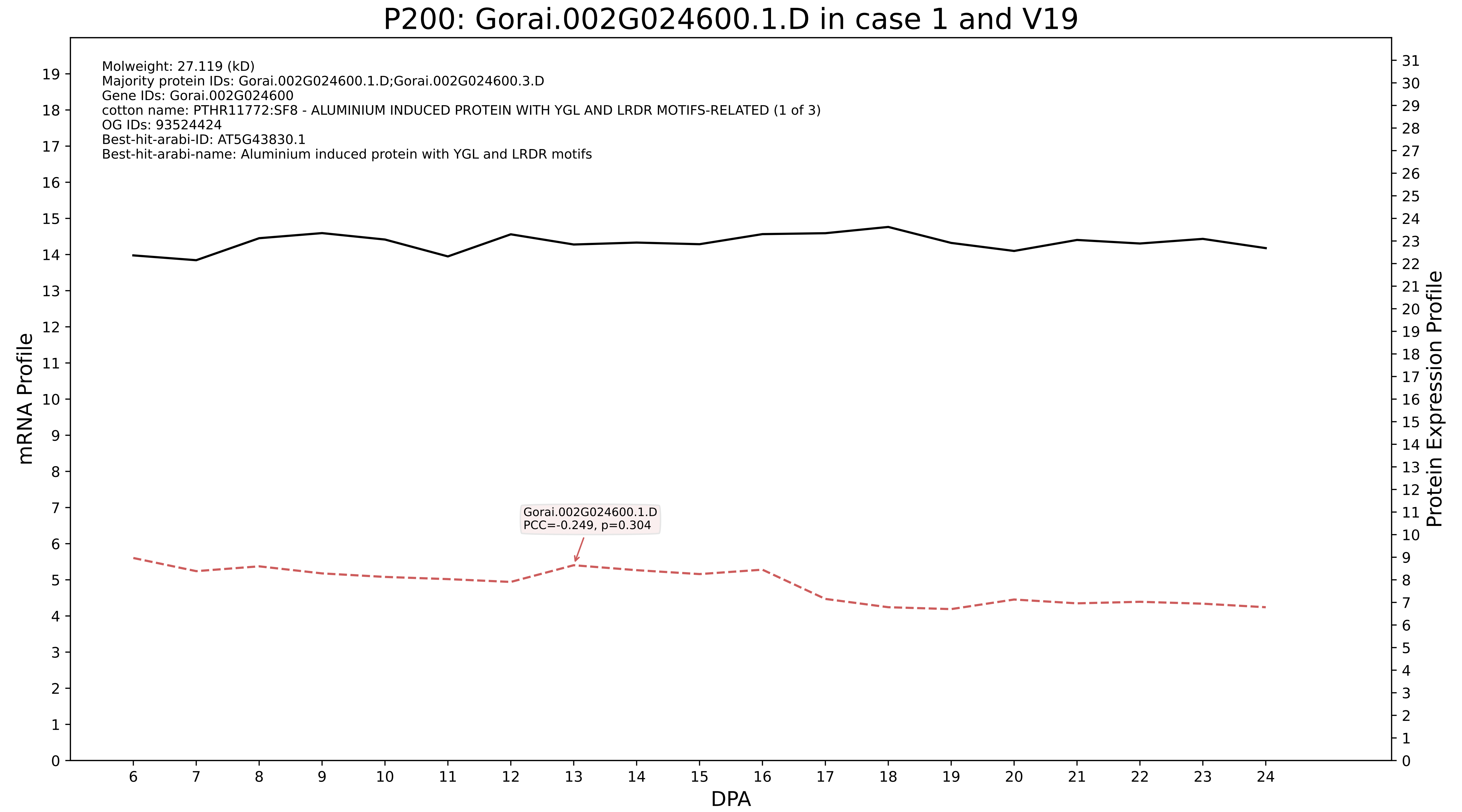
P200: Gorai.002G024600.1.D in case 1 and V19
Molweight: 27.119 (kD)
Majority protein IDs: Gorai.002G024600.1.D;Gorai.002G024600.3.D
Gene IDs: Gorai.002G024600
cotton name: PTHR11772:SF8 - ALUMINIUM INDUCED PROTEIN WITH YGL AND LRDR MOTIFS-RELATED (1 of 3)
OG IDs: 93524424
Best-hit-arabi-ID: AT5G43830.1
Best-hit-arabi-name: Aluminium induced protein with YGL and LRDR motifs
Gorai.002G024600.1.D
PCC=-0.249, p=0.304
mRNA Profile
Protein Expression Profile
DPA

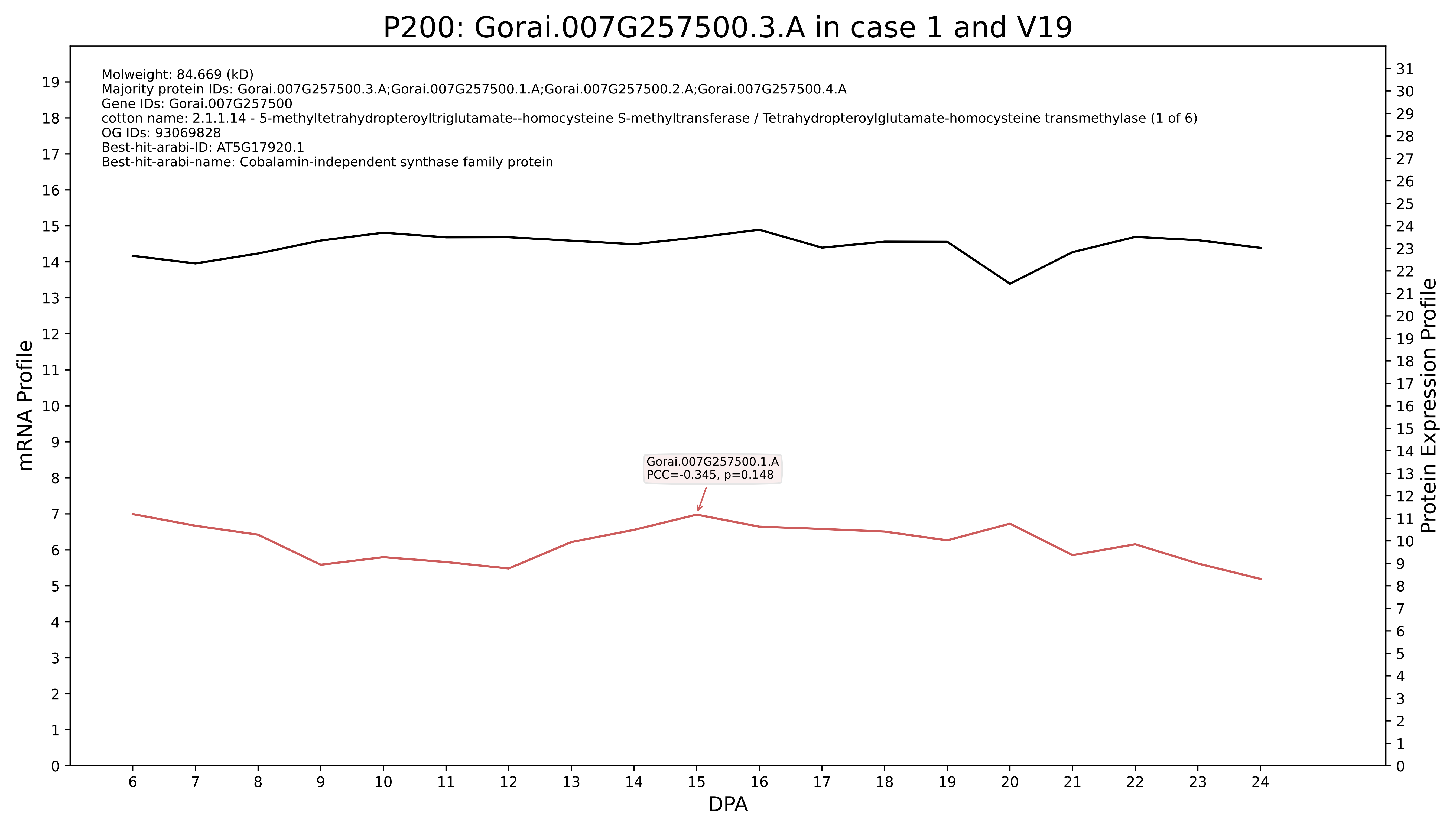
P200: Gorai.007G257500.3.A in case 1 and V19
Molweight: 84.669 (kD)
Majority protein IDs: Gorai.007G257500.3.A;Gorai.007G257500.1.A;Gorai.007G257500.2.A;Gorai.007G257500.4.A
Gene IDs: Gorai.007G257500
cotton name: 2.1.1.14 - 5-methyltetrahydropteroyltriglutamate--homocysteine S-methyltransferase / Tetrahydropteroylglutamate-homocysteine transmethylase (1 of 6)
OG IDs: 93069828
Best-hit-arabi-ID: AT5G17920.1
Best-hit-arabi-name: Cobalamin-independent synthase family protein
Gorai.007G257500.1.A
PCC=-0.345, p=0.148
mRNA Profile
Protein Expression Profile
DPA

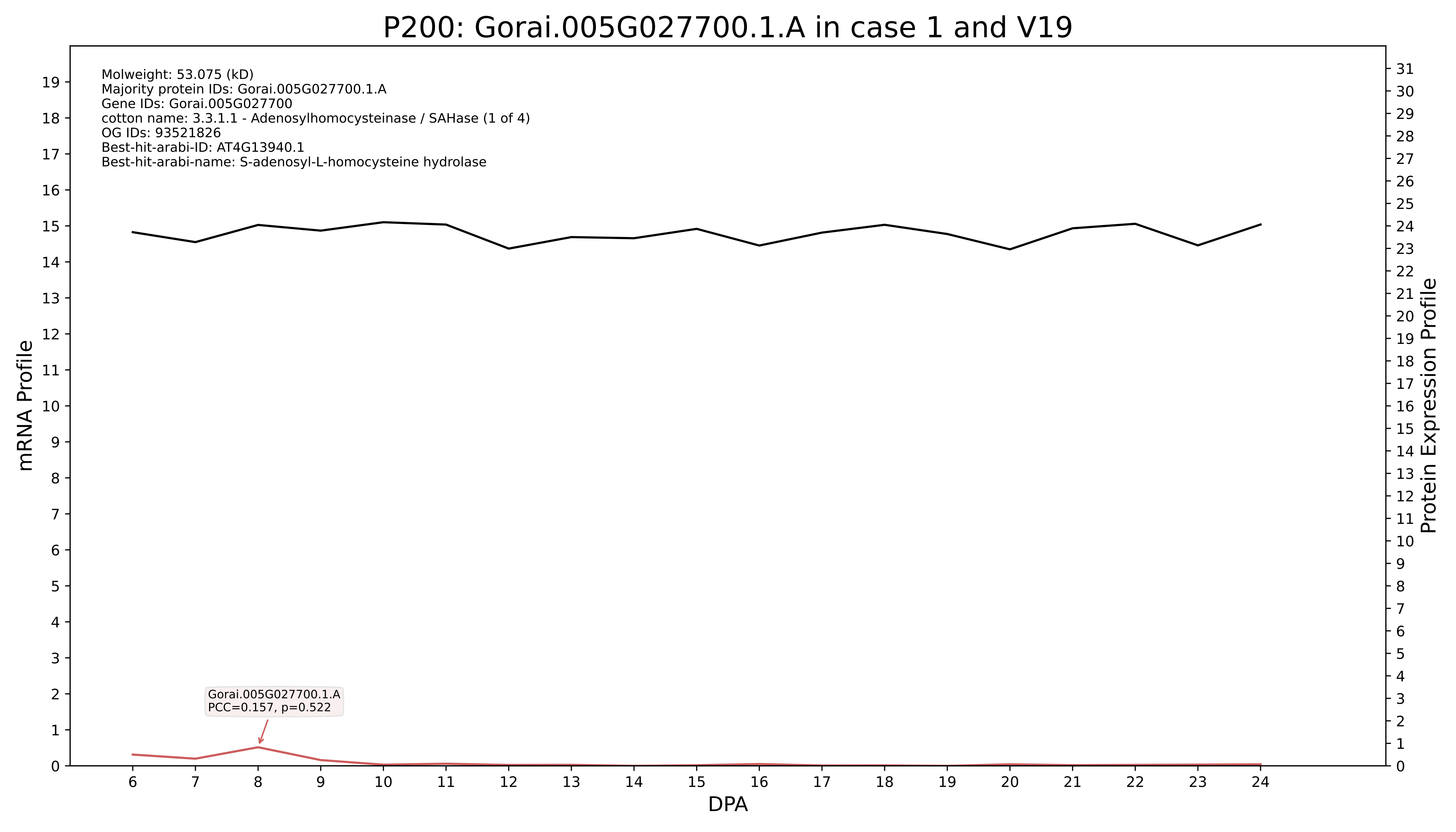

P200: Gorai.005G027700.1.A in case 1 and V19
Molweight: 53.075 (kD)
Majority protein IDs: Gorai.005G027700.1.A
Gene IDs: Gorai.005G027700
cotton name: 3.3.1.1 - Adenosylhomocysteinase / SAHase (1 of 4)
OG IDs: 93521826
Best-hit-arabi-ID: AT4G13940.1
Best-hit-arabi-name: S-adenosyl-L-homocysteine hydrolase
Gorai.005G027700.1.A
PCC=0.157, p=0.522
mRNA Profile
Protein Expression Profile
DPA

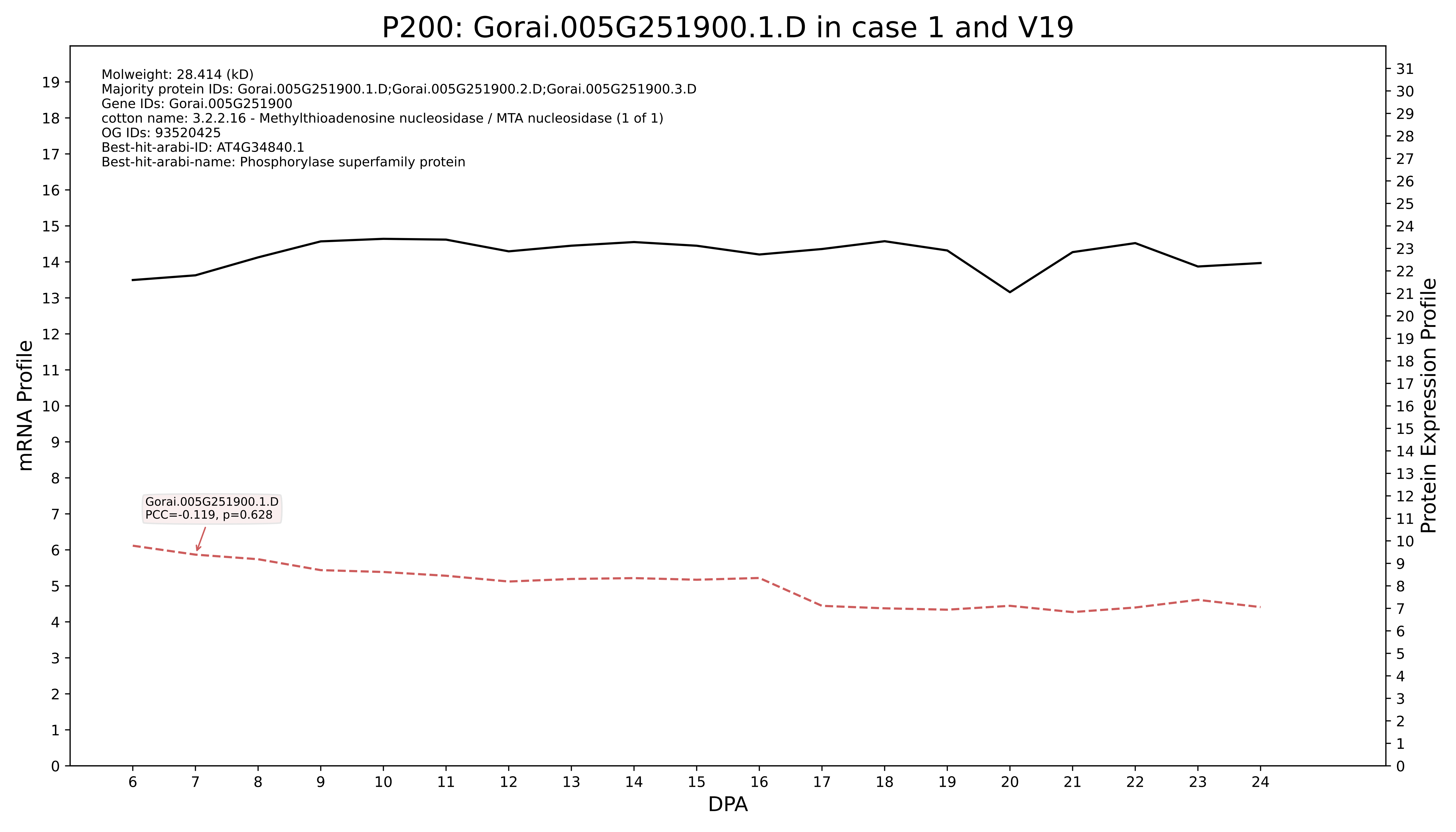
P200: Gorai.005G251900.1.D in case 1 and V19
Molweight: 28.414 (kD)
Majority protein IDs: Gorai.005G251900.1.D;Gorai.005G251900.2.D;Gorai.005G251900.3.D
Gene IDs: Gorai.005G251900
cotton name: 3.2.2.16 - Methylthioadenosine nucleosidase / MTA nucleosidase (1 of 1)
OG IDs: 93520425
Best-hit-arabi-ID: AT4G34840.1
Best-hit-arabi-name: Phosphorylase superfamily protein
Gorai.005G251900.1.D
PCC=-0.119, p=0.628
mRNA Profile
Protein Expression Profile
DPA

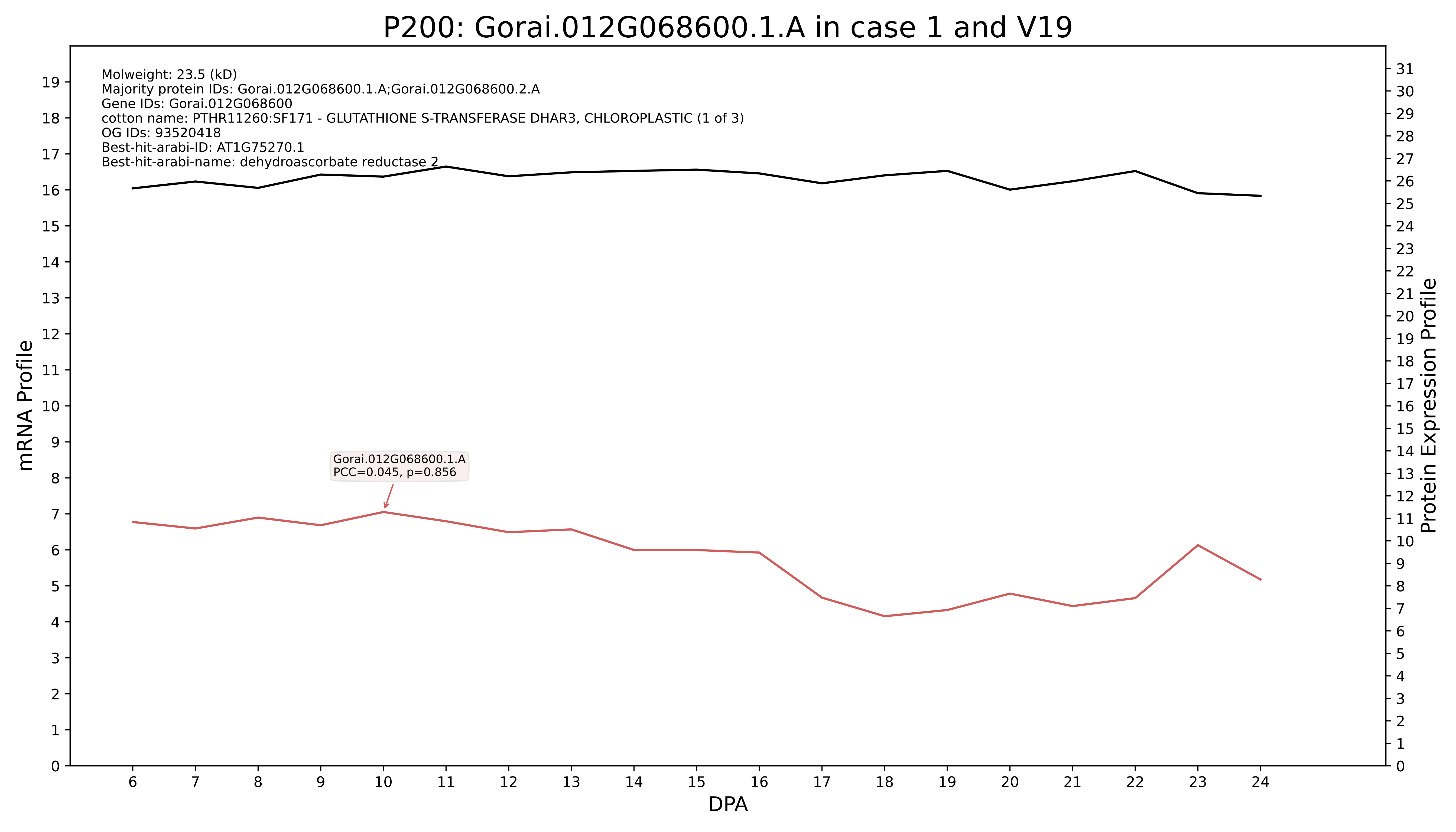

P200: Gorai.012G068600.1.A in case 1 and V19
Molweight: 23.5 (kD)
Majority protein IDs: Gorai.012G068600.1.A;Gorai.012G068600.2.A
Gene IDs: Gorai.012G068600
cotton name: PTHR11260:SF171 - GLUTATHIONE S-TRANSFERASE DHAR3, CHLOROPLASTIC (1 of 3)
OG IDs: 93520418
Best-hit-arabi-ID: AT1G75270.1
Best-hit-arabi-name: dehydroascorbate reductase 2
Gorai.012G068600.1.A
PCC=0.045, p=0.856
mRNA Profile
Protein Expression Profile
DPA

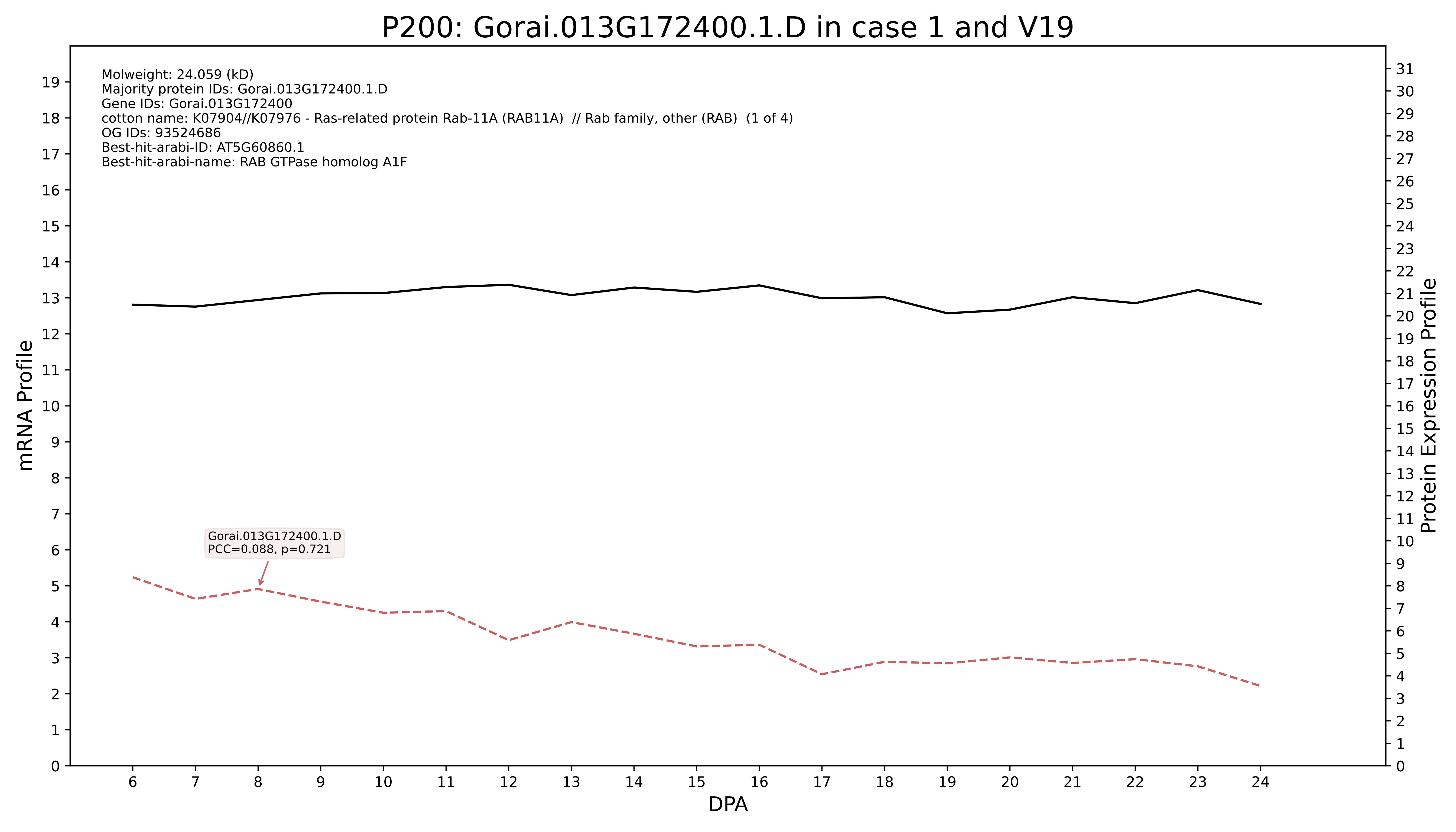
P200: Gorai.013G172400.1.D in case 1 and V19
Molweight: 24.059 (kD)
Majority protein IDs: Gorai.013G172400.1.D
Gene IDs: Gorai.013G172400
cotton name: K07904//K07976 - Ras-related protein Rab-11A (RAB11A) // Rab family, other (RAB) (1 of 4)
OG IDs: 93524686
Best-hit-arabi-ID: AT5G60860.1
Best-hit-arabi-name: RAB GTPase homolog A1F
Gorai.013G172400.1.D
PCC=0.088, p=0.721
mRNA Profile
Protein Expression Profile
DPA

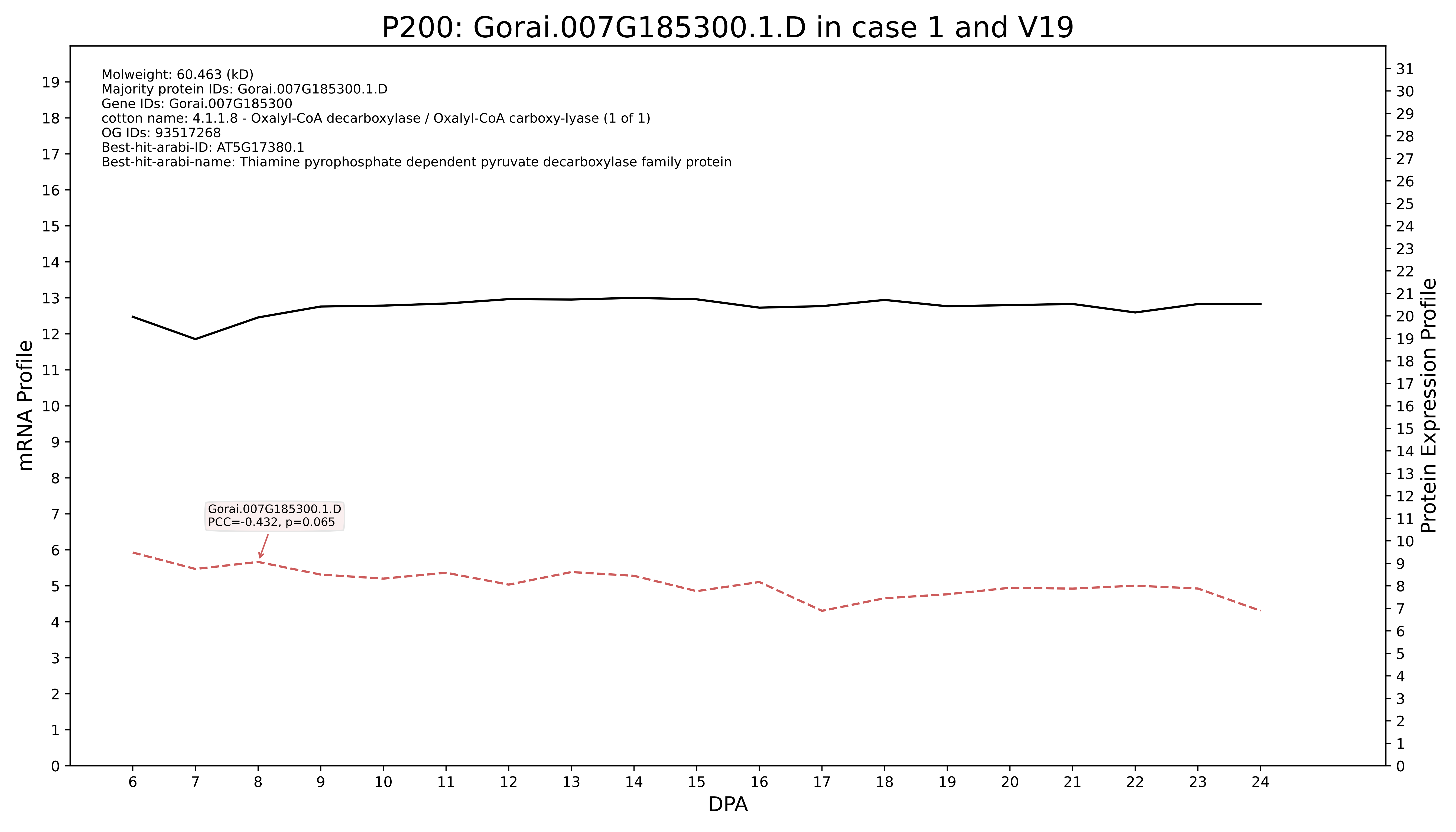
P200: Gorai.007G185300.1.D in case 1 and V19
Molweight: 60.463 (kD)
Majority protein IDs: Gorai.007G185300.1.D
Gene IDs: Gorai.007G185300
cotton name: 4.1.1.8 - Oxalyl-CoA decarboxylase / Oxalyl-CoA carboxy-lyase (1 of 1)
OG IDs: 93517268
Best-hit-arabi-ID: AT5G17380.1
Best-hit-arabi-name: Thiamine pyrophosphate dependent pyruvate decarboxylase family protein
Gorai.007G185300.1.D
PCC=-0.432, p=0.065
mRNA Profile
Protein Expression Profile
DPA

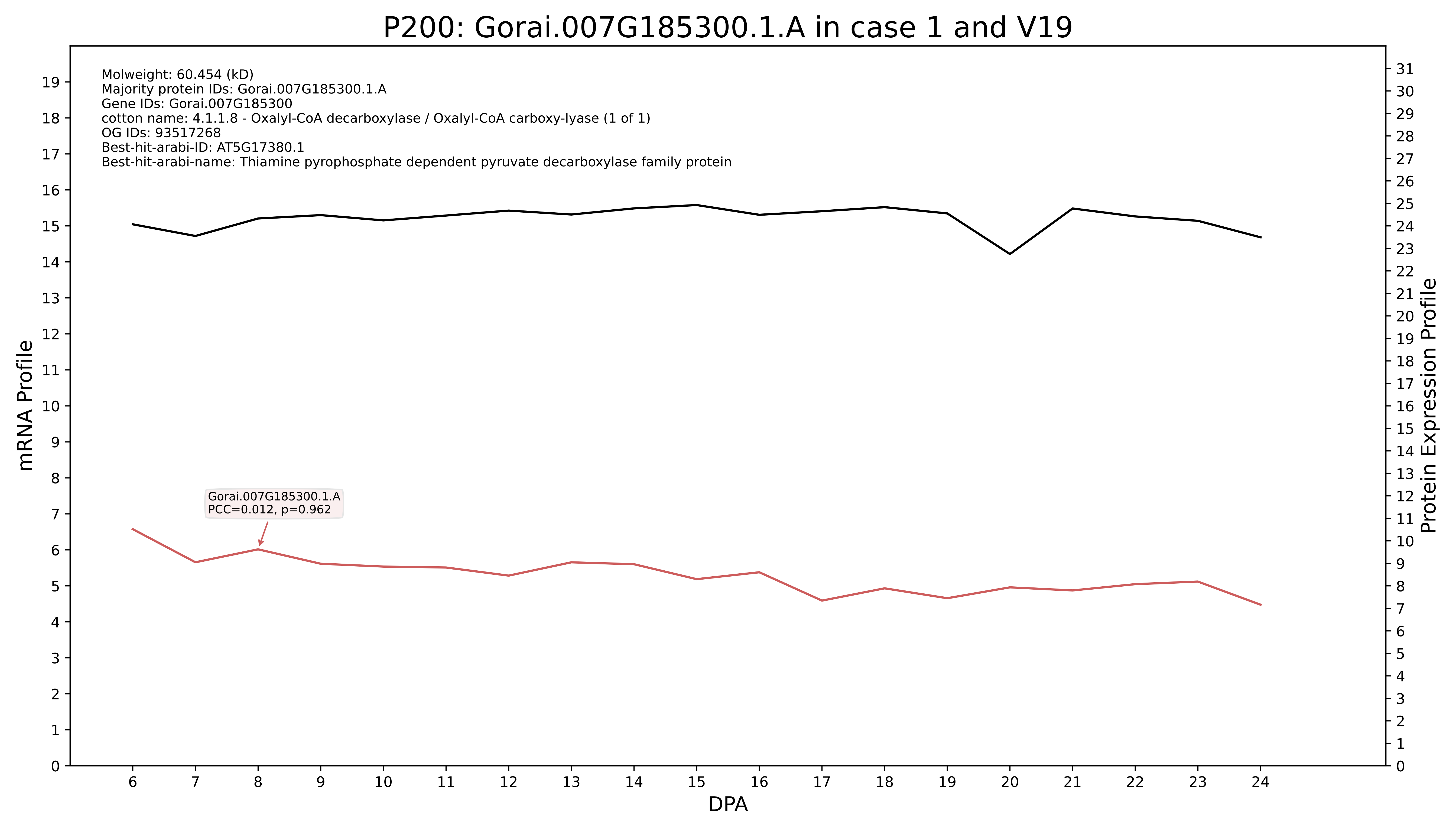
P200: Gorai.007G185300.1.A in case 1 and V19
Molweight: 60.454 (kD)
Majority protein IDs: Gorai.007G185300.1.A
Gene IDs: Gorai.007G185300
cotton name: 4.1.1.8 - Oxalyl-CoA decarboxylase / Oxalyl-CoA carboxy-lyase (1 of 1)
OG IDs: 93517268
Best-hit-arabi-ID: AT5G17380.1
Best-hit-arabi-name: Thiamine pyrophosphate dependent pyruvate decarboxylase family protein
Gorai.007G185300.1.A
PCC=0.012, p=0.962
mRNA Profile
Protein Expression Profile
DPA

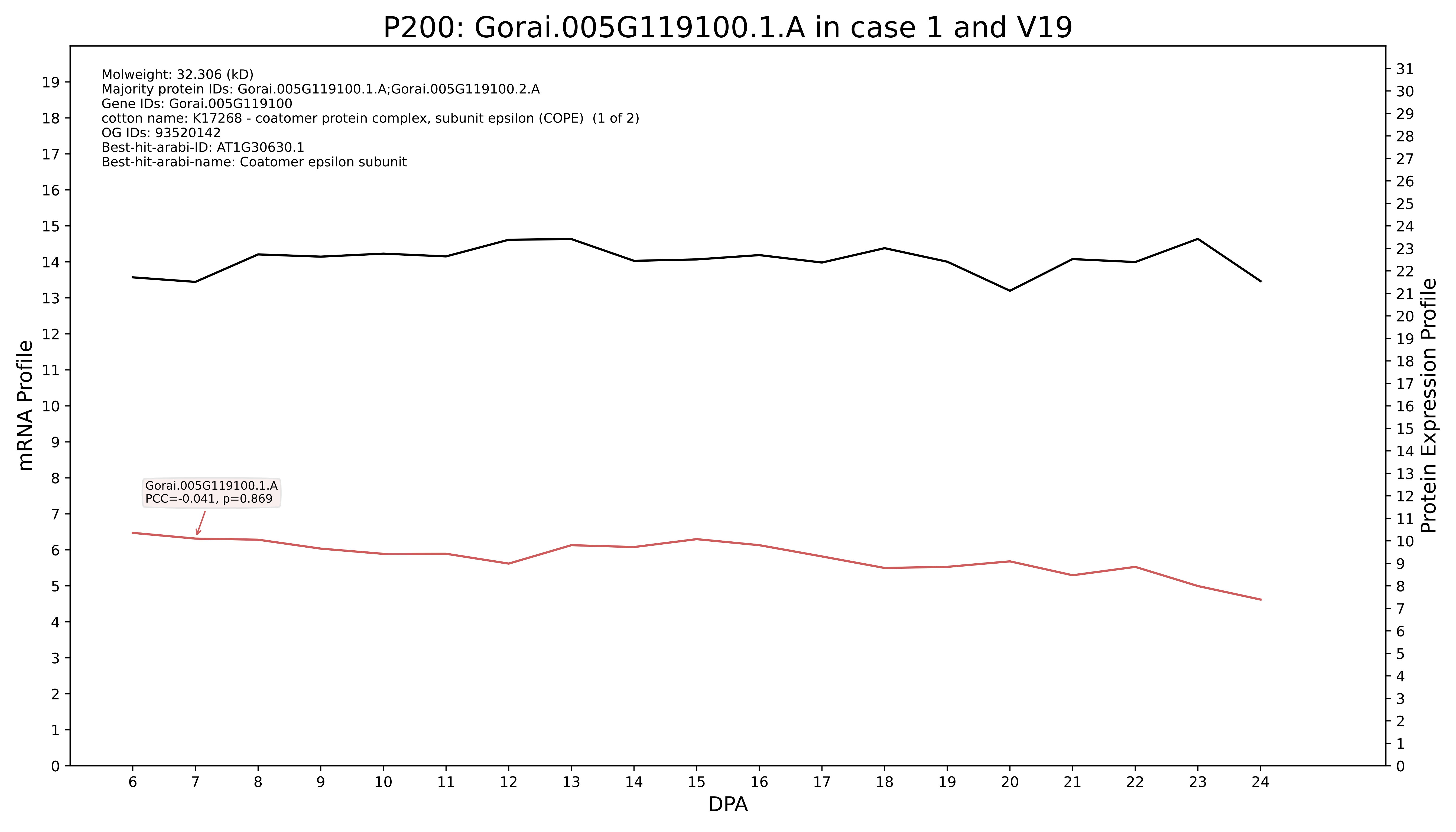
P200: Gorai.005G119100.1.A in case 1 and V19
Molweight: 32.306 (kD)
Majority protein IDs: Gorai.005G119100.1.A;Gorai.005G119100.2.A
Gene IDs: Gorai.005G119100
cotton name: K17268 - coatomer protein complex, subunit epsilon (COPE) (1 of 2)
OG IDs: 93520142
Best-hit-arabi-ID: AT1G30630.1
Best-hit-arabi-name: Coatomer epsilon subunit
Gorai.005G119100.1.A
PCC=-0.041, p=0.869
mRNA Profile
Protein Expression Profile
DPA
6
7
8
9
10
11
12
13
14
15
16
17
18
19
20
21
22
23
24

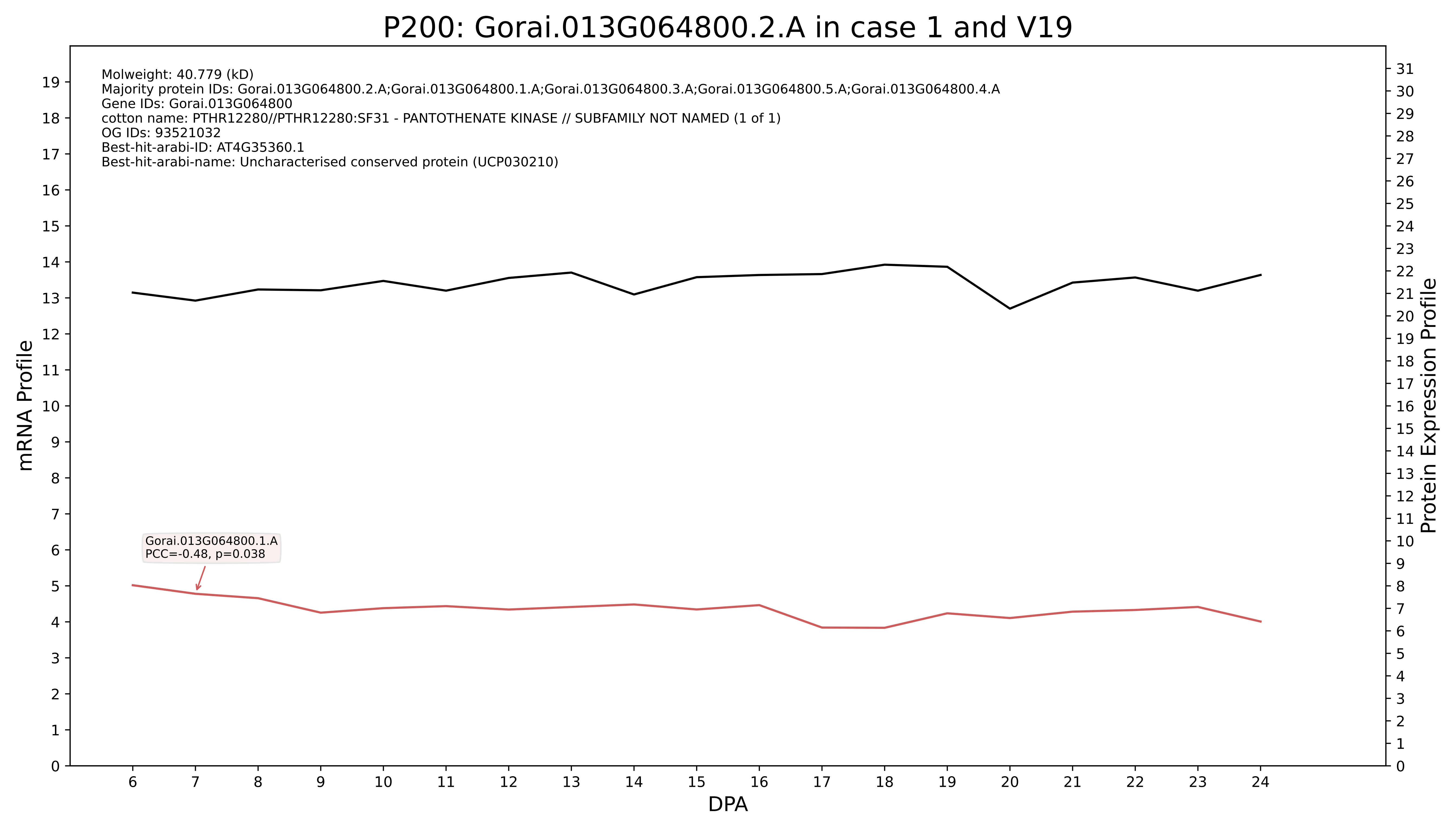
P200: Gorai.013G064800.2.A in case 1 and V19
Molweight: 40.779 (kD)
Majority protein IDs: Gorai.013G064800.2.A;Gorai.013G064800.1.A;Gorai.013G064800.3.A;Gorai.013G064800.5.A;Gorai.013G064800.4.A
Gene IDs: Gorai.013G064800
cotton name: PTHR12280//PTHR12280:SF31 - PANTOTHENATE KINASE // SUBFAMILY NOT NAMED (1 of 1)
OG IDs: 93521032
Best-hit-arabi-ID: AT4G35360.1
Best-hit-arabi-name: Uncharacterised conserved protein (UCP030210)
Gorai.013G064800.1.A
PCC=-0.48, p=0.038
mRNA Profile
Protein Expression Profile
DPA

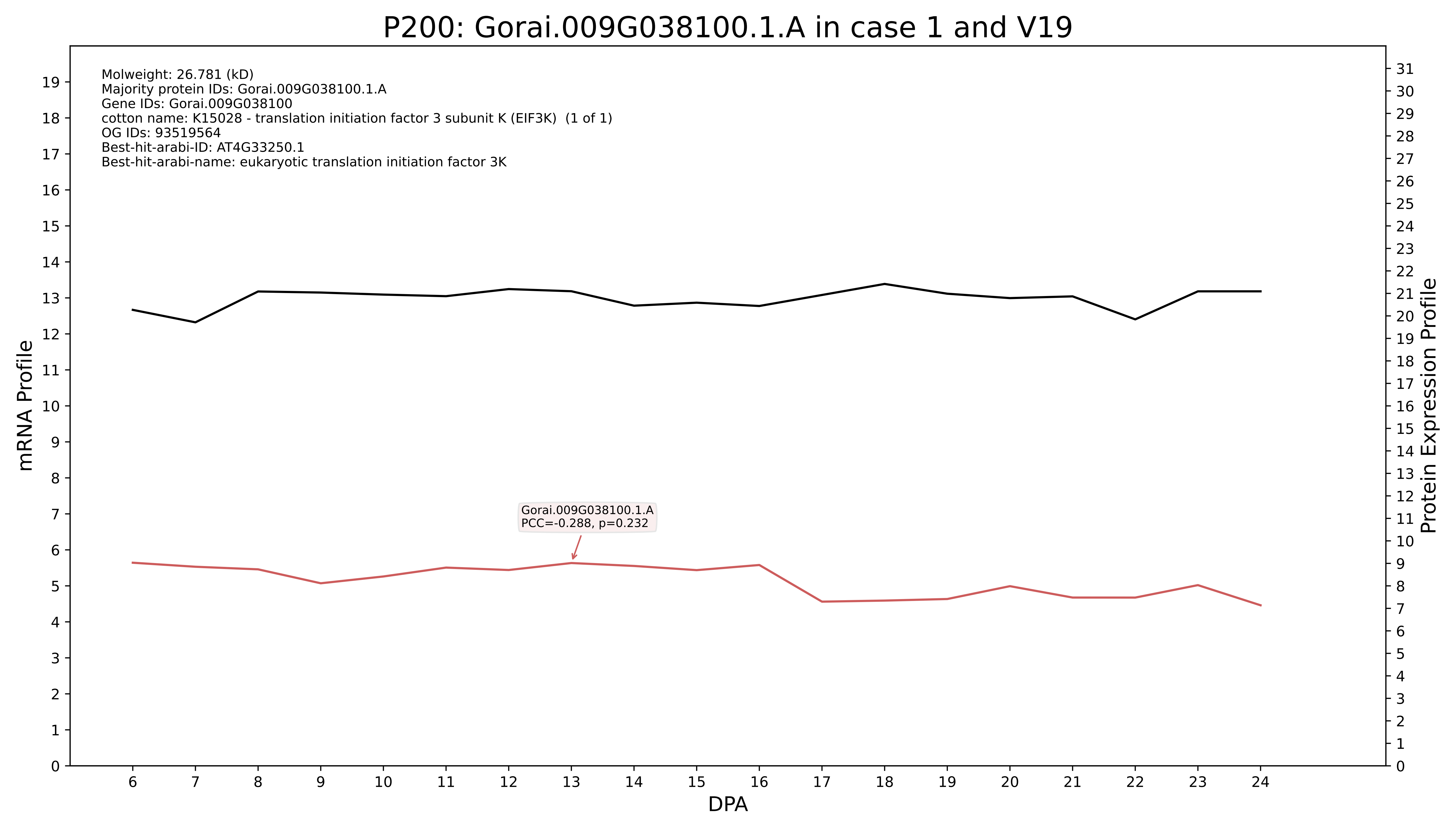
P200: Gorai.009G038100.1.A in case 1 and V19
Molweight: 26.781 (kD)
Majority protein IDs: Gorai.009G038100.1.A
Gene IDs: Gorai.009G038100
cotton name: K15028 - translation initiation factor 3 subunit K (EIF3K) (1 of 1)
OG IDs: 93519564
Best-hit-arabi-ID: AT4G33250.1
Best-hit-arabi-name: eukaryotic translation initiation factor 3K
Gorai.009G038100.1.A
PCC=-0.288, p=0.232
mRNA Profile
Protein Expression Profile
DPA

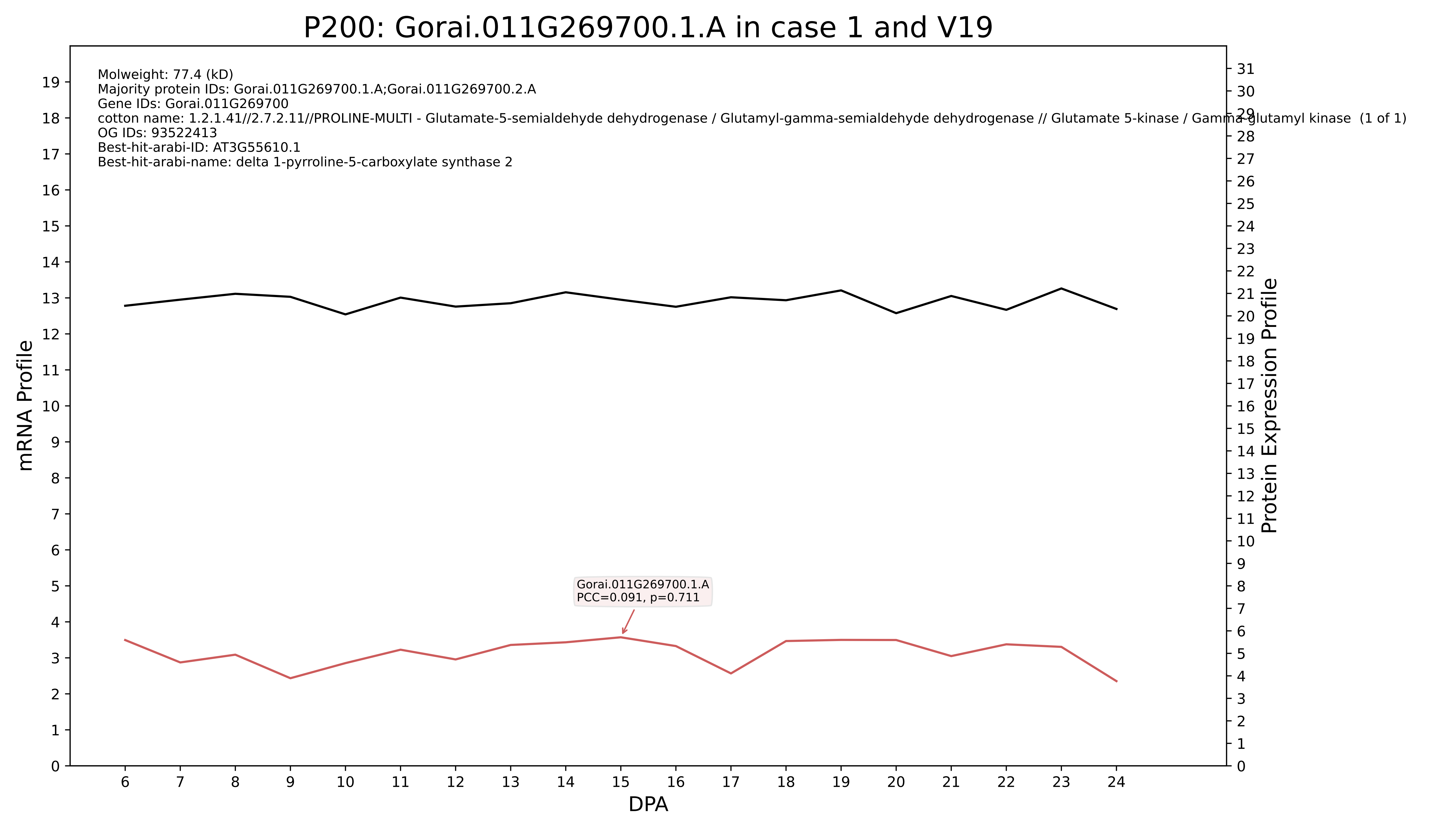

P200: Gorai.011G269700.1.A in case 1 and V19
Molweight: 77.4 (kD)
Majority protein IDs: Gorai.011G269700.1.A;Gorai.011G269700.2.A
Gene IDs: Gorai.011G269700
cotton name: 1.2.1.41//2.7.2.11//PROLINE-MULTI - Glutamate-5-semialdehyde dehydrogenase / Glutamyl-gamma-semialdehyde dehydrogenase // Glutamate 5-kinase / Gamma-glutamyl kinase (1 of 1)
OG IDs: 93522413
Best-hit-arabi-ID: AT3G55610.1
Best-hit-arabi-name: delta 1-pyrroline-5-carboxylate synthase 2
Gorai.011G269700.1.A
PCC=0.091, p=0.711
mRNA Profile
Protein Expression Profile
DPA

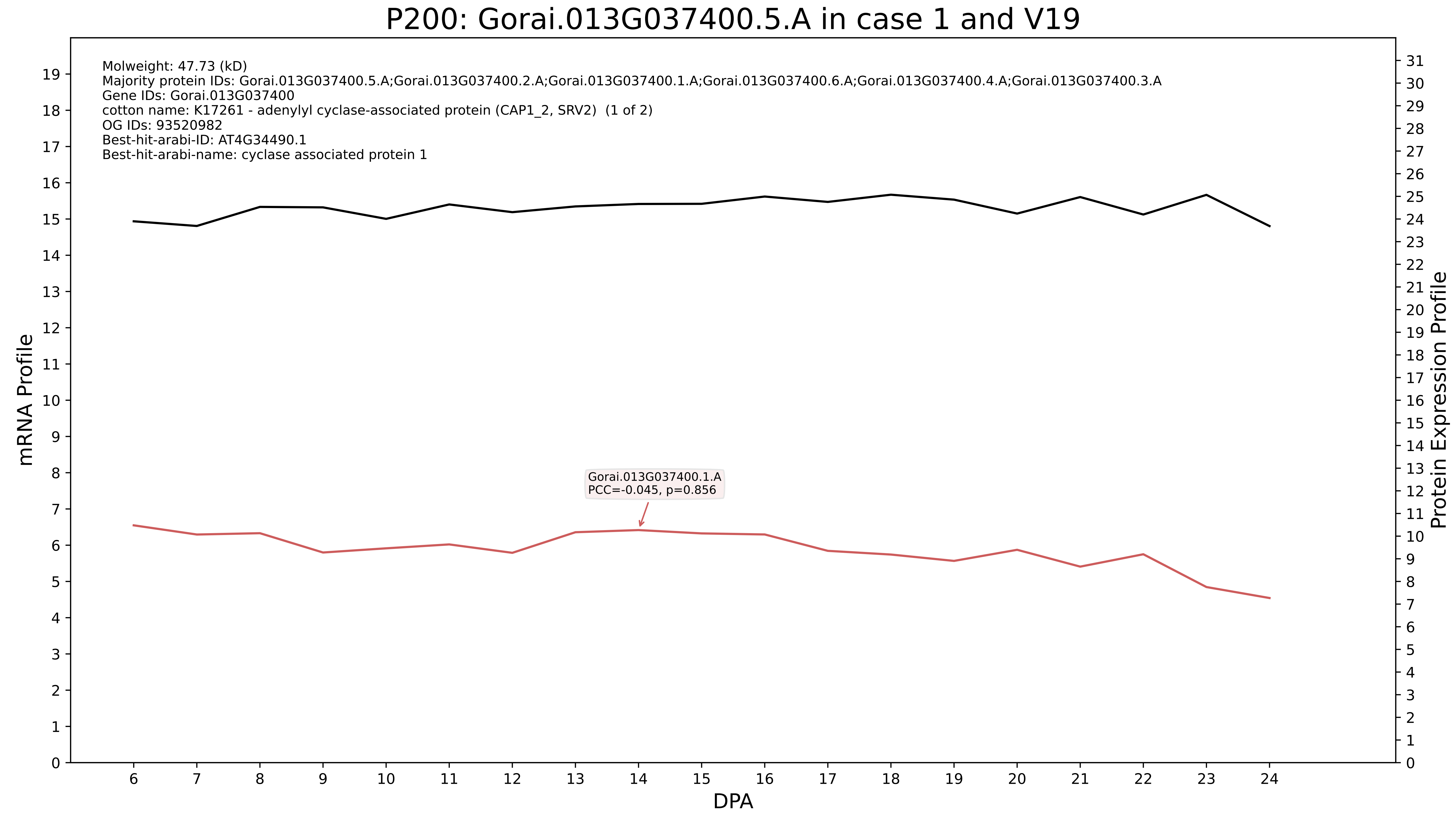

P200: Gorai.013G037400.5.A in case 1 and V19
Molweight: 47.73 (kD)
Majority protein IDs: Gorai.013G037400.5.A;Gorai.013G037400.2.A;Gorai.013G037400.1.A;Gorai.013G037400.6.A;Gorai.013G037400.4.A;Gorai.013G037400.3.A
Gene IDs: Gorai.013G037400
cotton name: K17261 - adenylyl cyclase-associated protein (CAP1_2, SRV2) (1 of 2)
OG IDs: 93520982
Best-hit-arabi-ID: AT4G34490.1
Best-hit-arabi-name: cyclase associated protein 1
Gorai.013G037400.1.A
PCC=-0.045, p=0.856
mRNA Profile
Protein Expression Profile
DPA

P200: Gorai.009G134500.1.A in case 1 and V19

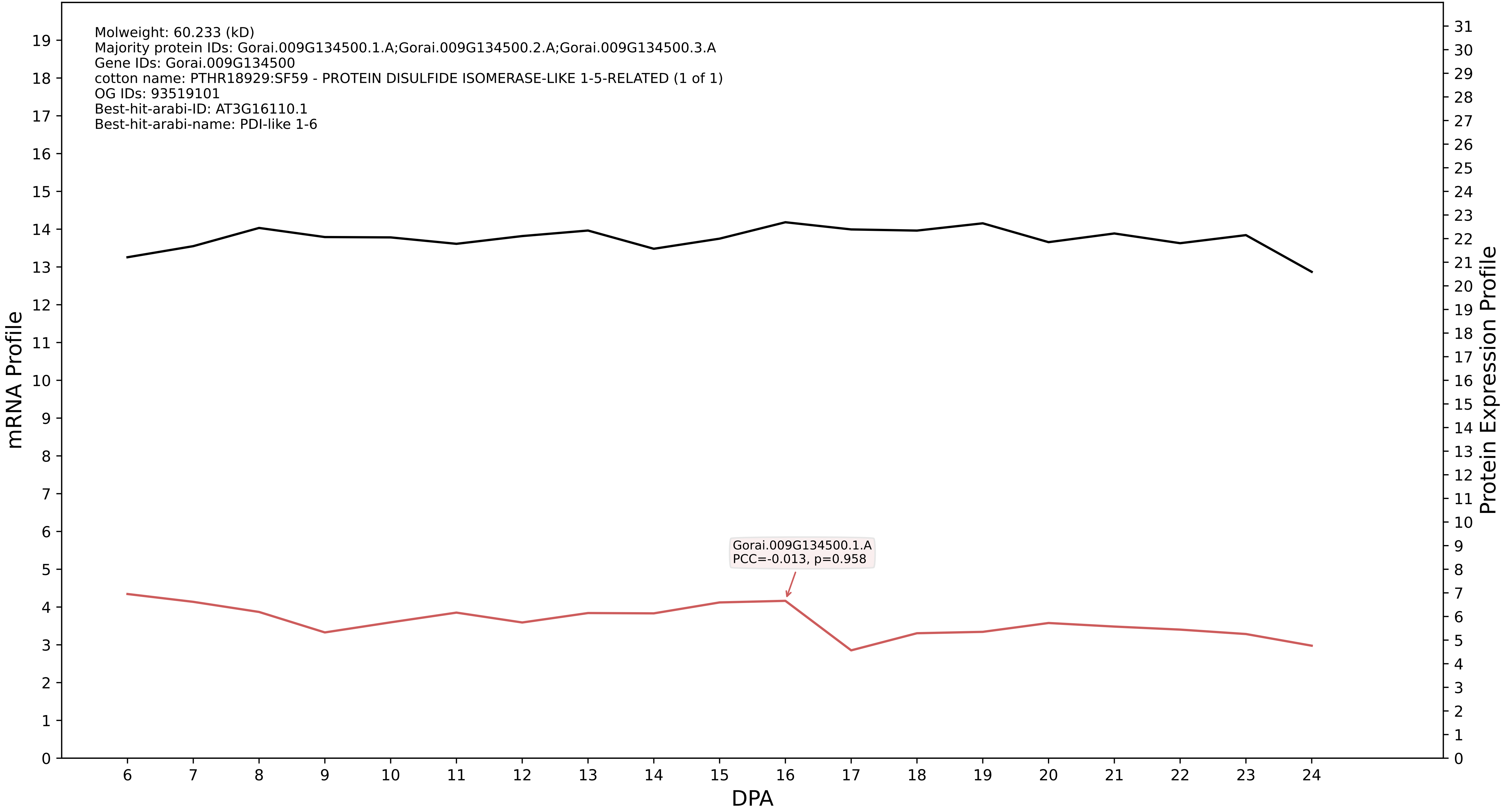

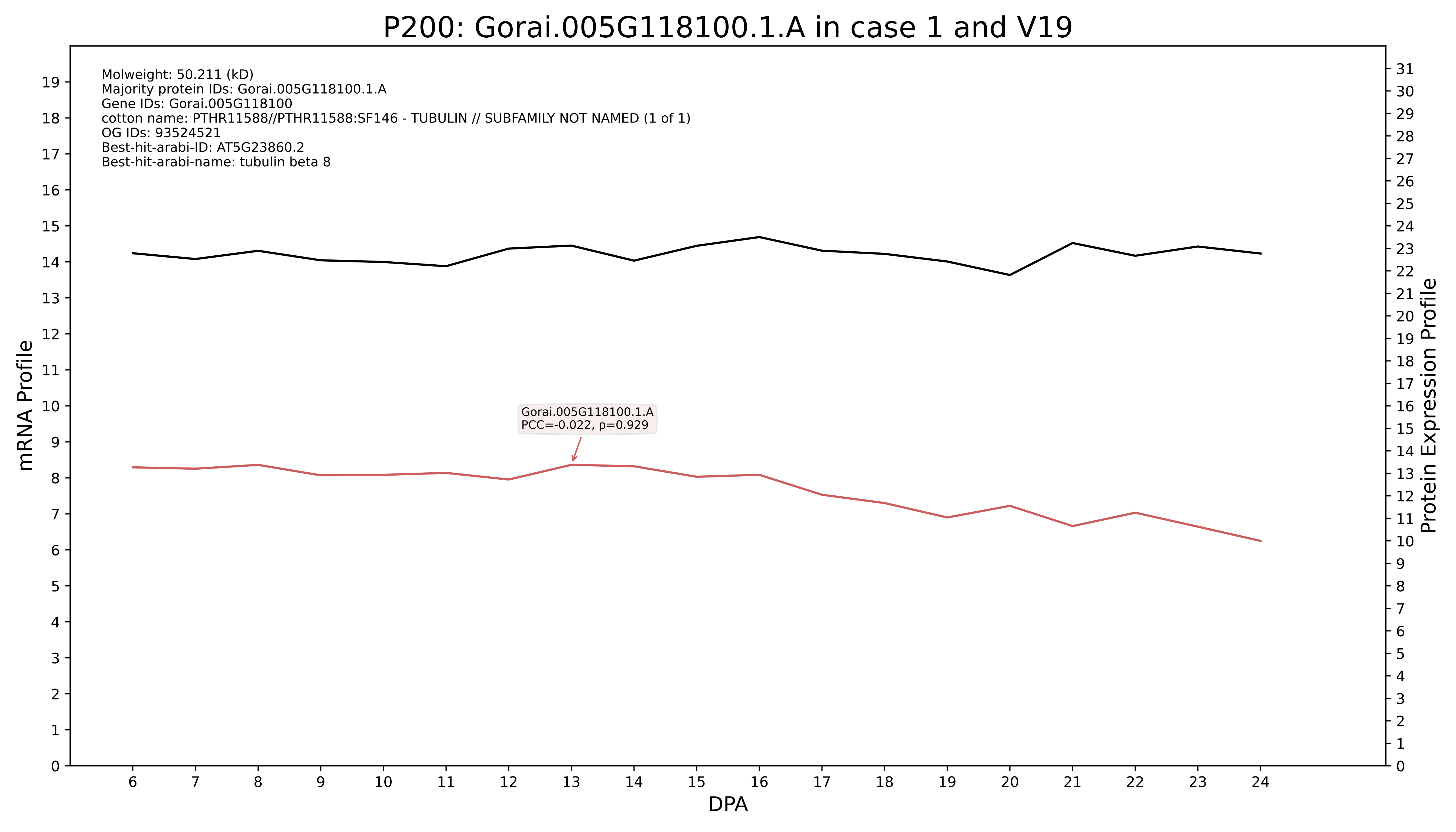
P200: Gorai.005G118100.1.A in case 1 and V19
Molweight: 50.211 (kD)
Majority protein IDs: Gorai.005G118100.1.A
Gene IDs: Gorai.005G118100
cotton name: PTHR11588//PTHR11588:SF146 - TUBULIN // SUBFAMILY NOT NAMED (1 of 1)
OG IDs: 93524521
Best-hit-arabi-ID: AT5G23860.2
Best-hit-arabi-name: tubulin beta 8
Gorai.005G118100.1.A
PCC=-0.022, p=0.929
mRNA Profile
Protein Expression Profile
DPA

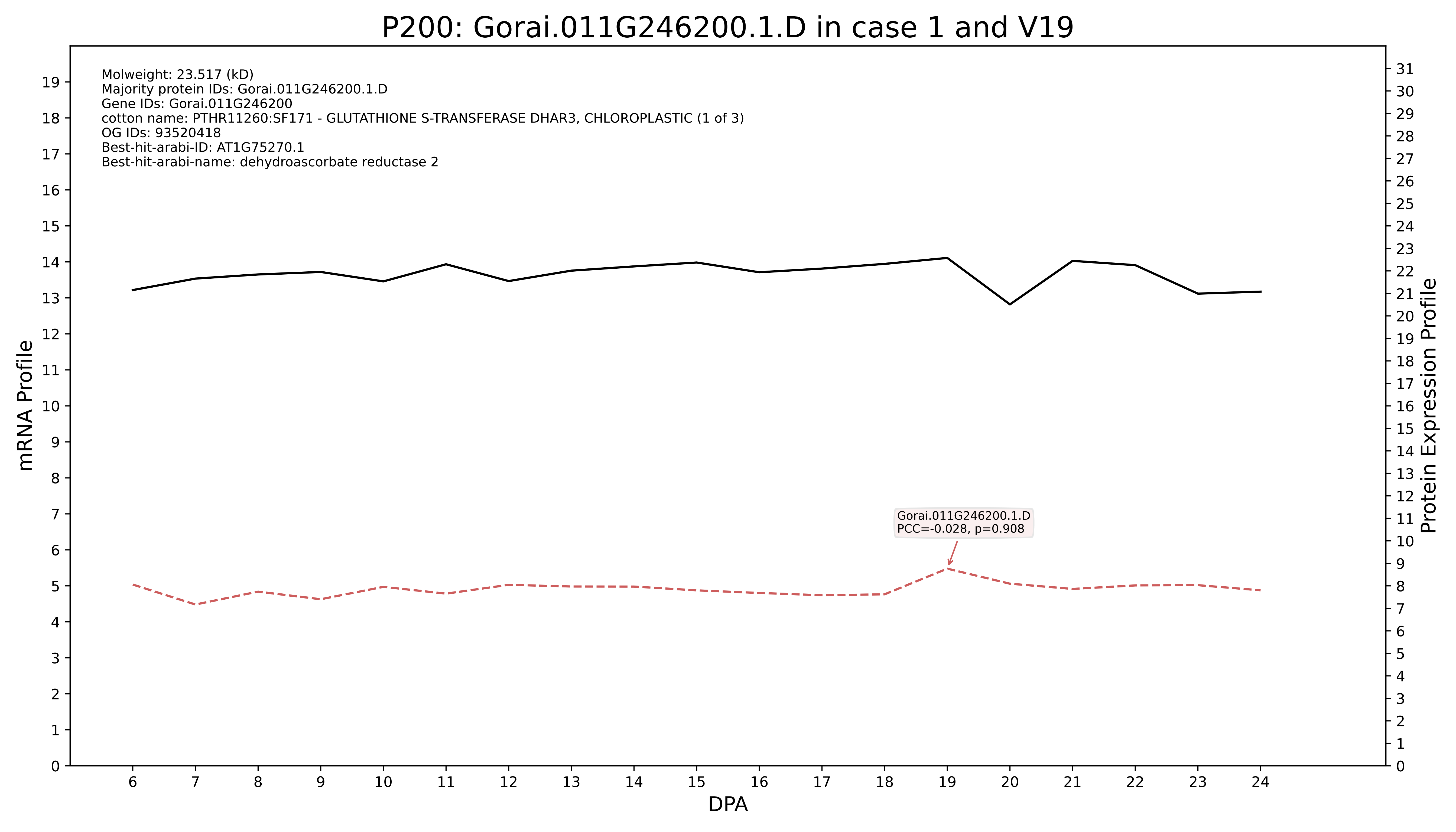
P200: Gorai.011G246200.1.D in case 1 and V19
Molweight: 23.517 (kD)
Majority protein IDs: Gorai.011G246200.1.D
Gene IDs: Gorai.011G246200
cotton name: PTHR11260:SF171 - GLUTATHIONE S-TRANSFERASE DHAR3, CHLOROPLASTIC (1 of 3)
OG IDs: 93520418
Best-hit-arabi-ID: AT1G75270.1
Best-hit-arabi-name: dehydroascorbate reductase 2
Gorai.011G246200.1.D
PCC=-0.028, p=0.908
mRNA Profile
Protein Expression Profile
DPA

P200: Gorai.005G208400.8.D in case 1 and V19

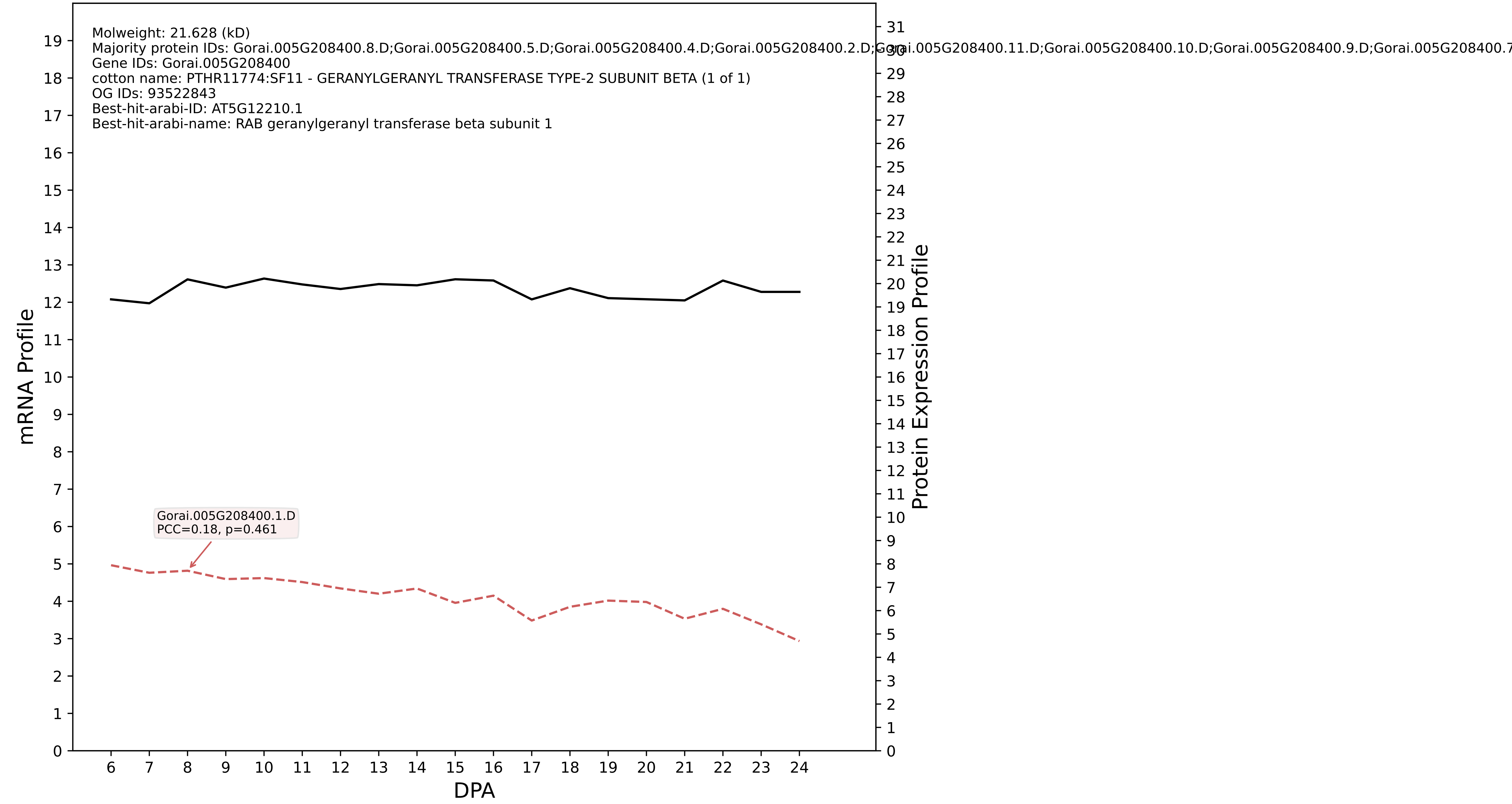

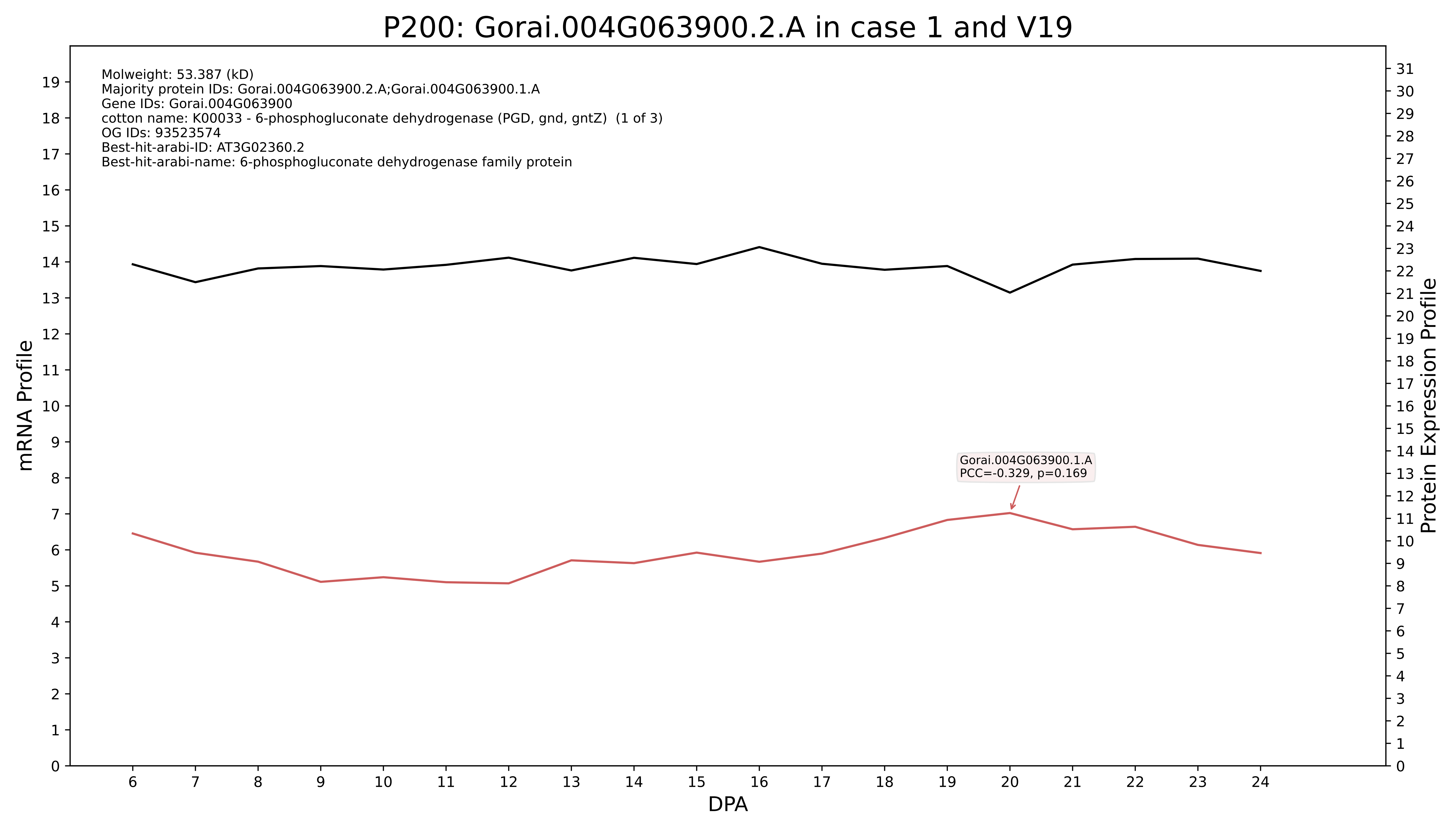
P200: Gorai.004G063900.2.A in case 1 and V19
Molweight: 53.387 (kD)
Majority protein IDs: Gorai.004G063900.2.A;Gorai.004G063900.1.A
Gene IDs: Gorai.004G063900
cotton name: K00033 - 6-phosphogluconate dehydrogenase (PGD, gnd, gntZ) (1 of 3)
OG IDs: 93523574
Best-hit-arabi-ID: AT3G02360.2
Best-hit-arabi-name: 6-phosphogluconate dehydrogenase family protein
Gorai.004G063900.1.A
PCC=-0.329, p=0.169
mRNA Profile
Protein Expression Profile
DPA
0
1
2
3
4
5
6
7
8
9
10
11
12
13
14
15
16
17
18
19
20
21
22
23
24
25
26
27
28
29
30
31

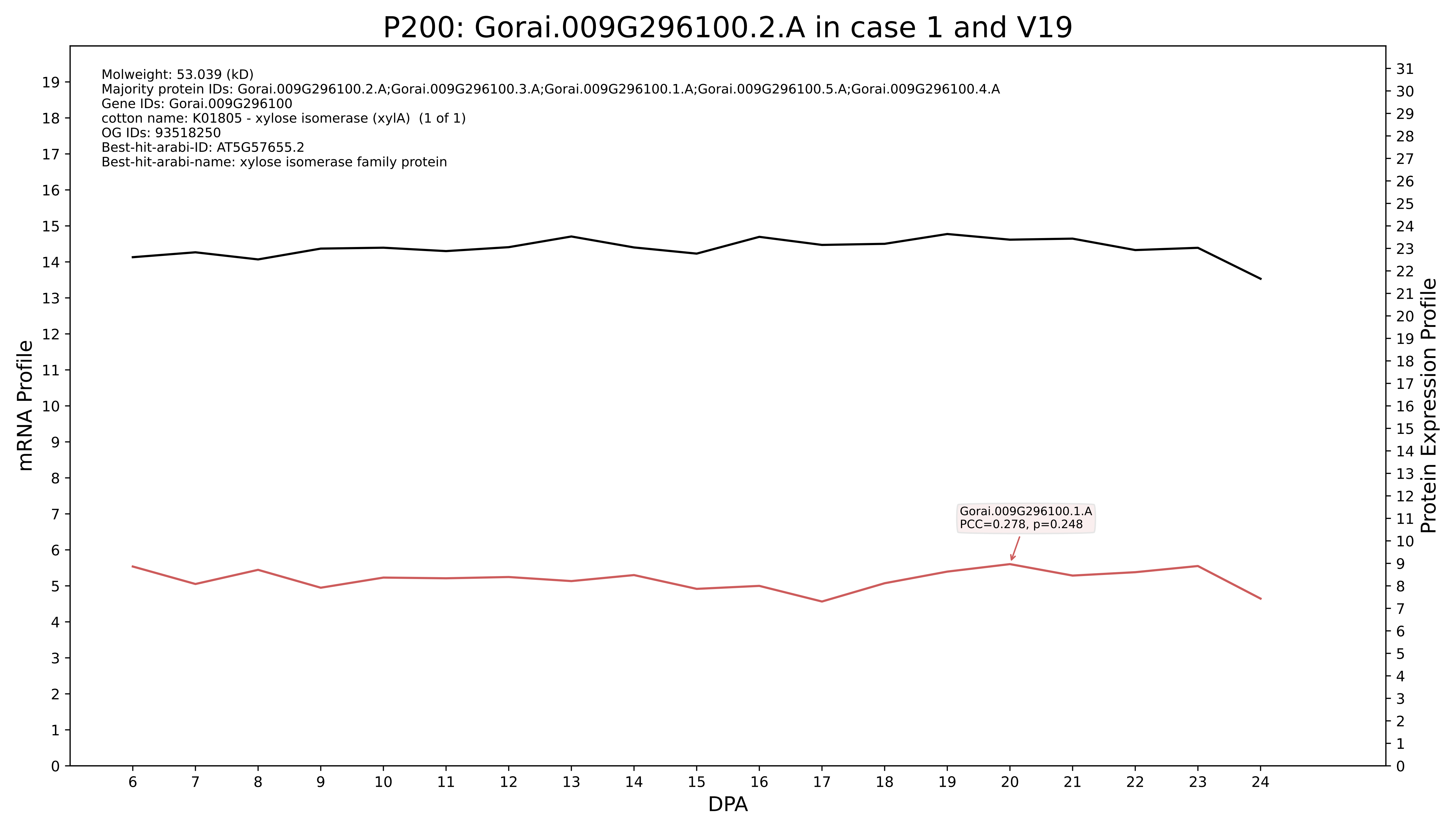
P200: Gorai.009G296100.2.A in case 1 and V19
Molweight: 53.039 (kD)
Majority protein IDs: Gorai.009G296100.2.A;Gorai.009G296100.3.A;Gorai.009G296100.1.A;Gorai.009G296100.5.A;Gorai.009G296100.4.A
Gene IDs: Gorai.009G296100
cotton name: K01805 - xylose isomerase (xylA) (1 of 1)
OG IDs: 93518250
Best-hit-arabi-ID: AT5G57655.2
Best-hit-arabi-name: xylose isomerase family protein
Gorai.009G296100.1.A
PCC=0.278, p=0.248
mRNA Profile
Protein Expression Profile
DPA
0
1
2
3
4
5
6
7
8
9
10
11
12
13
14
15
16
17
18
19
20
21
22
23
24
25
26
27
28
29
30
31

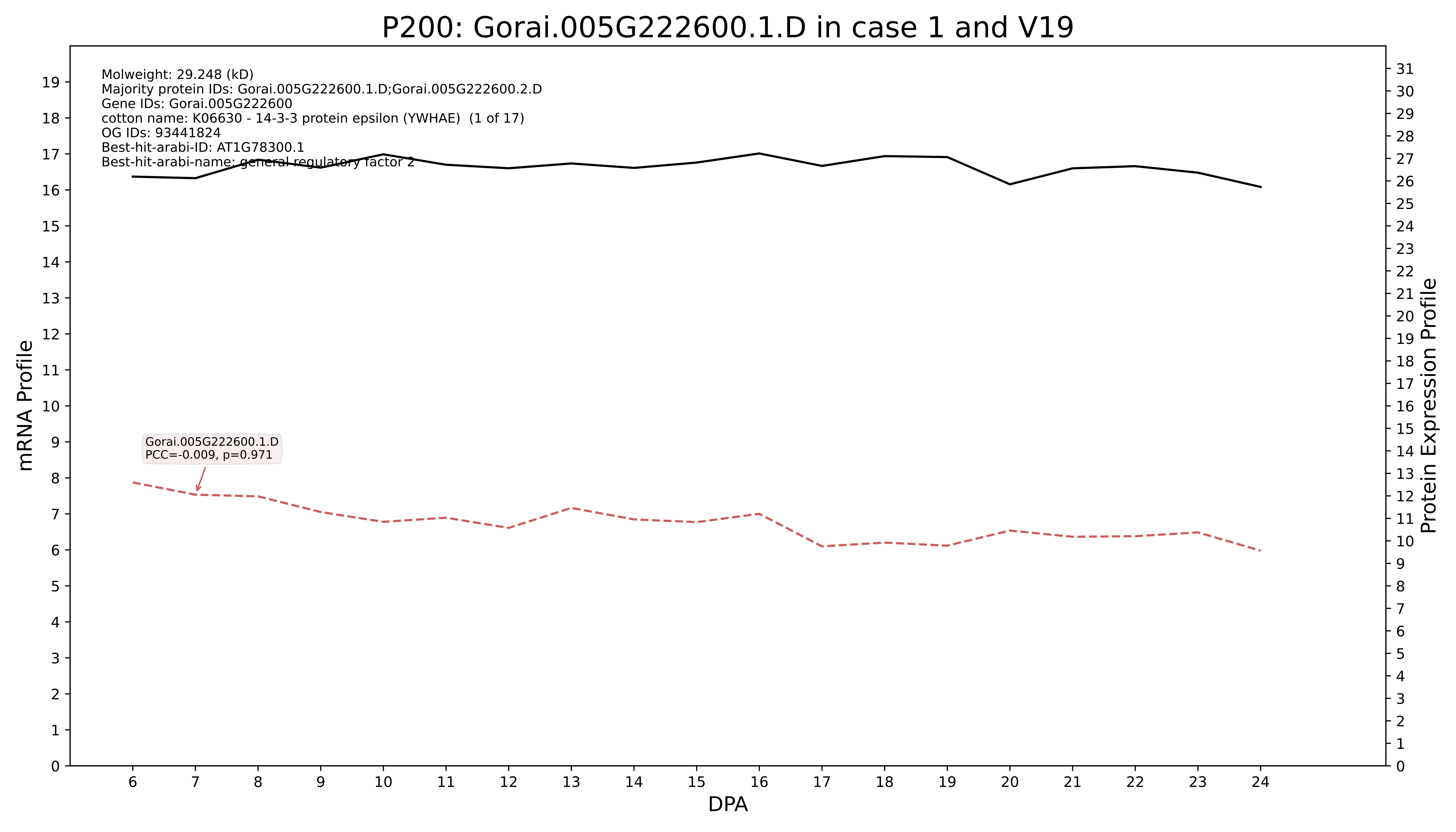
P200: Gorai.005G222600.1.D in case 1 and V19
Molweight: 29.248 (kD)
Majority protein IDs: Gorai.005G222600.1.D;Gorai.005G222600.2.D
Gene IDs: Gorai.005G222600
cotton name: K06630 - 14-3-3 protein epsilon (YWHAE) (1 of 17)
OG IDs: 93441824
Best-hit-arabi-ID: AT1G78300.1
Best-hit-arabi-name: general regulatory factor 2
Gorai.005G222600.1.D
PCC=-0.009, p=0.971
mRNA Profile
Protein Expression Profile
DPA

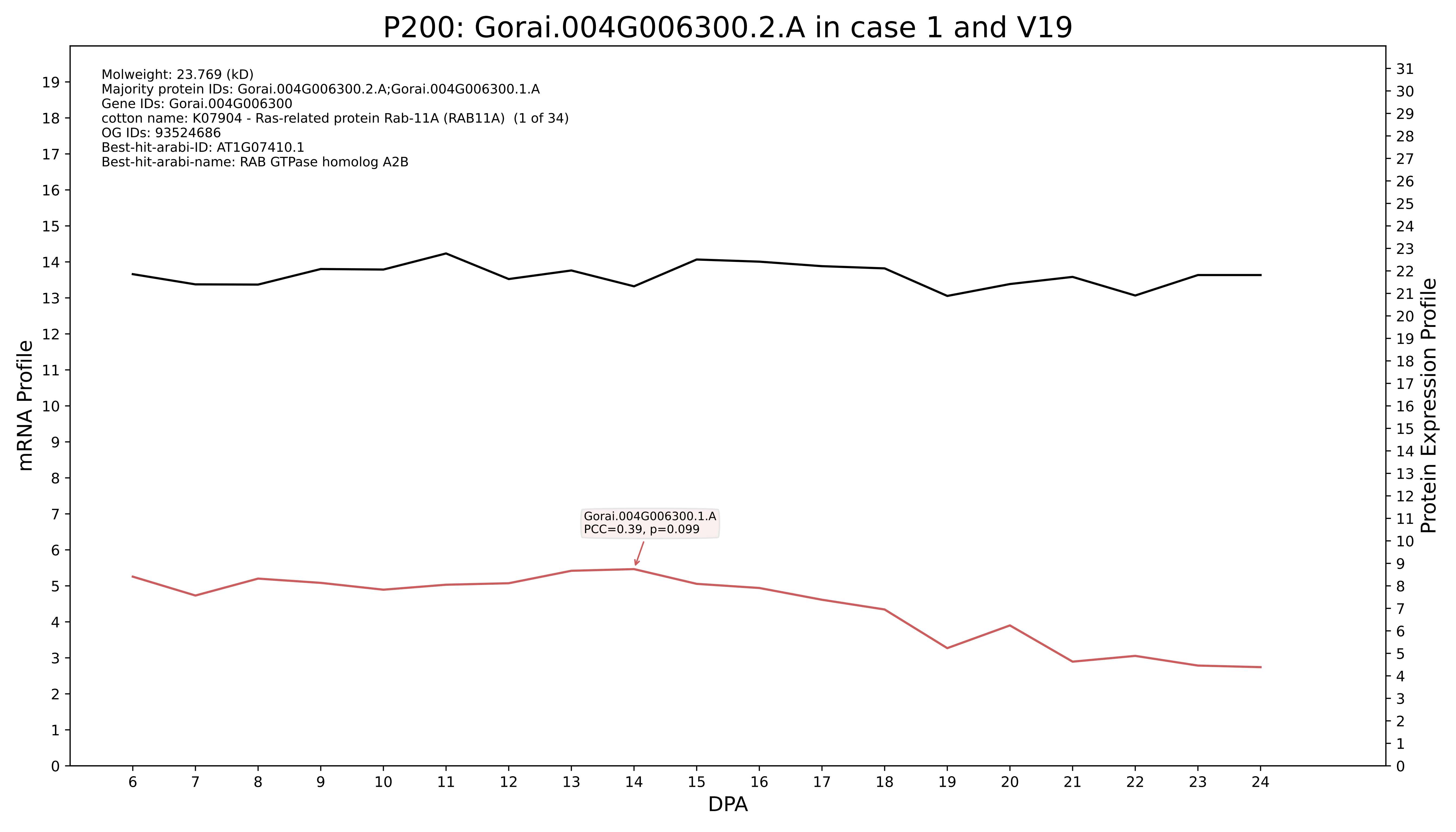

P200: Gorai.004G006300.2.A in case 1 and V19
Molweight: 23.769 (kD)
Majority protein IDs: Gorai.004G006300.2.A;Gorai.004G006300.1.A
Gene IDs: Gorai.004G006300
cotton name: K07904 - Ras-related protein Rab-11A (RAB11A) (1 of 34)
OG IDs: 93524686
Best-hit-arabi-ID: AT1G07410.1
Best-hit-arabi-name: RAB GTPase homolog A2B
Gorai.004G006300.1.A
PCC=0.39, p=0.099
mRNA Profile
Protein Expression Profile
DPA

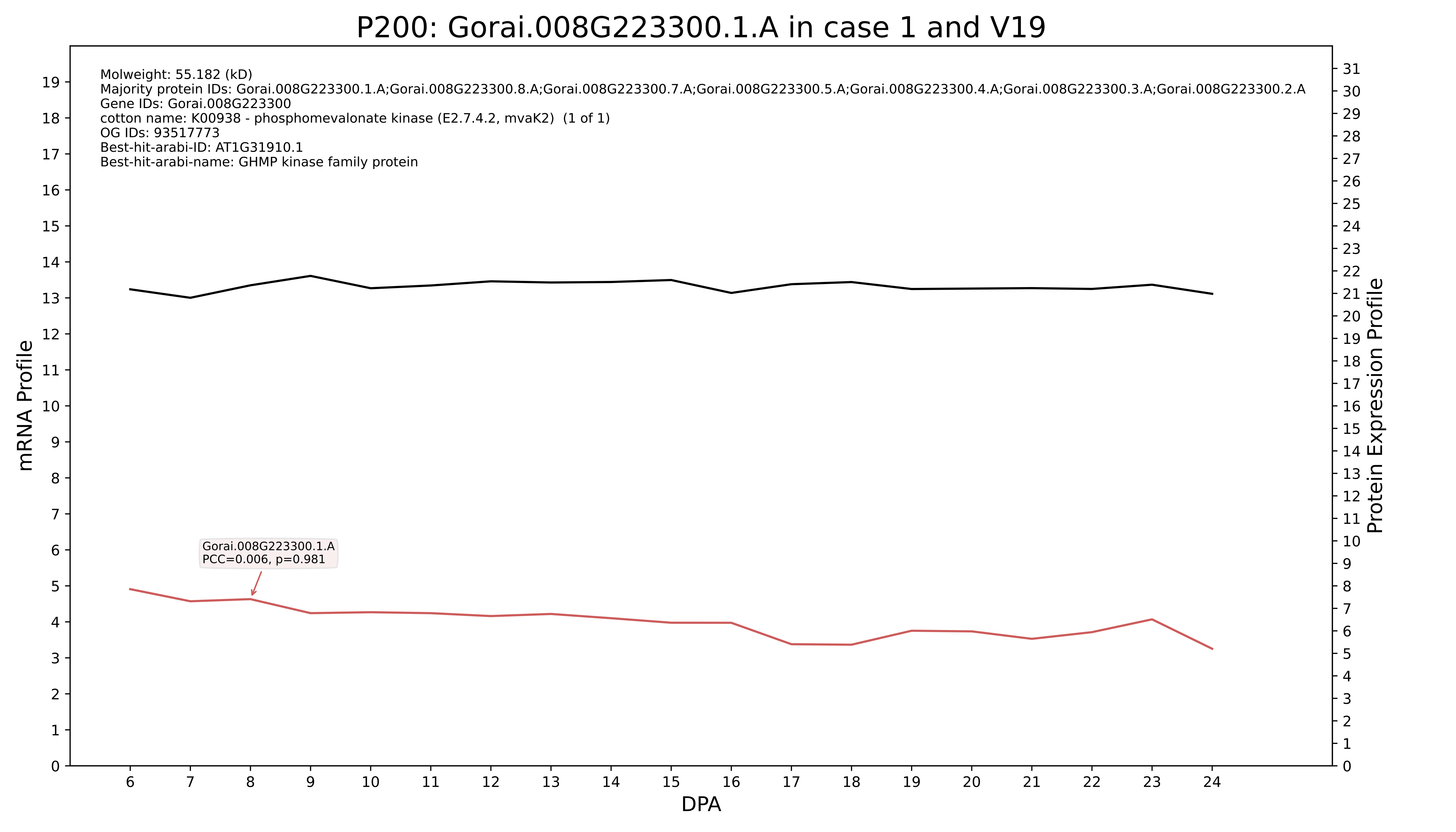

P200: Gorai.008G223300.1.A in case 1 and V19
Molweight: 55.182 (kD)
Majority protein IDs: Gorai.008G223300.1.A;Gorai.008G223300.8.A;Gorai.008G223300.7.A;Gorai.008G223300.5.A;Gorai.008G223300.4.A;Gorai.008G223300.3.A;Gorai.008G223300.2.A
Gene IDs: Gorai.008G223300
cotton name: K00938 - phosphomevalonate kinase (E2.7.4.2, mvaK2) (1 of 1)
OG IDs: 93517773
Best-hit-arabi-ID: AT1G31910.1
Best-hit-arabi-name: GHMP kinase family protein
Gorai.008G223300.1.A
PCC=0.006, p=0.981
mRNA Profile
Protein Expression Profile
DPA

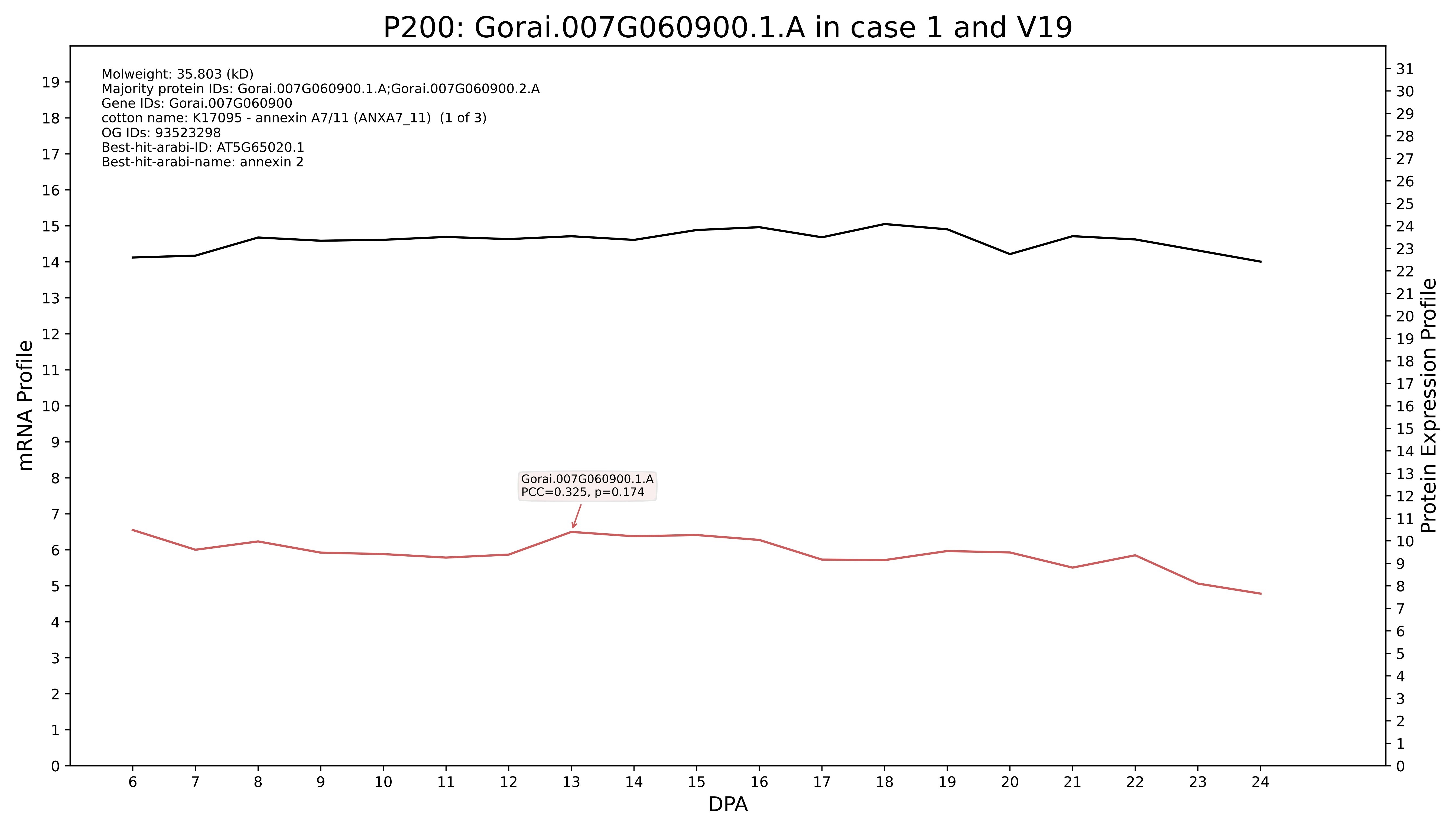
P200: Gorai.007G060900.1.A in case 1 and V19
Molweight: 35.803 (kD)
Majority protein IDs: Gorai.007G060900.1.A;Gorai.007G060900.2.A
Gene IDs: Gorai.007G060900
cotton name: K17095 - annexin A7/11 (ANXA7_11) (1 of 3)
OG IDs: 93523298
Best-hit-arabi-ID: AT5G65020.1
Best-hit-arabi-name: annexin 2
Gorai.007G060900.1.A
PCC=0.325, p=0.174
mRNA Profile
Protein Expression Profile
DPA

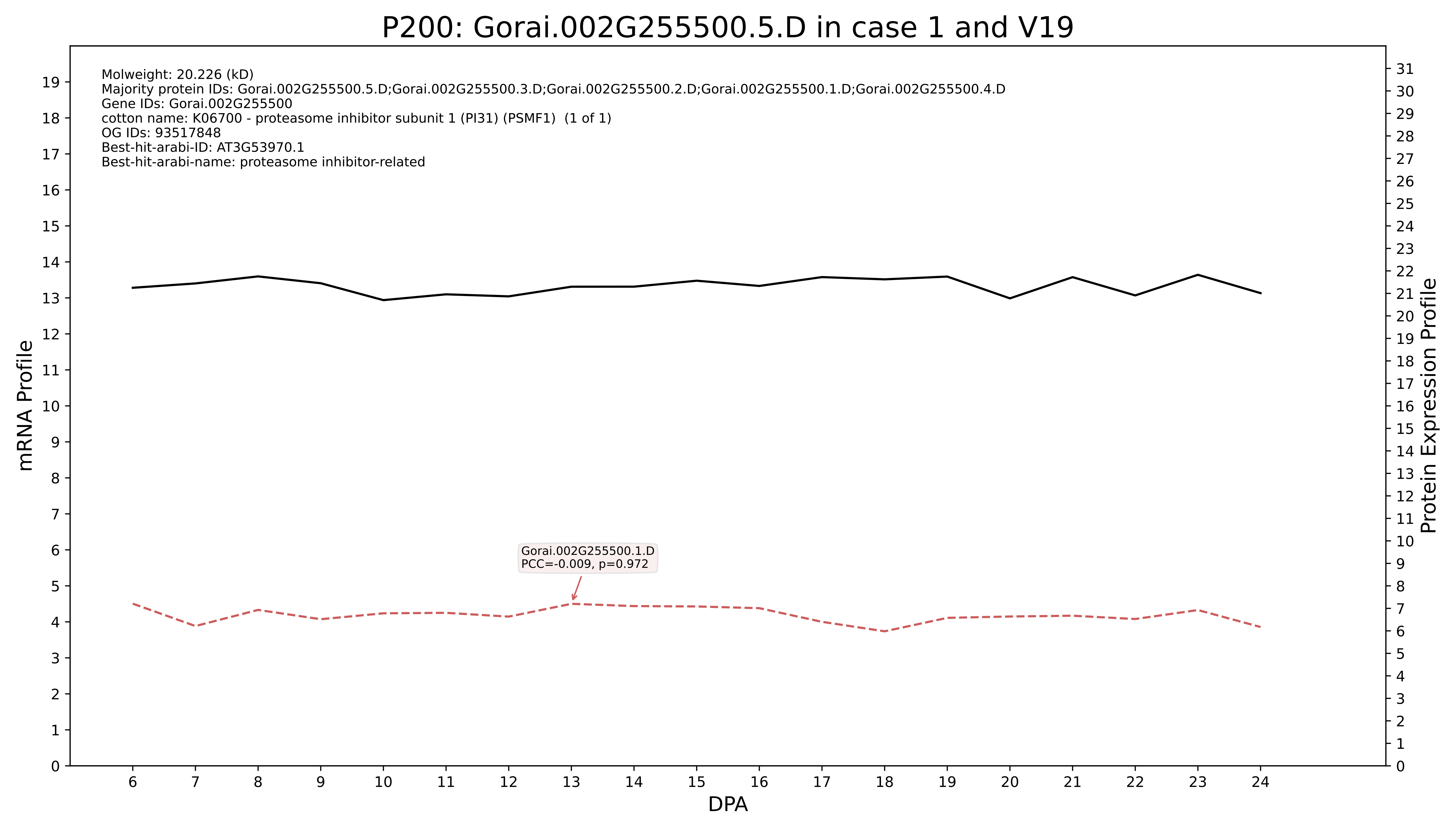

P200: Gorai.002G255500.5.D in case 1 and V19
Molweight: 20.226 (kD)
Majority protein IDs: Gorai.002G255500.5.D;Gorai.002G255500.3.D;Gorai.002G255500.2.D;Gorai.002G255500.1.D;Gorai.002G255500.4.D
Gene IDs: Gorai.002G255500
cotton name: K06700 - proteasome inhibitor subunit 1 (PI31) (PSMF1) (1 of 1)
OG IDs: 93517848
Best-hit-arabi-ID: AT3G53970.1
Best-hit-arabi-name: proteasome inhibitor-related
Gorai.002G255500.1.D
PCC=-0.009, p=0.972
mRNA Profile
Protein Expression Profile
DPA

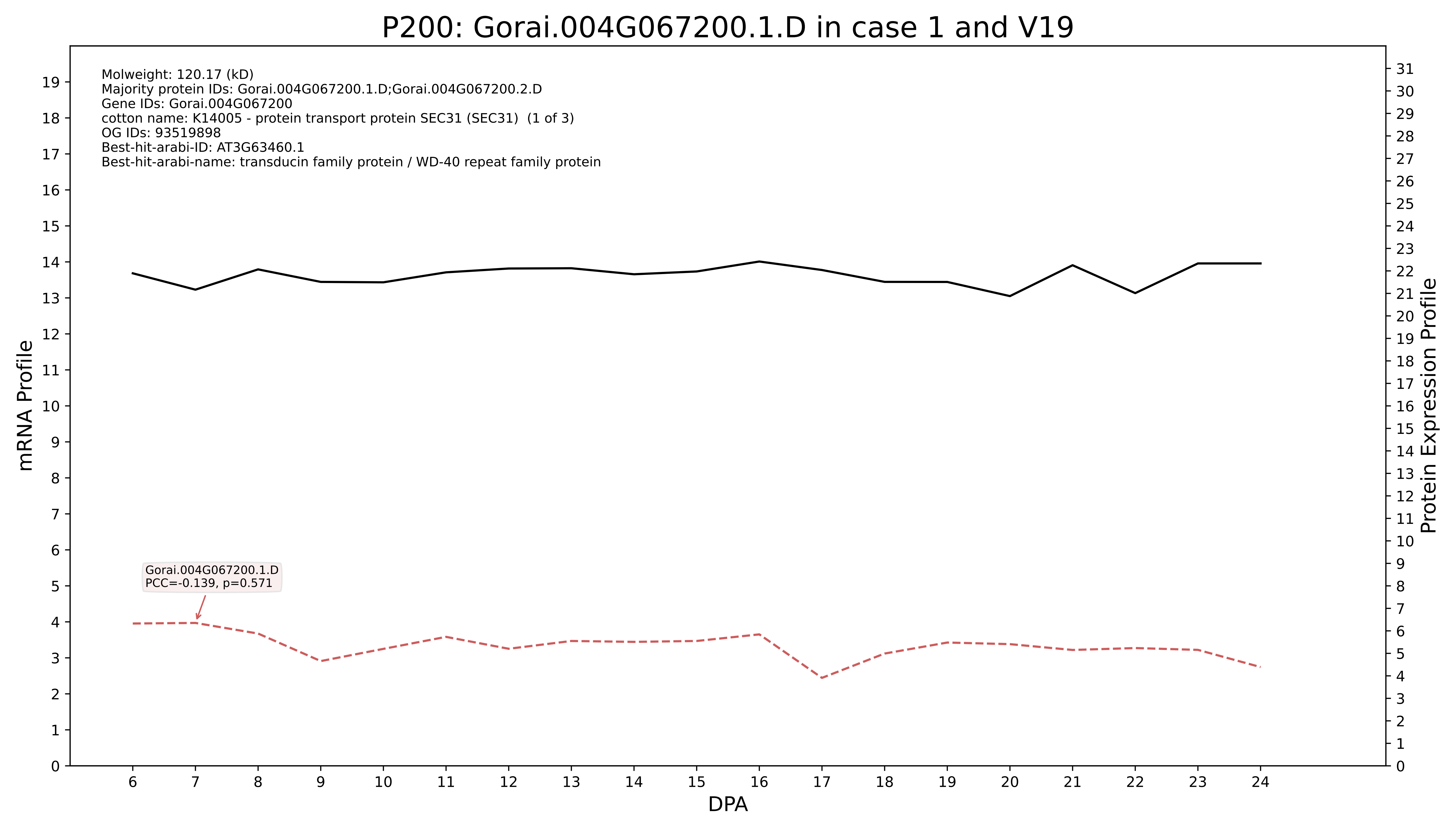
P200: Gorai.004G067200.1.D in case 1 and V19
Molweight: 120.17 (kD)
Majority protein IDs: Gorai.004G067200.1.D;Gorai.004G067200.2.D
Gene IDs: Gorai.004G067200
cotton name: K14005 - protein transport protein SEC31 (SEC31) (1 of 3)
OG IDs: 93519898
Best-hit-arabi-ID: AT3G63460.1
Best-hit-arabi-name: transducin family protein / WD-40 repeat family protein
Gorai.004G067200.1.D
PCC=-0.139, p=0.571
mRNA Profile
Protein Expression Profile
DPA

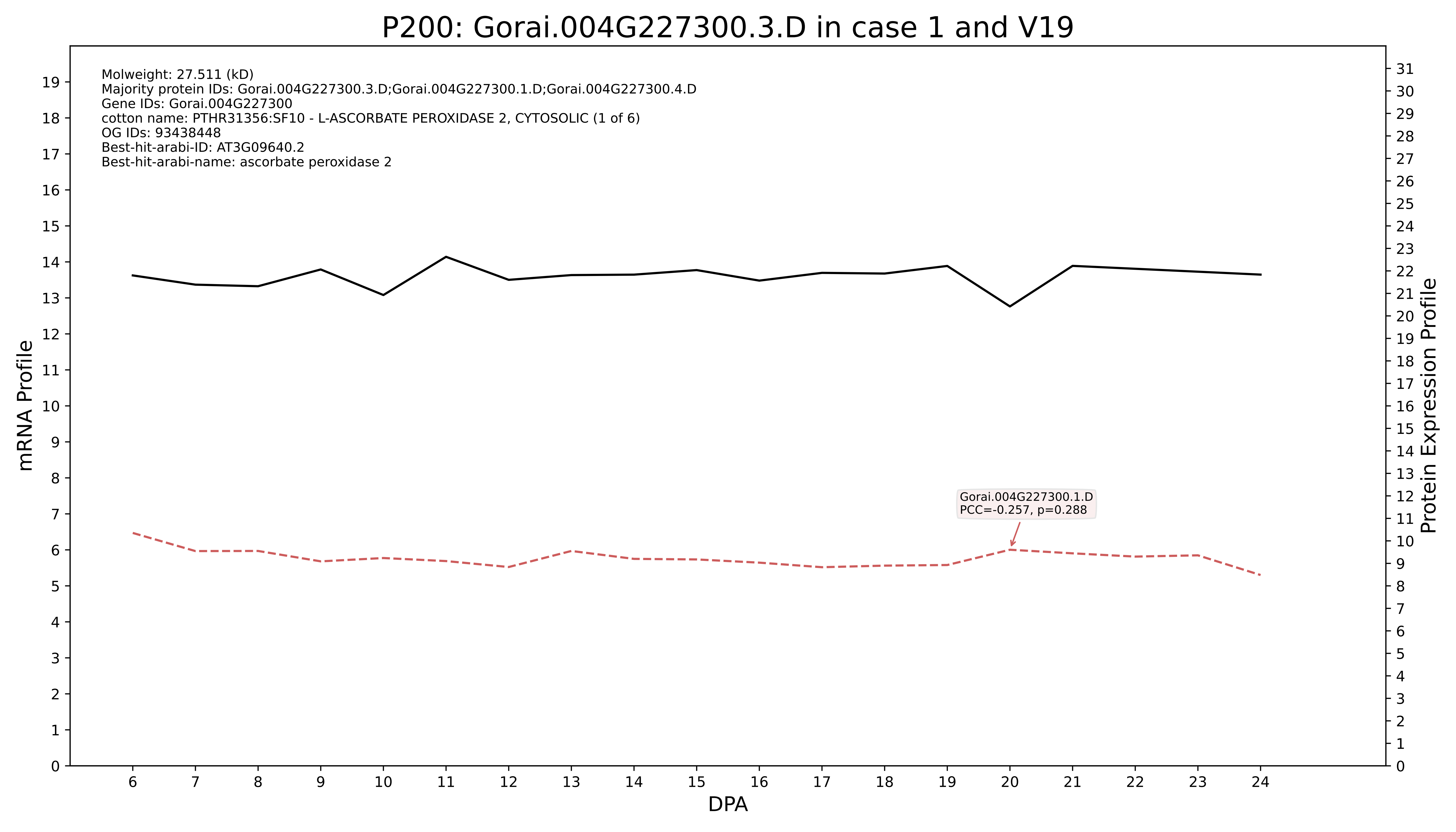

P200: Gorai.004G227300.3.D in case 1 and V19
Molweight: 27.511 (kD)
Majority protein IDs: Gorai.004G227300.3.D;Gorai.004G227300.1.D;Gorai.004G227300.4.D
Gene IDs: Gorai.004G227300
cotton name: PTHR31356:SF10 - L-ASCORBATE PEROXIDASE 2, CYTOSOLIC (1 of 6)
OG IDs: 93438448
Best-hit-arabi-ID: AT3G09640.2
Best-hit-arabi-name: ascorbate peroxidase 2
Gorai.004G227300.1.D
PCC=-0.257, p=0.288
mRNA Profile
Protein Expression Profile
DPA

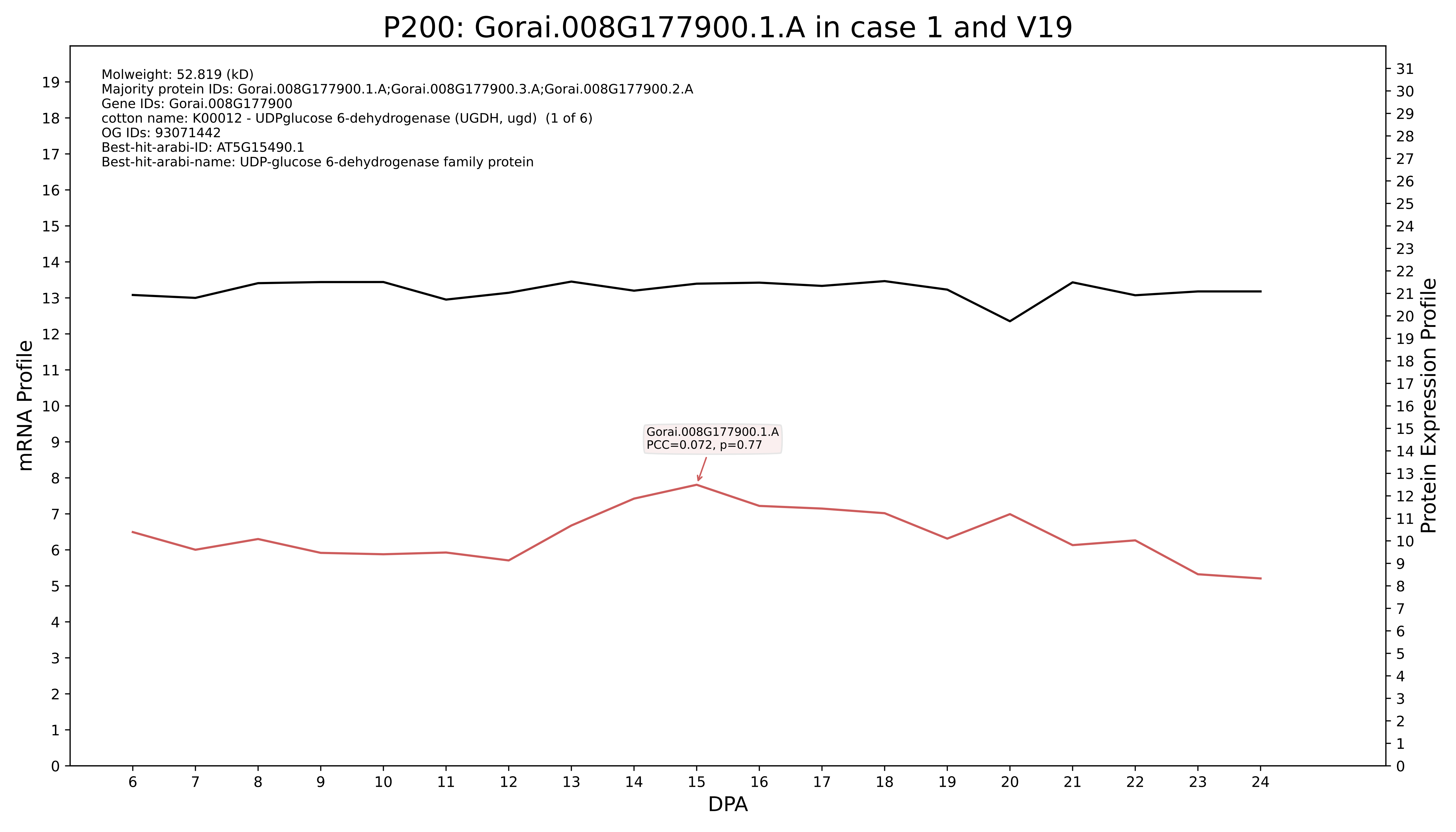

P200: Gorai.008G177900.1.A in case 1 and V19
Molweight: 52.819 (kD)
Majority protein IDs: Gorai.008G177900.1.A;Gorai.008G177900.3.A;Gorai.008G177900.2.A
Gene IDs: Gorai.008G177900
cotton name: K00012 - UDPglucose 6-dehydrogenase (UGDH, ugd) (1 of 6)
OG IDs: 93071442
Best-hit-arabi-ID: AT5G15490.1
Best-hit-arabi-name: UDP-glucose 6-dehydrogenase family protein
Gorai.008G177900.1.A
PCC=0.072, p=0.77
mRNA Profile
Protein Expression Profile
DPA

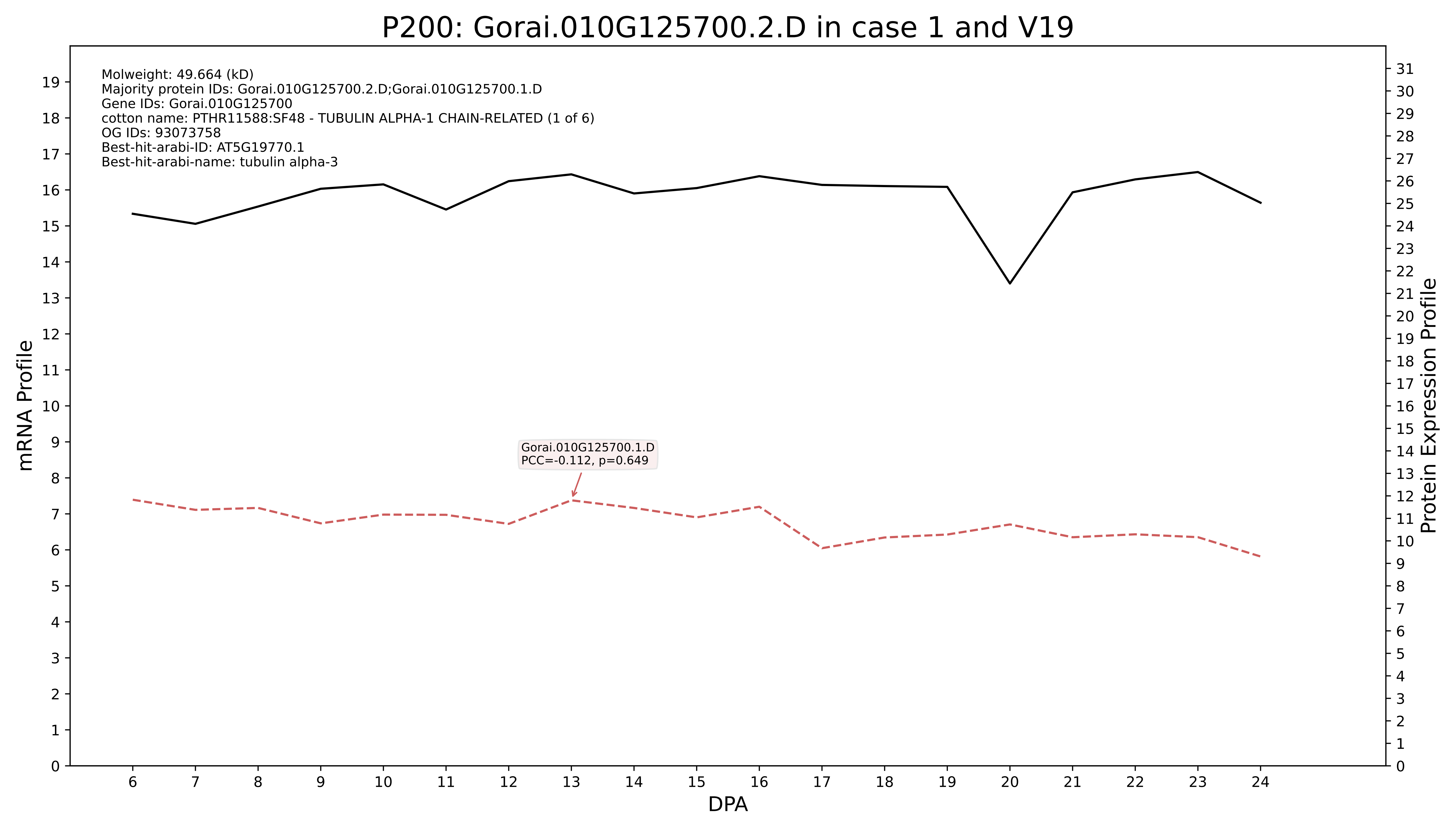
P200: Gorai.010G125700.2.D in case 1 and V19
Molweight: 49.664 (kD)
Majority protein IDs: Gorai.010G125700.2.D;Gorai.010G125700.1.D
Gene IDs: Gorai.010G125700
cotton name: PTHR11588:SF48 - TUBULIN ALPHA-1 CHAIN-RELATED (1 of 6)
OG IDs: 93073758
Best-hit-arabi-ID: AT5G19770.1
Best-hit-arabi-name: tubulin alpha-3
Gorai.010G125700.1.D
PCC=-0.112, p=0.649
mRNA Profile
Protein Expression Profile
DPA

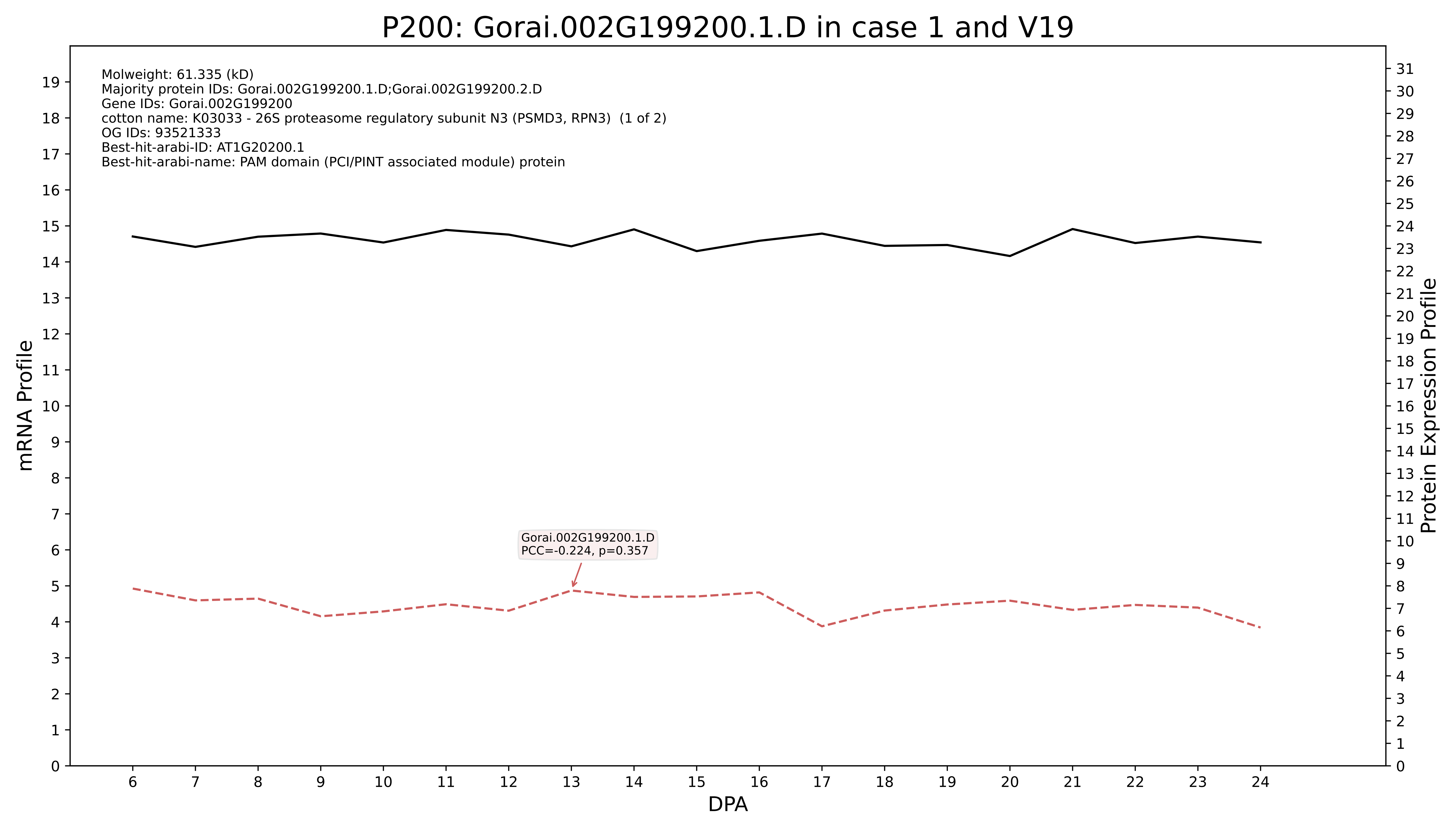
P200: Gorai.002G199200.1.D in case 1 and V19
Molweight: 61.335 (kD)
Majority protein IDs: Gorai.002G199200.1.D;Gorai.002G199200.2.D
Gene IDs: Gorai.002G199200
cotton name: K03033 - 26S proteasome regulatory subunit N3 (PSMD3, RPN3) (1 of 2)
OG IDs: 93521333
Best-hit-arabi-ID: AT1G20200.1
Best-hit-arabi-name: PAM domain (PCI/PINT associated module) protein
Gorai.002G199200.1.D
PCC=-0.224, p=0.357
mRNA Profile
Protein Expression Profile
DPA

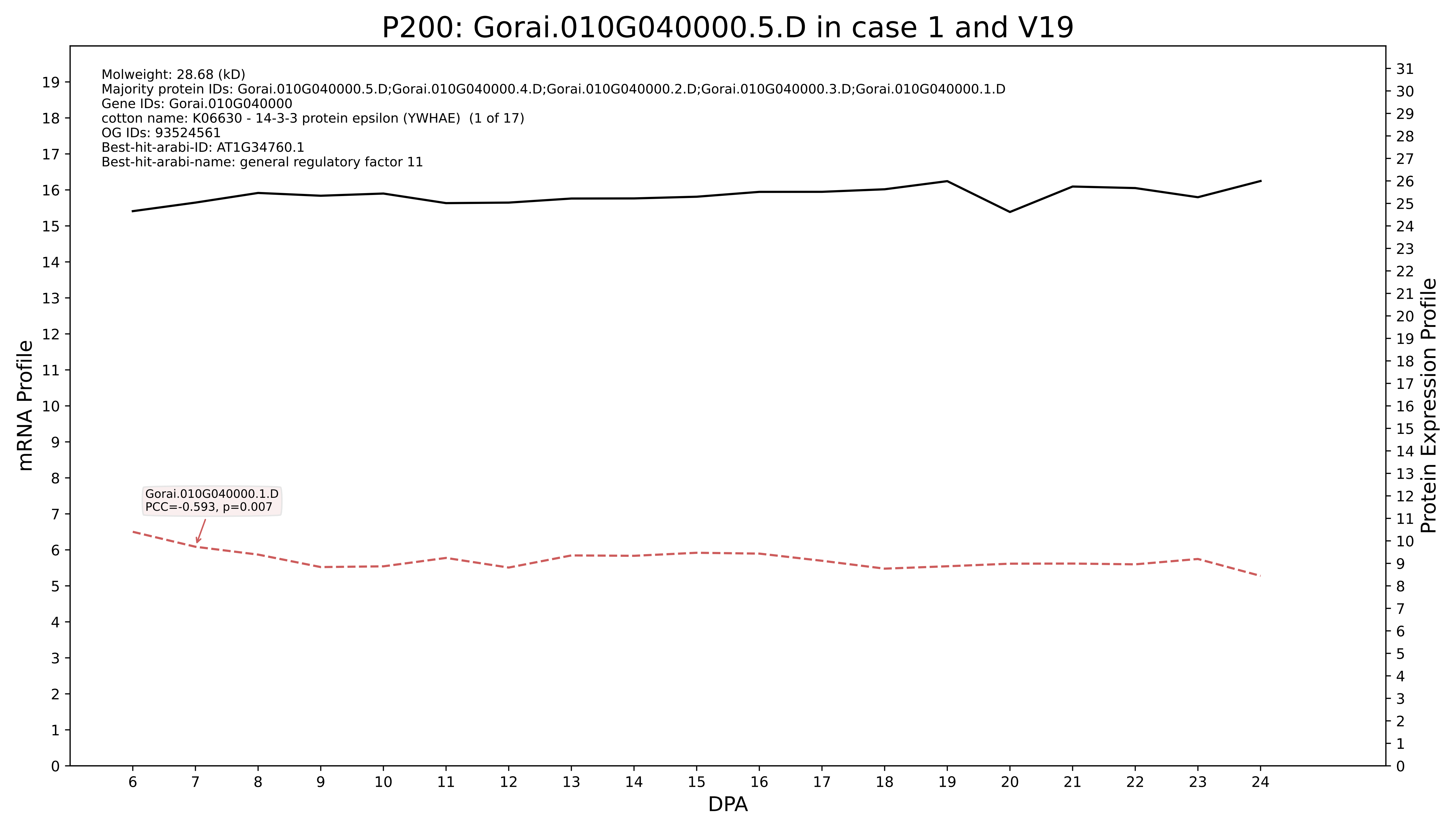
P200: Gorai.010G040000.5.D in case 1 and V19
Molweight: 28.68 (kD)
Majority protein IDs: Gorai.010G040000.5.D;Gorai.010G040000.4.D;Gorai.010G040000.2.D;Gorai.010G040000.3.D;Gorai.010G040000.1.D
Gene IDs: Gorai.010G040000
cotton name: K06630 - 14-3-3 protein epsilon (YWHAE) (1 of 17)
OG IDs: 93524561
Best-hit-arabi-ID: AT1G34760.1
Best-hit-arabi-name: general regulatory factor 11
Gorai.010G040000.1.D
PCC=-0.593, p=0.007
mRNA Profile
Protein Expression Profile
DPA
0
1
2
3
4
5
6
7
8
9
10
11
12
13
14
15
16
17
18
19
20
21
22
23
24
25
26
27
28
29
30
31

P200: Gorai.013G037600.1.D in case 1 and V19

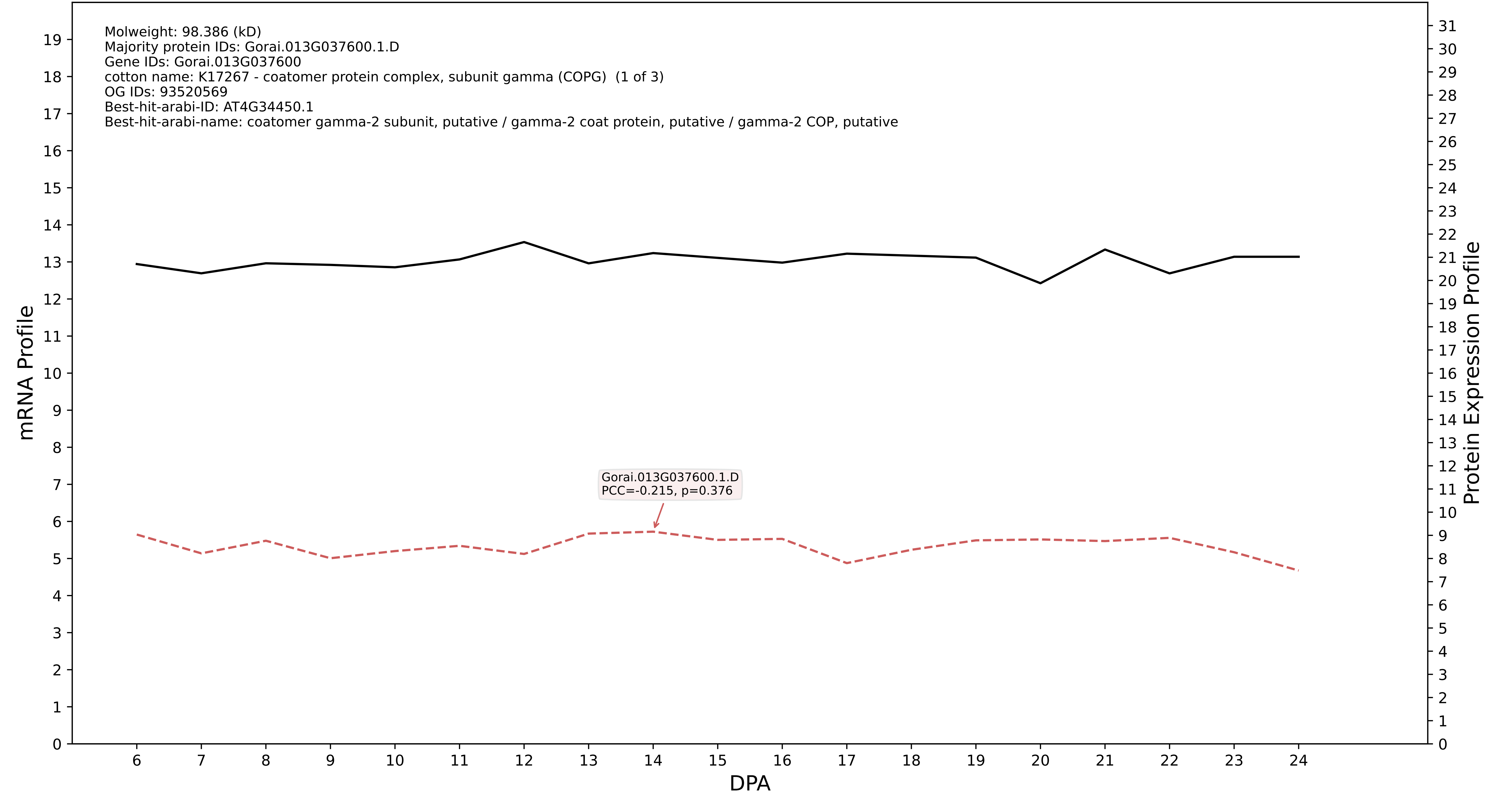

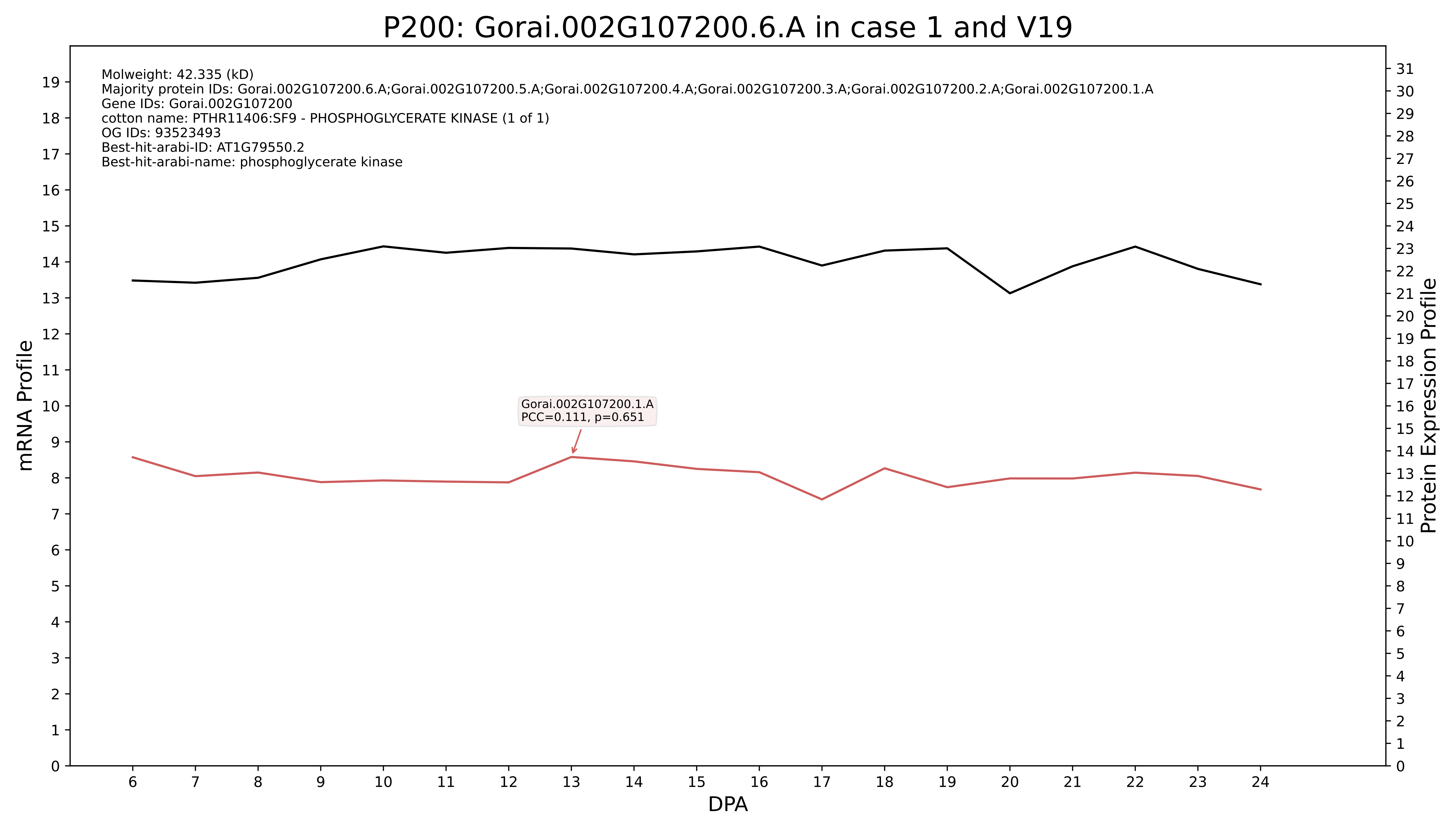
P200: Gorai.002G107200.6.A in case 1 and V19
Molweight: 42.335 (kD)
Majority protein IDs: Gorai.002G107200.6.A;Gorai.002G107200.5.A;Gorai.002G107200.4.A;Gorai.002G107200.3.A;Gorai.002G107200.2.A;Gorai.002G107200.1.A
Gene IDs: Gorai.002G107200
cotton name: PTHR11406:SF9 - PHOSPHOGLYCERATE KINASE (1 of 1)
OG IDs: 93523493
Best-hit-arabi-ID: AT1G79550.2
Best-hit-arabi-name: phosphoglycerate kinase
Gorai.002G107200.1.A
PCC=0.111, p=0.651
mRNA Profile
Protein Expression Profile
DPA

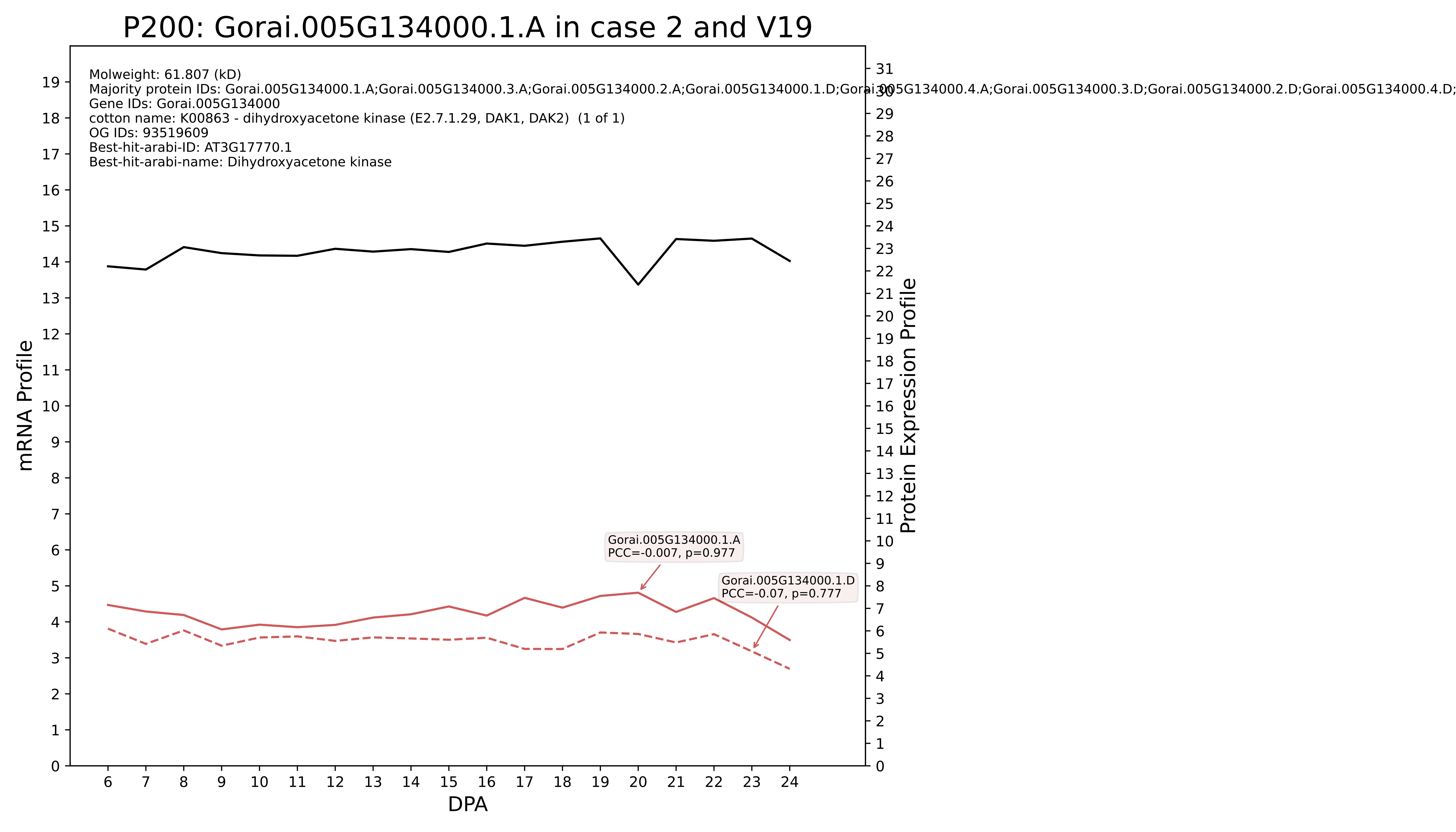
P200: Gorai.005G134000.1.A in case 2 and V19
Molweight: 61.807 (kD)
Majority protein IDs: Gorai.005G134000.1.A;Gorai.005G134000.3.A;Gorai.005G134000.2.A;Gorai.005G134000.1.D;Gorai.005G134000.4.A;Gorai.005G134000.3.D;Gorai.005G134000.2.D;Gorai.005G134000.4.D;
Gene IDs: Gorai.005G134000
cotton name: K00863 - dihydroxyacetone kinase (E2.7.1.29, DAK1, DAK2) (1 of 1)
OG IDs: 93519609
Best-hit-arabi-ID: AT3G17770.1
Best-hit-arabi-name: Dihydroxyacetone kinase
Gorai.005G134000.1.A
PCC=-0.007, p=0.977
Gorai.005G134000.1.D
PCC=-0.07, p=0.777
mRNA Profile
Protein Expression Profile
DPA

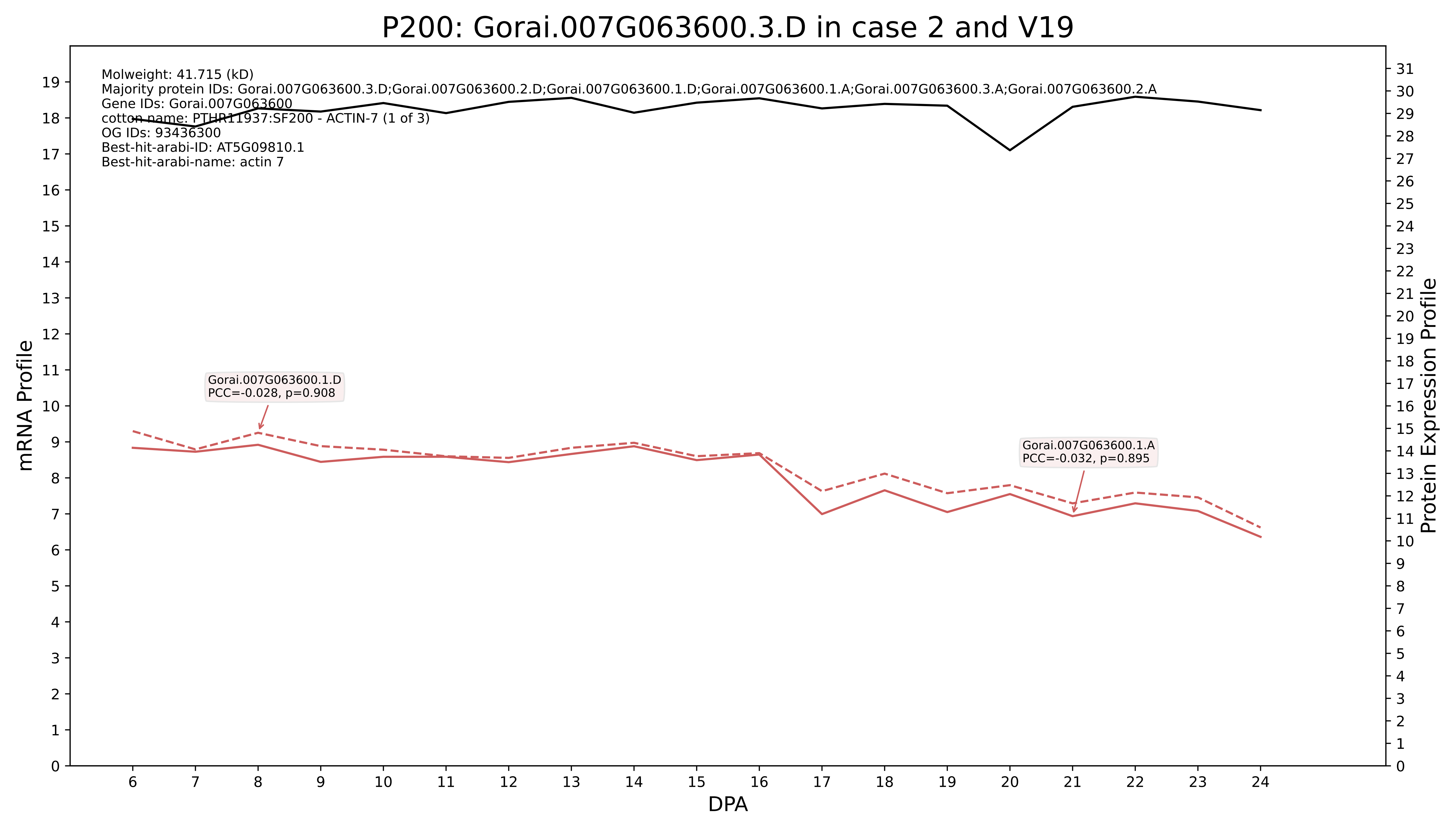

P200: Gorai.007G063600.3.D in case 2 and V19
Molweight: 41.715 (kD)
Majority protein IDs: Gorai.007G063600.3.D;Gorai.007G063600.2.D;Gorai.007G063600.1.D;Gorai.007G063600.1.A;Gorai.007G063600.3.A;Gorai.007G063600.2.A
Gene IDs: Gorai.007G063600
cotton-name: PTHR11937:SF200 - ACTIN-7 (1 of 3)
OG IDs: 93436300
Best-hit-arabi-ID: AT5G09810.1
Best-hit-arabi-name: actin 7
Gorai.007G063600.1.D
PCC=-0.028, p=0.908
Gorai.007G063600.1.A
PCC=-0.032, p=0.895
mRNA Profile
Protein Expression Profile
DPA
6
7
8
9
10
11
12
13
14
15
16
17
18
19
20
21
22
23
24

P200: Gorai.004G037900.1.D in case 2 and V19

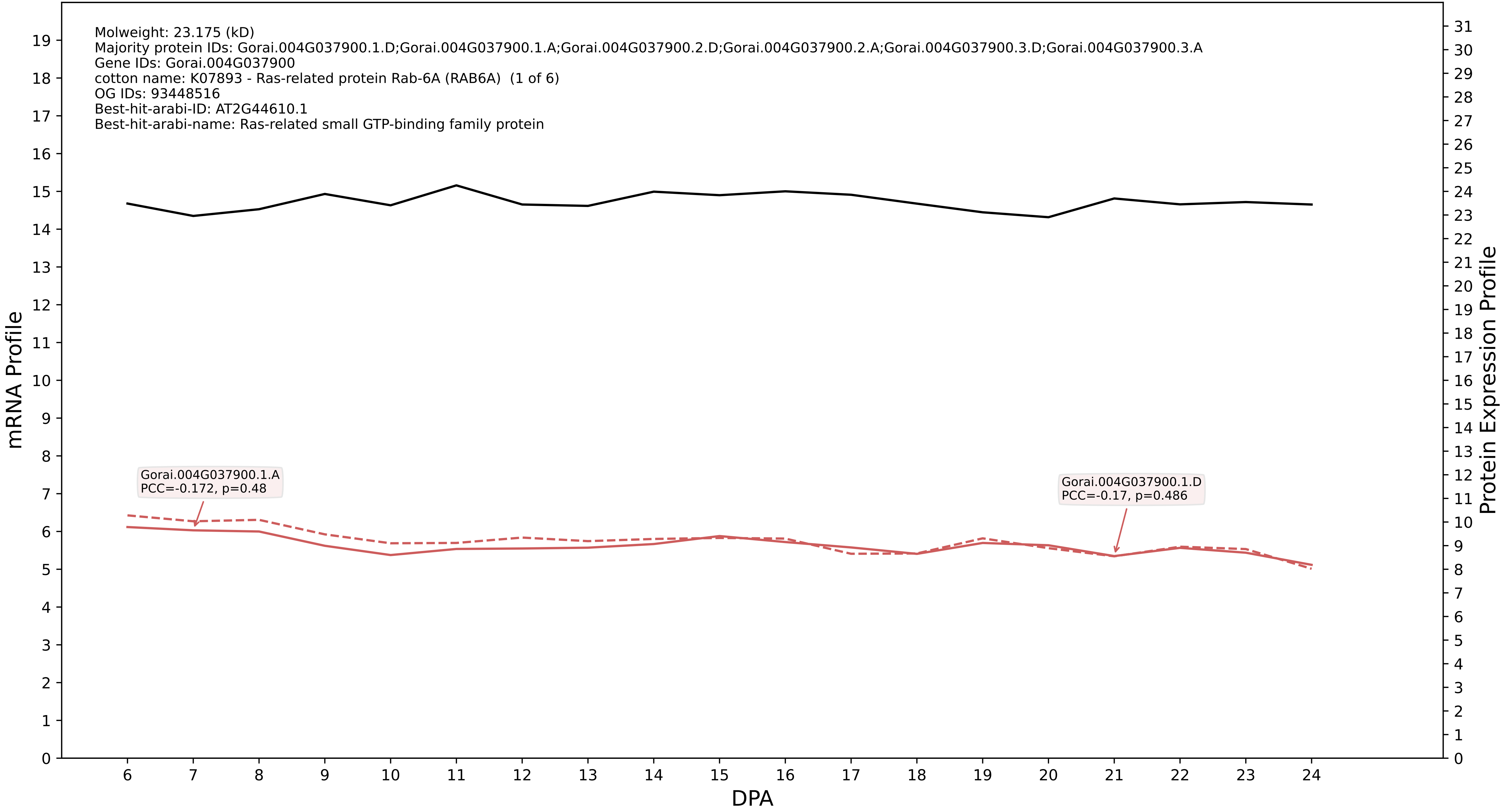

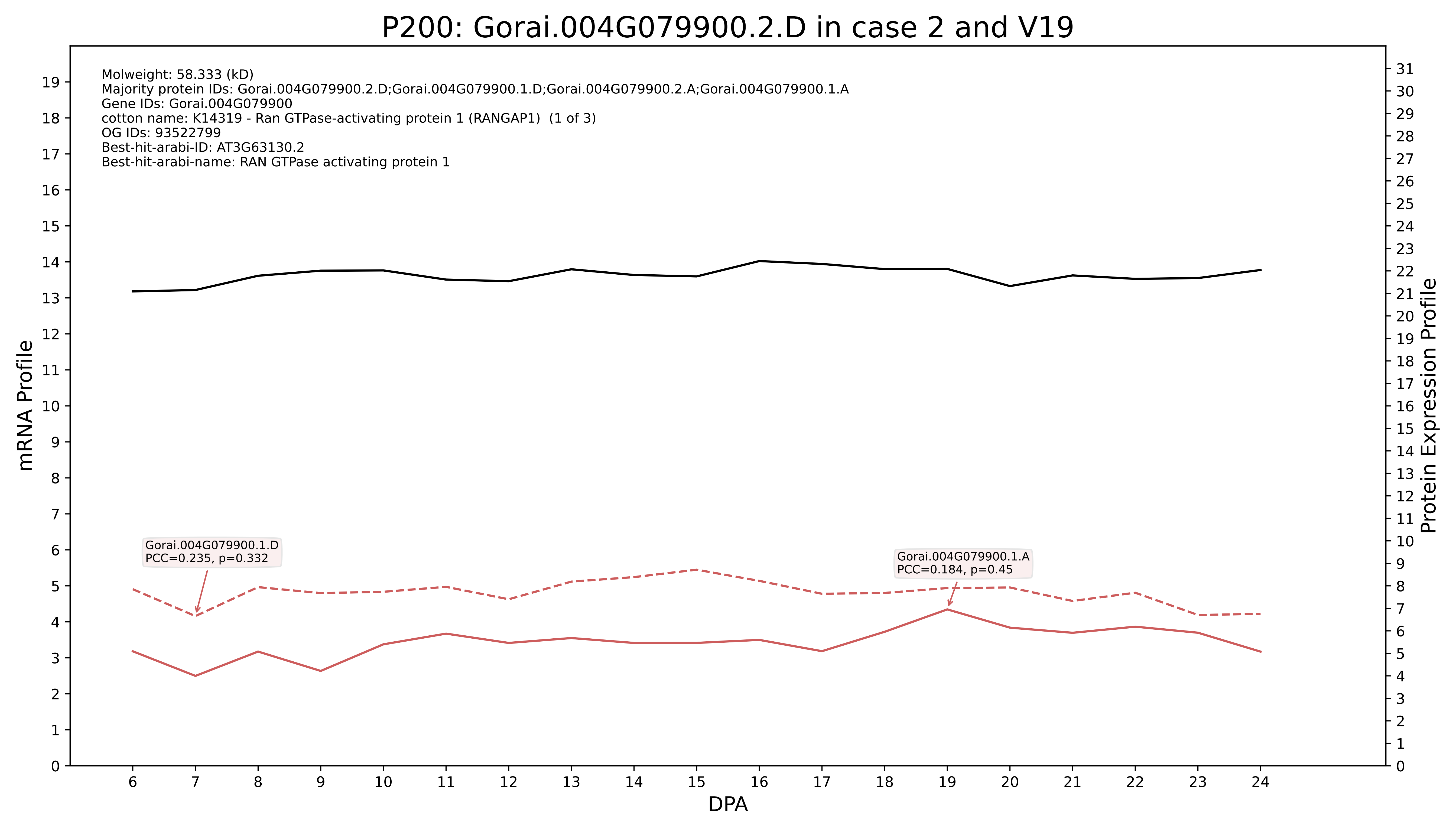
P200: Gorai.004G079900.2.D in case 2 and V19
Molweight: 58.333 (kD)
Majority protein IDs: Gorai.004G079900.2.D;Gorai.004G079900.1.D;Gorai.004G079900.2.A;Gorai.004G079900.1.A
Gene IDs: Gorai.004G079900
cotton name: K14319 - Ran GTPase-activating protein 1 (RANGAP1) (1 of 3)
OG IDs: 93522799
Best-hit-arabi-ID: AT3G63130.2
Best-hit-arabi-name: RAN GTPase activating protein 1
Gorai.004G079900.1.D
PCC=0.235, p=0.332
Gorai.004G079900.1.A
PCC=0.184, p=0.45
mRNA Profile
Protein Expression Profile
DPA

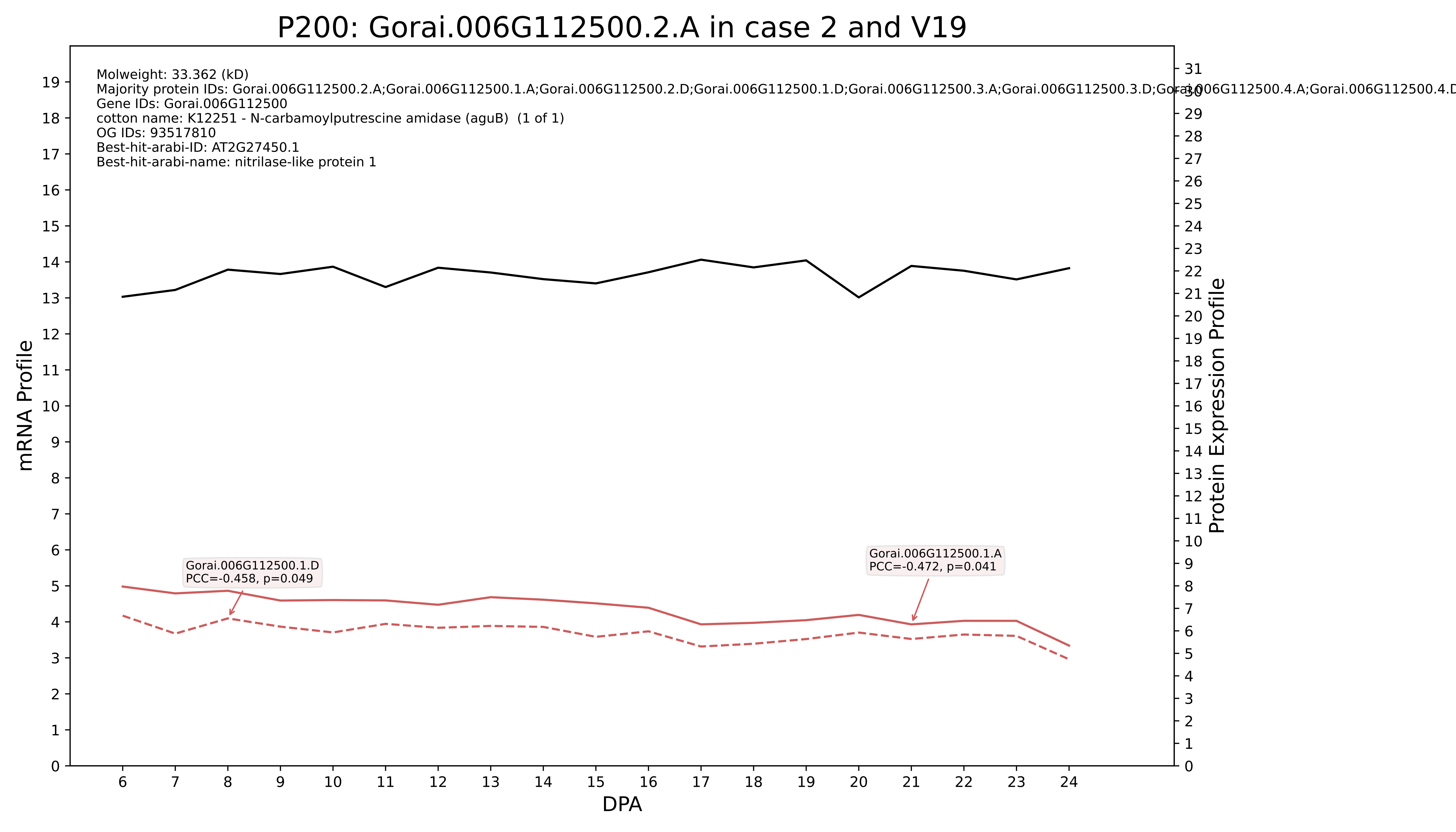
P200: Gorai.006G112500.2.A in case 2 and V19
Molweight: 33.362 (kD)
Majority protein IDs: Gorai.006G112500.2.A;Gorai.006G112500.1.A;Gorai.006G112500.2.D;Gorai.006G112500.1.D;Gorai.006G112500.3.A;Gorai.006G112500.3.D;Gorai.006G112500.4.A;Gorai.006G112500.4.D
Gene IDs: Gorai.006G112500
cotton name: K12251 - N-carbamoylputrescine amidase (aguB) (1 of 1)
OG IDs: 93517810
Best-hit-arabi-ID: AT2G27450.1
Best-hit-arabi-name: nitrilase-like protein 1
Gorai.006G112500.1.D
PCC=-0.458, p=0.049
Gorai.006G112500.1.A
PCC=-0.472, p=0.041
mRNA Profile
Protein Expression Profile
DPA

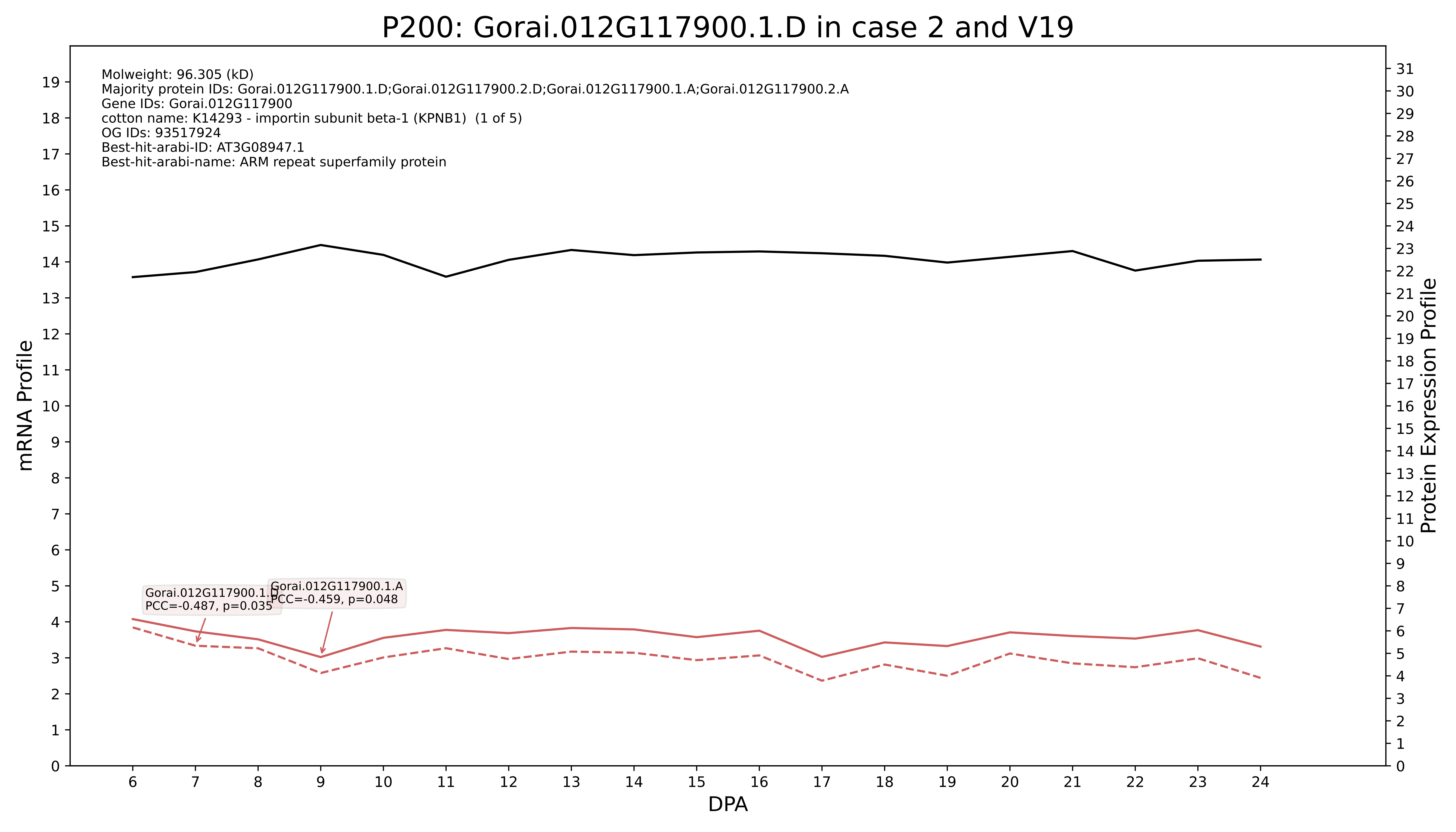
P200: Gorai.012G117900.1.D in case 2 and V19
Molweight: 96.305 (kD)
Majority protein IDs: Gorai.012G117900.1.D;Gorai.012G117900.2.D;Gorai.012G117900.1.A;Gorai.012G117900.2.A
Gene IDs: Gorai.012G117900
cotton name: K14293 - importin subunit beta-1 (KPNB1) (1 of 5)
OG IDs: 93517924
Best-hit-arabi-ID: AT3G08947.1
Best-hit-arabi-name: ARM repeat superfamily protein
Gorai.012G117900.1.D
PCC=-0.487, p=0.035
Gorai.012G117900.1.A
PCC=-0.459, p=0.048
mRNA Profile
Protein Expression Profile
DPA
0
1
2
3
4
5
6
7
8
9
10
11
12
13
14
15
16
17
18
19
20
21
22
23
24
25
26
27
28
29
30
31

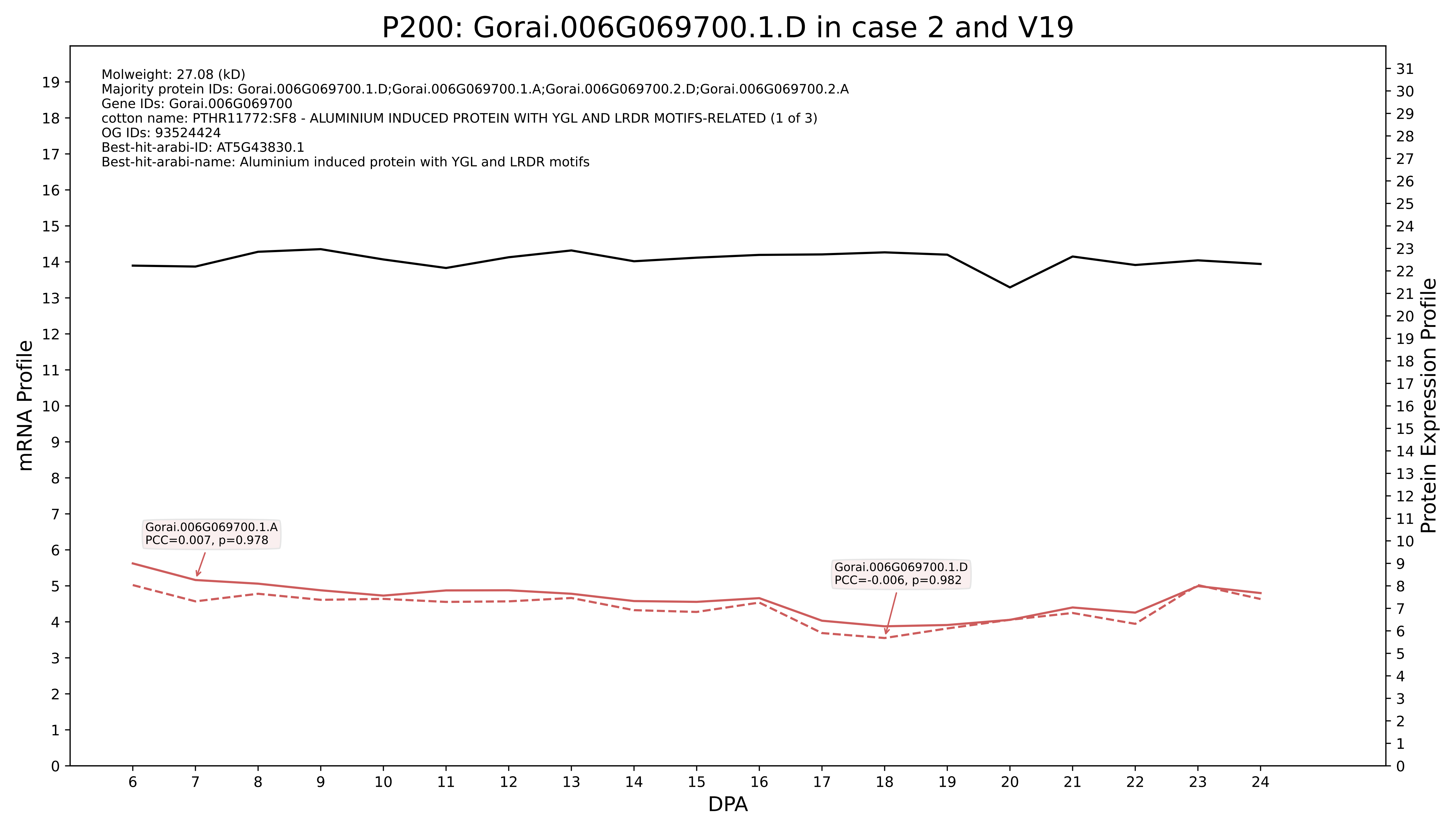
P200: Gorai.006G069700.1.D in case 2 and V19
Molweight: 27.08 (kD)
Majority protein IDs: Gorai.006G069700.1.D;Gorai.006G069700.1.A;Gorai.006G069700.2.D;Gorai.006G069700.2.A
Gene IDs: Gorai.006G069700
cotton name: PTHR11772:SF8 - ALUMINIUM INDUCED PROTEIN WITH YGL AND LRDR MOTIFS-RELATED (1 of 3)
OG IDs: 93524424
Best-hit-arabi-ID: AT5G43830.1
Best-hit-arabi-name: Aluminium induced protein with YGL and LRDR motifs
Gorai.006G069700.1.A
PCC=0.007, p=0.978
Gorai.006G069700.1.D
PCC=-0.006, p=0.982
mRNA Profile
Protein Expression Profile
DPA

P200: Gorai.002G236600.1.D in case 2 and V19

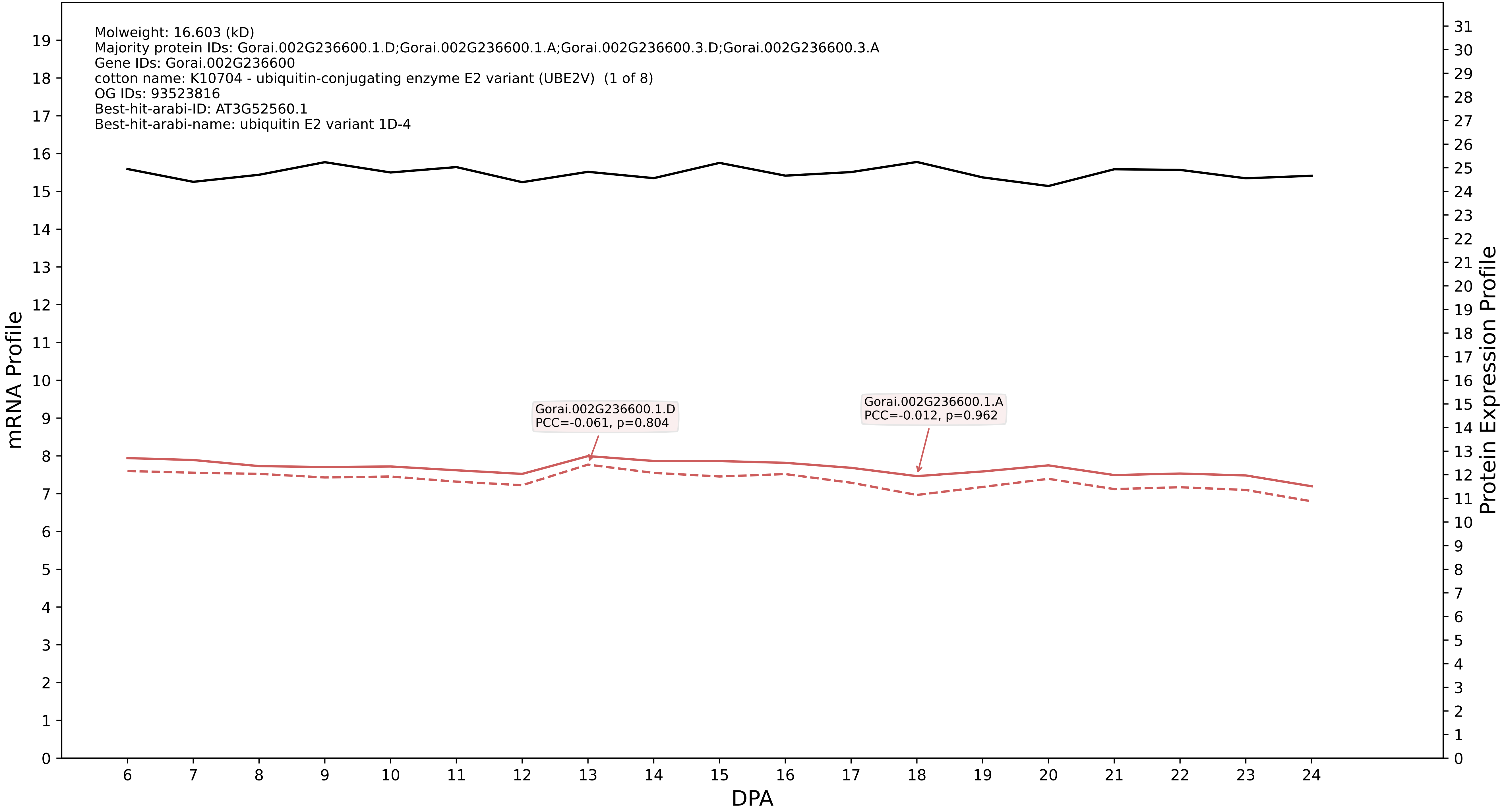

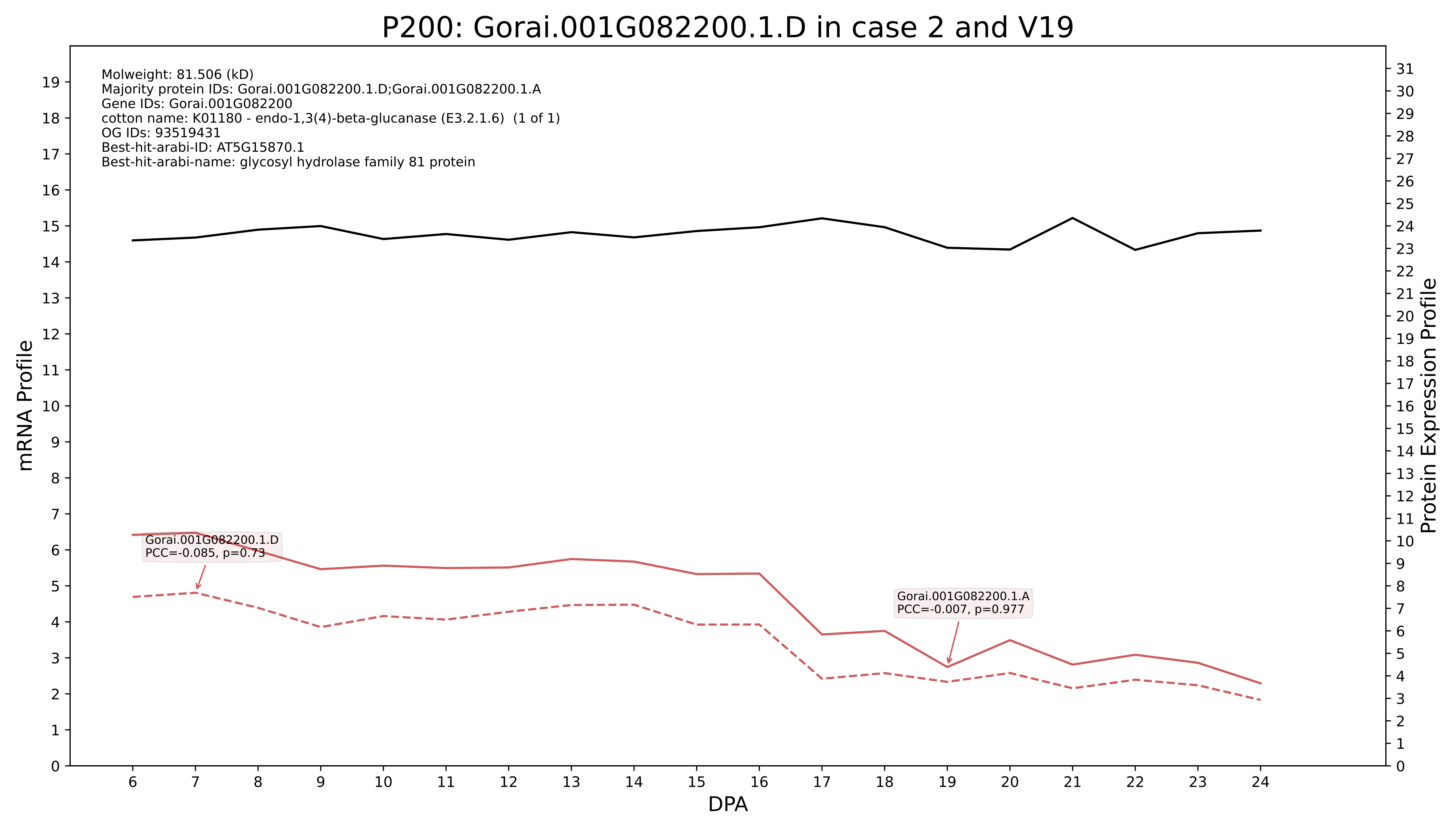
P200: Gorai.001G082200.1.D in case 2 and V19
Molweight: 81.506 (kD)
Majority protein IDs: Gorai.001G082200.1.D;Gorai.001G082200.1.A
Gene IDs: Gorai.001G082200
cotton name: K01180 - endo-1,3(4)-beta-glucanase (E3.2.1.6) (1 of 1)
OG IDs: 93519431
Best-hit-arabi-ID: AT5G15870.1
Best-hit-arabi-name: glycosyl hydrolase family 81 protein
Gorai.001G082200.1.D
PCC=-0.085, p=0.73
Gorai.001G082200.1.A
PCC=-0.007, p=0.977
mRNA Profile
Protein Expression Profile
DPA
0
1
2
3
4
5
6
7
8
9
10
11
12
13
14
15
16
17
18
19
20
21
22
23
24
25
26
27
28
29
30
31

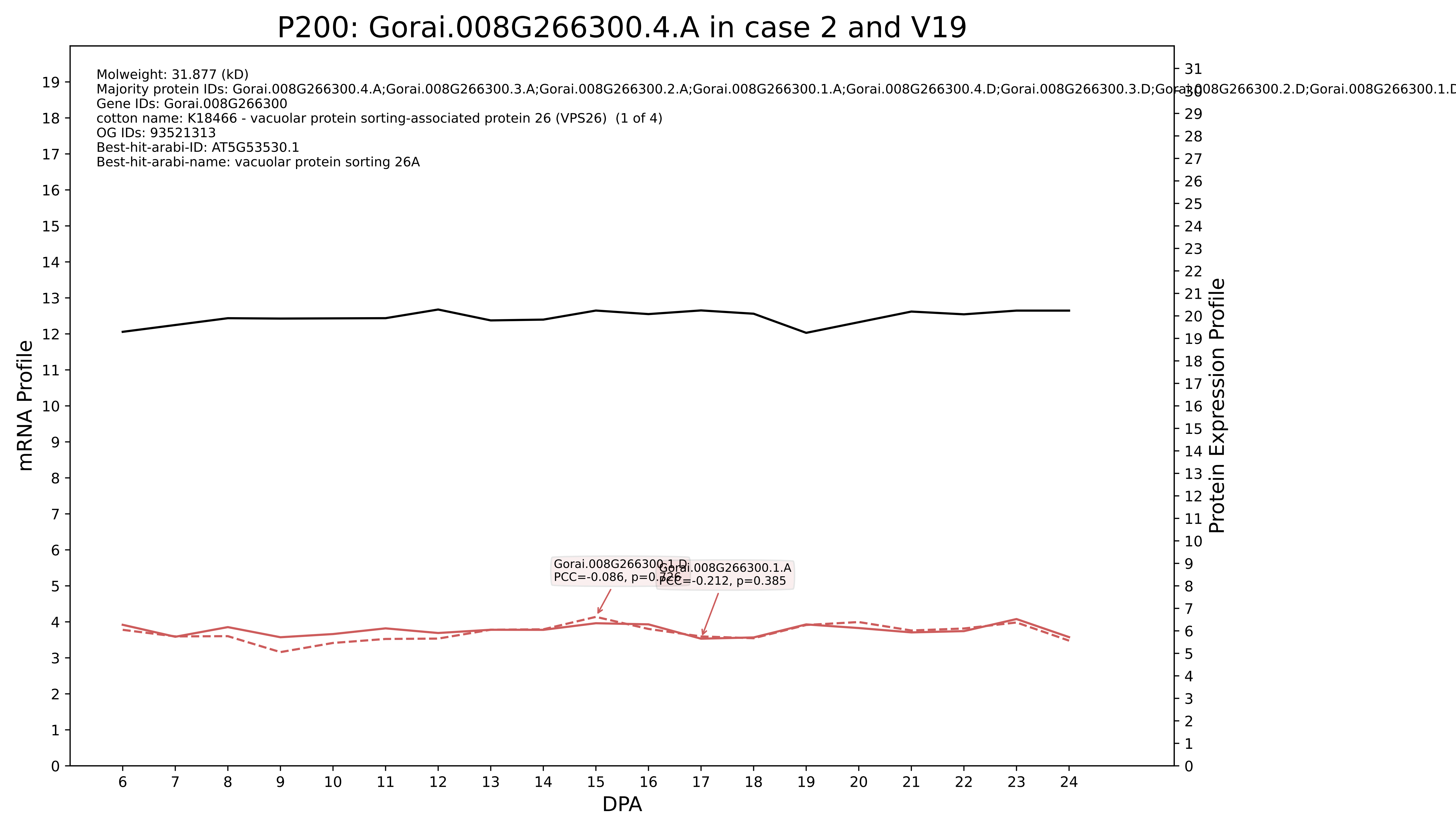
P200: Gorai.008G266300.4.A in case 2 and V19
Molweight: 31.877 (kD)
Majority protein IDs: Gorai.008G266300.4.A;Gorai.008G266300.3.A;Gorai.008G266300.2.A;Gorai.008G266300.1.A;Gorai.008G266300.4.D;Gorai.008G266300.3.D;Gorai.008G266300.2.D;Gorai.008G266300.1.D
Gene IDs: Gorai.008G266300
cotton name: K18466 - vacuolar protein sorting-associated protein 26 (VPS26) (1 of 4)
OG IDs: 93521313
Best-hit-arabi-ID: AT5G53530.1
Best-hit-arabi-name: vacuolar protein sorting 26A
Gorai.008G266300.1.D
PCC=-0.086, p=0.726
Gorai.008G266300.1.A
PCC=-0.212, p=0.385
mRNA Profile
Protein Expression Profile
DPA

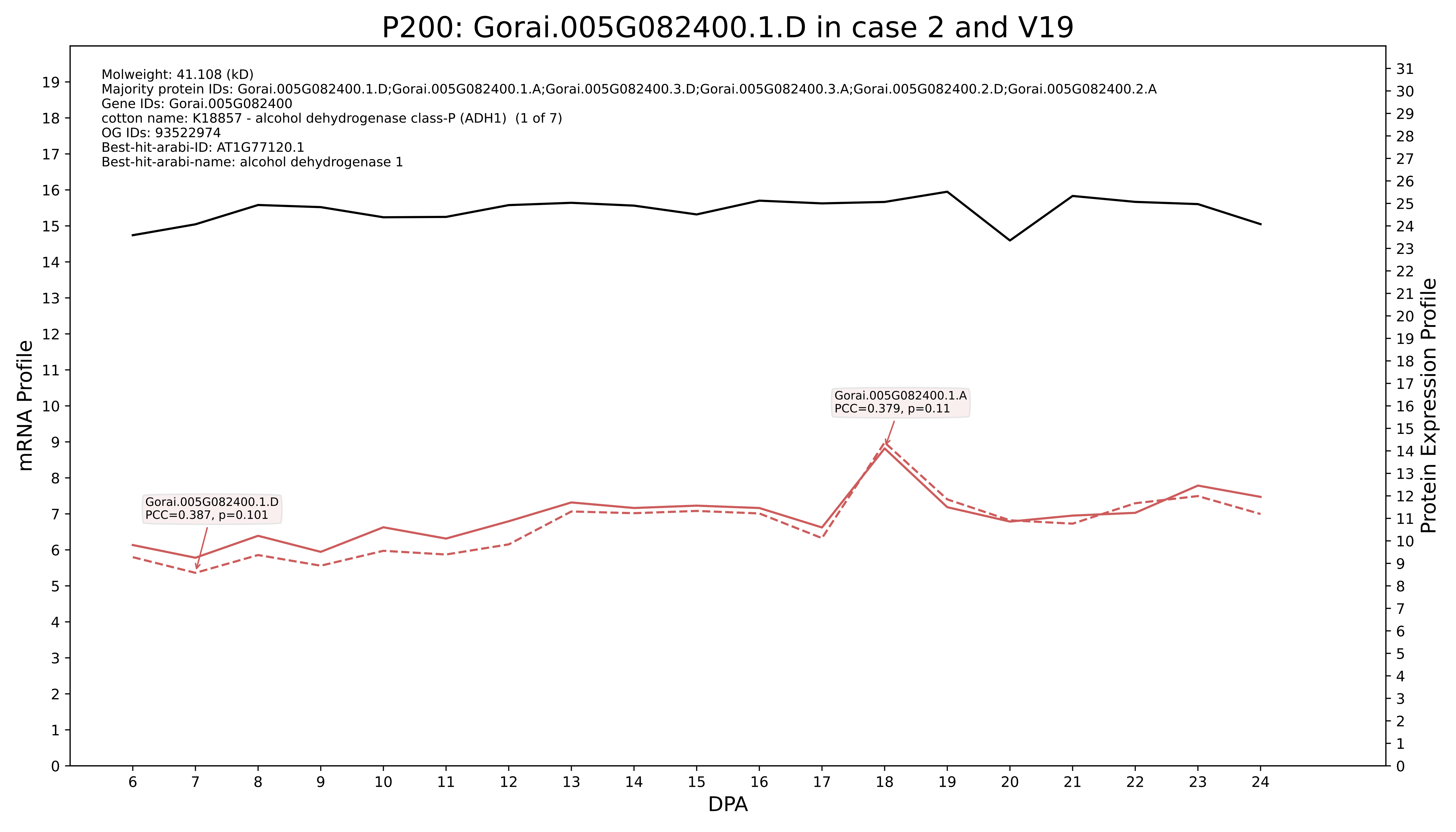
P200: Gorai.005G082400.1.D in case 2 and V19
Molweight: 41.108 (kD)
Majority protein IDs: Gorai.005G082400.1.D;Gorai.005G082400.1.A;Gorai.005G082400.3.D;Gorai.005G082400.3.A;Gorai.005G082400.2.D;Gorai.005G082400.2.A
Gene IDs: Gorai.005G082400
cotton name: K18857 - alcohol dehydrogenase class-P (ADH1) (1 of 7)
OG IDs: 93522974
Best-hit-arabi-ID: AT1G77120.1
Best-hit-arabi-name: alcohol dehydrogenase 1
Gorai.005G082400.1.A
PCC=0.379, p=0.11
Gorai.005G082400.1.D
PCC=0.387, p=0.101
mRNA Profile
Protein Expression Profile
DPA

P200: Gorai.009G224700.2.D in case 2 and V19

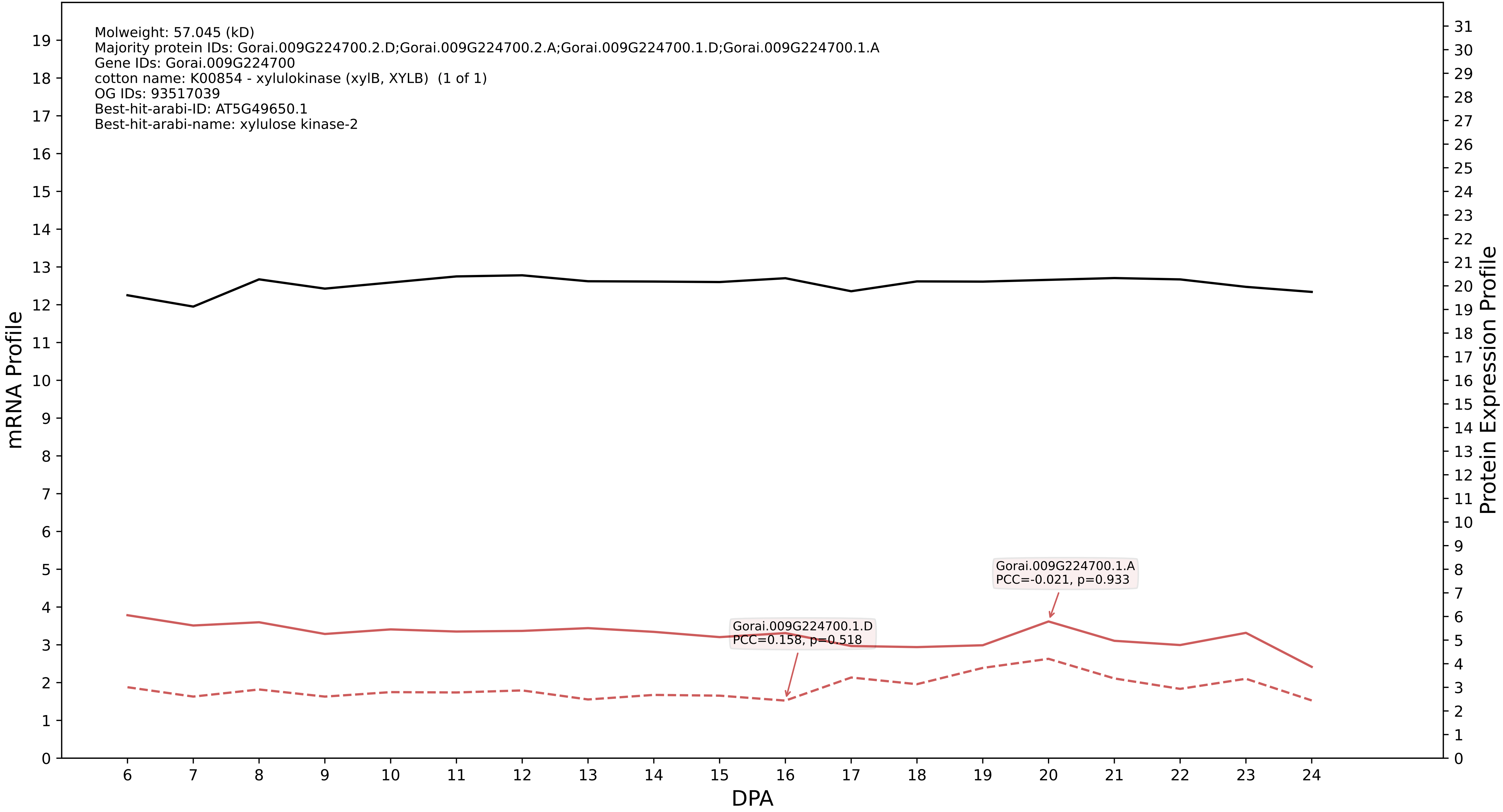

009G219000.7.D in case 2 and V19

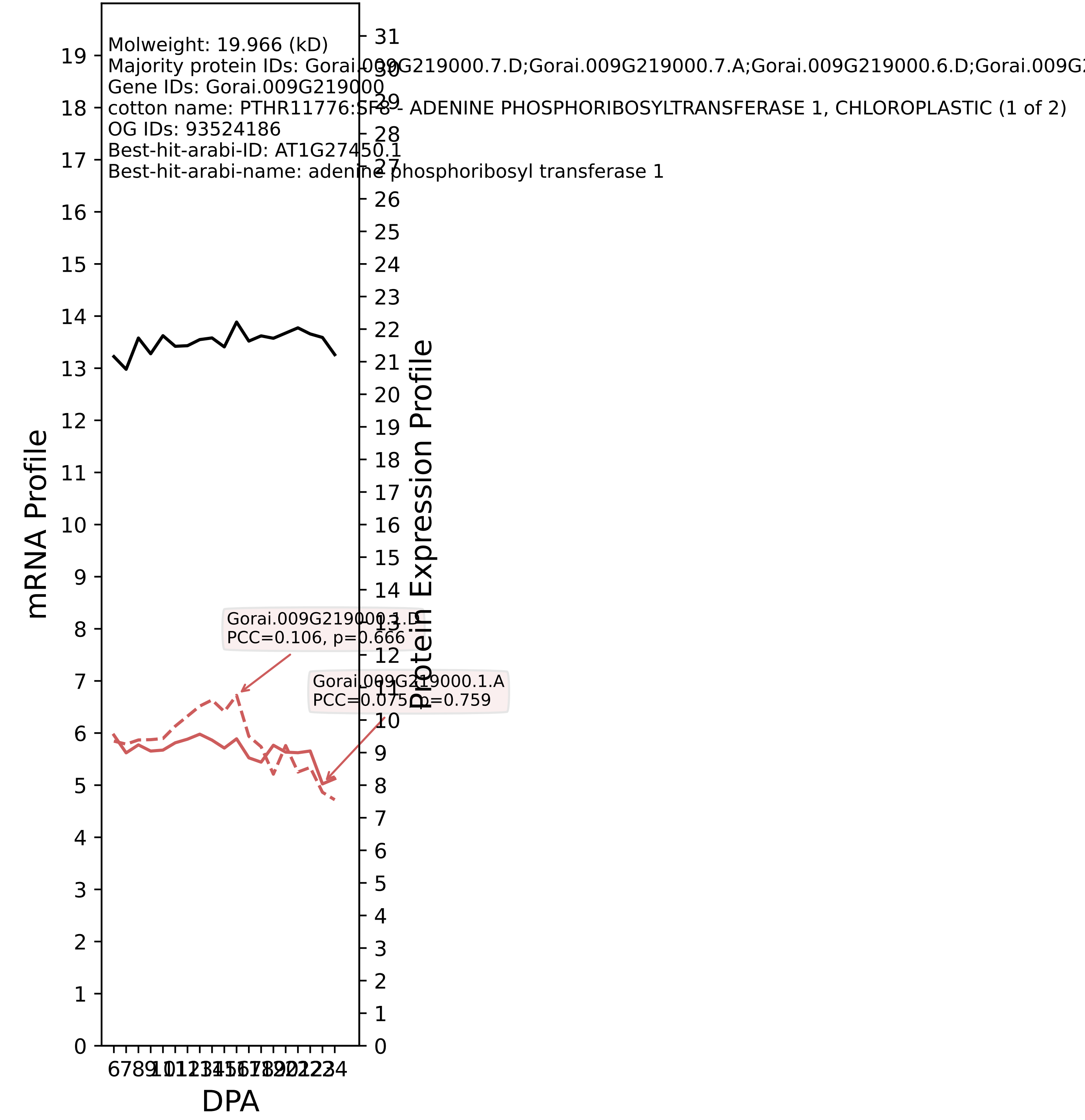

00: Gorai.006G132900.6.D in case 2 and V19

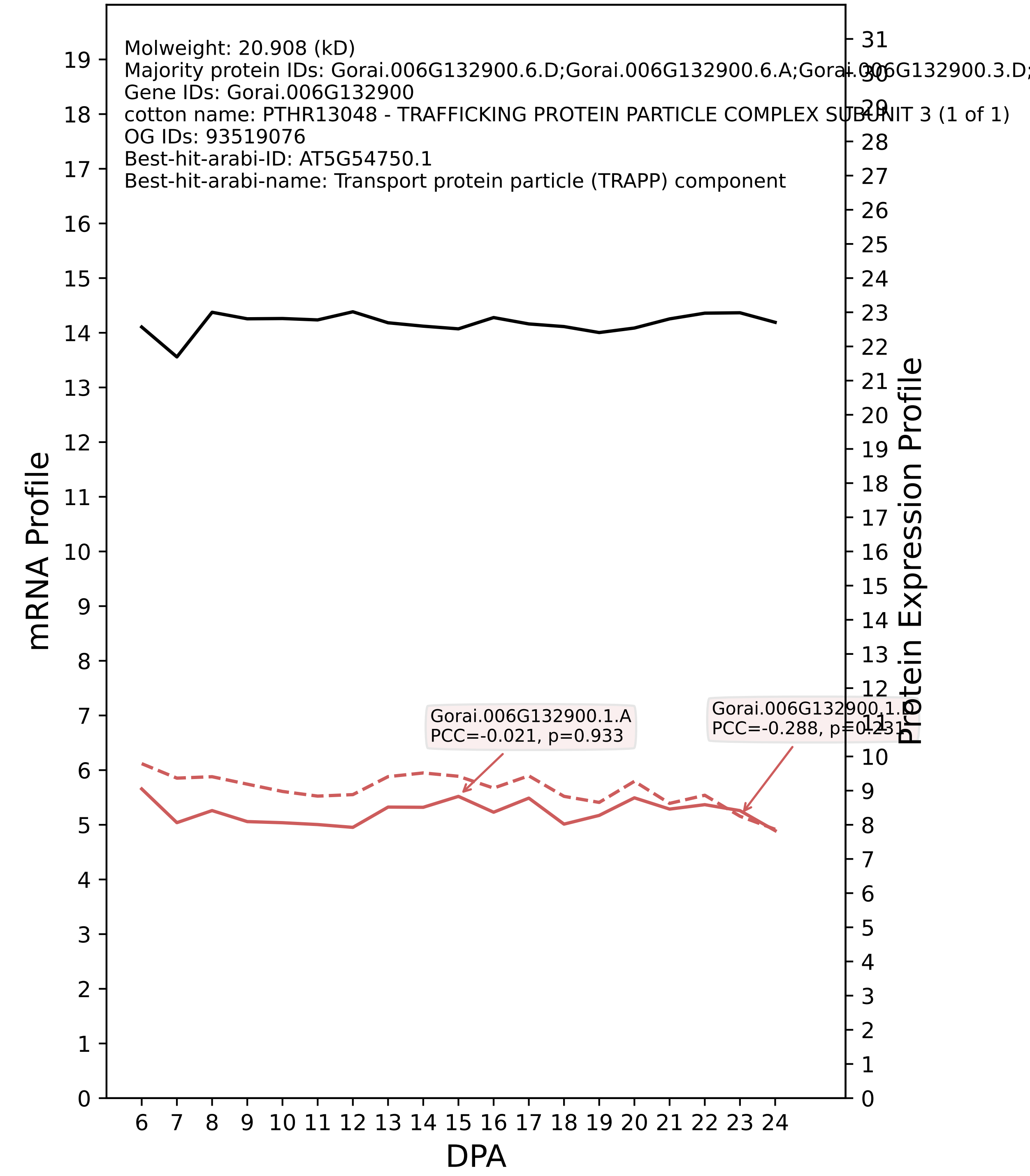

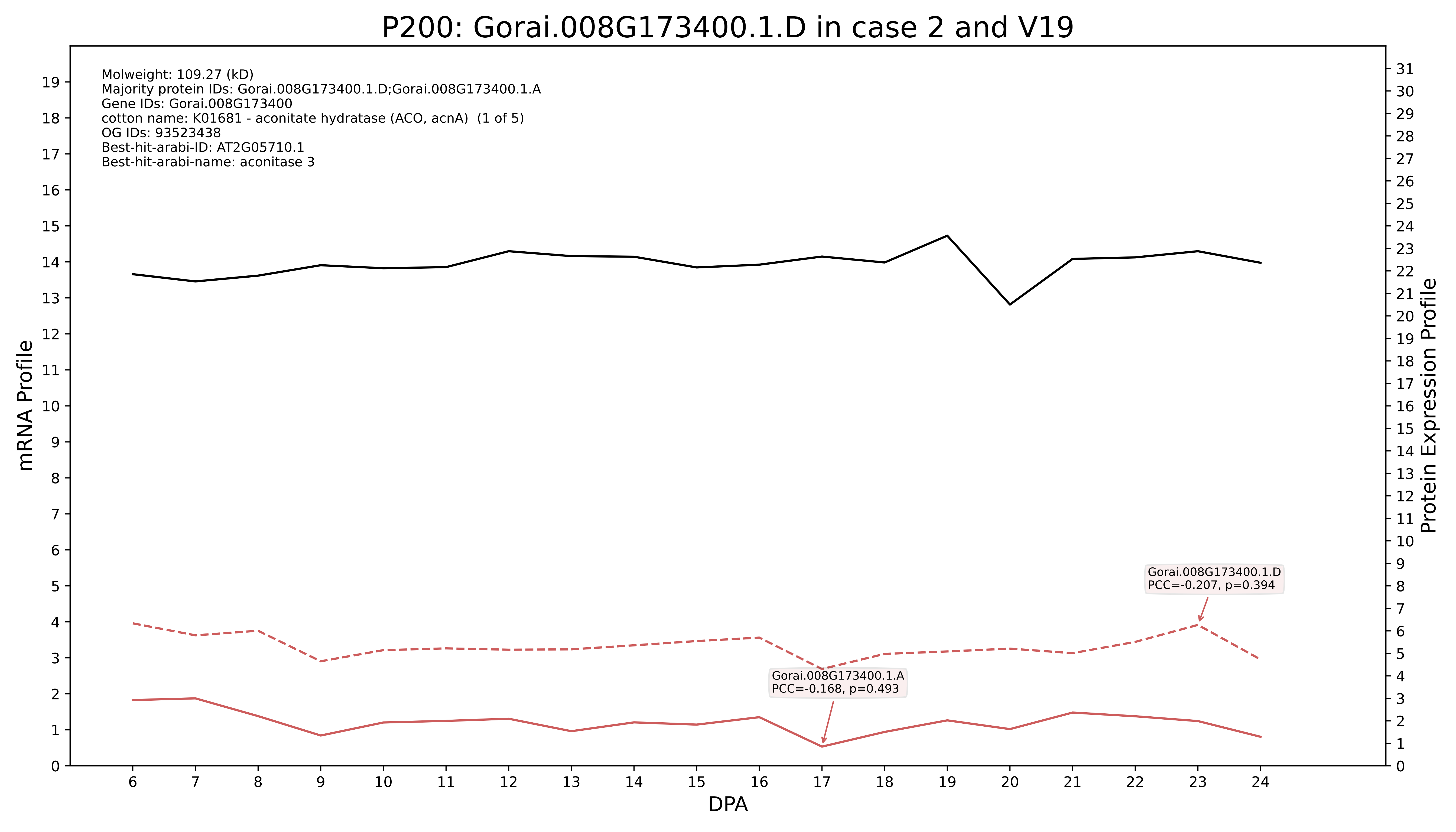
P200: Gorai.008G173400.1.D in case 2 and V19
Molweight: 109.27 (kD)
Majority protein IDs: Gorai.008G173400.1.D;Gorai.008G173400.1.A
Gene IDs: Gorai.008G173400
cotton name: K01681 - aconitate hydratase (ACO, acnA) (1 of 5)
OG IDs: 93523438
Best-hit-arabi-ID: AT2G05710.1
Best-hit-arabi-name: aconitase 3
Gorai.008G173400.1.D
PCC=-0.207, p=0.394
Gorai.008G173400.1.A
PCC=-0.168, p=0.493
mRNA Profile
Protein Expression Profile
DPA

P200: Gorai.002G268600.3.D in case 2 and V19

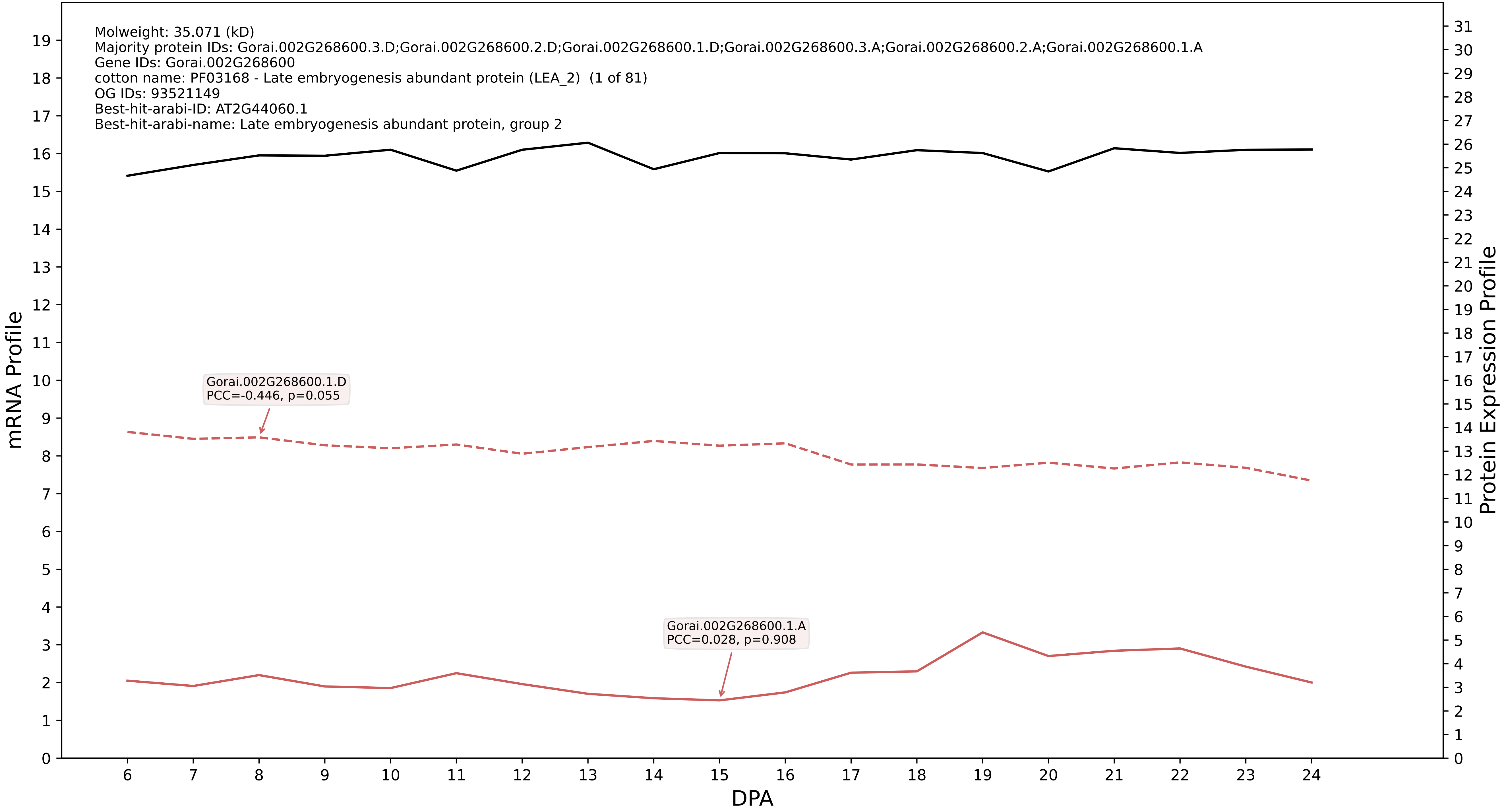

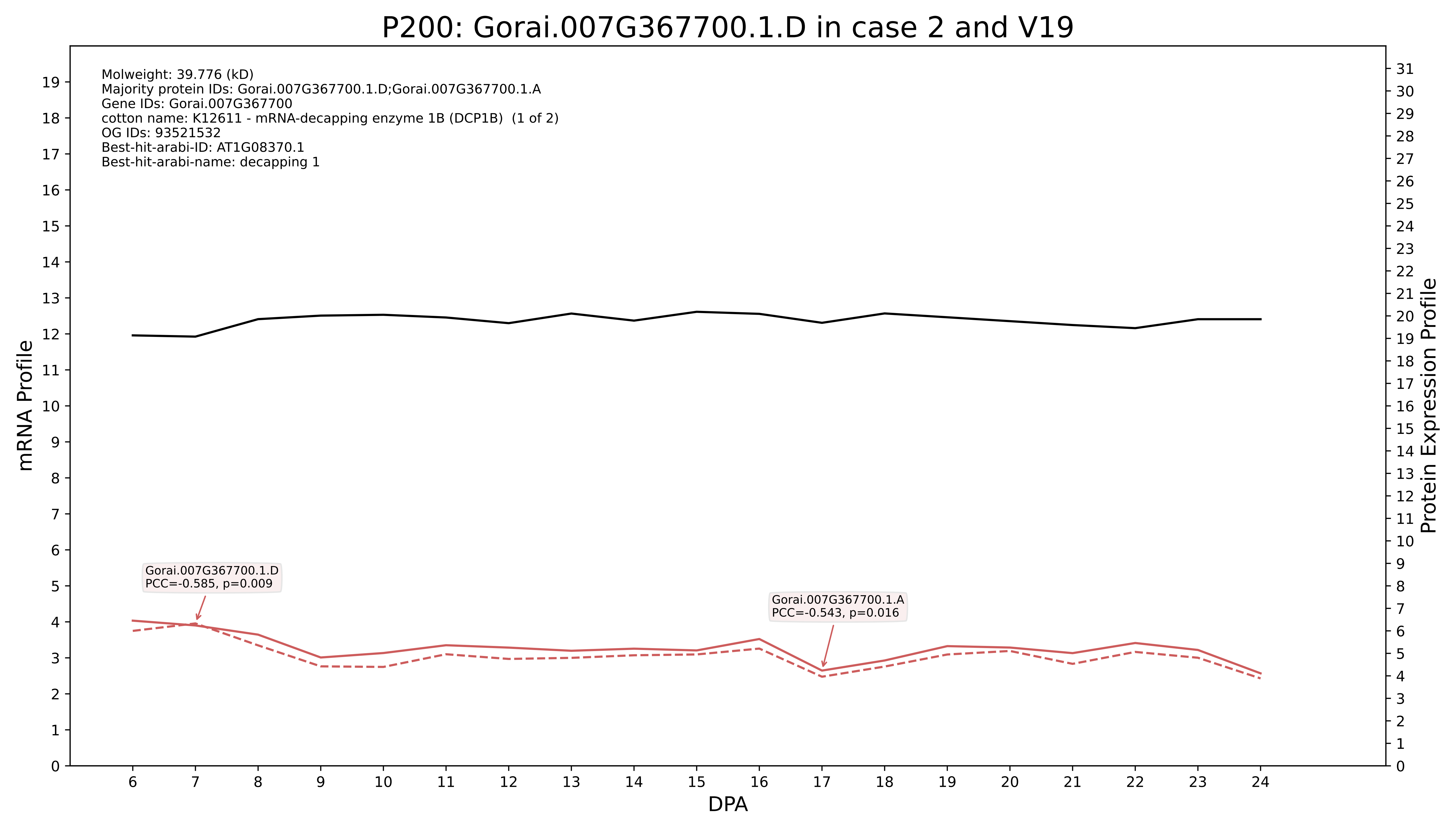
P200: Gorai.007G367700.1.D in case 2 and V19
Molweight: 39.776 (kD)
Majority protein IDs: Gorai.007G367700.1.D;Gorai.007G367700.1.A
Gene IDs: Gorai.007G367700
cotton name: K12611 - mRNA-decapping enzyme 1B (DCP1B) (1 of 2)
OG IDs: 93521532
Best-hit-arabi-ID: AT1G08370.1
Best-hit-arabi-name: decapping 1
Gorai.007G367700.1.D
PCC=-0.585, p=0.009
Gorai.007G367700.1.A
PCC=-0.543, p=0.016
mRNA Profile
Protein Expression Profile
DPA

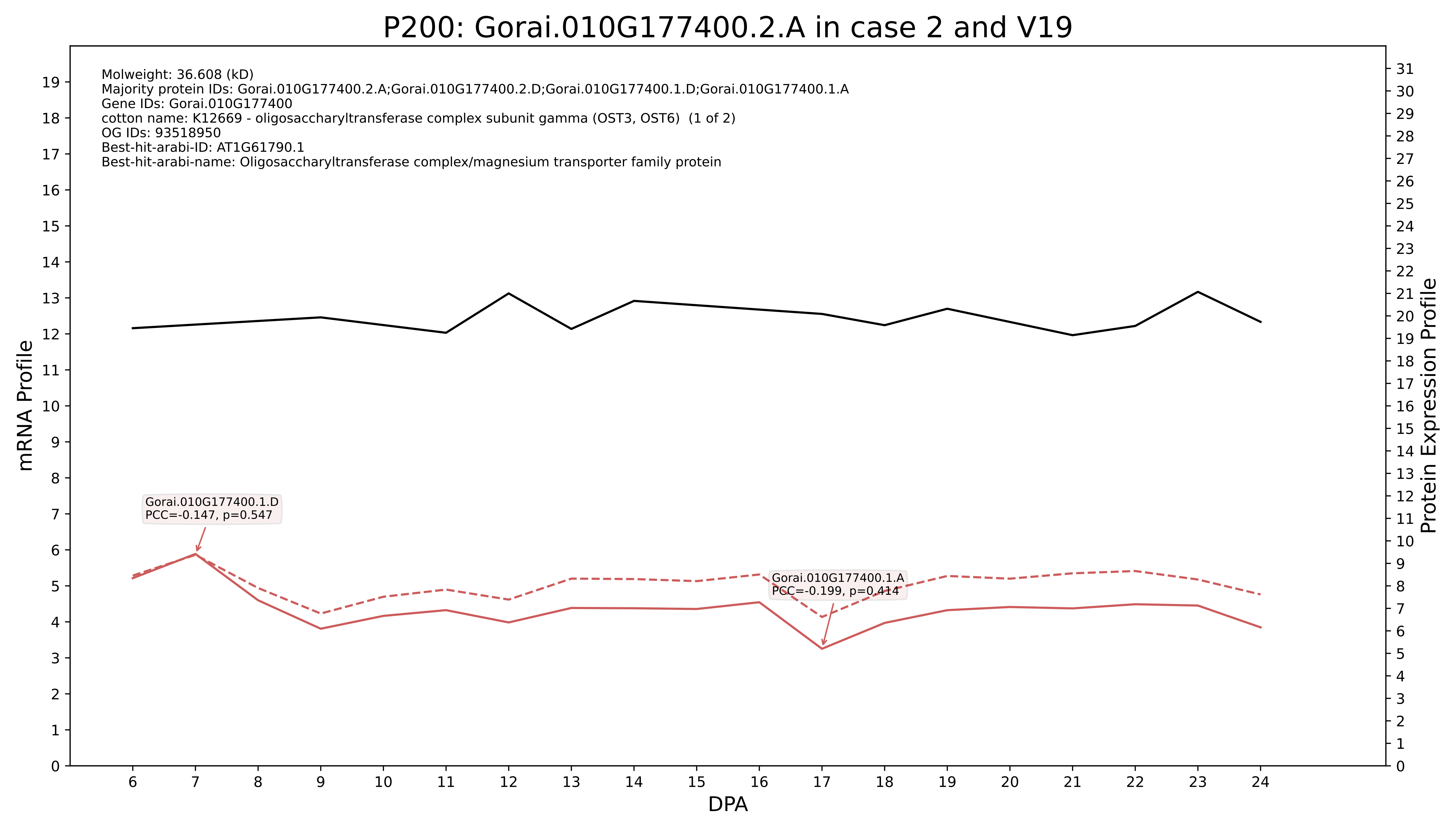

P200: Gorai.010G177400.2.A in case 2 and V19
Molweight: 36.608 (kD)
Majority protein IDs: Gorai.010G177400.2.A;Gorai.010G177400.2.D;Gorai.010G177400.1.D;Gorai.010G177400.1.A
Gene IDs: Gorai.010G177400
cotton name: K12669 - oligosaccharyltransferase complex subunit gamma (OST3, OST6) (1 of 2)
OG IDs: 93518950
Best-hit-arabi-ID: AT1G61790.1
Best-hit-arabi-name: Oligosaccharyltransferase complex/magnesium transporter family protein
Gorai.010G177400.1.D
PCC=-0.147, p=0.547
Gorai.010G177400.1.A
PCC=-0.199, p=0.414
mRNA Profile
Protein Expression Profile
DPA

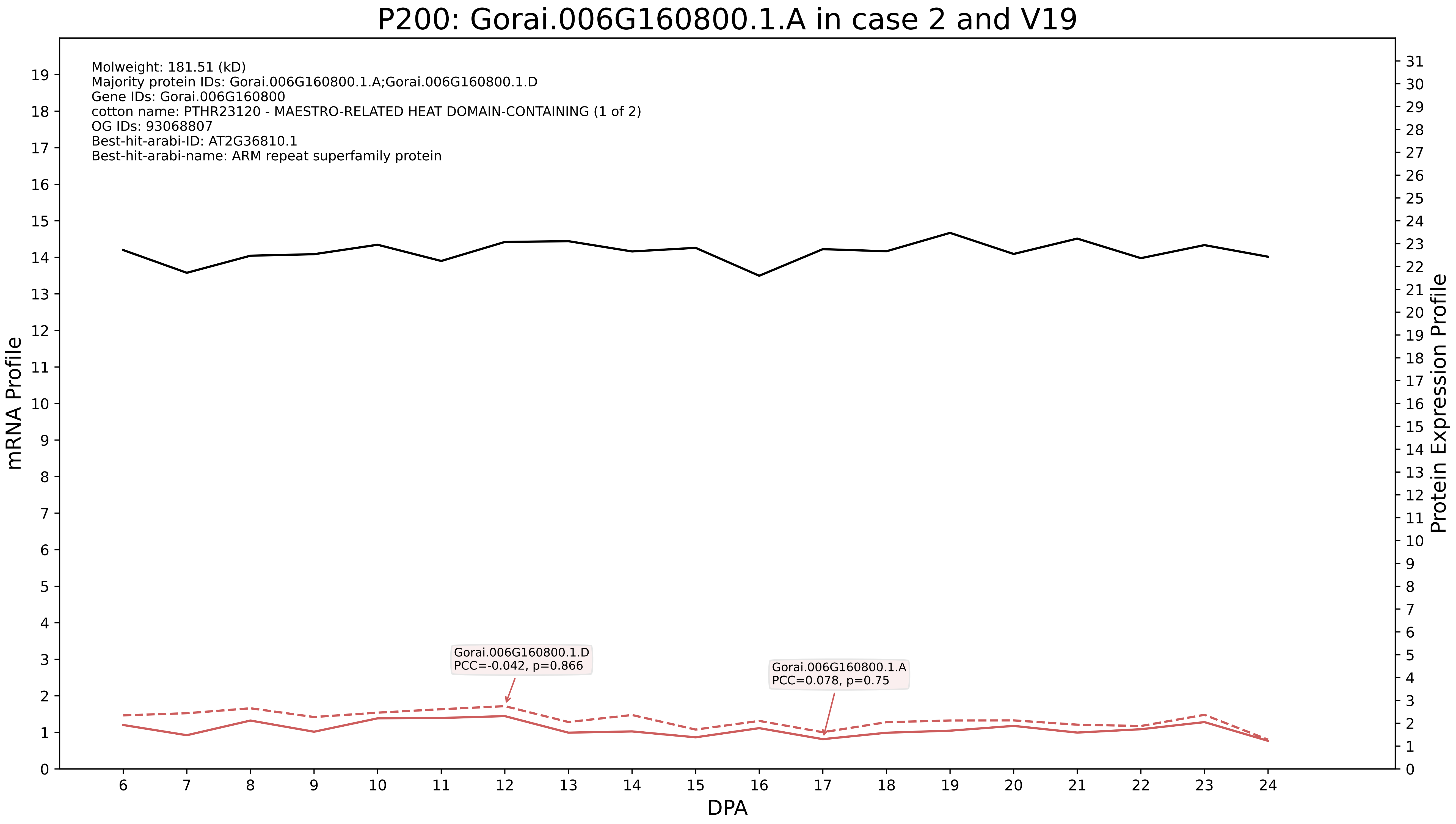
P200: Gorai.006G160800.1.A in case 2 and V19
Molweight: 181.51 (kD)
Majority protein IDs: Gorai.006G160800.1.A;Gorai.006G160800.1.D
Gene IDs: Gorai.006G160800
cotton name: PTHR23120 - MAESTRO-RELATED HEAT DOMAIN-CONTAINING (1 of 2)
OG IDs: 93068807
Best-hit-arabi-ID: AT2G36810.1
Best-hit-arabi-name: ARM repeat superfamily protein
Gorai.006G160800.1.D
PCC=-0.042, p=0.866
Gorai.006G160800.1.A
PCC=0.078, p=0.75
mRNA Profile
Protein Expression Profile
DPA

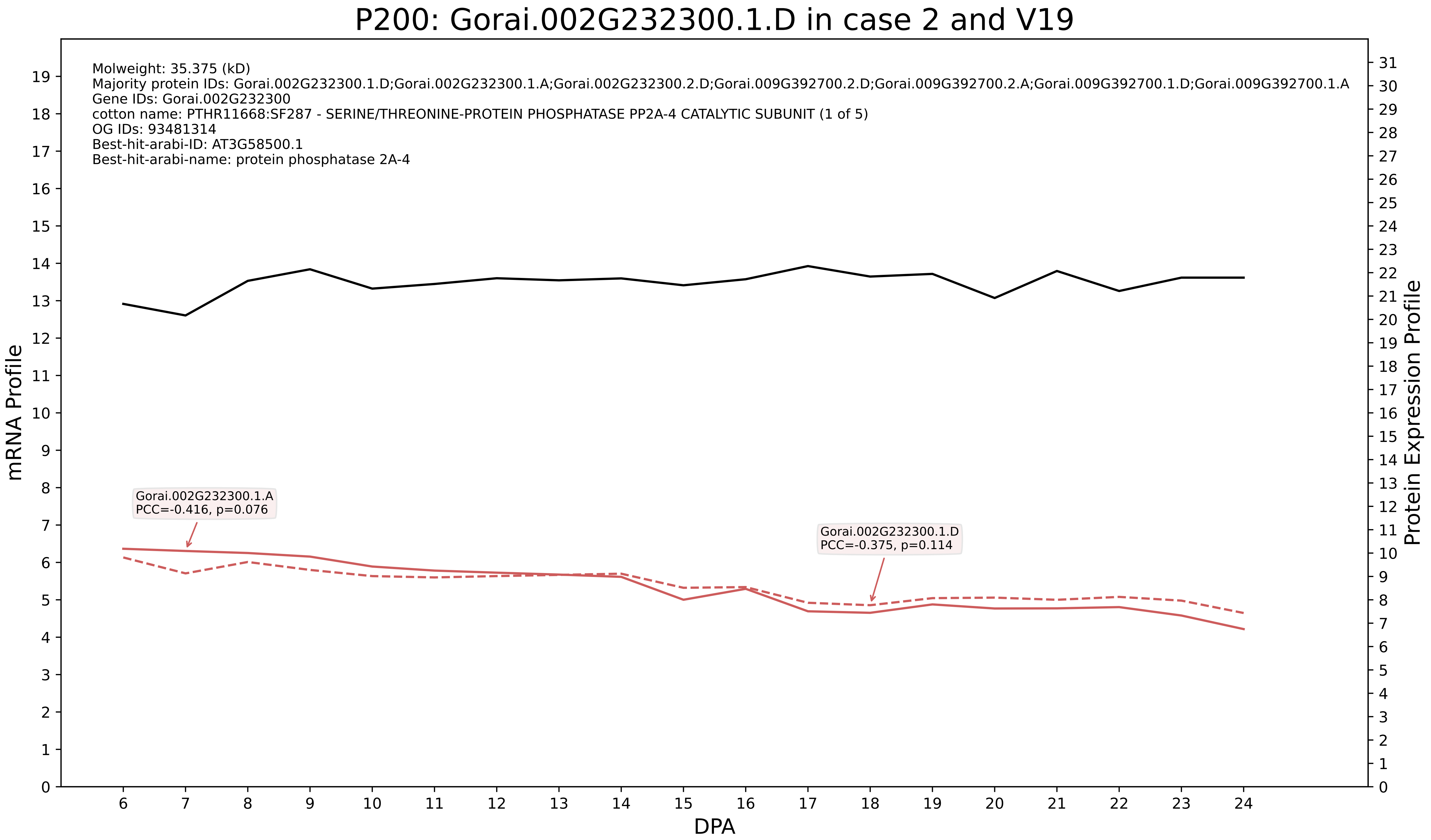
P200: Gorai.002G232300.1.D in case 2 and V19
Molweight: 35.375 (kD)
Majority protein IDs: Gorai.002G232300.1.D;Gorai.002G232300.1.A;Gorai.002G232300.2.D;Gorai.009G392700.2.D;Gorai.009G392700.2.A;Gorai.009G392700.1.D;Gorai.009G392700.1.A
Gene IDs: Gorai.002G232300
cotton name: PTHR11668:SF287 - SERINE/THREONINE-PROTEIN PHOSPHATASE PP2A-4 CATALYTIC SUBUNIT (1 of 5)
OG IDs: 93481314
Best-hit-arabi-ID: AT3G58500.1
Best-hit-arabi-name: protein phosphatase 2A-4
Gorai.002G232300.1.A
PCC=-0.416, p=0.076
Gorai.002G232300.1.D
PCC=-0.375, p=0.114
mRNA Profile
Protein Expression Profile
DPA
0
1
2
3
4
5
6
7
8
9
10
11
12
13
14
15
16
17
18
19
20
21
22
23
24
25
26
27
28
29
30
31

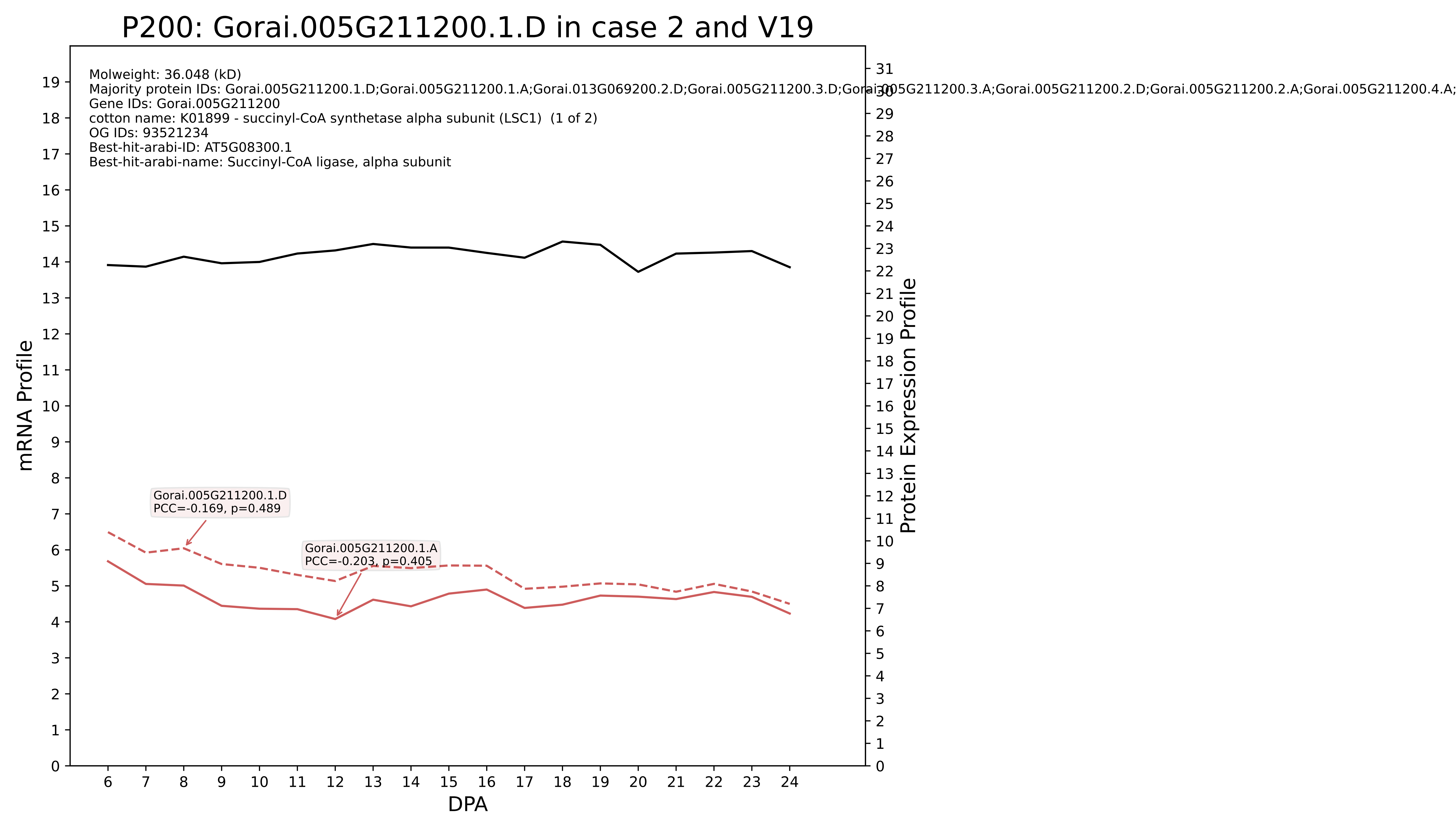

P200: Gorai.005G211200.1.D in case 2 and V19
Molweight: 36.048 (kD)
Majority protein IDs: Gorai.005G211200.1.D;Gorai.005G211200.1.A;Gorai.013G069200.2.D;Gorai.005G211200.3.D;Gorai.005G211200.3.A;Gorai.005G211200.2.D;Gorai.005G211200.2.A;Gorai.005G211200.4.A;
Gene IDs: Gorai.005G211200
cotton name: K01899 - succinyl-CoA synthetase alpha subunit (LSC1) (1 of 2)
OG IDs: 93521234
Best-hit-arabi-ID: AT5G08300.1
Best-hit-arabi-name: Succinyl-CoA ligase, alpha subunit
Gorai.005G211200.1.D
PCC=-0.169, p=0.489
Gorai.005G211200.1.A
PCC=-0.203, p=0.405
mRNA Profile
Protein Expression Profile
DPA

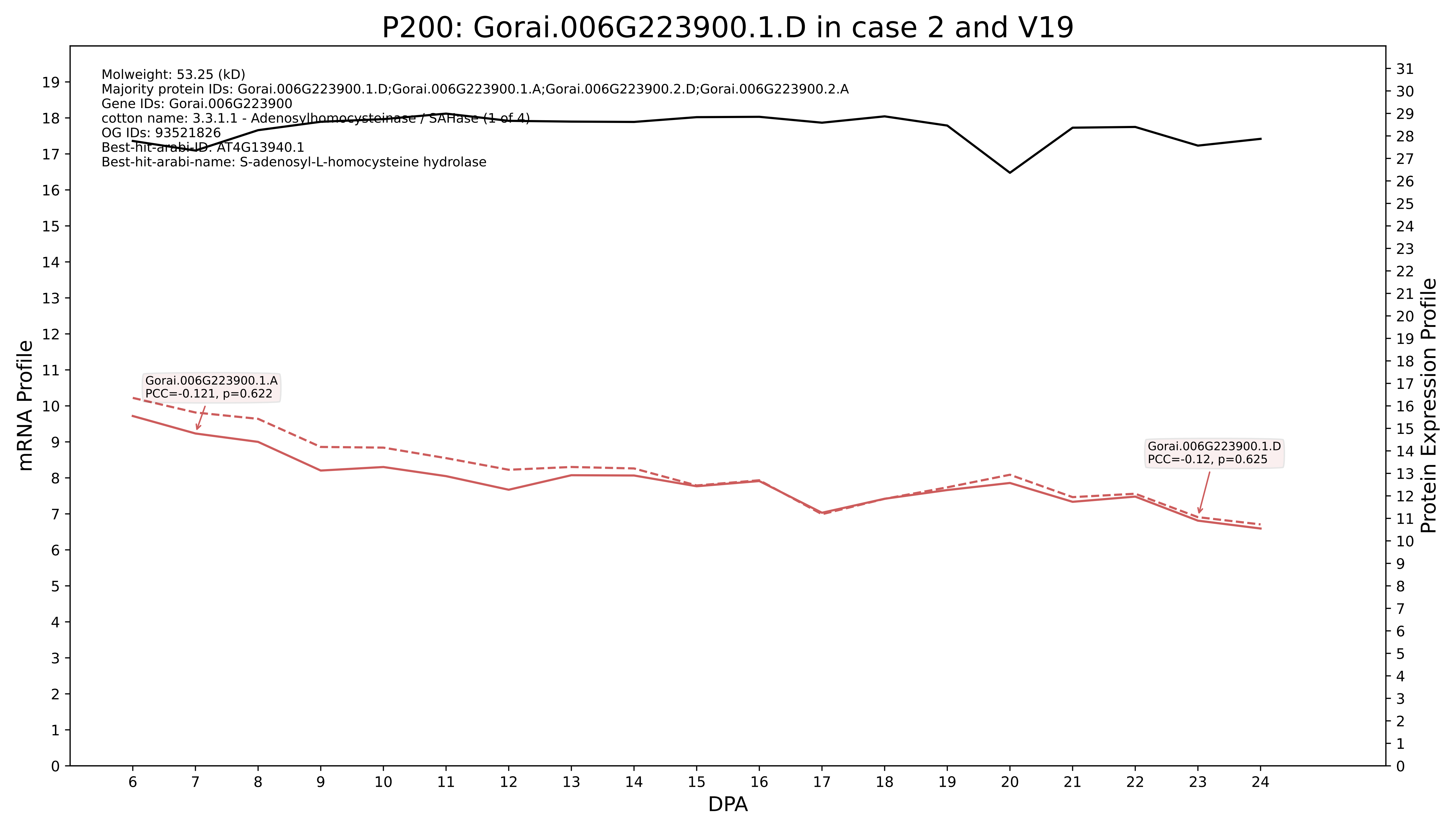
P200: Gorai.006G223900.1.D in case 2 and V19
Molweight: 53.25 (kD)
Majority protein IDs: Gorai.006G223900.1.D;Gorai.006G223900.1.A;Gorai.006G223900.2.D;Gorai.006G223900.2.A
Gene IDs: Gorai.006G223900
cotton name: 3.3.1.1 - Adenosylhomocysteinase / SAHase (1 of 4)
OG IDs: 93521826
Best-hit-arabi-ID: AT4G13940.1
Best-hit-arabi-name: S-adenosyl-L-homocysteine hydrolase
Gorai.006G223900.1.A
PCC=-0.121, p=0.622
Gorai.006G223900.1.D
PCC=-0.12, p=0.625
mRNA Profile
Protein Expression Profile
DPA

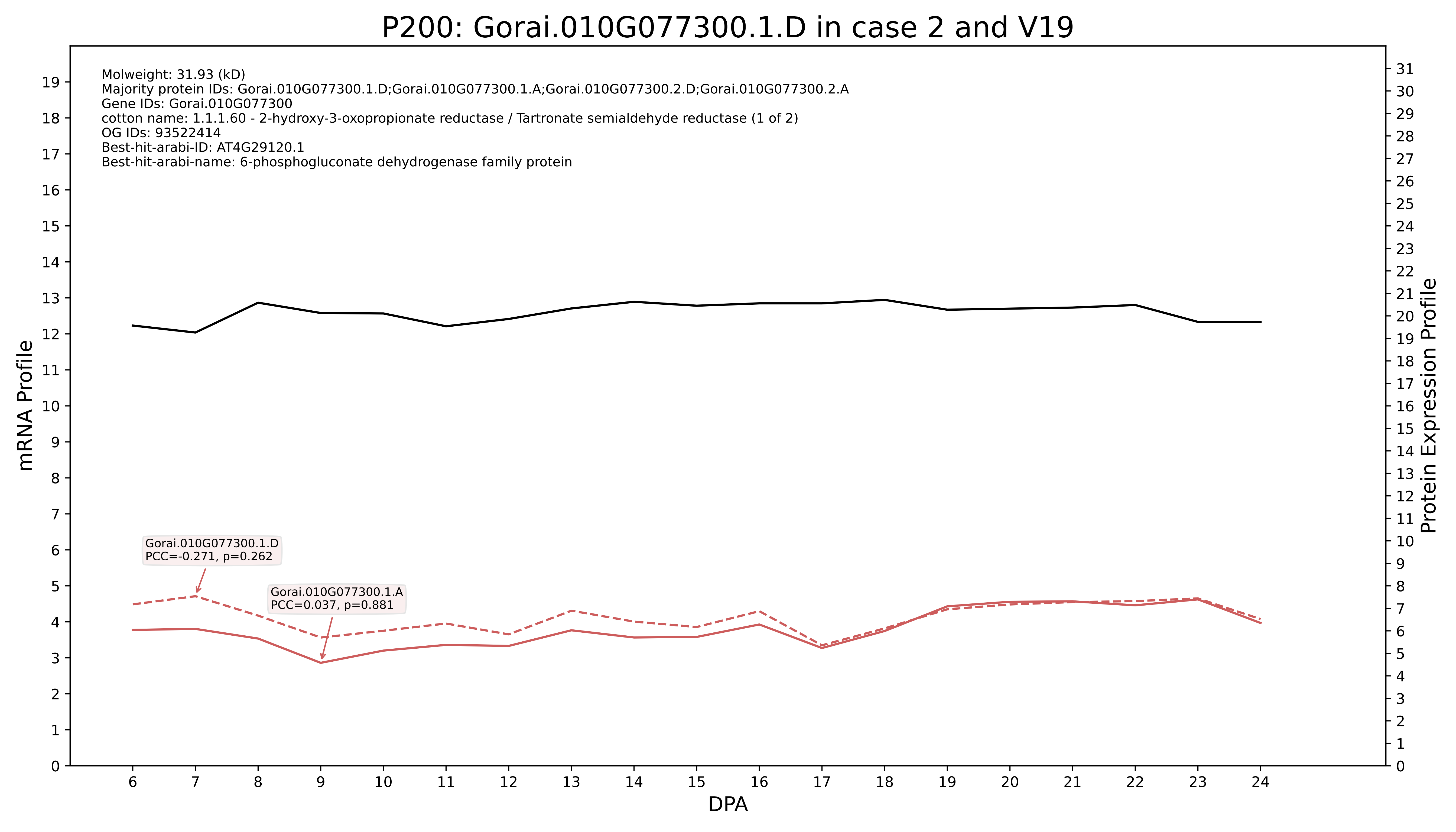
P200: Gorai.010G077300.1.D in case 2 and V19
Molweight: 31.93 (kD)
Majority protein IDs: Gorai.010G077300.1.D;Gorai.010G077300.1.A;Gorai.010G077300.2.D;Gorai.010G077300.2.A
Gene IDs: Gorai.010G077300
cotton name: 1.1.1.60 - 2-hydroxy-3-oxopropionate reductase / Tartronate semialdehyde reductase (1 of 2)
OG IDs: 93522414
Best-hit-arabi-ID: AT4G29120.1
Best-hit-arabi-name: 6-phosphogluconate dehydrogenase family protein
Gorai.010G077300.1.D
PCC=-0.271, p=0.262
Gorai.010G077300.1.A
PCC=0.037, p=0.881
mRNA Profile
Protein Expression Profile
DPA

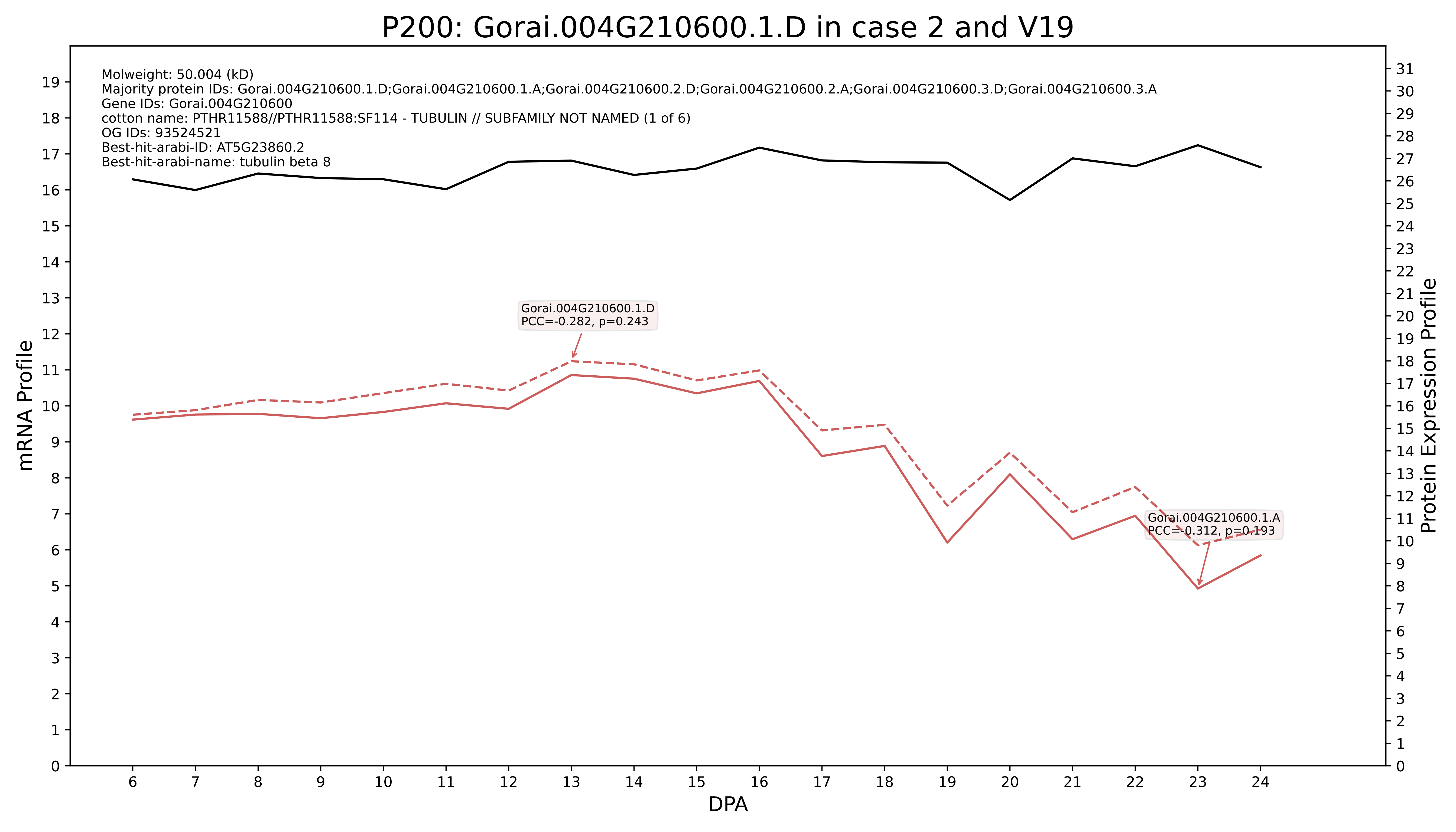
P200: Gorai.004G210600.1.D in case 2 and V19
Molweight: 50.004 (kD)
Majority protein IDs: Gorai.004G210600.1.D;Gorai.004G210600.1.A;Gorai.004G210600.2.D;Gorai.004G210600.2.A;Gorai.004G210600.3.D;Gorai.004G210600.3.A
Gene IDs: Gorai.004G210600
cotton name: PTHR11588//PTHR11588:SF114 - TUBULIN // SUBFAMILY NOT NAMED (1 of 6)
OG IDs: 93524521
Best-hit-arabi-ID: AT5G23860.2
Best-hit-arabi-name: tubulin beta 8
Gorai.004G210600.1.D
PCC=-0.282, p=0.243
Gorai.004G210600.1.A
PCC=-0.312, p=0.193
mRNA Profile
Protein Expression Profile
DPA

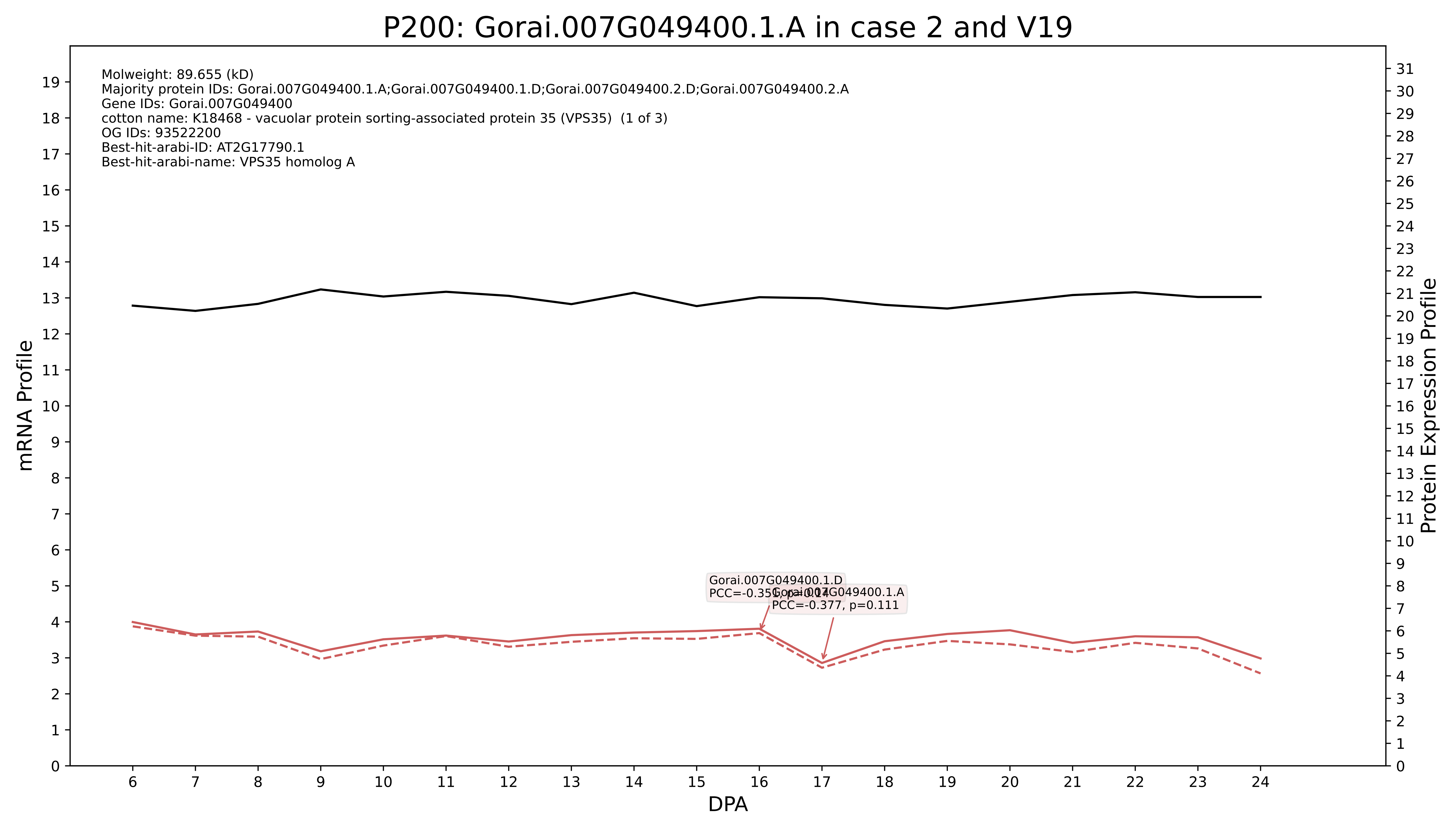
P200: Gorai.007G049400.1.A in case 2 and V19
Molweight: 89.655 (kD)
Majority protein IDs: Gorai.007G049400.1.A;Gorai.007G049400.1.D;Gorai.007G049400.2.D;Gorai.007G049400.2.A
Gene IDs: Gorai.007G049400
cotton name: K18468 - vacuolar protein sorting-associated protein 35 (VPS35) (1 of 3)
OG IDs: 93522200
Best-hit-arabi-ID: AT2G17790.1
Best-hit-arabi-name: VPS35 homolog A
Gorai.007G049400.1.D
Gorai.007G049400.1.A
PCC=-0.377, p=0.111
mRNA Profile
Protein Expression Profile
DPA
0
1
2
3
4
5
6
7
8
9
10
11
12
13
14
15
16
17
18
19
20
21
22
23
24
25
26
27
28
29
30
31

00: Gorai.003G094300.2.D in case 2 and V19

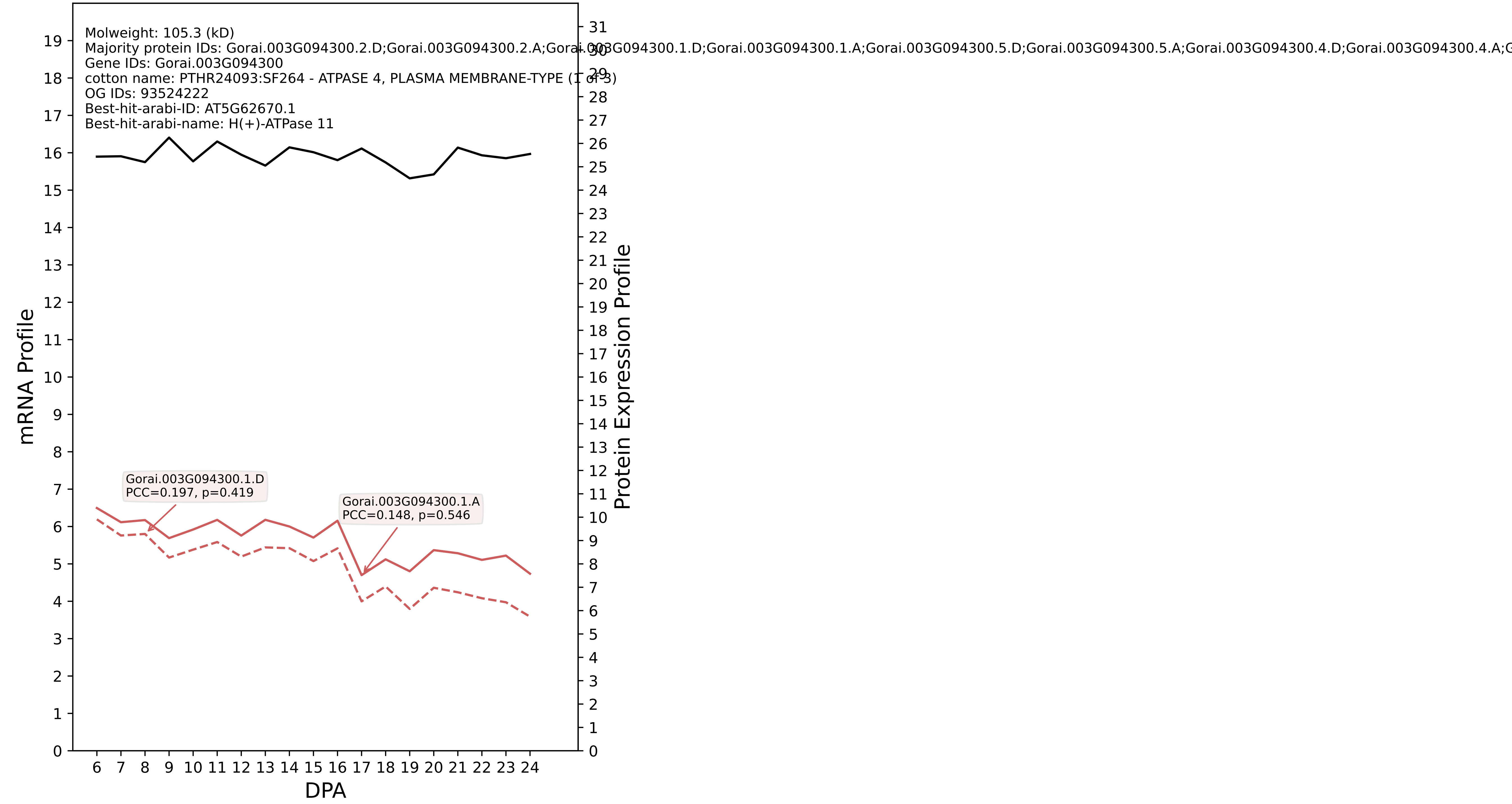

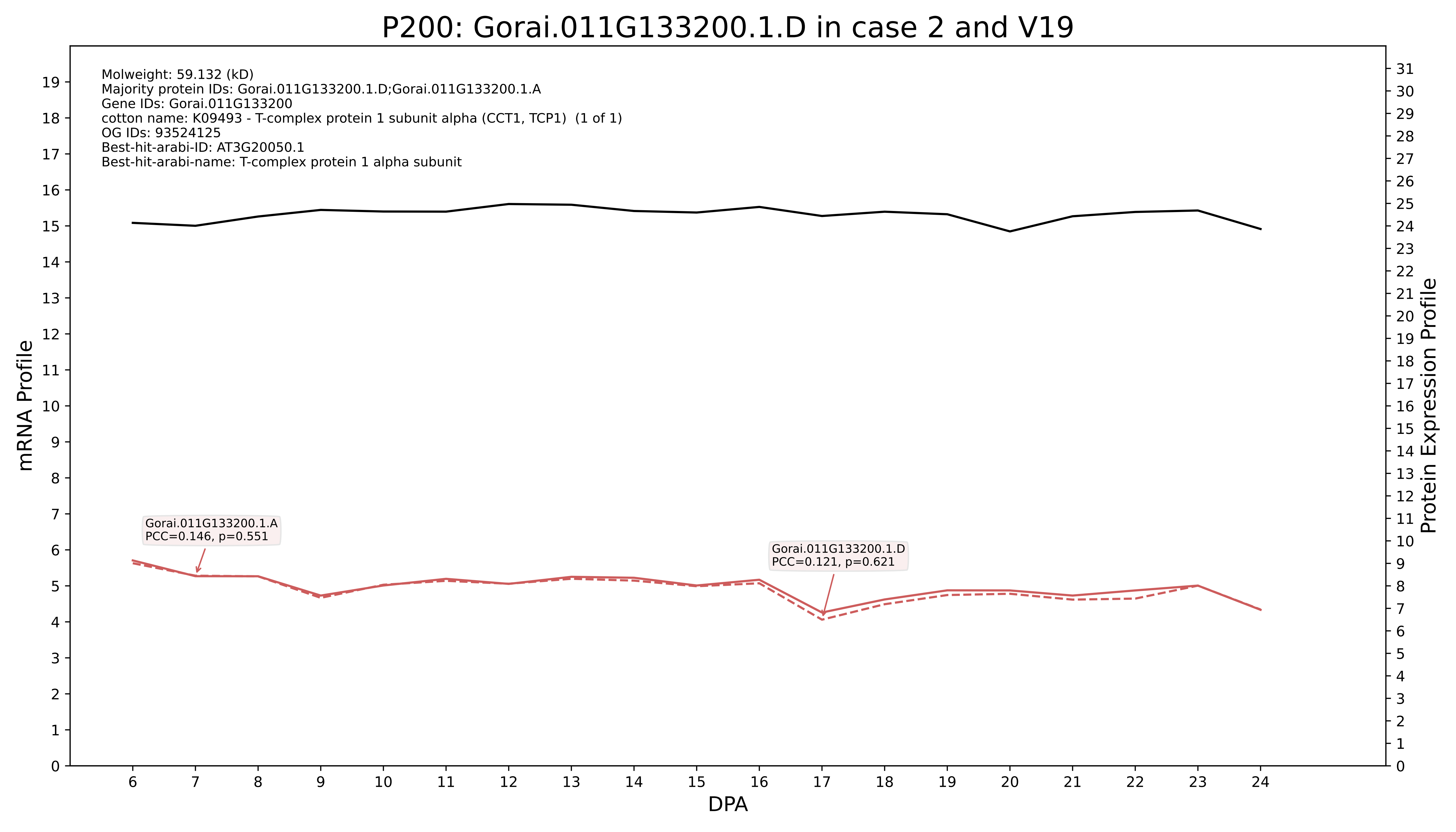

P200: Gorai.011G133200.1.D in case 2 and V19
Molweight: 59.132 (kD)
Majority protein IDs: Gorai.011G133200.1.D;Gorai.011G133200.1.A
Gene IDs: Gorai.011G133200
cotton name: K09493 - T-complex protein 1 subunit alpha (CCT1, TCP1) (1 of 1)
OG IDs: 93524125
Best-hit-arabi-ID: AT3G20050.1
Best-hit-arabi-name: T-complex protein 1 alpha subunit
Gorai.011G133200.1.A
PCC=0.146, p=0.551
Gorai.011G133200.1.D
PCC=0.121, p=0.621
mRNA Profile
Protein Expression Profile
DPA

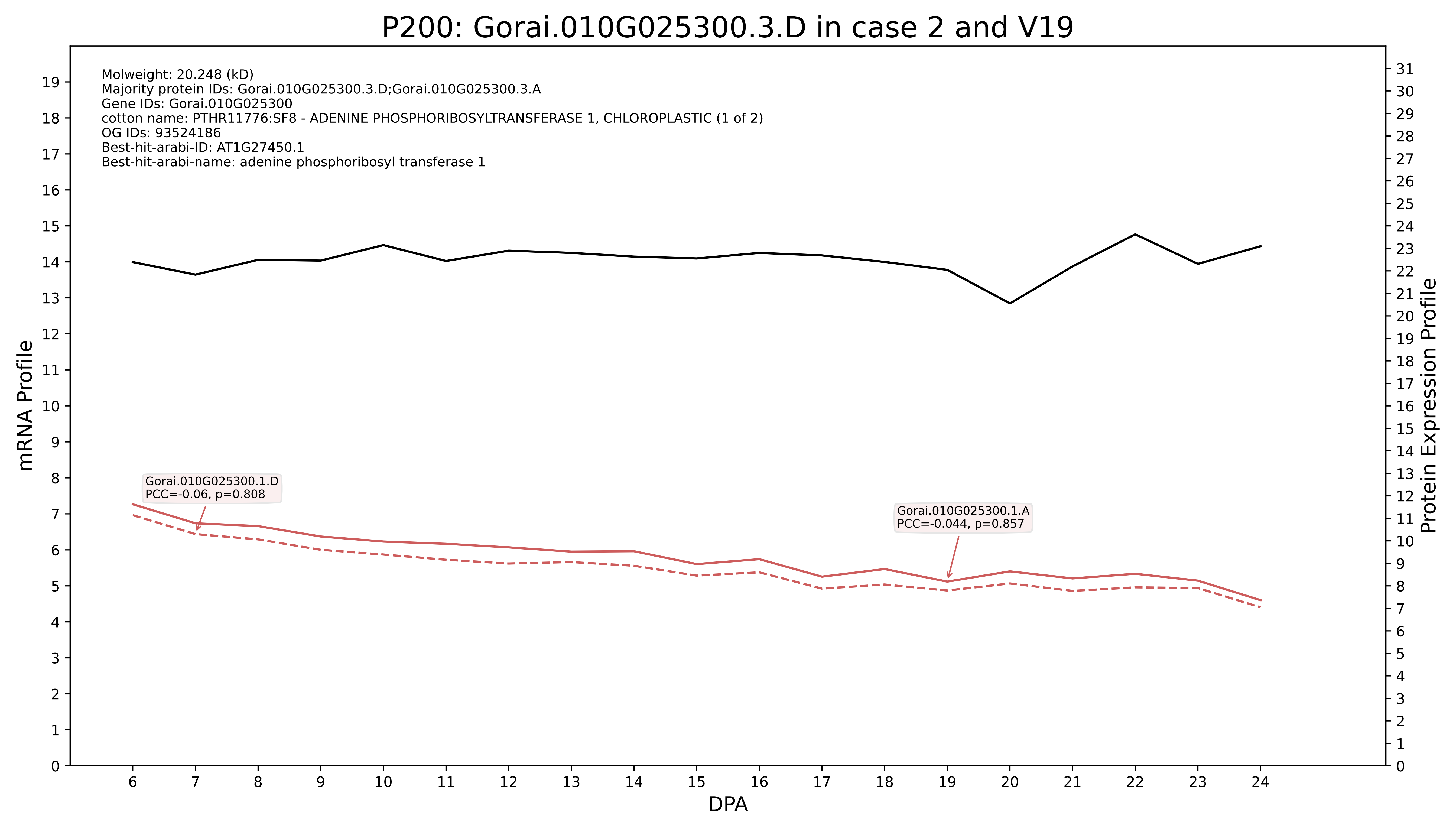
P200: Gorai.010G025300.3.D in case 2 and V19
Molweight: 20.248 (kD)
Majority protein IDs: Gorai.010G025300.3.D;Gorai.010G025300.3.A
Gene IDs: Gorai.010G025300
cotton name: PTHR11776:SF8 - ADENINE PHOSPHORIBOSYLTRANSFERASE 1, CHLOROPLASTIC (1 of 2)
OG IDs: 93524186
Best-hit-arabi-ID: AT1G27450.1
Best-hit-arabi-name: adenine phosphoribosyl transferase 1
Gorai.010G025300.1.D
PCC=-0.06, p=0.808
Gorai.010G025300.1.A
PCC=-0.044, p=0.857
mRNA Profile
Protein Expression Profile
DPA
0
1
2
3
4
5
6
7
8
9
10
11
12
13
14
15
16
17
18
19
20
21
22
23
24
25
26
27
28
29
30
31

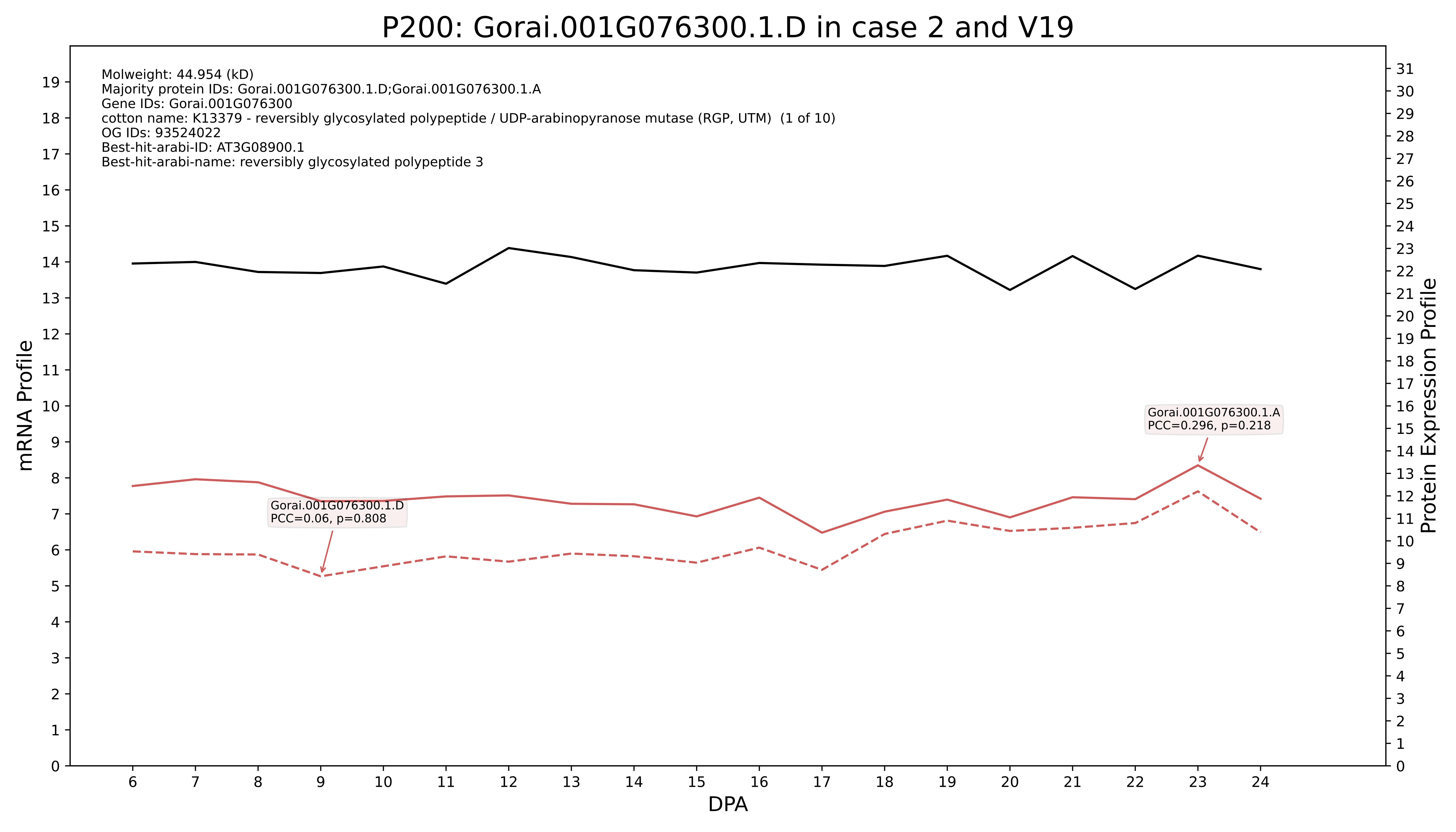
P200: Gorai.001G076300.1.D in case 2 and V19
Molweight: 44.954 (kD)
Majority protein IDs: Gorai.001G076300.1.D;Gorai.001G076300.1.A
Gene IDs: Gorai.001G076300
cotton name: K13379 - reversibly glycosylated polypeptide / UDP-arabinopyranose mutase (RGP, UTM) (1 of 10)
OG IDs: 93524022
Best-hit-arabi-ID: AT3G08900.1
Best-hit-arabi-name: reversibly glycosylated polypeptide 3
Gorai.001G076300.1.A
PCC=0.296, p=0.218
Gorai.001G076300.1.D
PCC=0.06, p=0.808
mRNA Profile
Protein Expression Profile
DPA
6
7
8
9
10
11
12
13
14
15
16
17
18
19
20
21
22
23
24

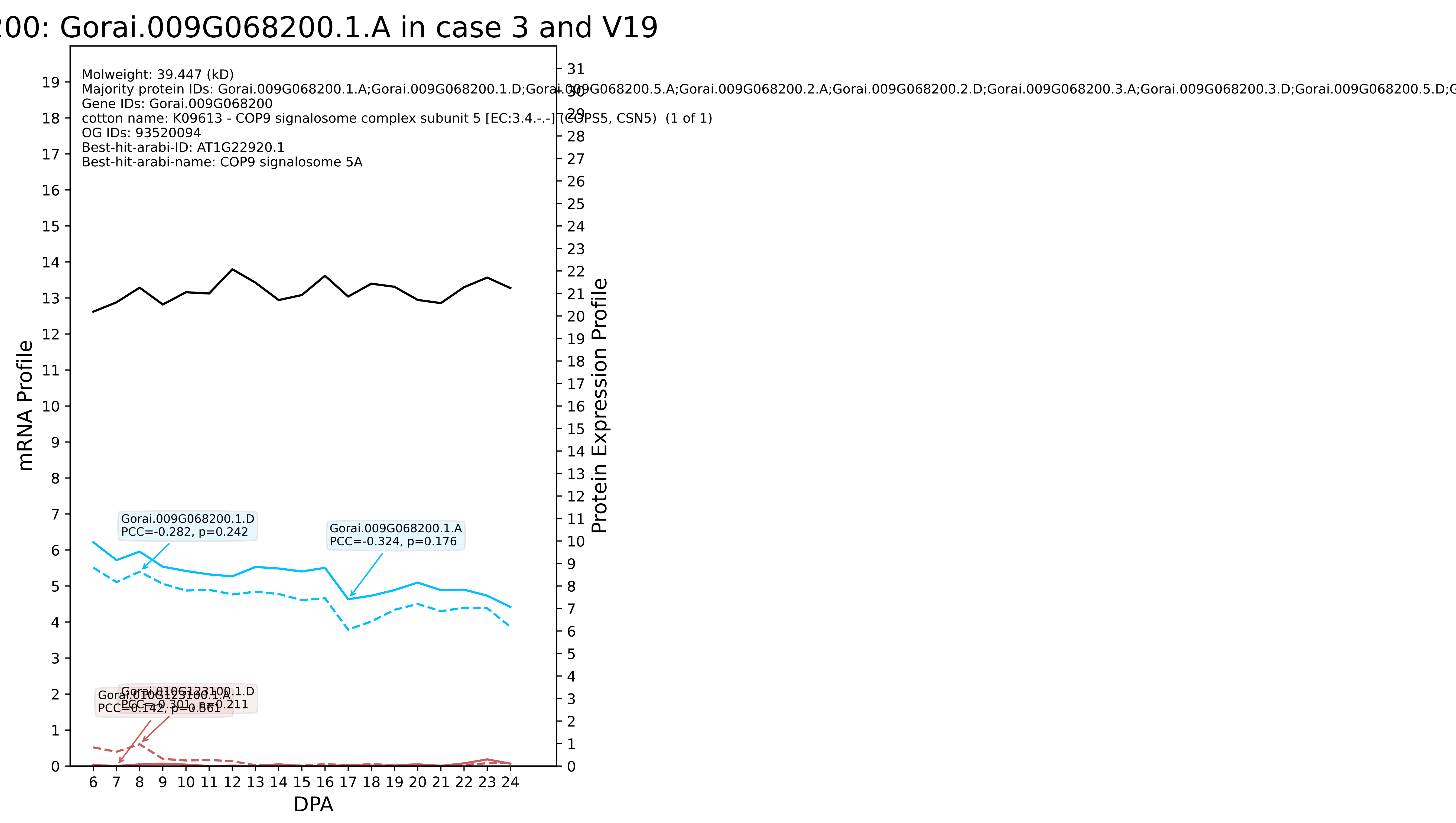
200: Gorai.009G068200.1.A in case 3 and V19
Molweight: 39.447 (kD)
Majority protein IDs: Gorai.009G068200.1.A;Gorai.009G068200.1.D;Gorai.009G068200.5.A;Gorai.009G068200.2.A;Gorai.009G068200.2.D;Gorai.009G068200.3.A;Gorai.009G068200.3.D;Gorai.009G068200.5.D;G
Gene IDs: Gorai.009G068200
cotton name: K09613 - COP9 signalosome complex subunit 5 [EC:3.4.-.-] (COPS5, CSN5) (1 of 1)
OG IDs: 93520094
Best-hit-arabi-ID: AT1G22920.1
Best-hit-arabi-name: COP9 signalosome 5A
Gorai.009G068200.1.D
PCC=-0.282, p=0.242
Gorai.009G068200.1.A
PCC=-0.324, p=0.176
mRNA Profile
Protein Expression Profile
DPA

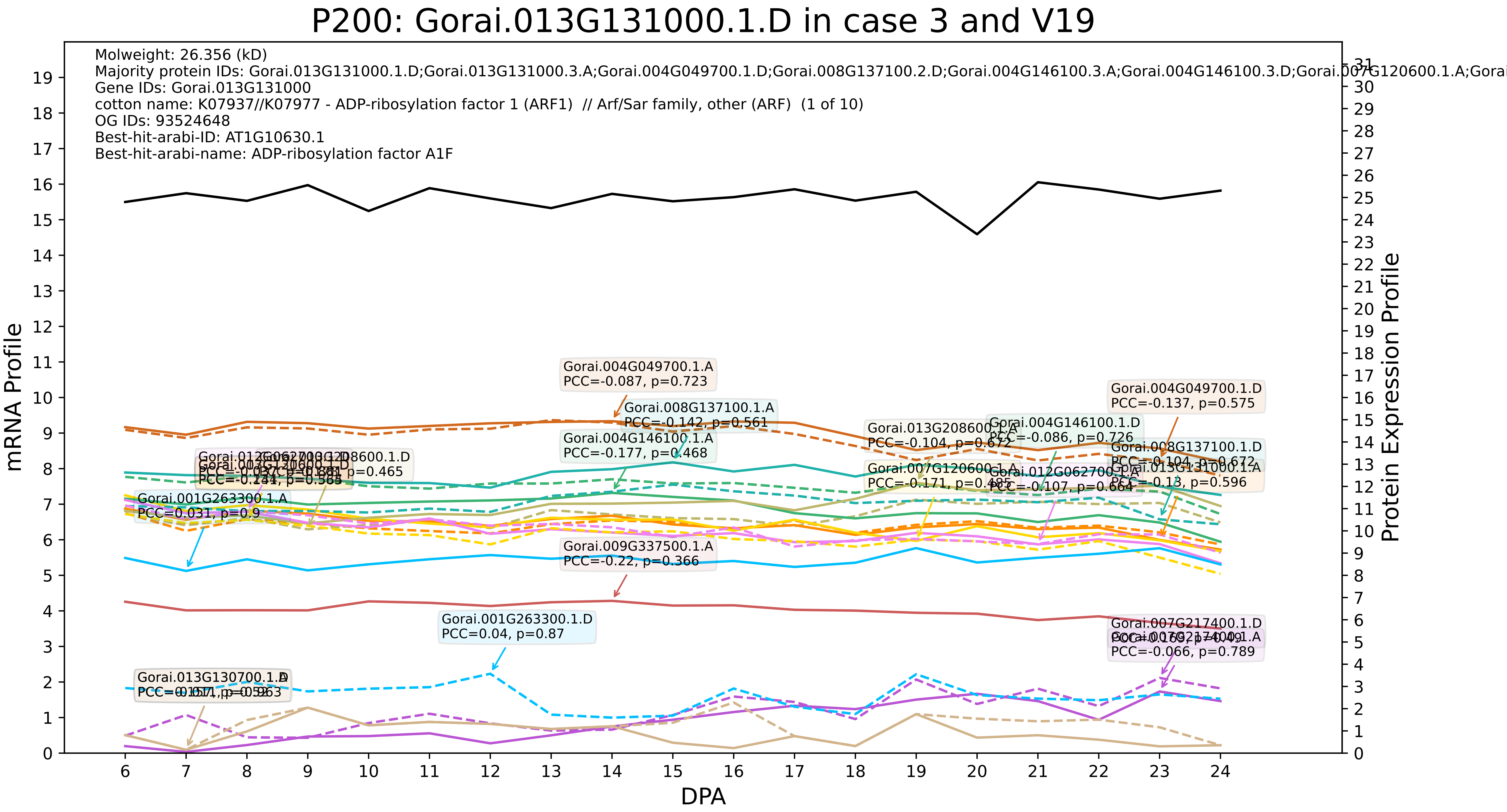
P200: Gorai.013G131000.1.D in case 3 and V19
Molweight: 26.356 (kD)
Majority protein IDs: Gorai.013G131000.1.D;Gorai.013G131000.3.A;Gorai.004G049700.1.D;Gorai.008G137100.2.D;Gorai.004G146100.3.A;Gorai.004G146100.3.D;Gorai.007G120600.1.A;Gorai
Gene IDs: Gorai.013G131000
cotton name: K07937//K07977 - ADP-ribosylation factor 1 (ARF1) // Arf/Sar family, other (ARF) (1 of 10)
OG IDs: 93524648
Best-hit-arabi-ID: AT1G10630.1
Best-hit-arabi-name: ADP-ribosylation factor A1F
mRNA Profile
Protein Expression Profile
DPA
Gorai.004G049700.1.A
PCC=-0.087, p=0.723
Gorai.008G137100.1.A
PCC=-0.142, p=0.561
Gorai.004G146100.1.A
PCC=-0.177, p=0.468
Gorai.004G049700.1.D
PCC=-0.137, p=0.575
Gorai.013G208600.1.A
PCC=-0.104, p=0.672
Gorai.004G146100.1.D
PCC=-0.086, p=0.726
Gorai.008G137100.1.D
PCC=-0.104, p=0.672
Gorai.013G131000.1.A
PCC=-0.13, p=0.596
Gorai.007G120600.1.A
PCC=-0.171, p=0.485
Gorai.012G062700.1.A
PCC=-0.107, p=0.664
Gorai.013G208600.1.D
p=0.465
Gorai.001G263300.1.A
PCC=0.031, p=0.9
Gorai.009G337500.1.A
PCC=-0.22, p=0.366
Gorai.001G263300.1.D
PCC=0.04, p=0.87
Gorai.007G217400.1.D
PCC=-0.066, p=0.789
Gorai.013G130700.1.D

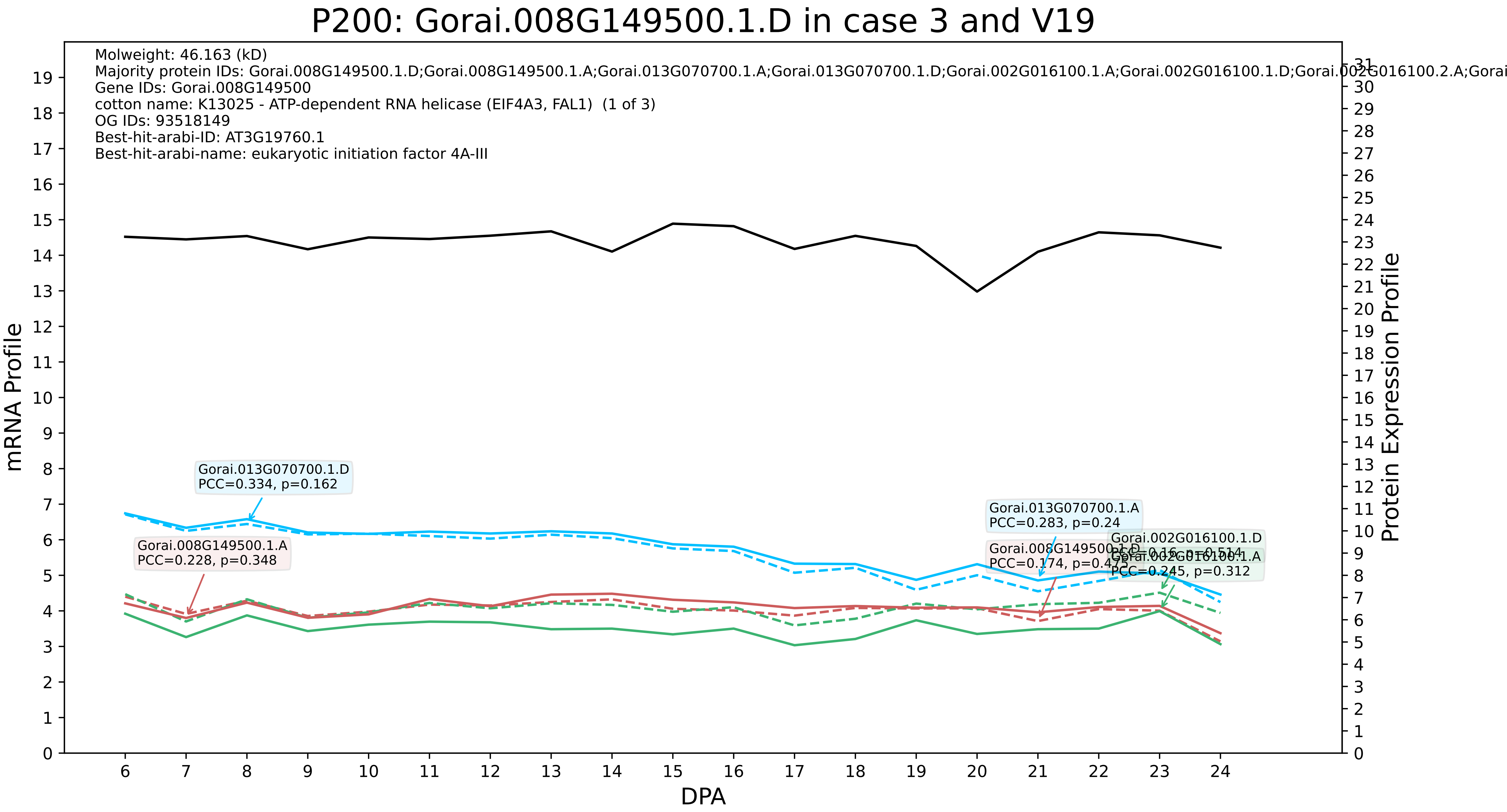

P200: Gorai.008G149500.1.D in case 3 and V19
Molweight: 46.163 (kD)
Majority protein IDs: Gorai.008G149500.1.D;Gorai.008G149500.1.A;Gorai.013G070700.1.A;Gorai.013G070700.1.D;Gorai.002G016100.1.A;Gorai.002G016100.1.D;Gorai.002G016100.2.A;Gorai
Gene IDs: Gorai.008G149500
cotton name: K13025 - ATP-dependent RNA helicase (EIF4A3, FAL1) (1 of 3)
OG IDs: 93518149
Best-hit-arabi-ID: AT3G19760.1
Best-hit-arabi-name: eukaryotic initiation factor 4A-III
Gorai.013G070700.1.D
PCC=0.334, p=0.162
Gorai.008G149500.1.A
PCC=0.228, p=0.348
Gorai.013G070700.1.A
PCC=0.283, p=0.24
Gorai.008G149500.1.D
PCC=0.174, p=0.475
Gorai.002G016100.1.D
PCC=0.16, p=0.514
Gorai.002G016100.1.A
PCC=0.245, p=0.312
mRNA Profile
Protein Expression Profile
DPA

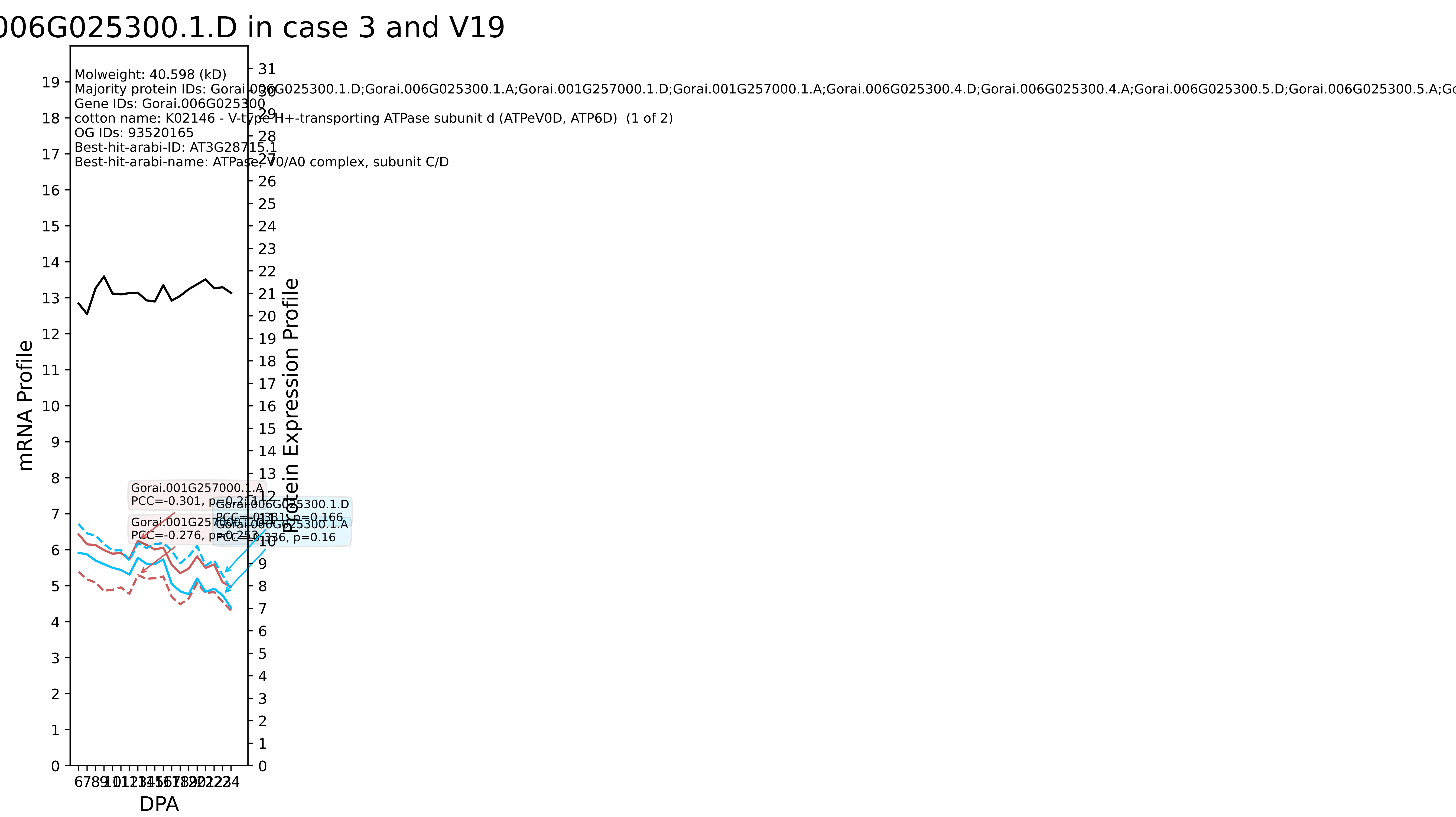

006G025300.1.D in case 3 and V19
Molweight: 40.598 (kD)
Majority protein IDs: Gorai.006G025300.1.D;Gorai.006G025300.1.A;Gorai.001G257000.1.D;Gorai.001G257000.1.A;Gorai.006G025300.4.D;Gorai.006G025300.4.A;Gorai.006G025300.5.D;Gorai.006G025300.5.A;Go
Gene IDs: Gorai.006G025300
cotton name: K02146 - V-type H+-transporting ATPase subunit d (ATPeV0D, ATP6D) (1 of 2)
OG IDs: 93520165
Best-hit-arabi-ID: AT3G28715.1
Best-hit-arabi-name: ATPase, V0/A0 complex, subunit C/D
Gorai.001G257000.1.A
PCC=-0.301, p=0.2
Gorai.006G025300.1.D
PCC=-0.331, p=0.166
Gorai.006G025300.1.A
PCC=-0.336, p=0.16
PCC=-0.276, p=0.253
mRNA Profile
Protein Expression Profile
DPA

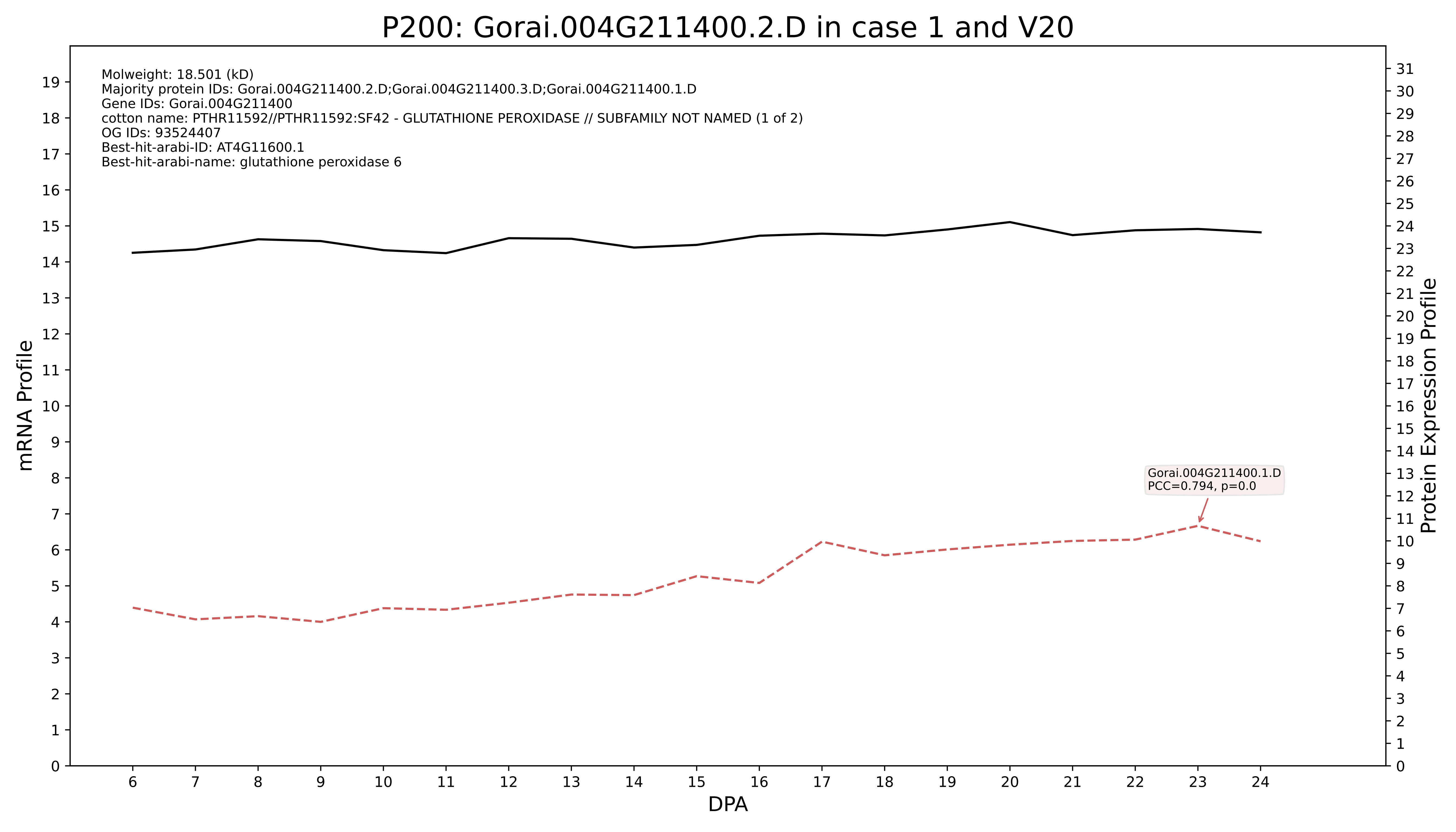
P200: Gorai.004G211400.2.D in case 1 and V20
Molweight: 18.501 (kD)
Majority protein IDs: Gorai.004G211400.2.D;Gorai.004G211400.3.D;Gorai.004G211400.1.D
Gene IDs: Gorai.004G211400
cotton name: PTHR11592//PTHR11592:SF42 - GLUTATHIONE PEROXIDASE // SUBFAMILY NOT NAMED (1 of 2)
OG IDs: 93524407
Best-hit-arabi-ID: AT4G11600.1
Best-hit-arabi-name: glutathione peroxidase 6
Gorai.004G211400.1.D
PCC=0.794, p=0.0
mRNA Profile
Protein Expression Profile
DPA

P200: Gorai.002G110500.5.A in case 1 and V20

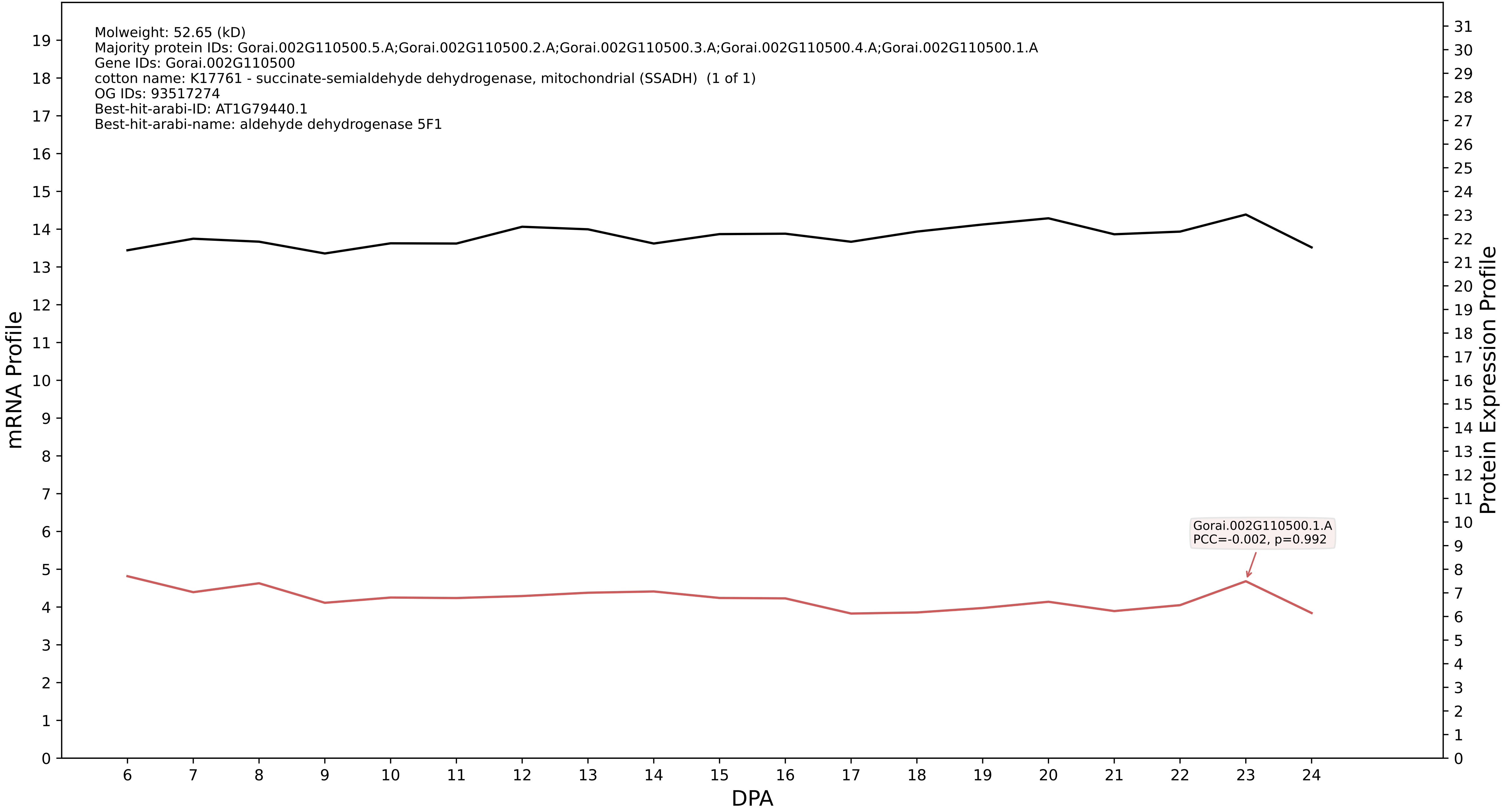

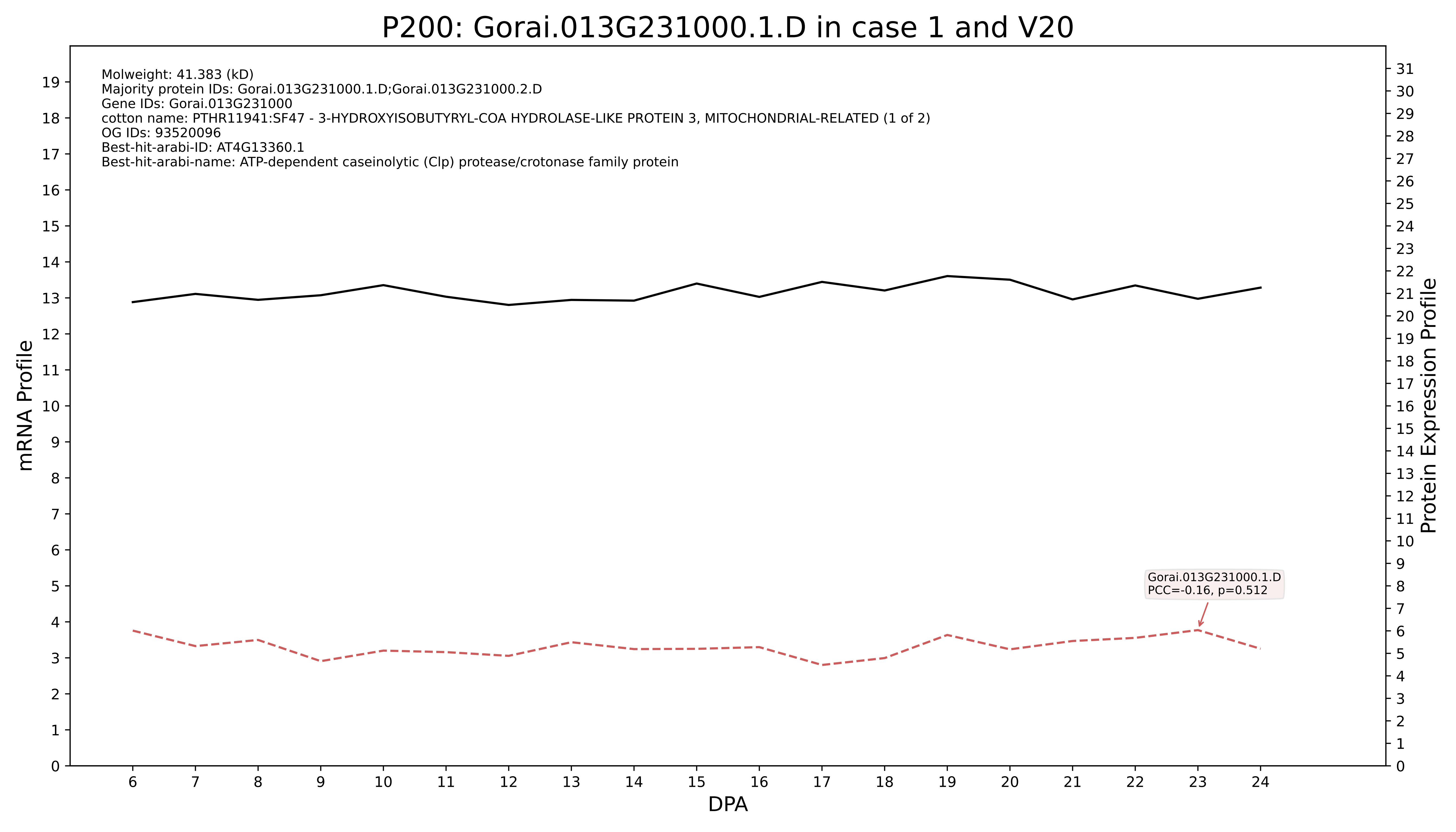
P200: Gorai.013G231000.1.D in case 1 and V20
Molweight: 41.383 (kD)
Majority protein IDs: Gorai.013G231000.1.D;Gorai.013G231000.2.D
Gene IDs: Gorai.013G231000
cotton name: PTHR11941:SF47 - 3-HYDROXYISOBUTYRYL-COA HYDROLASE-LIKE PROTEIN 3, MITOCHONDRIAL-RELATED (1 of 2)
OG IDs: 93520096
Best-hit-arabi-ID: AT4G13360.1
Best-hit-arabi-name: ATP-dependent caseinolytic (Clp) protease/crotonase family protein
Gorai.013G231000.1.D
PCC=-0.16, p=0.512
mRNA Profile
Protein Expression Profile
DPA
0
1
2
3
4
5
6
7
8
9
10
11
12
13
14
15
16
17
18
19
20
21
22
23
24
25
26
27
28
29
30
31

P200: Gorai.012G158800.1.D in case 1 and V20

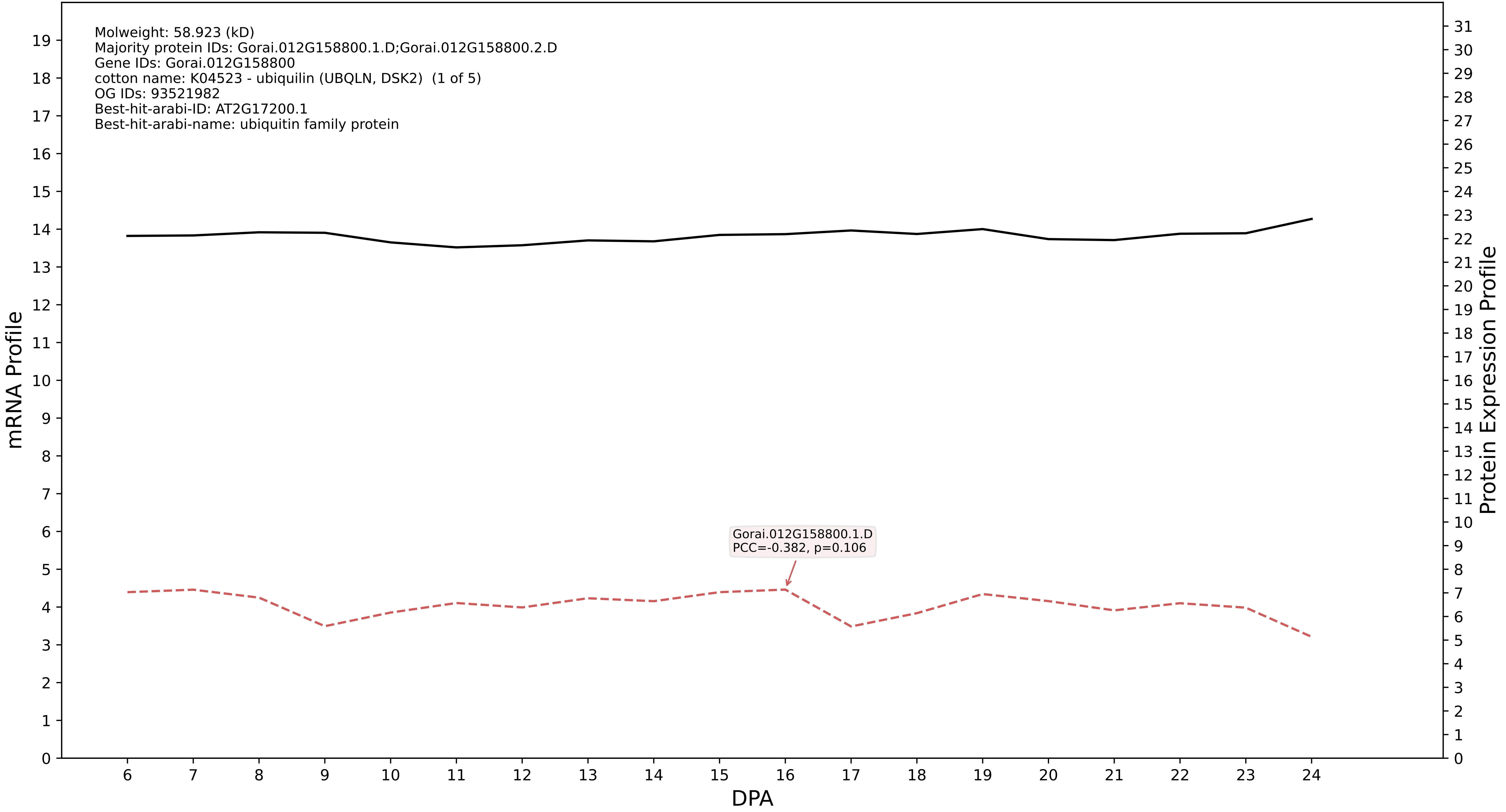

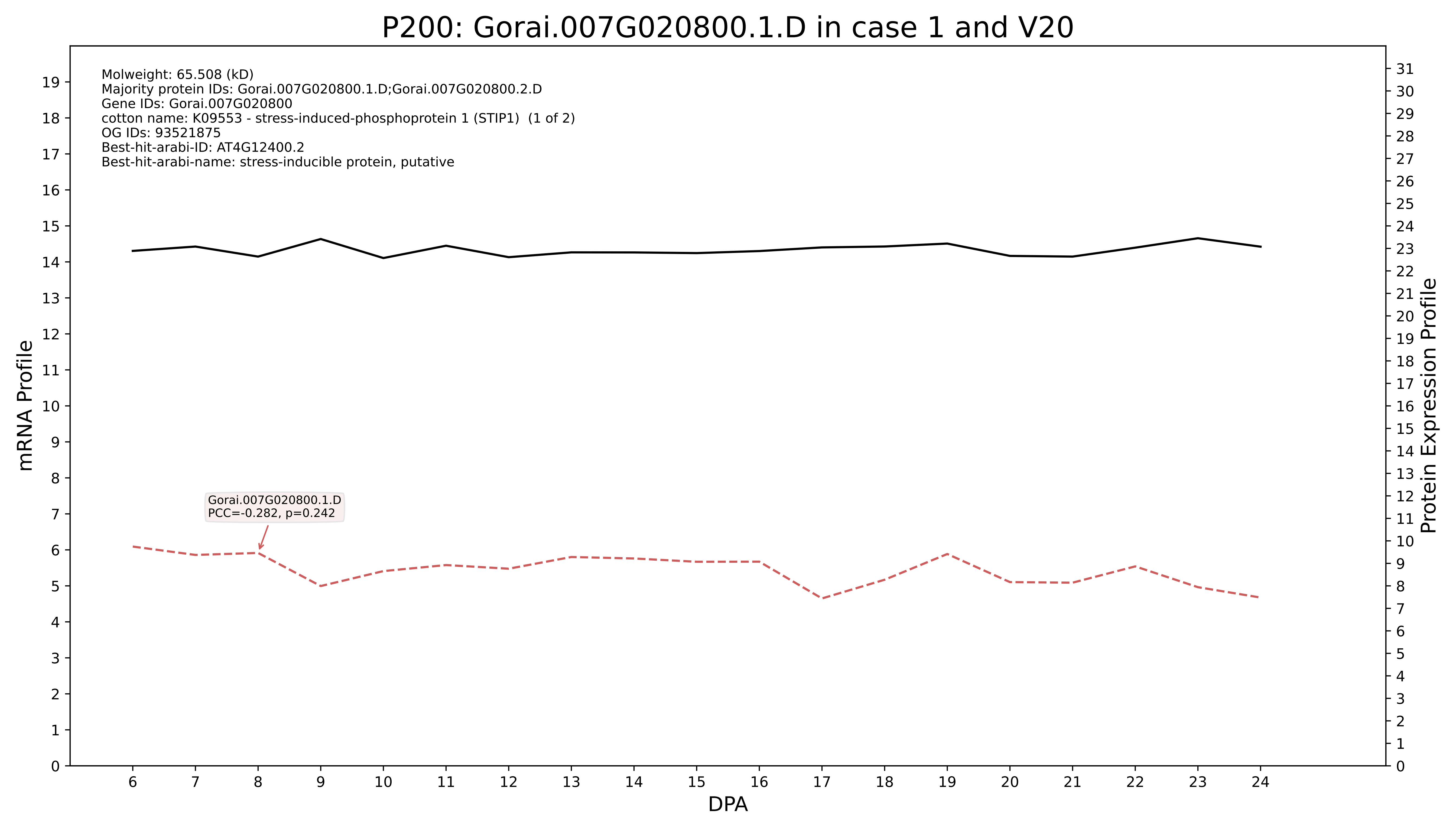
P200: Gorai.007G020800.1.D in case 1 and V20
Molweight: 65.508 (kD)
Majority protein IDs: Gorai.007G020800.1.D;Gorai.007G020800.2.D
Gene IDs: Gorai.007G020800
cotton name: K09553 - stress-induced-phosphoprotein 1 (STIP1) (1 of 2)
OG IDs: 93521875
Best-hit-arabi-ID: AT4G12400.2
Best-hit-arabi-name: stress-inducible protein, putative
Gorai.007G020800.1.D
PCC=-0.282, p=0.242
mRNA Profile
Protein Expression Profile
DPA

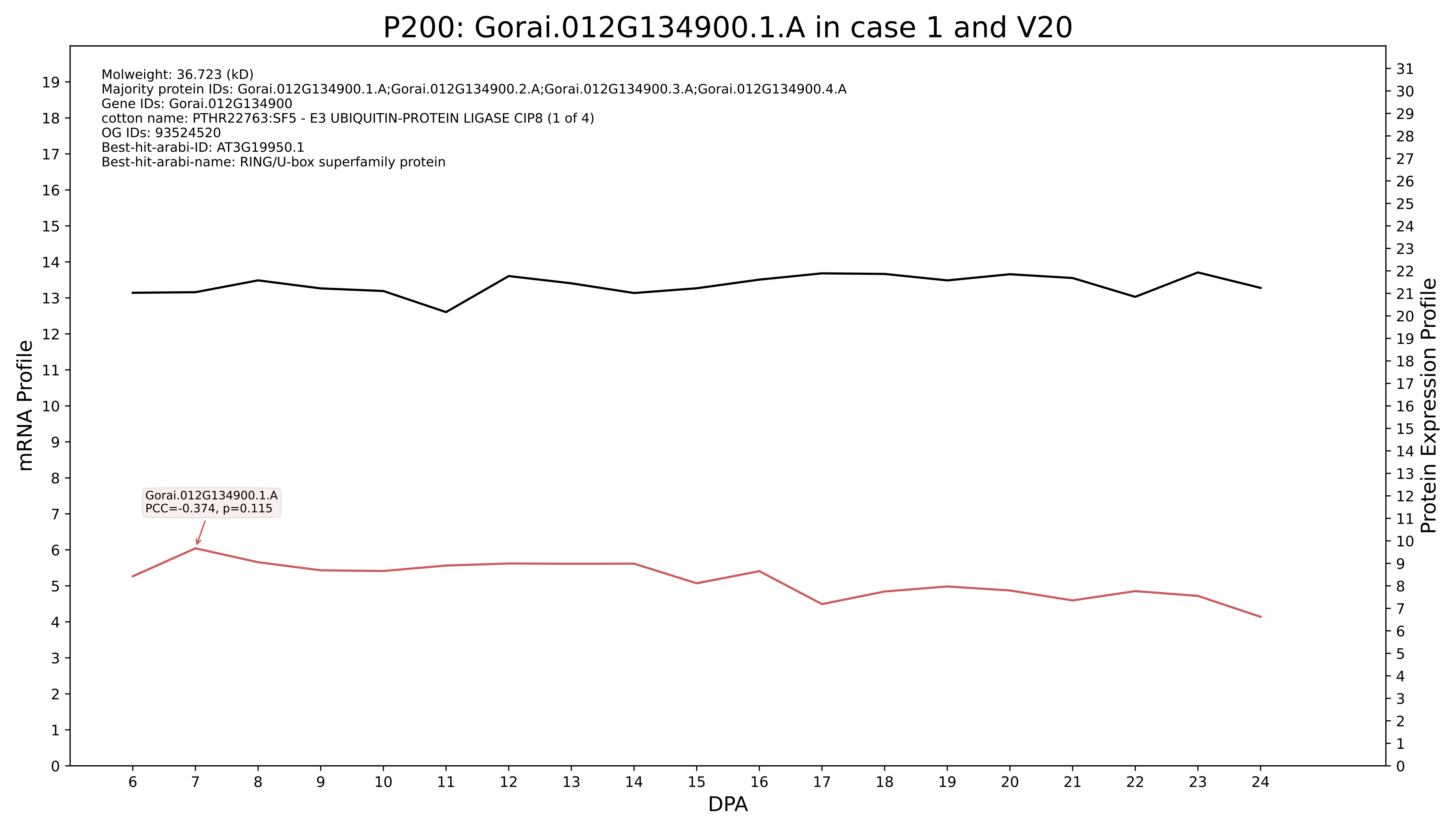
P200: Gorai.012G134900.1.A in case 1 and V20
Molweight: 36.723 (kD)
Majority protein IDs: Gorai.012G134900.1.A;Gorai.012G134900.2.A;Gorai.012G134900.3.A;Gorai.012G134900.4.A
Gene IDs: Gorai.012G134900
cotton name: PTHR22763:SF5 - E3 UBIQUITIN-PROTEIN LIGASE CIP8 (1 of 4)
OG IDs: 93524520
Best-hit-arabi-ID: AT3G19950.1
Best-hit-arabi-name: RING/U-box superfamily protein
Gorai.012G134900.1.A
PCC=-0.374, p=0.115
mRNA Profile
Protein Expression Profile
DPA

P200: Gorai.007G076000.3.D in case 1 and V20

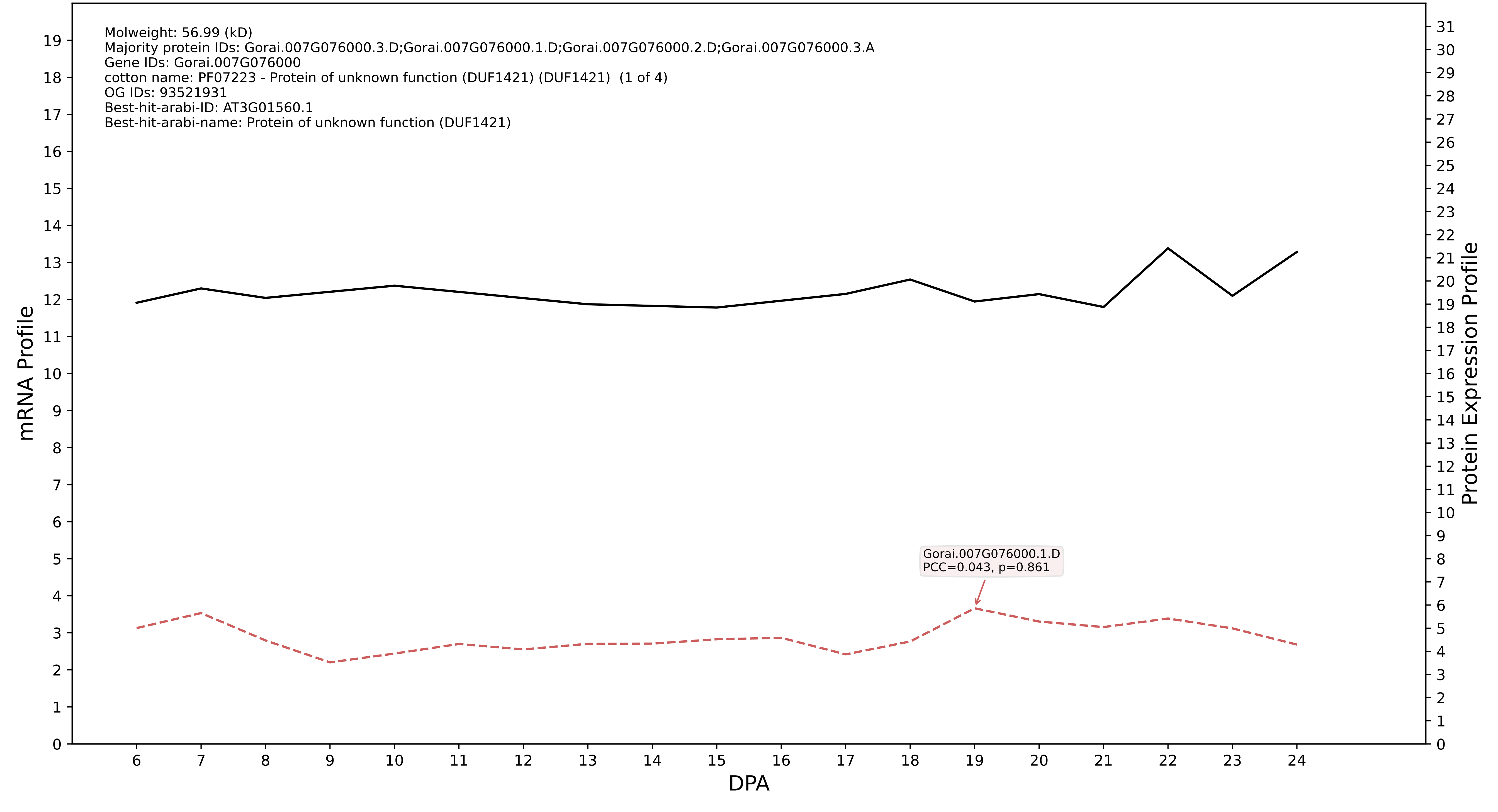

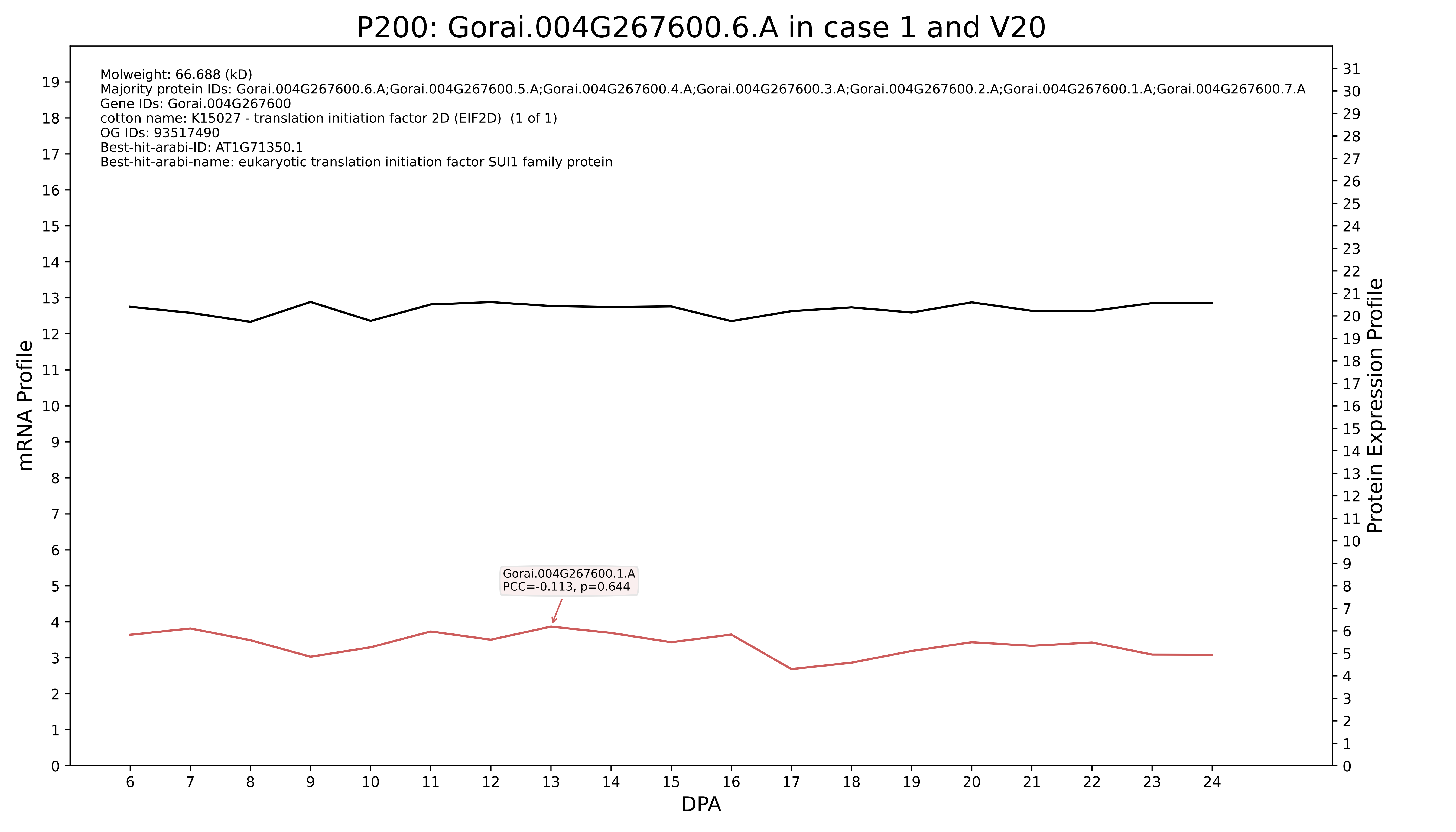

P200: Gorai.004G267600.6.A in case 1 and V20
Molweight: 66.688 (kD)
Majority protein IDs: Gorai.004G267600.6.A;Gorai.004G267600.5.A;Gorai.004G267600.4.A;Gorai.004G267600.3.A;Gorai.004G267600.2.A;Gorai.004G267600.1.A;Gorai.004G267600.7.A
Gene IDs: Gorai.004G267600
cotton name: K15027 - translation initiation factor 2D (EIF2D) (1 of 1)
OG IDs: 93517490
Best-hit-arabi-ID: AT1G71350.1
Best-hit-arabi-name: eukaryotic translation initiation factor SUI1 family protein
Gorai.004G267600.1.A
PCC=-0.113, p=0.644
mRNA Profile
Protein Expression Profile
DPA

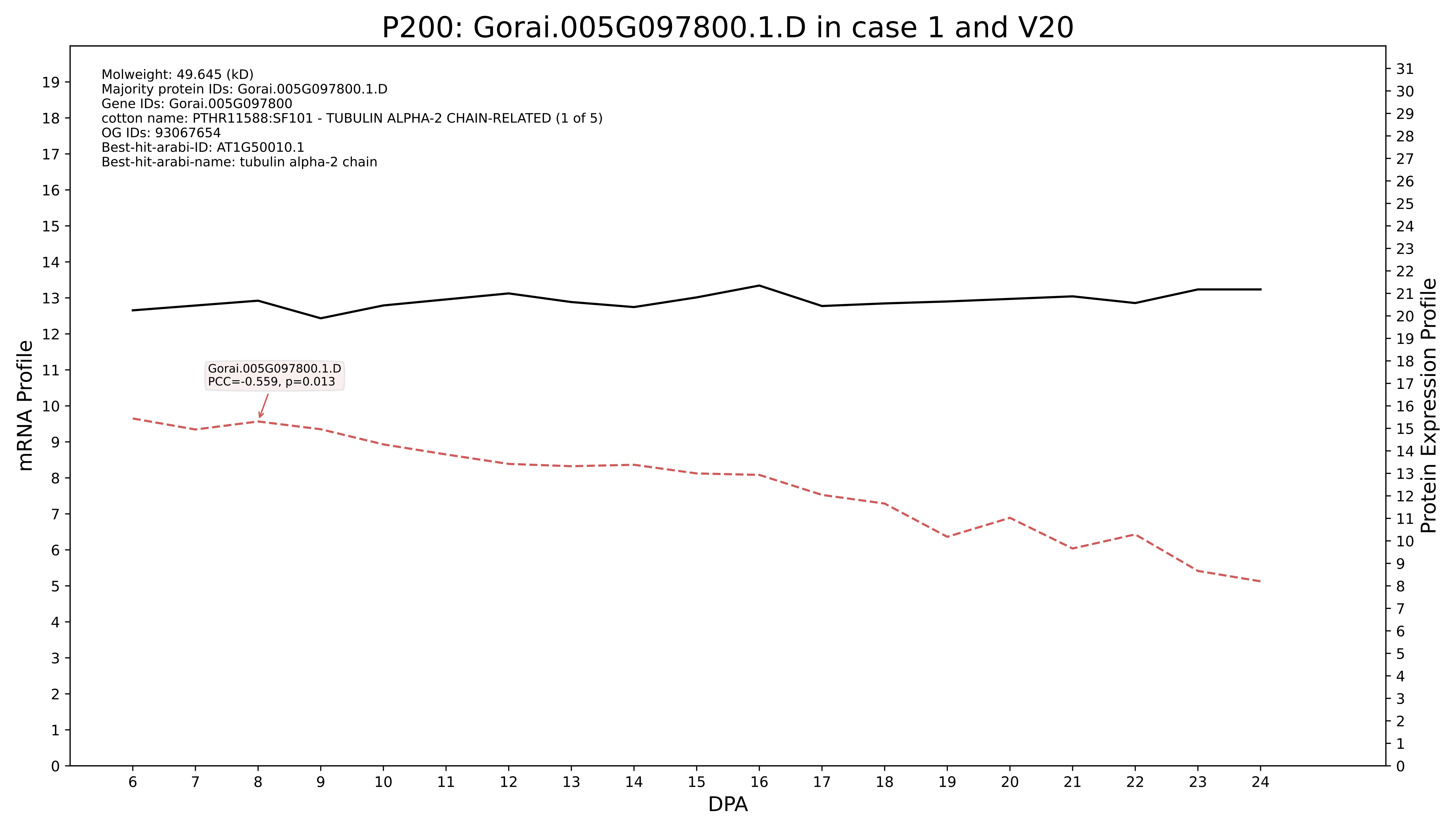
P200: Gorai.005G097800.1.D in case 1 and V20
Molweight: 49.645 (kD)
Majority protein IDs: Gorai.005G097800.1.D
Gene IDs: Gorai.005G097800
cotton name: PTHR11588:SF101 - TUBULIN ALPHA-2 CHAIN-RELATED (1 of 5)
OG IDs: 93067654
Best-hit-arabi-ID: AT1G50010.1
Best-hit-arabi-name: tubulin alpha-2 chain
Gorai.005G097800.1.D
PCC=-0.559, p=0.013
mRNA Profile
Protein Expression Profile
DPA

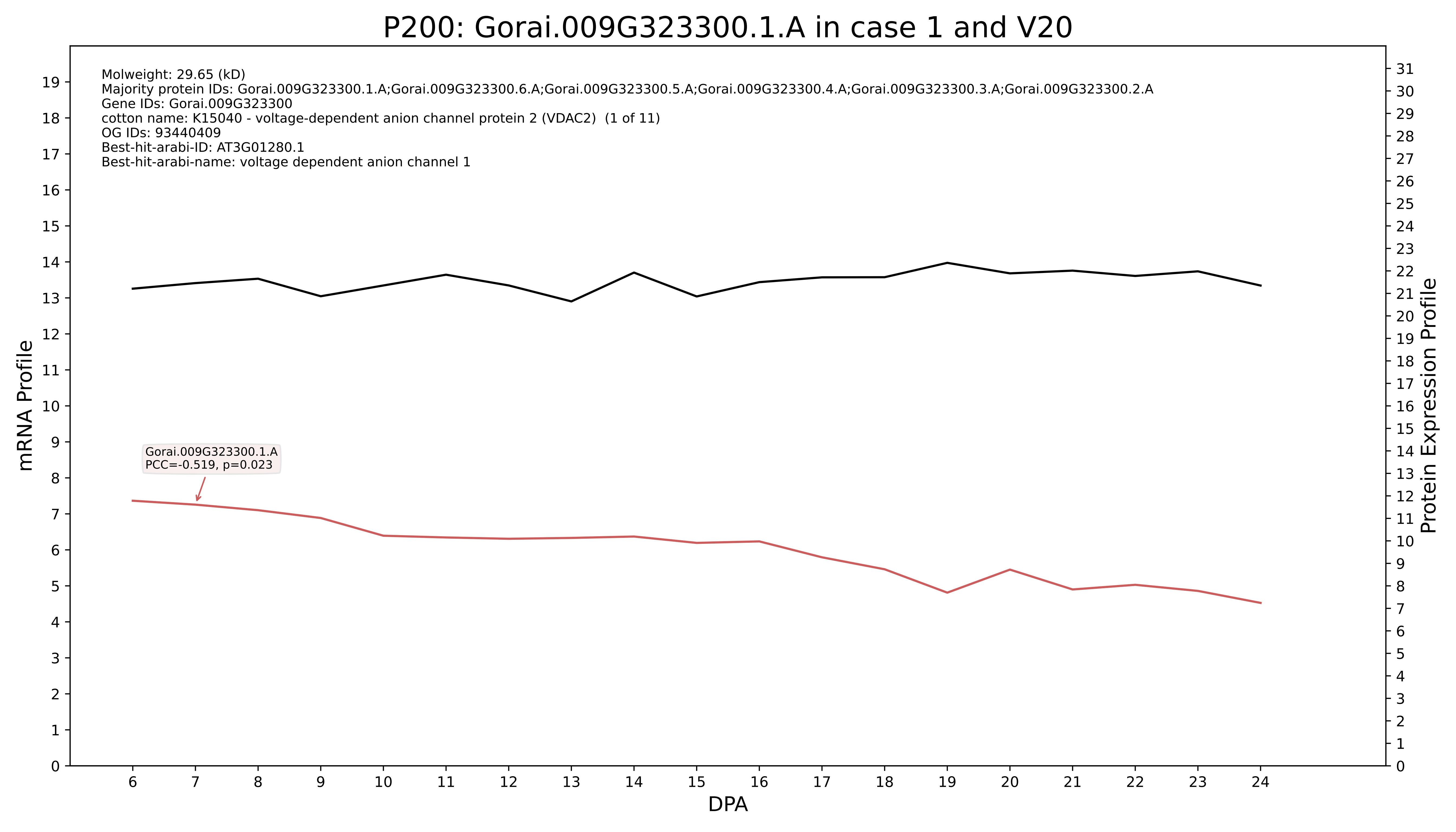
P200: Gorai.009G323300.1.A in case 1 and V20
Molweight: 29.65 (kD)
Majority protein IDs: Gorai.009G323300.1.A;Gorai.009G323300.6.A;Gorai.009G323300.5.A;Gorai.009G323300.4.A;Gorai.009G323300.3.A;Gorai.009G323300.2.A
Gene IDs: Gorai.009G323300
cotton name: K15040 - voltage-dependent anion channel protein 2 (VDAC2) (1 of 11)
OG IDs: 93440409
Best-hit-arabi-ID: AT3G01280.1
Best-hit-arabi-name: voltage dependent anion channel 1
Gorai.009G323300.1.A
PCC=-0.519, p=0.023
mRNA Profile
Protein Expression Profile
DPA

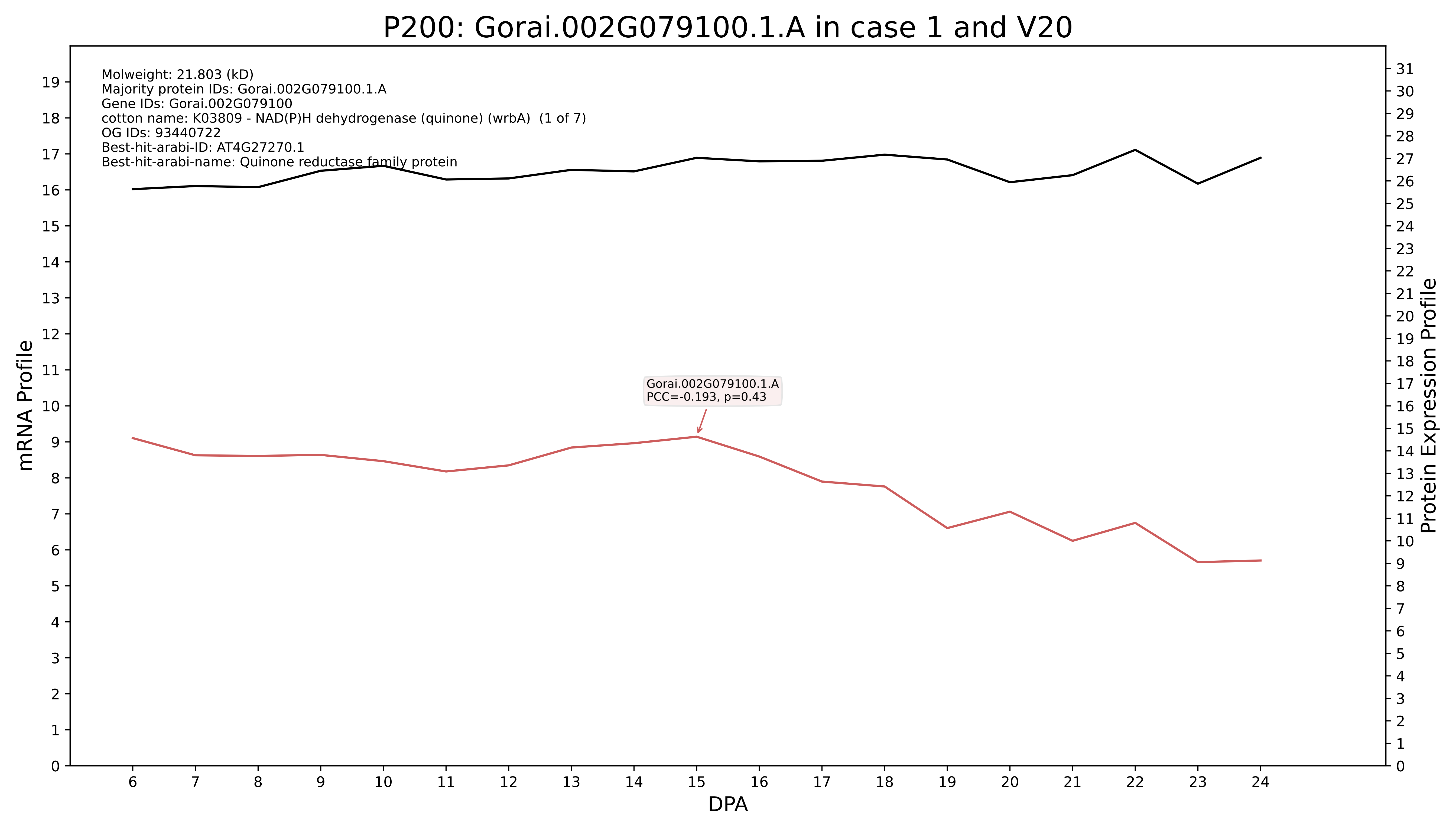
P200: Gorai.002G079100.1.A in case 1 and V20
Molweight: 21.803 (kD)
Majority protein IDs: Gorai.002G079100.1.A
Gene IDs: Gorai.002G079100
cotton name: K03809 - NAD(P)H dehydrogenase (quinone) (wrbA) (1 of 7)
OG IDs: 93440722
Best-hit-arabi-ID: AT4G27270.1
Best-hit-arabi-name: Quinone reductase family protein
Gorai.002G079100.1.A
PCC=-0.193, p=0.43
mRNA Profile
Protein Expression Profile
DPA
0
1
2
3
4
5
6
7
8
9
10
11
12
13
14
15
16
17
18
19
20
21
22
23
24
25
26
27
28
29
30
31

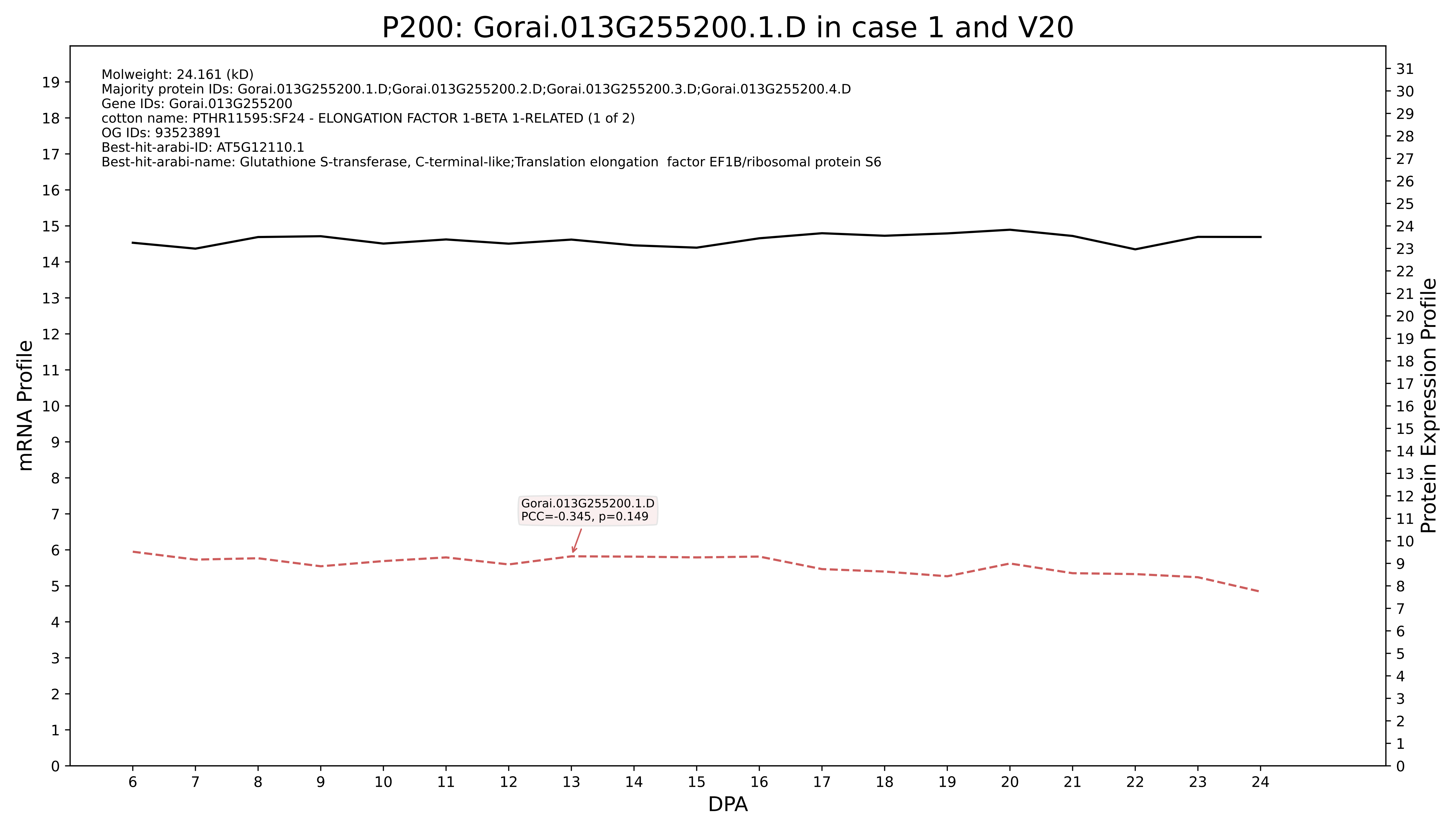
P200: Gorai.013G255200.1.D in case 1 and V20
Molweight: 24.161 (kD)
Majority protein IDs: Gorai.013G255200.1.D;Gorai.013G255200.2.D;Gorai.013G255200.3.D;Gorai.013G255200.4.D
Gene IDs: Gorai.013G255200
cotton name: PTHR11595:SF24 - ELONGATION FACTOR 1-BETA 1-RELATED (1 of 2)
OG IDs: 93523891
Best-hit-arabi-ID: AT5G12110.1
Best-hit-arabi-name: Glutathione S-transferase, C-terminal-like;Translation elongation factor EF1B/ribosomal protein S6
Gorai.013G255200.1.D
PCC=-0.345, p=0.149
mRNA Profile
Protein Expression Profile
DPA
0
1
2
3
4
5
6
7
8
9
10
11
12
13
14
15
16
17
18
19
20
21
22
23
24
25
26
27
28
29
30
31

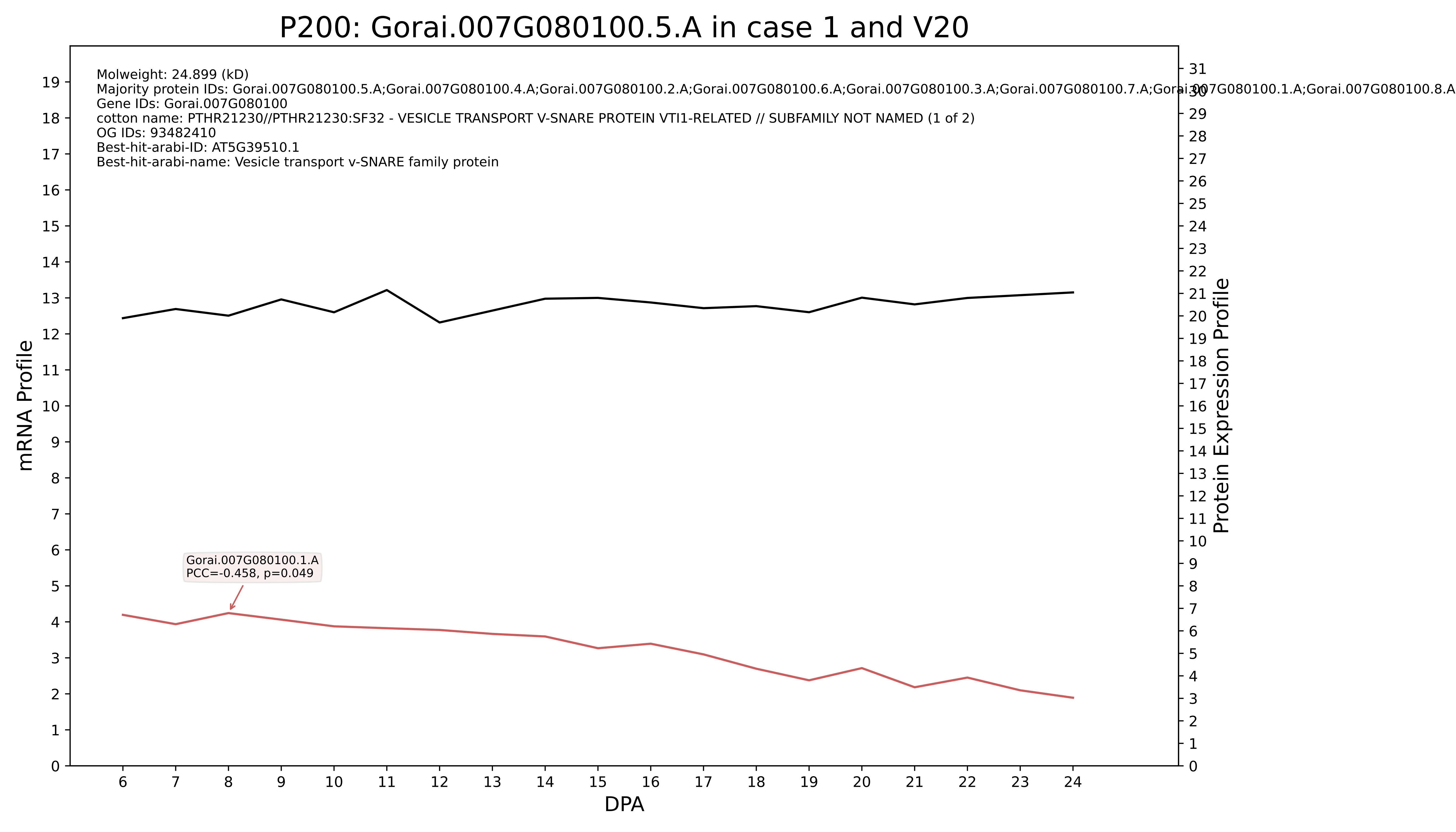
P200: Gorai.007G080100.5.A in case 1 and V20
Molweight: 24.899 (kD)
Majority protein IDs: Gorai.007G080100.5.A;Gorai.007G080100.4.A;Gorai.007G080100.2.A;Gorai.007G080100.6.A;Gorai.007G080100.3.A;Gorai.007G080100.7.A;Gorai.007G080100.1.A;Gorai.007G080100.8.A
Gene IDs: Gorai.007G080100
cotton name: PTHR21230//PTHR21230:SF32 - VESICLE TRANSPORT V-SNARE PROTEIN VTI1-RELATED // SUBFAMILY NOT NAMED (1 of 2)
OG IDs: 93482410
Best-hit-arabi-ID: AT5G39510.1
Best-hit-arabi-name: Vesicle transport v-SNARE family protein
Gorai.007G080100.1.A
PCC=-0.458, p=0.049
mRNA Profile
Protein Expression Profile
DPA

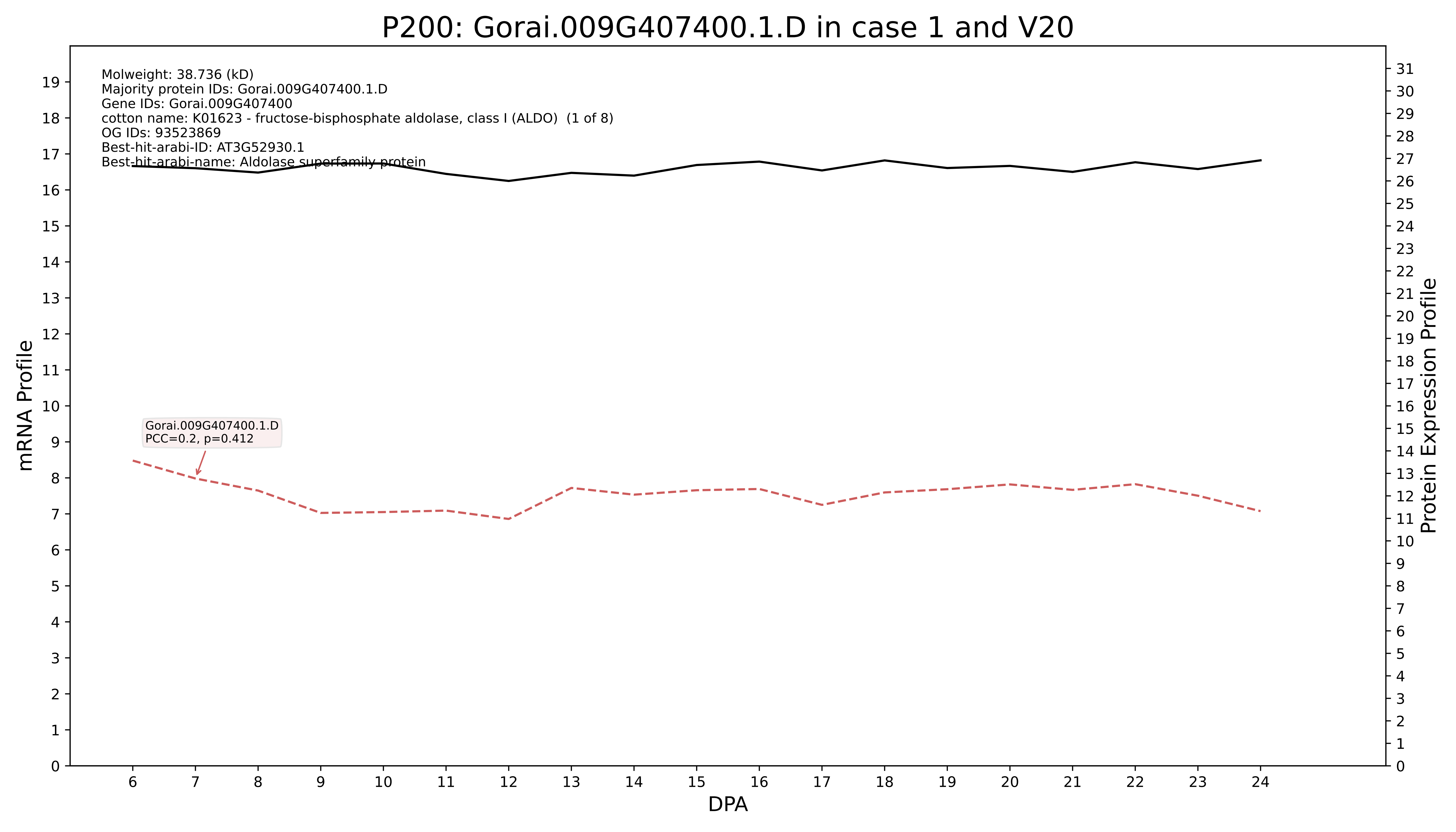
P200: Gorai.009G407400.1.D in case 1 and V20
Molweight: 38.736 (kD)
Majority protein IDs: Gorai.009G407400.1.D
Gene IDs: Gorai.009G407400
cotton name: K01623 - fructose-bisphosphate aldolase, class I (ALDO) (1 of 8)
OG IDs: 93523869
Best-hit-arabi-ID: AT3G52930.1
Best-hit-arabi-name: Aldolase superfamily protein
Gorai.009G407400.1.D
PCC=0.2, p=0.412
mRNA Profile
Protein Expression Profile
DPA
0
1
2
3
4
5
6
7
8
9
10
11
12
13
14
15
16
17
18
19
20
21
22
23
24
25
26
27
28
29
30
31

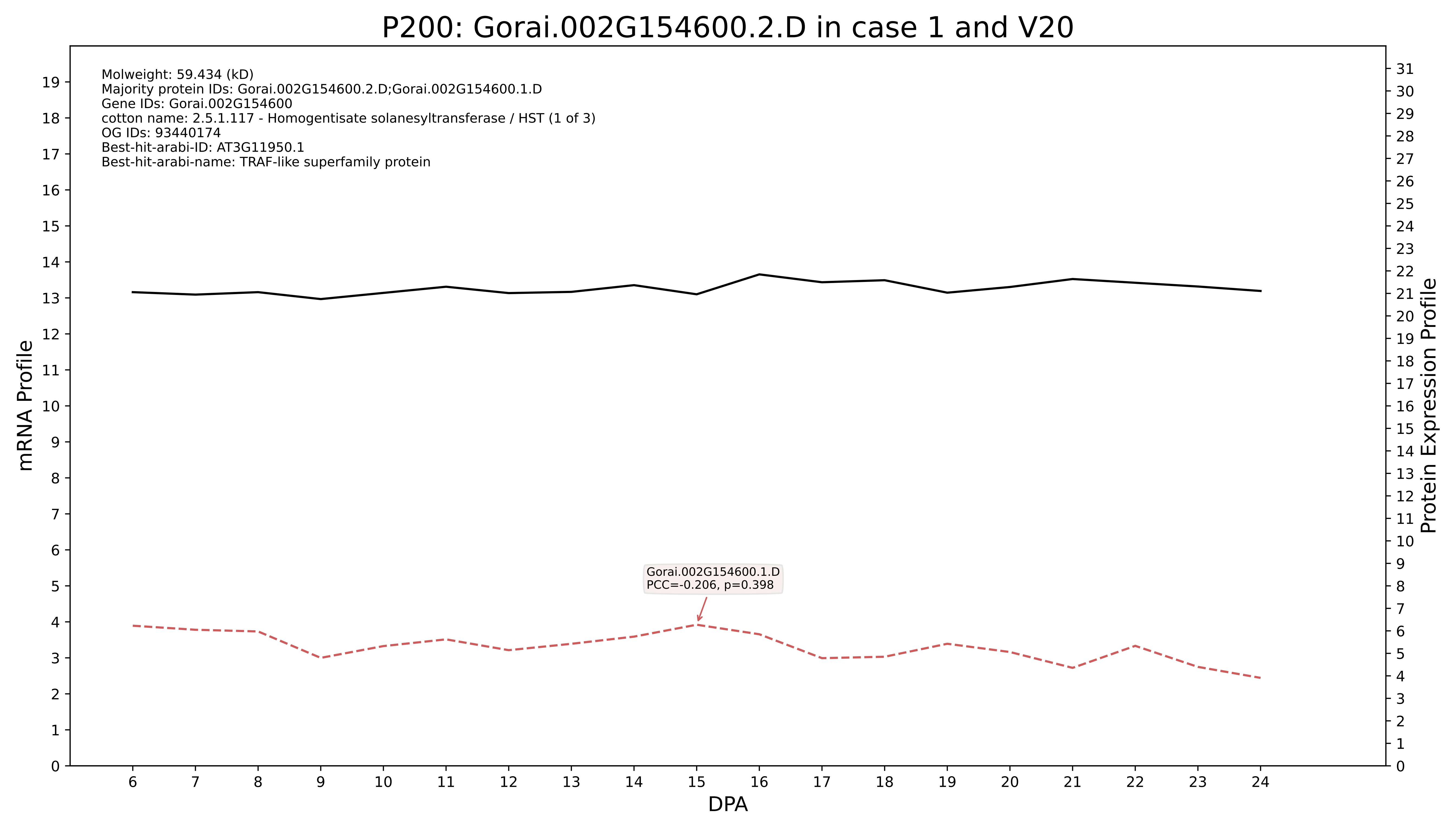
P200: Gorai.002G154600.2.D in case 1 and V20
Molweight: 59.434 (kD)
Majority protein IDs: Gorai.002G154600.2.D;Gorai.002G154600.1.D
Gene IDs: Gorai.002G154600
cotton name: 2.5.1.117 - Homogentisate solanesyltransferase / HST (1 of 3)
OG IDs: 93440174
Best-hit-arabi-ID: AT3G11950.1
Best-hit-arabi-name: TRAF-like superfamily protein
Gorai.002G154600.1.D
PCC=-0.206, p=0.398
mRNA Profile
Protein Expression Profile
DPA

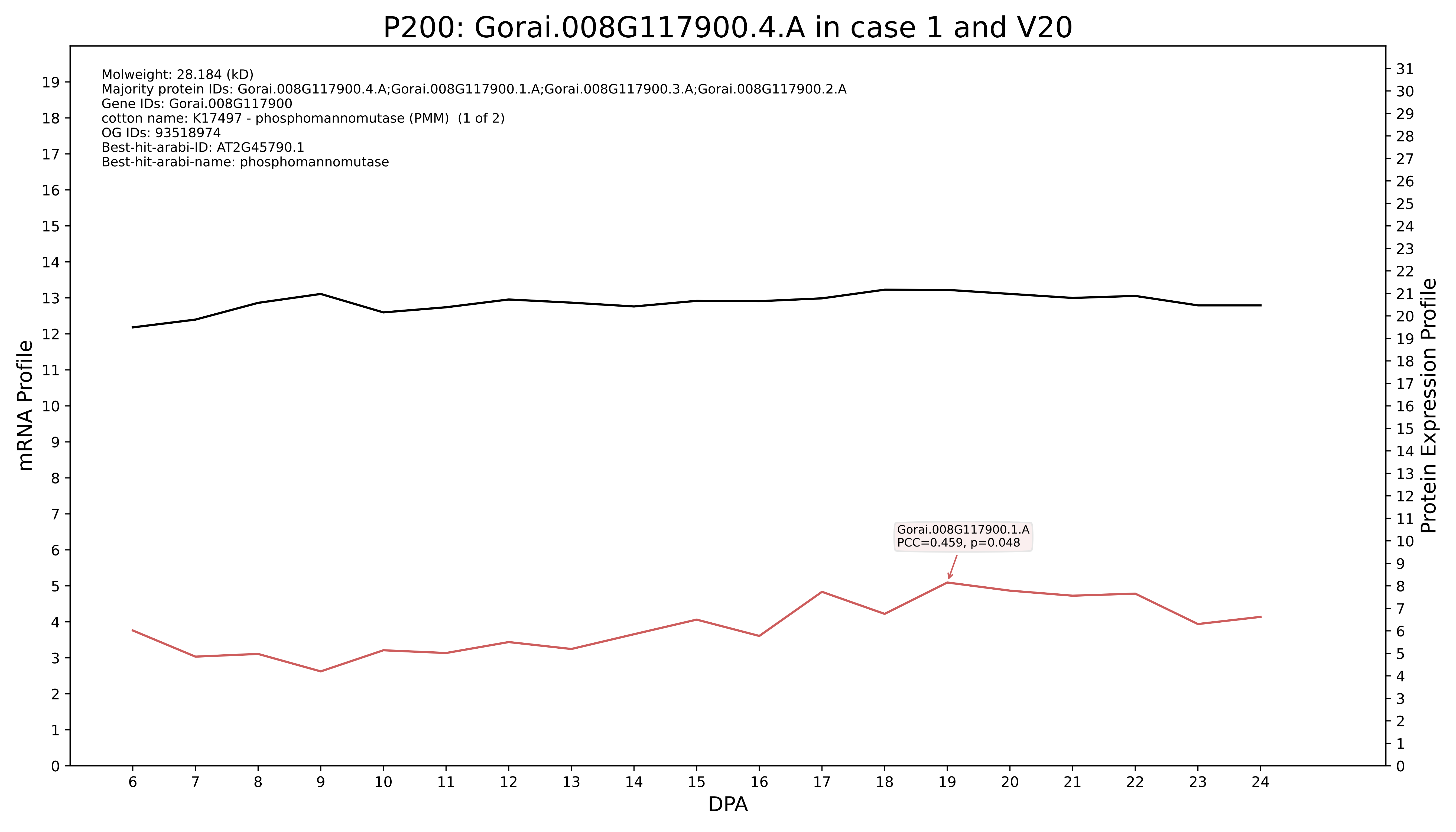
P200: Gorai.008G117900.4.A in case 1 and V20
Molweight: 28.184 (kD)
Majority protein IDs: Gorai.008G117900.4.A;Gorai.008G117900.1.A;Gorai.008G117900.3.A;Gorai.008G117900.2.A
Gene IDs: Gorai.008G117900
cotton name: K17497 - phosphomannomutase (PMM) (1 of 2)
OG IDs: 93518974
Best-hit-arabi-ID: AT2G45790.1
Best-hit-arabi-name: phosphomannomutase
Gorai.008G117900.1.A
PCC=0.459, p=0.048
mRNA Profile
Protein Expression Profile
DPA
0
1
2
3
4
5
6
7
8
9
10
11
12
13
14
15
16
17
18
19
20
21
22
23
24
25
26
27
28
29
30
31

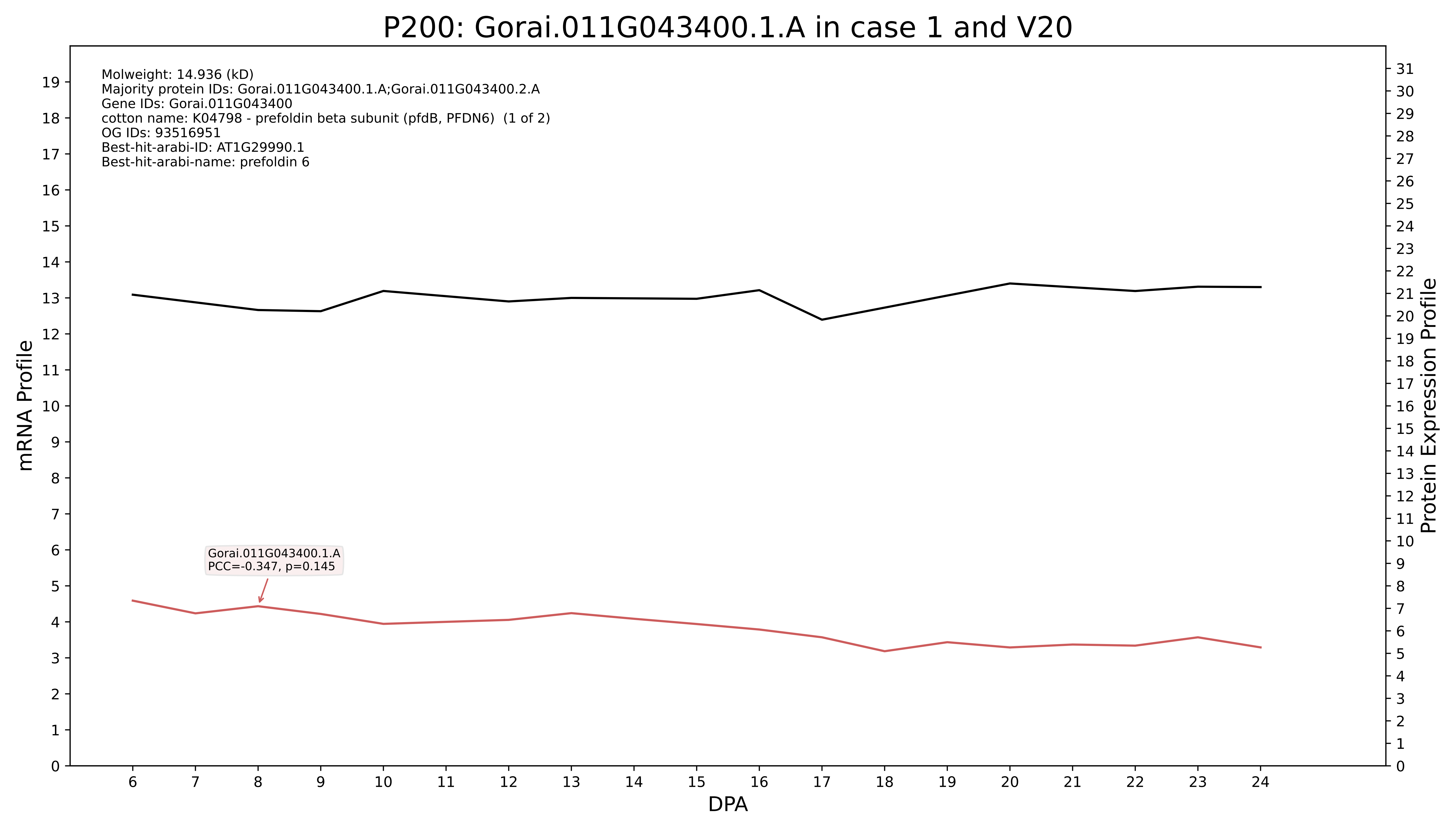
P200: Gorai.011G043400.1.A in case 1 and V20
Molweight: 14.936 (kD)
Majority protein IDs: Gorai.011G043400.1.A;Gorai.011G043400.2.A
Gene IDs: Gorai.011G043400
cotton name: K04798 - prefoldin beta subunit (pfdB, PFDN6) (1 of 2)
OG IDs: 93516951
Best-hit-arabi-ID: AT1G29990.1
Best-hit-arabi-name: prefoldin 6
Gorai.011G043400.1.A
PCC=-0.347, p=0.145
mRNA Profile
Protein Expression Profile
DPA
0
1
2
3
4
5
6
7
8
9
10
11
12
13
14
15
16
17
18
19
20
21
22
23
24
25
26
27
28
29
30
31

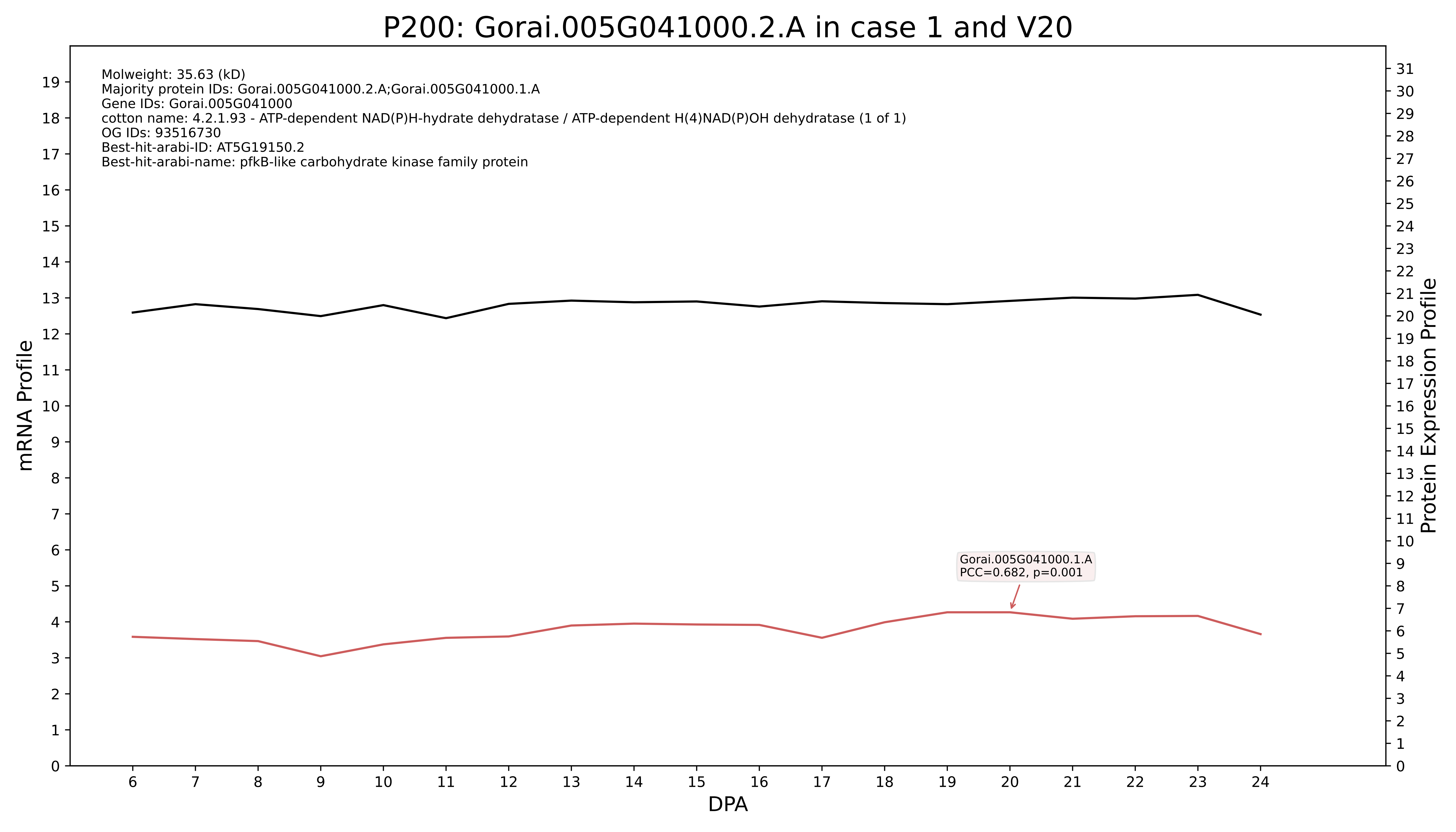
P200: Gorai.005G041000.2.A in case 1 and V20
Molweight: 35.63 (kD)
Majority protein IDs: Gorai.005G041000.2.A;Gorai.005G041000.1.A
Gene IDs: Gorai.005G041000
cotton name: 4.2.1.93 - ATP-dependent NAD(P)H-hydrate dehydratase / ATP-dependent H(4)NAD(P)OH dehydratase (1 of 1)
OG IDs: 93516730
Best-hit-arabi-ID: AT5G19150.2
Best-hit-arabi-name: pfkB-like carbohydrate kinase family protein
Gorai.005G041000.1.A
PCC=0.682, p=0.001
mRNA Profile
Protein Expression Profile
DPA
0
1
2
3
4
5
6
7
8
9
10
11
12
13
14
15
16
17
18
19
20
21
22
23
24
25
26
27
28
29
30
31

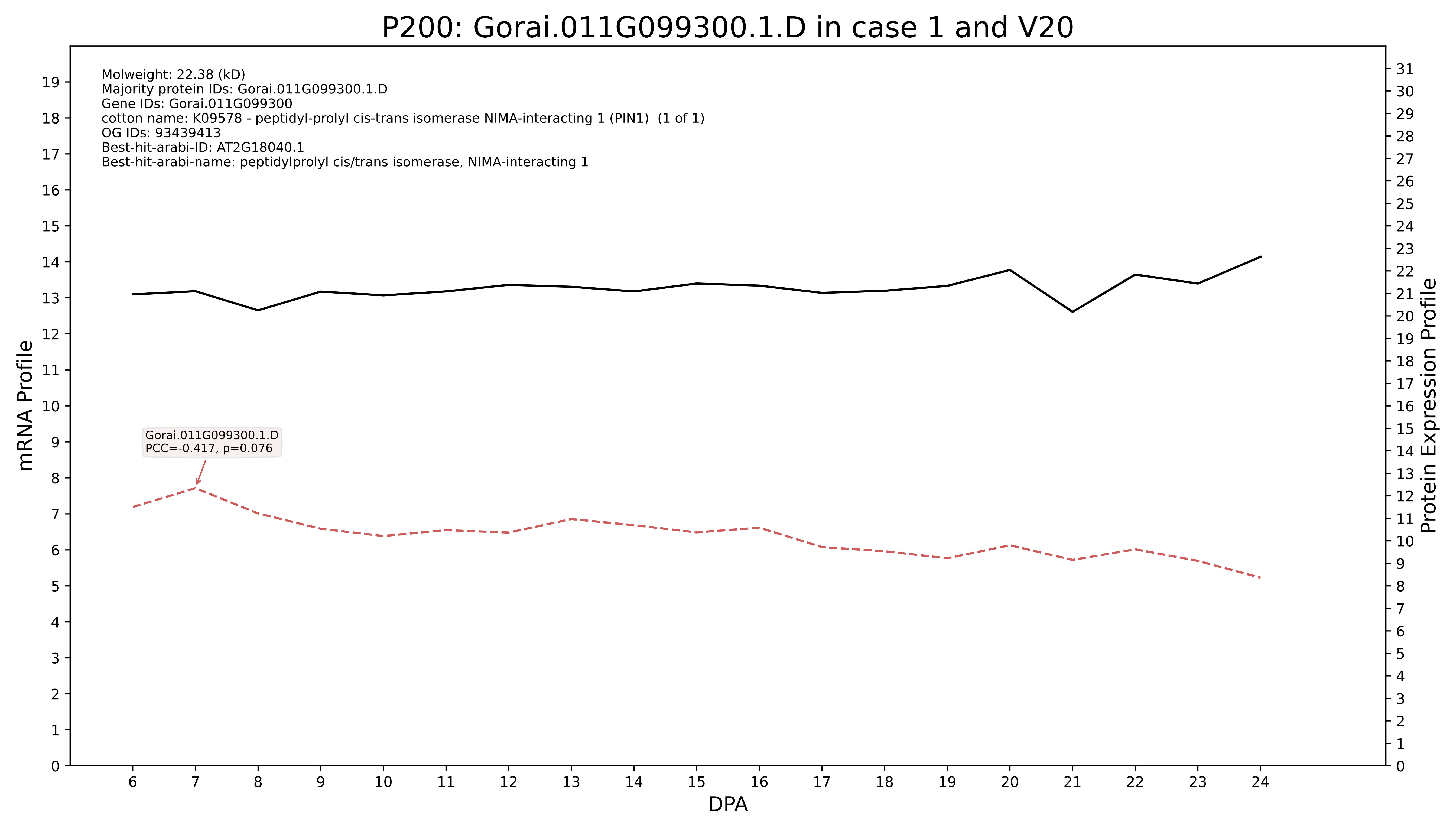

P200: Gorai.011G099300.1.D in case 1 and V20
Molweight: 22.38 (kD)
Majority protein IDs: Gorai.011G099300.1.D
Gene IDs: Gorai.011G099300
cotton name: K09578 - peptidyl-prolyl cis-trans isomerase NIMA-interacting 1 (PIN1) (1 of 1)
OG IDs: 93439413
Best-hit-arabi-ID: AT2G18040.1
Best-hit-arabi-name: peptidylprolyl cis/trans isomerase, NIMA-interacting 1
Gorai.011G099300.1.D
PCC=-0.417, p=0.076
mRNA Profile
Protein Expression Profile
DPA

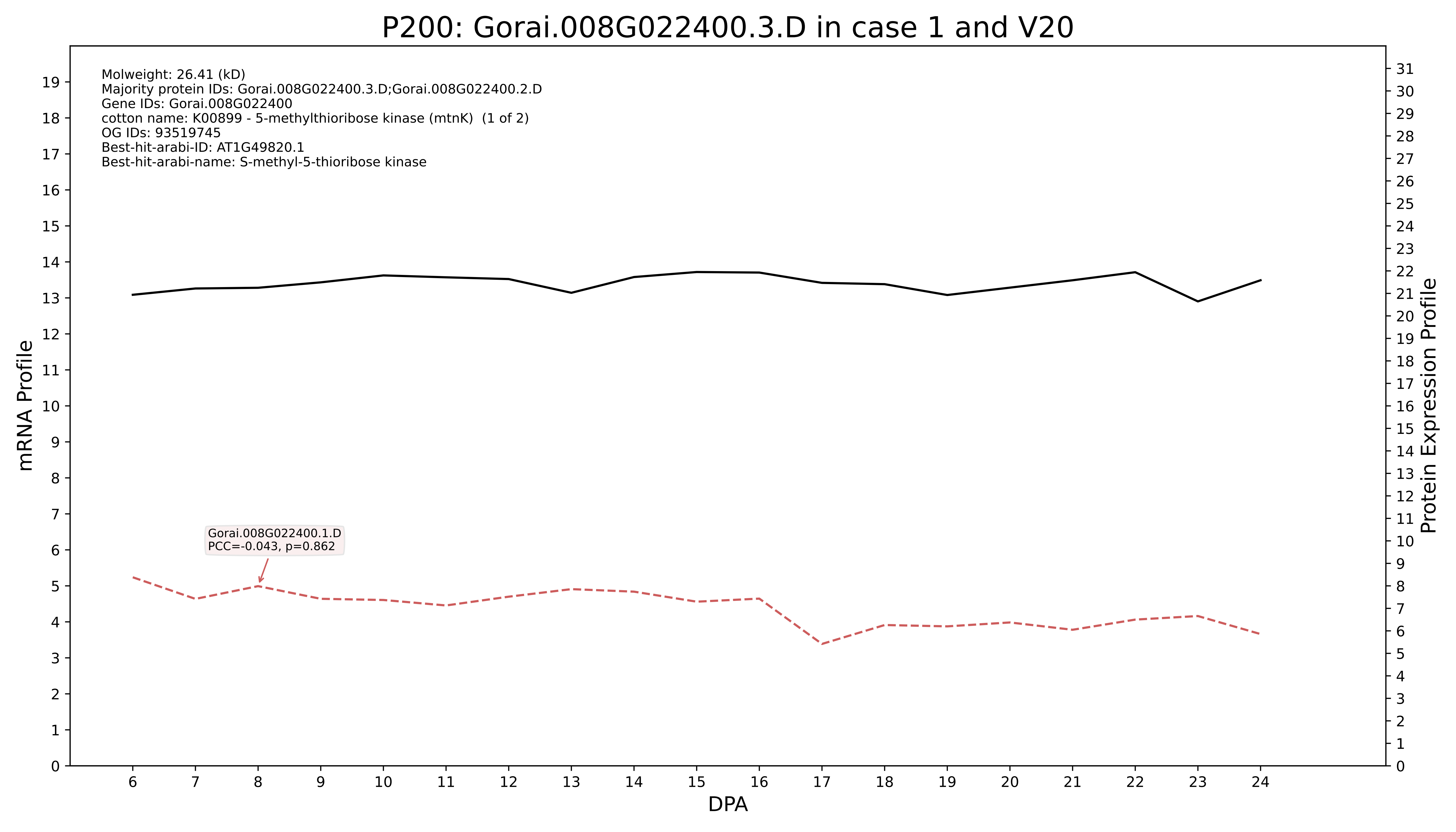
P200: Gorai.008G022400.3.D in case 1 and V20
Molweight: 26.41 (kD)
Majority protein IDs: Gorai.008G022400.3.D;Gorai.008G022400.2.D
Gene IDs: Gorai.008G022400
cotton name: K00899 - 5-methylthioribose kinase (mtnK) (1 of 2)
OG IDs: 93519745
Best-hit-arabi-ID: AT1G49820.1
Best-hit-arabi-name: S-methyl-5-thioribose kinase
Gorai.008G022400.1.D
PCC=-0.043, p=0.862
mRNA Profile
Protein Expression Profile
DPA

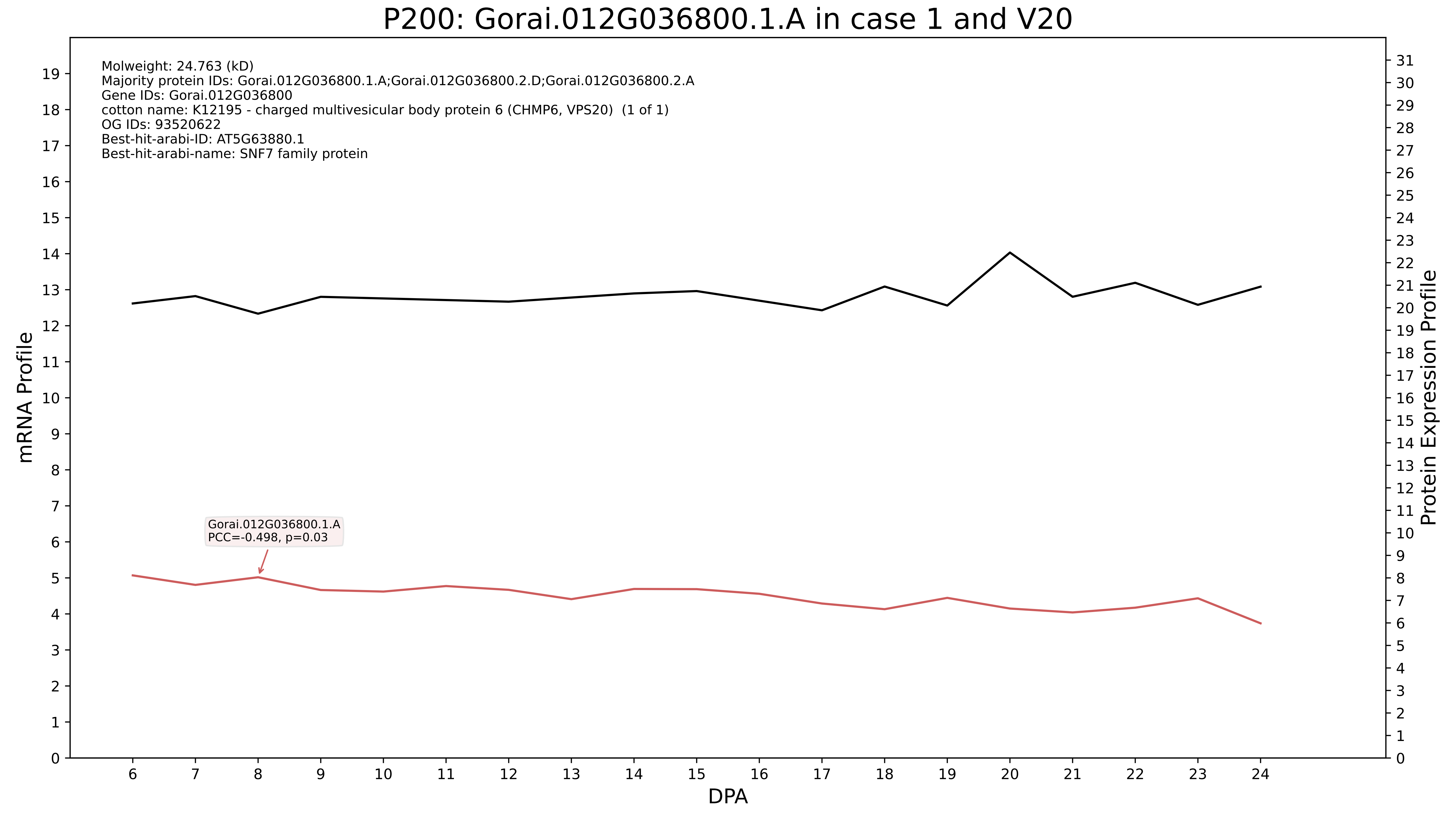
P200: Gorai.012G036800.1.A in case 1 and V20
Molweight: 24.763 (kD)
Majority protein IDs: Gorai.012G036800.1.A;Gorai.012G036800.2.D;Gorai.012G036800.2.A
Gene IDs: Gorai.012G036800
cotton name: K12195 - charged multivesicular body protein 6 (CHMP6, VPS20) (1 of 1)
OG IDs: 93520622
Best-hit-arabi-ID: AT5G63880.1
Best-hit-arabi-name: SNF7 family protein
Gorai.012G036800.1.A
PCC=-0.498, p=0.03
mRNA Profile
Protein Expression Profile
DPA

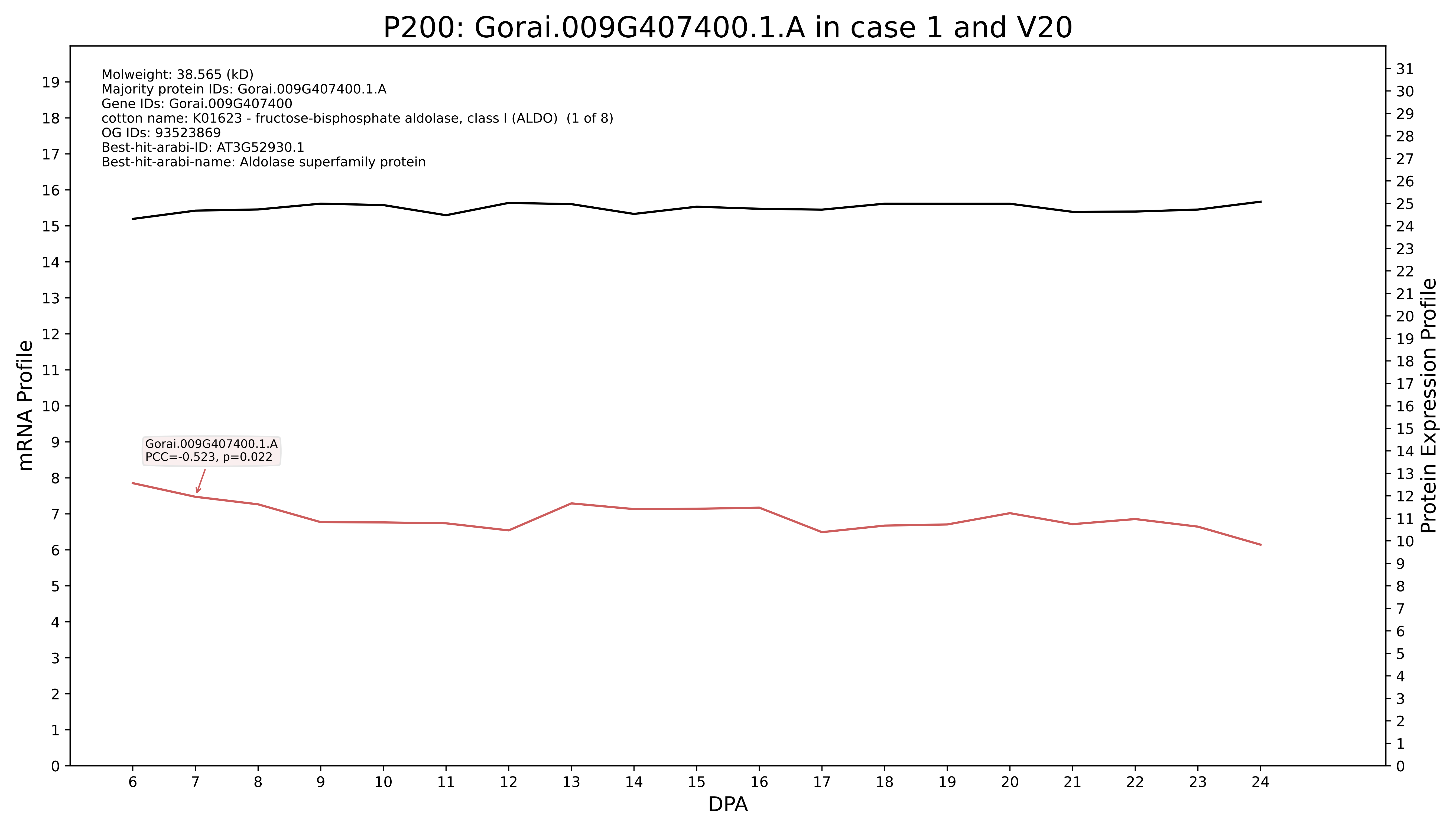
P200: Gorai.009G407400.1.A in case 1 and V20
Molweight: 38.565 (kD)
Majority protein IDs: Gorai.009G407400.1.A
Gene IDs: Gorai.009G407400
cotton name: K01623 - fructose-bisphosphate aldolase, class I (ALDO) (1 of 8)
OG IDs: 93523869
Best-hit-arabi-ID: AT3G52930.1
Best-hit-arabi-name: Aldolase superfamily protein
Gorai.009G407400.1.A
PCC=-0.523, p=0.022
mRNA Profile
Protein Expression Profile
DPA

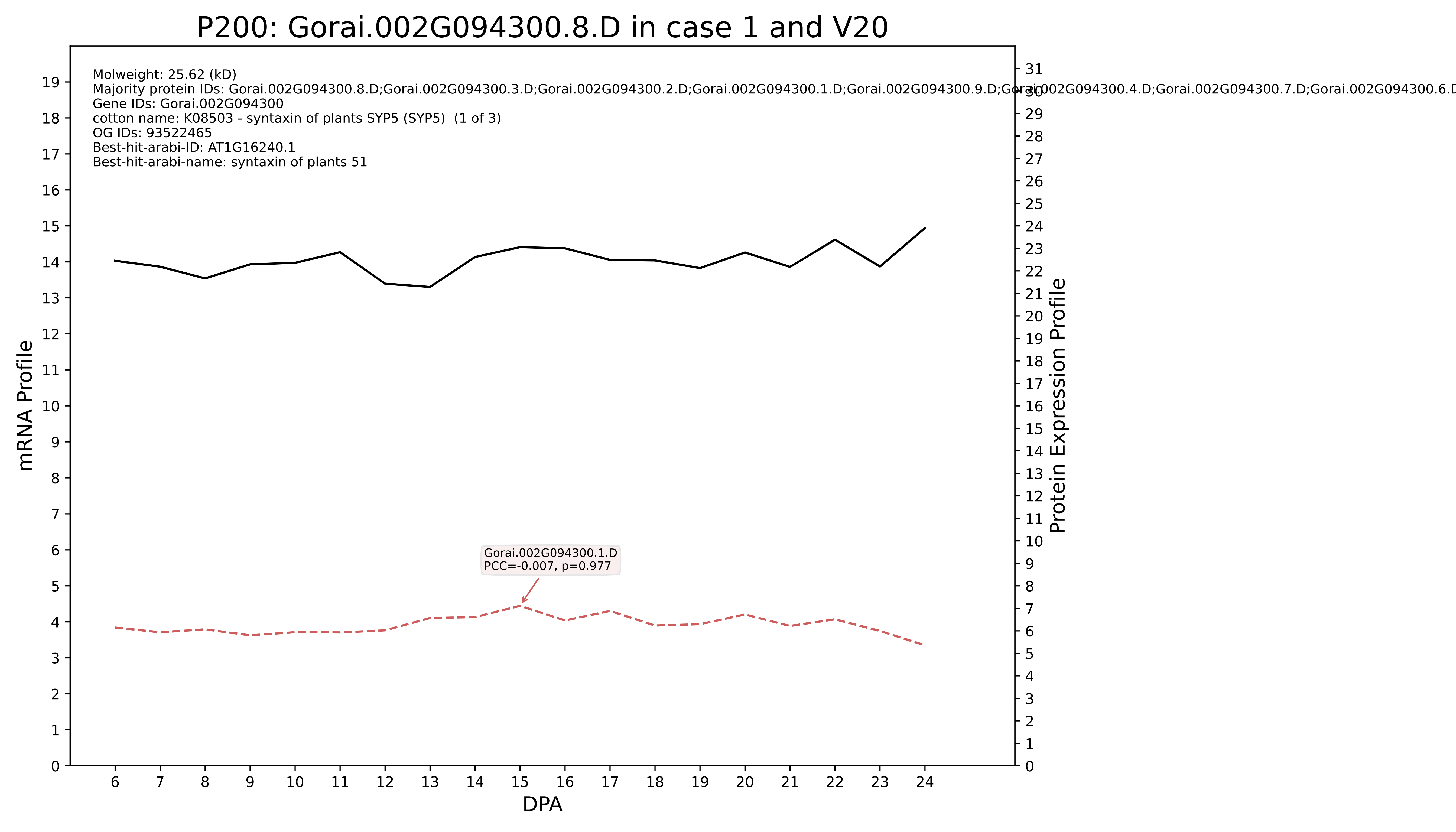
P200: Gorai.002G094300.8.D in case 1 and V20
Molweight: 25.62 (kD)
Majority protein IDs: Gorai.002G094300.8.D;Gorai.002G094300.3.D;Gorai.002G094300.2.D;Gorai.002G094300.1.D;Gorai.002G094300.9.D;Gorai.002G094300.4.D;Gorai.002G094300.7.D;Gorai.002G094300.6.D
Gene IDs: Gorai.002G094300
cotton name: K08503 - syntaxin of plants SYP5 (SYP5) (1 of 3)
OG IDs: 93522465
Best-hit-arabi-ID: AT1G16240.1
Best-hit-arabi-name: syntaxin of plants 51
Gorai.002G094300.1.D
PCC=-0.007, p=0.977
mRNA Profile
Protein Expression Profile
DPA

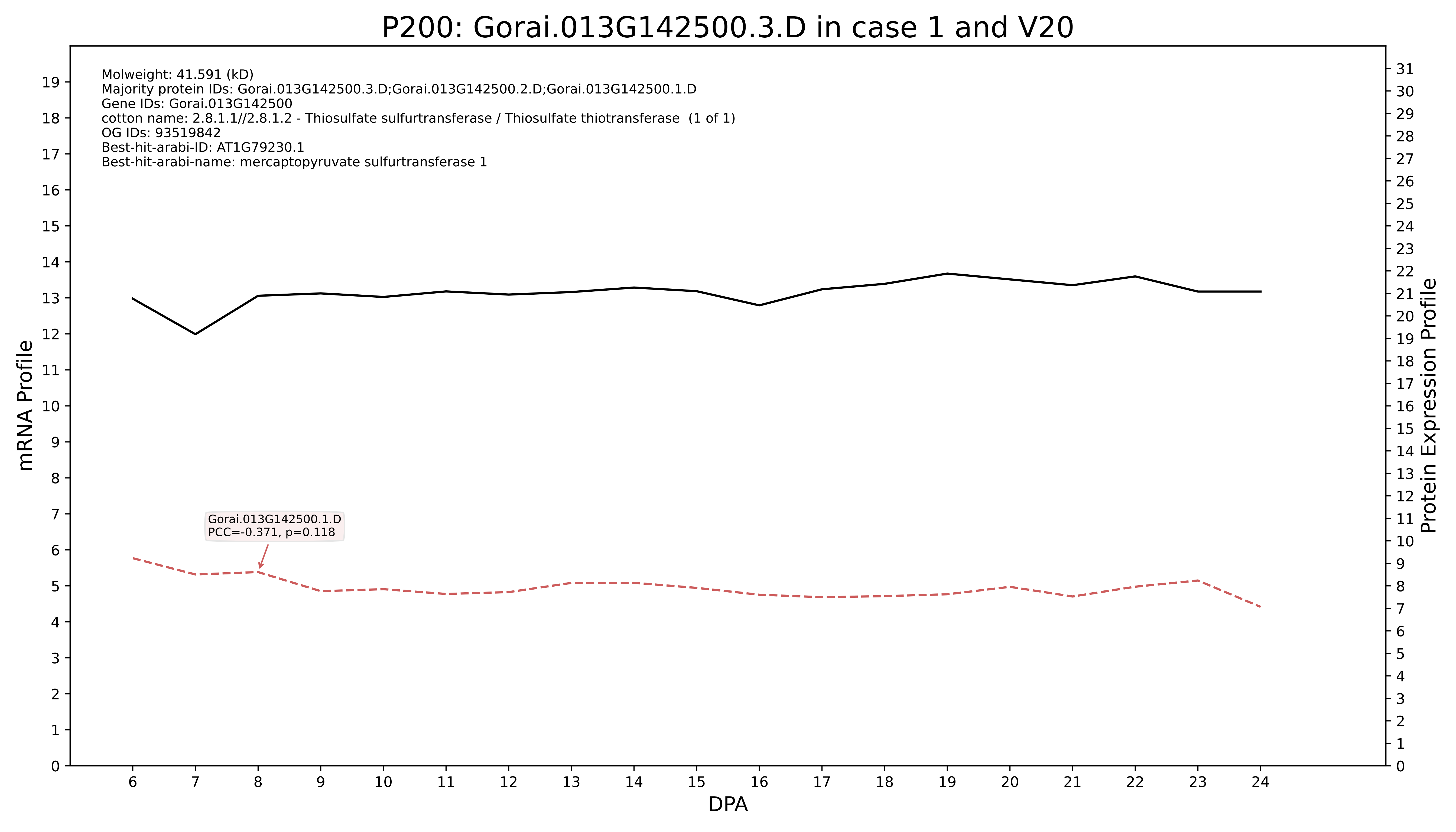

P200: Gorai.013G142500.3.D in case 1 and V20
Molweight: 41.591 (kD)
Majority protein IDs: Gorai.013G142500.3.D;Gorai.013G142500.2.D;Gorai.013G142500.1.D
Gene IDs: Gorai.013G142500
cotton name: 2.8.1.1//2.8.1.2 - Thiosulfate sulfurtransferase / Thiosulfate thiotransferase (1 of 1)
OG IDs: 93519842
Best-hit-arabi-ID: AT1G79230.1
Best-hit-arabi-name: mercaptopyruvate sulfurtransferase 1
Gorai.013G142500.1.D
PCC=-0.371, p=0.118
mRNA Profile
Protein Expression Profile
DPA

P200: Gorai.013G123700.1.D in case 1 and V20

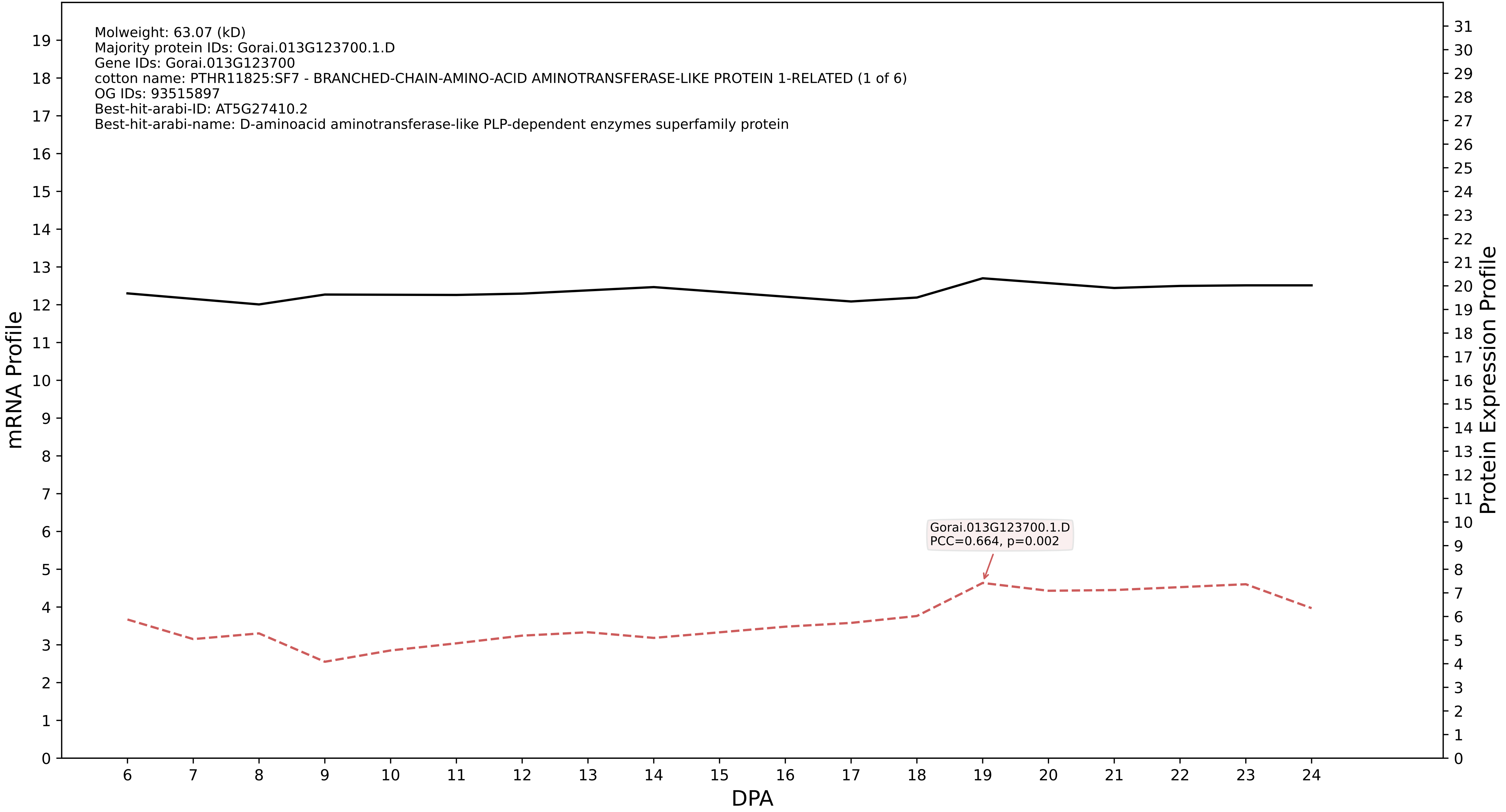

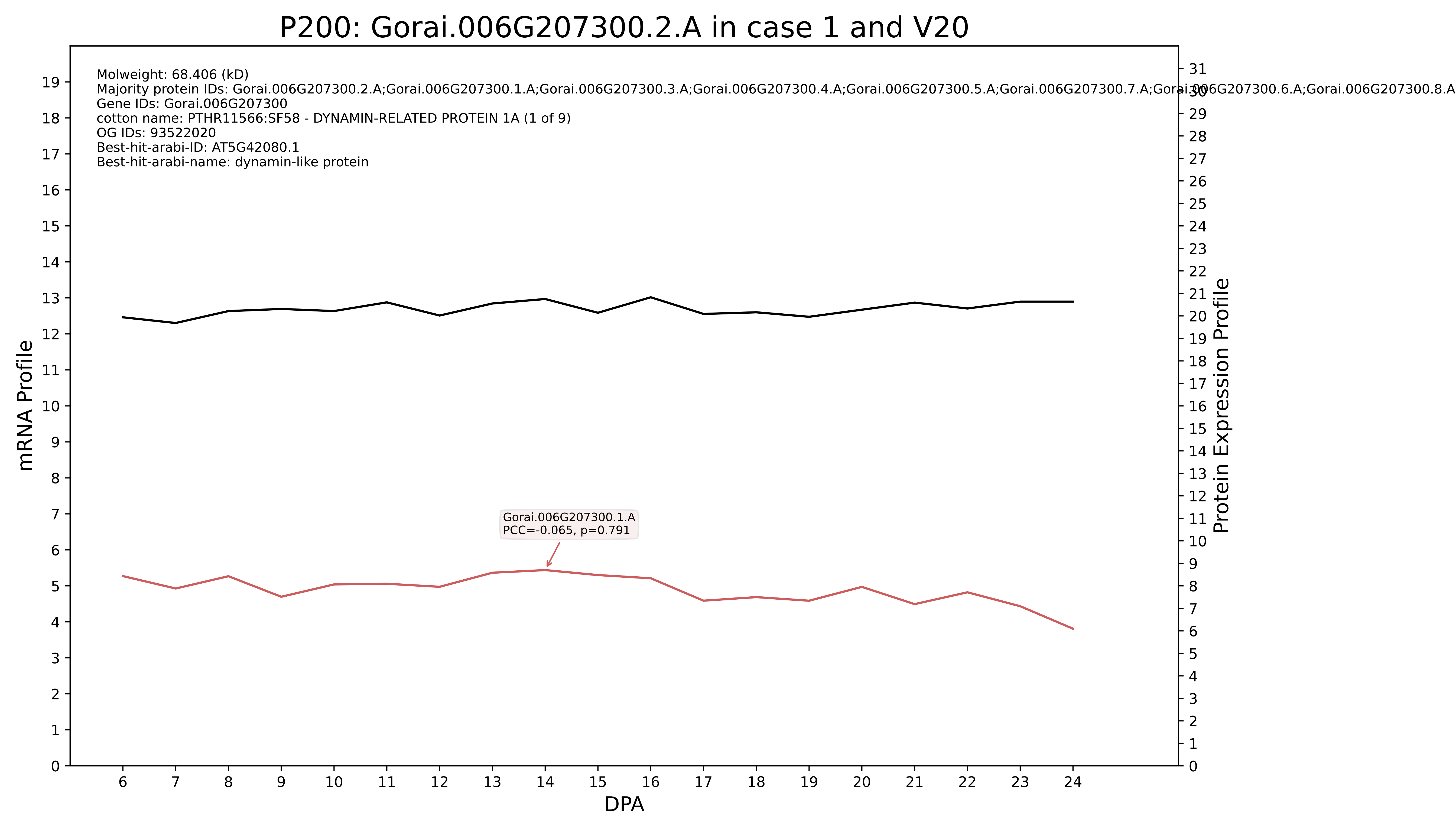
P200: Gorai.006G207300.2.A in case 1 and V20
Molweight: 68.406 (kD)
Majority protein IDs: Gorai.006G207300.2.A;Gorai.006G207300.1.A;Gorai.006G207300.3.A;Gorai.006G207300.4.A;Gorai.006G207300.5.A;Gorai.006G207300.7.A;Gorai.006G207300.6.A;Gorai.006G207300.8.A
Gene IDs: Gorai.006G207300
cotton name: PTHR11566:SF58 - DYNAMIN-RELATED PROTEIN 1A (1 of 9)
OG IDs: 93522020
Best-hit-arabi-ID: AT5G42080.1
Best-hit-arabi-name: dynamin-like protein
Gorai.006G207300.1.A
PCC=-0.065, p=0.791
mRNA Profile
Protein Expression Profile
DPA

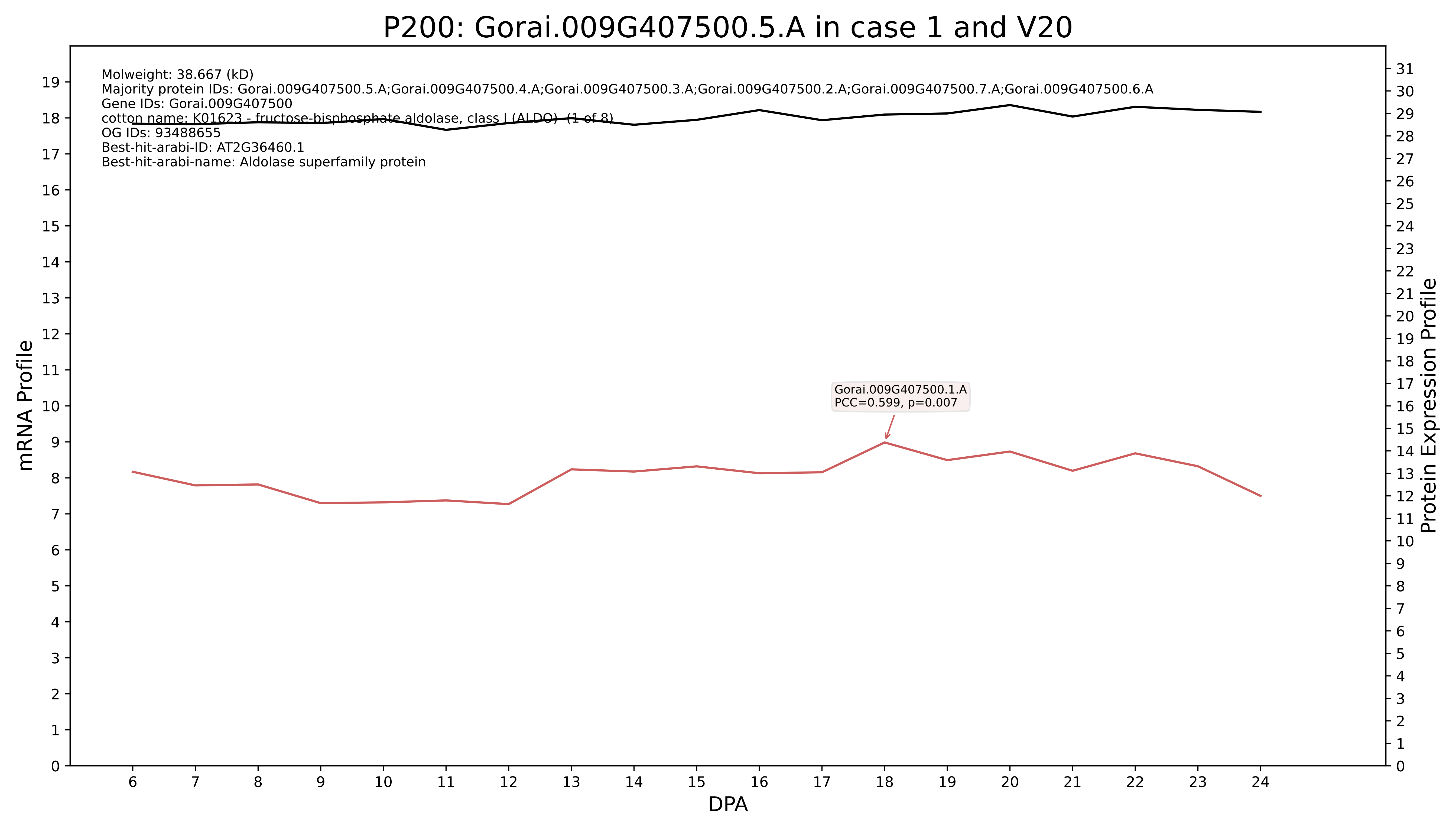

P200: Gorai.009G407500.5.A in case 1 and V20
Molweight: 38.667 (kD)
Majority protein IDs: Gorai.009G407500.5.A;Gorai.009G407500.4.A;Gorai.009G407500.3.A;Gorai.009G407500.2.A;Gorai.009G407500.7.A;Gorai.009G407500.6.A
Gene IDs: Gorai.009G407500
cotton name: K01623 - fructose-bisphosphate aldolase, class I (ALDO) (1 of 8)
OG IDs: 93488655
Best-hit-arabi-ID: AT2G36460.1
Best-hit-arabi-name: Aldolase superfamily protein
Gorai.009G407500.1.A
PCC=0.599, p=0.007
mRNA Profile
Protein Expression Profile
DPA

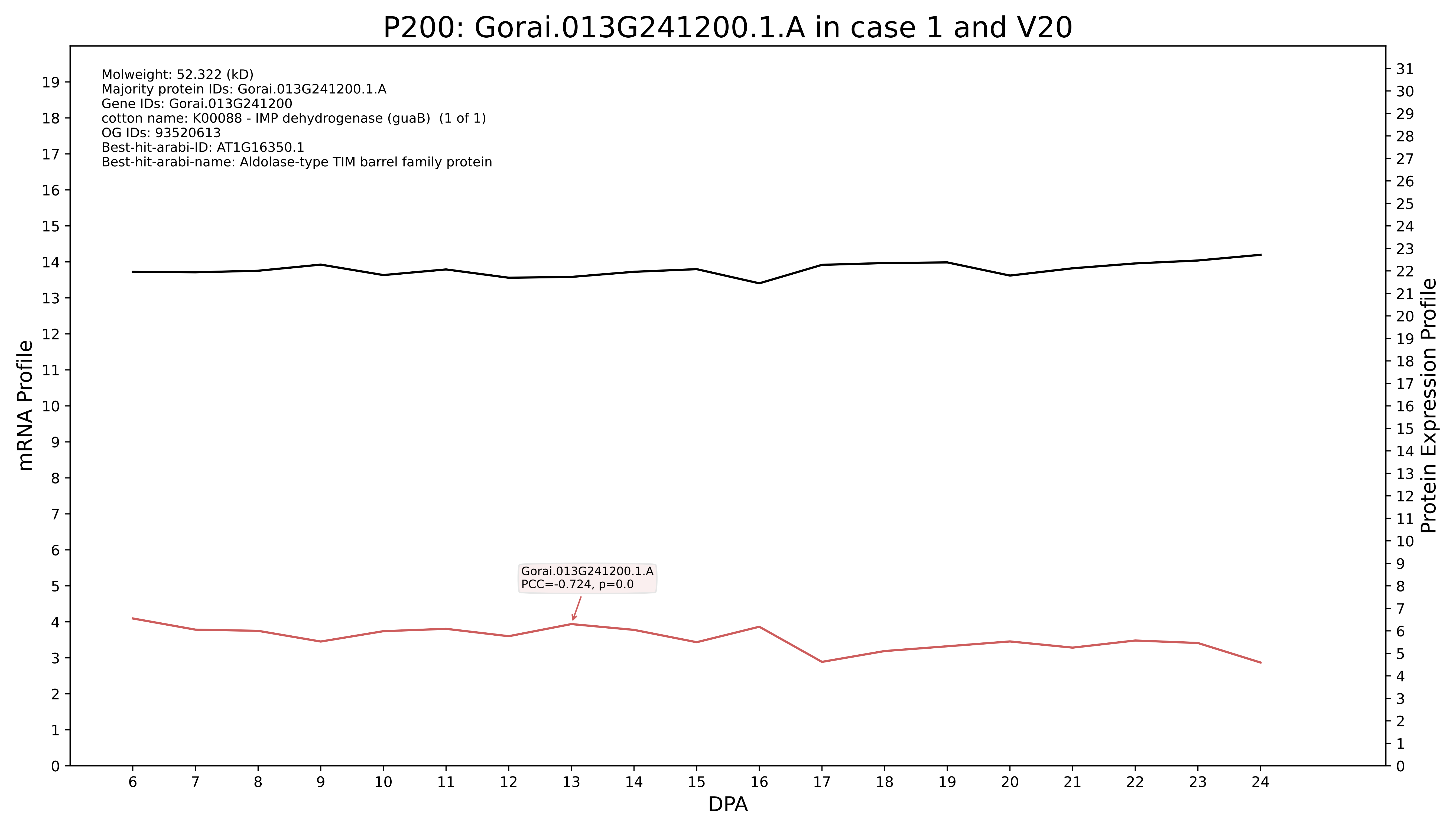
P200: Gorai.013G241200.1.A in case 1 and V20
Molweight: 52.322 (kD)
Majority protein IDs: Gorai.013G241200.1.A
Gene IDs: Gorai.013G241200
cotton name: K00088 - IMP dehydrogenase (guaB) (1 of 1)
OG IDs: 93520613
Best-hit-arabi-ID: AT1G16350.1
Best-hit-arabi-name: Aldolase-type TIM barrel family protein
Gorai.013G241200.1.A
PCC=-0.724, p=0.0
mRNA Profile
Protein Expression Profile
DPA

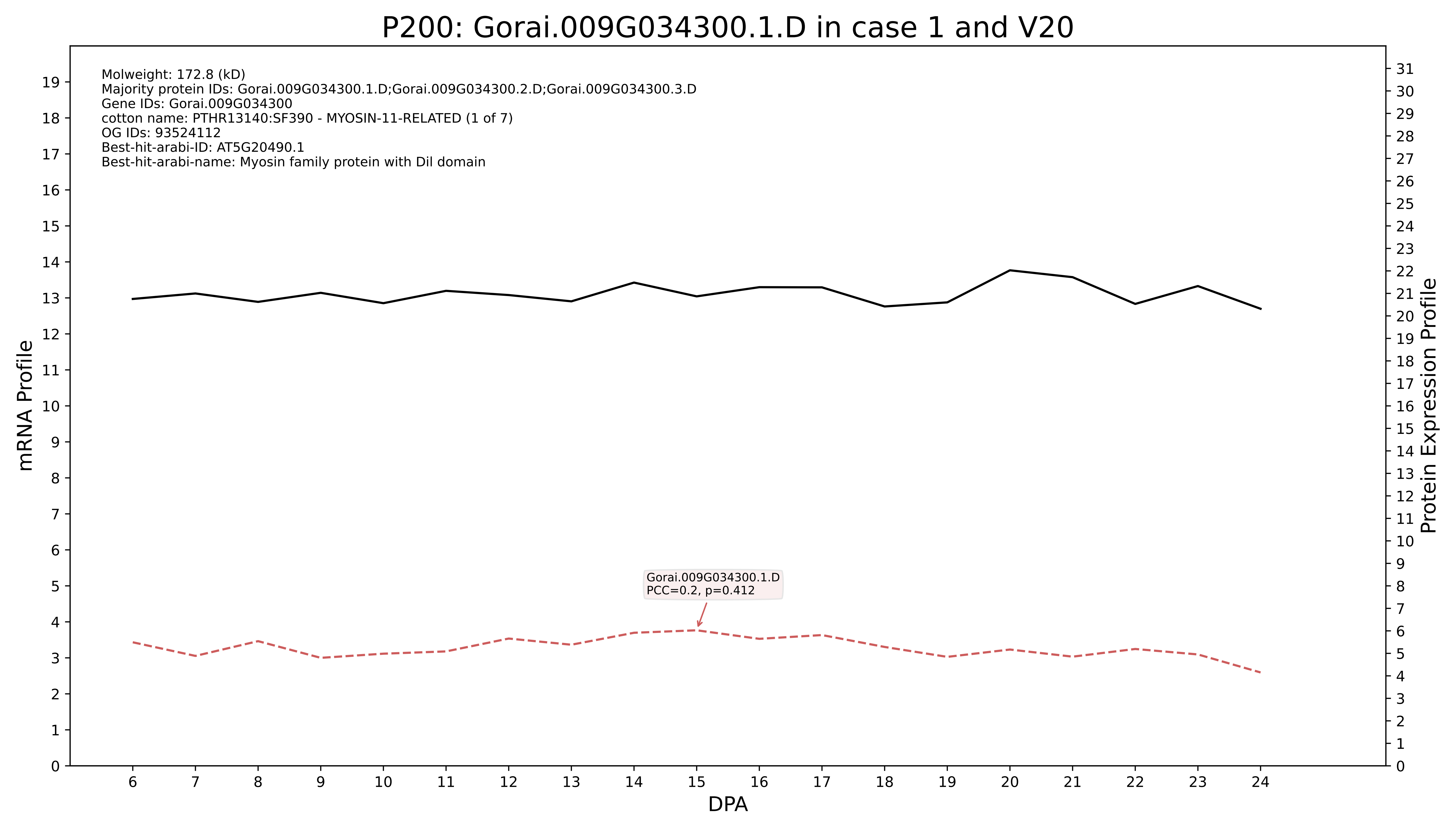
P200: Gorai.009G034300.1.D in case 1 and V20
Molweight: 172.8 (kD)
Majority protein IDs: Gorai.009G034300.1.D;Gorai.009G034300.2.D;Gorai.009G034300.3.D
Gene IDs: Gorai.009G034300
cotton name: PTHR13140:SF390 - MYOSIN-11-RELATED (1 of 7)
OG IDs: 93524112
Best-hit-arabi-ID: AT5G20490.1
Best-hit-arabi-name: Myosin family protein with Dil domain
Gorai.009G034300.1.D
PCC=0.2, p=0.412
mRNA Profile
Protein Expression Profile
DPA
0
1
2
3
4
5
6
7
8
9
10
11
12
13
14
15
16
17
18
19
20
21
22
23
24
25
26
27
28
29
30
31

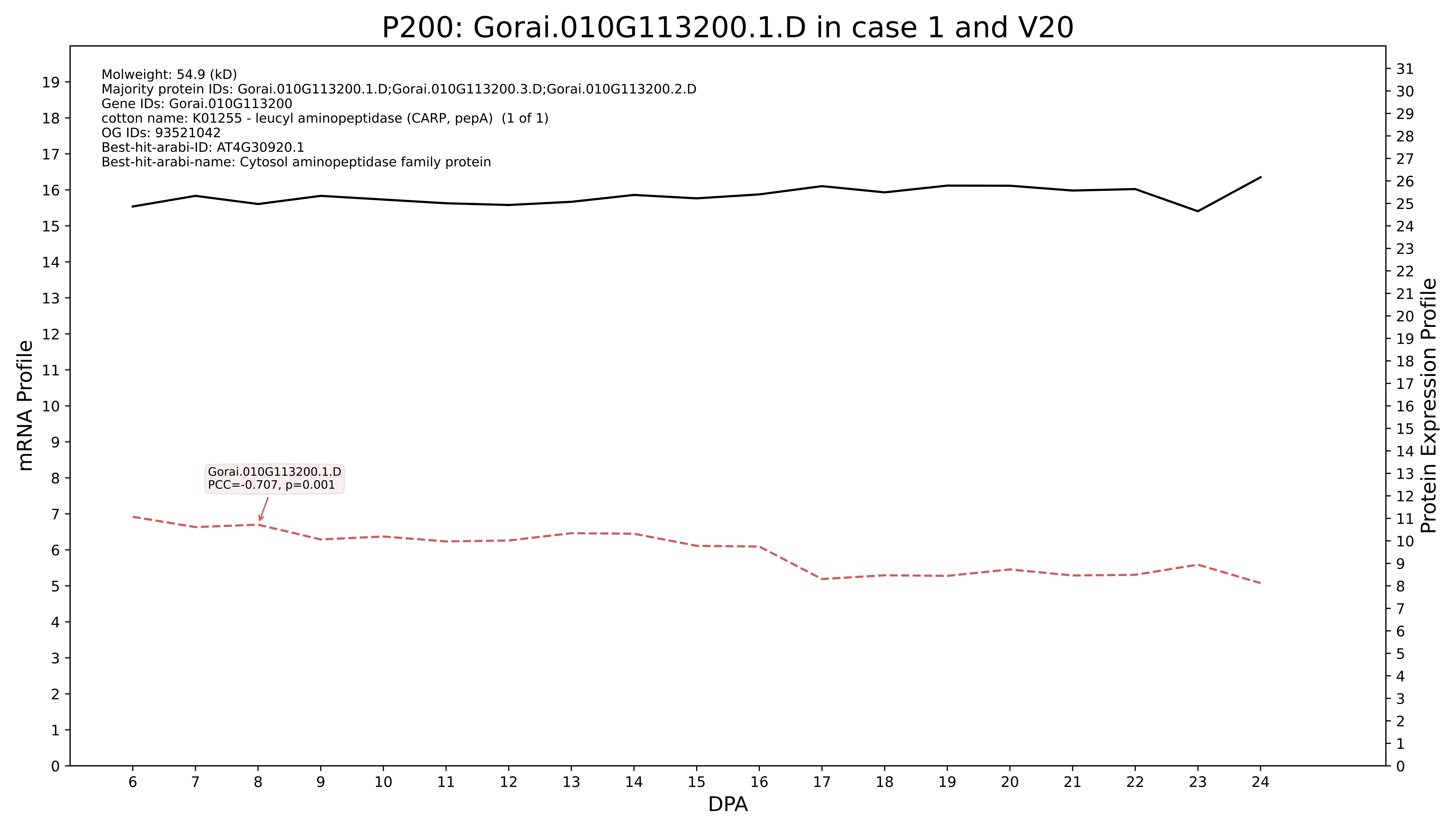
P200: Gorai.010G113200.1.D in case 1 and V20
Molweight: 54.9 (kD)
Majority protein IDs: Gorai.010G113200.1.D;Gorai.010G113200.3.D;Gorai.010G113200.2.D
Gene IDs: Gorai.010G113200
cotton name: K01255 - leucyl aminopeptidase (CARP, pepA) (1 of 1)
OG IDs: 93521042
Best-hit-arabi-ID: AT4G30920.1
Best-hit-arabi-name: Cytosol aminopeptidase family protein
Gorai.010G113200.1.D
PCC=-0.707, p=0.001
mRNA Profile
Protein Expression Profile
DPA
0
1
2
3
4
5
6
7
8
9
10
11
12
13
14
15
16
17
18
19
20
21
22
23
24
25
26
27
28
29
30
31

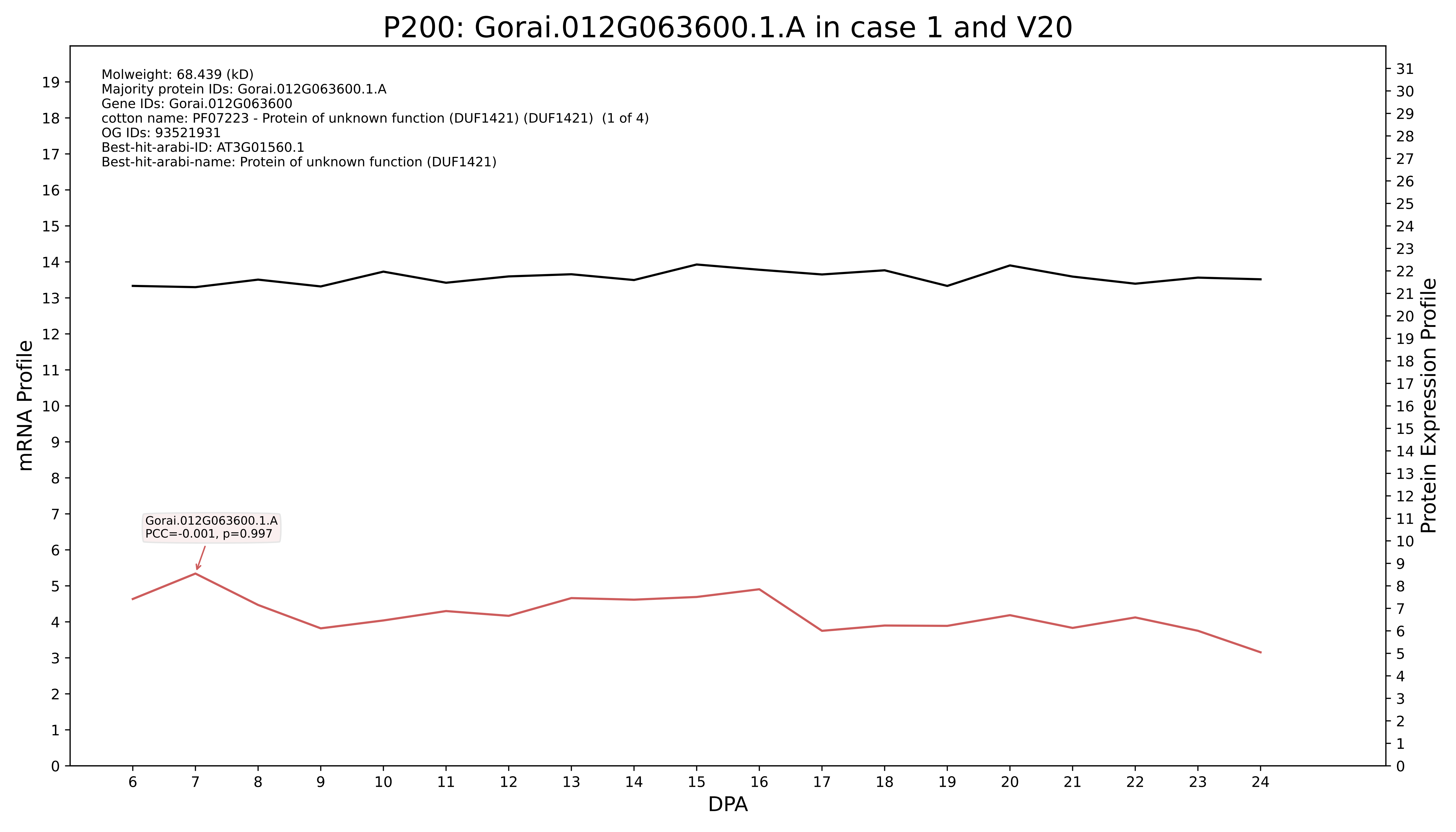

P200: Gorai.012G063600.1.A in case 1 and V20
Molweight: 68.439 (kD)
Majority protein IDs: Gorai.012G063600.1.A
Gene IDs: Gorai.012G063600
cotton name: PF07223 - Protein of unknown function (DUF1421) (DUF1421) (1 of 4)
OG IDs: 93521931
Best-hit-arabi-ID: AT3G01560.1
Best-hit-arabi-name: Protein of unknown function (DUF1421)
Gorai.012G063600.1.A
PCC=-0.001, p=0.997
mRNA Profile
Protein Expression Profile
DPA

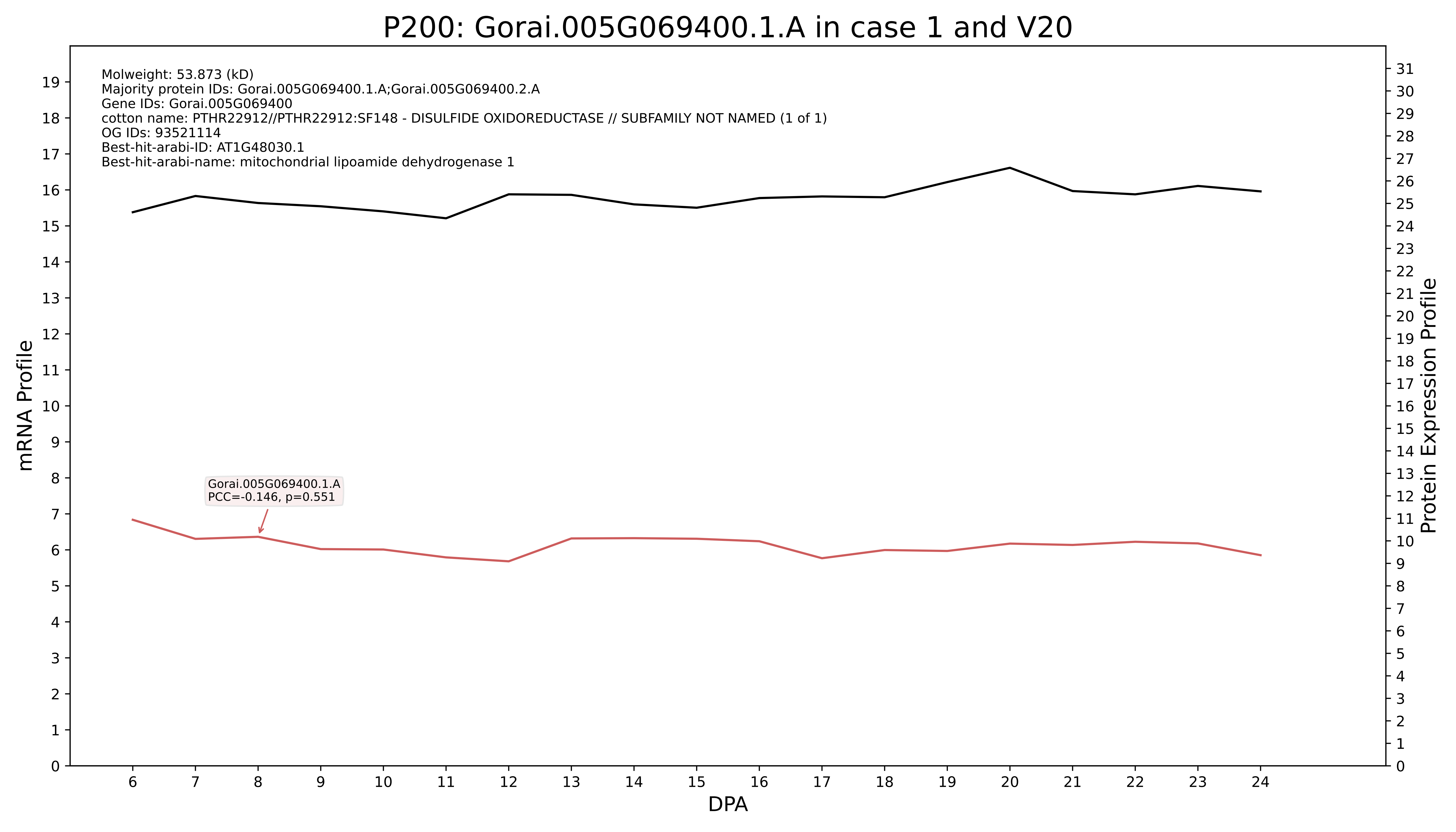

P200: Gorai.005G069400.1.A in case 1 and V20
Molweight: 53.873 (kD)
Majority protein IDs: Gorai.005G069400.1.A;Gorai.005G069400.2.A
Gene IDs: Gorai.005G069400
cotton name: PTHR22912//PTHR22912:SF148 - DISULFIDE OXIDOREDUCTASE // SUBFAMILY NOT NAMED (1 of 1)
OG IDs: 93521114
Best-hit-arabi-ID: AT1G48030.1
Best-hit-arabi-name: mitochondrial lipoamide dehydrogenase 1
Gorai.005G069400.1.A
PCC=-0.146, p=0.551
mRNA Profile
Protein Expression Profile
DPA

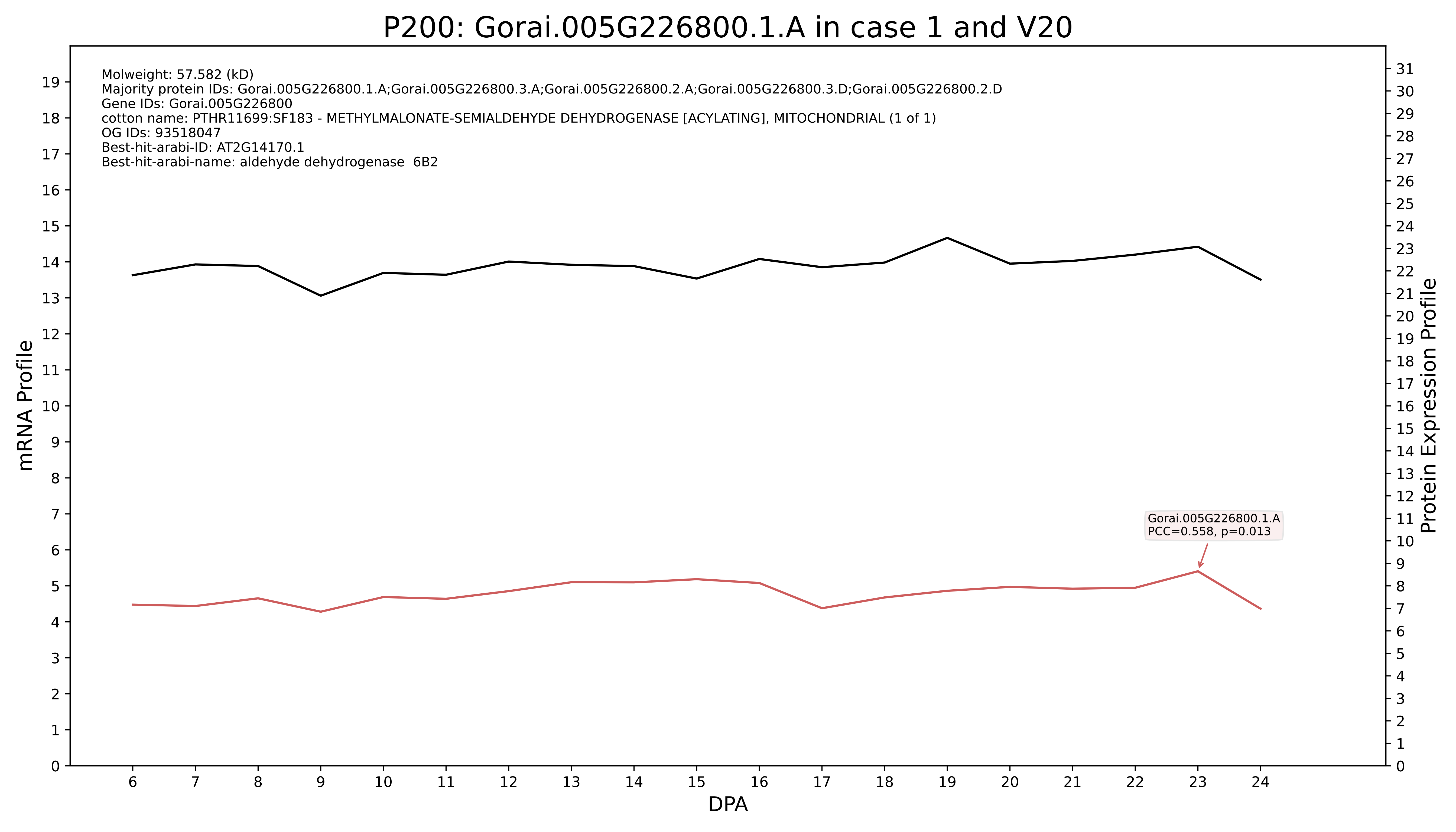

P200: Gorai.005G226800.1.A in case 1 and V20
Molweight: 57.582 (kD)
Majority protein IDs: Gorai.005G226800.1.A;Gorai.005G226800.3.A;Gorai.005G226800.2.A;Gorai.005G226800.3.D;Gorai.005G226800.2.D
Gene IDs: Gorai.005G226800
cotton name: PTHR11699:SF183 - METHYLMALONATE-SEMIALDEHYDE DEHYDROGENASE [ACYLATING], MITOCHONDRIAL (1 of 1)
OG IDs: 93518047
Best-hit-arabi-ID: AT2G14170.1
Best-hit-arabi-name: aldehyde dehydrogenase 6B2
Gorai.005G226800.1.A
PCC=0.558, p=0.013
mRNA Profile
Protein Expression Profile
DPA

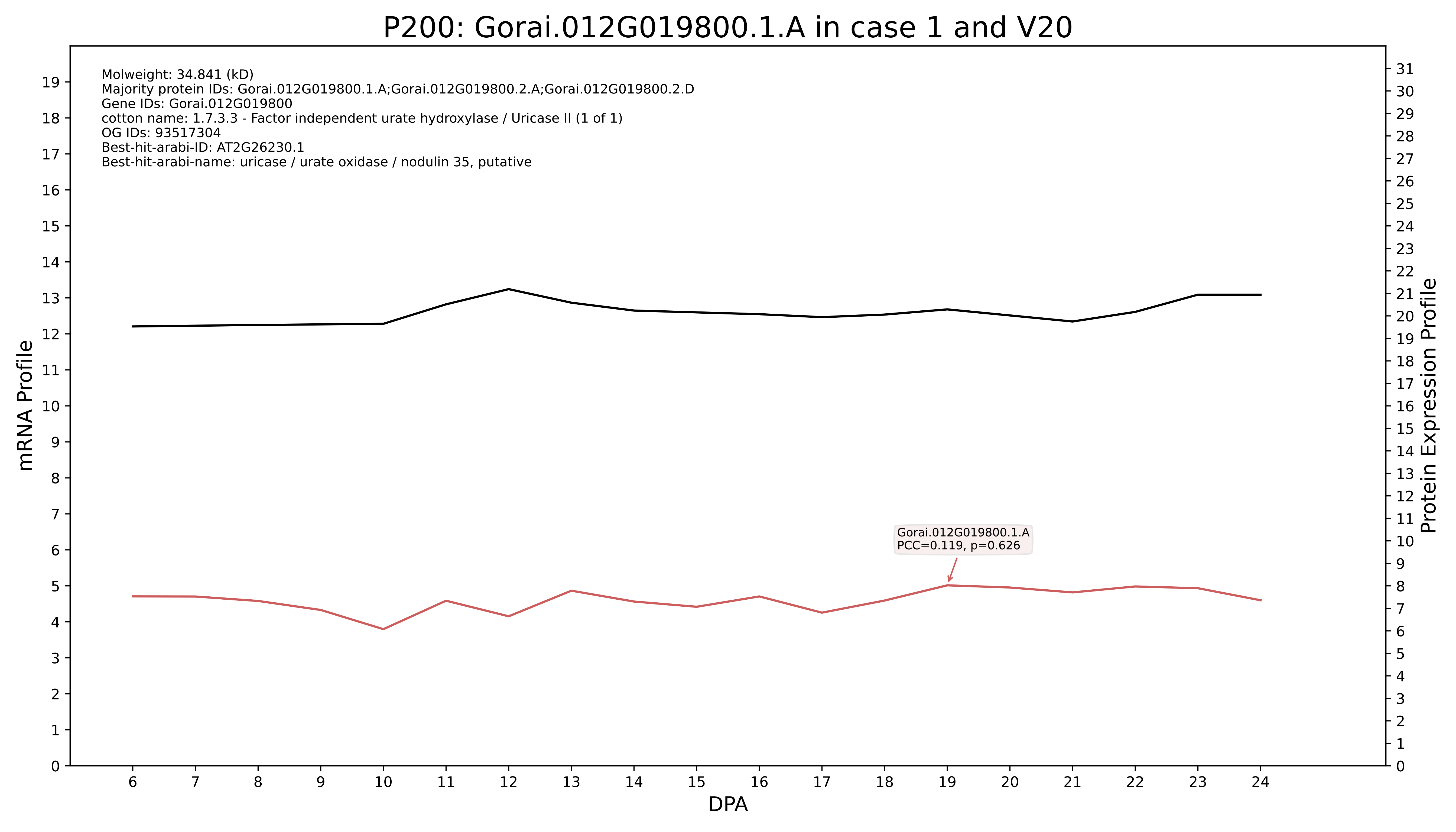
P200: Gorai.012G019800.1.A in case 1 and V20
Molweight: 34.841 (kD)
Majority protein IDs: Gorai.012G019800.1.A;Gorai.012G019800.2.A;Gorai.012G019800.2.D
Gene IDs: Gorai.012G019800
cotton name: 1.7.3.3 - Factor independent urate hydroxylase / Uricase II (1 of 1)
OG IDs: 93517304
Best-hit-arabi-ID: AT2G26230.1
Best-hit-arabi-name: uricase / urate oxidase / nodulin 35, putative
Gorai.012G019800.1.A
PCC=0.119, p=0.626
mRNA Profile
Protein Expression Profile
DPA
0
1
2
3
4
5
6
7
8
9
10
11
12
13
14
15
16
17
18
19
20
21
22
23
24
25
26
27
28
29
30
31

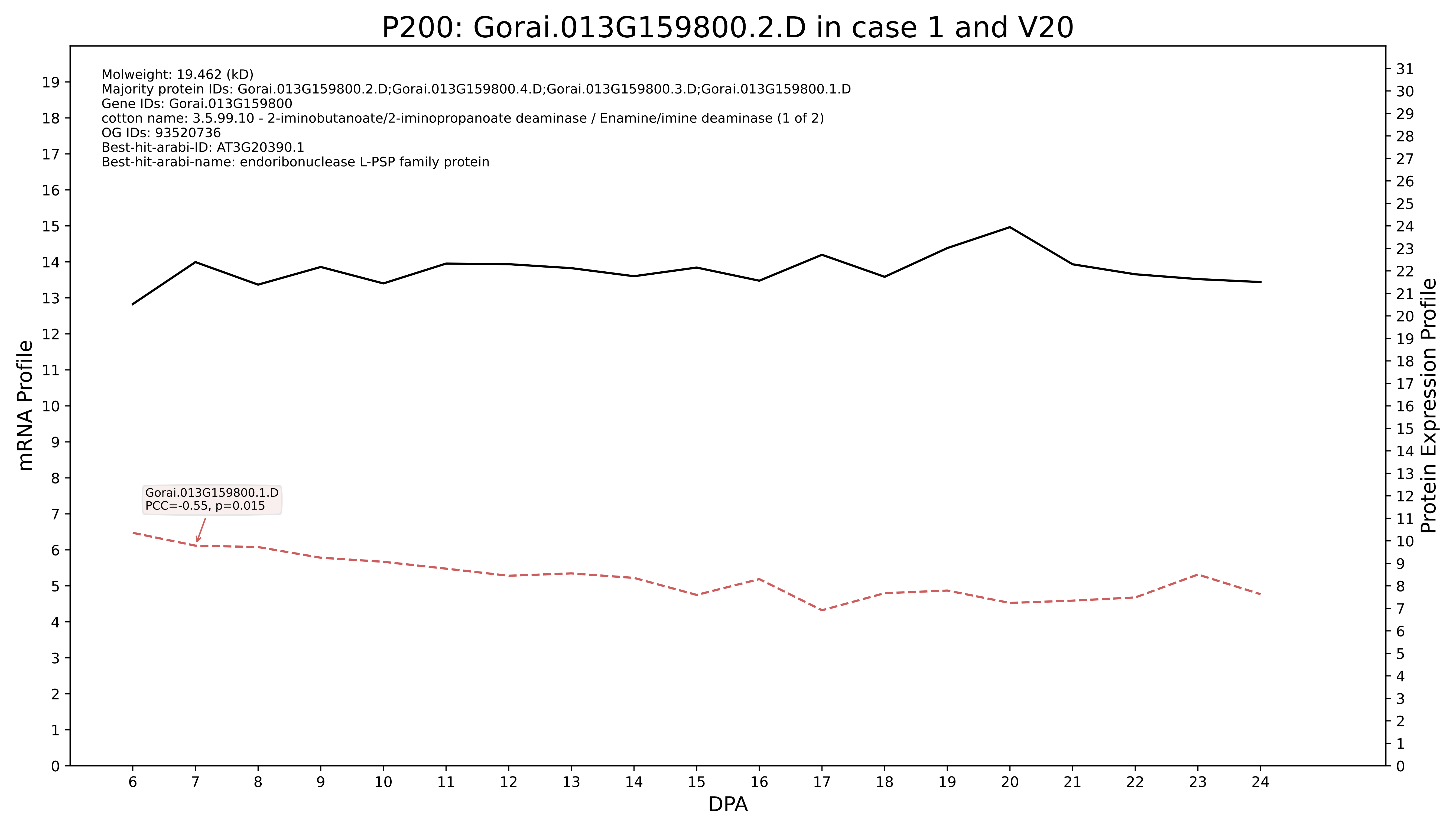

P200: Gorai.013G159800.2.D in case 1 and V20
Molweight: 19.462 (kD)
Majority protein IDs: Gorai.013G159800.2.D;Gorai.013G159800.4.D;Gorai.013G159800.3.D;Gorai.013G159800.1.D
Gene IDs: Gorai.013G159800
cotton name: 3.5.99.10 - 2-iminobutanoate/2-iminopropanoate deaminase / Enamine/imine deaminase (1 of 2)
OG IDs: 93520736
Best-hit-arabi-ID: AT3G20390.1
Best-hit-arabi-name: endoribonuclease L-PSP family protein
Gorai.013G159800.1.D
PCC=-0.55, p=0.015
mRNA Profile
Protein Expression Profile
DPA
0
1
2
3
4
5
6
7
8
9
10
11
12
13
14
15
16
17
18
19
20
21
22
23
24
25
26
27
28
29
30
31

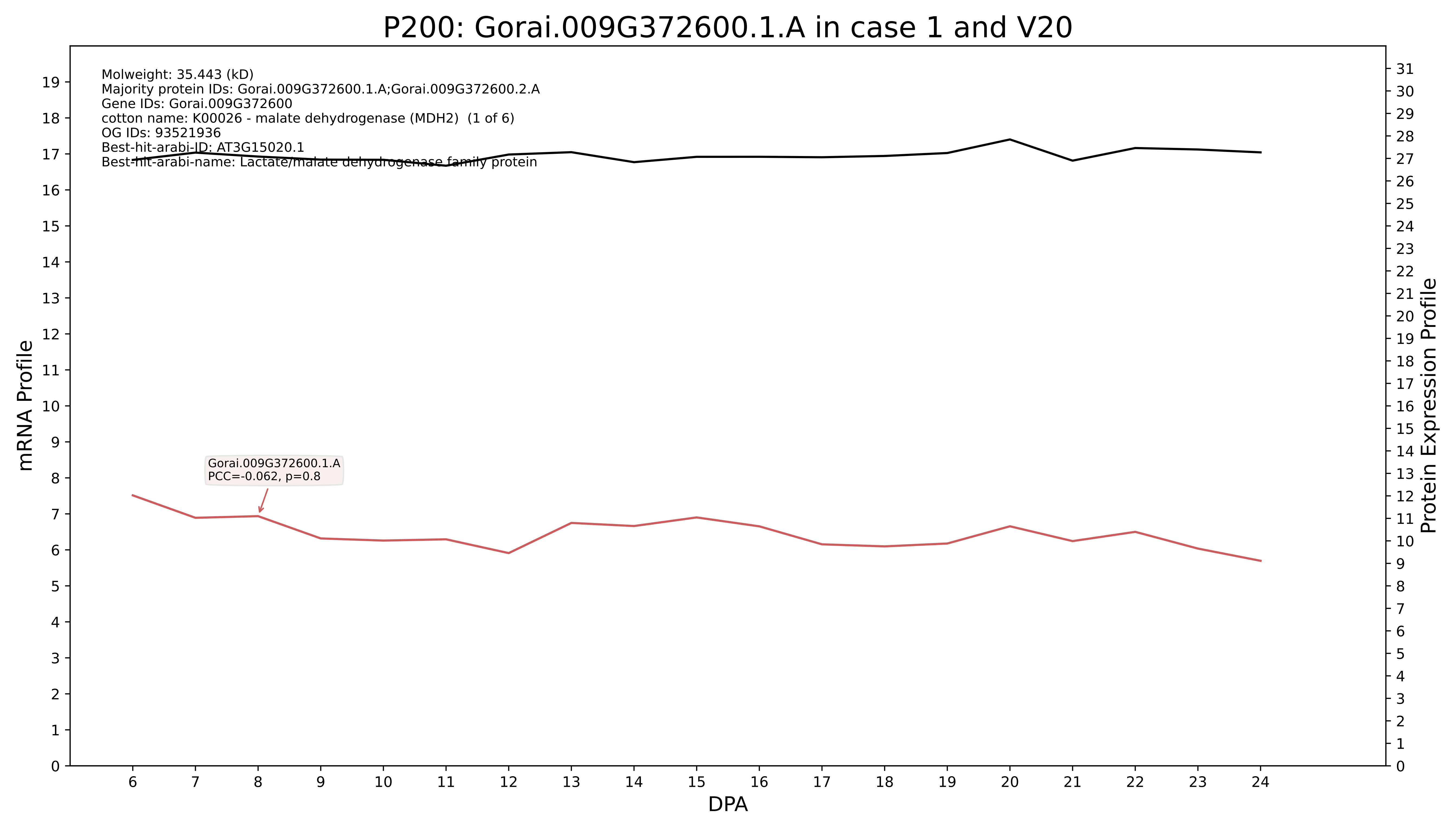
P200: Gorai.009G372600.1.A in case 1 and V20
Molweight: 35.443 (kD)
Majority protein IDs: Gorai.009G372600.1.A;Gorai.009G372600.2.A
Gene IDs: Gorai.009G372600
cotton name: K00026 - malate dehydrogenase (MDH2) (1 of 6)
OG IDs: 93521936
Best-hit-arabi-ID: AT3G15020.1
Best-hit-arabi-name: Lactate/malate dehydrogenase family protein
Gorai.009G372600.1.A
PCC=-0.062, p=0.8
mRNA Profile
Protein Expression Profile
DPA

P200: Gorai.013G022900.2.A in case 1 and V20

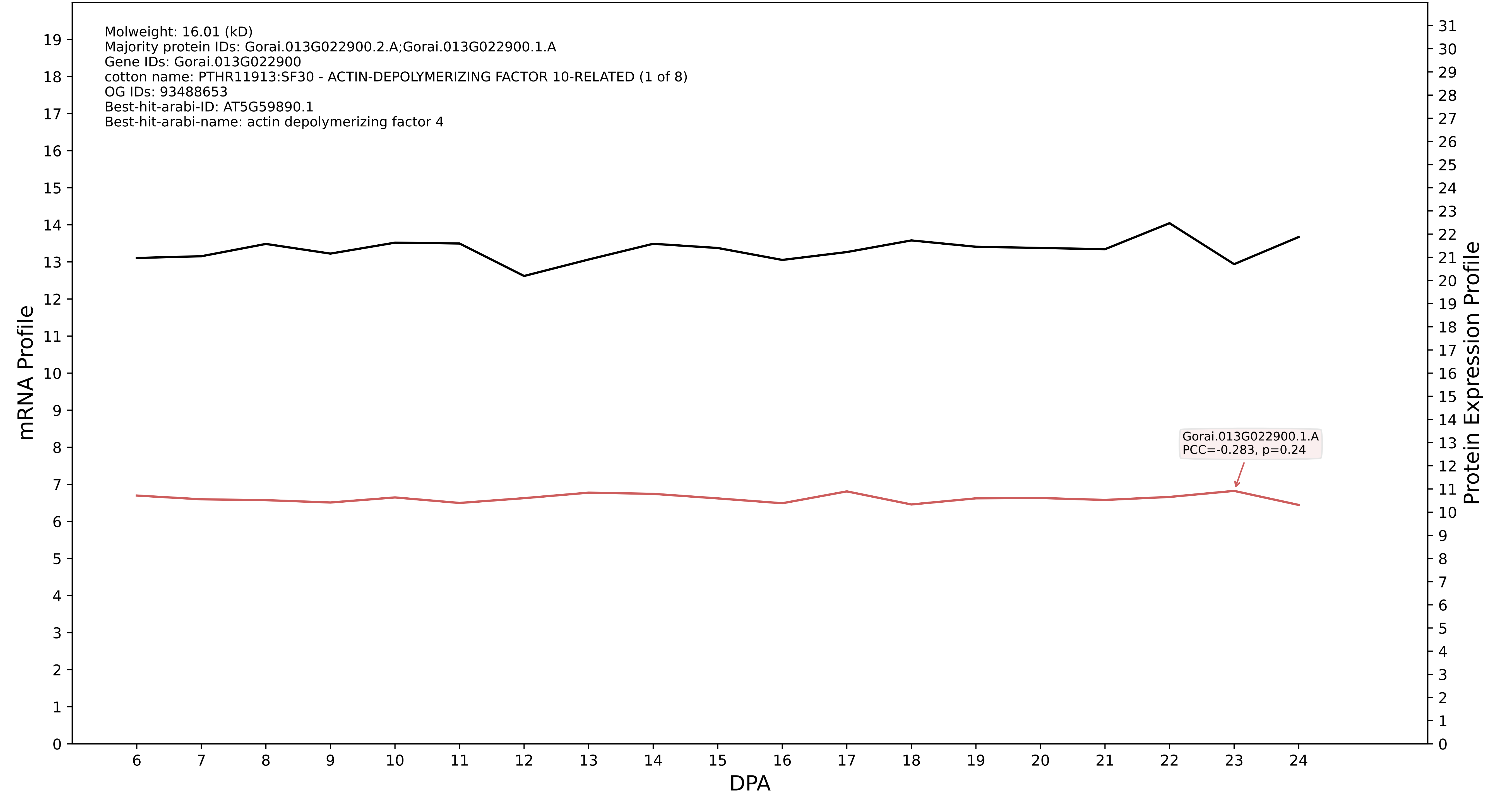

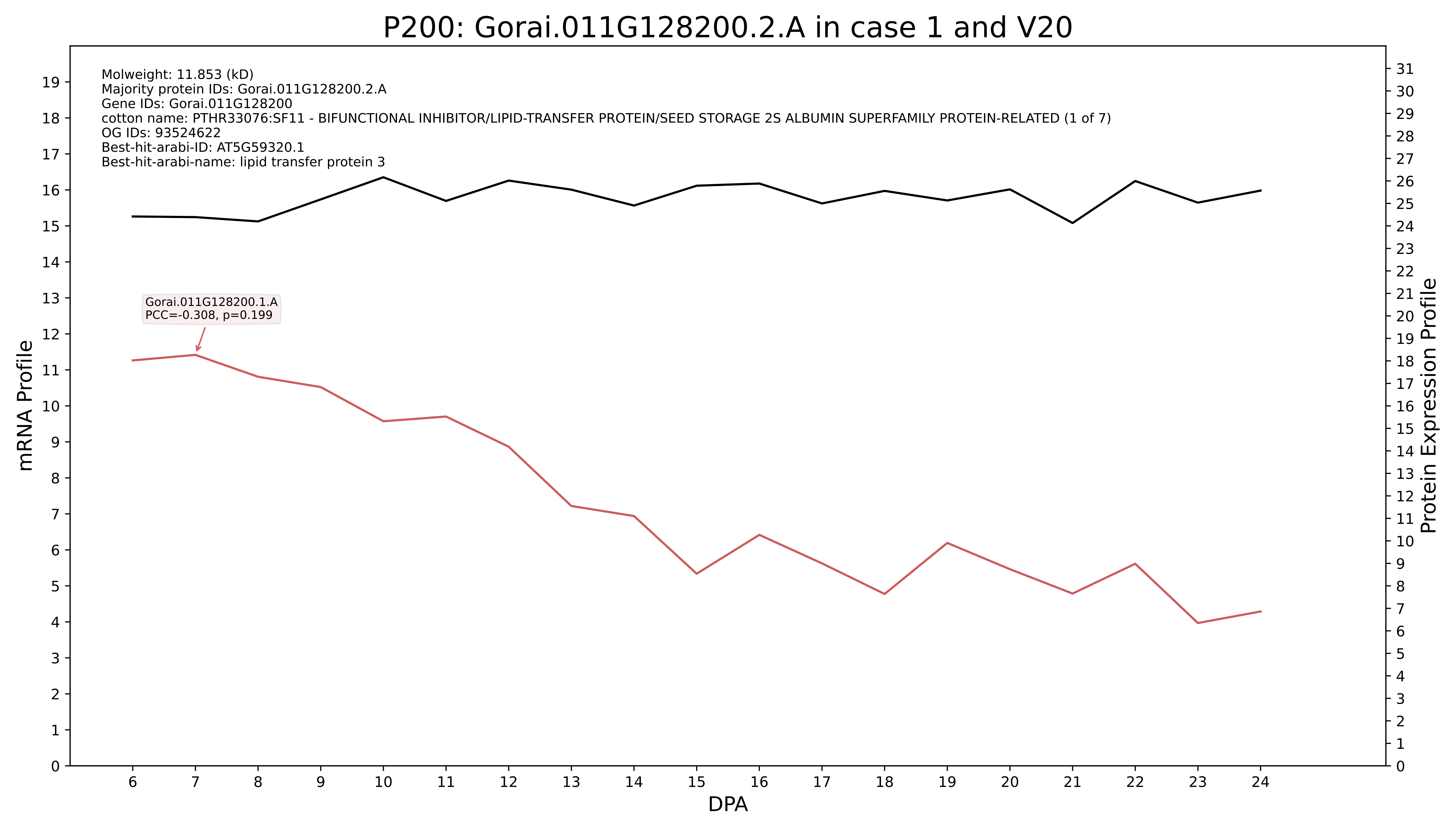
P200: Gorai.011G128200.2.A in case 1 and V20
Molweight: 11.853 (kD)
Majority protein IDs: Gorai.011G128200.2.A
Gene IDs: Gorai.011G128200
cotton name: PTHR33076:SF11 - BIFUNCTIONAL INHIBITOR/LIPID-TRANSFER PROTEIN/SEED STORAGE 2S ALBUMIN SUPERFAMILY PROTEIN-RELATED (1 of 7)
OG IDs: 93524622
Best-hit-arabi-ID: AT5G59320.1
Best-hit-arabi-name: lipid transfer protein 3
Gorai.011G128200.1.A
PCC=-0.308, p=0.199
mRNA Profile
Protein Expression Profile
DPA
0
1
2
3
4
5
6
7
8
9
10
11
12
13
14
15
16
17
18
19
20
21
22
23
24
25
26
27
28
29
30
31

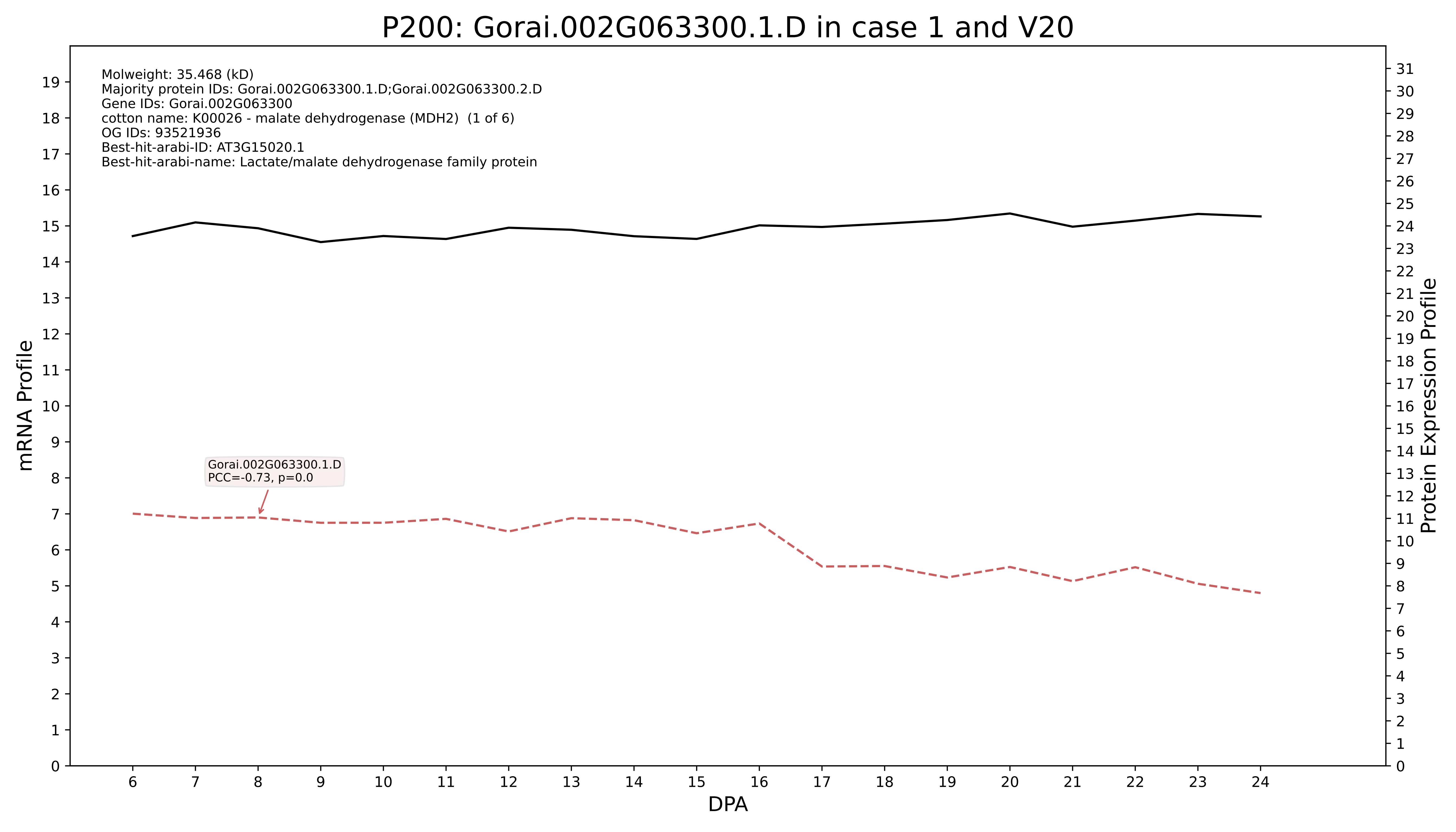

P200: Gorai.002G063300.1.D in case 1 and V20
Molweight: 35.468 (kD)
Majority protein IDs: Gorai.002G063300.1.D;Gorai.002G063300.2.D
Gene IDs: Gorai.002G063300
cotton name: K00026 - malate dehydrogenase (MDH2) (1 of 6)
OG IDs: 93521936
Best-hit-arabi-ID: AT3G15020.1
Best-hit-arabi-name: Lactate/malate dehydrogenase family protein
Gorai.002G063300.1.D
PCC=-0.73, p=0.0
mRNA Profile
Protein Expression Profile
DPA

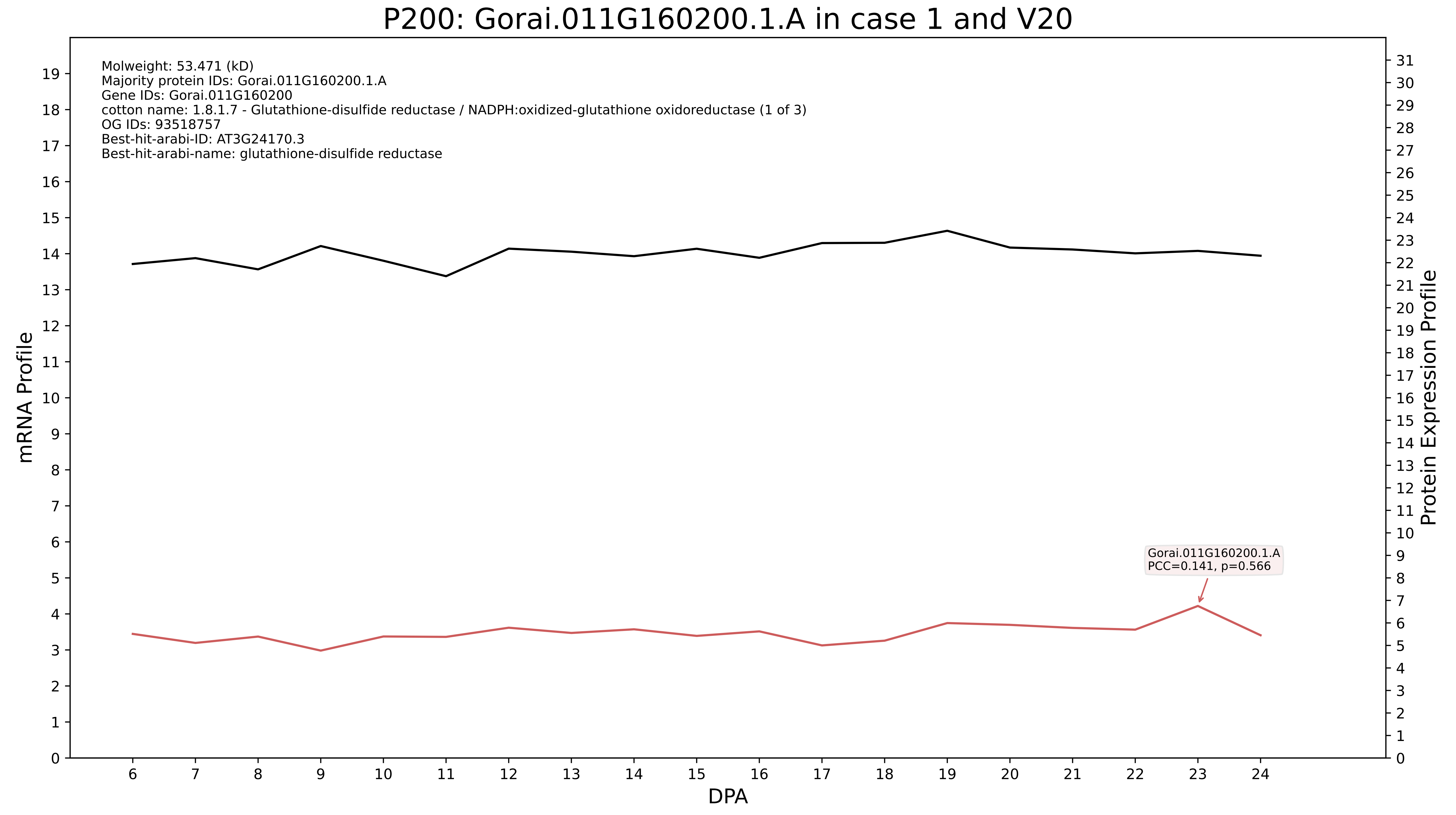
P200: Gorai.011G160200.1.A in case 1 and V20
Molweight: 53.471 (kD)
Majority protein IDs: Gorai.011G160200.1.A
Gene IDs: Gorai.011G160200
cotton name: 1.8.1.7 - Glutathione-disulfide reductase / NADPH:oxidized-glutathione oxidoreductase (1 of 3)
OG IDs: 93518757
Best-hit-arabi-ID: AT3G24170.3
Best-hit-arabi-name: glutathione-disulfide reductase
Gorai.011G160200.1.A
PCC=0.141, p=0.566
mRNA Profile
Protein Expression Profile
DPA

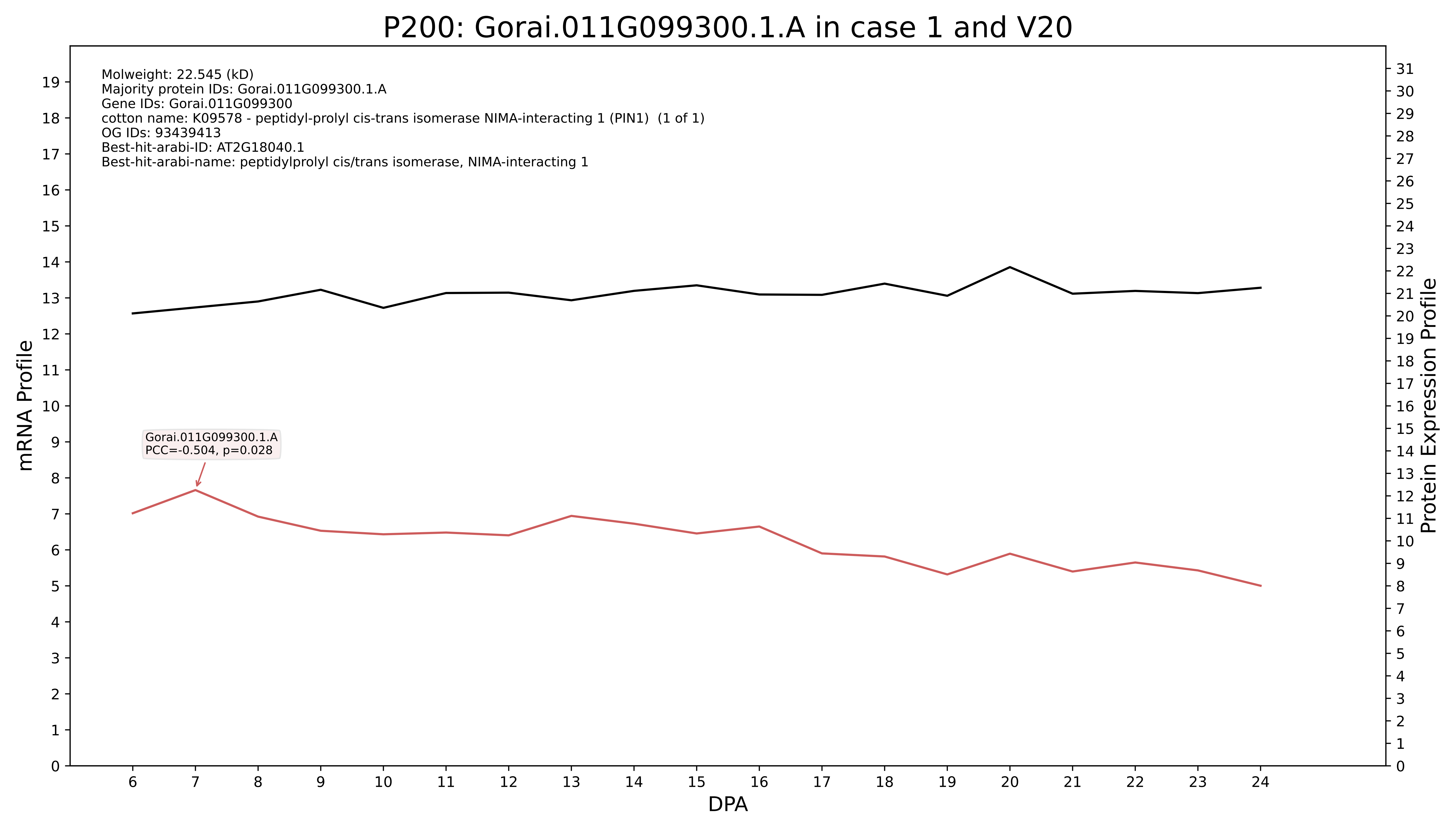
P200: Gorai.011G099300.1.A in case 1 and V20
Molweight: 22.545 (kD)
Majority protein IDs: Gorai.011G099300.1.A
Gene IDs: Gorai.011G099300
cotton name: K09578 - peptidyl-prolyl cis-trans isomerase NIMA-interacting 1 (PIN1) (1 of 1)
OG IDs: 93439413
Best-hit-arabi-ID: AT2G18040.1
Best-hit-arabi-name: peptidylprolyl cis/trans isomerase, NIMA-interacting 1
Gorai.011G099300.1.A
PCC=-0.504, p=0.028
mRNA Profile
Protein Expression Profile
DPA
0
1
2
3
4
5
6
7
8
9
10
11
12
13
14
15
16
17
18
19
20
21
22
23
24
25
26
27
28
29
30
31

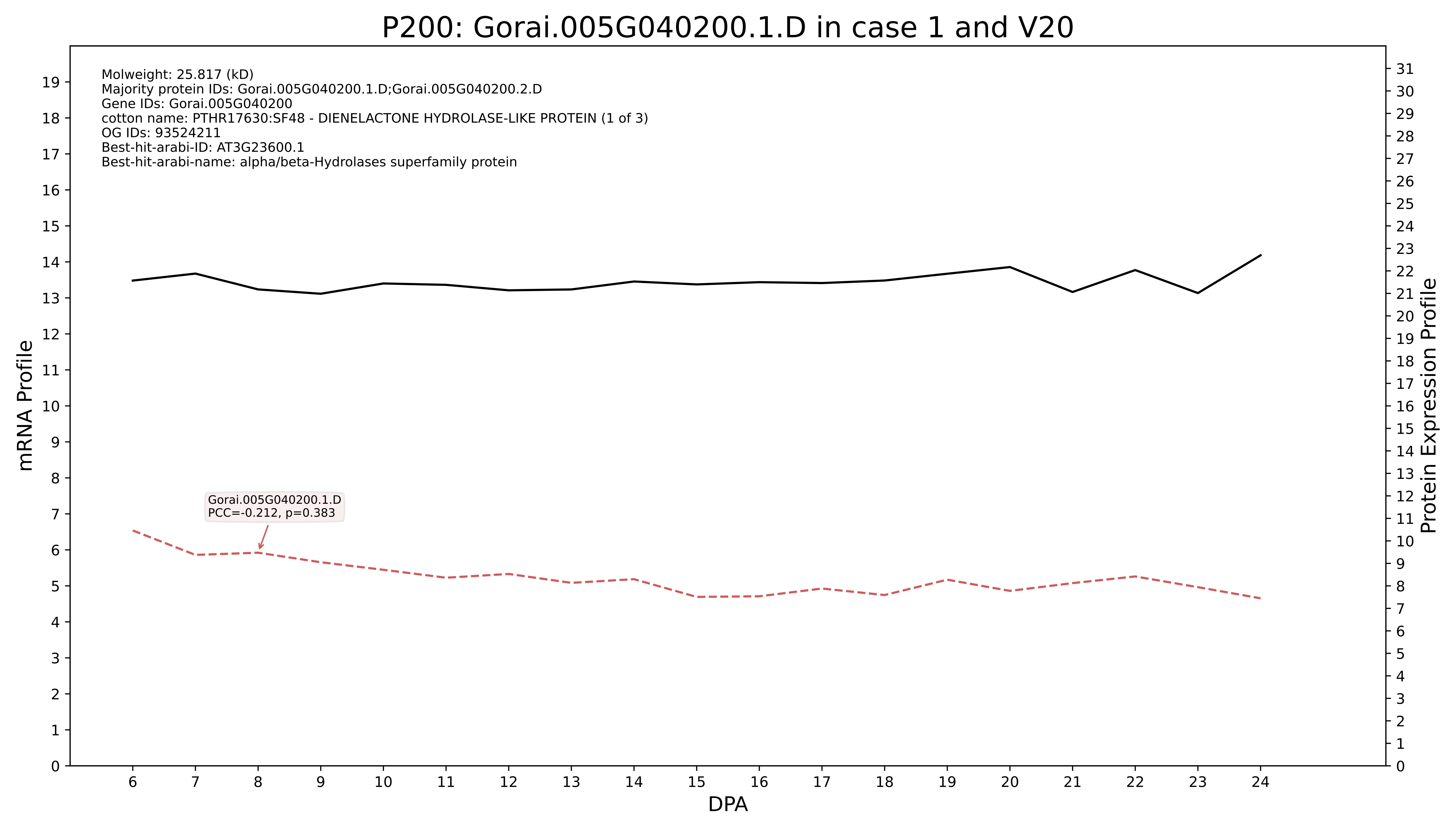

P200: Gorai.005G040200.1.D in case 1 and V20
Molweight: 25.817 (kD)
Majority protein IDs: Gorai.005G040200.1.D;Gorai.005G040200.2.D
Gene IDs: Gorai.005G040200
cotton name: PTHR17630:SF48 - DIENELACTONE HYDROLASE-LIKE PROTEIN (1 of 3)
OG IDs: 93524211
Best-hit-arabi-ID: AT3G23600.1
Best-hit-arabi-name: alpha/beta-Hydrolases superfamily protein
Gorai.005G040200.1.D
PCC=-0.212, p=0.383
mRNA Profile
Protein Expression Profile
DPA

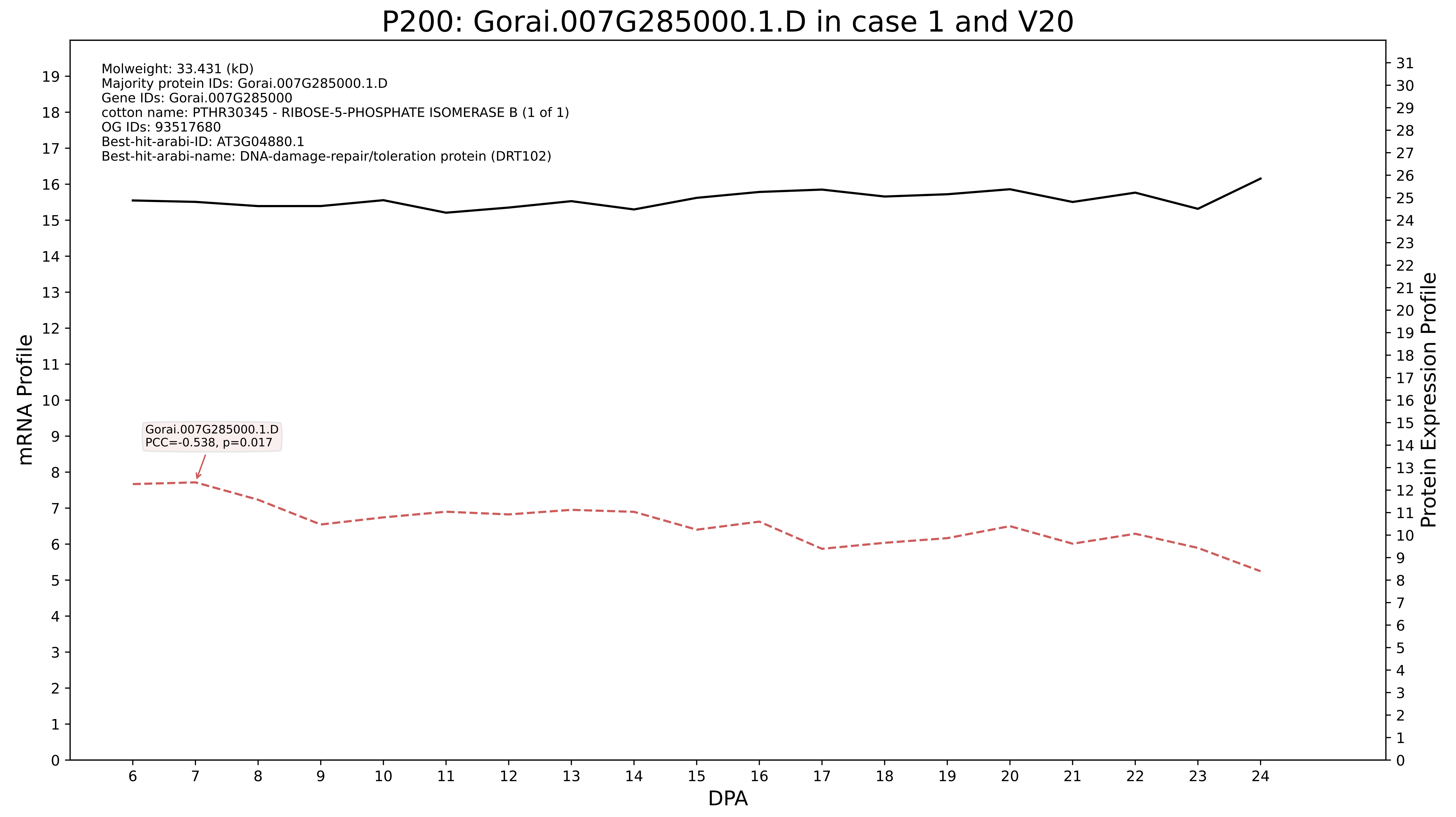
P200: Gorai.007G285000.1.D in case 1 and V20
Molweight: 33.431 (kD)
Majority protein IDs: Gorai.007G285000.1.D
Gene IDs: Gorai.007G285000
cotton name: PTHR30345 - RIBOSE-5-PHOSPHATE ISOMERASE B (1 of 1)
OG IDs: 93517680
Best-hit-arabi-ID: AT3G04880.1
Best-hit-arabi-name: DNA-damage-repair/toleration protein (DRT102)
Gorai.007G285000.1.D
PCC=-0.538, p=0.017
mRNA Profile
Protein Expression Profile
DPA
0
1
2
3
4
5
6
7
8
9
10
11
12
13
14
15
16
17
18
19
20
21
22
23
24
25
26
27
28
29
30
31

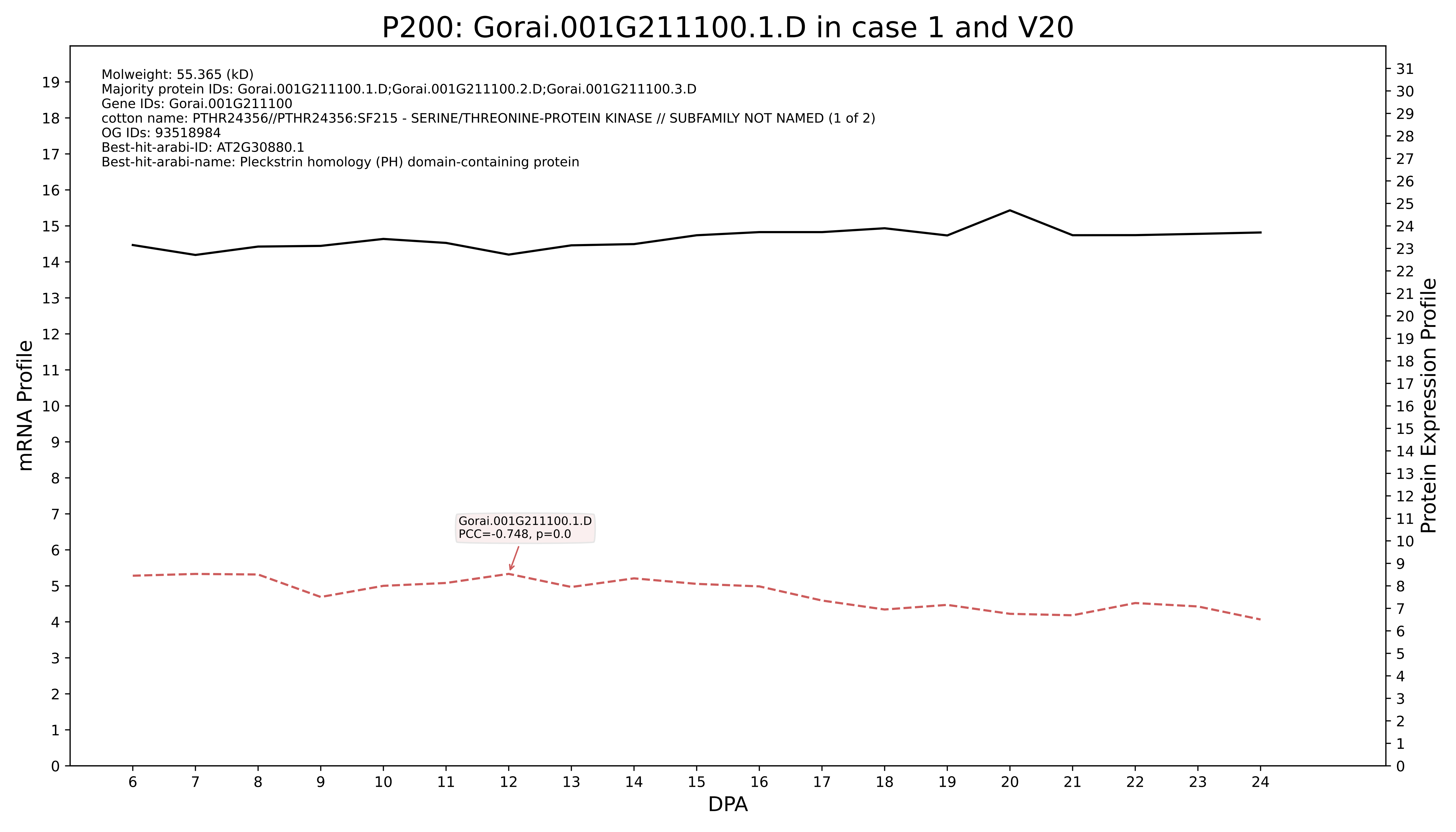

P200: Gorai.001G211100.1.D in case 1 and V20
Molweight: 55.365 (kD)
Majority protein IDs: Gorai.001G211100.1.D;Gorai.001G211100.2.D;Gorai.001G211100.3.D
Gene IDs: Gorai.001G211100
cotton name: PTHR24356//PTHR24356:SF215 - SERINE/THREONINE-PROTEIN KINASE // SUBFAMILY NOT NAMED (1 of 2)
OG IDs: 93518984
Best-hit-arabi-ID: AT2G30880.1
Best-hit-arabi-name: Pleckstrin homology (PH) domain-containing protein
Gorai.001G211100.1.D
PCC=-0.748, p=0.0
mRNA Profile
Protein Expression Profile
DPA

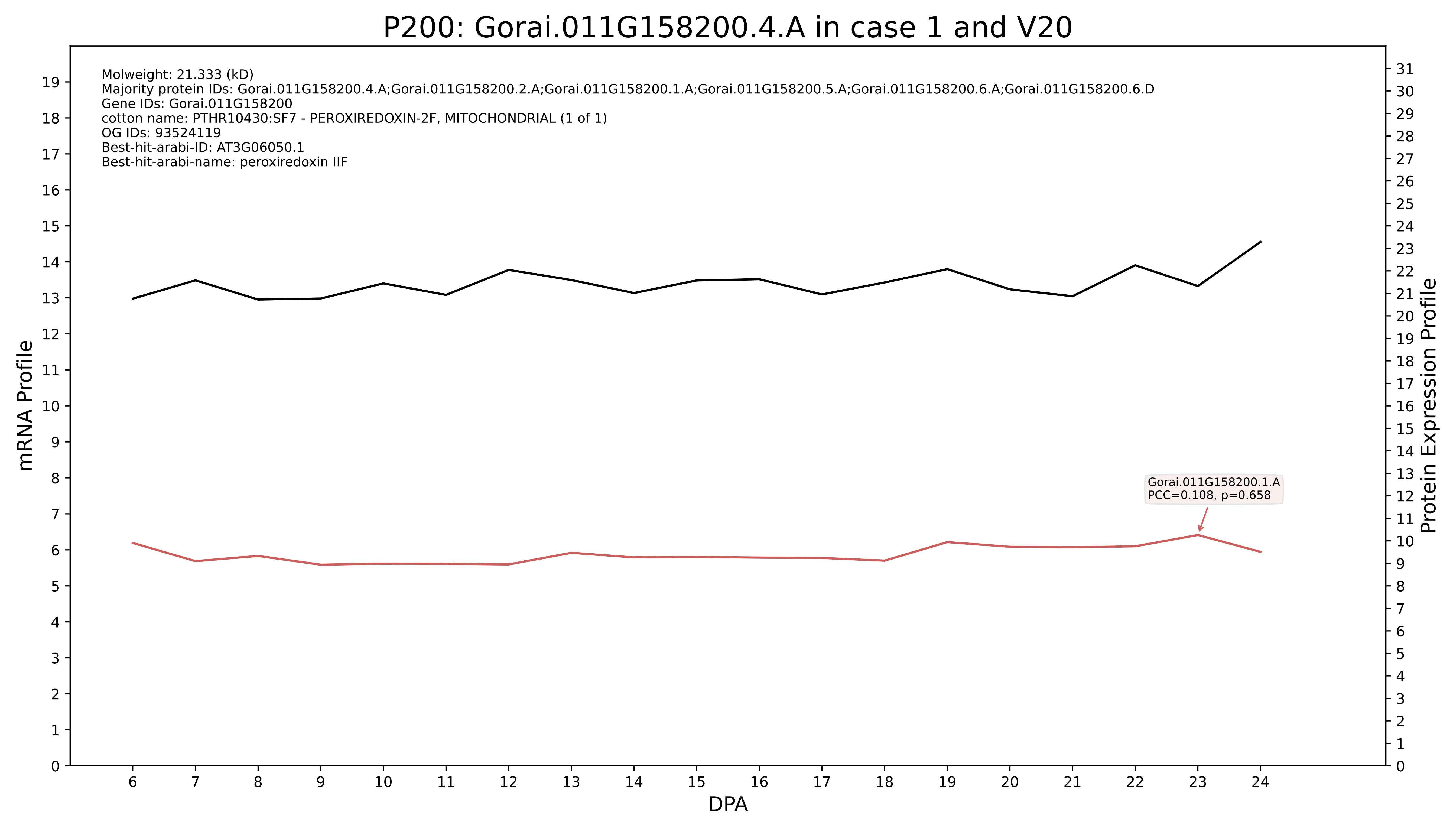
P200: Gorai.011G158200.4.A in case 1 and V20
Molweight: 21.333 (kD)
Majority protein IDs: Gorai.011G158200.4.A;Gorai.011G158200.2.A;Gorai.011G158200.1.A;Gorai.011G158200.5.A;Gorai.011G158200.6.A;Gorai.011G158200.6.D
Gene IDs: Gorai.011G158200
cotton name: PTHR10430:SF7 - PEROXIREDOXIN-2F, MITOCHONDRIAL (1 of 1)
OG IDs: 93524119
Best-hit-arabi-ID: AT3G06050.1
Best-hit-arabi-name: peroxiredoxin IIF
Gorai.011G158200.1.A
PCC=0.108, p=0.658
mRNA Profile
Protein Expression Profile
DPA

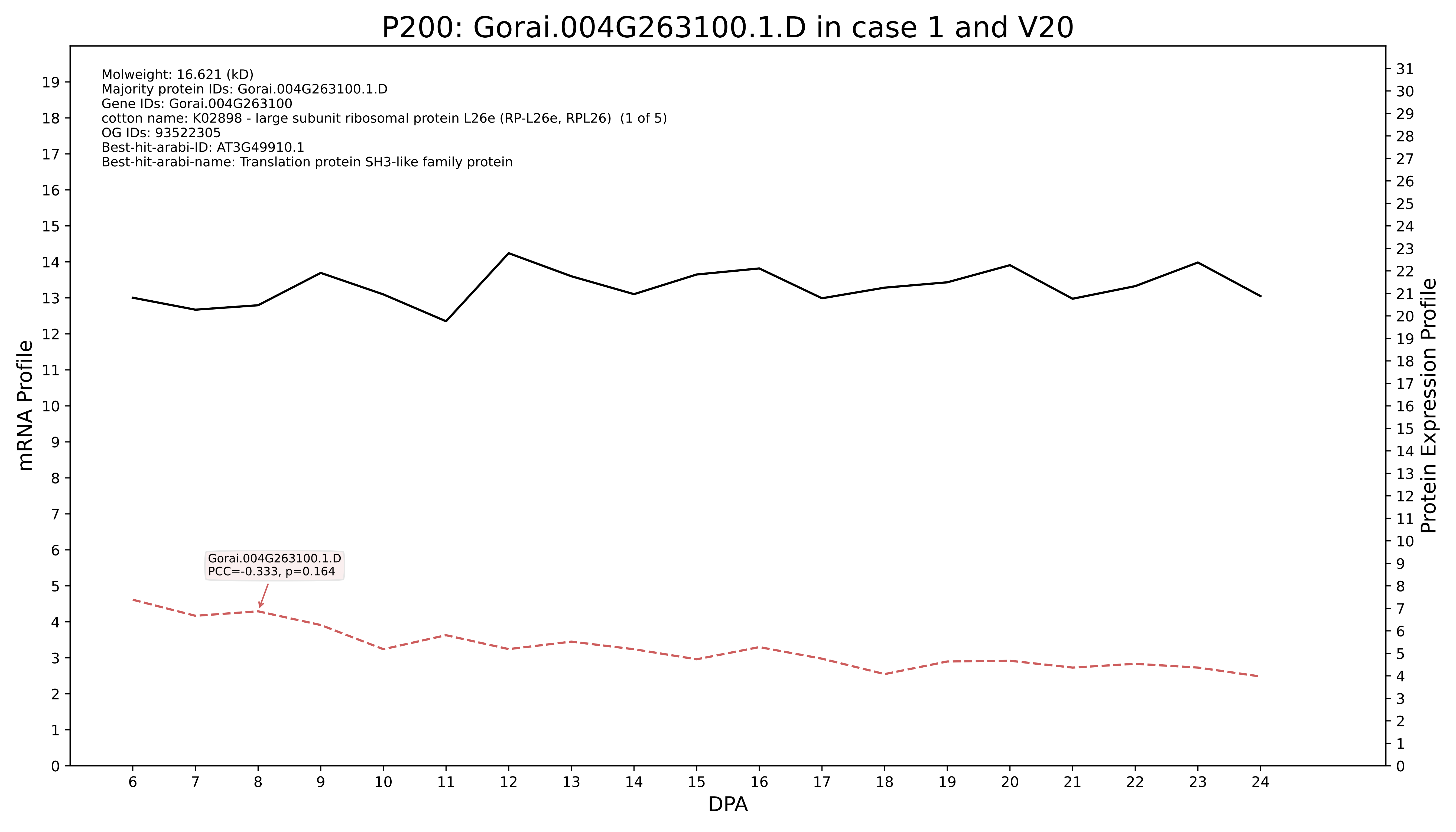
P200: Gorai.004G263100.1.D in case 1 and V20
Molweight: 16.621 (kD)
Majority protein IDs: Gorai.004G263100.1.D
Gene IDs: Gorai.004G263100
cotton name: K02898 - large subunit ribosomal protein L26e (RP-L26e, RPL26) (1 of 5)
OG IDs: 93522305
Best-hit-arabi-ID: AT3G49910.1
Best-hit-arabi-name: Translation protein SH3-like family protein
Gorai.004G263100.1.D
PCC=-0.333, p=0.164
mRNA Profile
Protein Expression Profile
DPA

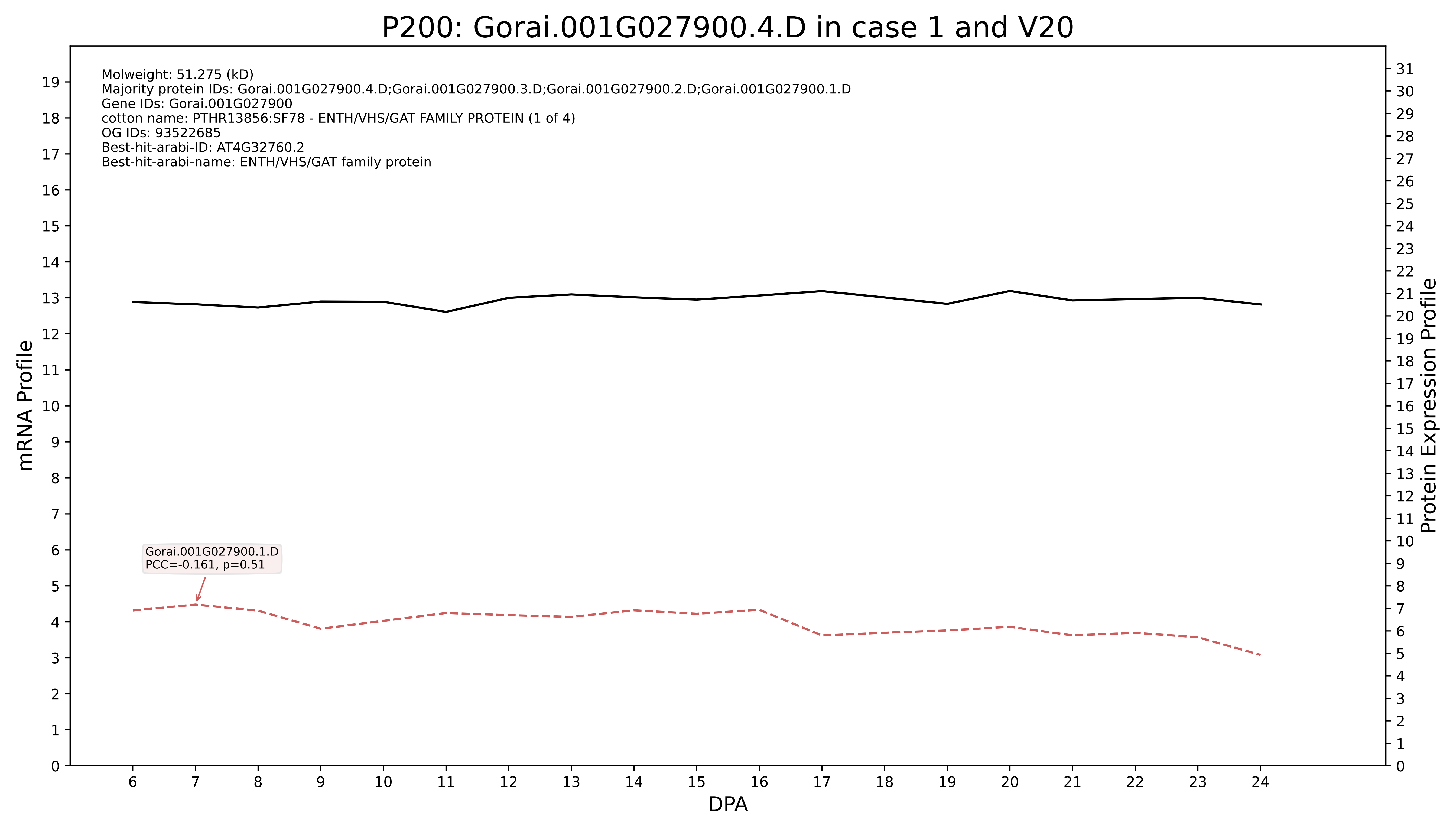
P200: Gorai.001G027900.4.D in case 1 and V20
Molweight: 51.275 (kD)
Majority protein IDs: Gorai.001G027900.4.D;Gorai.001G027900.3.D;Gorai.001G027900.2.D;Gorai.001G027900.1.D
Gene IDs: Gorai.001G027900
cotton name: PTHR13856:SF78 - ENTH/VHS/GAT FAMILY PROTEIN (1 of 4)
OG IDs: 93522685
Best-hit-arabi-ID: AT4G32760.2
Best-hit-arabi-name: ENTH/VHS/GAT family protein
Gorai.001G027900.1.D
PCC=-0.161, p=0.51
mRNA Profile
Protein Expression Profile
DPA
0
1
2
3
4
5
6
7
8
9
10
11
12
13
14
15
16
17
18
19
20
21
22
23
24
25
26
27
28
29
30
31

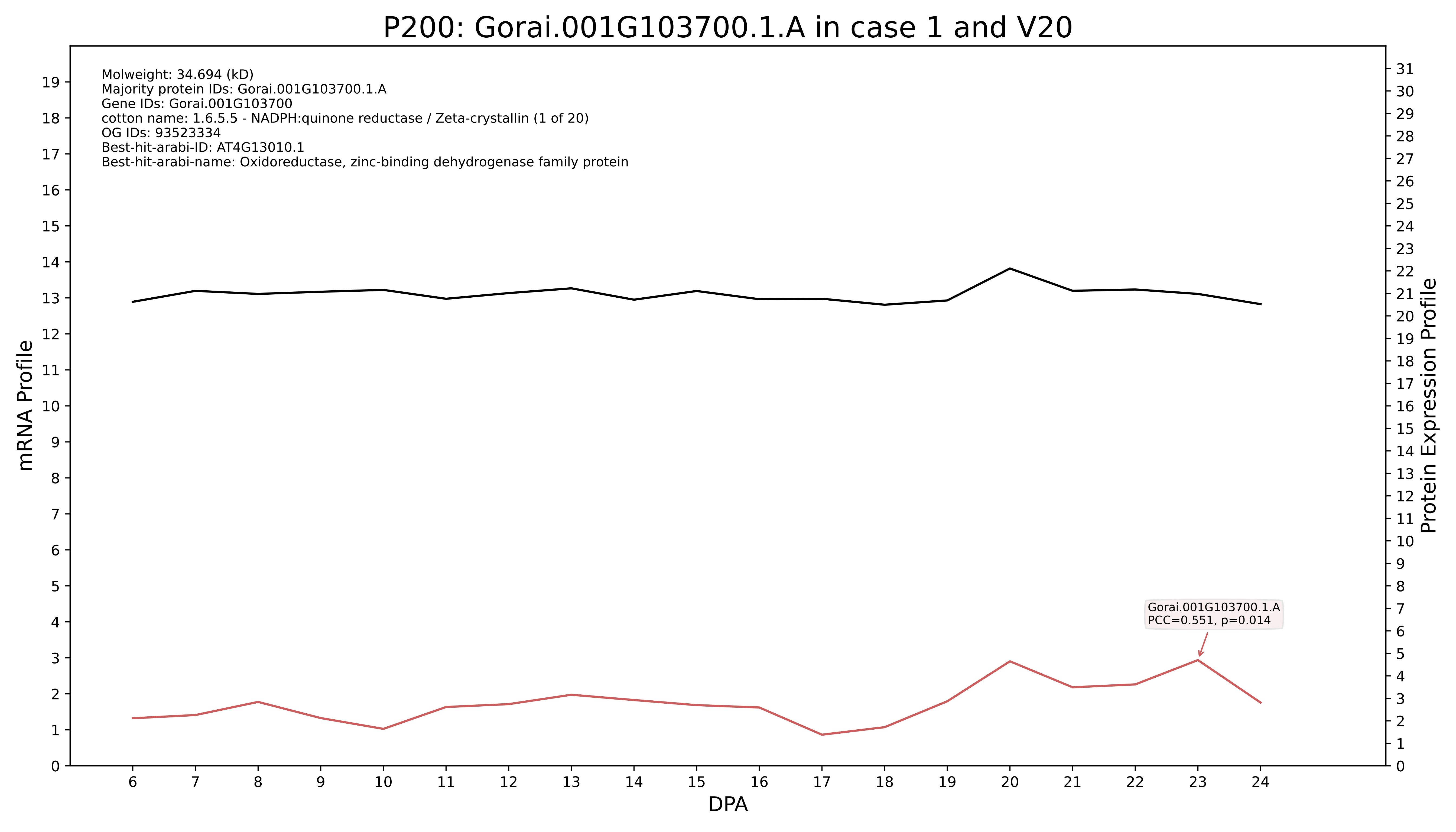
P200: Gorai.001G103700.1.A in case 1 and V20
Molweight: 34.694 (kD)
Majority protein IDs: Gorai.001G103700.1.A
Gene IDs: Gorai.001G103700
cotton name: 1.6.5.5 - NADPH:quinone reductase / Zeta-crystallin (1 of 20)
OG IDs: 93523334
Best-hit-arabi-ID: AT4G13010.1
Best-hit-arabi-name: Oxidoreductase, zinc-binding dehydrogenase family protein
Gorai.001G103700.1.A
PCC=0.551, p=0.014
mRNA Profile
Protein Expression Profile
DPA

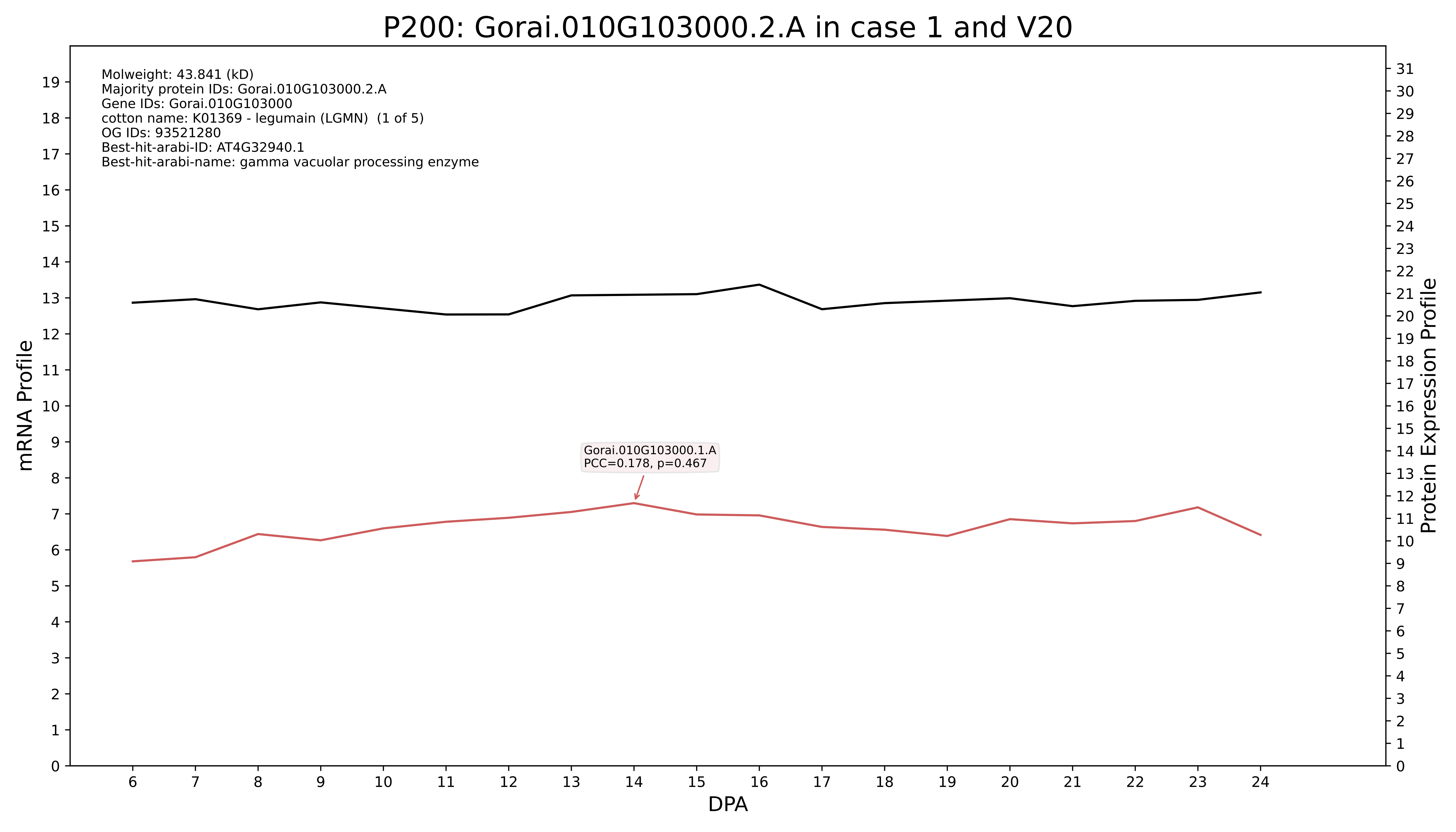
P200: Gorai.010G103000.2.A in case 1 and V20
Molweight: 43.841 (kD)
Majority protein IDs: Gorai.010G103000.2.A
Gene IDs: Gorai.010G103000
cotton name: K01369 - legumain (LGMN) (1 of 5)
OG IDs: 93521280
Best-hit-arabi-ID: AT4G32940.1
Best-hit-arabi-name: gamma vacuolar processing enzyme
Gorai.010G103000.1.A
PCC=0.178, p=0.467
mRNA Profile
Protein Expression Profile
DPA

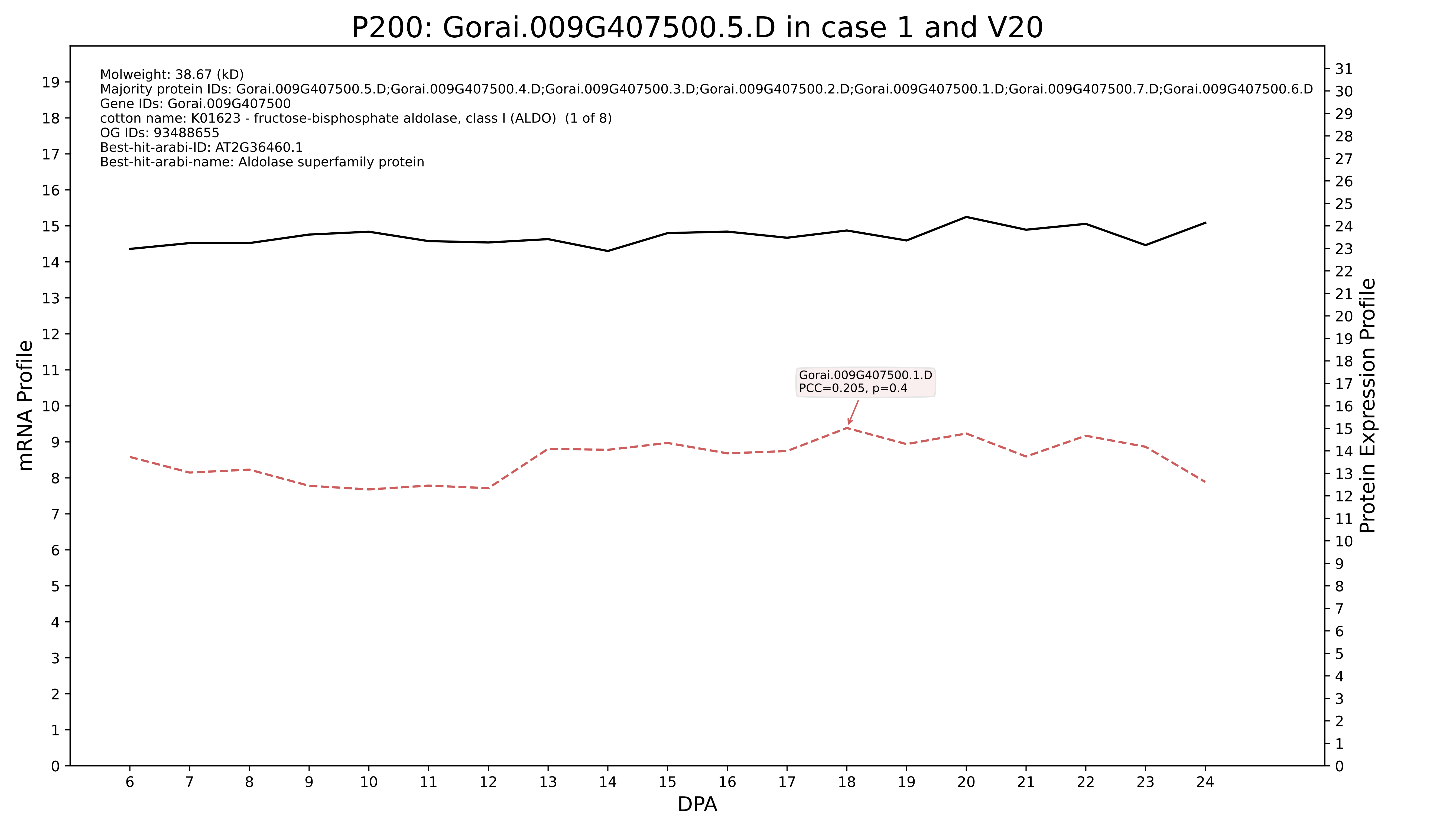
P200: Gorai.009G407500.5.D in case 1 and V20
Molweight: 38.67 (kD)
Majority protein IDs: Gorai.009G407500.5.D;Gorai.009G407500.4.D;Gorai.009G407500.3.D;Gorai.009G407500.2.D;Gorai.009G407500.1.D;Gorai.009G407500.7.D;Gorai.009G407500.6.D
Gene IDs: Gorai.009G407500
cotton name: K01623 - fructose-bisphosphate aldolase, class I (ALDO) (1 of 8)
OG IDs: 93488655
Best-hit-arabi-ID: AT2G36460.1
Best-hit-arabi-name: Aldolase superfamily protein
Gorai.009G407500.1.D
PCC=0.205, p=0.4
mRNA Profile
Protein Expression Profile
DPA

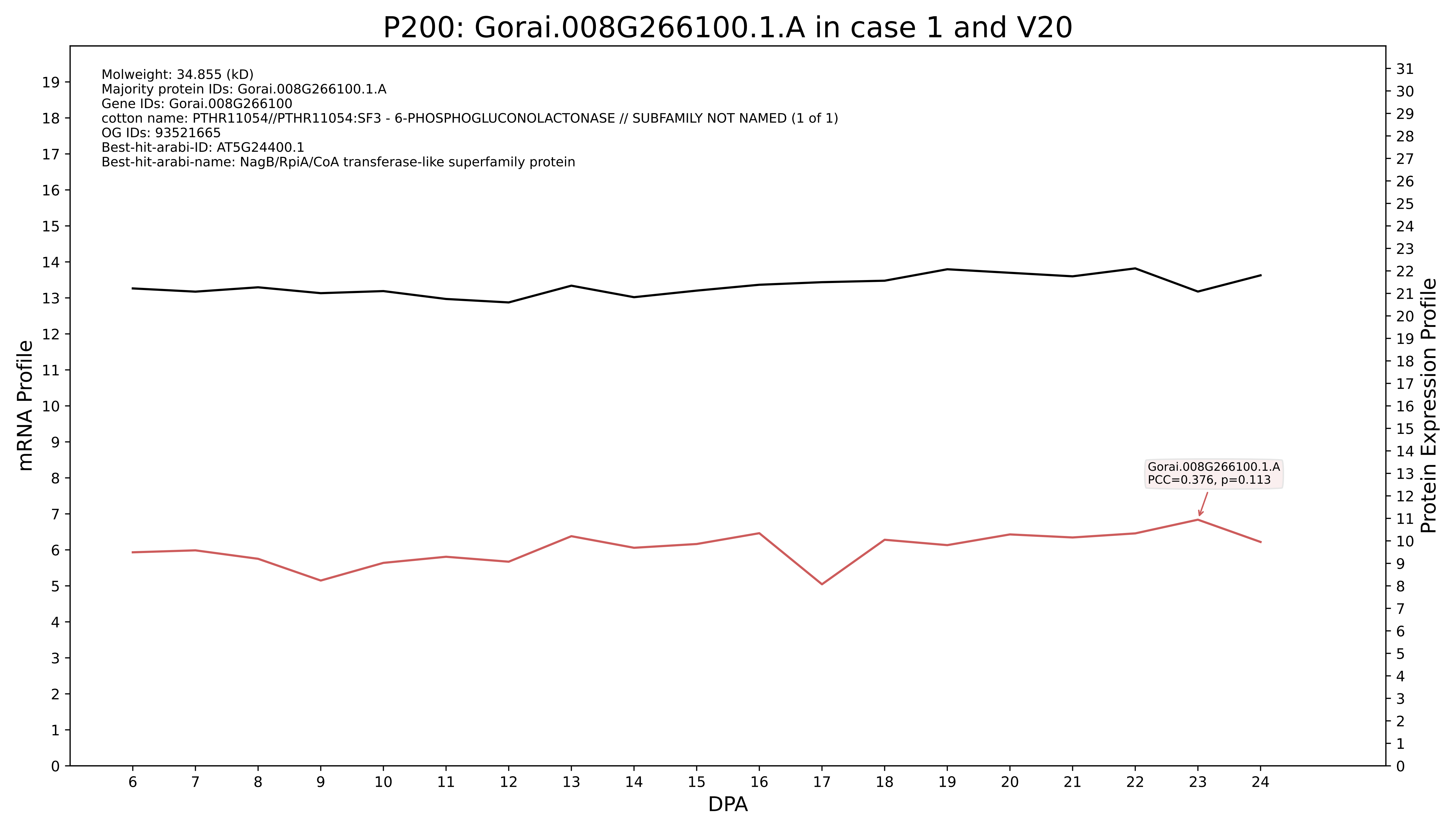
P200: Gorai.008G266100.1.A in case 1 and V20
Molweight: 34.855 (kD)
Majority protein IDs: Gorai.008G266100.1.A
Gene IDs: Gorai.008G266100
cotton name: PTHR11054//PTHR11054:SF3 - 6-PHOSPHOGLUCONOLACTONASE // SUBFAMILY NOT NAMED (1 of 1)
OG IDs: 93521665
Best-hit-arabi-ID: AT5G24400.1
Best-hit-arabi-name: NagB/RpiA/CoA transferase-like superfamily protein
Gorai.008G266100.1.A
PCC=0.376, p=0.113
mRNA Profile
Protein Expression Profile
DPA
0
1
2
3
4
5
6
7
8
9
10
11
12
13
14
15
16
17
18
19
20
21
22
23
24
25
26
27
28
29
30
31

P200: Gorai.008G175300.4.A in case 1 and V20

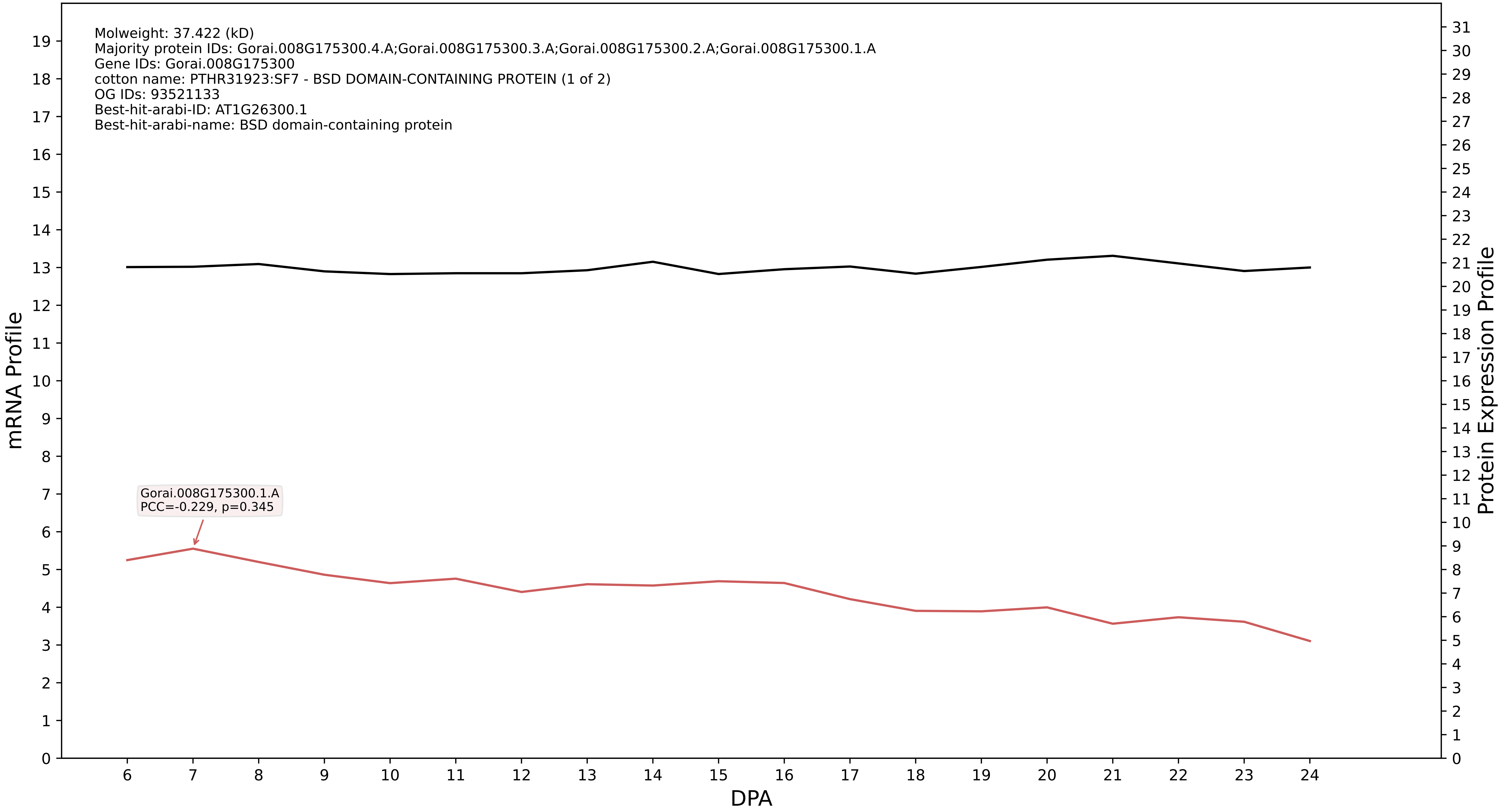

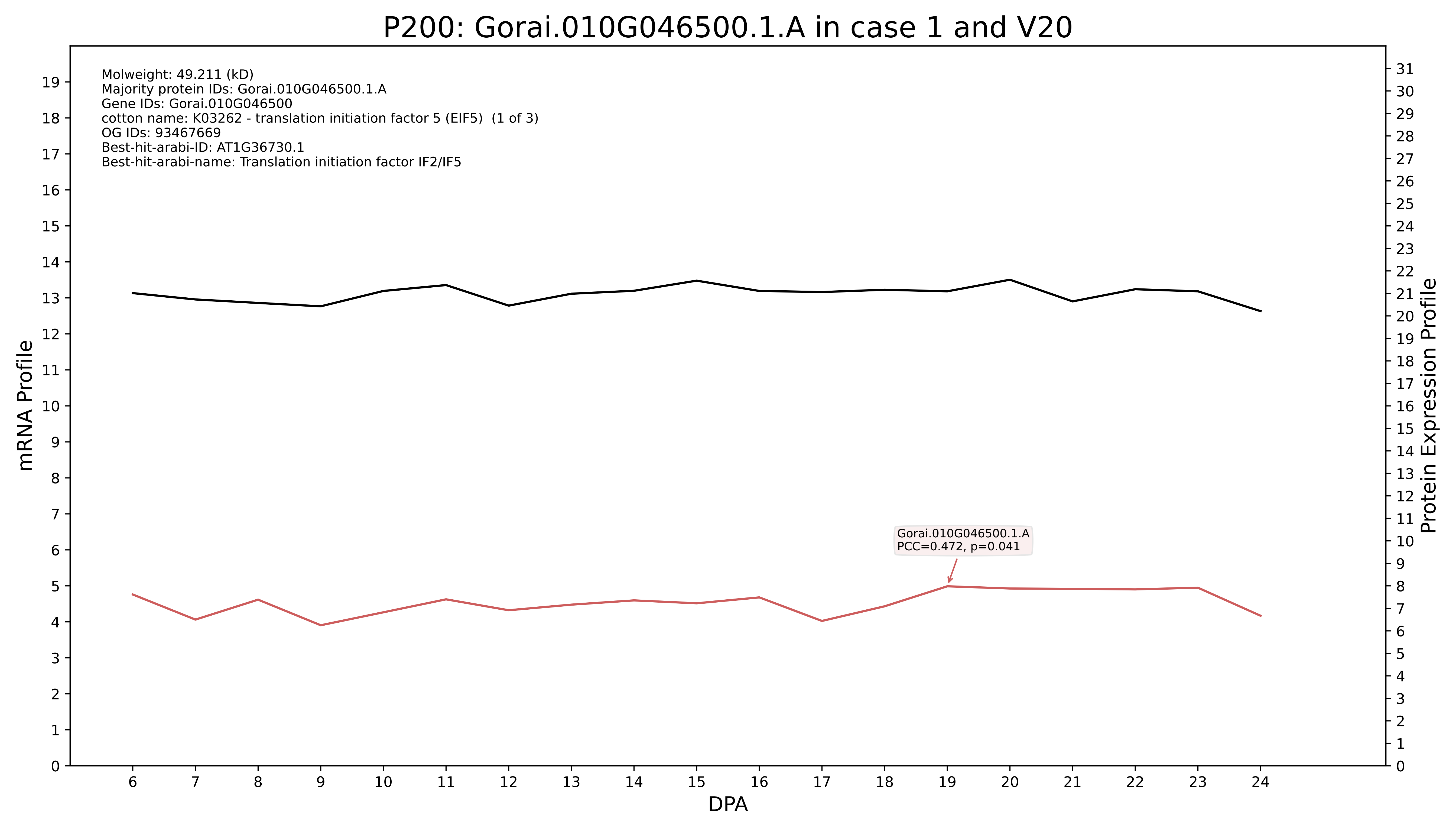

P200: Gorai.010G046500.1.A in case 1 and V20
Molweight: 49.211 (kD)
Majority protein IDs: Gorai.010G046500.1.A
Gene IDs: Gorai.010G046500
cotton name: K03262 - translation initiation factor 5 (EIF5) (1 of 3)
OG IDs: 93467669
Best-hit-arabi-ID: AT1G36730.1
Best-hit-arabi-name: Translation initiation factor IF2/IF5
Gorai.010G046500.1.A
PCC=0.472, p=0.041
mRNA Profile
Protein Expression Profile
DPA

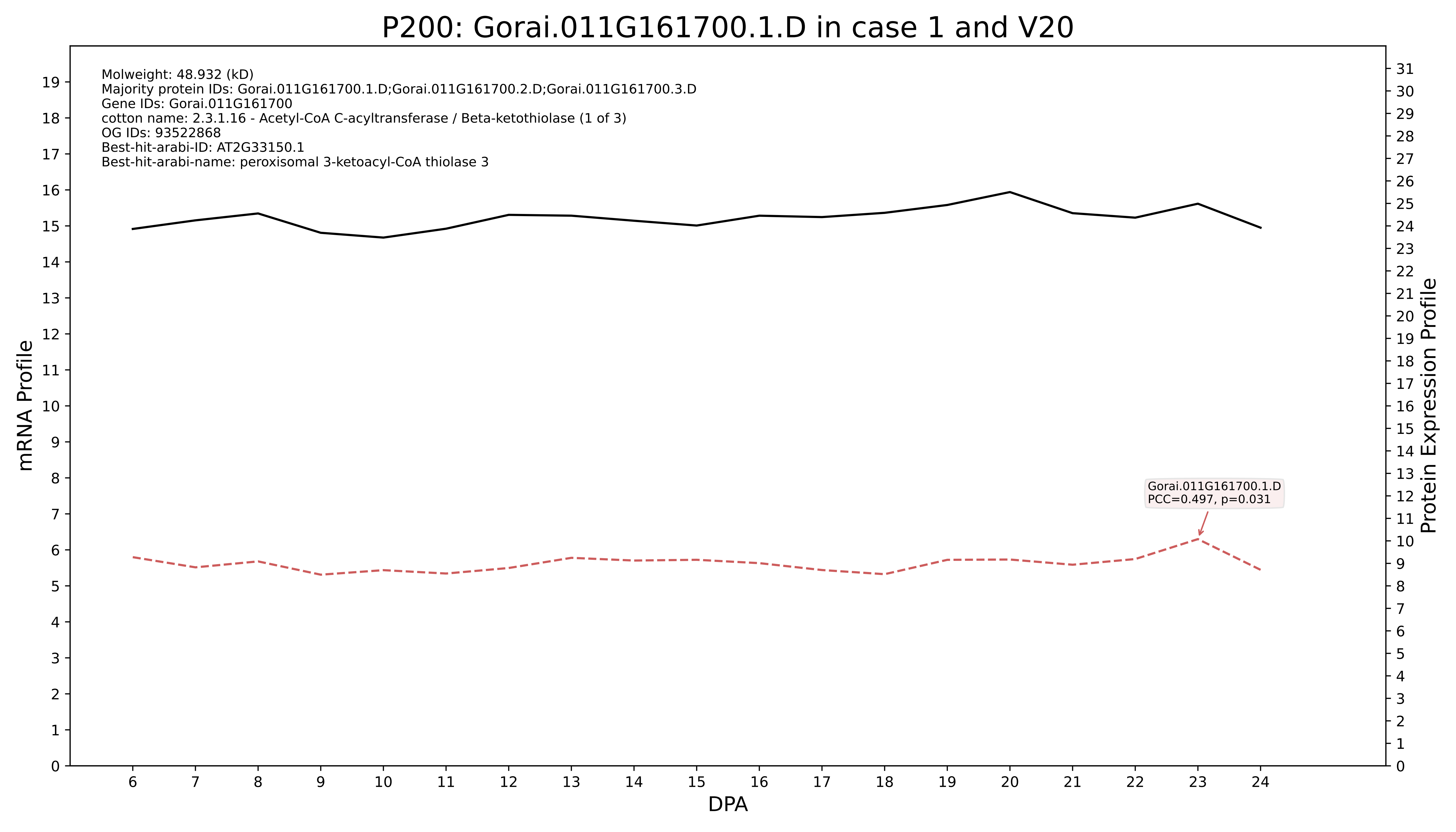
P200: Gorai.011G161700.1.D in case 1 and V20
Molweight: 48.932 (kD)
Majority protein IDs: Gorai.011G161700.1.D;Gorai.011G161700.2.D;Gorai.011G161700.3.D
Gene IDs: Gorai.011G161700
cotton name: 2.3.1.16 - Acetyl-CoA C-acyltransferase / Beta-ketothiolase (1 of 3)
OG IDs: 93522868
Best-hit-arabi-ID: AT2G33150.1
Best-hit-arabi-name: peroxisomal 3-ketoacyl-CoA thiolase 3
Gorai.011G161700.1.D
PCC=0.497, p=0.031
mRNA Profile
Protein Expression Profile
DPA

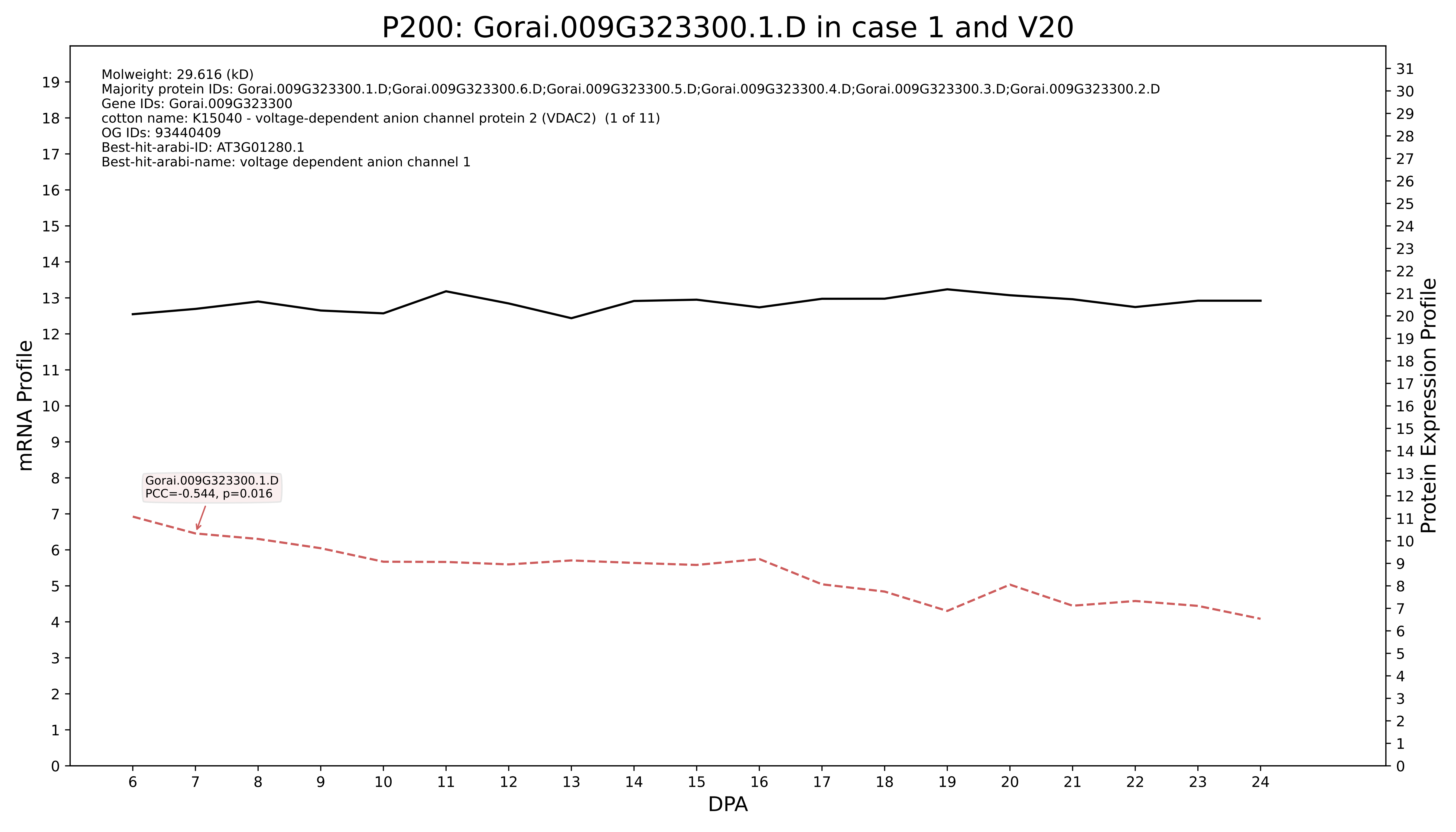
P200: Gorai.009G323300.1.D in case 1 and V20
Molweight: 29.616 (kD)
Majority protein IDs: Gorai.009G323300.1.D;Gorai.009G323300.6.D;Gorai.009G323300.5.D;Gorai.009G323300.4.D;Gorai.009G323300.3.D;Gorai.009G323300.2.D
Gene IDs: Gorai.009G323300
cotton name: K15040 - voltage-dependent anion channel protein 2 (VDAC2) (1 of 11)
OG IDs: 93440409
Best-hit-arabi-ID: AT3G01280.1
Best-hit-arabi-name: voltage dependent anion channel 1
Gorai.009G323300.1.D
PCC=-0.544, p=0.016
mRNA Profile
Protein Expression Profile
DPA
6
7
8
9
10
11
12
13
14
15
16
17
18
19
20
21
22
23
24

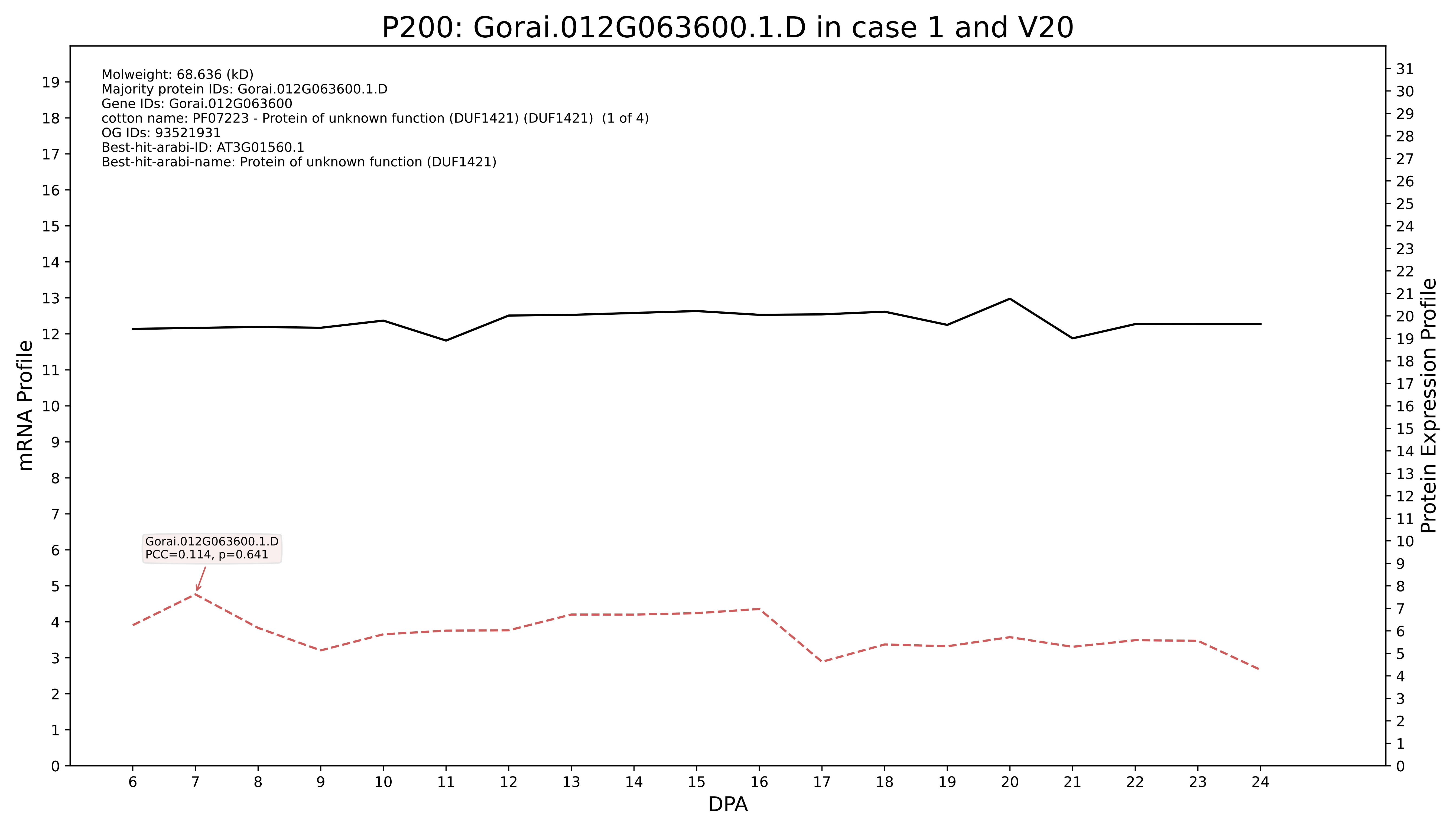
P200: Gorai.012G063600.1.D in case 1 and V20
Molweight: 68.636 (kD)
Majority protein IDs: Gorai.012G063600.1.D
Gene IDs: Gorai.012G063600
cotton name: PF07223 - Protein of unknown function (DUF1421) (DUF1421) (1 of 4)
OG IDs: 93521931
Best-hit-arabi-ID: AT3G01560.1
Best-hit-arabi-name: Protein of unknown function (DUF1421)
Gorai.012G063600.1.D
PCC=0.114, p=0.641
mRNA Profile
Protein Expression Profile
DPA
0
1
2
3
4
5
6
7
8
9
10
11
12
13
14
15
16
17
18
19
20
21
22
23
24
25
26
27
28
29
30
31

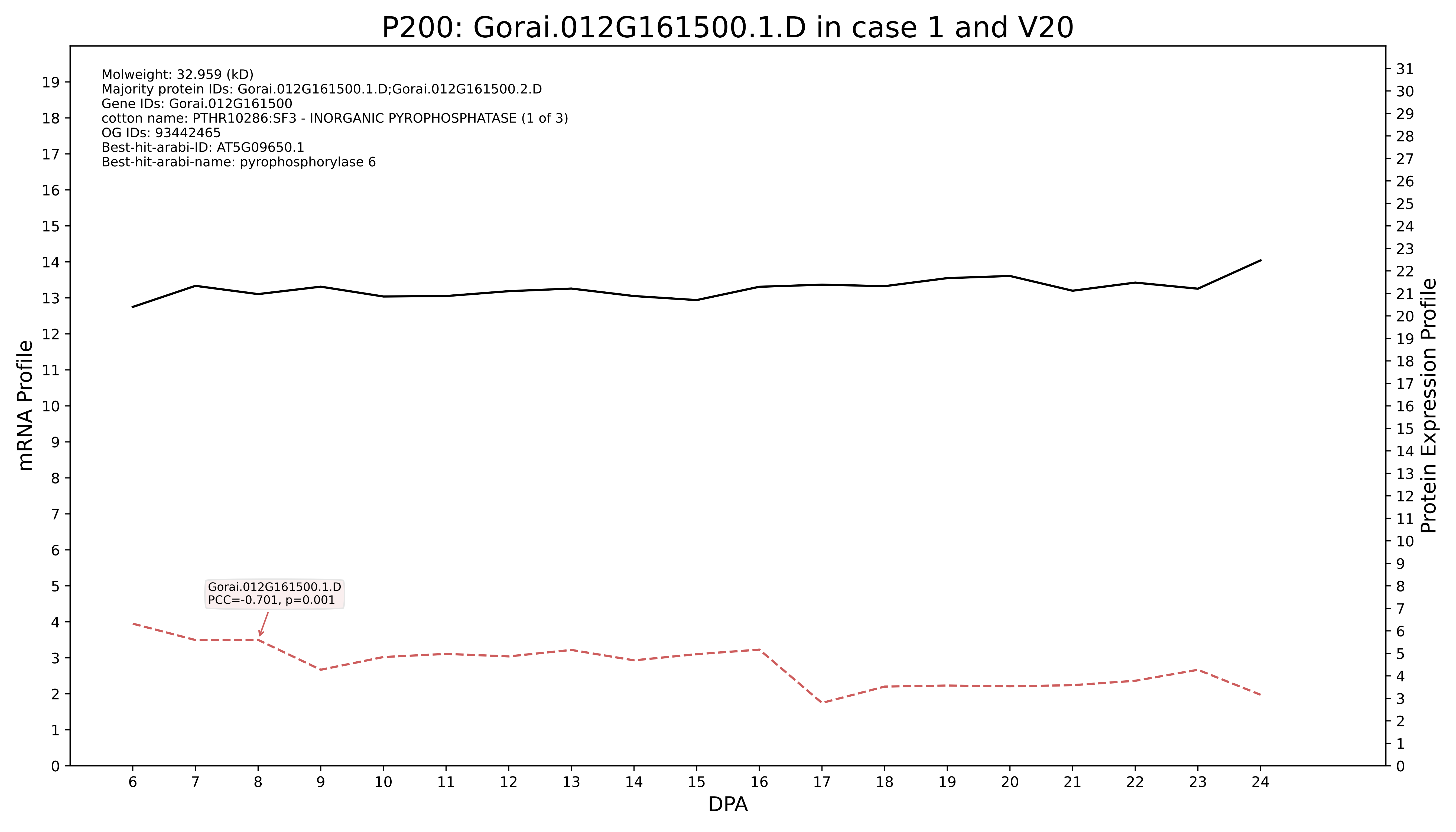
P200: Gorai.012G161500.1.D in case 1 and V20
Molweight: 32.959 (kD)
Majority protein IDs: Gorai.012G161500.1.D;Gorai.012G161500.2.D
Gene IDs: Gorai.012G161500
cotton name: PTHR10286:SF3 - INORGANIC PYROPHOSPHATASE (1 of 3)
OG IDs: 93442465
Best-hit-arabi-ID: AT5G09650.1
Best-hit-arabi-name: pyrophosphorylase 6
Gorai.012G161500.1.D
PCC=-0.701, p=0.001
mRNA Profile
Protein Expression Profile
DPA

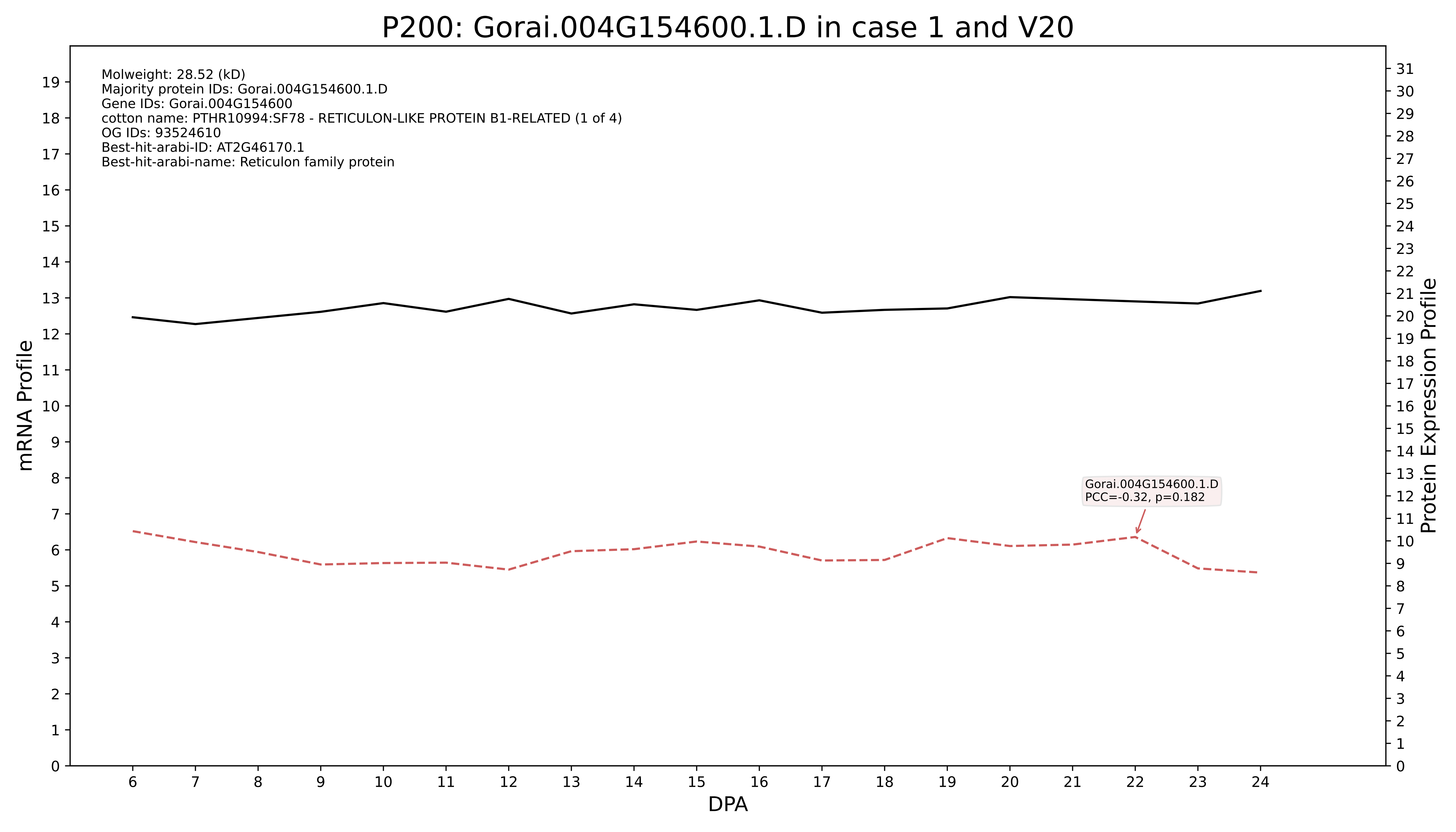
P200: Gorai.004G154600.1.D in case 1 and V20
Molweight: 28.52 (kD)
Majority protein IDs: Gorai.004G154600.1.D
Gene IDs: Gorai.004G154600
cotton name: PTHR10994:SF78 - RETICULON-LIKE PROTEIN B1-RELATED (1 of 4)
OG IDs: 93524610
Best-hit-arabi-ID: AT2G46170.1
Best-hit-arabi-name: Reticulon family protein
Gorai.004G154600.1.D
PCC=-0.32, p=0.182
mRNA Profile
Protein Expression Profile
DPA

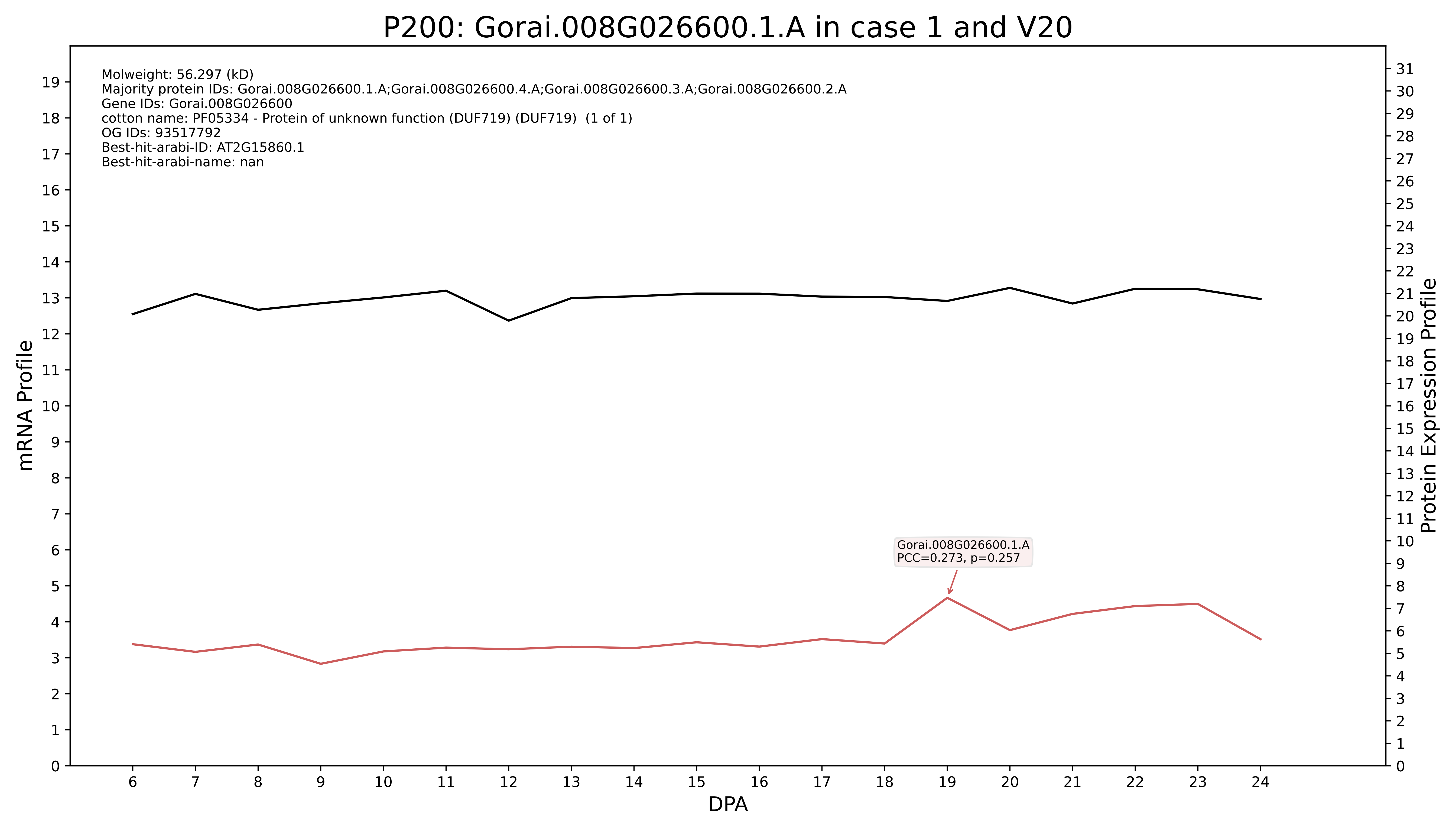
P200: Gorai.008G026600.1.A in case 1 and V20
Molweight: 56.297 (kD)
Majority protein IDs: Gorai.008G026600.1.A;Gorai.008G026600.4.A;Gorai.008G026600.3.A;Gorai.008G026600.2.A
Gene IDs: Gorai.008G026600
cotton name: PF05334 - Protein of unknown function (DUF719) (DUF719) (1 of 1)
OG IDs: 93517792
Best-hit-arabi-ID: AT2G15860.1
Best-hit-arabi-name: nan
Gorai.008G026600.1.A
PCC=0.273, p=0.257
mRNA Profile
Protein Expression Profile
DPA

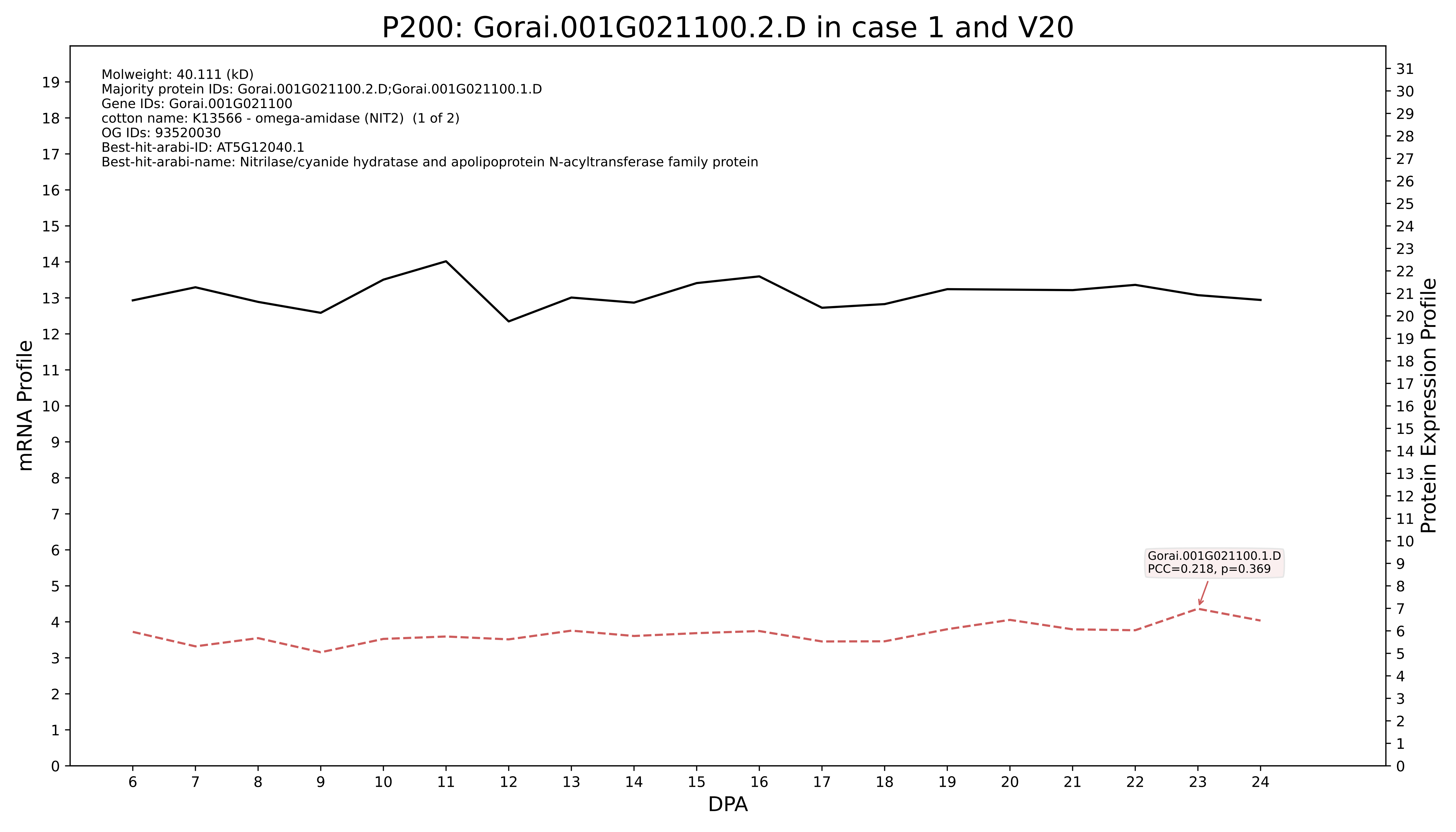

P200: Gorai.001G021100.2.D in case 1 and V20
Molweight: 40.111 (kD)
Majority protein IDs: Gorai.001G021100.2.D;Gorai.001G021100.1.D
Gene IDs: Gorai.001G021100
cotton name: K13566 - omega-amidase (NIT2) (1 of 2)
OG IDs: 93520030
Best-hit-arabi-ID: AT5G12040.1
Best-hit-arabi-name: Nitrilase/cyanide hydratase and apolipoprotein N-acyltransferase family protein
Gorai.001G021100.1.D
PCC=0.218, p=0.369
mRNA Profile
Protein Expression Profile
DPA

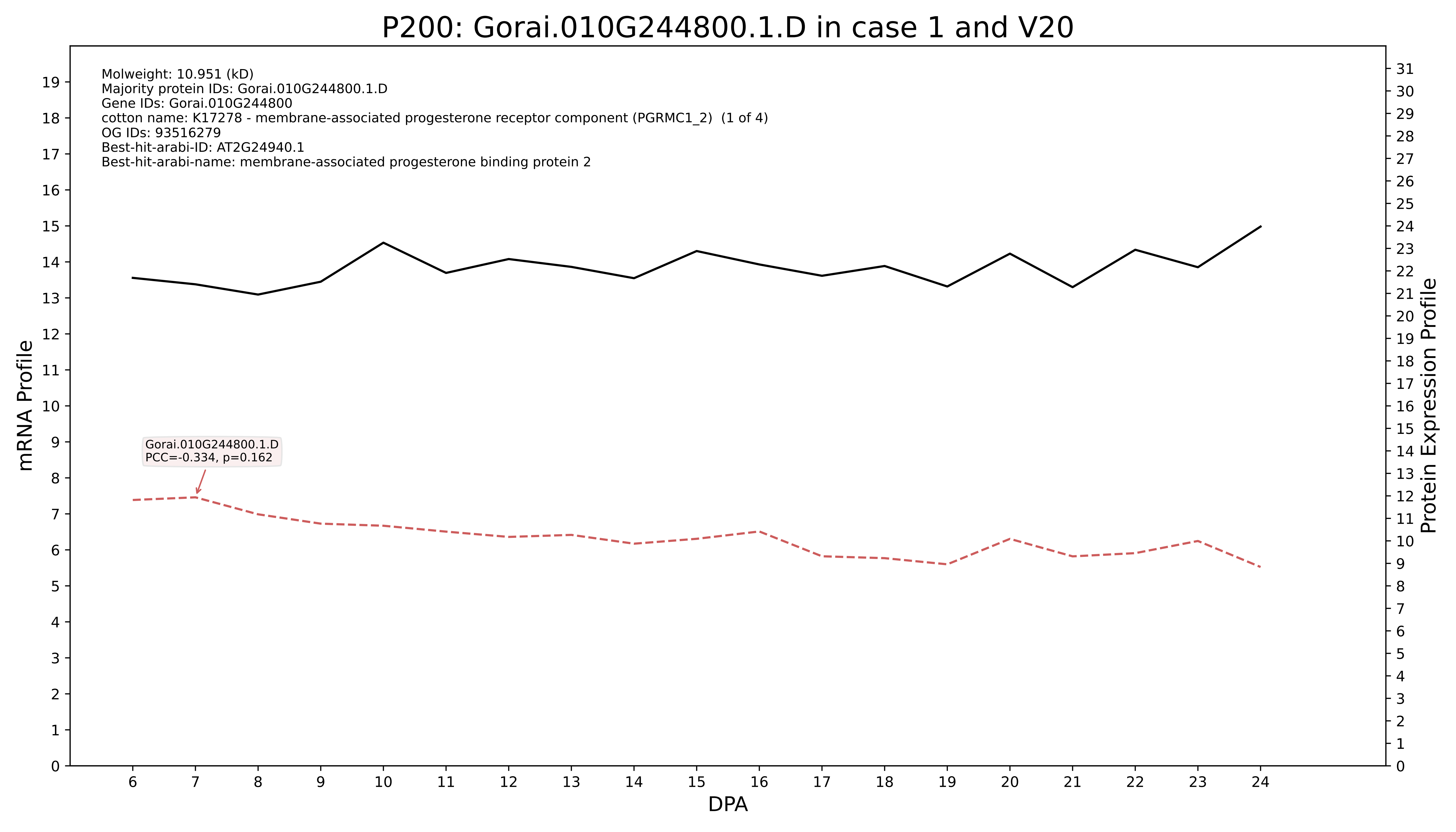

P200: Gorai.010G244800.1.D in case 1 and V20
Molweight: 10.951 (kD)
Majority protein IDs: Gorai.010G244800.1.D
Gene IDs: Gorai.010G244800
cotton name: K17278 - membrane-associated progesterone receptor component (PGRMC1_2) (1 of 4)
OG IDs: 93516279
Best-hit-arabi-ID: AT2G24940.1
Best-hit-arabi-name: membrane-associated progesterone binding protein 2
Gorai.010G244800.1.D
PCC=-0.334, p=0.162
mRNA Profile
Protein Expression Profile
DPA

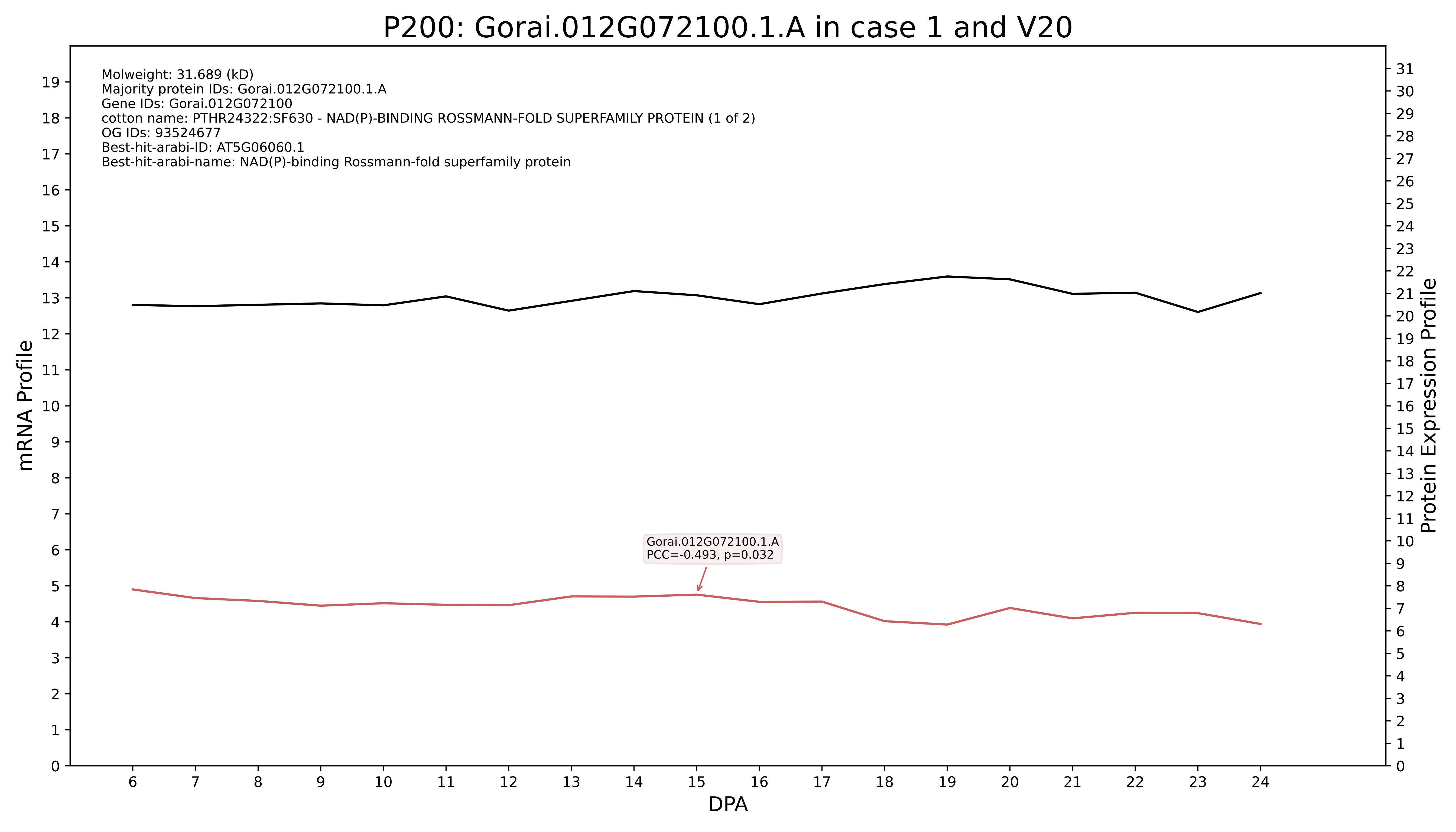
P200: Gorai.012G072100.1.A in case 1 and V20
Molweight: 31.689 (kD)
Majority protein IDs: Gorai.012G072100.1.A
Gene IDs: Gorai.012G072100
cotton name: PTHR24322:SF630 - NAD(P)-BINDING ROSSMANN-FOLD SUPERFAMILY PROTEIN (1 of 2)
OG IDs: 93524677
Best-hit-arabi-ID: AT5G06060.1
Best-hit-arabi-name: NAD(P)-binding Rossmann-fold superfamily protein
Gorai.012G072100.1.A
PCC=-0.493, p=0.032
mRNA Profile
Protein Expression Profile
DPA

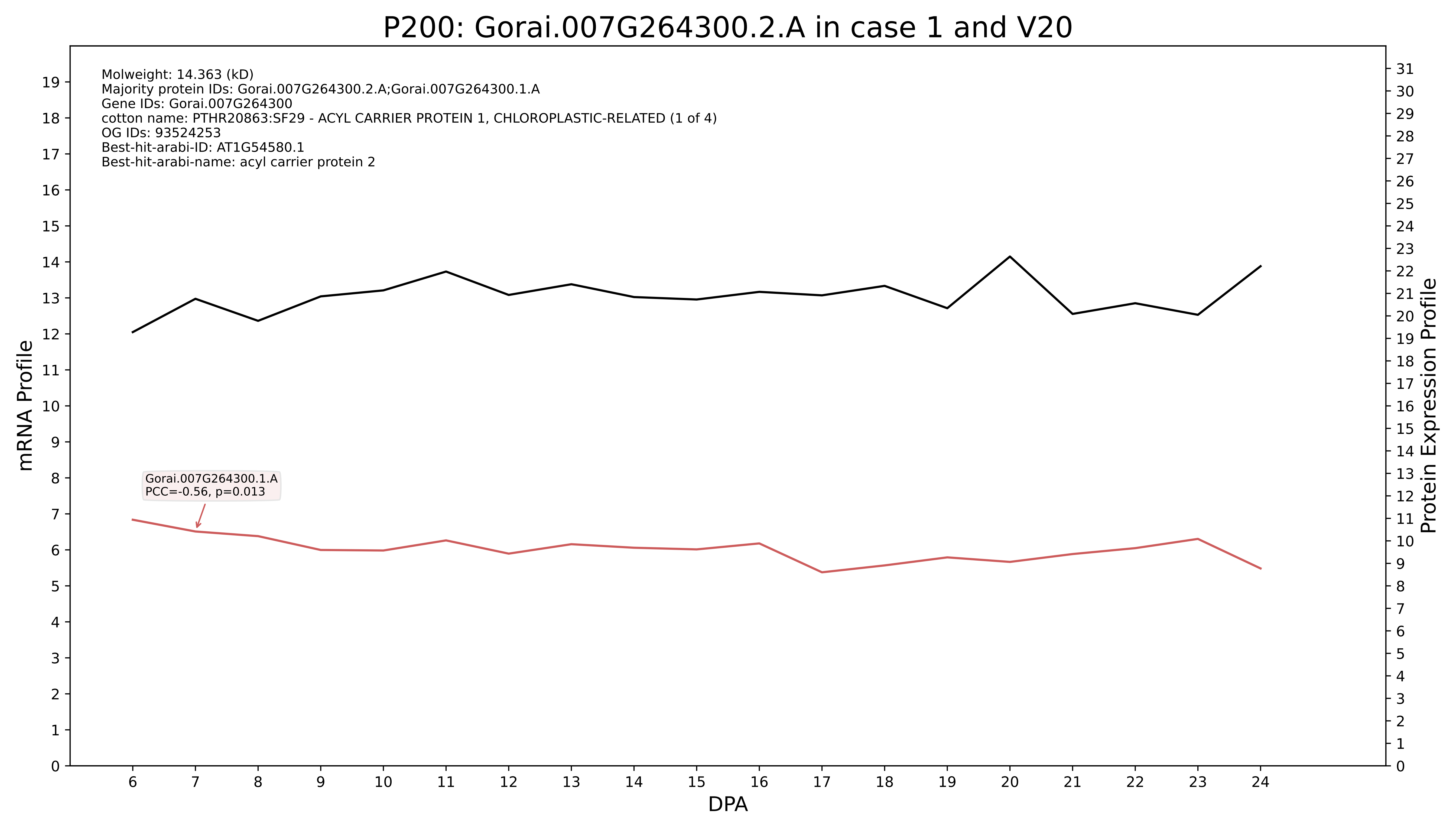
P200: Gorai.007G264300.2.A in case 1 and V20
Molweight: 14.363 (kD)
Majority protein IDs: Gorai.007G264300.2.A;Gorai.007G264300.1.A
Gene IDs: Gorai.007G264300
cotton name: PTHR20863:SF29 - ACYL CARRIER PROTEIN 1, CHLOROPLASTIC-RELATED (1 of 4)
OG IDs: 93524253
Best-hit-arabi-ID: AT1G54580.1
Best-hit-arabi-name: acyl carrier protein 2
Gorai.007G264300.1.A
PCC=-0.56, p=0.013
mRNA Profile
Protein Expression Profile
DPA

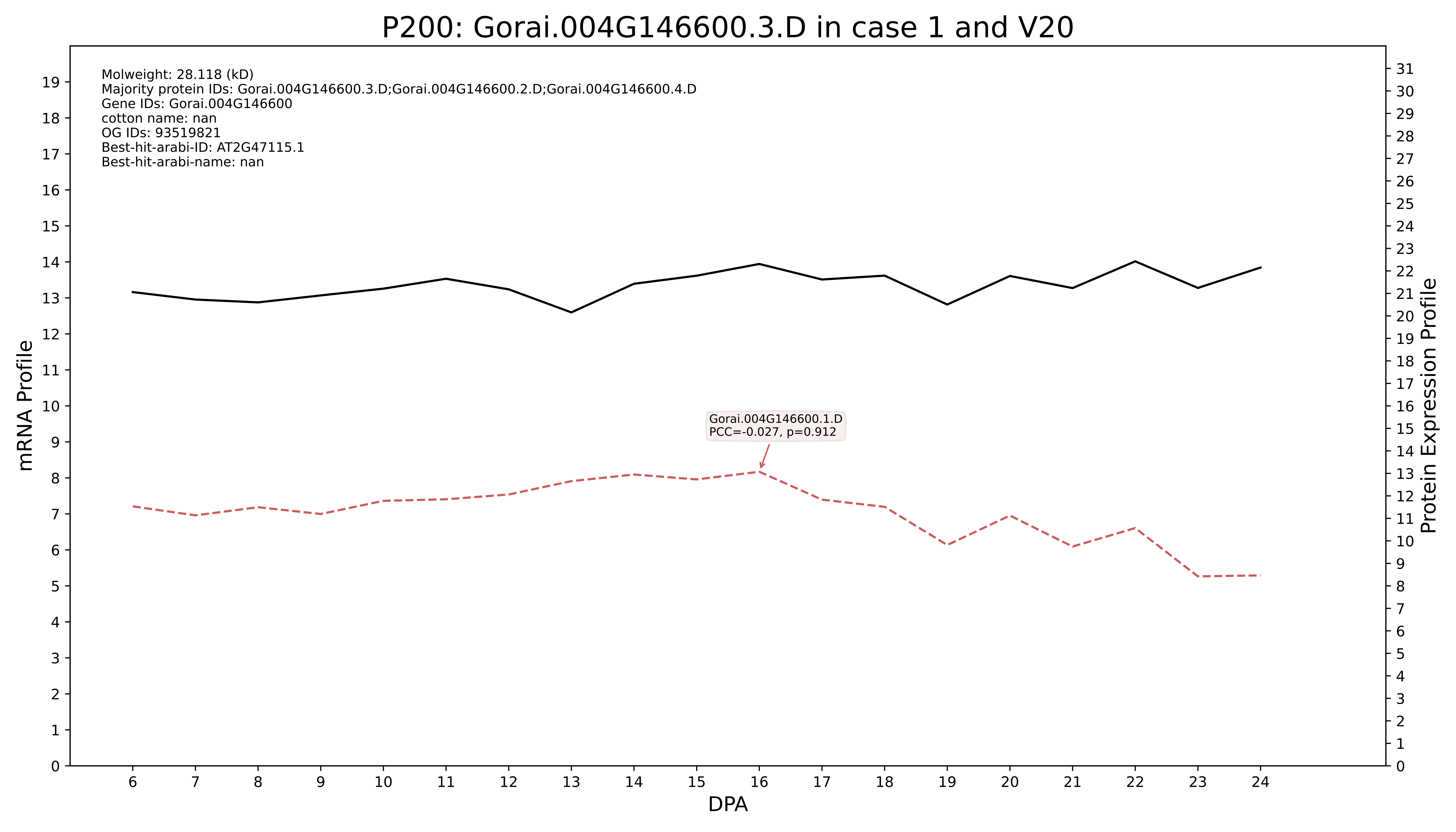

P200: Gorai.004G146600.3.D in case 1 and V20
Molweight: 28.118 (kD)
Majority protein IDs: Gorai.004G146600.3.D;Gorai.004G146600.2.D;Gorai.004G146600.4.D
Gene IDs: Gorai.004G146600
cotton name: nan
OG IDs: 93519821
Best-hit-arabi-ID: AT2G47115.1
Best-hit-arabi-name: nan
Gorai.004G146600.1.D
PCC=-0.027, p=0.912
mRNA Profile
Protein Expression Profile
DPA

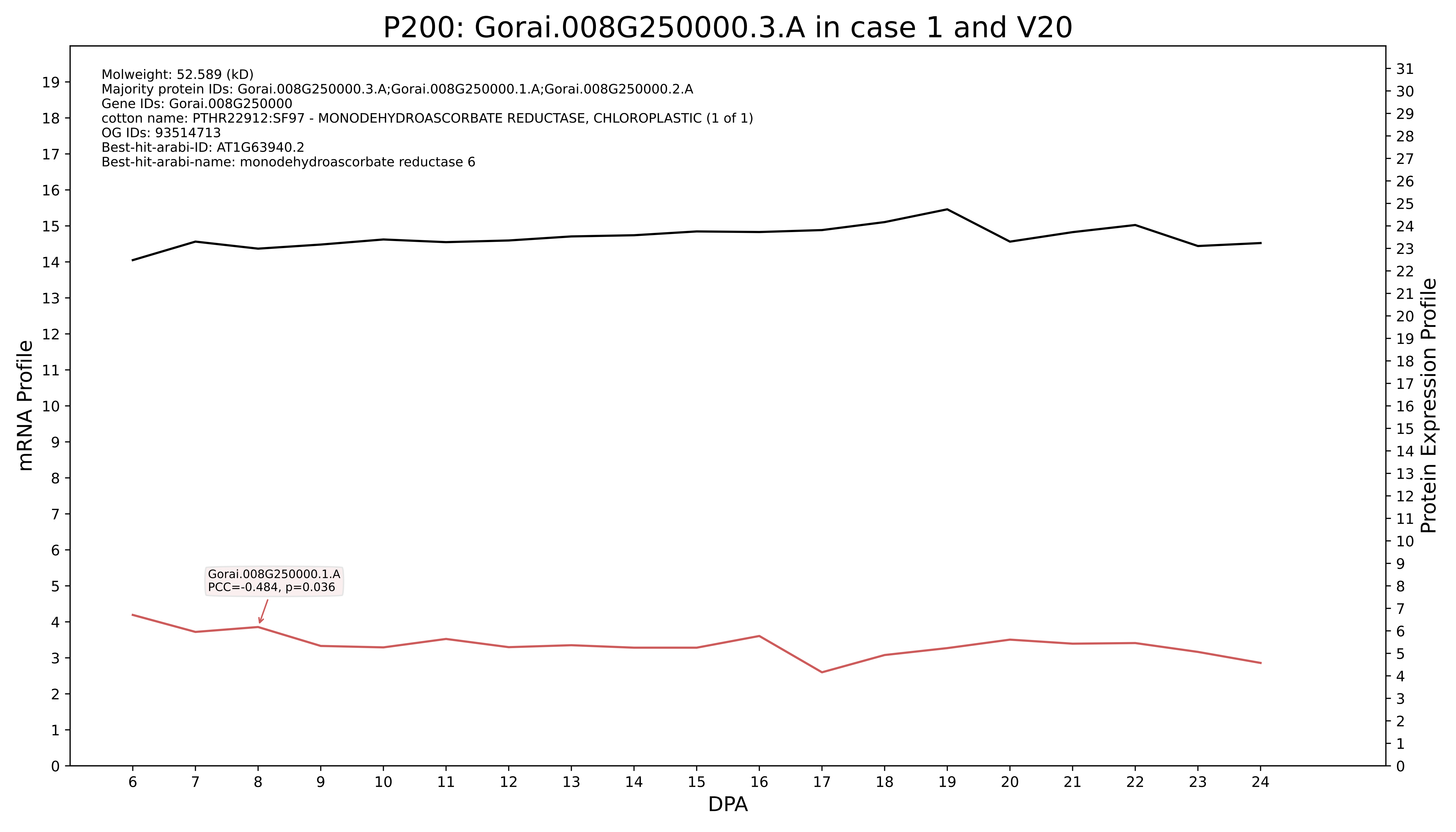

P200: Gorai.008G250000.3.A in case 1 and V20
Molweight: 52.589 (kD)
Majority protein IDs: Gorai.008G250000.3.A;Gorai.008G250000.1.A;Gorai.008G250000.2.A
Gene IDs: Gorai.008G250000
cotton name: PTHR22912:SF97 - MONODEHYDROASCORBATE REDUCTASE, CHLOROPLASTIC (1 of 1)
OG IDs: 93514713
Best-hit-arabi-ID: AT1G63940.2
Best-hit-arabi-name: monodehydroascorbate reductase 6
Gorai.008G250000.1.A
PCC=-0.484, p=0.036
mRNA Profile
Protein Expression Profile
DPA

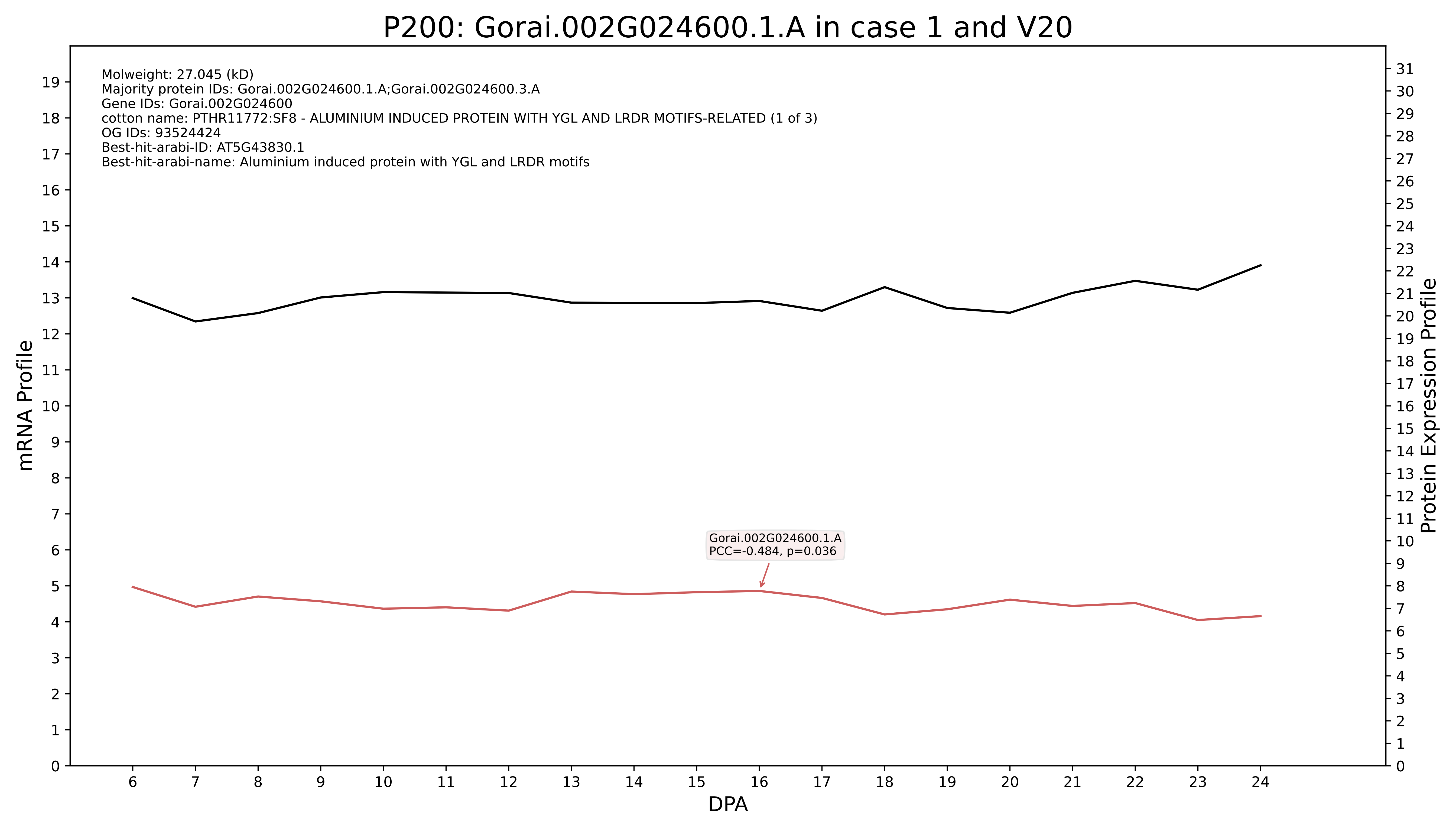
P200: Gorai.002G024600.1.A in case 1 and V20
Molweight: 27.045 (kD)
Majority protein IDs: Gorai.002G024600.1.A;Gorai.002G024600.3.A
Gene IDs: Gorai.002G024600
cotton name: PTHR11772:SF8 - ALUMINIUM INDUCED PROTEIN WITH YGL AND LRDR MOTIFS-RELATED (1 of 3)
OG IDs: 93524424
Best-hit-arabi-ID: AT5G43830.1
Best-hit-arabi-name: Aluminium induced protein with YGL and LRDR motifs
Gorai.002G024600.1.A
PCC=-0.484, p=0.036
mRNA Profile
Protein Expression Profile
DPA

P200: Gorai.003G182200.4.D in case 1 and V20

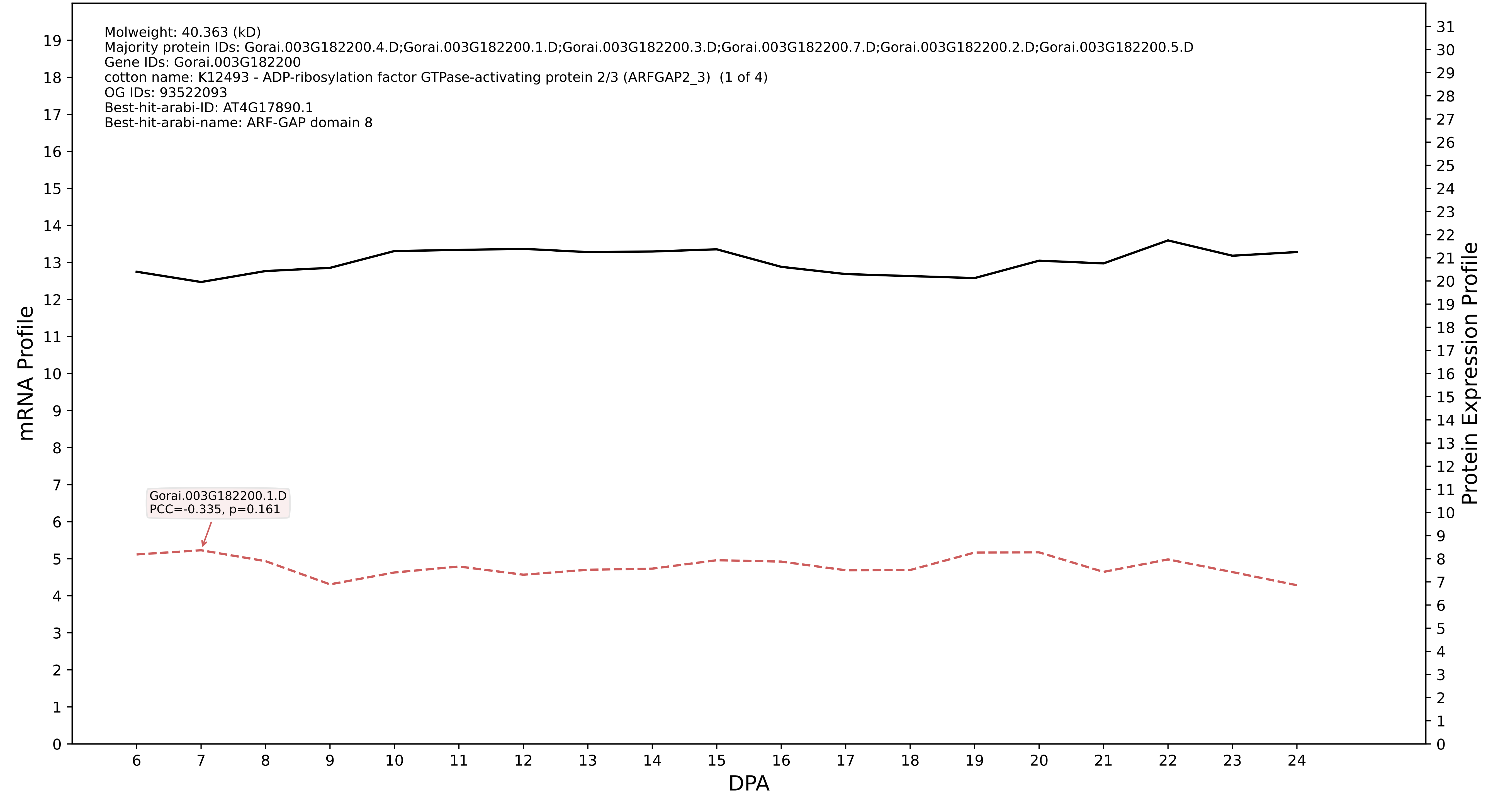

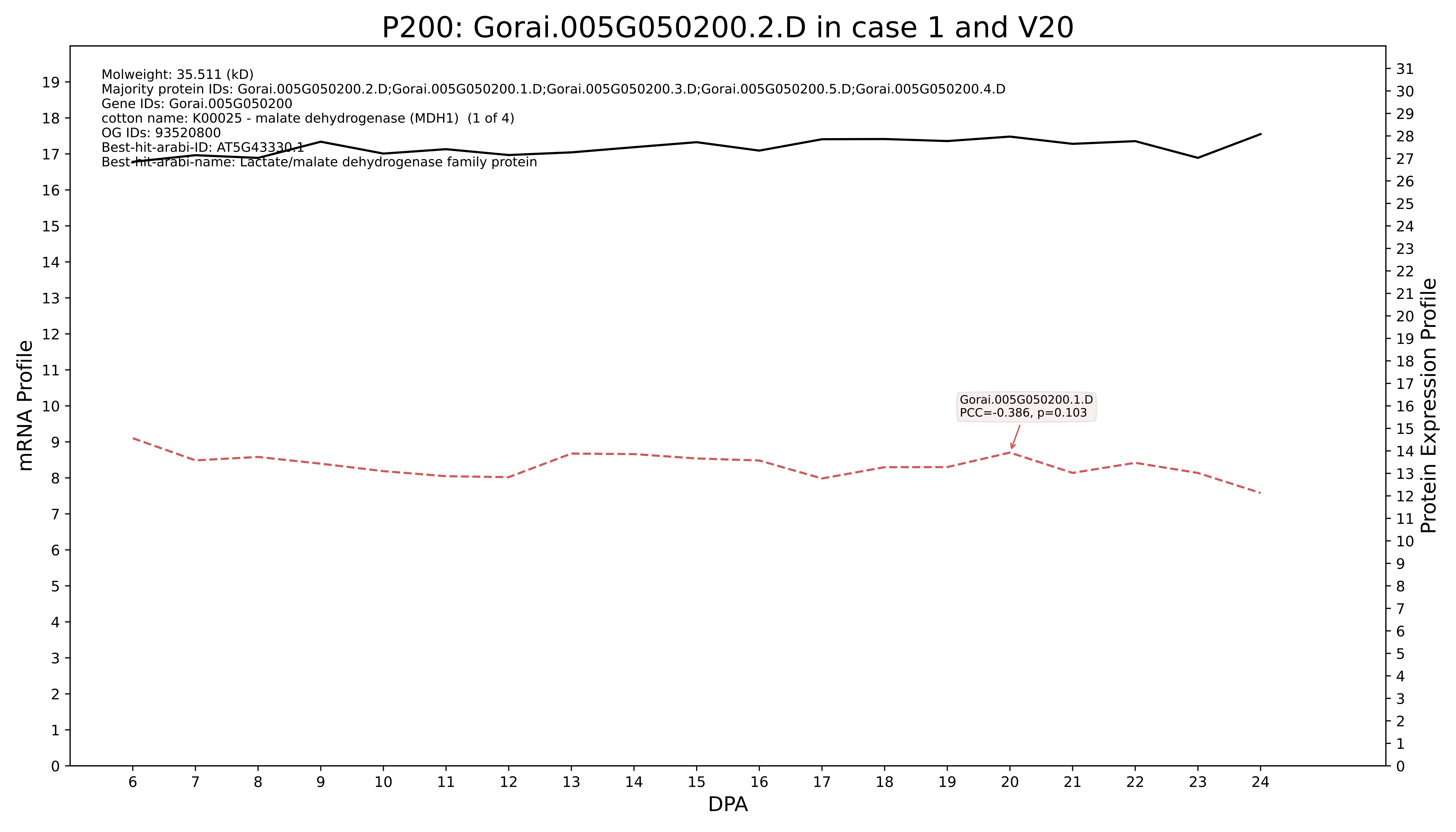

P200: Gorai.005G050200.2.D in case 1 and V20
Molweight: 35.511 (kD)
Majority protein IDs: Gorai.005G050200.2.D;Gorai.005G050200.1.D;Gorai.005G050200.3.D;Gorai.005G050200.5.D;Gorai.005G050200.4.D
Gene IDs: Gorai.005G050200
cotton name: K00025 - malate dehydrogenase (MDH1) (1 of 4)
OG IDs: 93520800
Best-hit-arabi-ID: AT5G43330.1
Best-hit-arabi-name: Lactate/malate dehydrogenase family protein
Gorai.005G050200.1.D
PCC=-0.386, p=0.103
mRNA Profile
Protein Expression Profile
DPA

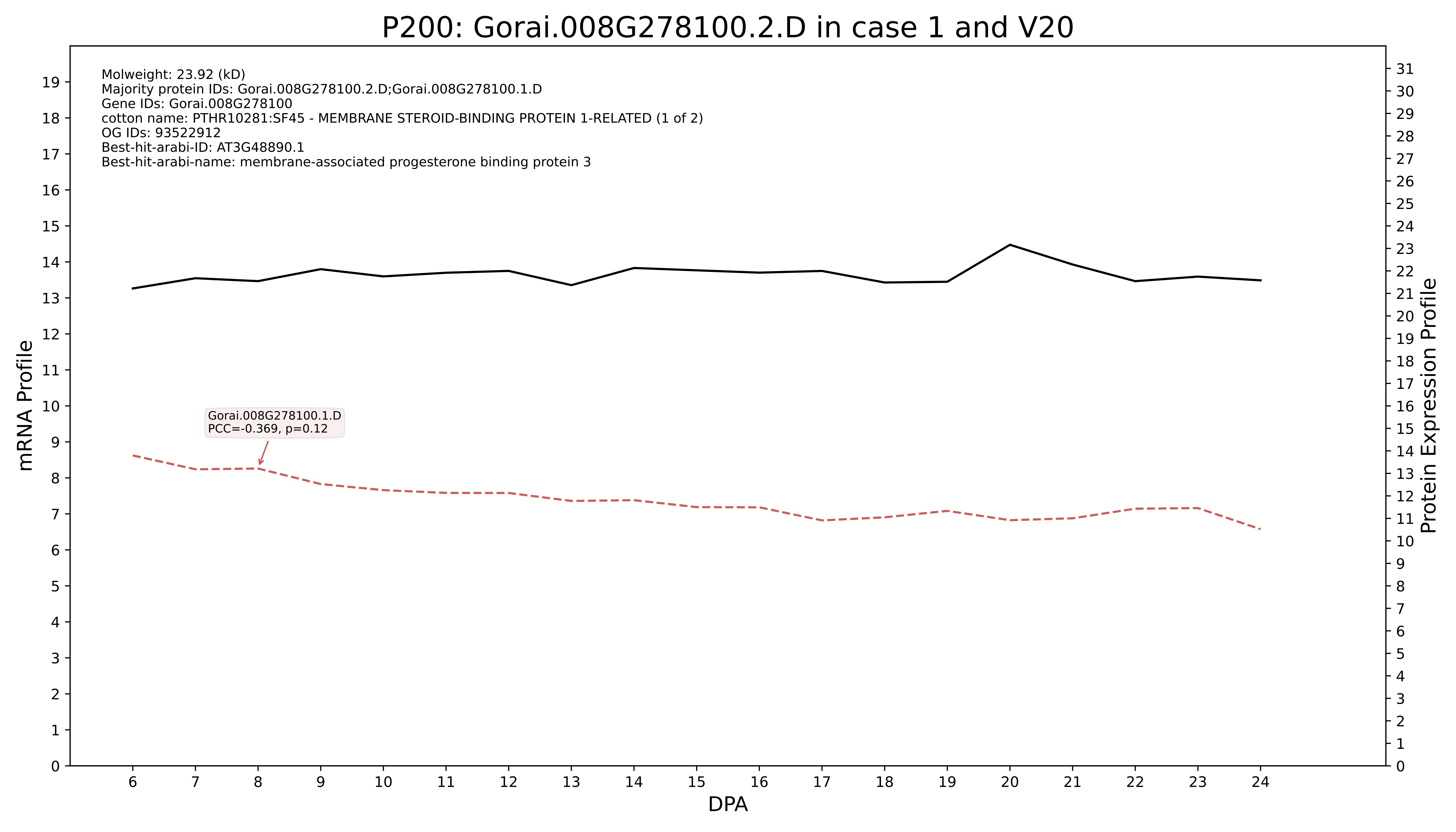
P200: Gorai.008G278100.2.D in case 1 and V20
Molweight: 23.92 (kD)
Majority protein IDs: Gorai.008G278100.2.D;Gorai.008G278100.1.D
Gene IDs: Gorai.008G278100
cotton name: PTHR10281:SF45 - MEMBRANE STEROID-BINDING PROTEIN 1-RELATED (1 of 2)
OG IDs: 93522912
Best-hit-arabi-ID: AT3G48890.1
Best-hit-arabi-name: membrane-associated progesterone binding protein 3
Gorai.008G278100.1.D
PCC=-0.369, p=0.12
mRNA Profile
Protein Expression Profile
DPA

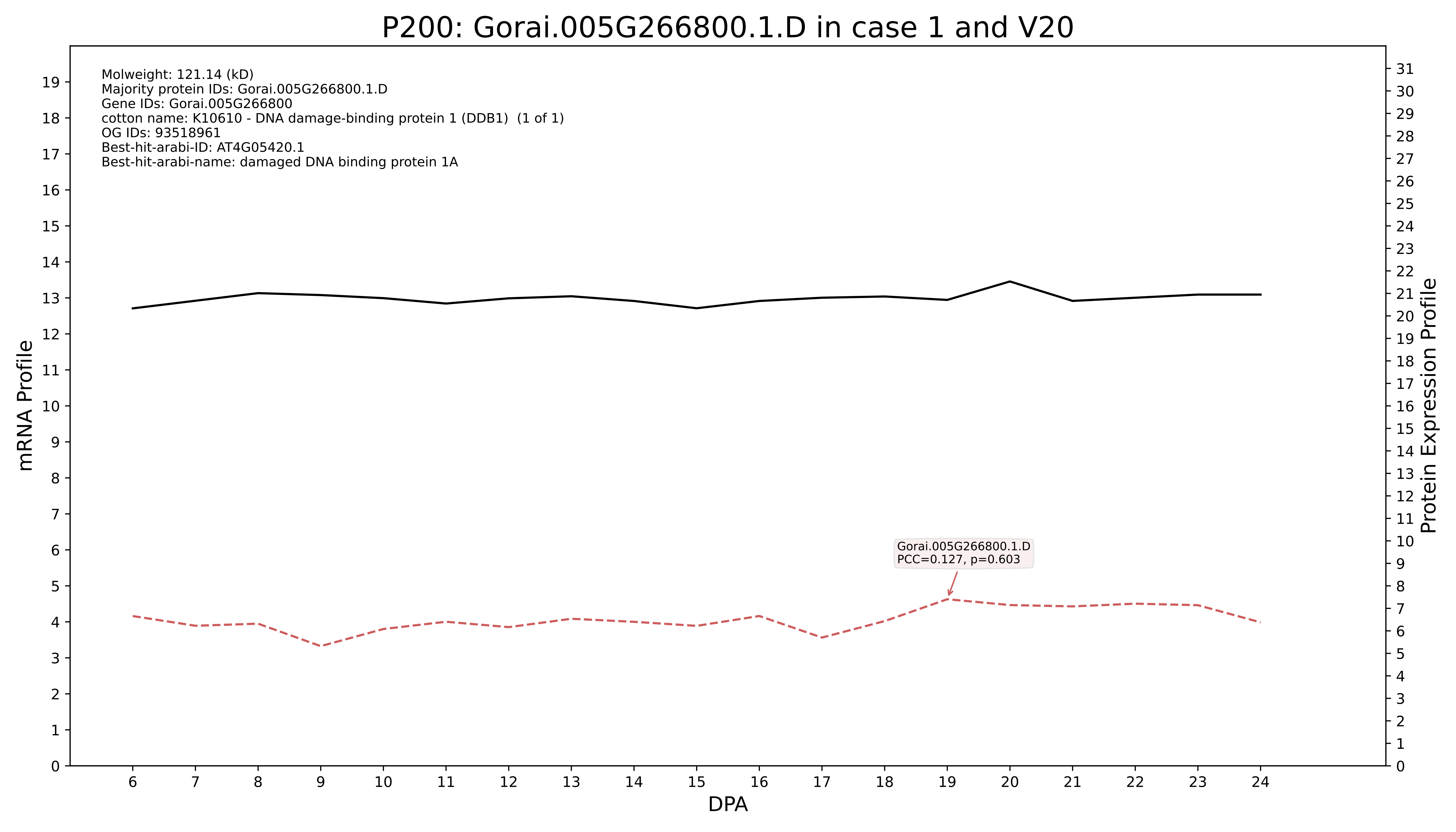
P200: Gorai.005G266800.1.D in case 1 and V20
Molweight: 121.14 (kD)
Majority protein IDs: Gorai.005G266800.1.D
Gene IDs: Gorai.005G266800
cotton name: K10610 - DNA damage-binding protein 1 (DDB1) (1 of 1)
OG IDs: 93518961
Best-hit-arabi-ID: AT4G05420.1
Best-hit-arabi-name: damaged DNA binding protein 1A
Gorai.005G266800.1.D
PCC=0.127, p=0.603
mRNA Profile
Protein Expression Profile
DPA

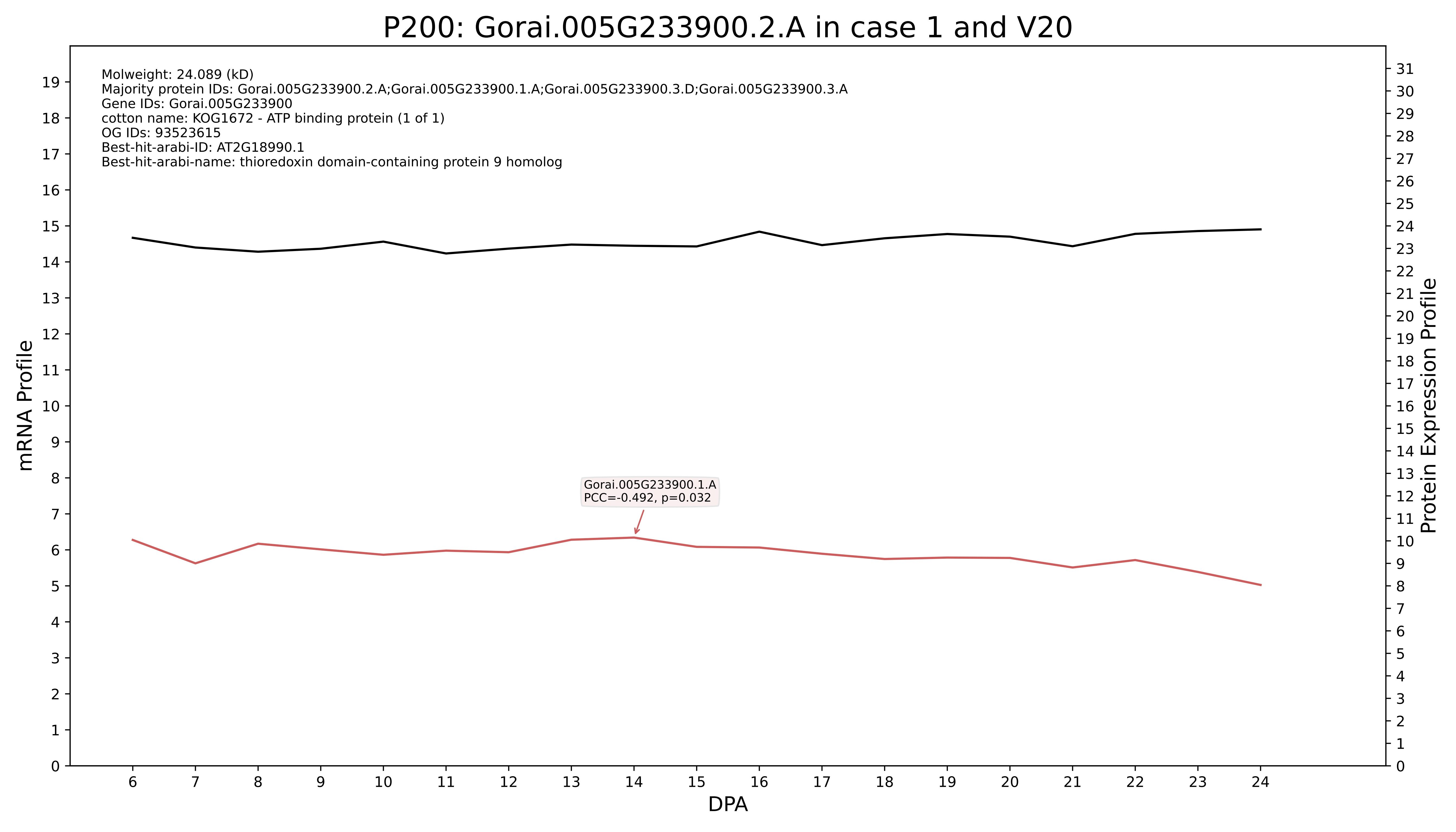

P200: Gorai.005G233900.2.A in case 1 and V20
Molweight: 24.089 (kD)
Majority protein IDs: Gorai.005G233900.2.A;Gorai.005G233900.1.A;Gorai.005G233900.3.D;Gorai.005G233900.3.A
Gene IDs: Gorai.005G233900
cotton name: KOG1672 - ATP binding protein (1 of 1)
OG IDs: 93523615
Best-hit-arabi-ID: AT2G18990.1
Best-hit-arabi-name: thioredoxin domain-containing protein 9 homolog
Gorai.005G233900.1.A
PCC=-0.492, p=0.032
mRNA Profile
Protein Expression Profile
DPA

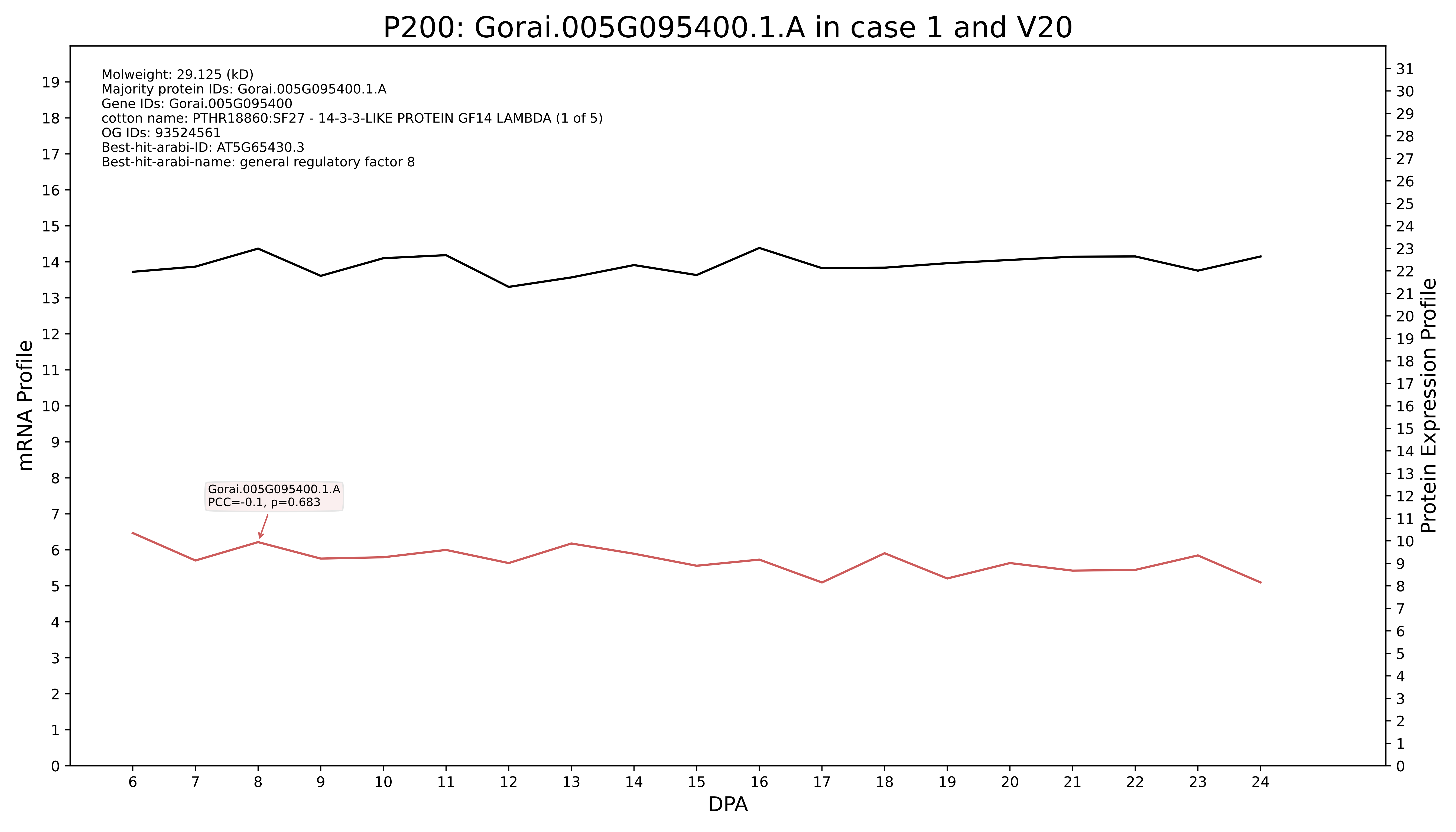
P200: Gorai.005G095400.1.A in case 1 and V20
Molweight: 29.125 (kD)
Majority protein IDs: Gorai.005G095400.1.A
Gene IDs: Gorai.005G095400
cotton name: PTHR18860:SF27 - 14-3-3-LIKE PROTEIN GF14 LAMBDA (1 of 5)
OG IDs: 93524561
Best-hit-arabi-ID: AT5G65430.3
Best-hit-arabi-name: general regulatory factor 8
Gorai.005G095400.1.A
PCC=-0.1, p=0.683
mRNA Profile
Protein Expression Profile
DPA

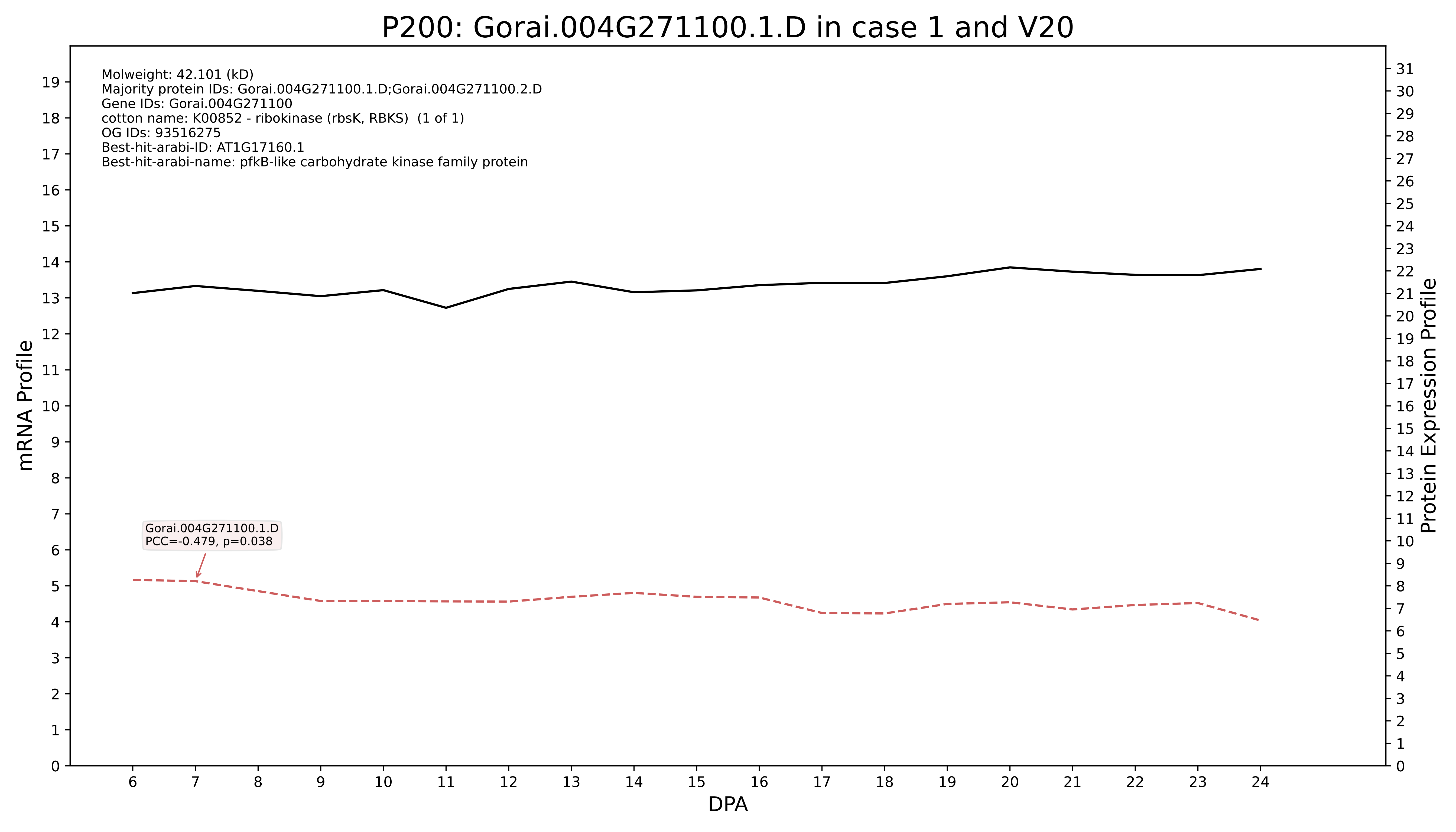
P200: Gorai.004G271100.1.D in case 1 and V20
Molweight: 42.101 (kD)
Majority protein IDs: Gorai.004G271100.1.D;Gorai.004G271100.2.D
Gene IDs: Gorai.004G271100
cotton name: K00852 - ribokinase (rbsK, RBKS) (1 of 1)
OG IDs: 93516275
Best-hit-arabi-ID: AT1G17160.1
Best-hit-arabi-name: pfkB-like carbohydrate kinase family protein
Gorai.004G271100.1.D
PCC=-0.479, p=0.038
mRNA Profile
Protein Expression Profile
DPA

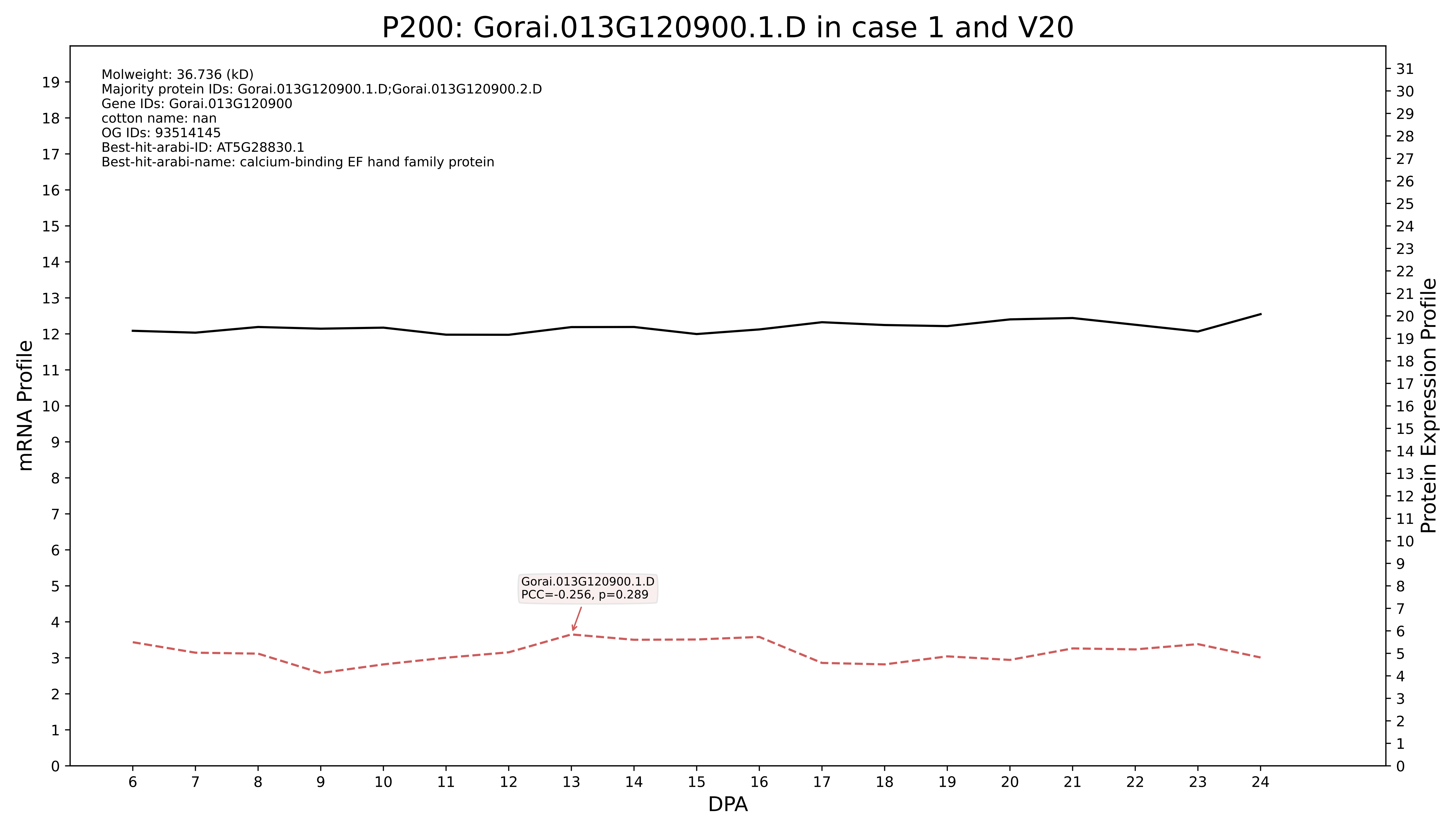
P200: Gorai.013G120900.1.D in case 1 and V20
Molweight: 36.736 (kD)
Majority protein IDs: Gorai.013G120900.1.D;Gorai.013G120900.2.D
Gene IDs: Gorai.013G120900
cotton name: nan
OG IDs: 93514145
Best-hit-arabi-ID: AT5G28830.1
Best-hit-arabi-name: calcium-binding EF hand family protein
Gorai.013G120900.1.D
PCC=-0.256, p=0.289
mRNA Profile
Protein Expression Profile
DPA

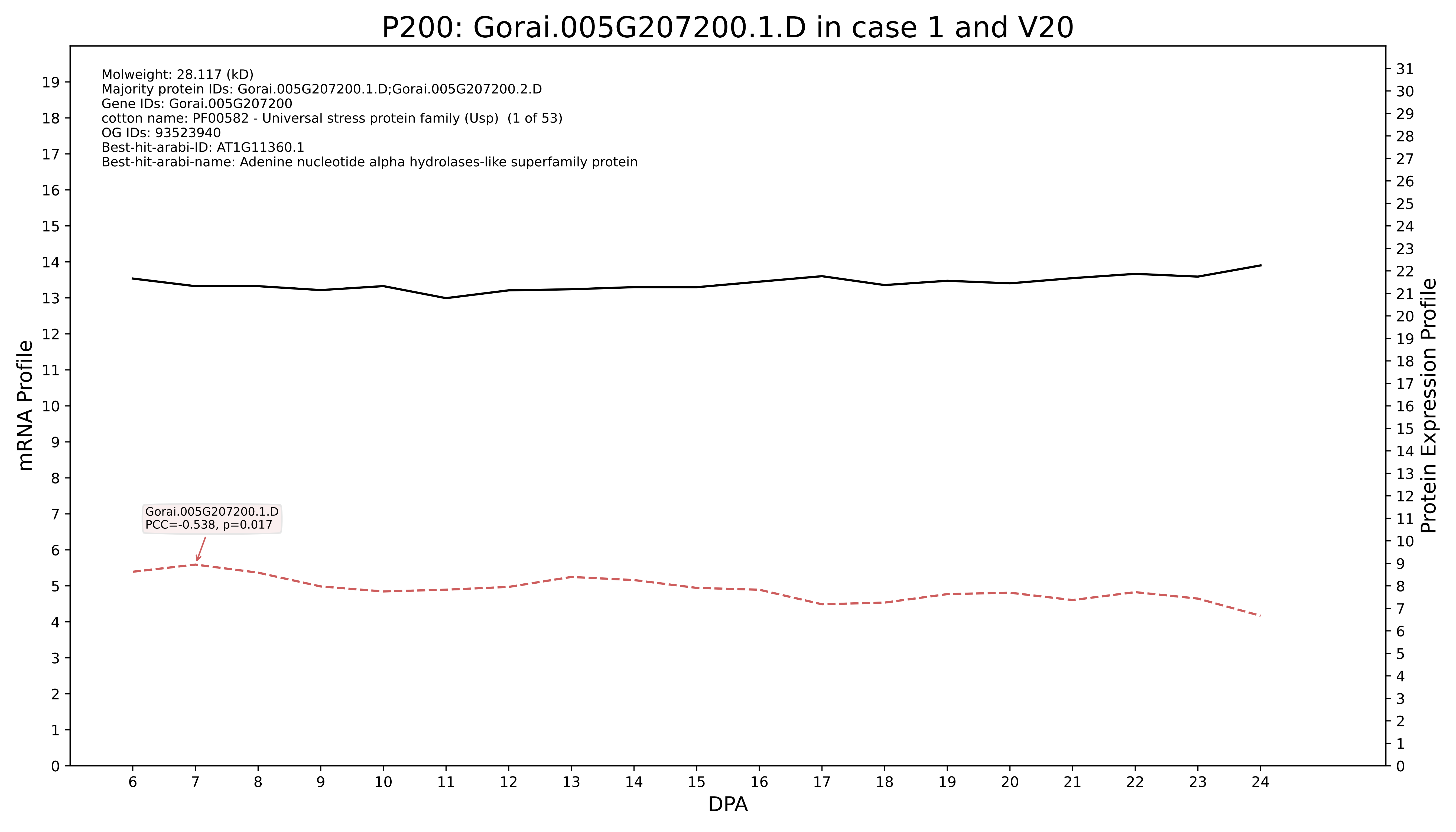

P200: Gorai.005G207200.1.D in case 1 and V20
Molweight: 28.117 (kD)
Majority protein IDs: Gorai.005G207200.1.D;Gorai.005G207200.2.D
Gene IDs: Gorai.005G207200
cotton name: PF00582 - Universal stress protein family (Usp) (1 of 53)
OG IDs: 93523940
Best-hit-arabi-ID: AT1G11360.1
Best-hit-arabi-name: Adenine nucleotide alpha hydrolases-like superfamily protein
Gorai.005G207200.1.D
PCC=-0.538, p=0.017
mRNA Profile
Protein Expression Profile
DPA

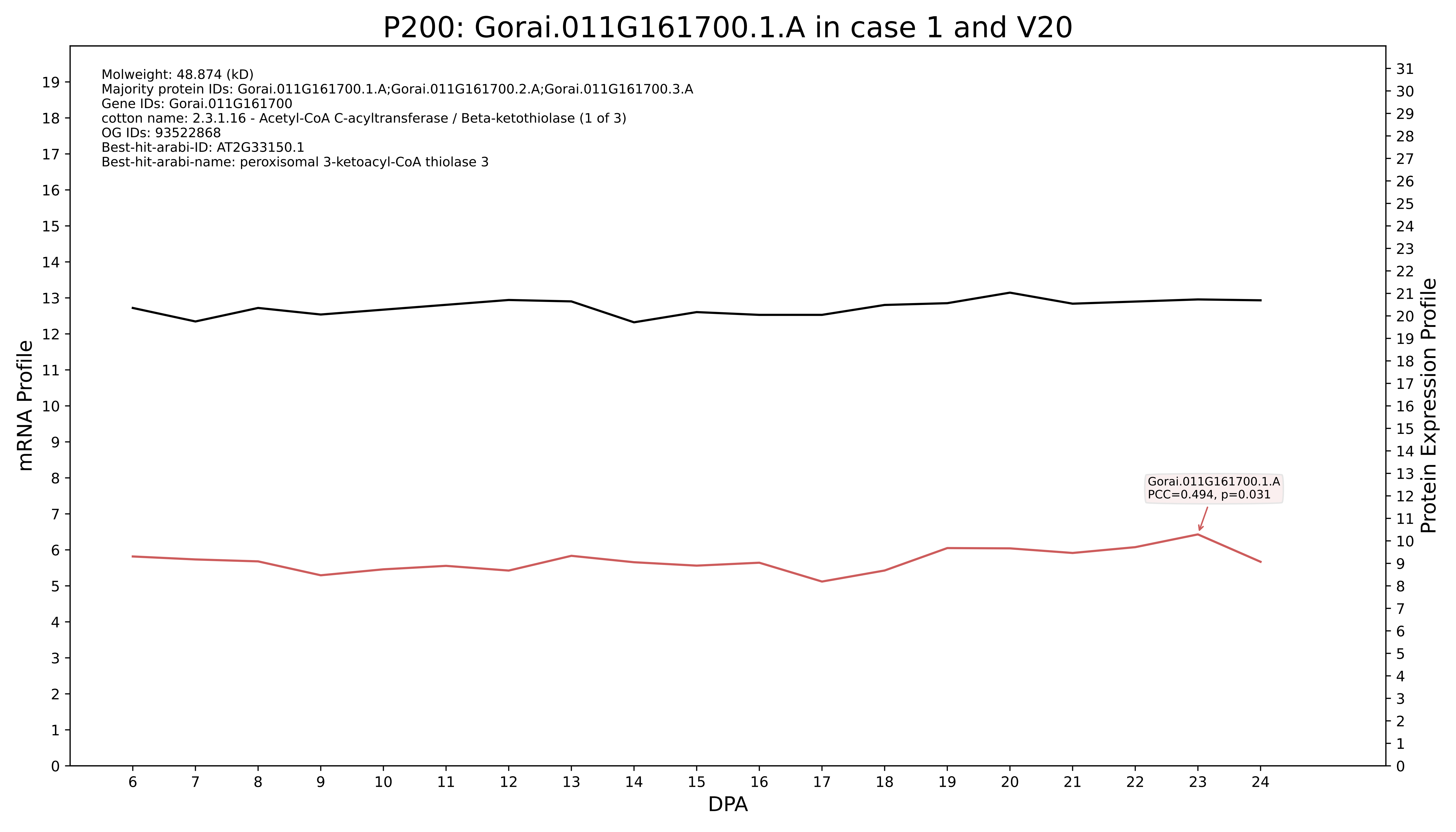
P200: Gorai.011G161700.1.A in case 1 and V20
Molweight: 48.874 (kD)
Majority protein IDs: Gorai.011G161700.1.A;Gorai.011G161700.2.A;Gorai.011G161700.3.A
Gene IDs: Gorai.011G161700
cotton name: 2.3.1.16 - Acetyl-CoA C-acyltransferase / Beta-ketothiolase (1 of 3)
OG IDs: 93522868
Best-hit-arabi-ID: AT2G33150.1
Best-hit-arabi-name: peroxisomal 3-ketoacyl-CoA thiolase 3
Gorai.011G161700.1.A
PCC=0.494, p=0.031
mRNA Profile
Protein Expression Profile
DPA

P200: Gorai.001G003700.1.D in case 1 and V20

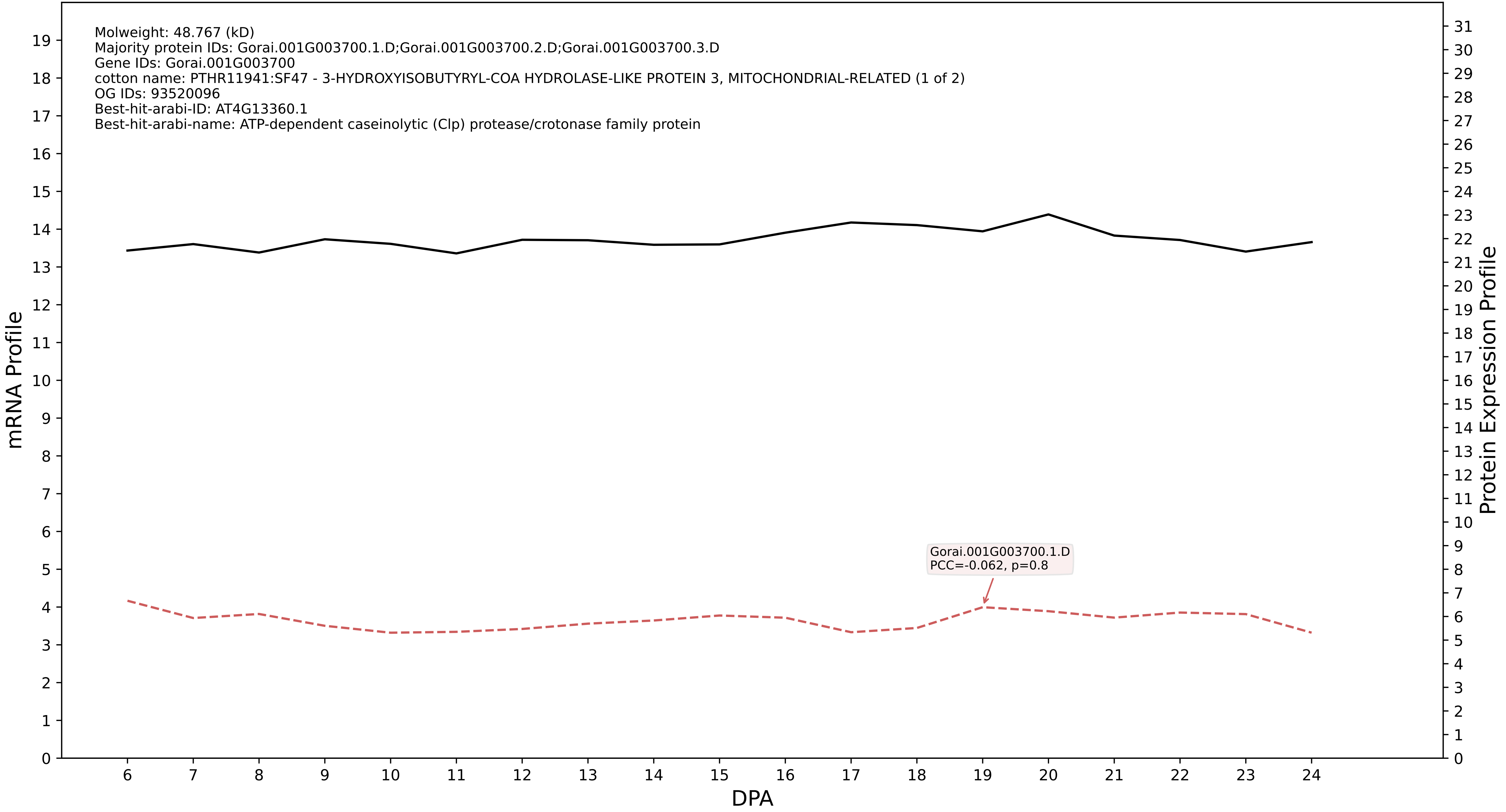

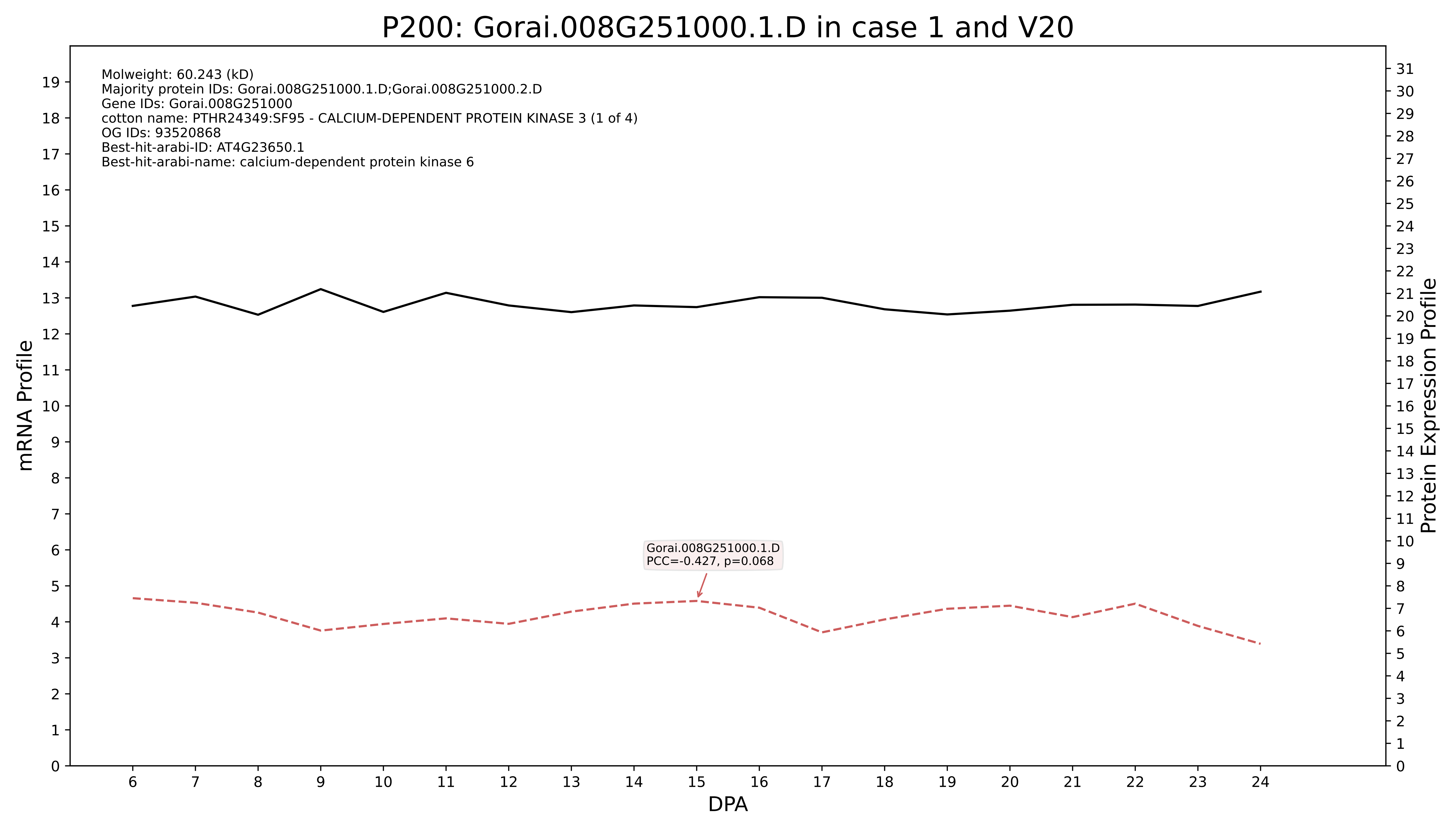
P200: Gorai.008G251000.1.D in case 1 and V20
Molweight: 60.243 (kD)
Majority protein IDs: Gorai.008G251000.1.D;Gorai.008G251000.2.D
Gene IDs: Gorai.008G251000
cotton name: PTHR24349:SF95 - CALCIUM-DEPENDENT PROTEIN KINASE 3 (1 of 4)
OG IDs: 93520868
Best-hit-arabi-ID: AT4G23650.1
Best-hit-arabi-name: calcium-dependent protein kinase 6
Gorai.008G251000.1.D
PCC=-0.427, p=0.068
mRNA Profile
Protein Expression Profile
DPA

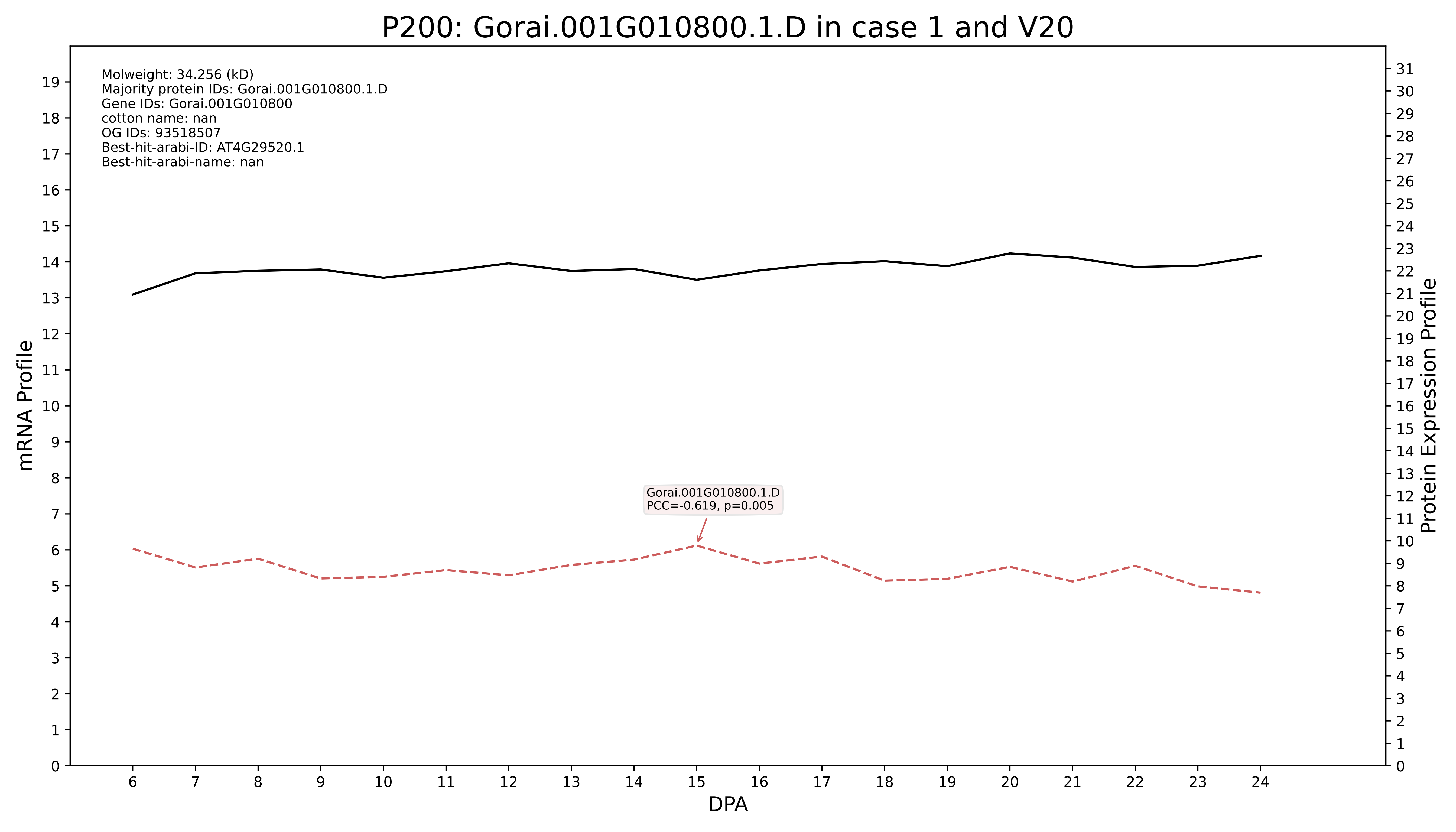

P200: Gorai.001G010800.1.D in case 1 and V20
Molweight: 34.256 (kD)
Majority protein IDs: Gorai.001G010800.1.D
Gene IDs: Gorai.001G010800
cotton name: nan
OG IDs: 93518507
Best-hit-arabi-ID: AT4G29520.1
Best-hit-arabi-name: nan
Gorai.001G010800.1.D
PCC=-0.619, p=0.005
mRNA Profile
Protein Expression Profile
DPA

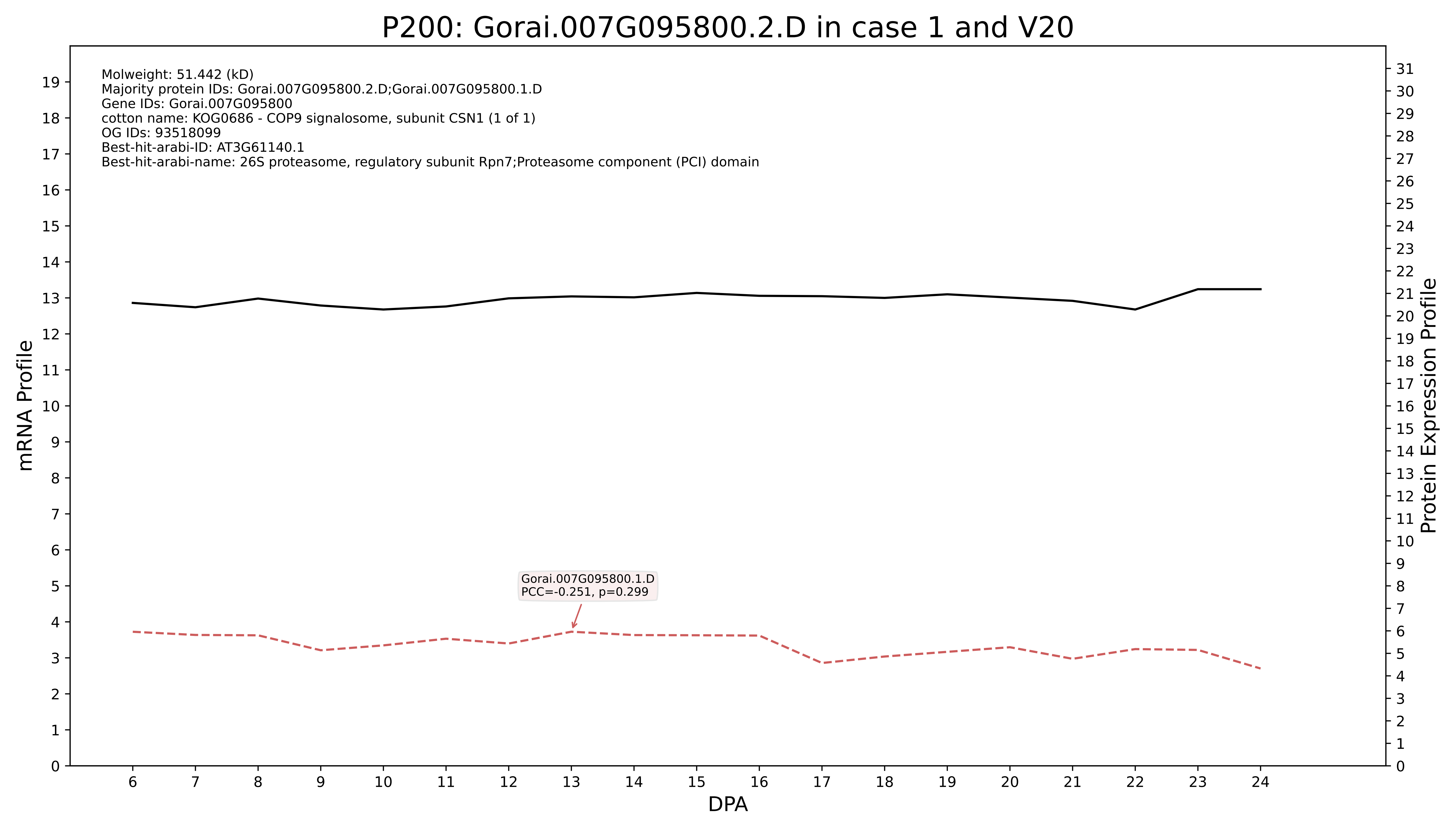
P200: Gorai.007G095800.2.D in case 1 and V20
Molweight: 51.442 (kD)
Majority protein IDs: Gorai.007G095800.2.D;Gorai.007G095800.1.D
Gene IDs: Gorai.007G095800
cotton name: KOG0686 - COP9 signalosome, subunit CSN1 (1 of 1)
OG IDs: 93518099
Best-hit-arabi-ID: AT3G61140.1
Best-hit-arabi-name: 26S proteasome, regulatory subunit Rpn7;Proteasome component (PCI) domain
Gorai.007G095800.1.D
PCC=-0.251, p=0.299
mRNA Profile
Protein Expression Profile
DPA

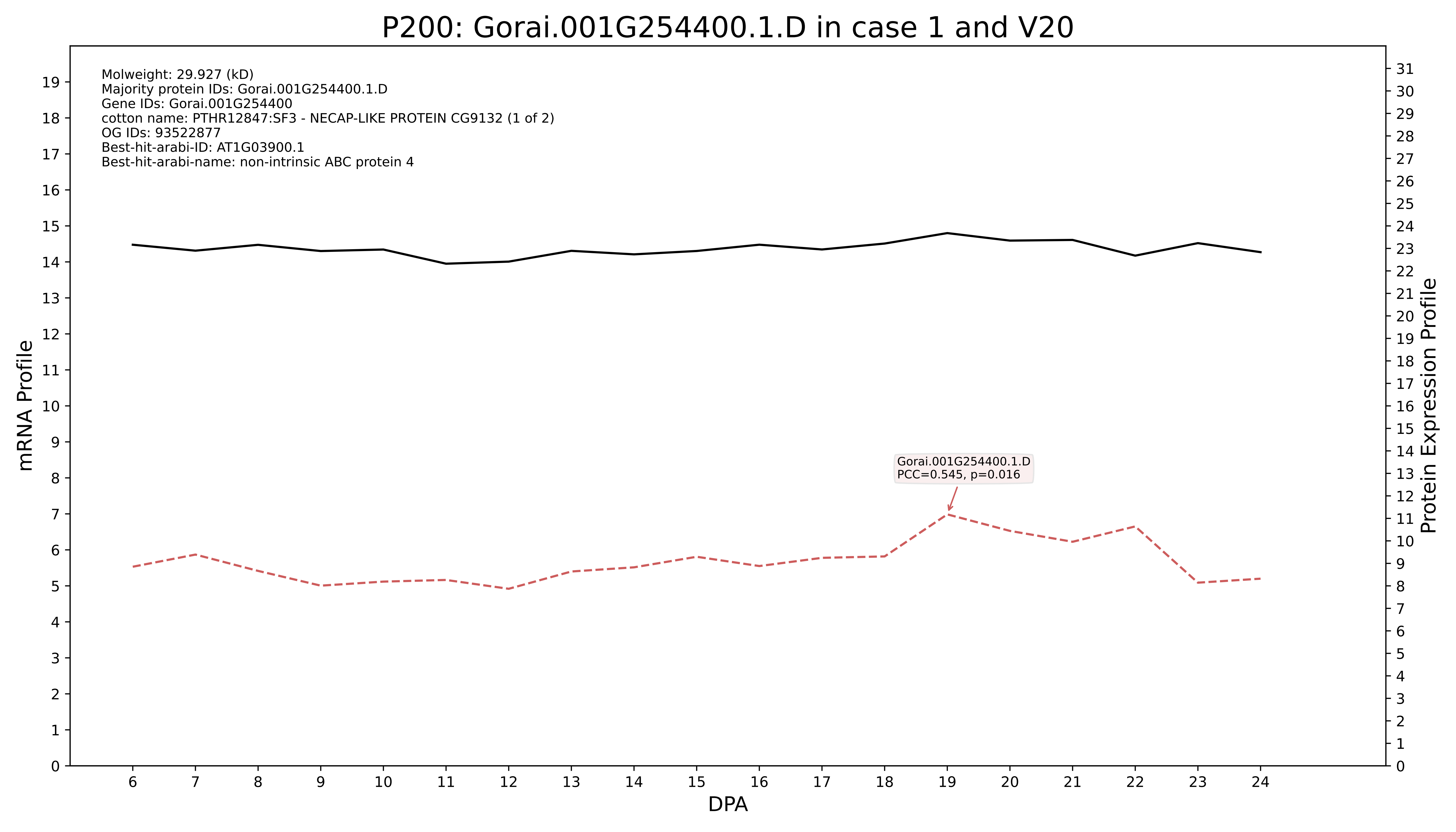

P200: Gorai.001G254400.1.D in case 1 and V20
Molweight: 29.927 (kD)
Majority protein IDs: Gorai.001G254400.1.D
Gene IDs: Gorai.001G254400
cotton name: PTHR12847:SF3 - NECAP-LIKE PROTEIN CG9132 (1 of 2)
OG IDs: 93522877
Best-hit-arabi-ID: AT1G03900.1
Best-hit-arabi-name: non-intrinsic ABC protein 4
Gorai.001G254400.1.D
PCC=0.545, p=0.016
mRNA Profile
Protein Expression Profile
DPA

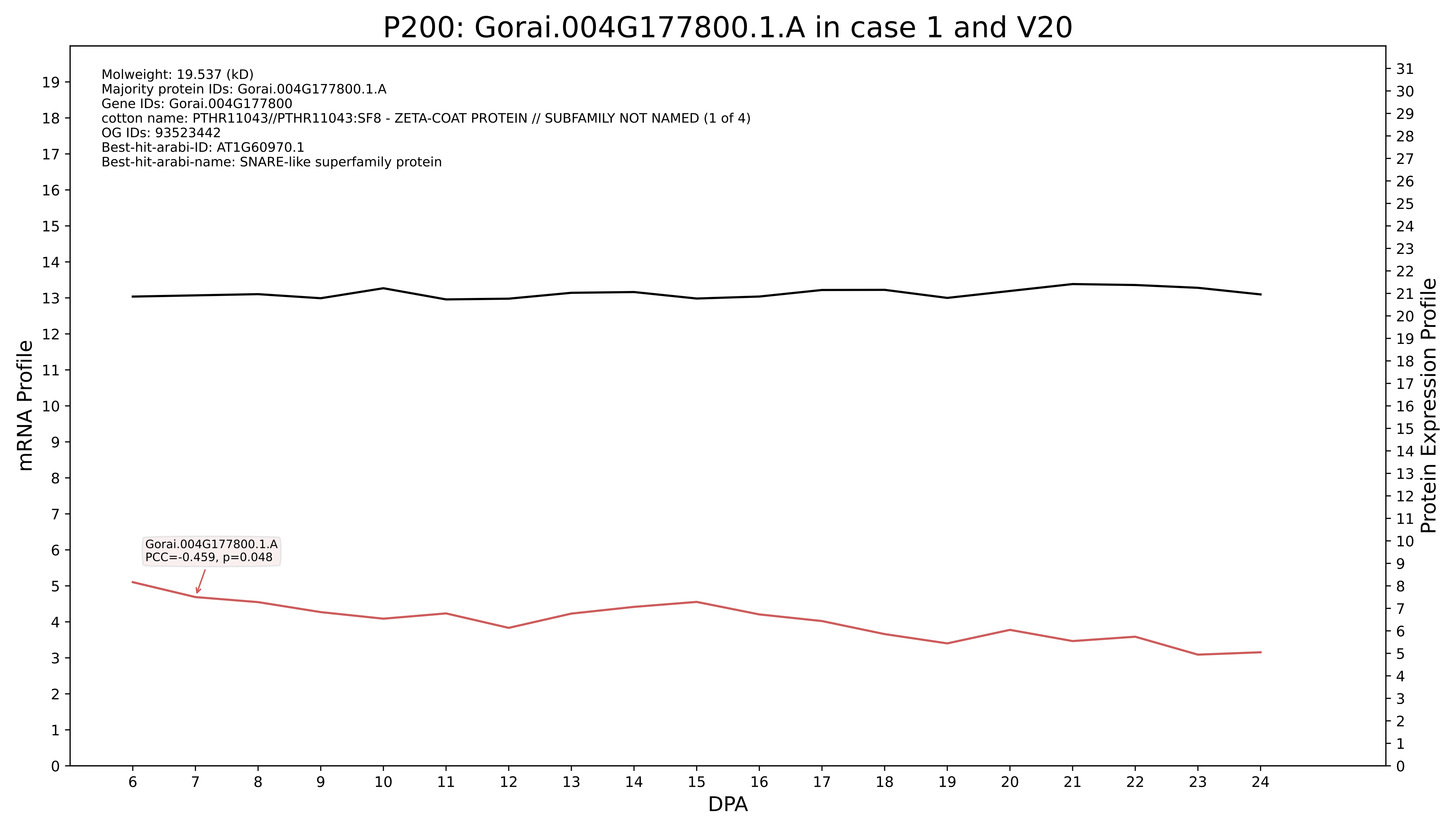
P200: Gorai.004G177800.1.A in case 1 and V20
Molweight: 19.537 (kD)
Majority protein IDs: Gorai.004G177800.1.A
Gene IDs: Gorai.004G177800
cotton name: PTHR11043//PTHR11043:SF8 - ZETA-COAT PROTEIN // SUBFAMILY NOT NAMED (1 of 4)
OG IDs: 93523442
Best-hit-arabi-ID: AT1G60970.1
Best-hit-arabi-name: SNARE-like superfamily protein
Gorai.004G177800.1.A
PCC=-0.459, p=0.048
mRNA Profile
Protein Expression Profile
DPA

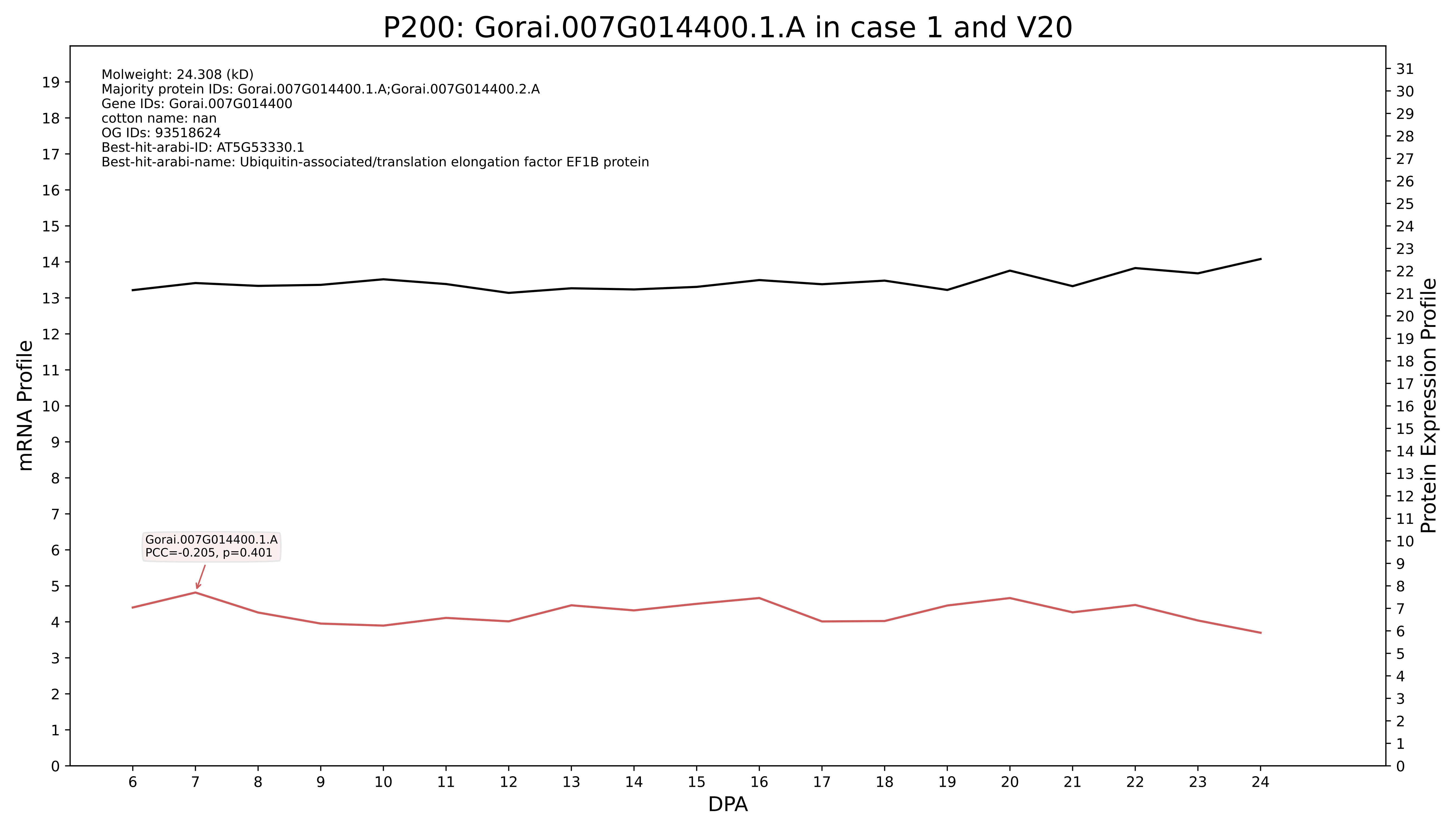

P200: Gorai.007G014400.1.A in case 1 and V20
Molweight: 24.308 (kD)
Majority protein IDs: Gorai.007G014400.1.A;Gorai.007G014400.2.A
Gene IDs: Gorai.007G014400
cotton name: nan
OG IDs: 93518624
Best-hit-arabi-ID: AT5G53330.1
Best-hit-arabi-name: Ubiquitin-associated/translation elongation factor EF1B protein
Gorai.007G014400.1.A
PCC=-0.205, p=0.401
mRNA Profile
Protein Expression Profile
DPA

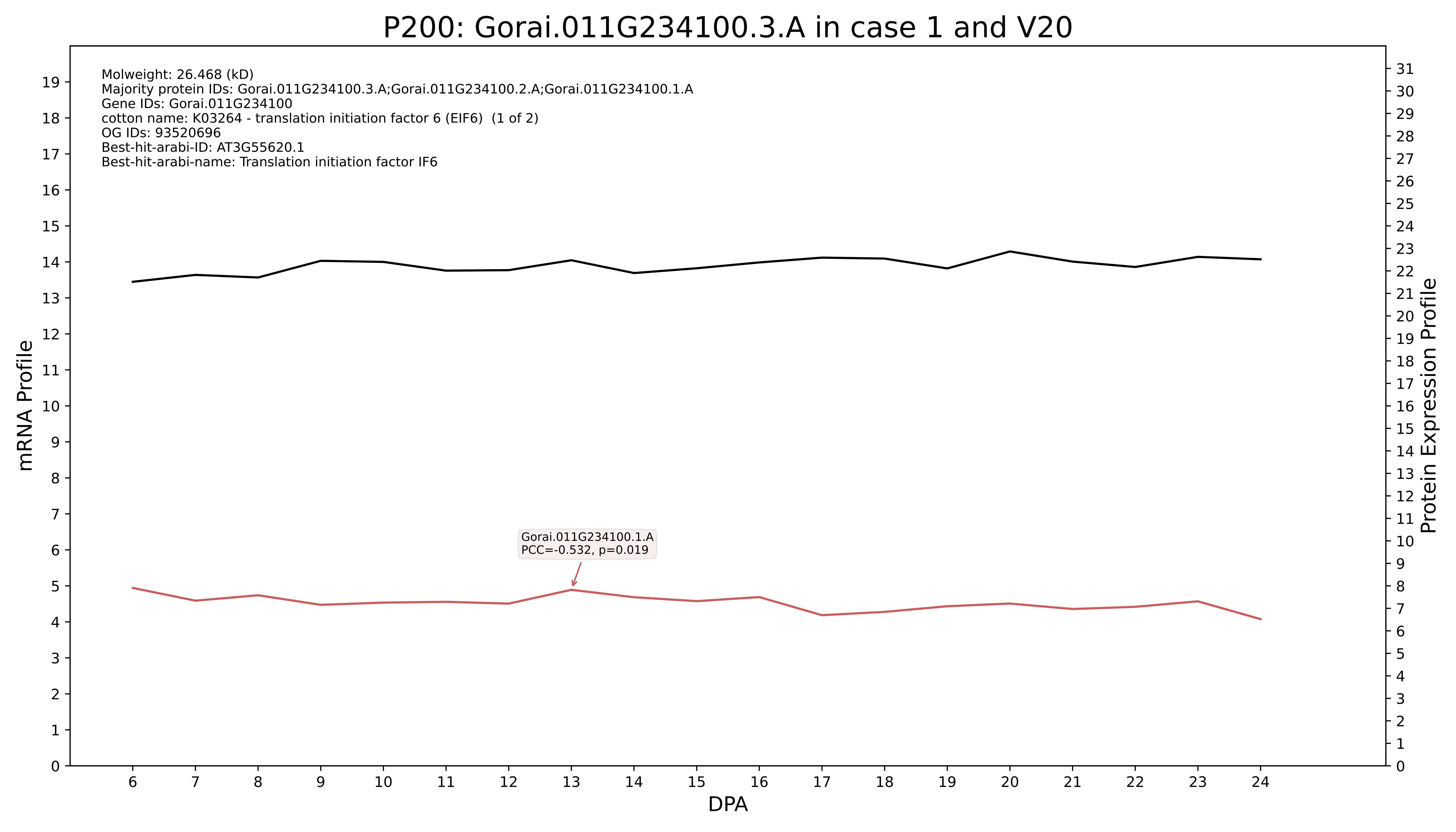

P200: Gorai.011G234100.3.A in case 1 and V20
Molweight: 26.468 (kD)
Majority protein IDs: Gorai.011G234100.3.A;Gorai.011G234100.2.A;Gorai.011G234100.1.A
Gene IDs: Gorai.011G234100
cotton name: K03264 - translation initiation factor 6 (EIF6) (1 of 2)
OG IDs: 93520696
Best-hit-arabi-ID: AT3G55620.1
Best-hit-arabi-name: Translation initiation factor IF6
Gorai.011G234100.1.A
PCC=-0.532, p=0.019
mRNA Profile
Protein Expression Profile
DPA

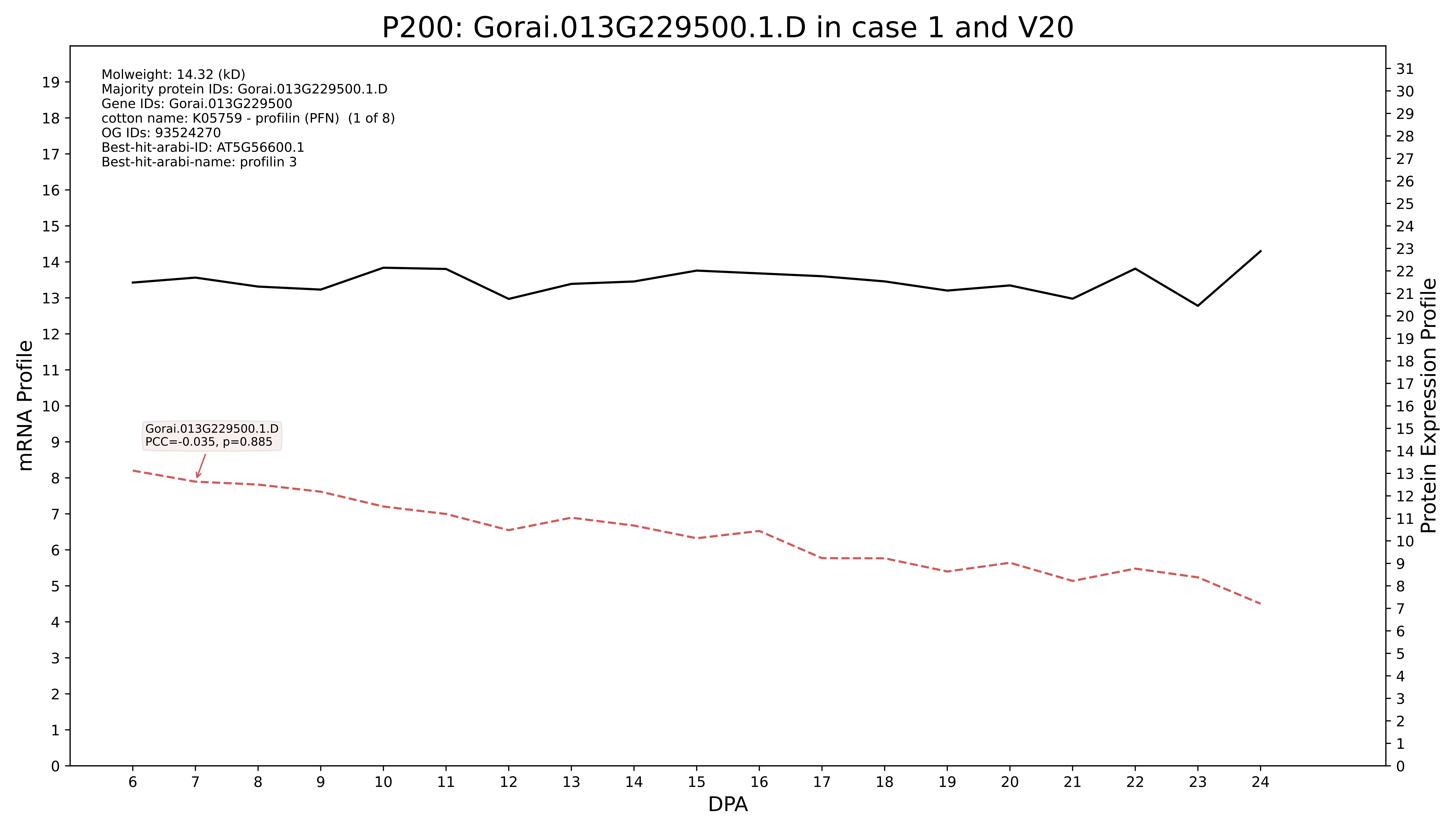
P200: Gorai.013G229500.1.D in case 1 and V20
Molweight: 14.32 (kD)
Majority protein IDs: Gorai.013G229500.1.D
Gene IDs: Gorai.013G229500
cotton name: K05759 - profilin (PFN) (1 of 8)
OG IDs: 93524270
Best-hit-arabi-ID: AT5G56600.1
Best-hit-arabi-name: profilin 3
Gorai.013G229500.1.D
PCC=-0.035, p=0.885
mRNA Profile
Protein Expression Profile
DPA
0
1
2
3
4
5
6
7
8
9
10
11
12
13
14
15
16
17
18
19
20
21
22
23
24
25
26
27
28
29
30
31

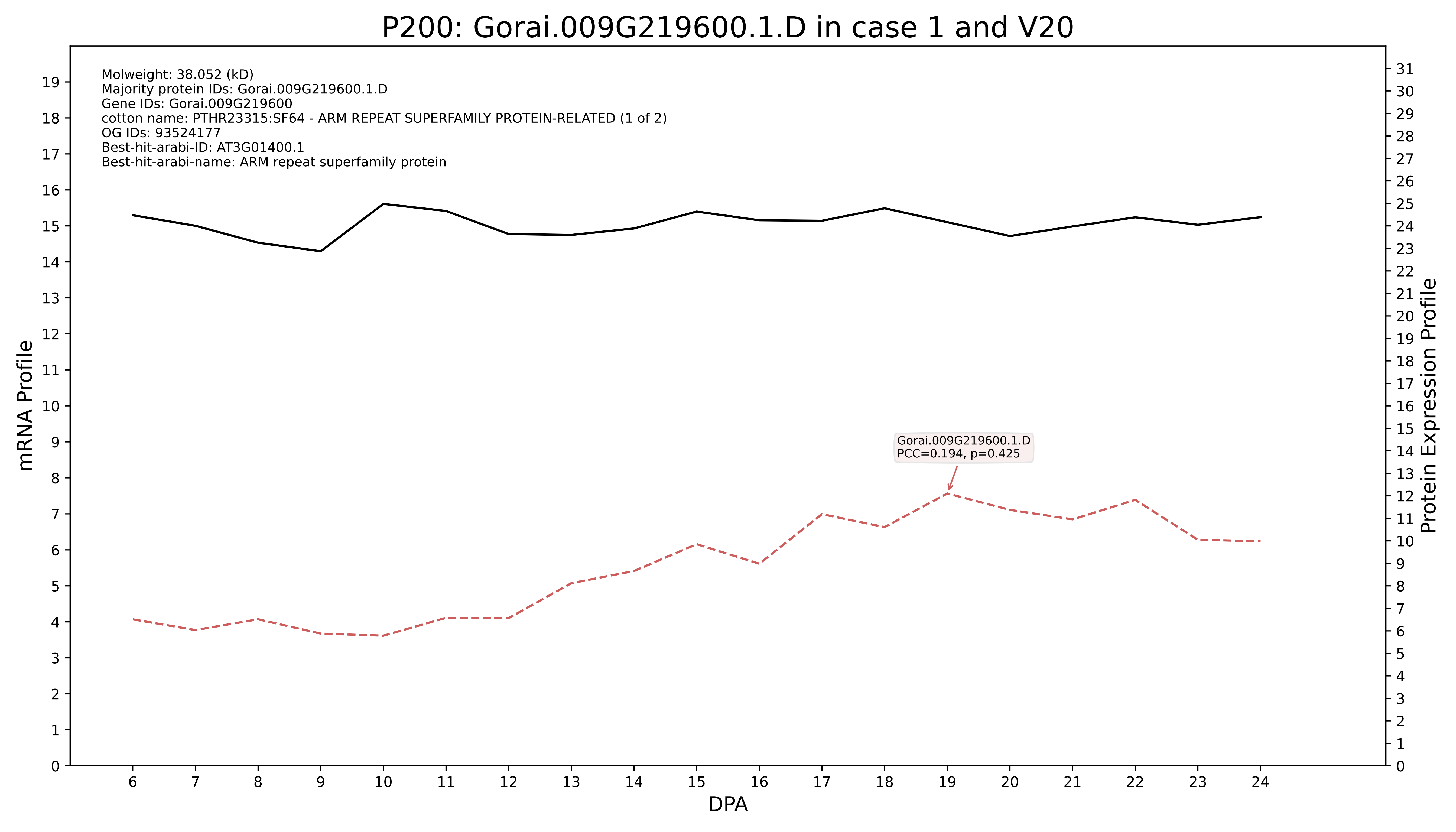
P200: Gorai.009G219600.1.D in case 1 and V20
Molweight: 38.052 (kD)
Majority protein IDs: Gorai.009G219600.1.D
Gene IDs: Gorai.009G219600
cotton name: PTHR23315:SF64 - ARM REPEAT SUPERFAMILY PROTEIN-RELATED (1 of 2)
OG IDs: 93524177
Best-hit-arabi-ID: AT3G01400.1
Best-hit-arabi-name: ARM repeat superfamily protein
Gorai.009G219600.1.D
PCC=0.194, p=0.425
mRNA Profile
Protein Expression Profile
DPA

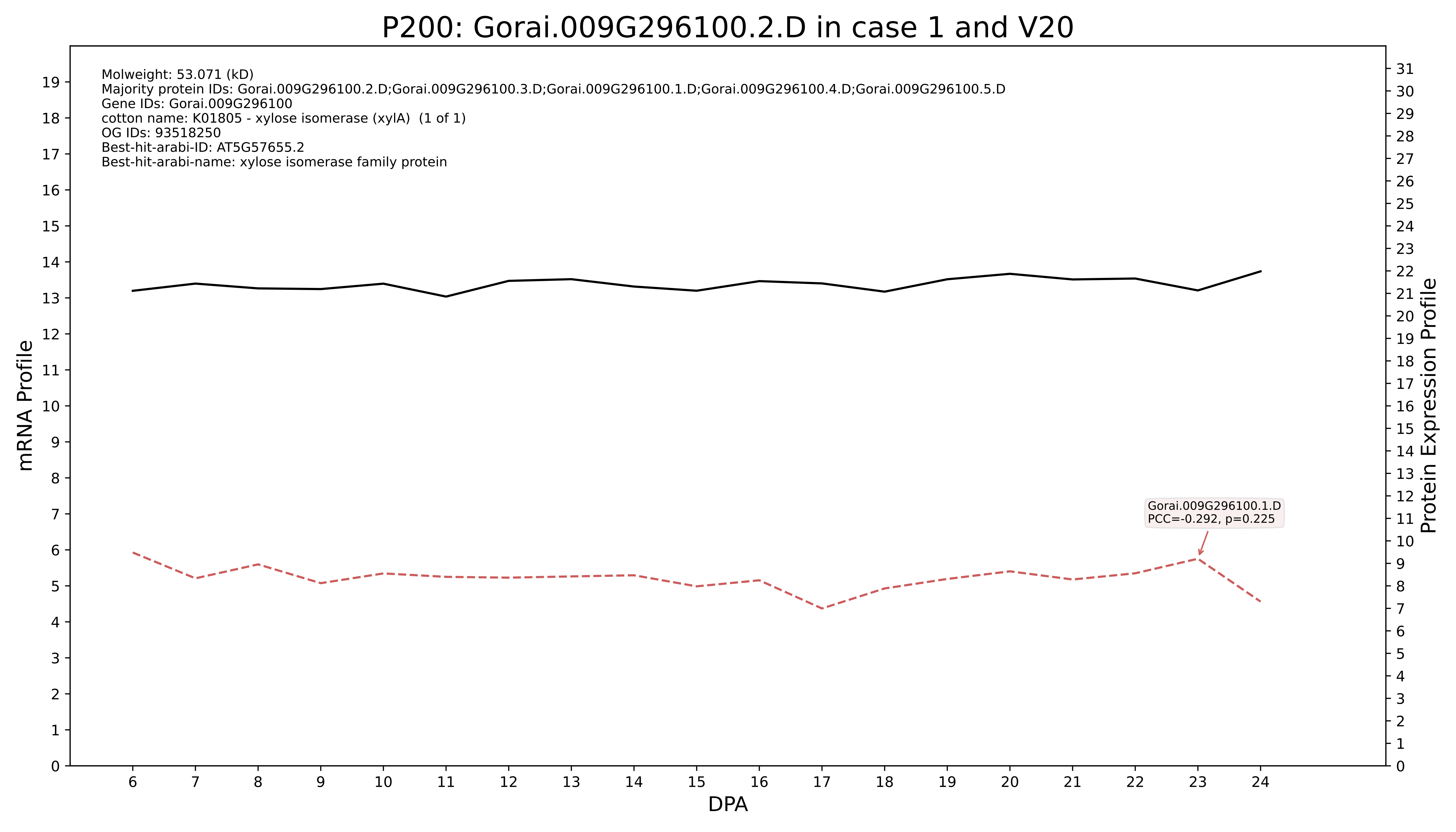
P200: Gorai.009G296100.2.D in case 1 and V20
Molweight: 53.071 (kD)
Majority protein IDs: Gorai.009G296100.2.D;Gorai.009G296100.3.D;Gorai.009G296100.1.D;Gorai.009G296100.4.D;Gorai.009G296100.5.D
Gene IDs: Gorai.009G296100
cotton name: K01805 - xylose isomerase (xylA) (1 of 1)
OG IDs: 93518250
Best-hit-arabi-ID: AT5G57655.2
Best-hit-arabi-name: xylose isomerase family protein
Gorai.009G296100.1.D
PCC=-0.292, p=0.225
mRNA Profile
Protein Expression Profile
DPA
0
1
2
3
4
5
6
7
8
9
10
11
12
13
14
15
16
17
18
19
20
21
22
23
24
25
26
27
28
29
30
31

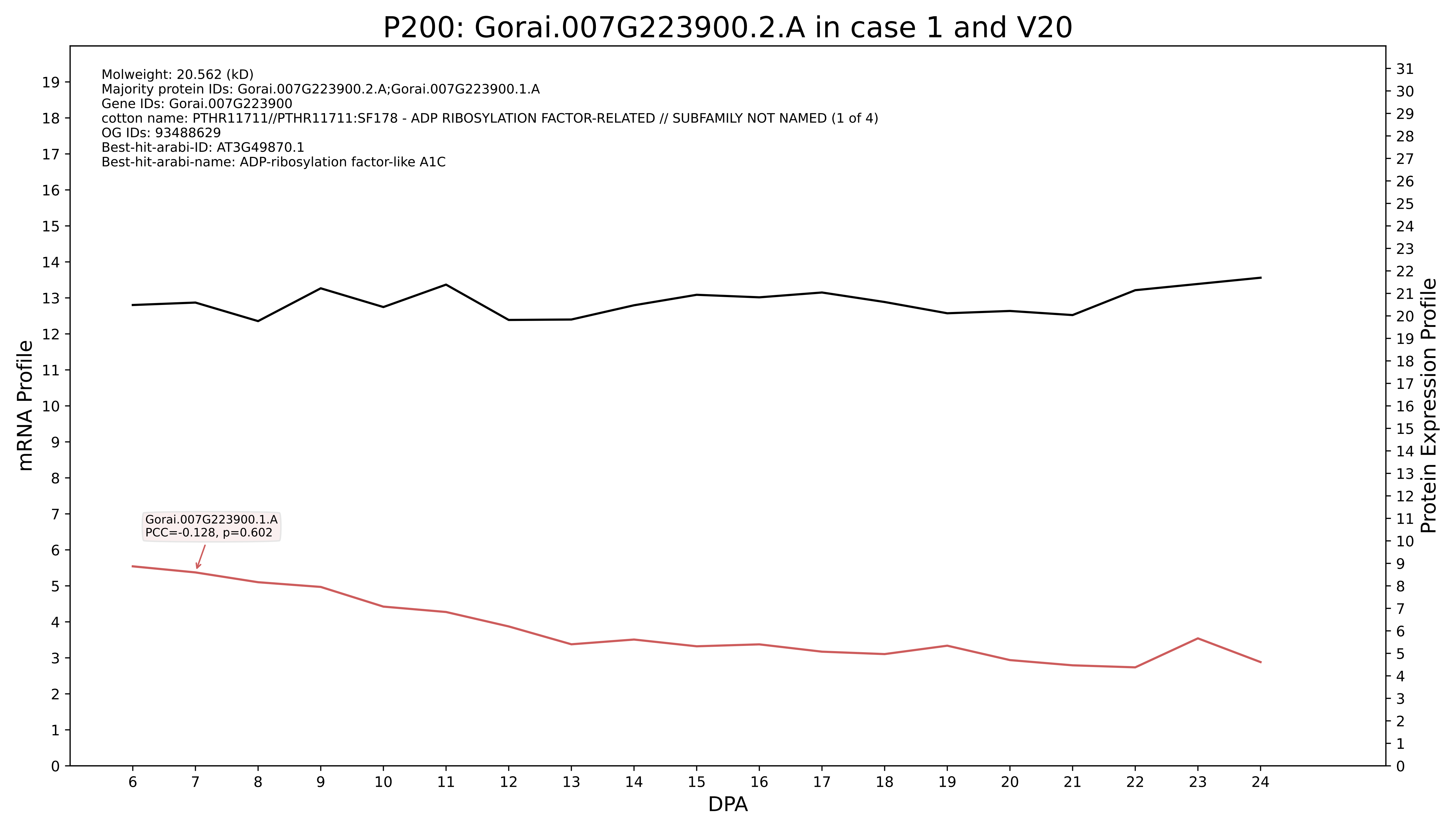
P200: Gorai.007G223900.2.A in case 1 and V20
Molweight: 20.562 (kD)
Majority protein IDs: Gorai.007G223900.2.A;Gorai.007G223900.1.A
Gene IDs: Gorai.007G223900
cotton name: PTHR11711//PTHR11711:SF178 - ADP RIBOSYLATION FACTOR-RELATED // SUBFAMILY NOT NAMED (1 of 4)
OG IDs: 93488629
Best-hit-arabi-ID: AT3G49870.1
Best-hit-arabi-name: ADP-ribosylation factor-like A1C
Gorai.007G223900.1.A
PCC=-0.128, p=0.602
mRNA Profile
Protein Expression Profile
DPA
0
1
2
3
4
5
6
7
8
9
10
11
12
13
14
15
16
17
18
19
20
21
22
23
24
25
26
27
28
29
30
31

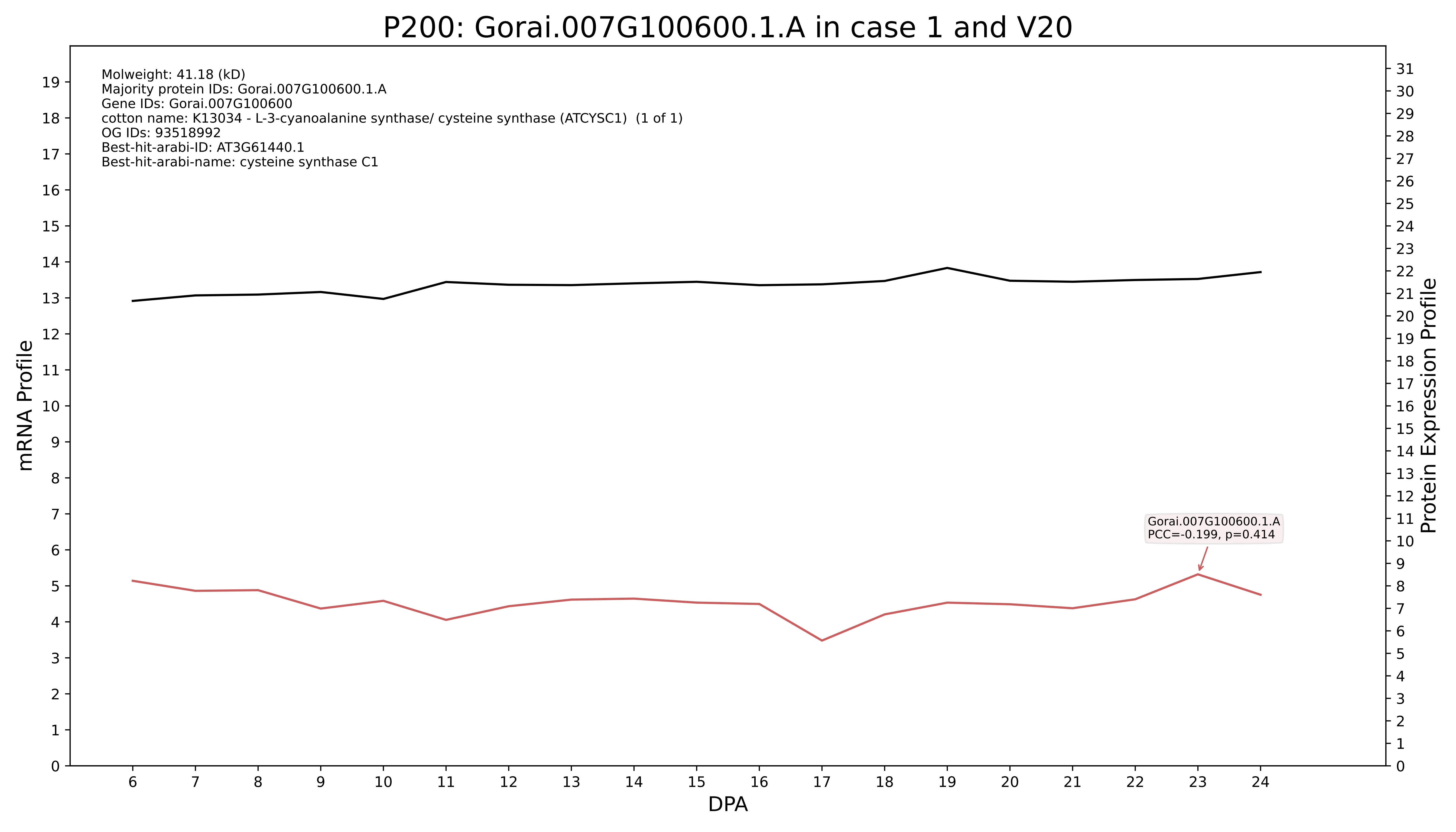
P200: Gorai.007G100600.1.A in case 1 and V20
Molweight: 41.18 (kD)
Majority protein IDs: Gorai.007G100600.1.A
Gene IDs: Gorai.007G100600
cotton name: K13034 - L-3-cyanoalanine synthase/ cysteine synthase (ATCYSC1) (1 of 1)
OG IDs: 93518992
Best-hit-arabi-ID: AT3G61440.1
Best-hit-arabi-name: cysteine synthase C1
Gorai.007G100600.1.A
PCC=-0.199, p=0.414
mRNA Profile
Protein Expression Profile
DPA

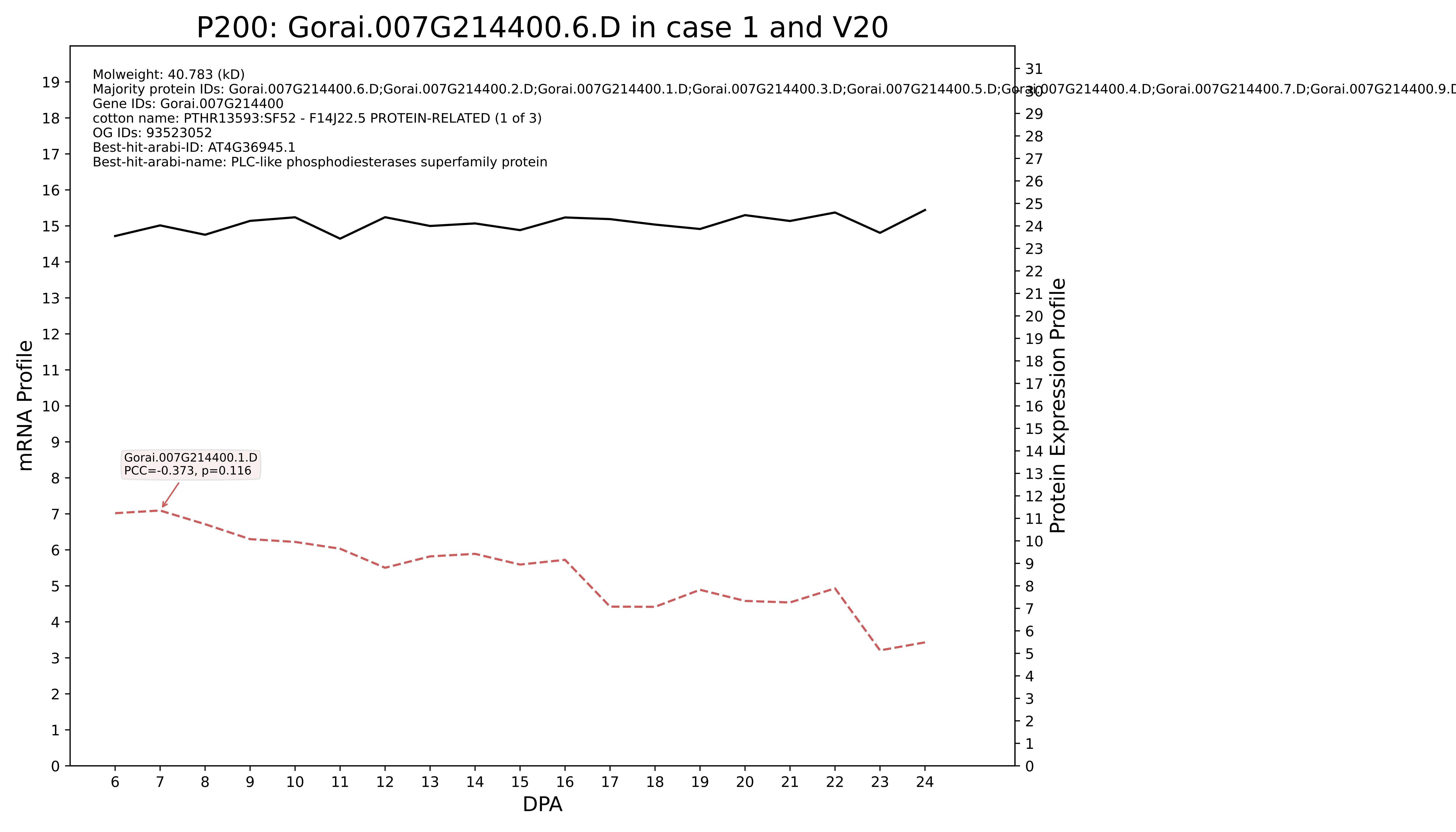
P200: Gorai.007G214400.6.D in case 1 and V20
Molweight: 40.783 (kD)
Majority protein IDs: Gorai.007G214400.6.D;Gorai.007G214400.2.D;Gorai.007G214400.1.D;Gorai.007G214400.3.D;Gorai.007G214400.5.D;Gorai.007G214400.4.D;Gorai.007G214400.7.D;Gorai.007G214400.9.D
Gene IDs: Gorai.007G214400
cotton name: PTHR13593:SF52 - F14J22.5 PROTEIN-RELATED (1 of 3)
OG IDs: 93523052
Best-hit-arabi-ID: AT4G36945.1
Best-hit-arabi-name: PLC-like phosphodiesterases superfamily protein
Gorai.007G214400.1.D
PCC=-0.373, p=0.116
mRNA Profile
Protein Expression Profile
DPA

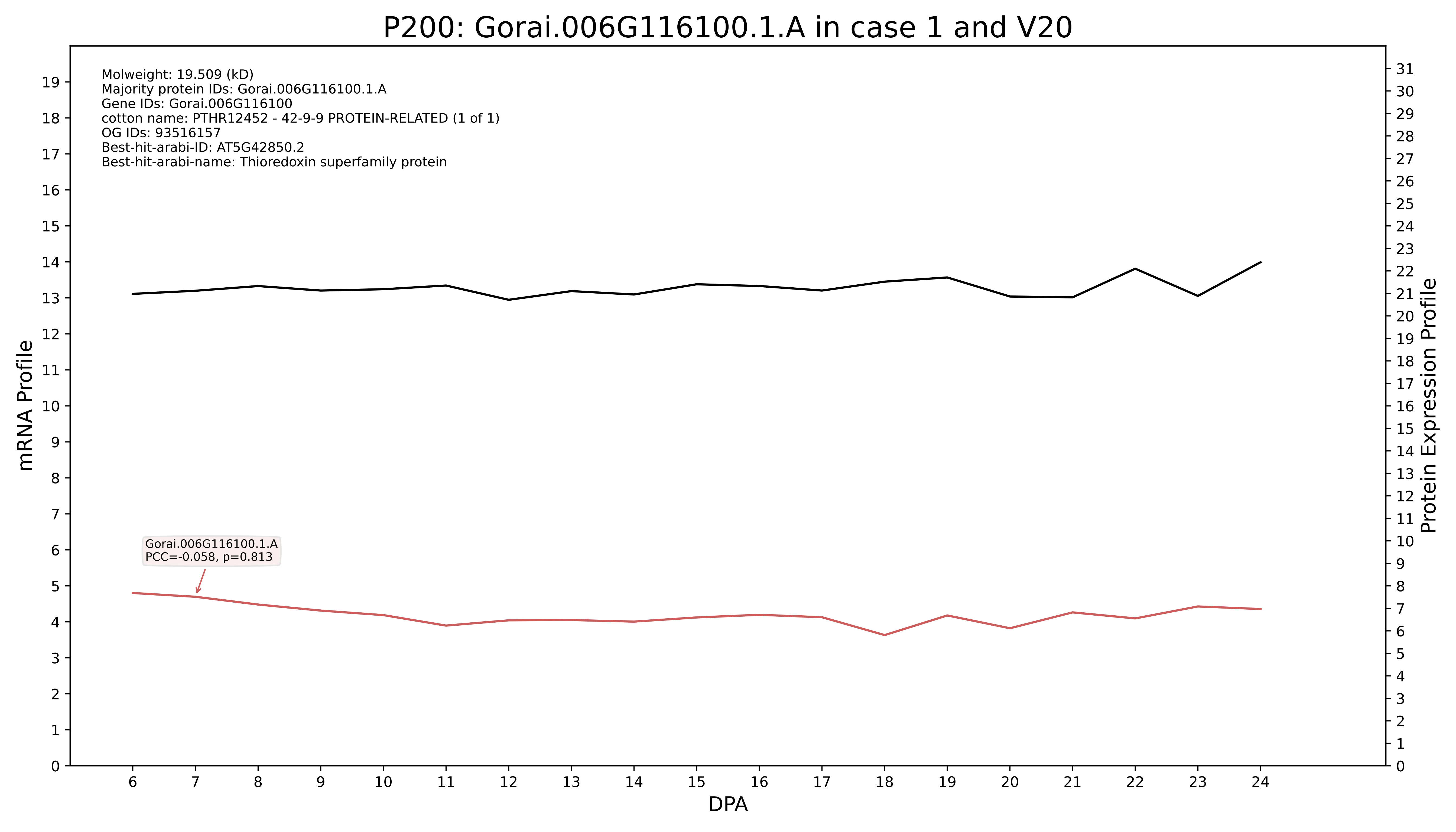

P200: Gorai.006G116100.1.A in case 1 and V20
Molweight: 19.509 (kD)
Majority protein IDs: Gorai.006G116100.1.A
Gene IDs: Gorai.006G116100
cotton name: PTHR12452 - 42-9-9 PROTEIN-RELATED (1 of 1)
OG IDs: 93516157
Best-hit-arabi-ID: AT5G42850.2
Best-hit-arabi-name: Thioredoxin superfamily protein
Gorai.006G116100.1.A
PCC=-0.058, p=0.813
mRNA Profile
Protein Expression Profile
DPA

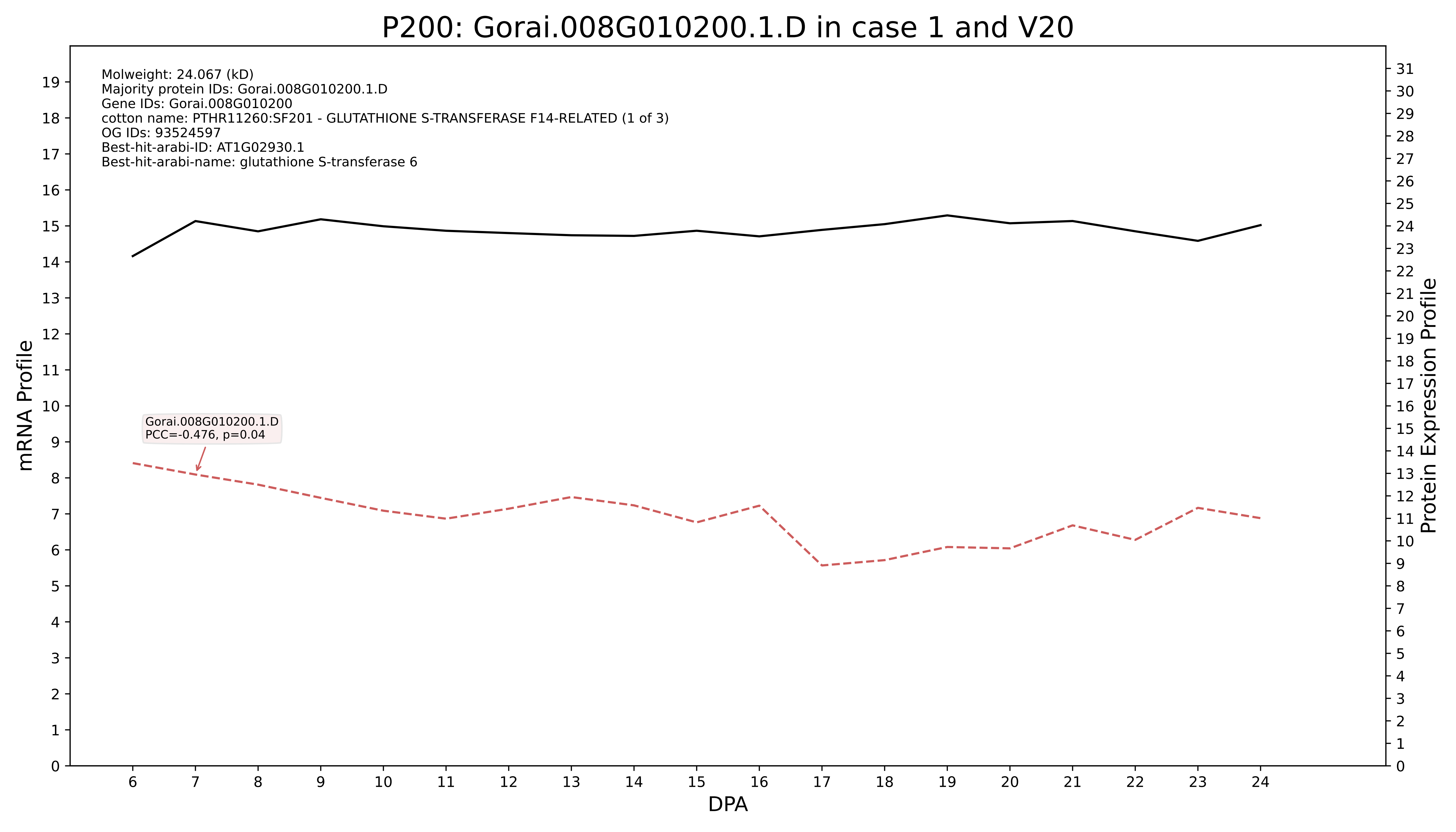
P200: Gorai.008G010200.1.D in case 1 and V20
Molweight: 24.067 (kD)
Majority protein IDs: Gorai.008G010200.1.D
Gene IDs: Gorai.008G010200
cotton name: PTHR11260:SF201 - GLUTATHIONE S-TRANSFERASE F14-RELATED (1 of 3)
OG IDs: 93524597
Best-hit-arabi-ID: AT1G02930.1
Best-hit-arabi-name: glutathione S-transferase 6
Gorai.008G010200.1.D
PCC=-0.476, p=0.04
mRNA Profile
Protein Expression Profile
DPA

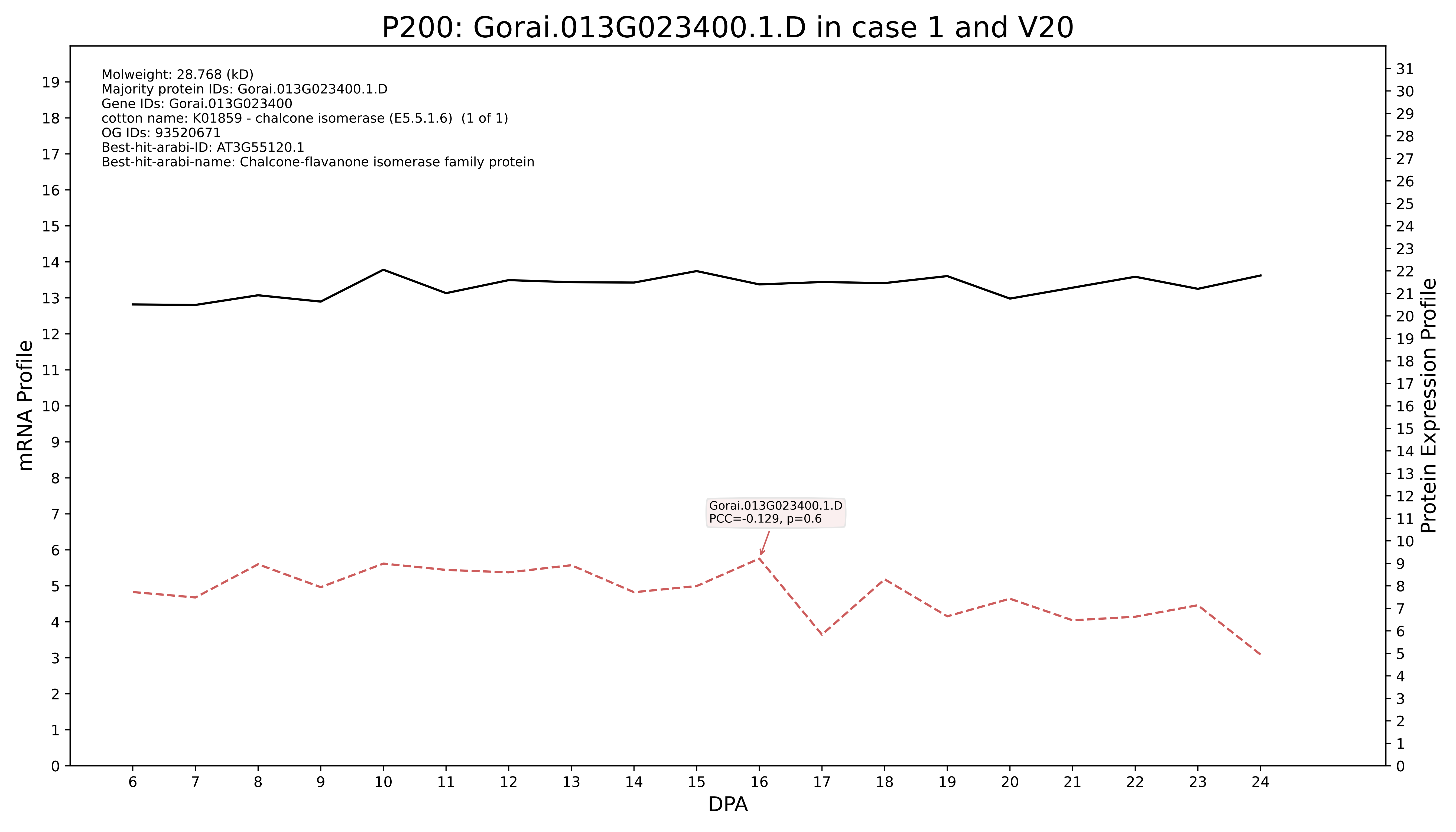
P200: Gorai.013G023400.1.D in case 1 and V20
Molweight: 28.768 (kD)
Majority protein IDs: Gorai.013G023400.1.D
Gene IDs: Gorai.013G023400
cotton name: K01859 - chalcone isomerase (E5.5.1.6) (1 of 1)
OG IDs: 93520671
Best-hit-arabi-ID: AT3G55120.1
Best-hit-arabi-name: Chalcone-flavanone isomerase family protein
Gorai.013G023400.1.D
PCC=-0.129, p=0.6
mRNA Profile
Protein Expression Profile
DPA

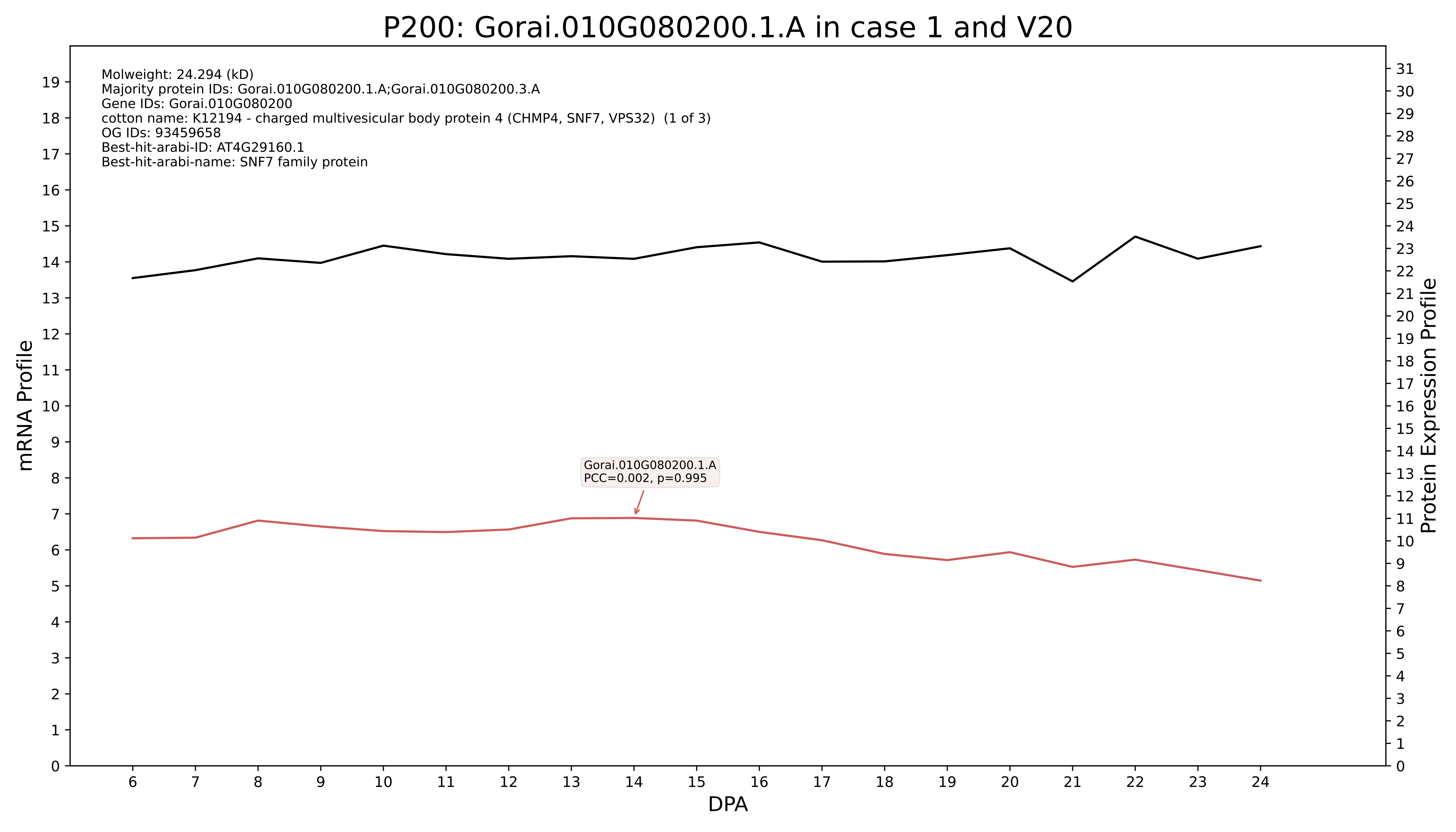

P200: Gorai.010G080200.1.A in case 1 and V20
Molweight: 24.294 (kD)
Majority protein IDs: Gorai.010G080200.1.A;Gorai.010G080200.3.A
Gene IDs: Gorai.010G080200
cotton name: K12194 - charged multivesicular body protein 4 (CHMP4, SNF7, VPS32) (1 of 3)
OG IDs: 93459658
Best-hit-arabi-ID: AT4G29160.1
Best-hit-arabi-name: SNF7 family protein
Gorai.010G080200.1.A
PCC=0.002, p=0.995
mRNA Profile
Protein Expression Profile
DPA

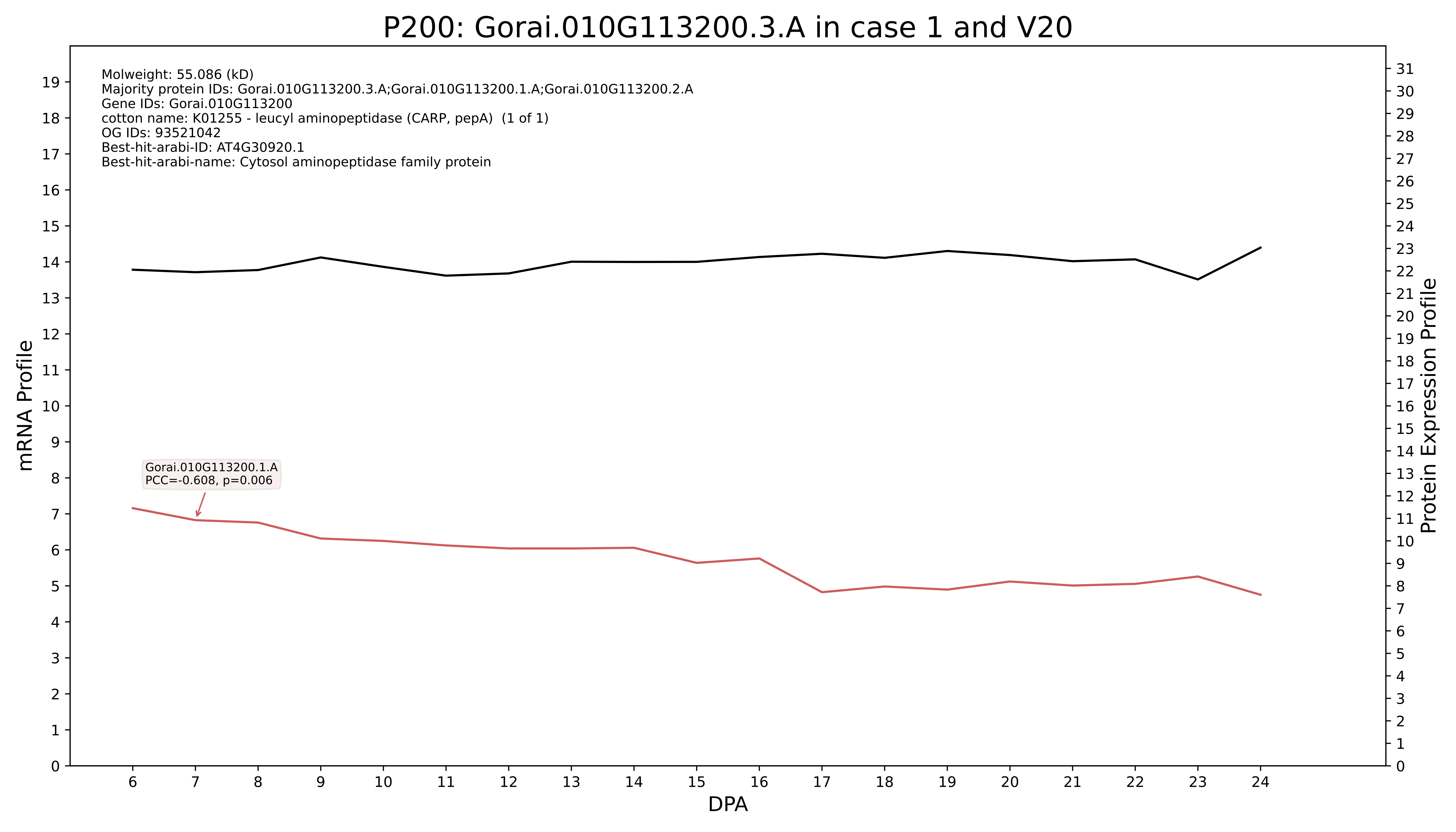

P200: Gorai.010G113200.3.A in case 1 and V20
Molweight: 55.086 (kD)
Majority protein IDs: Gorai.010G113200.3.A;Gorai.010G113200.1.A;Gorai.010G113200.2.A
Gene IDs: Gorai.010G113200
cotton name: K01255 - leucyl aminopeptidase (CARP, pepA) (1 of 1)
OG IDs: 93521042
Best-hit-arabi-ID: AT4G30920.1
Best-hit-arabi-name: Cytosol aminopeptidase family protein
Gorai.010G113200.1.A
PCC=-0.608, p=0.006
mRNA Profile
Protein Expression Profile
DPA

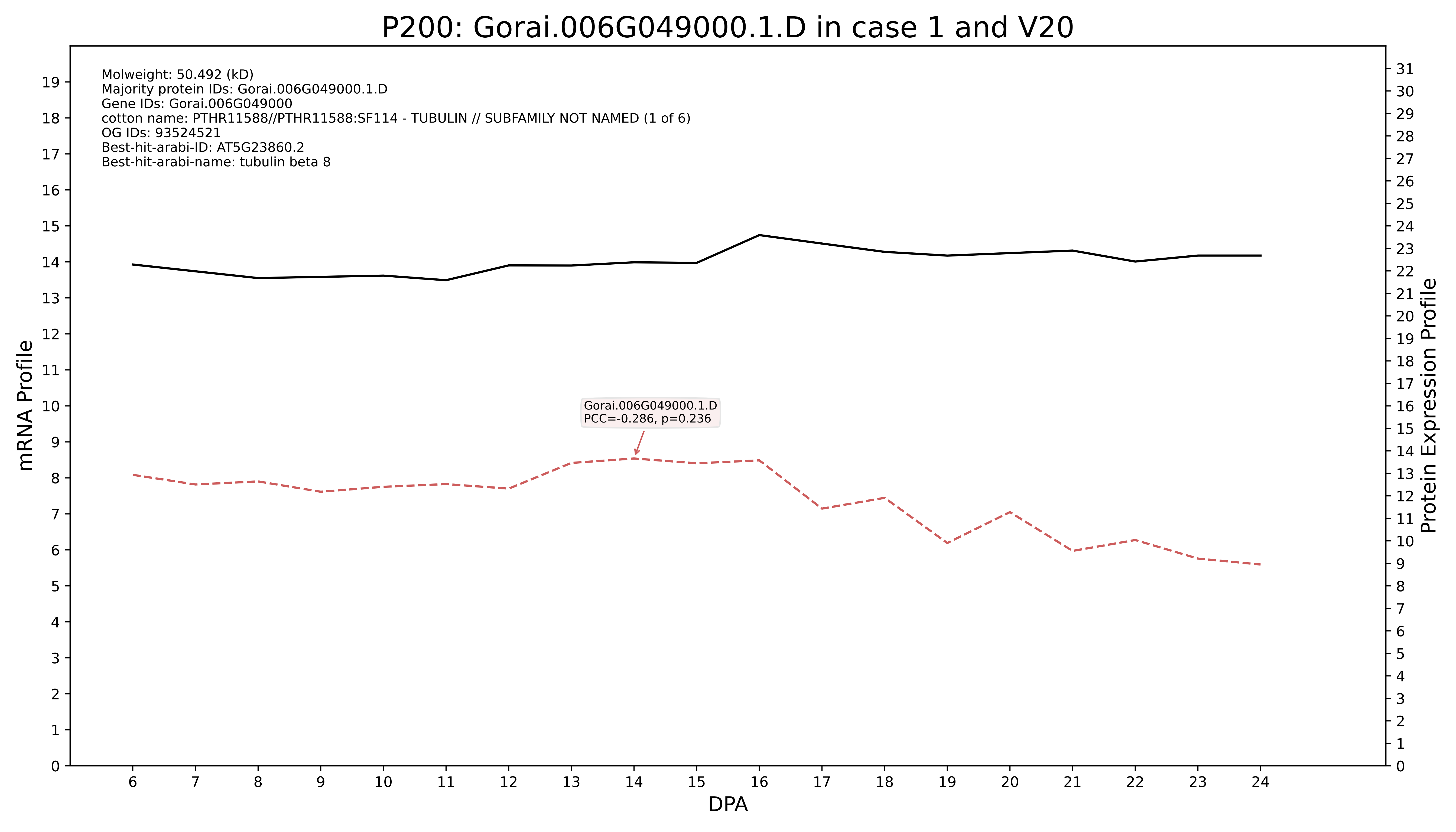
P200: Gorai.006G049000.1.D in case 1 and V20
Molweight: 50.492 (kD)
Majority protein IDs: Gorai.006G049000.1.D
Gene IDs: Gorai.006G049000
cotton name: PTHR11588//PTHR11588:SF114 - TUBULIN // SUBFAMILY NOT NAMED (1 of 6)
OG IDs: 93524521
Best-hit-arabi-ID: AT5G23860.2
Best-hit-arabi-name: tubulin beta 8
Gorai.006G049000.1.D
PCC=-0.286, p=0.236
mRNA Profile
Protein Expression Profile
DPA
6
7
8
9
10
11
12
13
14
15
16
17
18
19
20
21
22
23
24

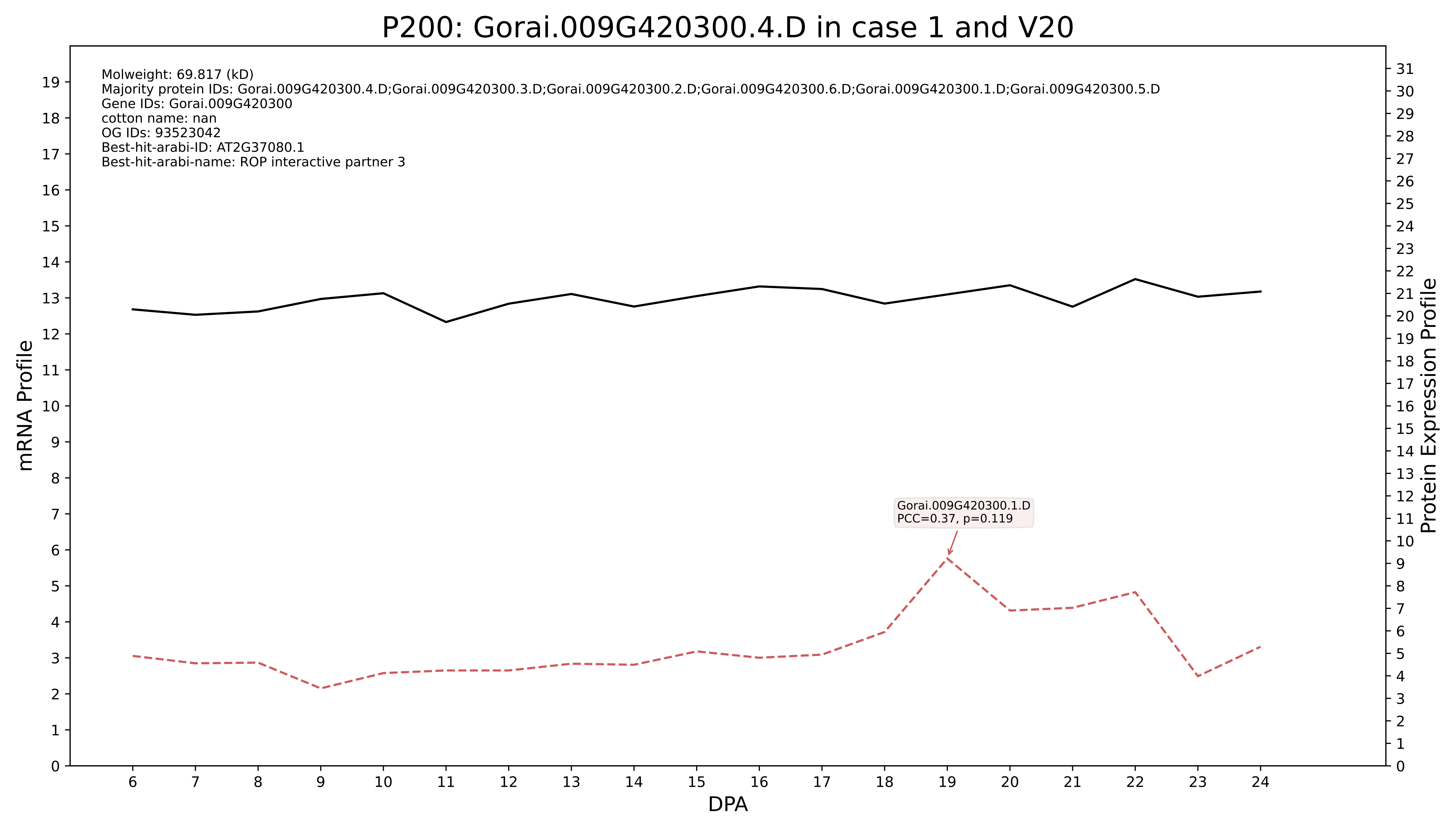
P200: Gorai.009G420300.4.D in case 1 and V20
Molweight: 69.817 (kD)
Majority protein IDs: Gorai.009G420300.4.D;Gorai.009G420300.3.D;Gorai.009G420300.2.D;Gorai.009G420300.6.D;Gorai.009G420300.1.D;Gorai.009G420300.5.D
Gene IDs: Gorai.009G420300
cotton name: nan
OG IDs: 93523042
Best-hit-arabi-ID: AT2G37080.1
Best-hit-arabi-name: ROP interactive partner 3
Gorai.009G420300.1.D
PCC=0.37, p=0.119
mRNA Profile
Protein Expression Profile
DPA

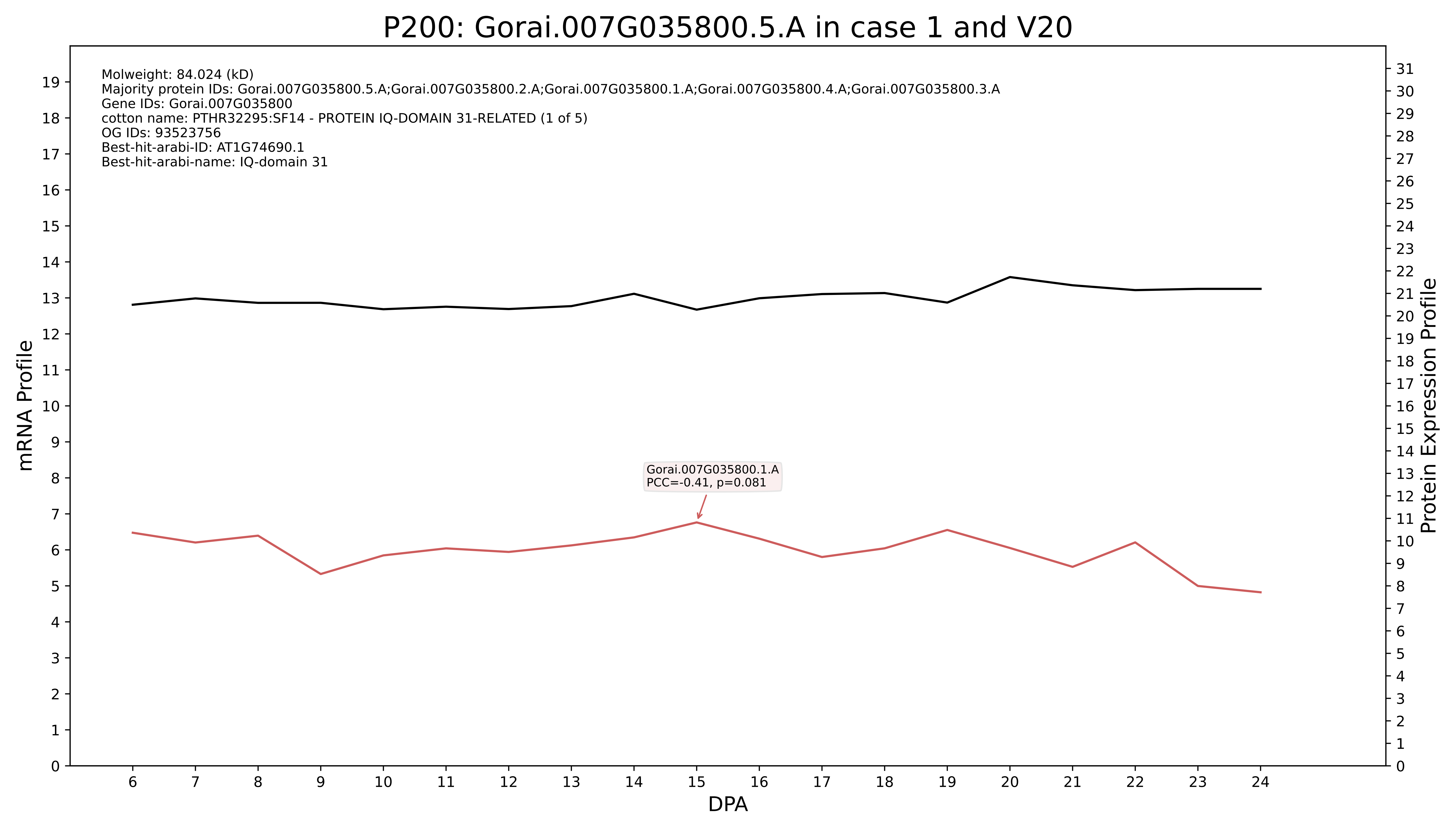
P200: Gorai.007G035800.5.A in case 1 and V20
Molweight: 84.024 (kD)
Majority protein IDs: Gorai.007G035800.5.A;Gorai.007G035800.2.A;Gorai.007G035800.1.A;Gorai.007G035800.4.A;Gorai.007G035800.3.A
Gene IDs: Gorai.007G035800
cotton name: PTHR32295:SF14 - PROTEIN IQ-DOMAIN 31-RELATED (1 of 5)
OG IDs: 93523756
Best-hit-arabi-ID: AT1G74690.1
Best-hit-arabi-name: IQ-domain 31
Gorai.007G035800.1.A
PCC=-0.41, p=0.081
mRNA Profile
Protein Expression Profile
DPA
6
7
8
9
10
11
12
13
14
15
16
17
18
19
20
21
22
23
24

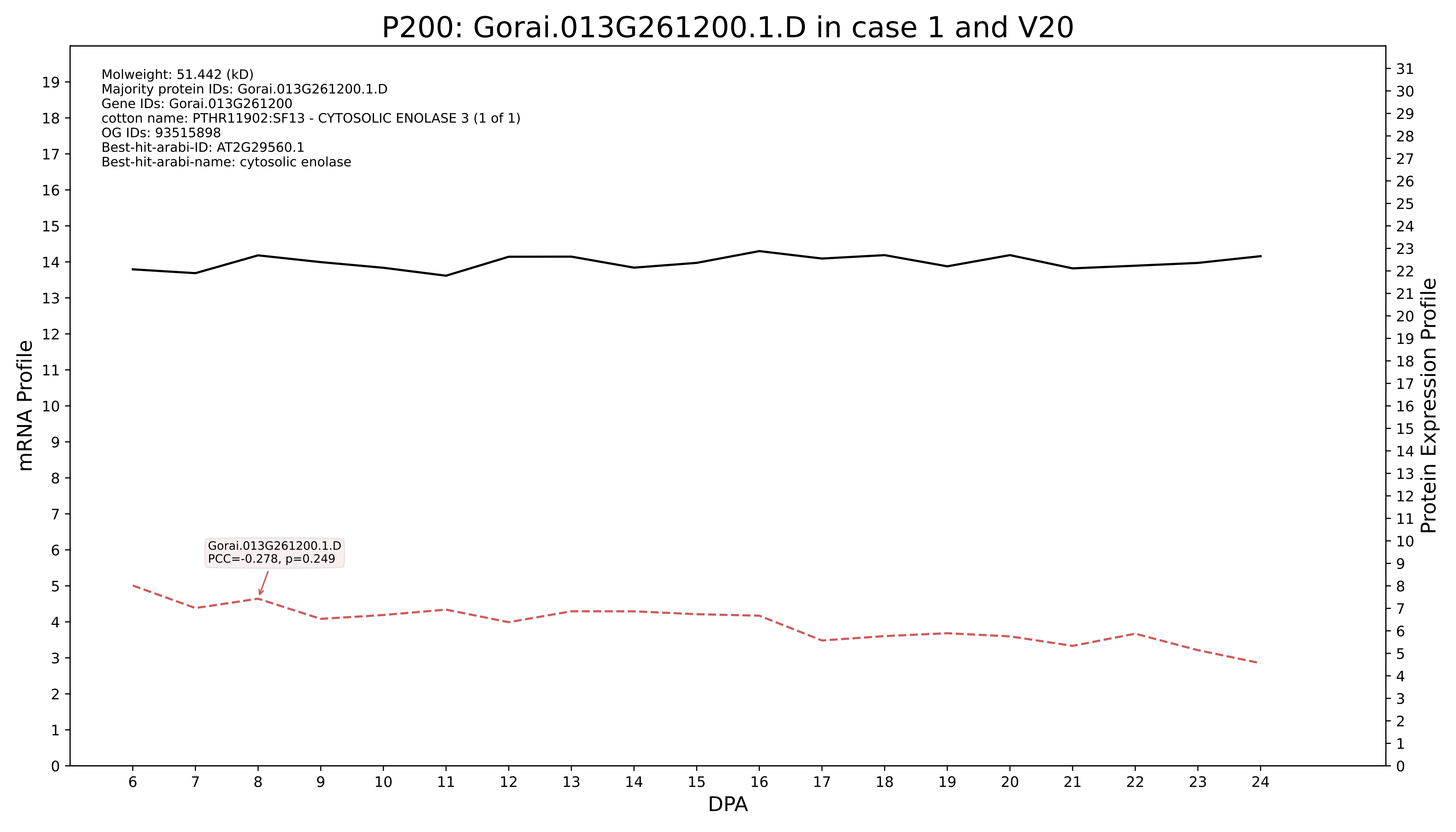

P200: Gorai.013G261200.1.D in case 1 and V20
Molweight: 51.442 (kD)
Majority protein IDs: Gorai.013G261200.1.D
Gene IDs: Gorai.013G261200
cotton name: PTHR11902:SF13 - CYTOSOLIC ENOLASE 3 (1 of 1)
OG IDs: 93515898
Best-hit-arabi-ID: AT2G29560.1
Best-hit-arabi-name: cytosolic enolase
Gorai.013G261200.1.D
PCC=-0.278, p=0.249
mRNA Profile
Protein Expression Profile
DPA

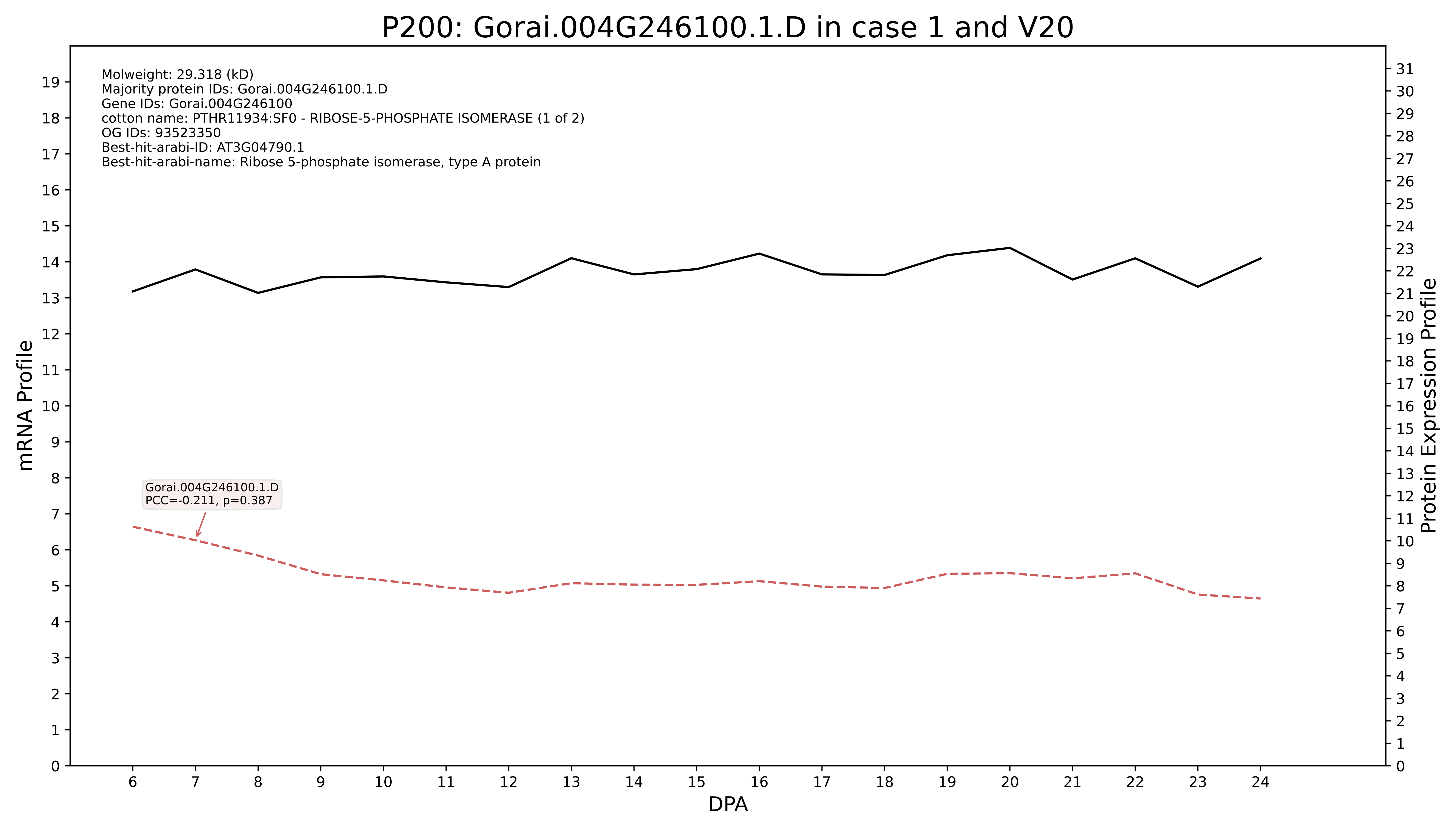
P200: Gorai.004G246100.1.D in case 1 and V20
Molweight: 29.318 (kD)
Majority protein IDs: Gorai.004G246100.1.D
Gene IDs: Gorai.004G246100
cotton name: PTHR11934:SF0 - RIBOSE-5-PHOSPHATE ISOMERASE (1 of 2)
OG IDs: 93523350
Best-hit-arabi-ID: AT3G04790.1
Best-hit-arabi-name: Ribose 5-phosphate isomerase, type A protein
Gorai.004G246100.1.D
PCC=-0.211, p=0.387
mRNA Profile
Protein Expression Profile
DPA

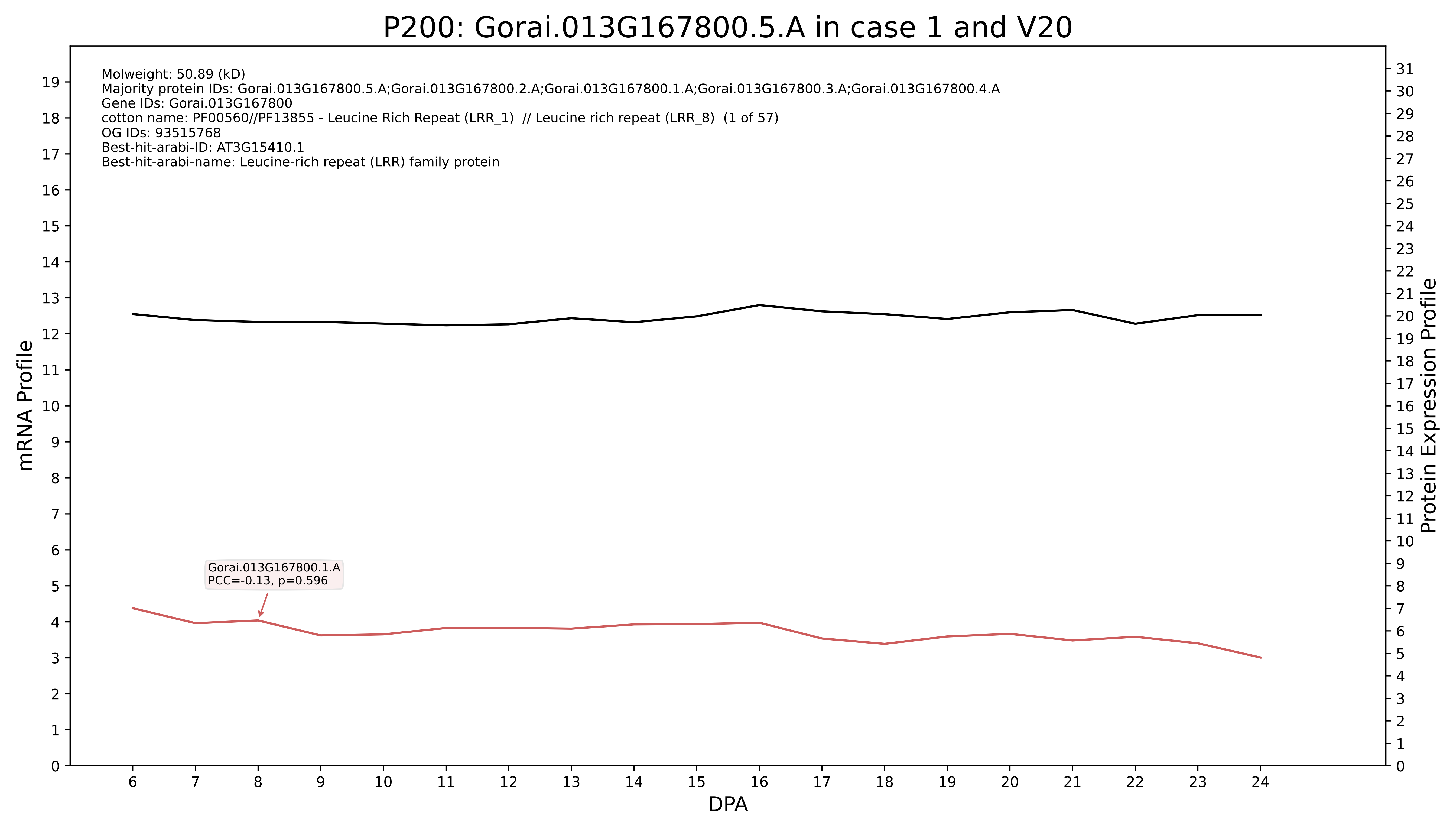
P200: Gorai.013G167800.5.A in case 1 and V20
Molweight: 50.89 (kD)
Majority protein IDs: Gorai.013G167800.5.A;Gorai.013G167800.2.A;Gorai.013G167800.1.A;Gorai.013G167800.3.A;Gorai.013G167800.4.A
Gene IDs: Gorai.013G167800
cotton name: PF00560//PF13855 - Leucine Rich Repeat (LRR_1) // Leucine rich repeat (LRR_8) (1 of 57)
OG IDs: 93515768
Best-hit-arabi-ID: AT3G15410.1
Best-hit-arabi-name: Leucine-rich repeat (LRR) family protein
Gorai.013G167800.1.A
PCC=-0.13, p=0.596
mRNA Profile
Protein Expression Profile
DPA

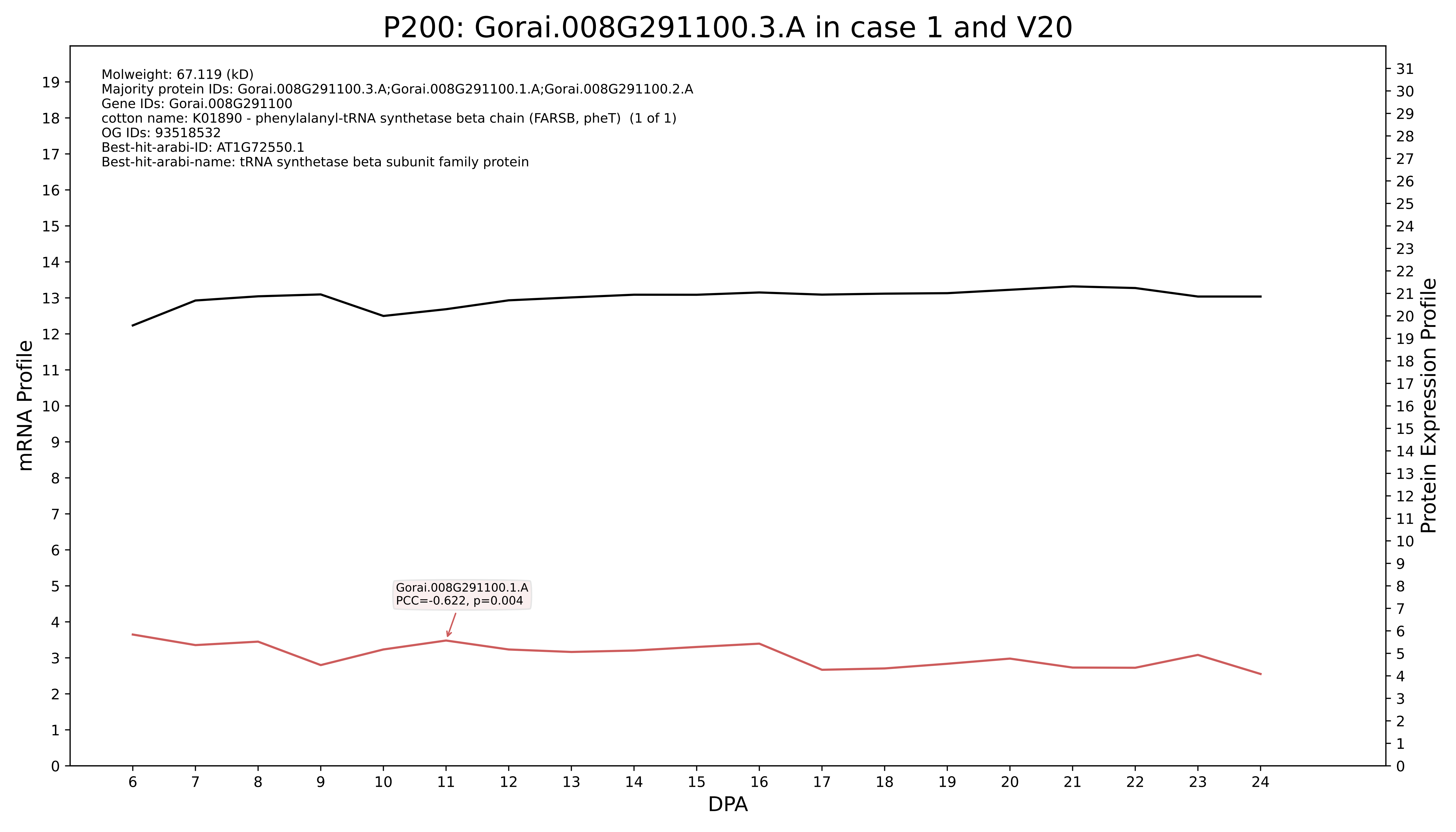
P200: Gorai.008G291100.3.A in case 1 and V20
Molweight: 67.119 (kD)
Majority protein IDs: Gorai.008G291100.3.A;Gorai.008G291100.1.A;Gorai.008G291100.2.A
Gene IDs: Gorai.008G291100
cotton name: K01890 - phenylalanyl-tRNA synthetase beta chain (FARSB, pheT) (1 of 1)
OG IDs: 93518532
Best-hit-arabi-ID: AT1G72550.1
Best-hit-arabi-name: tRNA synthetase beta subunit family protein
Gorai.008G291100.1.A
PCC=-0.622, p=0.004
mRNA Profile
Protein Expression Profile
DPA
0
1
2
3
4
5
6
7
8
9
10
11
12
13
14
15
16
17
18
19
20
21
22
23
24
25
26
27
28
29
30
31

P200: Gorai.012G027100.5.D in case 2 and V20

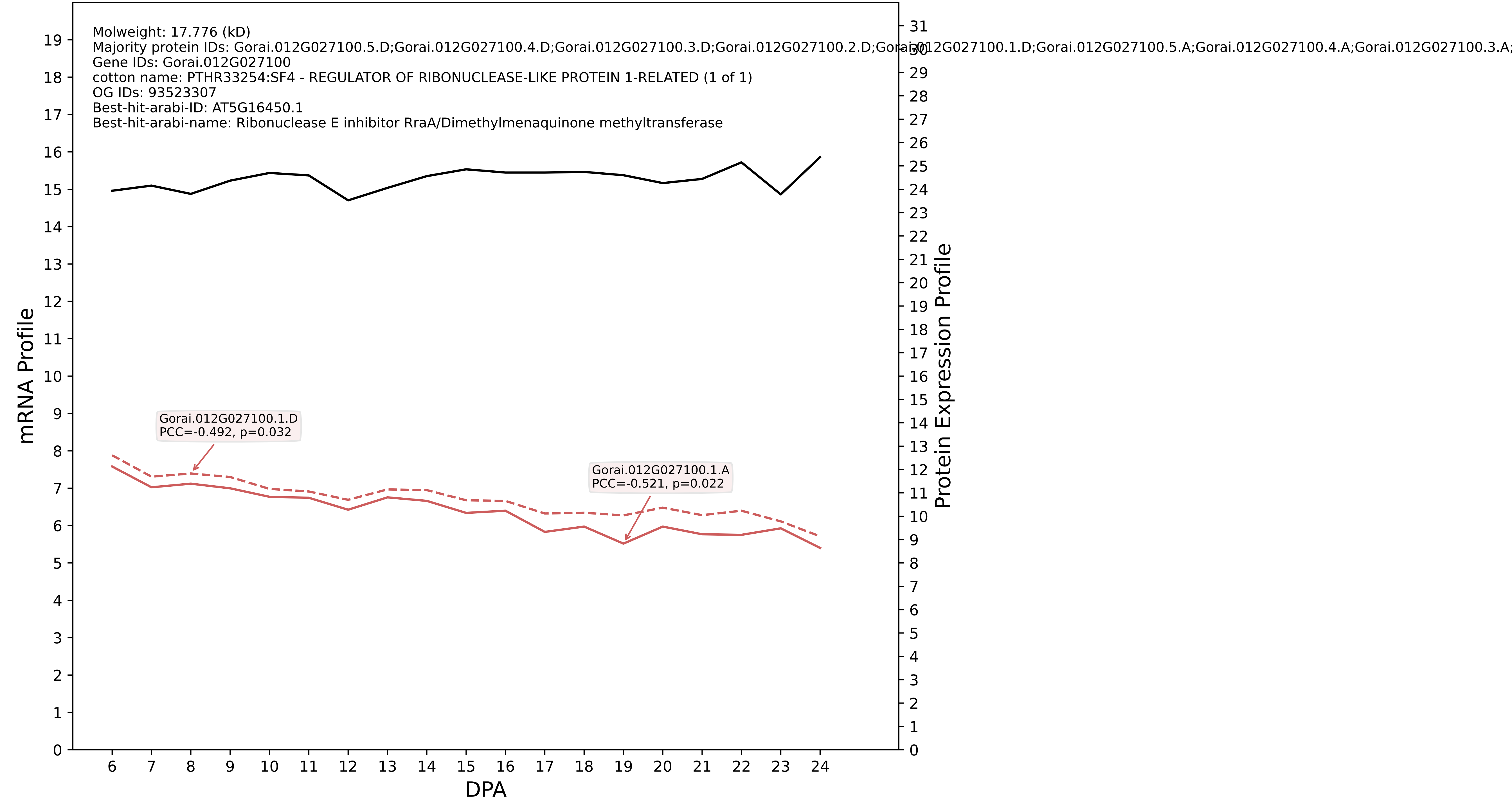

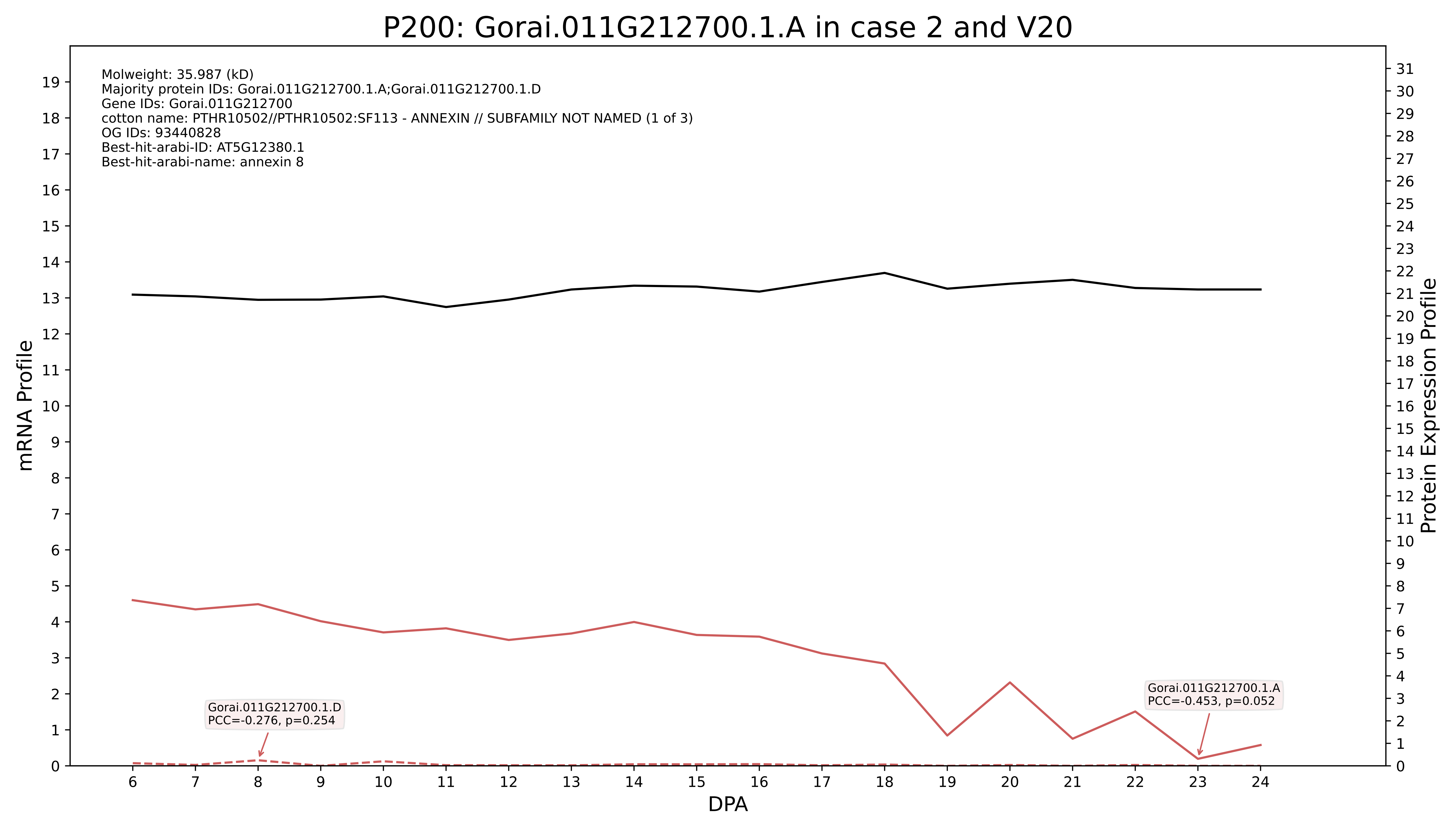
P200: Gorai.011G212700.1.A in case 2 and V20
Molweight: 35.987 (kD)
Majority protein IDs: Gorai.011G212700.1.A;Gorai.011G212700.1.D
Gene IDs: Gorai.011G212700
cotton name: PTHR10502//PTHR10502:SF113 - ANNEXIN // SUBFAMILY NOT NAMED (1 of 3)
OG IDs: 93440828
Best-hit-arabi-ID: AT5G12380.1
Best-hit-arabi-name: annexin 8
Gorai.011G212700.1.D
PCC=-0.276, p=0.254
Gorai.011G212700.1.A
PCC=-0.453, p=0.052
mRNA Profile
Protein Expression Profile
DPA

P200: Gorai.006G257100.1.D in case 2 and V20

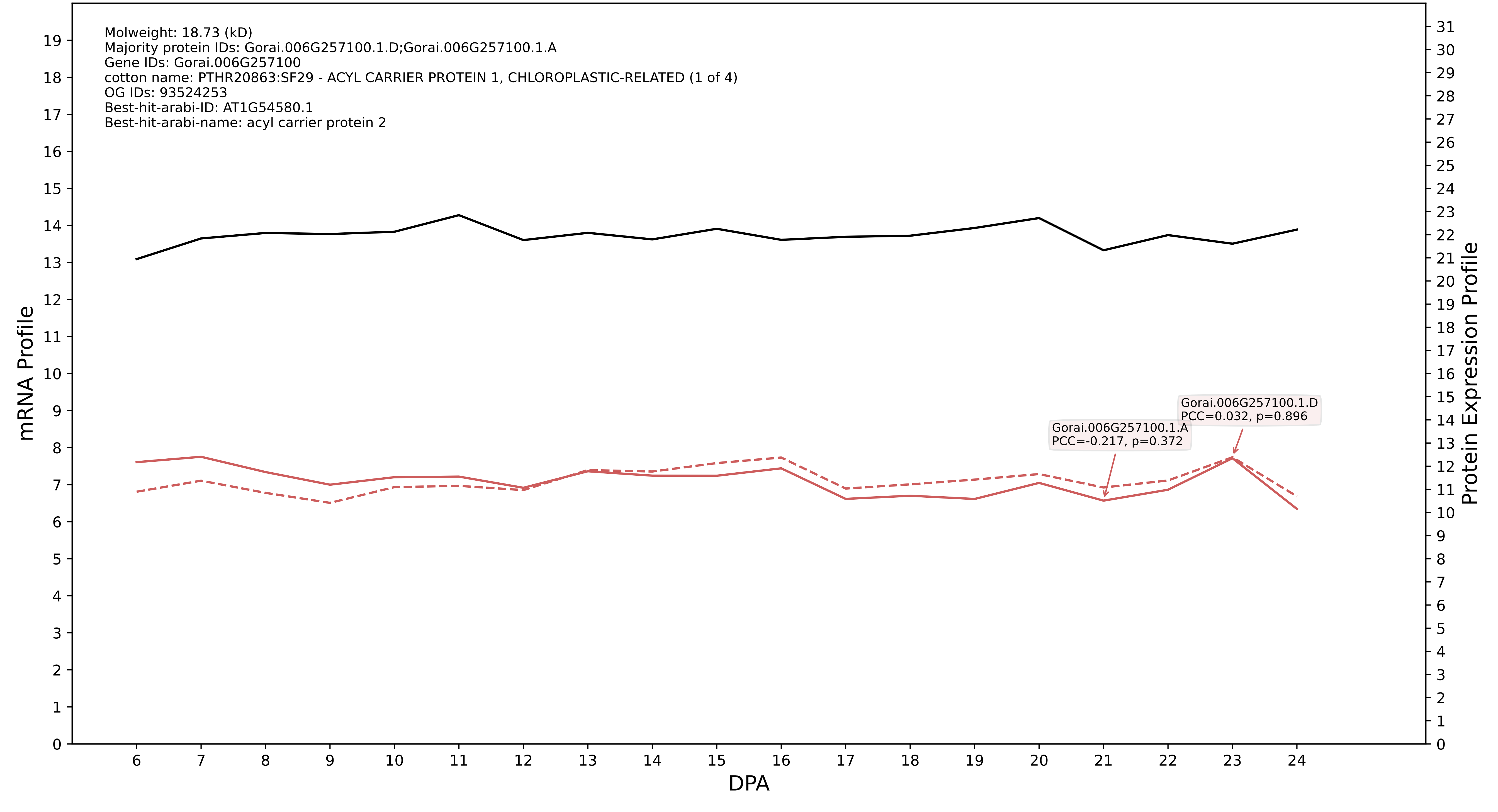

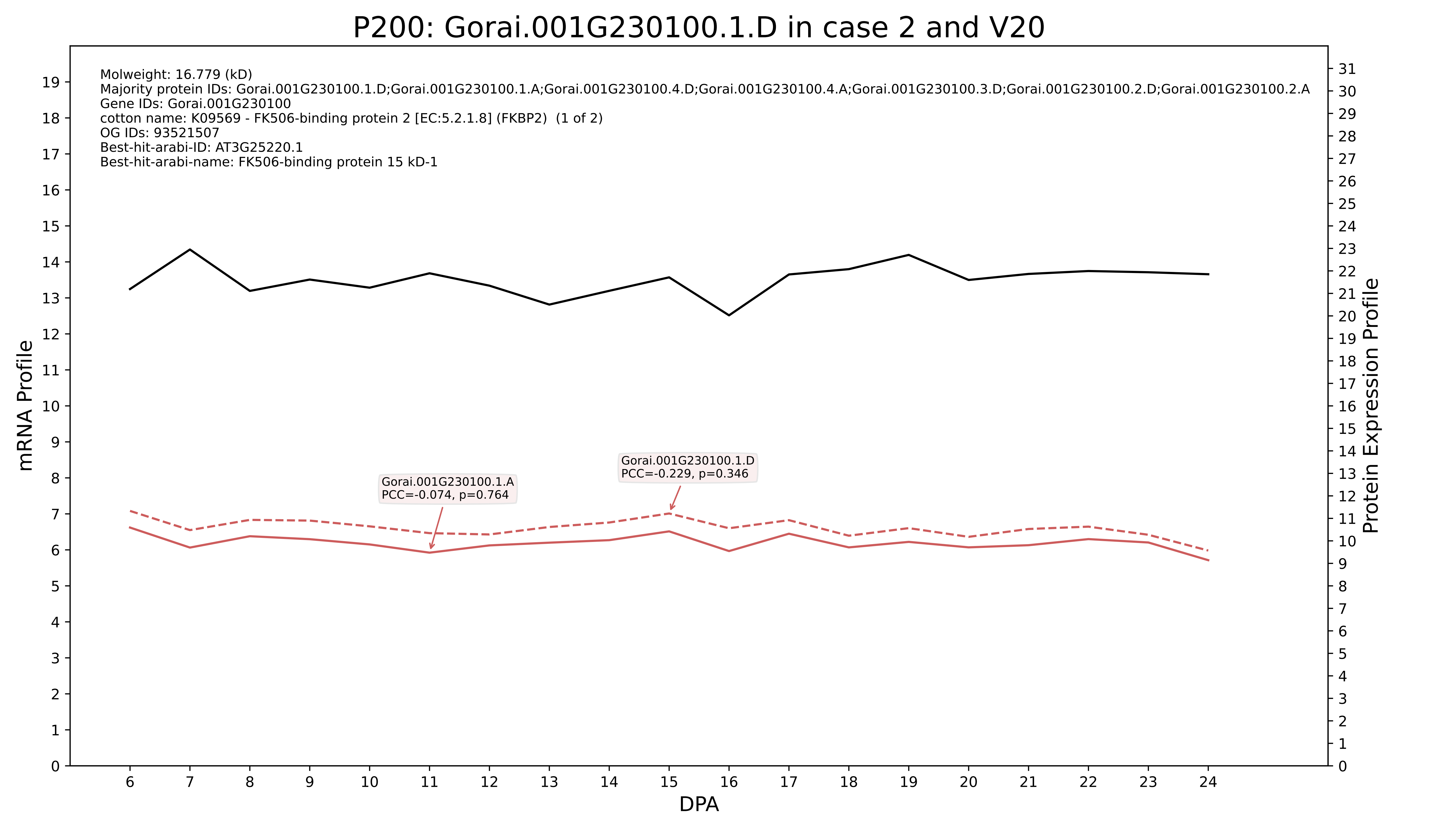
P200: Gorai.001G230100.1.D in case 2 and V20
Molweight: 16.779 (kD)
Majority protein IDs: Gorai.001G230100.1.D;Gorai.001G230100.1.A;Gorai.001G230100.4.D;Gorai.001G230100.4.A;Gorai.001G230100.3.D;Gorai.001G230100.2.D;Gorai.001G230100.2.A
Gene IDs: Gorai.001G230100
cotton name: K09569 - FK506-binding protein 2 [EC:5.2.1.8] (FKBP2) (1 of 2)
OG IDs: 93521507
Best-hit-arabi-ID: AT3G25220.1
Best-hit-arabi-name: FK506-binding protein 15 kD-1
Gorai.001G230100.1.A
PCC=-0.074, p=0.764
Gorai.001G230100.1.D
PCC=-0.229, p=0.346
mRNA Profile
Protein Expression Profile
DPA
0
1
2
3
4
5
6
7
8
9
10
11
12
13
14
15
16
17
18
19
20
21
22
23
24
25
26
27
28
29
30
31

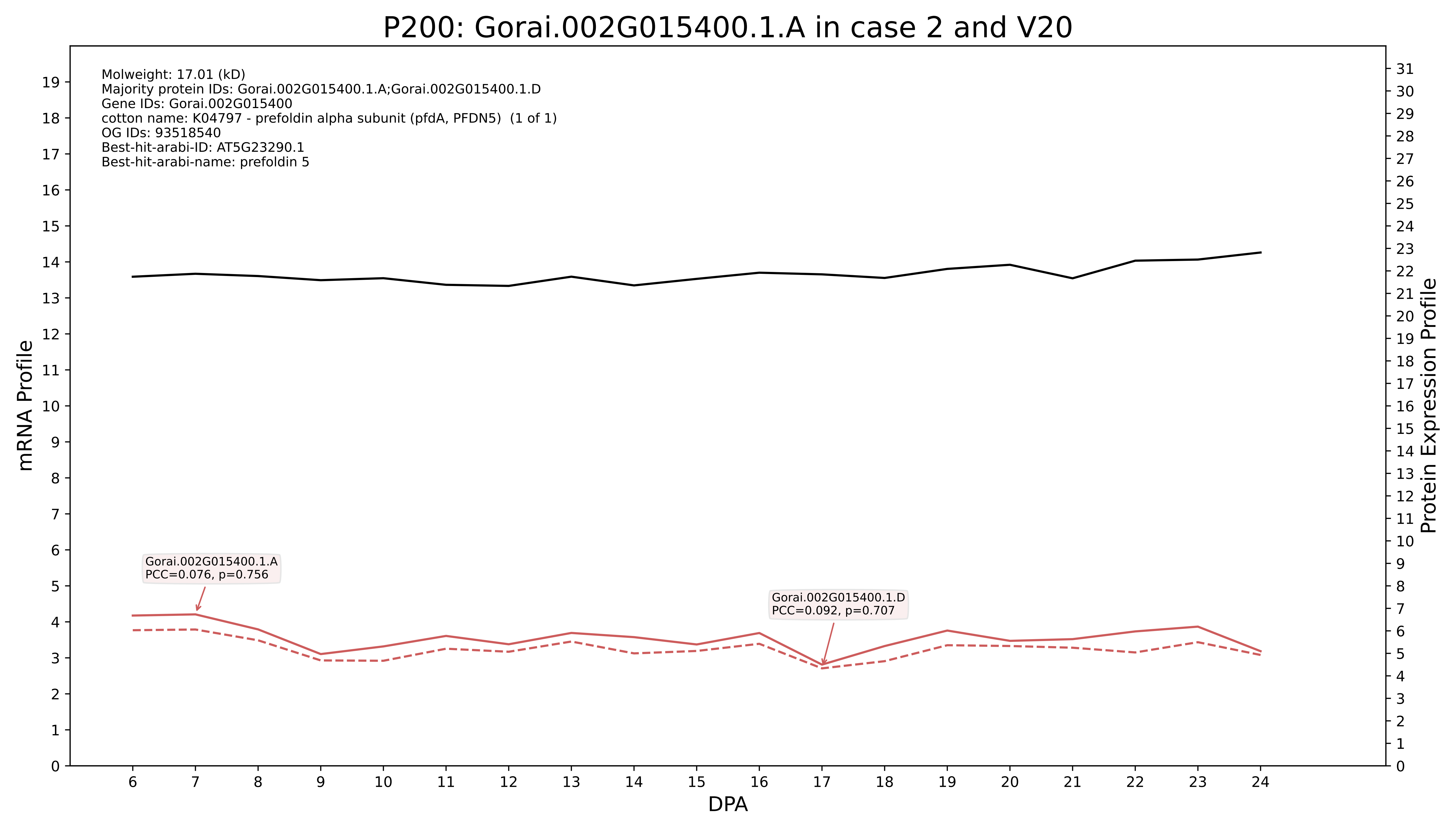
P200: Gorai.002G015400.1.A in case 2 and V20
Molweight: 17.01 (kD)
Majority protein IDs: Gorai.002G015400.1.A;Gorai.002G015400.1.D
Gene IDs: Gorai.002G015400
cotton name: K04797 - prefoldin alpha subunit (pfdA, PFDN5) (1 of 1)
OG IDs: 93518540
Best-hit-arabi-ID: AT5G23290.1
Best-hit-arabi-name: prefoldin 5
Gorai.002G015400.1.A
PCC=0.076, p=0.756
Gorai.002G015400.1.D
PCC=0.092, p=0.707
mRNA Profile
Protein Expression Profile
DPA
6
7
8
9
10
11
12
13
14
15
16
17
18
19
20
21
22
23
24

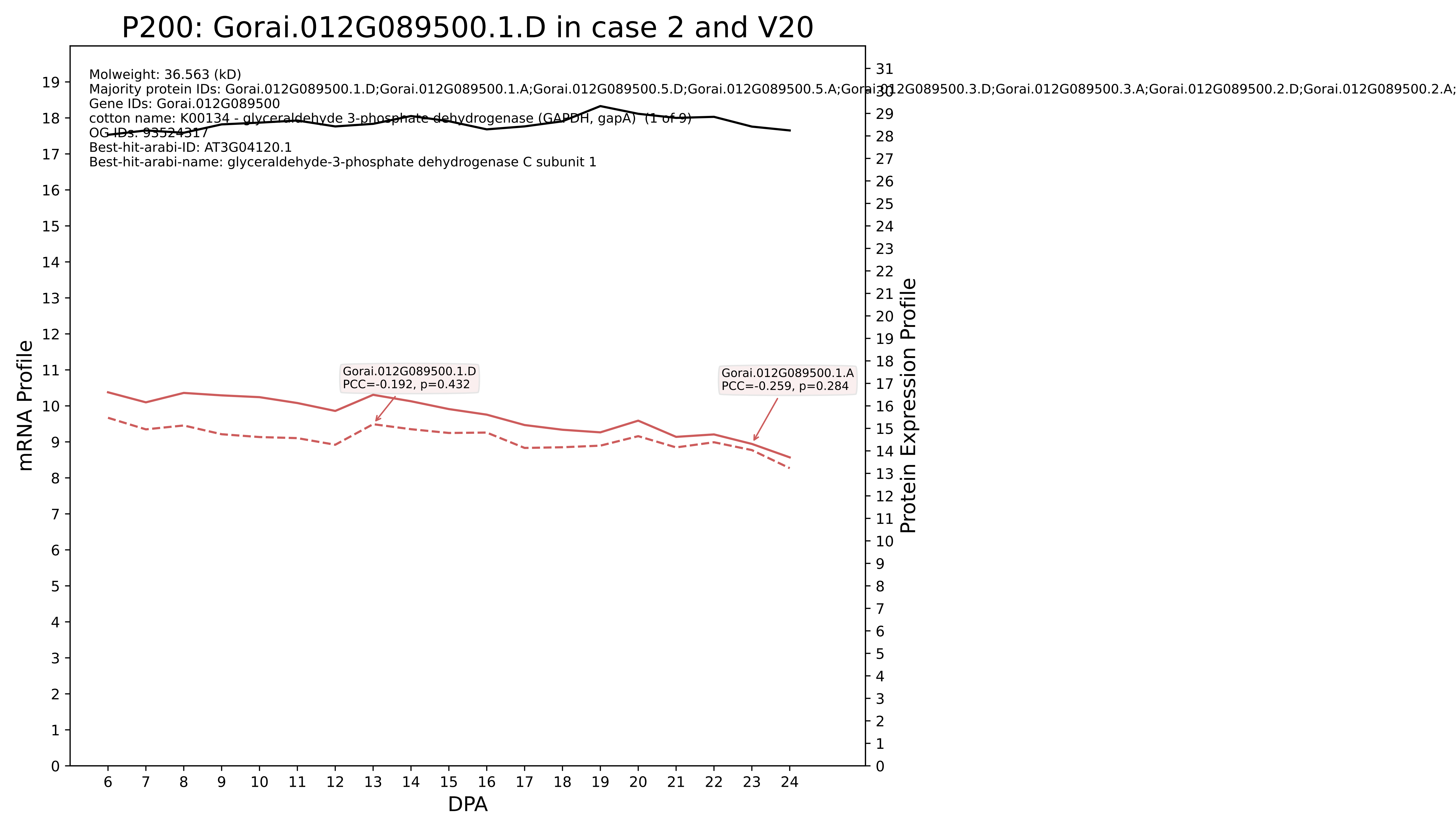
P200: Gorai.012G089500.1.D in case 2 and V20
Molweight: 36.563 (kD)
Majority protein IDs: Gorai.012G089500.1.D;Gorai.012G089500.1.A;Gorai.012G089500.5.D;Gorai.012G089500.5.A;Gorai.012G089500.3.D;Gorai.012G089500.3.A;Gorai.012G089500.2.D;Gorai.012G089500.2.A;
Gene IDs: Gorai.012G089500
cotton name: K00134 - glyceraldehyde 3-phosphate dehydrogenase (GAPDH, gapA) (1 of 9)
OG-IDs: 93524317
Best-hit-arabi-ID: AT3G04120.1
Best-hit-arabi-name: glyceraldehyde-3-phosphate dehydrogenase C subunit 1
Gorai.012G089500.1.D
PCC=-0.192, p=0.432
Gorai.012G089500.1.A
PCC=-0.259, p=0.284
mRNA Profile
Protein Expression Profile
DPA

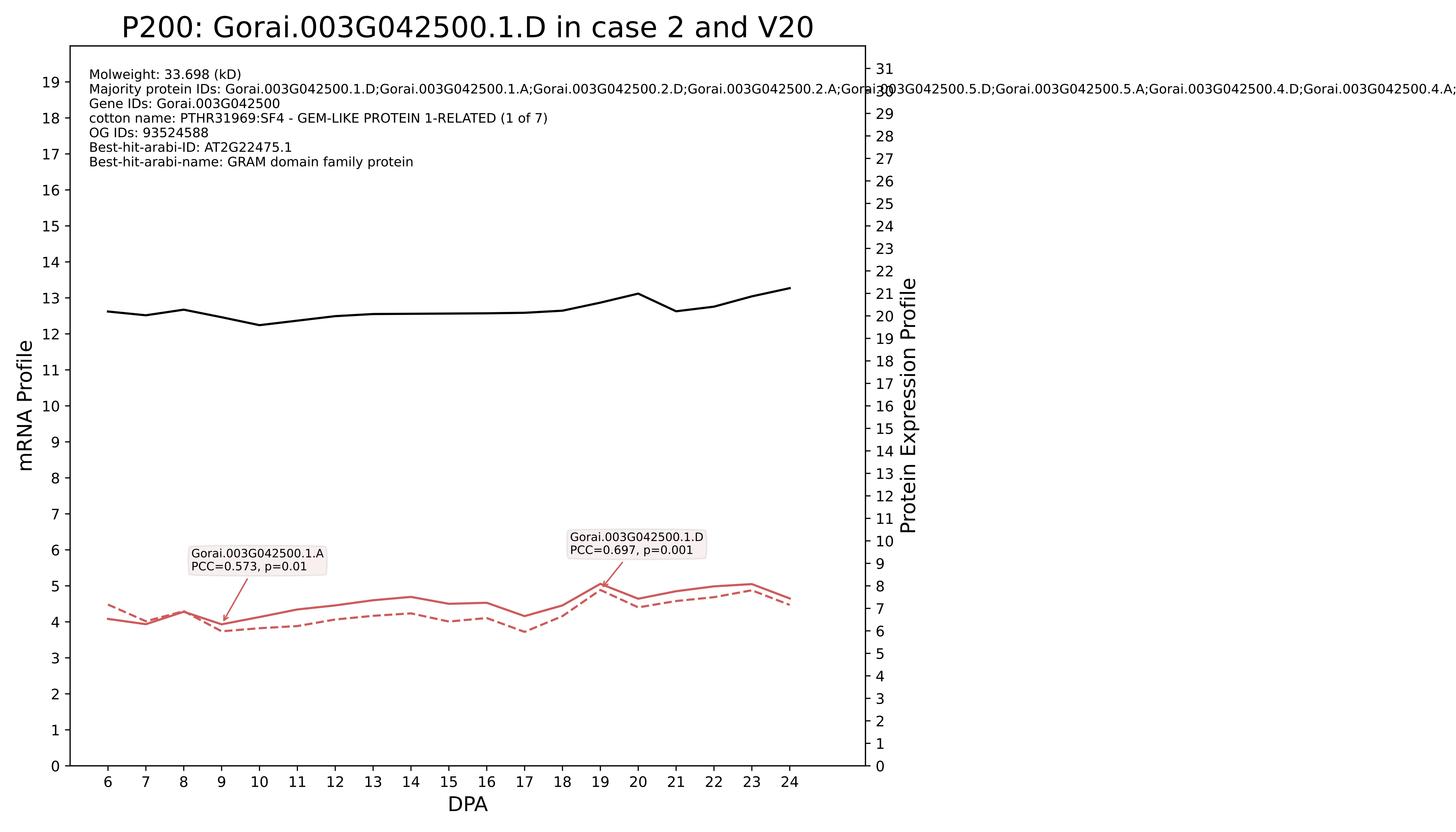
P200: Gorai.003G042500.1.D in case 2 and V20
Molweight: 33.698 (kD)
Majority protein IDs: Gorai.003G042500.1.D;Gorai.003G042500.1.A;Gorai.003G042500.2.D;Gorai.003G042500.2.A;Gorai.003G042500.5.D;Gorai.003G042500.5.A;Gorai.003G042500.4.D;Gorai.003G042500.4.A;
Gene IDs: Gorai.003G042500
cotton name: PTHR31969:SF4 - GEM-LIKE PROTEIN 1-RELATED (1 of 7)
OG IDs: 93524588
Best-hit-arabi-ID: AT2G22475.1
Best-hit-arabi-name: GRAM domain family protein
Gorai.003G042500.1.A
PCC=0.573, p=0.01
Gorai.003G042500.1.D
PCC=0.697, p=0.001
mRNA Profile
Protein Expression Profile
DPA

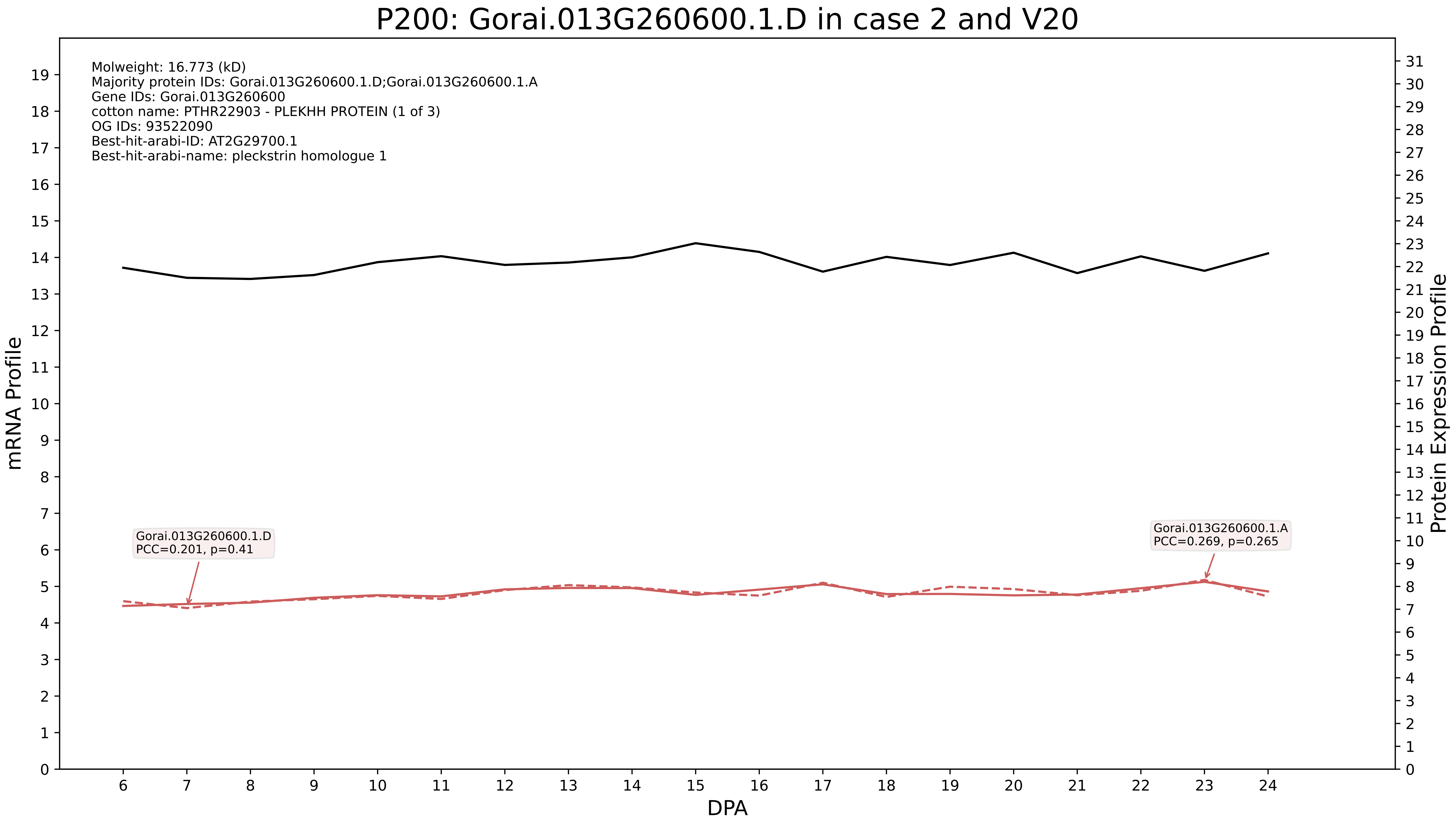
P200: Gorai.013G260600.1.D in case 2 and V20
Molweight: 16.773 (kD)
Majority protein IDs: Gorai.013G260600.1.D;Gorai.013G260600.1.A
Gene IDs: Gorai.013G260600
cotton name: PTHR22903 - PLEKHH PROTEIN (1 of 3)
OG IDs: 93522090
Best-hit-arabi-ID: AT2G29700.1
Best-hit-arabi-name: pleckstrin homologue 1
Gorai.013G260600.1.D
PCC=0.201, p=0.41
Gorai.013G260600.1.A
PCC=0.269, p=0.265
mRNA Profile
Protein Expression Profile
DPA

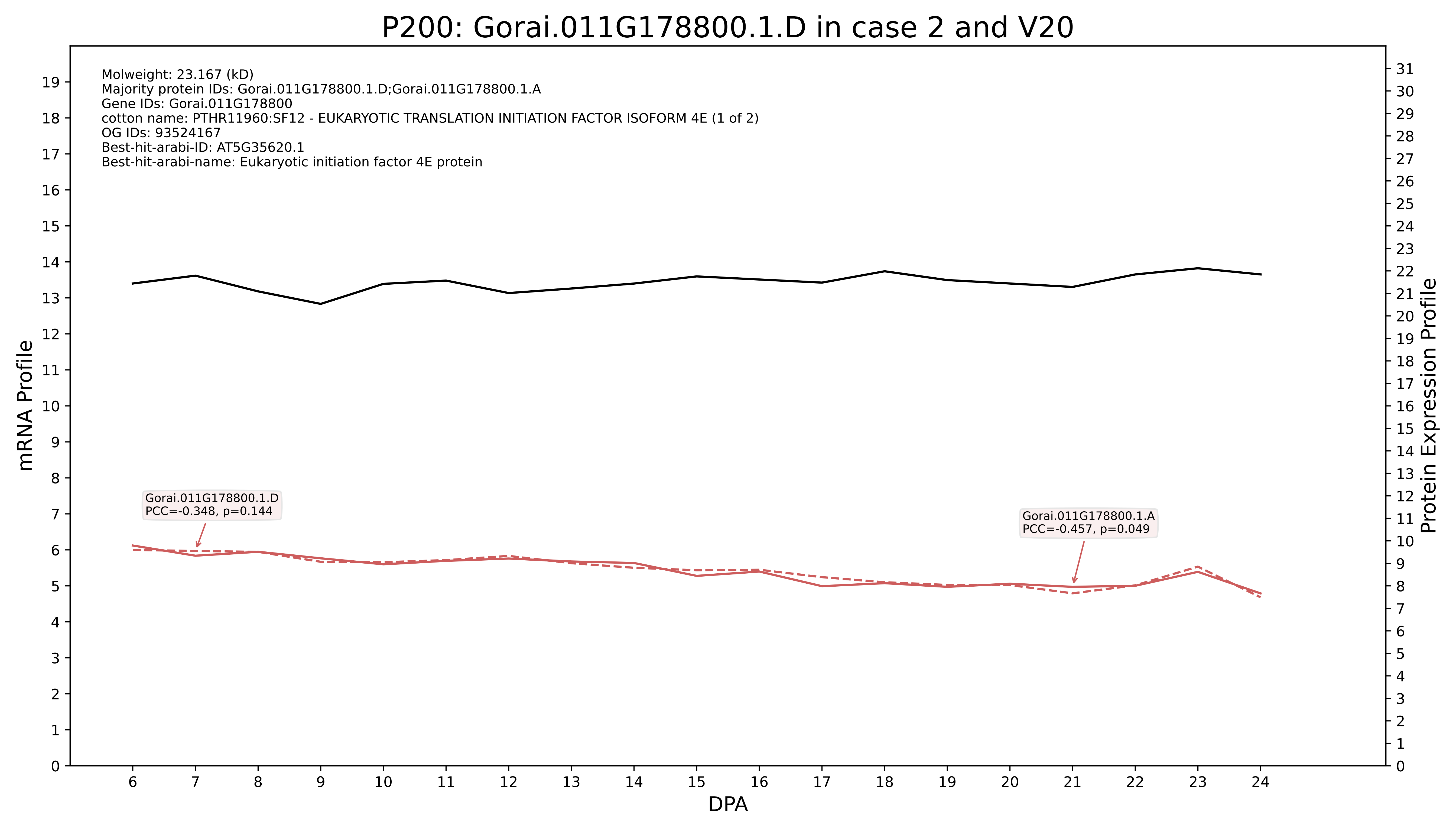
P200: Gorai.011G178800.1.D in case 2 and V20
Molweight: 23.167 (kD)
Majority protein IDs: Gorai.011G178800.1.D;Gorai.011G178800.1.A
Gene IDs: Gorai.011G178800
cotton name: PTHR11960:SF12 - EUKARYOTIC TRANSLATION INITIATION FACTOR ISOFORM 4E (1 of 2)
OG IDs: 93524167
Best-hit-arabi-ID: AT5G35620.1
Best-hit-arabi-name: Eukaryotic initiation factor 4E protein
Gorai.011G178800.1.D
PCC=-0.348, p=0.144
Gorai.011G178800.1.A
PCC=-0.457, p=0.049
mRNA Profile
Protein Expression Profile
DPA

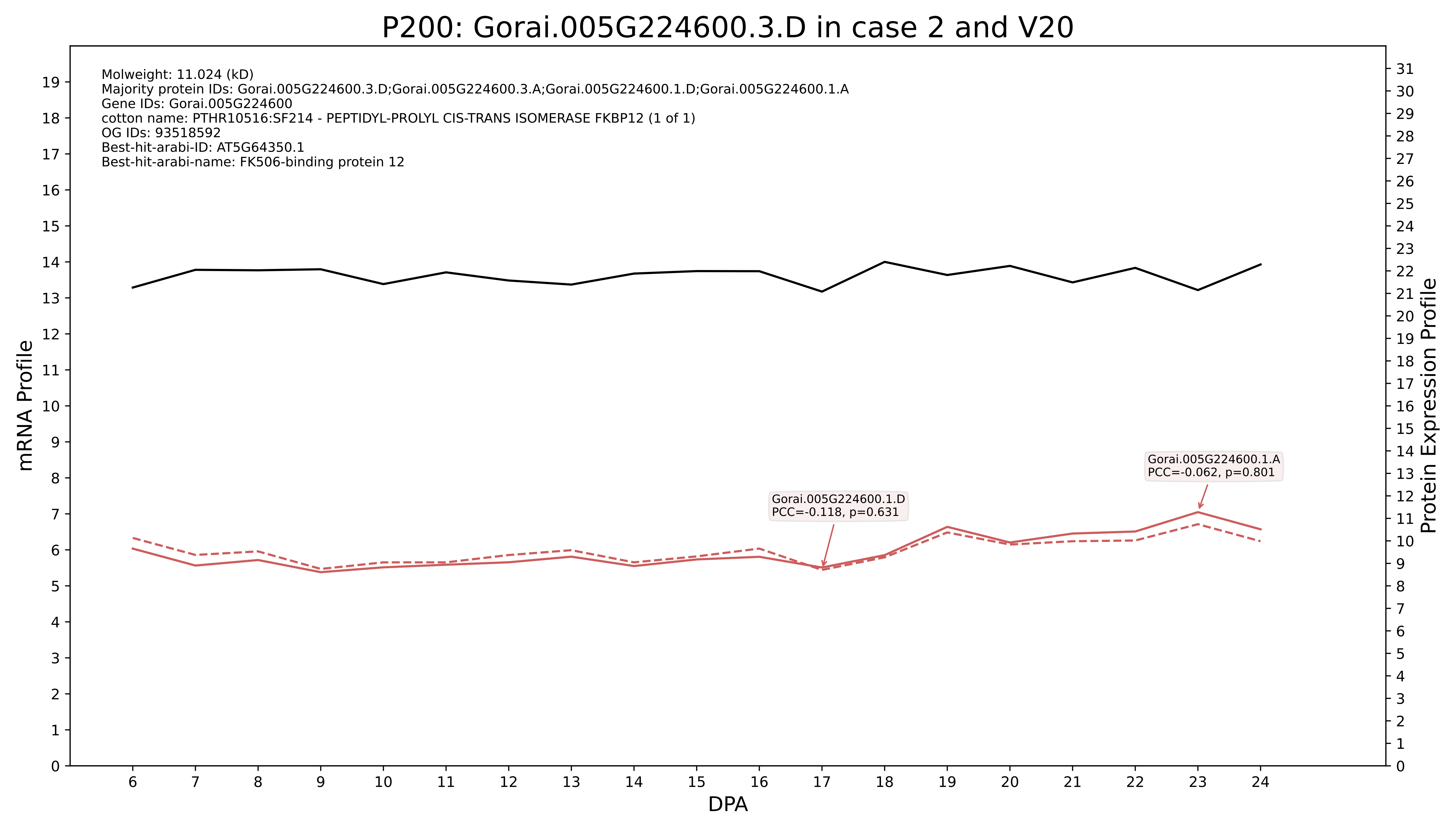
P200: Gorai.005G224600.3.D in case 2 and V20
Molweight: 11.024 (kD)
Majority protein IDs: Gorai.005G224600.3.D;Gorai.005G224600.3.A;Gorai.005G224600.1.D;Gorai.005G224600.1.A
Gene IDs: Gorai.005G224600
cotton name: PTHR10516:SF214 - PEPTIDYL-PROLYL CIS-TRANS ISOMERASE FKBP12 (1 of 1)
OG IDs: 93518592
Best-hit-arabi-ID: AT5G64350.1
Best-hit-arabi-name: FK506-binding protein 12
Gorai.005G224600.1.D
PCC=-0.118, p=0.631
Gorai.005G224600.1.A
PCC=-0.062, p=0.801
mRNA Profile
Protein Expression Profile
DPA

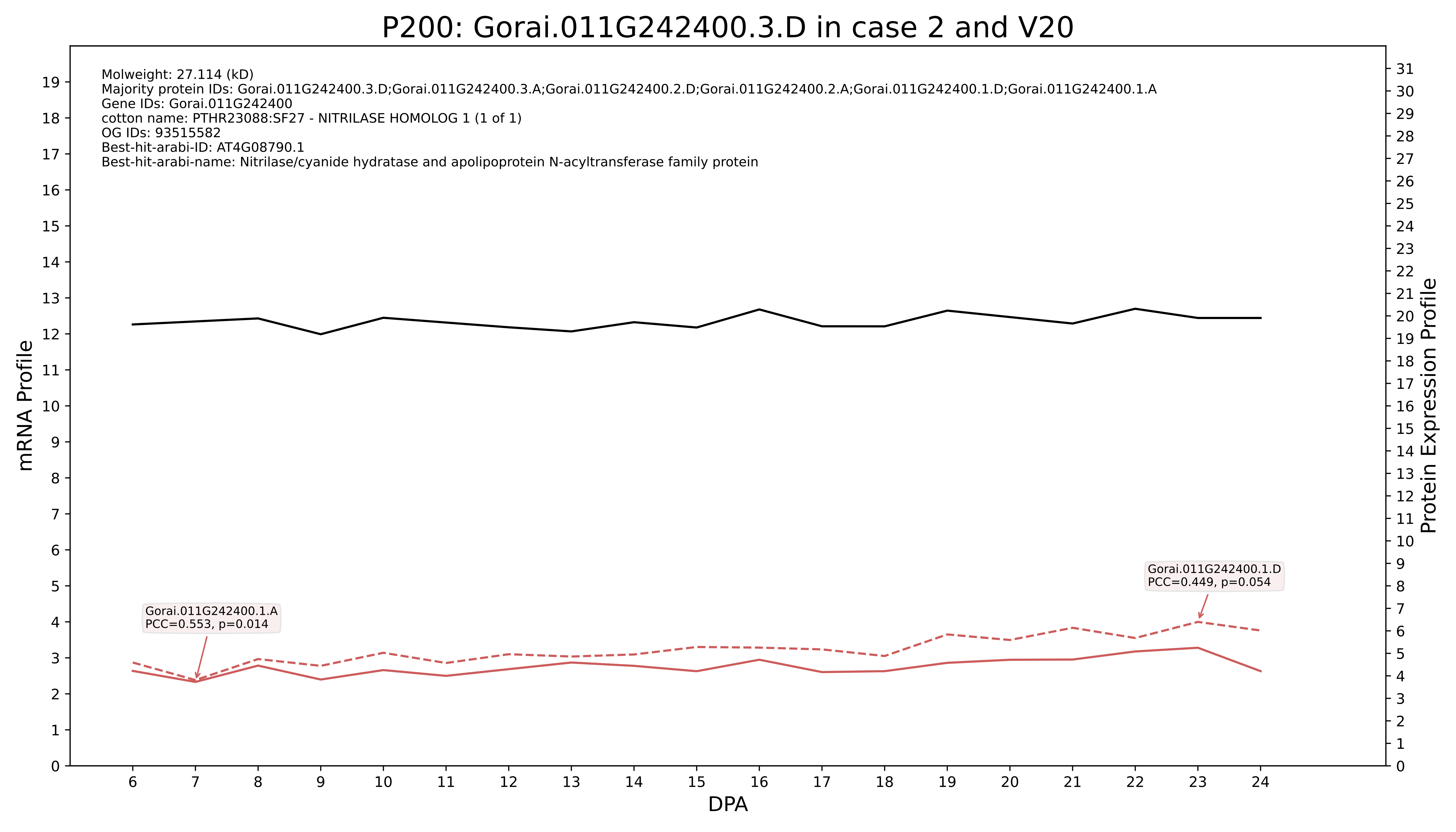
P200: Gorai.011G242400.3.D in case 2 and V20
Molweight: 27.114 (kD)
Majority protein IDs: Gorai.011G242400.3.D;Gorai.011G242400.3.A;Gorai.011G242400.2.D;Gorai.011G242400.2.A;Gorai.011G242400.1.D;Gorai.011G242400.1.A
Gene IDs: Gorai.011G242400
cotton name: PTHR23088:SF27 - NITRILASE HOMOLOG 1 (1 of 1)
OG IDs: 93515582
Best-hit-arabi-ID: AT4G08790.1
Best-hit-arabi-name: Nitrilase/cyanide hydratase and apolipoprotein N-acyltransferase family protein
Gorai.011G242400.1.A
PCC=0.553, p=0.014
Gorai.011G242400.1.D
PCC=0.449, p=0.054
mRNA Profile
Protein Expression Profile
DPA

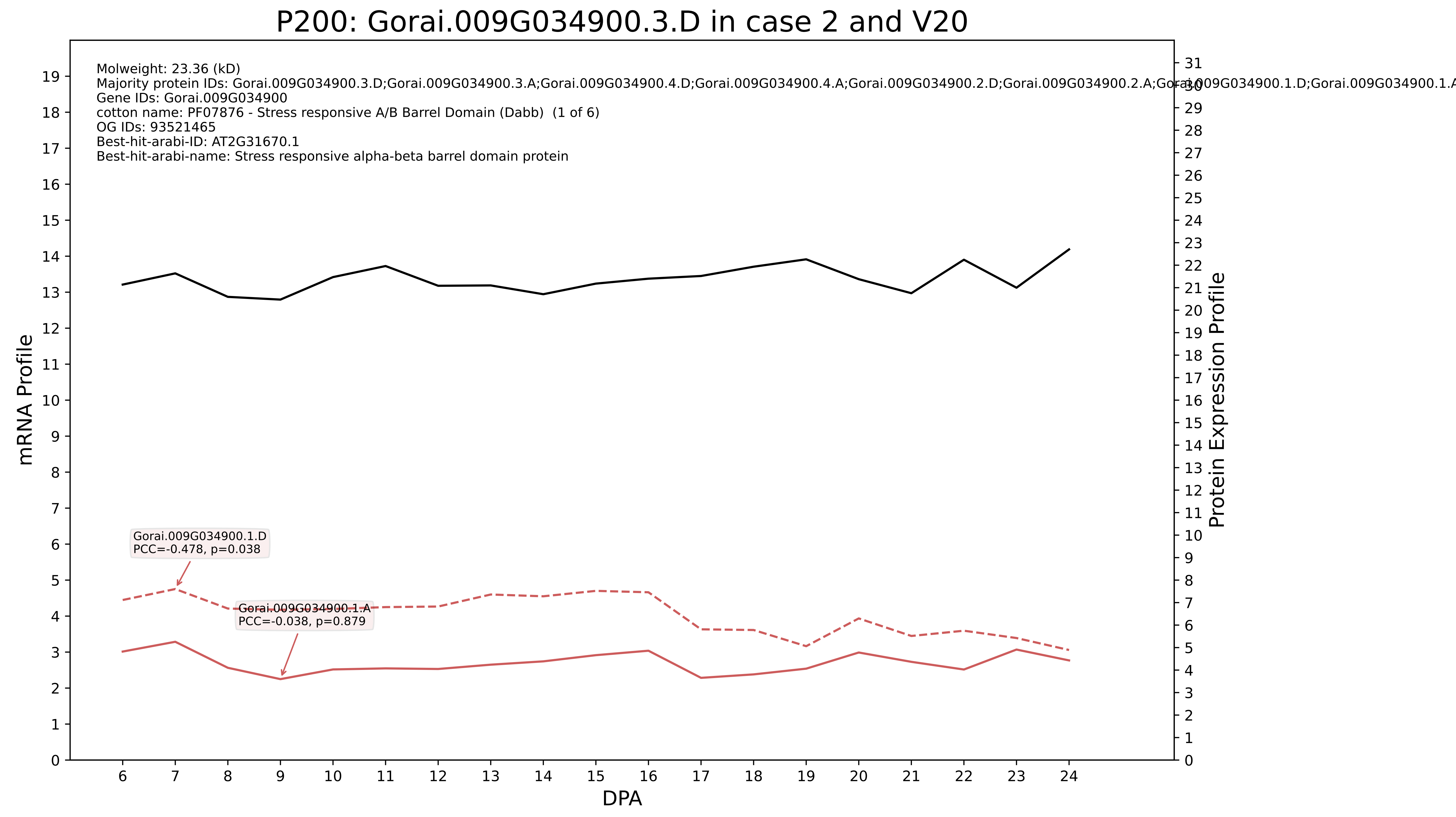
P200: Gorai.009G034900.3.D in case 2 and V20
Molweight: 23.36 (kD)
Majority protein IDs: Gorai.009G034900.3.D;Gorai.009G034900.3.A;Gorai.009G034900.4.D;Gorai.009G034900.4.A;Gorai.009G034900.2.D;Gorai.009G034900.2.A;Gorai.009G034900.1.D;Gorai.009G034900.1.A
Gene IDs: Gorai.009G034900
cotton name: PF07876 - Stress responsive A/B Barrel Domain (Dabb) (1 of 6)
OG IDs: 93521465
Best-hit-arabi-ID: AT2G31670.1
Best-hit-arabi-name: Stress responsive alpha-beta barrel domain protein
Gorai.009G034900.1.D
PCC=-0.478, p=0.038
Gorai.009G034900.1.A
PCC=-0.038, p=0.879
mRNA Profile
Protein Expression Profile
DPA

200: Gorai.007G275600.5.A in case 2 and V20

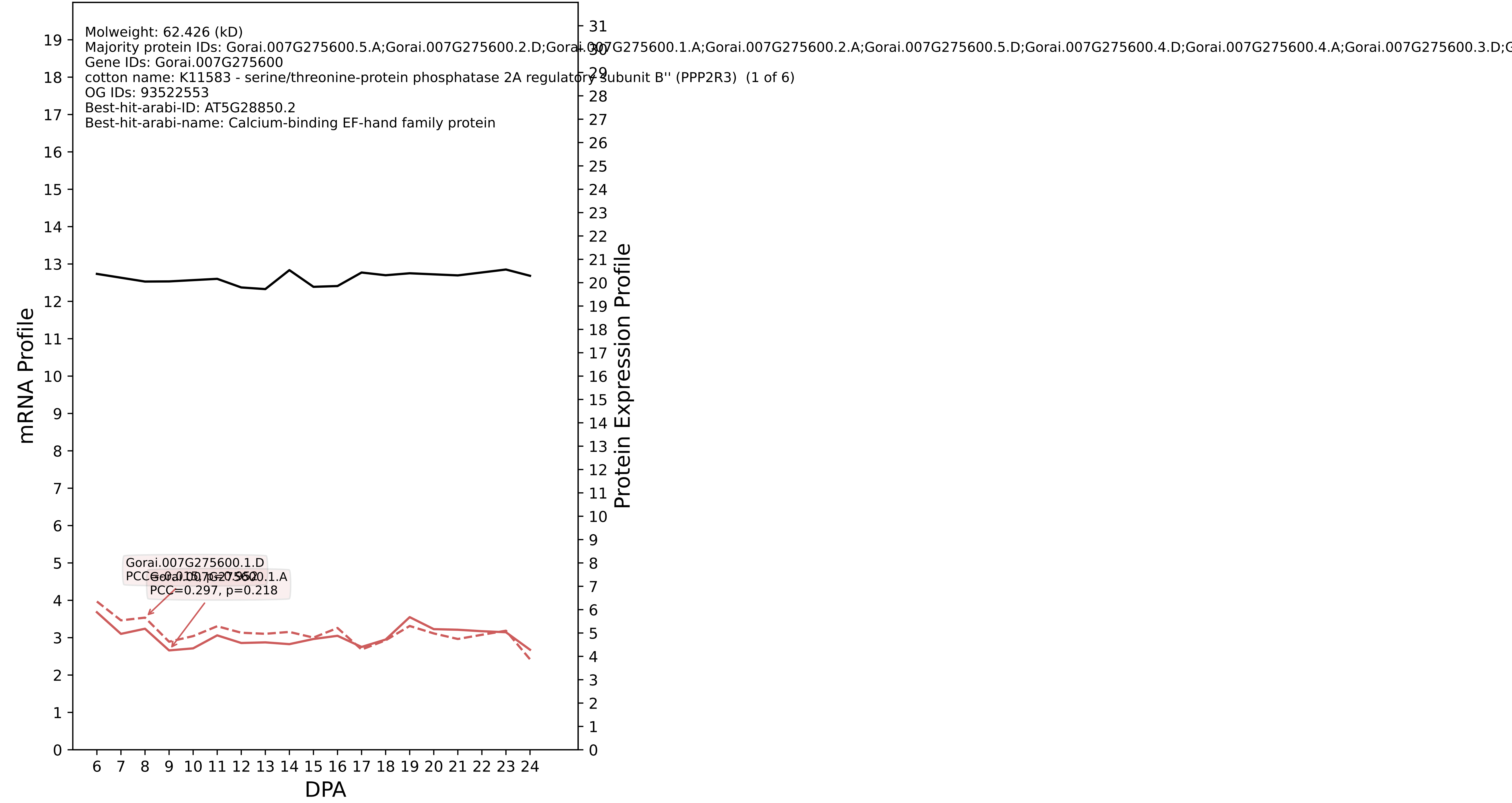

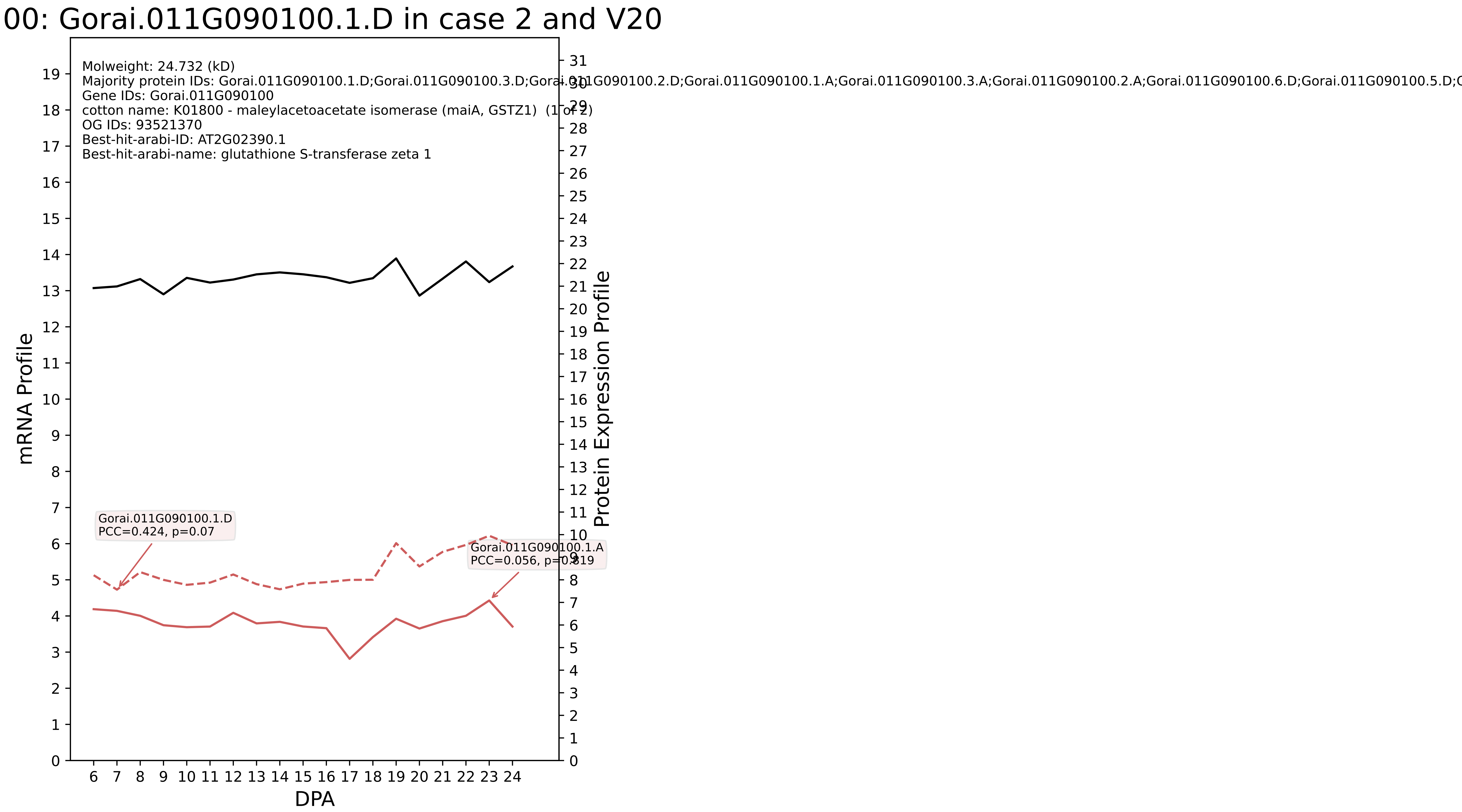
00: Gorai.011G090100.1.D in case 2 and V20
Molweight: 24.732 (kD)
Majority protein IDs: Gorai.011G090100.1.D;Gorai.011G090100.3.D;Gorai.011G090100.2.D;Gorai.011G090100.1.A;Gorai.011G090100.3.A;Gorai.011G090100.2.A;Gorai.011G090100.6.D;Gorai.011G090100.5.D;G
Gene IDs: Gorai.011G090100
cotton name: K01800 - maleylacetoacetate isomerase (maiA, GSTZ1) (1 of 2)
OG IDs: 93521370
Best-hit-arabi-ID: AT2G02390.1
Best-hit-arabi-name: glutathione S-transferase zeta 1
Gorai.011G090100.1.D
PCC=0.424, p=0.07
Gorai.011G090100.1.A
PCC=0.056, p=0.819
mRNA Profile
Protein Expression Profile
DPA

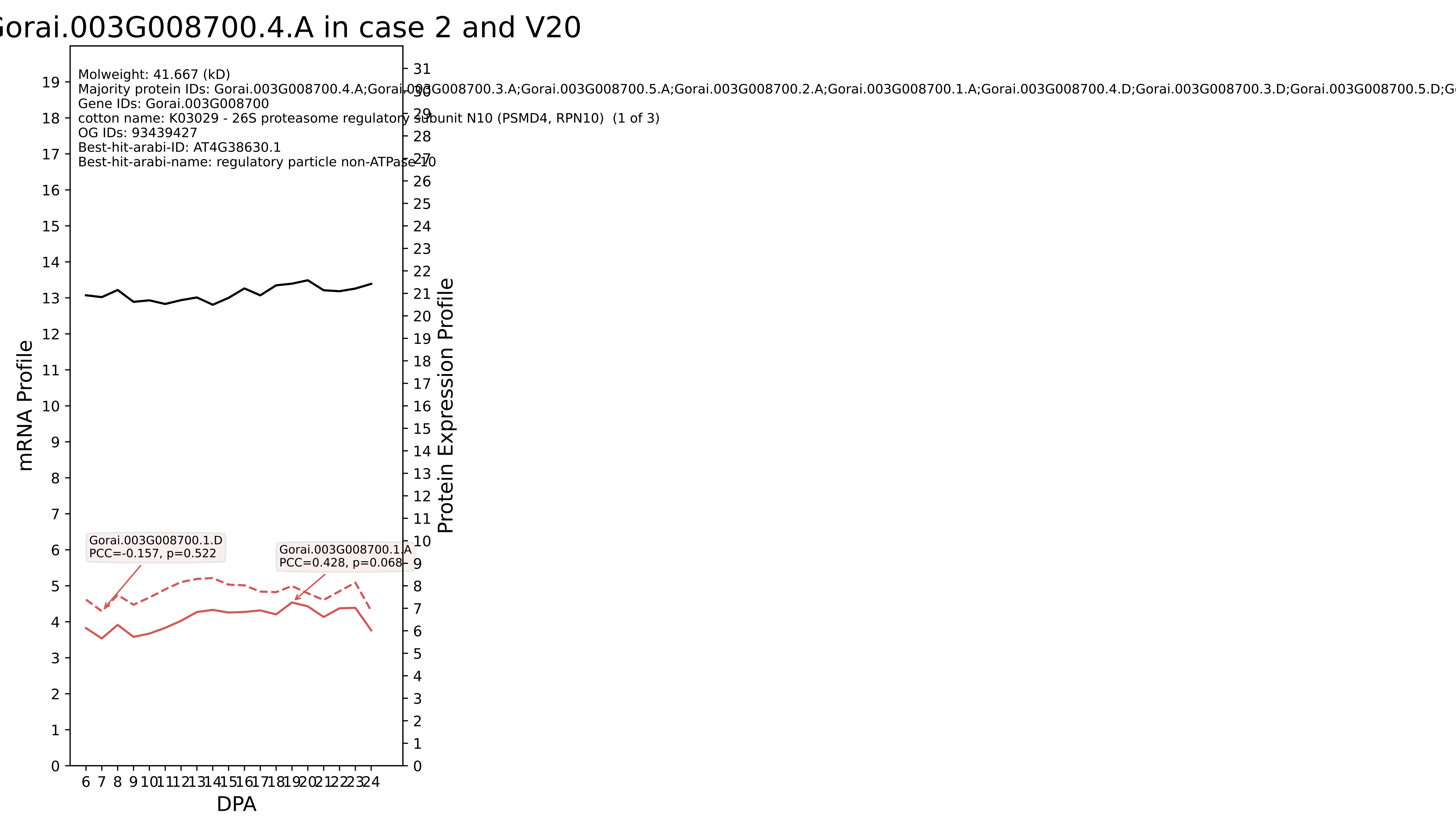

Gorai.003G008700.4.A in case 2 and V20
Molweight: 41.667 (kD)
Majority protein IDs: Gorai.003G008700.4.A;Gorai.003G008700.3.A;Gorai.003G008700.5.A;Gorai.003G008700.2.A;Gorai.003G008700.1.A;Gorai.003G008700.4.D;Gorai.003G008700.3.D;Gorai.003G008700.5.D;G
Gene IDs: Gorai.003G008700
cotton name: K03029 - 26S proteasome regulatory subunit N10 (PSMD4, RPN10) (1 of 3)
OG IDs: 93439427
Best-hit-arabi-ID: AT4G38630.1
Best-hit-arabi-name: regulatory particle non-ATPase 10
Gorai.003G008700.1.D
PCC=-0.157, p=0.522
Gorai.003G008700.1.A
PCC=0.428, p=0.068
mRNA Profile
Protein Expression Profile
DPA

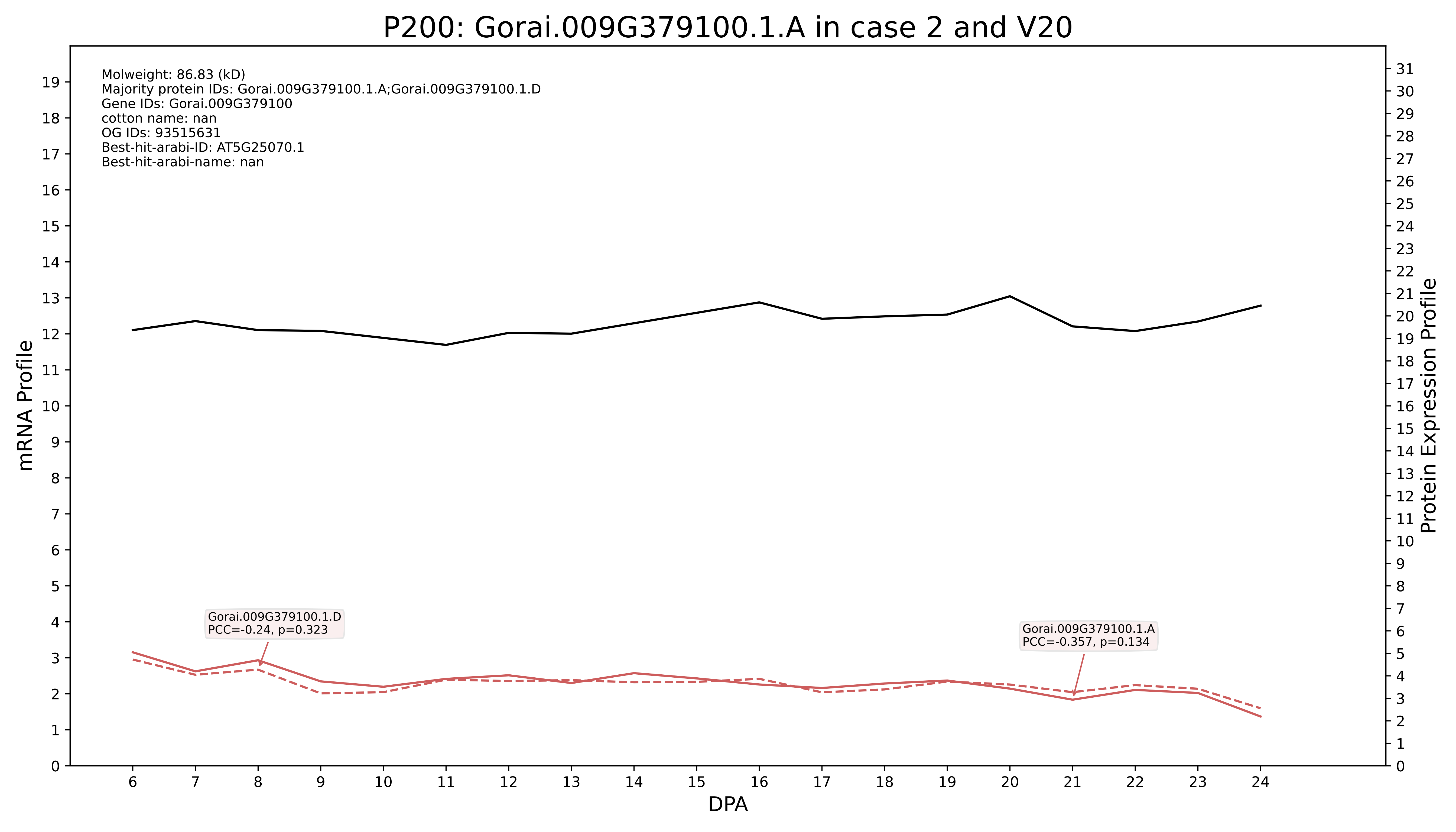
P200: Gorai.009G379100.1.A in case 2 and V20
Molweight: 86.83 (kD)
Majority protein IDs: Gorai.009G379100.1.A;Gorai.009G379100.1.D
Gene IDs: Gorai.009G379100
cotton name: nan
OG IDs: 93515631
Best-hit-arabi-ID: AT5G25070.1
Best-hit-arabi-name: nan
Gorai.009G379100.1.D
PCC=-0.24, p=0.323
Gorai.009G379100.1.A
PCC=-0.357, p=0.134
mRNA Profile
Protein Expression Profile
DPA
0
1
2
3
4
5
6
7
8
9
10
11
12
13
14
15
16
17
18
19
20
21
22
23
24
25
26
27
28
29
30
31

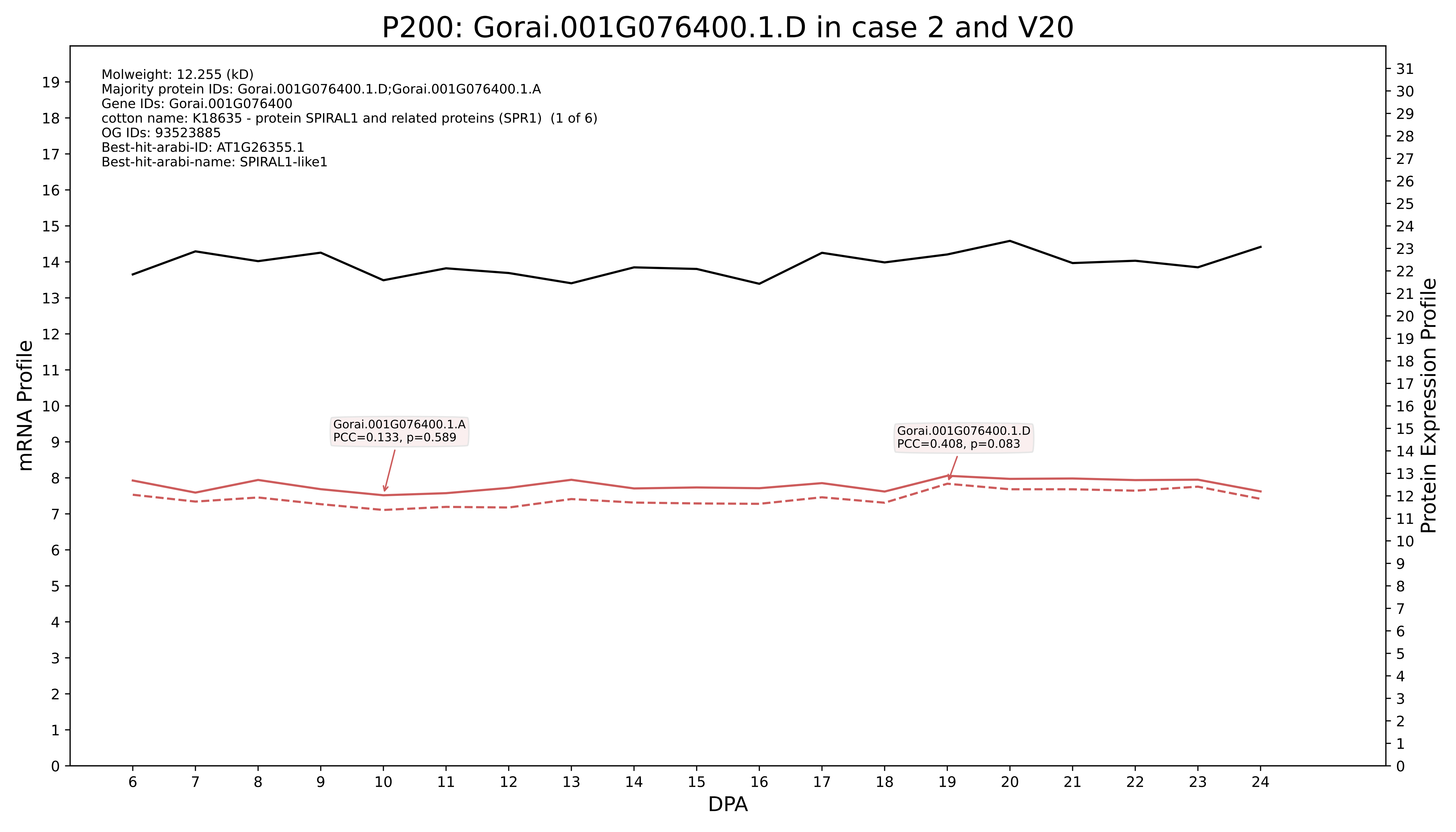

P200: Gorai.001G076400.1.D in case 2 and V20
Molweight: 12.255 (kD)
Majority protein IDs: Gorai.001G076400.1.D;Gorai.001G076400.1.A
Gene IDs: Gorai.001G076400
cotton name: K18635 - protein SPIRAL1 and related proteins (SPR1) (1 of 6)
OG IDs: 93523885
Best-hit-arabi-ID: AT1G26355.1
Best-hit-arabi-name: SPIRAL1-like1
Gorai.001G076400.1.A
PCC=0.133, p=0.589
Gorai.001G076400.1.D
PCC=0.408, p=0.083
mRNA Profile
Protein Expression Profile
DPA

P200: Gorai.011G014700.4.D in case 2 and V20

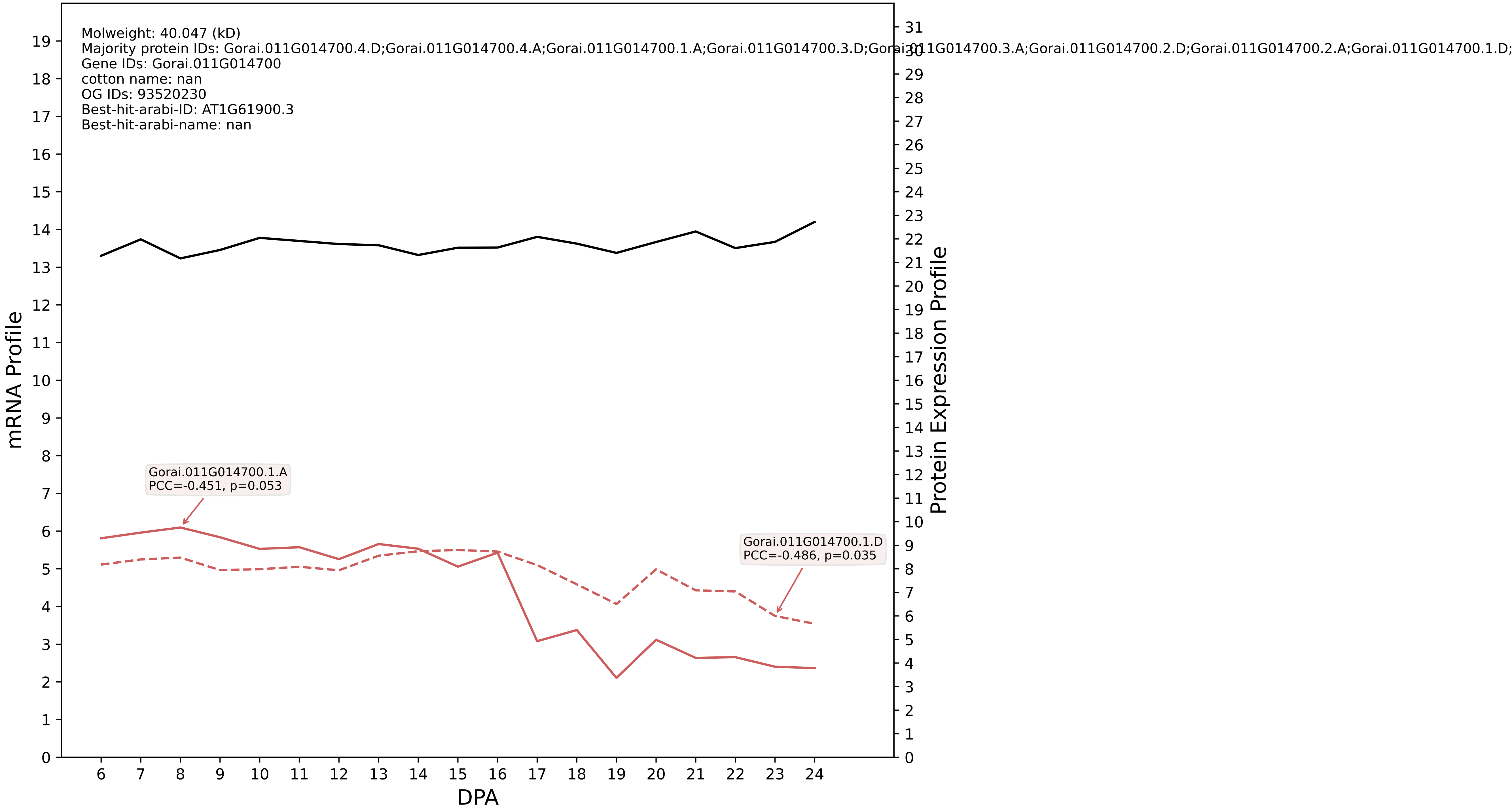

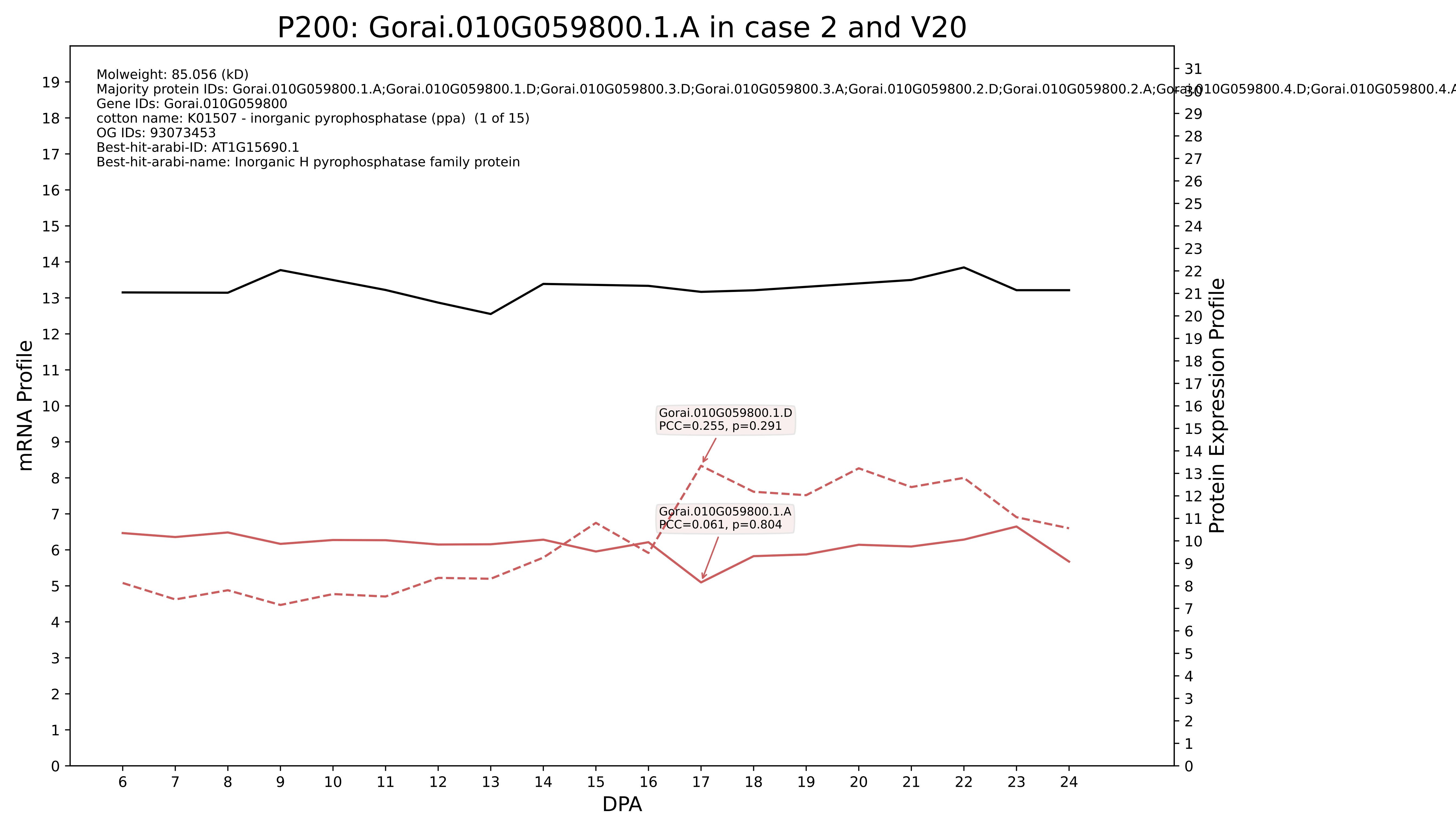
P200: Gorai.010G059800.1.A in case 2 and V20
Molweight: 85.056 (kD)
Majority protein IDs: Gorai.010G059800.1.A;Gorai.010G059800.1.D;Gorai.010G059800.3.D;Gorai.010G059800.3.A;Gorai.010G059800.2.D;Gorai.010G059800.2.A;Gorai.010G059800.4.D;Gorai.010G059800.4.A
Gene IDs: Gorai.010G059800
cotton name: K01507 - inorganic pyrophosphatase (ppa) (1 of 15)
OG IDs: 93073453
Best-hit-arabi-ID: AT1G15690.1
Best-hit-arabi-name: Inorganic H pyrophosphatase family protein
Gorai.010G059800.1.D
PCC=0.255, p=0.291
Gorai.010G059800.1.A
PCC=0.061, p=0.804
mRNA Profile
Protein Expression Profile
DPA

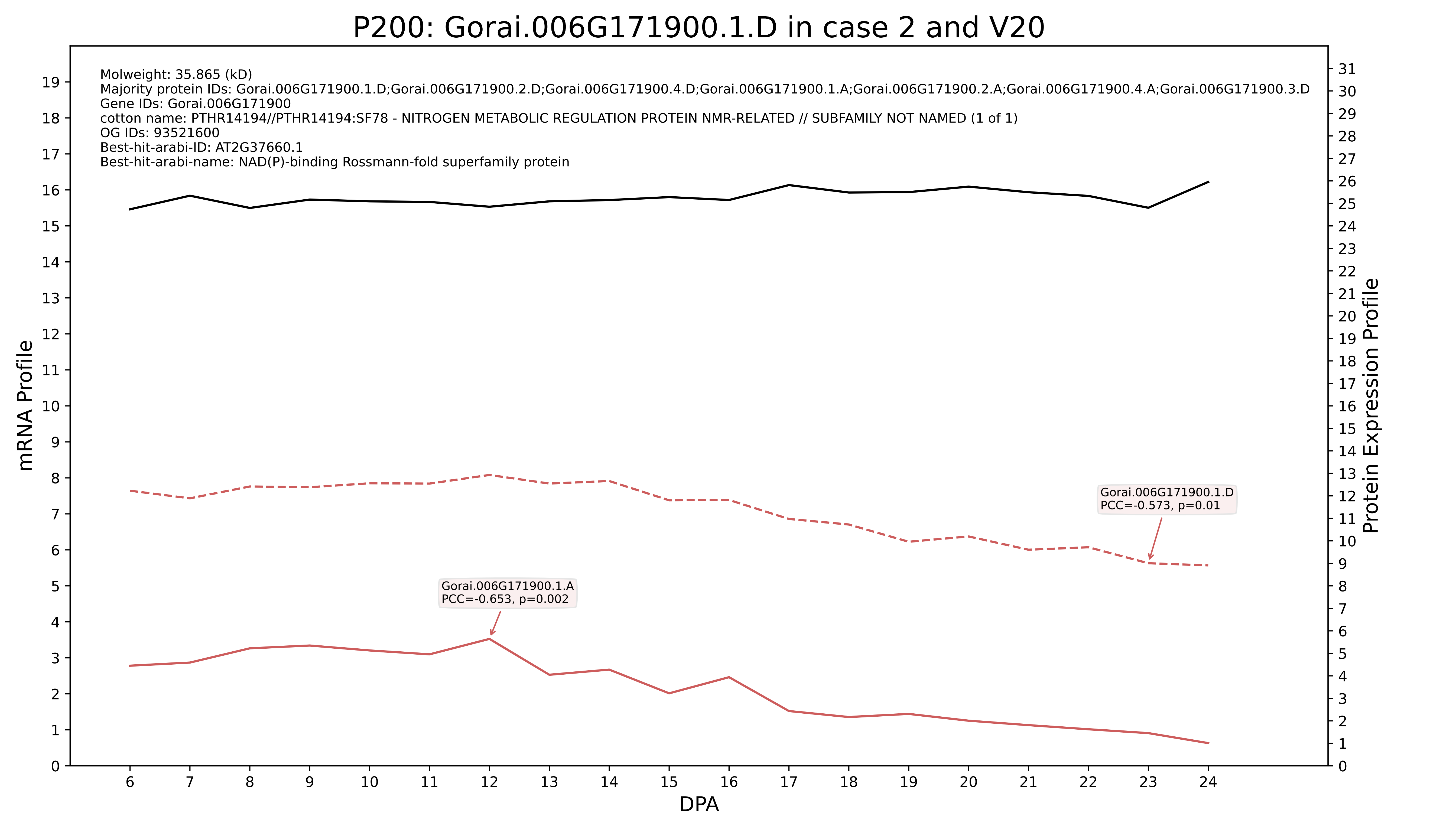

P200: Gorai.006G171900.1.D in case 2 and V20
Molweight: 35.865 (kD)
Majority protein IDs: Gorai.006G171900.1.D;Gorai.006G171900.2.D;Gorai.006G171900.4.D;Gorai.006G171900.1.A;Gorai.006G171900.2.A;Gorai.006G171900.4.A;Gorai.006G171900.3.D
Gene IDs: Gorai.006G171900
cotton name: PTHR14194//PTHR14194:SF78 - NITROGEN METABOLIC REGULATION PROTEIN NMR-RELATED // SUBFAMILY NOT NAMED (1 of 1)
OG IDs: 93521600
Best-hit-arabi-ID: AT2G37660.1
Best-hit-arabi-name: NAD(P)-binding Rossmann-fold superfamily protein
Gorai.006G171900.1.D
PCC=-0.573, p=0.01
Gorai.006G171900.1.A
PCC=-0.653, p=0.002
mRNA Profile
Protein Expression Profile
DPA

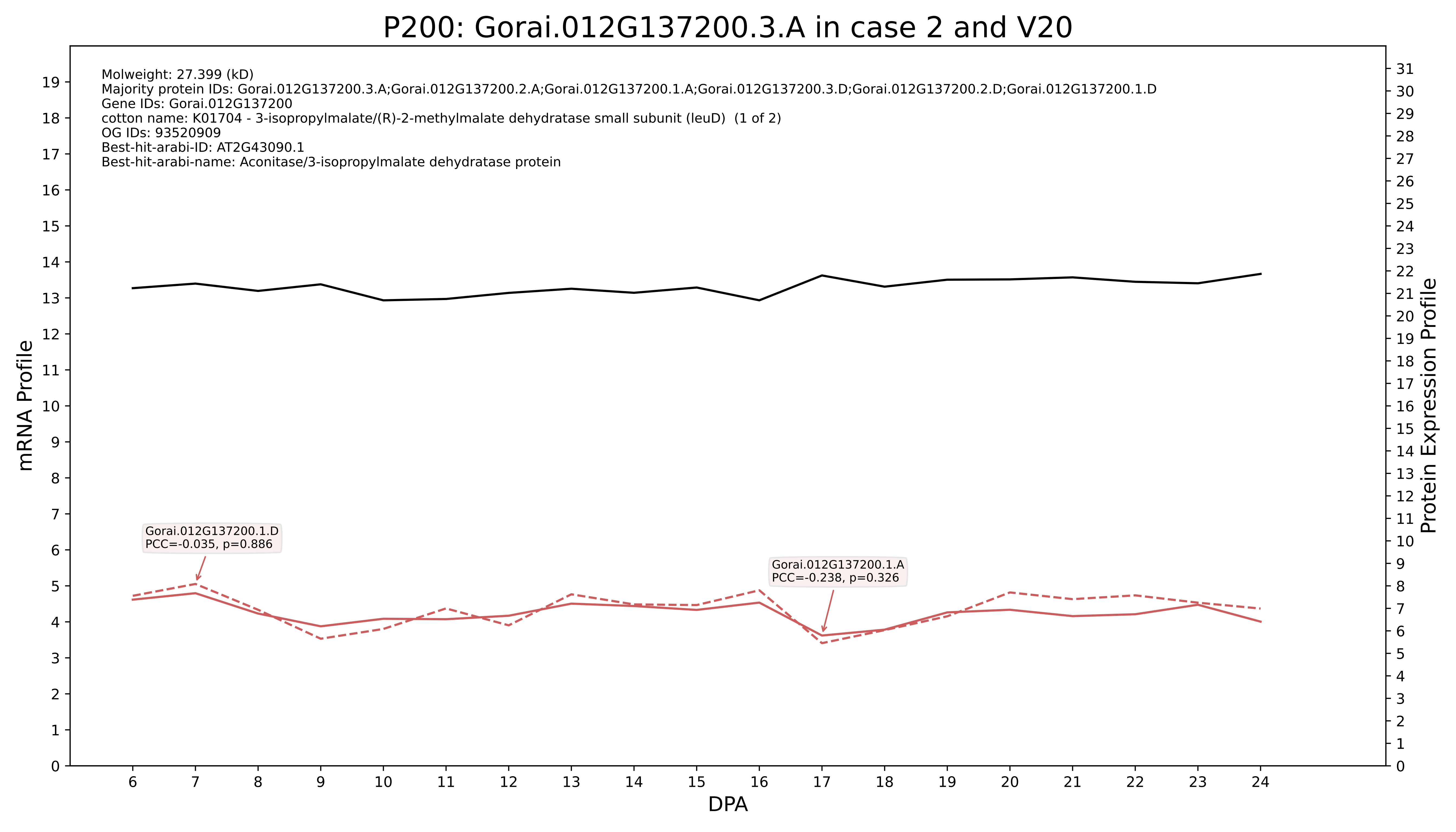

P200: Gorai.012G137200.3.A in case 2 and V20
Molweight: 27.399 (kD)
Majority protein IDs: Gorai.012G137200.3.A;Gorai.012G137200.2.A;Gorai.012G137200.1.A;Gorai.012G137200.3.D;Gorai.012G137200.2.D;Gorai.012G137200.1.D
Gene IDs: Gorai.012G137200
cotton name: K01704 - 3-isopropylmalate/(R)-2-methylmalate dehydratase small subunit (leuD) (1 of 2)
OG IDs: 93520909
Best-hit-arabi-ID: AT2G43090.1
Best-hit-arabi-name: Aconitase/3-isopropylmalate dehydratase protein
Gorai.012G137200.1.D
PCC=-0.035, p=0.886
Gorai.012G137200.1.A
PCC=-0.238, p=0.326
mRNA Profile
Protein Expression Profile
DPA

P200: Gorai.008G281900.1.A in case 2 and V20

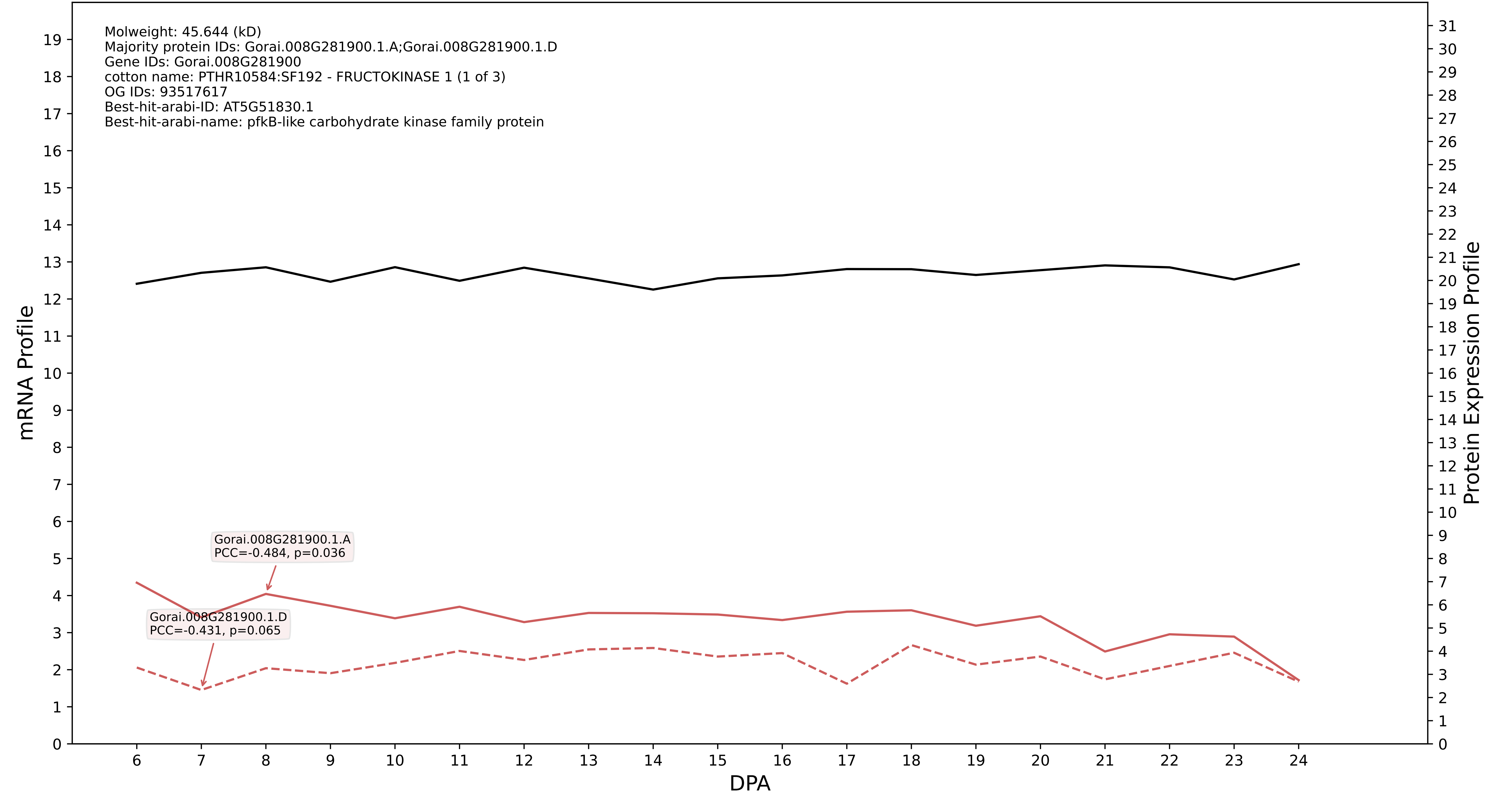

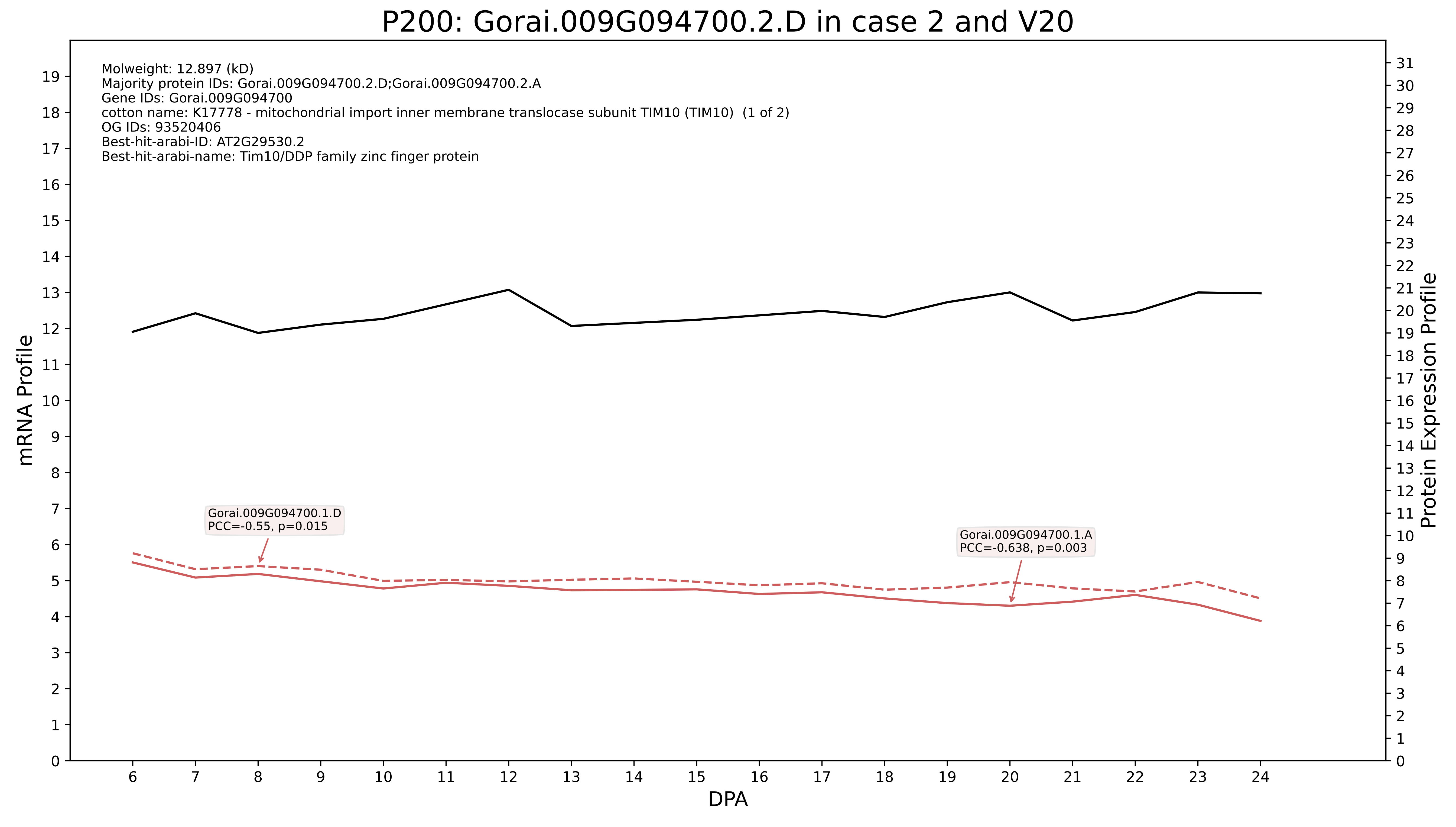
P200: Gorai.009G094700.2.D in case 2 and V20
Molweight: 12.897 (kD)
Majority protein IDs: Gorai.009G094700.2.D;Gorai.009G094700.2.A
Gene IDs: Gorai.009G094700
cotton name: K17778 - mitochondrial import inner membrane translocase subunit TIM10 (TIM10) (1 of 2)
OG IDs: 93520406
Best-hit-arabi-ID: AT2G29530.2
Best-hit-arabi-name: Tim10/DDP family zinc finger protein
Gorai.009G094700.1.D
PCC=-0.55, p=0.015
Gorai.009G094700.1.A
PCC=-0.638, p=0.003
mRNA Profile
Protein Expression Profile
DPA

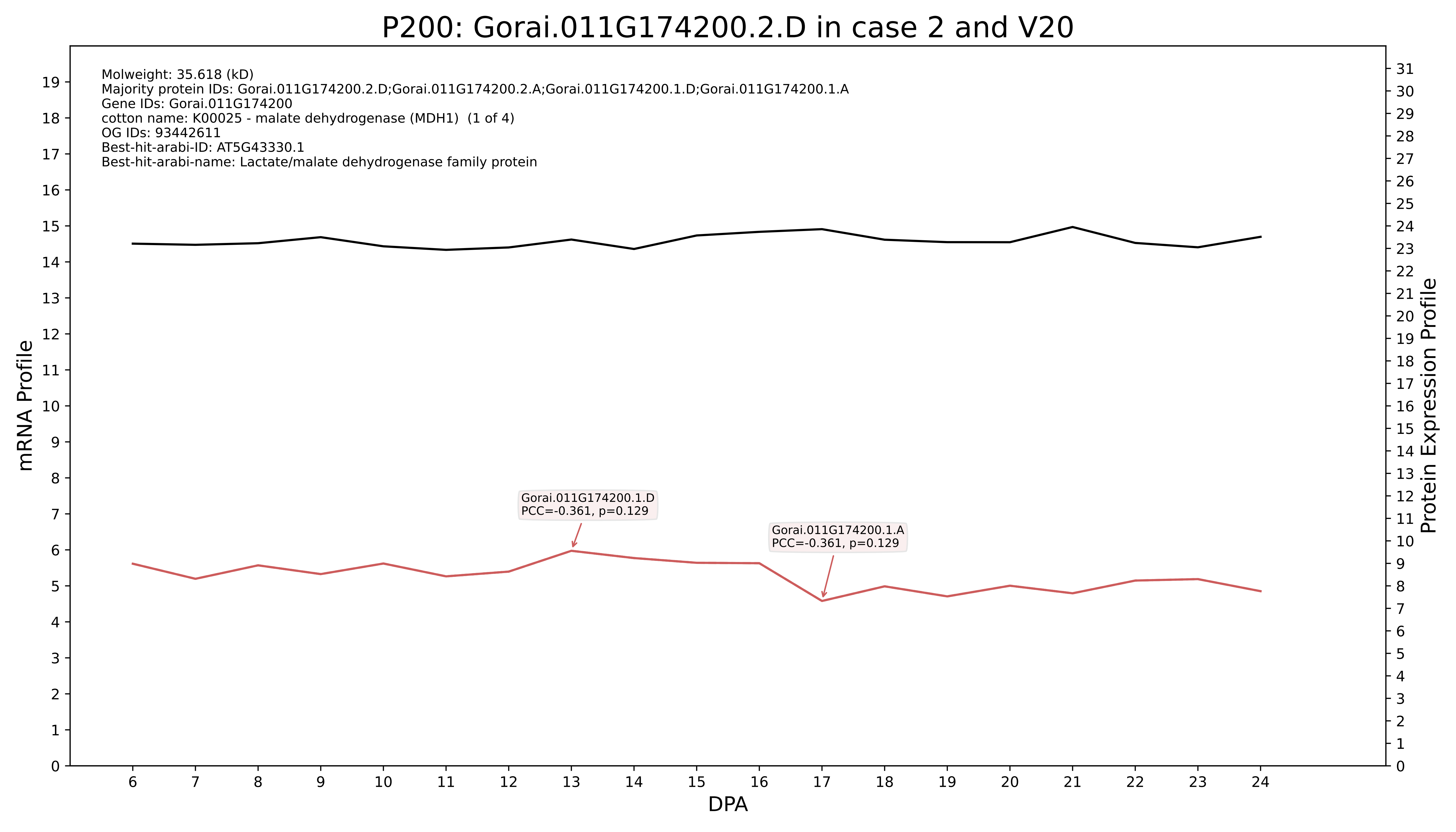

P200: Gorai.011G174200.2.D in case 2 and V20
Molweight: 35.618 (kD)
Majority protein IDs: Gorai.011G174200.2.D;Gorai.011G174200.2.A;Gorai.011G174200.1.D;Gorai.011G174200.1.A
Gene IDs: Gorai.011G174200
cotton name: K00025 - malate dehydrogenase (MDH1) (1 of 4)
OG IDs: 93442611
Best-hit-arabi-ID: AT5G43330.1
Best-hit-arabi-name: Lactate/malate dehydrogenase family protein
Gorai.011G174200.1.D
PCC=-0.361, p=0.129
Gorai.011G174200.1.A
PCC=-0.361, p=0.129
mRNA Profile
Protein Expression Profile
DPA

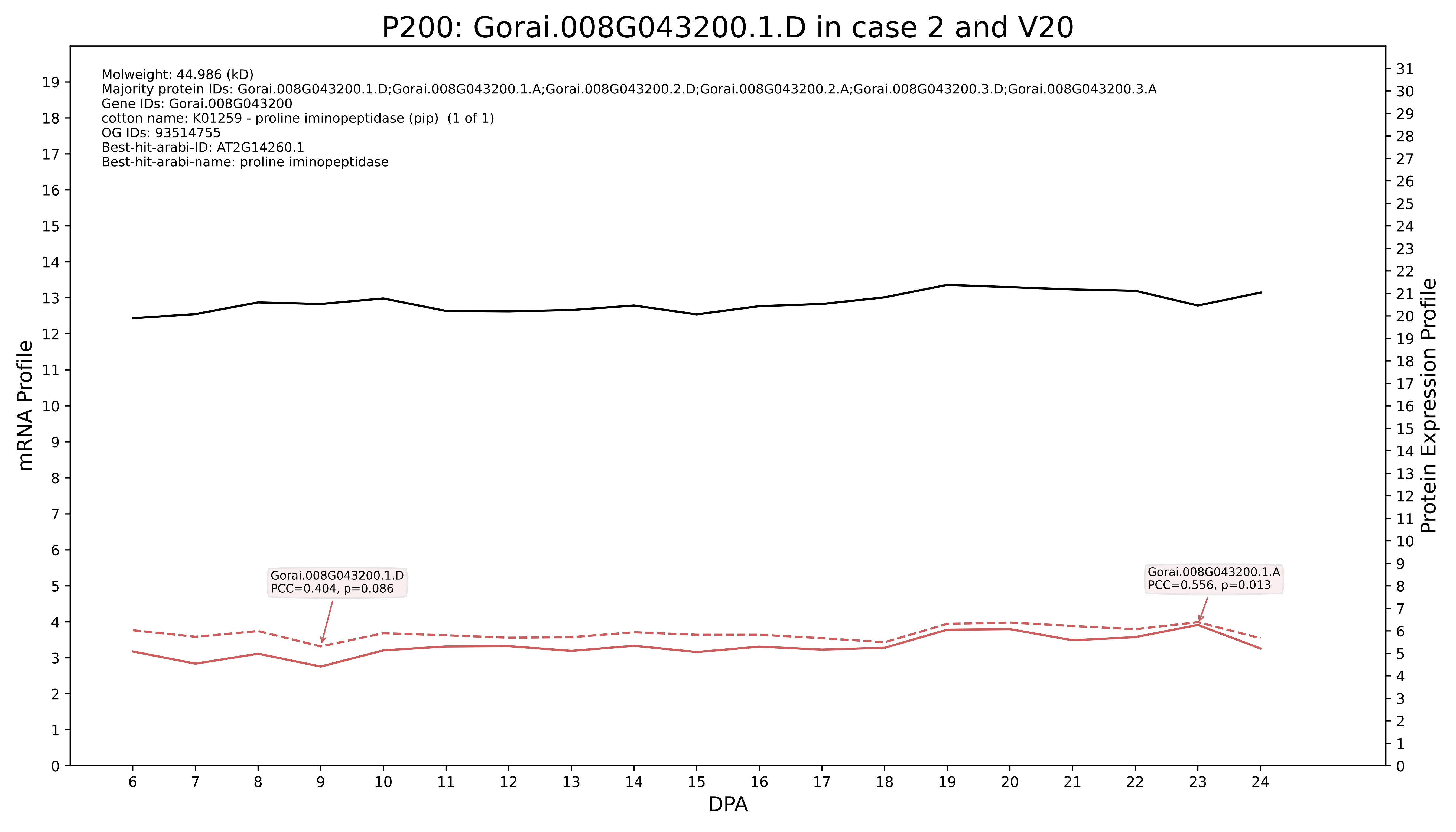
P200: Gorai.008G043200.1.D in case 2 and V20
Molweight: 44.986 (kD)
Majority protein IDs: Gorai.008G043200.1.D;Gorai.008G043200.1.A;Gorai.008G043200.2.D;Gorai.008G043200.2.A;Gorai.008G043200.3.D;Gorai.008G043200.3.A
Gene IDs: Gorai.008G043200
cotton name: K01259 - proline iminopeptidase (pip) (1 of 1)
OG IDs: 93514755
Best-hit-arabi-ID: AT2G14260.1
Best-hit-arabi-name: proline iminopeptidase
Gorai.008G043200.1.D
PCC=0.404, p=0.086
Gorai.008G043200.1.A
PCC=0.556, p=0.013
mRNA Profile
Protein Expression Profile
DPA

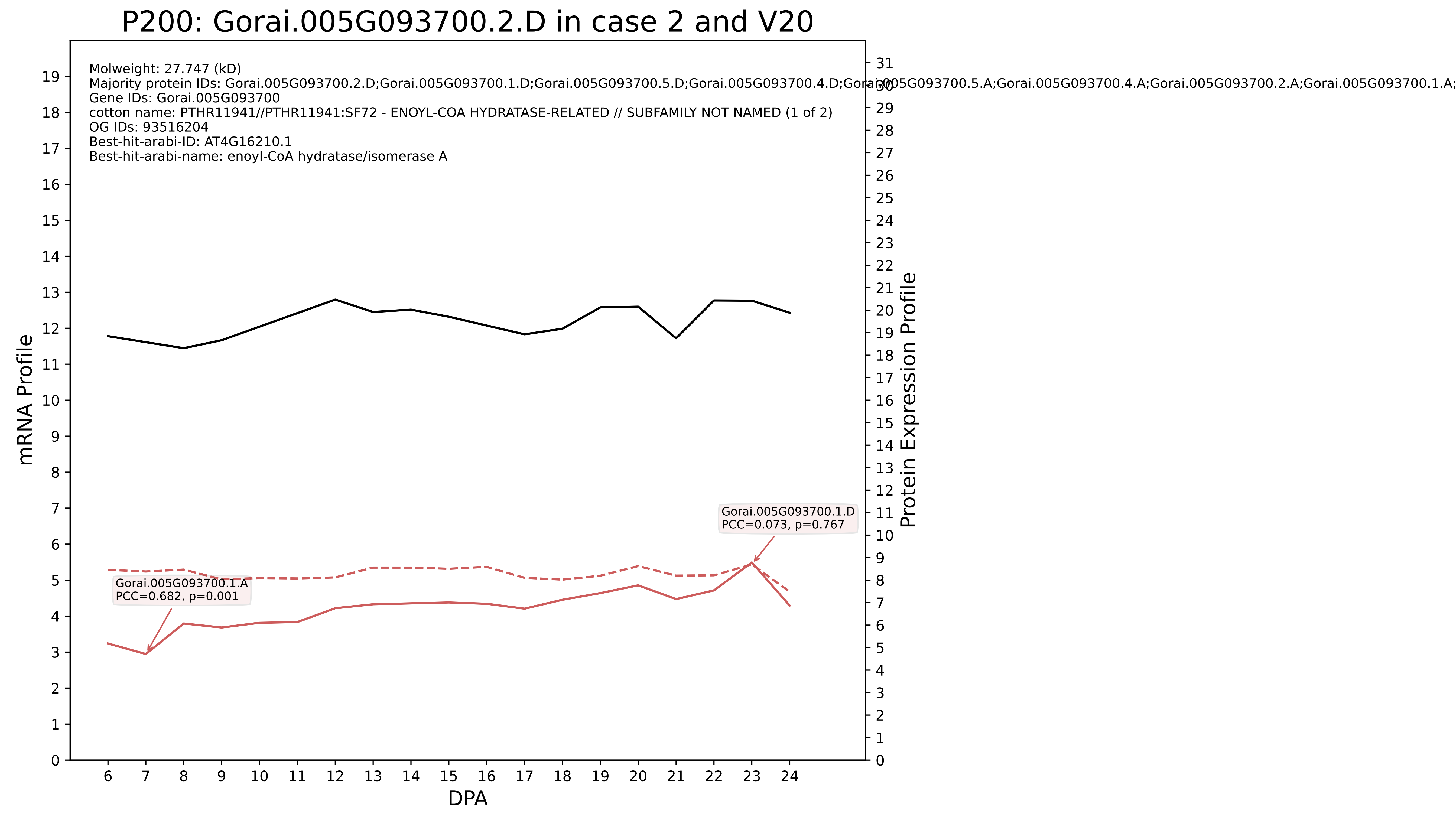
P200: Gorai.005G093700.2.D in case 2 and V20
Molweight: 27.747 (kD)
Majority protein IDs: Gorai.005G093700.2.D;Gorai.005G093700.1.D;Gorai.005G093700.5.D;Gorai.005G093700.4.D;Gorai.005G093700.5.A;Gorai.005G093700.4.A;Gorai.005G093700.2.A;Gorai.005G093700.1.A;
Gene IDs: Gorai.005G093700
cotton name: PTHR11941//PTHR11941:SF72 - ENOYL-COA HYDRATASE-RELATED // SUBFAMILY NOT NAMED (1 of 2)
OG IDs: 93516204
Best-hit-arabi-ID: AT4G16210.1
Best-hit-arabi-name: enoyl-CoA hydratase/isomerase A
Gorai.005G093700.1.D
PCC=0.073, p=0.767
Gorai.005G093700.1.A
PCC=0.682, p=0.001
mRNA Profile
Protein Expression Profile
DPA

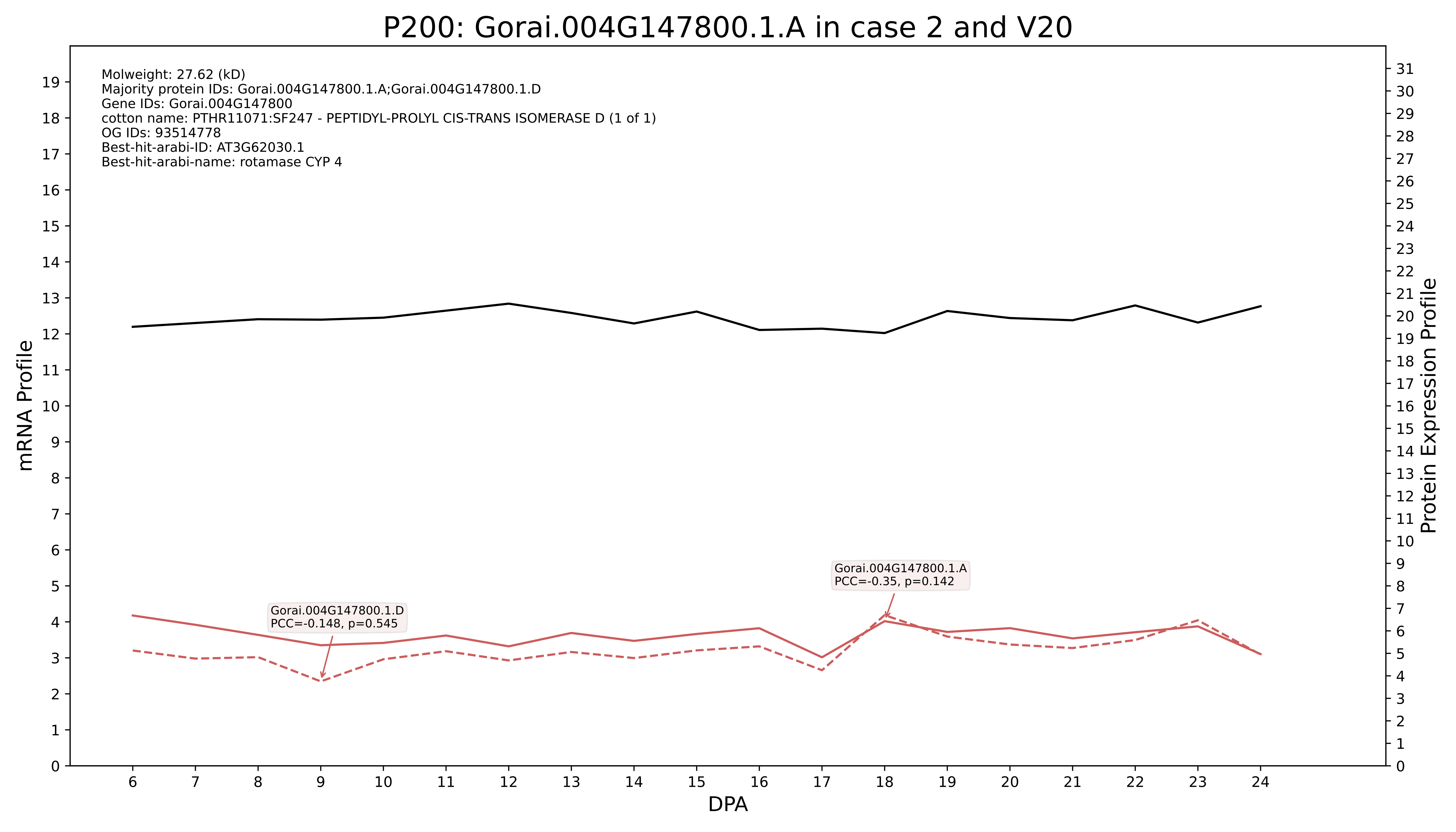
P200: Gorai.004G147800.1.A in case 2 and V20
Molweight: 27.62 (kD)
Majority protein IDs: Gorai.004G147800.1.A;Gorai.004G147800.1.D
Gene IDs: Gorai.004G147800
cotton name: PTHR11071:SF247 - PEPTIDYL-PROLYL CIS-TRANS ISOMERASE D (1 of 1)
OG IDs: 93514778
Best-hit-arabi-ID: AT3G62030.1
Best-hit-arabi-name: rotamase CYP 4
Gorai.004G147800.1.A
PCC=-0.35, p=0.142
Gorai.004G147800.1.D
PCC=-0.148, p=0.545
mRNA Profile
Protein Expression Profile
DPA
6
7
8
9
10
11
12
13
14
15
16
17
18
19
20
21
22
23
24
0
1
2
3
4
5
6
7
8
9
10
11
12
13
14
15
16
17
18
19
20
21
22
23
24
25
26
27
28
29
30
31

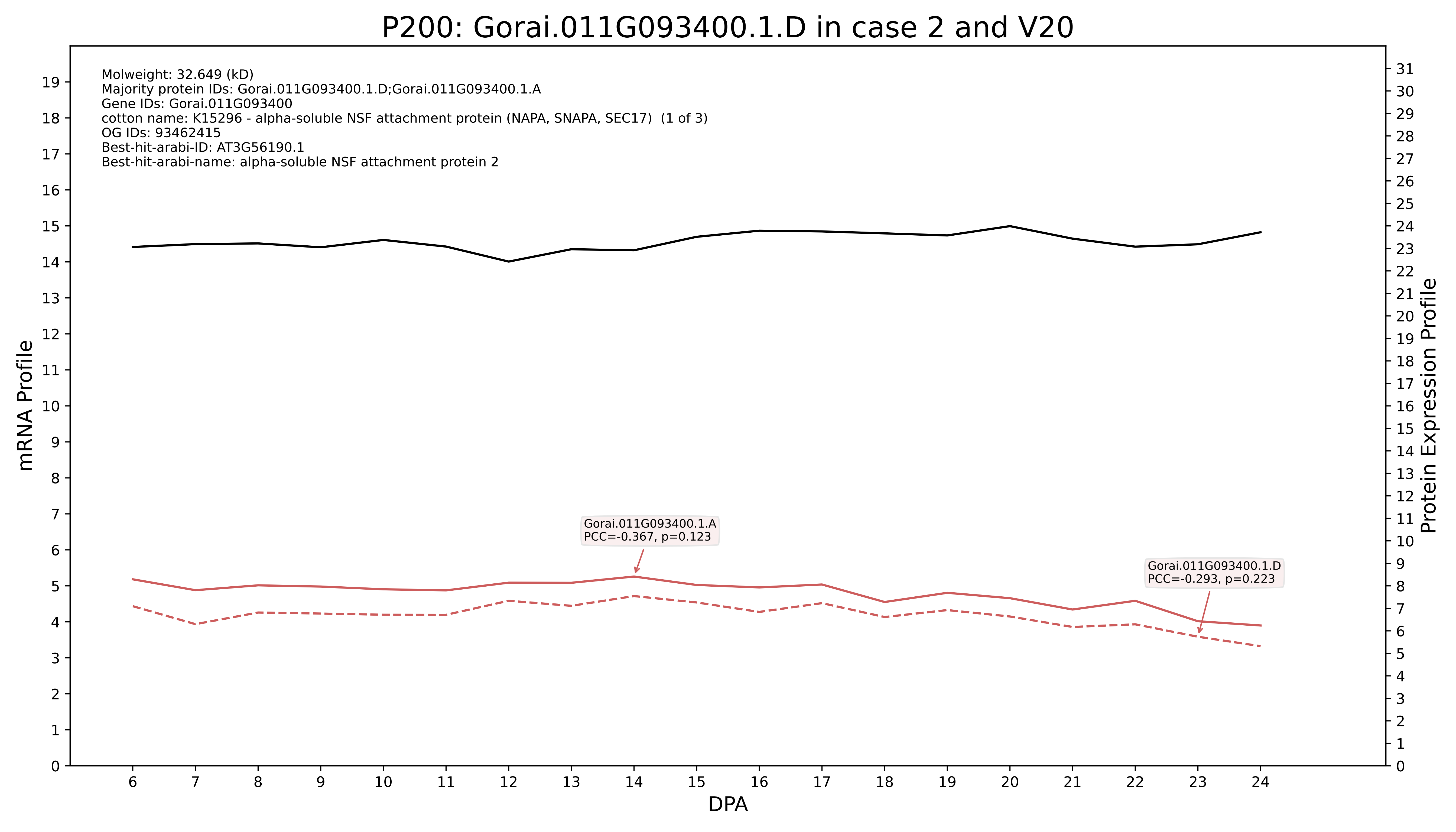
P200: Gorai.011G093400.1.D in case 2 and V20
Molweight: 32.649 (kD)
Majority protein IDs: Gorai.011G093400.1.D;Gorai.011G093400.1.A
Gene IDs: Gorai.011G093400
cotton name: K15296 - alpha-soluble NSF attachment protein (NAPA, SNAPA, SEC17) (1 of 3)
OG IDs: 93462415
Best-hit-arabi-ID: AT3G56190.1
Best-hit-arabi-name: alpha-soluble NSF attachment protein 2
Gorai.011G093400.1.A
PCC=-0.367, p=0.123
Gorai.011G093400.1.D
PCC=-0.293, p=0.223
mRNA Profile
Protein Expression Profile
DPA

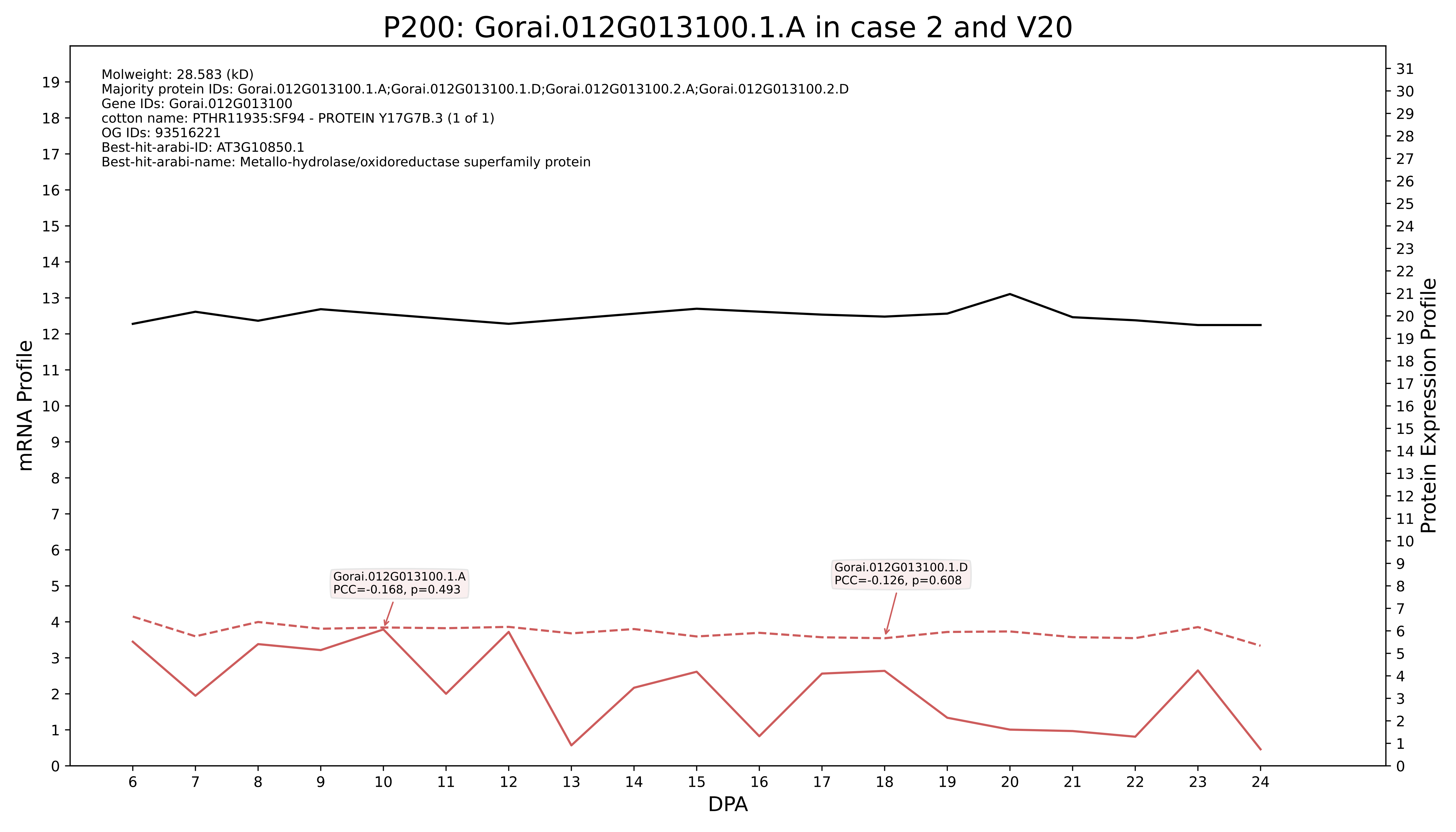
P200: Gorai.012G013100.1.A in case 2 and V20
Molweight: 28.583 (kD)
Majority protein IDs: Gorai.012G013100.1.A;Gorai.012G013100.1.D;Gorai.012G013100.2.A;Gorai.012G013100.2.D
Gene IDs: Gorai.012G013100
cotton name: PTHR11935:SF94 - PROTEIN Y17G7B.3 (1 of 1)
OG IDs: 93516221
Best-hit-arabi-ID: AT3G10850.1
Best-hit-arabi-name: Metallo-hydrolase/oxidoreductase superfamily protein
Gorai.012G013100.1.A
PCC=-0.168, p=0.493
Gorai.012G013100.1.D
PCC=-0.126, p=0.608
mRNA Profile
Protein Expression Profile
DPA

005G146400.7.D in case 2 and V20

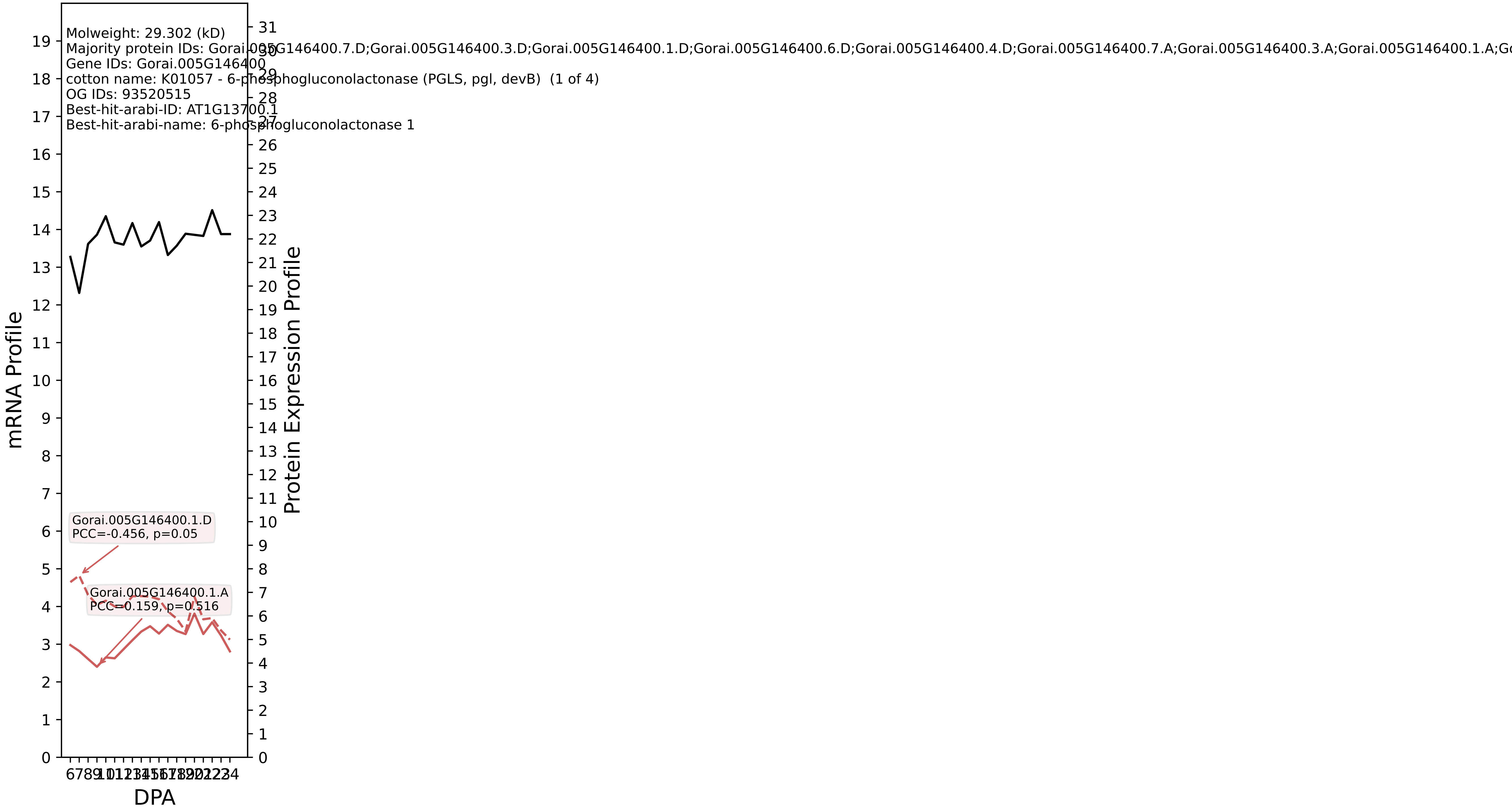

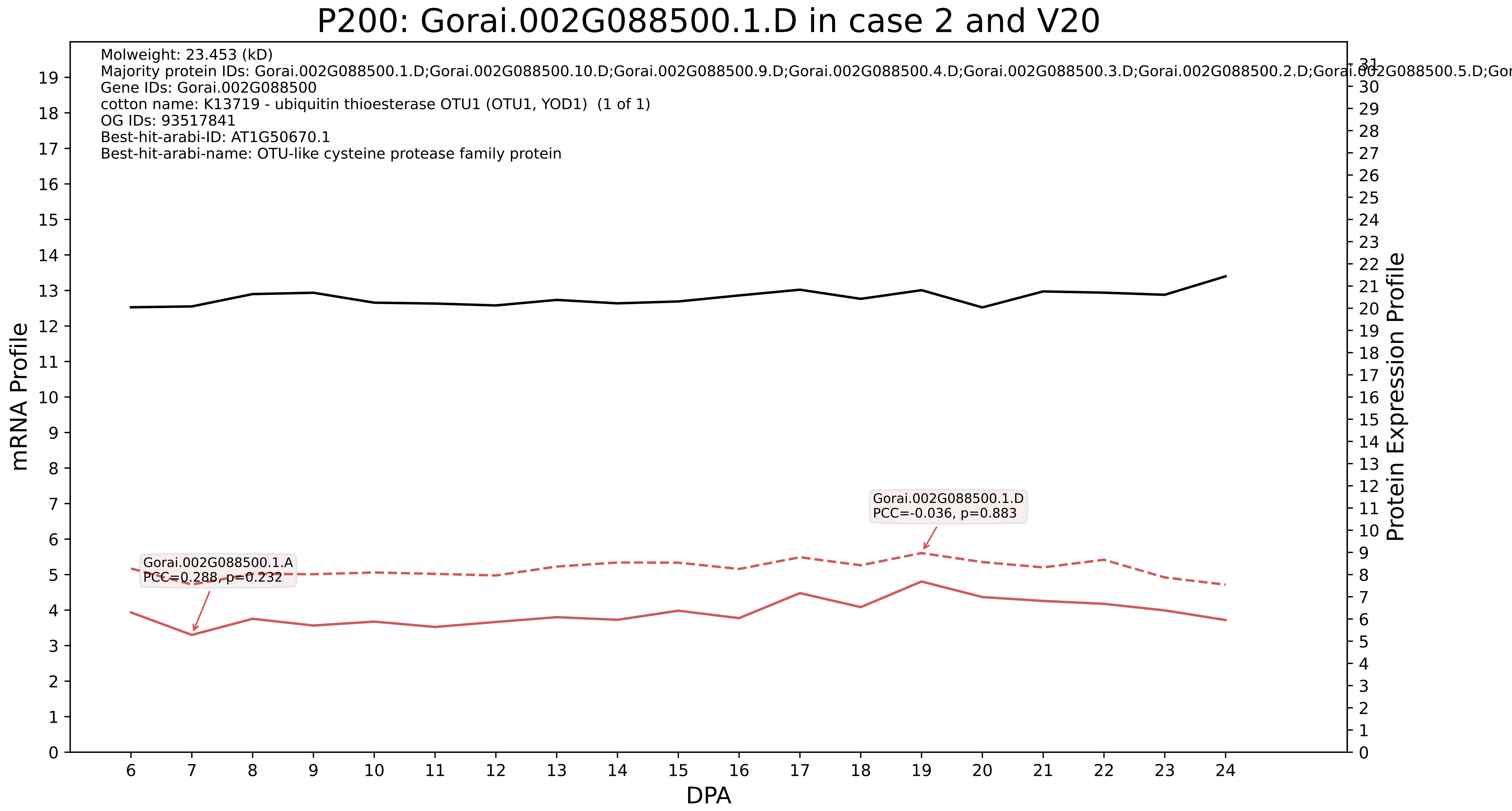
P200: Gorai.002G088500.1.D in case 2 and V20
Molweight: 23.453 (kD)
Majority protein IDs: Gorai.002G088500.1.D;Gorai.002G088500.10.D;Gorai.002G088500.9.D;Gorai.002G088500.4.D;Gorai.002G088500.3.D;Gorai.002G088500.2.D;Gorai.002G088500.5.D;Gor
Gene IDs: Gorai.002G088500
cotton name: K13719 - ubiquitin thioesterase OTU1 (OTU1, YOD1) (1 of 1)
OG IDs: 93517841
Best-hit-arabi-ID: AT1G50670.1
Best-hit-arabi-name: OTU-like cysteine protease family protein
Gorai.002G088500.1.D
PCC=-0.036, p=0.883
Gorai.002G088500.1.A
PCC=0.288, p=0.232
mRNA Profile
Protein Expression Profile
DPA

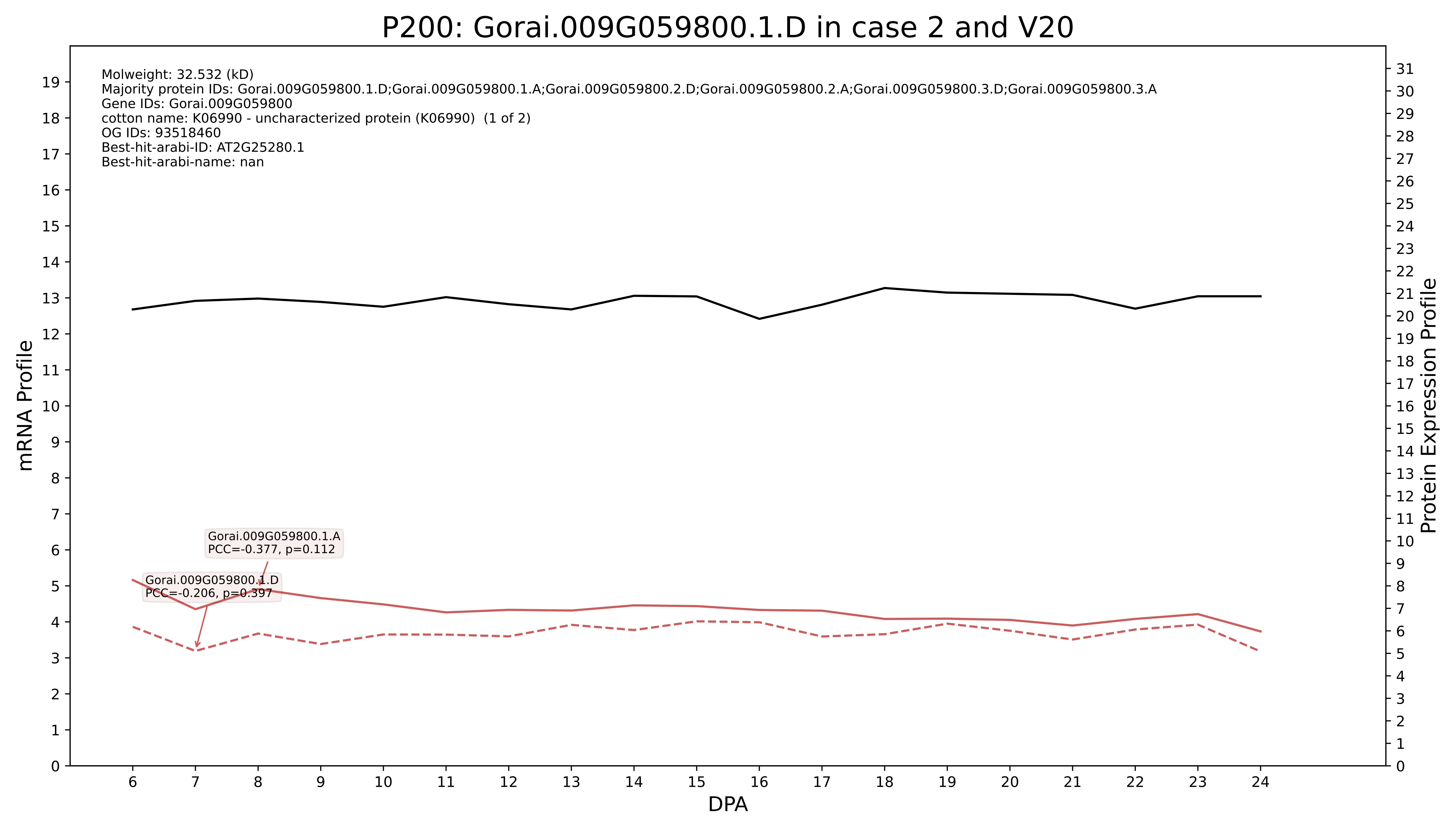
P200: Gorai.009G059800.1.D in case 2 and V20
Molweight: 32.532 (kD)
Majority protein IDs: Gorai.009G059800.1.D;Gorai.009G059800.1.A;Gorai.009G059800.2.D;Gorai.009G059800.2.A;Gorai.009G059800.3.D;Gorai.009G059800.3.A
Gene IDs: Gorai.009G059800
cotton name: K06990 - uncharacterized protein (K06990) (1 of 2)
OG IDs: 93518460
Best-hit-arabi-ID: AT2G25280.1
Best-hit-arabi-name: nan
Gorai.009G059800.1.A
PCC=-0.377, p=0.112
Gorai.009G059800.1.D
PCC=-0.206, p=0.397
mRNA Profile
Protein Expression Profile
DPA
0
1
2
3
4
5
6
7
8
9
10
11
12
13
14
15
16
17
18
19
20
21
22
23
24
25
26
27
28
29
30
31

P200: Gorai.013G120800.1.D in case 2 and V20

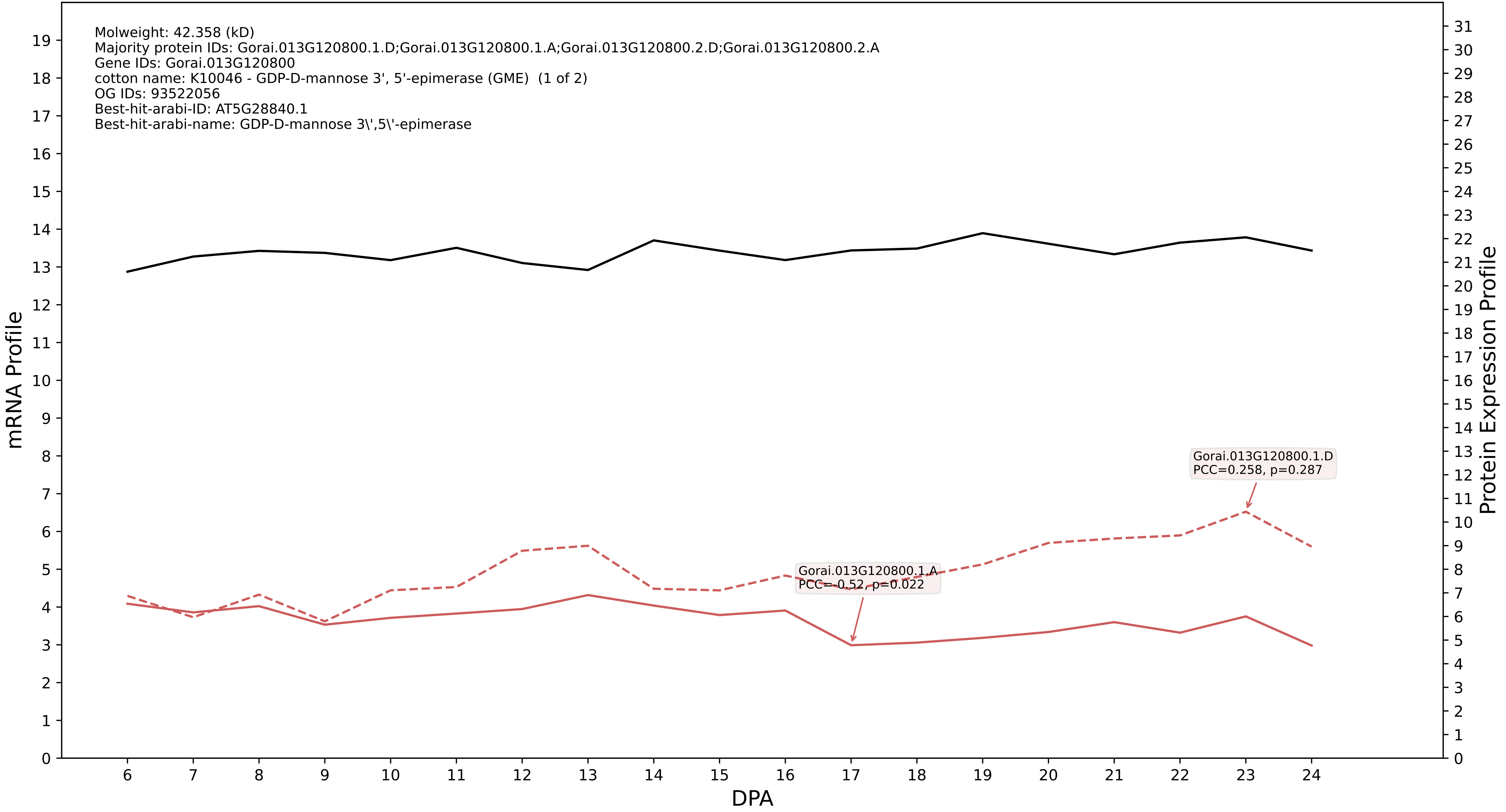

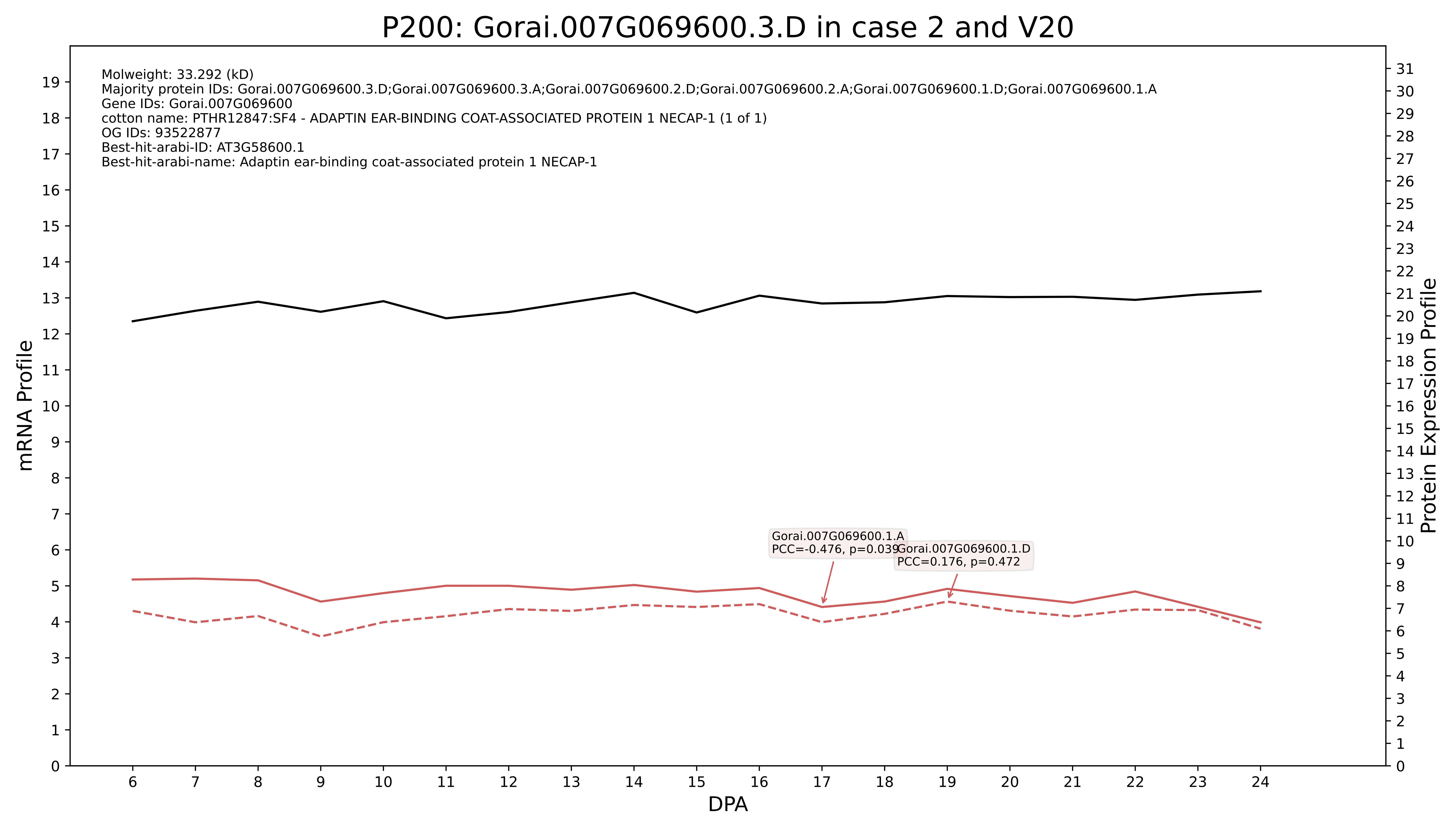
P200: Gorai.007G069600.3.D in case 2 and V20
Molweight: 33.292 (kD)
Majority protein IDs: Gorai.007G069600.3.D;Gorai.007G069600.3.A;Gorai.007G069600.2.D;Gorai.007G069600.2.A;Gorai.007G069600.1.D;Gorai.007G069600.1.A
Gene IDs: Gorai.007G069600
cotton name: PTHR12847:SF4 - ADAPTIN EAR-BINDING COAT-ASSOCIATED PROTEIN 1 NECAP-1 (1 of 1)
OG IDs: 93522877
Best-hit-arabi-ID: AT3G58600.1
Best-hit-arabi-name: Adaptin ear-binding coat-associated protein 1 NECAP-1
Gorai.007G069600.1.A
PCC=-0.476, p=0.039
Gorai.007G069600.1.D
PCC=0.176, p=0.472
mRNA Profile
Protein Expression Profile
DPA

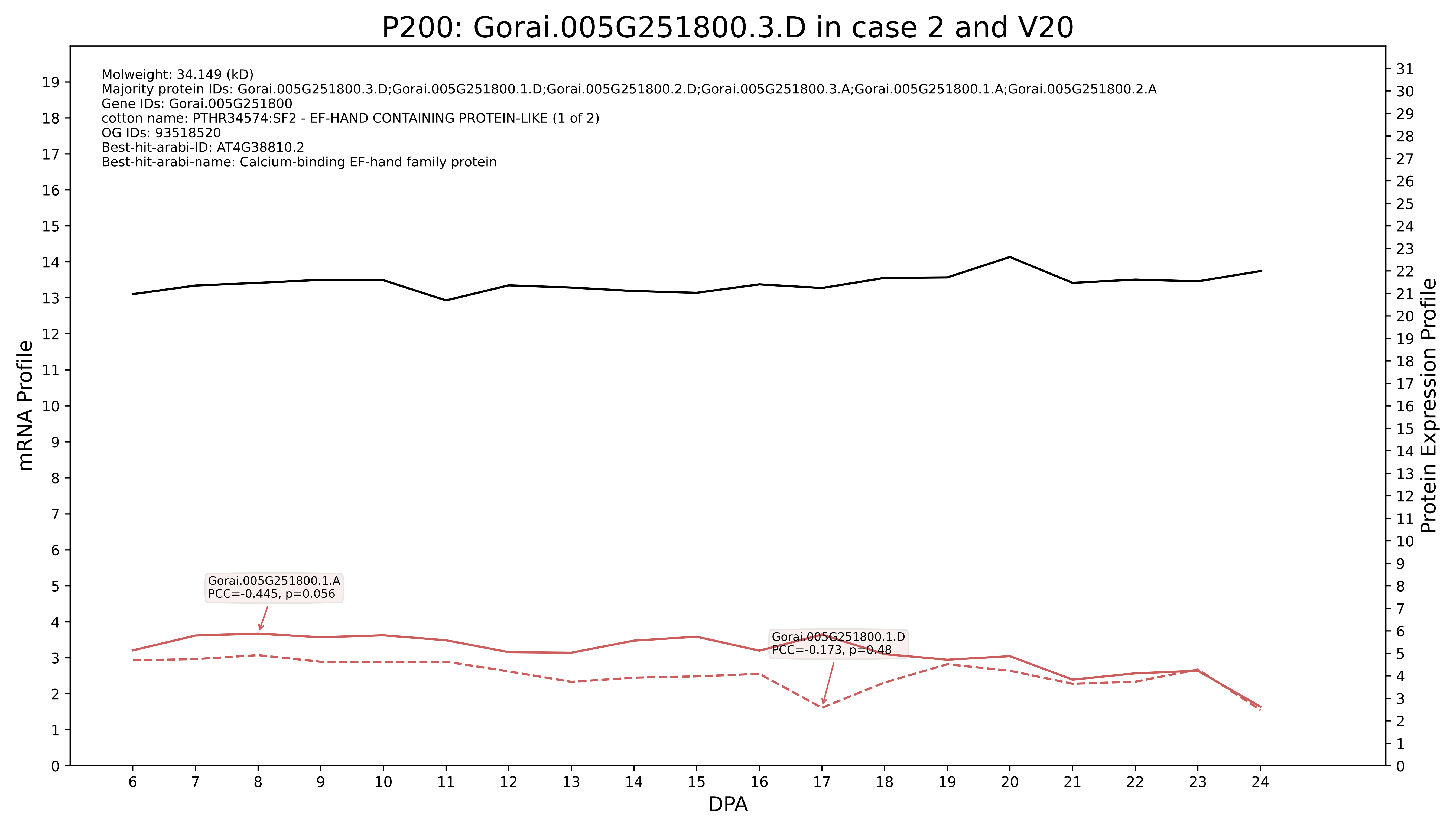

P200: Gorai.005G251800.3.D in case 2 and V20
Molweight: 34.149 (kD)
Majority protein IDs: Gorai.005G251800.3.D;Gorai.005G251800.1.D;Gorai.005G251800.2.D;Gorai.005G251800.3.A;Gorai.005G251800.1.A;Gorai.005G251800.2.A
Gene IDs: Gorai.005G251800
cotton name: PTHR34574:SF2 - EF-HAND CONTAINING PROTEIN-LIKE (1 of 2)
OG IDs: 93518520
Best-hit-arabi-ID: AT4G38810.2
Best-hit-arabi-name: Calcium-binding EF-hand family protein
Gorai.005G251800.1.A
PCC=-0.445, p=0.056
Gorai.005G251800.1.D
PCC=-0.173, p=0.48
mRNA Profile
Protein Expression Profile
DPA

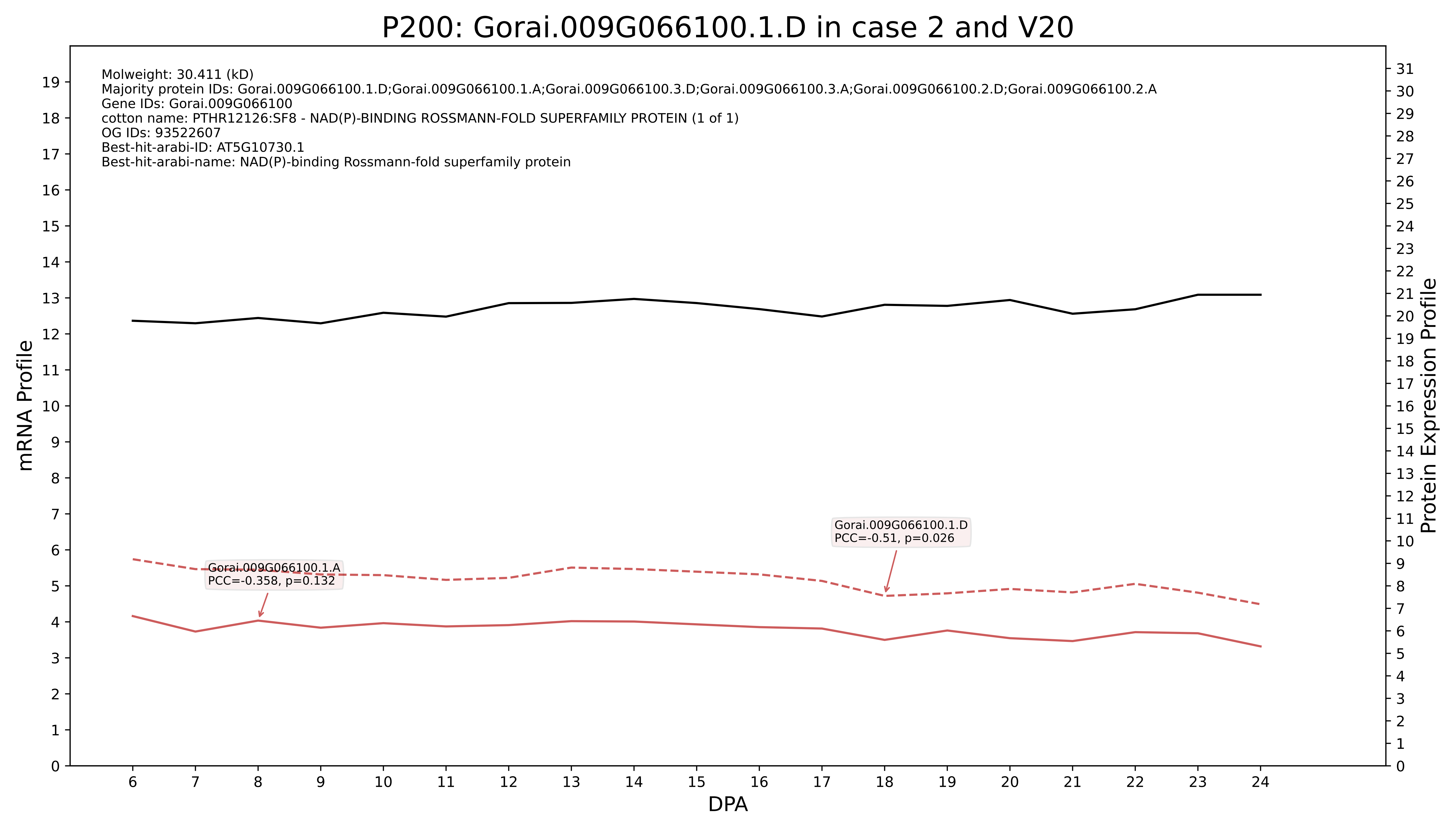
P200: Gorai.009G066100.1.D in case 2 and V20
Molweight: 30.411 (kD)
Majority protein IDs: Gorai.009G066100.1.D;Gorai.009G066100.1.A;Gorai.009G066100.3.D;Gorai.009G066100.3.A;Gorai.009G066100.2.D;Gorai.009G066100.2.A
Gene IDs: Gorai.009G066100
cotton name: PTHR12126:SF8 - NAD(P)-BINDING ROSSMANN-FOLD SUPERFAMILY PROTEIN (1 of 1)
OG IDs: 93522607
Best-hit-arabi-ID: AT5G10730.1
Best-hit-arabi-name: NAD(P)-binding Rossmann-fold superfamily protein
Gorai.009G066100.1.D
PCC=-0.51, p=0.026
Gorai.009G066100.1.A
PCC=-0.358, p=0.132
mRNA Profile
Protein Expression Profile
DPA
0
1
2
3
4
5
6
7
8
9
10
11
12
13
14
15
16
17
18
19
20
21
22
23
24
25
26
27
28
29
30
31

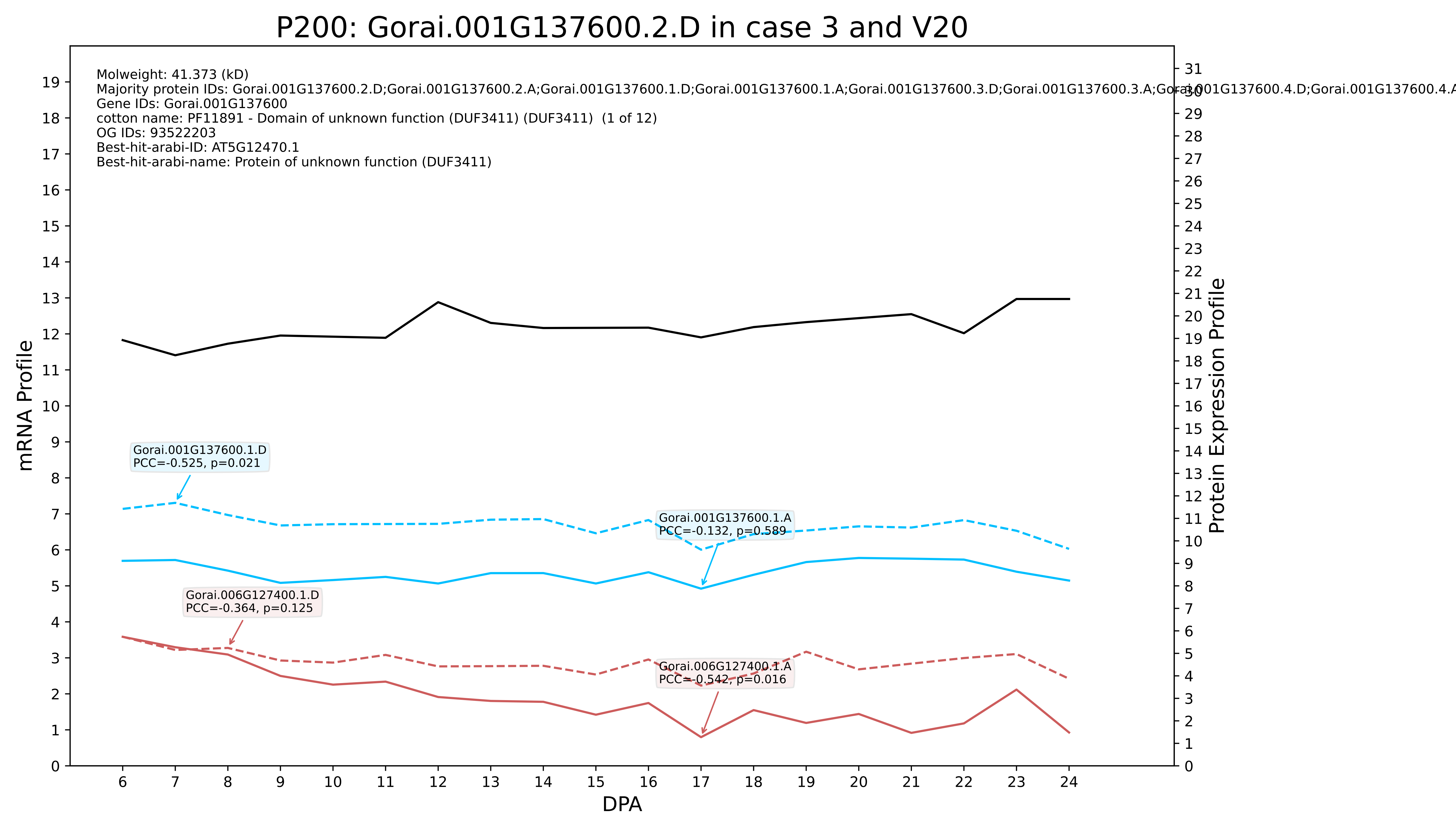
P200: Gorai.001G137600.2.D in case 3 and V20
Molweight: 41.373 (kD)
Majority protein IDs: Gorai.001G137600.2.D;Gorai.001G137600.2.A;Gorai.001G137600.1.D;Gorai.001G137600.1.A;Gorai.001G137600.3.D;Gorai.001G137600.3.A;Gorai.001G137600.4.D;Gorai.001G137600.4.A
Gene IDs: Gorai.001G137600
cotton name: PF11891 - Domain of unknown function (DUF3411) (DUF3411) (1 of 12)
OG IDs: 93522203
Best-hit-arabi-ID: AT5G12470.1
Best-hit-arabi-name: Protein of unknown function (DUF3411)
Gorai.001G137600.1.D
PCC=-0.525, p=0.021
Gorai.001G137600.1.A
PCC=-0.132, p=0.589
Gorai.006G127400.1.D
PCC=-0.364, p=0.125
Gorai.006G127400.1.A
PCC=-0.542, p=0.016
mRNA Profile
Protein Expression Profile
DPA

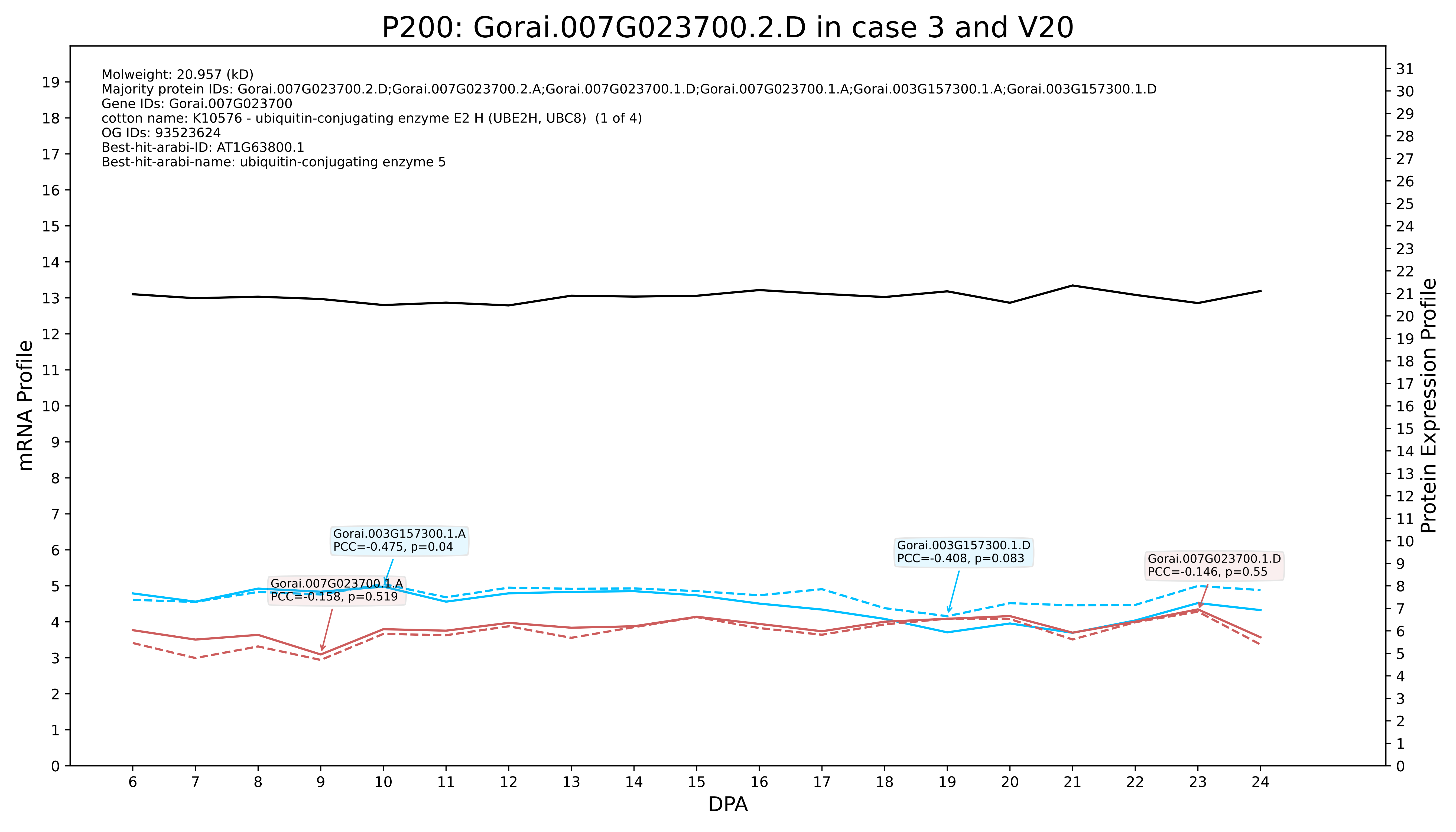

P200: Gorai.007G023700.2.D in case 3 and V20
Molweight: 20.957 (kD)
Majority protein IDs: Gorai.007G023700.2.D;Gorai.007G023700.2.A;Gorai.007G023700.1.D;Gorai.007G023700.1.A;Gorai.003G157300.1.A;Gorai.003G157300.1.D
Gene IDs: Gorai.007G023700
cotton name: K10576 - ubiquitin-conjugating enzyme E2 H (UBE2H, UBC8) (1 of 4)
OG IDs: 93523624
Best-hit-arabi-ID: AT1G63800.1
Best-hit-arabi-name: ubiquitin-conjugating enzyme 5
Gorai.003G157300.1.A
PCC=-0.475, p=0.04
Gorai.007G023700.1.A
PCC=-0.158, p=0.519
Gorai.003G157300.1.D
PCC=-0.408, p=0.083
Gorai.007G023700.1.D
PCC=-0.146, p=0.55
mRNA Profile
Protein Expression Profile
DPA

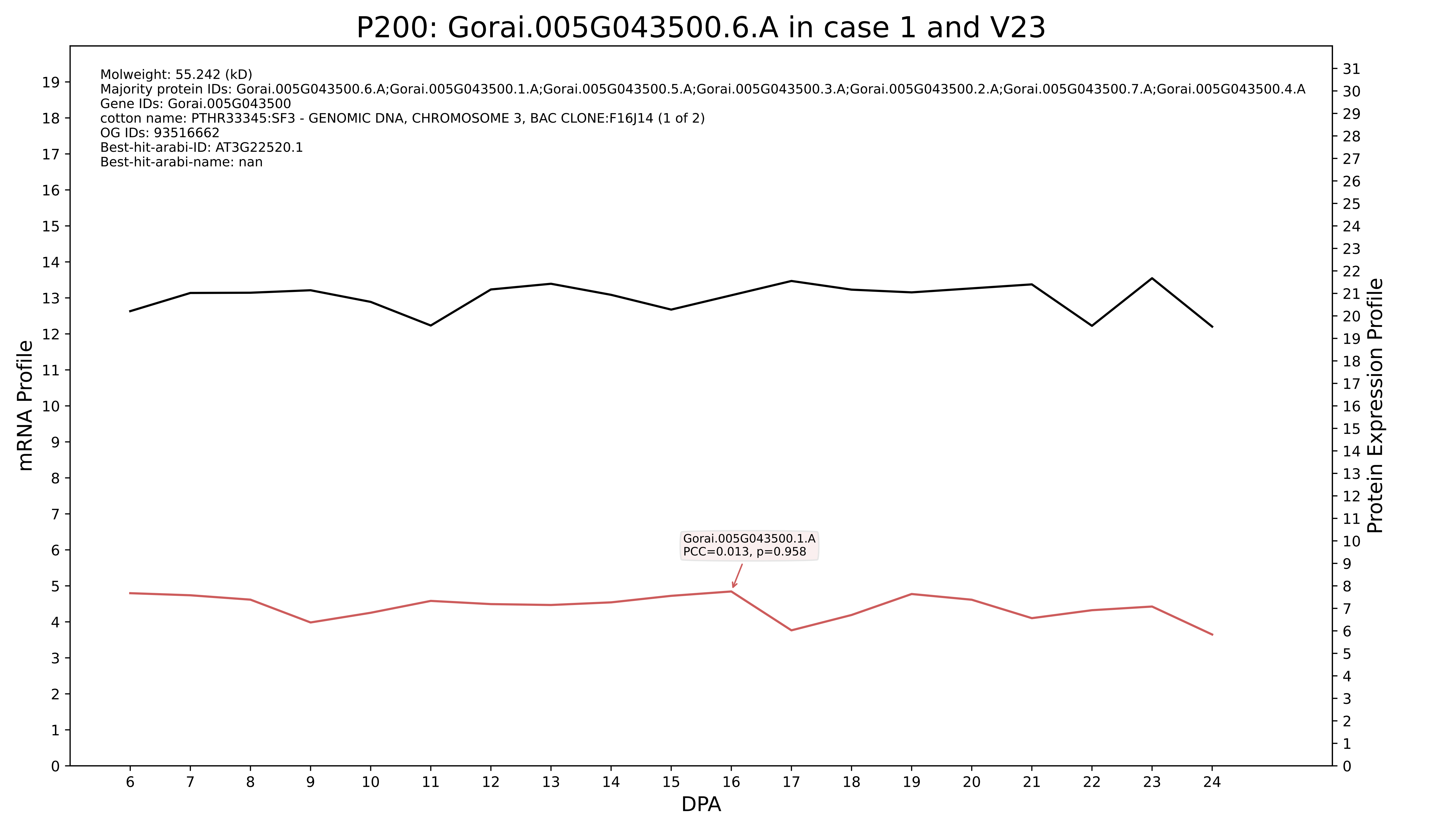
P200: Gorai.005G043500.6.A in case 1 and V23
Molweight: 55.242 (kD)
Majority protein IDs: Gorai.005G043500.6.A;Gorai.005G043500.1.A;Gorai.005G043500.5.A;Gorai.005G043500.3.A;Gorai.005G043500.2.A;Gorai.005G043500.7.A;Gorai.005G043500.4.A
Gene IDs: Gorai.005G043500
cotton name: PTHR33345:SF3 - GENOMIC DNA, CHROMOSOME 3, BAC CLONE:F16J14 (1 of 2)
OG IDs: 93516662
Best-hit-arabi-ID: AT3G22520.1
Best-hit-arabi-name: nan
Gorai.005G043500.1.A
PCC=0.013, p=0.958
mRNA Profile
Protein Expression Profile
DPA

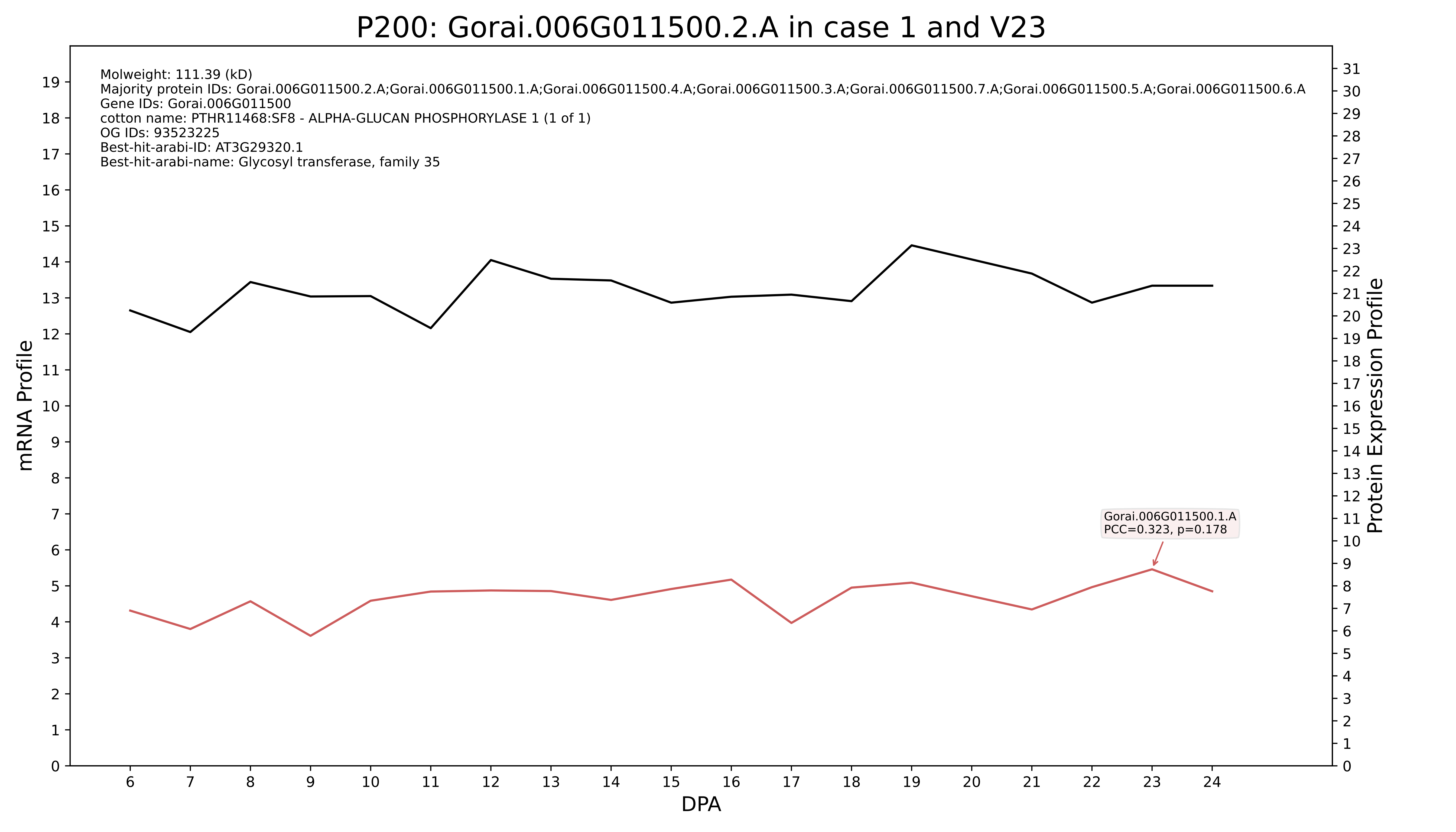
P200: Gorai.006G011500.2.A in case 1 and V23
Molweight: 111.39 (kD)
Majority protein IDs: Gorai.006G011500.2.A;Gorai.006G011500.1.A;Gorai.006G011500.4.A;Gorai.006G011500.3.A;Gorai.006G011500.7.A;Gorai.006G011500.5.A;Gorai.006G011500.6.A
Gene IDs: Gorai.006G011500
cotton name: PTHR11468:SF8 - ALPHA-GLUCAN PHOSPHORYLASE 1 (1 of 1)
OG IDs: 93523225
Best-hit-arabi-ID: AT3G29320.1
Best-hit-arabi-name: Glycosyl transferase, family 35
Gorai.006G011500.1.A
PCC=0.323, p=0.178
mRNA Profile
Protein Expression Profile
DPA

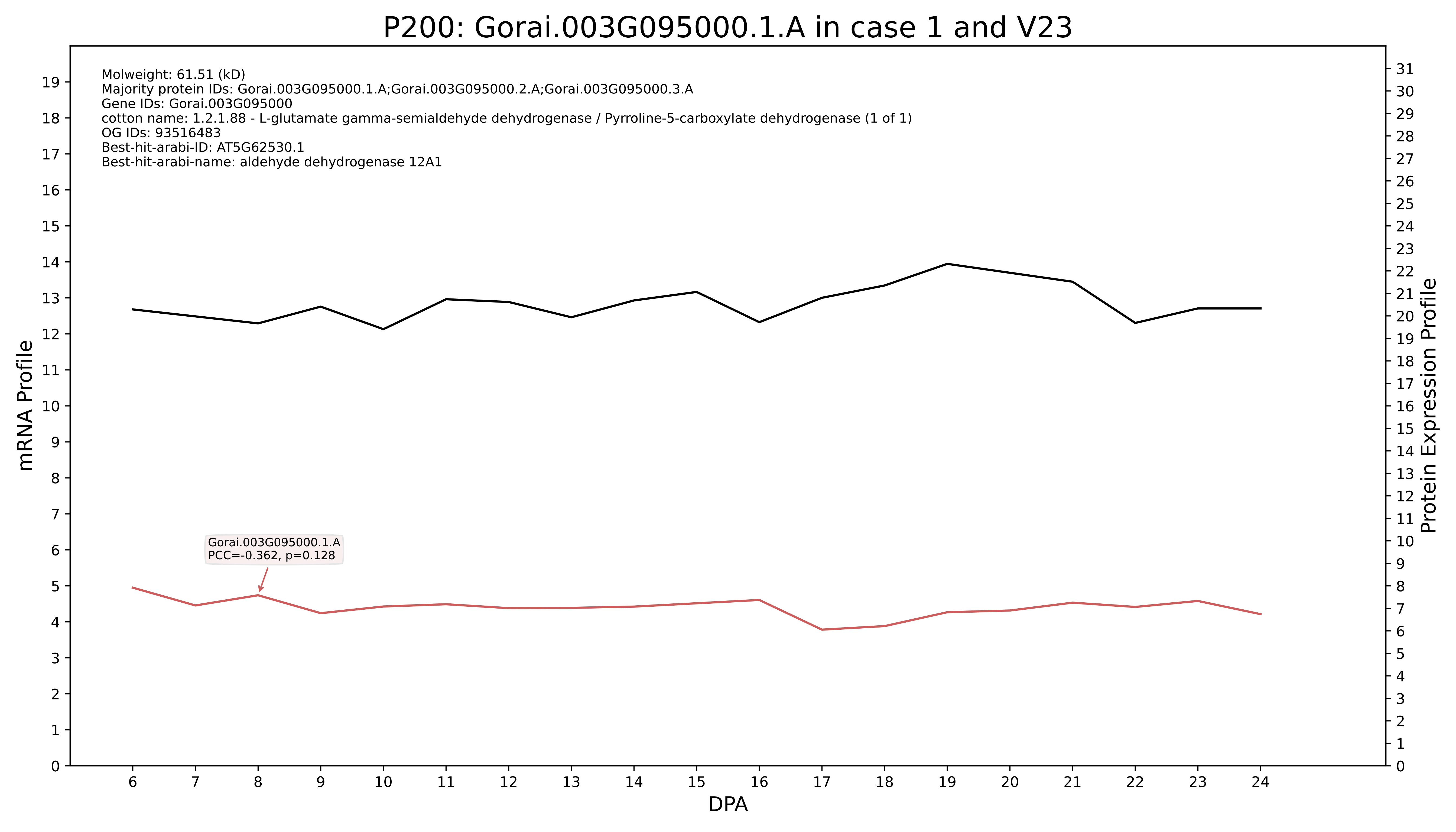
P200: Gorai.003G095000.1.A in case 1 and V23
Molweight: 61.51 (kD)
Majority protein IDs: Gorai.003G095000.1.A;Gorai.003G095000.2.A;Gorai.003G095000.3.A
Gene IDs: Gorai.003G095000
cotton name: 1.2.1.88 - L-glutamate gamma-semialdehyde dehydrogenase / Pyrroline-5-carboxylate dehydrogenase (1 of 1)
OG IDs: 93516483
Best-hit-arabi-ID: AT5G62530.1
Best-hit-arabi-name: aldehyde dehydrogenase 12A1
Gorai.003G095000.1.A
PCC=-0.362, p=0.128
mRNA Profile
Protein Expression Profile
DPA
6
7
8
9
10
11
12
13
14
15
16
17
18
19
20
21
22
23
24

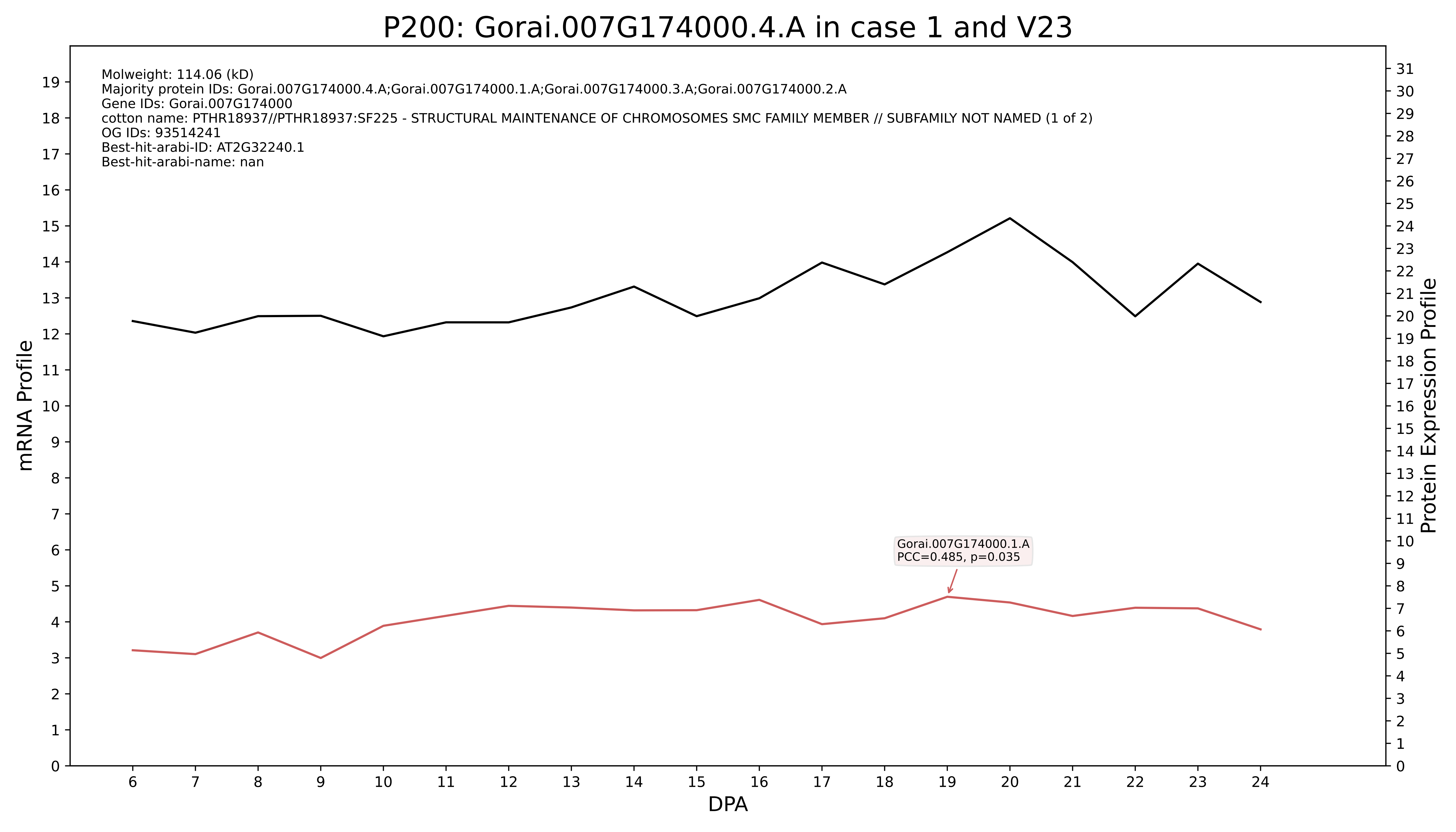
P200: Gorai.007G174000.4.A in case 1 and V23
Molweight: 114.06 (kD)
Majority protein IDs: Gorai.007G174000.4.A;Gorai.007G174000.1.A;Gorai.007G174000.3.A;Gorai.007G174000.2.A
Gene IDs: Gorai.007G174000
cotton name: PTHR18937//PTHR18937:SF225 - STRUCTURAL MAINTENANCE OF CHROMOSOMES SMC FAMILY MEMBER // SUBFAMILY NOT NAMED (1 of 2)
OG IDs: 93514241
Best-hit-arabi-ID: AT2G32240.1
Best-hit-arabi-name: nan
Gorai.007G174000.1.A
PCC=0.485, p=0.035
mRNA Profile
Protein Expression Profile
DPA

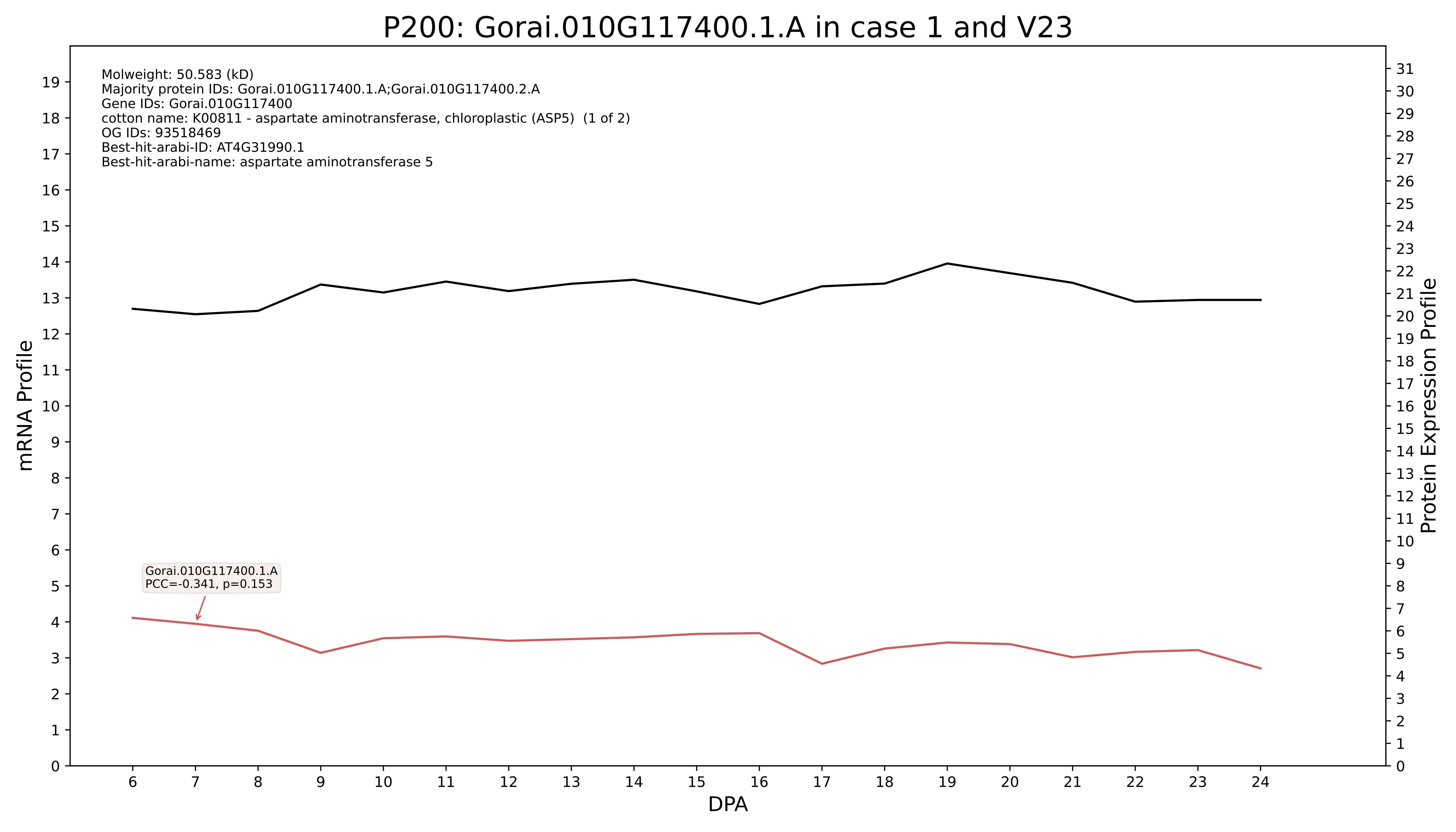
P200: Gorai.010G117400.1.A in case 1 and V23
Molweight: 50.583 (kD)
Majority protein IDs: Gorai.010G117400.1.A;Gorai.010G117400.2.A
Gene IDs: Gorai.010G117400
cotton name: K00811 - aspartate aminotransferase, chloroplastic (ASP5) (1 of 2)
OG IDs: 93518469
Best-hit-arabi-ID: AT4G31990.1
Best-hit-arabi-name: aspartate aminotransferase 5
Gorai.010G117400.1.A
PCC=-0.341, p=0.153
mRNA Profile
Protein Expression Profile
DPA

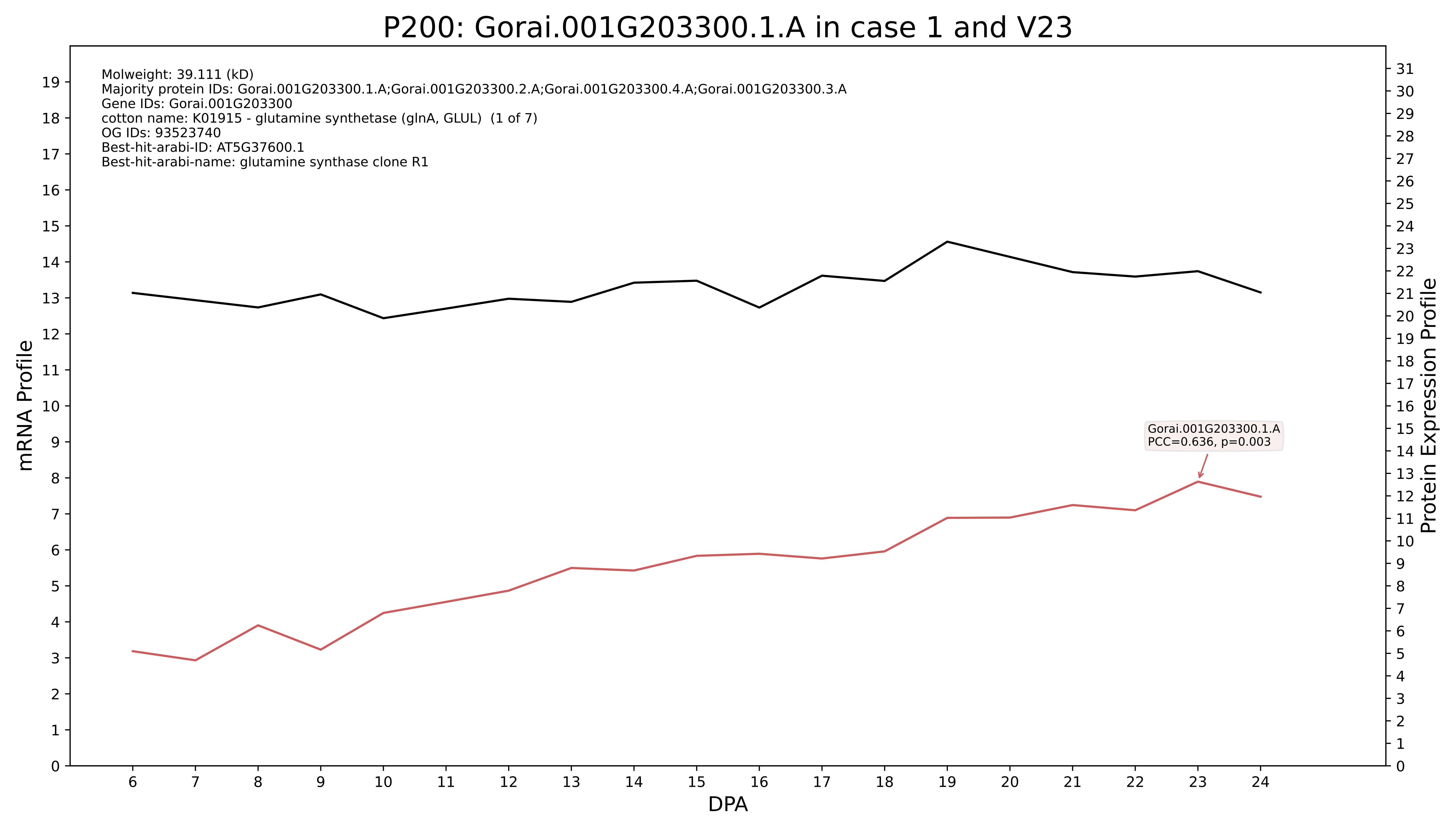

P200: Gorai.001G203300.1.A in case 1 and V23
Molweight: 39.111 (kD)
Majority protein IDs: Gorai.001G203300.1.A;Gorai.001G203300.2.A;Gorai.001G203300.4.A;Gorai.001G203300.3.A
Gene IDs: Gorai.001G203300
cotton name: K01915 - glutamine synthetase (glnA, GLUL) (1 of 7)
OG IDs: 93523740
Best-hit-arabi-ID: AT5G37600.1
Best-hit-arabi-name: glutamine synthase clone R1
Gorai.001G203300.1.A
PCC=0.636, p=0.003
mRNA Profile
Protein Expression Profile
DPA

P200: Gorai.004G065500.5.D in case 1 and V23

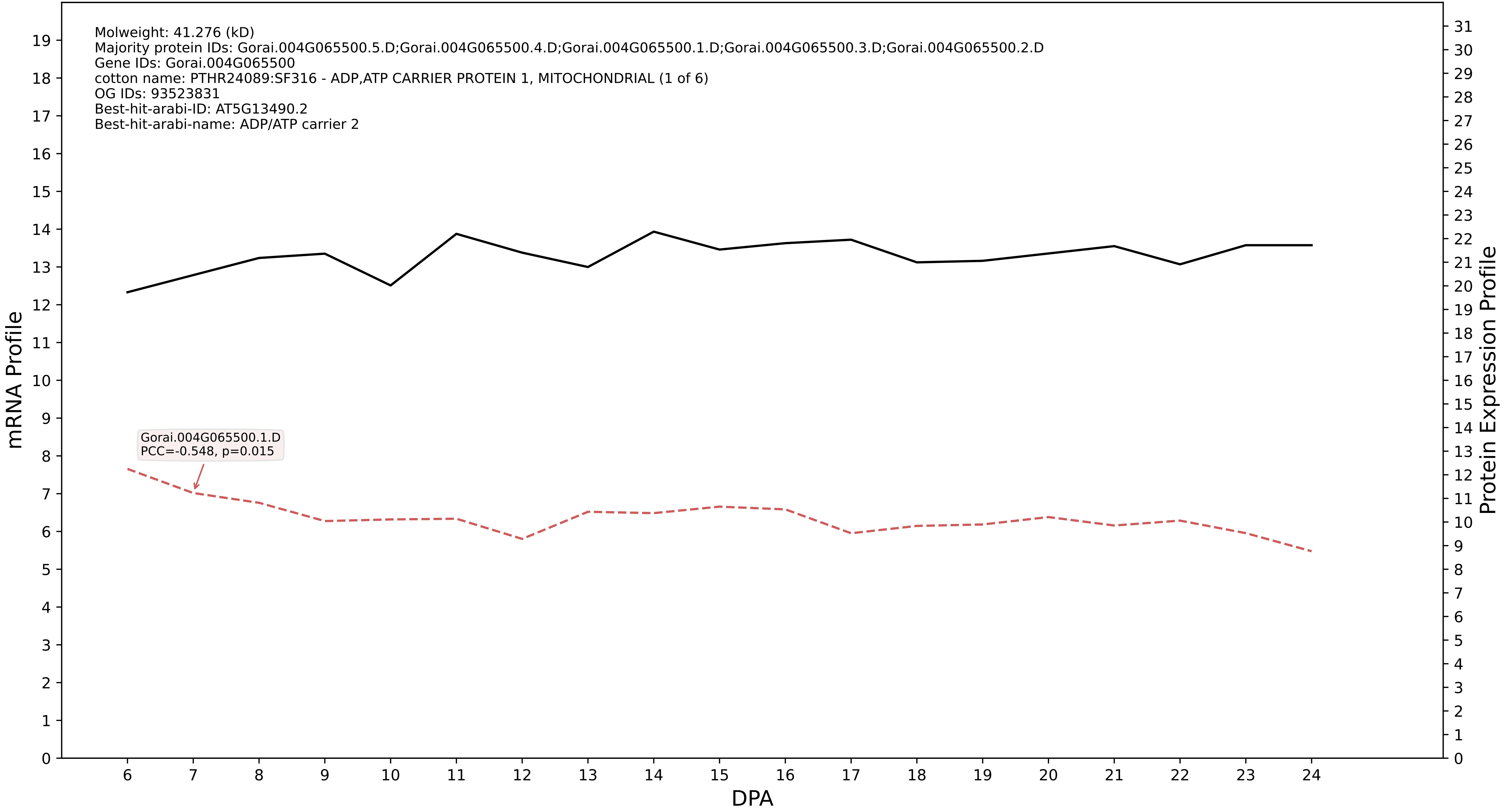

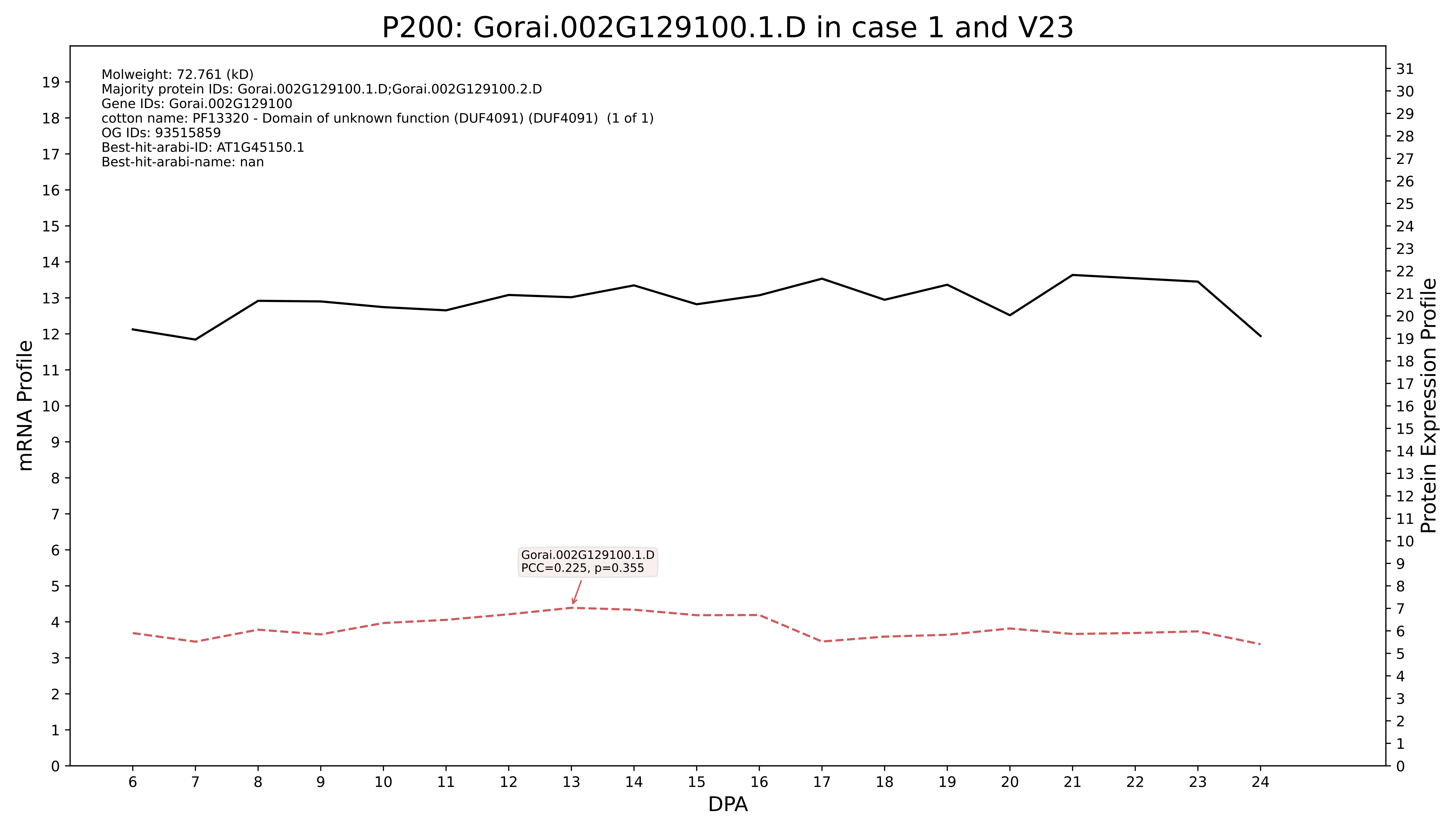
P200: Gorai.002G129100.1.D in case 1 and V23
Molweight: 72.761 (kD)
Majority protein IDs: Gorai.002G129100.1.D;Gorai.002G129100.2.D
Gene IDs: Gorai.002G129100
cotton name: PF13320 - Domain of unknown function (DUF4091) (DUF4091) (1 of 1)
OG IDs: 93515859
Best-hit-arabi-ID: AT1G45150.1
Best-hit-arabi-name: nan
Gorai.002G129100.1.D
PCC=0.225, p=0.355
mRNA Profile
Protein Expression Profile
DPA

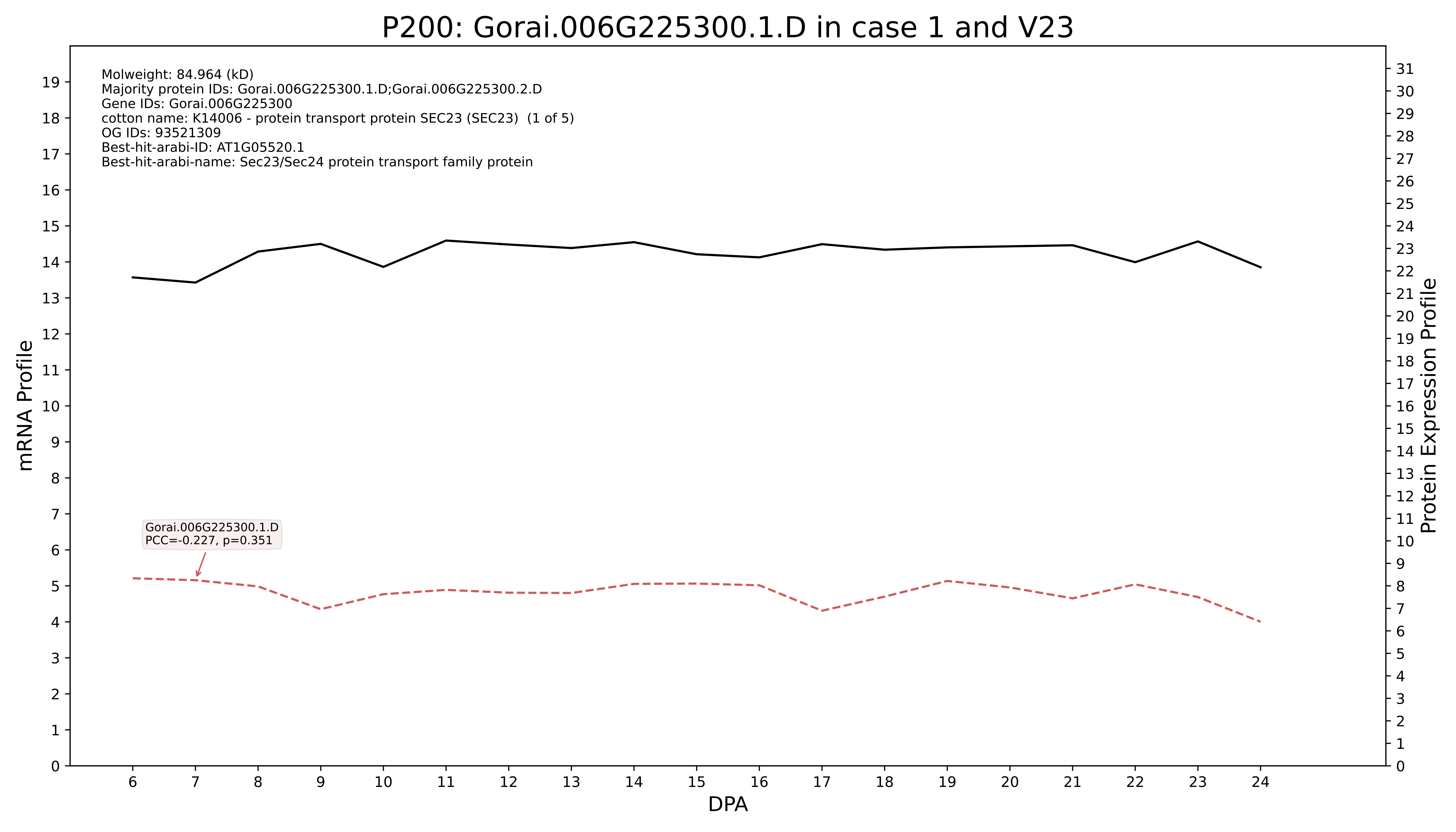
P200: Gorai.006G225300.1.D in case 1 and V23
Molweight: 84.964 (kD)
Majority protein IDs: Gorai.006G225300.1.D;Gorai.006G225300.2.D
Gene IDs: Gorai.006G225300
cotton name: K14006 - protein transport protein SEC23 (SEC23) (1 of 5)
OG IDs: 93521309
Best-hit-arabi-ID: AT1G05520.1
Best-hit-arabi-name: Sec23/Sec24 protein transport family protein
Gorai.006G225300.1.D
PCC=-0.227, p=0.351
mRNA Profile
Protein Expression Profile
DPA
0
1
2
3
4
5
6
7
8
9
10
11
12
13
14
15
16
17
18
19
20
21
22
23
24
25
26
27
28
29
30
31

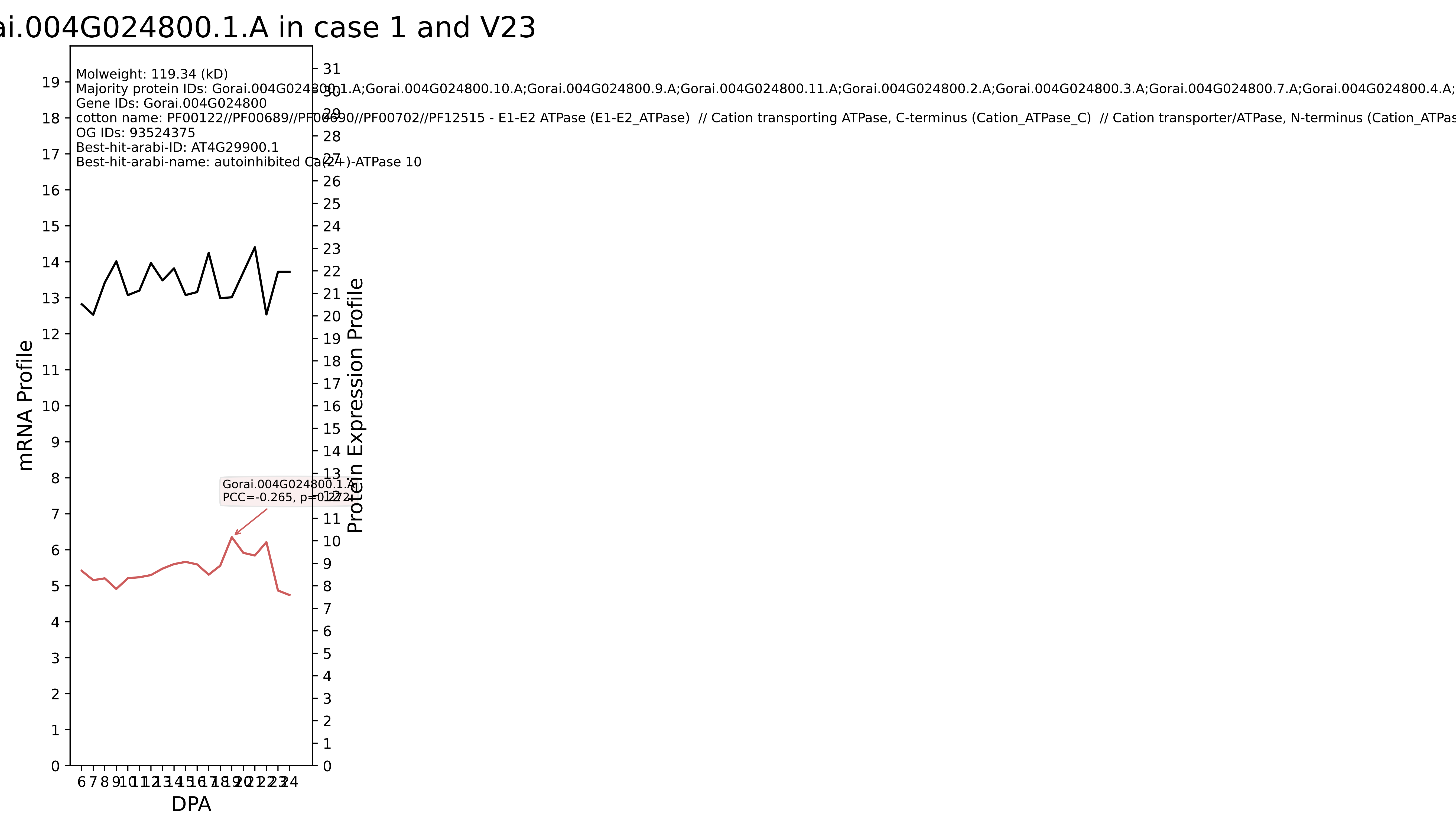
ai.004G024800.1.A in case 1 and V23
Molweight: 119.34 (kD)
Majority protein IDs: Gorai.004G024800.1.A;Gorai.004G024800.10.A;Gorai.004G024800.9.A;Gorai.004G024800.11.A;Gorai.004G024800.2.A;Gorai.004G024800.3.A;Gorai.004G024800.7.A;Gorai.004G024800.4.A;
Gene IDs: Gorai.004G024800
cotton name: PF00122//PF00689//PF00690//PF00702//PF12515 - E1-E2 ATPase (E1-E2_ATPase) // Cation transporting ATPase, C-terminus (Cation_ATPase_C) // Cation transporter/ATPase, N-terminus (Cation_ATPas
OG IDs: 93524375
Best-hit-arabi-ID: AT4G29900.1
Best-hit-arabi-name: autoinhibited Ca(2+)-ATPase 10
Gorai.004G024800.1.A
PCC=-0.265, p=0.272
mRNA Profile
Protein Expression Profile
DPA

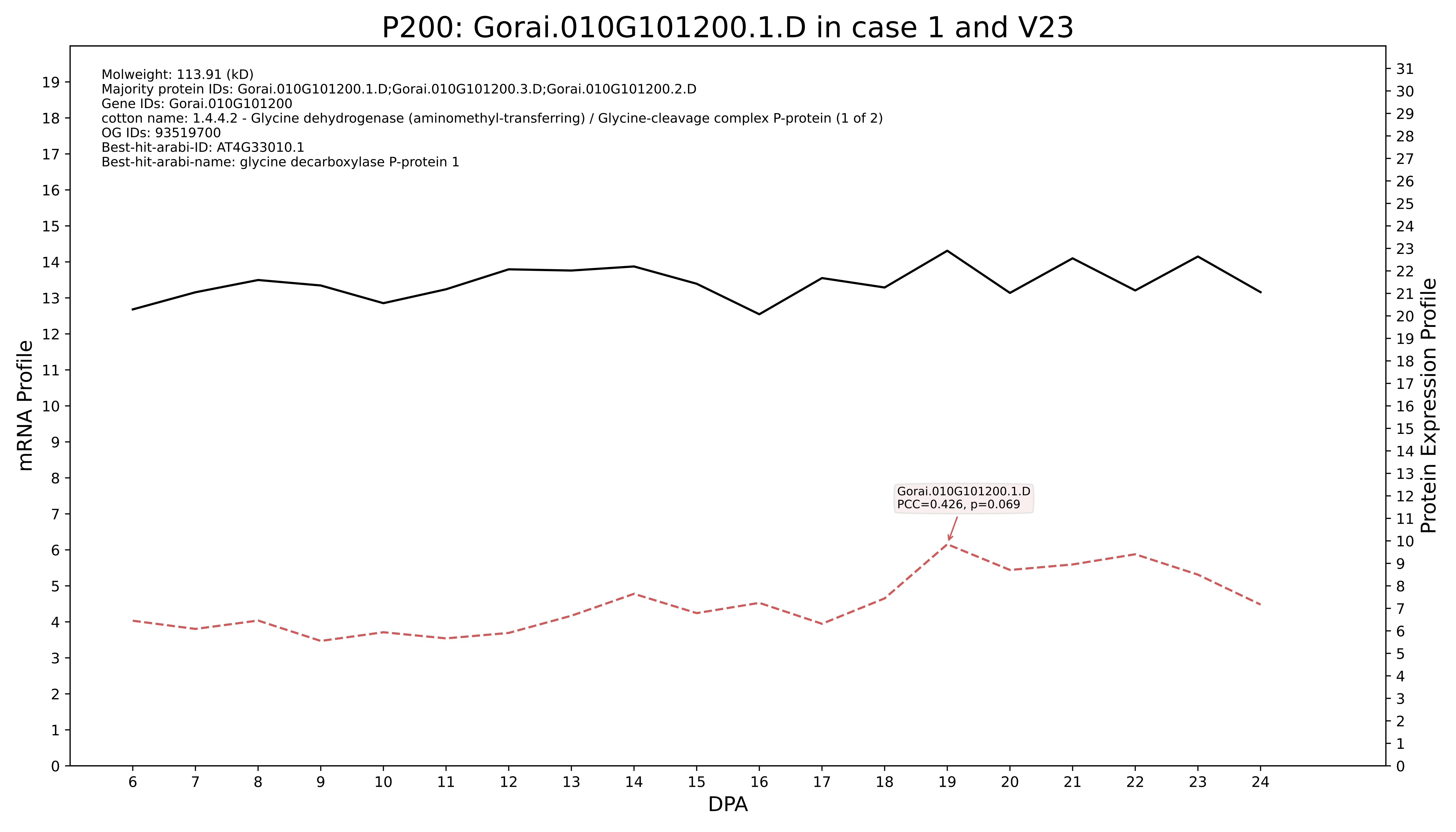
P200: Gorai.010G101200.1.D in case 1 and V23
Molweight: 113.91 (kD)
Majority protein IDs: Gorai.010G101200.1.D;Gorai.010G101200.3.D;Gorai.010G101200.2.D
Gene IDs: Gorai.010G101200
cotton name: 1.4.4.2 - Glycine dehydrogenase (aminomethyl-transferring) / Glycine-cleavage complex P-protein (1 of 2)
OG IDs: 93519700
Best-hit-arabi-ID: AT4G33010.1
Best-hit-arabi-name: glycine decarboxylase P-protein 1
Gorai.010G101200.1.D
PCC=0.426, p=0.069
mRNA Profile
Protein Expression Profile
DPA

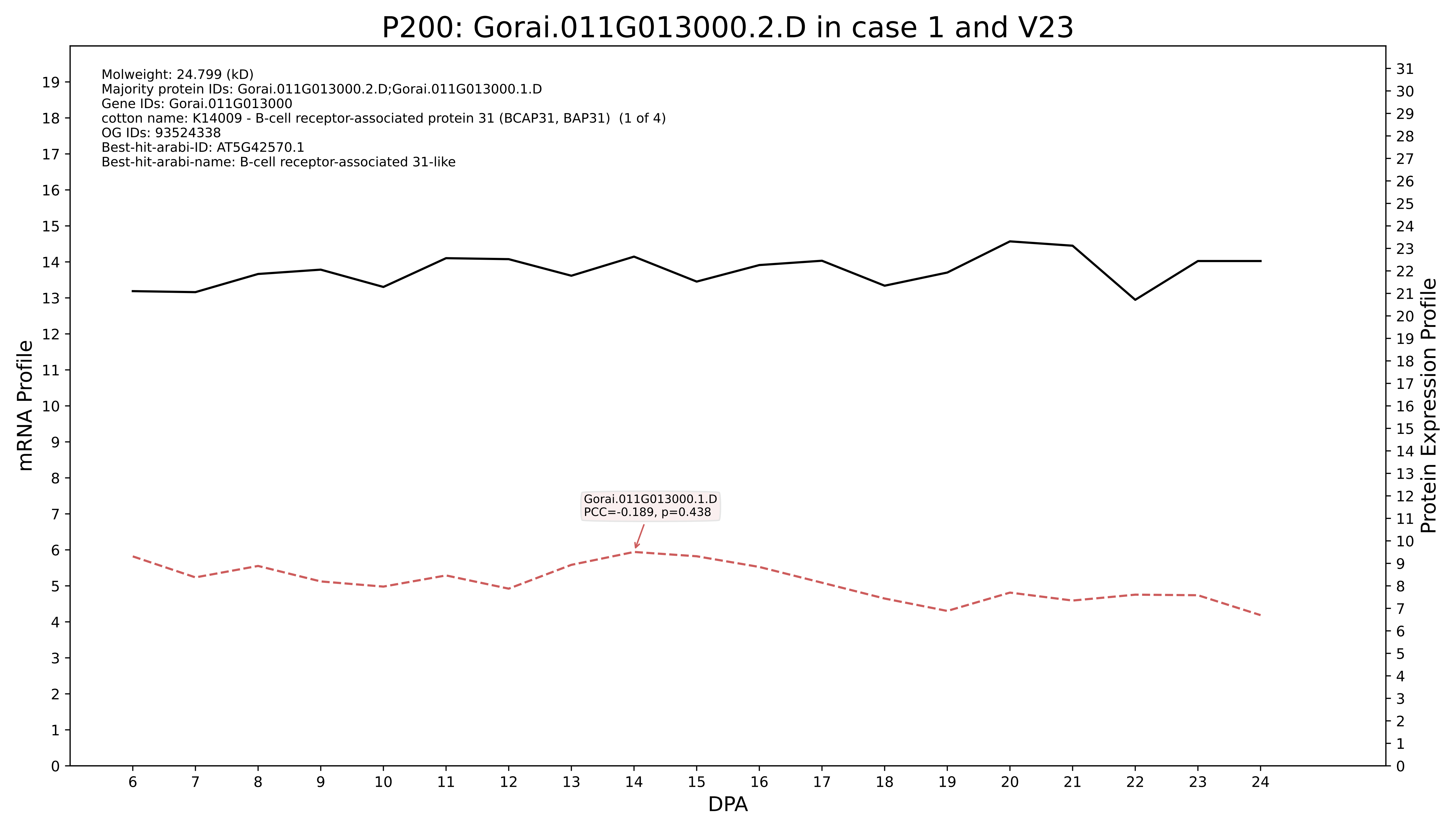
P200: Gorai.011G013000.2.D in case 1 and V23
Molweight: 24.799 (kD)
Majority protein IDs: Gorai.011G013000.2.D;Gorai.011G013000.1.D
Gene IDs: Gorai.011G013000
cotton name: K14009 - B-cell receptor-associated protein 31 (BCAP31, BAP31) (1 of 4)
OG IDs: 93524338
Best-hit-arabi-ID: AT5G42570.1
Best-hit-arabi-name: B-cell receptor-associated 31-like
Gorai.011G013000.1.D
PCC=-0.189, p=0.438
mRNA Profile
Protein Expression Profile
DPA
0
1
2
3
4
5
6
7
8
9
10
11
12
13
14
15
16
17
18
19
20
21
22
23
24
25
26
27
28
29
30
31

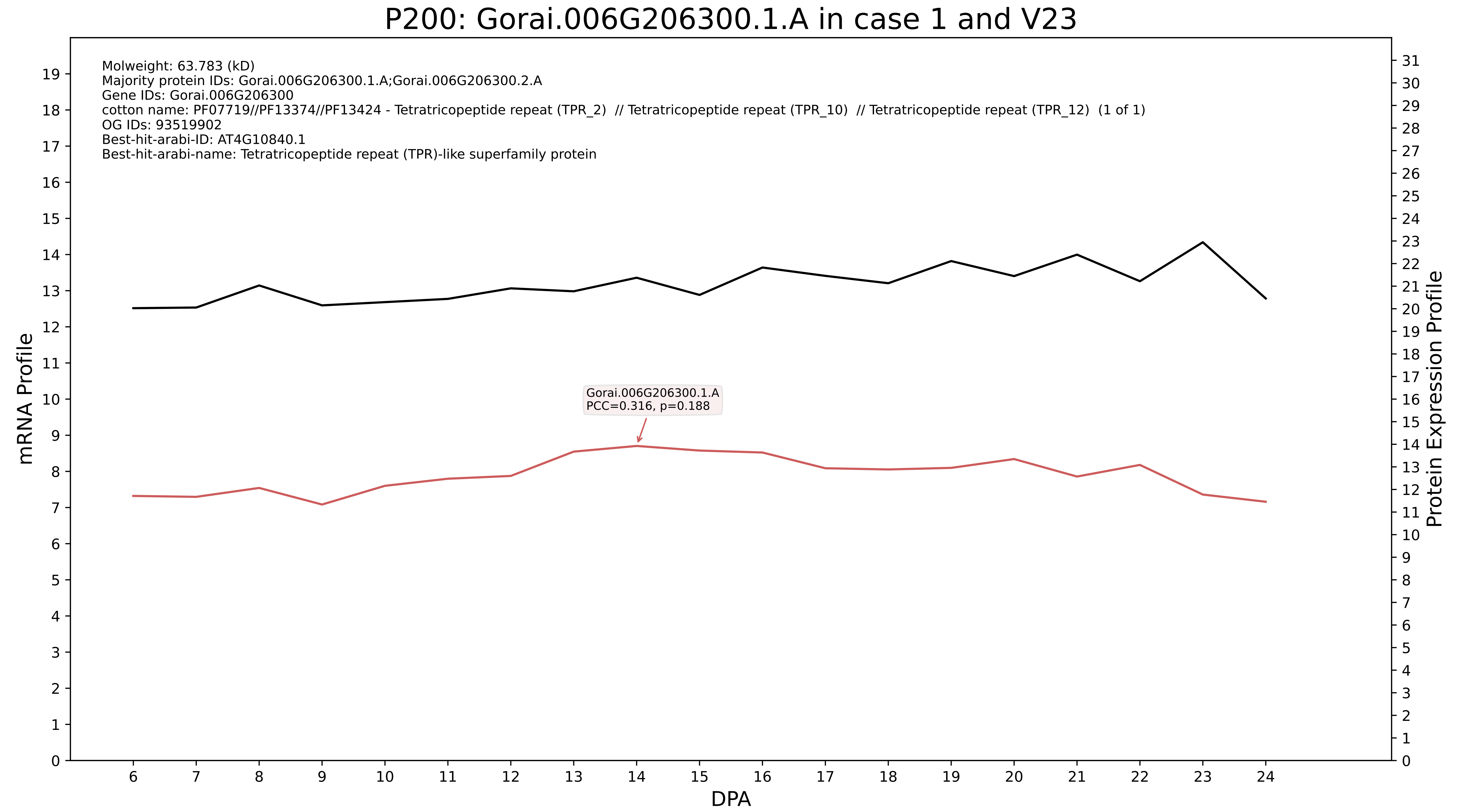
P200: Gorai.006G206300.1.A in case 1 and V23
Molweight: 63.783 (kD)
Majority protein IDs: Gorai.006G206300.1.A;Gorai.006G206300.2.A
Gene IDs: Gorai.006G206300
cotton name: PF07719//PF13374//PF13424 - Tetratricopeptide repeat (TPR_2) // Tetratricopeptide repeat (TPR_10) // Tetratricopeptide repeat (TPR_12) (1 of 1)
OG IDs: 93519902
Best-hit-arabi-ID: AT4G10840.1
Best-hit-arabi-name: Tetratricopeptide repeat (TPR)-like superfamily protein
Gorai.006G206300.1.A
PCC=0.316, p=0.188
mRNA Profile
Protein Expression Profile
DPA
0
1
2
3
4
5
6
7
8
9
10
11
12
13
14
15
16
17
18
19
20
21
22
23
24
25
26
27
28
29
30
31

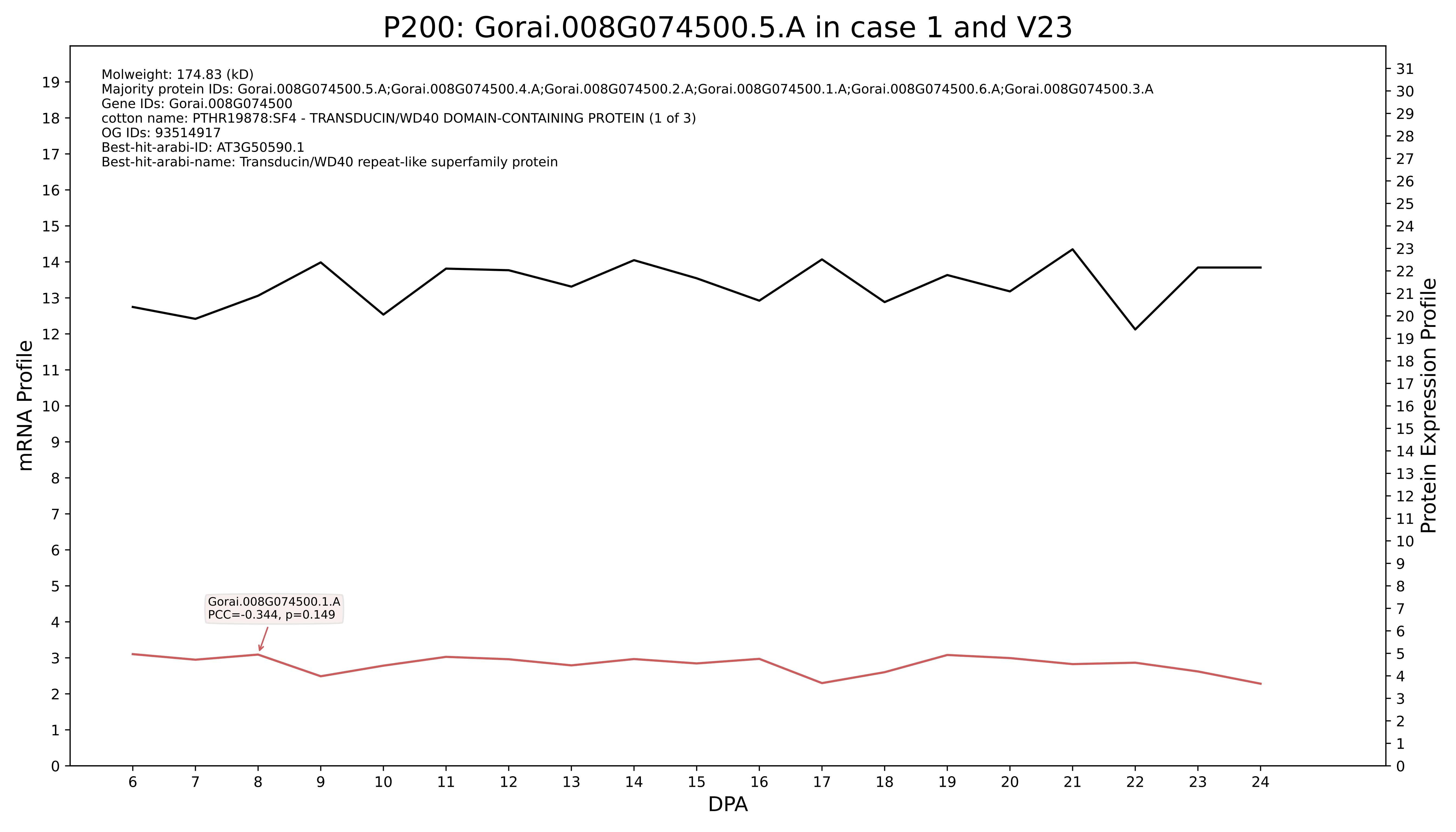

P200: Gorai.008G074500.5.A in case 1 and V23
Molweight: 174.83 (kD)
Majority protein IDs: Gorai.008G074500.5.A;Gorai.008G074500.4.A;Gorai.008G074500.2.A;Gorai.008G074500.1.A;Gorai.008G074500.6.A;Gorai.008G074500.3.A
Gene IDs: Gorai.008G074500
cotton name: PTHR19878:SF4 - TRANSDUCIN/WD40 DOMAIN-CONTAINING PROTEIN (1 of 3)
OG IDs: 93514917
Best-hit-arabi-ID: AT3G50590.1
Best-hit-arabi-name: Transducin/WD40 repeat-like superfamily protein
Gorai.008G074500.1.A
PCC=-0.344, p=0.149
mRNA Profile
Protein Expression Profile
DPA

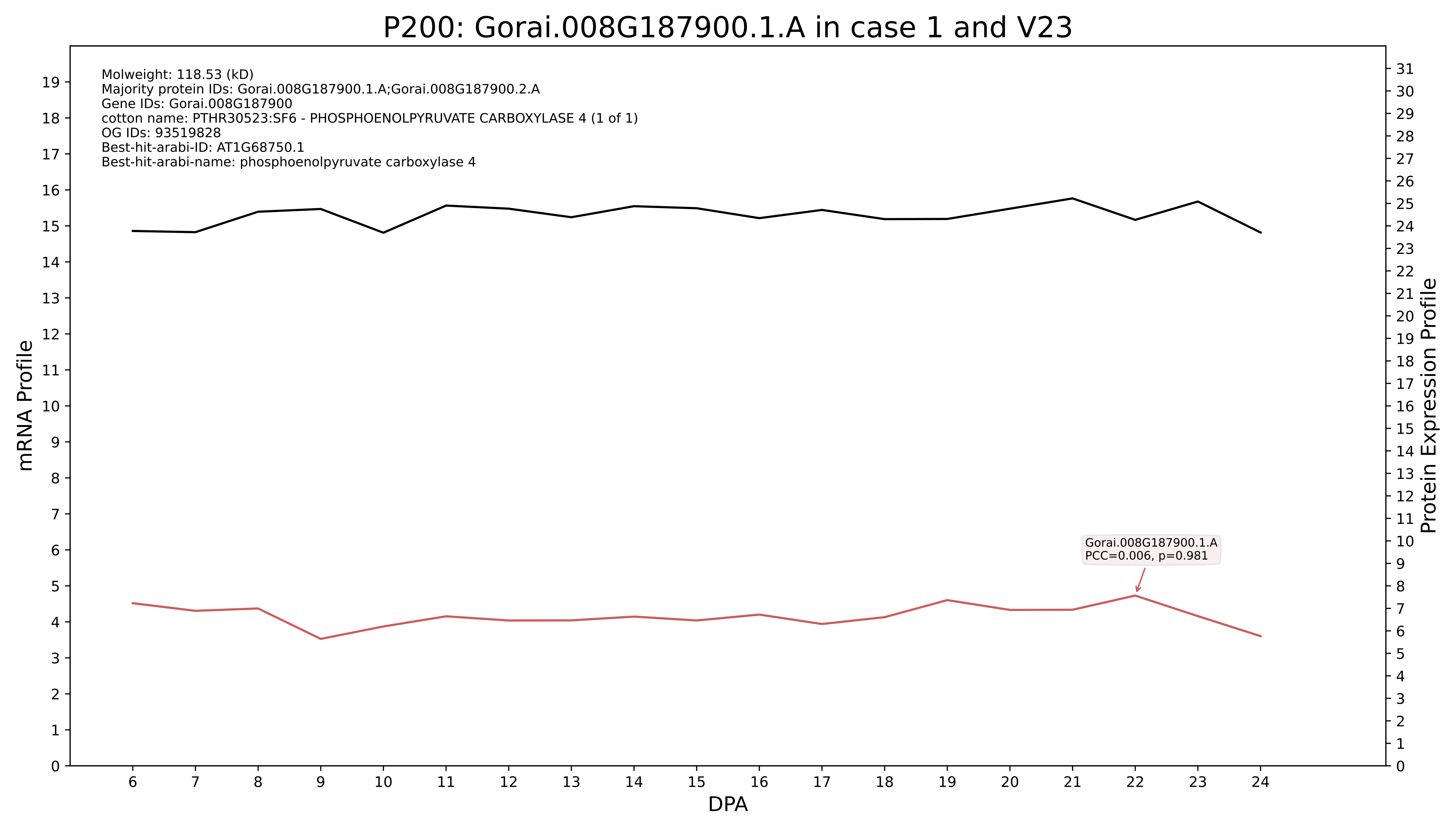
P200: Gorai.008G187900.1.A in case 1 and V23
Molweight: 118.53 (kD)
Majority protein IDs: Gorai.008G187900.1.A;Gorai.008G187900.2.A
Gene IDs: Gorai.008G187900
cotton name: PTHR30523:SF6 - PHOSPHOENOLPYRUVATE CARBOXYLASE 4 (1 of 1)
OG IDs: 93519828
Best-hit-arabi-ID: AT1G68750.1
Best-hit-arabi-name: phosphoenolpyruvate carboxylase 4
Gorai.008G187900.1.A
PCC=0.006, p=0.981
mRNA Profile
Protein Expression Profile
DPA

P200: Gorai.001G261400.1.A in case 1 and V23

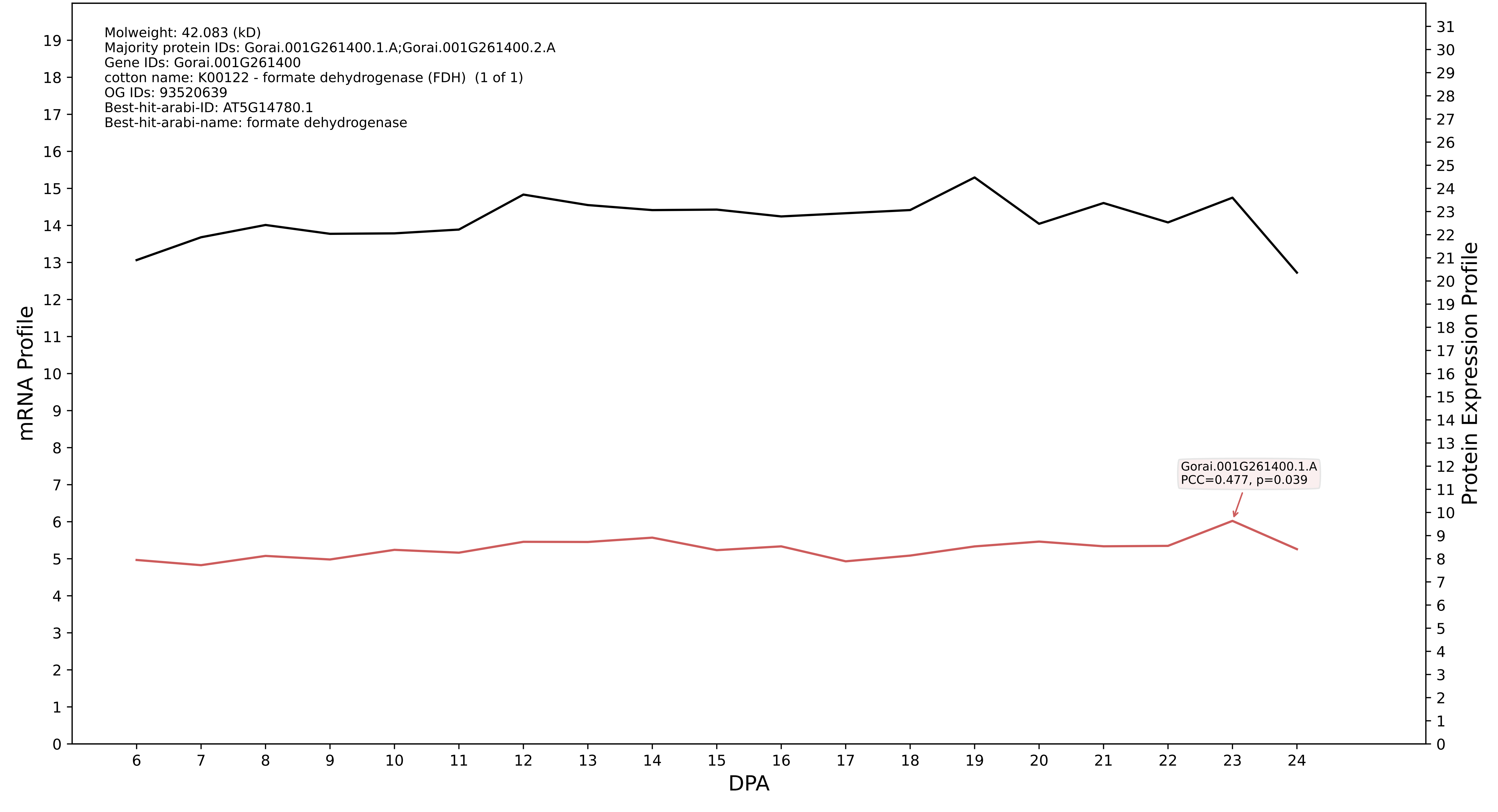

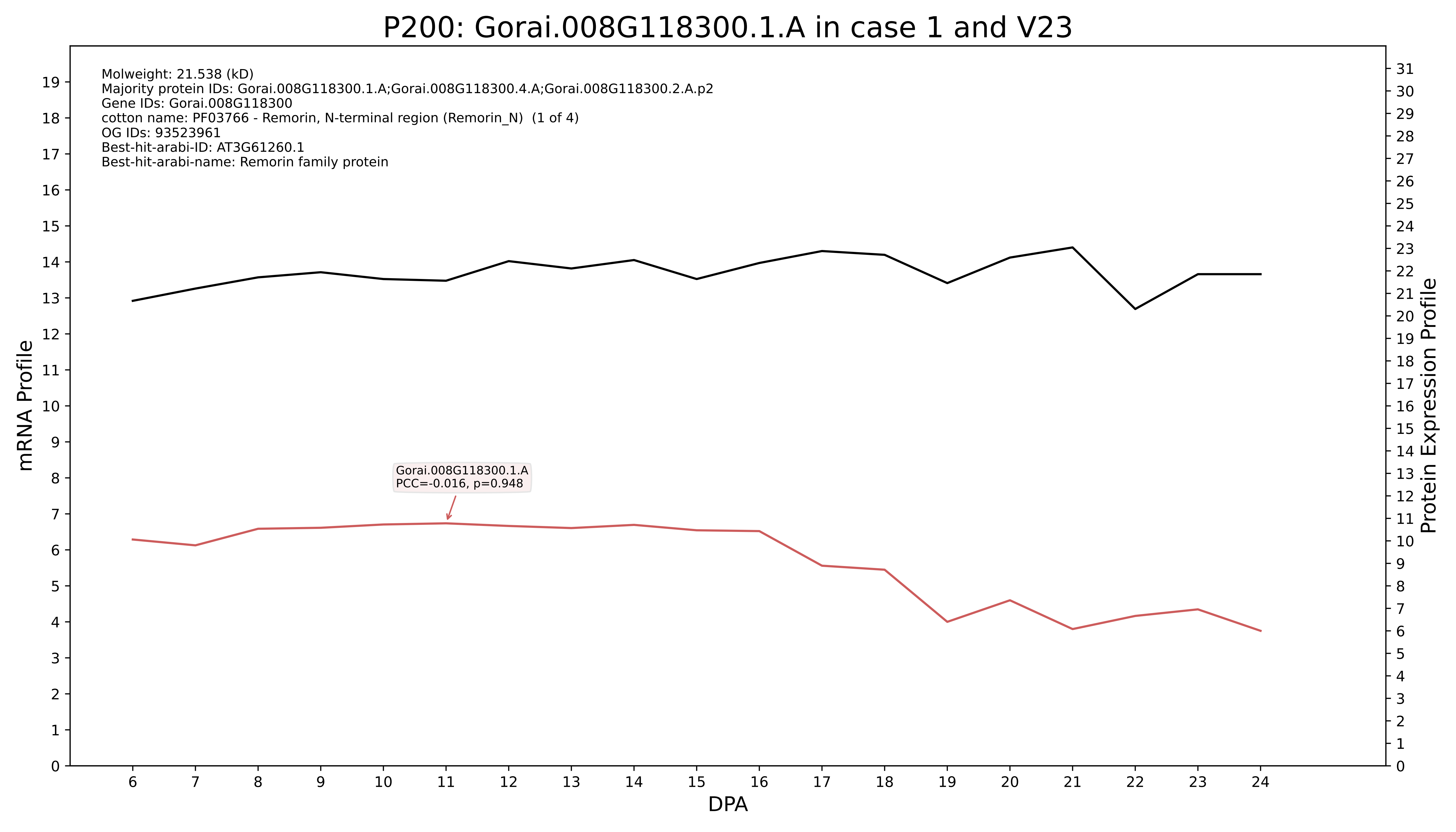
P200: Gorai.008G118300.1.A in case 1 and V23
Molweight: 21.538 (kD)
Majority protein IDs: Gorai.008G118300.1.A;Gorai.008G118300.4.A;Gorai.008G118300.2.A.p2
Gene IDs: Gorai.008G118300
cotton name: PF03766 - Remorin, N-terminal region (Remorin_N) (1 of 4)
OG IDs: 93523961
Best-hit-arabi-ID: AT3G61260.1
Best-hit-arabi-name: Remorin family protein
Gorai.008G118300.1.A
PCC=-0.016, p=0.948
mRNA Profile
Protein Expression Profile
DPA

P200: Gorai.013G202800.1.A in case 1 and V23

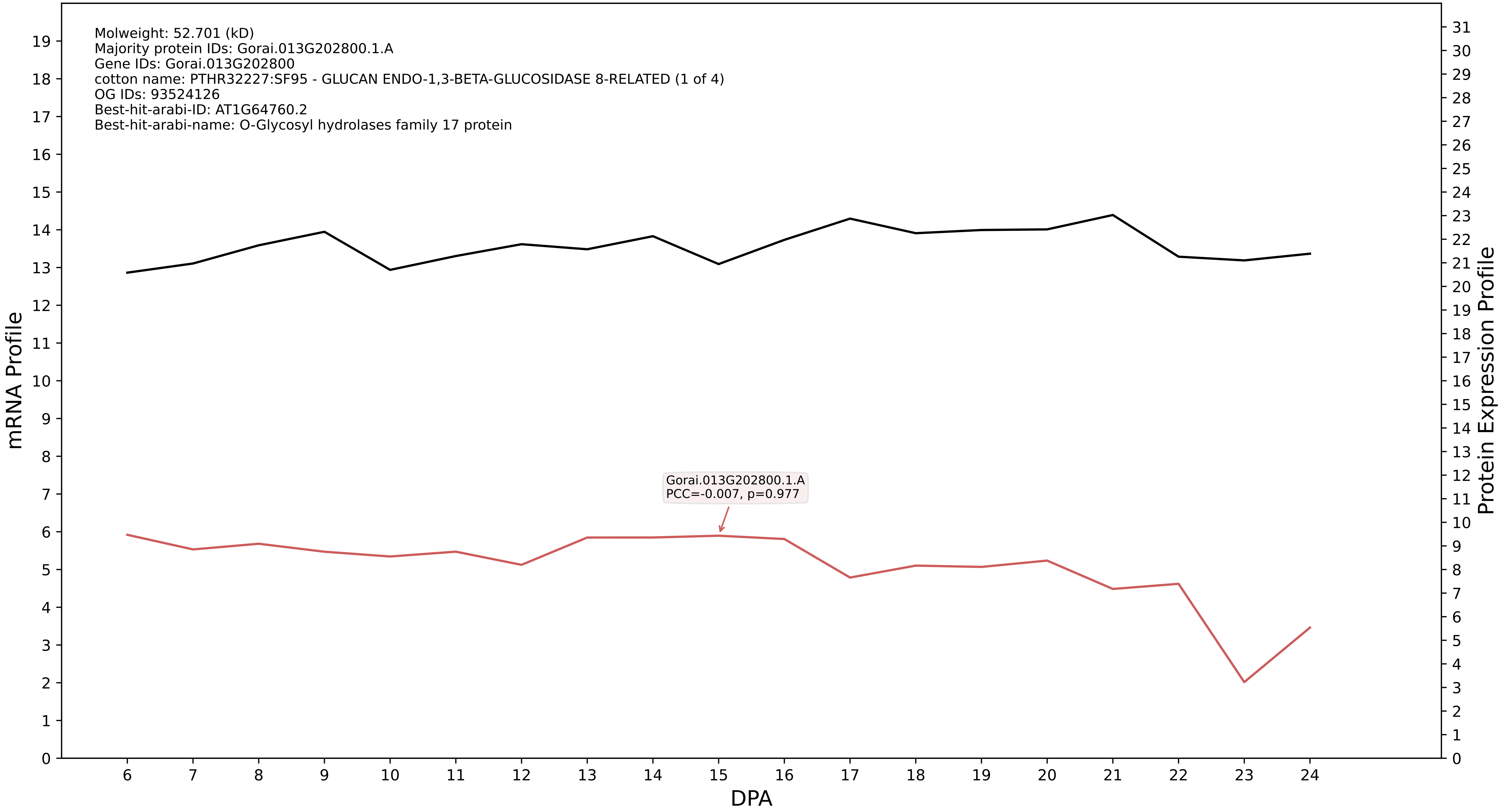

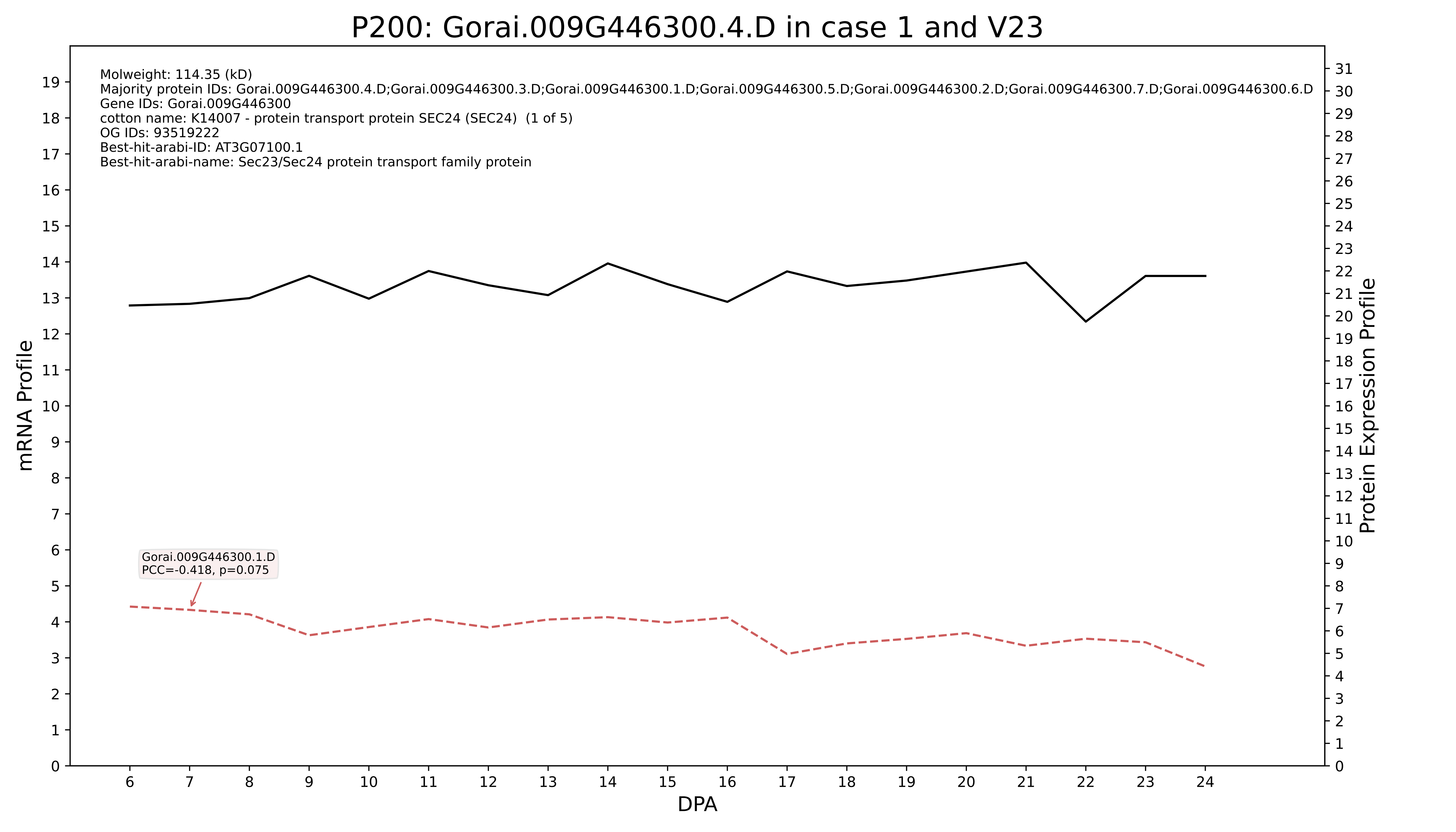
P200: Gorai.009G446300.4.D in case 1 and V23
Molweight: 114.35 (kD)
Majority protein IDs: Gorai.009G446300.4.D;Gorai.009G446300.3.D;Gorai.009G446300.1.D;Gorai.009G446300.5.D;Gorai.009G446300.2.D;Gorai.009G446300.7.D;Gorai.009G446300.6.D
Gene IDs: Gorai.009G446300
cotton name: K14007 - protein transport protein SEC24 (SEC24) (1 of 5)
OG IDs: 93519222
Best-hit-arabi-ID: AT3G07100.1
Best-hit-arabi-name: Sec23/Sec24 protein transport family protein
Gorai.009G446300.1.D
PCC=-0.418, p=0.075
mRNA Profile
Protein Expression Profile
DPA

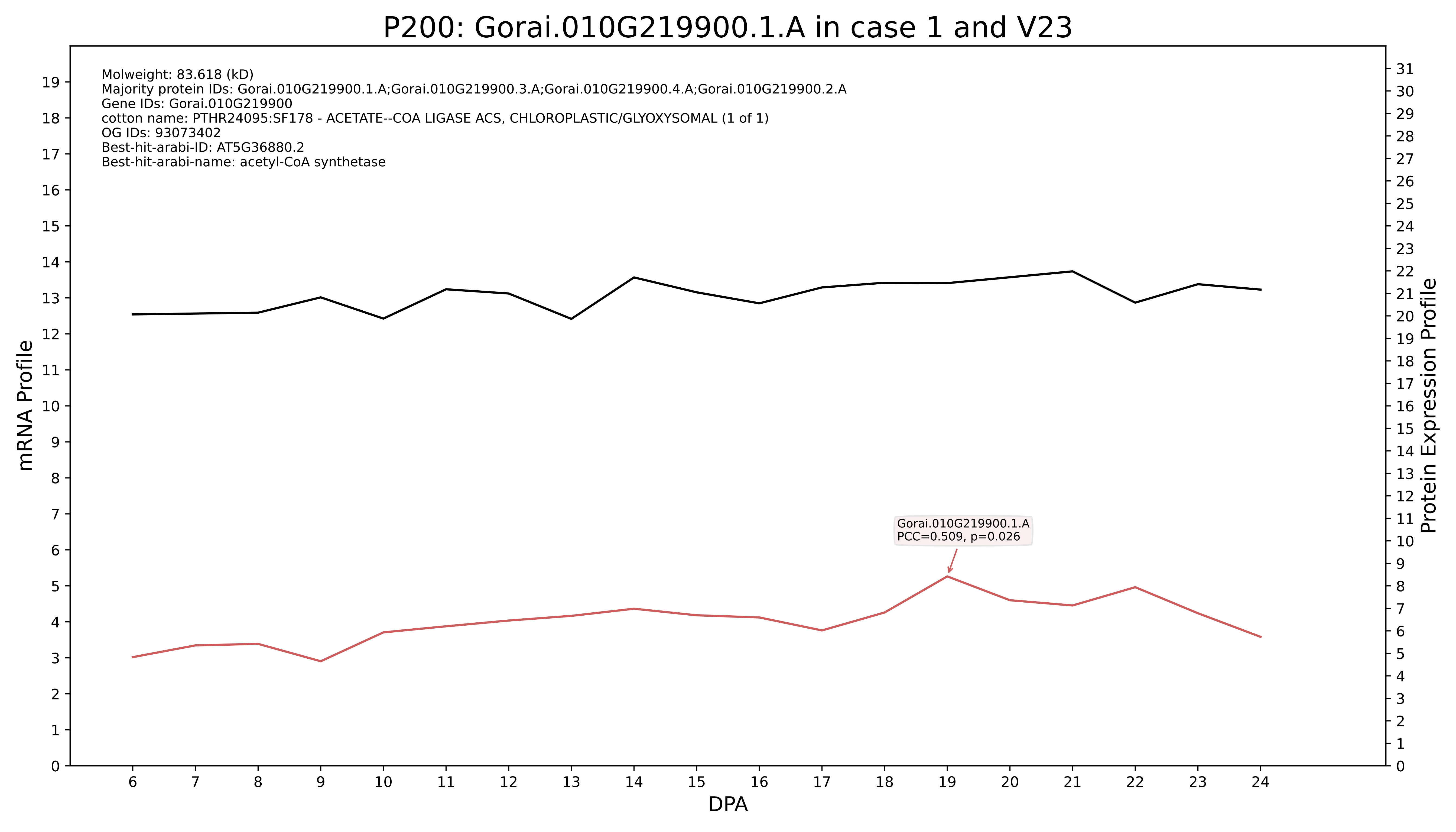
P200: Gorai.010G219900.1.A in case 1 and V23
Molweight: 83.618 (kD)
Majority protein IDs: Gorai.010G219900.1.A;Gorai.010G219900.3.A;Gorai.010G219900.4.A;Gorai.010G219900.2.A
Gene IDs: Gorai.010G219900
cotton name: PTHR24095:SF178 - ACETATE--COA LIGASE ACS, CHLOROPLASTIC/GLYOXYSOMAL (1 of 1)
OG IDs: 93073402
Best-hit-arabi-ID: AT5G36880.2
Best-hit-arabi-name: acetyl-CoA synthetase
Gorai.010G219900.1.A
PCC=0.509, p=0.026
mRNA Profile
Protein Expression Profile
DPA
0
1
2
3
4
5
6
7
8
9
10
11
12
13
14
15
16
17
18
19
20
21
22
23
24
25
26
27
28
29
30
31

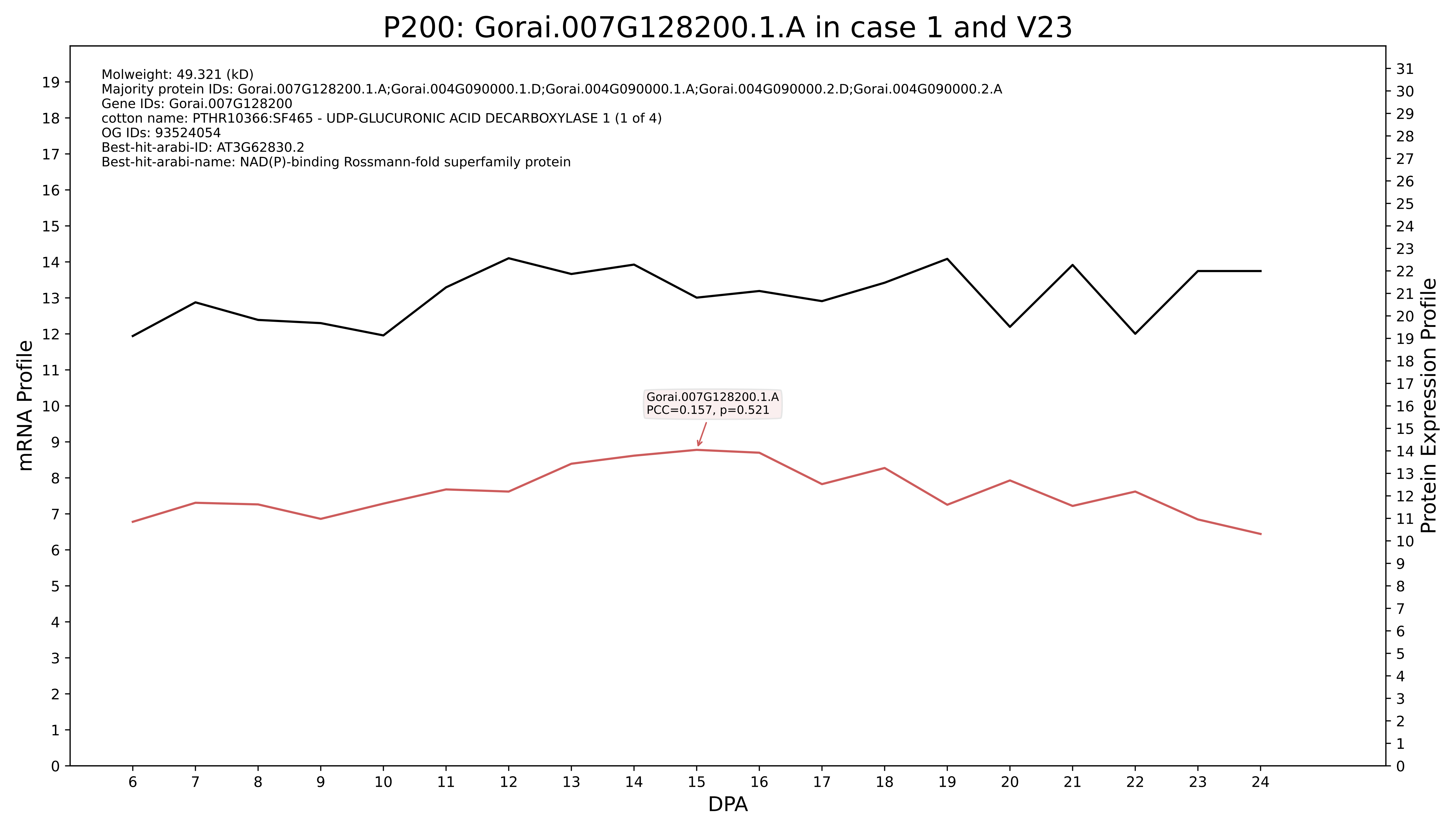
P200: Gorai.007G128200.1.A in case 1 and V23
Molweight: 49.321 (kD)
Majority protein IDs: Gorai.007G128200.1.A;Gorai.004G090000.1.D;Gorai.004G090000.1.A;Gorai.004G090000.2.D;Gorai.004G090000.2.A
Gene IDs: Gorai.007G128200
cotton name: PTHR10366:SF465 - UDP-GLUCURONIC ACID DECARBOXYLASE 1 (1 of 4)
OG IDs: 93524054
Best-hit-arabi-ID: AT3G62830.2
Best-hit-arabi-name: NAD(P)-binding Rossmann-fold superfamily protein
Gorai.007G128200.1.A
PCC=0.157, p=0.521
mRNA Profile
Protein Expression Profile
DPA

P200: Gorai.003G154700.3.A in case 1 and V23

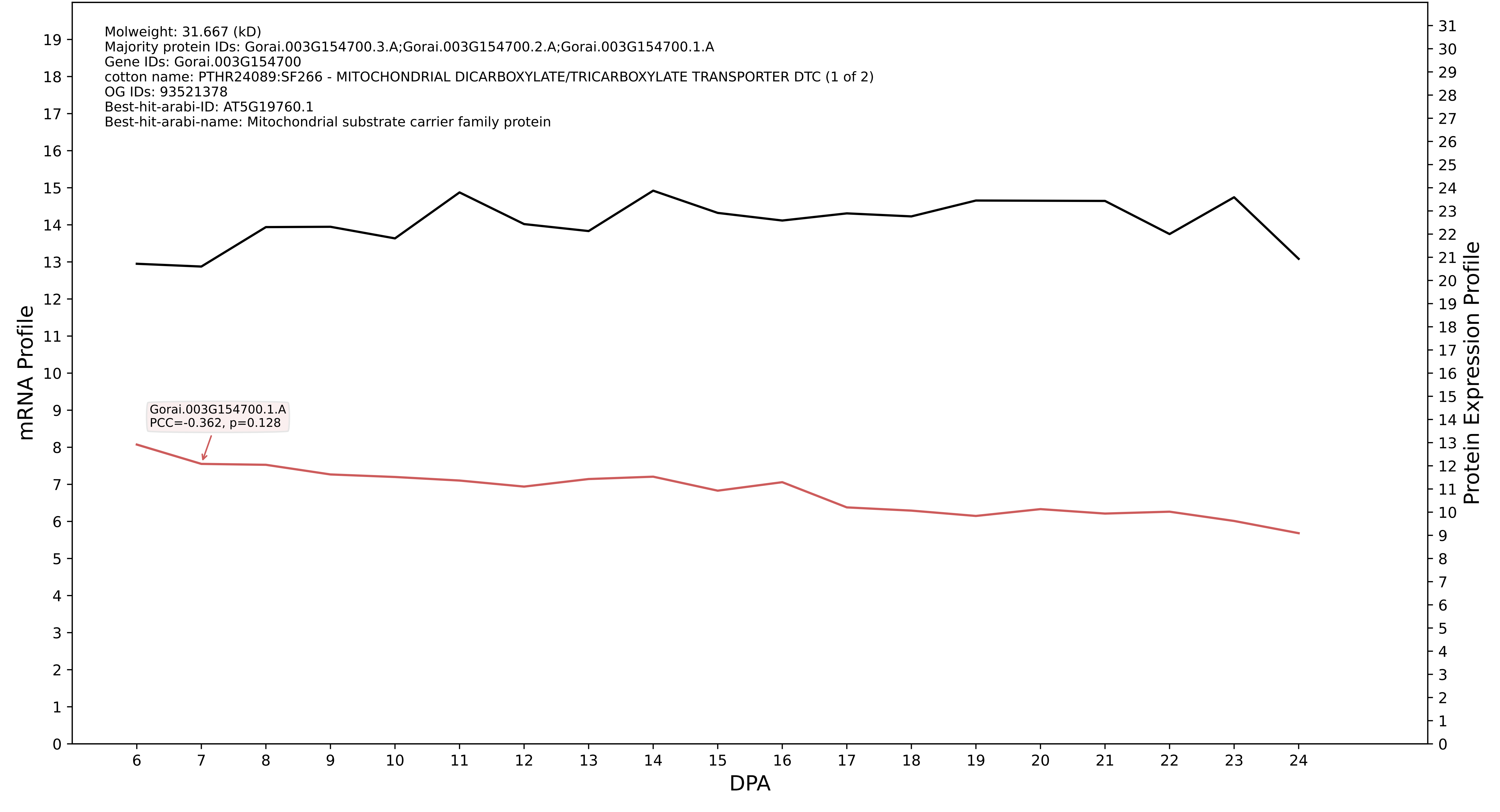

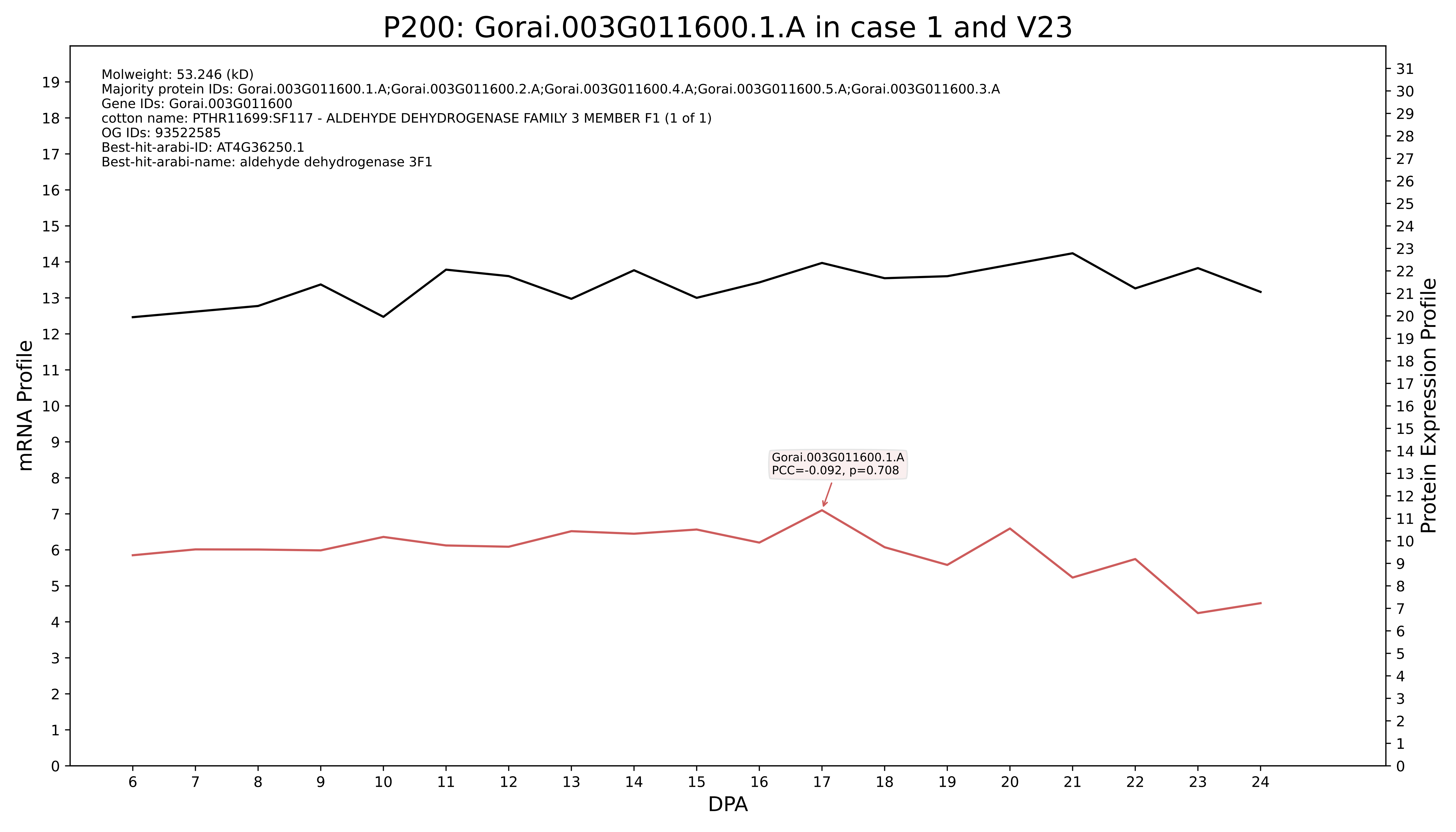
P200: Gorai.003G011600.1.A in case 1 and V23
Molweight: 53.246 (kD)
Majority protein IDs: Gorai.003G011600.1.A;Gorai.003G011600.2.A;Gorai.003G011600.4.A;Gorai.003G011600.5.A;Gorai.003G011600.3.A
Gene IDs: Gorai.003G011600
cotton name: PTHR11699:SF117 - ALDEHYDE DEHYDROGENASE FAMILY 3 MEMBER F1 (1 of 1)
OG IDs: 93522585
Best-hit-arabi-ID: AT4G36250.1
Best-hit-arabi-name: aldehyde dehydrogenase 3F1
Gorai.003G011600.1.A
PCC=-0.092, p=0.708
mRNA Profile
Protein Expression Profile
DPA

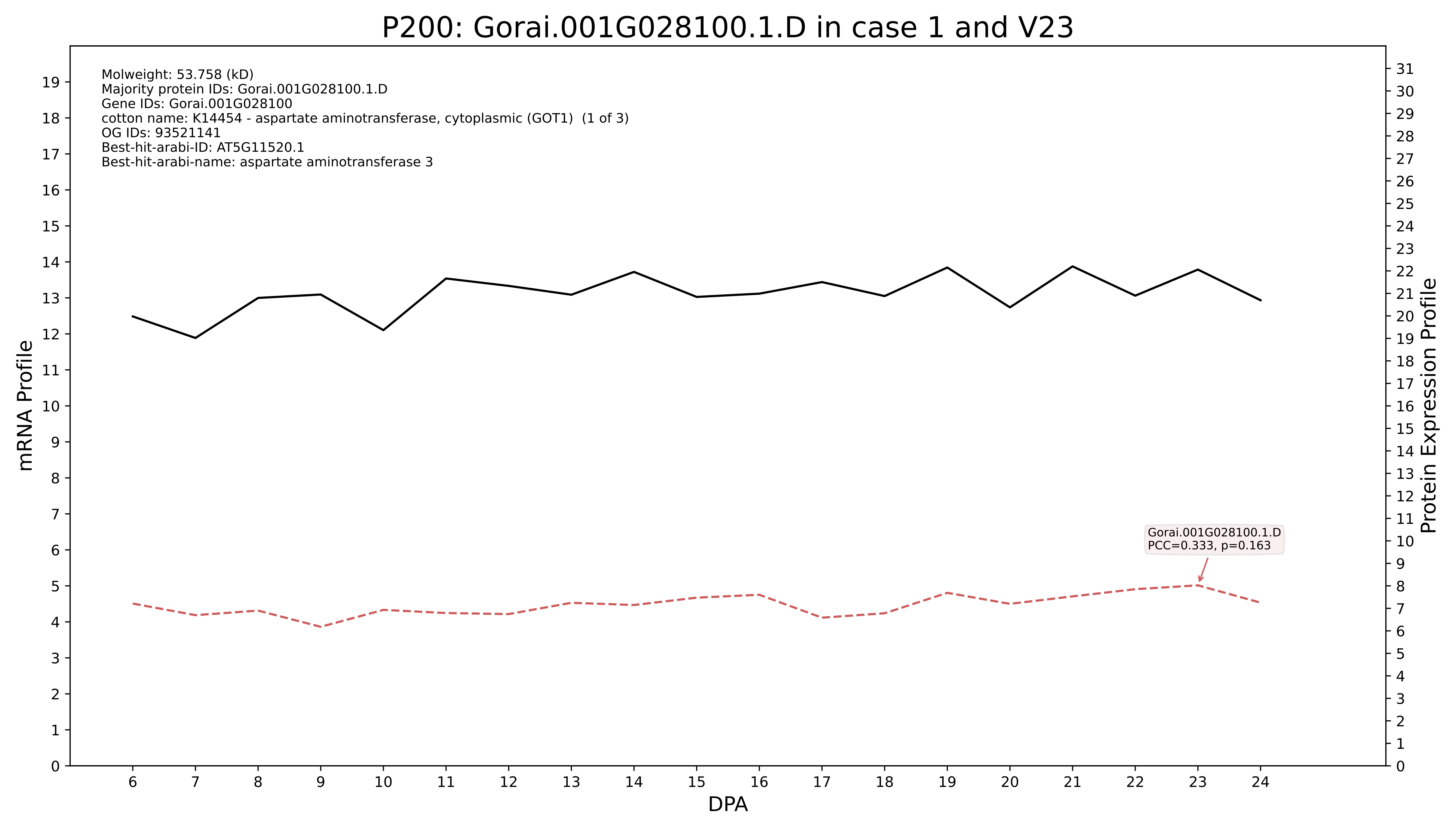

P200: Gorai.001G028100.1.D in case 1 and V23
Molweight: 53.758 (kD)
Majority protein IDs: Gorai.001G028100.1.D
Gene IDs: Gorai.001G028100
cotton name: K14454 - aspartate aminotransferase, cytoplasmic (GOT1) (1 of 3)
OG IDs: 93521141
Best-hit-arabi-ID: AT5G11520.1
Best-hit-arabi-name: aspartate aminotransferase 3
Gorai.001G028100.1.D
PCC=0.333, p=0.163
mRNA Profile
Protein Expression Profile
DPA

P200: Gorai.001G103800.1.D in case 1 and V23

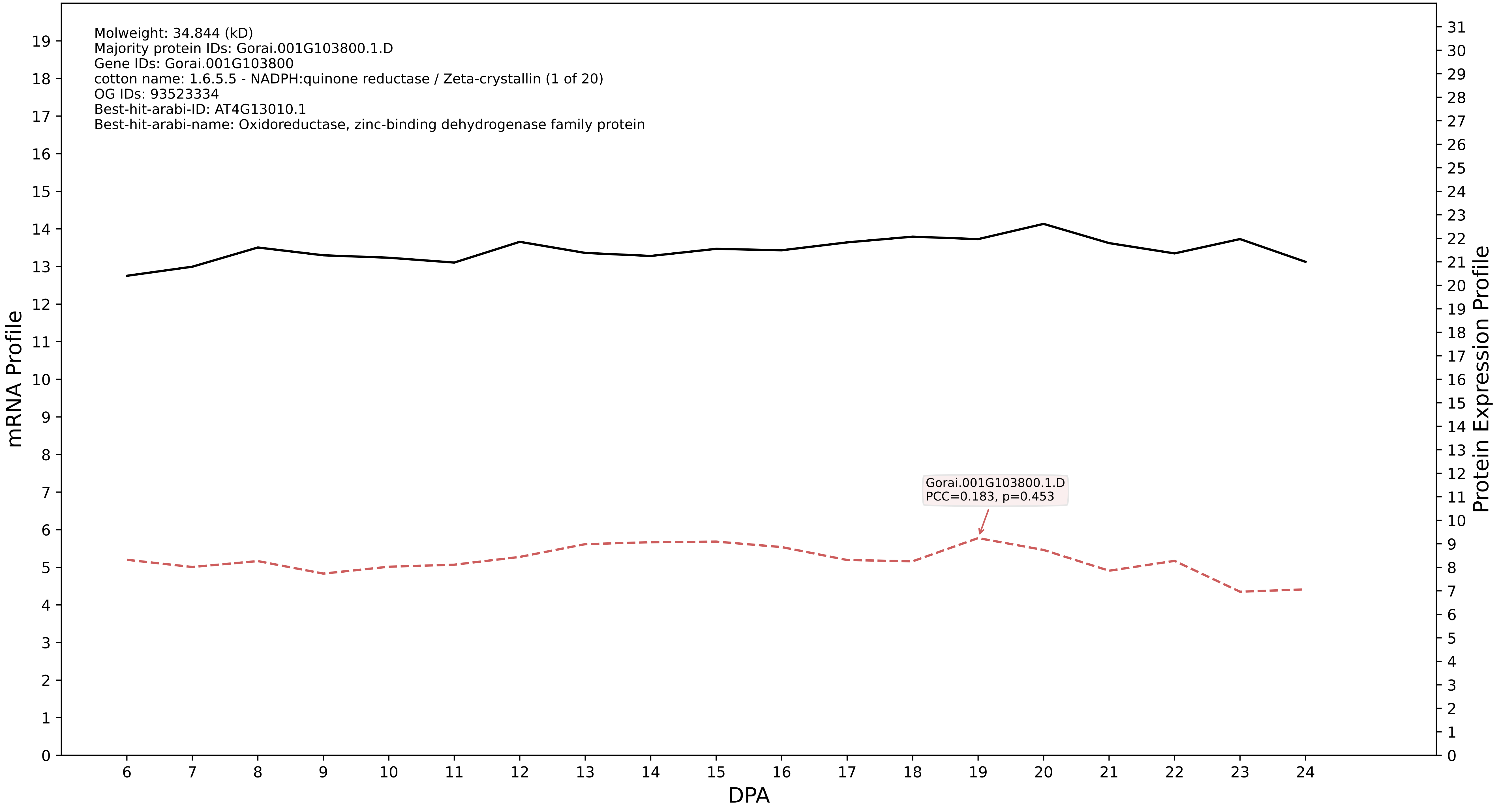

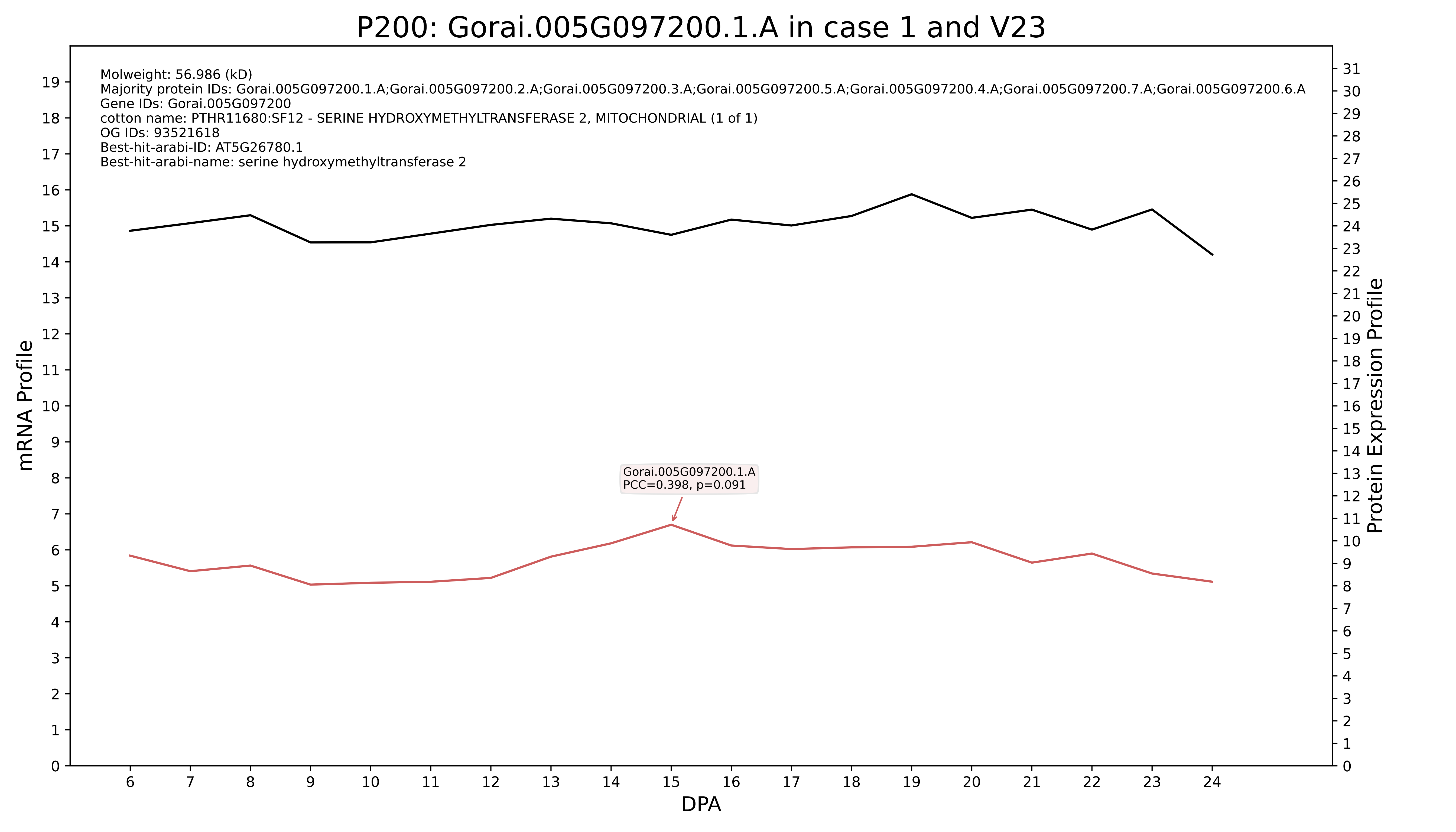
P200: Gorai.005G097200.1.A in case 1 and V23
Molweight: 56.986 (kD)
Majority protein IDs: Gorai.005G097200.1.A;Gorai.005G097200.2.A;Gorai.005G097200.3.A;Gorai.005G097200.5.A;Gorai.005G097200.4.A;Gorai.005G097200.7.A;Gorai.005G097200.6.A
Gene IDs: Gorai.005G097200
cotton name: PTHR11680:SF12 - SERINE HYDROXYMETHYLTRANSFERASE 2, MITOCHONDRIAL (1 of 1)
OG IDs: 93521618
Best-hit-arabi-ID: AT5G26780.1
Best-hit-arabi-name: serine hydroxymethyltransferase 2
Gorai.005G097200.1.A
PCC=0.398, p=0.091
mRNA Profile
Protein Expression Profile
DPA
6
7
8
9
10
11
12
13
14
15
16
17
18
19
20
21
22
23
24

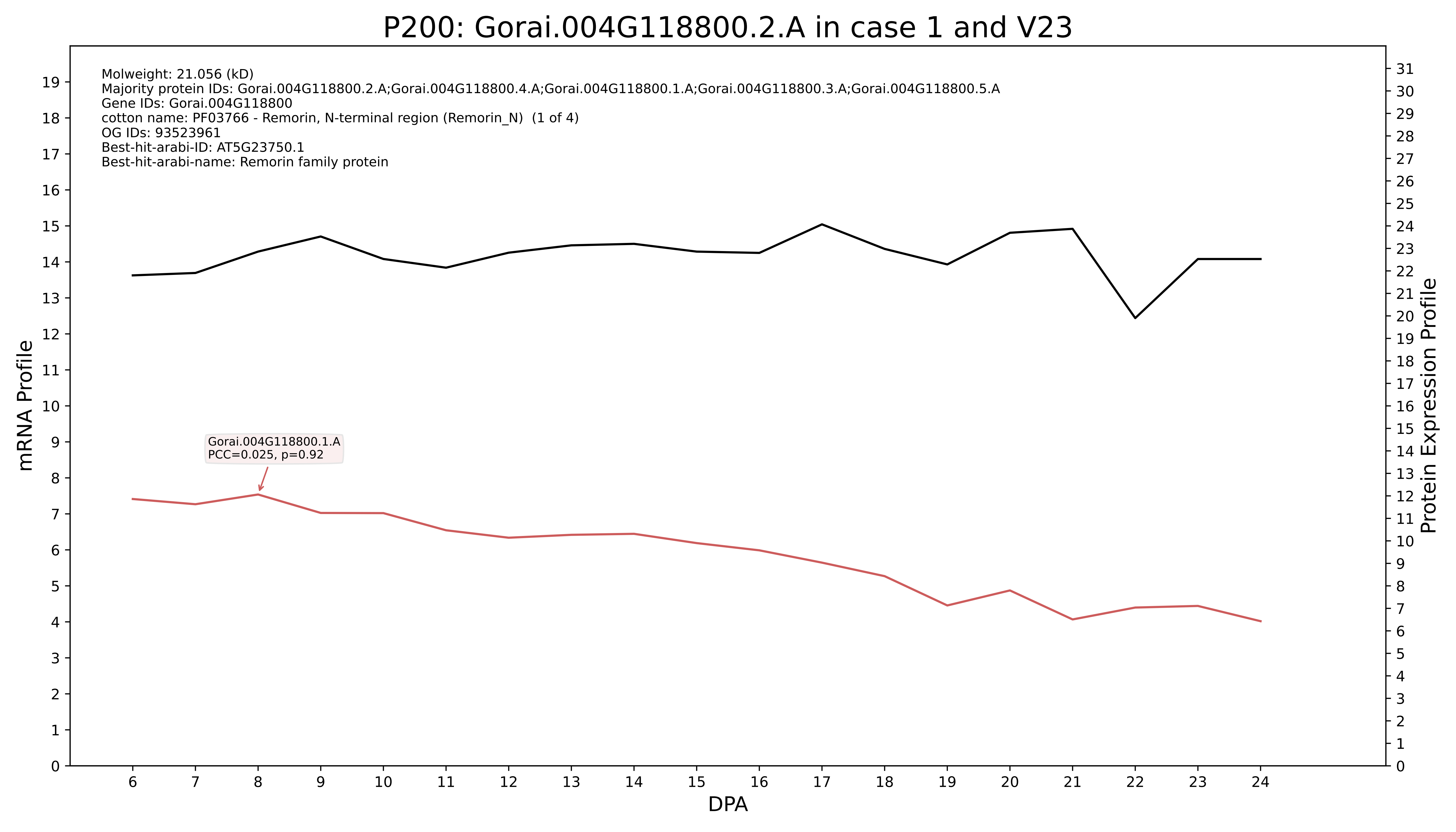
P200: Gorai.004G118800.2.A in case 1 and V23
Molweight: 21.056 (kD)
Majority protein IDs: Gorai.004G118800.2.A;Gorai.004G118800.4.A;Gorai.004G118800.1.A;Gorai.004G118800.3.A;Gorai.004G118800.5.A
Gene IDs: Gorai.004G118800
cotton name: PF03766 - Remorin, N-terminal region (Remorin_N) (1 of 4)
OG IDs: 93523961
Best-hit-arabi-ID: AT5G23750.1
Best-hit-arabi-name: Remorin family protein
Gorai.004G118800.1.A
PCC=0.025, p=0.92
mRNA Profile
Protein Expression Profile
DPA
0
1
2
3
4
5
6
7
8
9
10
11
12
13
14
15
16
17
18
19
20
21
22
23
24
25
26
27
28
29
30
31

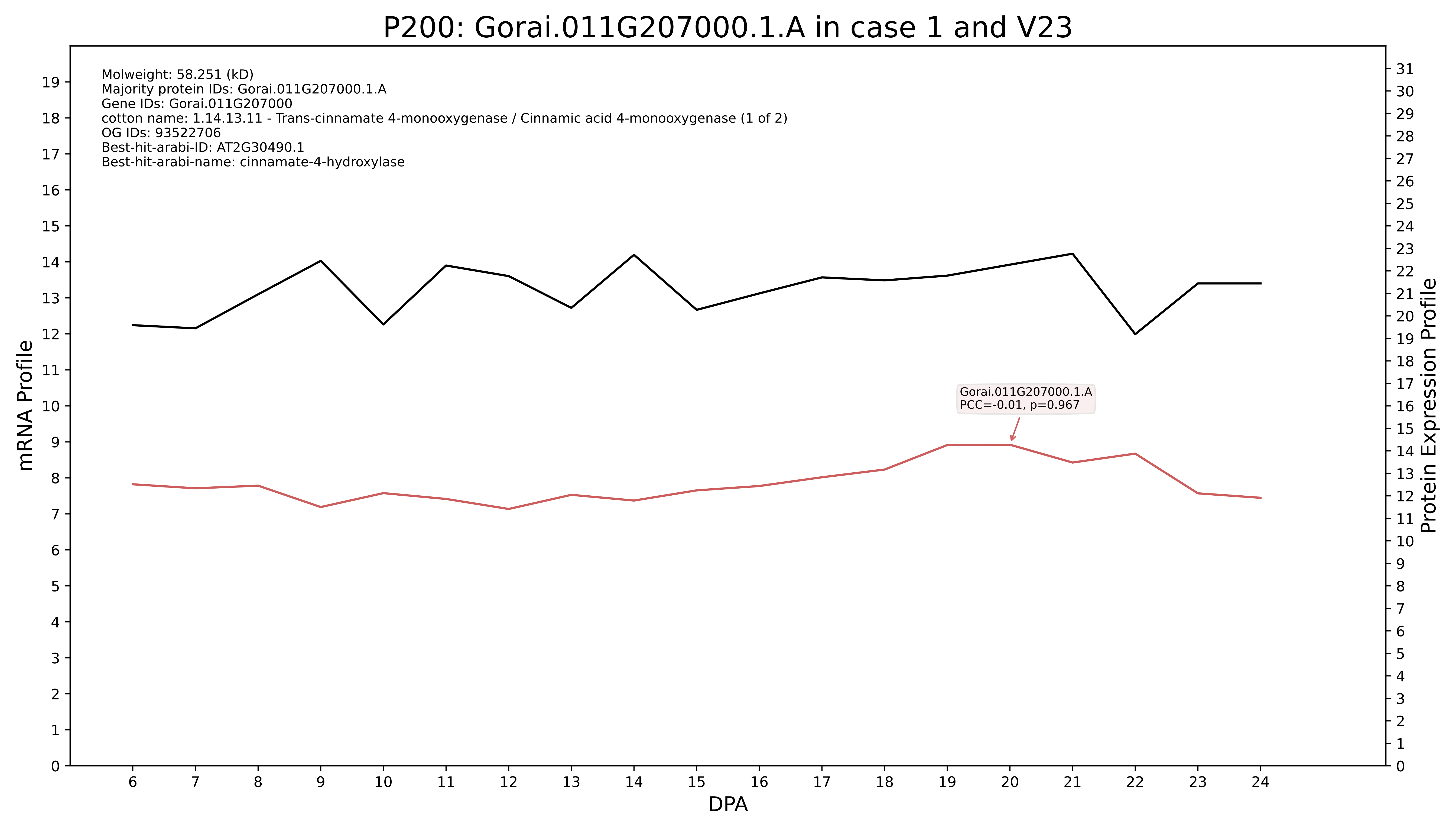
P200: Gorai.011G207000.1.A in case 1 and V23
Molweight: 58.251 (kD)
Majority protein IDs: Gorai.011G207000.1.A
Gene IDs: Gorai.011G207000
cotton name: 1.14.13.11 - Trans-cinnamate 4-monooxygenase / Cinnamic acid 4-monooxygenase (1 of 2)
OG IDs: 93522706
Best-hit-arabi-ID: AT2G30490.1
Best-hit-arabi-name: cinnamate-4-hydroxylase
Gorai.011G207000.1.A
PCC=-0.01, p=0.967
mRNA Profile
Protein Expression Profile
DPA
0
1
2
3
4
5
6
7
8
9
10
11
12
13
14
15
16
17
18
19
20
21
22
23
24
25
26
27
28
29
30
31

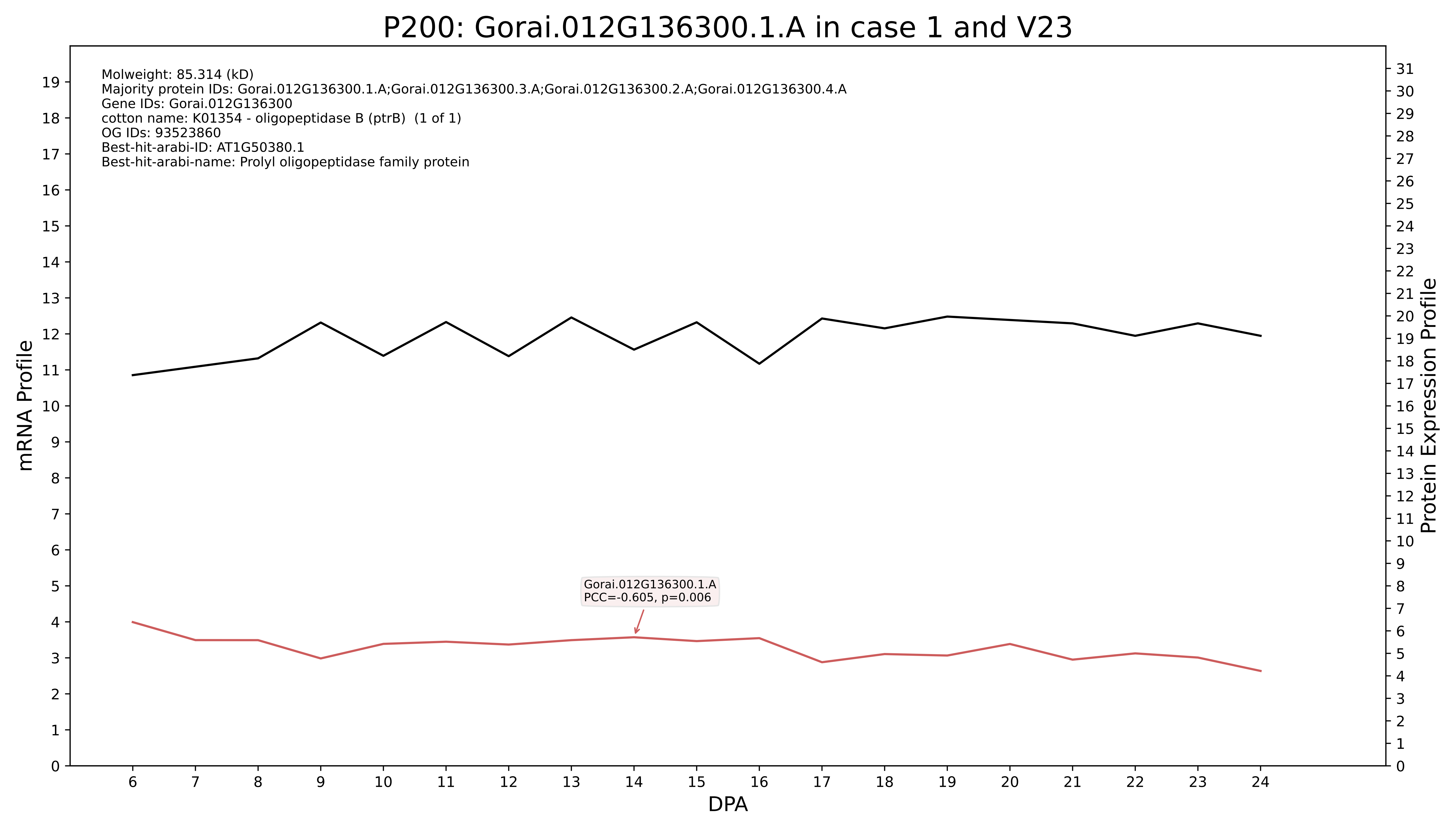
P200: Gorai.012G136300.1.A in case 1 and V23
Molweight: 85.314 (kD)
Majority protein IDs: Gorai.012G136300.1.A;Gorai.012G136300.3.A;Gorai.012G136300.2.A;Gorai.012G136300.4.A
Gene IDs: Gorai.012G136300
cotton name: K01354 - oligopeptidase B (ptrB) (1 of 1)
OG IDs: 93523860
Best-hit-arabi-ID: AT1G50380.1
Best-hit-arabi-name: Prolyl oligopeptidase family protein
Gorai.012G136300.1.A
PCC=-0.605, p=0.006
mRNA Profile
Protein Expression Profile
DPA

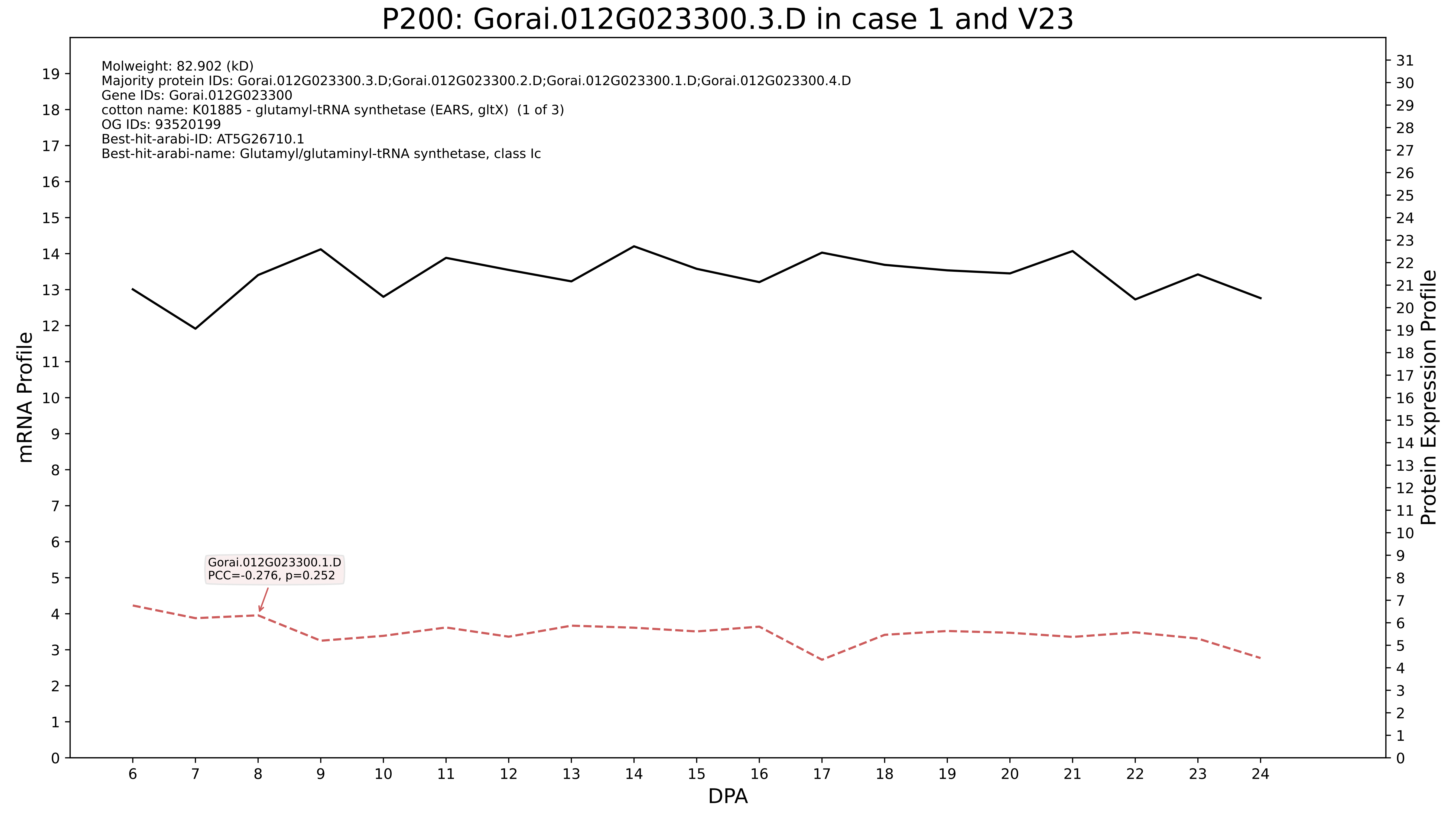
P200: Gorai.012G023300.3.D in case 1 and V23
Molweight: 82.902 (kD)
Majority protein IDs: Gorai.012G023300.3.D;Gorai.012G023300.2.D;Gorai.012G023300.1.D;Gorai.012G023300.4.D
Gene IDs: Gorai.012G023300
cotton name: K01885 - glutamyl-tRNA synthetase (EARS, gltX) (1 of 3)
OG IDs: 93520199
Best-hit-arabi-ID: AT5G26710.1
Best-hit-arabi-name: Glutamyl/glutaminyl-tRNA synthetase, class Ic
Gorai.012G023300.1.D
PCC=-0.276, p=0.252
mRNA Profile
Protein Expression Profile
DPA

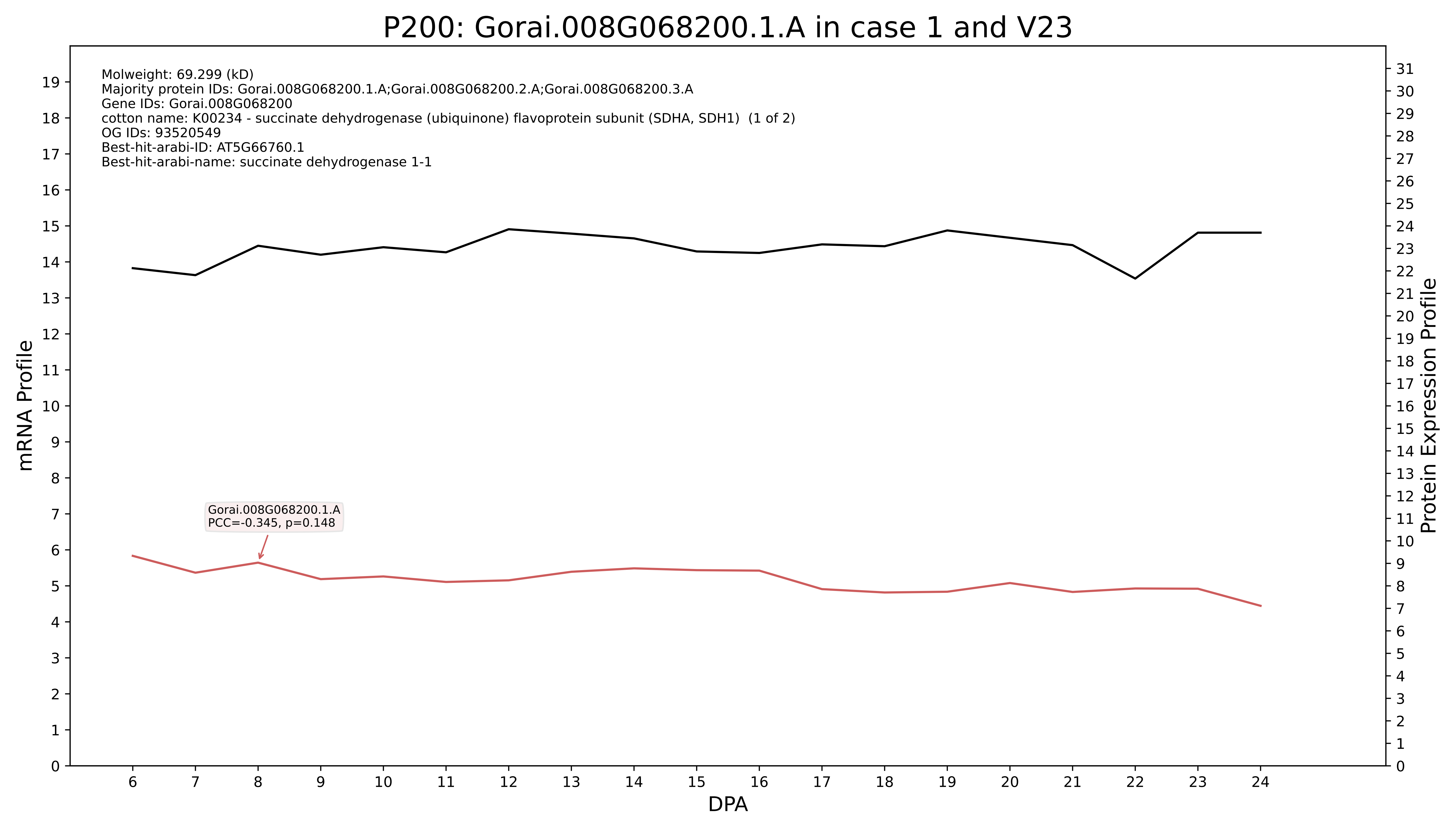

P200: Gorai.008G068200.1.A in case 1 and V23
Molweight: 69.299 (kD)
Majority protein IDs: Gorai.008G068200.1.A;Gorai.008G068200.2.A;Gorai.008G068200.3.A
Gene IDs: Gorai.008G068200
cotton name: K00234 - succinate dehydrogenase (ubiquinone) flavoprotein subunit (SDHA, SDH1) (1 of 2)
OG IDs: 93520549
Best-hit-arabi-ID: AT5G66760.1
Best-hit-arabi-name: succinate dehydrogenase 1-1
Gorai.008G068200.1.A
PCC=-0.345, p=0.148
mRNA Profile
Protein Expression Profile
DPA

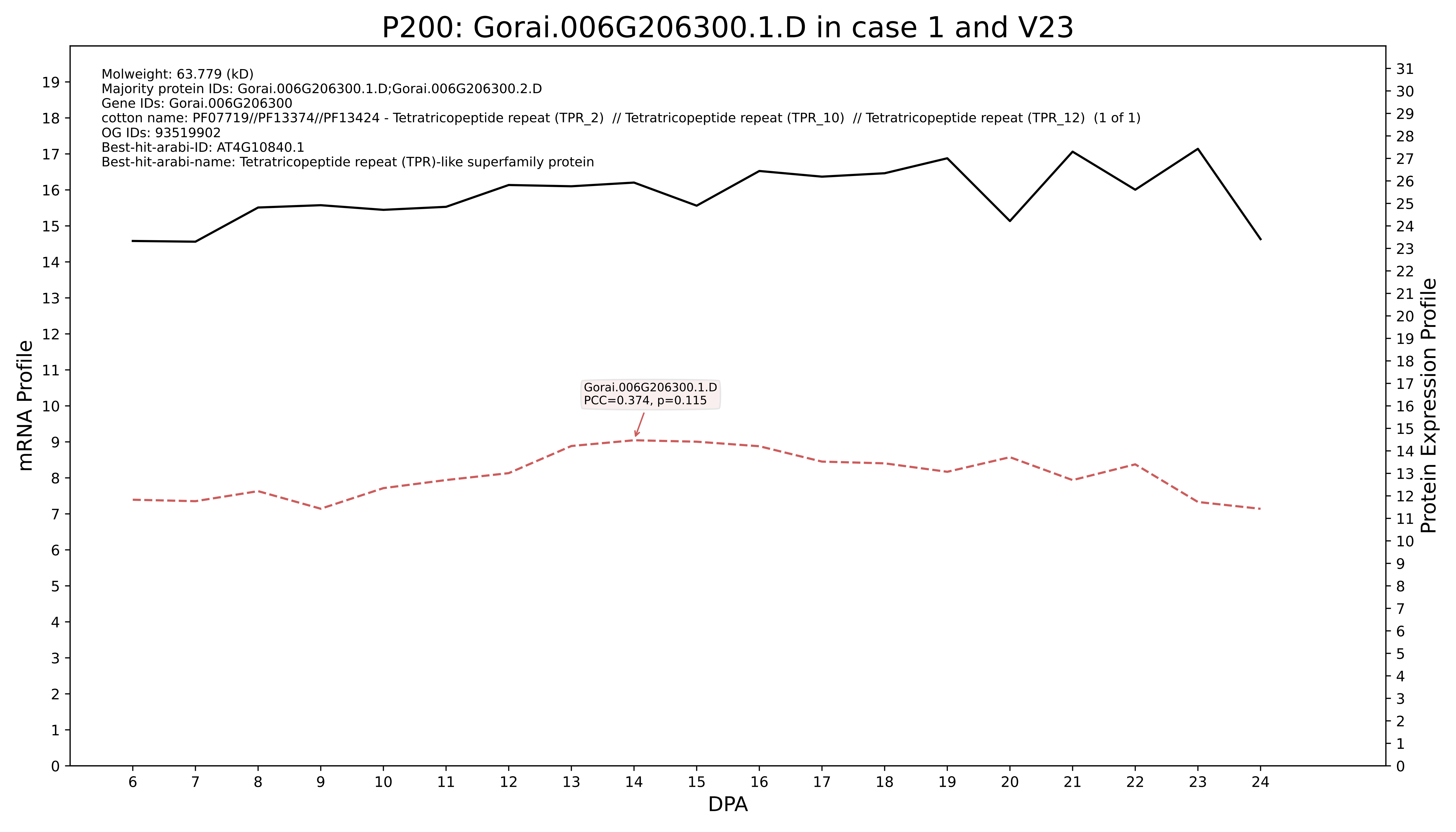
P200: Gorai.006G206300.1.D in case 1 and V23
Molweight: 63.779 (kD)
Majority protein IDs: Gorai.006G206300.1.D;Gorai.006G206300.2.D
Gene IDs: Gorai.006G206300
cotton name: PF07719//PF13374//PF13424 - Tetratricopeptide repeat (TPR_2) // Tetratricopeptide repeat (TPR_10) // Tetratricopeptide repeat (TPR_12) (1 of 1)
OG IDs: 93519902
Best-hit-arabi-ID: AT4G10840.1
Best-hit-arabi-name: Tetratricopeptide repeat (TPR)-like superfamily protein
Gorai.006G206300.1.D
PCC=0.374, p=0.115
mRNA Profile
Protein Expression Profile
DPA

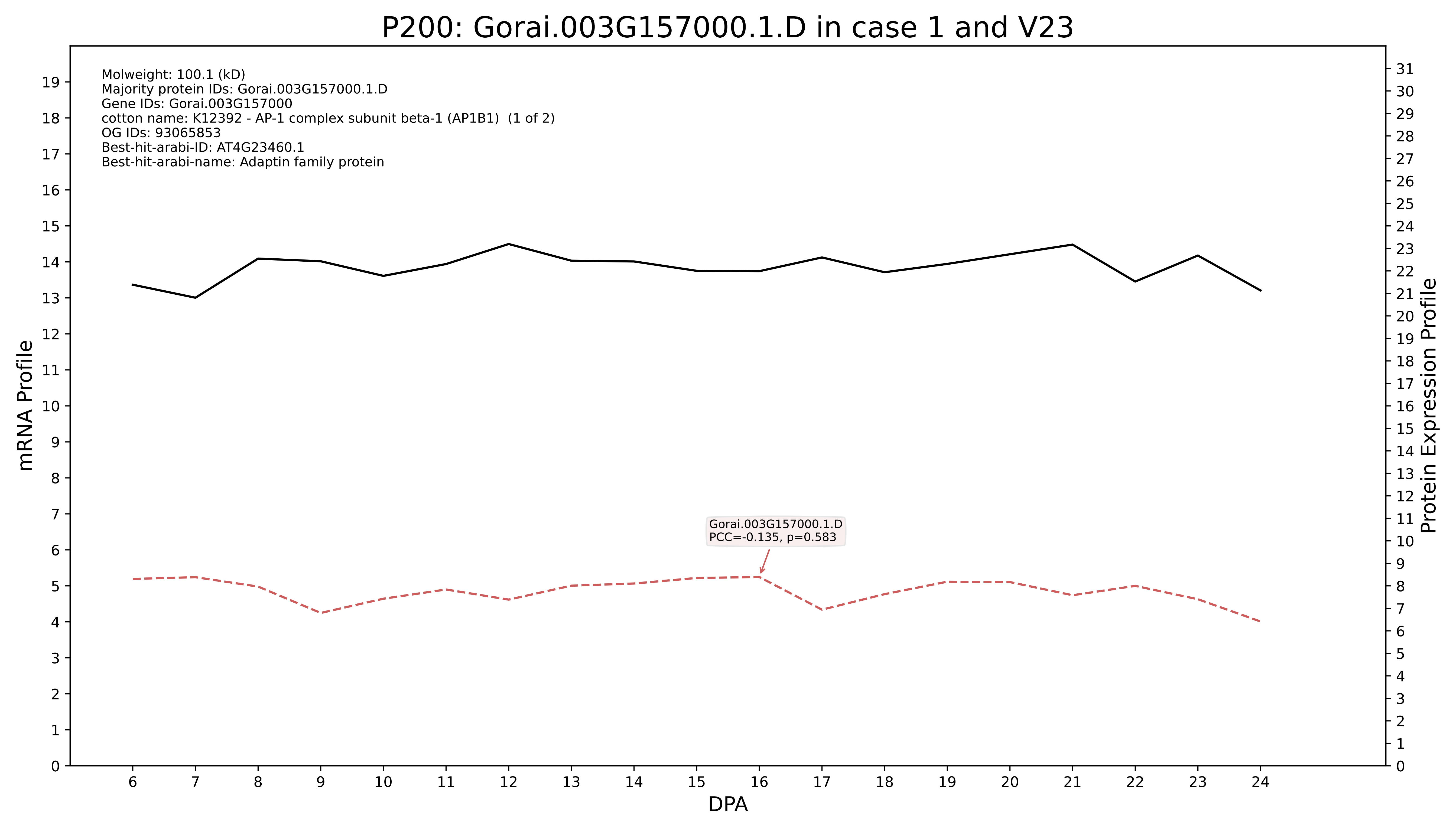
P200: Gorai.003G157000.1.D in case 1 and V23
Molweight: 100.1 (kD)
Majority protein IDs: Gorai.003G157000.1.D
Gene IDs: Gorai.003G157000
cotton name: K12392 - AP-1 complex subunit beta-1 (AP1B1) (1 of 2)
OG IDs: 93065853
Best-hit-arabi-ID: AT4G23460.1
Best-hit-arabi-name: Adaptin family protein
Gorai.003G157000.1.D
PCC=-0.135, p=0.583
mRNA Profile
Protein Expression Profile
DPA

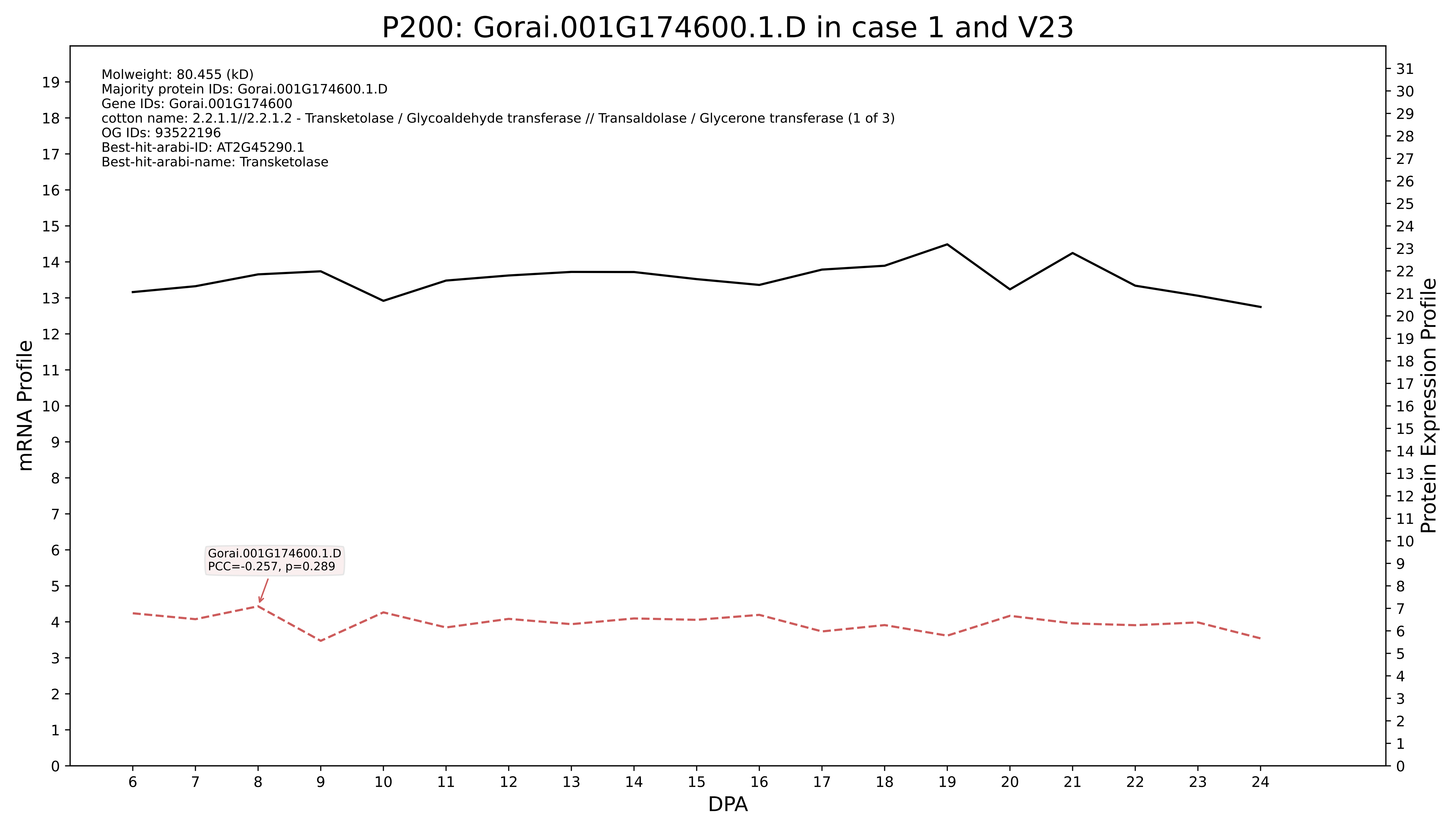
P200: Gorai.001G174600.1.D in case 1 and V23
Molweight: 80.455 (kD)
Majority protein IDs: Gorai.001G174600.1.D
Gene IDs: Gorai.001G174600
cotton name: 2.2.1.1//2.2.1.2 - Transketolase / Glycoaldehyde transferase // Transaldolase / Glycerone transferase (1 of 3)
OG IDs: 93522196
Best-hit-arabi-ID: AT2G45290.1
Best-hit-arabi-name: Transketolase
Gorai.001G174600.1.D
PCC=-0.257, p=0.289
mRNA Profile
Protein Expression Profile
DPA

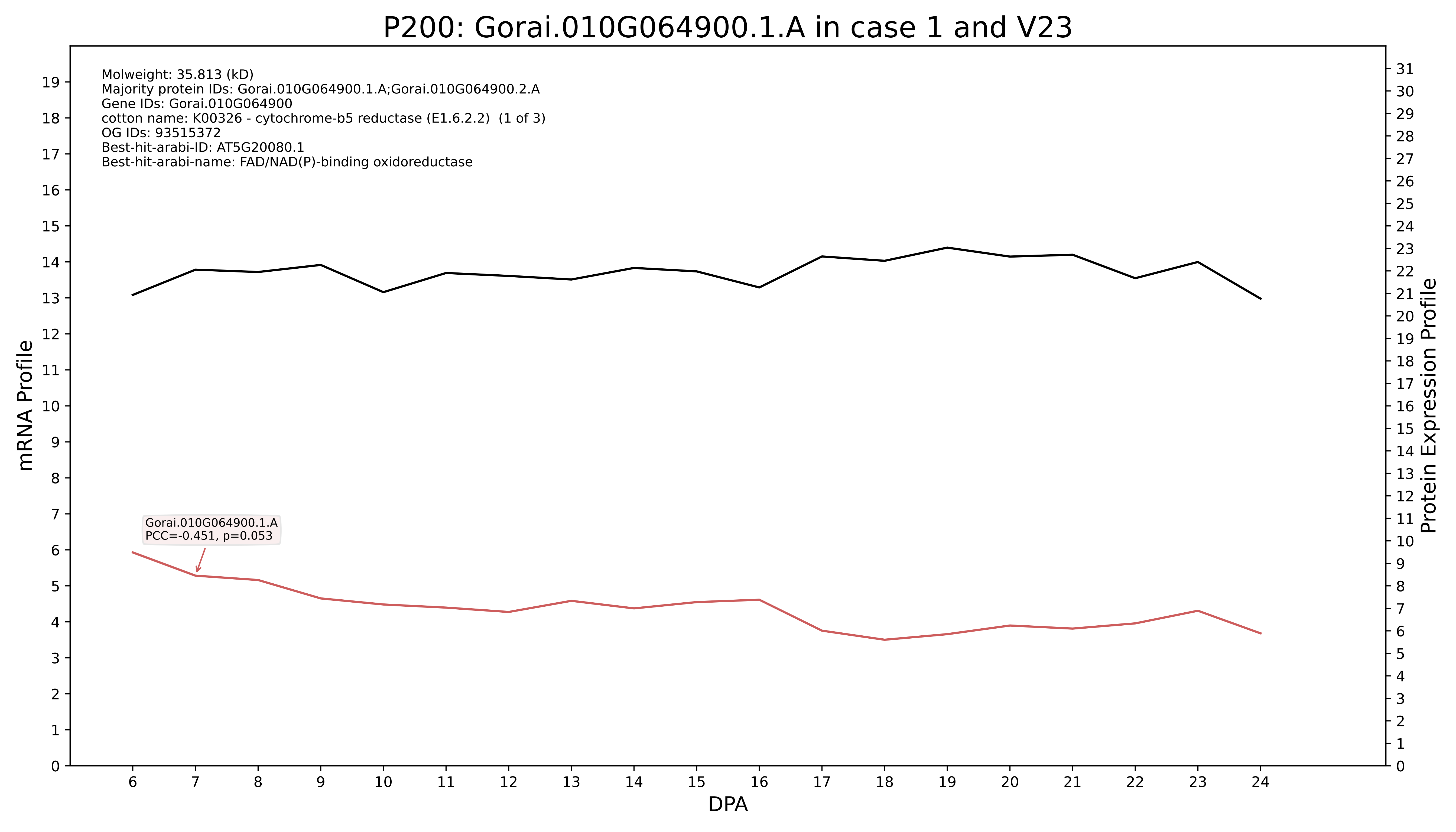
P200: Gorai.010G064900.1.A in case 1 and V23
Molweight: 35.813 (kD)
Majority protein IDs: Gorai.010G064900.1.A;Gorai.010G064900.2.A
Gene IDs: Gorai.010G064900
cotton name: K00326 - cytochrome-b5 reductase (E1.6.2.2) (1 of 3)
OG IDs: 93515372
Best-hit-arabi-ID: AT5G20080.1
Best-hit-arabi-name: FAD/NAD(P)-binding oxidoreductase
Gorai.010G064900.1.A
PCC=-0.451, p=0.053
mRNA Profile
Protein Expression Profile
DPA

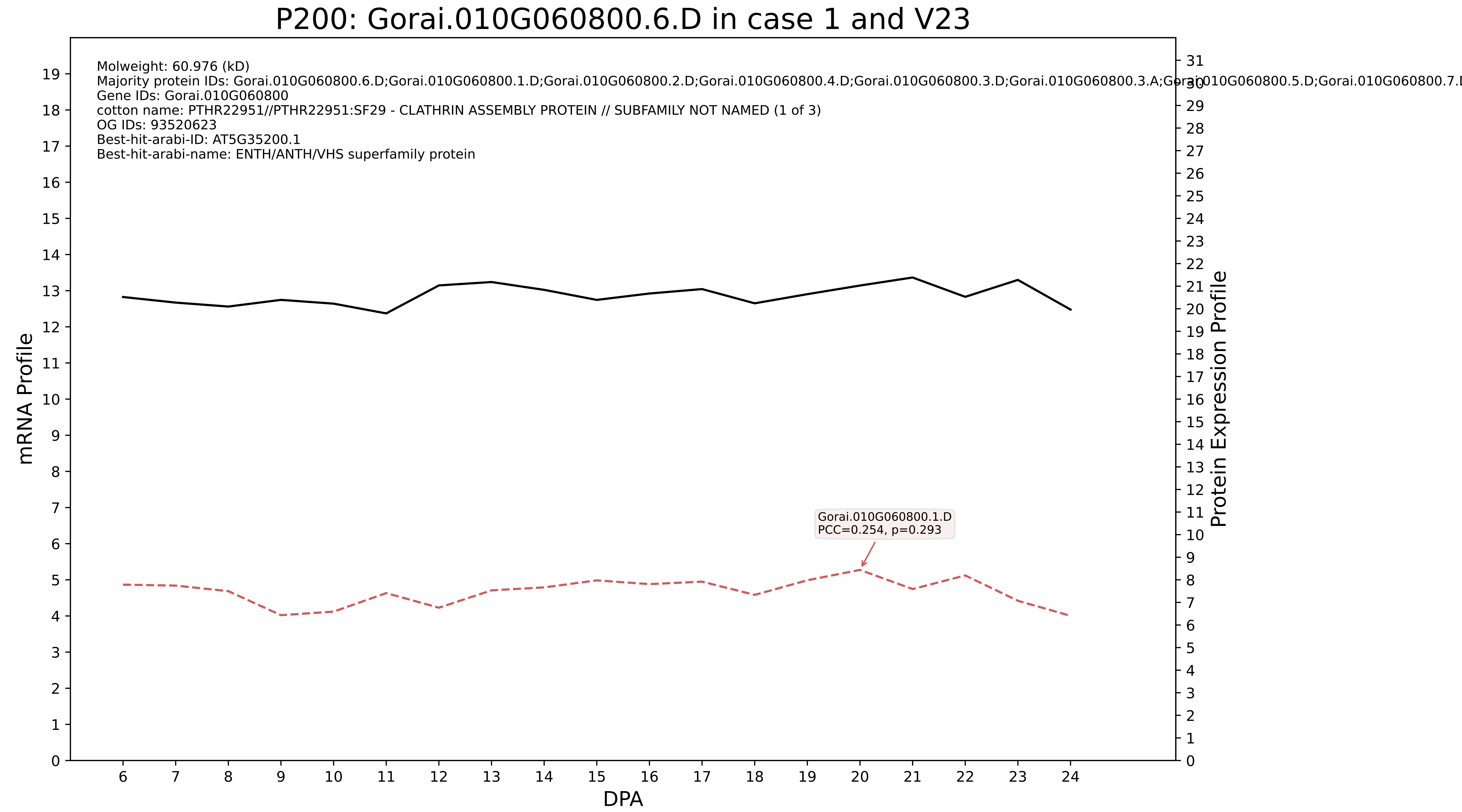
P200: Gorai.010G060800.6.D in case 1 and V23
Molweight: 60.976 (kD)
Majority protein IDs: Gorai.010G060800.6.D;Gorai.010G060800.1.D;Gorai.010G060800.2.D;Gorai.010G060800.4.D;Gorai.010G060800.3.D;Gorai.010G060800.3.A;Gorai.010G060800.5.D;Gorai.010G060800.7.D
Gene IDs: Gorai.010G060800
cotton name: PTHR22951//PTHR22951:SF29 - CLATHRIN ASSEMBLY PROTEIN // SUBFAMILY NOT NAMED (1 of 3)
OG IDs: 93520623
Best-hit-arabi-ID: AT5G35200.1
Best-hit-arabi-name: ENTH/ANTH/VHS superfamily protein
Gorai.010G060800.1.D
PCC=0.254, p=0.293
mRNA Profile
Protein Expression Profile
DPA

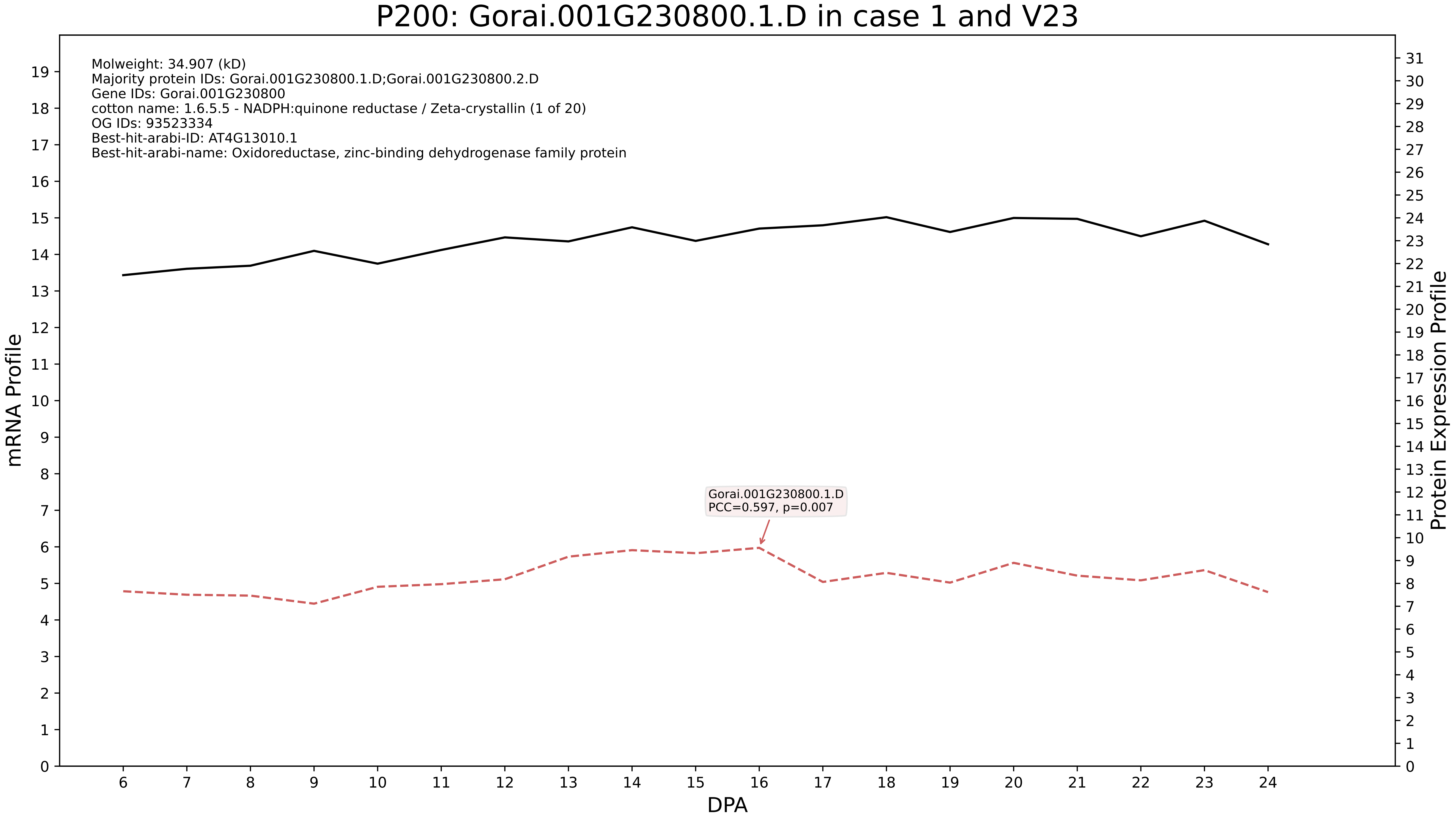
P200: Gorai.001G230800.1.D in case 1 and V23
Molweight: 34.907 (kD)
Majority protein IDs: Gorai.001G230800.1.D;Gorai.001G230800.2.D
Gene IDs: Gorai.001G230800
cotton name: 1.6.5.5 - NADPH:quinone reductase / Zeta-crystallin (1 of 20)
OG IDs: 93523334
Best-hit-arabi-ID: AT4G13010.1
Best-hit-arabi-name: Oxidoreductase, zinc-binding dehydrogenase family protein
Gorai.001G230800.1.D
PCC=0.597, p=0.007
mRNA Profile
Protein Expression Profile
DPA

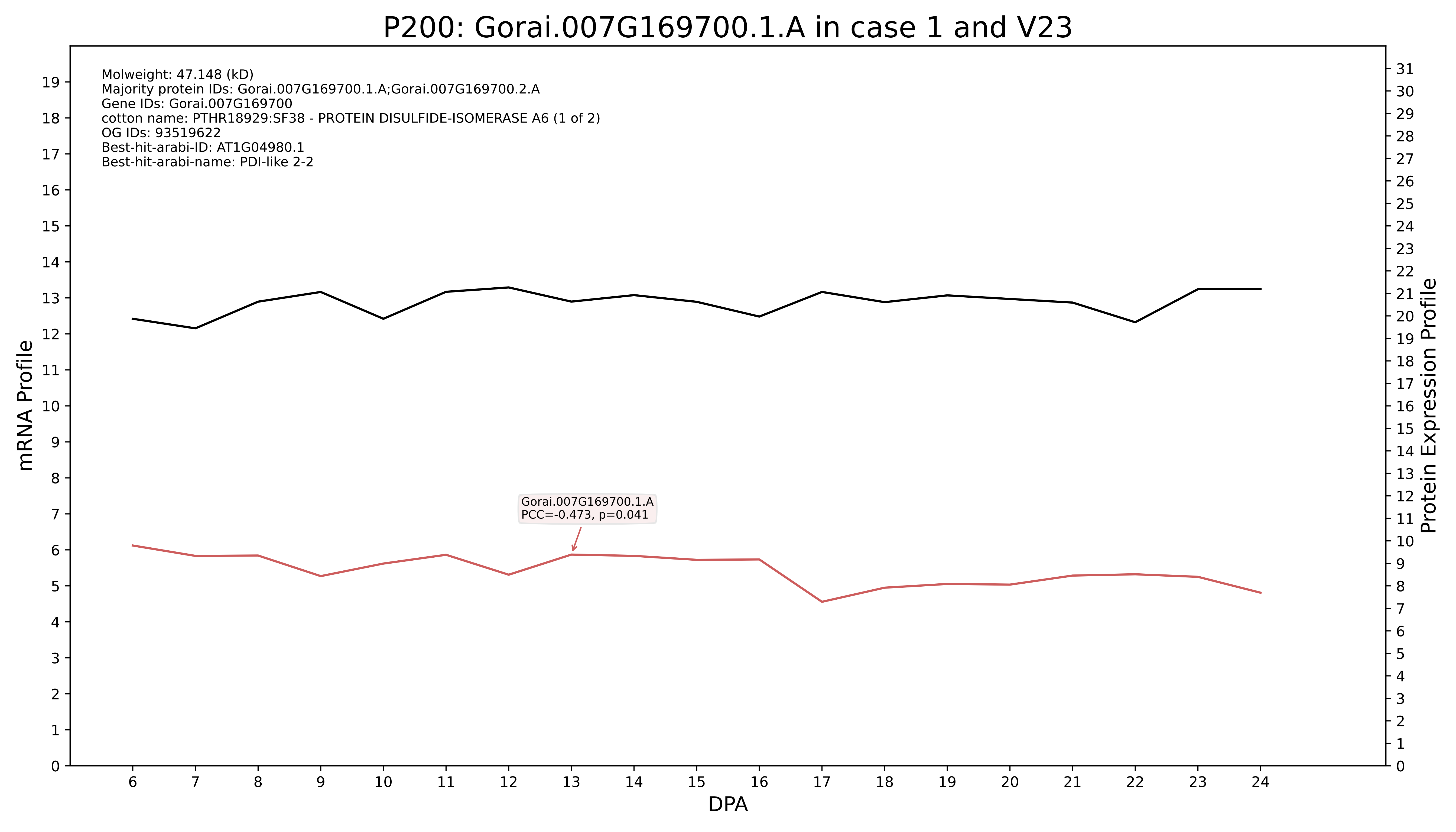
P200: Gorai.007G169700.1.A in case 1 and V23
Molweight: 47.148 (kD)
Majority protein IDs: Gorai.007G169700.1.A;Gorai.007G169700.2.A
Gene IDs: Gorai.007G169700
cotton name: PTHR18929:SF38 - PROTEIN DISULFIDE-ISOMERASE A6 (1 of 2)
OG IDs: 93519622
Best-hit-arabi-ID: AT1G04980.1
Best-hit-arabi-name: PDI-like 2-2
Gorai.007G169700.1.A
PCC=-0.473, p=0.041
mRNA Profile
Protein Expression Profile
DPA

P200: Gorai.007G308700.2.A in case 1 and V23

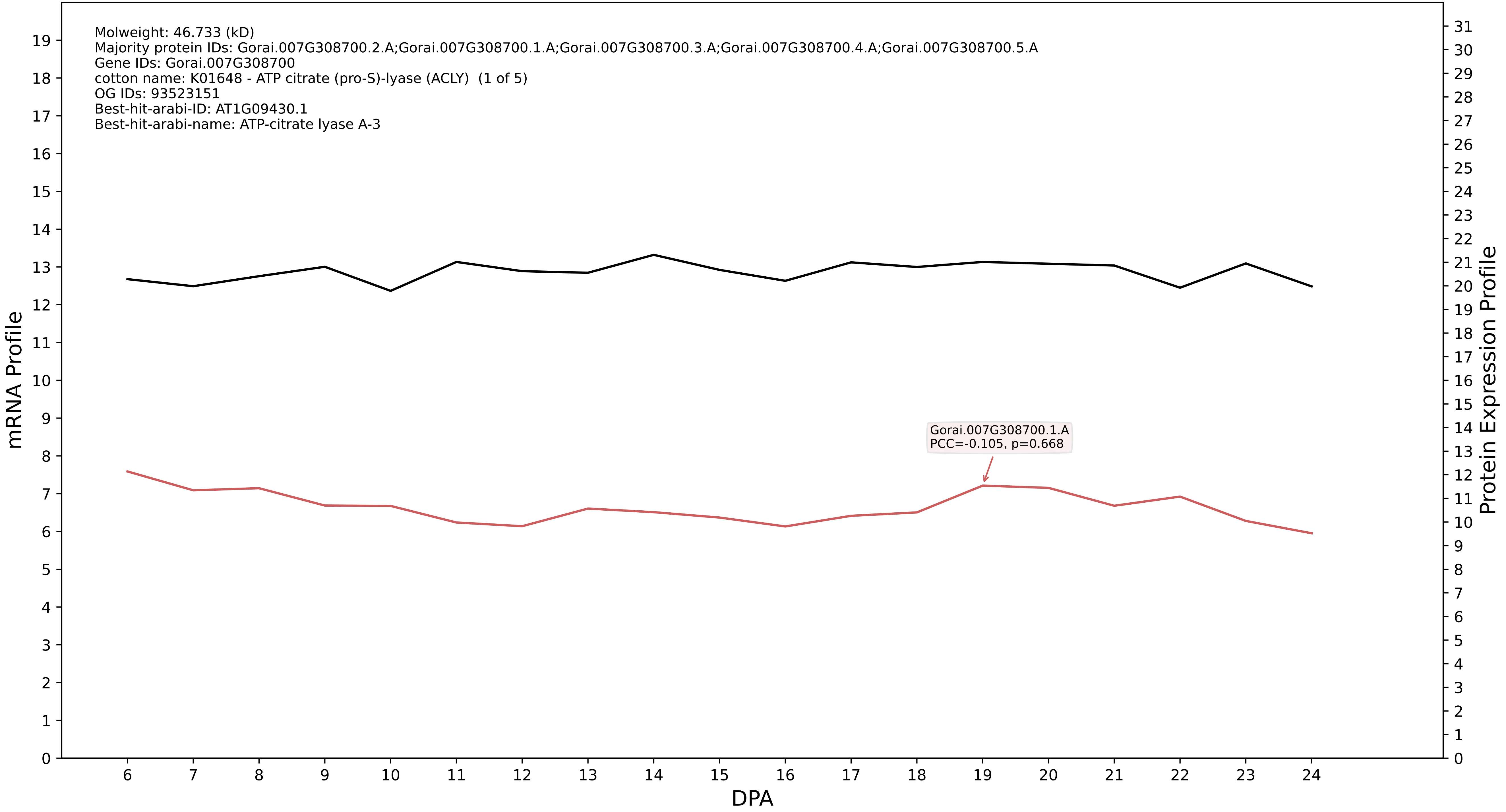

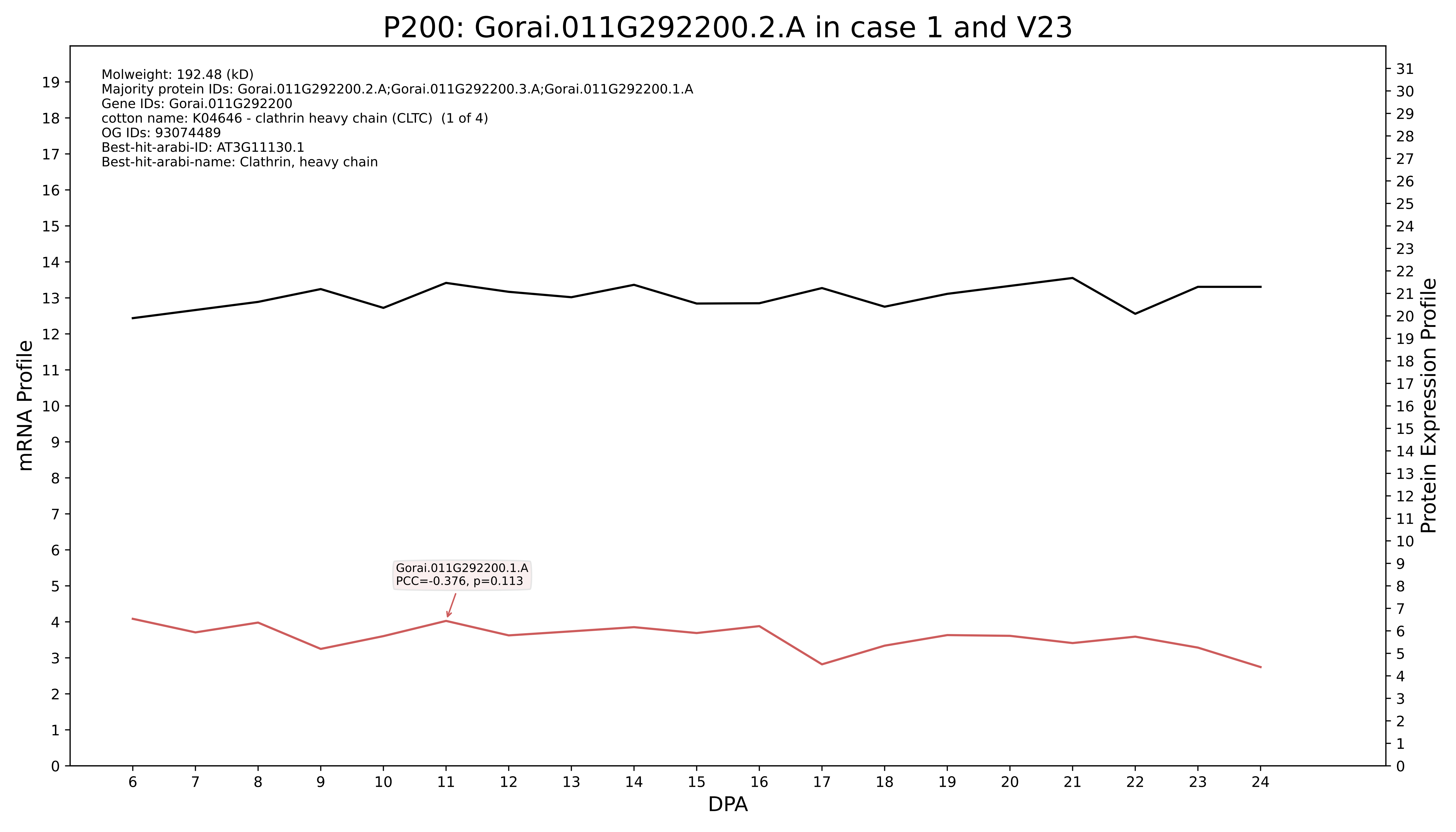

P200: Gorai.011G292200.2.A in case 1 and V23
Molweight: 192.48 (kD)
Majority protein IDs: Gorai.011G292200.2.A;Gorai.011G292200.3.A;Gorai.011G292200.1.A
Gene IDs: Gorai.011G292200
cotton name: K04646 - clathrin heavy chain (CLTC) (1 of 4)
OG IDs: 93074489
Best-hit-arabi-ID: AT3G11130.1
Best-hit-arabi-name: Clathrin, heavy chain
Gorai.011G292200.1.A
PCC=-0.376, p=0.113
mRNA Profile
Protein Expression Profile
DPA

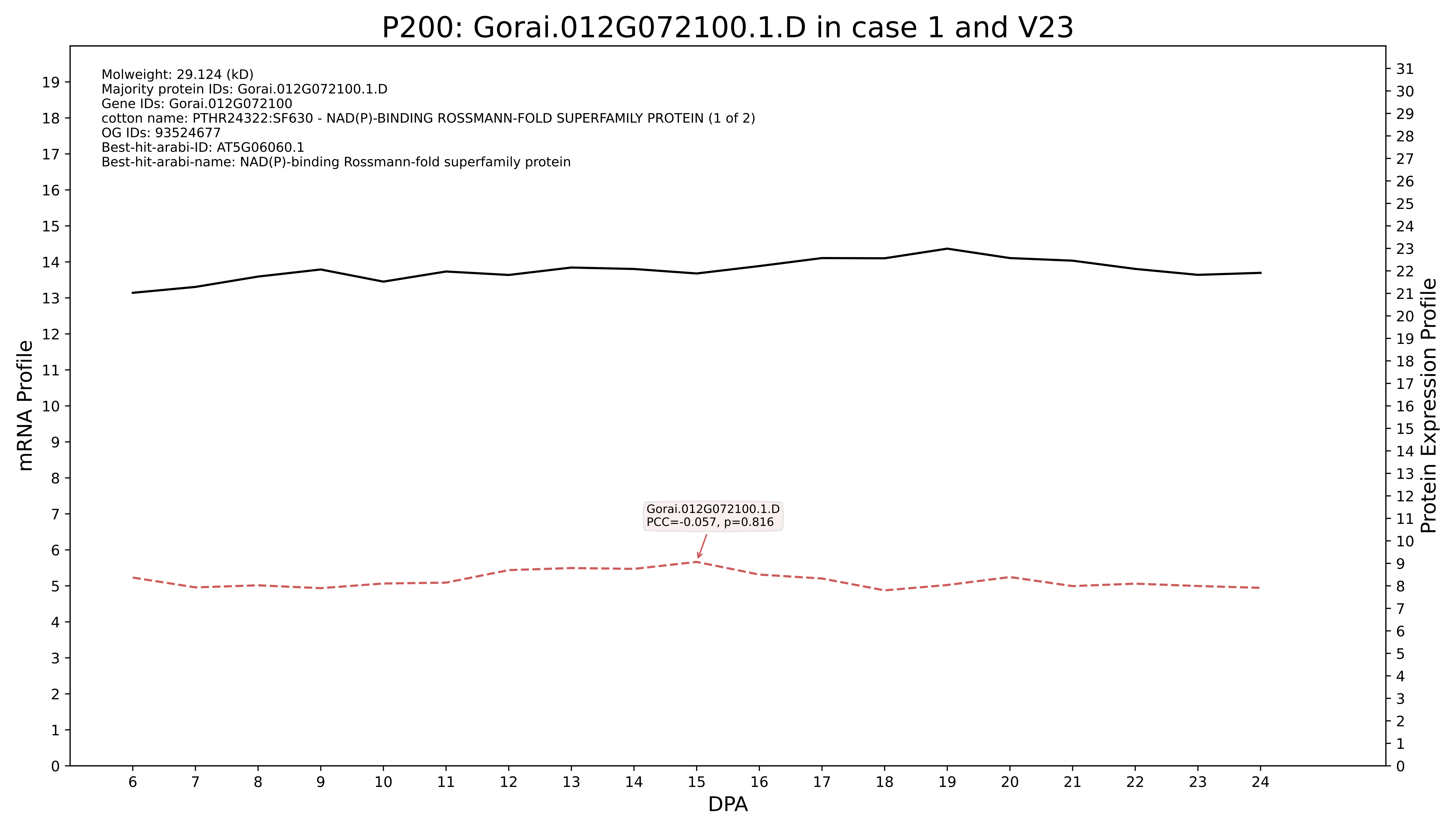
P200: Gorai.012G072100.1.D in case 1 and V23
Molweight: 29.124 (kD)
Majority protein IDs: Gorai.012G072100.1.D
Gene IDs: Gorai.012G072100
cotton name: PTHR24322:SF630 - NAD(P)-BINDING ROSSMANN-FOLD SUPERFAMILY PROTEIN (1 of 2)
OG IDs: 93524677
Best-hit-arabi-ID: AT5G06060.1
Best-hit-arabi-name: NAD(P)-binding Rossmann-fold superfamily protein
Gorai.012G072100.1.D
PCC=-0.057, p=0.816
mRNA Profile
Protein Expression Profile
DPA

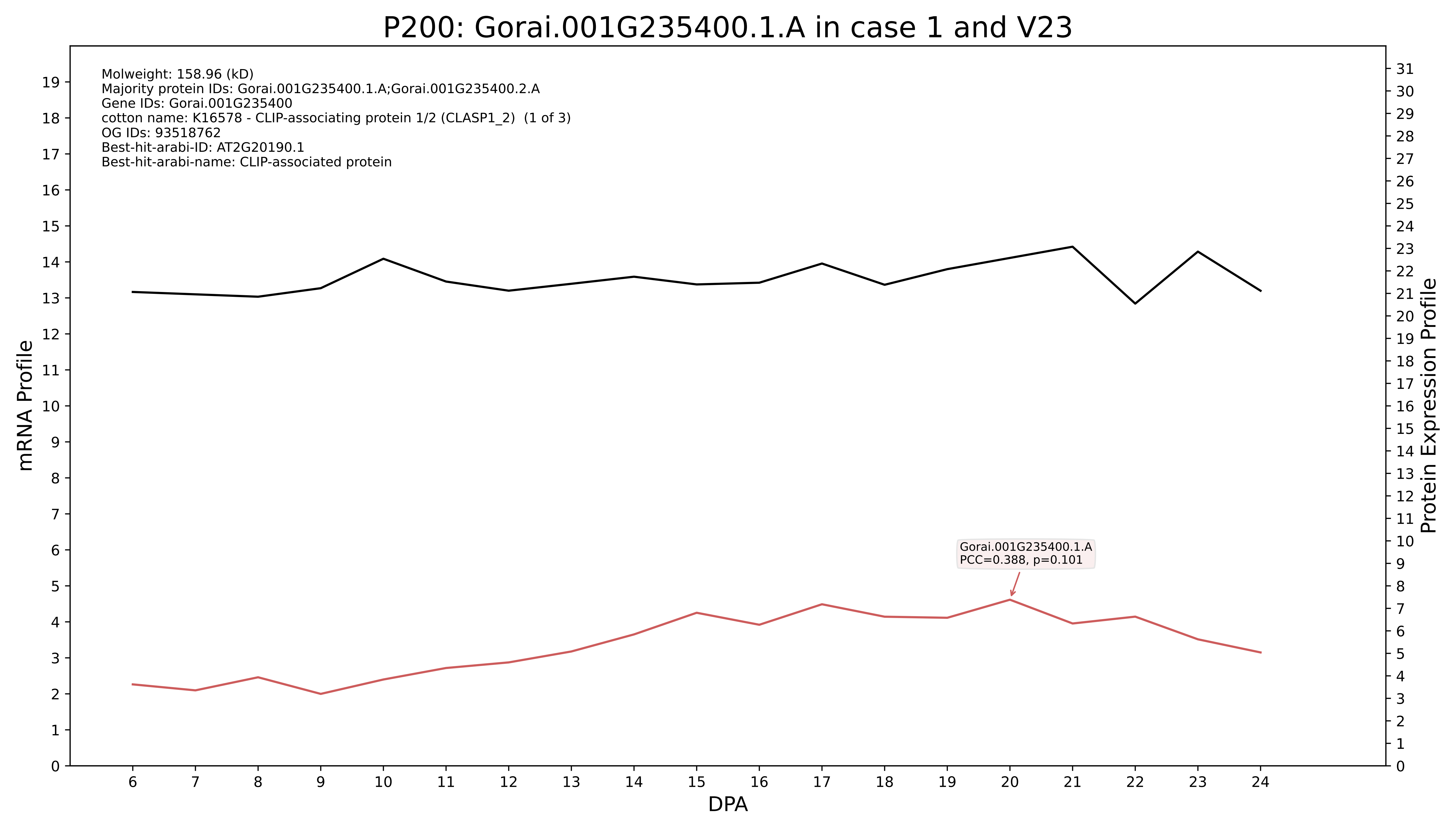
P200: Gorai.001G235400.1.A in case 1 and V23
Molweight: 158.96 (kD)
Majority protein IDs: Gorai.001G235400.1.A;Gorai.001G235400.2.A
Gene IDs: Gorai.001G235400
cotton name: K16578 - CLIP-associating protein 1/2 (CLASP1_2) (1 of 3)
OG IDs: 93518762
Best-hit-arabi-ID: AT2G20190.1
Best-hit-arabi-name: CLIP-associated protein
Gorai.001G235400.1.A
PCC=0.388, p=0.101
mRNA Profile
Protein Expression Profile
DPA

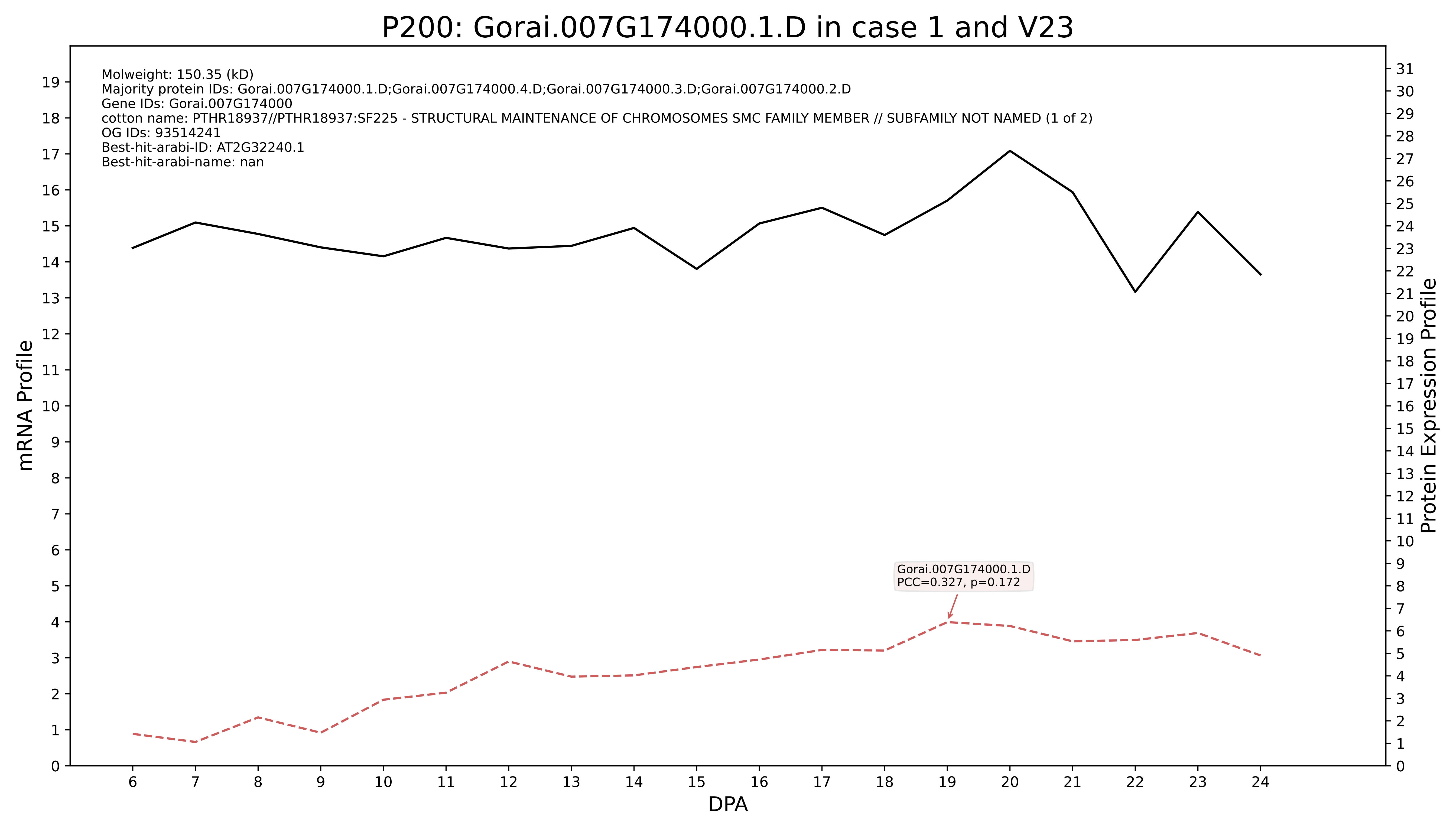
P200: Gorai.007G174000.1.D in case 1 and V23
Molweight: 150.35 (kD)
Majority protein IDs: Gorai.007G174000.1.D;Gorai.007G174000.4.D;Gorai.007G174000.3.D;Gorai.007G174000.2.D
Gene IDs: Gorai.007G174000
cotton name: PTHR18937//PTHR18937:SF225 - STRUCTURAL MAINTENANCE OF CHROMOSOMES SMC FAMILY MEMBER // SUBFAMILY NOT NAMED (1 of 2)
OG IDs: 93514241
Best-hit-arabi-ID: AT2G32240.1
Best-hit-arabi-name: nan
Gorai.007G174000.1.D
PCC=0.327, p=0.172
mRNA Profile
Protein Expression Profile
DPA

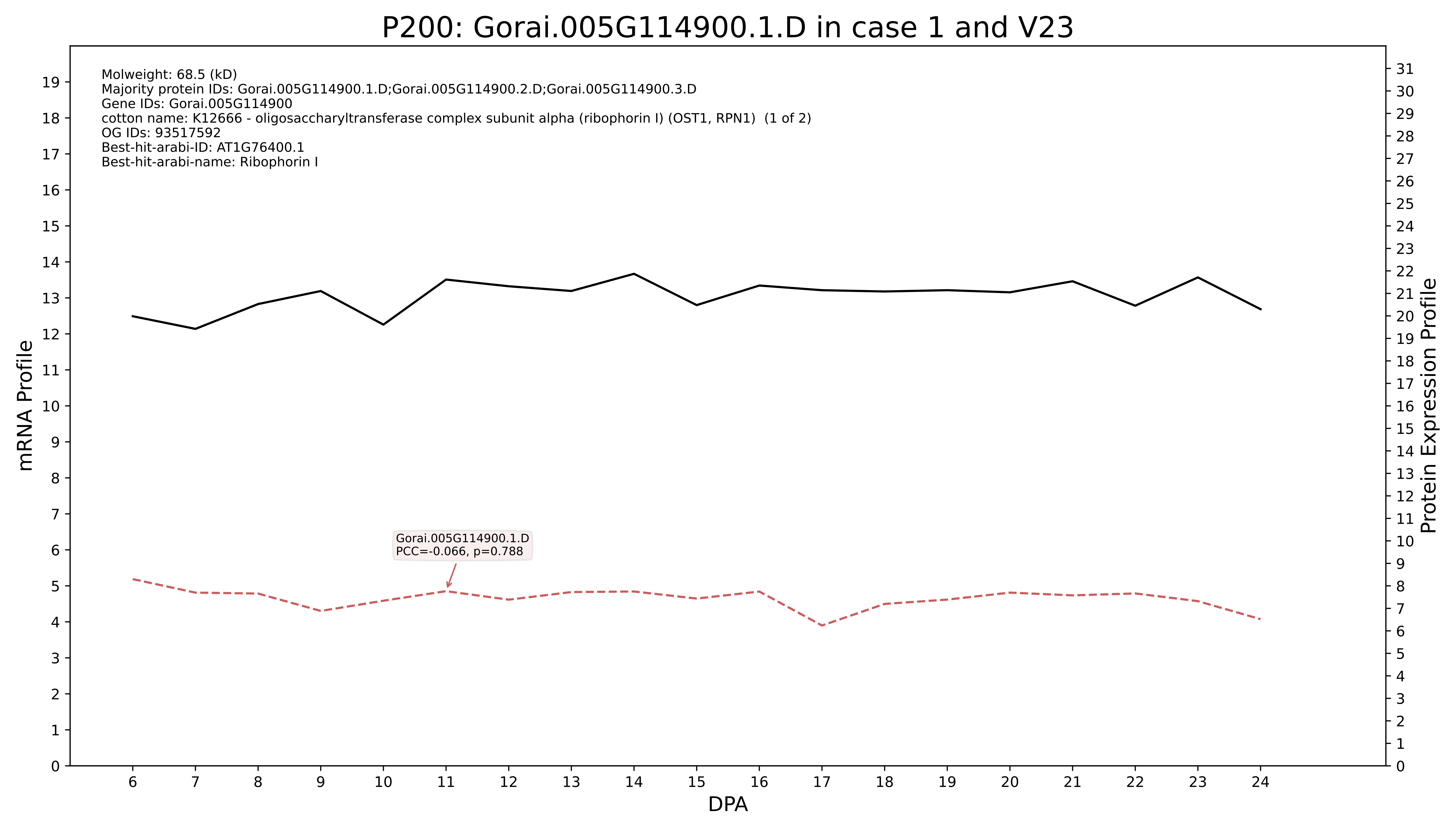
P200: Gorai.005G114900.1.D in case 1 and V23
Molweight: 68.5 (kD)
Majority protein IDs: Gorai.005G114900.1.D;Gorai.005G114900.2.D;Gorai.005G114900.3.D
Gene IDs: Gorai.005G114900
cotton name: K12666 - oligosaccharyltransferase complex subunit alpha (ribophorin I) (OST1, RPN1) (1 of 2)
OG IDs: 93517592
Best-hit-arabi-ID: AT1G76400.1
Best-hit-arabi-name: Ribophorin I
Gorai.005G114900.1.D
PCC=-0.066, p=0.788
mRNA Profile
Protein Expression Profile
DPA
0
1
2
3
4
5
6
7
8
9
10
11
12
13
14
15
16
17
18
19
20
21
22
23
24
25
26
27
28
29
30
31

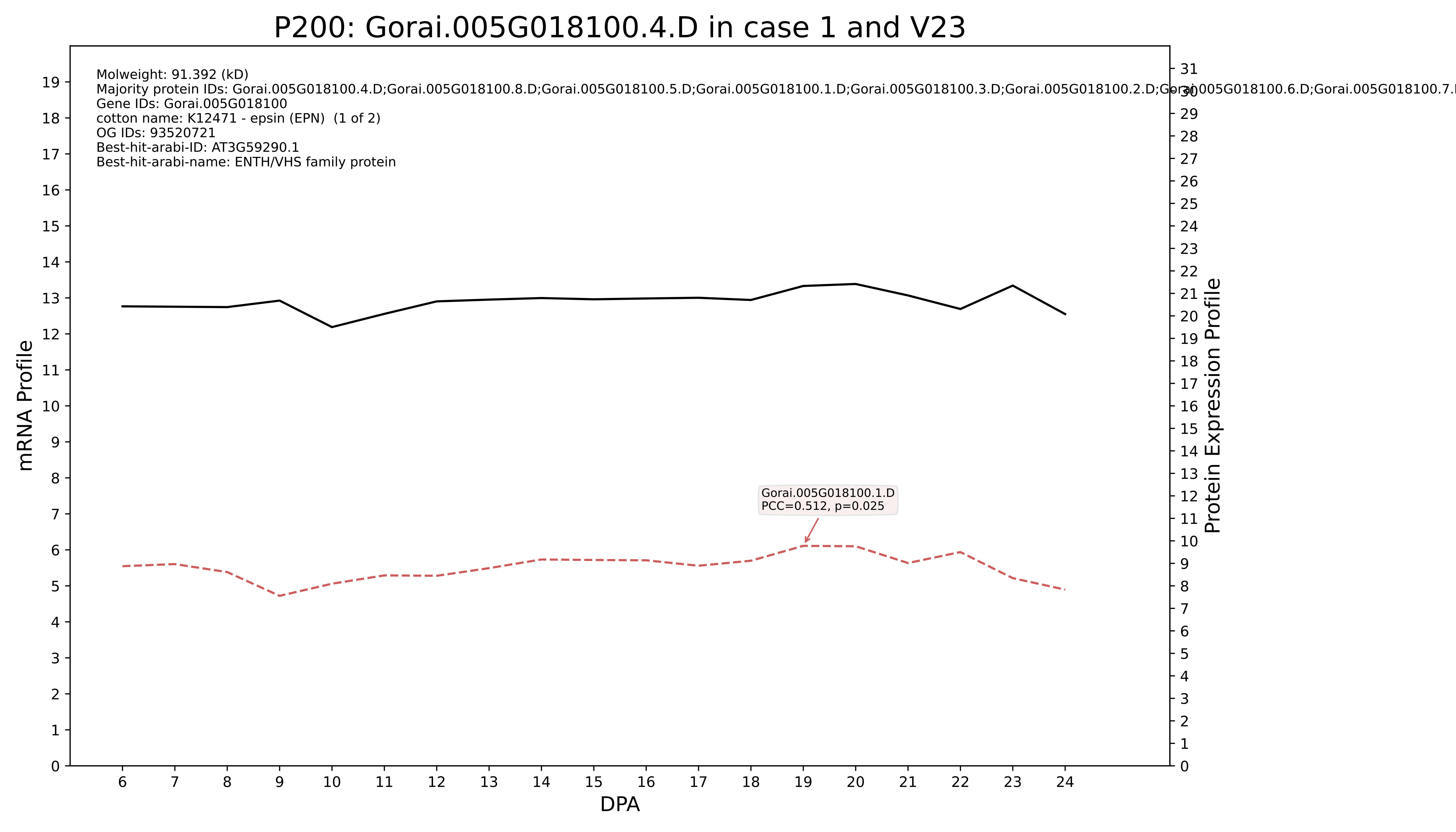
P200: Gorai.005G018100.4.D in case 1 and V23
Molweight: 91.392 (kD)
Majority protein IDs: Gorai.005G018100.4.D;Gorai.005G018100.8.D;Gorai.005G018100.5.D;Gorai.005G018100.1.D;Gorai.005G018100.3.D;Gorai.005G018100.2.D;Gorai.005G018100.6.D;Gorai.005G018100.7.D
Gene IDs: Gorai.005G018100
cotton name: K12471 - epsin (EPN) (1 of 2)
OG IDs: 93520721
Best-hit-arabi-ID: AT3G59290.1
Best-hit-arabi-name: ENTH/VHS family protein
Gorai.005G018100.1.D
PCC=0.512, p=0.025
mRNA Profile
Protein Expression Profile
DPA

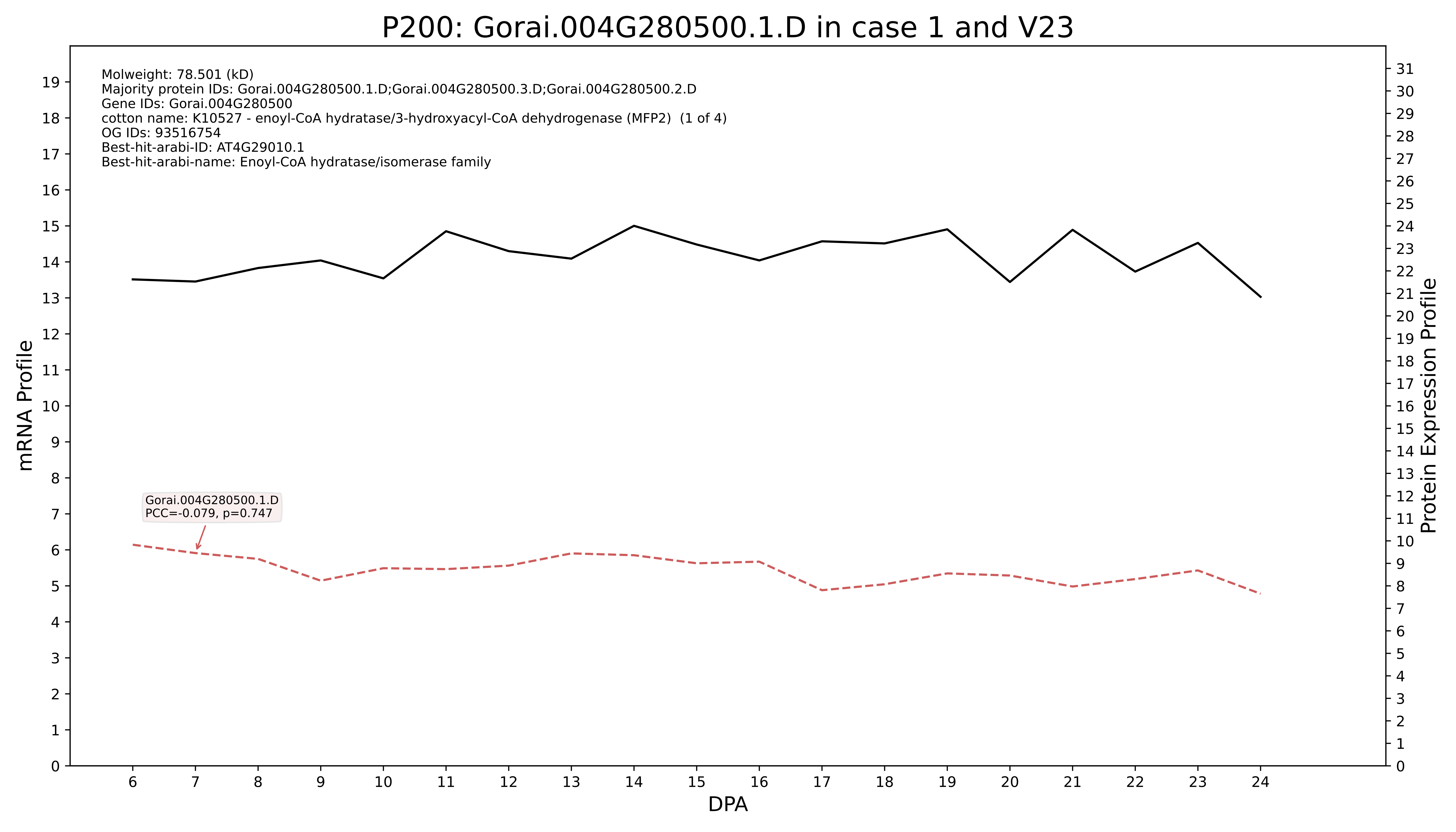

P200: Gorai.004G280500.1.D in case 1 and V23
Molweight: 78.501 (kD)
Majority protein IDs: Gorai.004G280500.1.D;Gorai.004G280500.3.D;Gorai.004G280500.2.D
Gene IDs: Gorai.004G280500
cotton name: K10527 - enoyl-CoA hydratase/3-hydroxyacyl-CoA dehydrogenase (MFP2) (1 of 4)
OG IDs: 93516754
Best-hit-arabi-ID: AT4G29010.1
Best-hit-arabi-name: Enoyl-CoA hydratase/isomerase family
Gorai.004G280500.1.D
PCC=-0.079, p=0.747
mRNA Profile
Protein Expression Profile
DPA

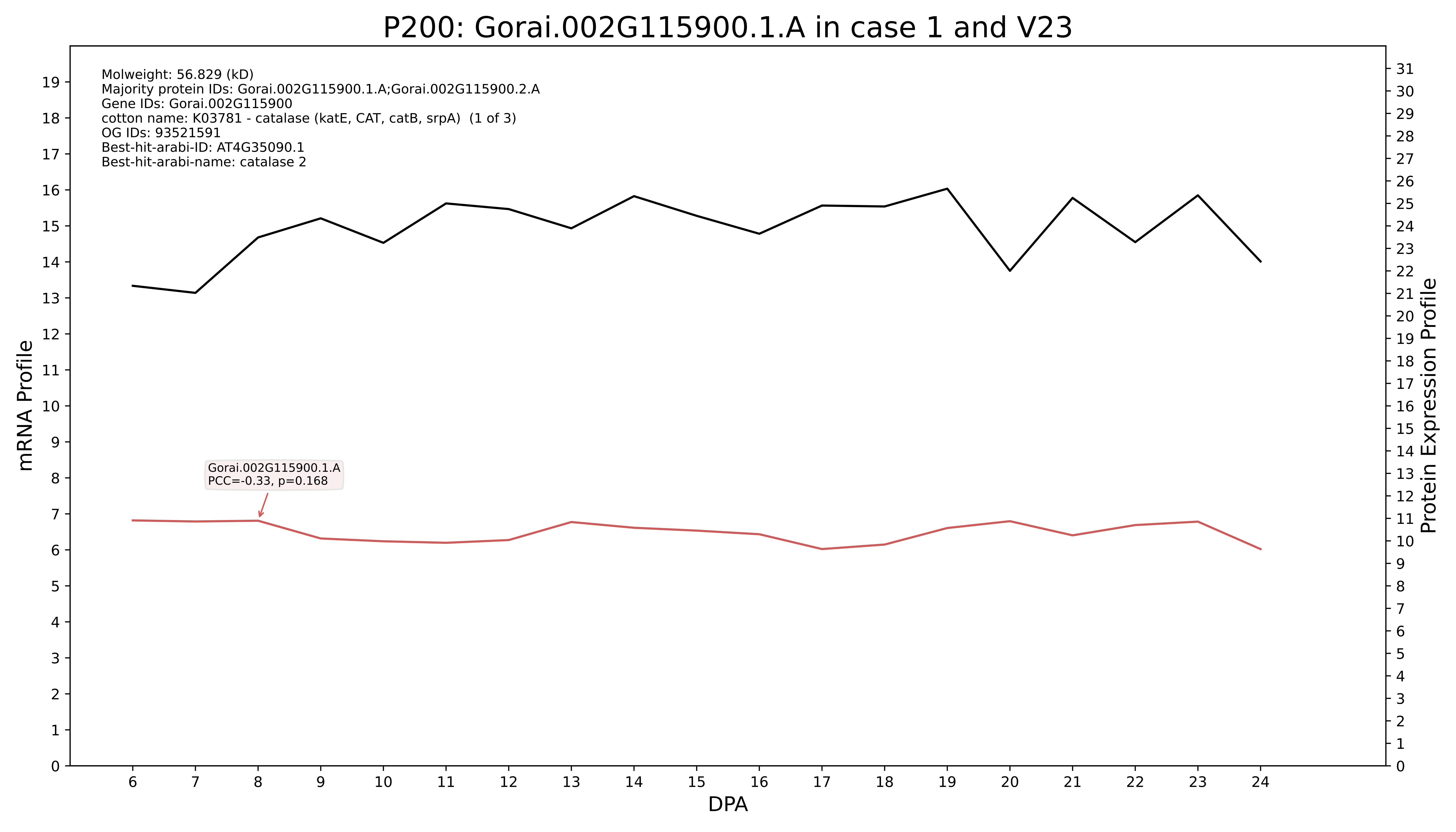

P200: Gorai.002G115900.1.A in case 1 and V23
Molweight: 56.829 (kD)
Majority protein IDs: Gorai.002G115900.1.A;Gorai.002G115900.2.A
Gene IDs: Gorai.002G115900
cotton name: K03781 - catalase (katE, CAT, catB, srpA) (1 of 3)
OG IDs: 93521591
Best-hit-arabi-ID: AT4G35090.1
Best-hit-arabi-name: catalase 2
Gorai.002G115900.1.A
PCC=-0.33, p=0.168
mRNA Profile
Protein Expression Profile
DPA

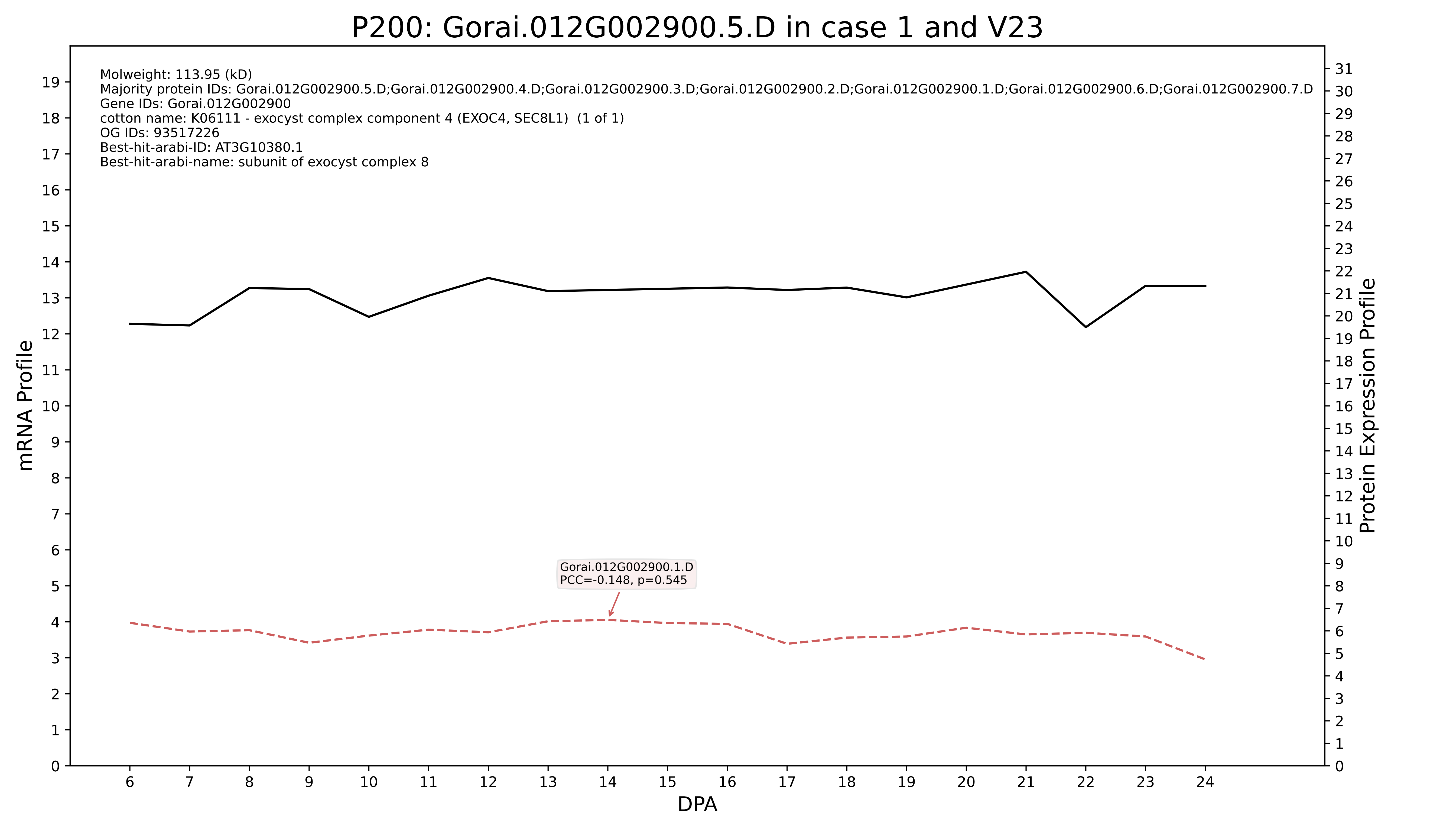
P200: Gorai.012G002900.5.D in case 1 and V23
Molweight: 113.95 (kD)
Majority protein IDs: Gorai.012G002900.5.D;Gorai.012G002900.4.D;Gorai.012G002900.3.D;Gorai.012G002900.2.D;Gorai.012G002900.1.D;Gorai.012G002900.6.D;Gorai.012G002900.7.D
Gene IDs: Gorai.012G002900
cotton name: K06111 - exocyst complex component 4 (EXOC4, SEC8L1) (1 of 1)
OG IDs: 93517226
Best-hit-arabi-ID: AT3G10380.1
Best-hit-arabi-name: subunit of exocyst complex 8
Gorai.012G002900.1.D
PCC=-0.148, p=0.545
mRNA Profile
Protein Expression Profile
DPA

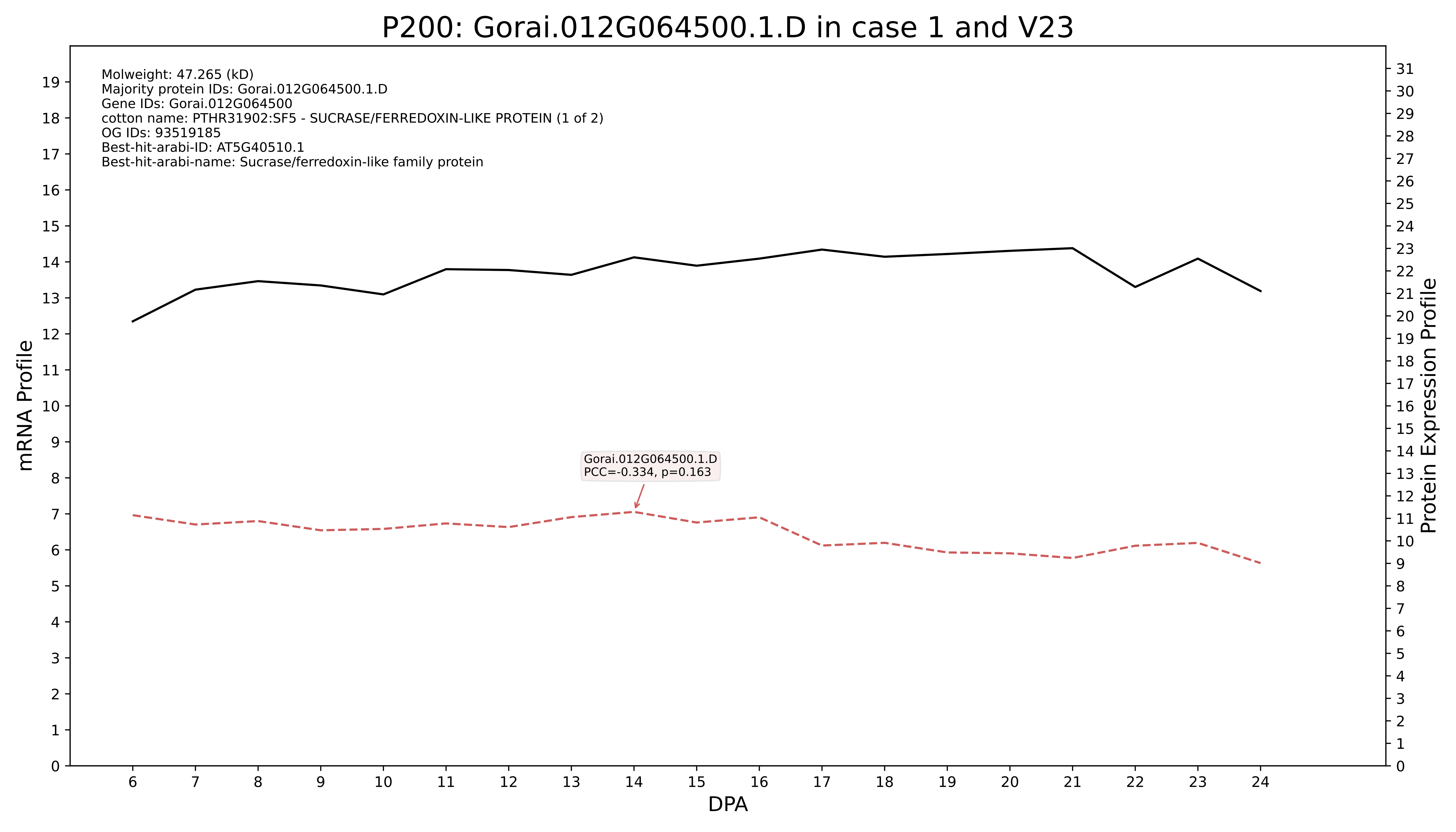

P200: Gorai.012G064500.1.D in case 1 and V23
Molweight: 47.265 (kD)
Majority protein IDs: Gorai.012G064500.1.D
Gene IDs: Gorai.012G064500
cotton name: PTHR31902:SF5 - SUCRASE/FERREDOXIN-LIKE PROTEIN (1 of 2)
OG IDs: 93519185
Best-hit-arabi-ID: AT5G40510.1
Best-hit-arabi-name: Sucrase/ferredoxin-like family protein
Gorai.012G064500.1.D
PCC=-0.334, p=0.163
mRNA Profile
Protein Expression Profile
DPA

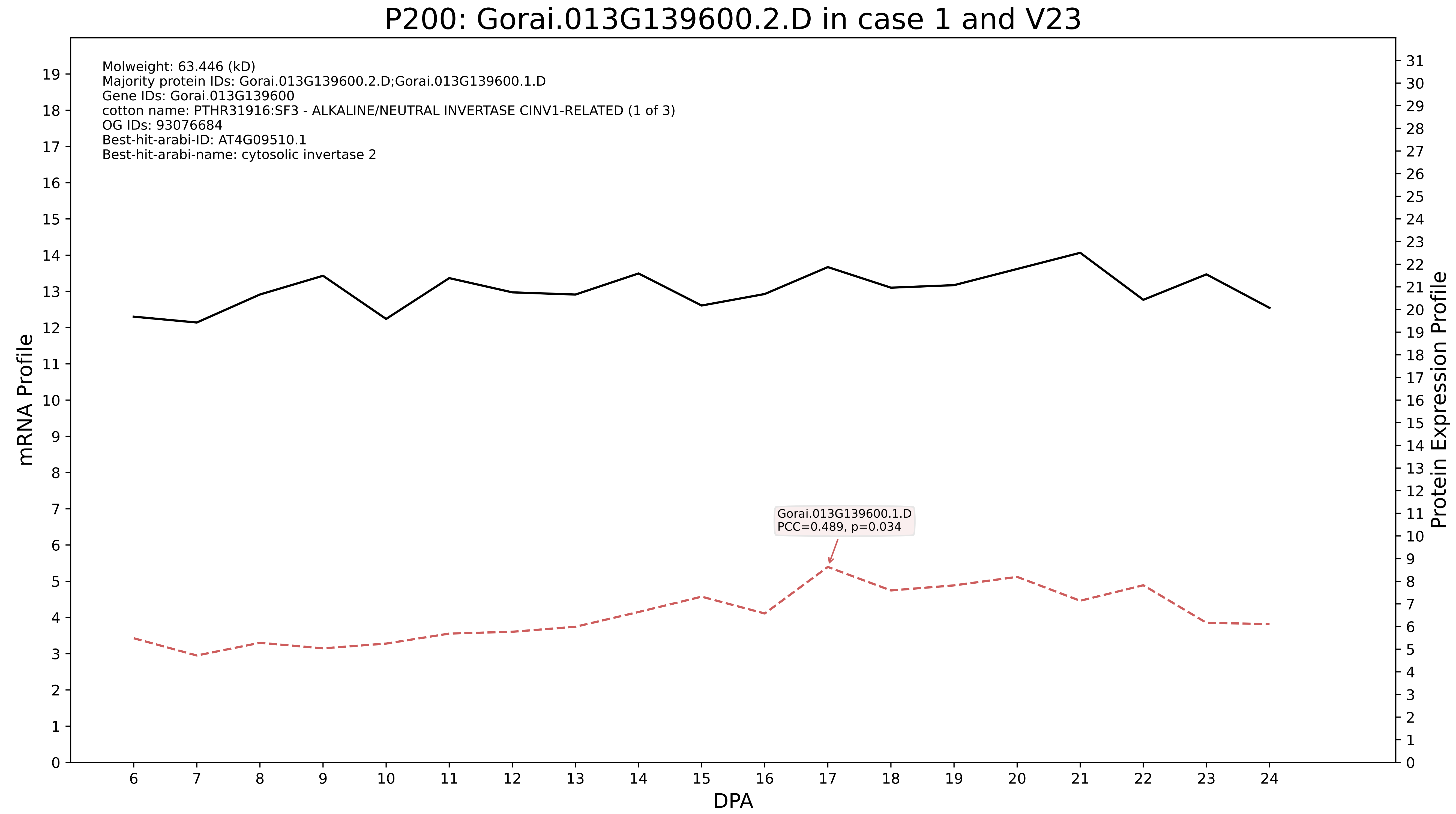
P200: Gorai.013G139600.2.D in case 1 and V23
Molweight: 63.446 (kD)
Majority protein IDs: Gorai.013G139600.2.D;Gorai.013G139600.1.D
Gene IDs: Gorai.013G139600
cotton name: PTHR31916:SF3 - ALKALINE/NEUTRAL INVERTASE CINV1-RELATED (1 of 3)
OG IDs: 93076684
Best-hit-arabi-ID: AT4G09510.1
Best-hit-arabi-name: cytosolic invertase 2
Gorai.013G139600.1.D
PCC=0.489, p=0.034
mRNA Profile
Protein Expression Profile
DPA
0
1
2
3
4
5
6
7
8
9
10
11
12
13
14
15
16
17
18
19
20
21
22
23
24
25
26
27
28
29
30
31

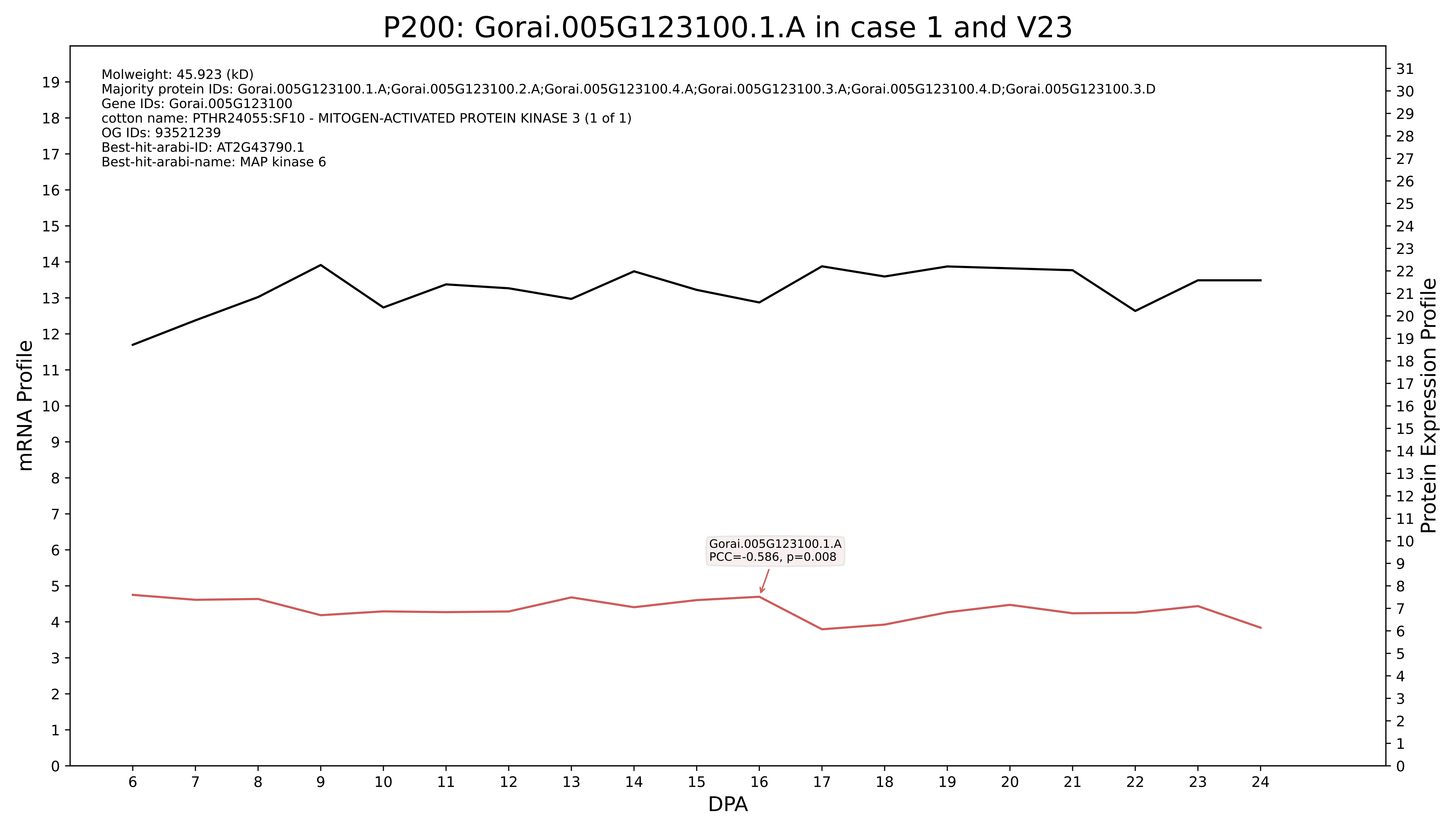

P200: Gorai.005G123100.1.A in case 1 and V23
Molweight: 45.923 (kD)
Majority protein IDs: Gorai.005G123100.1.A;Gorai.005G123100.2.A;Gorai.005G123100.4.A;Gorai.005G123100.3.A;Gorai.005G123100.4.D;Gorai.005G123100.3.D
Gene IDs: Gorai.005G123100
cotton name: PTHR24055:SF10 - MITOGEN-ACTIVATED PROTEIN KINASE 3 (1 of 1)
OG IDs: 93521239
Best-hit-arabi-ID: AT2G43790.1
Best-hit-arabi-name: MAP kinase 6
Gorai.005G123100.1.A
PCC=-0.586, p=0.008
mRNA Profile
Protein Expression Profile
DPA

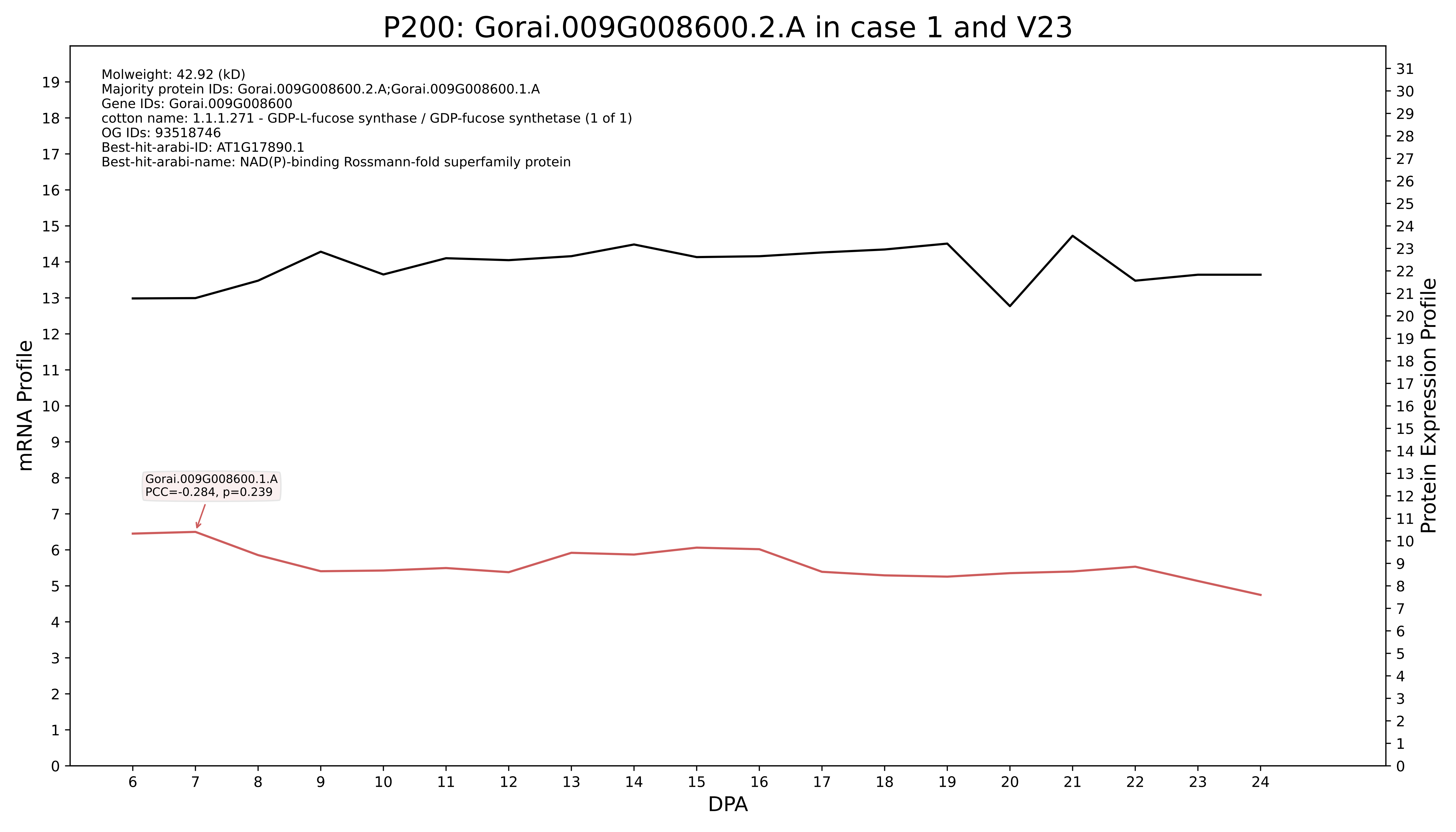
P200: Gorai.009G008600.2.A in case 1 and V23
Molweight: 42.92 (kD)
Majority protein IDs: Gorai.009G008600.2.A;Gorai.009G008600.1.A
Gene IDs: Gorai.009G008600
cotton name: 1.1.1.271 - GDP-L-fucose synthase / GDP-fucose synthetase (1 of 1)
OG IDs: 93518746
Best-hit-arabi-ID: AT1G17890.1
Best-hit-arabi-name: NAD(P)-binding Rossmann-fold superfamily protein
Gorai.009G008600.1.A
PCC=-0.284, p=0.239
mRNA Profile
Protein Expression Profile
DPA

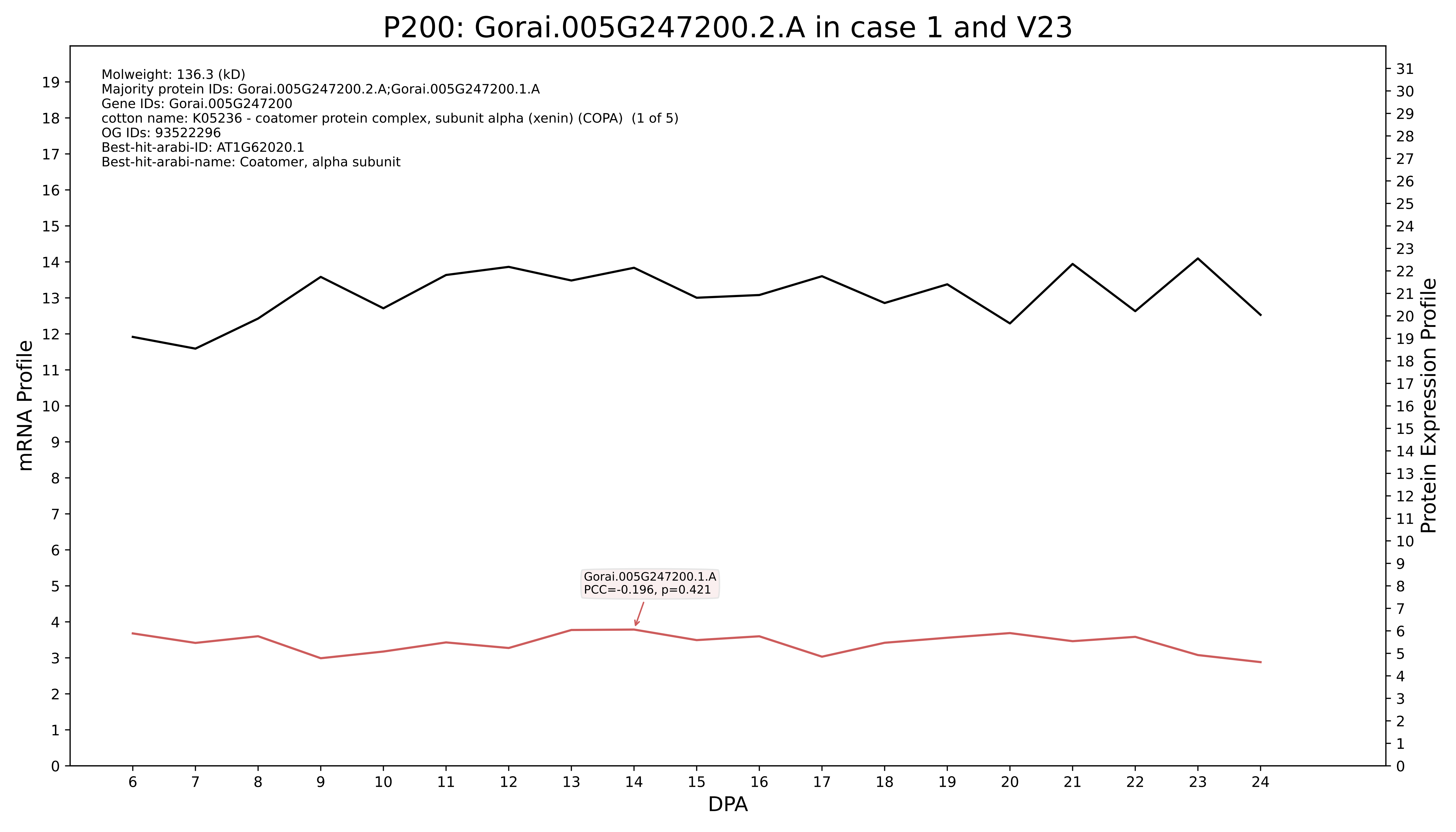

P200: Gorai.005G247200.2.A in case 1 and V23
Molweight: 136.3 (kD)
Majority protein IDs: Gorai.005G247200.2.A;Gorai.005G247200.1.A
Gene IDs: Gorai.005G247200
cotton name: K05236 - coatomer protein complex, subunit alpha (xenin) (COPA) (1 of 5)
OG IDs: 93522296
Best-hit-arabi-ID: AT1G62020.1
Best-hit-arabi-name: Coatomer, alpha subunit
Gorai.005G247200.1.A
PCC=-0.196, p=0.421
mRNA Profile
Protein Expression Profile
DPA

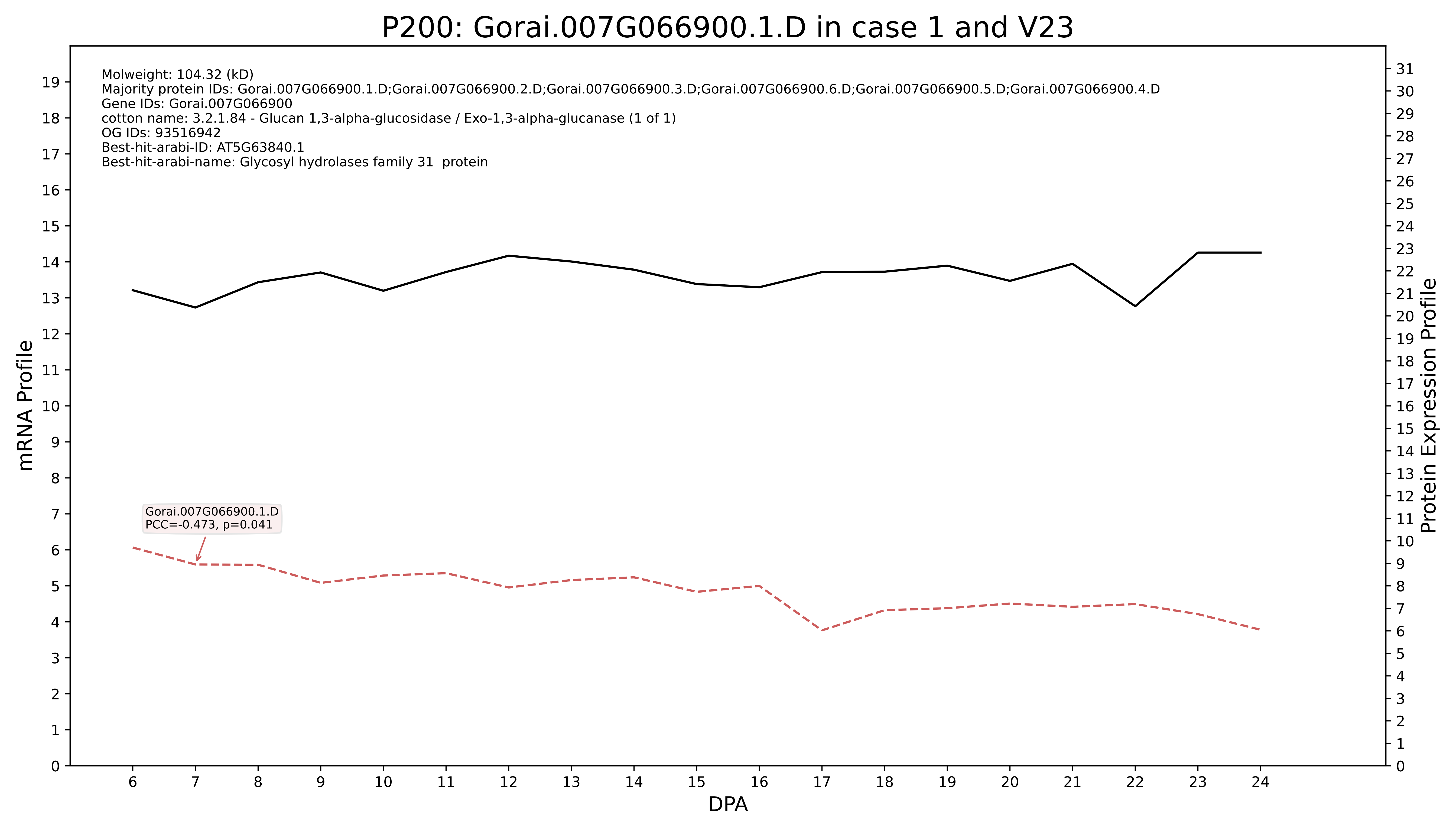
P200: Gorai.007G066900.1.D in case 1 and V23
Molweight: 104.32 (kD)
Majority protein IDs: Gorai.007G066900.1.D;Gorai.007G066900.2.D;Gorai.007G066900.3.D;Gorai.007G066900.6.D;Gorai.007G066900.5.D;Gorai.007G066900.4.D
Gene IDs: Gorai.007G066900
cotton name: 3.2.1.84 - Glucan 1,3-alpha-glucosidase / Exo-1,3-alpha-glucanase (1 of 1)
OG IDs: 93516942
Best-hit-arabi-ID: AT5G63840.1
Best-hit-arabi-name: Glycosyl hydrolases family 31 protein
Gorai.007G066900.1.D
PCC=-0.473, p=0.041
mRNA Profile
Protein Expression Profile
DPA

P200: Gorai.009G288200.1.A in case 1 and V23

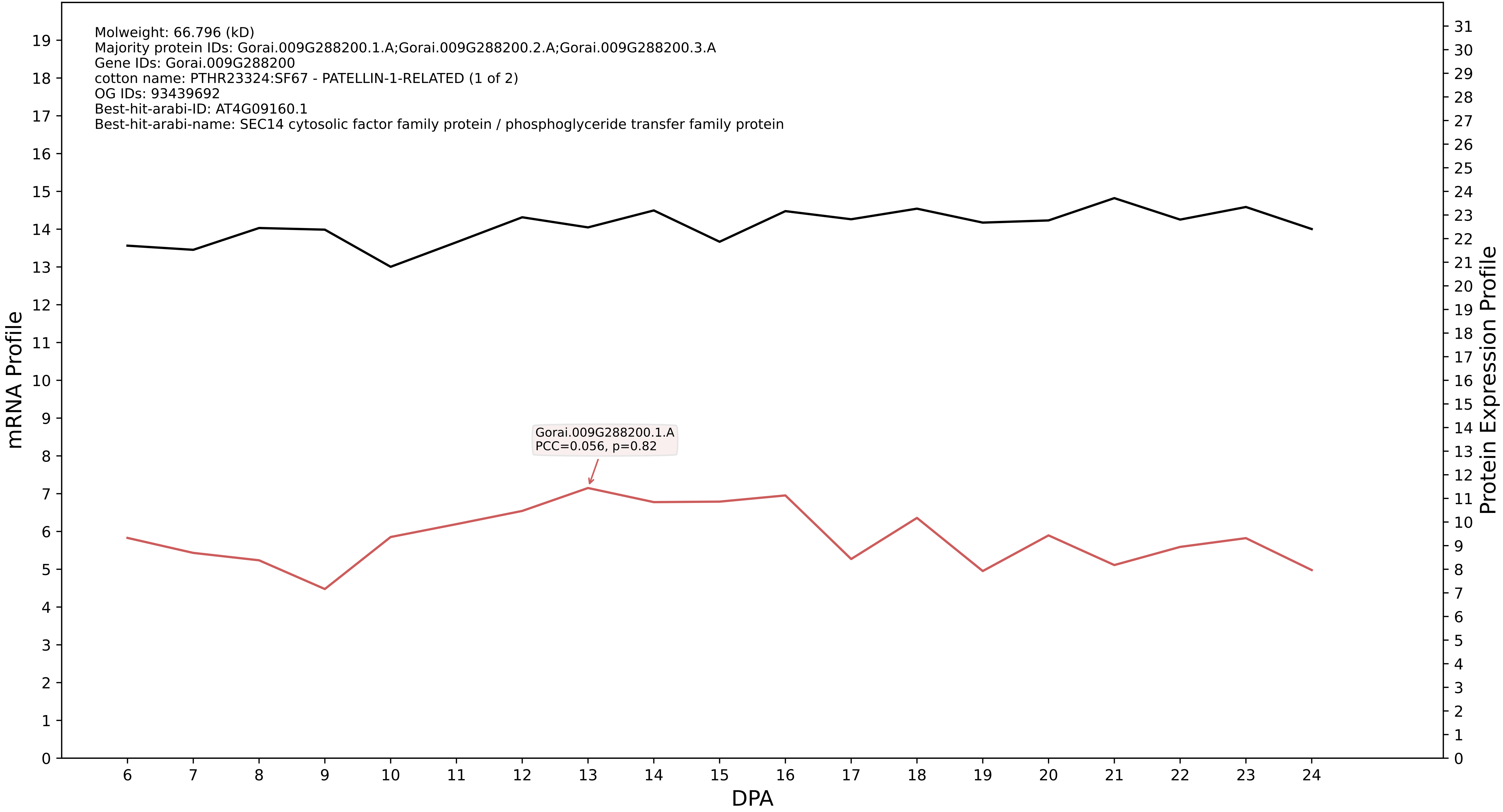

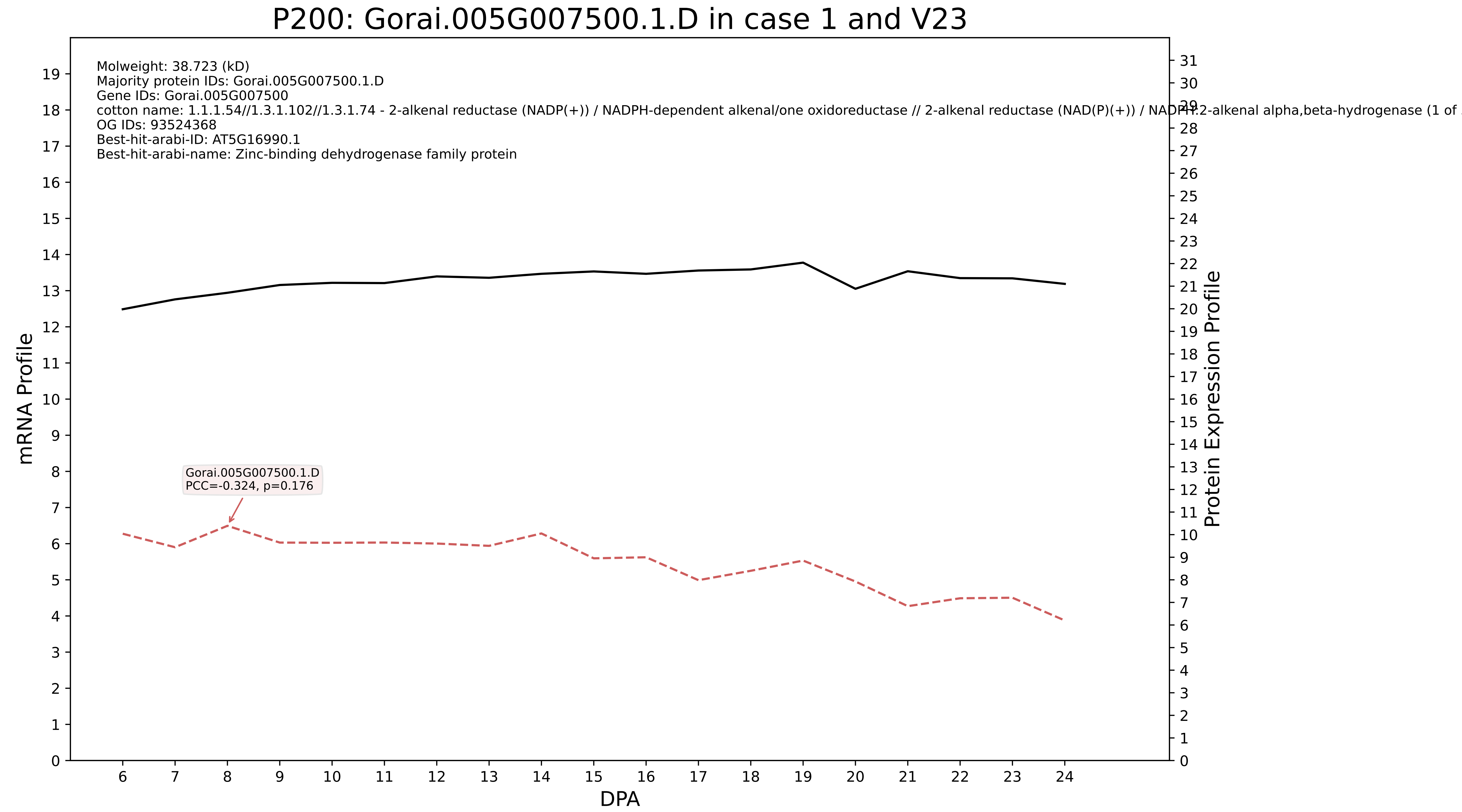
P200: Gorai.005G007500.1.D in case 1 and V23
Molweight: 38.723 (kD)
Majority protein IDs: Gorai.005G007500.1.D
Gene IDs: Gorai.005G007500
cotton name: 1.1.1.54//1.3.1.102//1.3.1.74 - 2-alkenal reductase (NADP(+)) / NADPH-dependent alkenal/one oxidoreductase // 2-alkenal reductase (NAD(P)(+)) / NADPH:2-alkenal alpha,beta-hydrogenase (1 of
OG IDs: 93524368
Best-hit-arabi-ID: AT5G16990.1
Best-hit-arabi-name: Zinc-binding dehydrogenase family protein
Gorai.005G007500.1.D
PCC=-0.324, p=0.176
mRNA Profile
Protein Expression Profile
DPA
0
1
2
3
4
5
6
7
8
9
10
11
12
13
14
15
16
17
18
19
20
21
22
23
24
25
26
27
28
29
30
31

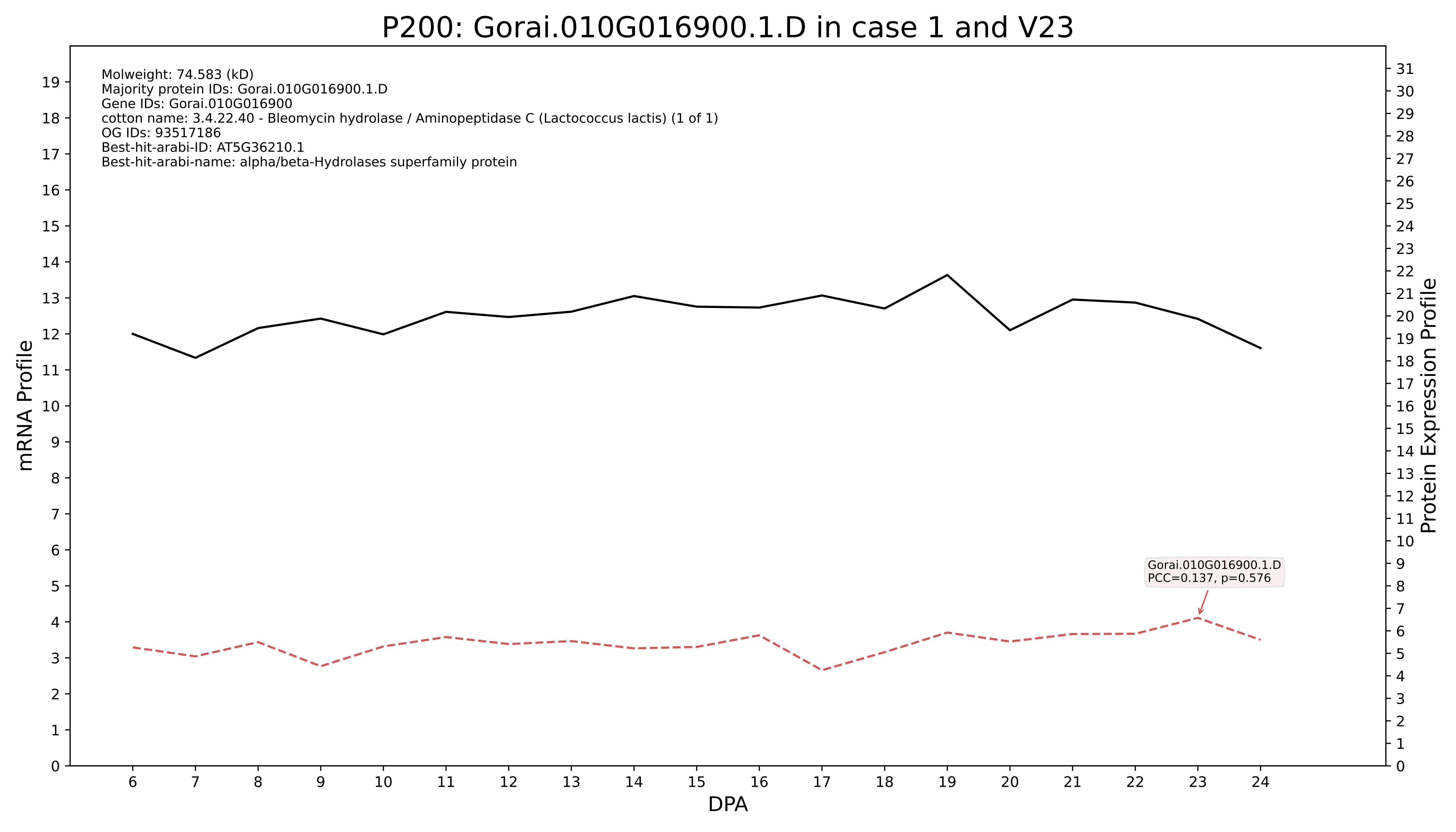
P200: Gorai.010G016900.1.D in case 1 and V23
Molweight: 74.583 (kD)
Majority protein IDs: Gorai.010G016900.1.D
Gene IDs: Gorai.010G016900
cotton name: 3.4.22.40 - Bleomycin hydrolase / Aminopeptidase C (Lactococcus lactis) (1 of 1)
OG IDs: 93517186
Best-hit-arabi-ID: AT5G36210.1
Best-hit-arabi-name: alpha/beta-Hydrolases superfamily protein
Gorai.010G016900.1.D
PCC=0.137, p=0.576
mRNA Profile
Protein Expression Profile
DPA

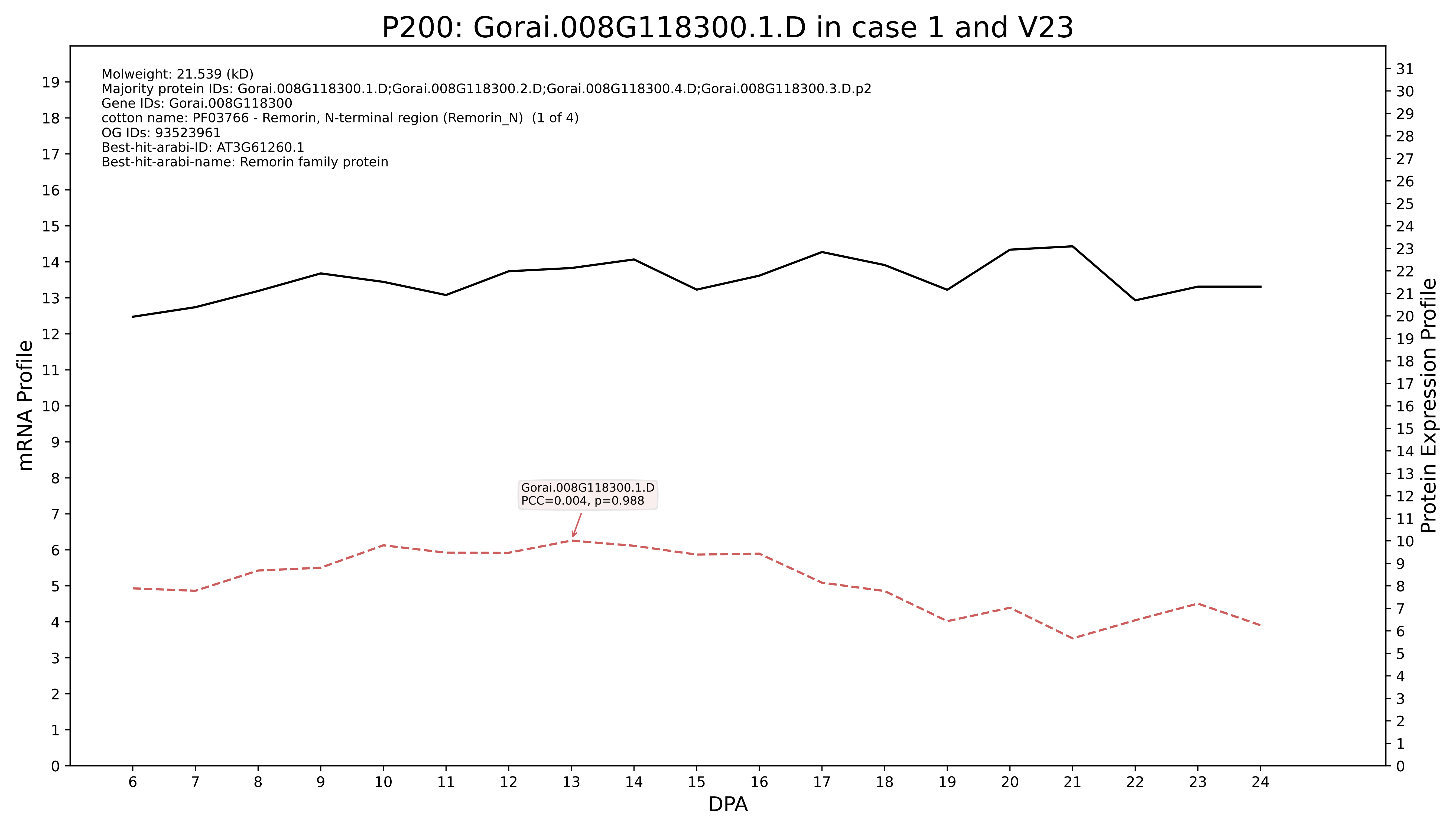
P200: Gorai.008G118300.1.D in case 1 and V23
Molweight: 21.539 (kD)
Majority protein IDs: Gorai.008G118300.1.D;Gorai.008G118300.2.D;Gorai.008G118300.4.D;Gorai.008G118300.3.D.p2
Gene IDs: Gorai.008G118300
cotton name: PF03766 - Remorin, N-terminal region (Remorin_N) (1 of 4)
OG IDs: 93523961
Best-hit-arabi-ID: AT3G61260.1
Best-hit-arabi-name: Remorin family protein
Gorai.008G118300.1.D
PCC=0.004, p=0.988
mRNA Profile
Protein Expression Profile
DPA

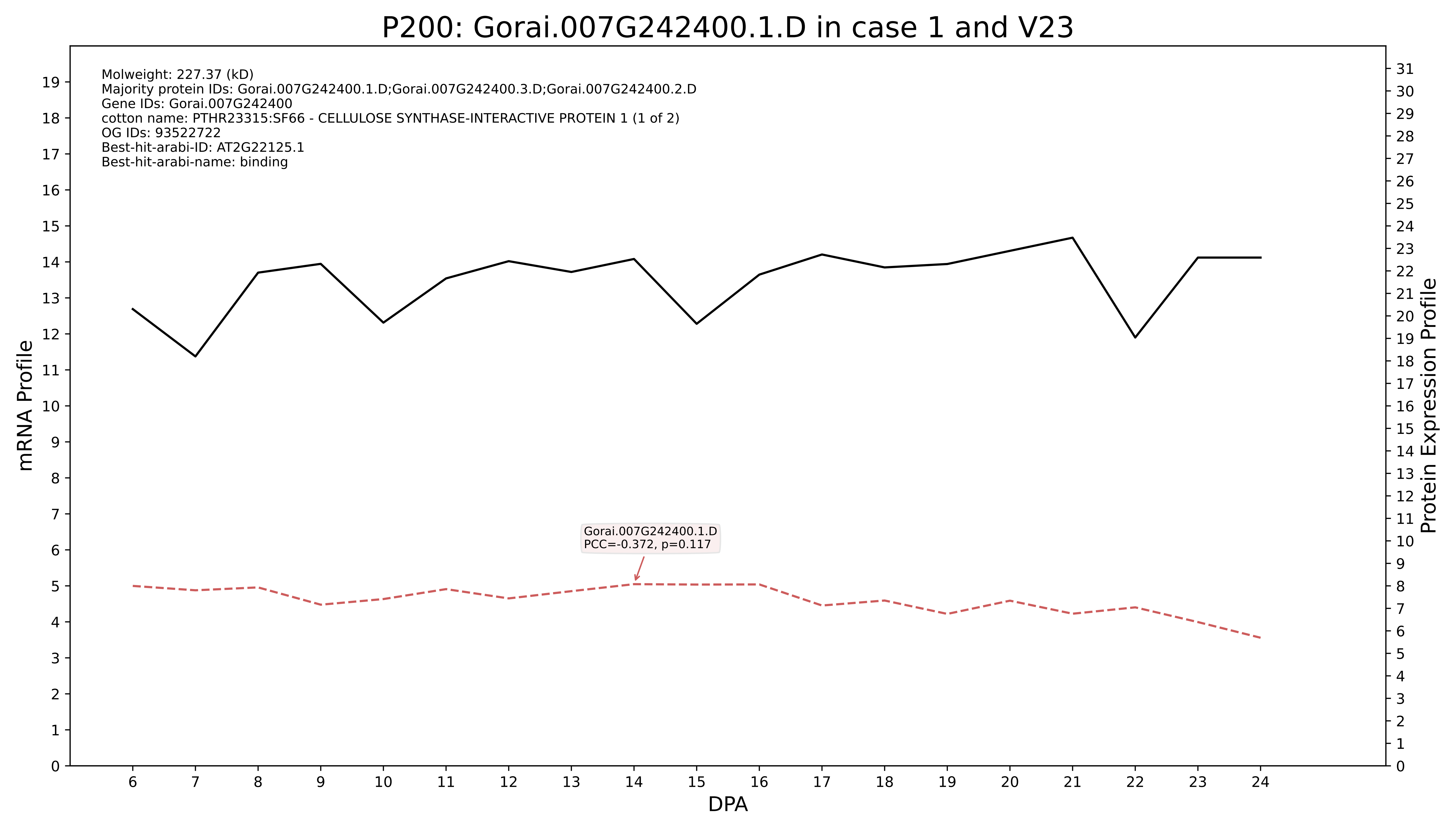
P200: Gorai.007G242400.1.D in case 1 and V23
Molweight: 227.37 (kD)
Majority protein IDs: Gorai.007G242400.1.D;Gorai.007G242400.3.D;Gorai.007G242400.2.D
Gene IDs: Gorai.007G242400
cotton name: PTHR23315:SF66 - CELLULOSE SYNTHASE-INTERACTIVE PROTEIN 1 (1 of 2)
OG IDs: 93522722
Best-hit-arabi-ID: AT2G22125.1
Best-hit-arabi-name: binding
Gorai.007G242400.1.D
PCC=-0.372, p=0.117
mRNA Profile
Protein Expression Profile
DPA

orai.009G395300.3.A in case 1 and V23

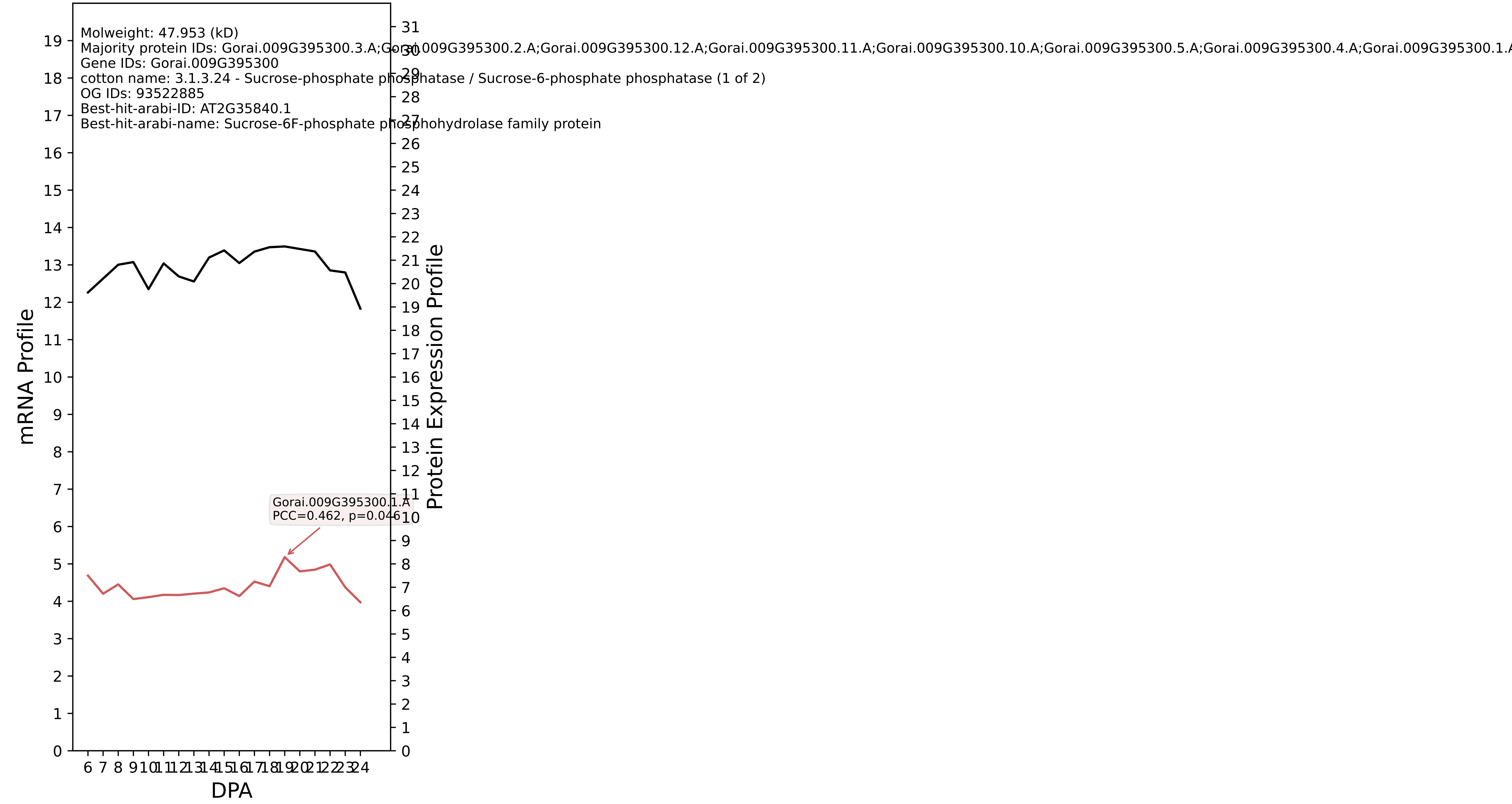

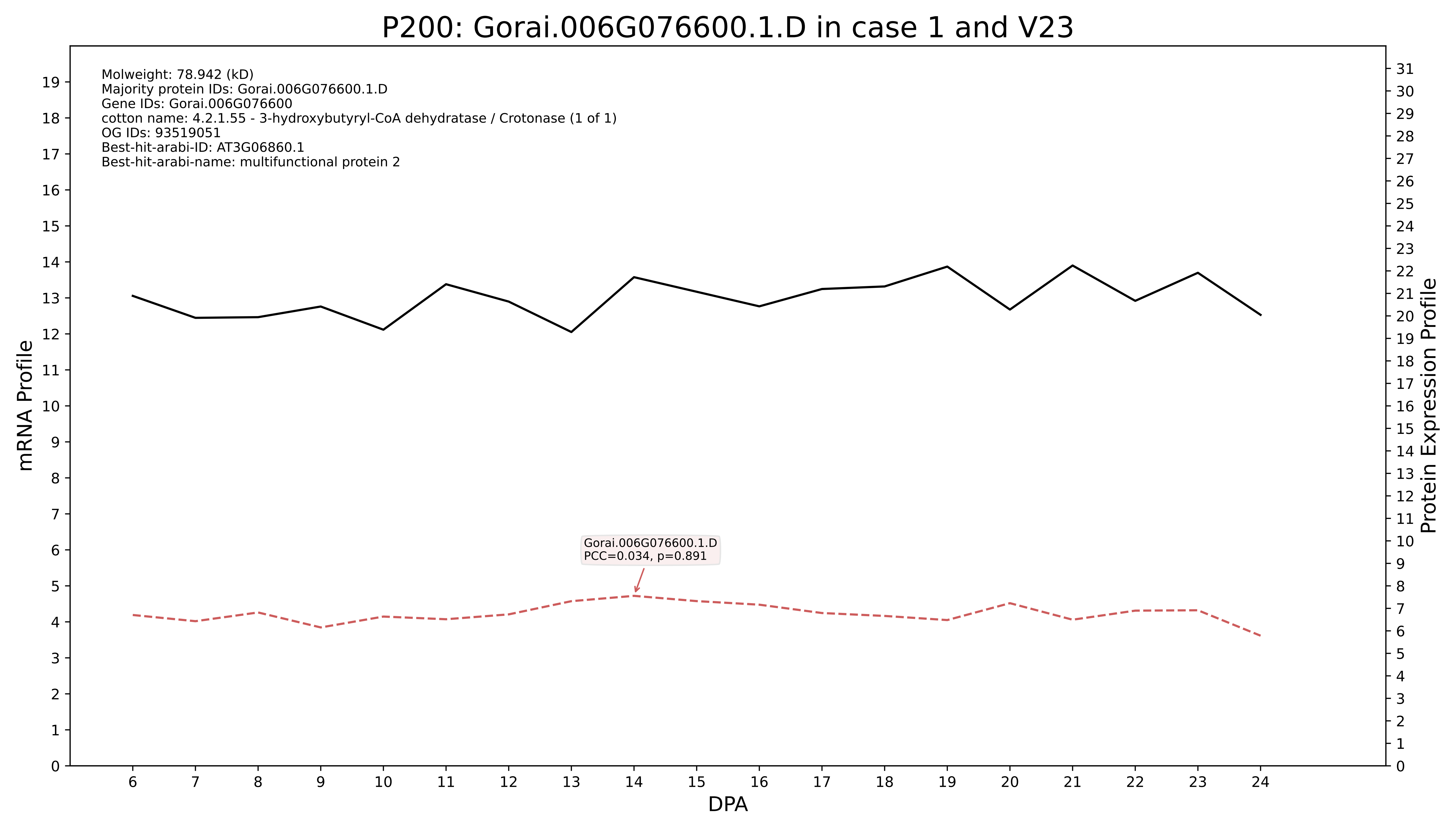

P200: Gorai.006G076600.1.D in case 1 and V23
Molweight: 78.942 (kD)
Majority protein IDs: Gorai.006G076600.1.D
Gene IDs: Gorai.006G076600
cotton name: 4.2.1.55 - 3-hydroxybutyryl-CoA dehydratase / Crotonase (1 of 1)
OG IDs: 93519051
Best-hit-arabi-ID: AT3G06860.1
Best-hit-arabi-name: multifunctional protein 2
Gorai.006G076600.1.D
PCC=0.034, p=0.891
mRNA Profile
Protein Expression Profile
DPA

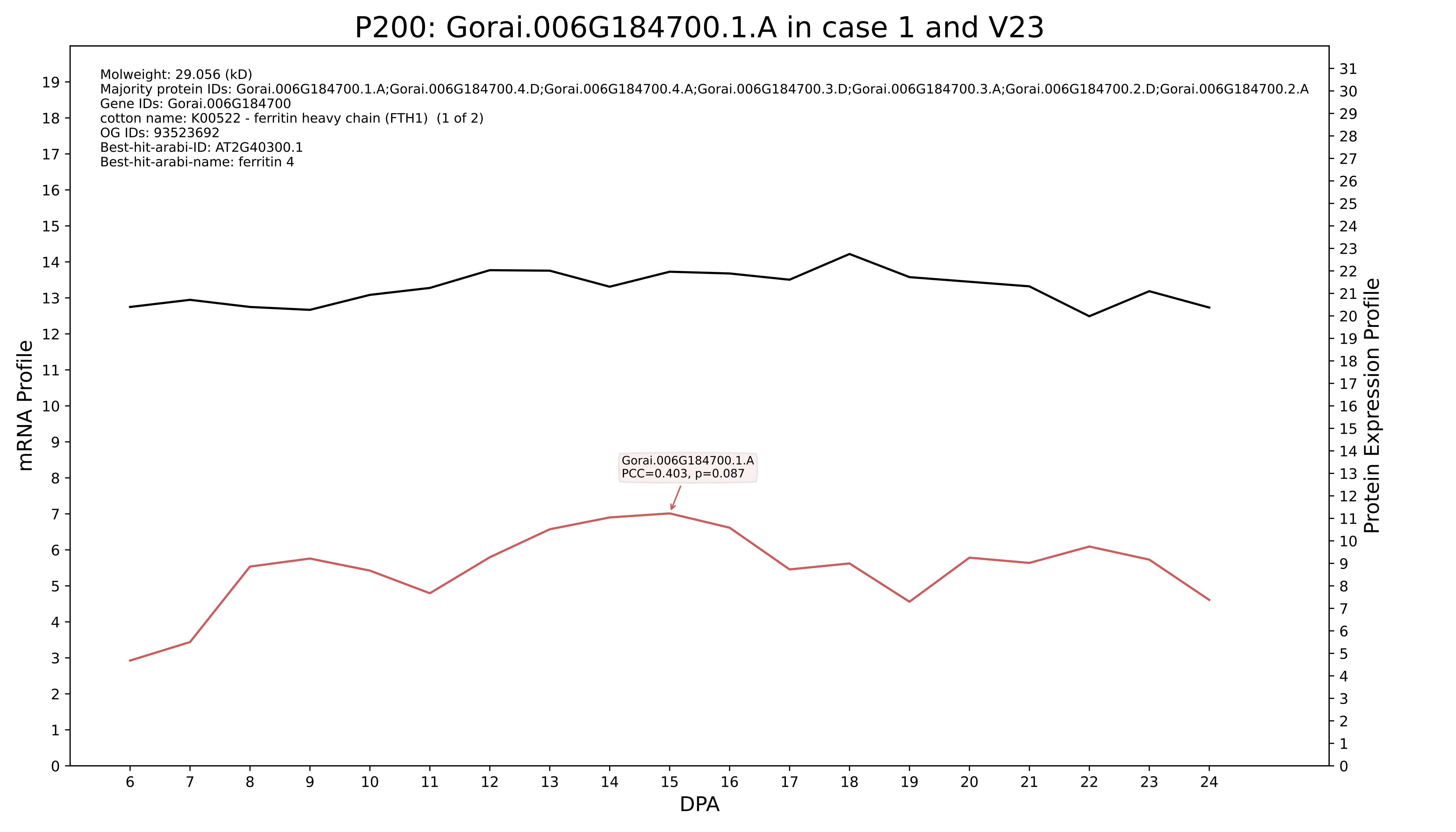

P200: Gorai.006G184700.1.A in case 1 and V23
Molweight: 29.056 (kD)
Majority protein IDs: Gorai.006G184700.1.A;Gorai.006G184700.4.D;Gorai.006G184700.4.A;Gorai.006G184700.3.D;Gorai.006G184700.3.A;Gorai.006G184700.2.D;Gorai.006G184700.2.A
Gene IDs: Gorai.006G184700
cotton name: K00522 - ferritin heavy chain (FTH1) (1 of 2)
OG IDs: 93523692
Best-hit-arabi-ID: AT2G40300.1
Best-hit-arabi-name: ferritin 4
Gorai.006G184700.1.A
PCC=0.403, p=0.087
mRNA Profile
Protein Expression Profile
DPA

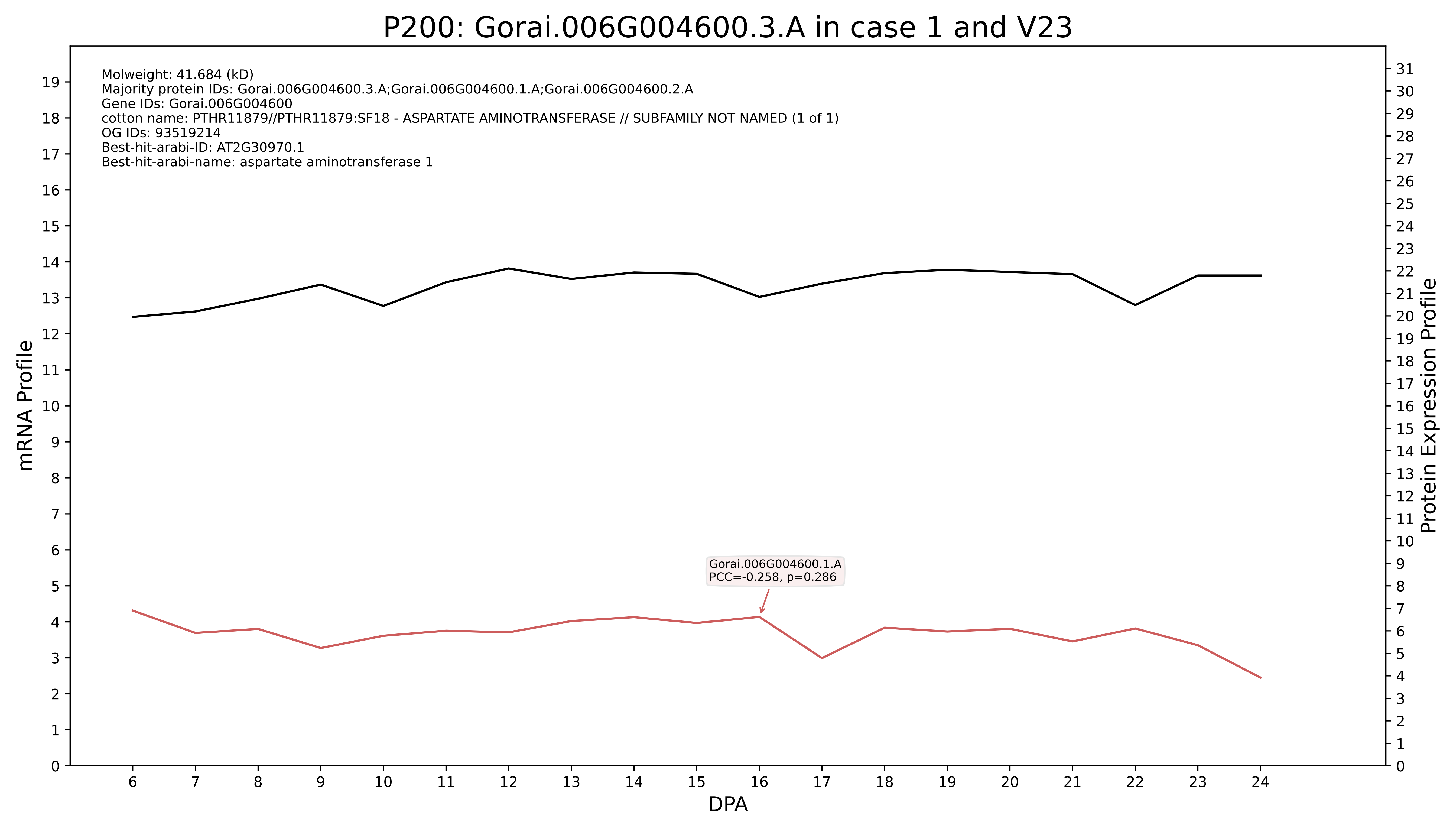
P200: Gorai.006G004600.3.A in case 1 and V23
Molweight: 41.684 (kD)
Majority protein IDs: Gorai.006G004600.3.A;Gorai.006G004600.1.A;Gorai.006G004600.2.A
Gene IDs: Gorai.006G004600
cotton name: PTHR11879//PTHR11879:SF18 - ASPARTATE AMINOTRANSFERASE // SUBFAMILY NOT NAMED (1 of 1)
OG IDs: 93519214
Best-hit-arabi-ID: AT2G30970.1
Best-hit-arabi-name: aspartate aminotransferase 1
Gorai.006G004600.1.A
PCC=-0.258, p=0.286
mRNA Profile
Protein Expression Profile
DPA

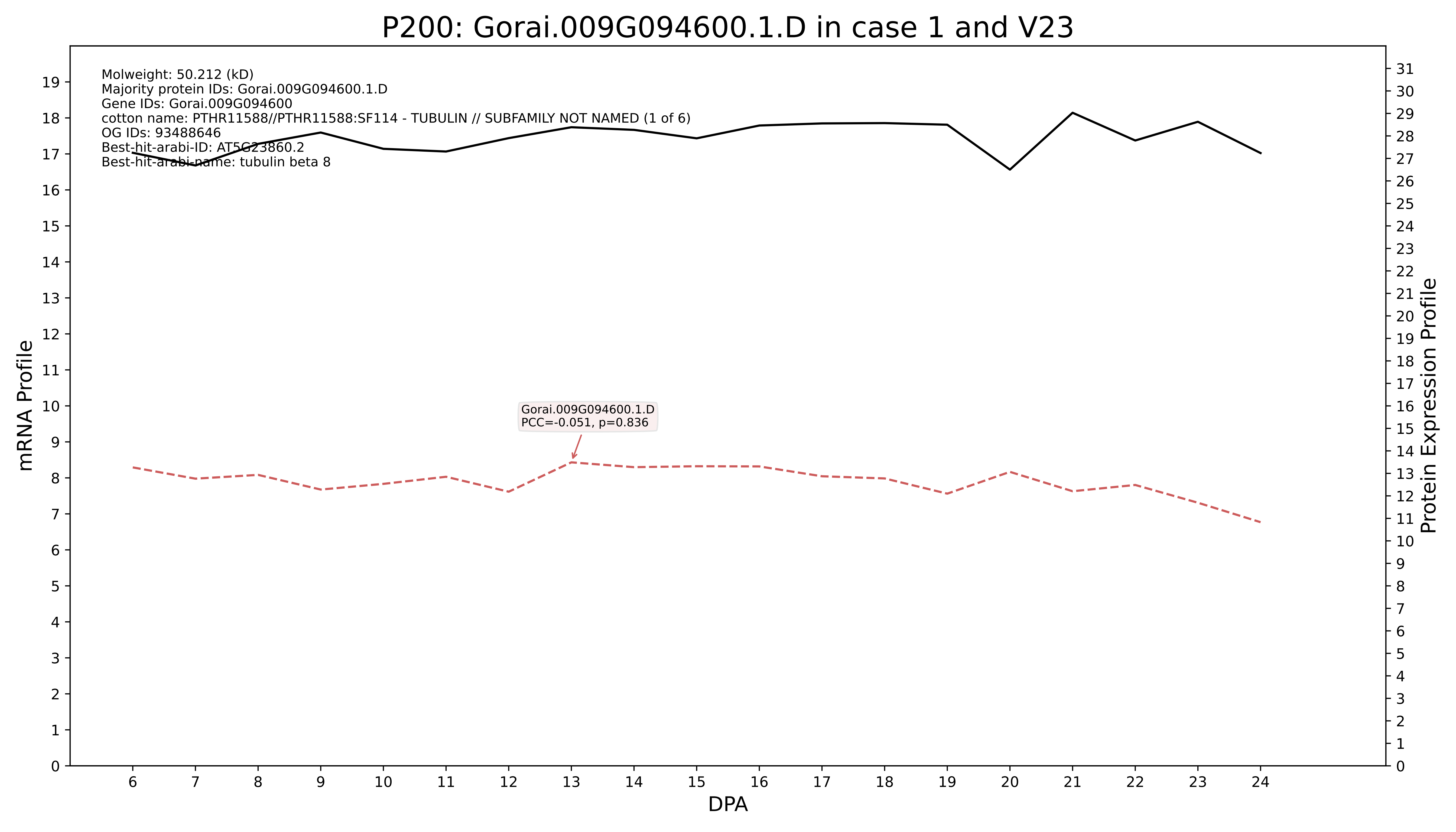
P200: Gorai.009G094600.1.D in case 1 and V23
Molweight: 50.212 (kD)
Majority protein IDs: Gorai.009G094600.1.D
Gene IDs: Gorai.009G094600
cotton name: PTHR11588//PTHR11588:SF114 - TUBULIN // SUBFAMILY NOT NAMED (1 of 6)
OG IDs: 93488646
Best-hit-arabi-ID: AT5G23860.2
Best-hit-arabi-name: tubulin beta 8
Gorai.009G094600.1.D
PCC=-0.051, p=0.836
mRNA Profile
Protein Expression Profile
DPA

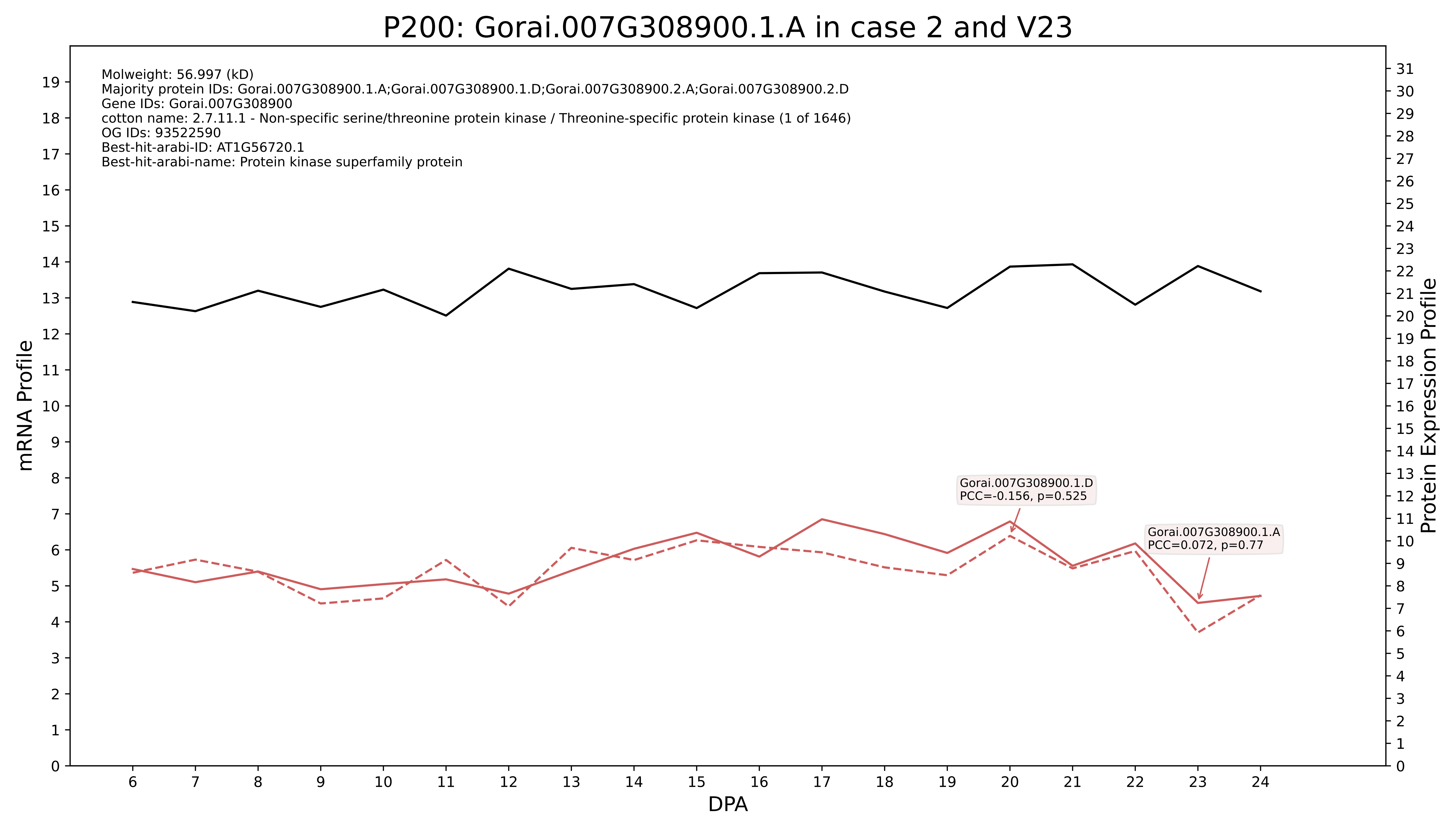

P200: Gorai.007G308900.1.A in case 2 and V23
Molweight: 56.997 (kD)
Majority protein IDs: Gorai.007G308900.1.A;Gorai.007G308900.1.D;Gorai.007G308900.2.A;Gorai.007G308900.2.D
Gene IDs: Gorai.007G308900
cotton name: 2.7.11.1 - Non-specific serine/threonine protein kinase / Threonine-specific protein kinase (1 of 1646)
OG IDs: 93522590
Best-hit-arabi-ID: AT1G56720.1
Best-hit-arabi-name: Protein kinase superfamily protein
Gorai.007G308900.1.D
PCC=-0.156, p=0.525
Gorai.007G308900.1.A
PCC=0.072, p=0.77
mRNA Profile
Protein Expression Profile
DPA

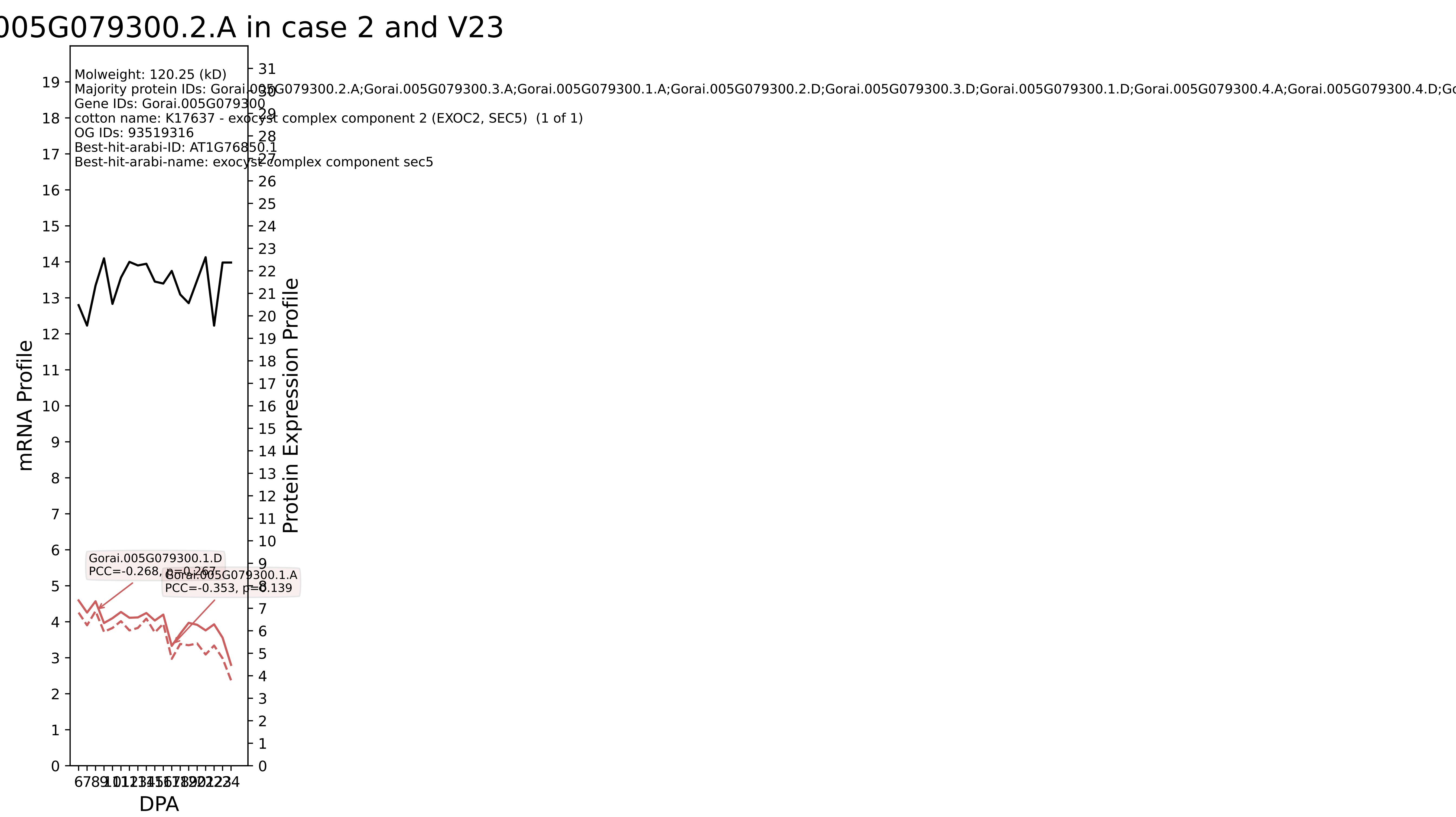
005G079300.2.A in case 2 and V23
Molweight: 120.25 (kD)
Majority protein IDs: Gorai.005G079300.2.A;Gorai.005G079300.3.A;Gorai.005G079300.1.A;Gorai.005G079300.2.D;Gorai.005G079300.3.D;Gorai.005G079300.1.D;Gorai.005G079300.4.A;Gorai.005G079300.4.D;Go
Gene IDs: Gorai.005G079300
cotton name: K17637 - exocyst complex component 2 (EXOC2, SEC5) (1 of 1)
OG IDs: 93519316
Best-hit-arabi-ID: AT1G76850.1
Best-hit-arabi-name: exocyst complex component sec5
Gorai.005G079300.1.D
PCC=-0.268, p=0.267
Gorai.005G079300.1.A
PCC=-0.353, p=0.139
mRNA Profile
Protein Expression Profile
DPA

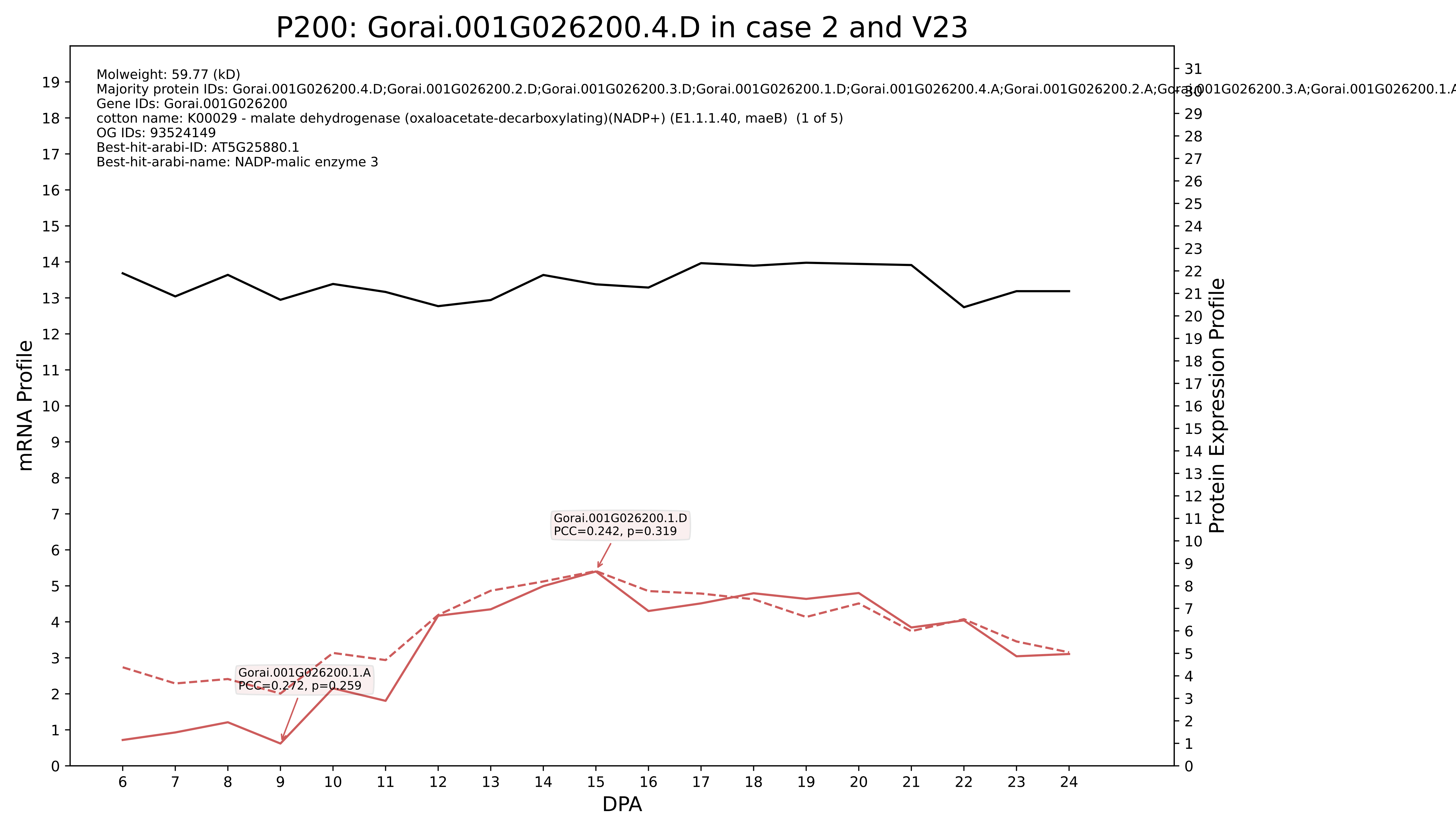
P200: Gorai.001G026200.4.D in case 2 and V23
Molweight: 59.77 (kD)
Majority protein IDs: Gorai.001G026200.4.D;Gorai.001G026200.2.D;Gorai.001G026200.3.D;Gorai.001G026200.1.D;Gorai.001G026200.4.A;Gorai.001G026200.2.A;Gorai.001G026200.3.A;Gorai.001G026200.1.A
Gene IDs: Gorai.001G026200
cotton name: K00029 - malate dehydrogenase (oxaloacetate-decarboxylating)(NADP+) (E1.1.1.40, maeB) (1 of 5)
OG IDs: 93524149
Best-hit-arabi-ID: AT5G25880.1
Best-hit-arabi-name: NADP-malic enzyme 3
Gorai.001G026200.1.D
PCC=0.242, p=0.319
Gorai.001G026200.1.A
PCC=0.272, p=0.259
mRNA Profile
Protein Expression Profile
DPA

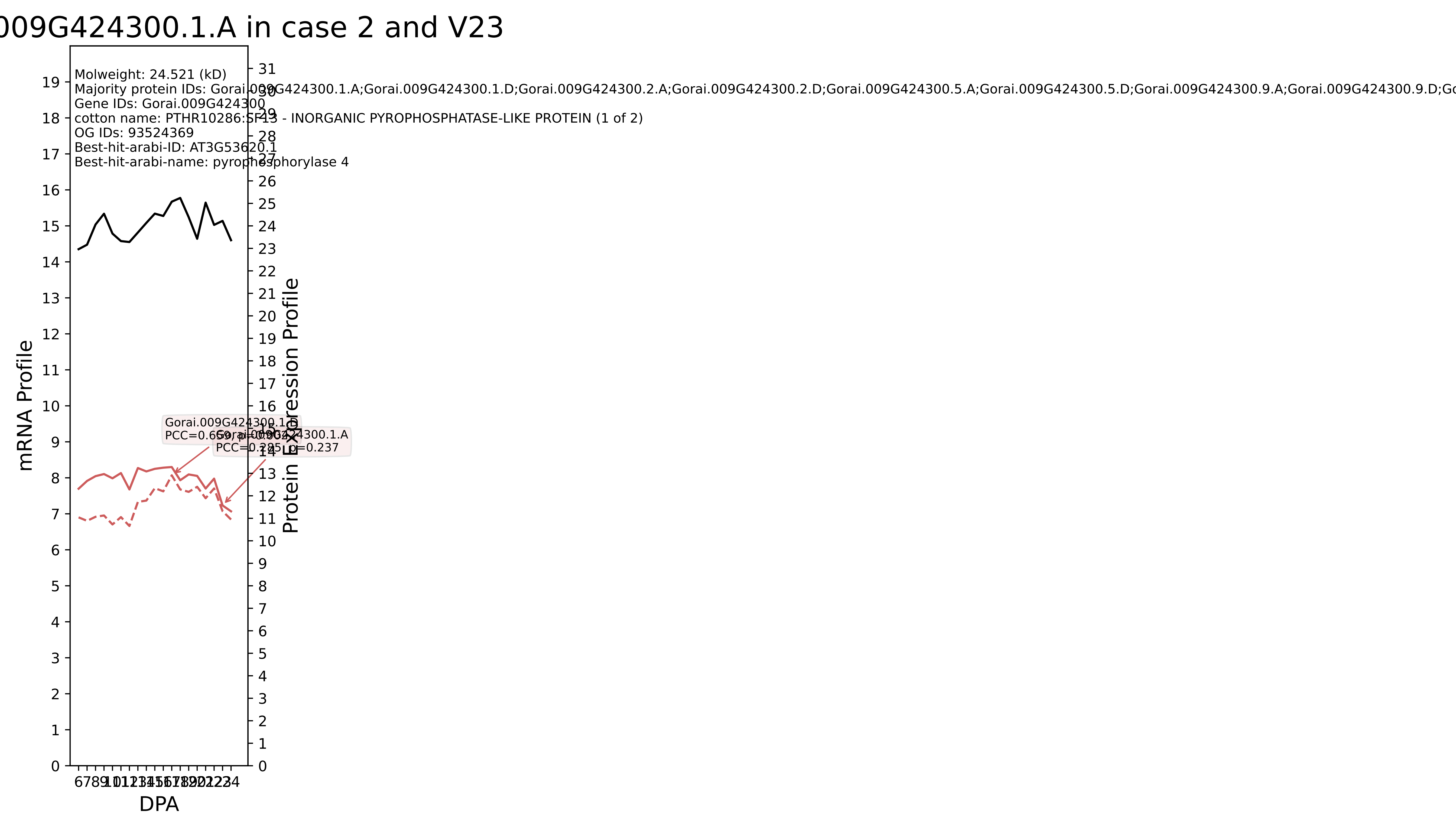
009G424300.1.A in case 2 and V23
Molweight: 24.521 (kD)
Majority protein IDs: Gorai.009G424300.1.A;Gorai.009G424300.1.D;Gorai.009G424300.2.A;Gorai.009G424300.2.D;Gorai.009G424300.5.A;Gorai.009G424300.5.D;Gorai.009G424300.9.A;Gorai.009G424300.9.D;Go
Gene IDs: Gorai.009G424300
cotton name: PTHR10286:SF13 - INORGANIC PYROPHOSPHATASE-LIKE PROTEIN (1 of 2)
OG IDs: 93524369
Best-hit-arabi-ID: AT3G53620.1
Best-hit-arabi-name: pyrophosphorylase 4
Gorai.009G424300.1
PCC=0.659, p=0.003
Gorai.009G424300.1.A
PCC=0.295, p=0.237
mRNA Profile
Protein Expression Profile
DPA

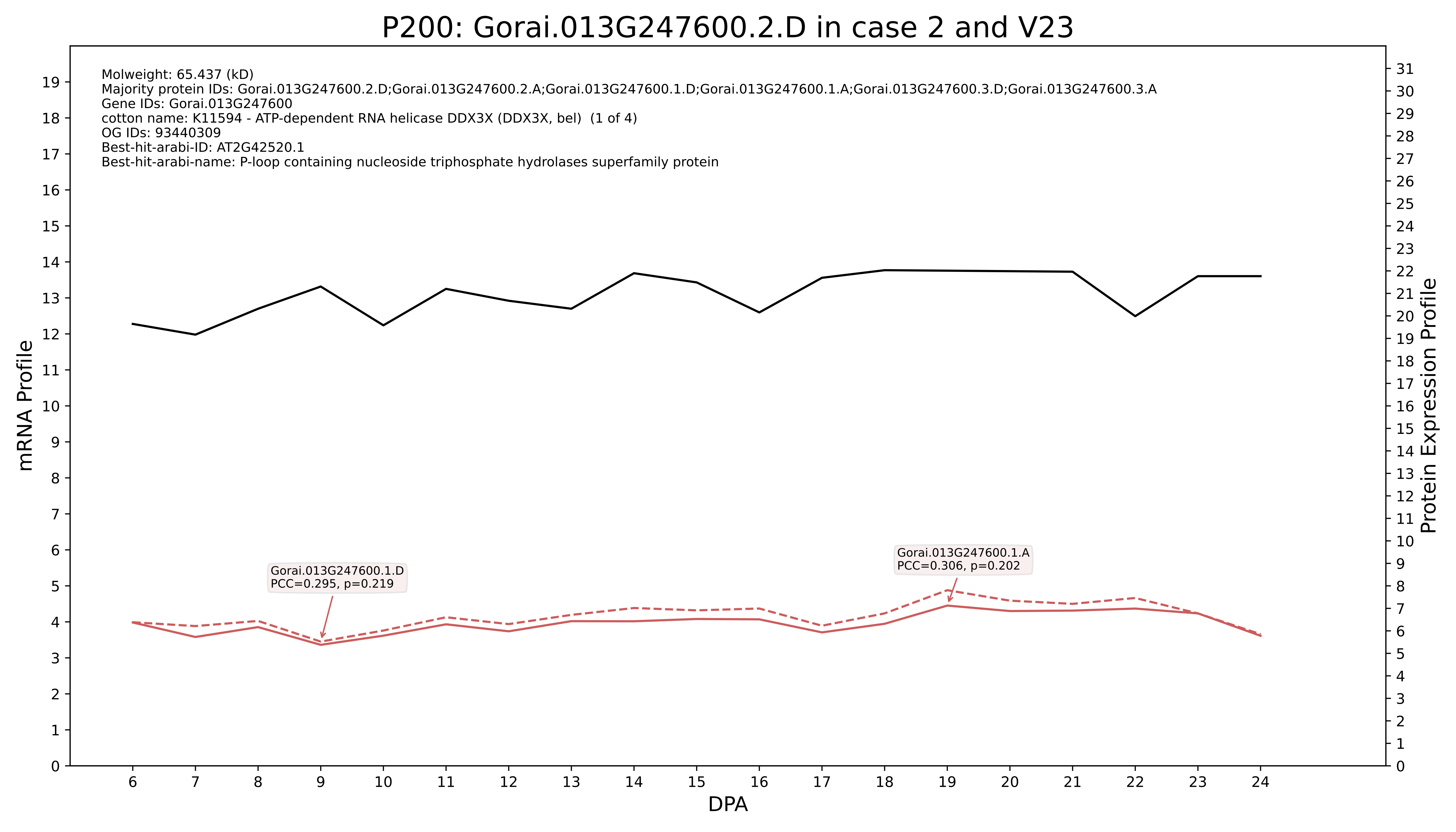
P200: Gorai.013G247600.2.D in case 2 and V23
Molweight: 65.437 (kD)
Majority protein IDs: Gorai.013G247600.2.D;Gorai.013G247600.2.A;Gorai.013G247600.1.D;Gorai.013G247600.1.A;Gorai.013G247600.3.D;Gorai.013G247600.3.A
Gene IDs: Gorai.013G247600
cotton name: K11594 - ATP-dependent RNA helicase DDX3X (DDX3X, bel) (1 of 4)
OG IDs: 93440309
Best-hit-arabi-ID: AT2G42520.1
Best-hit-arabi-name: P-loop containing nucleoside triphosphate hydrolases superfamily protein
Gorai.013G247600.1.D
PCC=0.295, p=0.219
Gorai.013G247600.1.A
PCC=0.306, p=0.202
mRNA Profile
Protein Expression Profile
DPA

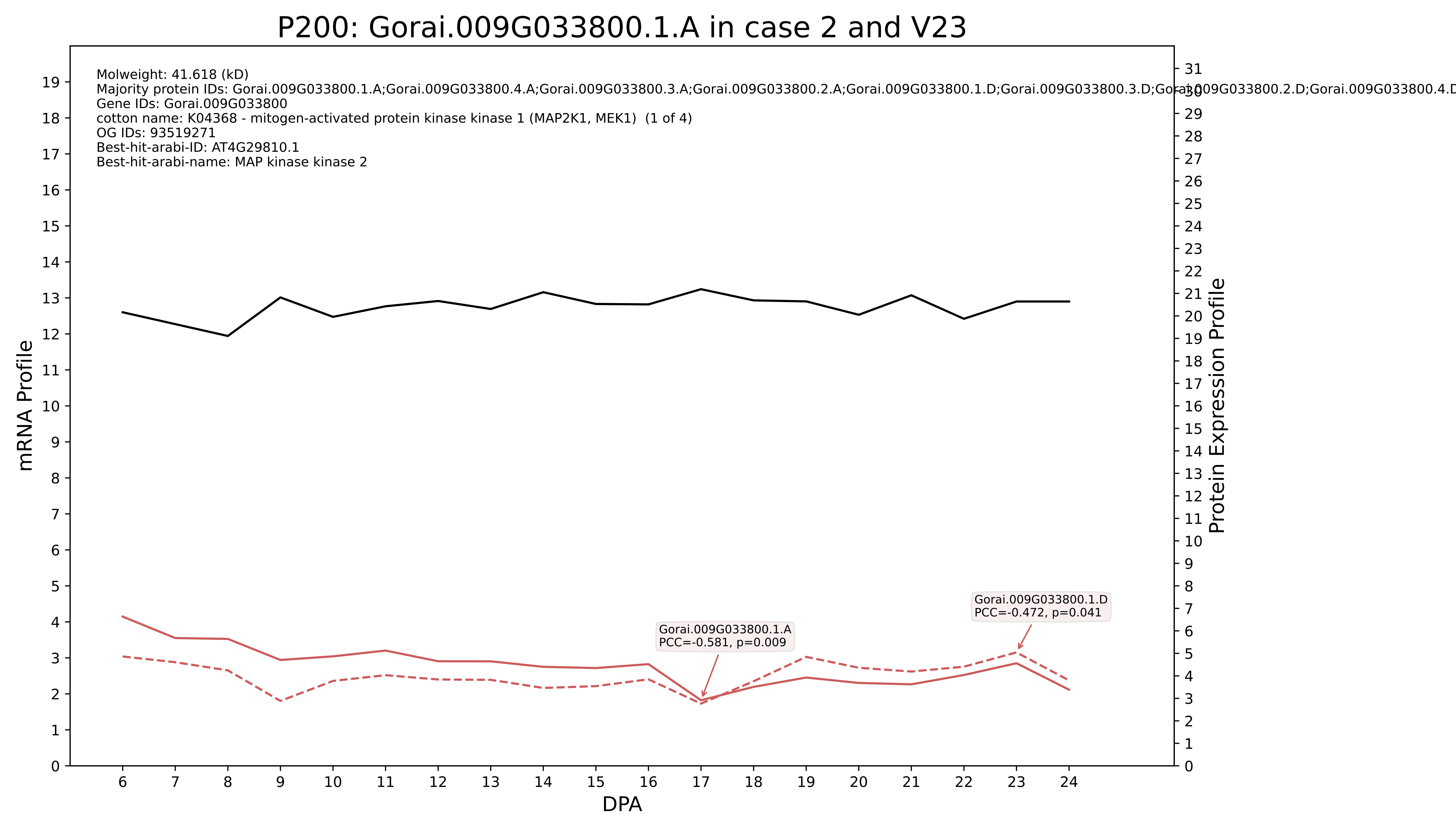
P200: Gorai.009G033800.1.A in case 2 and V23
Molweight: 41.618 (kD)
Majority protein IDs: Gorai.009G033800.1.A;Gorai.009G033800.4.A;Gorai.009G033800.3.A;Gorai.009G033800.2.A;Gorai.009G033800.1.D;Gorai.009G033800.3.D;Gorai.009G033800.2.D;Gorai.009G033800.4.D
Gene IDs: Gorai.009G033800
cotton name: K04368 - mitogen-activated protein kinase kinase 1 (MAP2K1, MEK1) (1 of 4)
OG IDs: 93519271
Best-hit-arabi-ID: AT4G29810.1
Best-hit-arabi-name: MAP kinase kinase 2
Gorai.009G033800.1.A
PCC=-0.581, p=0.009
Gorai.009G033800.1.D
PCC=-0.472, p=0.041
mRNA Profile
Protein Expression Profile
DPA

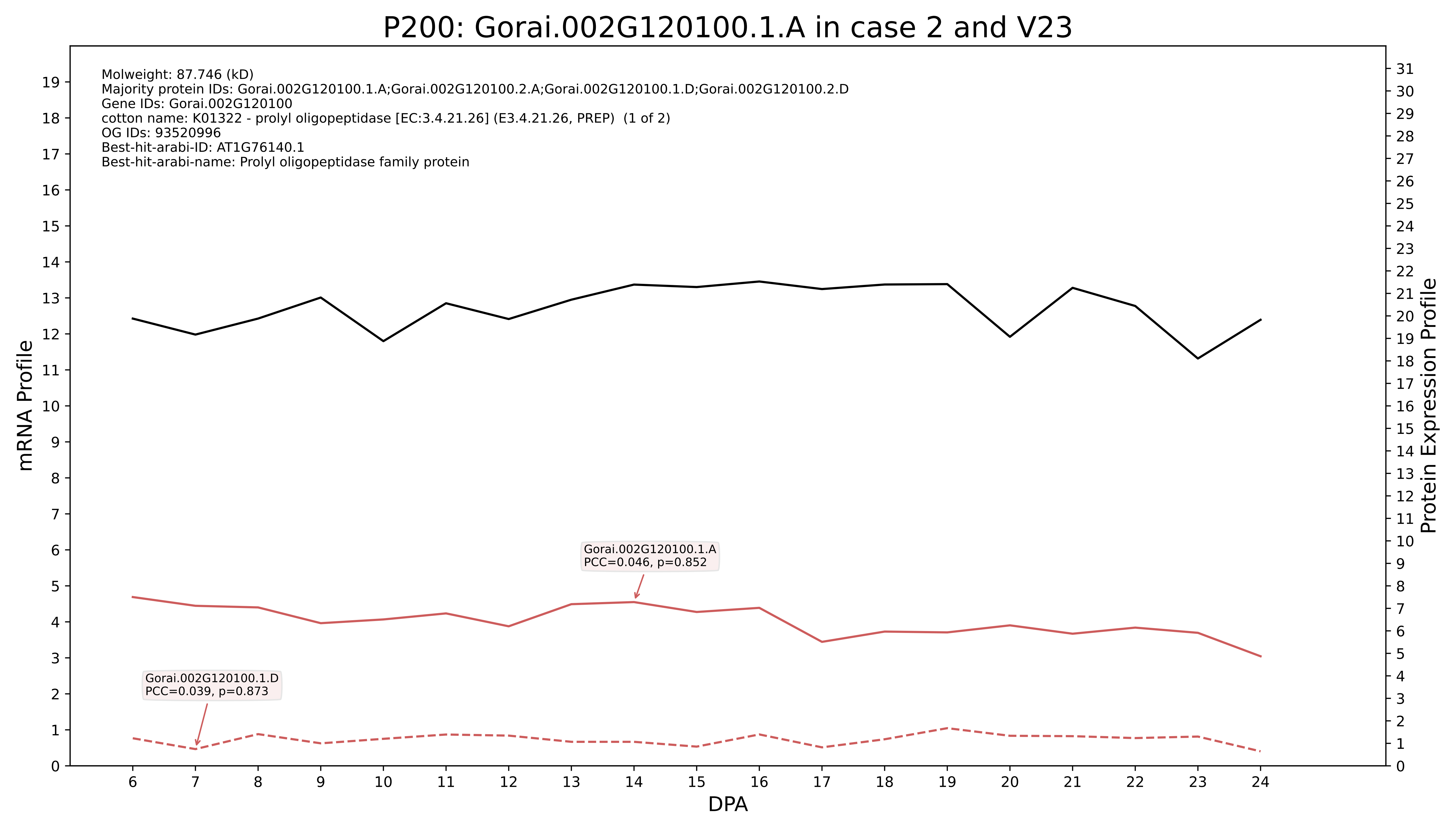
P200: Gorai.002G120100.1.A in case 2 and V23
Molweight: 87.746 (kD)
Majority protein IDs: Gorai.002G120100.1.A;Gorai.002G120100.2.A;Gorai.002G120100.1.D;Gorai.002G120100.2.D
Gene IDs: Gorai.002G120100
cotton name: K01322 - prolyl oligopeptidase [EC:3.4.21.26] (E3.4.21.26, PREP) (1 of 2)
OG IDs: 93520996
Best-hit-arabi-ID: AT1G76140.1
Best-hit-arabi-name: Prolyl oligopeptidase family protein
Gorai.002G120100.1.A
PCC=0.046, p=0.852
Gorai.002G120100.1.D
PCC=0.039, p=0.873
mRNA Profile
Protein Expression Profile
DPA

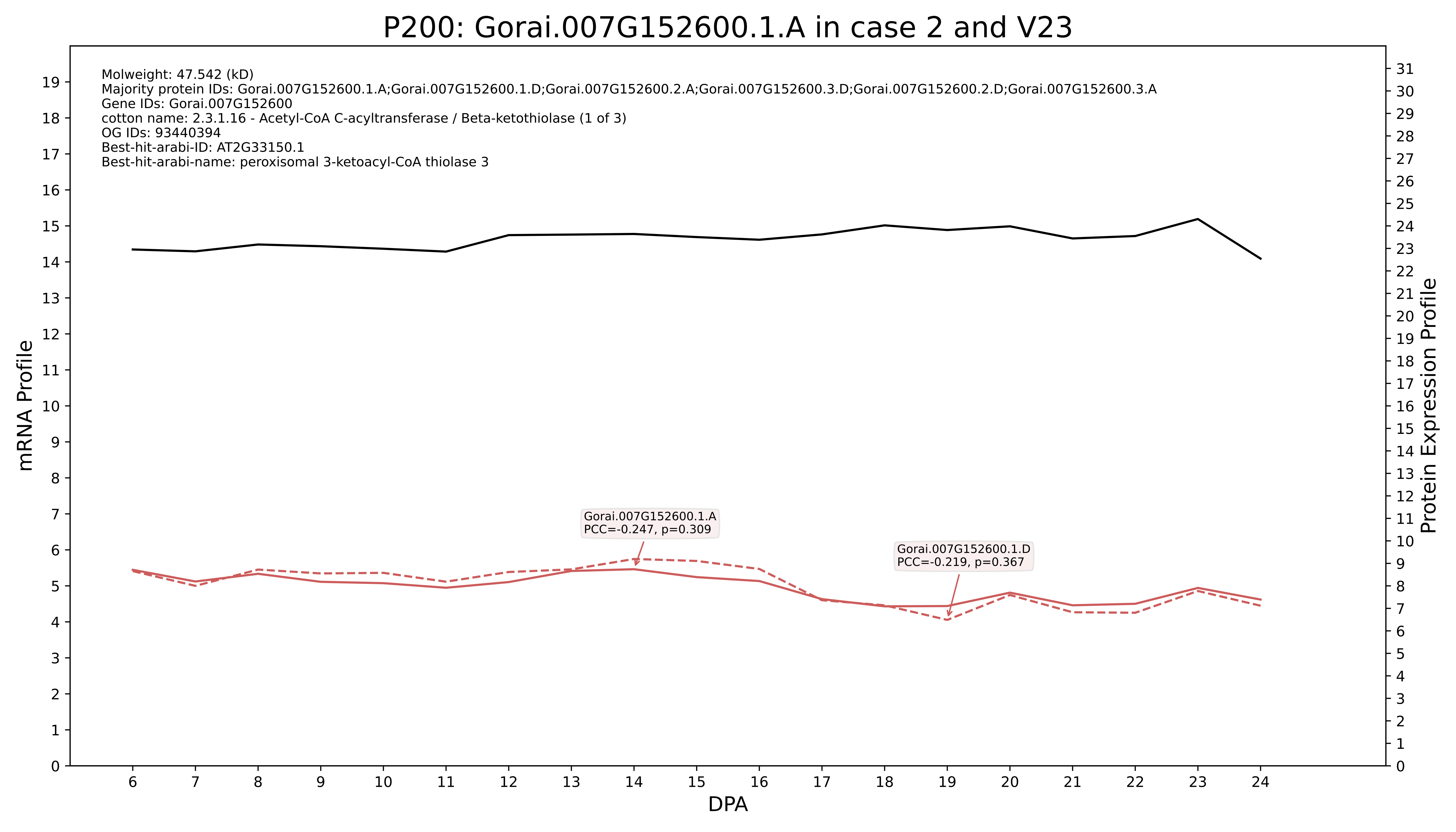
P200: Gorai.007G152600.1.A in case 2 and V23
Molweight: 47.542 (kD)
Majority protein IDs: Gorai.007G152600.1.A;Gorai.007G152600.1.D;Gorai.007G152600.2.A;Gorai.007G152600.3.D;Gorai.007G152600.2.D;Gorai.007G152600.3.A
Gene IDs: Gorai.007G152600
cotton name: 2.3.1.16 - Acetyl-CoA C-acyltransferase / Beta-ketothiolase (1 of 3)
OG IDs: 93440394
Best-hit-arabi-ID: AT2G33150.1
Best-hit-arabi-name: peroxisomal 3-ketoacyl-CoA thiolase 3
Gorai.007G152600.1.A
PCC=-0.247, p=0.309
Gorai.007G152600.1.D
PCC=-0.219, p=0.367
mRNA Profile
Protein Expression Profile
DPA

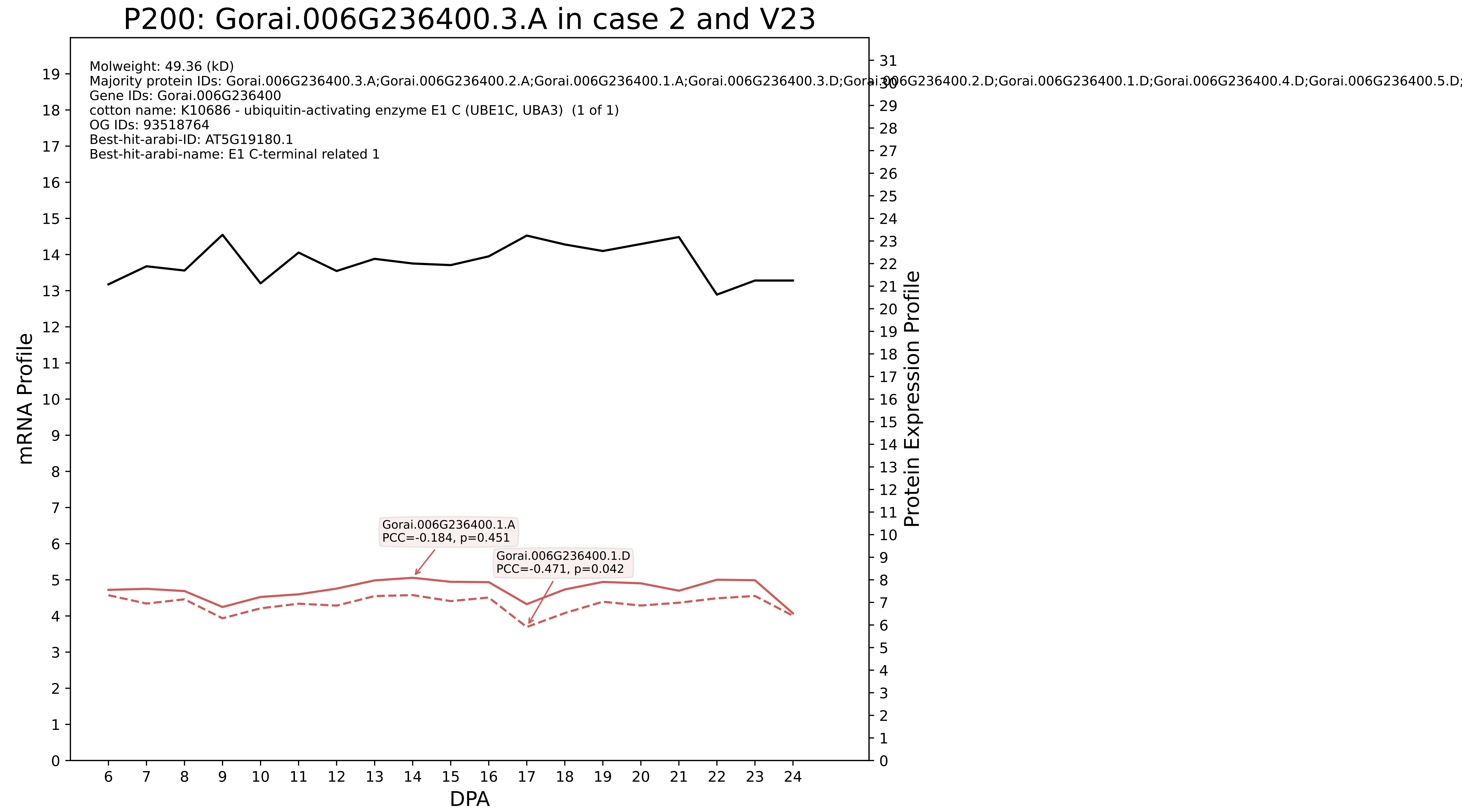
P200: Gorai.006G236400.3.A in case 2 and V23
Molweight: 49.36 (kD)
Majority protein IDs: Gorai.006G236400.3.A;Gorai.006G236400.2.A;Gorai.006G236400.1.A;Gorai.006G236400.3.D;Gorai.006G236400.2.D;Gorai.006G236400.1.D;Gorai.006G236400.4.D;Gorai.006G236400.5.D;
Gene IDs: Gorai.006G236400
cotton name: K10686 - ubiquitin-activating enzyme E1 C (UBE1C, UBA3) (1 of 1)
OG IDs: 93518764
Best-hit-arabi-ID: AT5G19180.1
Best-hit-arabi-name: E1 C-terminal related 1
Gorai.006G236400.1.A
PCC=-0.184, p=0.451
Gorai.006G236400.1.D
PCC=-0.471, p=0.042
mRNA Profile
Protein Expression Profile
DPA

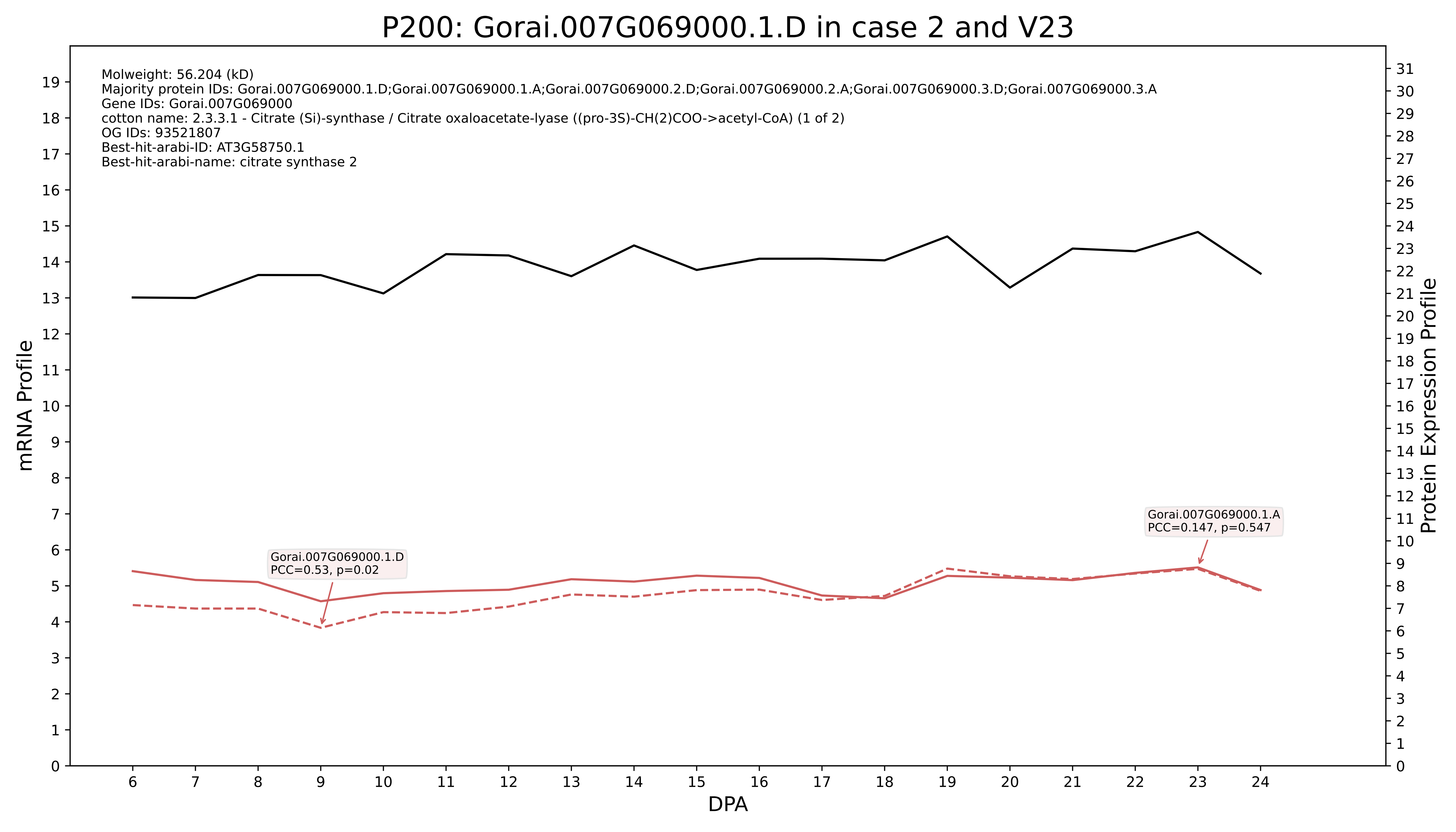
P200: Gorai.007G069000.1.D in case 2 and V23
Molweight: 56.204 (kD)
Majority protein IDs: Gorai.007G069000.1.D;Gorai.007G069000.1.A;Gorai.007G069000.2.D;Gorai.007G069000.2.A;Gorai.007G069000.3.D;Gorai.007G069000.3.A
Gene IDs: Gorai.007G069000
cotton name: 2.3.3.1 - Citrate (Si)-synthase / Citrate oxaloacetate-lyase ((pro-3S)-CH(2)COO->acetyl-CoA) (1 of 2)
OG IDs: 93521807
Best-hit-arabi-ID: AT3G58750.1
Best-hit-arabi-name: citrate synthase 2
Gorai.007G069000.1.D
PCC=0.53, p=0.02
Gorai.007G069000.1.A
PCC=0.147, p=0.547
mRNA Profile
Protein Expression Profile
DPA

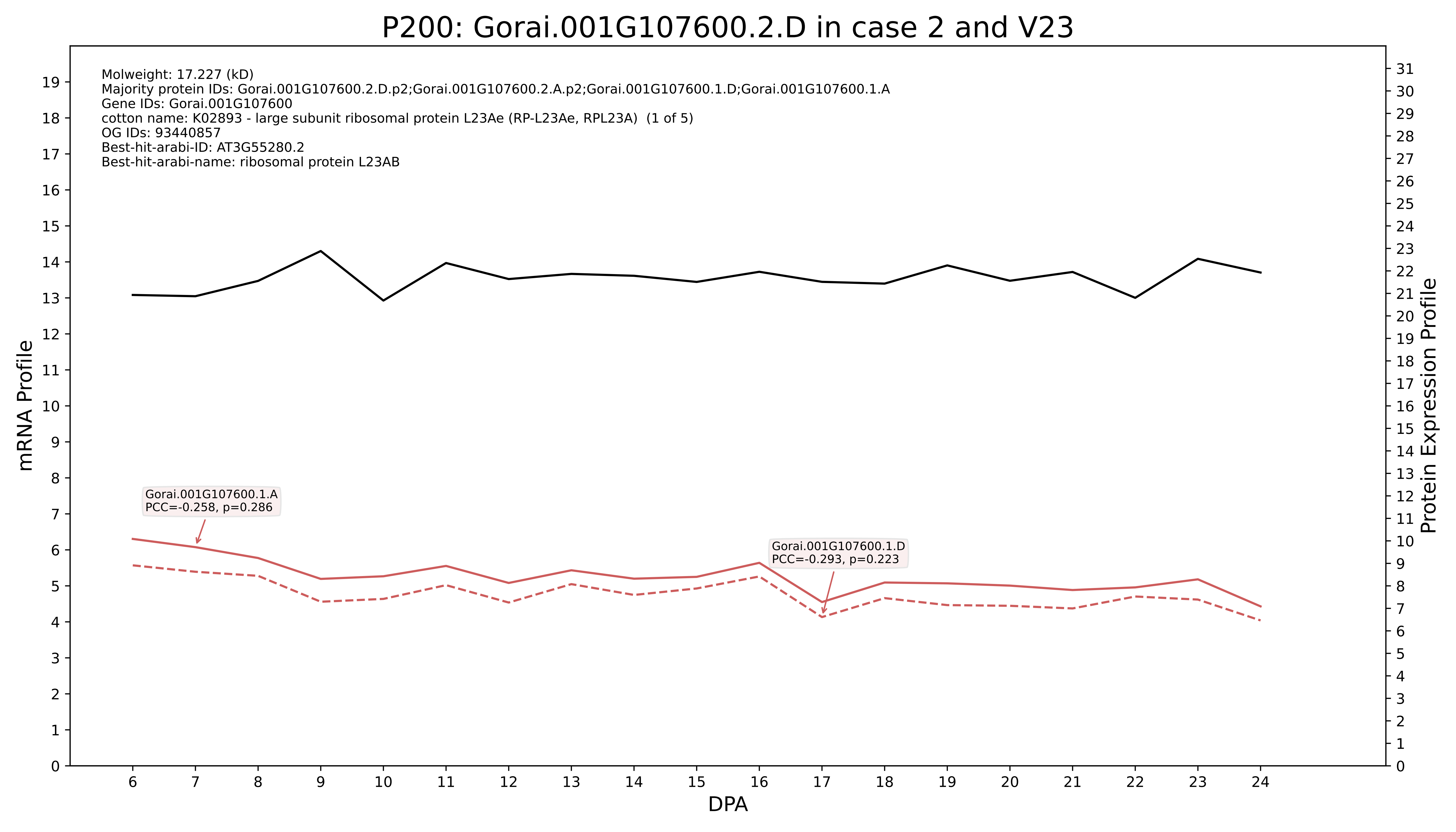
P200: Gorai.001G107600.2.D in case 2 and V23
Molweight: 17.227 (kD)
Majority protein IDs: Gorai.001G107600.2.D.p2;Gorai.001G107600.2.A.p2;Gorai.001G107600.1.D;Gorai.001G107600.1.A
Gene IDs: Gorai.001G107600
cotton name: K02893 - large subunit ribosomal protein L23Ae (RP-L23Ae, RPL23A) (1 of 5)
OG IDs: 93440857
Best-hit-arabi-ID: AT3G55280.2
Best-hit-arabi-name: ribosomal protein L23AB
Gorai.001G107600.1.A
PCC=-0.258, p=0.286
Gorai.001G107600.1.D
PCC=-0.293, p=0.223
mRNA Profile
Protein Expression Profile
DPA

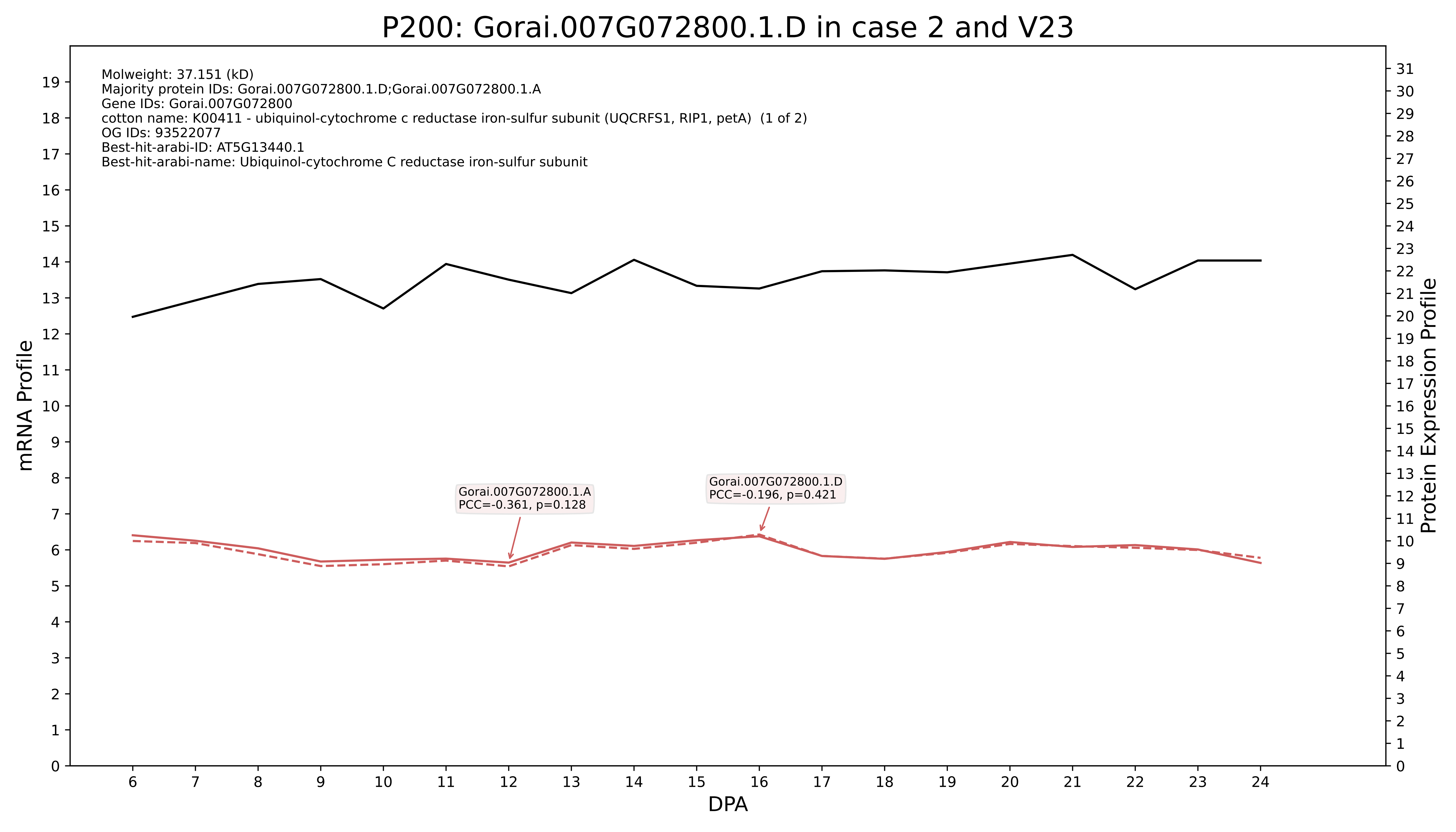
P200: Gorai.007G072800.1.D in case 2 and V23
Molweight: 37.151 (kD)
Majority protein IDs: Gorai.007G072800.1.D;Gorai.007G072800.1.A
Gene IDs: Gorai.007G072800
cotton name: K00411 - ubiquinol-cytochrome c reductase iron-sulfur subunit (UQCRFS1, RIP1, petA) (1 of 2)
OG IDs: 93522077
Best-hit-arabi-ID: AT5G13440.1
Best-hit-arabi-name: Ubiquinol-cytochrome C reductase iron-sulfur subunit
Gorai.007G072800.1.A
PCC=-0.361, p=0.128
Gorai.007G072800.1.D
PCC=-0.196, p=0.421
mRNA Profile
Protein Expression Profile
DPA
0
1
2
3
4
5
6
7
8
9
10
11
12
13
14
15
16
17
18
19
20
21
22
23
24
25
26
27
28
29
30
31

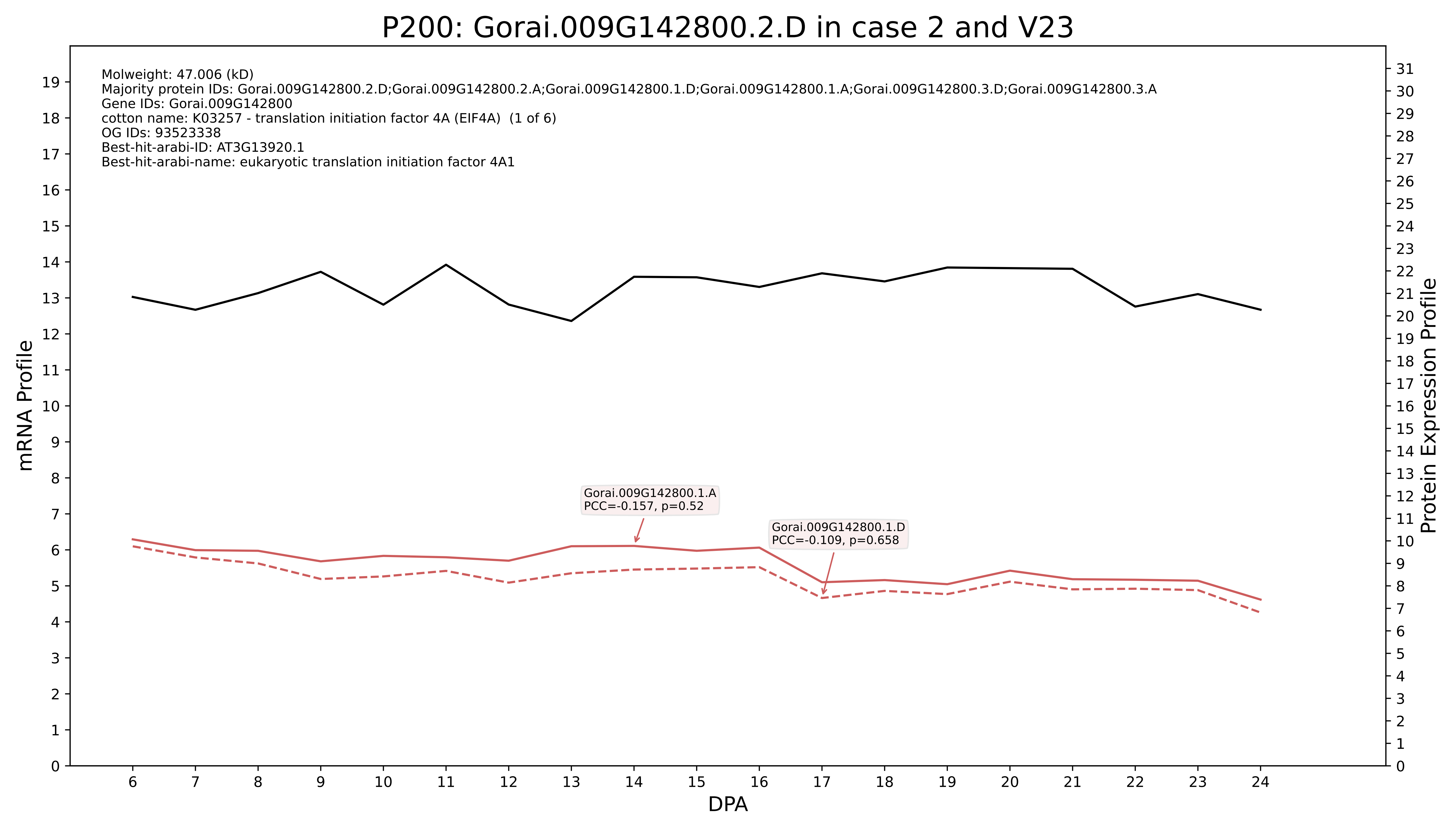
P200: Gorai.009G142800.2.D in case 2 and V23
Molweight: 47.006 (kD)
Majority protein IDs: Gorai.009G142800.2.D;Gorai.009G142800.2.A;Gorai.009G142800.1.D;Gorai.009G142800.1.A;Gorai.009G142800.3.D;Gorai.009G142800.3.A
Gene IDs: Gorai.009G142800
cotton name: K03257 - translation initiation factor 4A (EIF4A) (1 of 6)
OG IDs: 93523338
Best-hit-arabi-ID: AT3G13920.1
Best-hit-arabi-name: eukaryotic translation initiation factor 4A1
Gorai.009G142800.1.A
PCC=-0.157, p=0.52
Gorai.009G142800.1.D
PCC=-0.109, p=0.658
mRNA Profile
Protein Expression Profile
DPA

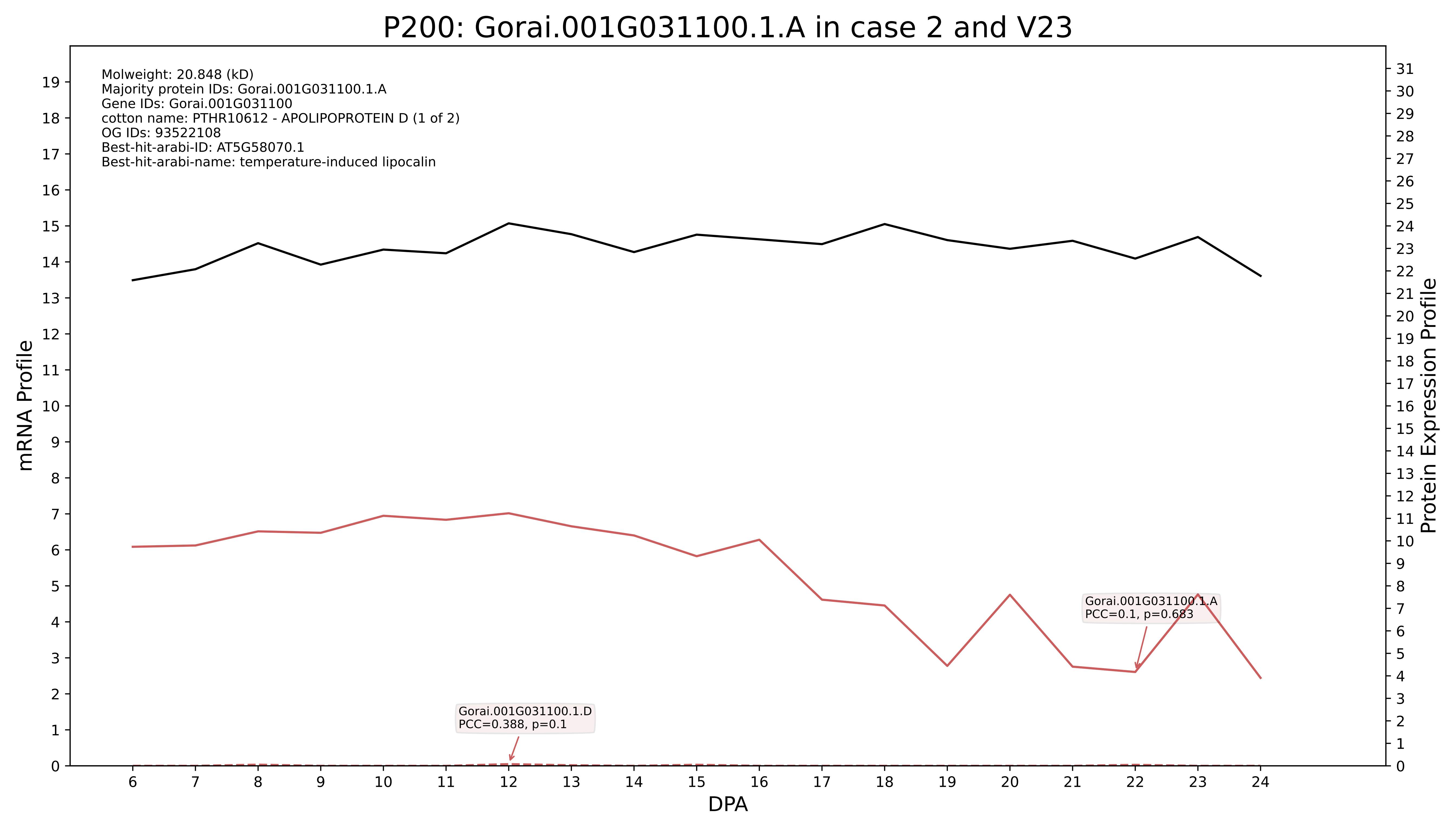

P200: Gorai.001G031100.1.A in case 2 and V23
Molweight: 20.848 (kD)
Majority protein IDs: Gorai.001G031100.1.A
Gene IDs: Gorai.001G031100
cotton name: PTHR10612 - APOLIPOPROTEIN D (1 of 2)
OG IDs: 93522108
Best-hit-arabi-ID: AT5G58070.1
Best-hit-arabi-name: temperature-induced lipocalin
Gorai.001G031100.1.A
PCC=0.1, p=0.683
Gorai.001G031100.1.D
PCC=0.388, p=0.1
mRNA Profile
Protein Expression Profile
DPA

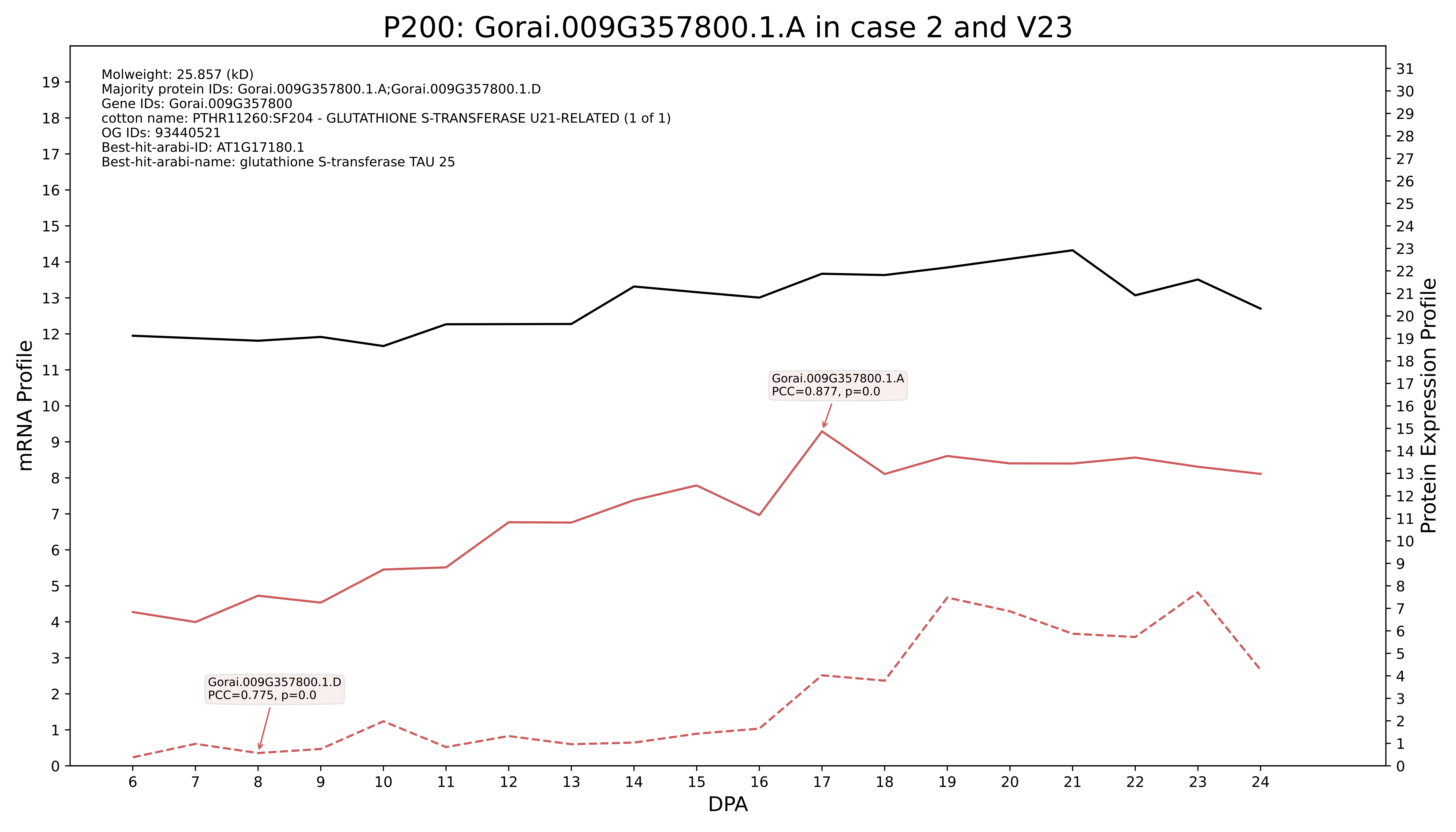
P200: Gorai.009G357800.1.A in case 2 and V23
Molweight: 25.857 (kD)
Majority protein IDs: Gorai.009G357800.1.A;Gorai.009G357800.1.D
Gene IDs: Gorai.009G357800
cotton name: PTHR11260:SF204 - GLUTATHIONE S-TRANSFERASE U21-RELATED (1 of 1)
OG IDs: 93440521
Best-hit-arabi-ID: AT1G17180.1
Best-hit-arabi-name: glutathione S-transferase TAU 25
Gorai.009G357800.1.A
PCC=0.877, p=0.0
Gorai.009G357800.1.D
PCC=0.775, p=0.0
mRNA Profile
Protein Expression Profile
DPA

P200: Gorai.007G351900.2.D in case 2 and V23

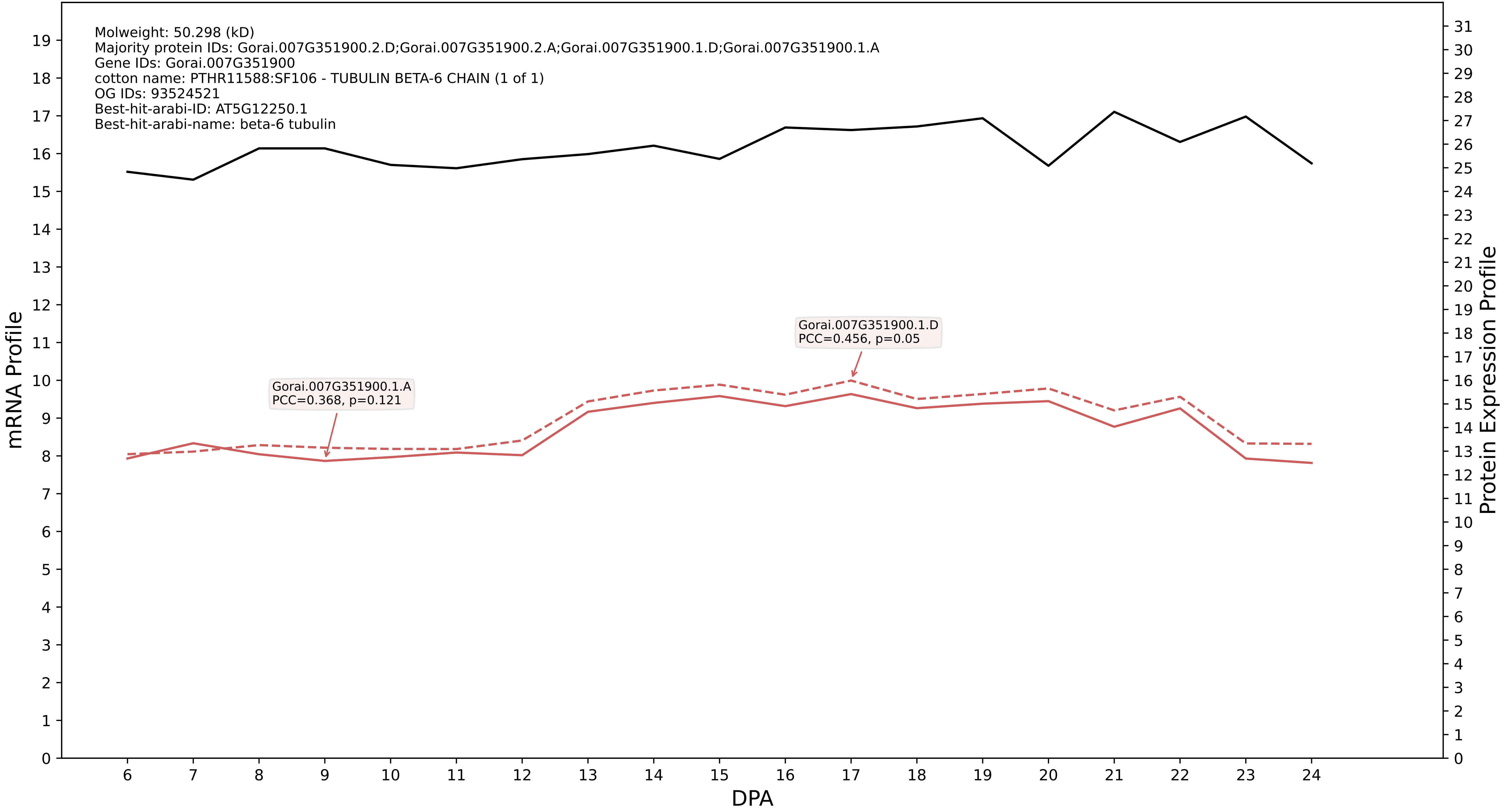

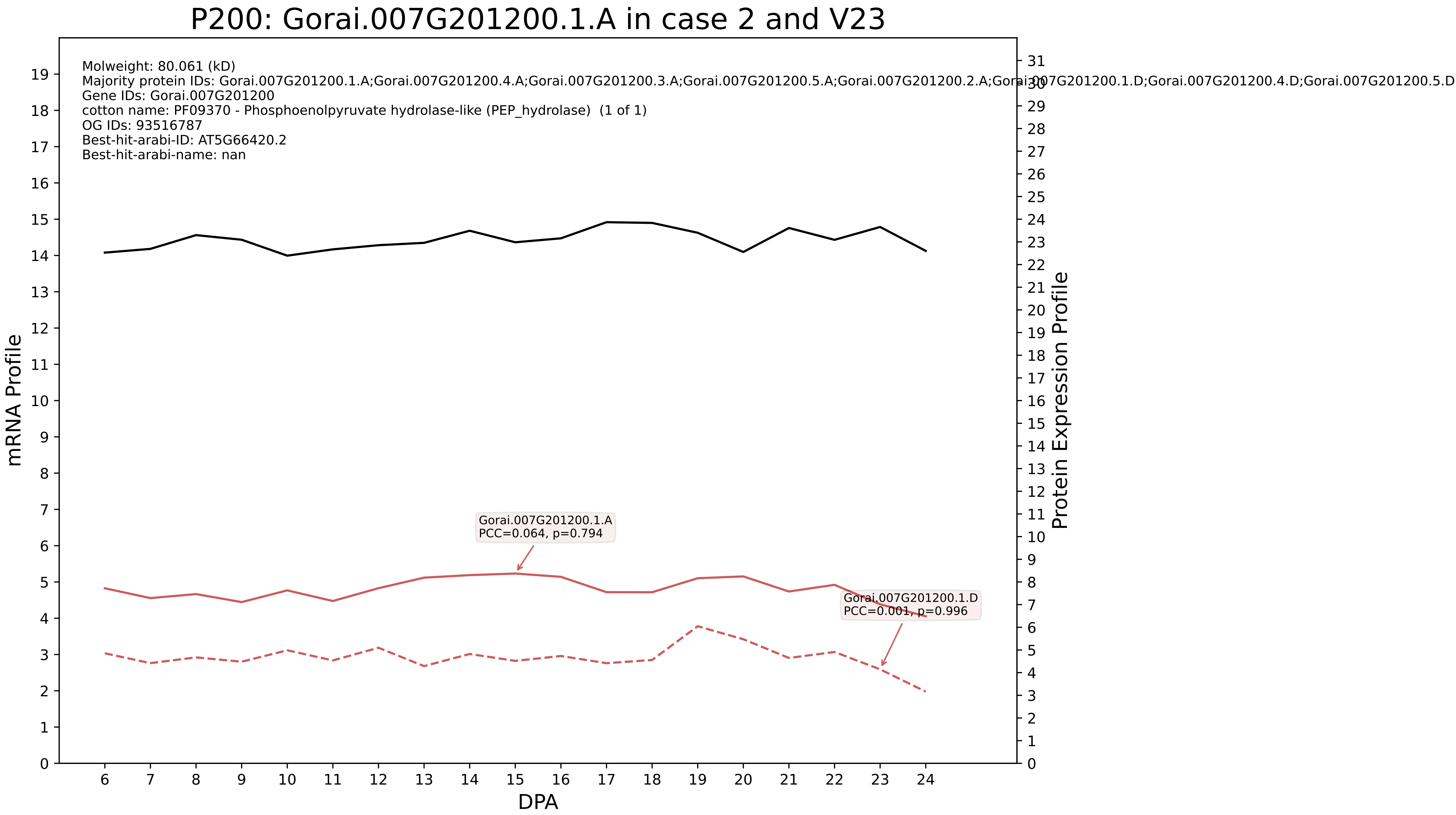

P200: Gorai.007G201200.1.A in case 2 and V23
Molweight: 80.061 (kD)
Majority protein IDs: Gorai.007G201200.1.A;Gorai.007G201200.4.A;Gorai.007G201200.3.A;Gorai.007G201200.5.A;Gorai.007G201200.2.A;Gorai.007G201200.1.D;Gorai.007G201200.4.D;Gorai.007G201200.5.D
Gene IDs: Gorai.007G201200
cotton name: PF09370 - Phosphoenolpyruvate hydrolase-like (PEP_hydrolase) (1 of 1)
OG IDs: 93516787
Best-hit-arabi-ID: AT5G66420.2
Best-hit-arabi-name: nan
Gorai.007G201200.1.A
PCC=0.064, p=0.794
Gorai.007G201200.1.D
PCC=0.001, p=0.996
mRNA Profile
Protein Expression Profile
DPA

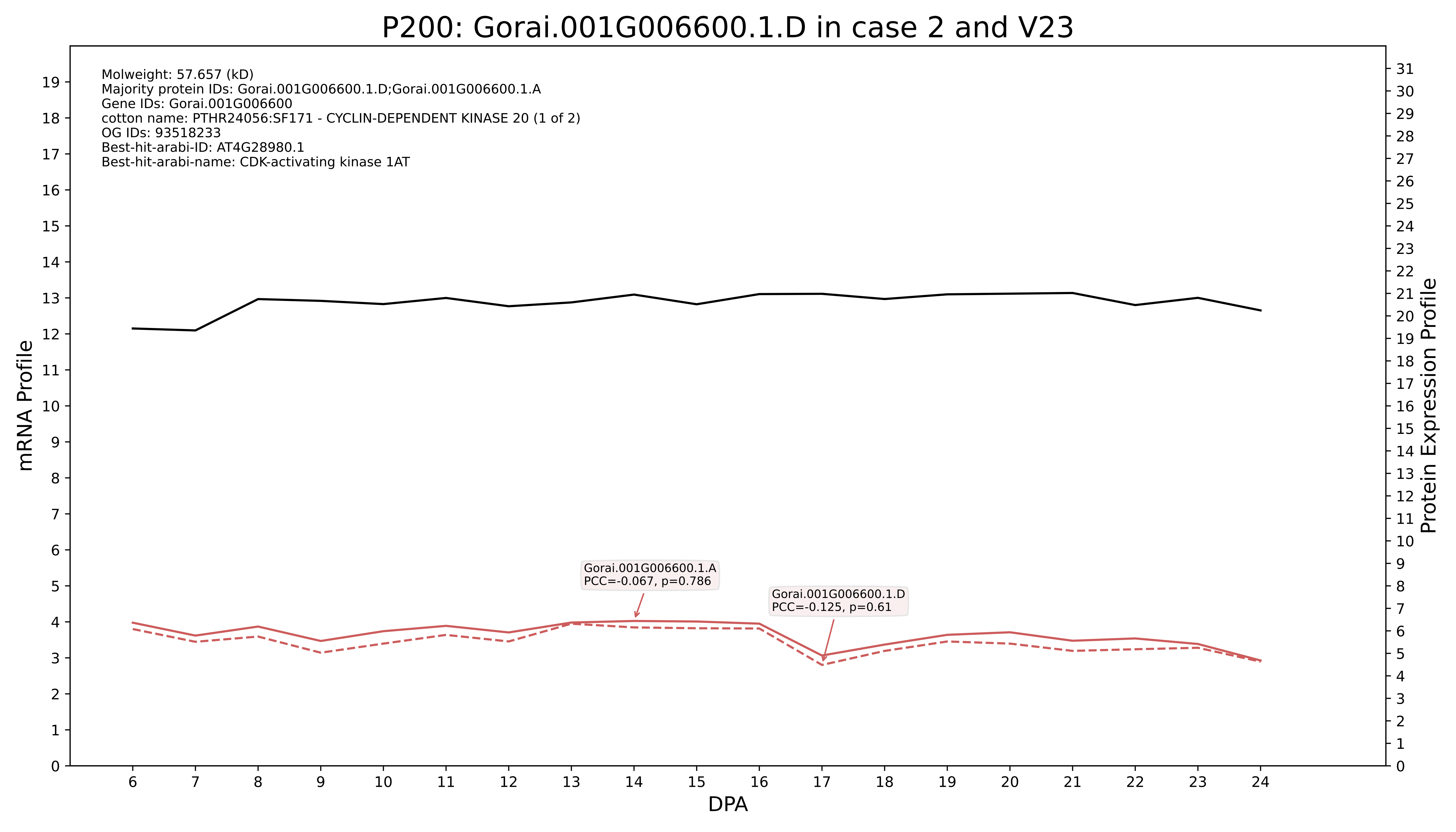
P200: Gorai.001G006600.1.D in case 2 and V23
Molweight: 57.657 (kD)
Majority protein IDs: Gorai.001G006600.1.D;Gorai.001G006600.1.A
Gene IDs: Gorai.001G006600
cotton name: PTHR24056:SF171 - CYCLIN-DEPENDENT KINASE 20 (1 of 2)
OG IDs: 93518233
Best-hit-arabi-ID: AT4G28980.1
Best-hit-arabi-name: CDK-activating kinase 1AT
Gorai.001G006600.1.A
PCC=-0.067, p=0.786
Gorai.001G006600.1.D
PCC=-0.125, p=0.61
mRNA Profile
Protein Expression Profile
DPA

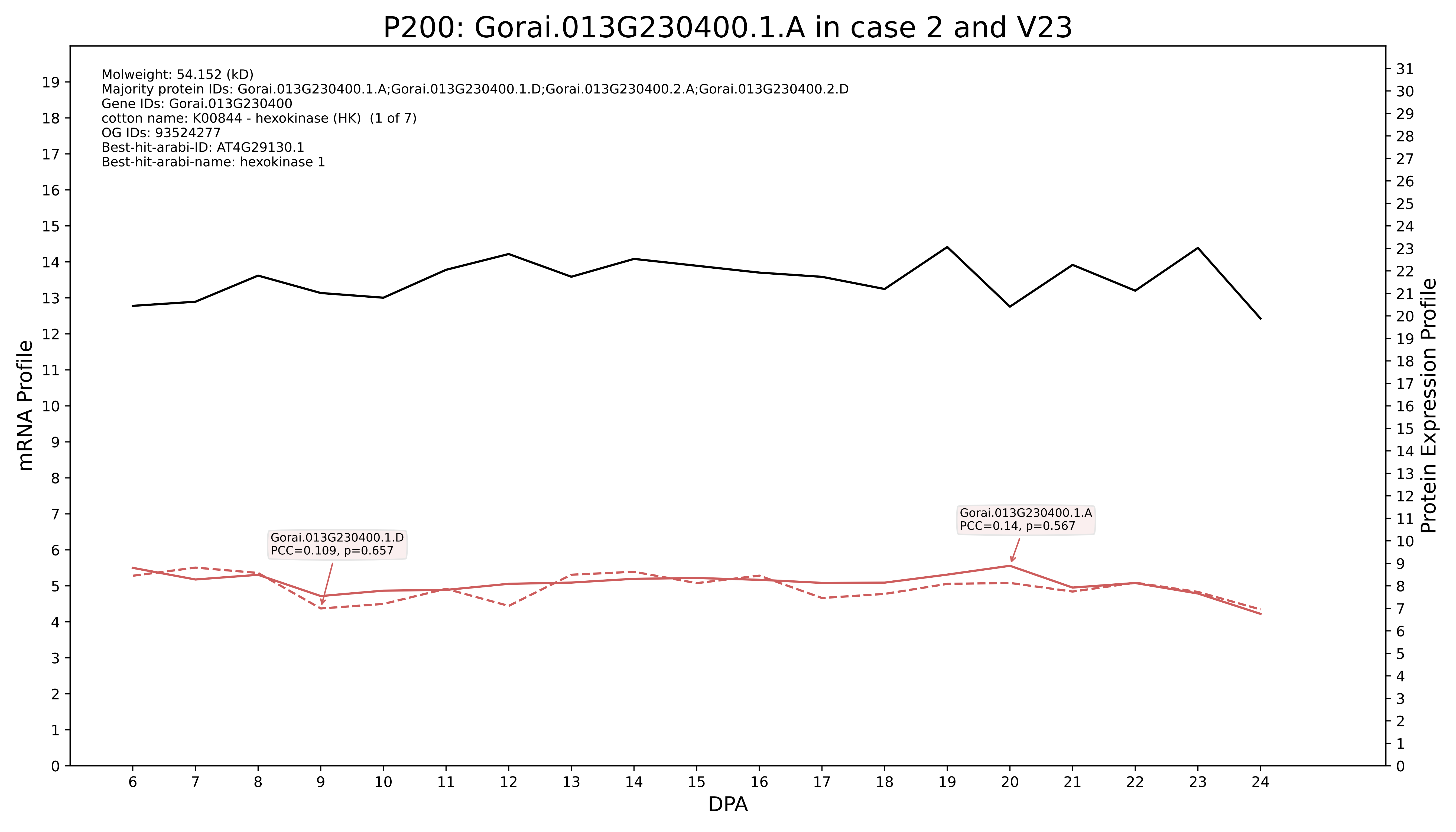
P200: Gorai.013G230400.1.A in case 2 and V23
Molweight: 54.152 (kD)
Majority protein IDs: Gorai.013G230400.1.A;Gorai.013G230400.1.D;Gorai.013G230400.2.A;Gorai.013G230400.2.D
Gene IDs: Gorai.013G230400
cotton name: K00844 - hexokinase (HK) (1 of 7)
OG IDs: 93524277
Best-hit-arabi-ID: AT4G29130.1
Best-hit-arabi-name: hexokinase 1
Gorai.013G230400.1.D
PCC=0.109, p=0.657
Gorai.013G230400.1.A
PCC=0.14, p=0.567
mRNA Profile
Protein Expression Profile
DPA

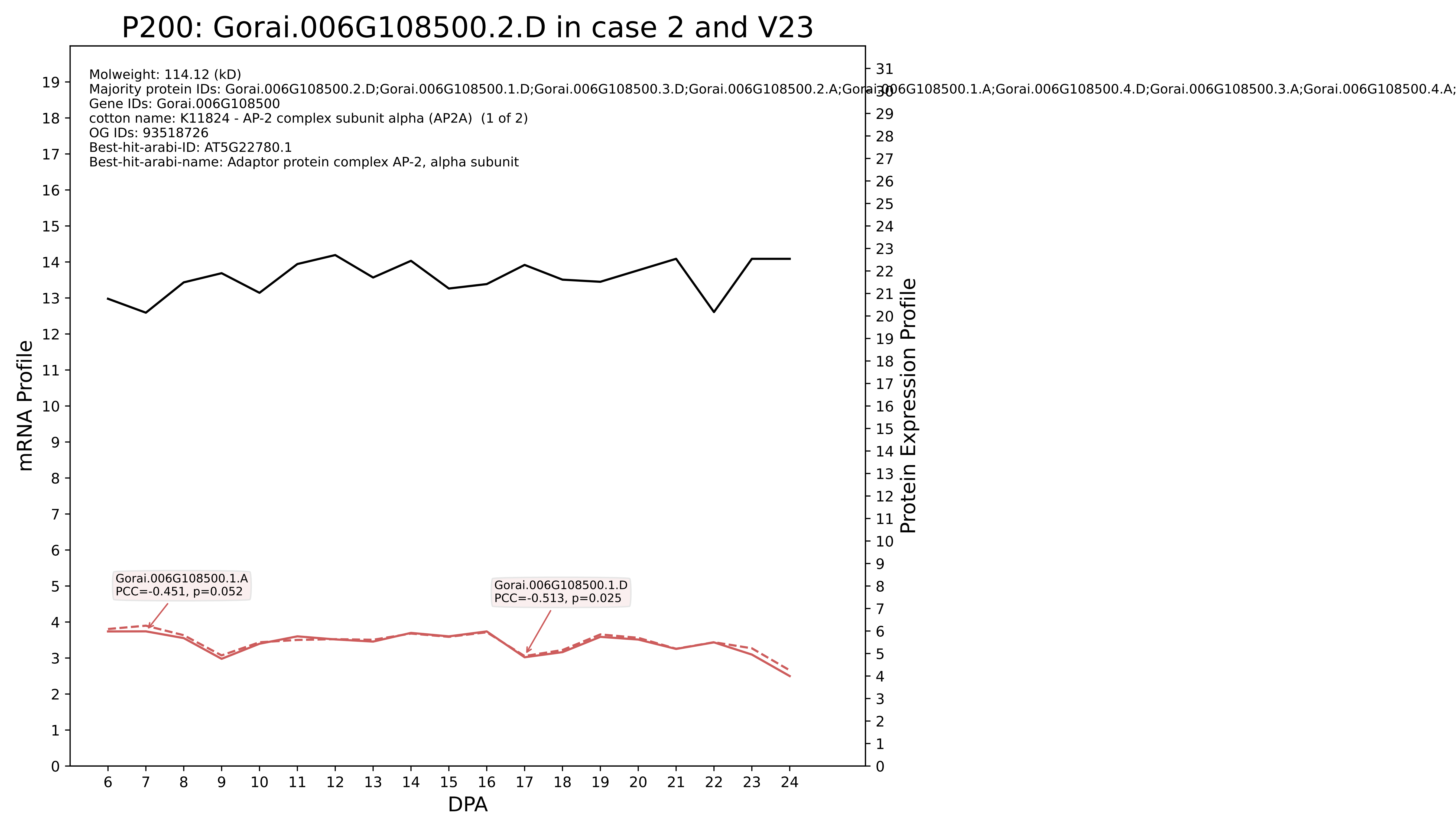
P200: Gorai.006G108500.2.D in case 2 and V23
Molweight: 114.12 (kD)
Majority protein IDs: Gorai.006G108500.2.D;Gorai.006G108500.1.D;Gorai.006G108500.3.D;Gorai.006G108500.2.A;Gorai.006G108500.1.A;Gorai.006G108500.4.D;Gorai.006G108500.3.A;Gorai.006G108500.4.A;
Gene IDs: Gorai.006G108500
cotton name: K11824 - AP-2 complex subunit alpha (AP2A) (1 of 2)
OG IDs: 93518726
Best-hit-arabi-ID: AT5G22780.1
Best-hit-arabi-name: Adaptor protein complex AP-2, alpha subunit
Gorai.006G108500.1.A
PCC=-0.451, p=0.052
Gorai.006G108500.1.D
PCC=-0.513, p=0.025
mRNA Profile
Protein Expression Profile
DPA

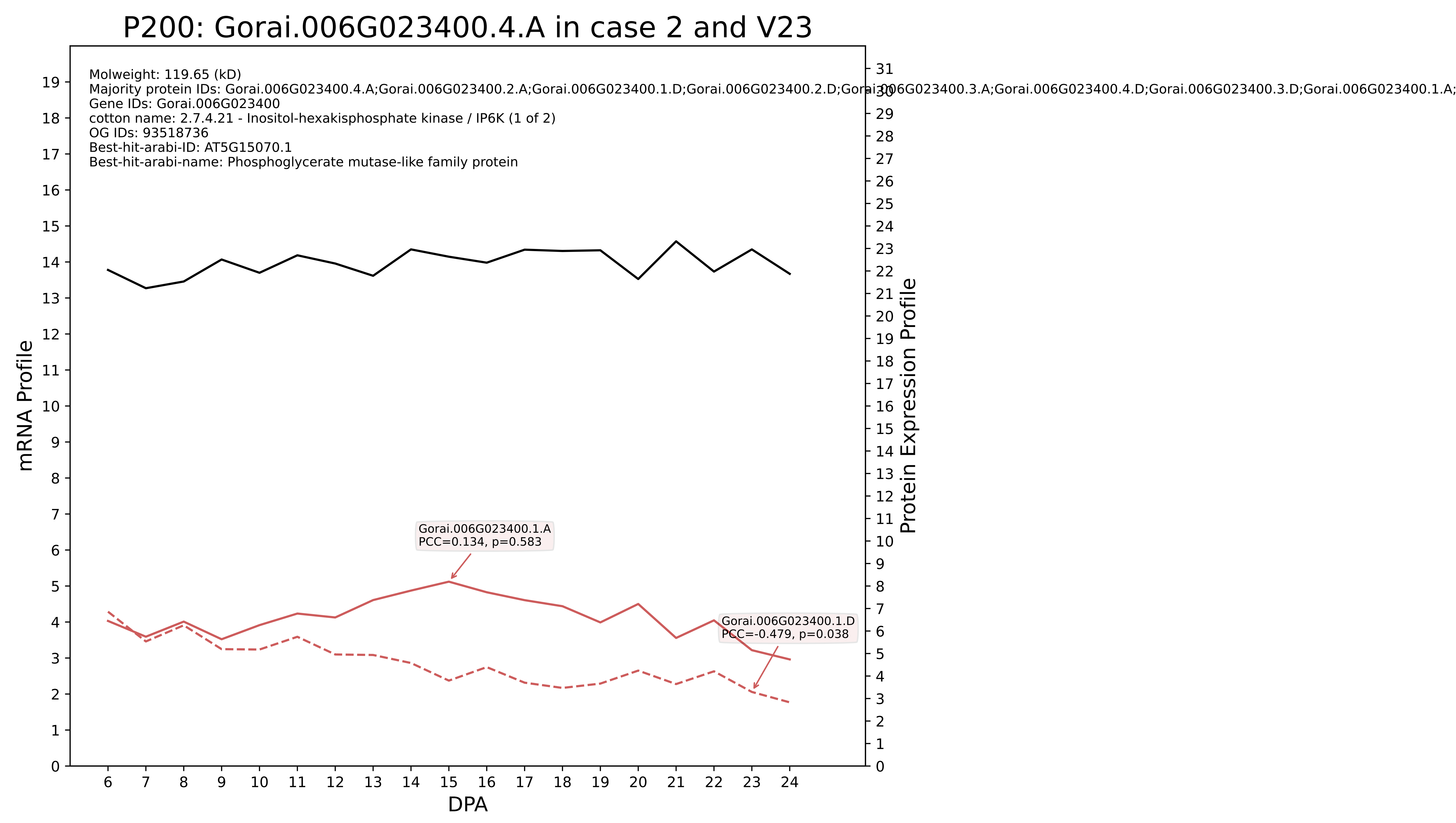
P200: Gorai.006G023400.4.A in case 2 and V23
Molweight: 119.65 (kD)
Majority protein IDs: Gorai.006G023400.4.A;Gorai.006G023400.2.A;Gorai.006G023400.1.D;Gorai.006G023400.2.D;Gorai.006G023400.3.A;Gorai.006G023400.4.D;Gorai.006G023400.3.D;Gorai.006G023400.1.A;
Gene IDs: Gorai.006G023400
cotton name: 2.7.4.21 - Inositol-hexakisphosphate kinase / IP6K (1 of 2)
OG IDs: 93518736
Best-hit-arabi-ID: AT5G15070.1
Best-hit-arabi-name: Phosphoglycerate mutase-like family protein
Gorai.006G023400.1.A
PCC=0.134, p=0.583
Gorai.006G023400.1.D
PCC=-0.479, p=0.038
mRNA Profile
Protein Expression Profile
DPA
6 7 8 9 10 11 12 13 14 15 16 17 18 19 20 21 22 23 24

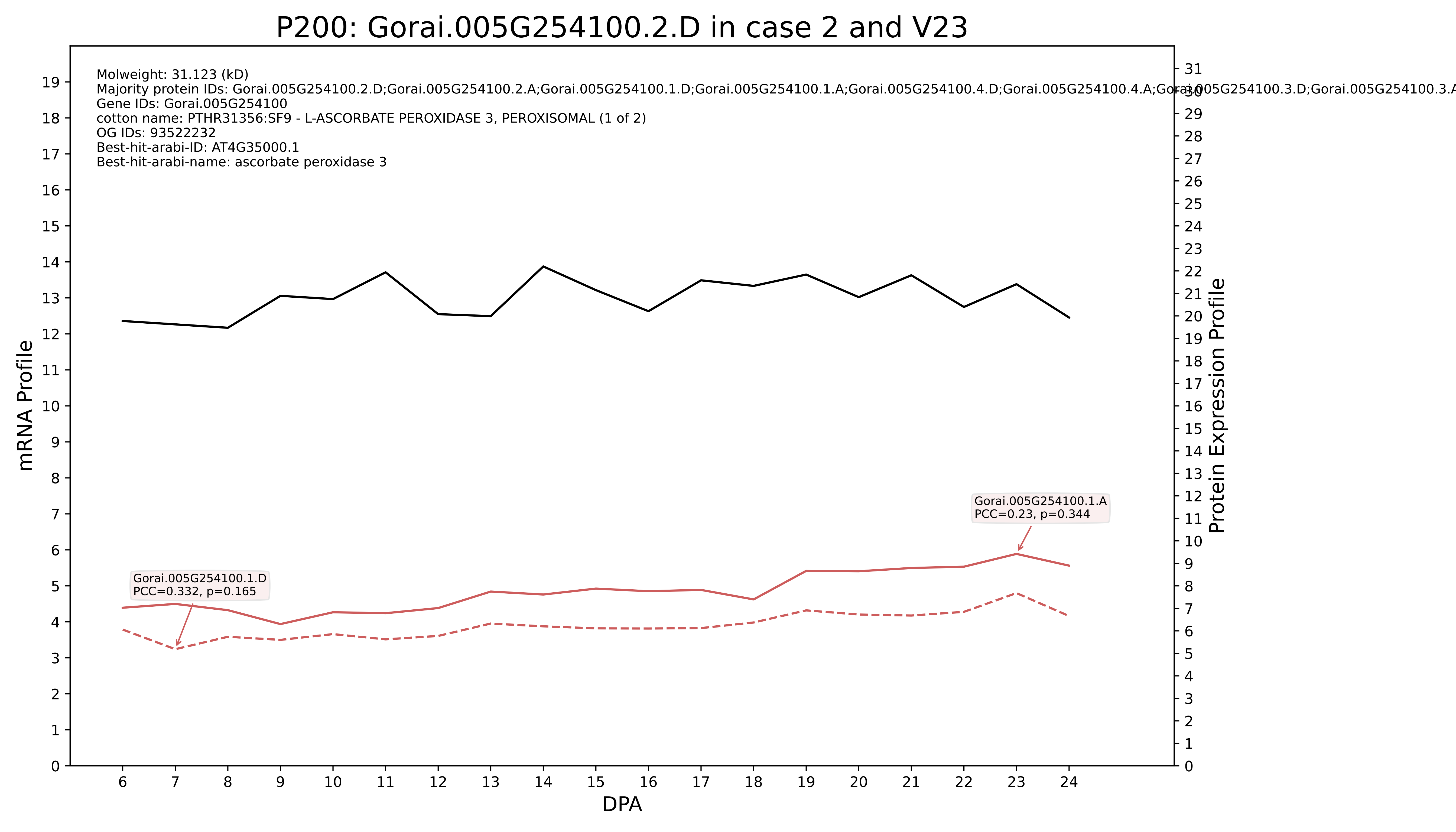
P200: Gorai.005G254100.2.D in case 2 and V23
Molweight: 31.123 (kD)
Majority protein IDs: Gorai.005G254100.2.D;Gorai.005G254100.2.A;Gorai.005G254100.1.D;Gorai.005G254100.1.A;Gorai.005G254100.4.D;Gorai.005G254100.4.A;Gorai.005G254100.3.D;Gorai.005G254100.3.A
Gene IDs: Gorai.005G254100
cotton name: PTHR31356:SF9 - L-ASCORBATE PEROXIDASE 3, PEROXISOMAL (1 of 2)
OG IDs: 93522232
Best-hit-arabi-ID: AT4G35000.1
Best-hit-arabi-name: ascorbate peroxidase 3
Gorai.005G254100.1.A
PCC=0.23, p=0.344
Gorai.005G254100.1.D
PCC=0.332, p=0.165
mRNA Profile
Protein Expression Profile
DPA

P200: Gorai.009G127800.2.A in case 2 and V23

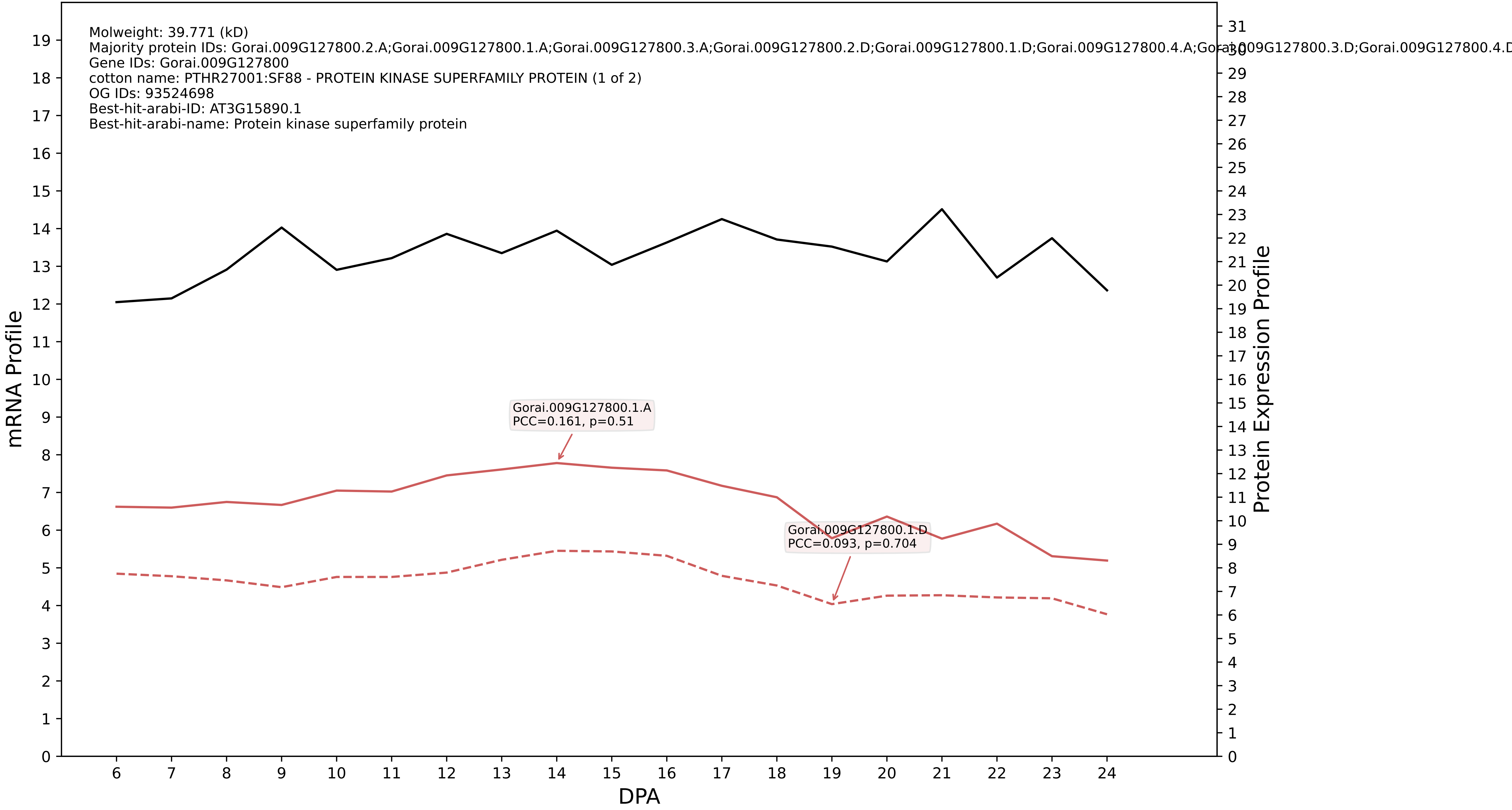

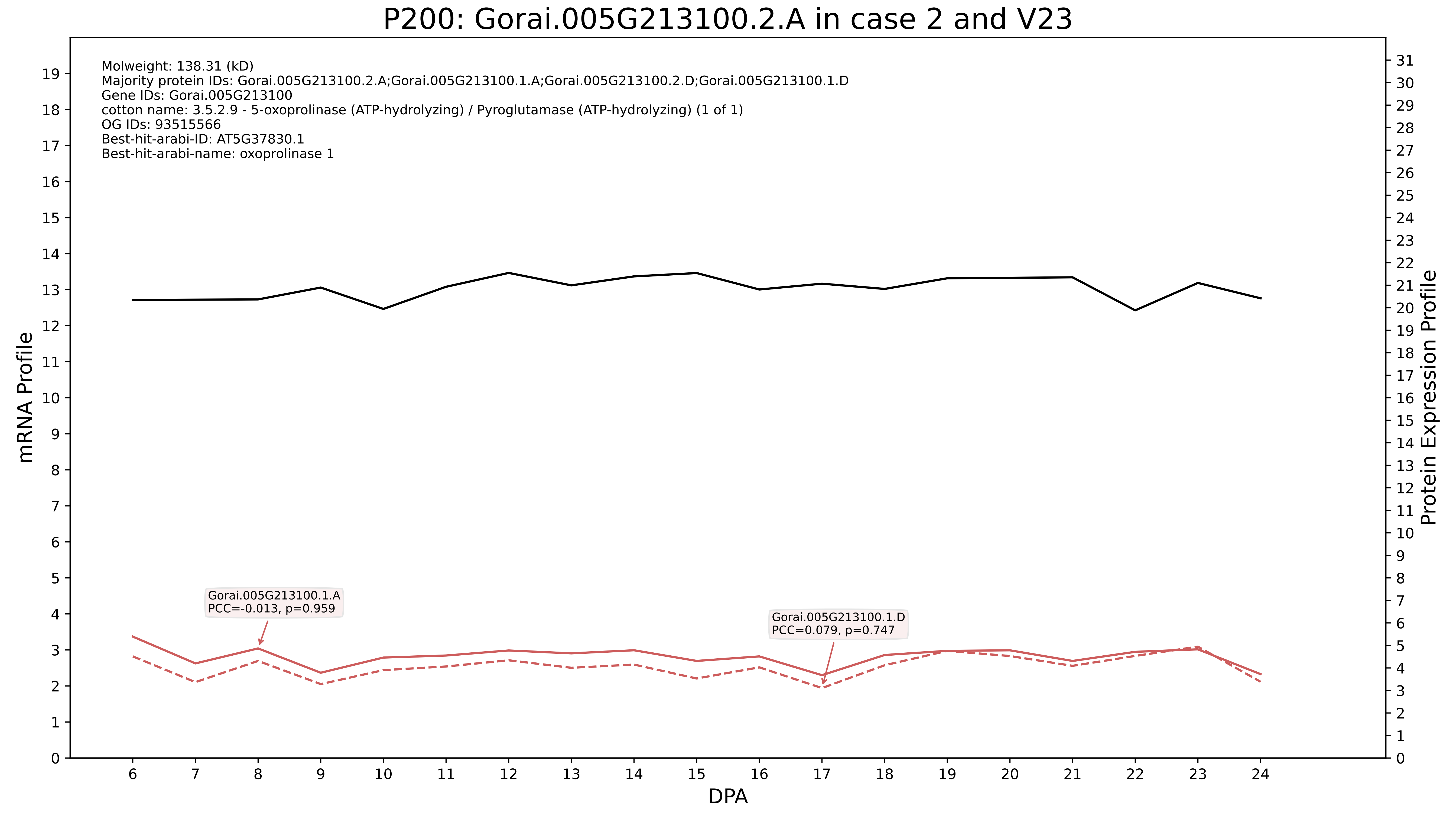
P200: Gorai.005G213100.2.A in case 2 and V23
Molweight: 138.31 (kD)
Majority protein IDs: Gorai.005G213100.2.A;Gorai.005G213100.1.A;Gorai.005G213100.2.D;Gorai.005G213100.1.D
Gene IDs: Gorai.005G213100
cotton name: 3.5.2.9 - 5-oxoprolinase (ATP-hydrolyzing) / Pyroglutamase (ATP-hydrolyzing) (1 of 1)
OG IDs: 93515566
Best-hit-arabi-ID: AT5G37830.1
Best-hit-arabi-name: oxoprolinase 1
Gorai.005G213100.1.A
PCC=-0.013, p=0.959
Gorai.005G213100.1.D
PCC=0.079, p=0.747
mRNA Profile
Protein Expression Profile
DPA

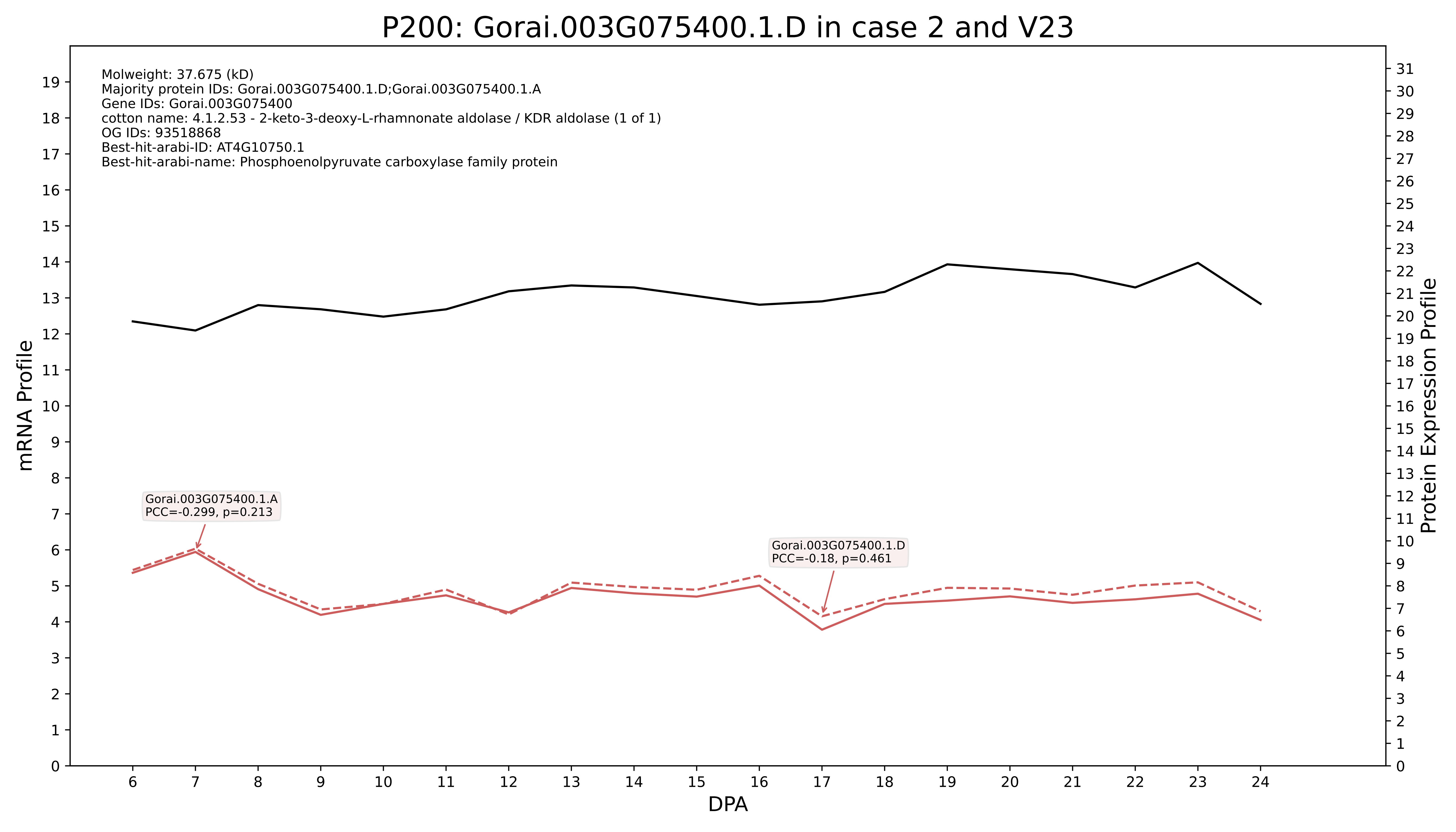

P200: Gorai.003G075400.1.D in case 2 and V23
Molweight: 37.675 (kD)
Majority protein IDs: Gorai.003G075400.1.D;Gorai.003G075400.1.A
Gene IDs: Gorai.003G075400
cotton name: 4.1.2.53 - 2-keto-3-deoxy-L-rhamnonate aldolase / KDR aldolase (1 of 1)
OG IDs: 93518868
Best-hit-arabi-ID: AT4G10750.1
Best-hit-arabi-name: Phosphoenolpyruvate carboxylase family protein
Gorai.003G075400.1.A
PCC=-0.299, p=0.213
Gorai.003G075400.1.D
PCC=-0.18, p=0.461
mRNA Profile
Protein Expression Profile
DPA

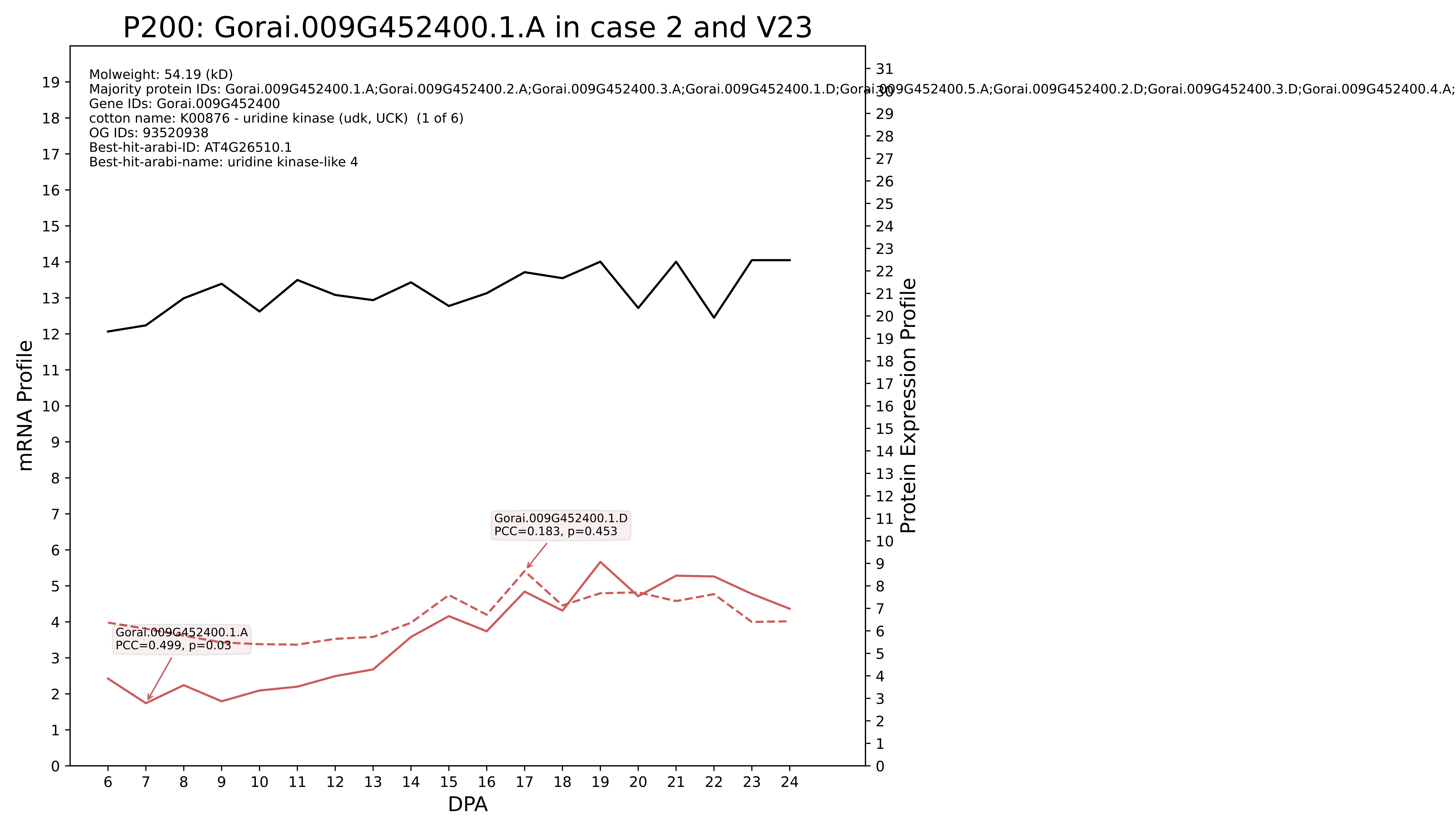
P200: Gorai.009G452400.1.A in case 2 and V23
Molweight: 54.19 (kD)
Majority protein IDs: Gorai.009G452400.1.A;Gorai.009G452400.2.A;Gorai.009G452400.3.A;Gorai.009G452400.1.D;Gorai.009G452400.5.A;Gorai.009G452400.2.D;Gorai.009G452400.3.D;Gorai.009G452400.4.A;
Gene IDs: Gorai.009G452400
cotton name: K00876 - uridine kinase (udk, UCK) (1 of 6)
OG IDs: 93520938
Best-hit-arabi-ID: AT4G26510.1
Best-hit-arabi-name: uridine kinase-like 4
Gorai.009G452400.1.D
PCC=0.183, p=0.453
Gorai.009G452400.1.A
PCC=0.499, p=0.03
mRNA Profile
Protein Expression Profile
DPA

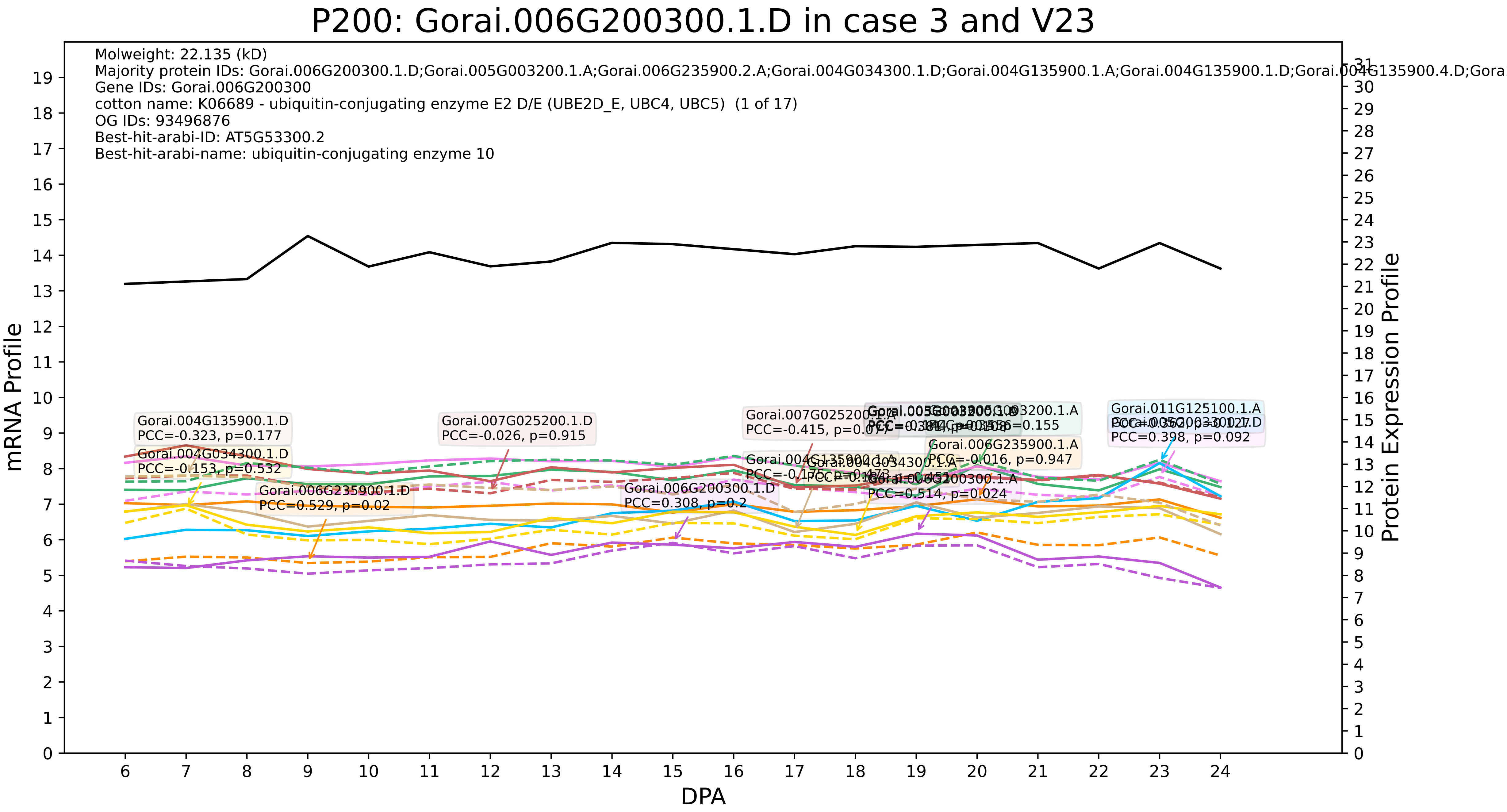
P200: Gorai.006G200300.1.D in case 3 and V23
Molweight: 22.135 (kD)
Majority protein IDs: Gorai.006G200300.1.D;Gorai.005G003200.1.A;Gorai.006G235900.2.A;Gorai.004G034300.1.D;Gorai.004G135900.1.A;Gorai.004G135900.1.D;Gorai.004G135900.4.D;Gorai
Gene IDs: Gorai.006G200300
cotton name: K06689 - ubiquitin-conjugating enzyme E2 D/E (UBE2D_E, UBC4, UBC5) (1 of 17)
OG IDs: 93496876
Best-hit-arabi-ID: AT5G53300.2
Best-hit-arabi-name: ubiquitin-conjugating enzyme 10
Gorai.004G135900.1.D
PCC=-0.323, p=0.177
Gorai.004G034300.1.D
PCC=-0.153, p=0.532
Gorai.006G235900.1.D
PCC=0.529, p=0.02
Gorai.007G025200.1.D
PCC=-0.026, p=0.915
Gorai.006G200300.1.D
PCC=0.308, p=0.2
PCC=-0.415, p=0.077
Gorai.006G235900.1.A
PCC=-0.016, p=0.947
PCC=0.514, p=0.024
Gorai.011G125100.1.A
PCC=0.398, p=0.092
mRNA Profile
Protein Expression Profile
DPA

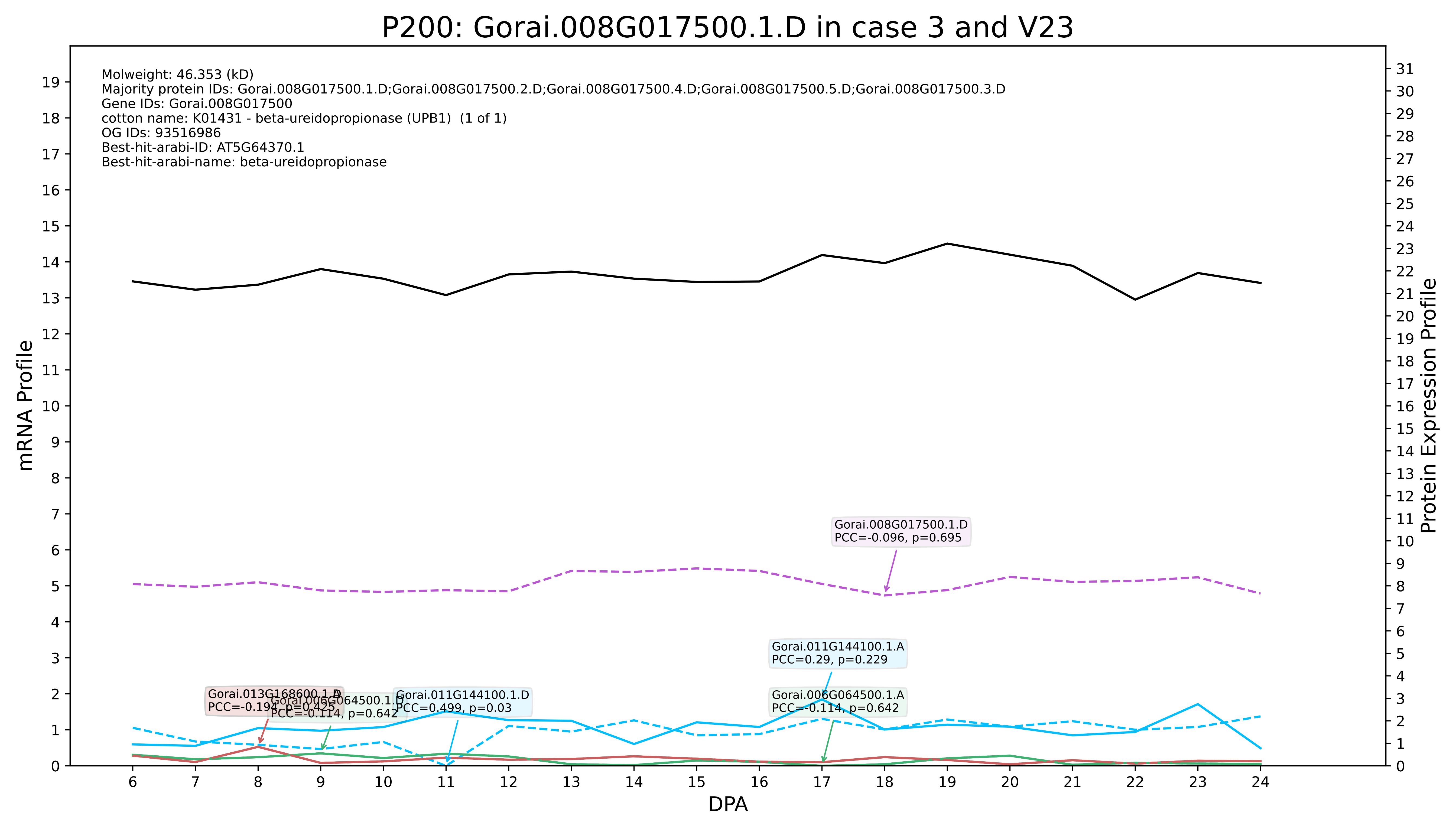
P200: Gorai.008G017500.1.D in case 3 and V23
Molweight: 46.353 (kD)
Majority protein IDs: Gorai.008G017500.1.D;Gorai.008G017500.2.D;Gorai.008G017500.4.D;Gorai.008G017500.5.D;Gorai.008G017500.3.D
Gene IDs: Gorai.008G017500
cotton name: K01431 - beta-ureidopropionase (UPB1) (1 of 1)
OG IDs: 93516986
Best-hit-arabi-ID: AT5G64370.1
Best-hit-arabi-name: beta-ureidopropionase
Gorai.008G017500.1.D
PCC=-0.096, p=0.695
Gorai.011G144100.1.A
PCC=0.29, p=0.229
Gorai.013G168600.1.D
PCC=-0.194, p=0.425
Gorai.006G064500.1.D
PCC=-0.114, p=0.642
Gorai.011G144100.1.D
PCC=0.499, p=0.03
Gorai.006G064500.1.A
PCC=-0.114, p=0.642
mRNA Profile
Protein Expression Profile
DPA
0
1
2
3
4
5
6
7
8
9
10
11
12
13
14
15
16
17
18
19
20
21
22
23
24
25
26
27
28
29
30
31

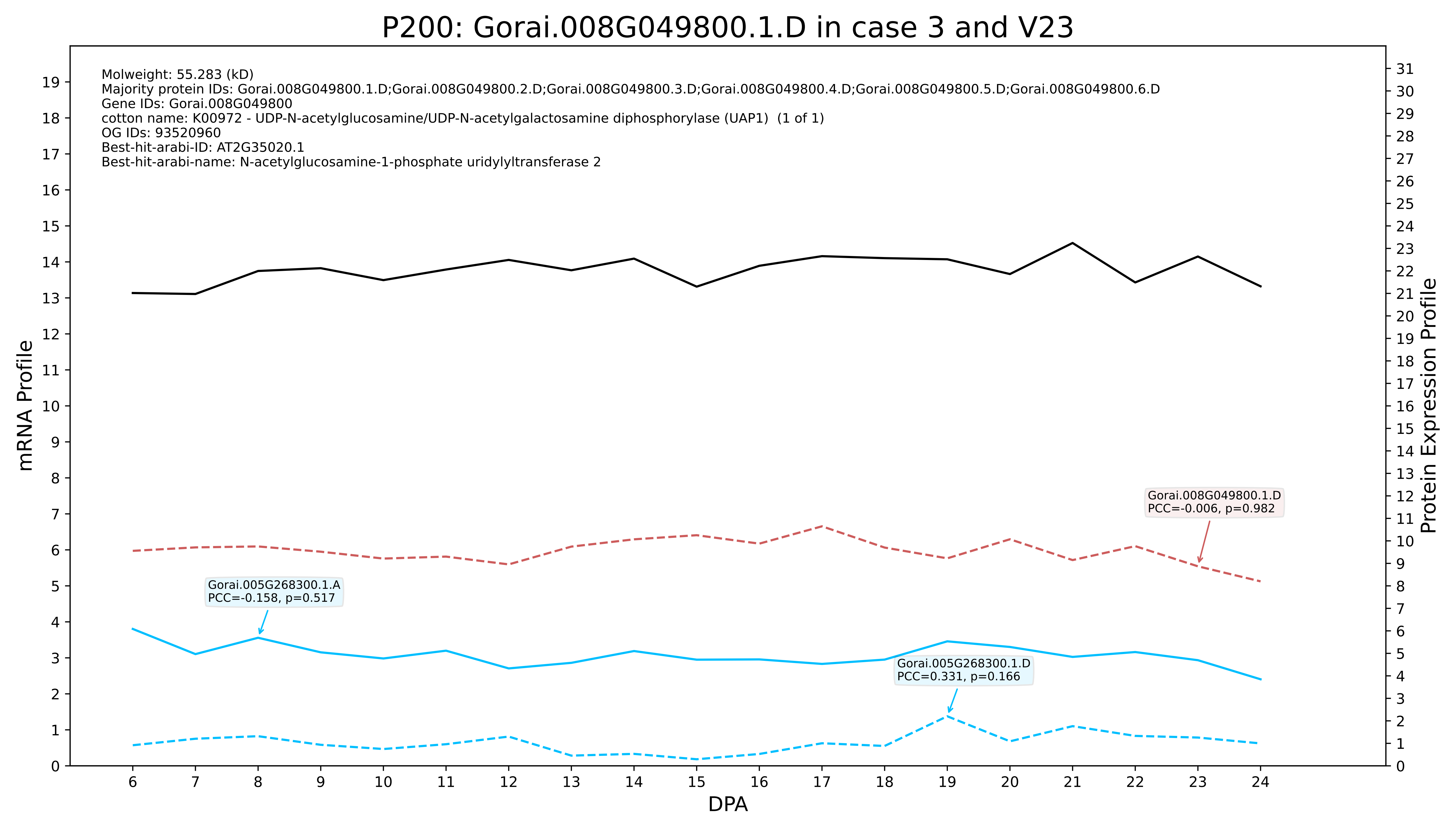

P200: Gorai.008G049800.1.D in case 3 and V23
Molweight: 55.283 (kD)
Majority protein IDs: Gorai.008G049800.1.D;Gorai.008G049800.2.D;Gorai.008G049800.3.D;Gorai.008G049800.4.D;Gorai.008G049800.5.D;Gorai.008G049800.6.D
Gene IDs: Gorai.008G049800
cotton name: K00972 - UDP-N-acetylglucosamine/UDP-N-acetylgalactosamine diphosphorylase (UAP1) (1 of 1)
OG IDs: 93520960
Best-hit-arabi-ID: AT2G35020.1
Best-hit-arabi-name: N-acetylglucosamine-1-phosphate uridylyltransferase 2
Gorai.008G049800.1.D
PCC=-0.006, p=0.982
Gorai.005G268300.1.A
PCC=-0.158, p=0.517
Gorai.005G268300.1.D
PCC=0.331, p=0.166
mRNA Profile
Protein Expression Profile
DPA

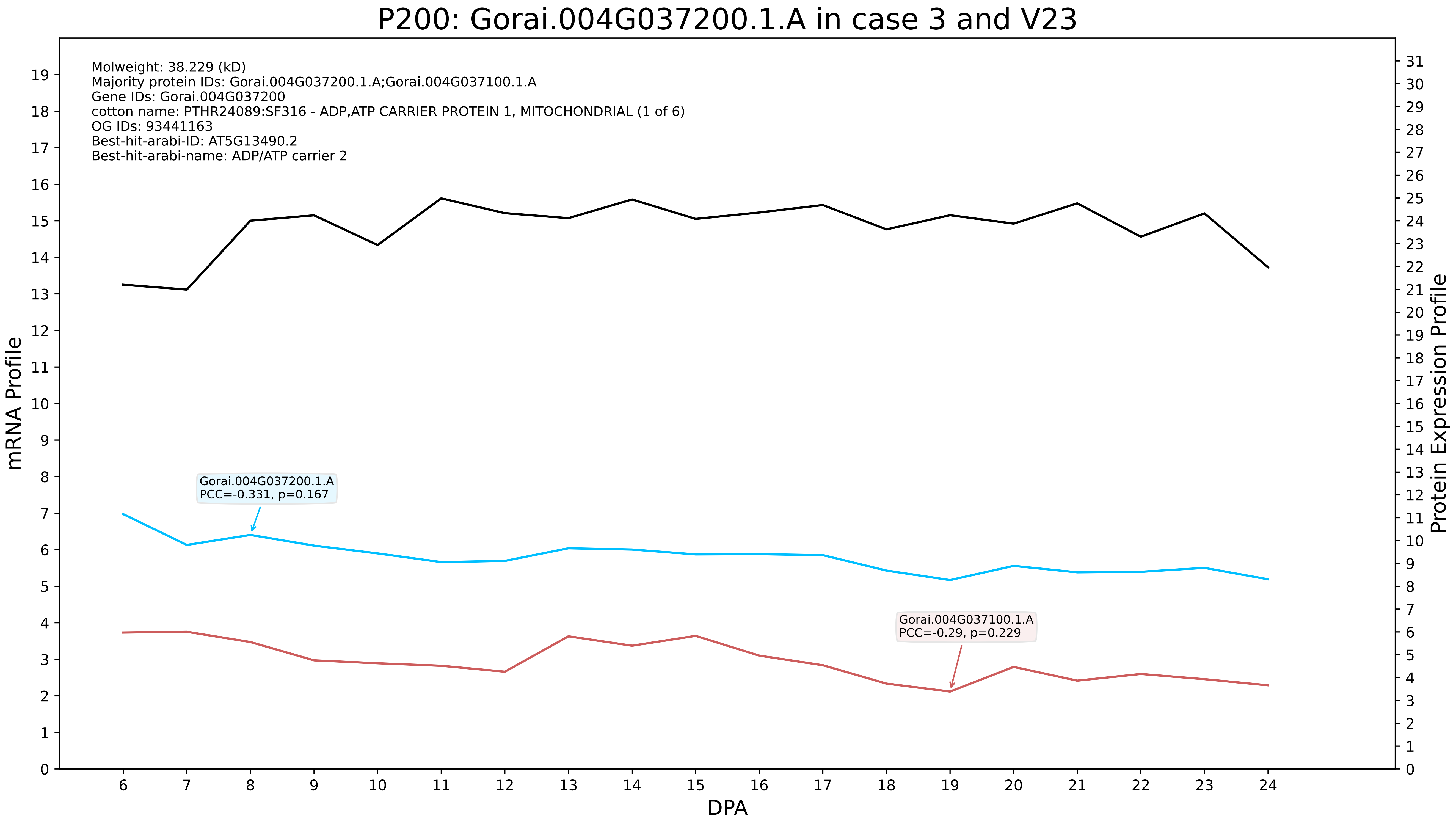
P200: Gorai.004G037200.1.A in case 3 and V23
Molweight: 38.229 (kD)
Majority protein IDs: Gorai.004G037200.1.A;Gorai.004G037100.1.A
Gene IDs: Gorai.004G037200
cotton name: PTHR24089:SF316 - ADP,ATP CARRIER PROTEIN 1, MITOCHONDRIAL (1 of 6)
OG IDs: 93441163
Best-hit-arabi-ID: AT5G13490.2
Best-hit-arabi-name: ADP/ATP carrier 2
Gorai.004G037200.1.A
PCC=-0.331, p=0.167
Gorai.004G037100.1.A
PCC=-0.29, p=0.229
mRNA Profile
Protein Expression Profile
DPA

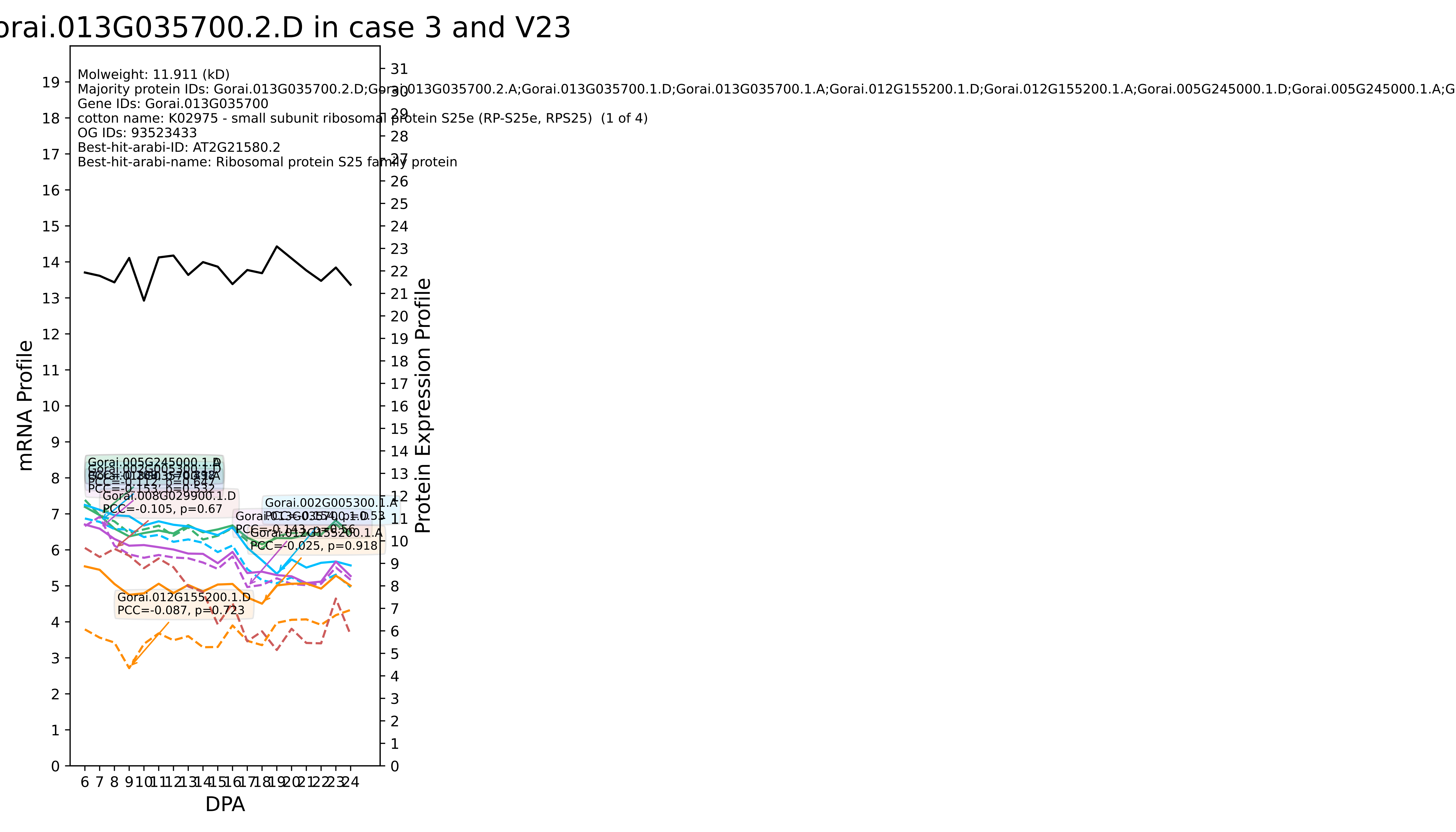
orai.013G035700.2.D in case 3 and V23
Molweight: 11.911 (kD)
Majority protein IDs: Gorai.013G035700.2.D;Gorai.013G035700.2.A;Gorai.013G035700.1.D;Gorai.013G035700.1.A;Gorai.012G155200.1.D;Gorai.012G155200.1.A;Gorai.005G245000.1.D;Gorai.005G245000.1.A;G
Gene IDs: Gorai.013G035700
cotton name: K02975 - small subunit ribosomal protein S25e (RP-S25e, RPS25) (1 of 4)
OG IDs: 93523433
Best-hit-arabi-ID: AT2G21580.2
Best-hit-arabi-name: Ribosomal protein S25 family protein
Gorai.005G245000.1.D
Gorai.002G005300.1.D
Gorai.008G029900.1.D
PCC=-0.105, p=0.67
Gorai.002G005300.1.A
Gorai.012G155200.1.A
PCC=-0.025, p=0.918
Gorai.012G155200.1.D
PCC=-0.087, p=0.723
mRNA Profile
Protein Expression Profile
DPA

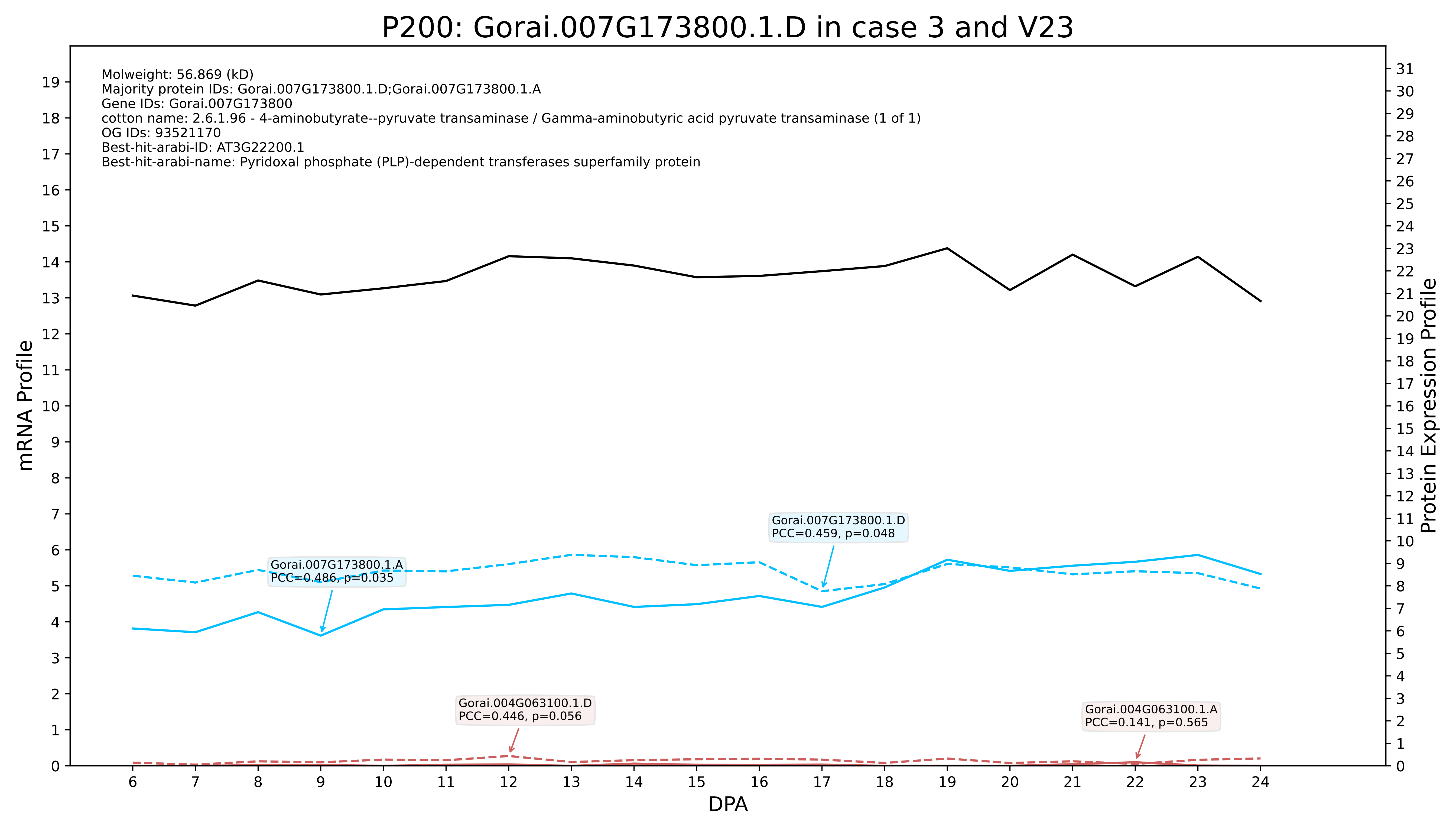

P200: Gorai.007G173800.1.D in case 3 and V23
Molweight: 56.869 (kD)
Majority protein IDs: Gorai.007G173800.1.D;Gorai.007G173800.1.A
Gene IDs: Gorai.007G173800
cotton name: 2.6.1.96 - 4-aminobutyrate--pyruvate transaminase / Gamma-aminobutyric acid pyruvate transaminase (1 of 1)
OG IDs: 93521170
Best-hit-arabi-ID: AT3G22200.1
Best-hit-arabi-name: Pyridoxal phosphate (PLP)-dependent transferases superfamily protein
Gorai.007G173800.1.D
PCC=0.459, p=0.048
Gorai.007G173800.1.A
PCC=0.486, p=0.035
Gorai.004G063100.1.D
PCC=0.446, p=0.056
Gorai.004G063100.1.A
PCC=0.141, p=0.565
mRNA Profile
Protein Expression Profile
DPA

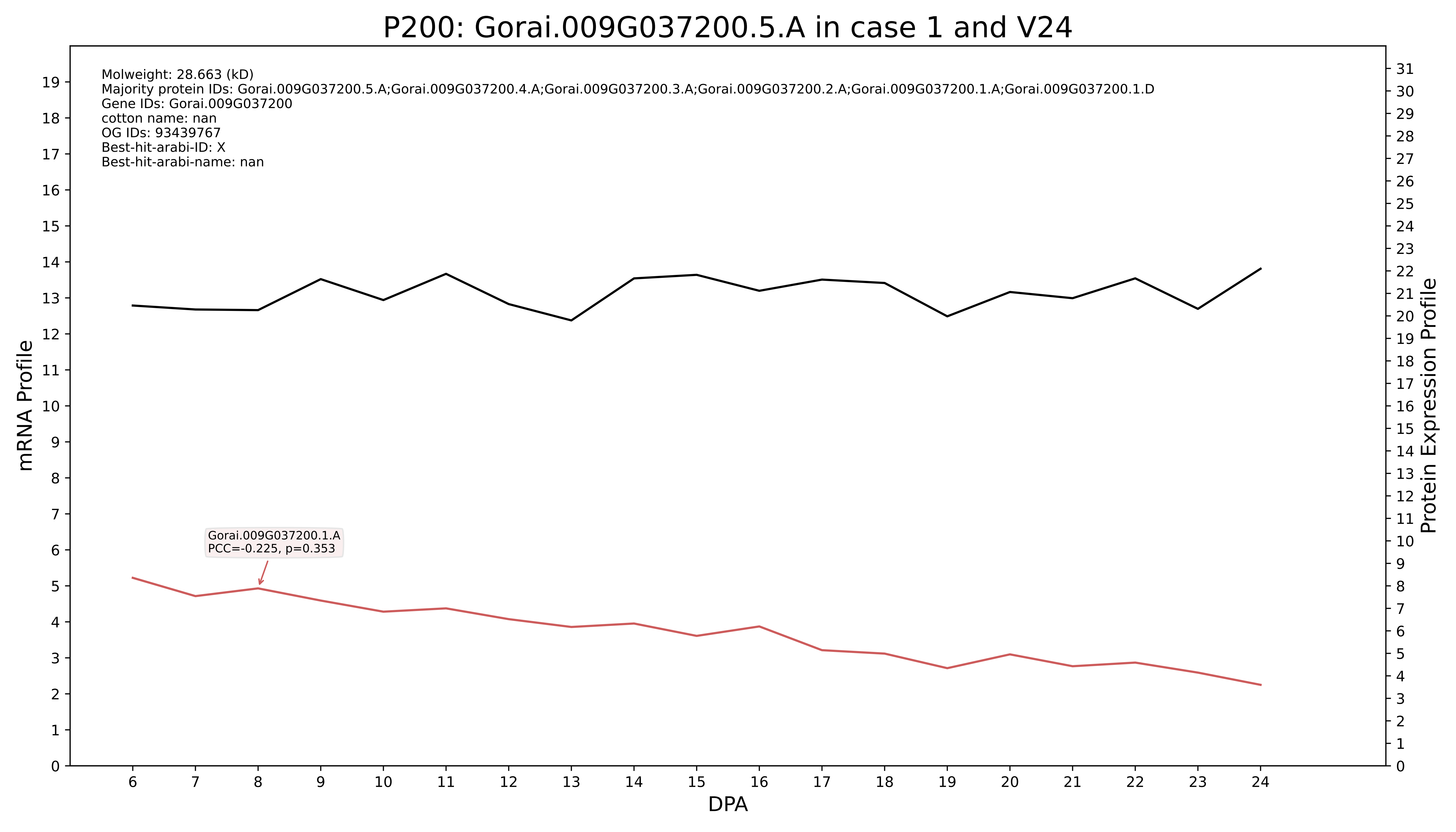

P200: Gorai.009G037200.5.A in case 1 and V24
Molweight: 28.663 (kD)
Majority protein IDs: Gorai.009G037200.5.A;Gorai.009G037200.4.A;Gorai.009G037200.3.A;Gorai.009G037200.2.A;Gorai.009G037200.1.A;Gorai.009G037200.1.D
Gene IDs: Gorai.009G037200
cotton name: nan
OG IDs: 93439767
Best-hit-arabi-ID: X
Best-hit-arabi-name: nan
Gorai.009G037200.1.A
PCC=-0.225, p=0.353
mRNA Profile
Protein Expression Profile
DPA

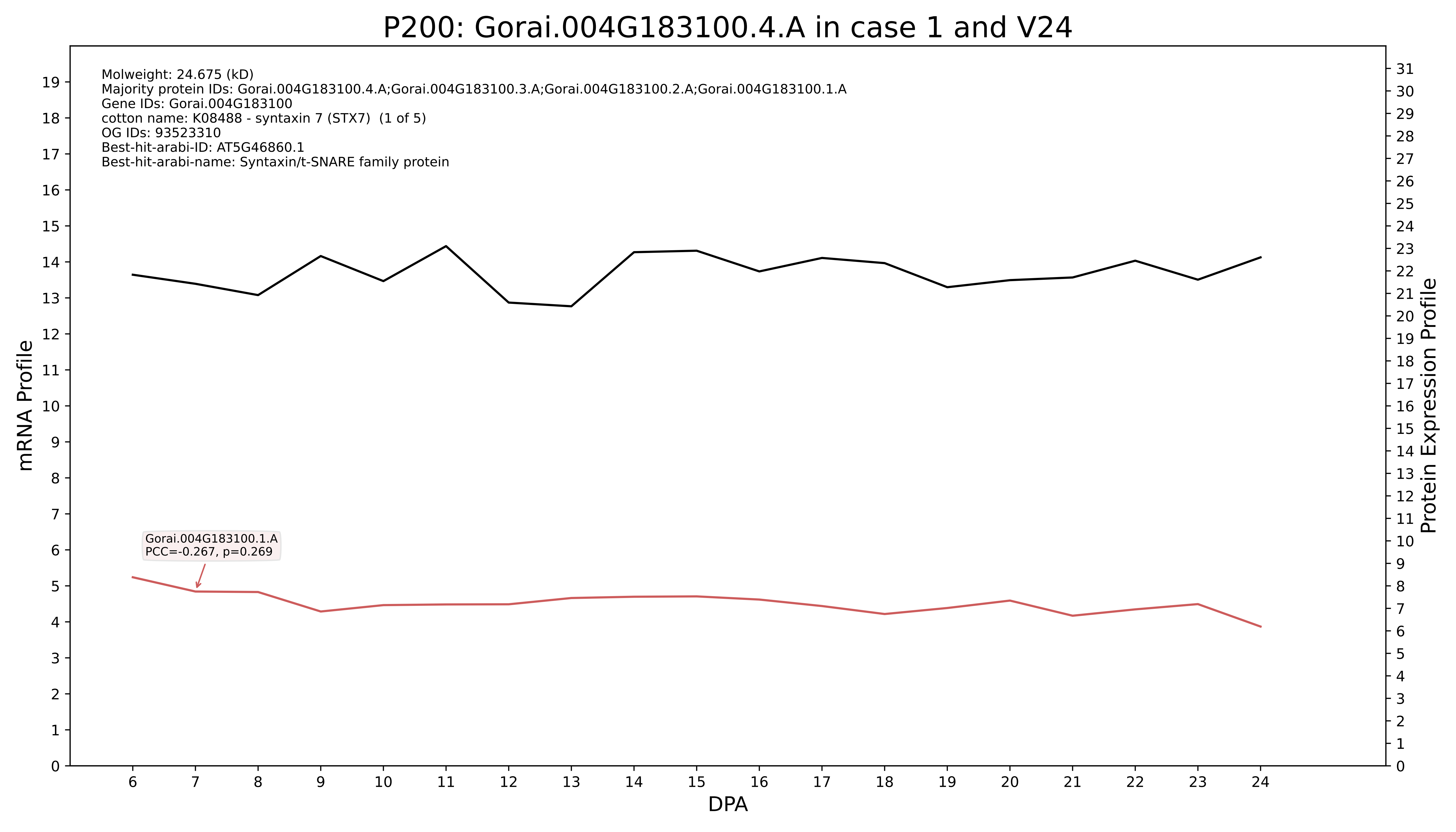

P200: Gorai.004G183100.4.A in case 1 and V24
Molweight: 24.675 (kD)
Majority protein IDs: Gorai.004G183100.4.A;Gorai.004G183100.3.A;Gorai.004G183100.2.A;Gorai.004G183100.1.A
Gene IDs: Gorai.004G183100
cotton name: K08488 - syntaxin 7 (STX7) (1 of 5)
OG IDs: 93523310
Best-hit-arabi-ID: AT5G46860.1
Best-hit-arabi-name: Syntaxin/t-SNARE family protein
Gorai.004G183100.1.A
PCC=-0.267, p=0.269
mRNA Profile
Protein Expression Profile
DPA

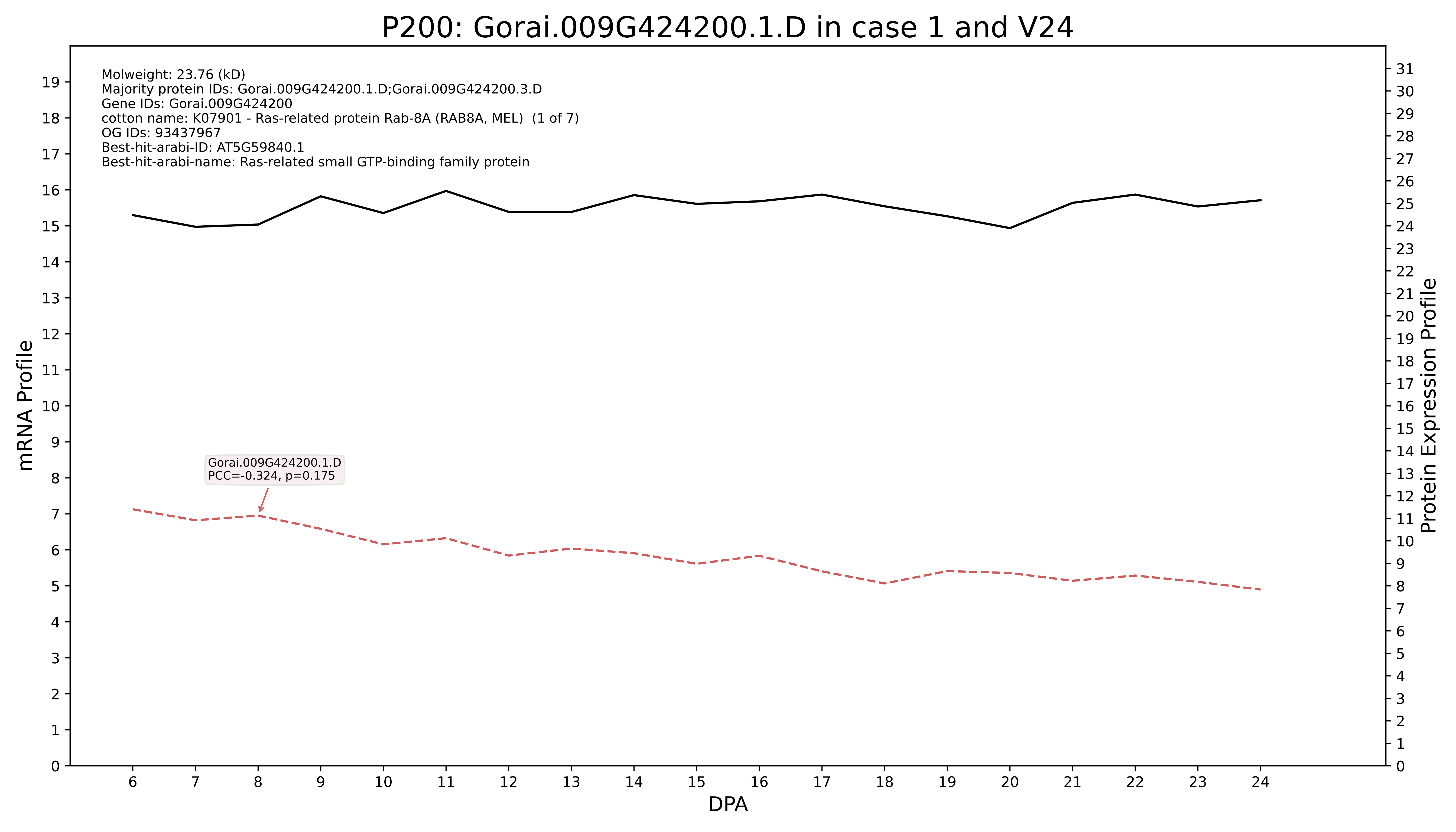
P200: Gorai.009G424200.1.D in case 1 and V24
Molweight: 23.76 (kD)
Majority protein IDs: Gorai.009G424200.1.D;Gorai.009G424200.3.D
Gene IDs: Gorai.009G424200
cotton name: K07901 - Ras-related protein Rab-8A (RAB8A, MEL) (1 of 7)
OG IDs: 93437967
Best-hit-arabi-ID: AT5G59840.1
Best-hit-arabi-name: Ras-related small GTP-binding family protein
Gorai.009G424200.1.D
PCC=-0.324, p=0.175
mRNA Profile
Protein Expression Profile
DPA
0
1
2
3
4
5
6
7
8
9
10
11
12
13
14
15
16
17
18
19
20
21
22
23
24
25
26
27
28
29
30
31

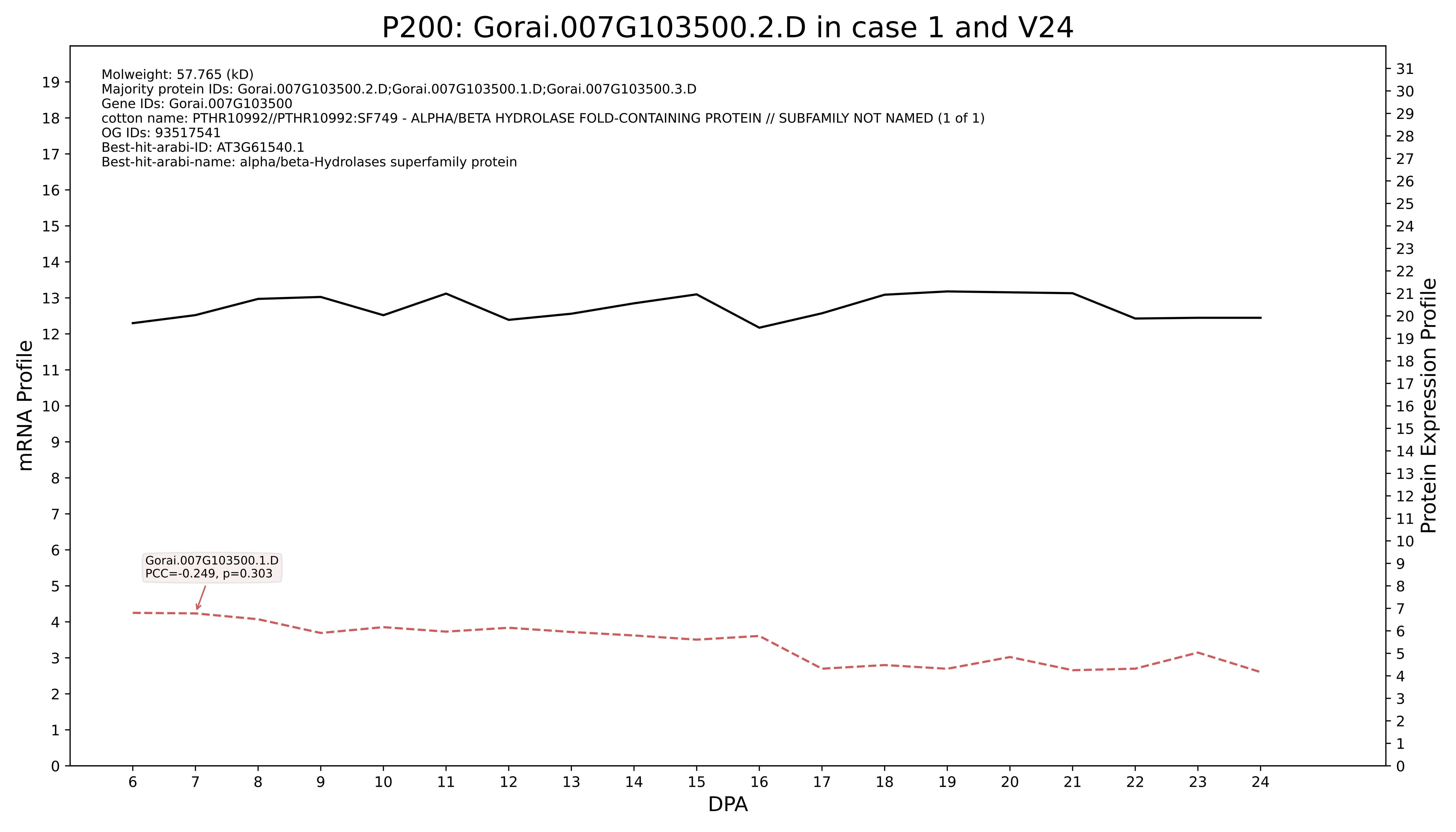
P200: Gorai.007G103500.2.D in case 1 and V24
Molweight: 57.765 (kD)
Majority protein IDs: Gorai.007G103500.2.D;Gorai.007G103500.1.D;Gorai.007G103500.3.D
Gene IDs: Gorai.007G103500
cotton name: PTHR10992//PTHR10992:SF749 - ALPHA/BETA HYDROLASE FOLD-CONTAINING PROTEIN // SUBFAMILY NOT NAMED (1 of 1)
OG IDs: 93517541
Best-hit-arabi-ID: AT3G61540.1
Best-hit-arabi-name: alpha/beta-Hydrolases superfamily protein
Gorai.007G103500.1.D
PCC=-0.249, p=0.303
mRNA Profile
Protein Expression Profile
DPA
0
1
2
3
4
5
6
7
8
9
10
11
12
13
14
15
16
17
18
19
20
21
22
23
24
25
26
27
28
29
30
31

P200: Gorai.009G254800.2.A in case 1 and V24

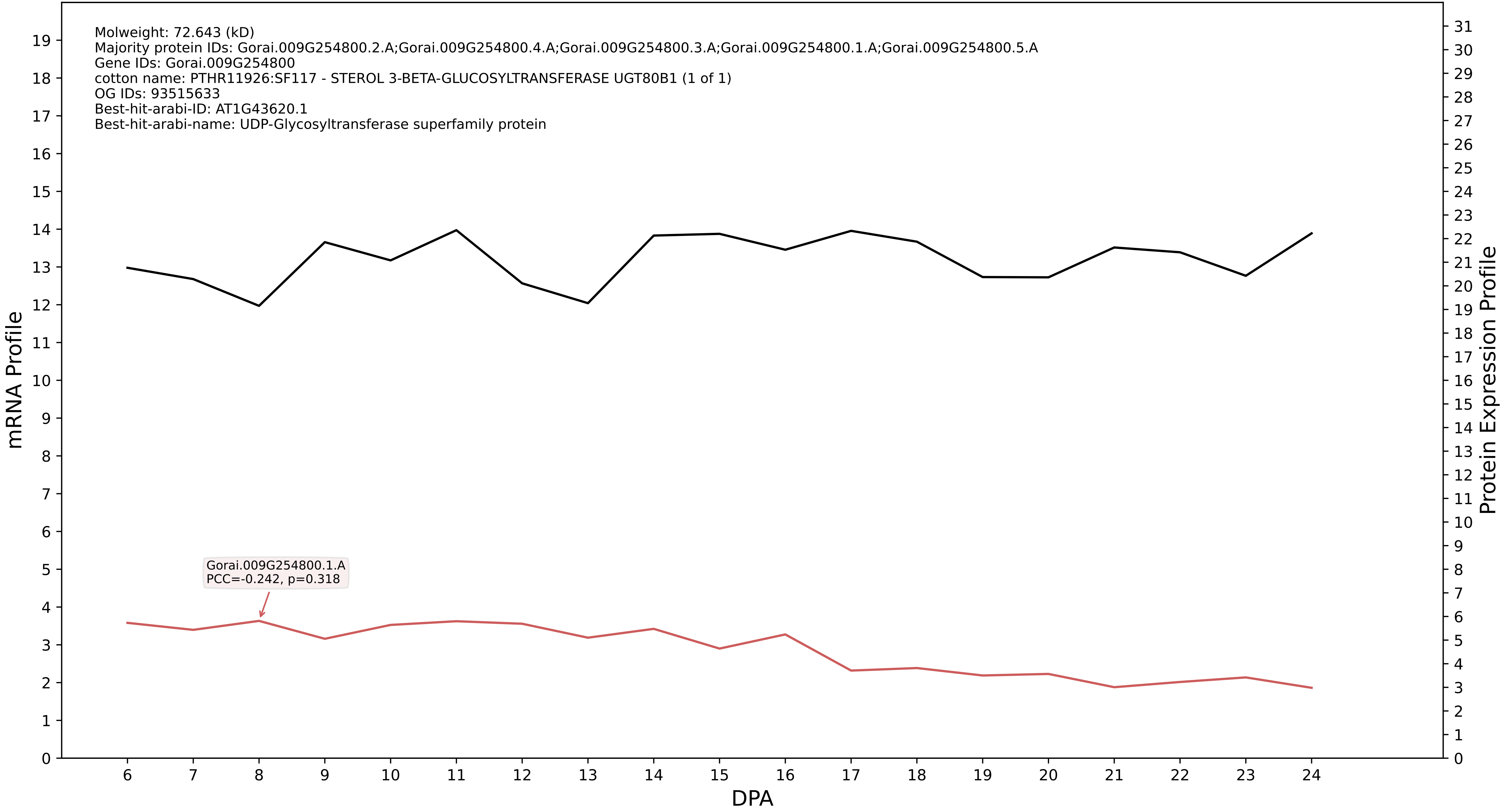

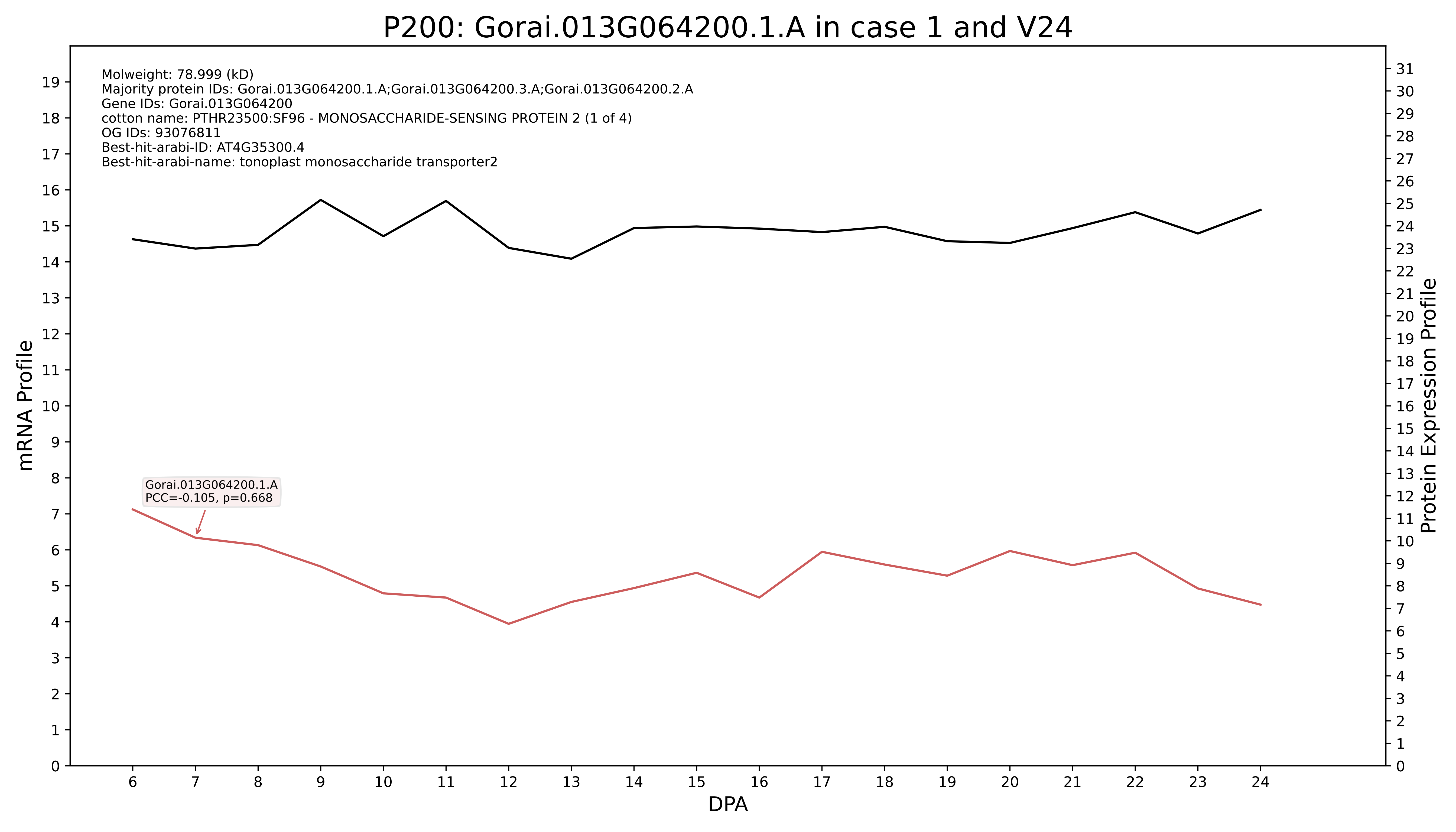
P200: Gorai.013G064200.1.A in case 1 and V24
Molweight: 78.999 (kD)
Majority protein IDs: Gorai.013G064200.1.A;Gorai.013G064200.3.A;Gorai.013G064200.2.A
Gene IDs: Gorai.013G064200
cotton name: PTHR23500:SF96 - MONOSACCHARIDE-SENSING PROTEIN 2 (1 of 4)
OG IDs: 93076811
Best-hit-arabi-ID: AT4G35300.4
Best-hit-arabi-name: tonoplast monosaccharide transporter2
Gorai.013G064200.1.A
PCC=-0.105, p=0.668
mRNA Profile
Protein Expression Profile
DPA
0
1
2
3
4
5
6
7
8
9
10
11
12
13
14
15
16
17
18
19
20
21
22
23
24
25
26
27
28
29
30
31

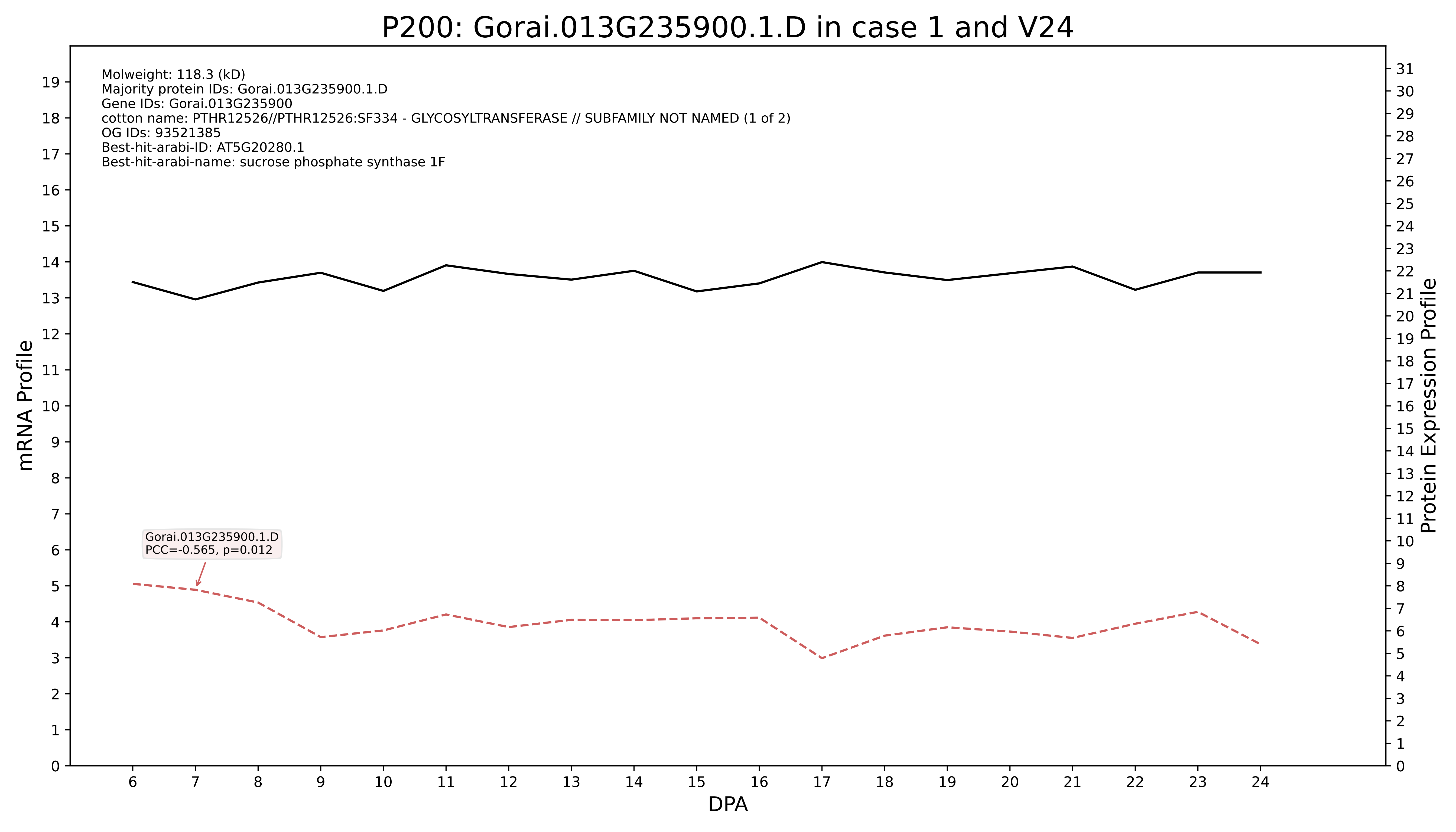
P200: Gorai.013G235900.1.D in case 1 and V24
Molweight: 118.3 (kD)
Majority protein IDs: Gorai.013G235900.1.D
Gene IDs: Gorai.013G235900
cotton name: PTHR12526//PTHR12526:SF334 - GLYCOSYLTRANSFERASE // SUBFAMILY NOT NAMED (1 of 2)
OG IDs: 93521385
Best-hit-arabi-ID: AT5G20280.1
Best-hit-arabi-name: sucrose phosphate synthase 1F
Gorai.013G235900.1.D
PCC=-0.565, p=0.012
mRNA Profile
Protein Expression Profile
DPA

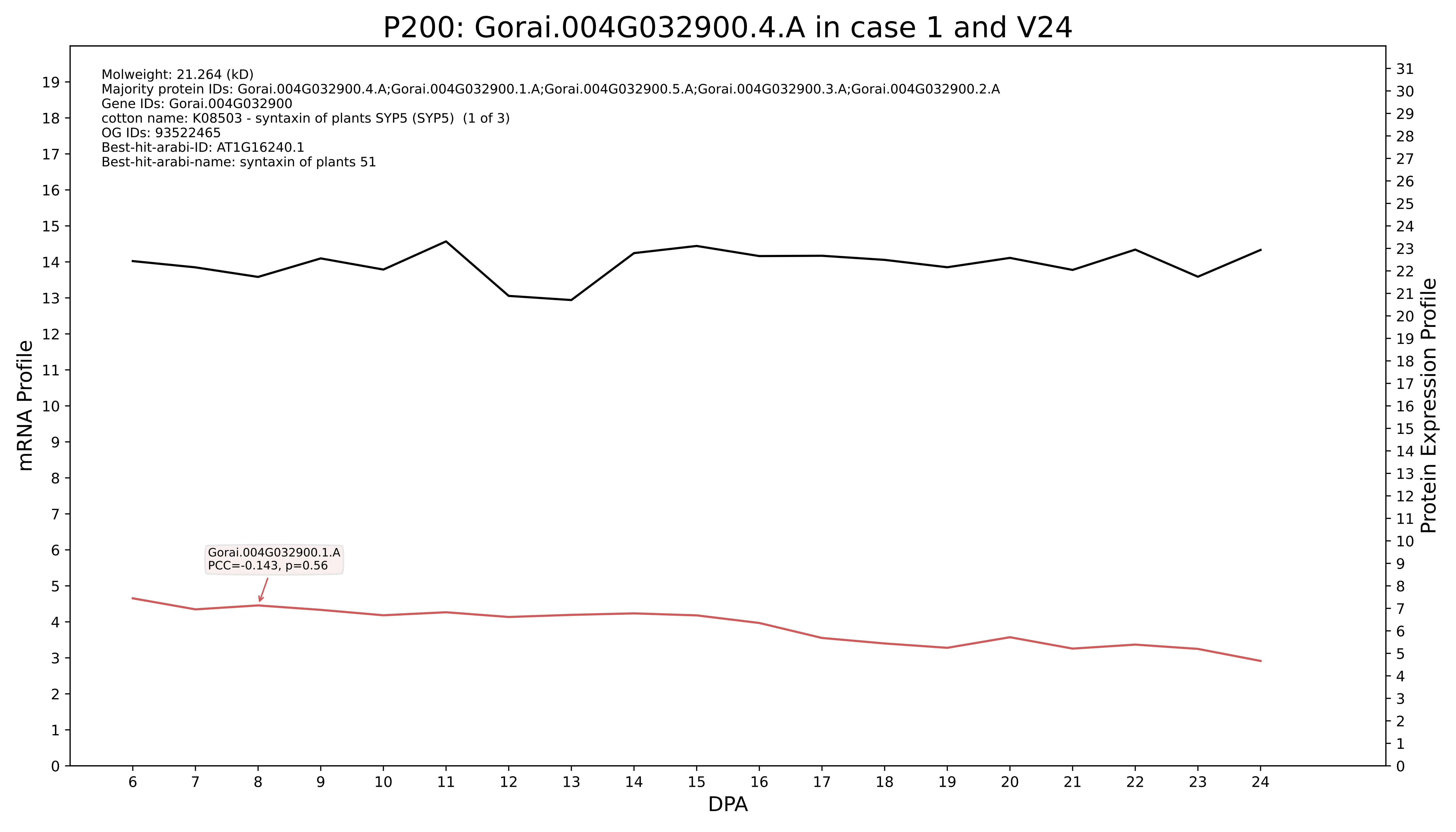
P200: Gorai.004G032900.4.A in case 1 and V24
Molweight: 21.264 (kD)
Majority protein IDs: Gorai.004G032900.4.A;Gorai.004G032900.1.A;Gorai.004G032900.5.A;Gorai.004G032900.3.A;Gorai.004G032900.2.A
Gene IDs: Gorai.004G032900
cotton name: K08503 - syntaxin of plants SYP5 (SYP5) (1 of 3)
OG IDs: 93522465
Best-hit-arabi-ID: AT1G16240.1
Best-hit-arabi-name: syntaxin of plants 51
Gorai.004G032900.1.A
PCC=-0.143, p=0.56
mRNA Profile
Protein Expression Profile
DPA

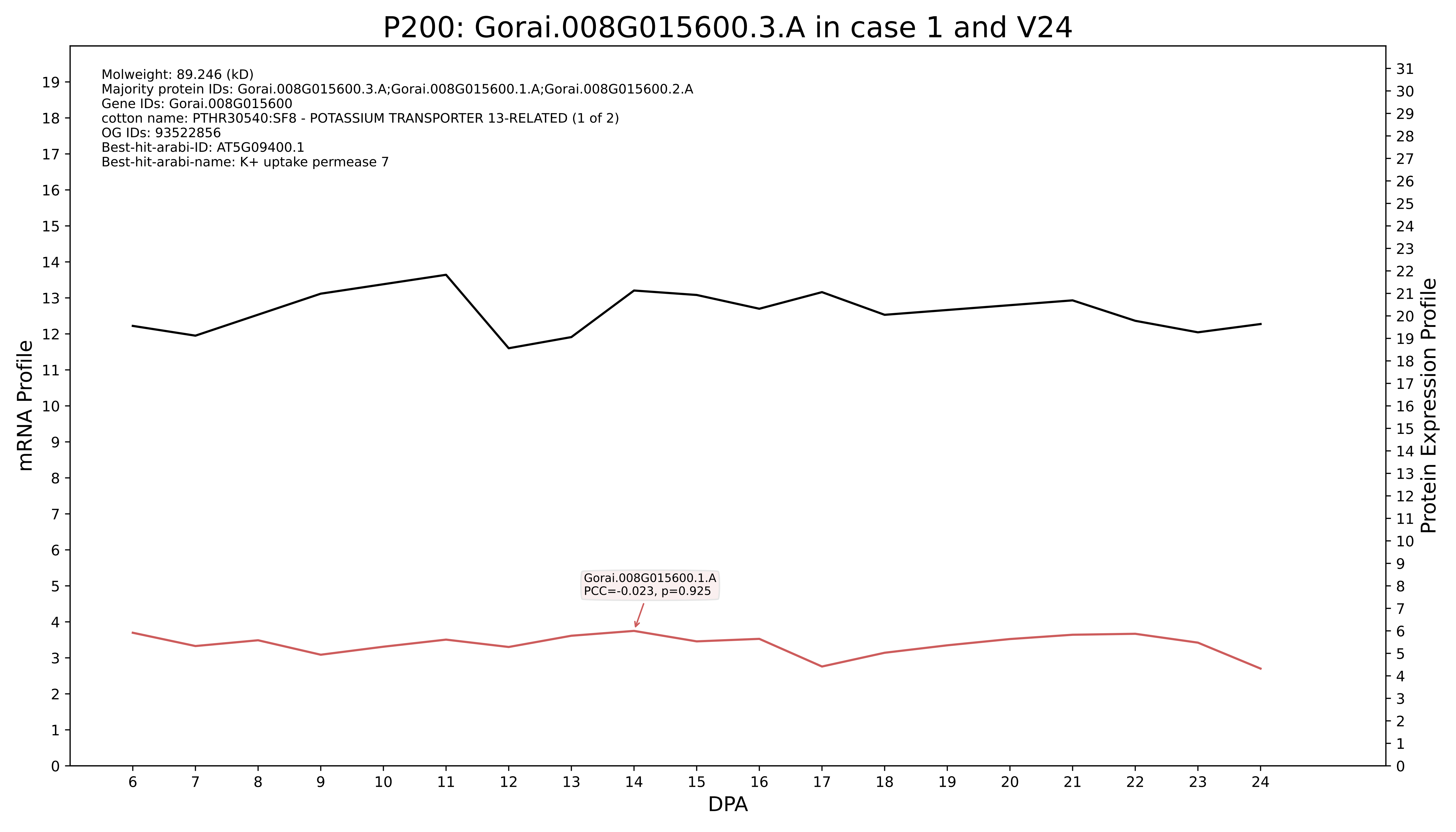
P200: Gorai.008G015600.3.A in case 1 and V24
Molweight: 89.246 (kD)
Majority protein IDs: Gorai.008G015600.3.A;Gorai.008G015600.1.A;Gorai.008G015600.2.A
Gene IDs: Gorai.008G015600
cotton name: PTHR30540:SF8 - POTASSIUM TRANSPORTER 13-RELATED (1 of 2)
OG IDs: 93522856
Best-hit-arabi-ID: AT5G09400.1
Best-hit-arabi-name: K+ uptake permease 7
Gorai.008G015600.1.A
PCC=-0.023, p=0.925
mRNA Profile
Protein Expression Profile
DPA

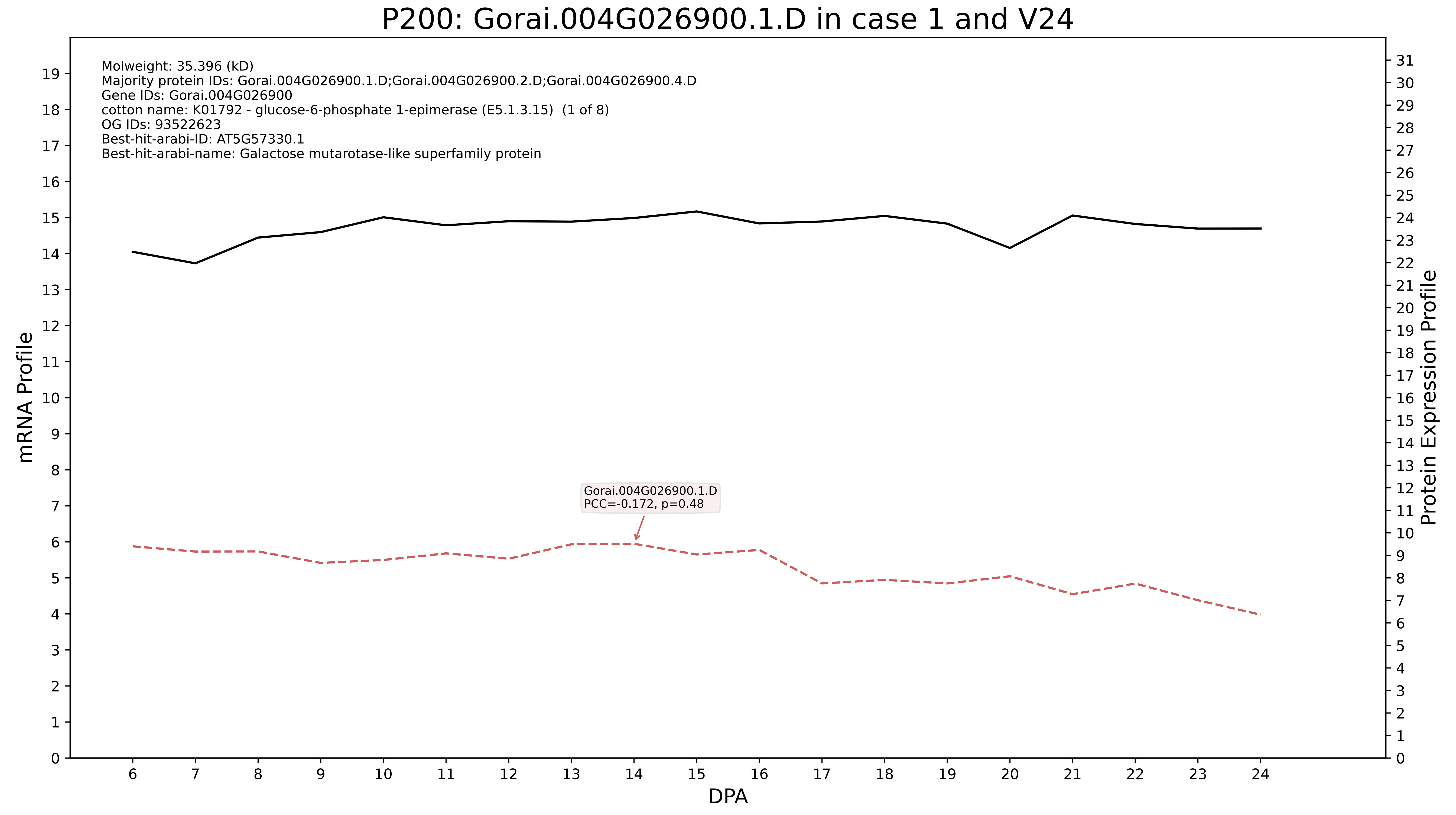
P200: Gorai.004G026900.1.D in case 1 and V24
Molweight: 35.396 (kD)
Majority protein IDs: Gorai.004G026900.1.D;Gorai.004G026900.2.D;Gorai.004G026900.4.D
Gene IDs: Gorai.004G026900
cotton name: K01792 - glucose-6-phosphate 1-epimerase (E5.1.3.15) (1 of 8)
OG IDs: 93522623
Best-hit-arabi-ID: AT5G57330.1
Best-hit-arabi-name: Galactose mutarotase-like superfamily protein
Gorai.004G026900.1.D
PCC=-0.172, p=0.48
mRNA Profile
Protein Expression Profile
DPA

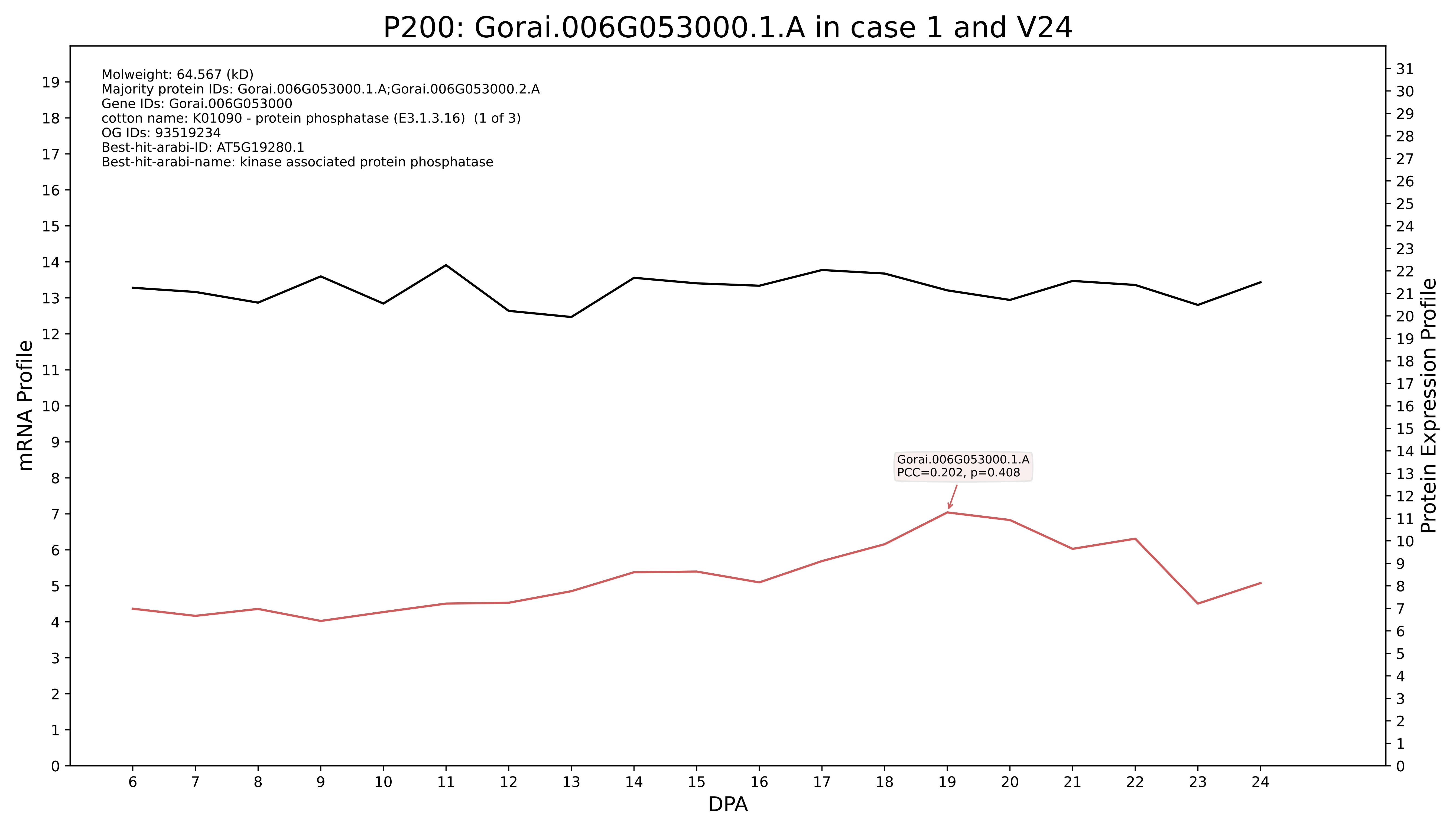
P200: Gorai.006G053000.1.A in case 1 and V24
Molweight: 64.567 (kD)
Majority protein IDs: Gorai.006G053000.1.A;Gorai.006G053000.2.A
Gene IDs: Gorai.006G053000
cotton name: K01090 - protein phosphatase (E3.1.3.16) (1 of 3)
OG IDs: 93519234
Best-hit-arabi-ID: AT5G19280.1
Best-hit-arabi-name: kinase associated protein phosphatase
Gorai.006G053000.1.A
PCC=0.202, p=0.408
mRNA Profile
Protein Expression Profile
DPA

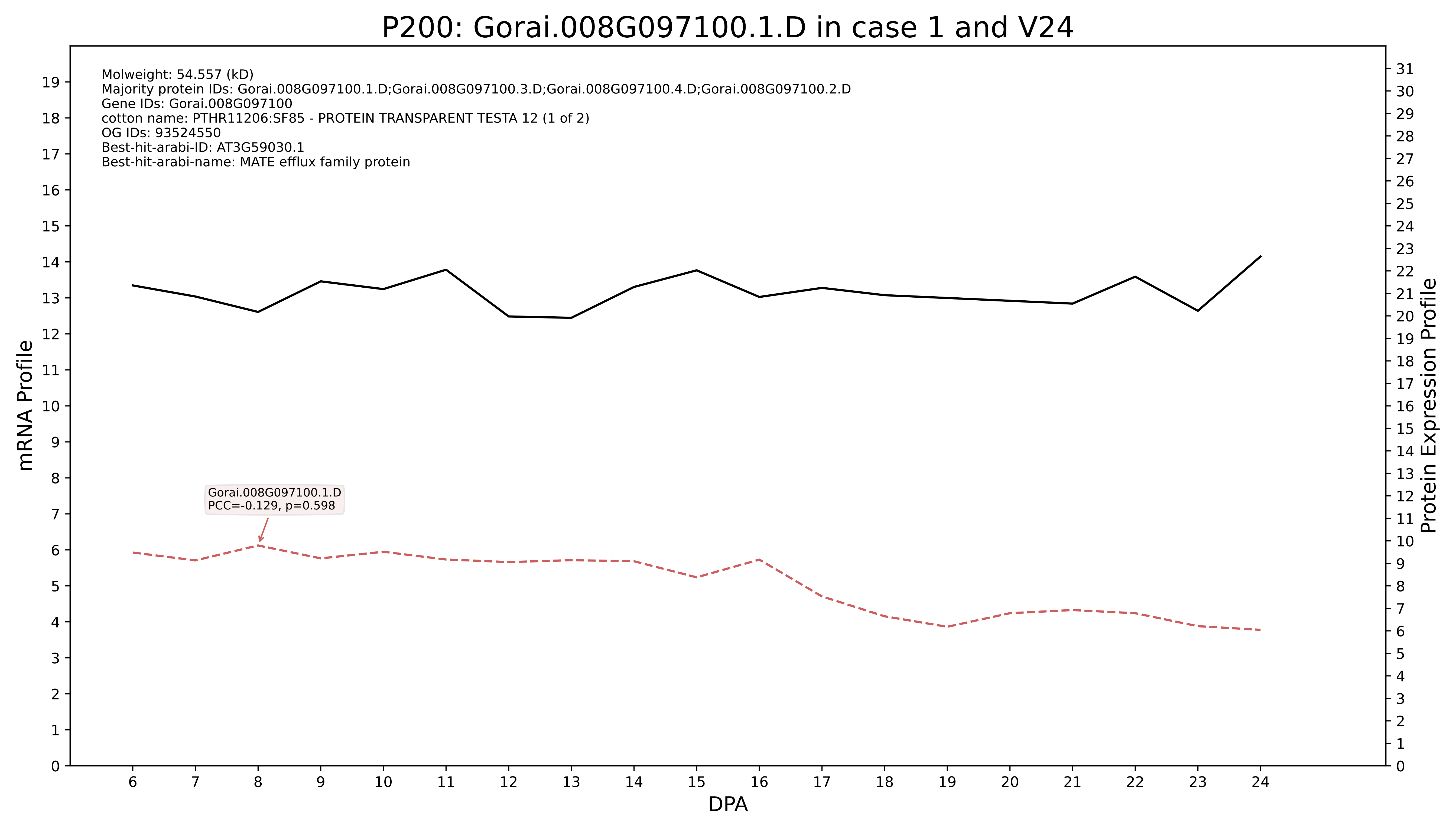

P200: Gorai.008G097100.1.D in case 1 and V24
Molweight: 54.557 (kD)
Majority protein IDs: Gorai.008G097100.1.D;Gorai.008G097100.3.D;Gorai.008G097100.4.D;Gorai.008G097100.2.D
Gene IDs: Gorai.008G097100
cotton name: PTHR11206:SF85 - PROTEIN TRANSPARENT TESTA 12 (1 of 2)
OG IDs: 93524550
Best-hit-arabi-ID: AT3G59030.1
Best-hit-arabi-name: MATE efflux family protein
Gorai.008G097100.1.D
PCC=-0.129, p=0.598
mRNA Profile
Protein Expression Profile
DPA

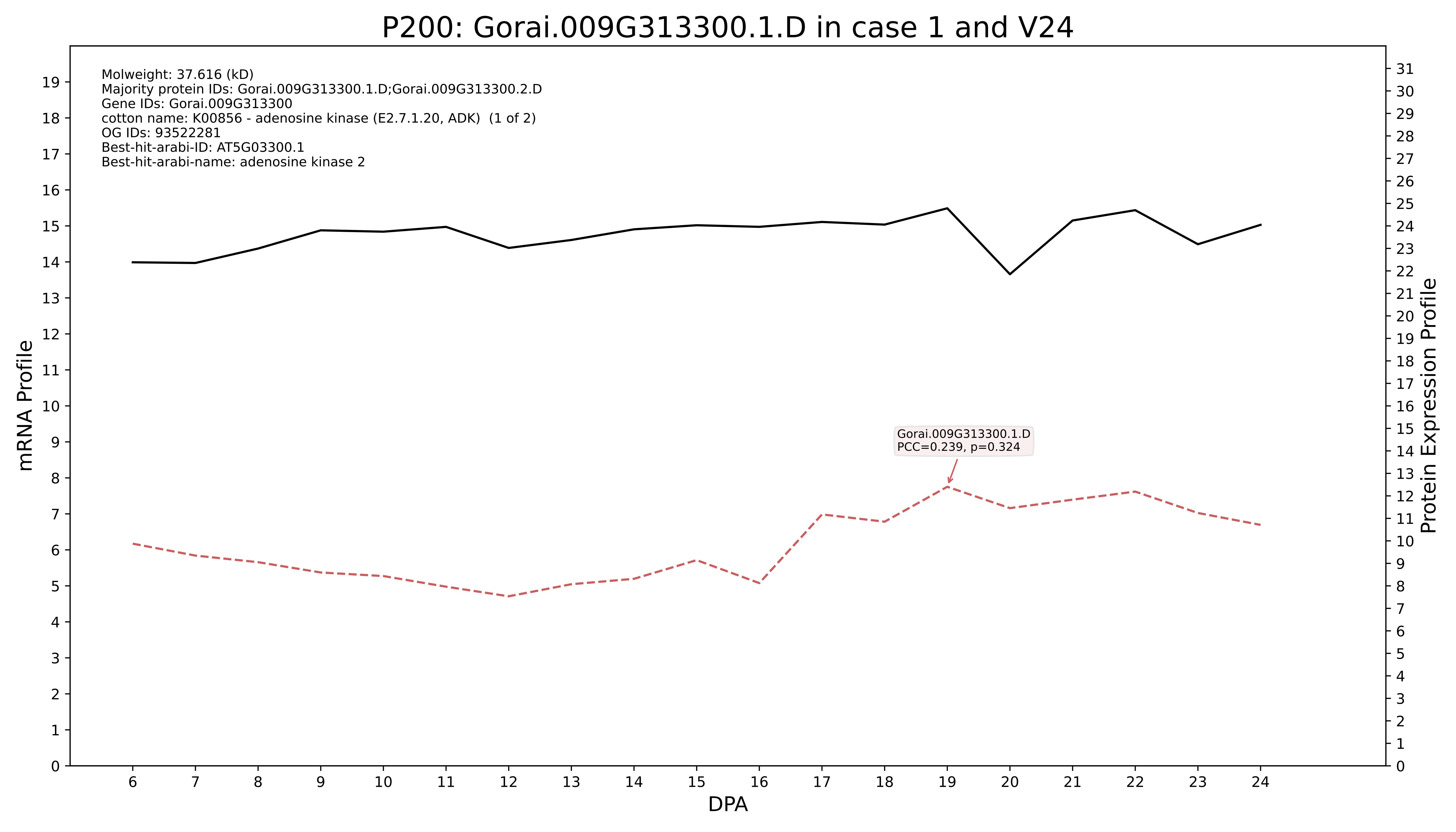
P200: Gorai.009G313300.1.D in case 1 and V24
Molweight: 37.616 (kD)
Majority protein IDs: Gorai.009G313300.1.D;Gorai.009G313300.2.D
Gene IDs: Gorai.009G313300
cotton name: K00856 - adenosine kinase (E2.7.1.20, ADK) (1 of 2)
OG IDs: 93522281
Best-hit-arabi-ID: AT5G03300.1
Best-hit-arabi-name: adenosine kinase 2
Gorai.009G313300.1.D
PCC=0.239, p=0.324
mRNA Profile
Protein Expression Profile
DPA

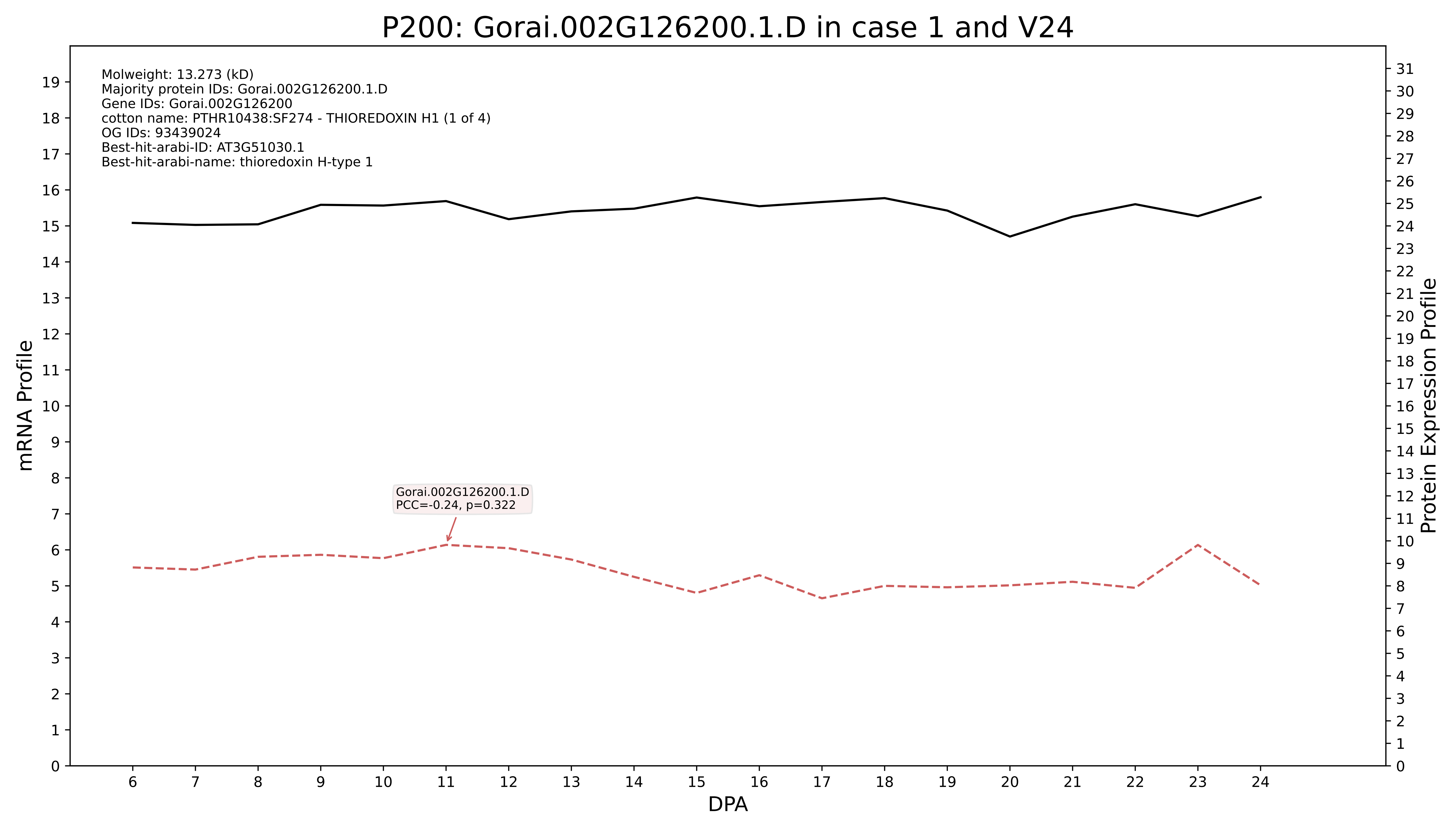

P200: Gorai.002G126200.1.D in case 1 and V24
Molweight: 13.273 (kD)
Majority protein IDs: Gorai.002G126200.1.D
Gene IDs: Gorai.002G126200
cotton name: PTHR10438:SF274 - THIOREDOXIN H1 (1 of 4)
OG IDs: 93439024
Best-hit-arabi-ID: AT3G51030.1
Best-hit-arabi-name: thioredoxin H-type 1
Gorai.002G126200.1.D
PCC=-0.24, p=0.322
mRNA Profile
Protein Expression Profile
DPA

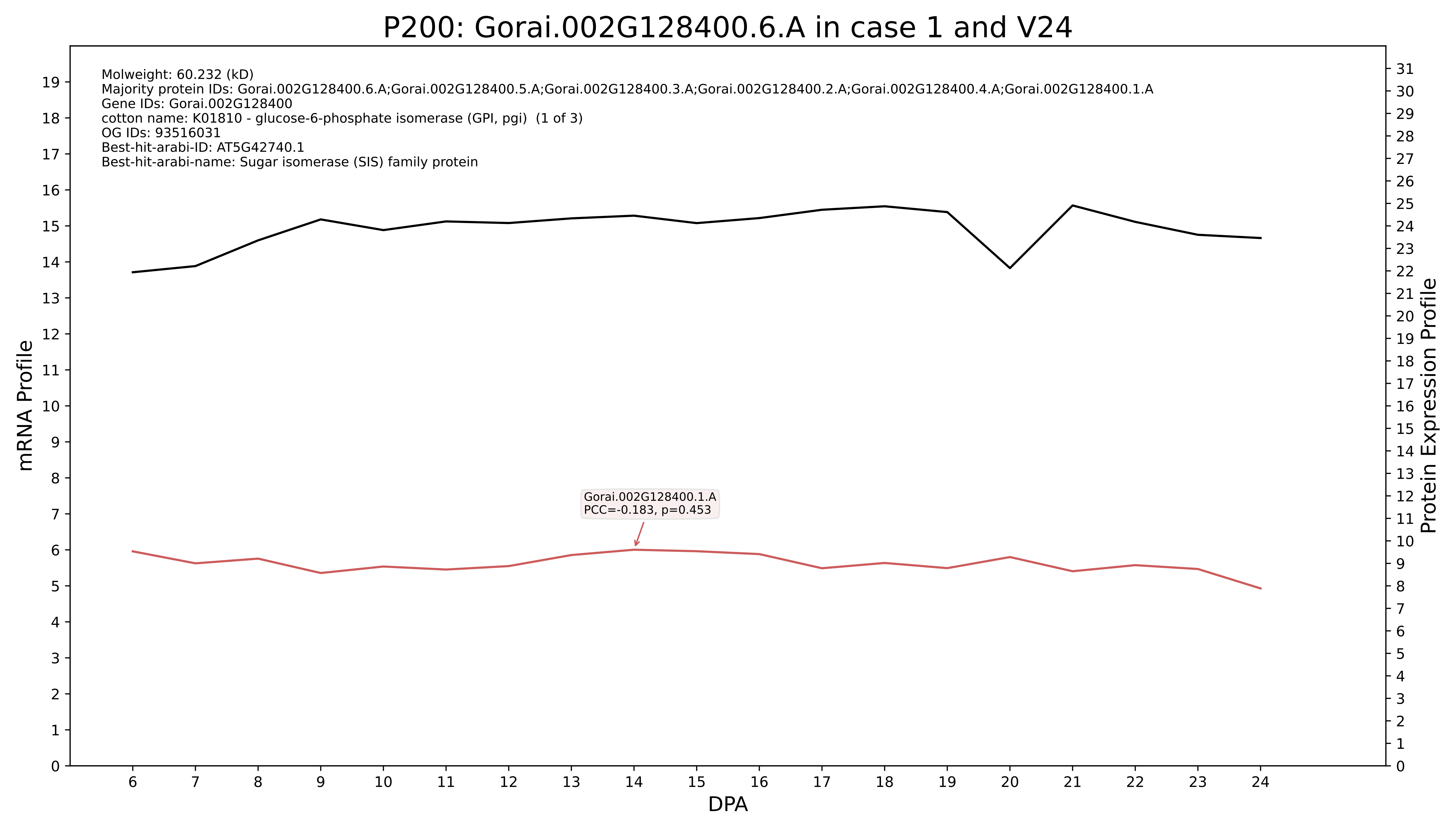
P200: Gorai.002G128400.6.A in case 1 and V24
Molweight: 60.232 (kD)
Majority protein IDs: Gorai.002G128400.6.A;Gorai.002G128400.5.A;Gorai.002G128400.3.A;Gorai.002G128400.2.A;Gorai.002G128400.4.A;Gorai.002G128400.1.A
Gene IDs: Gorai.002G128400
cotton name: K01810 - glucose-6-phosphate isomerase (GPI, pgi) (1 of 3)
OG IDs: 93516031
Best-hit-arabi-ID: AT5G42740.1
Best-hit-arabi-name: Sugar isomerase (SIS) family protein
Gorai.002G128400.1.A
PCC=-0.183, p=0.453
mRNA Profile
Protein Expression Profile
DPA

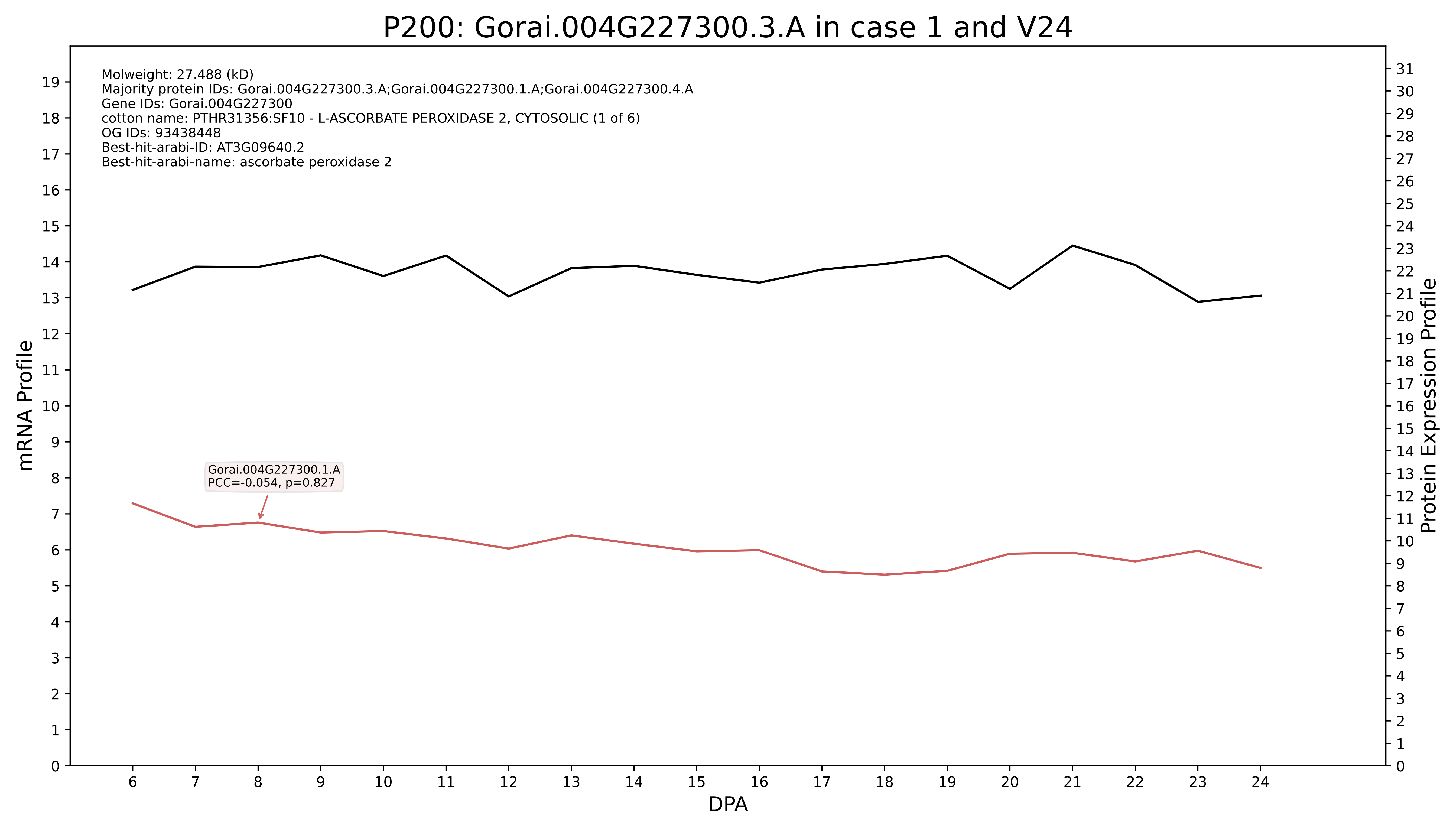
P200: Gorai.004G227300.3.A in case 1 and V24
Molweight: 27.488 (kD)
Majority protein IDs: Gorai.004G227300.3.A;Gorai.004G227300.1.A;Gorai.004G227300.4.A
Gene IDs: Gorai.004G227300
cotton name: PTHR31356:SF10 - L-ASCORBATE PEROXIDASE 2, CYTOSOLIC (1 of 6)
OG IDs: 93438448
Best-hit-arabi-ID: AT3G09640.2
Best-hit-arabi-name: ascorbate peroxidase 2
Gorai.004G227300.1.A
PCC=-0.054, p=0.827
mRNA Profile
Protein Expression Profile
DPA

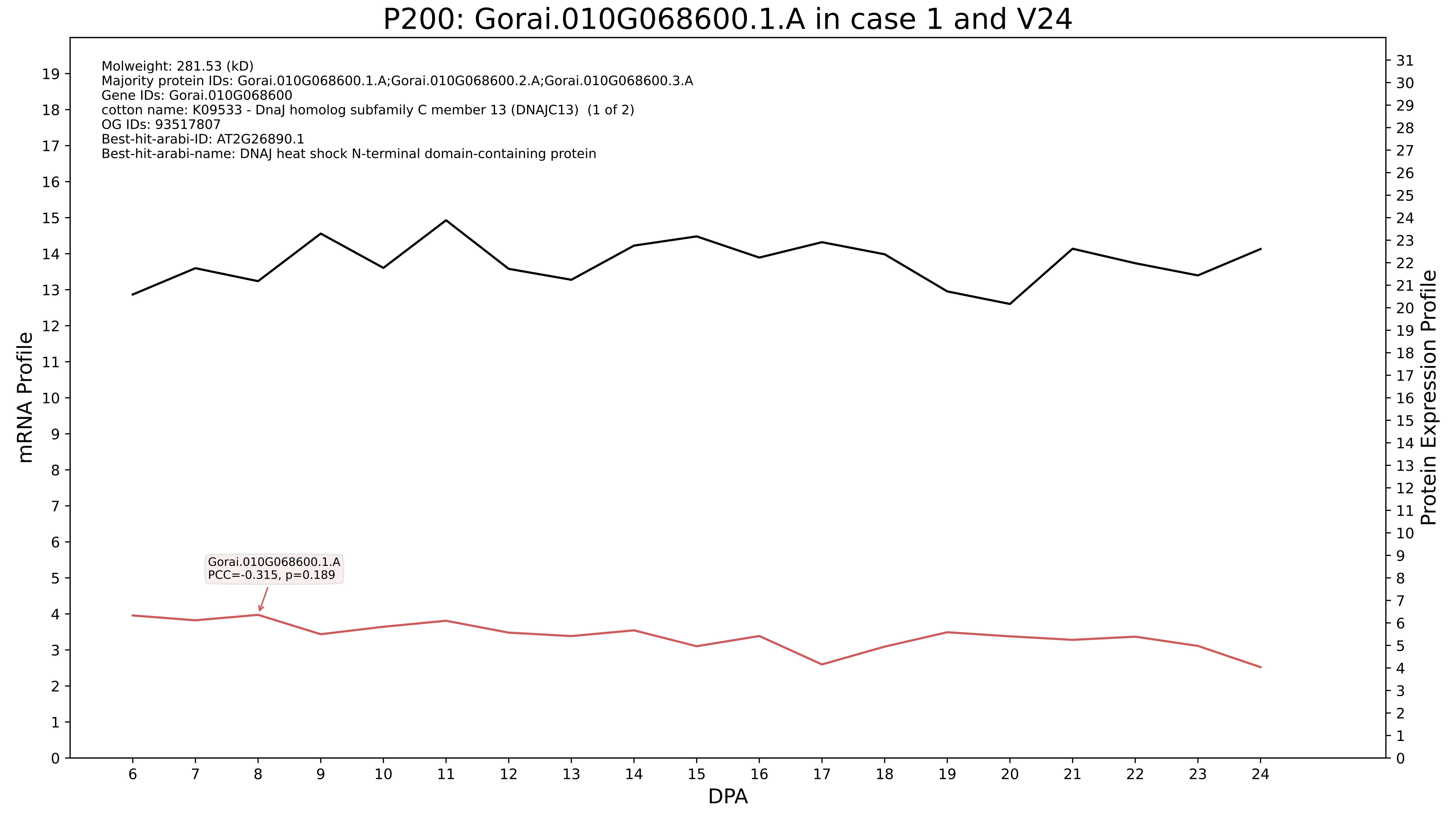
P200: Gorai.010G068600.1.A in case 1 and V24
Molweight: 281.53 (kD)
Majority protein IDs: Gorai.010G068600.1.A;Gorai.010G068600.2.A;Gorai.010G068600.3.A
Gene IDs: Gorai.010G068600
cotton name: K09533 - DnaJ homolog subfamily C member 13 (DNAJC13) (1 of 2)
OG IDs: 93517807
Best-hit-arabi-ID: AT2G26890.1
Best-hit-arabi-name: DNAJ heat shock N-terminal domain-containing protein
Gorai.010G068600.1.A
PCC=-0.315, p=0.189
mRNA Profile
Protein Expression Profile
DPA

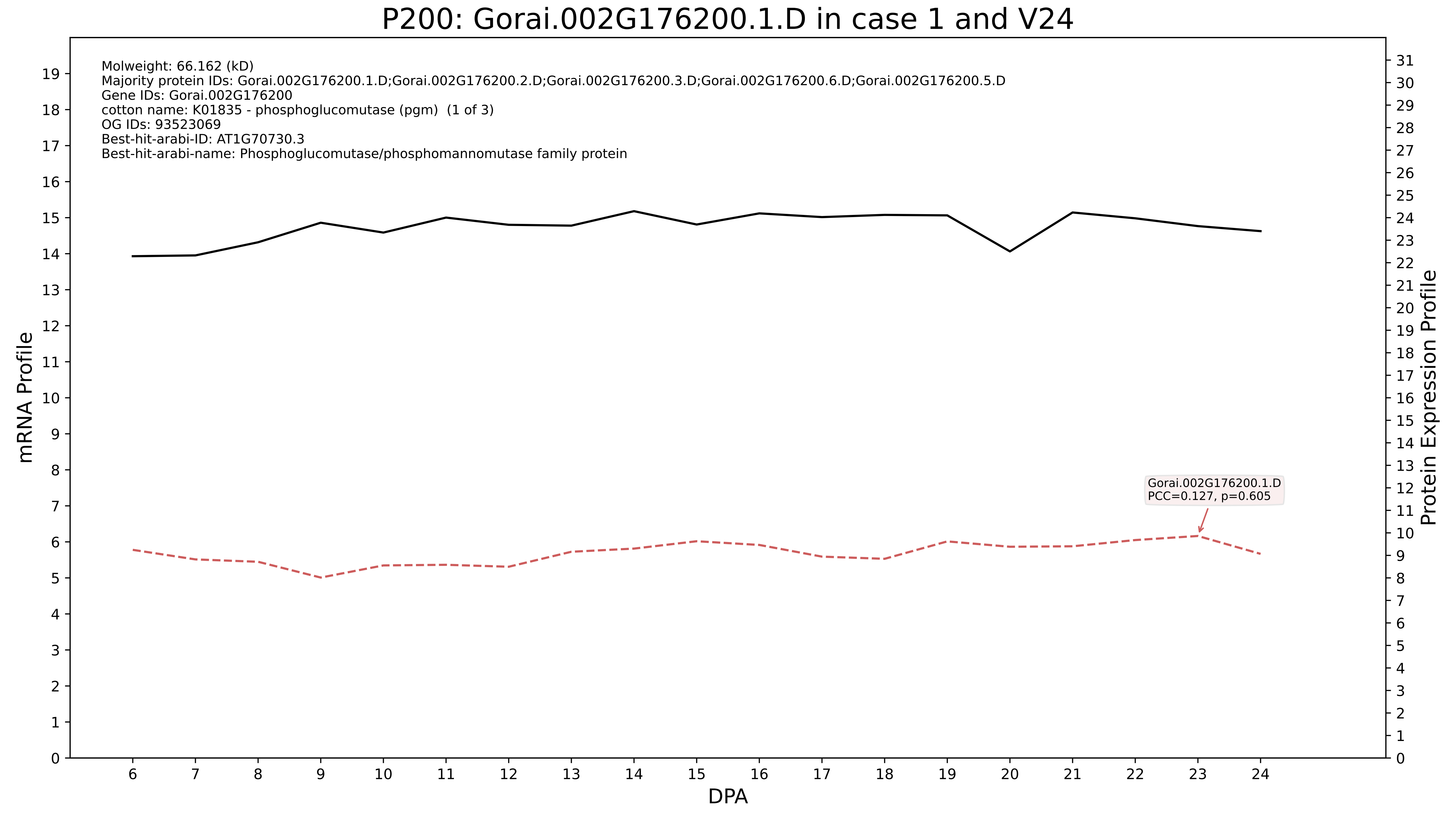
P200: Gorai.002G176200.1.D in case 1 and V24
Molweight: 66.162 (kD)
Majority protein IDs: Gorai.002G176200.1.D;Gorai.002G176200.2.D;Gorai.002G176200.3.D;Gorai.002G176200.6.D;Gorai.002G176200.5.D
Gene IDs: Gorai.002G176200
cotton name: K01835 - phosphoglucomutase (pgm) (1 of 3)
OG IDs: 93523069
Best-hit-arabi-ID: AT1G70730.3
Best-hit-arabi-name: Phosphoglucomutase/phosphomannomutase family protein
Gorai.002G176200.1.D
PCC=0.127, p=0.605
mRNA Profile
Protein Expression Profile
DPA

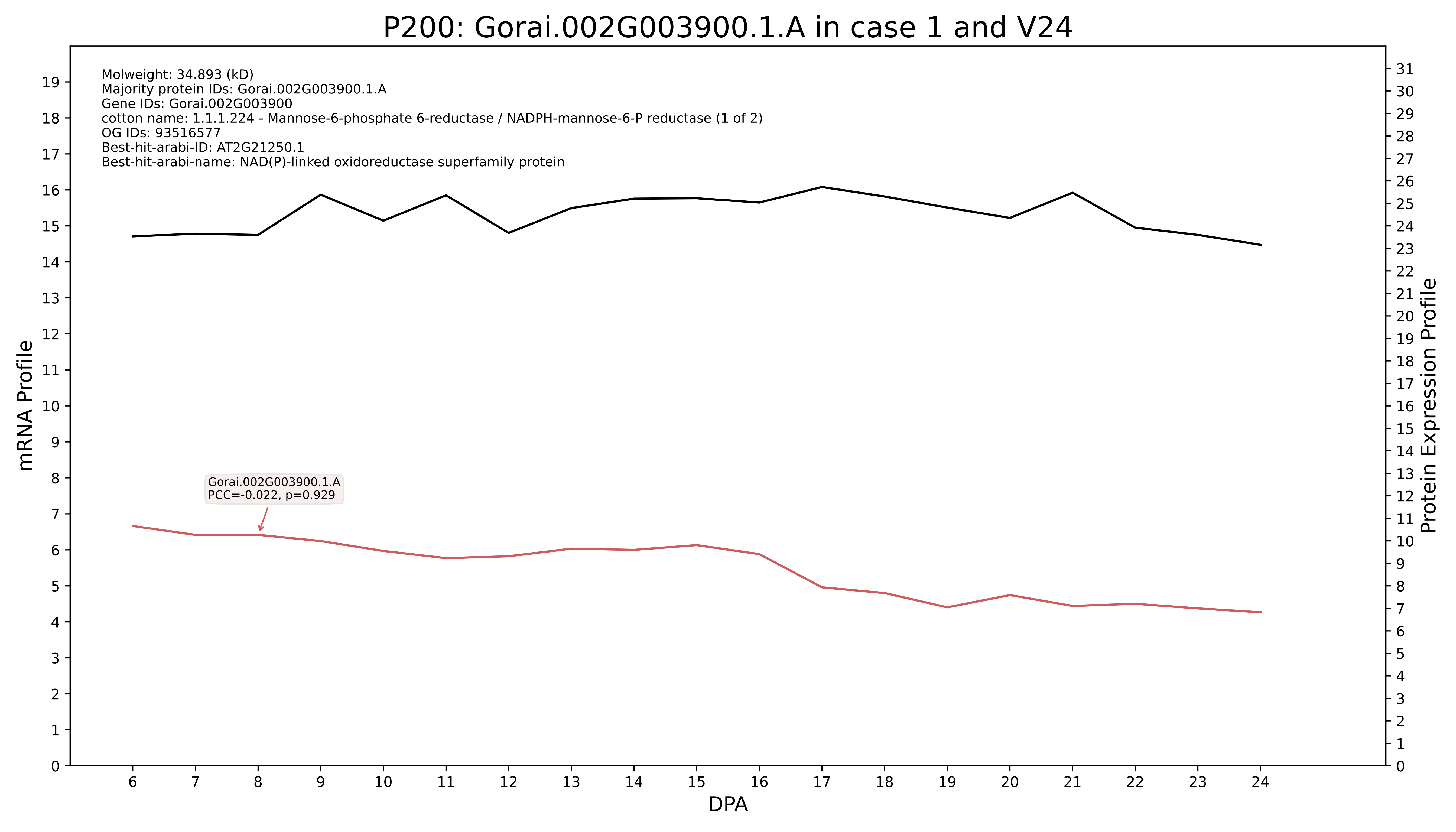
P200: Gorai.002G003900.1.A in case 1 and V24
Molweight: 34.893 (kD)
Majority protein IDs: Gorai.002G003900.1.A
Gene IDs: Gorai.002G003900
cotton name: 1.1.1.224 - Mannose-6-phosphate 6-reductase / NADPH-mannose-6-P reductase (1 of 2)
OG IDs: 93516577
Best-hit-arabi-ID: AT2G21250.1
Best-hit-arabi-name: NAD(P)-linked oxidoreductase superfamily protein
Gorai.002G003900.1.A
PCC=-0.022, p=0.929
mRNA Profile
Protein Expression Profile
DPA

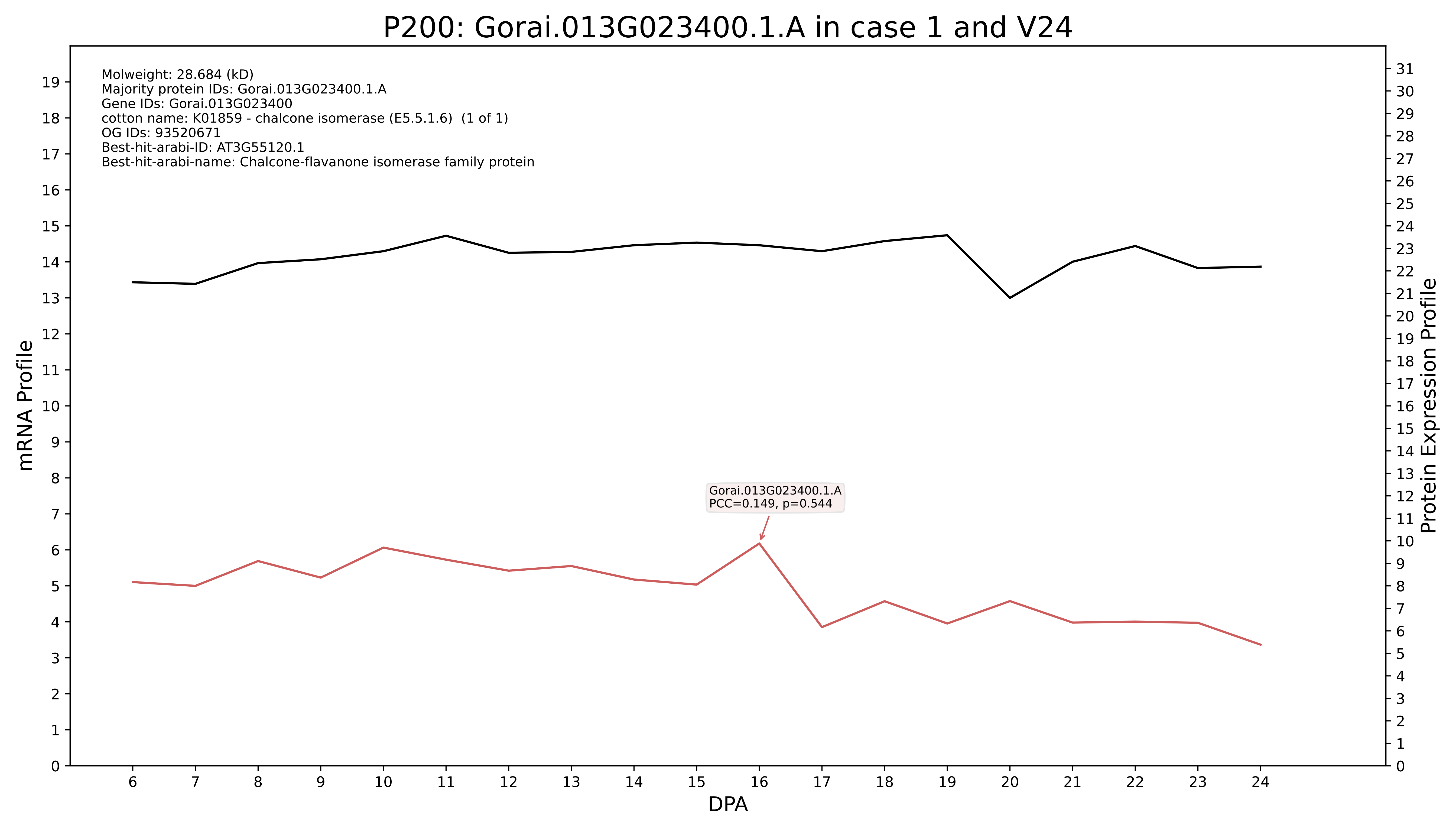

P200: Gorai.013G023400.1.A in case 1 and V24
Molweight: 28.684 (kD)
Majority protein IDs: Gorai.013G023400.1.A
Gene IDs: Gorai.013G023400
cotton name: K01859 - chalcone isomerase (E5.5.1.6) (1 of 1)
OG IDs: 93520671
Best-hit-arabi-ID: AT3G55120.1
Best-hit-arabi-name: Chalcone-flavanone isomerase family protein
Gorai.013G023400.1.A
PCC=0.149, p=0.544
mRNA Profile
Protein Expression Profile
DPA

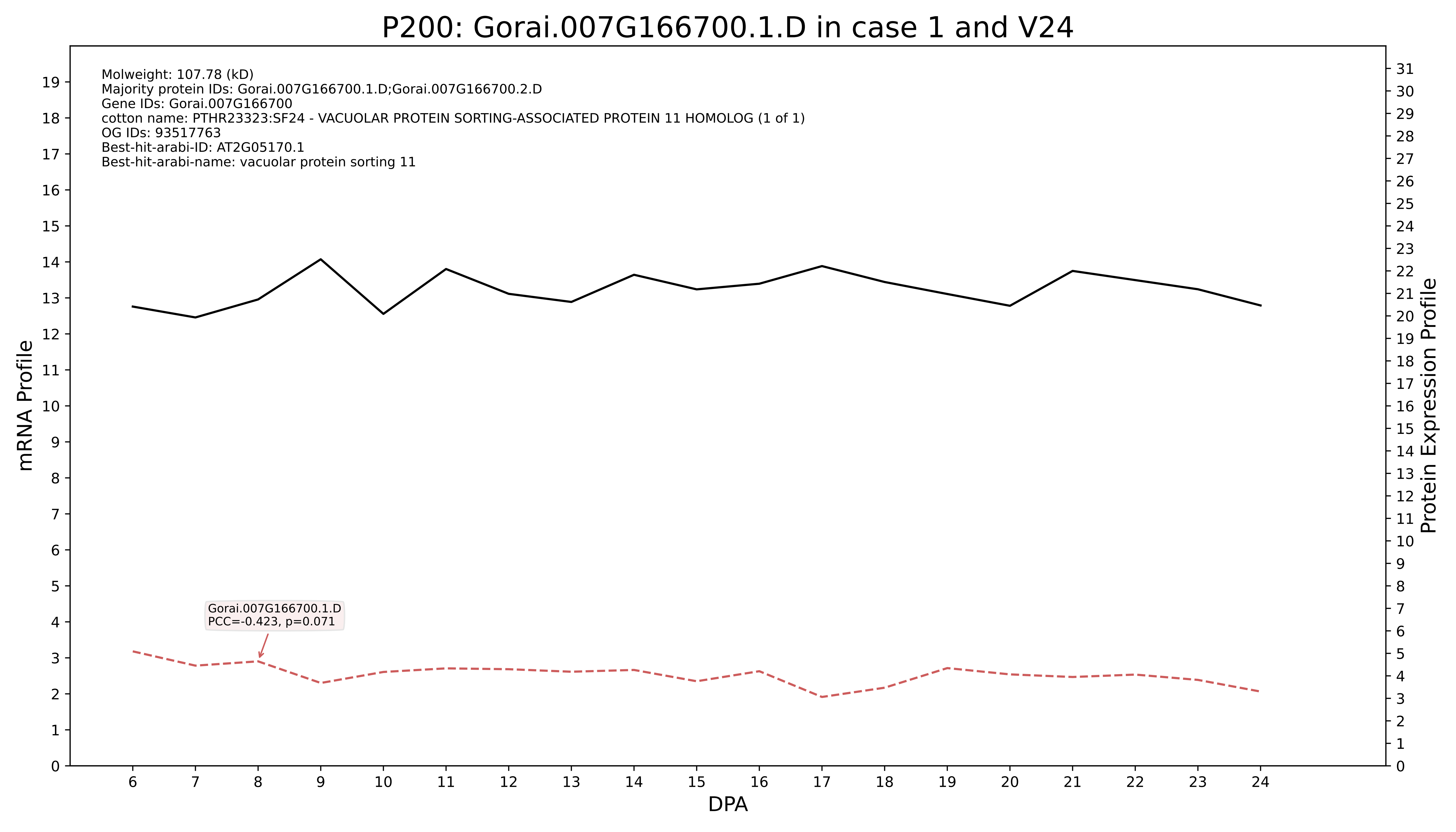
P200: Gorai.007G166700.1.D in case 1 and V24
Molweight: 107.78 (kD)
Majority protein IDs: Gorai.007G166700.1.D;Gorai.007G166700.2.D
Gene IDs: Gorai.007G166700
cotton name: PTHR23323:SF24 - VACUOLAR PROTEIN SORTING-ASSOCIATED PROTEIN 11 HOMOLOG (1 of 1)
OG IDs: 93517763
Best-hit-arabi-ID: AT2G05170.1
Best-hit-arabi-name: vacuolar protein sorting 11
Gorai.007G166700.1.D
PCC=-0.423, p=0.071
mRNA Profile
Protein Expression Profile
DPA

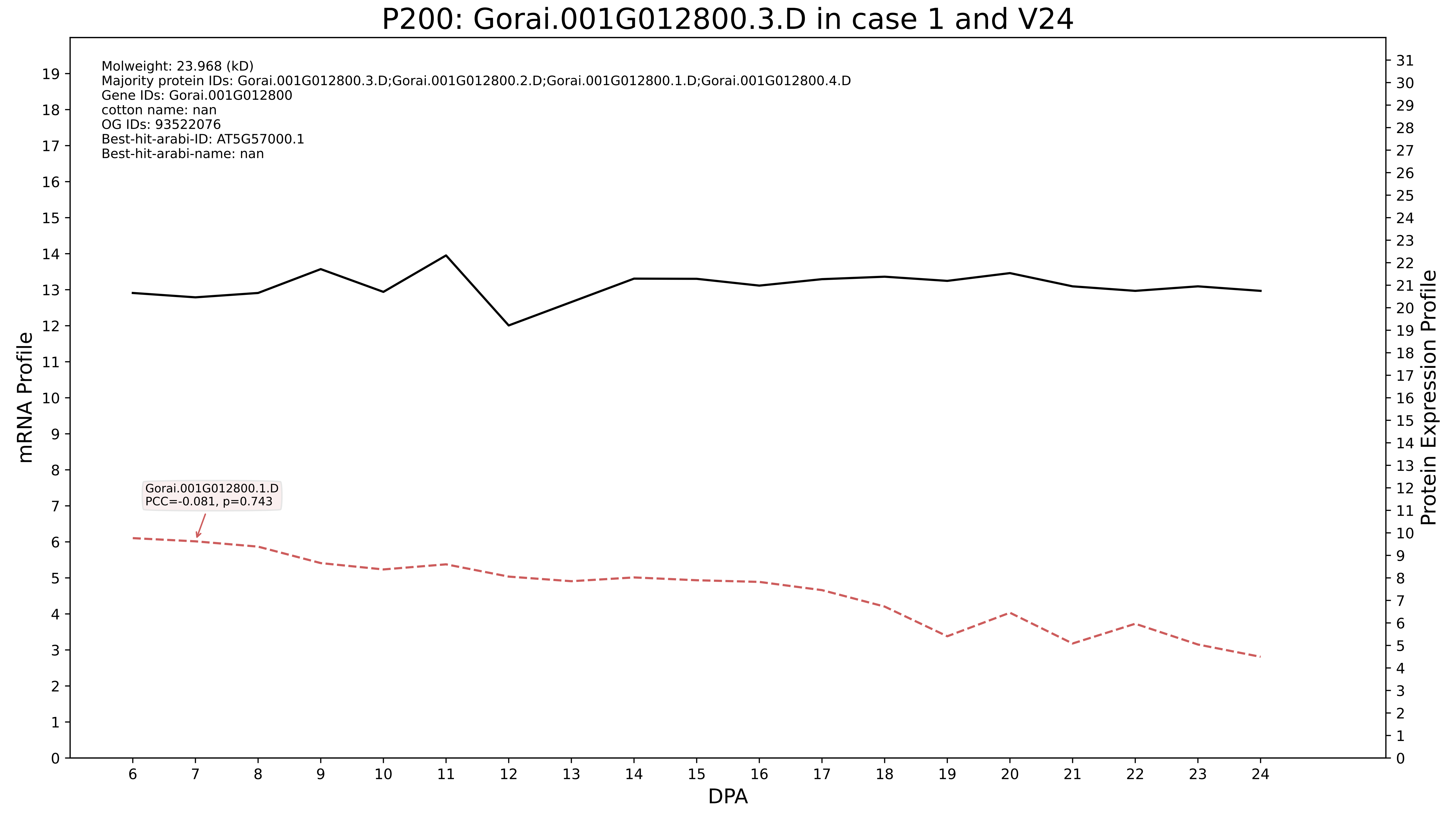
P200: Gorai.001G012800.3.D in case 1 and V24
Molweight: 23.968 (kD)
Majority protein IDs: Gorai.001G012800.3.D;Gorai.001G012800.2.D;Gorai.001G012800.1.D;Gorai.001G012800.4.D
Gene IDs: Gorai.001G012800
cotton name: nan
OG IDs: 93522076
Best-hit-arabi-ID: AT5G57000.1
Best-hit-arabi-name: nan
Gorai.001G012800.1.D
PCC=-0.081, p=0.743
mRNA Profile
Protein Expression Profile
DPA

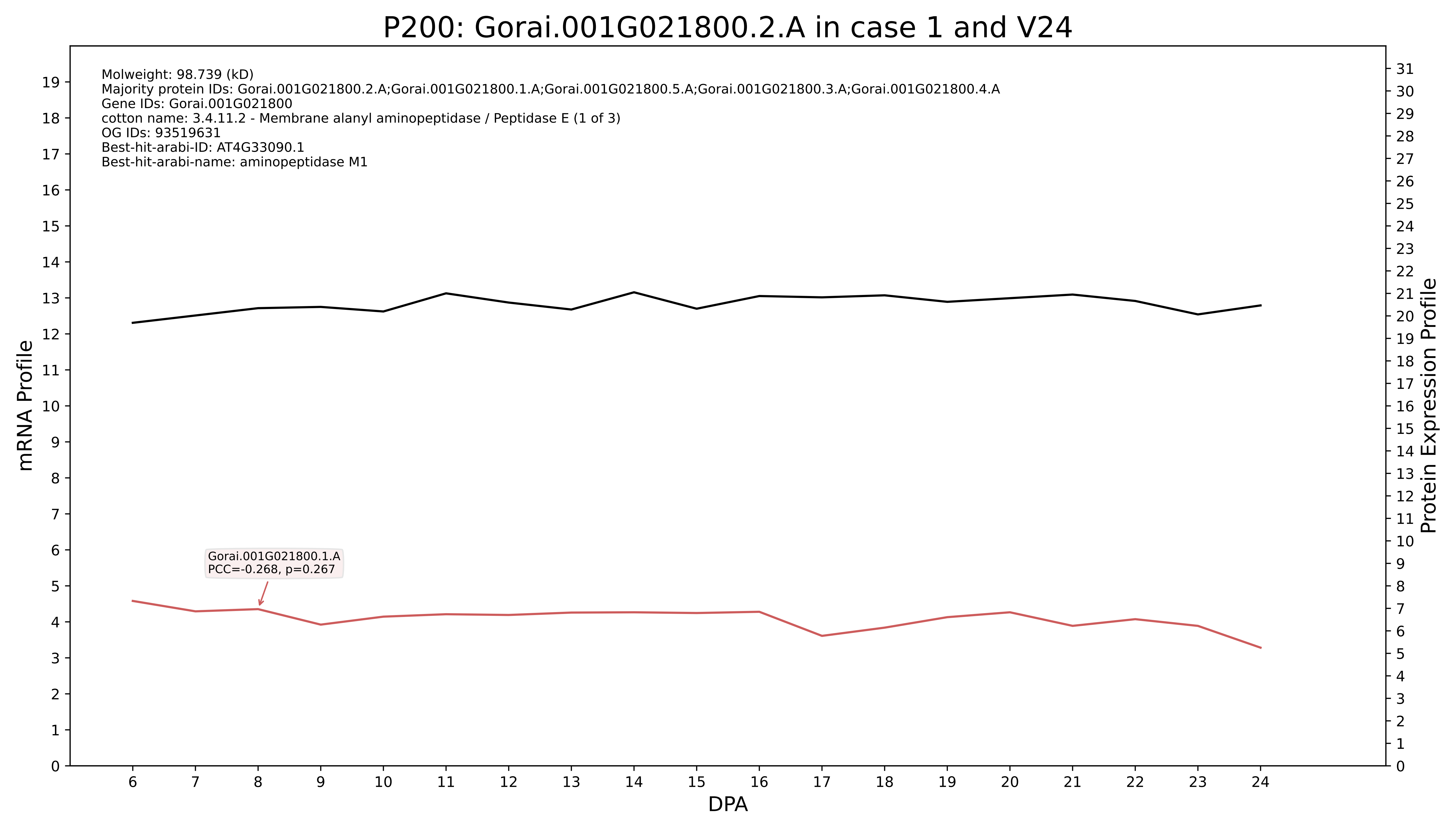
P200: Gorai.001G021800.2.A in case 1 and V24
Molweight: 98.739 (kD)
Majority protein IDs: Gorai.001G021800.2.A;Gorai.001G021800.1.A;Gorai.001G021800.5.A;Gorai.001G021800.3.A;Gorai.001G021800.4.A
Gene IDs: Gorai.001G021800
cotton name: 3.4.11.2 - Membrane alanyl aminopeptidase / Peptidase E (1 of 3)
OG IDs: 93519631
Best-hit-arabi-ID: AT4G33090.1
Best-hit-arabi-name: aminopeptidase M1
Gorai.001G021800.1.A
PCC=-0.268, p=0.267
mRNA Profile
Protein Expression Profile
DPA

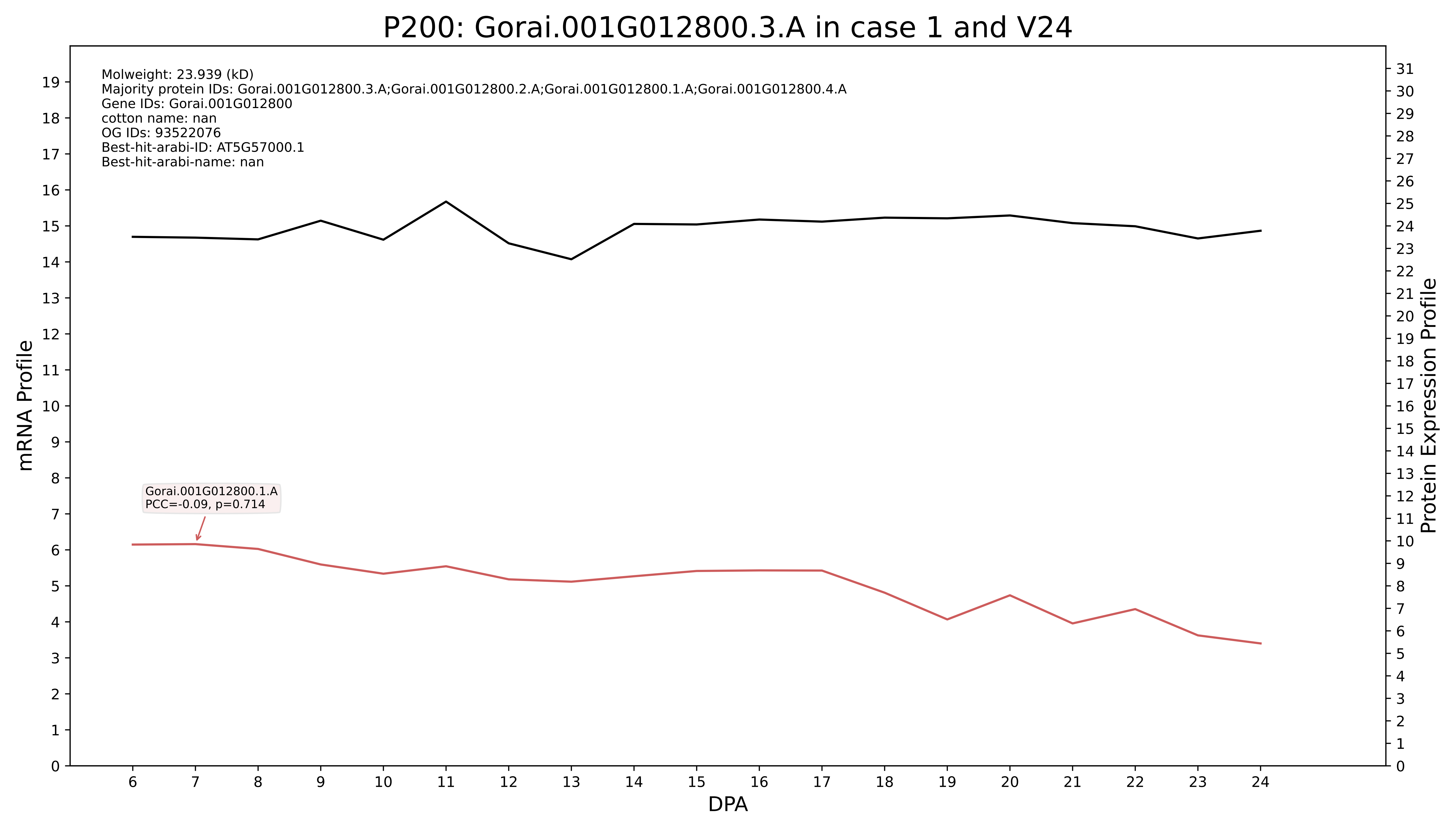
P200: Gorai.001G012800.3.A in case 1 and V24
Molweight: 23.939 (kD)
Majority protein IDs: Gorai.001G012800.3.A;Gorai.001G012800.2.A;Gorai.001G012800.1.A;Gorai.001G012800.4.A
Gene IDs: Gorai.001G012800
cotton name: nan
OG IDs: 93522076
Best-hit-arabi-ID: AT5G57000.1
Best-hit-arabi-name: nan
Gorai.001G012800.1.A
PCC=-0.09, p=0.714
mRNA Profile
Protein Expression Profile
DPA

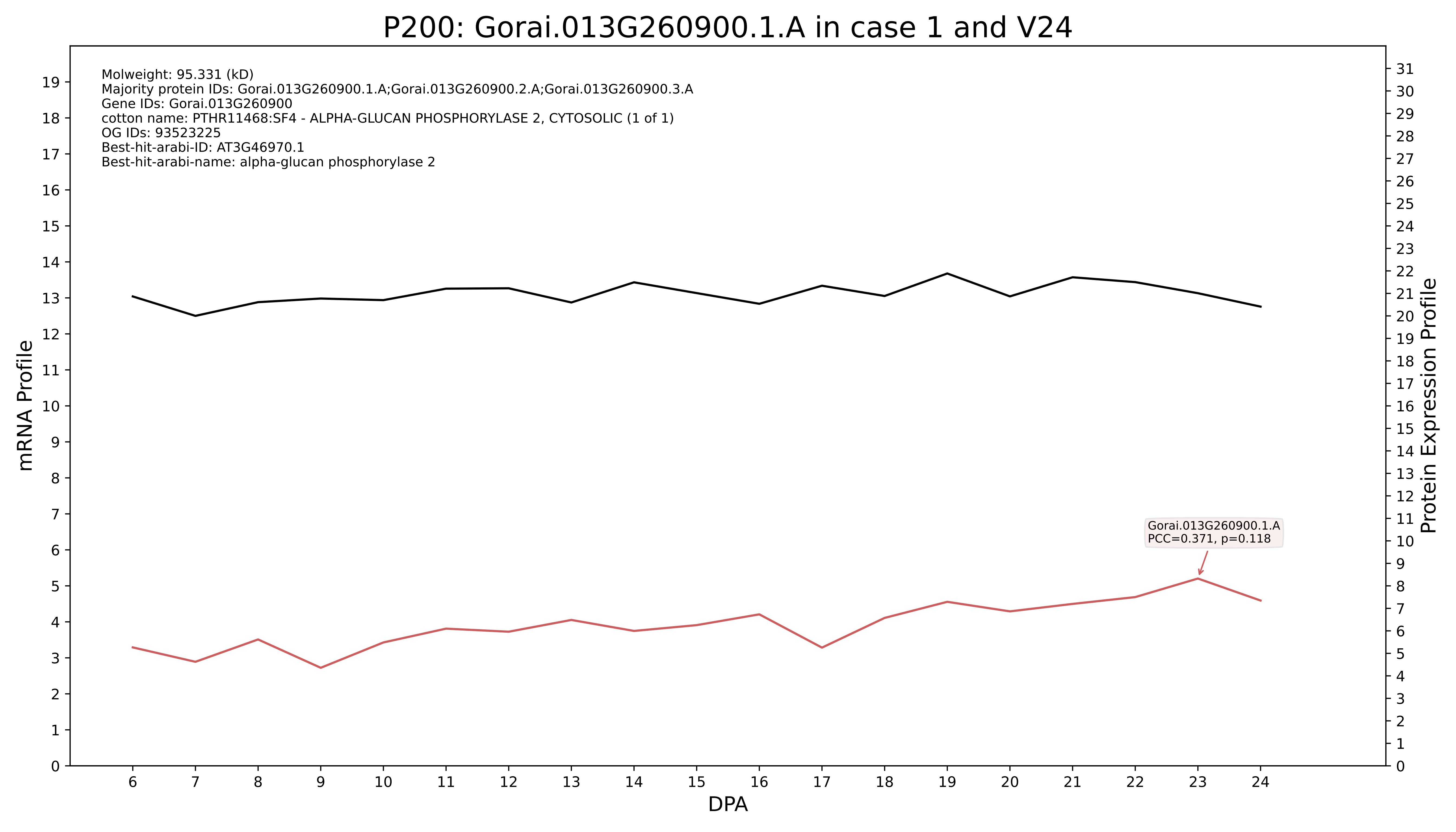
P200: Gorai.013G260900.1.A in case 1 and V24
Molweight: 95.331 (kD)
Majority protein IDs: Gorai.013G260900.1.A;Gorai.013G260900.2.A;Gorai.013G260900.3.A
Gene IDs: Gorai.013G260900
cotton name: PTHR11468:SF4 - ALPHA-GLUCAN PHOSPHORYLASE 2, CYTOSOLIC (1 of 1)
OG IDs: 93523225
Best-hit-arabi-ID: AT3G46970.1
Best-hit-arabi-name: alpha-glucan phosphorylase 2
Gorai.013G260900.1.A
PCC=0.371, p=0.118
mRNA Profile
Protein Expression Profile
DPA

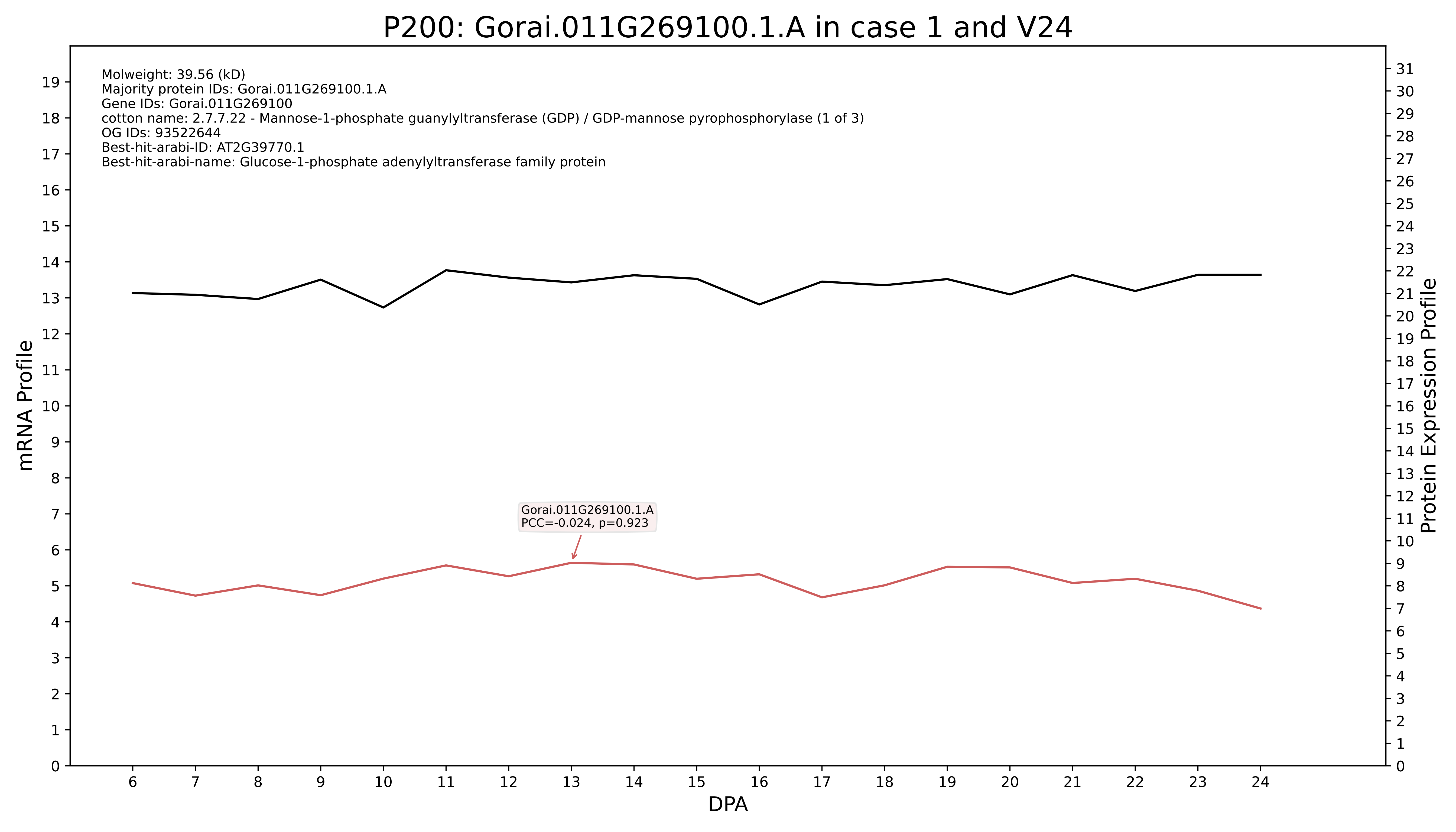
P200: Gorai.011G269100.1.A in case 1 and V24
Molweight: 39.56 (kD)
Majority protein IDs: Gorai.011G269100.1.A
Gene IDs: Gorai.011G269100
cotton name: 2.7.7.22 - Mannose-1-phosphate guanylyltransferase (GDP) / GDP-mannose pyrophosphorylase (1 of 3)
OG IDs: 93522644
Best-hit-arabi-ID: AT2G39770.1
Best-hit-arabi-name: Glucose-1-phosphate adenylyltransferase family protein
Gorai.011G269100.1.A
PCC=-0.024, p=0.923
mRNA Profile
Protein Expression Profile
DPA

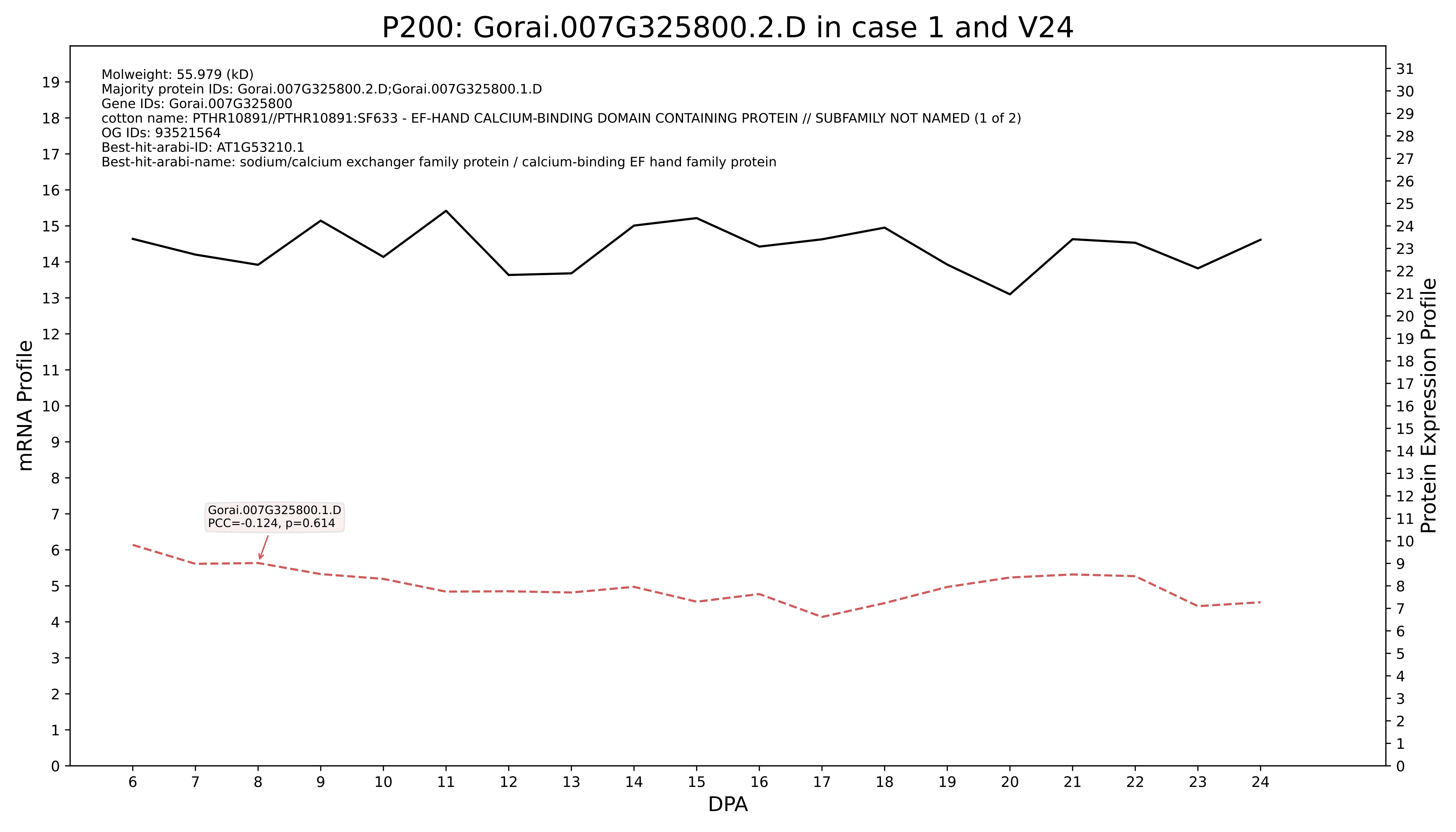
P200: Gorai.007G325800.2.D in case 1 and V24
Molweight: 55.979 (kD)
Majority protein IDs: Gorai.007G325800.2.D;Gorai.007G325800.1.D
Gene IDs: Gorai.007G325800
cotton name: PTHR10891//PTHR10891:SF633 - EF-HAND CALCIUM-BINDING DOMAIN CONTAINING PROTEIN // SUBFAMILY NOT NAMED (1 of 2)
OG IDs: 93521564
Best-hit-arabi-ID: AT1G53210.1
Best-hit-arabi-name: sodium/calcium exchanger family protein / calcium-binding EF hand family protein
Gorai.007G325800.1.D
PCC=-0.124, p=0.614
mRNA Profile
Protein Expression Profile
DPA

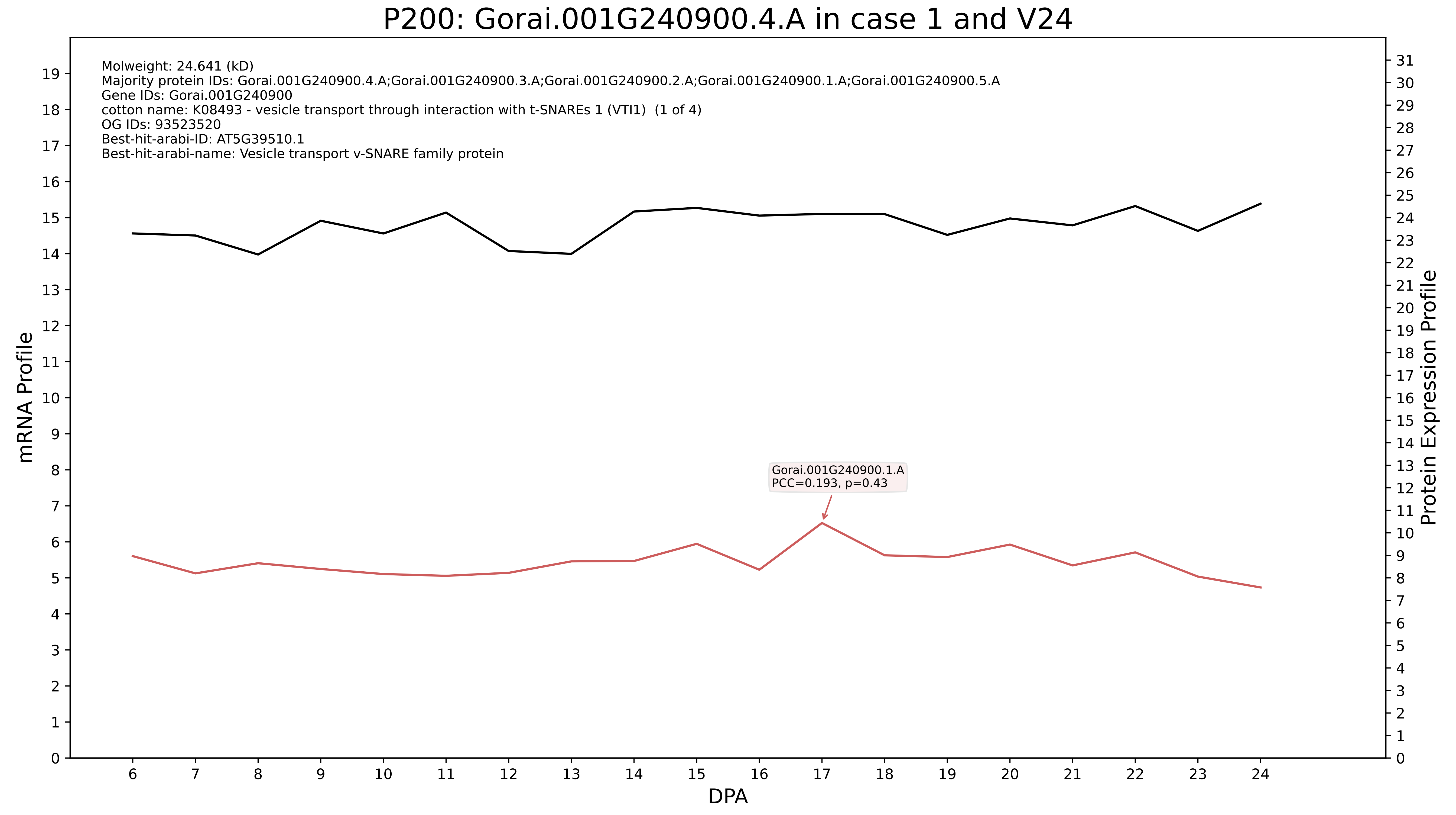

P200: Gorai.001G240900.4.A in case 1 and V24
Molweight: 24.641 (kD)
Majority protein IDs: Gorai.001G240900.4.A;Gorai.001G240900.3.A;Gorai.001G240900.2.A;Gorai.001G240900.1.A;Gorai.001G240900.5.A
Gene IDs: Gorai.001G240900
cotton name: K08493 - vesicle transport through interaction with t-SNAREs 1 (VTI1) (1 of 4)
OG IDs: 93523520
Best-hit-arabi-ID: AT5G39510.1
Best-hit-arabi-name: Vesicle transport v-SNARE family protein
Gorai.001G240900.1.A
PCC=0.193, p=0.43
mRNA Profile
Protein Expression Profile
DPA

P200: Gorai.008G101600.1.A in case 1 and V24

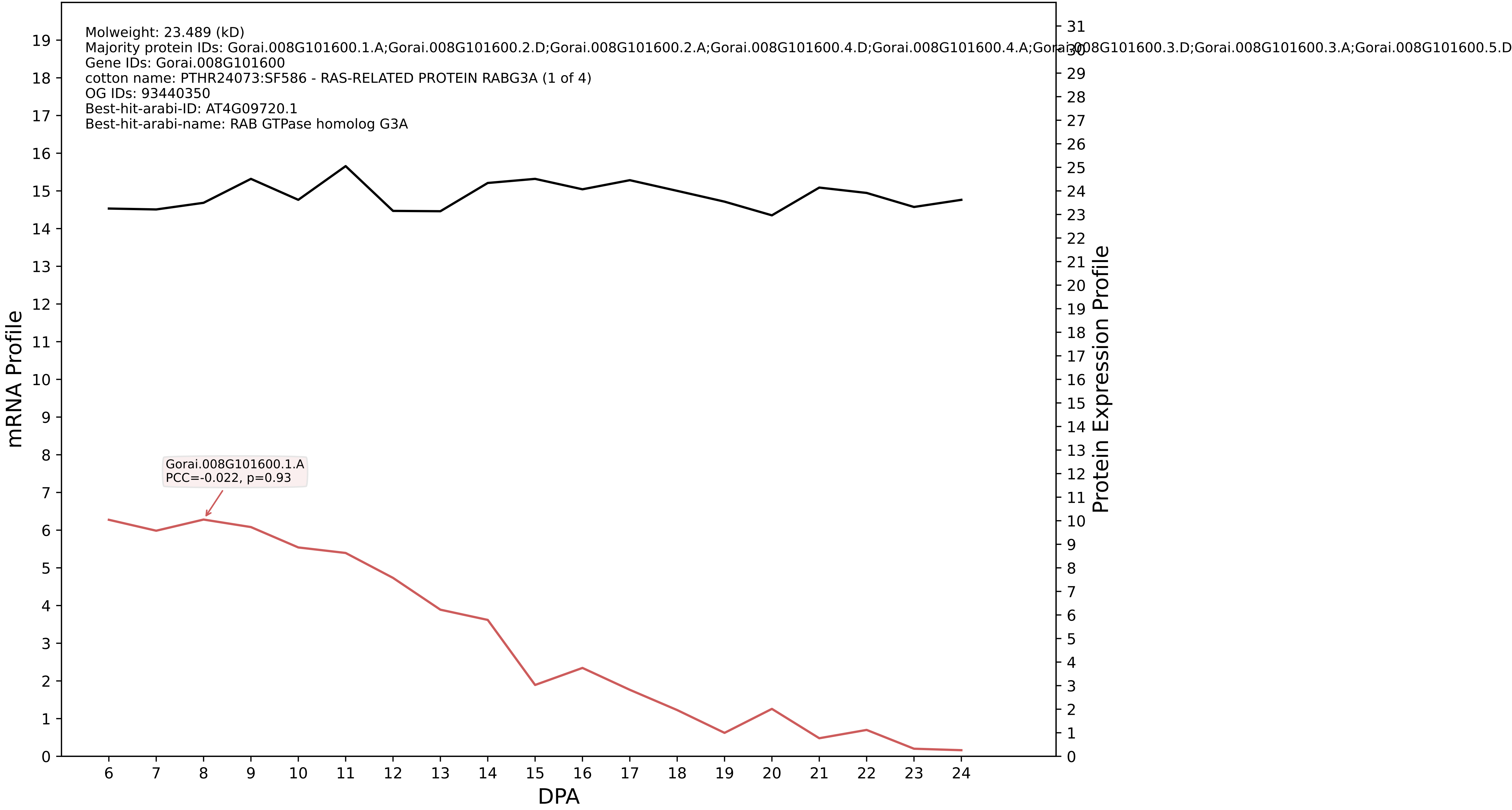

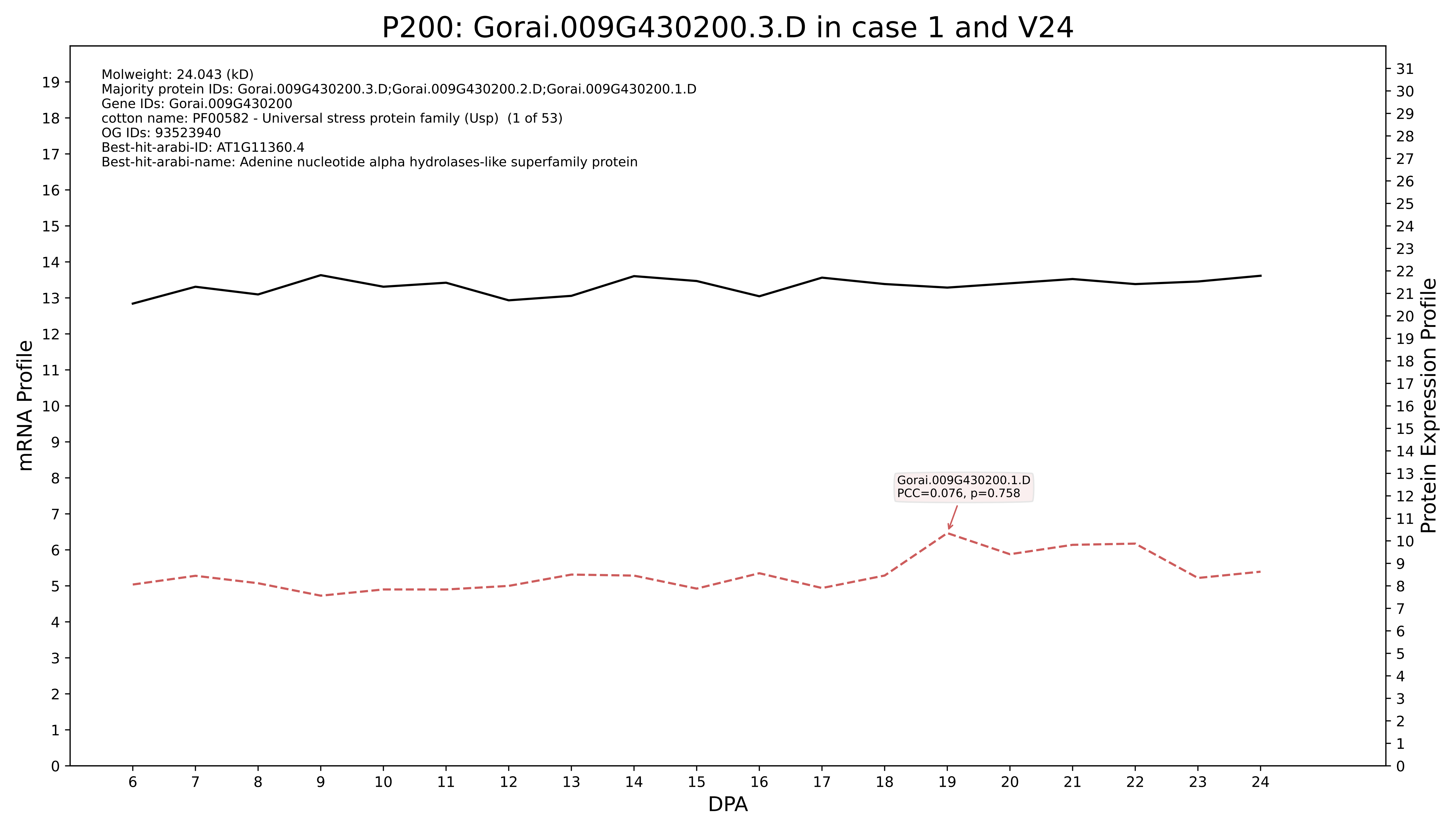

P200: Gorai.009G430200.3.D in case 1 and V24
Molweight: 24.043 (kD)
Majority protein IDs: Gorai.009G430200.3.D;Gorai.009G430200.2.D;Gorai.009G430200.1.D
Gene IDs: Gorai.009G430200
cotton name: PF00582 - Universal stress protein family (Usp) (1 of 53)
OG IDs: 93523940
Best-hit-arabi-ID: AT1G11360.4
Best-hit-arabi-name: Adenine nucleotide alpha hydrolases-like superfamily protein
Gorai.009G430200.1.D
PCC=0.076, p=0.758
mRNA Profile
Protein Expression Profile
DPA

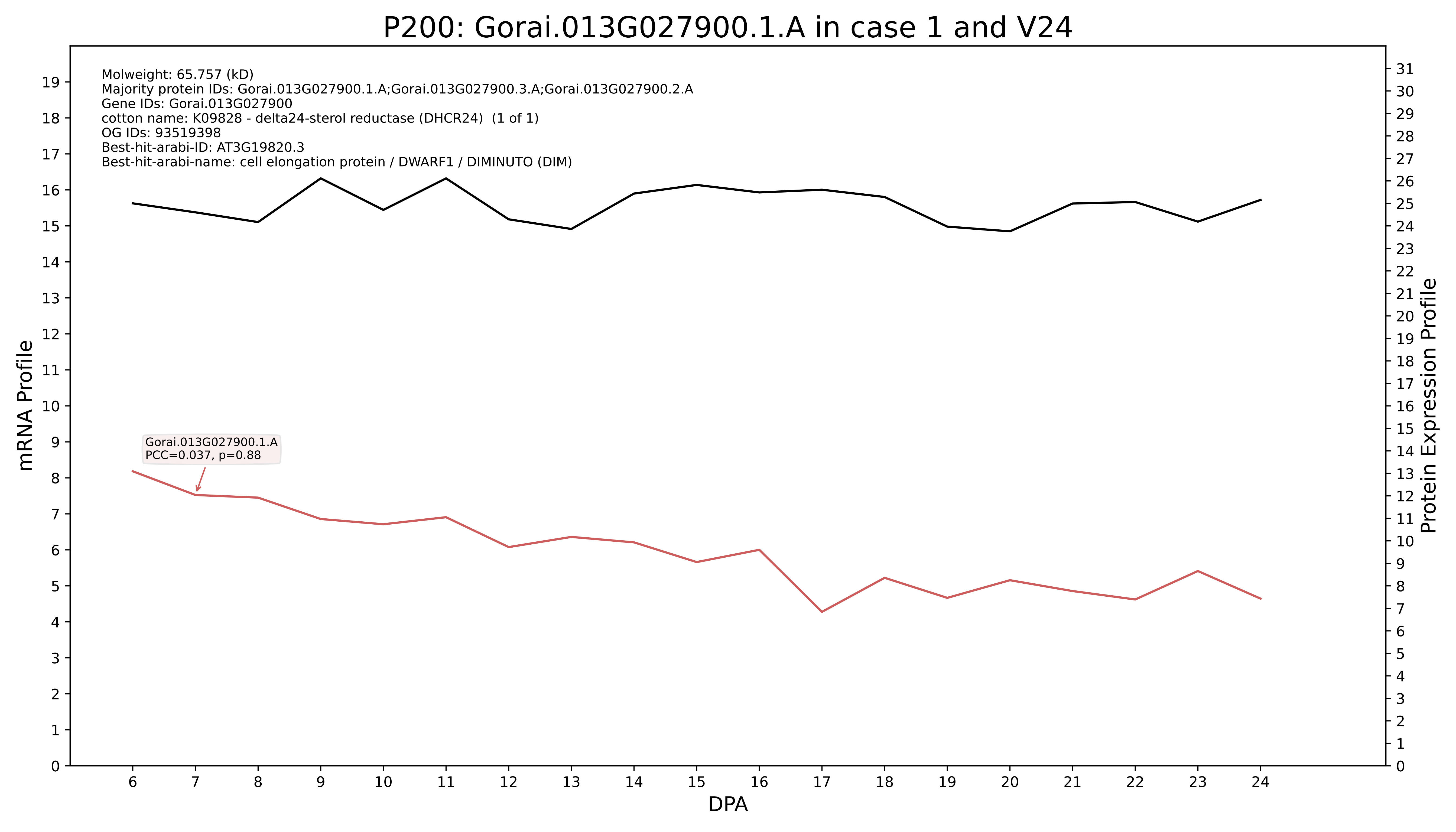
P200: Gorai.013G027900.1.A in case 1 and V24
Molweight: 65.757 (kD)
Majority protein IDs: Gorai.013G027900.1.A;Gorai.013G027900.3.A;Gorai.013G027900.2.A
Gene IDs: Gorai.013G027900
cotton name: K09828 - delta24-sterol reductase (DHCR24) (1 of 1)
OG IDs: 93519398
Best-hit-arabi-ID: AT3G19820.3
Best-hit-arabi-name: cell elongation protein / DWARF1 / DIMINUTO (DIM)
Gorai.013G027900.1.A
PCC=0.037, p=0.88
mRNA Profile
Protein Expression Profile
DPA
0
1
2
3
4
5
6
7
8
9
10
11
12
13
14
15
16
17
18
19
20
21
22
23
24
25
26
27
28
29
30
31

P200: Gorai.008G266200.8.D in case 1 and V24

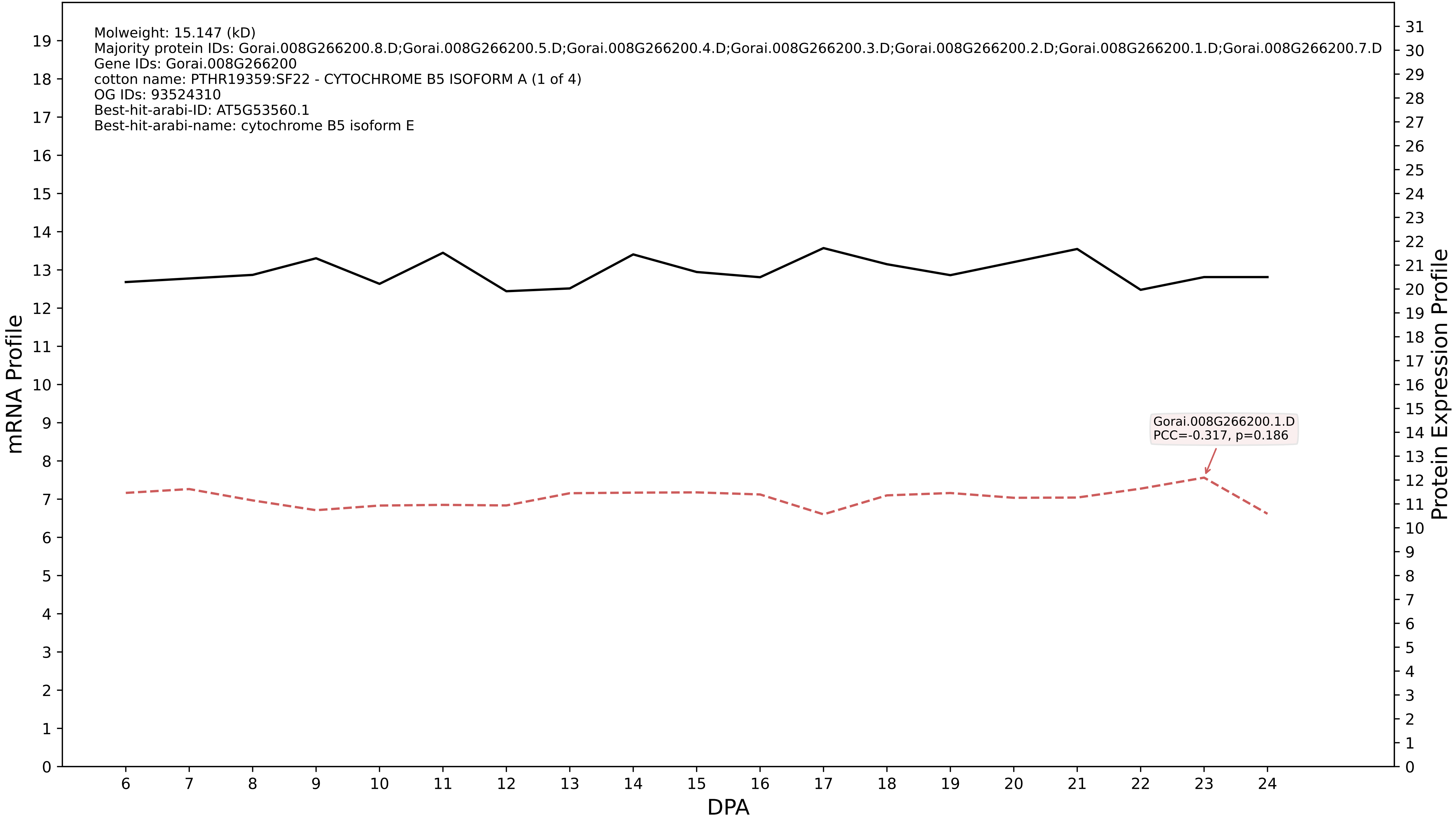

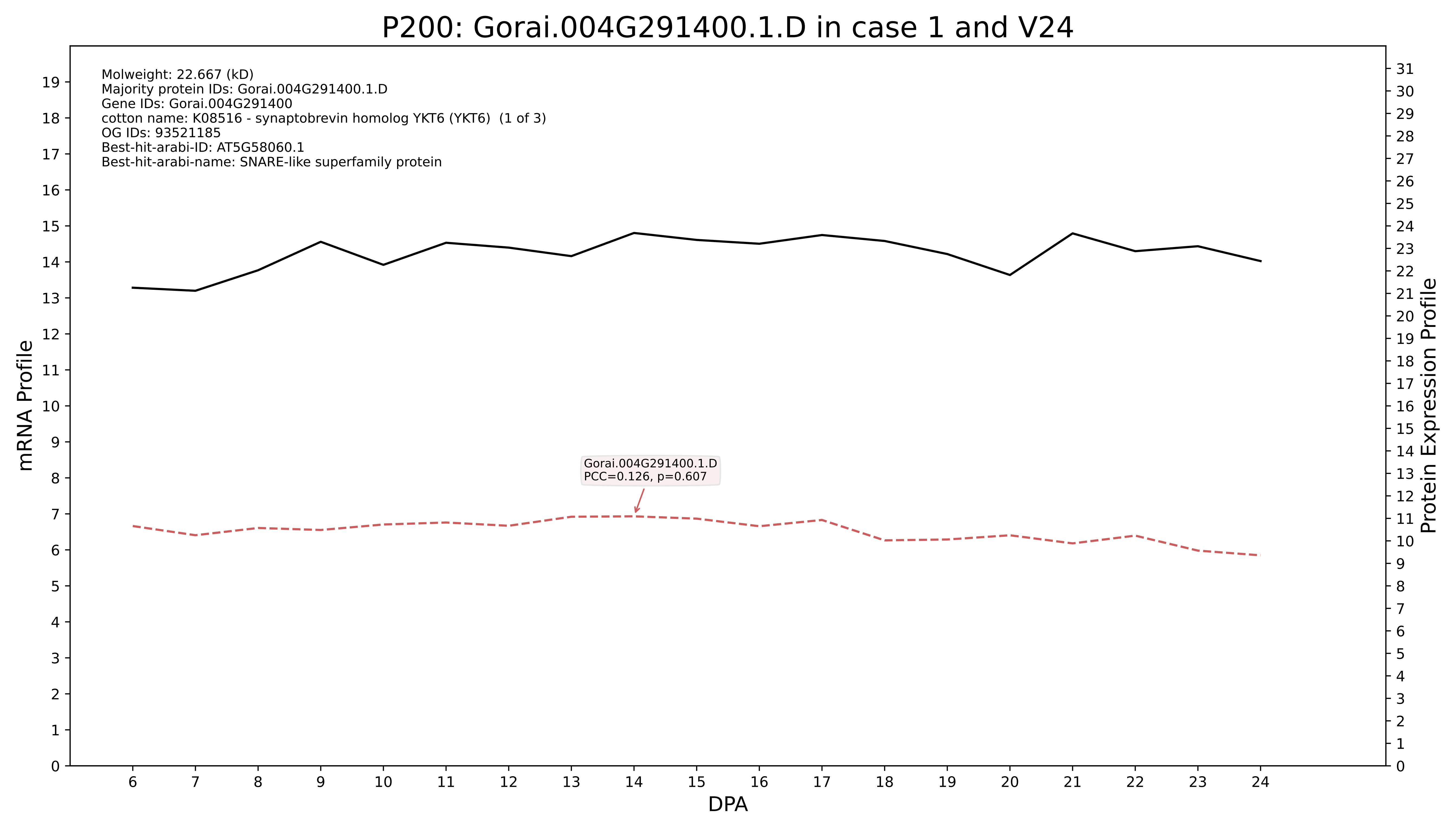
P200: Gorai.004G291400.1.D in case 1 and V24
Molweight: 22.667 (kD)
Majority protein IDs: Gorai.004G291400.1.D
Gene IDs: Gorai.004G291400
cotton name: K08516 - synaptobrevin homolog YKT6 (YKT6) (1 of 3)
OG IDs: 93521185
Best-hit-arabi-ID: AT5G58060.1
Best-hit-arabi-name: SNARE-like superfamily protein
Gorai.004G291400.1.D
PCC=0.126, p=0.607
mRNA Profile
Protein Expression Profile
DPA

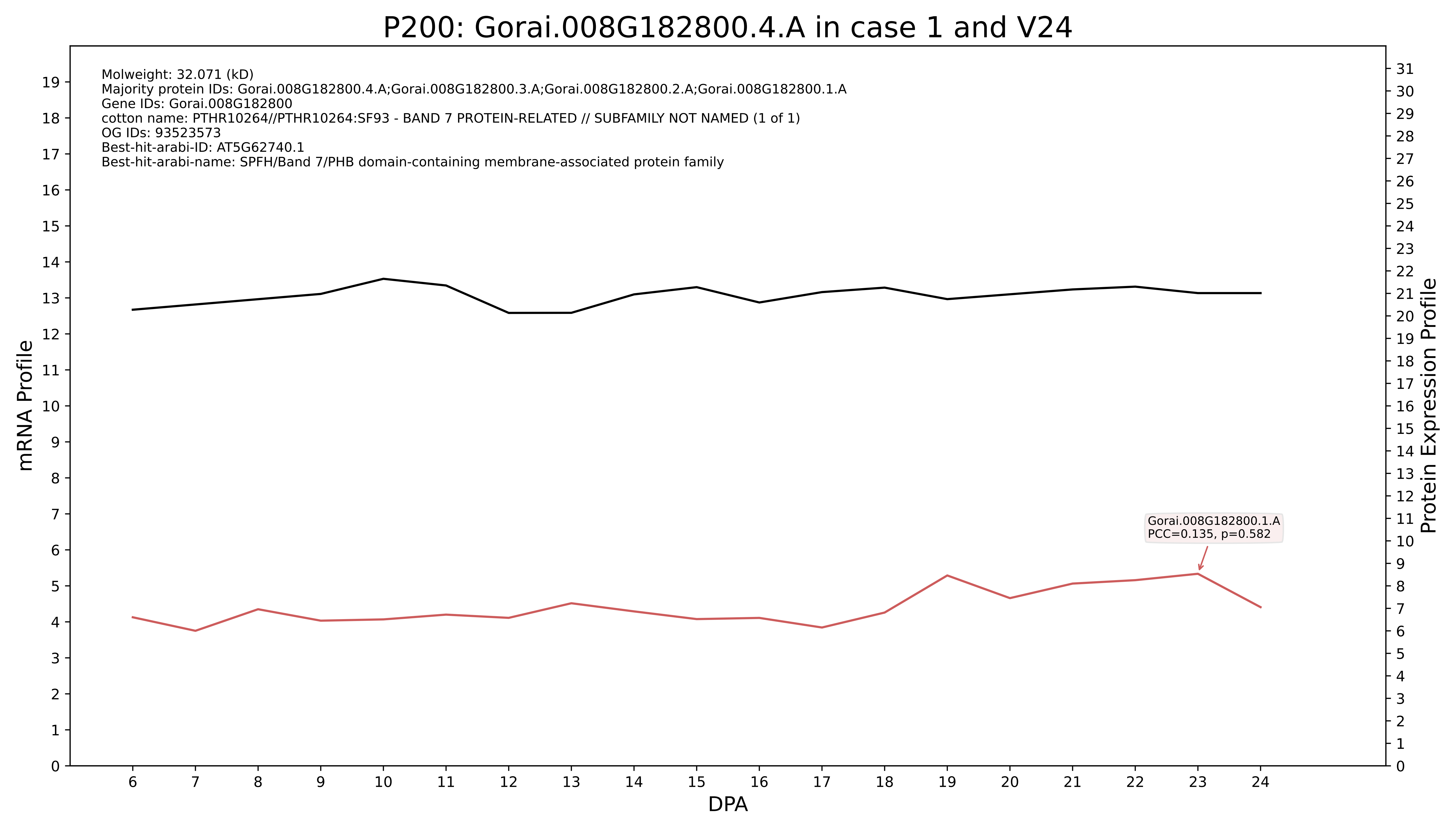
P200: Gorai.008G182800.4.A in case 1 and V24
Molweight: 32.071 (kD)
Majority protein IDs: Gorai.008G182800.4.A;Gorai.008G182800.3.A;Gorai.008G182800.2.A;Gorai.008G182800.1.A
Gene IDs: Gorai.008G182800
cotton name: PTHR10264//PTHR10264:SF93 - BAND 7 PROTEIN-RELATED // SUBFAMILY NOT NAMED (1 of 1)
OG IDs: 93523573
Best-hit-arabi-ID: AT5G62740.1
Best-hit-arabi-name: SPFH/Band 7/PHB domain-containing membrane-associated protein family
Gorai.008G182800.1.A
PCC=0.135, p=0.582
mRNA Profile
Protein Expression Profile
DPA

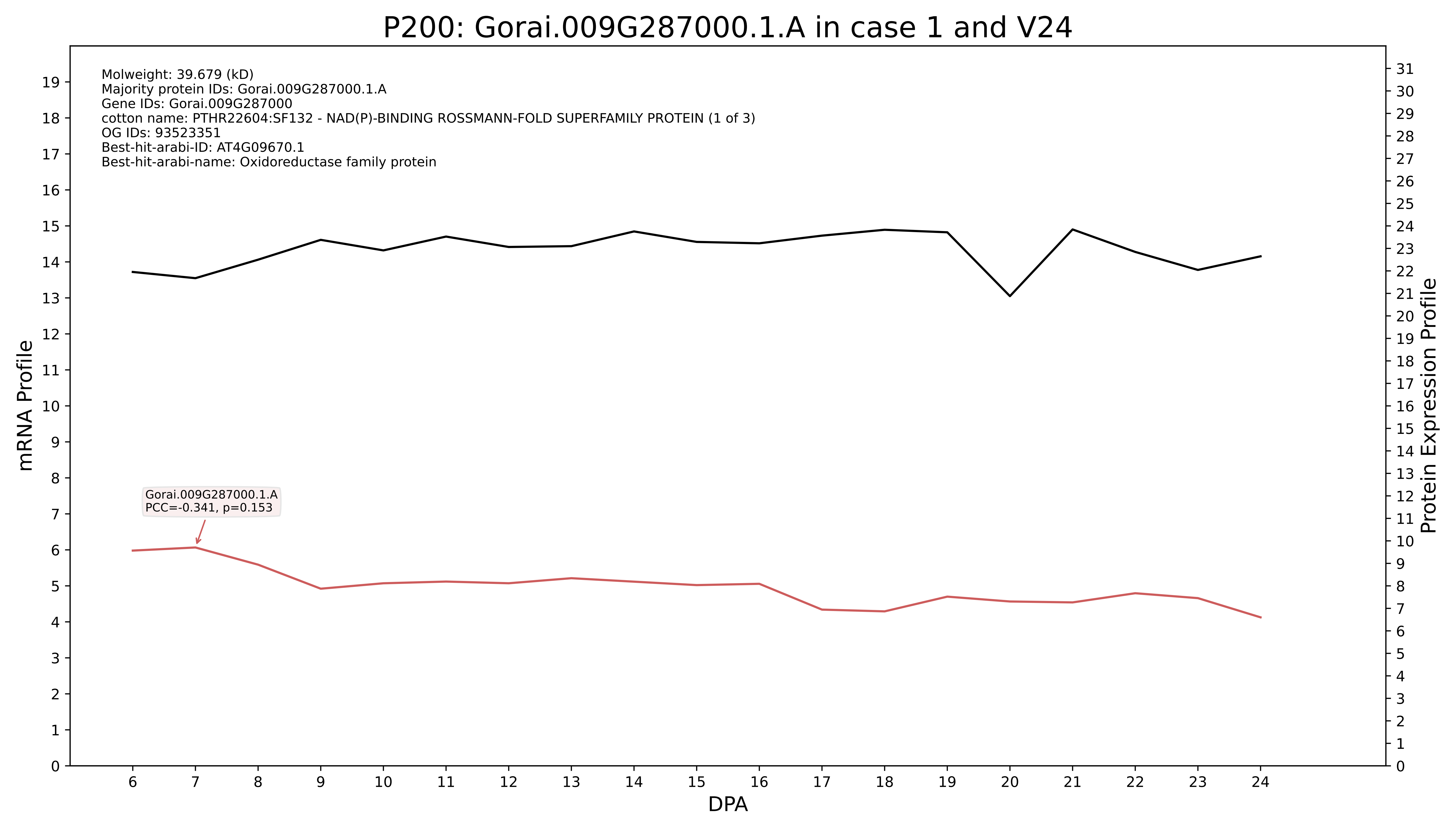

P200: Gorai.009G287000.1.A in case 1 and V24
Molweight: 39.679 (kD)
Majority protein IDs: Gorai.009G287000.1.A
Gene IDs: Gorai.009G287000
cotton name: PTHR22604:SF132 - NAD(P)-BINDING ROSSMANN-FOLD SUPERFAMILY PROTEIN (1 of 3)
OG IDs: 93523351
Best-hit-arabi-ID: AT4G09670.1
Best-hit-arabi-name: Oxidoreductase family protein
Gorai.009G287000.1.A
PCC=-0.341, p=0.153
mRNA Profile
Protein Expression Profile
DPA

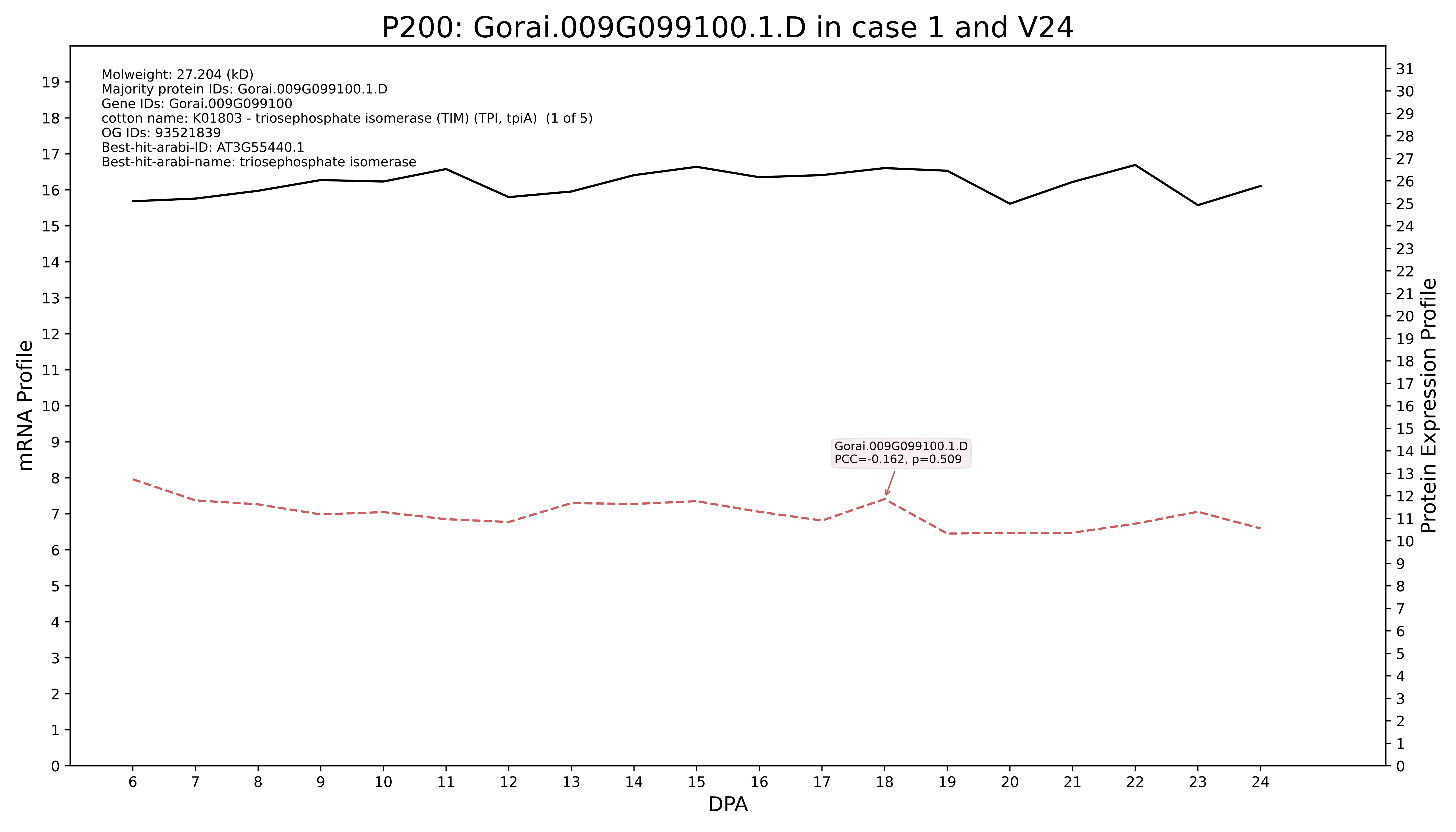
P200: Gorai.009G099100.1.D in case 1 and V24
Molweight: 27.204 (kD)
Majority protein IDs: Gorai.009G099100.1.D
Gene IDs: Gorai.009G099100
cotton name: K01803 - triosephosphate isomerase (TIM) (TPI, tpiA) (1 of 5)
OG IDs: 93521839
Best-hit-arabi-ID: AT3G55440.1
Best-hit-arabi-name: triosephosphate isomerase
Gorai.009G099100.1.D
PCC=-0.162, p=0.509
mRNA Profile
Protein Expression Profile
DPA

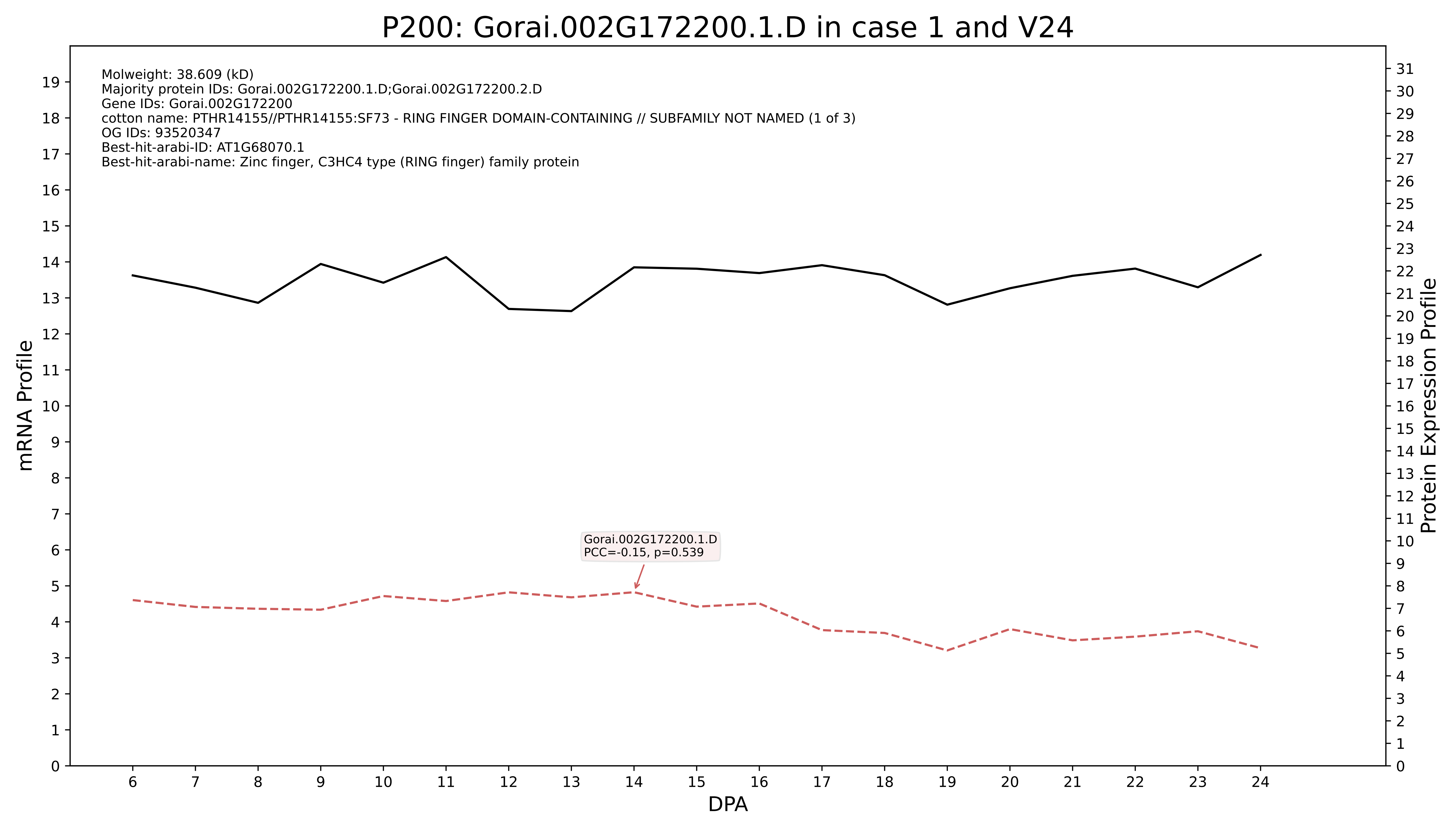

P200: Gorai.002G172200.1.D in case 1 and V24
Molweight: 38.609 (kD)
Majority protein IDs: Gorai.002G172200.1.D;Gorai.002G172200.2.D
Gene IDs: Gorai.002G172200
cotton name: PTHR14155//PTHR14155:SF73 - RING FINGER DOMAIN-CONTAINING // SUBFAMILY NOT NAMED (1 of 3)
OG IDs: 93520347
Best-hit-arabi-ID: AT1G68070.1
Best-hit-arabi-name: Zinc finger, C3HC4 type (RING finger) family protein
Gorai.002G172200.1.D
PCC=-0.15, p=0.539
mRNA Profile
Protein Expression Profile
DPA

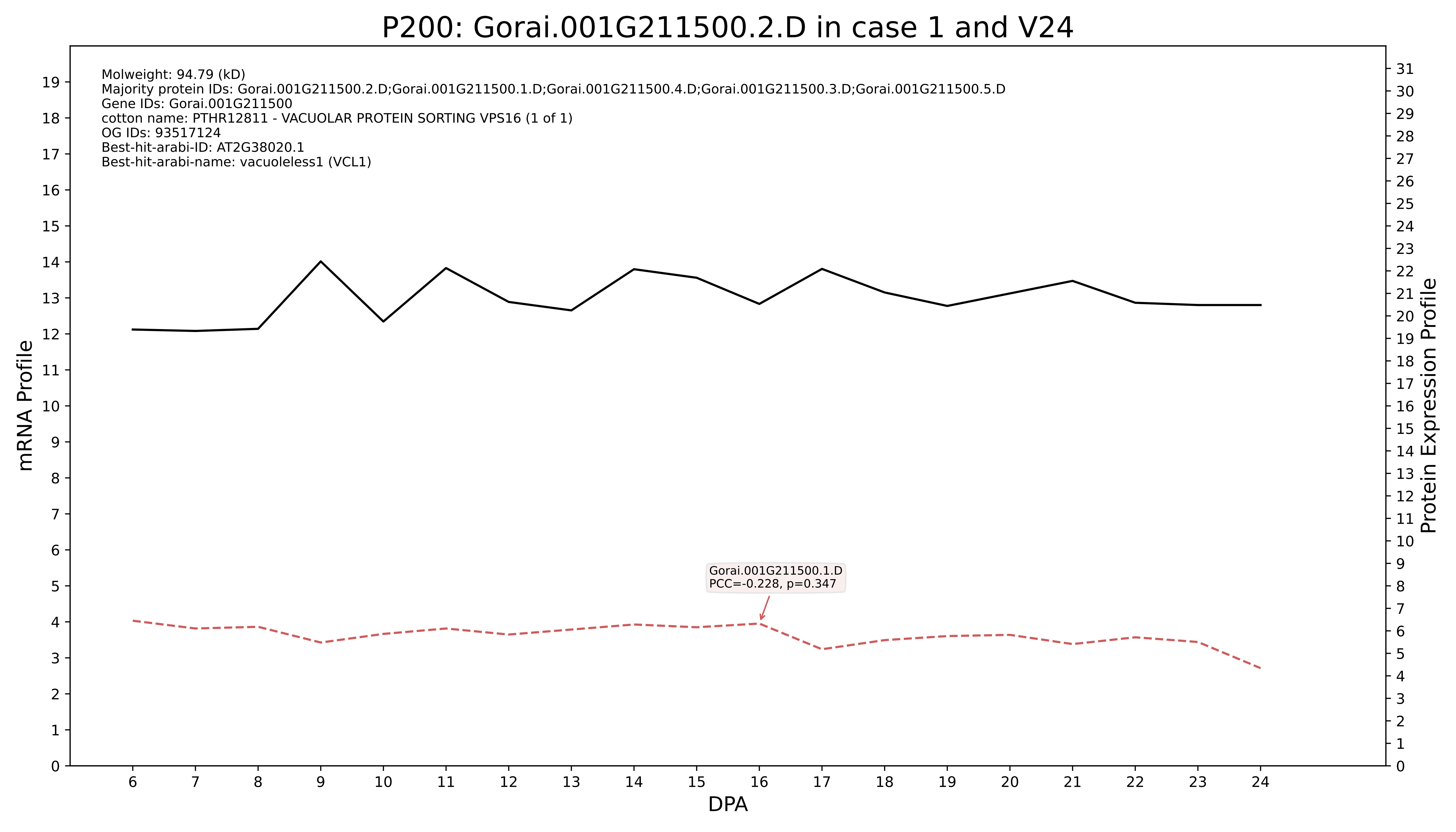
P200: Gorai.001G211500.2.D in case 1 and V24
Molweight: 94.79 (kD)
Majority protein IDs: Gorai.001G211500.2.D;Gorai.001G211500.1.D;Gorai.001G211500.4.D;Gorai.001G211500.3.D;Gorai.001G211500.5.D
Gene IDs: Gorai.001G211500
cotton name: PTHR12811 - VACUOLAR PROTEIN SORTING VPS16 (1 of 1)
OG IDs: 93517124
Best-hit-arabi-ID: AT2G38020.1
Best-hit-arabi-name: vacuoleless1 (VCL1)
Gorai.001G211500.1.D
PCC=-0.228, p=0.347
mRNA Profile
Protein Expression Profile
DPA

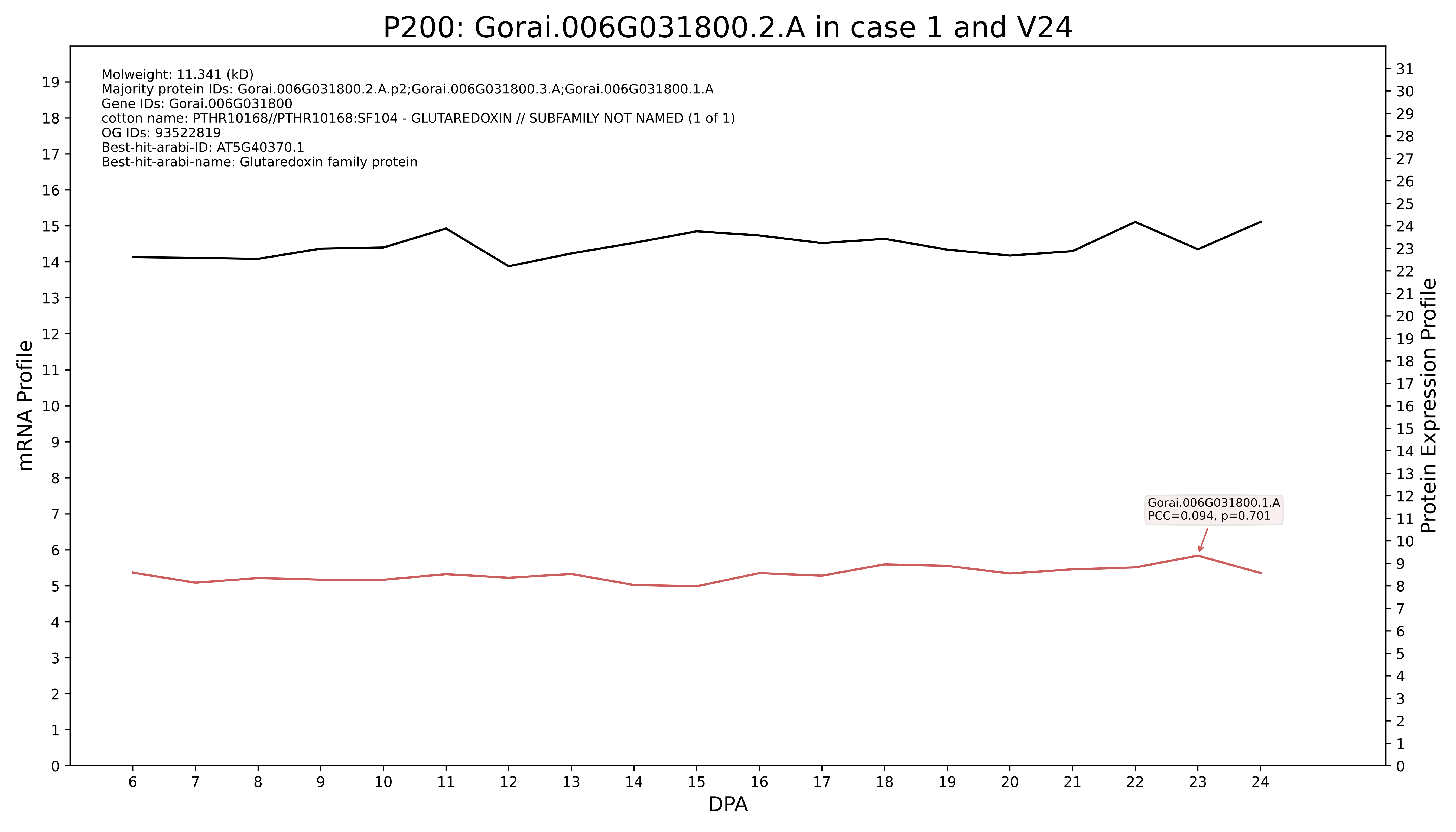
P200: Gorai.006G031800.2.A in case 1 and V24
Molweight: 11.341 (kD)
Majority protein IDs: Gorai.006G031800.2.A.p2;Gorai.006G031800.3.A;Gorai.006G031800.1.A
Gene IDs: Gorai.006G031800
cotton name: PTHR10168//PTHR10168:SF104 - GLUTAREDOXIN // SUBFAMILY NOT NAMED (1 of 1)
OG IDs: 93522819
Best-hit-arabi-ID: AT5G40370.1
Best-hit-arabi-name: Glutaredoxin family protein
Gorai.006G031800.1.A
PCC=0.094, p=0.701
mRNA Profile
Protein Expression Profile
DPA

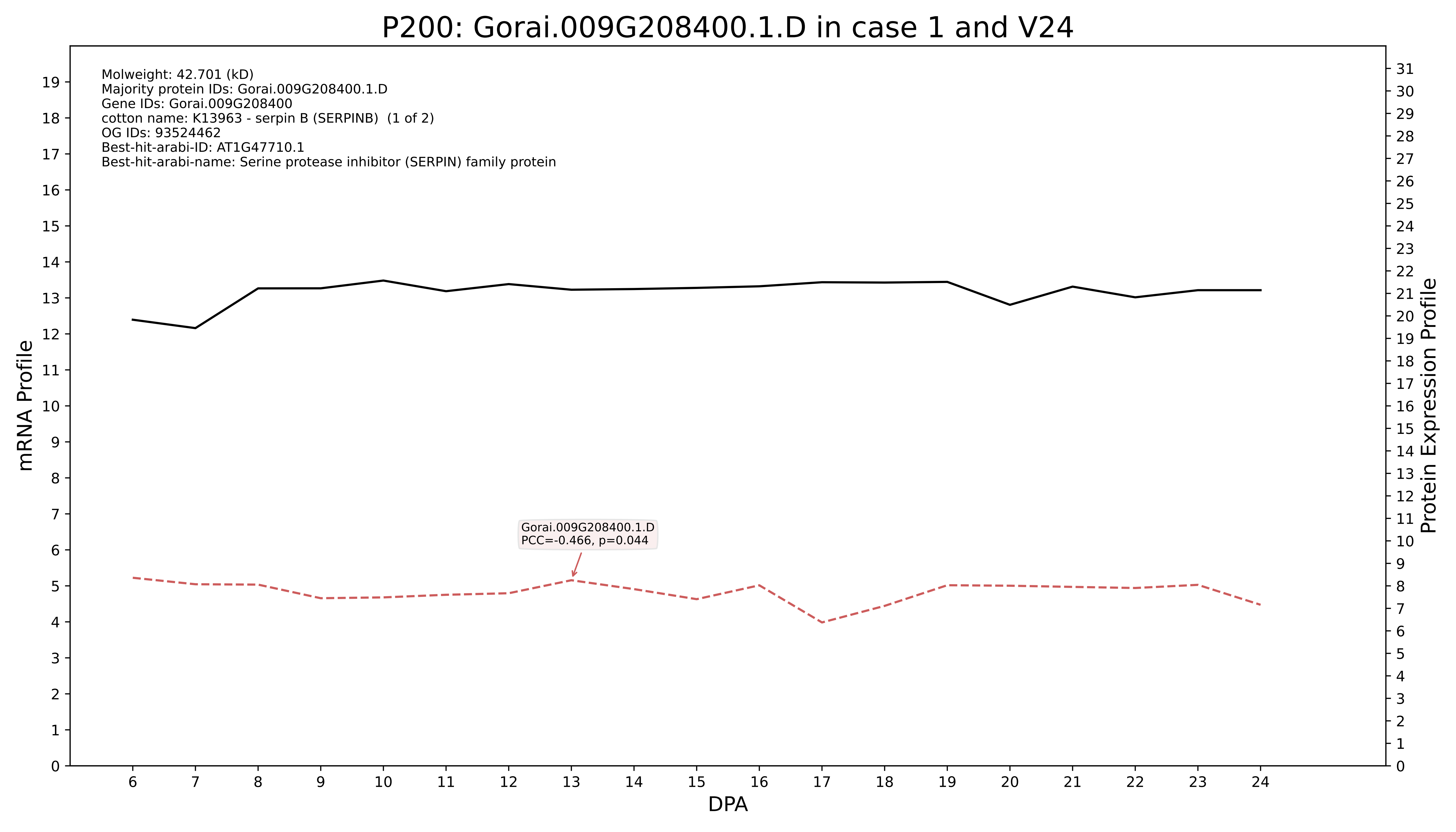
P200: Gorai.009G208400.1.D in case 1 and V24
Molweight: 42.701 (kD)
Majority protein IDs: Gorai.009G208400.1.D
Gene IDs: Gorai.009G208400
cotton name: K13963 - serpin B (SERPINB) (1 of 2)
OG IDs: 93524462
Best-hit-arabi-ID: AT1G47710.1
Best-hit-arabi-name: Serine protease inhibitor (SERPIN) family protein
Gorai.009G208400.1.D
PCC=-0.466, p=0.044
mRNA Profile
Protein Expression Profile
DPA
0
1
2
3
4
5
6
7
8
9
10
11
12
13
14
15
16
17
18
19
20
21
22
23
24
25
26
27
28
29
30
31

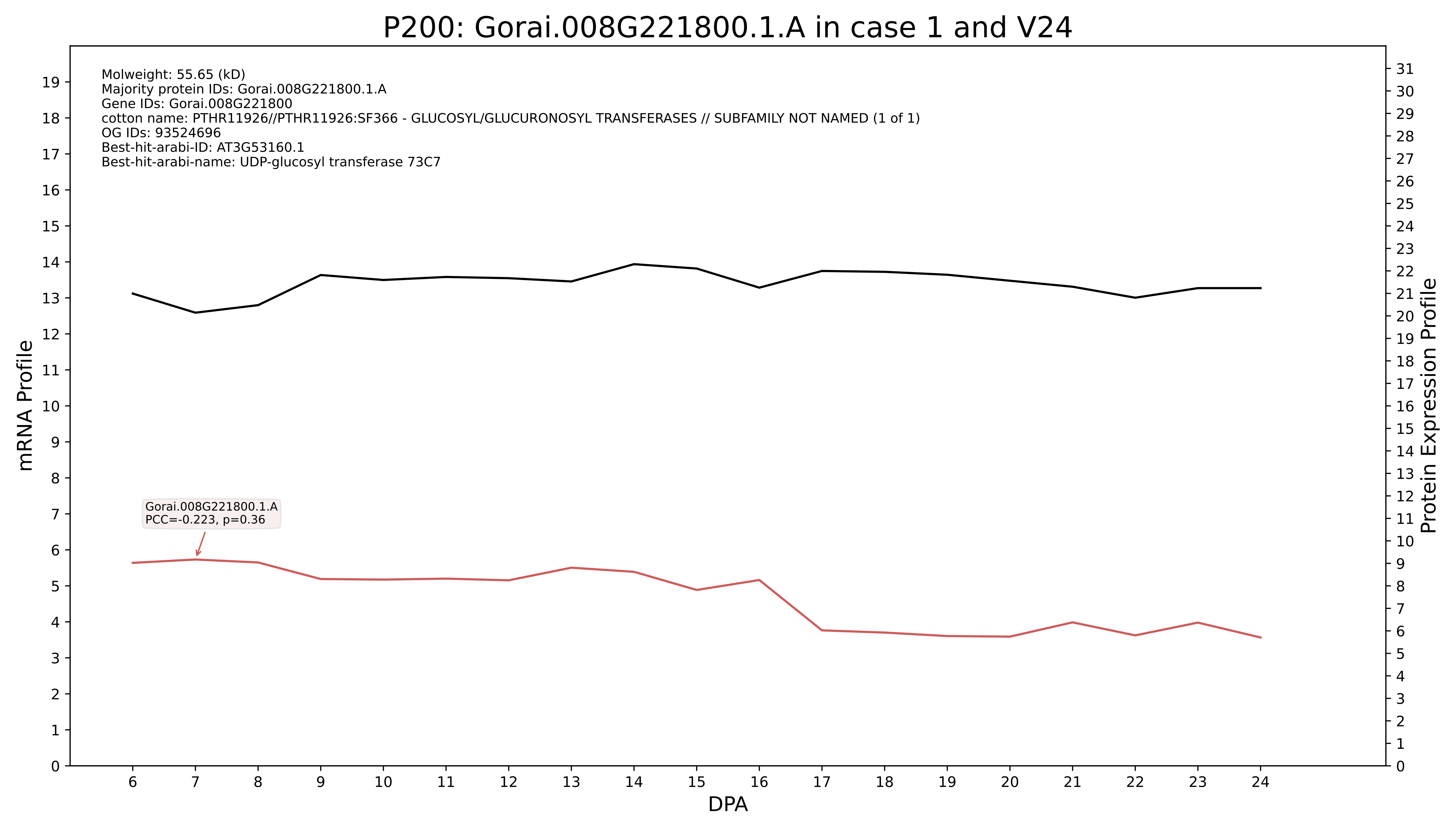
P200: Gorai.008G221800.1.A in case 1 and V24
Molweight: 55.65 (kD)
Majority protein IDs: Gorai.008G221800.1.A
Gene IDs: Gorai.008G221800
cotton name: PTHR11926//PTHR11926:SF366 - GLUCOSYL/GLUCURONOSYL TRANSFERASES // SUBFAMILY NOT NAMED (1 of 1)
OG IDs: 93524696
Best-hit-arabi-ID: AT3G53160.1
Best-hit-arabi-name: UDP-glucosyl transferase 73C7
Gorai.008G221800.1.A
PCC=-0.223, p=0.36
mRNA Profile
Protein Expression Profile
DPA
0
1
2
3
4
5
6
7
8
9
10
11
12
13
14
15
16
17
18
19
20
21
22
23
24
25
26
27
28
29
30
31

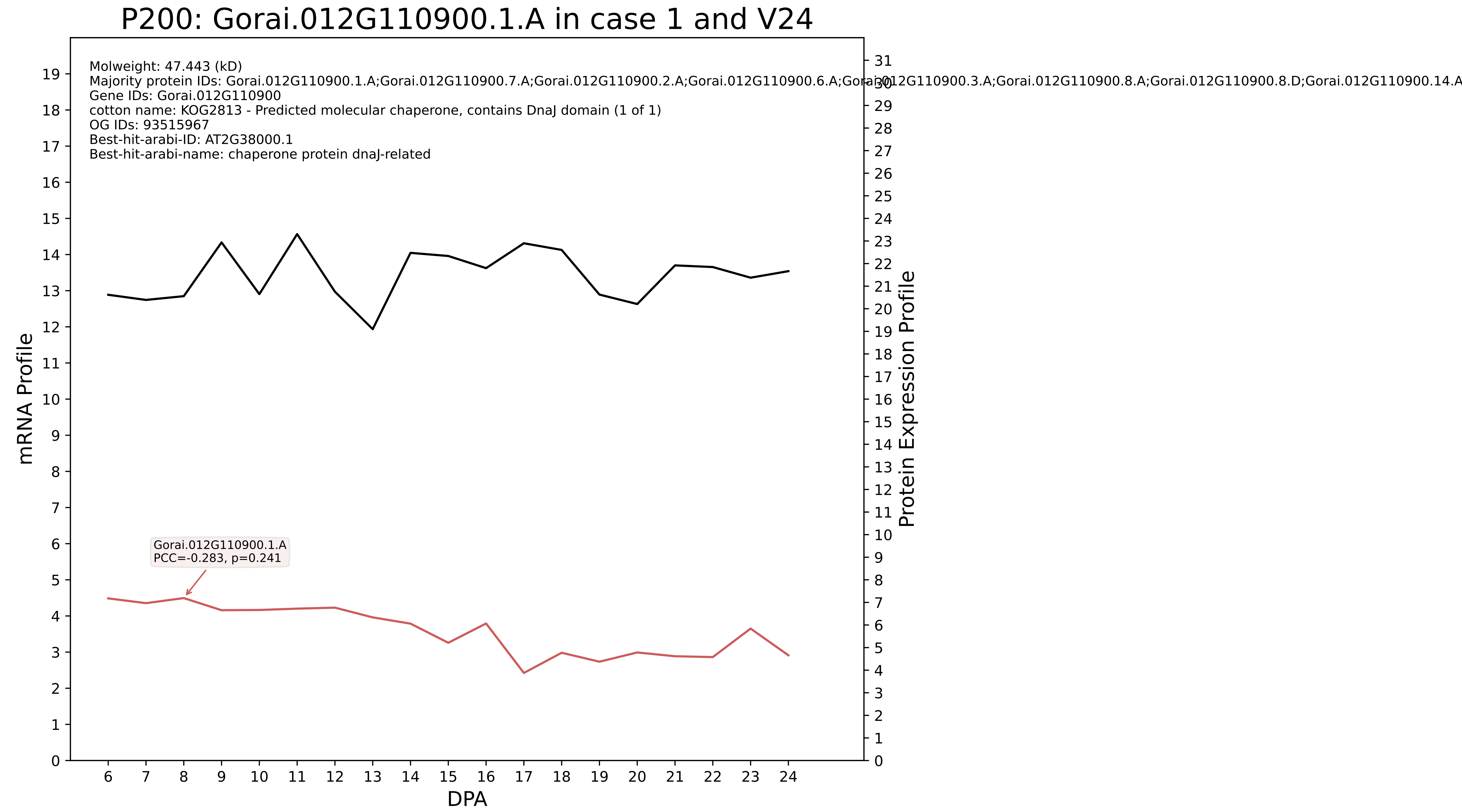
P200: Gorai.012G110900.1.A in case 1 and V24
Molweight: 47.443 (kD)
Majority protein IDs: Gorai.012G110900.1.A;Gorai.012G110900.7.A;Gorai.012G110900.2.A;Gorai.012G110900.6.A;Gorai.012G110900.3.A;Gorai.012G110900.8.A;Gorai.012G110900.8.D;Gorai.012G110900.14.A
Gene IDs: Gorai.012G110900
cotton name: KOG2813 - Predicted molecular chaperone, contains DnaJ domain (1 of 1)
OG IDs: 93515967
Best-hit-arabi-ID: AT2G38000.1
Best-hit-arabi-name: chaperone protein dnaJ-related
Gorai.012G110900.1.A
PCC=-0.283, p=0.241
mRNA Profile
Protein Expression Profile
DPA

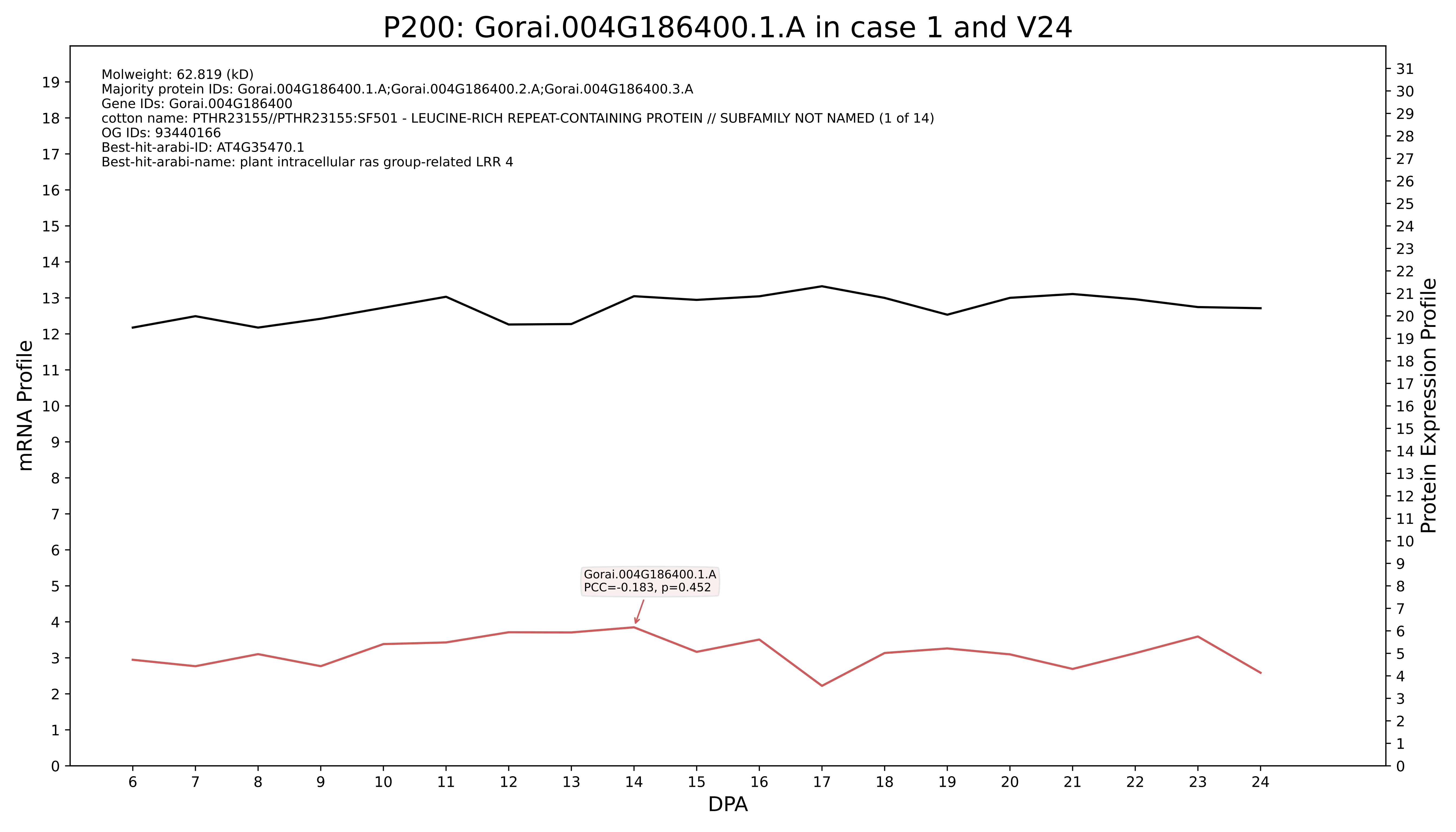

P200: Gorai.004G186400.1.A in case 1 and V24
Molweight: 62.819 (kD)
Majority protein IDs: Gorai.004G186400.1.A;Gorai.004G186400.2.A;Gorai.004G186400.3.A
Gene IDs: Gorai.004G186400
cotton name: PTHR23155//PTHR23155:SF501 - LEUCINE-RICH REPEAT-CONTAINING PROTEIN // SUBFAMILY NOT NAMED (1 of 14)
OG IDs: 93440166
Best-hit-arabi-ID: AT4G35470.1
Best-hit-arabi-name: plant intracellular ras group-related LRR 4
Gorai.004G186400.1.A
PCC=-0.183, p=0.452
mRNA Profile
Protein Expression Profile
DPA

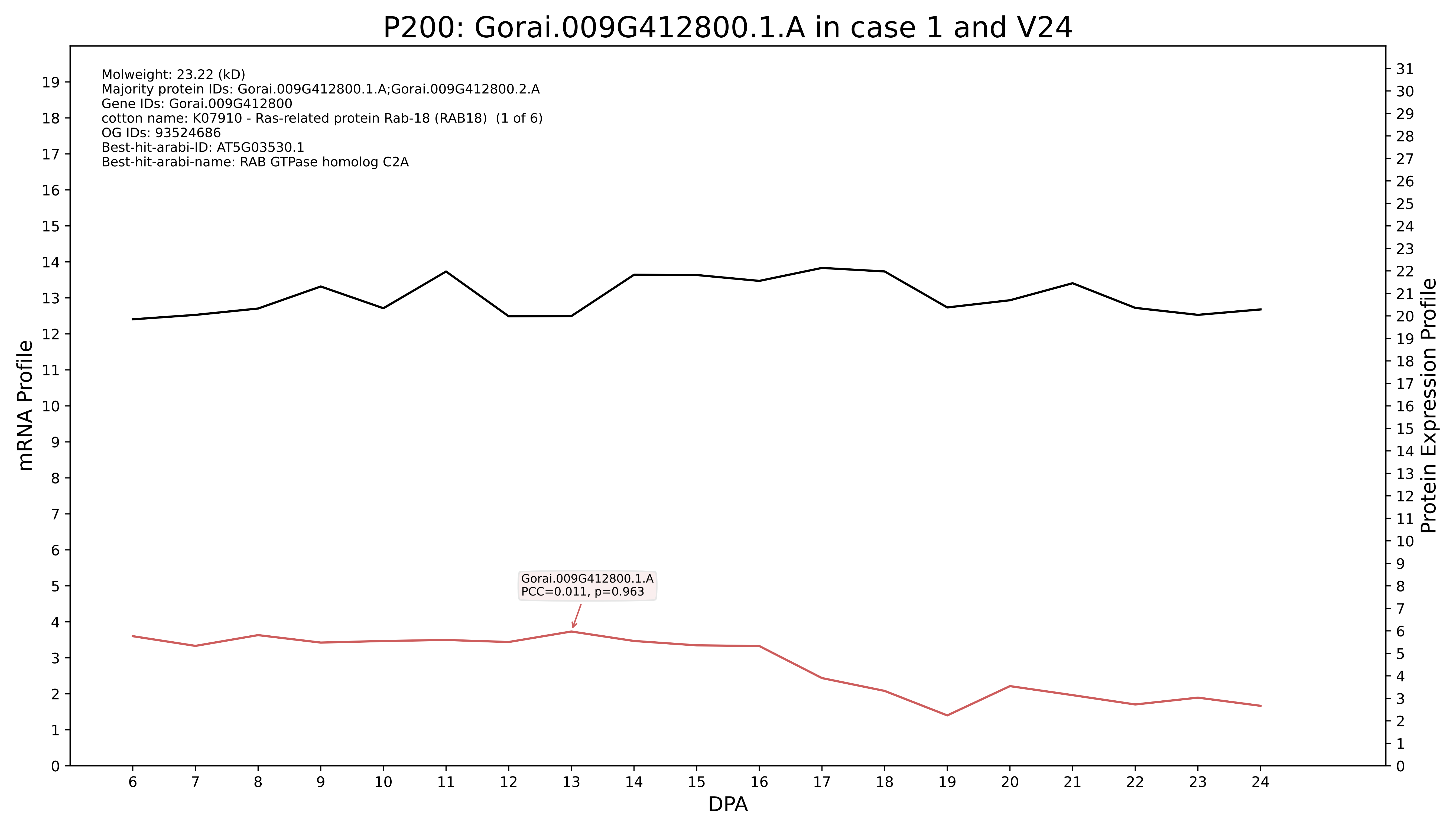
P200: Gorai.009G412800.1.A in case 1 and V24
Molweight: 23.22 (kD)
Majority protein IDs: Gorai.009G412800.1.A;Gorai.009G412800.2.A
Gene IDs: Gorai.009G412800
cotton name: K07910 - Ras-related protein Rab-18 (RAB18) (1 of 6)
OG IDs: 93524686
Best-hit-arabi-ID: AT5G03530.1
Best-hit-arabi-name: RAB GTPase homolog C2A
Gorai.009G412800.1.A
PCC=0.011, p=0.963
mRNA Profile
Protein Expression Profile
DPA

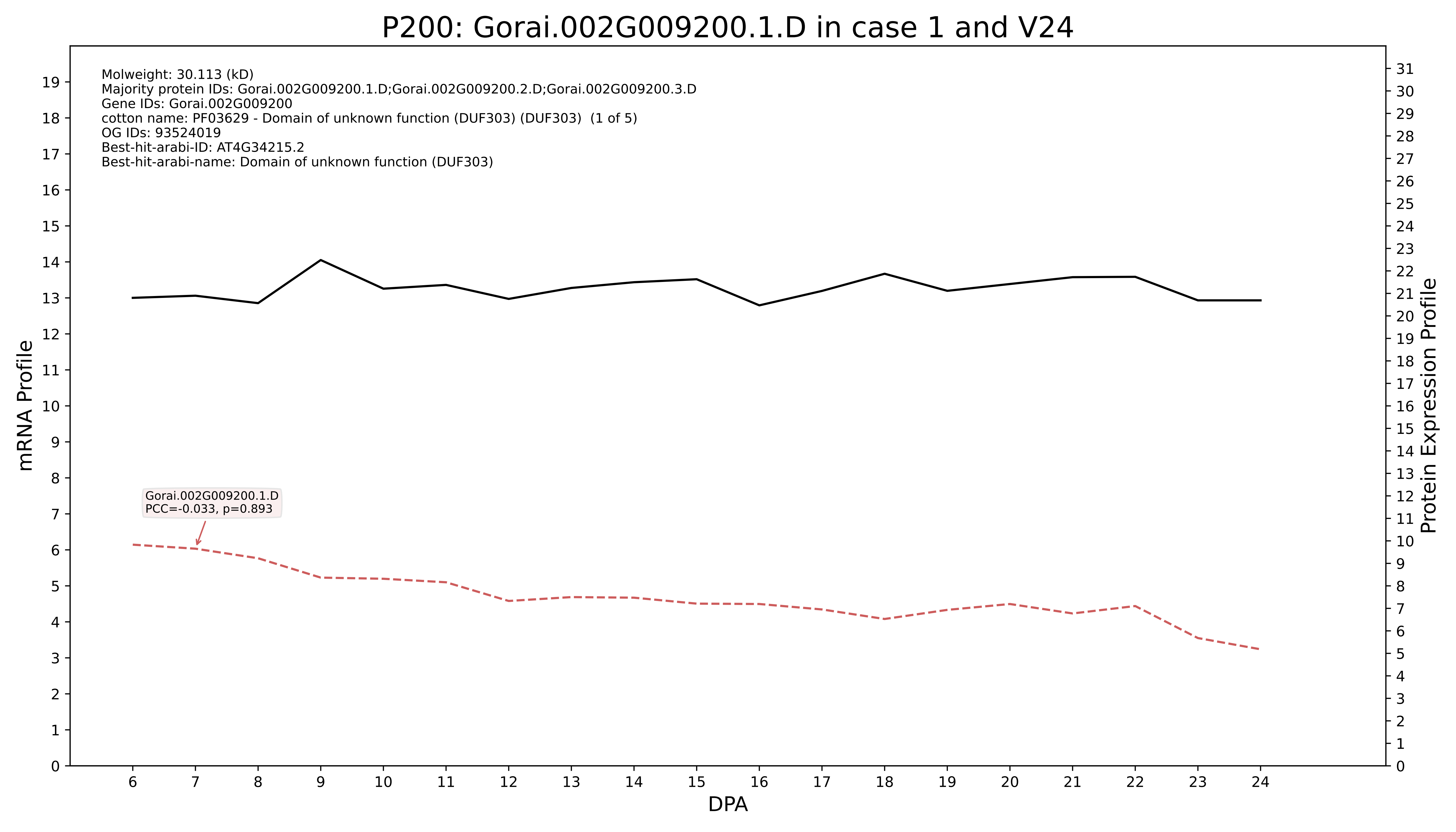
P200: Gorai.002G009200.1.D in case 1 and V24
Molweight: 30.113 (kD)
Majority protein IDs: Gorai.002G009200.1.D;Gorai.002G009200.2.D;Gorai.002G009200.3.D
Gene IDs: Gorai.002G009200
cotton name: PF03629 - Domain of unknown function (DUF303) (DUF303) (1 of 5)
OG IDs: 93524019
Best-hit-arabi-ID: AT4G34215.2
Best-hit-arabi-name: Domain of unknown function (DUF303)
Gorai.002G009200.1.D
PCC=-0.033, p=0.893
mRNA Profile
Protein Expression Profile
DPA
0
1
2
3
4
5
6
7
8
9
10
11
12
13
14
15
16
17
18
19
20
21
22
23
24
25
26
27
28
29
30
31

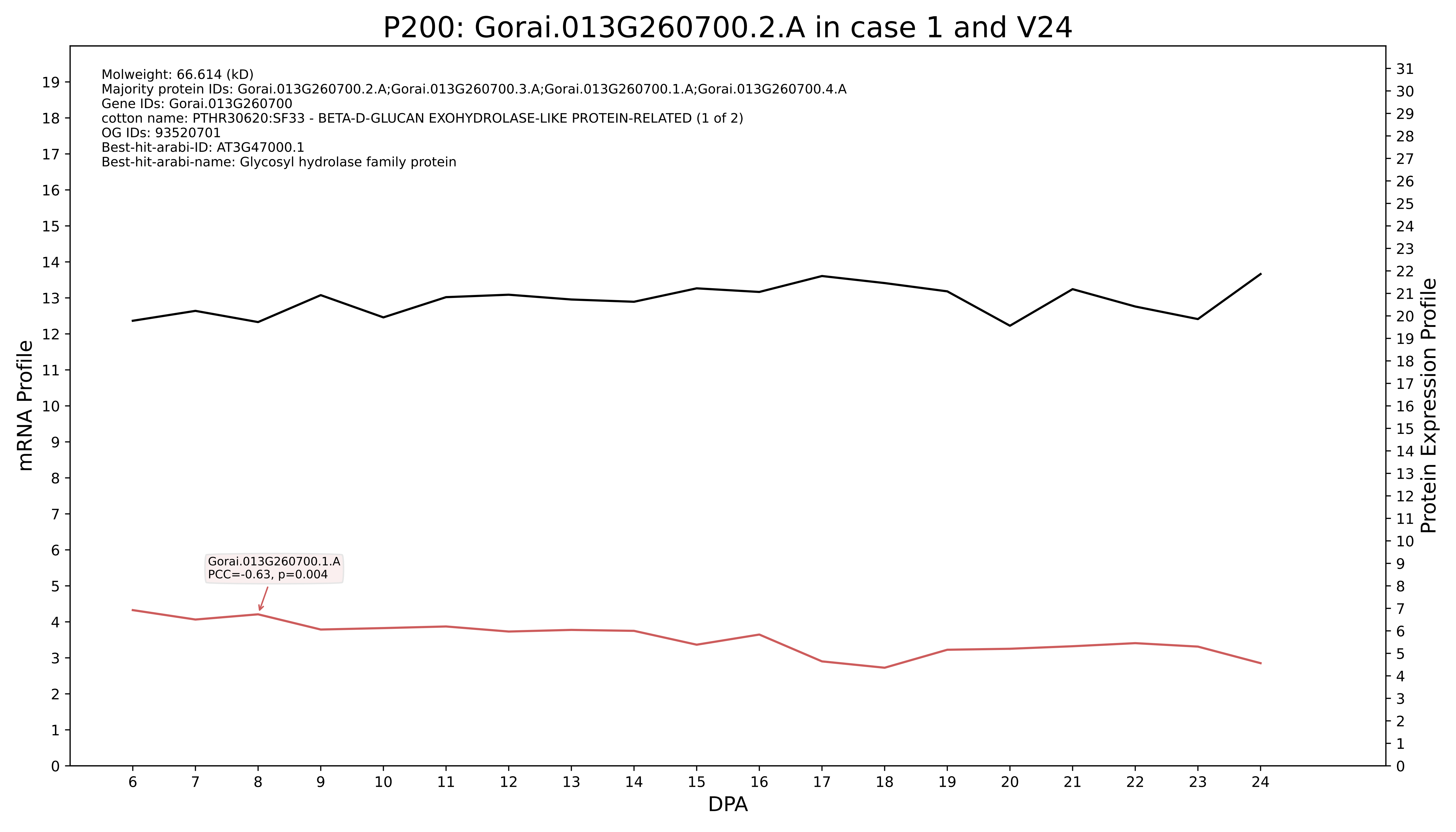
P200: Gorai.013G260700.2.A in case 1 and V24
Molweight: 66.614 (kD)
Majority protein IDs: Gorai.013G260700.2.A;Gorai.013G260700.3.A;Gorai.013G260700.1.A;Gorai.013G260700.4.A
Gene IDs: Gorai.013G260700
cotton name: PTHR30620:SF33 - BETA-D-GLUCAN EXOHYDROLASE-LIKE PROTEIN-RELATED (1 of 2)
OG IDs: 93520701
Best-hit-arabi-ID: AT3G47000.1
Best-hit-arabi-name: Glycosyl hydrolase family protein
Gorai.013G260700.1.A
PCC=-0.63, p=0.004
mRNA Profile
Protein Expression Profile
DPA

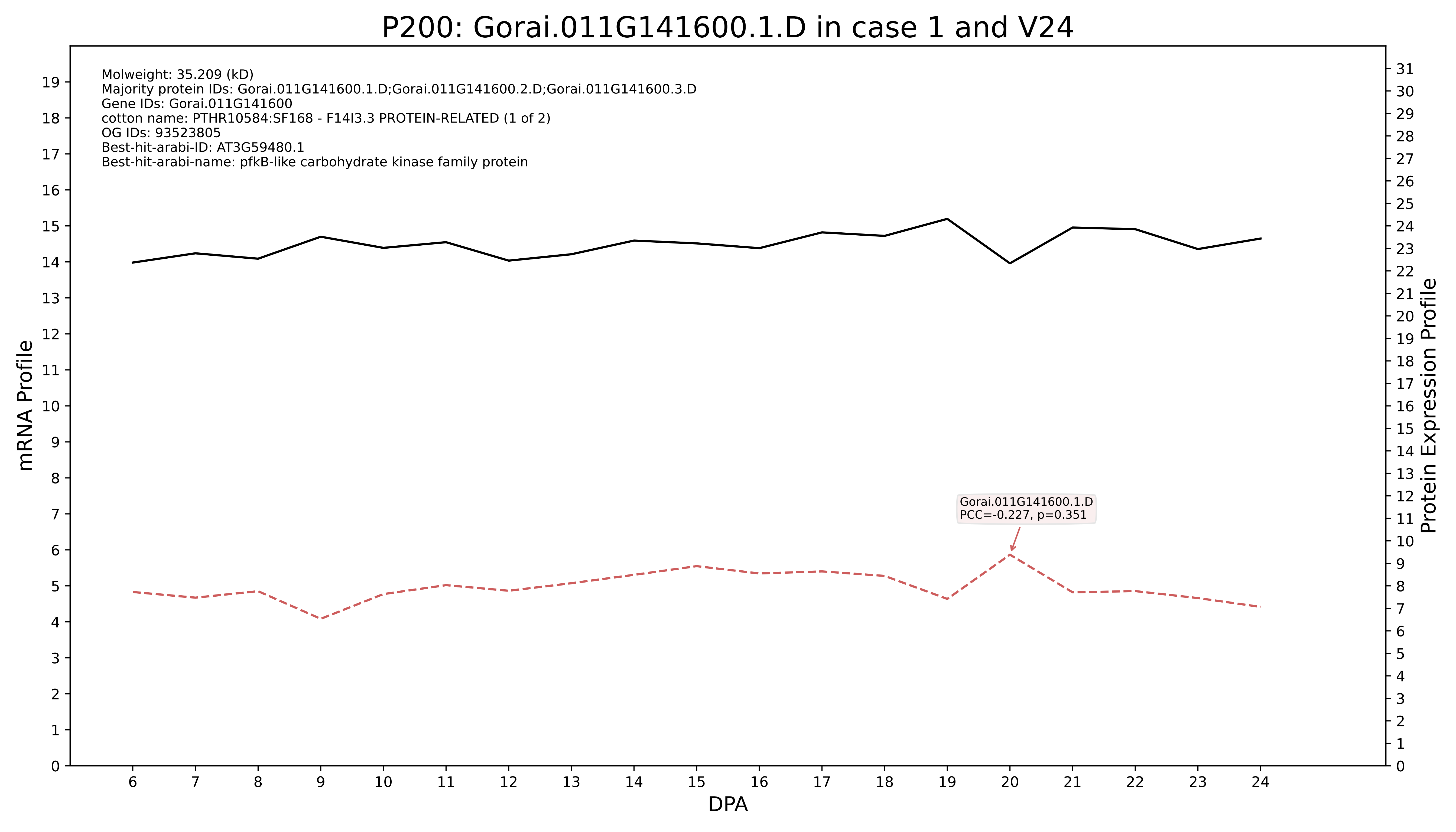
P200: Gorai.011G141600.1.D in case 1 and V24
Molweight: 35.209 (kD)
Majority protein IDs: Gorai.011G141600.1.D;Gorai.011G141600.2.D;Gorai.011G141600.3.D
Gene IDs: Gorai.011G141600
cotton name: PTHR10584:SF168 - F14I3.3 PROTEIN-RELATED (1 of 2)
OG IDs: 93523805
Best-hit-arabi-ID: AT3G59480.1
Best-hit-arabi-name: pfkB-like carbohydrate kinase family protein
Gorai.011G141600.1.D
PCC=-0.227, p=0.351
mRNA Profile
Protein Expression Profile
DPA

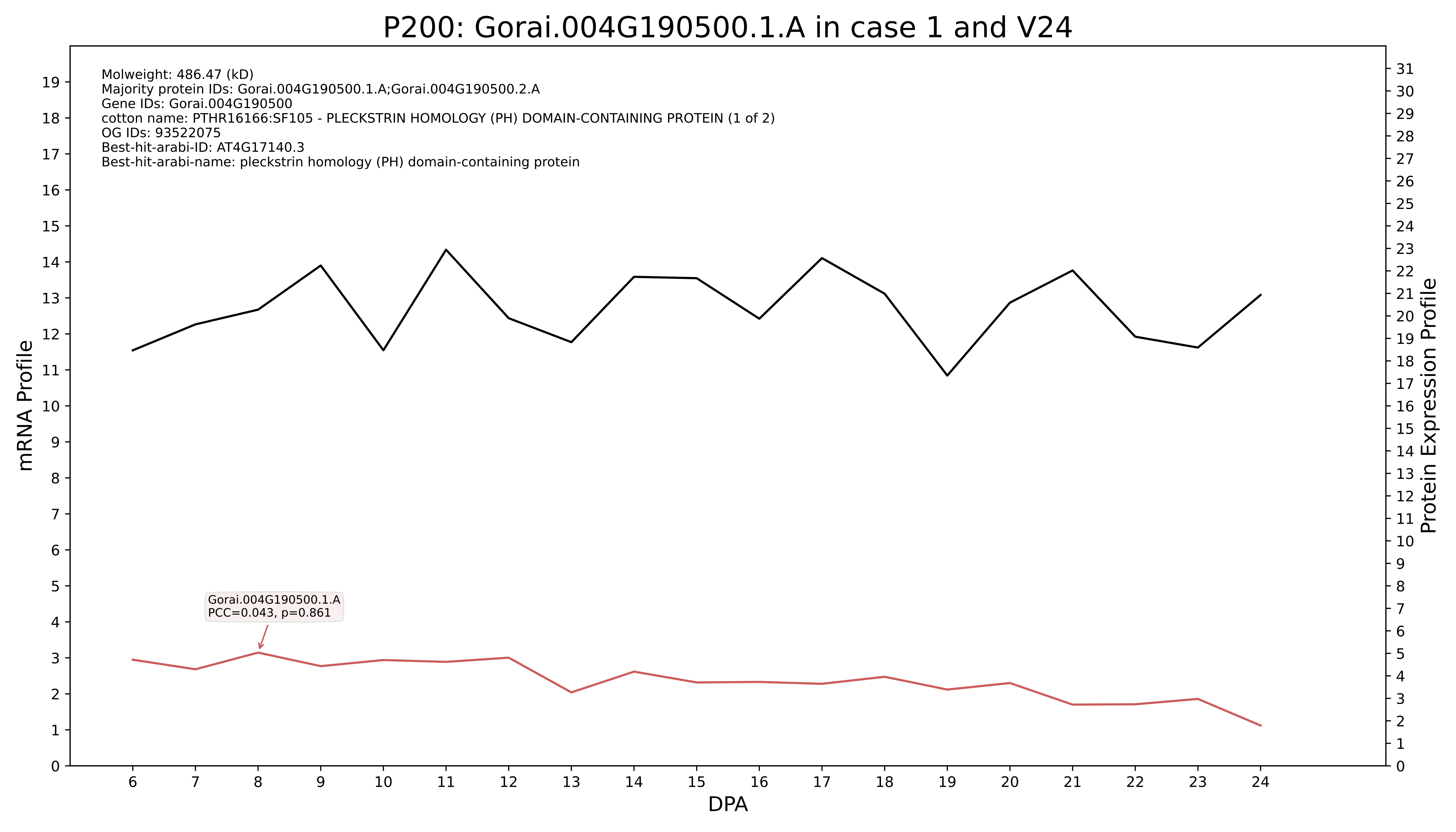
P200: Gorai.004G190500.1.A in case 1 and V24
Molweight: 486.47 (kD)
Majority protein IDs: Gorai.004G190500.1.A;Gorai.004G190500.2.A
Gene IDs: Gorai.004G190500
cotton name: PTHR16166:SF105 - PLECKSTRIN HOMOLOGY (PH) DOMAIN-CONTAINING PROTEIN (1 of 2)
OG IDs: 93522075
Best-hit-arabi-ID: AT4G17140.3
Best-hit-arabi-name: pleckstrin homology (PH) domain-containing protein
Gorai.004G190500.1.A
PCC=0.043, p=0.861
mRNA Profile
Protein Expression Profile
DPA

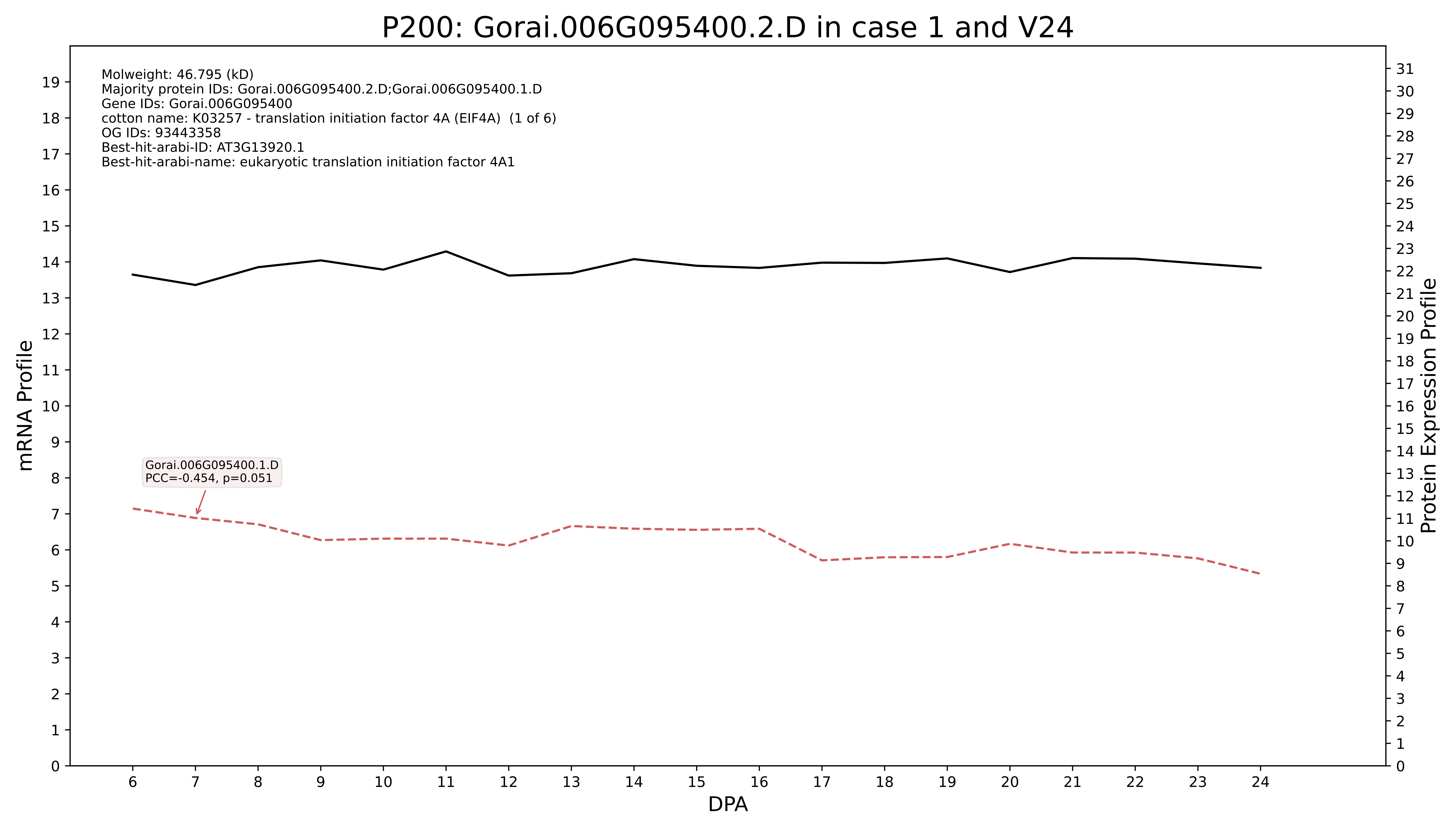

P200: Gorai.006G095400.2.D in case 1 and V24
Molweight: 46.795 (kD)
Majority protein IDs: Gorai.006G095400.2.D;Gorai.006G095400.1.D
Gene IDs: Gorai.006G095400
cotton name: K03257 - translation initiation factor 4A (EIF4A) (1 of 6)
OG IDs: 93443358
Best-hit-arabi-ID: AT3G13920.1
Best-hit-arabi-name: eukaryotic translation initiation factor 4A1
Gorai.006G095400.1.D
PCC=-0.454, p=0.051
mRNA Profile
Protein Expression Profile
DPA

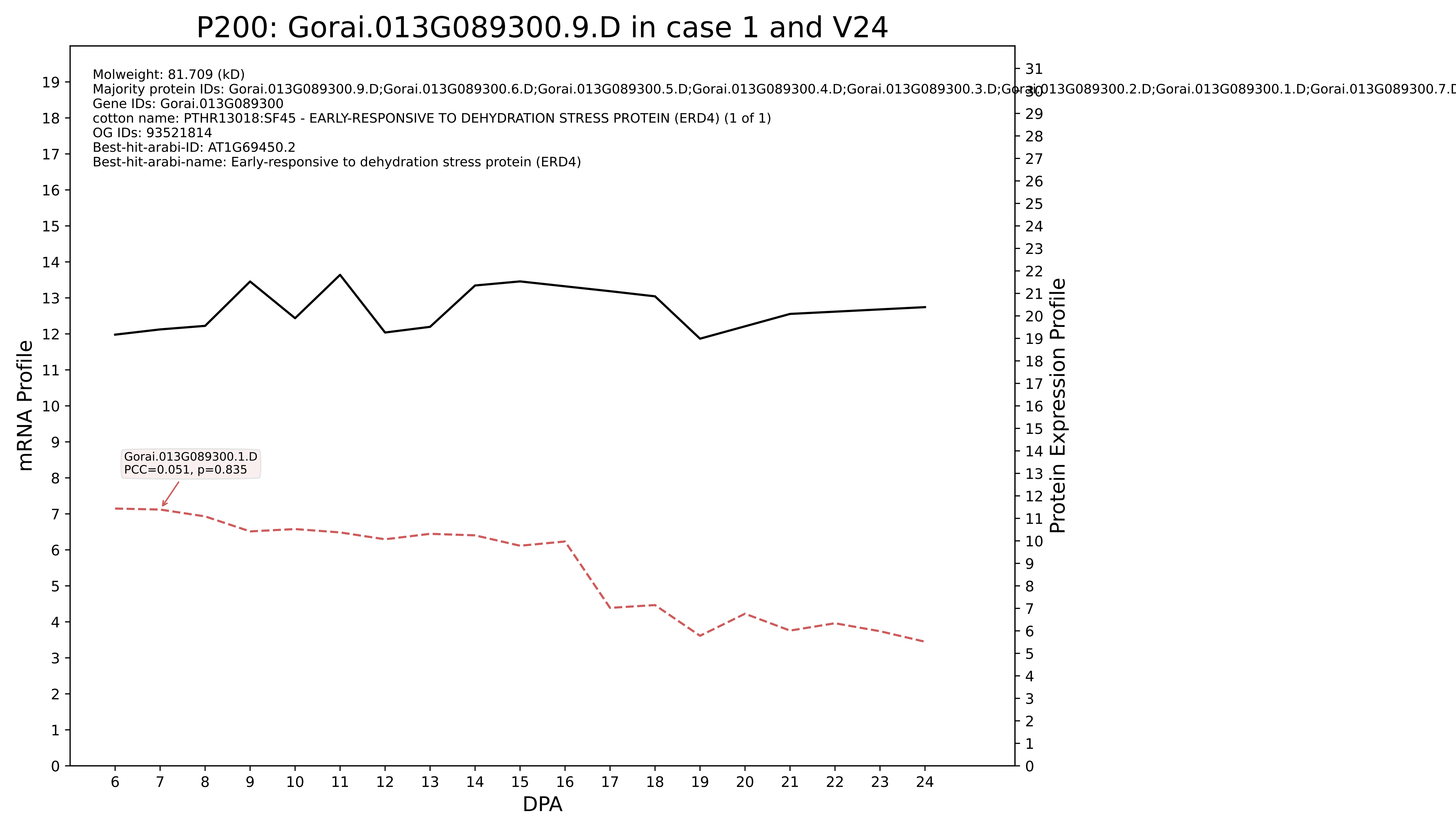
P200: Gorai.013G089300.9.D in case 1 and V24
Molweight: 81.709 (kD)
Majority protein IDs: Gorai.013G089300.9.D;Gorai.013G089300.6.D;Gorai.013G089300.5.D;Gorai.013G089300.4.D;Gorai.013G089300.3.D;Gorai.013G089300.2.D;Gorai.013G089300.1.D;Gorai.013G089300.7.D
Gene IDs: Gorai.013G089300
cotton name: PTHR13018:SF45 - EARLY-RESPONSIVE TO DEHYDRATION STRESS PROTEIN (ERD4) (1 of 1)
OG IDs: 93521814
Best-hit-arabi-ID: AT1G69450.2
Best-hit-arabi-name: Early-responsive to dehydration stress protein (ERD4)
Gorai.013G089300.1.D
PCC=0.051, p=0.835
mRNA Profile
Protein Expression Profile
DPA

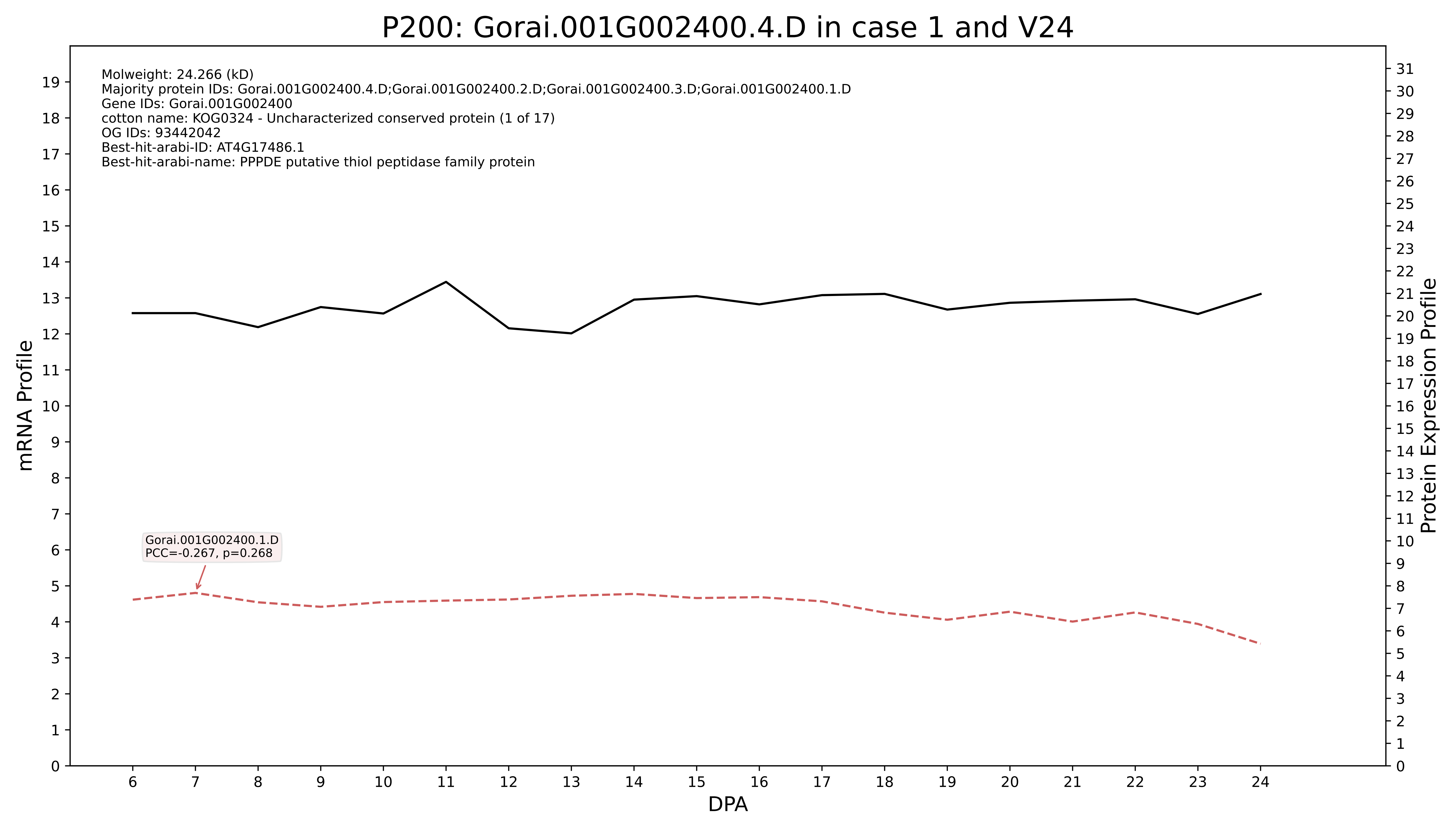
P200: Gorai.001G002400.4.D in case 1 and V24
Molweight: 24.266 (kD)
Majority protein IDs: Gorai.001G002400.4.D;Gorai.001G002400.2.D;Gorai.001G002400.3.D;Gorai.001G002400.1.D
Gene IDs: Gorai.001G002400
cotton name: KOG0324 - Uncharacterized conserved protein (1 of 17)
OG IDs: 93442042
Best-hit-arabi-ID: AT4G17486.1
Best-hit-arabi-name: PPPDE putative thiol peptidase family protein
Gorai.001G002400.1.D
PCC=-0.267, p=0.268
mRNA Profile
Protein Expression Profile
DPA

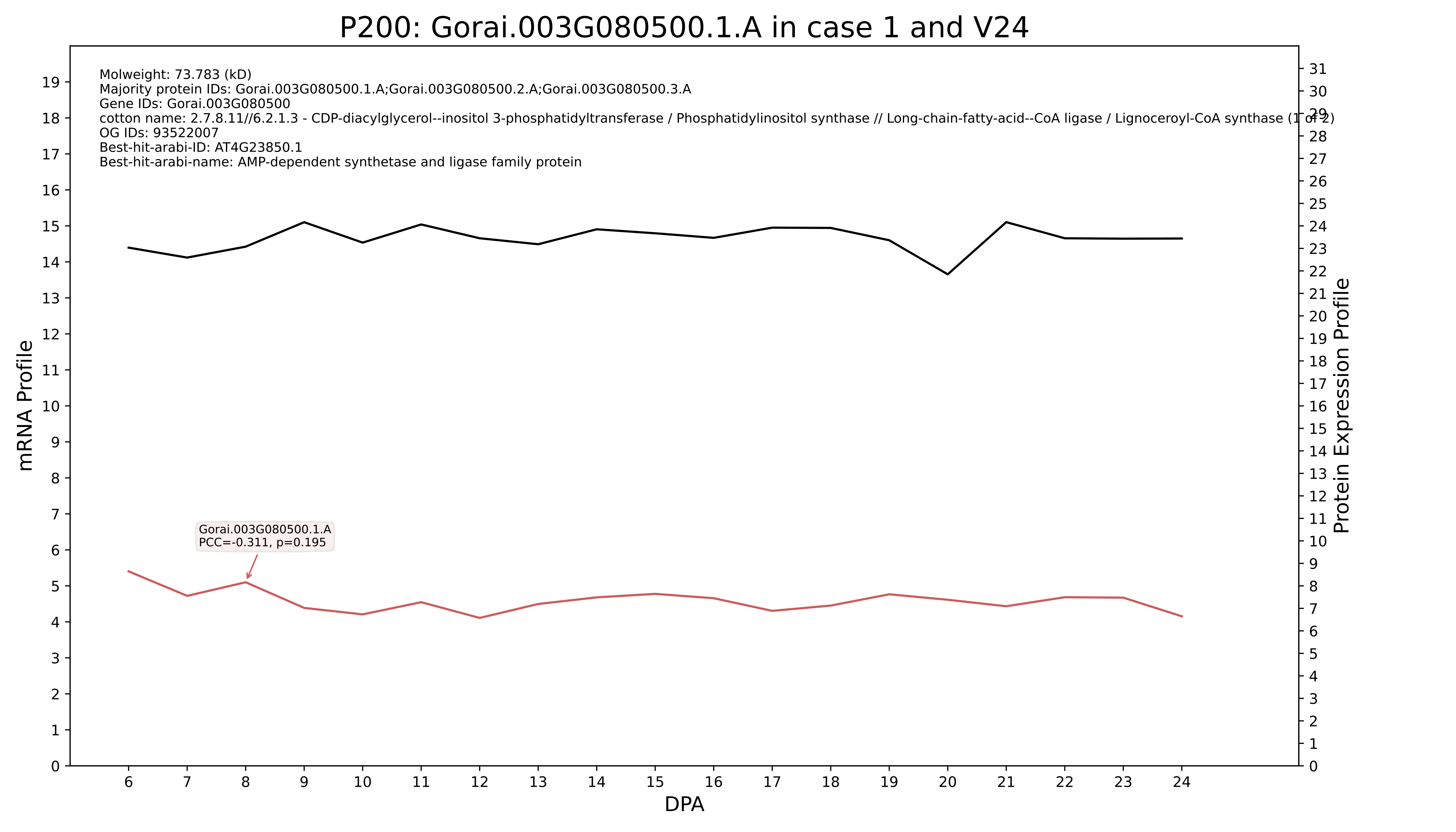

P200: Gorai.003G080500.1.A in case 1 and V24
Molweight: 73.783 (kD)
Majority protein IDs: Gorai.003G080500.1.A;Gorai.003G080500.2.A;Gorai.003G080500.3.A
Gene IDs: Gorai.003G080500
cotton name: 2.7.8.11//6.2.1.3 - CDP-diacylglycerol--inositol 3-phosphatidyltransferase / Phosphatidylinositol synthase // Long-chain-fatty-acid--CoA ligase / Lignoceroyl-CoA synthase (1 of 2)
OG IDs: 93522007
Best-hit-arabi-ID: AT4G23850.1
Best-hit-arabi-name: AMP-dependent synthetase and ligase family protein
Gorai.003G080500.1.A
PCC=-0.311, p=0.195
mRNA Profile
Protein Expression Profile
DPA

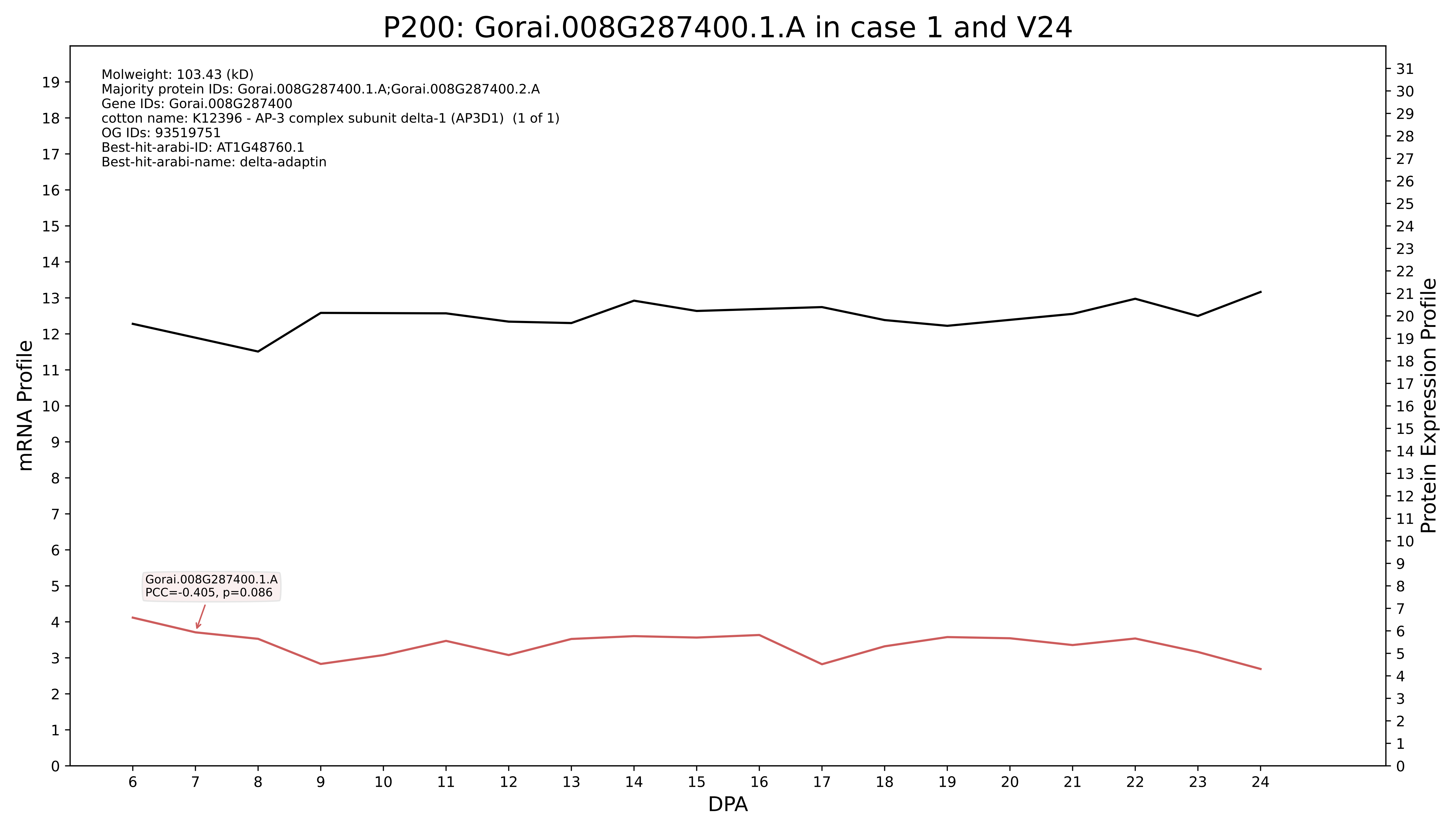
P200: Gorai.008G287400.1.A in case 1 and V24
Molweight: 103.43 (kD)
Majority protein IDs: Gorai.008G287400.1.A;Gorai.008G287400.2.A
Gene IDs: Gorai.008G287400
cotton name: K12396 - AP-3 complex subunit delta-1 (AP3D1) (1 of 1)
OG IDs: 93519751
Best-hit-arabi-ID: AT1G48760.1
Best-hit-arabi-name: delta-adaptin
Gorai.008G287400.1.A
PCC=-0.405, p=0.086
mRNA Profile
Protein Expression Profile
DPA

P200: Gorai.013G124500.2.D in case 1 and V24

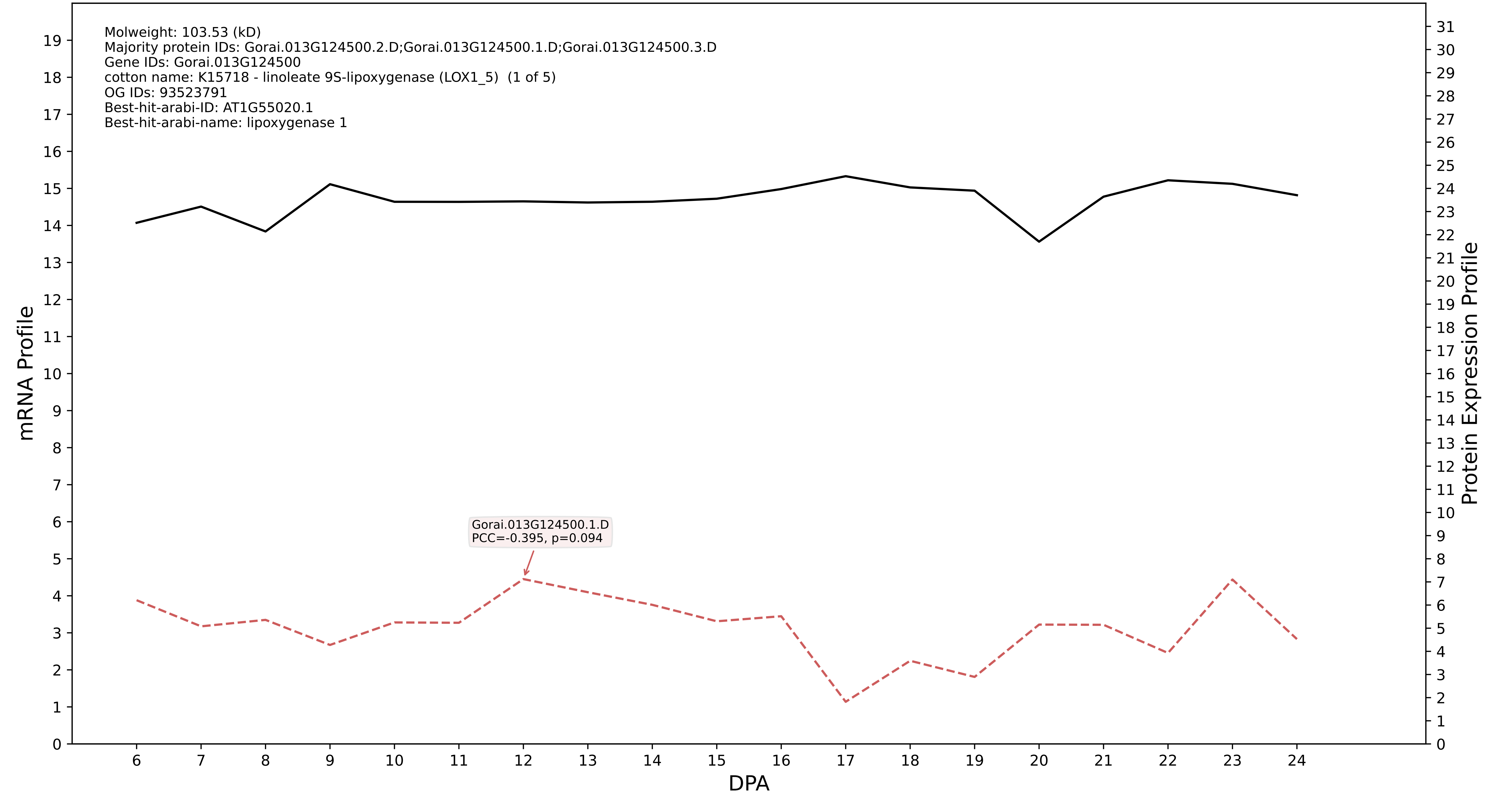

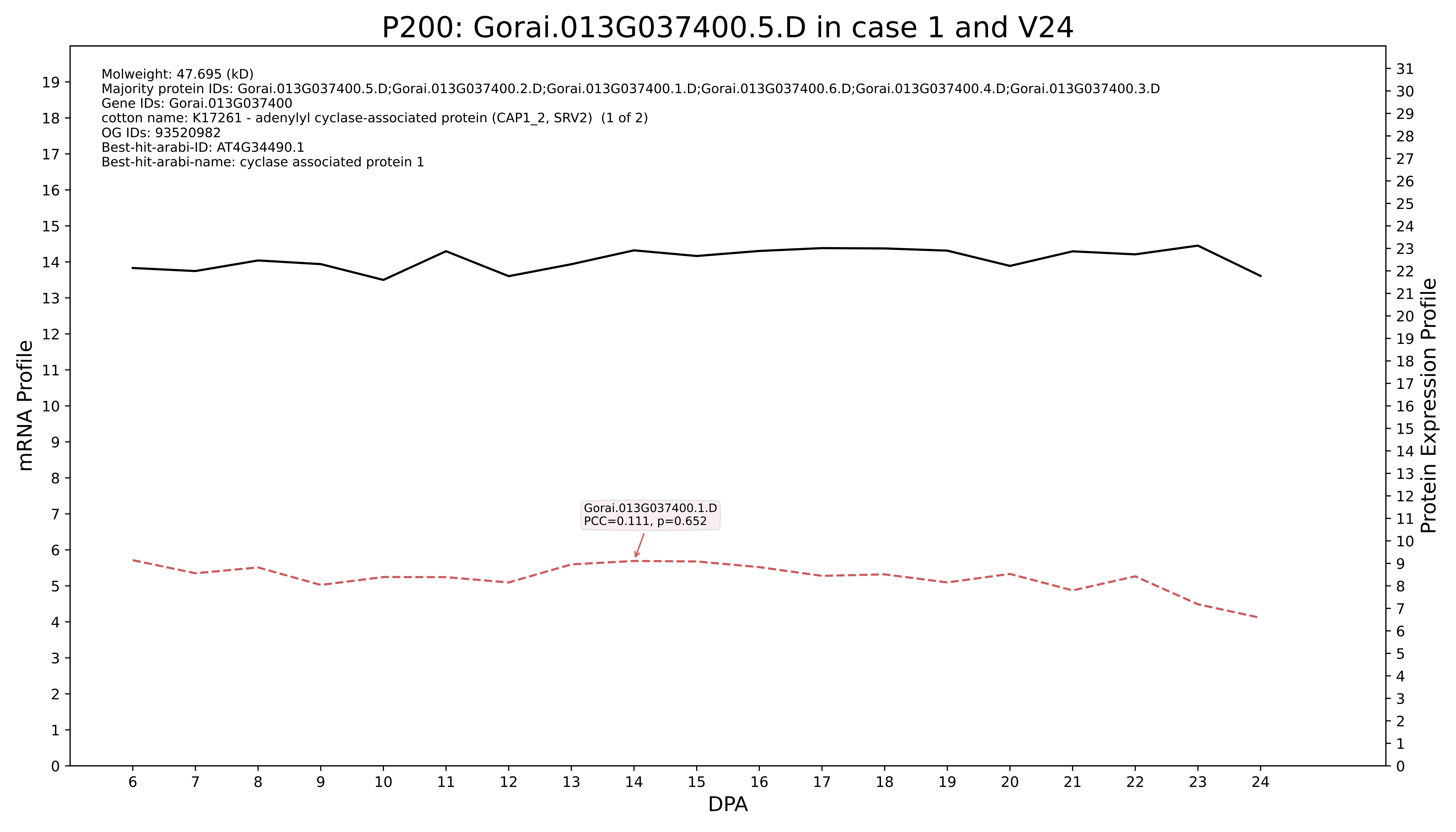
P200: Gorai.013G037400.5.D in case 1 and V24
Molweight: 47.695 (kD)
Majority protein IDs: Gorai.013G037400.5.D;Gorai.013G037400.2.D;Gorai.013G037400.1.D;Gorai.013G037400.6.D;Gorai.013G037400.4.D;Gorai.013G037400.3.D
Gene IDs: Gorai.013G037400
cotton name: K17261 - adenylyl cyclase-associated protein (CAP1_2, SRV2) (1 of 2)
OG IDs: 93520982
Best-hit-arabi-ID: AT4G34490.1
Best-hit-arabi-name: cyclase associated protein 1
Gorai.013G037400.1.D
PCC=0.111, p=0.652
mRNA Profile
Protein Expression Profile
DPA

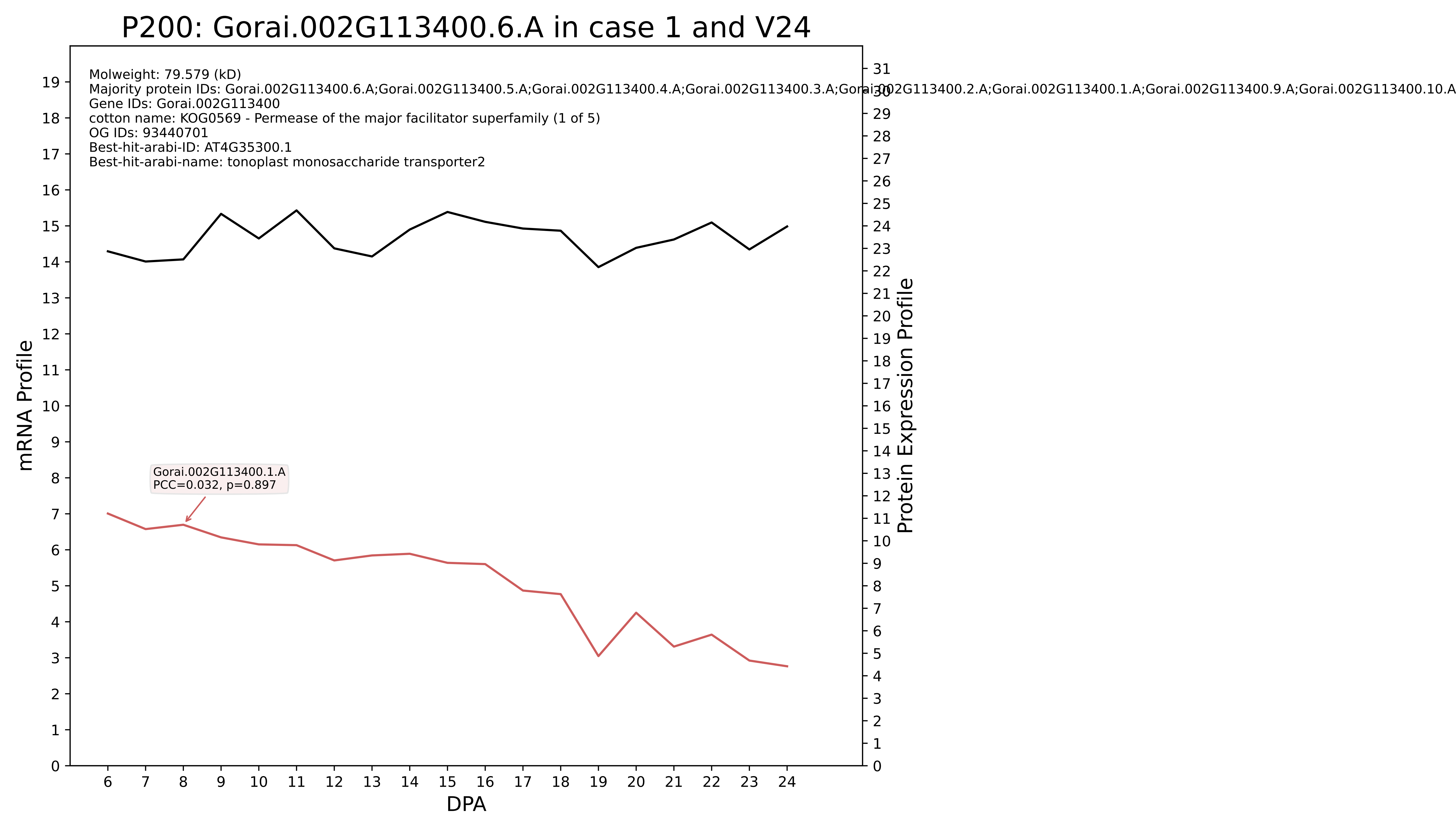
P200: Gorai.002G113400.6.A in case 1 and V24
Molweight: 79.579 (kD)
Majority protein IDs: Gorai.002G113400.6.A;Gorai.002G113400.5.A;Gorai.002G113400.4.A;Gorai.002G113400.3.A;Gorai.002G113400.2.A;Gorai.002G113400.1.A;Gorai.002G113400.9.A;Gorai.002G113400.10.A
Gene IDs: Gorai.002G113400
cotton name: KOG0569 - Permease of the major facilitator superfamily (1 of 5)
OG IDs: 93440701
Best-hit-arabi-ID: AT4G35300.1
Best-hit-arabi-name: tonoplast monosaccharide transporter2
Gorai.002G113400.1.A
PCC=0.032, p=0.897
mRNA Profile
Protein Expression Profile
DPA

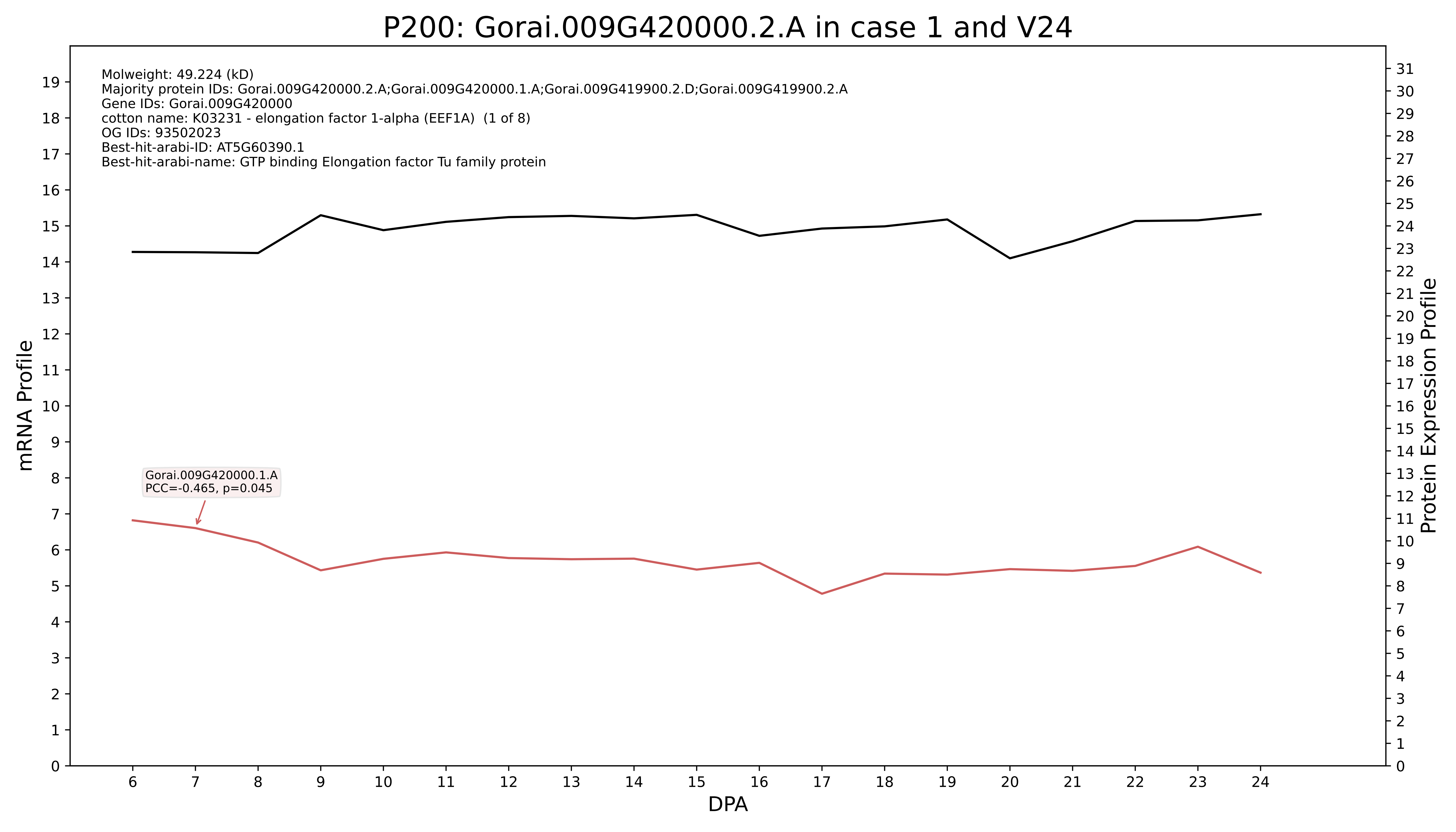
P200: Gorai.009G420000.2.A in case 1 and V24
Molweight: 49.224 (kD)
Majority protein IDs: Gorai.009G420000.2.A;Gorai.009G420000.1.A;Gorai.009G419900.2.D;Gorai.009G419900.2.A
Gene IDs: Gorai.009G420000
cotton name: K03231 - elongation factor 1-alpha (EEF1A) (1 of 8)
OG IDs: 93502023
Best-hit-arabi-ID: AT5G60390.1
Best-hit-arabi-name: GTP binding Elongation factor Tu family protein
Gorai.009G420000.1.A
PCC=-0.465, p=0.045
mRNA Profile
Protein Expression Profile
DPA

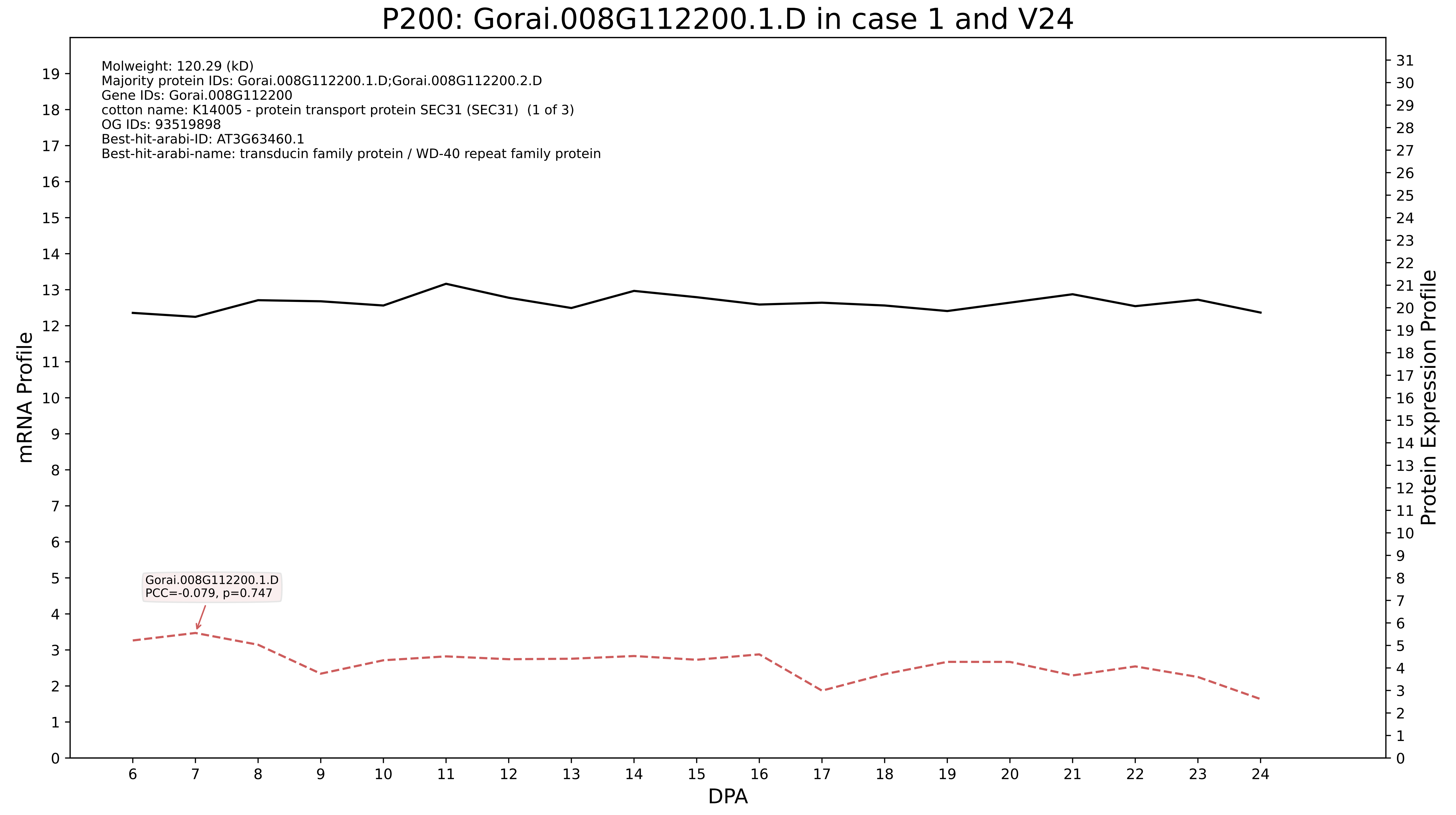
P200: Gorai.008G112200.1.D in case 1 and V24
Molweight: 120.29 (kD)
Majority protein IDs: Gorai.008G112200.1.D;Gorai.008G112200.2.D
Gene IDs: Gorai.008G112200
cotton name: K14005 - protein transport protein SEC31 (SEC31) (1 of 3)
OG IDs: 93519898
Best-hit-arabi-ID: AT3G63460.1
Best-hit-arabi-name: transducin family protein / WD-40 repeat family protein
Gorai.008G112200.1.D
PCC=-0.079, p=0.747
mRNA Profile
Protein Expression Profile
DPA

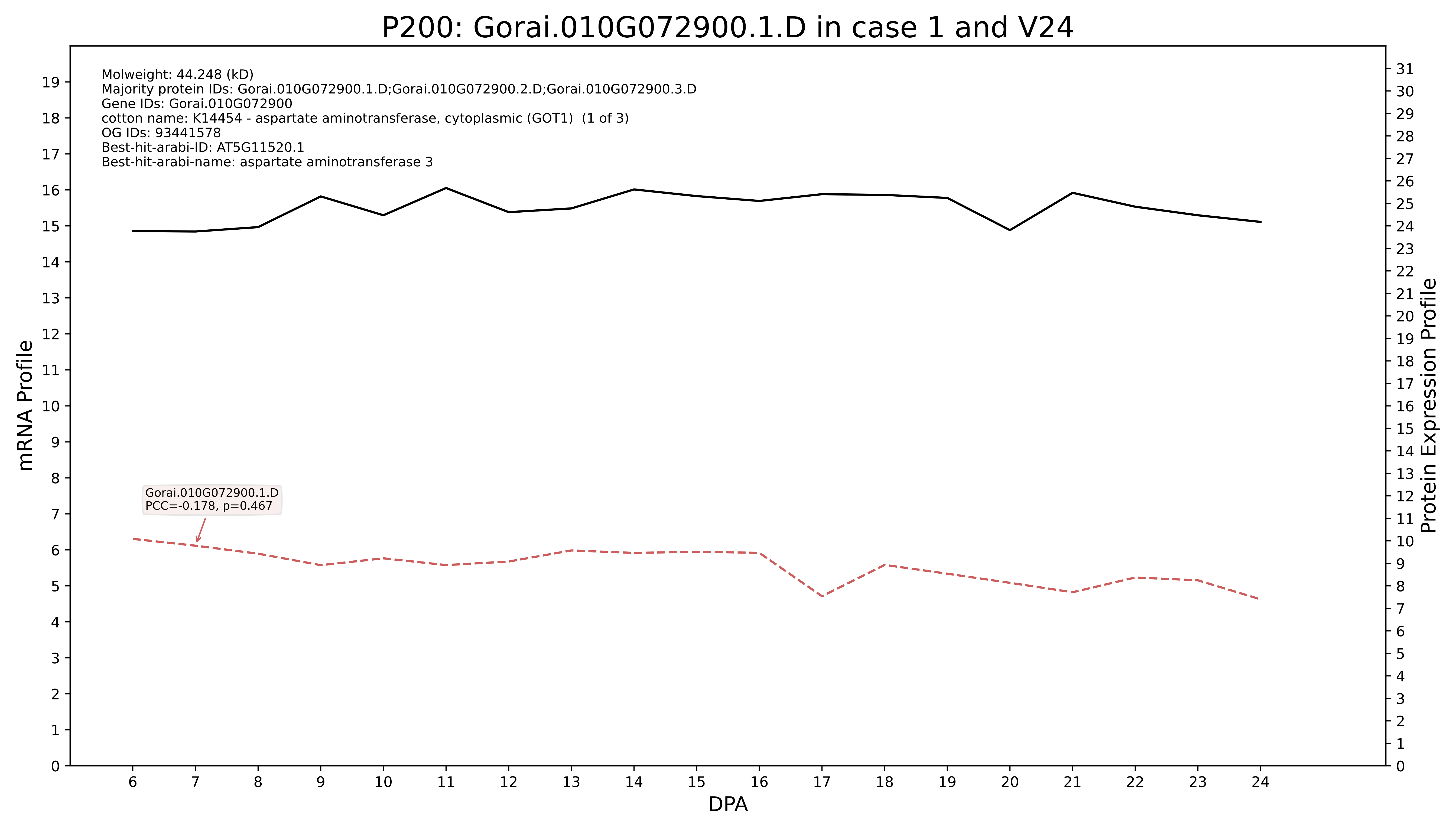

P200: Gorai.010G072900.1.D in case 1 and V24
Molweight: 44.248 (kD)
Majority protein IDs: Gorai.010G072900.1.D;Gorai.010G072900.2.D;Gorai.010G072900.3.D
Gene IDs: Gorai.010G072900
cotton name: K14454 - aspartate aminotransferase, cytoplasmic (GOT1) (1 of 3)
OG IDs: 93441578
Best-hit-arabi-ID: AT5G11520.1
Best-hit-arabi-name: aspartate aminotransferase 3
Gorai.010G072900.1.D
PCC=-0.178, p=0.467
mRNA Profile
Protein Expression Profile
DPA

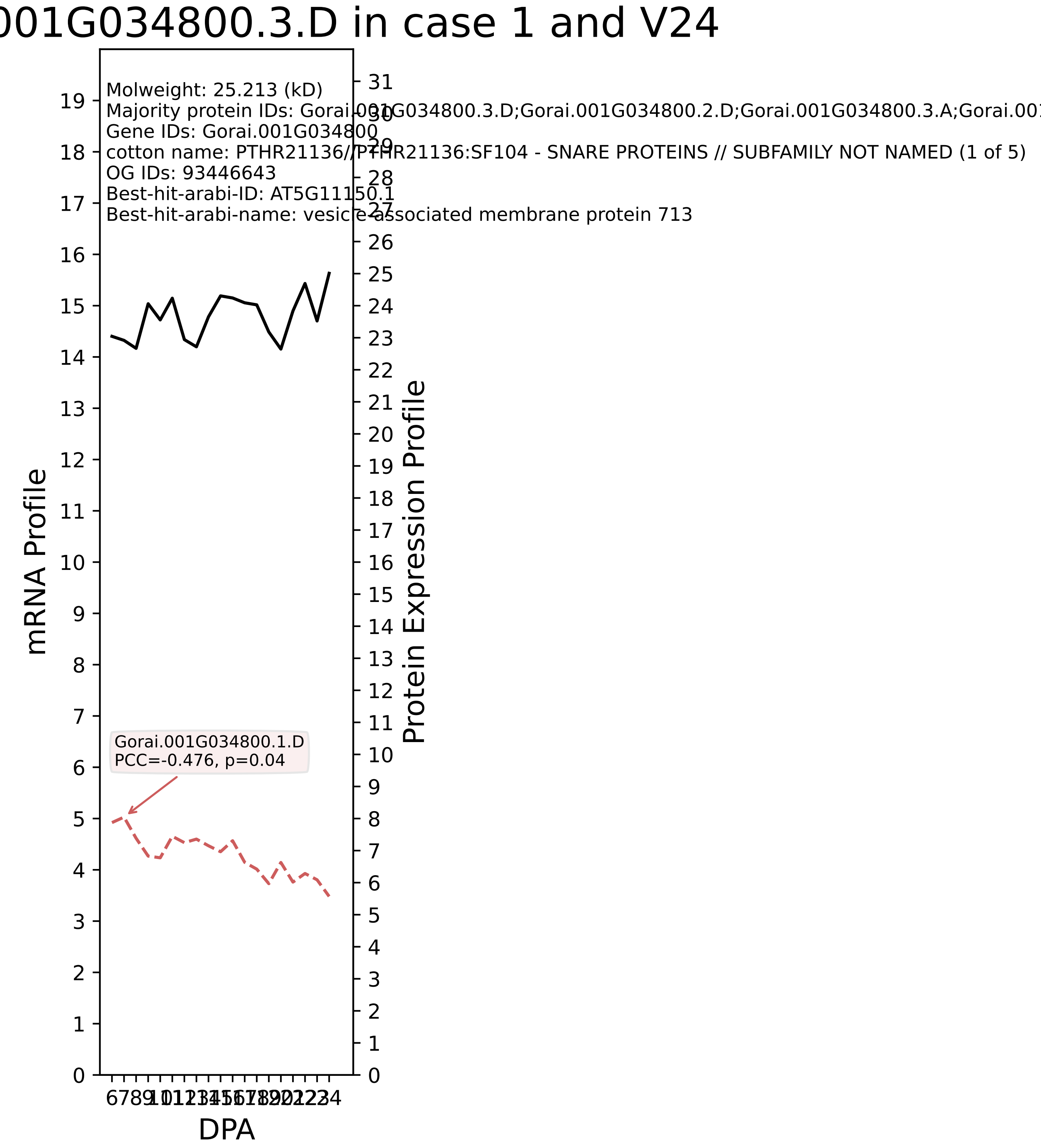

001G034800.3.D in case 1 and V24
Molweight: 25.213 (kD)
Majority protein IDs: Gorai.001G034800.3.D;Gorai.001G034800.2.D;Gorai.001G034800.3.A;Gorai.001G034800.2.A;Gorai.001G034800.6.D;Gorai.001G034800.4.D;Gorai.001G034800.1.D;Gorai.001G034800.5.D;G
Gene IDs: Gorai.001G034800
cotton name: PTHR21136//PTHR21136:SF104 - SNARE PROTEINS // SUBFAMILY NOT NAMED (1 of 5)
OG IDs: 93446643
Best-hit-arabi-ID: AT5G11150.1
Best-hit-arabi-name: vesicle-associated membrane protein 713
Gorai.001G034800.1.D
PCC=-0.476, p=0.04
mRNA Profile
Protein Expression Profile
DPA

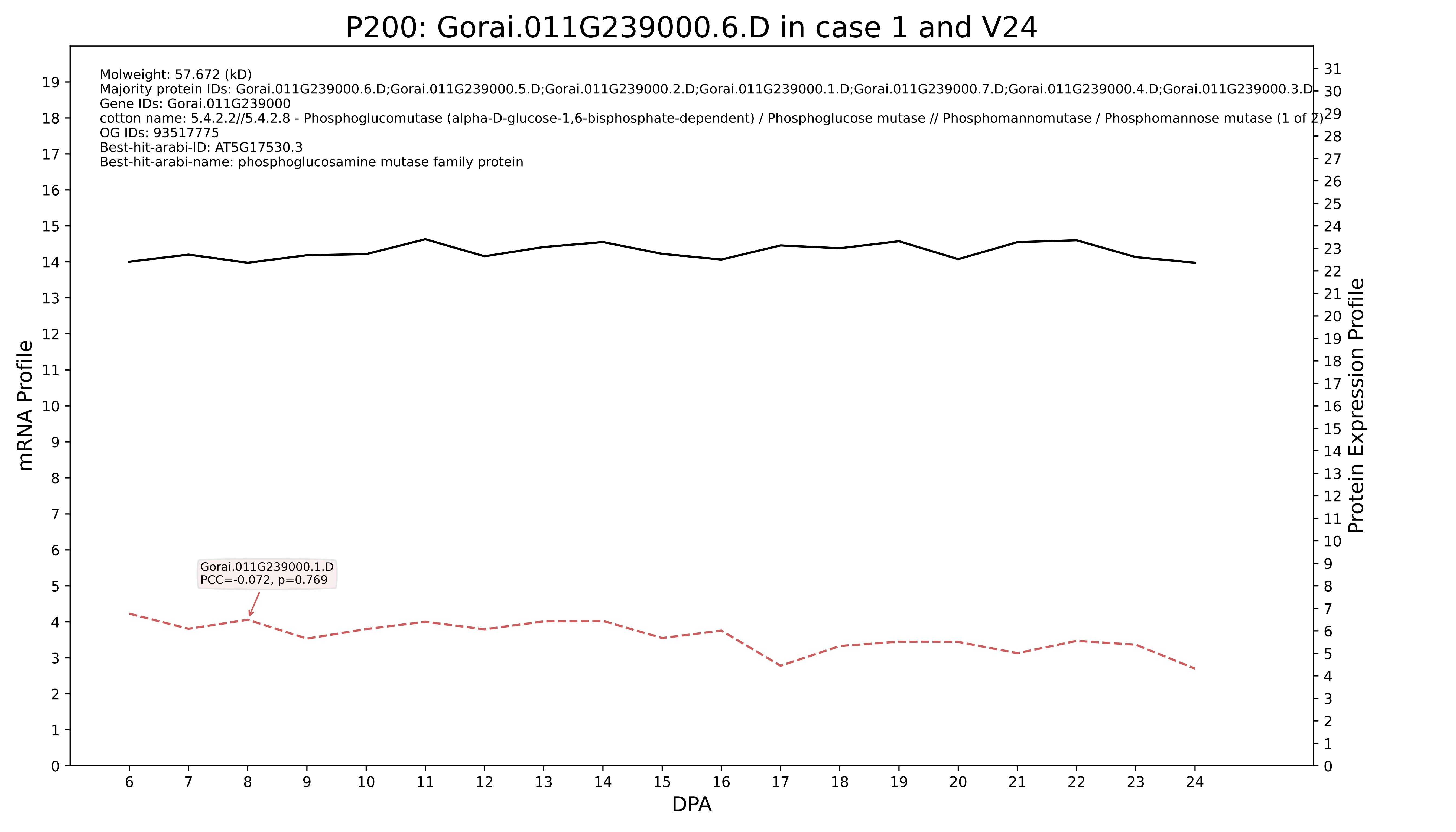
P200: Gorai.011G239000.6.D in case 1 and V24
Molweight: 57.672 (kD)
Majority protein IDs: Gorai.011G239000.6.D;Gorai.011G239000.5.D;Gorai.011G239000.2.D;Gorai.011G239000.1.D;Gorai.011G239000.7.D;Gorai.011G239000.4.D;Gorai.011G239000.3.D
Gene IDs: Gorai.011G239000
cotton name: 5.4.2.2//5.4.2.8 - Phosphoglucomutase (alpha-D-glucose-1,6-bisphosphate-dependent) / Phosphoglucose mutase // Phosphomannomutase / Phosphomannose mutase (1 of 2)
OG IDs: 93517775
Best-hit-arabi-ID: AT5G17530.3
Best-hit-arabi-name: phosphoglucosamine mutase family protein
Gorai.011G239000.1.D
PCC=-0.072, p=0.769
mRNA Profile
Protein Expression Profile
DPA
0
1
2
3
4
5
6
7
8
9
10
11
12
13
14
15
16
17
18
19
20
21
22
23
24
25
26
27
28
29
30
31

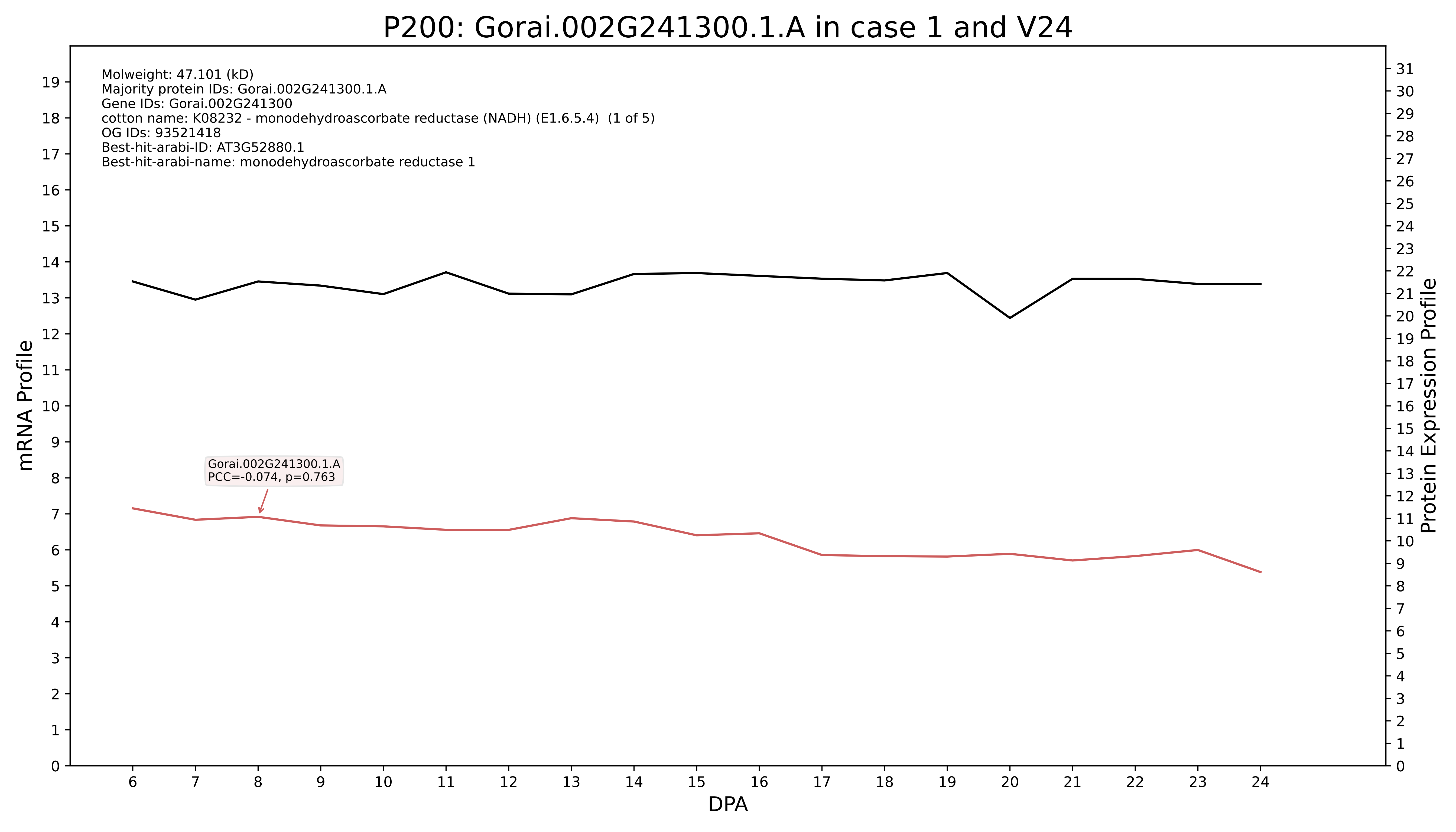

P200: Gorai.002G241300.1.A in case 1 and V24
Molweight: 47.101 (kD)
Majority protein IDs: Gorai.002G241300.1.A
Gene IDs: Gorai.002G241300
cotton name: K08232 - monodehydroascorbate reductase (NADH) (E1.6.5.4) (1 of 5)
OG IDs: 93521418
Best-hit-arabi-ID: AT3G52880.1
Best-hit-arabi-name: monodehydroascorbate reductase 1
Gorai.002G241300.1.A
PCC=-0.074, p=0.763
mRNA Profile
Protein Expression Profile
DPA

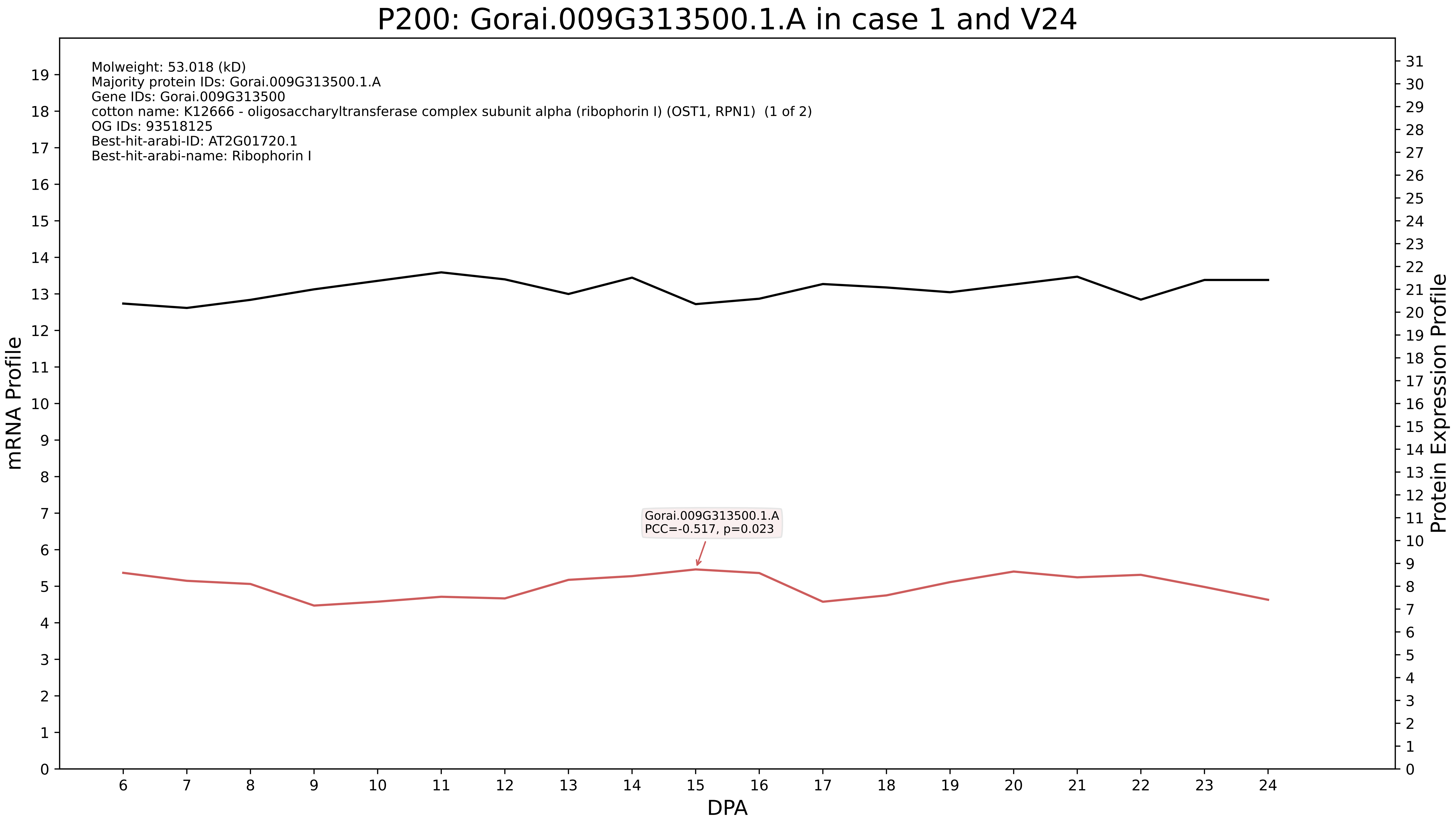
P200: Gorai.009G313500.1.A in case 1 and V24
Molweight: 53.018 (kD)
Majority protein IDs: Gorai.009G313500.1.A
Gene IDs: Gorai.009G313500
cotton name: K12666 - oligosaccharyltransferase complex subunit alpha (ribophorin I) (OST1, RPN1) (1 of 2)
OG IDs: 93518125
Best-hit-arabi-ID: AT2G01720.1
Best-hit-arabi-name: Ribophorin I
Gorai.009G313500.1.A
PCC=-0.517, p=0.023
mRNA Profile
Protein Expression Profile
DPA
0
1
2
3
4
5
6
7
8
9
10
11
12
13
14
15
16
17
18
19
20
21
22
23
24
25
26
27
28
29
30
31

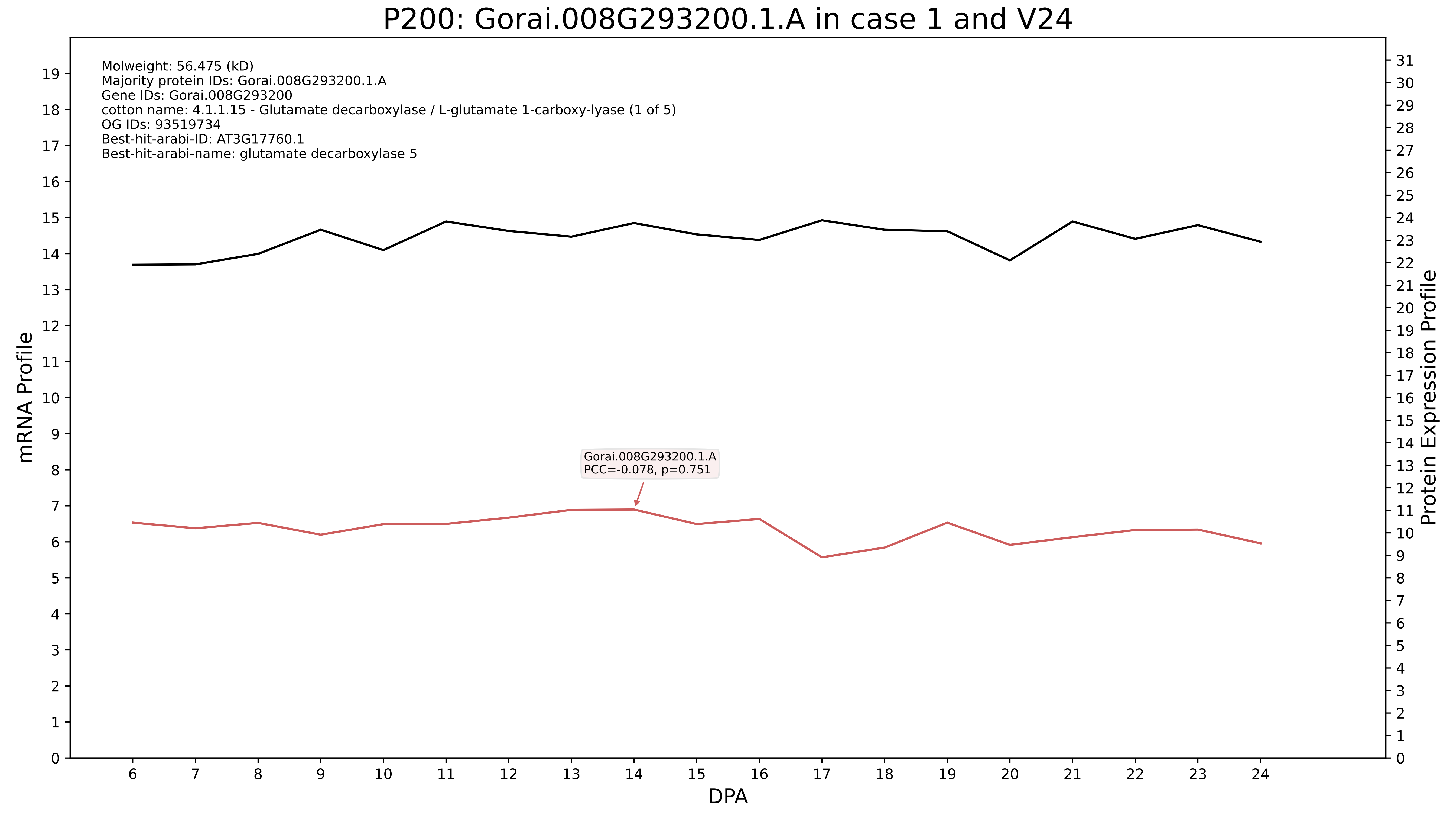

P200: Gorai.008G293200.1.A in case 1 and V24
Molweight: 56.475 (kD)
Majority protein IDs: Gorai.008G293200.1.A
Gene IDs: Gorai.008G293200
cotton name: 4.1.1.15 - Glutamate decarboxylase / L-glutamate 1-carboxy-lyase (1 of 5)
OG IDs: 93519734
Best-hit-arabi-ID: AT3G17760.1
Best-hit-arabi-name: glutamate decarboxylase 5
Gorai.008G293200.1.A
PCC=-0.078, p=0.751
mRNA Profile
Protein Expression Profile
DPA

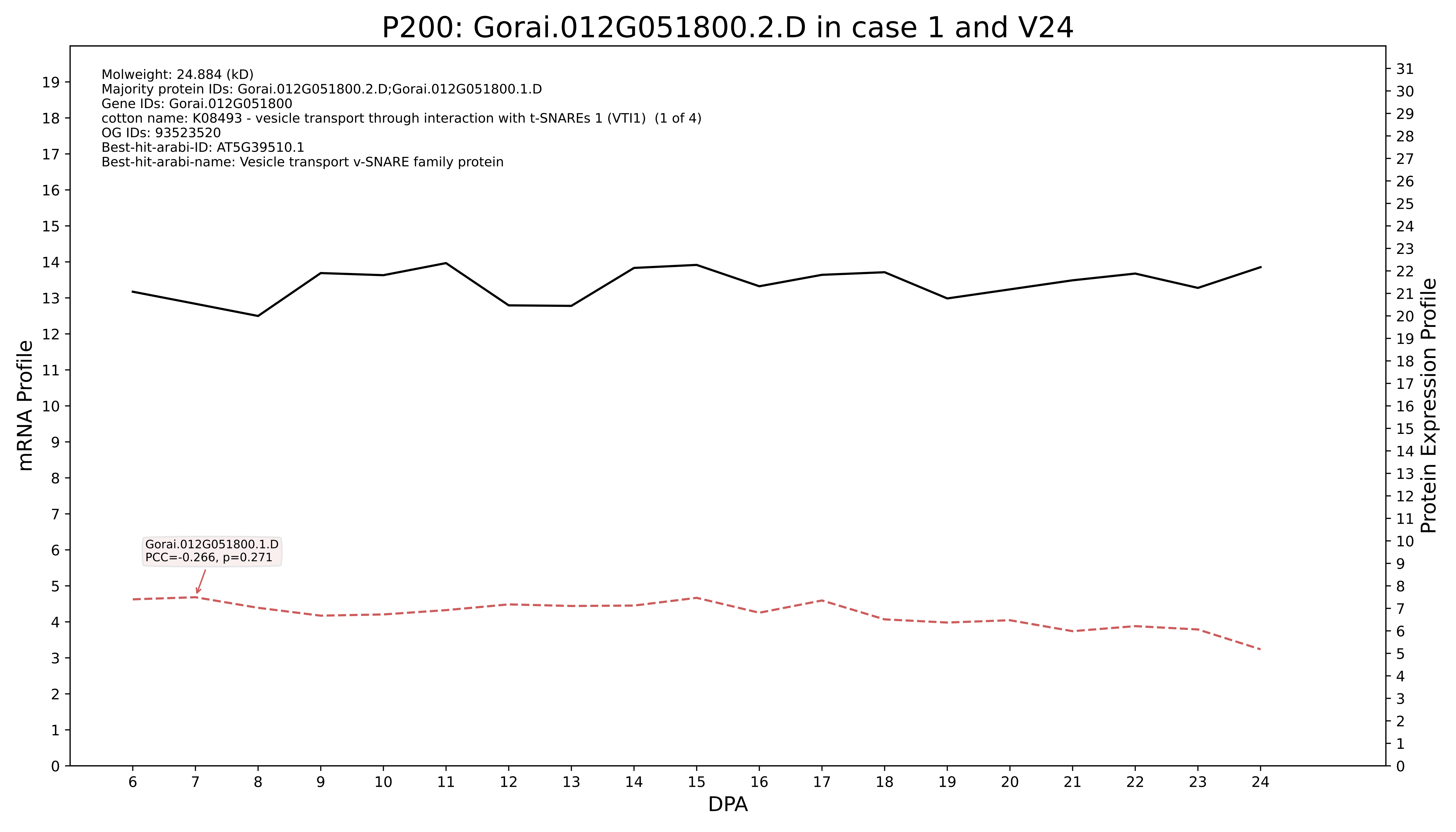

P200: Gorai.012G051800.2.D in case 1 and V24
Molweight: 24.884 (kD)
Majority protein IDs: Gorai.012G051800.2.D;Gorai.012G051800.1.D
Gene IDs: Gorai.012G051800
cotton name: K08493 - vesicle transport through interaction with t-SNAREs 1 (VTI1) (1 of 4)
OG IDs: 93523520
Best-hit-arabi-ID: AT5G39510.1
Best-hit-arabi-name: Vesicle transport v-SNARE family protein
Gorai.012G051800.1.D
PCC=-0.266, p=0.271
mRNA Profile
Protein Expression Profile
DPA

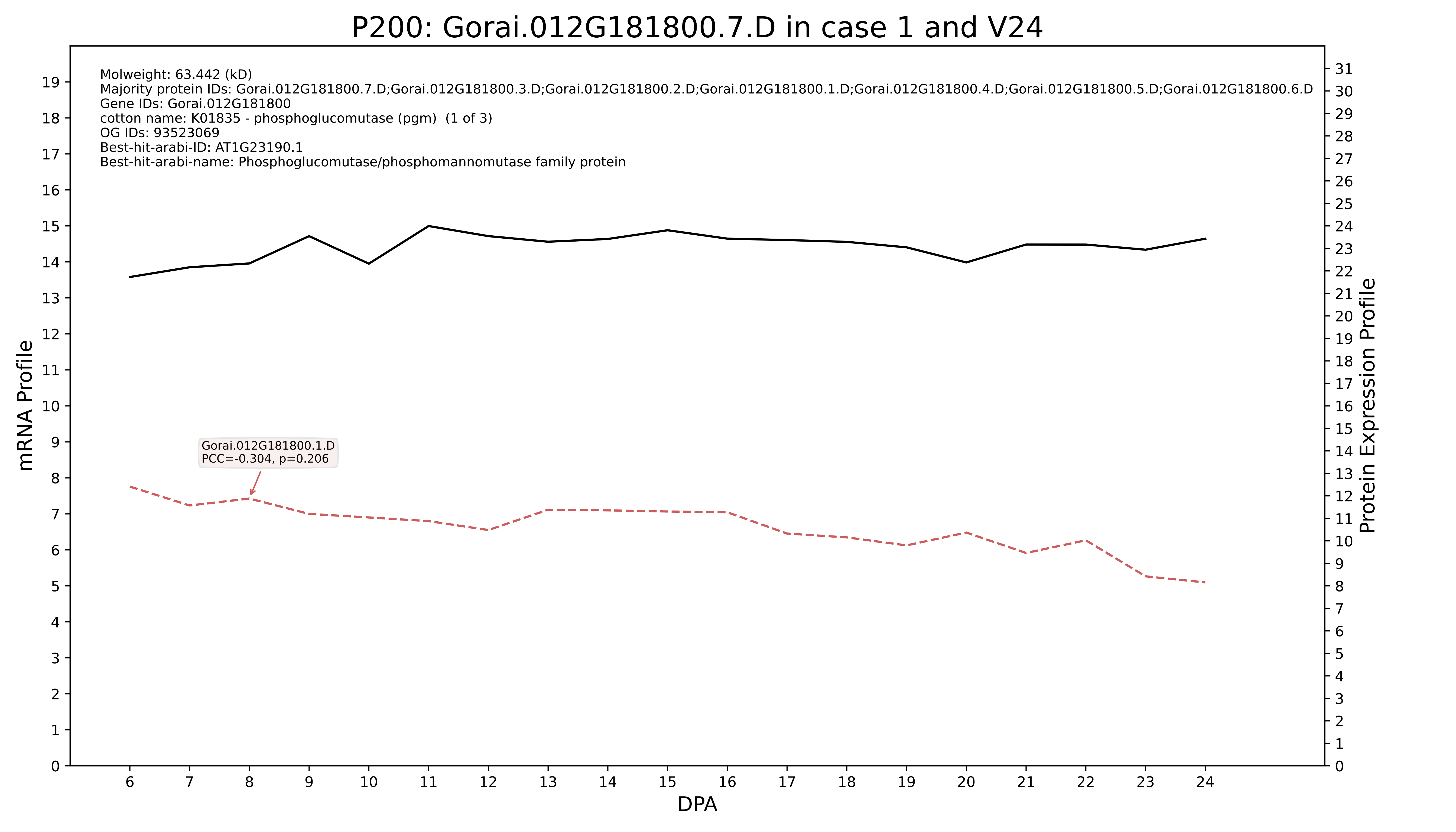
P200: Gorai.012G181800.7.D in case 1 and V24
Molweight: 63.442 (kD)
Majority protein IDs: Gorai.012G181800.7.D;Gorai.012G181800.3.D;Gorai.012G181800.2.D;Gorai.012G181800.1.D;Gorai.012G181800.4.D;Gorai.012G181800.5.D;Gorai.012G181800.6.D
Gene IDs: Gorai.012G181800
cotton name: K01835 - phosphoglucomutase (pgm) (1 of 3)
OG IDs: 93523069
Best-hit-arabi-ID: AT1G23190.1
Best-hit-arabi-name: Phosphoglucomutase/phosphomannomutase family protein
Gorai.012G181800.1.D
PCC=-0.304, p=0.206
mRNA Profile
Protein Expression Profile
DPA

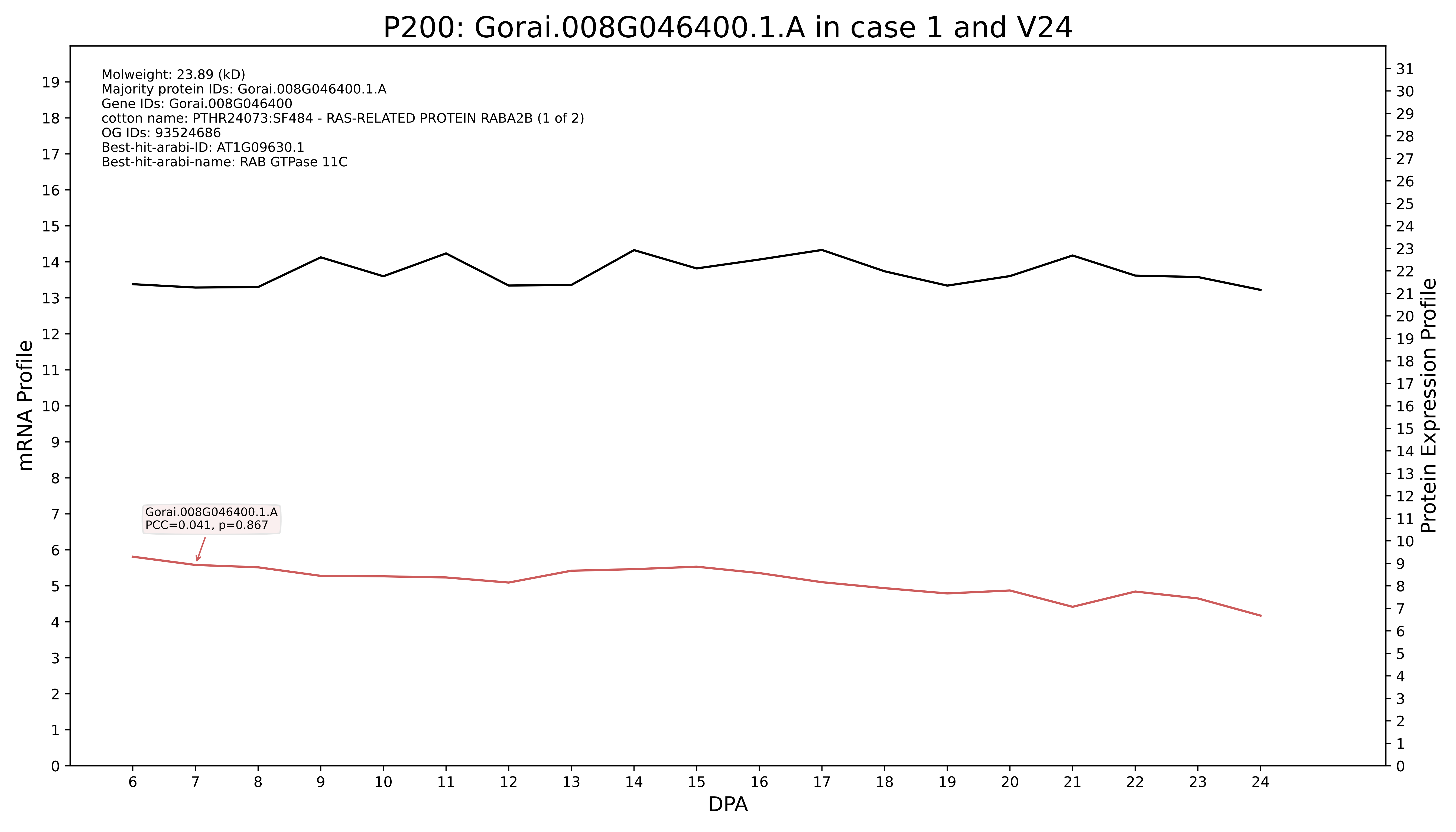

P200: Gorai.008G046400.1.A in case 1 and V24
Molweight: 23.89 (kD)
Majority protein IDs: Gorai.008G046400.1.A
Gene IDs: Gorai.008G046400
cotton name: PTHR24073:SF484 - RAS-RELATED PROTEIN RABA2B (1 of 2)
OG IDs: 93524686
Best-hit-arabi-ID: AT1G09630.1
Best-hit-arabi-name: RAB GTPase 11C
Gorai.008G046400.1.A
PCC=0.041, p=0.867
mRNA Profile
Protein Expression Profile
DPA

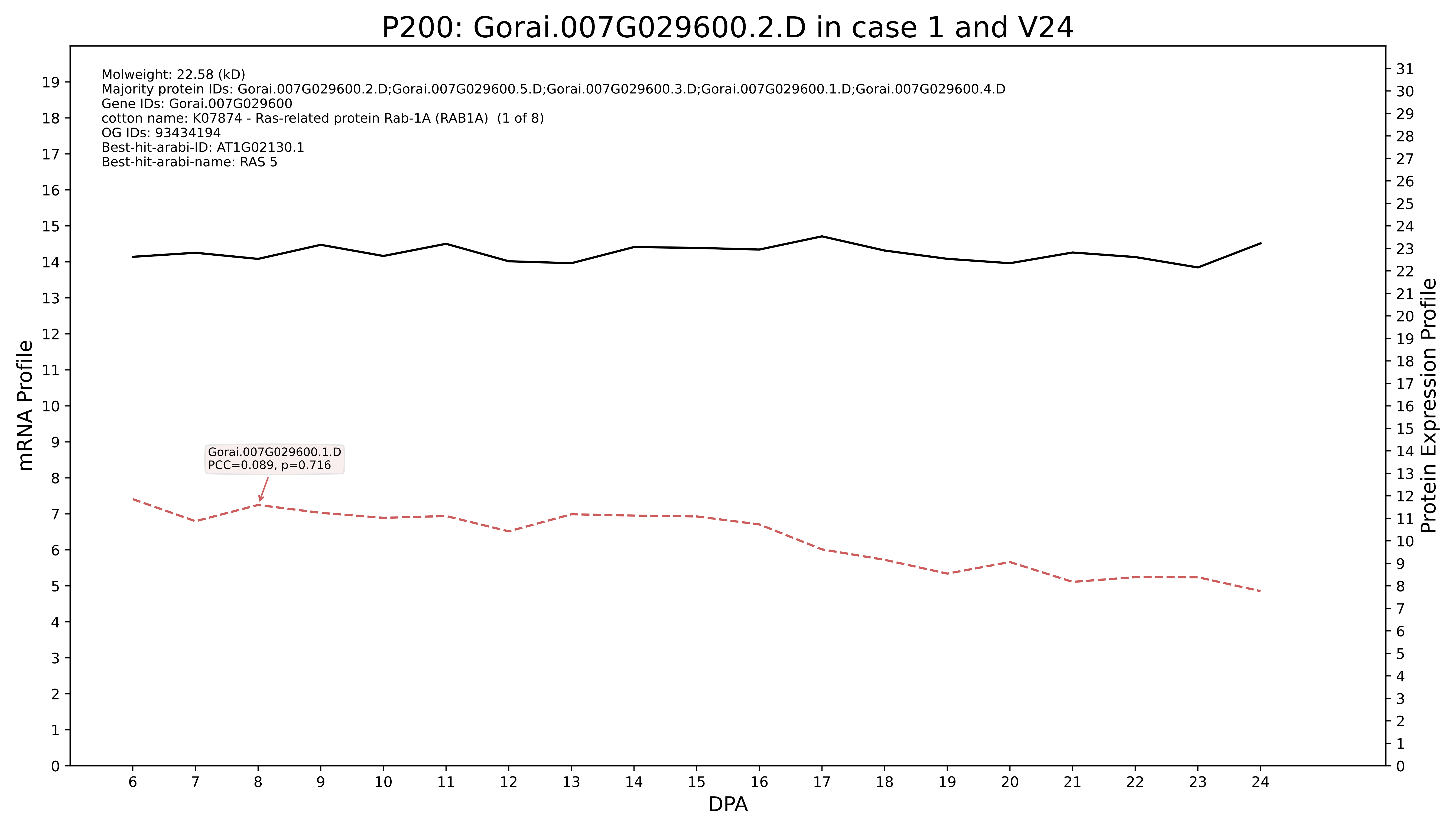

P200: Gorai.007G029600.2.D in case 1 and V24
Molweight: 22.58 (kD)
Majority protein IDs: Gorai.007G029600.2.D;Gorai.007G029600.5.D;Gorai.007G029600.3.D;Gorai.007G029600.1.D;Gorai.007G029600.4.D
Gene IDs: Gorai.007G029600
cotton name: K07874 - Ras-related protein Rab-1A (RAB1A) (1 of 8)
OG IDs: 93434194
Best-hit-arabi-ID: AT1G02130.1
Best-hit-arabi-name: RAS 5
Gorai.007G029600.1.D
PCC=0.089, p=0.716
mRNA Profile
Protein Expression Profile
DPA

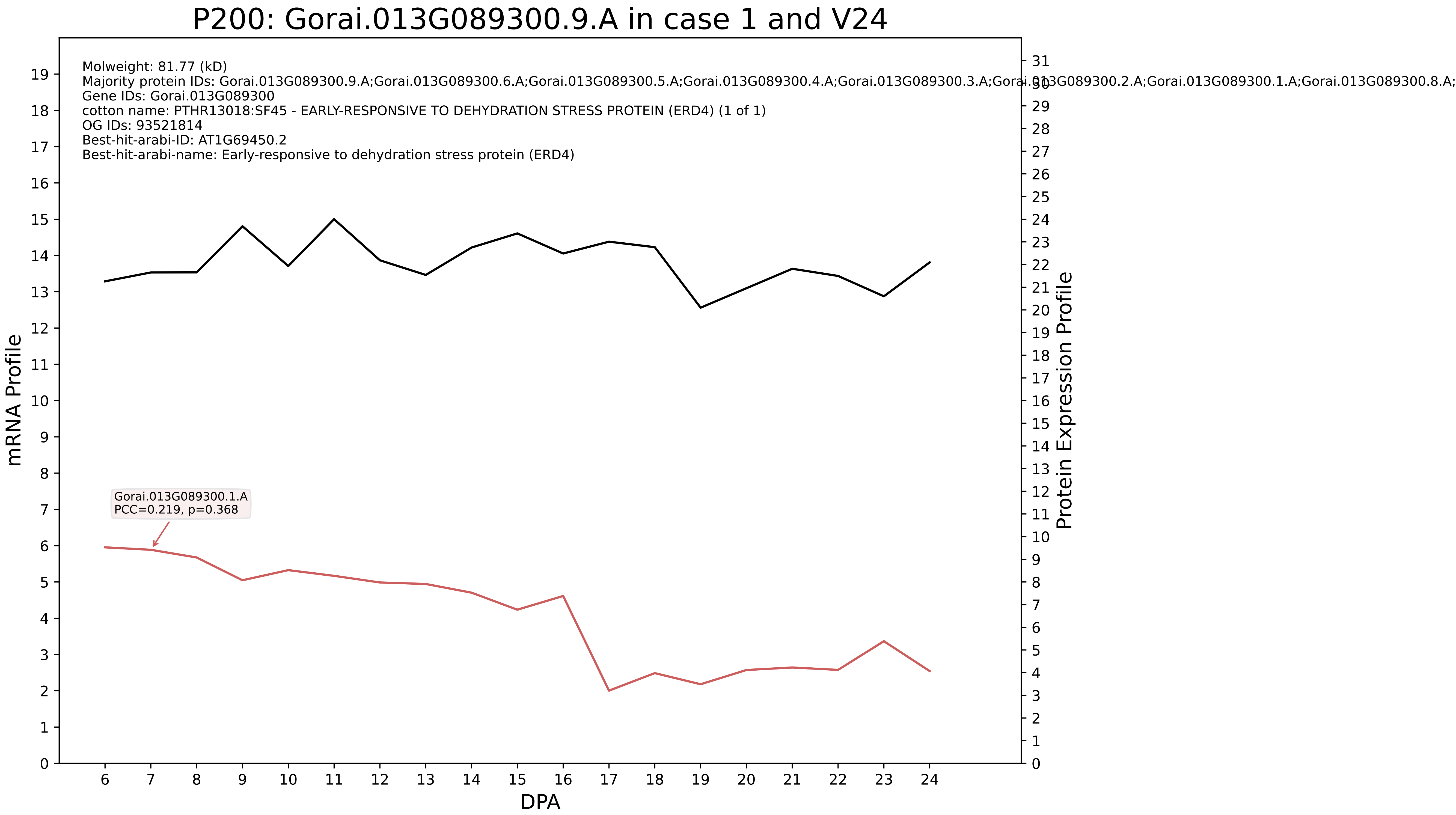
P200: Gorai.013G089300.9.A in case 1 and V24
Molweight: 81.77 (kD)
Majority protein IDs: Gorai.013G089300.9.A;Gorai.013G089300.6.A;Gorai.013G089300.5.A;Gorai.013G089300.4.A;Gorai.013G089300.3.A;Gorai.013G089300.2.A;Gorai.013G089300.1.A;Gorai.013G089300.8.A;
Gene IDs: Gorai.013G089300
cotton name: PTHR13018:SF45 - EARLY-RESPONSIVE TO DEHYDRATION STRESS PROTEIN (ERD4) (1 of 1)
OG IDs: 93521814
Best-hit-arabi-ID: AT1G69450.2
Best-hit-arabi-name: Early-responsive to dehydration stress protein (ERD4)
Gorai.013G089300.1.A
PCC=0.219, p=0.368
mRNA Profile
Protein Expression Profile
DPA

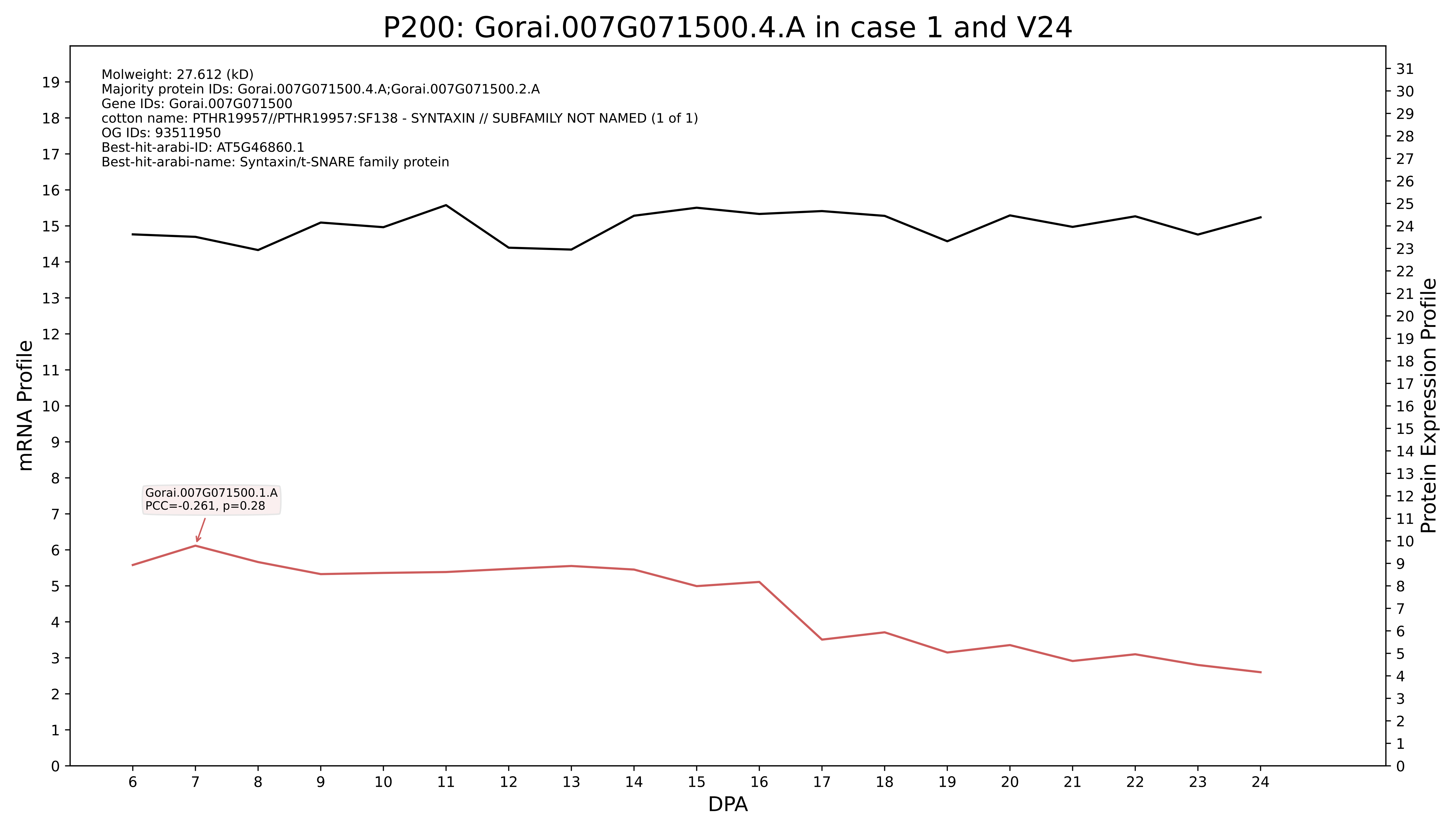
P200: Gorai.007G071500.4.A in case 1 and V24
Molweight: 27.612 (kD)
Majority protein IDs: Gorai.007G071500.4.A;Gorai.007G071500.2.A
Gene IDs: Gorai.007G071500
cotton name: PTHR19957//PTHR19957:SF138 - SYNTAXIN // SUBFAMILY NOT NAMED (1 of 1)
OG IDs: 93511950
Best-hit-arabi-ID: AT5G46860.1
Best-hit-arabi-name: Syntaxin/t-SNARE family protein
Gorai.007G071500.1.A
PCC=-0.261, p=0.28
mRNA Profile
Protein Expression Profile
DPA

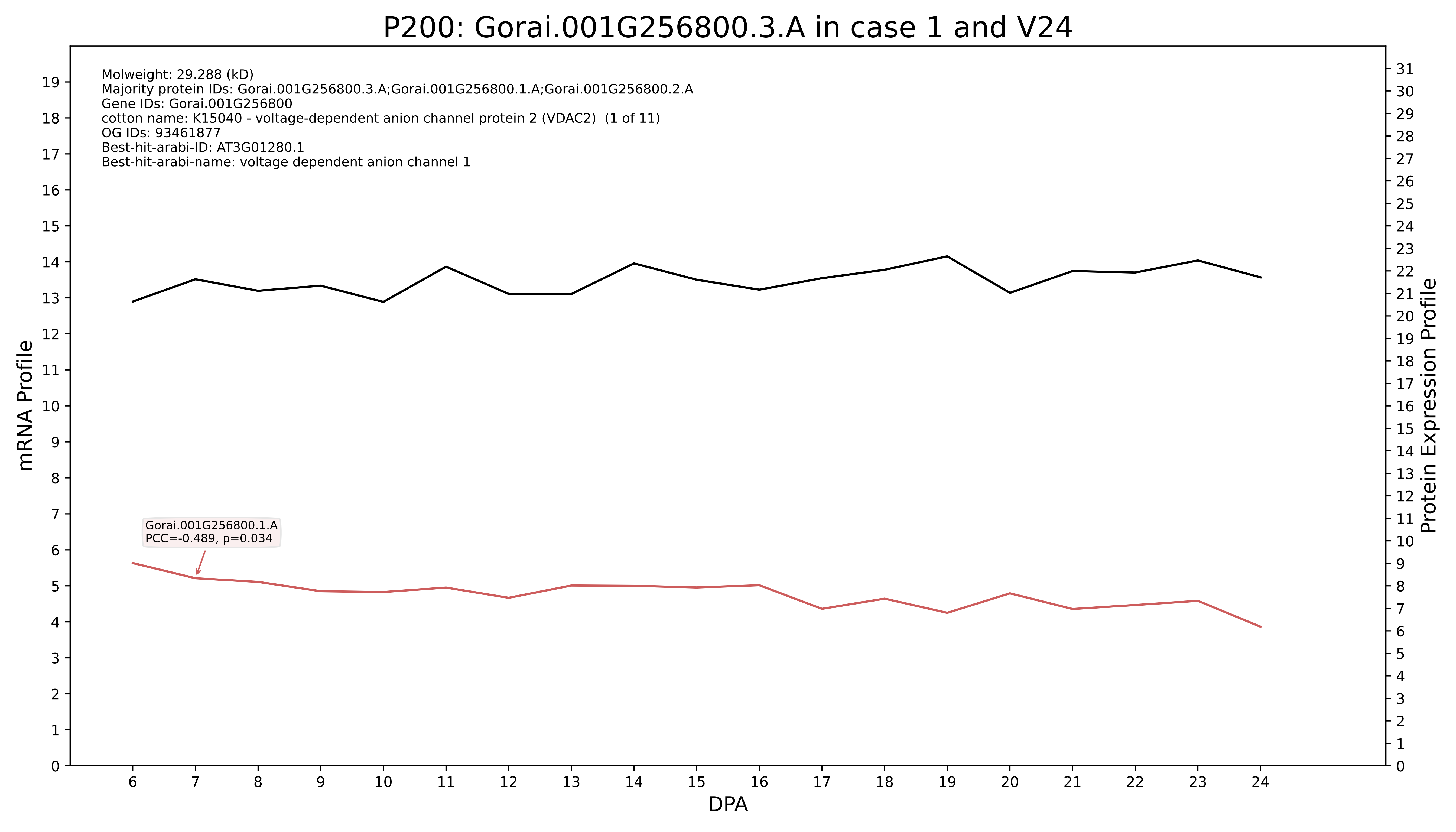
P200: Gorai.001G256800.3.A in case 1 and V24
Molweight: 29.288 (kD)
Majority protein IDs: Gorai.001G256800.3.A;Gorai.001G256800.1.A;Gorai.001G256800.2.A
Gene IDs: Gorai.001G256800
cotton name: K15040 - voltage-dependent anion channel protein 2 (VDAC2) (1 of 11)
OG IDs: 93461877
Best-hit-arabi-ID: AT3G01280.1
Best-hit-arabi-name: voltage dependent anion channel 1
Gorai.001G256800.1.A
PCC=-0.489, p=0.034
mRNA Profile
Protein Expression Profile
DPA

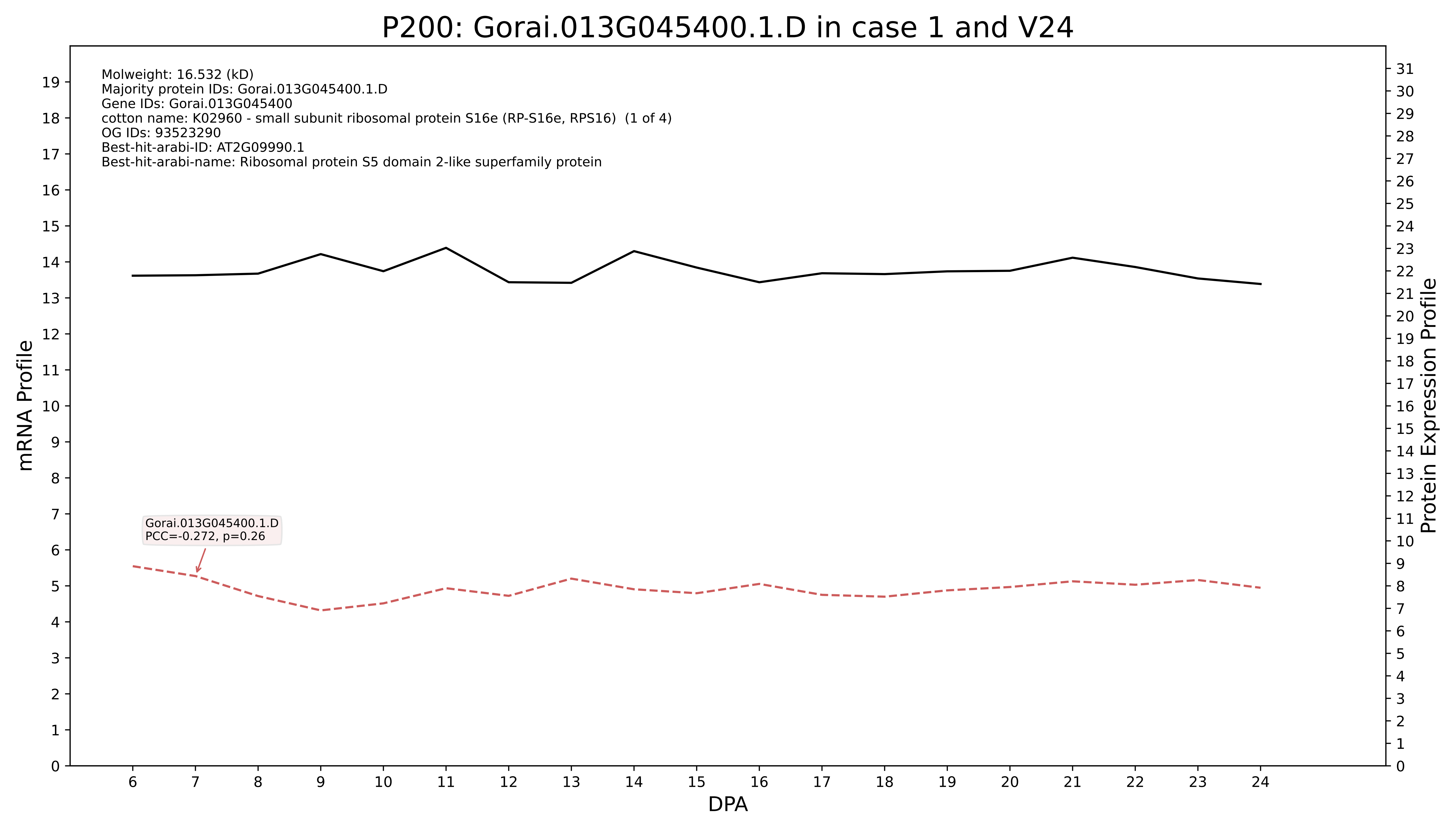
P200: Gorai.013G045400.1.D in case 1 and V24
Molweight: 16.532 (kD)
Majority protein IDs: Gorai.013G045400.1.D
Gene IDs: Gorai.013G045400
cotton name: K02960 - small subunit ribosomal protein S16e (RP-S16e, RPS16) (1 of 4)
OG IDs: 93523290
Best-hit-arabi-ID: AT2G09990.1
Best-hit-arabi-name: Ribosomal protein S5 domain 2-like superfamily protein
Gorai.013G045400.1.D
PCC=-0.272, p=0.26
mRNA Profile
Protein Expression Profile
DPA
0
1
2
3
4
5
6
7
8
9
10
11
12
13
14
15
16
17
18
19
20
21
22
23
24
25
26
27
28
29
30
31

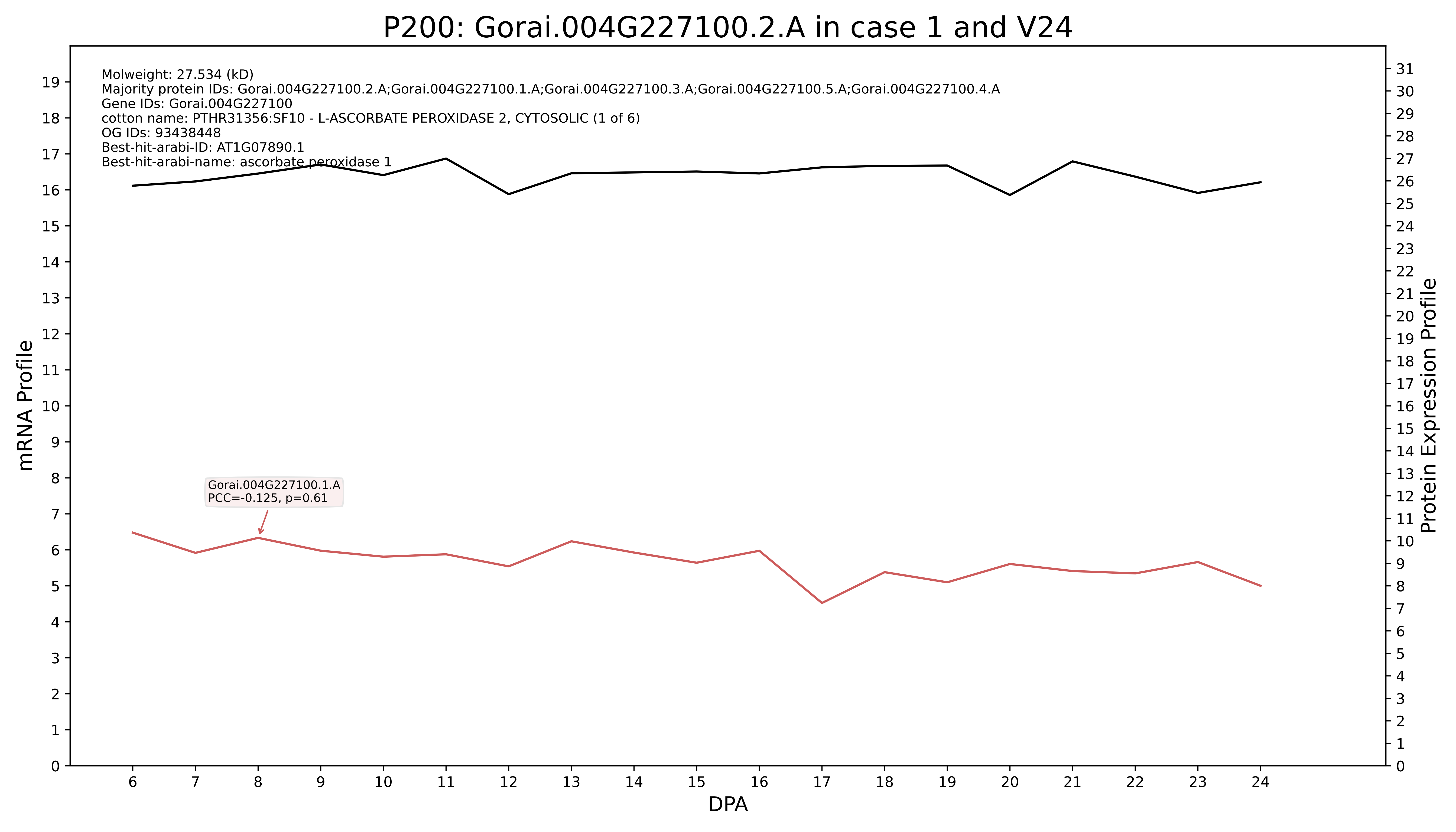
P200: Gorai.004G227100.2.A in case 1 and V24
Molweight: 27.534 (kD)
Majority protein IDs: Gorai.004G227100.2.A;Gorai.004G227100.1.A;Gorai.004G227100.3.A;Gorai.004G227100.5.A;Gorai.004G227100.4.A
Gene IDs: Gorai.004G227100
cotton name: PTHR31356:SF10 - L-ASCORBATE PEROXIDASE 2, CYTOSOLIC (1 of 6)
OG IDs: 93438448
Best-hit-arabi-ID: AT1G07890.1
Best-hit-arabi-name: ascorbate peroxidase 1
Gorai.004G227100.1.A
PCC=-0.125, p=0.61
mRNA Profile
Protein Expression Profile
DPA

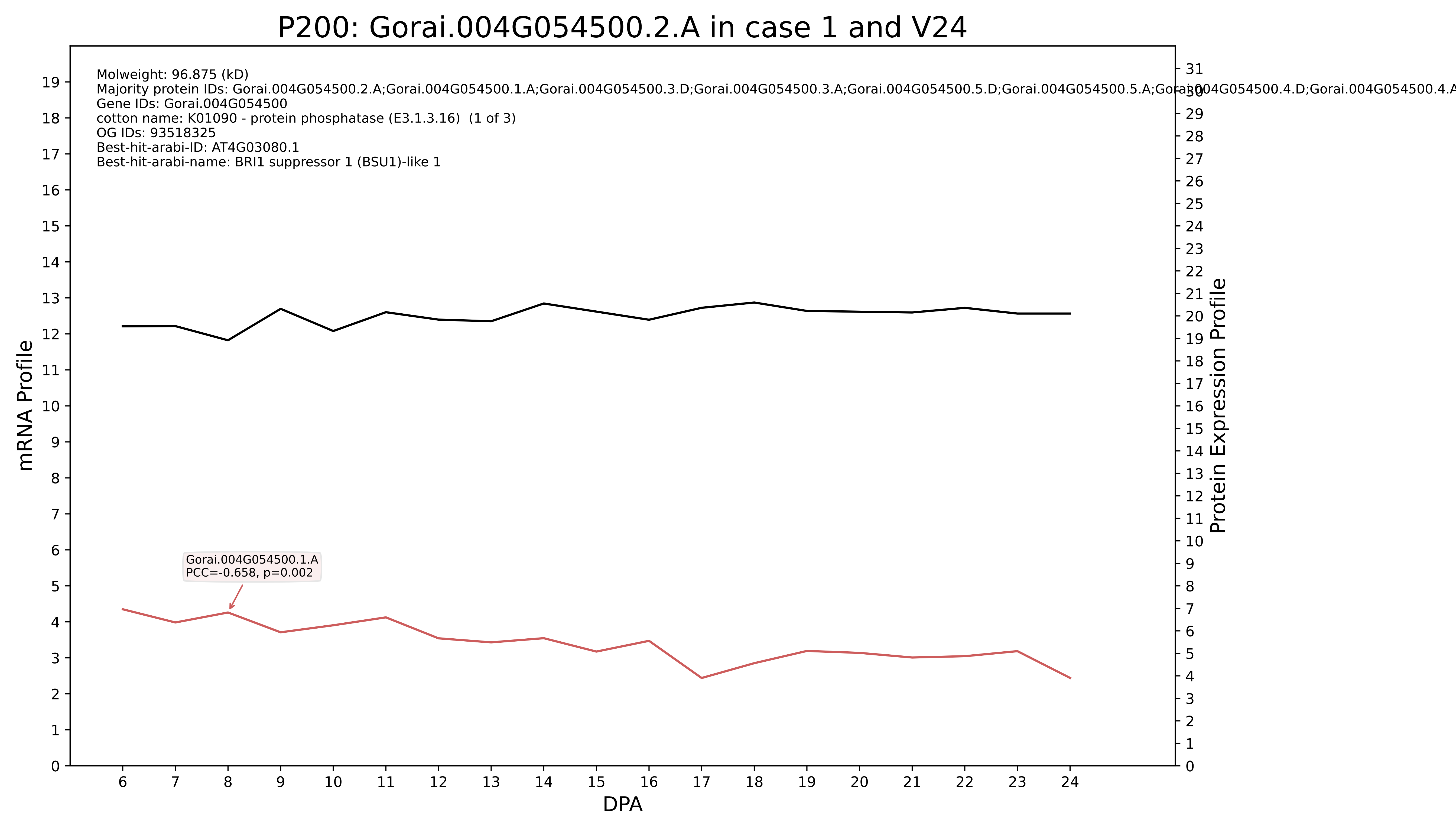
P200: Gorai.004G054500.2.A in case 1 and V24
Molweight: 96.875 (kD)
Majority protein IDs: Gorai.004G054500.2.A;Gorai.004G054500.1.A;Gorai.004G054500.3.D;Gorai.004G054500.3.A;Gorai.004G054500.5.D;Gorai.004G054500.5.A;Gorai.004G054500.4.D;Gorai.004G054500.4.A
Gene IDs: Gorai.004G054500
cotton name: K01090 - protein phosphatase (E3.1.3.16) (1 of 3)
OG IDs: 93518325
Best-hit-arabi-ID: AT4G03080.1
Best-hit-arabi-name: BRI1 suppressor 1 (BSU1)-like 1
Gorai.004G054500.1.A
PCC=-0.658, p=0.002
mRNA Profile
Protein Expression Profile
DPA

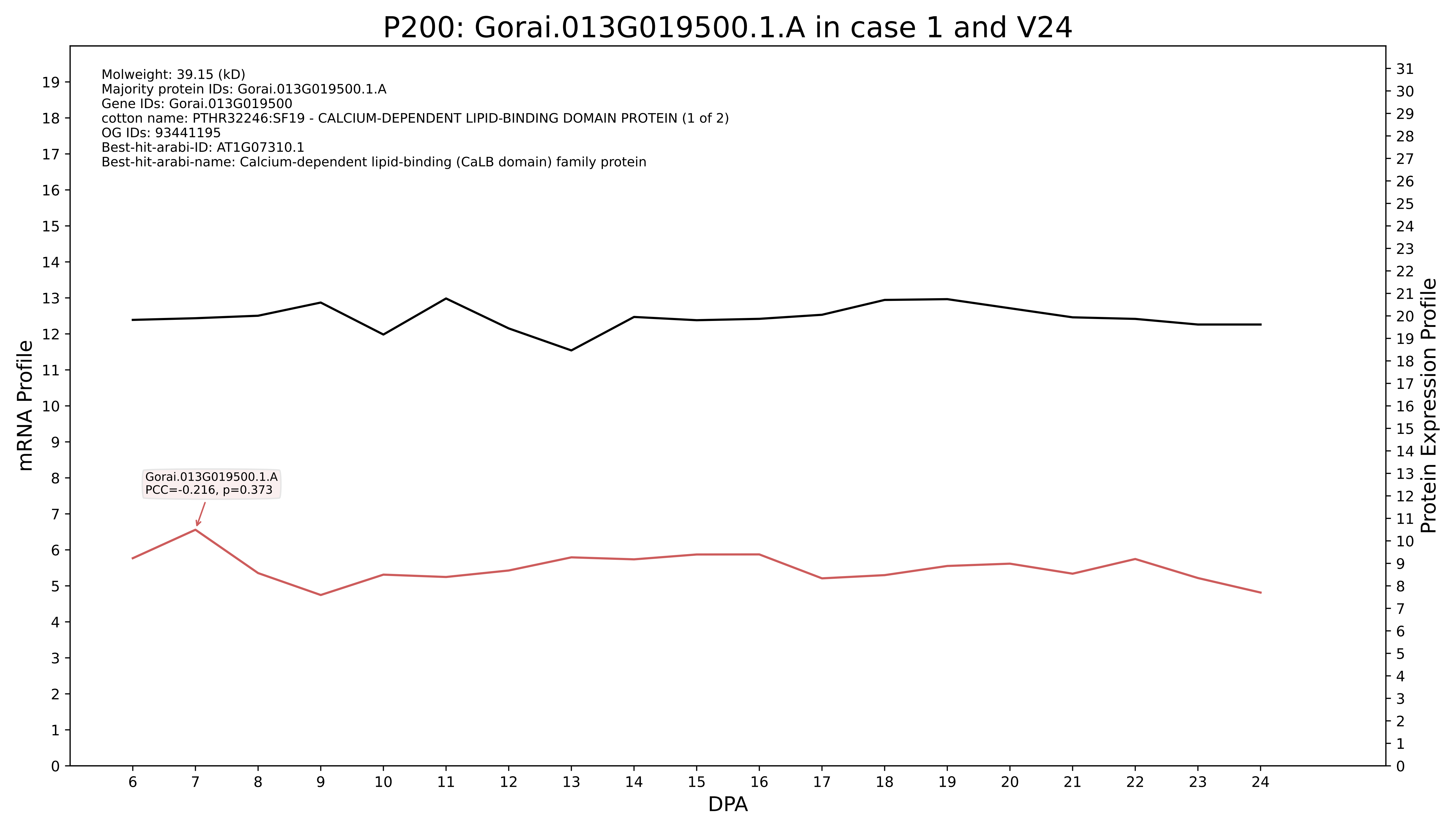
P200: Gorai.013G019500.1.A in case 1 and V24
Molweight: 39.15 (kD)
Majority protein IDs: Gorai.013G019500.1.A
Gene IDs: Gorai.013G019500
cotton name: PTHR32246:SF19 - CALCIUM-DEPENDENT LIPID-BINDING DOMAIN PROTEIN (1 of 2)
OG IDs: 93441195
Best-hit-arabi-ID: AT1G07310.1
Best-hit-arabi-name: Calcium-dependent lipid-binding (CaLB domain) family protein
Gorai.013G019500.1.A
PCC=-0.216, p=0.373
mRNA Profile
Protein Expression Profile
DPA

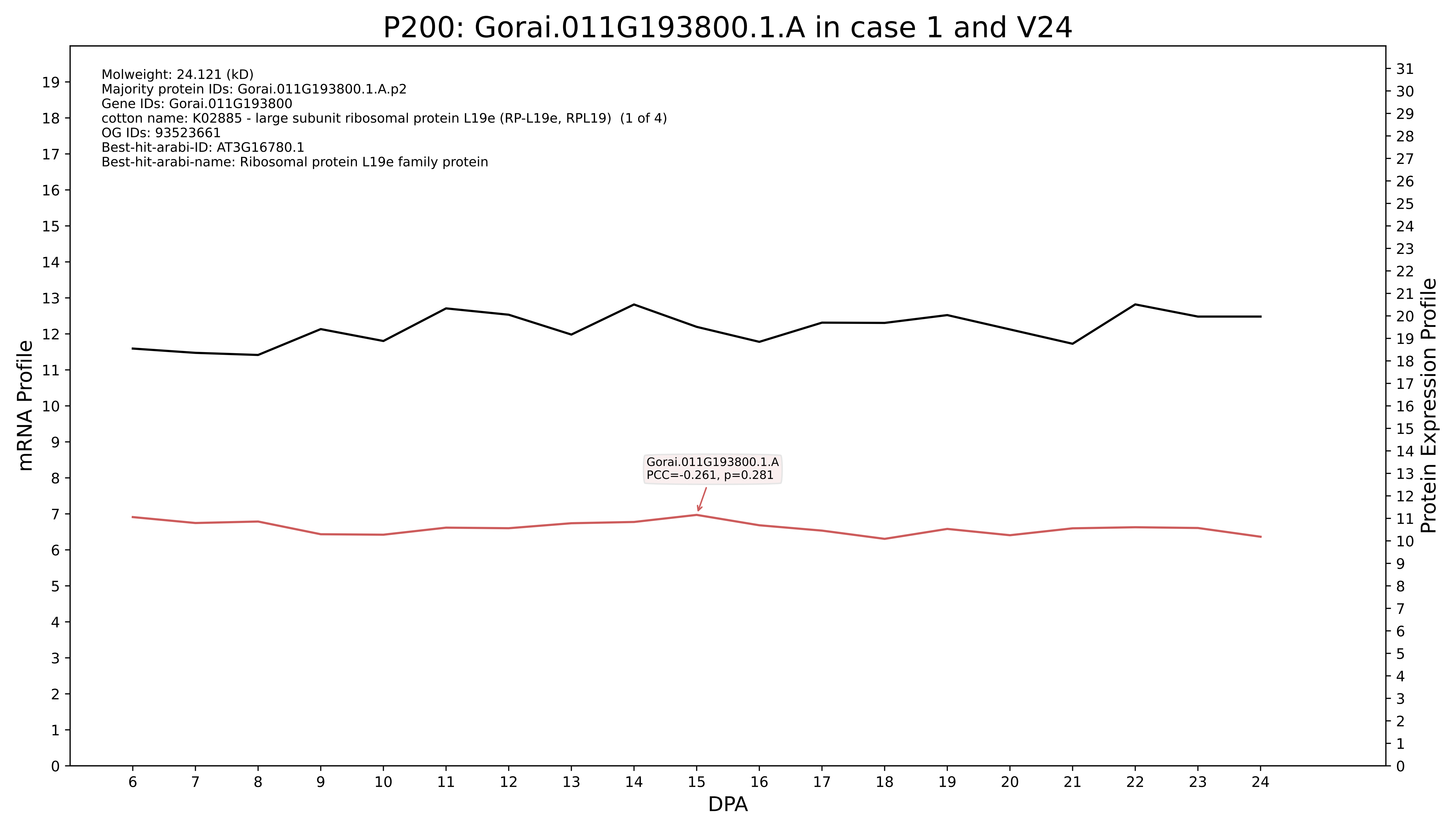
P200: Gorai.011G193800.1.A in case 1 and V24
Molweight: 24.121 (kD)
Majority protein IDs: Gorai.011G193800.1.A.p2
Gene IDs: Gorai.011G193800
cotton name: K02885 - large subunit ribosomal protein L19e (RP-L19e, RPL19) (1 of 4)
OG IDs: 93523661
Best-hit-arabi-ID: AT3G16780.1
Best-hit-arabi-name: Ribosomal protein L19e family protein
Gorai.011G193800.1.A
PCC=-0.261, p=0.281
mRNA Profile
Protein Expression Profile
DPA
6 7 8 9 10 11 12 13 14 15 16 17 18 19 20 21 22 23 24

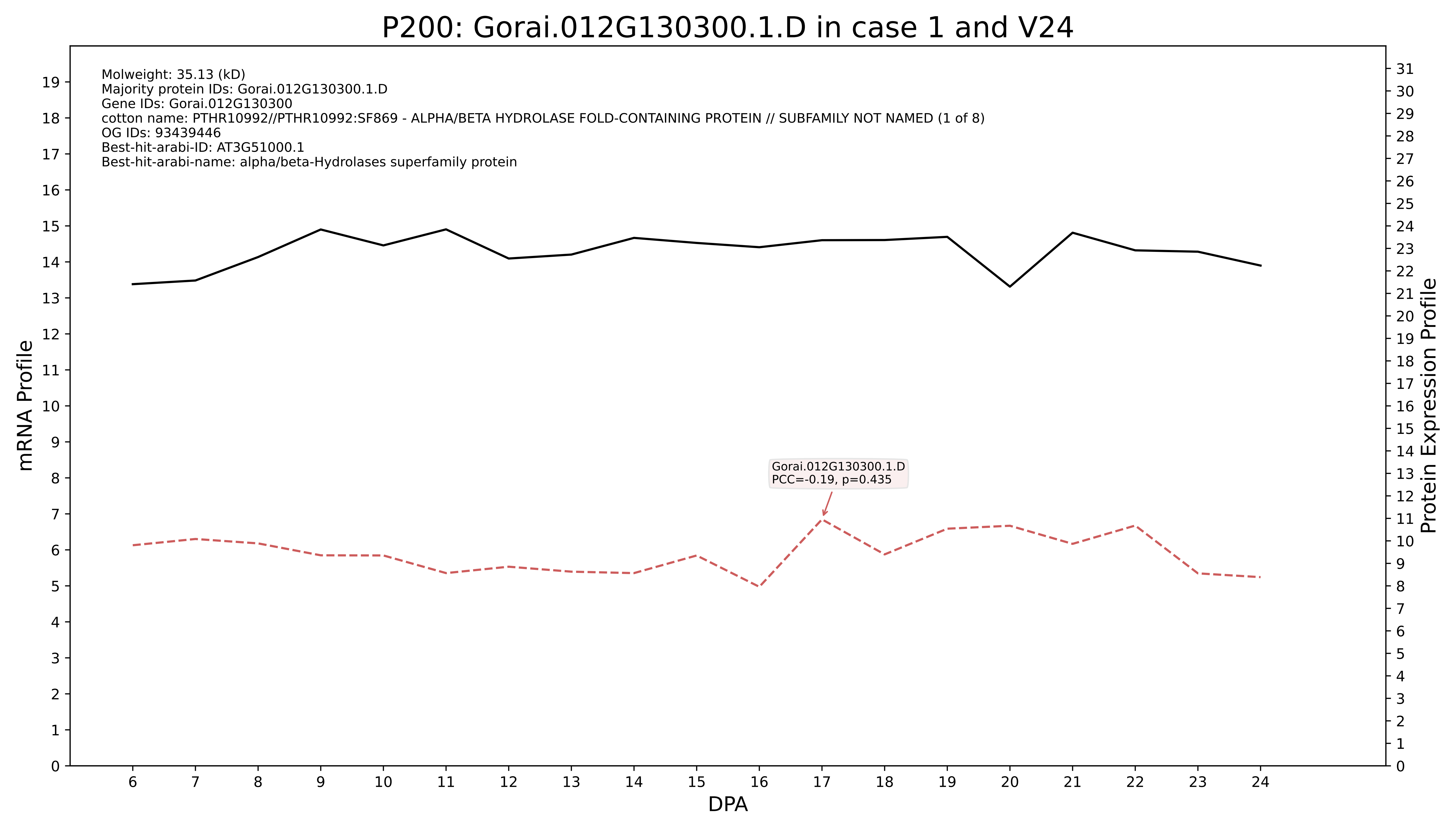
P200: Gorai.012G130300.1.D in case 1 and V24
Molweight: 35.13 (kD)
Majority protein IDs: Gorai.012G130300.1.D
Gene IDs: Gorai.012G130300
cotton name: PTHR10992//PTHR10992:SF869 - ALPHA/BETA HYDROLASE FOLD-CONTAINING PROTEIN // SUBFAMILY NOT NAMED (1 of 8)
OG IDs: 93439446
Best-hit-arabi-ID: AT3G51000.1
Best-hit-arabi-name: alpha/beta-Hydrolases superfamily protein
Gorai.012G130300.1.D
PCC=-0.19, p=0.435
mRNA Profile
Protein Expression Profile
DPA

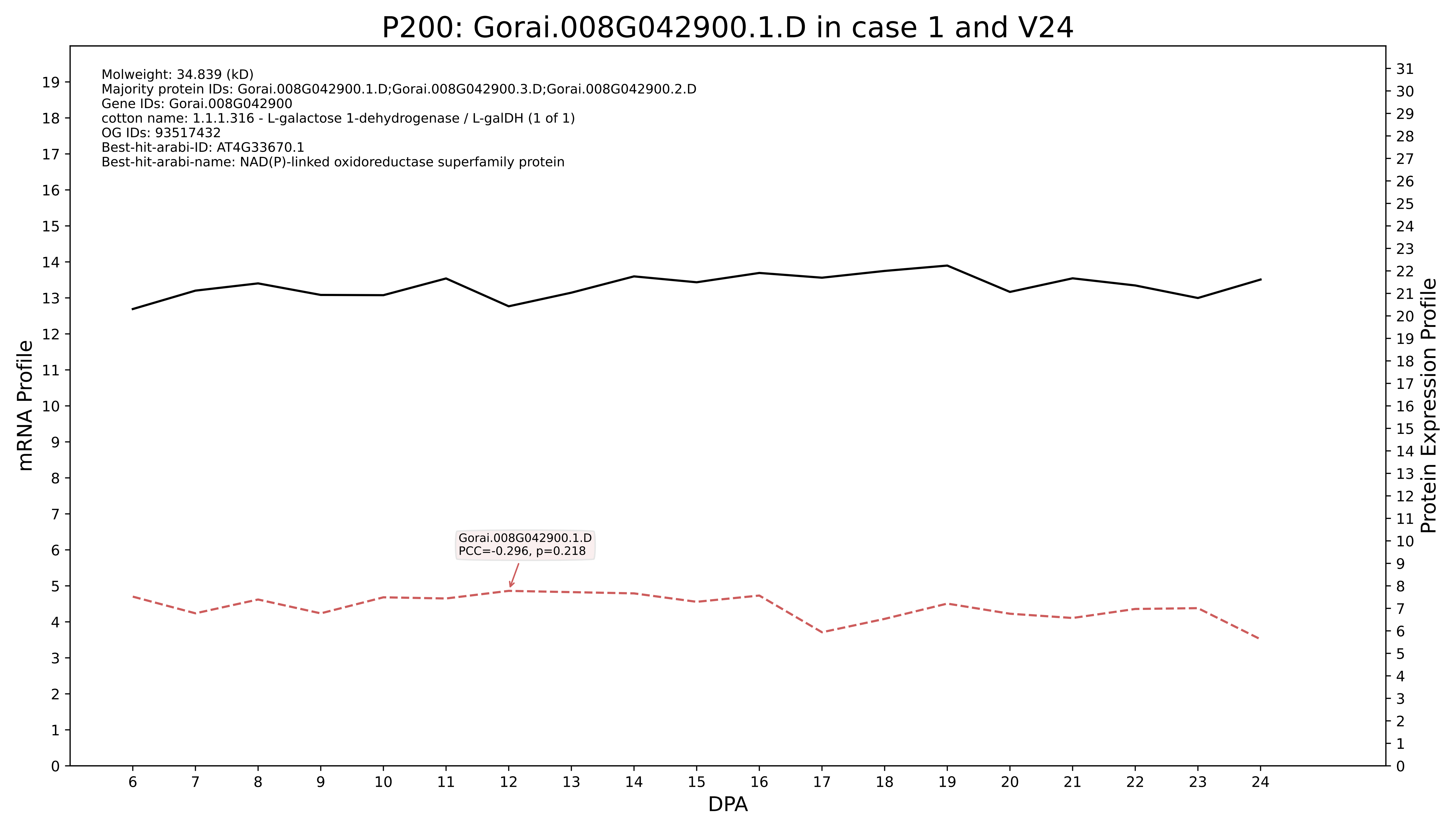
P200: Gorai.008G042900.1.D in case 1 and V24
Molweight: 34.839 (kD)
Majority protein IDs: Gorai.008G042900.1.D;Gorai.008G042900.3.D;Gorai.008G042900.2.D
Gene IDs: Gorai.008G042900
cotton name: 1.1.1.316 - L-galactose 1-dehydrogenase / L-galDH (1 of 1)
OG IDs: 93517432
Best-hit-arabi-ID: AT4G33670.1
Best-hit-arabi-name: NAD(P)-linked oxidoreductase superfamily protein
Gorai.008G042900.1.D
PCC=-0.296, p=0.218
mRNA Profile
Protein Expression Profile
DPA

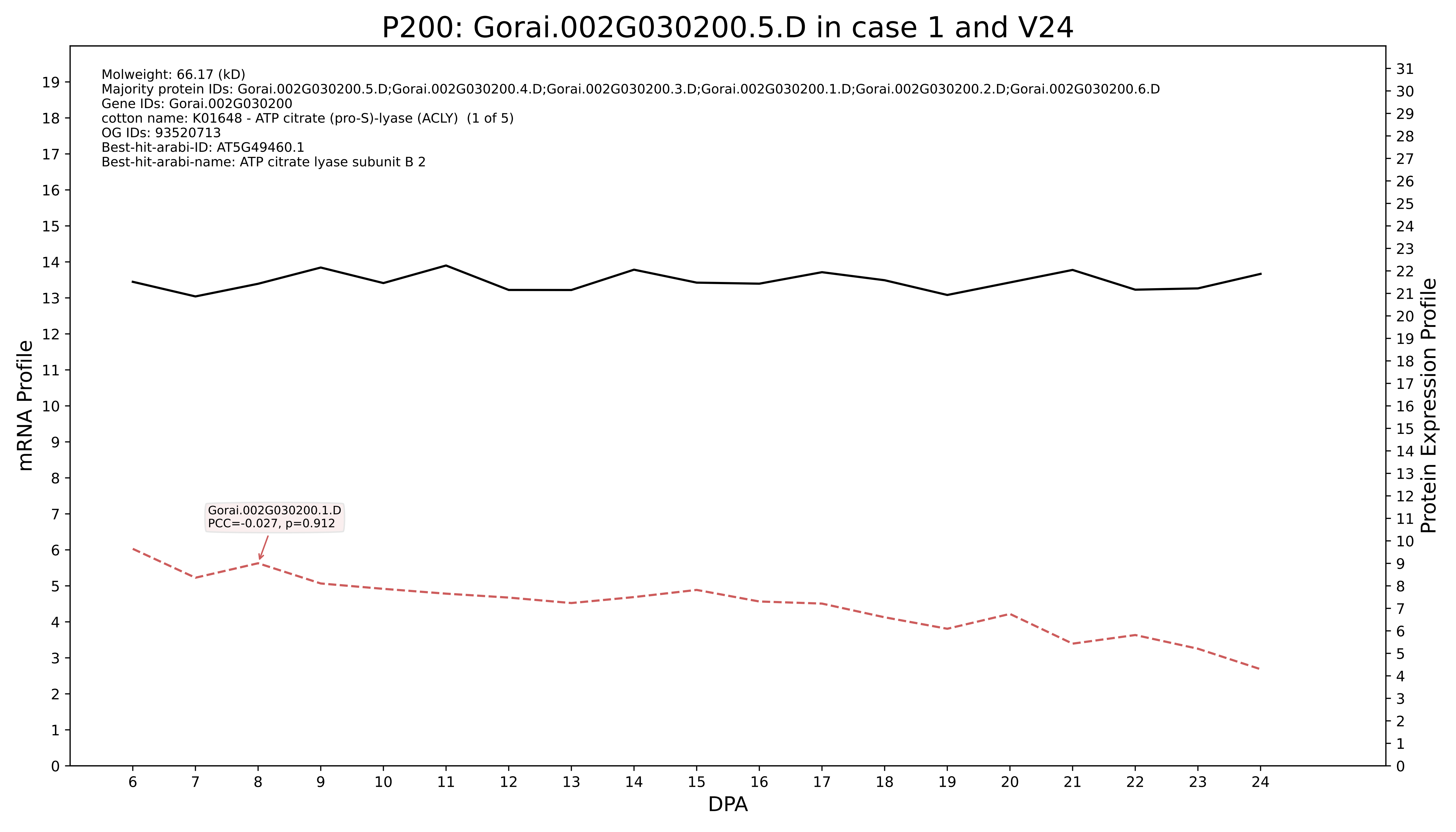
P200: Gorai.002G030200.5.D in case 1 and V24
Molweight: 66.17 (kD)
Majority protein IDs: Gorai.002G030200.5.D;Gorai.002G030200.4.D;Gorai.002G030200.3.D;Gorai.002G030200.1.D;Gorai.002G030200.2.D;Gorai.002G030200.6.D
Gene IDs: Gorai.002G030200
cotton name: K01648 - ATP citrate (pro-S)-lyase (ACLY) (1 of 5)
OG IDs: 93520713
Best-hit-arabi-ID: AT5G49460.1
Best-hit-arabi-name: ATP citrate lyase subunit B 2
Gorai.002G030200.1.D
PCC=-0.027, p=0.912
mRNA Profile
Protein Expression Profile
DPA

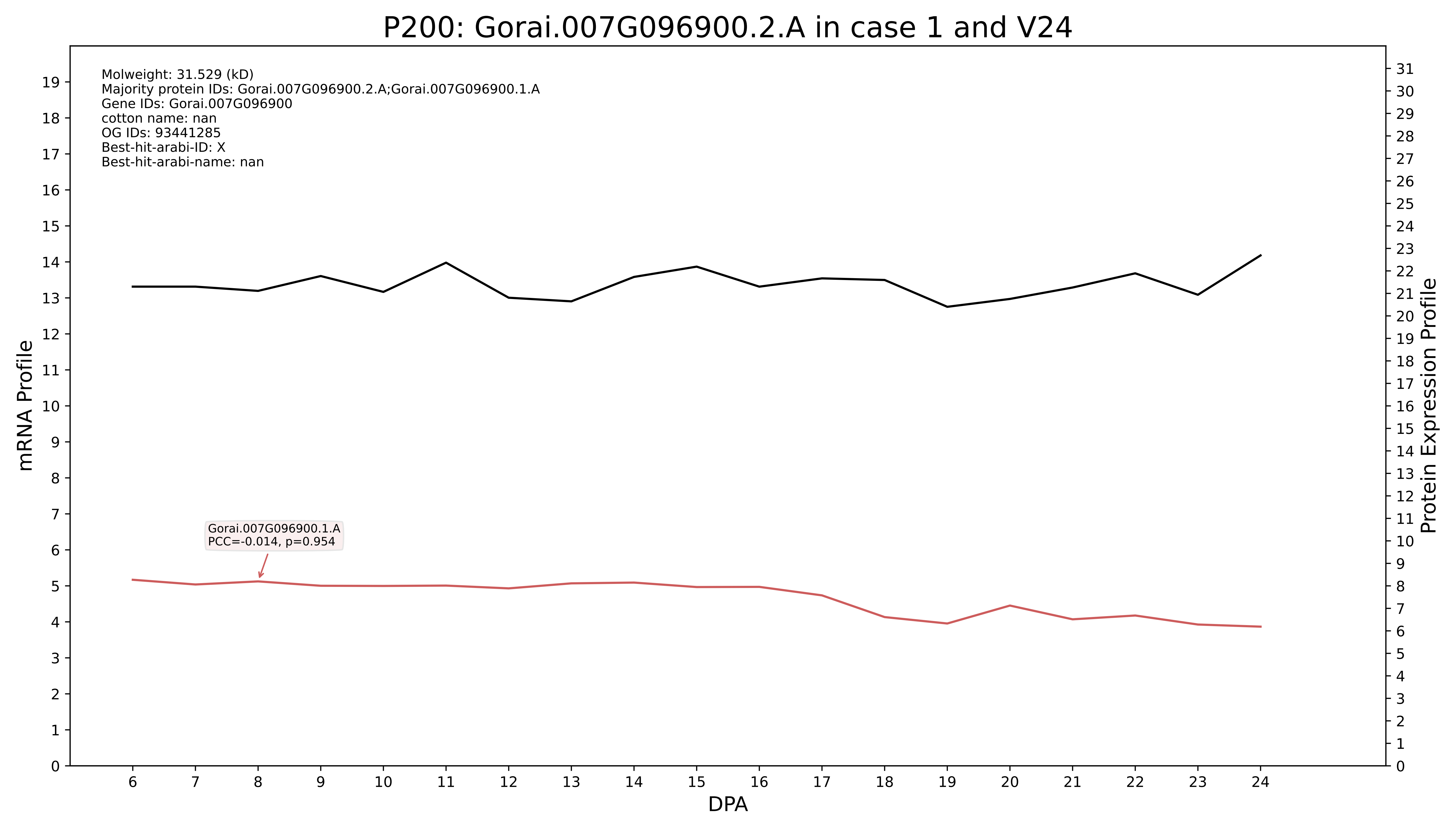

P200: Gorai.007G096900.2.A in case 1 and V24
Molweight: 31.529 (kD)
Majority protein IDs: Gorai.007G096900.2.A;Gorai.007G096900.1.A
Gene IDs: Gorai.007G096900
cotton name: nan
OG IDs: 93441285
Best-hit-arabi-ID: X
Best-hit-arabi-name: nan
Gorai.007G096900.1.A
PCC=-0.014, p=0.954
mRNA Profile
Protein Expression Profile
DPA
0
1
2
3
4
5
6
7
8
9
10
11
12
13
14
15
16
17
18
19
20
21
22
23
24
25
26
27
28
29
30
31

P200: Gorai.013G150400.2.D in case 1 and V24

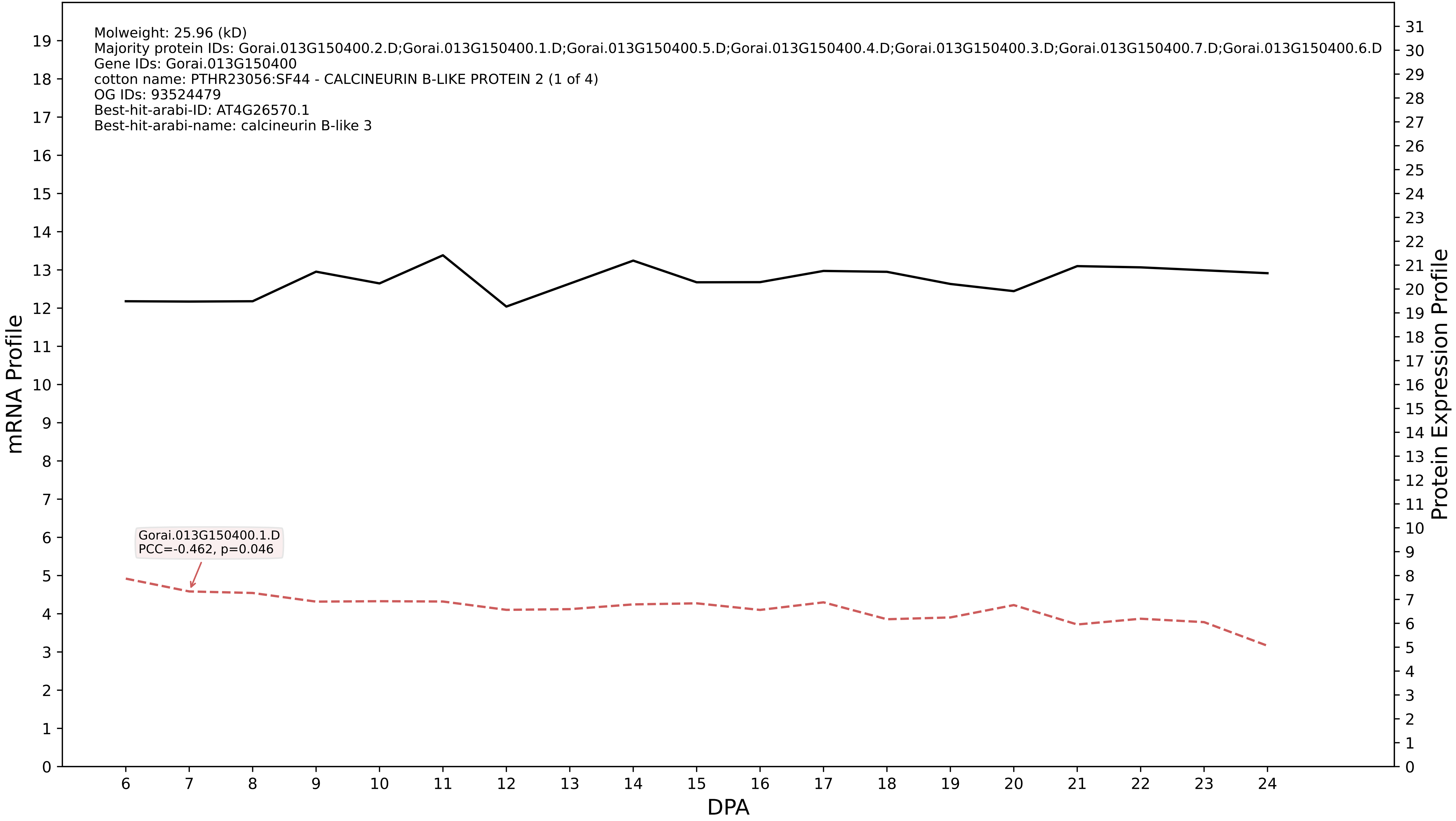

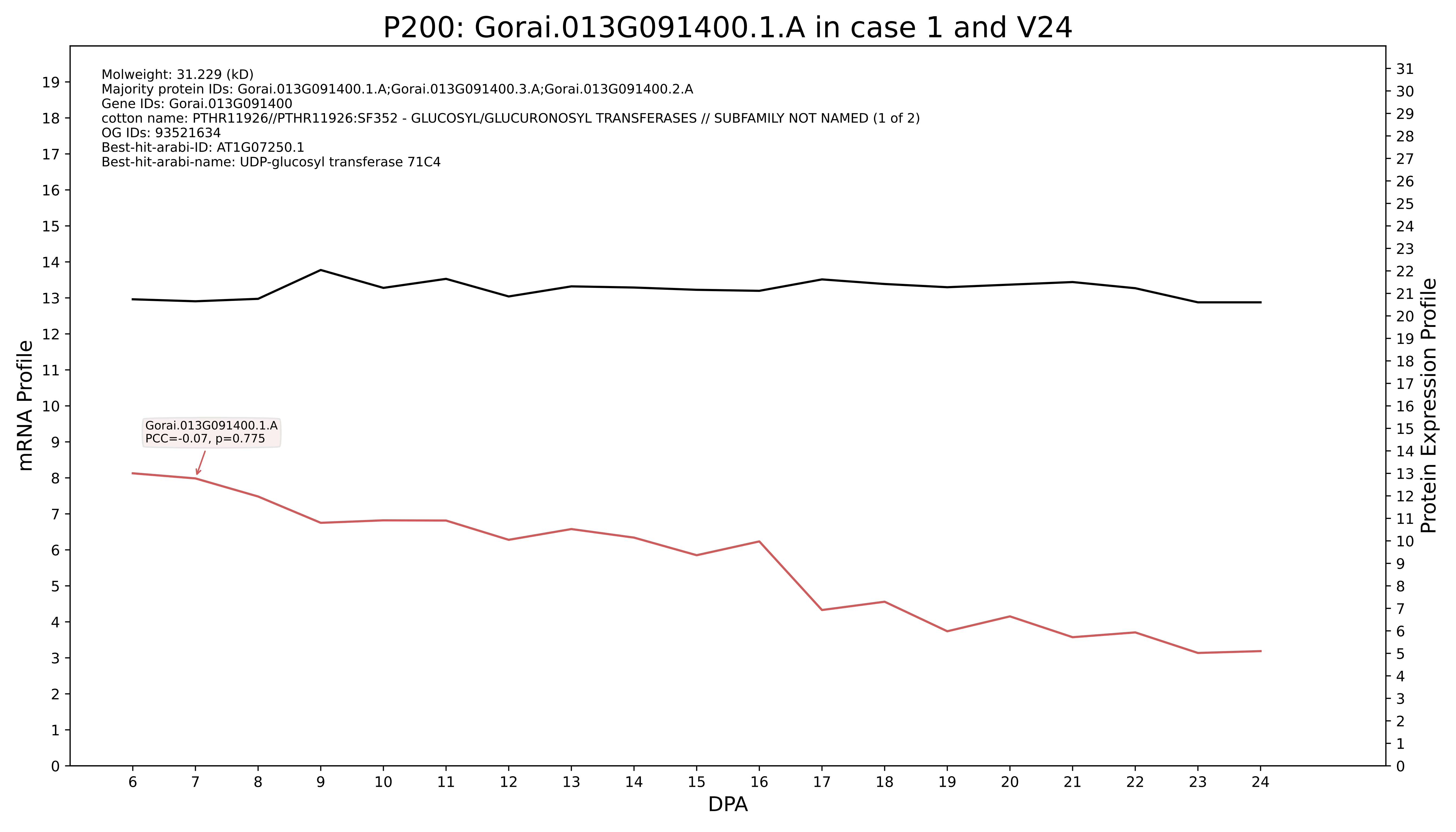
P200: Gorai.013G091400.1.A in case 1 and V24
Molweight: 31.229 (kD)
Majority protein IDs: Gorai.013G091400.1.A;Gorai.013G091400.3.A;Gorai.013G091400.2.A
Gene IDs: Gorai.013G091400
cotton name: PTHR11926//PTHR11926:SF352 - GLUCOSYL/GLUCURONOSYL TRANSFERASES // SUBFAMILY NOT NAMED (1 of 2)
OG IDs: 93521634
Best-hit-arabi-ID: AT1G07250.1
Best-hit-arabi-name: UDP-glucosyl transferase 71C4
Gorai.013G091400.1.A
PCC=-0.07, p=0.775
mRNA Profile
Protein Expression Profile
DPA

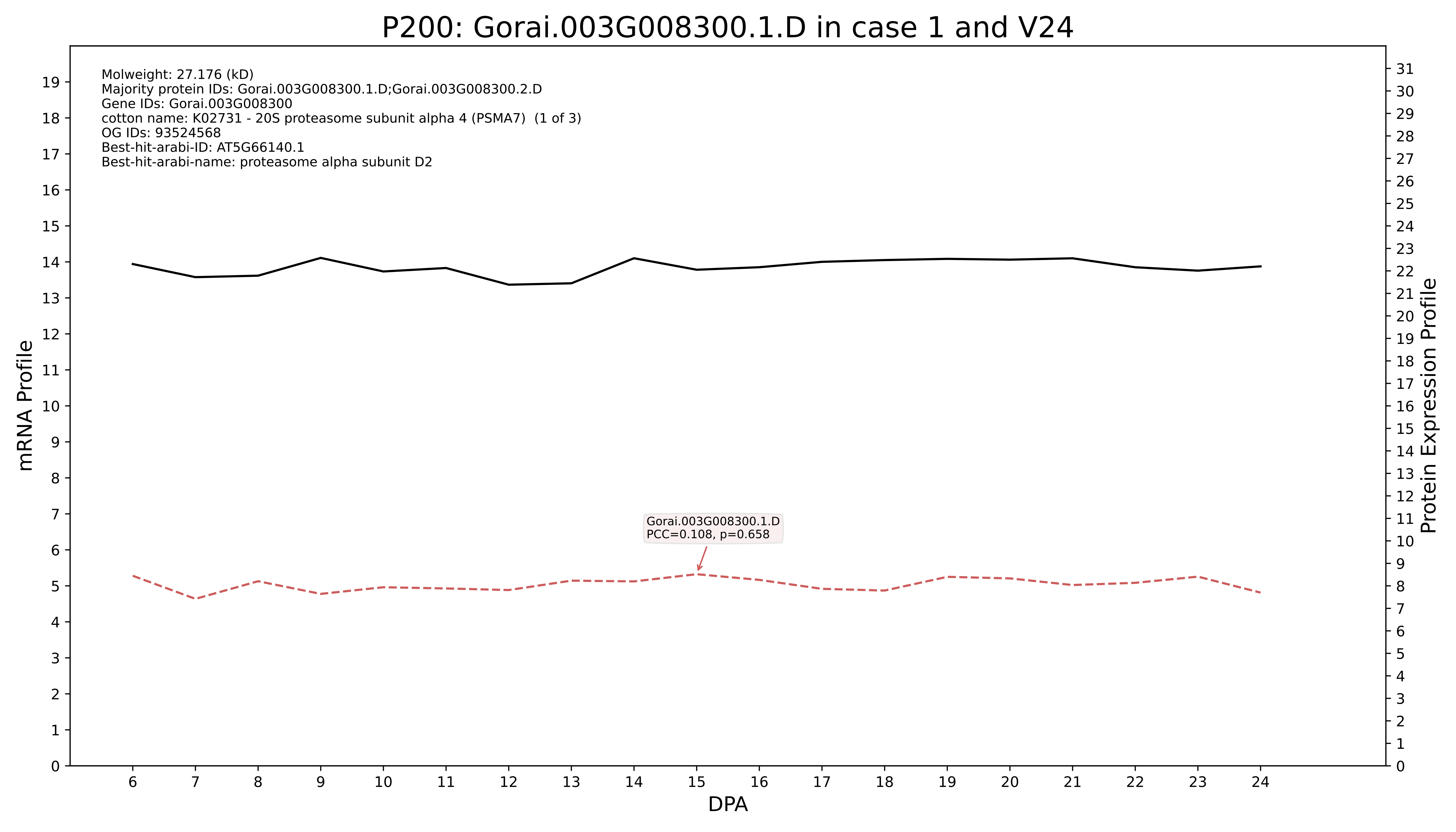
P200: Gorai.003G008300.1.D in case 1 and V24
Molweight: 27.176 (kD)
Majority protein IDs: Gorai.003G008300.1.D;Gorai.003G008300.2.D
Gene IDs: Gorai.003G008300
cotton name: K02731 - 20S proteasome subunit alpha 4 (PSMA7) (1 of 3)
OG IDs: 93524568
Best-hit-arabi-ID: AT5G66140.1
Best-hit-arabi-name: proteasome alpha subunit D2
Gorai.003G008300.1.D
PCC=0.108, p=0.658
mRNA Profile
Protein Expression Profile
DPA

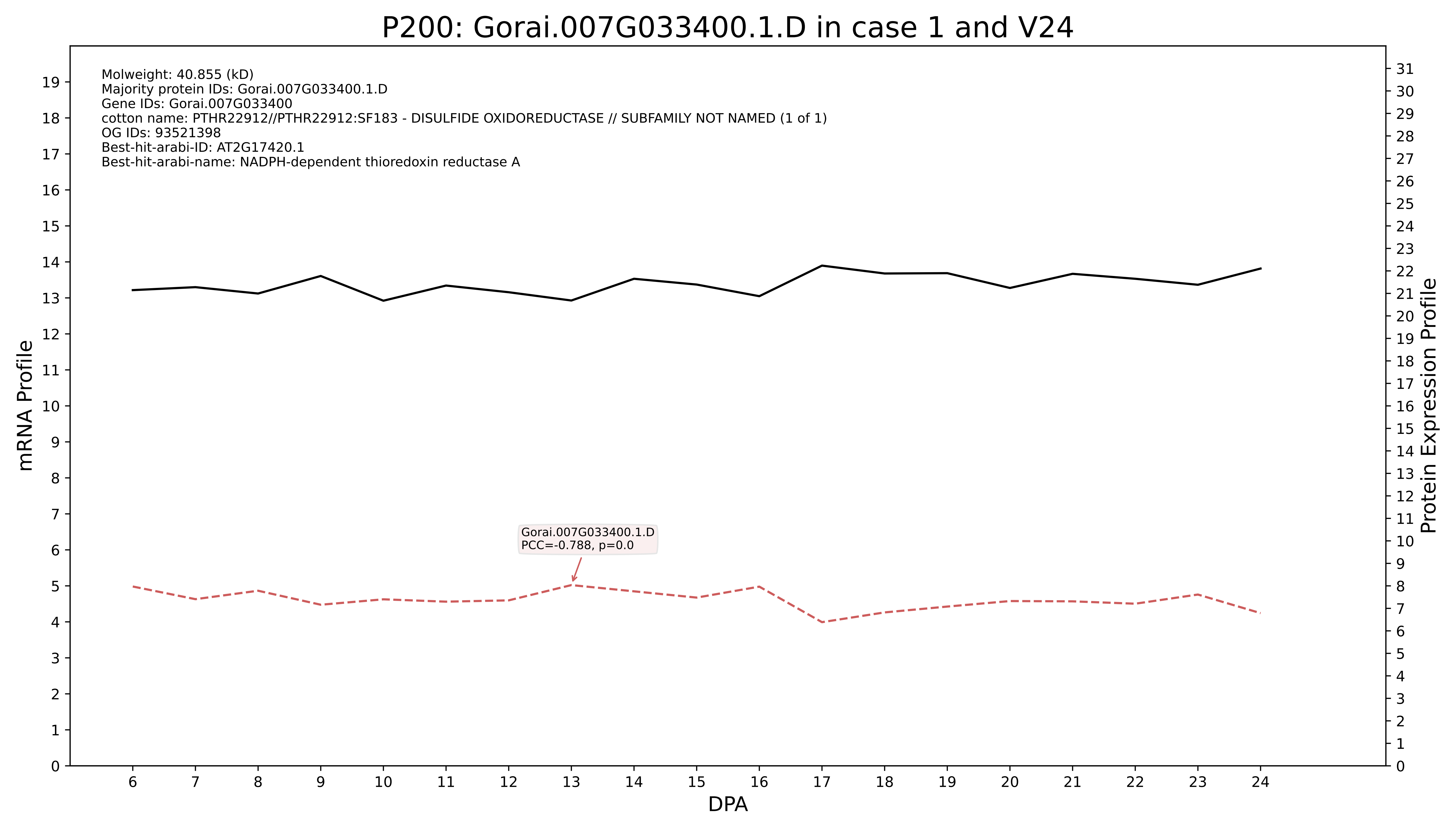
P200: Gorai.007G033400.1.D in case 1 and V24
Molweight: 40.855 (kD)
Majority protein IDs: Gorai.007G033400.1.D
Gene IDs: Gorai.007G033400
cotton name: PTHR22912//PTHR22912:SF183 - DISULFIDE OXIDOREDUCTASE // SUBFAMILY NOT NAMED (1 of 1)
OG IDs: 93521398
Best-hit-arabi-ID: AT2G17420.1
Best-hit-arabi-name: NADPH-dependent thioredoxin reductase A
Gorai.007G033400.1.D
PCC=-0.788, p=0.0
mRNA Profile
Protein Expression Profile
DPA

P200: Gorai.002G164400.2.D in case 1 and V24

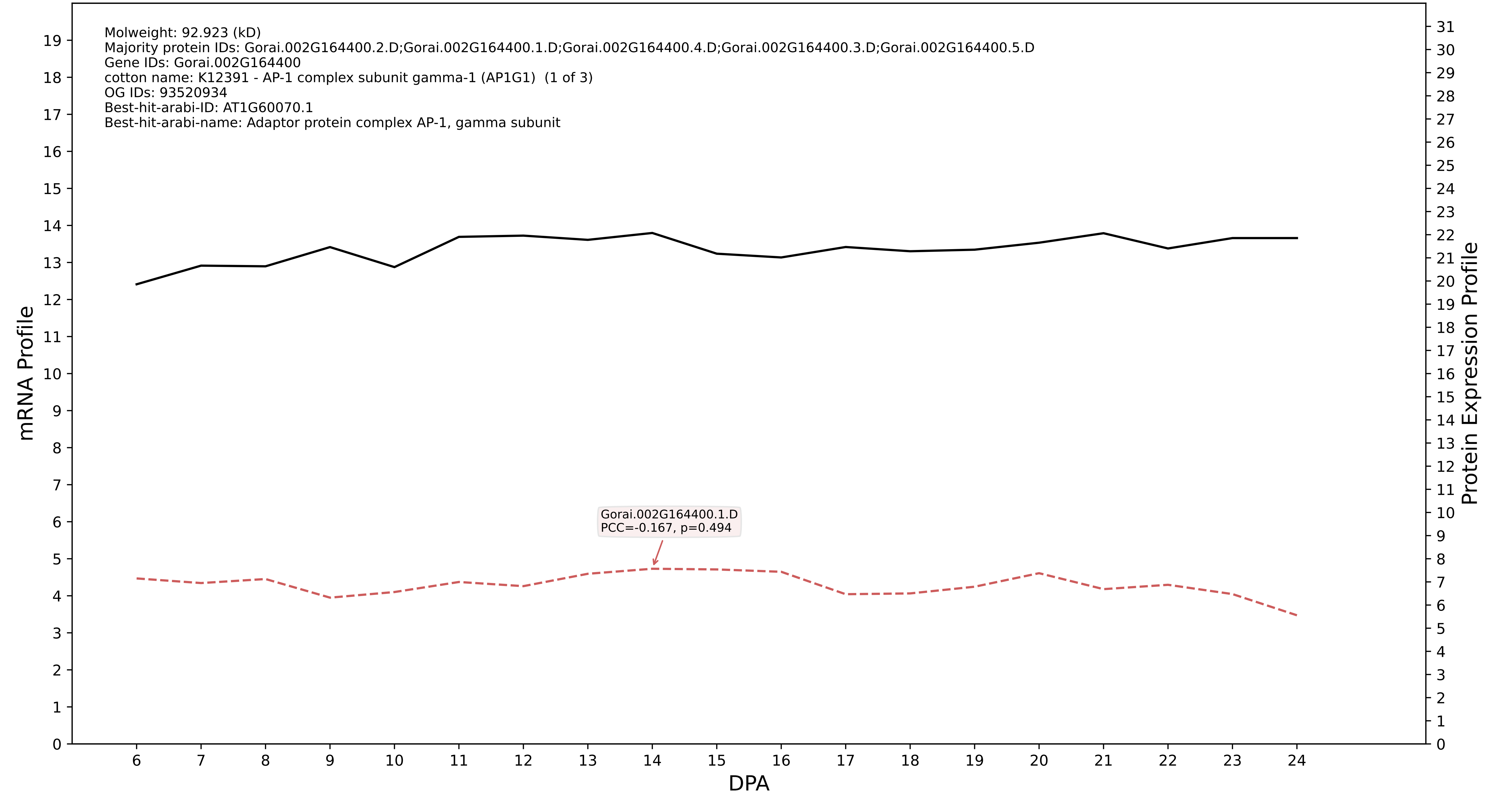

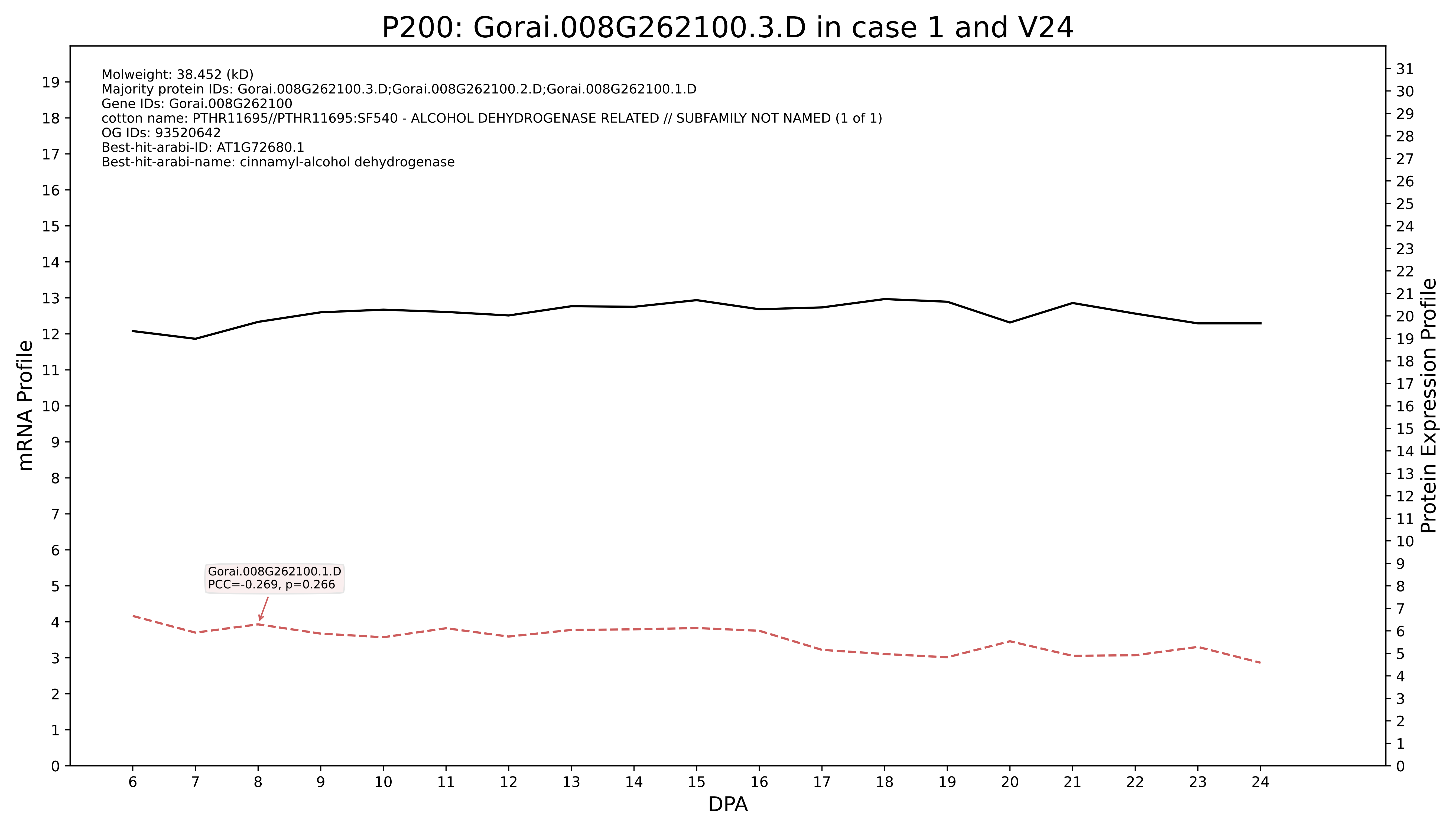
P200: Gorai.008G262100.3.D in case 1 and V24
Molweight: 38.452 (kD)
Majority protein IDs: Gorai.008G262100.3.D;Gorai.008G262100.2.D;Gorai.008G262100.1.D
Gene IDs: Gorai.008G262100
cotton name: PTHR11695//PTHR11695:SF540 - ALCOHOL DEHYDROGENASE RELATED // SUBFAMILY NOT NAMED (1 of 1)
OG IDs: 93520642
Best-hit-arabi-ID: AT1G72680.1
Best-hit-arabi-name: cinnamyl-alcohol dehydrogenase
Gorai.008G262100.1.D
PCC=-0.269, p=0.266
mRNA Profile
Protein Expression Profile
DPA

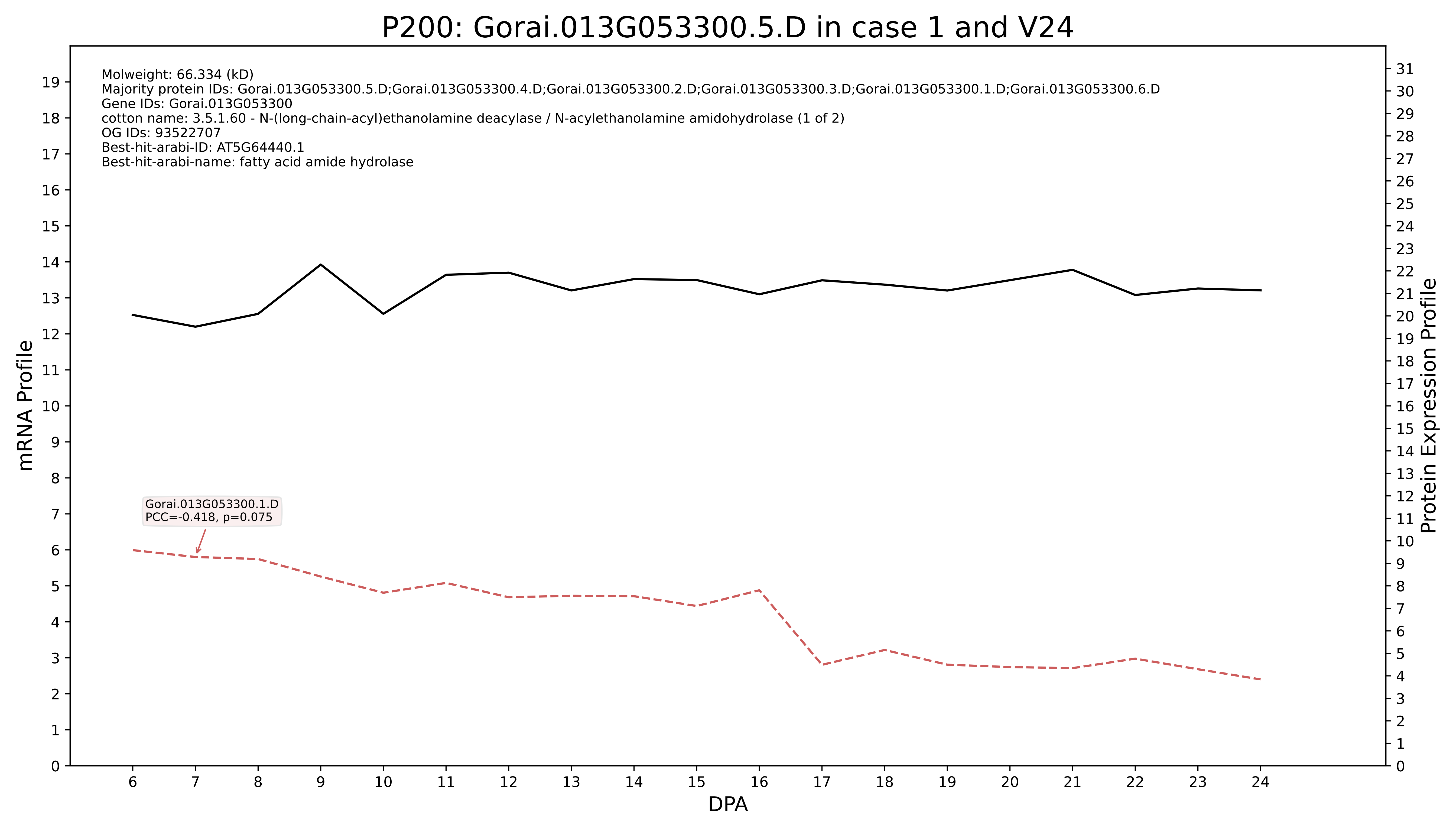

P200: Gorai.013G053300.5.D in case 1 and V24
Molweight: 66.334 (kD)
Majority protein IDs: Gorai.013G053300.5.D;Gorai.013G053300.4.D;Gorai.013G053300.2.D;Gorai.013G053300.3.D;Gorai.013G053300.1.D;Gorai.013G053300.6.D
Gene IDs: Gorai.013G053300
cotton name: 3.5.1.60 - N-(long-chain-acyl)ethanolamine deacylase / N-acylethanolamine amidohydrolase (1 of 2)
OG IDs: 93522707
Best-hit-arabi-ID: AT5G64440.1
Best-hit-arabi-name: fatty acid amide hydrolase
Gorai.013G053300.1.D
PCC=-0.418, p=0.075
mRNA Profile
Protein Expression Profile
DPA

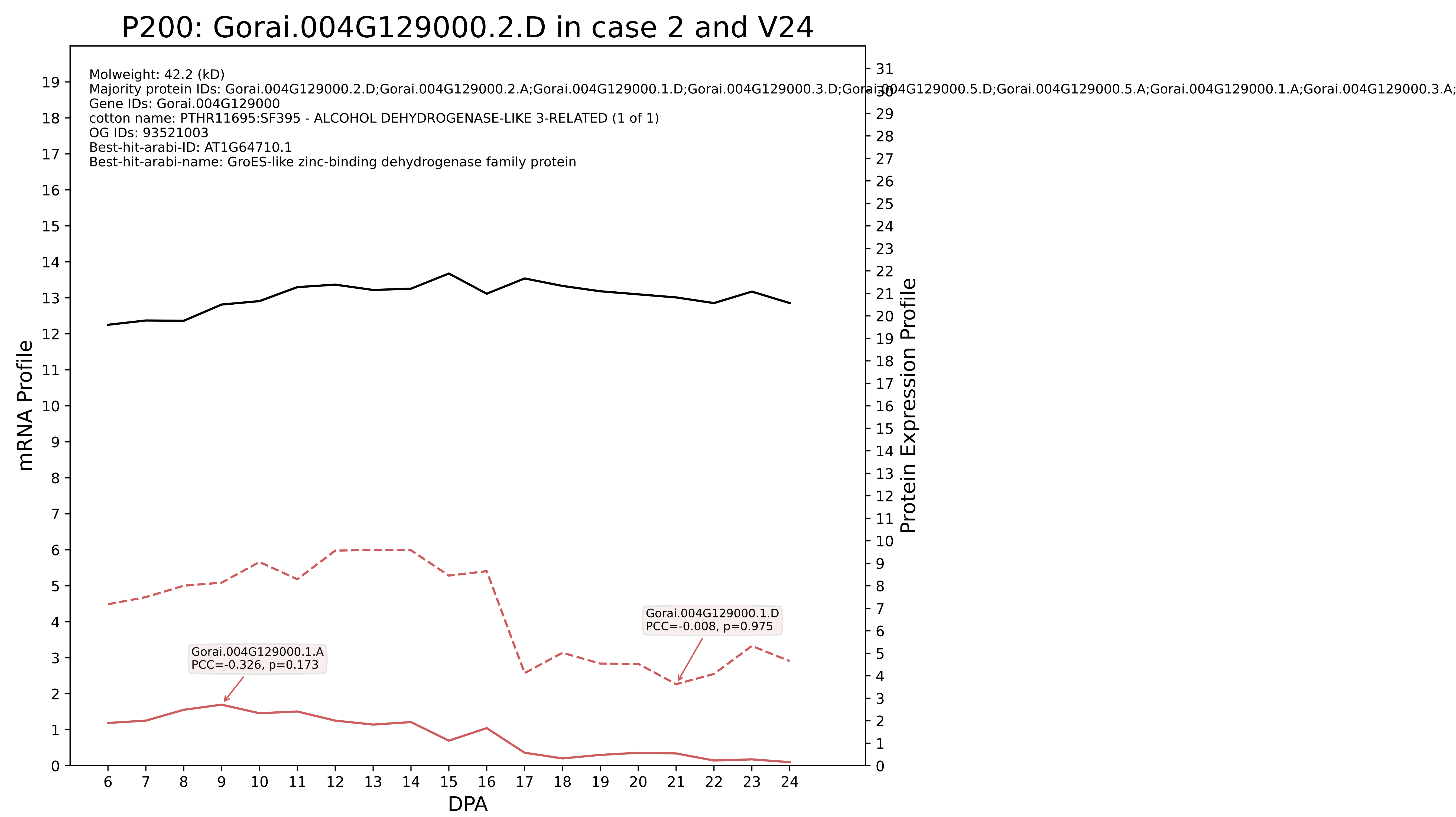
P200: Gorai.004G129000.2.D in case 2 and V24
Molweight: 42.2 (kD)
Majority protein IDs: Gorai.004G129000.2.D;Gorai.004G129000.2.A;Gorai.004G129000.1.D;Gorai.004G129000.3.D;Gorai.004G129000.5.D;Gorai.004G129000.5.A;Gorai.004G129000.1.A;Gorai.004G129000.3.A;
Gene IDs: Gorai.004G129000
cotton name: PTHR11695:SF395 - ALCOHOL DEHYDROGENASE-LIKE 3-RELATED (1 of 1)
OG IDs: 93521003
Best-hit-arabi-ID: AT1G64710.1
Best-hit-arabi-name: GroES-like zinc-binding dehydrogenase family protein
Gorai.004G129000.1.D
PCC=-0.008, p=0.975
Gorai.004G129000.1.A
PCC=-0.326, p=0.173
mRNA Profile
Protein Expression Profile
DPA

00: Gorai.013G116800.6.D in case 2 and V24

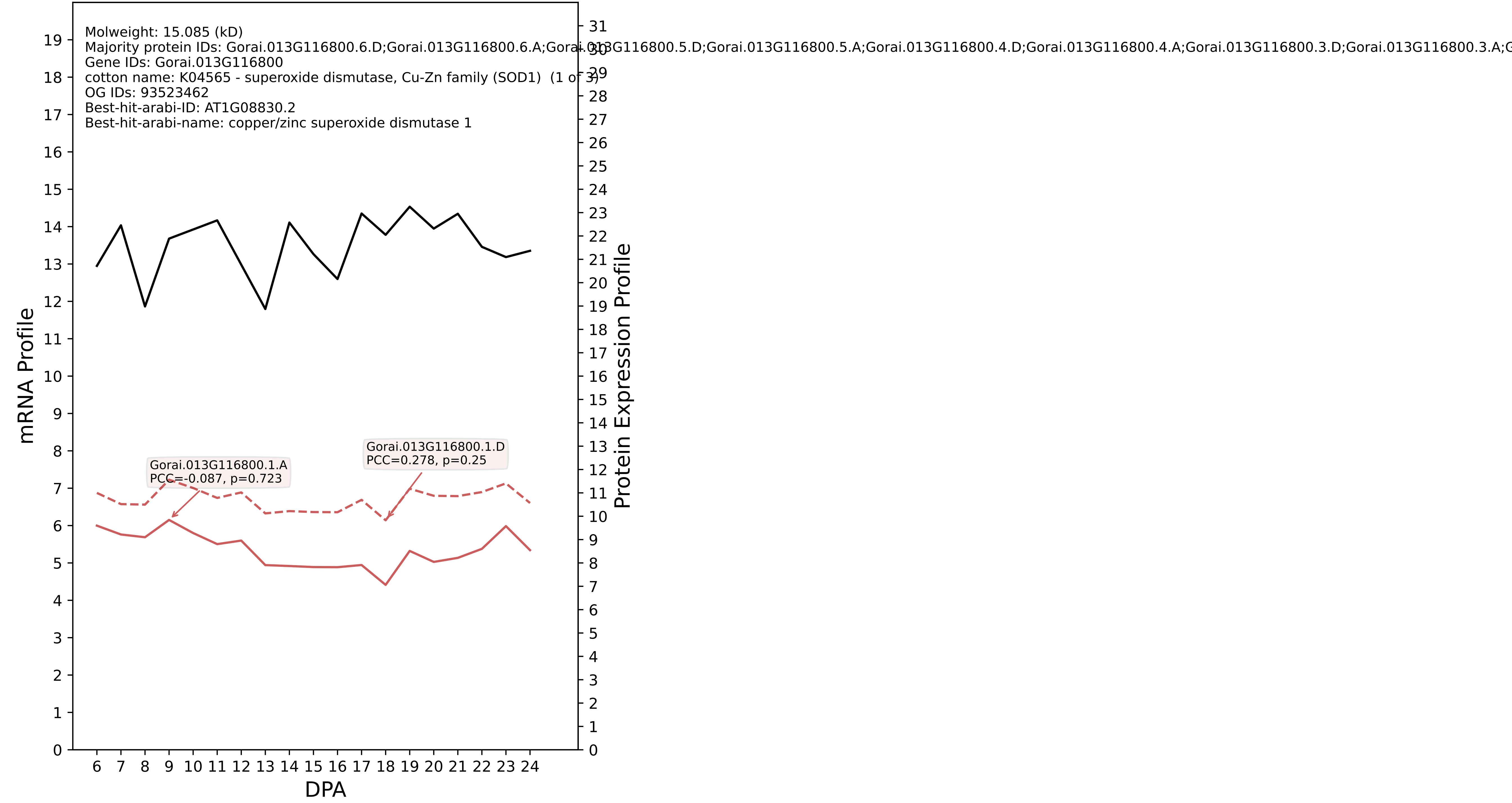

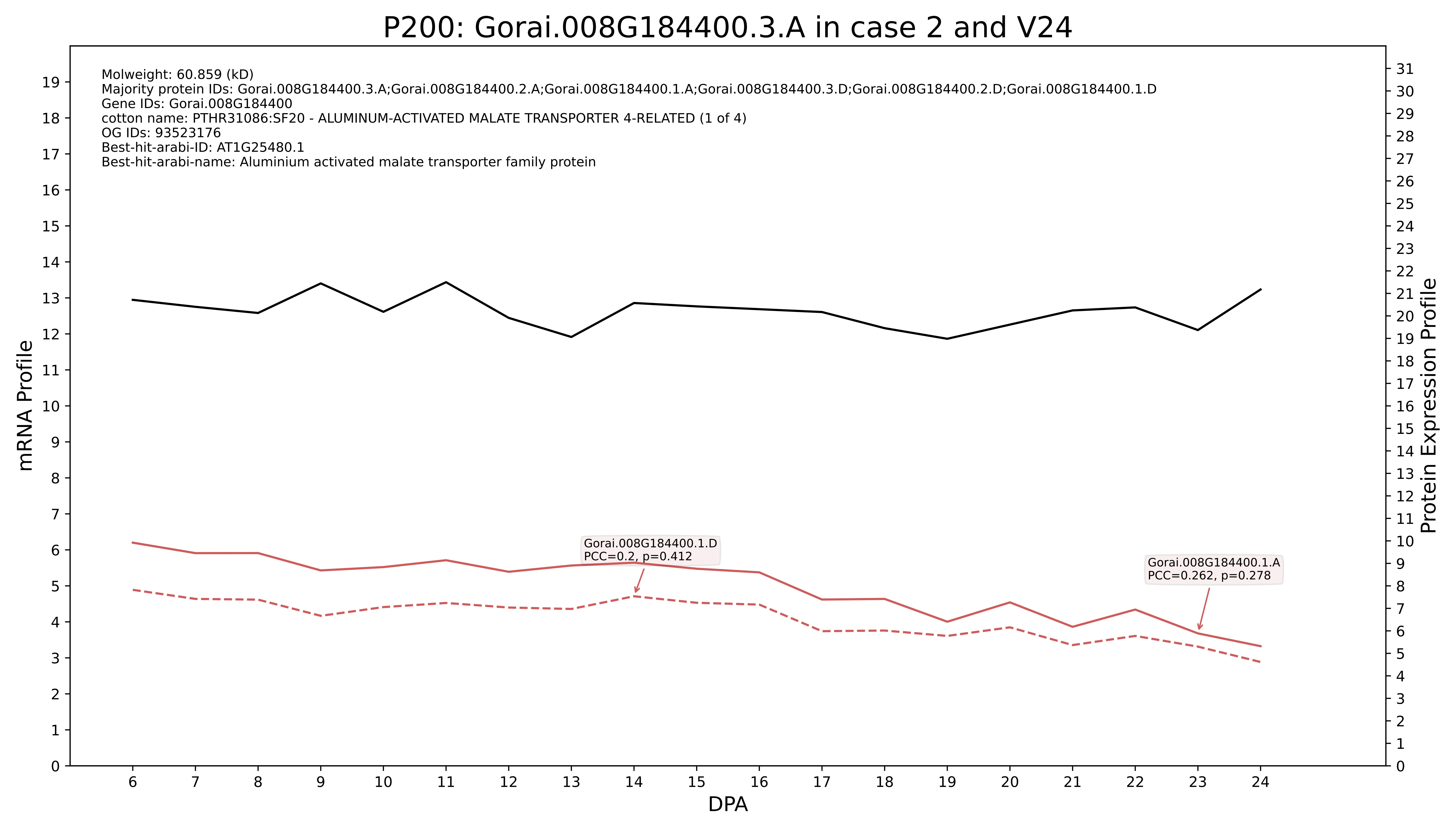
P200: Gorai.008G184400.3.A in case 2 and V24
Molweight: 60.859 (kD)
Majority protein IDs: Gorai.008G184400.3.A;Gorai.008G184400.2.A;Gorai.008G184400.1.A;Gorai.008G184400.3.D;Gorai.008G184400.2.D;Gorai.008G184400.1.D
Gene IDs: Gorai.008G184400
cotton name: PTHR31086:SF20 - ALUMINUM-ACTIVATED MALATE TRANSPORTER 4-RELATED (1 of 4)
OG IDs: 93523176
Best-hit-arabi-ID: AT1G25480.1
Best-hit-arabi-name: Aluminium activated malate transporter family protein
Gorai.008G184400.1.D
PCC=0.2, p=0.412
Gorai.008G184400.1.A
PCC=0.262, p=0.278
mRNA Profile
Protein Expression Profile
DPA

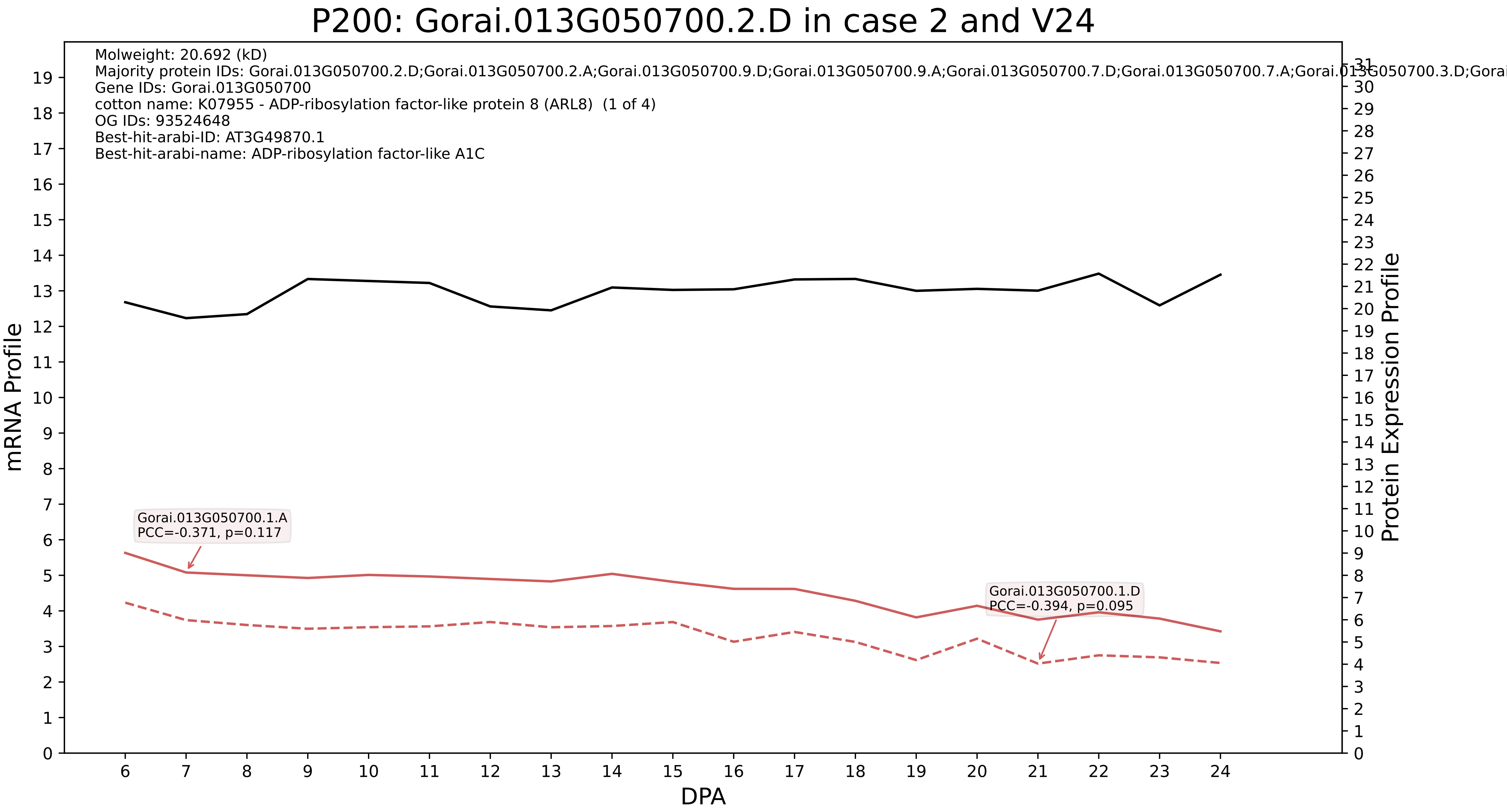

P200: Gorai.013G050700.2.D in case 2 and V24
Molweight: 20.692 (kD)
Majority protein IDs: Gorai.013G050700.2.D;Gorai.013G050700.2.A;Gorai.013G050700.9.D;Gorai.013G050700.9.A;Gorai.013G050700.7.D;Gorai.013G050700.7.A;Gorai.013G050700.3.D;Gorai
Gene IDs: Gorai.013G050700
cotton name: K07955 - ADP-ribosylation factor-like protein 8 (ARL8) (1 of 4)
OG IDs: 93524648
Best-hit-arabi-ID: AT3G49870.1
Best-hit-arabi-name: ADP-ribosylation factor-like A1C
Gorai.013G050700.1.A
PCC=-0.371, p=0.117
Gorai.013G050700.1.D
PCC=-0.394, p=0.095
mRNA Profile
Protein Expression Profile
DPA

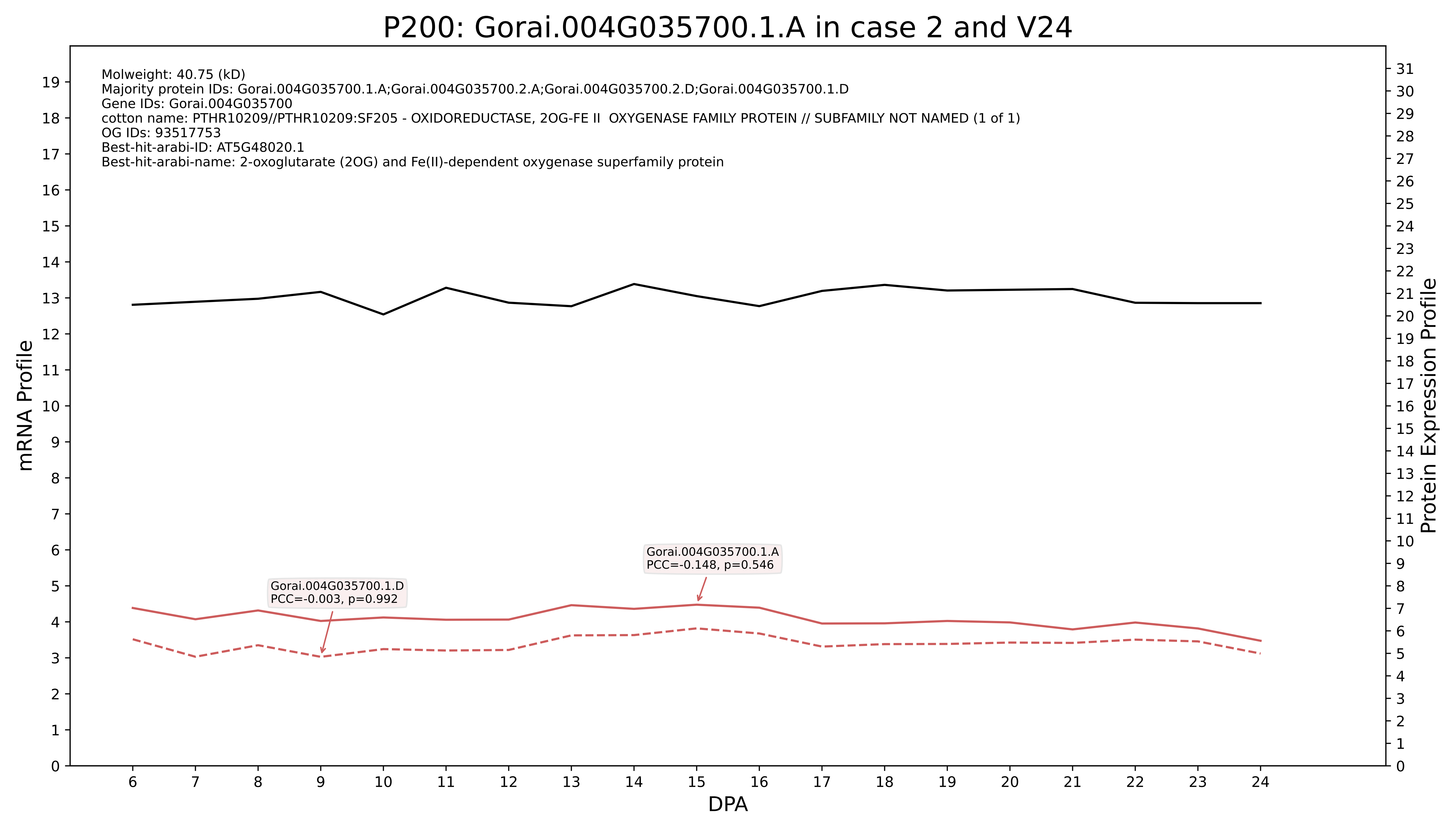
P200: Gorai.004G035700.1.A in case 2 and V24
Molweight: 40.75 (kD)
Majority protein IDs: Gorai.004G035700.1.A;Gorai.004G035700.2.A;Gorai.004G035700.2.D;Gorai.004G035700.1.D
Gene IDs: Gorai.004G035700
cotton name: PTHR10209//PTHR10209:SF205 - OXIDOREDUCTASE, 2OG-FE II OXYGENASE FAMILY PROTEIN // SUBFAMILY NOT NAMED (1 of 1)
OG IDs: 93517753
Best-hit-arabi-ID: AT5G48020.1
Best-hit-arabi-name: 2-oxoglutarate (2OG) and Fe(II)-dependent oxygenase superfamily protein
Gorai.004G035700.1.A
PCC=-0.148, p=0.546
Gorai.004G035700.1.D
PCC=-0.003, p=0.992
mRNA Profile
Protein Expression Profile
DPA

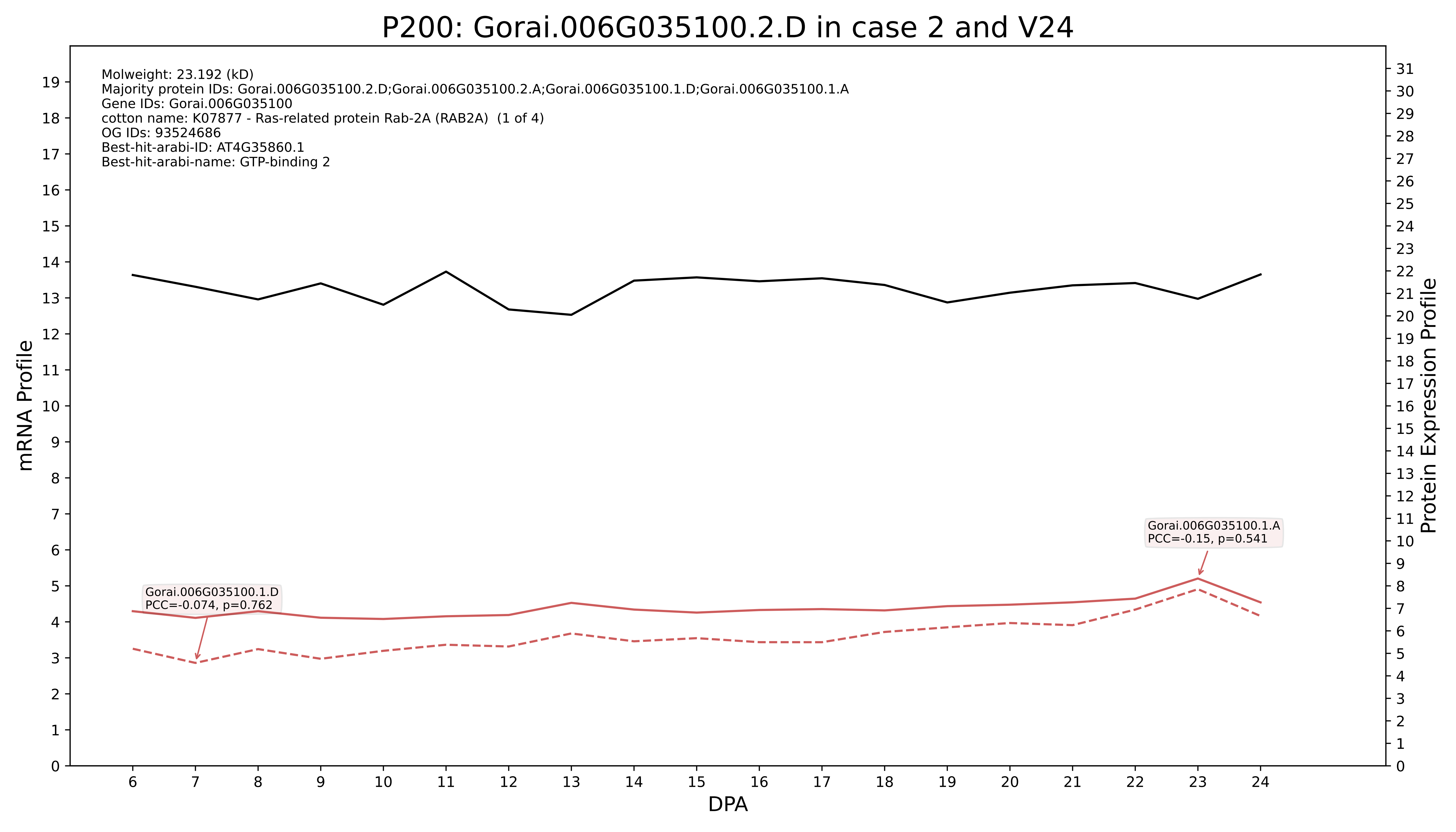
P200: Gorai.006G035100.2.D in case 2 and V24
Molweight: 23.192 (kD)
Majority protein IDs: Gorai.006G035100.2.D;Gorai.006G035100.2.A;Gorai.006G035100.1.D;Gorai.006G035100.1.A
Gene IDs: Gorai.006G035100
cotton name: K07877 - Ras-related protein Rab-2A (RAB2A) (1 of 4)
OG IDs: 93524686
Best-hit-arabi-ID: AT4G35860.1
Best-hit-arabi-name: GTP-binding 2
Gorai.006G035100.1.A
PCC=-0.15, p=0.541
Gorai.006G035100.1.D
PCC=-0.074, p=0.762
mRNA Profile
Protein Expression Profile
DPA

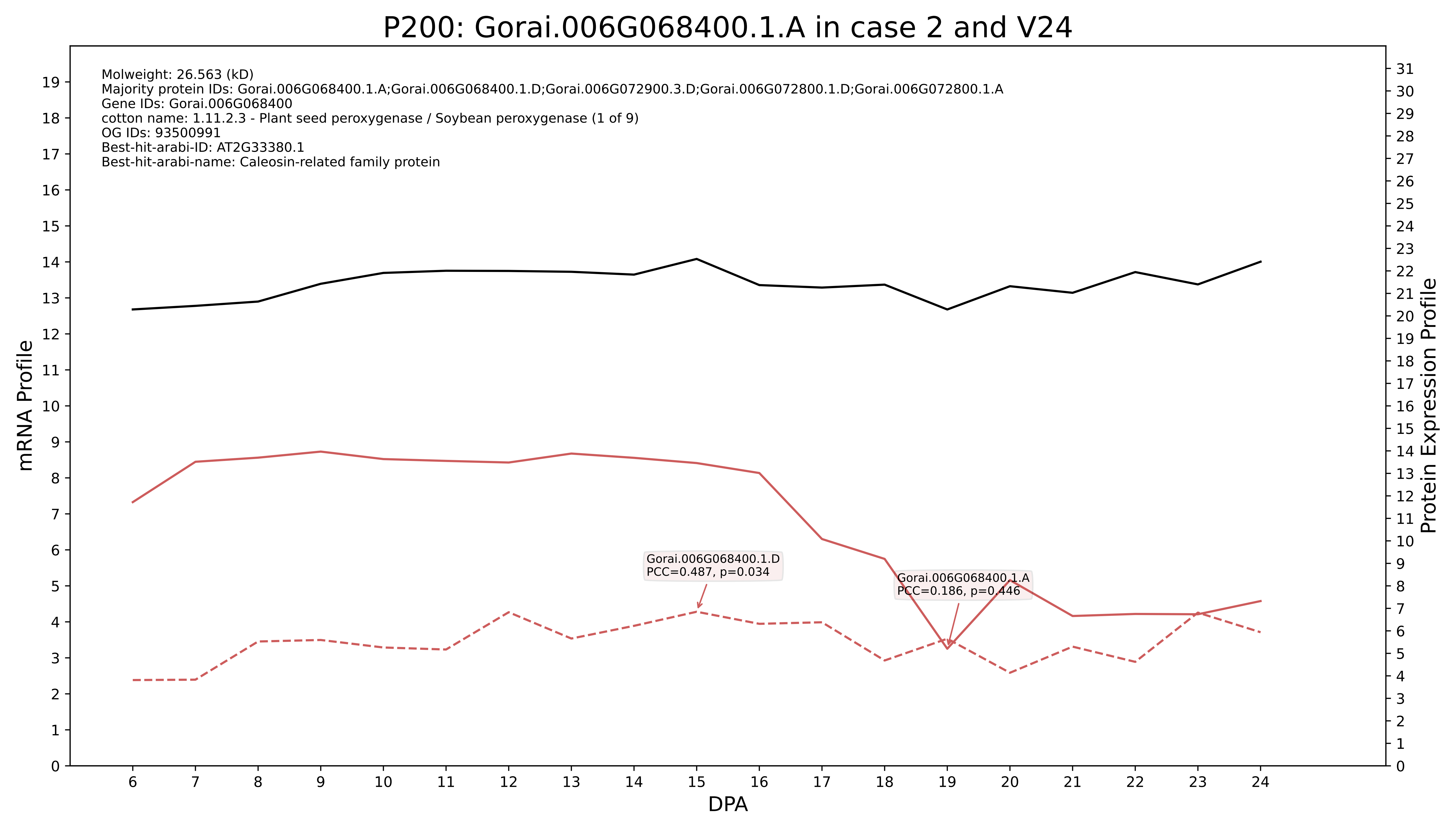
P200: Gorai.006G068400.1.A in case 2 and V24
Molweight: 26.563 (kD)
Majority protein IDs: Gorai.006G068400.1.A;Gorai.006G068400.1.D;Gorai.006G072900.3.D;Gorai.006G072800.1.D;Gorai.006G072800.1.A
Gene IDs: Gorai.006G068400
cotton name: 1.11.2.3 - Plant seed peroxygenase / Soybean peroxygenase (1 of 9)
OG IDs: 93500991
Best-hit-arabi-ID: AT2G33380.1
Best-hit-arabi-name: Caleosin-related family protein
Gorai.006G068400.1.D
PCC=0.487, p=0.034
Gorai.006G068400.1.A
PCC=0.186, p=0.446
mRNA Profile
Protein Expression Profile
DPA

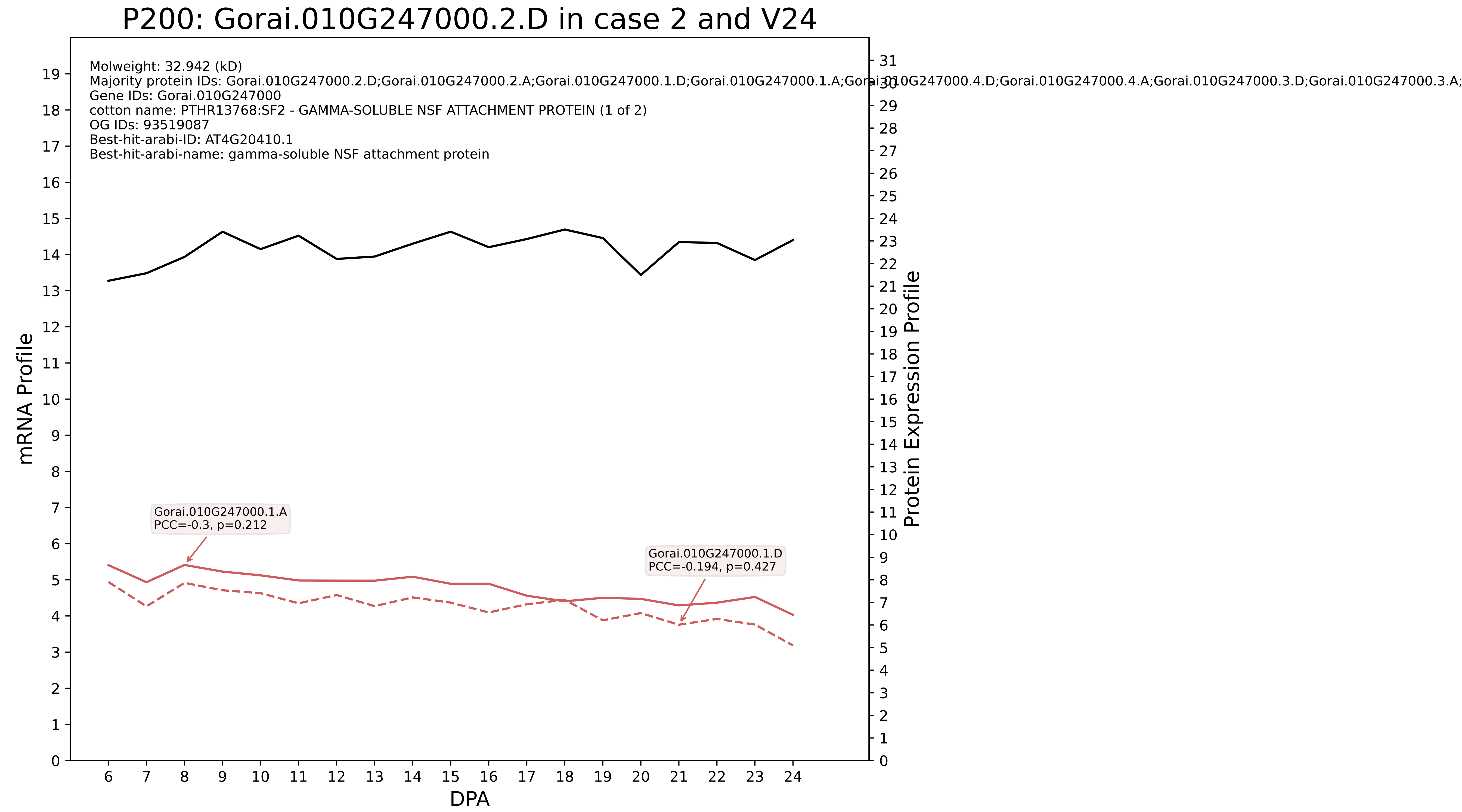
P200: Gorai.010G247000.2.D in case 2 and V24
Molweight: 32.942 (kD)
Majority protein IDs: Gorai.010G247000.2.D;Gorai.010G247000.2.A;Gorai.010G247000.1.D;Gorai.010G247000.1.A;Gorai.010G247000.4.D;Gorai.010G247000.4.A;Gorai.010G247000.3.D;Gorai.010G247000.3.A;
Gene IDs: Gorai.010G247000
cotton name: PTHR13768:SF2 - GAMMA-SOLUBLE NSF ATTACHMENT PROTEIN (1 of 2)
OG IDs: 93519087
Best-hit-arabi-ID: AT4G20410.1
Best-hit-arabi-name: gamma-soluble NSF attachment protein
Gorai.010G247000.1.A
PCC=-0.3, p=0.212
Gorai.010G247000.1.D
PCC=-0.194, p=0.427
mRNA Profile
Protein Expression Profile
DPA

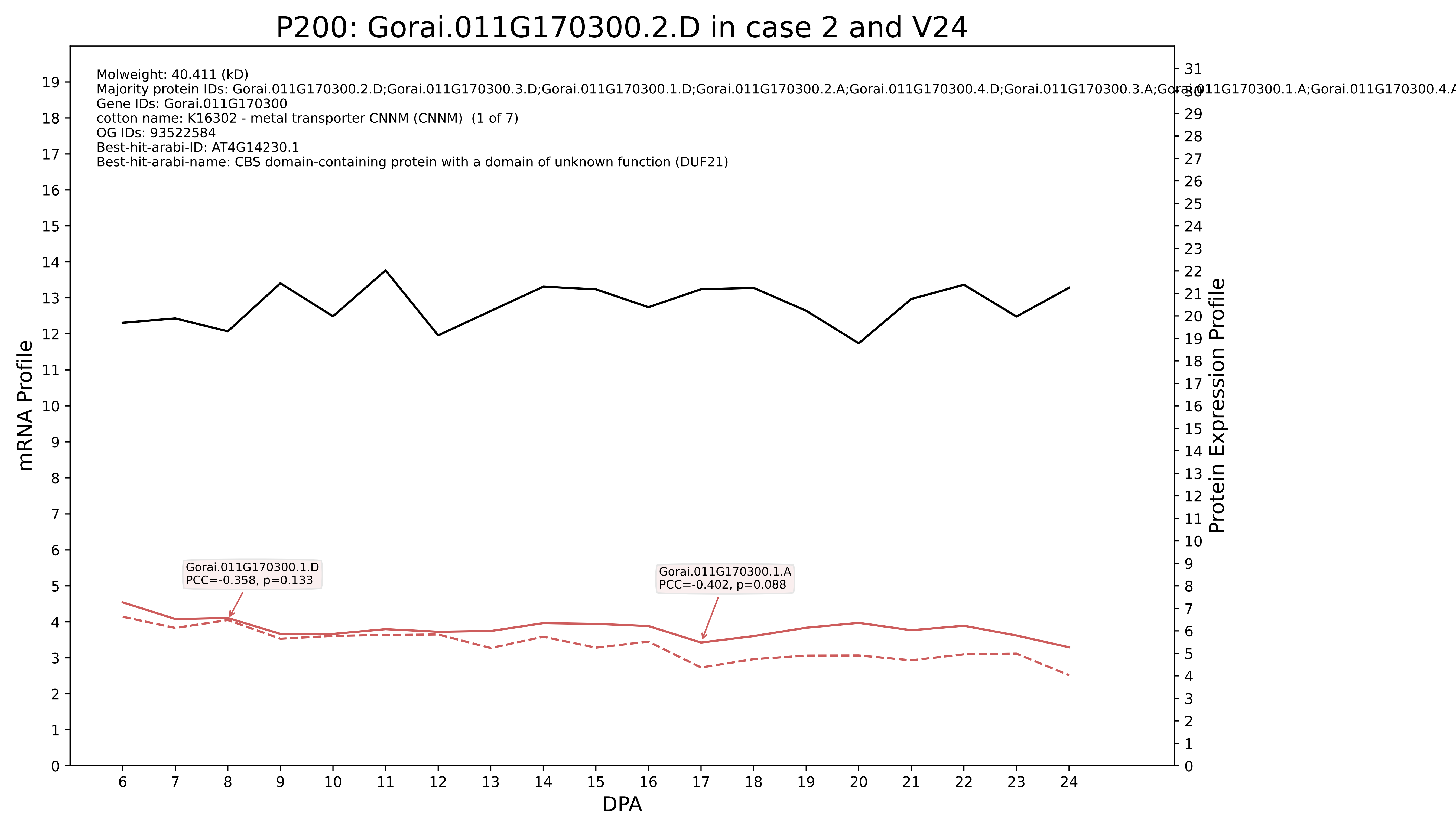

P200: Gorai.011G170300.2.D in case 2 and V24
Molweight: 40.411 (kD)
Majority protein IDs: Gorai.011G170300.2.D;Gorai.011G170300.3.D;Gorai.011G170300.1.D;Gorai.011G170300.2.A;Gorai.011G170300.4.D;Gorai.011G170300.3.A;Gorai.011G170300.1.A;Gorai.011G170300.4.A
Gene IDs: Gorai.011G170300
cotton name: K16302 - metal transporter CNNM (CNNM) (1 of 7)
OG IDs: 93522584
Best-hit-arabi-ID: AT4G14230.1
Best-hit-arabi-name: CBS domain-containing protein with a domain of unknown function (DUF21)
Gorai.011G170300.1.D
PCC=-0.358, p=0.133
Gorai.011G170300.1.A
PCC=-0.402, p=0.088
mRNA Profile
Protein Expression Profile
DPA

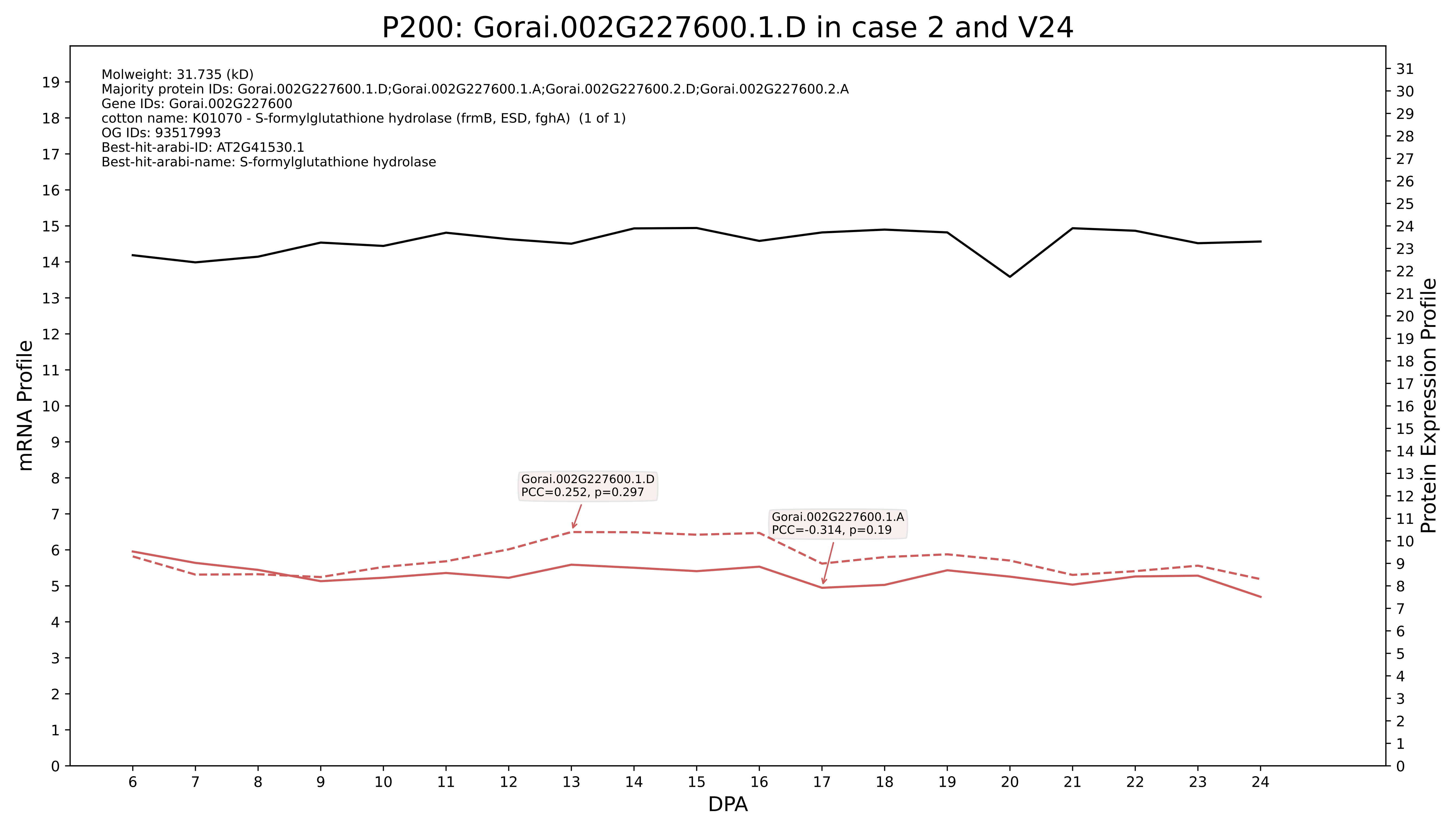
P200: Gorai.002G227600.1.D in case 2 and V24
Molweight: 31.735 (kD)
Majority protein IDs: Gorai.002G227600.1.D;Gorai.002G227600.1.A;Gorai.002G227600.2.D;Gorai.002G227600.2.A
Gene IDs: Gorai.002G227600
cotton name: K01070 - S-formylglutathione hydrolase (frmB, ESD, fghA) (1 of 1)
OG IDs: 93517993
Best-hit-arabi-ID: AT2G41530.1
Best-hit-arabi-name: S-formylglutathione hydrolase
Gorai.002G227600.1.D
PCC=0.252, p=0.297
Gorai.002G227600.1.A
PCC=-0.314, p=0.19
mRNA Profile
Protein Expression Profile
DPA

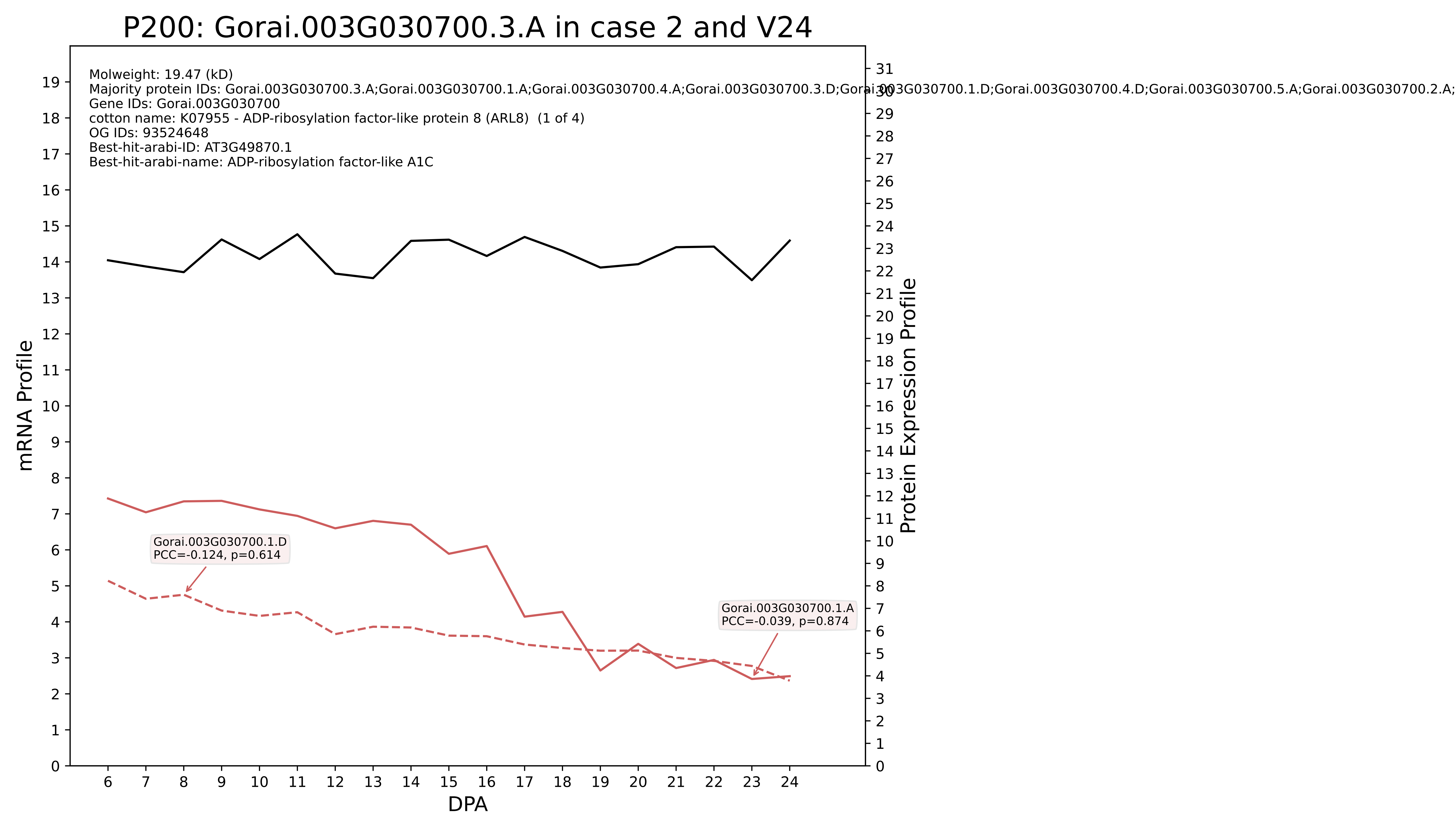

P200: Gorai.003G030700.3.A in case 2 and V24
Molweight: 19.47 (kD)
Majority protein IDs: Gorai.003G030700.3.A;Gorai.003G030700.1.A;Gorai.003G030700.4.A;Gorai.003G030700.3.D;Gorai.003G030700.1.D;Gorai.003G030700.4.D;Gorai.003G030700.5.A;Gorai.003G030700.2.A;
Gene IDs: Gorai.003G030700
cotton name: K07955 - ADP-ribosylation factor-like protein 8 (ARL8) (1 of 4)
OG IDs: 93524648
Best-hit-arabi-ID: AT3G49870.1
Best-hit-arabi-name: ADP-ribosylation factor-like A1C
Gorai.003G030700.1.D
PCC=-0.124, p=0.614
Gorai.003G030700.1.A
PCC=-0.039, p=0.874
mRNA Profile
Protein Expression Profile
DPA

P200: Gorai.009G182900.1.A in case 2 and V24

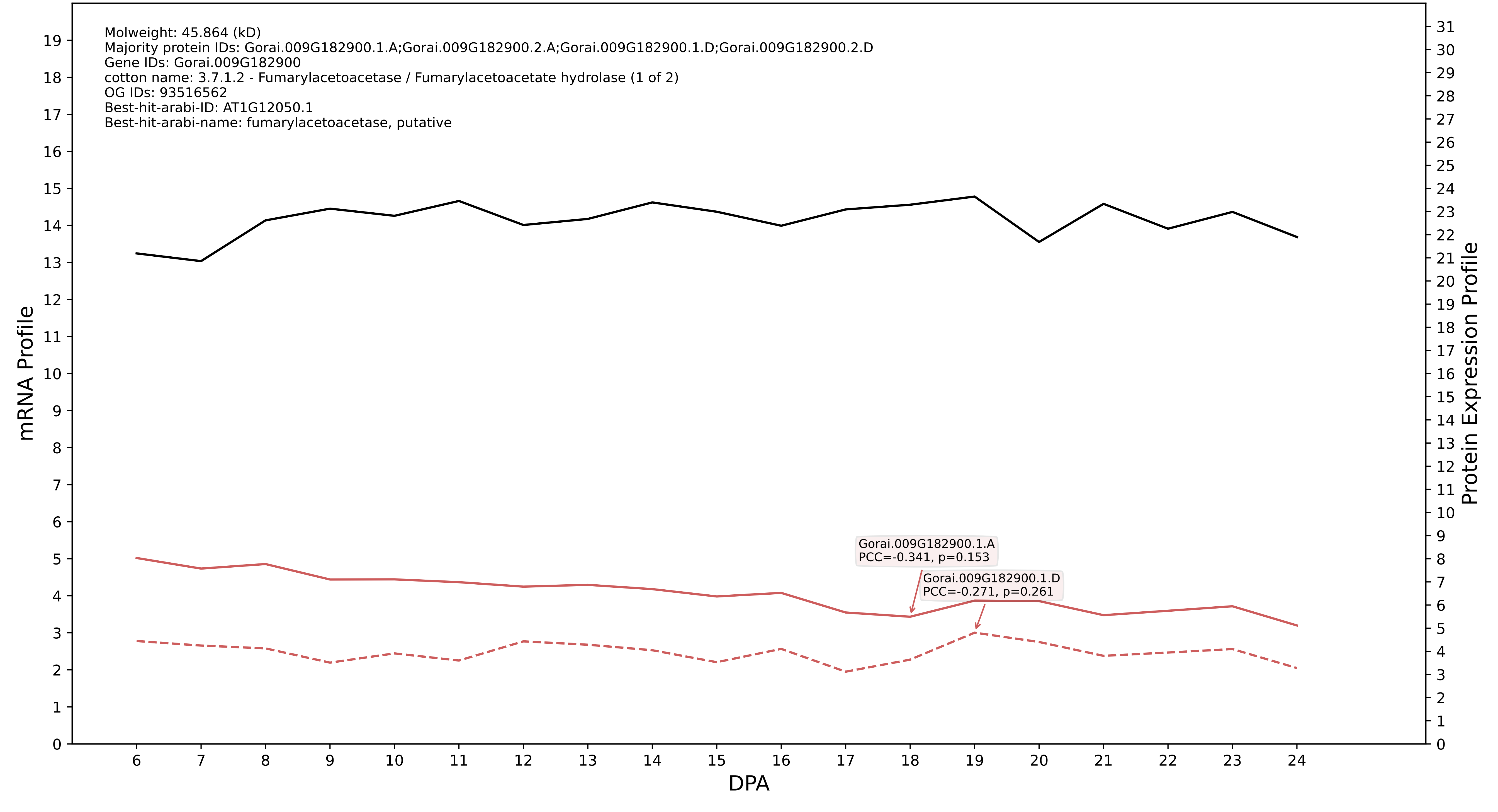

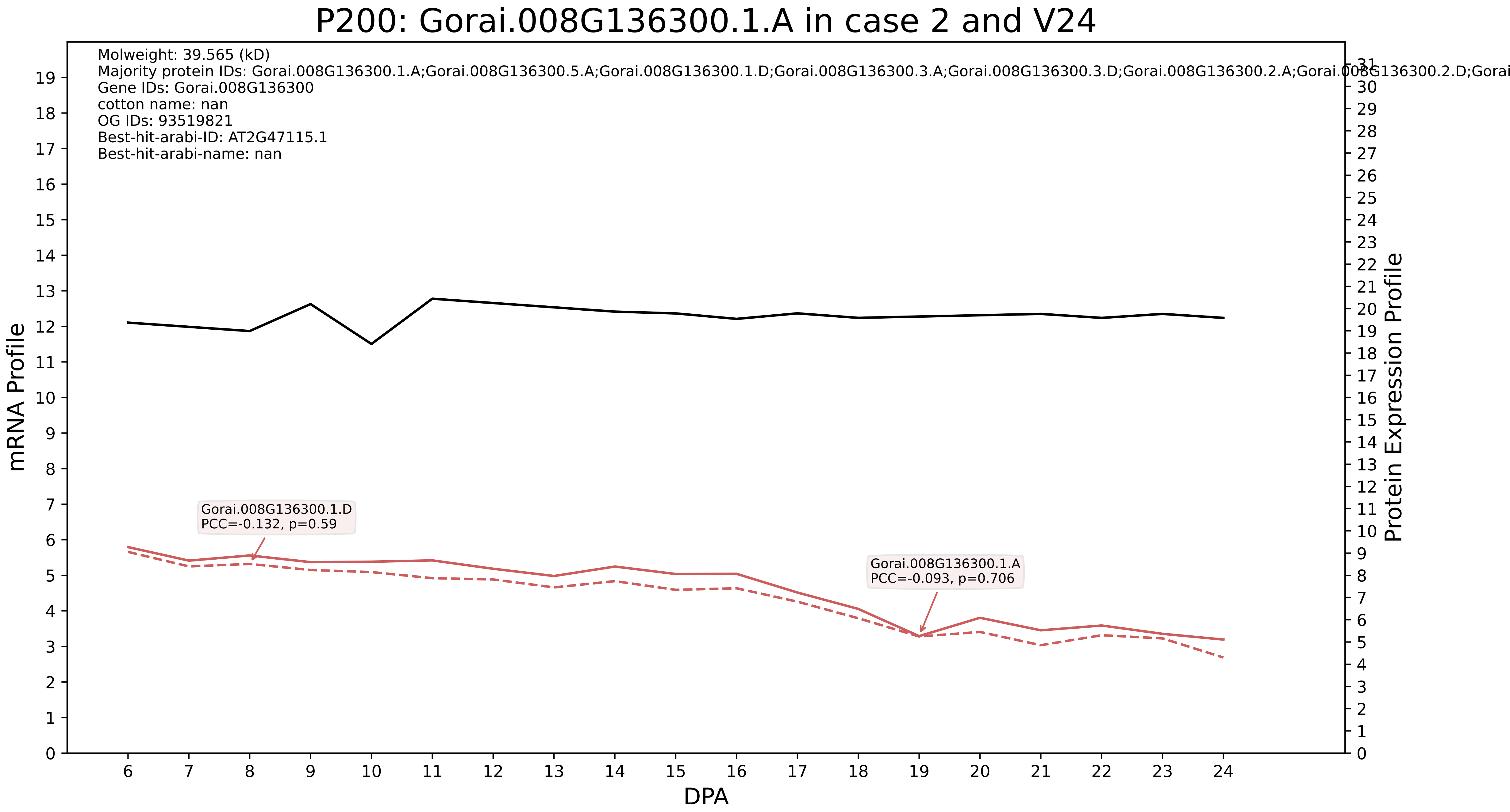
P200: Gorai.008G136300.1.A in case 2 and V24
Molweight: 39.565 (kD)
Majority protein IDs: Gorai.008G136300.1.A;Gorai.008G136300.5.A;Gorai.008G136300.1.D;Gorai.008G136300.3.A;Gorai.008G136300.3.D;Gorai.008G136300.2.A;Gorai.008G136300.2.D;Gorai
Gene IDs: Gorai.008G136300
cotton name: nan
OG IDs: 93519821
Best-hit-arabi-ID: AT2G47115.1
Best-hit-arabi-name: nan
Gorai.008G136300.1.D
PCC=-0.132, p=0.59
Gorai.008G136300.1.A
PCC=-0.093, p=0.706
mRNA Profile
Protein Expression Profile
DPA
0
1
2
3
4
5
6
7
8
9
10
11
12
13
14
15
16
17
18
19
0
1
2
3
4
5
6
7
8
9
10
11
12
13
14
15
16
17
18
19
20
21
22
23
24
25
26
27
28
29
30
31
6
7
8
9
10
11
12
13
14
15
16
17
18
19
20
21
22
23
24

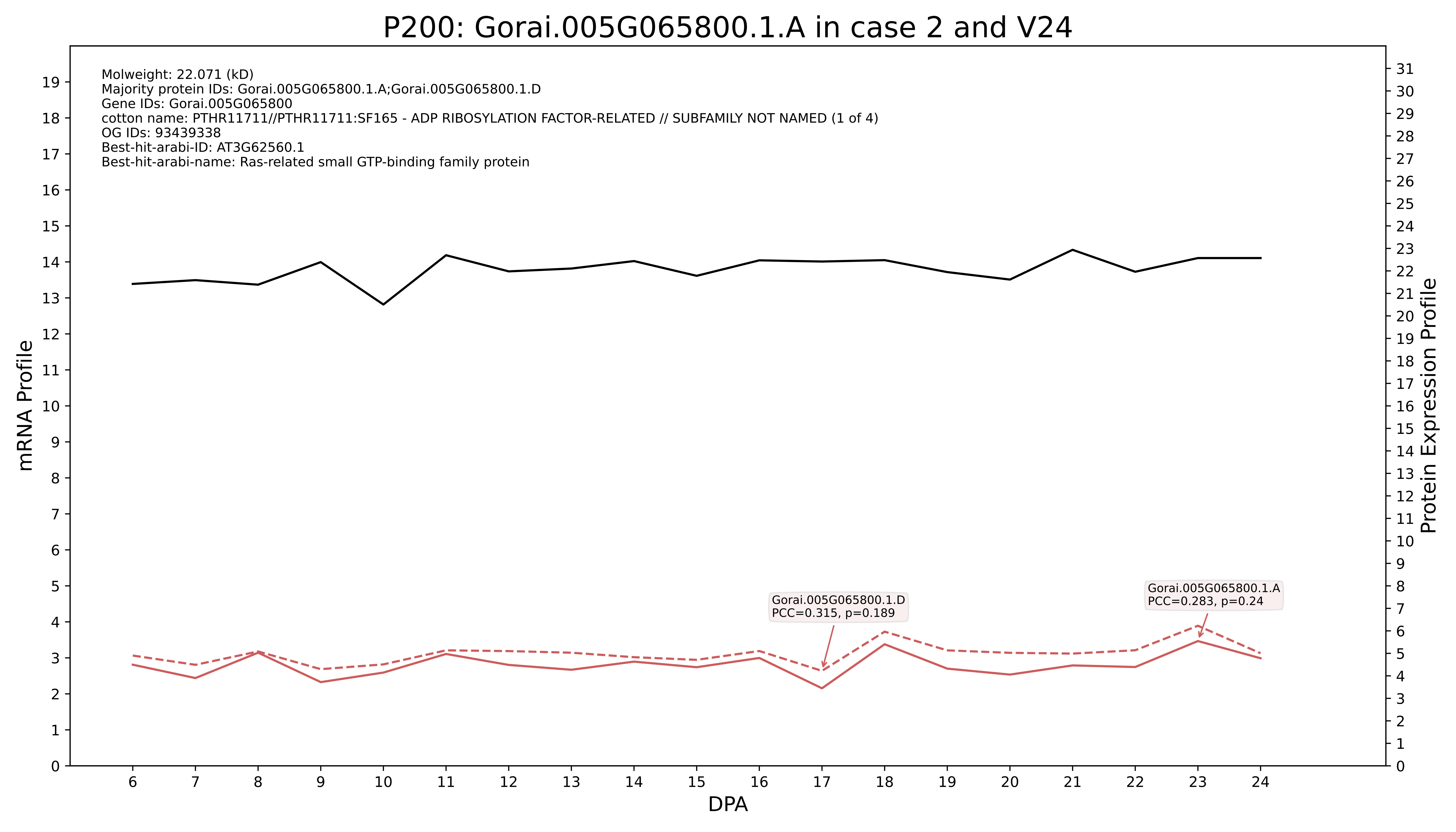
P200: Gorai.005G065800.1.A in case 2 and V24
Molweight: 22.071 (kD)
Majority protein IDs: Gorai.005G065800.1.A;Gorai.005G065800.1.D
Gene IDs: Gorai.005G065800
cotton name: PTHR11711//PTHR11711:SF165 - ADP RIBOSYLATION FACTOR-RELATED // SUBFAMILY NOT NAMED (1 of 4)
OG IDs: 93439338
Best-hit-arabi-ID: AT3G62560.1
Best-hit-arabi-name: Ras-related small GTP-binding family protein
Gorai.005G065800.1.D
PCC=0.315, p=0.189
Gorai.005G065800.1.A
PCC=0.283, p=0.24
mRNA Profile
Protein Expression Profile
DPA

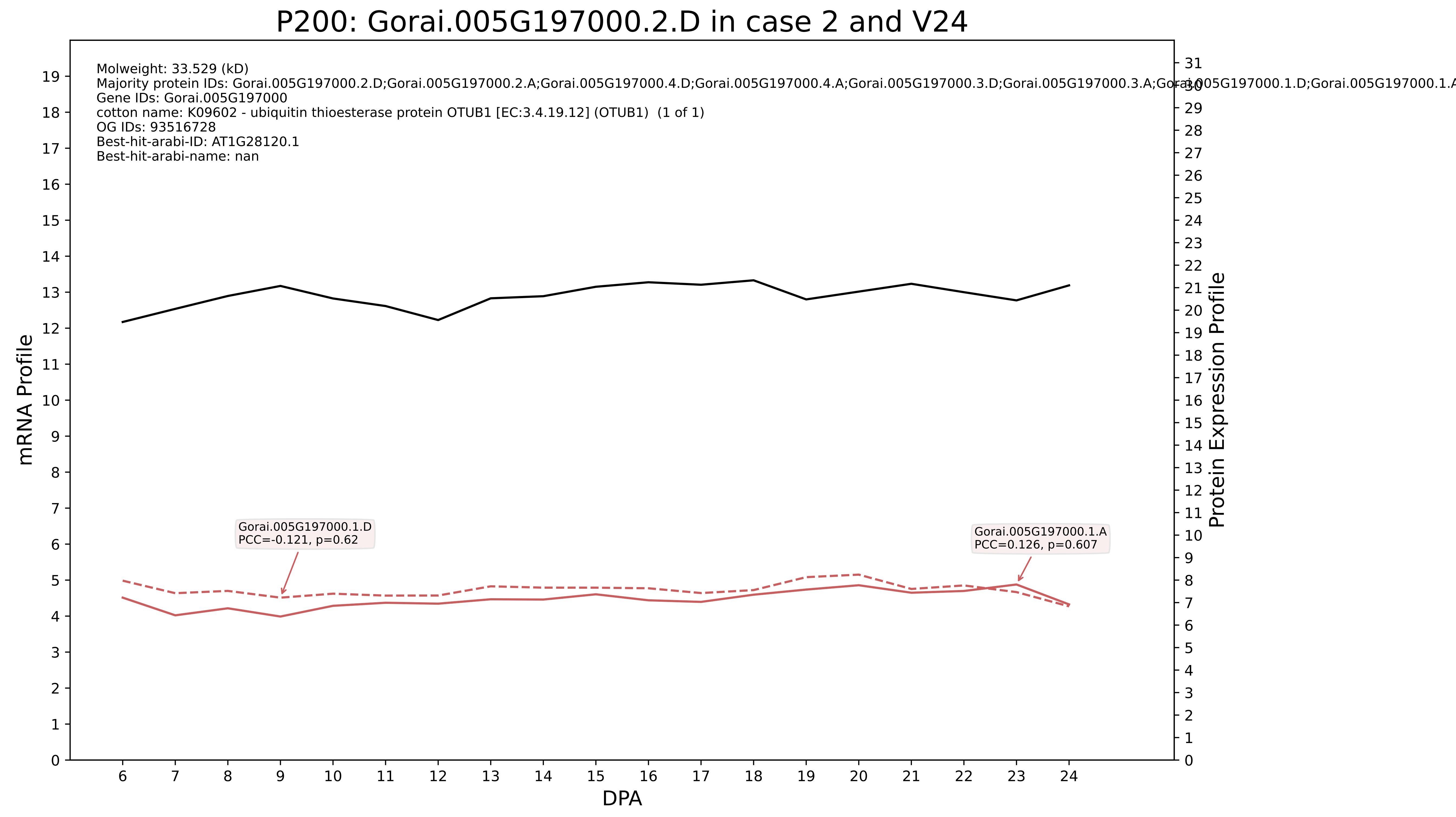
P200: Gorai.005G197000.2.D in case 2 and V24
Molweight: 33.529 (kD)
Majority protein IDs: Gorai.005G197000.2.D;Gorai.005G197000.2.A;Gorai.005G197000.4.D;Gorai.005G197000.4.A;Gorai.005G197000.3.D;Gorai.005G197000.3.A;Gorai.005G197000.1.D;Gorai.005G197000.1.A
Gene IDs: Gorai.005G197000
cotton name: K09602 - ubiquitin thioesterase protein OTUB1 [EC:3.4.19.12] (OTUB1) (1 of 1)
OG IDs: 93516728
Best-hit-arabi-ID: AT1G28120.1
Best-hit-arabi-name: nan
Gorai.005G197000.1.D
PCC=-0.121, p=0.62
Gorai.005G197000.1.A
PCC=0.126, p=0.607
mRNA Profile
Protein Expression Profile
DPA

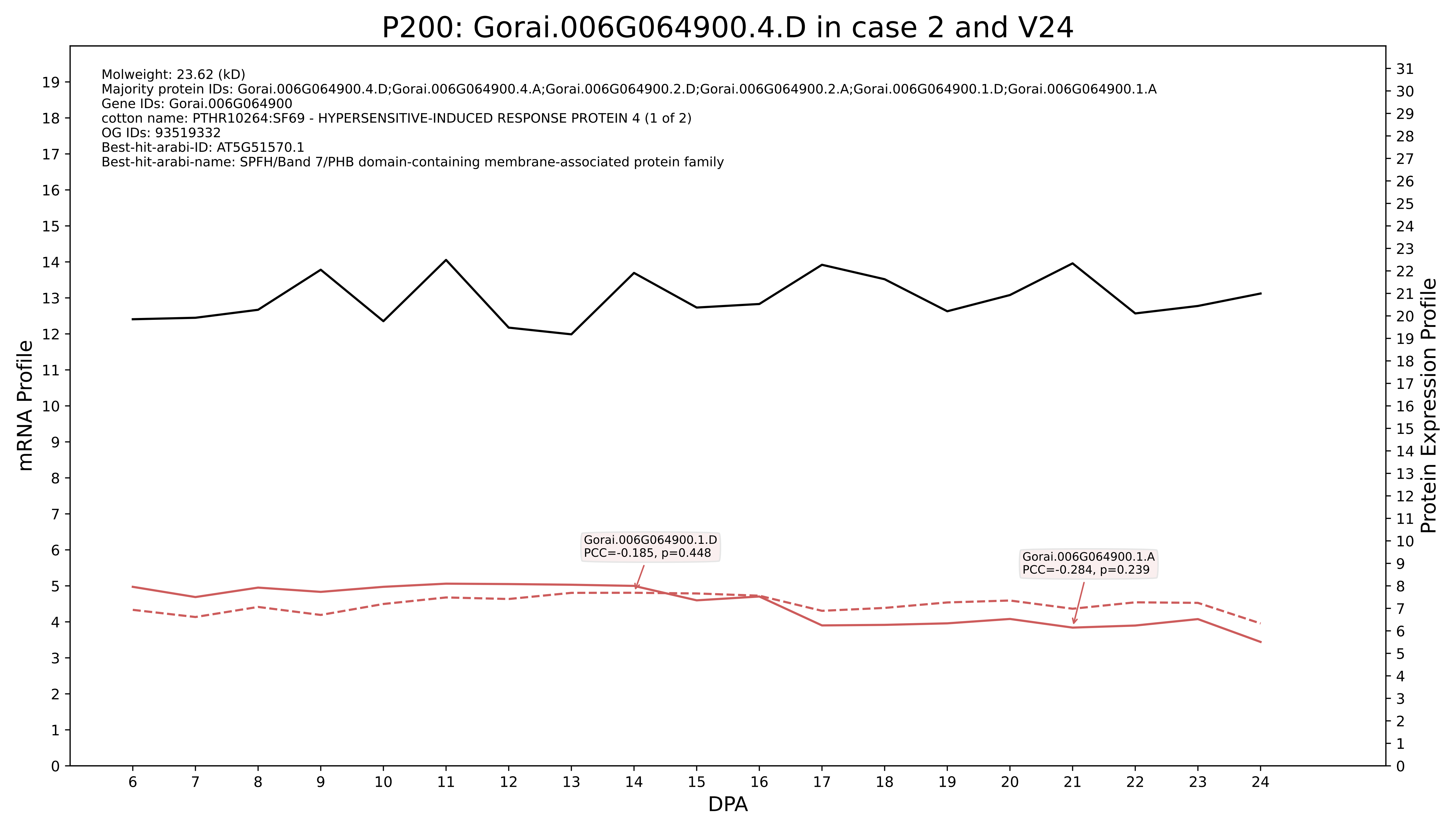
P200: Gorai.006G064900.4.D in case 2 and V24
Molweight: 23.62 (kD)
Majority protein IDs: Gorai.006G064900.4.D;Gorai.006G064900.4.A;Gorai.006G064900.2.D;Gorai.006G064900.2.A;Gorai.006G064900.1.D;Gorai.006G064900.1.A
Gene IDs: Gorai.006G064900
cotton name: PTHR10264:SF69 - HYPERSENSITIVE-INDUCED RESPONSE PROTEIN 4 (1 of 2)
OG IDs: 93519332
Best-hit-arabi-ID: AT5G51570.1
Best-hit-arabi-name: SPFH/Band 7/PHB domain-containing membrane-associated protein family
Gorai.006G064900.1.D
PCC=-0.185, p=0.448
Gorai.006G064900.1.A
PCC=-0.284, p=0.239
mRNA Profile
Protein Expression Profile
DPA

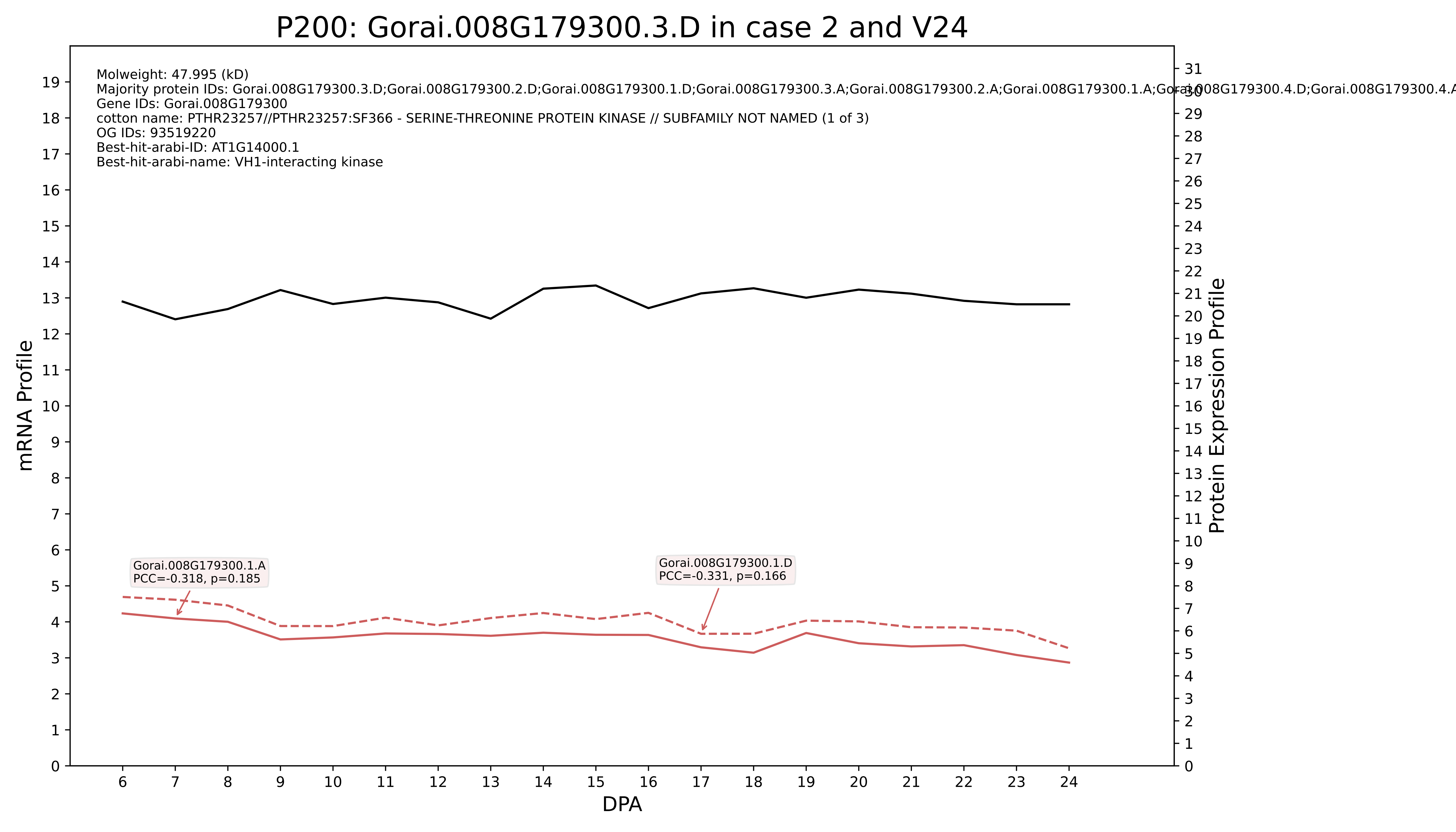

P200: Gorai.008G179300.3.D in case 2 and V24
Molweight: 47.995 (kD)
Majority protein IDs: Gorai.008G179300.3.D;Gorai.008G179300.2.D;Gorai.008G179300.1.D;Gorai.008G179300.3.A;Gorai.008G179300.2.A;Gorai.008G179300.1.A;Gorai.008G179300.4.D;Gorai.008G179300.4.A
Gene IDs: Gorai.008G179300
cotton name: PTHR23257//PTHR23257:SF366 - SERINE-THREONINE PROTEIN KINASE // SUBFAMILY NOT NAMED (1 of 3)
OG IDs: 93519220
Best-hit-arabi-ID: AT1G14000.1
Best-hit-arabi-name: VH1-interacting kinase
Gorai.008G179300.1.A
PCC=-0.318, p=0.185
Gorai.008G179300.1.D
PCC=-0.331, p=0.166
mRNA Profile
Protein Expression Profile
DPA

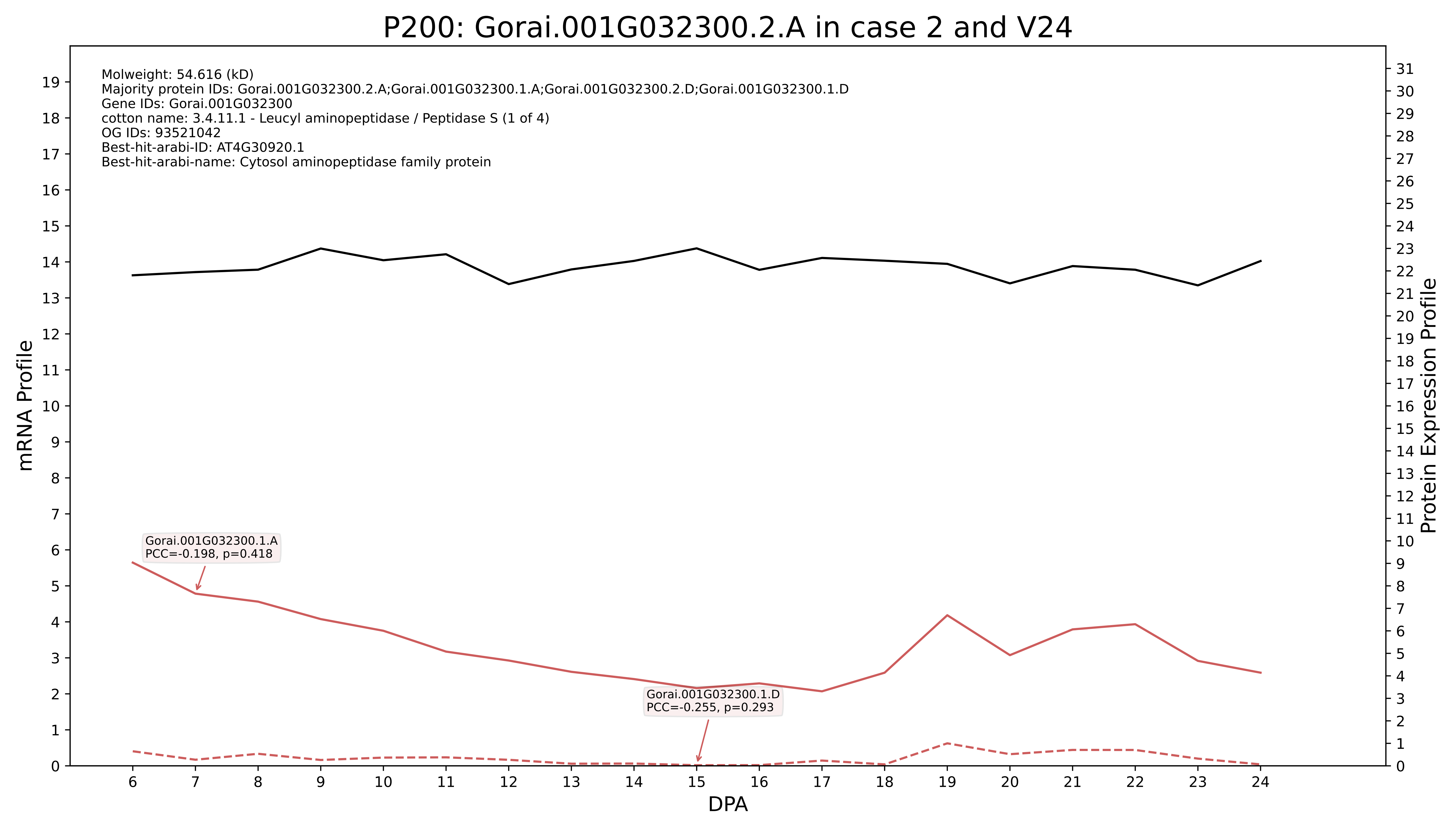
P200: Gorai.001G032300.2.A in case 2 and V24
Molweight: 54.616 (kD)
Majority protein IDs: Gorai.001G032300.2.A;Gorai.001G032300.1.A;Gorai.001G032300.2.D;Gorai.001G032300.1.D
Gene IDs: Gorai.001G032300
cotton name: 3.4.11.1 - Leucyl aminopeptidase / Peptidase S (1 of 4)
OG IDs: 93521042
Best-hit-arabi-ID: AT4G30920.1
Best-hit-arabi-name: Cytosol aminopeptidase family protein
Gorai.001G032300.1.A
PCC=-0.198, p=0.418
Gorai.001G032300.1.D
PCC=-0.255, p=0.293
mRNA Profile
Protein Expression Profile
DPA

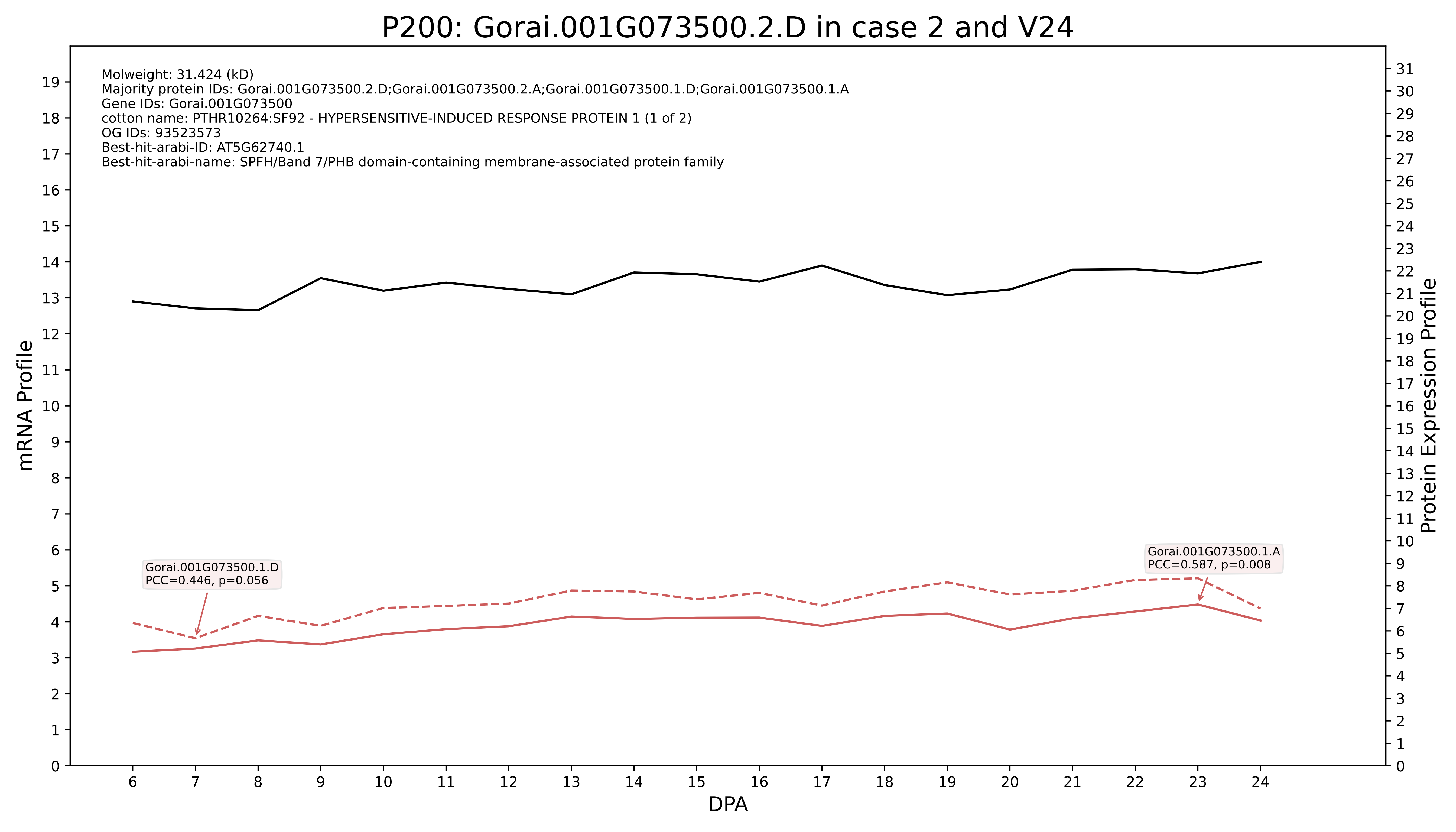

P200: Gorai.001G073500.2.D in case 2 and V24
Molweight: 31.424 (kD)
Majority protein IDs: Gorai.001G073500.2.D;Gorai.001G073500.2.A;Gorai.001G073500.1.D;Gorai.001G073500.1.A
Gene IDs: Gorai.001G073500
cotton name: PTHR10264:SF92 - HYPERSENSITIVE-INDUCED RESPONSE PROTEIN 1 (1 of 2)
OG IDs: 93523573
Best-hit-arabi-ID: AT5G62740.1
Best-hit-arabi-name: SPFH/Band 7/PHB domain-containing membrane-associated protein family
Gorai.001G073500.1.D
PCC=0.446, p=0.056
Gorai.001G073500.1.A
PCC=0.587, p=0.008
mRNA Profile
Protein Expression Profile
DPA

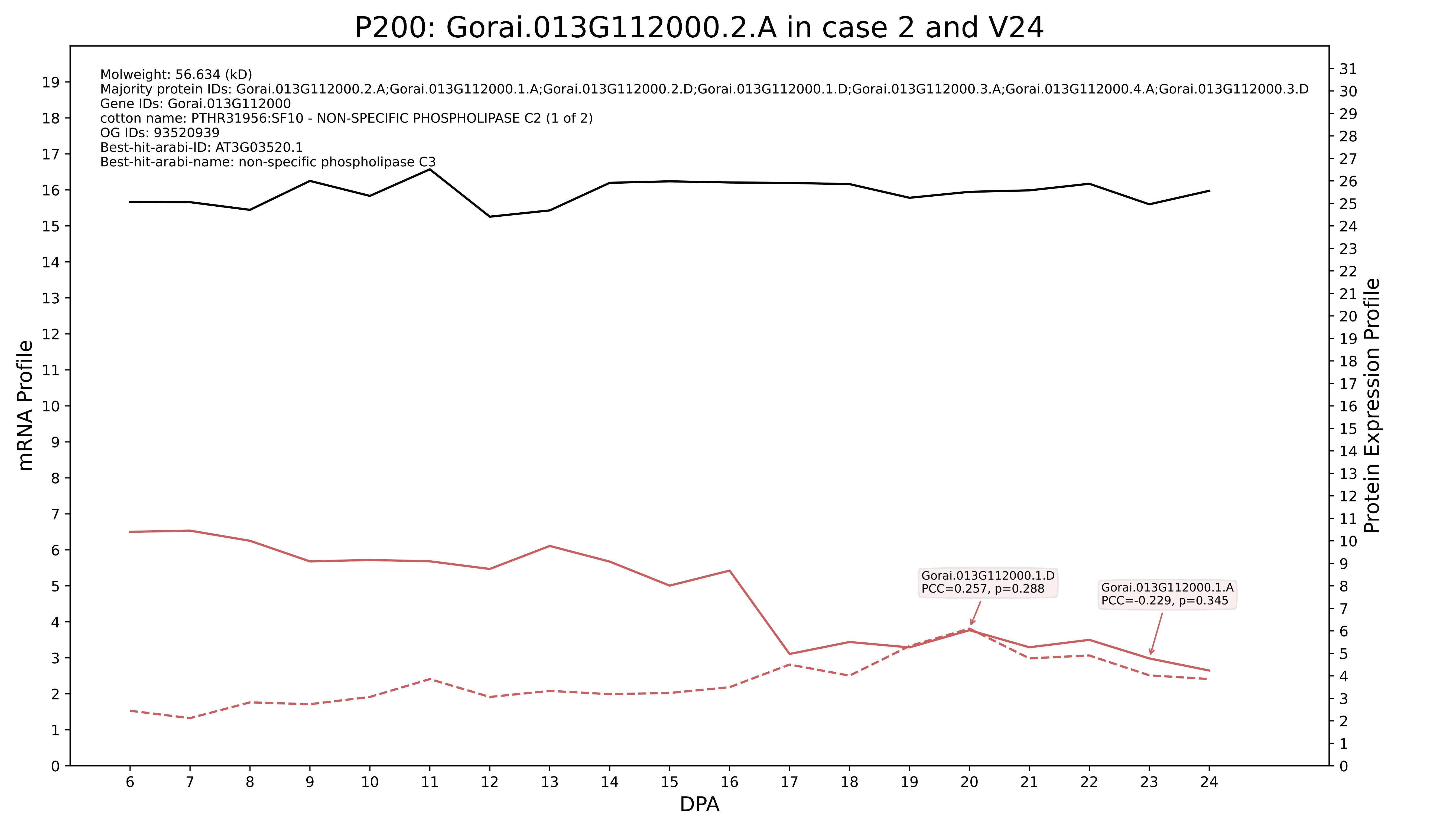

P200: Gorai.013G112000.2.A in case 2 and V24
Molweight: 56.634 (kD)
Majority protein IDs: Gorai.013G112000.2.A;Gorai.013G112000.1.A;Gorai.013G112000.2.D;Gorai.013G112000.1.D;Gorai.013G112000.3.A;Gorai.013G112000.4.A;Gorai.013G112000.3.D
Gene IDs: Gorai.013G112000
cotton name: PTHR31956:SF10 - NON-SPECIFIC PHOSPHOLIPASE C2 (1 of 2)
OG IDs: 93520939
Best-hit-arabi-ID: AT3G03520.1
Best-hit-arabi-name: non-specific phospholipase C3
Gorai.013G112000.1.D
PCC=0.257, p=0.288
Gorai.013G112000.1.A
PCC=-0.229, p=0.345
mRNA Profile
Protein Expression Profile
DPA

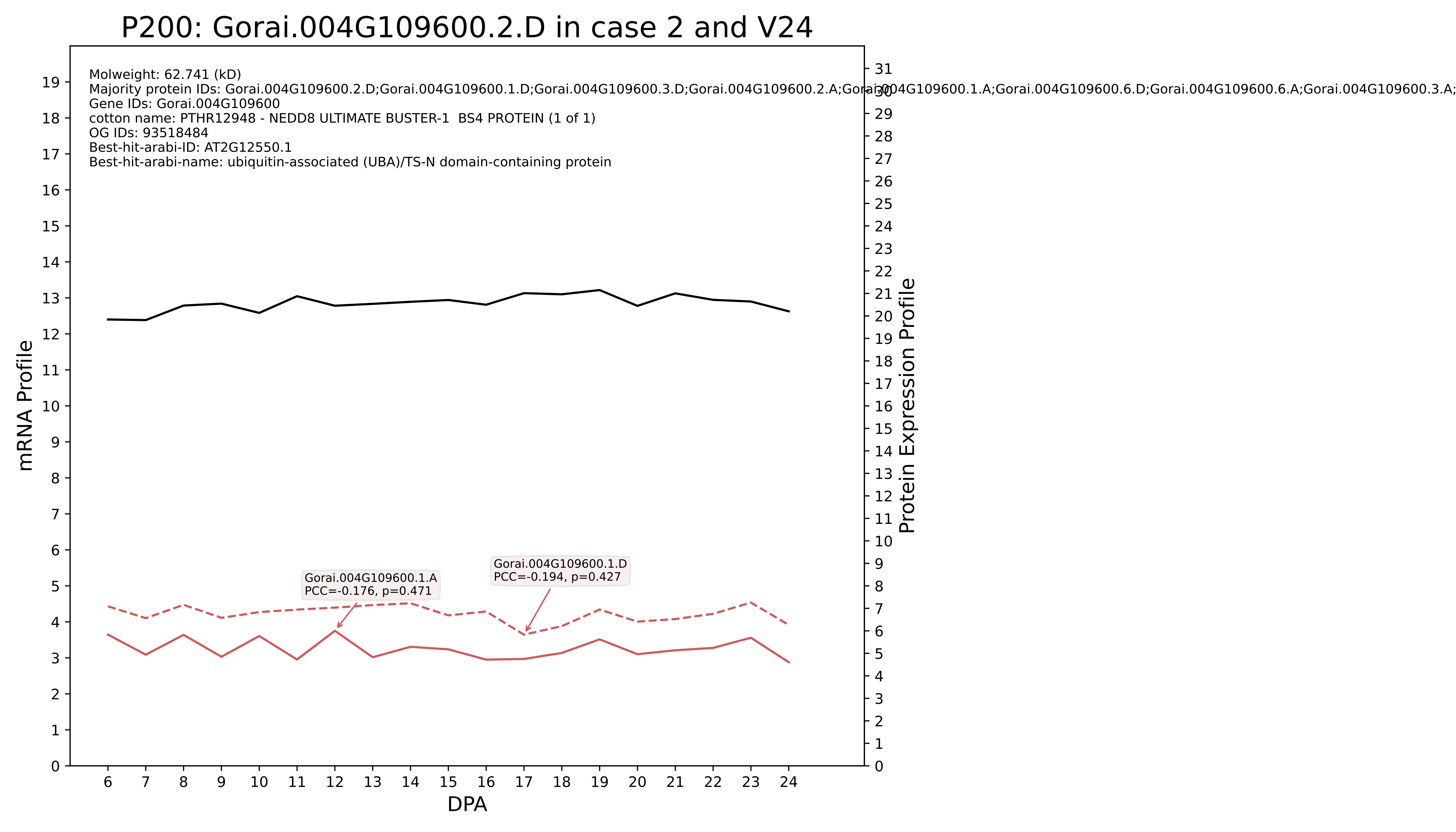

P200: Gorai.004G109600.2.D in case 2 and V24
Molweight: 62.741 (kD)
Majority protein IDs: Gorai.004G109600.2.D;Gorai.004G109600.1.D;Gorai.004G109600.3.D;Gorai.004G109600.2.A;Gorai.004G109600.1.A;Gorai.004G109600.6.D;Gorai.004G109600.6.A;Gorai.004G109600.3.A;
Gene IDs: Gorai.004G109600
cotton name: PTHR12948 - NEDD8 ULTIMATE BUSTER-1 BS4 PROTEIN (1 of 1)
OG IDs: 93518484
Best-hit-arabi-ID: AT2G12550.1
Best-hit-arabi-name: ubiquitin-associated (UBA)/TS-N domain-containing protein
Gorai.004G109600.1.A
PCC=-0.176, p=0.471
Gorai.004G109600.1.D
PCC=-0.194, p=0.427
mRNA Profile
Protein Expression Profile
DPA

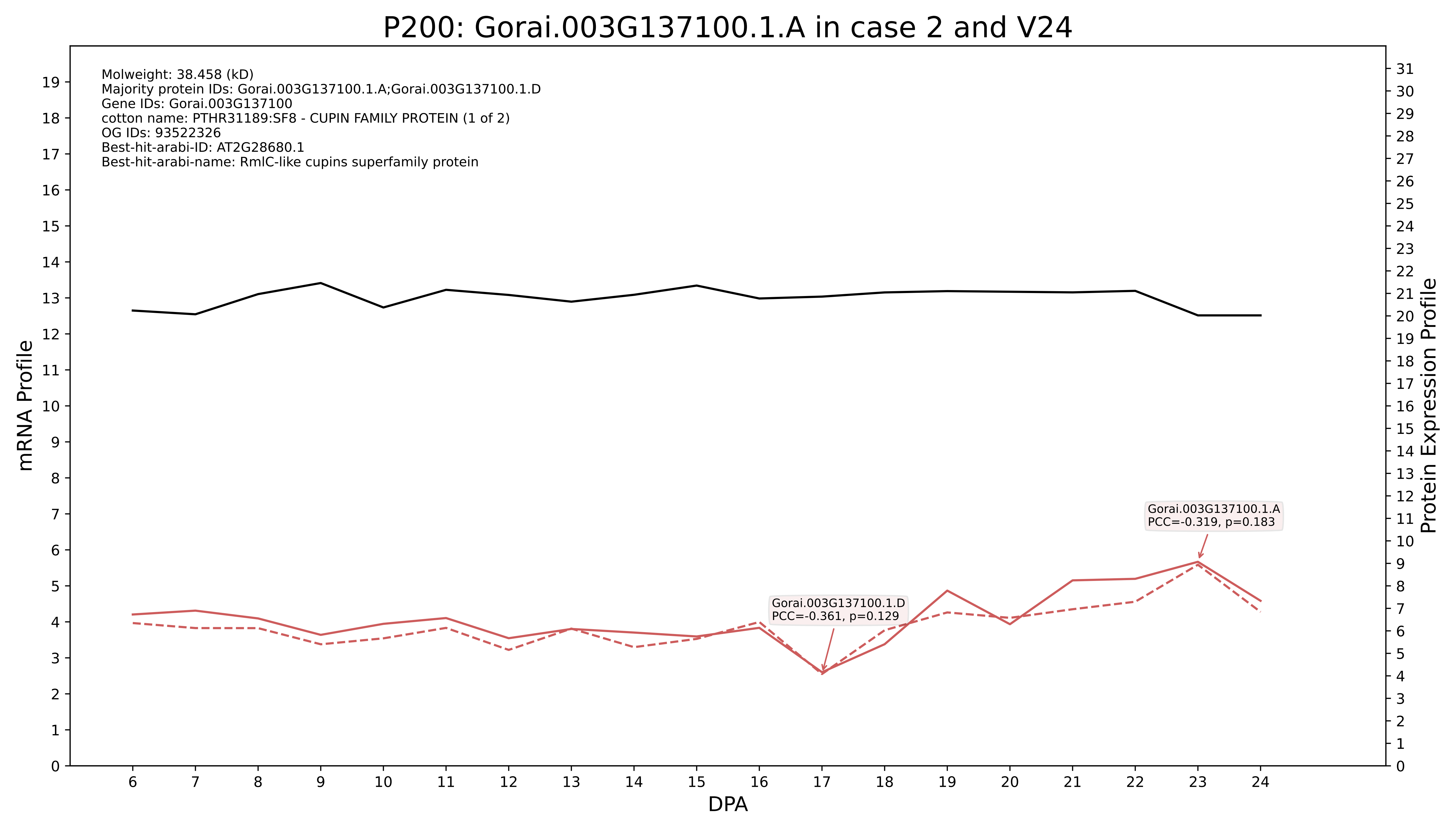
P200: Gorai.003G137100.1.A in case 2 and V24
Molweight: 38.458 (kD)
Majority protein IDs: Gorai.003G137100.1.A;Gorai.003G137100.1.D
Gene IDs: Gorai.003G137100
cotton name: PTHR31189:SF8 - CUPIN FAMILY PROTEIN (1 of 2)
OG IDs: 93522326
Best-hit-arabi-ID: AT2G28680.1
Best-hit-arabi-name: RmlC-like cupins superfamily protein
Gorai.003G137100.1.A
PCC=-0.319, p=0.183
Gorai.003G137100.1.D
PCC=-0.361, p=0.129
mRNA Profile
Protein Expression Profile
DPA

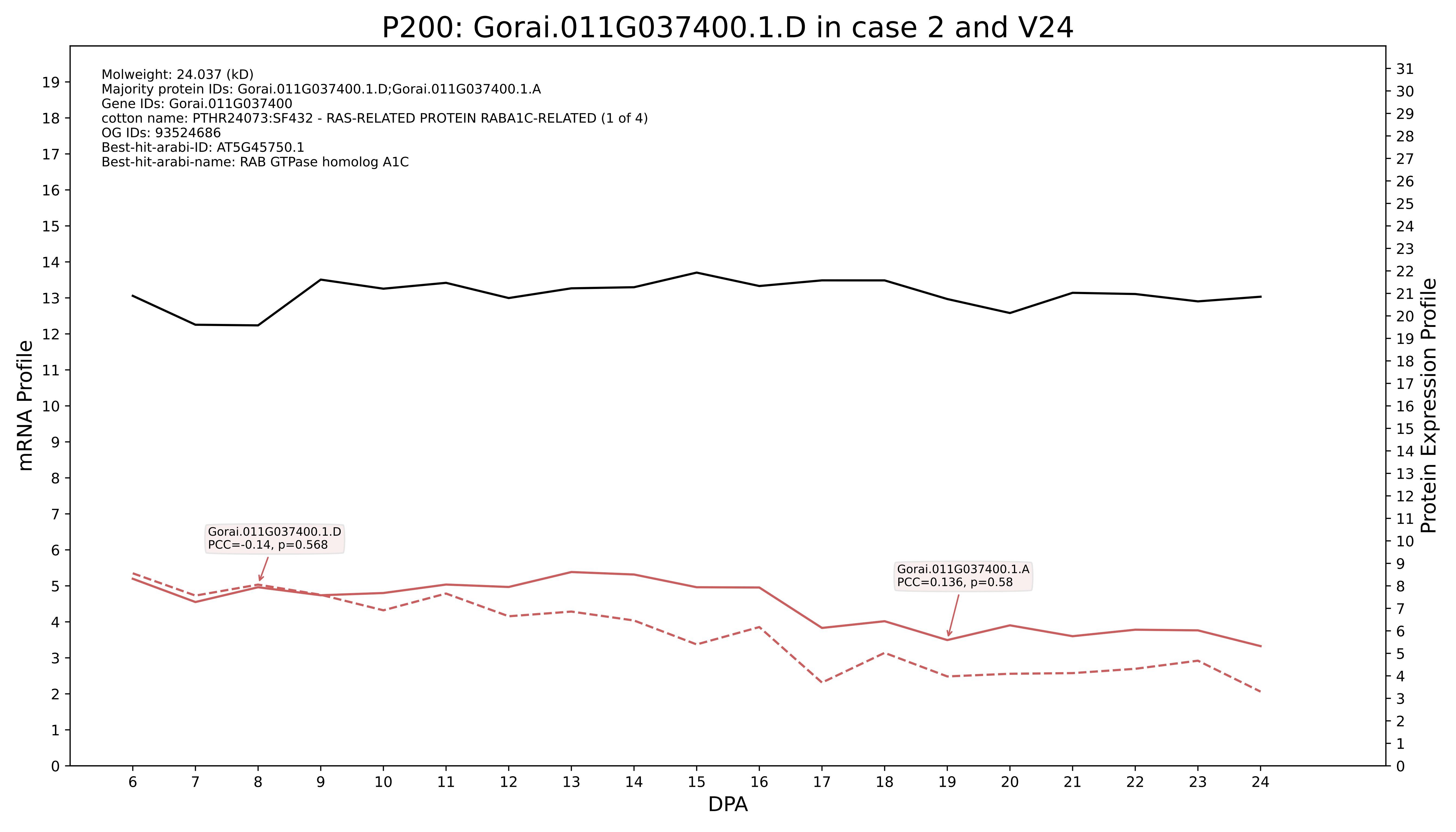

P200: Gorai.011G037400.1.D in case 2 and V24
Molweight: 24.037 (kD)
Majority protein IDs: Gorai.011G037400.1.D;Gorai.011G037400.1.A
Gene IDs: Gorai.011G037400
cotton name: PTHR24073:SF432 - RAS-RELATED PROTEIN RABA1C-RELATED (1 of 4)
OG IDs: 93524686
Best-hit-arabi-ID: AT5G45750.1
Best-hit-arabi-name: RAB GTPase homolog A1C
Gorai.011G037400.1.D
PCC=-0.14, p=0.568
Gorai.011G037400.1.A
PCC=0.136, p=0.58
mRNA Profile
Protein Expression Profile
DPA

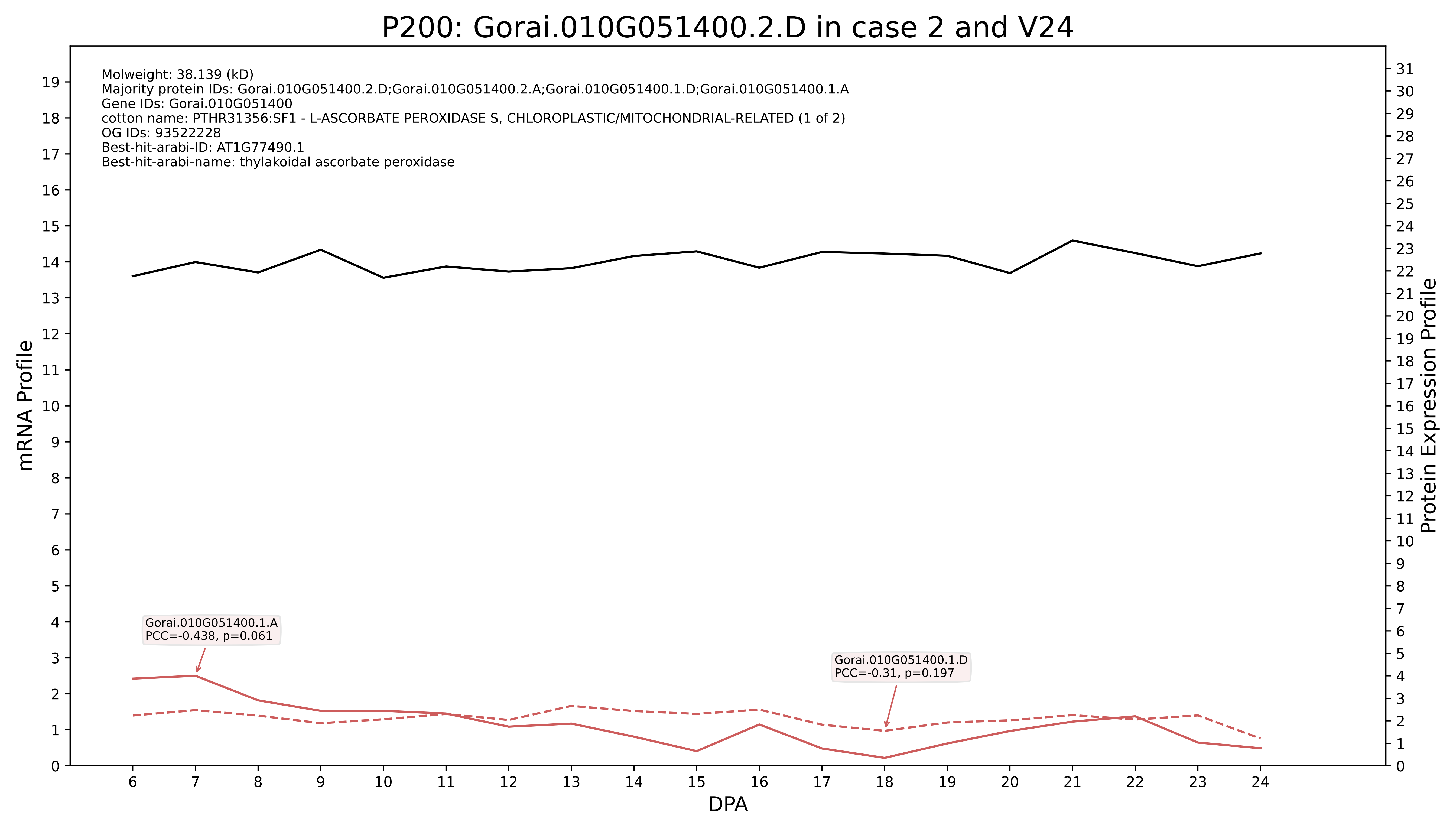
P200: Gorai.010G051400.2.D in case 2 and V24
Molweight: 38.139 (kD)
Majority protein IDs: Gorai.010G051400.2.D;Gorai.010G051400.2.A;Gorai.010G051400.1.D;Gorai.010G051400.1.A
Gene IDs: Gorai.010G051400
cotton name: PTHR31356:SF1 - L-ASCORBATE PEROXIDASE S, CHLOROPLASTIC/MITOCHONDRIAL-RELATED (1 of 2)
OG IDs: 93522228
Best-hit-arabi-ID: AT1G77490.1
Best-hit-arabi-name: thylakoidal ascorbate peroxidase
Gorai.010G051400.1.A
PCC=-0.438, p=0.061
Gorai.010G051400.1.D
PCC=-0.31, p=0.197
mRNA Profile
Protein Expression Profile
DPA

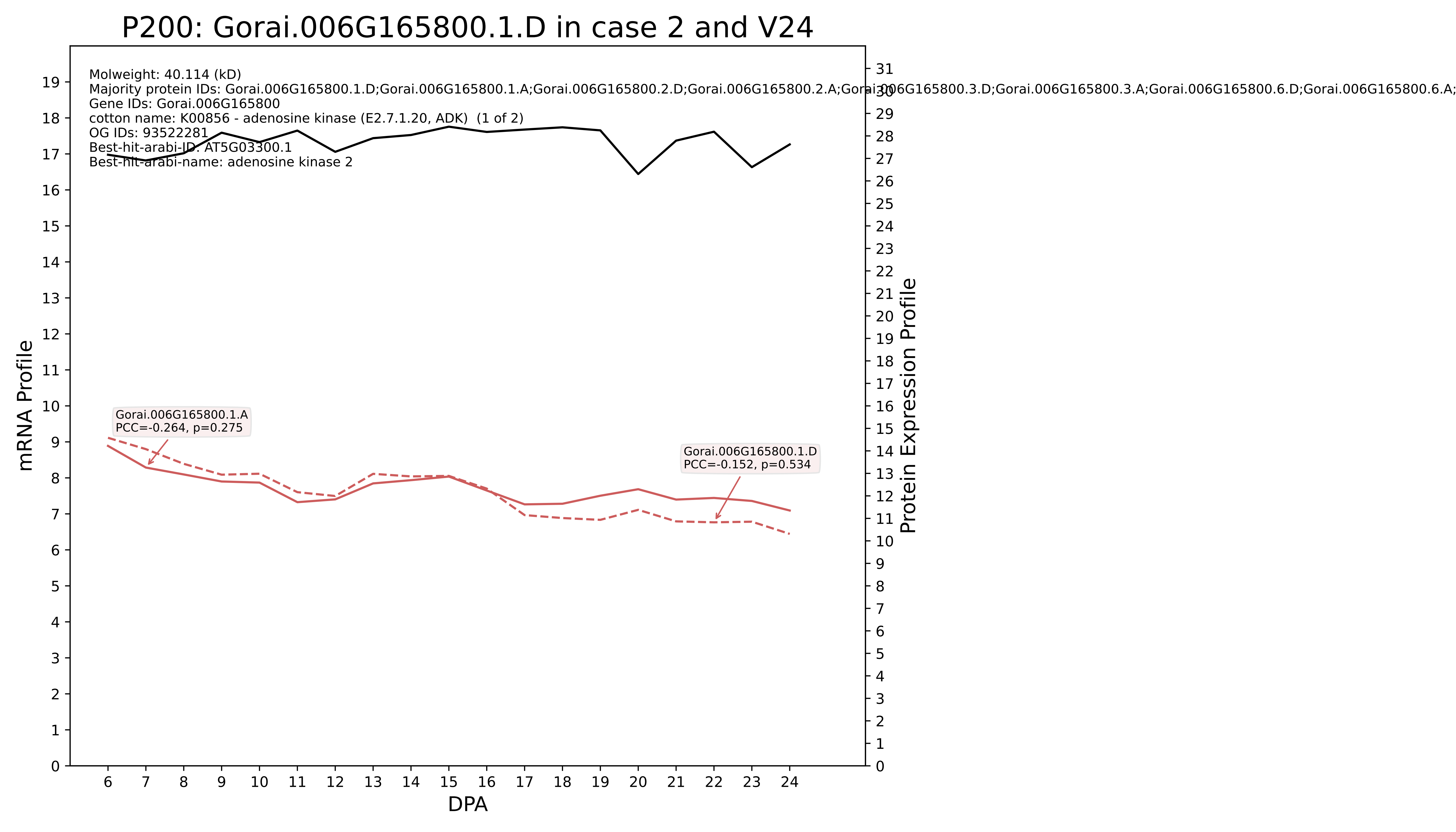
P200: Gorai.006G165800.1.D in case 2 and V24
Molweight: 40.114 (kD)
Majority protein IDs: Gorai.006G165800.1.D;Gorai.006G165800.1.A;Gorai.006G165800.2.D;Gorai.006G165800.2.A;Gorai.006G165800.3.D;Gorai.006G165800.3.A;Gorai.006G165800.6.D;Gorai.006G165800.6.A;
Gene IDs: Gorai.006G165800
cotton name: K00856 - adenosine kinase (E2.7.1.20, ADK) (1 of 2)
OG IDs: 93522281
Best-hit-arabi-ID: AT5G03300.1
Best-hit-arabi-name: adenosine kinase 2
Gorai.006G165800.1.A
PCC=-0.264, p=0.275
Gorai.006G165800.1.D
PCC=-0.152, p=0.534
mRNA Profile
Protein Expression Profile
DPA

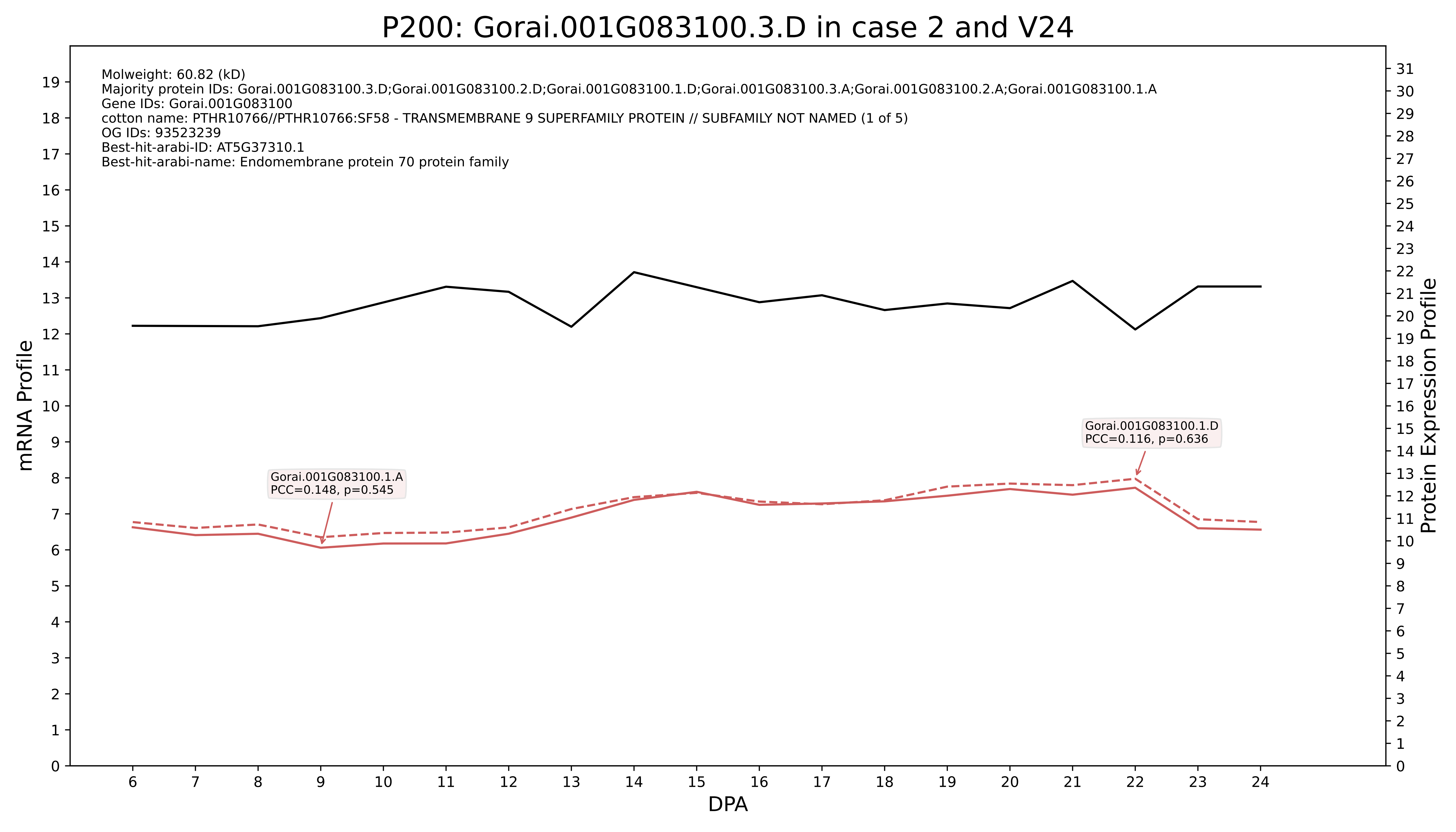
P200: Gorai.001G083100.3.D in case 2 and V24
Molweight: 60.82 (kD)
Majority protein IDs: Gorai.001G083100.3.D;Gorai.001G083100.2.D;Gorai.001G083100.1.D;Gorai.001G083100.3.A;Gorai.001G083100.2.A;Gorai.001G083100.1.A
Gene IDs: Gorai.001G083100
cotton name: PTHR10766//PTHR10766:SF58 - TRANSMEMBRANE 9 SUPERFAMILY PROTEIN // SUBFAMILY NOT NAMED (1 of 5)
OG IDs: 93523239
Best-hit-arabi-ID: AT5G37310.1
Best-hit-arabi-name: Endomembrane protein 70 protein family
Gorai.001G083100.1.A
PCC=0.148, p=0.545
Gorai.001G083100.1.D
PCC=0.116, p=0.636
mRNA Profile
Protein Expression Profile
DPA

P200: Gorai.002G268400.1.A in case 2 and V24

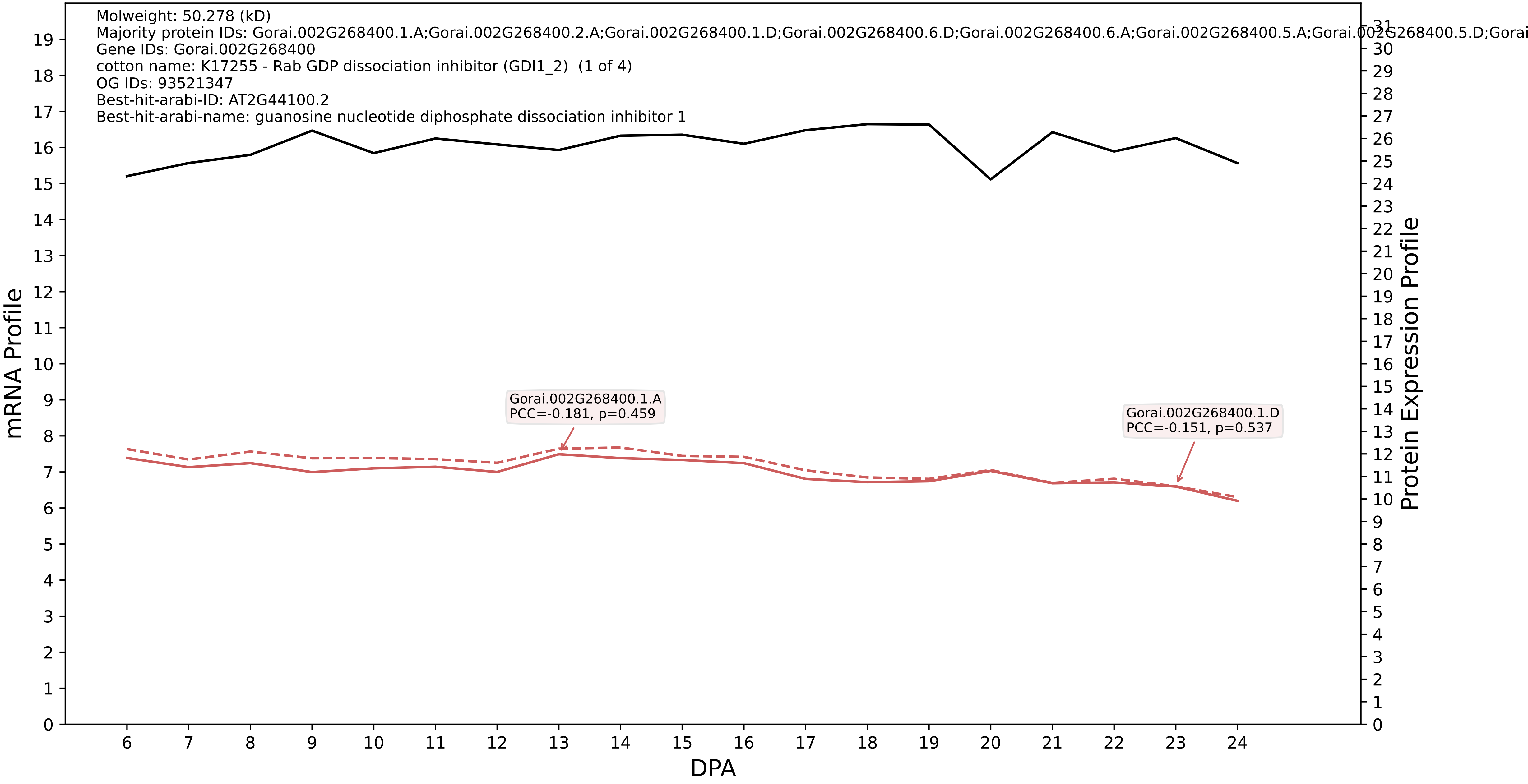

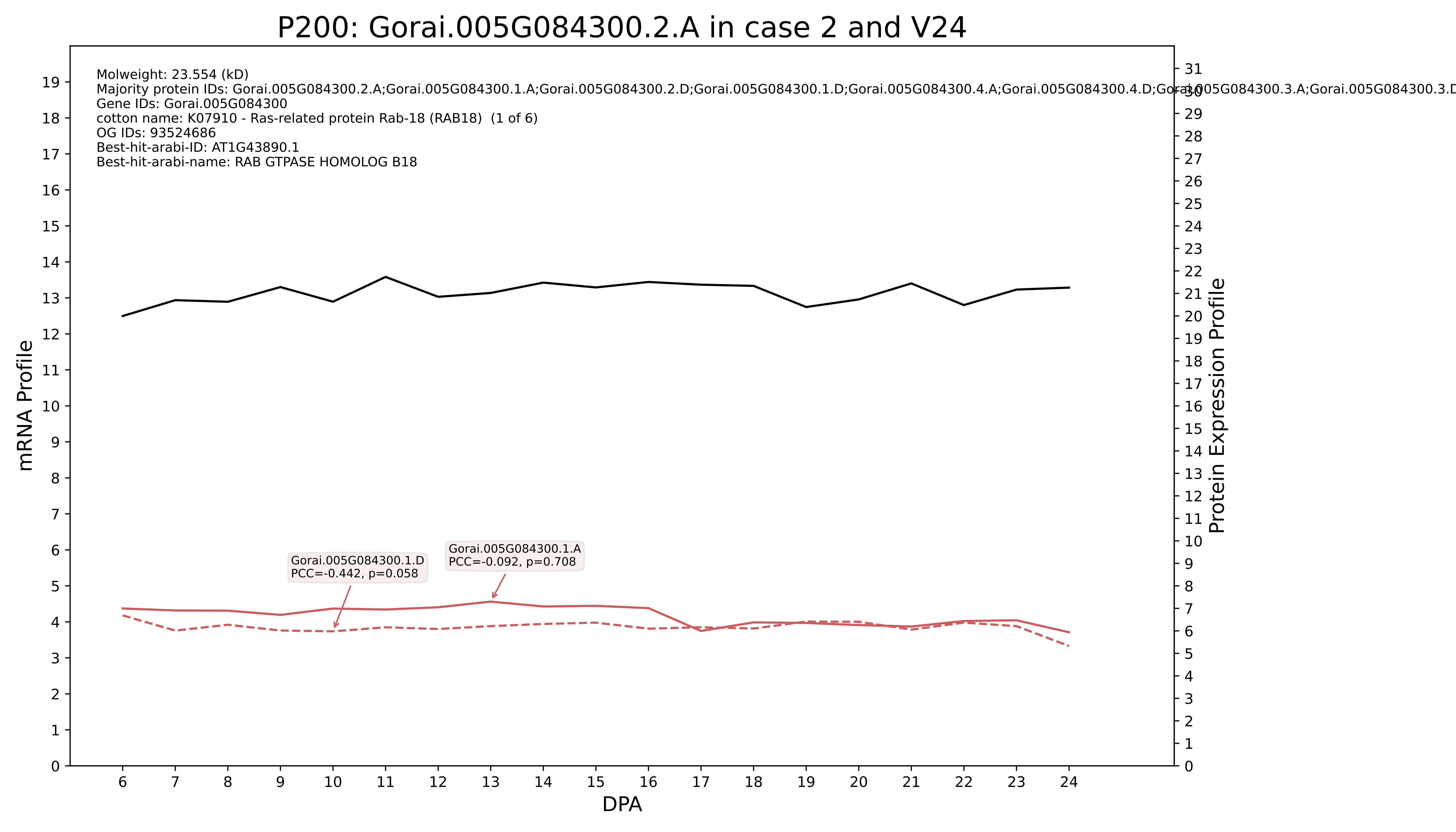
P200: Gorai.005G084300.2.A in case 2 and V24
Molweight: 23.554 (kD)
Majority protein IDs: Gorai.005G084300.2.A;Gorai.005G084300.1.A;Gorai.005G084300.2.D;Gorai.005G084300.1.D;Gorai.005G084300.4.A;Gorai.005G084300.4.D;Gorai.005G084300.3.A;Gorai.005G084300.3.D
Gene IDs: Gorai.005G084300
cotton name: K07910 - Ras-related protein Rab-18 (RAB18) (1 of 6)
OG IDs: 93524686
Best-hit-arabi-ID: AT1G43890.1
Best-hit-arabi-name: RAB GTPASE HOMOLOG B18
Gorai.005G084300.1.D
PCC=-0.442, p=0.058
Gorai.005G084300.1.A
PCC=-0.092, p=0.708
mRNA Profile
Protein Expression Profile
DPA

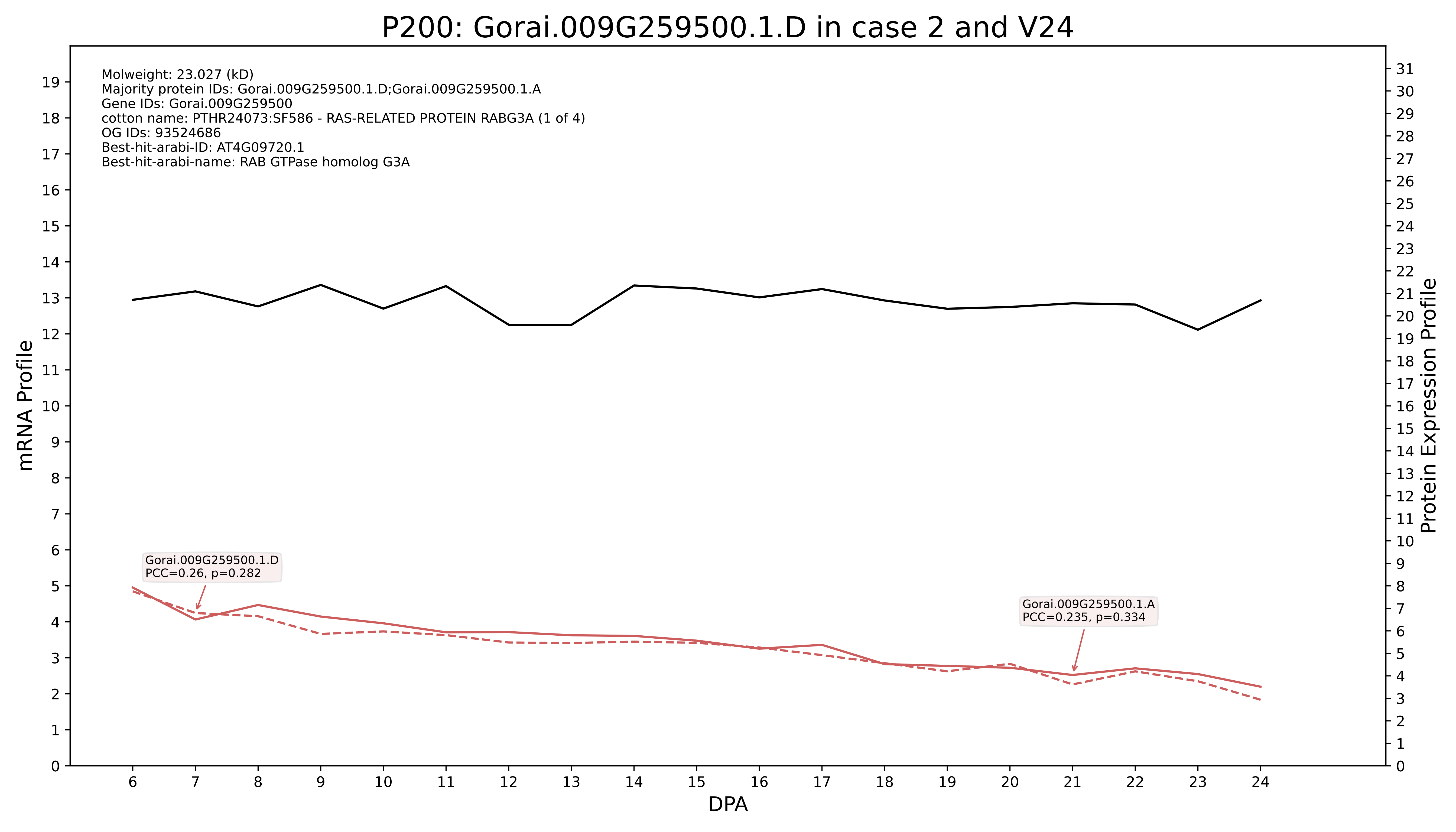
P200: Gorai.009G259500.1.D in case 2 and V24
Molweight: 23.027 (kD)
Majority protein IDs: Gorai.009G259500.1.D;Gorai.009G259500.1.A
Gene IDs: Gorai.009G259500
cotton name: PTHR24073:SF586 - RAS-RELATED PROTEIN RABG3A (1 of 4)
OG IDs: 93524686
Best-hit-arabi-ID: AT4G09720.1
Best-hit-arabi-name: RAB GTPase homolog G3A
Gorai.009G259500.1.D
PCC=0.26, p=0.282
Gorai.009G259500.1.A
PCC=0.235, p=0.334
mRNA Profile
Protein Expression Profile
DPA

P200: Gorai.009G185200.1.D in case 2 and V24

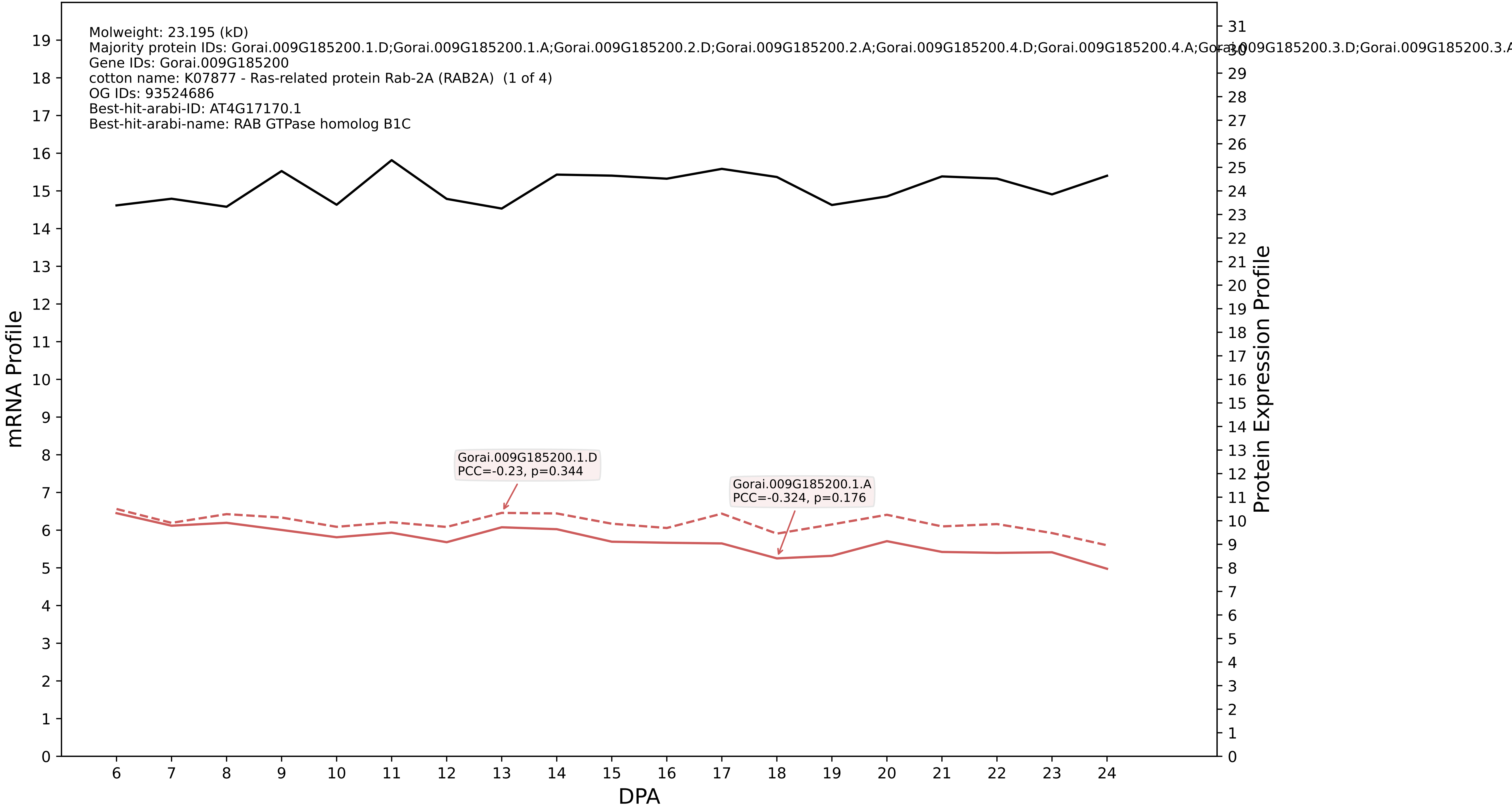

P200: Gorai.007G198800.1.D in case 2 and V24

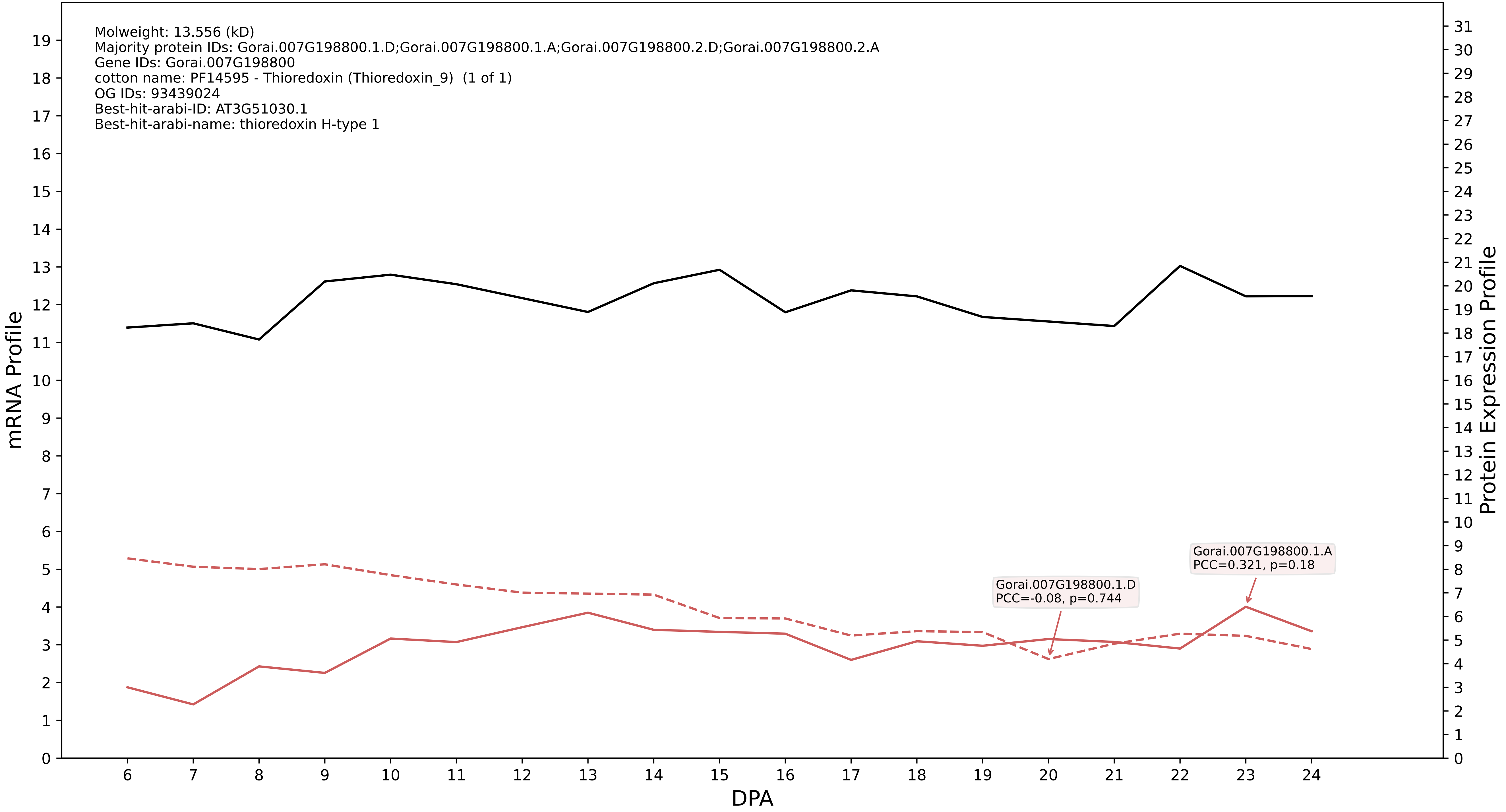

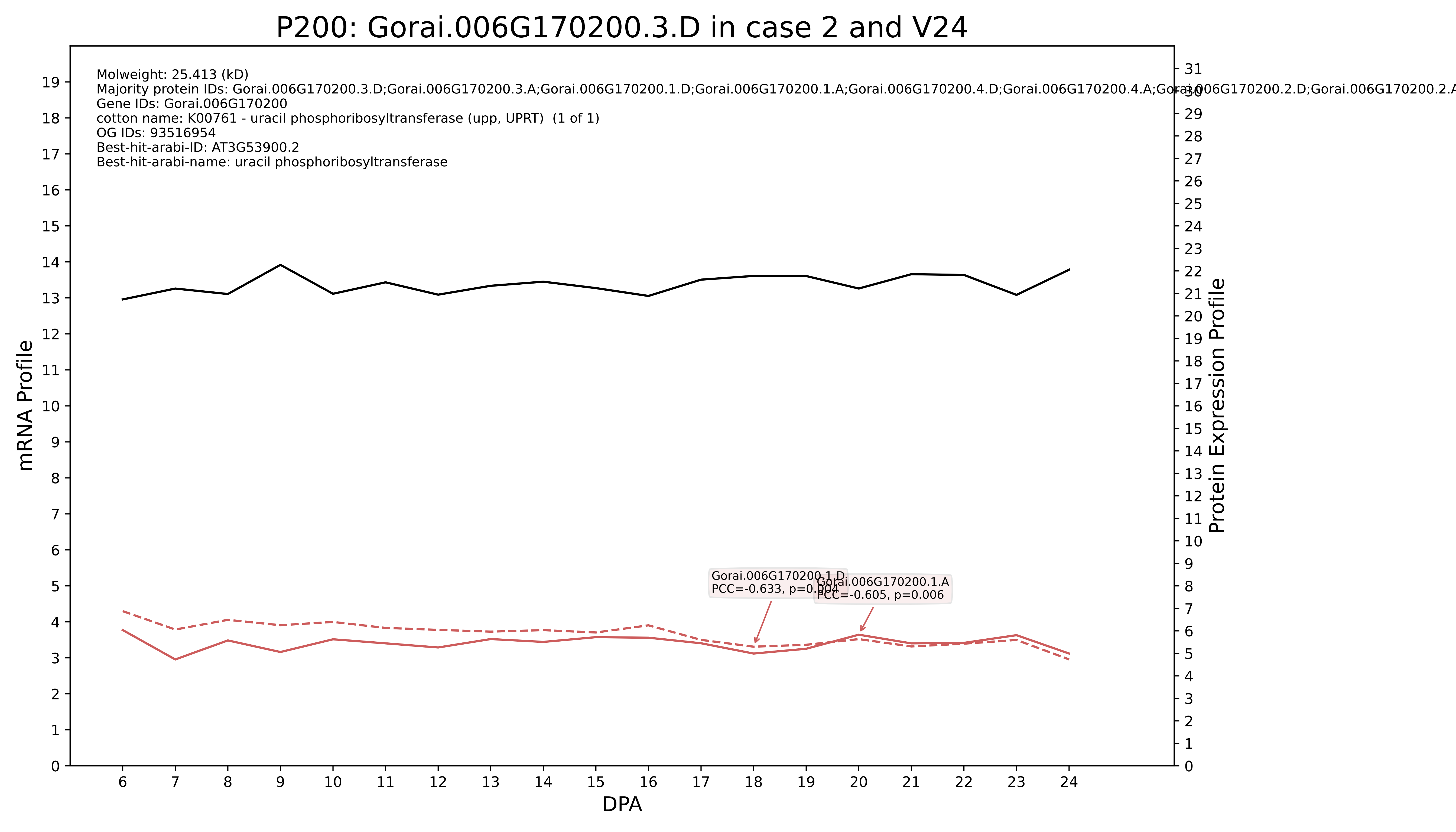
P200: Gorai.006G170200.3.D in case 2 and V24
Molweight: 25.413 (kD)
Majority protein IDs: Gorai.006G170200.3.D;Gorai.006G170200.3.A;Gorai.006G170200.1.D;Gorai.006G170200.1.A;Gorai.006G170200.4.D;Gorai.006G170200.4.A;Gorai.006G170200.2.D;Gorai.006G170200.2.A
Gene IDs: Gorai.006G170200
cotton name: K00761 - uracil phosphoribosyltransferase (upp, UPRT) (1 of 1)
OG IDs: 93516954
Best-hit-arabi-ID: AT3G53900.2
Best-hit-arabi-name: uracil phosphoribosyltransferase
Gorai.006G170200.1.D
PCC=-0.633, p=0.004
Gorai.006G170200.1.A
PCC=-0.605, p=0.006
mRNA Profile
Protein Expression Profile
DPA

P200: Gorai.011G237500.4.D in case 2 and V24

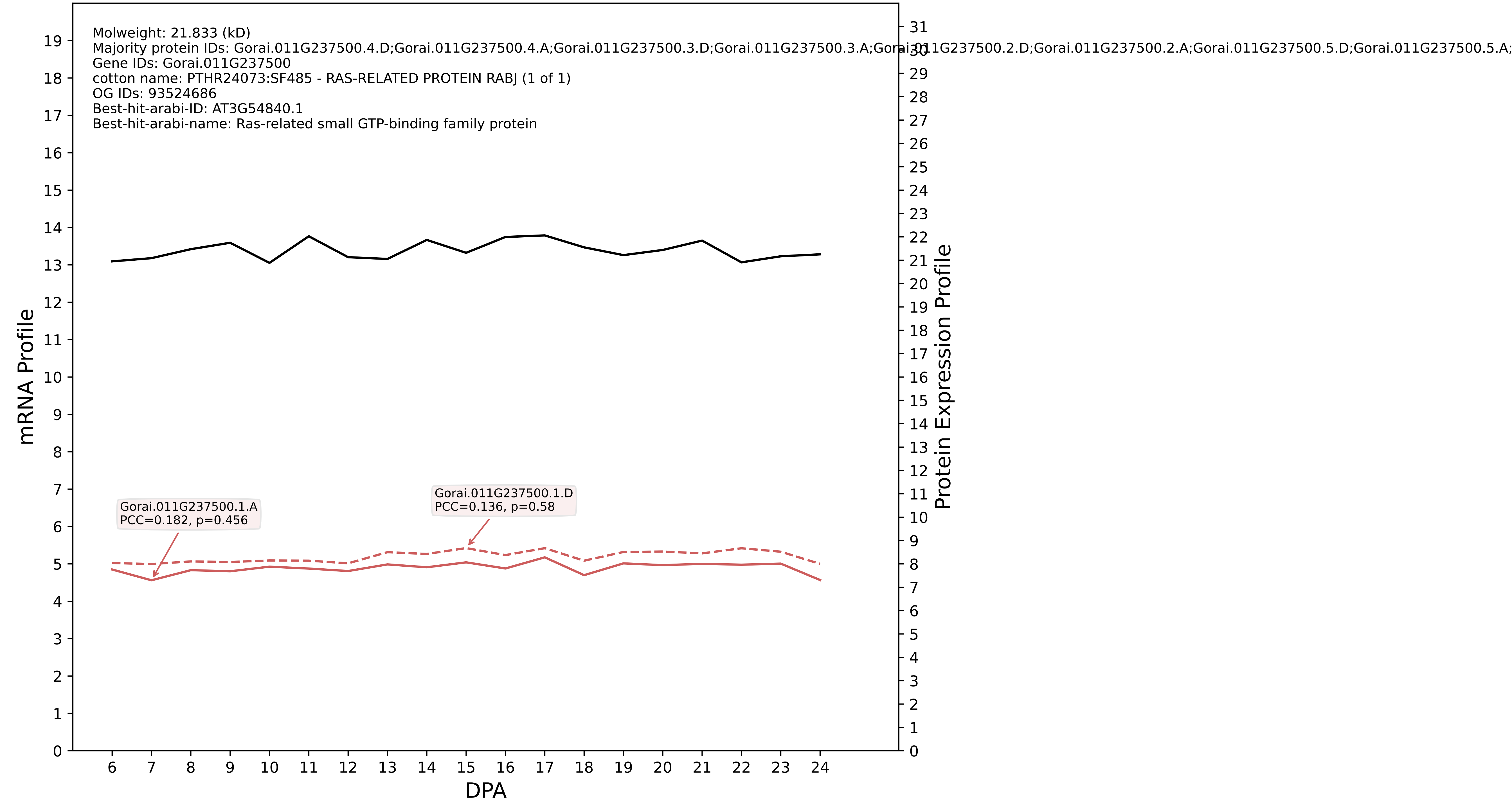

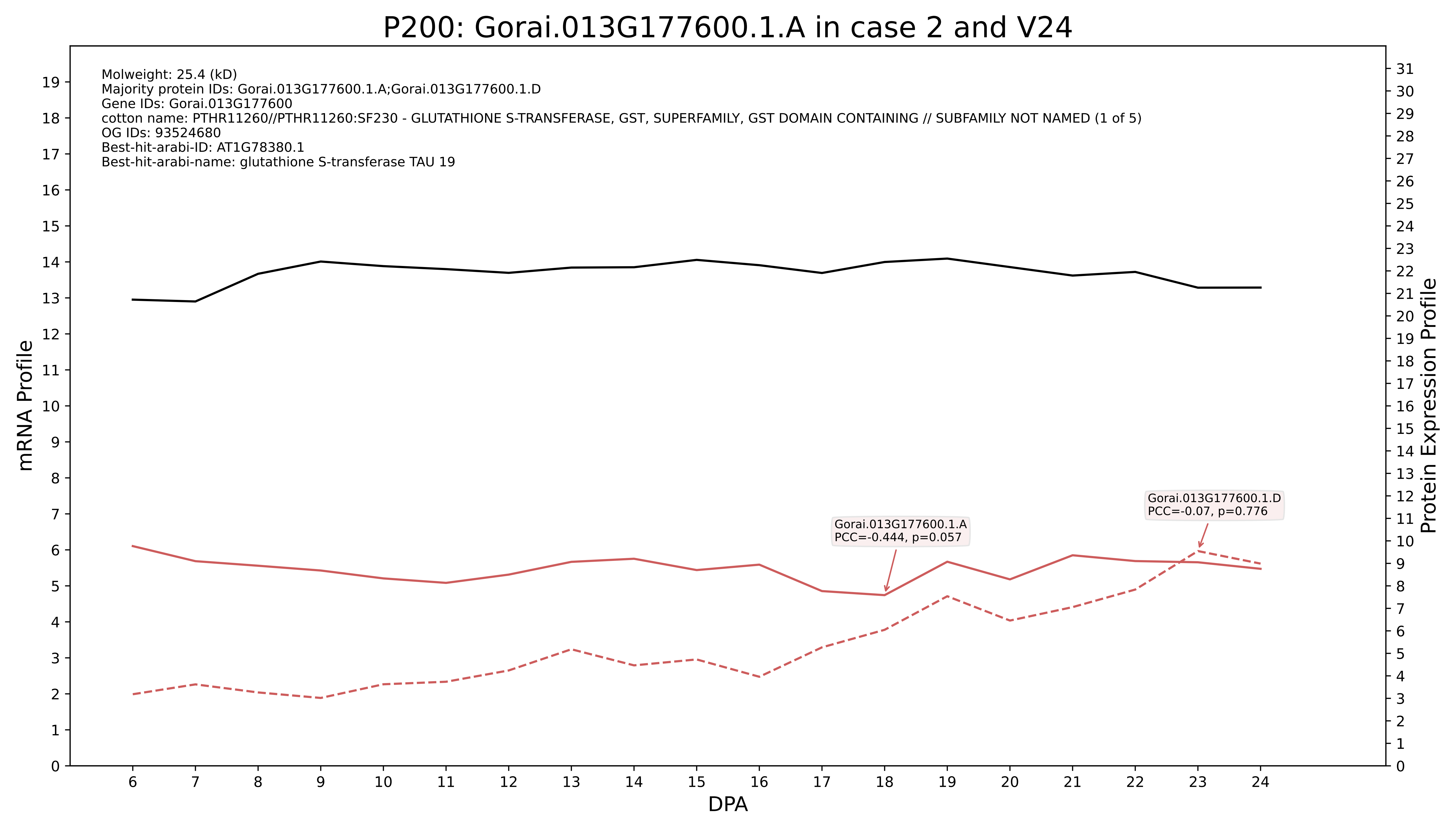
P200: Gorai.013G177600.1.A in case 2 and V24
Molweight: 25.4 (kD)
Majority protein IDs: Gorai.013G177600.1.A;Gorai.013G177600.1.D
Gene IDs: Gorai.013G177600
cotton name: PTHR11260//PTHR11260:SF230 - GLUTATHIONE S-TRANSFERASE, GST, SUPERFAMILY, GST DOMAIN CONTAINING // SUBFAMILY NOT NAMED (1 of 5)
OG IDs: 93524680
Best-hit-arabi-ID: AT1G78380.1
Best-hit-arabi-name: glutathione S-transferase TAU 19
Gorai.013G177600.1.A
PCC=-0.444, p=0.057
Gorai.013G177600.1.D
PCC=-0.07, p=0.776
mRNA Profile
Protein Expression Profile
DPA

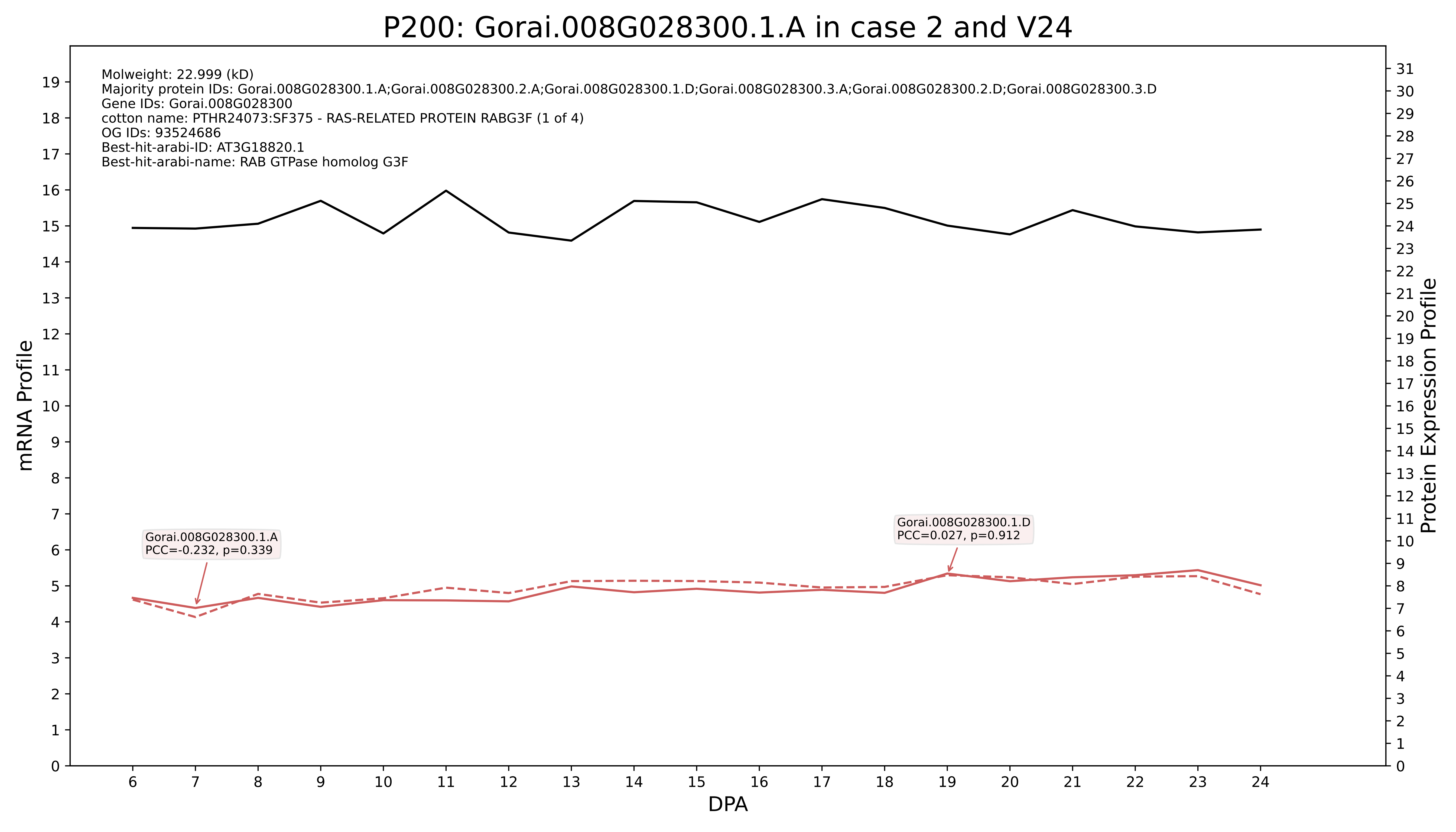
P200: Gorai.008G028300.1.A in case 2 and V24
Molweight: 22.999 (kD)
Majority protein IDs: Gorai.008G028300.1.A;Gorai.008G028300.2.A;Gorai.008G028300.1.D;Gorai.008G028300.3.A;Gorai.008G028300.2.D;Gorai.008G028300.3.D
Gene IDs: Gorai.008G028300
cotton name: PTHR24073:SF375 - RAS-RELATED PROTEIN RABG3F (1 of 4)
OG IDs: 93524686
Best-hit-arabi-ID: AT3G18820.1
Best-hit-arabi-name: RAB GTPase homolog G3F
Gorai.008G028300.1.A
PCC=-0.232, p=0.339
Gorai.008G028300.1.D
PCC=0.027, p=0.912
mRNA Profile
Protein Expression Profile
DPA

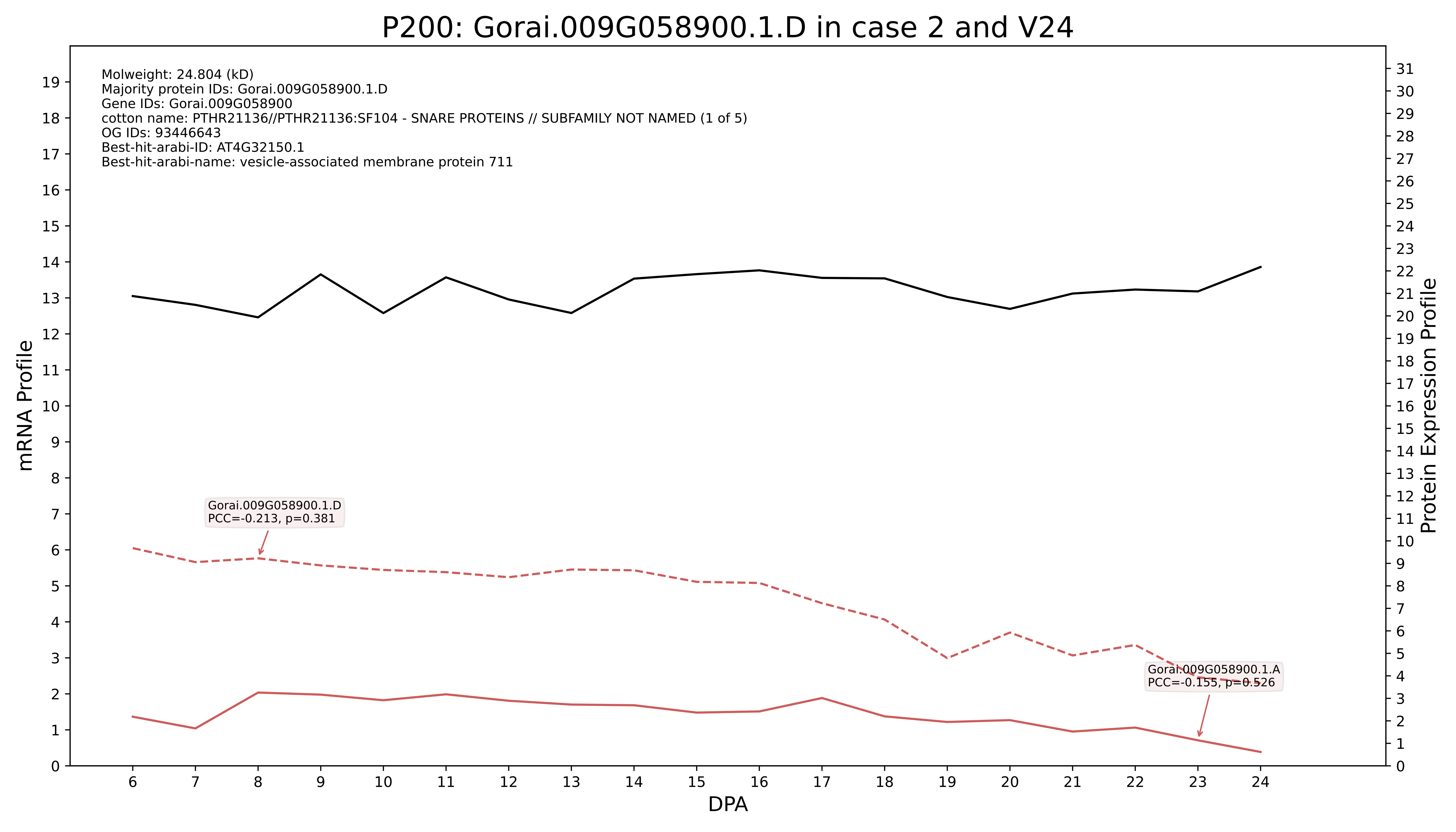
P200: Gorai.009G058900.1.D in case 2 and V24
Molweight: 24.804 (kD)
Majority protein IDs: Gorai.009G058900.1.D
Gene IDs: Gorai.009G058900
cotton name: PTHR21136//PTHR21136:SF104 - SNARE PROTEINS // SUBFAMILY NOT NAMED (1 of 5)
OG IDs: 93446643
Best-hit-arabi-ID: AT4G32150.1
Best-hit-arabi-name: vesicle-associated membrane protein 711
Gorai.009G058900.1.D
PCC=-0.213, p=0.381
Gorai.009G058900.1.A
PCC=-0.155, p=0.526
mRNA Profile
Protein Expression Profile
DPA

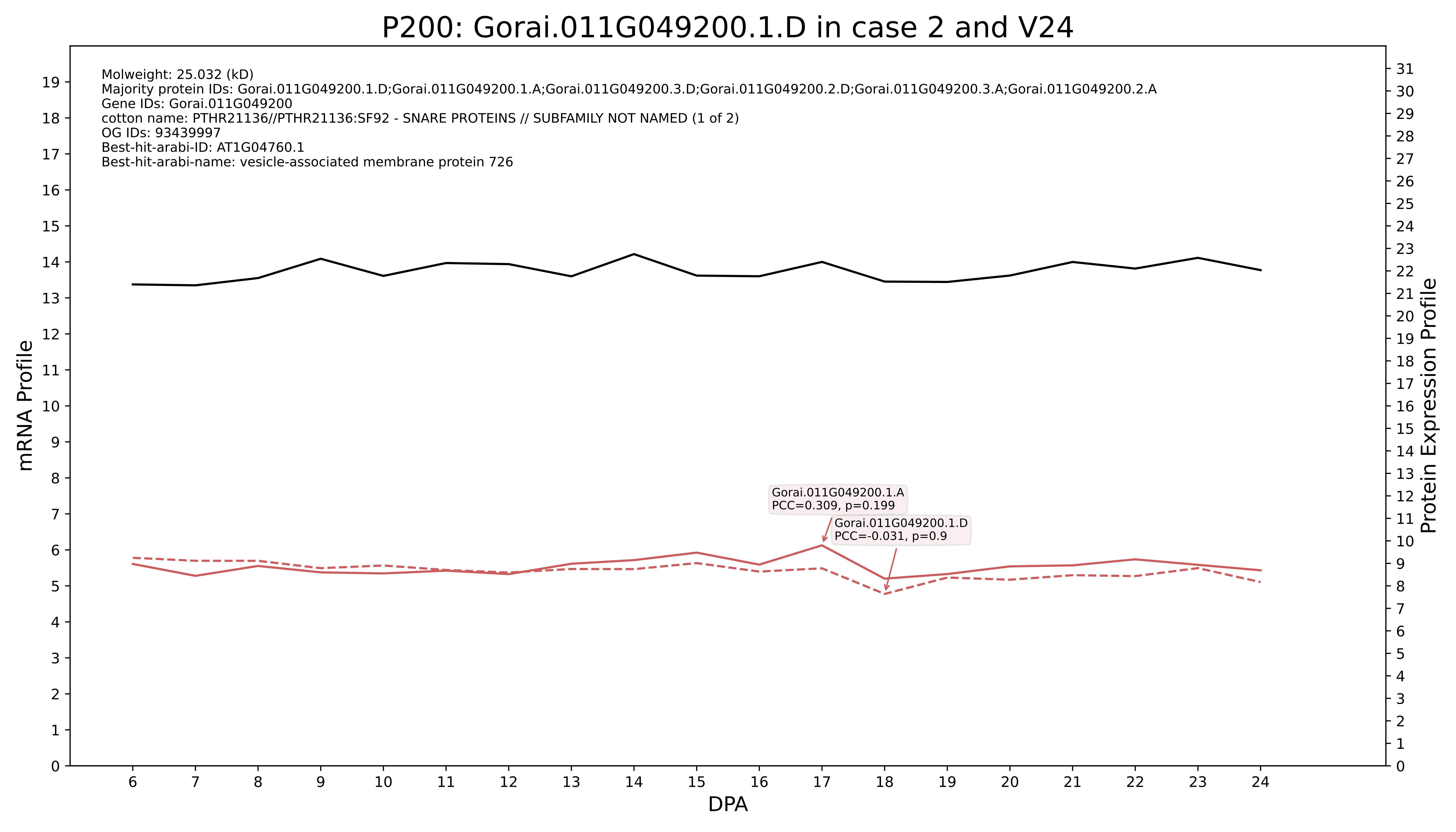

P200: Gorai.011G049200.1.D in case 2 and V24
Molweight: 25.032 (kD)
Majority protein IDs: Gorai.011G049200.1.D;Gorai.011G049200.1.A;Gorai.011G049200.3.D;Gorai.011G049200.2.D;Gorai.011G049200.3.A;Gorai.011G049200.2.A
Gene IDs: Gorai.011G049200
cotton name: PTHR21136//PTHR21136:SF92 - SNARE PROTEINS // SUBFAMILY NOT NAMED (1 of 2)
OG IDs: 93439997
Best-hit-arabi-ID: AT1G04760.1
Best-hit-arabi-name: vesicle-associated membrane protein 726
Gorai.011G049200.1.A
PCC=0.309, p=0.199
Gorai.011G049200.1.D
PCC=-0.031, p=0.9
mRNA Profile
Protein Expression Profile
DPA

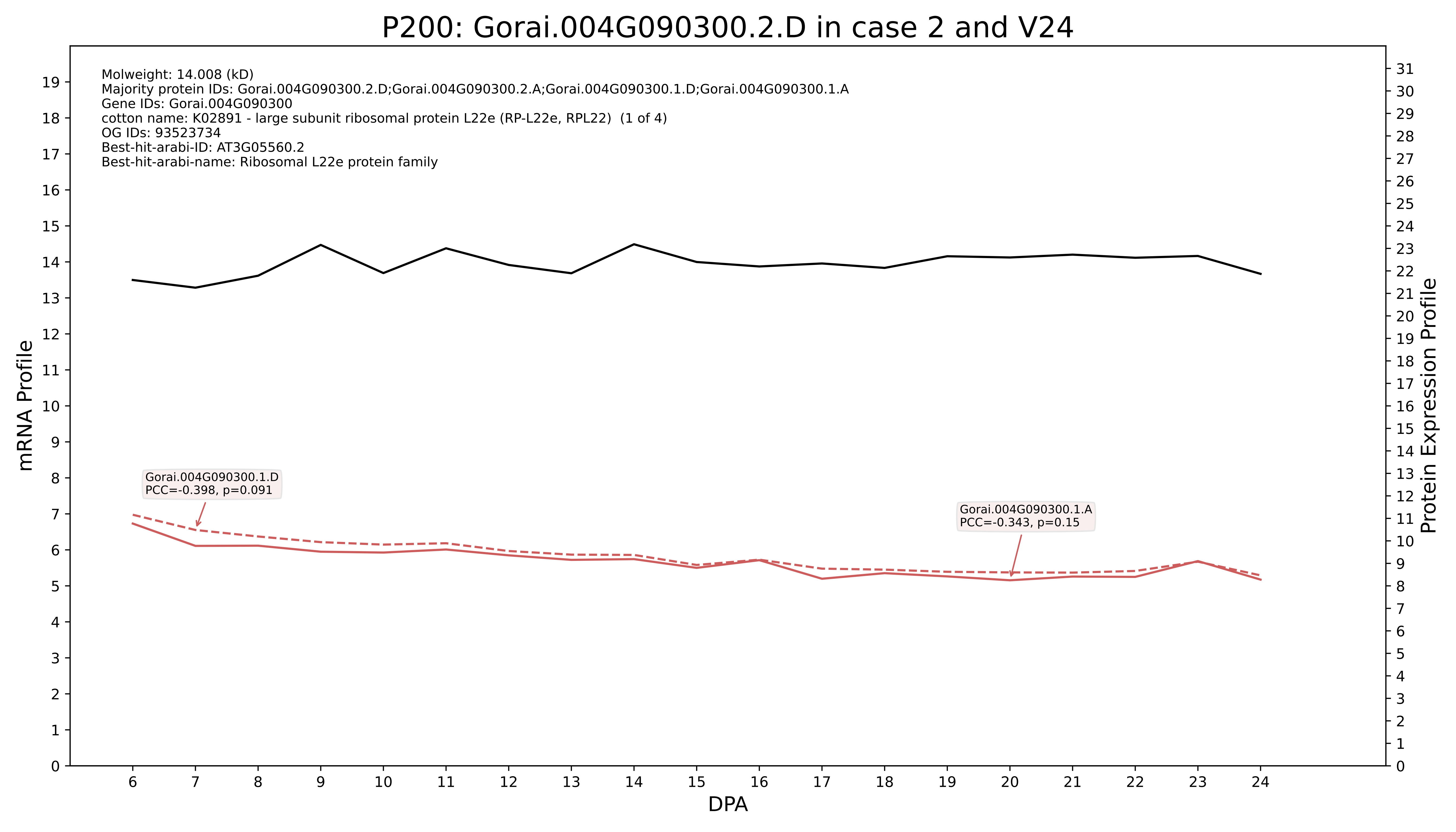

P200: Gorai.004G090300.2.D in case 2 and V24
Molweight: 14.008 (kD)
Majority protein IDs: Gorai.004G090300.2.D;Gorai.004G090300.2.A;Gorai.004G090300.1.D;Gorai.004G090300.1.A
Gene IDs: Gorai.004G090300
cotton name: K02891 - large subunit ribosomal protein L22e (RP-L22e, RPL22) (1 of 4)
OG IDs: 93523734
Best-hit-arabi-ID: AT3G05560.2
Best-hit-arabi-name: Ribosomal L22e protein family
Gorai.004G090300.1.D
PCC=-0.398, p=0.091
Gorai.004G090300.1.A
PCC=-0.343, p=0.15
mRNA Profile
Protein Expression Profile
DPA

P200: Gorai.009G119700.1.D in case 2 and V24

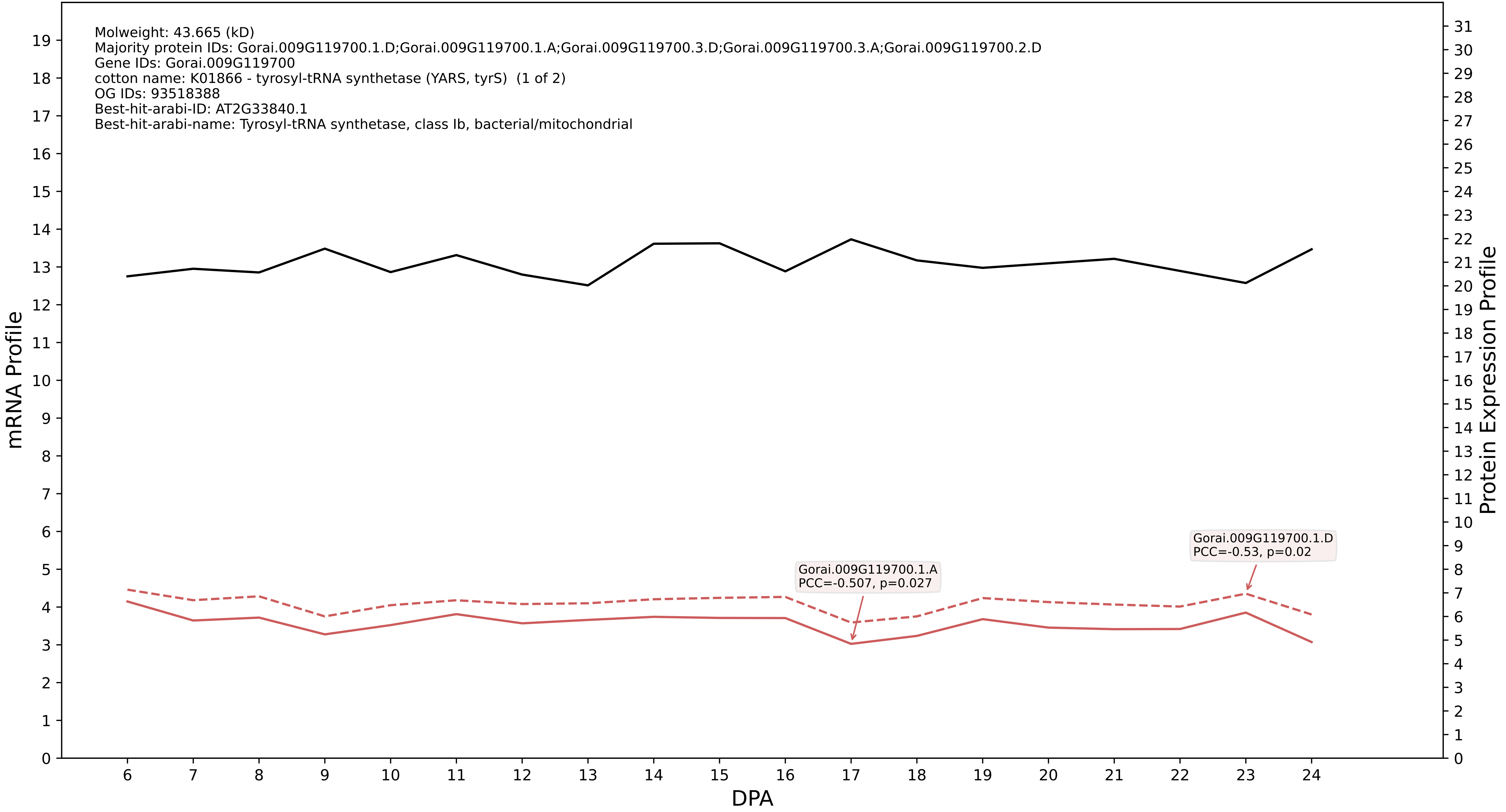

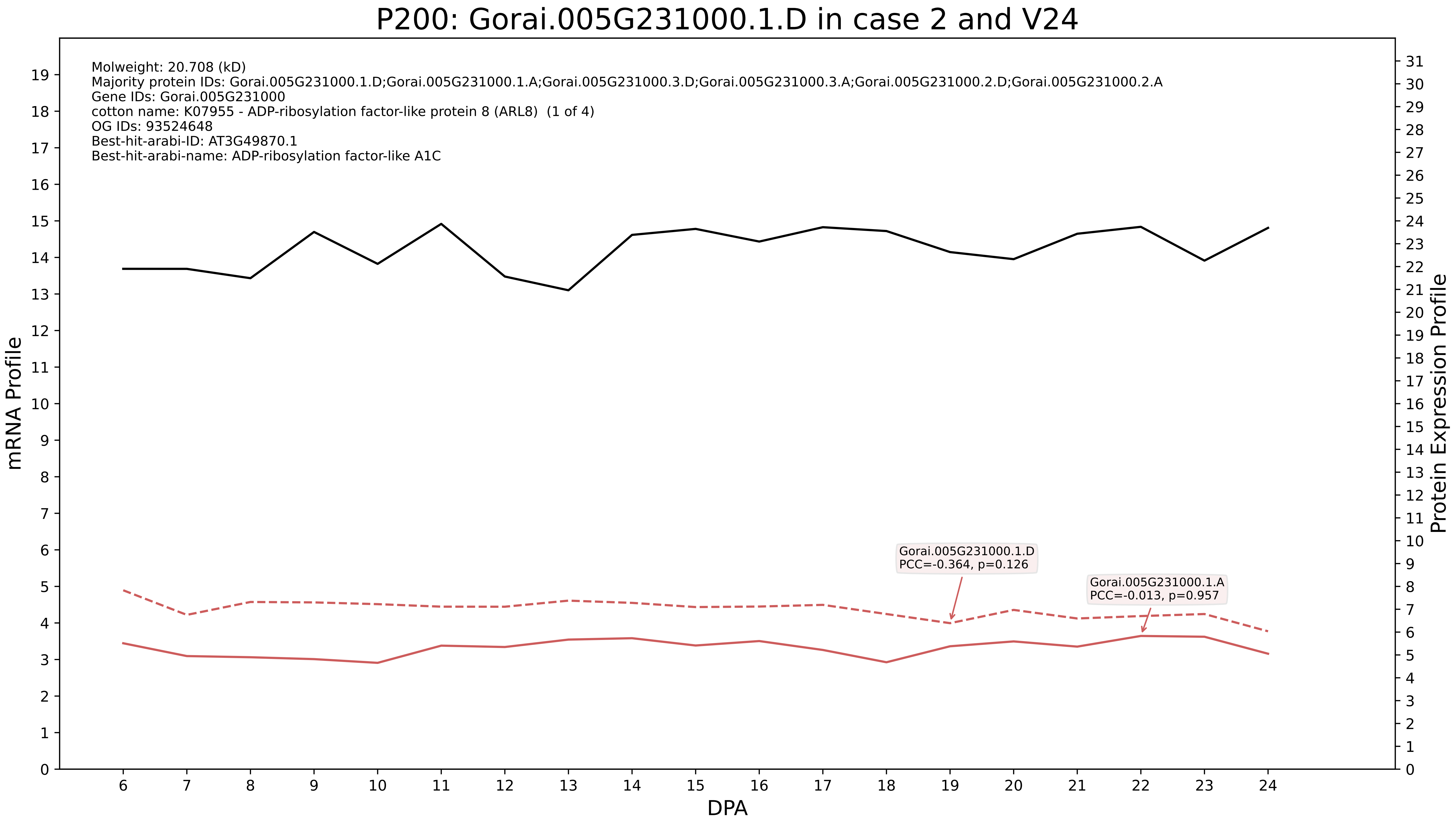
P200: Gorai.005G231000.1.D in case 2 and V24
Molweight: 20.708 (kD)
Majority protein IDs: Gorai.005G231000.1.D;Gorai.005G231000.1.A;Gorai.005G231000.3.D;Gorai.005G231000.3.A;Gorai.005G231000.2.D;Gorai.005G231000.2.A
Gene IDs: Gorai.005G231000
cotton name: K07955 - ADP-ribosylation factor-like protein 8 (ARL8) (1 of 4)
OG IDs: 93524648
Best-hit-arabi-ID: AT3G49870.1
Best-hit-arabi-name: ADP-ribosylation factor-like A1C
Gorai.005G231000.1.D
PCC=-0.364, p=0.126
Gorai.005G231000.1.A
PCC=-0.013, p=0.957
mRNA Profile
Protein Expression Profile
DPA
0
1
2
3
4
5
6
7
8
9
10
11
12
13
14
15
16
17
18
19
20
21
22
23
24
25
26
27
28
29
30
31

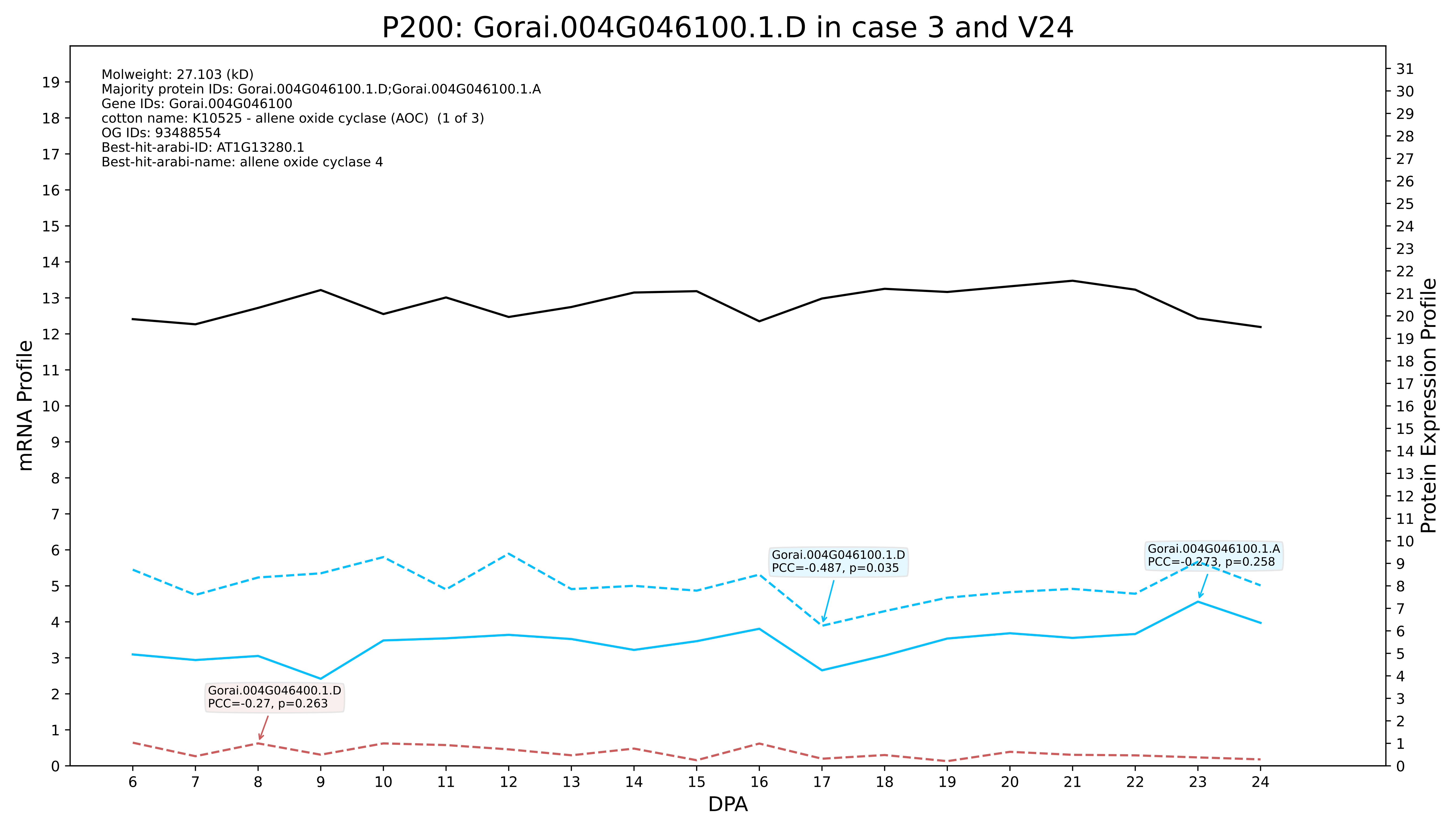

P200: Gorai.004G046100.1.D in case 3 and V24
Molweight: 27.103 (kD)
Majority protein IDs: Gorai.004G046100.1.D;Gorai.004G046100.1.A
Gene IDs: Gorai.004G046100
cotton name: K10525 - allene oxide cyclase (AOC) (1 of 3)
OG IDs: 93488554
Best-hit-arabi-ID: AT1G13280.1
Best-hit-arabi-name: allene oxide cyclase 4
Gorai.004G046100.1.D
PCC=-0.487, p=0.035
Gorai.004G046100.1.A
PCC=-0.273, p=0.258
Gorai.004G046400.1.D
PCC=-0.27, p=0.263
mRNA Profile
Protein Expression Profile
DPA

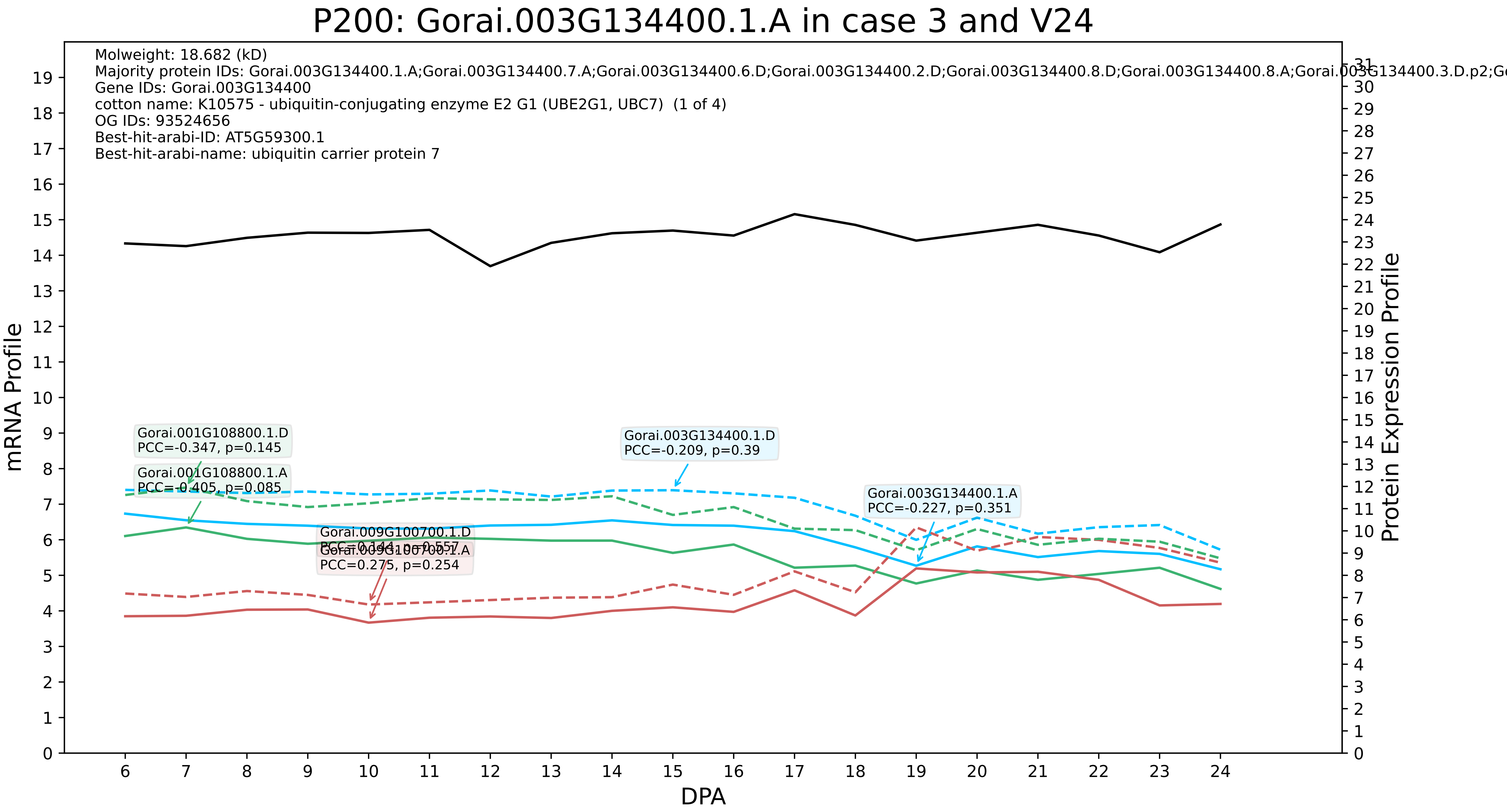

P200: Gorai.003G134400.1.A in case 3 and V24
Molweight: 18.682 (kD)
Majority protein IDs: Gorai.003G134400.1.A;Gorai.003G134400.7.A;Gorai.003G134400.6.D;Gorai.003G134400.2.D;Gorai.003G134400.8.D;Gorai.003G134400.8.A;Gorai.003G134400.3.D.p2;G
Gene IDs: Gorai.003G134400
cotton name: K10575 - ubiquitin-conjugating enzyme E2 G1 (UBE2G1, UBC7) (1 of 4)
OG IDs: 93524656
Best-hit-arabi-ID: AT5G59300.1
Best-hit-arabi-name: ubiquitin carrier protein 7
Gorai.001G108800.1.D
PCC=-0.347, p=0.145
Gorai.001G108800.1.A
PCC=-0.405, p=0.085
Gorai.003G134400.1.D
PCC=-0.209, p=0.39
Gorai.003G134400.1.A
PCC=-0.227, p=0.351
Gorai.009G100700.1.D
PCC=0.144, p=0.557
Gorai.009G100700.1.A
PCC=0.275, p=0.254
mRNA Profile
Protein Expression Profile
DPA

P200: Gorai.006G159400.1.D in case 3 and V24

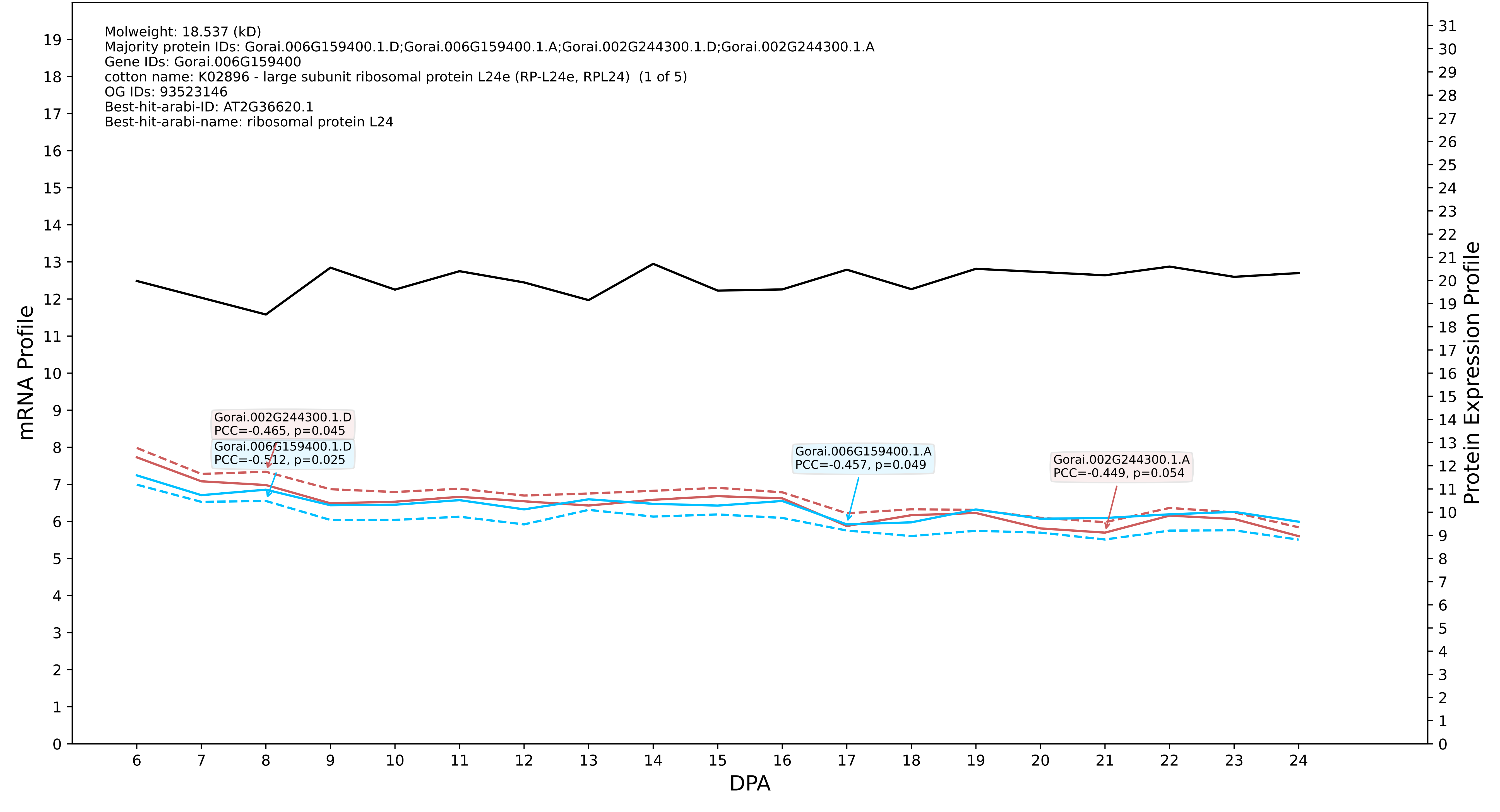

P200: Gorai.004G188200.1.D in case 3 and V24

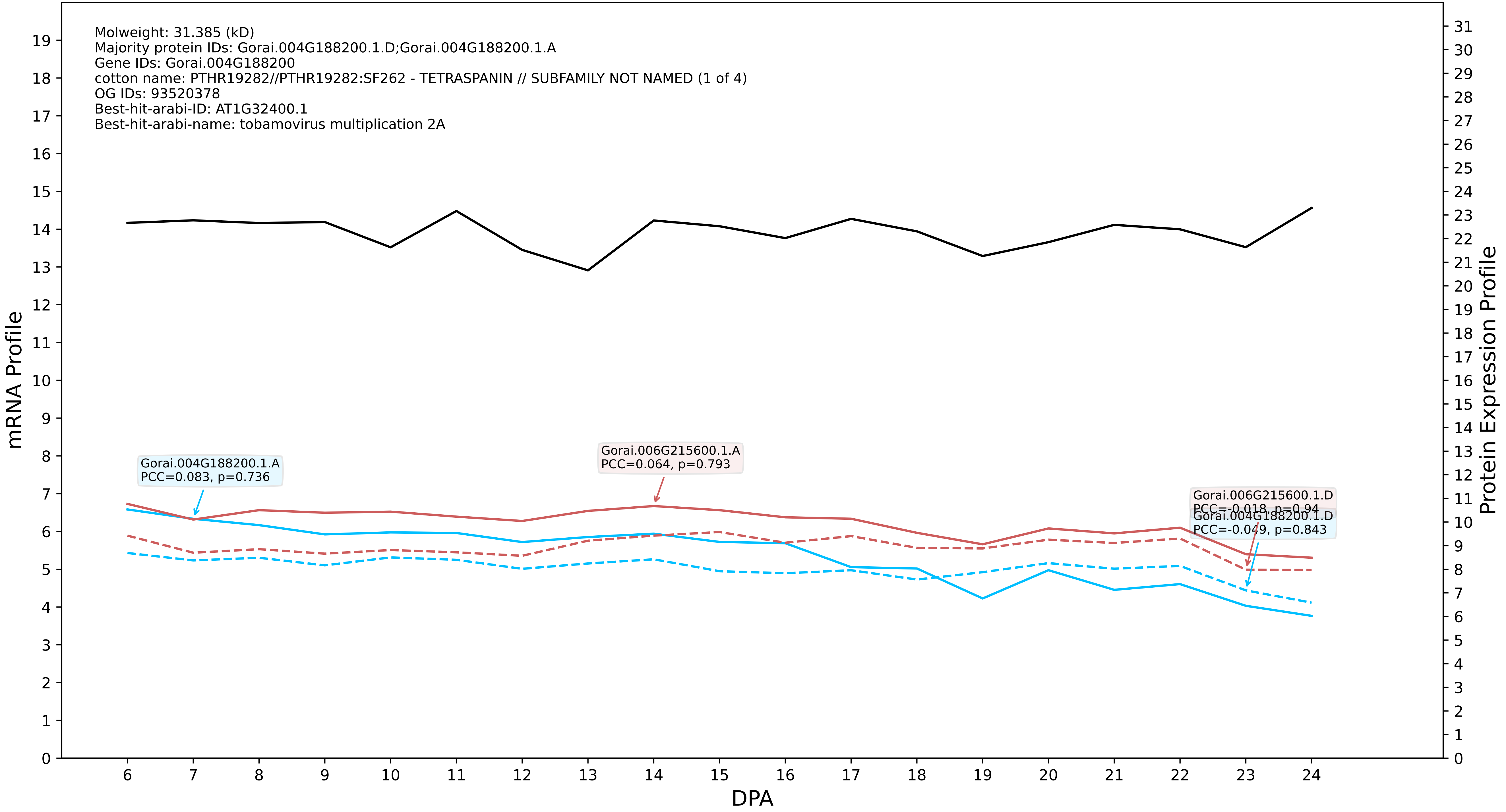

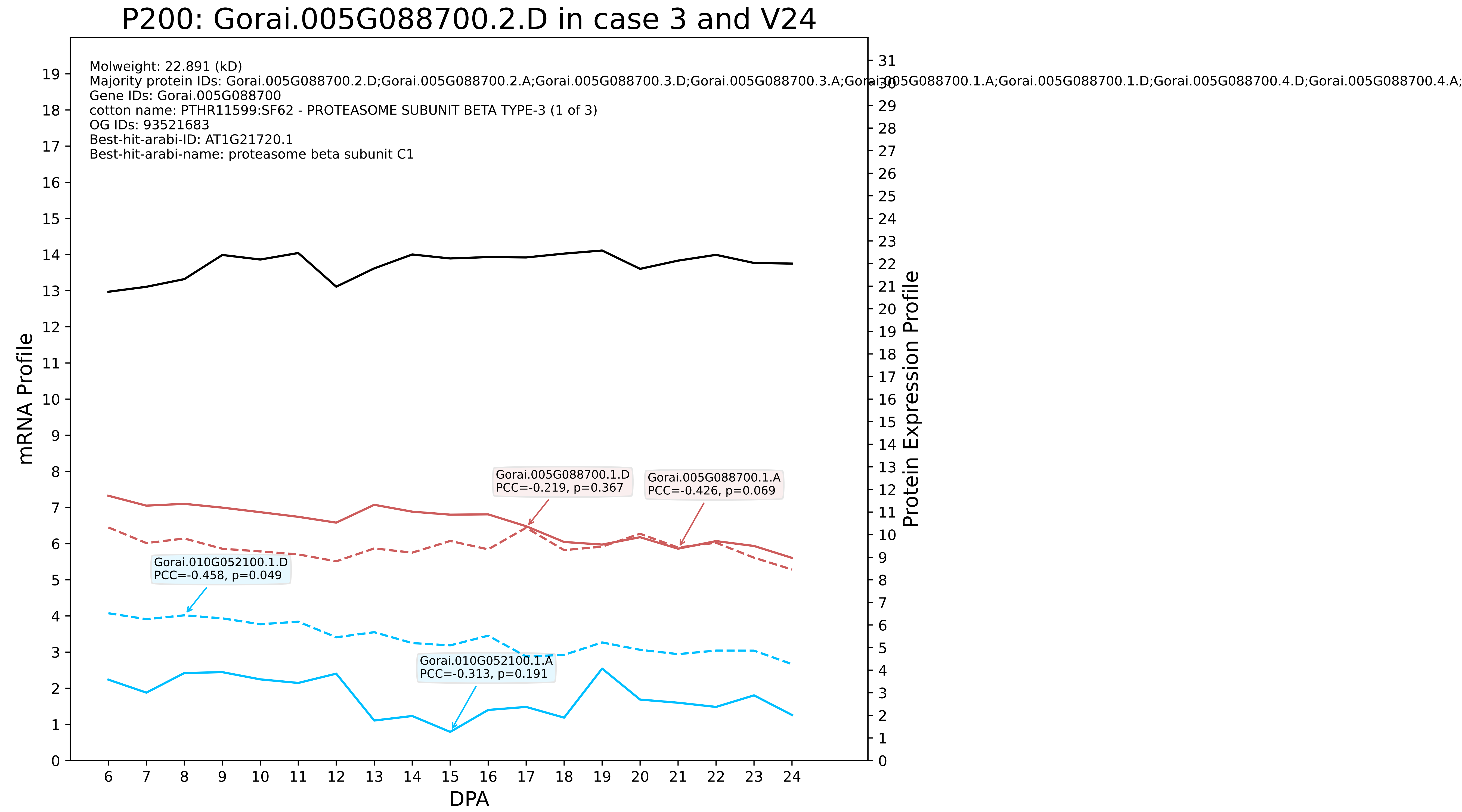

P200: Gorai.005G088700.2.D in case 3 and V24
Molweight: 22.891 (kD)
Majority protein IDs: Gorai.005G088700.2.D;Gorai.005G088700.2.A;Gorai.005G088700.3.D;Gorai.005G088700.3.A;Gorai.005G088700.1.A;Gorai.005G088700.1.D;Gorai.005G088700.4.D;Gorai.005G088700.4.A;
Gene IDs: Gorai.005G088700
cotton name: PTHR11599:SF62 - PROTEASOME SUBUNIT BETA TYPE-3 (1 of 3)
OG IDs: 93521683
Best-hit-arabi-ID: AT1G21720.1
Best-hit-arabi-name: proteasome beta subunit C1
Gorai.005G088700.1.D
PCC=-0.219, p=0.367
Gorai.005G088700.1.A
PCC=-0.426, p=0.069
Gorai.010G052100.1.D
PCC=-0.458, p=0.049
Gorai.010G052100.1.A
PCC=-0.313, p=0.191
mRNA Profile
Protein Expression Profile
DPA

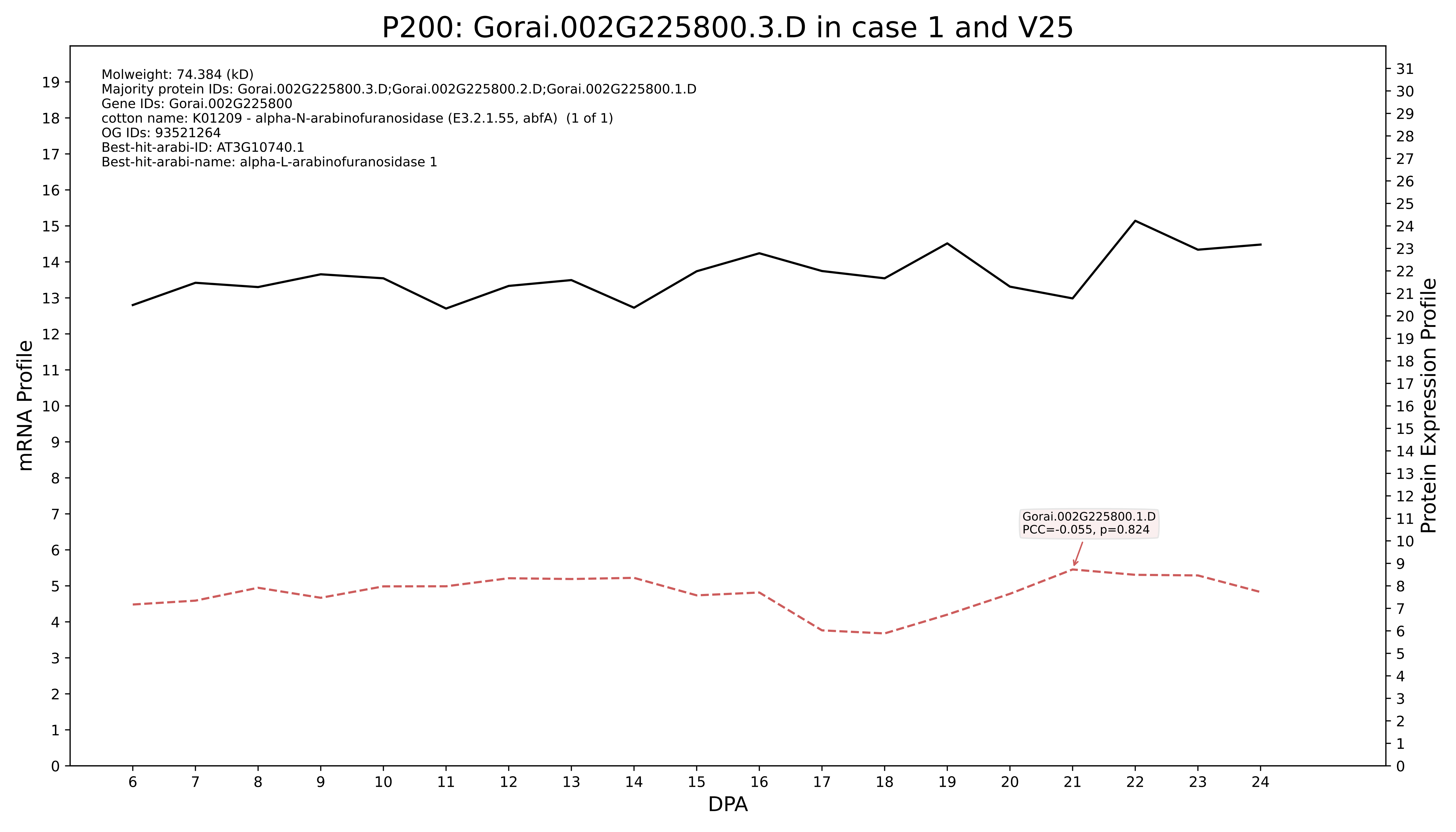
P200: Gorai.002G225800.3.D in case 1 and V25
Molweight: 74.384 (kD)
Majority protein IDs: Gorai.002G225800.3.D;Gorai.002G225800.2.D;Gorai.002G225800.1.D
Gene IDs: Gorai.002G225800
cotton name: K01209 - alpha-N-arabinofuranosidase (E3.2.1.55, abfA) (1 of 1)
OG IDs: 93521264
Best-hit-arabi-ID: AT3G10740.1
Best-hit-arabi-name: alpha-L-arabinofuranosidase 1
Gorai.002G225800.1.D
PCC=-0.055, p=0.824
mRNA Profile
Protein Expression Profile
DPA

P200: Gorai.001G230800.1.A in case 1 and V25

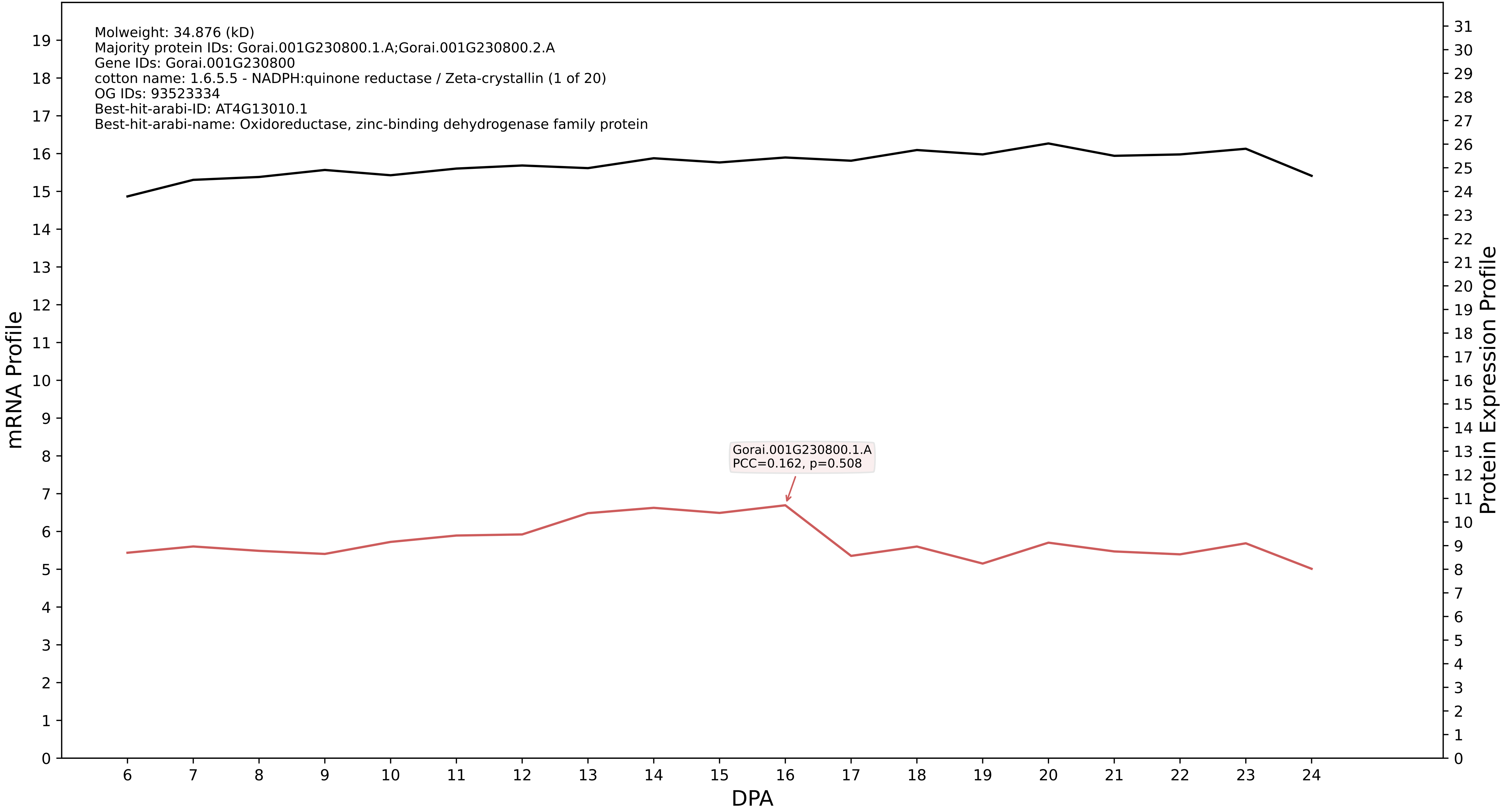

P200: Gorai.004G211800.1.A in case 1 and V25

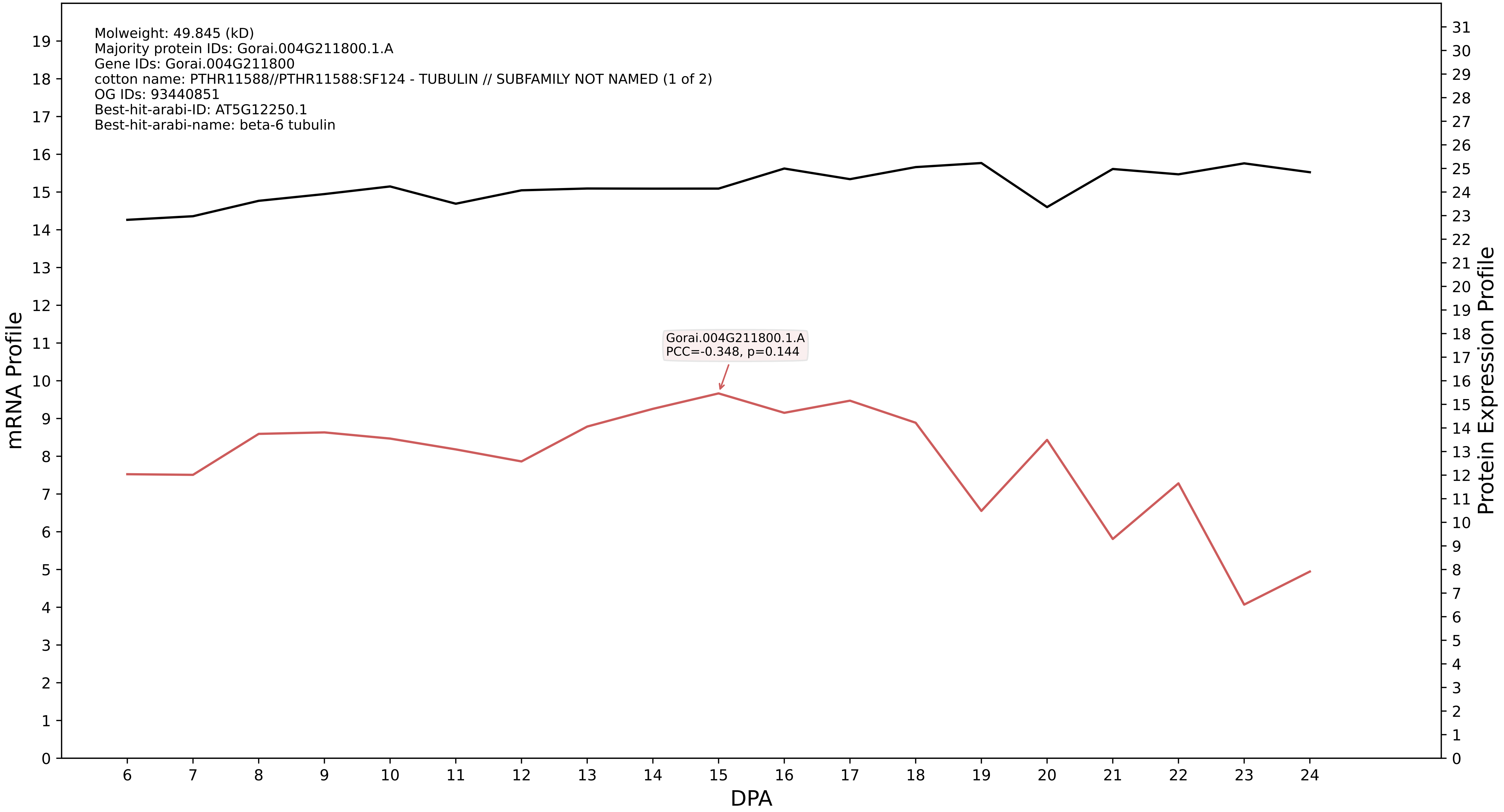

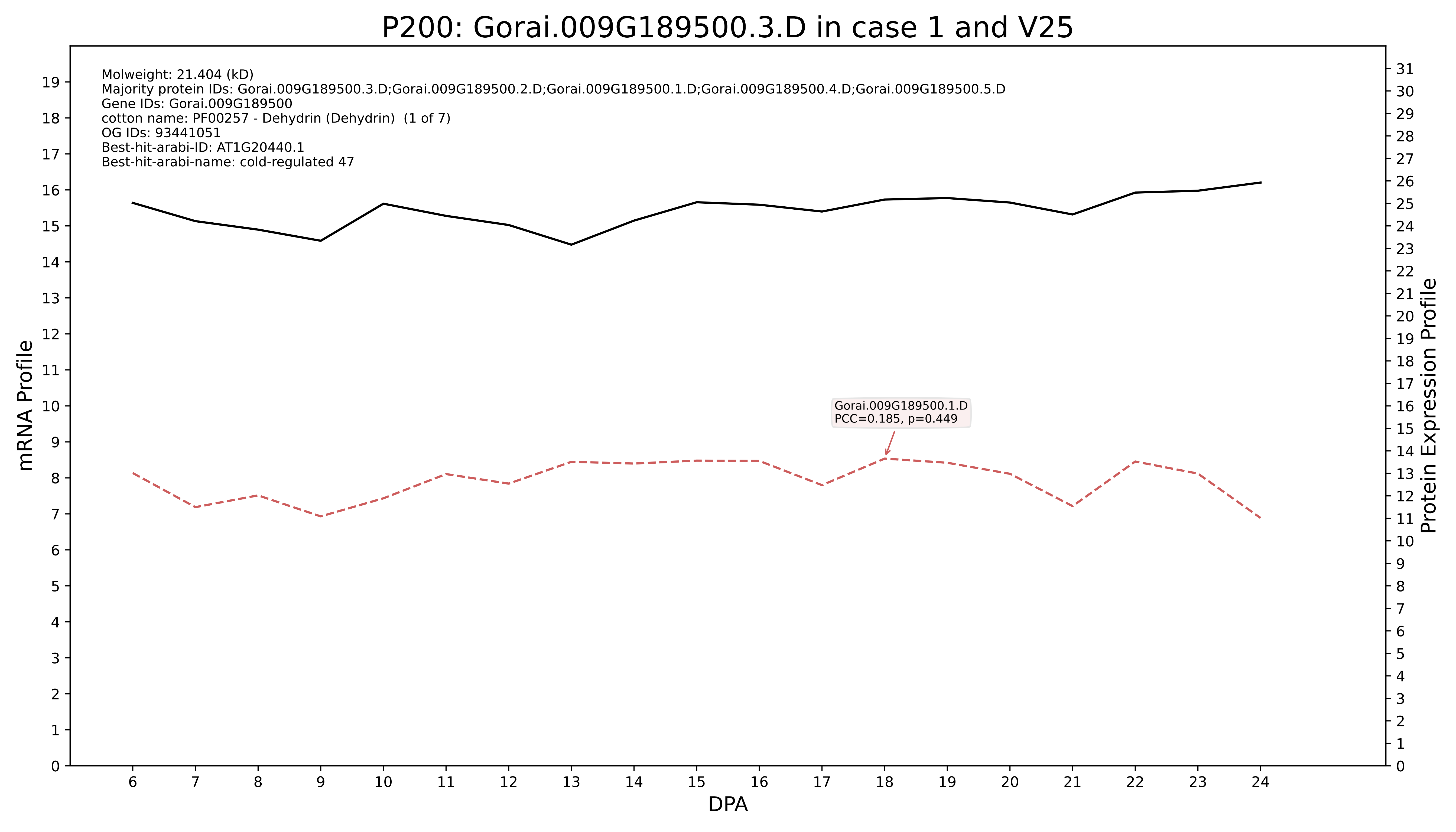

P200: Gorai.009G189500.3.D in case 1 and V25
Molweight: 21.404 (kD)
Majority protein IDs: Gorai.009G189500.3.D;Gorai.009G189500.2.D;Gorai.009G189500.1.D;Gorai.009G189500.4.D;Gorai.009G189500.5.D
Gene IDs: Gorai.009G189500
cotton name: PF00257 - Dehydrin (Dehydrin) (1 of 7)
OG IDs: 93441051
Best-hit-arabi-ID: AT1G20440.1
Best-hit-arabi-name: cold-regulated 47
Gorai.009G189500.1.D
PCC=0.185, p=0.449
mRNA Profile
Protein Expression Profile
DPA

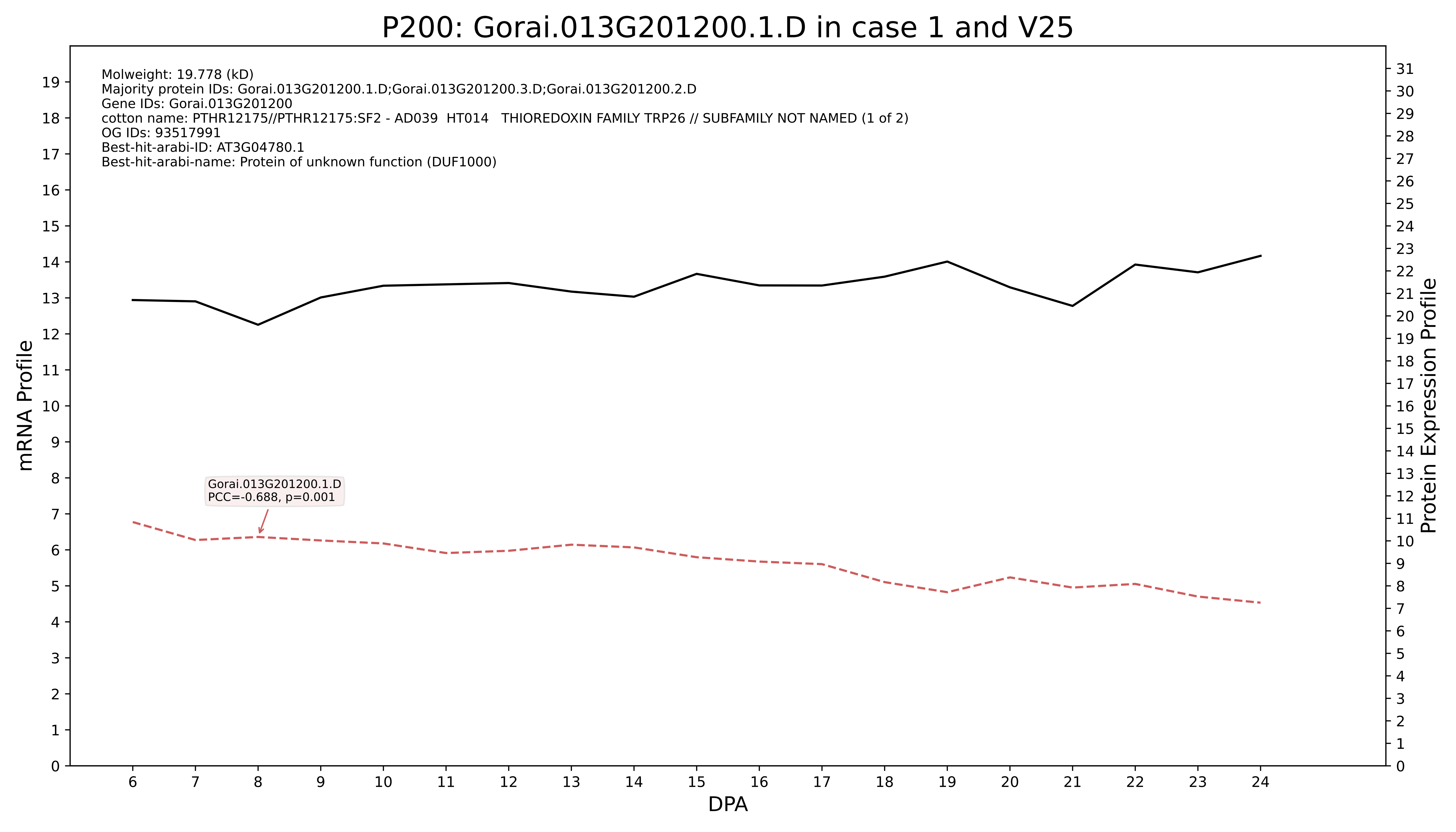
P200: Gorai.013G201200.1.D in case 1 and V25
Molweight: 19.778 (kD)
Majority protein IDs: Gorai.013G201200.1.D;Gorai.013G201200.3.D;Gorai.013G201200.2.D
Gene IDs: Gorai.013G201200
cotton name: PTHR12175//PTHR12175:SF2 - AD039 HT014 THIOREDOXIN FAMILY TRP26 // SUBFAMILY NOT NAMED (1 of 2)
OG IDs: 93517991
Best-hit-arabi-ID: AT3G04780.1
Best-hit-arabi-name: Protein of unknown function (DUF1000)
Gorai.013G201200.1.D
PCC=-0.688, p=0.001
mRNA Profile
Protein Expression Profile
DPA

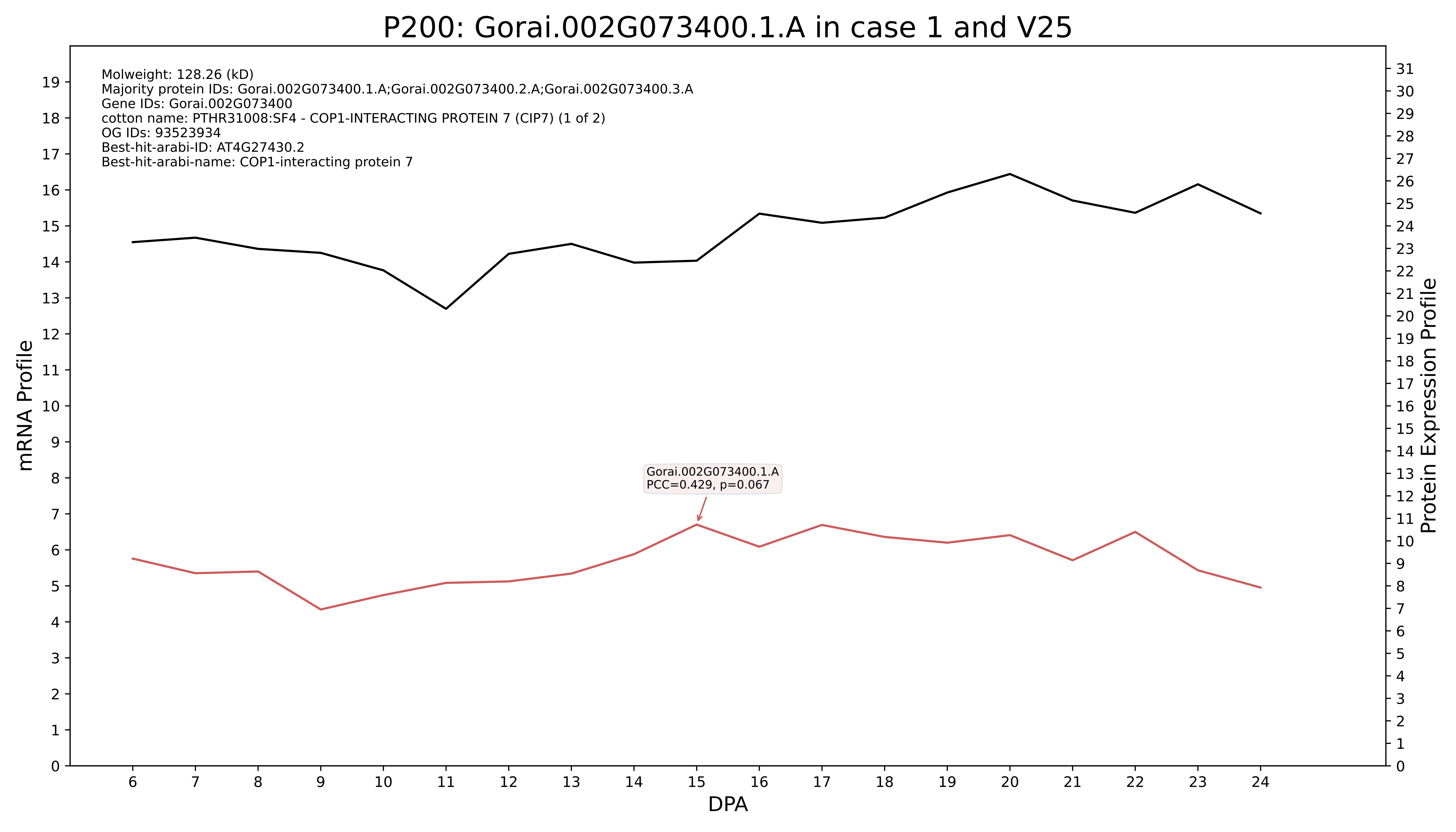
P200: Gorai.002G073400.1.A in case 1 and V25
Molweight: 128.26 (kD)
Majority protein IDs: Gorai.002G073400.1.A;Gorai.002G073400.2.A;Gorai.002G073400.3.A
Gene IDs: Gorai.002G073400
cotton name: PTHR31008:SF4 - COP1-INTERACTING PROTEIN 7 (CIP7) (1 of 2)
OG IDs: 93523934
Best-hit-arabi-ID: AT4G27430.2
Best-hit-arabi-name: COP1-interacting protein 7
Gorai.002G073400.1.A
PCC=0.429, p=0.067
mRNA Profile
Protein Expression Profile
DPA

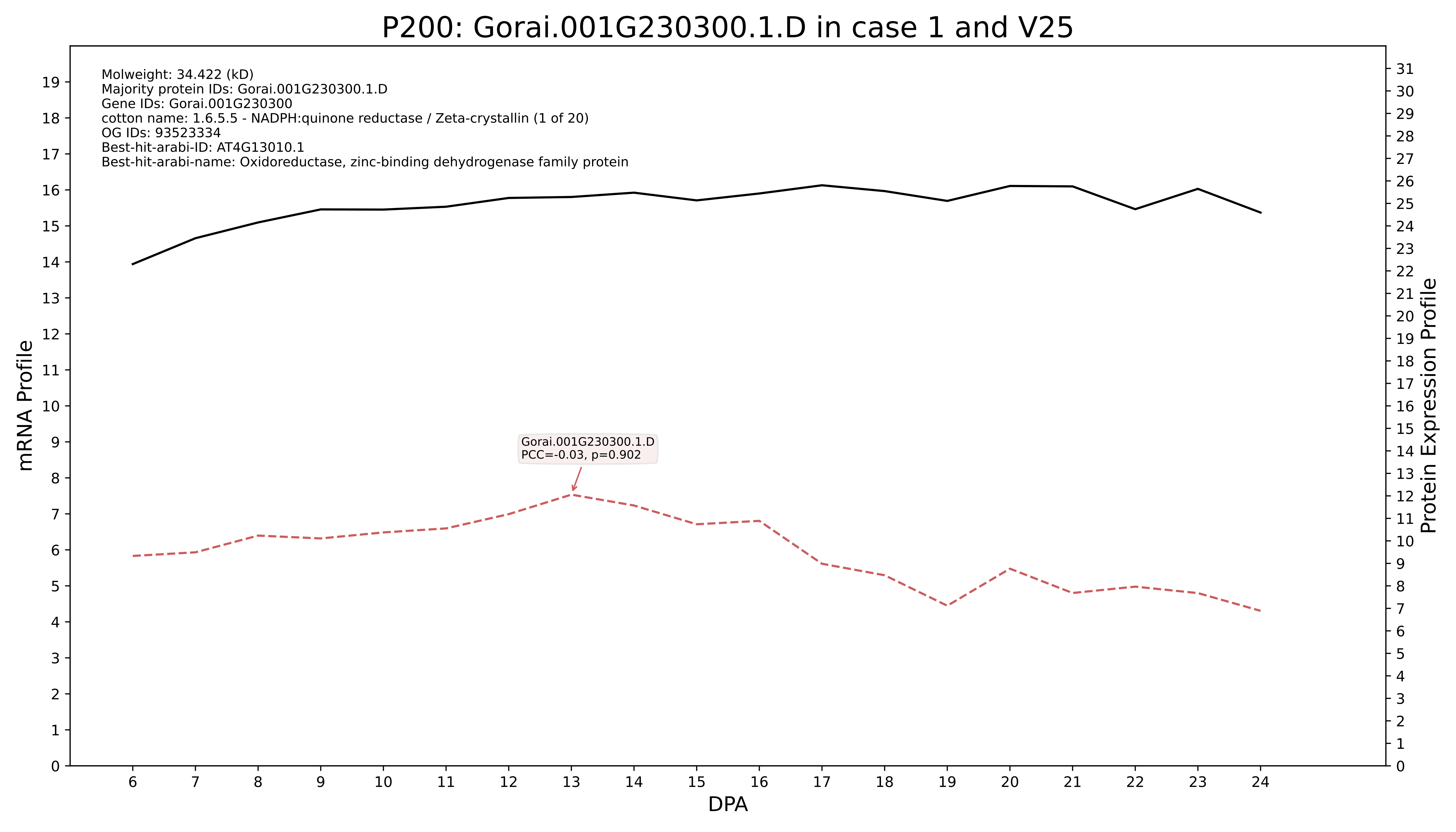

P200: Gorai.001G230300.1.D in case 1 and V25
Molweight: 34.422 (kD)
Majority protein IDs: Gorai.001G230300.1.D
Gene IDs: Gorai.001G230300
cotton name: 1.6.5.5 - NADPH:quinone reductase / Zeta-crystallin (1 of 20)
OG IDs: 93523334
Best-hit-arabi-ID: AT4G13010.1
Best-hit-arabi-name: Oxidoreductase, zinc-binding dehydrogenase family protein
Gorai.001G230300.1.D
PCC=-0.03, p=0.902
mRNA Profile
Protein Expression Profile
DPA

P200: Gorai.011G089300.1.A in case 1 and V25

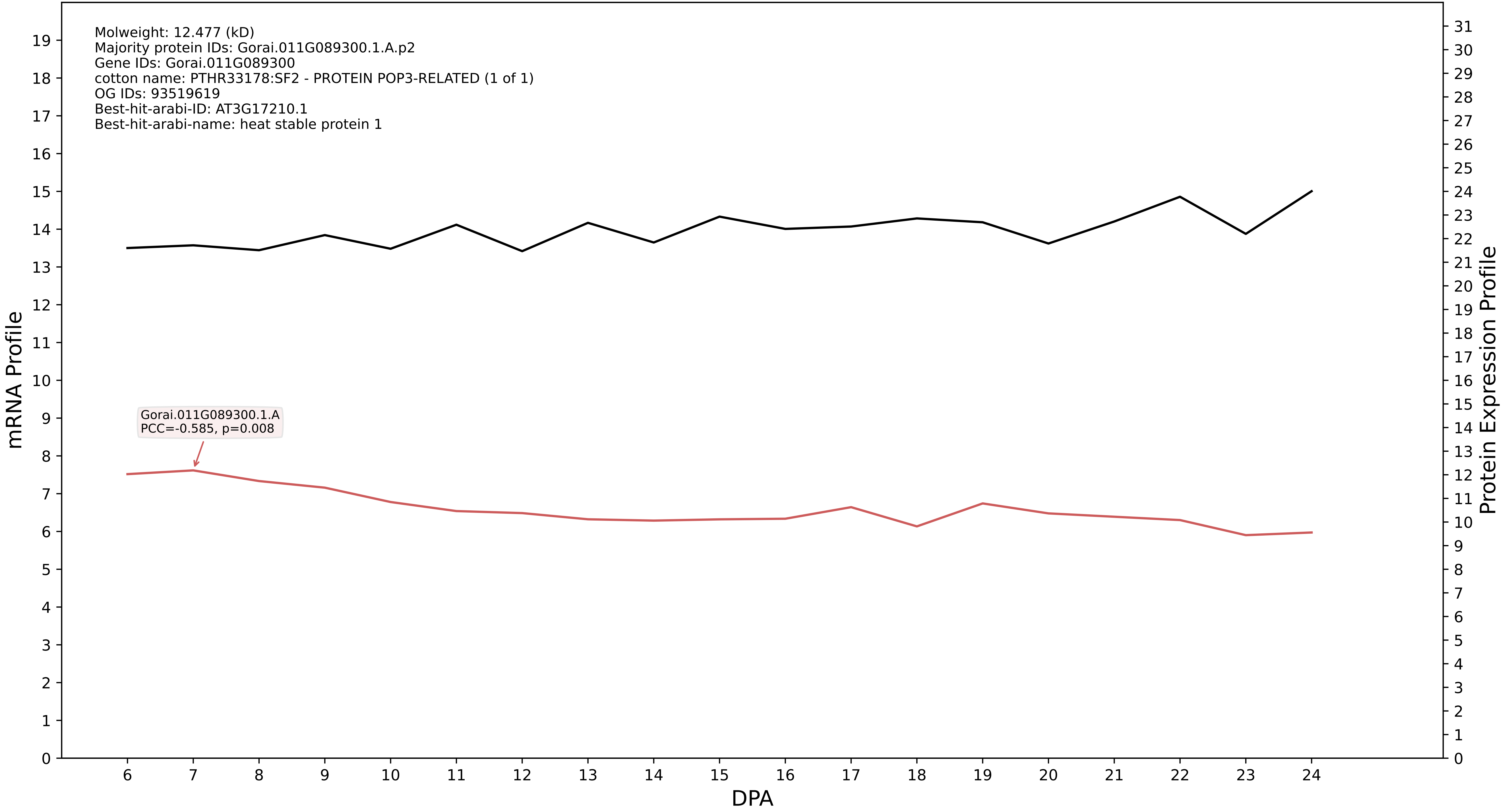

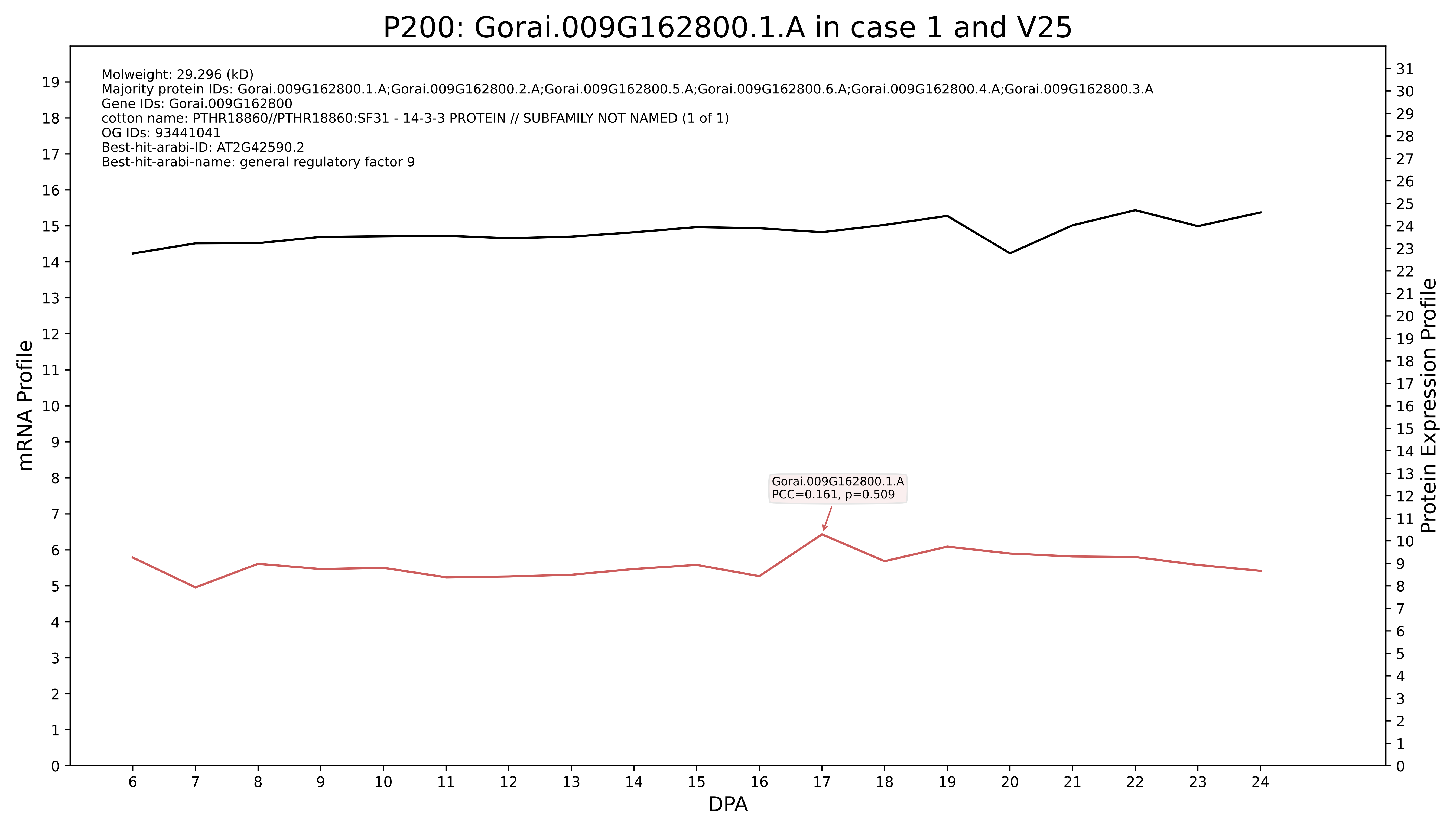
P200: Gorai.009G162800.1.A in case 1 and V25
Molweight: 29.296 (kD)
Majority protein IDs: Gorai.009G162800.1.A;Gorai.009G162800.2.A;Gorai.009G162800.5.A;Gorai.009G162800.6.A;Gorai.009G162800.4.A;Gorai.009G162800.3.A
Gene IDs: Gorai.009G162800
cotton name: PTHR18860//PTHR18860:SF31 - 14-3-3 PROTEIN // SUBFAMILY NOT NAMED (1 of 1)
OG IDs: 93441041
Best-hit-arabi-ID: AT2G42590.2
Best-hit-arabi-name: general regulatory factor 9
Gorai.009G162800.1.A
PCC=0.161, p=0.509
mRNA Profile
Protein Expression Profile
DPA

P200: Gorai.010G038400.3.D in case 1 and V25

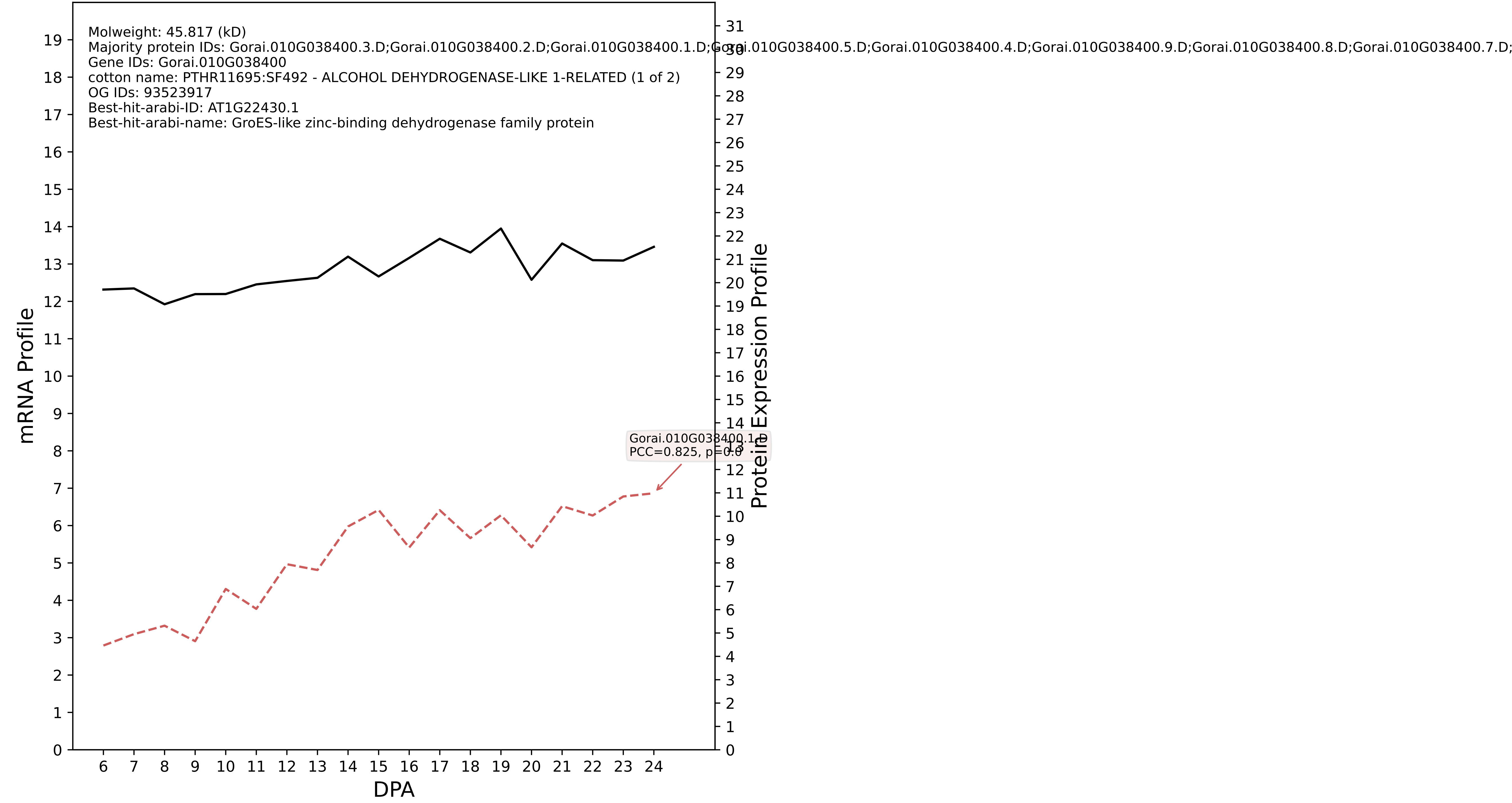

P200: Gorai.013G229500.1.A in case 1 and V25

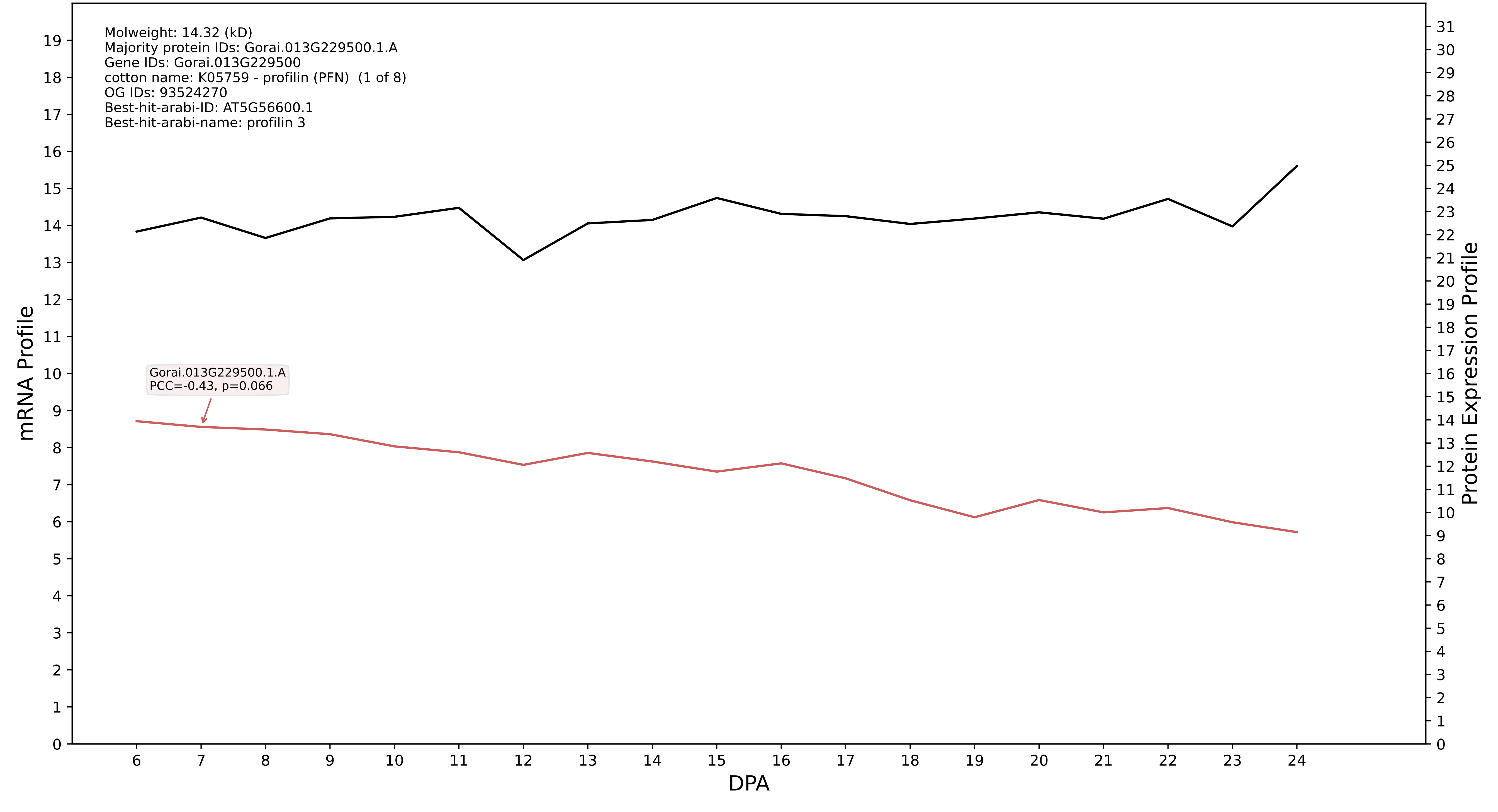

P200: Gorai.002G192900.2.D in case 1 and V25

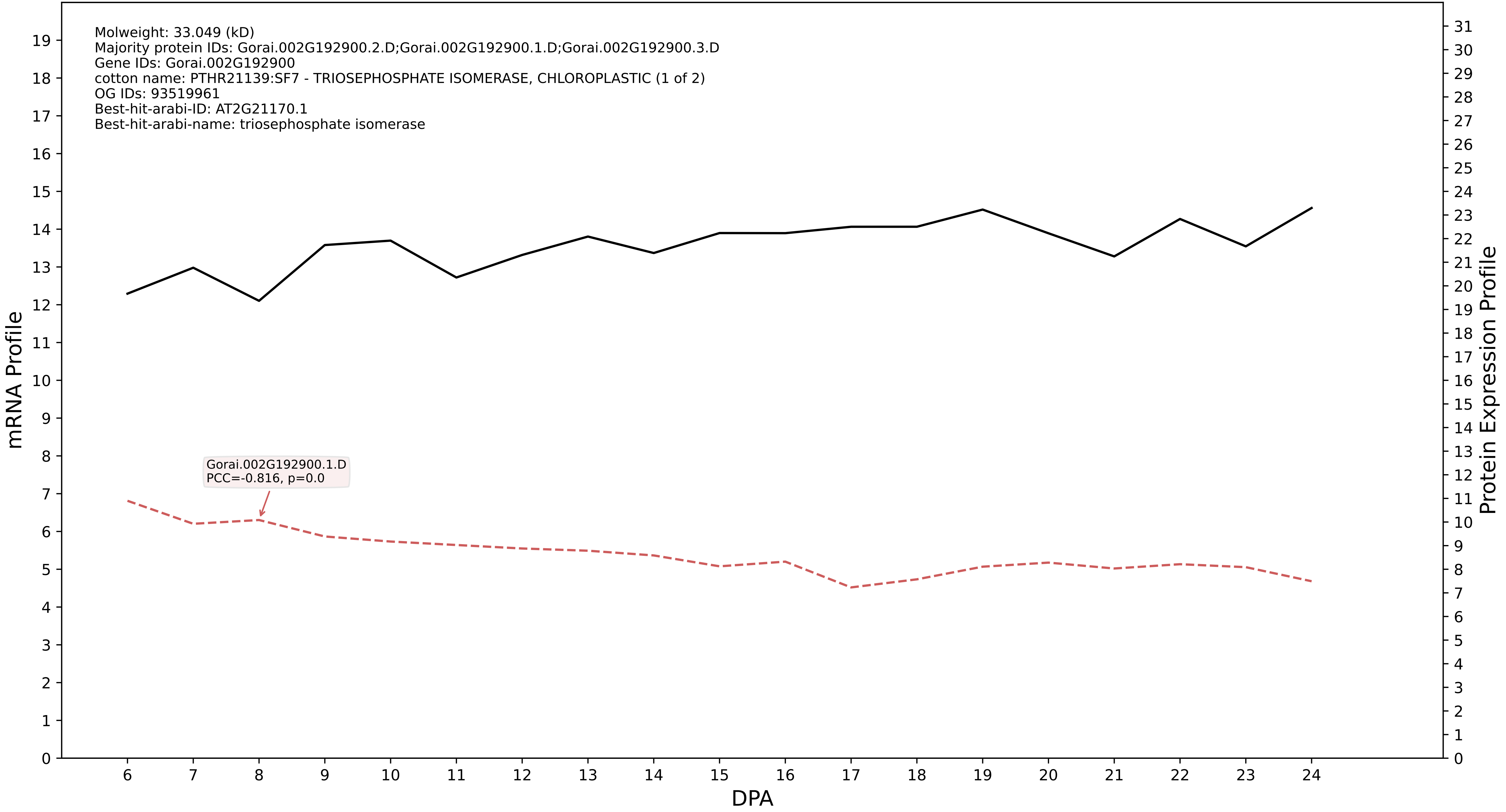

P200: Gorai.004G282400.1.A in case 1 and V25

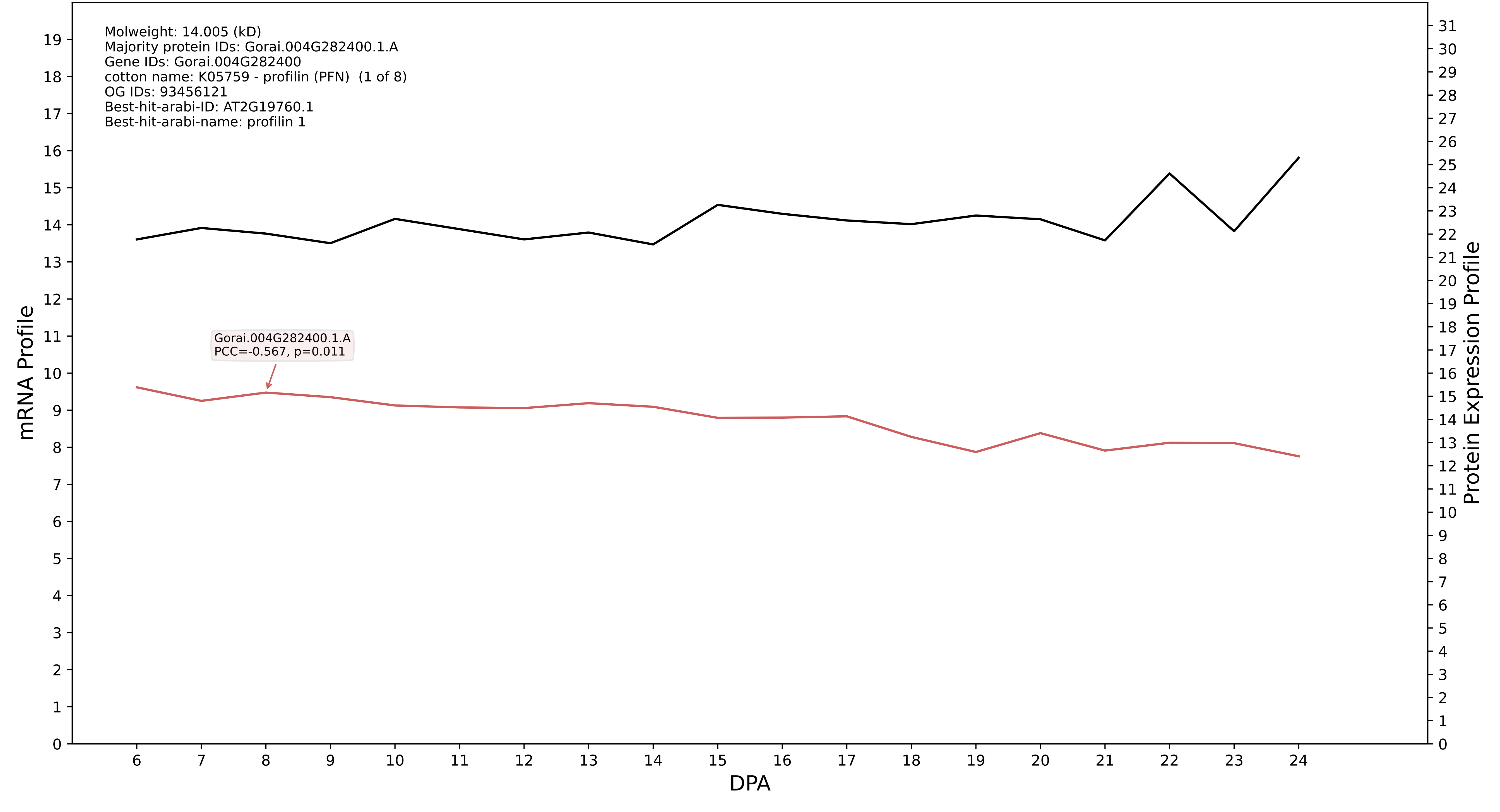

P200: Gorai.011G261700.1.D in case 1 and V25

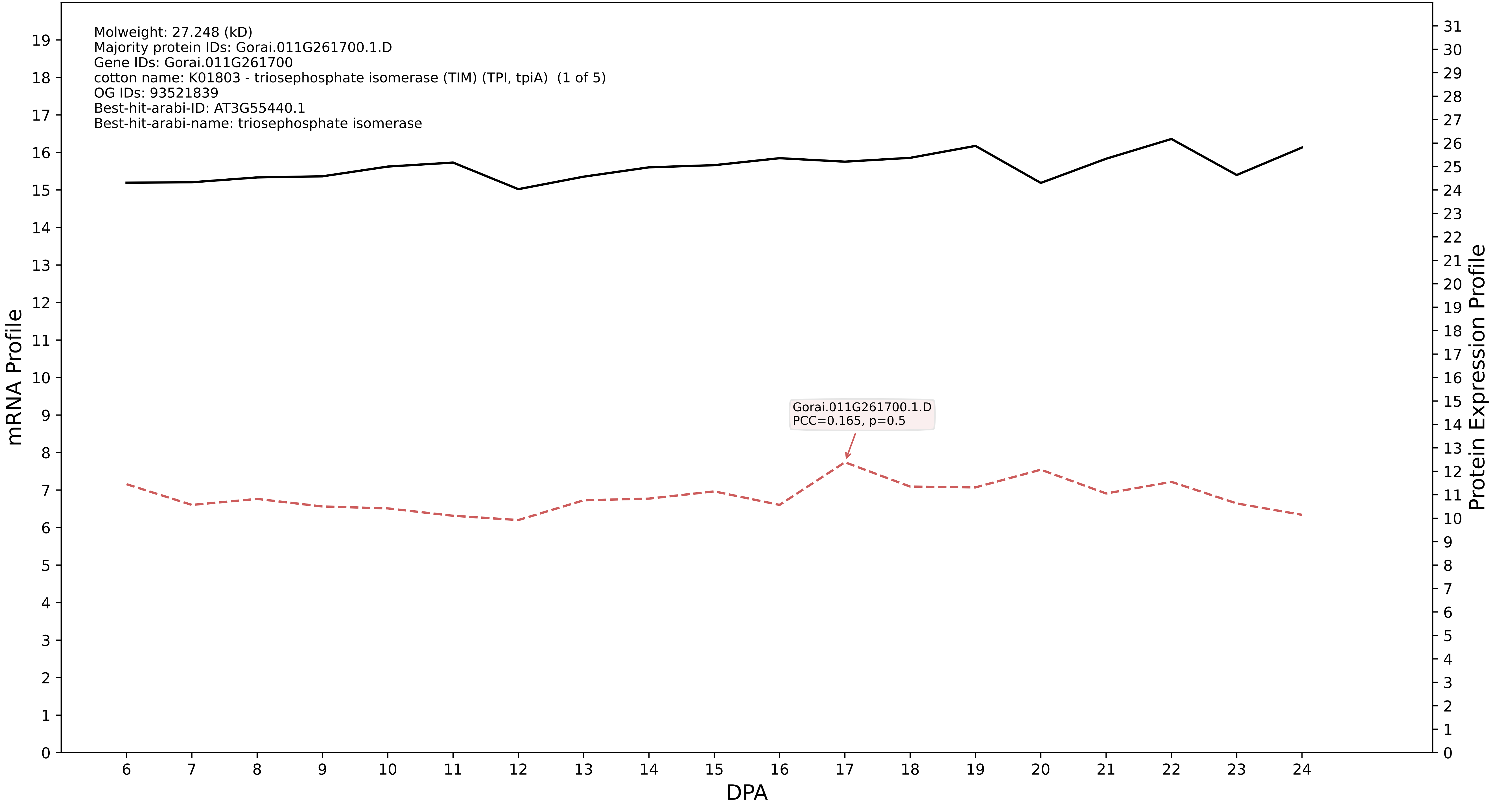

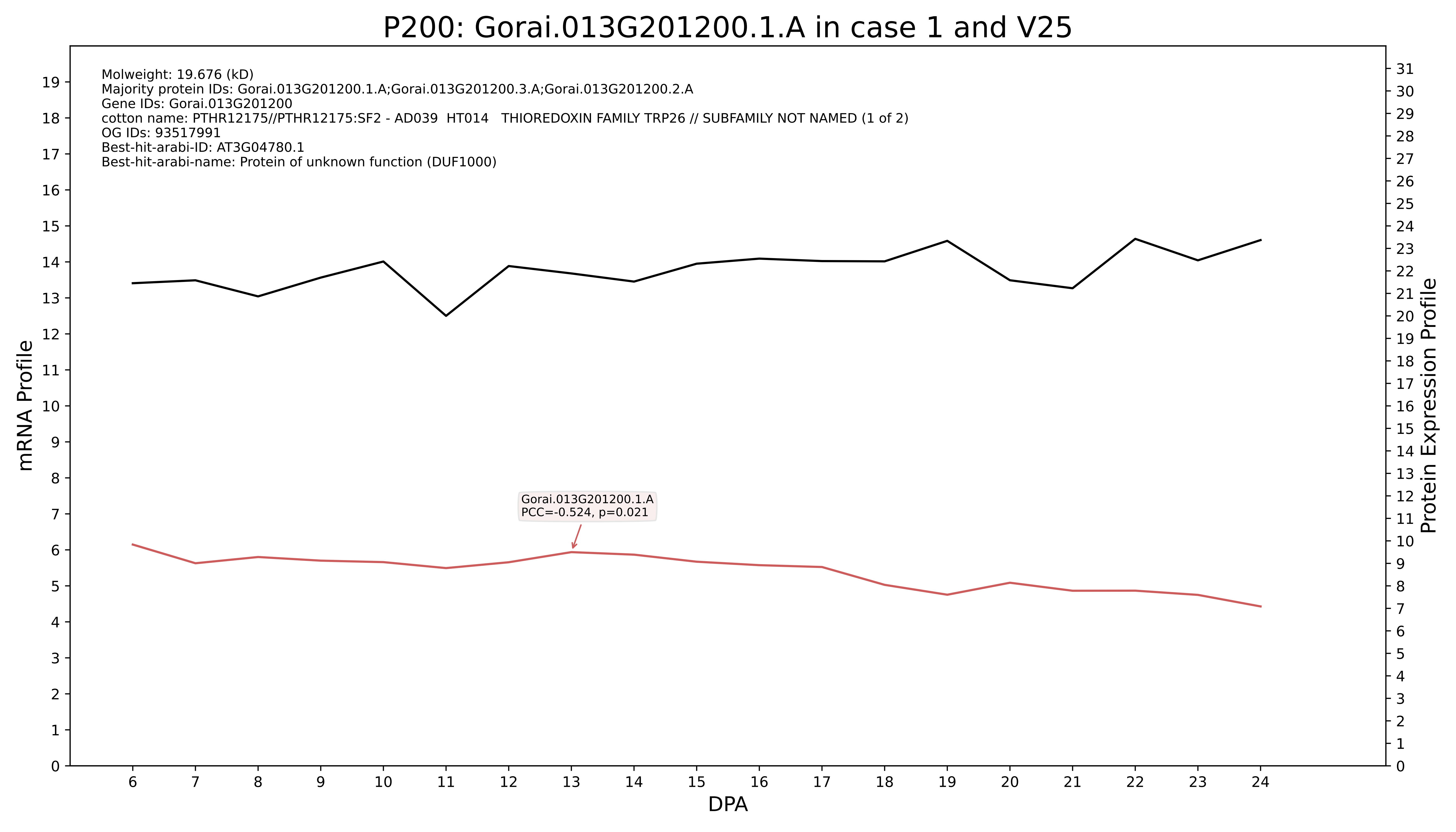
P200: Gorai.013G201200.1.A in case 1 and V25
Molweight: 19.676 (kD)
Majority protein IDs: Gorai.013G201200.1.A;Gorai.013G201200.3.A;Gorai.013G201200.2.A
Gene IDs: Gorai.013G201200
cotton name: PTHR12175//PTHR12175:SF2 - AD039 HT014 THIOREDOXIN FAMILY TRP26 // SUBFAMILY NOT NAMED (1 of 2)
OG IDs: 93517991
Best-hit-arabi-ID: AT3G04780.1
Best-hit-arabi-name: Protein of unknown function (DUF1000)
Gorai.013G201200.1.A
PCC=-0.524, p=0.021
mRNA Profile
Protein Expression Profile
DPA

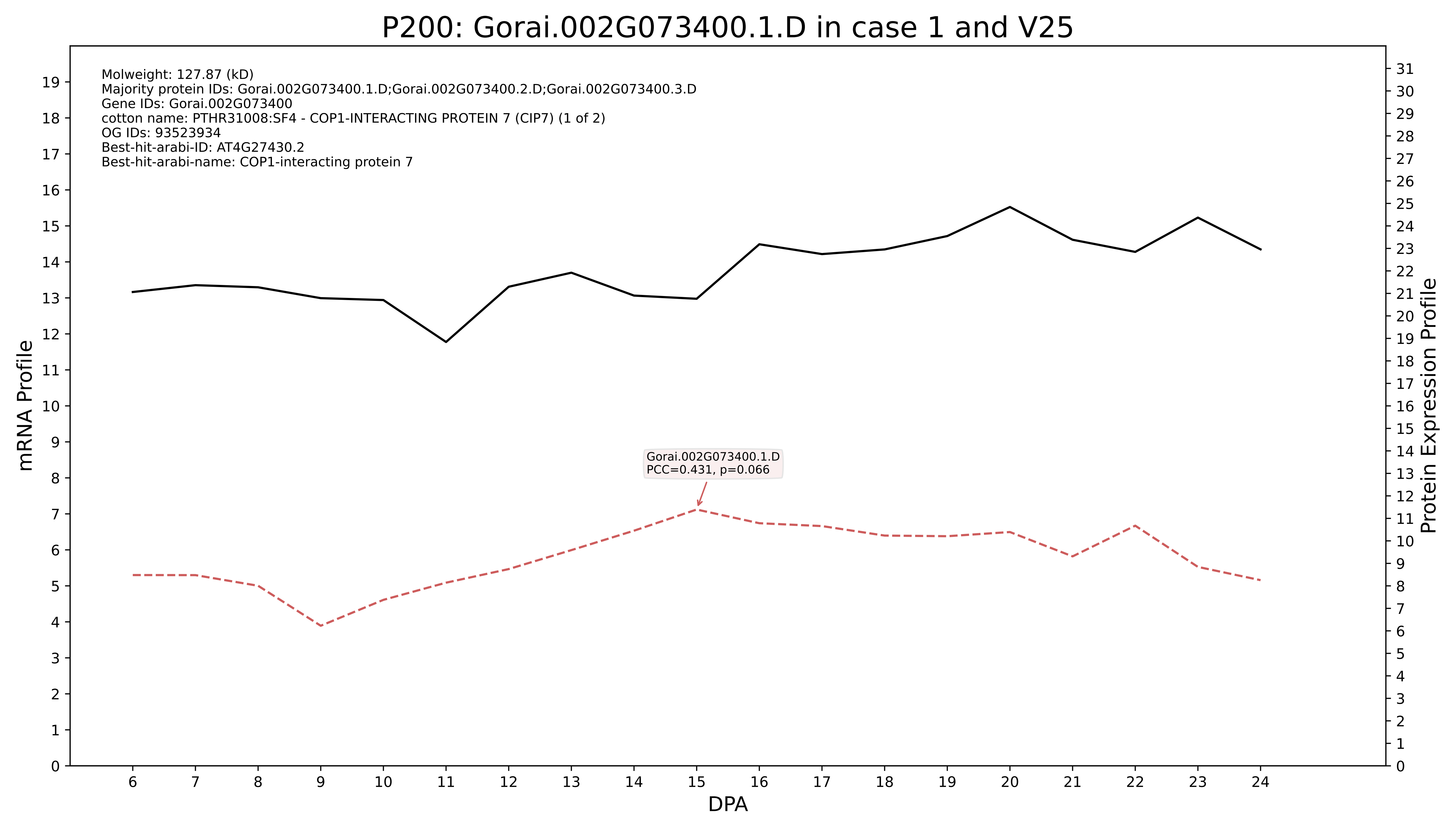
P200: Gorai.002G073400.1.D in case 1 and V25
Molweight: 127.87 (kD)
Majority protein IDs: Gorai.002G073400.1.D;Gorai.002G073400.2.D;Gorai.002G073400.3.D
Gene IDs: Gorai.002G073400
cotton name: PTHR31008:SF4 - COP1-INTERACTING PROTEIN 7 (CIP7) (1 of 2)
OG IDs: 93523934
Best-hit-arabi-ID: AT4G27430.2
Best-hit-arabi-name: COP1-interacting protein 7
Gorai.002G073400.1.D
PCC=0.431, p=0.066
mRNA Profile
Protein Expression Profile
DPA

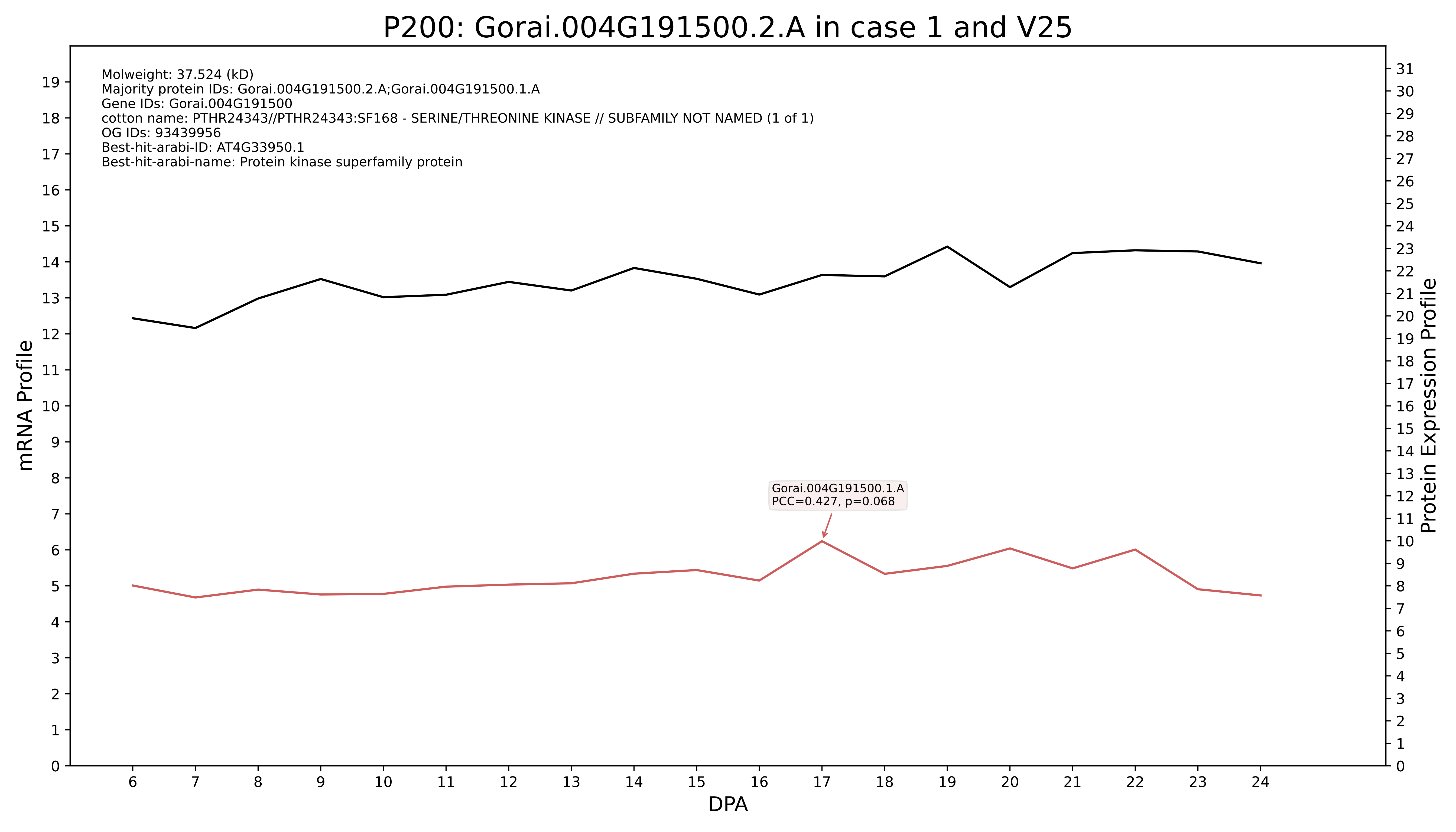
P200: Gorai.004G191500.2.A in case 1 and V25
Molweight: 37.524 (kD)
Majority protein IDs: Gorai.004G191500.2.A;Gorai.004G191500.1.A
Gene IDs: Gorai.004G191500
cotton name: PTHR24343//PTHR24343:SF168 - SERINE/THREONINE KINASE // SUBFAMILY NOT NAMED (1 of 1)
OG IDs: 93439956
Best-hit-arabi-ID: AT4G33950.1
Best-hit-arabi-name: Protein kinase superfamily protein
Gorai.004G191500.1.A
PCC=0.427, p=0.068
mRNA Profile
Protein Expression Profile
DPA

P200: Gorai.003G053100.2.D in case 1 and V25

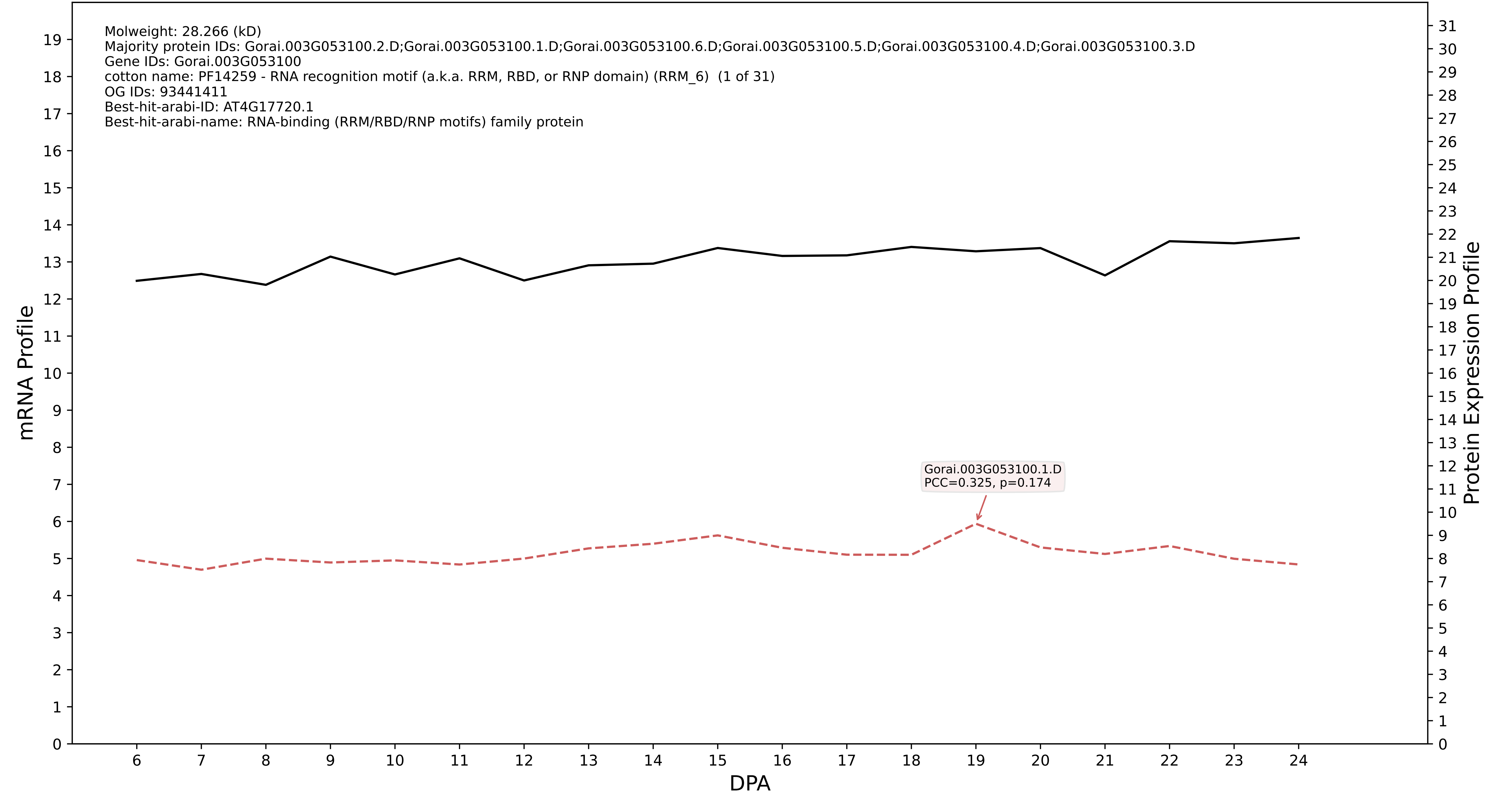

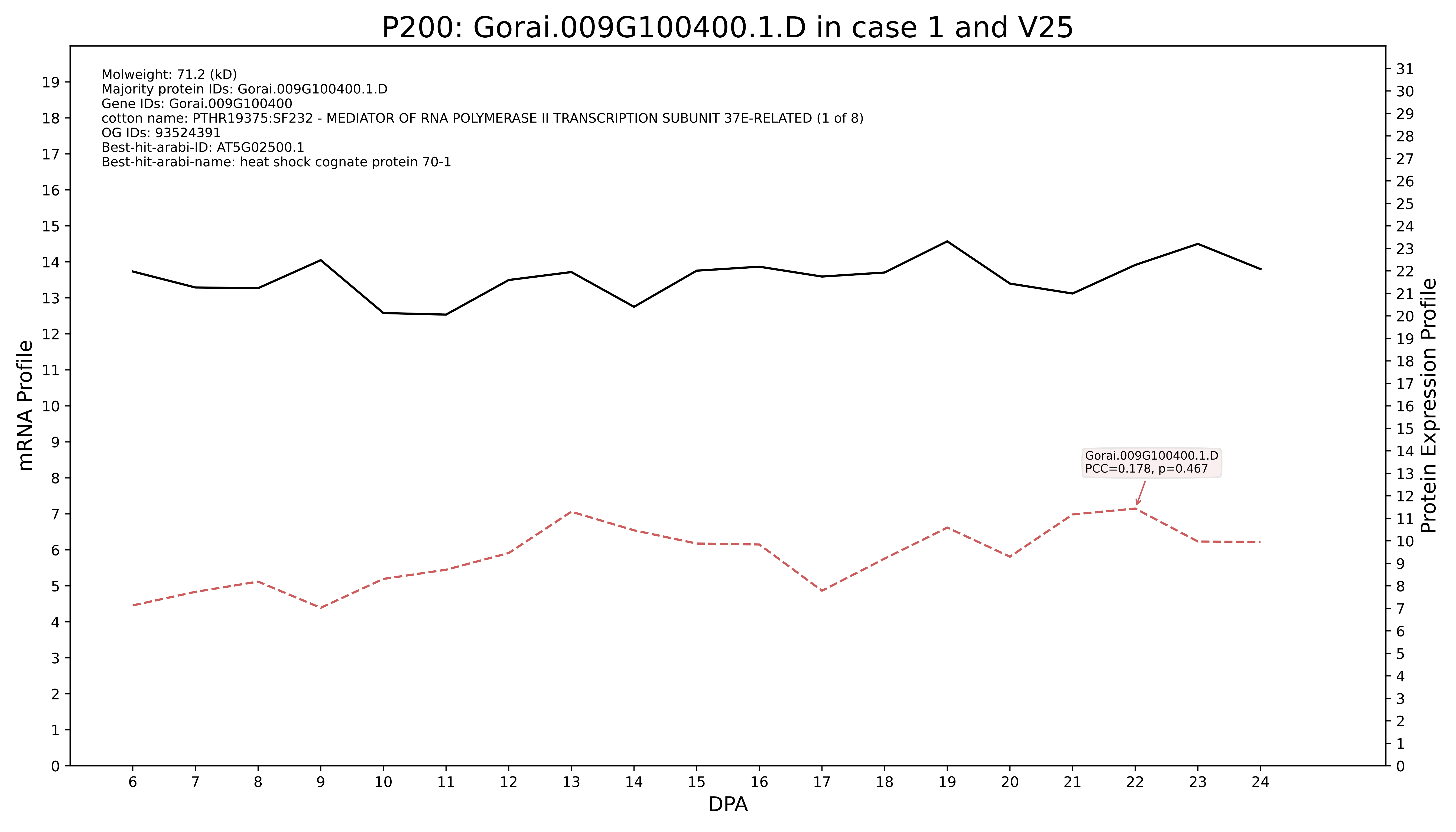

P200: Gorai.009G100400.1.D in case 1 and V25
Molweight: 71.2 (kD)
Majority protein IDs: Gorai.009G100400.1.D
Gene IDs: Gorai.009G100400
cotton name: PTHR19375:SF232 - MEDIATOR OF RNA POLYMERASE II TRANSCRIPTION SUBUNIT 37E-RELATED (1 of 8)
OG IDs: 93524391
Best-hit-arabi-ID: AT5G02500.1
Best-hit-arabi-name: heat shock cognate protein 70-1
Gorai.009G100400.1.D
PCC=0.178, p=0.467
mRNA Profile
Protein Expression Profile
DPA

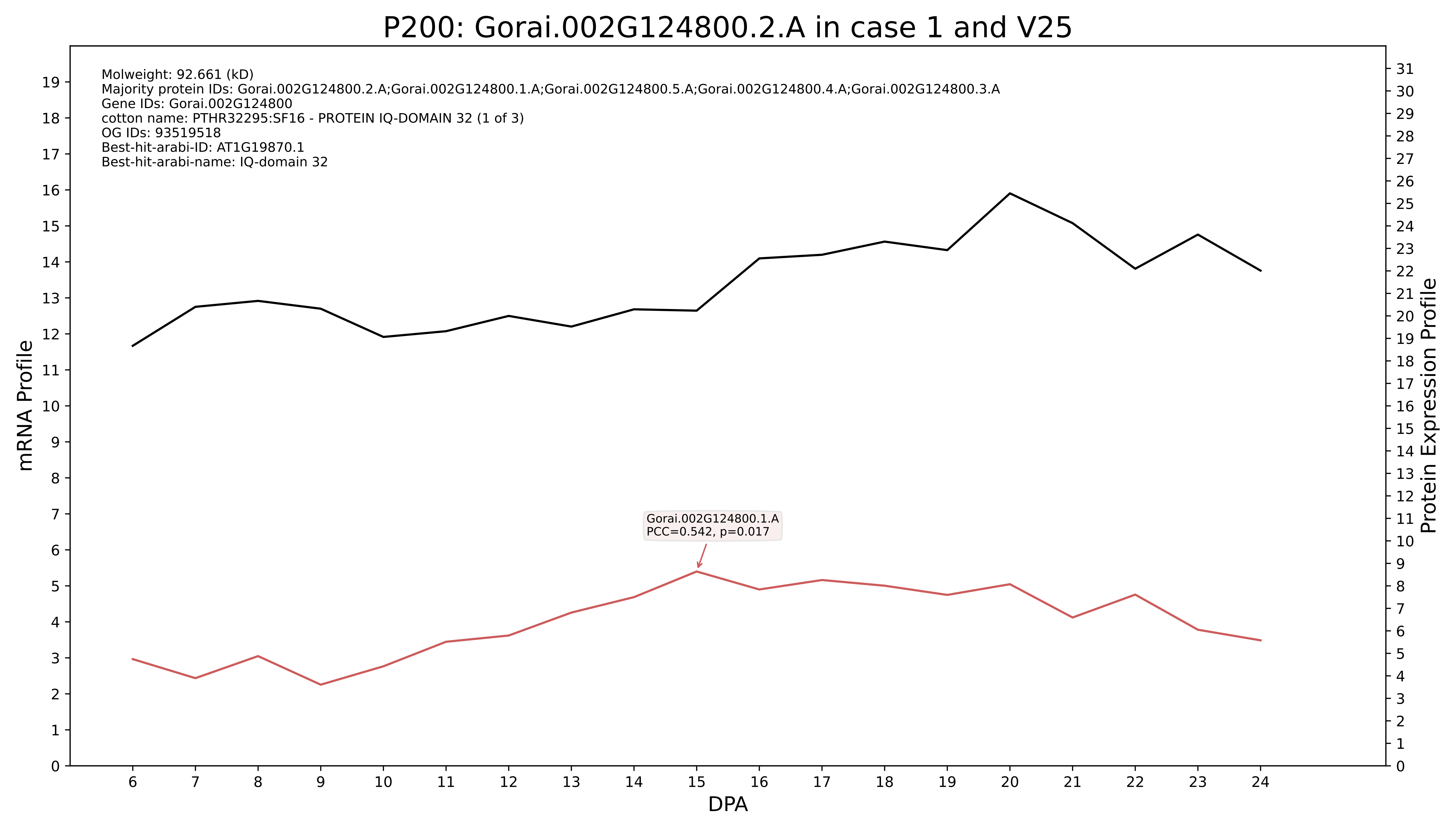
P200: Gorai.002G124800.2.A in case 1 and V25
Molweight: 92.661 (kD)
Majority protein IDs: Gorai.002G124800.2.A;Gorai.002G124800.1.A;Gorai.002G124800.5.A;Gorai.002G124800.4.A;Gorai.002G124800.3.A
Gene IDs: Gorai.002G124800
cotton name: PTHR32295:SF16 - PROTEIN IQ-DOMAIN 32 (1 of 3)
OG IDs: 93519518
Best-hit-arabi-ID: AT1G19870.1
Best-hit-arabi-name: IQ-domain 32
Gorai.002G124800.1.A
PCC=0.542, p=0.017
mRNA Profile
Protein Expression Profile
DPA

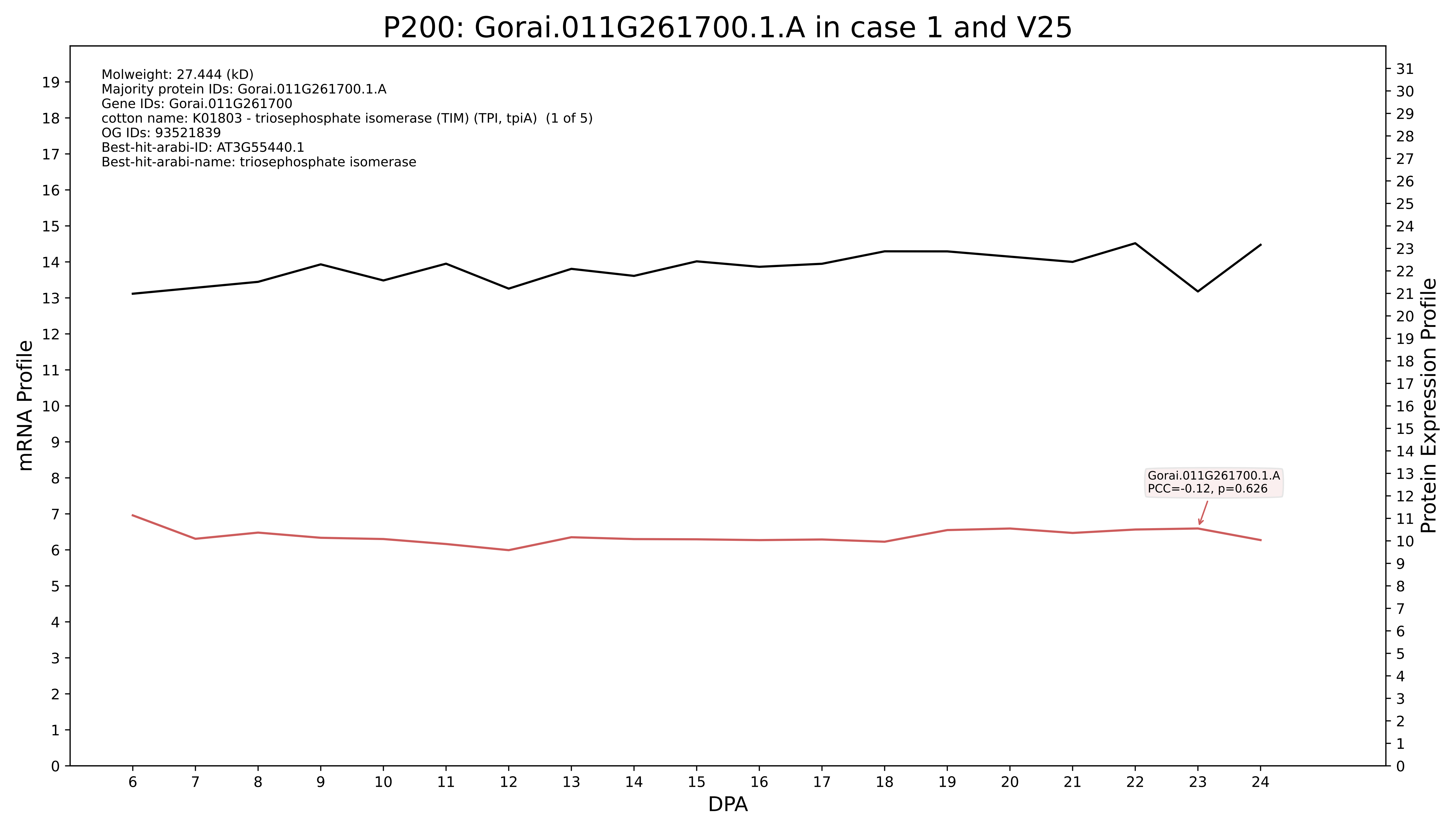
P200: Gorai.011G261700.1.A in case 1 and V25
Molweight: 27.444 (kD)
Majority protein IDs: Gorai.011G261700.1.A
Gene IDs: Gorai.011G261700
cotton name: K01803 - triosephosphate isomerase (TIM) (TPI, tpiA) (1 of 5)
OG IDs: 93521839
Best-hit-arabi-ID: AT3G55440.1
Best-hit-arabi-name: triosephosphate isomerase
Gorai.011G261700.1.A
PCC=-0.12, p=0.626
mRNA Profile
Protein Expression Profile
DPA

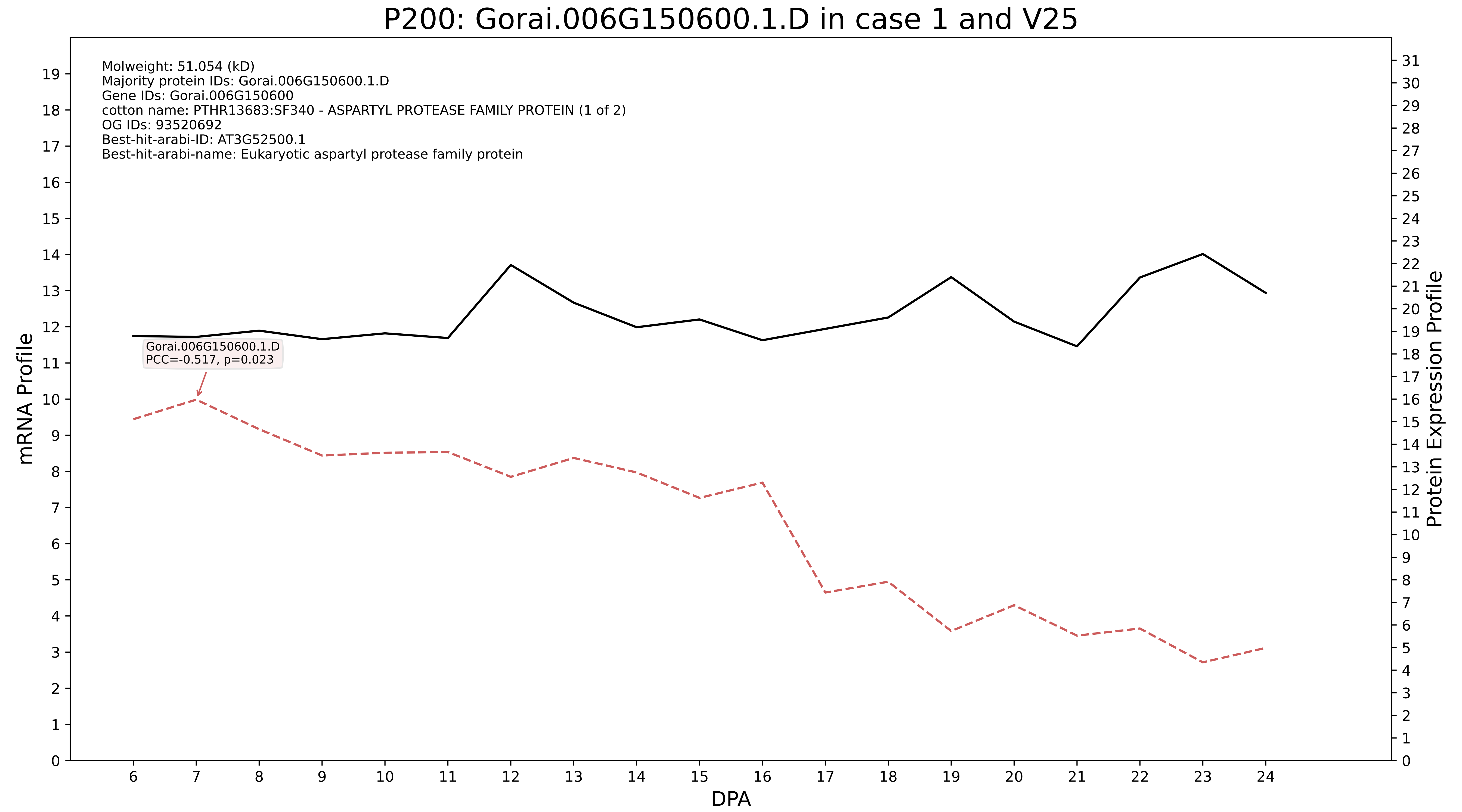

P200: Gorai.006G150600.1.D in case 1 and V25
Molweight: 51.054 (kD)
Majority protein IDs: Gorai.006G150600.1.D
Gene IDs: Gorai.006G150600
cotton name: PTHR13683:SF340 - ASPARTYL PROTEASE FAMILY PROTEIN (1 of 2)
OG IDs: 93520692
Best-hit-arabi-ID: AT3G52500.1
Best-hit-arabi-name: Eukaryotic aspartyl protease family protein
Gorai.006G150600.1.D
PCC=-0.517, p=0.023
mRNA Profile
Protein Expression Profile
DPA

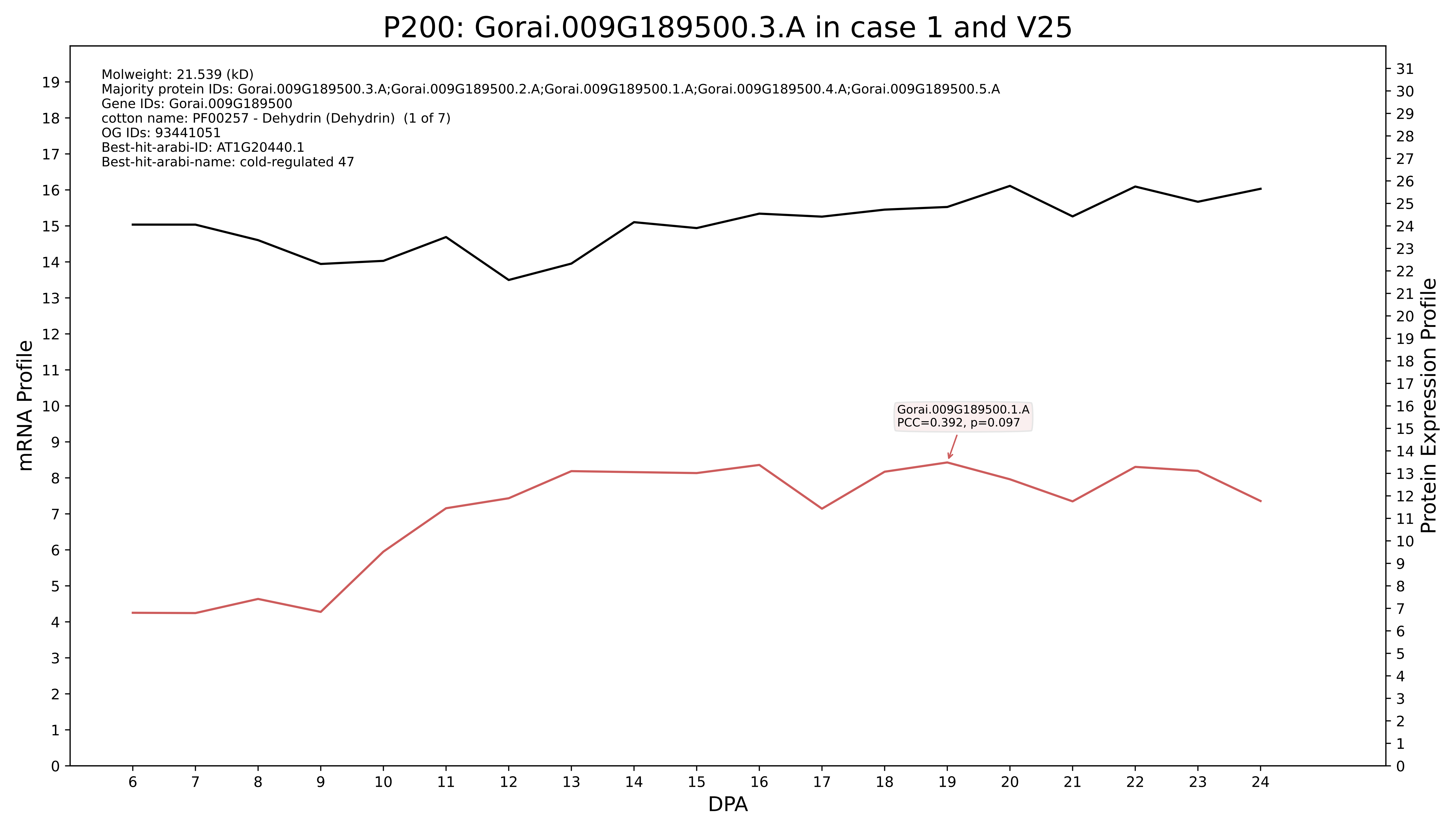
P200: Gorai.009G189500.3.A in case 1 and V25
Molweight: 21.539 (kD)
Majority protein IDs: Gorai.009G189500.3.A;Gorai.009G189500.2.A;Gorai.009G189500.1.A;Gorai.009G189500.4.A;Gorai.009G189500.5.A
Gene IDs: Gorai.009G189500
cotton name: PF00257 - Dehydrin (Dehydrin) (1 of 7)
OG IDs: 93441051
Best-hit-arabi-ID: AT1G20440.1
Best-hit-arabi-name: cold-regulated 47
Gorai.009G189500.1.A
PCC=0.392, p=0.097
mRNA Profile
Protein Expression Profile
DPA

P200: Gorai.008G217100.1.D in case 1 and V25

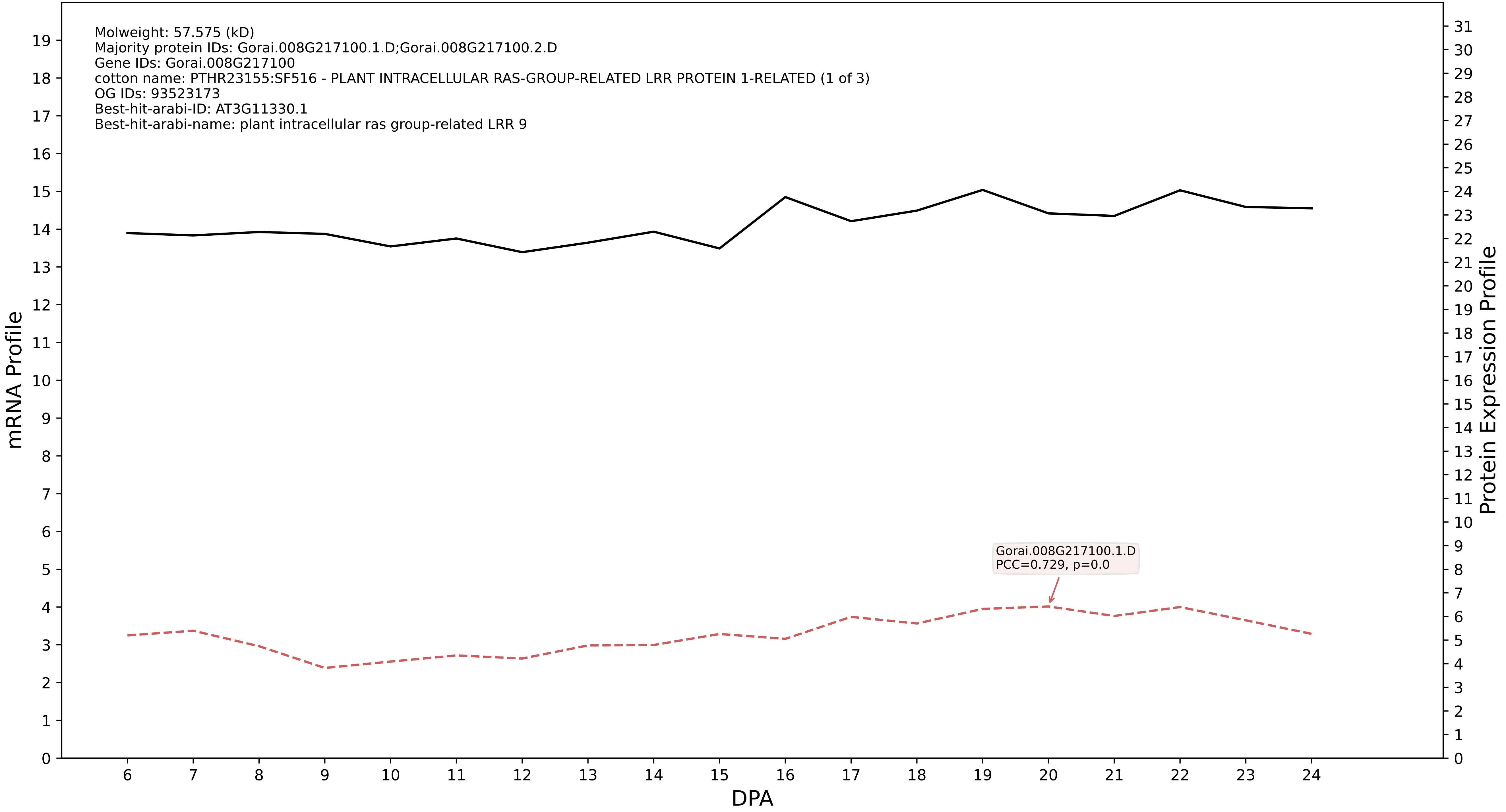

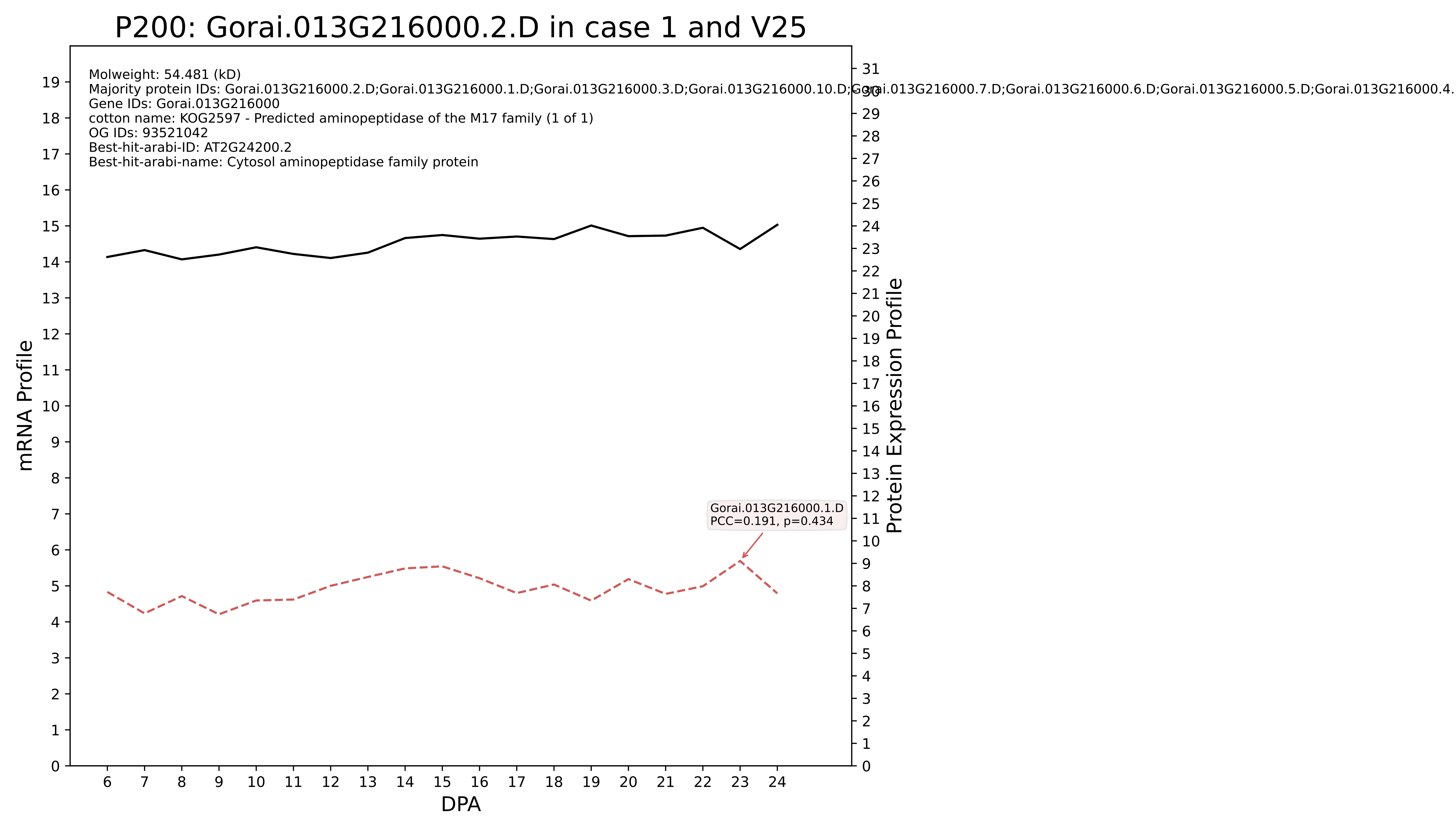
P200: Gorai.013G216000.2.D in case 1 and V25
Molweight: 54.481 (kD)
Majority protein IDs: Gorai.013G216000.2.D;Gorai.013G216000.1.D;Gorai.013G216000.3.D;Gorai.013G216000.10.D;Gorai.013G216000.7.D;Gorai.013G216000.6.D;Gorai.013G216000.5.D;Gorai.013G216000.4.
Gene IDs: Gorai.013G216000
cotton name: KOG2597 - Predicted aminopeptidase of the M17 family (1 of 1)
OG IDs: 93521042
Best-hit-arabi-ID: AT2G24200.2
Best-hit-arabi-name: Cytosol aminopeptidase family protein
Gorai.013G216000.1.D
PCC=0.191, p=0.434
mRNA Profile
Protein Expression Profile
DPA

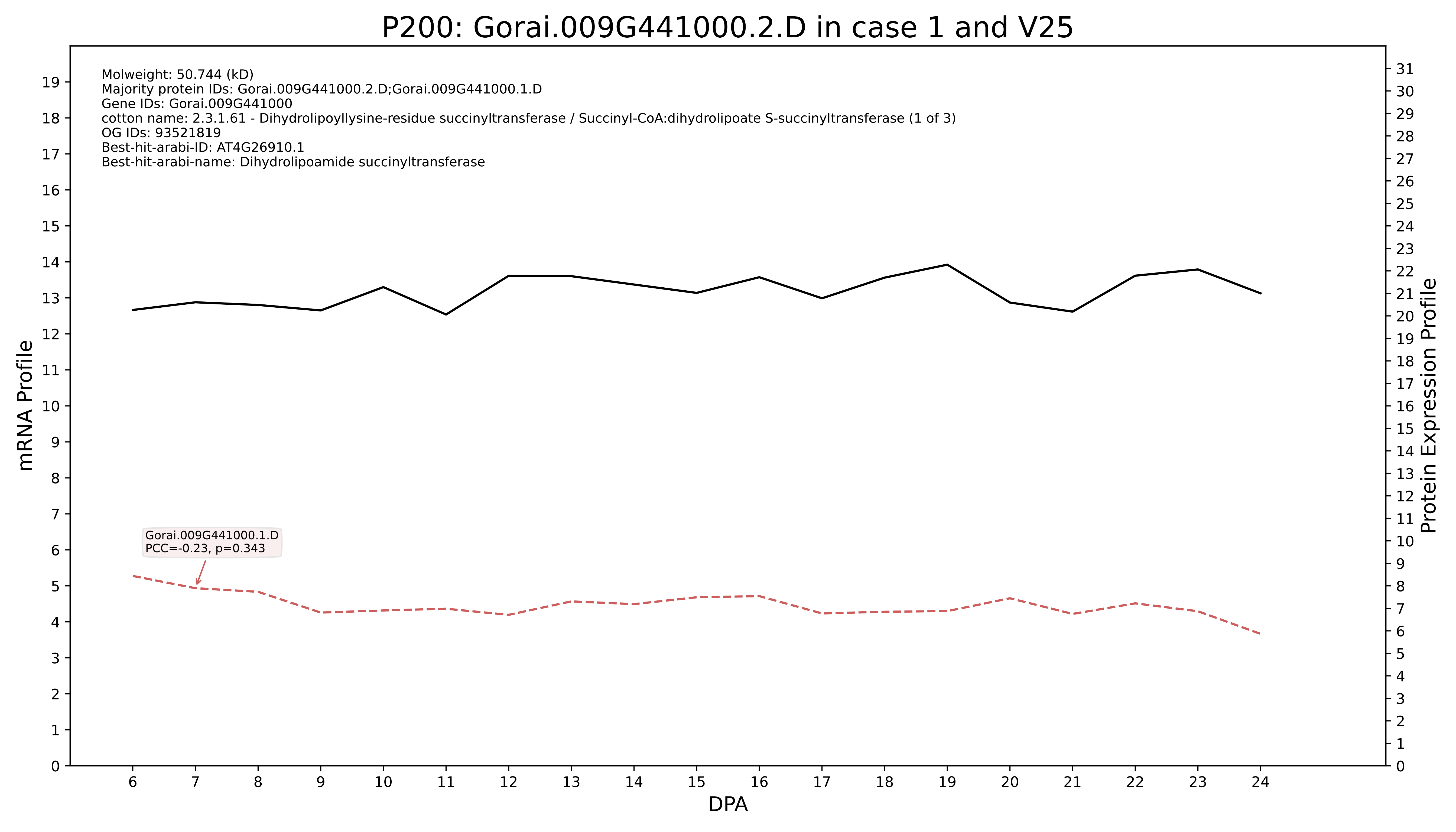

P200: Gorai.009G441000.2.D in case 1 and V25
Molweight: 50.744 (kD)
Majority protein IDs: Gorai.009G441000.2.D;Gorai.009G441000.1.D
Gene IDs: Gorai.009G441000
cotton name: 2.3.1.61 - Dihydrolipoyllysine-residue succinyltransferase / Succinyl-CoA:dihydrolipoate S-succinyltransferase (1 of 3)
OG IDs: 93521819
Best-hit-arabi-ID: AT4G26910.1
Best-hit-arabi-name: Dihydrolipoamide succinyltransferase
Gorai.009G441000.1.D
PCC=-0.23, p=0.343
mRNA Profile
Protein Expression Profile
DPA

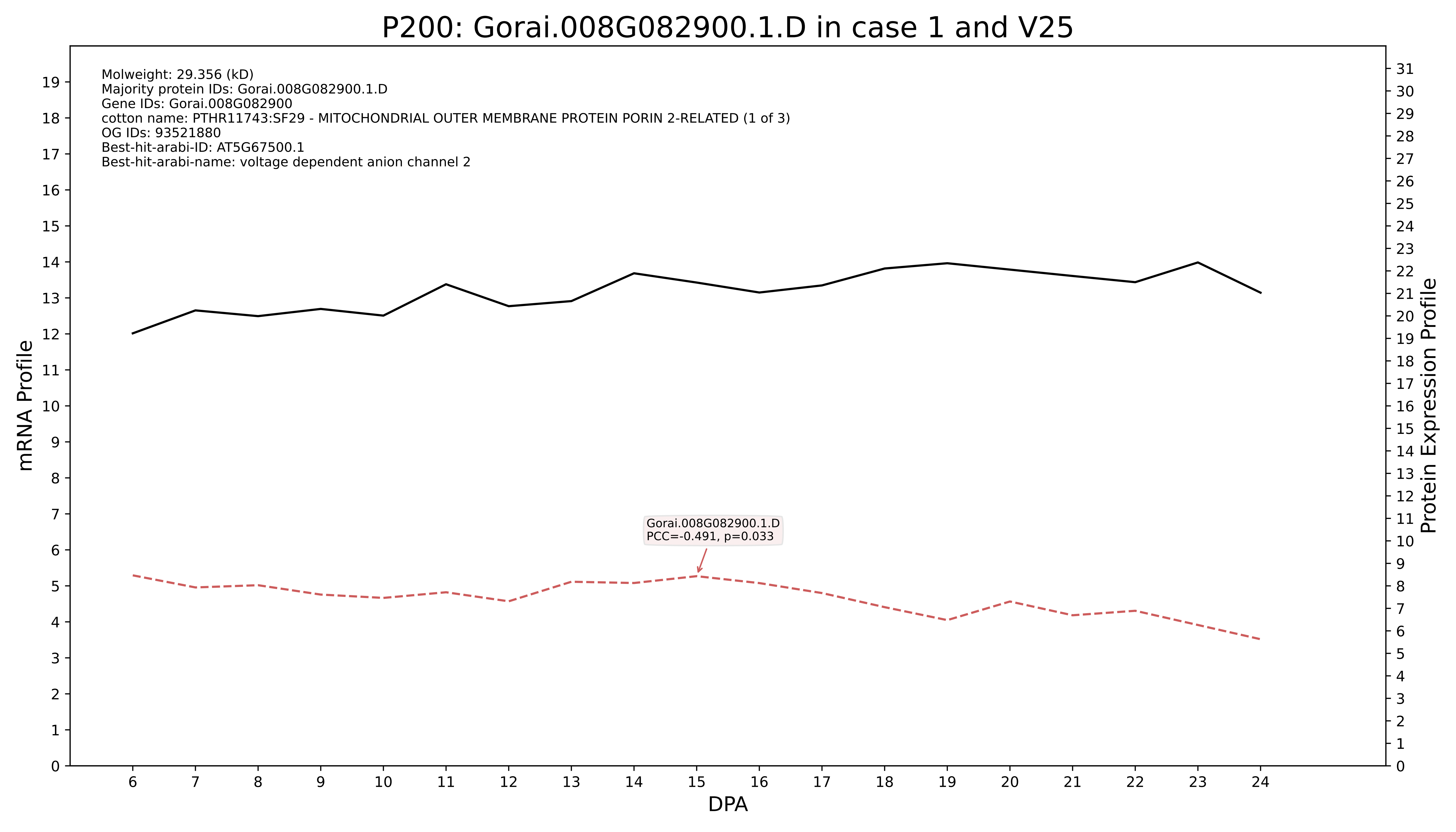
P200: Gorai.008G082900.1.D in case 1 and V25
Molweight: 29.356 (kD)
Majority protein IDs: Gorai.008G082900.1.D
Gene IDs: Gorai.008G082900
cotton name: PTHR11743:SF29 - MITOCHONDRIAL OUTER MEMBRANE PROTEIN PORIN 2-RELATED (1 of 3)
OG IDs: 93521880
Best-hit-arabi-ID: AT5G67500.1
Best-hit-arabi-name: voltage dependent anion channel 2
Gorai.008G082900.1.D
PCC=-0.491, p=0.033
mRNA Profile
Protein Expression Profile
DPA

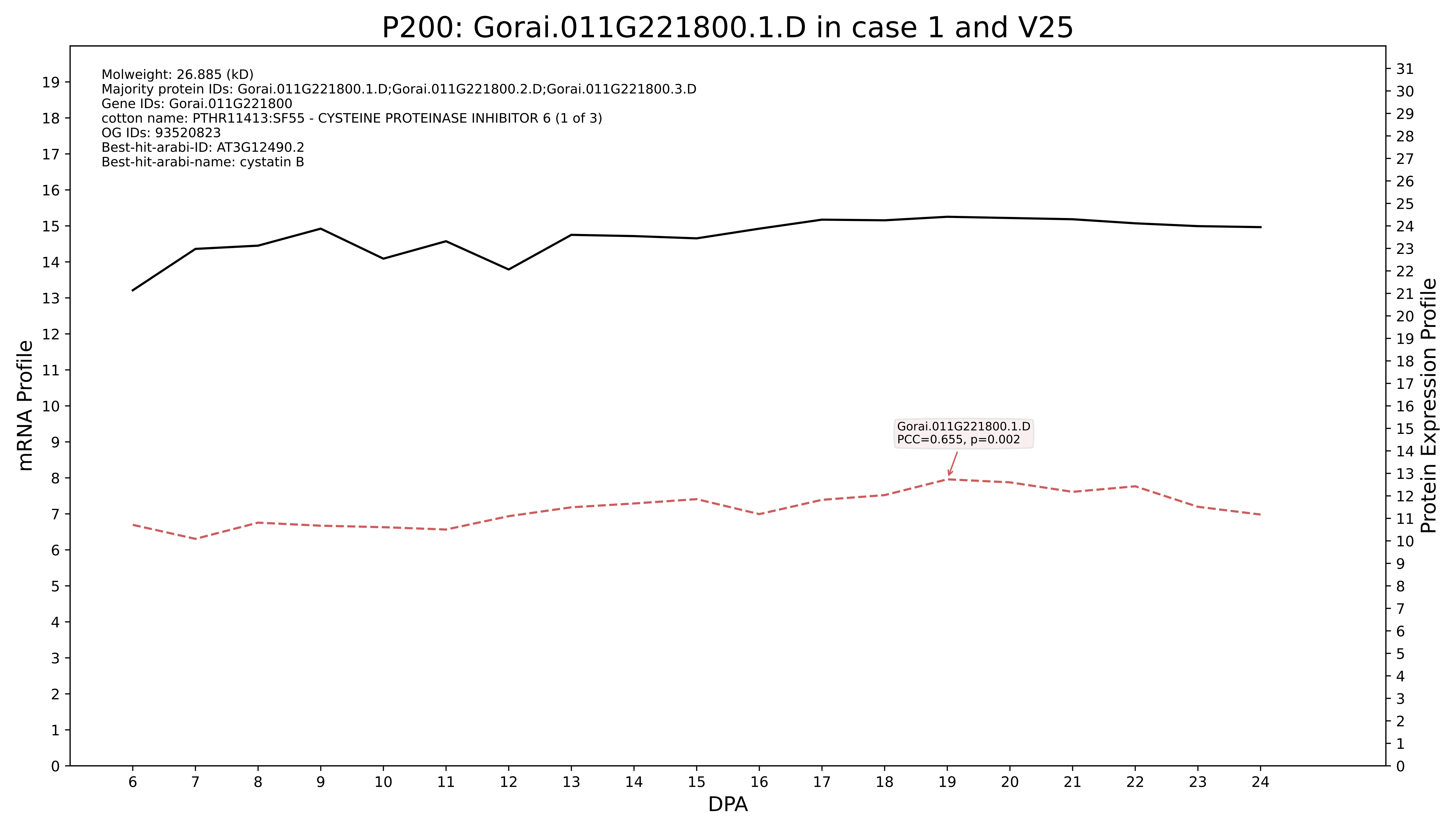
P200: Gorai.011G221800.1.D in case 1 and V25
Molweight: 26.885 (kD)
Majority protein IDs: Gorai.011G221800.1.D;Gorai.011G221800.2.D;Gorai.011G221800.3.D
Gene IDs: Gorai.011G221800
cotton name: PTHR11413:SF55 - CYSTEINE PROTEINASE INHIBITOR 6 (1 of 3)
OG IDs: 93520823
Best-hit-arabi-ID: AT3G12490.2
Best-hit-arabi-name: cystatin B
Gorai.011G221800.1.D
PCC=0.655, p=0.002
mRNA Profile
Protein Expression Profile
DPA

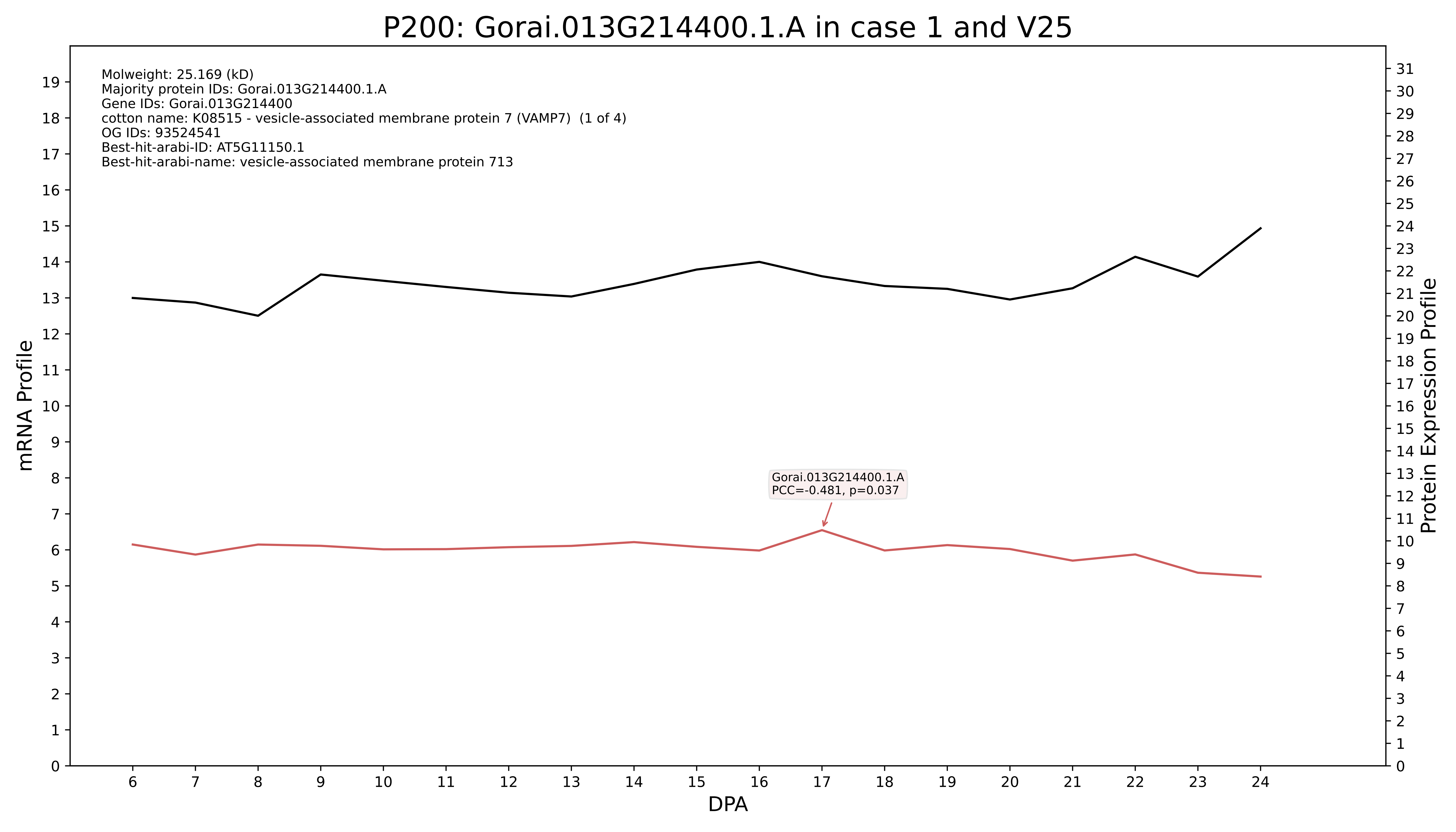
P200: Gorai.013G214400.1.A in case 1 and V25
Molweight: 25.169 (kD)
Majority protein IDs: Gorai.013G214400.1.A
Gene IDs: Gorai.013G214400
cotton name: K08515 - vesicle-associated membrane protein 7 (VAMP7) (1 of 4)
OG IDs: 93524541
Best-hit-arabi-ID: AT5G11150.1
Best-hit-arabi-name: vesicle-associated membrane protein 713
Gorai.013G214400.1.A
PCC=-0.481, p=0.037
mRNA Profile
Protein Expression Profile
DPA
0
1
2
3
4
5
6
7
8
9
10
11
12
13
14
15
16
17
18
19
20
21
22
23
24
25
26
27
28
29
30
31

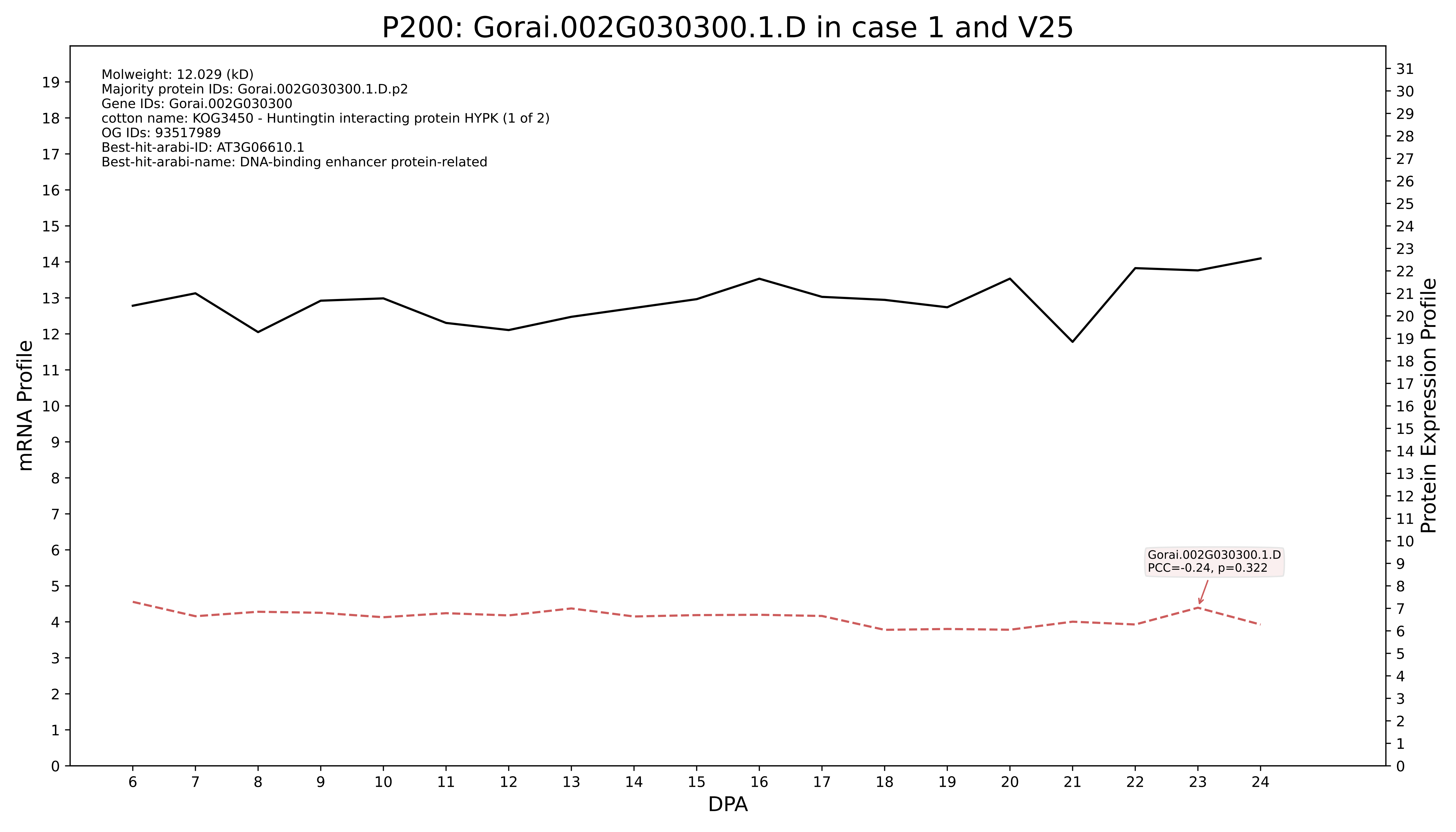

P200: Gorai.002G030300.1.D in case 1 and V25
Molweight: 12.029 (kD)
Majority protein IDs: Gorai.002G030300.1.D.p2
Gene IDs: Gorai.002G030300
cotton name: KOG3450 - Huntingtin interacting protein HYPK (1 of 2)
OG IDs: 93517989
Best-hit-arabi-ID: AT3G06610.1
Best-hit-arabi-name: DNA-binding enhancer protein-related
Gorai.002G030300.1.D
PCC=-0.24, p=0.322
mRNA Profile
Protein Expression Profile
DPA

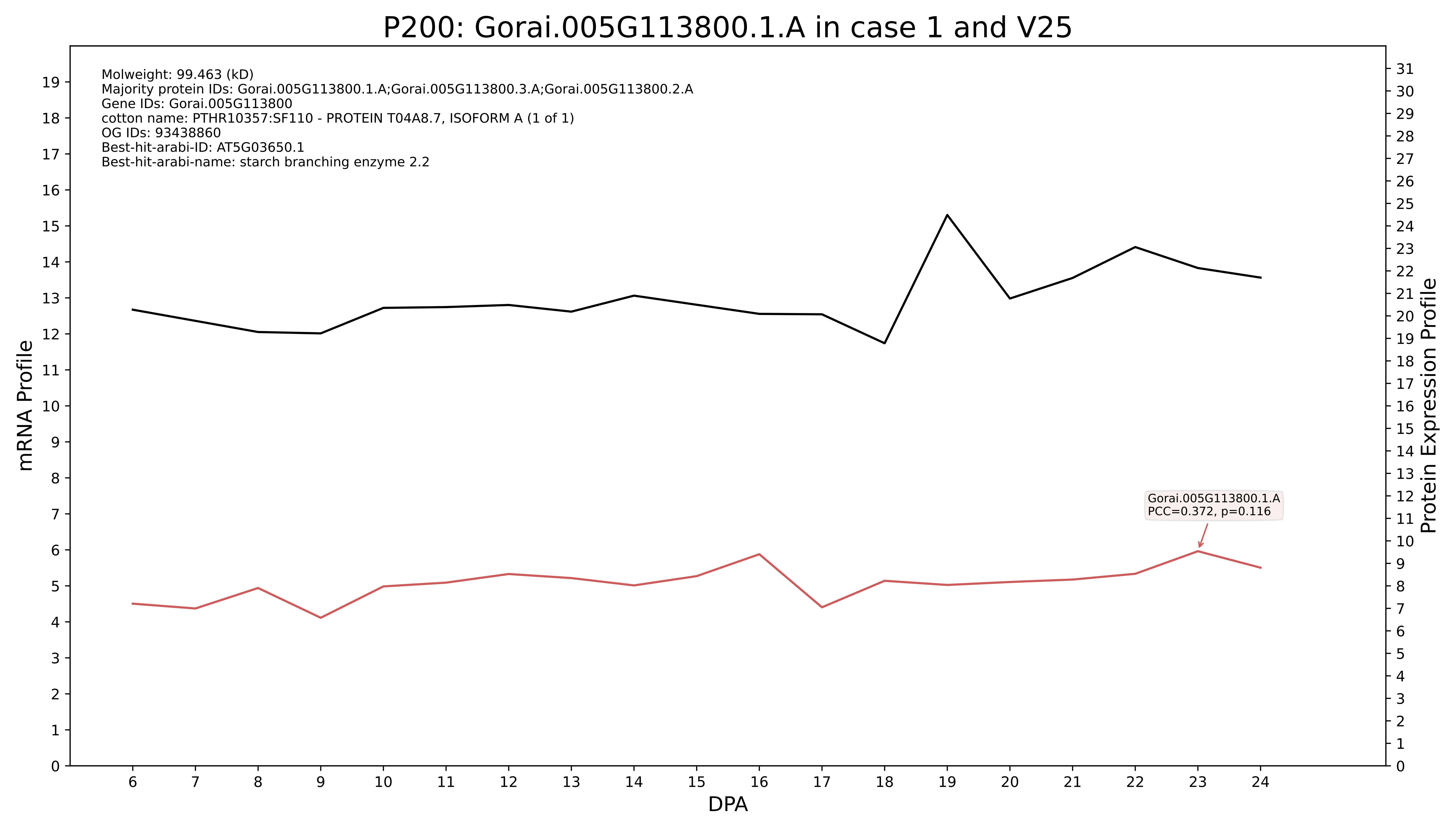

P200: Gorai.005G113800.1.A in case 1 and V25
Molweight: 99.463 (kD)
Majority protein IDs: Gorai.005G113800.1.A;Gorai.005G113800.3.A;Gorai.005G113800.2.A
Gene IDs: Gorai.005G113800
cotton name: PTHR10357:SF110 - PROTEIN T04A8.7, ISOFORM A (1 of 1)
OG IDs: 93438860
Best-hit-arabi-ID: AT5G03650.1
Best-hit-arabi-name: starch branching enzyme 2.2
Gorai.005G113800.1.A
PCC=0.372, p=0.116
mRNA Profile
Protein Expression Profile
DPA

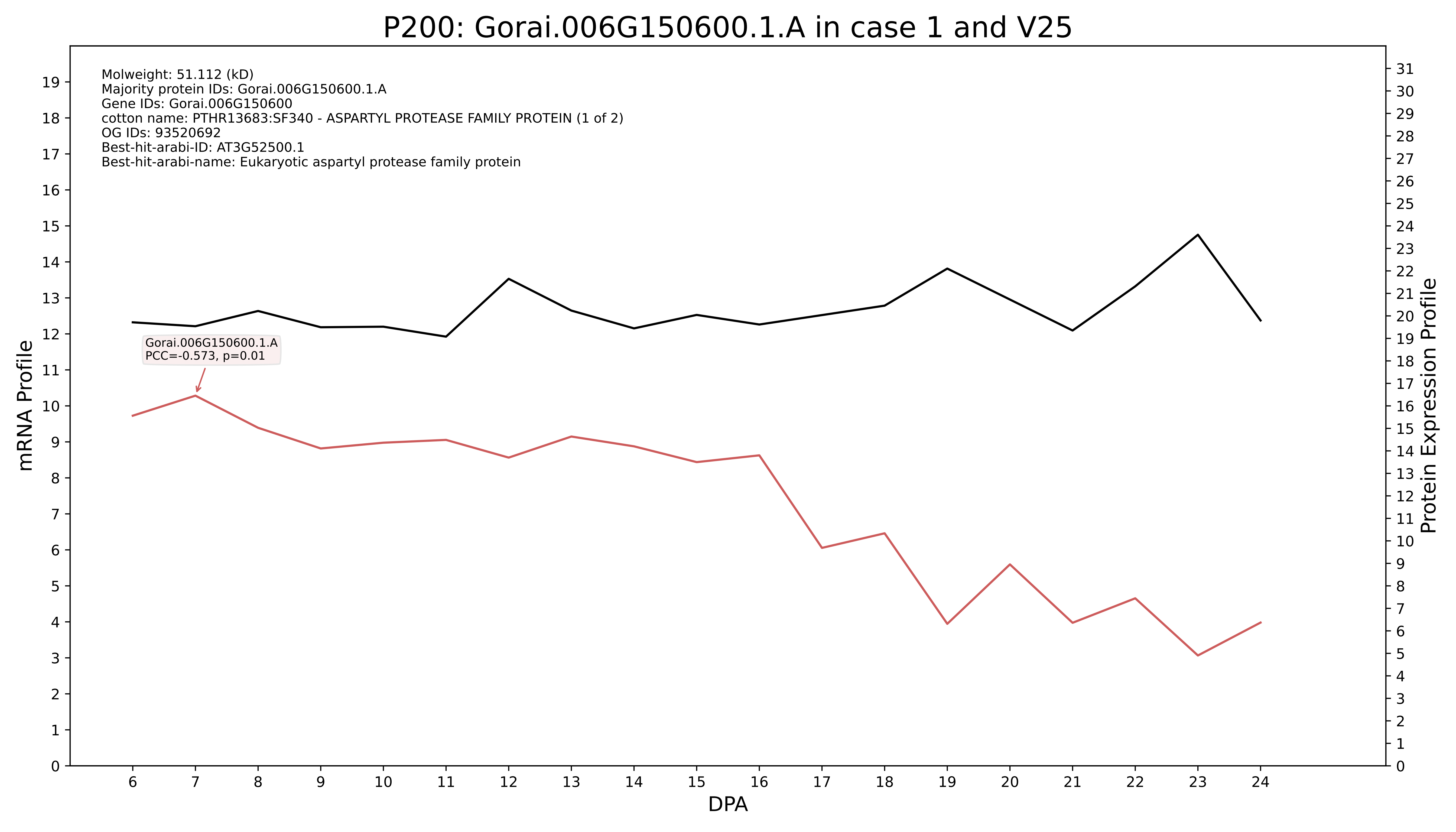
P200: Gorai.006G150600.1.A in case 1 and V25
Molweight: 51.112 (kD)
Majority protein IDs: Gorai.006G150600.1.A
Gene IDs: Gorai.006G150600
cotton name: PTHR13683:SF340 - ASPARTYL PROTEASE FAMILY PROTEIN (1 of 2)
OG IDs: 93520692
Best-hit-arabi-ID: AT3G52500.1
Best-hit-arabi-name: Eukaryotic aspartyl protease family protein
Gorai.006G150600.1.A
PCC=-0.573, p=0.01
mRNA Profile
Protein Expression Profile
DPA

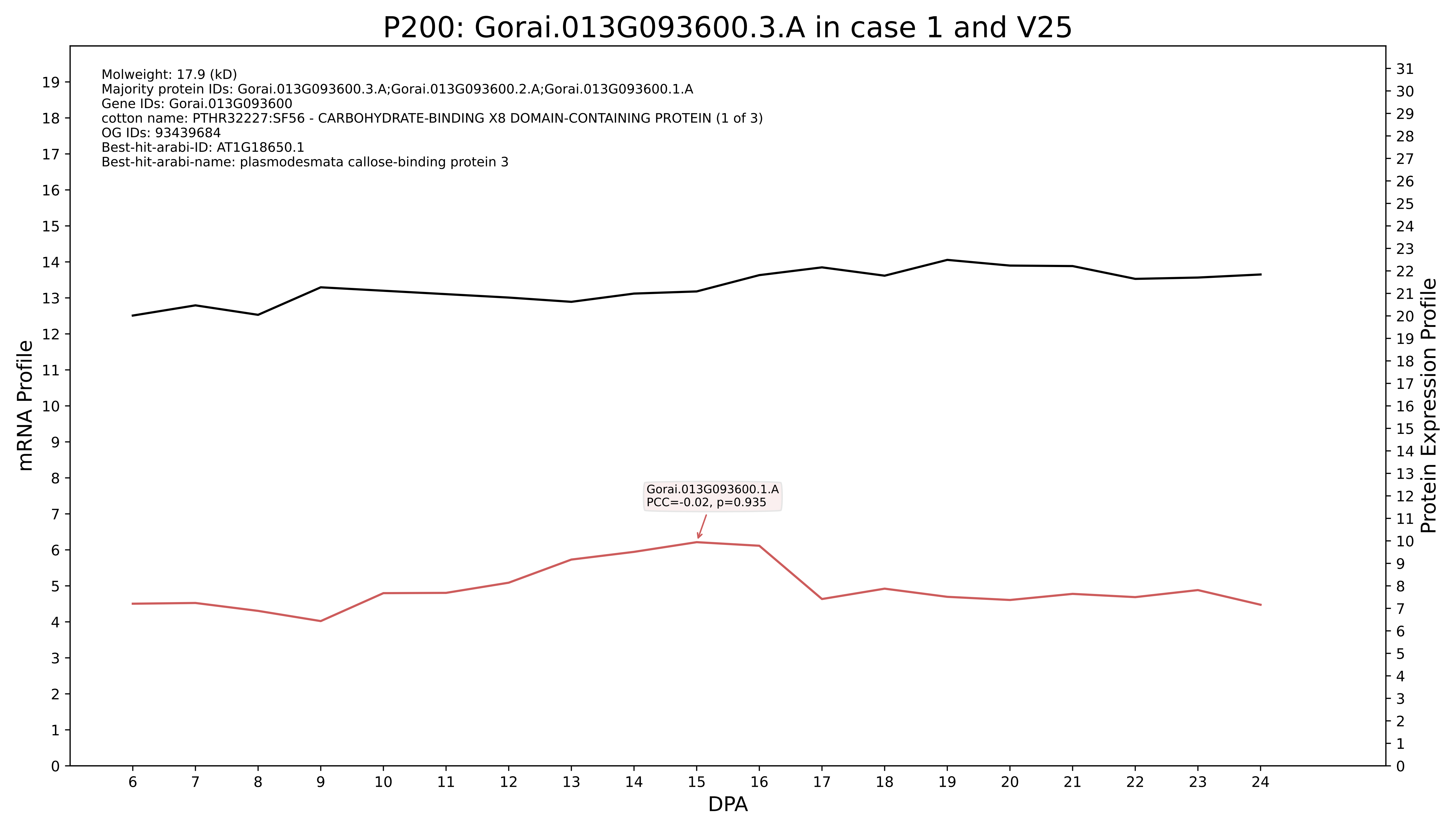
P200: Gorai.013G093600.3.A in case 1 and V25
Molweight: 17.9 (kD)
Majority protein IDs: Gorai.013G093600.3.A;Gorai.013G093600.2.A;Gorai.013G093600.1.A
Gene IDs: Gorai.013G093600
cotton name: PTHR32227:SF56 - CARBOHYDRATE-BINDING X8 DOMAIN-CONTAINING PROTEIN (1 of 3)
OG IDs: 93439684
Best-hit-arabi-ID: AT1G18650.1
Best-hit-arabi-name: plasmodesmata callose-binding protein 3
Gorai.013G093600.1.A
PCC=-0.02, p=0.935
mRNA Profile
Protein Expression Profile
DPA

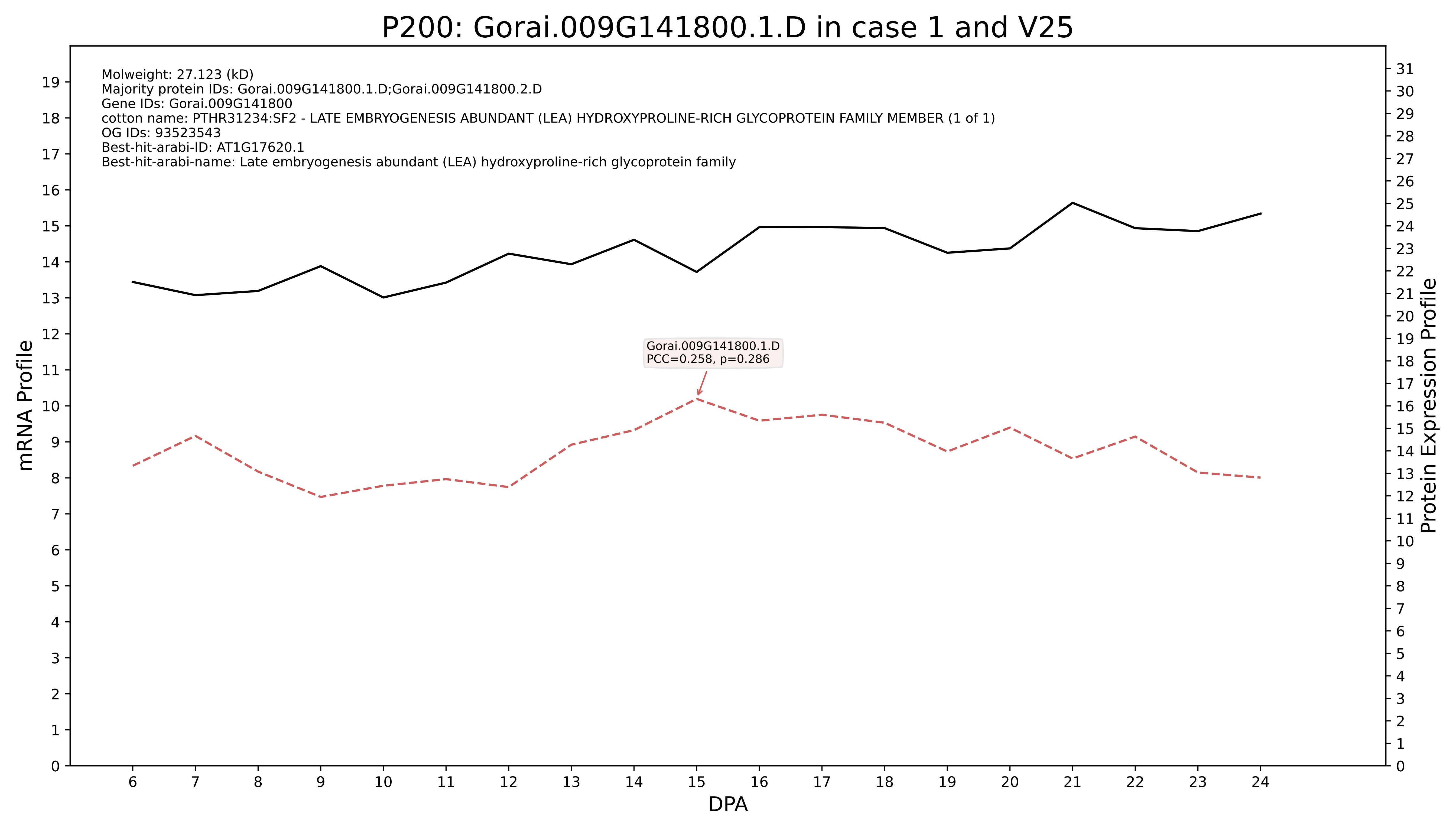
P200: Gorai.009G141800.1.D in case 1 and V25
Molweight: 27.123 (kD)
Majority protein IDs: Gorai.009G141800.1.D;Gorai.009G141800.2.D
Gene IDs: Gorai.009G141800
cotton name: PTHR31234:SF2 - LATE EMBRYOGENESIS ABUNDANT (LEA) HYDROXYPROLINE-RICH GLYCOPROTEIN FAMILY MEMBER (1 of 1)
OG IDs: 93523543
Best-hit-arabi-ID: AT1G17620.1
Best-hit-arabi-name: Late embryogenesis abundant (LEA) hydroxyproline-rich glycoprotein family
Gorai.009G141800.1.D
PCC=0.258, p=0.286
mRNA Profile
Protein Expression Profile
DPA

P200: Gorai.009G175900.1.D in case 1 and V25

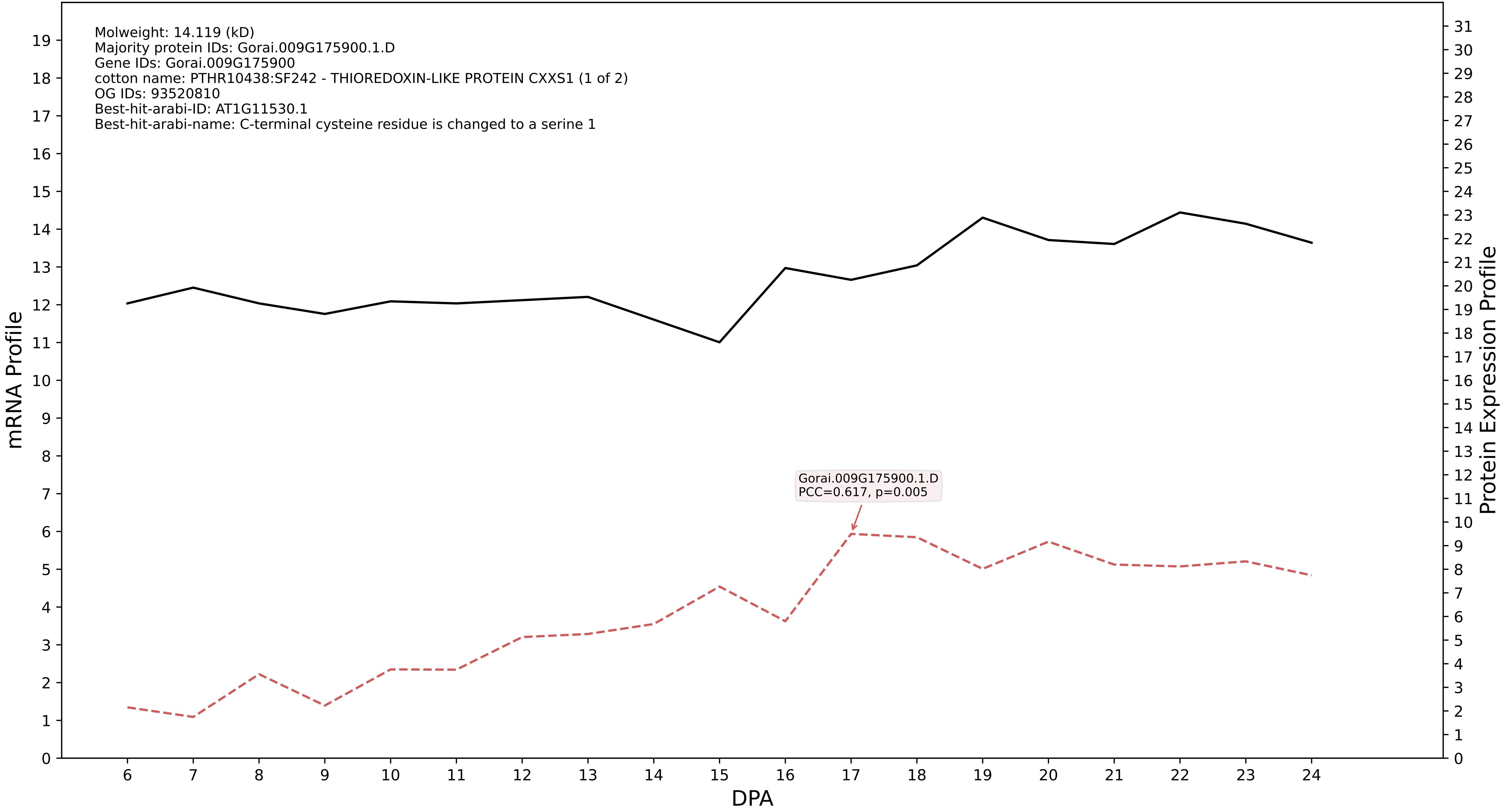

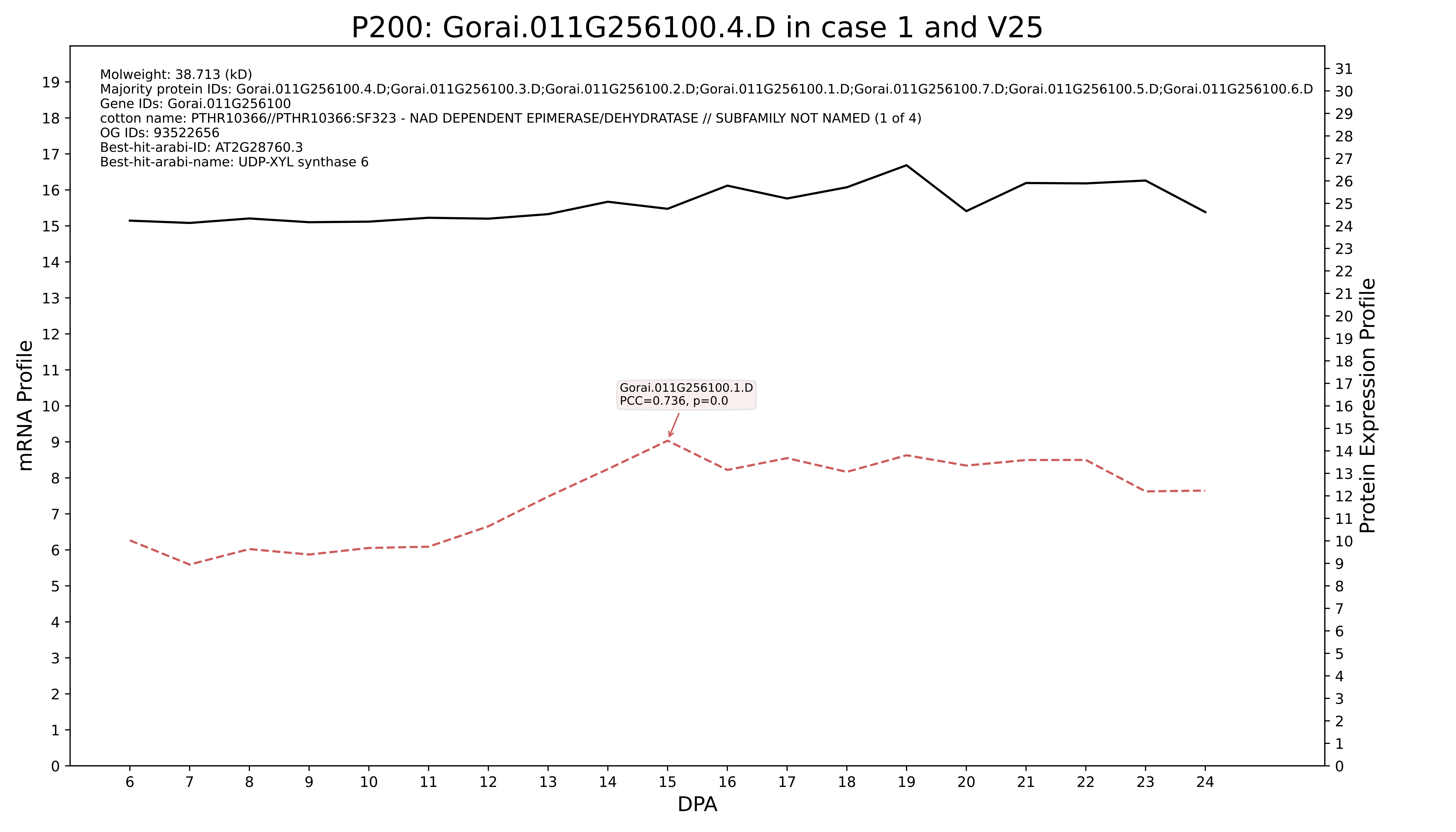
P200: Gorai.011G256100.4.D in case 1 and V25
Molweight: 38.713 (kD)
Majority protein IDs: Gorai.011G256100.4.D;Gorai.011G256100.3.D;Gorai.011G256100.2.D;Gorai.011G256100.1.D;Gorai.011G256100.7.D;Gorai.011G256100.5.D;Gorai.011G256100.6.D
Gene IDs: Gorai.011G256100
cotton name: PTHR10366//PTHR10366:SF323 - NAD DEPENDENT EPIMERASE/DEHYDRATASE // SUBFAMILY NOT NAMED (1 of 4)
OG IDs: 93522656
Best-hit-arabi-ID: AT2G28760.3
Best-hit-arabi-name: UDP-XYL synthase 6
Gorai.011G256100.1.D
PCC=0.736, p=0.0
mRNA Profile
Protein Expression Profile
DPA
0
1
2
3
4
5
6
7
8
9
10
11
12
13
14
15
16
17
18
19
20
21
22
23
24
25
26
27
28
29
30
31

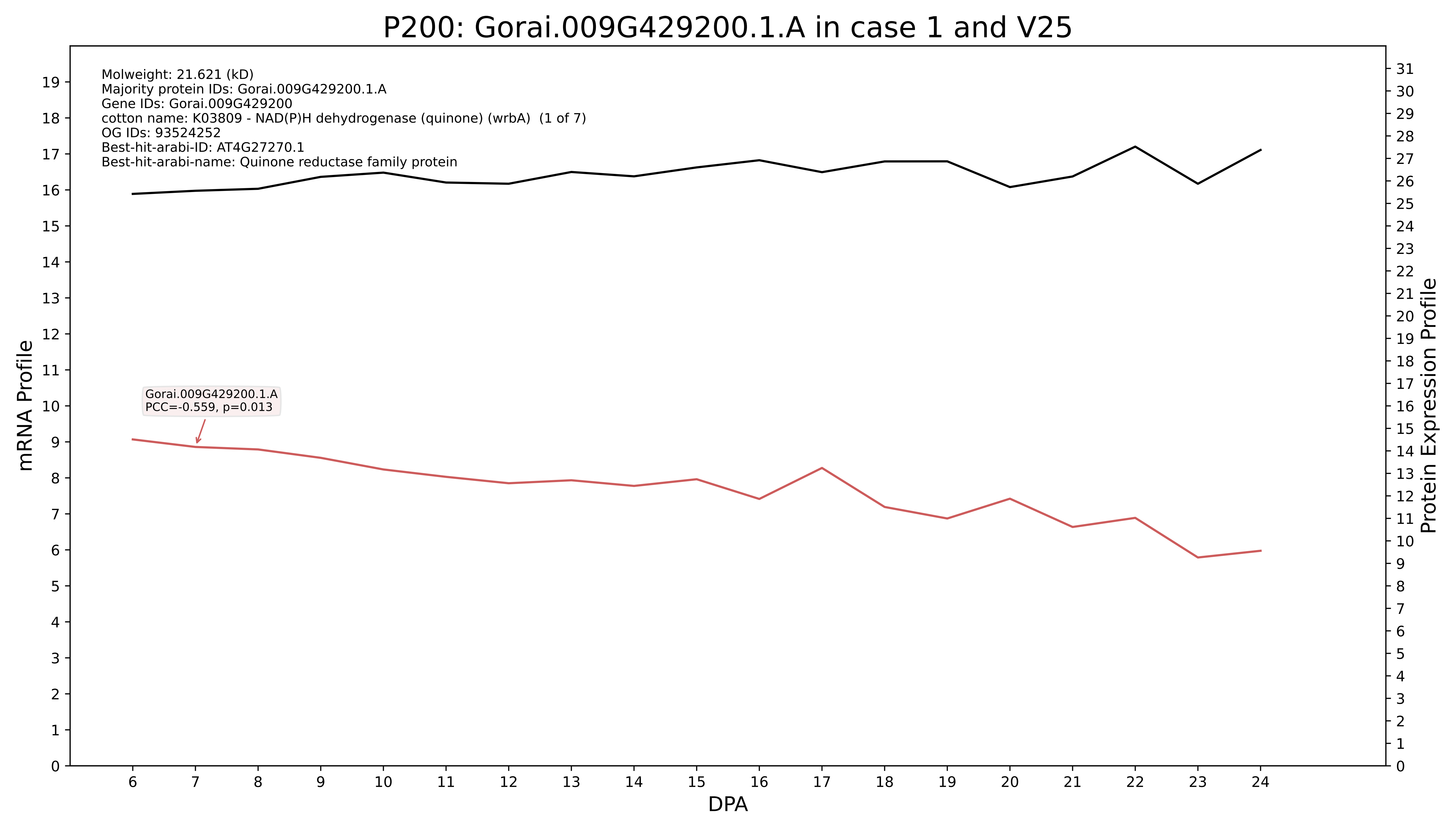
P200: Gorai.009G429200.1.A in case 1 and V25
Molweight: 21.621 (kD)
Majority protein IDs: Gorai.009G429200.1.A
Gene IDs: Gorai.009G429200
cotton name: K03809 - NAD(P)H dehydrogenase (quinone) (wrbA) (1 of 7)
OG IDs: 93524252
Best-hit-arabi-ID: AT4G27270.1
Best-hit-arabi-name: Quinone reductase family protein
Gorai.009G429200.1.A
PCC=-0.559, p=0.013
mRNA Profile
Protein Expression Profile
DPA
0
1
2
3
4
5
6
7
8
9
10
11
12
13
14
15
16
17
18
19
20
21
22
23
24
25
26
27
28
29
30
31

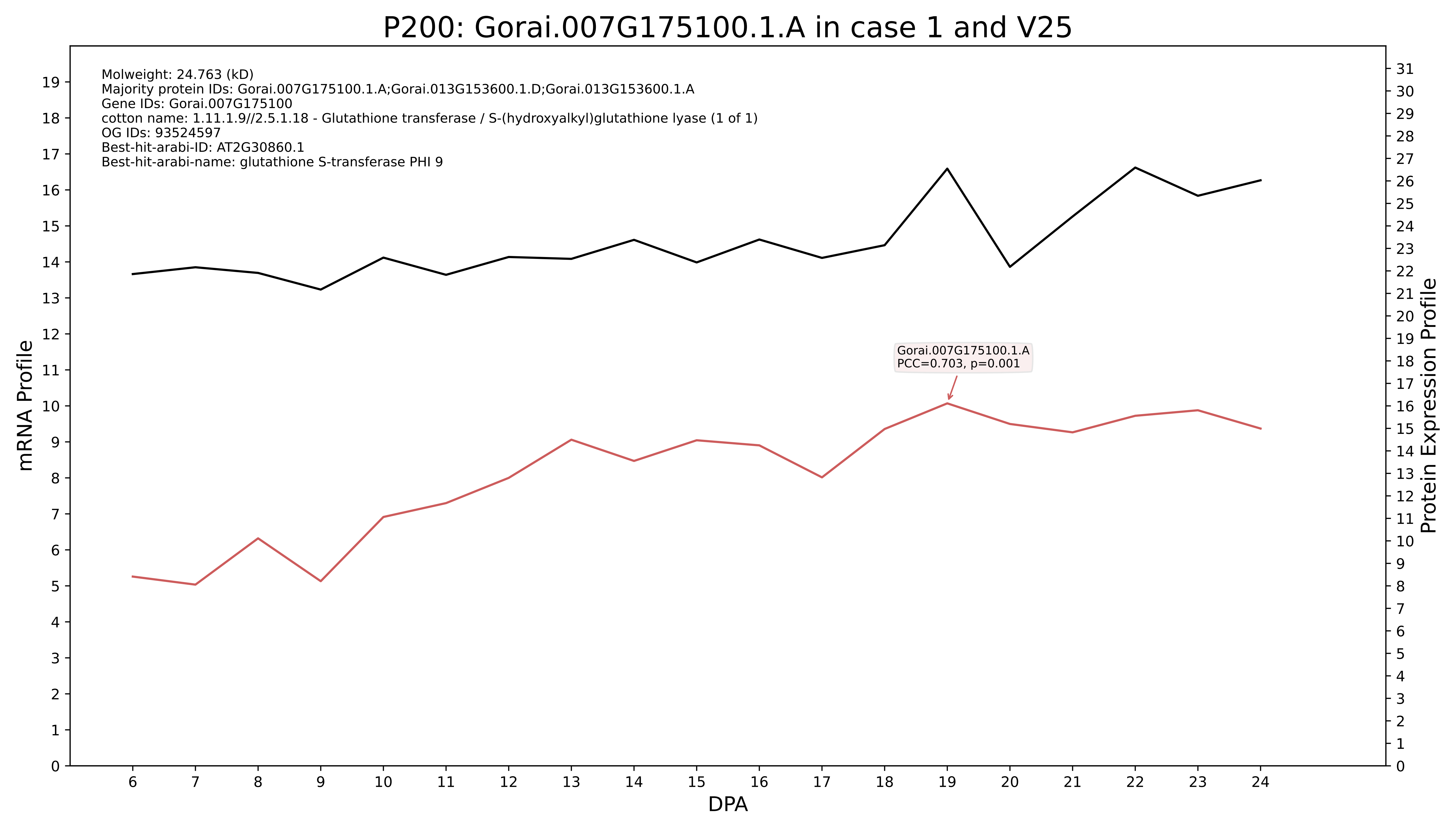

P200: Gorai.007G175100.1.A in case 1 and V25
Molweight: 24.763 (kD)
Majority protein IDs: Gorai.007G175100.1.A;Gorai.013G153600.1.D;Gorai.013G153600.1.A
Gene IDs: Gorai.007G175100
cotton name: 1.11.1.9//2.5.1.18 - Glutathione transferase / S-(hydroxyalkyl)glutathione lyase (1 of 1)
OG IDs: 93524597
Best-hit-arabi-ID: AT2G30860.1
Best-hit-arabi-name: glutathione S-transferase PHI 9
Gorai.007G175100.1.A
PCC=0.703, p=0.001
mRNA Profile
Protein Expression Profile
DPA

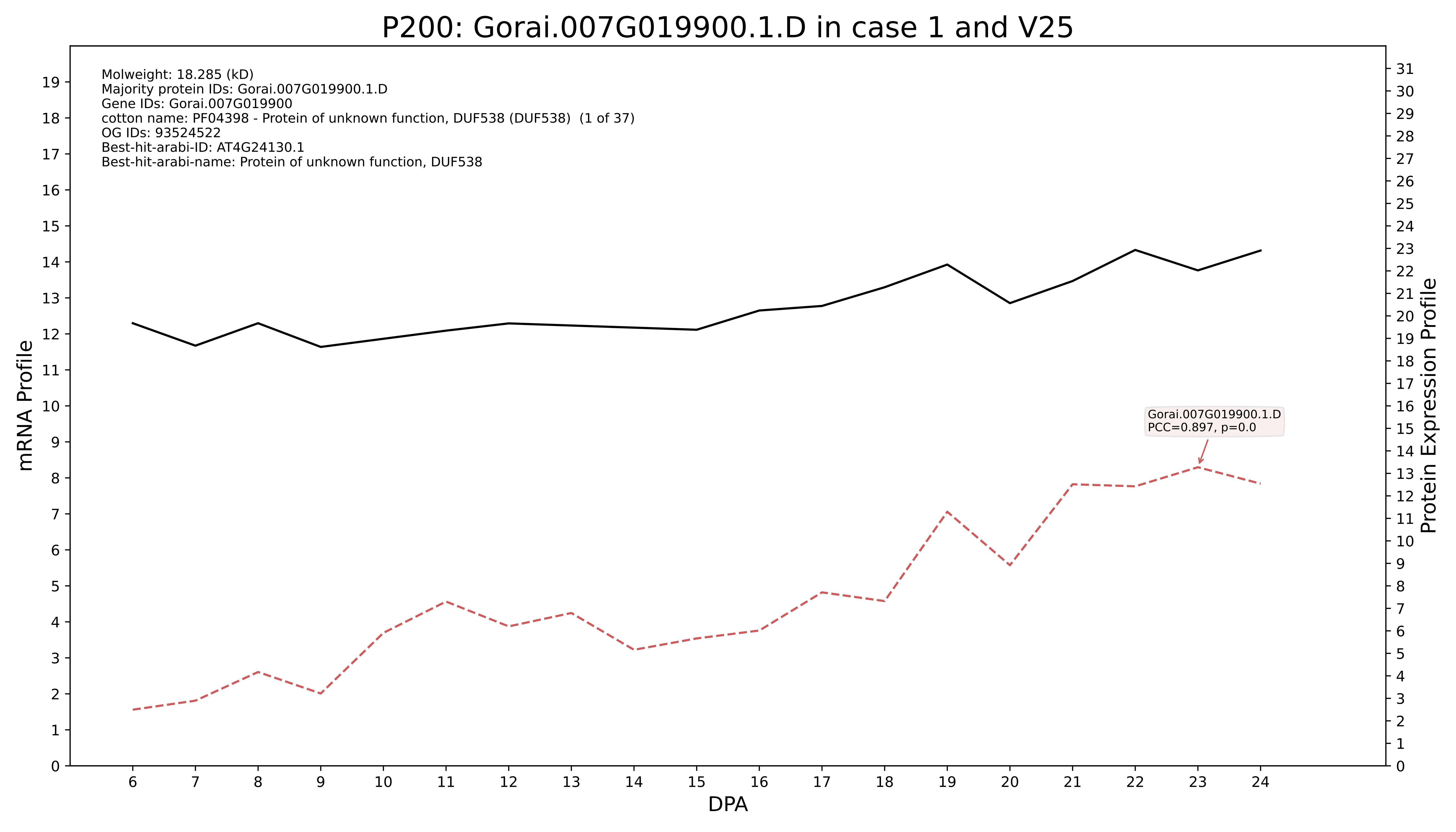
P200: Gorai.007G019900.1.D in case 1 and V25
Molweight: 18.285 (kD)
Majority protein IDs: Gorai.007G019900.1.D
Gene IDs: Gorai.007G019900
cotton name: PF04398 - Protein of unknown function, DUF538 (DUF538) (1 of 37)
OG IDs: 93524522
Best-hit-arabi-ID: AT4G24130.1
Best-hit-arabi-name: Protein of unknown function, DUF538
Gorai.007G019900.1.D
PCC=0.897, p=0.0
mRNA Profile
Protein Expression Profile
DPA

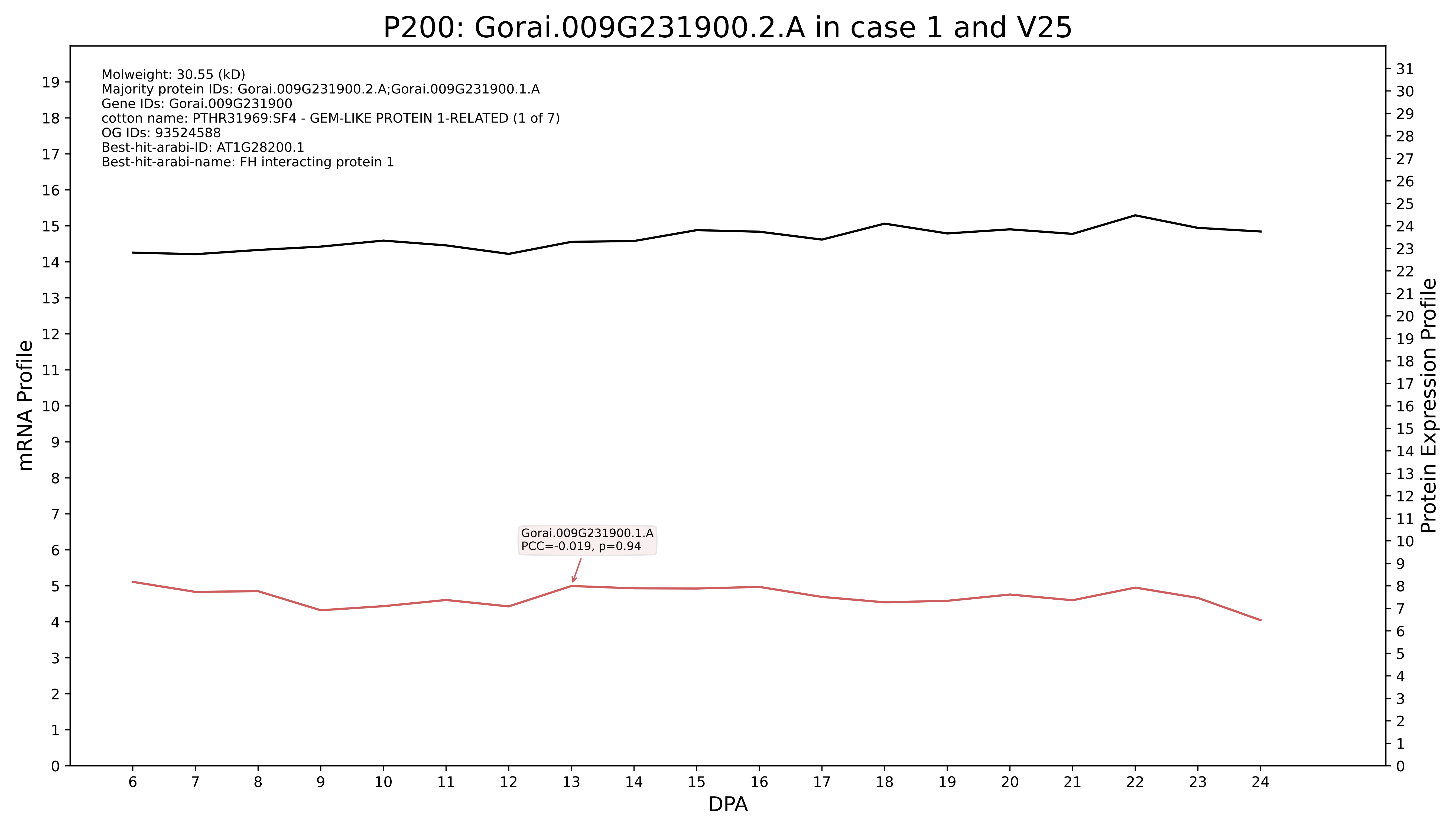
P200: Gorai.009G231900.2.A in case 1 and V25
Molweight: 30.55 (kD)
Majority protein IDs: Gorai.009G231900.2.A;Gorai.009G231900.1.A
Gene IDs: Gorai.009G231900
cotton name: PTHR31969:SF4 - GEM-LIKE PROTEIN 1-RELATED (1 of 7)
OG IDs: 93524588
Best-hit-arabi-ID: AT1G28200.1
Best-hit-arabi-name: FH interacting protein 1
Gorai.009G231900.1.A
PCC=-0.019, p=0.94
mRNA Profile
Protein Expression Profile
DPA

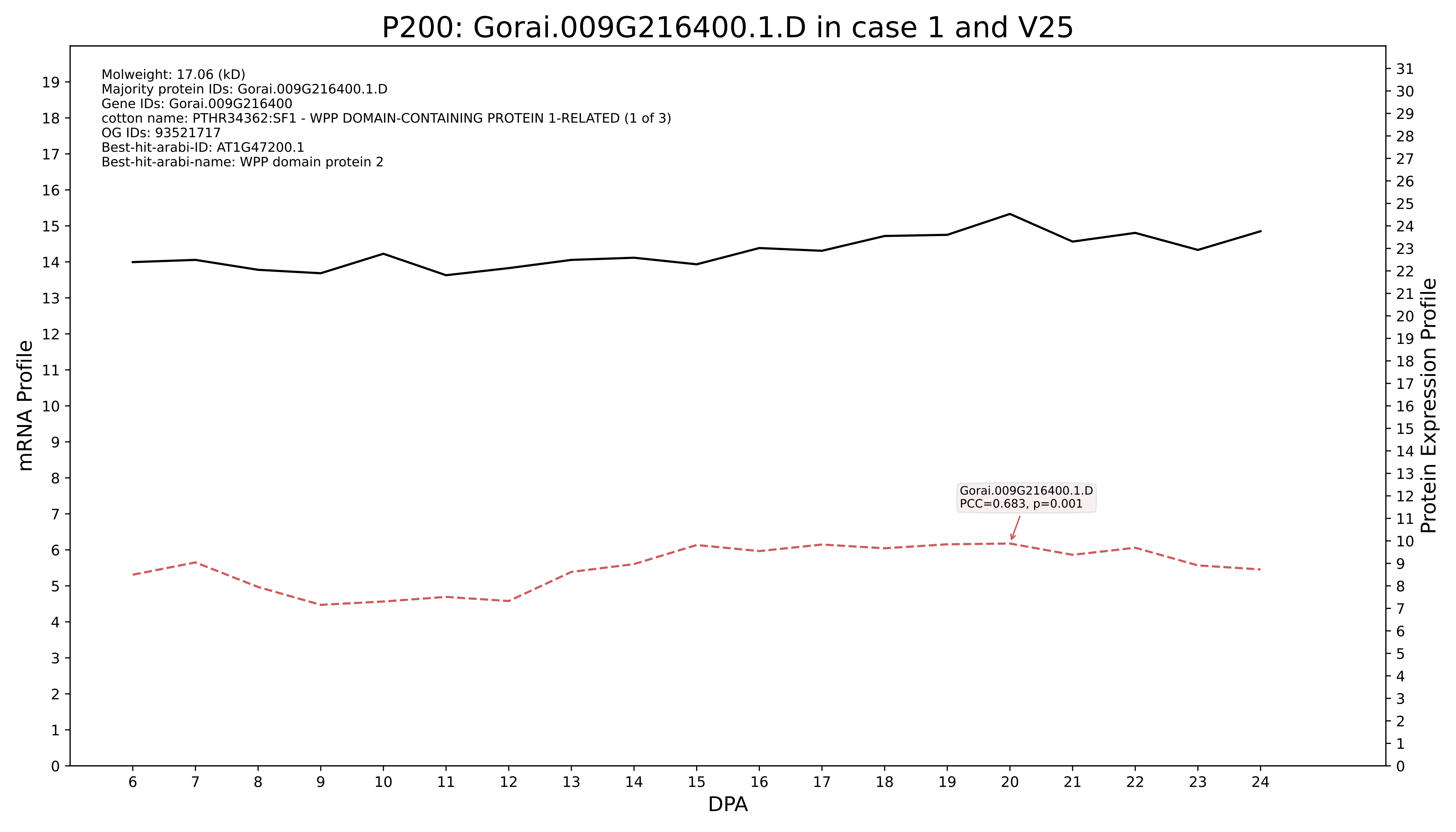
P200: Gorai.009G216400.1.D in case 1 and V25
Molweight: 17.06 (kD)
Majority protein IDs: Gorai.009G216400.1.D
Gene IDs: Gorai.009G216400
cotton name: PTHR34362:SF1 - WPP DOMAIN-CONTAINING PROTEIN 1-RELATED (1 of 3)
OG IDs: 93521717
Best-hit-arabi-ID: AT1G47200.1
Best-hit-arabi-name: WPP domain protein 2
Gorai.009G216400.1.D
PCC=0.683, p=0.001
mRNA Profile
Protein Expression Profile
DPA

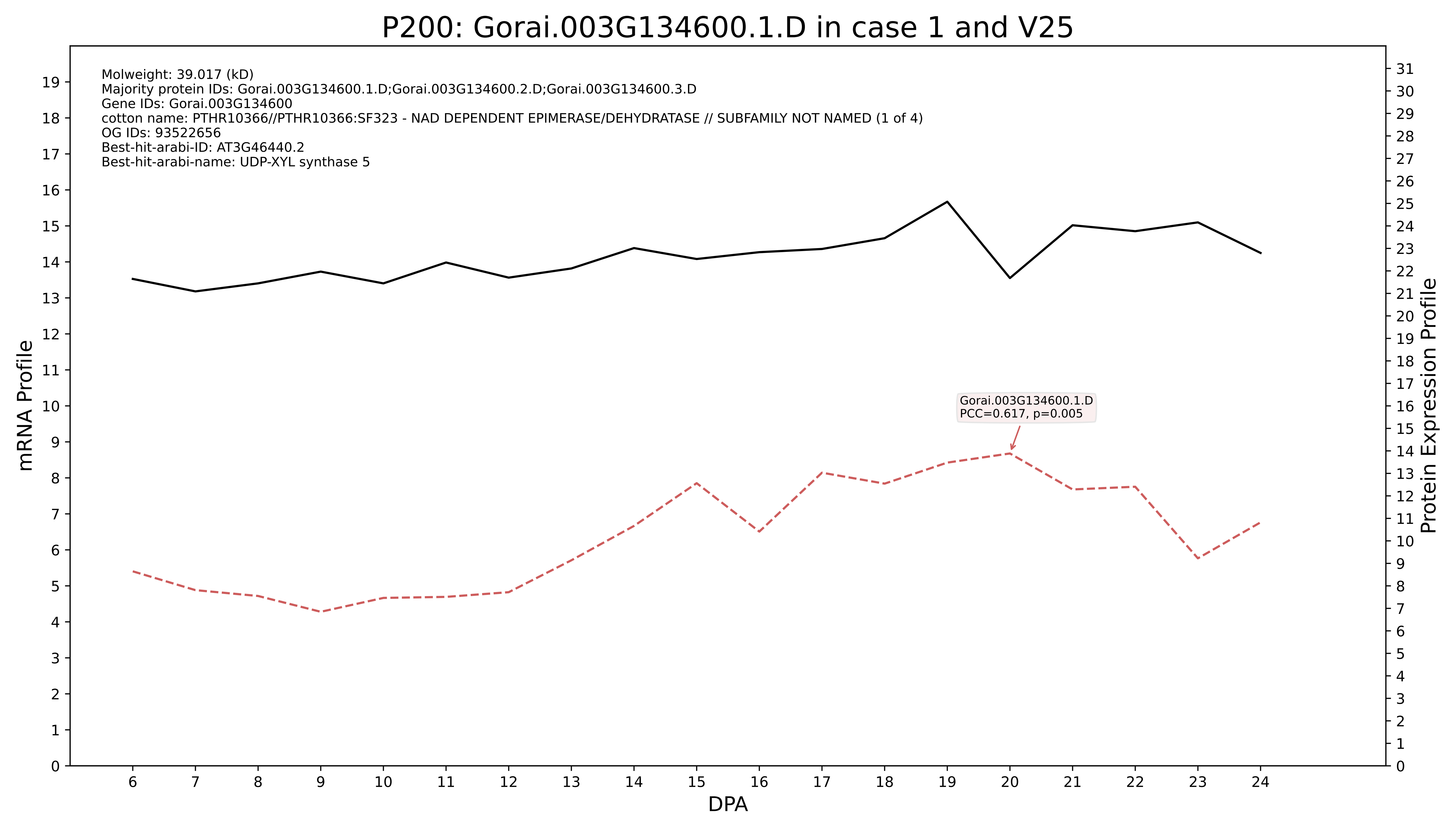
P200: Gorai.003G134600.1.D in case 1 and V25
Molweight: 39.017 (kD)
Majority protein IDs: Gorai.003G134600.1.D;Gorai.003G134600.2.D;Gorai.003G134600.3.D
Gene IDs: Gorai.003G134600
cotton name: PTHR10366//PTHR10366:SF323 - NAD DEPENDENT EPIMERASE/DEHYDRATASE // SUBFAMILY NOT NAMED (1 of 4)
OG IDs: 93522656
Best-hit-arabi-ID: AT3G46440.2
Best-hit-arabi-name: UDP-XYL synthase 5
Gorai.003G134600.1.D
PCC=0.617, p=0.005
mRNA Profile
Protein Expression Profile
DPA

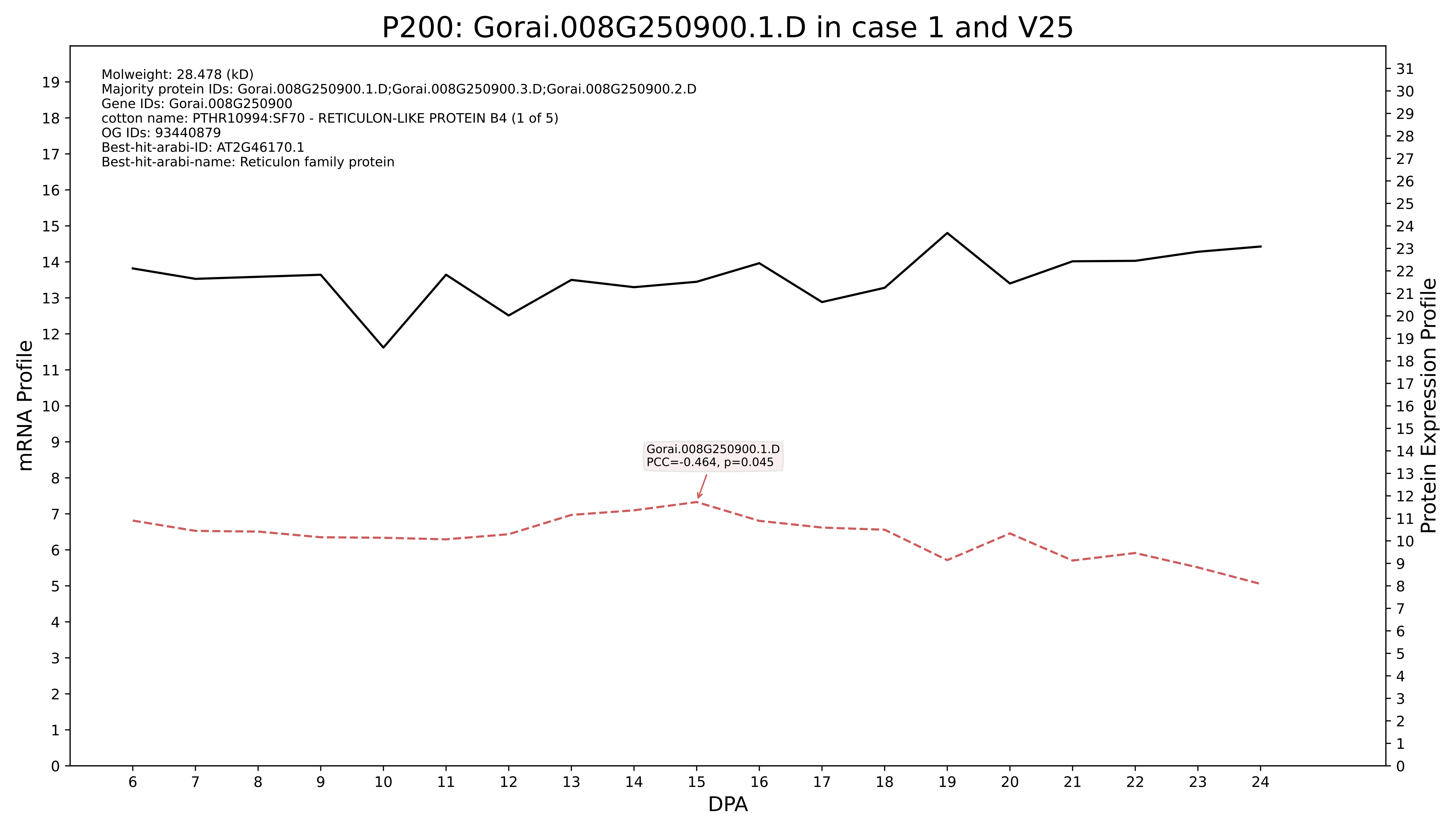
P200: Gorai.008G250900.1.D in case 1 and V25
Molweight: 28.478 (kD)
Majority protein IDs: Gorai.008G250900.1.D;Gorai.008G250900.3.D;Gorai.008G250900.2.D
Gene IDs: Gorai.008G250900
cotton name: PTHR10994:SF70 - RETICULON-LIKE PROTEIN B4 (1 of 5)
OG IDs: 93440879
Best-hit-arabi-ID: AT2G46170.1
Best-hit-arabi-name: Reticulon family protein
Gorai.008G250900.1.D
PCC=-0.464, p=0.045
mRNA Profile
Protein Expression Profile
DPA

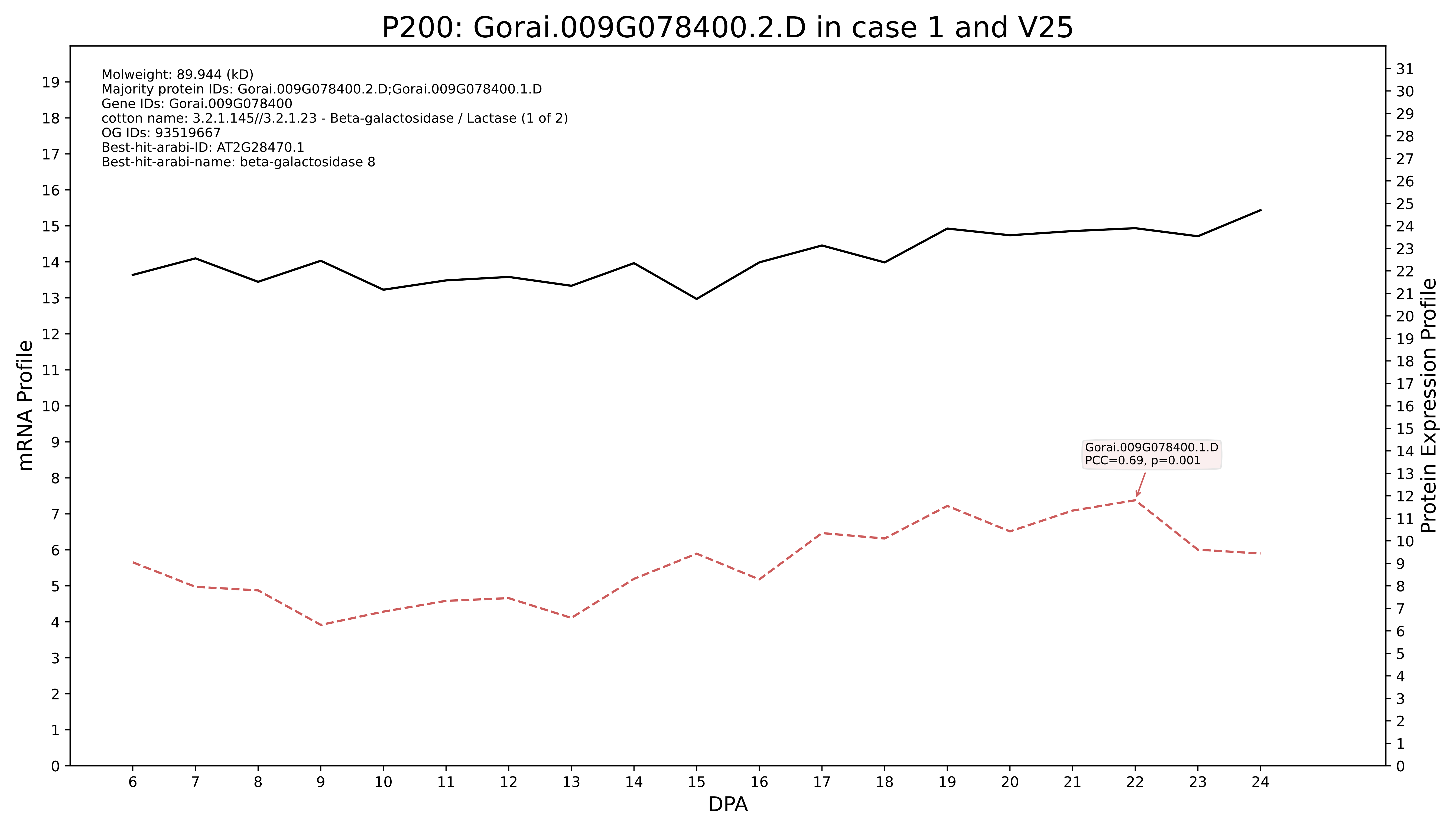
P200: Gorai.009G078400.2.D in case 1 and V25
Molweight: 89.944 (kD)
Majority protein IDs: Gorai.009G078400.2.D;Gorai.009G078400.1.D
Gene IDs: Gorai.009G078400
cotton name: 3.2.1.145//3.2.1.23 - Beta-galactosidase / Lactase (1 of 2)
OG IDs: 93519667
Best-hit-arabi-ID: AT2G28470.1
Best-hit-arabi-name: beta-galactosidase 8
Gorai.009G078400.1.D
PCC=0.69, p=0.001
mRNA Profile
Protein Expression Profile
DPA

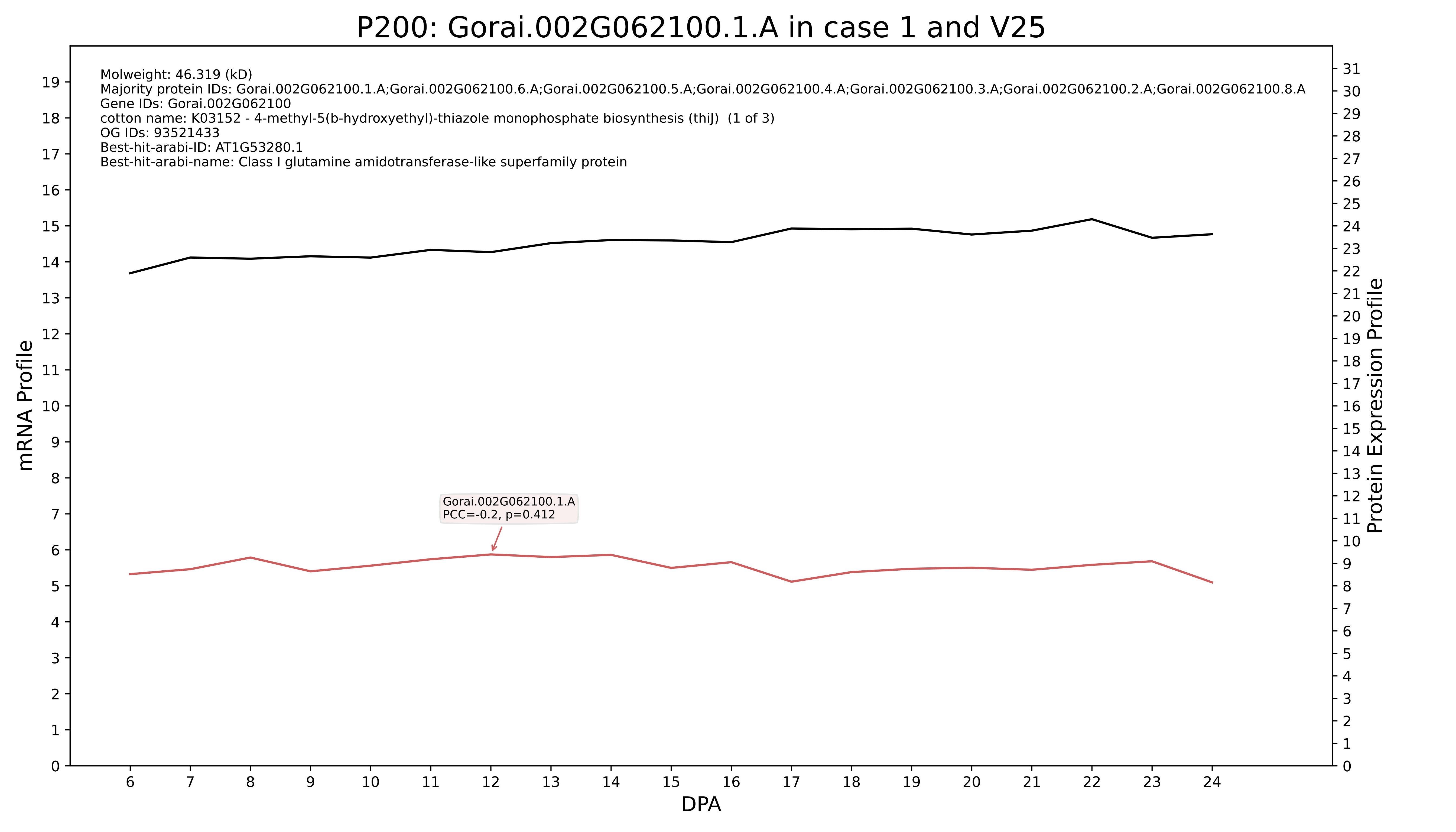
P200: Gorai.002G062100.1.A in case 1 and V25
Molweight: 46.319 (kD)
Majority protein IDs: Gorai.002G062100.1.A;Gorai.002G062100.6.A;Gorai.002G062100.5.A;Gorai.002G062100.4.A;Gorai.002G062100.3.A;Gorai.002G062100.2.A;Gorai.002G062100.8.A
Gene IDs: Gorai.002G062100
cotton name: K03152 - 4-methyl-5(b-hydroxyethyl)-thiazole monophosphate biosynthesis (thiJ) (1 of 3)
OG IDs: 93521433
Best-hit-arabi-ID: AT1G53280.1
Best-hit-arabi-name: Class I glutamine amidotransferase-like superfamily protein
Gorai.002G062100.1.A
PCC=-0.2, p=0.412
mRNA Profile
Protein Expression Profile
DPA

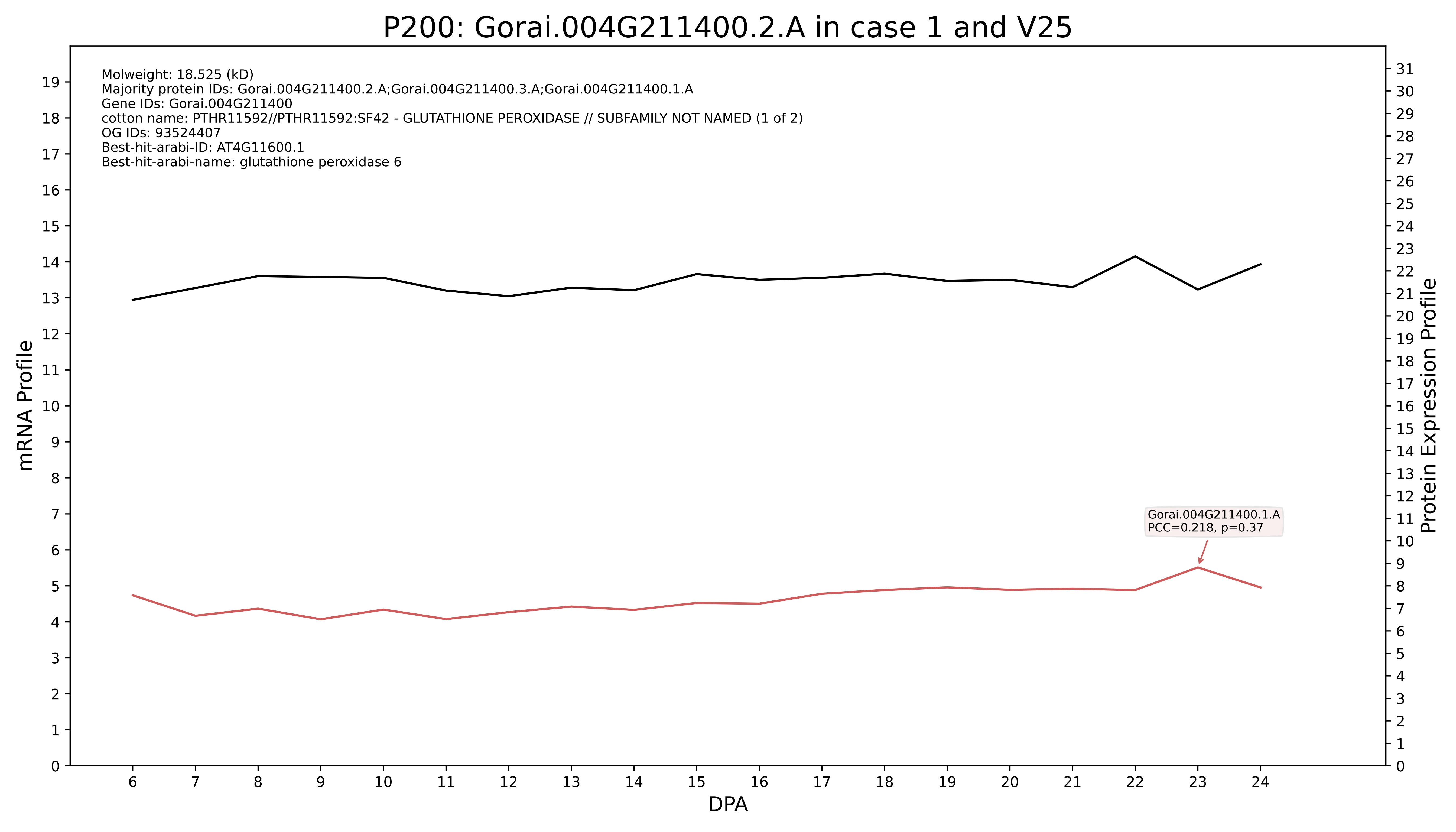
P200: Gorai.004G211400.2.A in case 1 and V25
Molweight: 18.525 (kD)
Majority protein IDs: Gorai.004G211400.2.A;Gorai.004G211400.3.A;Gorai.004G211400.1.A
Gene IDs: Gorai.004G211400
cotton name: PTHR11592//PTHR11592:SF42 - GLUTATHIONE PEROXIDASE // SUBFAMILY NOT NAMED (1 of 2)
OG IDs: 93524407
Best-hit-arabi-ID: AT4G11600.1
Best-hit-arabi-name: glutathione peroxidase 6
Gorai.004G211400.1.A
PCC=0.218, p=0.37
mRNA Profile
Protein Expression Profile
DPA
6 7 8 9 10 11 12 13 14 15 16 17 18 19 20 21 22 23 24
0 1 2 3 4 5 6 7 8 9 10 11 12 13 14 15 16 17 18 19
0 1 2 3 4 5 6 7 8 9 10 11 12 13 14 15 16 17 18 19 20 21 22 23 24 25 26 27 28 29 30 31

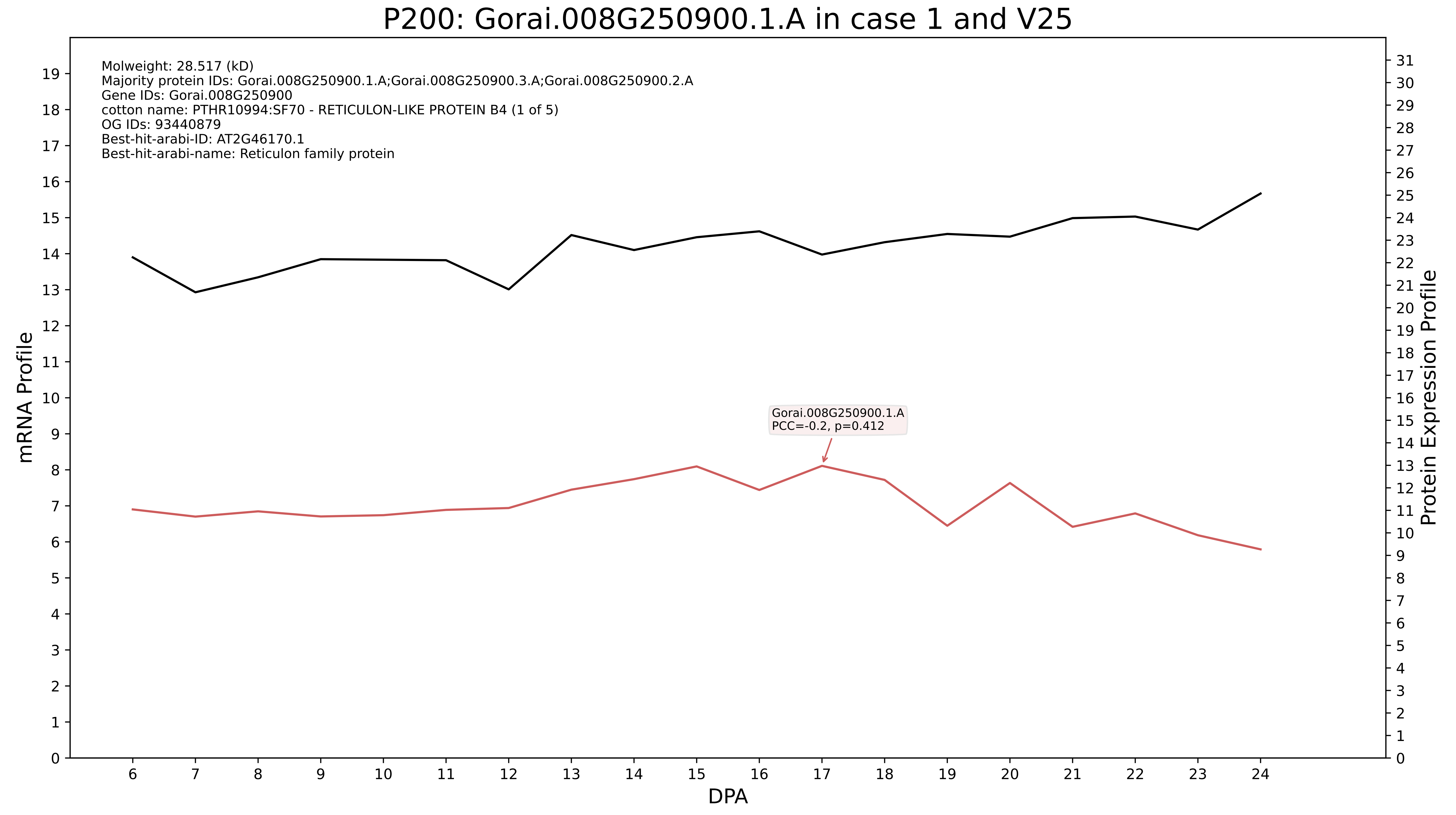

P200: Gorai.008G250900.1.A in case 1 and V25
Molweight: 28.517 (kD)
Majority protein IDs: Gorai.008G250900.1.A;Gorai.008G250900.3.A;Gorai.008G250900.2.A
Gene IDs: Gorai.008G250900
cotton name: PTHR10994:SF70 - RETICULON-LIKE PROTEIN B4 (1 of 5)
OG IDs: 93440879
Best-hit-arabi-ID: AT2G46170.1
Best-hit-arabi-name: Reticulon family protein
Gorai.008G250900.1.A
PCC=-0.2, p=0.412
mRNA Profile
Protein Expression Profile
DPA

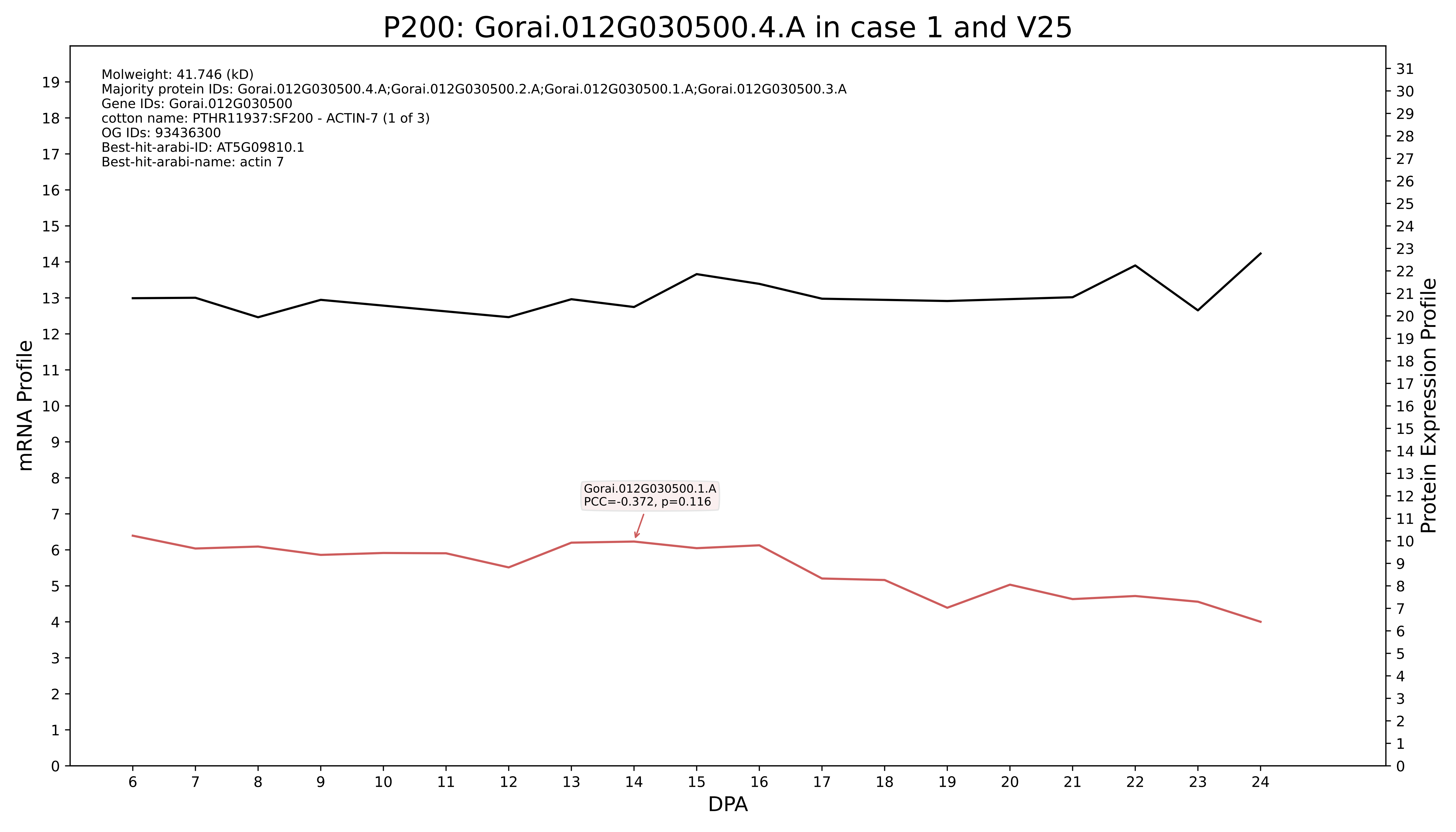
P200: Gorai.012G030500.4.A in case 1 and V25
Molweight: 41.746 (kD)
Majority protein IDs: Gorai.012G030500.4.A;Gorai.012G030500.2.A;Gorai.012G030500.1.A;Gorai.012G030500.3.A
Gene IDs: Gorai.012G030500
cotton name: PTHR11937:SF200 - ACTIN-7 (1 of 3)
OG IDs: 93436300
Best-hit-arabi-ID: AT5G09810.1
Best-hit-arabi-name: actin 7
Gorai.012G030500.1.A
PCC=-0.372, p=0.116
mRNA Profile
Protein Expression Profile
DPA

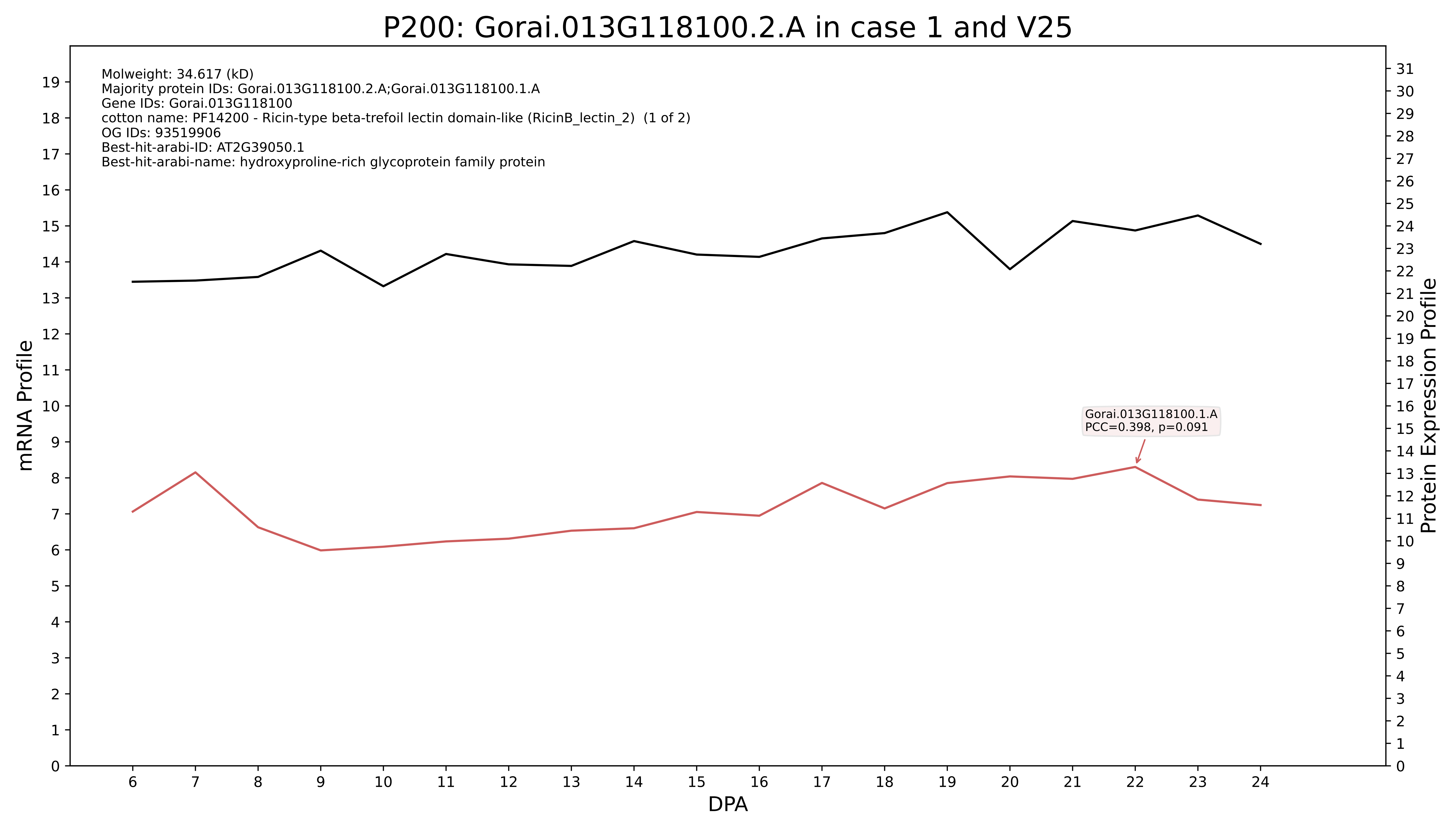
P200: Gorai.013G118100.2.A in case 1 and V25
Molweight: 34.617 (kD)
Majority protein IDs: Gorai.013G118100.2.A;Gorai.013G118100.1.A
Gene IDs: Gorai.013G118100
cotton name: PF14200 - Ricin-type beta-trefoil lectin domain-like (RicinB_lectin_2) (1 of 2)
OG IDs: 93519906
Best-hit-arabi-ID: AT2G39050.1
Best-hit-arabi-name: hydroxyproline-rich glycoprotein family protein
Gorai.013G118100.1.A
PCC=0.398, p=0.091
mRNA Profile
Protein Expression Profile
DPA

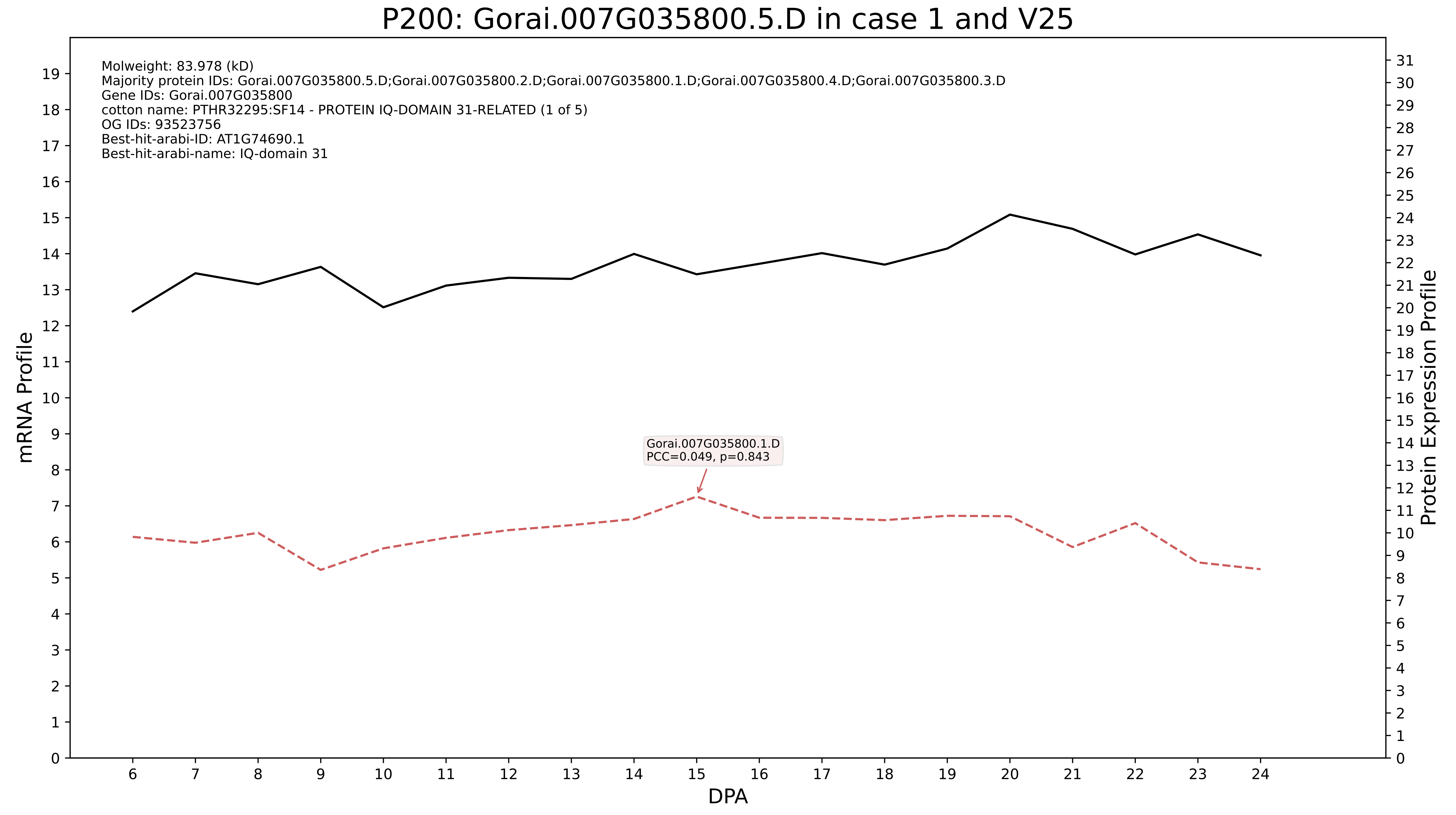
P200: Gorai.007G035800.5.D in case 1 and V25
Molweight: 83.978 (kD)
Majority protein IDs: Gorai.007G035800.5.D;Gorai.007G035800.2.D;Gorai.007G035800.1.D;Gorai.007G035800.4.D;Gorai.007G035800.3.D
Gene IDs: Gorai.007G035800
cotton name: PTHR32295:SF14 - PROTEIN IQ-DOMAIN 31-RELATED (1 of 5)
OG IDs: 93523756
Best-hit-arabi-ID: AT1G74690.1
Best-hit-arabi-name: IQ-domain 31
Gorai.007G035800.1.D
PCC=0.049, p=0.843
mRNA Profile
Protein Expression Profile
DPA
6
7
8
9
10
11
12
13
14
15
16
17
18
19
20
21
22
23
24

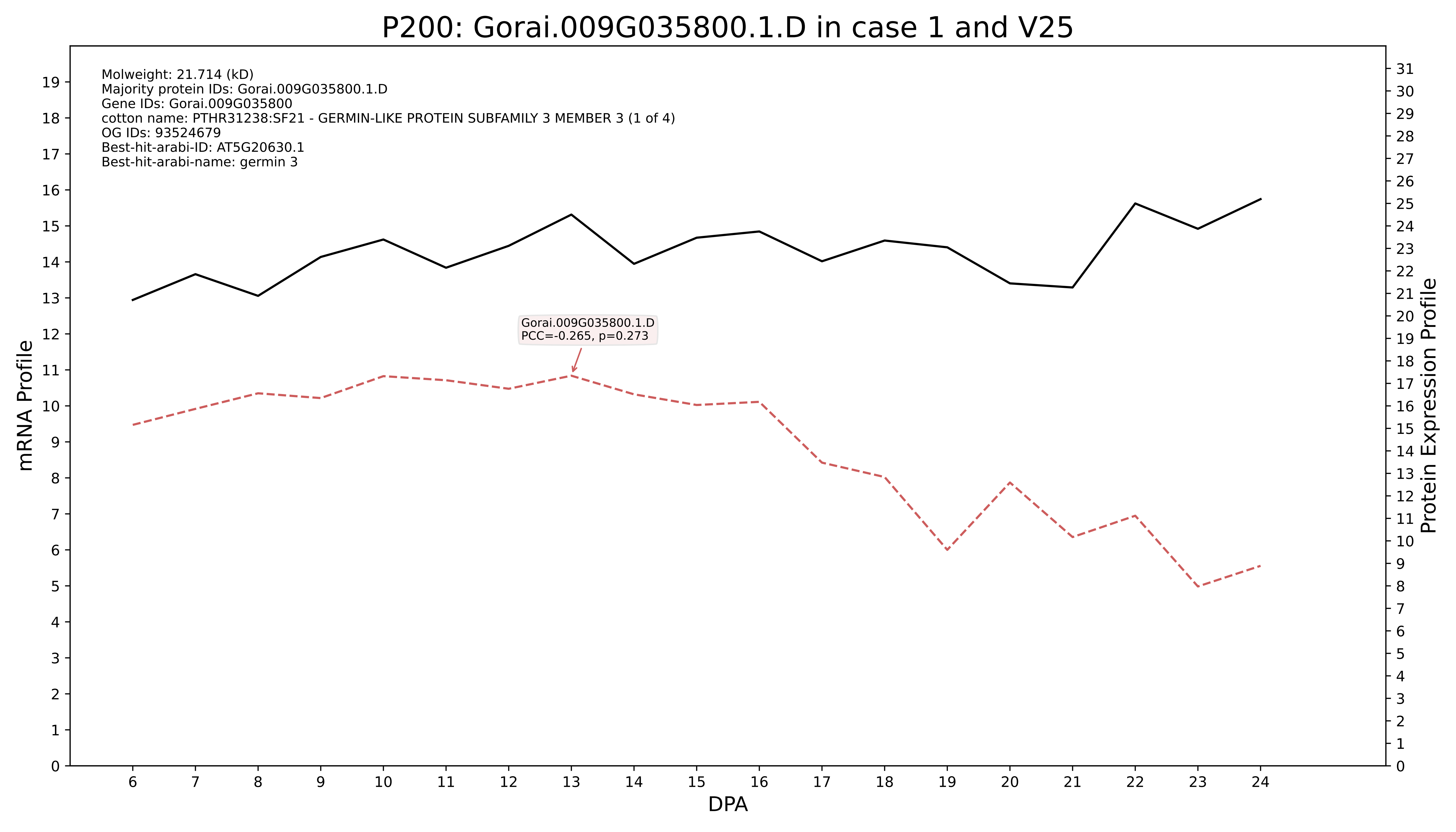
P200: Gorai.009G035800.1.D in case 1 and V25
Molweight: 21.714 (kD)
Majority protein IDs: Gorai.009G035800.1.D
Gene IDs: Gorai.009G035800
cotton name: PTHR31238:SF21 - GERMIN-LIKE PROTEIN SUBFAMILY 3 MEMBER 3 (1 of 4)
OG IDs: 93524679
Best-hit-arabi-ID: AT5G20630.1
Best-hit-arabi-name: germin 3
Gorai.009G035800.1.D
PCC=-0.265, p=0.273
mRNA Profile
Protein Expression Profile
DPA

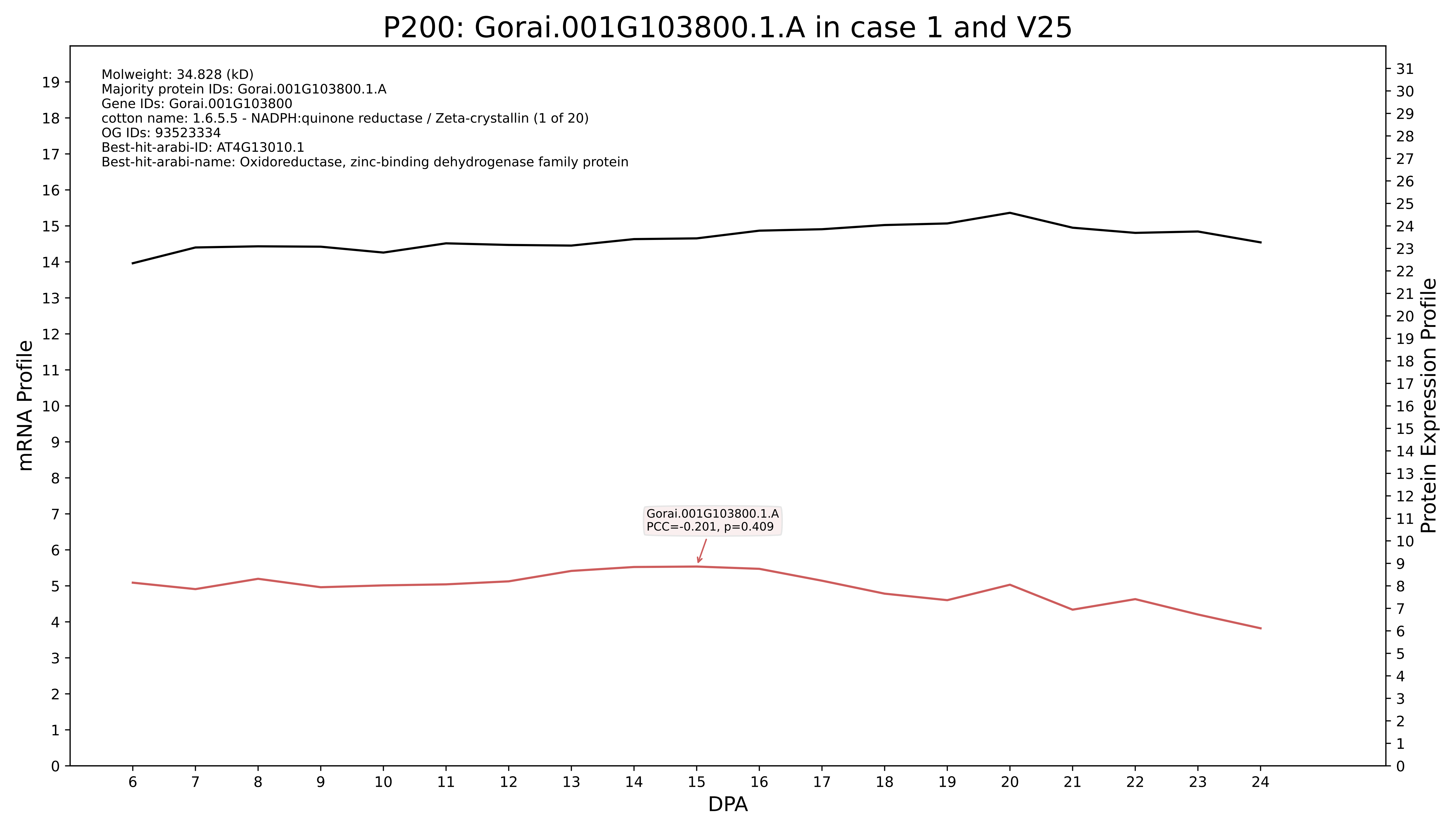
P200: Gorai.001G103800.1.A in case 1 and V25
Molweight: 34.828 (kD)
Majority protein IDs: Gorai.001G103800.1.A
Gene IDs: Gorai.001G103800
cotton name: 1.6.5.5 - NADPH:quinone reductase / Zeta-crystallin (1 of 20)
OG IDs: 93523334
Best-hit-arabi-ID: AT4G13010.1
Best-hit-arabi-name: Oxidoreductase, zinc-binding dehydrogenase family protein
Gorai.001G103800.1.A
PCC=-0.201, p=0.409
mRNA Profile
Protein Expression Profile
DPA

P200: Gorai.007G047400.2.D in case 1 and V25

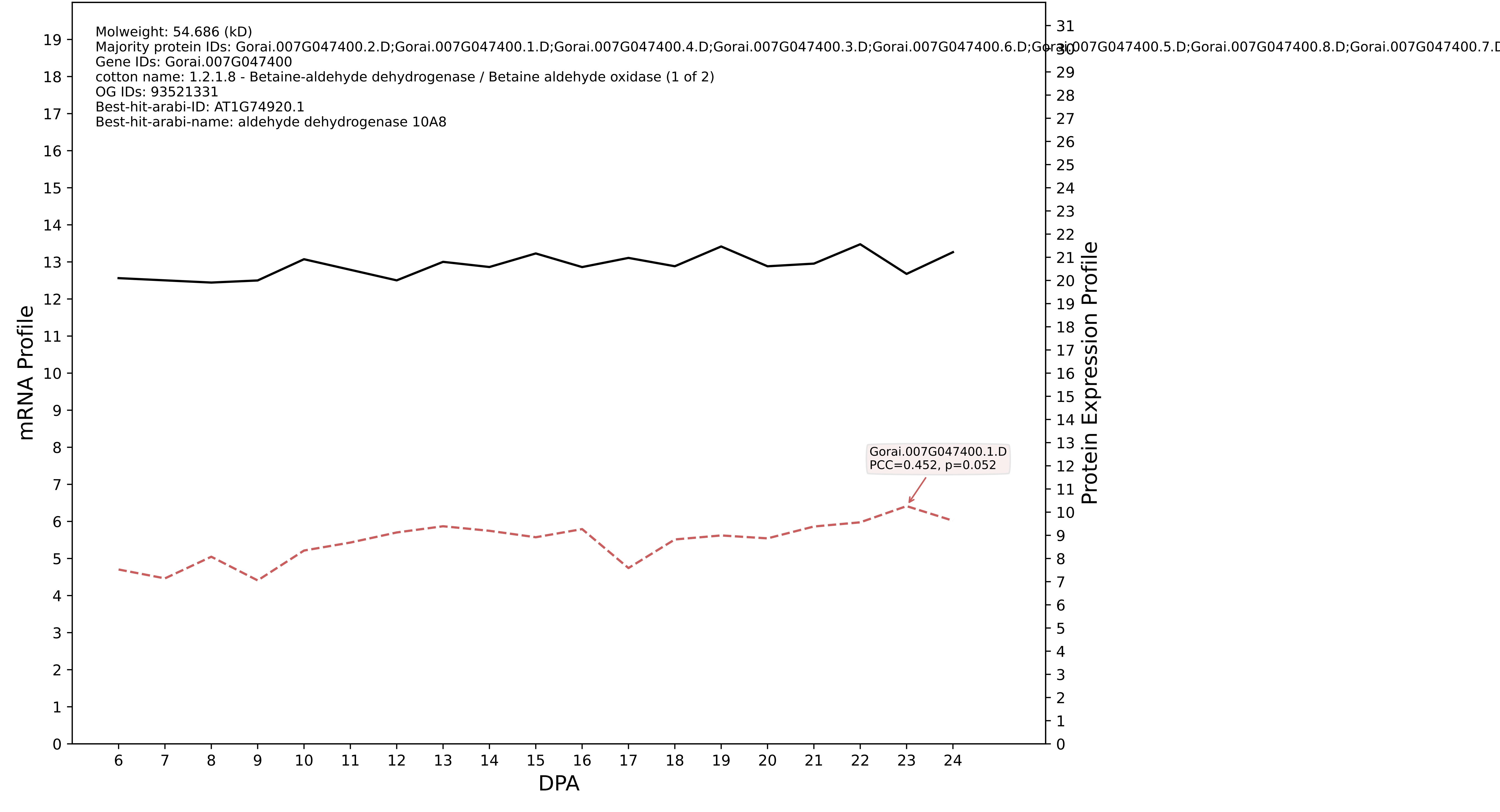

P200: Gorai.009G429200.1.D in case 1 and V25

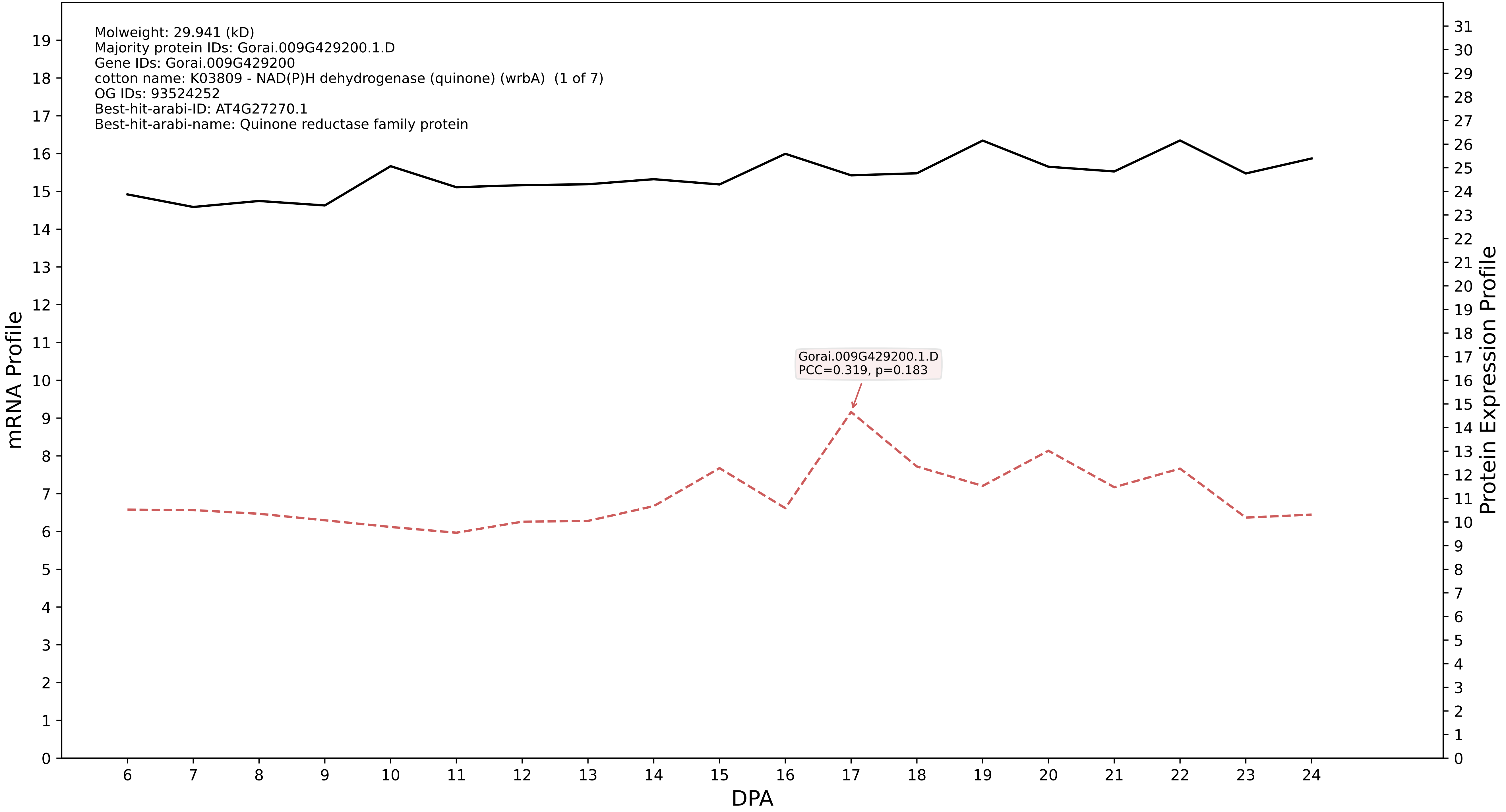

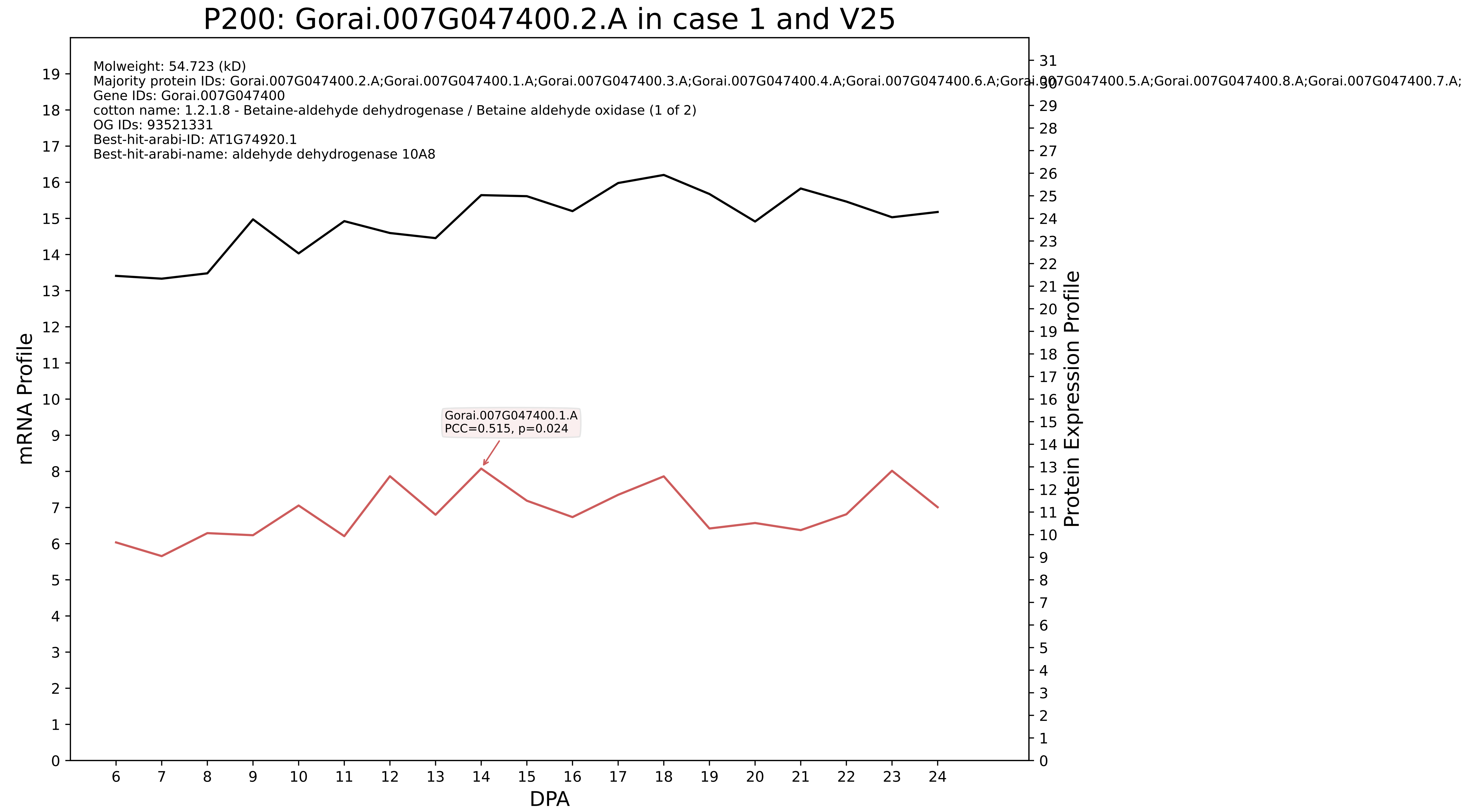
P200: Gorai.007G047400.2.A in case 1 and V25
Molweight: 54.723 (kD)
Majority protein IDs: Gorai.007G047400.2.A;Gorai.007G047400.1.A;Gorai.007G047400.3.A;Gorai.007G047400.4.A;Gorai.007G047400.6.A;Gorai.007G047400.5.A;Gorai.007G047400.8.A;Gorai.007G047400.7.A;
Gene IDs: Gorai.007G047400
cotton name: 1.2.1.8 - Betaine-aldehyde dehydrogenase / Betaine aldehyde oxidase (1 of 2)
OG IDs: 93521331
Best-hit-arabi-ID: AT1G74920.1
Best-hit-arabi-name: aldehyde dehydrogenase 10A8
Gorai.007G047400.1.A
PCC=0.515, p=0.024
mRNA Profile
Protein Expression Profile
DPA

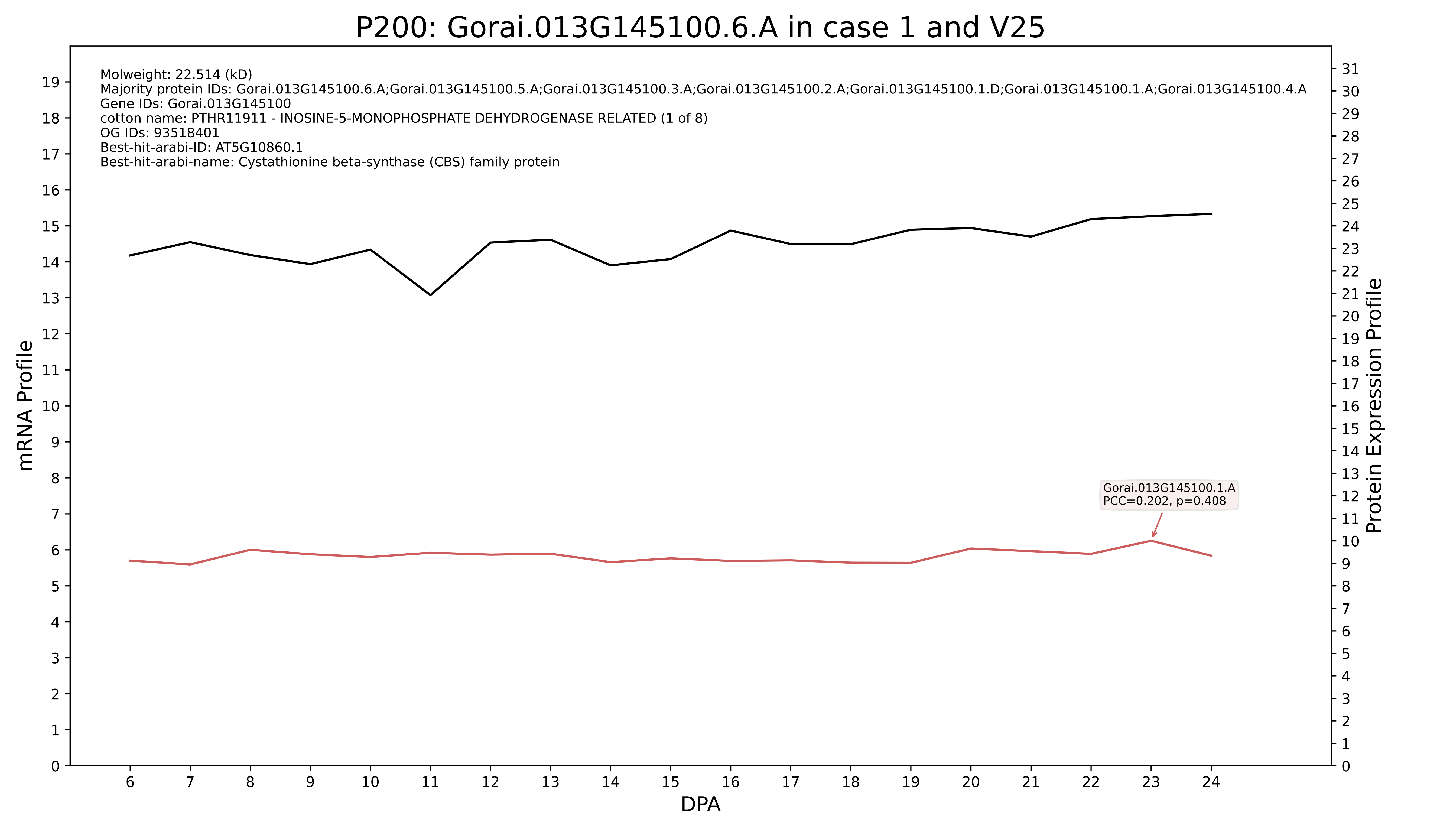
P200: Gorai.013G145100.6.A in case 1 and V25
Molweight: 22.514 (kD)
Majority protein IDs: Gorai.013G145100.6.A;Gorai.013G145100.5.A;Gorai.013G145100.3.A;Gorai.013G145100.2.A;Gorai.013G145100.1.D;Gorai.013G145100.1.A;Gorai.013G145100.4.A
Gene IDs: Gorai.013G145100
cotton name: PTHR11911 - INOSINE-5-MONOPHOSPHATE DEHYDROGENASE RELATED (1 of 8)
OG IDs: 93518401
Best-hit-arabi-ID: AT5G10860.1
Best-hit-arabi-name: Cystathionine beta-synthase (CBS) family protein
Gorai.013G145100.1.A
PCC=0.202, p=0.408
mRNA Profile
Protein Expression Profile
DPA

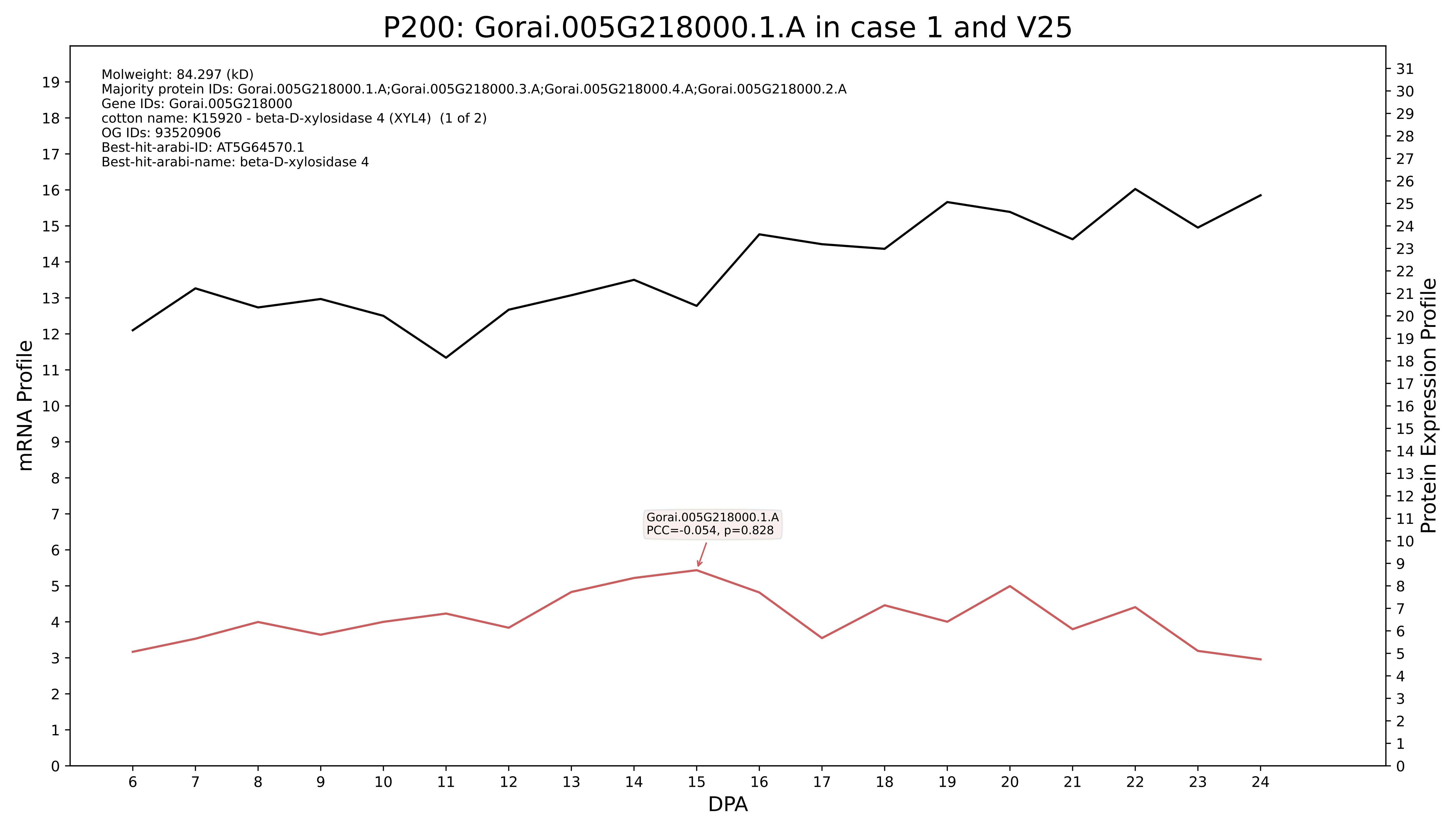

P200: Gorai.005G218000.1.A in case 1 and V25
Molweight: 84.297 (kD)
Majority protein IDs: Gorai.005G218000.1.A;Gorai.005G218000.3.A;Gorai.005G218000.4.A;Gorai.005G218000.2.A
Gene IDs: Gorai.005G218000
cotton name: K15920 - beta-D-xylosidase 4 (XYL4) (1 of 2)
OG IDs: 93520906
Best-hit-arabi-ID: AT5G64570.1
Best-hit-arabi-name: beta-D-xylosidase 4
Gorai.005G218000.1.A
PCC=-0.054, p=0.828
mRNA Profile
Protein Expression Profile
DPA

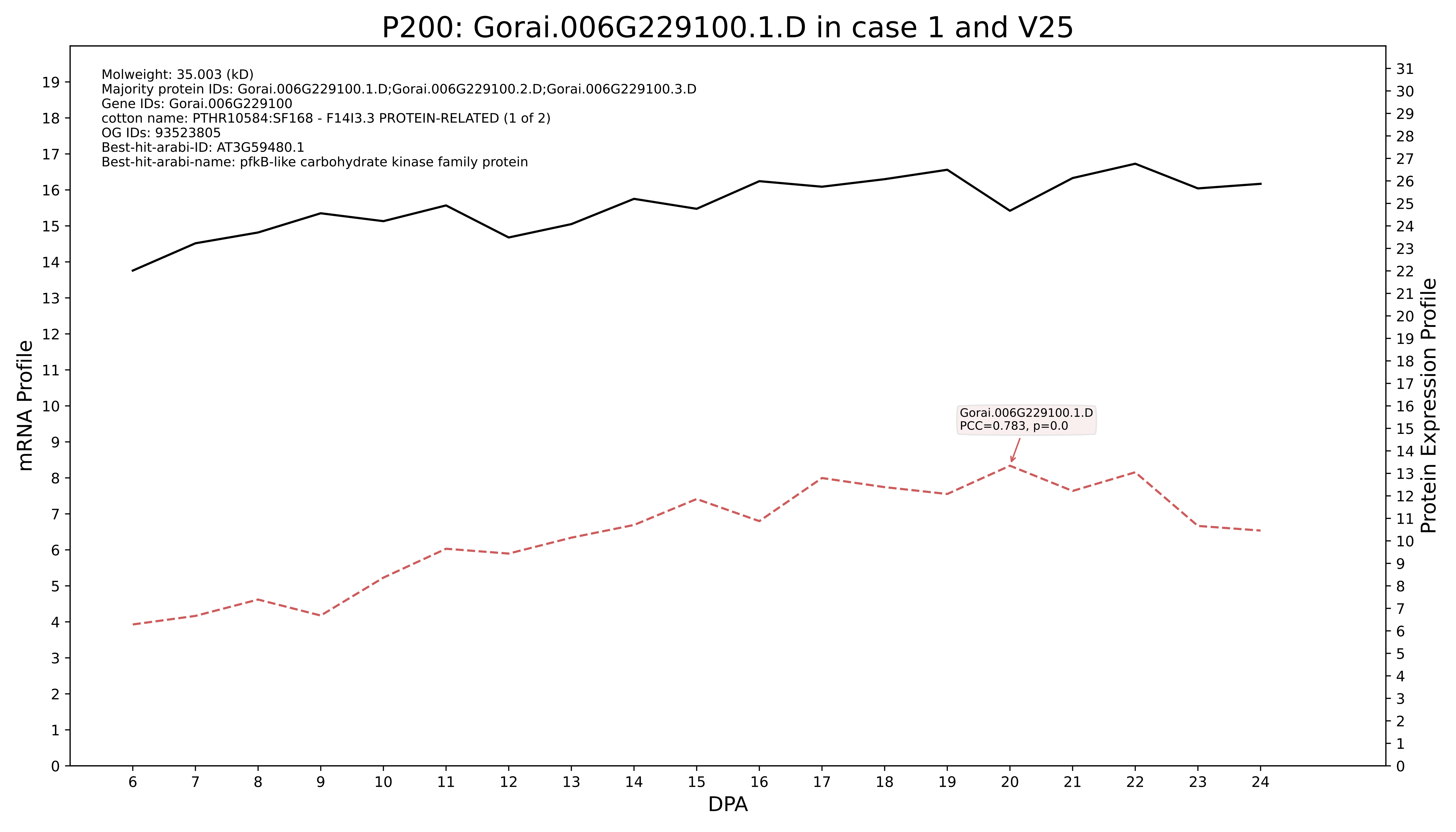
P200: Gorai.006G229100.1.D in case 1 and V25
Molweight: 35.003 (kD)
Majority protein IDs: Gorai.006G229100.1.D;Gorai.006G229100.2.D;Gorai.006G229100.3.D
Gene IDs: Gorai.006G229100
cotton name: PTHR10584:SF168 - F14I3.3 PROTEIN-RELATED (1 of 2)
OG IDs: 93523805
Best-hit-arabi-ID: AT3G59480.1
Best-hit-arabi-name: pfkB-like carbohydrate kinase family protein
Gorai.006G229100.1.D
PCC=0.783, p=0.0
mRNA Profile
Protein Expression Profile
DPA

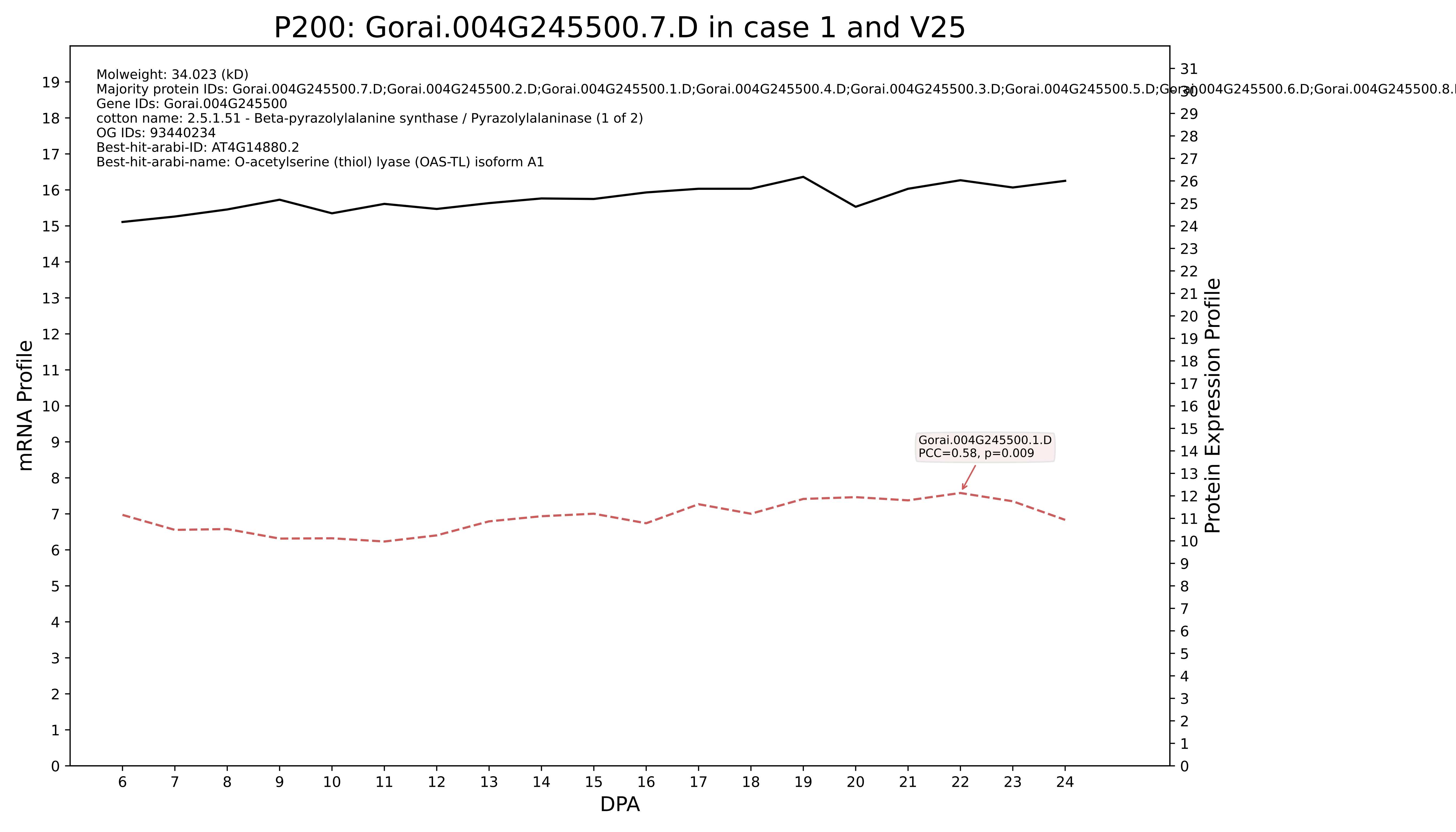
P200: Gorai.004G245500.7.D in case 1 and V25
Molweight: 34.023 (kD)
Majority protein IDs: Gorai.004G245500.7.D;Gorai.004G245500.2.D;Gorai.004G245500.1.D;Gorai.004G245500.4.D;Gorai.004G245500.3.D;Gorai.004G245500.5.D;Gorai.004G245500.6.D;Gorai.004G245500.8.D
Gene IDs: Gorai.004G245500
cotton name: 2.5.1.51 - Beta-pyrazolylalanine synthase / Pyrazolylalaninase (1 of 2)
OG IDs: 93440234
Best-hit-arabi-ID: AT4G14880.2
Best-hit-arabi-name: O-acetylserine (thiol) lyase (OAS-TL) isoform A1
Gorai.004G245500.1.D
PCC=0.58, p=0.009
mRNA Profile
Protein Expression Profile
DPA

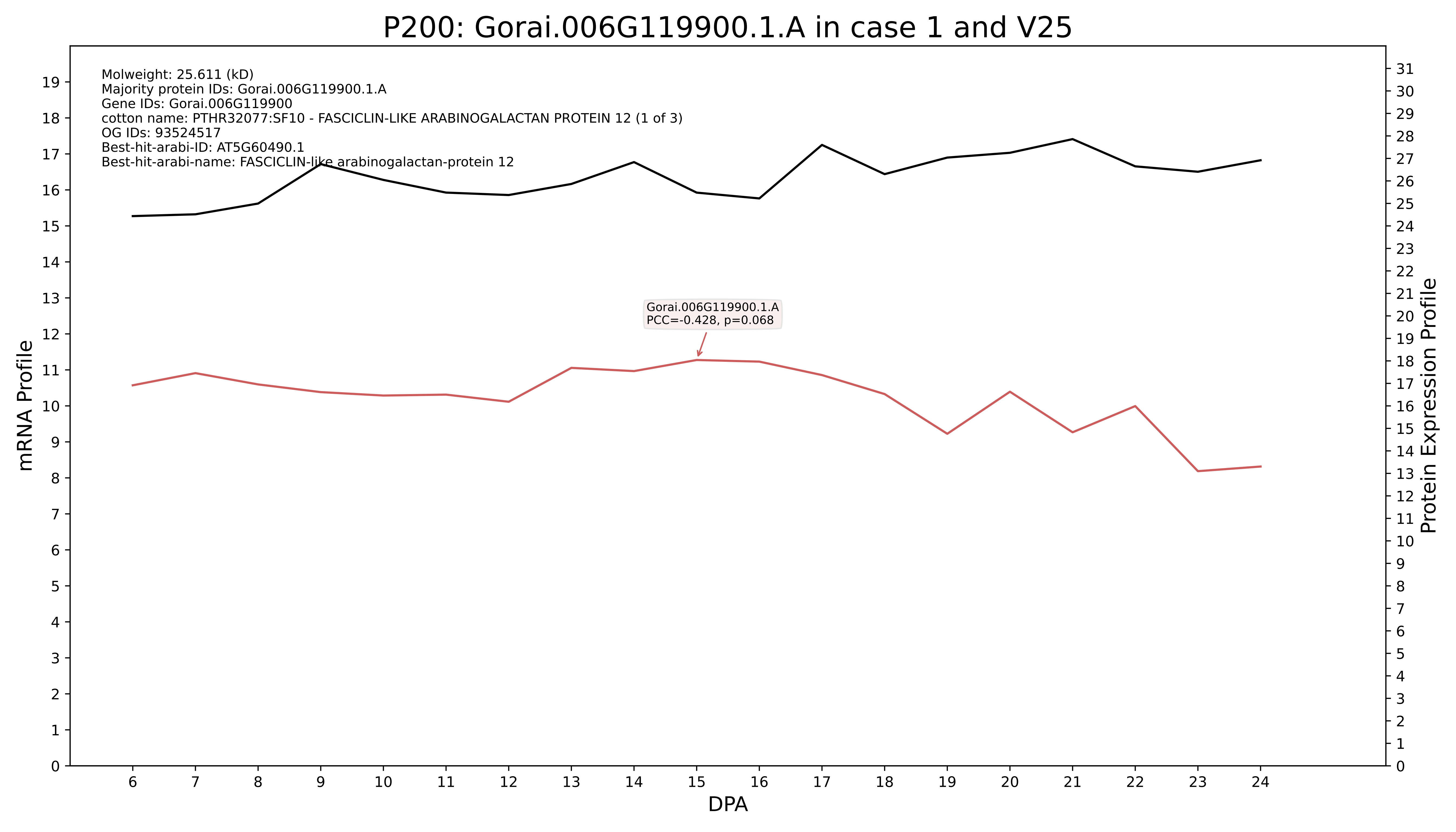
P200: Gorai.006G119900.1.A in case 1 and V25
Molweight: 25.611 (kD)
Majority protein IDs: Gorai.006G119900.1.A
Gene IDs: Gorai.006G119900
cotton name: PTHR32077:SF10 - FASCICLIN-LIKE ARABINOGALACTAN PROTEIN 12 (1 of 3)
OG IDs: 93524517
Best-hit-arabi-ID: AT5G60490.1
Best-hit-arabi-name: FASCICLIN-like arabinogalactan-protein 12
Gorai.006G119900.1.A
PCC=-0.428, p=0.068
mRNA Profile
Protein Expression Profile
DPA
0
1
2
3
4
5
6
7
8
9
10
11
12
13
14
15
16
17
18
19
20
21
22
23
24
25
26
27
28
29
30
31

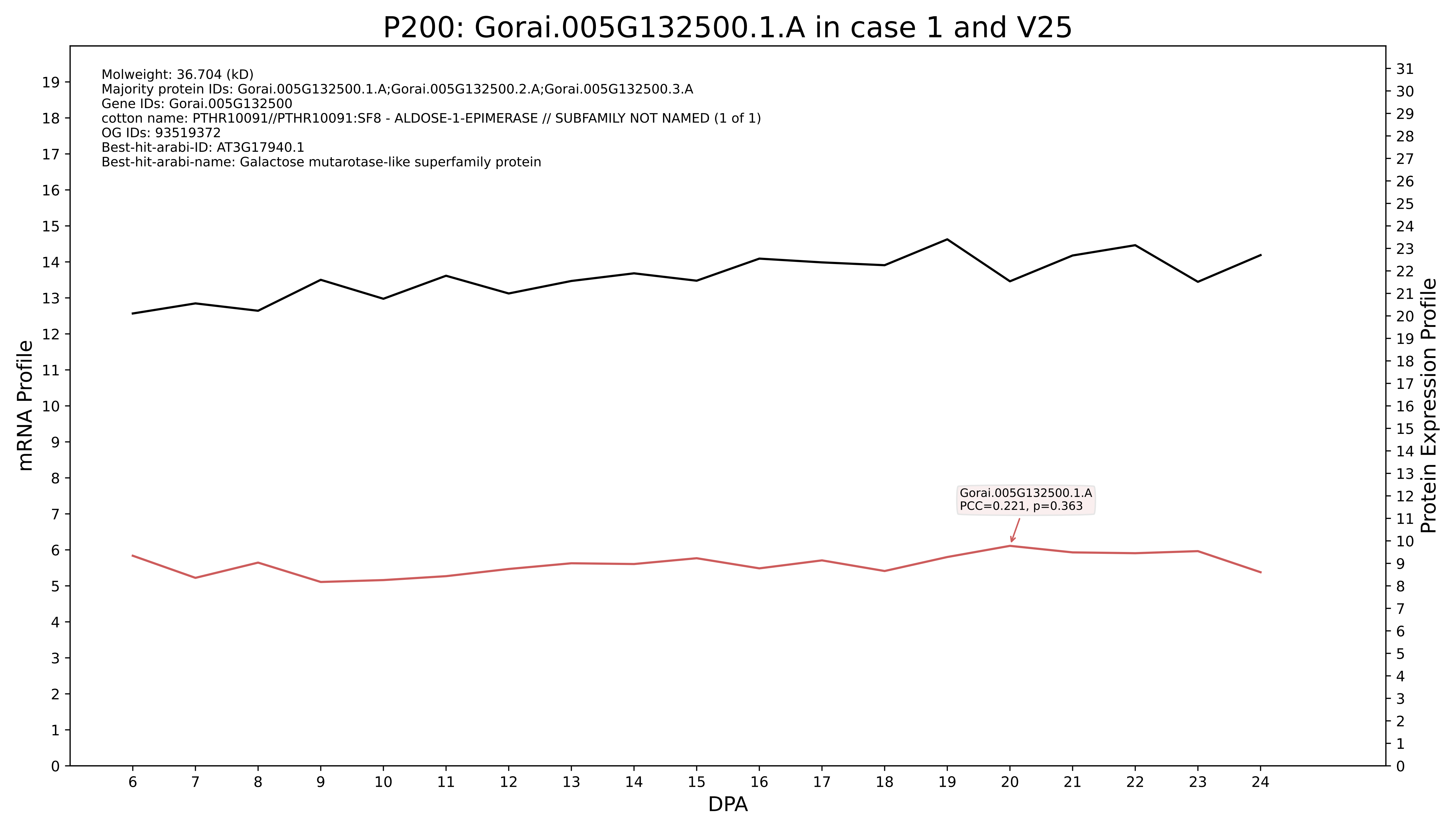
P200: Gorai.005G132500.1.A in case 1 and V25
Molweight: 36.704 (kD)
Majority protein IDs: Gorai.005G132500.1.A;Gorai.005G132500.2.A;Gorai.005G132500.3.A
Gene IDs: Gorai.005G132500
cotton name: PTHR10091//PTHR10091:SF8 - ALDOSE-1-EPIMERASE // SUBFAMILY NOT NAMED (1 of 1)
OG IDs: 93519372
Best-hit-arabi-ID: AT3G17940.1
Best-hit-arabi-name: Galactose mutarotase-like superfamily protein
Gorai.005G132500.1.A
PCC=0.221, p=0.363
mRNA Profile
Protein Expression Profile
DPA
6
7
8
9
10
11
12
13
14
15
16
17
18
19
20
21
22
23
24

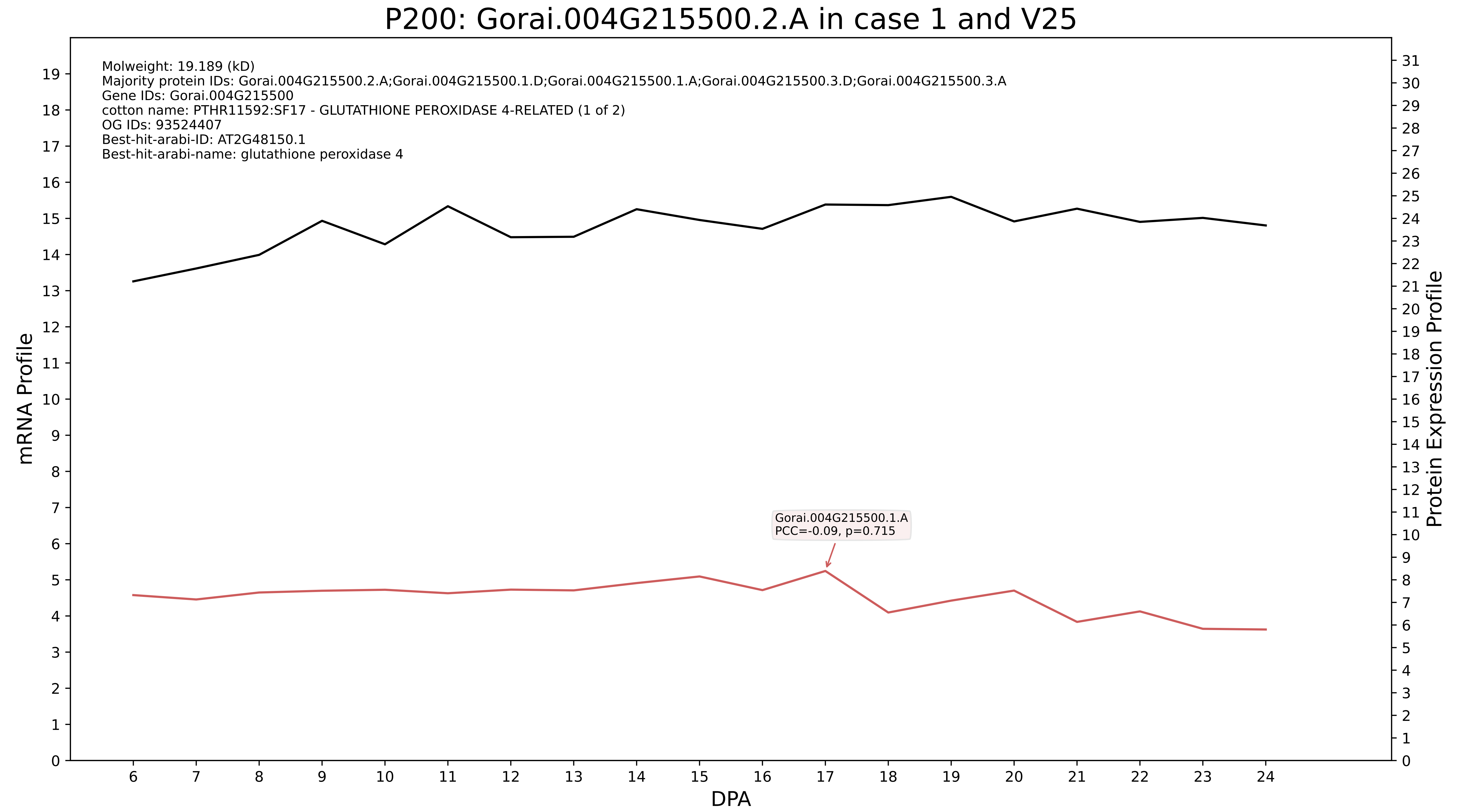
P200: Gorai.004G215500.2.A in case 1 and V25
Molweight: 19.189 (kD)
Majority protein IDs: Gorai.004G215500.2.A;Gorai.004G215500.1.D;Gorai.004G215500.1.A;Gorai.004G215500.3.D;Gorai.004G215500.3.A
Gene IDs: Gorai.004G215500
cotton name: PTHR11592:SF17 - GLUTATHIONE PEROXIDASE 4-RELATED (1 of 2)
OG IDs: 93524407
Best-hit-arabi-ID: AT2G48150.1
Best-hit-arabi-name: glutathione peroxidase 4
Gorai.004G215500.1.A
PCC=-0.09, p=0.715
mRNA Profile
Protein Expression Profile
DPA
0
1
2
3
4
5
6
7
8
9
10
11
12
13
14
15
16
17
18
19
20
21
22
23
24
25
26
27
28
29
30
31

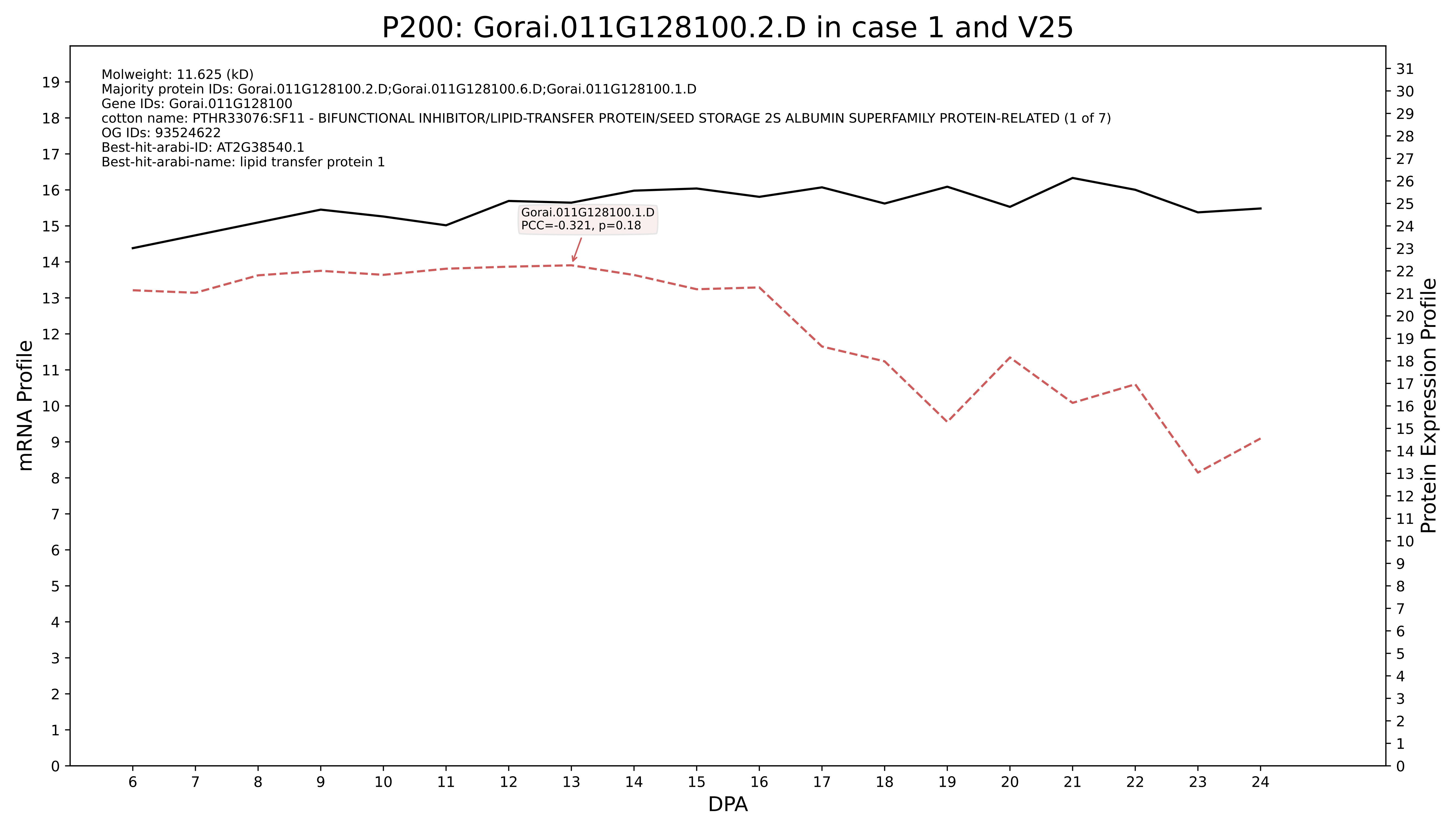
P200: Gorai.011G128100.2.D in case 1 and V25
Molweight: 11.625 (kD)
Majority protein IDs: Gorai.011G128100.2.D;Gorai.011G128100.6.D;Gorai.011G128100.1.D
Gene IDs: Gorai.011G128100
cotton name: PTHR33076:SF11 - BIFUNCTIONAL INHIBITOR/LIPID-TRANSFER PROTEIN/SEED STORAGE 2S ALBUMIN SUPERFAMILY PROTEIN-RELATED (1 of 7)
OG IDs: 93524622
Best-hit-arabi-ID: AT2G38540.1
Best-hit-arabi-name: lipid transfer protein 1
Gorai.011G128100.1.D
PCC=-0.321, p=0.18
mRNA Profile
Protein Expression Profile
DPA

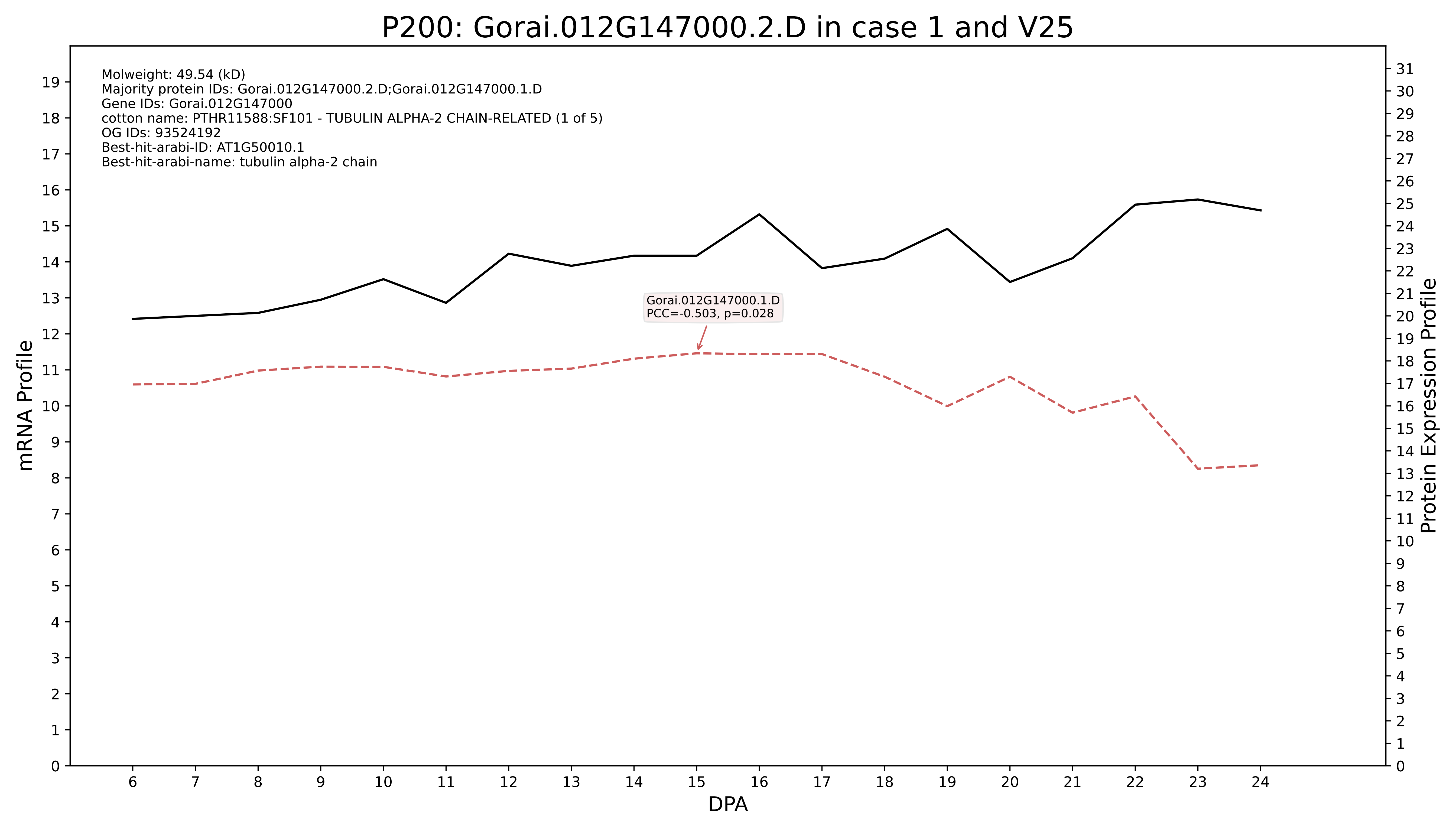
P200: Gorai.012G147000.2.D in case 1 and V25
Molweight: 49.54 (kD)
Majority protein IDs: Gorai.012G147000.2.D;Gorai.012G147000.1.D
Gene IDs: Gorai.012G147000
cotton name: PTHR11588:SF101 - TUBULIN ALPHA-2 CHAIN-RELATED (1 of 5)
OG IDs: 93524192
Best-hit-arabi-ID: AT1G50010.1
Best-hit-arabi-name: tubulin alpha-2 chain
Gorai.012G147000.1.D
PCC=-0.503, p=0.028
mRNA Profile
Protein Expression Profile
DPA

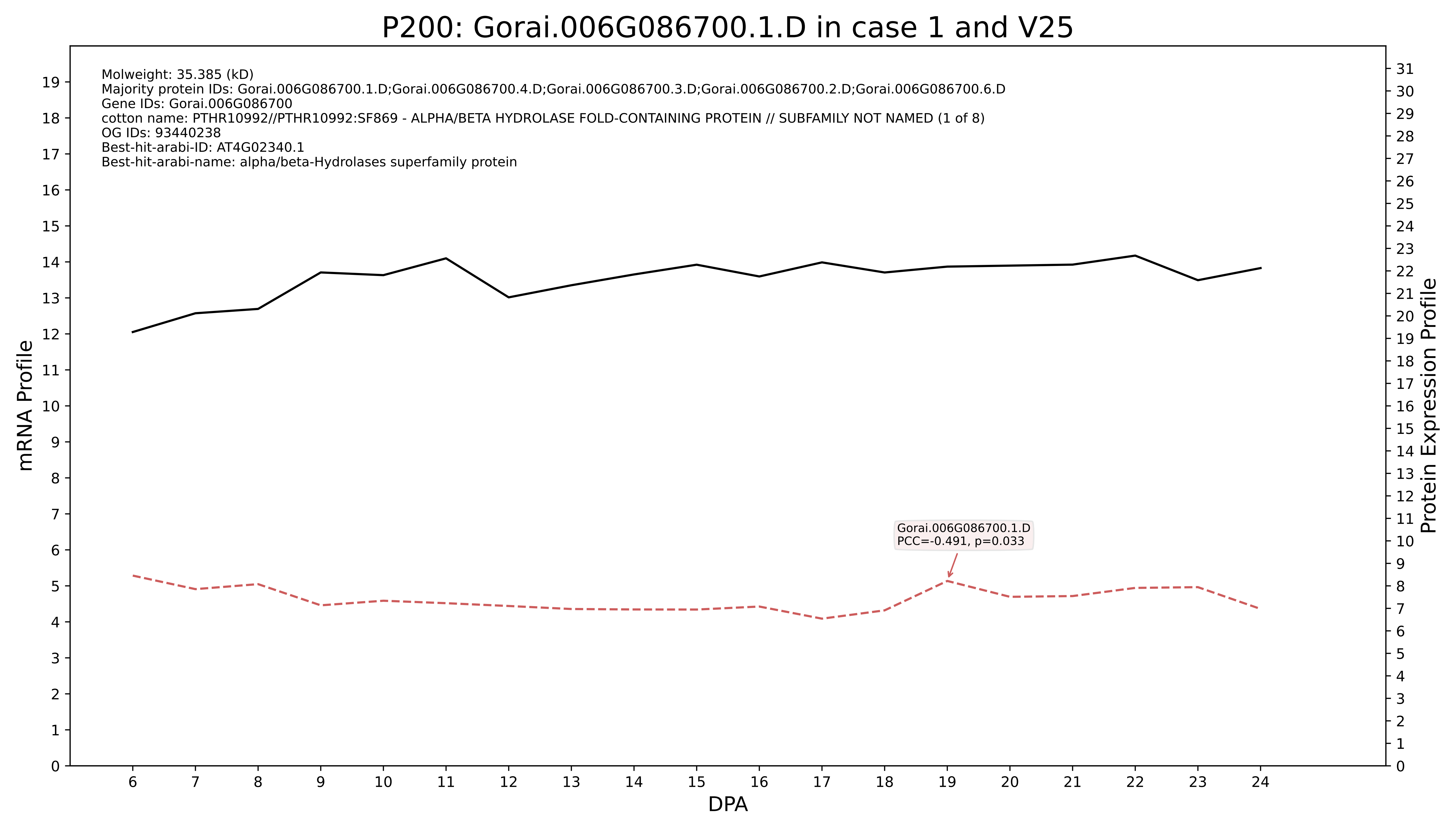

P200: Gorai.006G086700.1.D in case 1 and V25
Molweight: 35.385 (kD)
Majority protein IDs: Gorai.006G086700.1.D;Gorai.006G086700.4.D;Gorai.006G086700.3.D;Gorai.006G086700.2.D;Gorai.006G086700.6.D
Gene IDs: Gorai.006G086700
cotton name: PTHR10992//PTHR10992:SF869 - ALPHA/BETA HYDROLASE FOLD-CONTAINING PROTEIN // SUBFAMILY NOT NAMED (1 of 8)
OG IDs: 93440238
Best-hit-arabi-ID: AT4G02340.1
Best-hit-arabi-name: alpha/beta-Hydrolases superfamily protein
Gorai.006G086700.1.D
PCC=-0.491, p=0.033
mRNA Profile
Protein Expression Profile
DPA

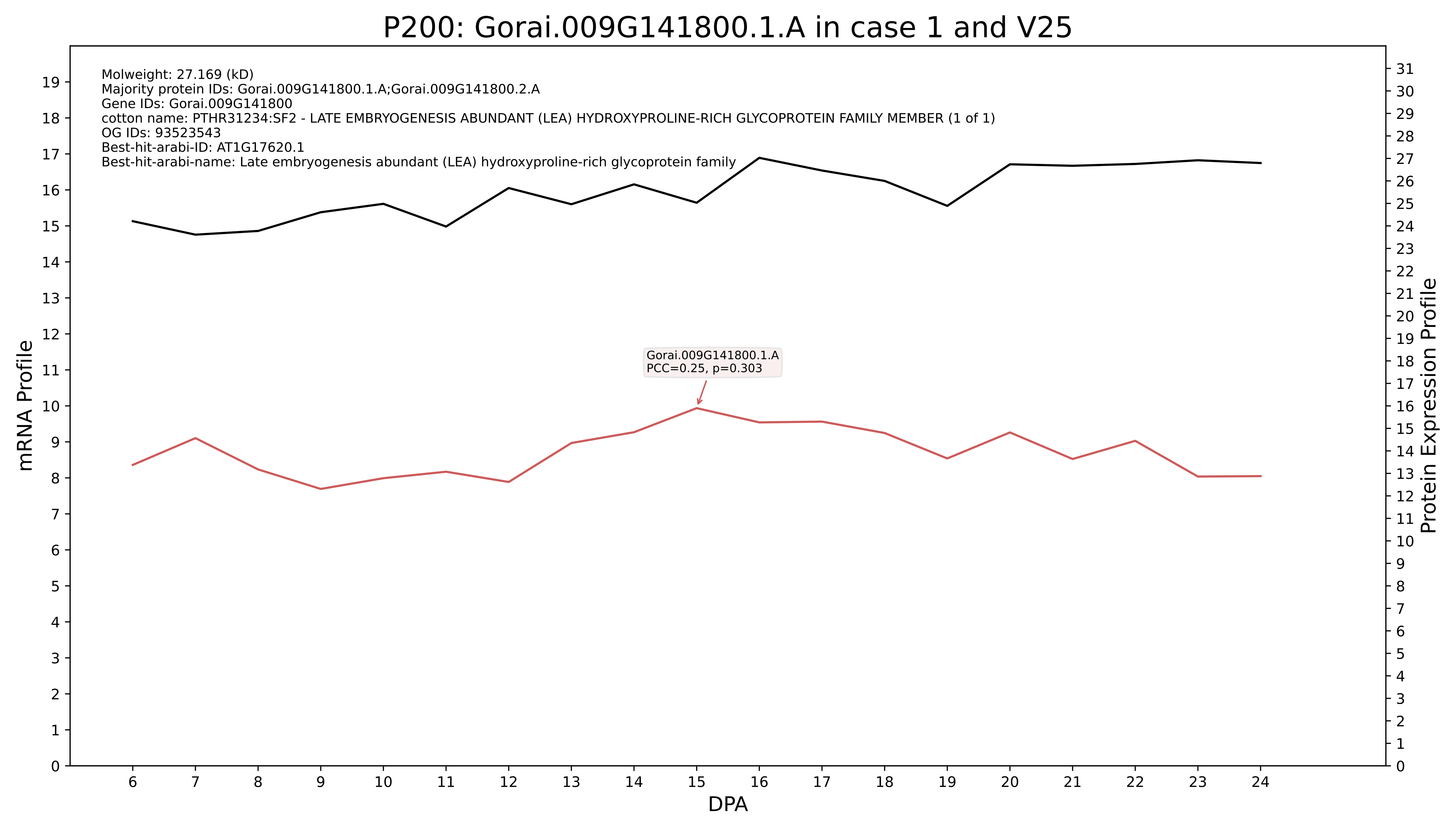

P200: Gorai.009G141800.1.A in case 1 and V25
Molweight: 27.169 (kD)
Majority protein IDs: Gorai.009G141800.1.A;Gorai.009G141800.2.A
Gene IDs: Gorai.009G141800
cotton name: PTHR31234:SF2 - LATE EMBRYOGENESIS ABUNDANT (LEA) HYDROXYPROLINE-RICH GLYCOPROTEIN FAMILY MEMBER (1 of 1)
OG IDs: 93523543
Best-hit-arabi-ID: AT1G17620.1
Best-hit-arabi-name: Late embryogenesis abundant (LEA) hydroxyproline-rich glycoprotein family
Gorai.009G141800.1.A
PCC=0.25, p=0.303
mRNA Profile
Protein Expression Profile
DPA

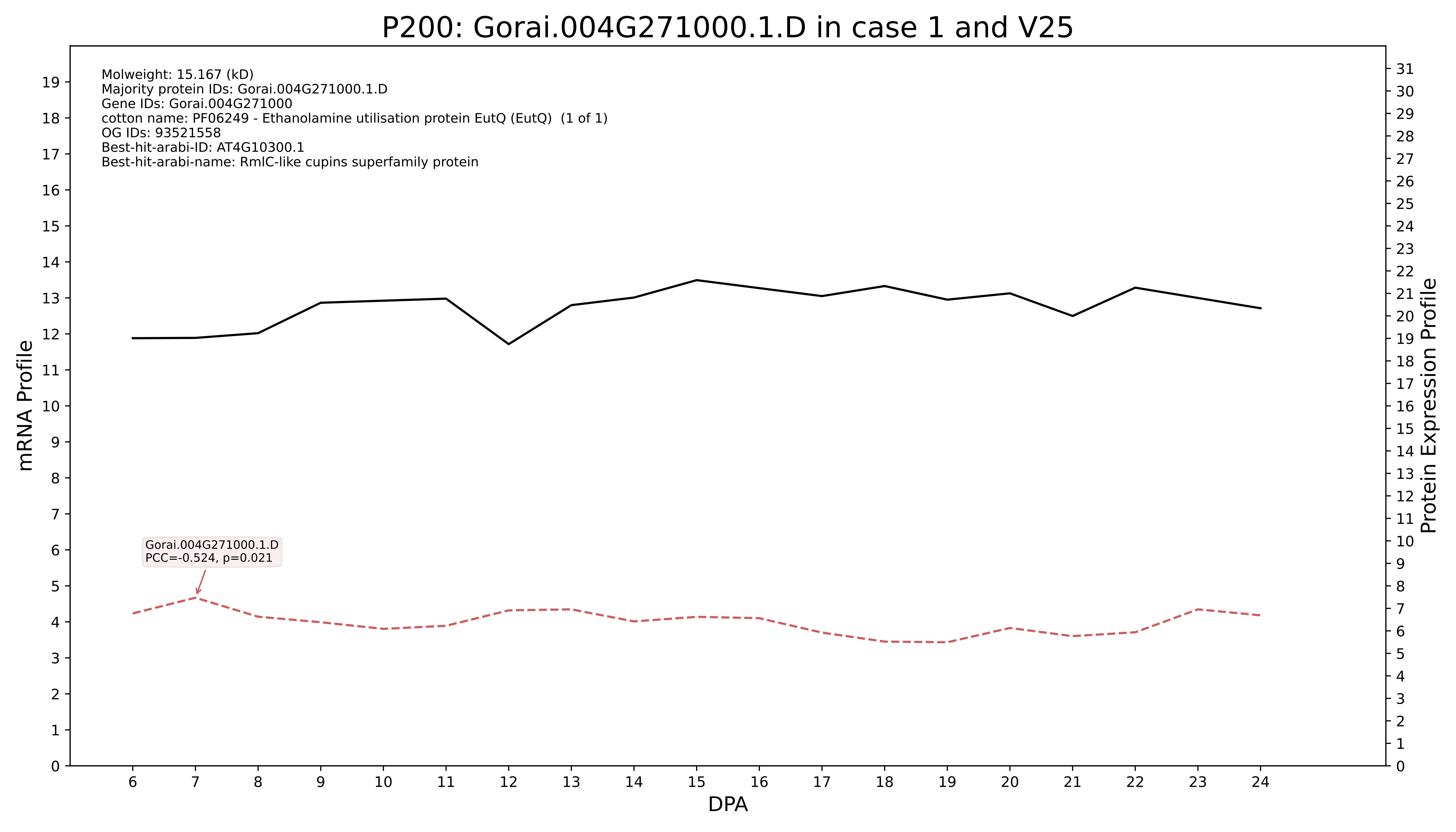
P200: Gorai.004G271000.1.D in case 1 and V25
Molweight: 15.167 (kD)
Majority protein IDs: Gorai.004G271000.1.D
Gene IDs: Gorai.004G271000
cotton name: PF06249 - Ethanolamine utilisation protein EutQ (EutQ) (1 of 1)
OG IDs: 93521558
Best-hit-arabi-ID: AT4G10300.1
Best-hit-arabi-name: RmlC-like cupins superfamily protein
Gorai.004G271000.1.D
PCC=-0.524, p=0.021
mRNA Profile
Protein Expression Profile
DPA

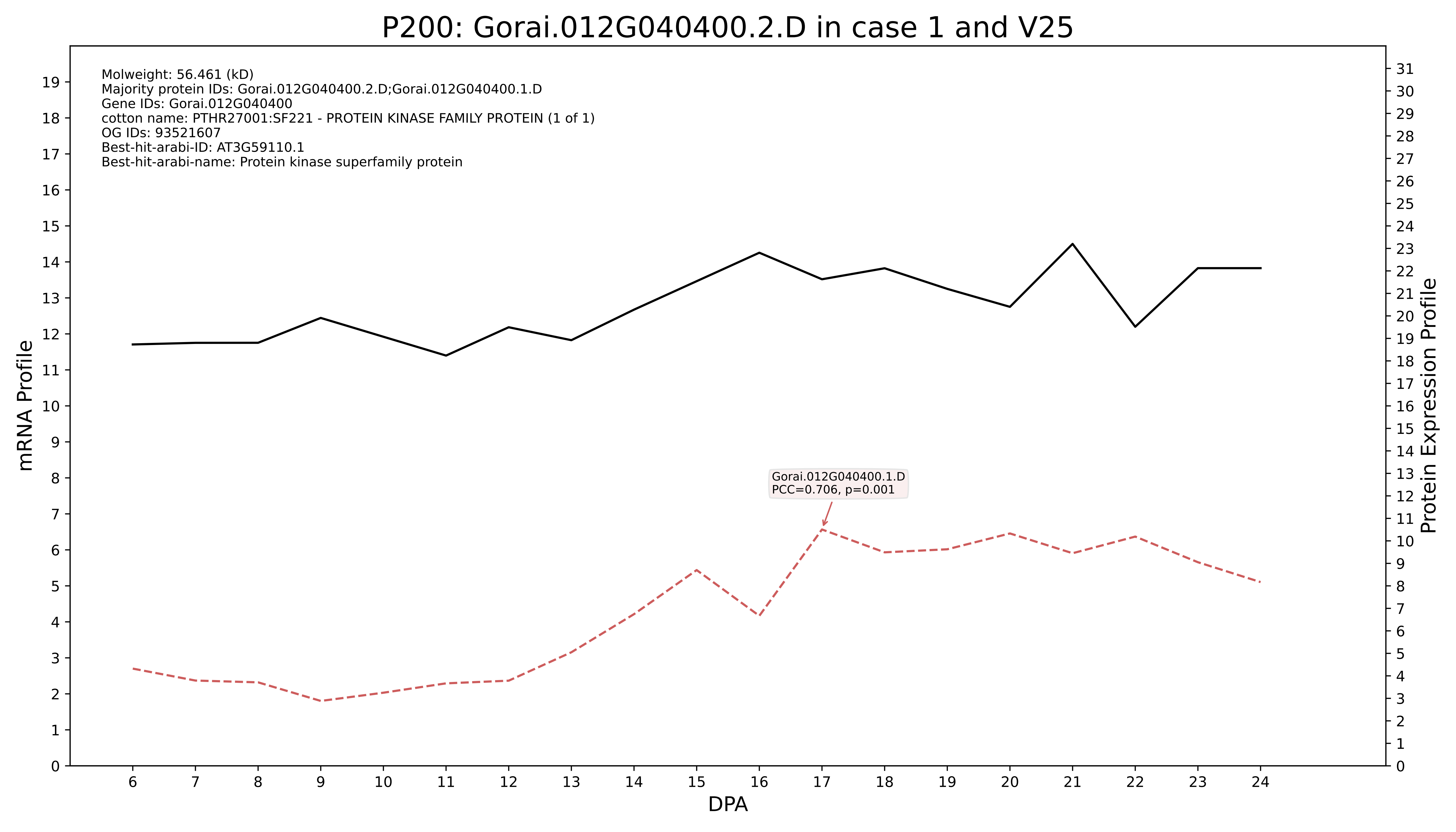
P200: Gorai.012G040400.2.D in case 1 and V25
Molweight: 56.461 (kD)
Majority protein IDs: Gorai.012G040400.2.D;Gorai.012G040400.1.D
Gene IDs: Gorai.012G040400
cotton name: PTHR27001:SF221 - PROTEIN KINASE FAMILY PROTEIN (1 of 1)
OG IDs: 93521607
Best-hit-arabi-ID: AT3G59110.1
Best-hit-arabi-name: Protein kinase superfamily protein
Gorai.012G040400.1.D
PCC=0.706, p=0.001
mRNA Profile
Protein Expression Profile
DPA

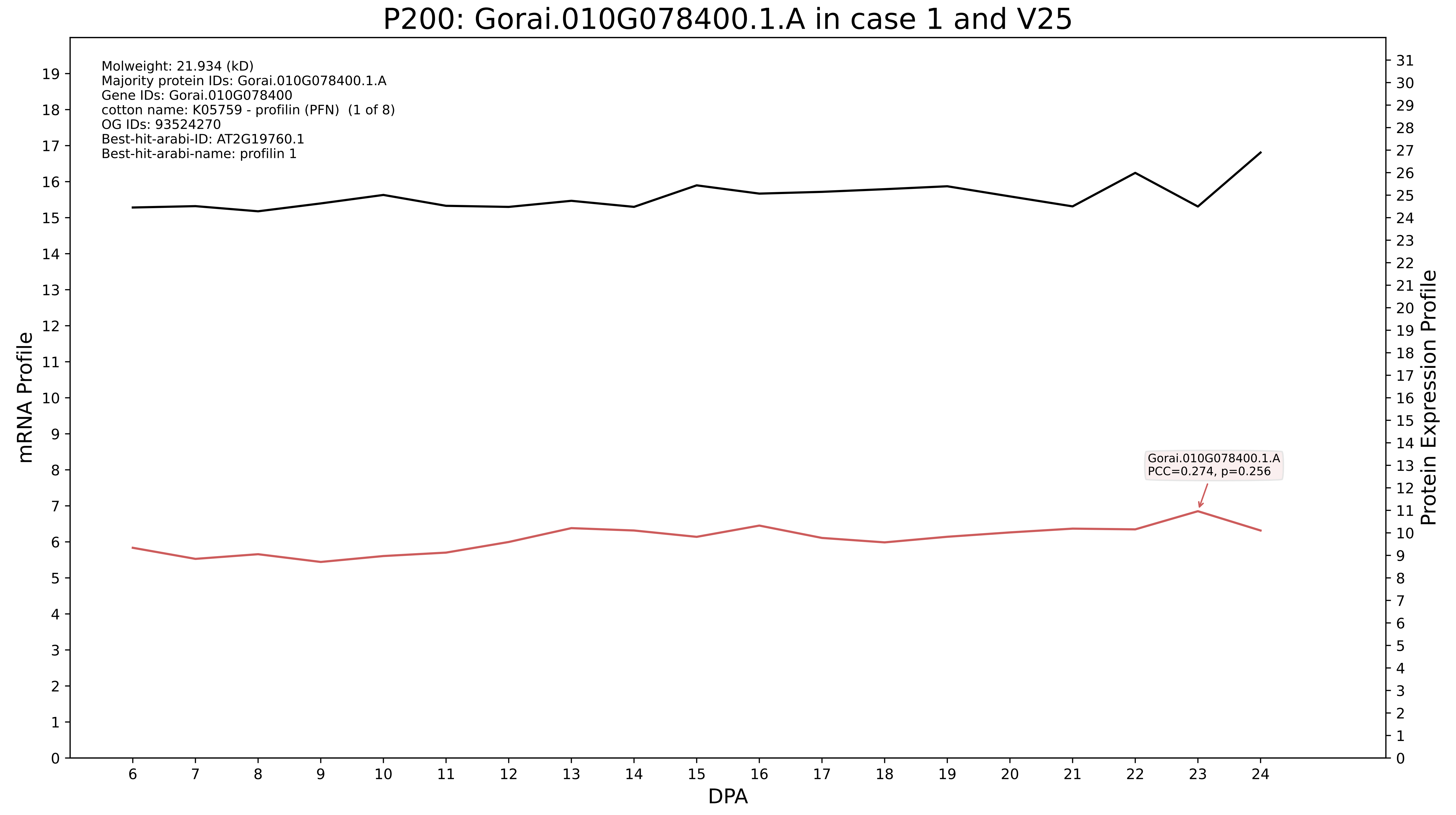
P200: Gorai.010G078400.1.A in case 1 and V25
Molweight: 21.934 (kD)
Majority protein IDs: Gorai.010G078400.1.A
Gene IDs: Gorai.010G078400
cotton name: K05759 - profilin (PFN) (1 of 8)
OG IDs: 93524270
Best-hit-arabi-ID: AT2G19760.1
Best-hit-arabi-name: profilin 1
Gorai.010G078400.1.A
PCC=0.274, p=0.256
mRNA Profile
Protein Expression Profile
DPA
0
1
2
3
4
5
6
7
8
9
10
11
12
13
14
15
16
17
18
19
20
21
22
23
24
25
26
27
28
29
30
31

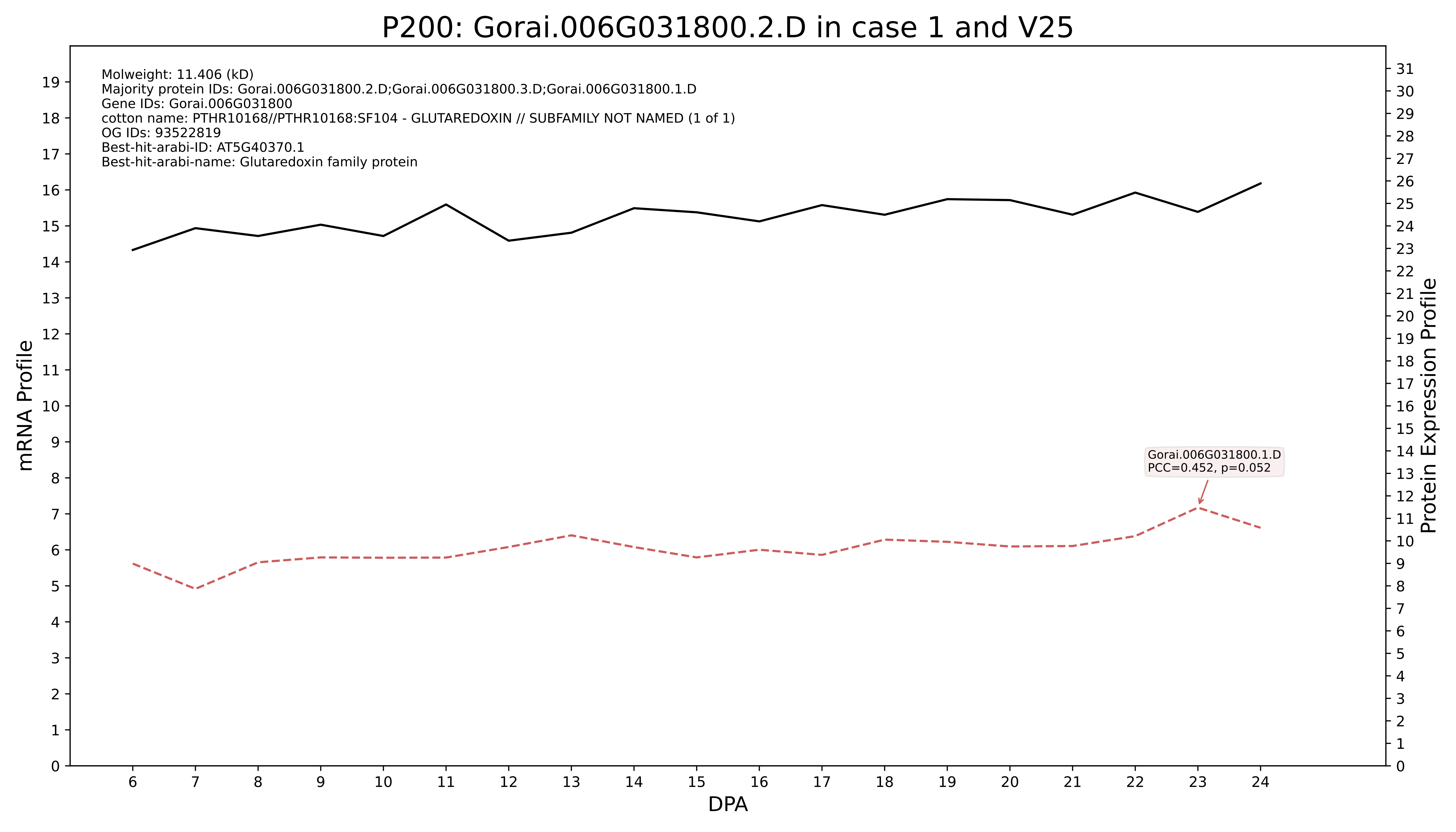
P200: Gorai.006G031800.2.D in case 1 and V25
Molweight: 11.406 (kD)
Majority protein IDs: Gorai.006G031800.2.D;Gorai.006G031800.3.D;Gorai.006G031800.1.D
Gene IDs: Gorai.006G031800
cotton name: PTHR10168//PTHR10168:SF104 - GLUTAREDOXIN // SUBFAMILY NOT NAMED (1 of 1)
OG IDs: 93522819
Best-hit-arabi-ID: AT5G40370.1
Best-hit-arabi-name: Glutaredoxin family protein
Gorai.006G031800.1.D
PCC=0.452, p=0.052
mRNA Profile
Protein Expression Profile
DPA

P200: Gorai.007G157500.1.D in case 1 and V25

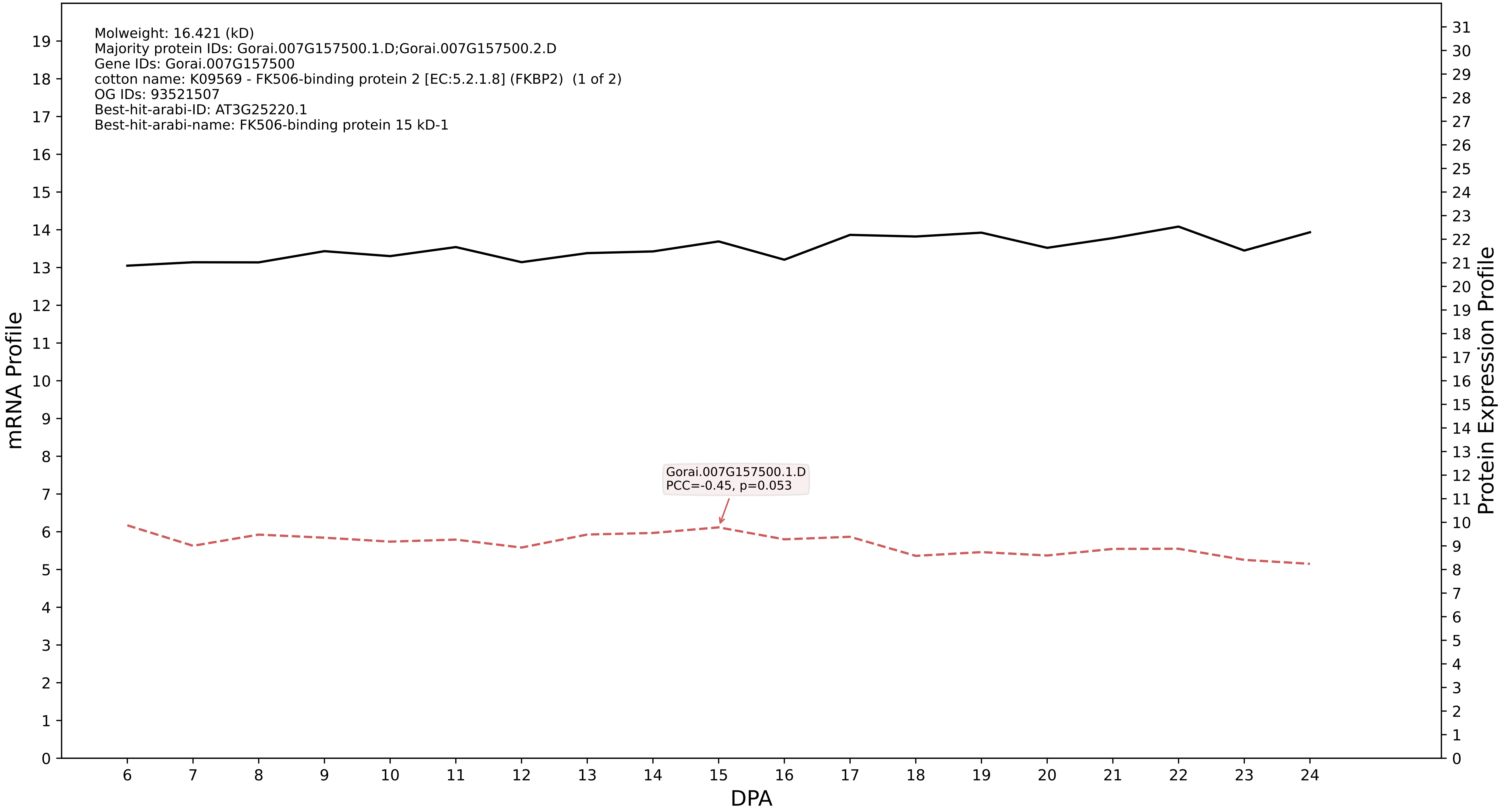

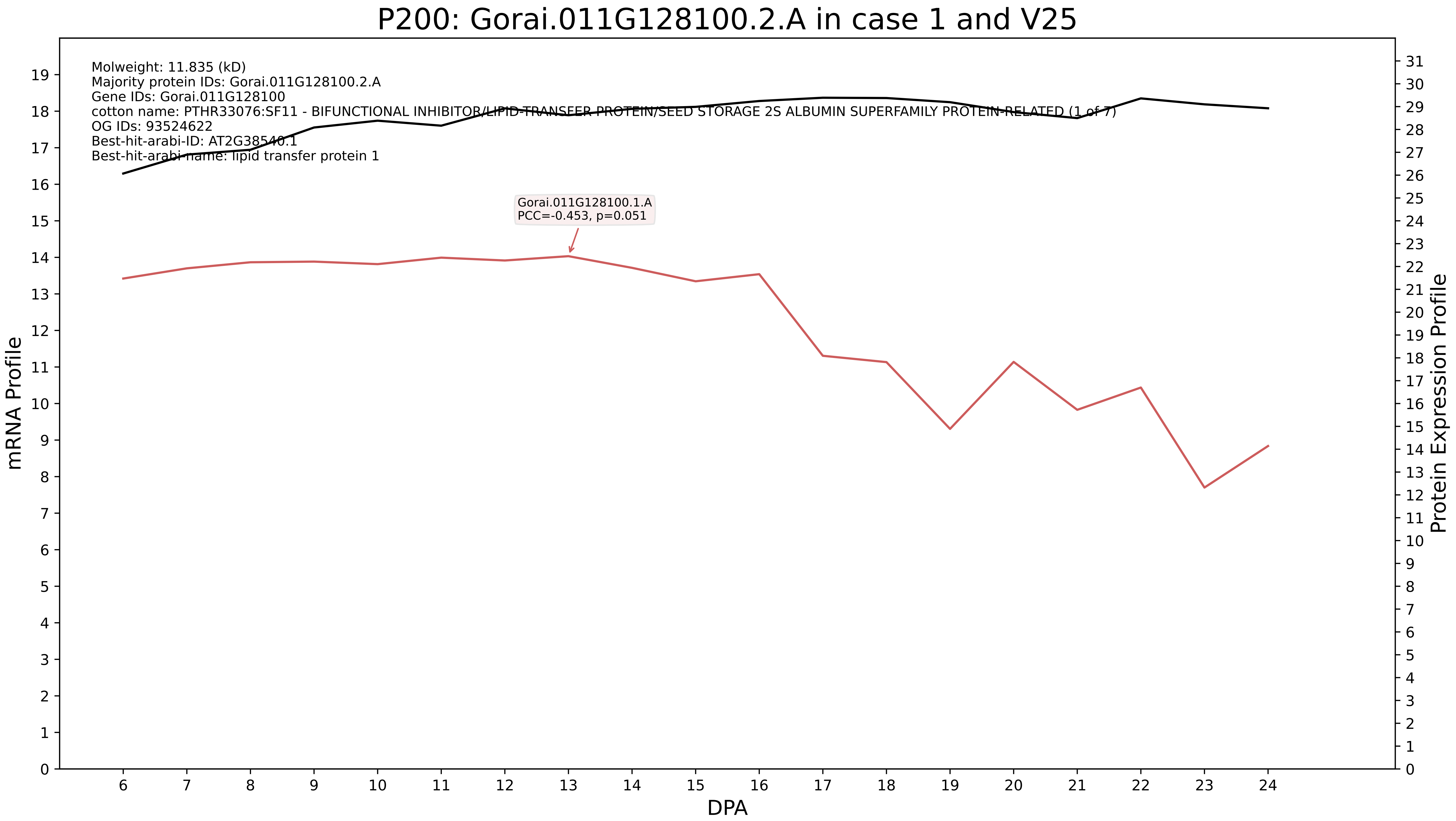
P200: Gorai.011G128100.2.A in case 1 and V25
Molweight: 11.835 (kD)
Majority protein IDs: Gorai.011G128100.2.A
Gene IDs: Gorai.011G128100
cotton name: PTHR33076:SF11 - BIFUNCTIONAL INHIBITOR/LIPID-TRANSFER PROTEIN/SEED STORAGE 2S ALBUMIN SUPERFAMILY PROTEIN-RELATED (1 of 7)
OG IDs: 93524622
Best-hit-arabi-ID: AT2G38540.1
Best-hit-arabi-name: lipid transfer protein 1
Gorai.011G128100.1.A
PCC=-0.453, p=0.051
mRNA Profile
Protein Expression Profile
DPA
0
1
2
3
4
5
6
7
8
9
10
11
12
13
14
15
16
17
18
19
20
21
22
23
24
25
26
27
28
29
30
31

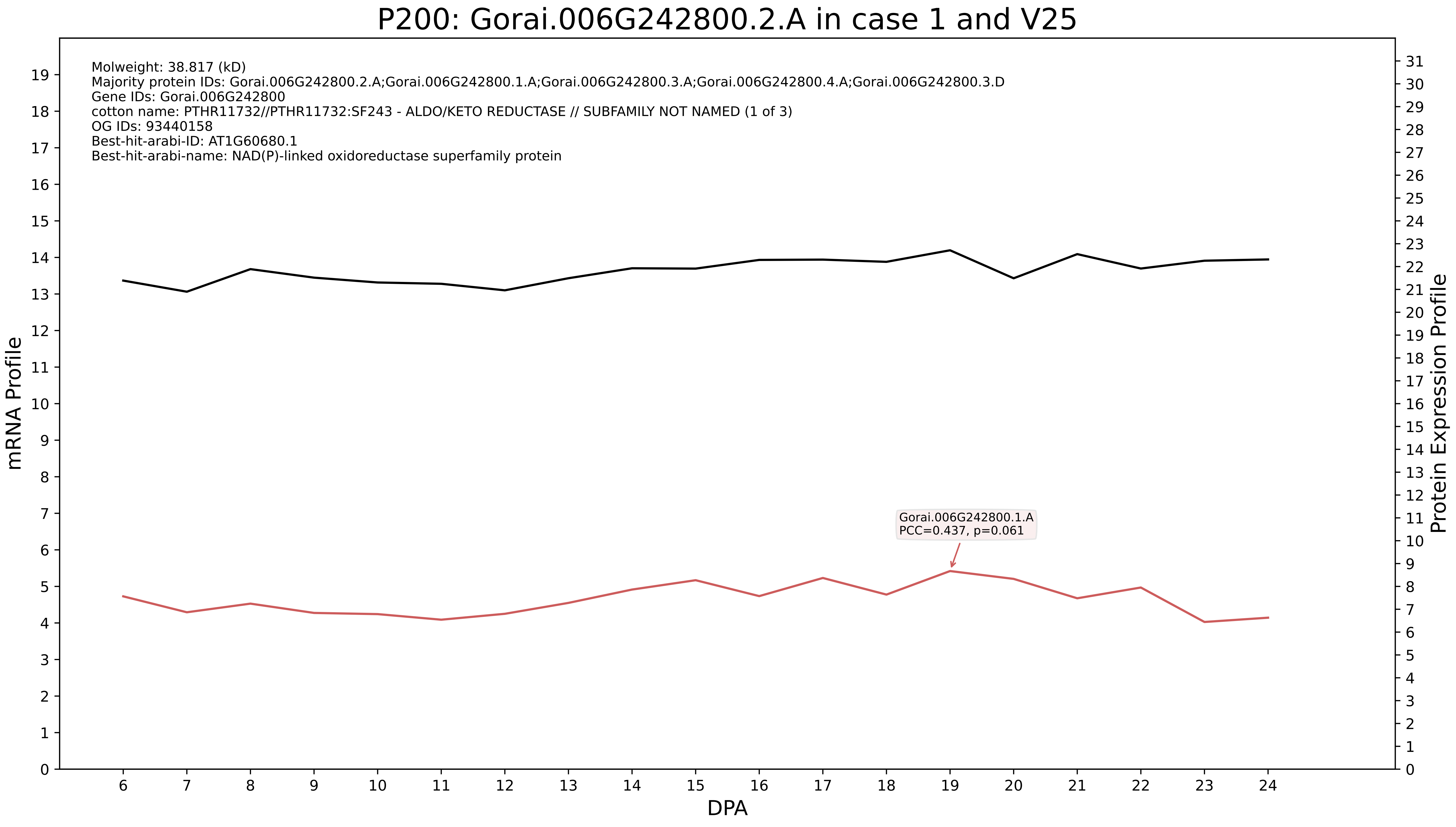
P200: Gorai.006G242800.2.A in case 1 and V25
Molweight: 38.817 (kD)
Majority protein IDs: Gorai.006G242800.2.A;Gorai.006G242800.1.A;Gorai.006G242800.3.A;Gorai.006G242800.4.A;Gorai.006G242800.3.D
Gene IDs: Gorai.006G242800
cotton name: PTHR11732//PTHR11732:SF243 - ALDO/KETO REDUCTASE // SUBFAMILY NOT NAMED (1 of 3)
OG IDs: 93440158
Best-hit-arabi-ID: AT1G60680.1
Best-hit-arabi-name: NAD(P)-linked oxidoreductase superfamily protein
Gorai.006G242800.1.A
PCC=0.437, p=0.061
mRNA Profile
Protein Expression Profile
DPA

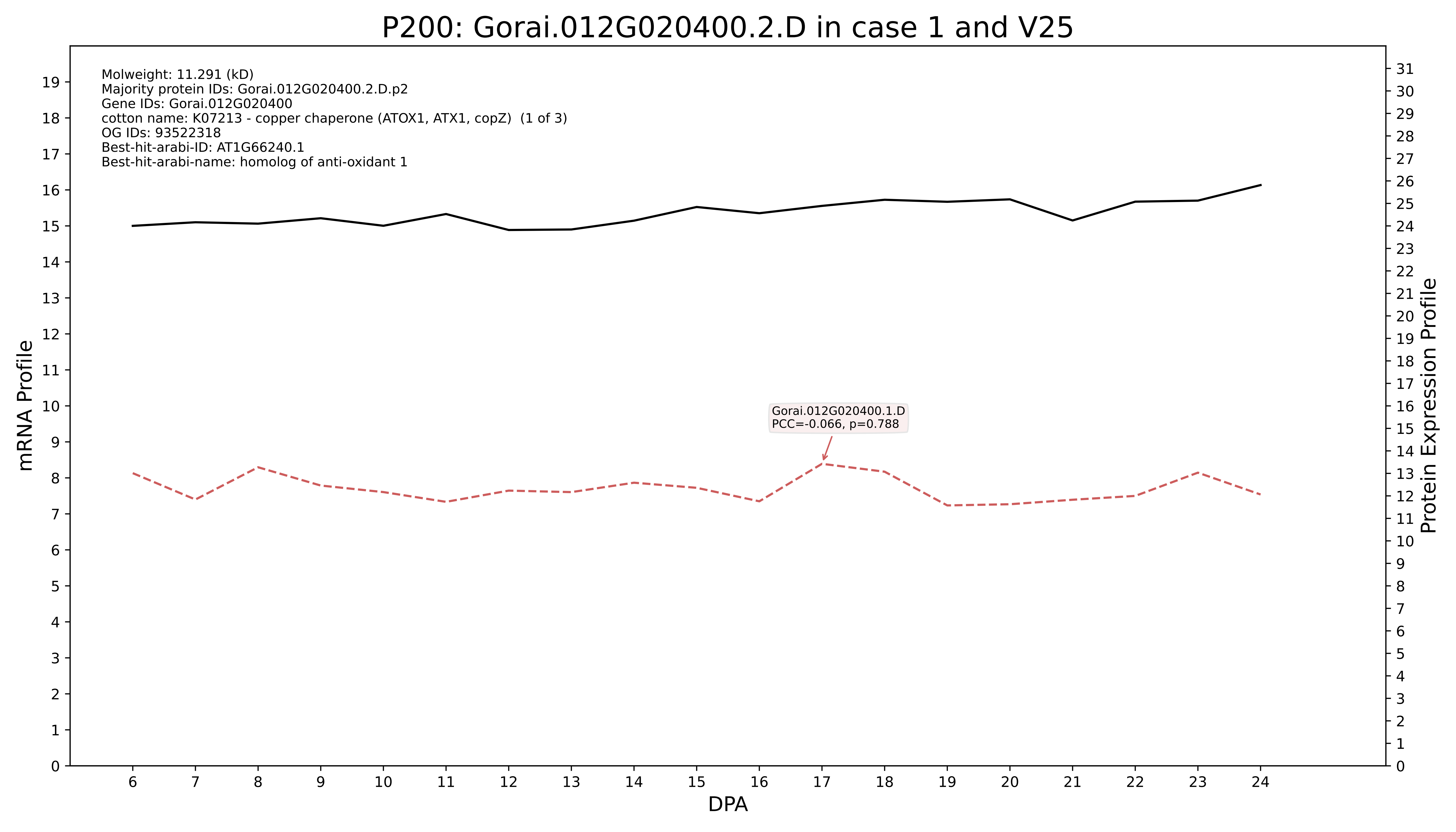

P200: Gorai.012G020400.2.D in case 1 and V25
Molweight: 11.291 (kD)
Majority protein IDs: Gorai.012G020400.2.D.p2
Gene IDs: Gorai.012G020400
cotton name: K07213 - copper chaperone (ATOX1, ATX1, copZ) (1 of 3)
OG IDs: 93522318
Best-hit-arabi-ID: AT1G66240.1
Best-hit-arabi-name: homolog of anti-oxidant 1
Gorai.012G020400.1.D
PCC=-0.066, p=0.788
mRNA Profile
Protein Expression Profile
DPA

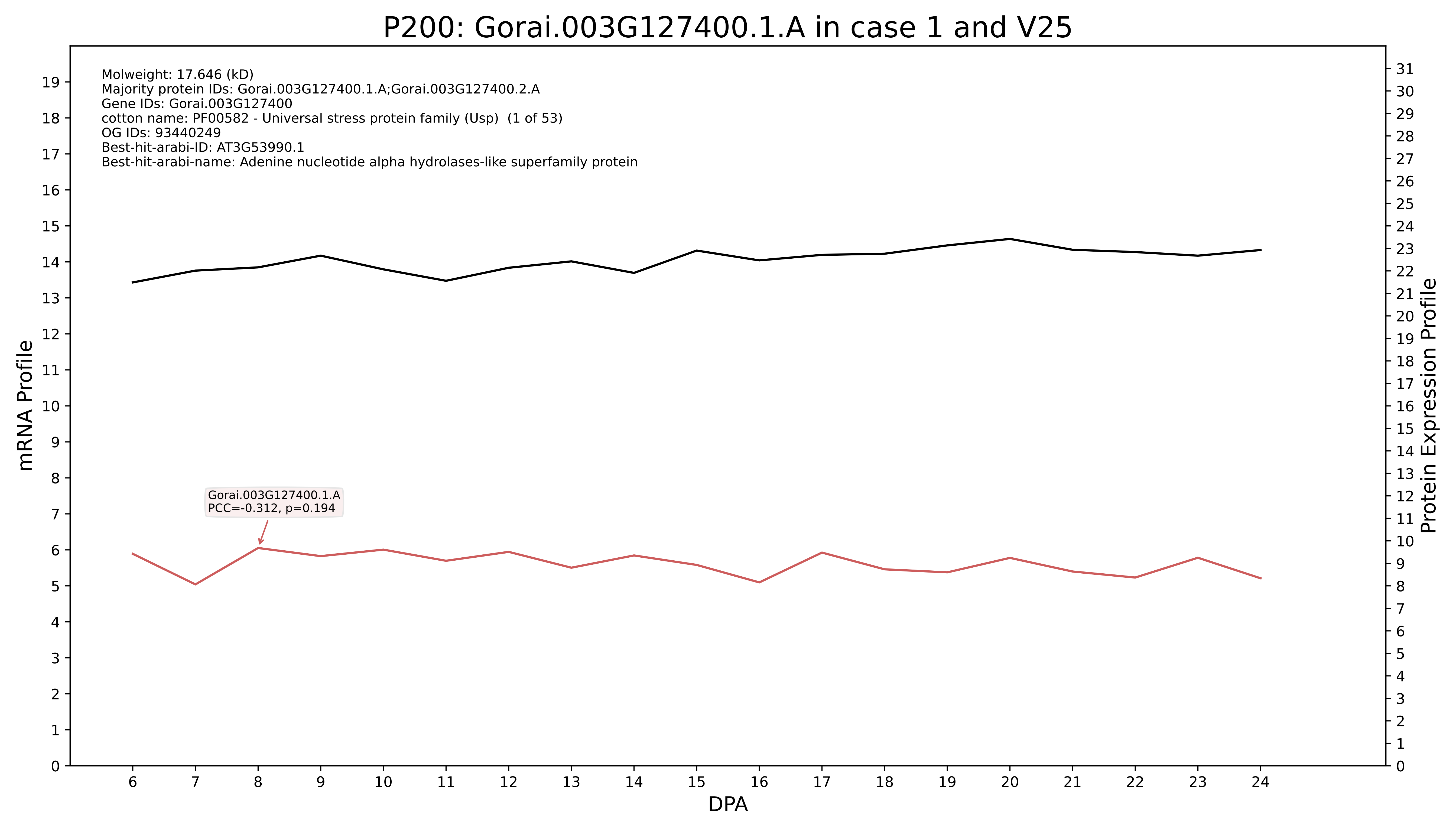
P200: Gorai.003G127400.1.A in case 1 and V25
Molweight: 17.646 (kD)
Majority protein IDs: Gorai.003G127400.1.A;Gorai.003G127400.2.A
Gene IDs: Gorai.003G127400
cotton name: PF00582 - Universal stress protein family (Usp) (1 of 53)
OG IDs: 93440249
Best-hit-arabi-ID: AT3G53990.1
Best-hit-arabi-name: Adenine nucleotide alpha hydrolases-like superfamily protein
Gorai.003G127400.1.A
PCC=-0.312, p=0.194
mRNA Profile
Protein Expression Profile
DPA

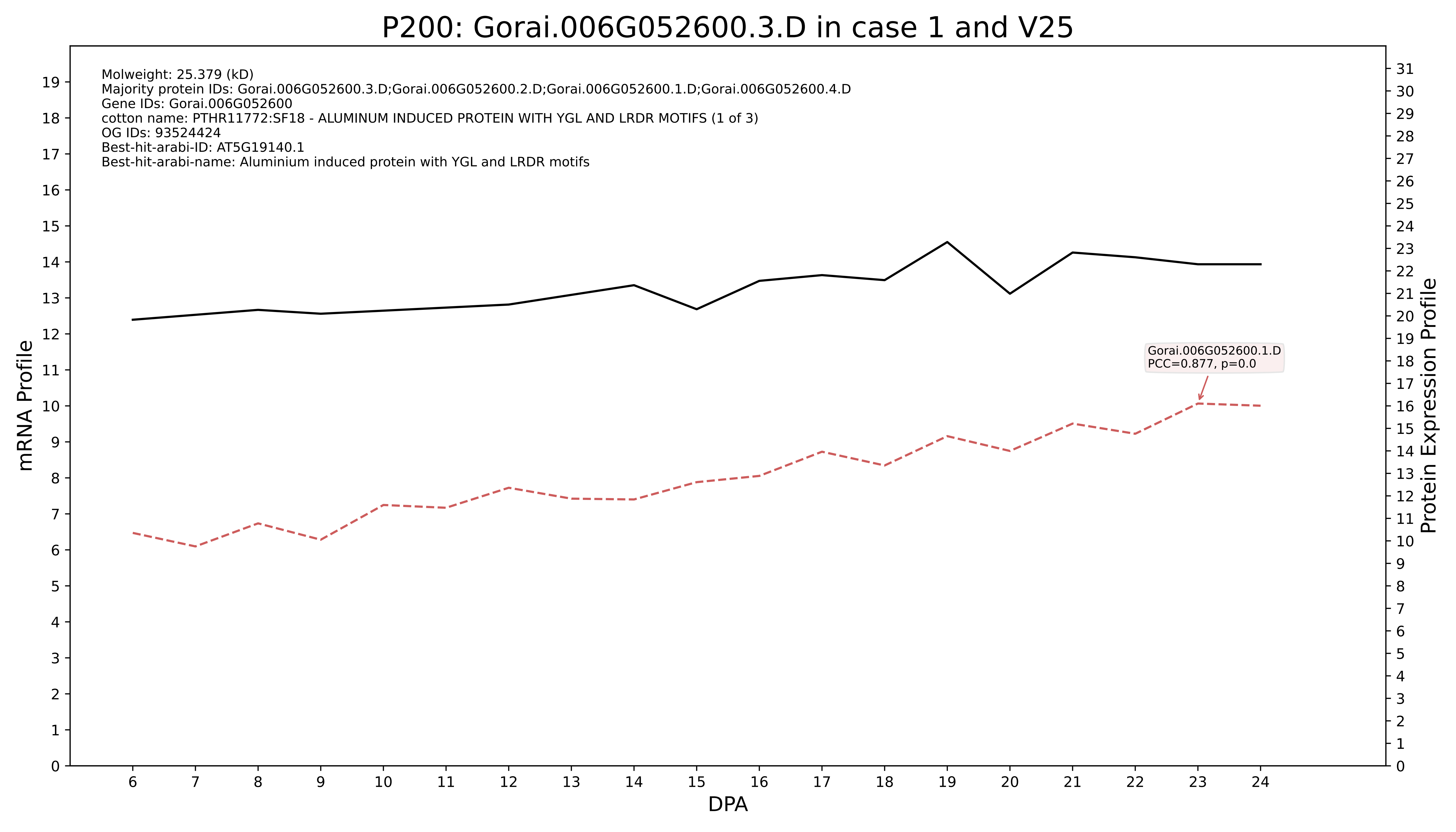

P200: Gorai.006G052600.3.D in case 1 and V25
Molweight: 25.379 (kD)
Majority protein IDs: Gorai.006G052600.3.D;Gorai.006G052600.2.D;Gorai.006G052600.1.D;Gorai.006G052600.4.D
Gene IDs: Gorai.006G052600
cotton name: PTHR11772:SF18 - ALUMINUM INDUCED PROTEIN WITH YGL AND LRDR MOTIFS (1 of 3)
OG IDs: 93524424
Best-hit-arabi-ID: AT5G19140.1
Best-hit-arabi-name: Aluminium induced protein with YGL and LRDR motifs
Gorai.006G052600.1.D
PCC=0.877, p=0.0
mRNA Profile
Protein Expression Profile
DPA

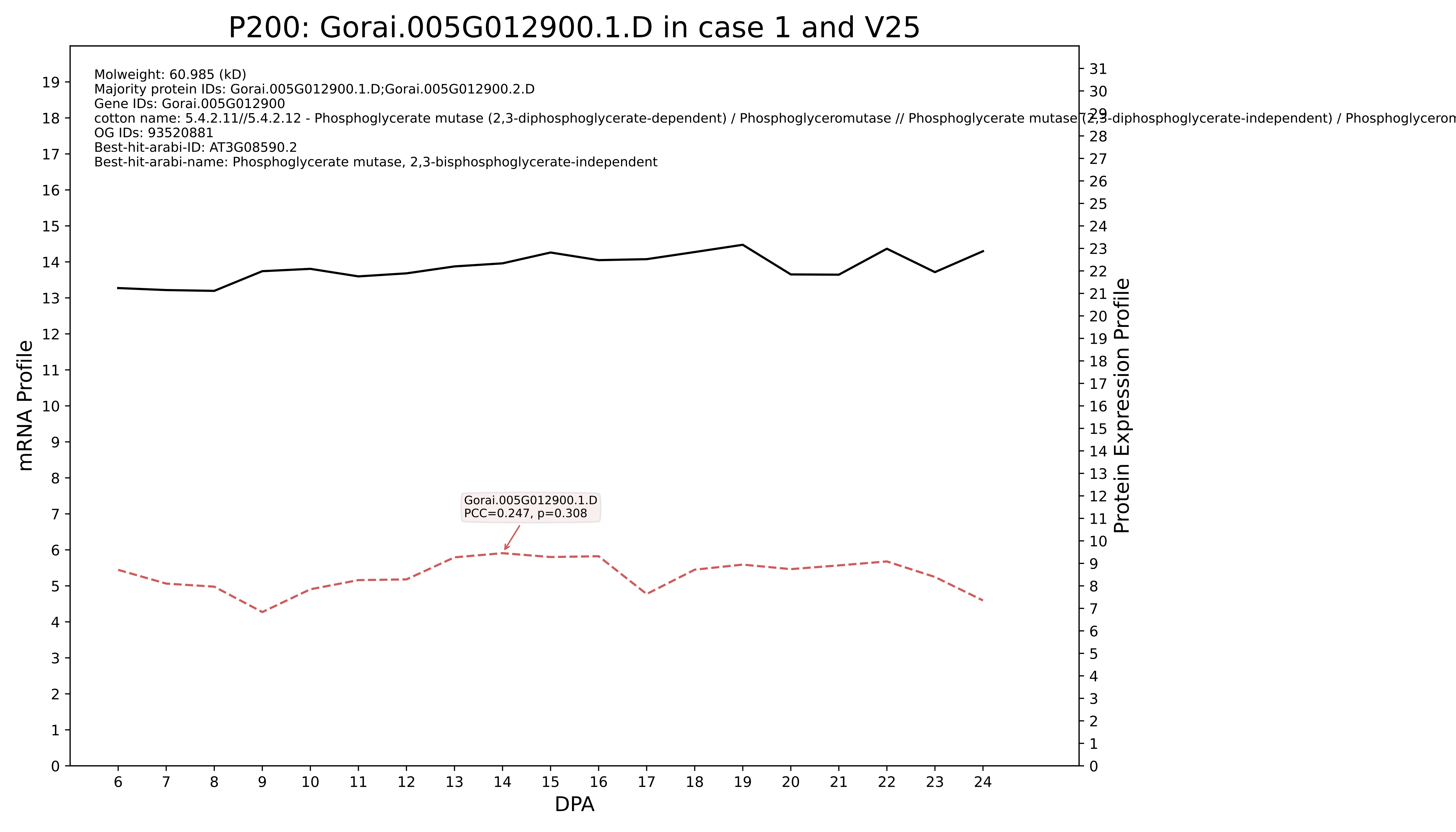
P200: Gorai.005G012900.1.D in case 1 and V25
Molweight: 60.985 (kD)
Majority protein IDs: Gorai.005G012900.1.D;Gorai.005G012900.2.D
Gene IDs: Gorai.005G012900
cotton name: 5.4.2.11//5.4.2.12 - Phosphoglycerate mutase (2,3-diphosphoglycerate-dependent) / Phosphoglyceromutase // Phosphoglycerate mutase (2,3-diphosphoglycerate-independent) / Phosphoglyceron
OG IDs: 93520881
Best-hit-arabi-ID: AT3G08590.2
Best-hit-arabi-name: Phosphoglycerate mutase, 2,3-bisphosphoglycerate-independent
Gorai.005G012900.1.D
PCC=0.247, p=0.308
mRNA Profile
Protein Expression Profile
DPA
0 1 2 3 4 5 6 7 8 9 10 11 12 13 14 15 16 17 18 19
0 1 2 3 4 5 6 7 8 9 10 11 12 13 14 15 16 17 18 19 20 21 22 23 24 25 26 27 28 29 30 31
6 7 8 9 10 11 12 13 14 15 16 17 18 19 20 21 22 23 24

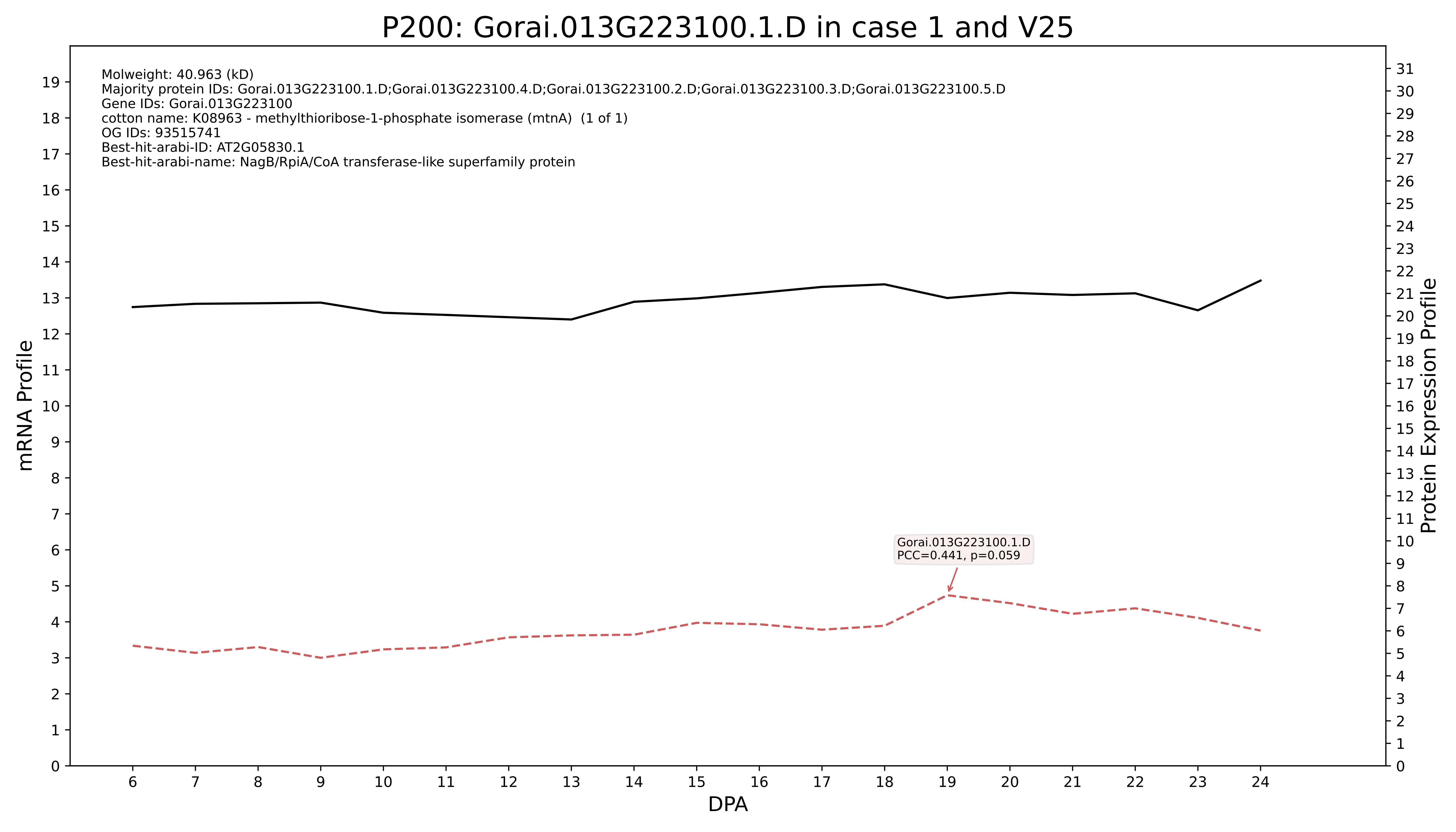
P200: Gorai.013G223100.1.D in case 1 and V25
Molweight: 40.963 (kD)
Majority protein IDs: Gorai.013G223100.1.D;Gorai.013G223100.4.D;Gorai.013G223100.2.D;Gorai.013G223100.3.D;Gorai.013G223100.5.D
Gene IDs: Gorai.013G223100
cotton name: K08963 - methylthioribose-1-phosphate isomerase (mtnA) (1 of 1)
OG IDs: 93515741
Best-hit-arabi-ID: AT2G05830.1
Best-hit-arabi-name: NagB/RpiA/CoA transferase-like superfamily protein
Gorai.013G223100.1.D
PCC=0.441, p=0.059
mRNA Profile
Protein Expression Profile
DPA

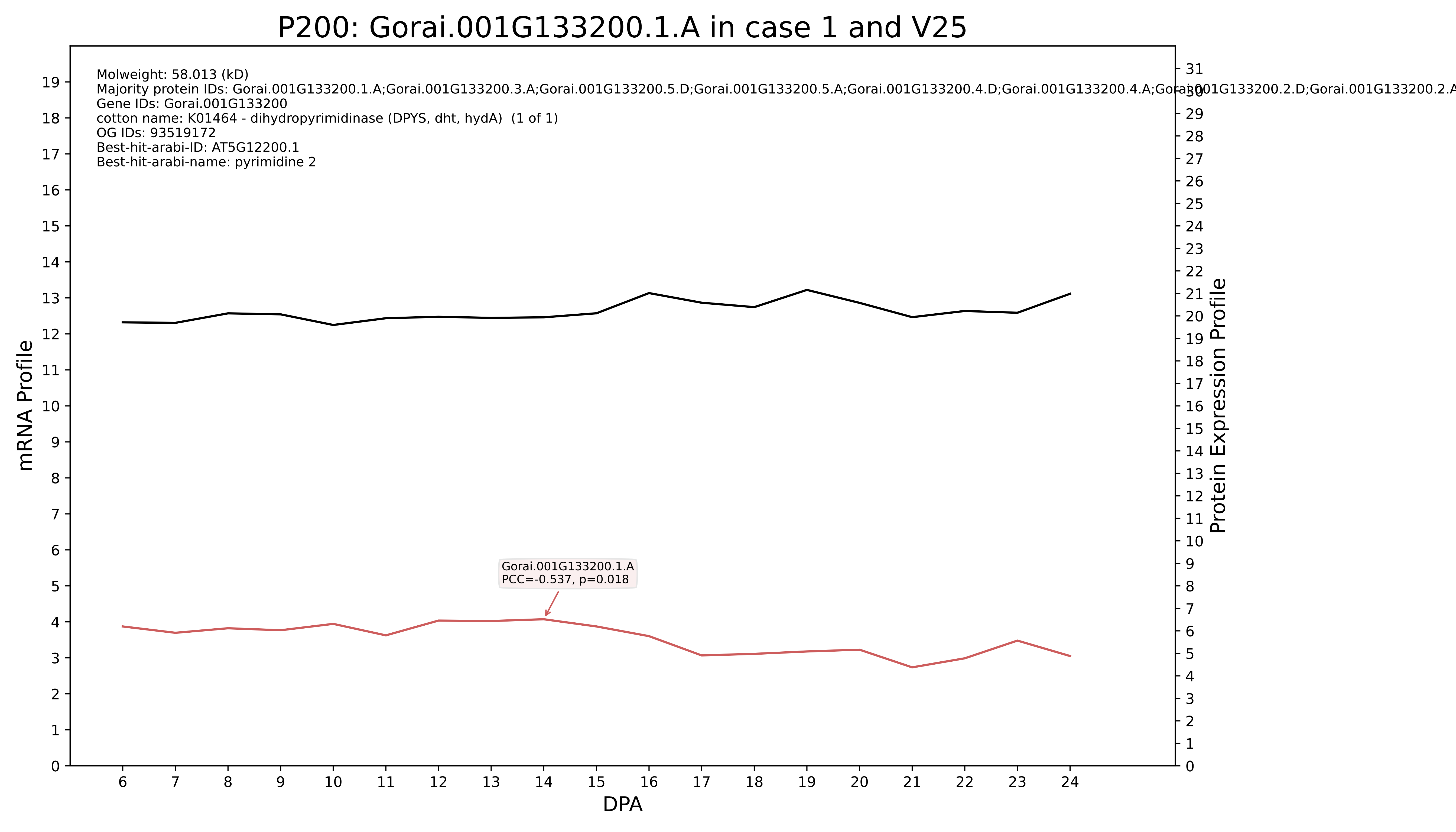
P200: Gorai.001G133200.1.A in case 1 and V25
Molweight: 58.013 (kD)
Majority protein IDs: Gorai.001G133200.1.A;Gorai.001G133200.3.A;Gorai.001G133200.5.D;Gorai.001G133200.5.A;Gorai.001G133200.4.D;Gorai.001G133200.4.A;Gorai.001G133200.2.D;Gorai.001G133200.2.A
Gene IDs: Gorai.001G133200
cotton name: K01464 - dihydropyrimidinase (DPYS, dht, hydA) (1 of 1)
OG IDs: 93519172
Best-hit-arabi-ID: AT5G12200.1
Best-hit-arabi-name: pyrimidine 2
Gorai.001G133200.1.A
PCC=-0.537, p=0.018
mRNA Profile
Protein Expression Profile
DPA
0
1
2
3
4
5
6
7
8
9
10
11
12
13
14
15
16
17
18
19
20
21
22
23
24
25
26
27
28
29
30
31

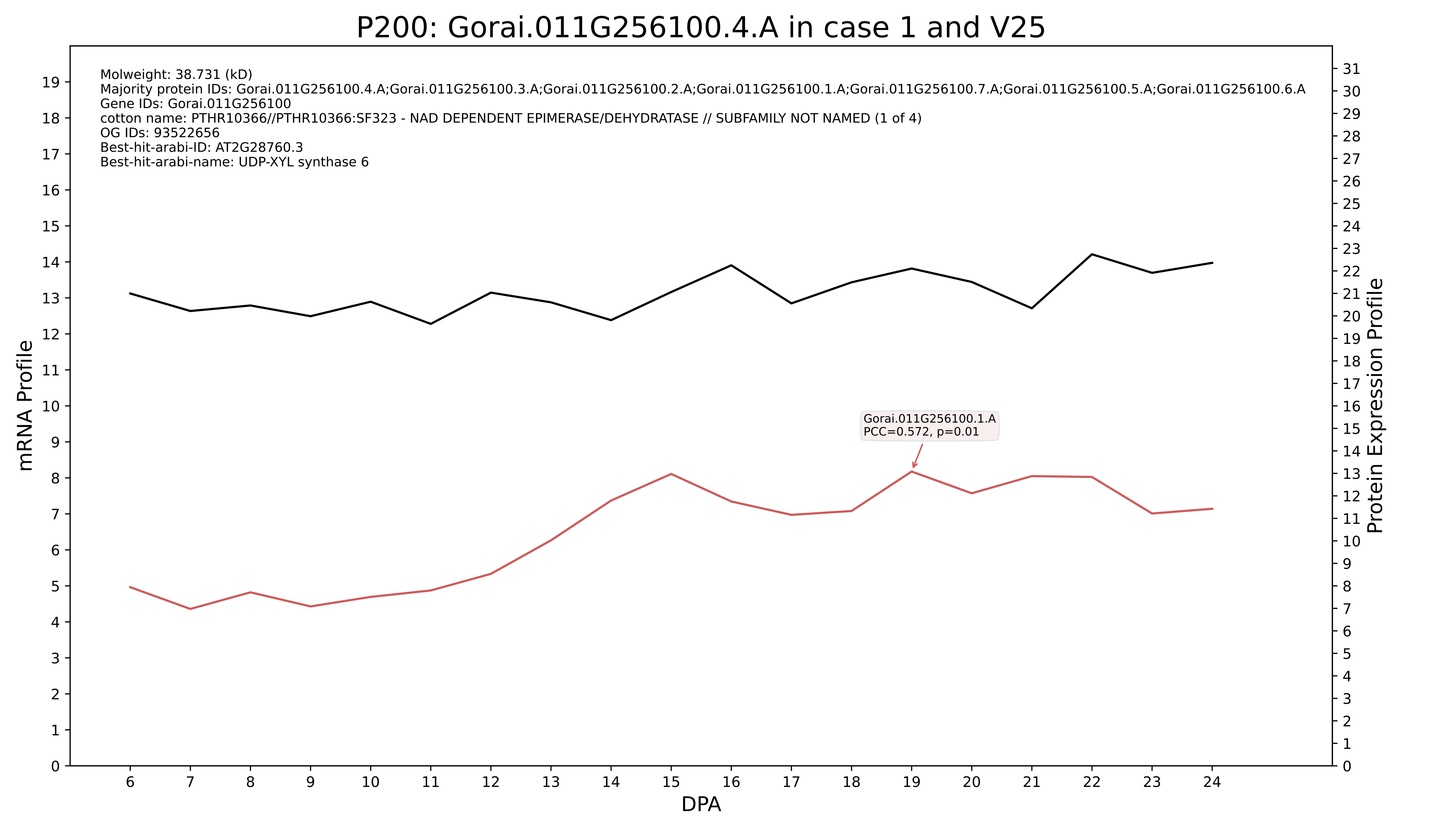

P200: Gorai.011G256100.4.A in case 1 and V25
Molweight: 38.731 (kD)
Majority protein IDs: Gorai.011G256100.4.A;Gorai.011G256100.3.A;Gorai.011G256100.2.A;Gorai.011G256100.1.A;Gorai.011G256100.7.A;Gorai.011G256100.5.A;Gorai.011G256100.6.A
Gene IDs: Gorai.011G256100
cotton name: PTHR10366//PTHR10366:SF323 - NAD DEPENDENT EPIMERASE/DEHYDRATASE // SUBFAMILY NOT NAMED (1 of 4)
OG IDs: 93522656
Best-hit-arabi-ID: AT2G28760.3
Best-hit-arabi-name: UDP-XYL synthase 6
Gorai.011G256100.1.A
PCC=0.572, p=0.01
mRNA Profile
Protein Expression Profile
DPA

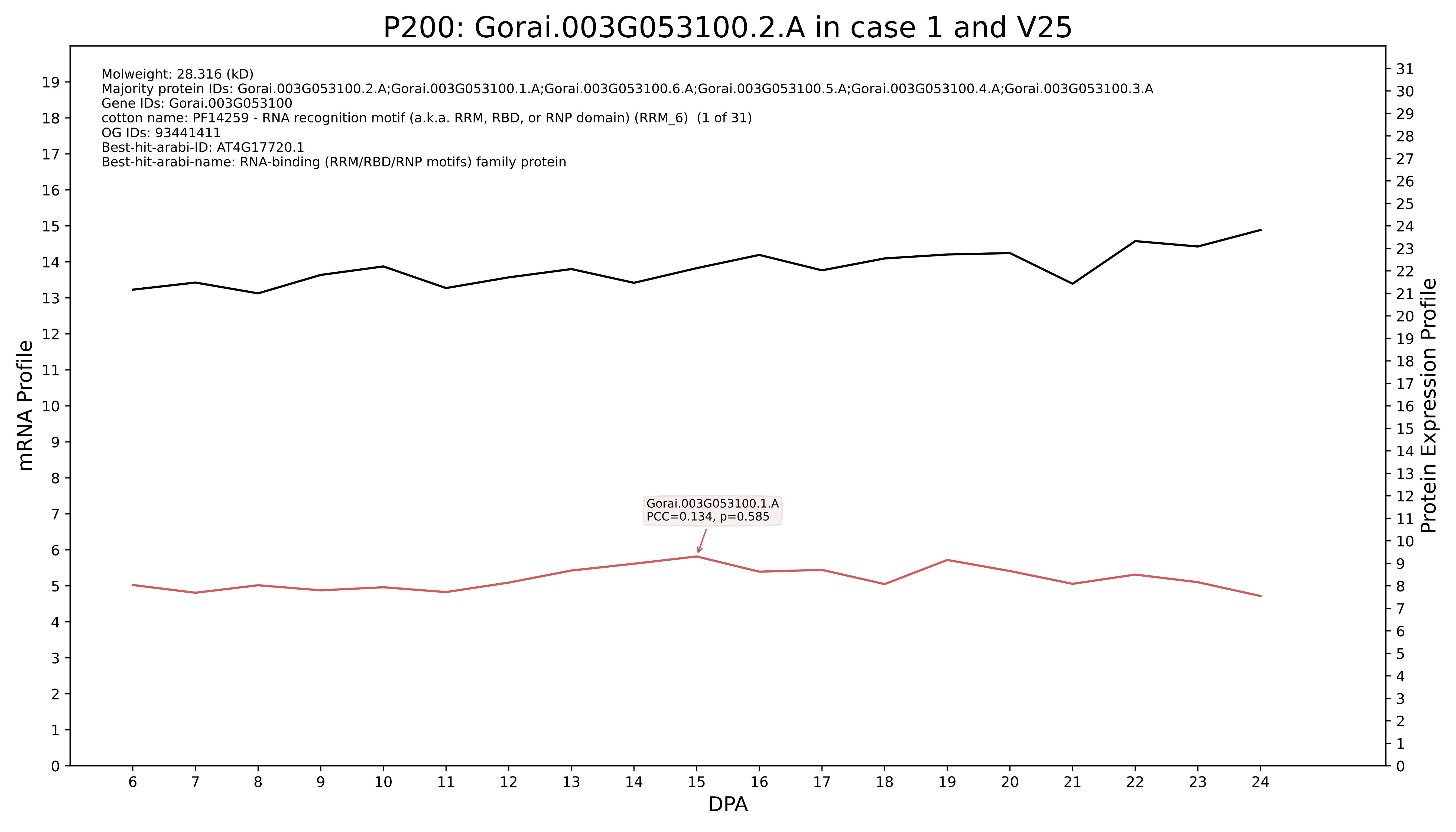
P200: Gorai.003G053100.2.A in case 1 and V25
Molweight: 28.316 (kD)
Majority protein IDs: Gorai.003G053100.2.A;Gorai.003G053100.1.A;Gorai.003G053100.6.A;Gorai.003G053100.5.A;Gorai.003G053100.4.A;Gorai.003G053100.3.A
Gene IDs: Gorai.003G053100
cotton name: PF14259 - RNA recognition motif (a.k.a. RRM, RBD, or RNP domain) (RRM_6) (1 of 31)
OG IDs: 93441411
Best-hit-arabi-ID: AT4G17720.1
Best-hit-arabi-name: RNA-binding (RRM/RBD/RNP motifs) family protein
Gorai.003G053100.1.A
PCC=0.134, p=0.585
mRNA Profile
Protein Expression Profile
DPA

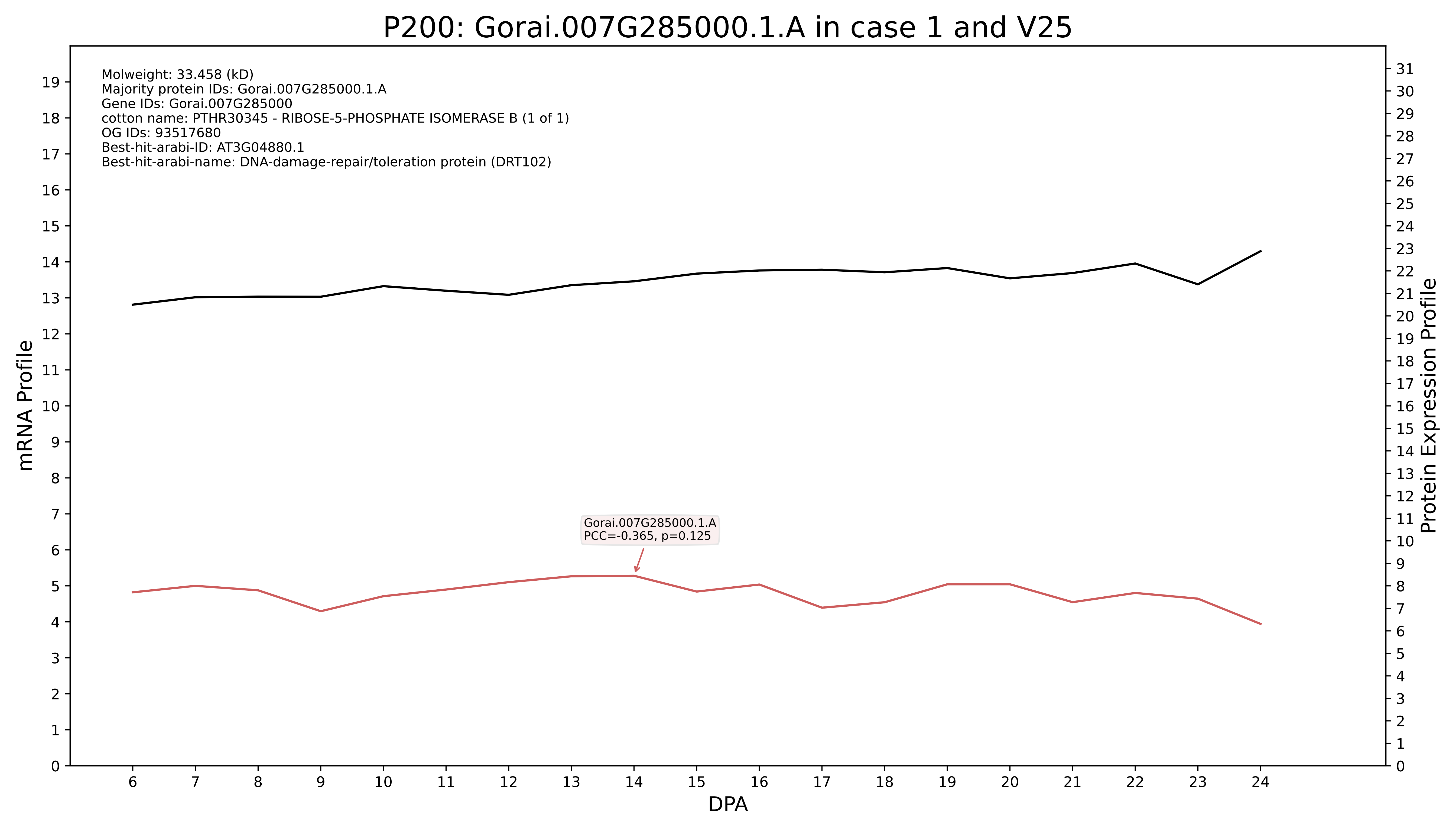
P200: Gorai.007G285000.1.A in case 1 and V25
Molweight: 33.458 (kD)
Majority protein IDs: Gorai.007G285000.1.A
Gene IDs: Gorai.007G285000
cotton name: PTHR30345 - RIBOSE-5-PHOSPHATE ISOMERASE B (1 of 1)
OG IDs: 93517680
Best-hit-arabi-ID: AT3G04880.1
Best-hit-arabi-name: DNA-damage-repair/toleration protein (DRT102)
Gorai.007G285000.1.A
PCC=-0.365, p=0.125
mRNA Profile
Protein Expression Profile
DPA

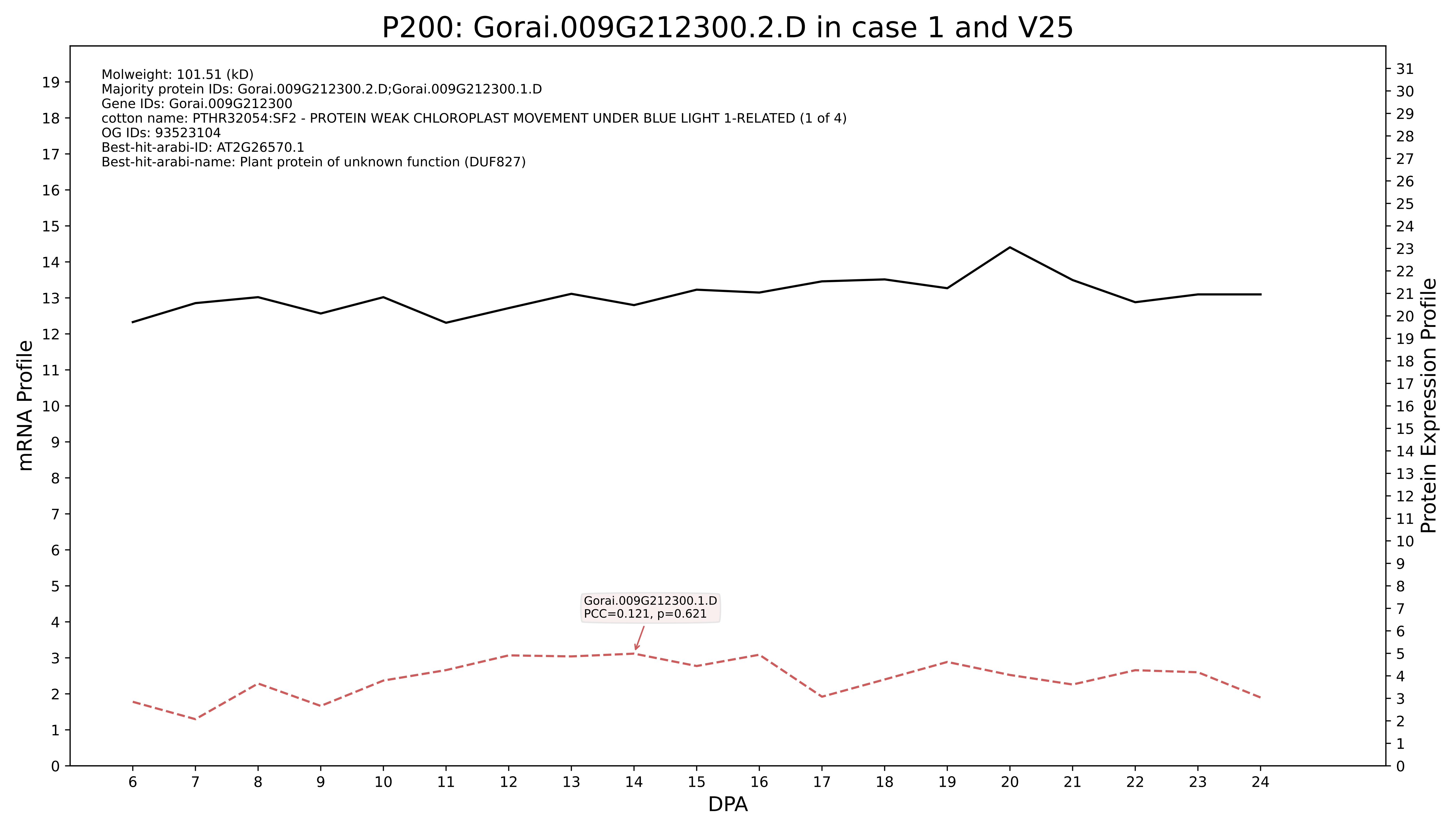
P200: Gorai.009G212300.2.D in case 1 and V25
Molweight: 101.51 (kD)
Majority protein IDs: Gorai.009G212300.2.D;Gorai.009G212300.1.D
Gene IDs: Gorai.009G212300
cotton name: PTHR32054:SF2 - PROTEIN WEAK CHLOROPLAST MOVEMENT UNDER BLUE LIGHT 1-RELATED (1 of 4)
OG IDs: 93523104
Best-hit-arabi-ID: AT2G26570.1
Best-hit-arabi-name: Plant protein of unknown function (DUF827)
Gorai.009G212300.1.D
PCC=0.121, p=0.621
mRNA Profile
Protein Expression Profile
DPA

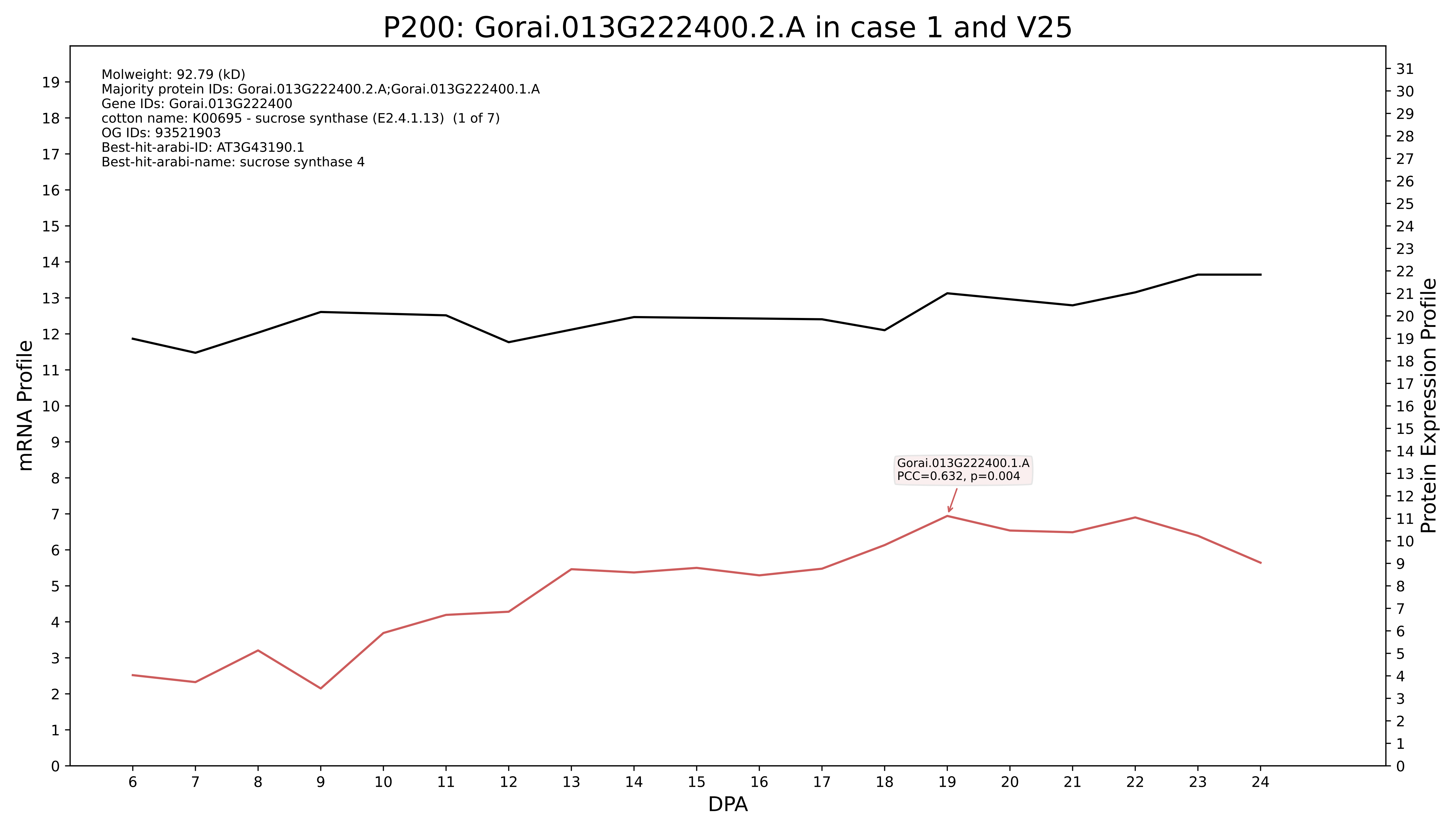
P200: Gorai.013G222400.2.A in case 1 and V25
Molweight: 92.79 (kD)
Majority protein IDs: Gorai.013G222400.2.A;Gorai.013G222400.1.A
Gene IDs: Gorai.013G222400
cotton name: K00695 - sucrose synthase (E2.4.1.13) (1 of 7)
OG IDs: 93521903
Best-hit-arabi-ID: AT3G43190.1
Best-hit-arabi-name: sucrose synthase 4
Gorai.013G222400.1.A
PCC=0.632, p=0.004
mRNA Profile
Protein Expression Profile
DPA

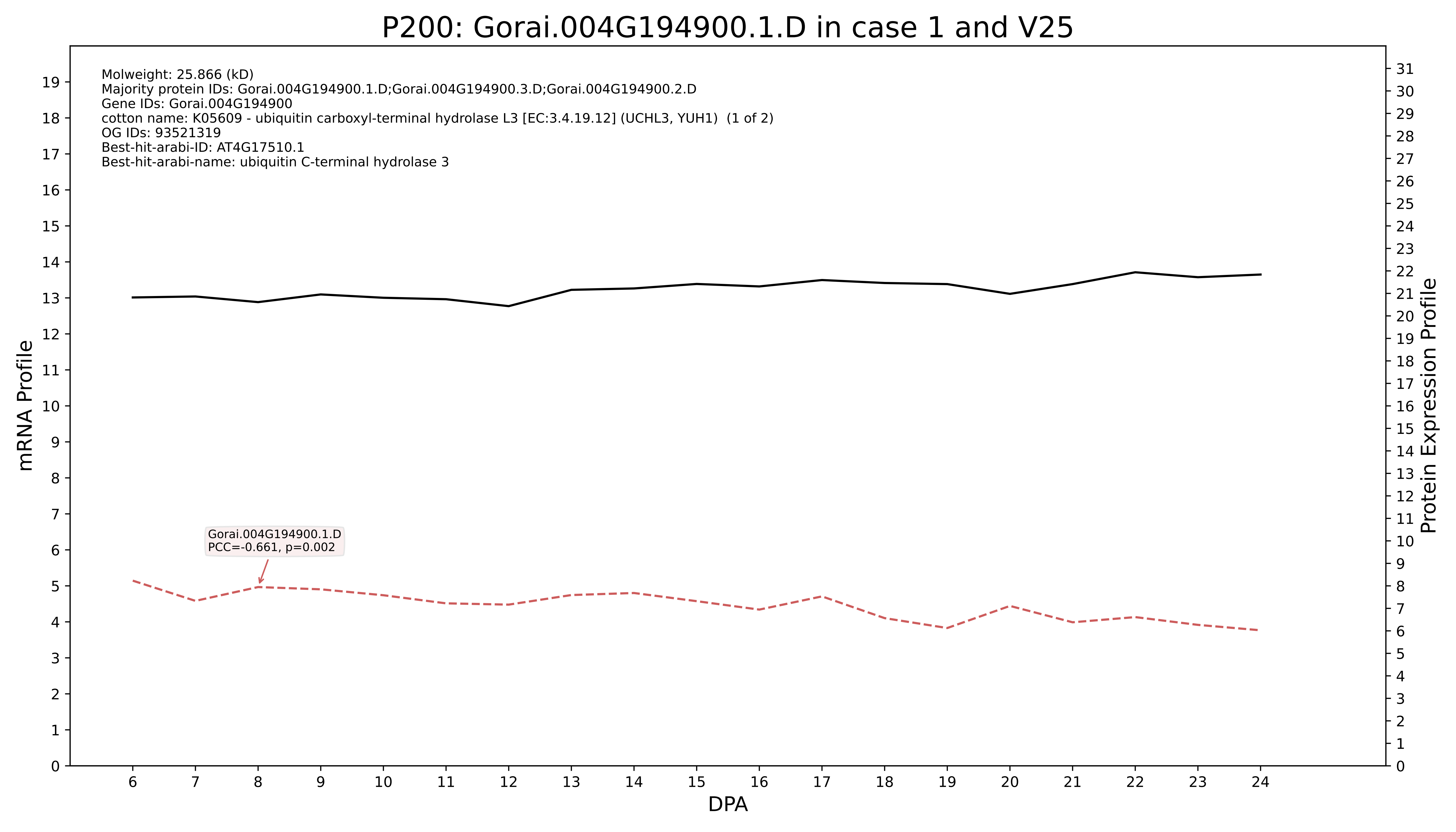
P200: Gorai.004G194900.1.D in case 1 and V25
Molweight: 25.866 (kD)
Majority protein IDs: Gorai.004G194900.1.D;Gorai.004G194900.3.D;Gorai.004G194900.2.D
Gene IDs: Gorai.004G194900
cotton name: K05609 - ubiquitin carboxyl-terminal hydrolase L3 [EC:3.4.19.12] (UCHL3, YUH1) (1 of 2)
OG IDs: 93521319
Best-hit-arabi-ID: AT4G17510.1
Best-hit-arabi-name: ubiquitin C-terminal hydrolase 3
Gorai.004G194900.1.D
PCC=-0.661, p=0.002
mRNA Profile
Protein Expression Profile
DPA

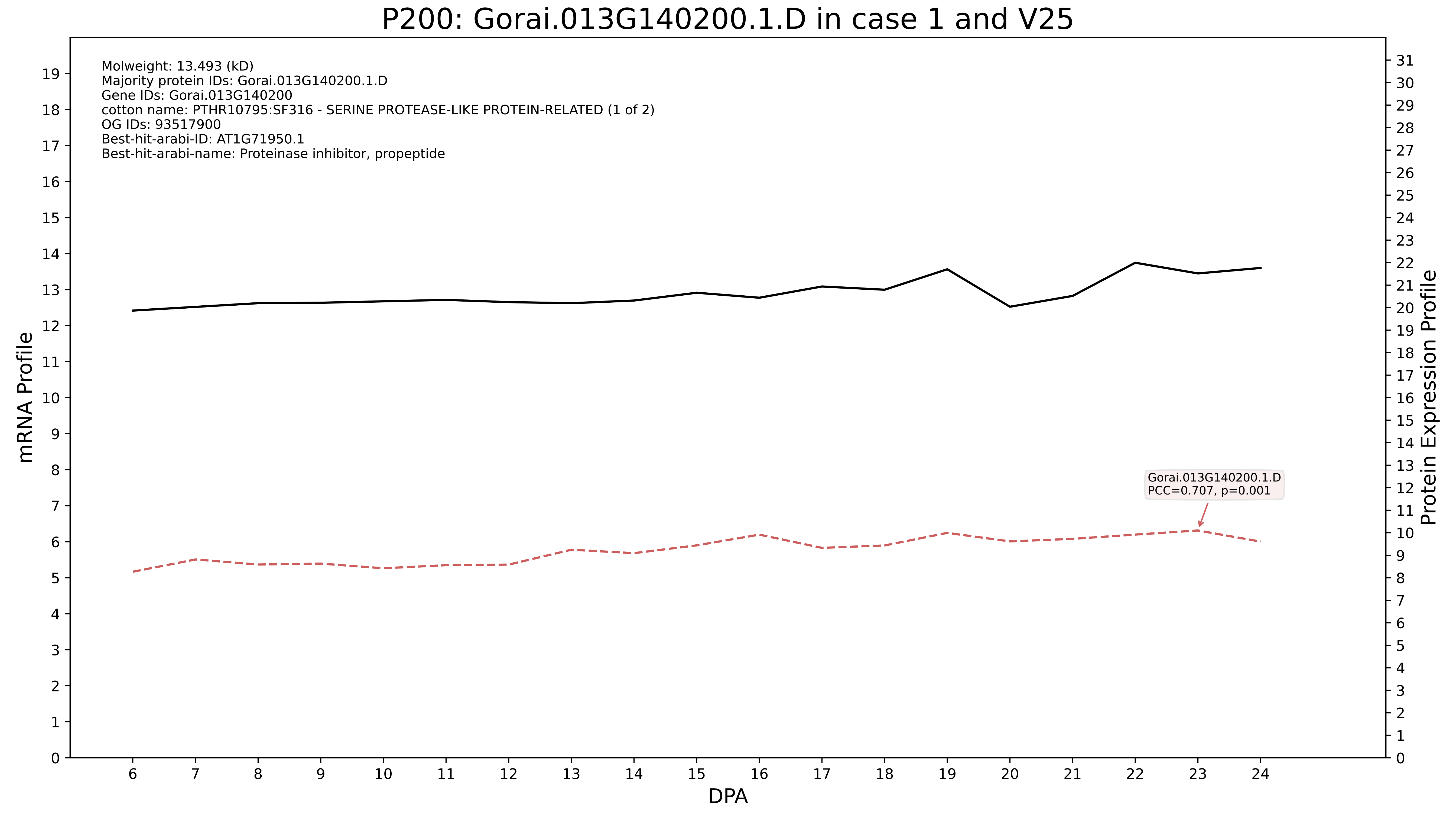
P200: Gorai.013G140200.1.D in case 1 and V25
Molweight: 13.493 (kD)
Majority protein IDs: Gorai.013G140200.1.D
Gene IDs: Gorai.013G140200
cotton name: PTHR10795:SF316 - SERINE PROTEASE-LIKE PROTEIN-RELATED (1 of 2)
OG IDs: 93517900
Best-hit-arabi-ID: AT1G71950.1
Best-hit-arabi-name: Proteinase inhibitor, propeptide
Gorai.013G140200.1.D
PCC=0.707, p=0.001
mRNA Profile
Protein Expression Profile
DPA

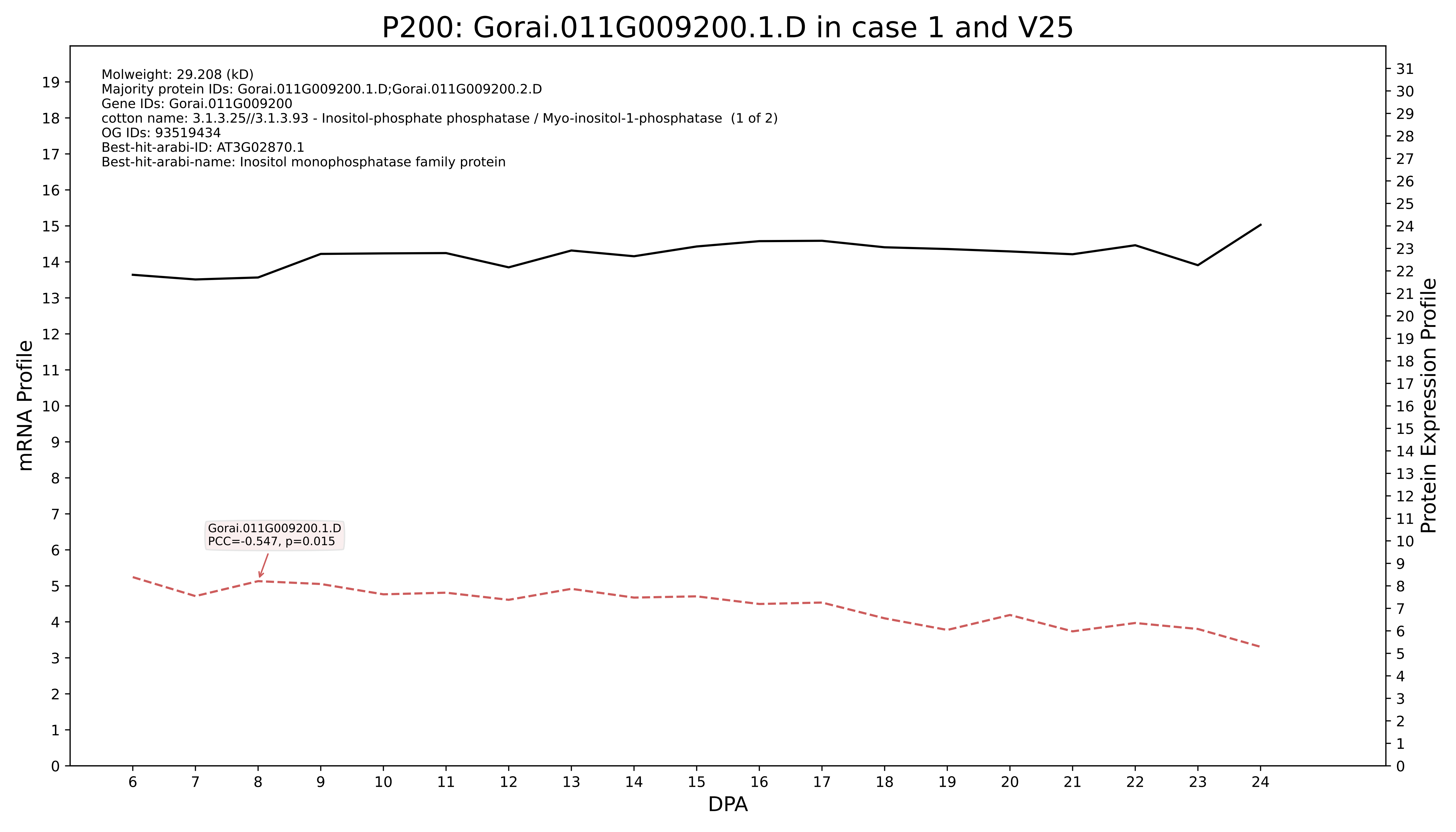
P200: Gorai.011G009200.1.D in case 1 and V25
Molweight: 29.208 (kD)
Majority protein IDs: Gorai.011G009200.1.D;Gorai.011G009200.2.D
Gene IDs: Gorai.011G009200
cotton name: 3.1.3.25//3.1.3.93 - Inositol-phosphate phosphatase / Myo-inositol-1-phosphatase (1 of 2)
OG IDs: 93519434
Best-hit-arabi-ID: AT3G02870.1
Best-hit-arabi-name: Inositol monophosphatase family protein
Gorai.011G009200.1.D
PCC=-0.547, p=0.015
mRNA Profile
Protein Expression Profile
DPA
0
1
2
3
4
5
6
7
8
9
10
11
12
13
14
15
16
17
18
19
20
21
22
23
24
25
26
27
28
29
30
31

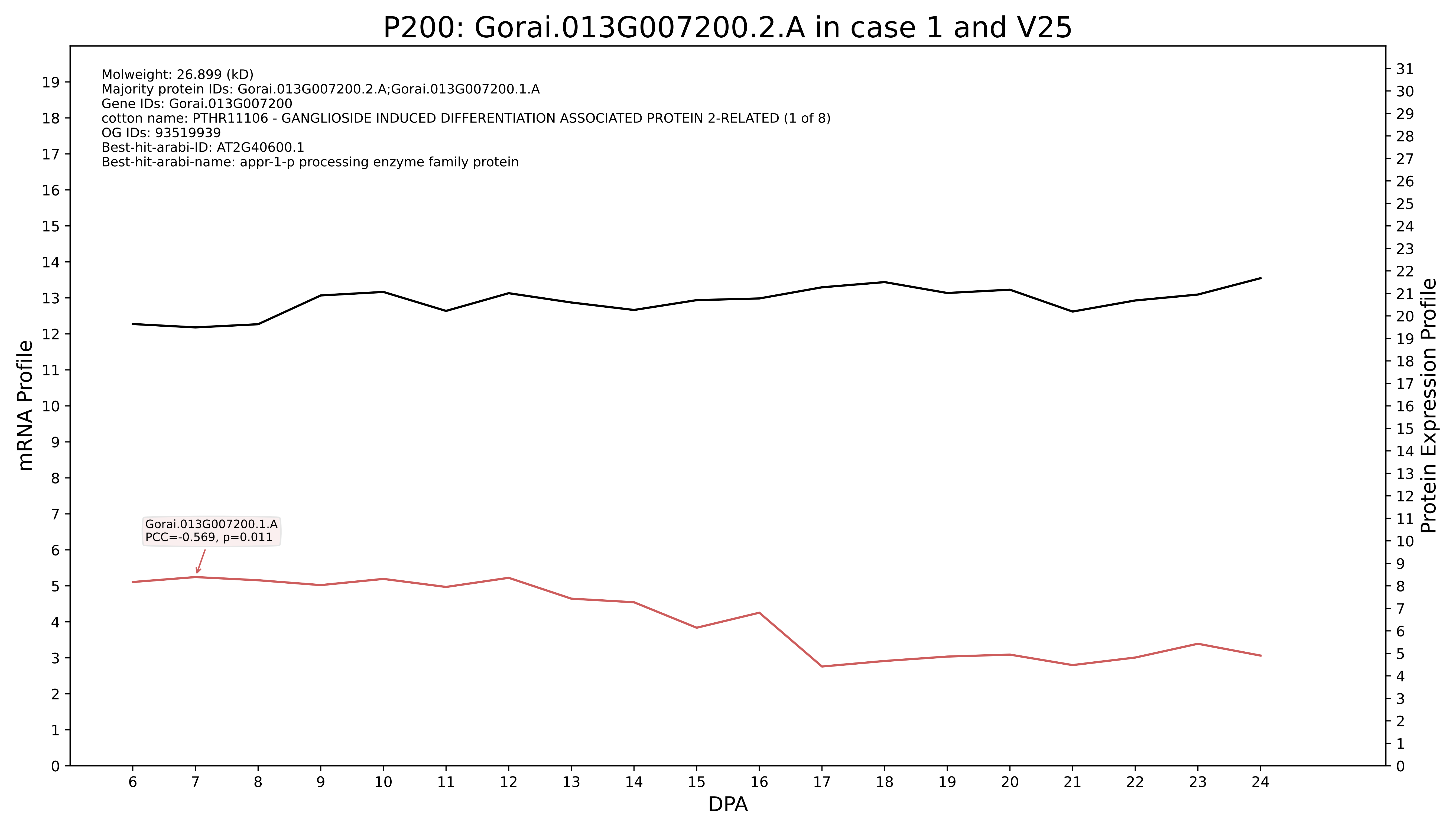

P200: Gorai.013G007200.2.A in case 1 and V25
Molweight: 26.899 (kD)
Majority protein IDs: Gorai.013G007200.2.A;Gorai.013G007200.1.A
Gene IDs: Gorai.013G007200
cotton name: PTHR11106 - GANGLIOSIDE INDUCED DIFFERENTIATION ASSOCIATED PROTEIN 2-RELATED (1 of 8)
OG IDs: 93519939
Best-hit-arabi-ID: AT2G40600.1
Best-hit-arabi-name: appr-1-p processing enzyme family protein
Gorai.013G007200.1.A
PCC=-0.569, p=0.011
mRNA Profile
Protein Expression Profile
DPA

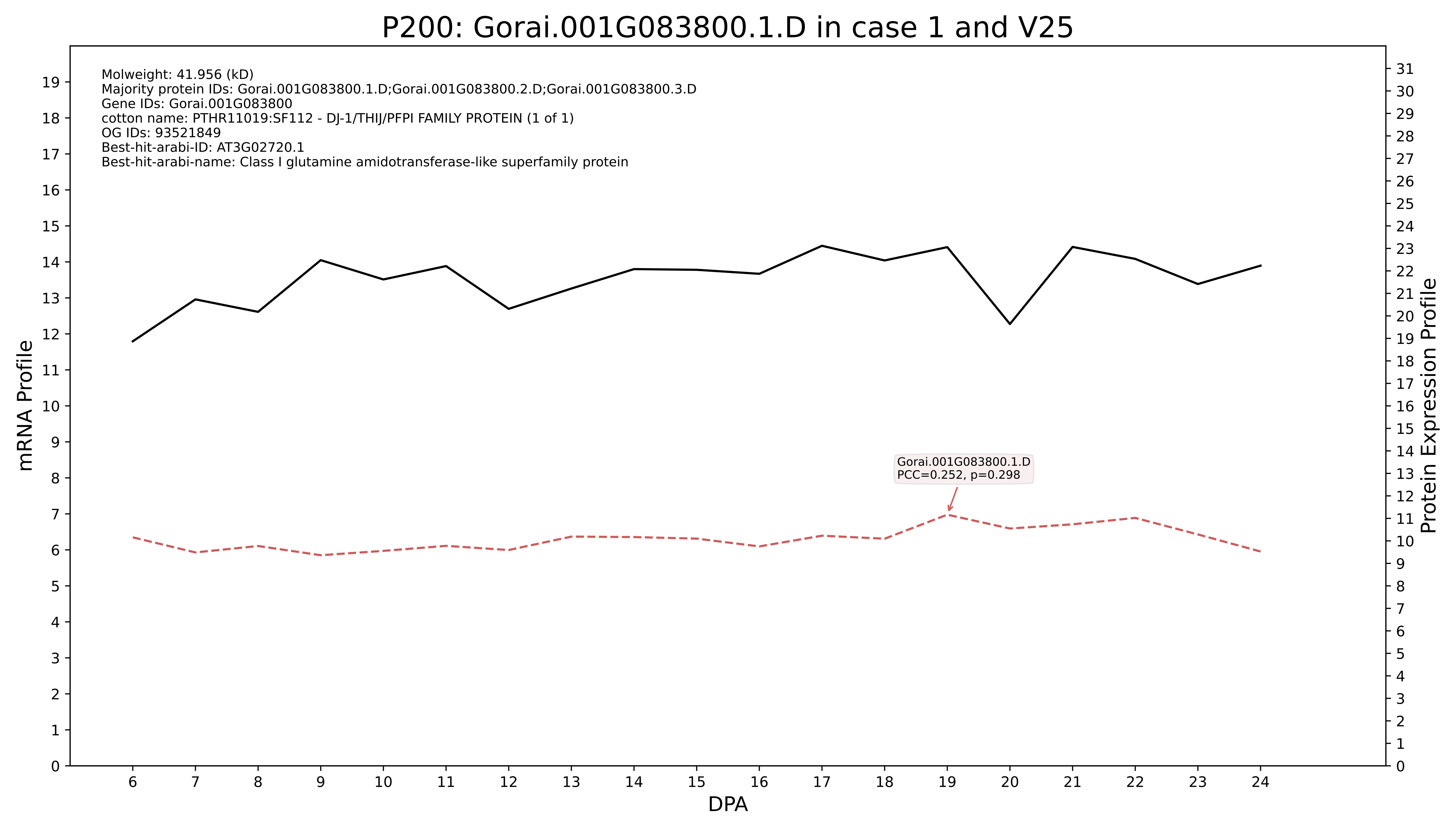

P200: Gorai.001G083800.1.D in case 1 and V25
Molweight: 41.956 (kD)
Majority protein IDs: Gorai.001G083800.1.D;Gorai.001G083800.2.D;Gorai.001G083800.3.D
Gene IDs: Gorai.001G083800
cotton name: PTHR11019:SF112 - DJ-1/THIJ/PFPI FAMILY PROTEIN (1 of 1)
OG IDs: 93521849
Best-hit-arabi-ID: AT3G02720.1
Best-hit-arabi-name: Class I glutamine amidotransferase-like superfamily protein
Gorai.001G083800.1.D
PCC=0.252, p=0.298
mRNA Profile
Protein Expression Profile
DPA

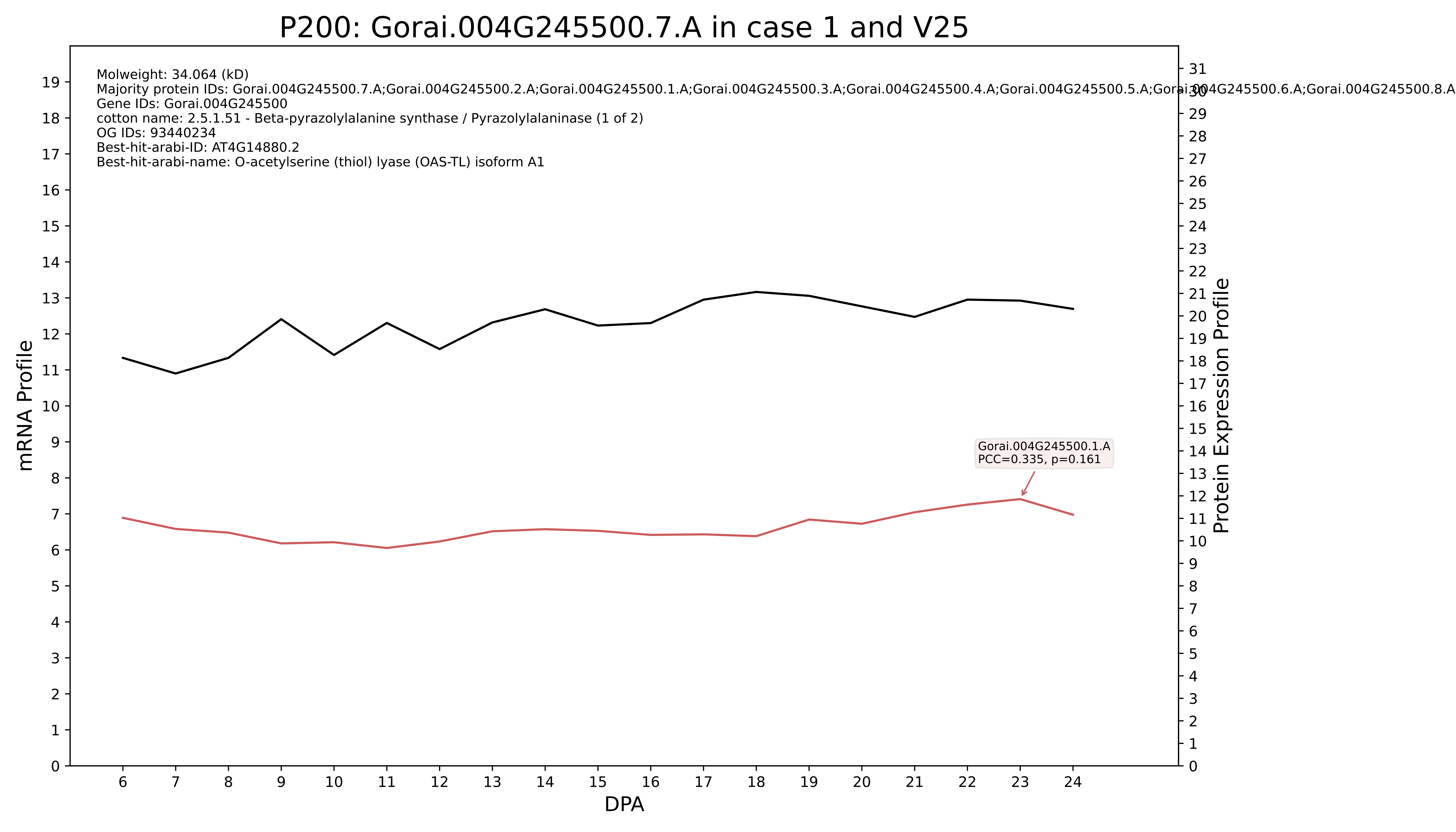

P200: Gorai.004G245500.7.A in case 1 and V25
Molweight: 34.064 (kD)
Majority protein IDs: Gorai.004G245500.7.A;Gorai.004G245500.2.A;Gorai.004G245500.1.A;Gorai.004G245500.3.A;Gorai.004G245500.4.A;Gorai.004G245500.5.A;Gorai.004G245500.6.A;Gorai.004G245500.8.A
Gene IDs: Gorai.004G245500
cotton name: 2.5.1.51 - Beta-pyrazolylalanine synthase / Pyrazolylalaninase (1 of 2)
OG IDs: 93440234
Best-hit-arabi-ID: AT4G14880.2
Best-hit-arabi-name: O-acetylserine (thiol) lyase (OAS-TL) isoform A1
Gorai.004G245500.1.A
PCC=0.335, p=0.161
mRNA Profile
Protein Expression Profile
DPA

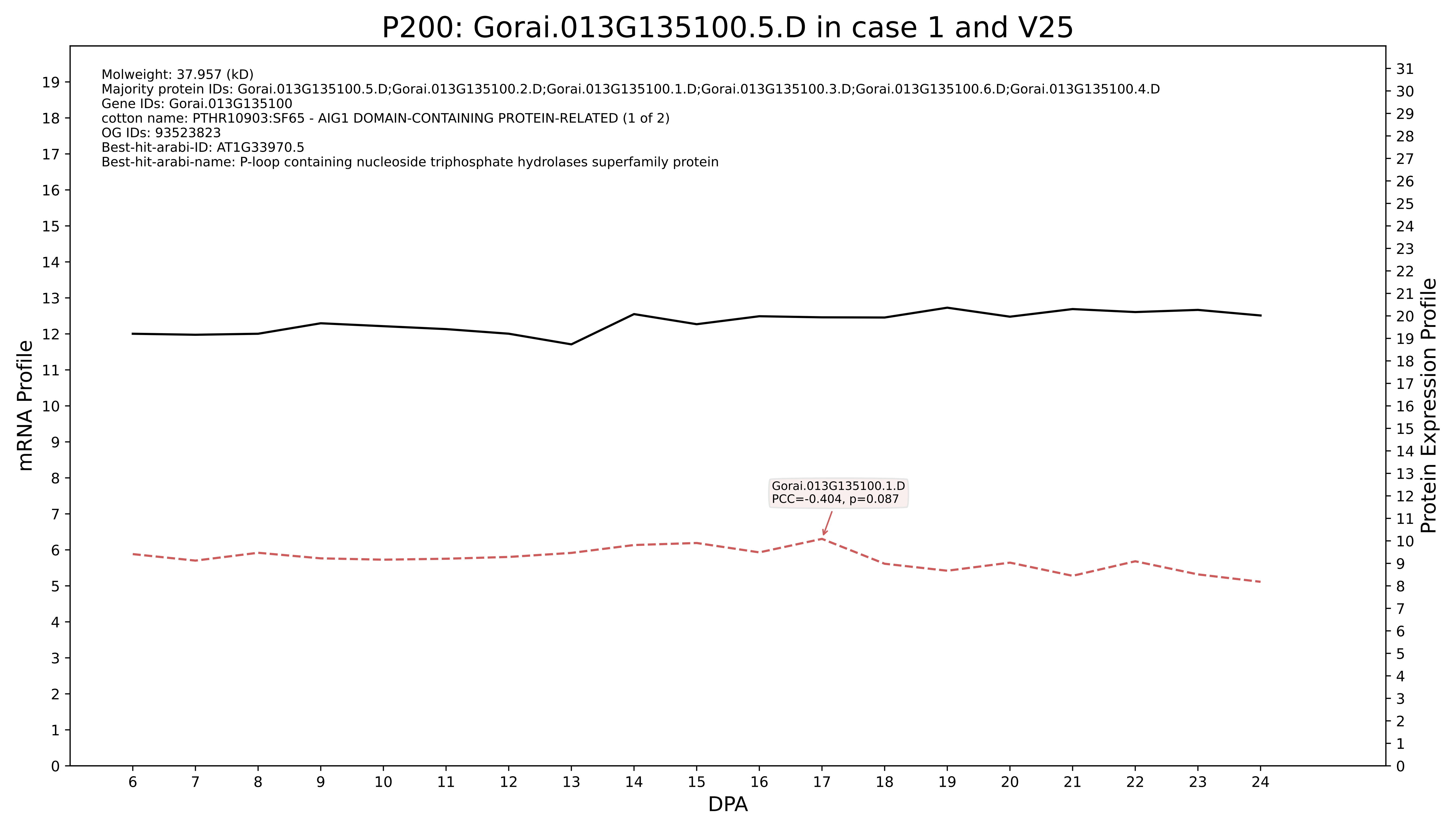

P200: Gorai.013G135100.5.D in case 1 and V25
Molweight: 37.957 (kD)
Majority protein IDs: Gorai.013G135100.5.D;Gorai.013G135100.2.D;Gorai.013G135100.1.D;Gorai.013G135100.3.D;Gorai.013G135100.6.D;Gorai.013G135100.4.D
Gene IDs: Gorai.013G135100
cotton name: PTHR10903:SF65 - AIG1 DOMAIN-CONTAINING PROTEIN-RELATED (1 of 2)
OG IDs: 93523823
Best-hit-arabi-ID: AT1G33970.5
Best-hit-arabi-name: P-loop containing nucleoside triphosphate hydrolases superfamily protein
Gorai.013G135100.1.D
PCC=-0.404, p=0.087
mRNA Profile
Protein Expression Profile
DPA

P200: Gorai.008G010200.1.A in case 1 and V25

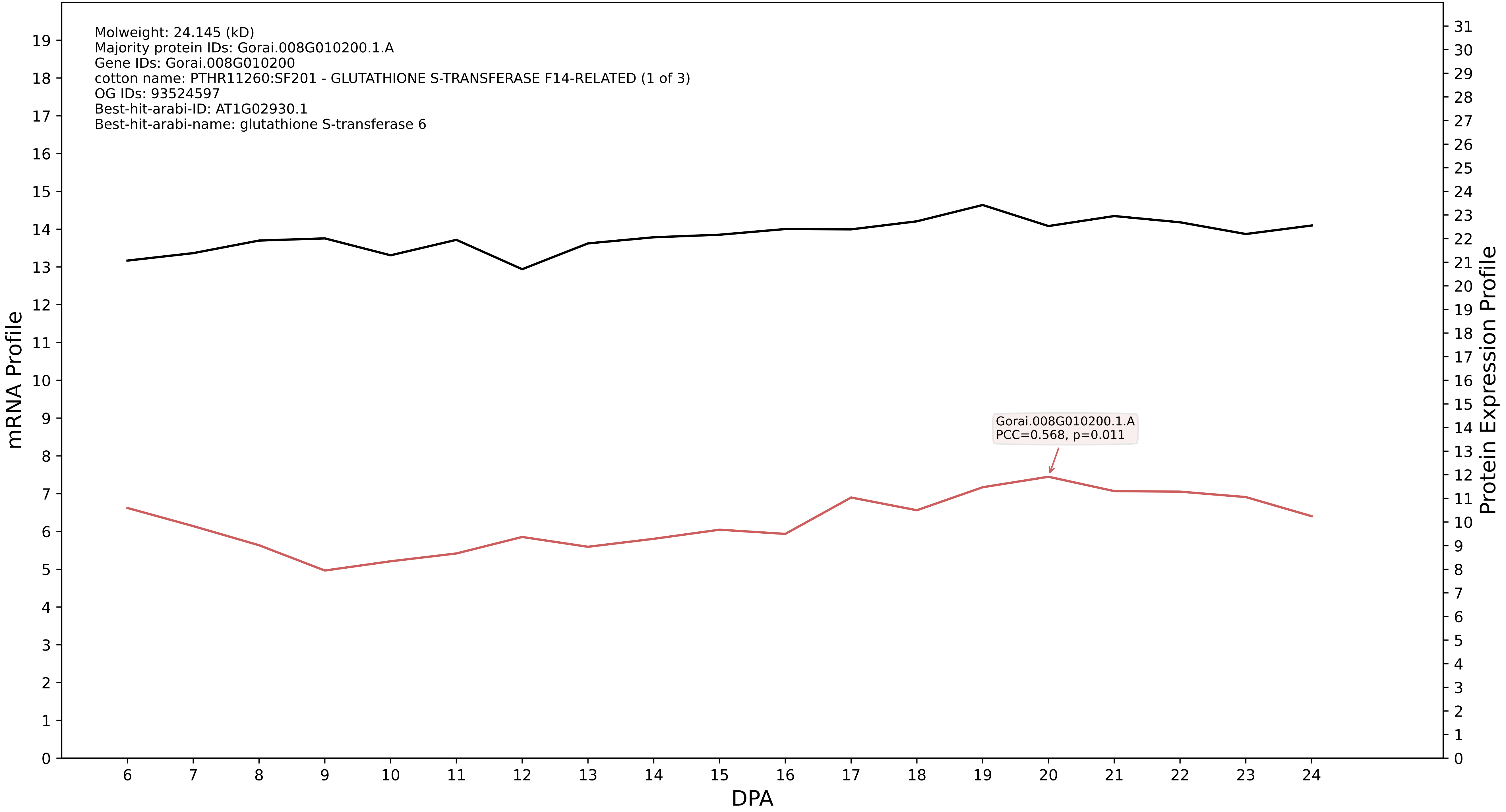

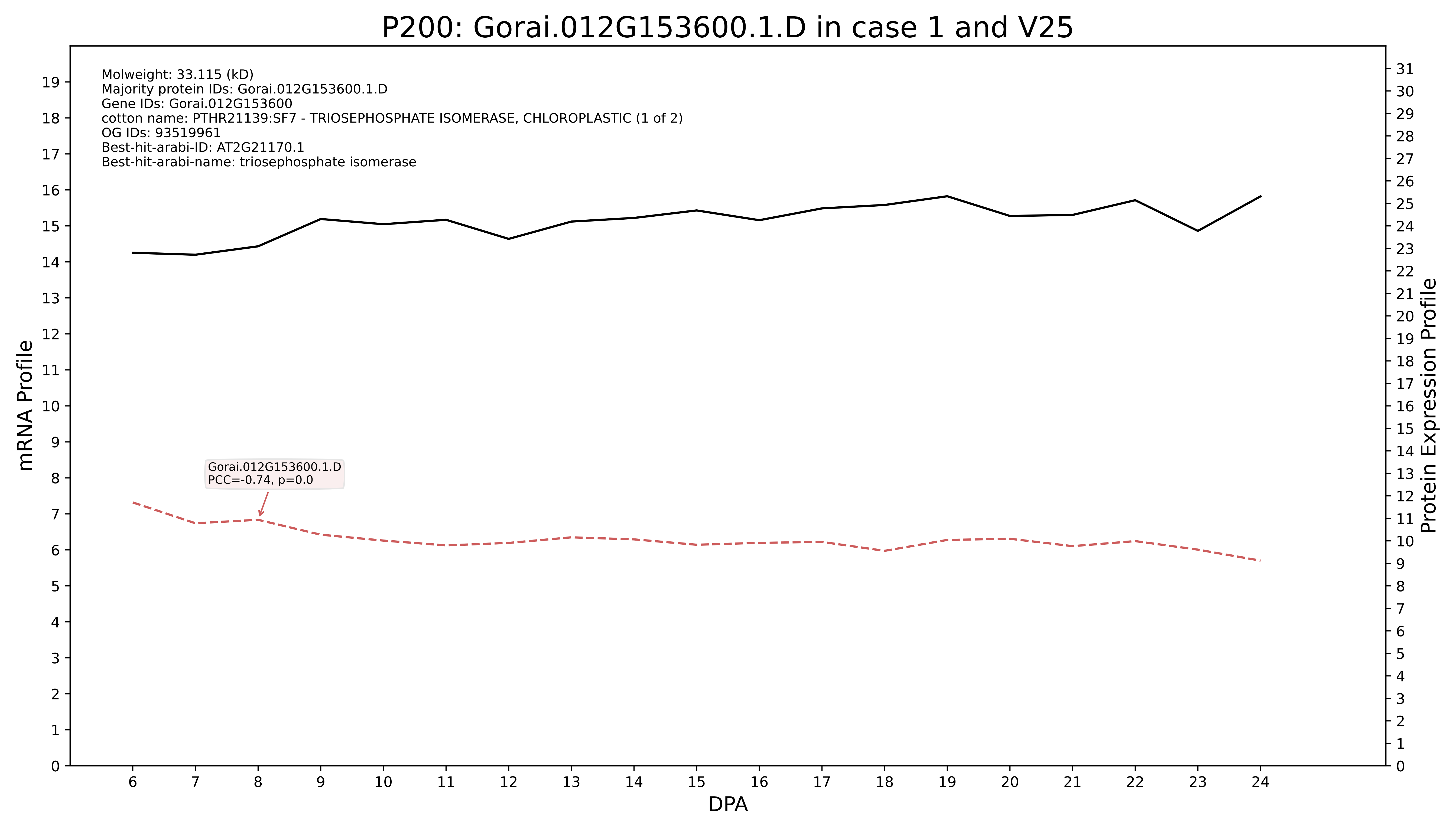
P200: Gorai.012G153600.1.D in case 1 and V25
Molweight: 33.115 (kD)
Majority protein IDs: Gorai.012G153600.1.D
Gene IDs: Gorai.012G153600
cotton name: PTHR21139:SF7 - TRIOSEPHOSPHATE ISOMERASE, CHLOROPLASTIC (1 of 2)
OG IDs: 93519961
Best-hit-arabi-ID: AT2G21170.1
Best-hit-arabi-name: triosephosphate isomerase
Gorai.012G153600.1.D
PCC=-0.74, p=0.0
mRNA Profile
Protein Expression Profile
DPA

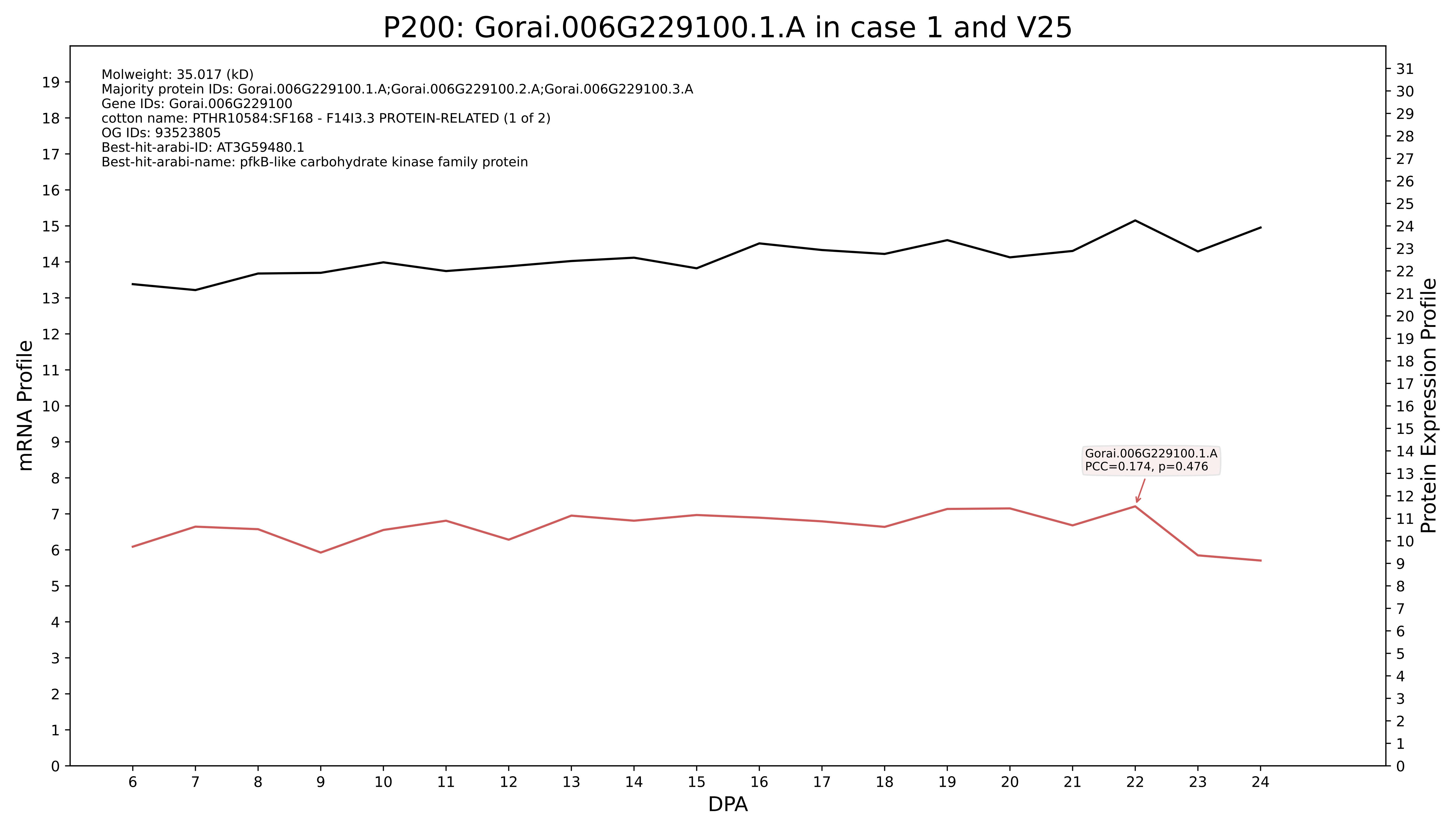

P200: Gorai.006G229100.1.A in case 1 and V25
Molweight: 35.017 (kD)
Majority protein IDs: Gorai.006G229100.1.A;Gorai.006G229100.2.A;Gorai.006G229100.3.A
Gene IDs: Gorai.006G229100
cotton name: PTHR10584:SF168 - F14I3.3 PROTEIN-RELATED (1 of 2)
OG IDs: 93523805
Best-hit-arabi-ID: AT3G59480.1
Best-hit-arabi-name: pfkB-like carbohydrate kinase family protein
Gorai.006G229100.1.A
PCC=0.174, p=0.476
mRNA Profile
Protein Expression Profile
DPA

P200: Gorai.009G293300.1.A in case 1 and V25

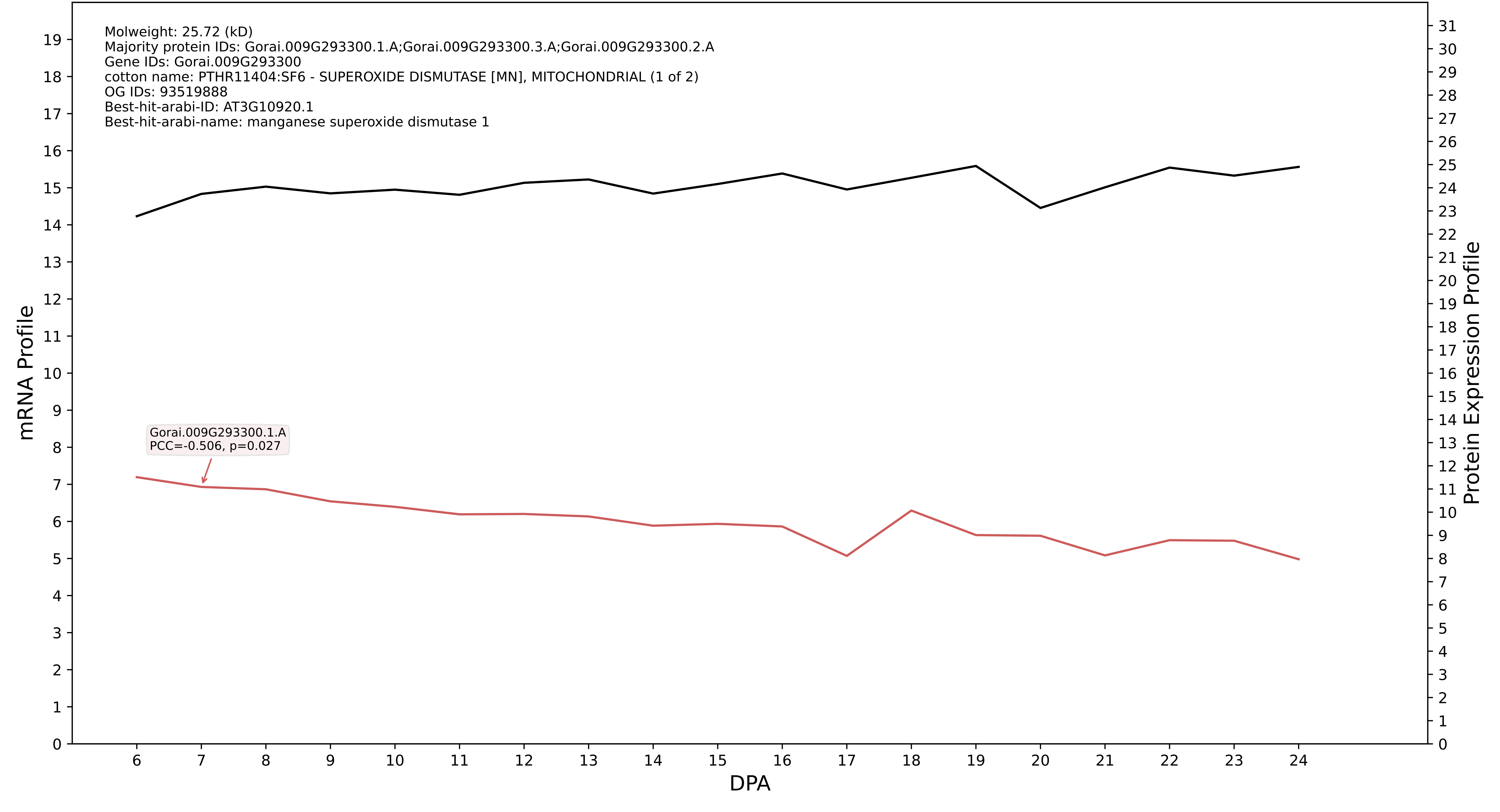

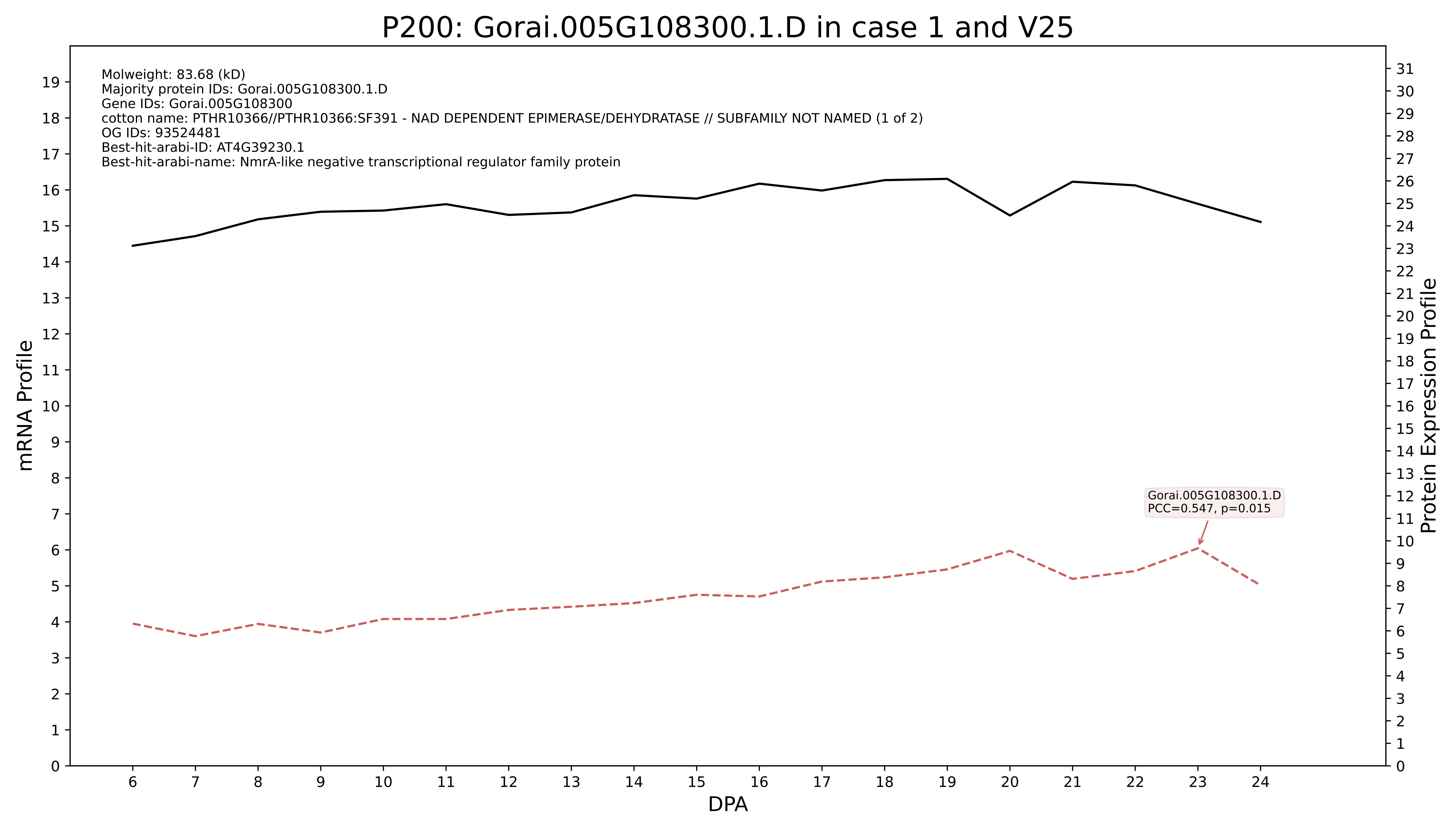
P200: Gorai.005G108300.1.D in case 1 and V25
Molweight: 83.68 (kD)
Majority protein IDs: Gorai.005G108300.1.D
Gene IDs: Gorai.005G108300
cotton name: PTHR10366//PTHR10366:SF391 - NAD DEPENDENT EPIMERASE/DEHYDRATASE // SUBFAMILY NOT NAMED (1 of 2)
OG IDs: 93524481
Best-hit-arabi-ID: AT4G39230.1
Best-hit-arabi-name: NmrA-like negative transcriptional regulator family protein
Gorai.005G108300.1.D
PCC=0.547, p=0.015
mRNA Profile
Protein Expression Profile
DPA

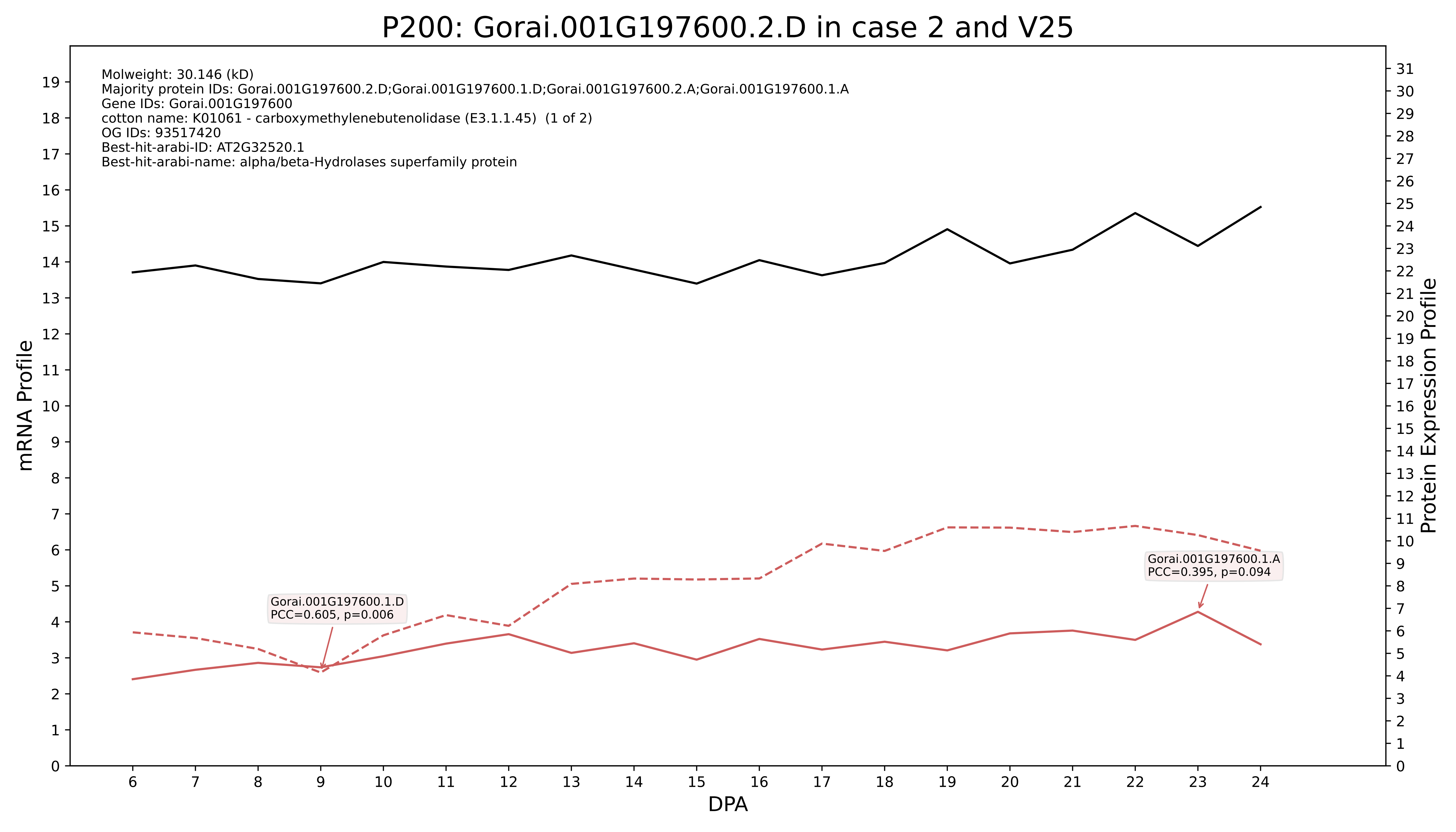

P200: Gorai.001G197600.2.D in case 2 and V25
Molweight: 30.146 (kD)
Majority protein IDs: Gorai.001G197600.2.D;Gorai.001G197600.1.D;Gorai.001G197600.2.A;Gorai.001G197600.1.A
Gene IDs: Gorai.001G197600
cotton name: K01061 - carboxymethylenebutenolidase (E3.1.1.45) (1 of 2)
OG IDs: 93517420
Best-hit-arabi-ID: AT2G32520.1
Best-hit-arabi-name: alpha/beta-Hydrolases superfamily protein
Gorai.001G197600.1.D
PCC=0.605, p=0.006
Gorai.001G197600.1.A
PCC=0.395, p=0.094
mRNA Profile
Protein Expression Profile
DPA

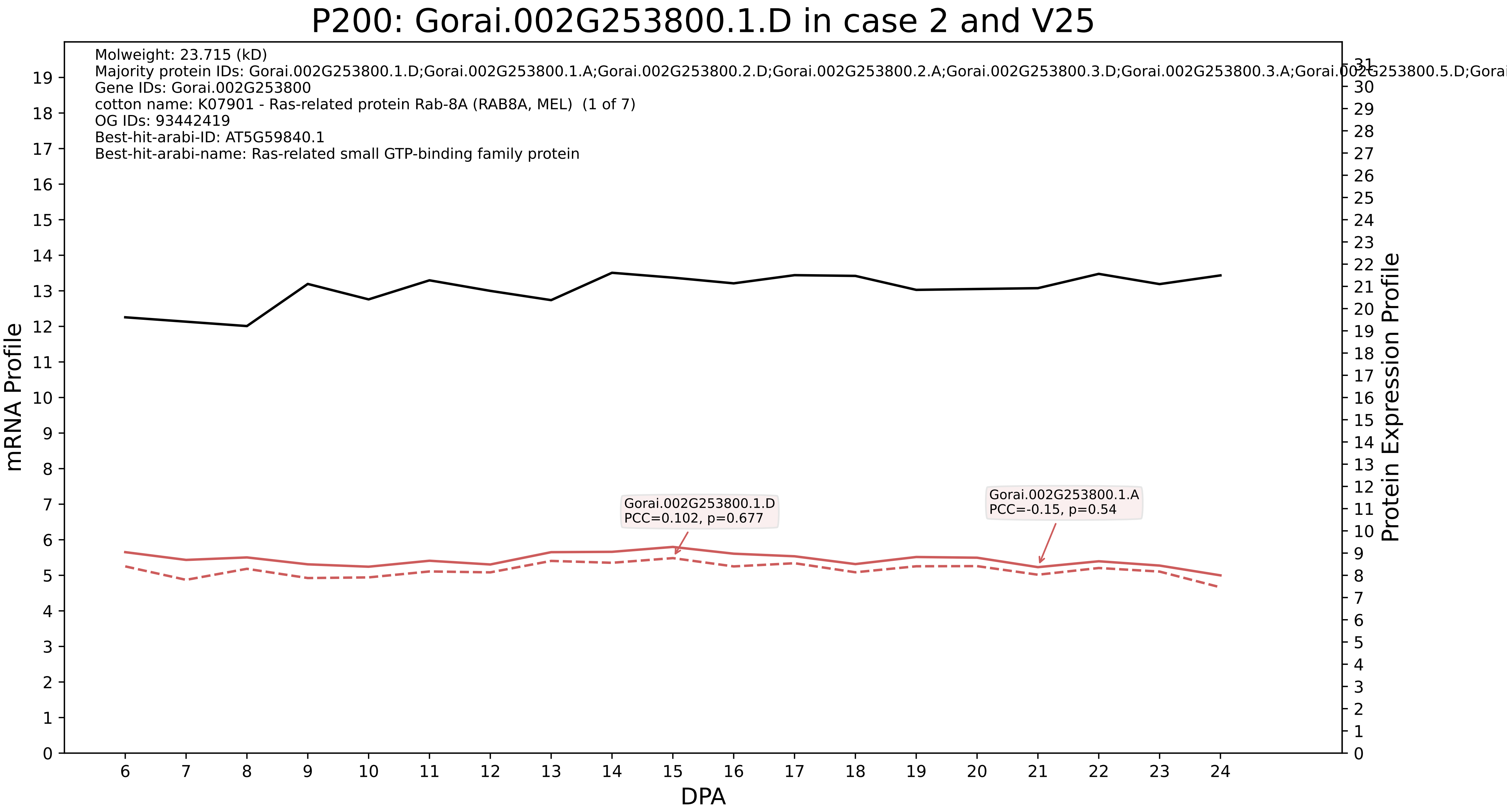
P200: Gorai.002G253800.1.D in case 2 and V25
Molweight: 23.715 (kD)
Majority protein IDs: Gorai.002G253800.1.D;Gorai.002G253800.1.A;Gorai.002G253800.2.D;Gorai.002G253800.2.A;Gorai.002G253800.3.D;Gorai.002G253800.3.A;Gorai.002G253800.5.D;Gorai
Gene IDs: Gorai.002G253800
cotton name: K07901 - Ras-related protein Rab-8A (RAB8A, MEL) (1 of 7)
OG IDs: 93442419
Best-hit-arabi-ID: AT5G59840.1
Best-hit-arabi-name: Ras-related small GTP-binding family protein
Gorai.002G253800.1.D
PCC=0.102, p=0.677
Gorai.002G253800.1.A
PCC=-0.15, p=0.54
mRNA Profile
Protein Expression Profile
DPA

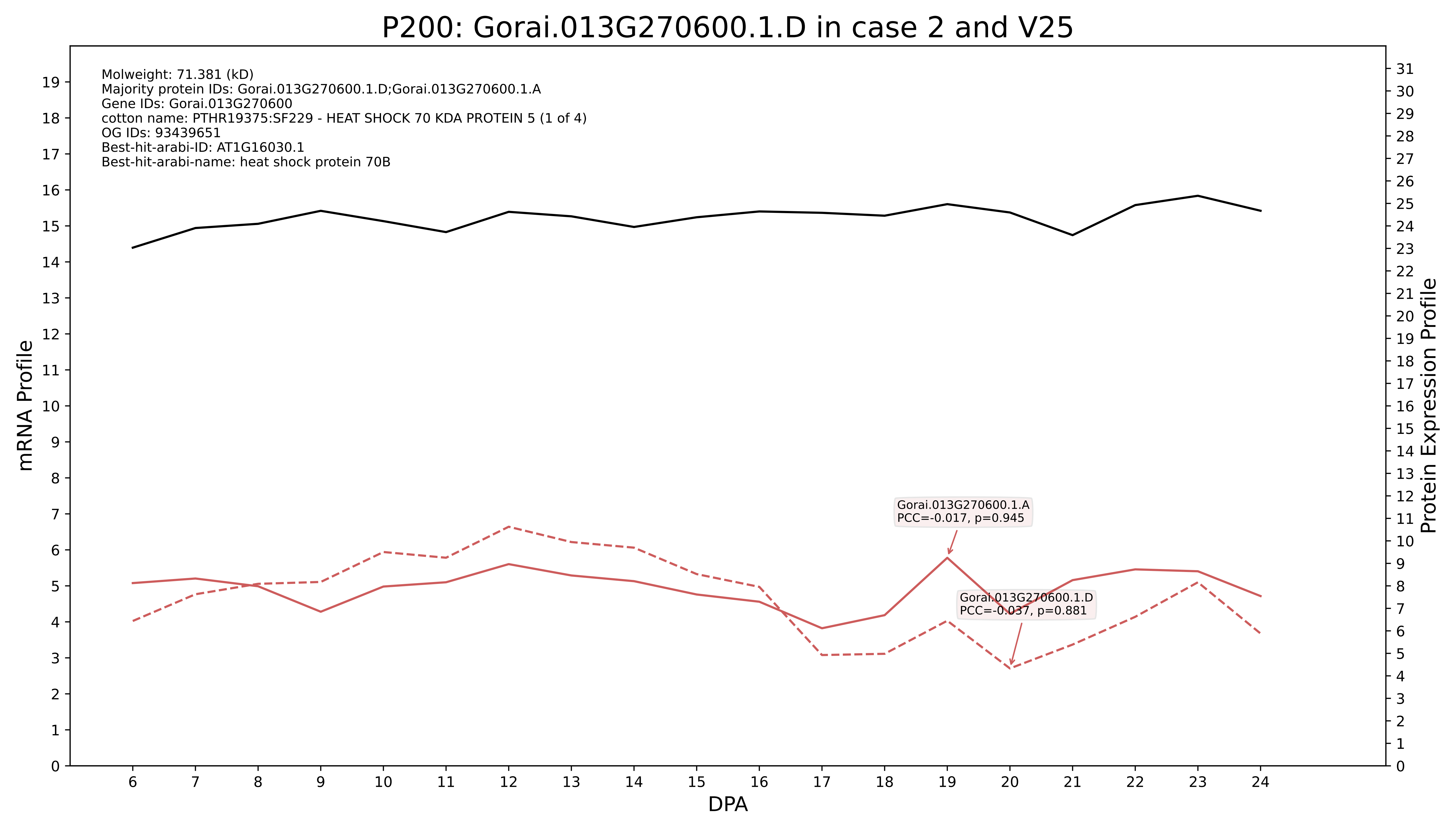
P200: Gorai.013G270600.1.D in case 2 and V25
Molweight: 71.381 (kD)
Majority protein IDs: Gorai.013G270600.1.D;Gorai.013G270600.1.A
Gene IDs: Gorai.013G270600
cotton name: PTHR19375:SF229 - HEAT SHOCK 70 KDA PROTEIN 5 (1 of 4)
OG IDs: 93439651
Best-hit-arabi-ID: AT1G16030.1
Best-hit-arabi-name: heat shock protein 70B
Gorai.013G270600.1.A
PCC=-0.017, p=0.945
Gorai.013G270600.1.D
PCC=-0.037, p=0.881
mRNA Profile
Protein Expression Profile
DPA

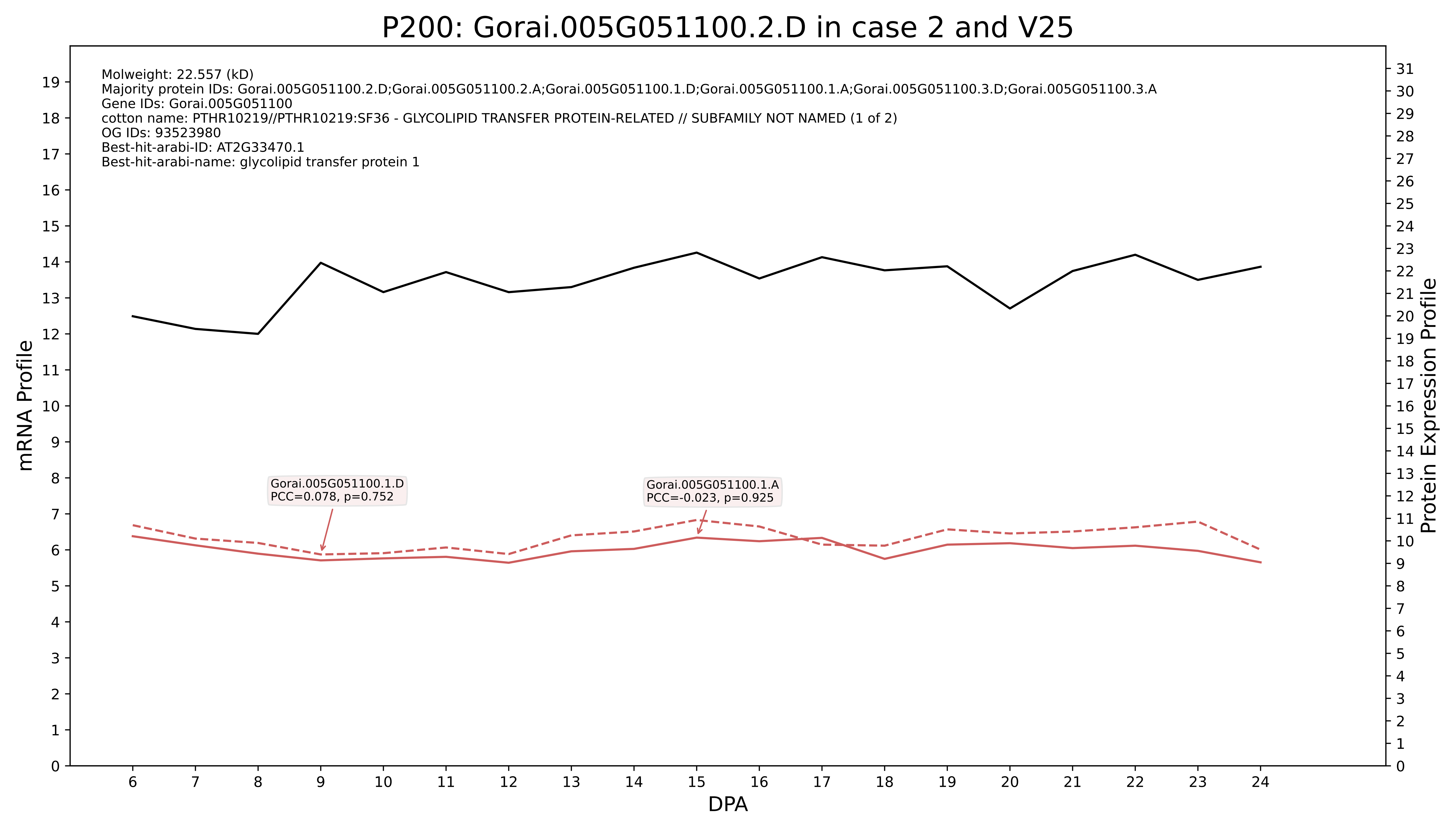

P200: Gorai.005G051100.2.D in case 2 and V25
Molweight: 22.557 (kD)
Majority protein IDs: Gorai.005G051100.2.D;Gorai.005G051100.2.A;Gorai.005G051100.1.D;Gorai.005G051100.1.A;Gorai.005G051100.3.D;Gorai.005G051100.3.A
Gene IDs: Gorai.005G051100
cotton name: PTHR10219//PTHR10219:SF36 - GLYCOLIPID TRANSFER PROTEIN-RELATED // SUBFAMILY NOT NAMED (1 of 2)
OG IDs: 93523980
Best-hit-arabi-ID: AT2G33470.1
Best-hit-arabi-name: glycolipid transfer protein 1
Gorai.005G051100.1.D
PCC=0.078, p=0.752
Gorai.005G051100.1.A
PCC=-0.023, p=0.925
mRNA Profile
Protein Expression Profile
DPA

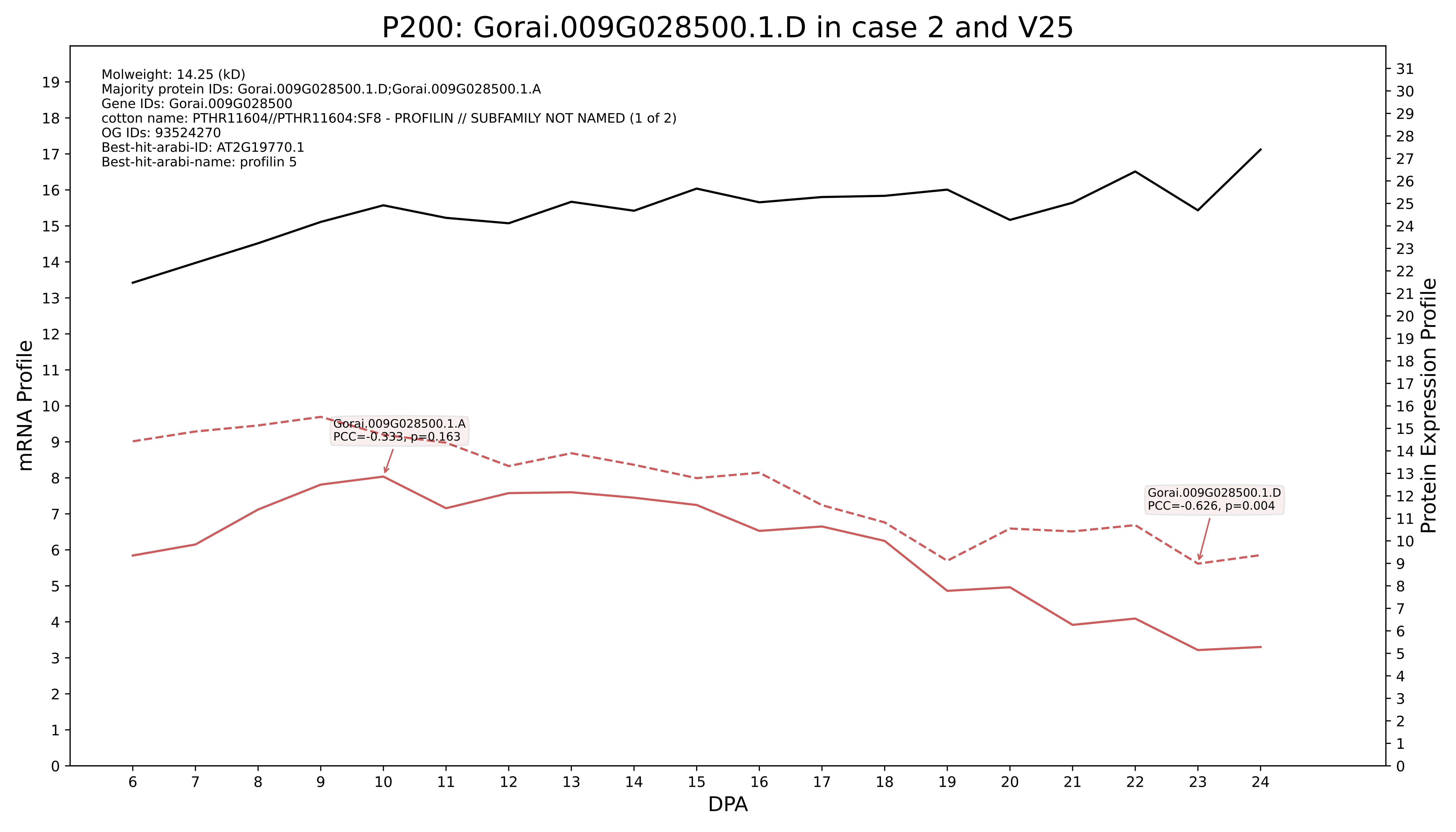
P200: Gorai.009G028500.1.D in case 2 and V25
Molweight: 14.25 (kD)
Majority protein IDs: Gorai.009G028500.1.D;Gorai.009G028500.1.A
Gene IDs: Gorai.009G028500
cotton name: PTHR11604//PTHR11604:SF8 - PROFILIN // SUBFAMILY NOT NAMED (1 of 2)
OG IDs: 93524270
Best-hit-arabi-ID: AT2G19770.1
Best-hit-arabi-name: profilin 5
Gorai.009G028500.1.A
PCC=-0.333, p=0.163
Gorai.009G028500.1.D
PCC=-0.626, p=0.004
mRNA Profile
Protein Expression Profile
DPA
0
1
2
3
4
5
6
7
8
9
10
11
12
13
14
15
16
17
18
19
20
21
22
23
24
25
26
27
28
29
30
31

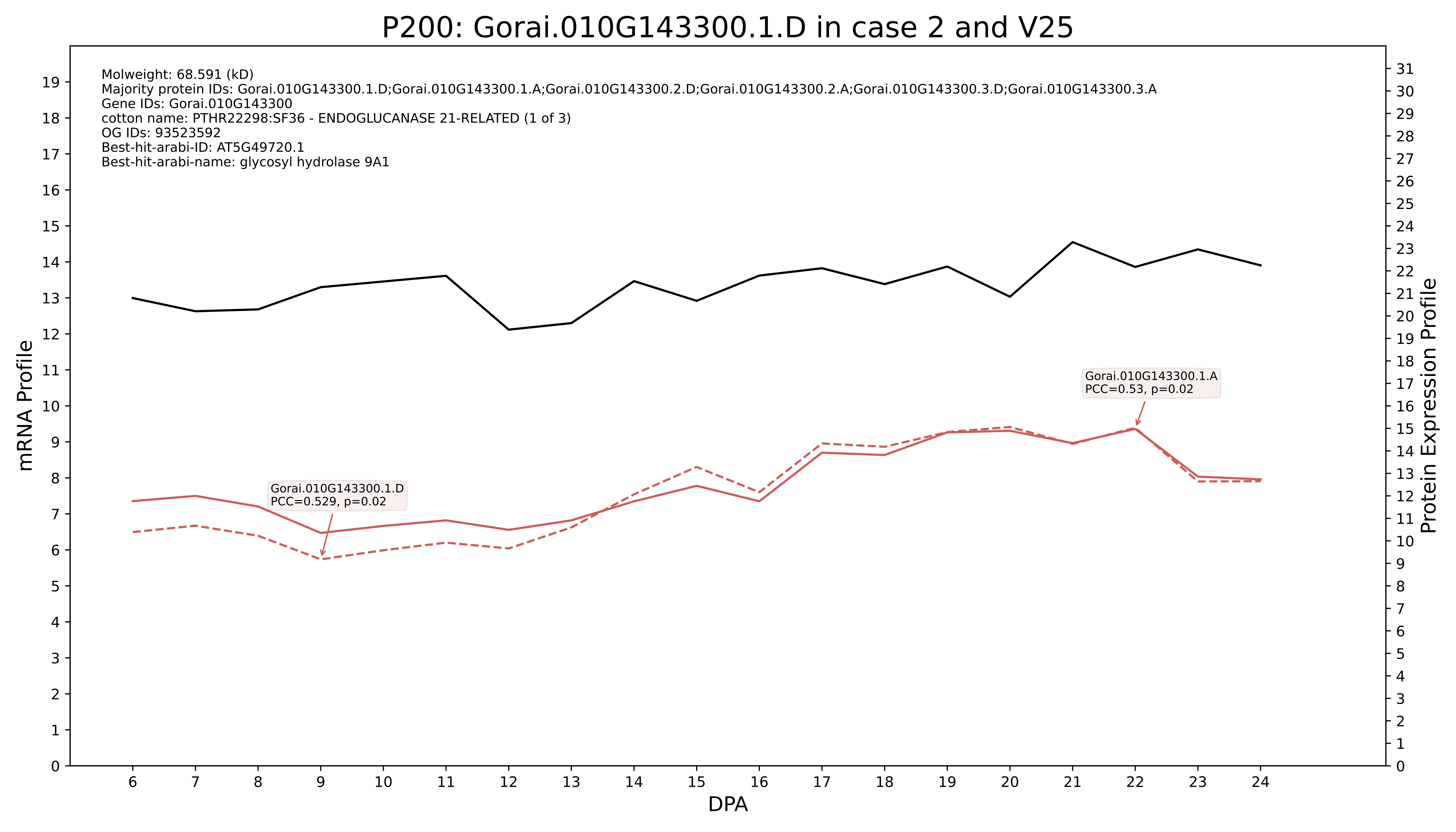
P200: Gorai.010G143300.1.D in case 2 and V25
Molweight: 68.591 (kD)
Majority protein IDs: Gorai.010G143300.1.D;Gorai.010G143300.1.A;Gorai.010G143300.2.D;Gorai.010G143300.2.A;Gorai.010G143300.3.D;Gorai.010G143300.3.A
Gene IDs: Gorai.010G143300
cotton name: PTHR22298:SF36 - ENDOGLUCANASE 21-RELATED (1 of 3)
OG IDs: 93523592
Best-hit-arabi-ID: AT5G49720.1
Best-hit-arabi-name: glycosyl hydrolase 9A1
Gorai.010G143300.1.A
PCC=0.53, p=0.02
Gorai.010G143300.1.D
PCC=0.529, p=0.02
mRNA Profile
Protein Expression Profile
DPA

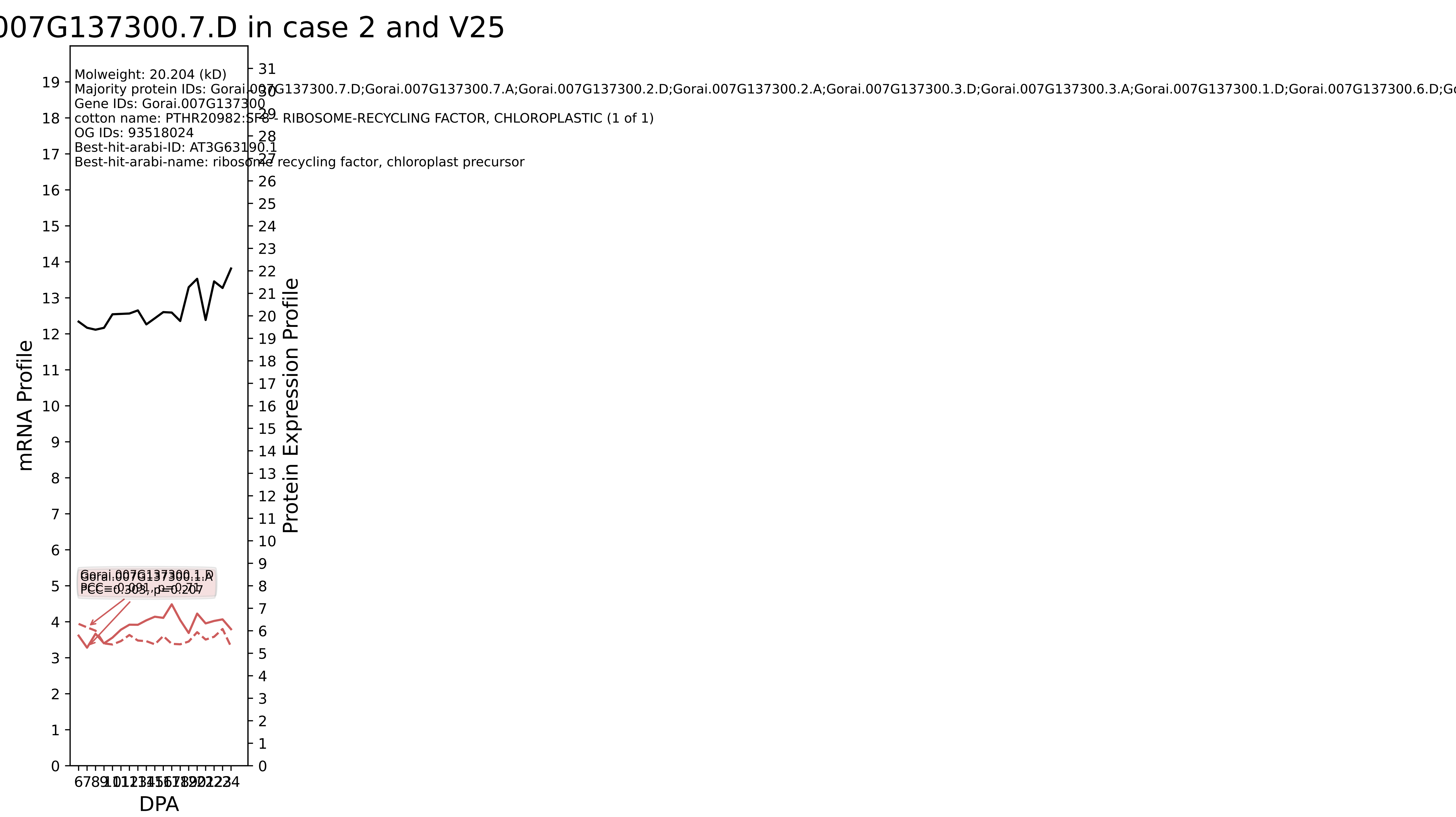

007G137300.7.D in case 2 and V25
Molweight: 20.204 (kD)
Majority protein IDs: Gorai.007G137300.7.D;Gorai.007G137300.7.A;Gorai.007G137300.2.D;Gorai.007G137300.2.A;Gorai.007G137300.3.D;Gorai.007G137300.3.A;Gorai.007G137300.1.D;Gorai.007G137300.6.D;G
Gene IDs: Gorai.007G137300
cotton name: PTHR20982:SF8 - RIBOSOME-RECYCLING FACTOR, CHLOROPLASTIC (1 of 1)
OG IDs: 93518024
Best-hit-arabi-ID: AT3G63190.1
Best-hit-arabi-name: ribosome recycling factor, chloroplast precursor
mRNA Profile
Protein Expression Profile
DPA

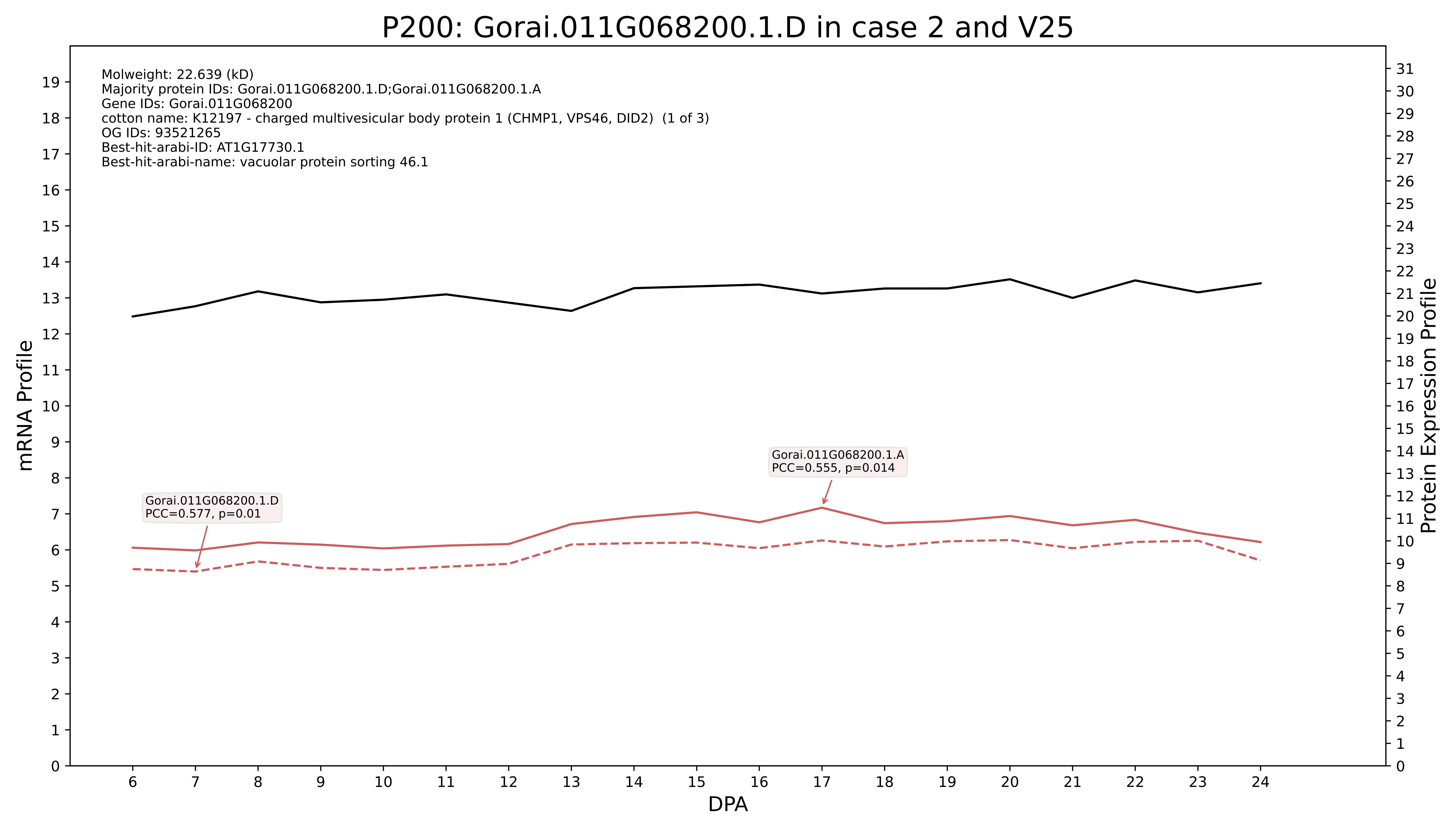
P200: Gorai.011G068200.1.D in case 2 and V25
Molweight: 22.639 (kD)
Majority protein IDs: Gorai.011G068200.1.D;Gorai.011G068200.1.A
Gene IDs: Gorai.011G068200
cotton name: K12197 - charged multivesicular body protein 1 (CHMP1, VPS46, DID2) (1 of 3)
OG IDs: 93521265
Best-hit-arabi-ID: AT1G17730.1
Best-hit-arabi-name: vacuolar protein sorting 46.1
Gorai.011G068200.1.A
PCC=0.555, p=0.014
Gorai.011G068200.1.D
PCC=0.577, p=0.01
mRNA Profile
Protein Expression Profile
DPA

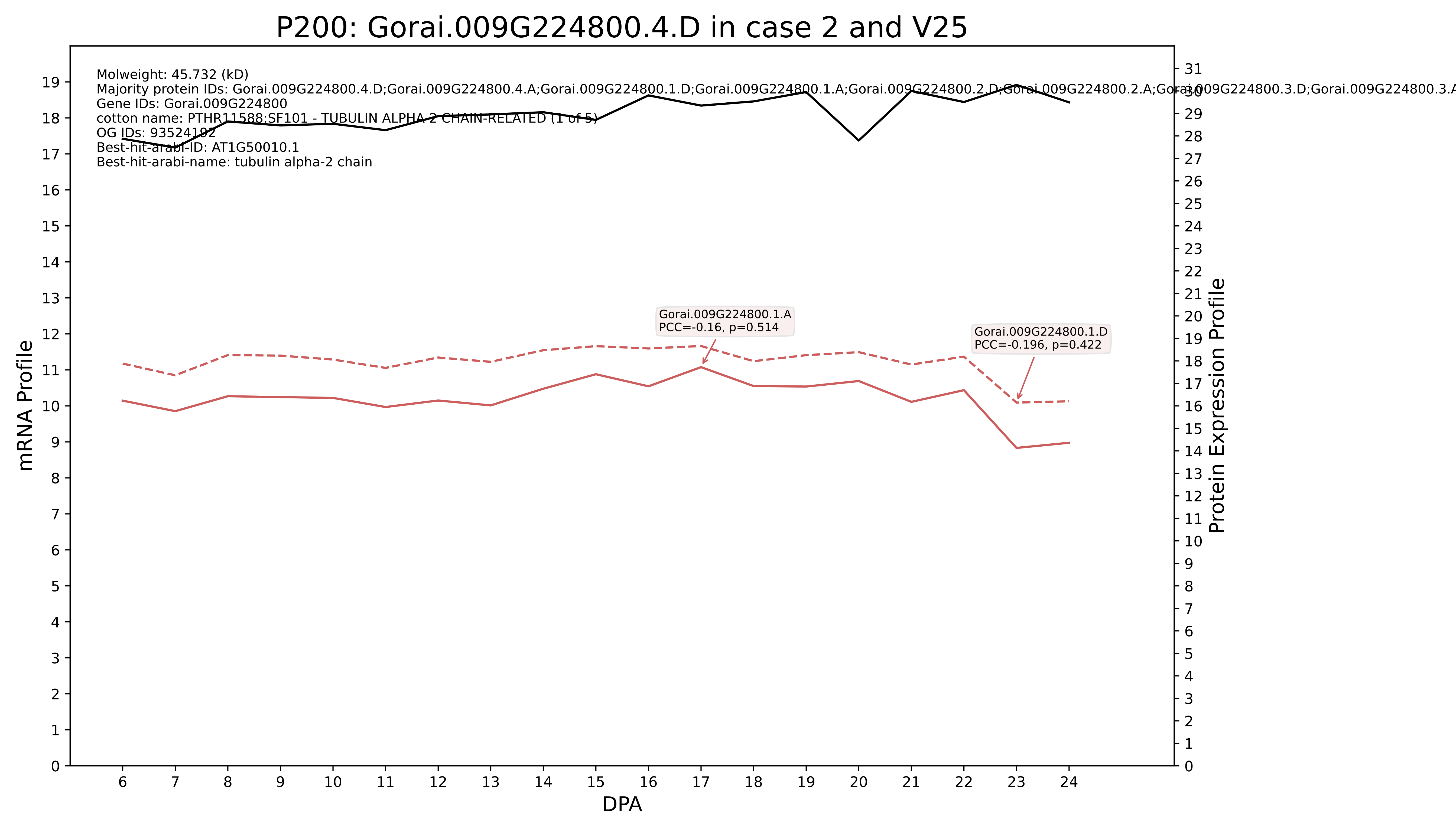
P200: Gorai.009G224800.4.D in case 2 and V25
Molweight: 45.732 (kD)
Majority protein IDs: Gorai.009G224800.4.D;Gorai.009G224800.4.A;Gorai.009G224800.1.D;Gorai.009G224800.1.A;Gorai.009G224800.2.D;Gorai.009G224800.2.A;Gorai.009G224800.3.D;Gorai.009G224800.3.A
Gene IDs: Gorai.009G224800
cotton name: PTHR11588:SF101 - TUBULIN ALPHA-2 CHAIN-RELATED (1 of 5)
OG IDs: 93524192
Best-hit-arabi-ID: AT1G50010.1
Best-hit-arabi-name: tubulin alpha-2 chain
Gorai.009G224800.1.A
PCC=-0.16, p=0.514
Gorai.009G224800.1.D
PCC=-0.196, p=0.422
mRNA Profile
Protein Expression Profile
DPA

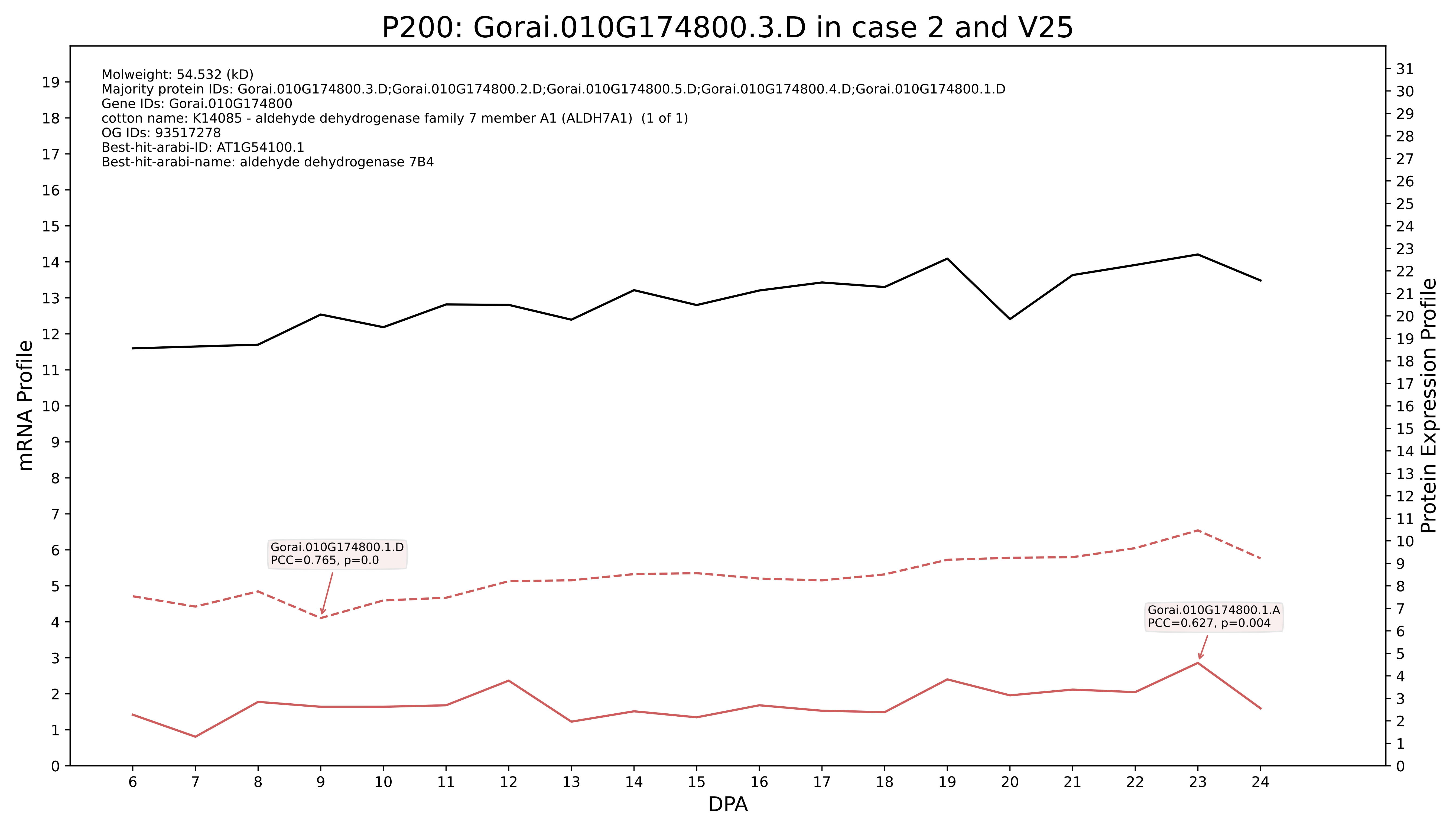
P200: Gorai.010G174800.3.D in case 2 and V25
Molweight: 54.532 (kD)
Majority protein IDs: Gorai.010G174800.3.D;Gorai.010G174800.2.D;Gorai.010G174800.5.D;Gorai.010G174800.4.D;Gorai.010G174800.1.D
Gene IDs: Gorai.010G174800
cotton name: K14085 - aldehyde dehydrogenase family 7 member A1 (ALDH7A1) (1 of 1)
OG IDs: 93517278
Best-hit-arabi-ID: AT1G54100.1
Best-hit-arabi-name: aldehyde dehydrogenase 7B4
Gorai.010G174800.1.D
PCC=0.765, p=0.0
Gorai.010G174800.1.A
PCC=0.627, p=0.004
mRNA Profile
Protein Expression Profile
DPA

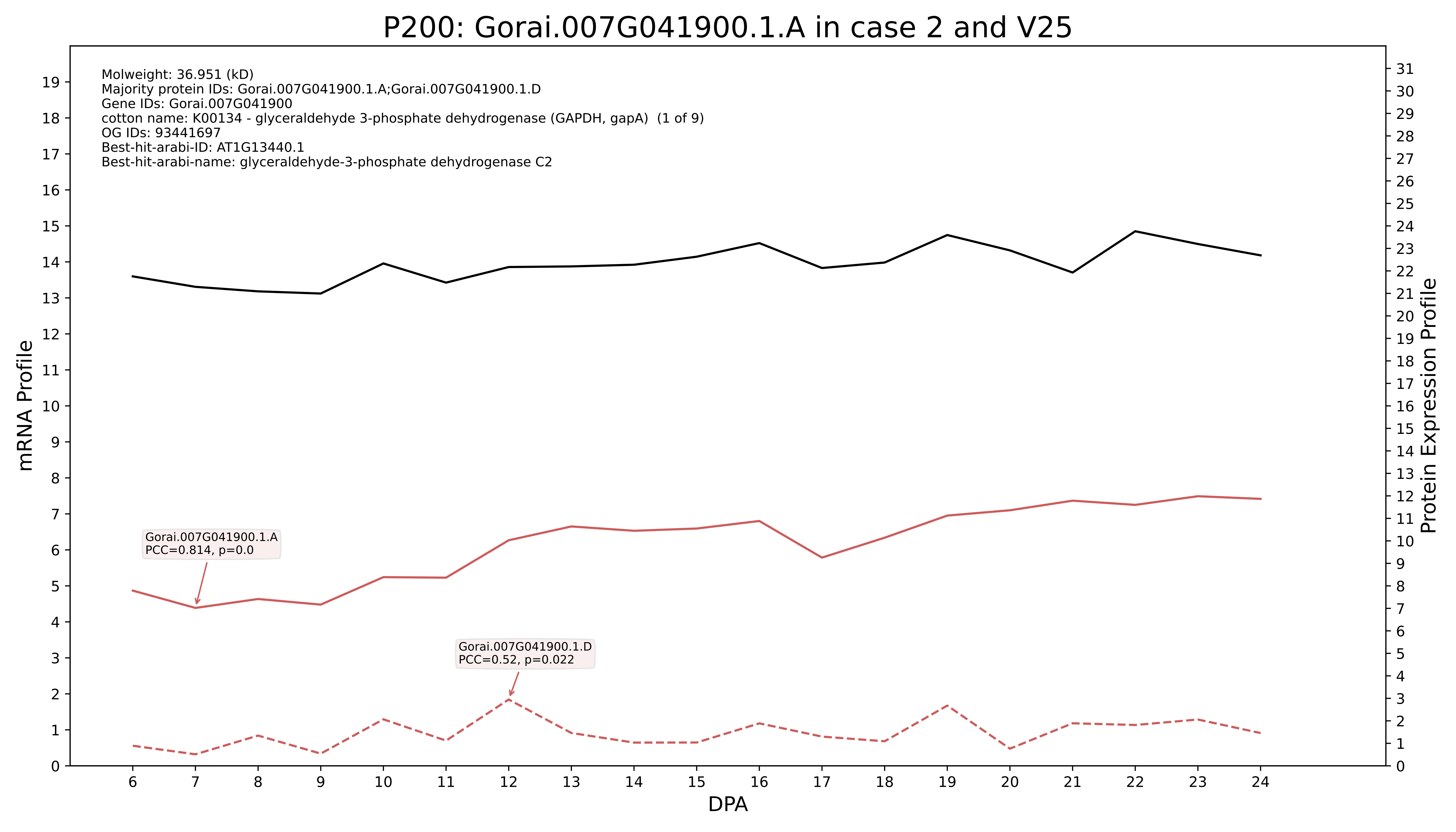
P200: Gorai.007G041900.1.A in case 2 and V25
Molweight: 36.951 (kD)
Majority protein IDs: Gorai.007G041900.1.A;Gorai.007G041900.1.D
Gene IDs: Gorai.007G041900
cotton name: K00134 - glyceraldehyde 3-phosphate dehydrogenase (GAPDH, gapA) (1 of 9)
OG IDs: 93441697
Best-hit-arabi-ID: AT1G13440.1
Best-hit-arabi-name: glyceraldehyde-3-phosphate dehydrogenase C2
Gorai.007G041900.1.A
PCC=0.814, p=0.0
Gorai.007G041900.1.D
PCC=0.52, p=0.022
mRNA Profile
Protein Expression Profile
DPA
6
7
8
9
10
11
12
13
14
15
16
17
18
19
20
21
22
23
24

P200: Gorai.011G128300.1.D in case 2 and V25

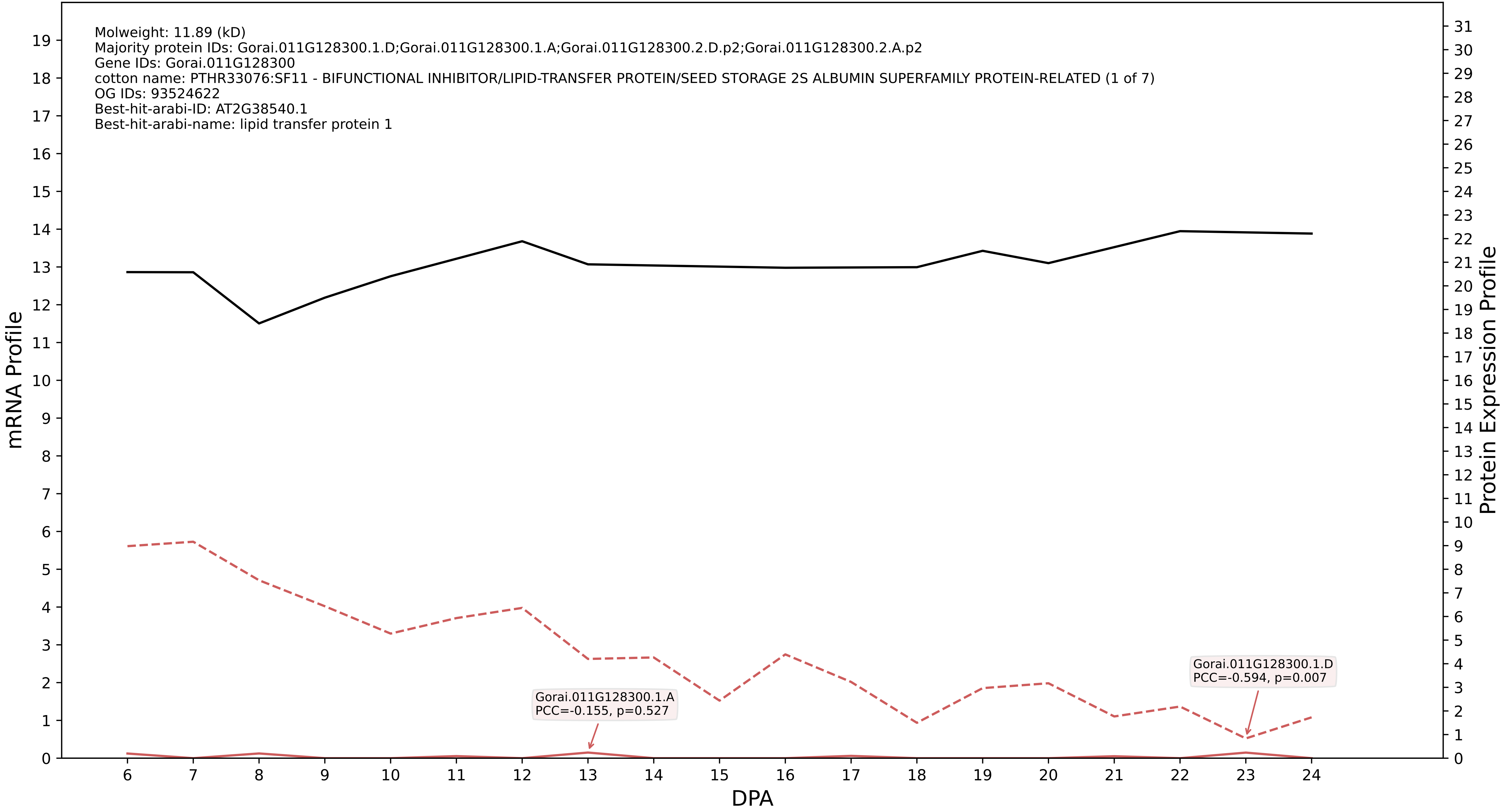

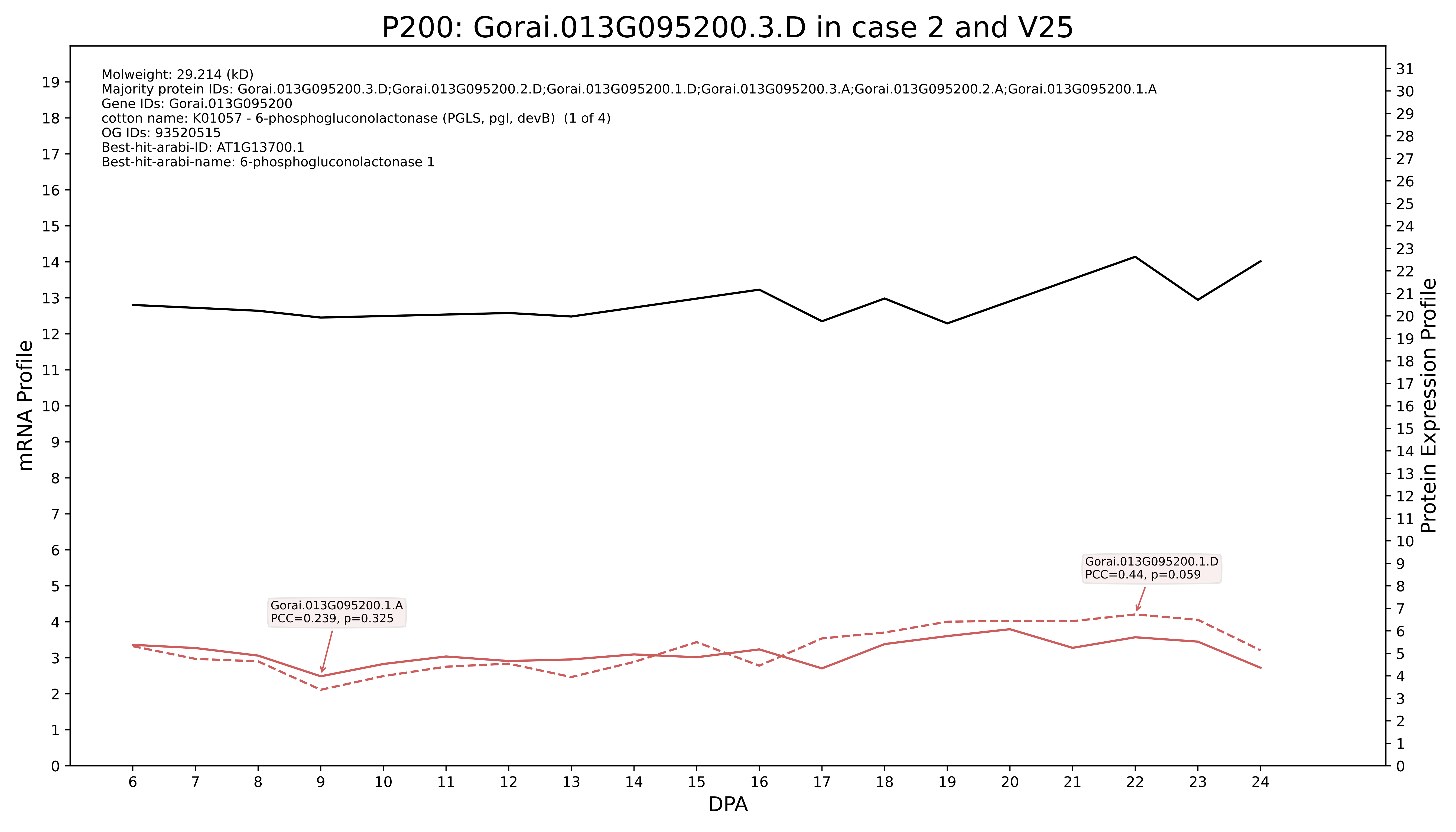
P200: Gorai.013G095200.3.D in case 2 and V25
Molweight: 29.214 (kD)
Majority protein IDs: Gorai.013G095200.3.D;Gorai.013G095200.2.D;Gorai.013G095200.1.D;Gorai.013G095200.3.A;Gorai.013G095200.2.A;Gorai.013G095200.1.A
Gene IDs: Gorai.013G095200
cotton name: K01057 - 6-phosphogluconolactonase (PGLS, pgl, devB) (1 of 4)
OG IDs: 93520515
Best-hit-arabi-ID: AT1G13700.1
Best-hit-arabi-name: 6-phosphogluconolactonase 1
Gorai.013G095200.1.D
PCC=0.44, p=0.059
Gorai.013G095200.1.A
PCC=0.239, p=0.325
mRNA Profile
Protein Expression Profile
DPA

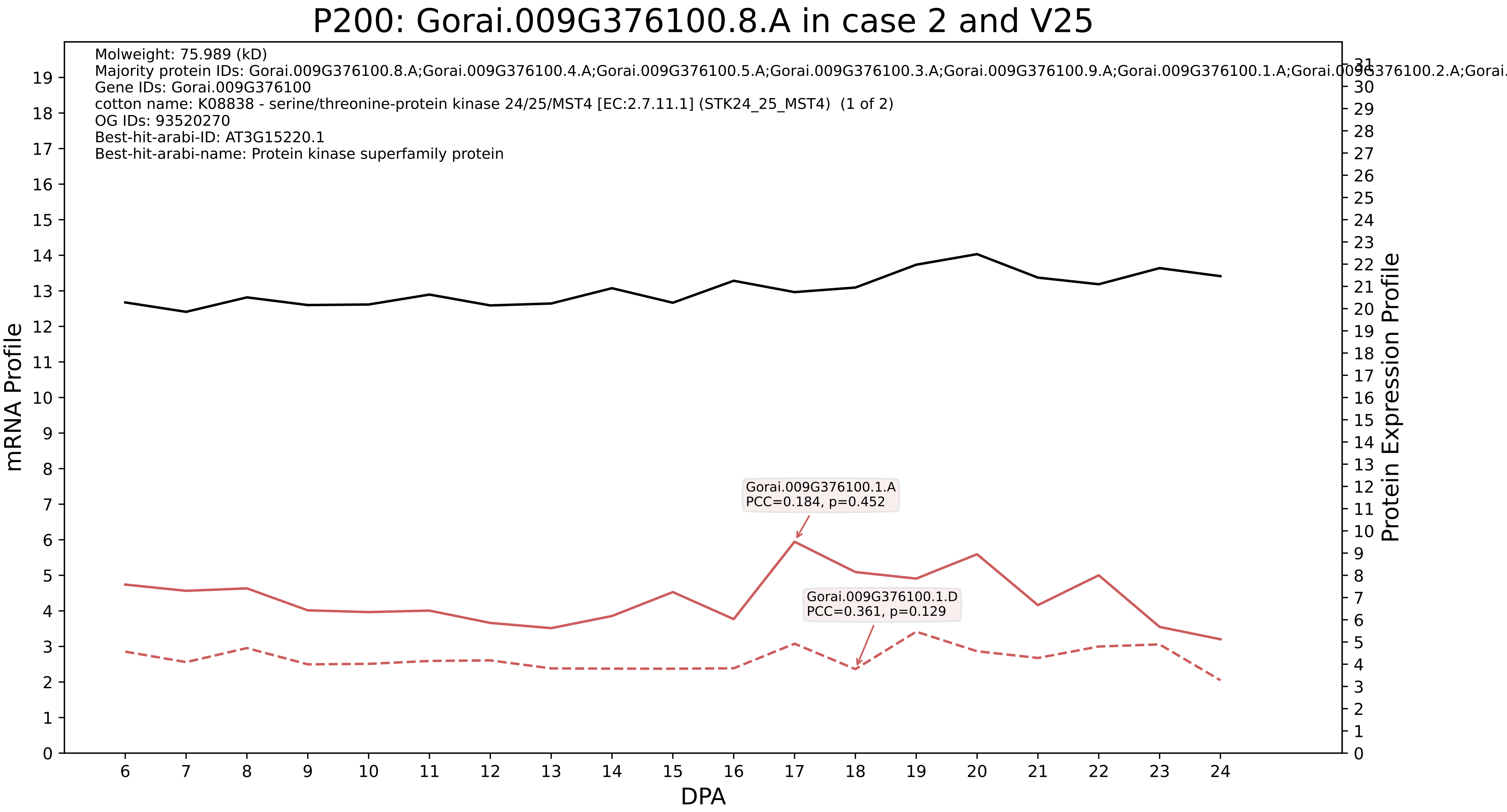
P200: Gorai.009G376100.8.A in case 2 and V25
Molweight: 75.989 (kD)
Majority protein IDs: Gorai.009G376100.8.A;Gorai.009G376100.4.A;Gorai.009G376100.5.A;Gorai.009G376100.3.A;Gorai.009G376100.9.A;Gorai.009G376100.1.A;Gorai.009G376100.2.A;Gorai.
Gene IDs: Gorai.009G376100
cotton name: K08838 - serine/threonine-protein kinase 24/25/MST4 [EC:2.7.11.1] (STK24_25_MST4) (1 of 2)
OG IDs: 93520270
Best-hit-arabi-ID: AT3G15220.1
Best-hit-arabi-name: Protein kinase superfamily protein
Gorai.009G376100.1.A
PCC=0.184, p=0.452
Gorai.009G376100.1.D
PCC=0.361, p=0.129
mRNA Profile
Protein Expression Profile
DPA

P200: Gorai.007G021300.1.A in case 2 and V25

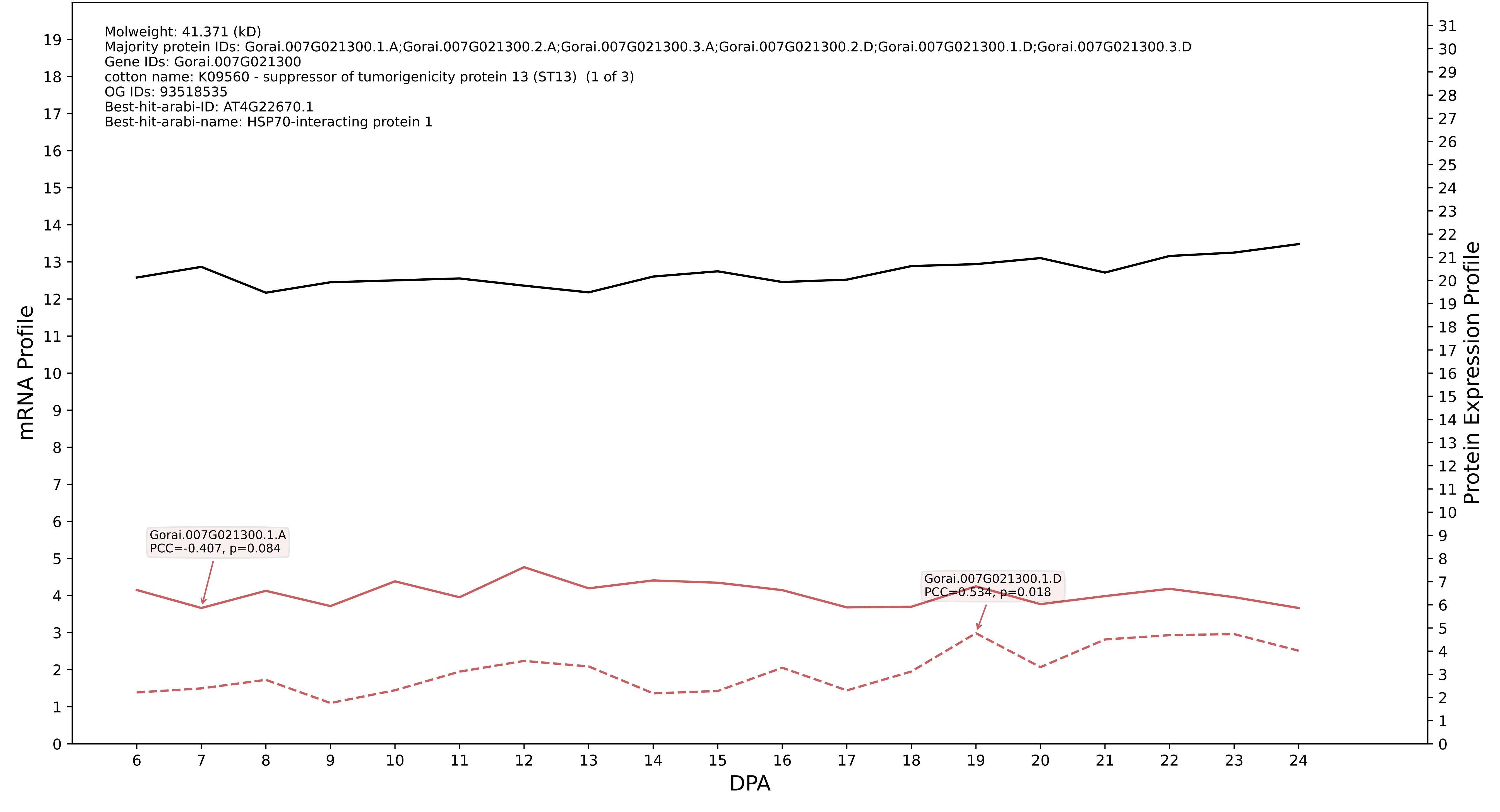

00: Gorai.010G053700.3.D in case 2 and V25

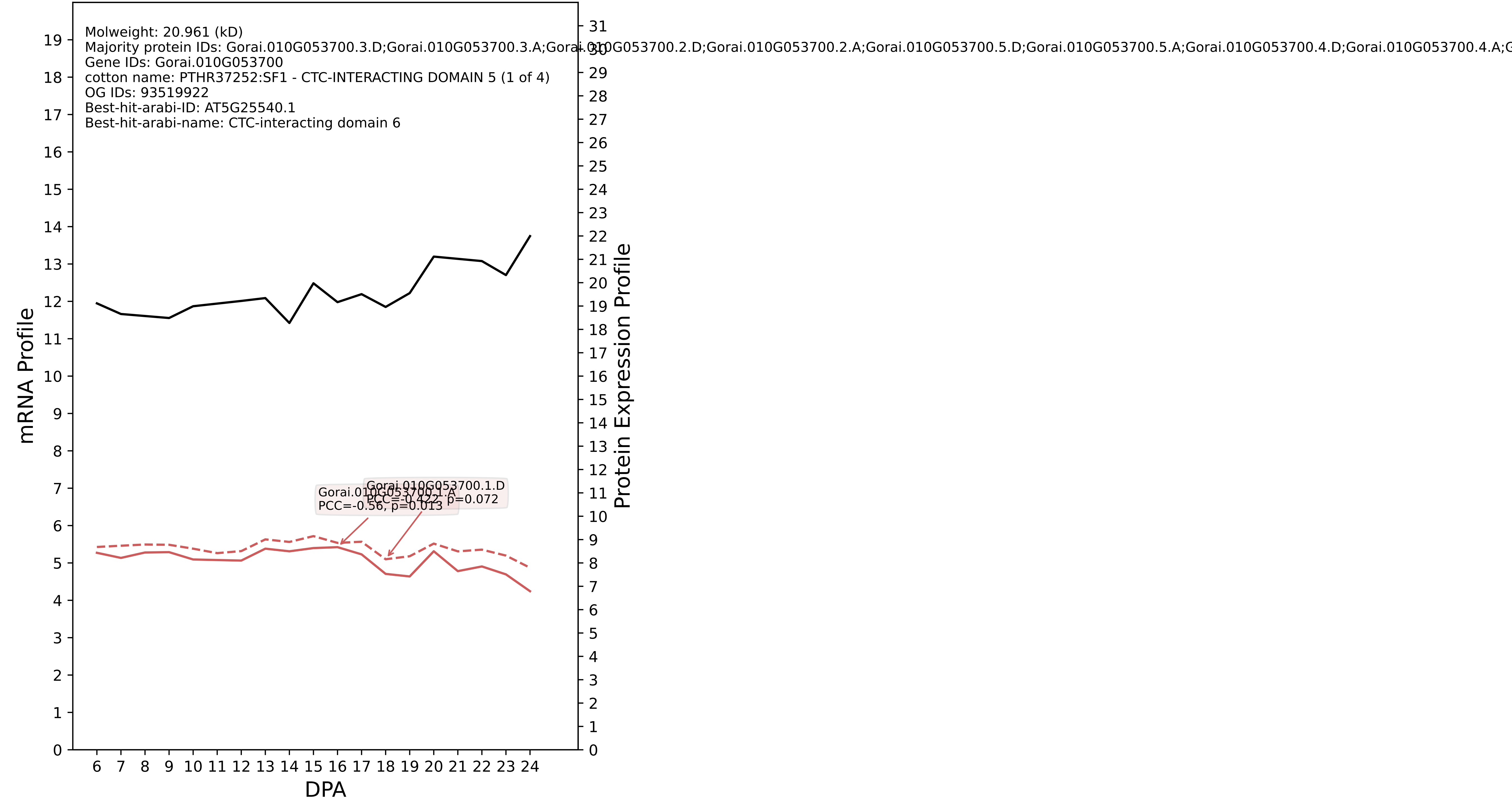

P200: Gorai.009G279800.1.A in case 2 and V25

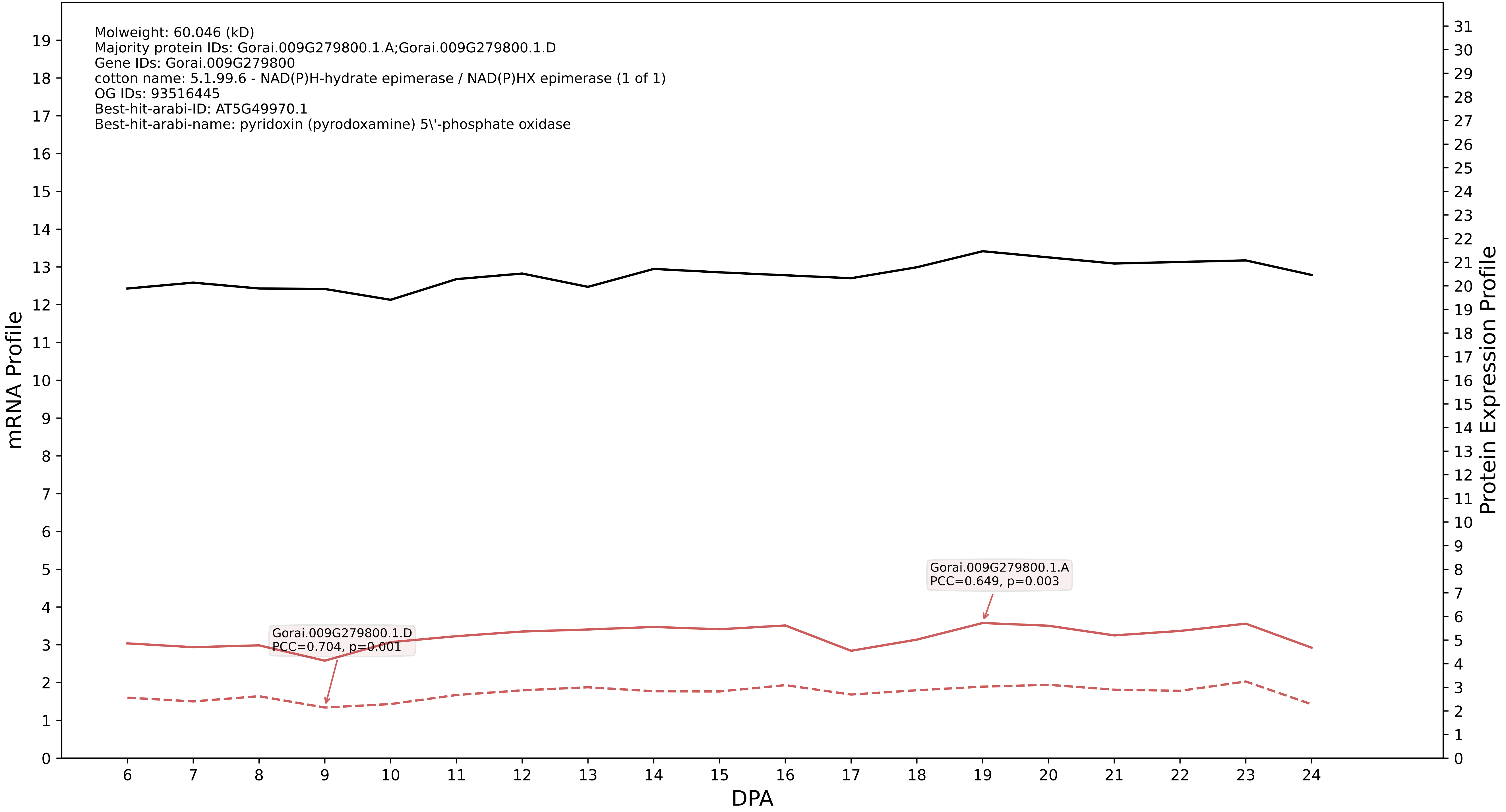

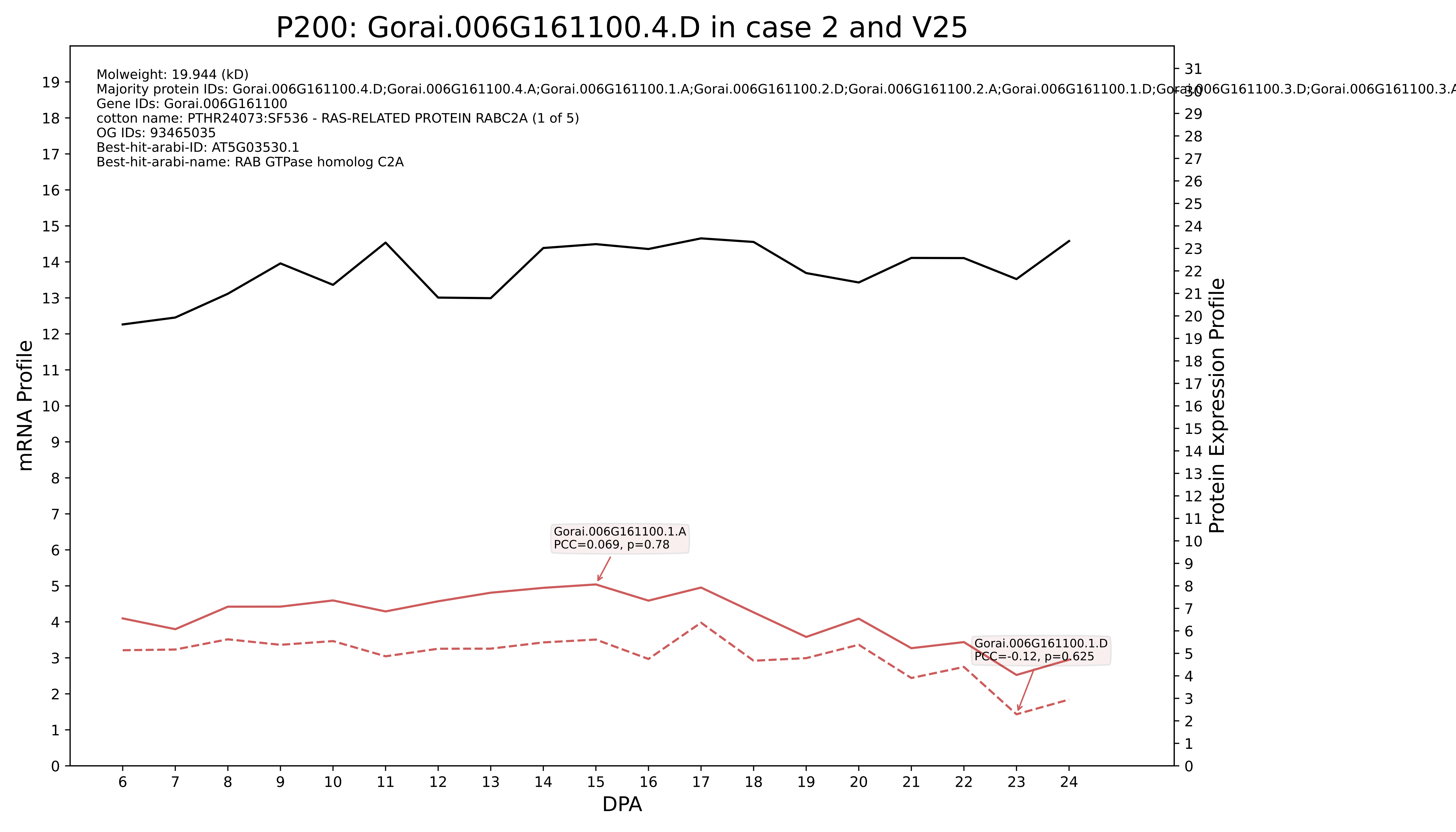
P200: Gorai.006G161100.4.D in case 2 and V25
Molweight: 19.944 (kD)
Majority protein IDs: Gorai.006G161100.4.D;Gorai.006G161100.4.A;Gorai.006G161100.1.A;Gorai.006G161100.2.D;Gorai.006G161100.2.A;Gorai.006G161100.1.D;Gorai.006G161100.3.D;Gorai.006G161100.3.A
Gene IDs: Gorai.006G161100
cotton name: PTHR24073:SF536 - RAS-RELATED PROTEIN RABC2A (1 of 5)
OG IDs: 93465035
Best-hit-arabi-ID: AT5G03530.1
Best-hit-arabi-name: RAB GTPase homolog C2A
Gorai.006G161100.1.A
PCC=0.069, p=0.78
Gorai.006G161100.1.D
PCC=-0.12, p=0.625
mRNA Profile
Protein Expression Profile
DPA

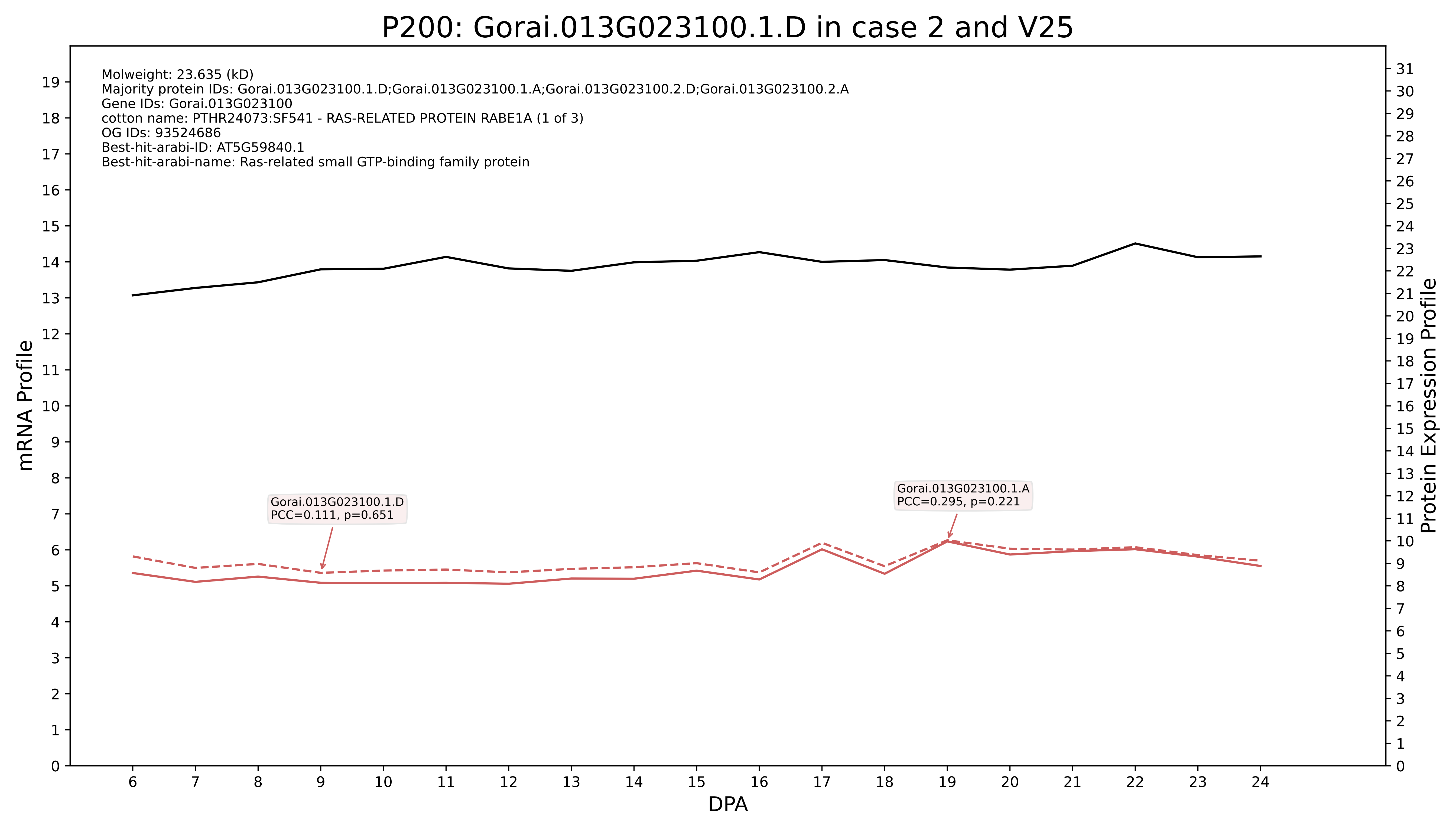
P200: Gorai.013G023100.1.D in case 2 and V25
Molweight: 23.635 (kD)
Majority protein IDs: Gorai.013G023100.1.D;Gorai.013G023100.1.A;Gorai.013G023100.2.D;Gorai.013G023100.2.A
Gene IDs: Gorai.013G023100
cotton name: PTHR24073:SF541 - RAS-RELATED PROTEIN RABE1A (1 of 3)
OG IDs: 93524686
Best-hit-arabi-ID: AT5G59840.1
Best-hit-arabi-name: Ras-related small GTP-binding family protein
Gorai.013G023100.1.D
PCC=0.111, p=0.651
Gorai.013G023100.1.A
PCC=0.295, p=0.221
mRNA Profile
Protein Expression Profile
DPA

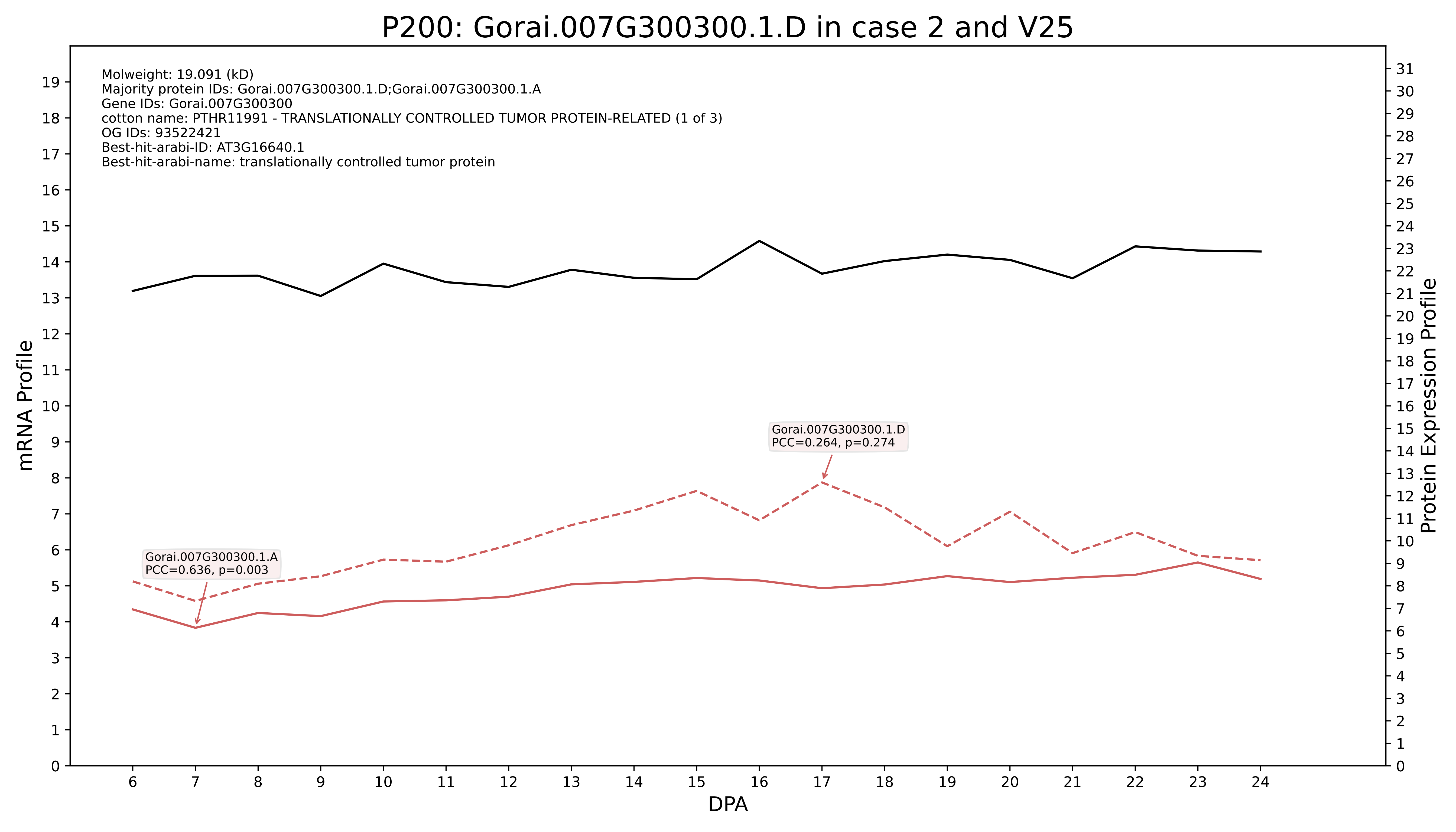
P200: Gorai.007G300300.1.D in case 2 and V25
Molweight: 19.091 (kD)
Majority protein IDs: Gorai.007G300300.1.D;Gorai.007G300300.1.A
Gene IDs: Gorai.007G300300
cotton name: PTHR11991 - TRANSLATIONALLY CONTROLLED TUMOR PROTEIN-RELATED (1 of 3)
OG IDs: 93522421
Best-hit-arabi-ID: AT3G16640.1
Best-hit-arabi-name: translationally controlled tumor protein
Gorai.007G300300.1.D
PCC=0.264, p=0.274
Gorai.007G300300.1.A
PCC=0.636, p=0.003
mRNA Profile
Protein Expression Profile
DPA

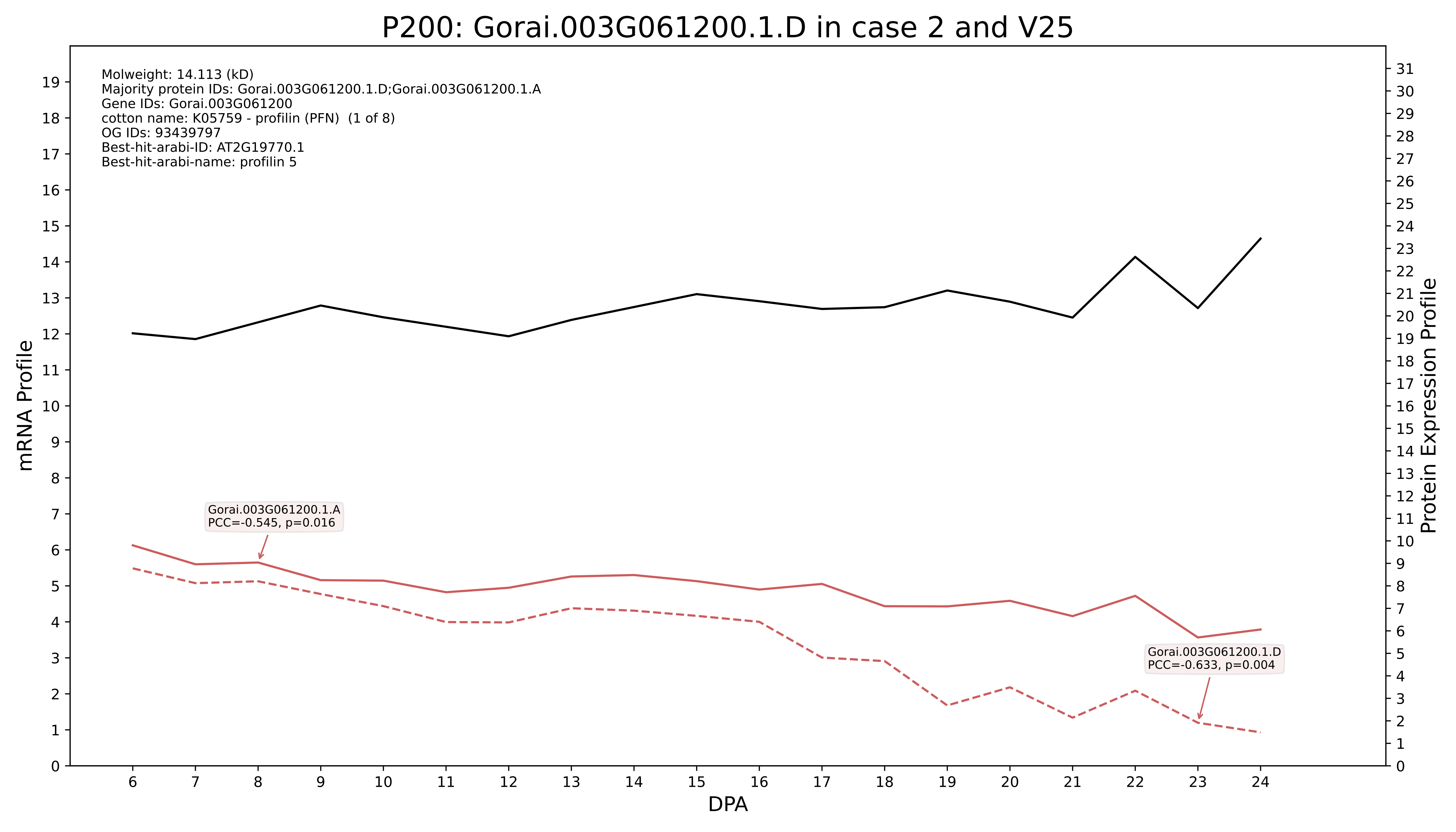
P200: Gorai.003G061200.1.D in case 2 and V25
Molweight: 14.113 (kD)
Majority protein IDs: Gorai.003G061200.1.D;Gorai.003G061200.1.A
Gene IDs: Gorai.003G061200
cotton name: K05759 - profilin (PFN) (1 of 8)
OG IDs: 93439797
Best-hit-arabi-ID: AT2G19770.1
Best-hit-arabi-name: profilin 5
Gorai.003G061200.1.A
PCC=-0.545, p=0.016
Gorai.003G061200.1.D
PCC=-0.633, p=0.004
mRNA Profile
Protein Expression Profile
DPA
0
1
2
3
4
5
6
7
8
9
10
11
12
13
14
15
16
17
18
19
20
21
22
23
24
25
26
27
28
29
30
31

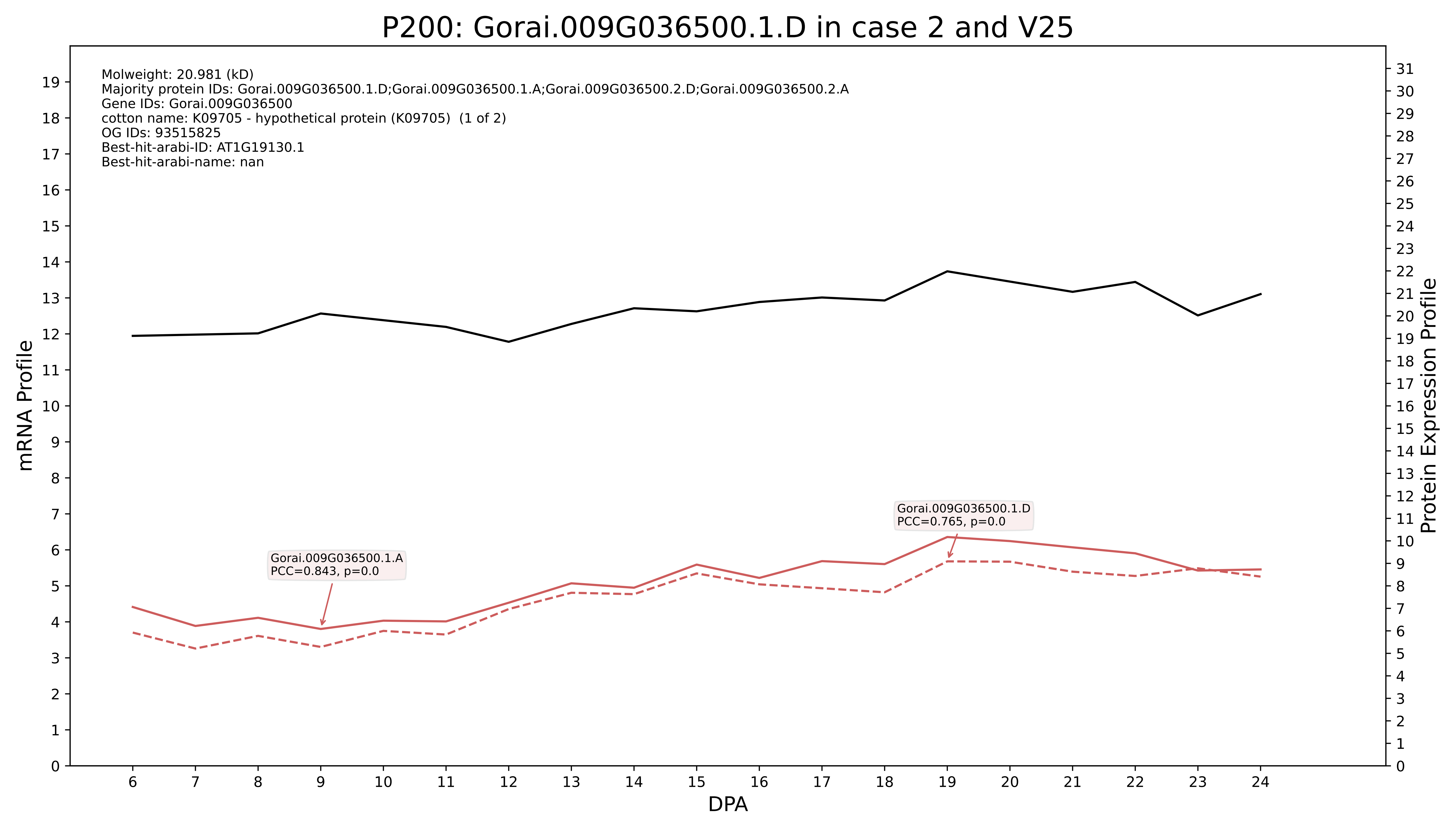
P200: Gorai.009G036500.1.D in case 2 and V25
Molweight: 20.981 (kD)
Majority protein IDs: Gorai.009G036500.1.D;Gorai.009G036500.1.A;Gorai.009G036500.2.D;Gorai.009G036500.2.A
Gene IDs: Gorai.009G036500
cotton name: K09705 - hypothetical protein (K09705) (1 of 2)
OG IDs: 93515825
Best-hit-arabi-ID: AT1G19130.1
Best-hit-arabi-name: nan
Gorai.009G036500.1.D
PCC=0.765, p=0.0
Gorai.009G036500.1.A
PCC=0.843, p=0.0
mRNA Profile
Protein Expression Profile
DPA

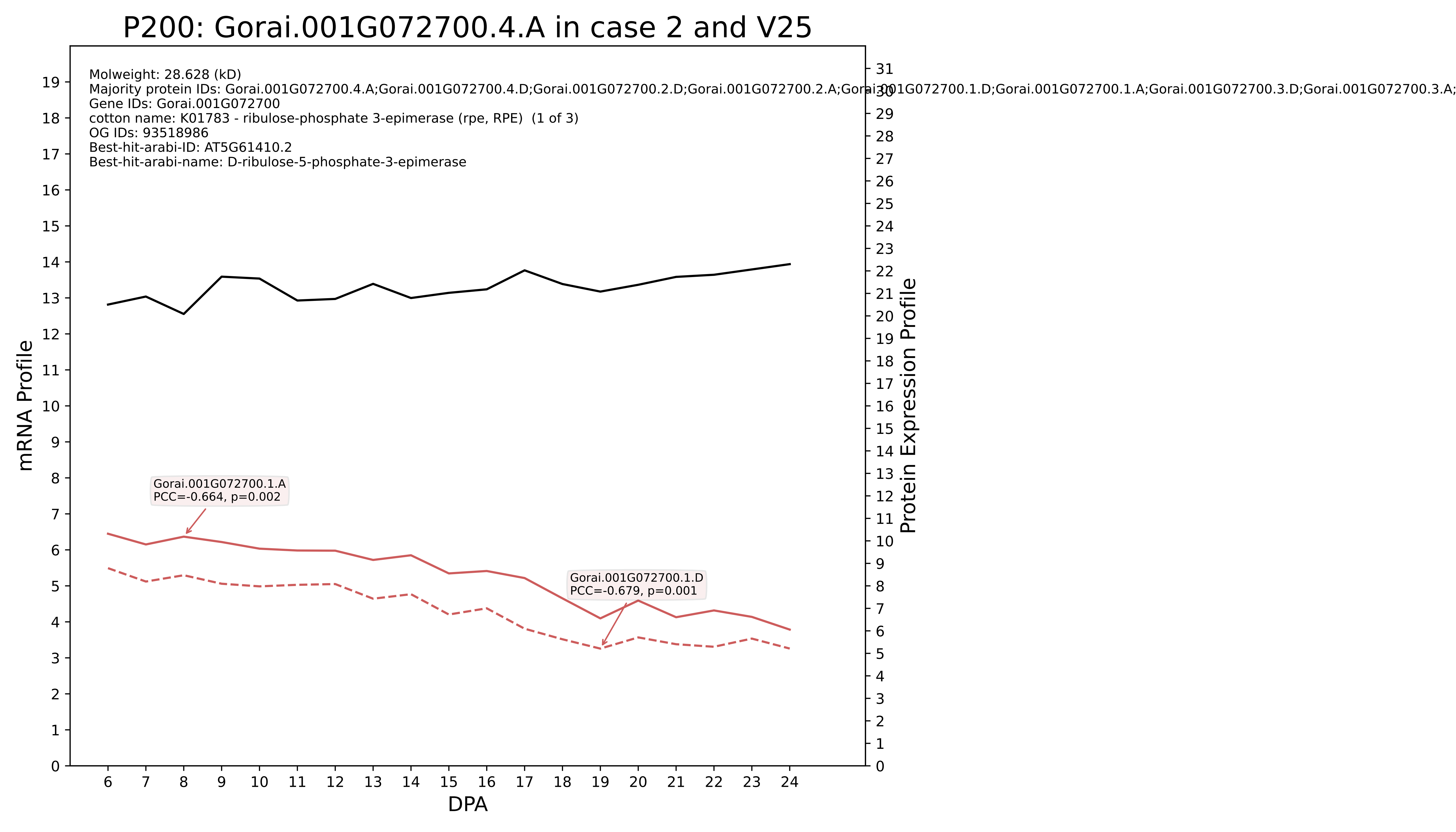
P200: Gorai.001G072700.4.A in case 2 and V25
Molweight: 28.628 (kD)
Majority protein IDs: Gorai.001G072700.4.A;Gorai.001G072700.4.D;Gorai.001G072700.2.D;Gorai.001G072700.2.A;Gorai.001G072700.1.D;Gorai.001G072700.1.A;Gorai.001G072700.3.D;Gorai.001G072700.3.A;
Gene IDs: Gorai.001G072700
cotton name: K01783 - ribulose-phosphate 3-epimerase (rpe, RPE) (1 of 3)
OG IDs: 93518986
Best-hit-arabi-ID: AT5G61410.2
Best-hit-arabi-name: D-ribulose-5-phosphate-3-epimerase
Gorai.001G072700.1.A
PCC=-0.664, p=0.002
Gorai.001G072700.1.D
PCC=-0.679, p=0.001
mRNA Profile
Protein Expression Profile
DPA

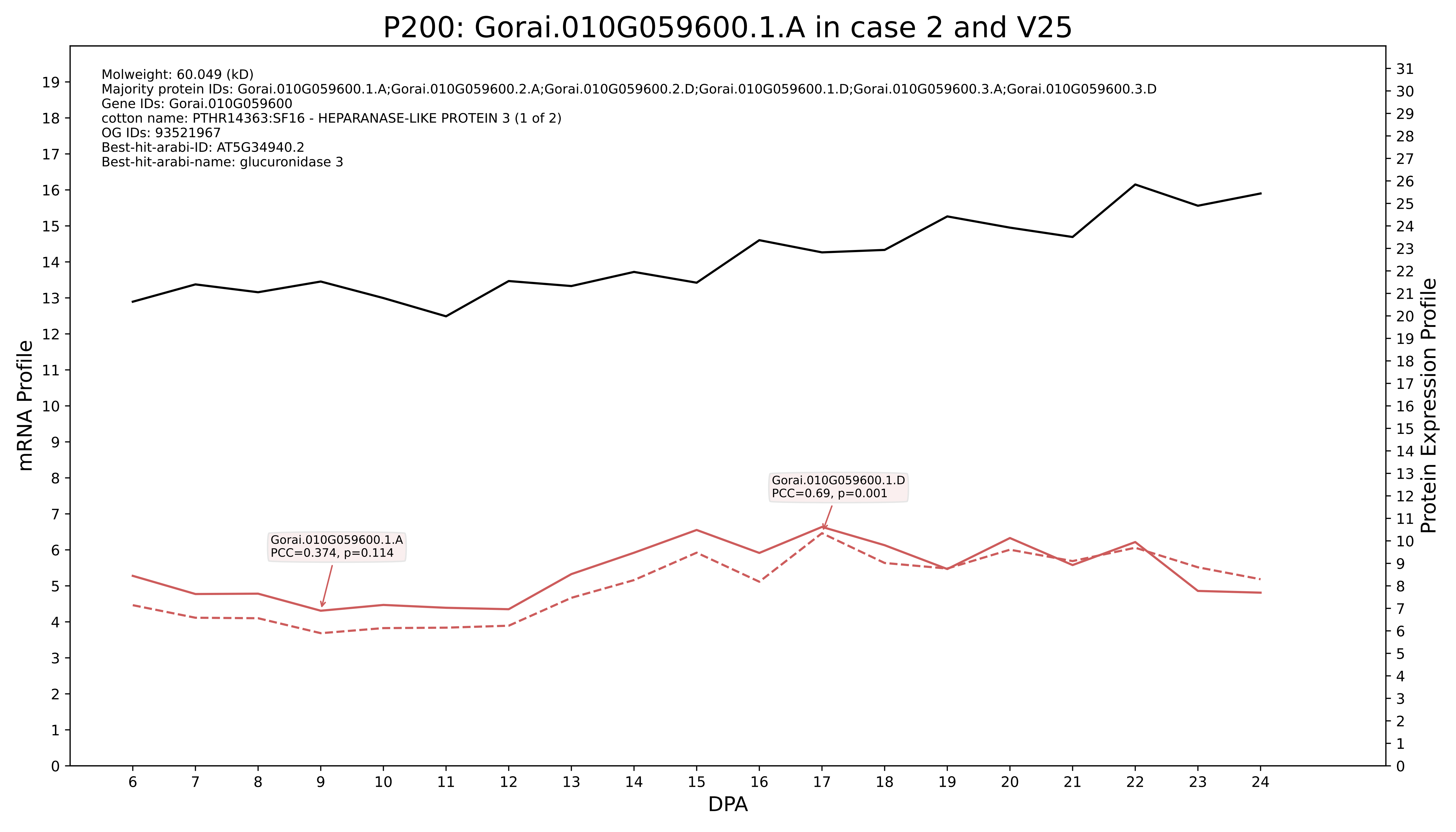
P200: Gorai.010G059600.1.A in case 2 and V25
Molweight: 60.049 (kD)
Majority protein IDs: Gorai.010G059600.1.A;Gorai.010G059600.2.A;Gorai.010G059600.2.D;Gorai.010G059600.1.D;Gorai.010G059600.3.A;Gorai.010G059600.3.D
Gene IDs: Gorai.010G059600
cotton name: PTHR14363:SF16 - HEPARANASE-LIKE PROTEIN 3 (1 of 2)
OG IDs: 93521967
Best-hit-arabi-ID: AT5G34940.2
Best-hit-arabi-name: glucuronidase 3
Gorai.010G059600.1.D
PCC=0.69, p=0.001
Gorai.010G059600.1.A
PCC=0.374, p=0.114
mRNA Profile
Protein Expression Profile
DPA

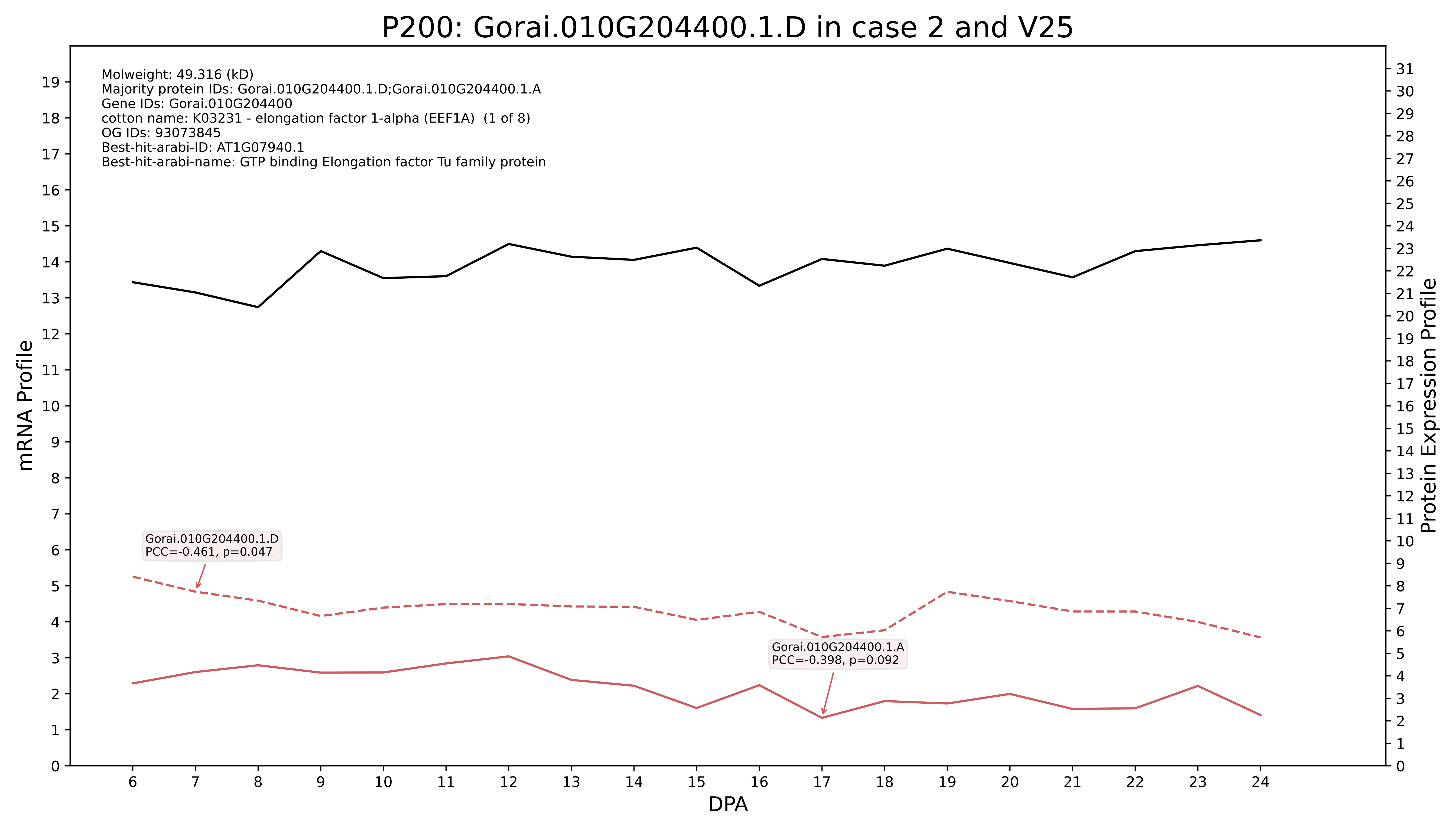
P200: Gorai.010G204400.1.D in case 2 and V25
Molweight: 49.316 (kD)
Majority protein IDs: Gorai.010G204400.1.D;Gorai.010G204400.1.A
Gene IDs: Gorai.010G204400
cotton name: K03231 - elongation factor 1-alpha (EEF1A) (1 of 8)
OG IDs: 93073845
Best-hit-arabi-ID: AT1G07940.1
Best-hit-arabi-name: GTP binding Elongation factor Tu family protein
Gorai.010G204400.1.D
PCC=-0.461, p=0.047
Gorai.010G204400.1.A
PCC=-0.398, p=0.092
mRNA Profile
Protein Expression Profile
DPA
0
1
2
3
4
5
6
7
8
9
10
11
12
13
14
15
16
17
18
19
20
21
22
23
24
25
26
27
28
29
30
31

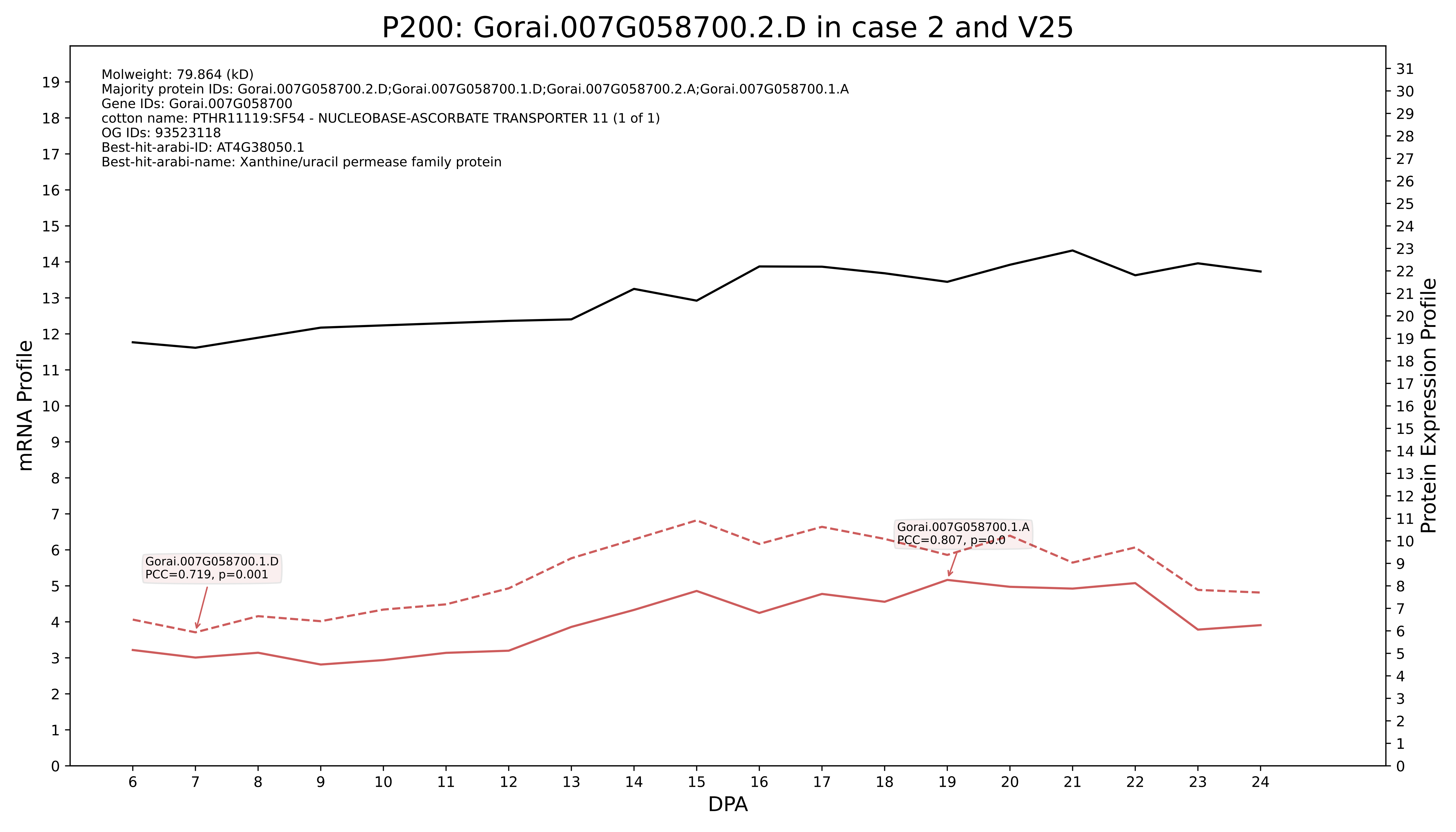
P200: Gorai.007G058700.2.D in case 2 and V25
Molweight: 79.864 (kD)
Majority protein IDs: Gorai.007G058700.2.D;Gorai.007G058700.1.D;Gorai.007G058700.2.A;Gorai.007G058700.1.A
Gene IDs: Gorai.007G058700
cotton name: PTHR11119:SF54 - NUCLEOBASE-ASCORBATE TRANSPORTER 11 (1 of 1)
OG IDs: 93523118
Best-hit-arabi-ID: AT4G38050.1
Best-hit-arabi-name: Xanthine/uracil permease family protein
Gorai.007G058700.1.D
PCC=0.719, p=0.001
Gorai.007G058700.1.A
PCC=0.807, p=0.0
mRNA Profile
Protein Expression Profile
DPA

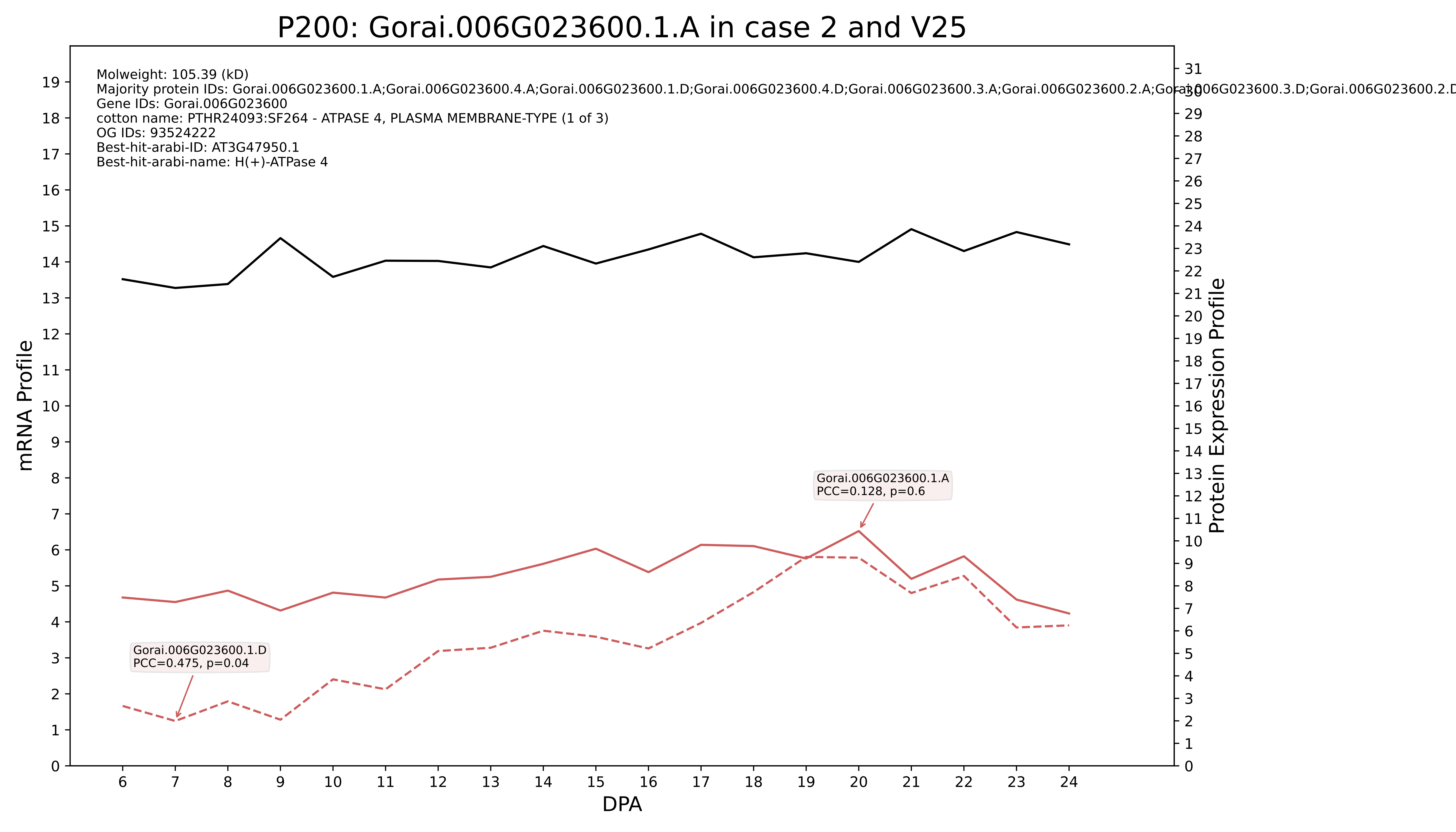

P200: Gorai.006G023600.1.A in case 2 and V25
Molweight: 105.39 (kD)
Majority protein IDs: Gorai.006G023600.1.A;Gorai.006G023600.4.A;Gorai.006G023600.1.D;Gorai.006G023600.4.D;Gorai.006G023600.3.A;Gorai.006G023600.2.A;Gorai.006G023600.3.D;Gorai.006G023600.2.D
Gene IDs: Gorai.006G023600
cotton name: PTHR24093:SF264 - ATPASE 4, PLASMA MEMBRANE-TYPE (1 of 3)
OG IDs: 93524222
Best-hit-arabi-ID: AT3G47950.1
Best-hit-arabi-name: H(+)-ATPase 4
Gorai.006G023600.1.A
PCC=0.128, p=0.6
Gorai.006G023600.1.D
PCC=0.475, p=0.04
mRNA Profile
Protein Expression Profile
DPA

P200: Gorai.007G310200.1.A in case 2 and V25

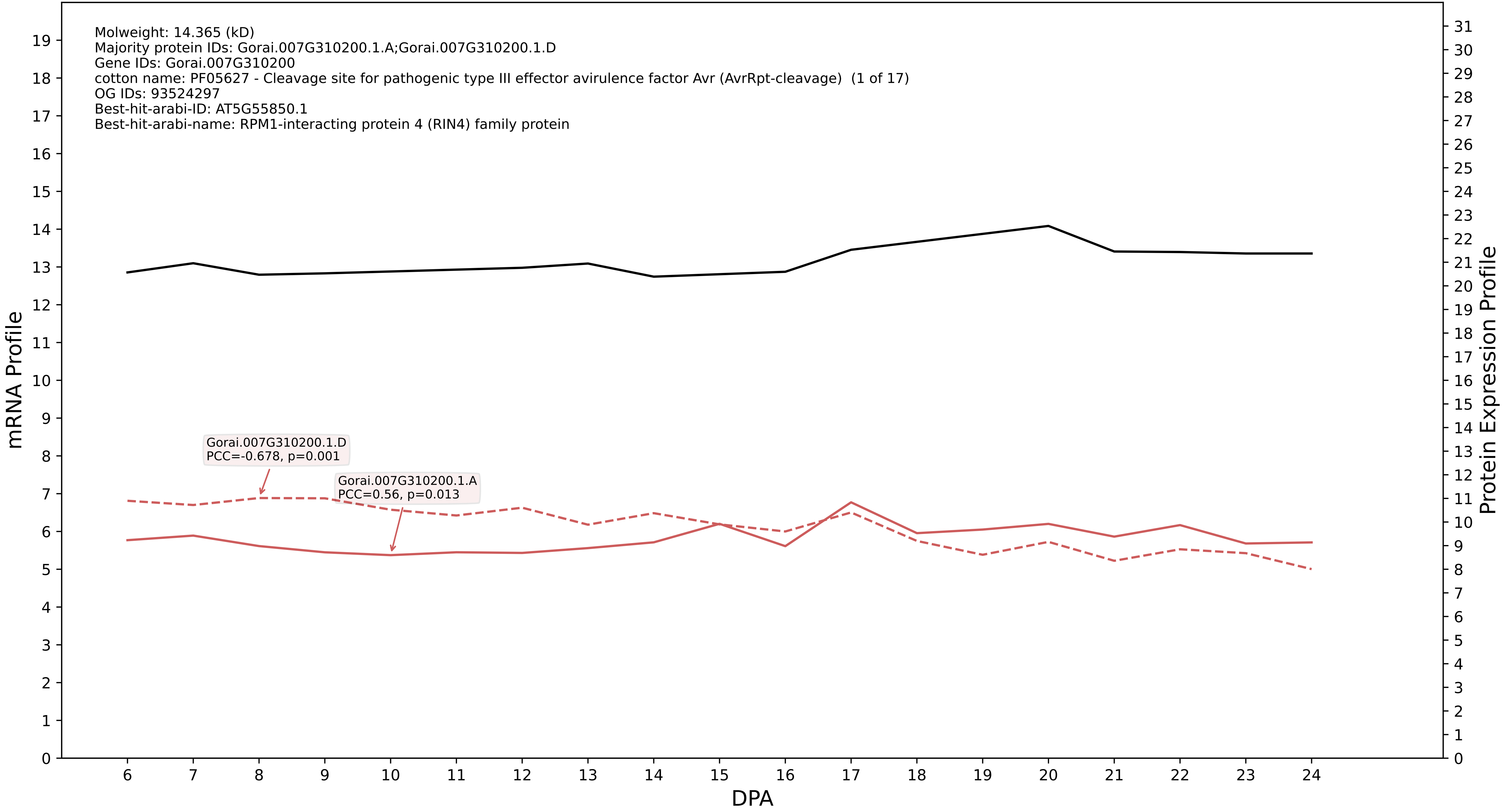

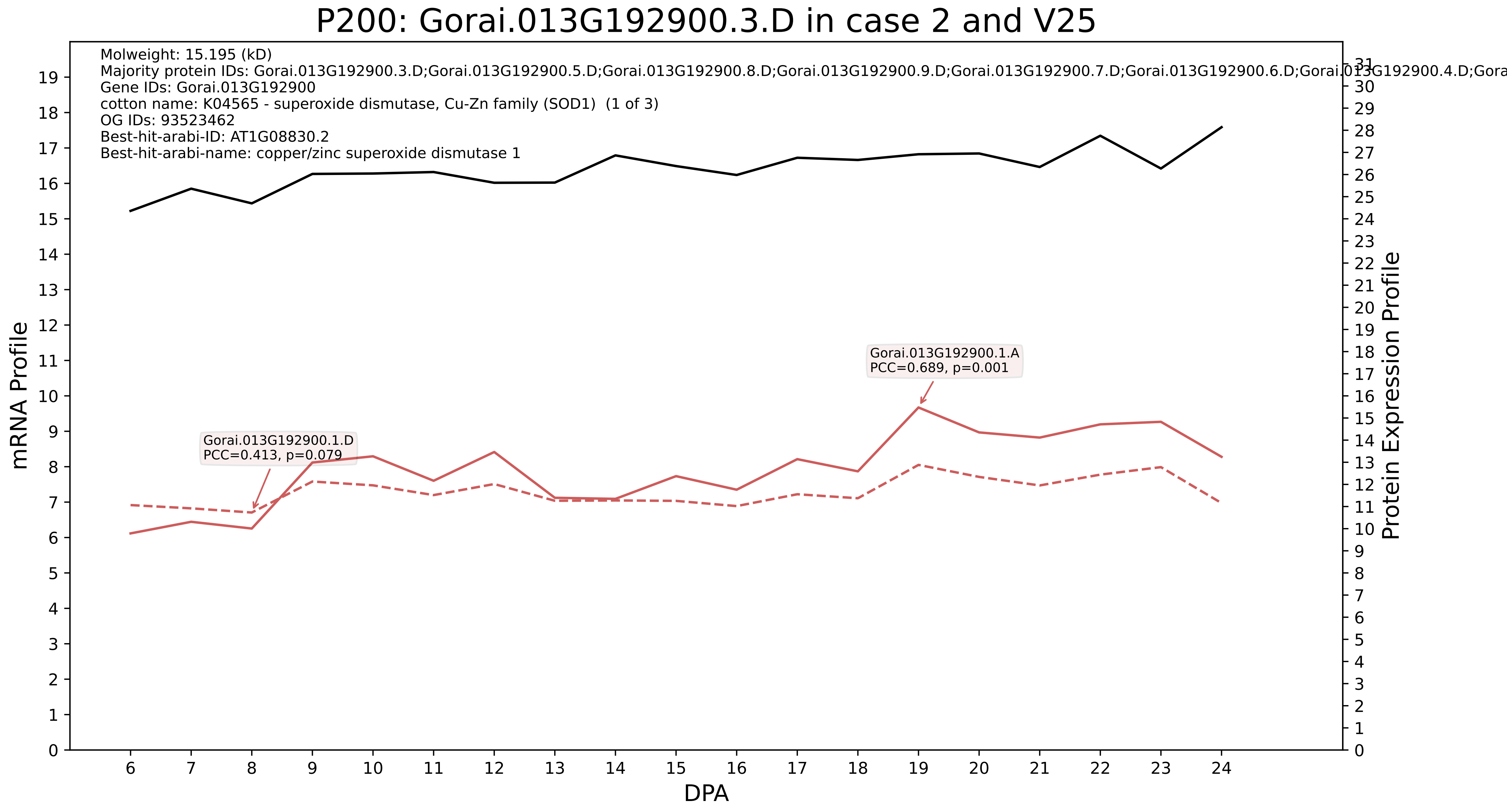

P200: Gorai.013G192900.3.D in case 2 and V25
Molweight: 15.195 (kD)
Majority protein IDs: Gorai.013G192900.3.D;Gorai.013G192900.5.D;Gorai.013G192900.8.D;Gorai.013G192900.9.D;Gorai.013G192900.7.D;Gorai.013G192900.6.D;Gorai.013G192900.4.D;Gora
Gene IDs: Gorai.013G192900
cotton name: K04565 - superoxide dismutase, Cu-Zn family (SOD1) (1 of 3)
OG IDs: 93523462
Best-hit-arabi-ID: AT1G08830.2
Best-hit-arabi-name: copper/zinc superoxide dismutase 1
Gorai.013G192900.1.A
PCC=0.689, p=0.001
Gorai.013G192900.1.D
PCC=0.413, p=0.079
mRNA Profile
Protein Expression Profile
DPA

P200: Gorai.013G195300.1.D in case 2 and V25

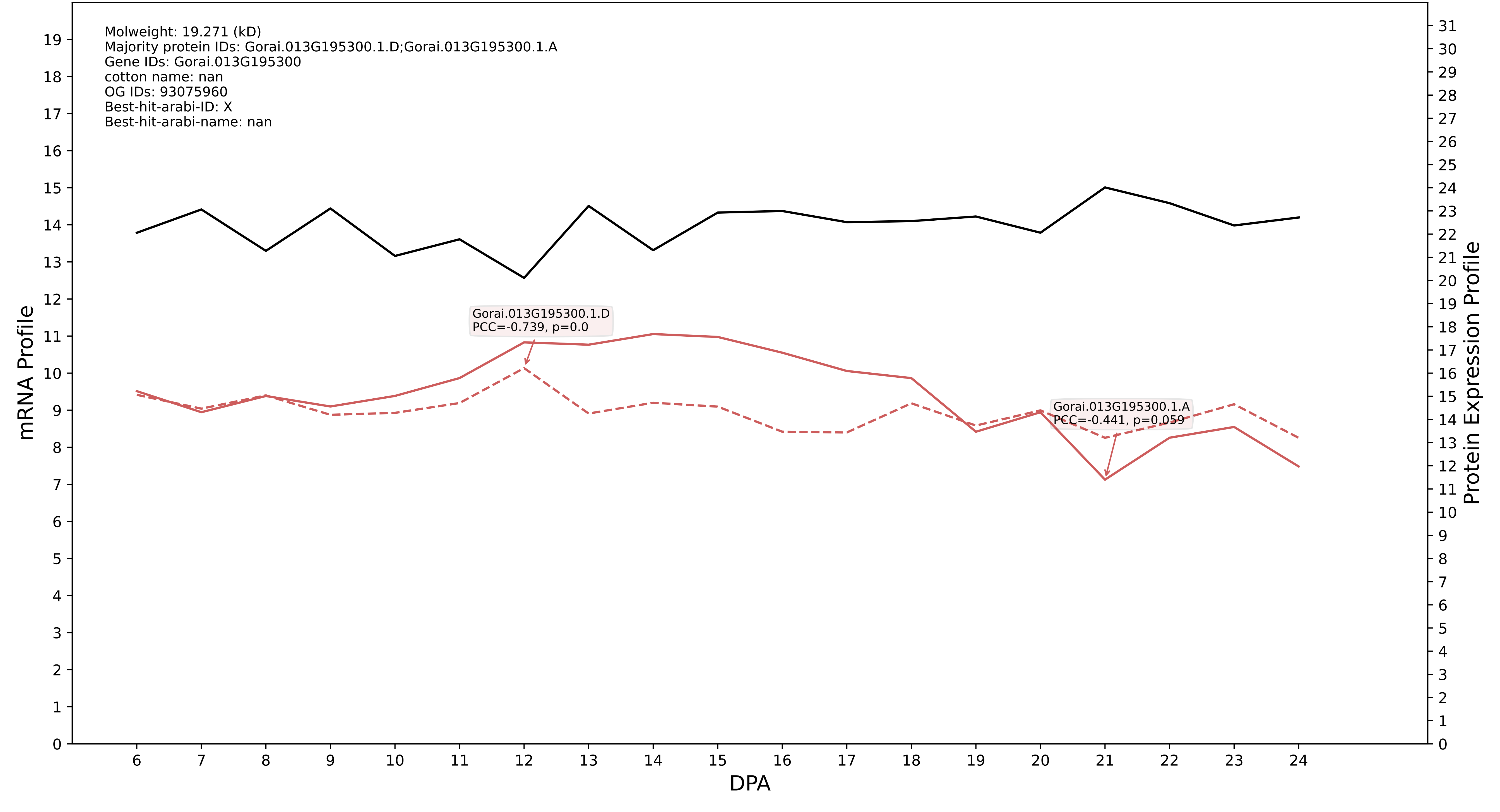

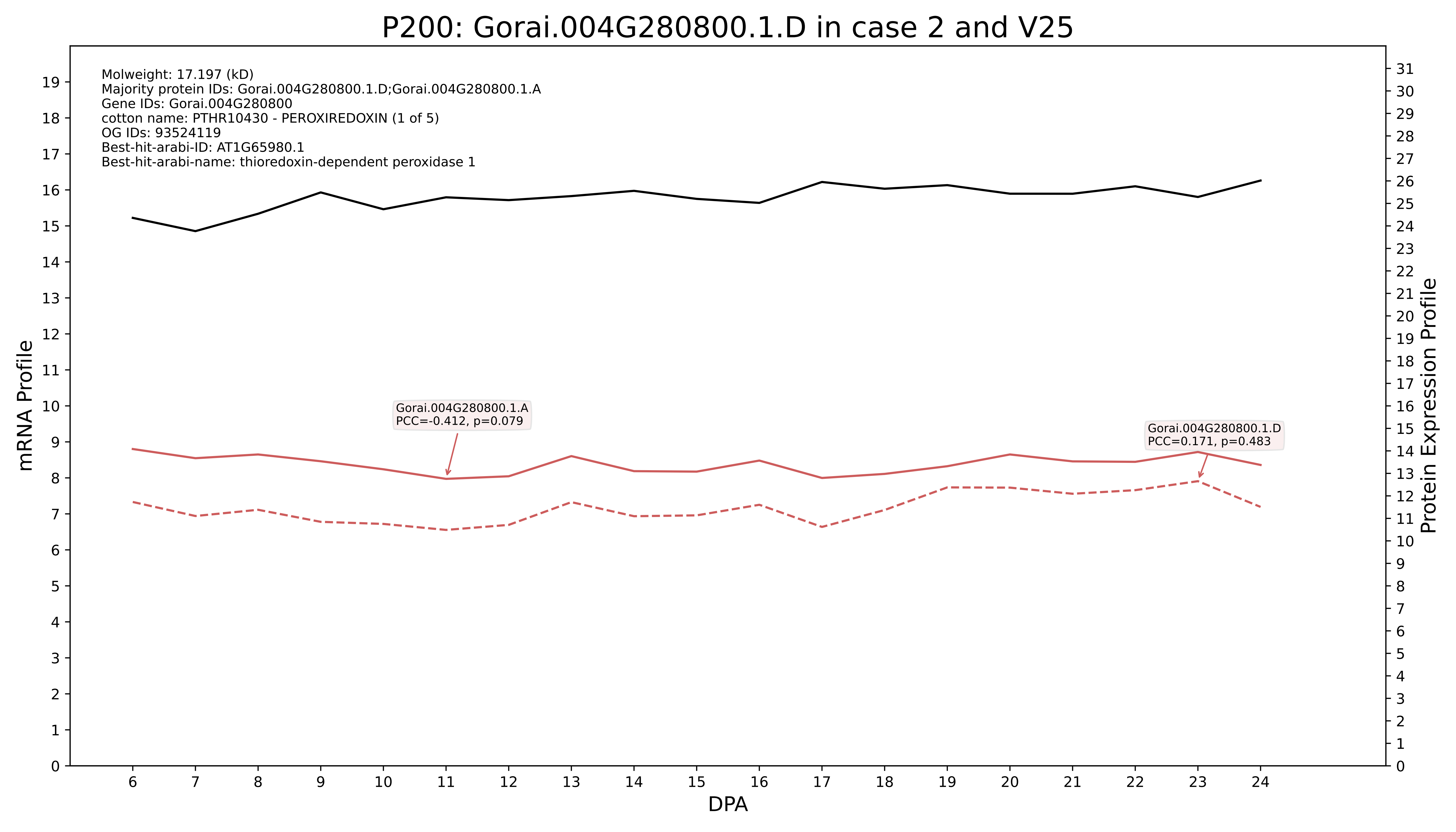
P200: Gorai.004G280800.1.D in case 2 and V25
Molweight: 17.197 (kD)
Majority protein IDs: Gorai.004G280800.1.D;Gorai.004G280800.1.A
Gene IDs: Gorai.004G280800
cotton name: PTHR10430 - PEROXIREDOXIN (1 of 5)
OG IDs: 93524119
Best-hit-arabi-ID: AT1G65980.1
Best-hit-arabi-name: thioredoxin-dependent peroxidase 1
Gorai.004G280800.1.A
PCC=-0.412, p=0.079
Gorai.004G280800.1.D
PCC=0.171, p=0.483
mRNA Profile
Protein Expression Profile
DPA

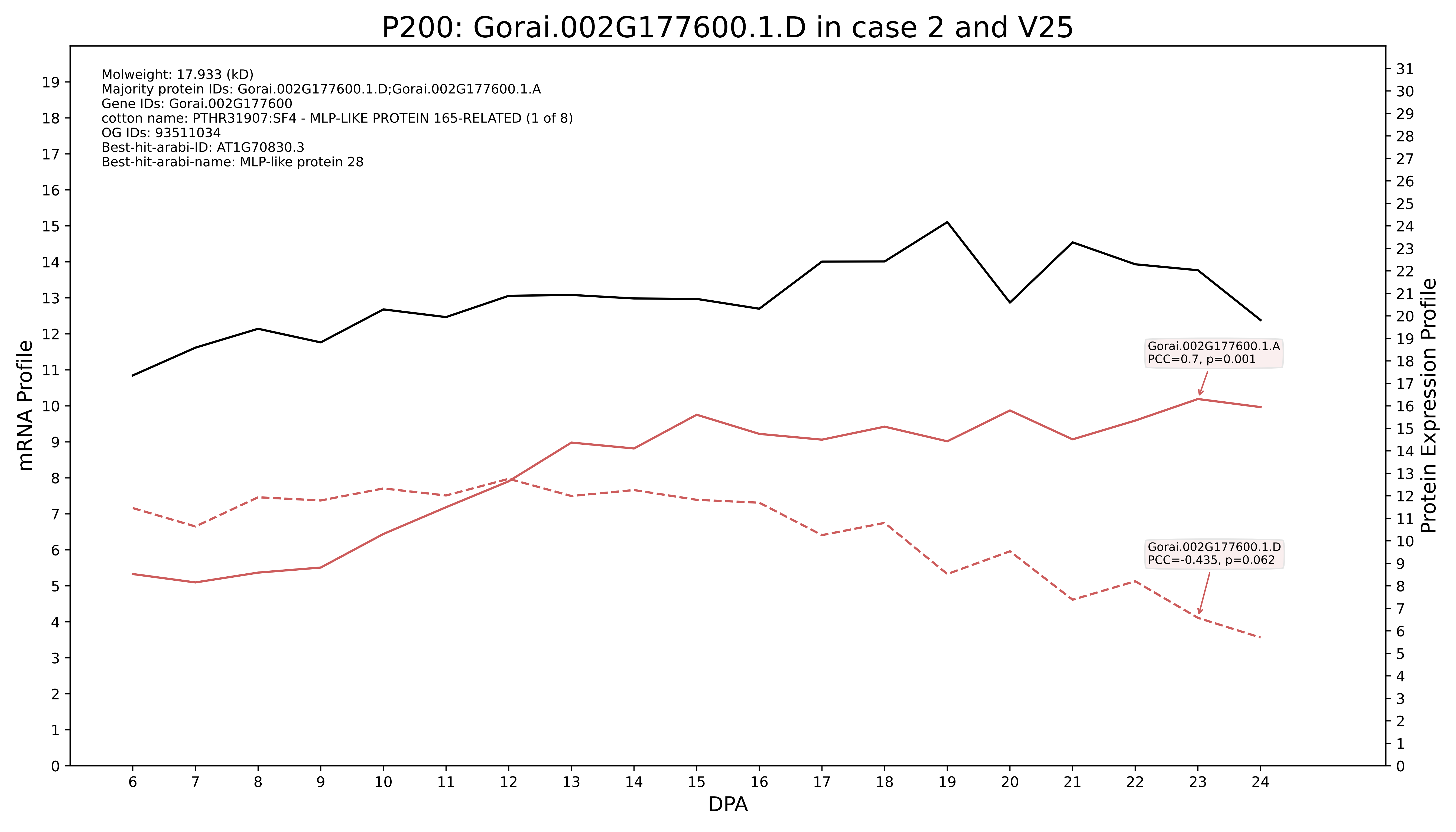
P200: Gorai.002G177600.1.D in case 2 and V25
Molweight: 17.933 (kD)
Majority protein IDs: Gorai.002G177600.1.D;Gorai.002G177600.1.A
Gene IDs: Gorai.002G177600
cotton name: PTHR31907:SF4 - MLP-LIKE PROTEIN 165-RELATED (1 of 8)
OG IDs: 93511034
Best-hit-arabi-ID: AT1G70830.3
Best-hit-arabi-name: MLP-like protein 28
Gorai.002G177600.1.A
PCC=0.7, p=0.001
Gorai.002G177600.1.D
PCC=-0.435, p=0.062
mRNA Profile
Protein Expression Profile
DPA

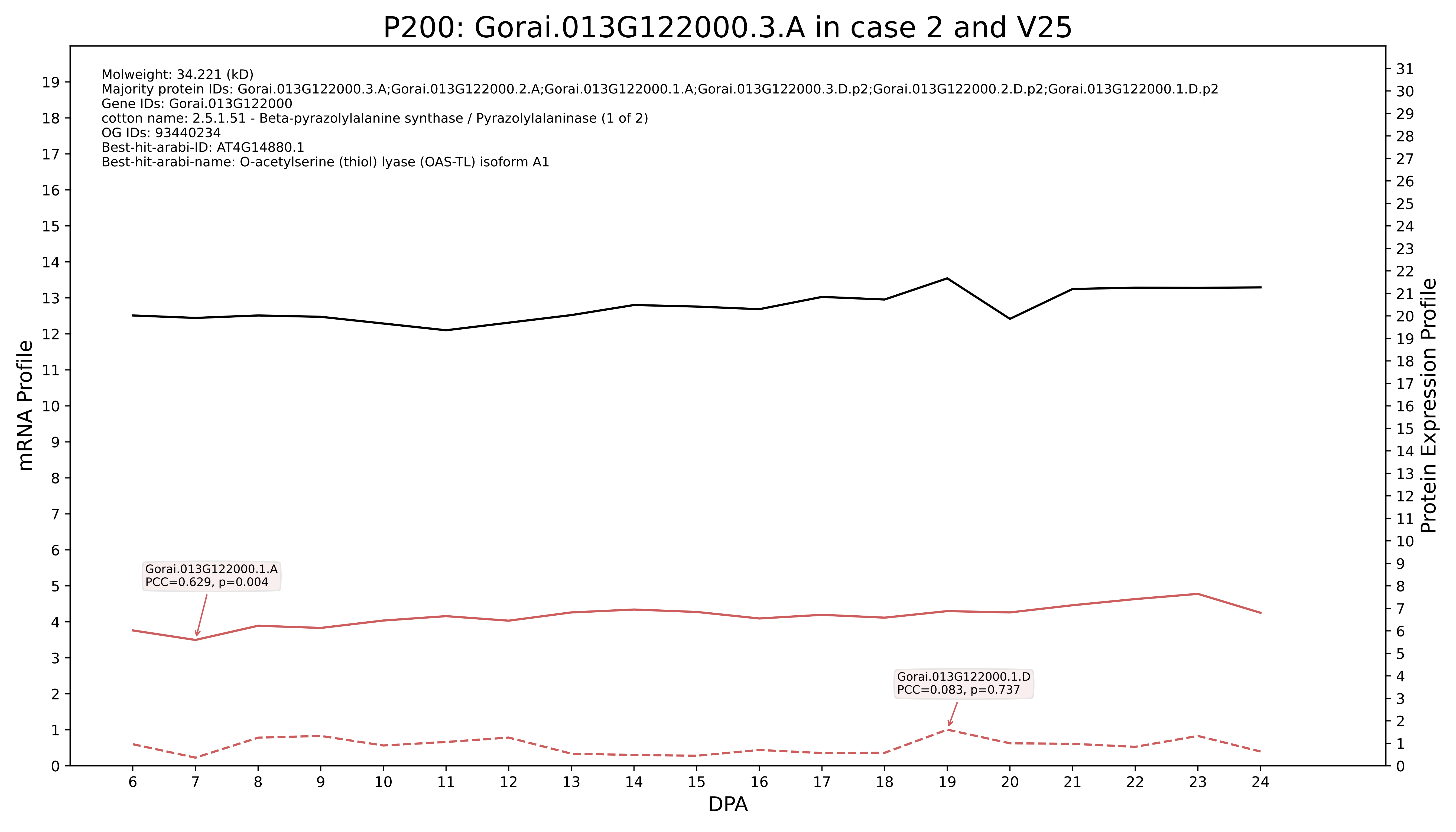

P200: Gorai.013G122000.3.A in case 2 and V25
Molweight: 34.221 (kD)
Majority protein IDs: Gorai.013G122000.3.A;Gorai.013G122000.2.A;Gorai.013G122000.1.A;Gorai.013G122000.3.D.p2;Gorai.013G122000.2.D.p2;Gorai.013G122000.1.D.p2
Gene IDs: Gorai.013G122000
cotton name: 2.5.1.51 - Beta-pyrazolylalanine synthase / Pyrazolylalaninase (1 of 2)
OG IDs: 93440234
Best-hit-arabi-ID: AT4G14880.1
Best-hit-arabi-name: O-acetylserine (thiol) lyase (OAS-TL) isoform A1
Gorai.013G122000.1.A
PCC=0.629, p=0.004
Gorai.013G122000.1.D
PCC=0.083, p=0.737
mRNA Profile
Protein Expression Profile
DPA

002G222900.3.D in case 2 and V25

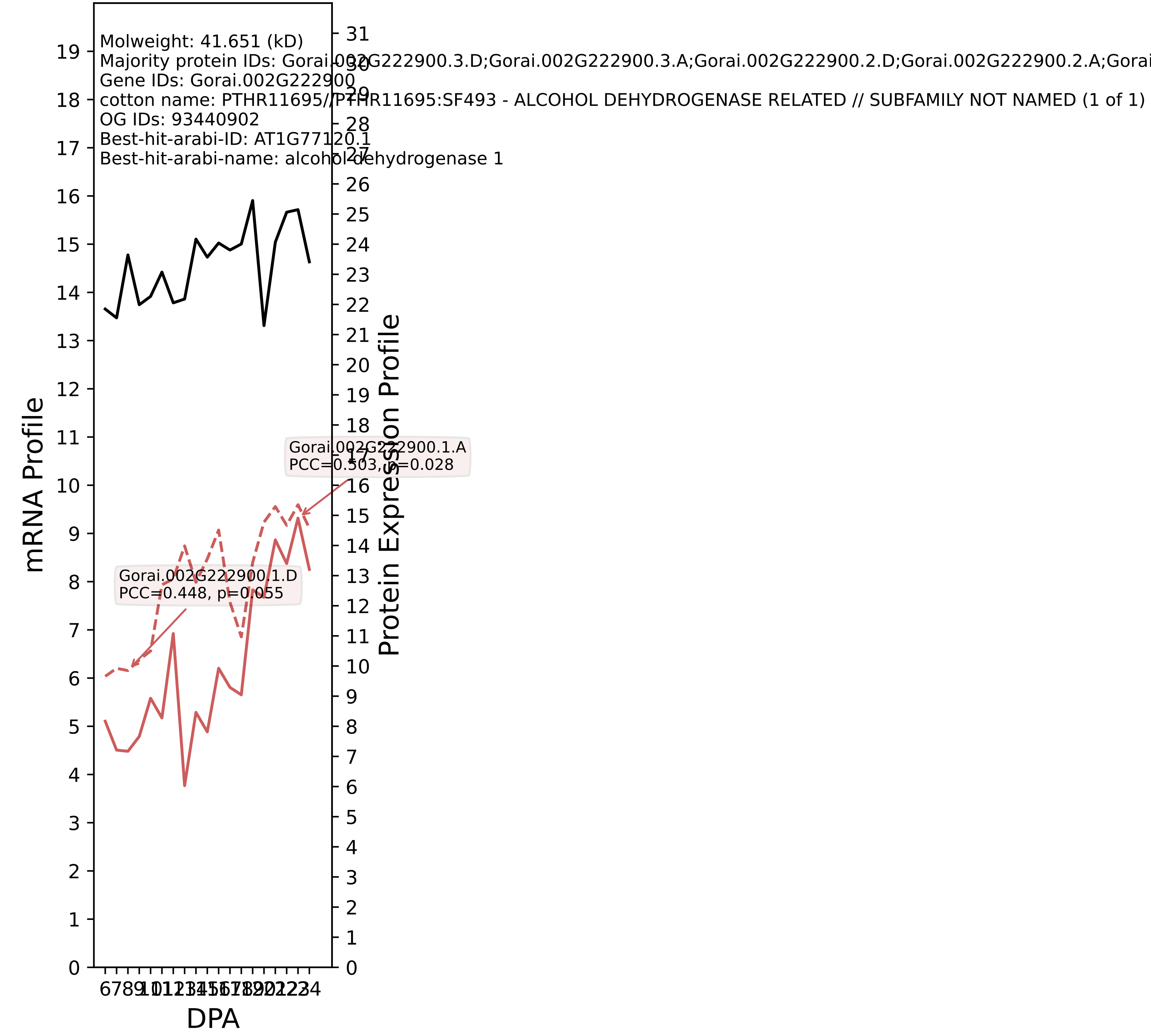

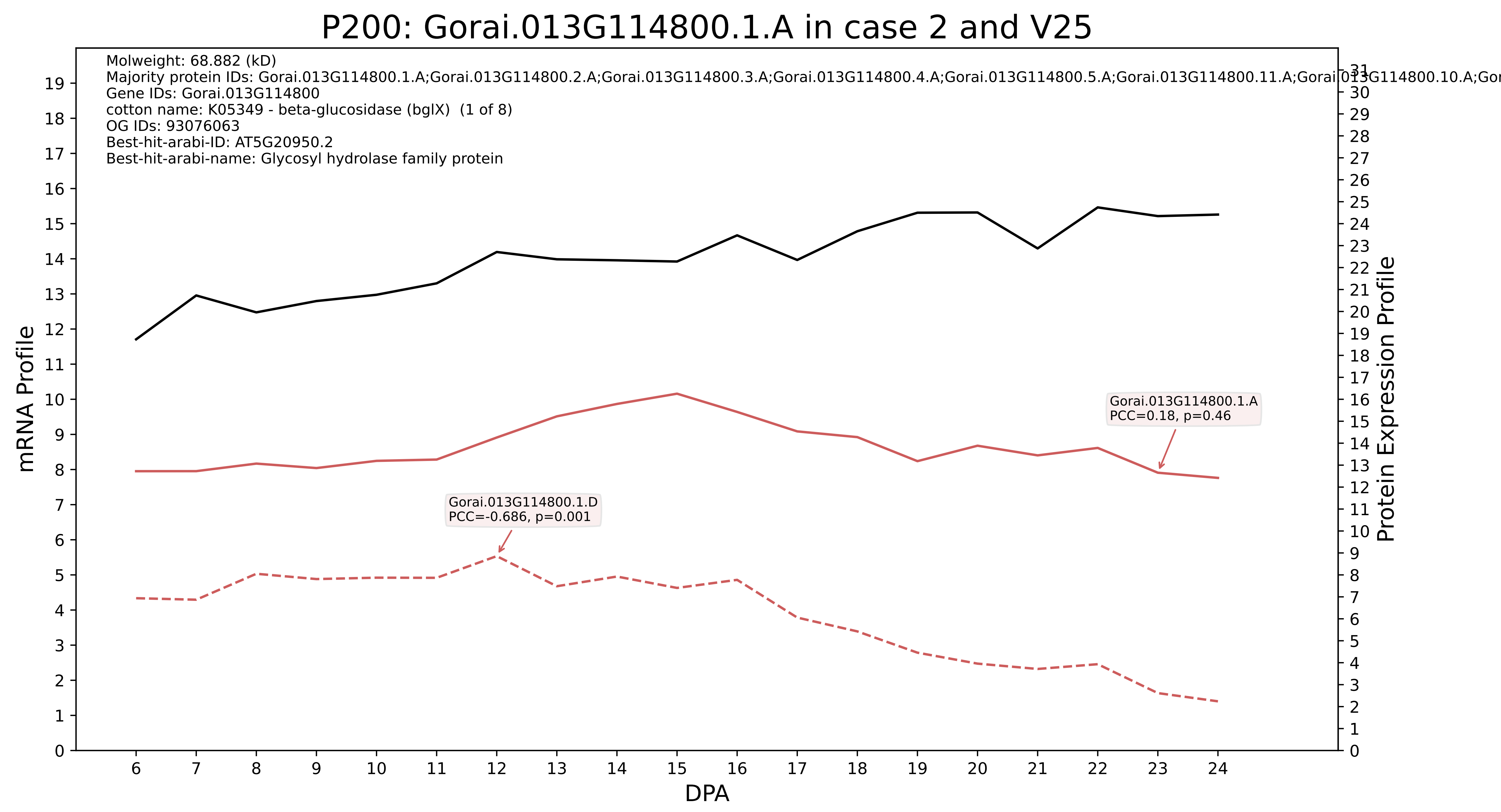

P200: Gorai.013G114800.1.A in case 2 and V25
Molweight: 68.882 (kD)
Majority protein IDs: Gorai.013G114800.1.A;Gorai.013G114800.2.A;Gorai.013G114800.3.A;Gorai.013G114800.4.A;Gorai.013G114800.5.A;Gorai.013G114800.11.A;Gorai.013G114800.10.A;Gor
Gene IDs: Gorai.013G114800
cotton name: K05349 - beta-glucosidase (bglX) (1 of 8)
OG IDs: 93076063
Best-hit-arabi-ID: AT5G20950.2
Best-hit-arabi-name: Glycosyl hydrolase family protein
Gorai.013G114800.1.A
PCC=0.18, p=0.46
Gorai.013G114800.1.D
PCC=-0.686, p=0.001
mRNA Profile
Protein Expression Profile
DPA

P200: Gorai.006G120500.2.D in case 2 and V25

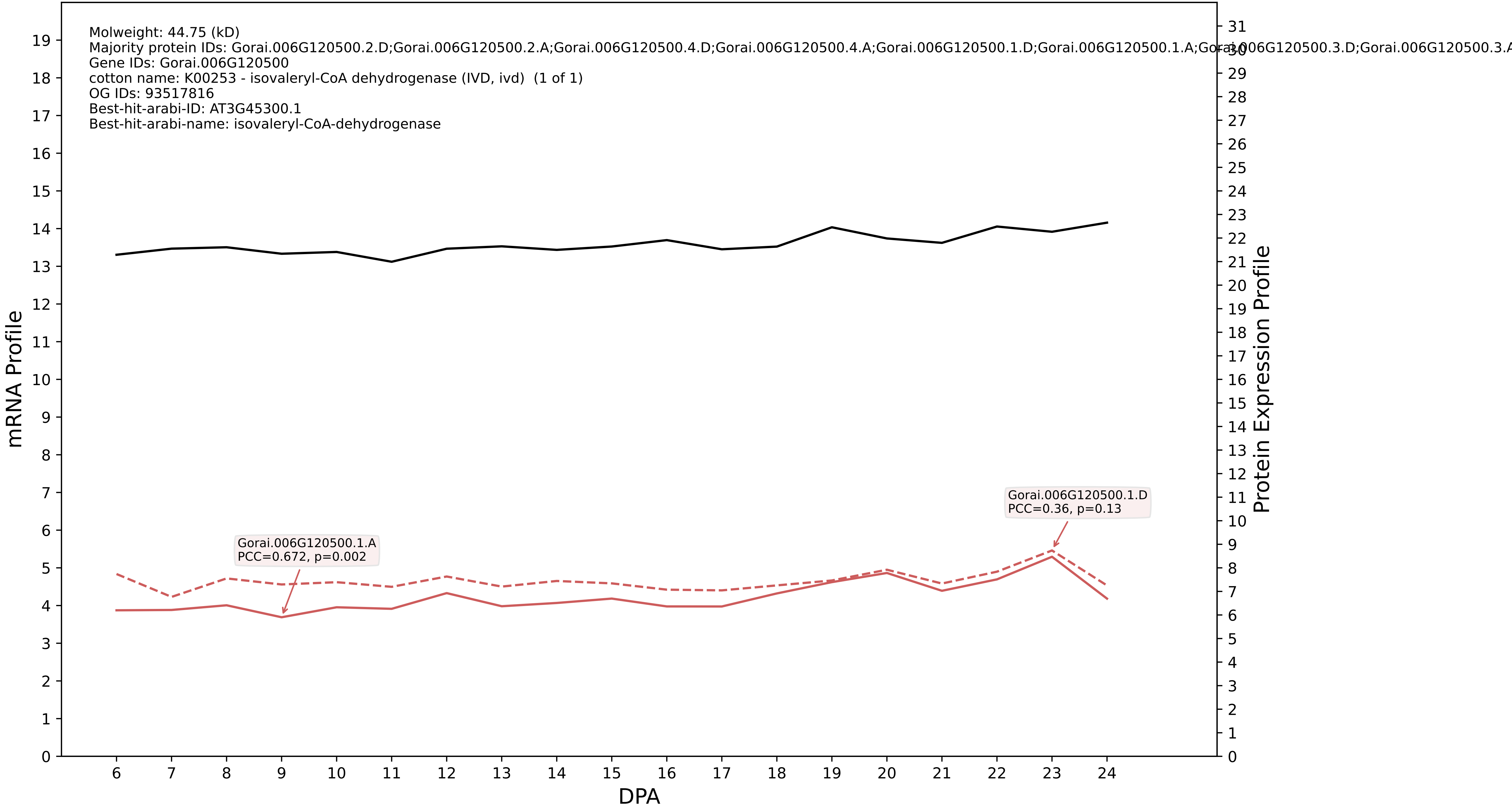

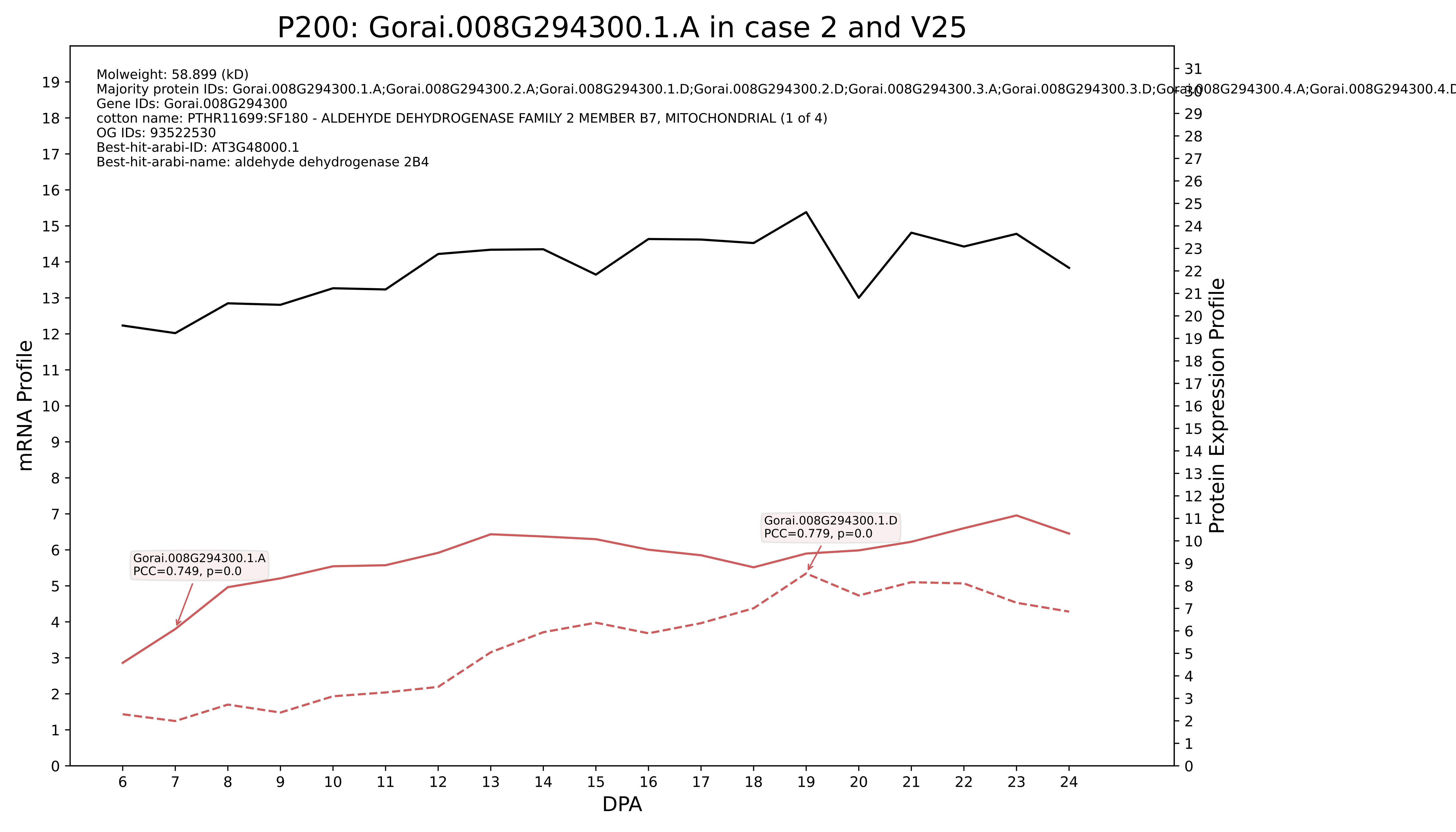
P200: Gorai.008G294300.1.A in case 2 and V25
Molweight: 58.899 (kD)
Majority protein IDs: Gorai.008G294300.1.A;Gorai.008G294300.2.A;Gorai.008G294300.1.D;Gorai.008G294300.2.D;Gorai.008G294300.3.A;Gorai.008G294300.3.D;Gorai.008G294300.4.A;Gorai.008G294300.4.D
Gene IDs: Gorai.008G294300
cotton name: PTHR11699:SF180 - ALDEHYDE DEHYDROGENASE FAMILY 2 MEMBER B7, MITOCHONDRIAL (1 of 4)
OG IDs: 93522530
Best-hit-arabi-ID: AT3G48000.1
Best-hit-arabi-name: aldehyde dehydrogenase 2B4
Gorai.008G294300.1.D
PCC=0.779, p=0.0
Gorai.008G294300.1.A
PCC=0.749, p=0.0
mRNA Profile
Protein Expression Profile
DPA

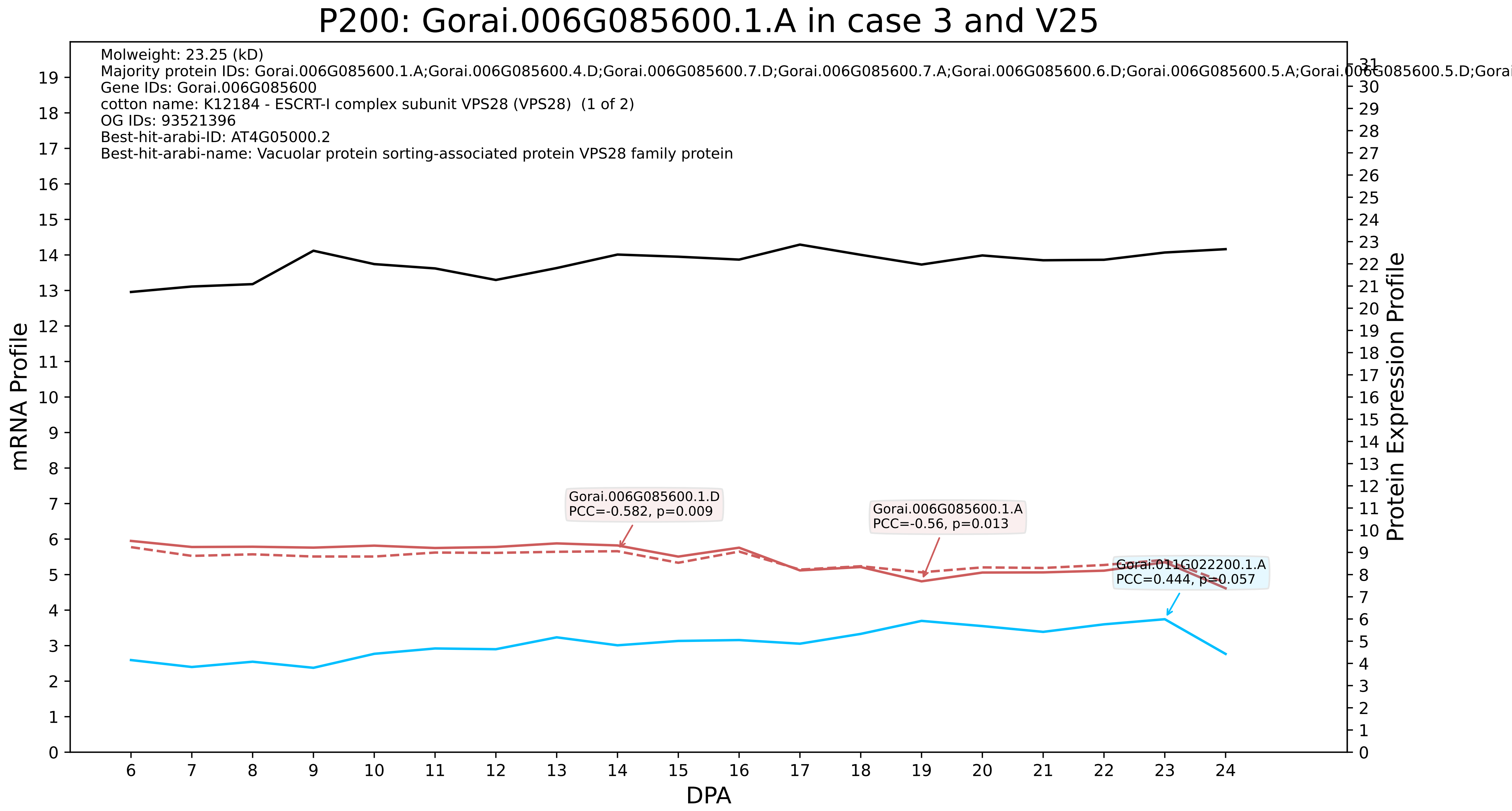
P200: Gorai.006G085600.1.A in case 3 and V25
Molweight: 23.25 (kD)
Majority protein IDs: Gorai.006G085600.1.A;Gorai.006G085600.4.D;Gorai.006G085600.7.D;Gorai.006G085600.7.A;Gorai.006G085600.6.D;Gorai.006G085600.5.A;Gorai.006G085600.5.D;Gorai
Gene IDs: Gorai.006G085600
cotton name: K12184 - ESCRT-I complex subunit VPS28 (VPS28) (1 of 2)
OG IDs: 93521396
Best-hit-arabi-ID: AT4G05000.2
Best-hit-arabi-name: Vacuolar protein sorting-associated protein VPS28 family protein
Gorai.006G085600.1.D
PCC=-0.582, p=0.009
Gorai.006G085600.1.A
PCC=-0.56, p=0.013
Gorai.011G022200.1.A
PCC=0.444, p=0.057
mRNA Profile
Protein Expression Profile
DPA

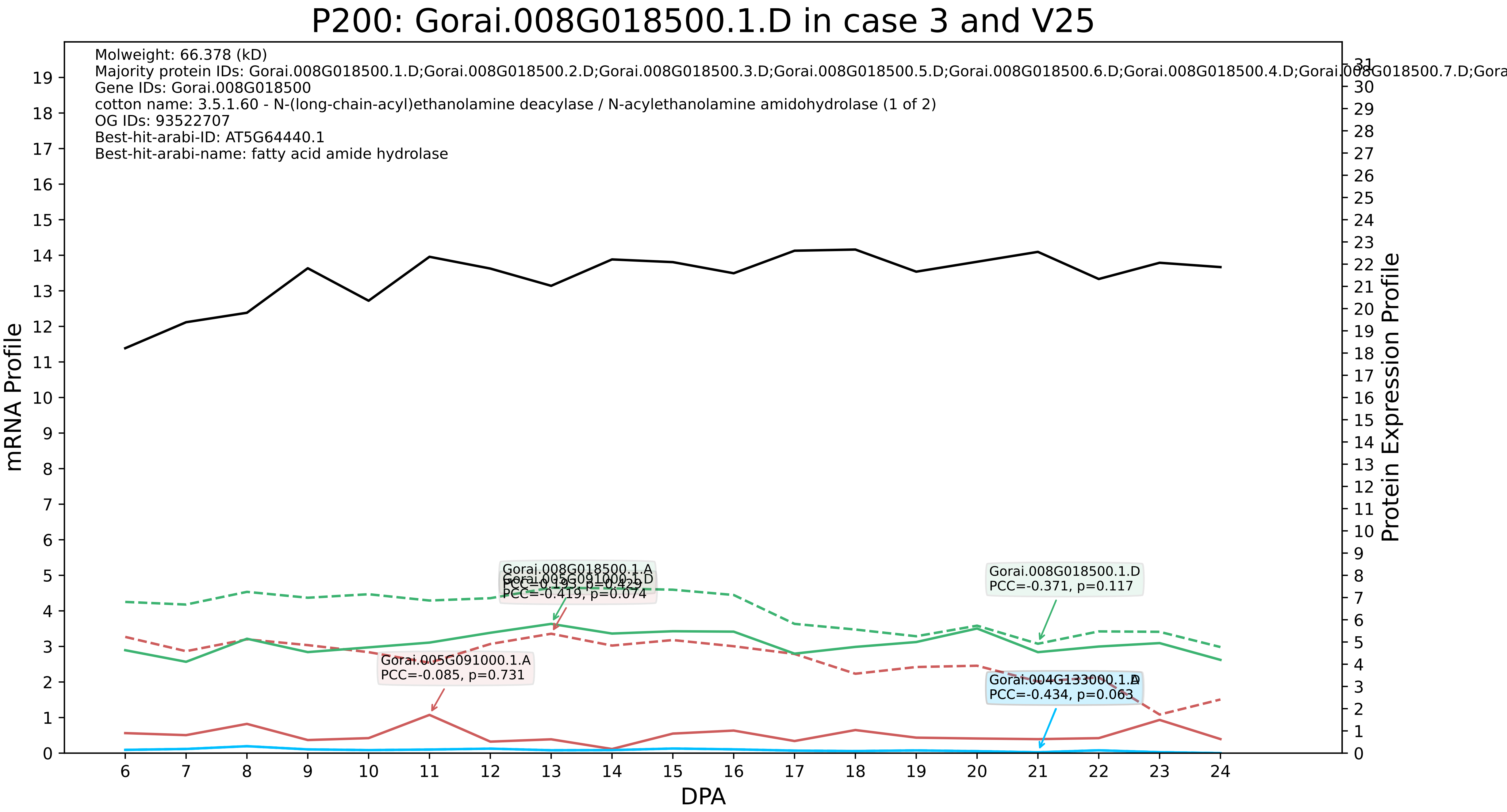
P200: Gorai.008G018500.1.D in case 3 and V25
Molweight: 66.378 (kD)
Majority protein IDs: Gorai.008G018500.1.D;Gorai.008G018500.2.D;Gorai.008G018500.3.D;Gorai.008G018500.5.D;Gorai.008G018500.6.D;Gorai.008G018500.4.D;Gorai.008G018500.7.D;Gora
Gene IDs: Gorai.008G018500
cotton name: 3.5.1.60 - N-(long-chain-acyl)ethanolamine deacylase / N-acylethanolamine amidohydrolase (1 of 2)
OG IDs: 93522707
Best-hit-arabi-ID: AT5G64440.1
Best-hit-arabi-name: fatty acid amide hydrolase
Gorai.008G018500.1.A
PCC=0.193, p=0.429
Gorai.005G091000.1.D
PCC=-0.419, p=0.074
Gorai.008G018500.1.D
PCC=-0.371, p=0.117
Gorai.005G091000.1.A
PCC=-0.085, p=0.731
Gorai.004G133000.1.D
PCC=-0.434, p=0.063
mRNA Profile
Protein Expression Profile
DPA

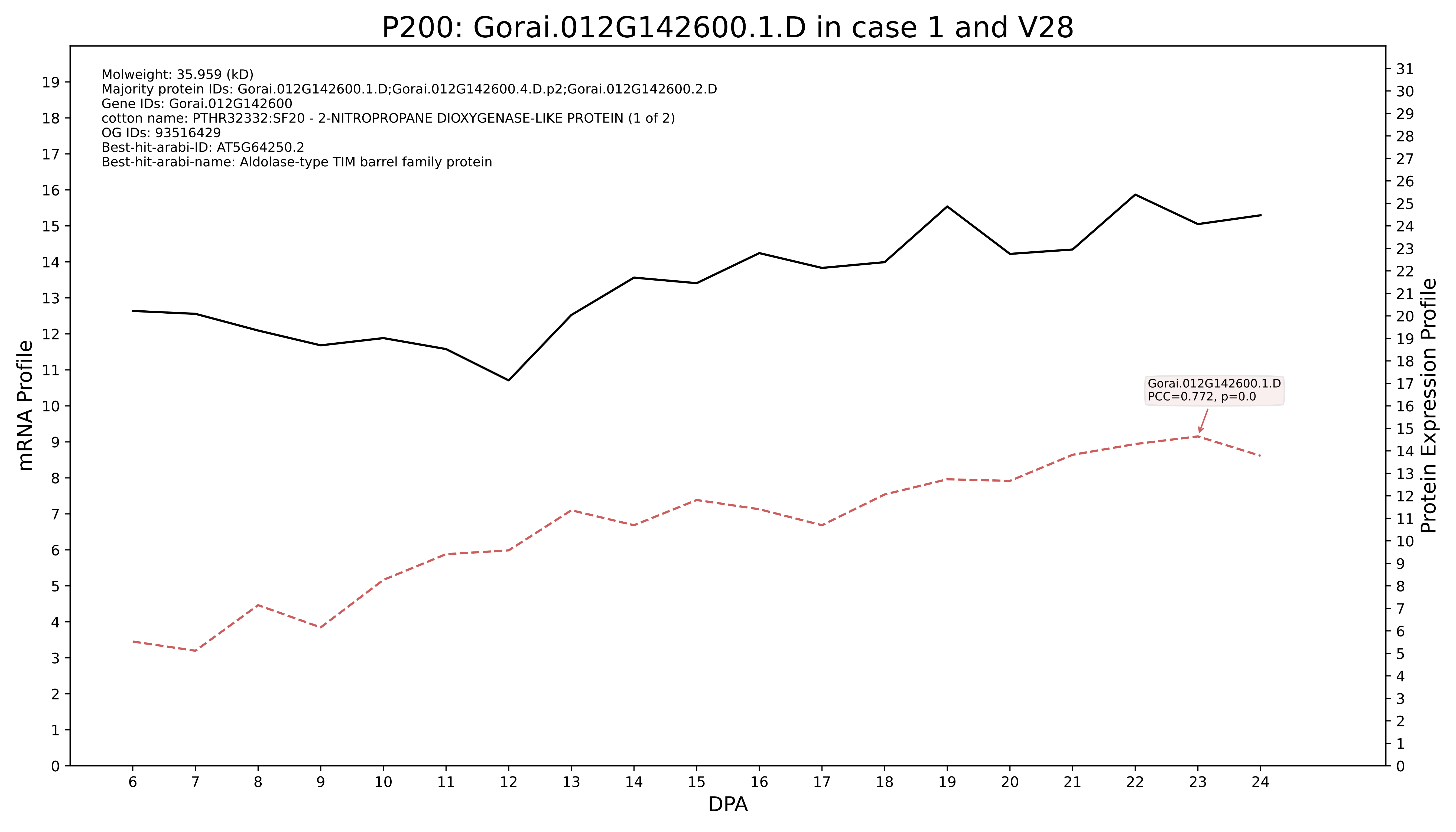
P200: Gorai.012G142600.1.D in case 1 and V28
Molweight: 35.959 (kD)
Majority protein IDs: Gorai.012G142600.1.D;Gorai.012G142600.4.D.p2;Gorai.012G142600.2.D
Gene IDs: Gorai.012G142600
cotton name: PTHR32332:SF20 - 2-NITROPROPANE DIOXYGENASE-LIKE PROTEIN (1 of 2)
OG IDs: 93516429
Best-hit-arabi-ID: AT5G64250.2
Best-hit-arabi-name: Aldolase-type TIM barrel family protein
Gorai.012G142600.1.D
PCC=0.772, p=0.0
mRNA Profile
Protein Expression Profile
DPA

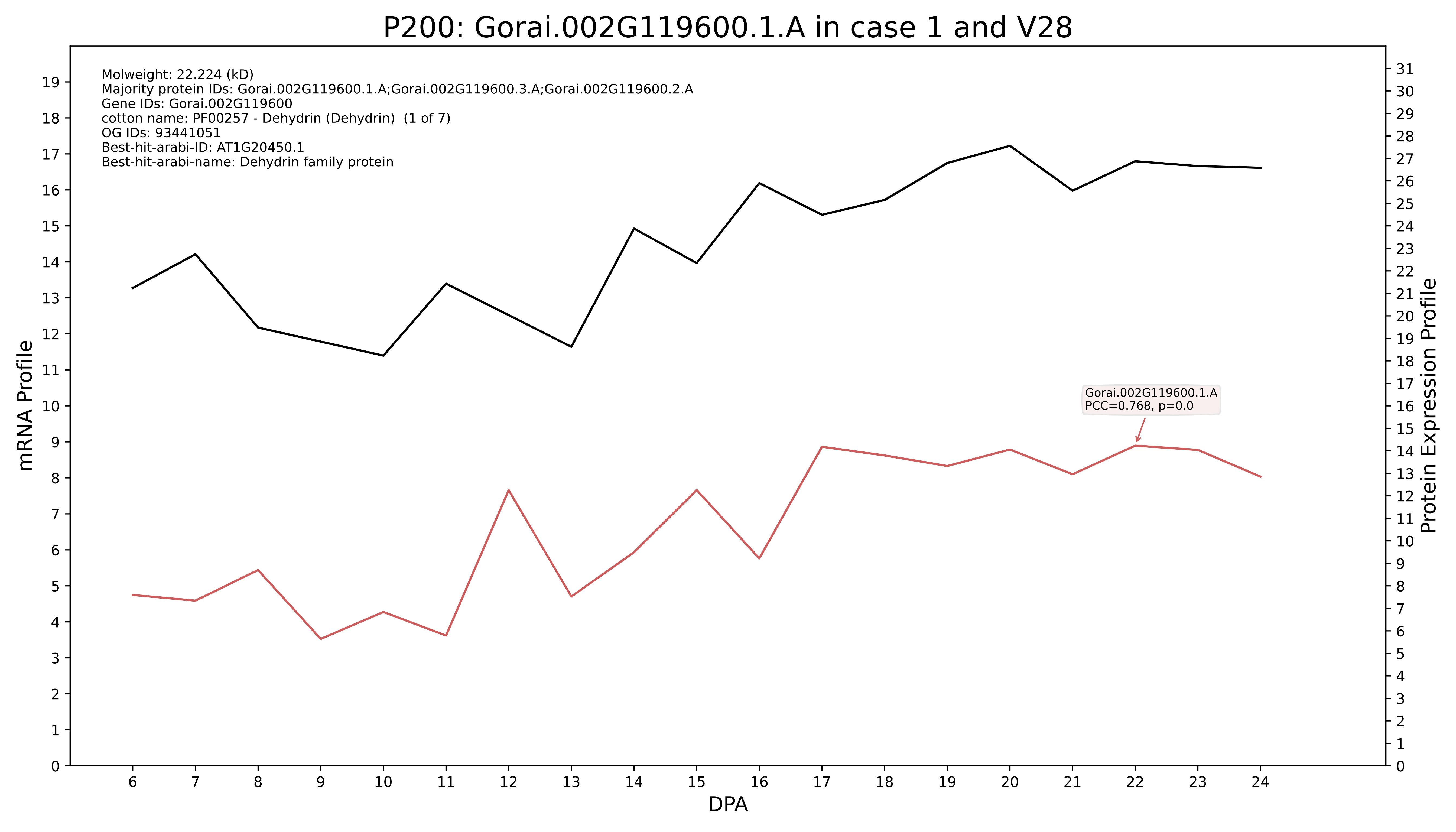
P200: Gorai.002G119600.1.A in case 1 and V28
Molweight: 22.224 (kD)
Majority protein IDs: Gorai.002G119600.1.A;Gorai.002G119600.3.A;Gorai.002G119600.2.A
Gene IDs: Gorai.002G119600
cotton name: PF00257 - Dehydrin (Dehydrin) (1 of 7)
OG IDs: 93441051
Best-hit-arabi-ID: AT1G20450.1
Best-hit-arabi-name: Dehydrin family protein
Gorai.002G119600.1.A
PCC=0.768, p=0.0
mRNA Profile
Protein Expression Profile
DPA

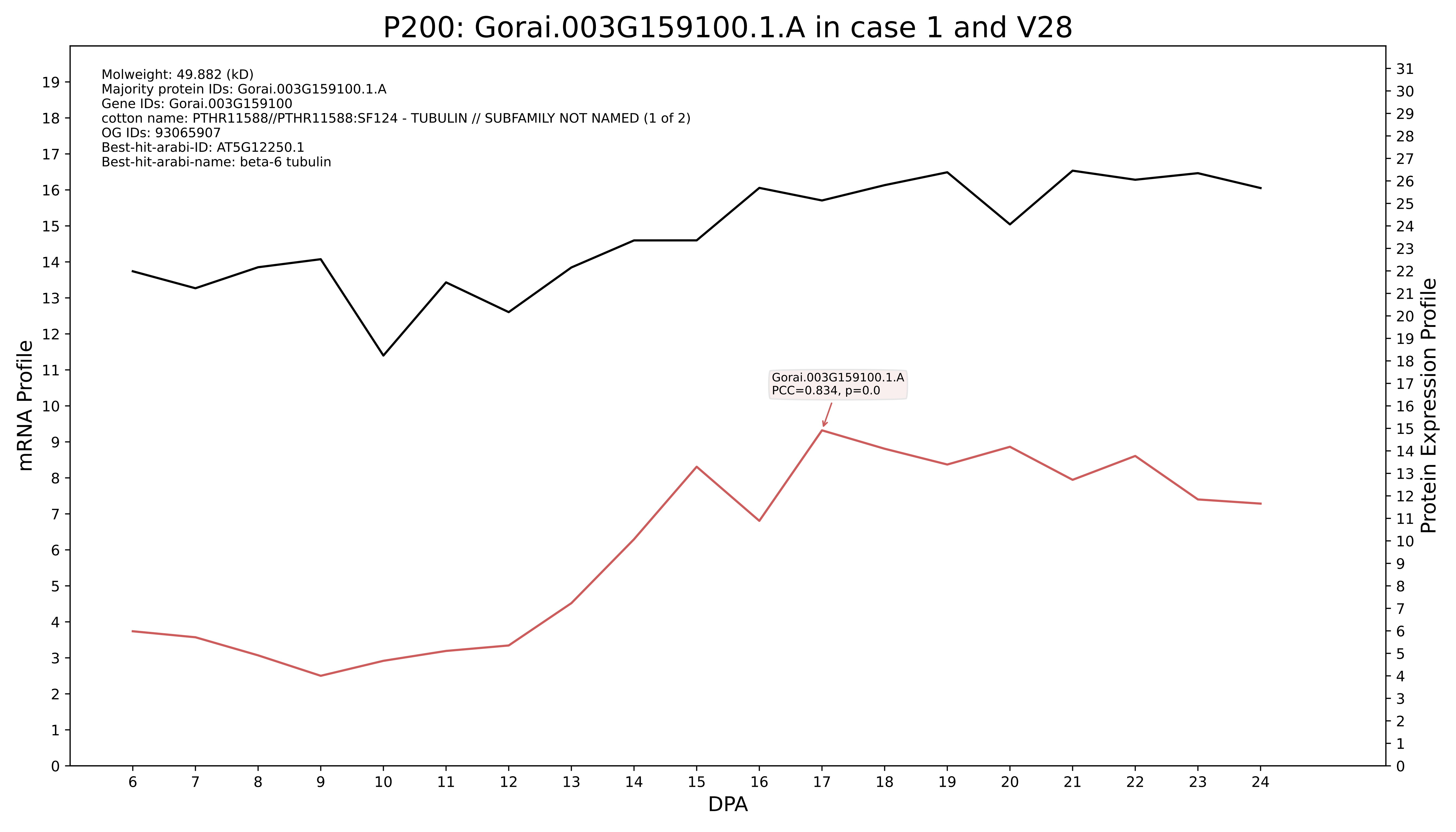
P200: Gorai.003G159100.1.A in case 1 and V28
Molweight: 49.882 (kD)
Majority protein IDs: Gorai.003G159100.1.A
Gene IDs: Gorai.003G159100
cotton name: PTHR11588//PTHR11588:SF124 - TUBULIN // SUBFAMILY NOT NAMED (1 of 2)
OG IDs: 93065907
Best-hit-arabi-ID: AT5G12250.1
Best-hit-arabi-name: beta-6 tubulin
Gorai.003G159100.1.A
PCC=0.834, p=0.0
mRNA Profile
Protein Expression Profile
DPA

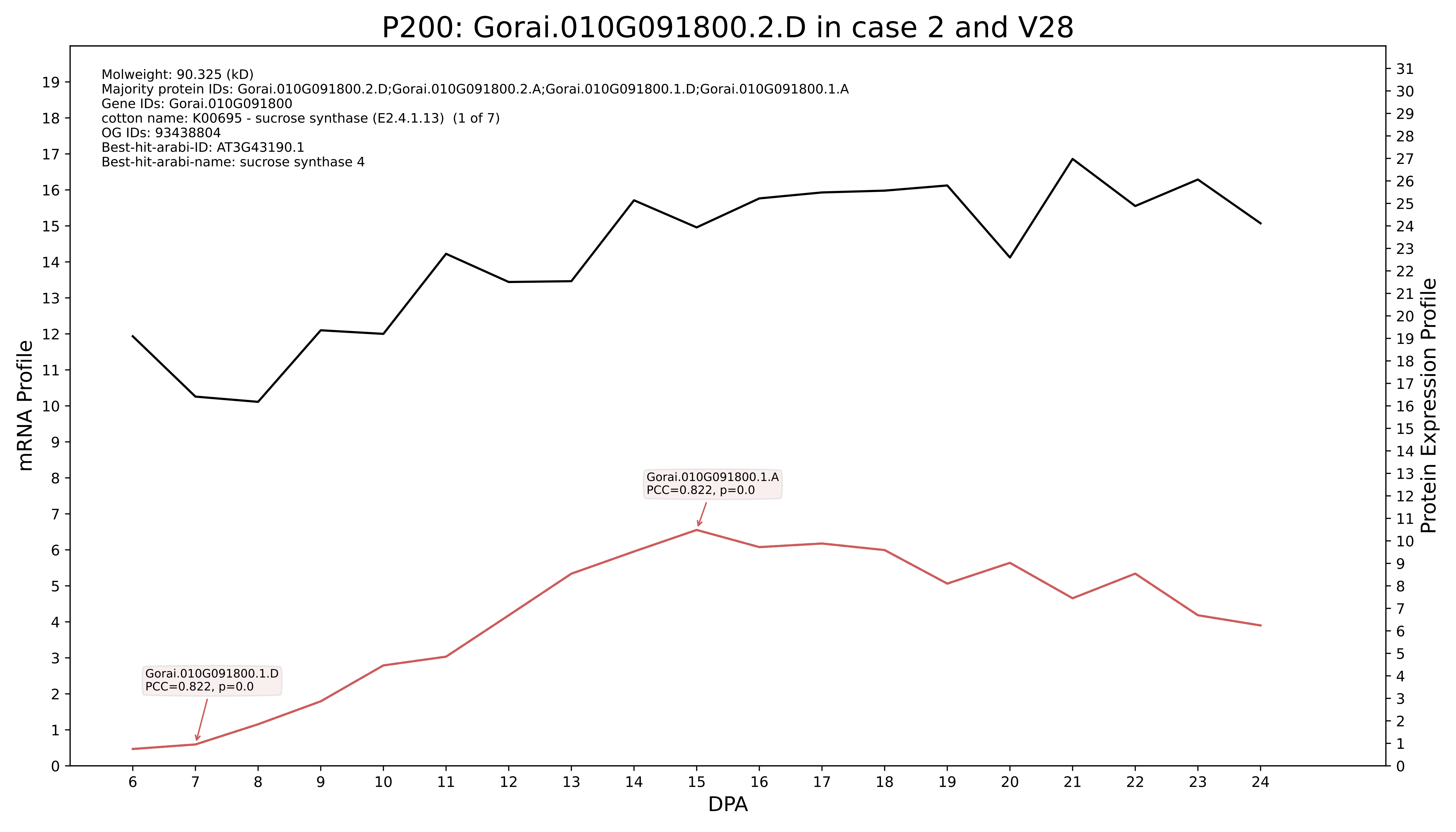

P200: Gorai.010G091800.2.D in case 2 and V28
Molweight: 90.325 (kD)
Majority protein IDs: Gorai.010G091800.2.D;Gorai.010G091800.2.A;Gorai.010G091800.1.D;Gorai.010G091800.1.A
Gene IDs: Gorai.010G091800
cotton name: K00695 - sucrose synthase (E2.4.1.13) (1 of 7)
OG IDs: 93438804
Best-hit-arabi-ID: AT3G43190.1
Best-hit-arabi-name: sucrose synthase 4
Gorai.010G091800.1.A
PCC=0.822, p=0.0
Gorai.010G091800.1.D
PCC=0.822, p=0.0
mRNA Profile
Protein Expression Profile
DPA

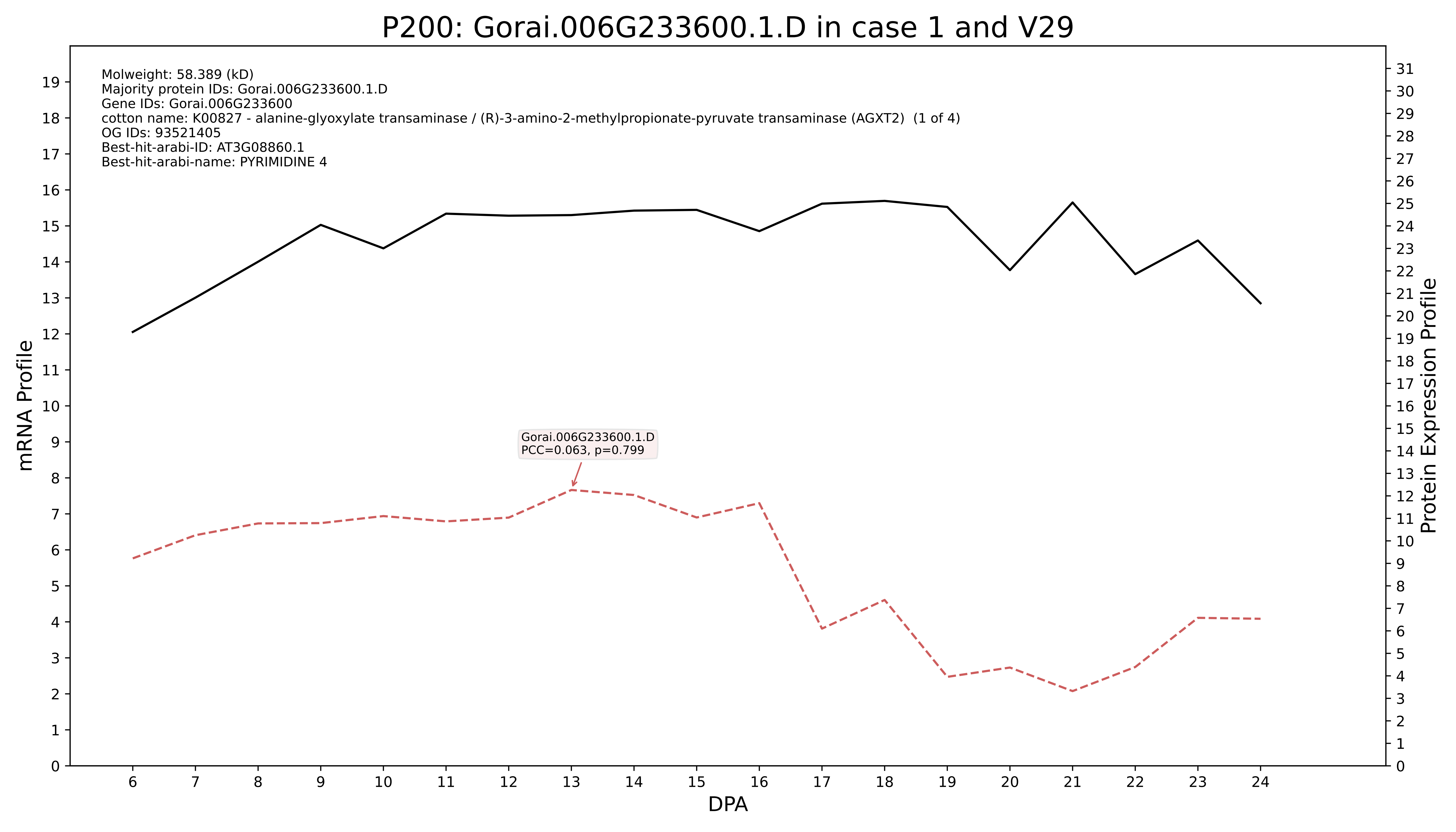

P200: Gorai.006G233600.1.D in case 1 and V29
Molweight: 58.389 (kD)
Majority protein IDs: Gorai.006G233600.1.D
Gene IDs: Gorai.006G233600
cotton name: K00827 - alanine-glyoxylate transaminase / (R)-3-amino-2-methylpropionate-pyruvate transaminase (AGXT2) (1 of 4)
OG IDs: 93521405
Best-hit-arabi-ID: AT3G08860.1
Best-hit-arabi-name: PYRIMIDINE 4
Gorai.006G233600.1.D
PCC=0.063, p=0.799
mRNA Profile
Protein Expression Profile
DPA

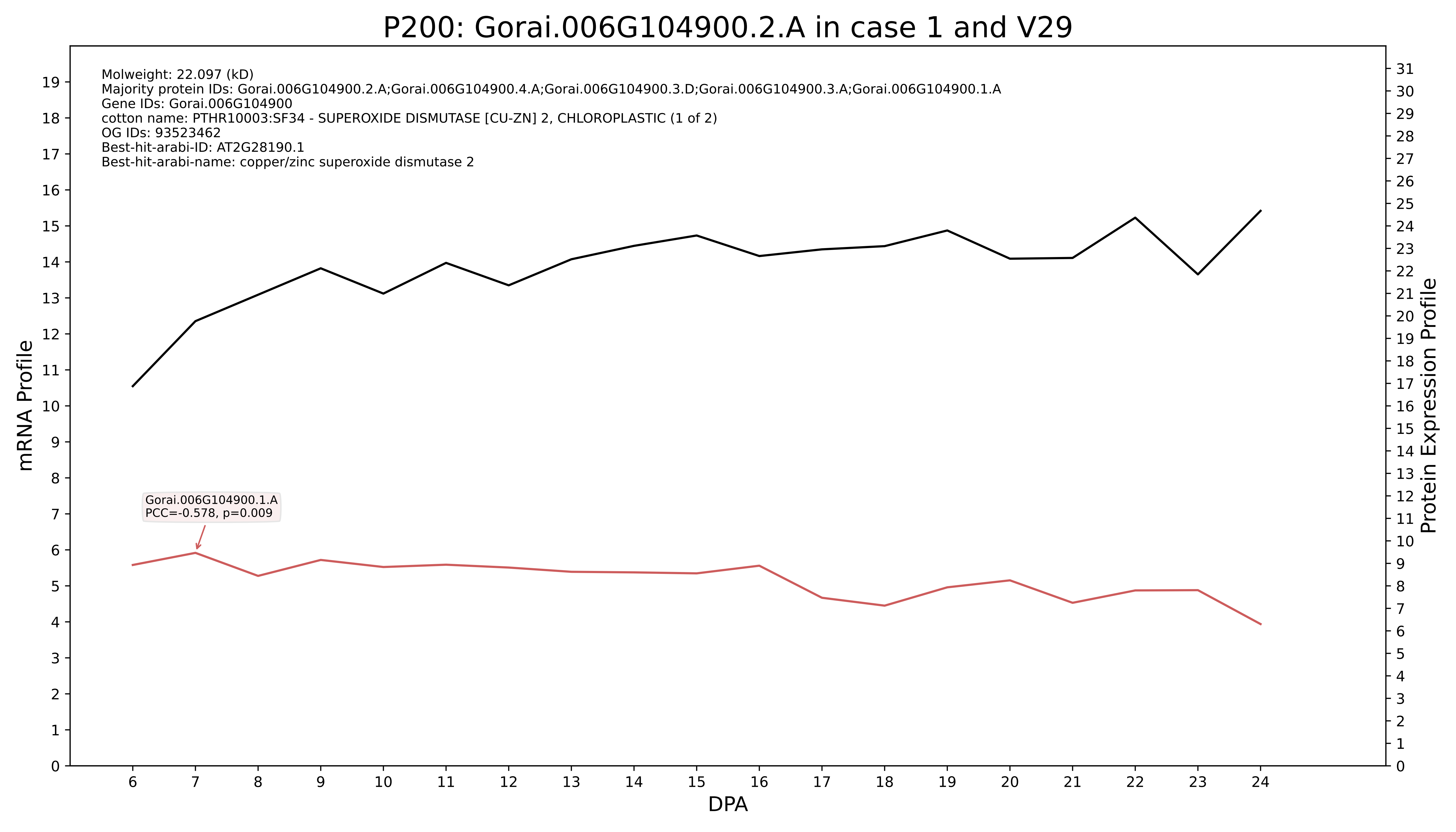
P200: Gorai.006G104900.2.A in case 1 and V29
Molweight: 22.097 (kD)
Majority protein IDs: Gorai.006G104900.2.A;Gorai.006G104900.4.A;Gorai.006G104900.3.D;Gorai.006G104900.3.A;Gorai.006G104900.1.A
Gene IDs: Gorai.006G104900
cotton name: PTHR10003:SF34 - SUPEROXIDE DISMUTASE [CU-ZN] 2, CHLOROPLASTIC (1 of 2)
OG IDs: 93523462
Best-hit-arabi-ID: AT2G28190.1
Best-hit-arabi-name: copper/zinc superoxide dismutase 2
Gorai.006G104900.1.A
PCC=-0.578, p=0.009
mRNA Profile
Protein Expression Profile
DPA

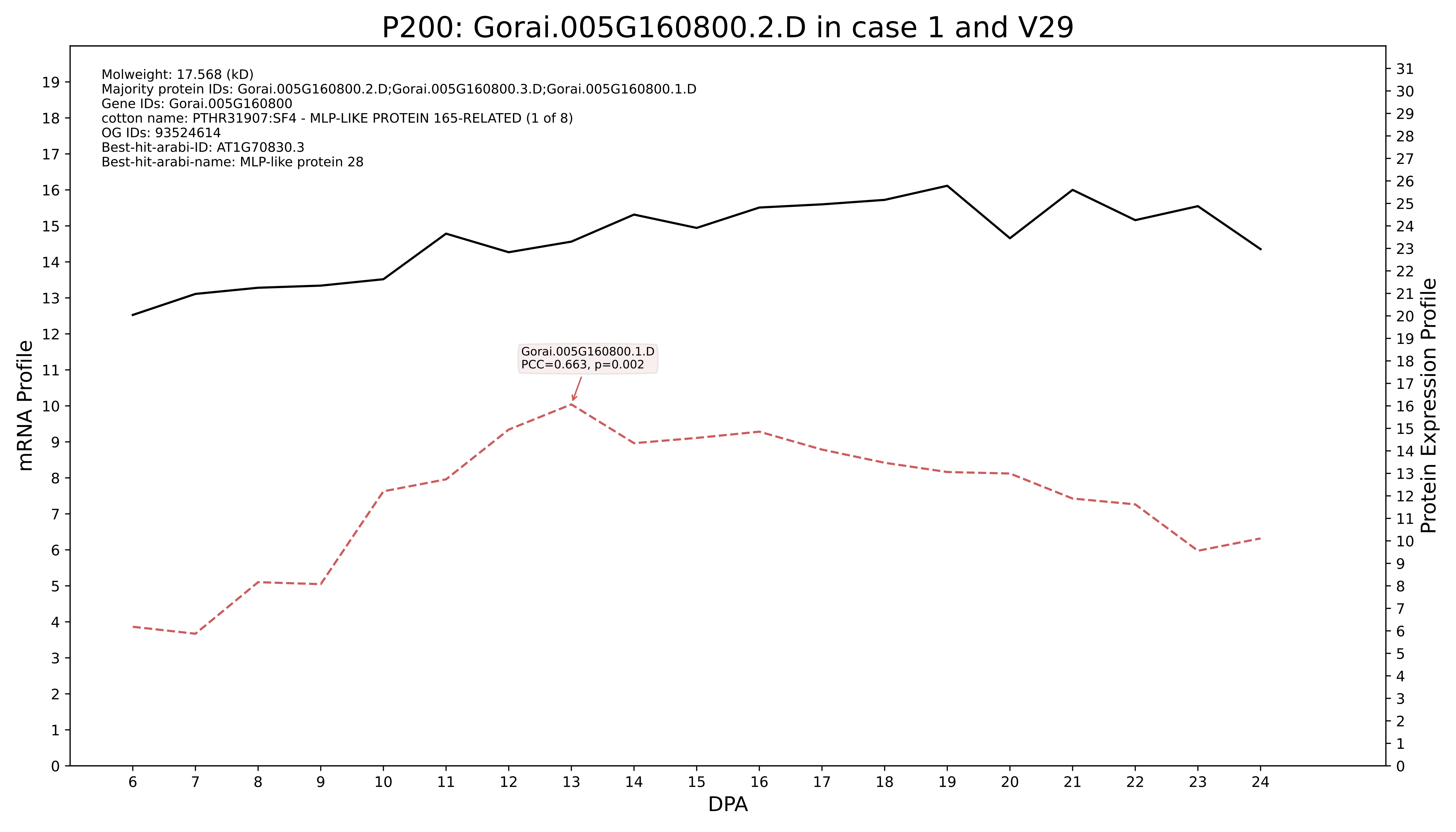
P200: Gorai.005G160800.2.D in case 1 and V29
Molweight: 17.568 (kD)
Majority protein IDs: Gorai.005G160800.2.D;Gorai.005G160800.3.D;Gorai.005G160800.1.D
Gene IDs: Gorai.005G160800
cotton name: PTHR31907:SF4 - MLP-LIKE PROTEIN 165-RELATED (1 of 8)
OG IDs: 93524614
Best-hit-arabi-ID: AT1G70830.3
Best-hit-arabi-name: MLP-like protein 28
Gorai.005G160800.1.D
PCC=0.663, p=0.002
mRNA Profile
Protein Expression Profile
DPA

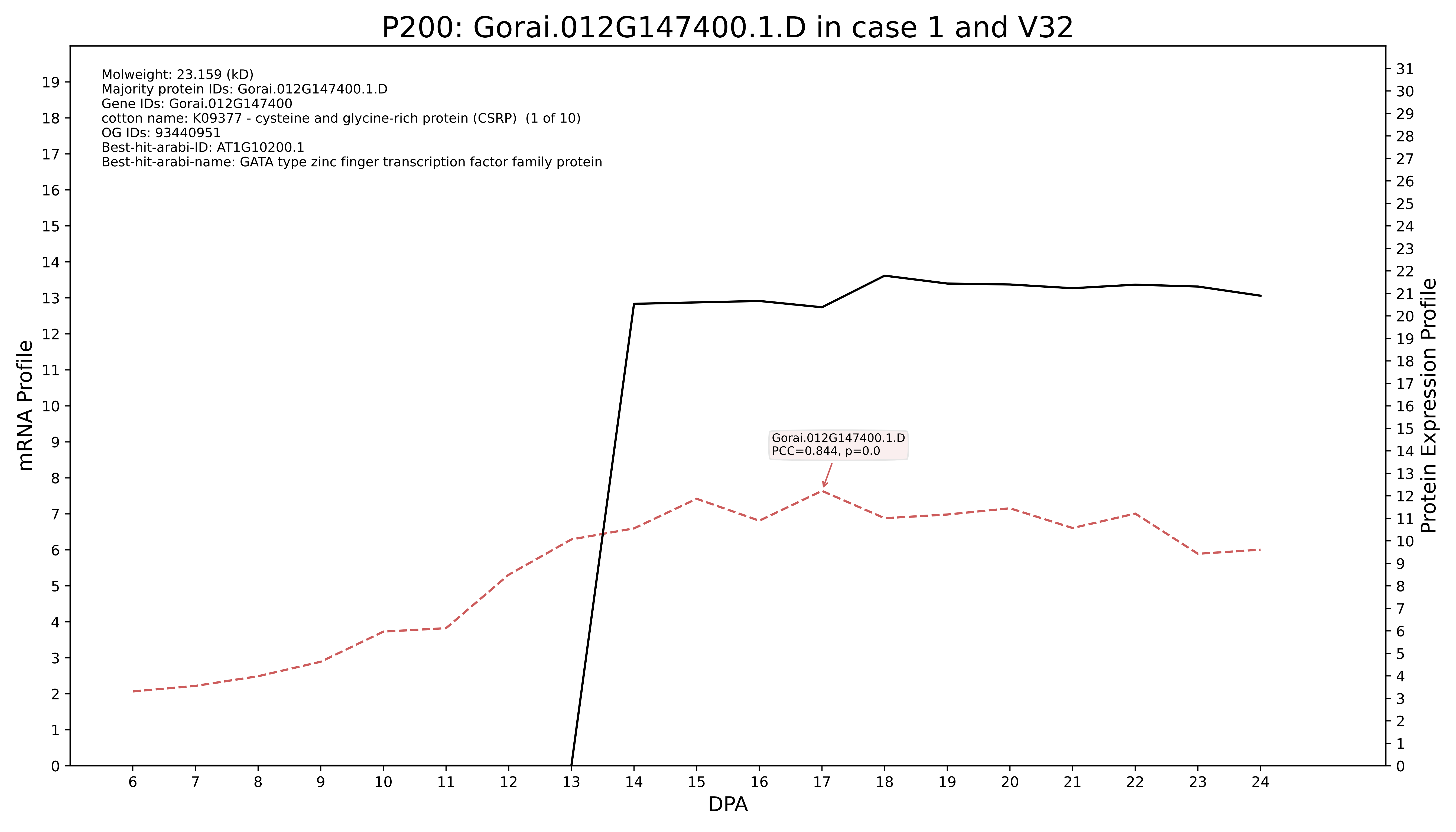

P200: Gorai.012G147400.1.D in case 1 and V32
Molweight: 23.159 (kD)
Majority protein IDs: Gorai.012G147400.1.D
Gene IDs: Gorai.012G147400
cotton name: K09377 - cysteine and glycine-rich protein (CSRP) (1 of 10)
OG IDs: 93440951
Best-hit-arabi-ID: AT1G10200.1
Best-hit-arabi-name: GATA type zinc finger transcription factor family protein
Gorai.012G147400.1.D
PCC=0.844, p=0.0
mRNA Profile
Protein Expression Profile
DPA

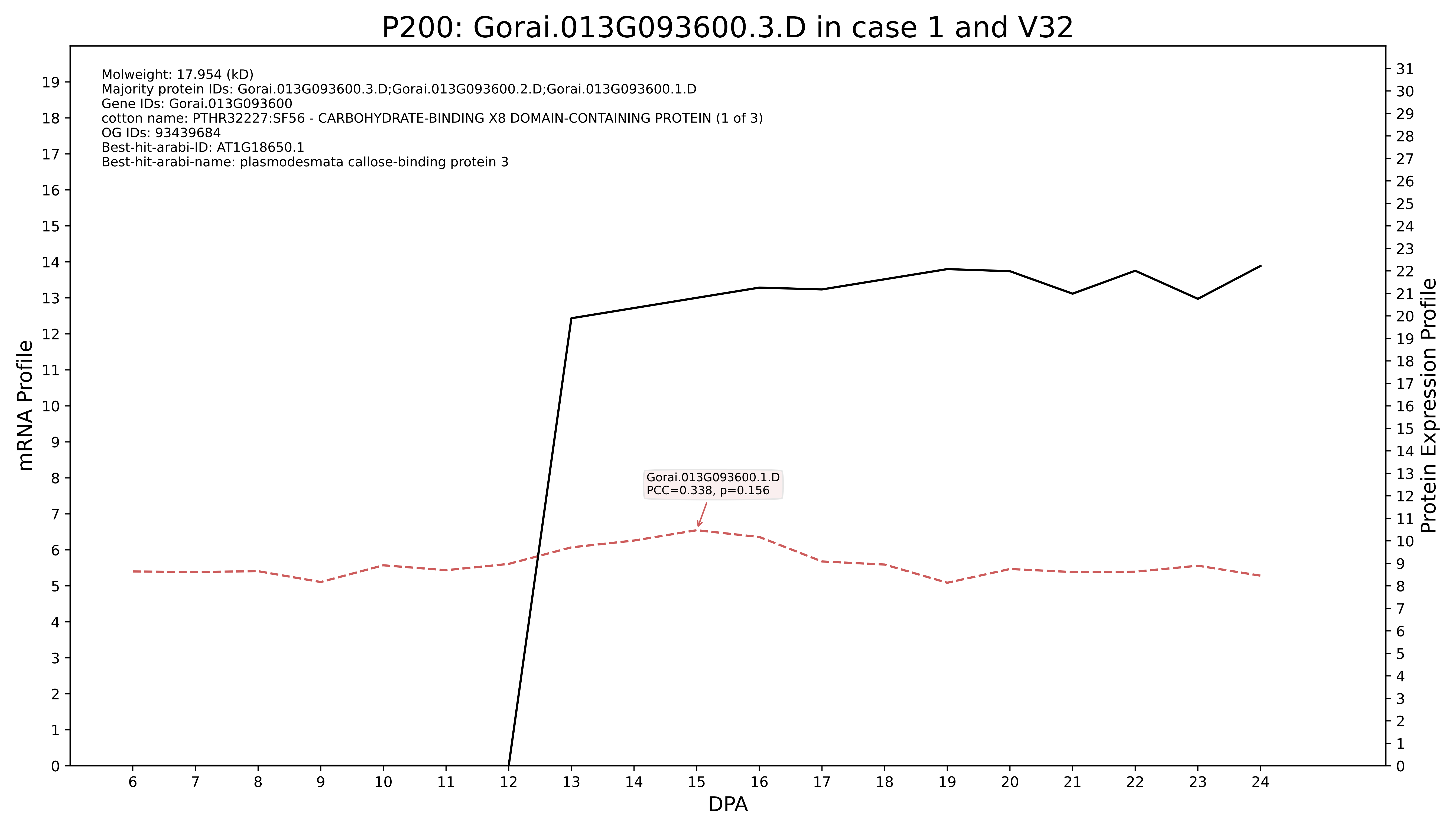
P200: Gorai.013G093600.3.D in case 1 and V32
Molweight: 17.954 (kD)
Majority protein IDs: Gorai.013G093600.3.D;Gorai.013G093600.2.D;Gorai.013G093600.1.D
Gene IDs: Gorai.013G093600
cotton name: PTHR32227:SF56 - CARBOHYDRATE-BINDING X8 DOMAIN-CONTAINING PROTEIN (1 of 3)
OG IDs: 93439684
Best-hit-arabi-ID: AT1G18650.1
Best-hit-arabi-name: plasmodesmata callose-binding protein 3
Gorai.013G093600.1.D
PCC=0.338, p=0.156
mRNA Profile
Protein Expression Profile
DPA

P200: Gorai.012G061400.1.A in case 1 and V32

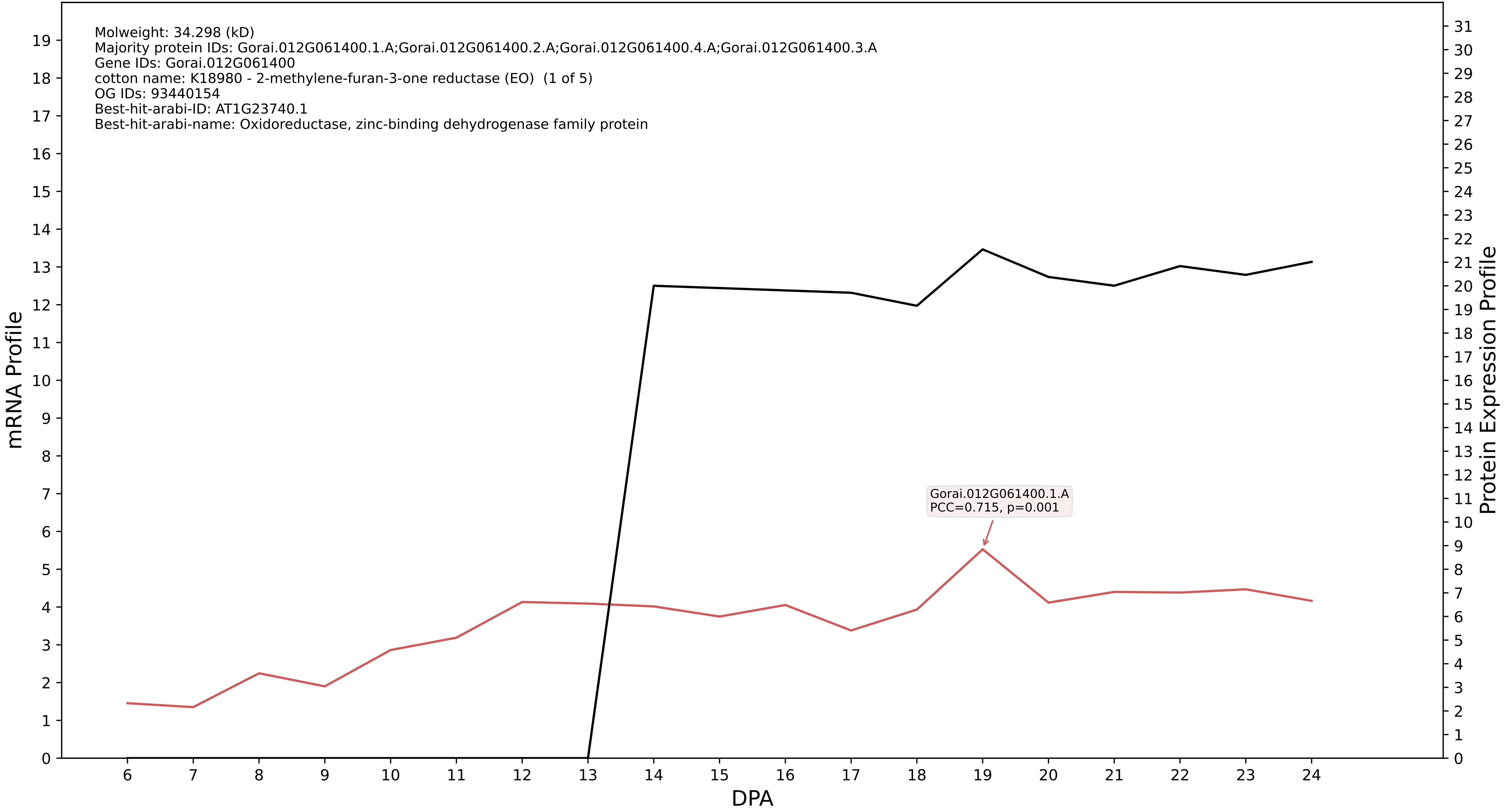

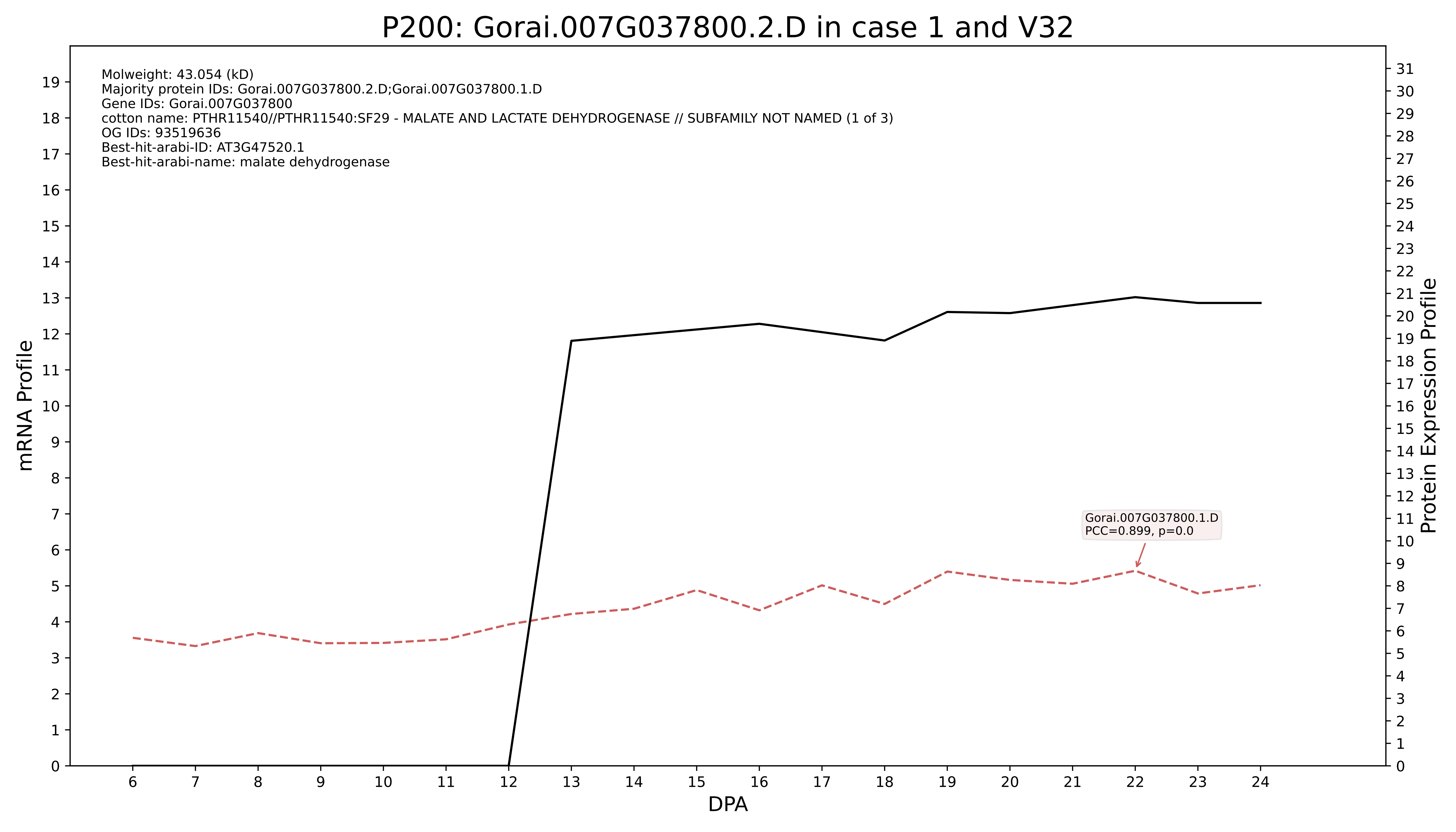
P200: Gorai.007G037800.2.D in case 1 and V32
Molweight: 43.054 (kD)
Majority protein IDs: Gorai.007G037800.2.D;Gorai.007G037800.1.D
Gene IDs: Gorai.007G037800
cotton name: PTHR11540//PTHR11540:SF29 - MALATE AND LACTATE DEHYDROGENASE // SUBFAMILY NOT NAMED (1 of 3)
OG IDs: 93519636
Best-hit-arabi-ID: AT3G47520.1
Best-hit-arabi-name: malate dehydrogenase
Gorai.007G037800.1.D
PCC=0.899, p=0.0
mRNA Profile
Protein Expression Profile
DPA

P200: Gorai.005G043900.1.A in case 1 and V32

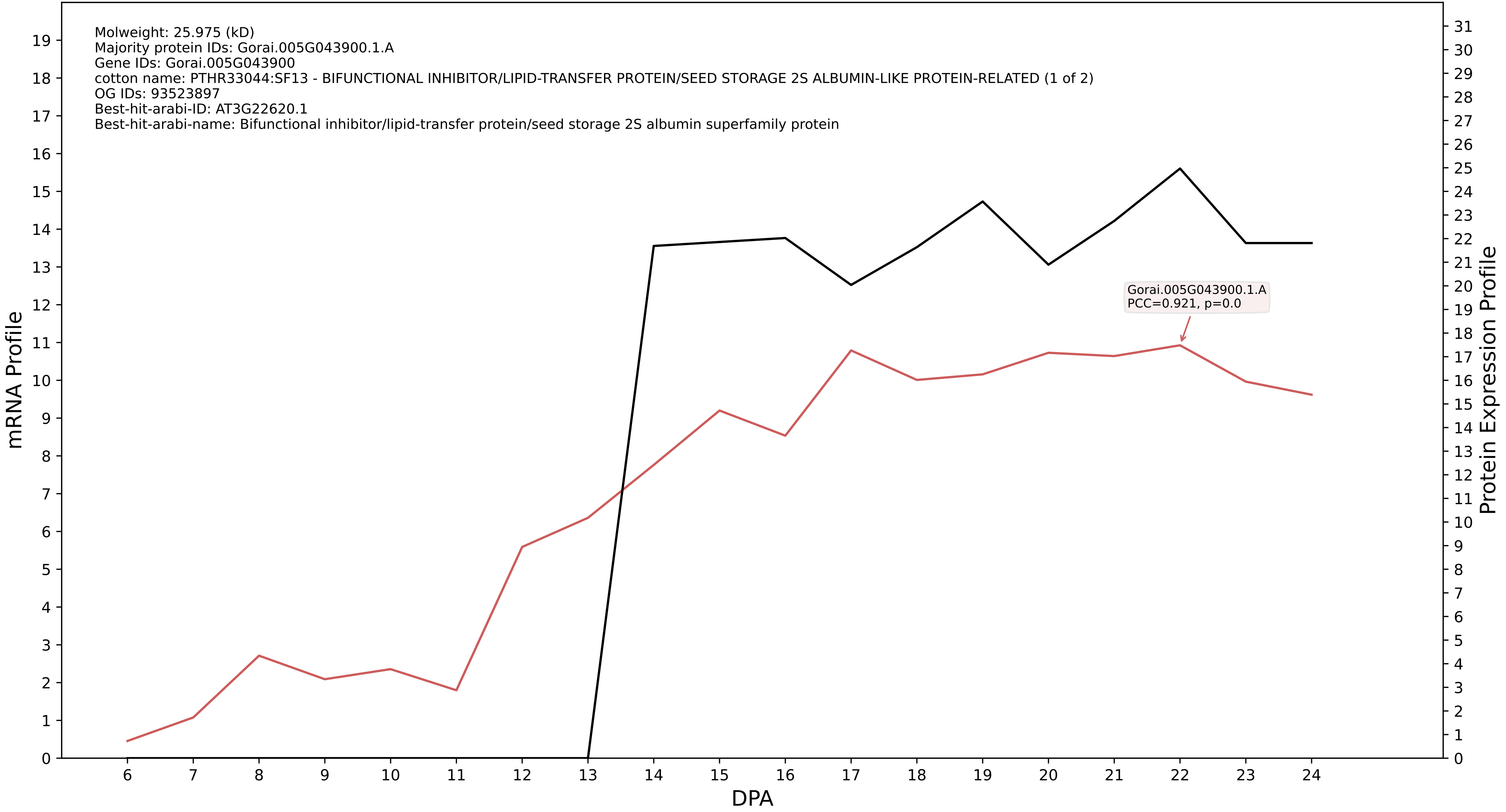

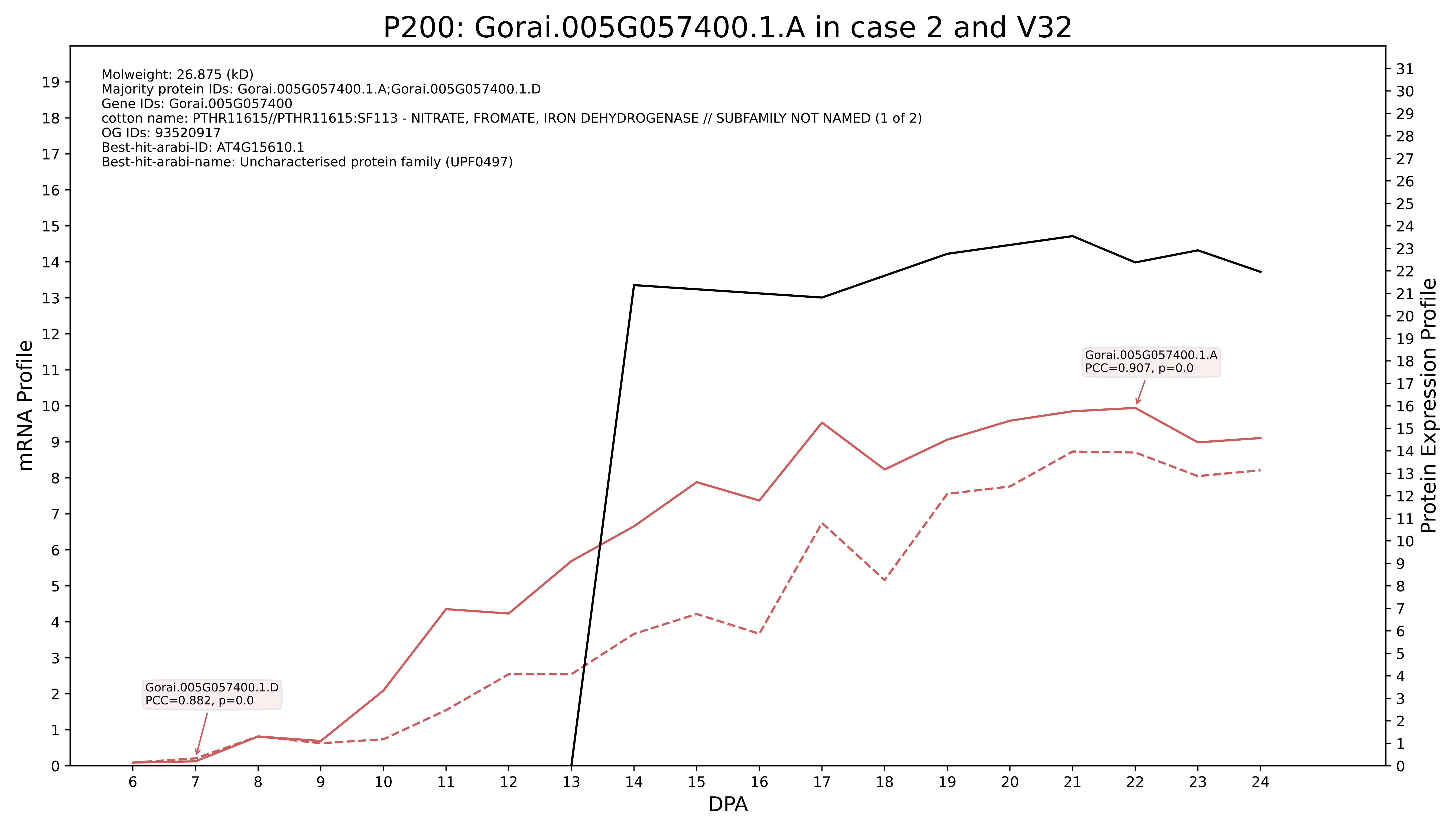
P200: Gorai.005G057400.1.A in case 2 and V32
Molweight: 26.875 (kD)
Majority protein IDs: Gorai.005G057400.1.A;Gorai.005G057400.1.D
Gene IDs: Gorai.005G057400
cotton name: PTHR11615//PTHR11615:SF113 - NITRATE, FROMATE, IRON DEHYDROGENASE // SUBFAMILY NOT NAMED (1 of 2)
OG IDs: 93520917
Best-hit-arabi-ID: AT4G15610.1
Best-hit-arabi-name: Uncharacterised protein family (UPF0497)
Gorai.005G057400.1.A
PCC=0.907, p=0.0
Gorai.005G057400.1.D
PCC=0.882, p=0.0
mRNA Profile
Protein Expression Profile
DPA

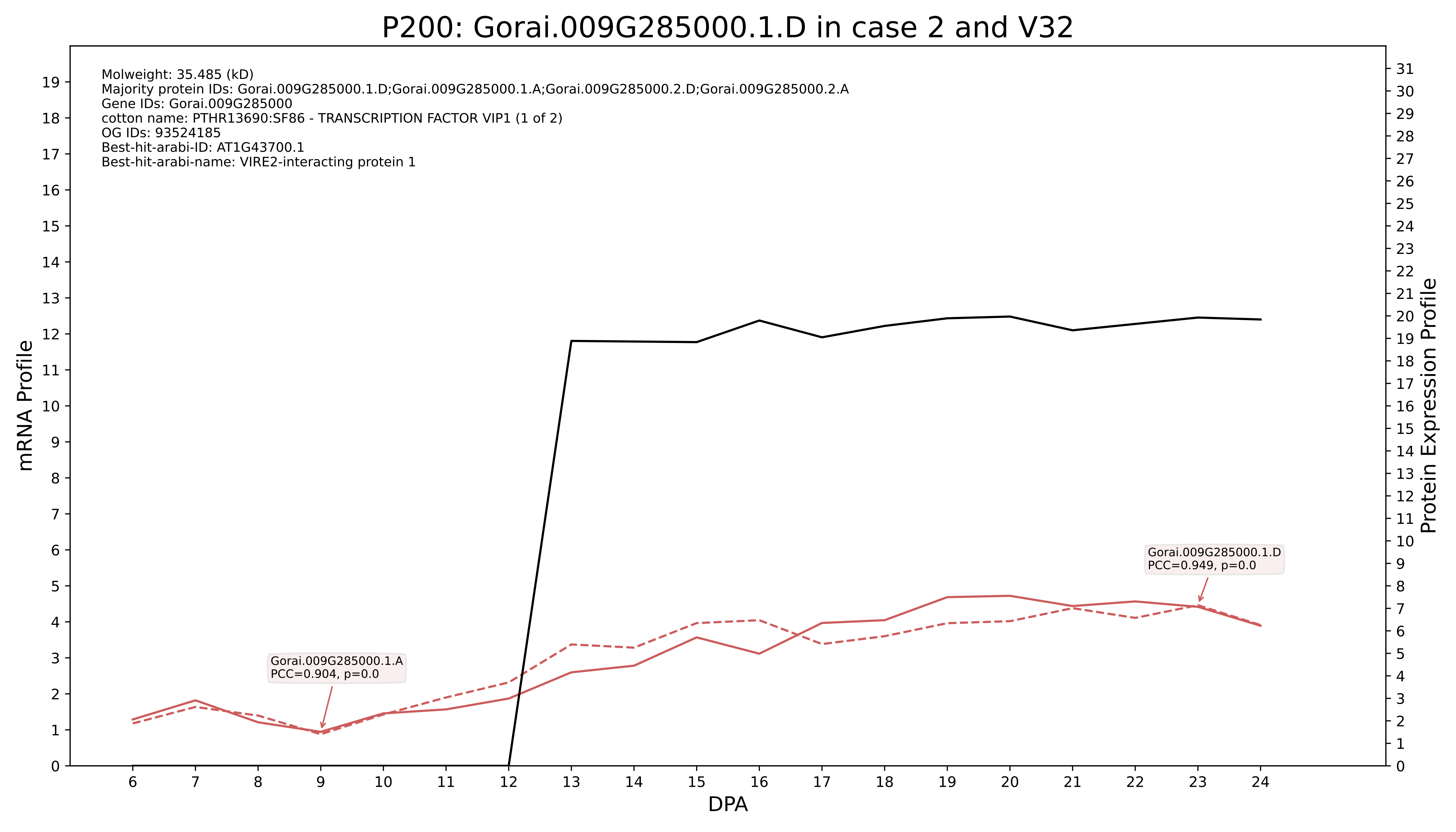

P200: Gorai.009G285000.1.D in case 2 and V32
Molweight: 35.485 (kD)
Majority protein IDs: Gorai.009G285000.1.D;Gorai.009G285000.1.A;Gorai.009G285000.2.D;Gorai.009G285000.2.A
Gene IDs: Gorai.009G285000
cotton name: PTHR13690:SF86 - TRANSCRIPTION FACTOR VIP1 (1 of 2)
OG IDs: 93524185
Best-hit-arabi-ID: AT1G43700.1
Best-hit-arabi-name: VIRE2-interacting protein 1
Gorai.009G285000.1.D
PCC=0.949, p=0.0
Gorai.009G285000.1.A
PCC=0.904, p=0.0
mRNA Profile
Protein Expression Profile
DPA

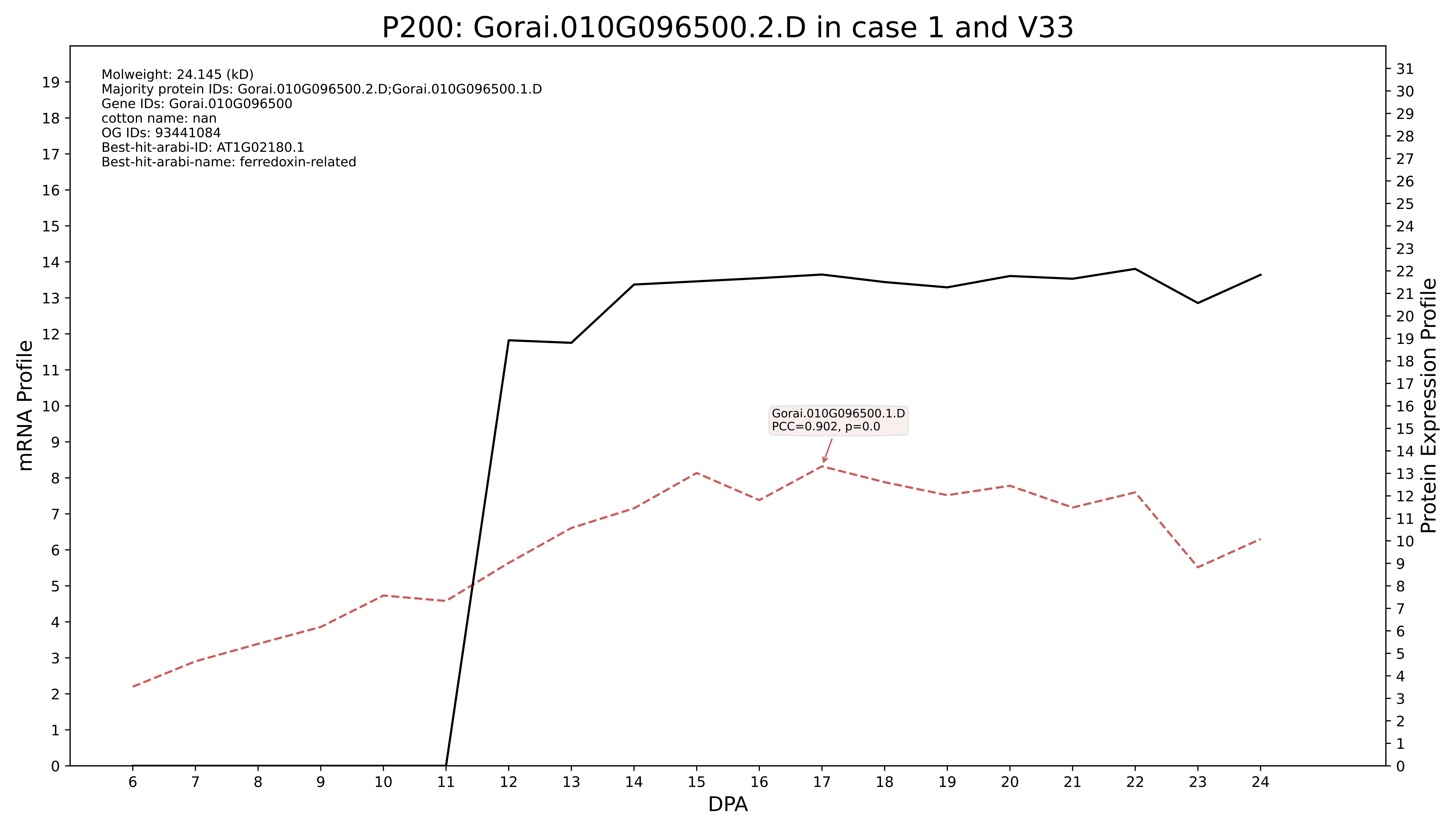

P200: Gorai.010G096500.2.D in case 1 and V33
Molweight: 24.145 (kD)
Majority protein IDs: Gorai.010G096500.2.D;Gorai.010G096500.1.D
Gene IDs: Gorai.010G096500
cotton name: nan
OG IDs: 93441084
Best-hit-arabi-ID: AT1G02180.1
Best-hit-arabi-name: ferredoxin-related
Gorai.010G096500.1.D
PCC=0.902, p=0.0
mRNA Profile
Protein Expression Profile
DPA
0 1 2 3 4 5 6 7 8 9 10 11 12 13 14 15 16 17 18 19
0 1 2 3 4 5 6 7 8 9 10 11 12 13 14 15 16 17 18 19 20 21 22 23 24 25 26 27 28 29 30 31
6 7 8 9 10 11 12 13 14 15 16 17 18 19 20 21 22 23 24

P200: Gorai.008G082900.1.A in case 1 and V33

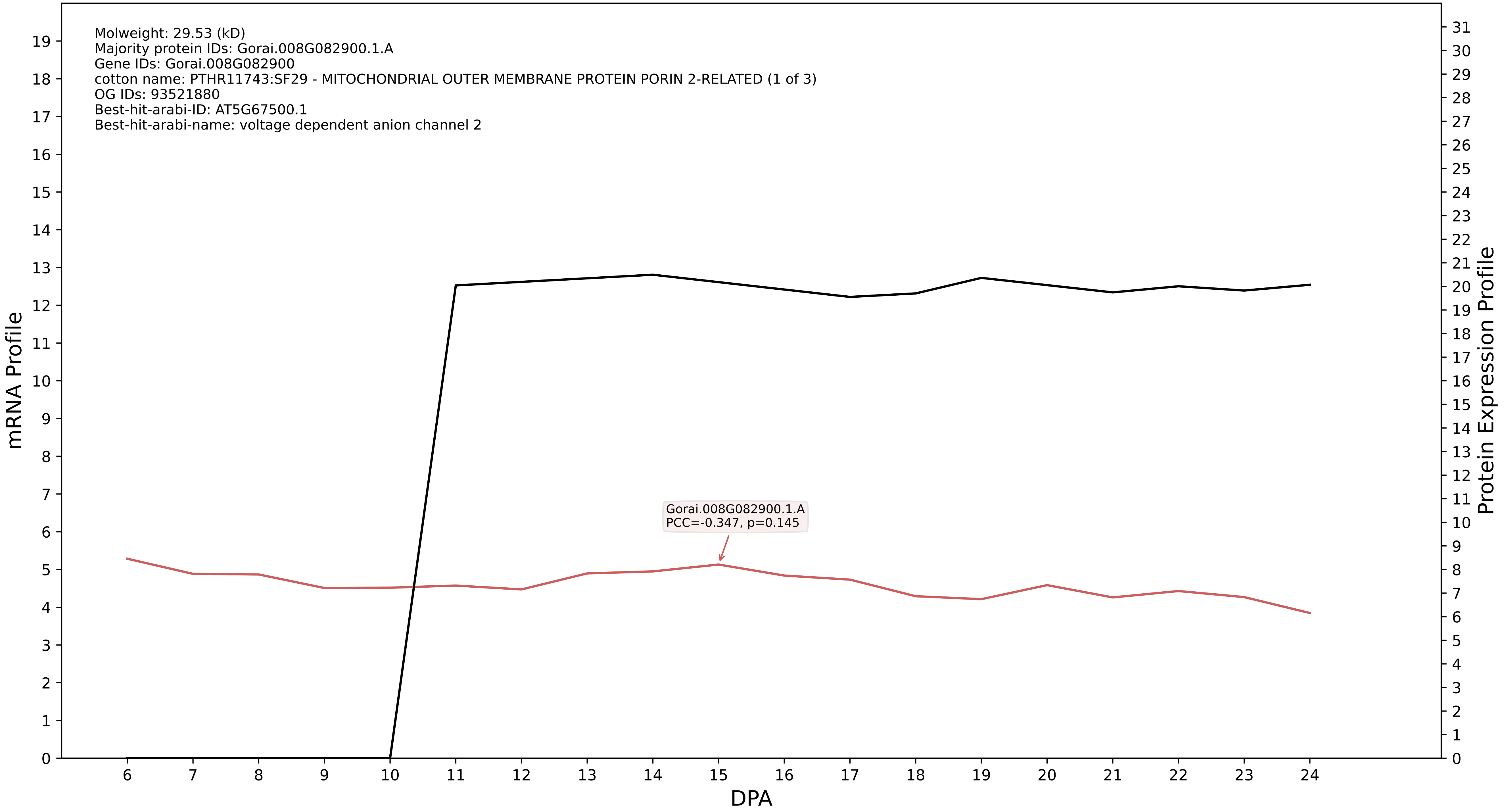

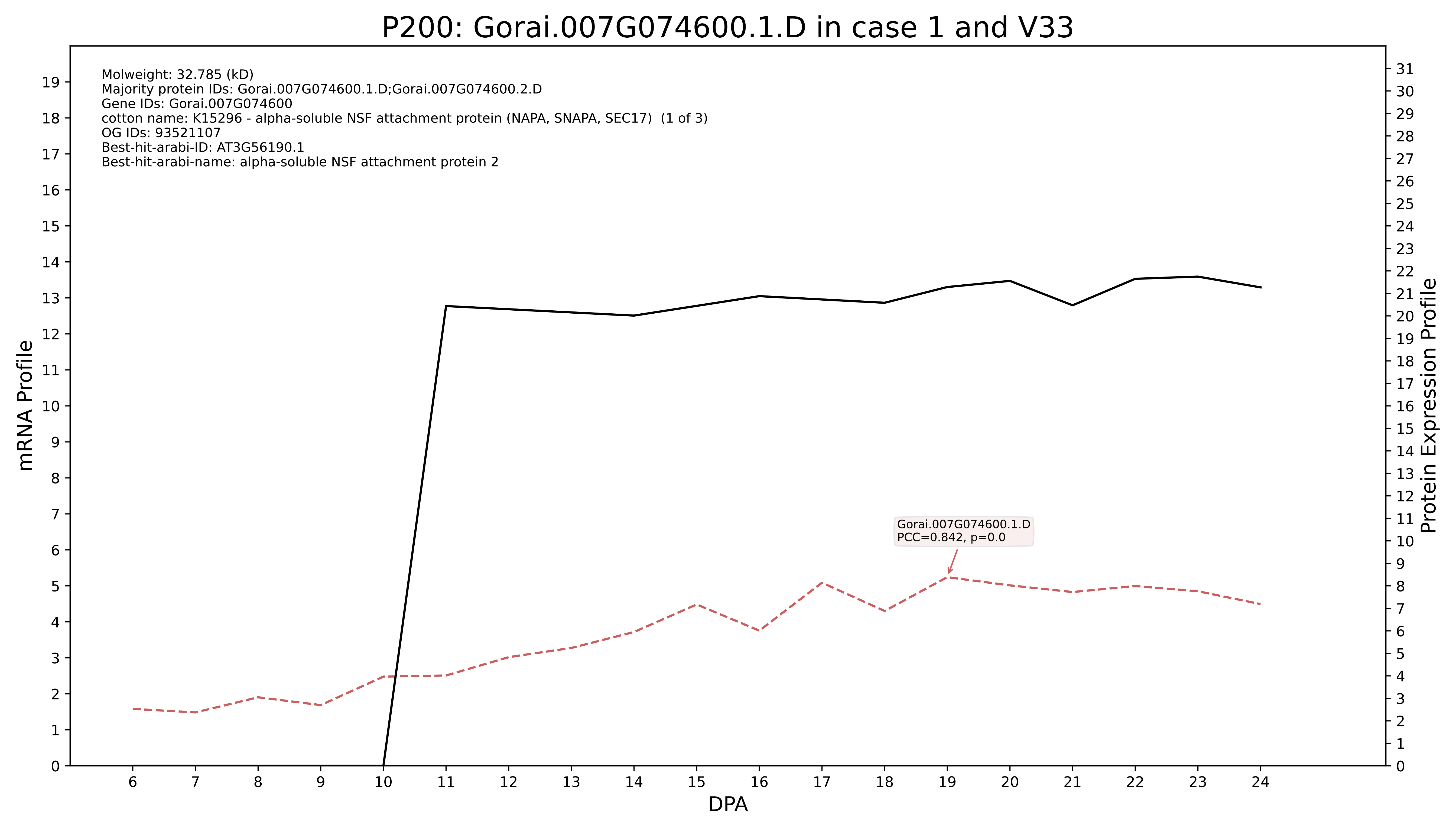
P200: Gorai.007G074600.1.D in case 1 and V33
Molweight: 32.785 (kD)
Majority protein IDs: Gorai.007G074600.1.D;Gorai.007G074600.2.D
Gene IDs: Gorai.007G074600
cotton name: K15296 - alpha-soluble NSF attachment protein (NAPA, SNAPA, SEC17) (1 of 3)
OG IDs: 93521107
Best-hit-arabi-ID: AT3G56190.1
Best-hit-arabi-name: alpha-soluble NSF attachment protein 2
Gorai.007G074600.1.D
PCC=0.842, p=0.0
mRNA Profile
Protein Expression Profile
DPA

P200: Gorai.013G156800.2.D in case 1 and V33

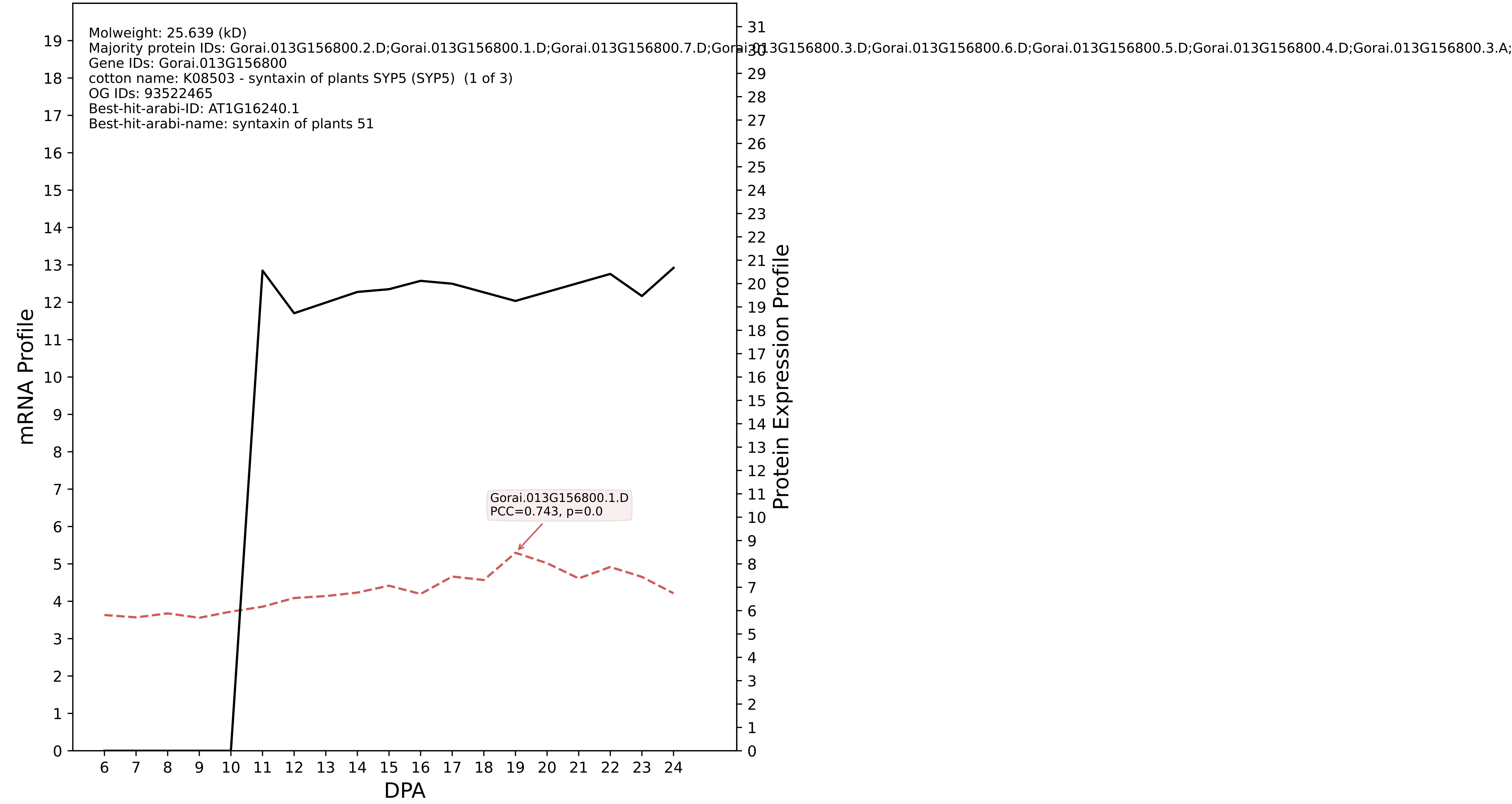

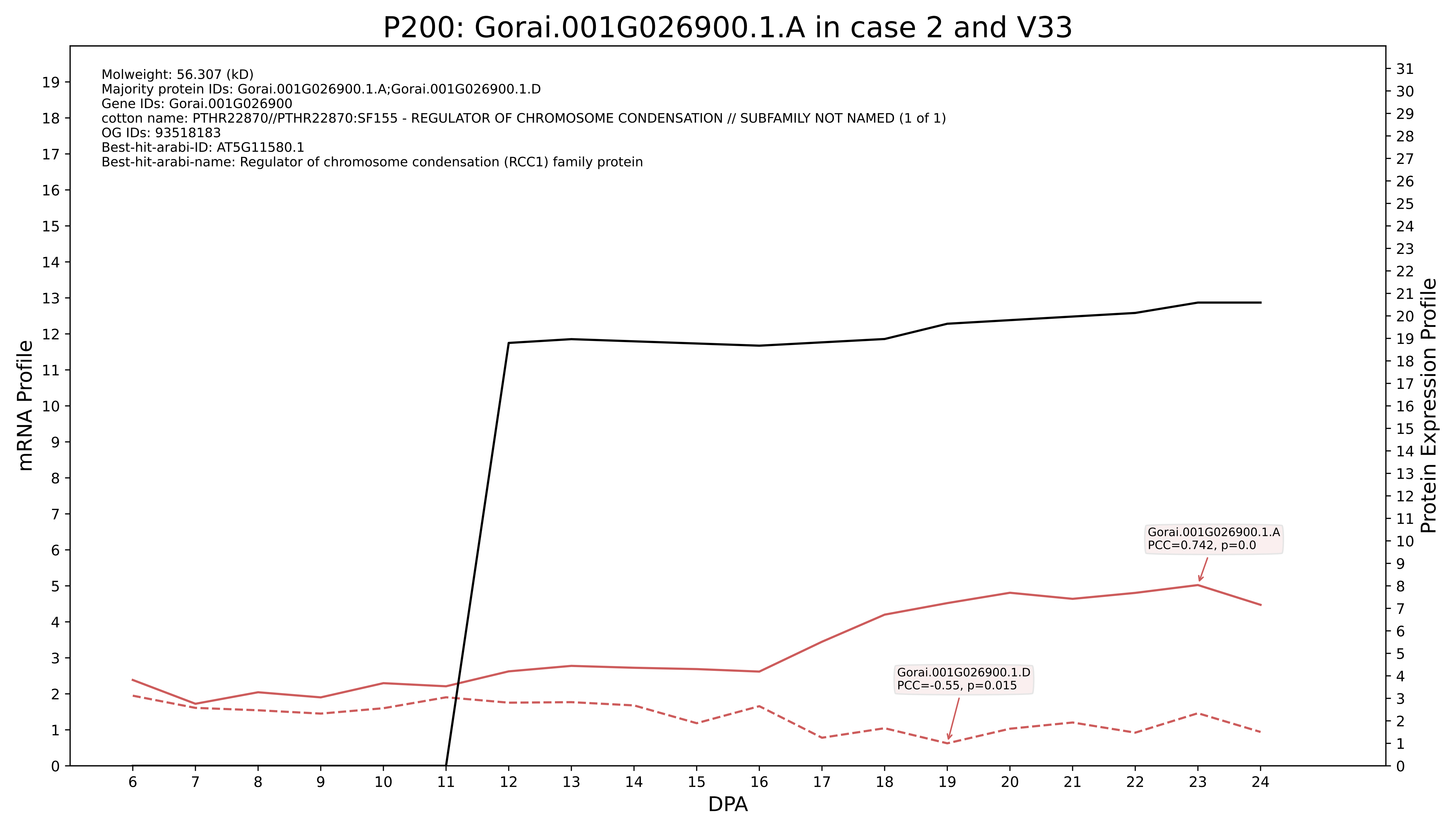

P200: Gorai.001G026900.1.A in case 2 and V33
Molweight: 56.307 (kD)
Majority protein IDs: Gorai.001G026900.1.A;Gorai.001G026900.1.D
Gene IDs: Gorai.001G026900
cotton name: PTHR22870//PTHR22870:SF155 - REGULATOR OF CHROMOSOME CONDENSATION // SUBFAMILY NOT NAMED (1 of 1)
OG IDs: 93518183
Best-hit-arabi-ID: AT5G11580.1
Best-hit-arabi-name: Regulator of chromosome condensation (RCC1) family protein
Gorai.001G026900.1.A
PCC=0.742, p=0.0
Gorai.001G026900.1.D
PCC=-0.55, p=0.015
mRNA Profile
Protein Expression Profile
DPA

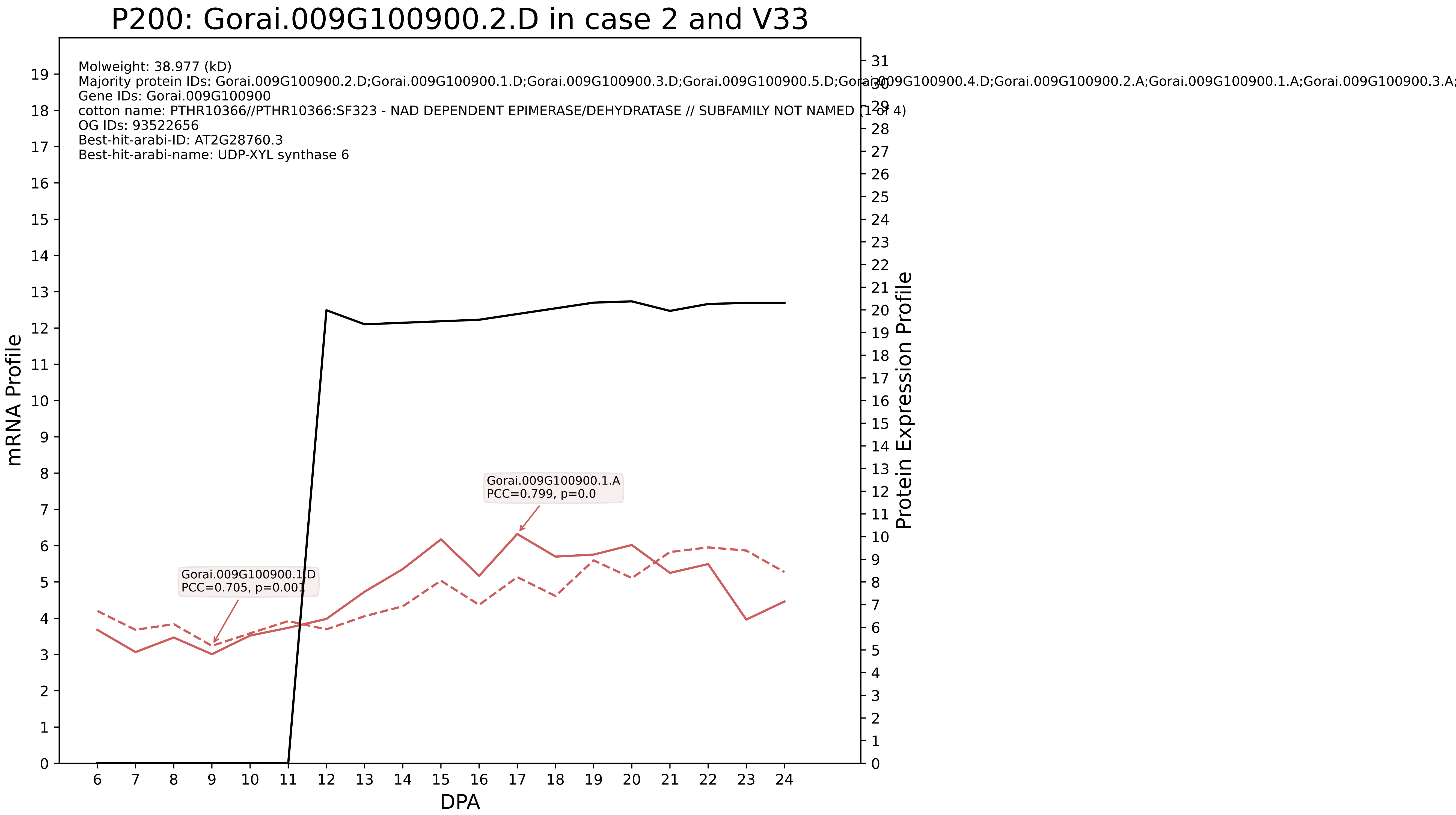
P200: Gorai.009G100900.2.D in case 2 and V33
Molweight: 38.977 (kD)
Majority protein IDs: Gorai.009G100900.2.D;Gorai.009G100900.1.D;Gorai.009G100900.3.D;Gorai.009G100900.5.D;Gorai.009G100900.4.D;Gorai.009G100900.2.A;Gorai.009G100900.1.A;Gorai.009G100900.3.A;
Gene IDs: Gorai.009G100900
cotton name: PTHR10366//PTHR10366:SF323 - NAD DEPENDENT EPIMERASE/DEHYDRATASE // SUBFAMILY NOT NAMED (1 of 4)
OG IDs: 93522656
Best-hit-arabi-ID: AT2G28760.3
Best-hit-arabi-name: UDP-XYL synthase 6
Gorai.009G100900.1.A
PCC=0.799, p=0.0
Gorai.009G100900.1.D
PCC=0.705, p=0.001
mRNA Profile
Protein Expression Profile
DPA

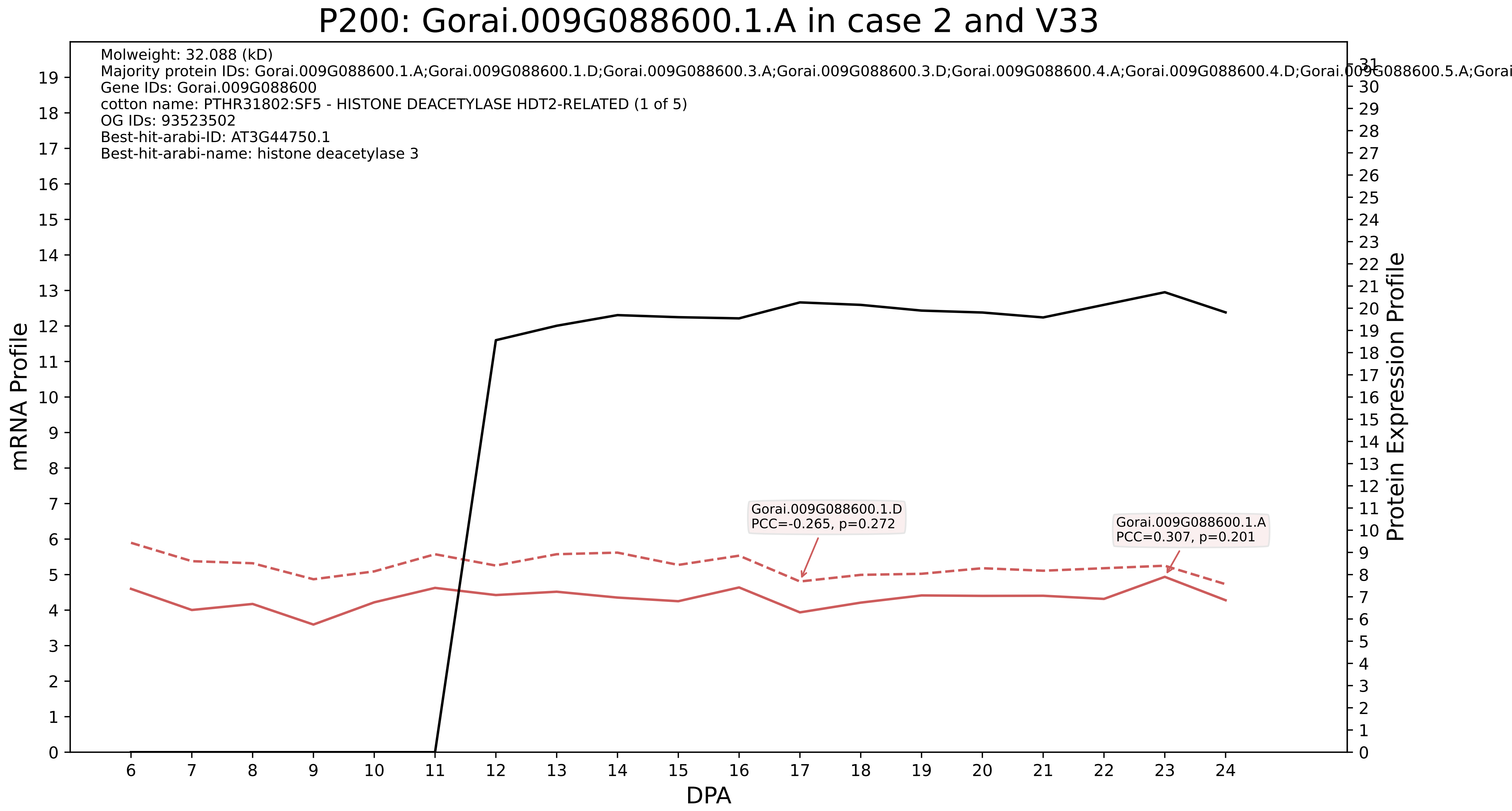
P200: Gorai.009G088600.1.A in case 2 and V33
Molweight: 32.088 (kD)
Majority protein IDs: Gorai.009G088600.1.A;Gorai.009G088600.1.D;Gorai.009G088600.3.A;Gorai.009G088600.3.D;Gorai.009G088600.4.A;Gorai.009G088600.4.D;Gorai.009G088600.5.A;Gorai
Gene IDs: Gorai.009G088600
cotton name: PTHR31802:SF5 - HISTONE DEACETYLASE HDT2-RELATED (1 of 5)
OG IDs: 93523502
Best-hit-arabi-ID: AT3G44750.1
Best-hit-arabi-name: histone deacetylase 3
Gorai.009G088600.1.D
PCC=-0.265, p=0.272
Gorai.009G088600.1.A
PCC=0.307, p=0.201
mRNA Profile
Protein Expression Profile
DPA

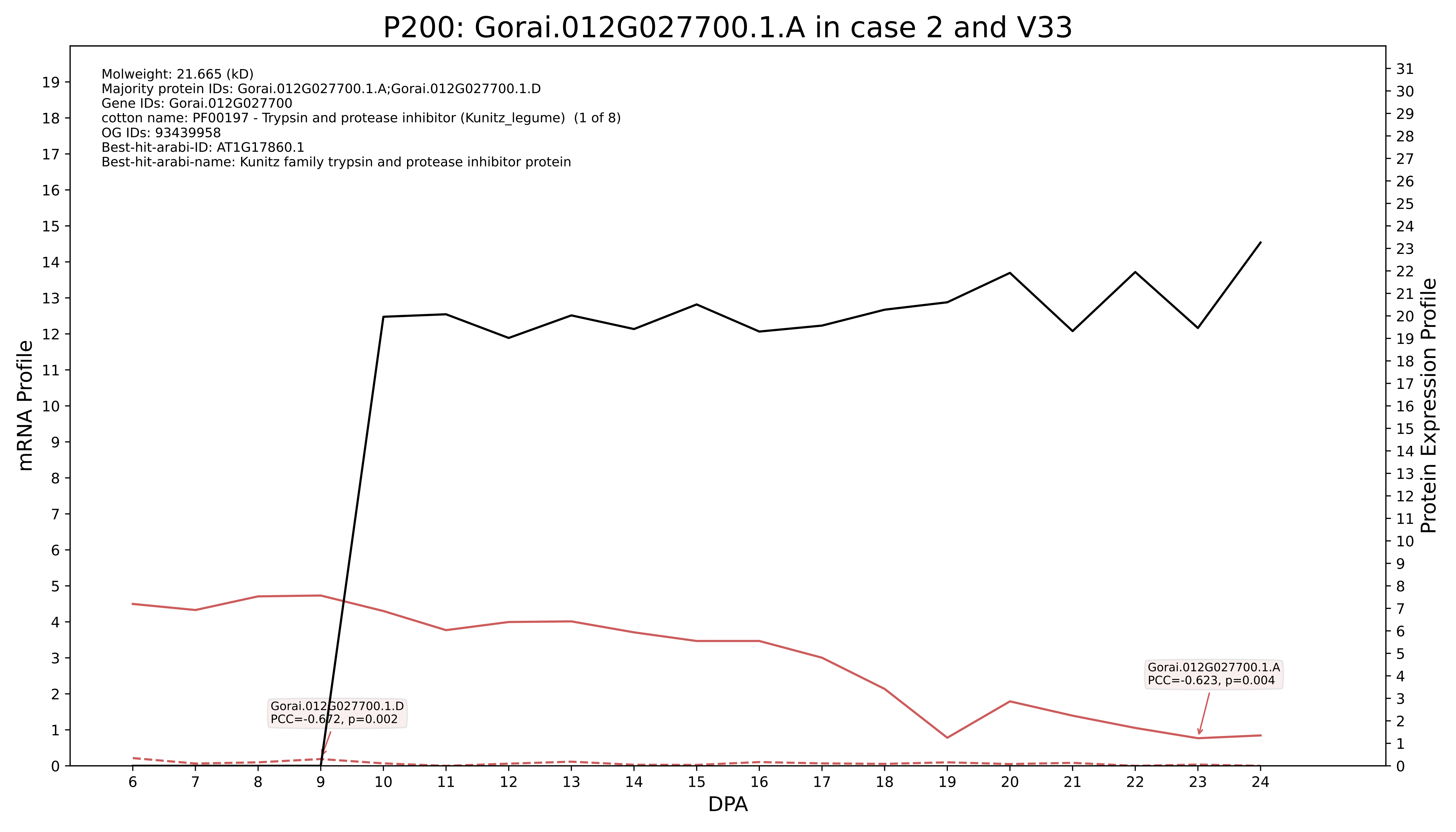
P200: Gorai.012G027700.1.A in case 2 and V33
Molweight: 21.665 (kD)
Majority protein IDs: Gorai.012G027700.1.A;Gorai.012G027700.1.D
Gene IDs: Gorai.012G027700
cotton name: PF00197 - Trypsin and protease inhibitor (Kunitz_legume) (1 of 8)
OG IDs: 93439958
Best-hit-arabi-ID: AT1G17860.1
Best-hit-arabi-name: Kunitz family trypsin and protease inhibitor protein
Gorai.012G027700.1.D
PCC=-0.672, p=0.002
Gorai.012G027700.1.A
PCC=-0.623, p=0.004
mRNA Profile
Protein Expression Profile
DPA

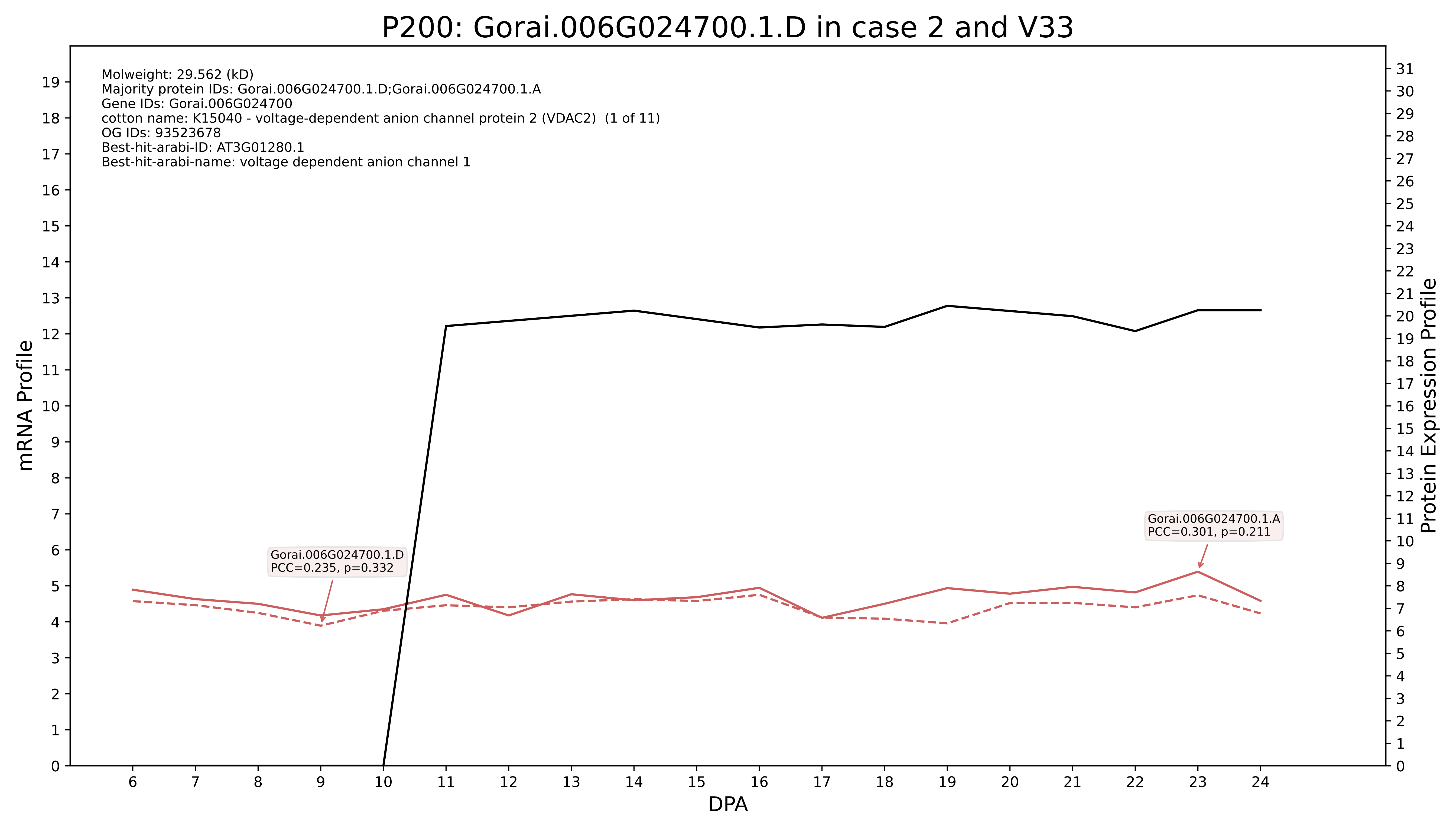
P200: Gorai.006G024700.1.D in case 2 and V33
Molweight: 29.562 (kD)
Majority protein IDs: Gorai.006G024700.1.D;Gorai.006G024700.1.A
Gene IDs: Gorai.006G024700
cotton name: K15040 - voltage-dependent anion channel protein 2 (VDAC2) (1 of 11)
OG IDs: 93523678
Best-hit-arabi-ID: AT3G01280.1
Best-hit-arabi-name: voltage dependent anion channel 1
Gorai.006G024700.1.D
PCC=0.235, p=0.332
Gorai.006G024700.1.A
PCC=0.301, p=0.211
mRNA Profile
Protein Expression Profile
DPA

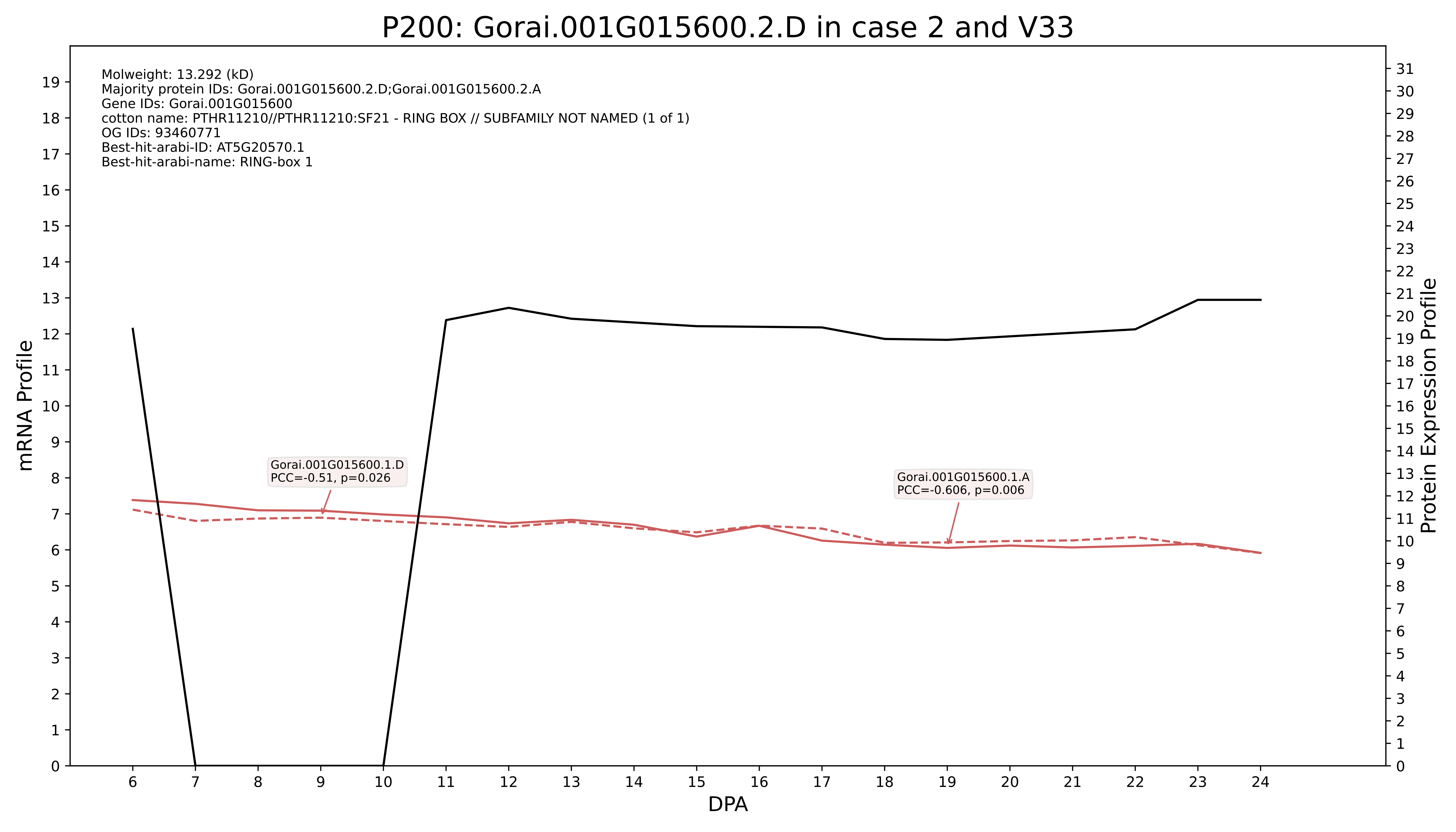
P200: Gorai.001G015600.2.D in case 2 and V33
Molweight: 13.292 (kD)
Majority protein IDs: Gorai.001G015600.2.D;Gorai.001G015600.2.A
Gene IDs: Gorai.001G015600
cotton name: PTHR11210//PTHR11210:SF21 - RING BOX // SUBFAMILY NOT NAMED (1 of 1)
OG IDs: 93460771
Best-hit-arabi-ID: AT5G20570.1
Best-hit-arabi-name: RING-box 1
Gorai.001G015600.1.D
PCC=-0.51, p=0.026
Gorai.001G015600.1.A
PCC=-0.606, p=0.006
mRNA Profile
Protein Expression Profile
DPA
0
1
2
3
4
5
6
7
8
9
10
11
12
13
14
15
16
17
18
19
20
21
22
23
24
25
26
27
28
29
30
31

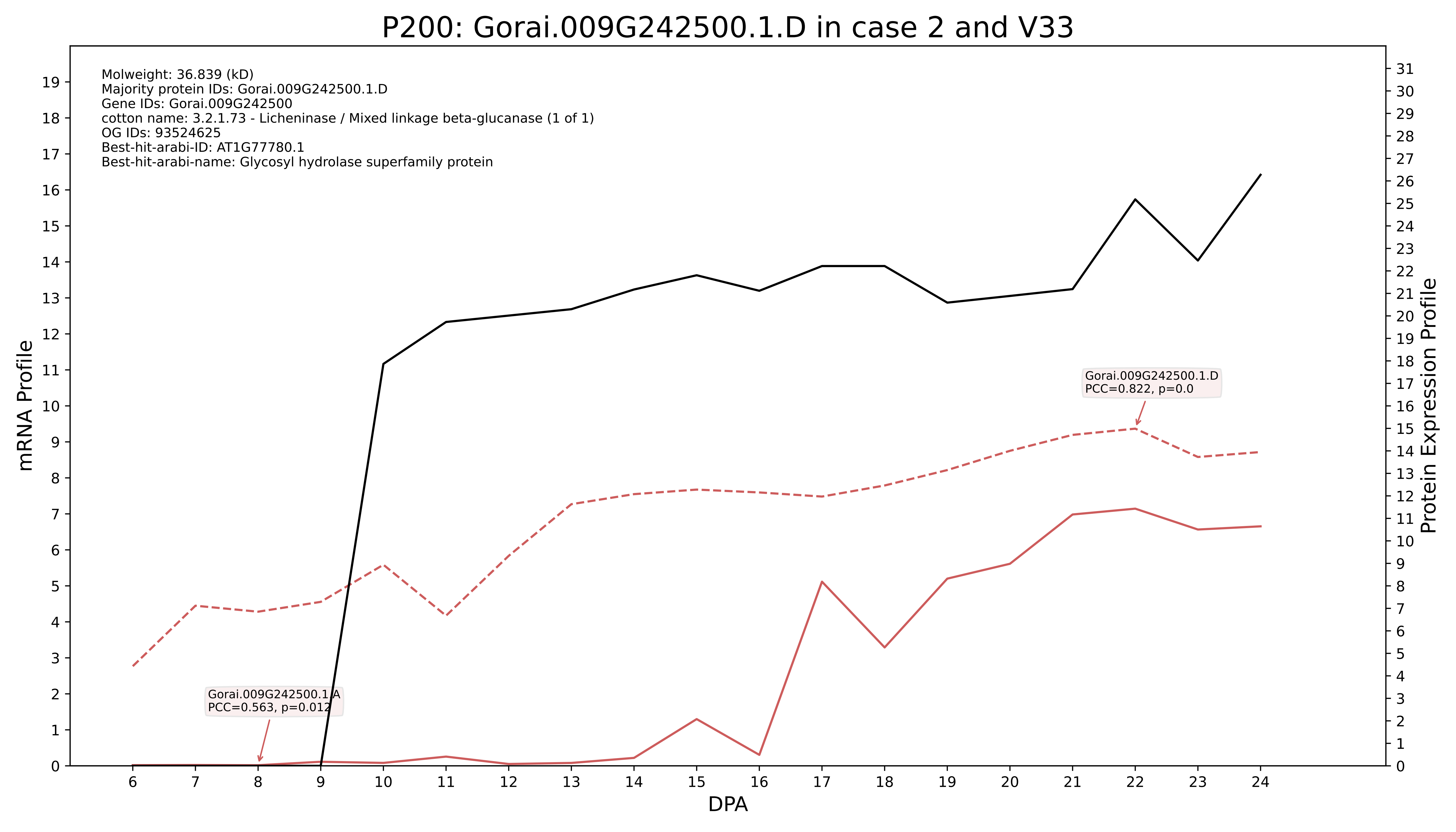

P200: Gorai.009G242500.1.D in case 2 and V33
Molweight: 36.839 (kD)
Majority protein IDs: Gorai.009G242500.1.D
Gene IDs: Gorai.009G242500
cotton name: 3.2.1.73 - Licheninase / Mixed linkage beta-glucanase (1 of 1)
OG IDs: 93524625
Best-hit-arabi-ID: AT1G77780.1
Best-hit-arabi-name: Glycosyl hydrolase superfamily protein
Gorai.009G242500.1.D
PCC=0.822, p=0.0
Gorai.009G242500.1.A
PCC=0.563, p=0.012
mRNA Profile
Protein Expression Profile
DPA

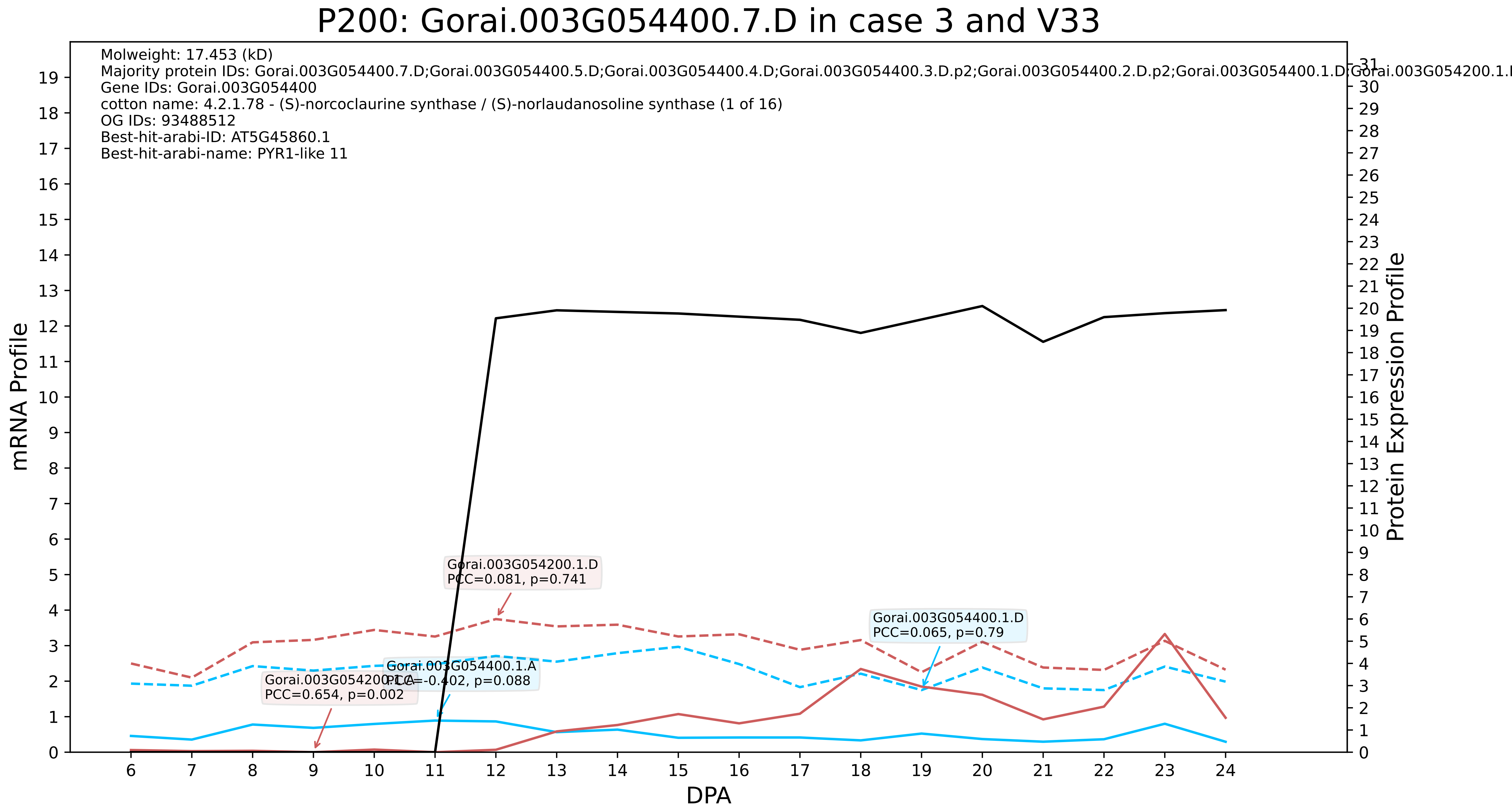

P200: Gorai.003G054400.7.D in case 3 and V33
Molweight: 17.453 (kD)
Majority protein IDs: Gorai.003G054400.7.D;Gorai.003G054400.5.D;Gorai.003G054400.4.D;Gorai.003G054400.3.D.p2;Gorai.003G054400.2.D.p2;Gorai.003G054400.1.D;Gorai.003G054200.1.D
Gene IDs: Gorai.003G054400
cotton name: 4.2.1.78 - (S)-norcoclaurine synthase / (S)-norlaudanosoline synthase (1 of 16)
OG IDs: 93488512
Best-hit-arabi-ID: AT5G45860.1
Best-hit-arabi-name: PYR1-like 11
Gorai.003G054200.1.D
PCC=0.081, p=0.741
Gorai.003G054400.1.D
PCC=0.065, p=0.79
Gorai.003G054400.1.A
PCC=-0.402, p=0.088
Gorai.003G054200.1.A
PCC=0.654, p=0.002
mRNA Profile
Protein Expression Profile
DPA

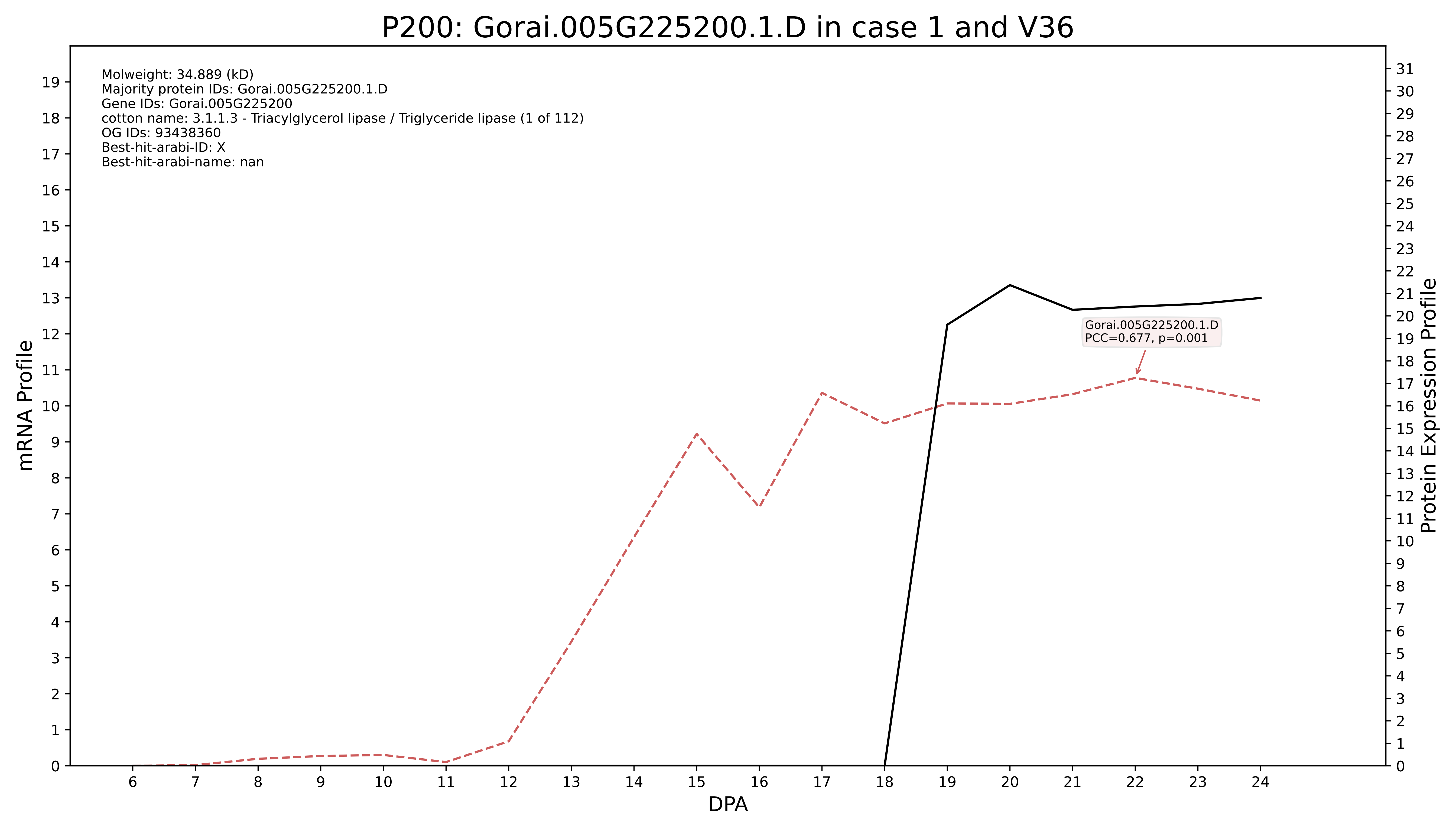
P200: Gorai.005G225200.1.D in case 1 and V36
Molweight: 34.889 (kD)
Majority protein IDs: Gorai.005G225200.1.D
Gene IDs: Gorai.005G225200
cotton name: 3.1.1.3 - Triacylglycerol lipase / Triglyceride lipase (1 of 112)
OG IDs: 93438360
Best-hit-arabi-ID: X
Best-hit-arabi-name: nan
Gorai.005G225200.1.D
PCC=0.677, p=0.001
mRNA Profile
Protein Expression Profile
DPA

P200: Gorai.009G040600.1.A in case 1 and V36

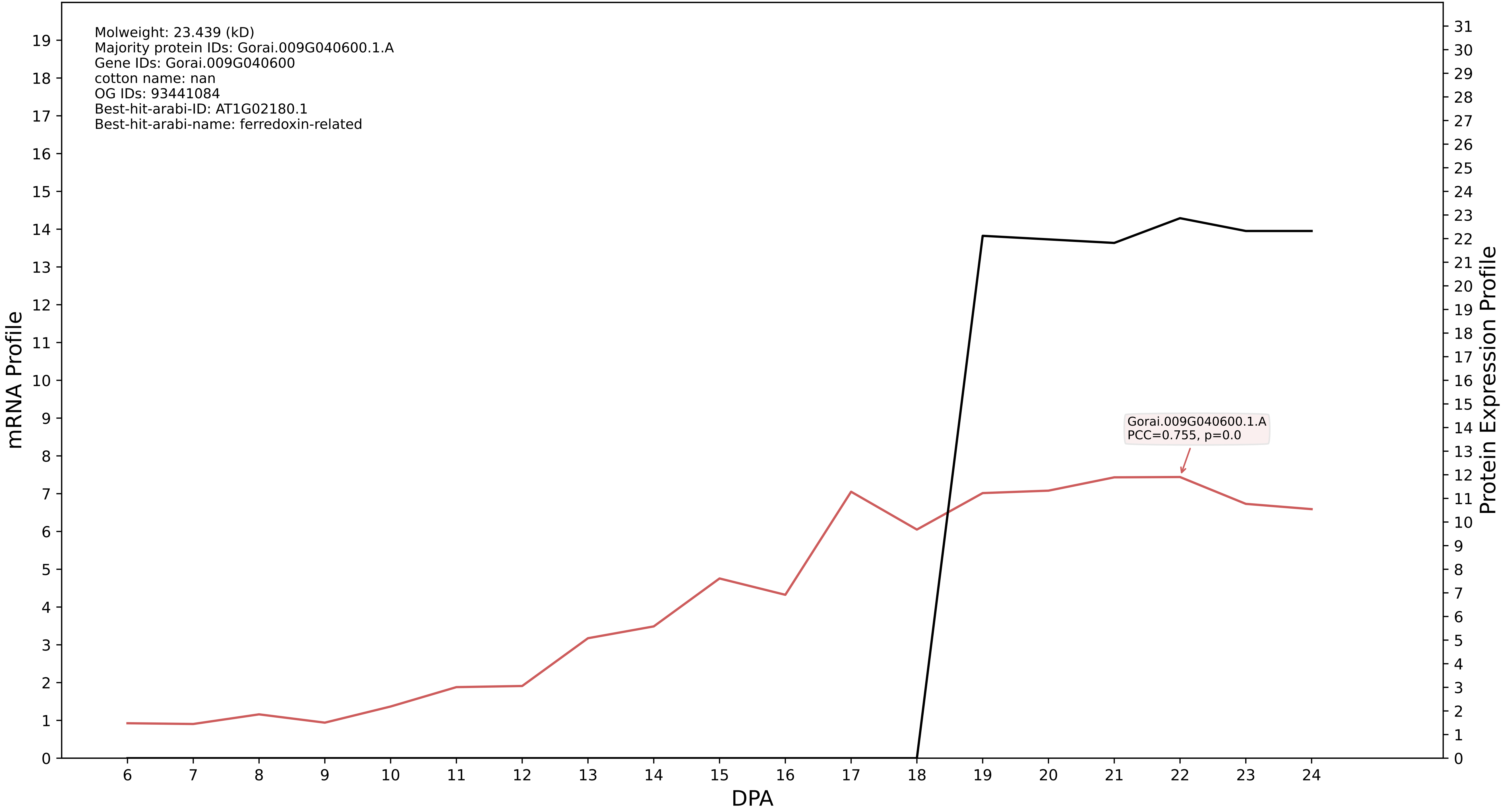

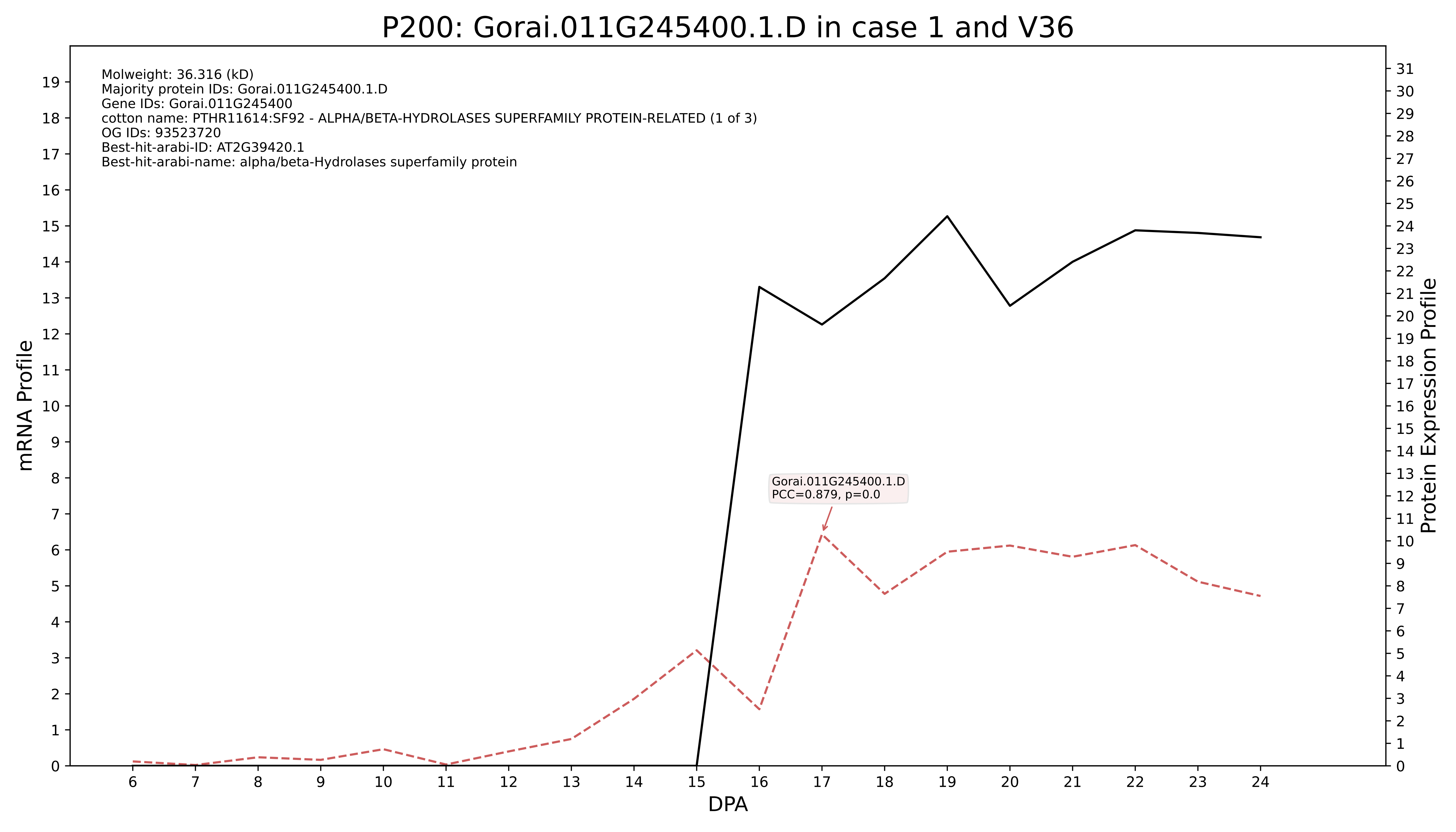

P200: Gorai.011G245400.1.D in case 1 and V36
Molweight: 36.316 (kD)
Majority protein IDs: Gorai.011G245400.1.D
Gene IDs: Gorai.011G245400
cotton name: PTHR11614:SF92 - ALPHA/BETA-HYDROLASES SUPERFAMILY PROTEIN-RELATED (1 of 3)
OG IDs: 93523720
Best-hit-arabi-ID: AT2G39420.1
Best-hit-arabi-name: alpha/beta-Hydrolases superfamily protein
Gorai.011G245400.1.D
PCC=0.879, p=0.0
mRNA Profile
Protein Expression Profile
DPA

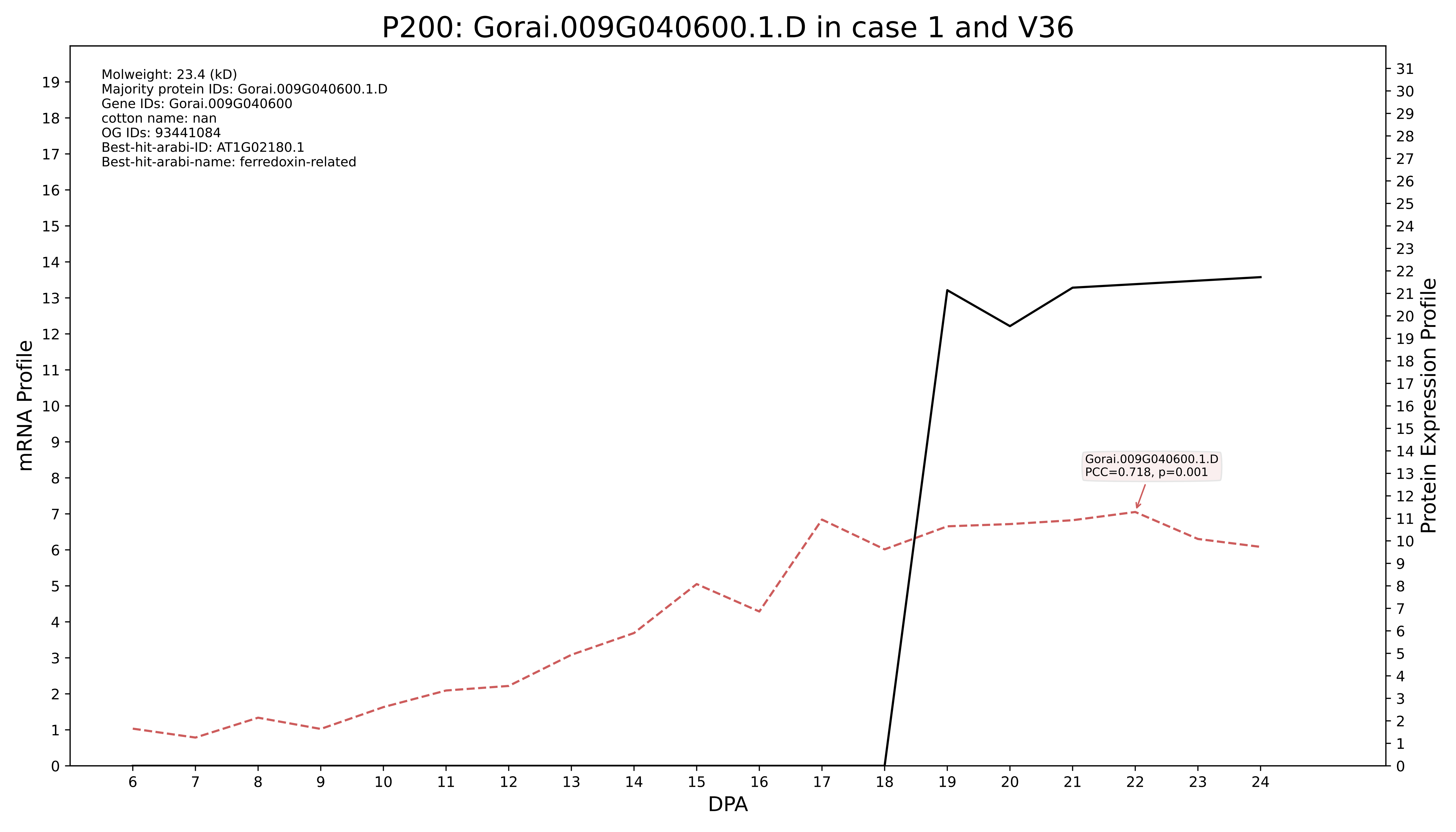

P200: Gorai.009G040600.1.D in case 1 and V36
Molweight: 23.4 (kD)
Majority protein IDs: Gorai.009G040600.1.D
Gene IDs: Gorai.009G040600
cotton name: nan
OG IDs: 93441084
Best-hit-arabi-ID: AT1G02180.1
Best-hit-arabi-name: ferredoxin-related
Gorai.009G040600.1.D
PCC=0.718, p=0.001
mRNA Profile
Protein Expression Profile
DPA

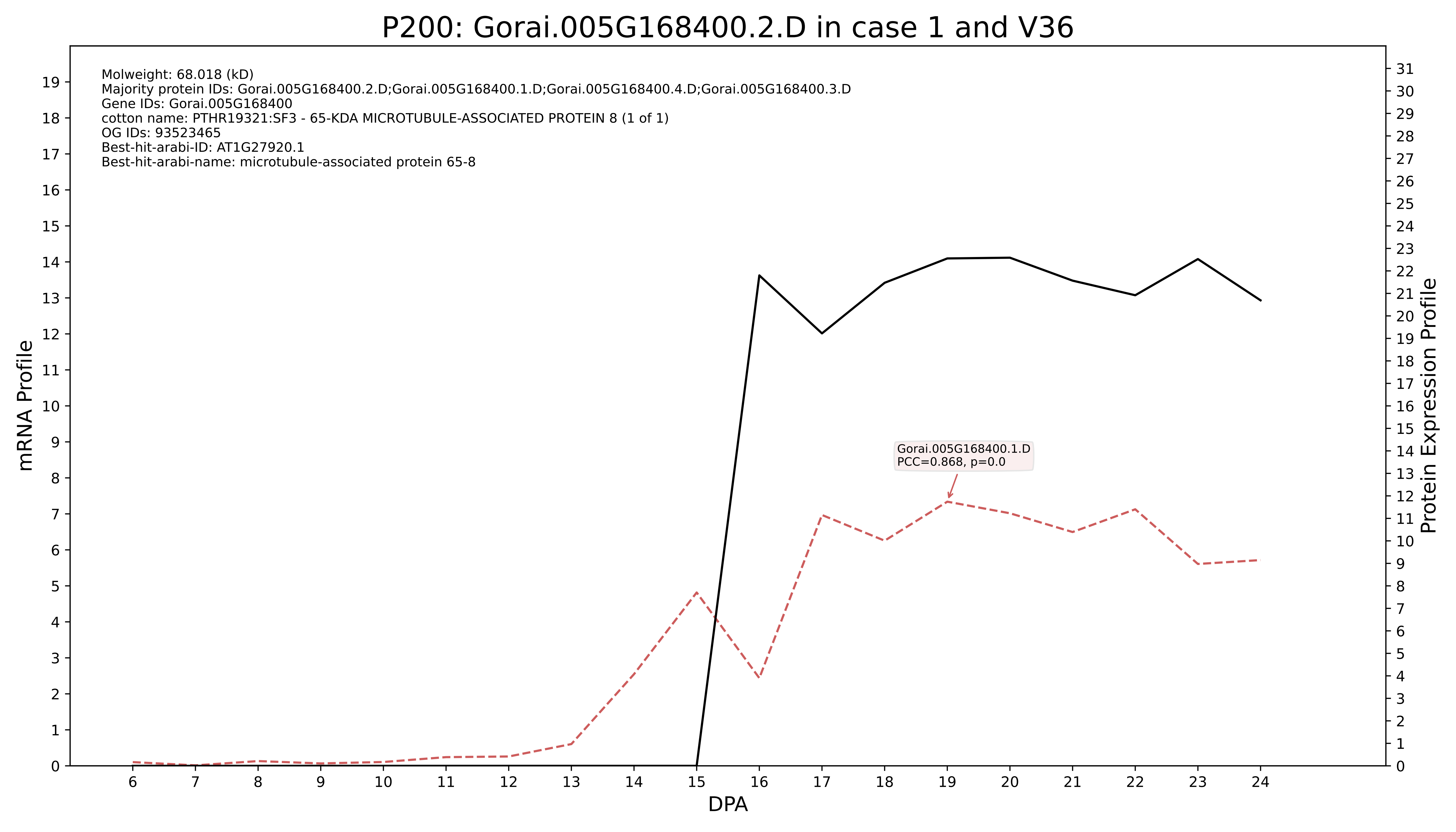
P200: Gorai.005G168400.2.D in case 1 and V36
Molweight: 68.018 (kD)
Majority protein IDs: Gorai.005G168400.2.D;Gorai.005G168400.1.D;Gorai.005G168400.4.D;Gorai.005G168400.3.D
Gene IDs: Gorai.005G168400
cotton name: PTHR19321:SF3 - 65-KDA MICROTUBULE-ASSOCIATED PROTEIN 8 (1 of 1)
OG IDs: 93523465
Best-hit-arabi-ID: AT1G27920.1
Best-hit-arabi-name: microtubule-associated protein 65-8
Gorai.005G168400.1.D
PCC=0.868, p=0.0
mRNA Profile
Protein Expression Profile
DPA

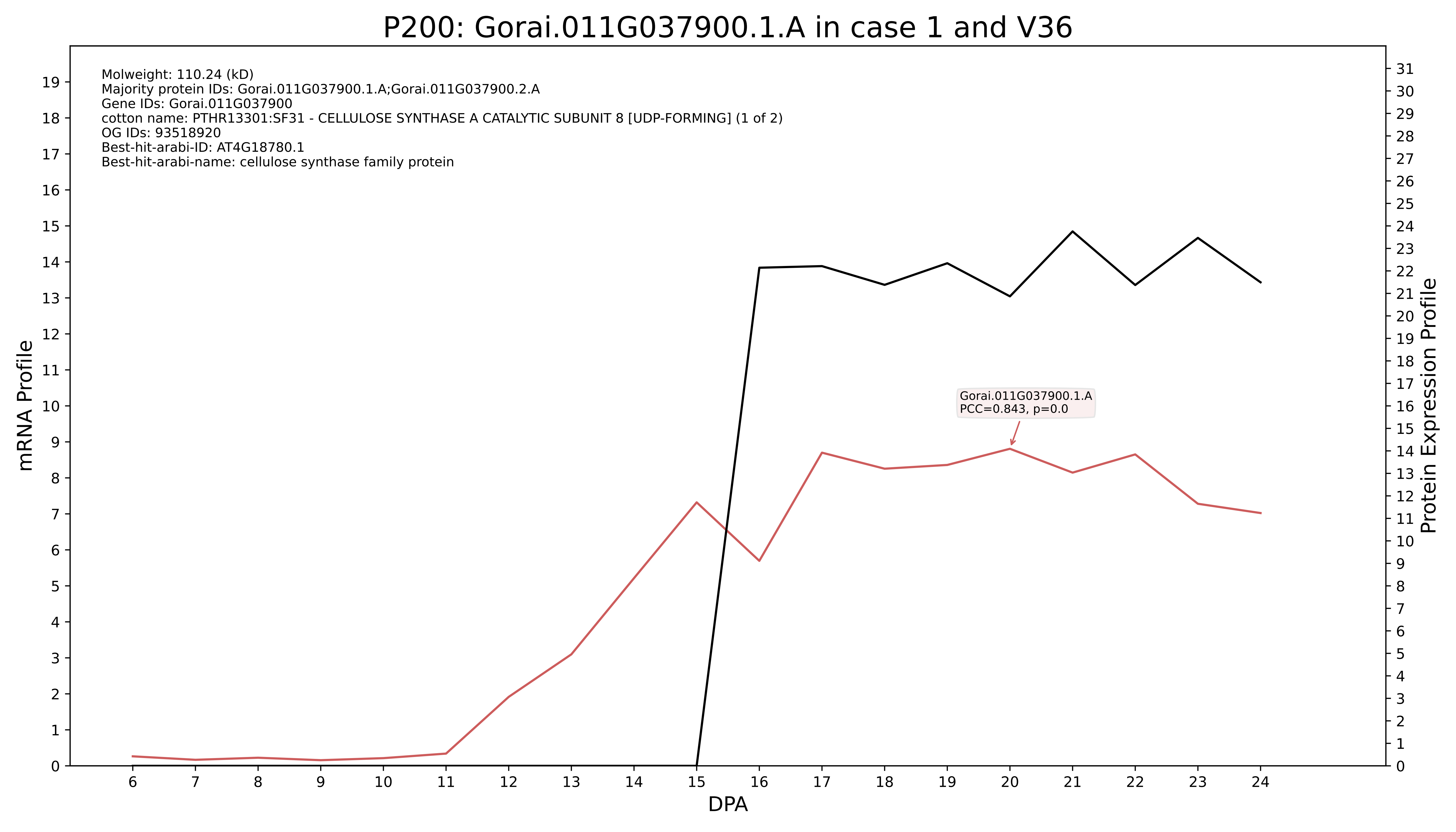

P200: Gorai.011G037900.1.A in case 1 and V36
Molweight: 110.24 (kD)
Majority protein IDs: Gorai.011G037900.1.A;Gorai.011G037900.2.A
Gene IDs: Gorai.011G037900
cotton name: PTHR13301:SF31 - CELLULOSE SYNTHASE A CATALYTIC SUBUNIT 8 [UDP-FORMING] (1 of 2)
OG IDs: 93518920
Best-hit-arabi-ID: AT4G18780.1
Best-hit-arabi-name: cellulose synthase family protein
Gorai.011G037900.1.A
PCC=0.843, p=0.0
mRNA Profile
Protein Expression Profile
DPA

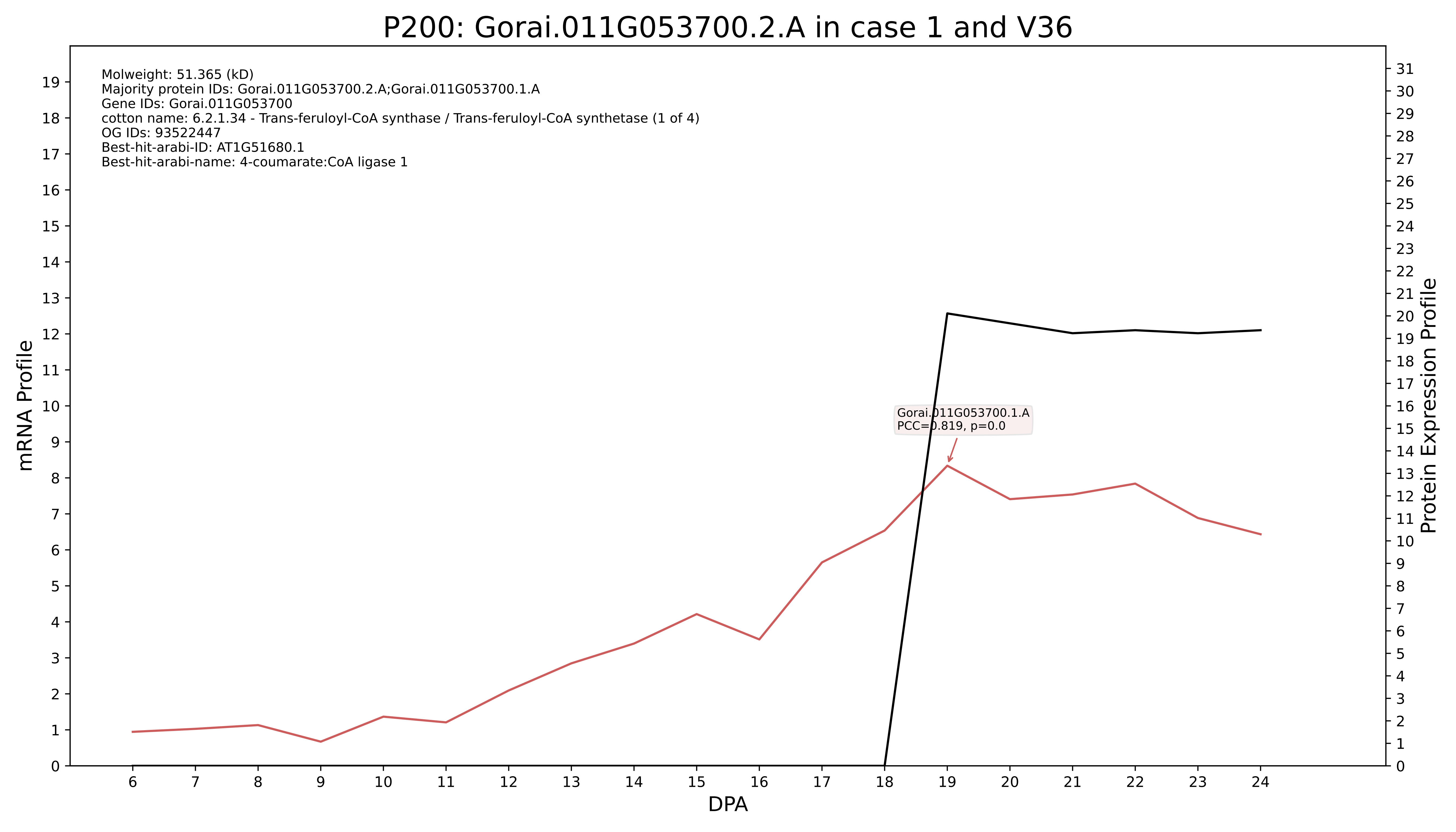
P200: Gorai.011G053700.2.A in case 1 and V36
Molweight: 51.365 (kD)
Majority protein IDs: Gorai.011G053700.2.A;Gorai.011G053700.1.A
Gene IDs: Gorai.011G053700
cotton name: 6.2.1.34 - Trans-feruloyl-CoA synthase / Trans-feruloyl-CoA synthetase (1 of 4)
OG IDs: 93522447
Best-hit-arabi-ID: AT1G51680.1
Best-hit-arabi-name: 4-coumarate:CoA ligase 1
Gorai.011G053700.1.A
PCC=0.819, p=0.0
mRNA Profile
Protein Expression Profile
DPA

P200: Gorai.003G108700.3.D in case 1 and V36

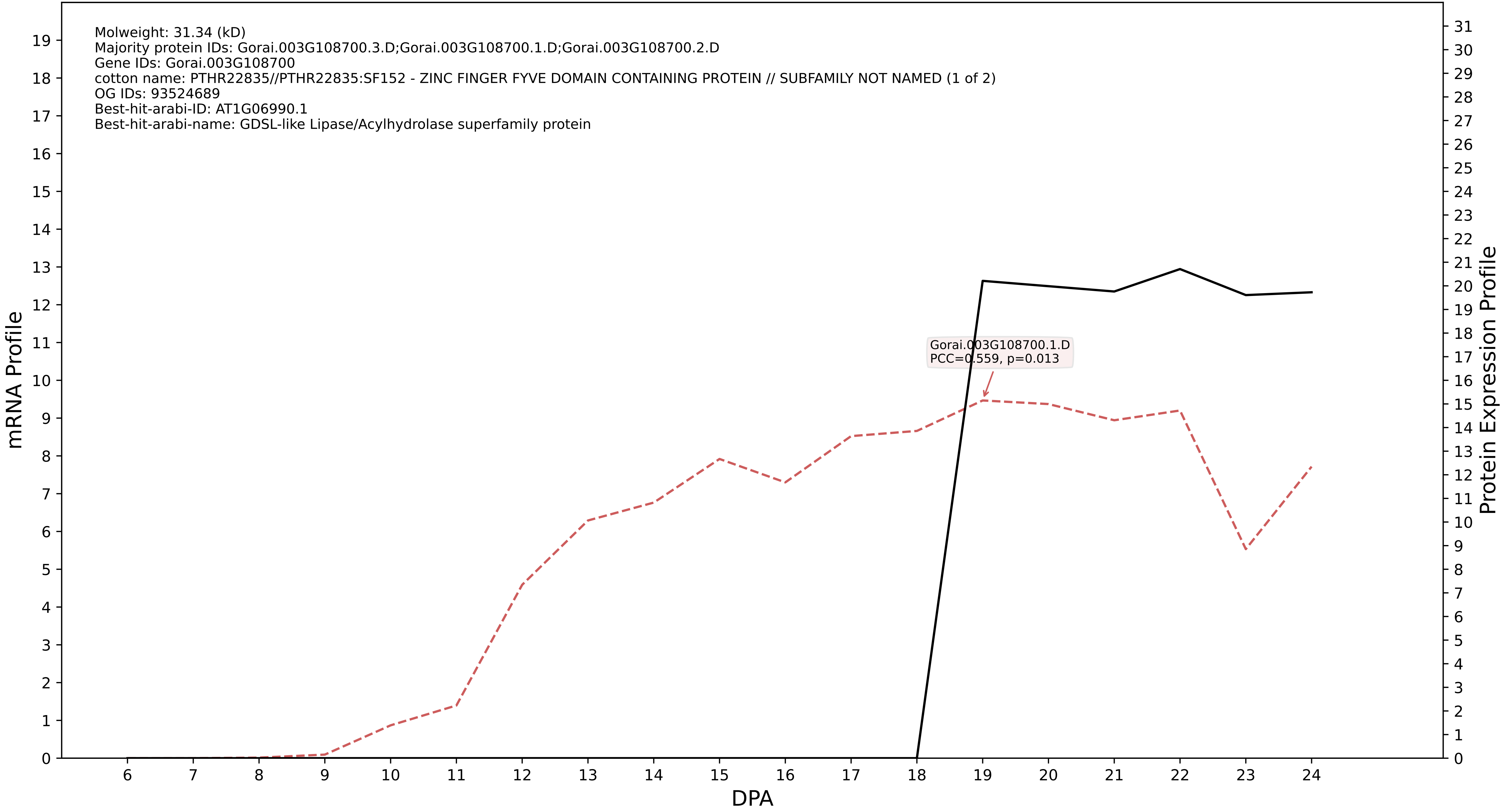

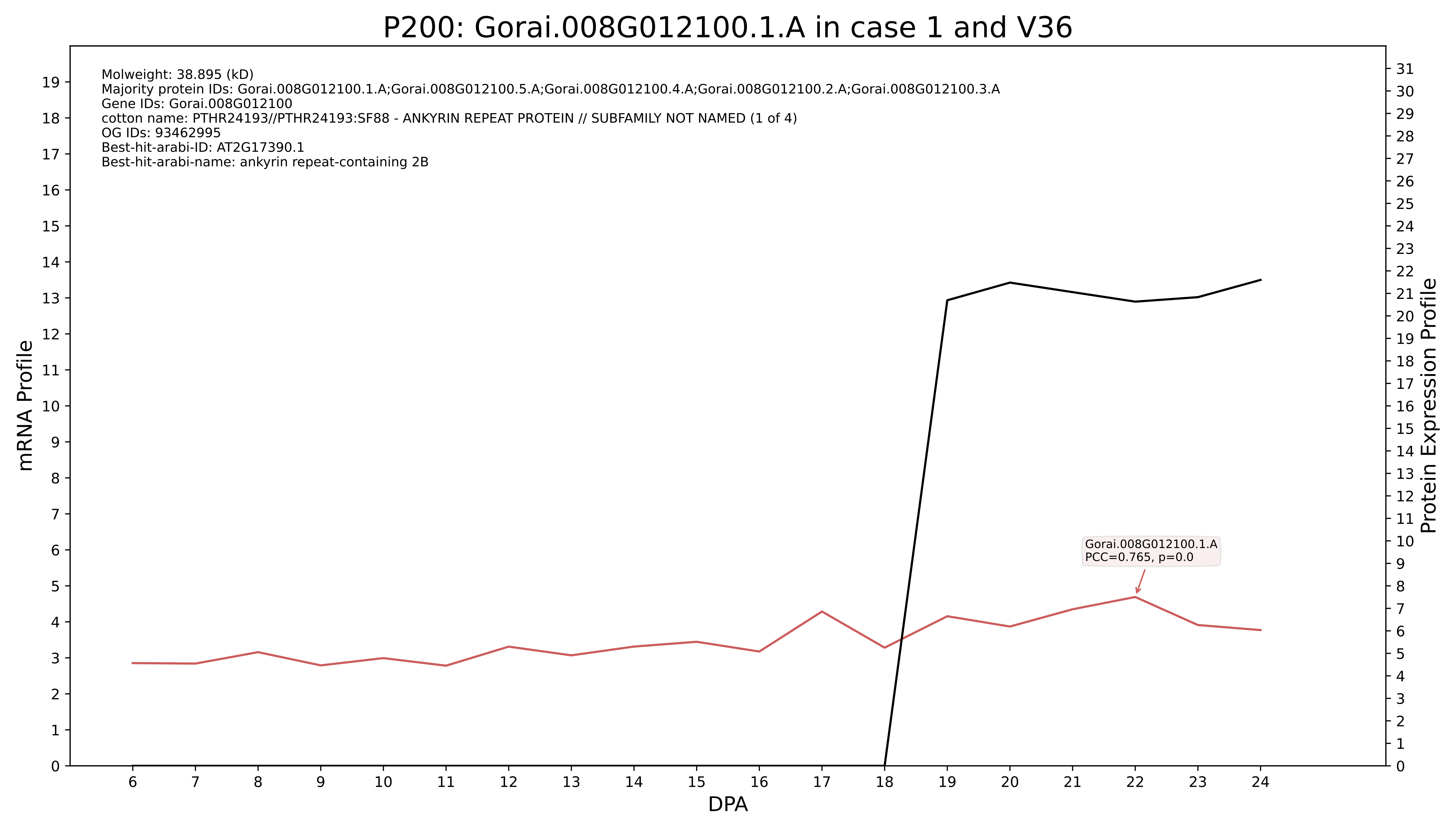
P200: Gorai.008G012100.1.A in case 1 and V36
Molweight: 38.895 (kD)
Majority protein IDs: Gorai.008G012100.1.A;Gorai.008G012100.5.A;Gorai.008G012100.4.A;Gorai.008G012100.2.A;Gorai.008G012100.3.A
Gene IDs: Gorai.008G012100
cotton name: PTHR24193//PTHR24193:SF88 - ANKYRIN REPEAT PROTEIN // SUBFAMILY NOT NAMED (1 of 4)
OG IDs: 93462995
Best-hit-arabi-ID: AT2G17390.1
Best-hit-arabi-name: ankyrin repeat-containing 2B
Gorai.008G012100.1.A
PCC=0.765, p=0.0
mRNA Profile
Protein Expression Profile
DPA

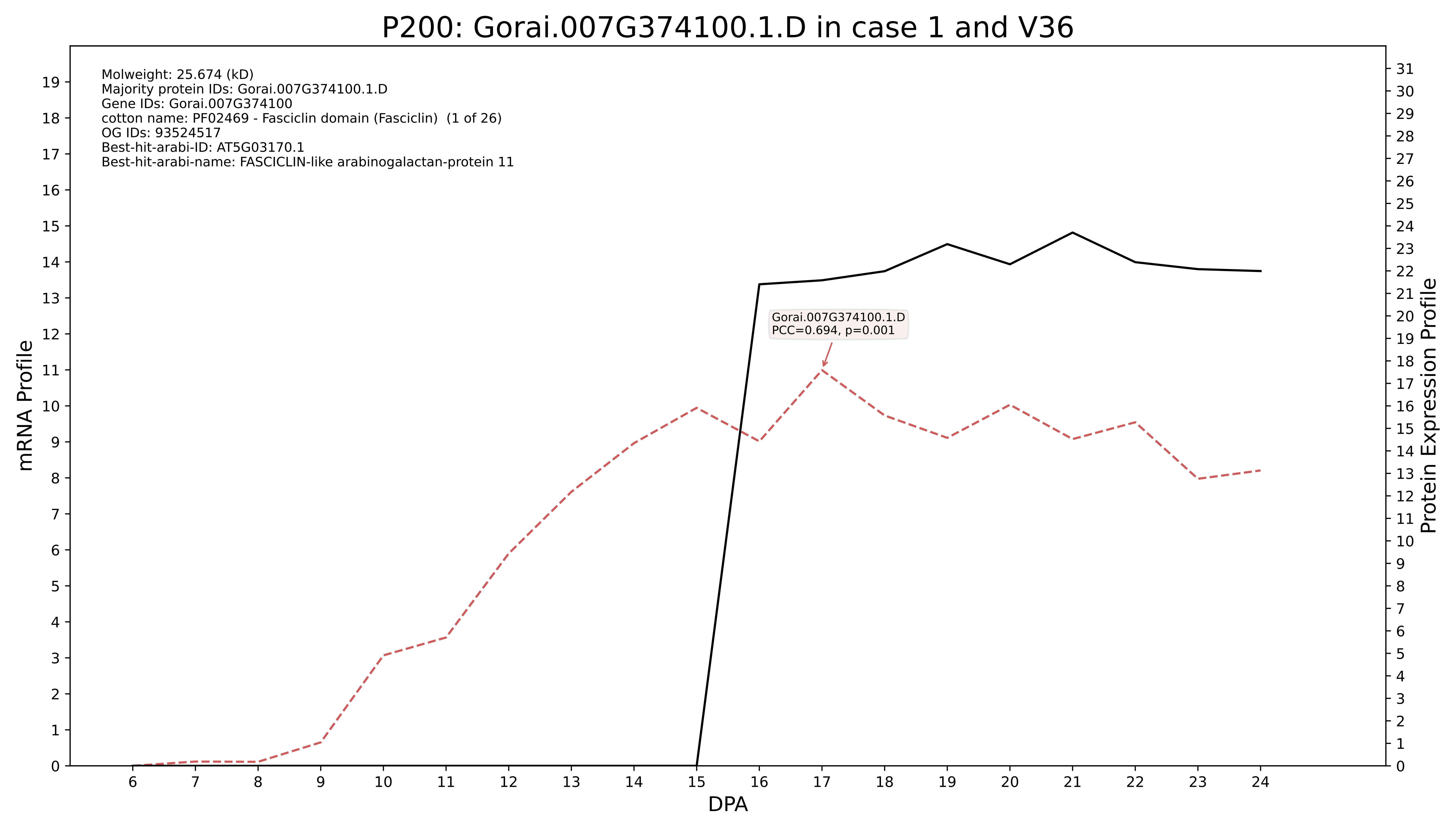

P200: Gorai.007G374100.1.D in case 1 and V36
Molweight: 25.674 (kD)
Majority protein IDs: Gorai.007G374100.1.D
Gene IDs: Gorai.007G374100
cotton name: PF02469 - Fasciclin domain (Fasciclin) (1 of 26)
OG IDs: 93524517
Best-hit-arabi-ID: AT5G03170.1
Best-hit-arabi-name: FASCICLIN-like arabinogalactan-protein 11
Gorai.007G374100.1.D
PCC=0.694, p=0.001
mRNA Profile
Protein Expression Profile
DPA

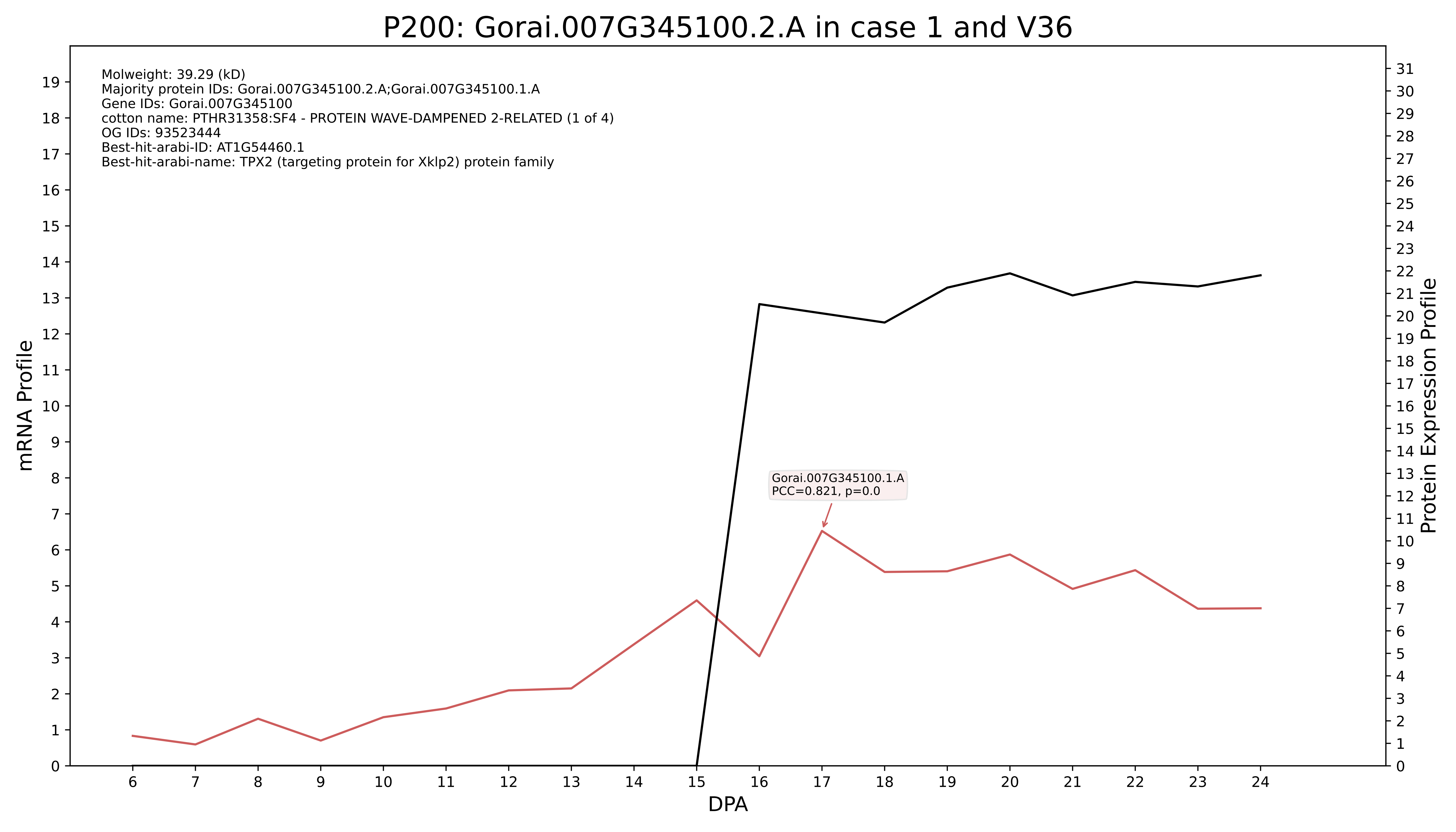
P200: Gorai.007G345100.2.A in case 1 and V36
Molweight: 39.29 (kD)
Majority protein IDs: Gorai.007G345100.2.A;Gorai.007G345100.1.A
Gene IDs: Gorai.007G345100
cotton name: PTHR31358:SF4 - PROTEIN WAVE-DAMPENED 2-RELATED (1 of 4)
OG IDs: 93523444
Best-hit-arabi-ID: AT1G54460.1
Best-hit-arabi-name: TPX2 (targeting protein for Xklp2) protein family
Gorai.007G345100.1.A
PCC=0.821, p=0.0
mRNA Profile
Protein Expression Profile
DPA

P200: Gorai.007G090100.1.D in case 1 and V36

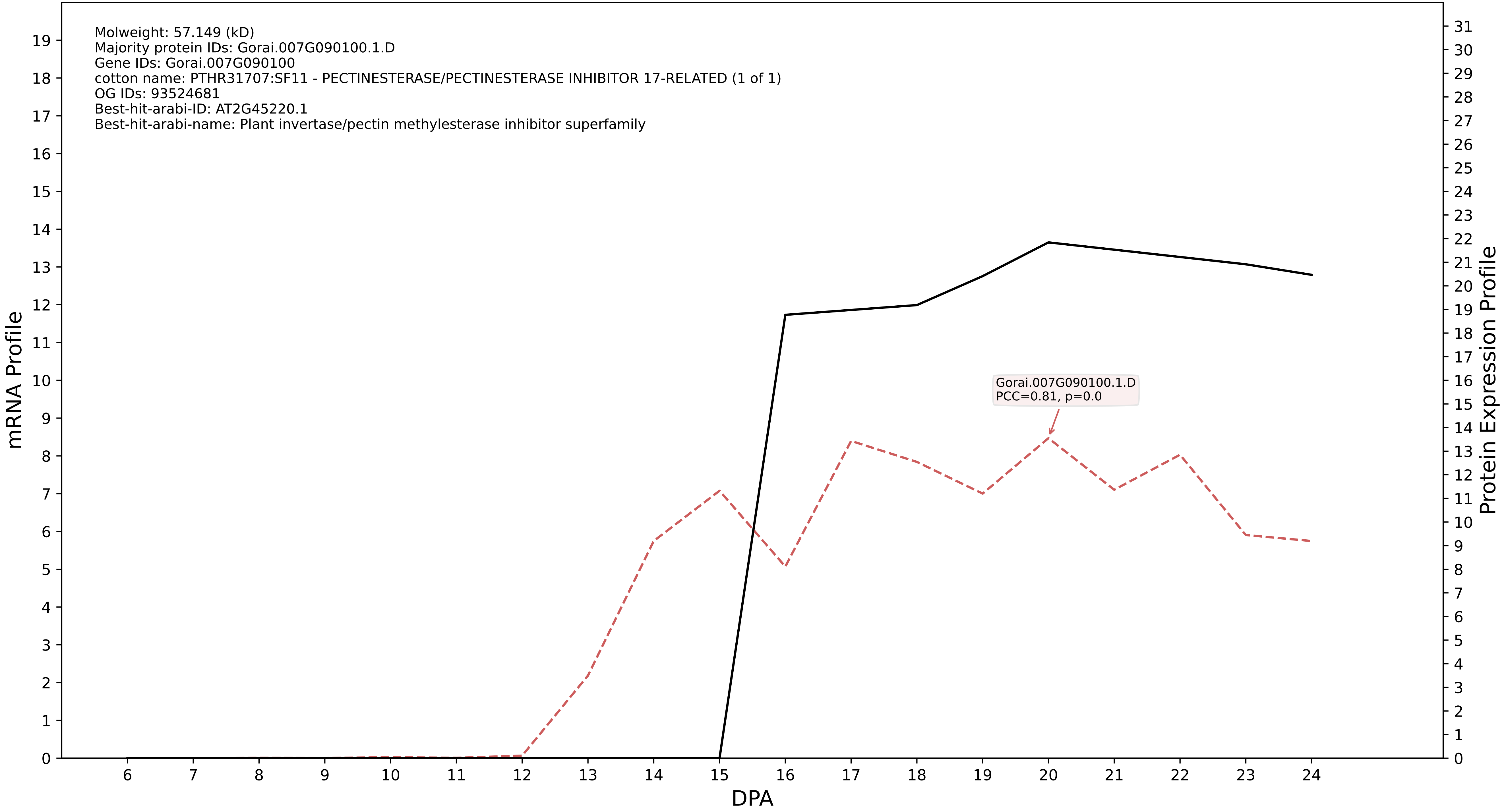

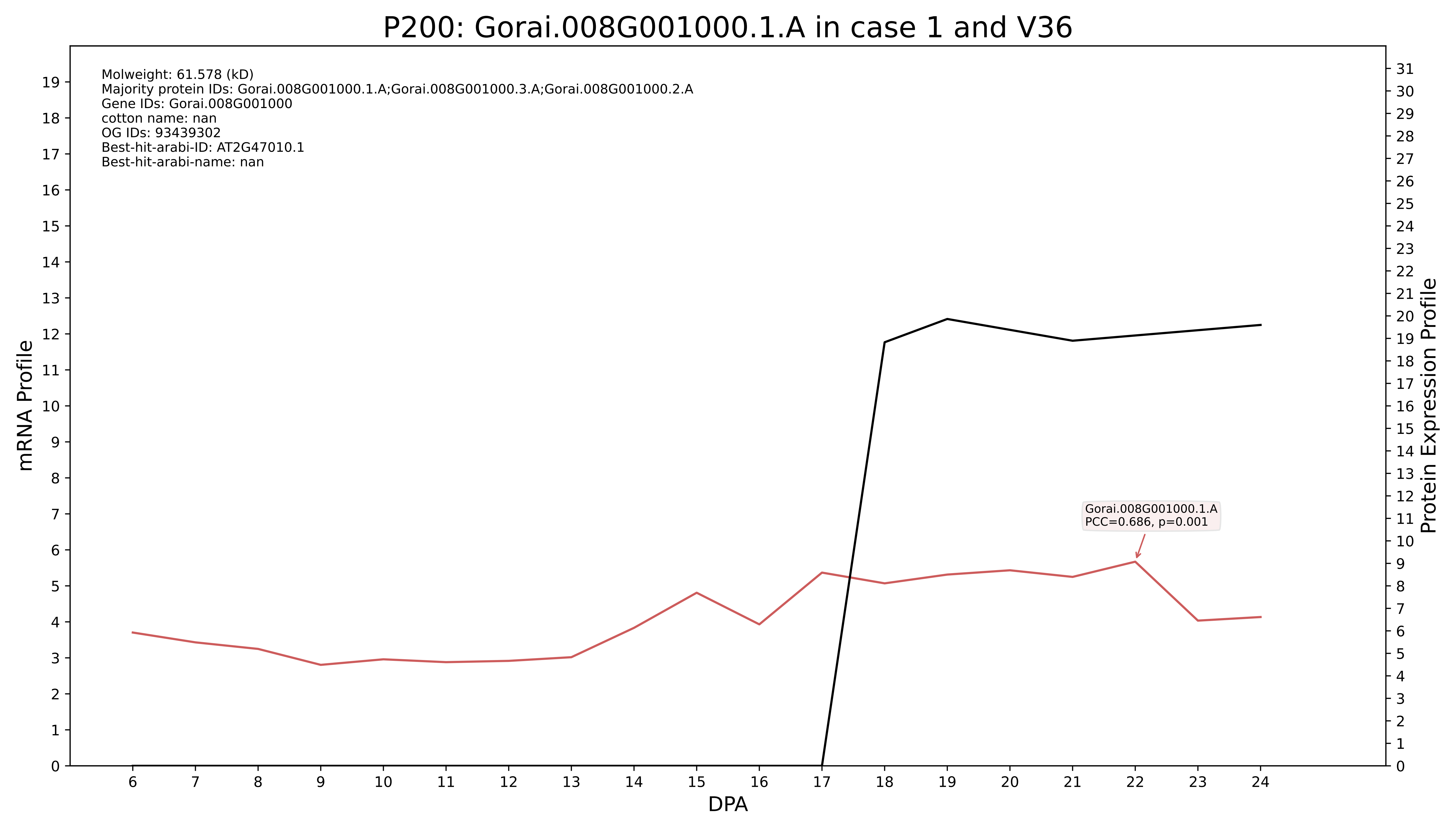
P200: Gorai.008G001000.1.A in case 1 and V36
Molweight: 61.578 (kD)
Majority protein IDs: Gorai.008G001000.1.A;Gorai.008G001000.3.A;Gorai.008G001000.2.A
Gene IDs: Gorai.008G001000
cotton name: nan
OG IDs: 93439302
Best-hit-arabi-ID: AT2G47010.1
Best-hit-arabi-name: nan
Gorai.008G001000.1.A
PCC=0.686, p=0.001
mRNA Profile
Protein Expression Profile
DPA

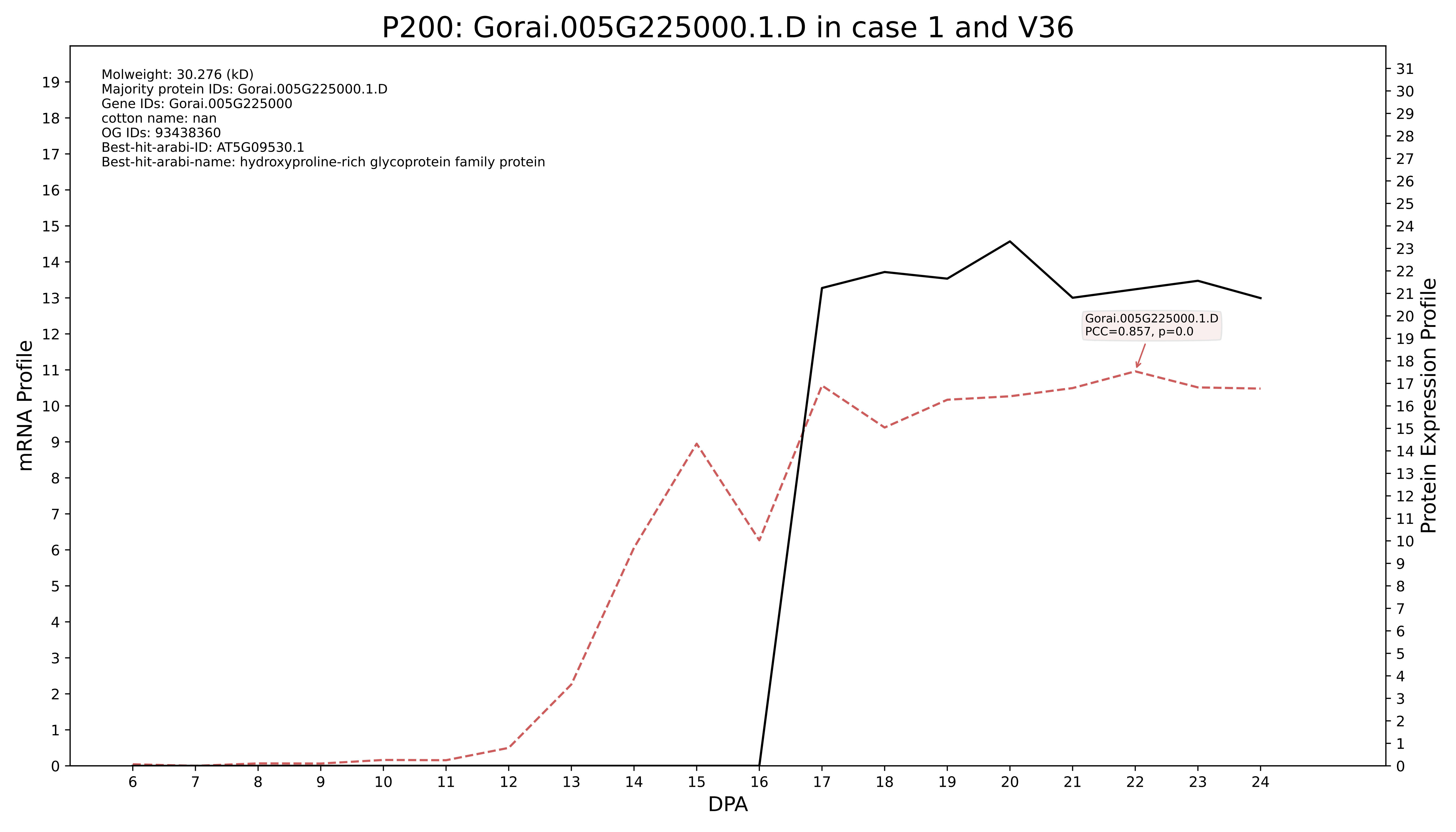
P200: Gorai.005G225000.1.D in case 1 and V36
Molweight: 30.276 (kD)
Majority protein IDs: Gorai.005G225000.1.D
Gene IDs: Gorai.005G225000
cotton name: nan
OG IDs: 93438360
Best-hit-arabi-ID: AT5G09530.1
Best-hit-arabi-name: hydroxyproline-rich glycoprotein family protein
Gorai.005G225000.1.D
PCC=0.857, p=0.0
mRNA Profile
Protein Expression Profile
DPA

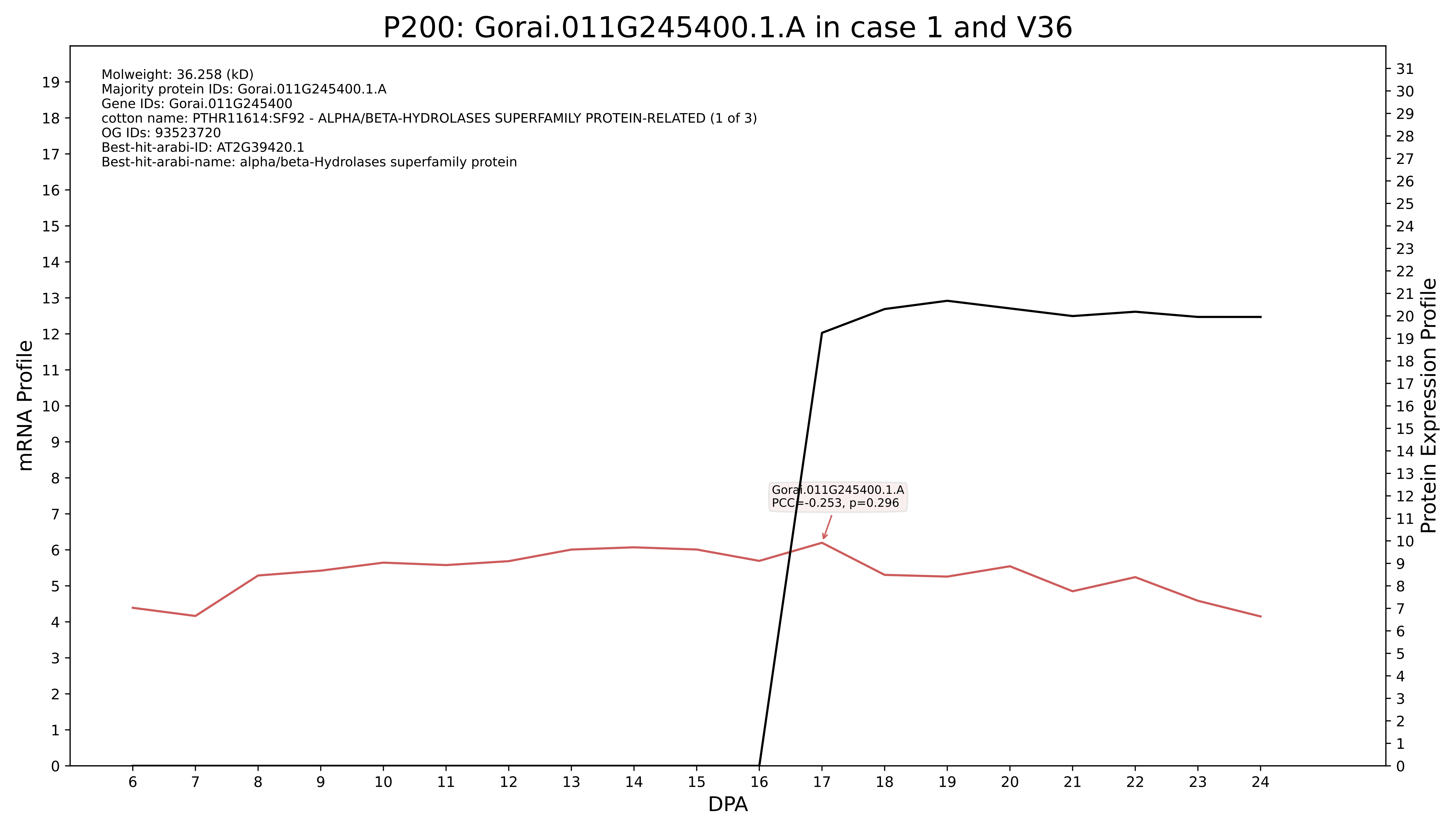
P200: Gorai.011G245400.1.A in case 1 and V36
Molweight: 36.258 (kD)
Majority protein IDs: Gorai.011G245400.1.A
Gene IDs: Gorai.011G245400
cotton name: PTHR11614:SF92 - ALPHA/BETA-HYDROLASES SUPERFAMILY PROTEIN-RELATED (1 of 3)
OG IDs: 93523720
Best-hit-arabi-ID: AT2G39420.1
Best-hit-arabi-name: alpha/beta-Hydrolases superfamily protein
Gorai.011G245400.1.A
PCC=-0.253, p=0.296
mRNA Profile
Protein Expression Profile
DPA
0
1
2
3
4
5
6
7
8
9
10
11
12
13
14
15
16
17
18
19
20
21
22
23
24
25
26
27
28
29
30
31

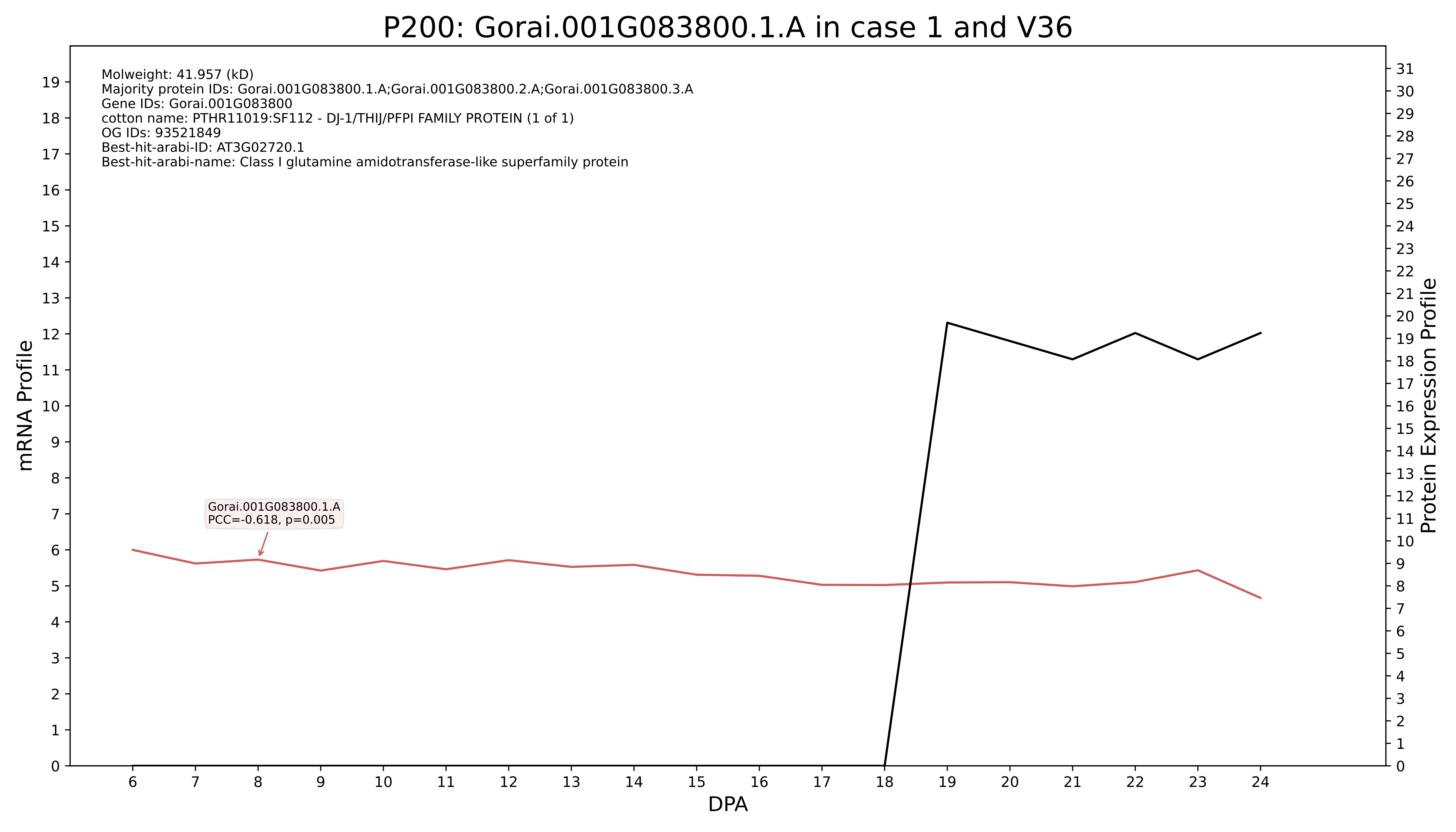

P200: Gorai.001G083800.1.A in case 1 and V36
Molweight: 41.957 (kD)
Majority protein IDs: Gorai.001G083800.1.A;Gorai.001G083800.2.A;Gorai.001G083800.3.A
Gene IDs: Gorai.001G083800
cotton name: PTHR11019:SF112 - DJ-1/THIJ/PFPI FAMILY PROTEIN (1 of 1)
OG IDs: 93521849
Best-hit-arabi-ID: AT3G02720.1
Best-hit-arabi-name: Class I glutamine amidotransferase-like superfamily protein
Gorai.001G083800.1.A
PCC=-0.618, p=0.005
mRNA Profile
Protein Expression Profile
DPA

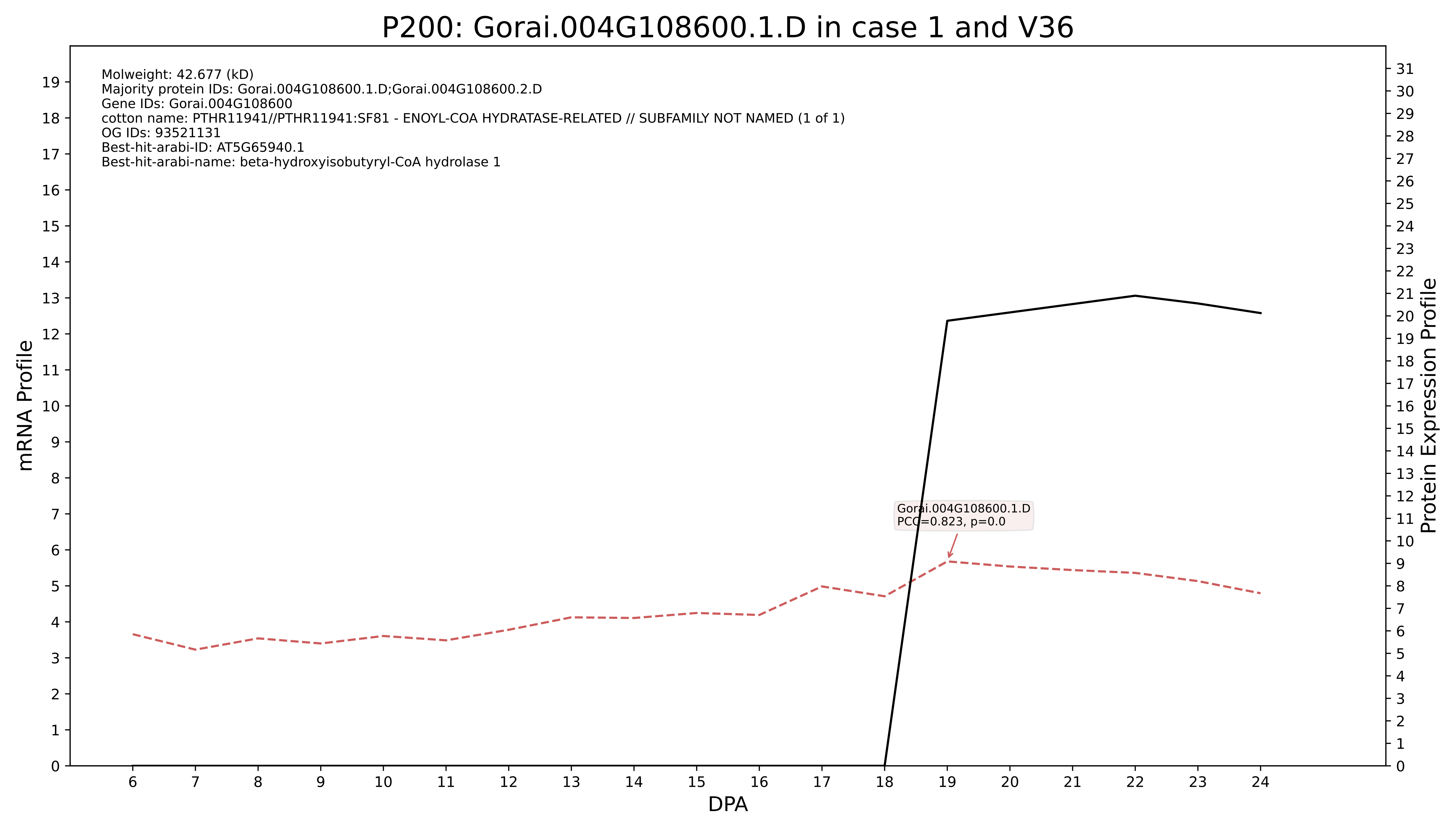
P200: Gorai.004G108600.1.D in case 1 and V36
Molweight: 42.677 (kD)
Majority protein IDs: Gorai.004G108600.1.D;Gorai.004G108600.2.D
Gene IDs: Gorai.004G108600
cotton name: PTHR11941//PTHR11941:SF81 - ENOYL-COA HYDRATASE-RELATED // SUBFAMILY NOT NAMED (1 of 1)
OG IDs: 93521131
Best-hit-arabi-ID: AT5G65940.1
Best-hit-arabi-name: beta-hydroxyisobutyryl-CoA hydrolase 1
Gorai.004G108600.1.D
PCC=0.823, p=0.0
mRNA Profile
Protein Expression Profile
DPA

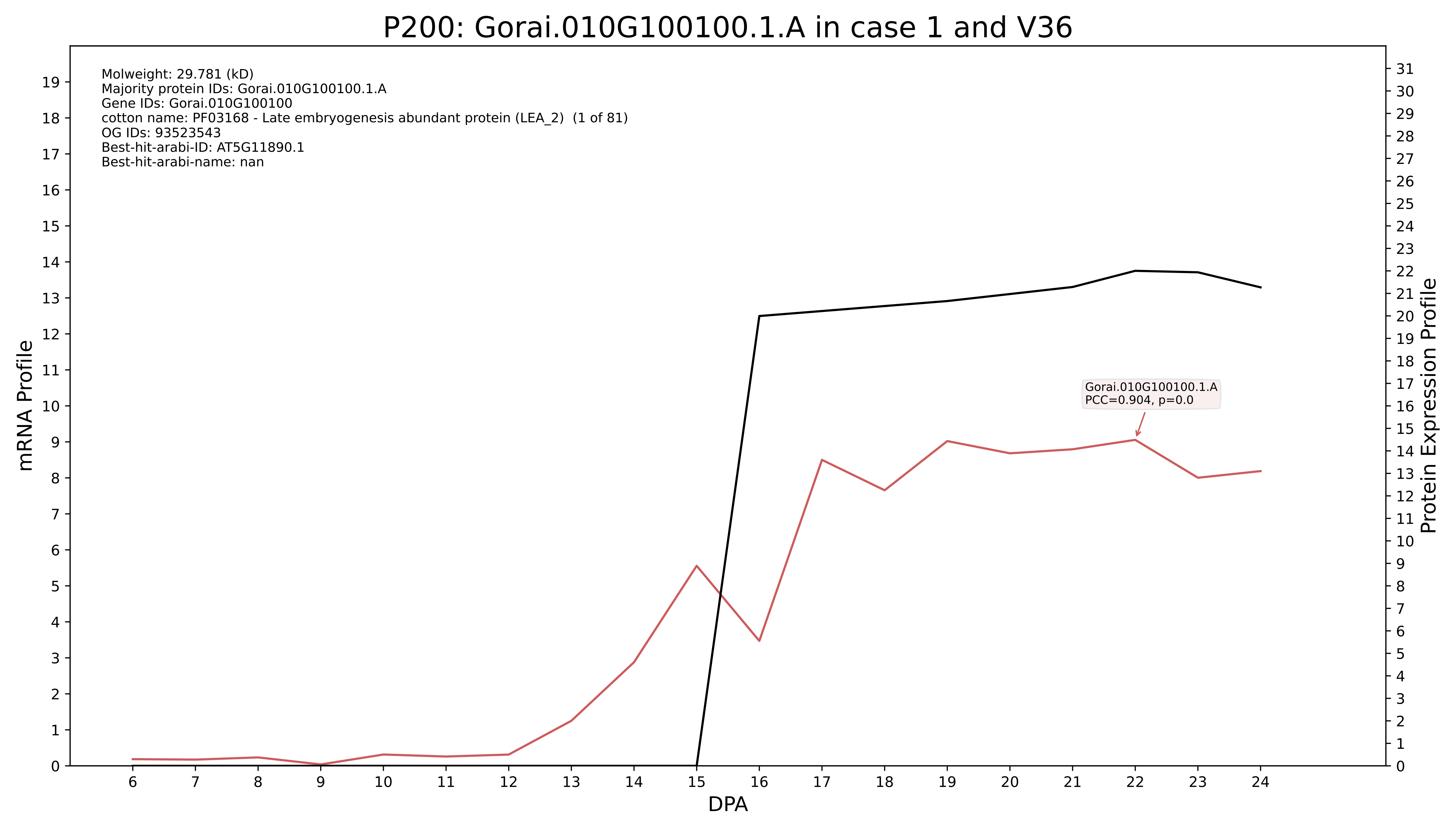
P200: Gorai.010G100100.1.A in case 1 and V36
Molweight: 29.781 (kD)
Majority protein IDs: Gorai.010G100100.1.A
Gene IDs: Gorai.010G100100
cotton name: PF03168 - Late embryogenesis abundant protein (LEA_2) (1 of 81)
OG IDs: 93523543
Best-hit-arabi-ID: AT5G11890.1
Best-hit-arabi-name: nan
Gorai.010G100100.1.A
PCC=0.904, p=0.0
mRNA Profile
Protein Expression Profile
DPA

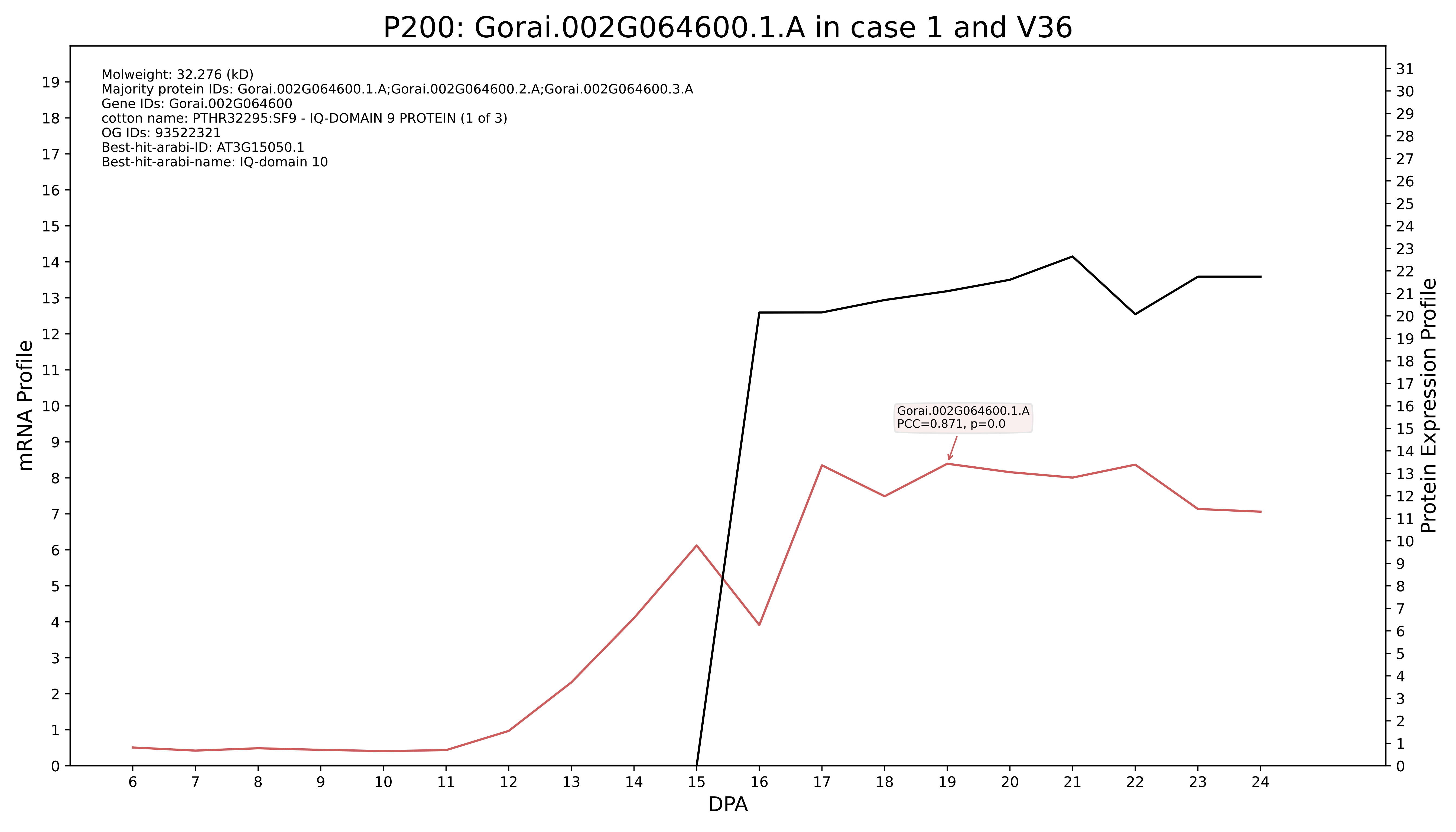
P200: Gorai.002G064600.1.A in case 1 and V36
Molweight: 32.276 (kD)
Majority protein IDs: Gorai.002G064600.1.A;Gorai.002G064600.2.A;Gorai.002G064600.3.A
Gene IDs: Gorai.002G064600
cotton name: PTHR32295:SF9 - IQ-DOMAIN 9 PROTEIN (1 of 3)
OG IDs: 93522321
Best-hit-arabi-ID: AT3G15050.1
Best-hit-arabi-name: IQ-domain 10
Gorai.002G064600.1.A
PCC=0.871, p=0.0
mRNA Profile
Protein Expression Profile
DPA

P200: Gorai.006G046000.1.D in case 1 and V36

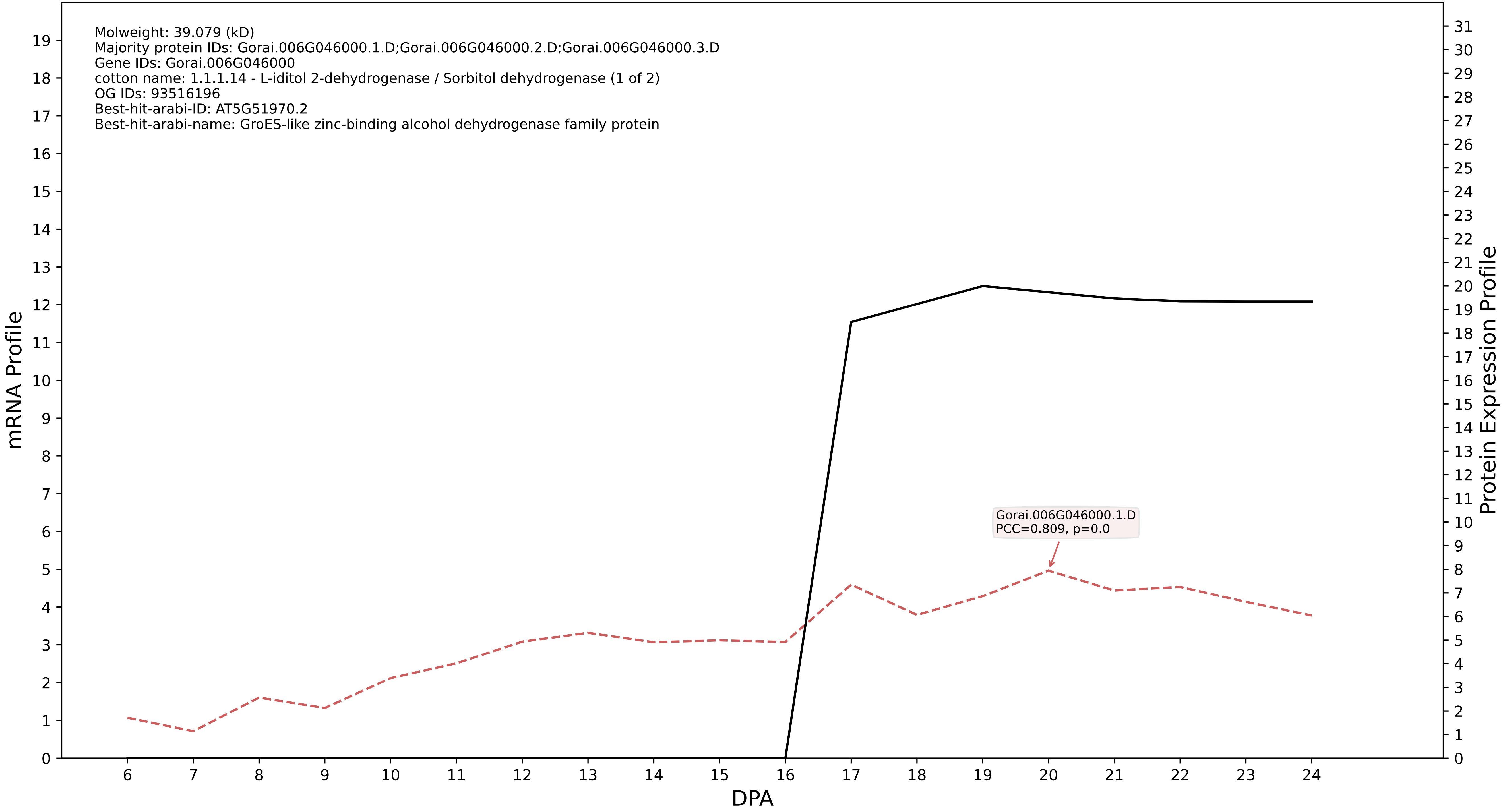

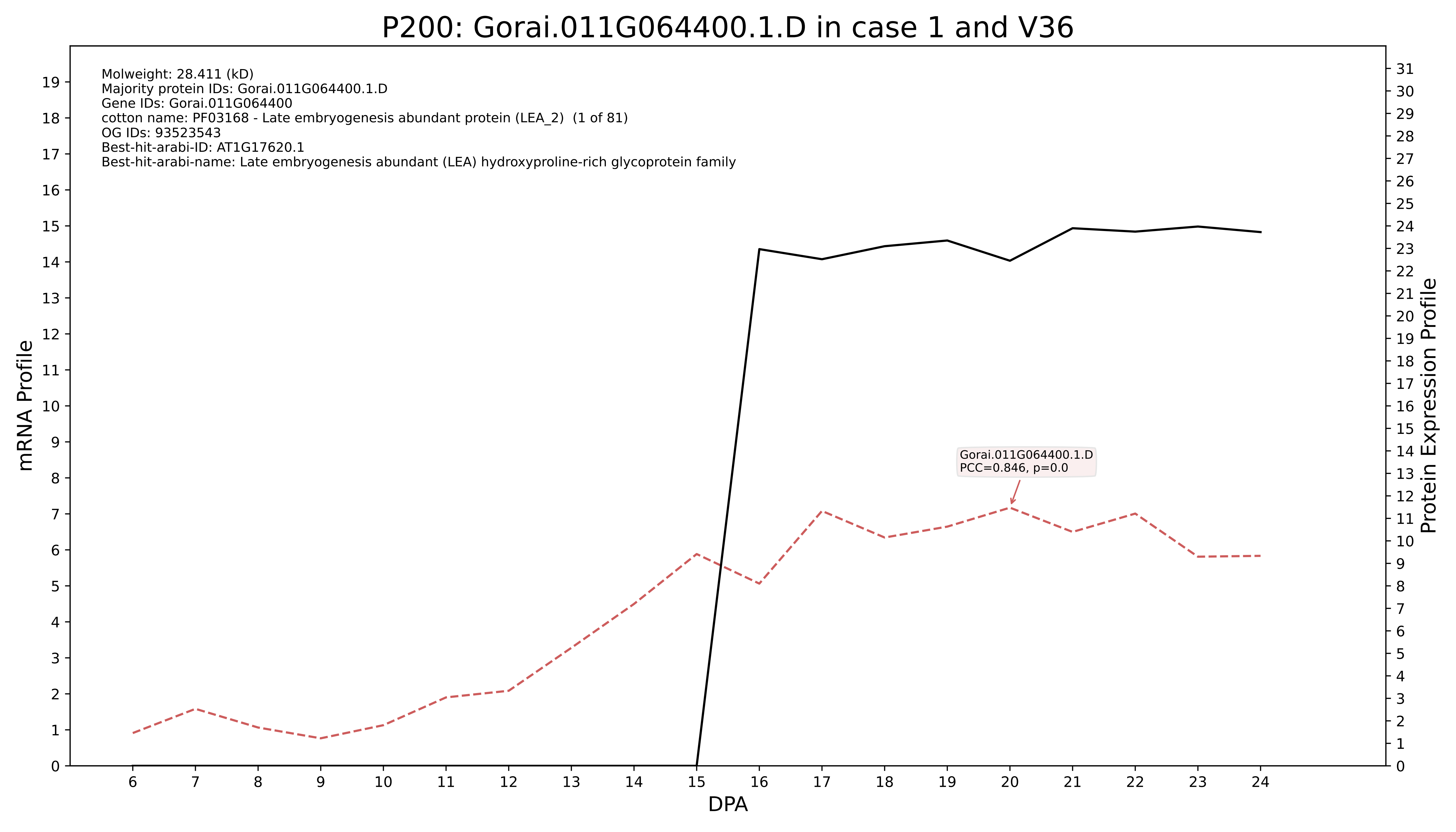

P200: Gorai.011G064400.1.D in case 1 and V36
Molweight: 28.411 (kD)
Majority protein IDs: Gorai.011G064400.1.D
Gene IDs: Gorai.011G064400
cotton name: PF03168 - Late embryogenesis abundant protein (LEA_2) (1 of 81)
OG IDs: 93523543
Best-hit-arabi-ID: AT1G17620.1
Best-hit-arabi-name: Late embryogenesis abundant (LEA) hydroxyproline-rich glycoprotein family
Gorai.011G064400.1.D
PCC=0.846, p=0.0
mRNA Profile
Protein Expression Profile
DPA

P200: Gorai.004G052900.1.A in case 1 and V36

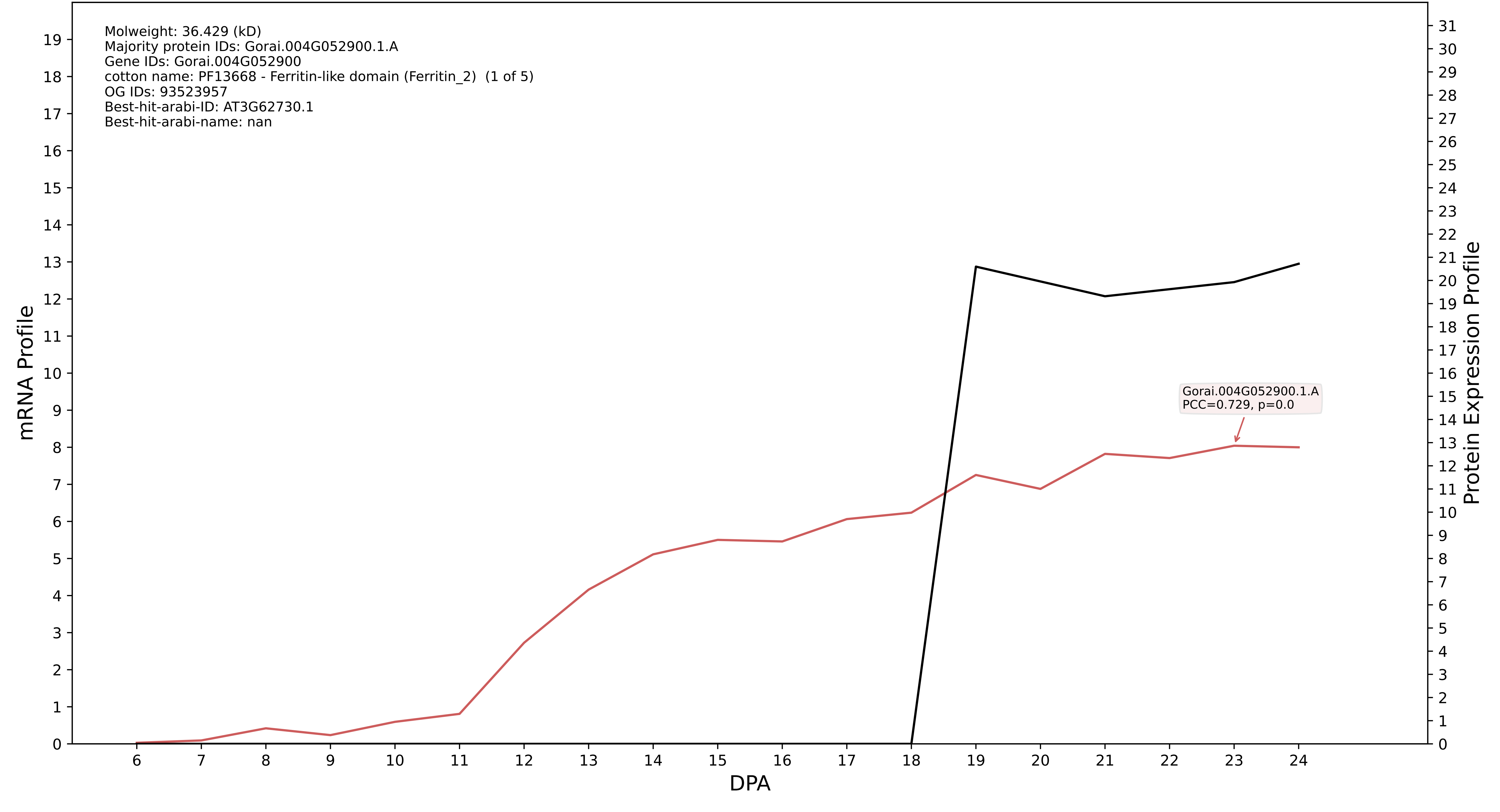

P200: Gorai.007G090100.1.A in case 1 and V36

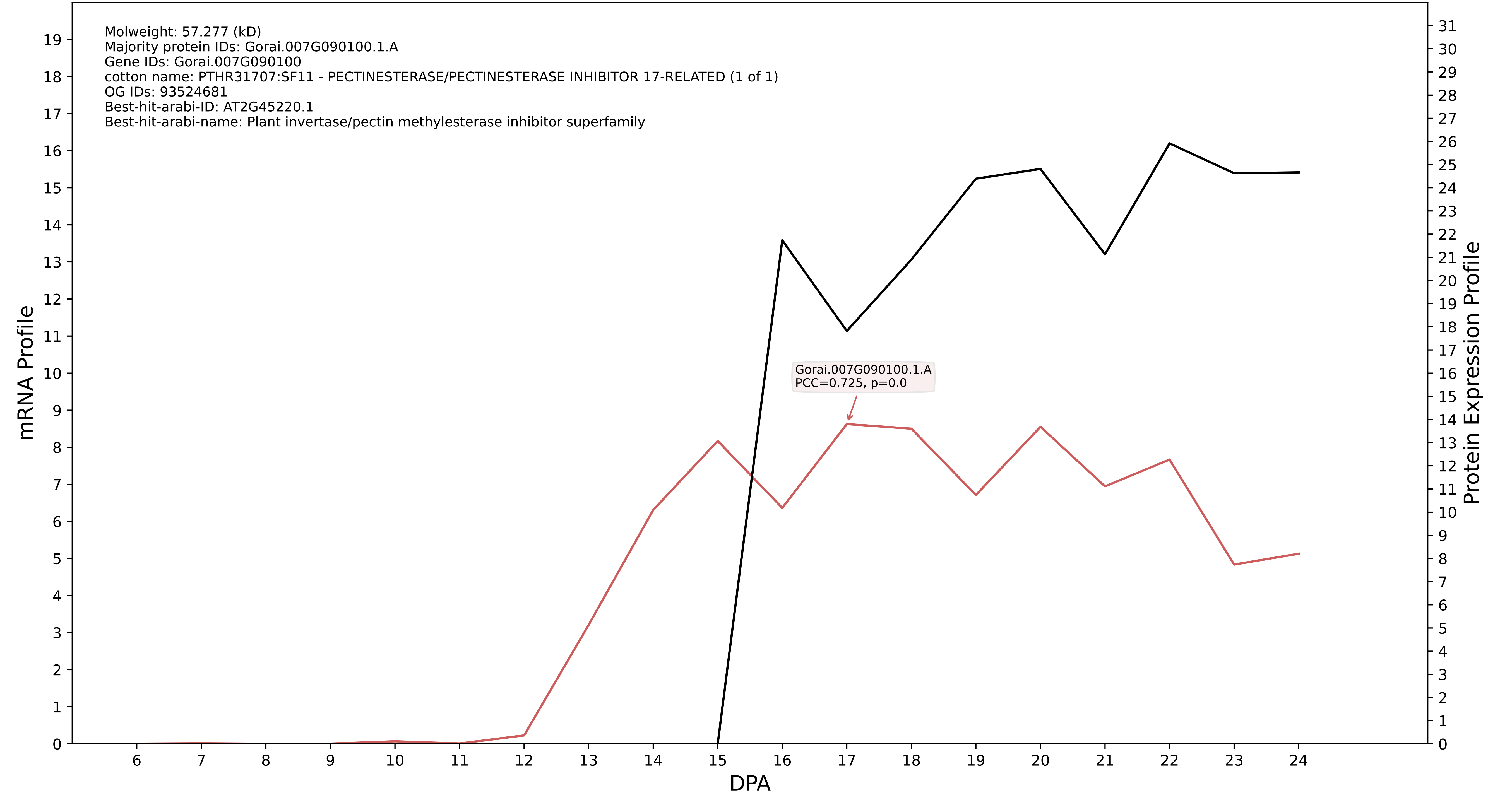

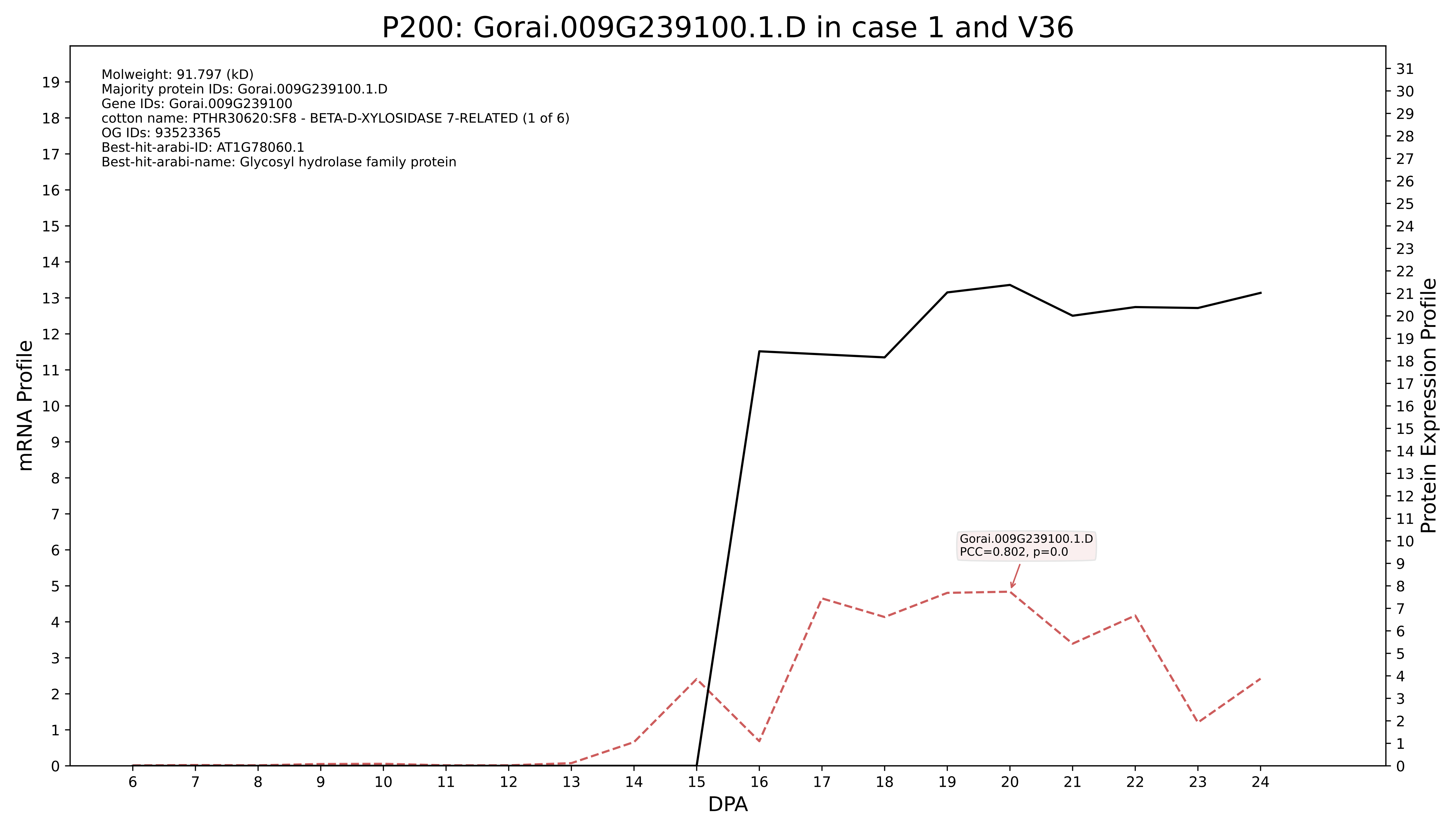
P200: Gorai.009G239100.1.D in case 1 and V36
Molweight: 91.797 (kD)
Majority protein IDs: Gorai.009G239100.1.D
Gene IDs: Gorai.009G239100
cotton name: PTHR30620:SF8 - BETA-D-XYLOSIDASE 7-RELATED (1 of 6)
OG IDs: 93523365
Best-hit-arabi-ID: AT1G78060.1
Best-hit-arabi-name: Glycosyl hydrolase family protein
Gorai.009G239100.1.D
PCC=0.802, p=0.0
mRNA Profile
Protein Expression Profile
DPA
0
1
2
3
4
5
6
7
8
9
10
11
12
13
14
15
16
17
18
19
20
21
22
23
24
25
26
27
28
29
30
31

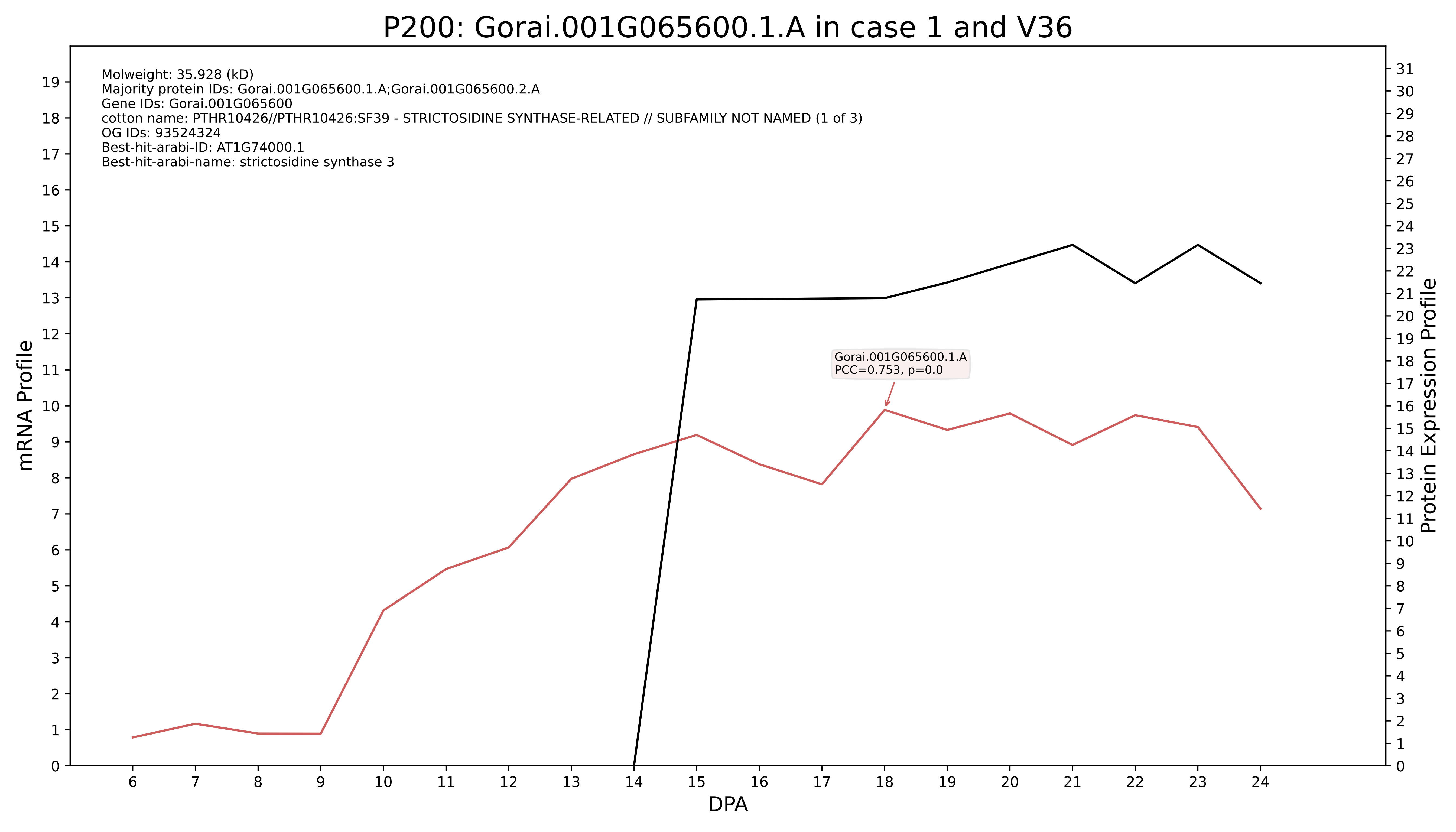
P200: Gorai.001G065600.1.A in case 1 and V36
Molweight: 35.928 (kD)
Majority protein IDs: Gorai.001G065600.1.A;Gorai.001G065600.2.A
Gene IDs: Gorai.001G065600
cotton name: PTHR10426//PTHR10426:SF39 - STRICTOSIDINE SYNTHASE-RELATED // SUBFAMILY NOT NAMED (1 of 3)
OG IDs: 93524324
Best-hit-arabi-ID: AT1G74000.1
Best-hit-arabi-name: strictosidine synthase 3
Gorai.001G065600.1.A
PCC=0.753, p=0.0
mRNA Profile
Protein Expression Profile
DPA
0
1
2
3
4
5
6
7
8
9
10
11
12
13
14
15
16
17
18
19
20
21
22
23
24
25
26
27
28
29
30
31

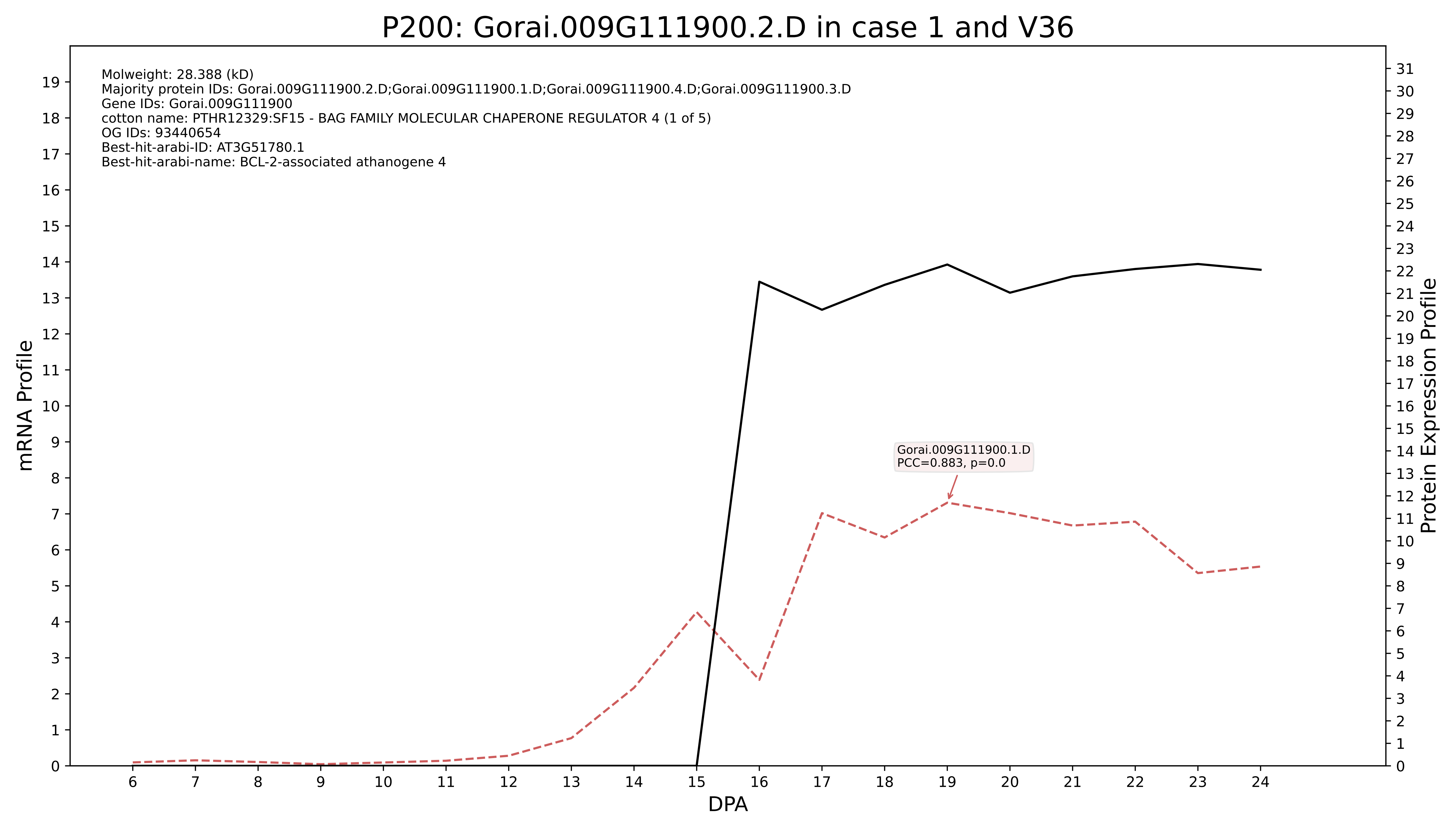
P200: Gorai.009G111900.2.D in case 1 and V36
Molweight: 28.388 (kD)
Majority protein IDs: Gorai.009G111900.2.D;Gorai.009G111900.1.D;Gorai.009G111900.4.D;Gorai.009G111900.3.D
Gene IDs: Gorai.009G111900
cotton name: PTHR12329:SF15 - BAG FAMILY MOLECULAR CHAPERONE REGULATOR 4 (1 of 5)
OG IDs: 93440654
Best-hit-arabi-ID: AT3G51780.1
Best-hit-arabi-name: BCL-2-associated athanogene 4
Gorai.009G111900.1.D
PCC=0.883, p=0.0
mRNA Profile
Protein Expression Profile
DPA

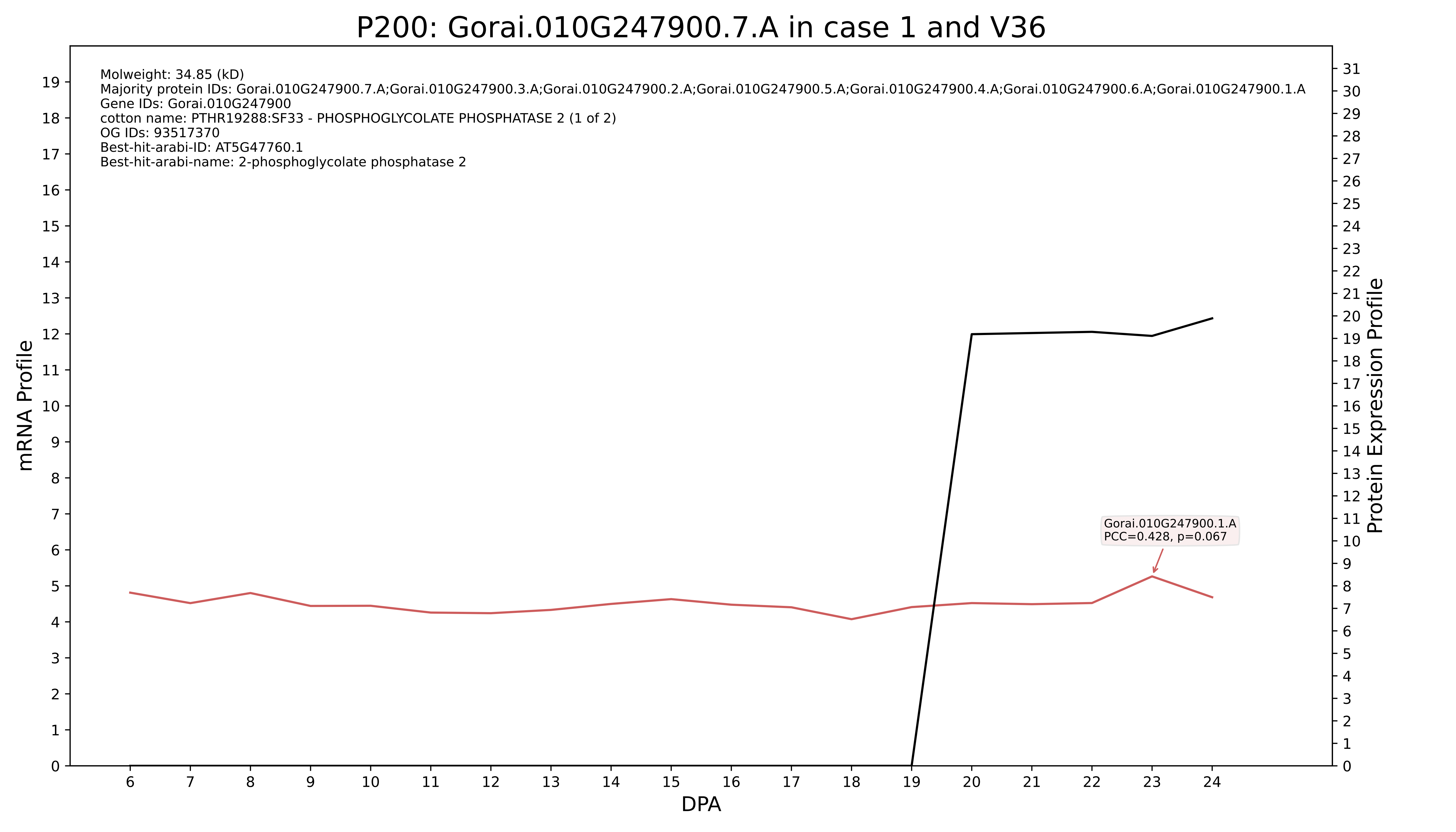

P200: Gorai.010G247900.7.A in case 1 and V36
Molweight: 34.85 (kD)
Majority protein IDs: Gorai.010G247900.7.A;Gorai.010G247900.3.A;Gorai.010G247900.2.A;Gorai.010G247900.5.A;Gorai.010G247900.4.A;Gorai.010G247900.6.A;Gorai.010G247900.1.A
Gene IDs: Gorai.010G247900
cotton name: PTHR19288:SF33 - PHOSPHOGLYCOLATE PHOSPHATASE 2 (1 of 2)
OG IDs: 93517370
Best-hit-arabi-ID: AT5G47760.1
Best-hit-arabi-name: 2-phosphoglycolate phosphatase 2
Gorai.010G247900.1.A
PCC=0.428, p=0.067
mRNA Profile
Protein Expression Profile
DPA

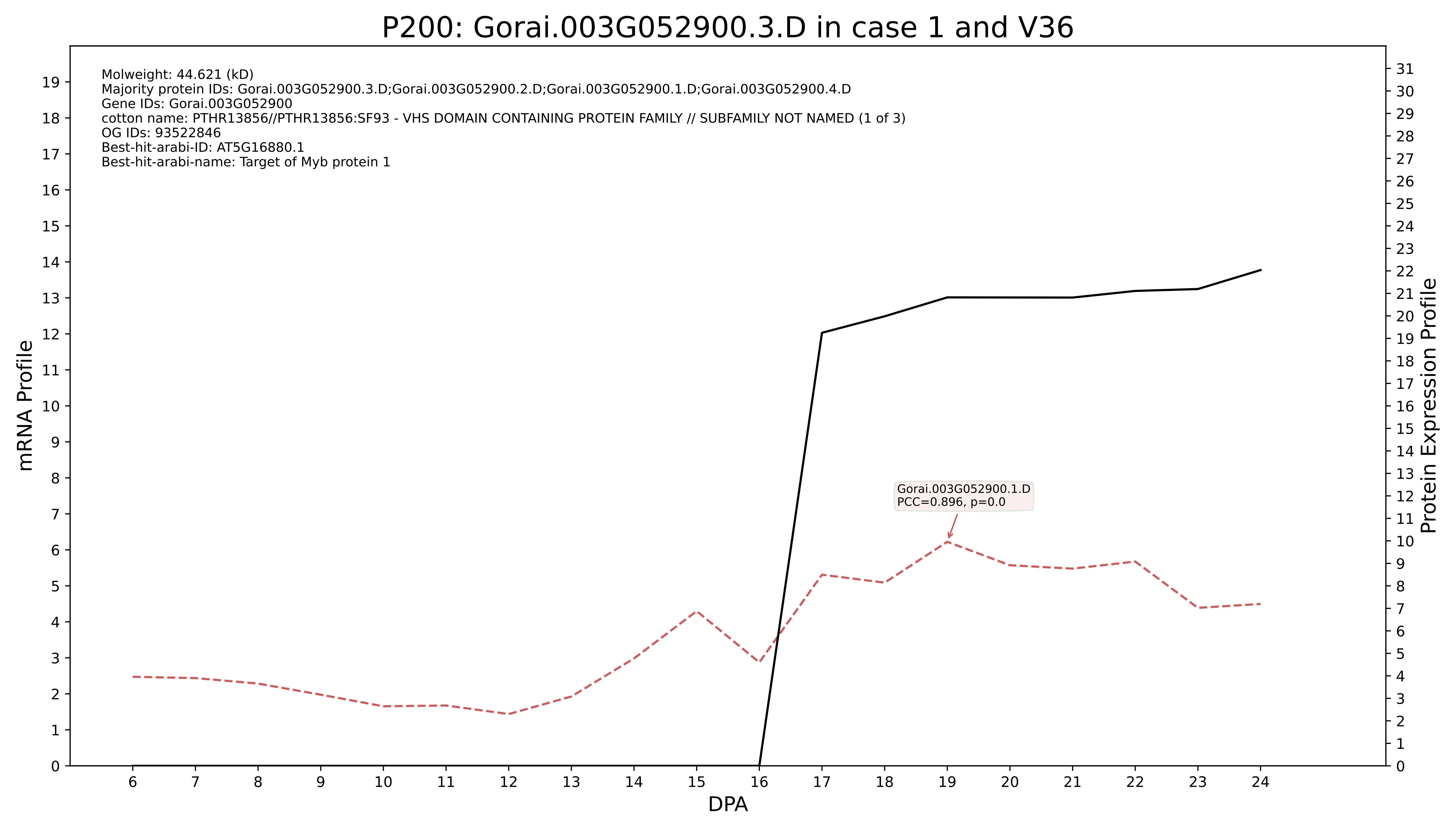

P200: Gorai.003G052900.3.D in case 1 and V36
Molweight: 44.621 (kD)
Majority protein IDs: Gorai.003G052900.3.D;Gorai.003G052900.2.D;Gorai.003G052900.1.D;Gorai.003G052900.4.D
Gene IDs: Gorai.003G052900
cotton name: PTHR13856//PTHR13856:SF93 - VHS DOMAIN CONTAINING PROTEIN FAMILY // SUBFAMILY NOT NAMED (1 of 3)
OG IDs: 93522846
Best-hit-arabi-ID: AT5G16880.1
Best-hit-arabi-name: Target of Myb protein 1
Gorai.003G052900.1.D
PCC=0.896, p=0.0
mRNA Profile
Protein Expression Profile
DPA

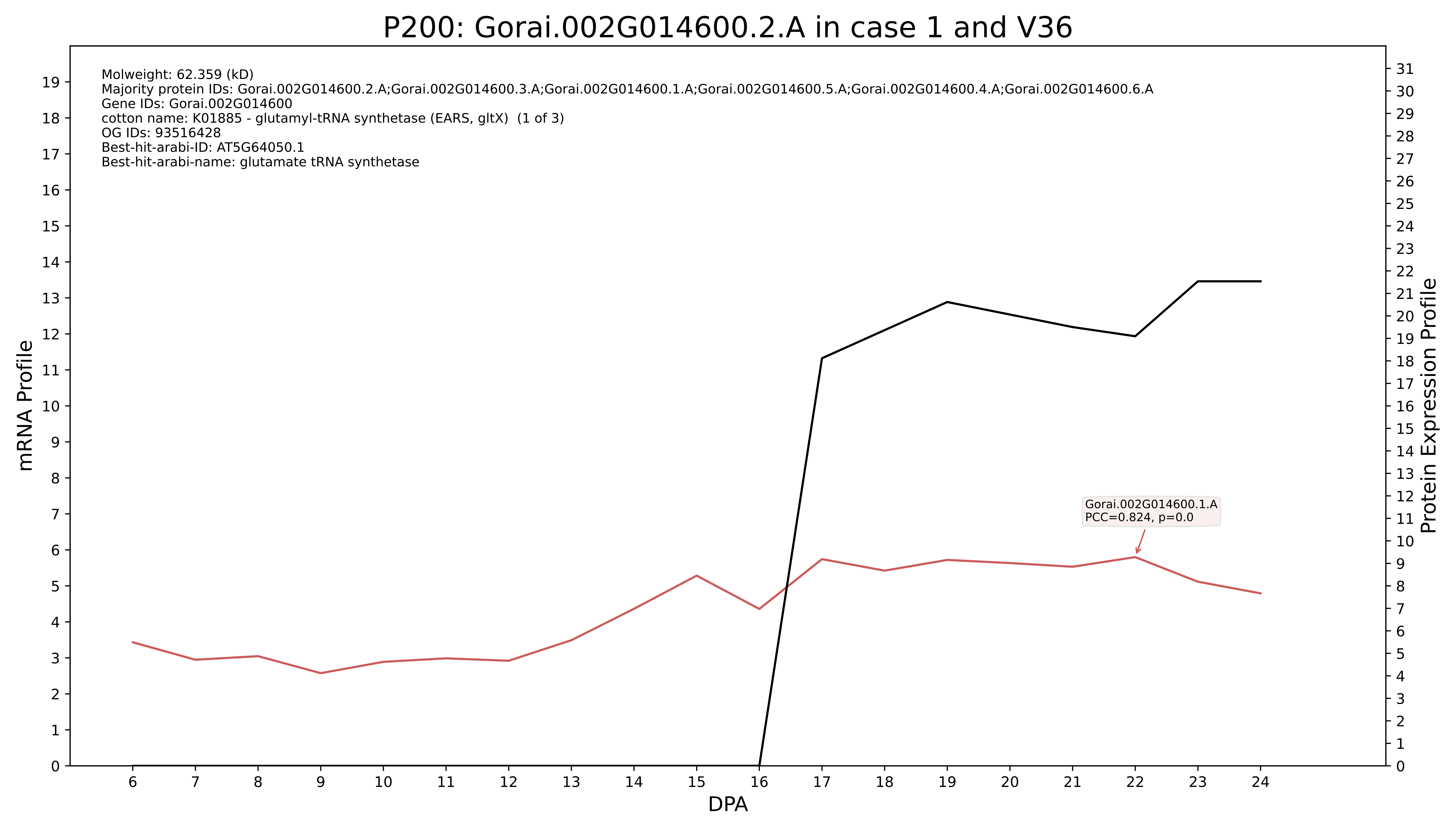
P200: Gorai.002G014600.2.A in case 1 and V36
Molweight: 62.359 (kD)
Majority protein IDs: Gorai.002G014600.2.A;Gorai.002G014600.3.A;Gorai.002G014600.1.A;Gorai.002G014600.5.A;Gorai.002G014600.4.A;Gorai.002G014600.6.A
Gene IDs: Gorai.002G014600
cotton name: K01885 - glutamyl-tRNA synthetase (EARS, gltX) (1 of 3)
OG IDs: 93516428
Best-hit-arabi-ID: AT5G64050.1
Best-hit-arabi-name: glutamate tRNA synthetase
Gorai.002G014600.1.A
PCC=0.824, p=0.0
mRNA Profile
Protein Expression Profile
DPA
0
1
2
3
4
5
6
7
8
9
10
11
12
13
14
15
16
17
18
19
20
21
22
23
24
25
26
27
28
29
30
31

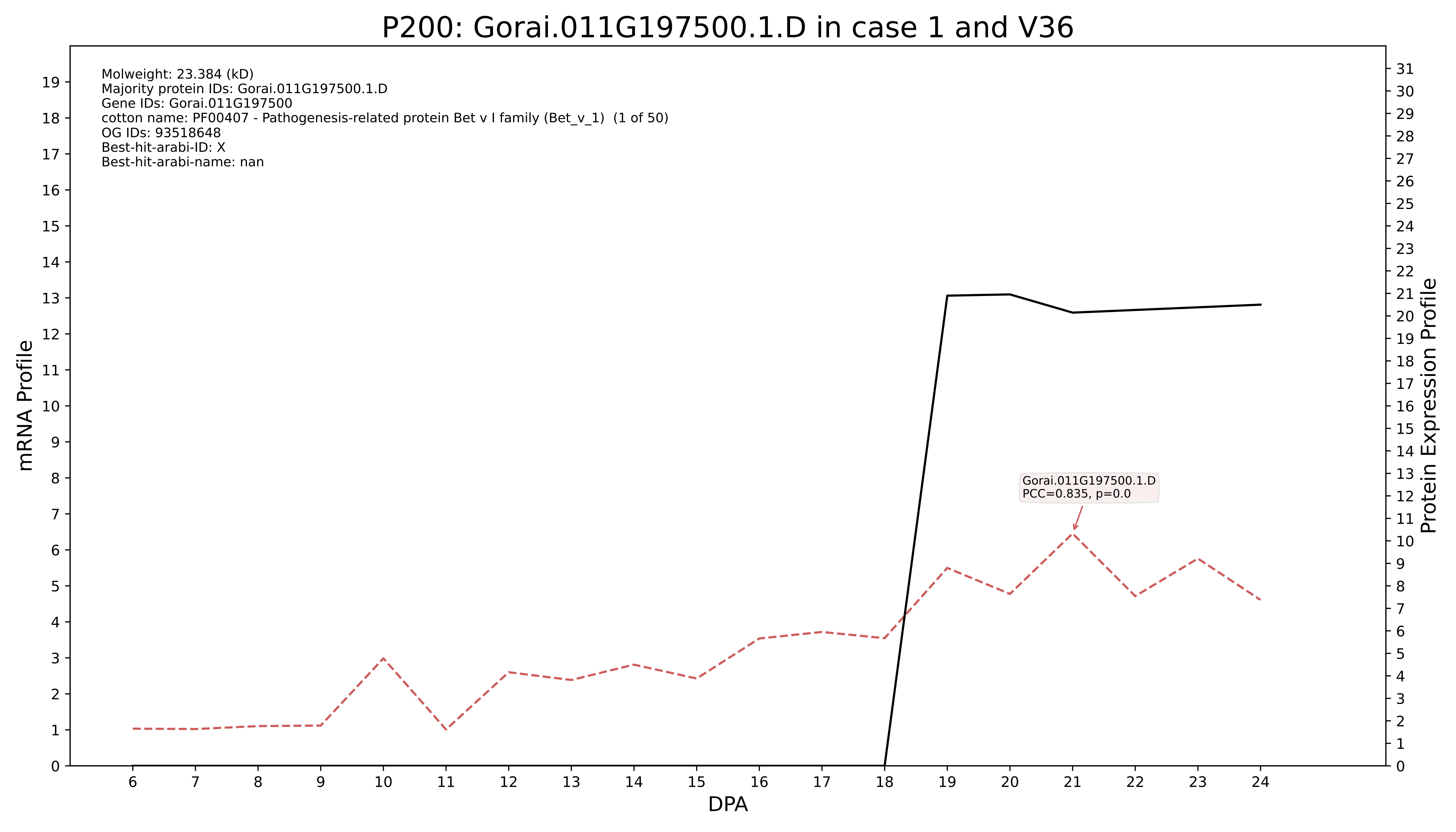

P200: Gorai.011G197500.1.D in case 1 and V36
Molweight: 23.384 (kD)
Majority protein IDs: Gorai.011G197500.1.D
Gene IDs: Gorai.011G197500
cotton name: PF00407 - Pathogenesis-related protein Bet v I family (Bet_v_1) (1 of 50)
OG IDs: 93518648
Best-hit-arabi-ID: X
Best-hit-arabi-name: nan
Gorai.011G197500.1.D
PCC=0.835, p=0.0
mRNA Profile
Protein Expression Profile
DPA

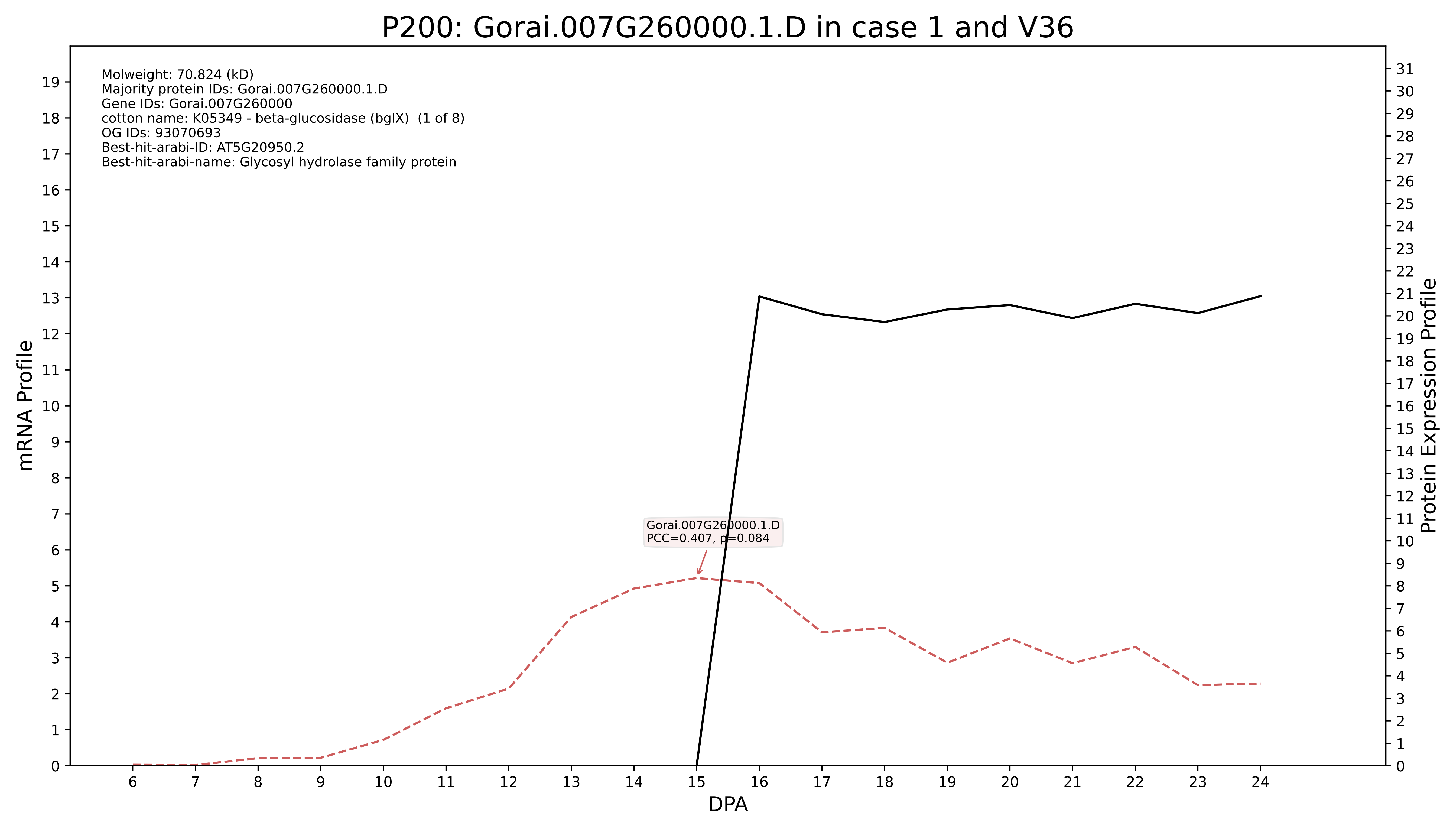
P200: Gorai.007G260000.1.D in case 1 and V36
Molweight: 70.824 (kD)
Majority protein IDs: Gorai.007G260000.1.D
Gene IDs: Gorai.007G260000
cotton name: K05349 - beta-glucosidase (bglX) (1 of 8)
OG IDs: 93070693
Best-hit-arabi-ID: AT5G20950.2
Best-hit-arabi-name: Glycosyl hydrolase family protein
Gorai.007G260000.1.D
PCC=0.407, p=0.084
mRNA Profile
Protein Expression Profile
DPA

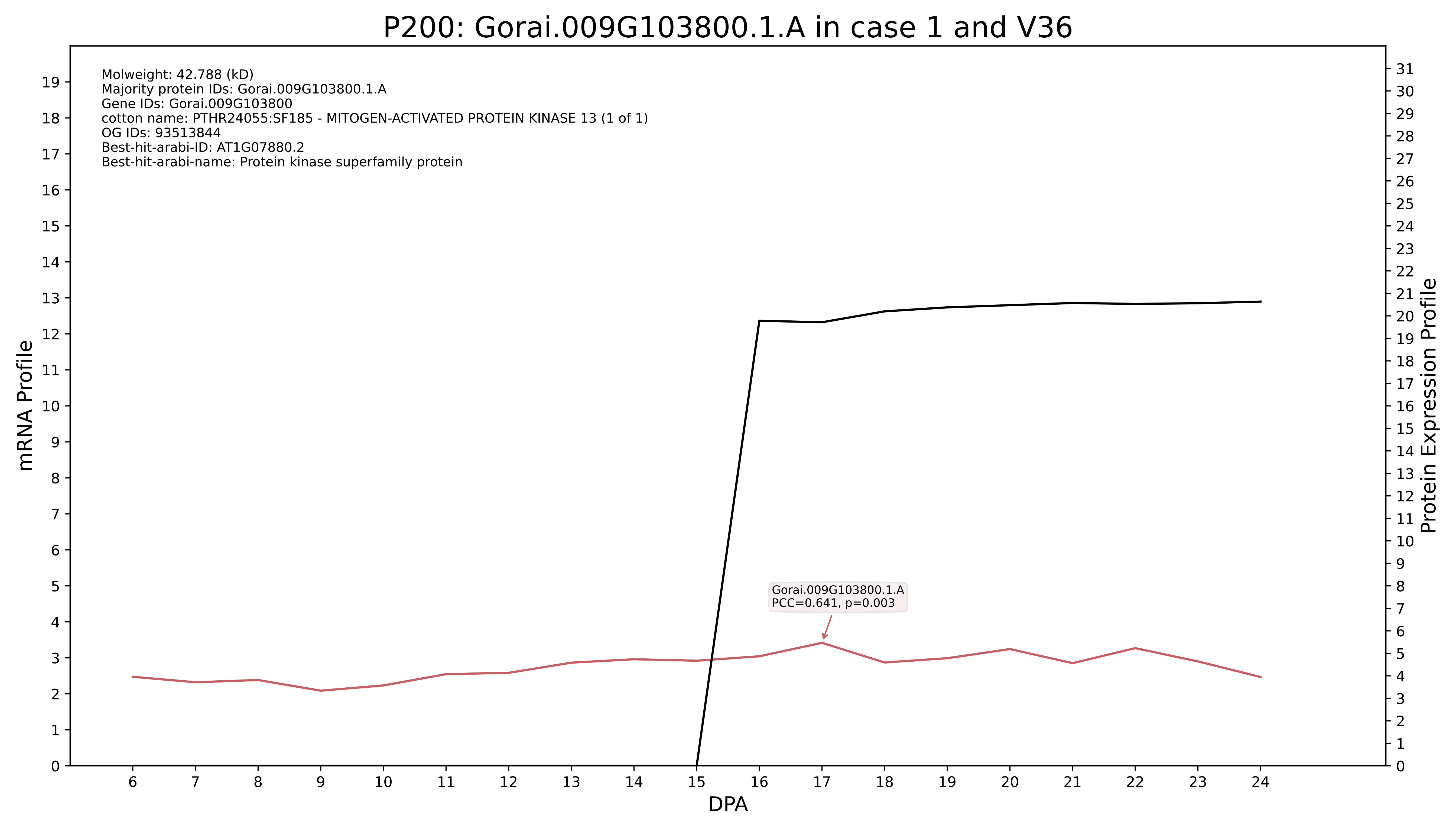
P200: Gorai.009G103800.1.A in case 1 and V36
Molweight: 42.788 (kD)
Majority protein IDs: Gorai.009G103800.1.A
Gene IDs: Gorai.009G103800
cotton name: PTHR24055:SF185 - MITOGEN-ACTIVATED PROTEIN KINASE 13 (1 of 1)
OG IDs: 93513844
Best-hit-arabi-ID: AT1G07880.2
Best-hit-arabi-name: Protein kinase superfamily protein
Gorai.009G103800.1.A
PCC=0.641, p=0.003
mRNA Profile
Protein Expression Profile
DPA

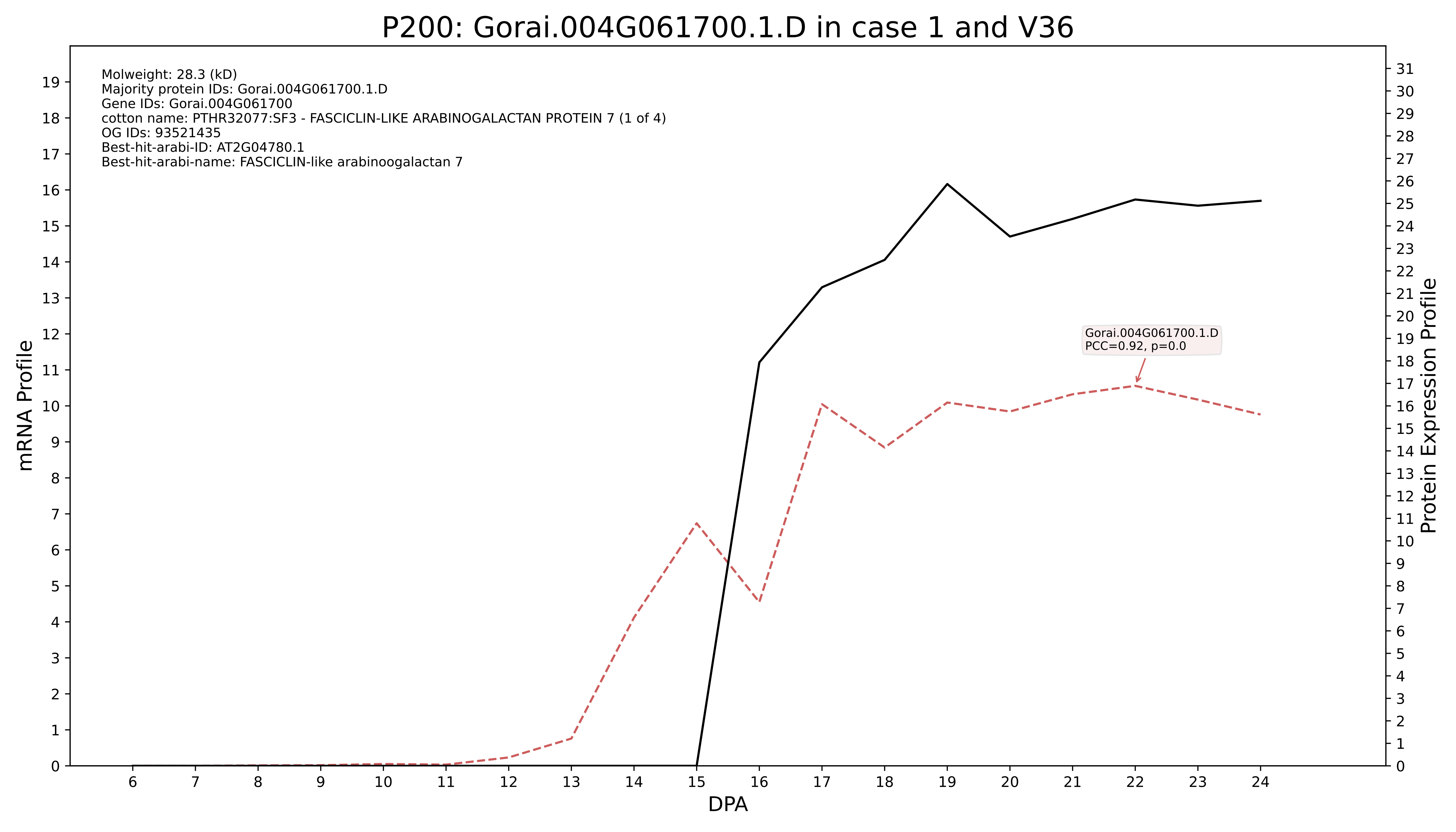

P200: Gorai.004G061700.1.D in case 1 and V36
Molweight: 28.3 (kD)
Majority protein IDs: Gorai.004G061700.1.D
Gene IDs: Gorai.004G061700
cotton name: PTHR32077:SF3 - FASCICLIN-LIKE ARABINOGALACTAN PROTEIN 7 (1 of 4)
OG IDs: 93521435
Best-hit-arabi-ID: AT2G04780.1
Best-hit-arabi-name: FASCICLIN-like arabinoogalactan 7
Gorai.004G061700.1.D
PCC=0.92, p=0.0
mRNA Profile
Protein Expression Profile
DPA

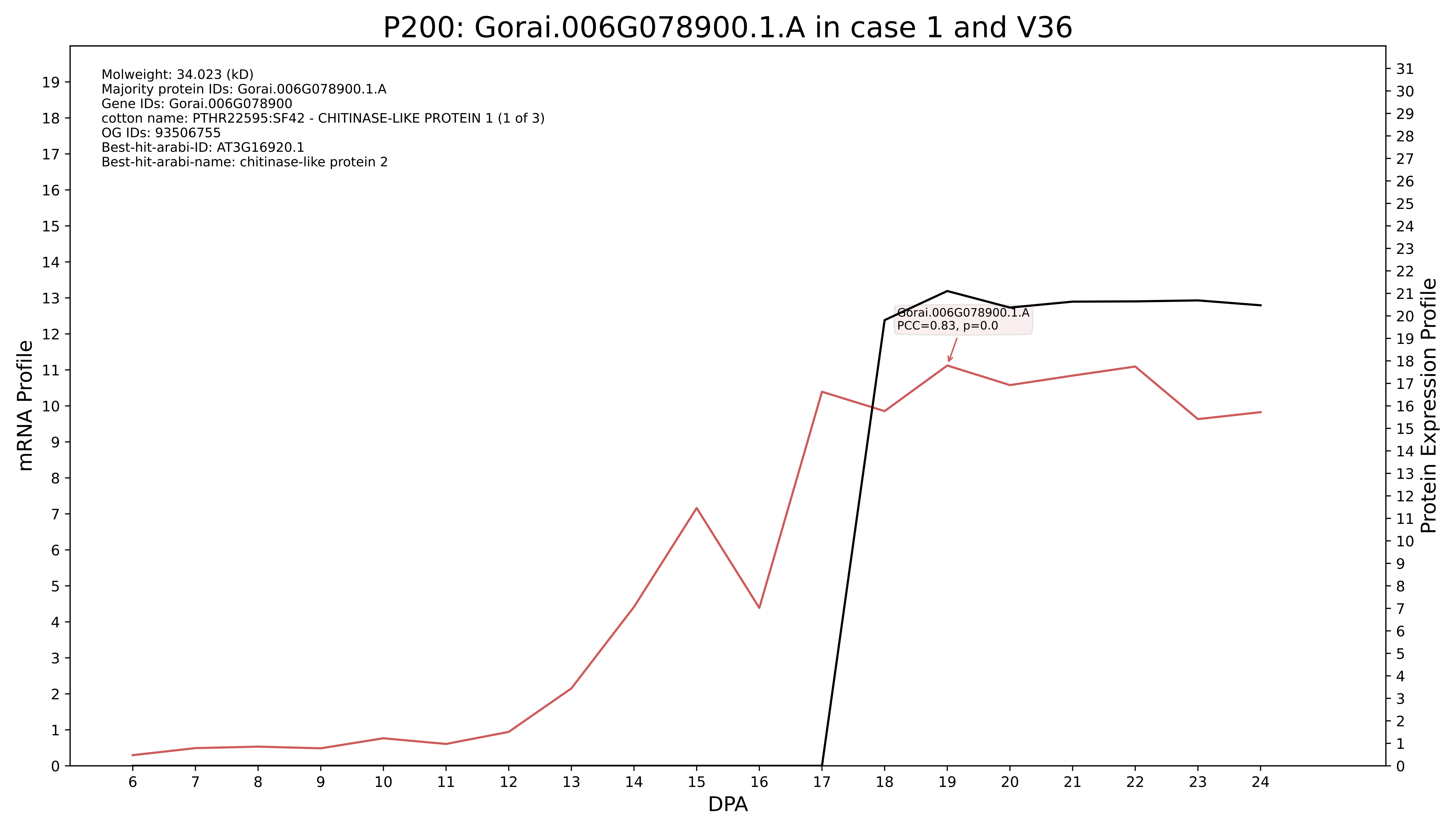
P200: Gorai.006G078900.1.A in case 1 and V36
Molweight: 34.023 (kD)
Majority protein IDs: Gorai.006G078900.1.A
Gene IDs: Gorai.006G078900
cotton name: PTHR22595:SF42 - CHITINASE-LIKE PROTEIN 1 (1 of 3)
OG IDs: 93506755
Best-hit-arabi-ID: AT3G16920.1
Best-hit-arabi-name: chitinase-like protein 2
Gorai.006G078900.1.A
PCC=0.83, p=0.0
mRNA Profile
Protein Expression Profile
DPA
0
1
2
3
4
5
6
7
8
9
10
11
12
13
14
15
16
17
18
19
20
21
22
23
24
25
26
27
28
29
30
31

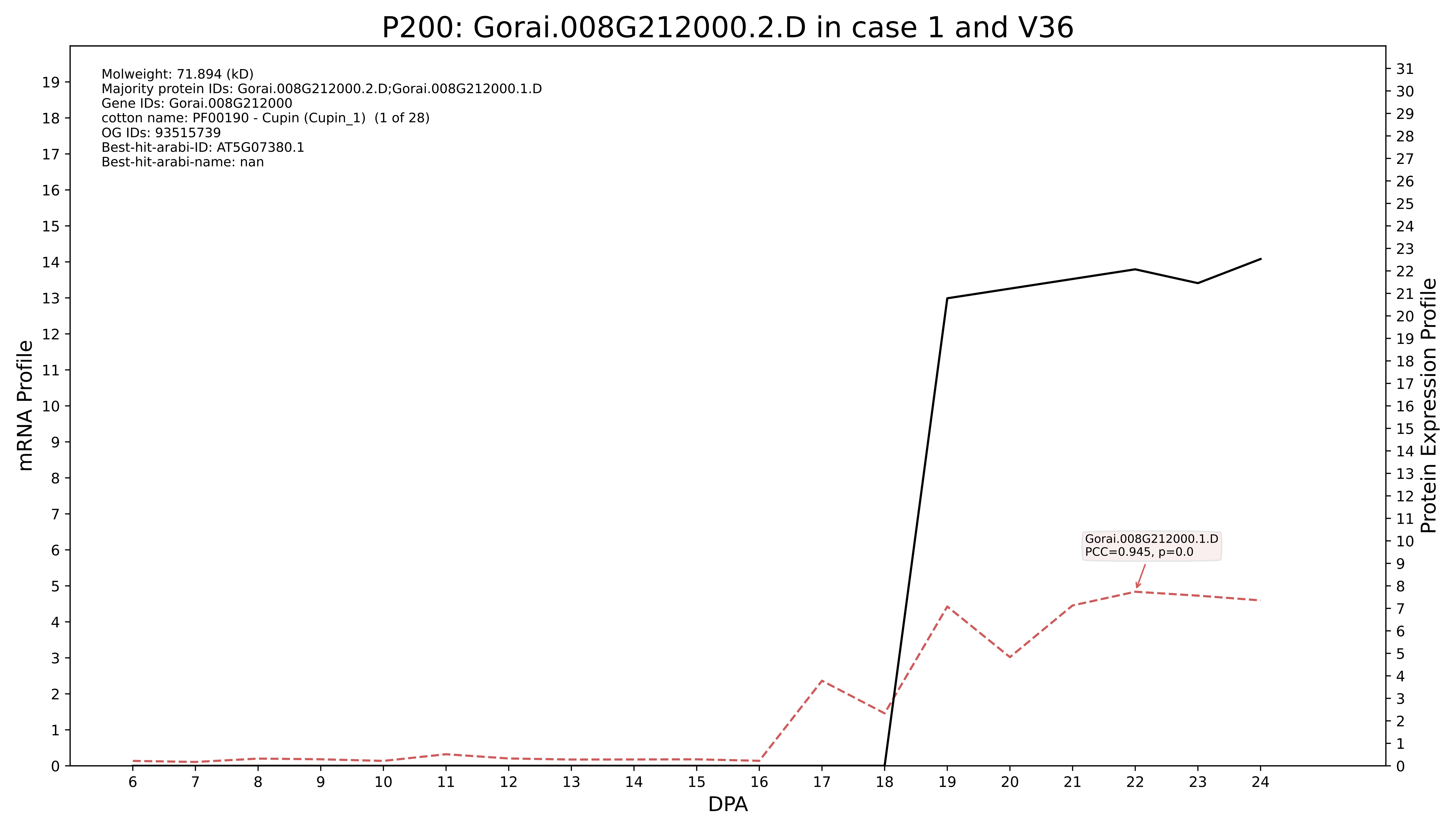
P200: Gorai.008G212000.2.D in case 1 and V36
Molweight: 71.894 (kD)
Majority protein IDs: Gorai.008G212000.2.D;Gorai.008G212000.1.D
Gene IDs: Gorai.008G212000
cotton name: PF00190 - Cupin (Cupin_1) (1 of 28)
OG IDs: 93515739
Best-hit-arabi-ID: AT5G07380.1
Best-hit-arabi-name: nan
Gorai.008G212000.1.D
PCC=0.945, p=0.0
mRNA Profile
Protein Expression Profile
DPA

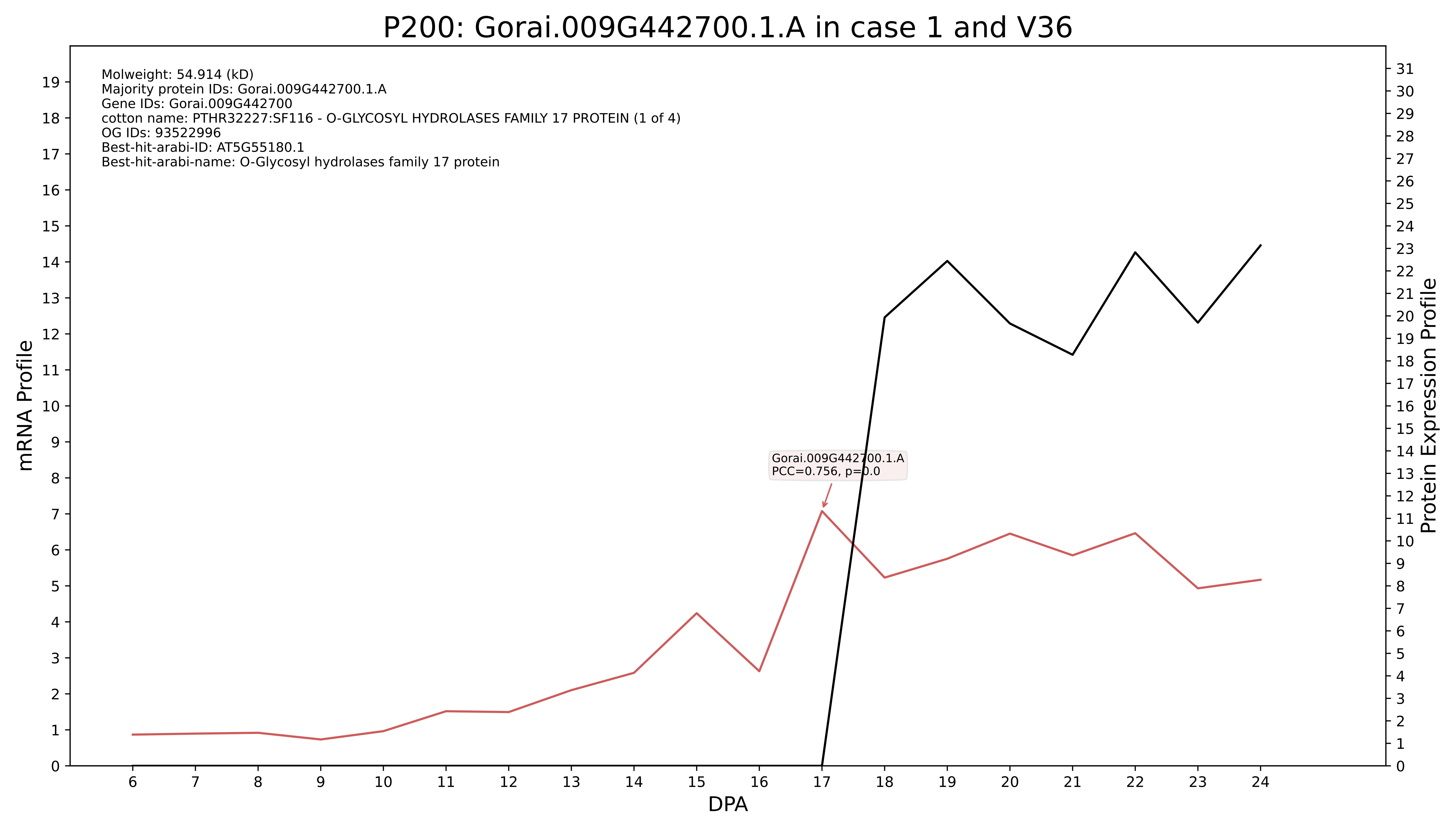
P200: Gorai.009G442700.1.A in case 1 and V36
Molweight: 54.914 (kD)
Majority protein IDs: Gorai.009G442700.1.A
Gene IDs: Gorai.009G442700
cotton name: PTHR32227:SF116 - O-GLYCOSYL HYDROLASES FAMILY 17 PROTEIN (1 of 4)
OG IDs: 93522996
Best-hit-arabi-ID: AT5G55180.1
Best-hit-arabi-name: O-Glycosyl hydrolases family 17 protein
Gorai.009G442700.1.A
PCC=0.756, p=0.0
mRNA Profile
Protein Expression Profile
DPA

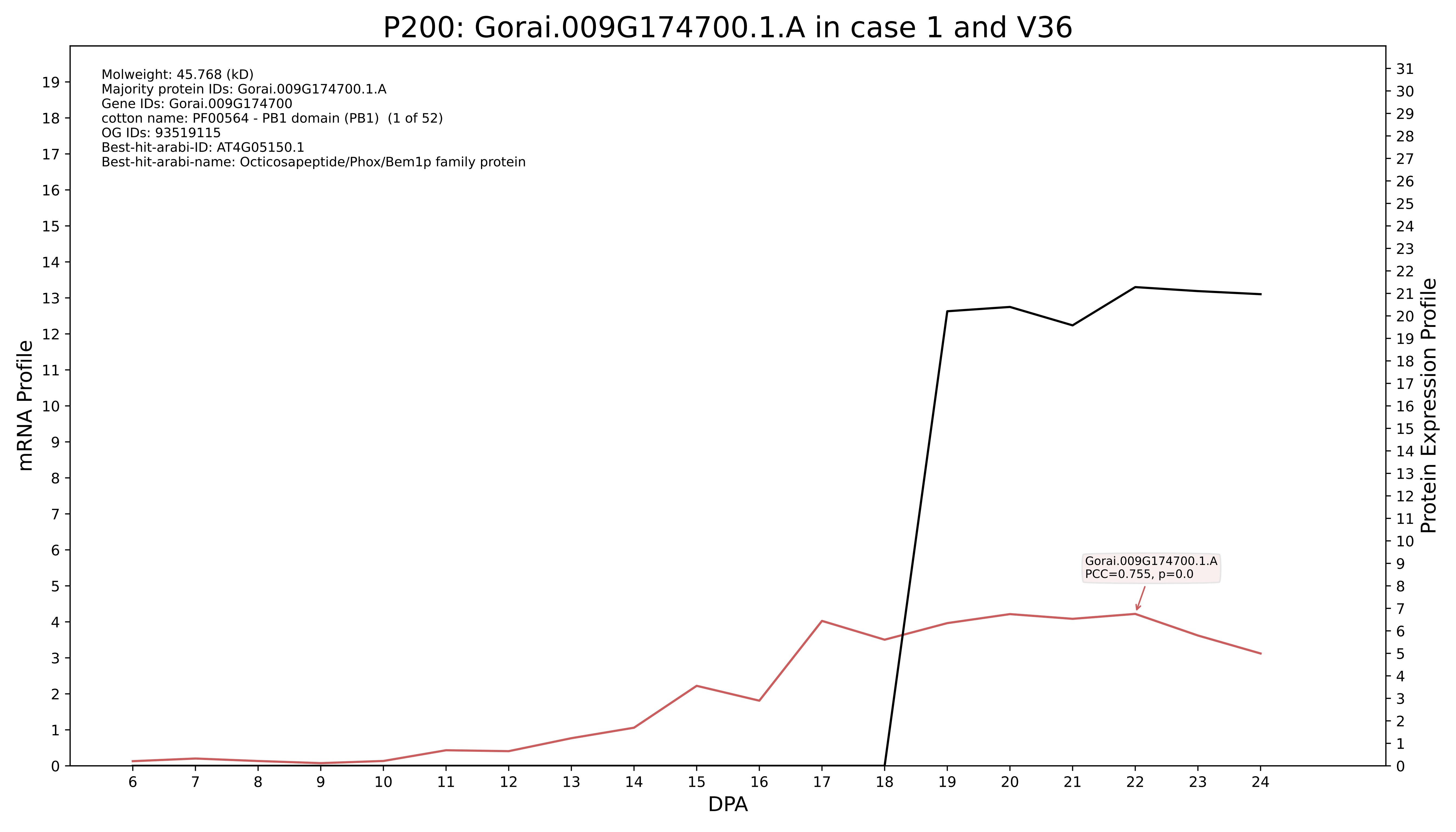

P200: Gorai.009G174700.1.A in case 1 and V36
Molweight: 45.768 (kD)
Majority protein IDs: Gorai.009G174700.1.A
Gene IDs: Gorai.009G174700
cotton name: PF00564 - PB1 domain (PB1) (1 of 52)
OG IDs: 93519115
Best-hit-arabi-ID: AT4G05150.1
Best-hit-arabi-name: Octicosapeptide/Phox/Bem1p family protein
Gorai.009G174700.1.A
PCC=0.755, p=0.0
mRNA Profile
Protein Expression Profile
DPA

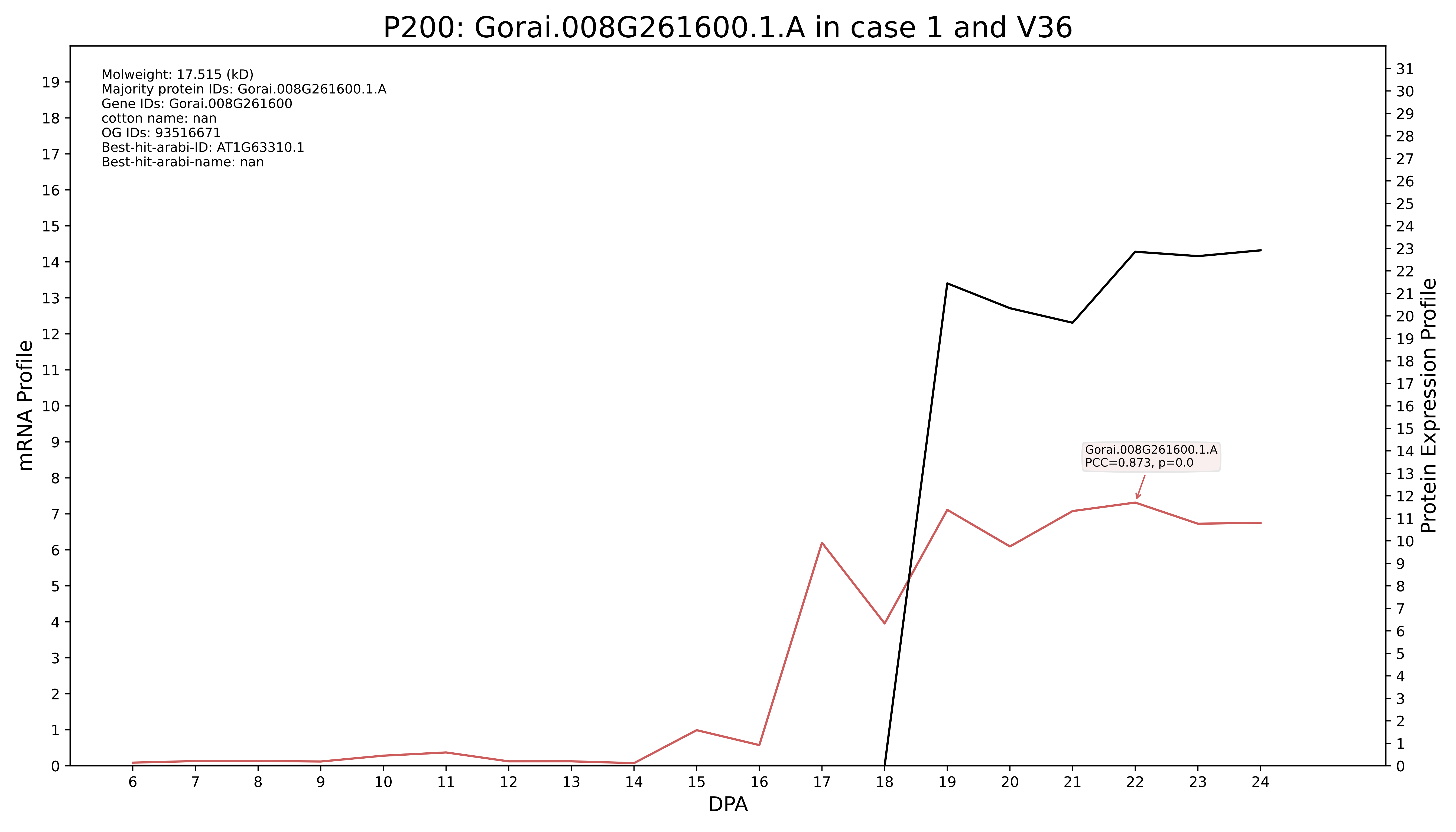
P200: Gorai.008G261600.1.A in case 1 and V36
Molweight: 17.515 (kD)
Majority protein IDs: Gorai.008G261600.1.A
Gene IDs: Gorai.008G261600
cotton name: nan
OG IDs: 93516671
Best-hit-arabi-ID: AT1G63310.1
Best-hit-arabi-name: nan
Gorai.008G261600.1.A
PCC=0.873, p=0.0
mRNA Profile
Protein Expression Profile
DPA
6
7
8
9
10
11
12
13
14
15
16
17
18
19
20
21
22
23
24

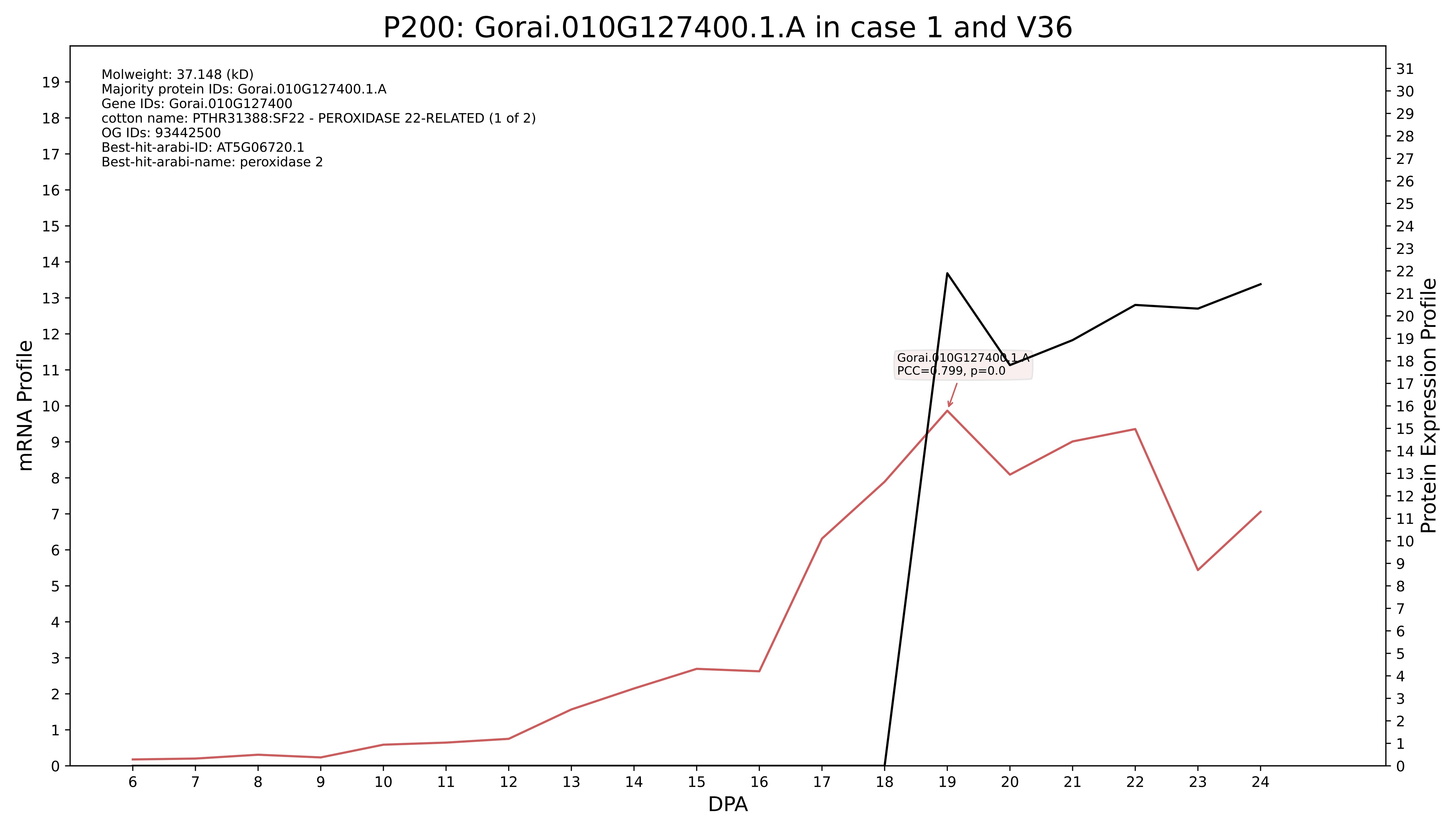

P200: Gorai.010G127400.1.A in case 1 and V36
Molweight: 37.148 (kD)
Majority protein IDs: Gorai.010G127400.1.A
Gene IDs: Gorai.010G127400
cotton name: PTHR31388:SF22 - PEROXIDASE 22-RELATED (1 of 2)
OG IDs: 93442500
Best-hit-arabi-ID: AT5G06720.1
Best-hit-arabi-name: peroxidase 2
Gorai.010G127400.1.A
PCC=0.799, p=0.0
mRNA Profile
Protein Expression Profile
DPA

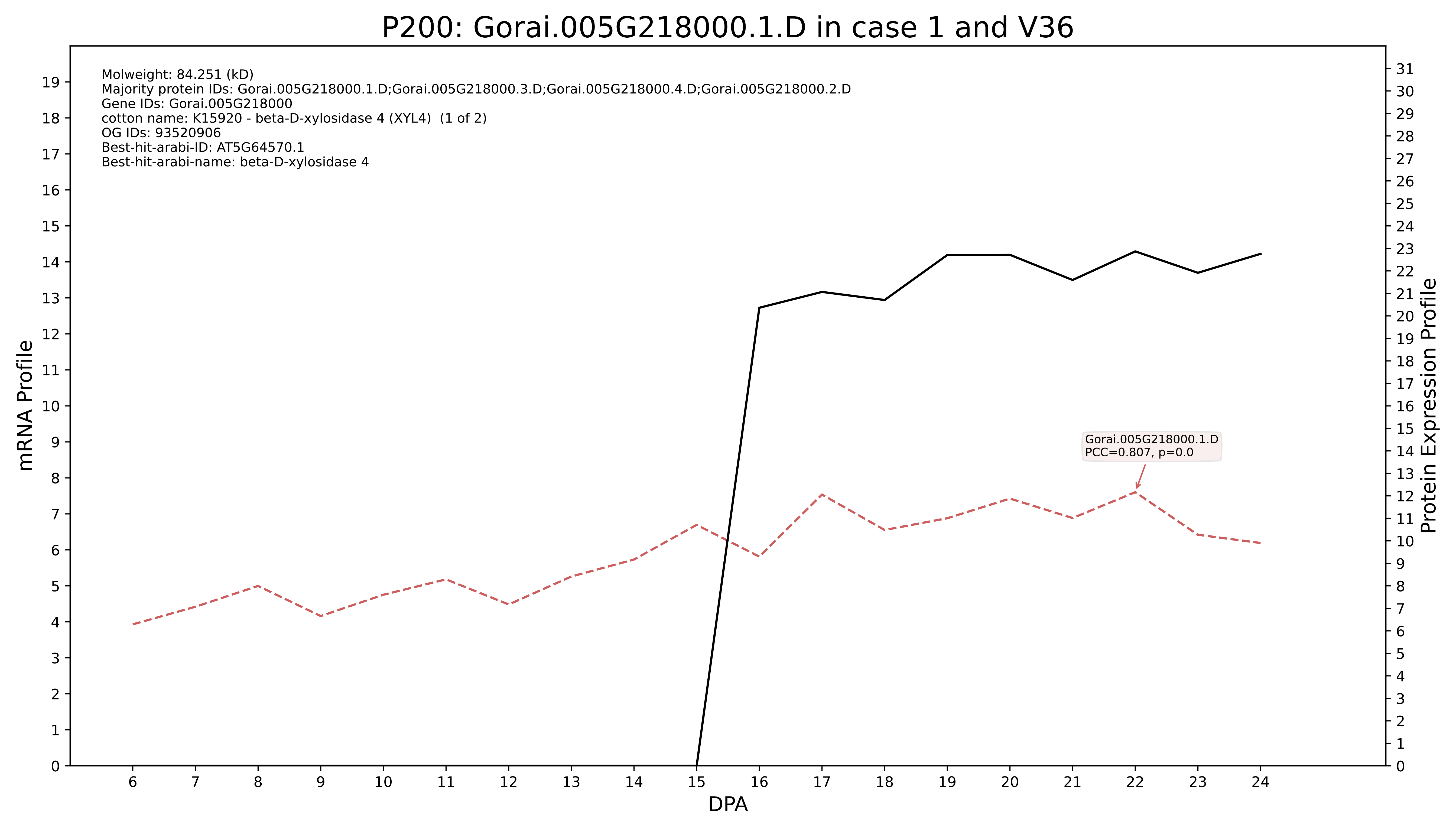
P200: Gorai.005G218000.1.D in case 1 and V36
Molweight: 84.251 (kD)
Majority protein IDs: Gorai.005G218000.1.D;Gorai.005G218000.3.D;Gorai.005G218000.4.D;Gorai.005G218000.2.D
Gene IDs: Gorai.005G218000
cotton name: K15920 - beta-D-xylosidase 4 (XYL4) (1 of 2)
OG IDs: 93520906
Best-hit-arabi-ID: AT5G64570.1
Best-hit-arabi-name: beta-D-xylosidase 4
Gorai.005G218000.1.D
PCC=0.807, p=0.0
mRNA Profile
Protein Expression Profile
DPA

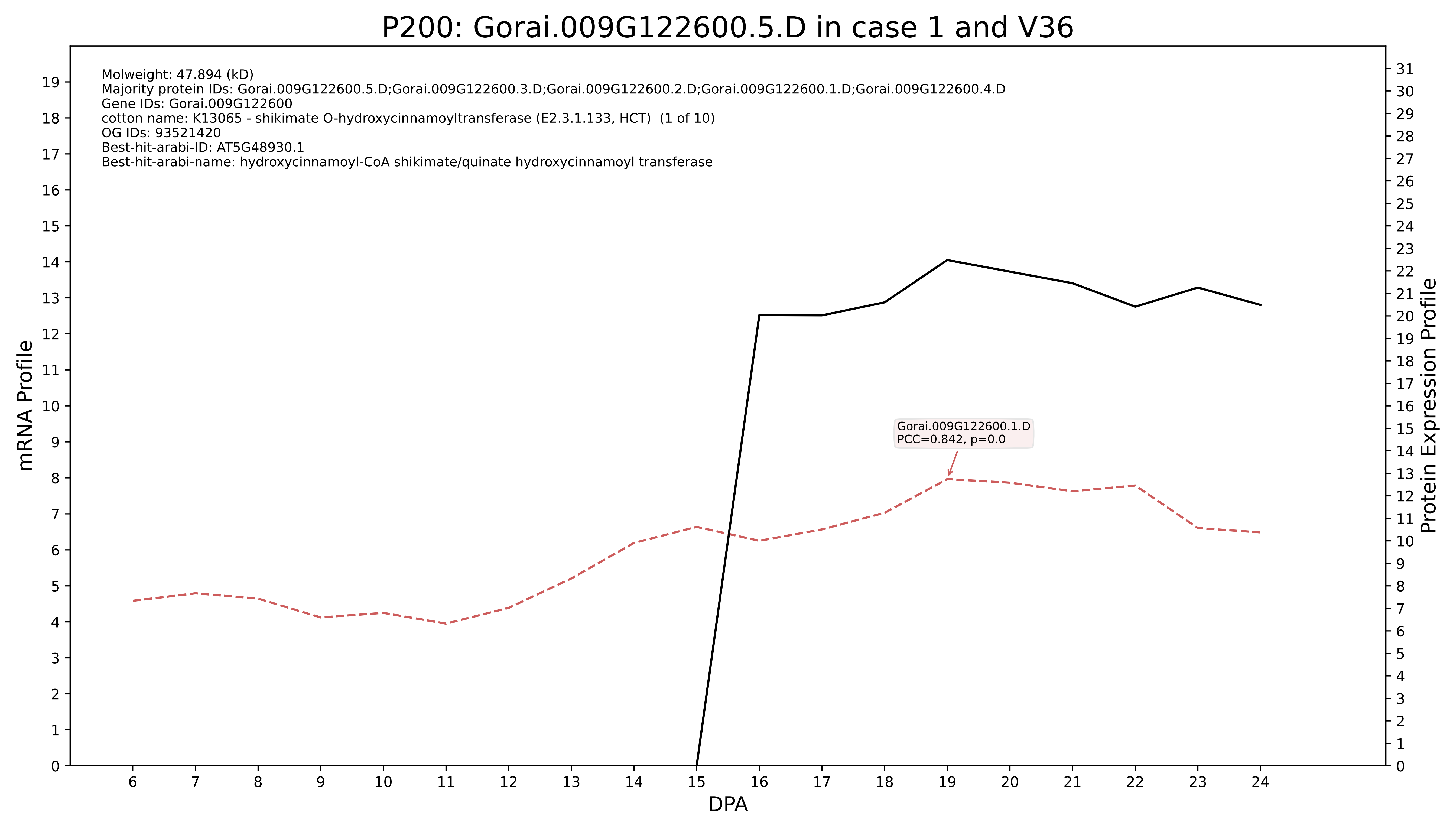
P200: Gorai.009G122600.5.D in case 1 and V36
Molweight: 47.894 (kD)
Majority protein IDs: Gorai.009G122600.5.D;Gorai.009G122600.3.D;Gorai.009G122600.2.D;Gorai.009G122600.1.D;Gorai.009G122600.4.D
Gene IDs: Gorai.009G122600
cotton name: K13065 - shikimate O-hydroxycinnamoyltransferase (E2.3.1.133, HCT) (1 of 10)
OG IDs: 93521420
Best-hit-arabi-ID: AT5G48930.1
Best-hit-arabi-name: hydroxycinnamoyl-CoA shikimate/quinate hydroxycinnamoyl transferase
Gorai.009G122600.1.D
PCC=0.842, p=0.0
mRNA Profile
Protein Expression Profile
DPA

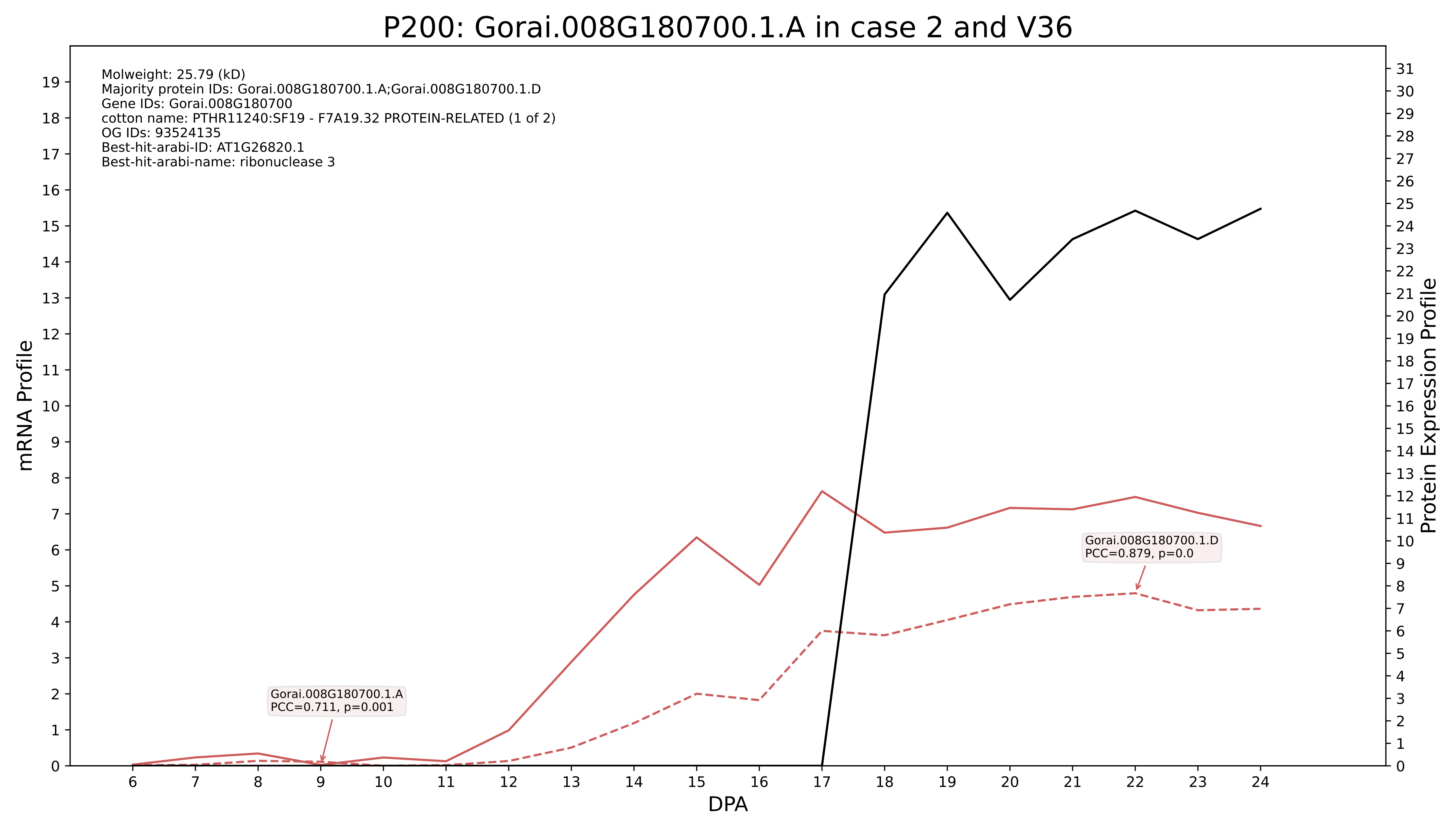

P200: Gorai.008G180700.1.A in case 2 and V36
Molweight: 25.79 (kD)
Majority protein IDs: Gorai.008G180700.1.A;Gorai.008G180700.1.D
Gene IDs: Gorai.008G180700
cotton name: PTHR11240:SF19 - F7A19.32 PROTEIN-RELATED (1 of 2)
OG IDs: 93524135
Best-hit-arabi-ID: AT1G26820.1
Best-hit-arabi-name: ribonuclease 3
Gorai.008G180700.1.D
PCC=0.879, p=0.0
Gorai.008G180700.1.A
PCC=0.711, p=0.001
mRNA Profile
Protein Expression Profile
DPA

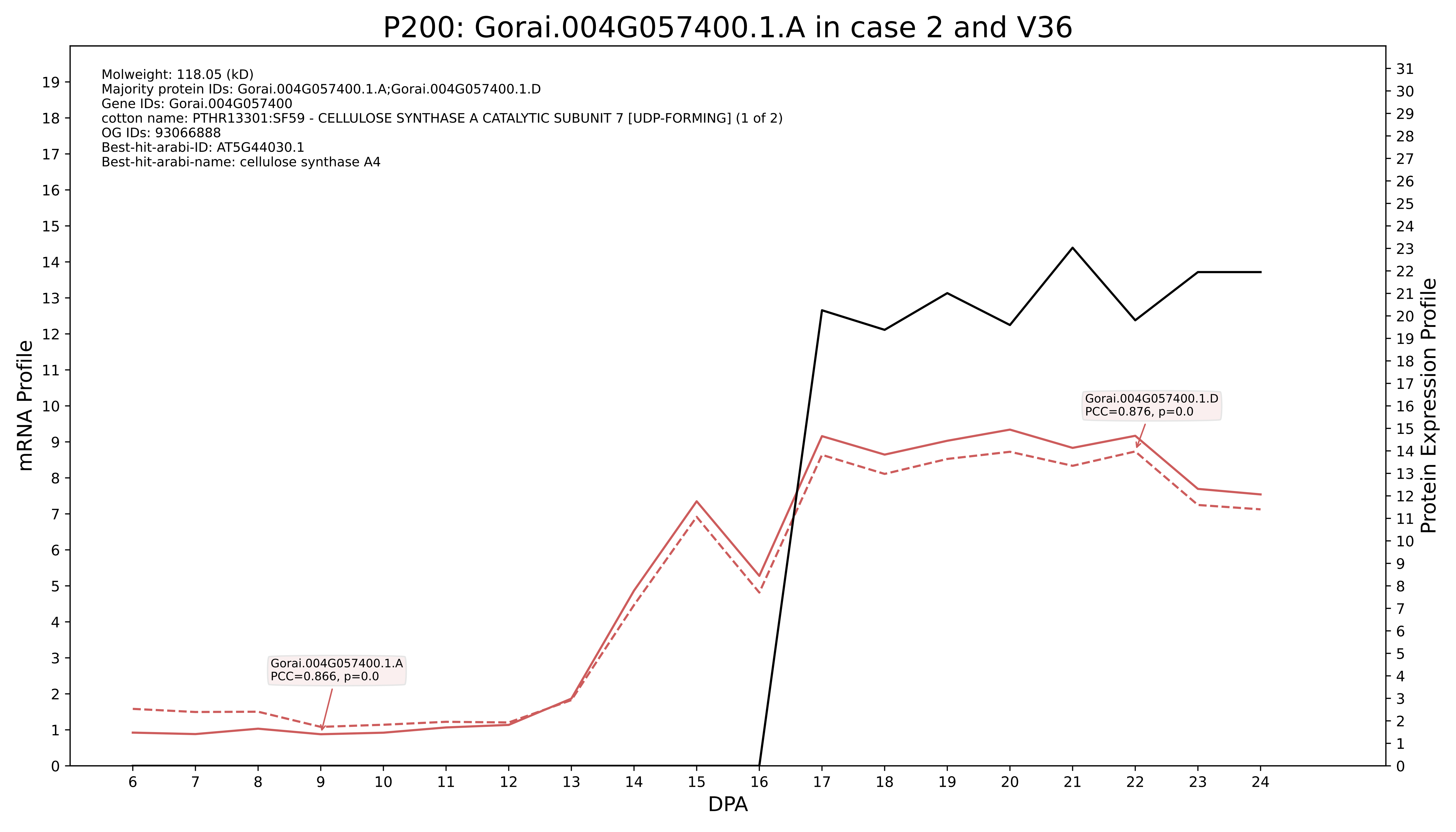
P200: Gorai.004G057400.1.A in case 2 and V36
Molweight: 118.05 (kD)
Majority protein IDs: Gorai.004G057400.1.A;Gorai.004G057400.1.D
Gene IDs: Gorai.004G057400
cotton name: PTHR13301:SF59 - CELLULOSE SYNTHASE A CATALYTIC SUBUNIT 7 [UDP-FORMING] (1 of 2)
OG IDs: 93066888
Best-hit-arabi-ID: AT5G44030.1
Best-hit-arabi-name: cellulose synthase A4
Gorai.004G057400.1.D
PCC=0.876, p=0.0
Gorai.004G057400.1.A
PCC=0.866, p=0.0
mRNA Profile
Protein Expression Profile
DPA

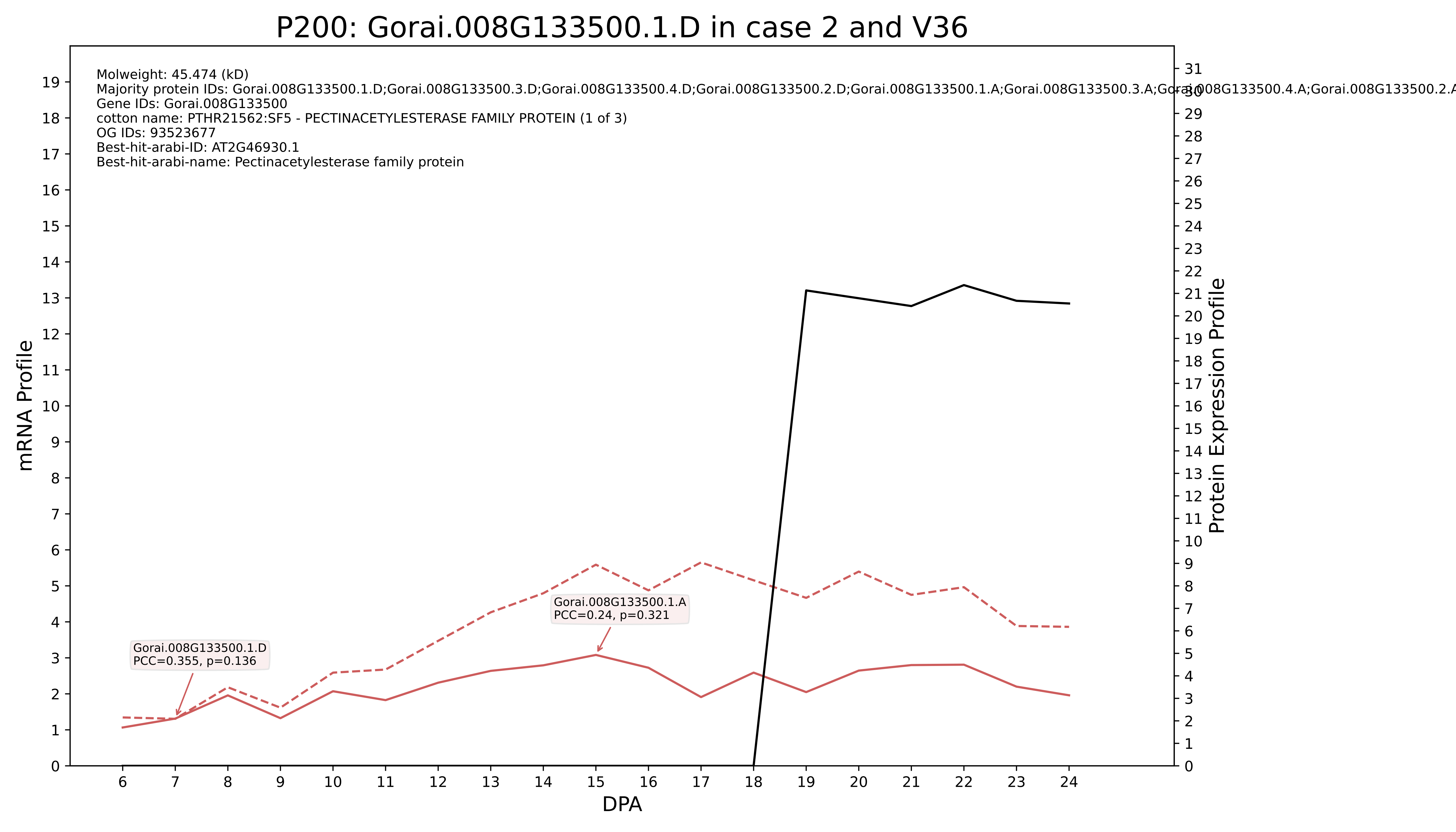
P200: Gorai.008G133500.1.D in case 2 and V36
Molweight: 45.474 (kD)
Majority protein IDs: Gorai.008G133500.1.D;Gorai.008G133500.3.D;Gorai.008G133500.4.D;Gorai.008G133500.2.D;Gorai.008G133500.1.A;Gorai.008G133500.3.A;Gorai.008G133500.4.A;Gorai.008G133500.2.A
Gene IDs: Gorai.008G133500
cotton name: PTHR21562:SF5 - PECTINACETYLESTERASE FAMILY PROTEIN (1 of 3)
OG IDs: 93523677
Best-hit-arabi-ID: AT2G46930.1
Best-hit-arabi-name: Pectinacetylesterase family protein
Gorai.008G133500.1.A
PCC=0.24, p=0.321
Gorai.008G133500.1.D
PCC=0.355, p=0.136
mRNA Profile
Protein Expression Profile
DPA

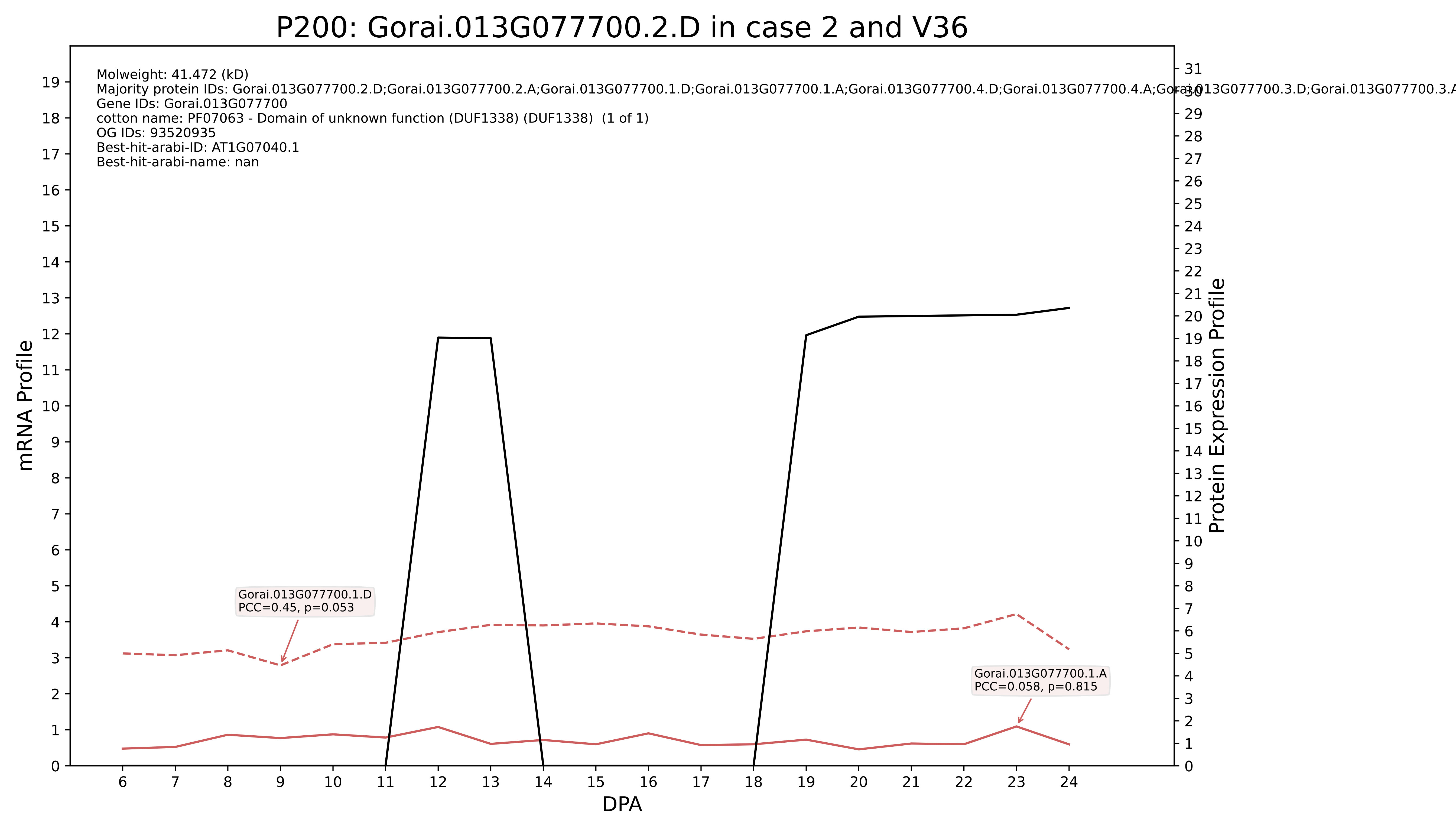
P200: Gorai.013G077700.2.D in case 2 and V36
Molweight: 41.472 (kD)
Majority protein IDs: Gorai.013G077700.2.D;Gorai.013G077700.2.A;Gorai.013G077700.1.D;Gorai.013G077700.1.A;Gorai.013G077700.4.D;Gorai.013G077700.4.A;Gorai.013G077700.3.D;Gorai.013G077700.3.A
Gene IDs: Gorai.013G077700
cotton name: PF07063 - Domain of unknown function (DUF1338) (DUF1338) (1 of 1)
OG IDs: 93520935
Best-hit-arabi-ID: AT1G07040.1
Best-hit-arabi-name: nan
Gorai.013G077700.1.D
PCC=0.45, p=0.053
Gorai.013G077700.1.A
PCC=0.058, p=0.815
mRNA Profile
Protein Expression Profile
DPA

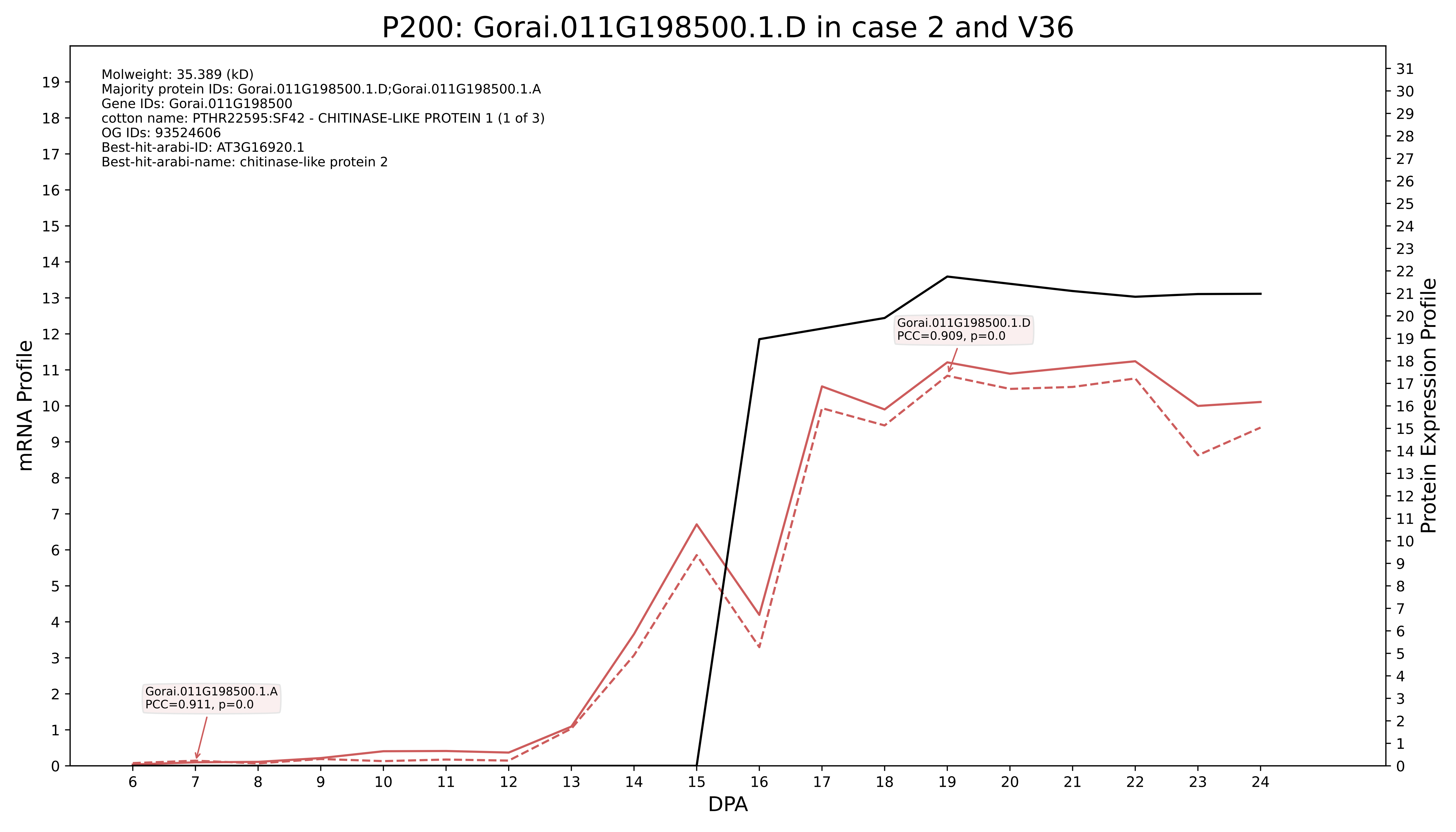
P200: Gorai.011G198500.1.D in case 2 and V36
Molweight: 35.389 (kD)
Majority protein IDs: Gorai.011G198500.1.D;Gorai.011G198500.1.A
Gene IDs: Gorai.011G198500
cotton name: PTHR22595:SF42 - CHITINASE-LIKE PROTEIN 1 (1 of 3)
OG IDs: 93524606
Best-hit-arabi-ID: AT3G16920.1
Best-hit-arabi-name: chitinase-like protein 2
Gorai.011G198500.1.D
PCC=0.909, p=0.0
Gorai.011G198500.1.A
PCC=0.911, p=0.0
mRNA Profile
Protein Expression Profile
DPA

P200: Gorai.003G119000.2.A in case 2 and V36

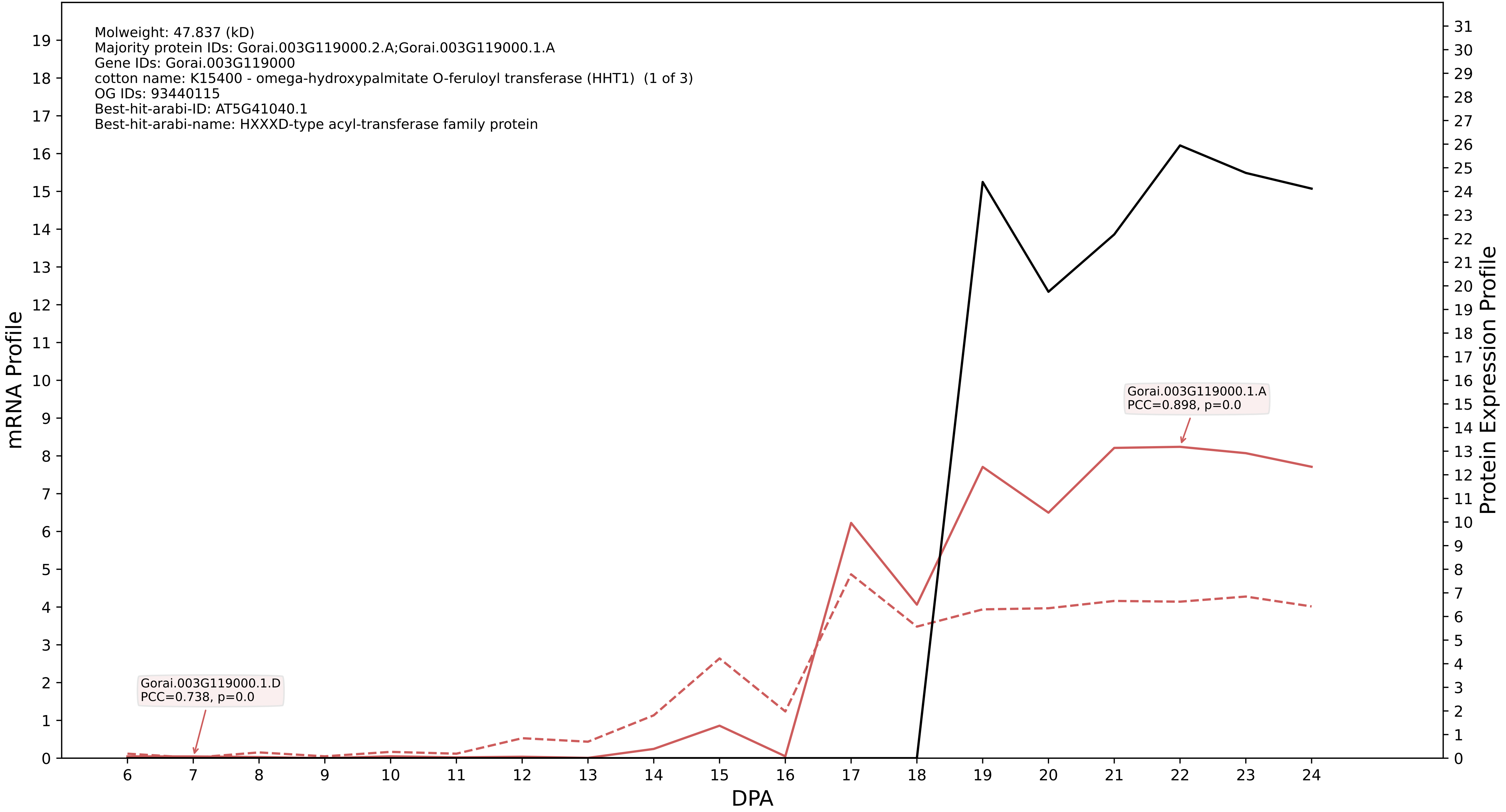

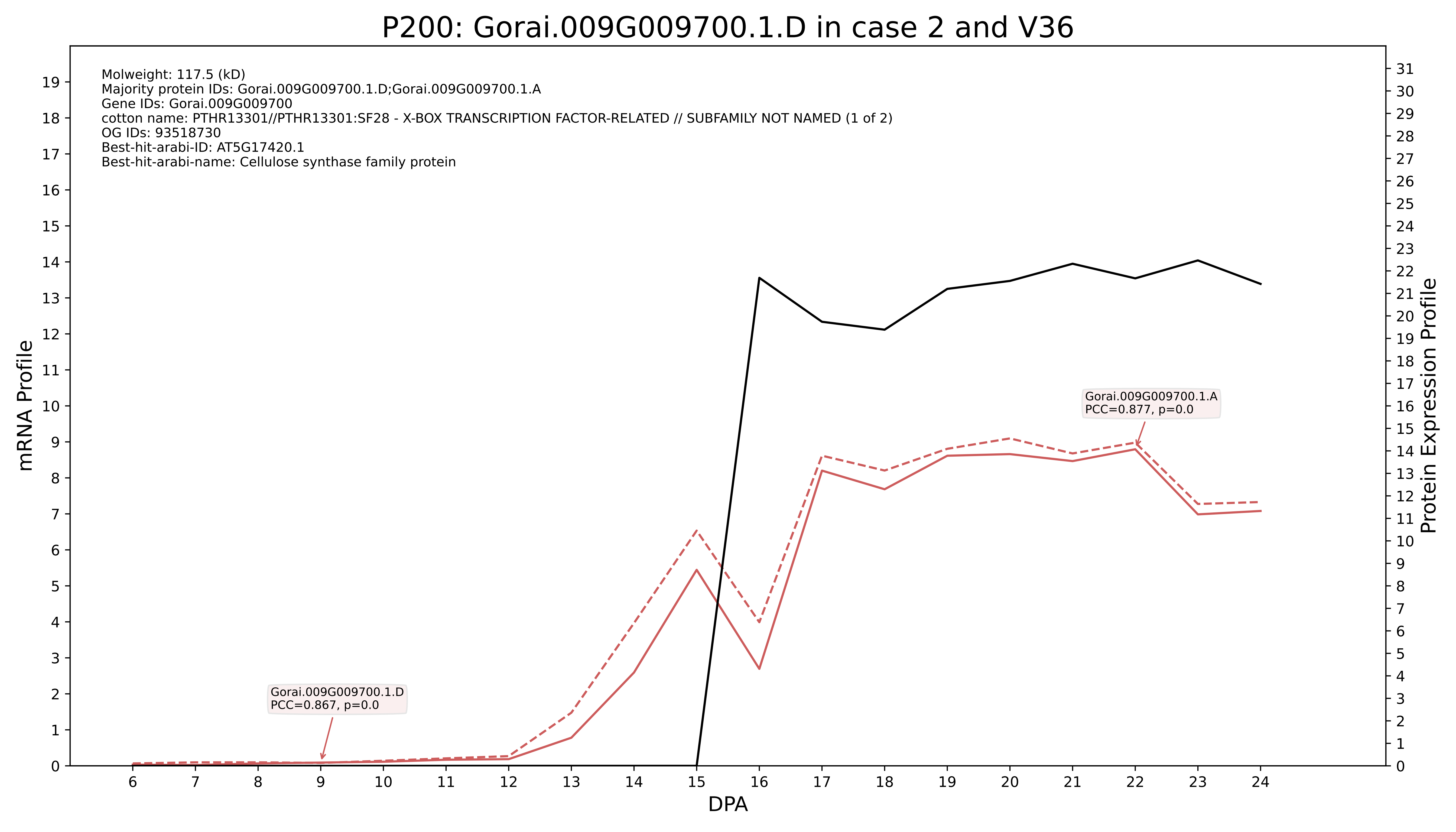

P200: Gorai.009G009700.1.D in case 2 and V36
Molweight: 117.5 (kD)
Majority protein IDs: Gorai.009G009700.1.D;Gorai.009G009700.1.A
Gene IDs: Gorai.009G009700
cotton name: PTHR13301//PTHR13301:SF28 - X-BOX TRANSCRIPTION FACTOR-RELATED // SUBFAMILY NOT NAMED (1 of 2)
OG IDs: 93518730
Best-hit-arabi-ID: AT5G17420.1
Best-hit-arabi-name: Cellulose synthase family protein
Gorai.009G009700.1.A
PCC=0.877, p=0.0
Gorai.009G009700.1.D
PCC=0.867, p=0.0
mRNA Profile
Protein Expression Profile
DPA

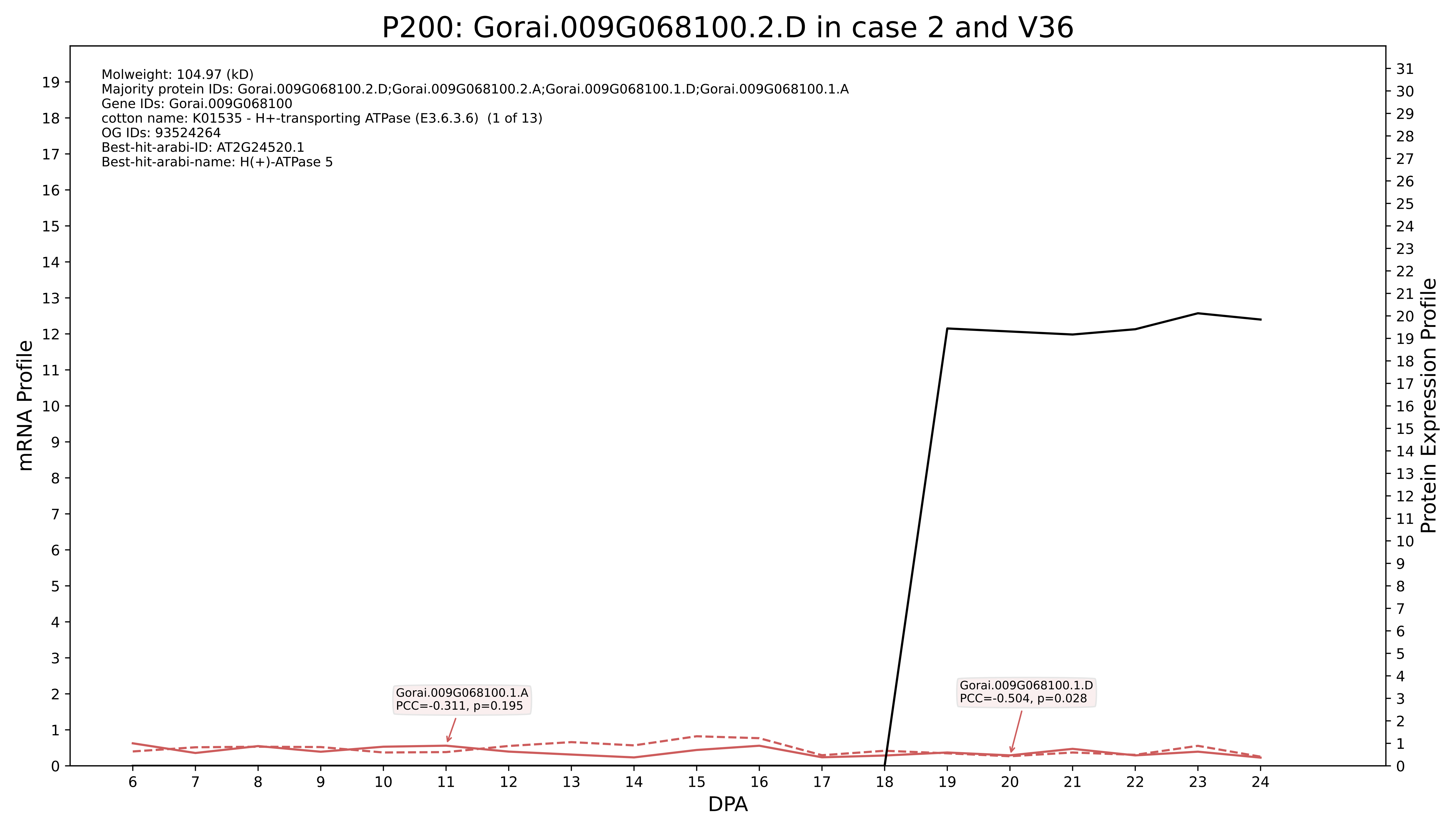

P200: Gorai.009G068100.2.D in case 2 and V36
Molweight: 104.97 (kD)
Majority protein IDs: Gorai.009G068100.2.D;Gorai.009G068100.2.A;Gorai.009G068100.1.D;Gorai.009G068100.1.A
Gene IDs: Gorai.009G068100
cotton name: K01535 - H+-transporting ATPase (E3.6.3.6) (1 of 13)
OG IDs: 93524264
Best-hit-arabi-ID: AT2G24520.1
Best-hit-arabi-name: H(+)-ATPase 5
Gorai.009G068100.1.A
PCC=-0.311, p=0.195
Gorai.009G068100.1.D
PCC=-0.504, p=0.028
mRNA Profile
Protein Expression Profile
DPA

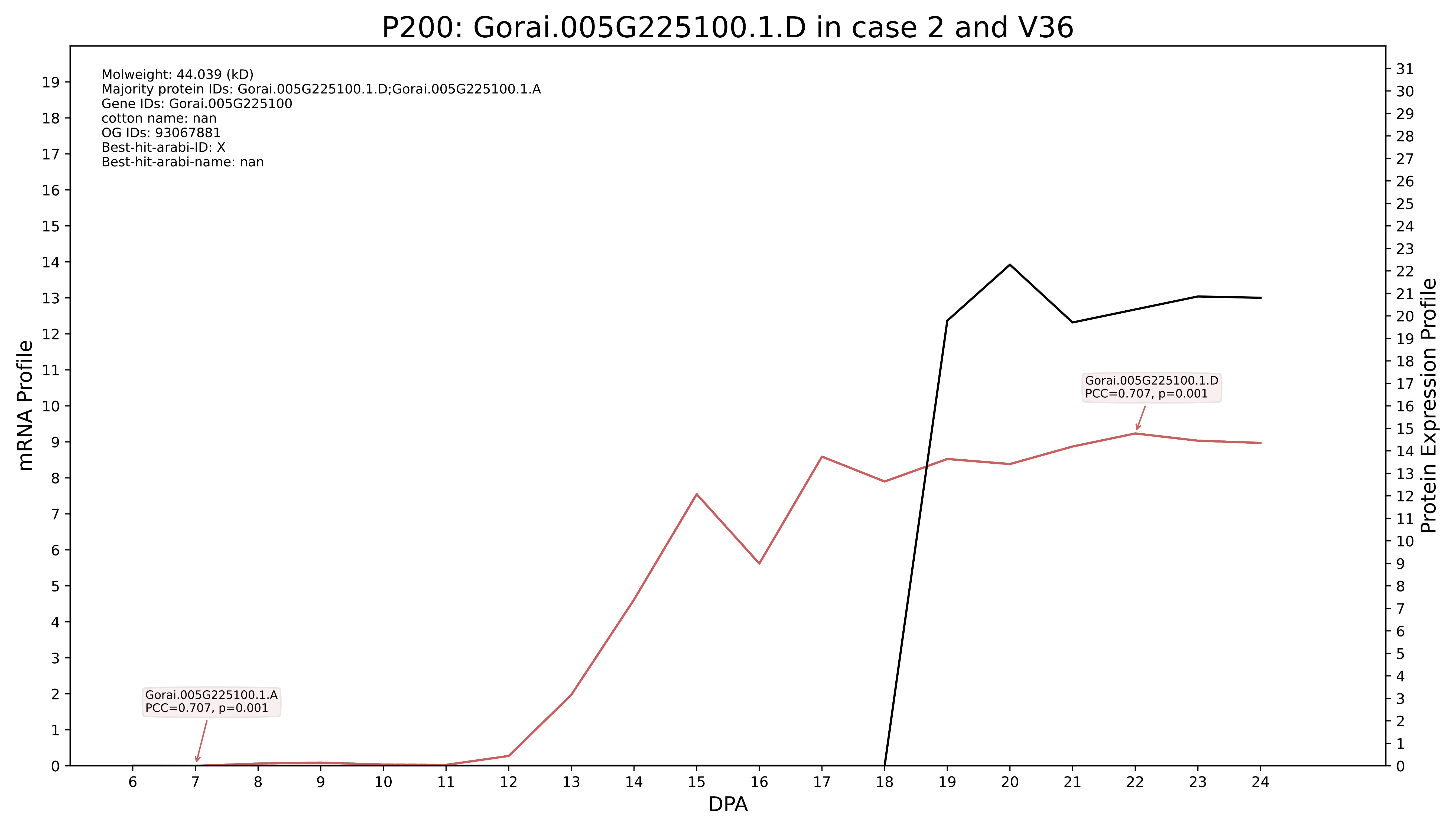
P200: Gorai.005G225100.1.D in case 2 and V36
Molweight: 44.039 (kD)
Majority protein IDs: Gorai.005G225100.1.D;Gorai.005G225100.1.A
Gene IDs: Gorai.005G225100
cotton name: nan
OG IDs: 93067881
Best-hit-arabi-ID: X
Best-hit-arabi-name: nan
Gorai.005G225100.1.D
PCC=0.707, p=0.001
Gorai.005G225100.1.A
PCC=0.707, p=0.001
mRNA Profile
Protein Expression Profile
DPA

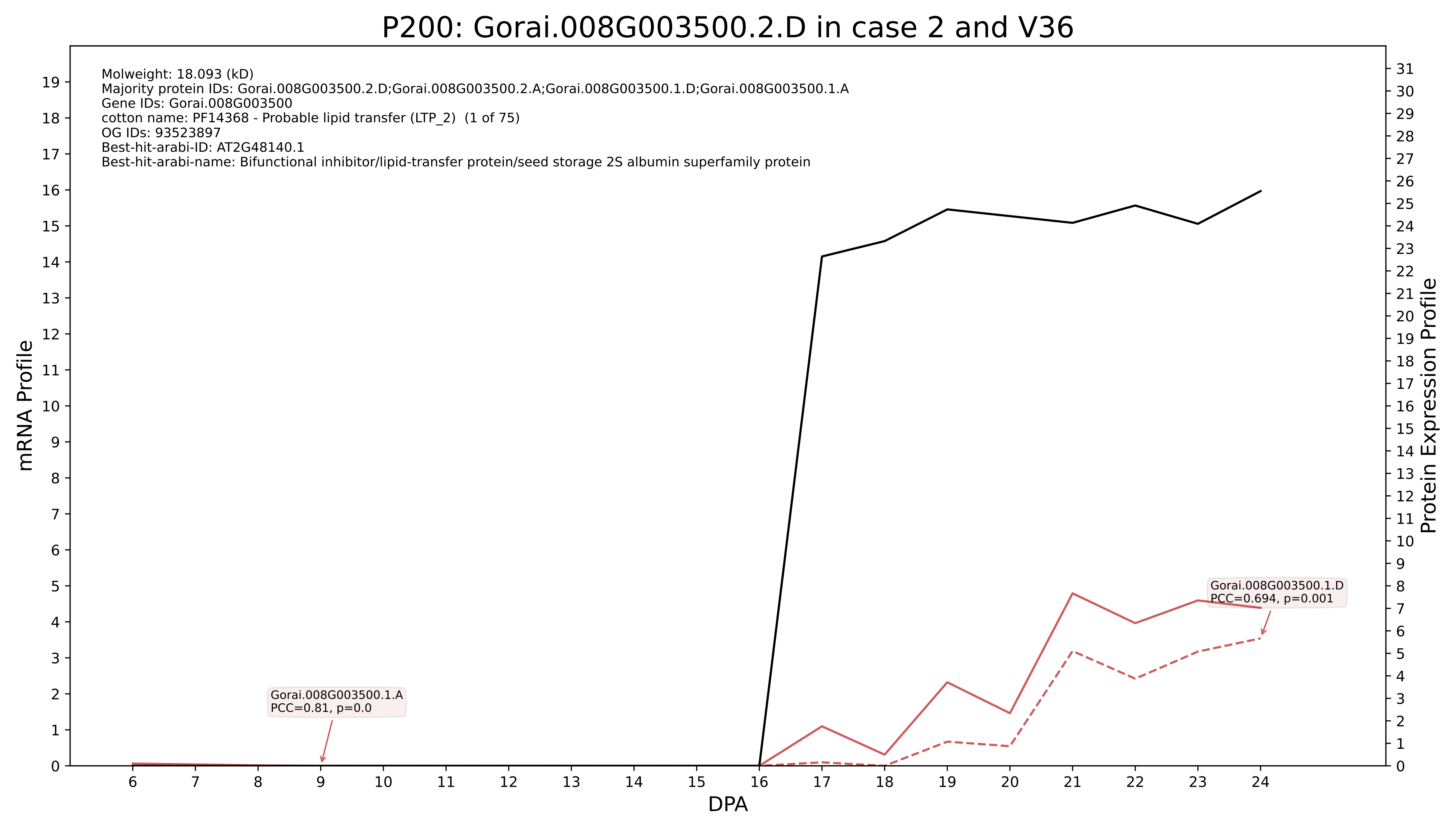
P200: Gorai.008G003500.2.D in case 2 and V36
Molweight: 18.093 (kD)
Majority protein IDs: Gorai.008G003500.2.D;Gorai.008G003500.2.A;Gorai.008G003500.1.D;Gorai.008G003500.1.A
Gene IDs: Gorai.008G003500
cotton name: PF14368 - Probable lipid transfer (LTP_2) (1 of 75)
OG IDs: 93523897
Best-hit-arabi-ID: AT2G48140.1
Best-hit-arabi-name: Bifunctional inhibitor/lipid-transfer protein/seed storage 2S albumin superfamily protein
Gorai.008G003500.1.A
PCC=0.81, p=0.0
Gorai.008G003500.1.D
PCC=0.694, p=0.001
mRNA Profile
Protein Expression Profile
DPA

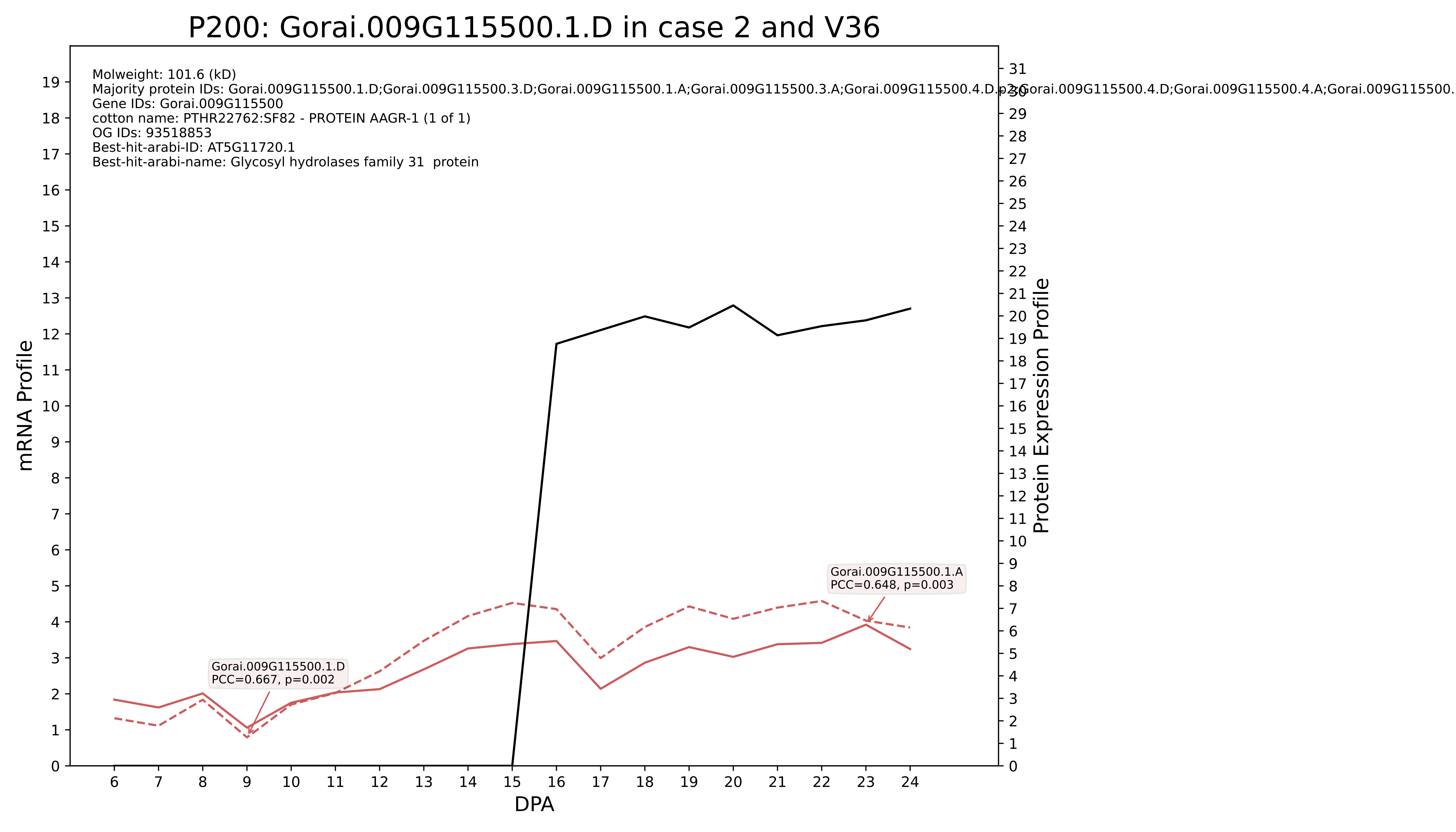

P200: Gorai.009G115500.1.D in case 2 and V36
Molweight: 101.6 (kD)
Majority protein IDs: Gorai.009G115500.1.D;Gorai.009G115500.3.D;Gorai.009G115500.1.A;Gorai.009G115500.3.A;Gorai.009G115500.4.D.p2;Gorai.009G115500.4.D;Gorai.009G115500.4.A;Gorai.009G115500.
Gene IDs: Gorai.009G115500
cotton name: PTHR22762:SF82 - PROTEIN AAGR-1 (1 of 1)
OG IDs: 93518853
Best-hit-arabi-ID: AT5G11720.1
Best-hit-arabi-name: Glycosyl hydrolases family 31 protein
Gorai.009G115500.1.D
PCC=0.667, p=0.002
Gorai.009G115500.1.A
PCC=0.648, p=0.003
mRNA Profile
Protein Expression Profile
DPA

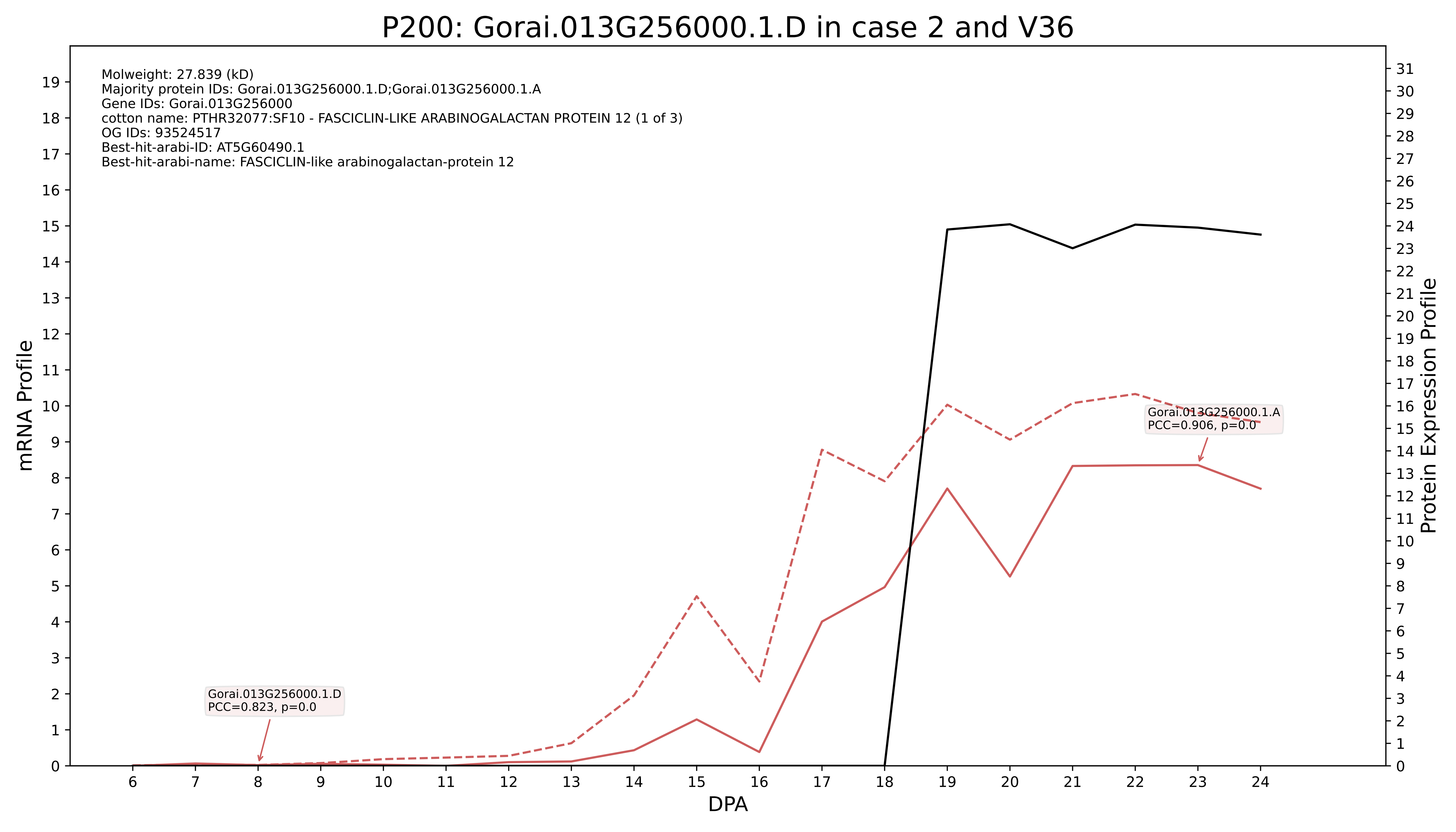
P200: Gorai.013G256000.1.D in case 2 and V36
Molweight: 27.839 (kD)
Majority protein IDs: Gorai.013G256000.1.D;Gorai.013G256000.1.A
Gene IDs: Gorai.013G256000
cotton name: PTHR32077:SF10 - FASCICLIN-LIKE ARABINOGALACTAN PROTEIN 12 (1 of 3)
OG IDs: 93524517
Best-hit-arabi-ID: AT5G60490.1
Best-hit-arabi-name: FASCICLIN-like arabinogalactan-protein 12
Gorai.013G256000.1.A
PCC=0.906, p=0.0
Gorai.013G256000.1.D
PCC=0.823, p=0.0
mRNA Profile
Protein Expression Profile
DPA

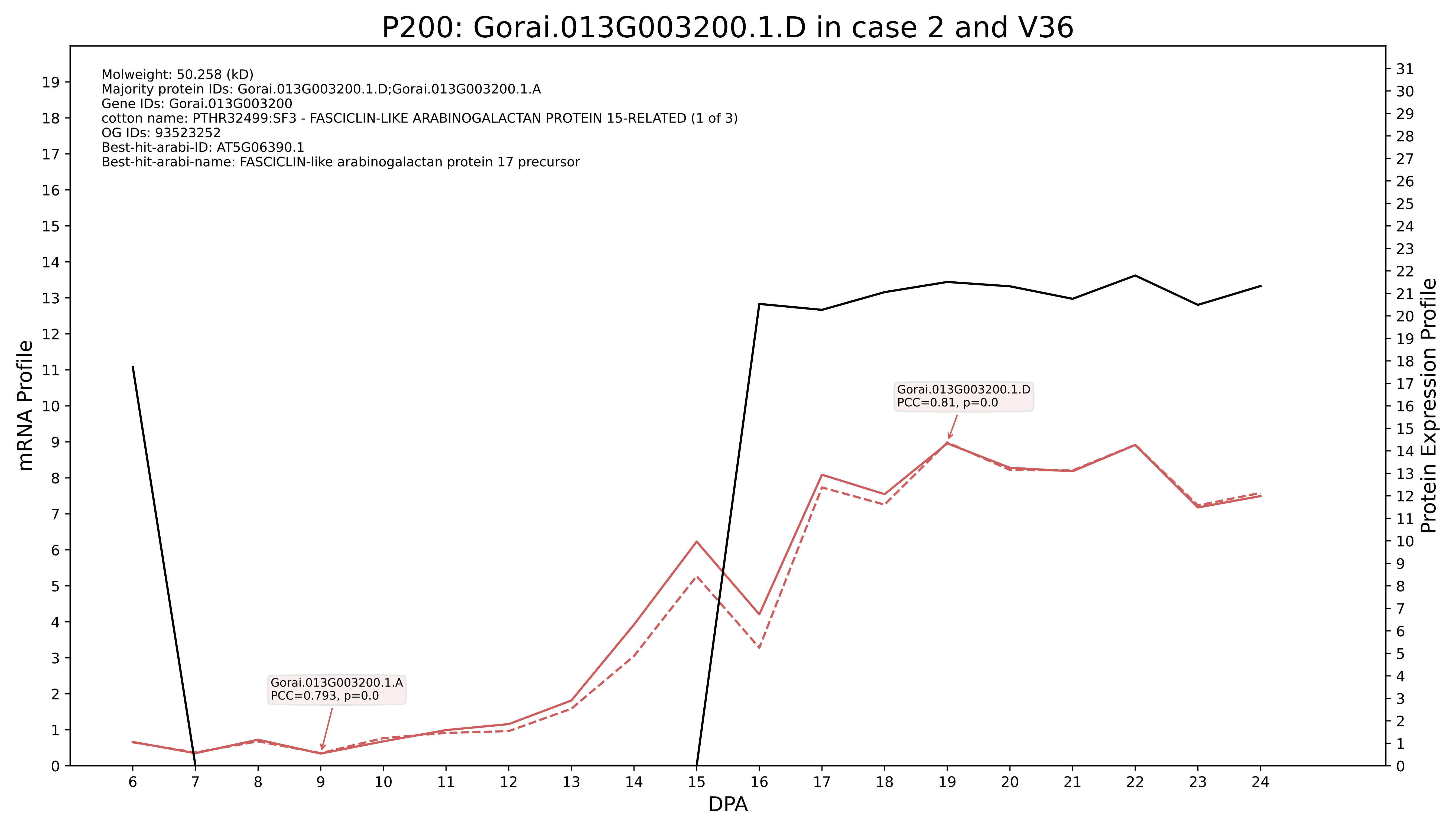

P200: Gorai.013G003200.1.D in case 2 and V36
Molweight: 50.258 (kD)
Majority protein IDs: Gorai.013G003200.1.D;Gorai.013G003200.1.A
Gene IDs: Gorai.013G003200
cotton name: PTHR32499:SF3 - FASCICLIN-LIKE ARABINOGALACTAN PROTEIN 15-RELATED (1 of 3)
OG IDs: 93523252
Best-hit-arabi-ID: AT5G06390.1
Best-hit-arabi-name: FASCICLIN-like arabinogalactan protein 17 precursor
Gorai.013G003200.1.D
PCC=0.81, p=0.0
Gorai.013G003200.1.A
PCC=0.793, p=0.0
mRNA Profile
Protein Expression Profile
DPA

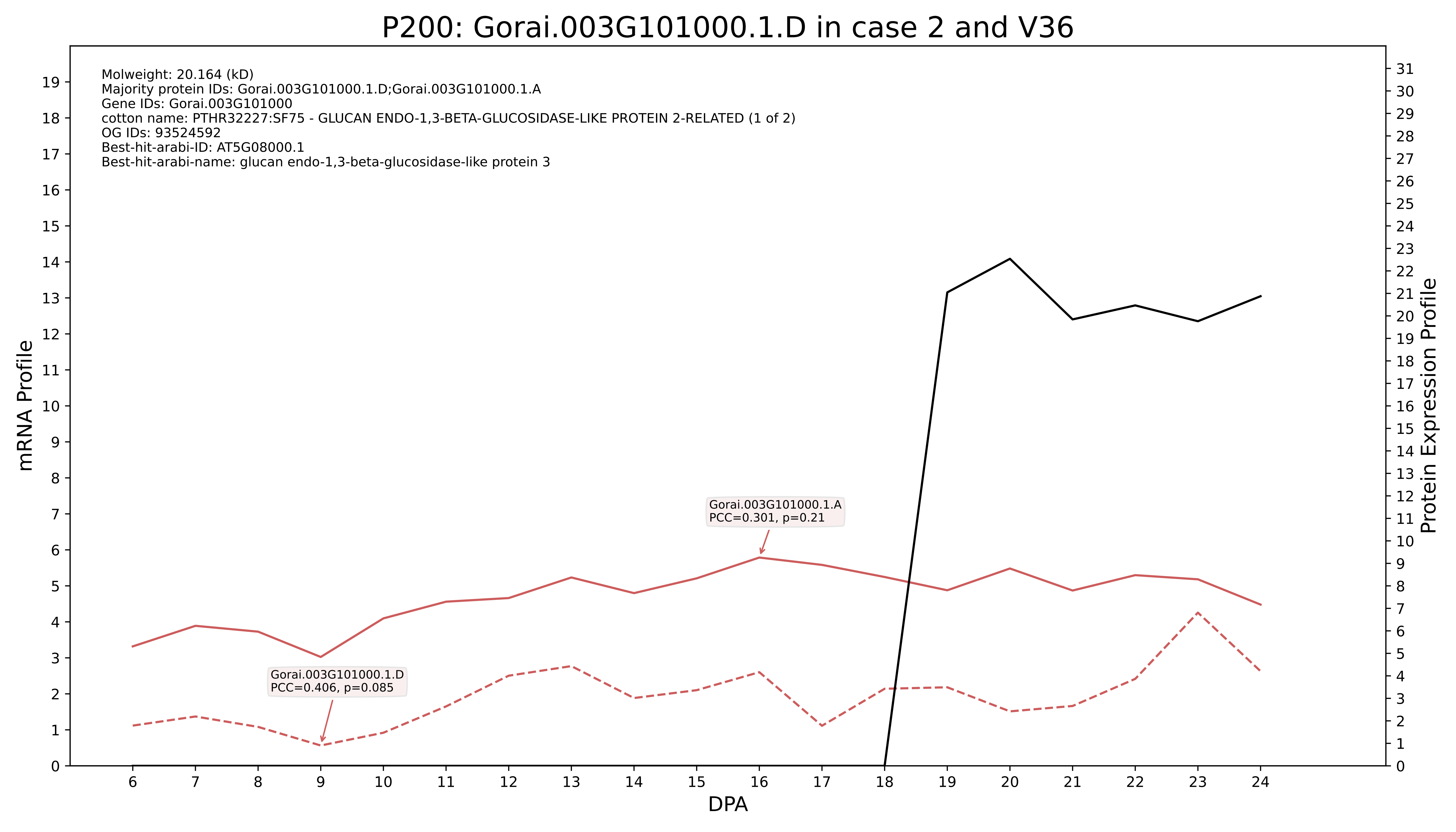
P200: Gorai.003G101000.1.D in case 2 and V36
Molweight: 20.164 (kD)
Majority protein IDs: Gorai.003G101000.1.D;Gorai.003G101000.1.A
Gene IDs: Gorai.003G101000
cotton name: PTHR32227:SF75 - GLUCAN ENDO-1,3-BETA-GLUCOSIDASE-LIKE PROTEIN 2-RELATED (1 of 2)
OG IDs: 93524592
Best-hit-arabi-ID: AT5G08000.1
Best-hit-arabi-name: glucan endo-1,3-beta-glucosidase-like protein 3
Gorai.003G101000.1.A
PCC=0.301, p=0.21
Gorai.003G101000.1.D
PCC=0.406, p=0.085
mRNA Profile
Protein Expression Profile
DPA
0
1
2
3
4
5
6
7
8
9
10
11
12
13
14
15
16
17
18
19
20
21
22
23
24
25
26
27
28
29
30
31

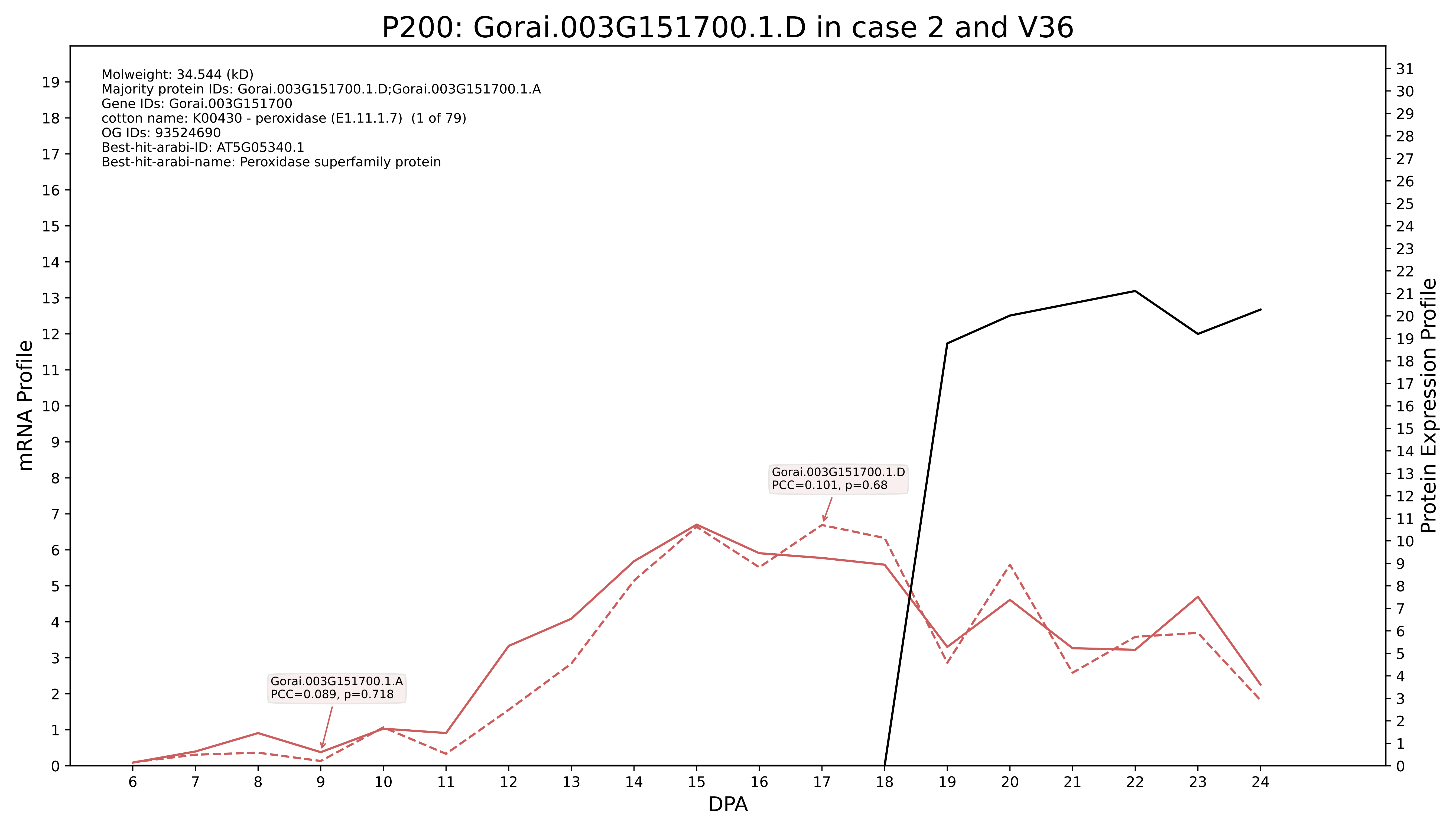
P200: Gorai.003G151700.1.D in case 2 and V36
Molweight: 34.544 (kD)
Majority protein IDs: Gorai.003G151700.1.D;Gorai.003G151700.1.A
Gene IDs: Gorai.003G151700
cotton name: K00430 - peroxidase (E1.11.1.7) (1 of 79)
OG IDs: 93524690
Best-hit-arabi-ID: AT5G05340.1
Best-hit-arabi-name: Peroxidase superfamily protein
Gorai.003G151700.1.D
PCC=0.101, p=0.68
Gorai.003G151700.1.A
PCC=0.089, p=0.718
mRNA Profile
Protein Expression Profile
DPA

P200: Gorai.007G176400.2.D in case 2 and V36

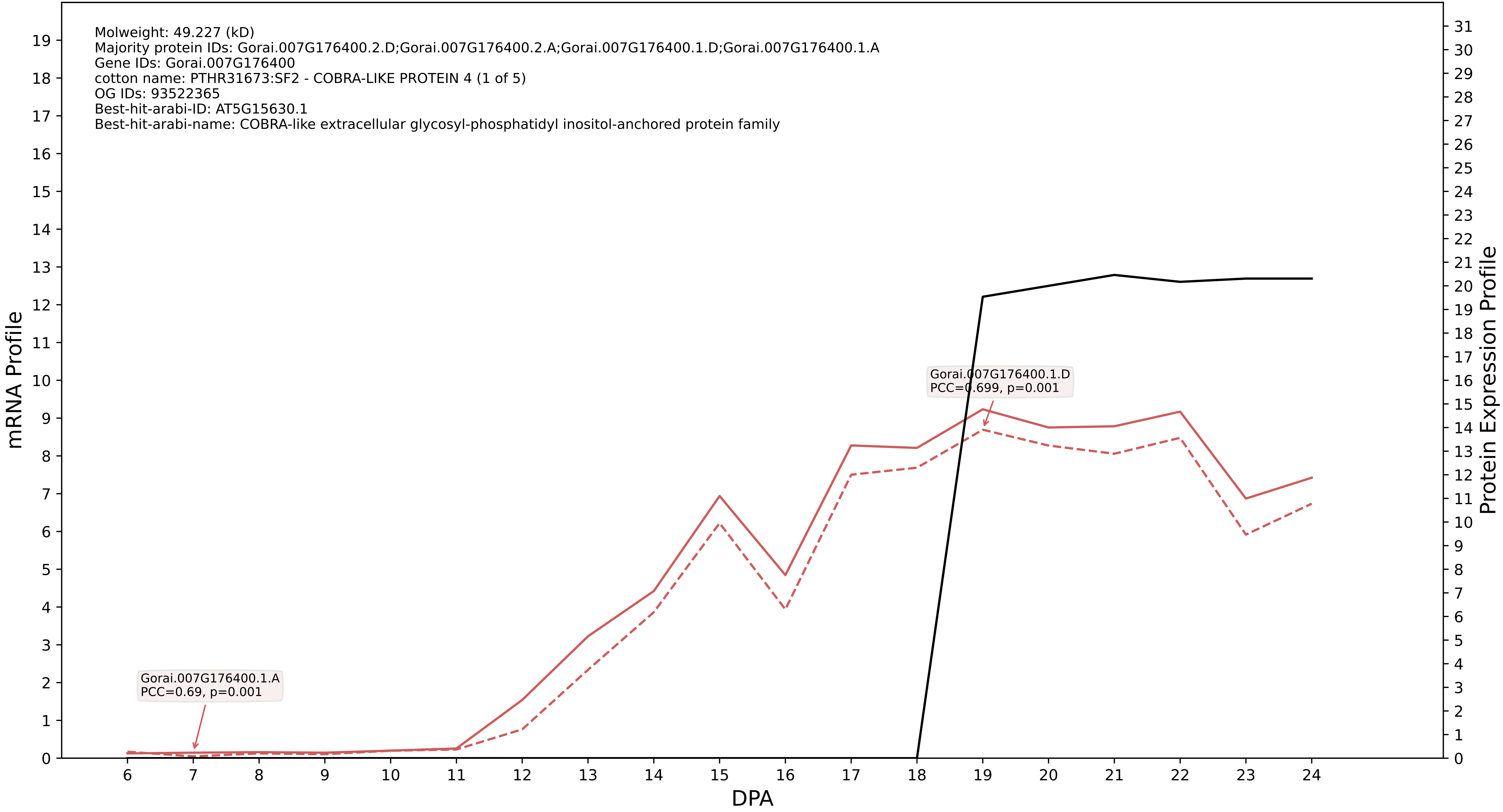

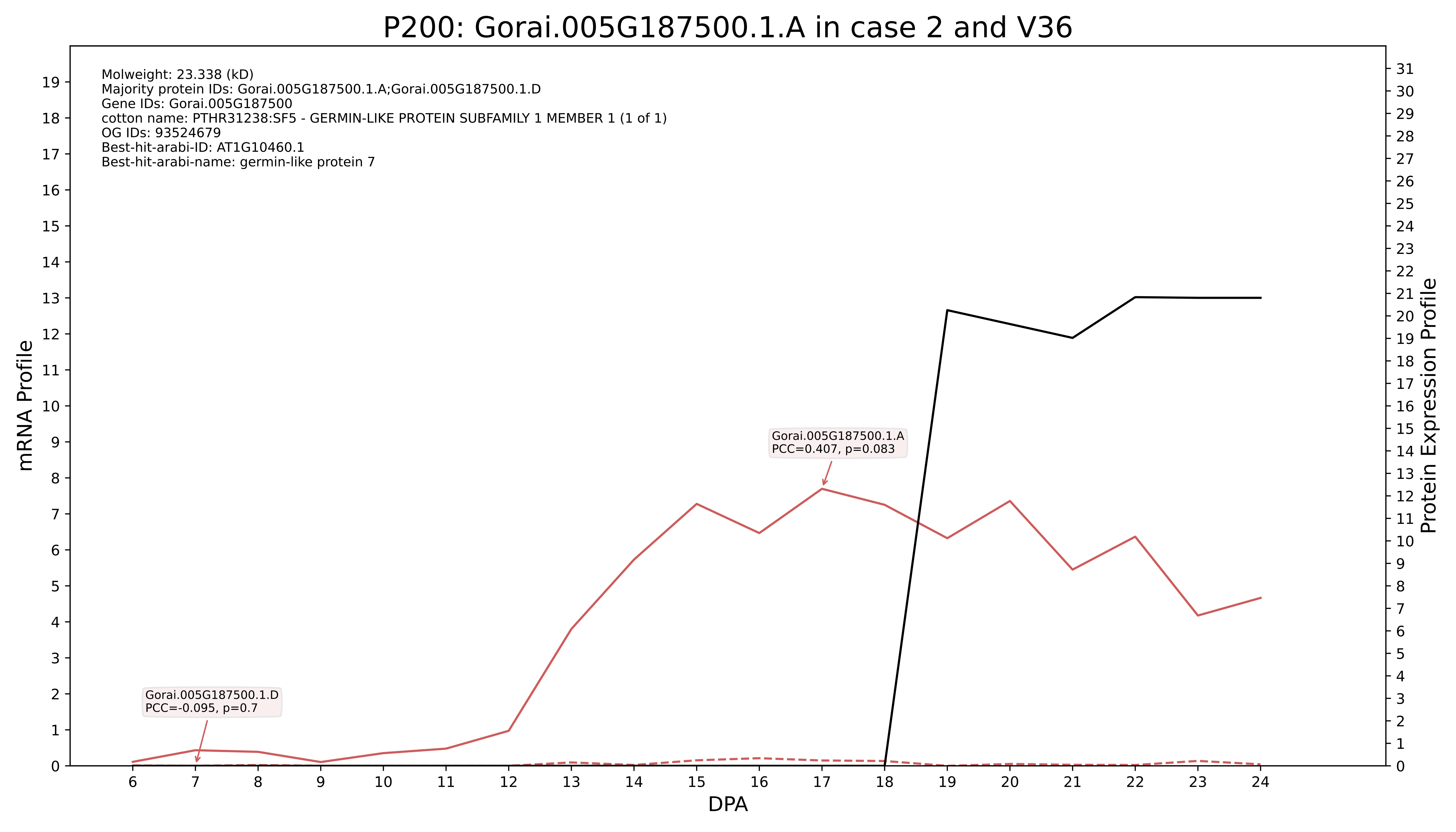

P200: Gorai.005G187500.1.A in case 2 and V36
Molweight: 23.338 (kD)
Majority protein IDs: Gorai.005G187500.1.A;Gorai.005G187500.1.D
Gene IDs: Gorai.005G187500
cotton name: PTHR31238:SF5 - GERMIN-LIKE PROTEIN SUBFAMILY 1 MEMBER 1 (1 of 1)
OG IDs: 93524679
Best-hit-arabi-ID: AT1G10460.1
Best-hit-arabi-name: germin-like protein 7
Gorai.005G187500.1.A
PCC=0.407, p=0.083
Gorai.005G187500.1.D
PCC=-0.095, p=0.7
mRNA Profile
Protein Expression Profile
DPA

200: Gorai.007G262600.2.A in case 2 and V36

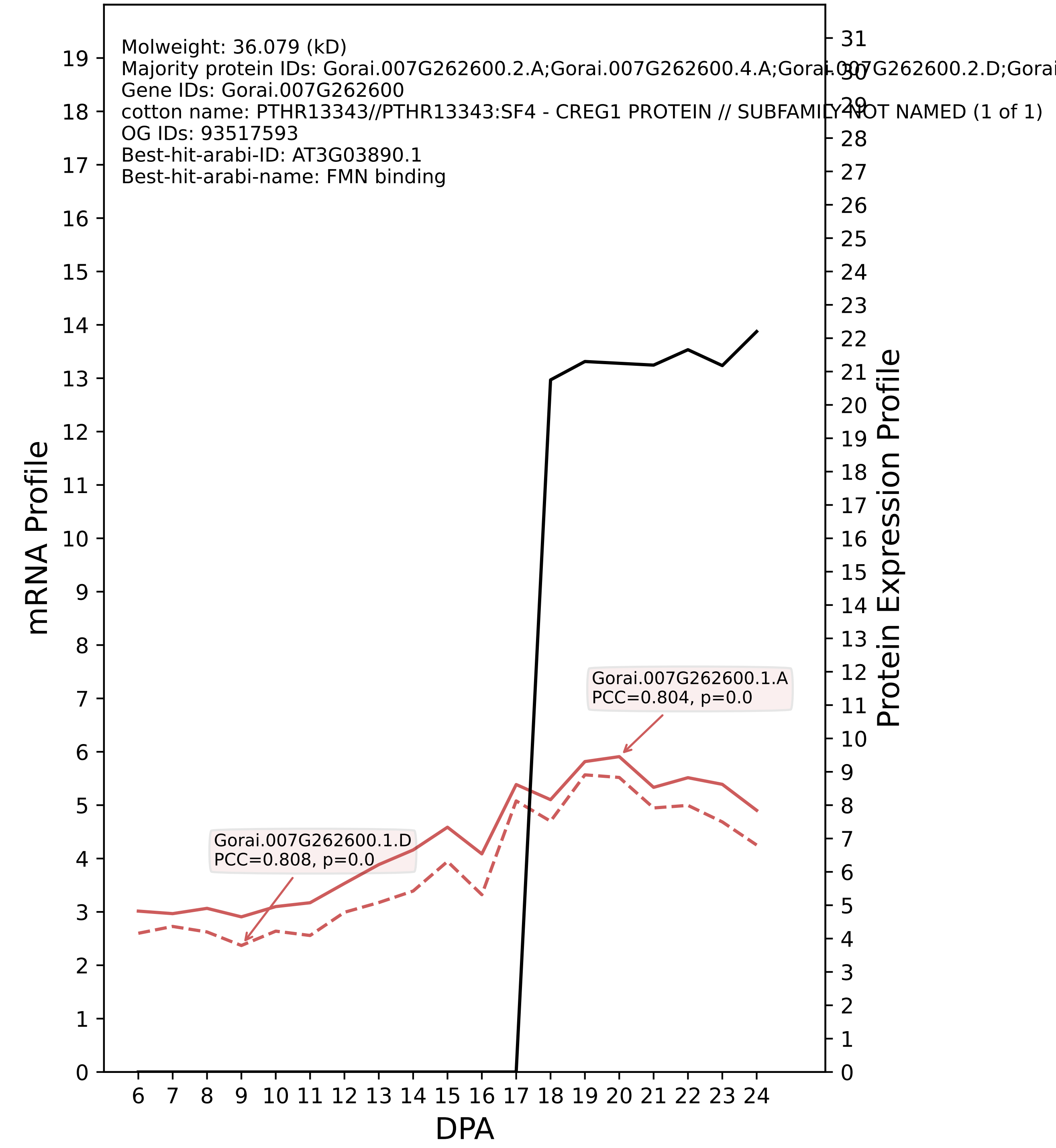

P200: Gorai.008G161800.2.D in case 2 and V36

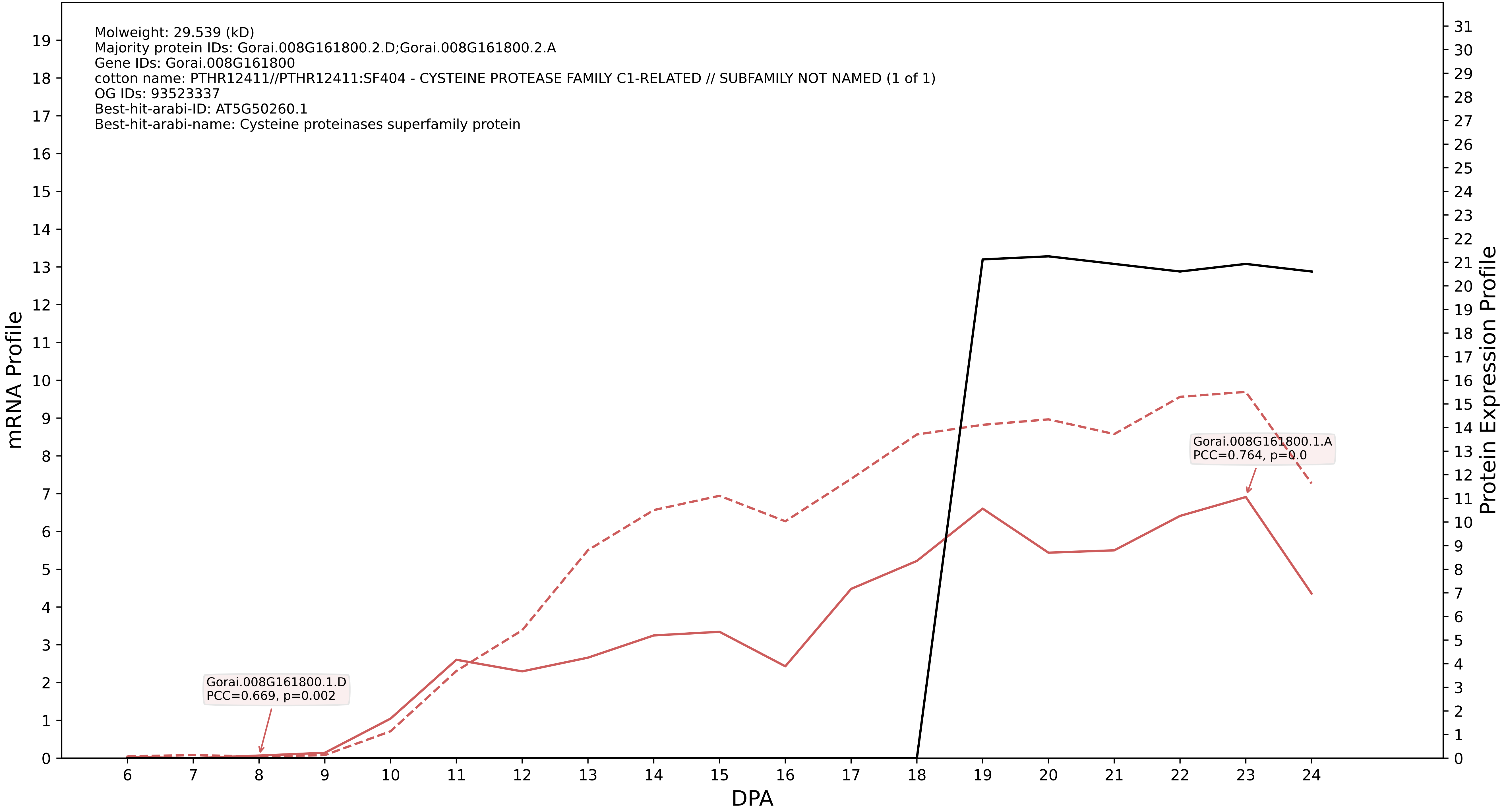

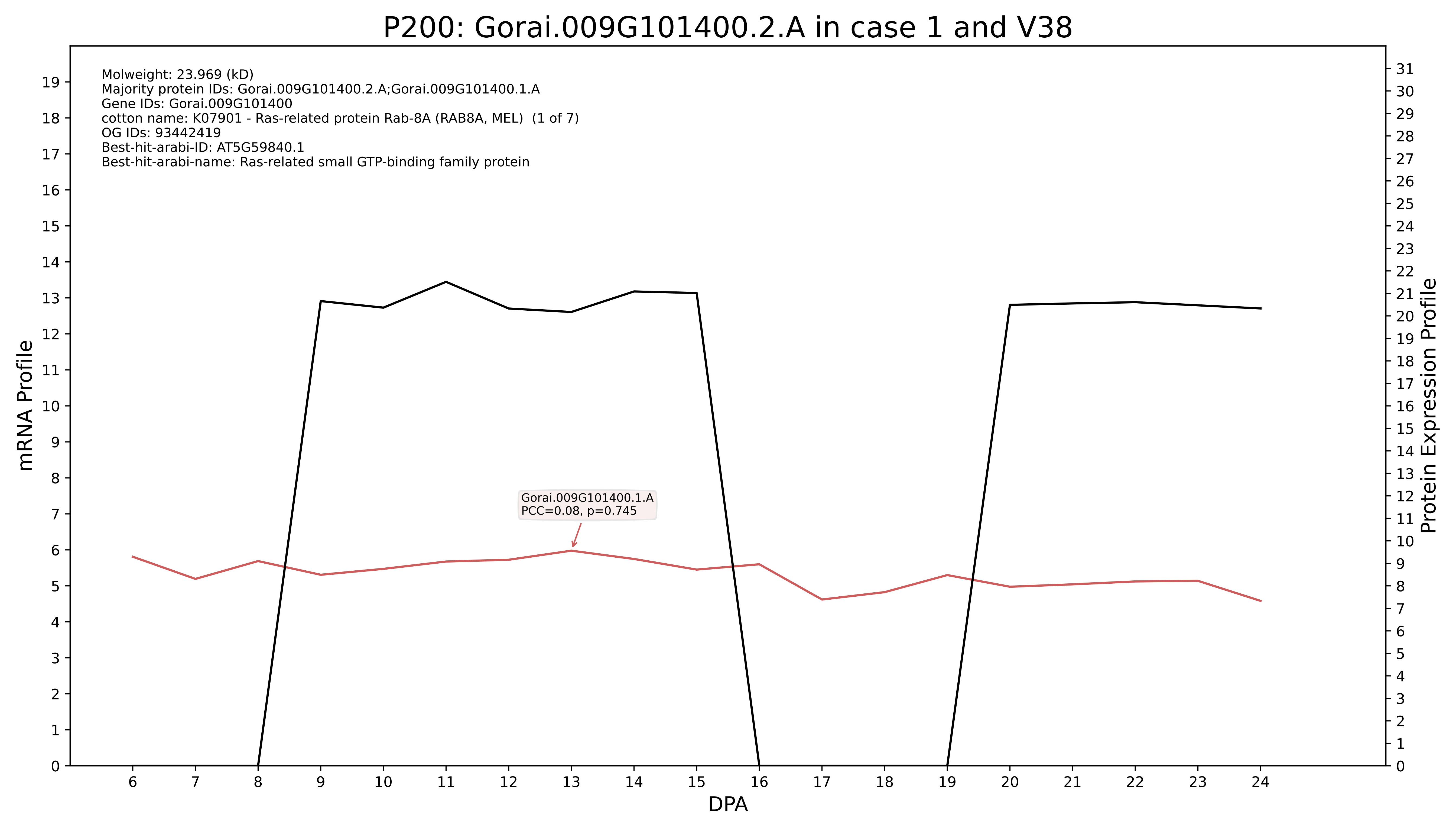
P200: Gorai.009G101400.2.A in case 1 and V38
Molweight: 23.969 (kD)
Majority protein IDs: Gorai.009G101400.2.A;Gorai.009G101400.1.A
Gene IDs: Gorai.009G101400
cotton name: K07901 - Ras-related protein Rab-8A (RAB8A, MEL) (1 of 7)
OG IDs: 93442419
Best-hit-arabi-ID: AT5G59840.1
Best-hit-arabi-name: Ras-related small GTP-binding family protein
Gorai.009G101400.1.A
PCC=0.08, p=0.745
mRNA Profile
Protein Expression Profile
DPA

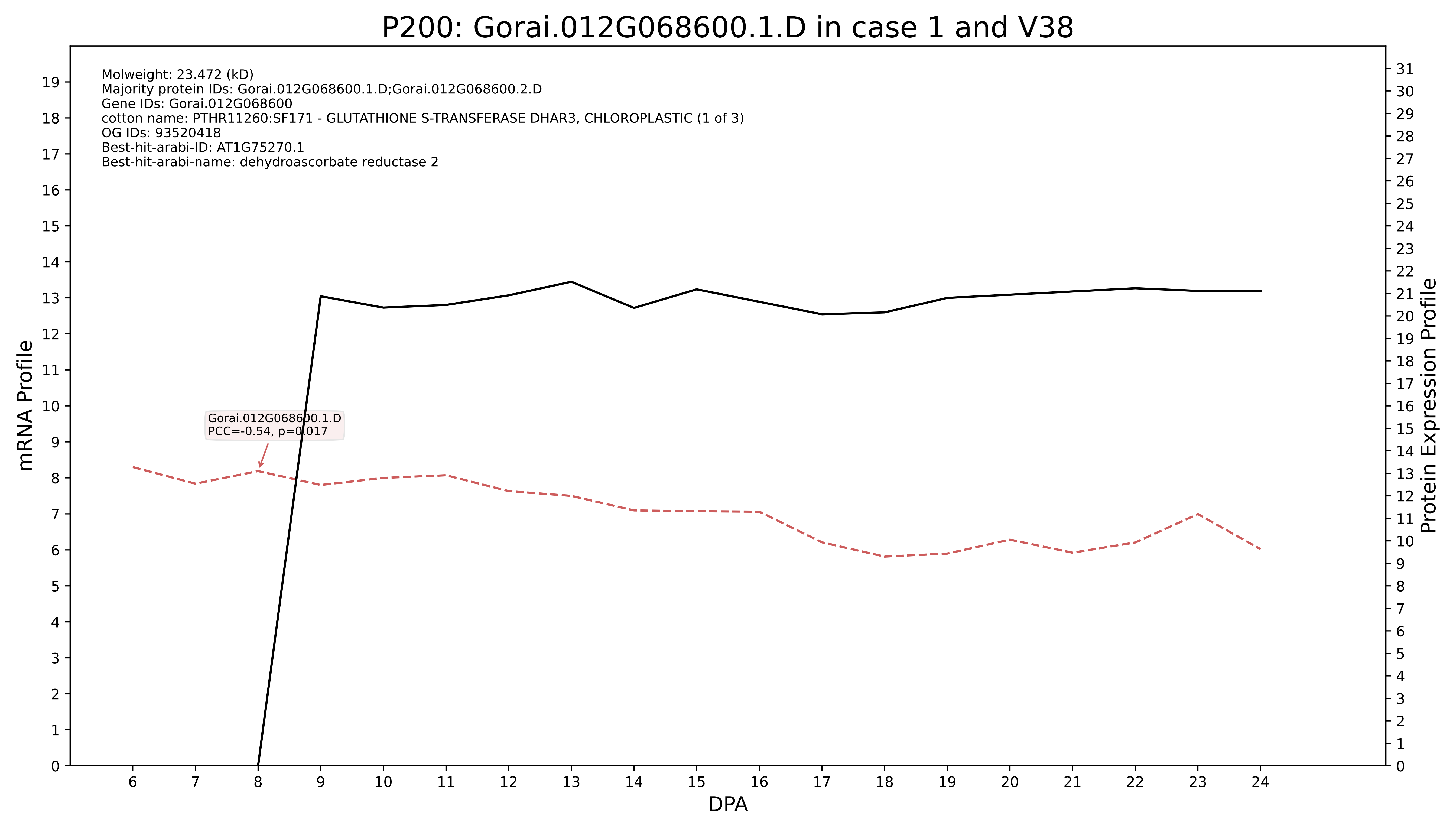
P200: Gorai.012G068600.1.D in case 1 and V38
Molweight: 23.472 (kD)
Majority protein IDs: Gorai.012G068600.1.D;Gorai.012G068600.2.D
Gene IDs: Gorai.012G068600
cotton name: PTHR11260:SF171 - GLUTATHIONE S-TRANSFERASE DHAR3, CHLOROPLASTIC (1 of 3)
OG IDs: 93520418
Best-hit-arabi-ID: AT1G75270.1
Best-hit-arabi-name: dehydroascorbate reductase 2
Gorai.012G068600.1.D
PCC=-0.54, p=0.017
mRNA Profile
Protein Expression Profile
DPA

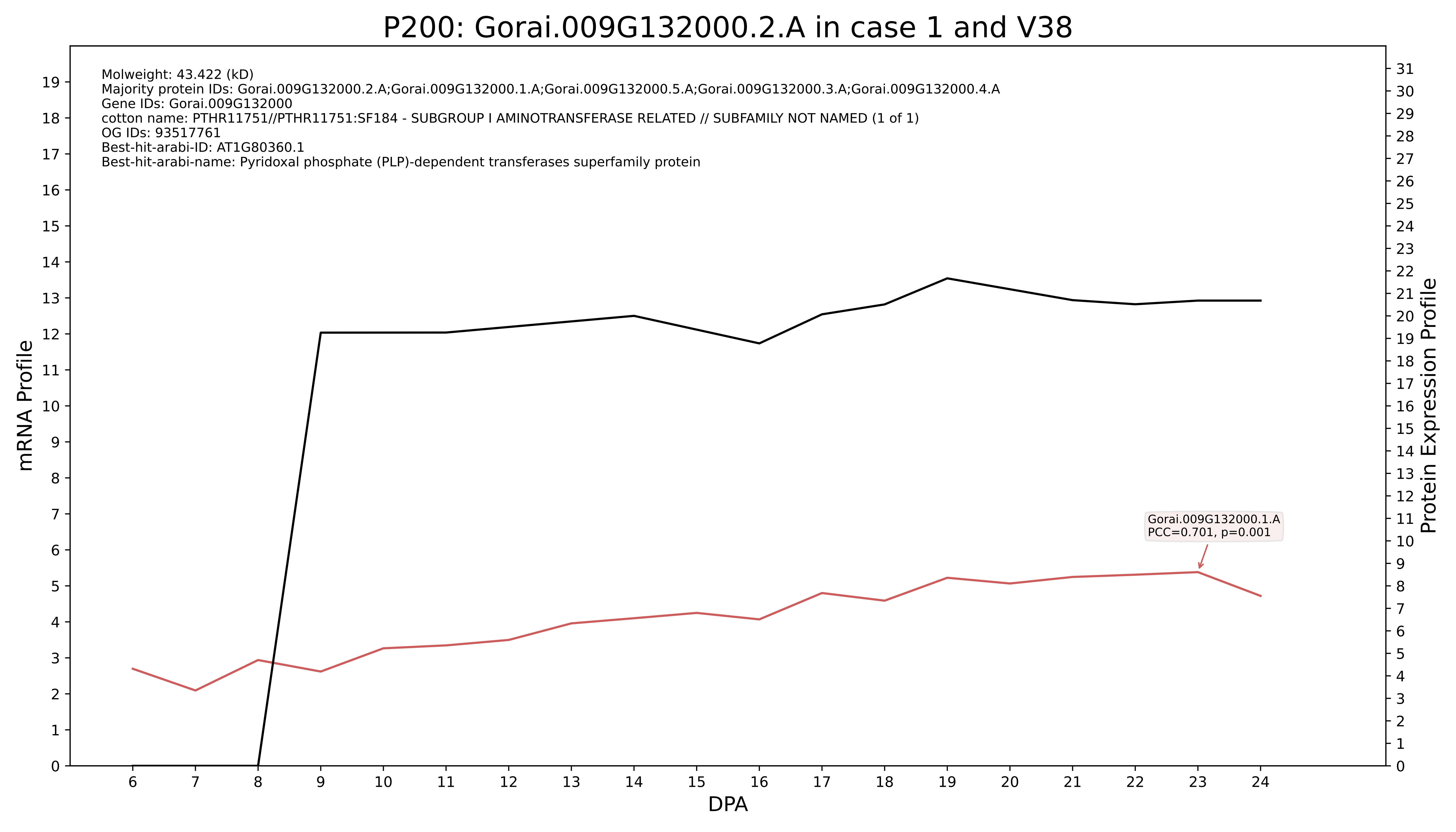
P200: Gorai.009G132000.2.A in case 1 and V38
Molweight: 43.422 (kD)
Majority protein IDs: Gorai.009G132000.2.A;Gorai.009G132000.1.A;Gorai.009G132000.5.A;Gorai.009G132000.3.A;Gorai.009G132000.4.A
Gene IDs: Gorai.009G132000
cotton name: PTHR11751//PTHR11751:SF184 - SUBGROUP I AMINOTRANSFERASE RELATED // SUBFAMILY NOT NAMED (1 of 1)
OG IDs: 93517761
Best-hit-arabi-ID: AT1G80360.1
Best-hit-arabi-name: Pyridoxal phosphate (PLP)-dependent transferases superfamily protein
Gorai.009G132000.1.A
PCC=0.701, p=0.001
mRNA Profile
Protein Expression Profile
DPA

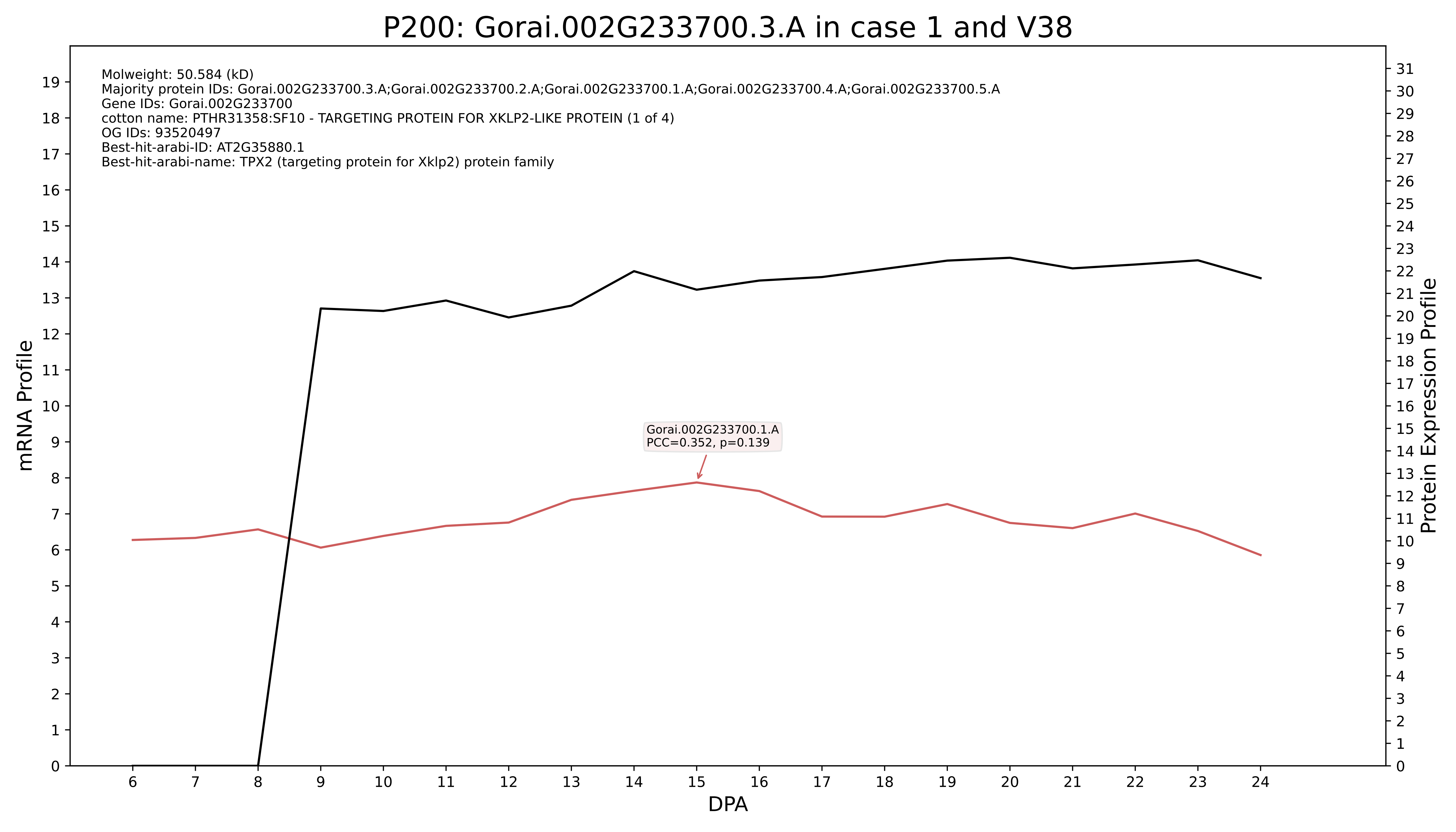
P200: Gorai.002G233700.3.A in case 1 and V38
Molweight: 50.584 (kD)
Majority protein IDs: Gorai.002G233700.3.A;Gorai.002G233700.2.A;Gorai.002G233700.1.A;Gorai.002G233700.4.A;Gorai.002G233700.5.A
Gene IDs: Gorai.002G233700
cotton name: PTHR31358:SF10 - TARGETING PROTEIN FOR XKLP2-LIKE PROTEIN (1 of 4)
OG IDs: 93520497
Best-hit-arabi-ID: AT2G35880.1
Best-hit-arabi-name: TPX2 (targeting protein for Xklp2) protein family
Gorai.002G233700.1.A
PCC=0.352, p=0.139
mRNA Profile
Protein Expression Profile
DPA

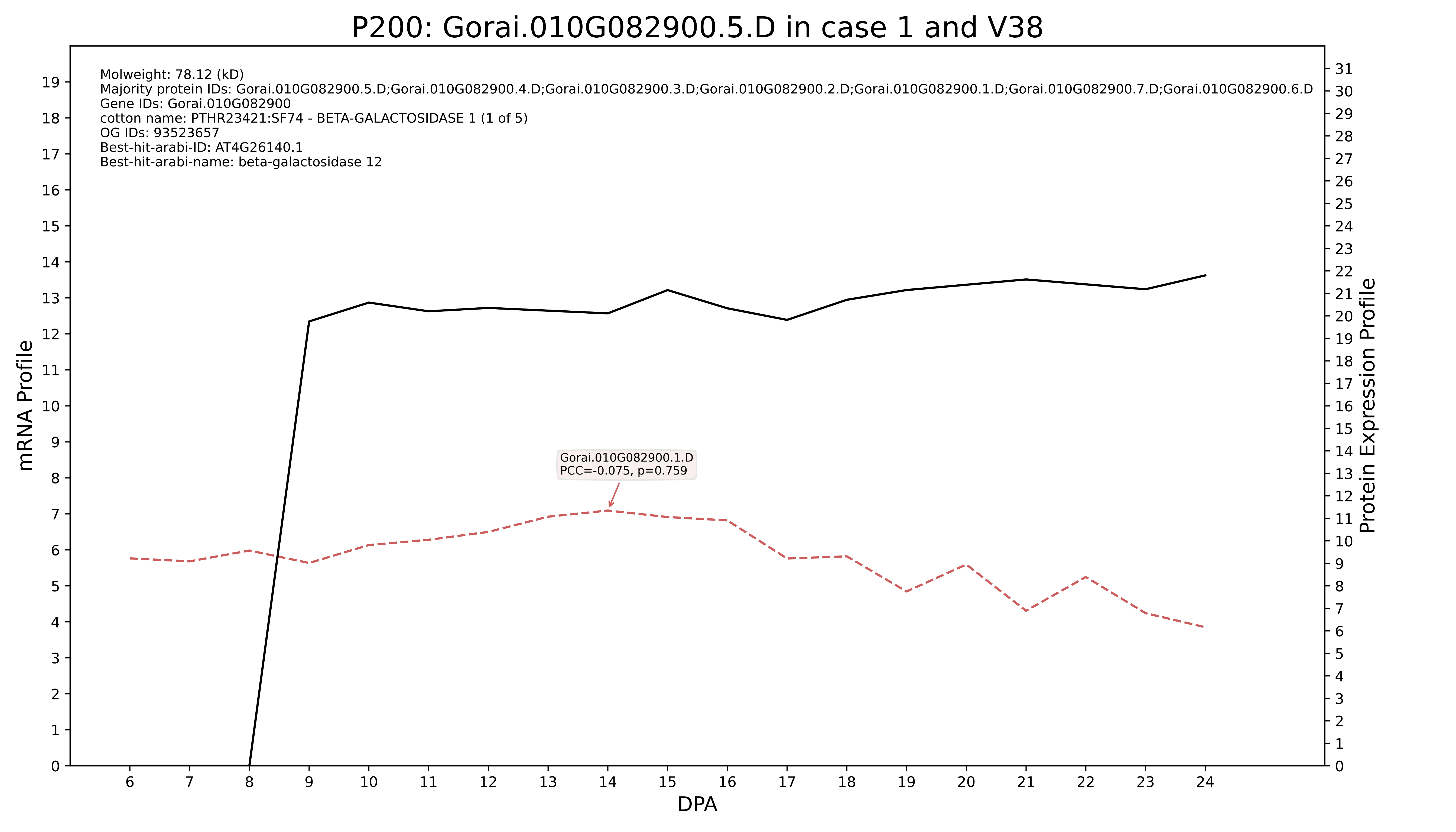
P200: Gorai.010G082900.5.D in case 1 and V38
Molweight: 78.12 (kD)
Majority protein IDs: Gorai.010G082900.5.D;Gorai.010G082900.4.D;Gorai.010G082900.3.D;Gorai.010G082900.2.D;Gorai.010G082900.1.D;Gorai.010G082900.7.D;Gorai.010G082900.6.D
Gene IDs: Gorai.010G082900
cotton name: PTHR23421:SF74 - BETA-GALACTOSIDASE 1 (1 of 5)
OG IDs: 93523657
Best-hit-arabi-ID: AT4G26140.1
Best-hit-arabi-name: beta-galactosidase 12
Gorai.010G082900.1.D
PCC=-0.075, p=0.759
mRNA Profile
Protein Expression Profile
DPA

P200: Gorai.011G193800.1.D in case 1 and V38

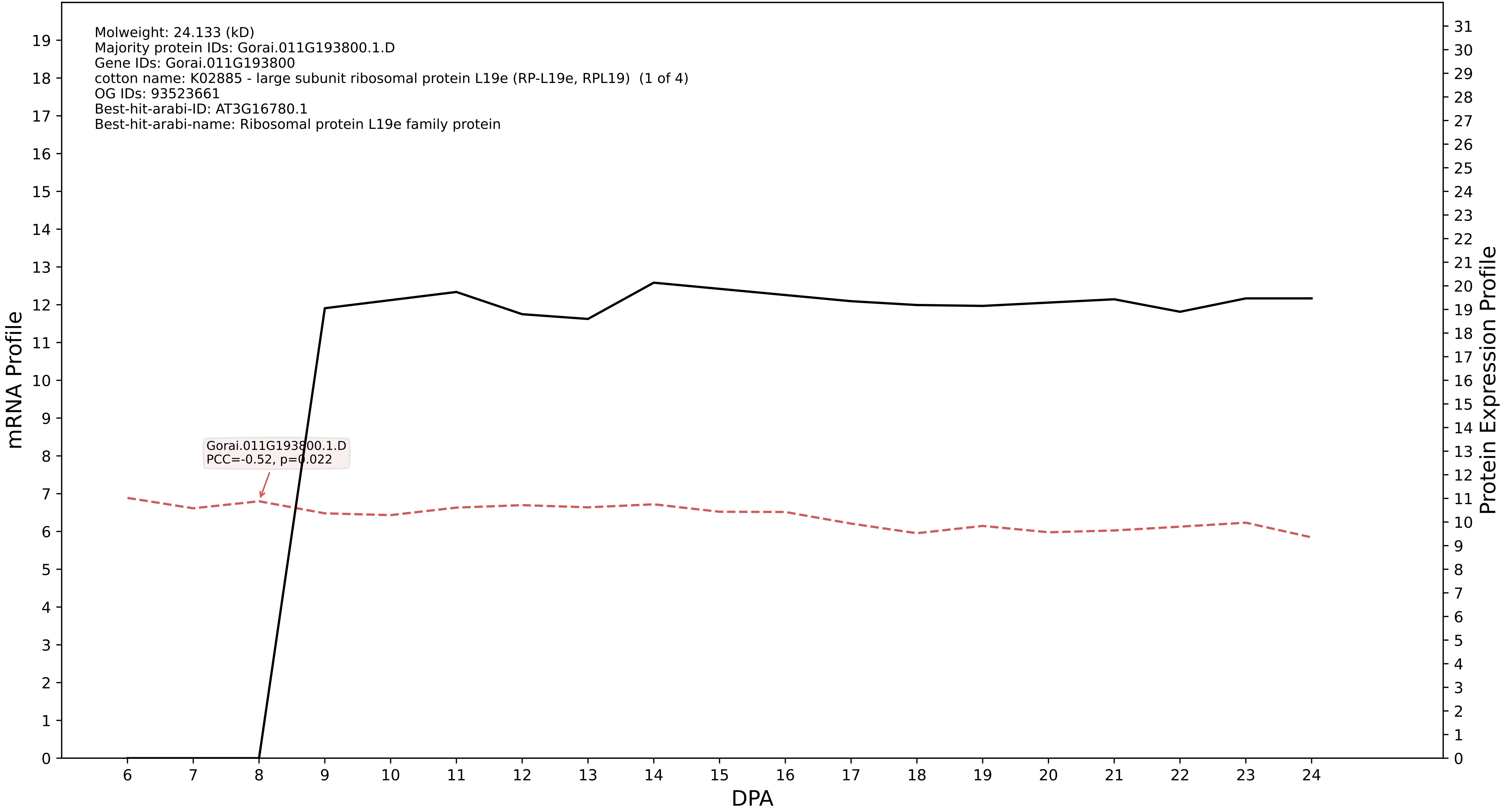

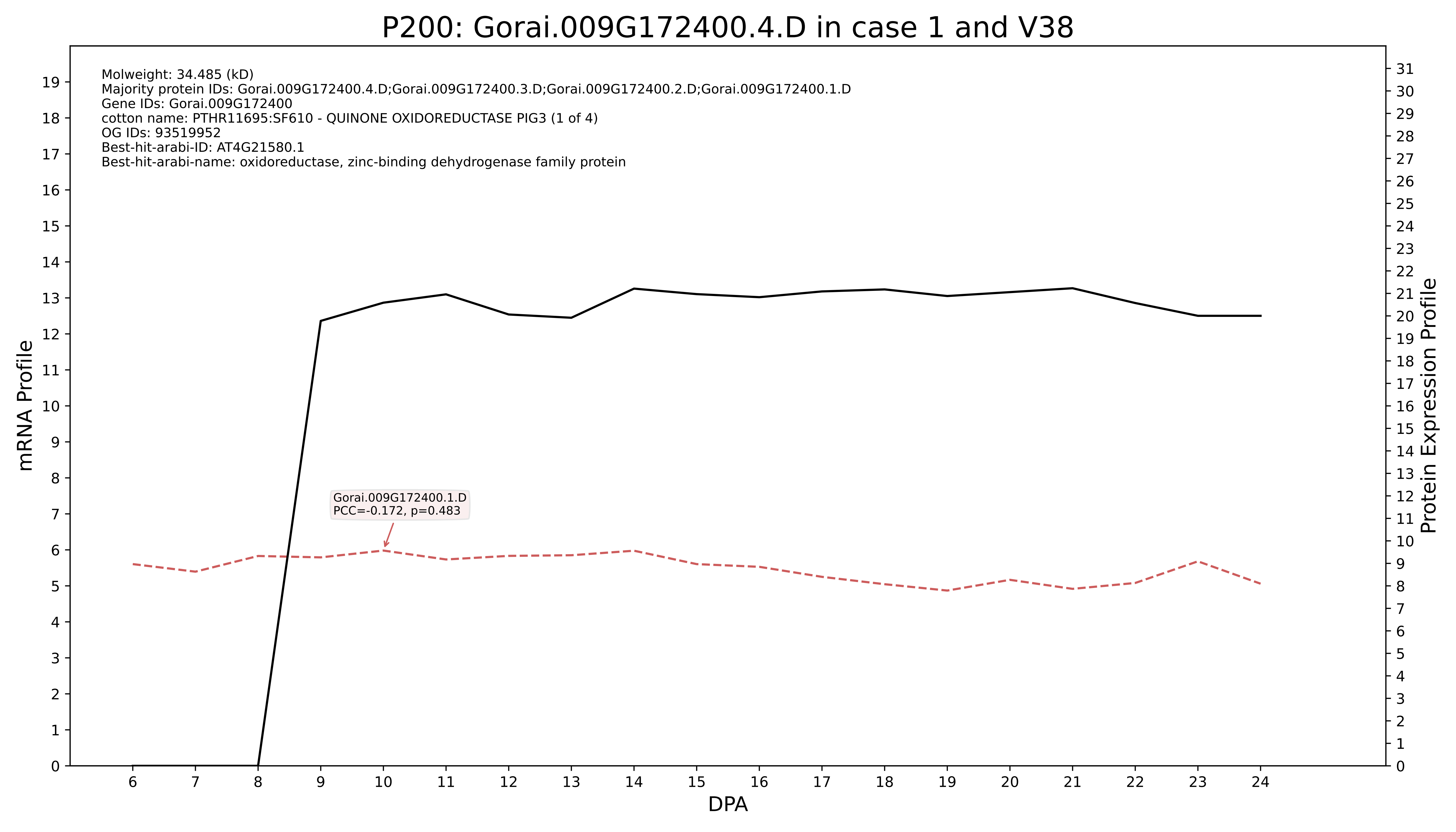
P200: Gorai.009G172400.4.D in case 1 and V38
Molweight: 34.485 (kD)
Majority protein IDs: Gorai.009G172400.4.D;Gorai.009G172400.3.D;Gorai.009G172400.2.D;Gorai.009G172400.1.D
Gene IDs: Gorai.009G172400
cotton name: PTHR11695:SF610 - QUINONE OXIDOREDUCTASE PIG3 (1 of 4)
OG IDs: 93519952
Best-hit-arabi-ID: AT4G21580.1
Best-hit-arabi-name: oxidoreductase, zinc-binding dehydrogenase family protein
Gorai.009G172400.1.D
PCC=-0.172, p=0.483
mRNA Profile
Protein Expression Profile
DPA

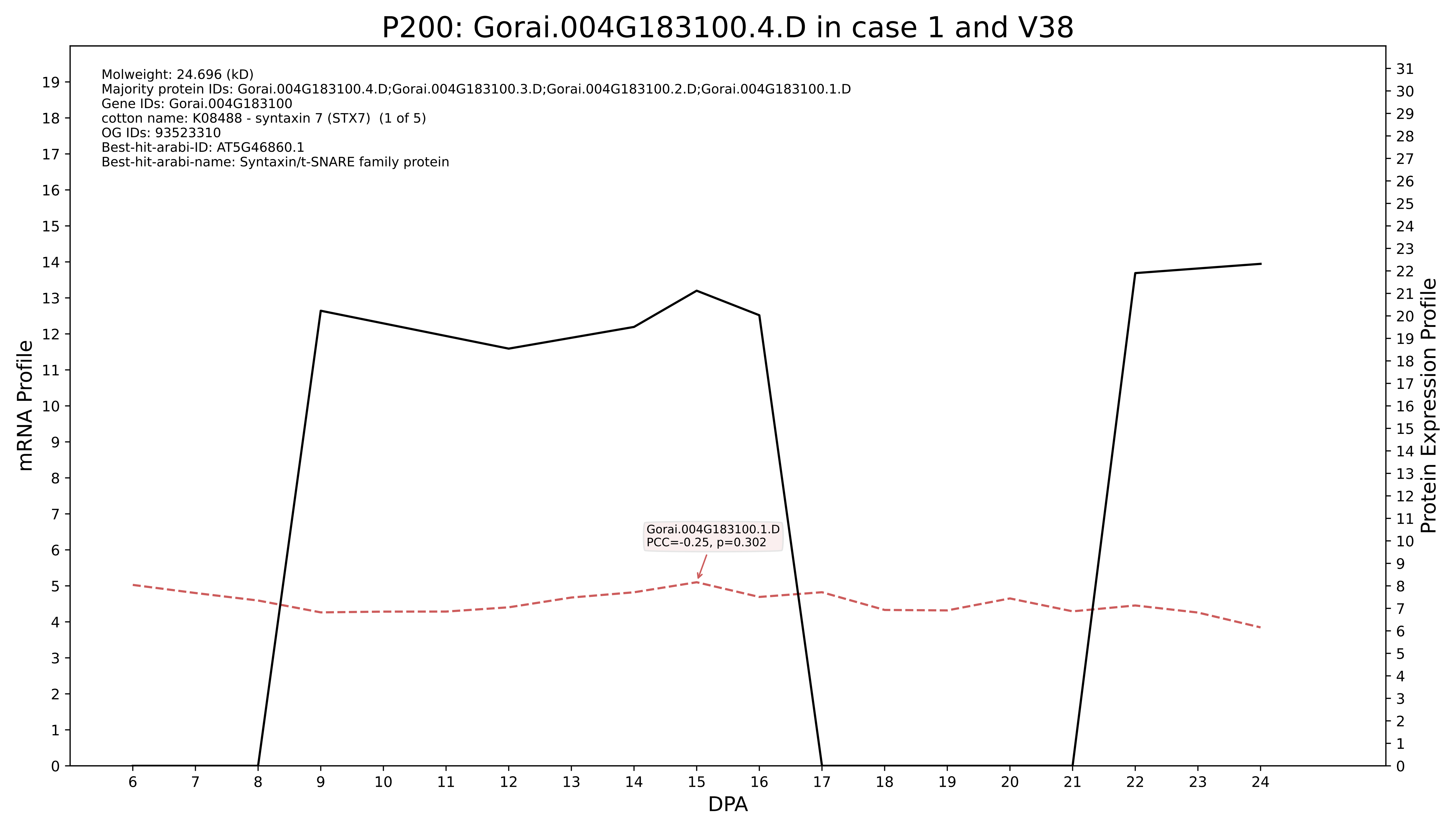
P200: Gorai.004G183100.4.D in case 1 and V38
Molweight: 24.696 (kD)
Majority protein IDs: Gorai.004G183100.4.D;Gorai.004G183100.3.D;Gorai.004G183100.2.D;Gorai.004G183100.1.D
Gene IDs: Gorai.004G183100
cotton name: K08488 - syntaxin 7 (STX7) (1 of 5)
OG IDs: 93523310
Best-hit-arabi-ID: AT5G46860.1
Best-hit-arabi-name: Syntaxin/t-SNARE family protein
Gorai.004G183100.1.D
PCC=-0.25, p=0.302
mRNA Profile
Protein Expression Profile
DPA

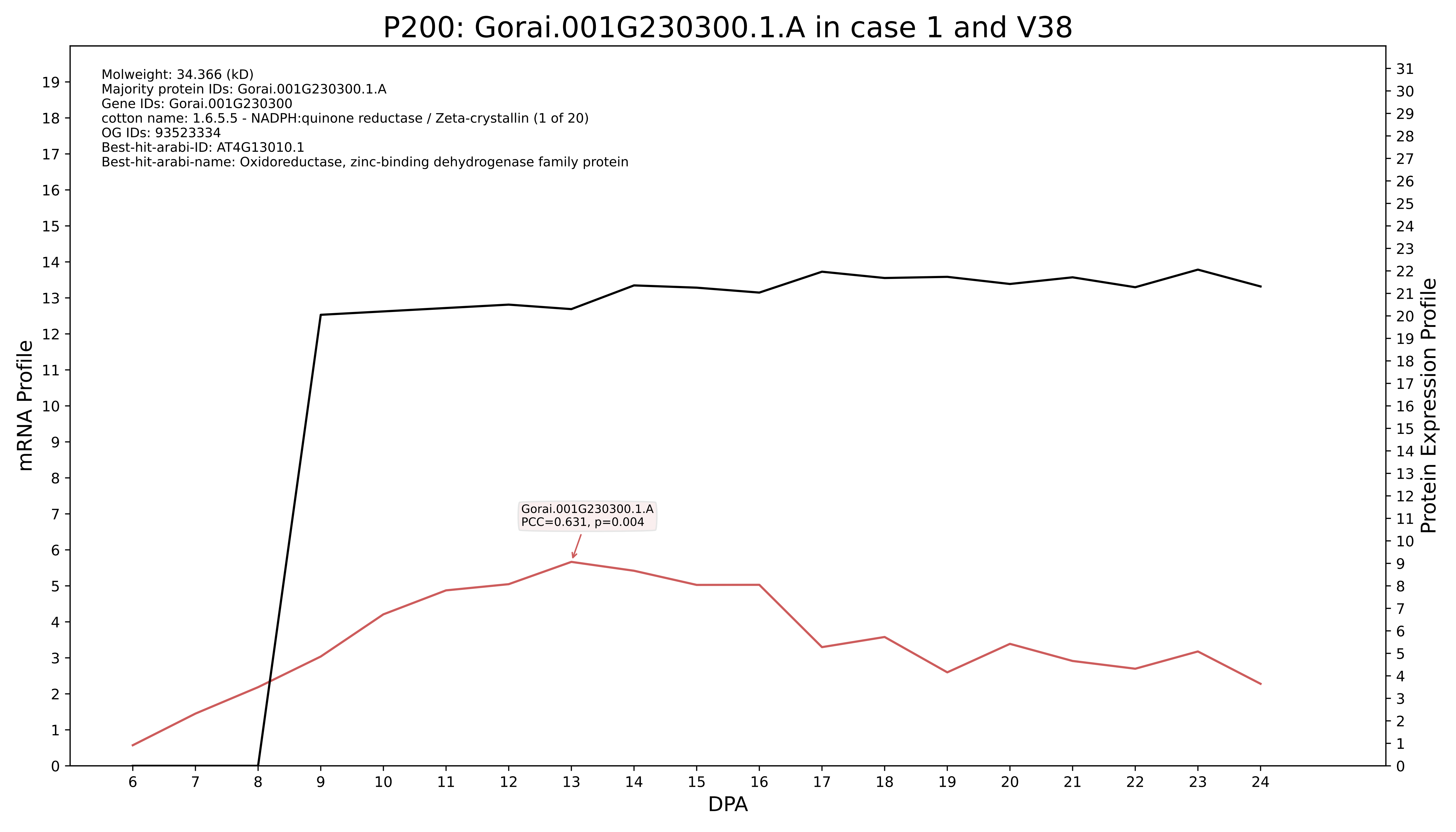
P200: Gorai.001G230300.1.A in case 1 and V38
Molweight: 34.366 (kD)
Majority protein IDs: Gorai.001G230300.1.A
Gene IDs: Gorai.001G230300
cotton name: 1.6.5.5 - NADPH:quinone reductase / Zeta-crystallin (1 of 20)
OG IDs: 93523334
Best-hit-arabi-ID: AT4G13010.1
Best-hit-arabi-name: Oxidoreductase, zinc-binding dehydrogenase family protein
Gorai.001G230300.1.A
PCC=0.631, p=0.004
mRNA Profile
Protein Expression Profile
DPA
0 1 2 3 4 5 6 7 8 9 10 11 12 13 14 15 16 17 18 19
0 1 2 3 4 5 6 7 8 9 10 11 12 13 14 15 16 17 18 19 20 21 22 23 24 25 26 27 28 29 30 31
6 7 8 9 10 11 12 13 14 15 16 17 18 19 20 21 22 23 24

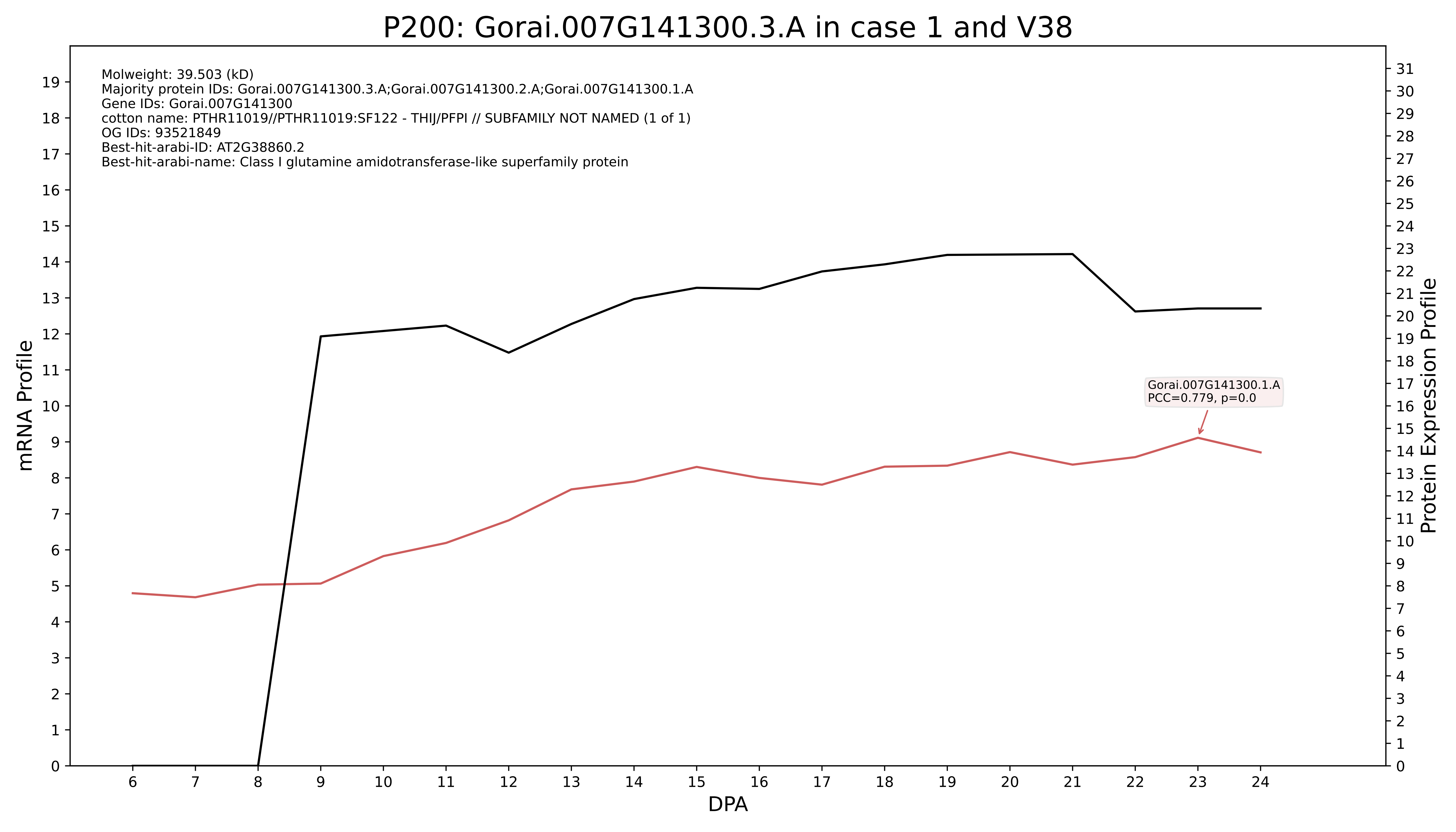
P200: Gorai.007G141300.3.A in case 1 and V38
Molweight: 39.503 (kD)
Majority protein IDs: Gorai.007G141300.3.A;Gorai.007G141300.2.A;Gorai.007G141300.1.A
Gene IDs: Gorai.007G141300
cotton name: PTHR11019//PTHR11019:SF122 - THIJ/PFPI // SUBFAMILY NOT NAMED (1 of 1)
OG IDs: 93521849
Best-hit-arabi-ID: AT2G38860.2
Best-hit-arabi-name: Class I glutamine amidotransferase-like superfamily protein
Gorai.007G141300.1.A
PCC=0.779, p=0.0
mRNA Profile
Protein Expression Profile
DPA

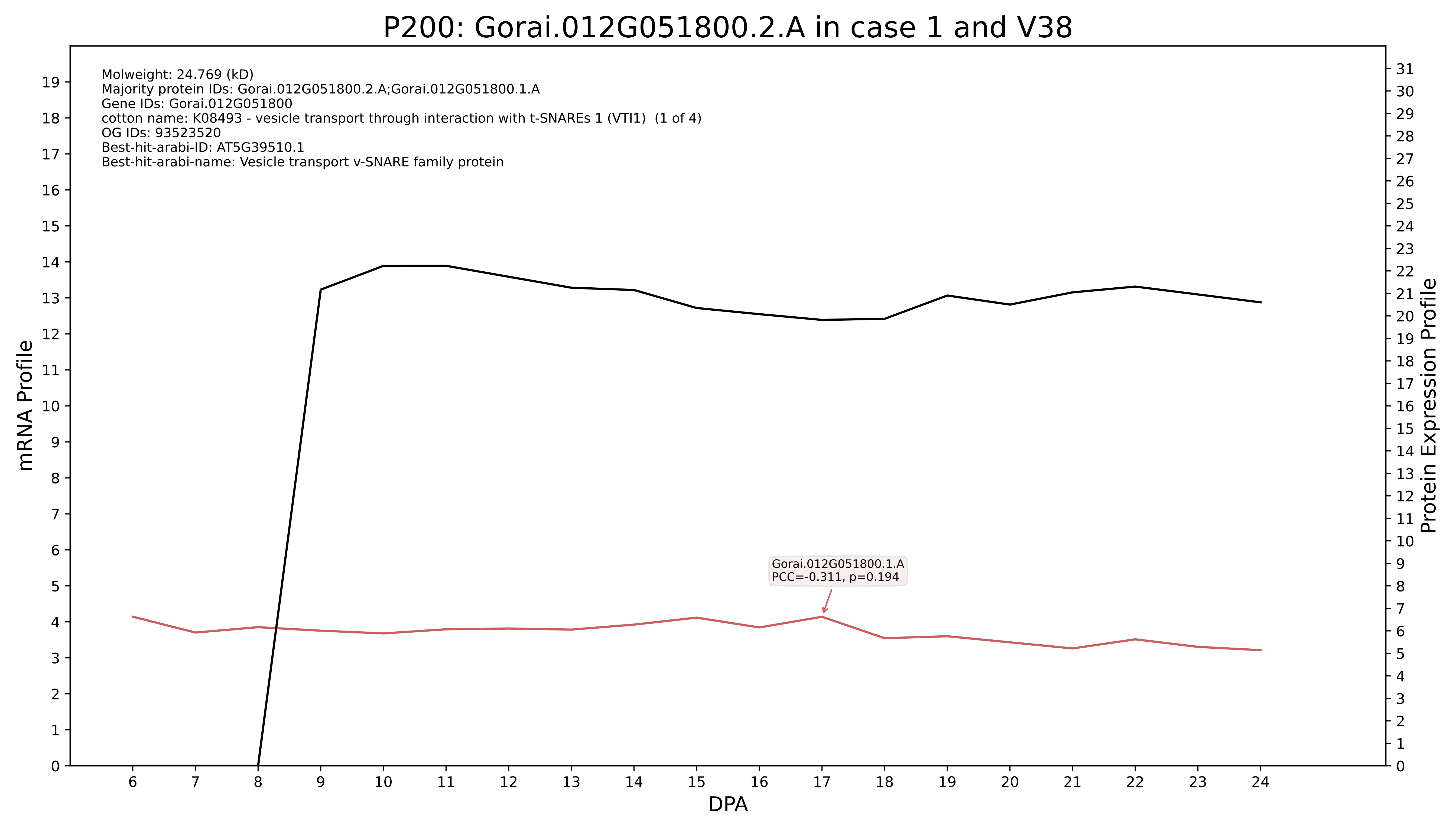

P200: Gorai.012G051800.2.A in case 1 and V38
Molweight: 24.769 (kD)
Majority protein IDs: Gorai.012G051800.2.A;Gorai.012G051800.1.A
Gene IDs: Gorai.012G051800
cotton name: K08493 - vesicle transport through interaction with t-SNAREs 1 (VTI1) (1 of 4)
OG IDs: 93523520
Best-hit-arabi-ID: AT5G39510.1
Best-hit-arabi-name: Vesicle transport v-SNARE family protein
Gorai.012G051800.1.A
PCC=-0.311, p=0.194
mRNA Profile
Protein Expression Profile
DPA

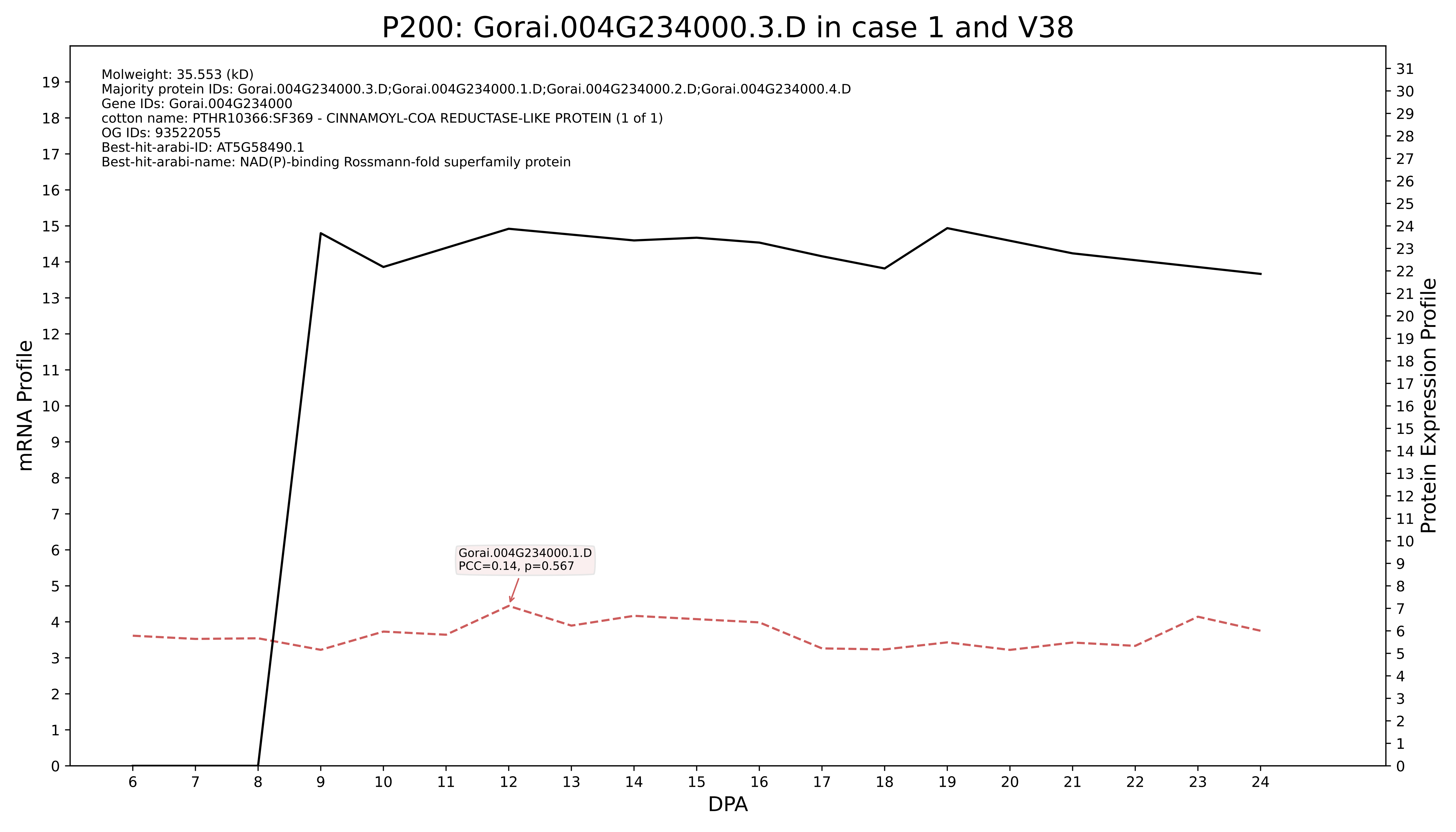

P200: Gorai.004G234000.3.D in case 1 and V38
Molweight: 35.553 (kD)
Majority protein IDs: Gorai.004G234000.3.D;Gorai.004G234000.1.D;Gorai.004G234000.2.D;Gorai.004G234000.4.D
Gene IDs: Gorai.004G234000
cotton name: PTHR10366:SF369 - CINNAMOYL-COA REDUCTASE-LIKE PROTEIN (1 of 1)
OG IDs: 93522055
Best-hit-arabi-ID: AT5G58490.1
Best-hit-arabi-name: NAD(P)-binding Rossmann-fold superfamily protein
Gorai.004G234000.1.D
PCC=0.14, p=0.567
mRNA Profile
Protein Expression Profile
DPA

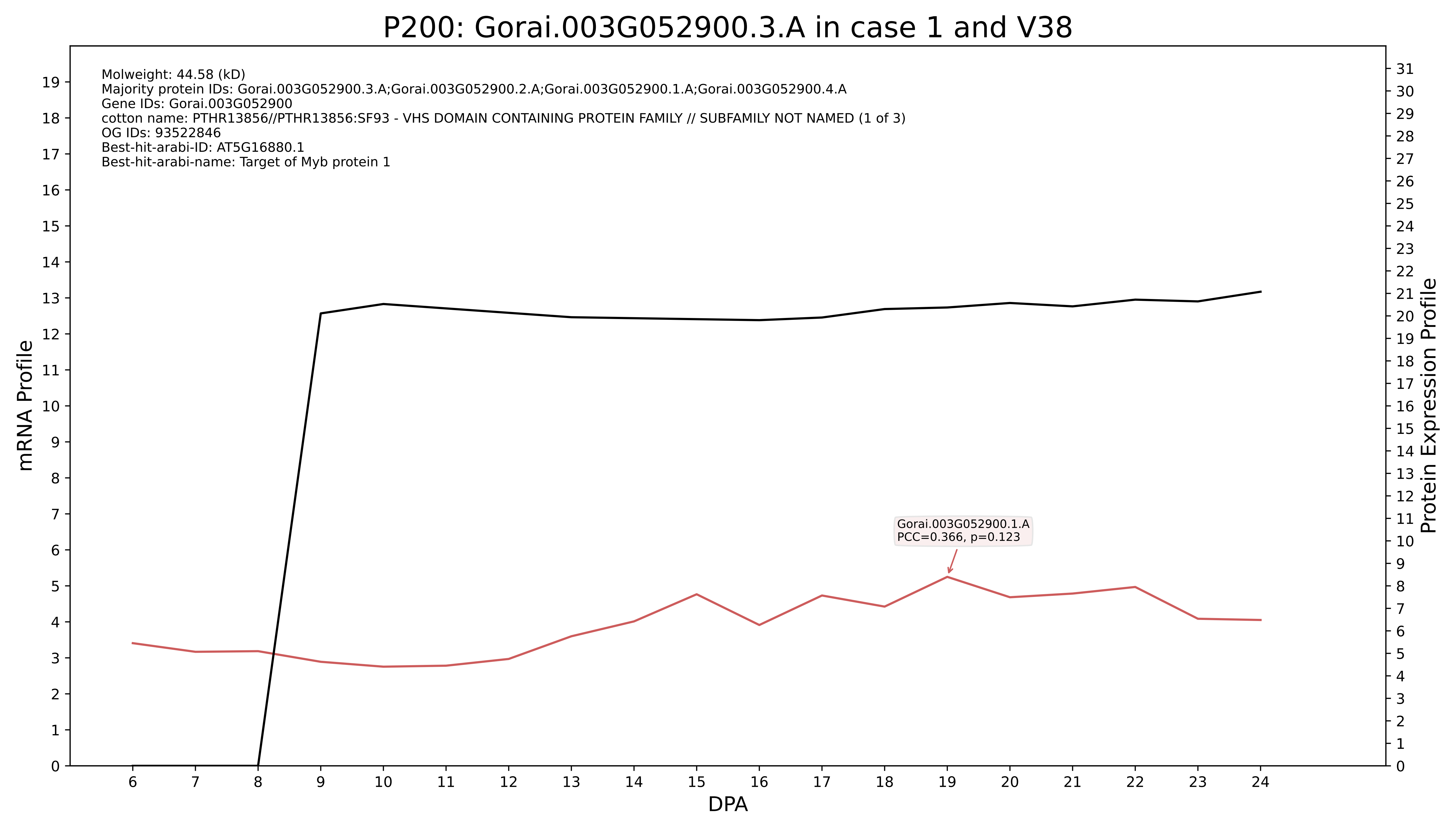

P200: Gorai.003G052900.3.A in case 1 and V38
Molweight: 44.58 (kD)
Majority protein IDs: Gorai.003G052900.3.A;Gorai.003G052900.2.A;Gorai.003G052900.1.A;Gorai.003G052900.4.A
Gene IDs: Gorai.003G052900
cotton name: PTHR13856//PTHR13856:SF93 - VHS DOMAIN CONTAINING PROTEIN FAMILY // SUBFAMILY NOT NAMED (1 of 3)
OG IDs: 93522846
Best-hit-arabi-ID: AT5G16880.1
Best-hit-arabi-name: Target of Myb protein 1
Gorai.003G052900.1.A
PCC=0.366, p=0.123
mRNA Profile
Protein Expression Profile
DPA

P200: Gorai.013G145300.6.A in case 1 and V38

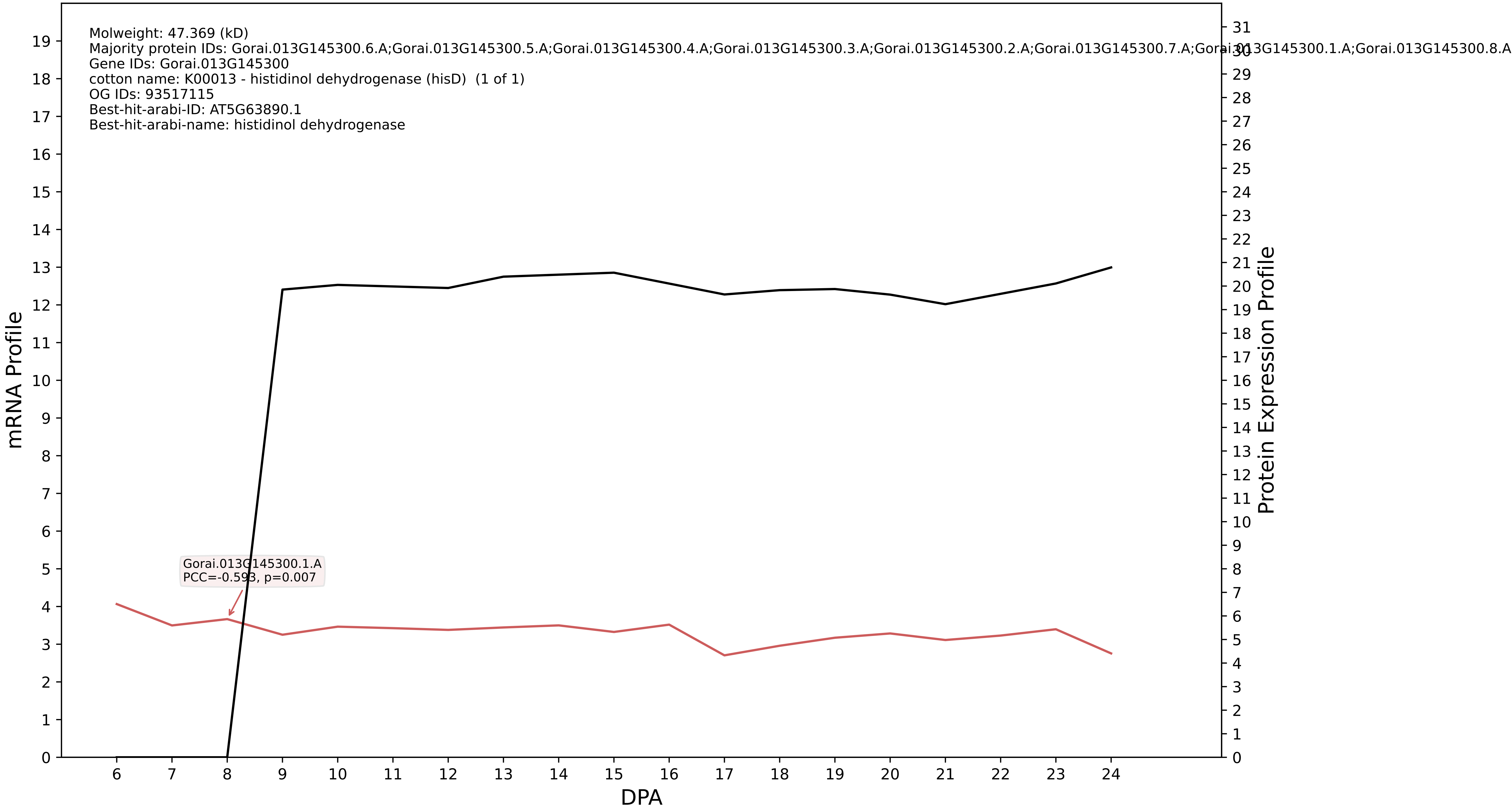

P200: Gorai.013G234400.1.D in case 1 and V38

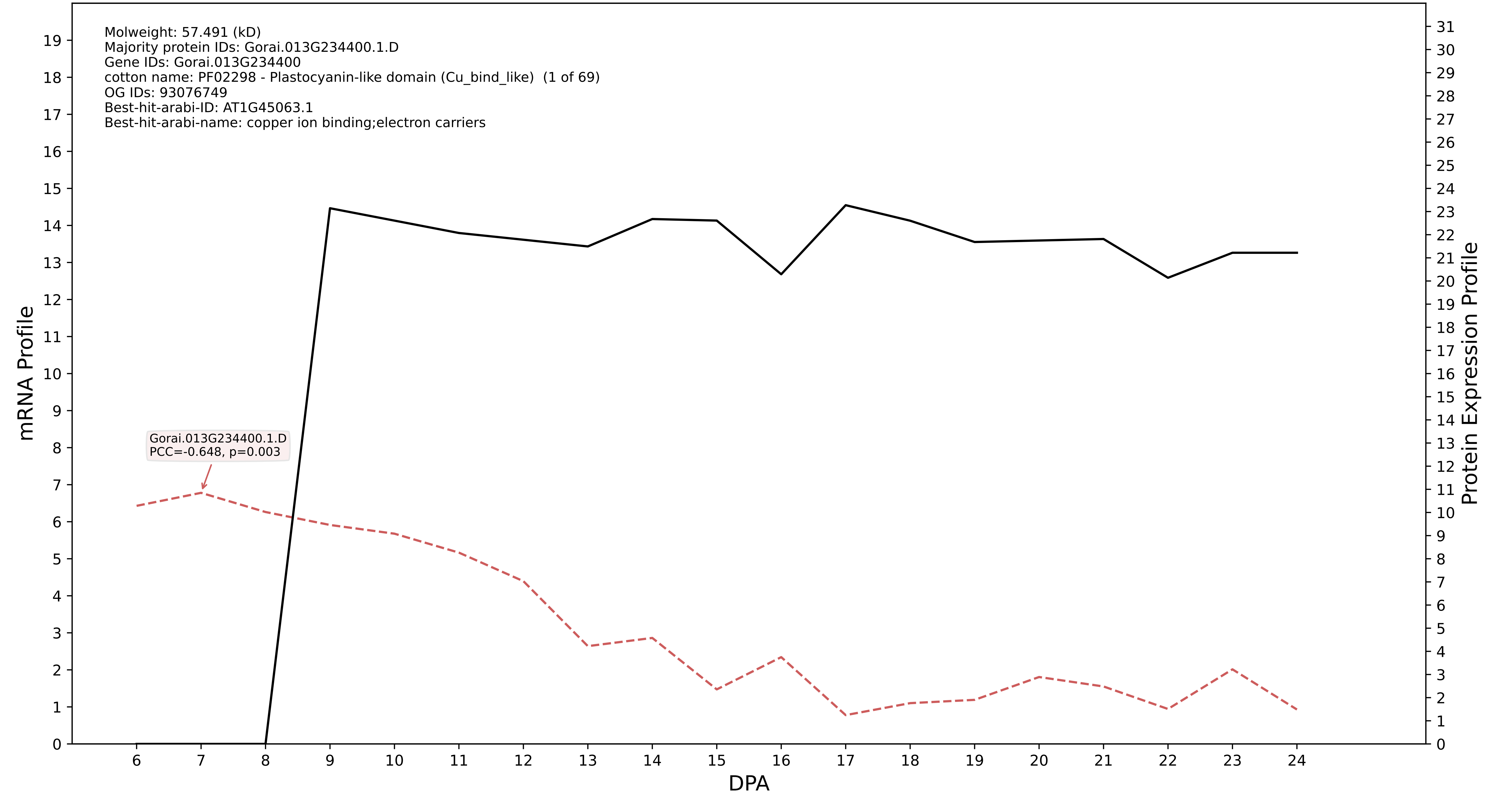

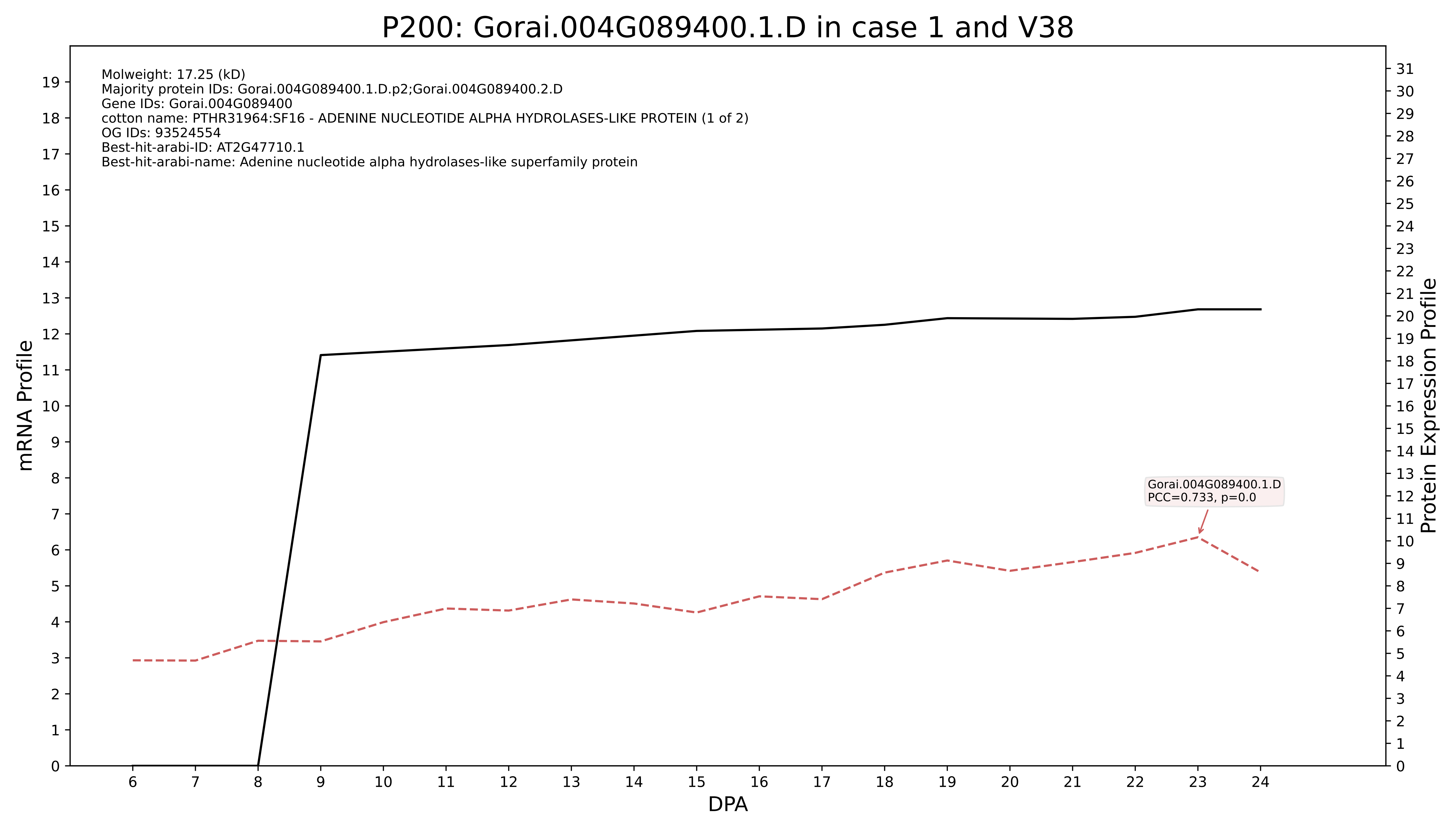
P200: Gorai.004G089400.1.D in case 1 and V38
Molweight: 17.25 (kD)
Majority protein IDs: Gorai.004G089400.1.D.p2;Gorai.004G089400.2.D
Gene IDs: Gorai.004G089400
cotton name: PTHR31964:SF16 - ADENINE NUCLEOTIDE ALPHA HYDROLASES-LIKE PROTEIN (1 of 2)
OG IDs: 93524554
Best-hit-arabi-ID: AT2G47710.1
Best-hit-arabi-name: Adenine nucleotide alpha hydrolases-like superfamily protein
Gorai.004G089400.1.D
PCC=0.733, p=0.0
mRNA Profile
Protein Expression Profile
DPA

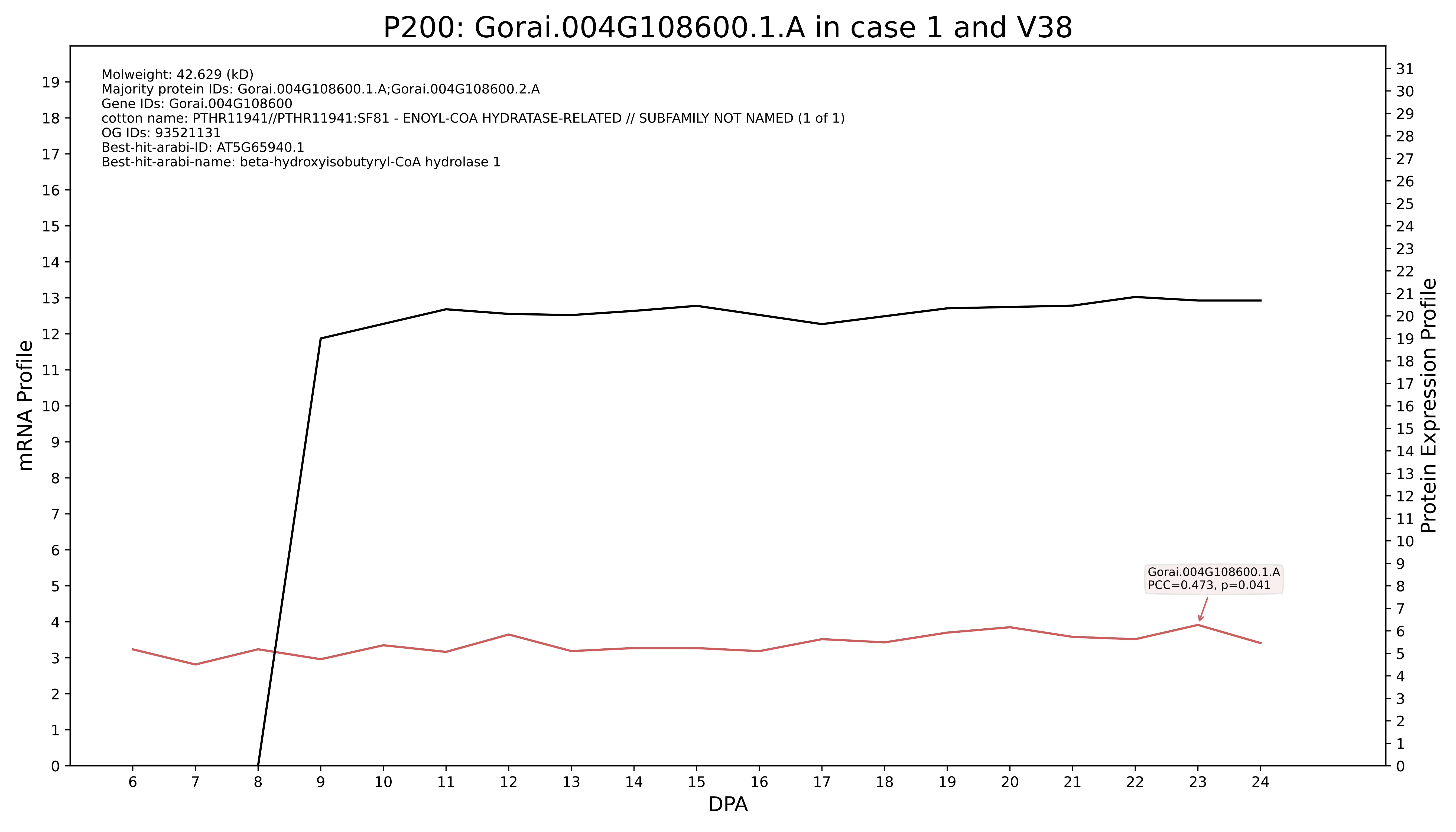

P200: Gorai.004G108600.1.A in case 1 and V38
Molweight: 42.629 (kD)
Majority protein IDs: Gorai.004G108600.1.A;Gorai.004G108600.2.A
Gene IDs: Gorai.004G108600
cotton name: PTHR11941//PTHR11941:SF81 - ENOYL-COA HYDRATASE-RELATED // SUBFAMILY NOT NAMED (1 of 1)
OG IDs: 93521131
Best-hit-arabi-ID: AT5G65940.1
Best-hit-arabi-name: beta-hydroxyisobutyryl-CoA hydrolase 1
Gorai.004G108600.1.A
PCC=0.473, p=0.041
mRNA Profile
Protein Expression Profile
DPA

P200: Gorai.013G231000.1.A in case 1 and V38

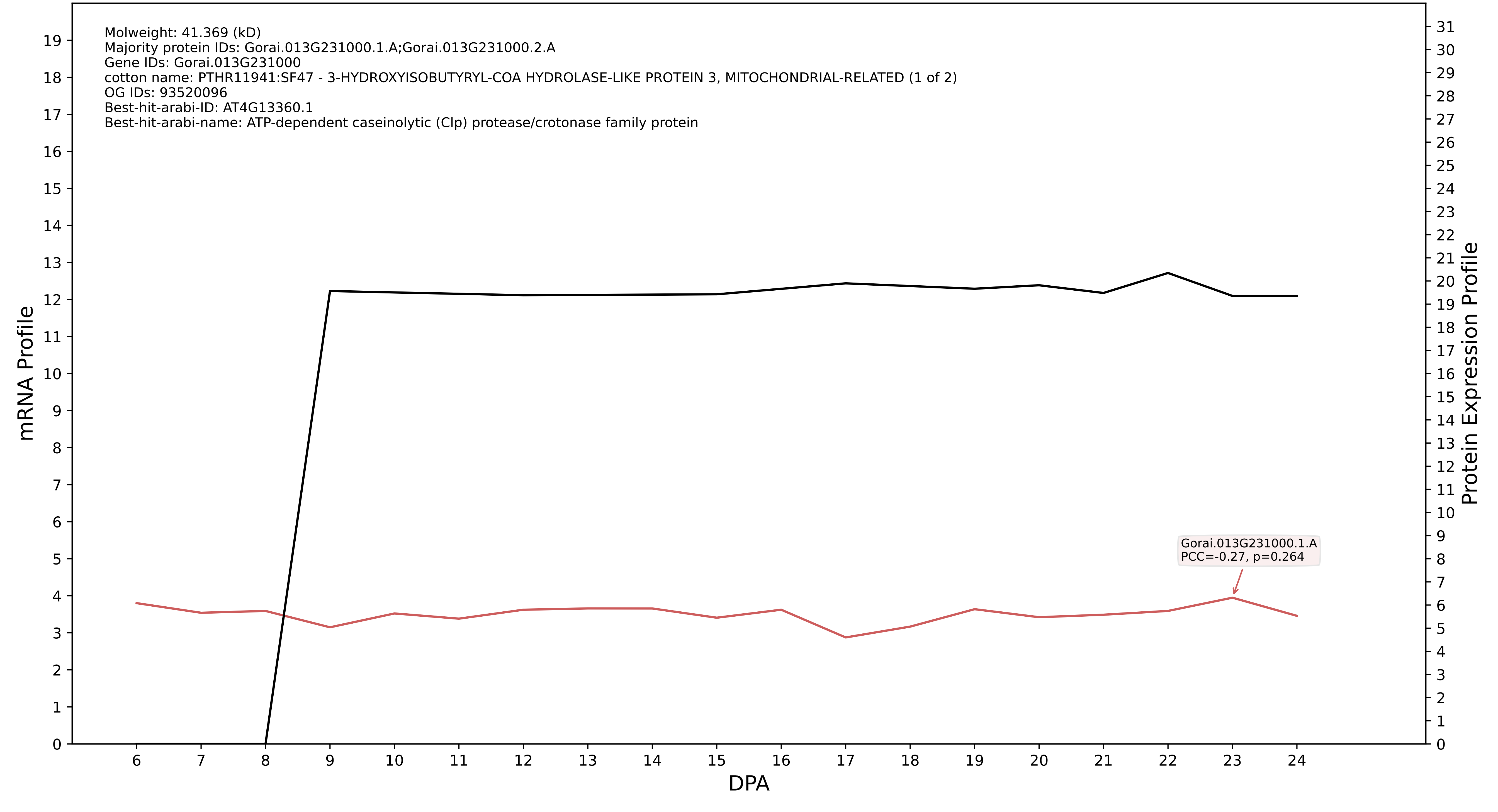

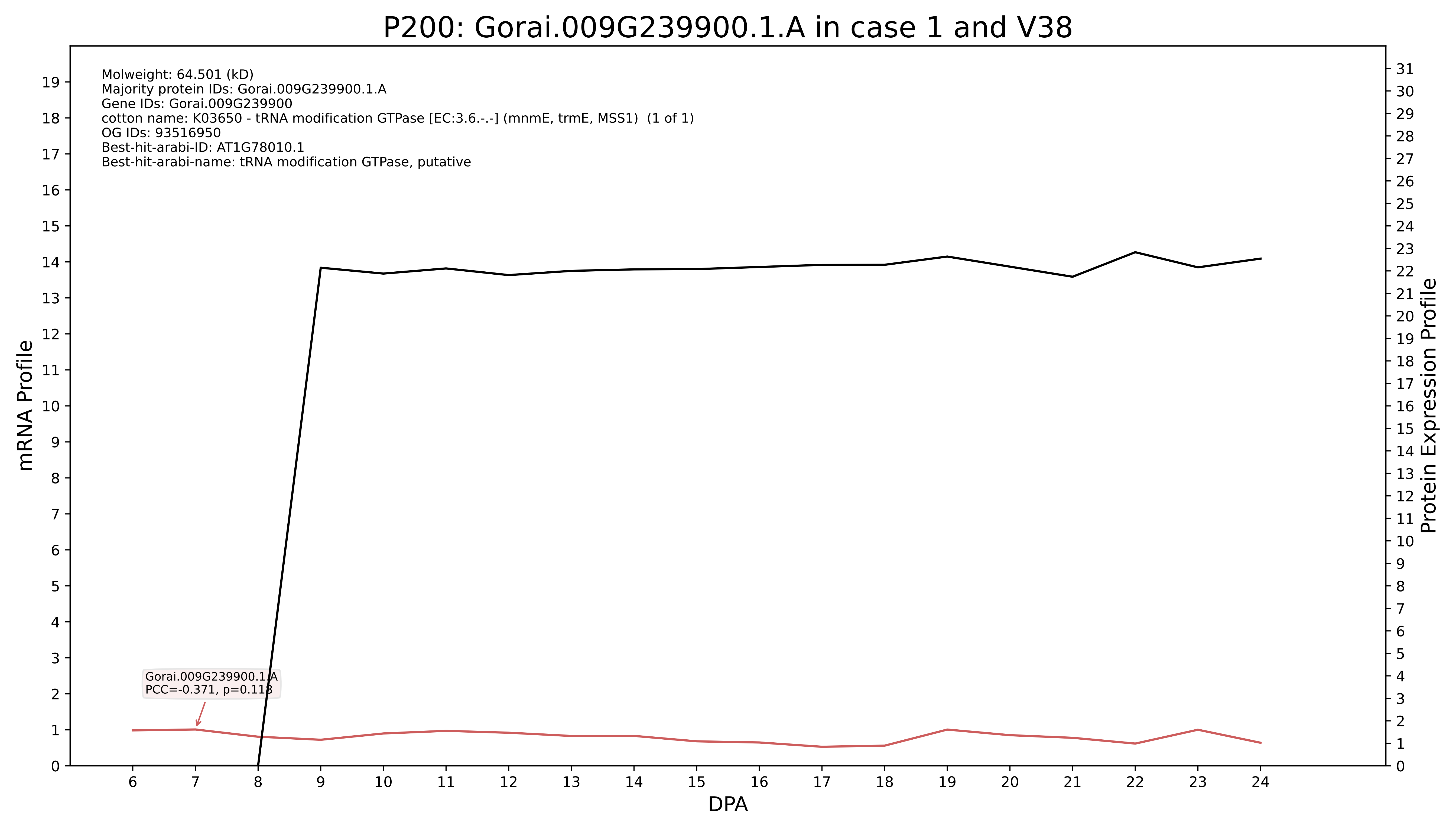
P200: Gorai.009G239900.1.A in case 1 and V38
Molweight: 64.501 (kD)
Majority protein IDs: Gorai.009G239900.1.A
Gene IDs: Gorai.009G239900
cotton name: K03650 - tRNA modification GTPase [EC:3.6.-.-] (mnmE, trmE, MSS1) (1 of 1)
OG IDs: 93516950
Best-hit-arabi-ID: AT1G78010.1
Best-hit-arabi-name: tRNA modification GTPase, putative
Gorai.009G239900.1.A
PCC=-0.371, p=0.118
mRNA Profile
Protein Expression Profile
DPA

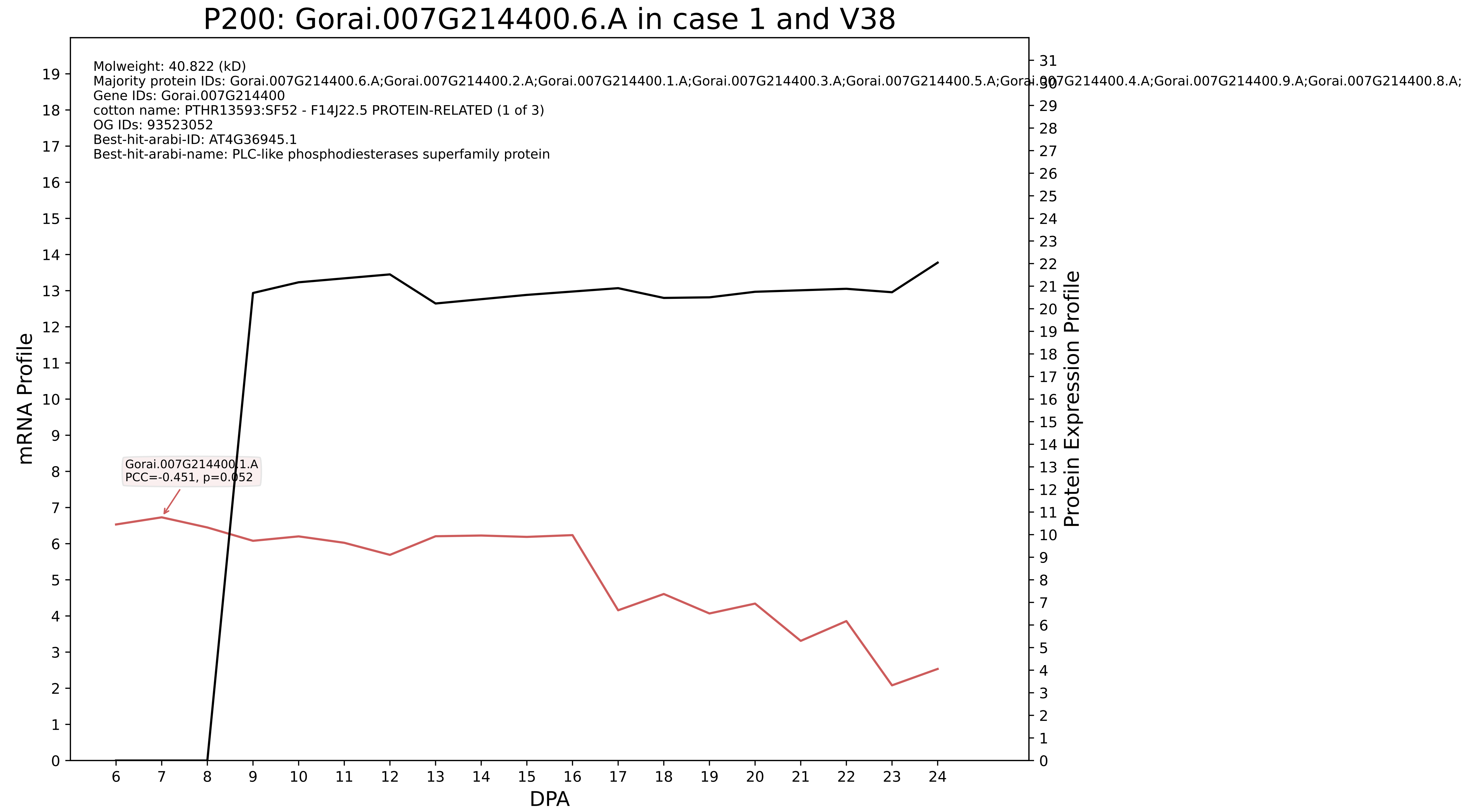
P200: Gorai.007G214400.6.A in case 1 and V38
Molweight: 40.822 (kD)
Majority protein IDs: Gorai.007G214400.6.A;Gorai.007G214400.2.A;Gorai.007G214400.1.A;Gorai.007G214400.3.A;Gorai.007G214400.5.A;Gorai.007G214400.4.A;Gorai.007G214400.9.A;Gorai.007G214400.8.A;
Gene IDs: Gorai.007G214400
cotton name: PTHR13593:SF52 - F14J22.5 PROTEIN-RELATED (1 of 3)
OG IDs: 93523052
Best-hit-arabi-ID: AT4G36945.1
Best-hit-arabi-name: PLC-like phosphodiesterases superfamily protein
Gorai.007G214400.1.A
PCC=-0.451, p=0.052
mRNA Profile
Protein Expression Profile
DPA

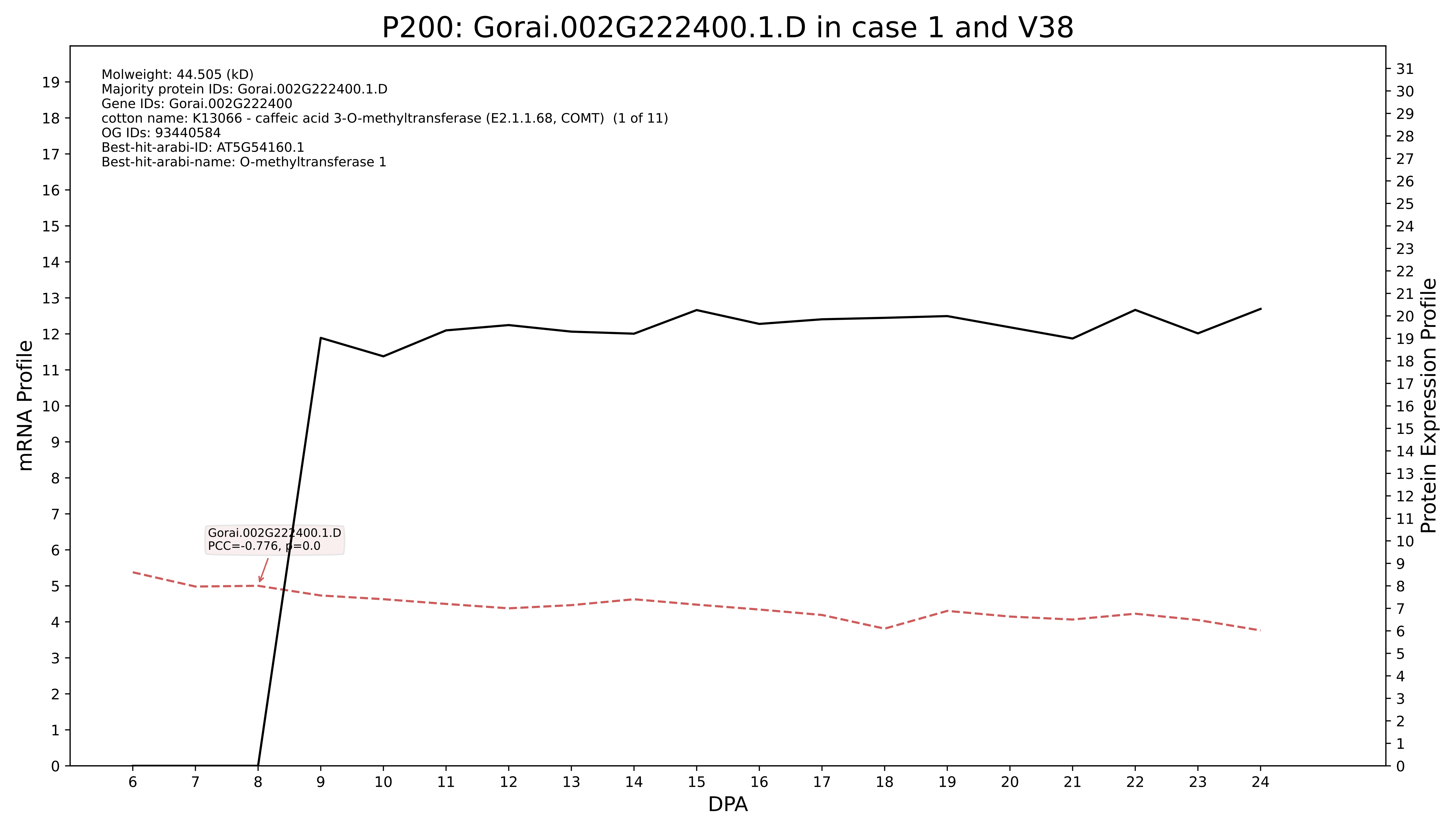

P200: Gorai.002G222400.1.D in case 1 and V38
Molweight: 44.505 (kD)
Majority protein IDs: Gorai.002G222400.1.D
Gene IDs: Gorai.002G222400
cotton name: K13066 - caffeic acid 3-O-methyltransferase (E2.1.1.68, COMT) (1 of 11)
OG IDs: 93440584
Best-hit-arabi-ID: AT5G54160.1
Best-hit-arabi-name: O-methyltransferase 1
Gorai.002G222400.1.D
PCC=-0.776, p=0.0
mRNA Profile
Protein Expression Profile
DPA
0
1
2
3
4
5
6
7
8
9
10
11
12
13
14
15
16
17
18
19
20
21
22
23
24
25
26
27
28
29
30
31

P200: Gorai.005G145800.4.D in case 2 and V38

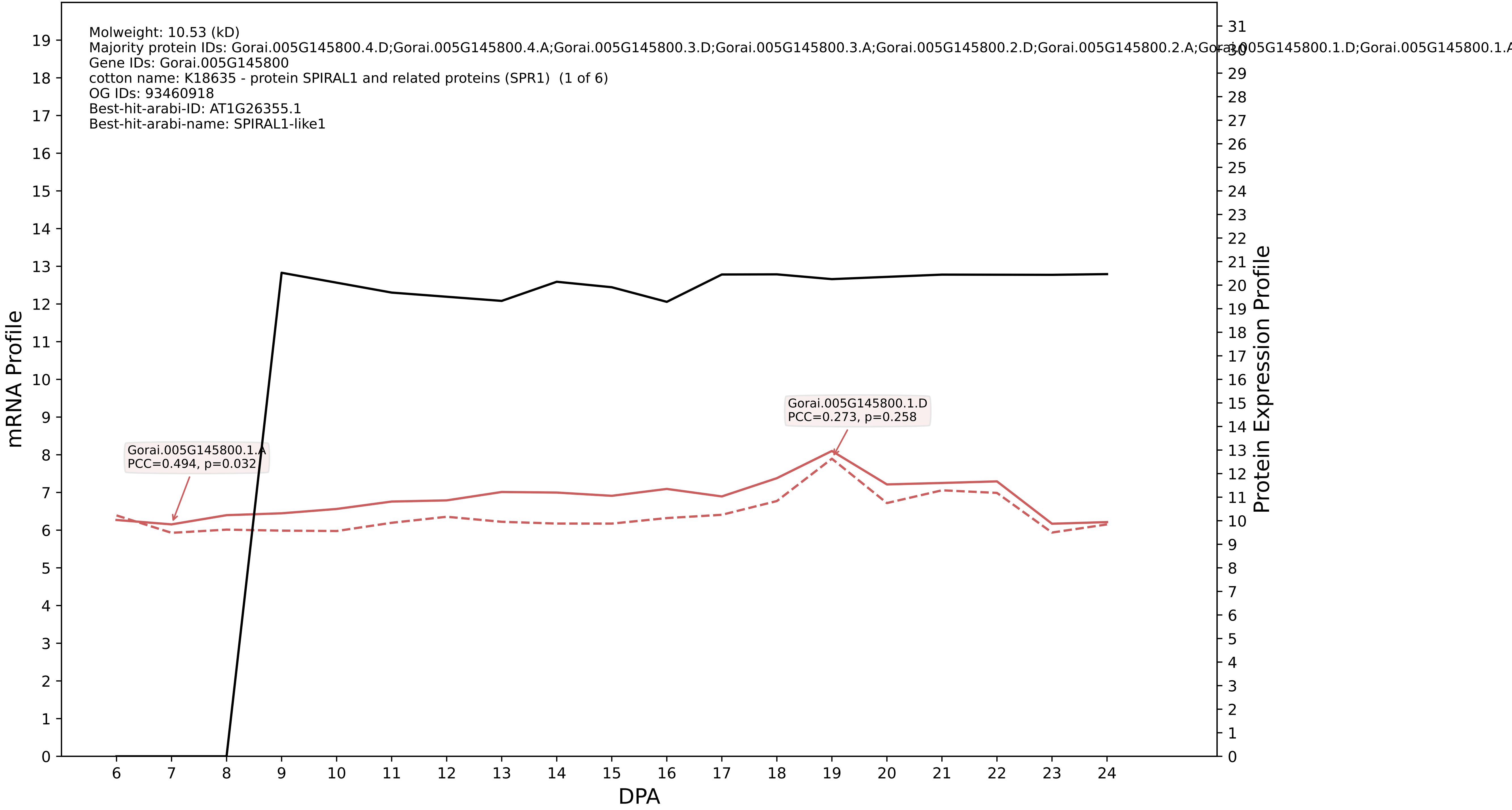

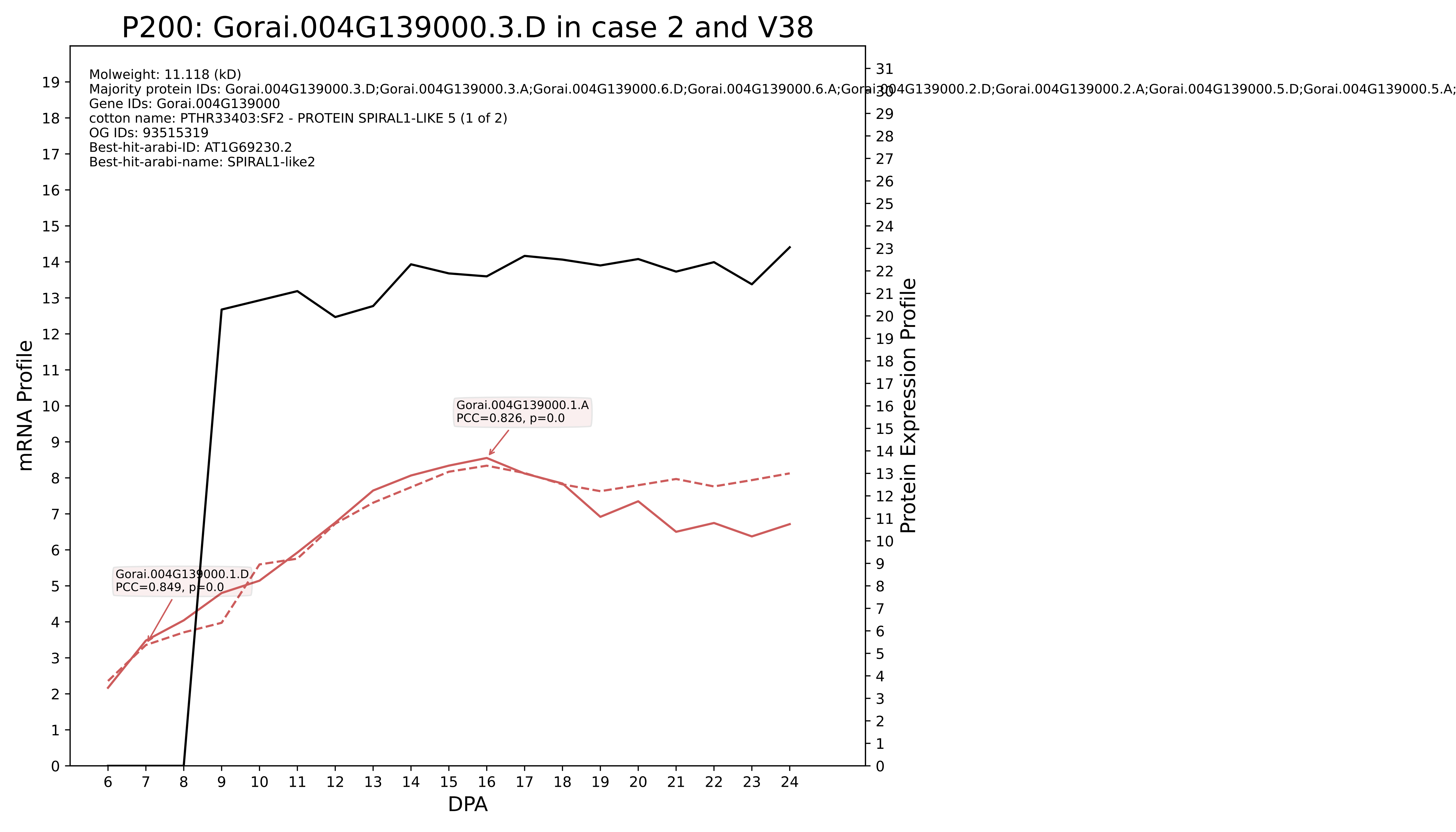
P200: Gorai.004G139000.3.D in case 2 and V38
Molweight: 11.118 (kD)
Majority protein IDs: Gorai.004G139000.3.D;Gorai.004G139000.3.A;Gorai.004G139000.6.D;Gorai.004G139000.6.A;Gorai.004G139000.2.D;Gorai.004G139000.2.A;Gorai.004G139000.5.D;Gorai.004G139000.5.A;
Gene IDs: Gorai.004G139000
cotton name: PTHR33403:SF2 - PROTEIN SPIRAL1-LIKE 5 (1 of 2)
OG IDs: 93515319
Best-hit-arabi-ID: AT1G69230.2
Best-hit-arabi-name: SPIRAL1-like2
Gorai.004G139000.1.A
PCC=0.826, p=0.0
Gorai.004G139000.1.D
PCC=0.849, p=0.0
mRNA Profile
Protein Expression Profile
DPA

P200: Gorai.011G230400.1.D in case 2 and V38

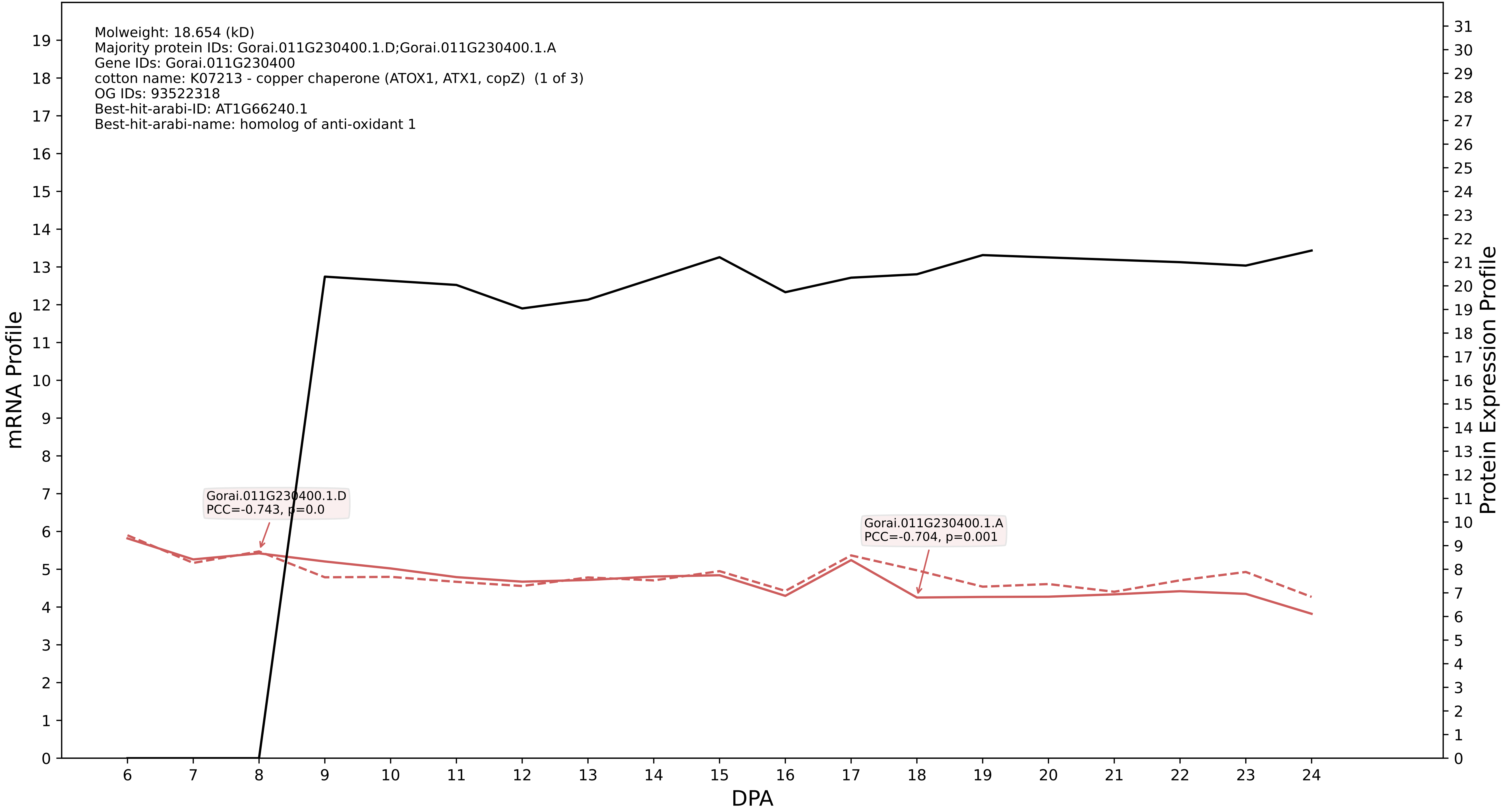

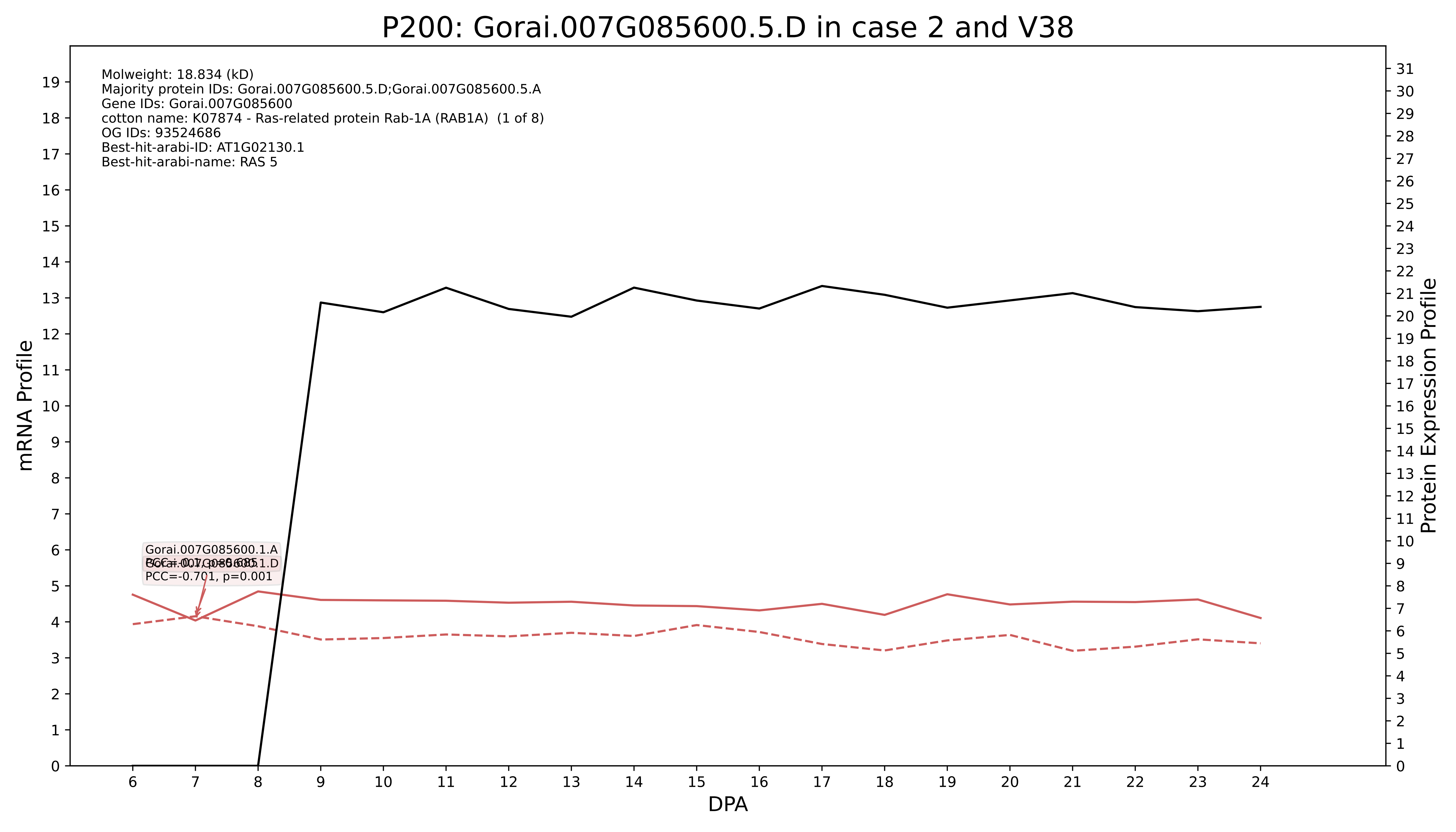
P200: Gorai.007G085600.5.D in case 2 and V38
Molweight: 18.834 (kD)
Majority protein IDs: Gorai.007G085600.5.D;Gorai.007G085600.5.A
Gene IDs: Gorai.007G085600
cotton name: K07874 - Ras-related protein Rab-1A (RAB1A) (1 of 8)
OG IDs: 93524686
Best-hit-arabi-ID: AT1G02130.1
Best-hit-arabi-name: RAS 5
Gorai.007G085600.1.A
Gorai.007G085600.1.D
PCC=-0.701, p=0.001
mRNA Profile
Protein Expression Profile
DPA
6
7
8
9
10
11
12
13
14
15
16
17
18
19
20
21
22
23
24

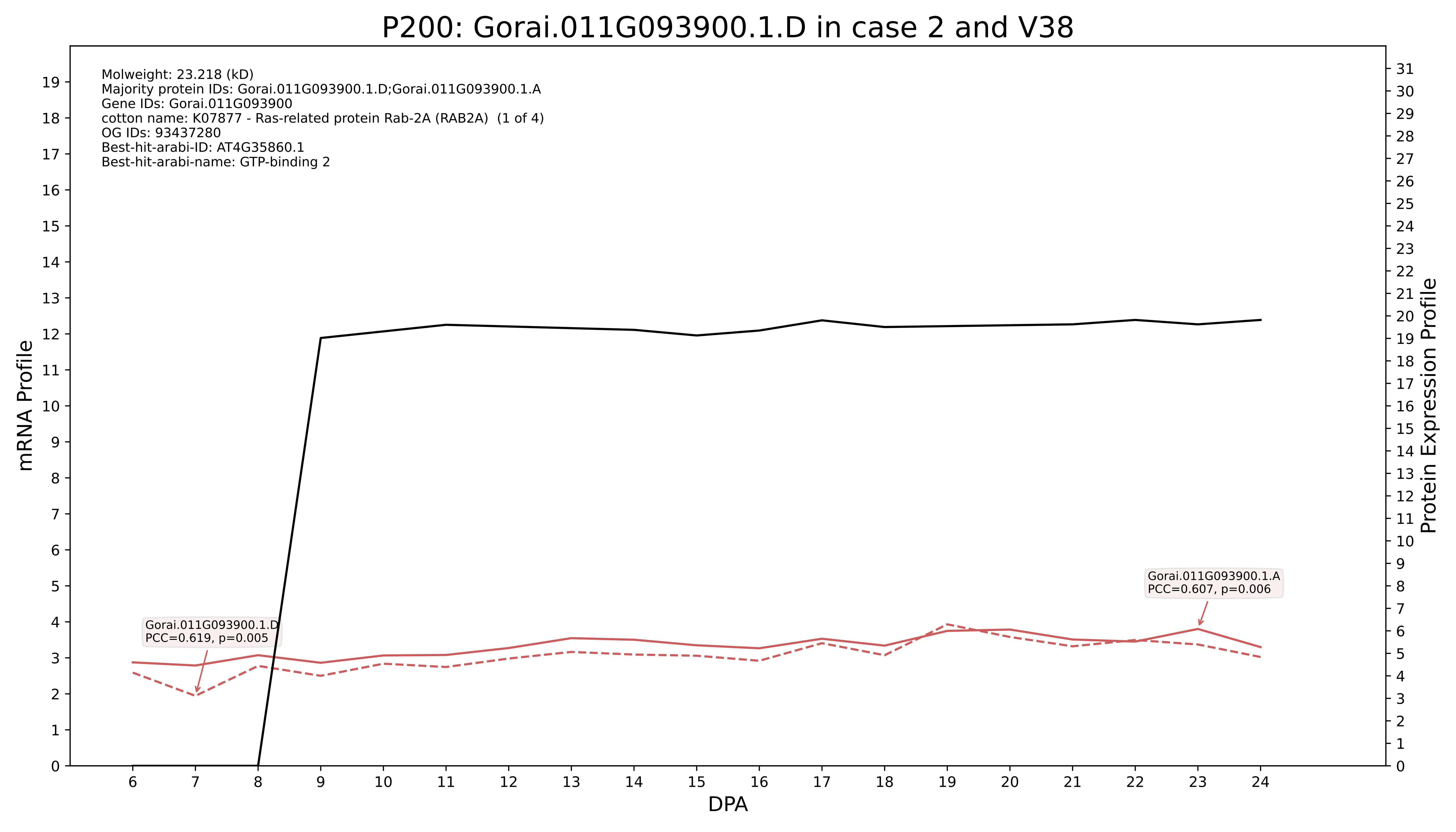
P200: Gorai.011G093900.1.D in case 2 and V38
Molweight: 23.218 (kD)
Majority protein IDs: Gorai.011G093900.1.D;Gorai.011G093900.1.A
Gene IDs: Gorai.011G093900
cotton name: K07877 - Ras-related protein Rab-2A (RAB2A) (1 of 4)
OG IDs: 93437280
Best-hit-arabi-ID: AT4G35860.1
Best-hit-arabi-name: GTP-binding 2
Gorai.011G093900.1.D
PCC=0.619, p=0.005
Gorai.011G093900.1.A
PCC=0.607, p=0.006
mRNA Profile
Protein Expression Profile
DPA

P200: Gorai.009G193300.1.D in case 3 and V38

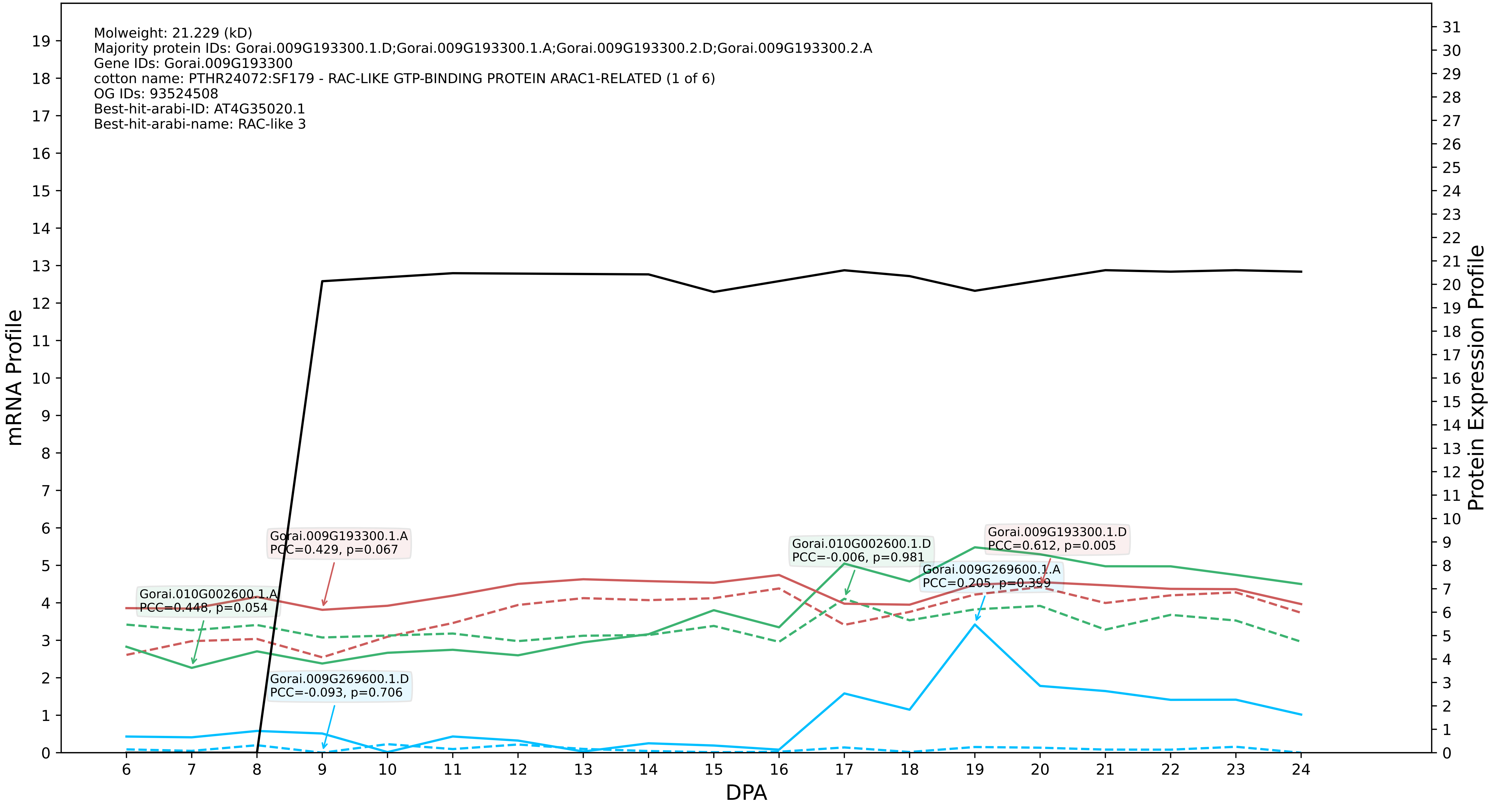

Supplementary Figure S12. Profiles of paired mRNAs and proteins (S200).

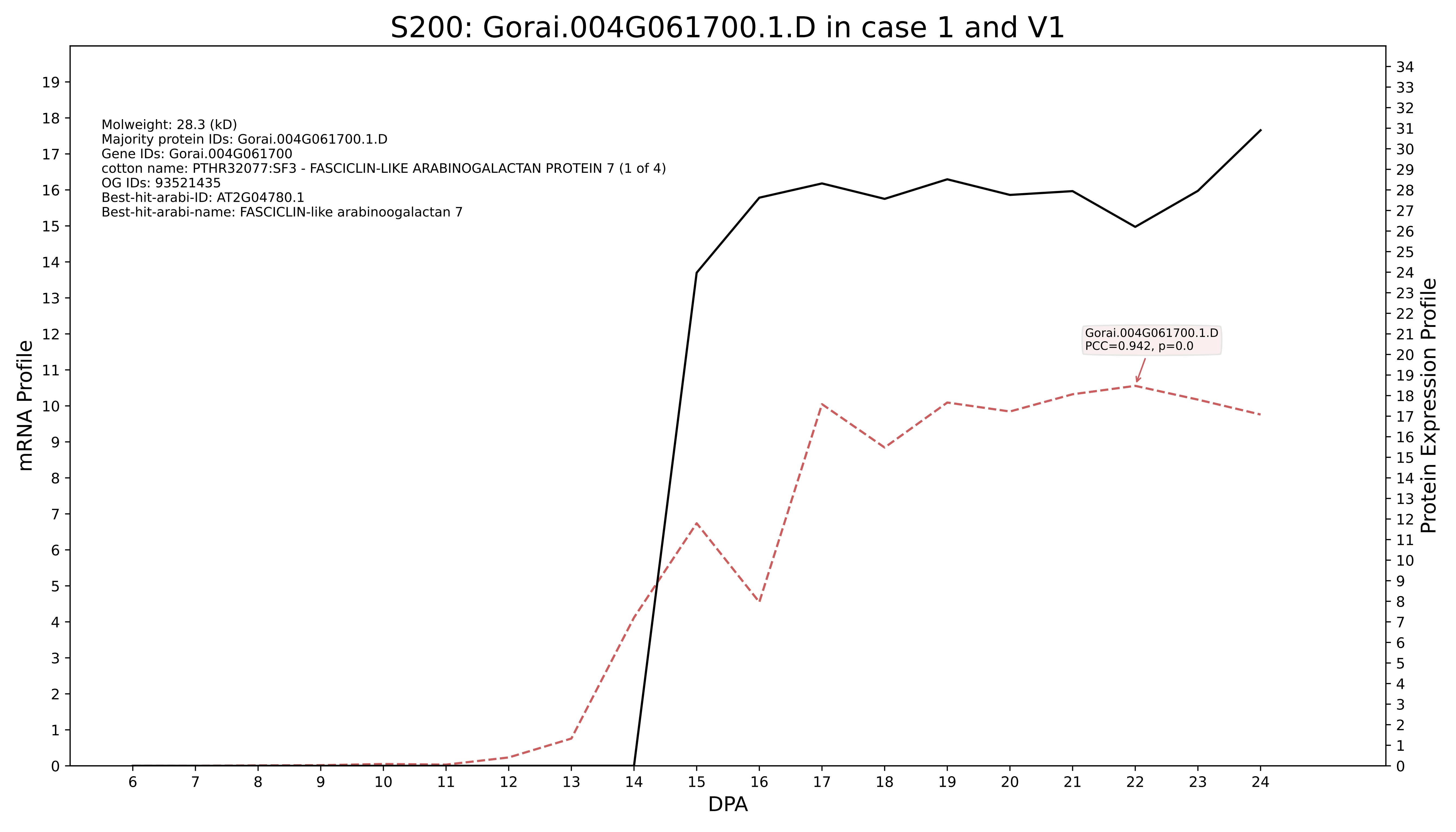
S200: Gorai.004G061700.1.D in case 1 and V1
Molweight: 28.3 (kD)
Majority protein IDs: Gorai.004G061700.1.D
Gene IDs: Gorai.004G061700
cotton name: PTHR32077:SF3 - FASCICLIN-LIKE ARABINOGALACTAN PROTEIN 7 (1 of 4)
OG IDs: 93521435
Best-hit-arabi-ID: AT2G04780.1
Best-hit-arabi-name: FASCICLIN-like arabinoogalactan 7
Gorai.004G061700.1.D
PCC=0.942, p=0.0
mRNA Profile
Protein Expression Profile
DPA

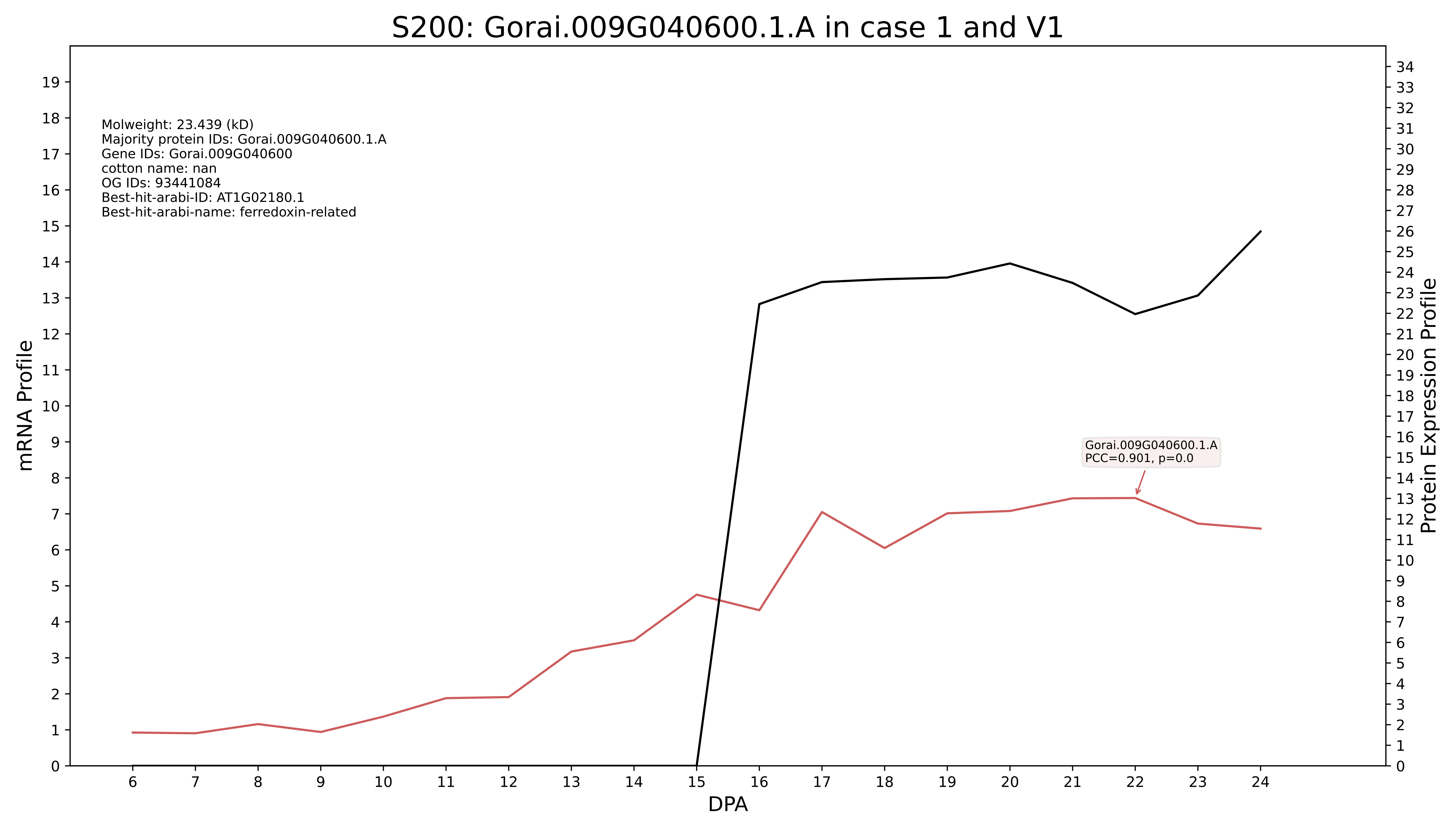
S200: Gorai.009G040600.1.A in case 1 and V1
Molweight: 23.439 (kD)
Majority protein IDs: Gorai.009G040600.1.A
Gene IDs: Gorai.009G040600
cotton name: nan
OG IDs: 93441084
Best-hit-arabi-ID: AT1G02180.1
Best-hit-arabi-name: ferredoxin-related
Gorai.009G040600.1.A
PCC=0.901, p=0.0
mRNA Profile
Protein Expression Profile
DPA

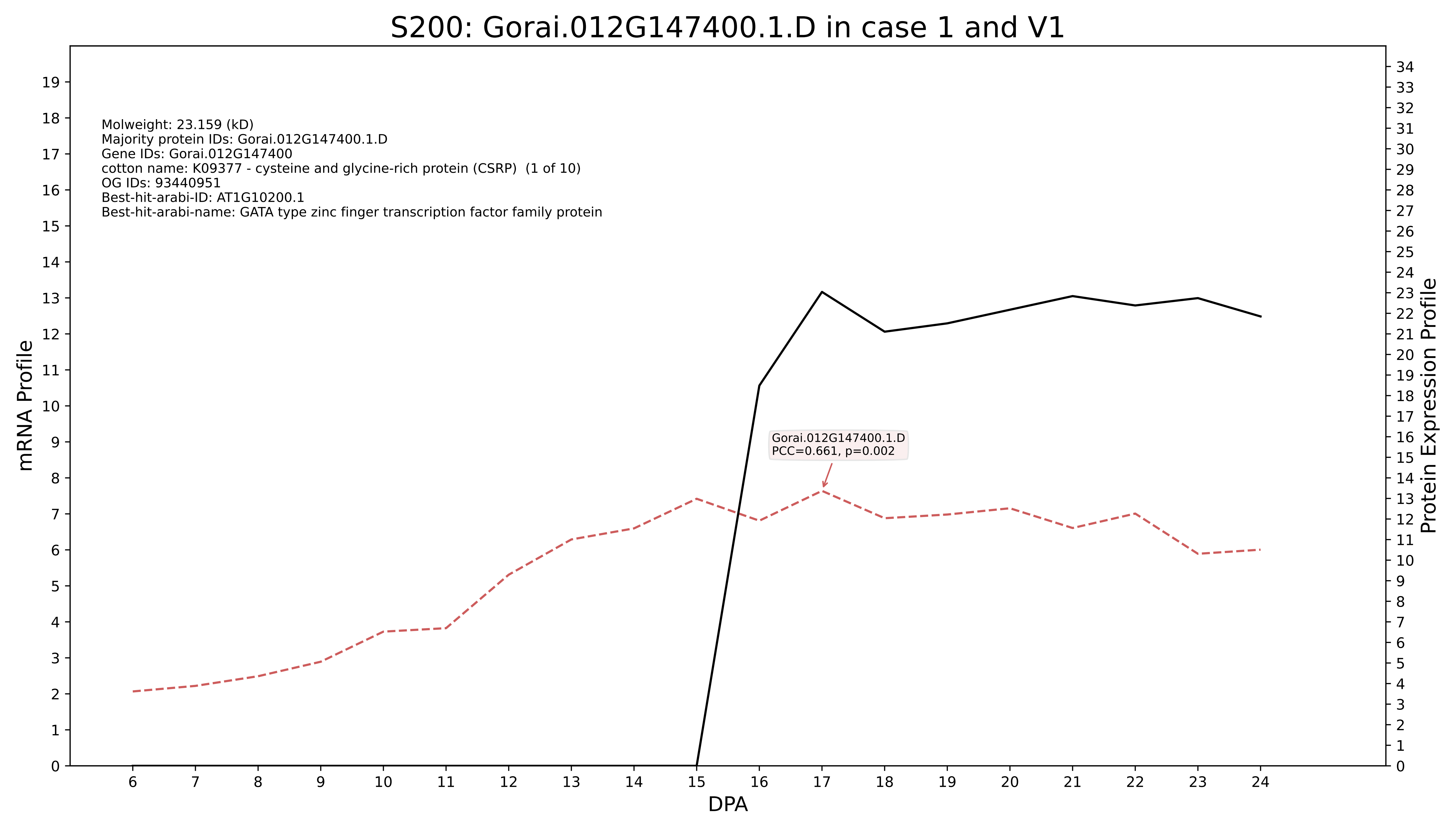

S200: Gorai.012G147400.1.D in case 1 and V1
Molweight: 23.159 (kD)
Majority protein IDs: Gorai.012G147400.1.D
Gene IDs: Gorai.012G147400
cotton name: K09377 - cysteine and glycine-rich protein (CSRP) (1 of 10)
OG IDs: 93440951
Best-hit-arabi-ID: AT1G10200.1
Best-hit-arabi-name: GATA type zinc finger transcription factor family protein
Gorai.012G147400.1.D
PCC=0.661, p=0.002
mRNA Profile
Protein Expression Profile
DPA

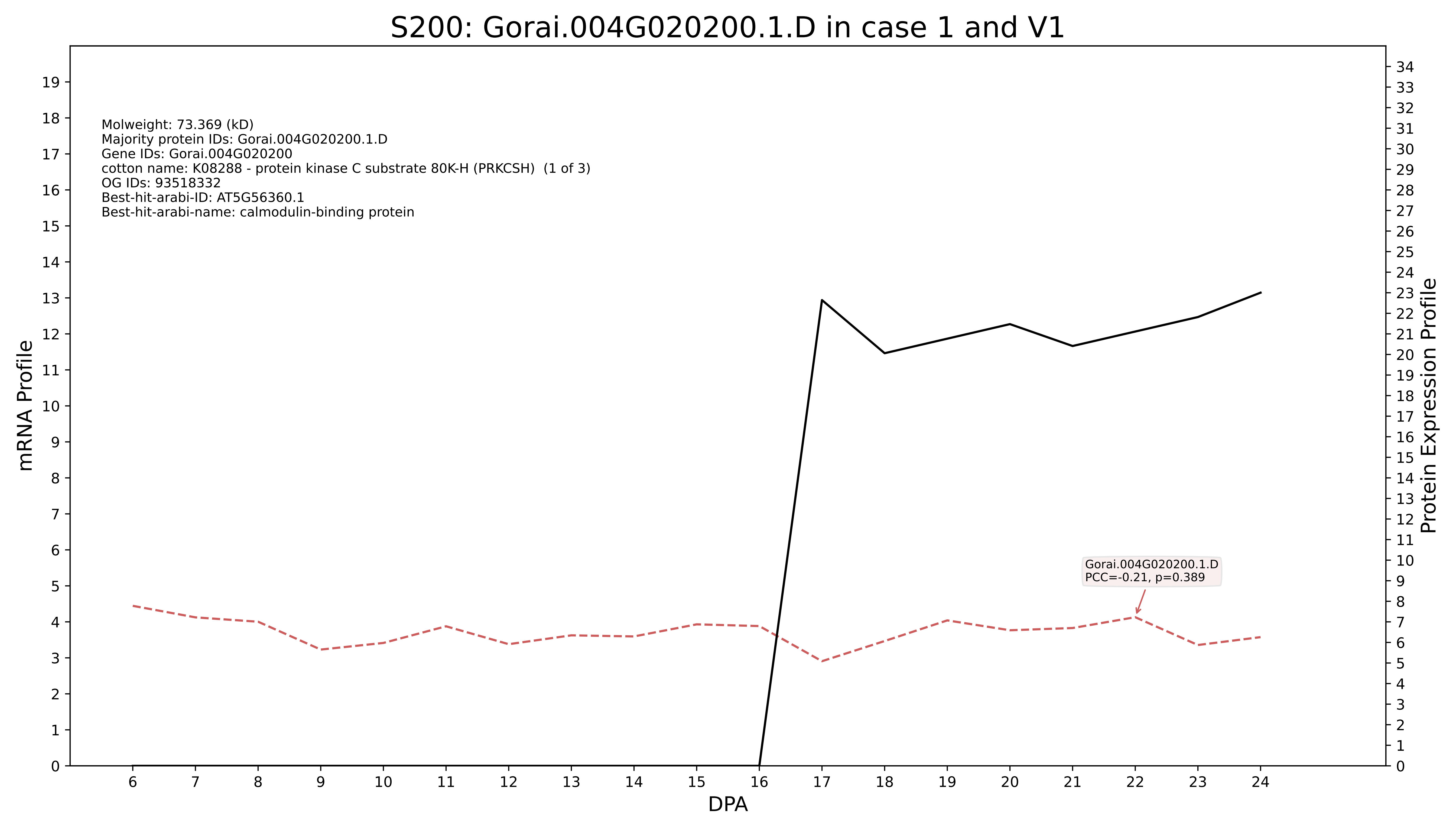

S200: Gorai.004G020200.1.D in case 1 and V1
Molweight: 73.369 (kD)
Majority protein IDs: Gorai.004G020200.1.D
Gene IDs: Gorai.004G020200
cotton name: K08288 - protein kinase C substrate 80K-H (PRKCSH) (1 of 3)
OG IDs: 93518332
Best-hit-arabi-ID: AT5G56360.1
Best-hit-arabi-name: calmodulin-binding protein
Gorai.004G020200.1.D
PCC=-0.21, p=0.389
mRNA Profile
Protein Expression Profile
DPA

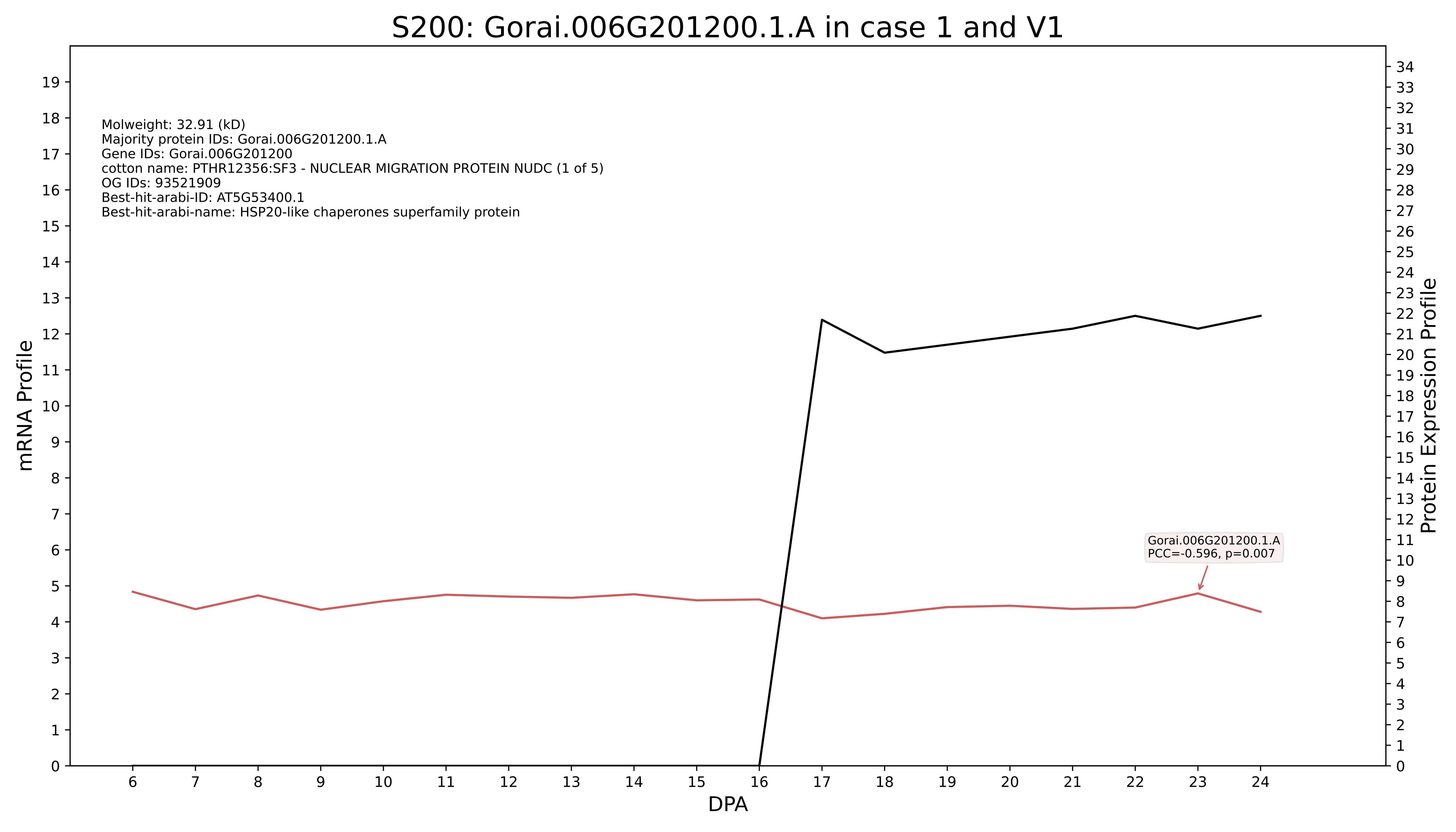

S200: Gorai.006G201200.1.A in case 1 and V1
Molweight: 32.91 (kD)
Majority protein IDs: Gorai.006G201200.1.A
Gene IDs: Gorai.006G201200
cotton name: PTHR12356:SF3 - NUCLEAR MIGRATION PROTEIN NUDC (1 of 5)
OG IDs: 93521909
Best-hit-arabi-ID: AT5G53400.1
Best-hit-arabi-name: HSP20-like chaperones superfamily protein
Gorai.006G201200.1.A
PCC=-0.596, p=0.007
mRNA Profile
Protein Expression Profile
DPA

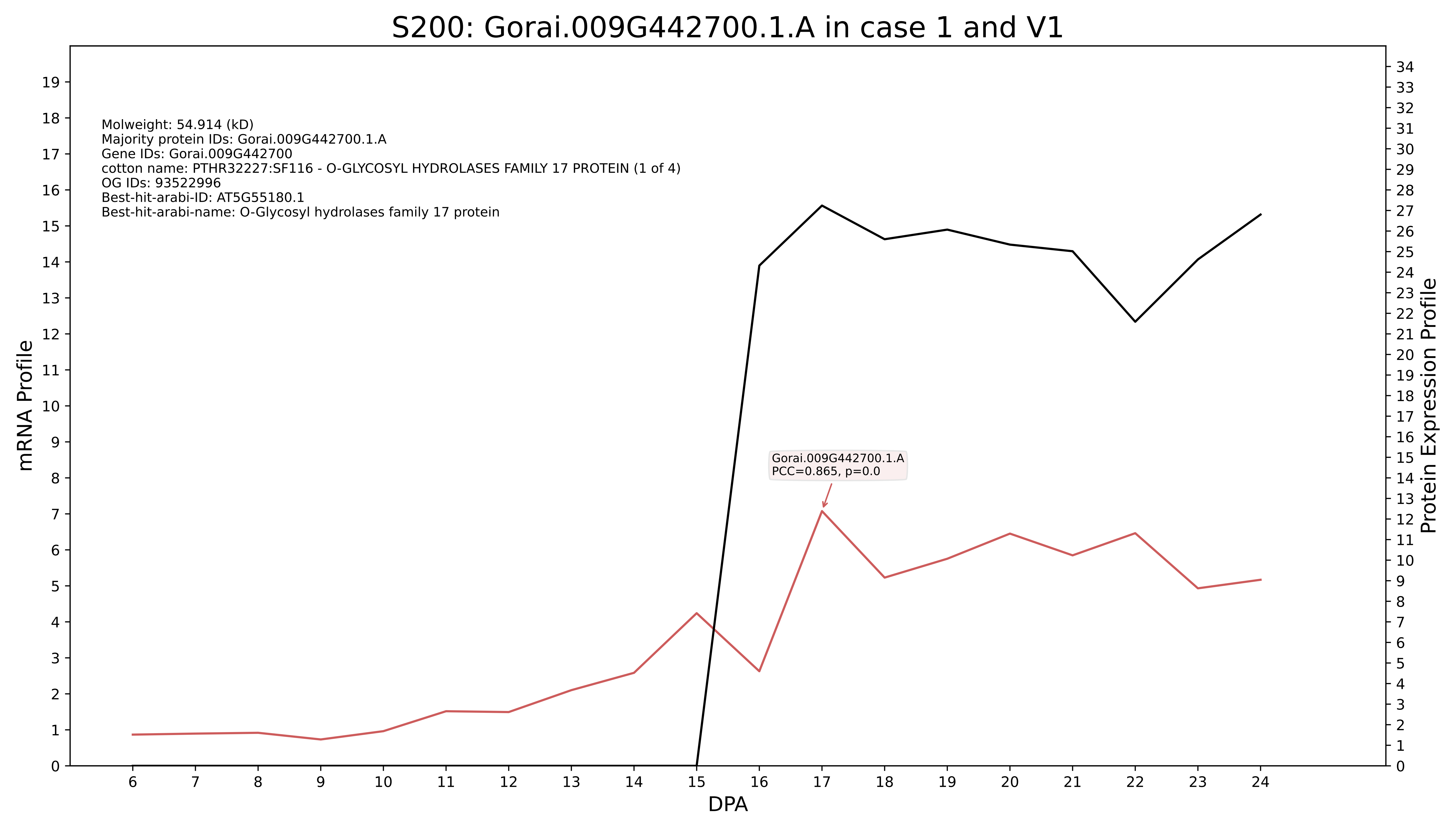
S200: Gorai.009G442700.1.A in case 1 and V1
Molweight: 54.914 (kD)
Majority protein IDs: Gorai.009G442700.1.A
Gene IDs: Gorai.009G442700
cotton name: PTHR32227:SF116 - O-GLYCOSYL HYDROLASES FAMILY 17 PROTEIN (1 of 4)
OG IDs: 93522996
Best-hit-arabi-ID: AT5G55180.1
Best-hit-arabi-name: O-Glycosyl hydrolases family 17 protein
Gorai.009G442700.1.A
PCC=0.865, p=0.0
mRNA Profile
Protein Expression Profile
DPA

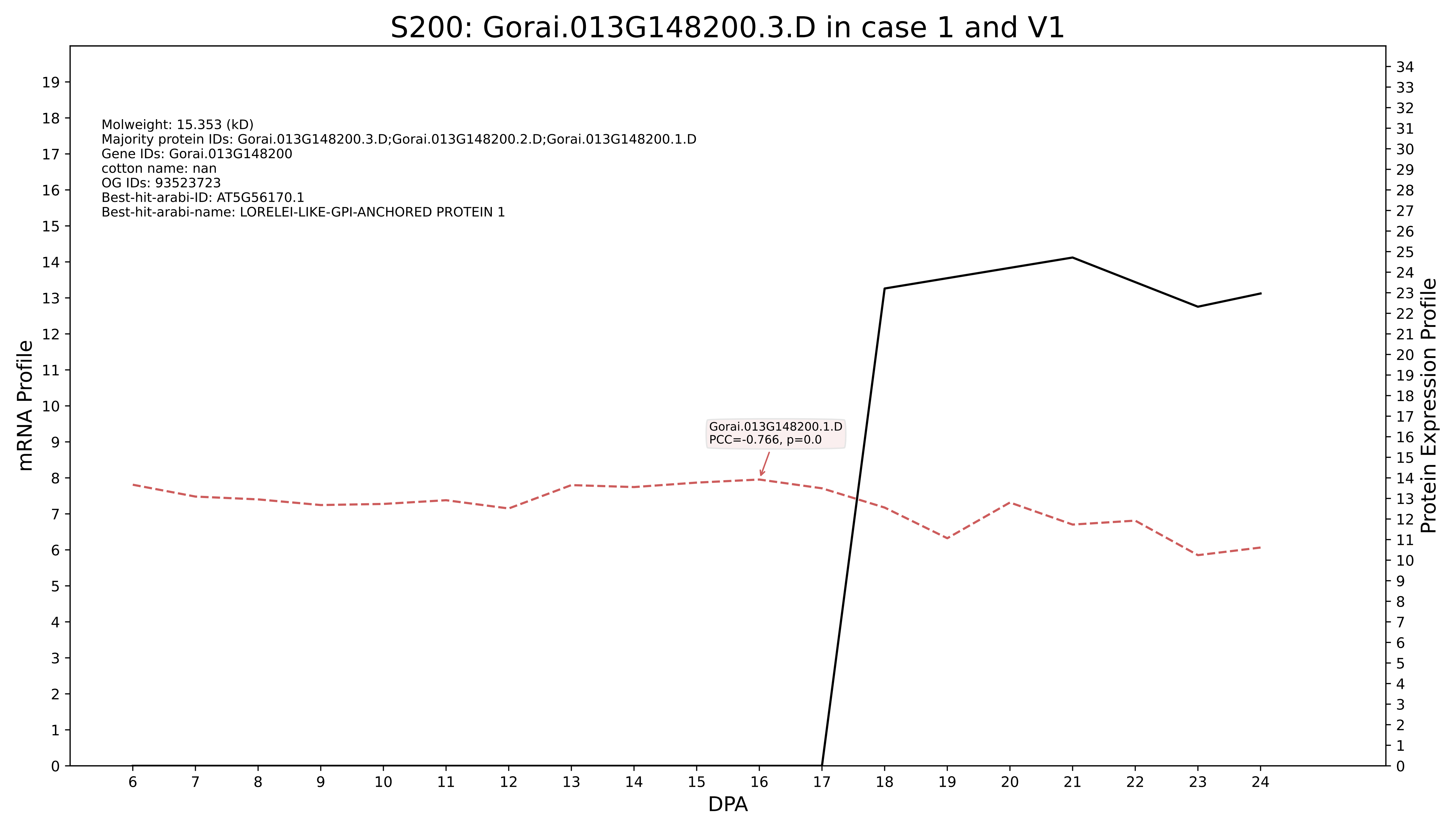
S200: Gorai.013G148200.3.D in case 1 and V1
Molweight: 15.353 (kD)
Majority protein IDs: Gorai.013G148200.3.D;Gorai.013G148200.2.D;Gorai.013G148200.1.D
Gene IDs: Gorai.013G148200
cotton name: nan
OG IDs: 93523723
Best-hit-arabi-ID: AT5G56170.1
Best-hit-arabi-name: LORELEI-LIKE-GPI-ANCHORED PROTEIN 1
Gorai.013G148200.1.D
PCC=-0.766, p=0.0
mRNA Profile
Protein Expression Profile
DPA

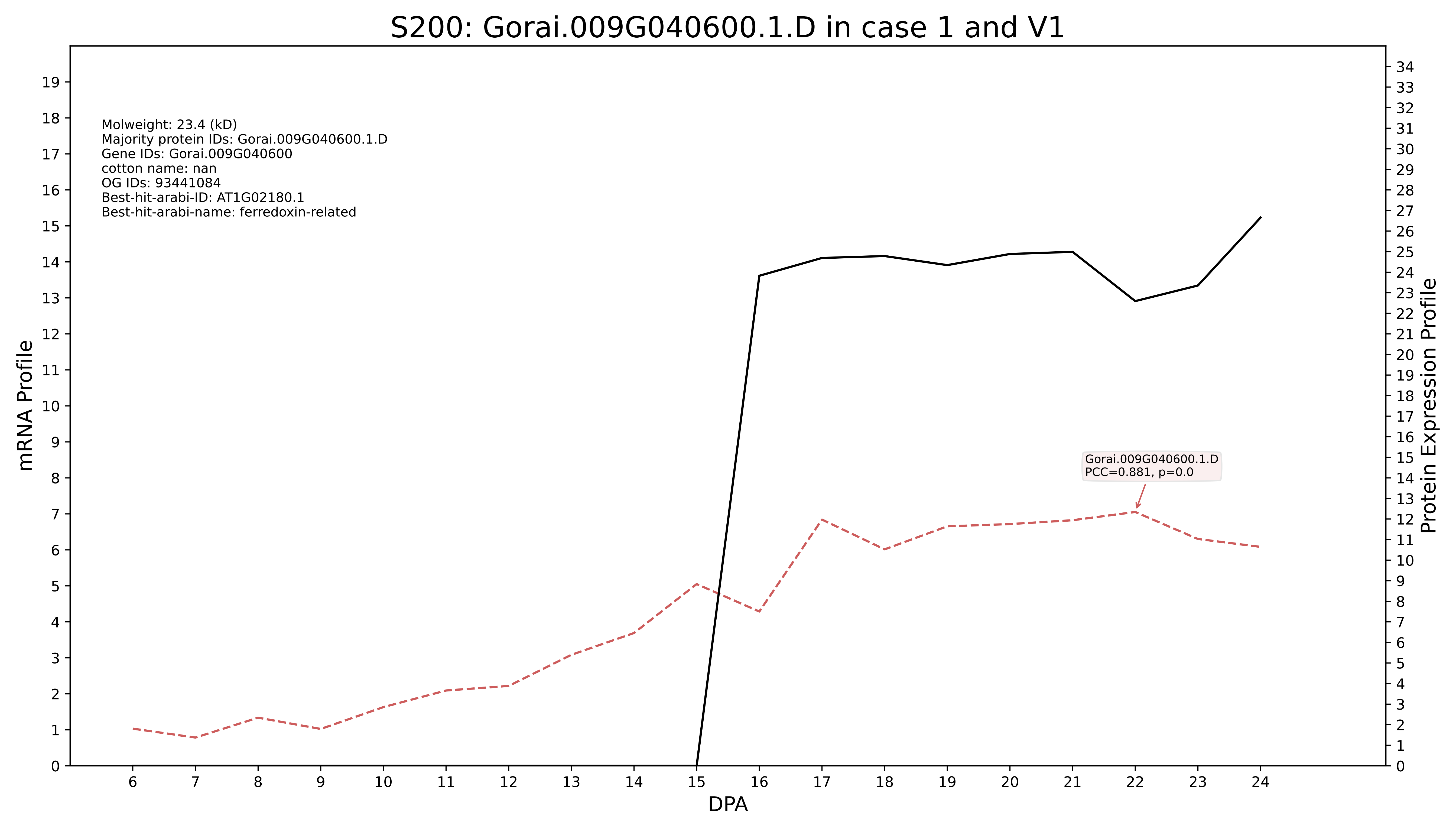
S200: Gorai.009G040600.1.D in case 1 and V1
Molweight: 23.4 (kD)
Majority protein IDs: Gorai.009G040600.1.D
Gene IDs: Gorai.009G040600
cotton name: nan
OG IDs: 93441084
Best-hit-arabi-ID: AT1G02180.1
Best-hit-arabi-name: ferredoxin-related
Gorai.009G040600.1.D
PCC=0.881, p=0.0
mRNA Profile
Protein Expression Profile
DPA
0
1
2
3
4
5
6
7
8
9
10
11
12
13
14
15
16
17
18
19
20
21
22
23
24
25
26
27
28
29
30
31
32
33
34

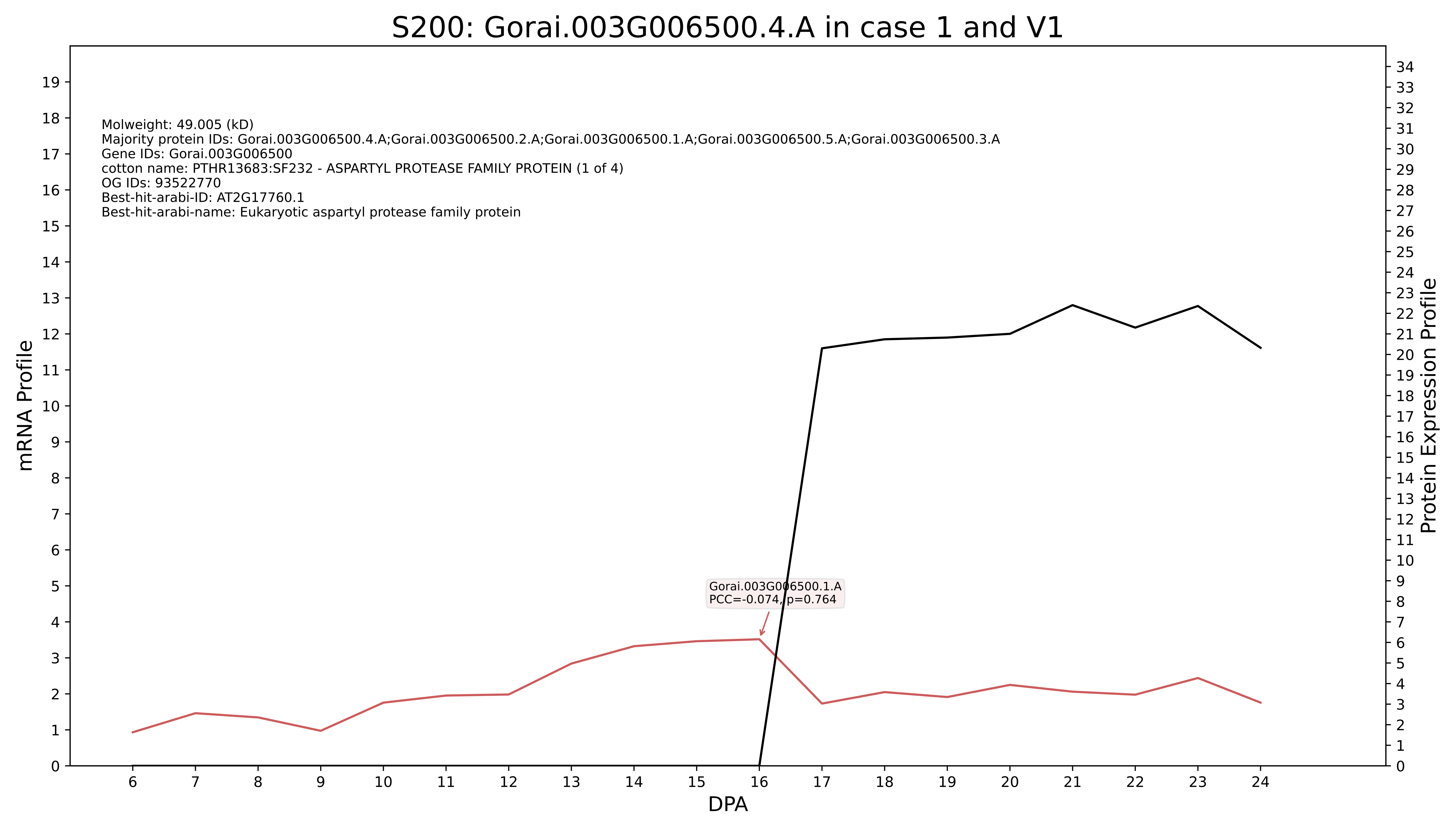

S200: Gorai.003G006500.4.A in case 1 and V1
Molweight: 49.005 (kD)
Majority protein IDs: Gorai.003G006500.4.A;Gorai.003G006500.2.A;Gorai.003G006500.1.A;Gorai.003G006500.5.A;Gorai.003G006500.3.A
Gene IDs: Gorai.003G006500
cotton name: PTHR13683:SF232 - ASPARTYL PROTEASE FAMILY PROTEIN (1 of 4)
OG IDs: 93522770
Best-hit-arabi-ID: AT2G17760.1
Best-hit-arabi-name: Eukaryotic aspartyl protease family protein
Gorai.003G006500.1.A
PCC=-0.074, p=0.764
mRNA Profile
Protein Expression Profile
DPA

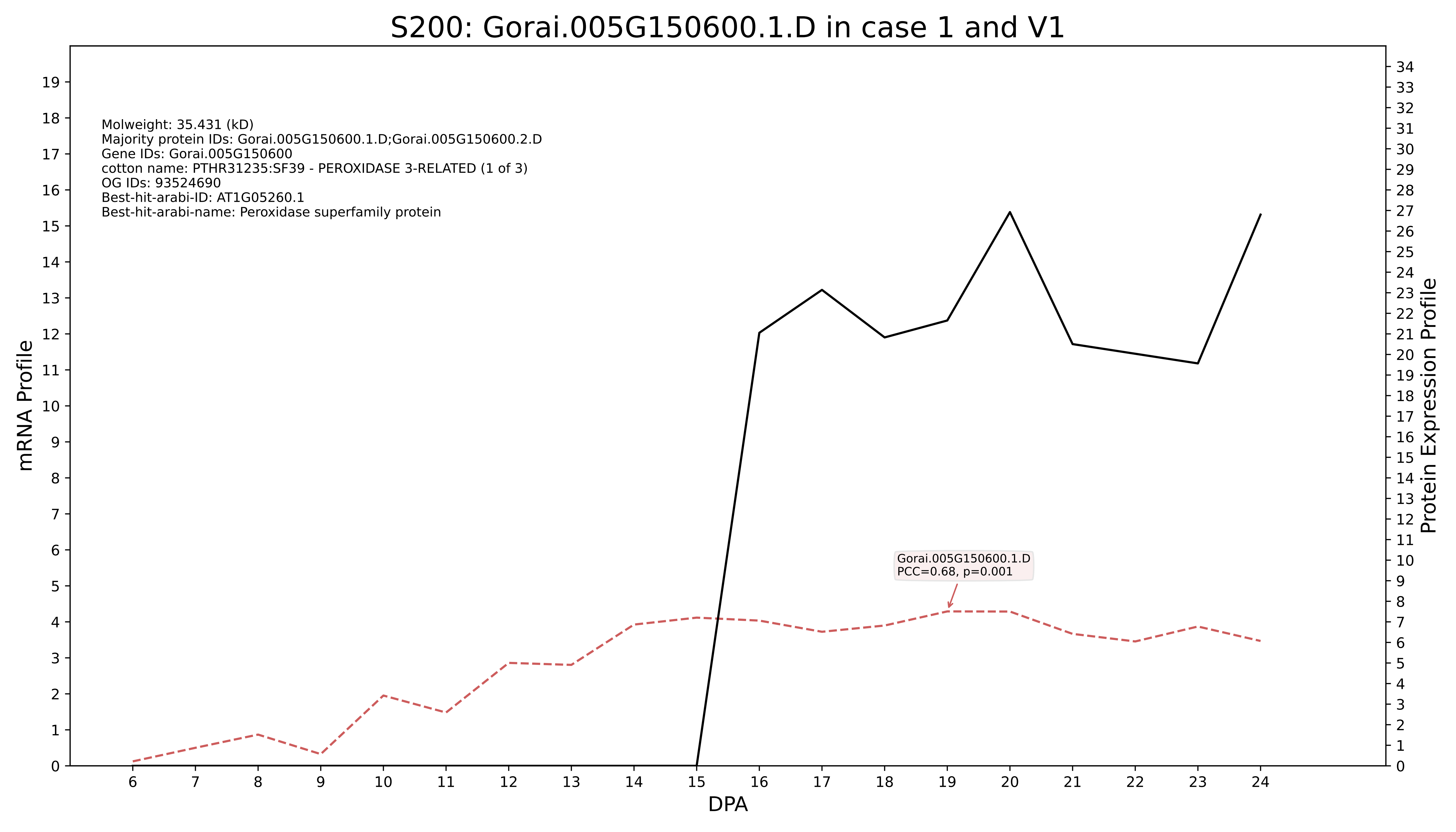

S200: Gorai.005G150600.1.D in case 1 and V1
Molweight: 35.431 (kD)
Majority protein IDs: Gorai.005G150600.1.D;Gorai.005G150600.2.D
Gene IDs: Gorai.005G150600
cotton name: PTHR31235:SF39 - PEROXIDASE 3-RELATED (1 of 3)
OG IDs: 93524690
Best-hit-arabi-ID: AT1G05260.1
Best-hit-arabi-name: Peroxidase superfamily protein
Gorai.005G150600.1.D
PCC=0.68, p=0.001
mRNA Profile
Protein Expression Profile
DPA

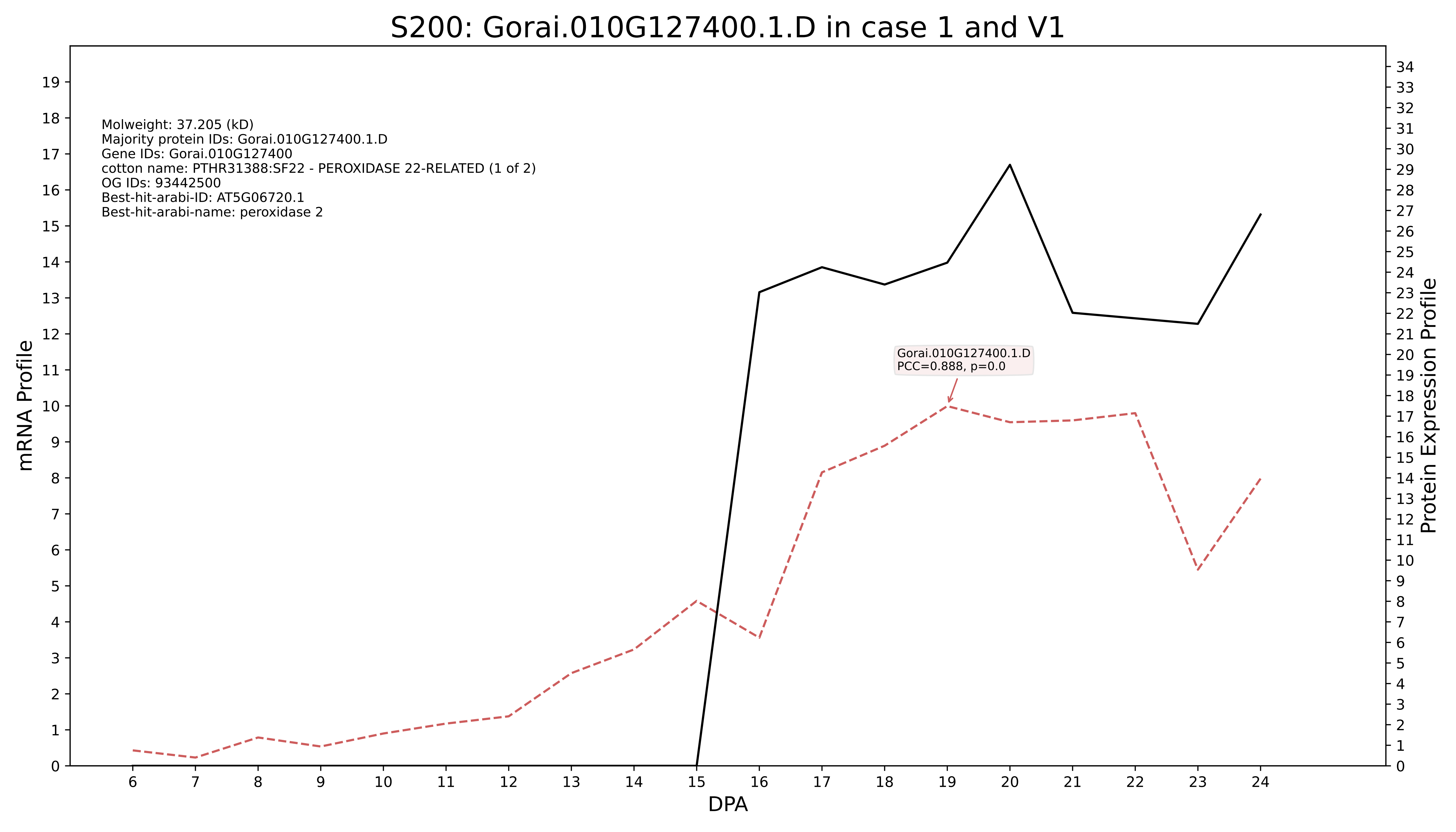
S200: Gorai.010G127400.1.D in case 1 and V1
Molweight: 37.205 (kD)
Majority protein IDs: Gorai.010G127400.1.D
Gene IDs: Gorai.010G127400
cotton name: PTHR31388:SF22 - PEROXIDASE 22-RELATED (1 of 2)
OG IDs: 93442500
Best-hit-arabi-ID: AT5G06720.1
Best-hit-arabi-name: peroxidase 2
Gorai.010G127400.1.D
PCC=0.888, p=0.0
mRNA Profile
Protein Expression Profile
DPA

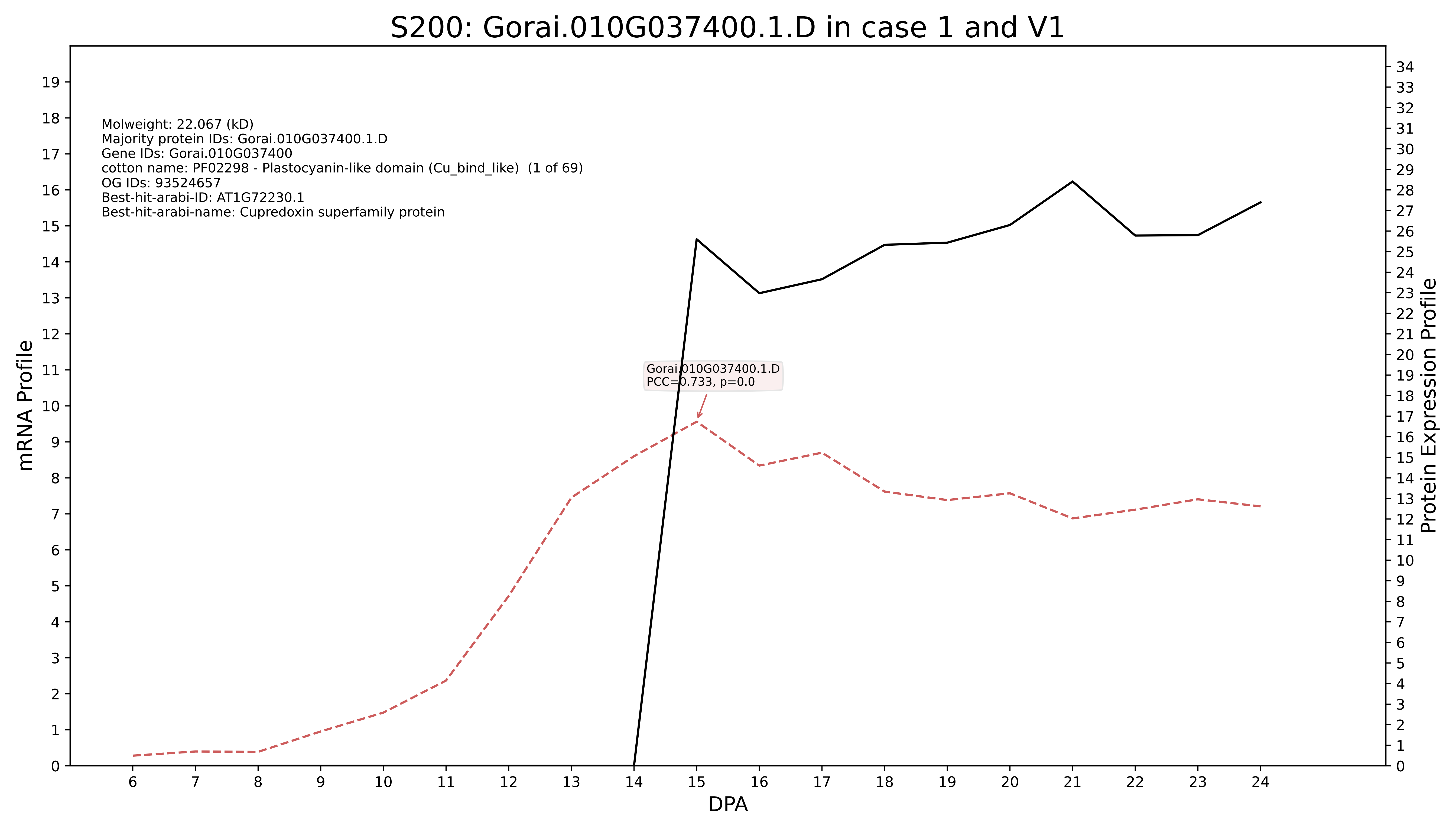
S200: Gorai.010G037400.1.D in case 1 and V1
Molweight: 22.067 (kD)
Majority protein IDs: Gorai.010G037400.1.D
Gene IDs: Gorai.010G037400
cotton name: PF02298 - Plastocyanin-like domain (Cu_bind_like) (1 of 69)
OG IDs: 93524657
Best-hit-arabi-ID: AT1G72230.1
Best-hit-arabi-name: Cupredoxin superfamily protein
Gorai.010G037400.1.D
PCC=0.733, p=0.0
mRNA Profile
Protein Expression Profile
DPA
0
1
2
3
4
5
6
7
8
9
10
11
12
13
14
15
16
17
18
19
20
21
22
23
24
25
26
27
28
29
30
31
32
33
34

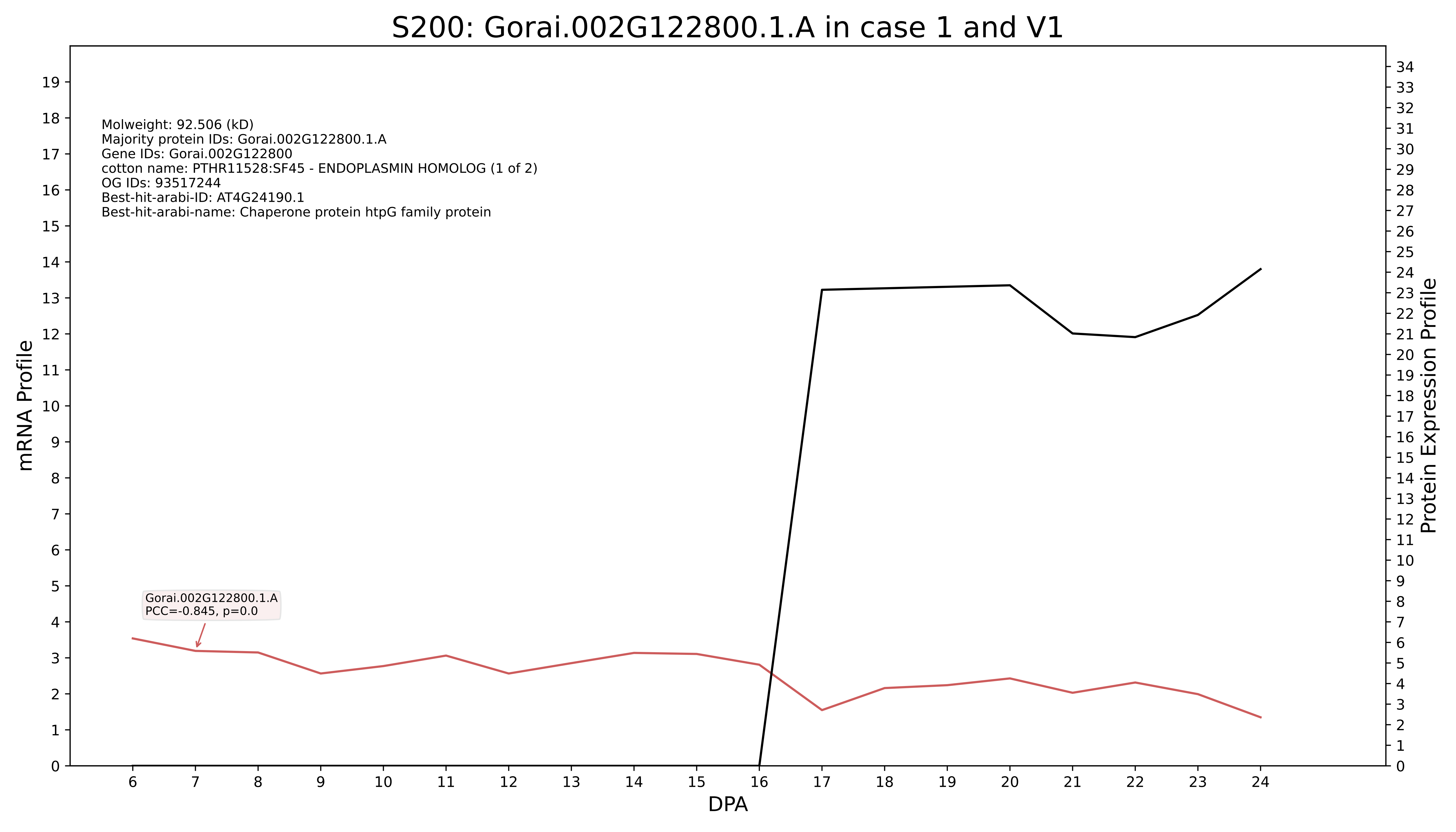
S200: Gorai.002G122800.1.A in case 1 and V1
Molweight: 92.506 (kD)
Majority protein IDs: Gorai.002G122800.1.A
Gene IDs: Gorai.002G122800
cotton name: PTHR11528:SF45 - ENDOPLASMIN HOMOLOG (1 of 2)
OG IDs: 93517244
Best-hit-arabi-ID: AT4G24190.1
Best-hit-arabi-name: Chaperone protein htpG family protein
Gorai.002G122800.1.A
PCC=-0.845, p=0.0
mRNA Profile
Protein Expression Profile
DPA

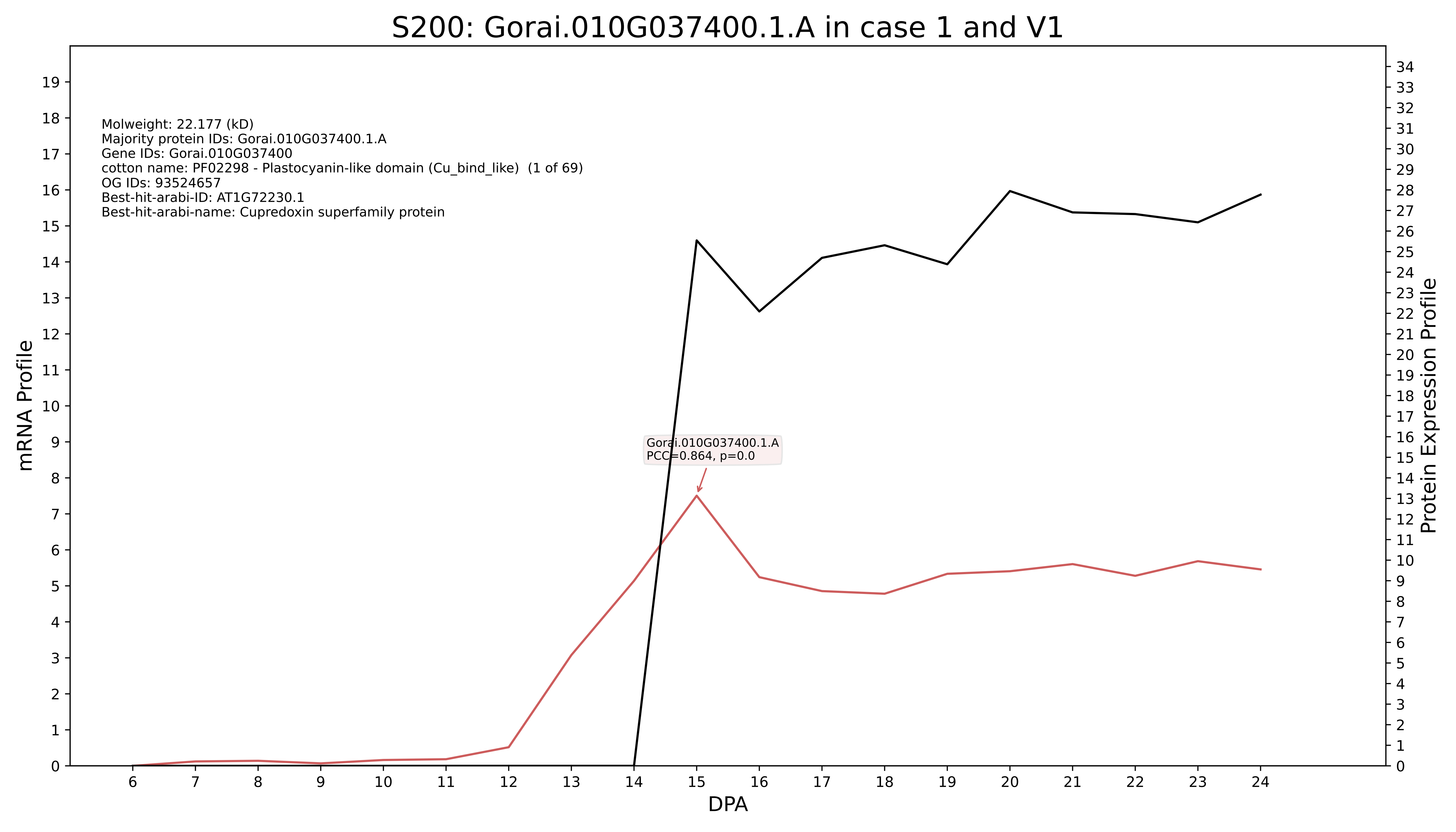
S200: Gorai.010G037400.1.A in case 1 and V1
Molweight: 22.177 (kD)
Majority protein IDs: Gorai.010G037400.1.A
Gene IDs: Gorai.010G037400
cotton name: PF02298 - Plastocyanin-like domain (Cu_bind_like) (1 of 69)
OG IDs: 93524657
Best-hit-arabi-ID: AT1G72230.1
Best-hit-arabi-name: Cupredoxin superfamily protein
Gorai.010G037400.1.A
PCC=0.864, p=0.0
mRNA Profile
Protein Expression Profile
DPA

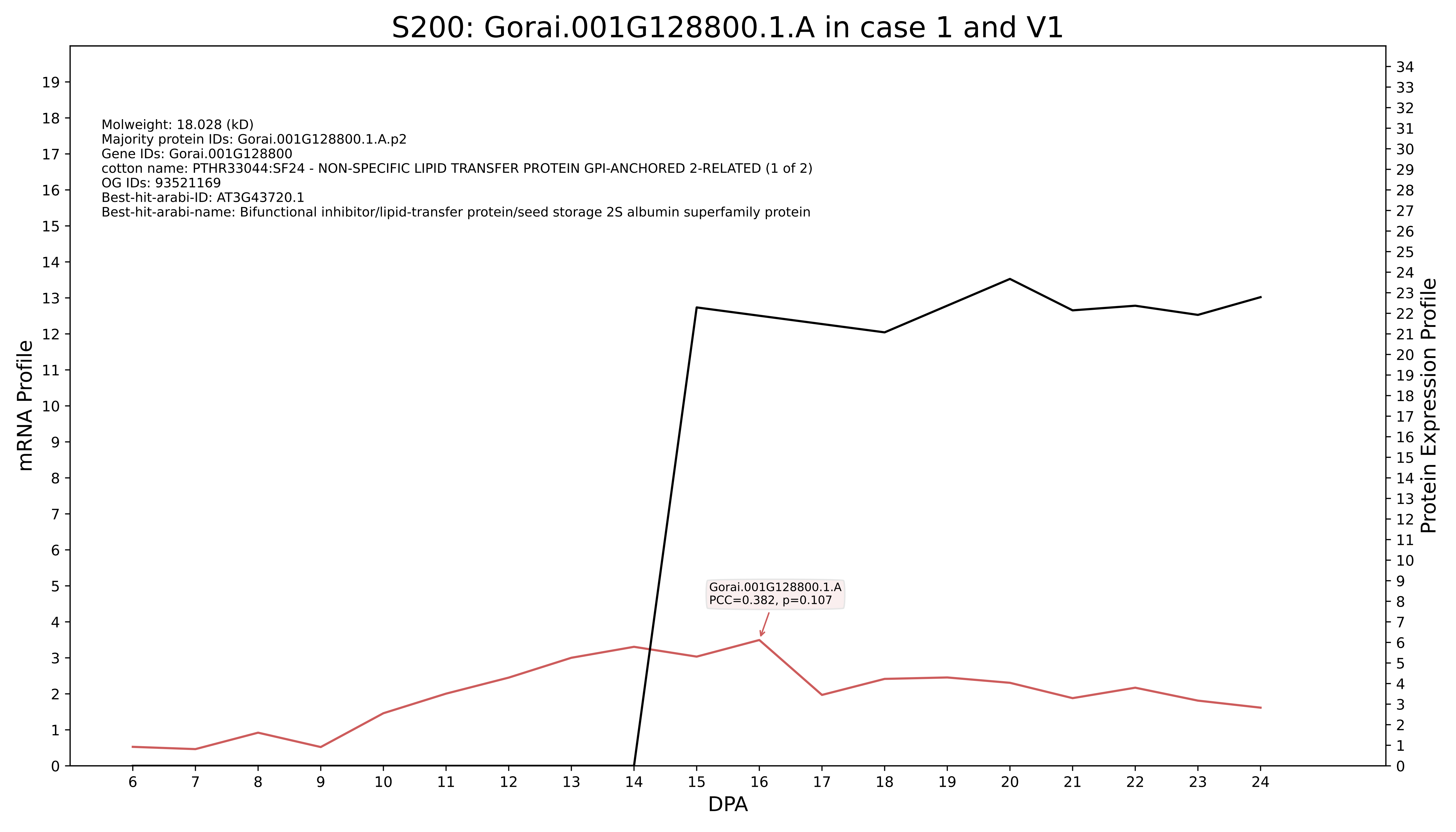

S200: Gorai.001G128800.1.A in case 1 and V1
Molweight: 18.028 (kD)
Majority protein IDs: Gorai.001G128800.1.A.p2
Gene IDs: Gorai.001G128800
cotton name: PTHR33044:SF24 - NON-SPECIFIC LIPID TRANSFER PROTEIN GPI-ANCHORED 2-RELATED (1 of 2)
OG IDs: 93521169
Best-hit-arabi-ID: AT3G43720.1
Best-hit-arabi-name: Bifunctional inhibitor/lipid-transfer protein/seed storage 2S albumin superfamily protein
Gorai.001G128800.1.A
PCC=0.382, p=0.107
mRNA Profile
Protein Expression Profile
DPA

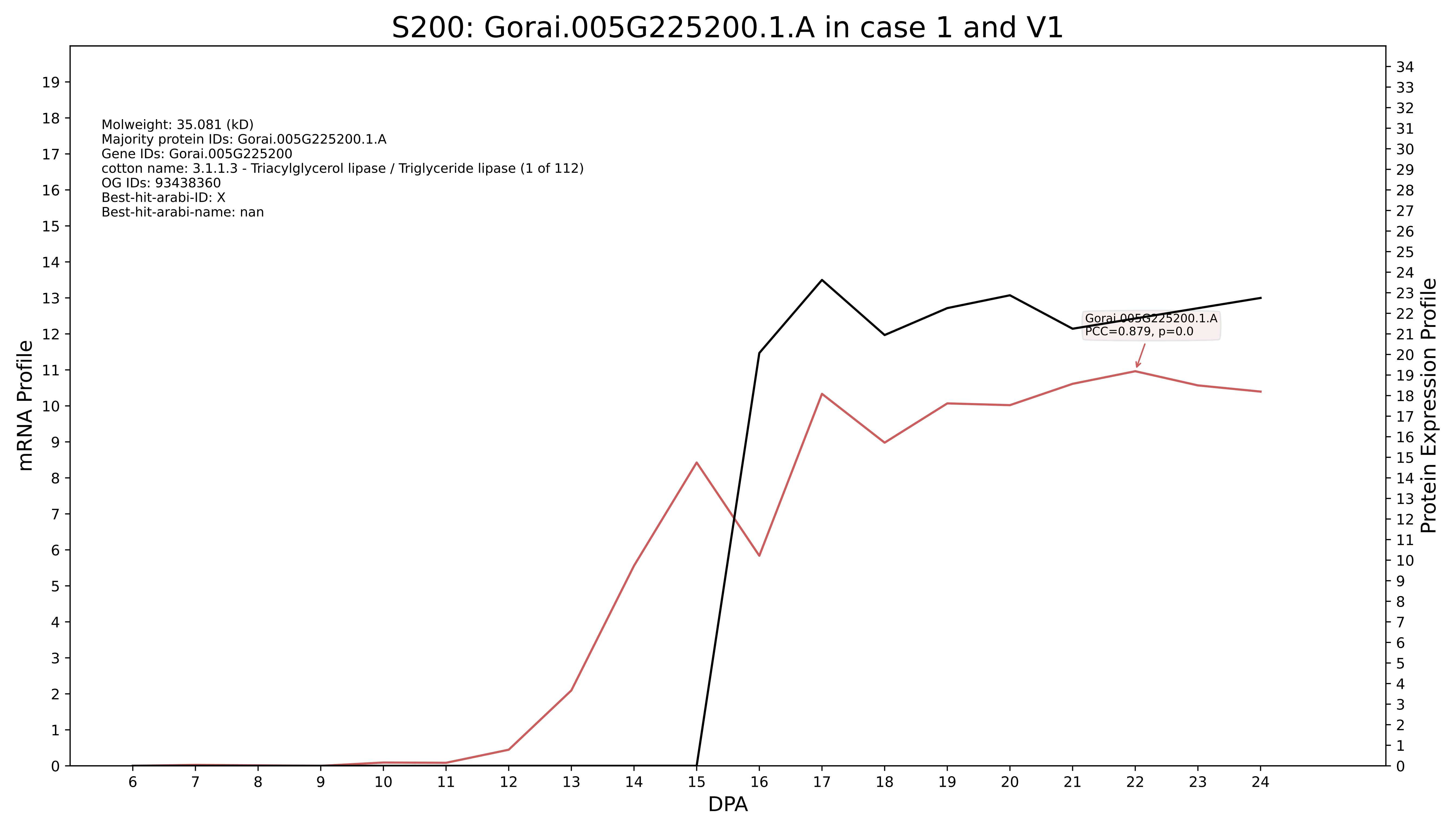
S200: Gorai.005G225200.1.A in case 1 and V1
Molweight: 35.081 (kD)
Majority protein IDs: Gorai.005G225200.1.A
Gene IDs: Gorai.005G225200
cotton name: 3.1.1.3 - Triacylglycerol lipase / Triglyceride lipase (1 of 112)
OG IDs: 93438360
Best-hit-arabi-ID: X
Best-hit-arabi-name: nan
Gorai.005G225200.1.A
PCC=0.879, p=0.0
mRNA Profile
Protein Expression Profile
DPA

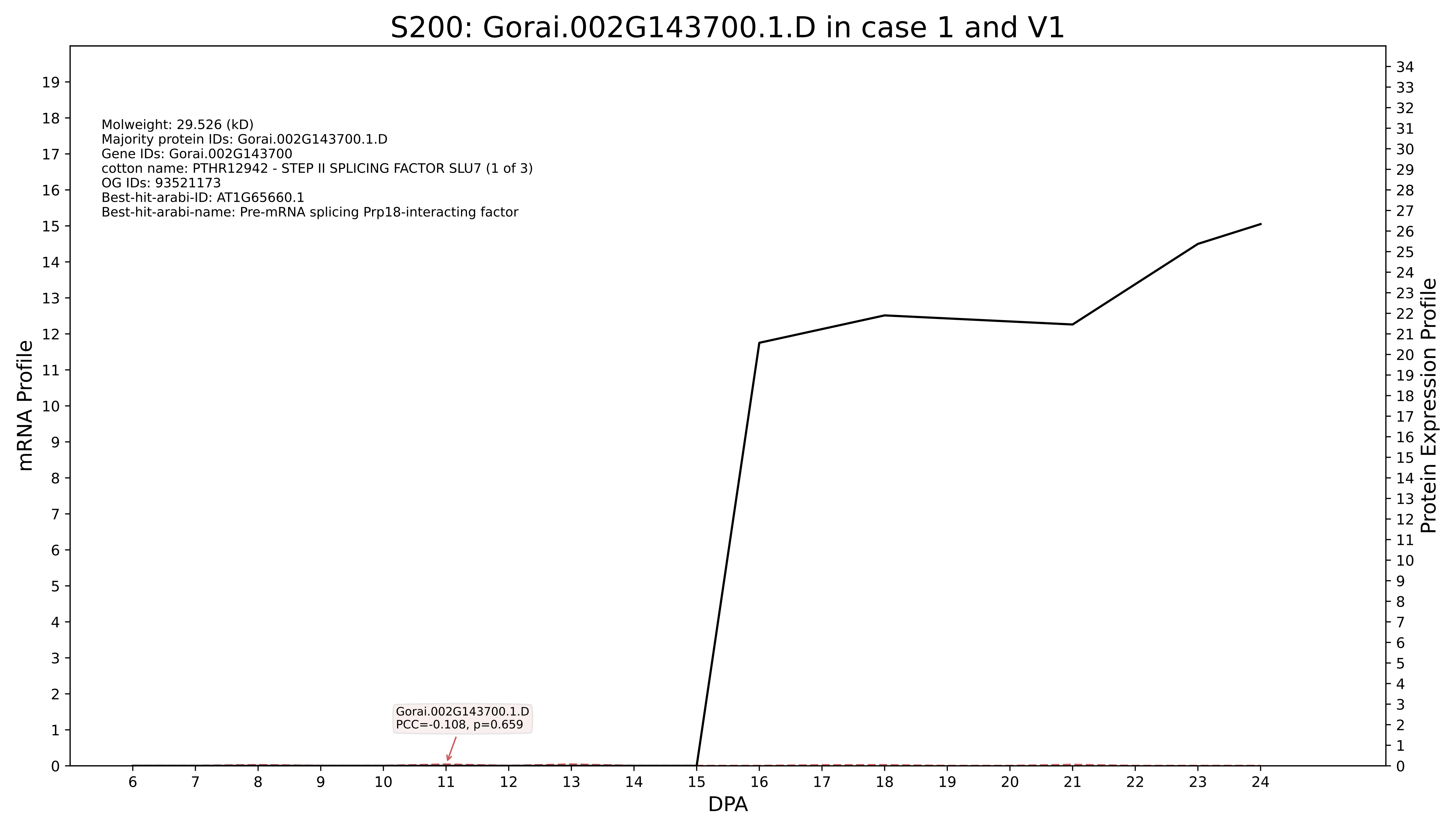
S200: Gorai.002G143700.1.D in case 1 and V1
Molweight: 29.526 (kD)
Majority protein IDs: Gorai.002G143700.1.D
Gene IDs: Gorai.002G143700
cotton name: PTHR12942 - STEP II SPLICING FACTOR SLU7 (1 of 3)
OG IDs: 93521173
Best-hit-arabi-ID: AT1G65660.1
Best-hit-arabi-name: Pre-mRNA splicing Prp18-interacting factor
Gorai.002G143700.1.D
PCC=-0.108, p=0.659
mRNA Profile
Protein Expression Profile
DPA
6 7 8 9 10 11 12 13 14 15 16 17 18 19 20 21 22 23 24

S200: Gorai.009G239100.1.D in case 1 and V1

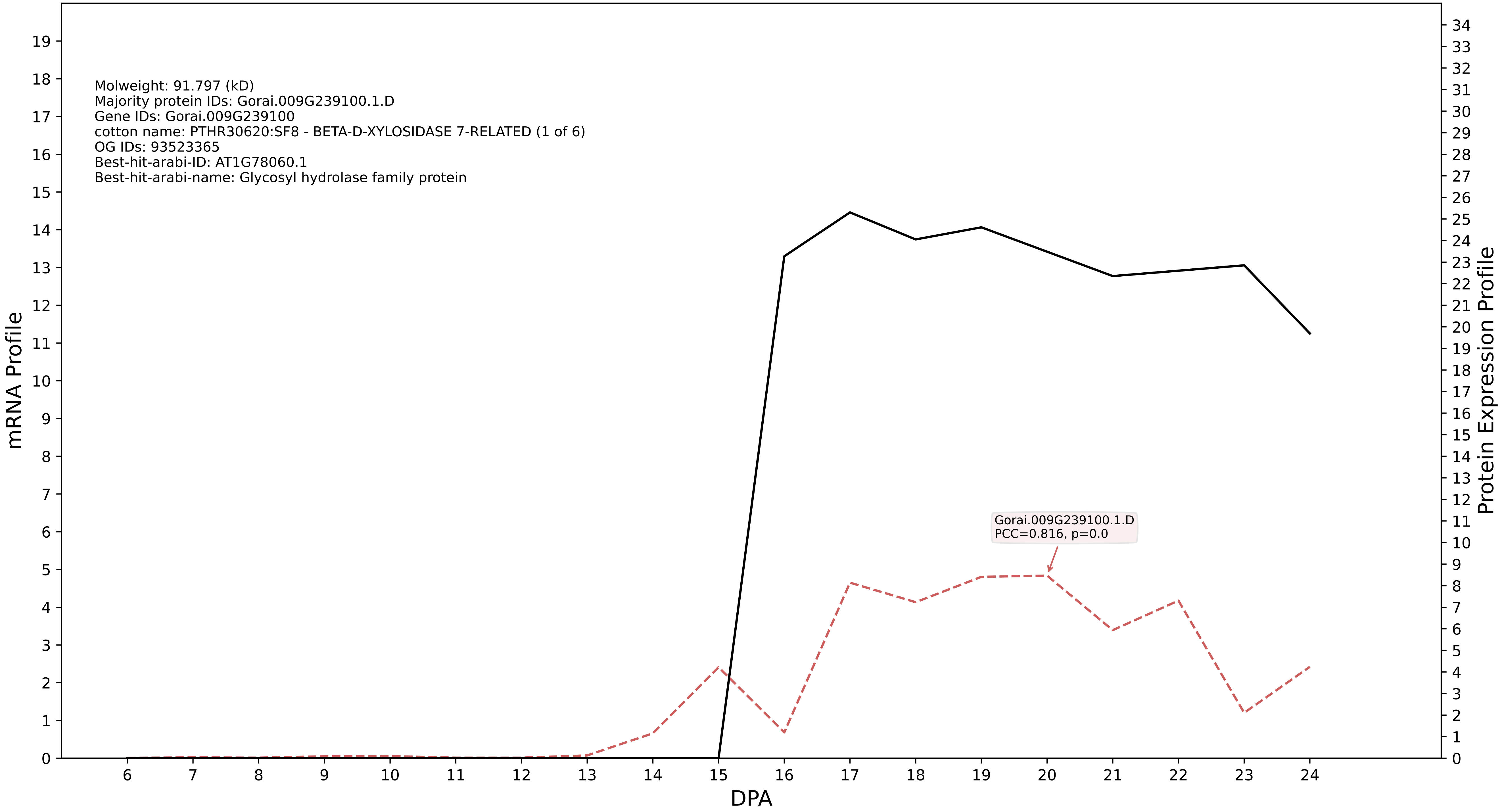

S200: Gorai.013G003200.1.D in case 2 and V1

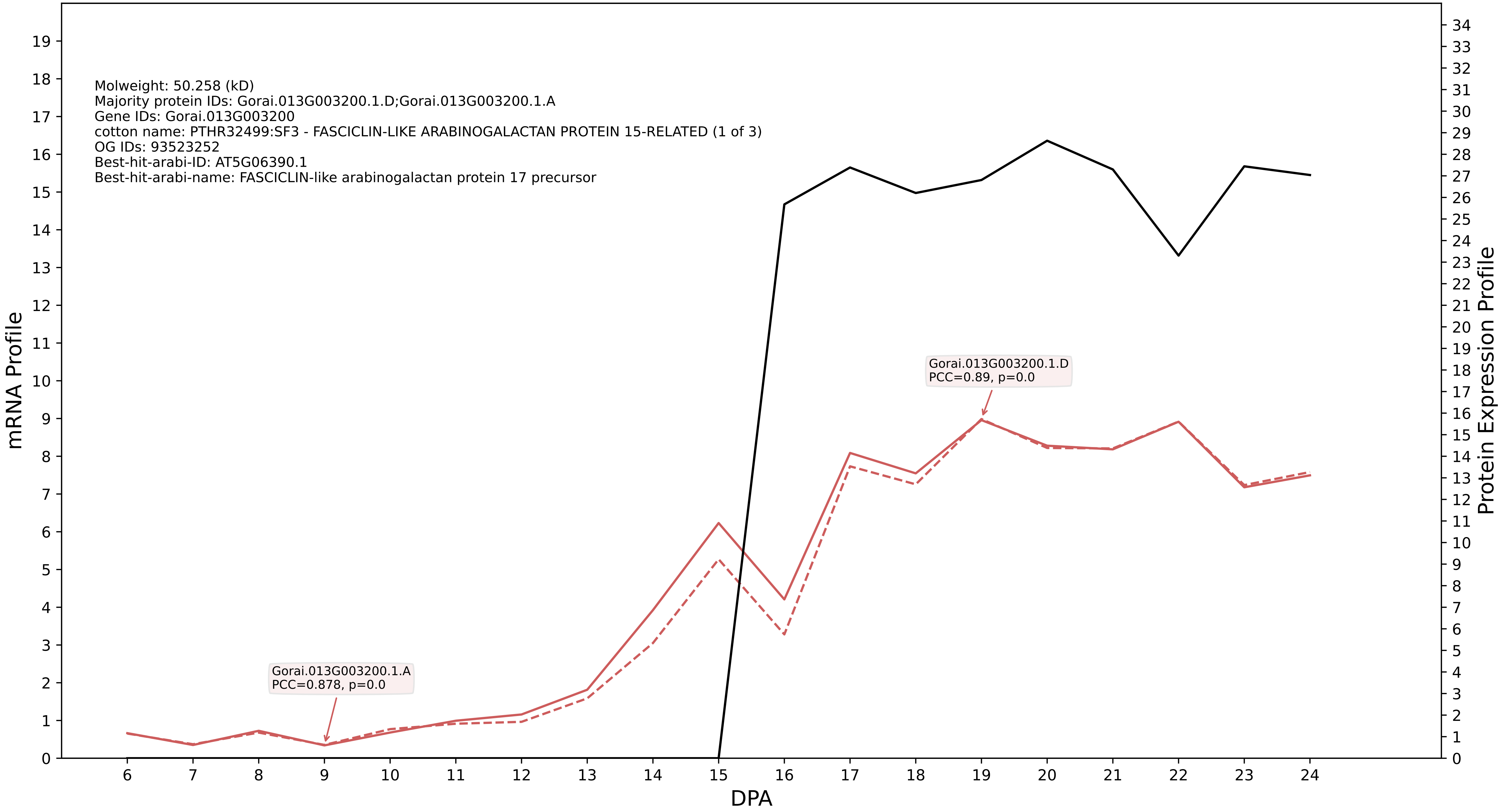

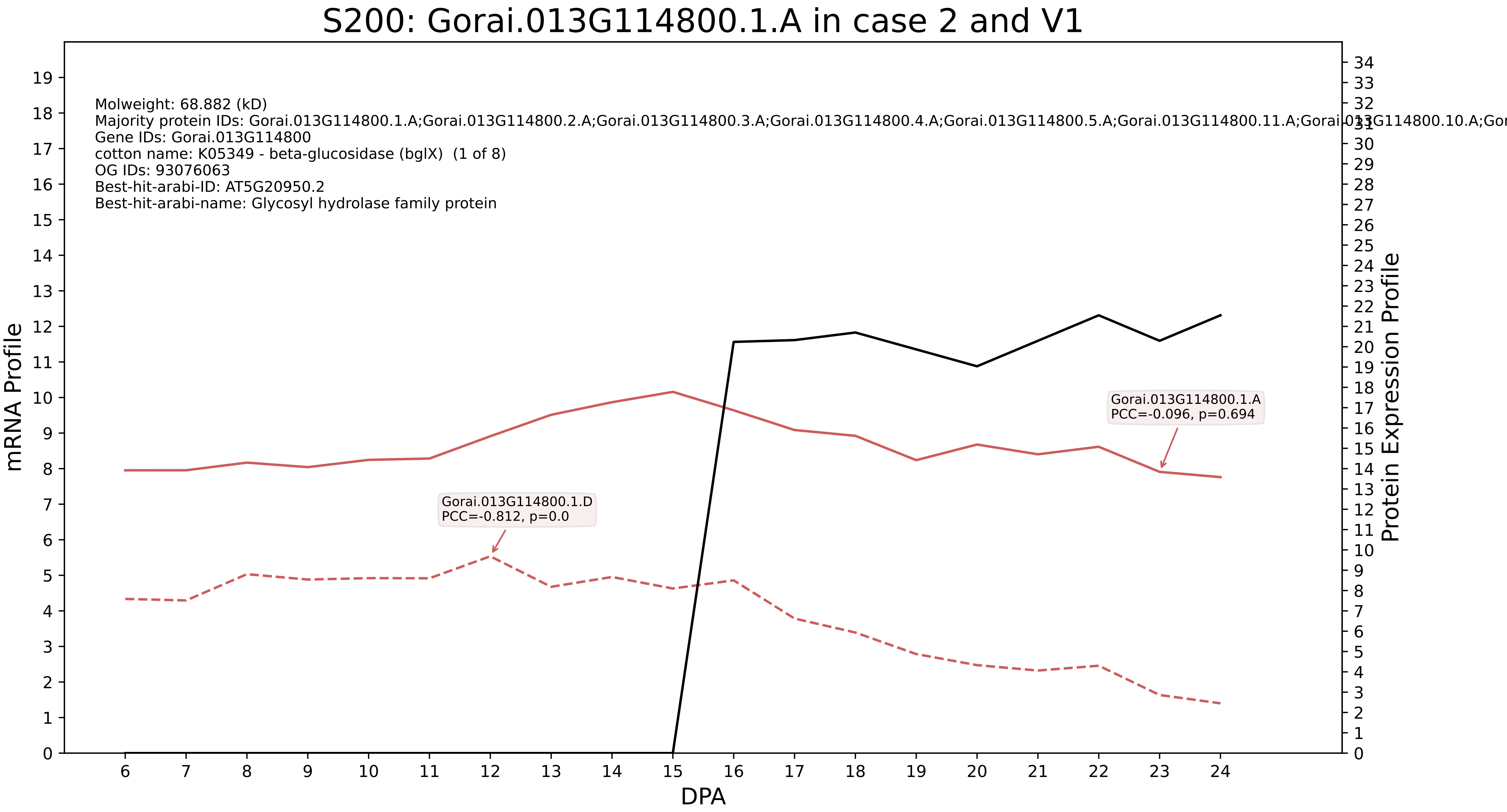

S200: Gorai.013G114800.1.A in case 2 and V1
Molweight: 68.882 (kD)
Majority protein IDs: Gorai.013G114800.1.A;Gorai.013G114800.2.A;Gorai.013G114800.3.A;Gorai.013G114800.4.A;Gorai.013G114800.5.A;Gorai.013G114800.11.A;Gorai.013G114800.10.A;Gor
Gene IDs: Gorai.013G114800
cotton name: K05349 - beta-glucosidase (bglX) (1 of 8)
OG IDs: 93076063
Best-hit-arabi-ID: AT5G20950.2
Best-hit-arabi-name: Glycosyl hydrolase family protein
Gorai.013G114800.1.A
PCC=-0.096, p=0.694
Gorai.013G114800.1.D
PCC=-0.812, p=0.0
mRNA Profile
Protein Expression Profile
DPA

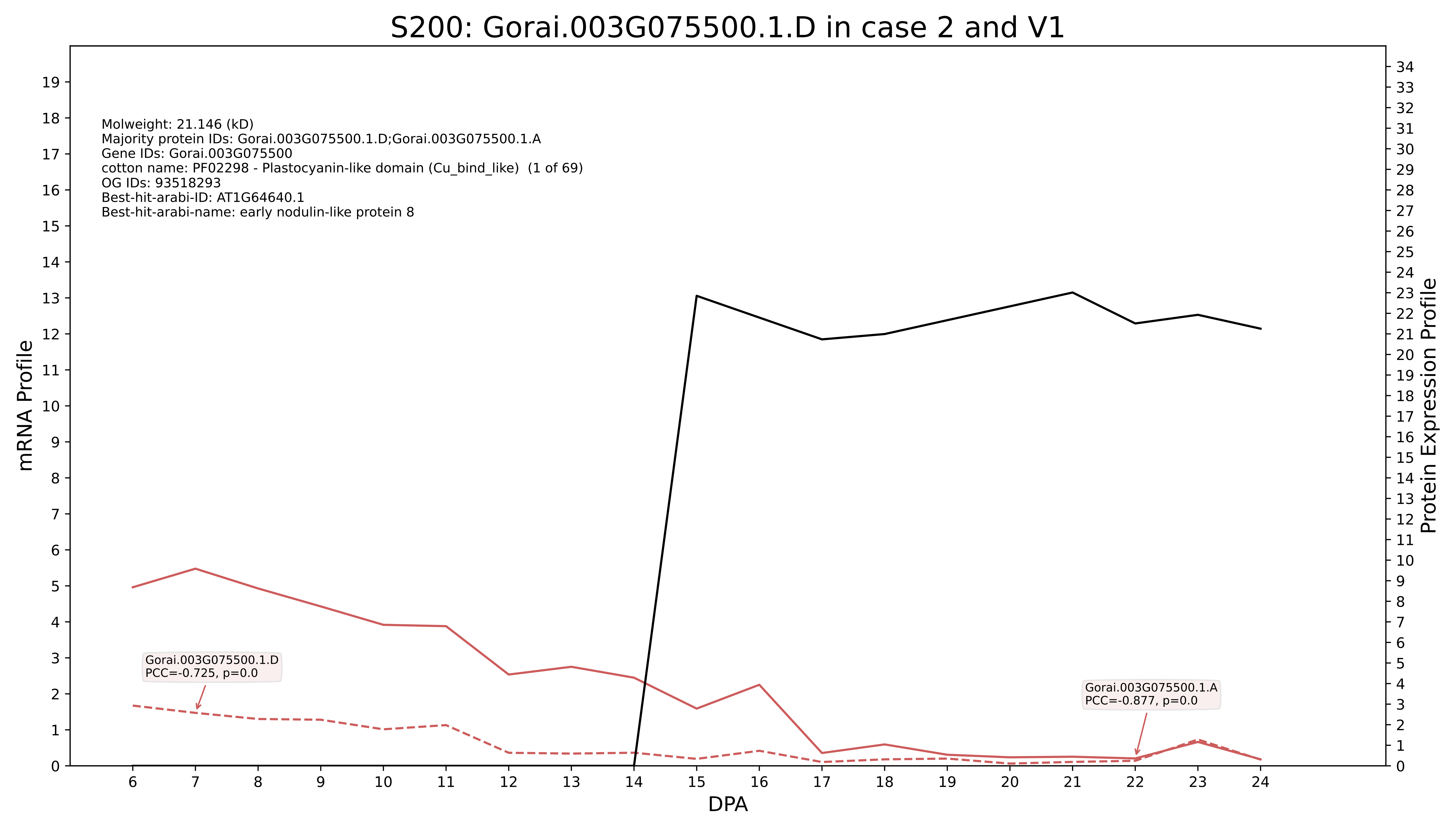

S200: Gorai.003G075500.1.D in case 2 and V1
Molweight: 21.146 (kD)
Majority protein IDs: Gorai.003G075500.1.D;Gorai.003G075500.1.A
Gene IDs: Gorai.003G075500
cotton name: PF02298 - Plastocyanin-like domain (Cu_bind_like) (1 of 69)
OG IDs: 93518293
Best-hit-arabi-ID: AT1G64640.1
Best-hit-arabi-name: early nodulin-like protein 8
Gorai.003G075500.1.D
PCC=-0.725, p=0.0
Gorai.003G075500.1.A
PCC=-0.877, p=0.0
mRNA Profile
Protein Expression Profile
DPA

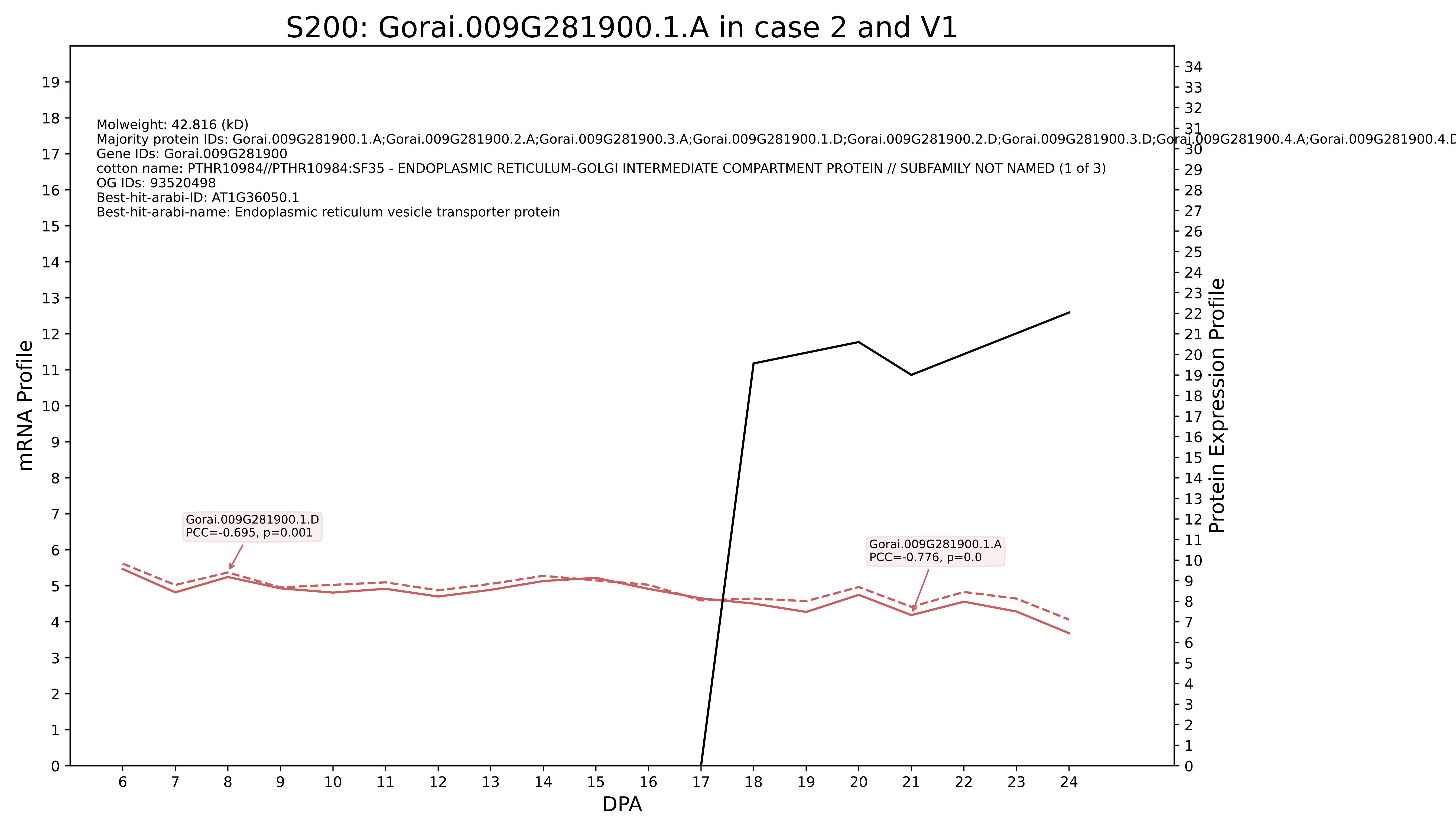
S200: Gorai.009G281900.1.A in case 2 and V1
Molweight: 42.816 (kD)
Majority protein IDs: Gorai.009G281900.1.A;Gorai.009G281900.2.A;Gorai.009G281900.3.A;Gorai.009G281900.1.D;Gorai.009G281900.2.D;Gorai.009G281900.3.D;Gorai.009G281900.4.A;Gorai.009G281900.4.D
Gene IDs: Gorai.009G281900
cotton name: PTHR10984//PTHR10984:SF35 - ENDOPLASMIC RETICULUM-GOLGI INTERMEDIATE COMPARTMENT PROTEIN // SUBFAMILY NOT NAMED (1 of 3)
OG IDs: 93520498
Best-hit-arabi-ID: AT1G36050.1
Best-hit-arabi-name: Endoplasmic reticulum vesicle transporter protein
Gorai.009G281900.1.D
PCC=-0.695, p=0.001
Gorai.009G281900.1.A
PCC=-0.776, p=0.0
mRNA Profile
Protein Expression Profile
DPA

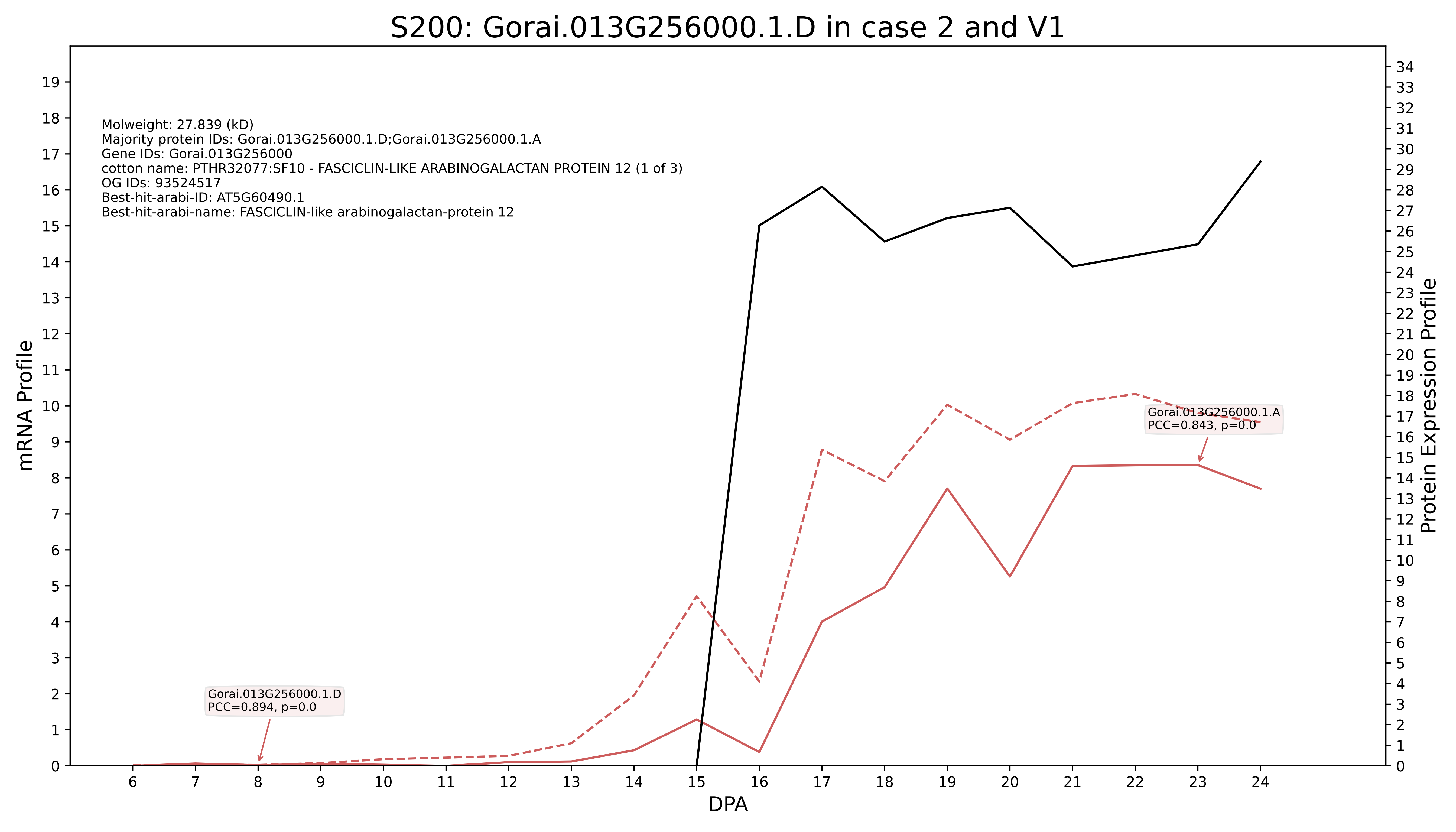
S200: Gorai.013G256000.1.D in case 2 and V1
Molweight: 27.839 (kD)
Majority protein IDs: Gorai.013G256000.1.D;Gorai.013G256000.1.A
Gene IDs: Gorai.013G256000
cotton name: PTHR32077:SF10 - FASCICLIN-LIKE ARABINOGALACTAN PROTEIN 12 (1 of 3)
OG IDs: 93524517
Best-hit-arabi-ID: AT5G60490.1
Best-hit-arabi-name: FASCICLIN-like arabinogalactan-protein 12
Gorai.013G256000.1.A
PCC=0.843, p=0.0
Gorai.013G256000.1.D
PCC=0.894, p=0.0
mRNA Profile
Protein Expression Profile
DPA

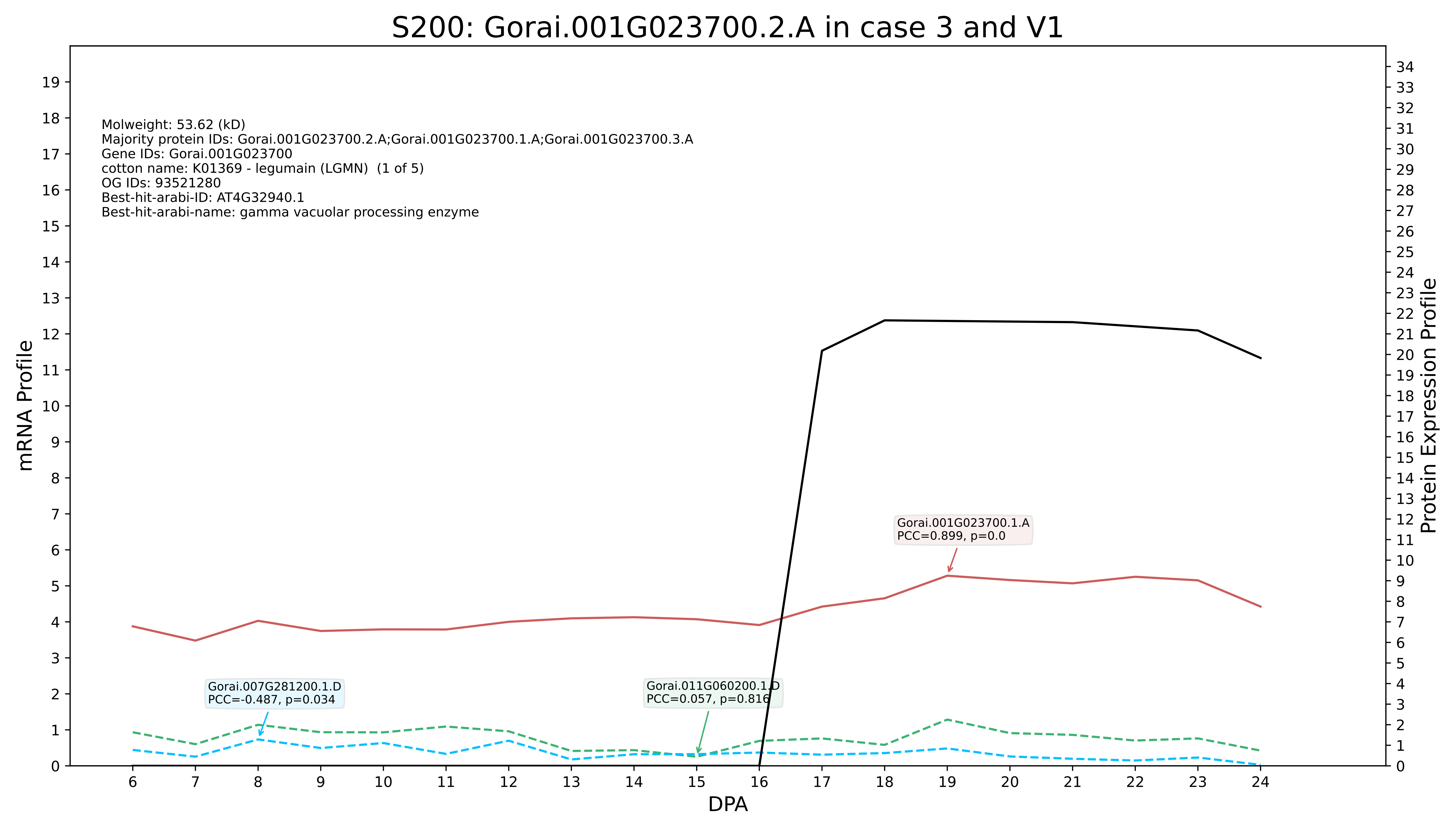
S200: Gorai.001G023700.2.A in case 3 and V1
Molweight: 53.62 (kD)
Majority protein IDs: Gorai.001G023700.2.A;Gorai.001G023700.1.A;Gorai.001G023700.3.A
Gene IDs: Gorai.001G023700
cotton name: K01369 - legumain (LGMN) (1 of 5)
OG IDs: 93521280
Best-hit-arabi-ID: AT4G32940.1
Best-hit-arabi-name: gamma vacuolar processing enzyme
Gorai.001G023700.1.A
PCC=0.899, p=0.0
Gorai.007G281200.1.D
PCC=-0.487, p=0.034
Gorai.011G060200.1.D
PCC=0.057, p=0.816
mRNA Profile
Protein Expression Profile
DPA

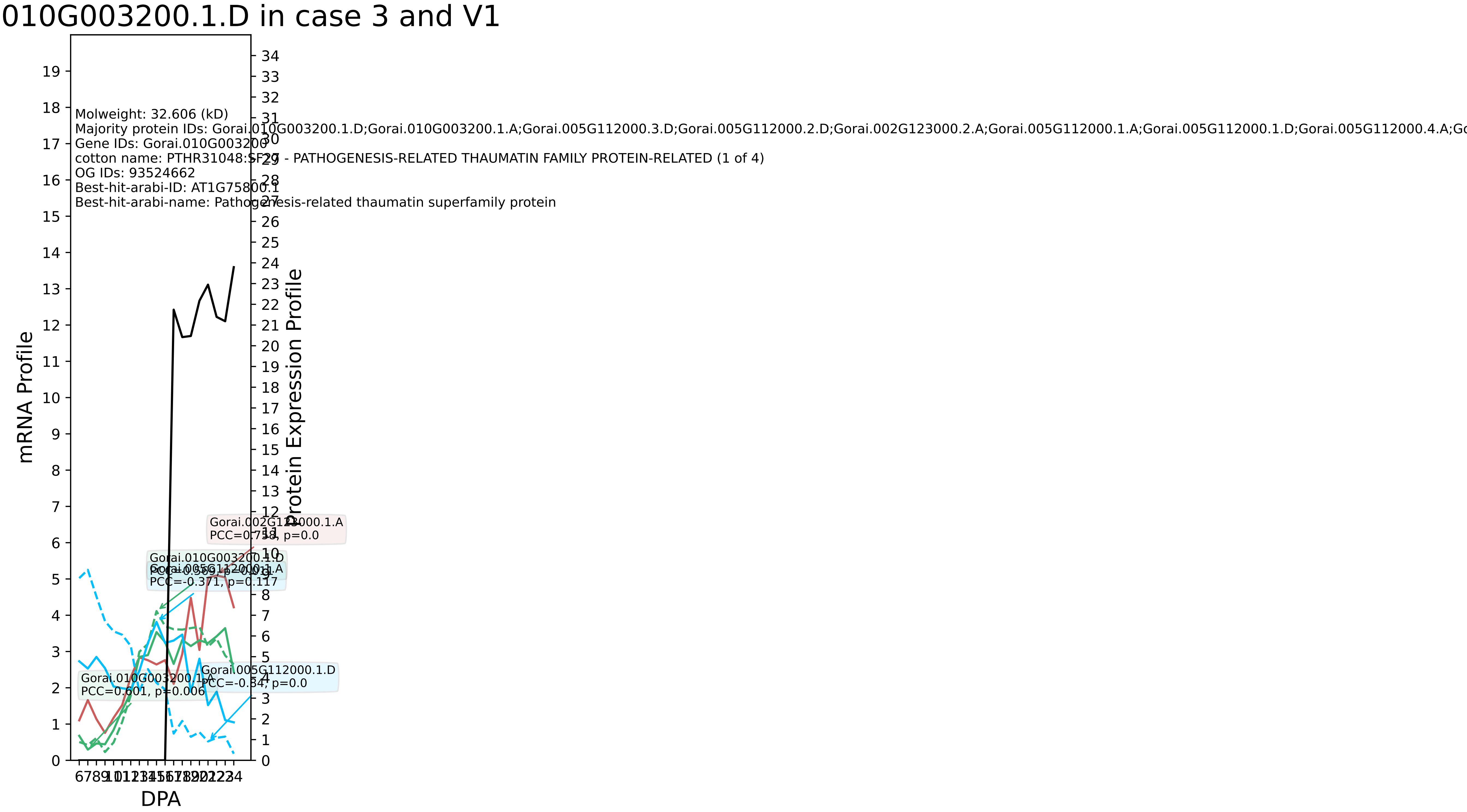
010G003200.1.D in case 3 and V1
Molweight: 32.606 (kD)
Majority protein IDs: Gorai.010G003200.1.D;Gorai.010G003200.1.A;Gorai.005G112000.3.D;Gorai.005G112000.2.D;Gorai.002G123000.2.A;Gorai.005G112000.1.A;Gorai.005G112000.1.D;Gorai.005G112000.4.A;Go
Gene IDs: Gorai.010G003200
cotton name: PTHR31048:SF27 - PATHOGENESIS-RELATED THAUMATIN FAMILY PROTEIN-RELATED (1 of 4)
OG IDs: 93524662
Best-hit-arabi-ID: AT1G75800.1
Best-hit-arabi-name: Pathogenesis-related thaumatin superfamily protein
Gorai.002G123000.1.A
PCC=0.758, p=0.0
Gorai.010G003200.1.D
Gorai.005G112000.1.A
PCC=-0.371, p=0.117
Gorai.010G003200.1.A
PCC=0.601, p=0.006
Gorai.005G112000.1.D
PCC=-0.84, p=0.0
mRNA Profile
Protein Expression Profile
DPA

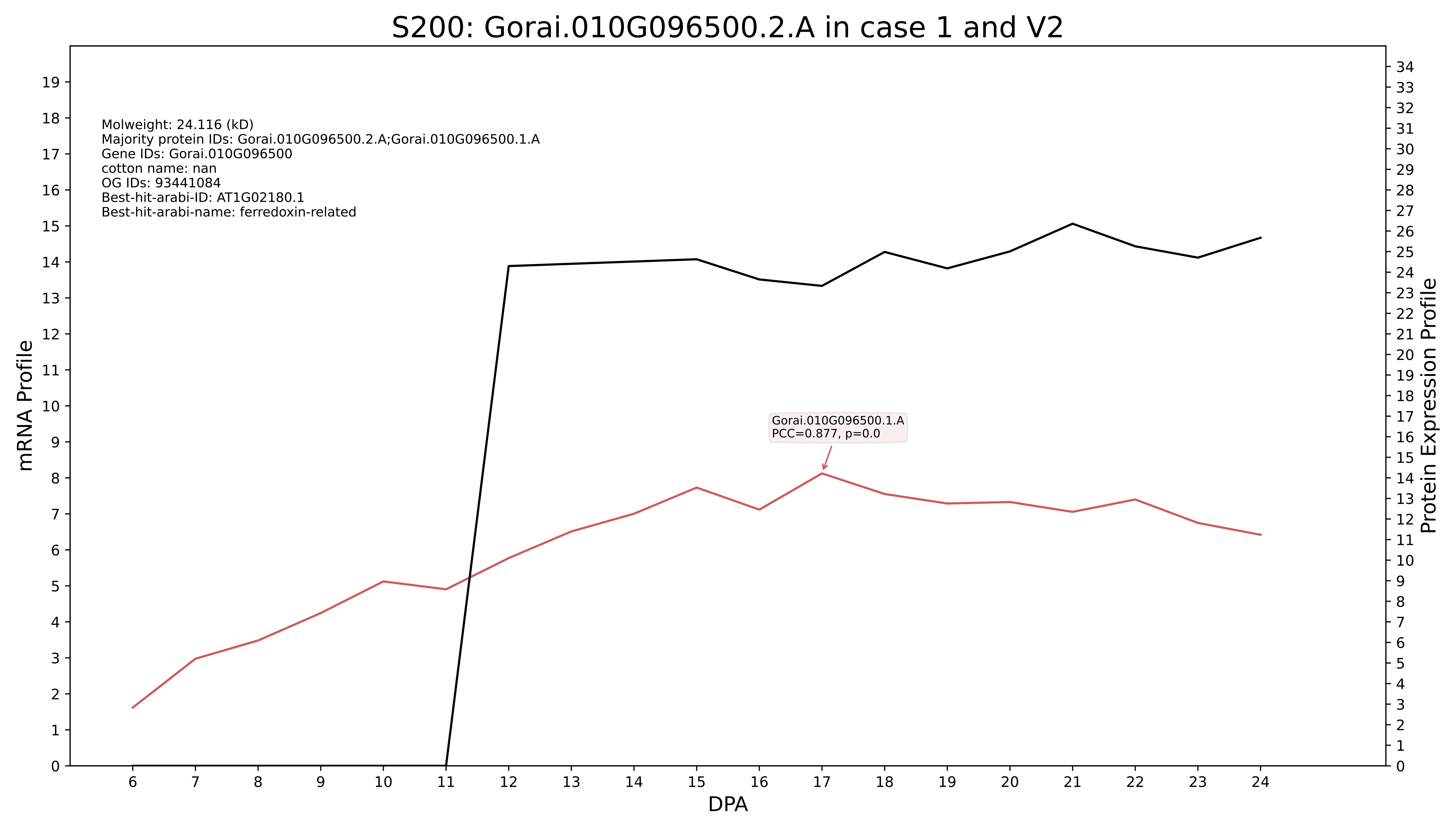
S200: Gorai.010G096500.2.A in case 1 and V2
Molweight: 24.116 (kD)
Majority protein IDs: Gorai.010G096500.2.A;Gorai.010G096500.1.A
Gene IDs: Gorai.010G096500
cotton name: nan
OG IDs: 93441084
Best-hit-arabi-ID: AT1G02180.1
Best-hit-arabi-name: ferredoxin-related
Gorai.010G096500.1.A
PCC=0.877, p=0.0
mRNA Profile
Protein Expression Profile
DPA
0
1
2
3
4
5
6
7
8
9
10
11
12
13
14
15
16
17
18
19
20
21
22
23
24
25
26
27
28
29
30
31
32
33
34

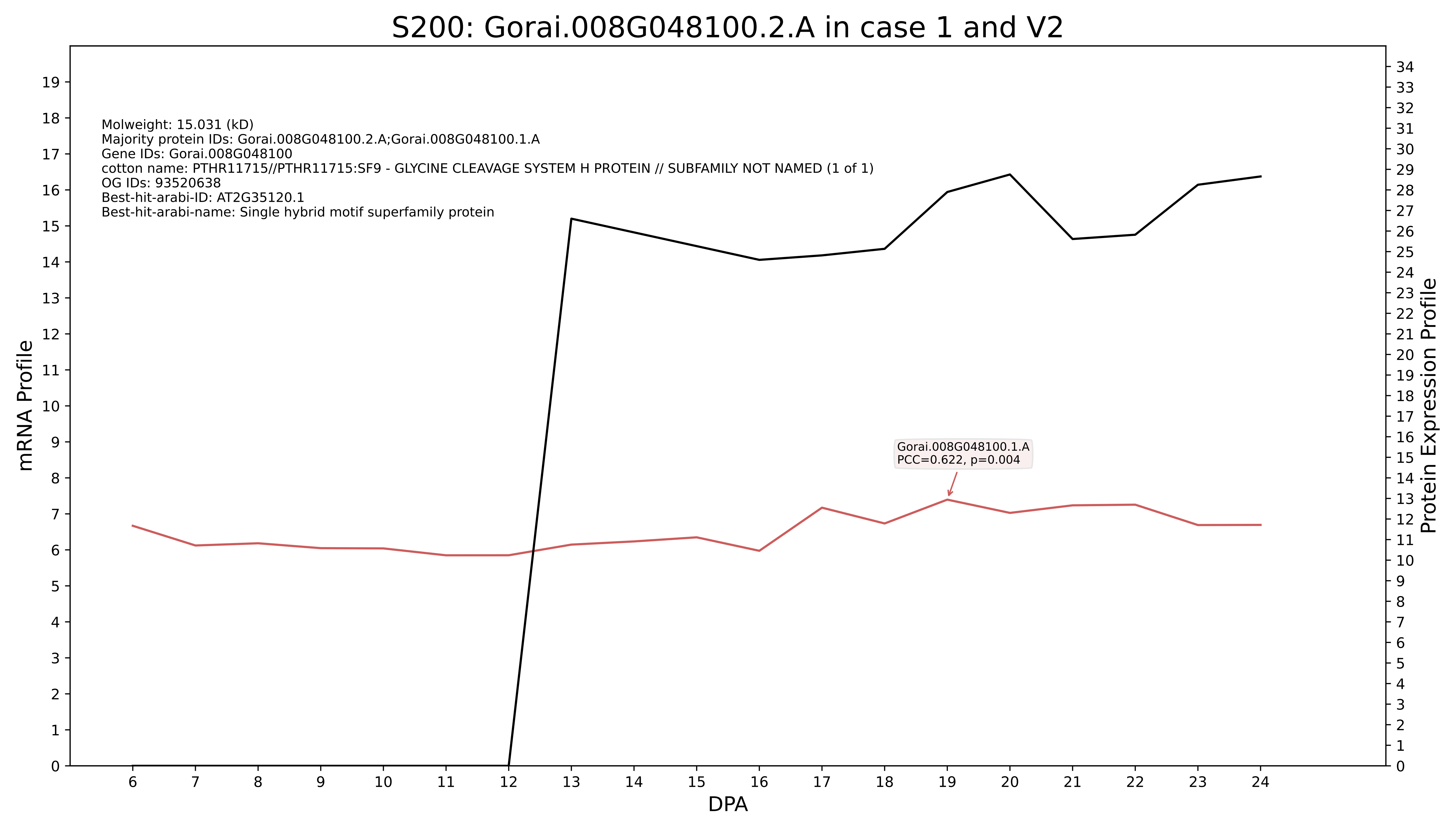
S200: Gorai.008G048100.2.A in case 1 and V2
Molweight: 15.031 (kD)
Majority protein IDs: Gorai.008G048100.2.A;Gorai.008G048100.1.A
Gene IDs: Gorai.008G048100
cotton name: PTHR11715//PTHR11715:SF9 - GLYCINE CLEAVAGE SYSTEM H PROTEIN // SUBFAMILY NOT NAMED (1 of 1)
OG IDs: 93520638
Best-hit-arabi-ID: AT2G35120.1
Best-hit-arabi-name: Single hybrid motif superfamily protein
Gorai.008G048100.1.A
PCC=0.622, p=0.004
mRNA Profile
Protein Expression Profile
DPA

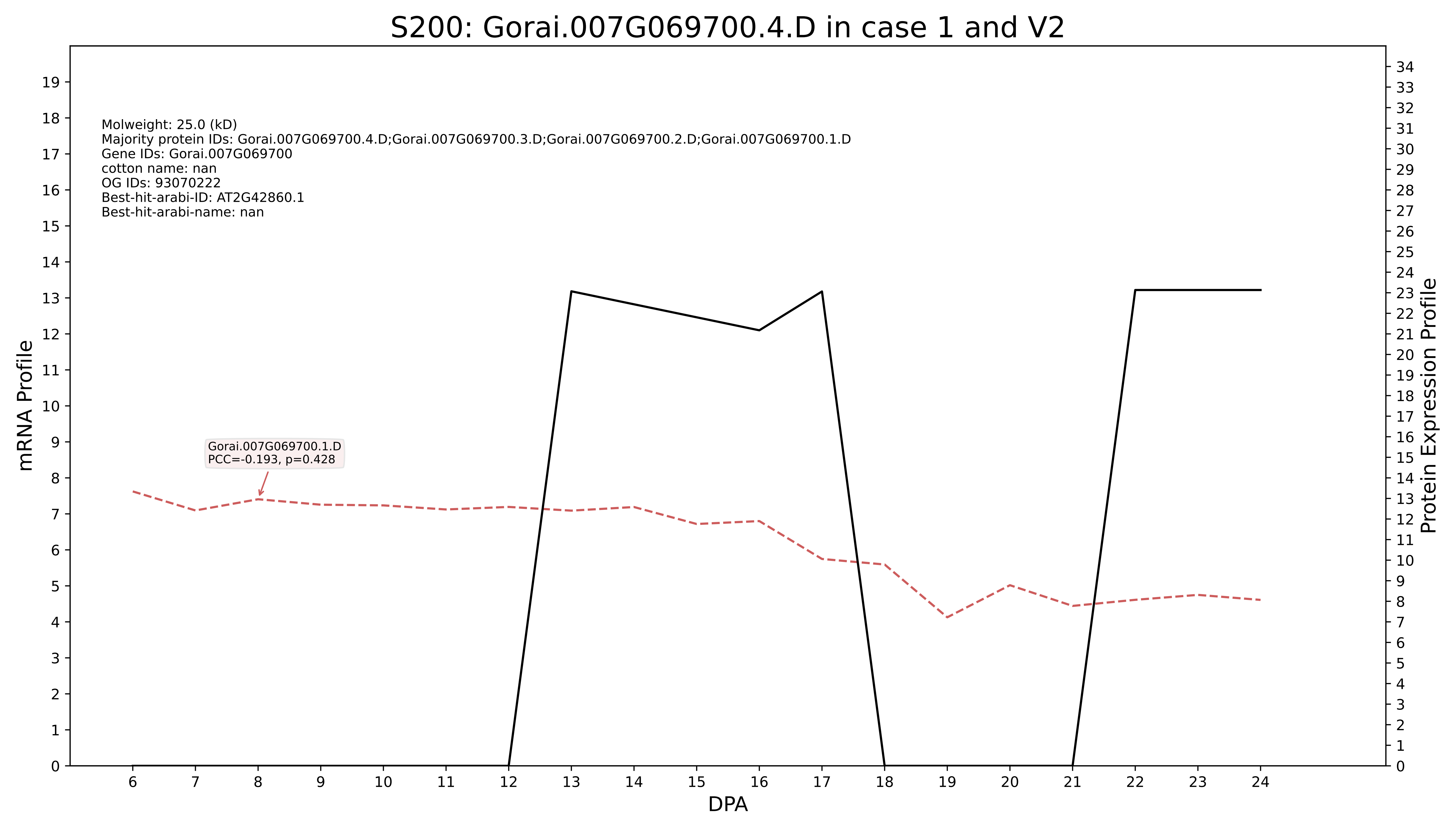
S200: Gorai.007G069700.4.D in case 1 and V2
Molweight: 25.0 (kD)
Majority protein IDs: Gorai.007G069700.4.D;Gorai.007G069700.3.D;Gorai.007G069700.2.D;Gorai.007G069700.1.D
Gene IDs: Gorai.007G069700
cotton name: nan
OG IDs: 93070222
Best-hit-arabi-ID: AT2G42860.1
Best-hit-arabi-name: nan
Gorai.007G069700.1.D
PCC=-0.193, p=0.428
mRNA Profile
Protein Expression Profile
DPA

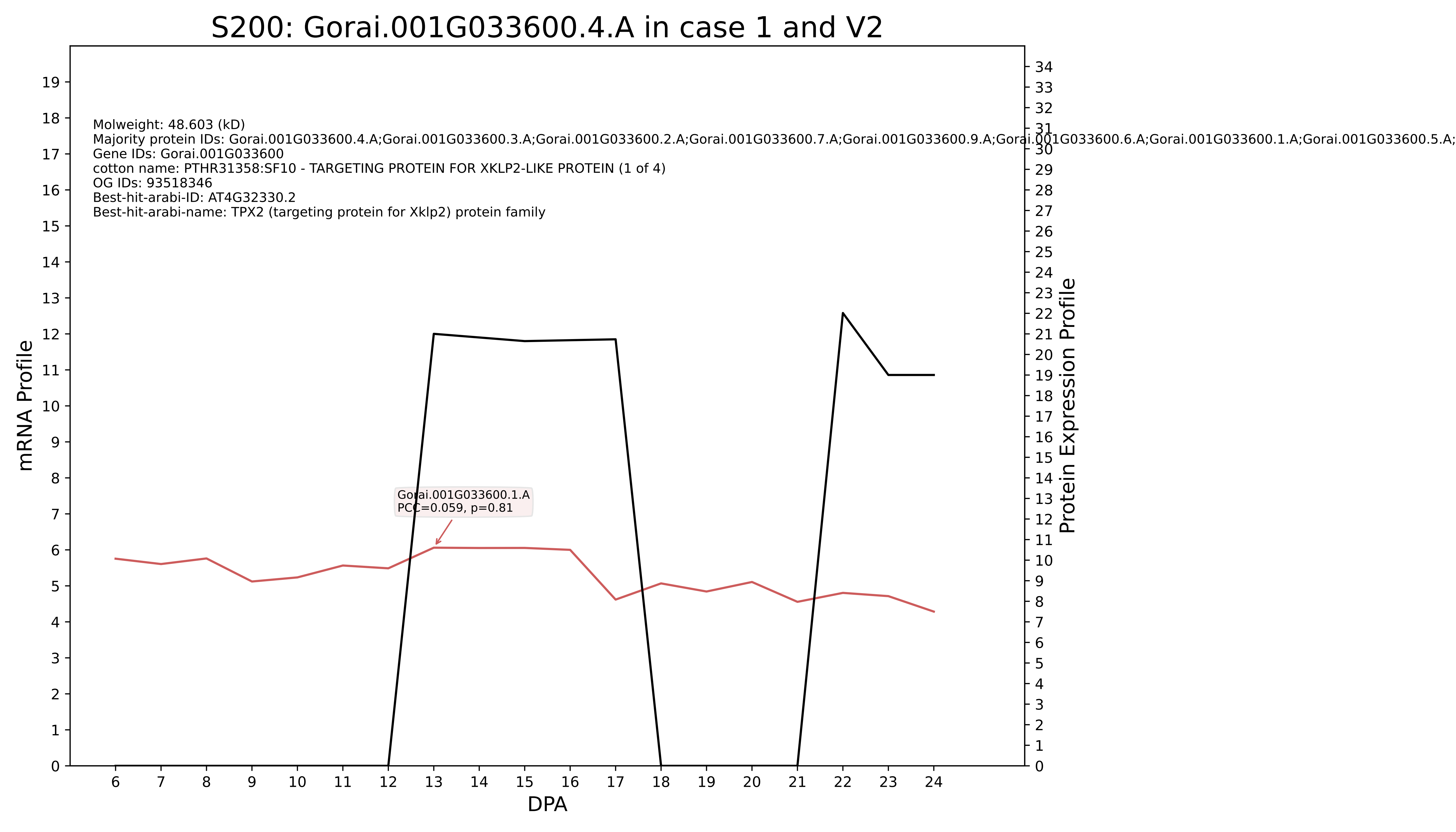
S200: Gorai.001G033600.4.A in case 1 and V2
Molweight: 48.603 (kD)
Majority protein IDs: Gorai.001G033600.4.A;Gorai.001G033600.3.A;Gorai.001G033600.2.A;Gorai.001G033600.7.A;Gorai.001G033600.9.A;Gorai.001G033600.6.A;Gorai.001G033600.1.A;Gorai.001G033600.5.A;
Gene IDs: Gorai.001G033600
cotton name: PTHR31358:SF10 - TARGETING PROTEIN FOR XKLP2-LIKE PROTEIN (1 of 4)
OG IDs: 93518346
Best-hit-arabi-ID: AT4G32330.2
Best-hit-arabi-name: TPX2 (targeting protein for Xklp2) protein family
Gorai.001G033600.1.A
PCC=0.059, p=0.81
mRNA Profile
Protein Expression Profile
DPA

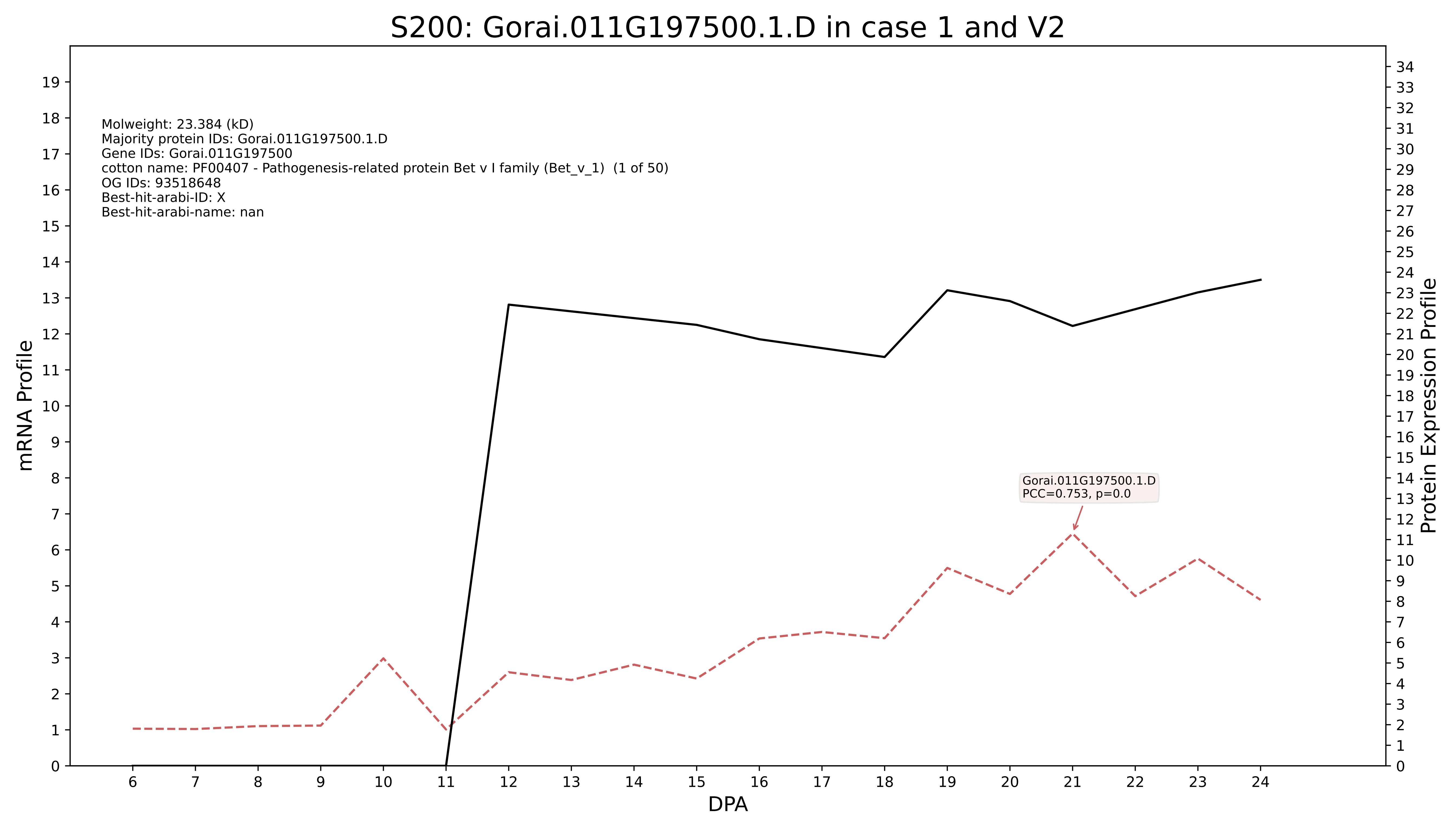
S200: Gorai.011G197500.1.D in case 1 and V2
Molweight: 23.384 (kD)
Majority protein IDs: Gorai.011G197500.1.D
Gene IDs: Gorai.011G197500
cotton name: PF00407 - Pathogenesis-related protein Bet v I family (Bet_v_1) (1 of 50)
OG IDs: 93518648
Best-hit-arabi-ID: X
Best-hit-arabi-name: nan
Gorai.011G197500.1.D
PCC=0.753, p=0.0
mRNA Profile
Protein Expression Profile
DPA
0
1
2
3
4
5
6
7
8
9
10
11
12
13
14
15
16
17
18
19
20
21
22
23
24
25
26
27
28
29
30
31
32
33
34

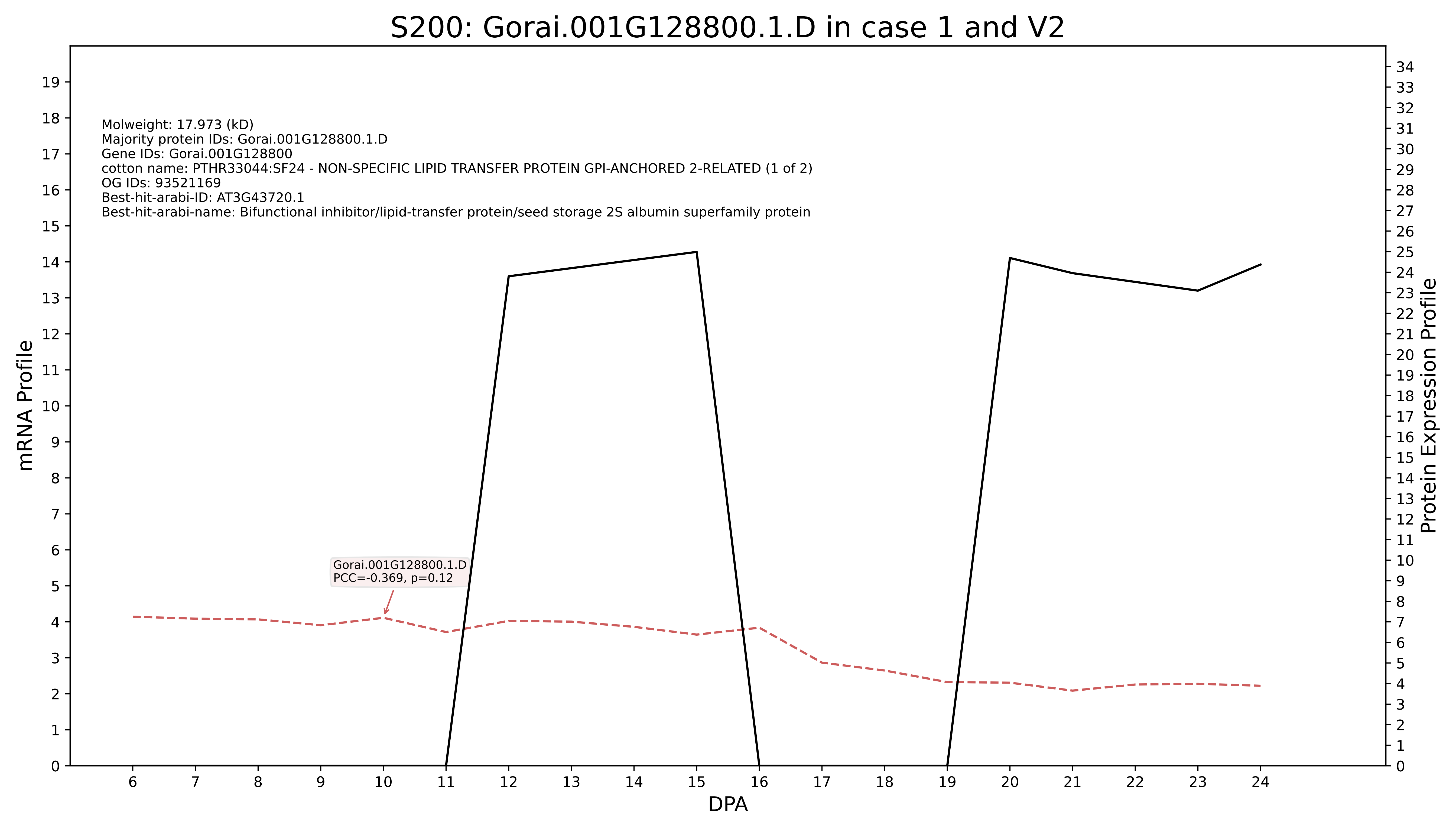
S200: Gorai.001G128800.1.D in case 1 and V2
Molweight: 17.973 (kD)
Majority protein IDs: Gorai.001G128800.1.D
Gene IDs: Gorai.001G128800
cotton name: PTHR33044:SF24 - NON-SPECIFIC LIPID TRANSFER PROTEIN GPI-ANCHORED 2-RELATED (1 of 2)
OG IDs: 93521169
Best-hit-arabi-ID: AT3G43720.1
Best-hit-arabi-name: Bifunctional inhibitor/lipid-transfer protein/seed storage 2S albumin superfamily protein
Gorai.001G128800.1.D
PCC=-0.369, p=0.12
mRNA Profile
Protein Expression Profile
DPA

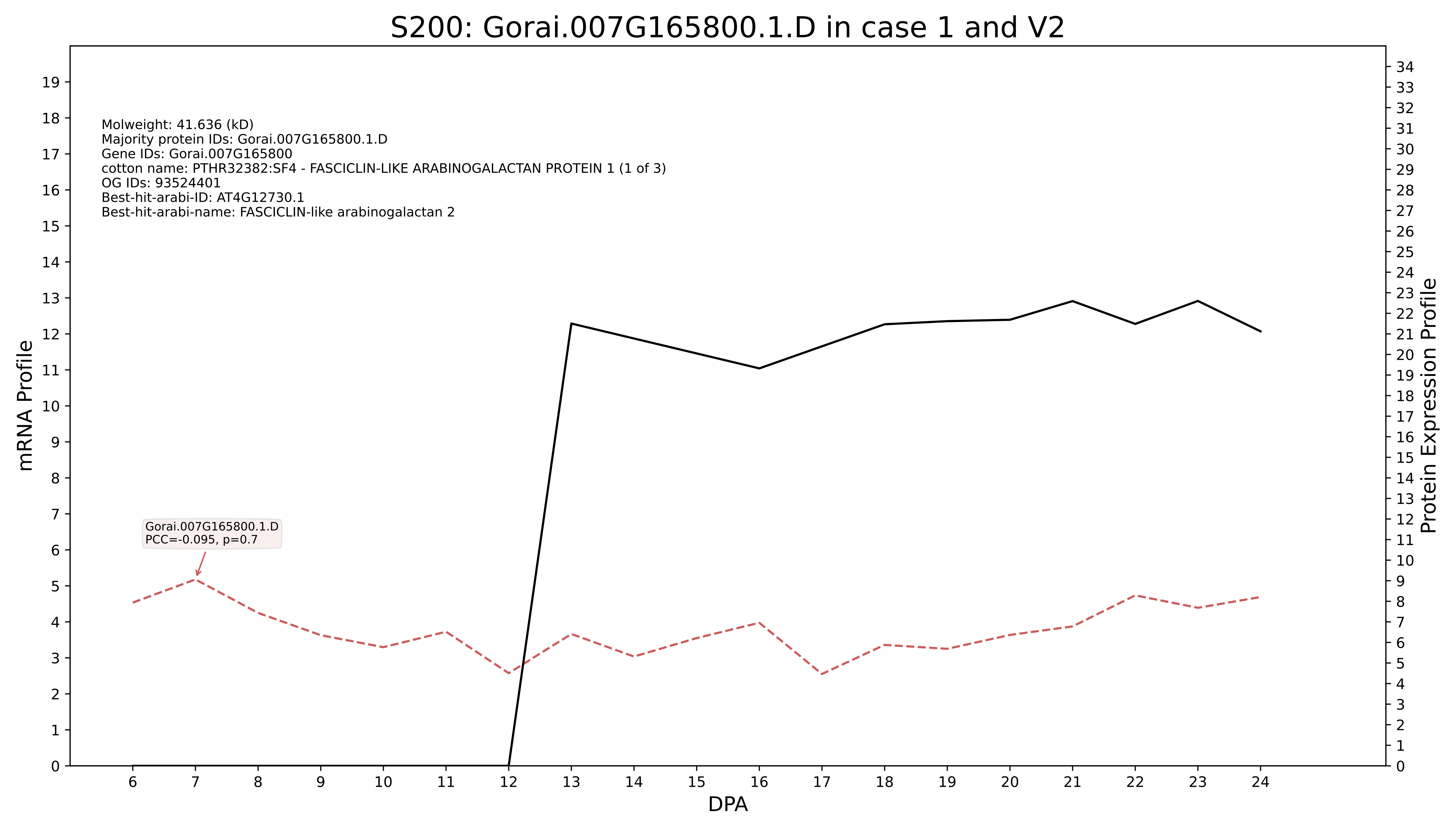

S200: Gorai.007G165800.1.D in case 1 and V2
Molweight: 41.636 (kD)
Majority protein IDs: Gorai.007G165800.1.D
Gene IDs: Gorai.007G165800
cotton name: PTHR32382:SF4 - FASCICLIN-LIKE ARABINOGALACTAN PROTEIN 1 (1 of 3)
OG IDs: 93524401
Best-hit-arabi-ID: AT4G12730.1
Best-hit-arabi-name: FASCICLIN-like arabinogalactan 2
Gorai.007G165800.1.D
PCC=-0.095, p=0.7
mRNA Profile
Protein Expression Profile
DPA

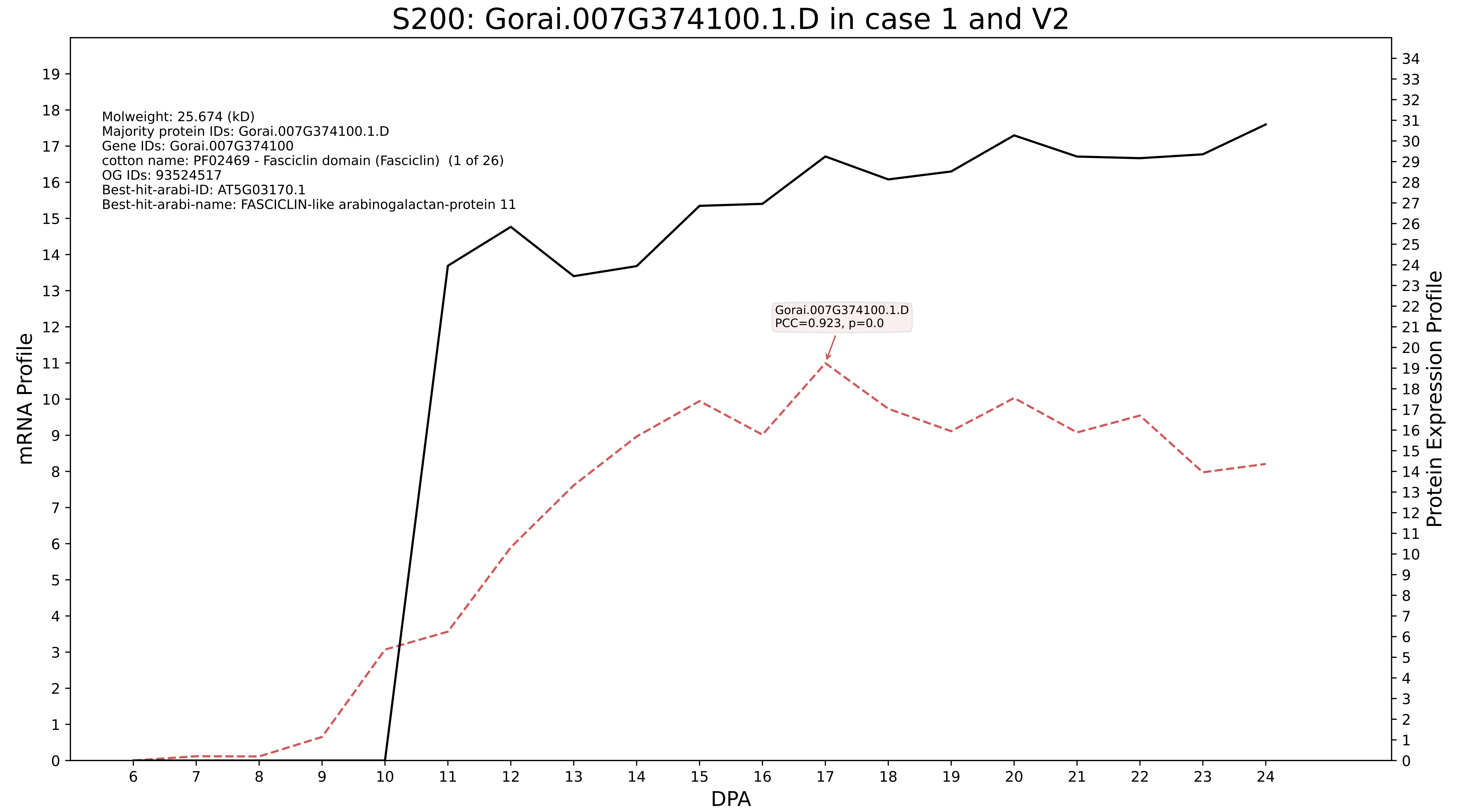
S200: Gorai.007G374100.1.D in case 1 and V2
Molweight: 25.674 (kD)
Majority protein IDs: Gorai.007G374100.1.D
Gene IDs: Gorai.007G374100
cotton name: PF02469 - Fasciclin domain (Fasciclin) (1 of 26)
OG IDs: 93524517
Best-hit-arabi-ID: AT5G03170.1
Best-hit-arabi-name: FASCICLIN-like arabinogalactan-protein 11
Gorai.007G374100.1.D
PCC=0.923, p=0.0
mRNA Profile
Protein Expression Profile
DPA

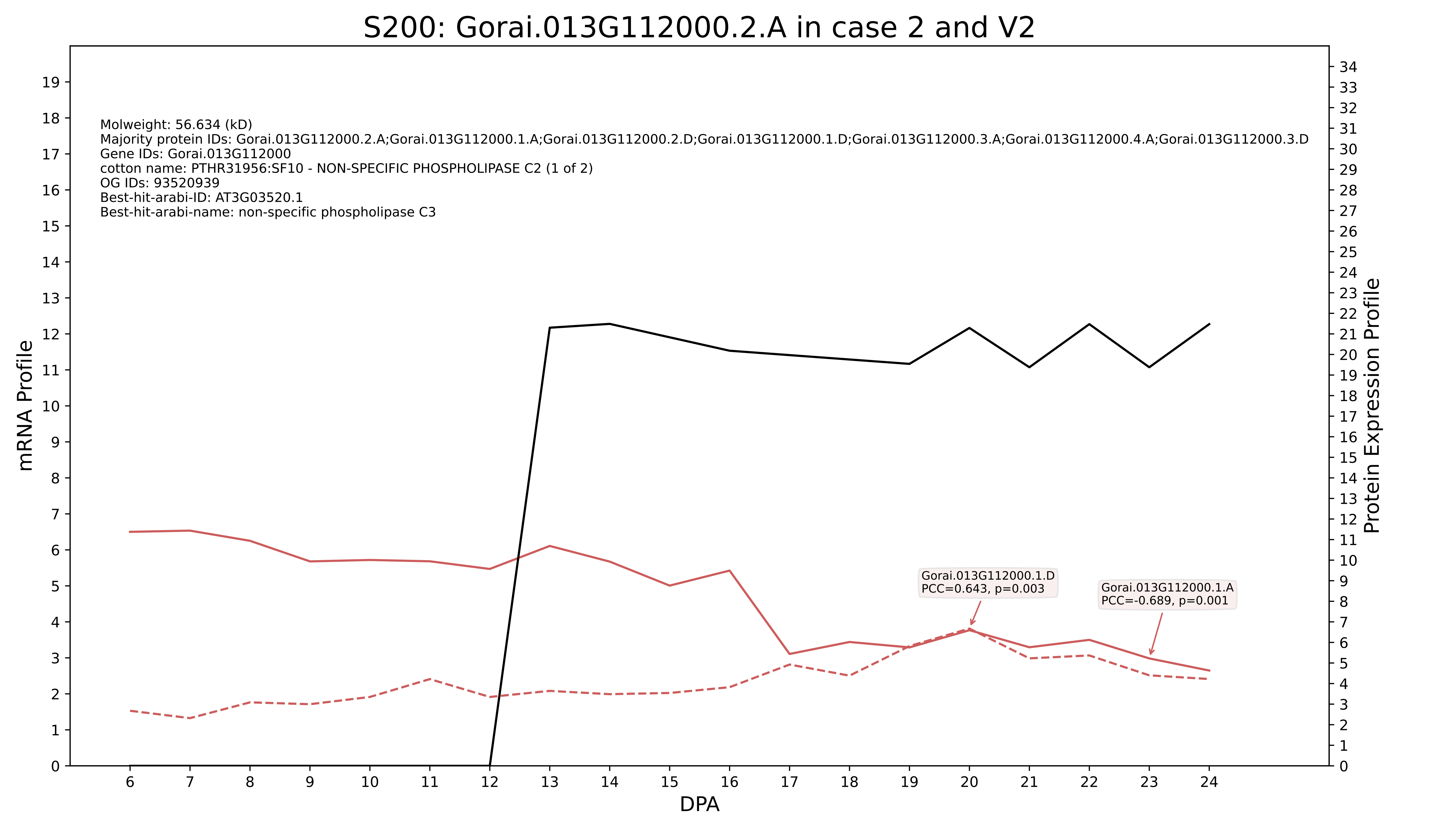

S200: Gorai.013G112000.2.A in case 2 and V2
Molweight: 56.634 (kD)
Majority protein IDs: Gorai.013G112000.2.A;Gorai.013G112000.1.A;Gorai.013G112000.2.D;Gorai.013G112000.1.D;Gorai.013G112000.3.A;Gorai.013G112000.4.A;Gorai.013G112000.3.D
Gene IDs: Gorai.013G112000
cotton name: PTHR31956:SF10 - NON-SPECIFIC PHOSPHOLIPASE C2 (1 of 2)
OG IDs: 93520939
Best-hit-arabi-ID: AT3G03520.1
Best-hit-arabi-name: non-specific phospholipase C3
Gorai.013G112000.1.D
PCC=0.643, p=0.003
Gorai.013G112000.1.A
PCC=-0.689, p=0.001
mRNA Profile
Protein Expression Profile
DPA

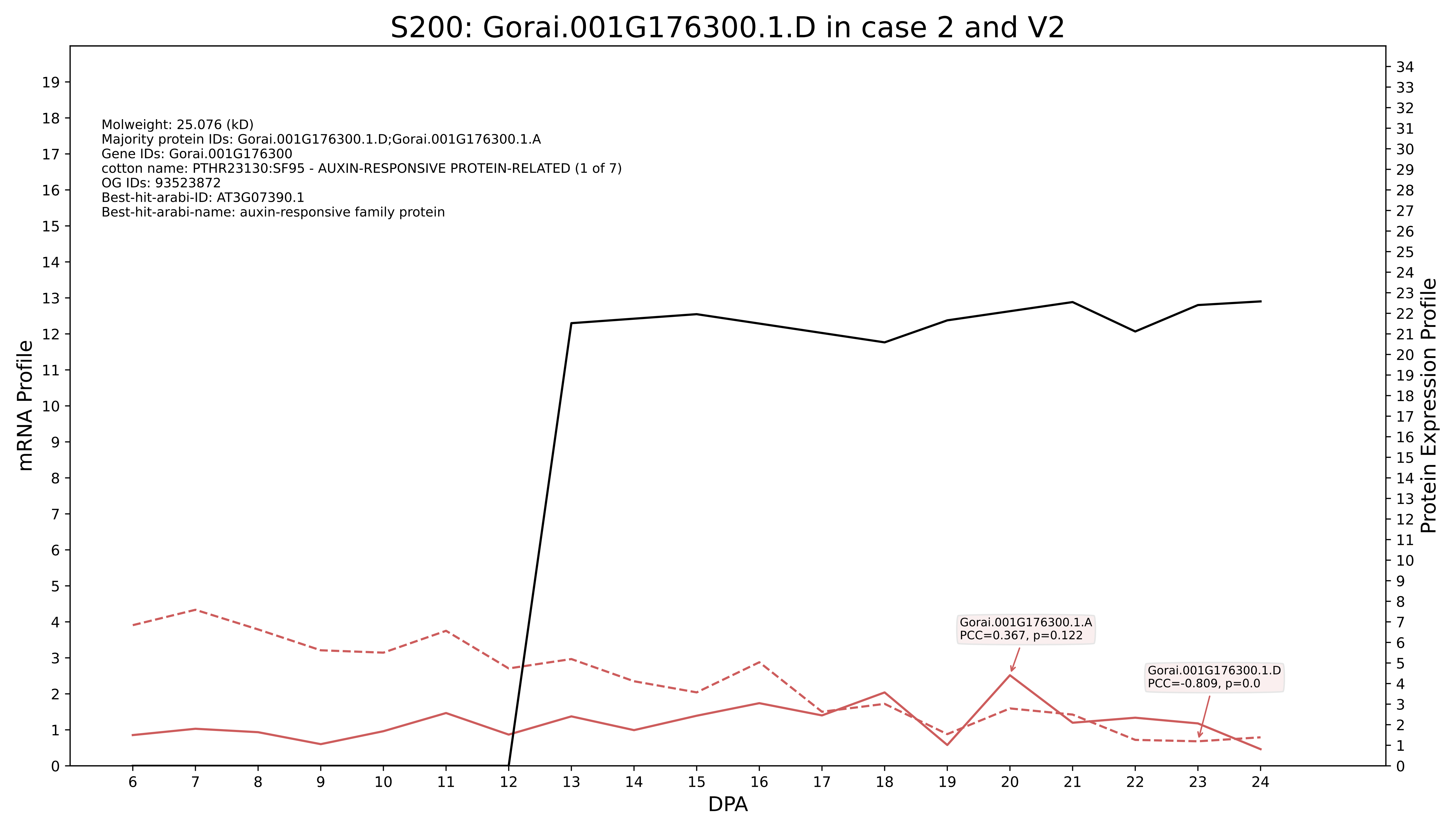
S200: Gorai.001G176300.1.D in case 2 and V2
Molweight: 25.076 (kD)
Majority protein IDs: Gorai.001G176300.1.D;Gorai.001G176300.1.A
Gene IDs: Gorai.001G176300
cotton name: PTHR23130:SF95 - AUXIN-RESPONSIVE PROTEIN-RELATED (1 of 7)
OG IDs: 93523872
Best-hit-arabi-ID: AT3G07390.1
Best-hit-arabi-name: auxin-responsive family protein
Gorai.001G176300.1.A
PCC=0.367, p=0.122
Gorai.001G176300.1.D
PCC=-0.809, p=0.0
mRNA Profile
Protein Expression Profile
DPA

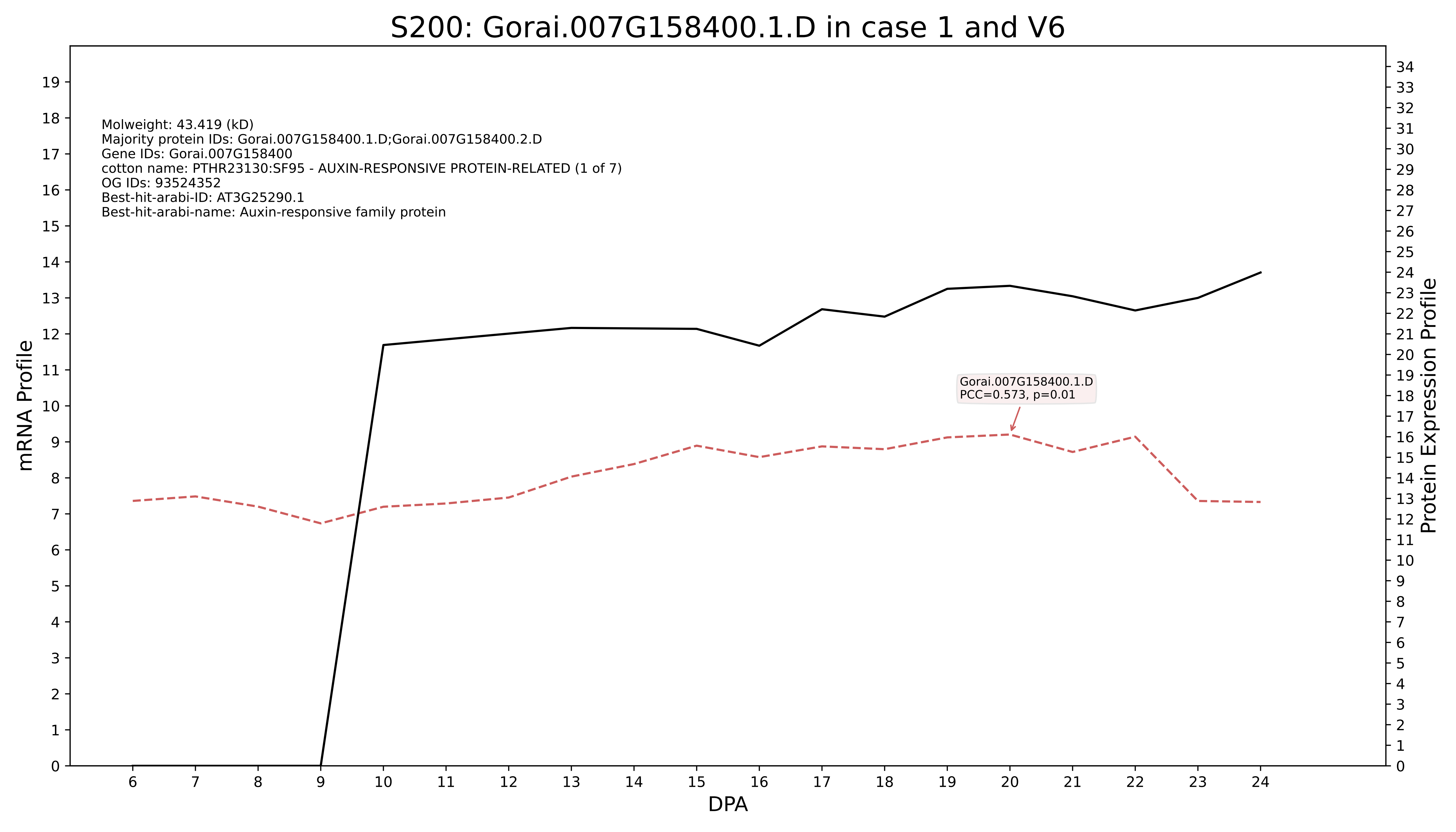

S200: Gorai.007G158400.1.D in case 1 and V6
Molweight: 43.419 (kD)
Majority protein IDs: Gorai.007G158400.1.D;Gorai.007G158400.2.D
Gene IDs: Gorai.007G158400
cotton name: PTHR23130:SF95 - AUXIN-RESPONSIVE PROTEIN-RELATED (1 of 7)
OG IDs: 93524352
Best-hit-arabi-ID: AT3G25290.1
Best-hit-arabi-name: Auxin-responsive family protein
Gorai.007G158400.1.D
PCC=0.573, p=0.01
mRNA Profile
Protein Expression Profile
DPA

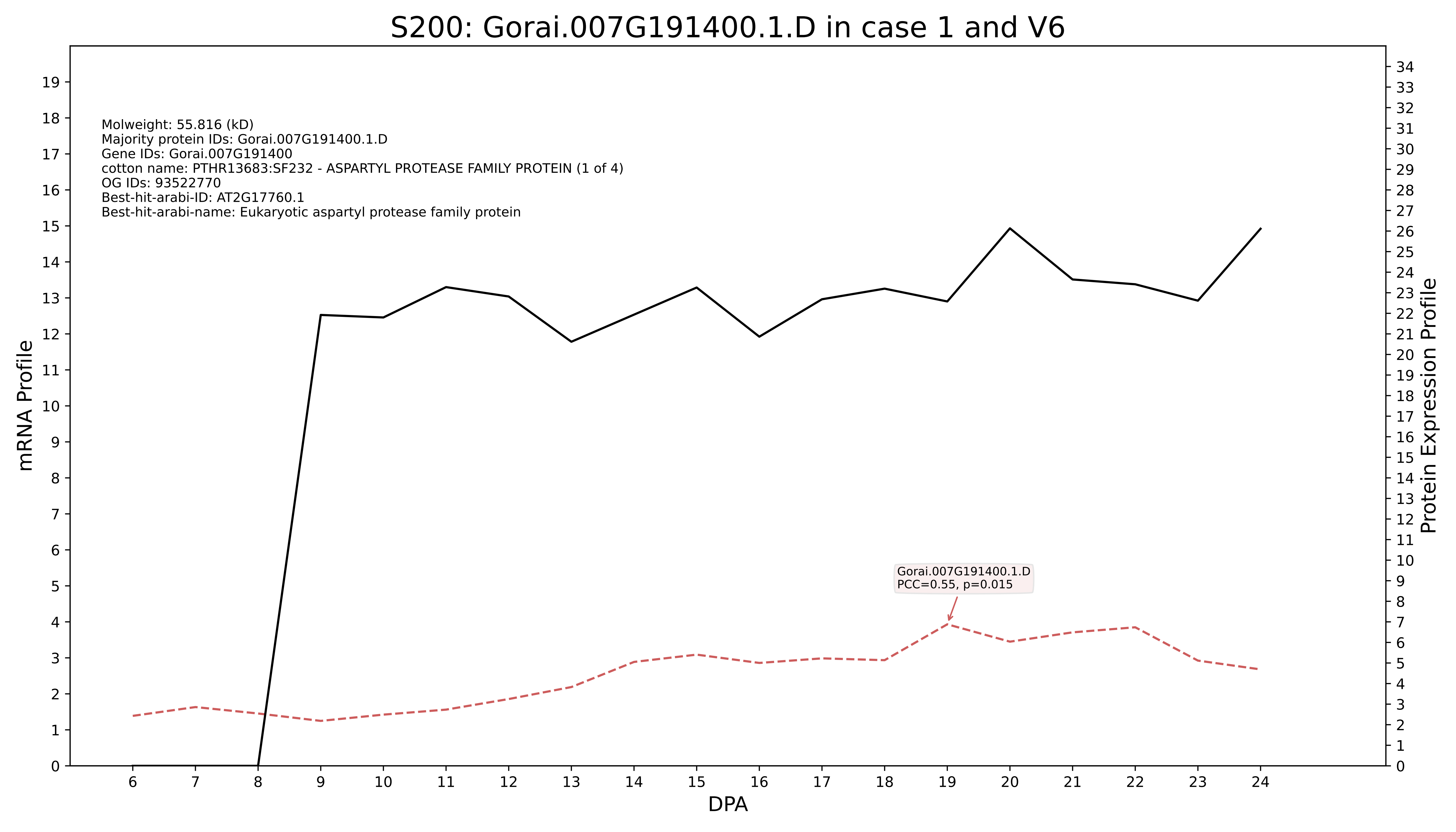
S200: Gorai.007G191400.1.D in case 1 and V6
Molweight: 55.816 (kD)
Majority protein IDs: Gorai.007G191400.1.D
Gene IDs: Gorai.007G191400
cotton name: PTHR13683:SF232 - ASPARTYL PROTEASE FAMILY PROTEIN (1 of 4)
OG IDs: 93522770
Best-hit-arabi-ID: AT2G17760.1
Best-hit-arabi-name: Eukaryotic aspartyl protease family protein
Gorai.007G191400.1.D
PCC=0.55, p=0.015
mRNA Profile
Protein Expression Profile
DPA

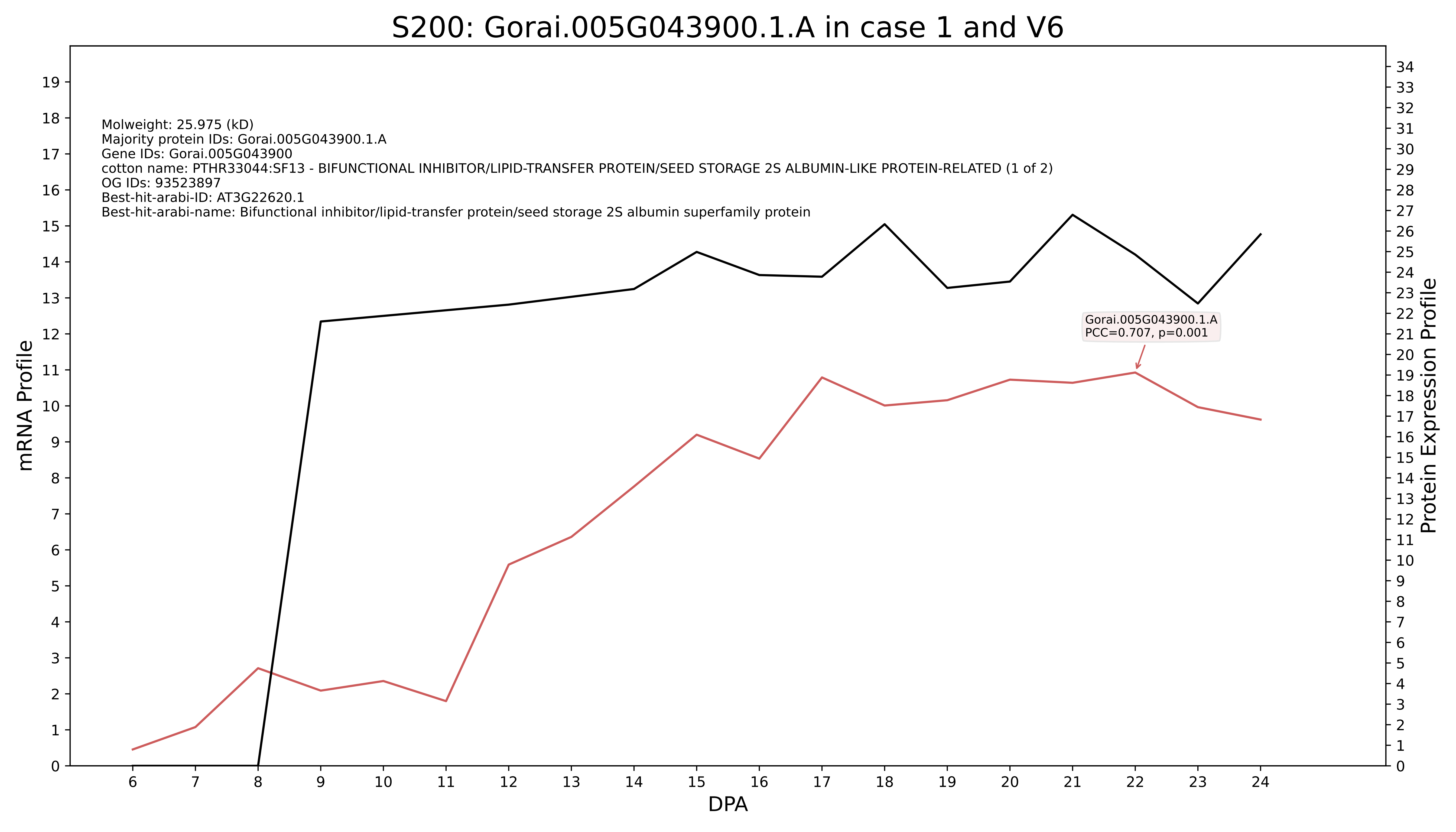

S200: Gorai.005G043900.1.A in case 1 and V6
Molweight: 25.975 (kD)
Majority protein IDs: Gorai.005G043900.1.A
Gene IDs: Gorai.005G043900
cotton name: PTHR33044:SF13 - BIFUNCTIONAL INHIBITOR/LIPID-TRANSFER PROTEIN/SEED STORAGE 2S ALBUMIN-LIKE PROTEIN-RELATED (1 of 2)
OG IDs: 93523897
Best-hit-arabi-ID: AT3G22620.1
Best-hit-arabi-name: Bifunctional inhibitor/lipid-transfer protein/seed storage 2S albumin superfamily protein
Gorai.005G043900.1.A
PCC=0.707, p=0.001
mRNA Profile
Protein Expression Profile
DPA

S200: Gorai.002G124800.2.A in case 1 and V6

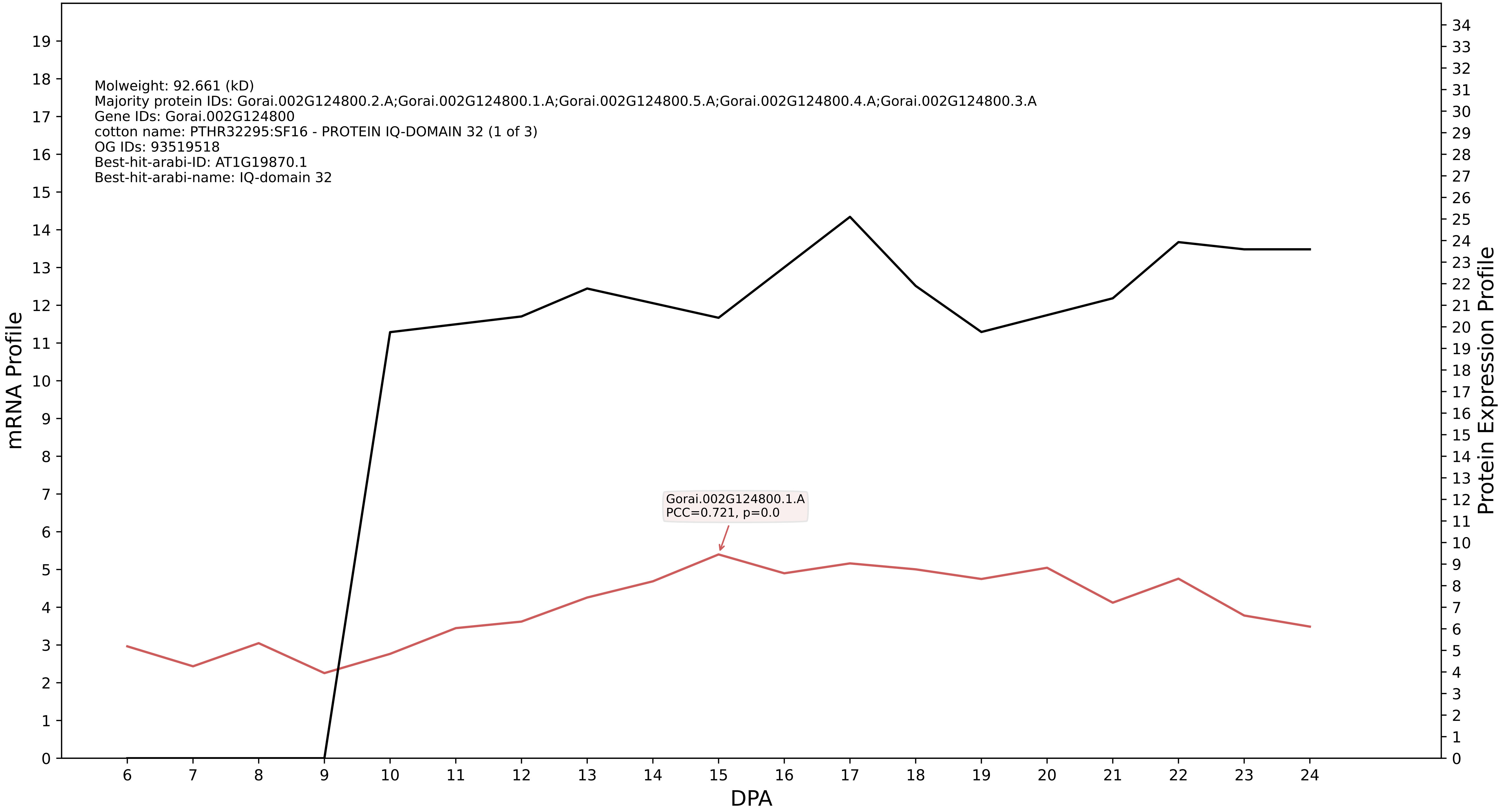

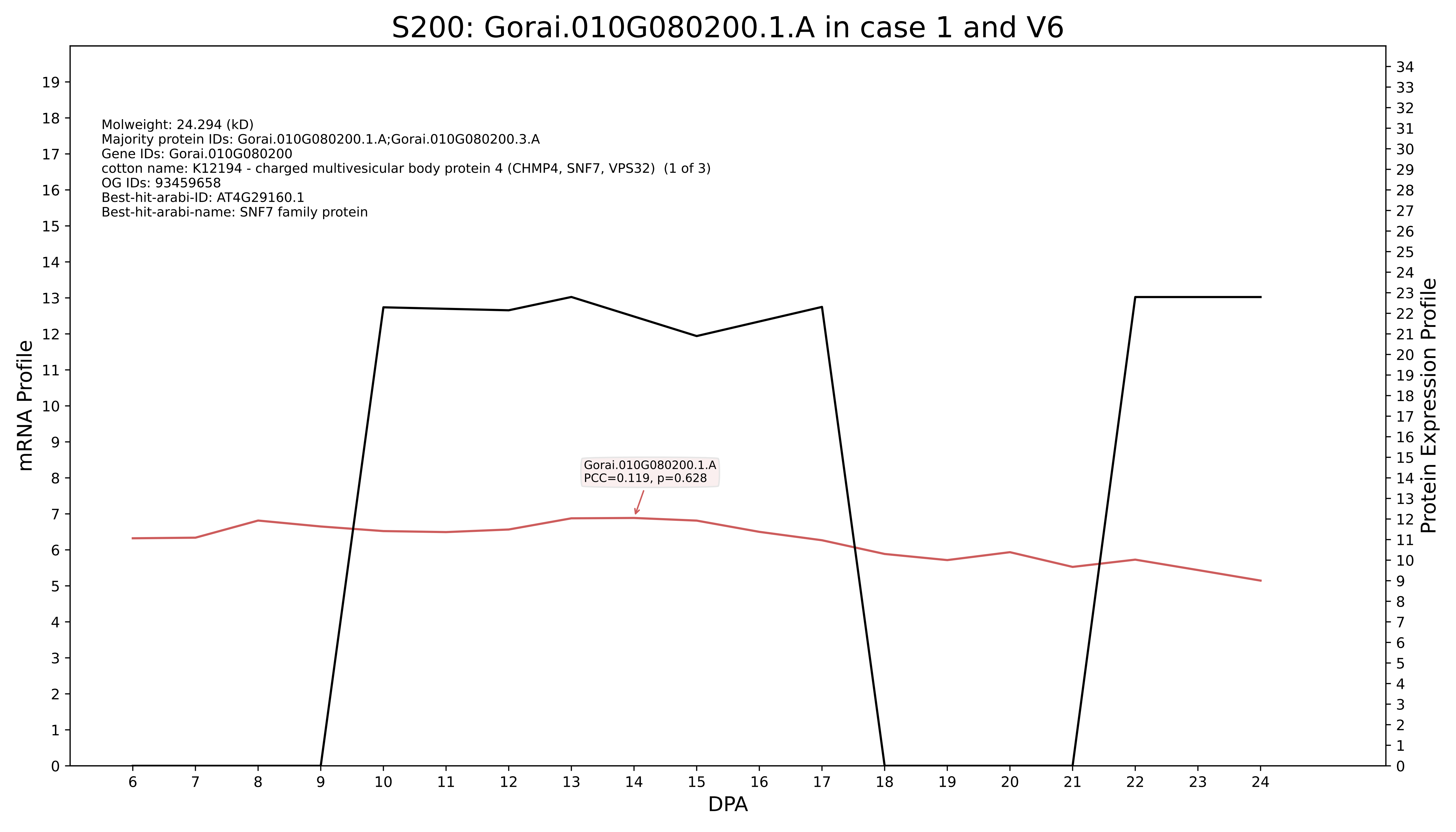

S200: Gorai.010G080200.1.A in case 1 and V6
Molweight: 24.294 (kD)
Majority protein IDs: Gorai.010G080200.1.A;Gorai.010G080200.3.A
Gene IDs: Gorai.010G080200
cotton name: K12194 - charged multivesicular body protein 4 (CHMP4, SNF7, VPS32) (1 of 3)
OG IDs: 93459658
Best-hit-arabi-ID: AT4G29160.1
Best-hit-arabi-name: SNF7 family protein
Gorai.010G080200.1.A
PCC=0.119, p=0.628
mRNA Profile
Protein Expression Profile
DPA

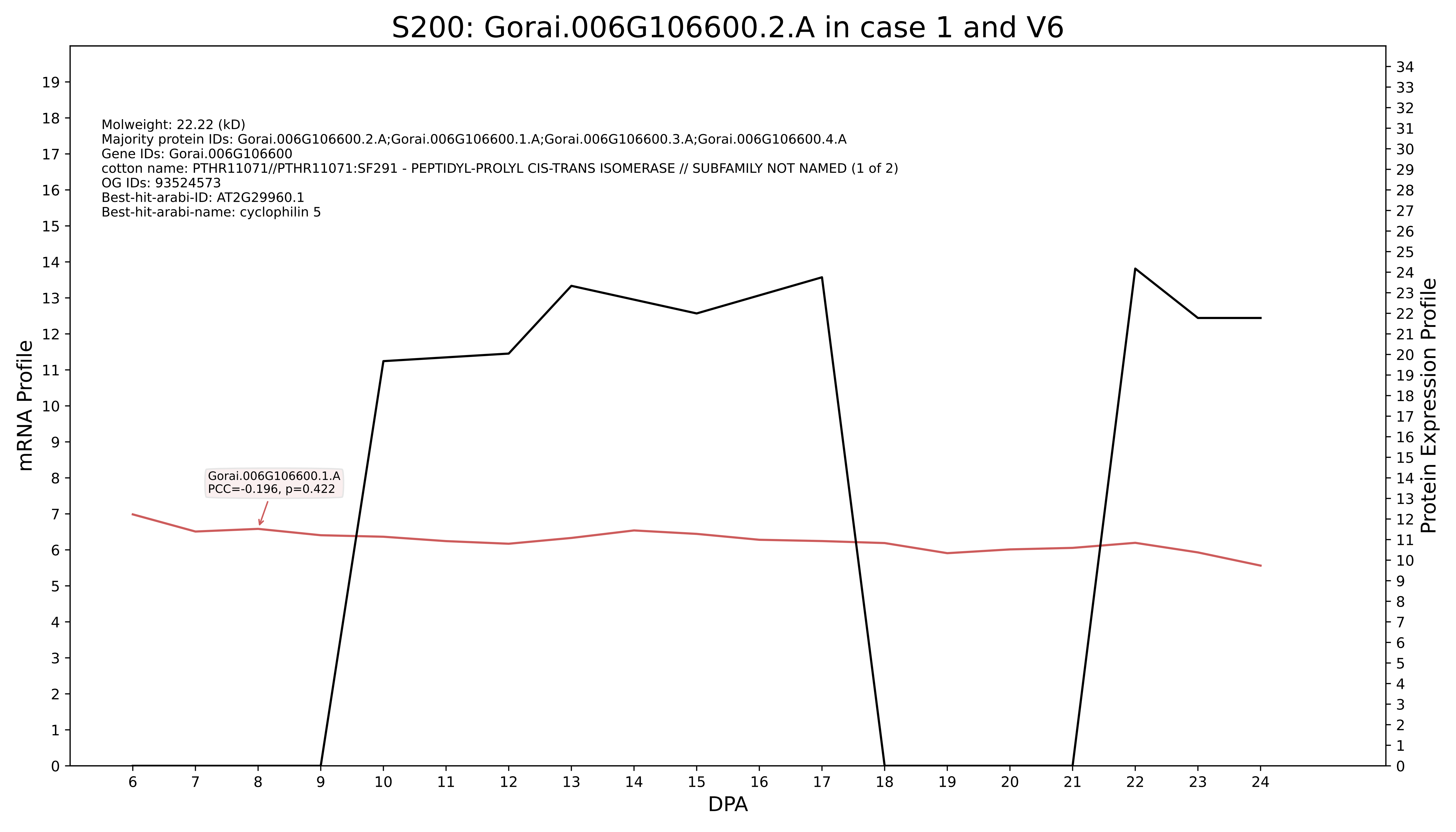
S200: Gorai.006G106600.2.A in case 1 and V6
Molweight: 22.22 (kD)
Majority protein IDs: Gorai.006G106600.2.A;Gorai.006G106600.1.A;Gorai.006G106600.3.A;Gorai.006G106600.4.A
Gene IDs: Gorai.006G106600
cotton name: PTHR11071//PTHR11071:SF291 - PEPTIDYL-PROLYL CIS-TRANS ISOMERASE // SUBFAMILY NOT NAMED (1 of 2)
OG IDs: 93524573
Best-hit-arabi-ID: AT2G29960.1
Best-hit-arabi-name: cyclophilin 5
Gorai.006G106600.1.A
PCC=-0.196, p=0.422
mRNA Profile
Protein Expression Profile
DPA

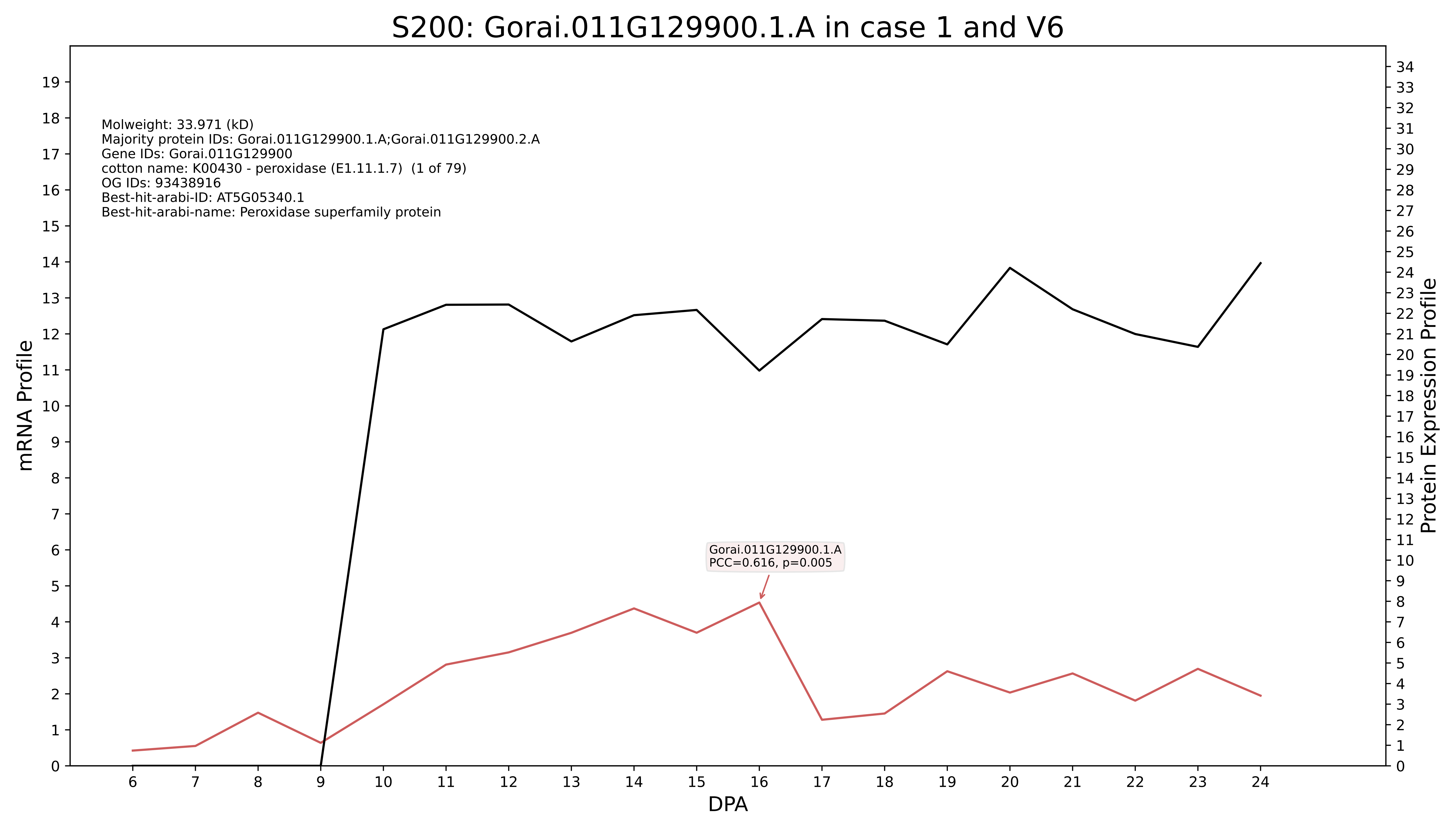
S200: Gorai.011G129900.1.A in case 1 and V6
Molweight: 33.971 (kD)
Majority protein IDs: Gorai.011G129900.1.A;Gorai.011G129900.2.A
Gene IDs: Gorai.011G129900
cotton name: K00430 - peroxidase (E1.11.1.7) (1 of 79)
OG IDs: 93438916
Best-hit-arabi-ID: AT5G05340.1
Best-hit-arabi-name: Peroxidase superfamily protein
Gorai.011G129900.1.A
PCC=0.616, p=0.005
mRNA Profile
Protein Expression Profile
DPA

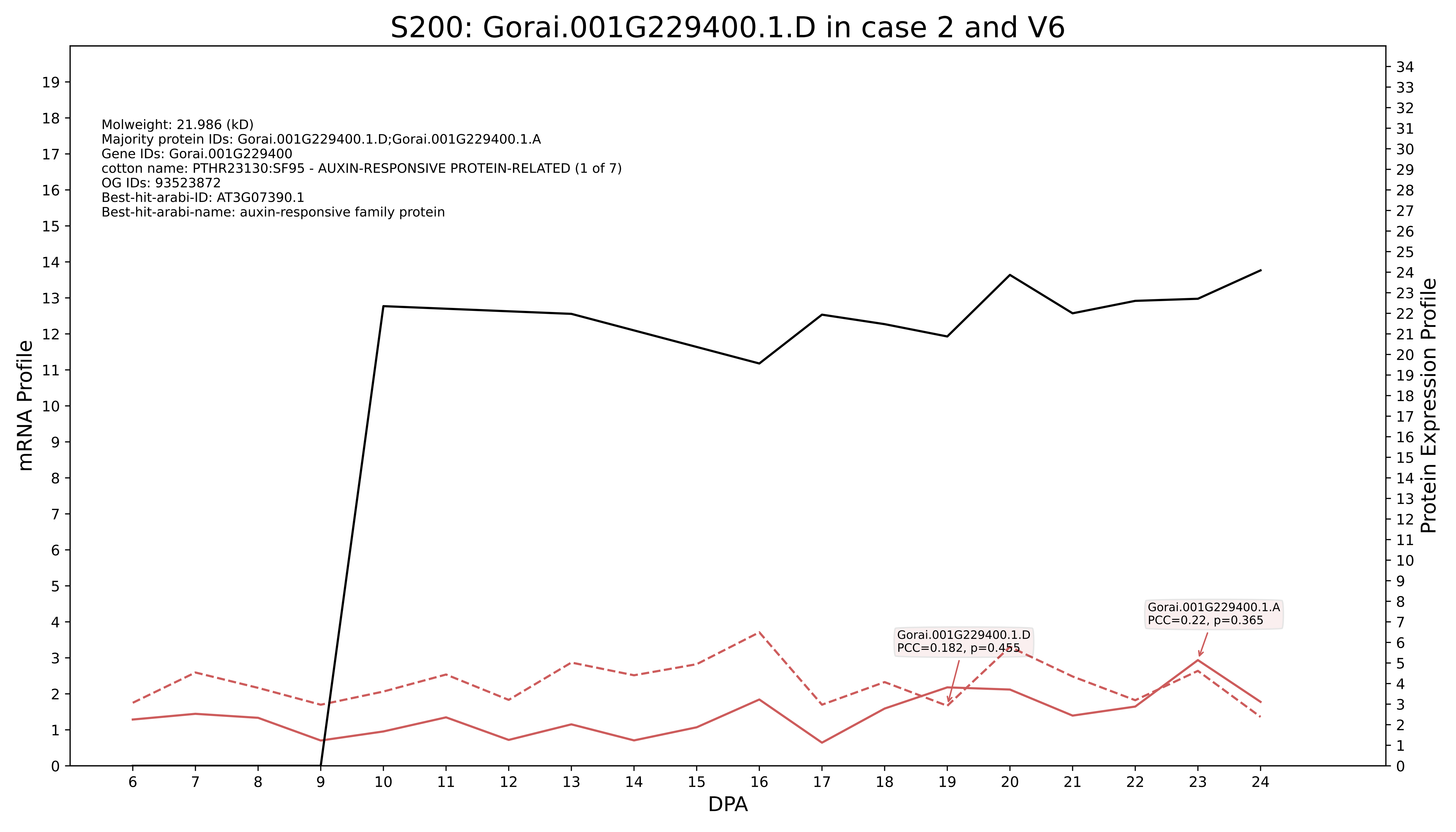

S200: Gorai.001G229400.1.D in case 2 and V6
Molweight: 21.986 (kD)
Majority protein IDs: Gorai.001G229400.1.D;Gorai.001G229400.1.A
Gene IDs: Gorai.001G229400
cotton name: PTHR23130:SF95 - AUXIN-RESPONSIVE PROTEIN-RELATED (1 of 7)
OG IDs: 93523872
Best-hit-arabi-ID: AT3G07390.1
Best-hit-arabi-name: auxin-responsive family protein
Gorai.001G229400.1.D
PCC=0.182, p=0.455
Gorai.001G229400.1.A
PCC=0.22, p=0.365
mRNA Profile
Protein Expression Profile
DPA

S200: Gorai.004G061700.1.A in case 1 and V7

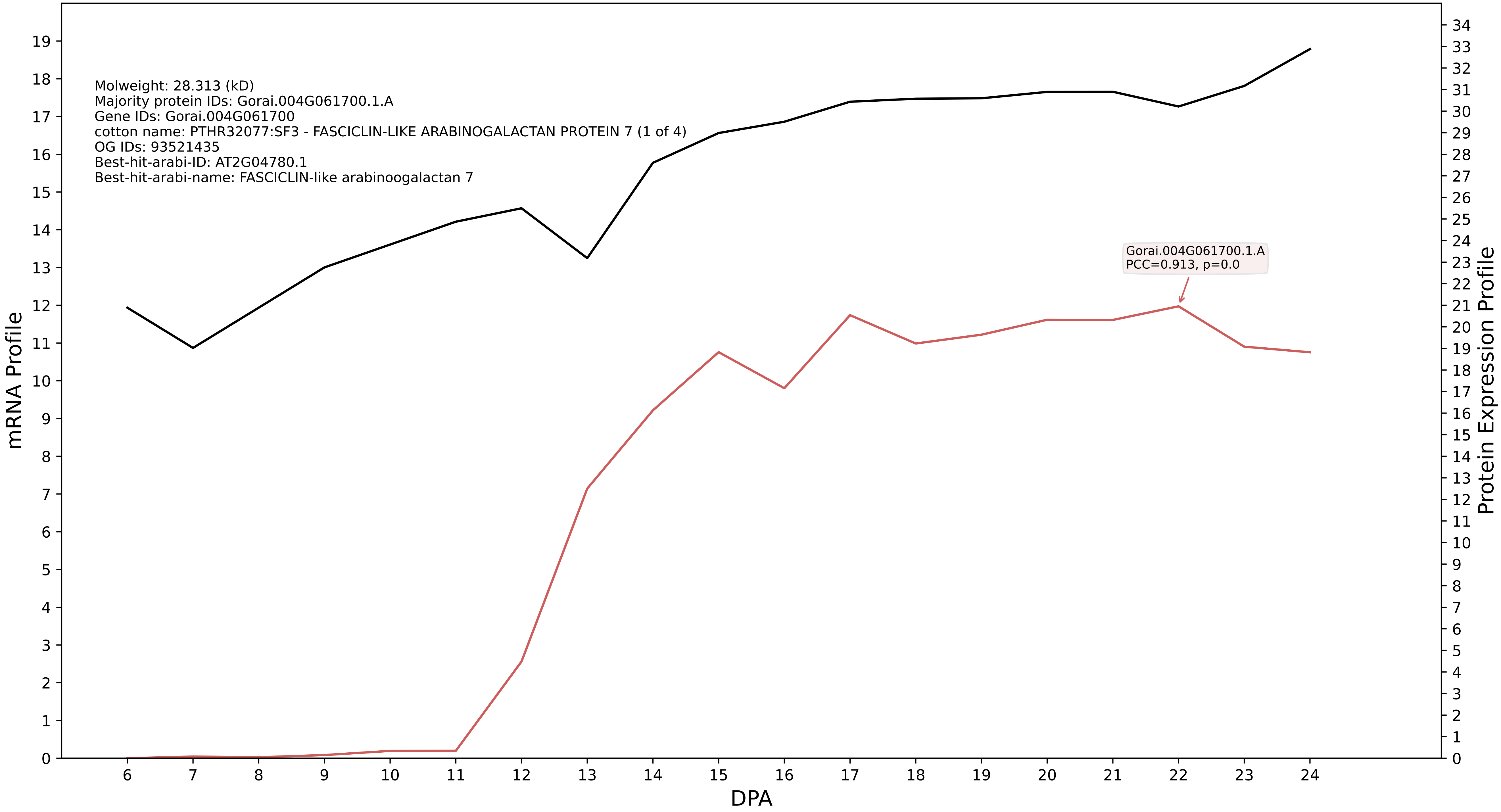

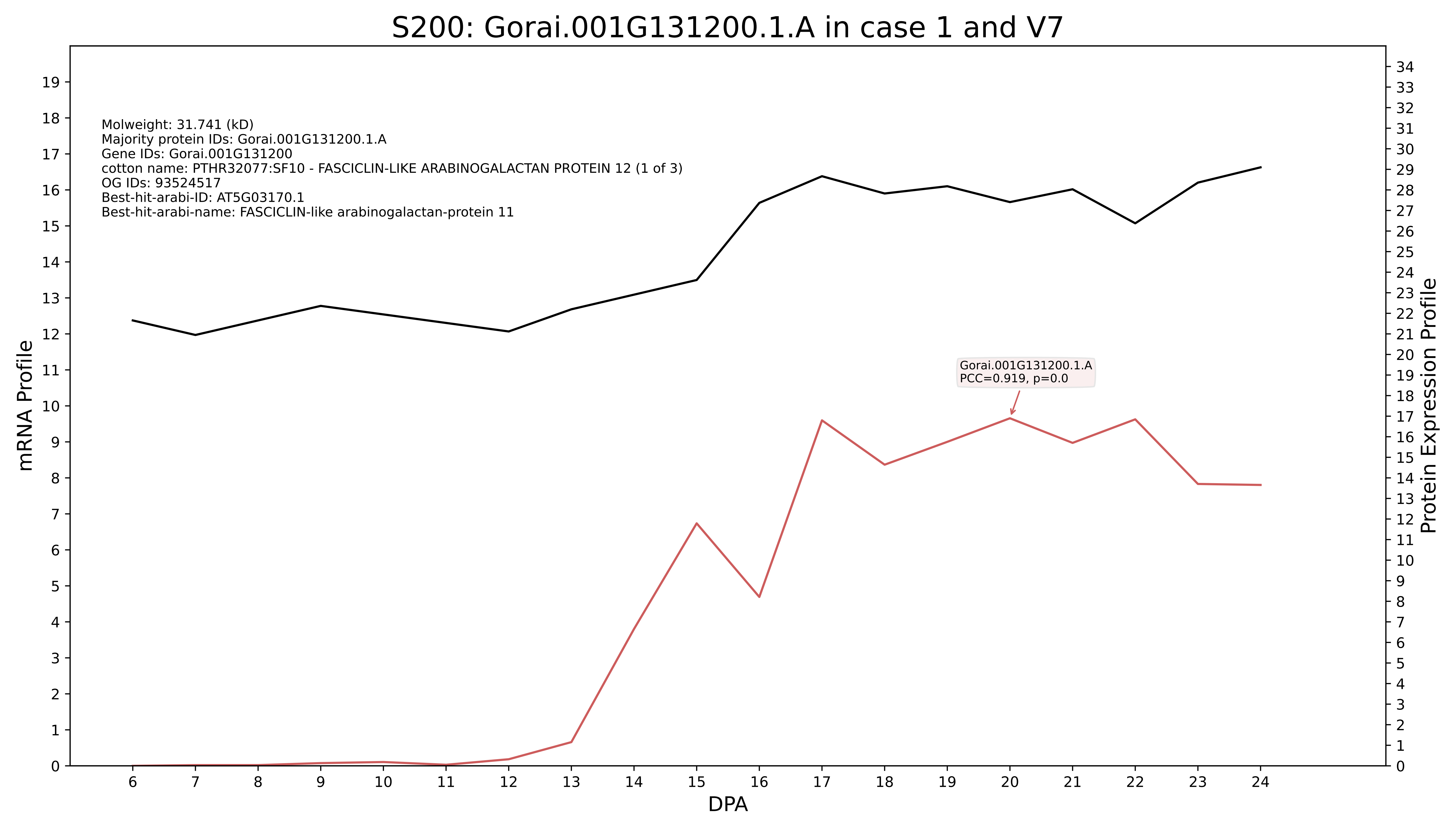
S200: Gorai.001G131200.1.A in case 1 and V7
Molweight: 31.741 (kD)
Majority protein IDs: Gorai.001G131200.1.A
Gene IDs: Gorai.001G131200
cotton name: PTHR32077:SF10 - FASCICLIN-LIKE ARABINOGALACTAN PROTEIN 12 (1 of 3)
OG IDs: 93524517
Best-hit-arabi-ID: AT5G03170.1
Best-hit-arabi-name: FASCICLIN-like arabinogalactan-protein 11
Gorai.001G131200.1.A
PCC=0.919, p=0.0
mRNA Profile
Protein Expression Profile
DPA

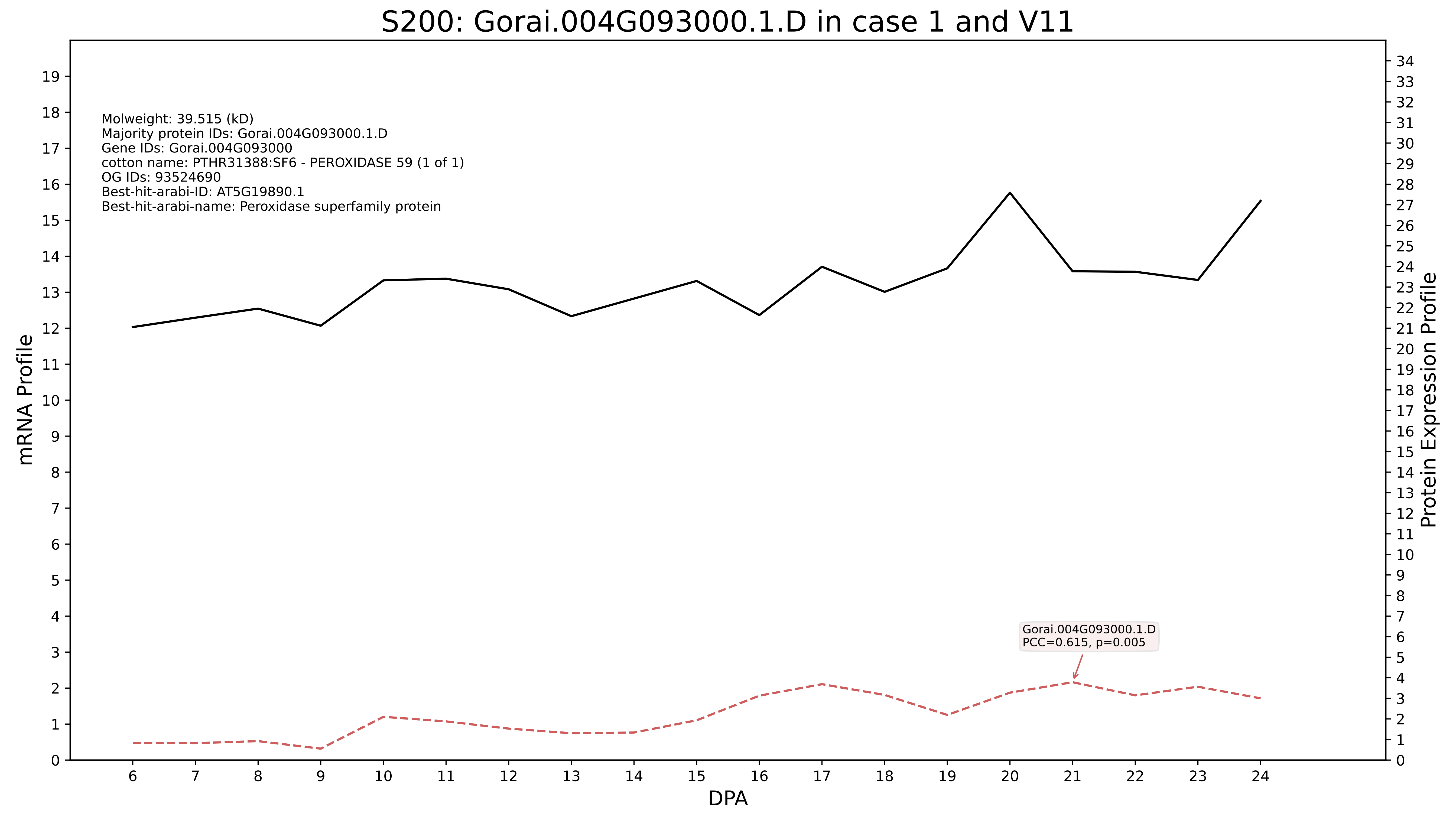

S200: Gorai.004G093000.1.D in case 1 and V11
Molweight: 39.515 (kD)
Majority protein IDs: Gorai.004G093000.1.D
Gene IDs: Gorai.004G093000
cotton name: PTHR31388:SF6 - PEROXIDASE 59 (1 of 1)
OG IDs: 93524690
Best-hit-arabi-ID: AT5G19890.1
Best-hit-arabi-name: Peroxidase superfamily protein
Gorai.004G093000.1.D
PCC=0.615, p=0.005
mRNA Profile
Protein Expression Profile
DPA

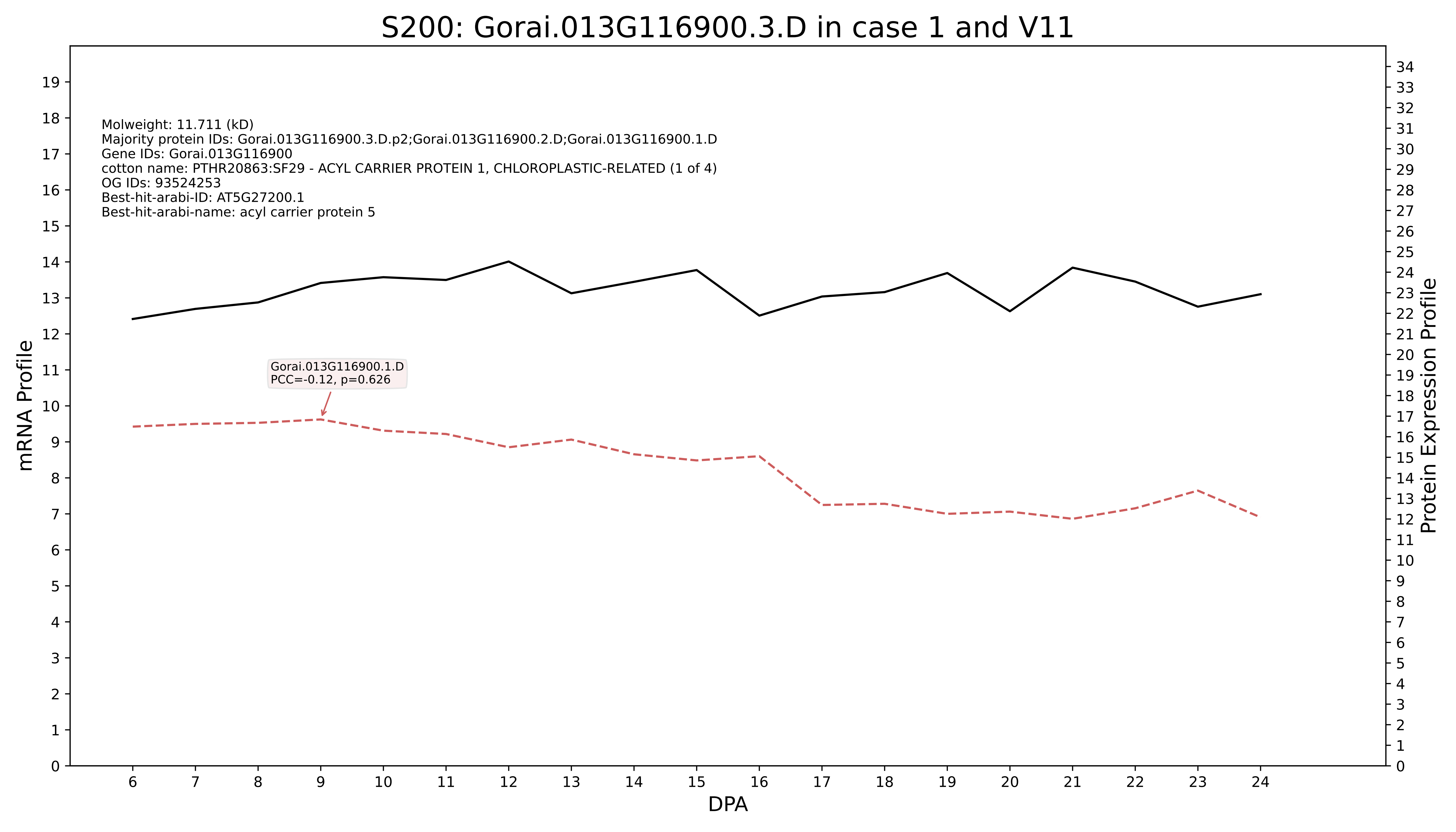
S200: Gorai.013G116900.3.D in case 1 and V11
Molweight: 11.711 (kD)
Majority protein IDs: Gorai.013G116900.3.D.p2;Gorai.013G116900.2.D;Gorai.013G116900.1.D
Gene IDs: Gorai.013G116900
cotton name: PTHR20863:SF29 - ACYL CARRIER PROTEIN 1, CHLOROPLASTIC-RELATED (1 of 4)
OG IDs: 93524253
Best-hit-arabi-ID: AT5G27200.1
Best-hit-arabi-name: acyl carrier protein 5
Gorai.013G116900.1.D
PCC=-0.12, p=0.626
mRNA Profile
Protein Expression Profile
DPA

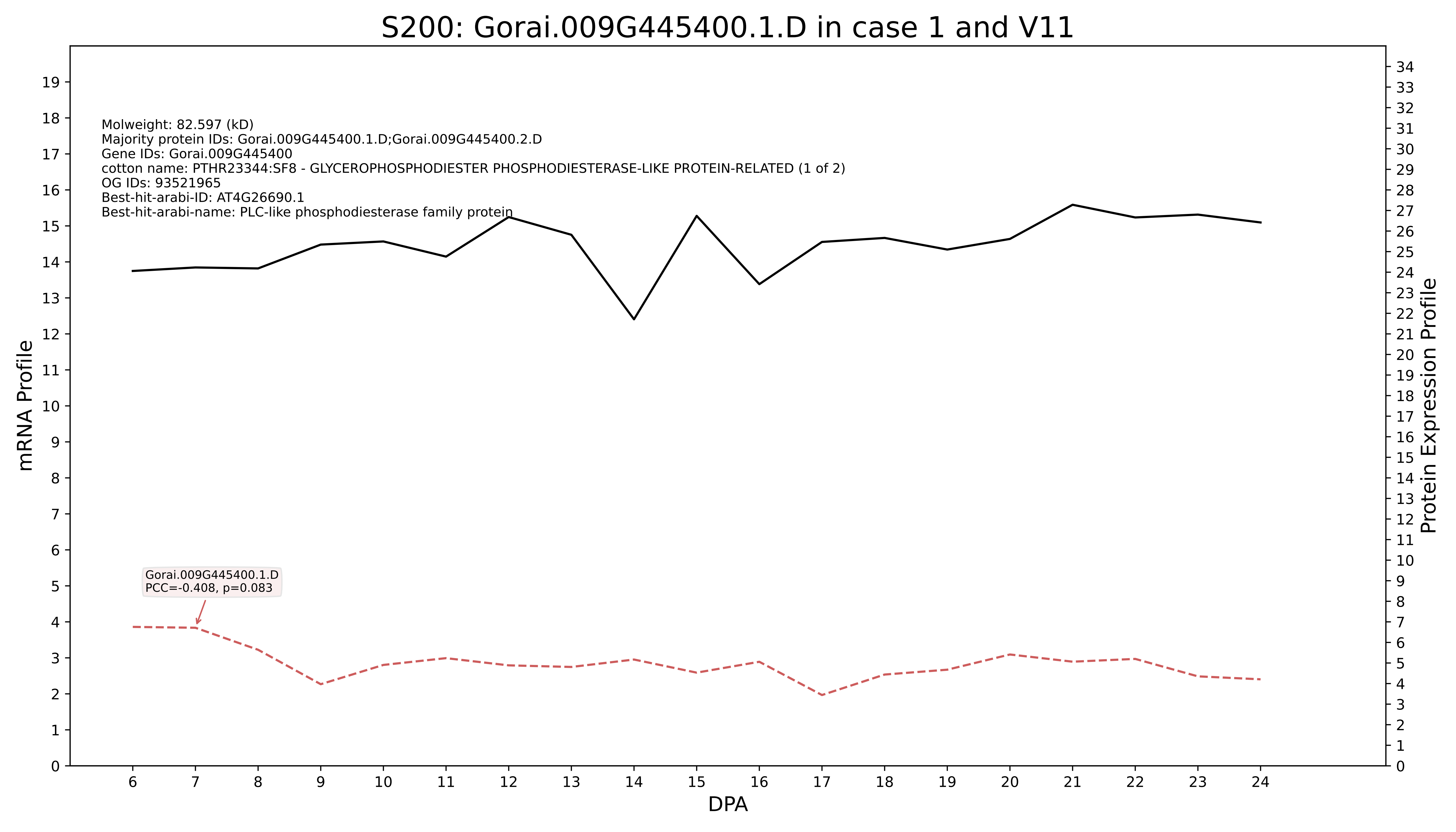
S200: Gorai.009G445400.1.D in case 1 and V11
Molweight: 82.597 (kD)
Majority protein IDs: Gorai.009G445400.1.D;Gorai.009G445400.2.D
Gene IDs: Gorai.009G445400
cotton name: PTHR23344:SF8 - GLYCEROPHOSPHODIESTER PHOSPHODIESTERASE-LIKE PROTEIN-RELATED (1 of 2)
OG IDs: 93521965
Best-hit-arabi-ID: AT4G26690.1
Best-hit-arabi-name: PLC-like phosphodiesterase family protein
Gorai.009G445400.1.D
PCC=-0.408, p=0.083
mRNA Profile
Protein Expression Profile
DPA
6
7
8
9
10
11
12
13
14
15
16
17
18
19
20
21
22
23
24

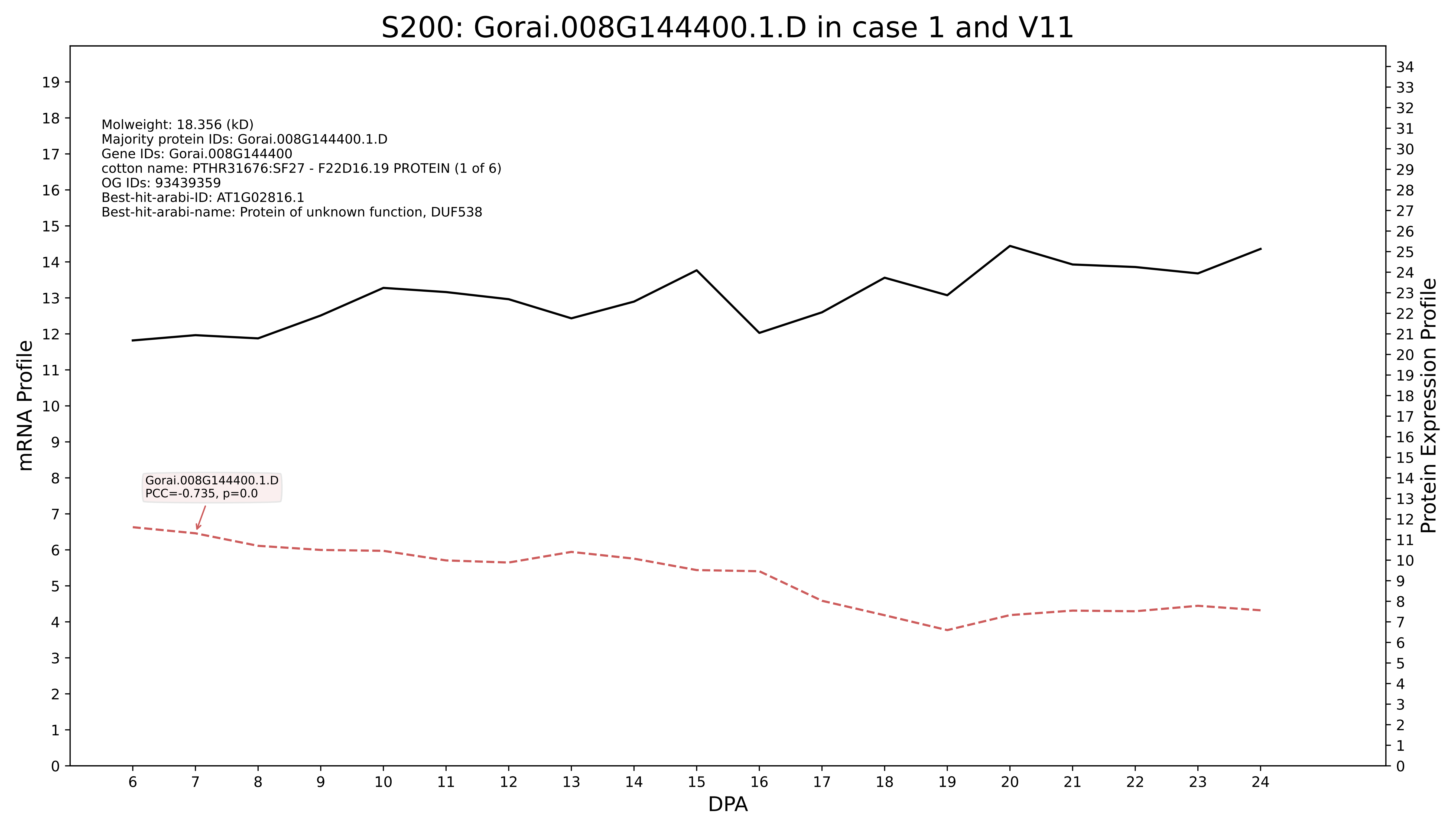

S200: Gorai.008G144400.1.D in case 1 and V11
Molweight: 18.356 (kD)
Majority protein IDs: Gorai.008G144400.1.D
Gene IDs: Gorai.008G144400
cotton name: PTHR31676:SF27 - F22D16.19 PROTEIN (1 of 6)
OG IDs: 93439359
Best-hit-arabi-ID: AT1G02816.1
Best-hit-arabi-name: Protein of unknown function, DUF538
Gorai.008G144400.1.D
PCC=-0.735, p=0.0
mRNA Profile
Protein Expression Profile
DPA

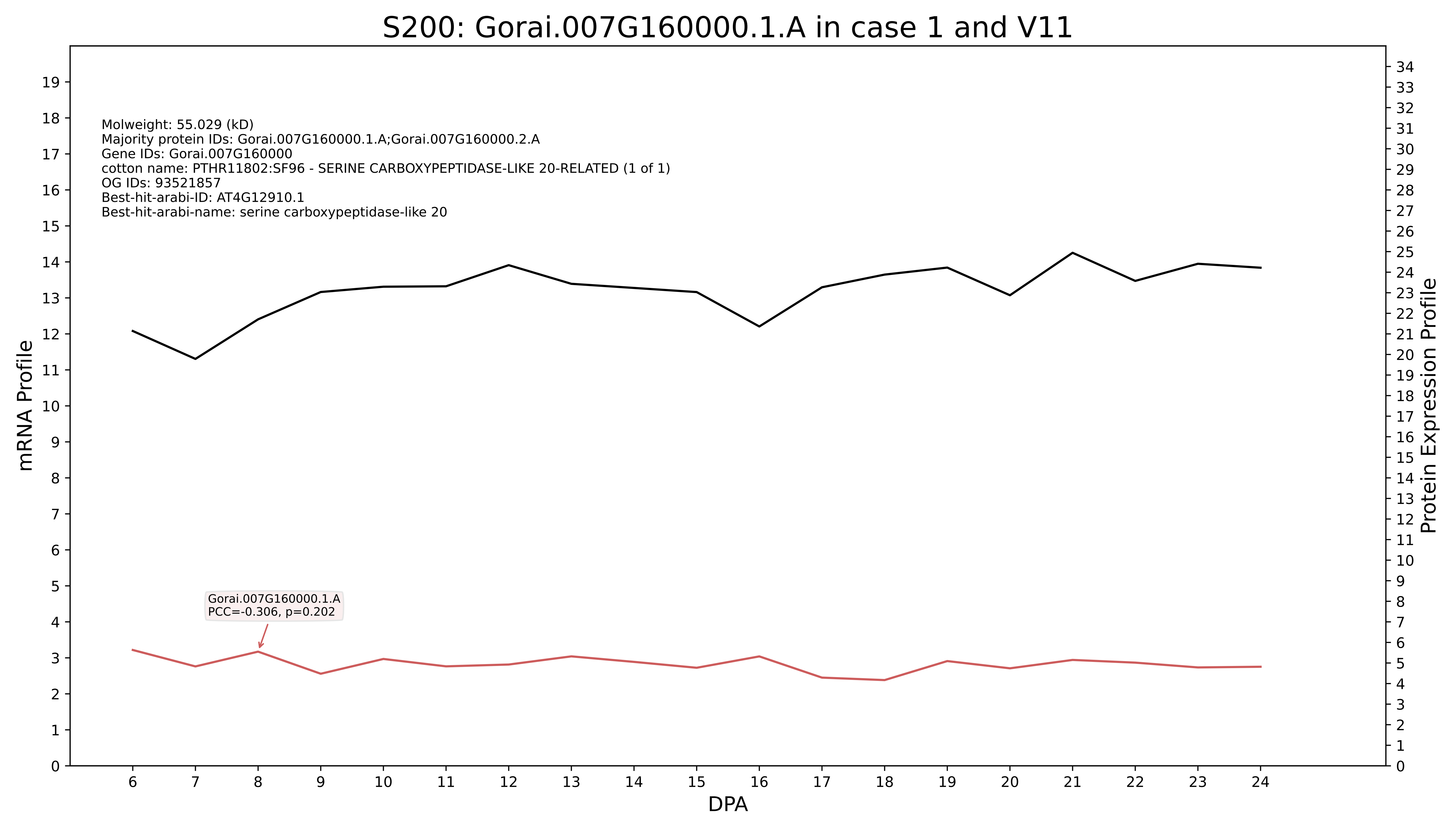

S200: Gorai.007G160000.1.A in case 1 and V11
Molweight: 55.029 (kD)
Majority protein IDs: Gorai.007G160000.1.A;Gorai.007G160000.2.A
Gene IDs: Gorai.007G160000
cotton name: PTHR11802:SF96 - SERINE CARBOXYPEPTIDASE-LIKE 20-RELATED (1 of 1)
OG IDs: 93521857
Best-hit-arabi-ID: AT4G12910.1
Best-hit-arabi-name: serine carboxypeptidase-like 20
Gorai.007G160000.1.A
PCC=-0.306, p=0.202
mRNA Profile
Protein Expression Profile
DPA

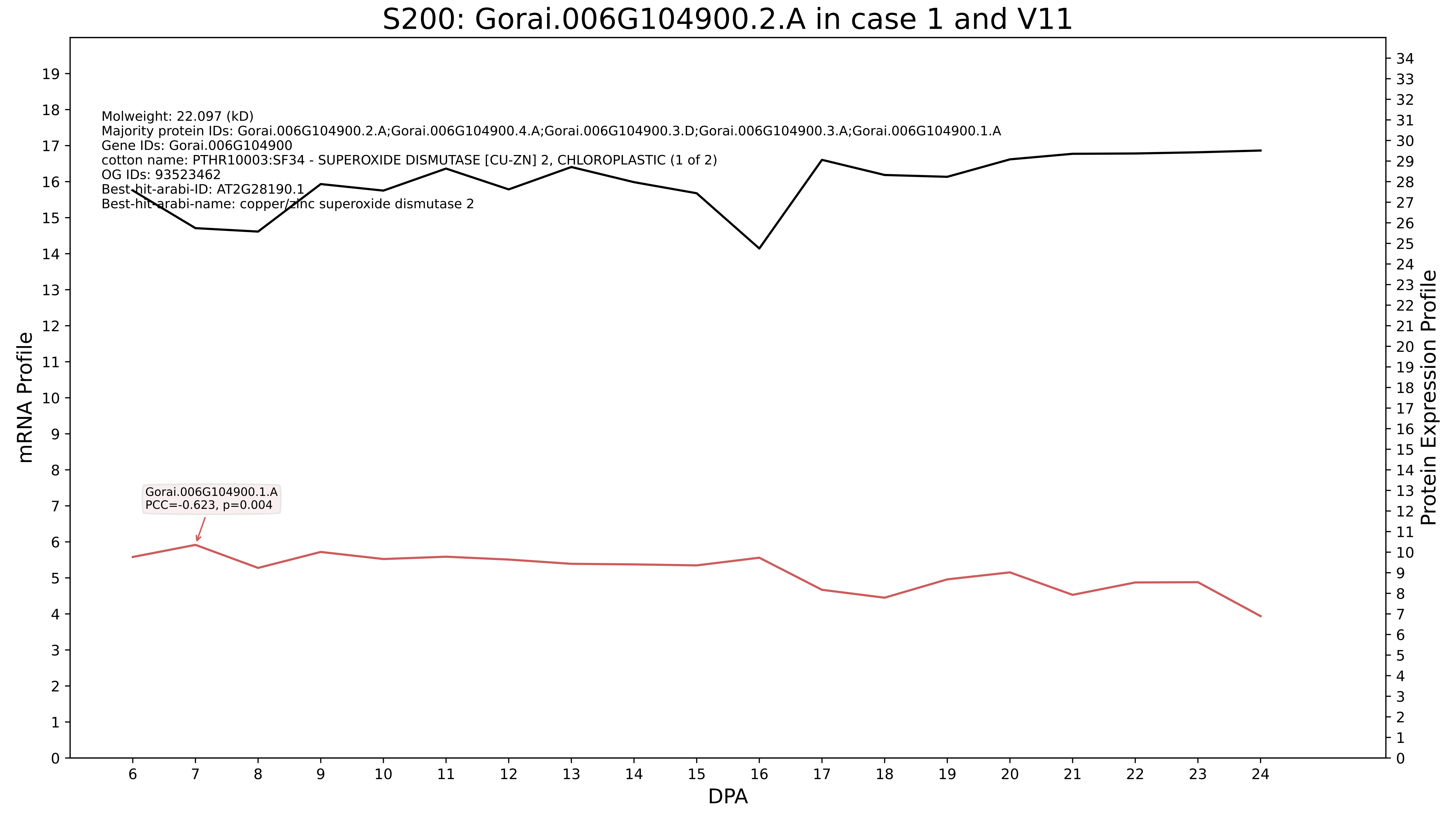
S200: Gorai.006G104900.2.A in case 1 and V11
Molweight: 22.097 (kD)
Majority protein IDs: Gorai.006G104900.2.A;Gorai.006G104900.4.A;Gorai.006G104900.3.D;Gorai.006G104900.3.A;Gorai.006G104900.1.A
Gene IDs: Gorai.006G104900
cotton name: PTHR10003:SF34 - SUPEROXIDE DISMUTASE [CU-ZN] 2, CHLOROPLASTIC (1 of 2)
OG IDs: 93523462
Best-hit-arabi-ID: AT2G28190.1
Best-hit-arabi-name: copper/zinc superoxide dismutase 2
Gorai.006G104900.1.A
PCC=-0.623, p=0.004
mRNA Profile
Protein Expression Profile
DPA

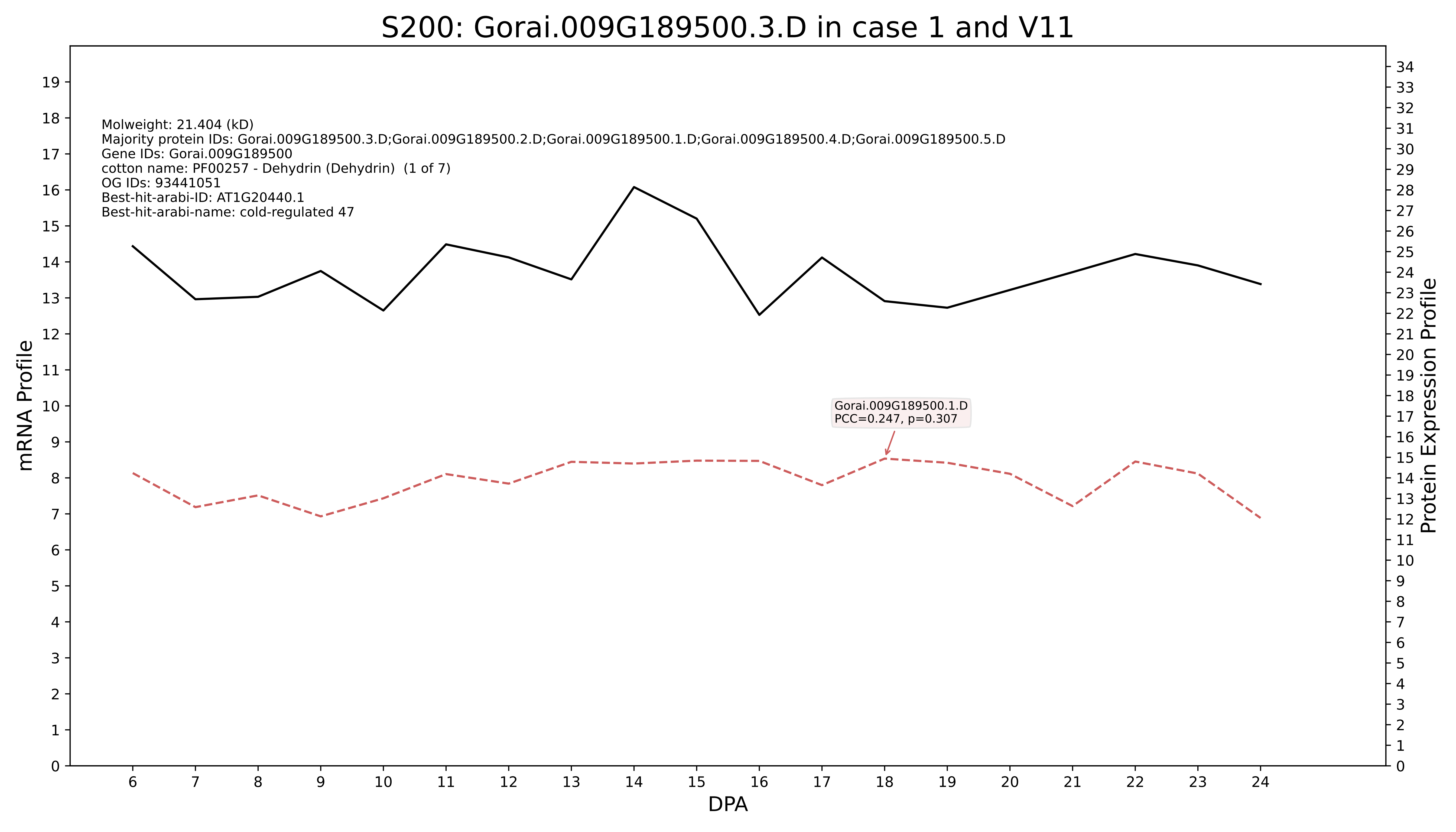
S200: Gorai.009G189500.3.D in case 1 and V11
Molweight: 21.404 (kD)
Majority protein IDs: Gorai.009G189500.3.D;Gorai.009G189500.2.D;Gorai.009G189500.1.D;Gorai.009G189500.4.D;Gorai.009G189500.5.D
Gene IDs: Gorai.009G189500
cotton name: PF00257 - Dehydrin (Dehydrin) (1 of 7)
OG IDs: 93441051
Best-hit-arabi-ID: AT1G20440.1
Best-hit-arabi-name: cold-regulated 47
Gorai.009G189500.1.D
PCC=0.247, p=0.307
mRNA Profile
Protein Expression Profile
DPA

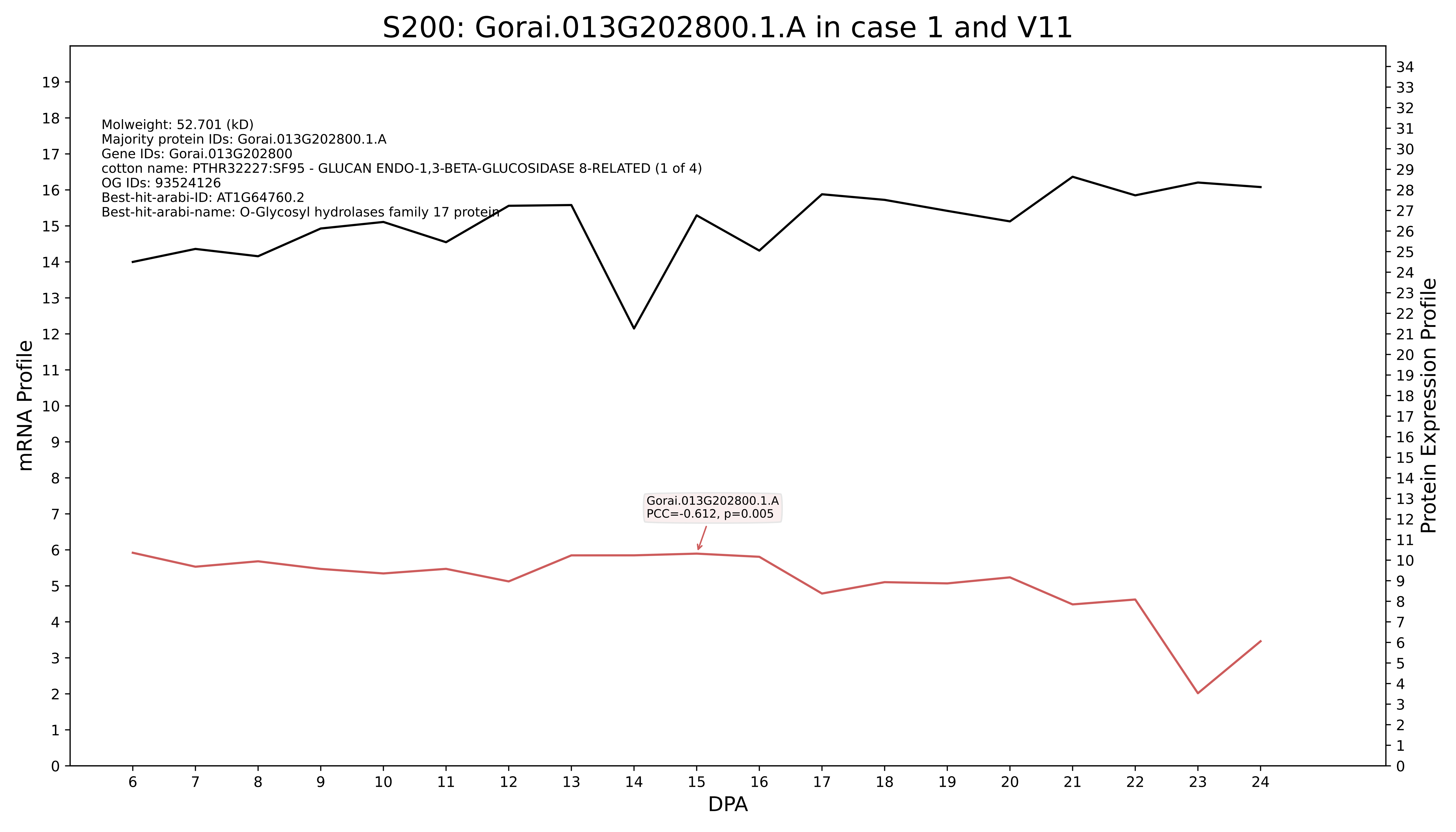
S200: Gorai.013G202800.1.A in case 1 and V11
Molweight: 52.701 (kD)
Majority protein IDs: Gorai.013G202800.1.A
Gene IDs: Gorai.013G202800
cotton name: PTHR32227:SF95 - GLUCAN ENDO-1,3-BETA-GLUCOSIDASE 8-RELATED (1 of 4)
OG IDs: 93524126
Best-hit-arabi-ID: AT1G64760.2
Best-hit-arabi-name: O-Glycosyl hydrolases family 17 protein
Gorai.013G202800.1.A
PCC=-0.612, p=0.005
mRNA Profile
Protein Expression Profile
DPA

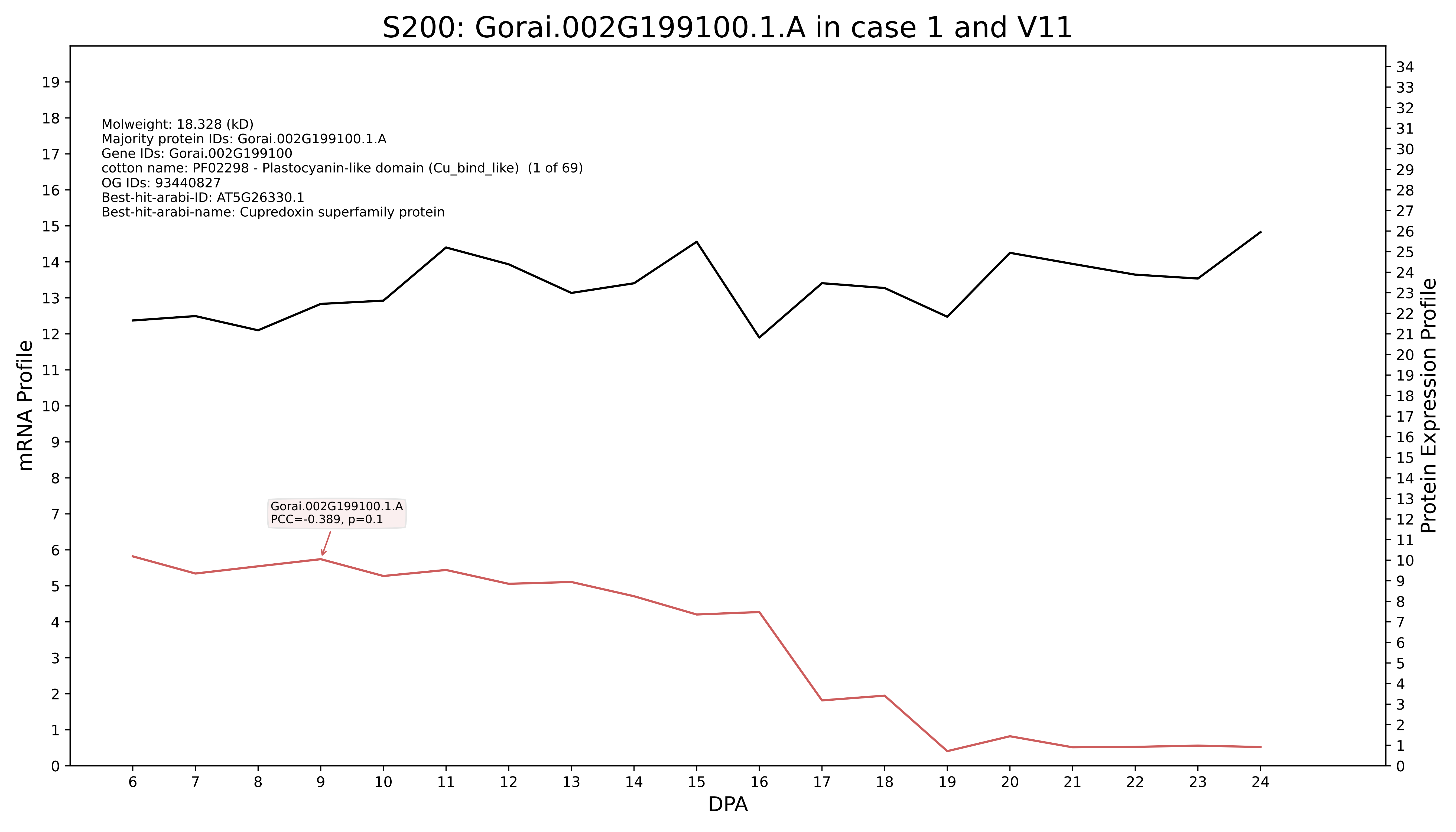
S200: Gorai.002G199100.1.A in case 1 and V11
Molweight: 18.328 (kD)
Majority protein IDs: Gorai.002G199100.1.A
Gene IDs: Gorai.002G199100
cotton name: PF02298 - Plastocyanin-like domain (Cu_bind_like) (1 of 69)
OG IDs: 93440827
Best-hit-arabi-ID: AT5G26330.1
Best-hit-arabi-name: Cupredoxin superfamily protein
Gorai.002G199100.1.A
PCC=-0.389, p=0.1
mRNA Profile
Protein Expression Profile
DPA

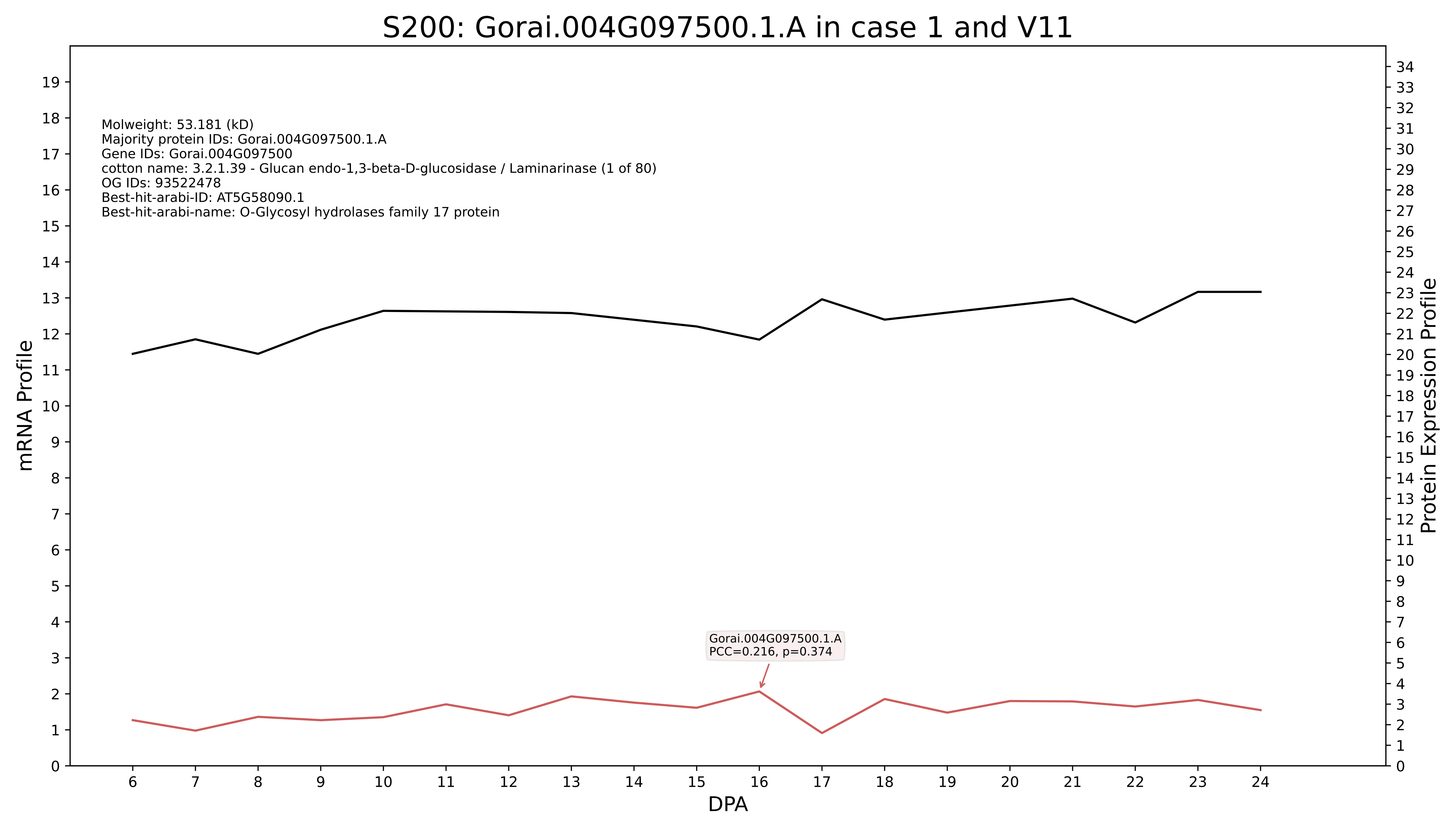
S200: Gorai.004G097500.1.A in case 1 and V11
Molweight: 53.181 (kD)
Majority protein IDs: Gorai.004G097500.1.A
Gene IDs: Gorai.004G097500
cotton name: 3.2.1.39 - Glucan endo-1,3-beta-D-glucosidase / Laminarinase (1 of 80)
OG IDs: 93522478
Best-hit-arabi-ID: AT5G58090.1
Best-hit-arabi-name: O-Glycosyl hydrolases family 17 protein
Gorai.004G097500.1.A
PCC=0.216, p=0.374
mRNA Profile
Protein Expression Profile
DPA
0
1
2
3
4
5
6
7
8
9
10
11
12
13
14
15
16
17
18
19
20
21
22
23
24
25
26
27
28
29
30
31
32
33
34

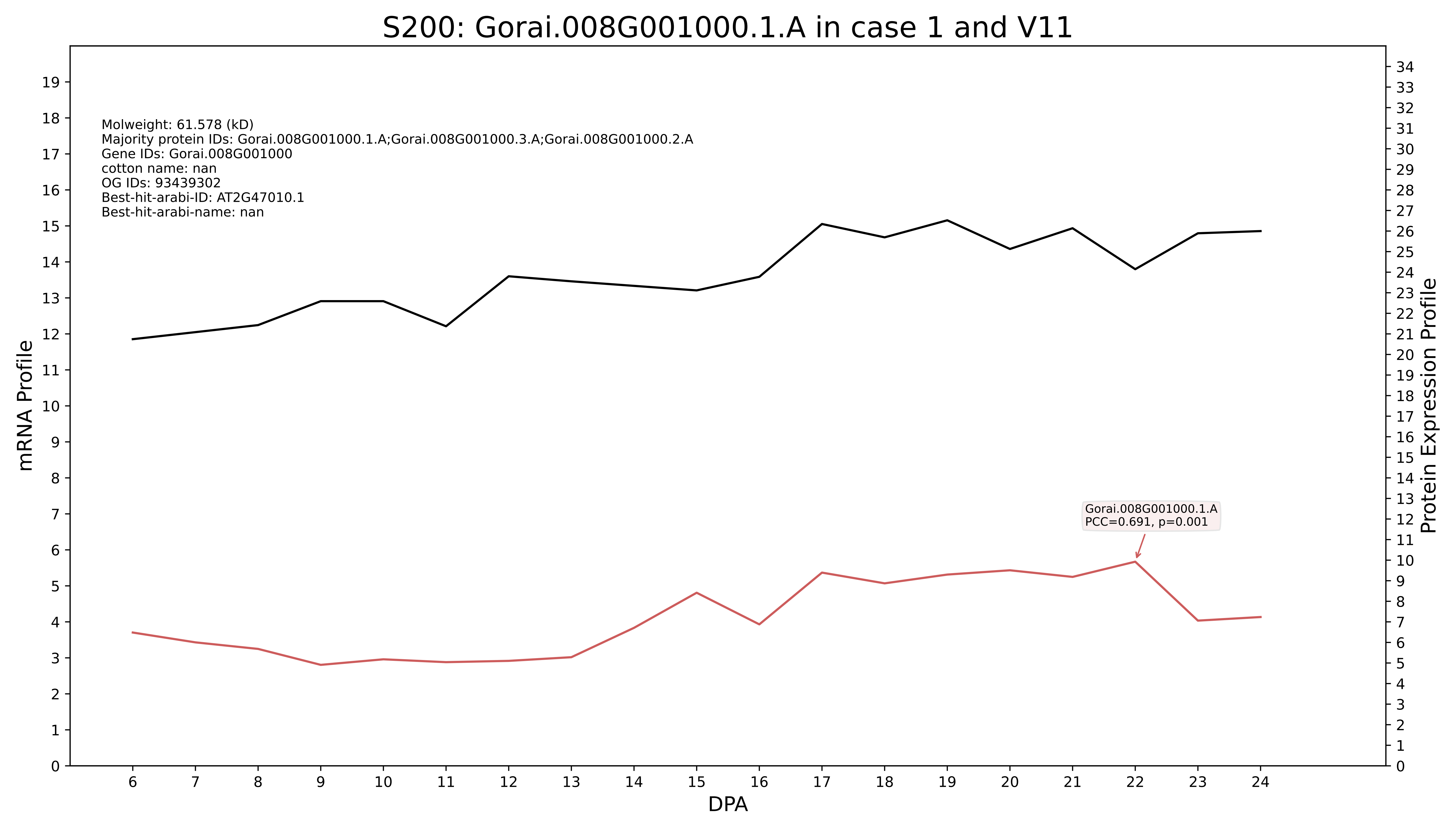
S200: Gorai.008G001000.1.A in case 1 and V11
Molweight: 61.578 (kD)
Majority protein IDs: Gorai.008G001000.1.A;Gorai.008G001000.3.A;Gorai.008G001000.2.A
Gene IDs: Gorai.008G001000
cotton name: nan
OG IDs: 93439302
Best-hit-arabi-ID: AT2G47010.1
Best-hit-arabi-name: nan
Gorai.008G001000.1.A
PCC=0.691, p=0.001
mRNA Profile
Protein Expression Profile
DPA
0
1
2
3
4
5
6
7
8
9
10
11
12
13
14
15
16
17
18
19
20
21
22
23
24
25
26
27
28
29
30
31
32
33
34

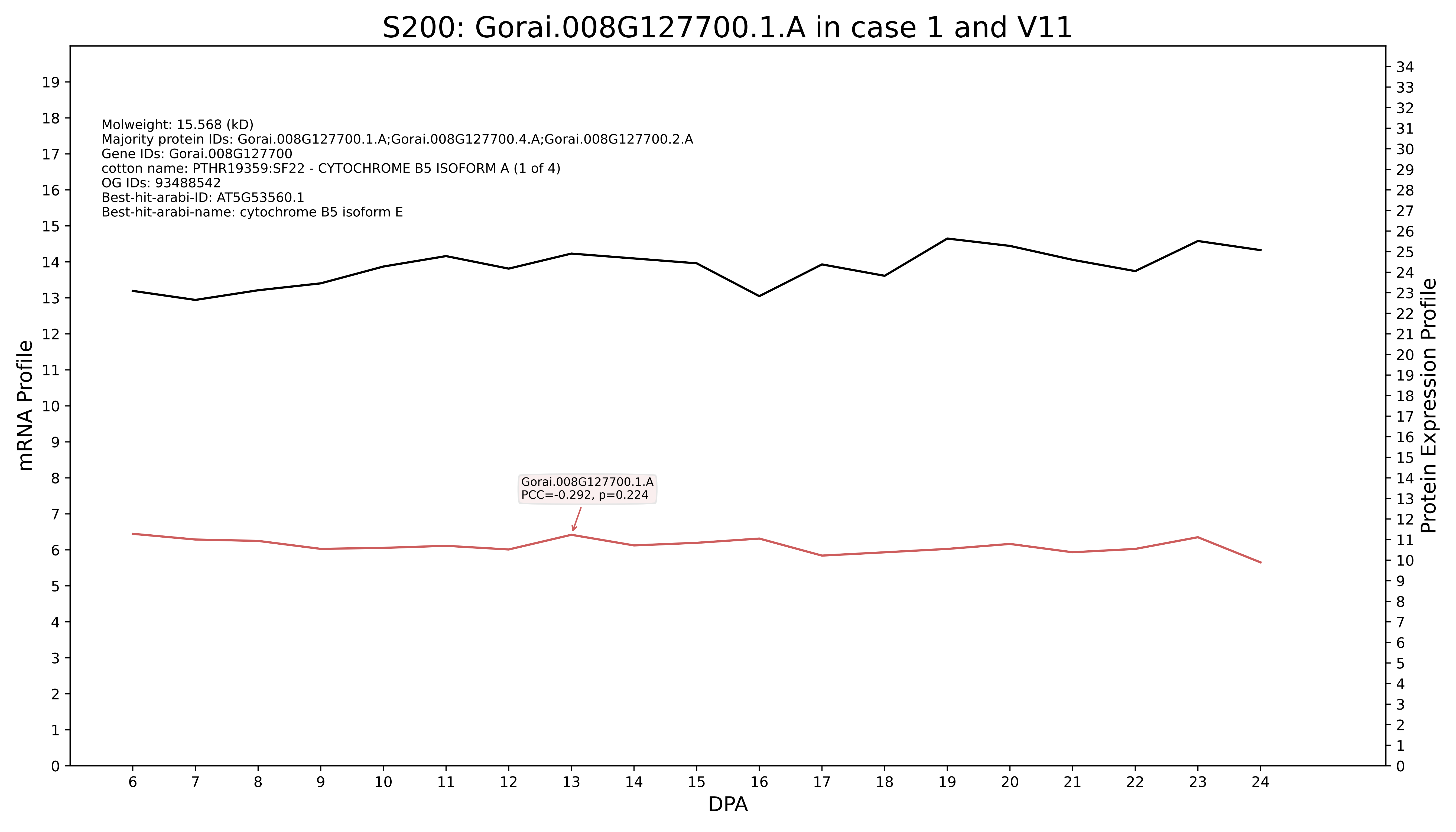
S200: Gorai.008G127700.1.A in case 1 and V11
Molweight: 15.568 (kD)
Majority protein IDs: Gorai.008G127700.1.A;Gorai.008G127700.4.A;Gorai.008G127700.2.A
Gene IDs: Gorai.008G127700
cotton name: PTHR19359:SF22 - CYTOCHROME B5 ISOFORM A (1 of 4)
OG IDs: 93488542
Best-hit-arabi-ID: AT5G53560.1
Best-hit-arabi-name: cytochrome B5 isoform E
Gorai.008G127700.1.A
PCC=-0.292, p=0.224
mRNA Profile
Protein Expression Profile
DPA

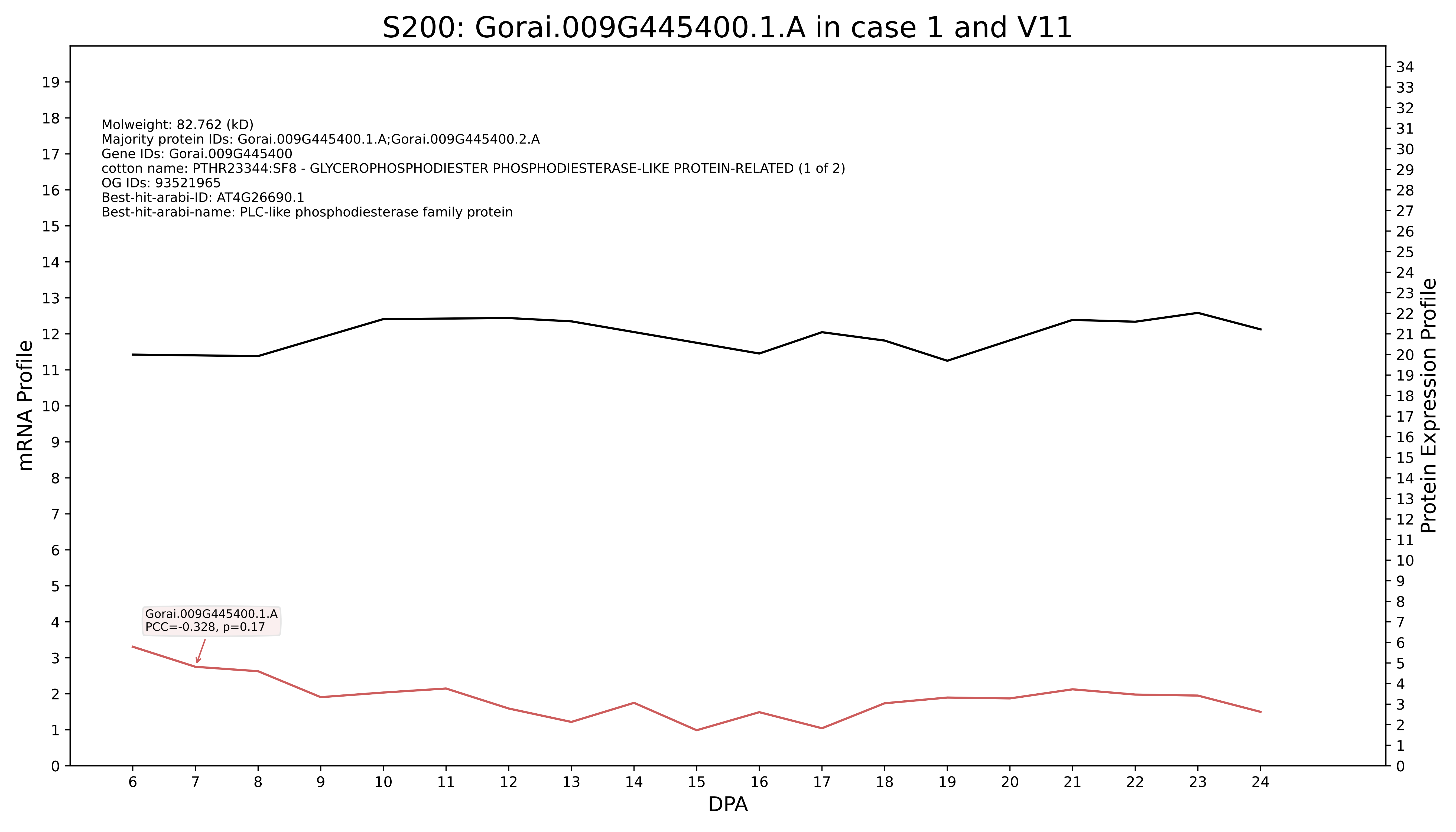
S200: Gorai.009G445400.1.A in case 1 and V11
Molweight: 82.762 (kD)
Majority protein IDs: Gorai.009G445400.1.A;Gorai.009G445400.2.A
Gene IDs: Gorai.009G445400
cotton name: PTHR23344:SF8 - GLYCEROPHOSPHODIESTER PHOSPHODIESTERASE-LIKE PROTEIN-RELATED (1 of 2)
OG IDs: 93521965
Best-hit-arabi-ID: AT4G26690.1
Best-hit-arabi-name: PLC-like phosphodiesterase family protein
Gorai.009G445400.1.A
PCC=-0.328, p=0.17
mRNA Profile
Protein Expression Profile
DPA

S200: Gorai.011G228300.1.A in case 1 and V11

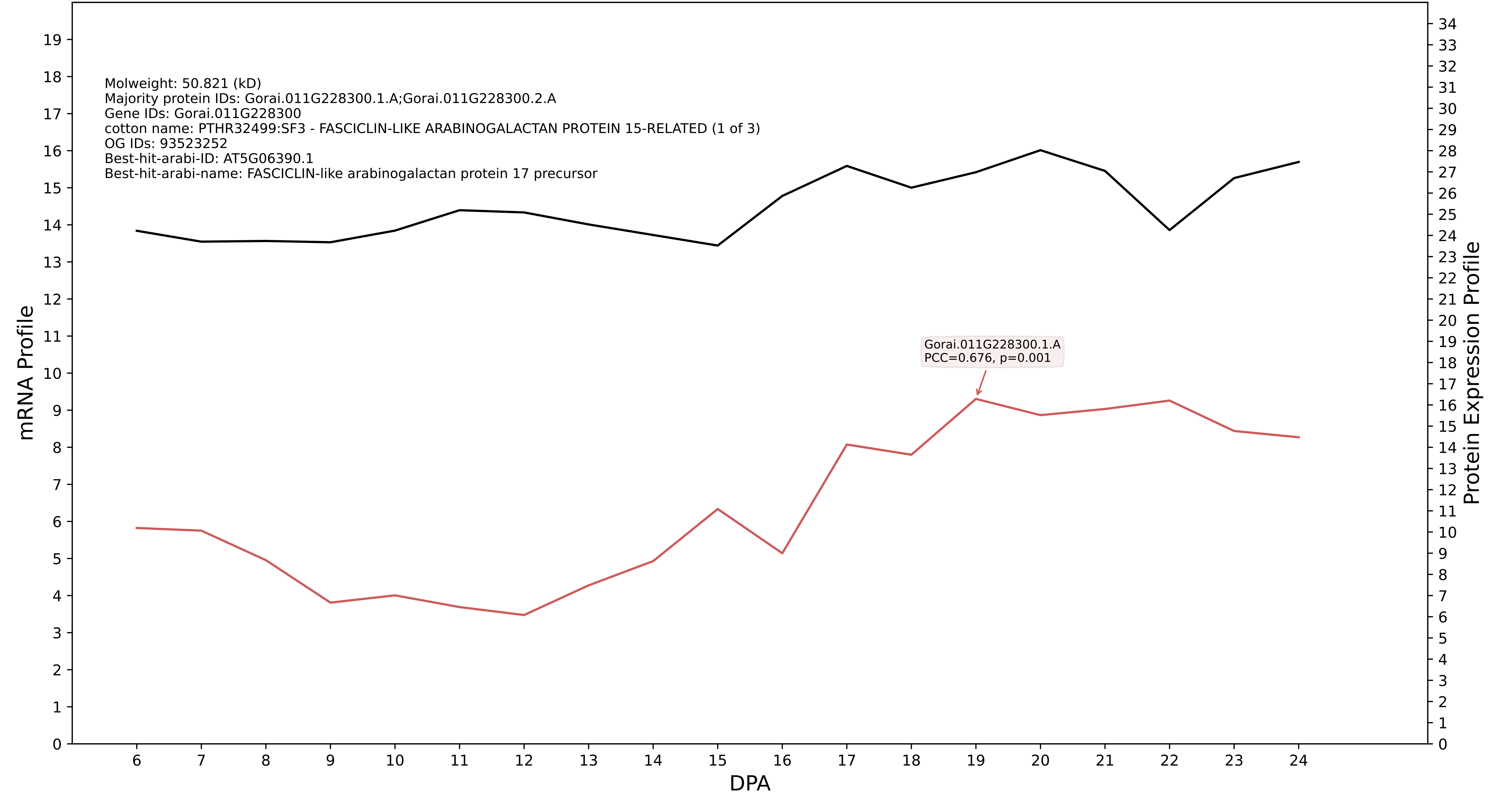

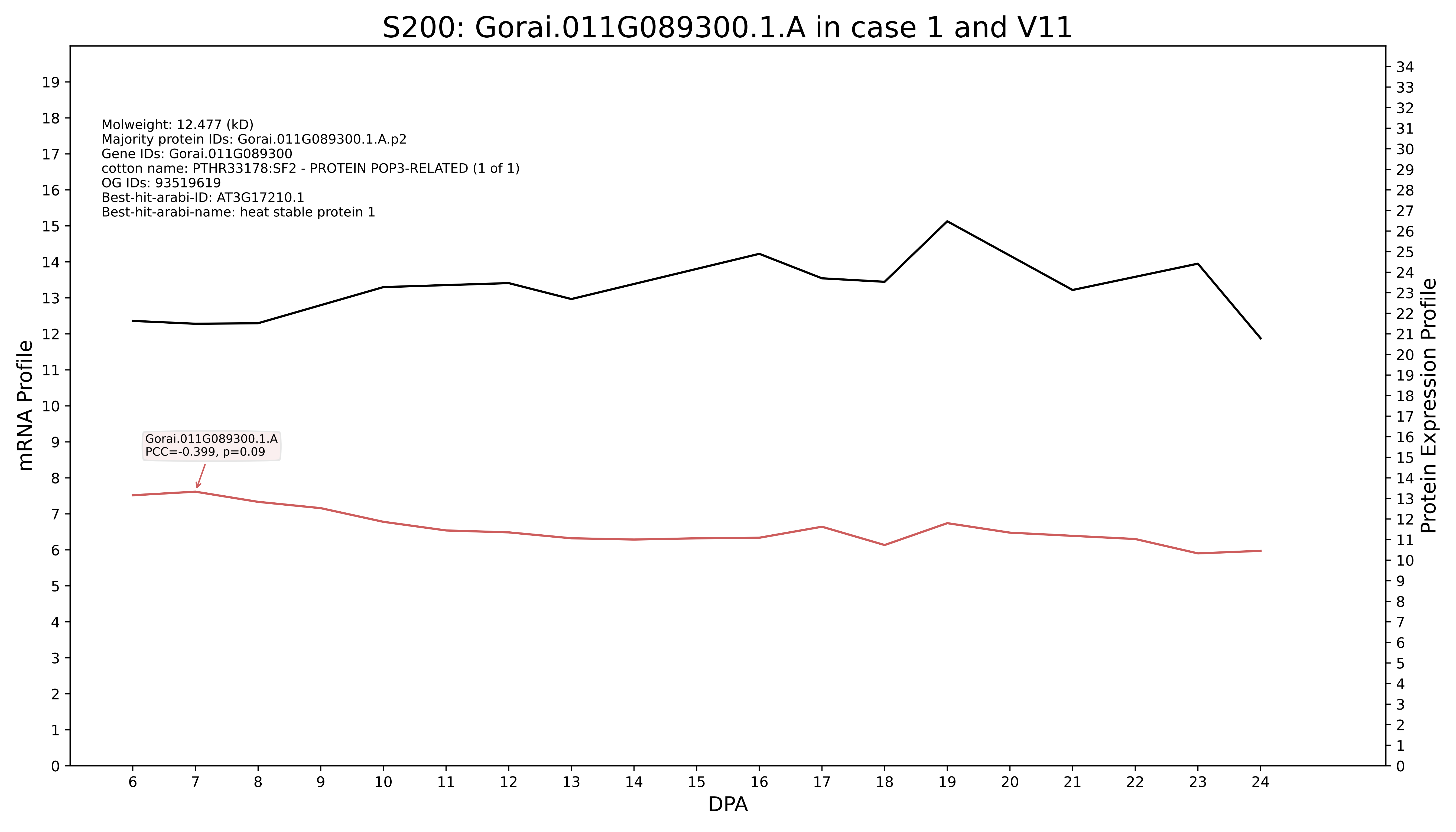
S200: Gorai.011G089300.1.A in case 1 and V11
Molweight: 12.477 (kD)
Majority protein IDs: Gorai.011G089300.1.A.p2
Gene IDs: Gorai.011G089300
cotton name: PTHR33178:SF2 - PROTEIN POP3-RELATED (1 of 1)
OG IDs: 93519619
Best-hit-arabi-ID: AT3G17210.1
Best-hit-arabi-name: heat stable protein 1
Gorai.011G089300.1.A
PCC=-0.399, p=0.09
mRNA Profile
Protein Expression Profile
DPA
0
1
2
3
4
5
6
7
8
9
10
11
12
13
14
15
16
17
18
19
20
21
22
23
24
25
26
27
28
29
30
31
32
33
34

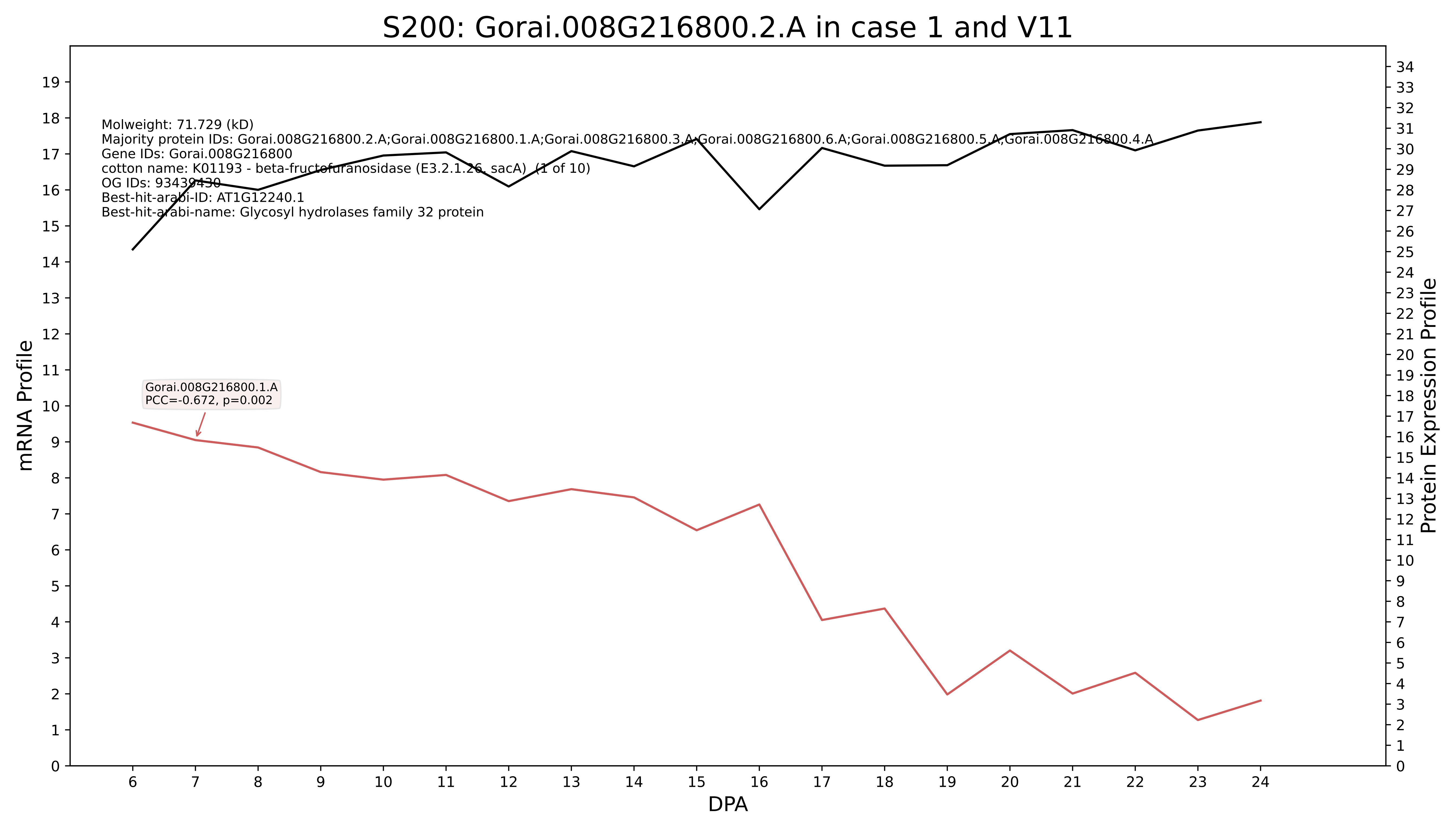

S200: Gorai.008G216800.2.A in case 1 and V11
Molweight: 71.729 (kD)
Majority protein IDs: Gorai.008G216800.2.A;Gorai.008G216800.1.A;Gorai.008G216800.3.A;Gorai.008G216800.6.A;Gorai.008G216800.5.A;Gorai.008G216800.4.A
Gene IDs: Gorai.008G216800
cotton name: K01193 - beta-fructofuranosidase (E3.2.1.26, sacA) (1 of 10)
OG IDs: 93439430
Best-hit-arabi-ID: AT1G12240.1
Best-hit-arabi-name: Glycosyl hydrolases family 32 protein
Gorai.008G216800.1.A
PCC=-0.672, p=0.002
mRNA Profile
Protein Expression Profile
DPA

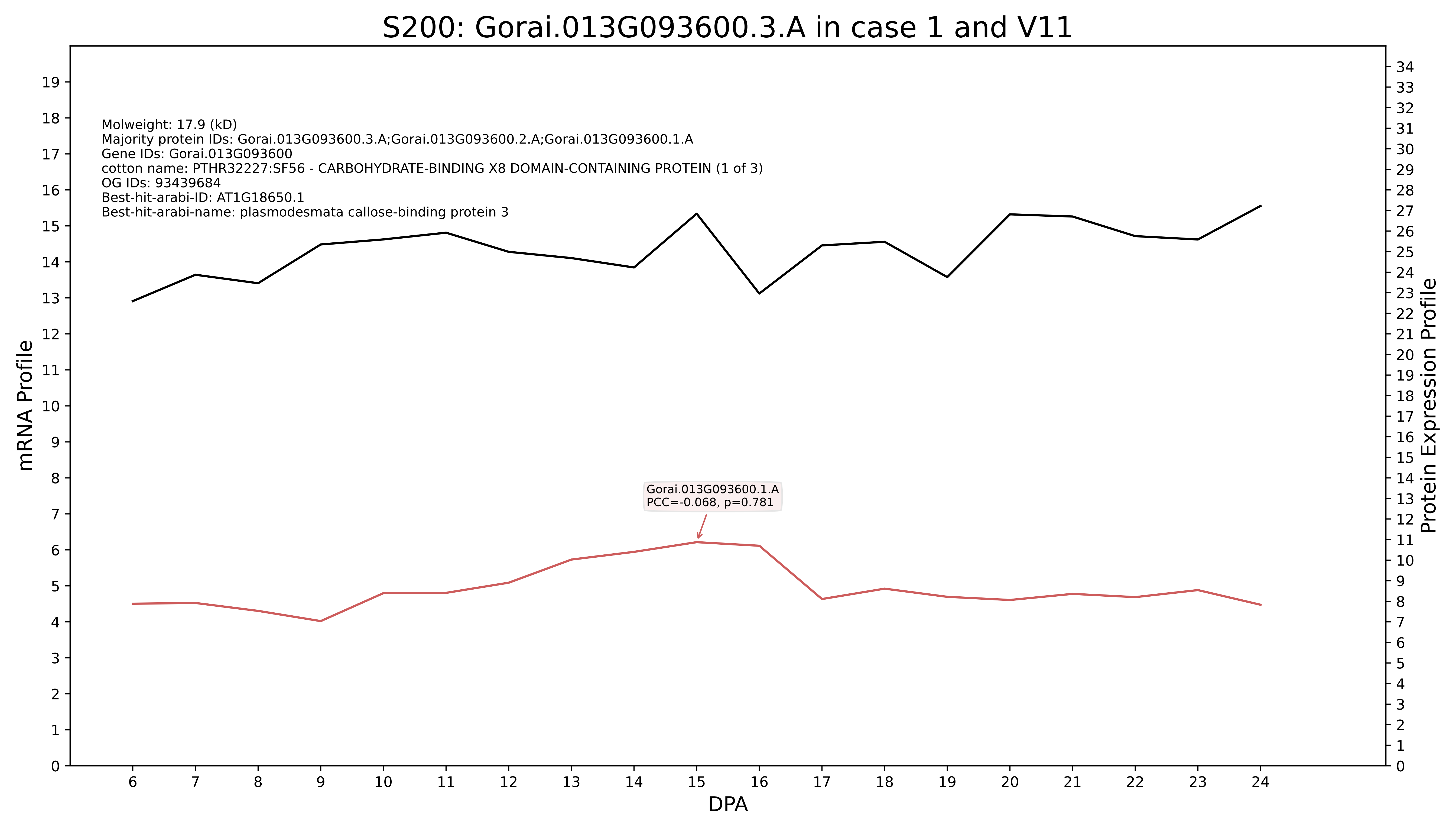
S200: Gorai.013G093600.3.A in case 1 and V11
Molweight: 17.9 (kD)
Majority protein IDs: Gorai.013G093600.3.A;Gorai.013G093600.2.A;Gorai.013G093600.1.A
Gene IDs: Gorai.013G093600
cotton name: PTHR32227:SF56 - CARBOHYDRATE-BINDING X8 DOMAIN-CONTAINING PROTEIN (1 of 3)
OG IDs: 93439684
Best-hit-arabi-ID: AT1G18650.1
Best-hit-arabi-name: plasmodesmata callose-binding protein 3
Gorai.013G093600.1.A
PCC=-0.068, p=0.781
mRNA Profile
Protein Expression Profile
DPA

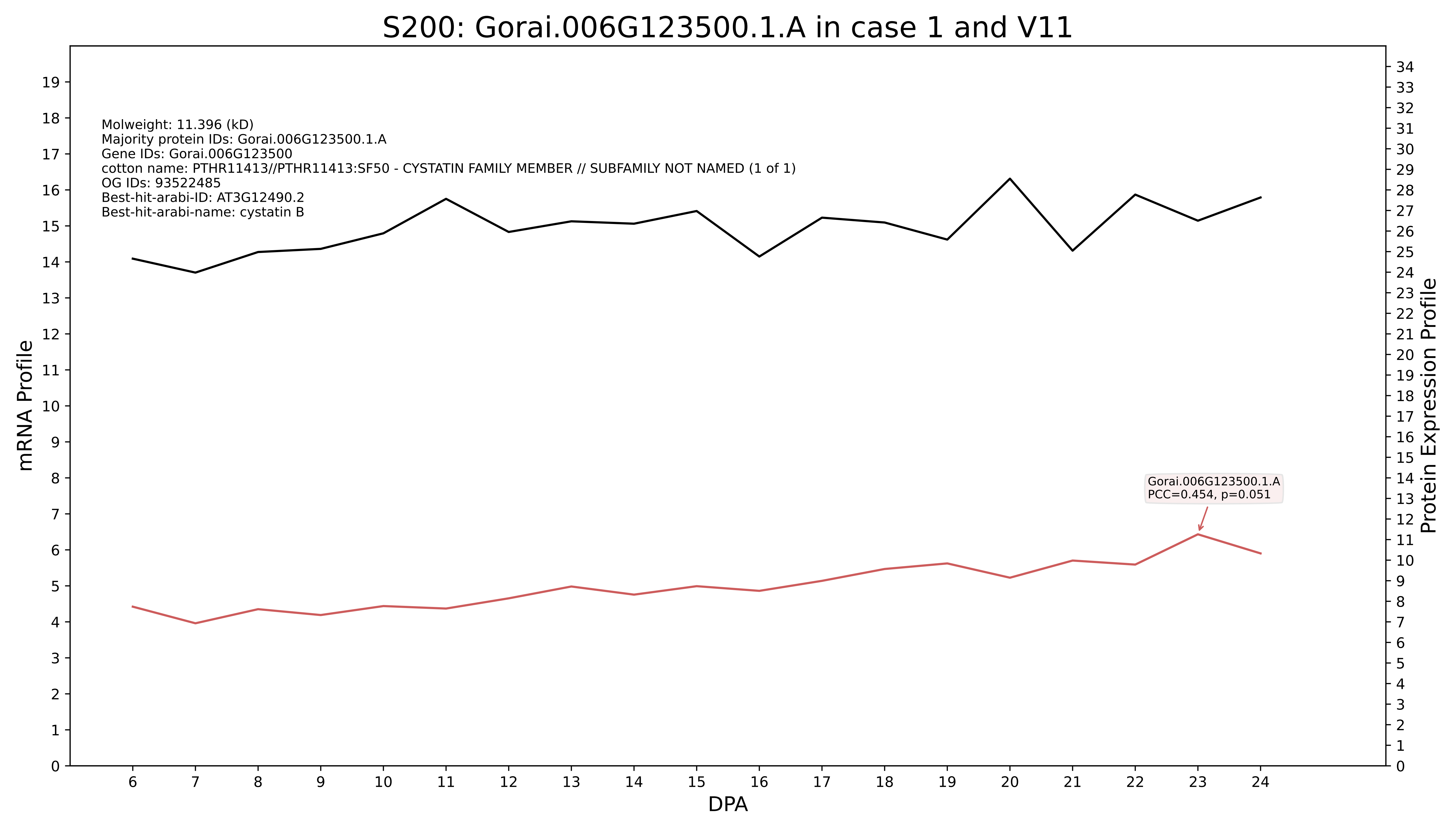
S200: Gorai.006G123500.1.A in case 1 and V11
Molweight: 11.396 (kD)
Majority protein IDs: Gorai.006G123500.1.A
Gene IDs: Gorai.006G123500
cotton name: PTHR11413//PTHR11413:SF50 - CYSTATIN FAMILY MEMBER // SUBFAMILY NOT NAMED (1 of 1)
OG IDs: 93522485
Best-hit-arabi-ID: AT3G12490.2
Best-hit-arabi-name: cystatin B
Gorai.006G123500.1.A
PCC=0.454, p=0.051
mRNA Profile
Protein Expression Profile
DPA

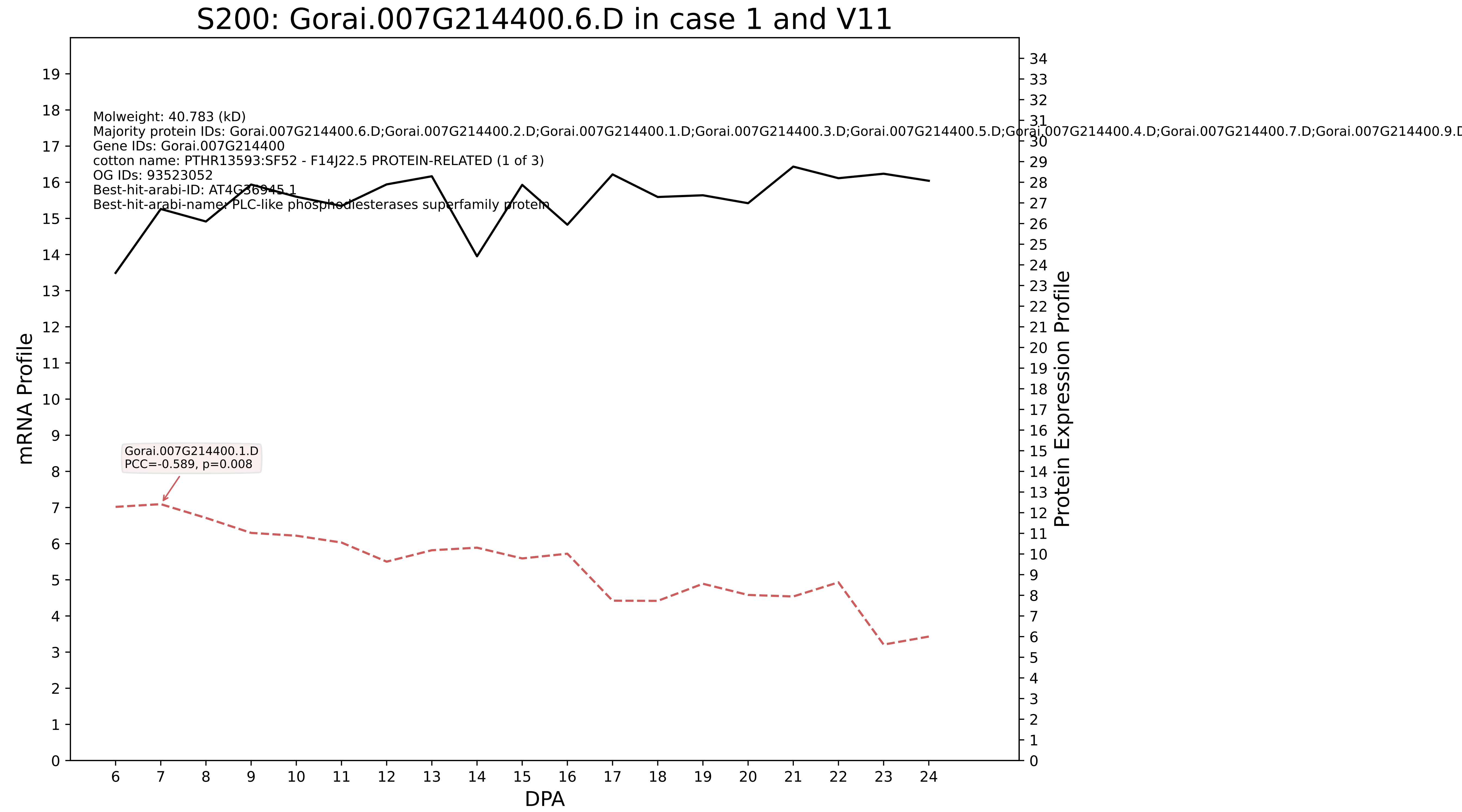
S200: Gorai.007G214400.6.D in case 1 and V11
Molweight: 40.783 (kD)
Majority protein IDs: Gorai.007G214400.6.D;Gorai.007G214400.2.D;Gorai.007G214400.1.D;Gorai.007G214400.3.D;Gorai.007G214400.5.D;Gorai.007G214400.4.D;Gorai.007G214400.7.D;Gorai.007G214400.9.D
Gene IDs: Gorai.007G214400
cotton name: PTHR13593:SF52 - F14J22.5 PROTEIN-RELATED (1 of 3)
OG IDs: 93523052
Best-hit-arabi-ID: AT4G36945.1
Best-hit-arabi-name: PLC-like phosphodiesterases superfamily protein
Gorai.007G214400.1.D
PCC=-0.589, p=0.008
mRNA Profile
Protein Expression Profile
DPA

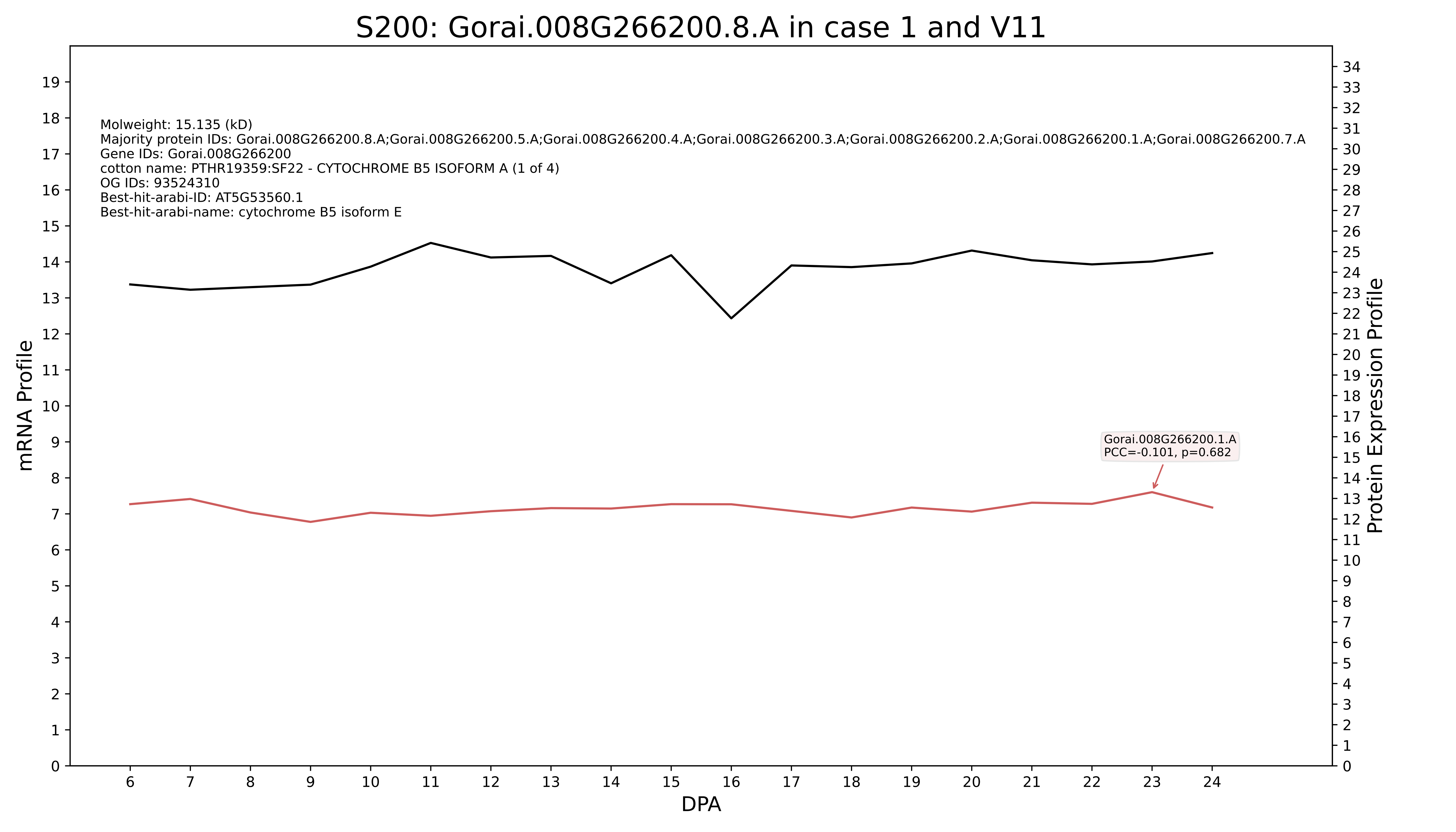
S200: Gorai.008G266200.8.A in case 1 and V11
Molweight: 15.135 (kD)
Majority protein IDs: Gorai.008G266200.8.A;Gorai.008G266200.5.A;Gorai.008G266200.4.A;Gorai.008G266200.3.A;Gorai.008G266200.2.A;Gorai.008G266200.1.A;Gorai.008G266200.7.A
Gene IDs: Gorai.008G266200
cotton name: PTHR19359:SF22 - CYTOCHROME B5 ISOFORM A (1 of 4)
OG IDs: 93524310
Best-hit-arabi-ID: AT5G53560.1
Best-hit-arabi-name: cytochrome B5 isoform E
Gorai.008G266200.1.A
PCC=-0.101, p=0.682
mRNA Profile
Protein Expression Profile
DPA

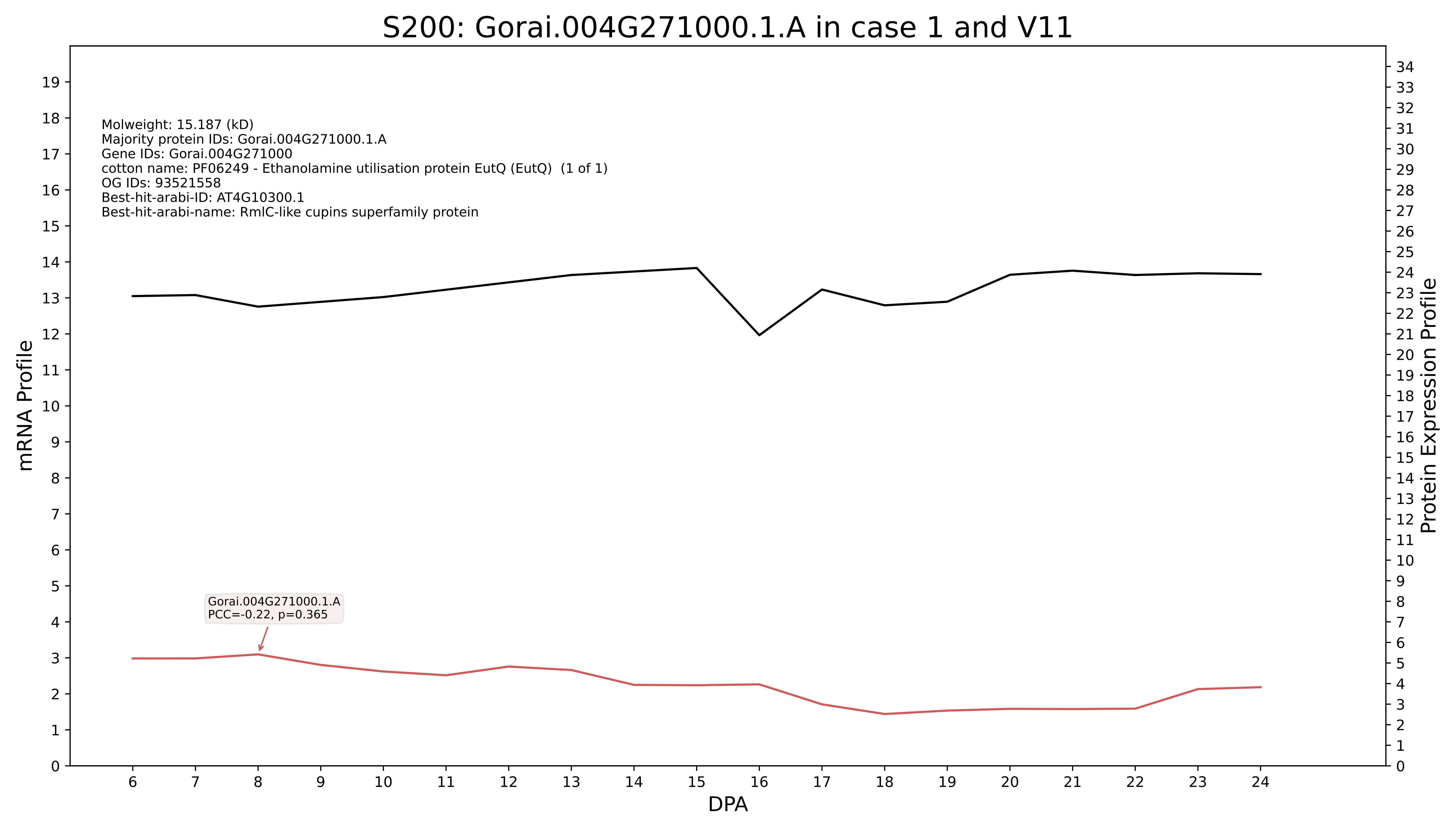

S200: Gorai.004G271000.1.A in case 1 and V11
Molweight: 15.187 (kD)
Majority protein IDs: Gorai.004G271000.1.A
Gene IDs: Gorai.004G271000
cotton name: PF06249 - Ethanolamine utilisation protein EutQ (EutQ) (1 of 1)
OG IDs: 93521558
Best-hit-arabi-ID: AT4G10300.1
Best-hit-arabi-name: RmlC-like cupins superfamily protein
Gorai.004G271000.1.A
PCC=-0.22, p=0.365
mRNA Profile
Protein Expression Profile
DPA

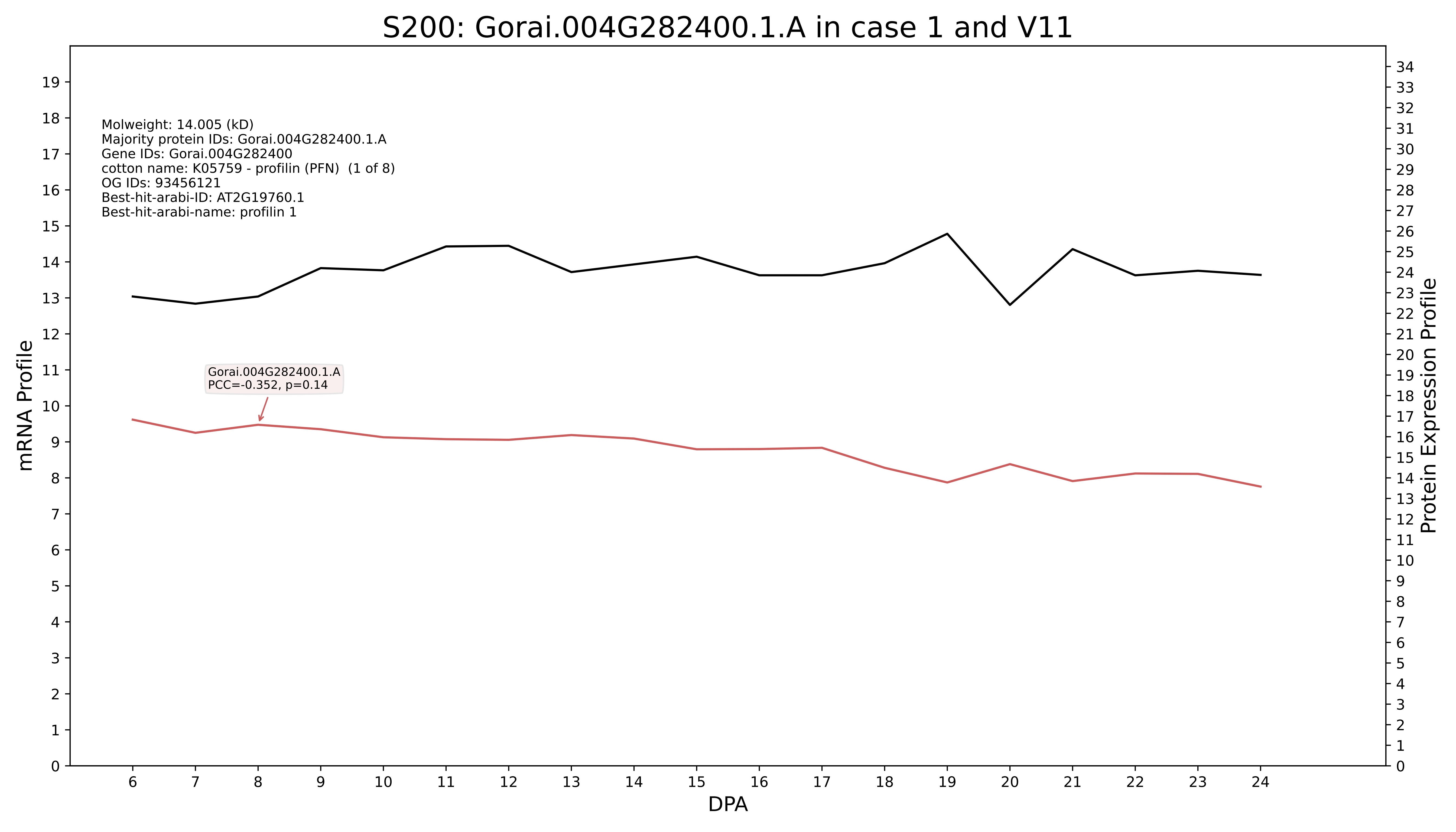

S200: Gorai.004G282400.1.A in case 1 and V11
Molweight: 14.005 (kD)
Majority protein IDs: Gorai.004G282400.1.A
Gene IDs: Gorai.004G282400
cotton name: K05759 - profilin (PFN) (1 of 8)
OG IDs: 93456121
Best-hit-arabi-ID: AT2G19760.1
Best-hit-arabi-name: profilin 1
Gorai.004G282400.1.A
PCC=-0.352, p=0.14
mRNA Profile
Protein Expression Profile
DPA

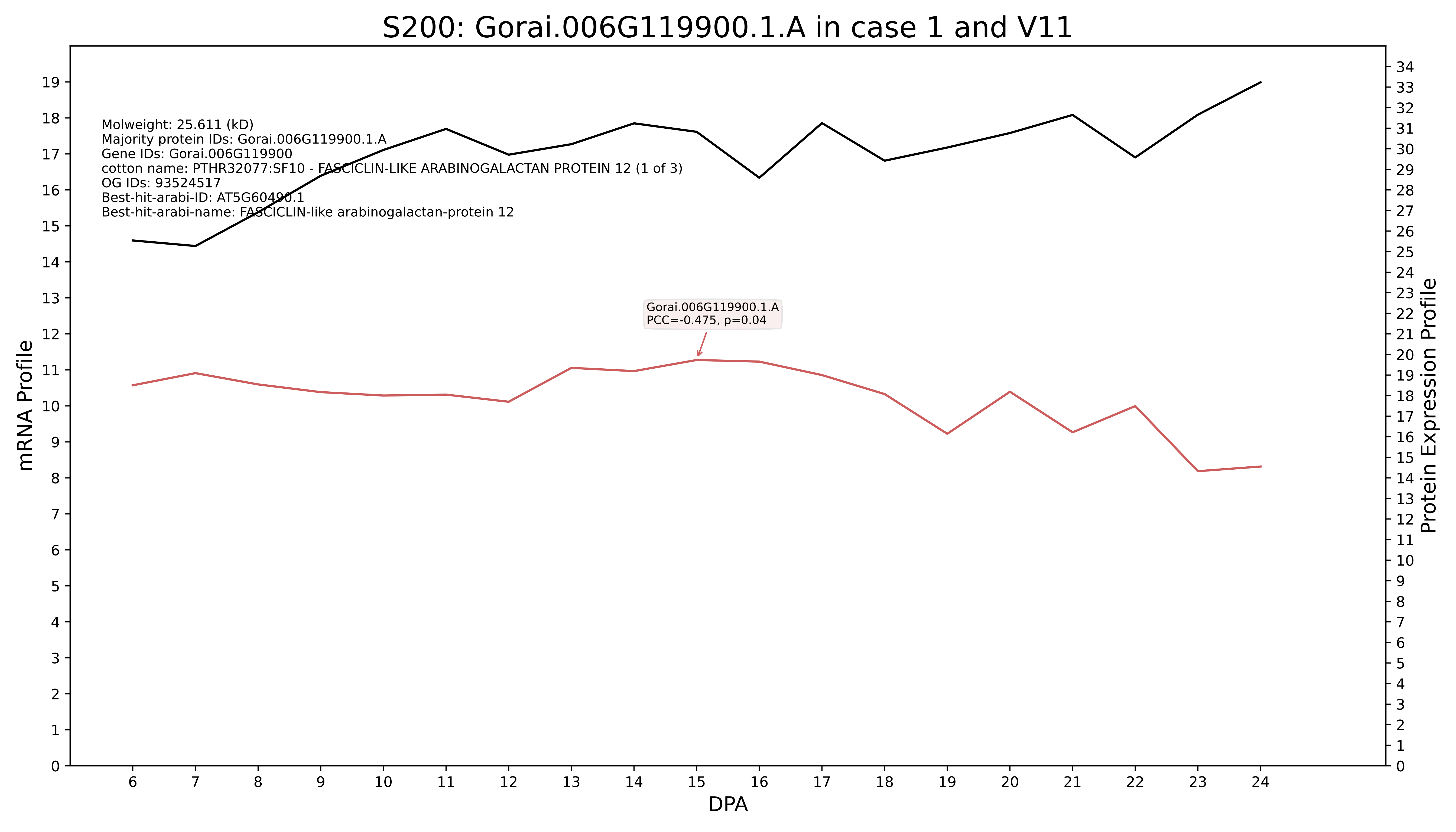
S200: Gorai.006G119900.1.A in case 1 and V11
Molweight: 25.611 (kD)
Majority protein IDs: Gorai.006G119900.1.A
Gene IDs: Gorai.006G119900
cotton name: PTHR32077:SF10 - FASCICLIN-LIKE ARABINOGALACTAN PROTEIN 12 (1 of 3)
OG IDs: 93524517
Best-hit-arabi-ID: AT5G60490.1
Best-hit-arabi-name: FASCICLIN-like arabinogalactan-protein 12
Gorai.006G119900.1.A
PCC=-0.475, p=0.04
mRNA Profile
Protein Expression Profile
DPA

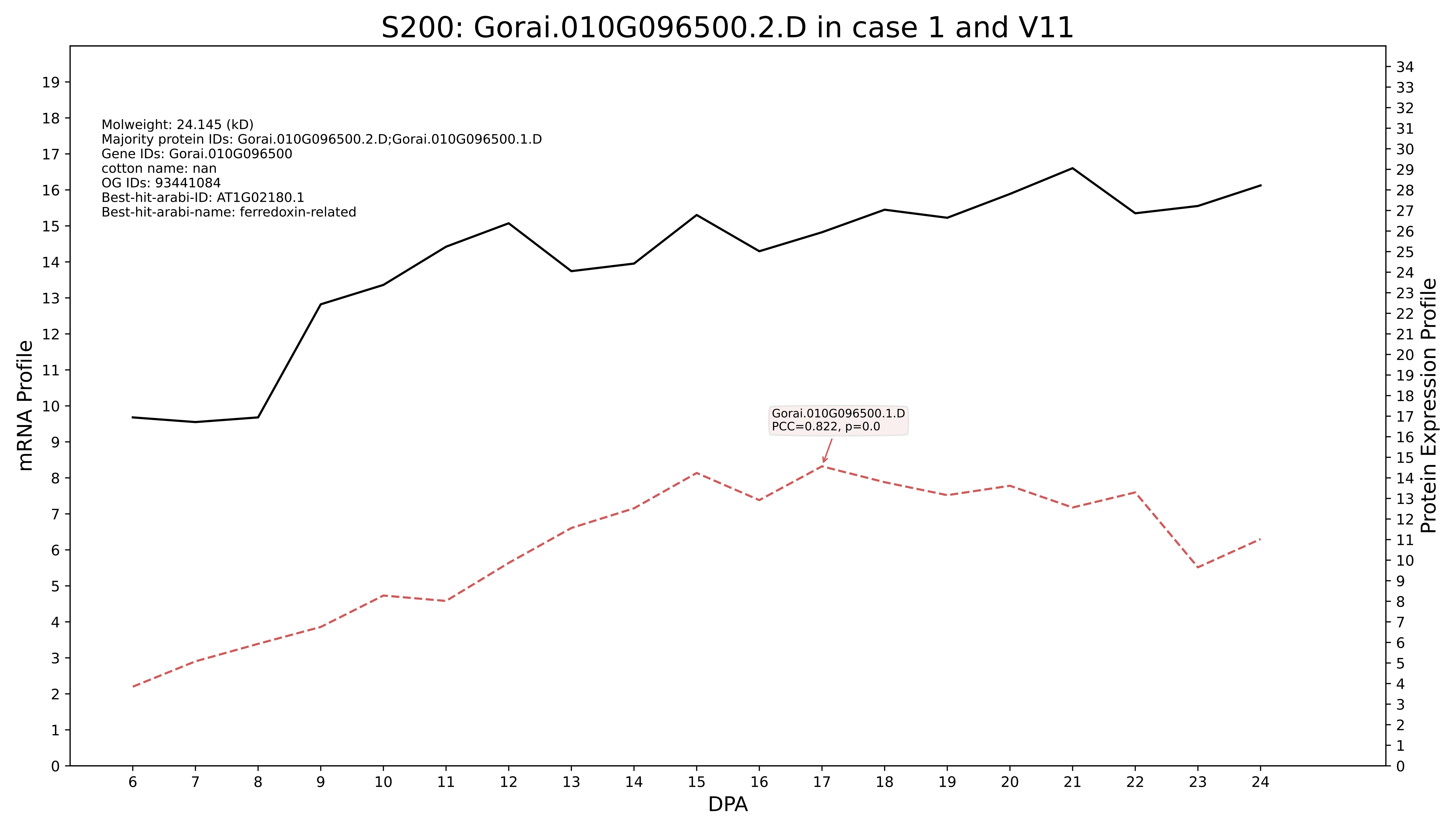
S200: Gorai.010G096500.2.D in case 1 and V11
Molweight: 24.145 (kD)
Majority protein IDs: Gorai.010G096500.2.D;Gorai.010G096500.1.D
Gene IDs: Gorai.010G096500
cotton name: nan
OG IDs: 93441084
Best-hit-arabi-ID: AT1G02180.1
Best-hit-arabi-name: ferredoxin-related
Gorai.010G096500.1.D
PCC=0.822, p=0.0
mRNA Profile
Protein Expression Profile
DPA
6 7 8 9 10 11 12 13 14 15 16 17 18 19 20 21 22 23 24
0 1 2 3 4 5 6 7 8 9 10 11 12 13 14 15 16 17 18 19
0 1 2 3 4 5 6 7 8 9 10 11 12 13 14 15 16 17 18 19 20 21 22 23 24 25 26 27 28 29 30 31 32 33 34

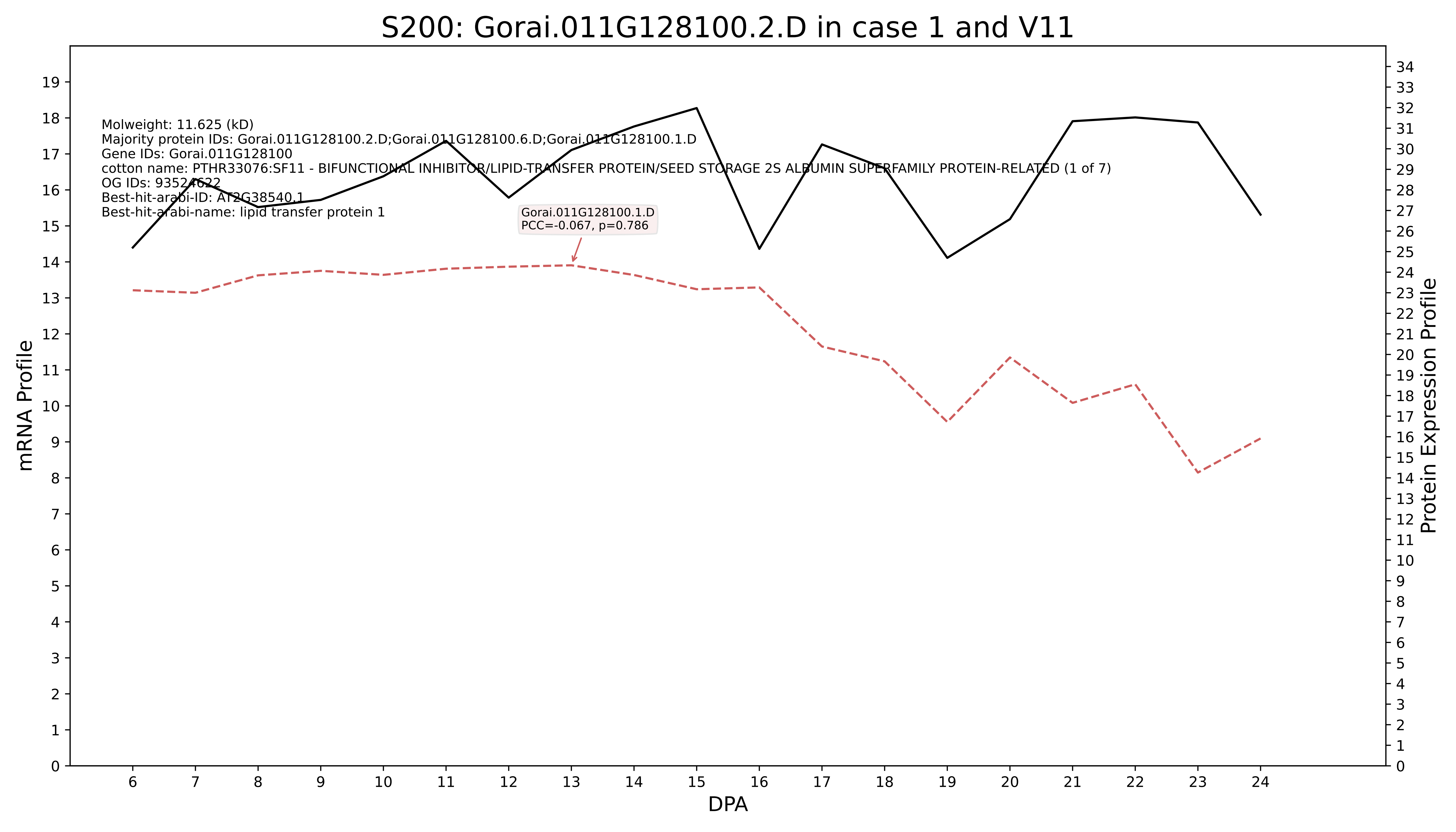
S200: Gorai.011G128100.2.D in case 1 and V11
Molweight: 11.625 (kD)
Majority protein IDs: Gorai.011G128100.2.D;Gorai.011G128100.6.D;Gorai.011G128100.1.D
Gene IDs: Gorai.011G128100
cotton name: PTHR33076:SF11 - BIFUNCTIONAL INHIBITOR/LIPID-TRANSFER PROTEIN/SEED STORAGE 2S ALBUMIN SUPERFAMILY PROTEIN-RELATED (1 of 7)
OG IDs: 93524622
Best-hit-arabi-ID: AT2G38540.1
Best-hit-arabi-name: lipid transfer protein 1
Gorai.011G128100.1.D
PCC=-0.067, p=0.786
mRNA Profile
Protein Expression Profile
DPA

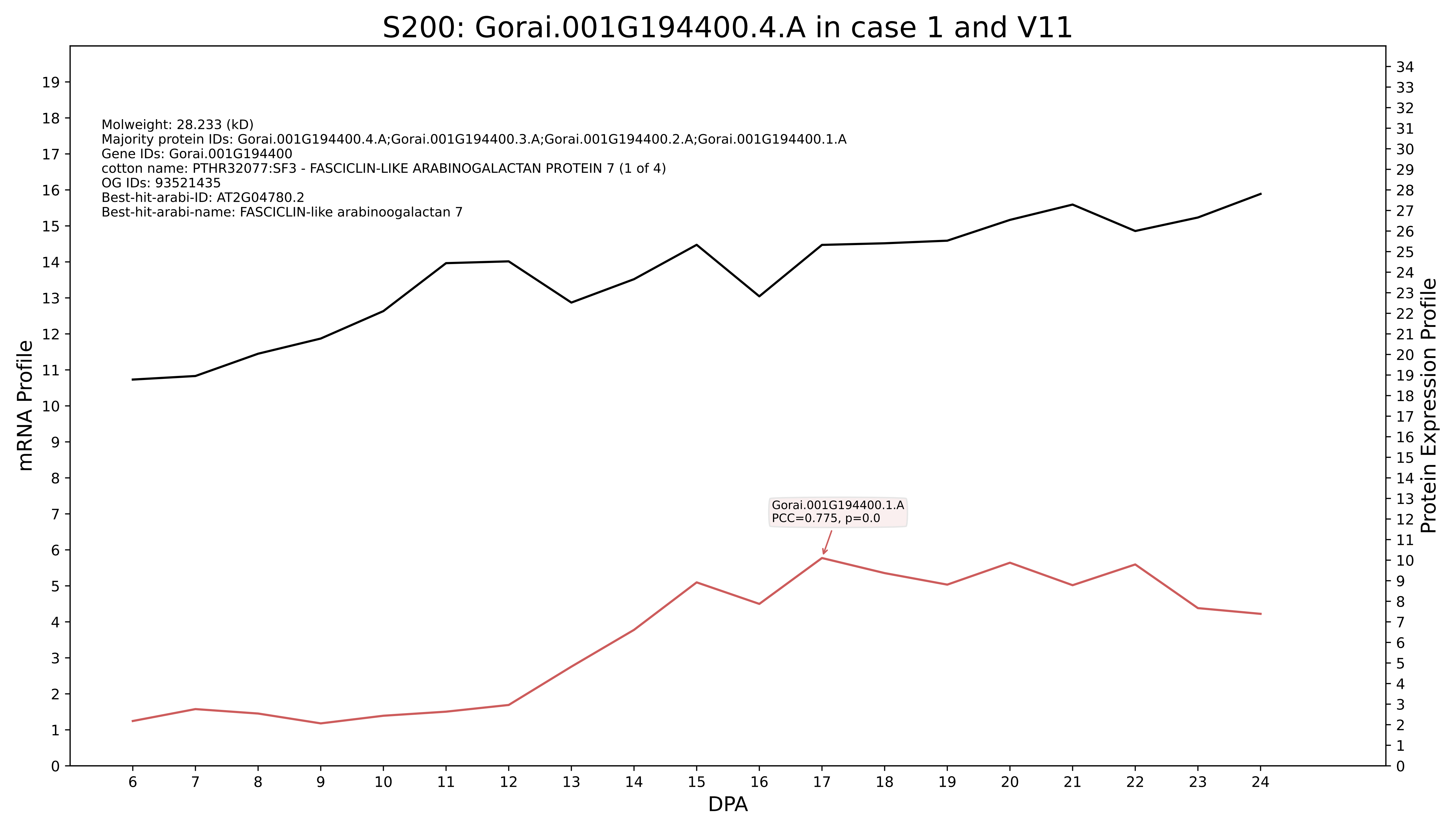

S200: Gorai.001G194400.4.A in case 1 and V11
Molweight: 28.233 (kD)
Majority protein IDs: Gorai.001G194400.4.A;Gorai.001G194400.3.A;Gorai.001G194400.2.A;Gorai.001G194400.1.A
Gene IDs: Gorai.001G194400
cotton name: PTHR32077:SF3 - FASCICLIN-LIKE ARABINOGALACTAN PROTEIN 7 (1 of 4)
OG IDs: 93521435
Best-hit-arabi-ID: AT2G04780.2
Best-hit-arabi-name: FASCICLIN-like arabinoogalactan 7
Gorai.001G194400.1.A
PCC=0.775, p=0.0
mRNA Profile
Protein Expression Profile
DPA
0
1
2
3
4
5
6
7
8
9
10
11
12
13
14
15
16
17
18
19
20
21
22
23
24
25
26
27
28
29
30
31
32
33
34

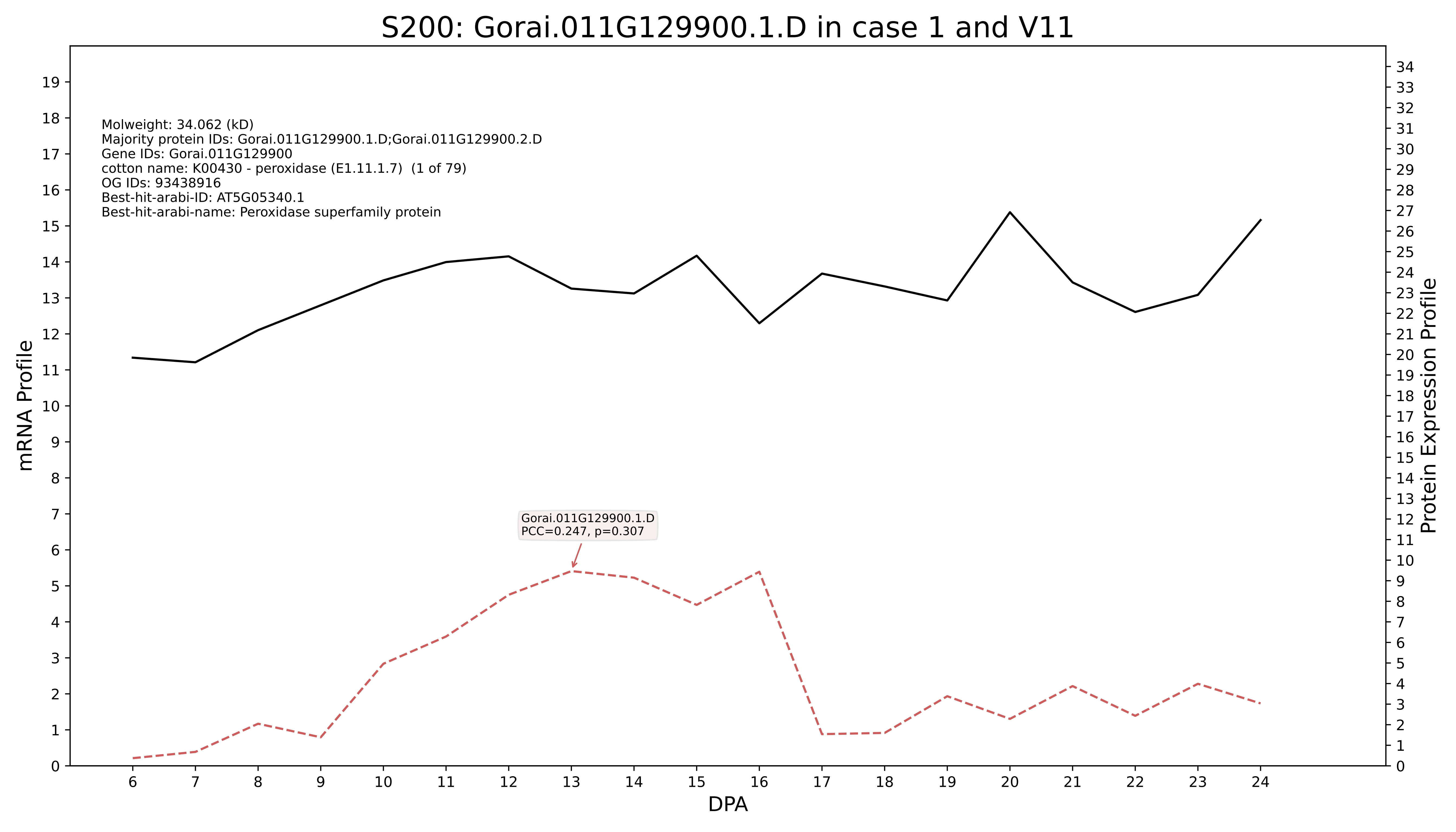
S200: Gorai.011G129900.1.D in case 1 and V11
Molweight: 34.062 (kD)
Majority protein IDs: Gorai.011G129900.1.D;Gorai.011G129900.2.D
Gene IDs: Gorai.011G129900
cotton name: K00430 - peroxidase (E1.11.1.7) (1 of 79)
OG IDs: 93438916
Best-hit-arabi-ID: AT5G05340.1
Best-hit-arabi-name: Peroxidase superfamily protein
Gorai.011G129900.1.D
PCC=0.247, p=0.307
mRNA Profile
Protein Expression Profile
DPA

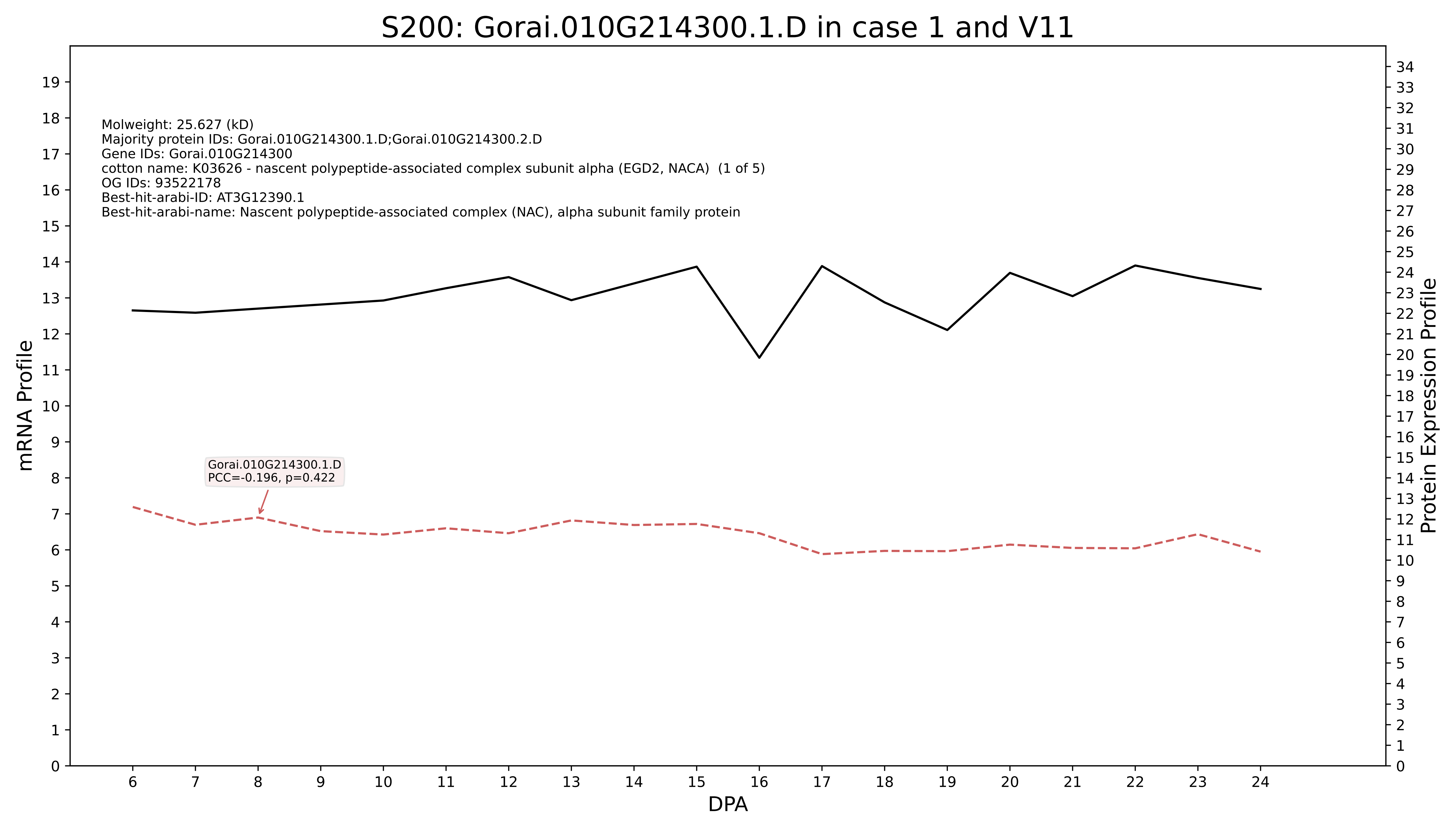
S200: Gorai.010G214300.1.D in case 1 and V11
Molweight: 25.627 (kD)
Majority protein IDs: Gorai.010G214300.1.D;Gorai.010G214300.2.D
Gene IDs: Gorai.010G214300
cotton name: K03626 - nascent polypeptide-associated complex subunit alpha (EGD2, NACA) (1 of 5)
OG IDs: 93522178
Best-hit-arabi-ID: AT3G12390.1
Best-hit-arabi-name: Nascent polypeptide-associated complex (NAC), alpha subunit family protein
Gorai.010G214300.1.D
PCC=-0.196, p=0.422
mRNA Profile
Protein Expression Profile
DPA

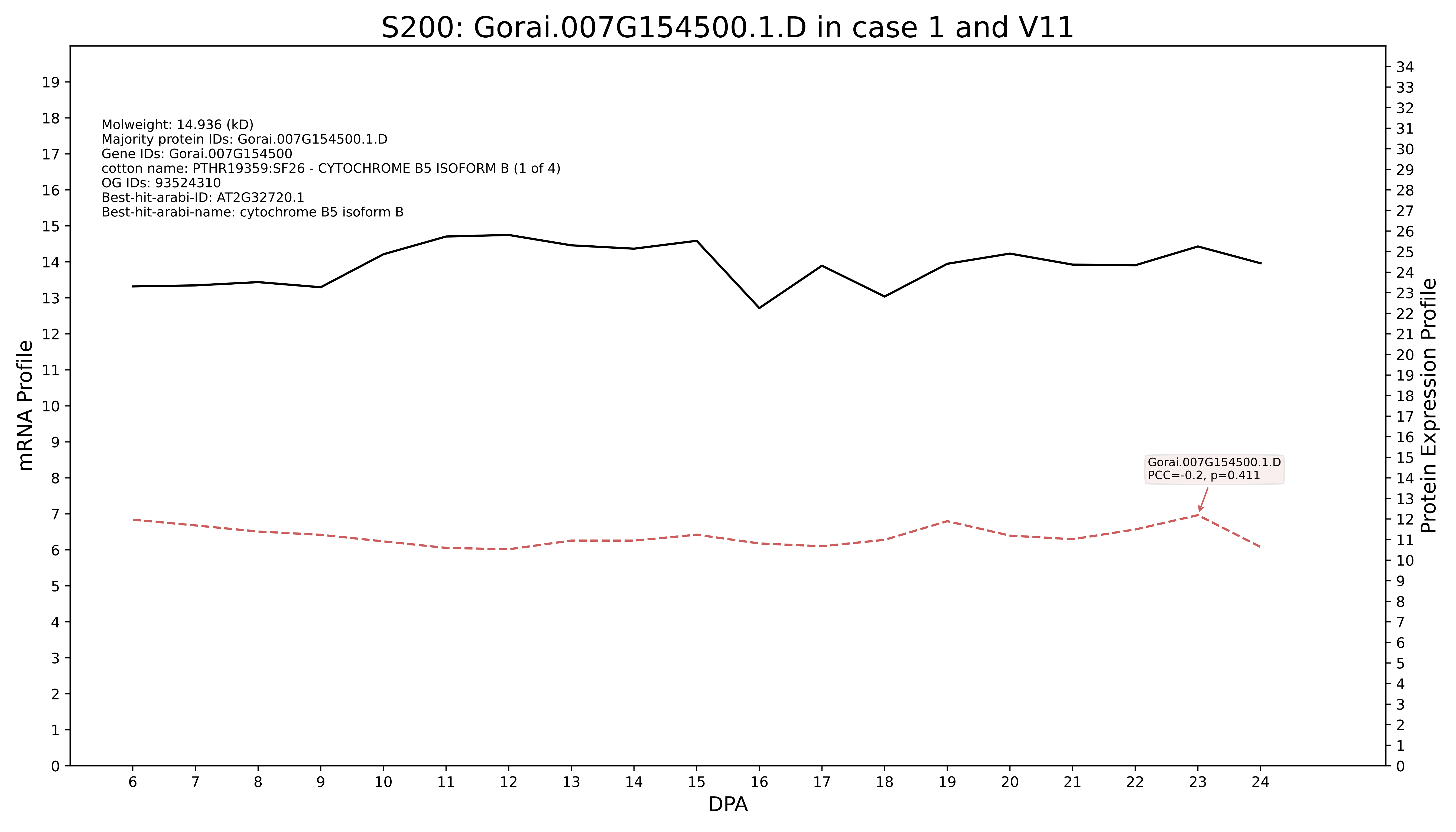

S200: Gorai.007G154500.1.D in case 1 and V11
Molweight: 14.936 (kD)
Majority protein IDs: Gorai.007G154500.1.D
Gene IDs: Gorai.007G154500
cotton name: PTHR19359:SF26 - CYTOCHROME B5 ISOFORM B (1 of 4)
OG IDs: 93524310
Best-hit-arabi-ID: AT2G32720.1
Best-hit-arabi-name: cytochrome B5 isoform B
Gorai.007G154500.1.D
PCC=-0.2, p=0.411
mRNA Profile
Protein Expression Profile
DPA

S200: Gorai.001G219000.2.A in case 1 and V11

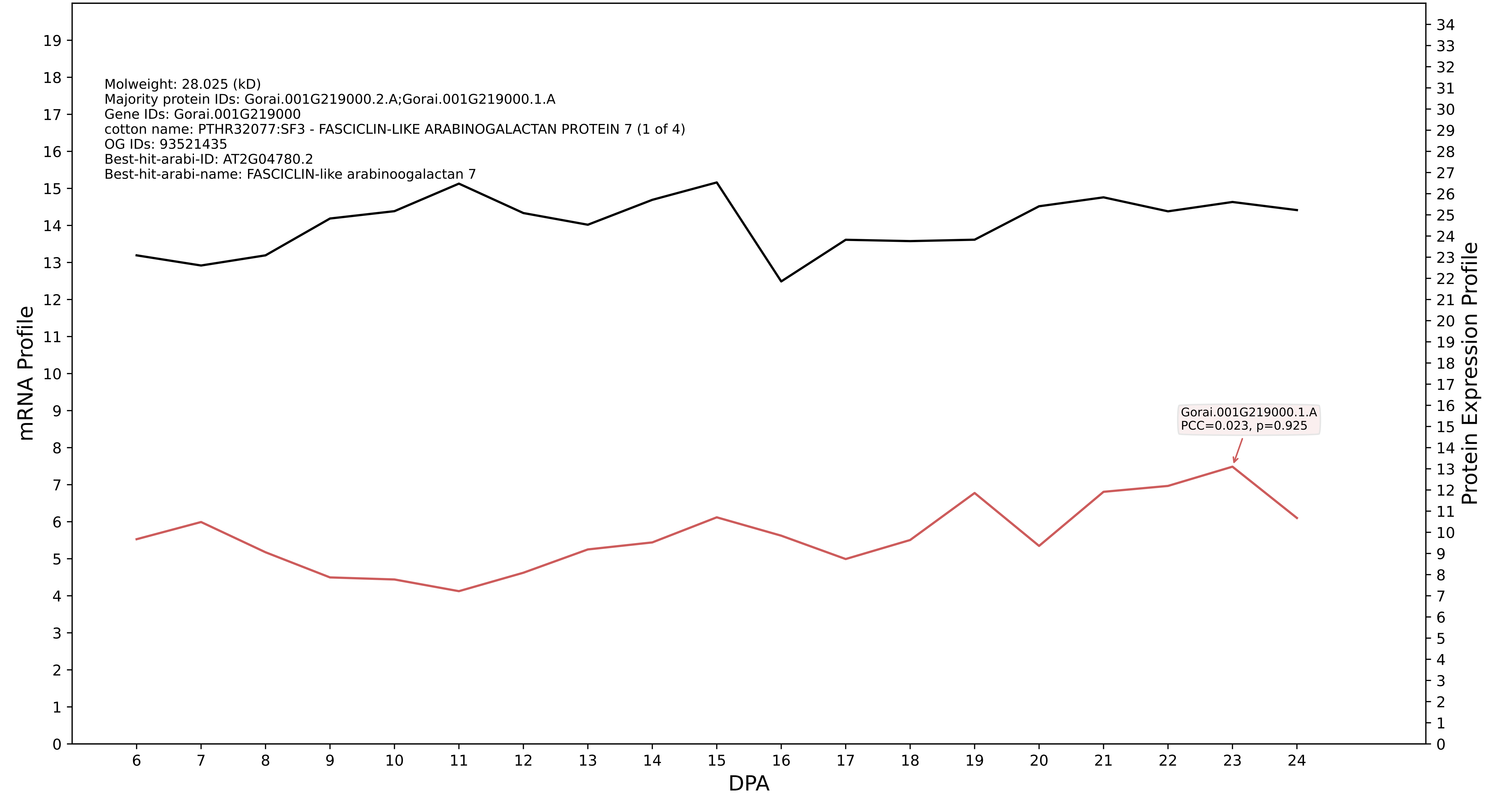

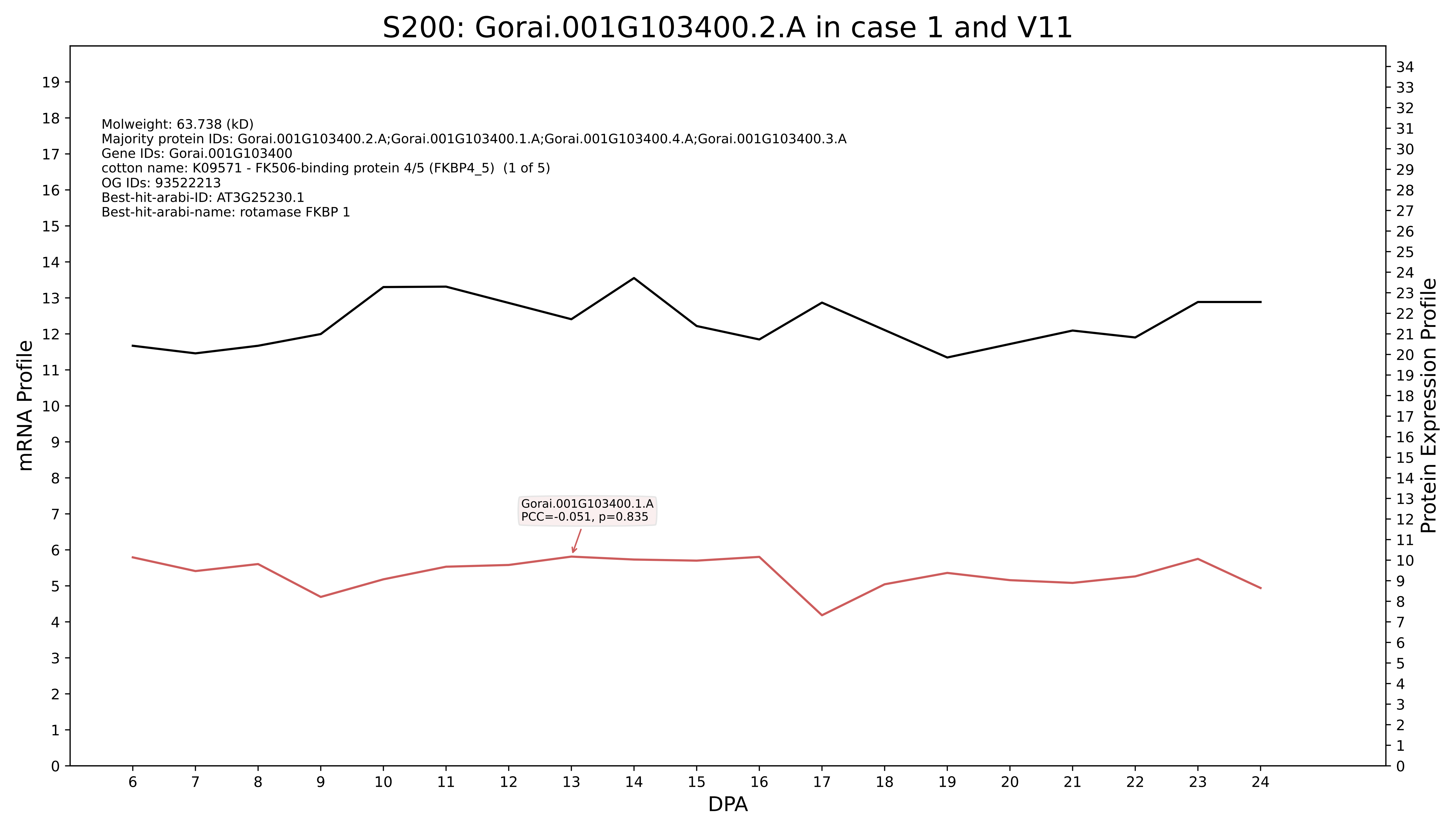
S200: Gorai.001G103400.2.A in case 1 and V11
Molweight: 63.738 (kD)
Majority protein IDs: Gorai.001G103400.2.A;Gorai.001G103400.1.A;Gorai.001G103400.4.A;Gorai.001G103400.3.A
Gene IDs: Gorai.001G103400
cotton name: K09571 - FK506-binding protein 4/5 (FKBP4_5) (1 of 5)
OG IDs: 93522213
Best-hit-arabi-ID: AT3G25230.1
Best-hit-arabi-name: rotamase FKBP 1
Gorai.001G103400.1.A
PCC=-0.051, p=0.835
mRNA Profile
Protein Expression Profile
DPA

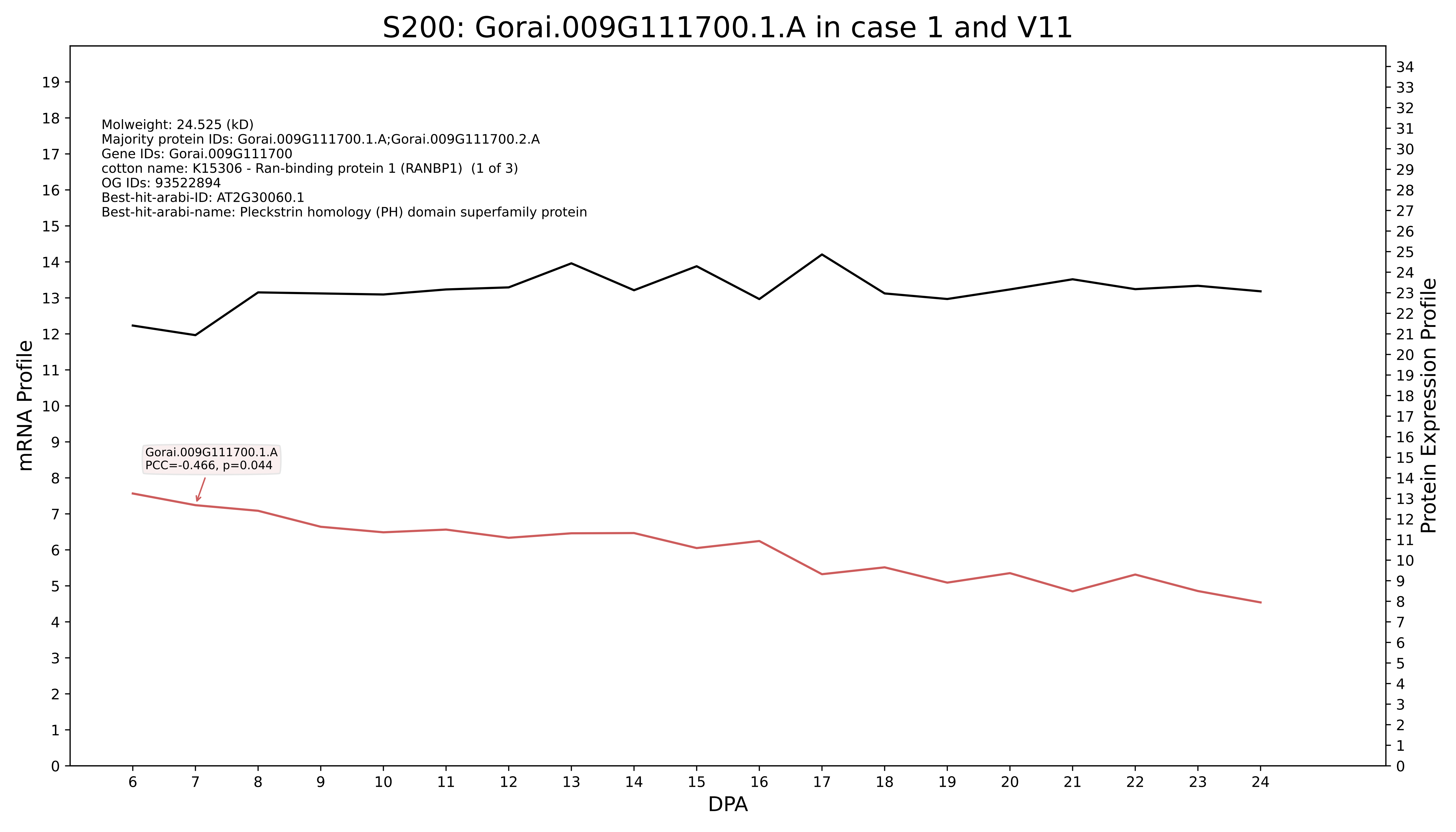
S200: Gorai.009G111700.1.A in case 1 and V11
Molweight: 24.525 (kD)
Majority protein IDs: Gorai.009G111700.1.A;Gorai.009G111700.2.A
Gene IDs: Gorai.009G111700
cotton name: K15306 - Ran-binding protein 1 (RANBP1) (1 of 3)
OG IDs: 93522894
Best-hit-arabi-ID: AT2G30060.1
Best-hit-arabi-name: Pleckstrin homology (PH) domain superfamily protein
Gorai.009G111700.1.A
PCC=-0.466, p=0.044
mRNA Profile
Protein Expression Profile
DPA

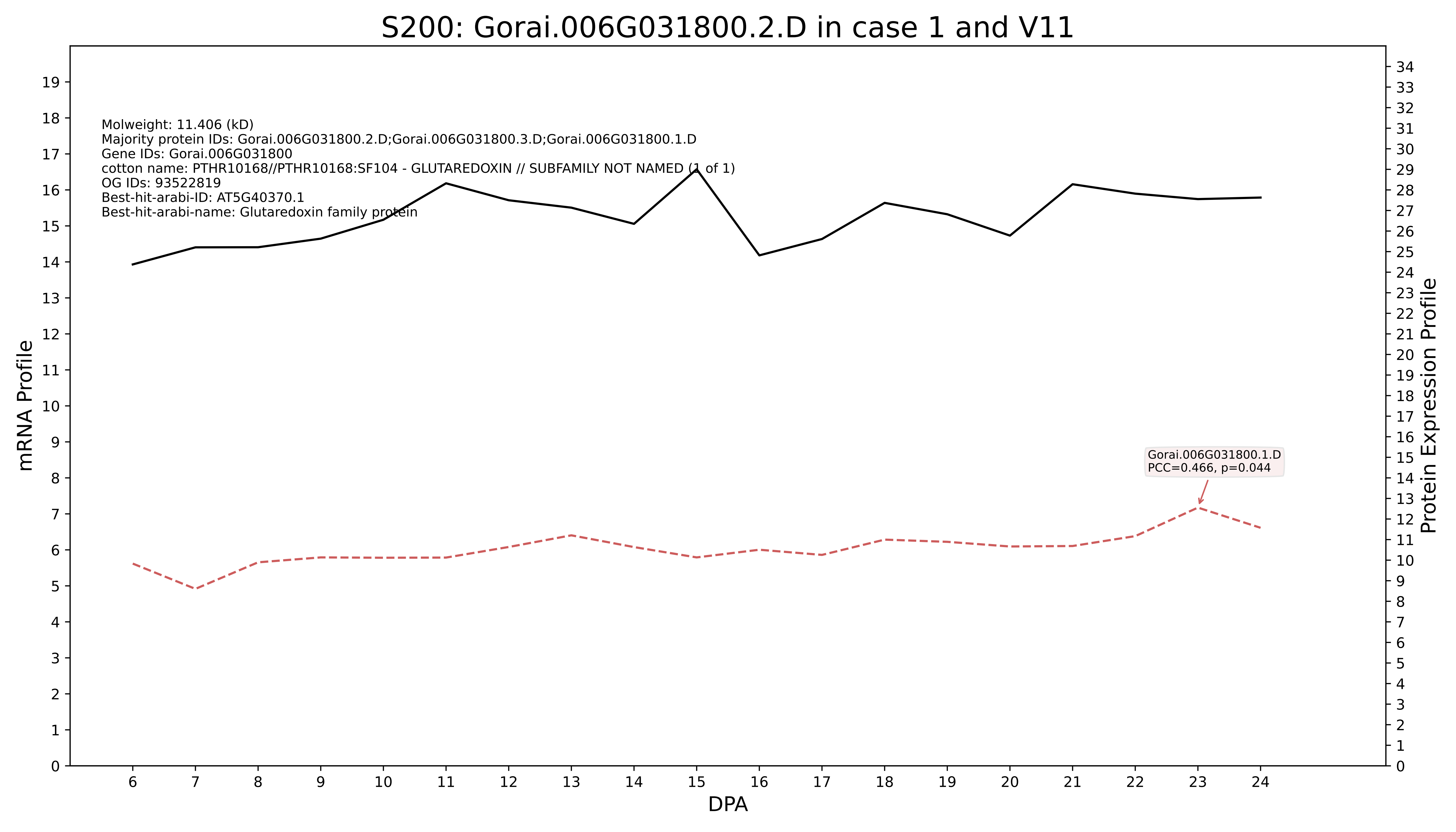
S200: Gorai.006G031800.2.D in case 1 and V11
Molweight: 11.406 (kD)
Majority protein IDs: Gorai.006G031800.2.D;Gorai.006G031800.3.D;Gorai.006G031800.1.D
Gene IDs: Gorai.006G031800
cotton name: PTHR10168//PTHR10168:SF104 - GLUTAREDOXIN // SUBFAMILY NOT NAMED (1 of 1)
OG IDs: 93522819
Best-hit-arabi-ID: AT5G40370.1
Best-hit-arabi-name: Glutaredoxin family protein
Gorai.006G031800.1.D
PCC=0.466, p=0.044
mRNA Profile
Protein Expression Profile
DPA

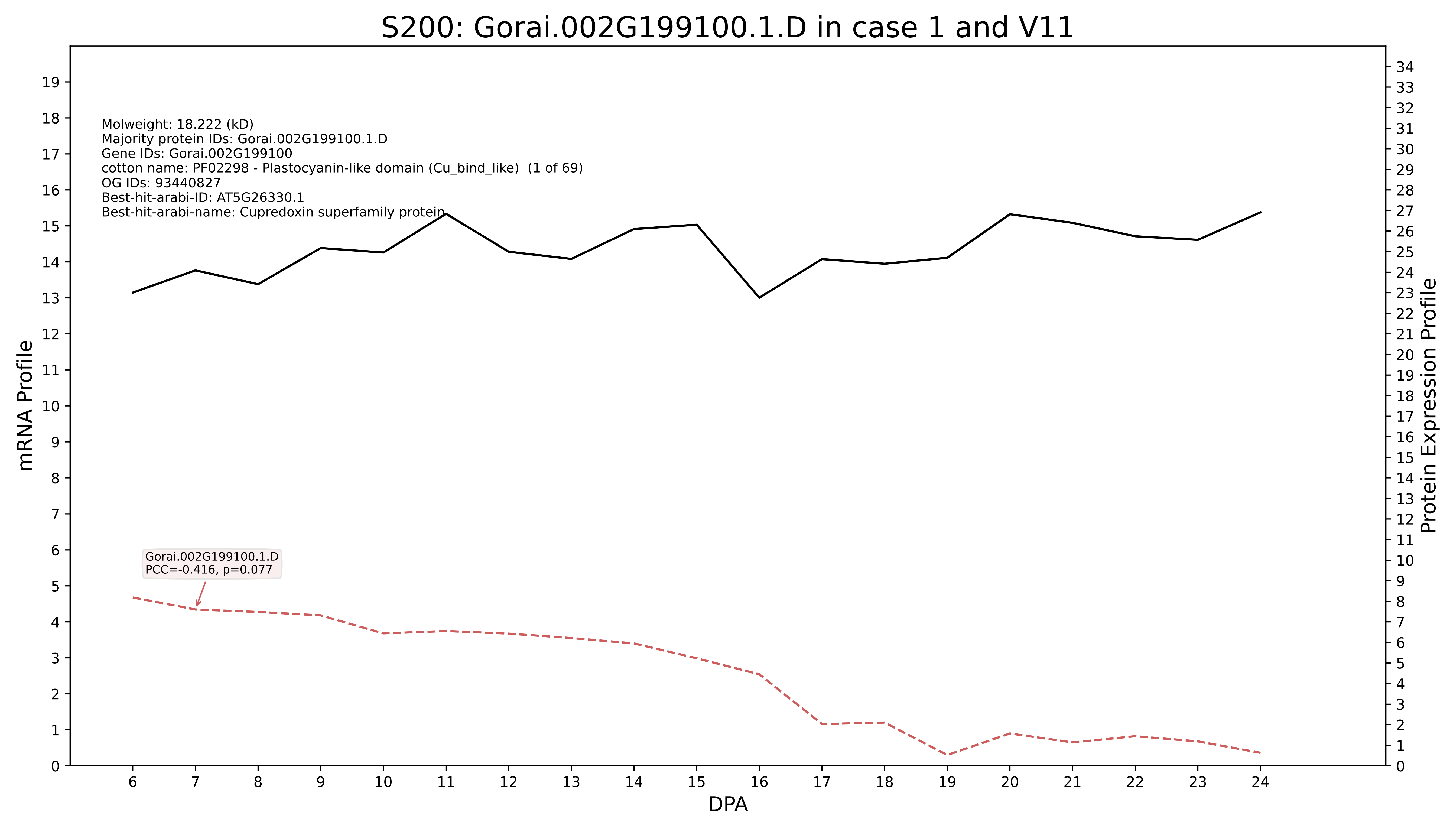
S200: Gorai.002G199100.1.D in case 1 and V11
Molweight: 18.222 (kD)
Majority protein IDs: Gorai.002G199100.1.D
Gene IDs: Gorai.002G199100
cotton name: PF02298 - Plastocyanin-like domain (Cu_bind_like) (1 of 69)
OG IDs: 93440827
Best-hit-arabi-ID: AT5G26330.1
Best-hit-arabi-name: Cupredoxin superfamily protein
Gorai.002G199100.1.D
PCC=-0.416, p=0.077
mRNA Profile
Protein Expression Profile
DPA
0
1
2
3
4
5
6
7
8
9
10
11
12
13
14
15
16
17
18
19
20
21
22
23
24
25
26
27
28
29
30
31
32
33
34

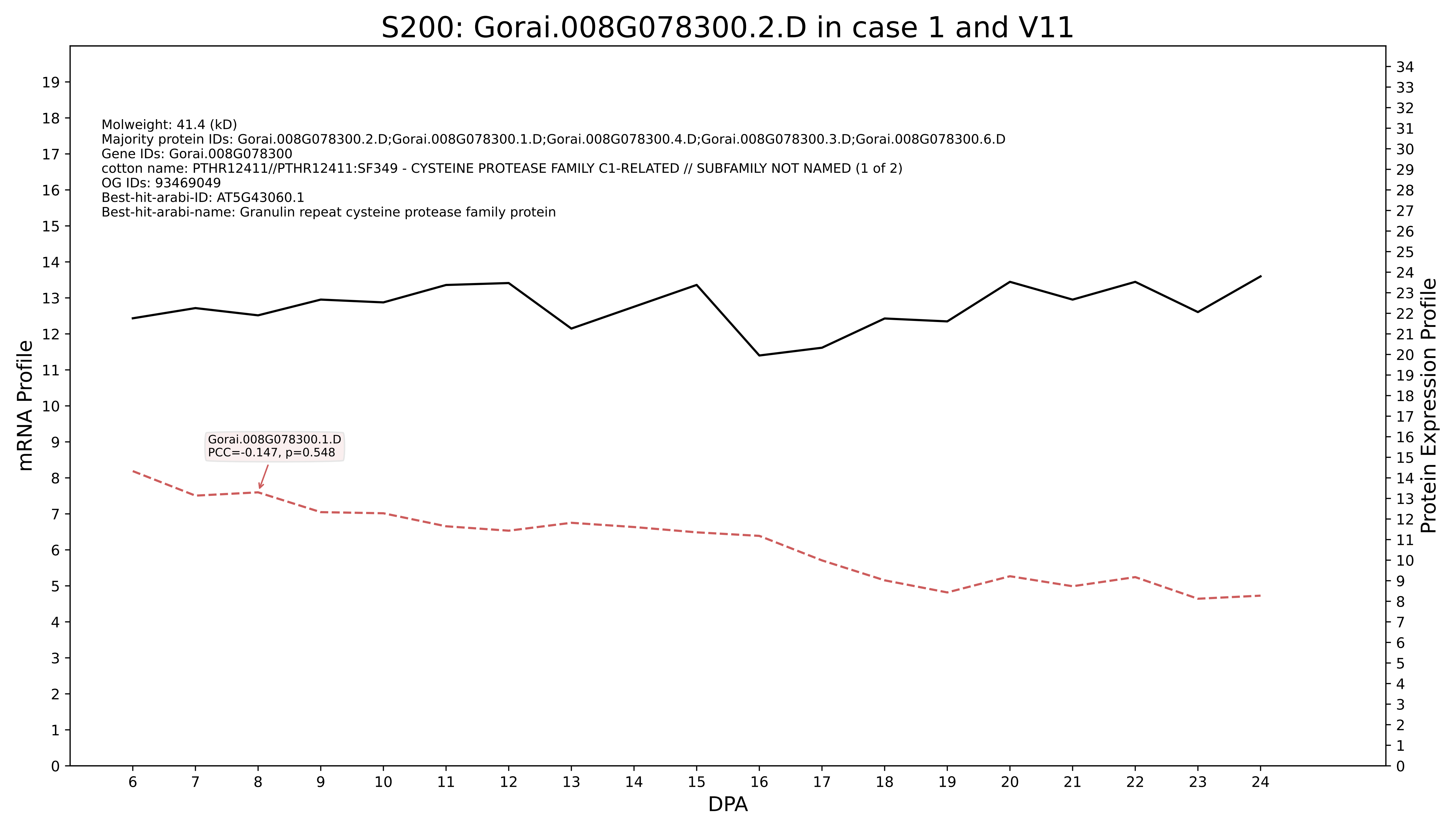
S200: Gorai.008G078300.2.D in case 1 and V11
Molweight: 41.4 (kD)
Majority protein IDs: Gorai.008G078300.2.D;Gorai.008G078300.1.D;Gorai.008G078300.4.D;Gorai.008G078300.3.D;Gorai.008G078300.6.D
Gene IDs: Gorai.008G078300
cotton name: PTHR12411//PTHR12411:SF349 - CYSTEINE PROTEASE FAMILY C1-RELATED // SUBFAMILY NOT NAMED (1 of 2)
OG IDs: 93469049
Best-hit-arabi-ID: AT5G43060.1
Best-hit-arabi-name: Granulin repeat cysteine protease family protein
Gorai.008G078300.1.D
PCC=-0.147, p=0.548
mRNA Profile
Protein Expression Profile
DPA

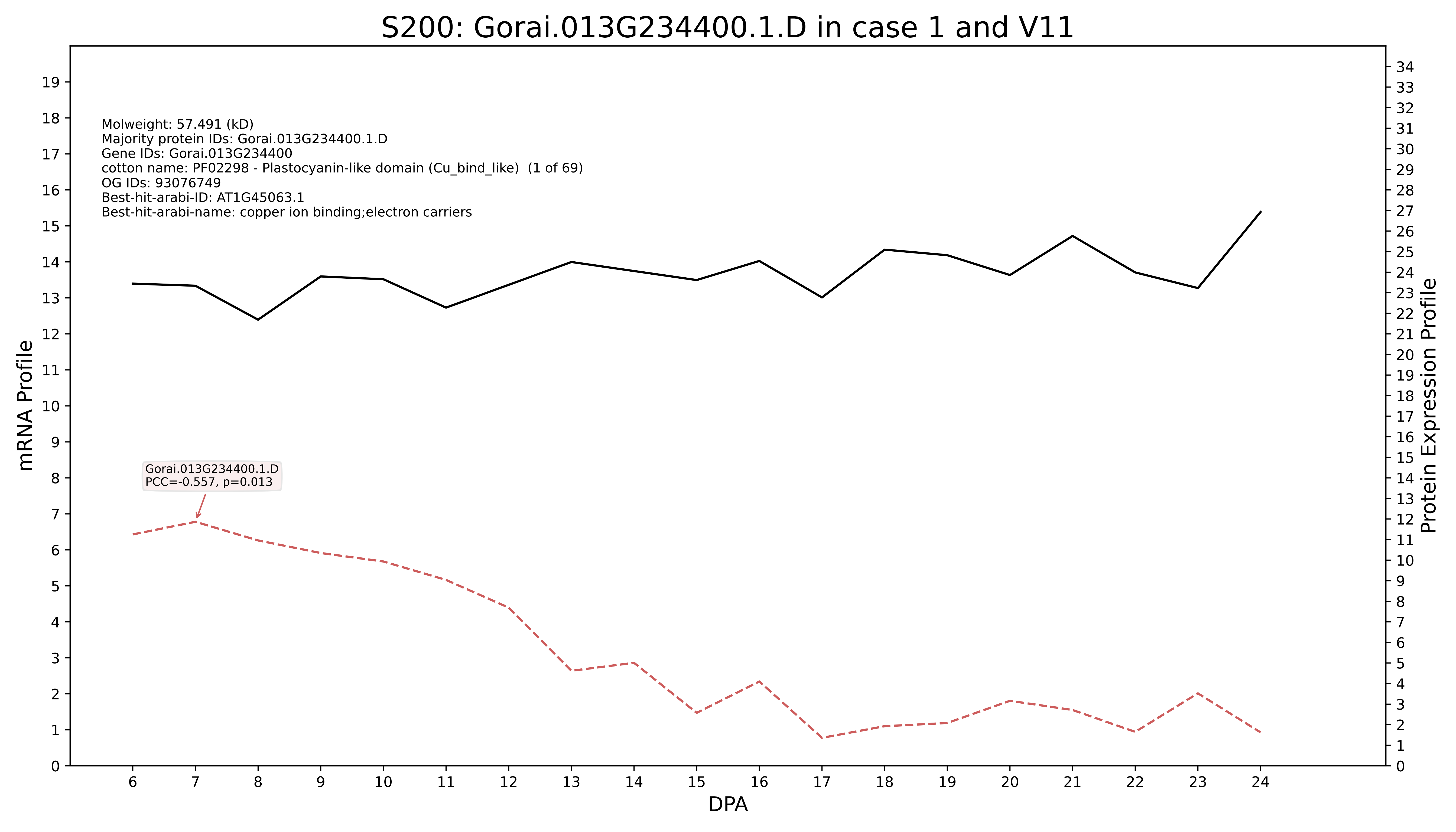
S200: Gorai.013G234400.1.D in case 1 and V11
Molweight: 57.491 (kD)
Majority protein IDs: Gorai.013G234400.1.D
Gene IDs: Gorai.013G234400
cotton name: PF02298 - Plastocyanin-like domain (Cu_bind_like) (1 of 69)
OG IDs: 93076749
Best-hit-arabi-ID: AT1G45063.1
Best-hit-arabi-name: copper ion binding;electron carriers
Gorai.013G234400.1.D
PCC=-0.557, p=0.013
mRNA Profile
Protein Expression Profile
DPA
0
1
2
3
4
5
6
7
8
9
10
11
12
13
14
15
16
17
18
19
20
21
22
23
24
25
26
27
28
29
30
31
32
33
34

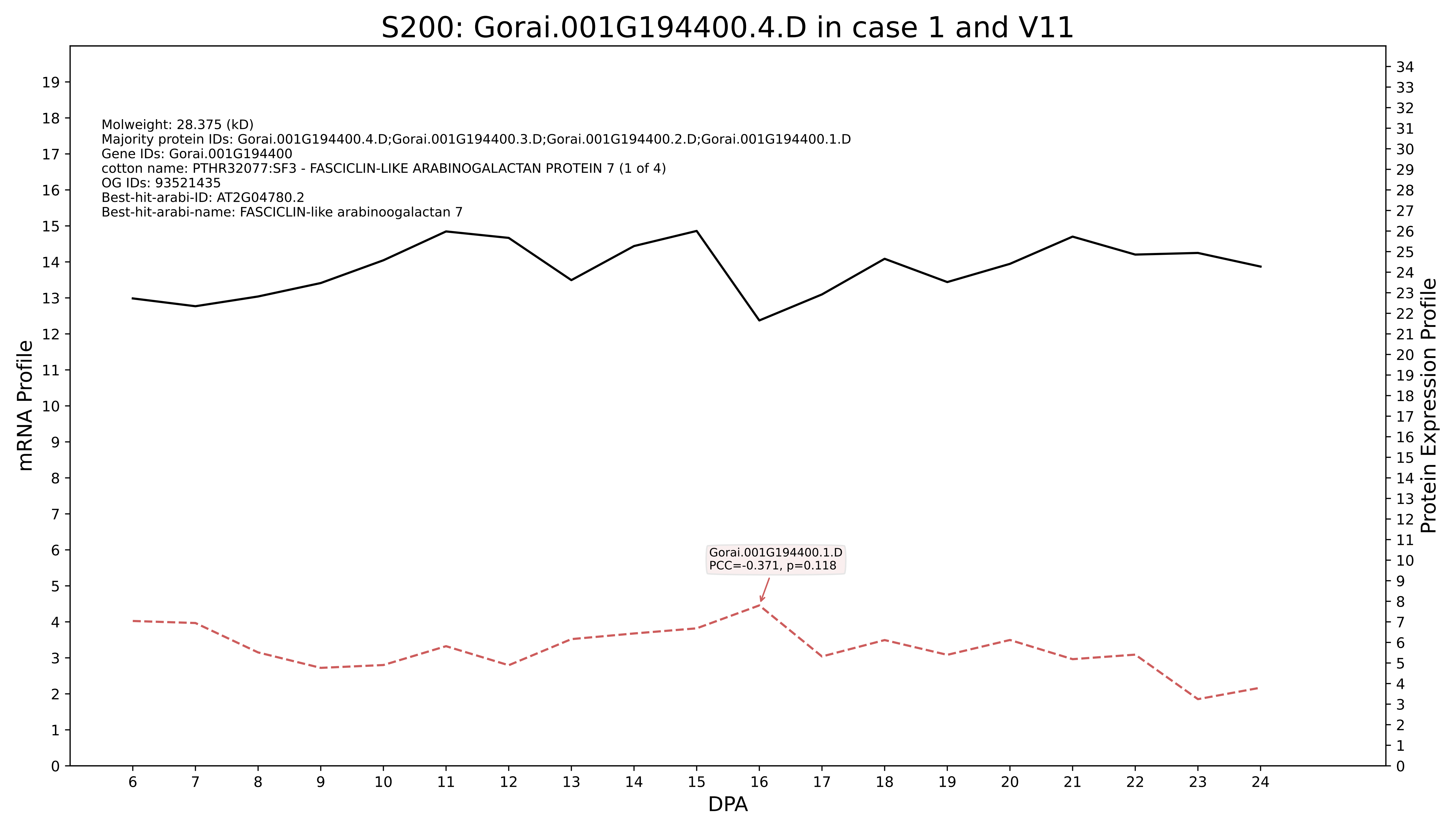
S200: Gorai.001G194400.4.D in case 1 and V11
Molweight: 28.375 (kD)
Majority protein IDs: Gorai.001G194400.4.D;Gorai.001G194400.3.D;Gorai.001G194400.2.D;Gorai.001G194400.1.D
Gene IDs: Gorai.001G194400
cotton name: PTHR32077:SF3 - FASCICLIN-LIKE ARABINOGALACTAN PROTEIN 7 (1 of 4)
OG IDs: 93521435
Best-hit-arabi-ID: AT2G04780.2
Best-hit-arabi-name: FASCICLIN-like arabinoogalactan 7
Gorai.001G194400.1.D
PCC=-0.371, p=0.118
mRNA Profile
Protein Expression Profile
DPA

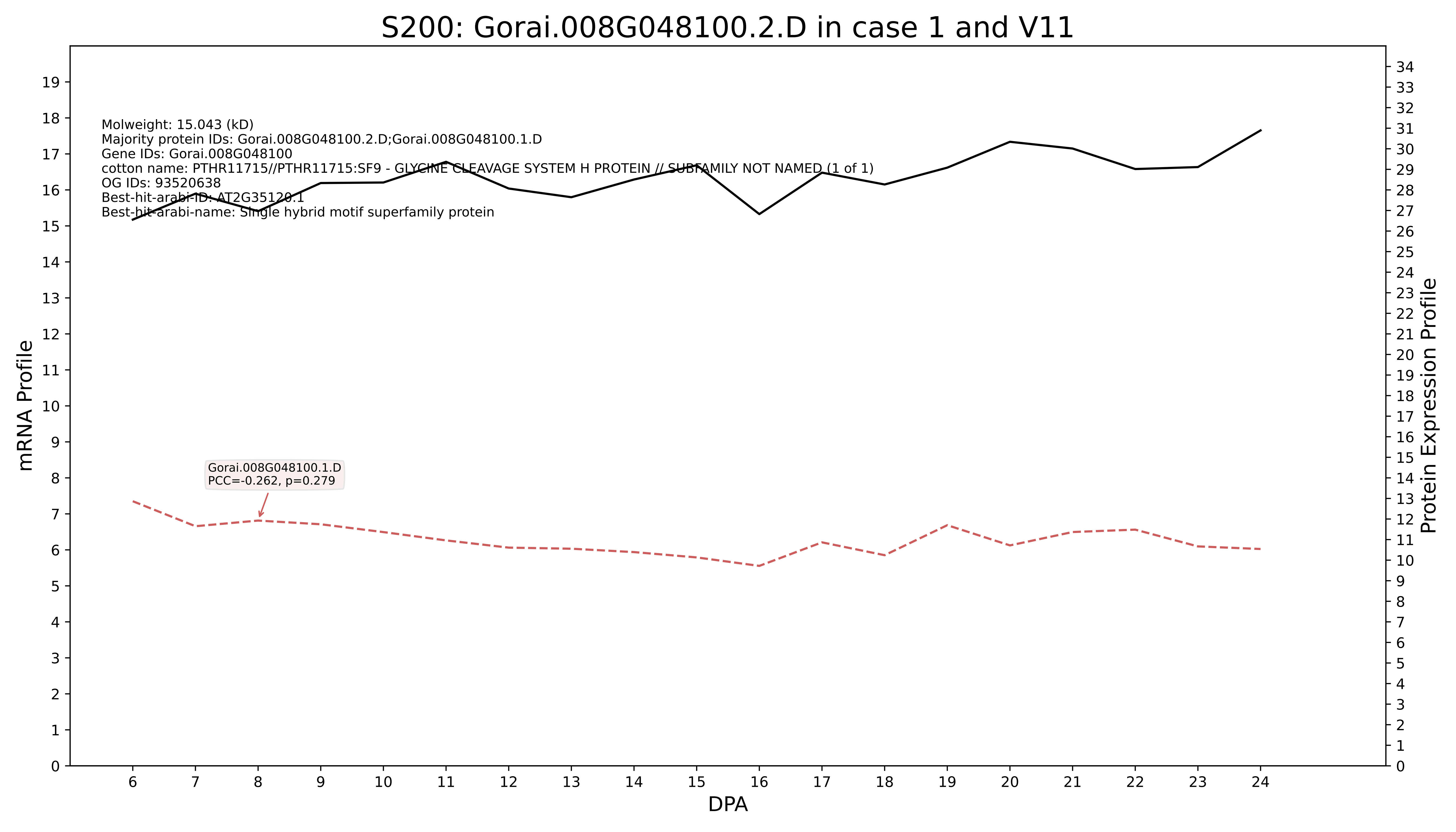

S200: Gorai.008G048100.2.D in case 1 and V11
Molweight: 15.043 (kD)
Majority protein IDs: Gorai.008G048100.2.D;Gorai.008G048100.1.D
Gene IDs: Gorai.008G048100
cotton name: PTHR11715//PTHR11715:SF9 - GLYCINE CLEAVAGE SYSTEM H PROTEIN // SUBFAMILY NOT NAMED (1 of 1)
OG IDs: 93520638
Best-hit-arabi-ID: AT2G35120.1
Best-hit-arabi-name: Single hybrid motif superfamily protein
Gorai.008G048100.1.D
PCC=-0.262, p=0.279
mRNA Profile
Protein Expression Profile
DPA

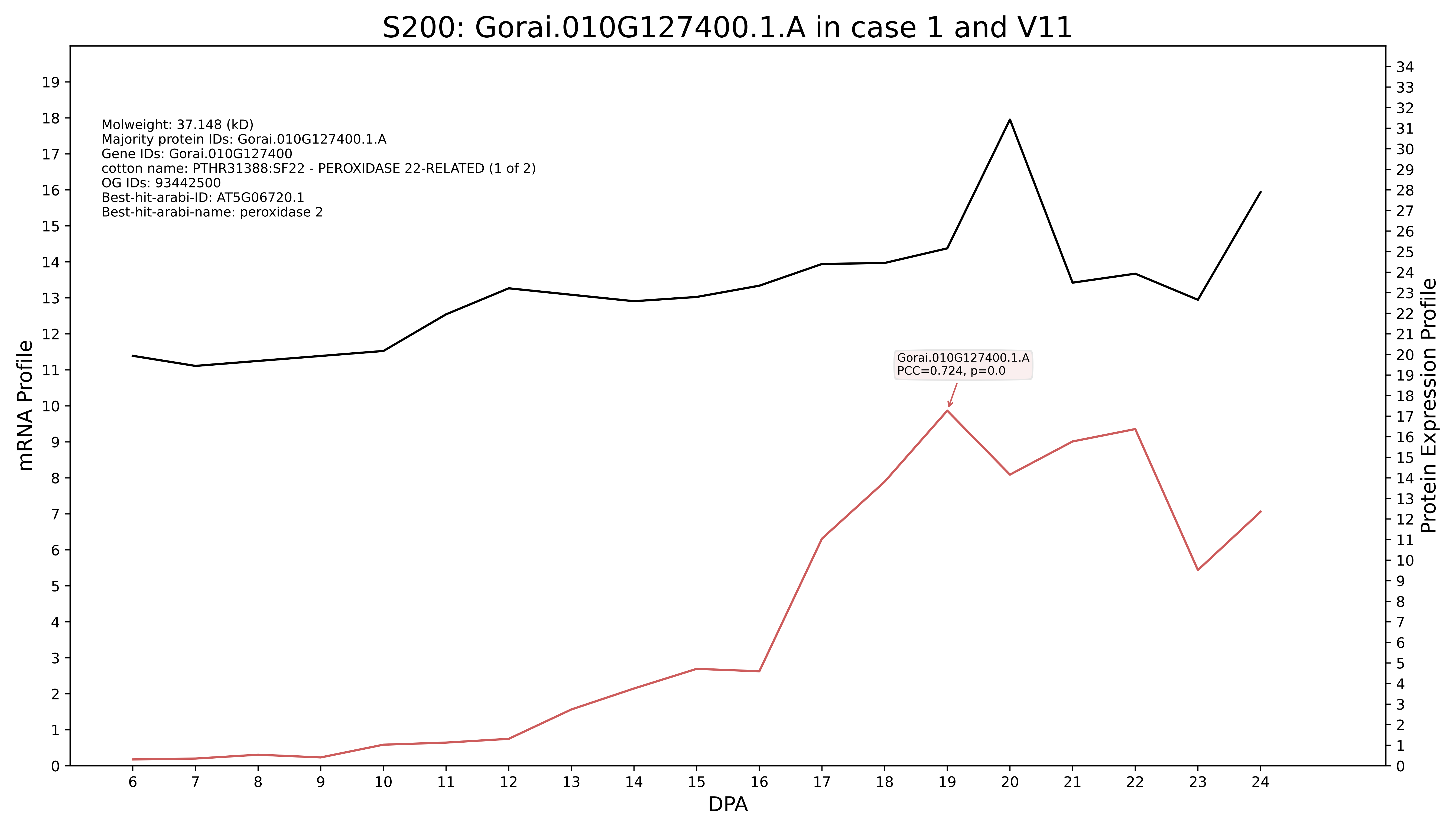

S200: Gorai.010G127400.1.A in case 1 and V11
Molweight: 37.148 (kD)
Majority protein IDs: Gorai.010G127400.1.A
Gene IDs: Gorai.010G127400
cotton name: PTHR31388:SF22 - PEROXIDASE 22-RELATED (1 of 2)
OG IDs: 93442500
Best-hit-arabi-ID: AT5G06720.1
Best-hit-arabi-name: peroxidase 2
Gorai.010G127400.1.A
PCC=0.724, p=0.0
mRNA Profile
Protein Expression Profile
DPA

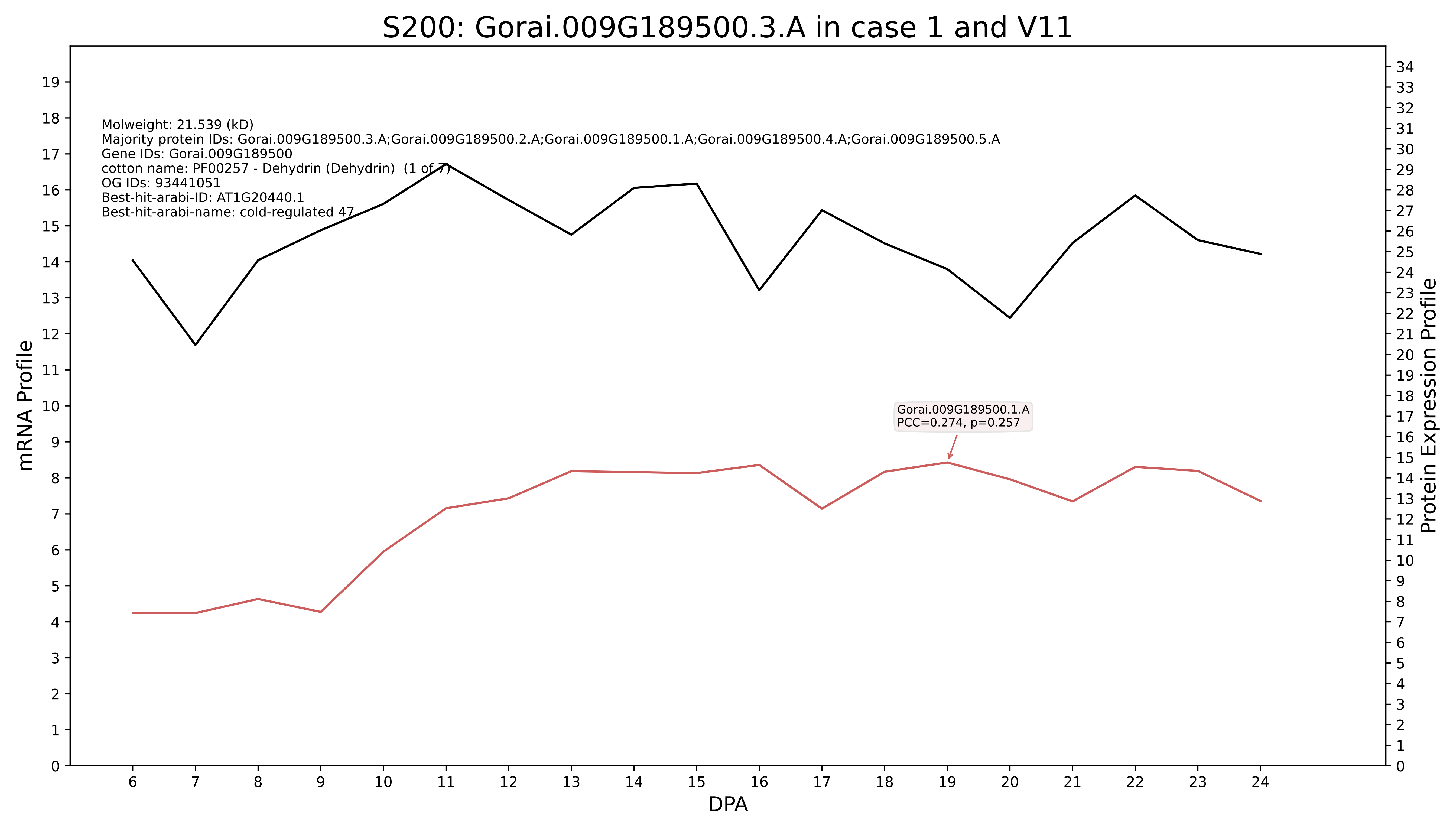
S200: Gorai.009G189500.3.A in case 1 and V11
Molweight: 21.539 (kD)
Majority protein IDs: Gorai.009G189500.3.A;Gorai.009G189500.2.A;Gorai.009G189500.1.A;Gorai.009G189500.4.A;Gorai.009G189500.5.A
Gene IDs: Gorai.009G189500
cotton name: PF00257 - Dehydrin (Dehydrin) (1 of 7)
OG IDs: 93441051
Best-hit-arabi-ID: AT1G20440.1
Best-hit-arabi-name: cold-regulated 47
Gorai.009G189500.1.A
PCC=0.274, p=0.257
mRNA Profile
Protein Expression Profile
DPA

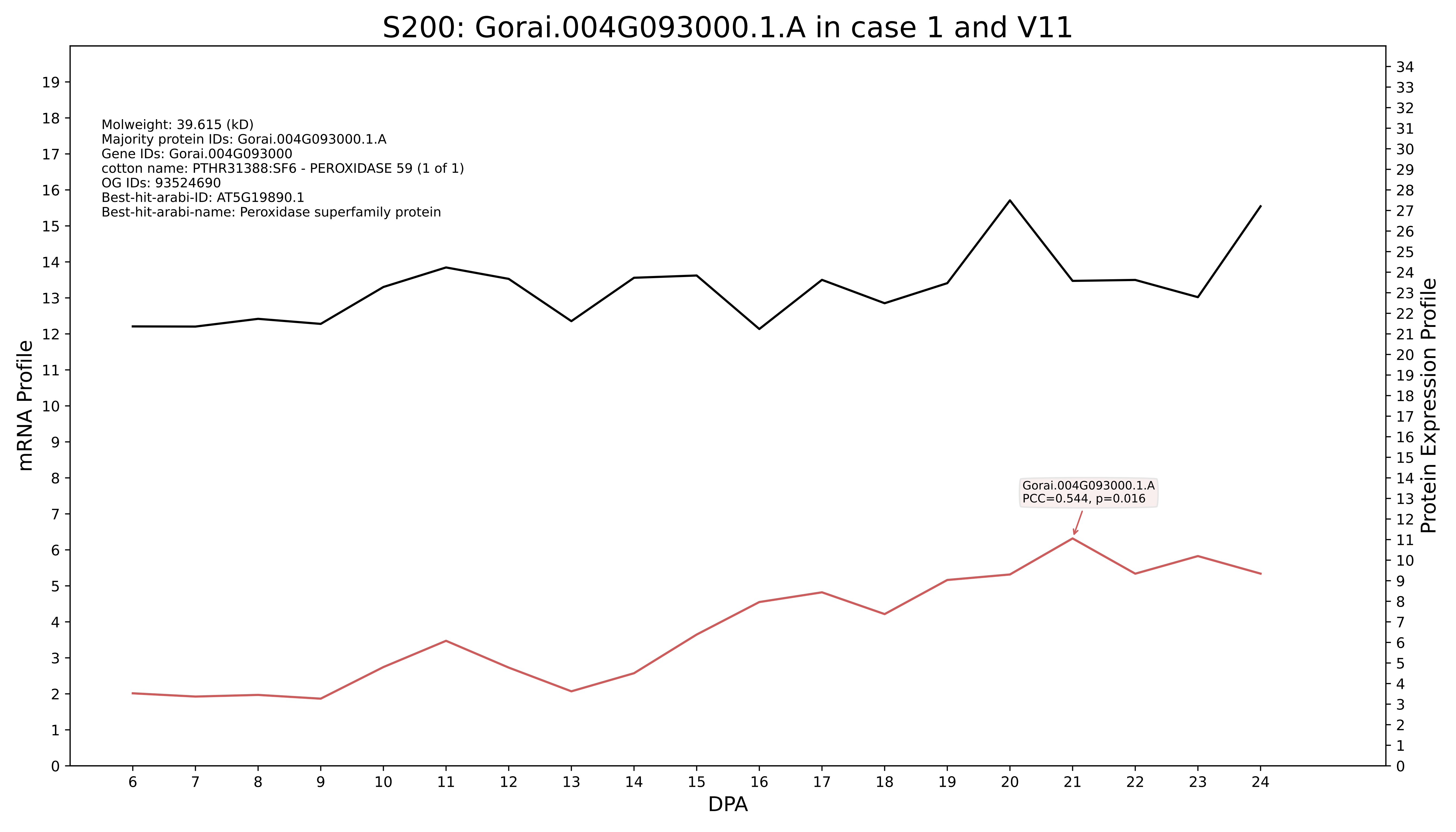

S200: Gorai.004G093000.1.A in case 1 and V11
Molweight: 39.615 (kD)
Majority protein IDs: Gorai.004G093000.1.A
Gene IDs: Gorai.004G093000
cotton name: PTHR31388:SF6 - PEROXIDASE 59 (1 of 1)
OG IDs: 93524690
Best-hit-arabi-ID: AT5G19890.1
Best-hit-arabi-name: Peroxidase superfamily protein
Gorai.004G093000.1.A
PCC=0.544, p=0.016
mRNA Profile
Protein Expression Profile
DPA

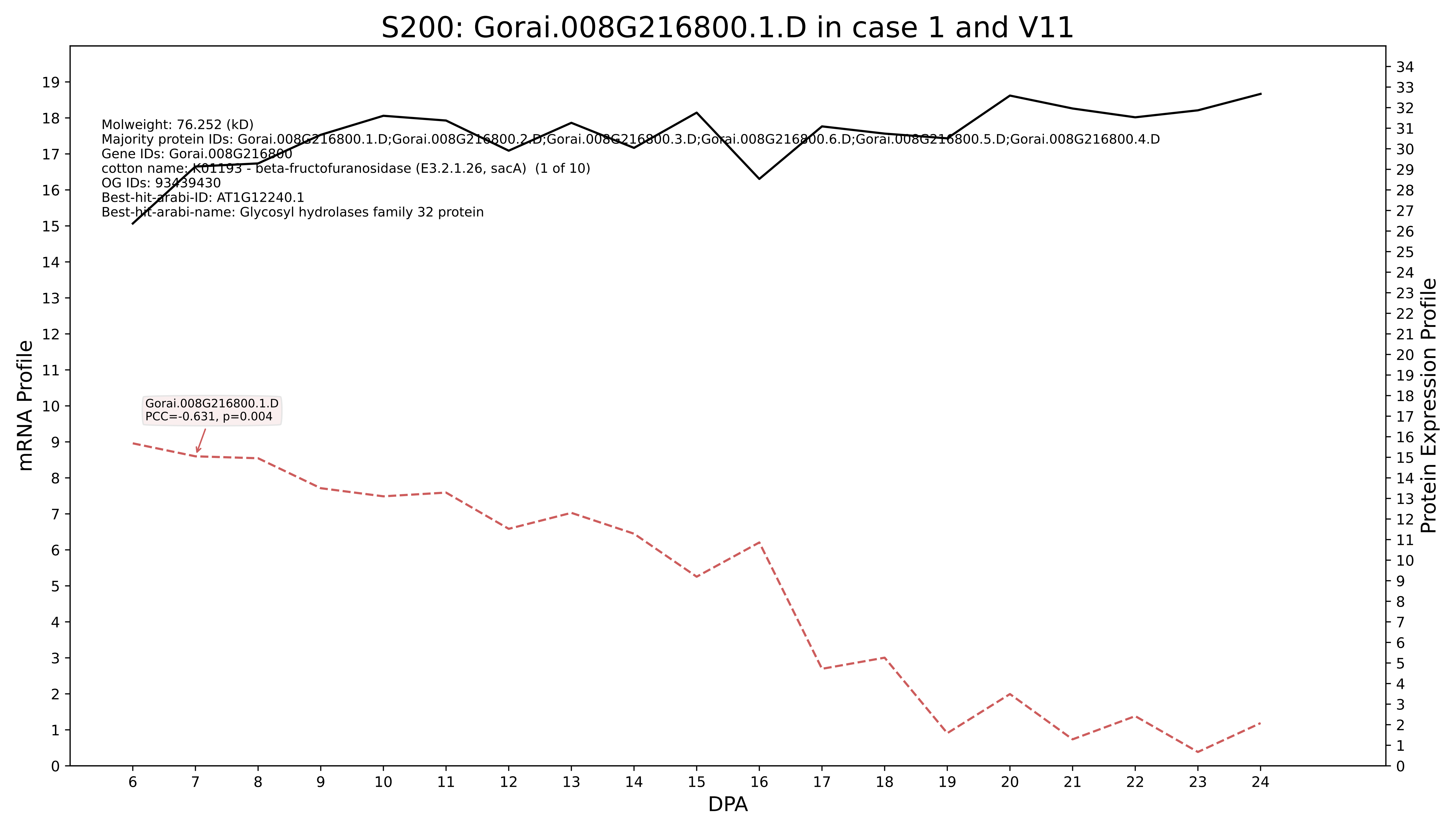
S200: Gorai.008G216800.1.D in case 1 and V11
Molweight: 76.252 (kD)
Majority protein IDs: Gorai.008G216800.1.D;Gorai.008G216800.2.D;Gorai.008G216800.3.D;Gorai.008G216800.6.D;Gorai.008G216800.5.D;Gorai.008G216800.4.D
Gene IDs: Gorai.008G216800
cotton name: K01193 - beta-fructofuranosidase (E3.2.1.26, sacA) (1 of 10)
OG IDs: 93439430
Best-hit-arabi-ID: AT1G12240.1
Best-hit-arabi-name: Glycosyl hydrolases family 32 protein
Gorai.008G216800.1.D
PCC=-0.631, p=0.004
mRNA Profile
Protein Expression Profile
DPA

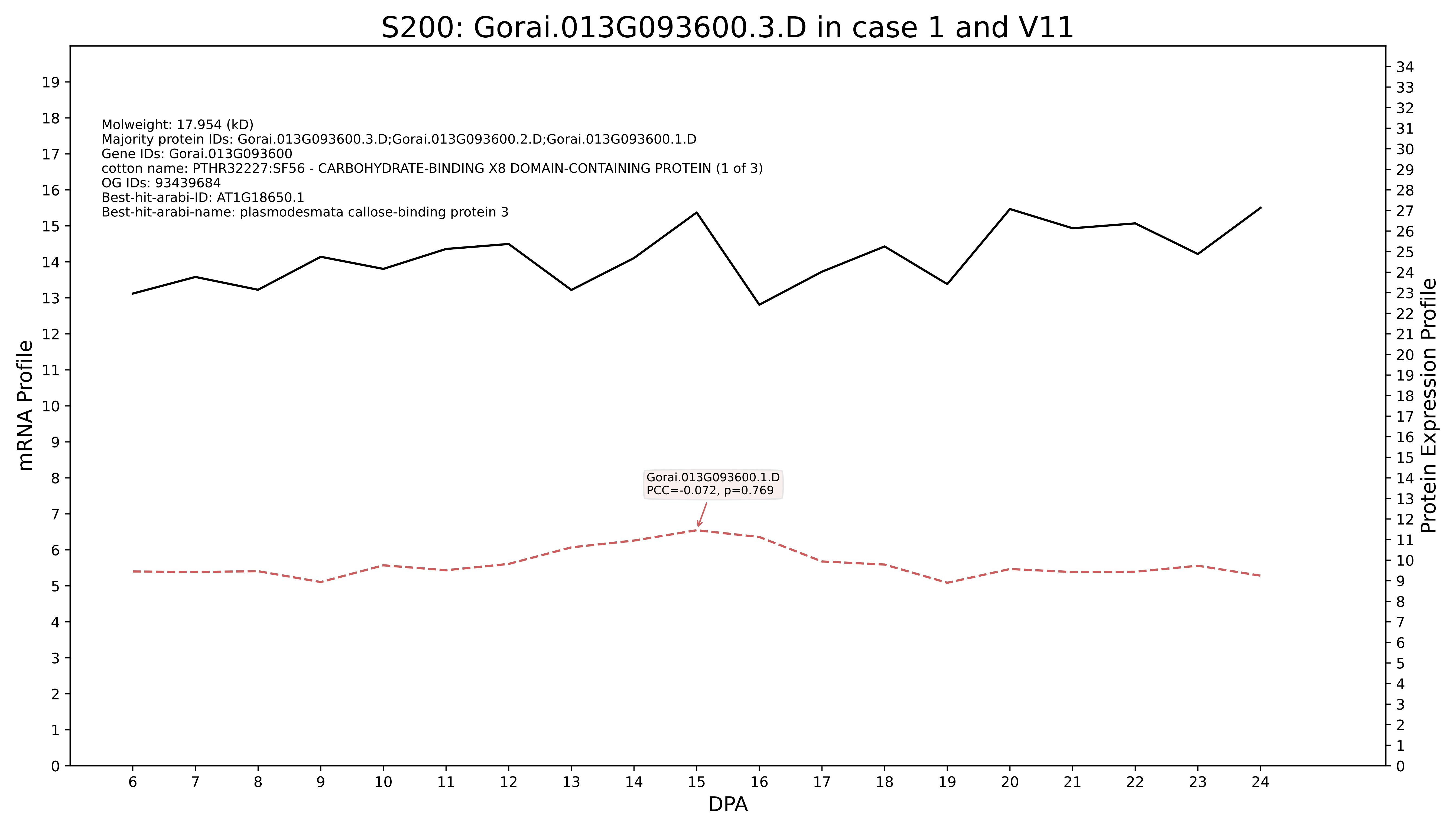
S200: Gorai.013G093600.3.D in case 1 and V11
Molweight: 17.954 (kD)
Majority protein IDs: Gorai.013G093600.3.D;Gorai.013G093600.2.D;Gorai.013G093600.1.D
Gene IDs: Gorai.013G093600
cotton name: PTHR32227:SF56 - CARBOHYDRATE-BINDING X8 DOMAIN-CONTAINING PROTEIN (1 of 3)
OG IDs: 93439684
Best-hit-arabi-ID: AT1G18650.1
Best-hit-arabi-name: plasmodesmata callose-binding protein 3
Gorai.013G093600.1.D
PCC=-0.072, p=0.769
mRNA Profile
Protein Expression Profile
DPA

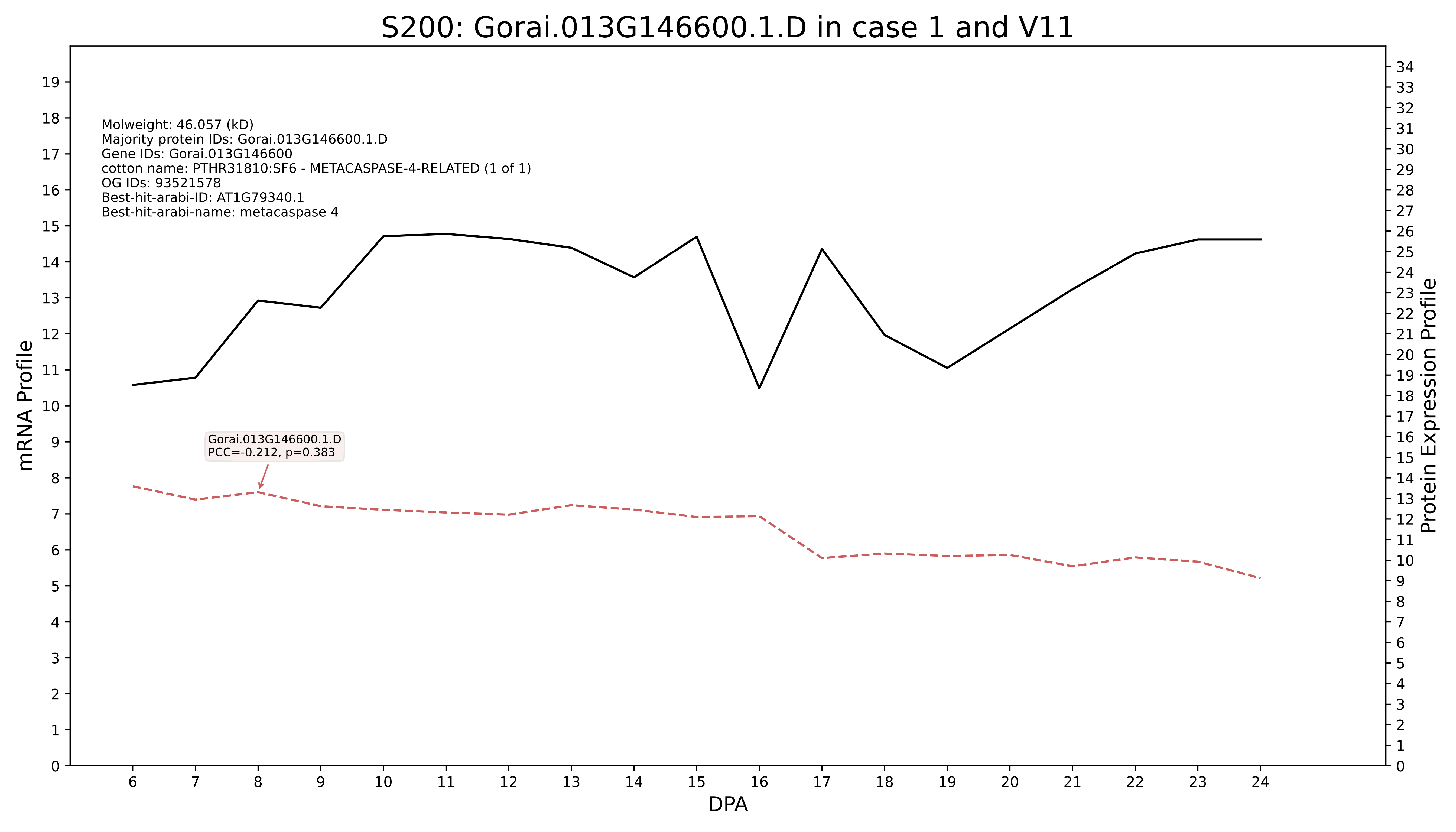
S200: Gorai.013G146600.1.D in case 1 and V11
Molweight: 46.057 (kD)
Majority protein IDs: Gorai.013G146600.1.D
Gene IDs: Gorai.013G146600
cotton name: PTHR31810:SF6 - METACASPASE-4-RELATED (1 of 1)
OG IDs: 93521578
Best-hit-arabi-ID: AT1G79340.1
Best-hit-arabi-name: metacaspase 4
Gorai.013G146600.1.D
PCC=-0.212, p=0.383
mRNA Profile
Protein Expression Profile
DPA
0
1
2
3
4
5
6
7
8
9
10
11
12
13
14
15
16
17
18
19
20
21
22
23
24
25
26
27
28
29
30
31
32
33
34

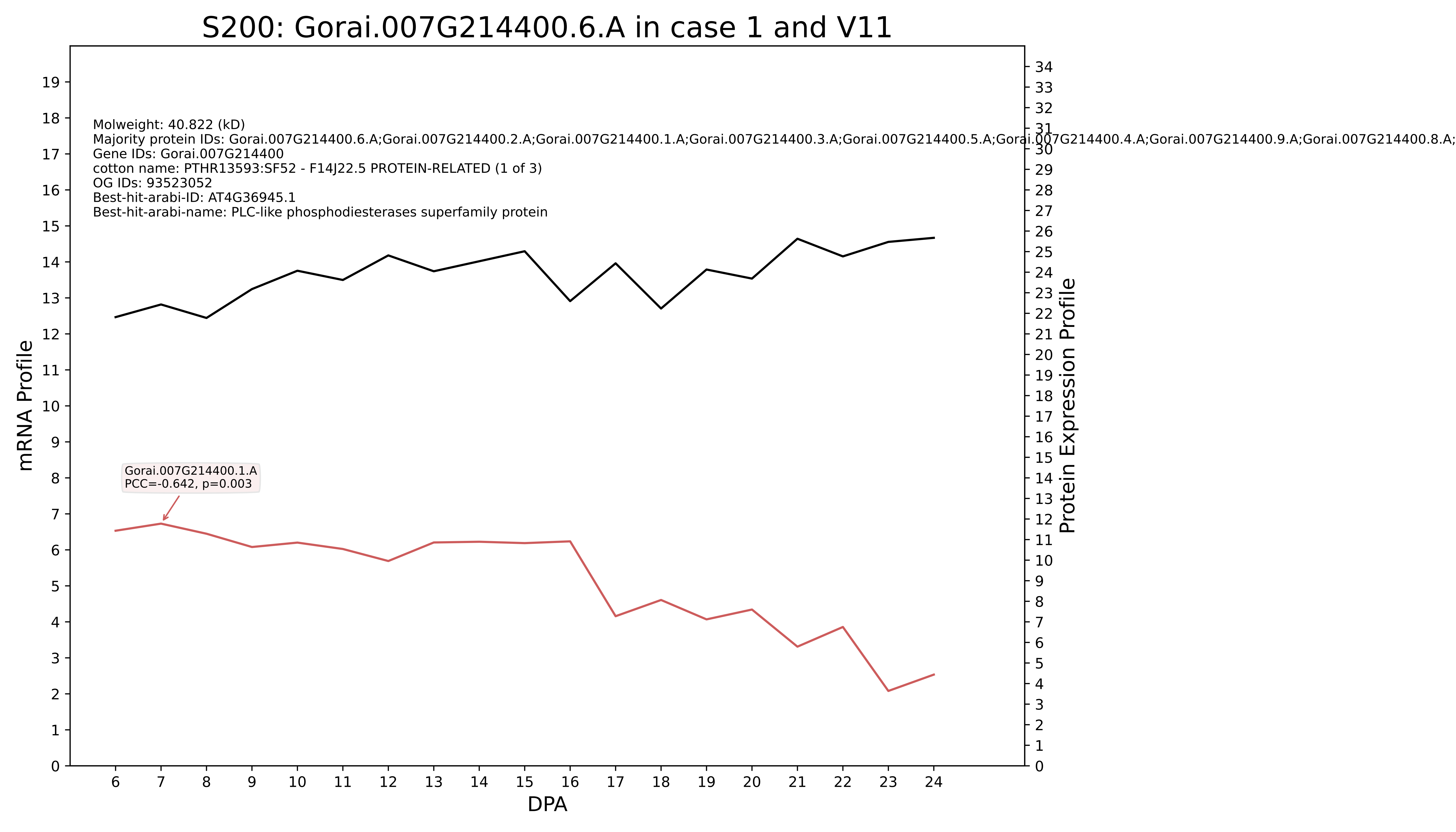
S200: Gorai.007G214400.6.A in case 1 and V11
Molweight: 40.822 (kD)
Majority protein IDs: Gorai.007G214400.6.A;Gorai.007G214400.2.A;Gorai.007G214400.1.A;Gorai.007G214400.3.A;Gorai.007G214400.5.A;Gorai.007G214400.4.A;Gorai.007G214400.9.A;Gorai.007G214400.8.A;
Gene IDs: Gorai.007G214400
cotton name: PTHR13593:SF52 - F14J22.5 PROTEIN-RELATED (1 of 3)
OG IDs: 93523052
Best-hit-arabi-ID: AT4G36945.1
Best-hit-arabi-name: PLC-like phosphodiesterases superfamily protein
Gorai.007G214400.1.A
PCC=-0.642, p=0.003
mRNA Profile
Protein Expression Profile
DPA

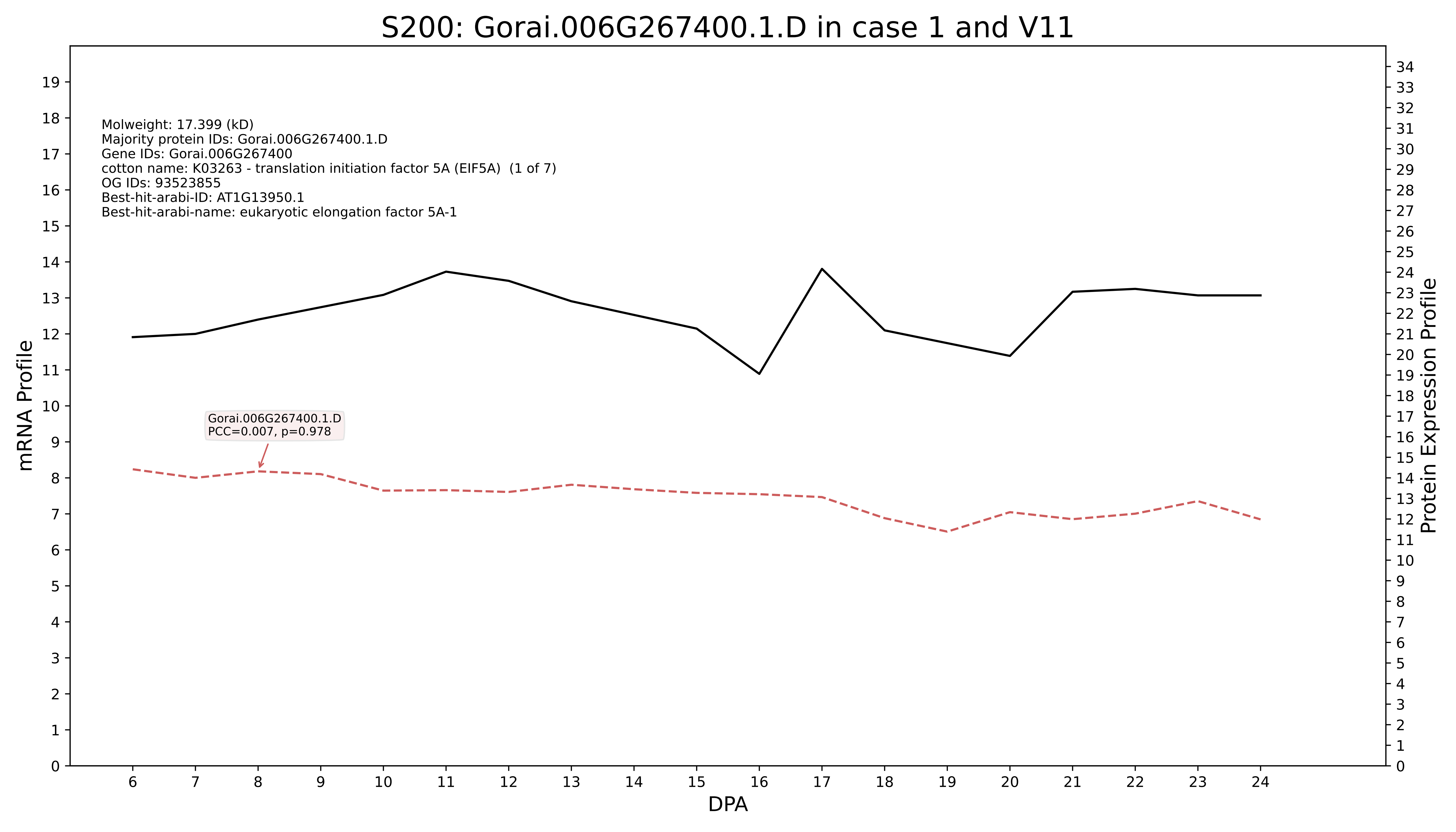
S200: Gorai.006G267400.1.D in case 1 and V11
Molweight: 17.399 (kD)
Majority protein IDs: Gorai.006G267400.1.D
Gene IDs: Gorai.006G267400
cotton name: K03263 - translation initiation factor 5A (EIF5A) (1 of 7)
OG IDs: 93523855
Best-hit-arabi-ID: AT1G13950.1
Best-hit-arabi-name: eukaryotic elongation factor 5A-1
Gorai.006G267400.1.D
PCC=0.007, p=0.978
mRNA Profile
Protein Expression Profile
DPA

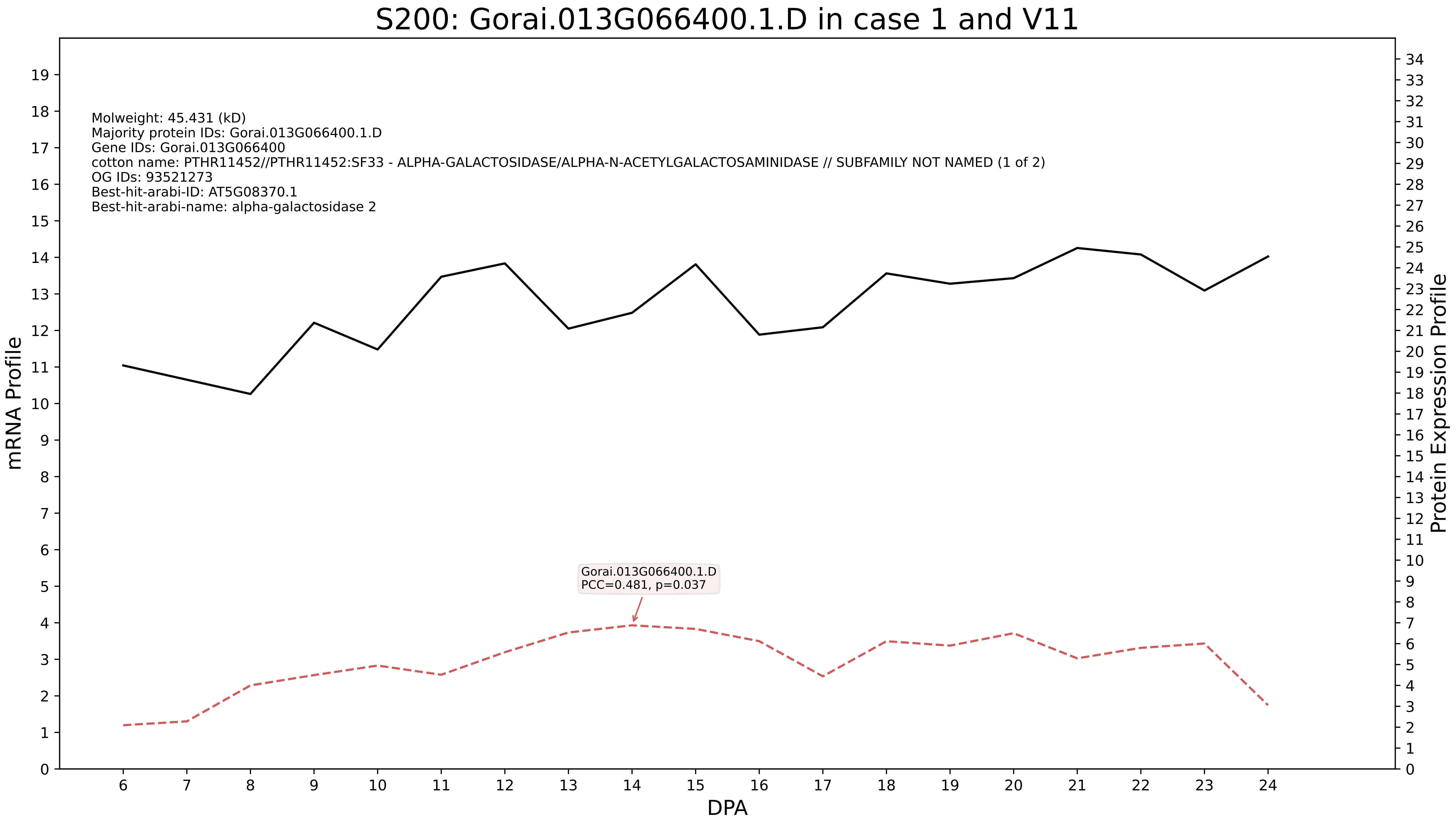
S200: Gorai.013G066400.1.D in case 1 and V11
Molweight: 45.431 (kD)
Majority protein IDs: Gorai.013G066400.1.D
Gene IDs: Gorai.013G066400
cotton name: PTHR11452//PTHR11452:SF33 - ALPHA-GALACTOSIDASE/ALPHA-N-ACETYLGALACTOSAMINIDASE // SUBFAMILY NOT NAMED (1 of 2)
OG IDs: 93521273
Best-hit-arabi-ID: AT5G08370.1
Best-hit-arabi-name: alpha-galactosidase 2
Gorai.013G066400.1.D
PCC=0.481, p=0.037
mRNA Profile
Protein Expression Profile
DPA
0
1
2
3
4
5
6
7
8
9
10
11
12
13
14
15
16
17
18
19
20
21
22
23
24
25
26
27
28
29
30
31
32
33
34

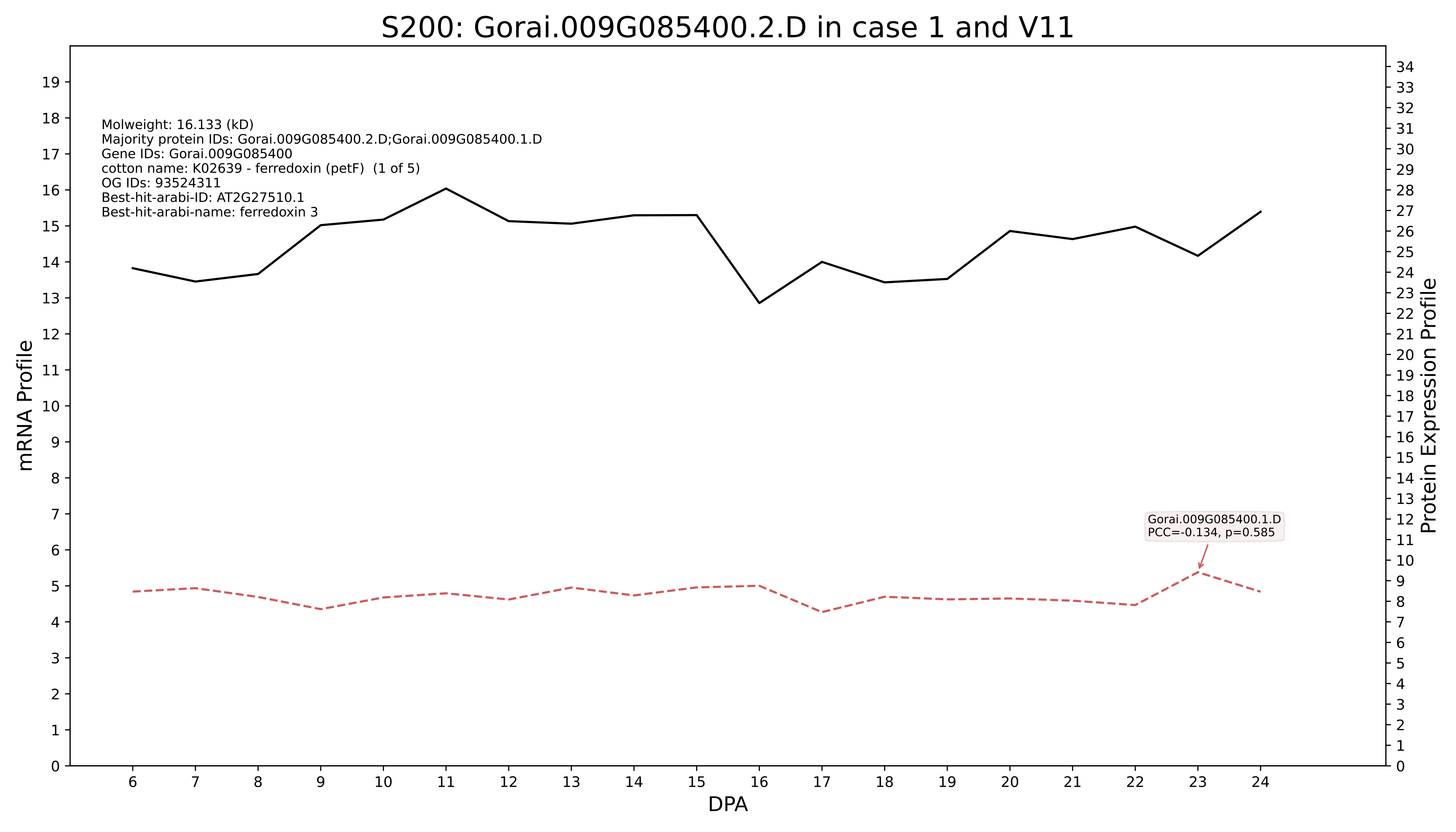
S200: Gorai.009G085400.2.D in case 1 and V11
Molweight: 16.133 (kD)
Majority protein IDs: Gorai.009G085400.2.D;Gorai.009G085400.1.D
Gene IDs: Gorai.009G085400
cotton name: K02639 - ferredoxin (petF) (1 of 5)
OG IDs: 93524311
Best-hit-arabi-ID: AT2G27510.1
Best-hit-arabi-name: ferredoxin 3
Gorai.009G085400.1.D
PCC=-0.134, p=0.585
mRNA Profile
Protein Expression Profile
DPA

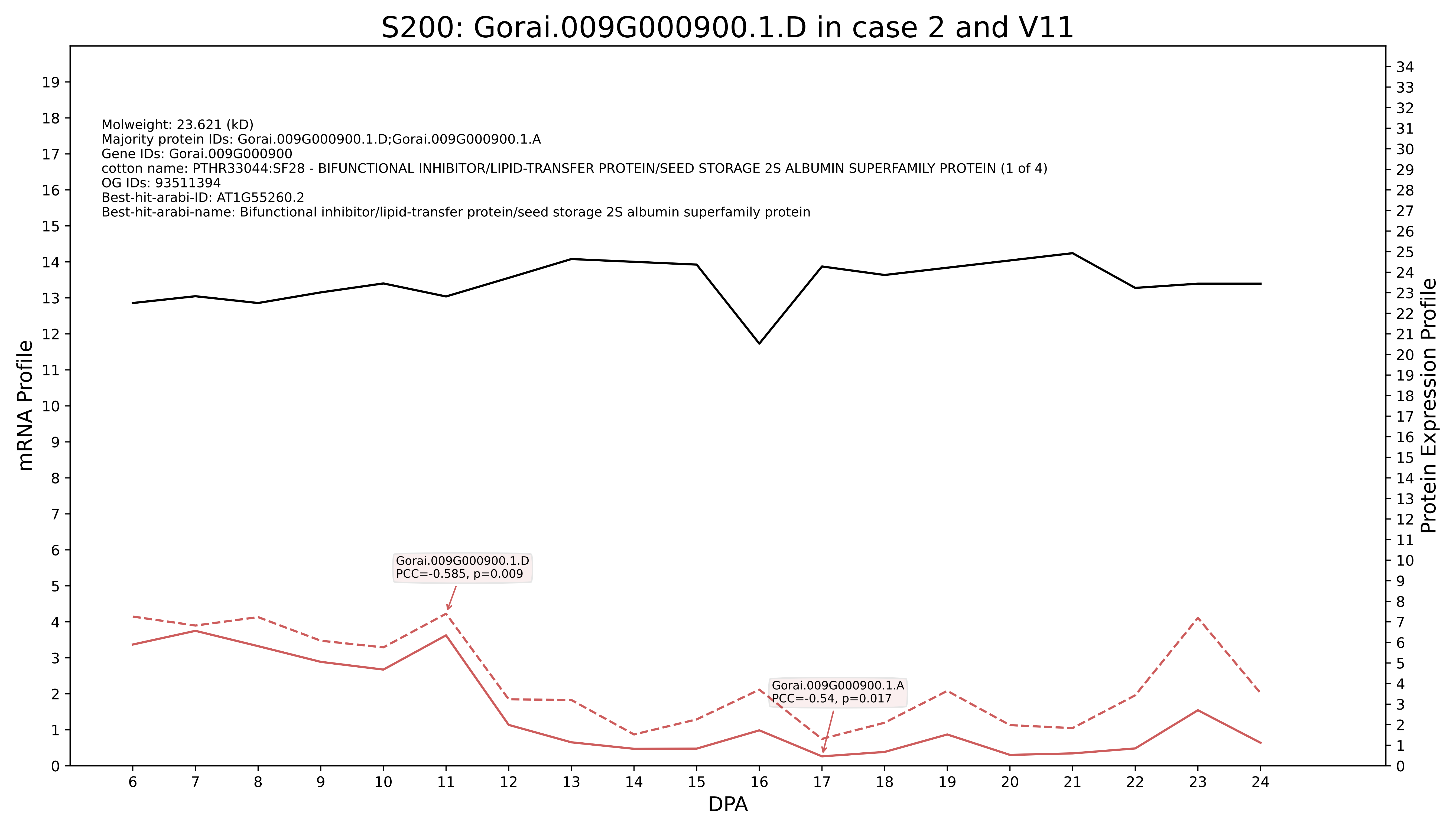
S200: Gorai.009G000900.1.D in case 2 and V11
Molweight: 23.621 (kD)
Majority protein IDs: Gorai.009G000900.1.D;Gorai.009G000900.1.A
Gene IDs: Gorai.009G000900
cotton name: PTHR33044:SF28 - BIFUNCTIONAL INHIBITOR/LIPID-TRANSFER PROTEIN/SEED STORAGE 2S ALBUMIN SUPERFAMILY PROTEIN (1 of 4)
OG IDs: 93511394
Best-hit-arabi-ID: AT1G55260.2
Best-hit-arabi-name: Bifunctional inhibitor/lipid-transfer protein/seed storage 2S albumin superfamily protein
Gorai.009G000900.1.D
PCC=-0.585, p=0.009
Gorai.009G000900.1.A
PCC=-0.54, p=0.017
mRNA Profile
Protein Expression Profile
DPA

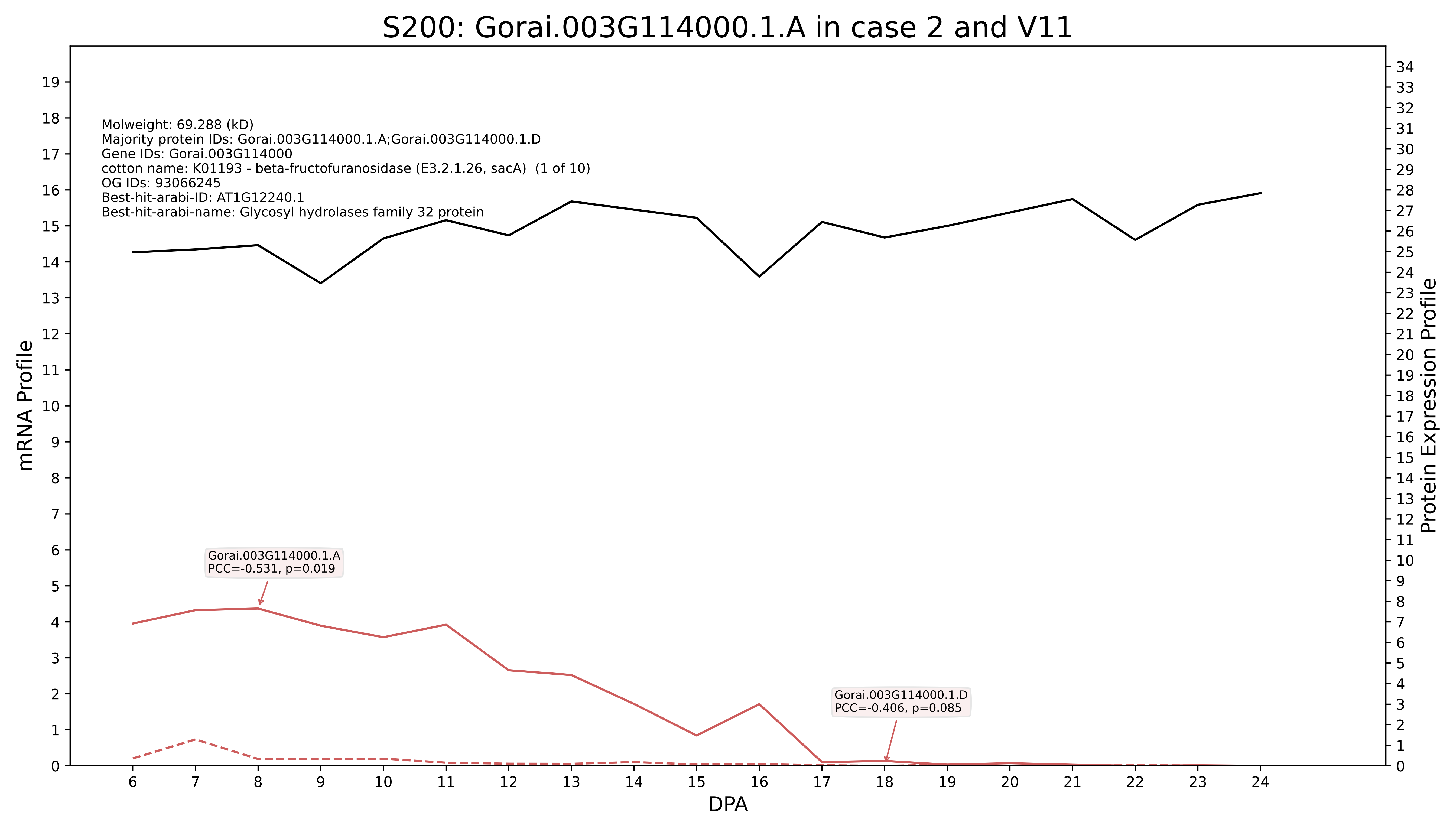
S200: Gorai.003G114000.1.A in case 2 and V11
Molweight: 69.288 (kD)
Majority protein IDs: Gorai.003G114000.1.A;Gorai.003G114000.1.D
Gene IDs: Gorai.003G114000
cotton name: K01193 - beta-fructofuranosidase (E3.2.1.26, sacA) (1 of 10)
OG IDs: 93066245
Best-hit-arabi-ID: AT1G12240.1
Best-hit-arabi-name: Glycosyl hydrolases family 32 protein
Gorai.003G114000.1.A
PCC=-0.531, p=0.019
Gorai.003G114000.1.D
PCC=-0.406, p=0.085
mRNA Profile
Protein Expression Profile
DPA

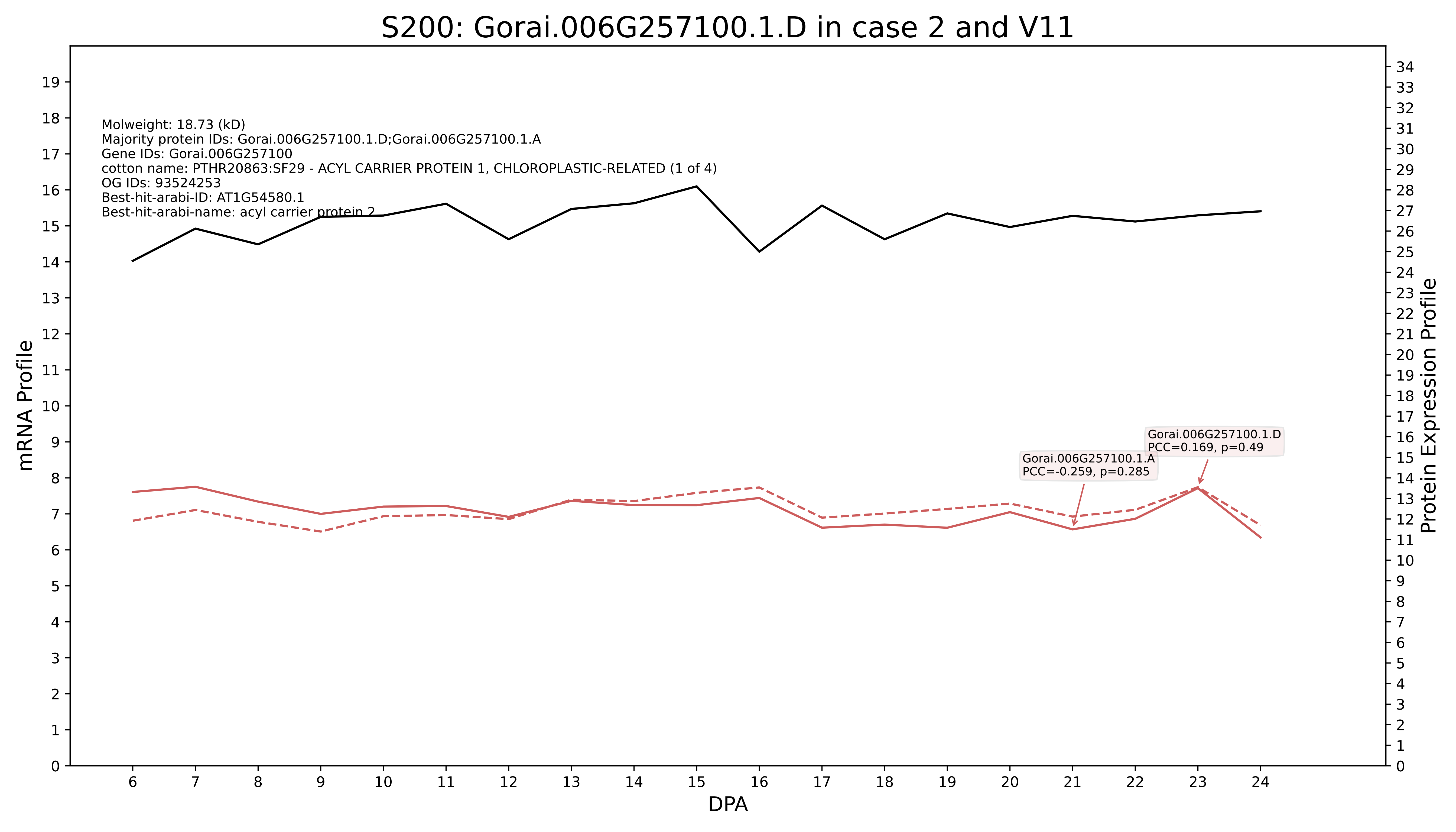
S200: Gorai.006G257100.1.D in case 2 and V11
Molweight: 18.73 (kD)
Majority protein IDs: Gorai.006G257100.1.D;Gorai.006G257100.1.A
Gene IDs: Gorai.006G257100
cotton name: PTHR20863:SF29 - ACYL CARRIER PROTEIN 1, CHLOROPLASTIC-RELATED (1 of 4)
OG IDs: 93524253
Best-hit-arabi-ID: AT1G54580.1
Best-hit-arabi-name: acyl carrier protein 2
Gorai.006G257100.1.A
PCC=-0.259, p=0.285
Gorai.006G257100.1.D
PCC=0.169, p=0.49
mRNA Profile
Protein Expression Profile
DPA
6
7
8
9
10
11
12
13
14
15
16
17
18
19
20
21
22
23
24

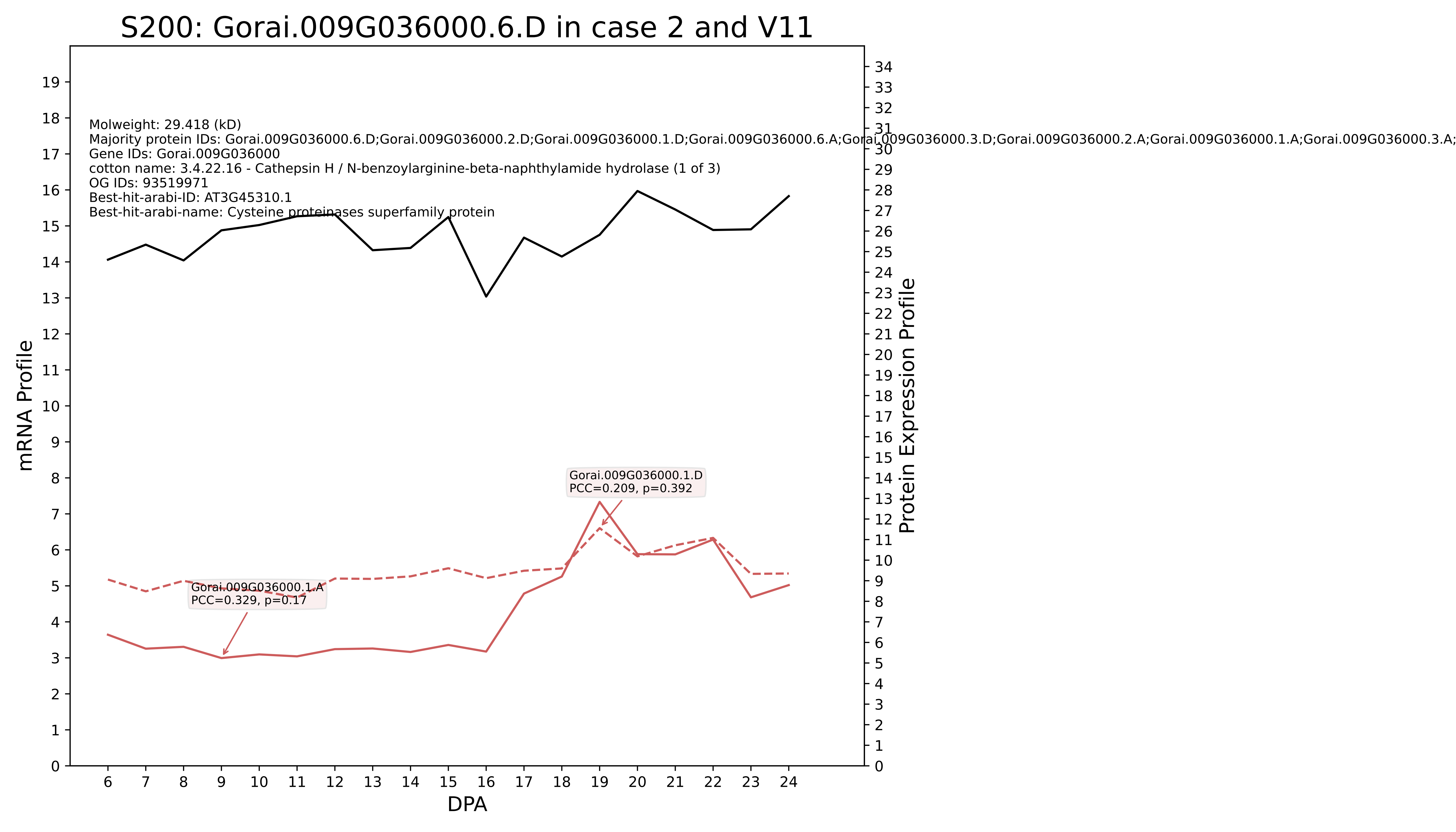

S200: Gorai.009G036000.6.D in case 2 and V11
Molweight: 29.418 (kD)
Majority protein IDs: Gorai.009G036000.6.D;Gorai.009G036000.2.D;Gorai.009G036000.1.D;Gorai.009G036000.6.A;Gorai.009G036000.3.D;Gorai.009G036000.2.A;Gorai.009G036000.1.A;Gorai.009G036000.3.A;
Gene IDs: Gorai.009G036000
cotton name: 3.4.22.16 - Cathepsin H / N-benzoylarginine-beta-naphthylamide hydrolase (1 of 3)
OG IDs: 93519971
Best-hit-arabi-ID: AT3G45310.1
Best-hit-arabi-name: Cysteine proteinases superfamily protein
Gorai.009G036000.1.D
PCC=0.209, p=0.392
Gorai.009G036000.1.A
PCC=0.329, p=0.17
mRNA Profile
Protein Expression Profile
DPA

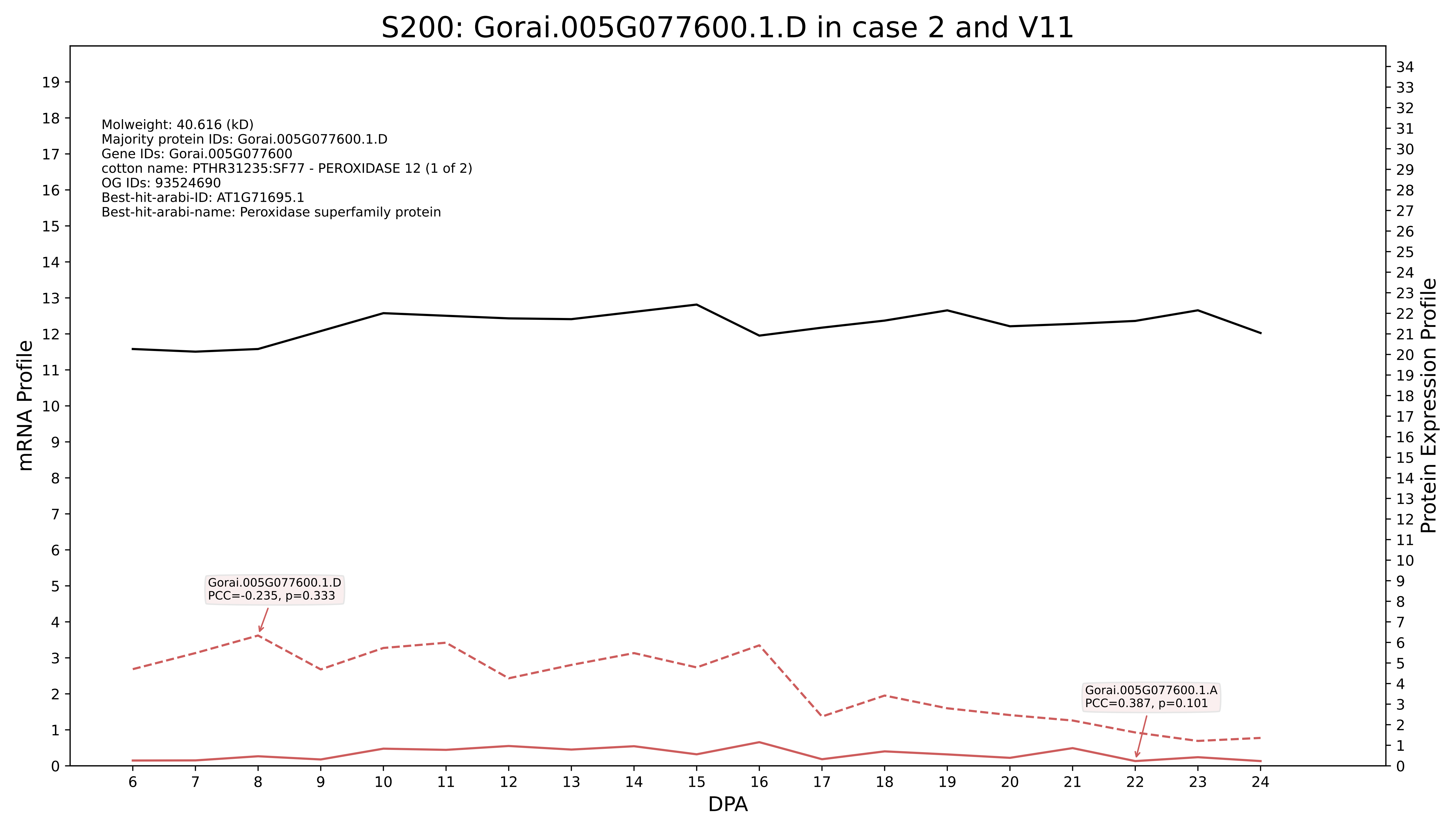
S200: Gorai.005G077600.1.D in case 2 and V11
Molweight: 40.616 (kD)
Majority protein IDs: Gorai.005G077600.1.D
Gene IDs: Gorai.005G077600
cotton name: PTHR31235:SF77 - PEROXIDASE 12 (1 of 2)
OG IDs: 93524690
Best-hit-arabi-ID: AT1G71695.1
Best-hit-arabi-name: Peroxidase superfamily protein
Gorai.005G077600.1.D
PCC=-0.235, p=0.333
Gorai.005G077600.1.A
PCC=0.387, p=0.101
mRNA Profile
Protein Expression Profile
DPA

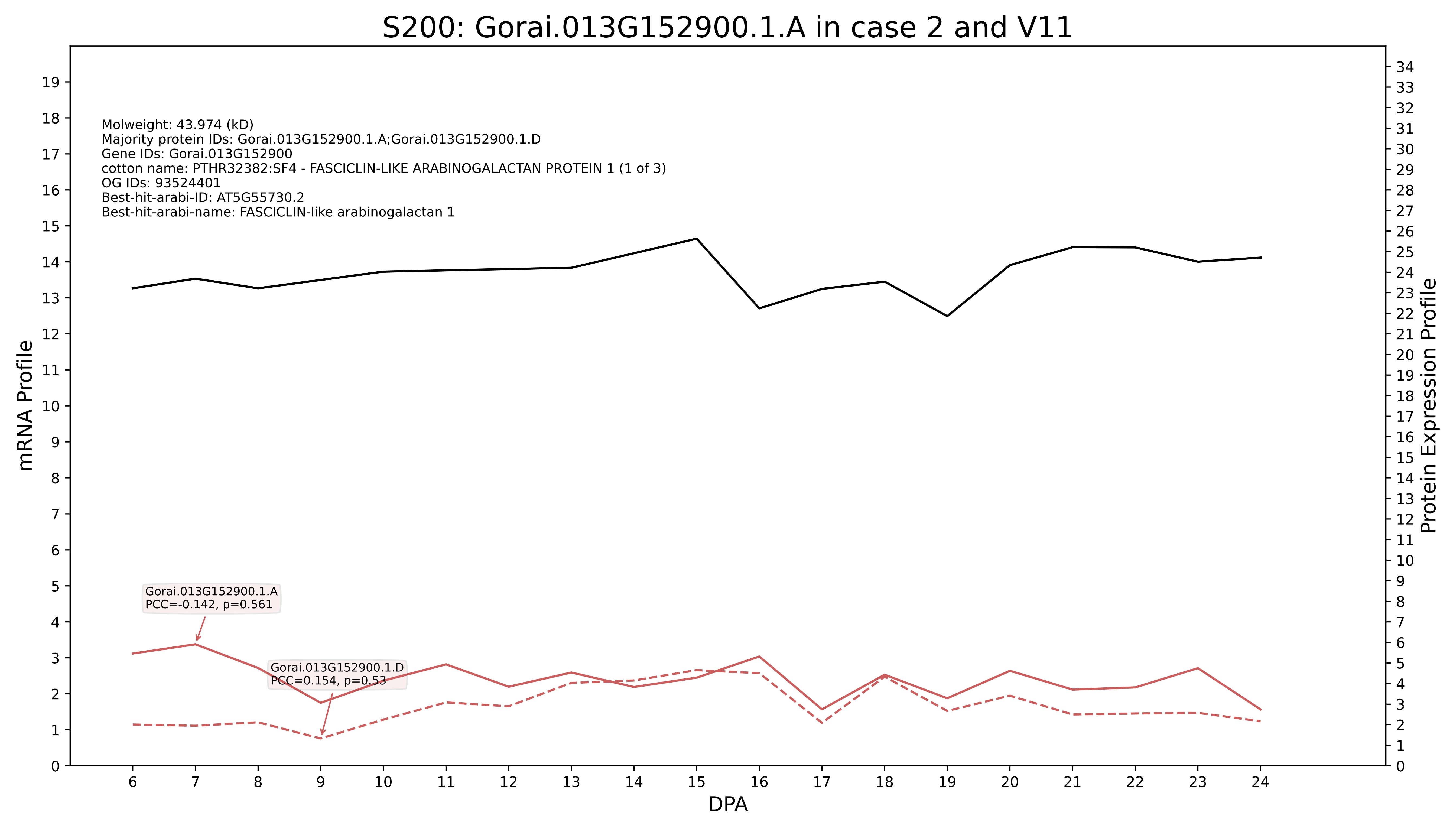

S200: Gorai.013G152900.1.A in case 2 and V11
Molweight: 43.974 (kD)
Majority protein IDs: Gorai.013G152900.1.A;Gorai.013G152900.1.D
Gene IDs: Gorai.013G152900
cotton name: PTHR32382:SF4 - FASCICLIN-LIKE ARABINOGALACTAN PROTEIN 1 (1 of 3)
OG IDs: 93524401
Best-hit-arabi-ID: AT5G55730.2
Best-hit-arabi-name: FASCICLIN-like arabinogalactan 1
Gorai.013G152900.1.A
PCC=-0.142, p=0.561
Gorai.013G152900.1.D
PCC=0.154, p=0.53
mRNA Profile
Protein Expression Profile
DPA

S200: Gorai.010G159600.5.D in case 2 and V11

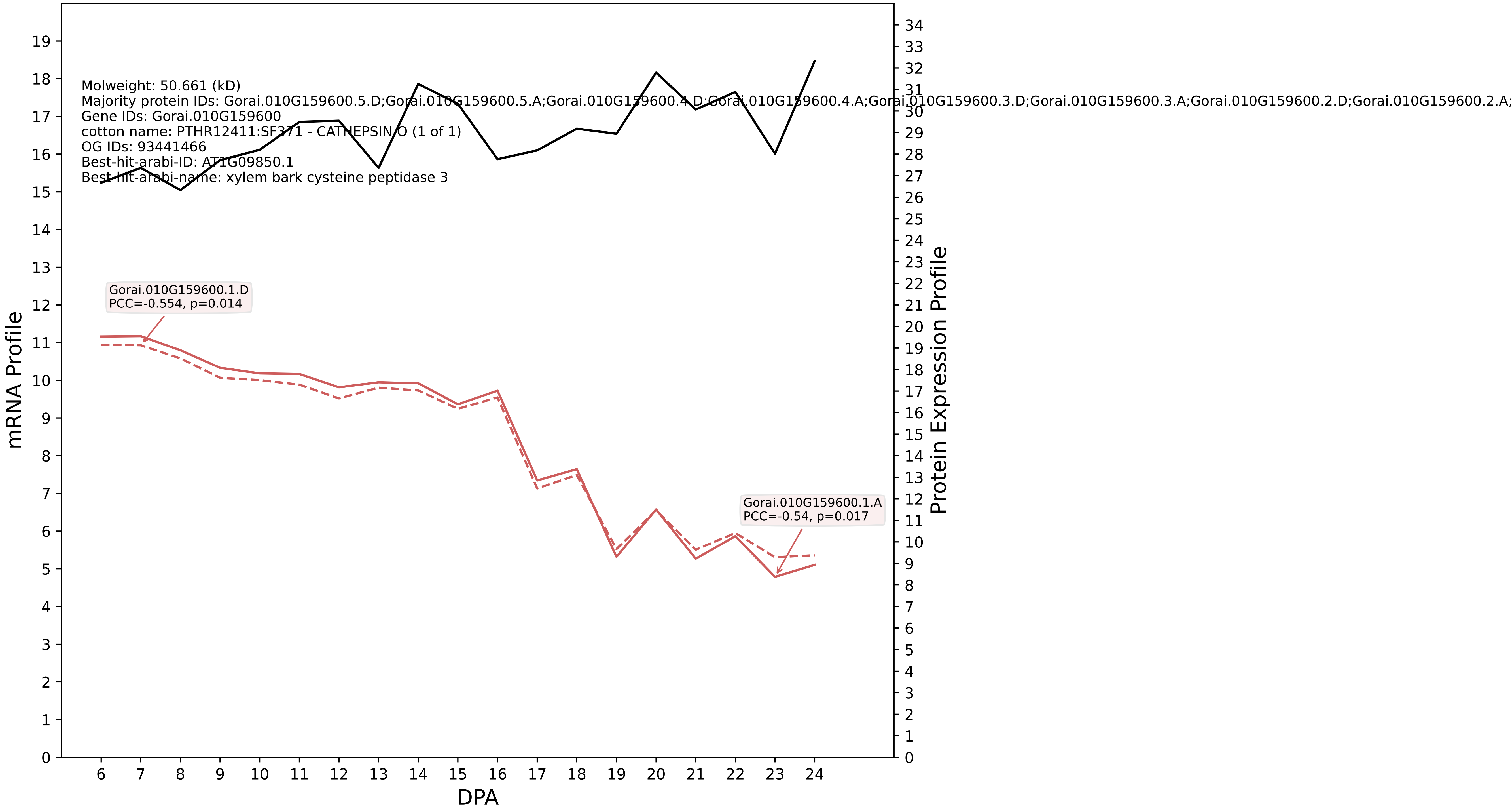

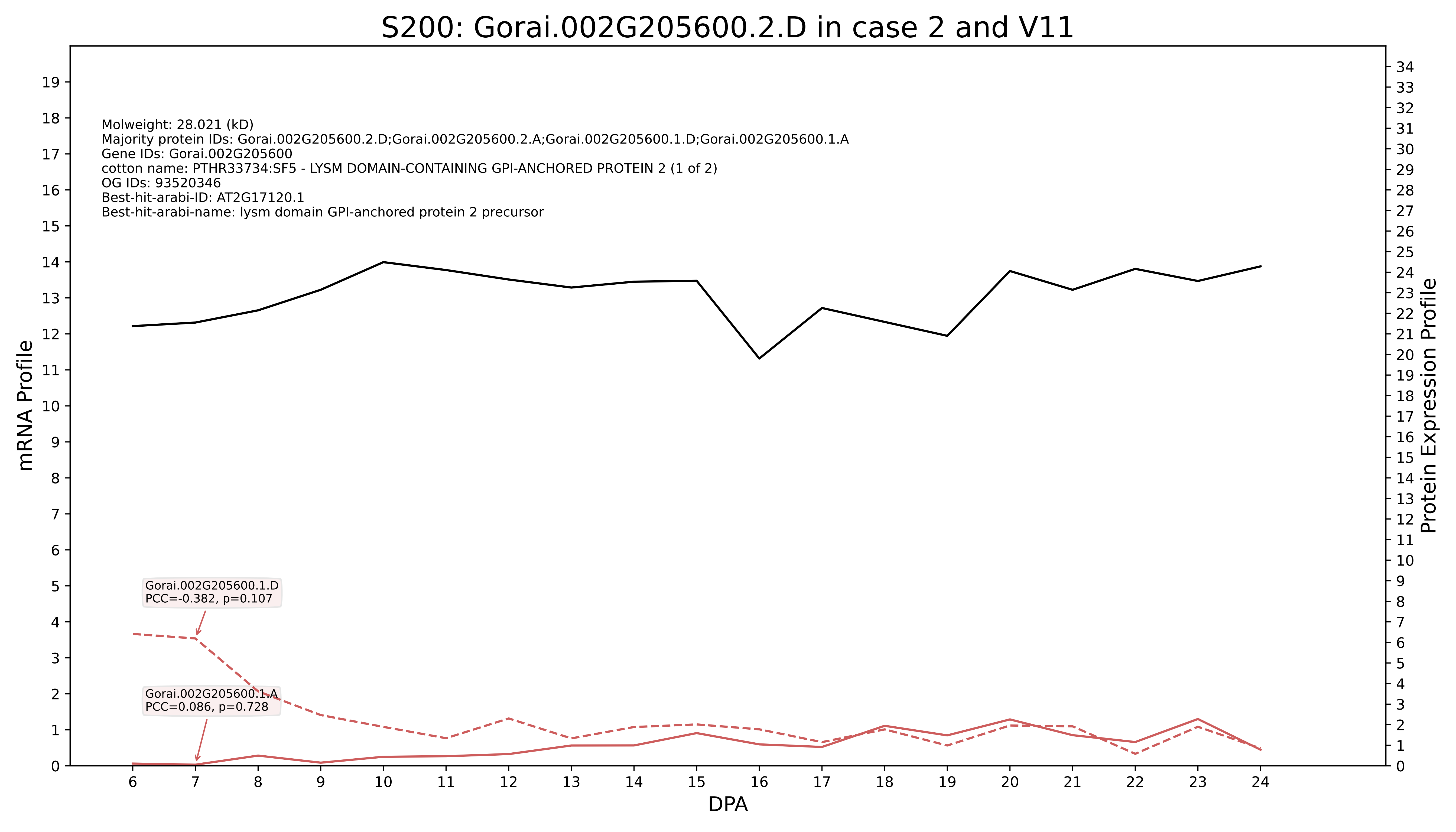
S200: Gorai.002G205600.2.D in case 2 and V11
Molweight: 28.021 (kD)
Majority protein IDs: Gorai.002G205600.2.D;Gorai.002G205600.2.A;Gorai.002G205600.1.D;Gorai.002G205600.1.A
Gene IDs: Gorai.002G205600
cotton name: PTHR33734:SF5 - LYSM DOMAIN-CONTAINING GPI-ANCHORED PROTEIN 2 (1 of 2)
OG IDs: 93520346
Best-hit-arabi-ID: AT2G17120.1
Best-hit-arabi-name: lysm domain GPI-anchored protein 2 precursor
Gorai.002G205600.1.D
PCC=-0.382, p=0.107
Gorai.002G205600.1.A
PCC=0.086, p=0.728
mRNA Profile
Protein Expression Profile
DPA

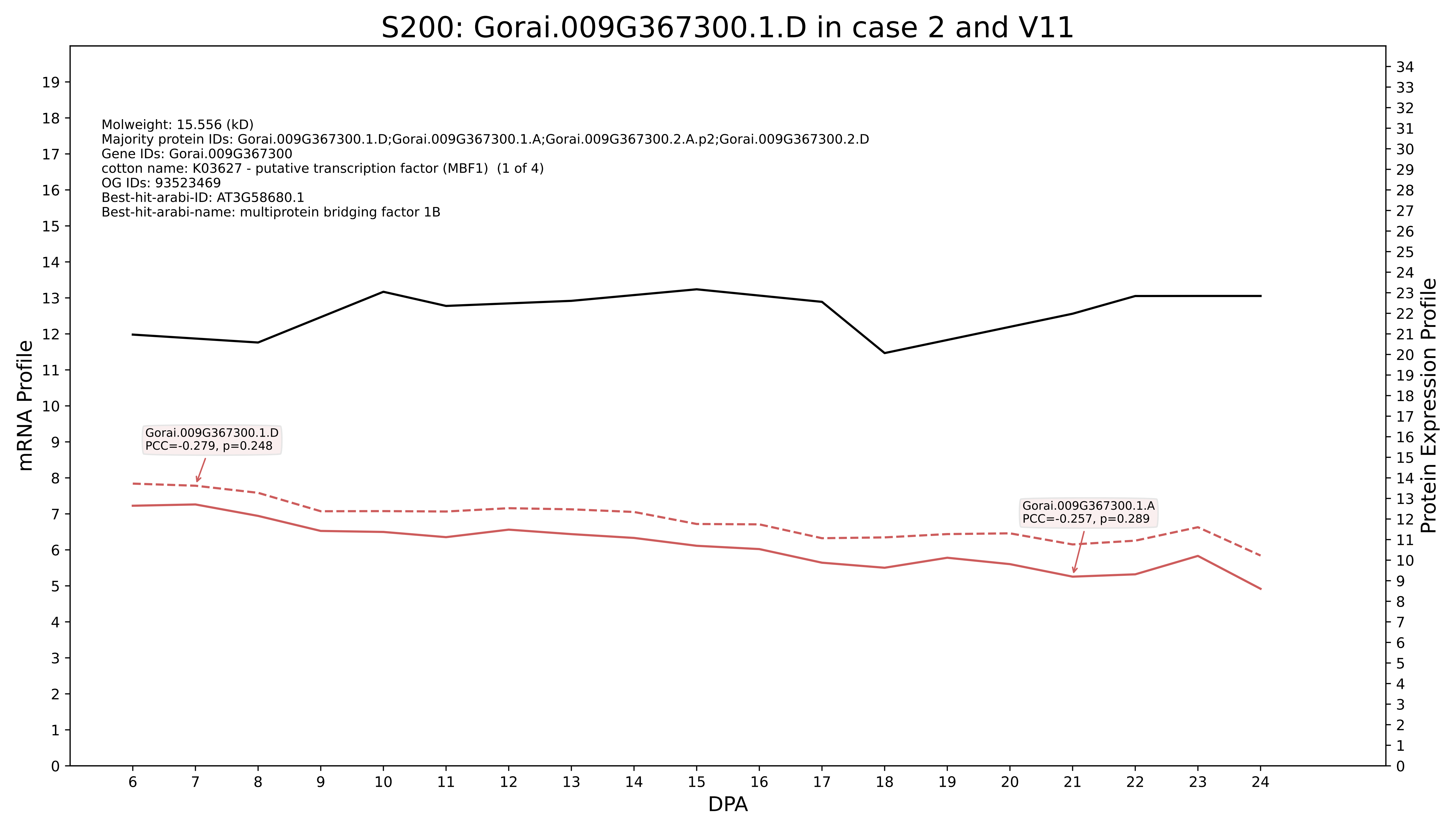
S200: Gorai.009G367300.1.D in case 2 and V11
Molweight: 15.556 (kD)
Majority protein IDs: Gorai.009G367300.1.D;Gorai.009G367300.1.A;Gorai.009G367300.2.A.p2;Gorai.009G367300.2.D
Gene IDs: Gorai.009G367300
cotton name: K03627 - putative transcription factor (MBF1) (1 of 4)
OG IDs: 93523469
Best-hit-arabi-ID: AT3G58680.1
Best-hit-arabi-name: multiprotein bridging factor 1B
Gorai.009G367300.1.D
PCC=-0.279, p=0.248
Gorai.009G367300.1.A
PCC=-0.257, p=0.289
mRNA Profile
Protein Expression Profile
DPA

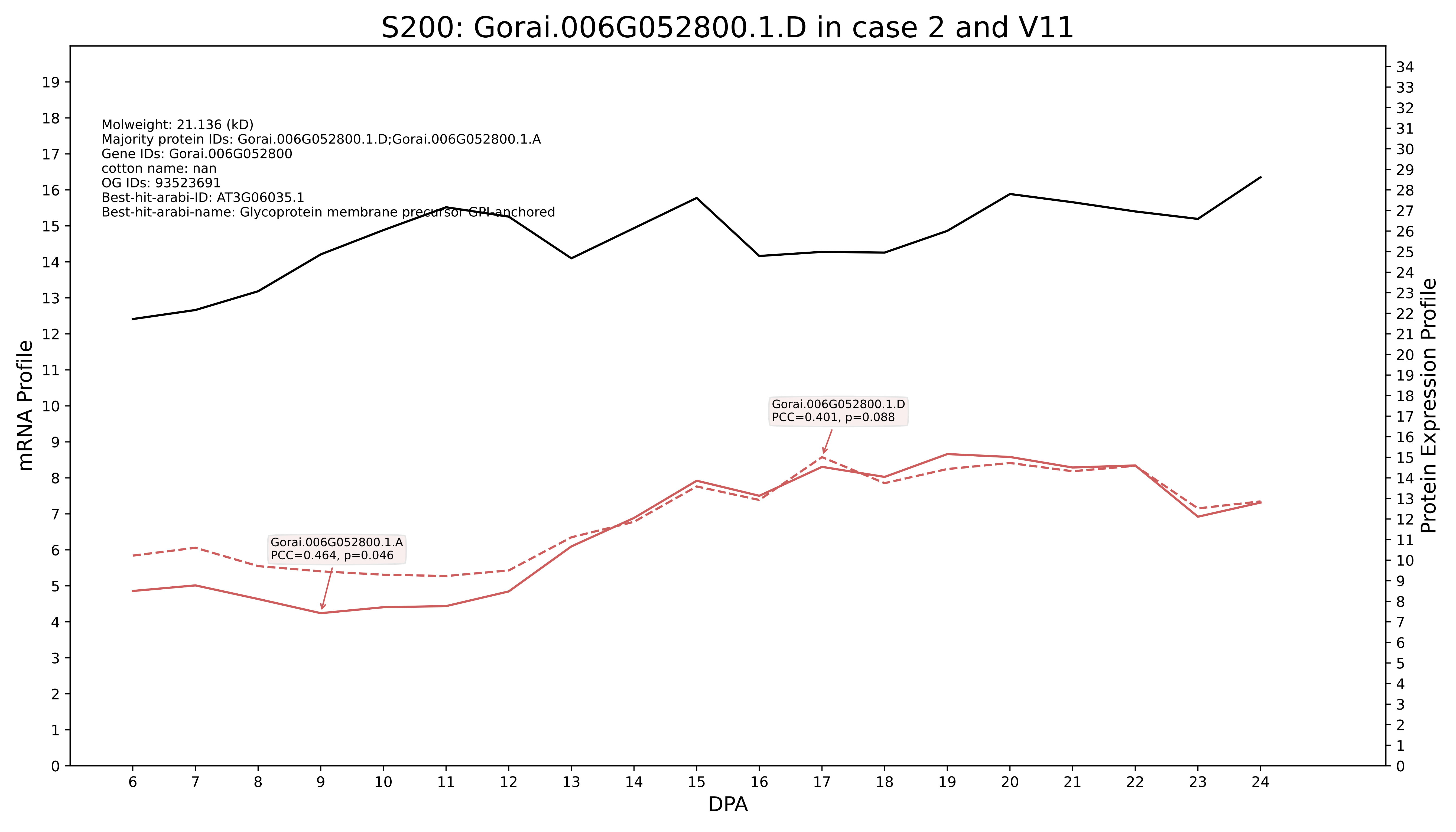
S200: Gorai.006G052800.1.D in case 2 and V11
Molweight: 21.136 (kD)
Majority protein IDs: Gorai.006G052800.1.D;Gorai.006G052800.1.A
Gene IDs: Gorai.006G052800
cotton name: nan
OG IDs: 93523691
Best-hit-arabi-ID: AT3G06035.1
Best-hit-arabi-name: Glycoprotein membrane precursor GPI-anchored
Gorai.006G052800.1.D
PCC=0.401, p=0.088
Gorai.006G052800.1.A
PCC=0.464, p=0.046
mRNA Profile
Protein Expression Profile
DPA

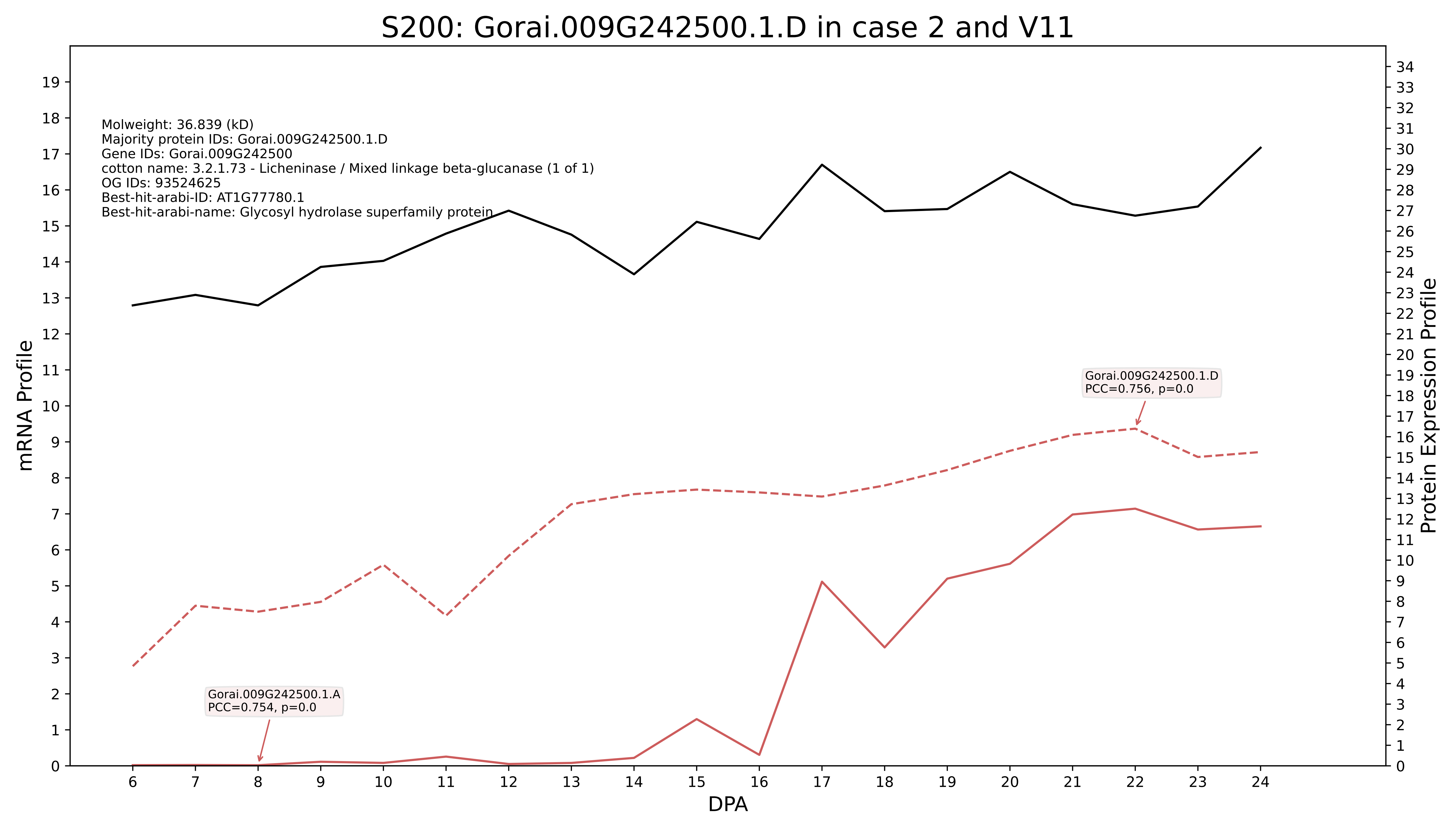

S200: Gorai.009G242500.1.D in case 2 and V11
Molweight: 36.839 (kD)
Majority protein IDs: Gorai.009G242500.1.D
Gene IDs: Gorai.009G242500
cotton name: 3.2.1.73 - Licheninase / Mixed linkage beta-glucanase (1 of 1)
OG IDs: 93524625
Best-hit-arabi-ID: AT1G77780.1
Best-hit-arabi-name: Glycosyl hydrolase superfamily protein
Gorai.009G242500.1.D
PCC=0.756, p=0.0
Gorai.009G242500.1.A
PCC=0.754, p=0.0
mRNA Profile
Protein Expression Profile
DPA

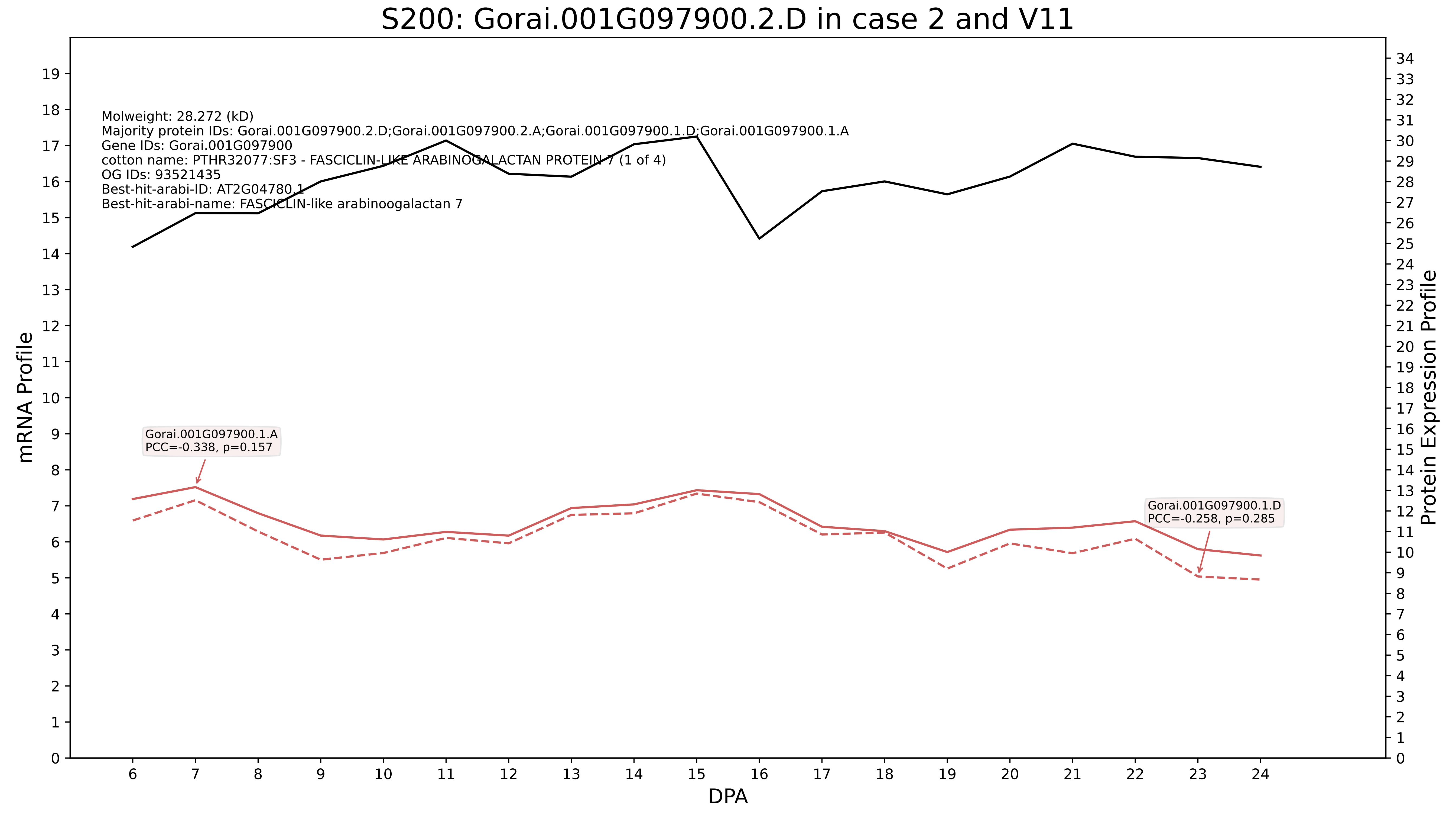
S200: Gorai.001G097900.2.D in case 2 and V11
Molweight: 28.272 (kD)
Majority protein IDs: Gorai.001G097900.2.D;Gorai.001G097900.2.A;Gorai.001G097900.1.D;Gorai.001G097900.1.A
Gene IDs: Gorai.001G097900
cotton name: PTHR32077:SF3 - FASCICLIN-LIKE ARABINOGALACTAN PROTEIN 7 (1 of 4)
OG IDs: 93521435
Best-hit-arabi-ID: AT2G04780.1
Best-hit-arabi-name: FASCICLIN-like arabinoogalactan 7
Gorai.001G097900.1.A
PCC=-0.338, p=0.157
Gorai.001G097900.1.D
PCC=-0.258, p=0.285
mRNA Profile
Protein Expression Profile
DPA
0
1
2
3
4
5
6
7
8
9
10
11
12
13
14
15
16
17
18
19
20
21
22
23
24
25
26
27
28
29
30
31
32
33
34

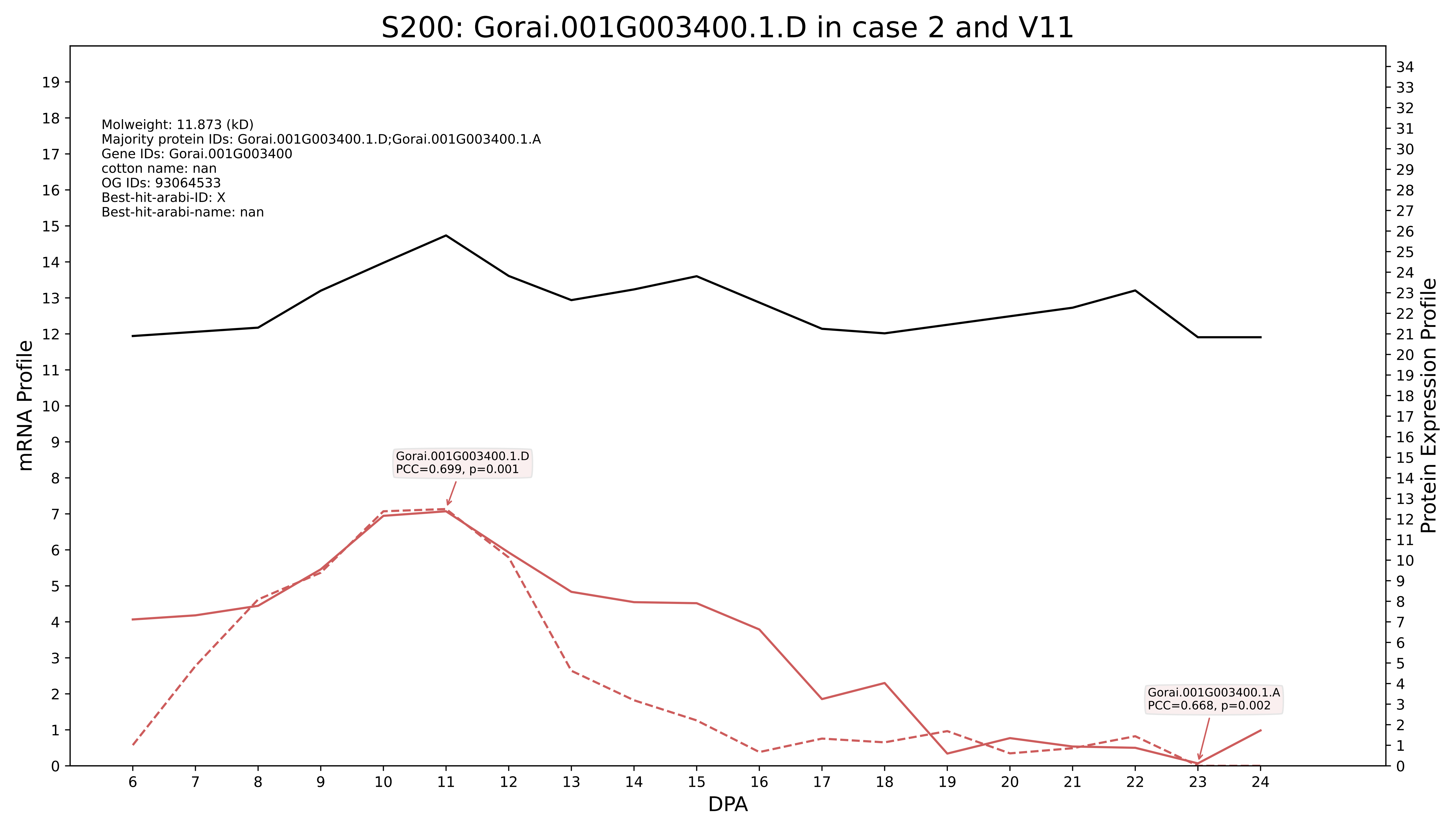
S200: Gorai.001G003400.1.D in case 2 and V11
Molweight: 11.873 (kD)
Majority protein IDs: Gorai.001G003400.1.D;Gorai.001G003400.1.A
Gene IDs: Gorai.001G003400
cotton name: nan
OG IDs: 93064533
Best-hit-arabi-ID: X
Best-hit-arabi-name: nan
Gorai.001G003400.1.D
PCC=0.699, p=0.001
Gorai.001G003400.1.A
PCC=0.668, p=0.002
mRNA Profile
Protein Expression Profile
DPA

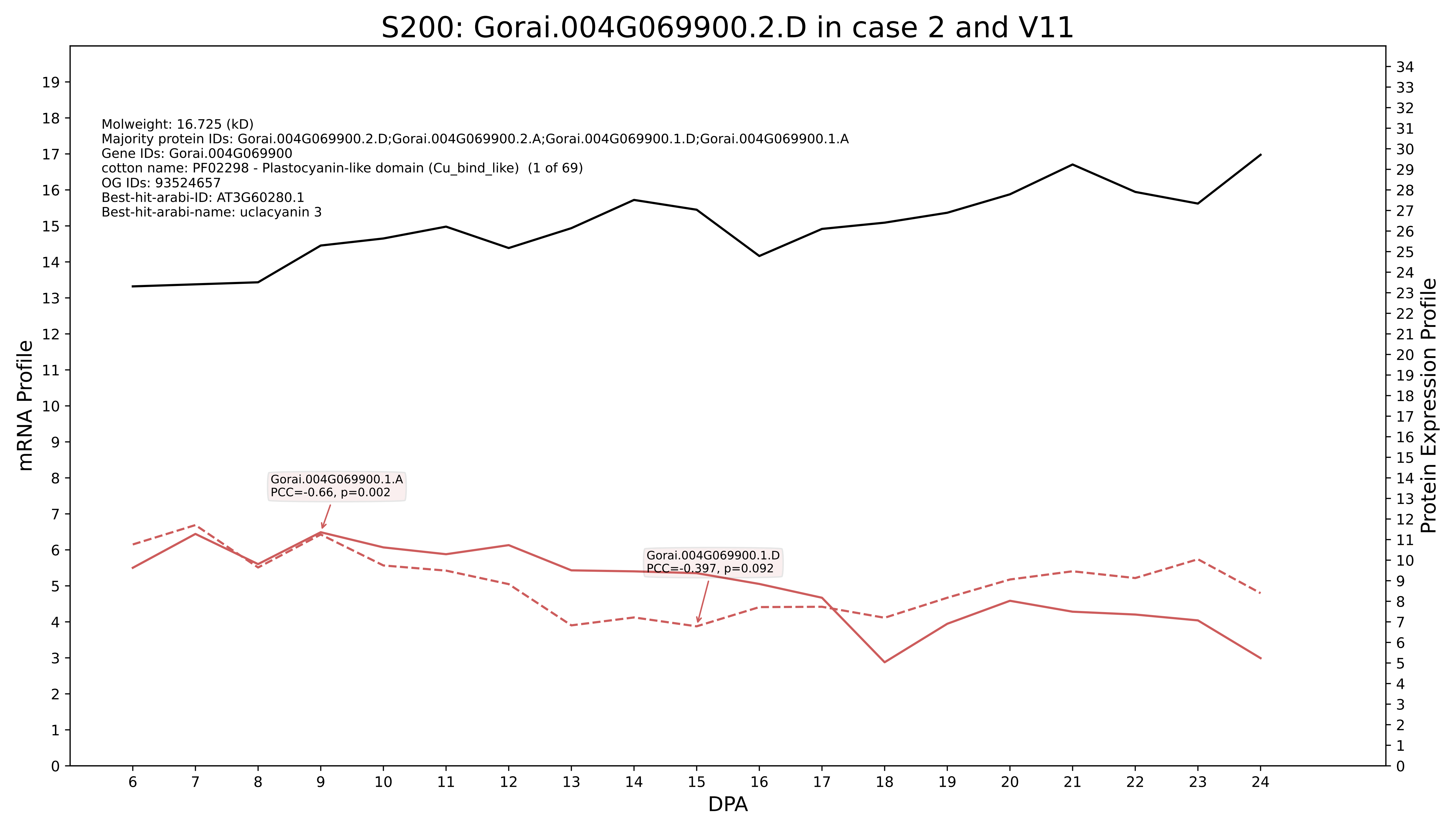
S200: Gorai.004G069900.2.D in case 2 and V11
Molweight: 16.725 (kD)
Majority protein IDs: Gorai.004G069900.2.D;Gorai.004G069900.2.A;Gorai.004G069900.1.D;Gorai.004G069900.1.A
Gene IDs: Gorai.004G069900
cotton name: PF02298 - Plastocyanin-like domain (Cu_bind_like) (1 of 69)
OG IDs: 93524657
Best-hit-arabi-ID: AT3G60280.1
Best-hit-arabi-name: uclacyanin 3
Gorai.004G069900.1.A
PCC=-0.66, p=0.002
Gorai.004G069900.1.D
PCC=-0.397, p=0.092
mRNA Profile
Protein Expression Profile
DPA

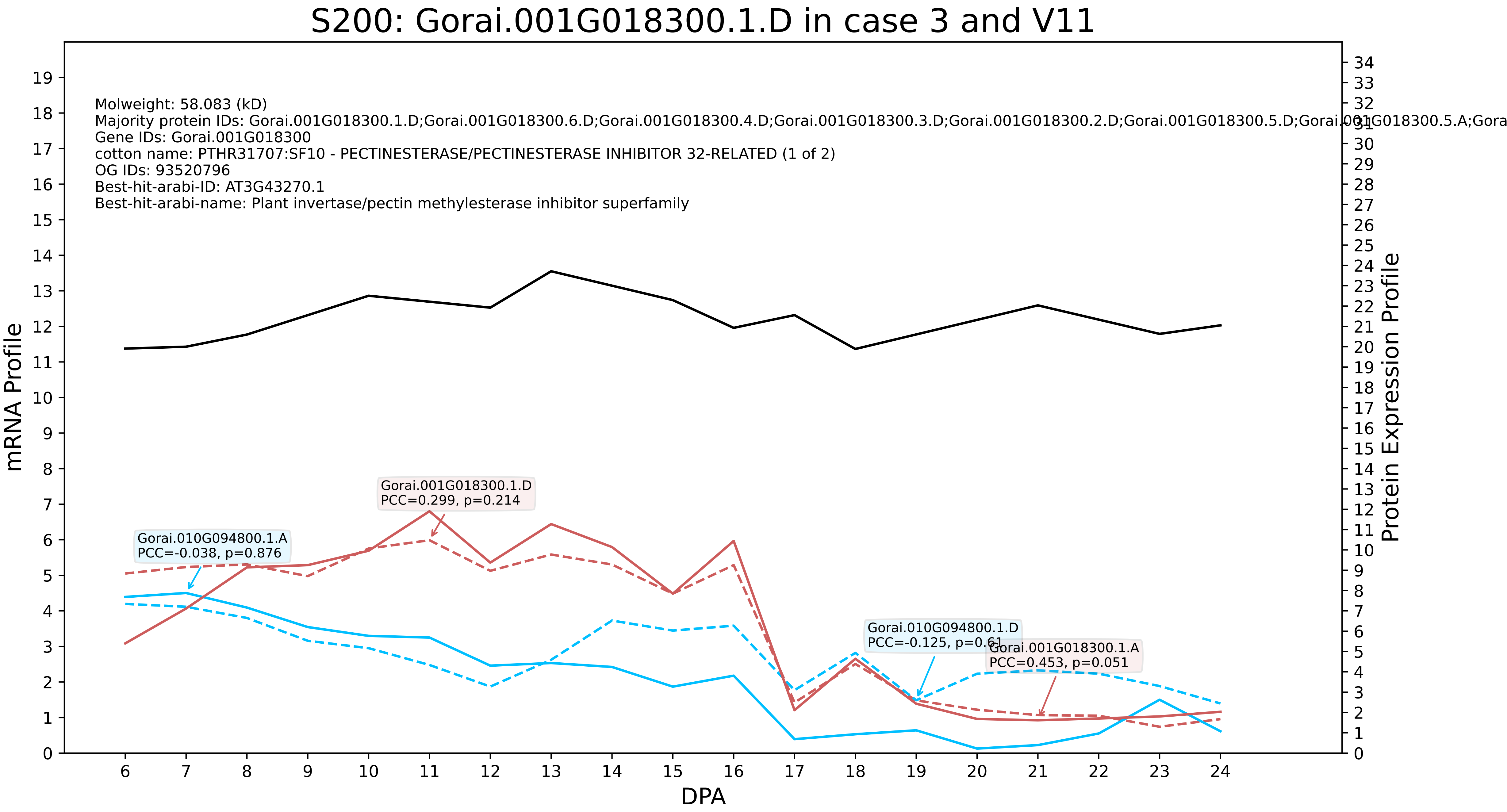
S200: Gorai.001G018300.1.D in case 3 and V11
Molweight: 58.083 (kD)
Majority protein IDs: Gorai.001G018300.1.D;Gorai.001G018300.6.D;Gorai.001G018300.4.D;Gorai.001G018300.3.D;Gorai.001G018300.2.D;Gorai.001G018300.5.D;Gorai.001G018300.5.A;Gora
Gene IDs: Gorai.001G018300
cotton name: PTHR31707:SF10 - PECTINESTERASE/PECTINESTERASE INHIBITOR 32-RELATED (1 of 2)
OG IDs: 93520796
Best-hit-arabi-ID: AT3G43270.1
Best-hit-arabi-name: Plant invertase/pectin methylesterase inhibitor superfamily
Gorai.001G018300.1.D
PCC=0.299, p=0.214
Gorai.010G094800.1.A
PCC=-0.038, p=0.876
Gorai.010G094800.1.D
PCC=-0.125, p=0.61
Gorai.001G018300.1.A
PCC=0.453, p=0.051
mRNA Profile
Protein Expression Profile
DPA

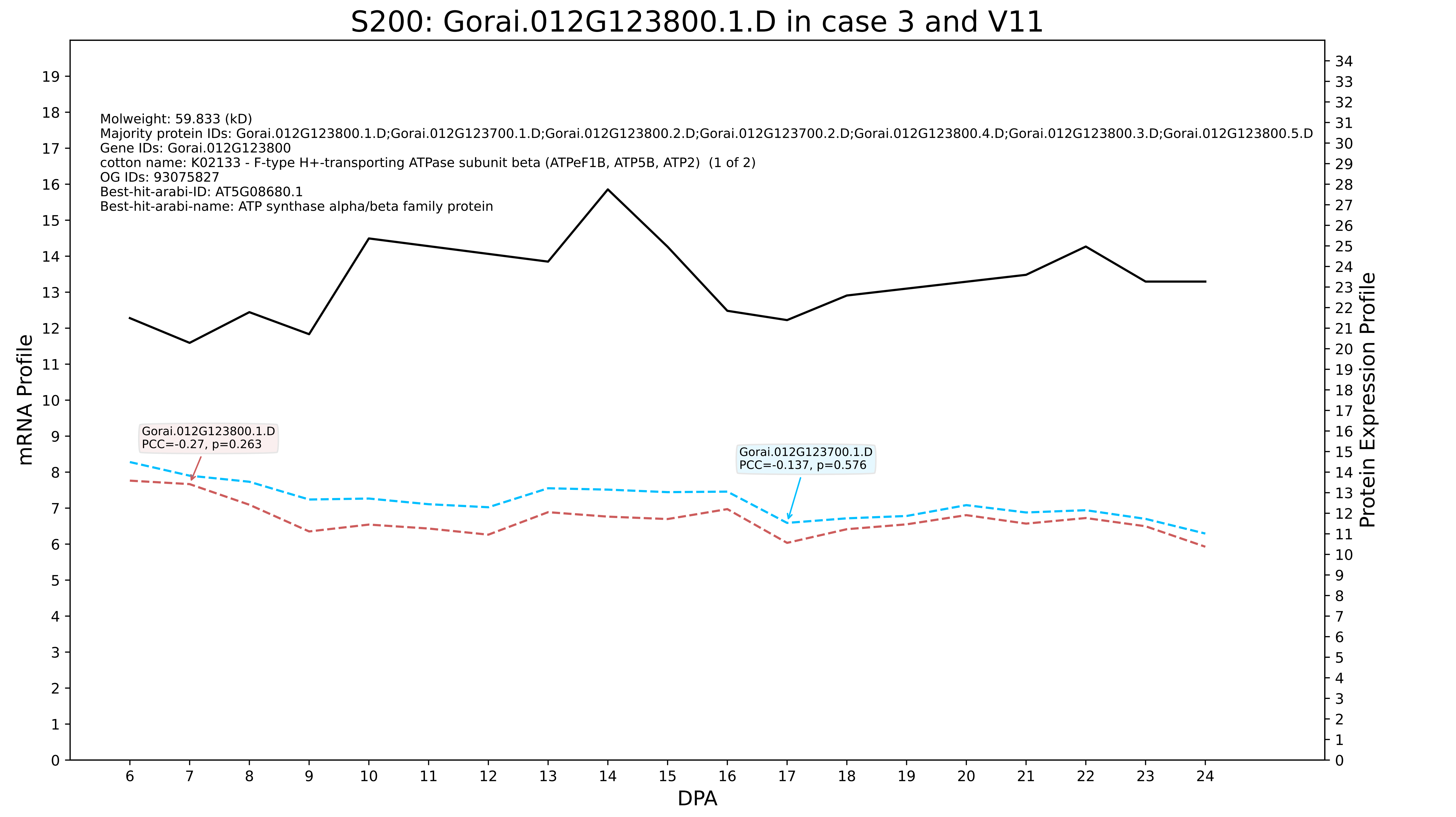
S200: Gorai.012G123800.1.D in case 3 and V11
Molweight: 59.833 (kD)
Majority protein IDs: Gorai.012G123800.1.D;Gorai.012G123700.1.D;Gorai.012G123800.2.D;Gorai.012G123700.2.D;Gorai.012G123800.4.D;Gorai.012G123800.3.D;Gorai.012G123800.5.D
Gene IDs: Gorai.012G123800
cotton name: K02133 - F-type H+-transporting ATPase subunit beta (ATPeF1B, ATP5B, ATP2) (1 of 2)
OG IDs: 93075827
Best-hit-arabi-ID: AT5G08680.1
Best-hit-arabi-name: ATP synthase alpha/beta family protein
Gorai.012G123800.1.D
PCC=-0.27, p=0.263
Gorai.012G123700.1.D
PCC=-0.137, p=0.576
mRNA Profile
Protein Expression Profile
DPA

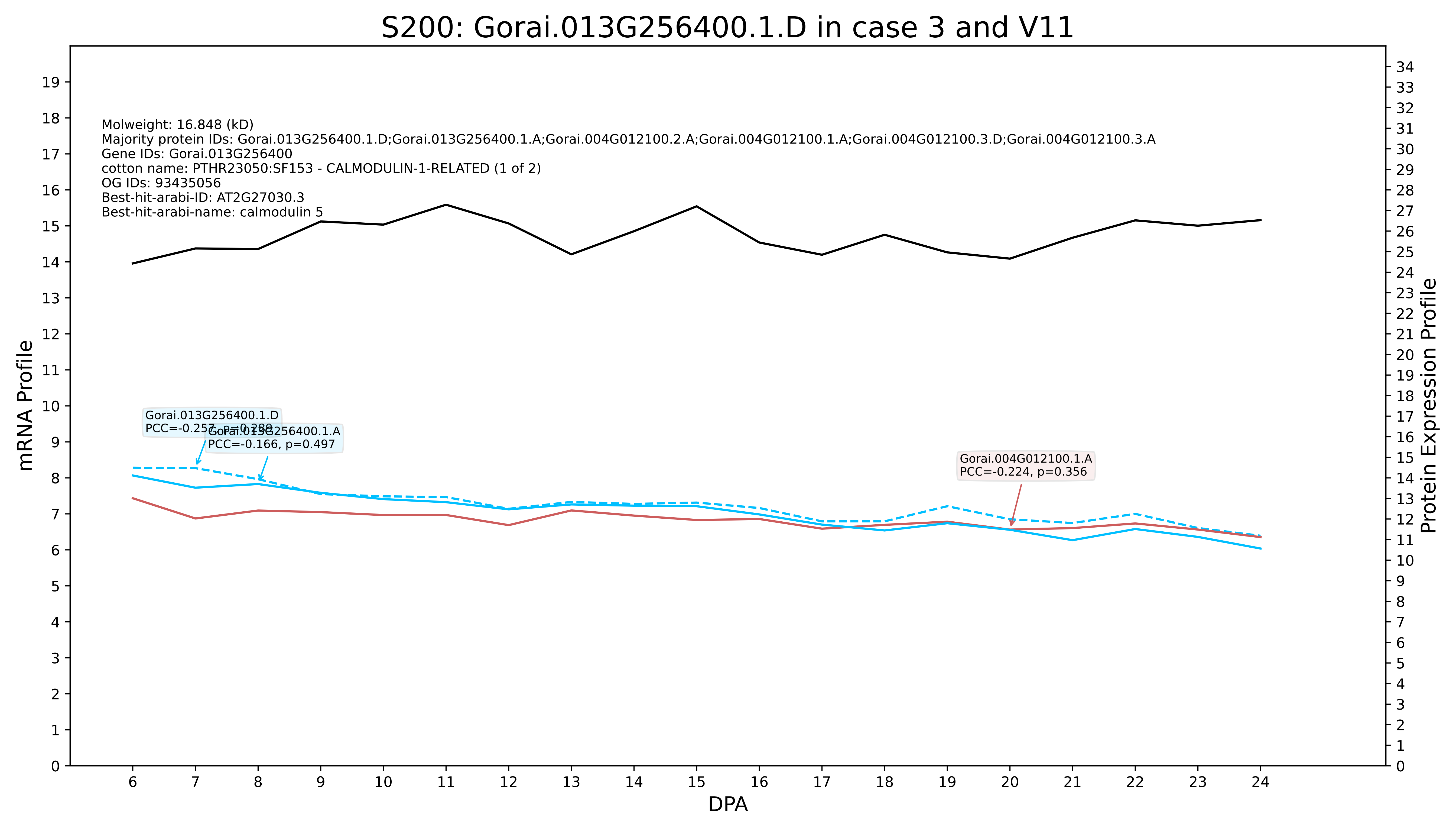

S200: Gorai.013G256400.1.D in case 3 and V11
Molweight: 16.848 (kD)
Majority protein IDs: Gorai.013G256400.1.D;Gorai.013G256400.1.A;Gorai.004G012100.2.A;Gorai.004G012100.1.A;Gorai.004G012100.3.D;Gorai.004G012100.3.A
Gene IDs: Gorai.013G256400
cotton name: PTHR23050:SF153 - CALMODULIN-1-RELATED (1 of 2)
OG IDs: 93435056
Best-hit-arabi-ID: AT2G27030.3
Best-hit-arabi-name: calmodulin 5
Gorai.013G256400.1.D
PCC=-0.257, p=0.289
Gorai.013G256400.1.A
PCC=-0.166, p=0.497
Gorai.004G012100.1.A
PCC=-0.224, p=0.356
mRNA Profile
Protein Expression Profile
DPA

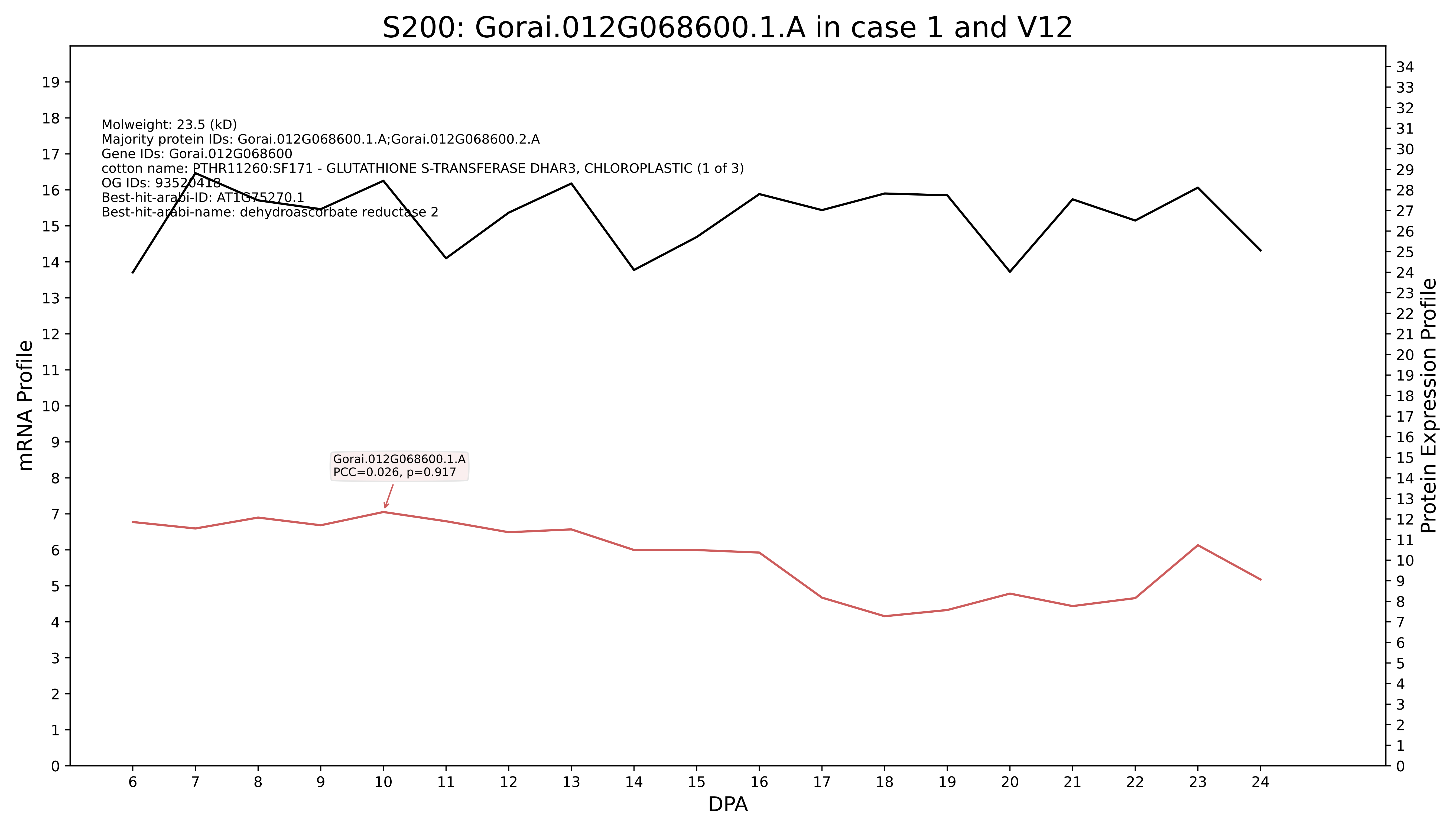
S200: Gorai.012G068600.1.A in case 1 and V12
Molweight: 23.5 (kD)
Majority protein IDs: Gorai.012G068600.1.A;Gorai.012G068600.2.A
Gene IDs: Gorai.012G068600
cotton name: PTHR11260:SF171 - GLUTATHIONE S-TRANSFERASE DHAR3, CHLOROPLASTIC (1 of 3)
OG IDs: 93520418
Best-hit-arabi-ID: AT1G75270.1
Best-hit-arabi-name: dehydroascorbate reductase 2
Gorai.012G068600.1.A
PCC=0.026, p=0.917
mRNA Profile
Protein Expression Profile
DPA

S200: Gorai.002G107200.6.D in case 1 and V12

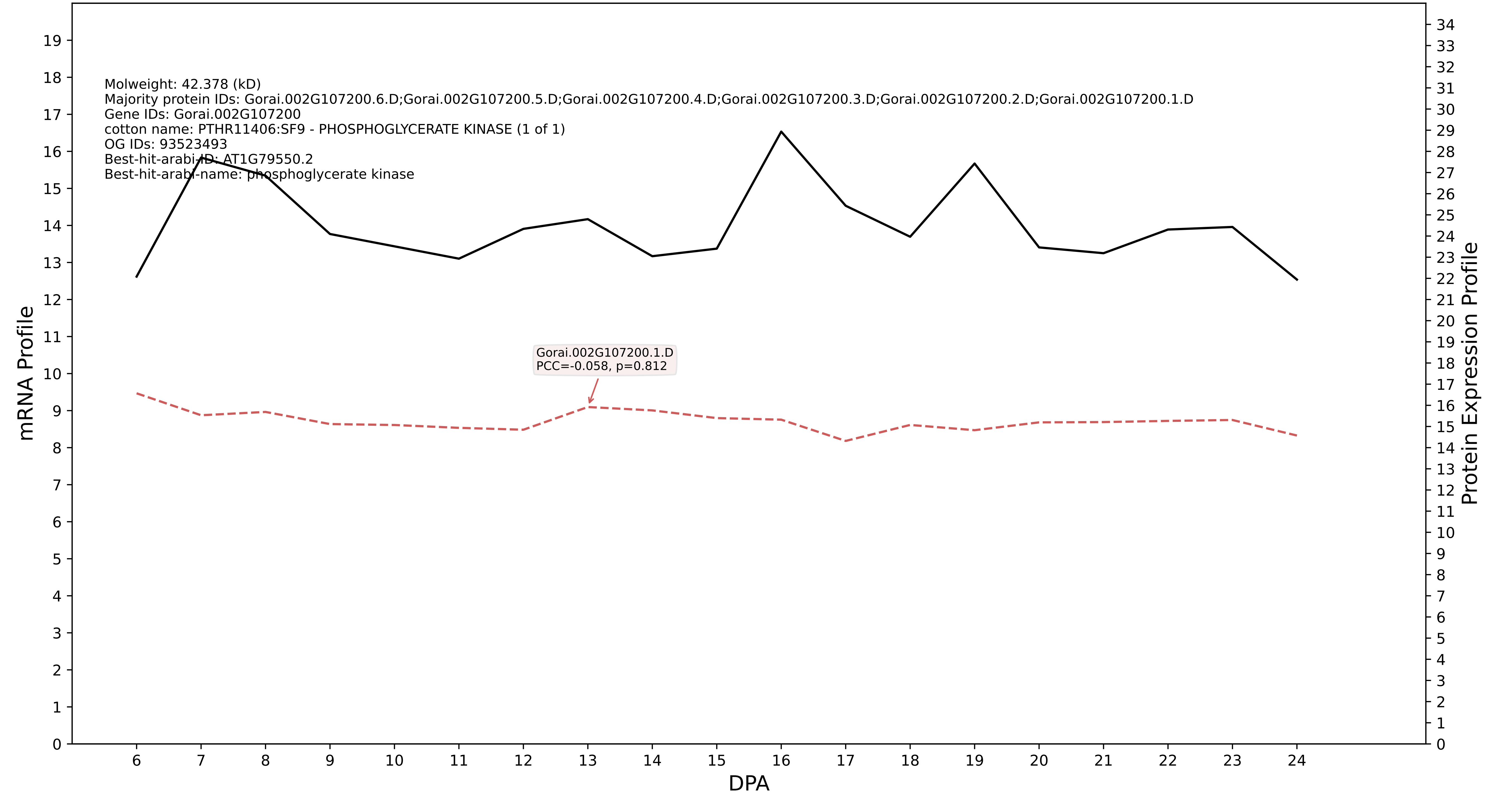

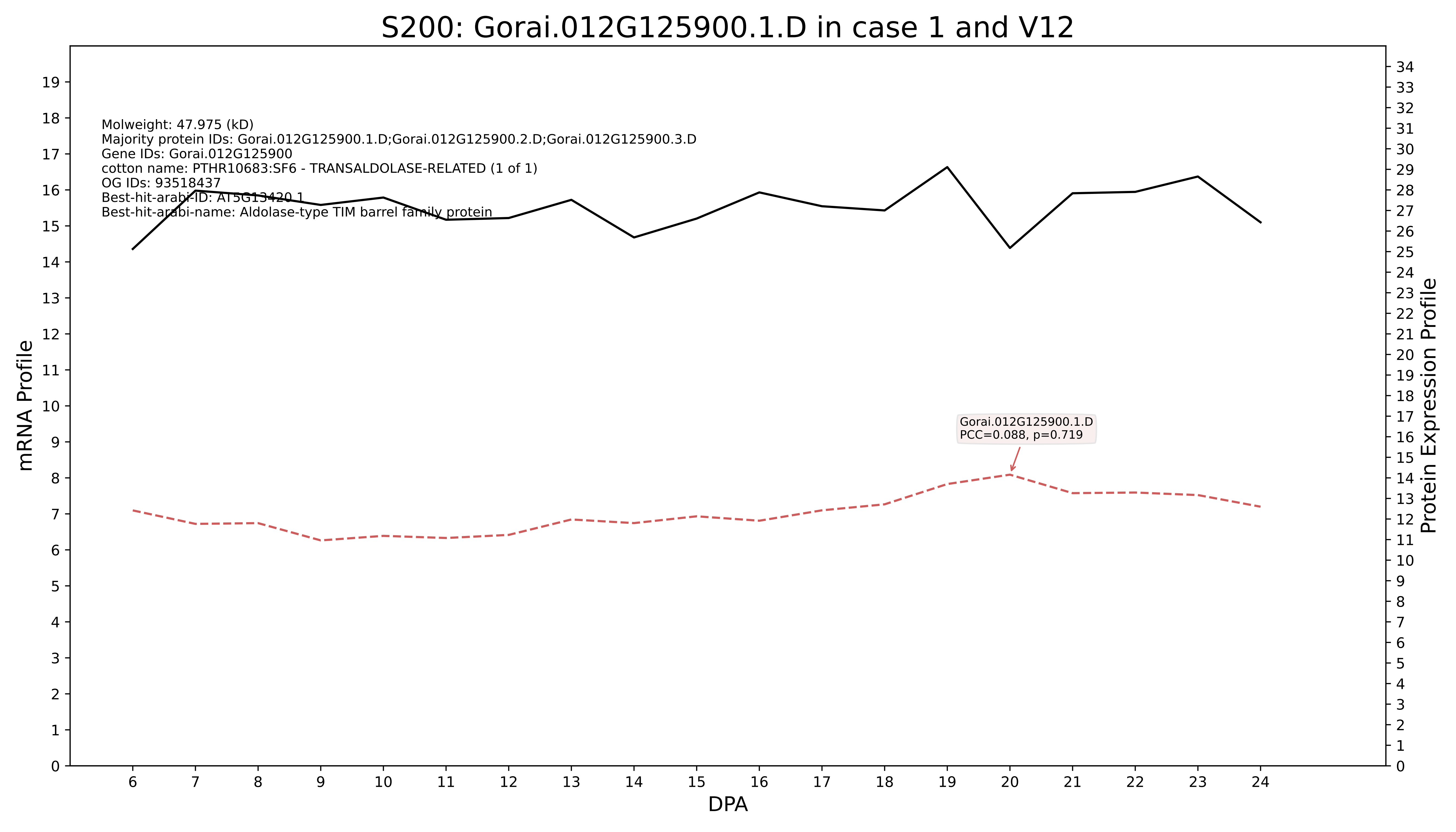
S200: Gorai.012G125900.1.D in case 1 and V12
Molweight: 47.975 (kD)
Majority protein IDs: Gorai.012G125900.1.D;Gorai.012G125900.2.D;Gorai.012G125900.3.D
Gene IDs: Gorai.012G125900
cotton name: PTHR10683:SF6 - TRANSALDOLASE-RELATED (1 of 1)
OG IDs: 93518437
Best-hit-arabi-ID: AT5G13420.1
Best-hit-arabi-name: Aldolase-type TIM barrel family protein
Gorai.012G125900.1.D
PCC=0.088, p=0.719
mRNA Profile
Protein Expression Profile
DPA

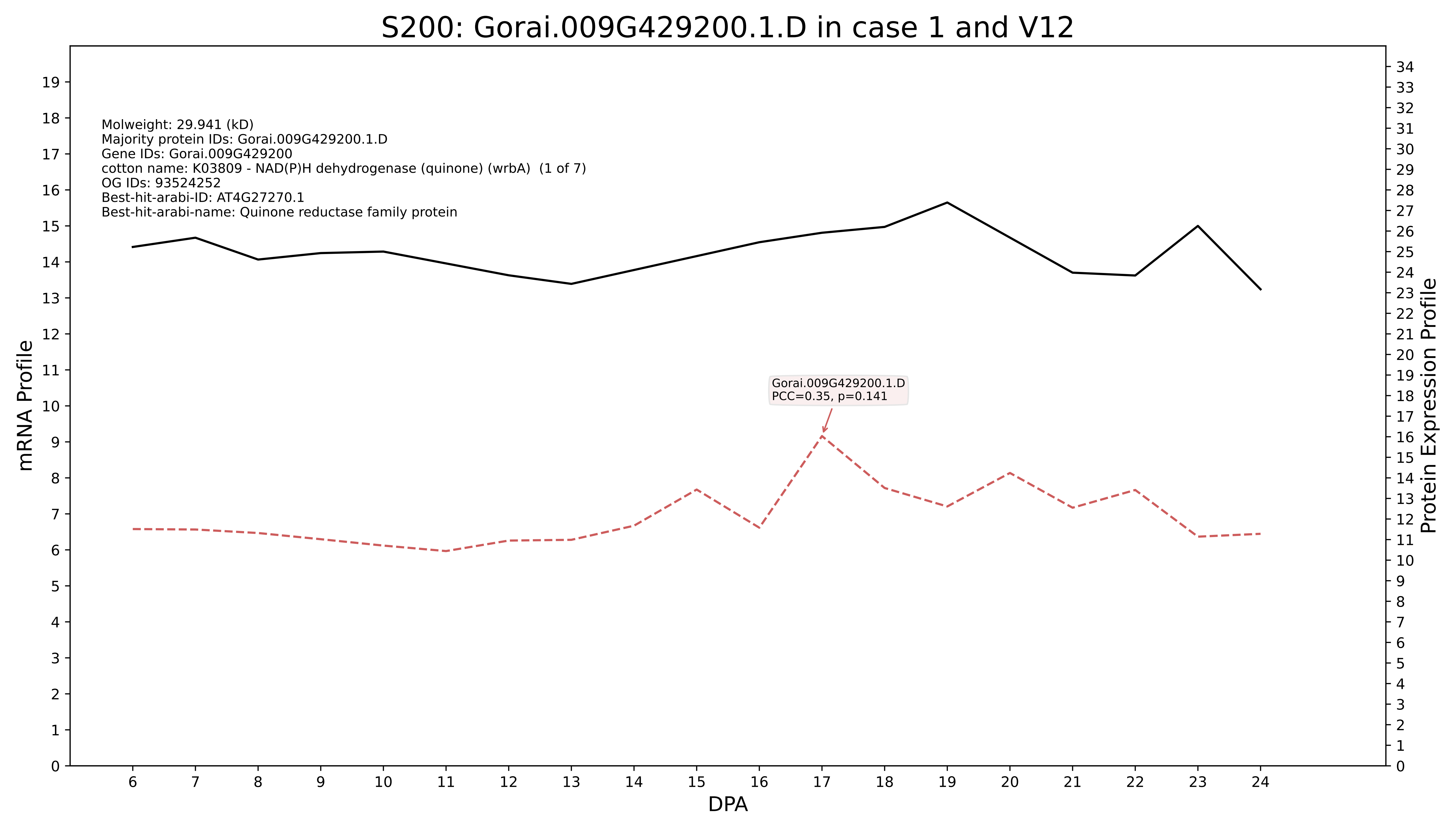
S200: Gorai.009G429200.1.D in case 1 and V12
Molweight: 29.941 (kD)
Majority protein IDs: Gorai.009G429200.1.D
Gene IDs: Gorai.009G429200
cotton name: K03809 - NAD(P)H dehydrogenase (quinone) (wrbA) (1 of 7)
OG IDs: 93524252
Best-hit-arabi-ID: AT4G27270.1
Best-hit-arabi-name: Quinone reductase family protein
Gorai.009G429200.1.D
PCC=0.35, p=0.141
mRNA Profile
Protein Expression Profile
DPA
0 1 2 3 4 5 6 7 8 9 10 11 12 13 14 15 16 17 18 19
0 1 2 3 4 5 6 7 8 9 10 11 12 13 14 15 16 17 18 19 20 21 22 23 24 25 26 27 28 29 30 31 32 33 34
6 7 8 9 10 11 12 13 14 15 16 17 18 19 20 21 22 23 24

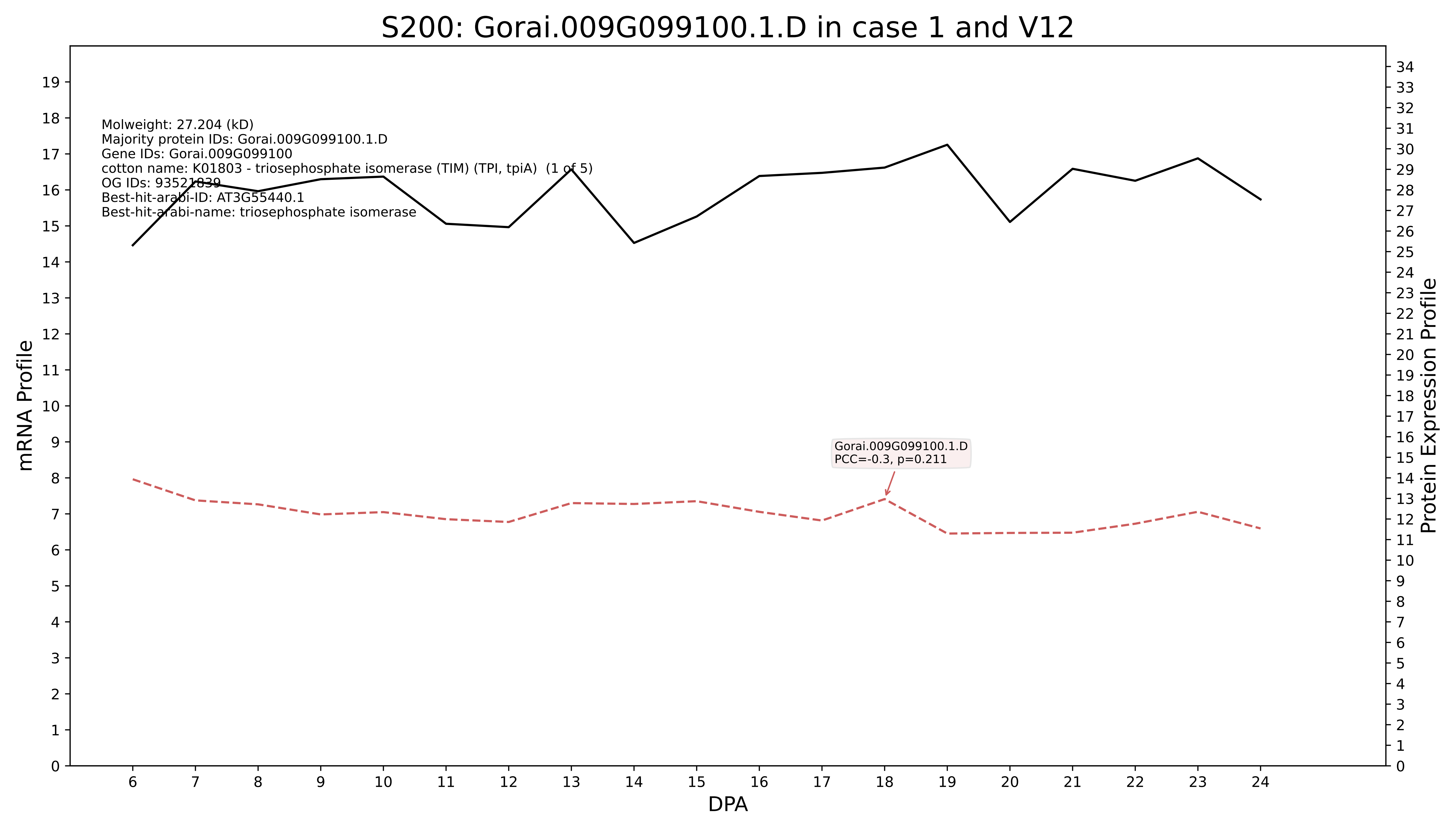
S200: Gorai.009G099100.1.D in case 1 and V12
Molweight: 27.204 (kD)
Majority protein IDs: Gorai.009G099100.1.D
Gene IDs: Gorai.009G099100
cotton name: K01803 - triosephosphate isomerase (TIM) (TPI, tpiA) (1 of 5)
OG IDs: 93521839
Best-hit-arabi-ID: AT3G55440.1
Best-hit-arabi-name: triosephosphate isomerase
Gorai.009G099100.1.D
PCC=-0.3, p=0.211
mRNA Profile
Protein Expression Profile
DPA
0
1
2
3
4
5
6
7
8
9
10
11
12
13
14
15
16
17
18
19
20
21
22
23
24
25
26
27
28
29
30
31
32
33
34

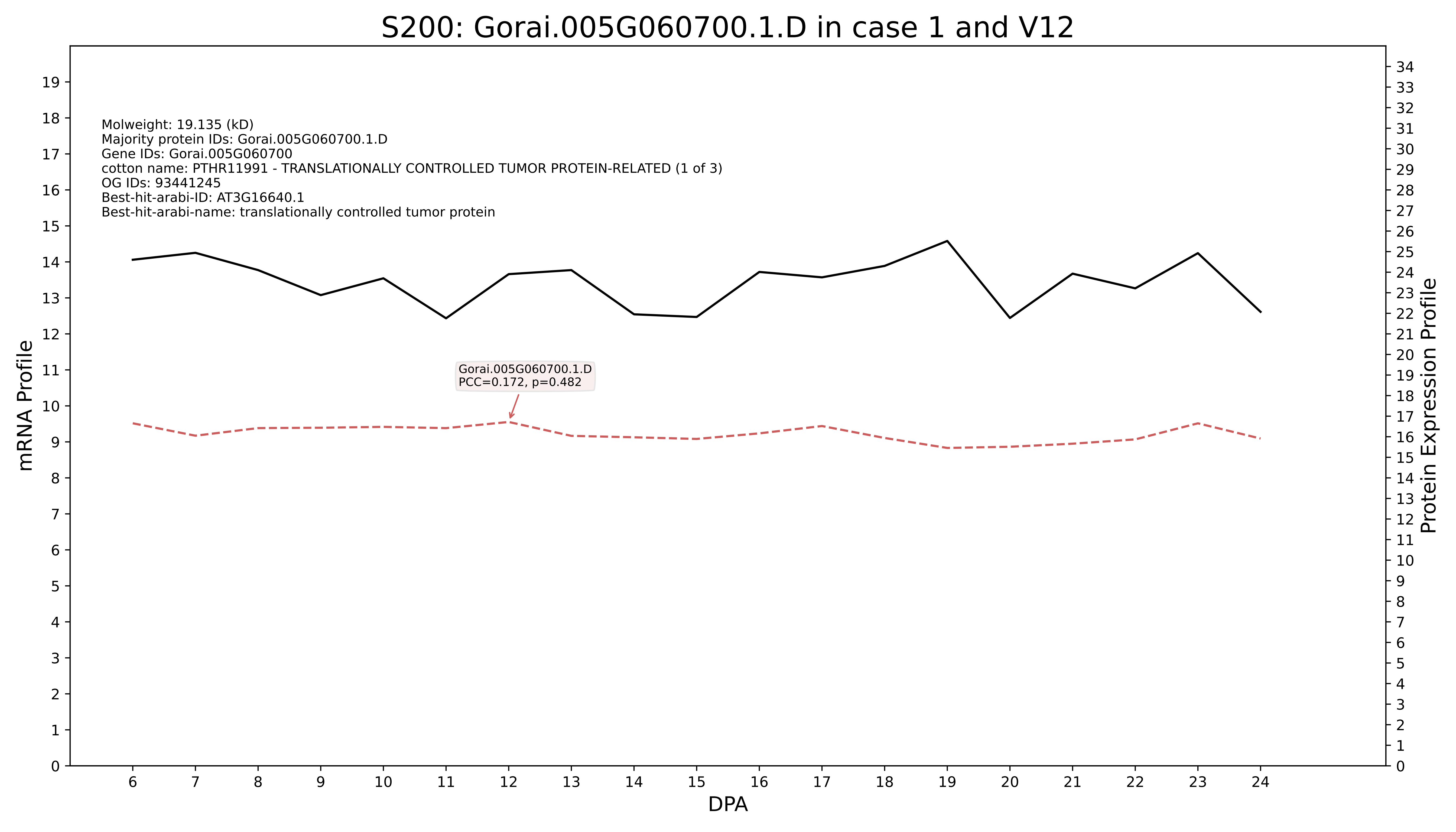
S200: Gorai.005G060700.1.D in case 1 and V12
Molweight: 19.135 (kD)
Majority protein IDs: Gorai.005G060700.1.D
Gene IDs: Gorai.005G060700
cotton name: PTHR11991 - TRANSLATIONALLY CONTROLLED TUMOR PROTEIN-RELATED (1 of 3)
OG IDs: 93441245
Best-hit-arabi-ID: AT3G16640.1
Best-hit-arabi-name: translationally controlled tumor protein
Gorai.005G060700.1.D
PCC=0.172, p=0.482
mRNA Profile
Protein Expression Profile
DPA

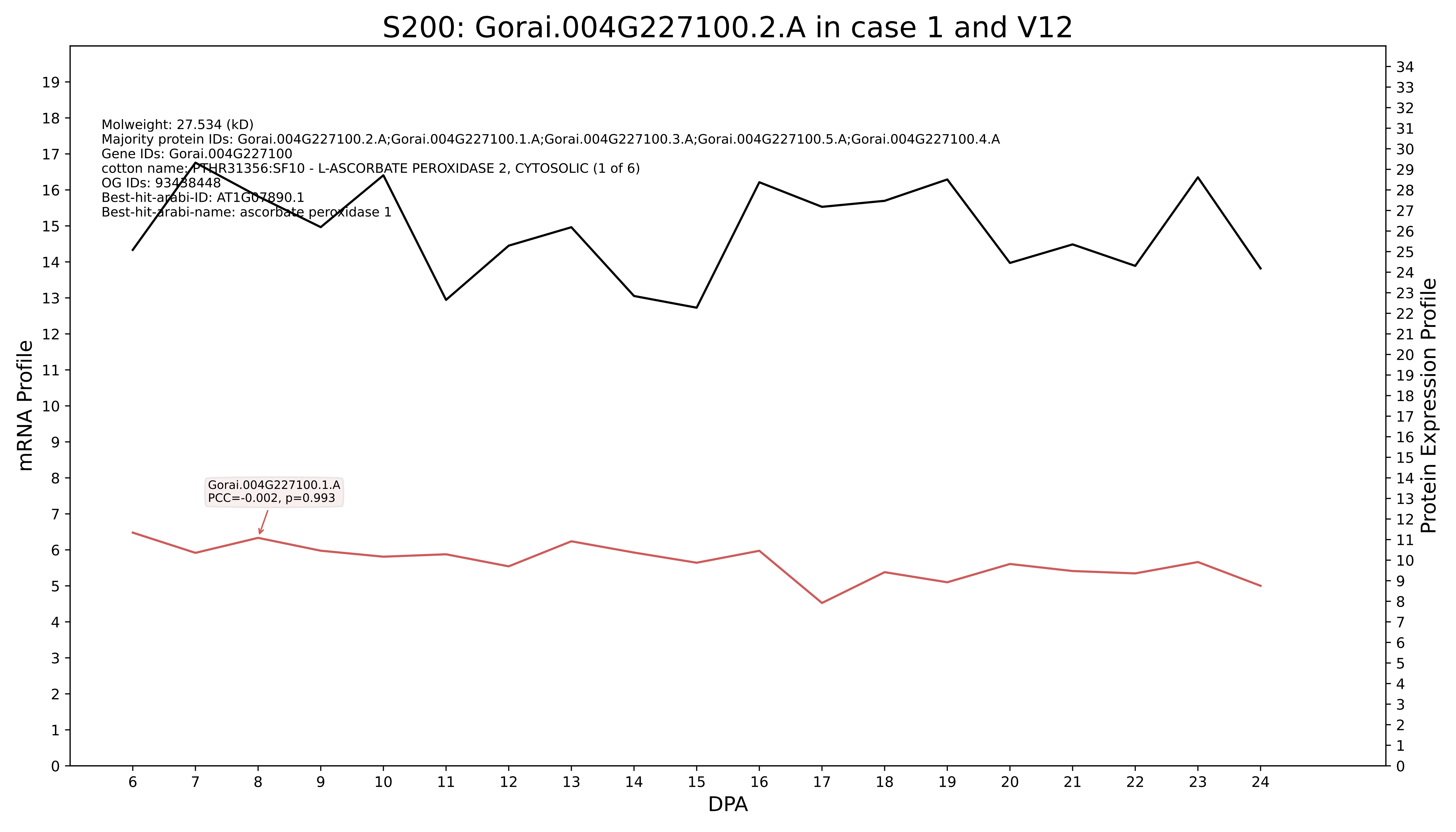

S200: Gorai.004G227100.2.A in case 1 and V12
Molweight: 27.534 (kD)
Majority protein IDs: Gorai.004G227100.2.A;Gorai.004G227100.1.A;Gorai.004G227100.3.A;Gorai.004G227100.5.A;Gorai.004G227100.4.A
Gene IDs: Gorai.004G227100
cotton name: PTHR31356:SF10 - L-ASCORBATE PEROXIDASE 2, CYTOSOLIC (1 of 6)
OG IDs: 93438448
Best-hit-arabi-ID: AT1G07890.1
Best-hit-arabi-name: ascorbate peroxidase 1
Gorai.004G227100.1.A
PCC=-0.002, p=0.993
mRNA Profile
Protein Expression Profile
DPA

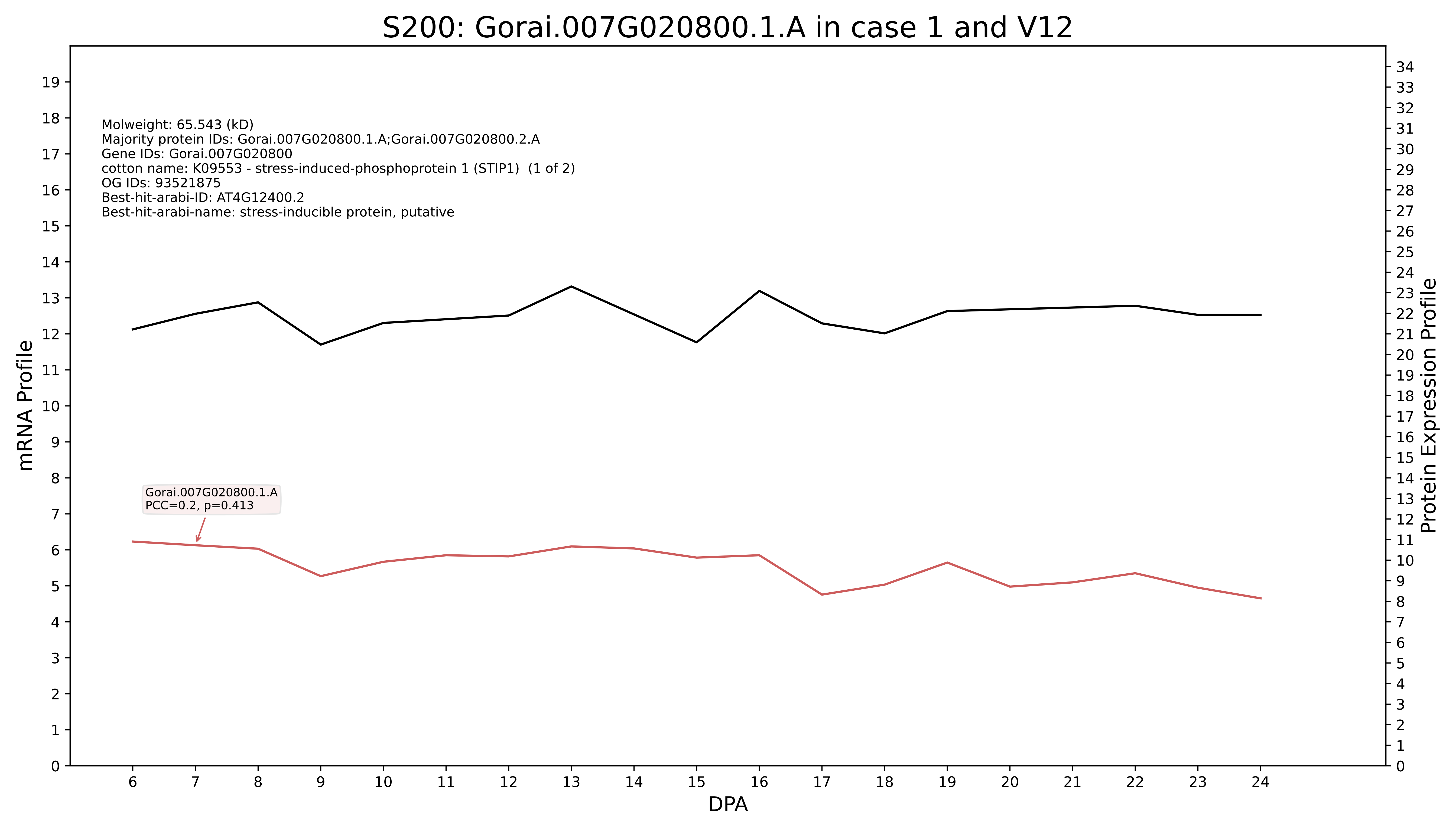
S200: Gorai.007G020800.1.A in case 1 and V12
Molweight: 65.543 (kD)
Majority protein IDs: Gorai.007G020800.1.A;Gorai.007G020800.2.A
Gene IDs: Gorai.007G020800
cotton name: K09553 - stress-induced-phosphoprotein 1 (STIP1) (1 of 2)
OG IDs: 93521875
Best-hit-arabi-ID: AT4G12400.2
Best-hit-arabi-name: stress-inducible protein, putative
Gorai.007G020800.1.A
PCC=0.2, p=0.413
mRNA Profile
Protein Expression Profile
DPA

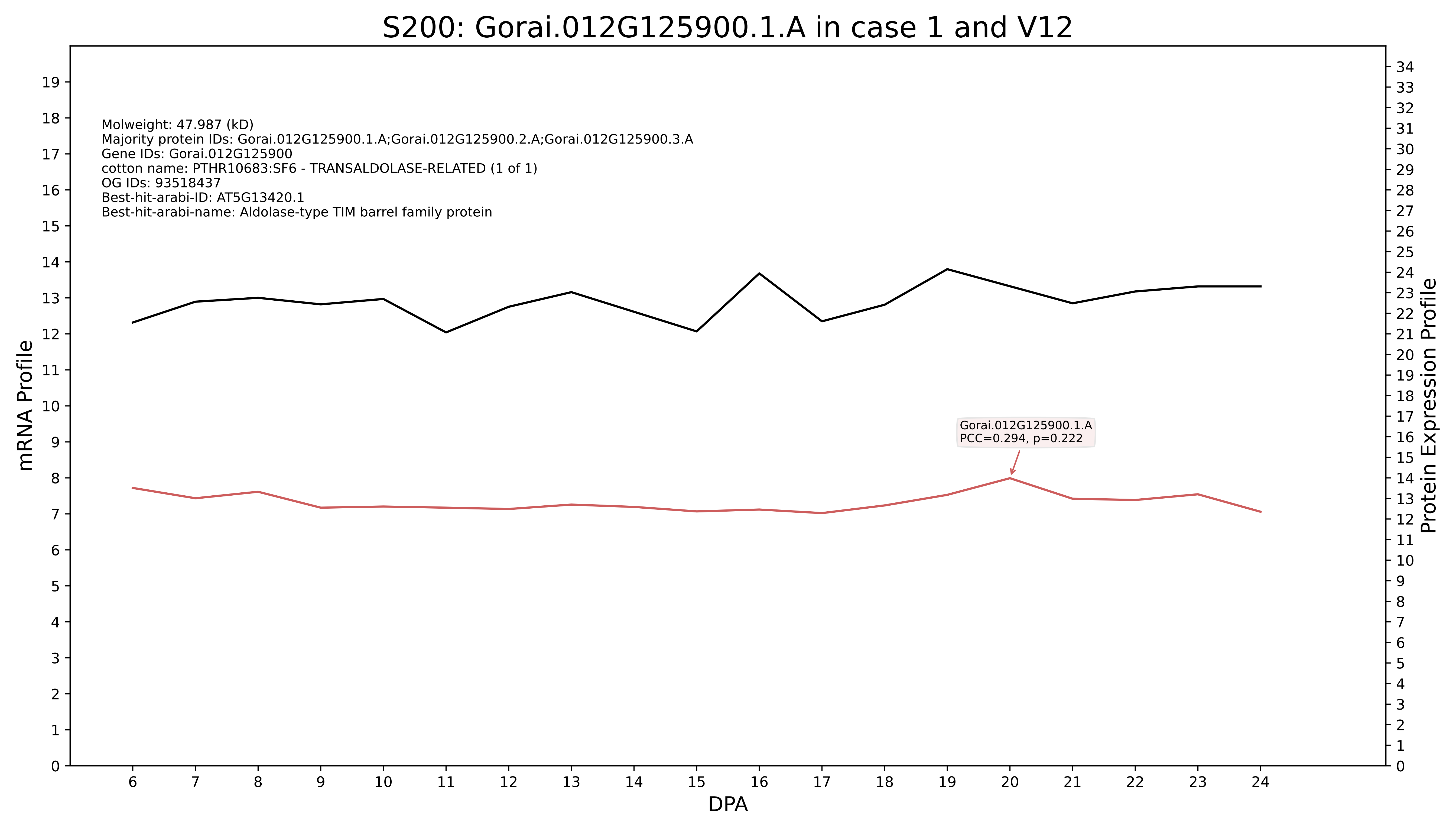
S200: Gorai.012G125900.1.A in case 1 and V12
Molweight: 47.987 (kD)
Majority protein IDs: Gorai.012G125900.1.A;Gorai.012G125900.2.A;Gorai.012G125900.3.A
Gene IDs: Gorai.012G125900
cotton name: PTHR10683:SF6 - TRANSALDOLASE-RELATED (1 of 1)
OG IDs: 93518437
Best-hit-arabi-ID: AT5G13420.1
Best-hit-arabi-name: Aldolase-type TIM barrel family protein
Gorai.012G125900.1.A
PCC=0.294, p=0.222
mRNA Profile
Protein Expression Profile
DPA

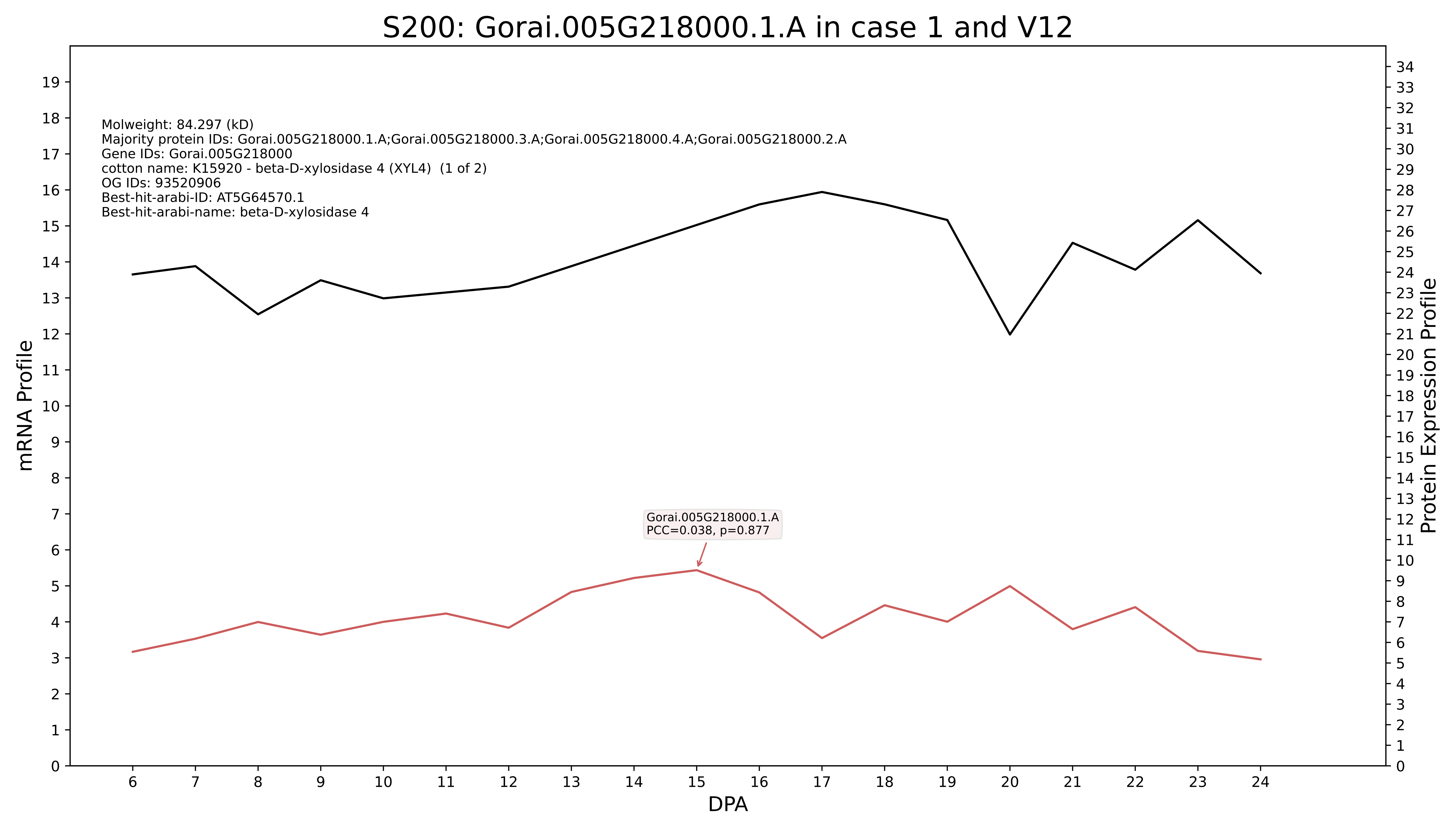
S200: Gorai.005G218000.1.A in case 1 and V12
Molweight: 84.297 (kD)
Majority protein IDs: Gorai.005G218000.1.A;Gorai.005G218000.3.A;Gorai.005G218000.4.A;Gorai.005G218000.2.A
Gene IDs: Gorai.005G218000
cotton name: K15920 - beta-D-xylosidase 4 (XYL4) (1 of 2)
OG IDs: 93520906
Best-hit-arabi-ID: AT5G64570.1
Best-hit-arabi-name: beta-D-xylosidase 4
Gorai.005G218000.1.A
PCC=0.038, p=0.877
mRNA Profile
Protein Expression Profile
DPA

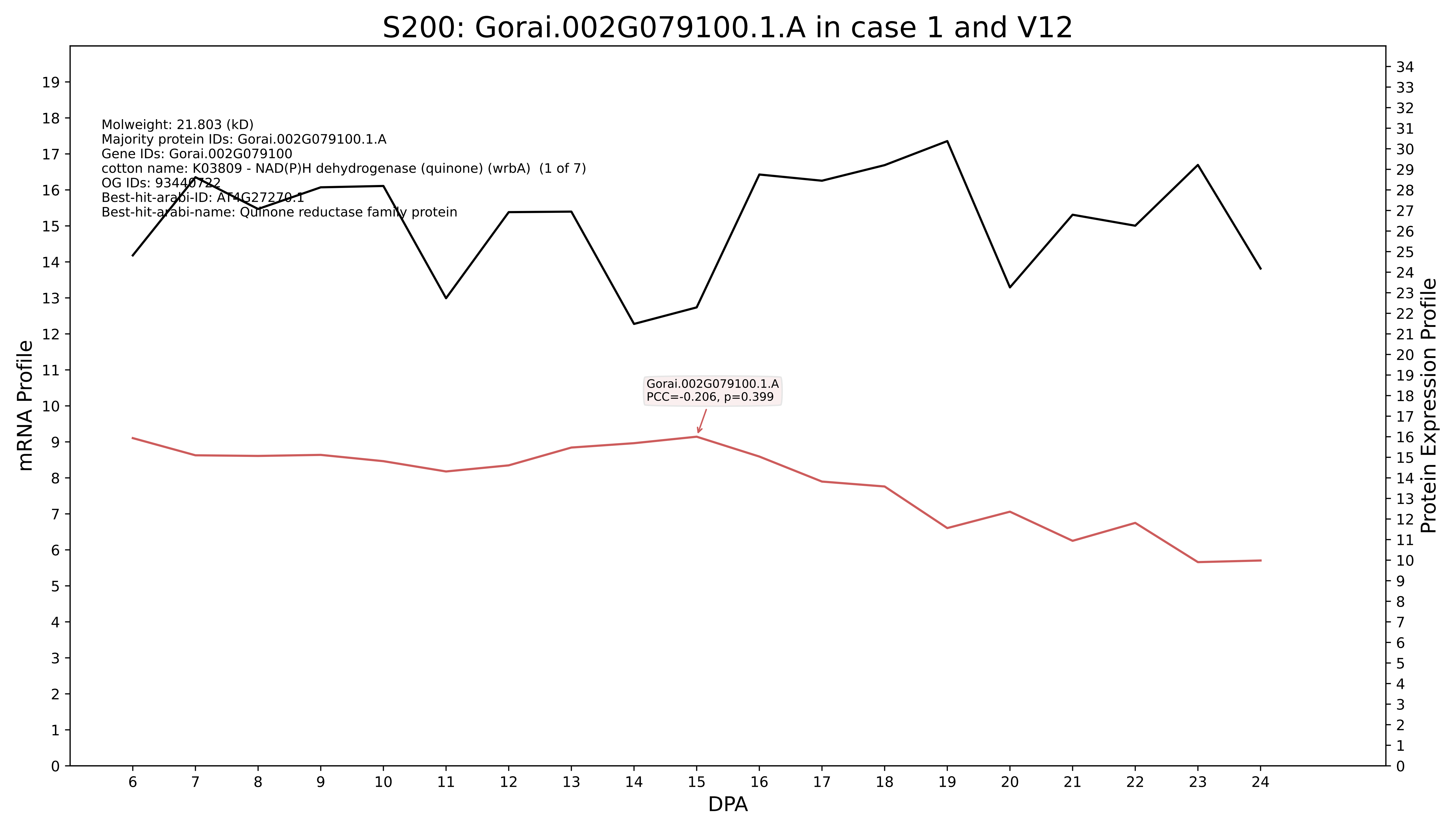

S200: Gorai.002G079100.1.A in case 1 and V12
Molweight: 21.803 (kD)
Majority protein IDs: Gorai.002G079100.1.A
Gene IDs: Gorai.002G079100
cotton name: K03809 - NAD(P)H dehydrogenase (quinone) (wrbA) (1 of 7)
OG IDs: 93440722
Best-hit-arabi-ID: AT4G27270.1
Best-hit-arabi-name: Quinone reductase family protein
Gorai.002G079100.1.A
PCC=-0.206, p=0.399
mRNA Profile
Protein Expression Profile
DPA
0
1
2
3
4
5
6
7
8
9
10
11
12
13
14
15
16
17
18
19
20
21
22
23
24
25
26
27
28
29
30
31
32
33
34

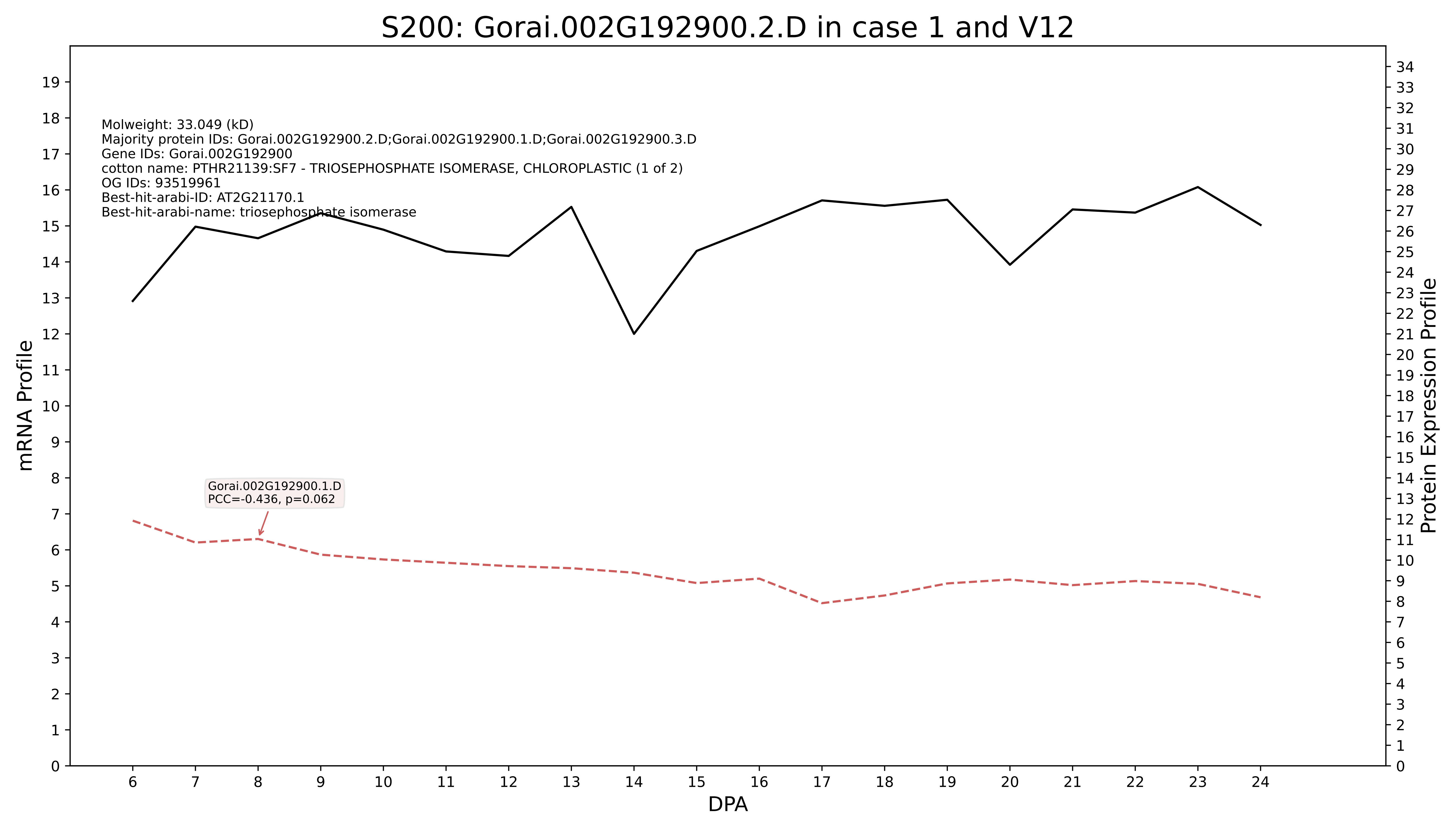
S200: Gorai.002G192900.2.D in case 1 and V12
Molweight: 33.049 (kD)
Majority protein IDs: Gorai.002G192900.2.D;Gorai.002G192900.1.D;Gorai.002G192900.3.D
Gene IDs: Gorai.002G192900
cotton name: PTHR21139:SF7 - TRIOSEPHOSPHATE ISOMERASE, CHLOROPLASTIC (1 of 2)
OG IDs: 93519961
Best-hit-arabi-ID: AT2G21170.1
Best-hit-arabi-name: triosephosphate isomerase
Gorai.002G192900.1.D
PCC=-0.436, p=0.062
mRNA Profile
Protein Expression Profile
DPA
0
1
2
3
4
5
6
7
8
9
10
11
12
13
14
15
16
17
18
19
20
21
22
23
24
25
26
27
28
29
30
31
32
33
34

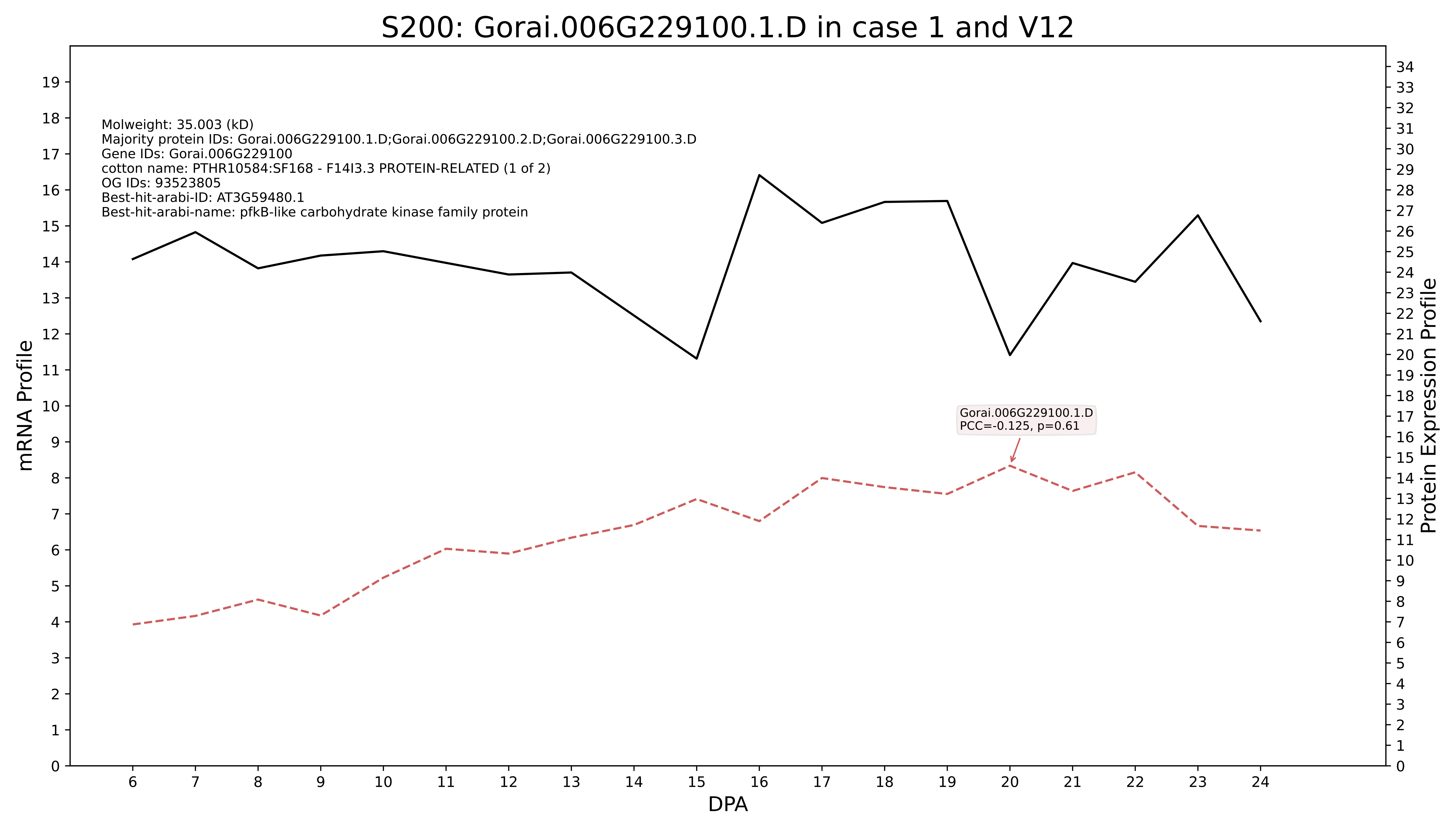
S200: Gorai.006G229100.1.D in case 1 and V12
Molweight: 35.003 (kD)
Majority protein IDs: Gorai.006G229100.1.D;Gorai.006G229100.2.D;Gorai.006G229100.3.D
Gene IDs: Gorai.006G229100
cotton name: PTHR10584:SF168 - F14I3.3 PROTEIN-RELATED (1 of 2)
OG IDs: 93523805
Best-hit-arabi-ID: AT3G59480.1
Best-hit-arabi-name: pfkB-like carbohydrate kinase family protein
Gorai.006G229100.1.D
PCC=-0.125, p=0.61
mRNA Profile
Protein Expression Profile
DPA

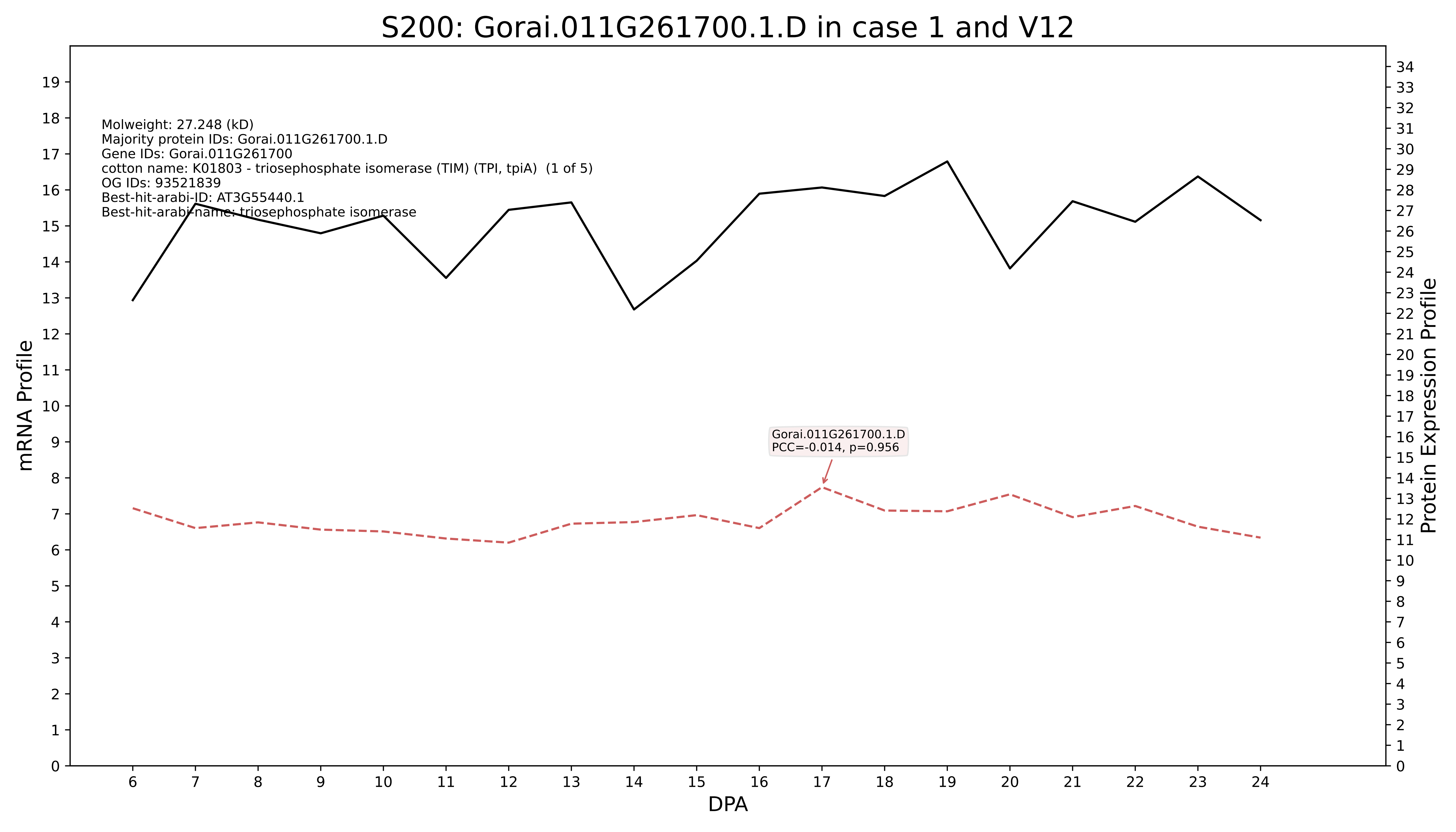
S200: Gorai.011G261700.1.D in case 1 and V12
Molweight: 27.248 (kD)
Majority protein IDs: Gorai.011G261700.1.D
Gene IDs: Gorai.011G261700
cotton name: K01803 - triosephosphate isomerase (TIM) (TPI, tpiA) (1 of 5)
OG IDs: 93521839
Best-hit-arabi-ID: AT3G55440.1
Best-hit-arabi-name: triosephosphate isomerase
Gorai.011G261700.1.D
PCC=-0.014, p=0.956
mRNA Profile
Protein Expression Profile
DPA

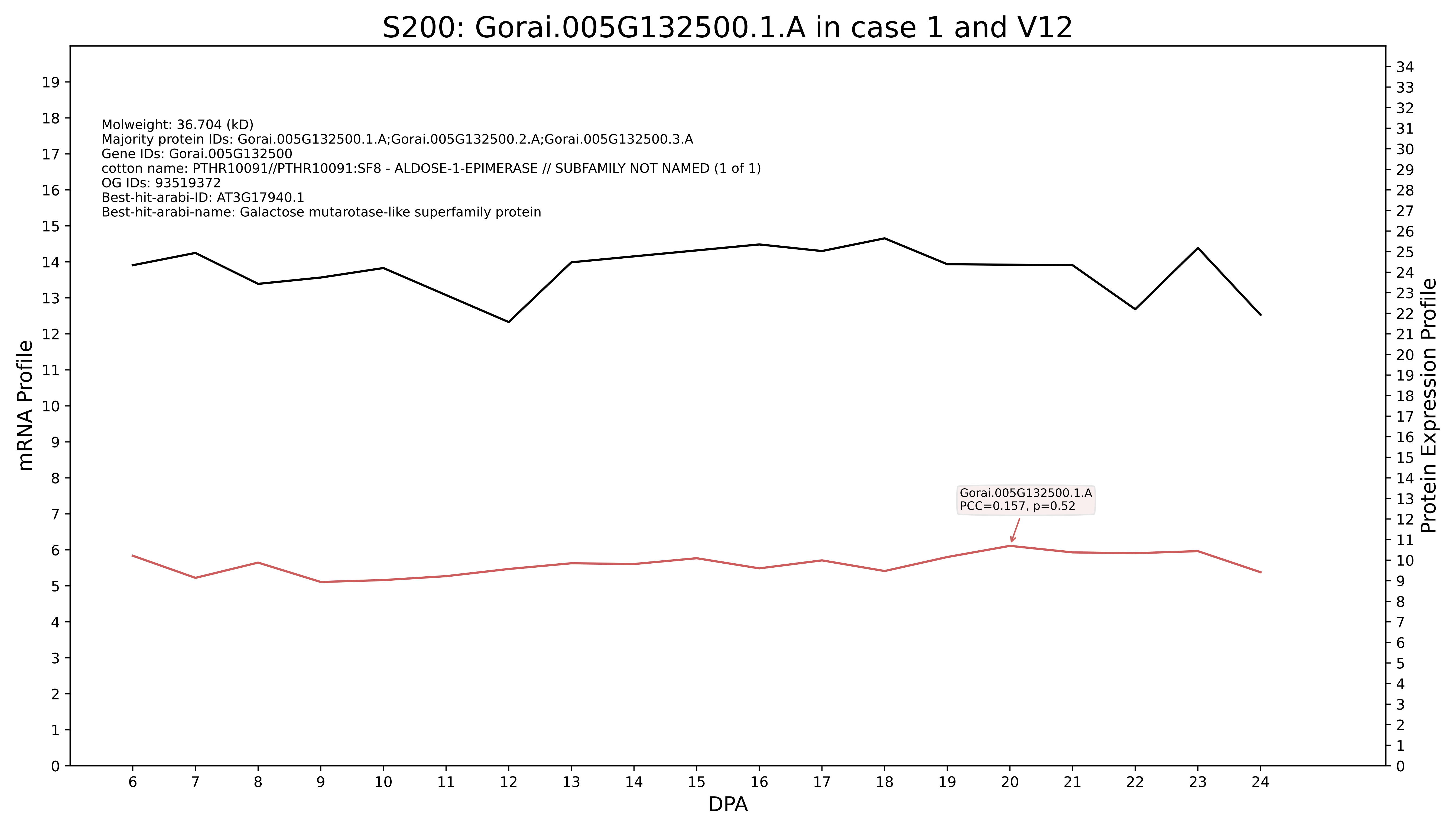
S200: Gorai.005G132500.1.A in case 1 and V12
Molweight: 36.704 (kD)
Majority protein IDs: Gorai.005G132500.1.A;Gorai.005G132500.2.A;Gorai.005G132500.3.A
Gene IDs: Gorai.005G132500
cotton name: PTHR10091//PTHR10091:SF8 - ALDOSE-1-EPIMERASE // SUBFAMILY NOT NAMED (1 of 1)
OG IDs: 93519372
Best-hit-arabi-ID: AT3G17940.1
Best-hit-arabi-name: Galactose mutarotase-like superfamily protein
Gorai.005G132500.1.A
PCC=0.157, p=0.52
mRNA Profile
Protein Expression Profile
DPA
0
1
2
3
4
5
6
7
8
9
10
11
12
13
14
15
16
17
18
19
0
1
2
3
4
5
6
7
8
9
10
11
12
13
14
15
16
17
18
19
20
21
22
23
24
25
26
27
28
29
30
31
32
33
34
6
7
8
9
10
11
12
13
14
15
16
17
18
19
20
21
22
23
24

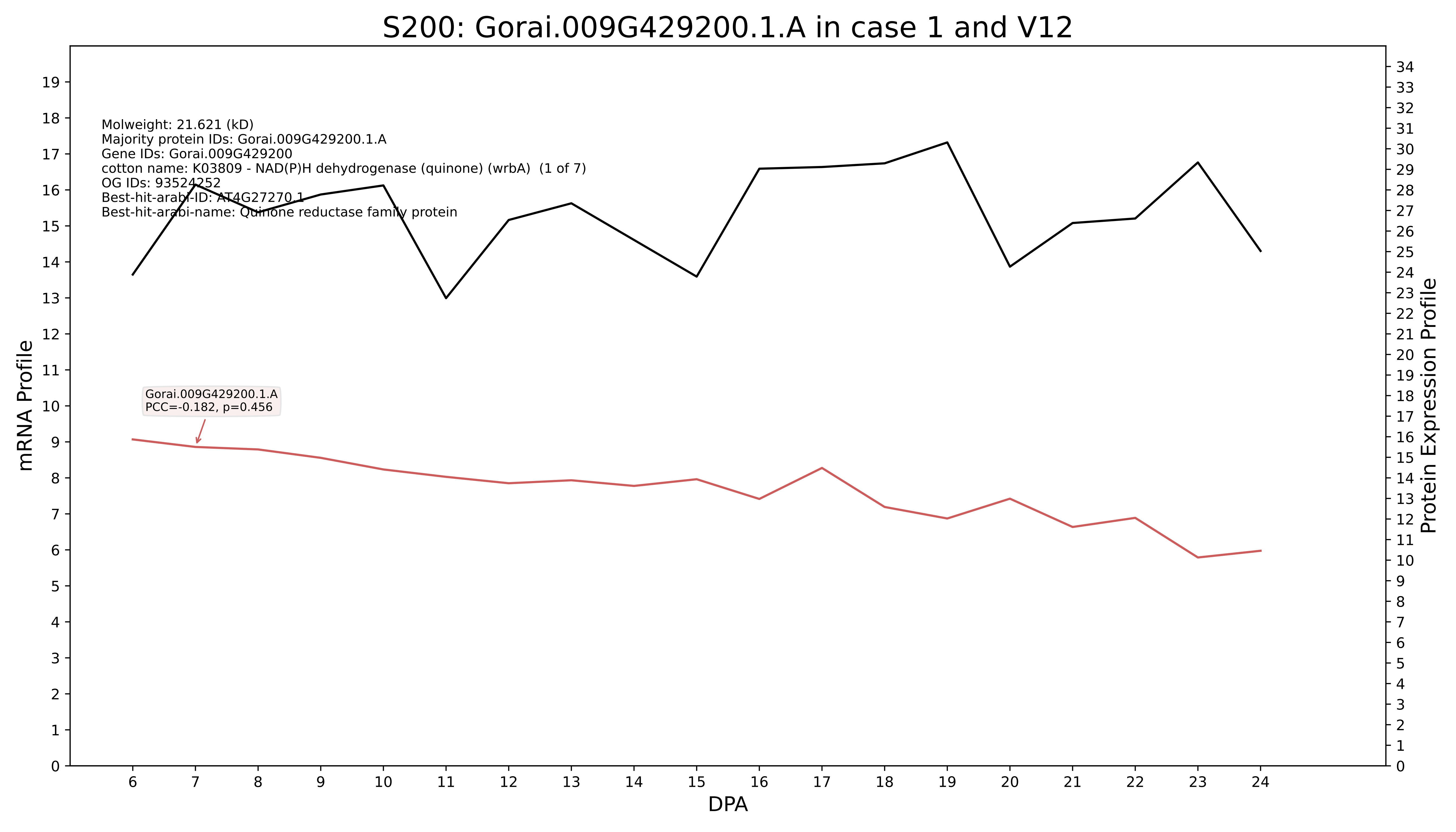

S200: Gorai.009G429200.1.A in case 1 and V12
Molweight: 21.621 (kD)
Majority protein IDs: Gorai.009G429200.1.A
Gene IDs: Gorai.009G429200
cotton name: K03809 - NAD(P)H dehydrogenase (quinone) (wrbA) (1 of 7)
OG IDs: 93524252
Best-hit-arabi-ID: AT4G27270.1
Best-hit-arabi-name: Quinone reductase family protein
Gorai.009G429200.1.A
PCC=-0.182, p=0.456
mRNA Profile
Protein Expression Profile
DPA

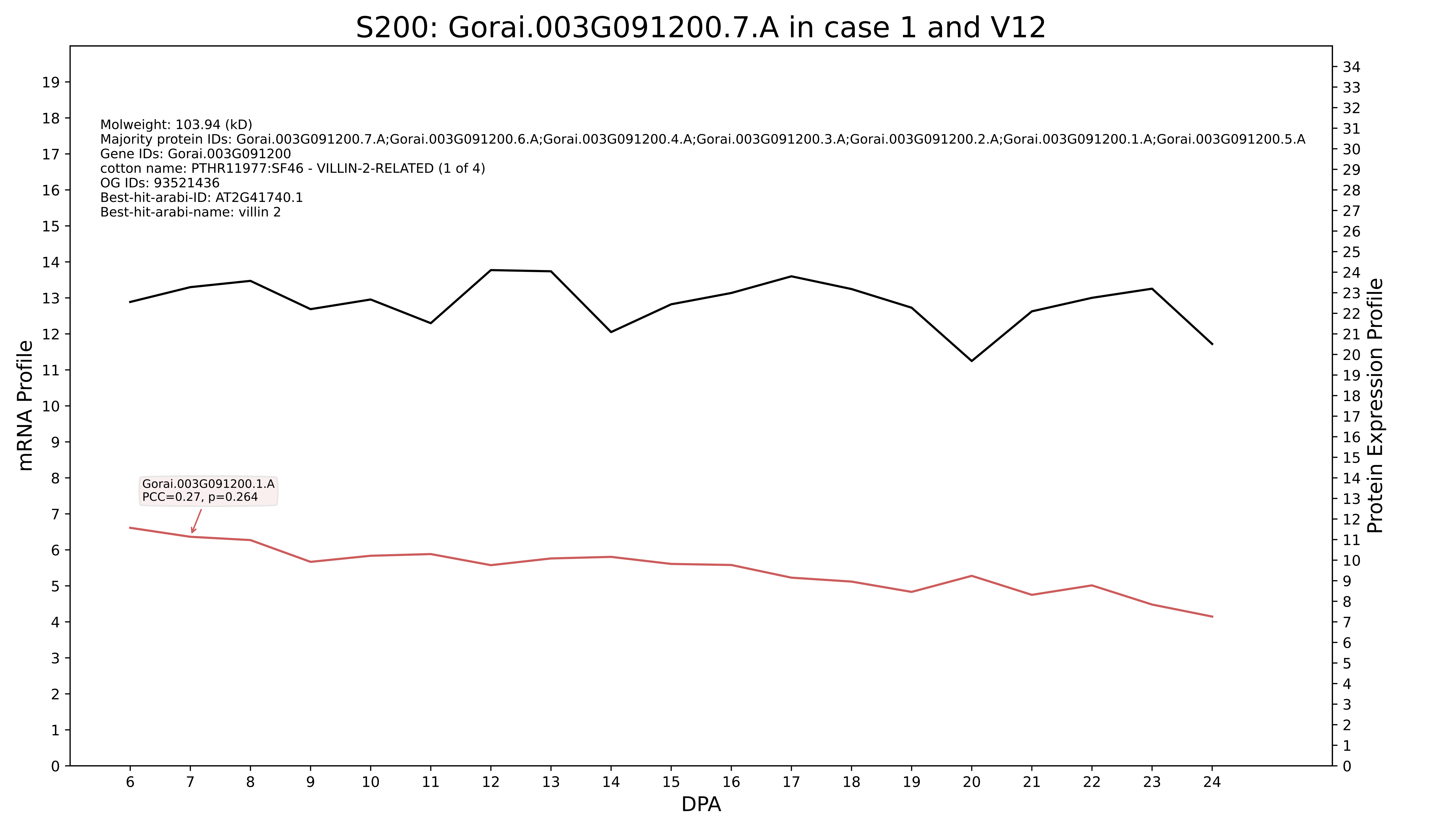
S200: Gorai.003G091200.7.A in case 1 and V12
Molweight: 103.94 (kD)
Majority protein IDs: Gorai.003G091200.7.A;Gorai.003G091200.6.A;Gorai.003G091200.4.A;Gorai.003G091200.3.A;Gorai.003G091200.2.A;Gorai.003G091200.1.A;Gorai.003G091200.5.A
Gene IDs: Gorai.003G091200
cotton name: PTHR11977:SF46 - VILLIN-2-RELATED (1 of 4)
OG IDs: 93521436
Best-hit-arabi-ID: AT2G41740.1
Best-hit-arabi-name: villin 2
Gorai.003G091200.1.A
PCC=0.27, p=0.264
mRNA Profile
Protein Expression Profile
DPA
0
1
2
3
4
5
6
7
8
9
10
11
12
13
14
15
16
17
18
19
20
21
22
23
24
25
26
27
28
29
30
31
32
33
34

S200: Gorai.002G243100.1.A in case 1 and V12

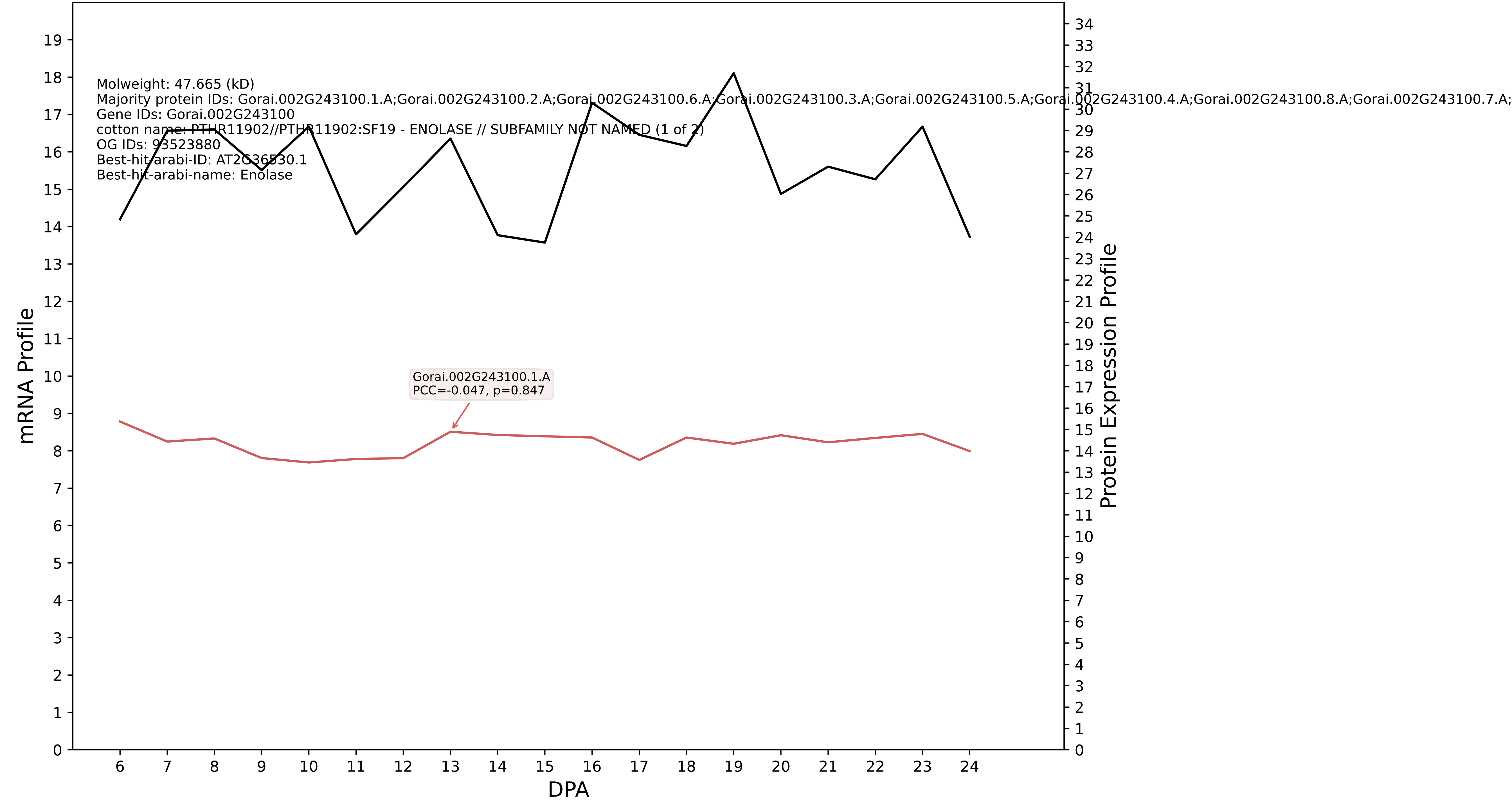

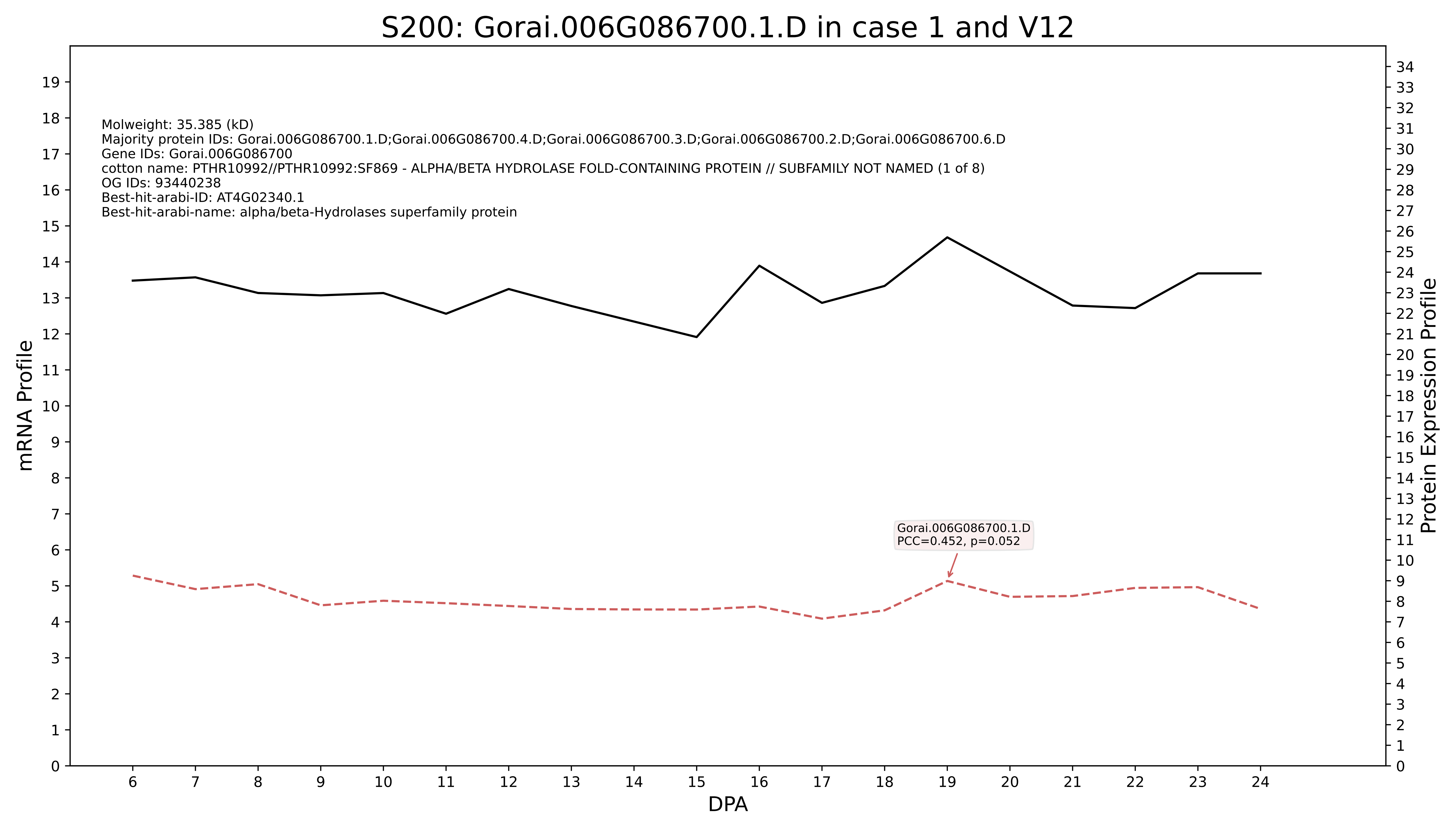
S200: Gorai.006G086700.1.D in case 1 and V12
Molweight: 35.385 (kD)
Majority protein IDs: Gorai.006G086700.1.D;Gorai.006G086700.4.D;Gorai.006G086700.3.D;Gorai.006G086700.2.D;Gorai.006G086700.6.D
Gene IDs: Gorai.006G086700
cotton name: PTHR10992//PTHR10992:SF869 - ALPHA/BETA HYDROLASE FOLD-CONTAINING PROTEIN // SUBFAMILY NOT NAMED (1 of 8)
OG IDs: 93440238
Best-hit-arabi-ID: AT4G02340.1
Best-hit-arabi-name: alpha/beta-Hydrolases superfamily protein
Gorai.006G086700.1.D
PCC=0.452, p=0.052
mRNA Profile
Protein Expression Profile
DPA

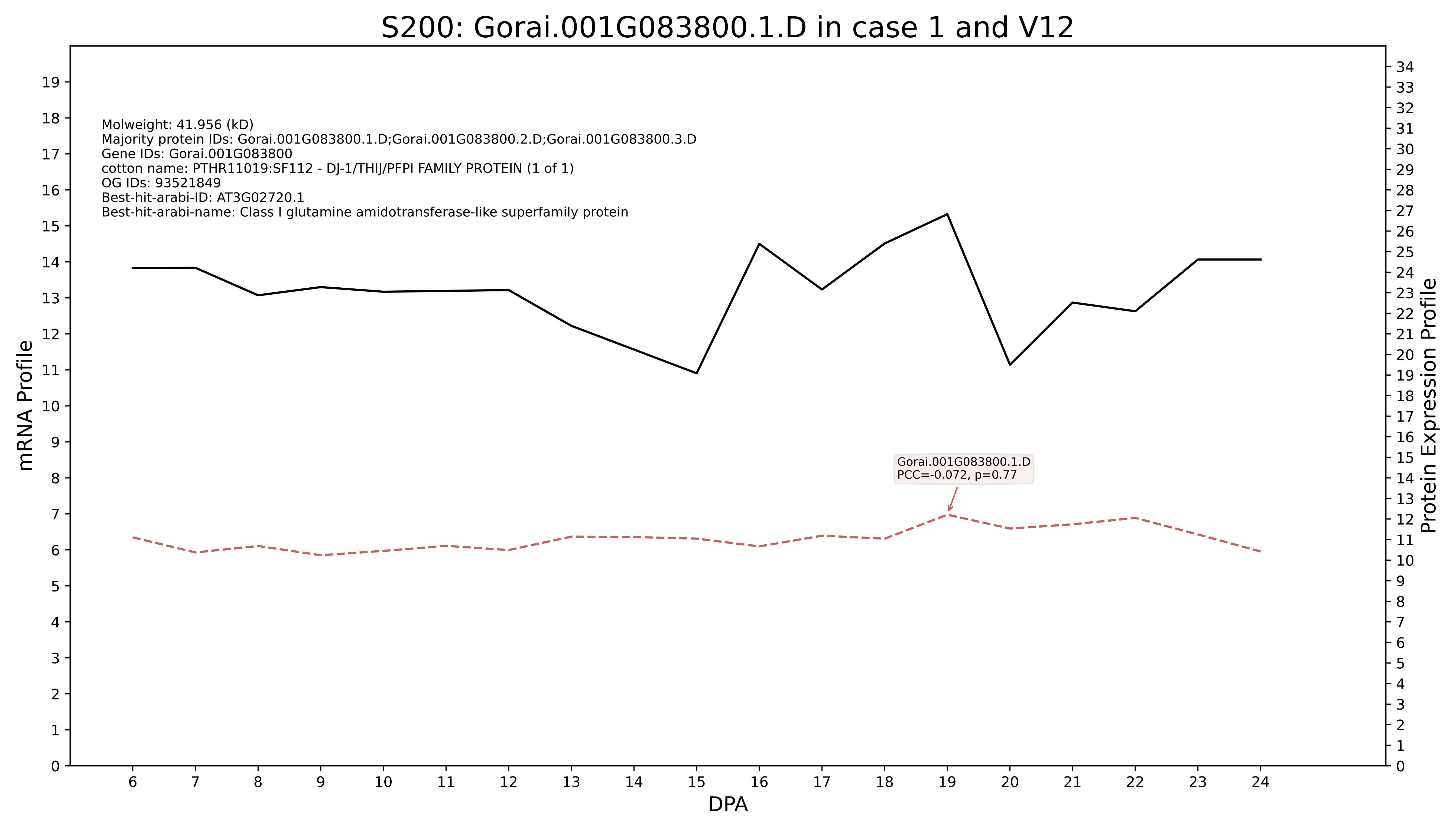
S200: Gorai.001G083800.1.D in case 1 and V12
Molweight: 41.956 (kD)
Majority protein IDs: Gorai.001G083800.1.D;Gorai.001G083800.2.D;Gorai.001G083800.3.D
Gene IDs: Gorai.001G083800
cotton name: PTHR11019:SF112 - DJ-1/THIJ/PFPI FAMILY PROTEIN (1 of 1)
OG IDs: 93521849
Best-hit-arabi-ID: AT3G02720.1
Best-hit-arabi-name: Class I glutamine amidotransferase-like superfamily protein
Gorai.001G083800.1.D
PCC=-0.072, p=0.77
mRNA Profile
Protein Expression Profile
DPA

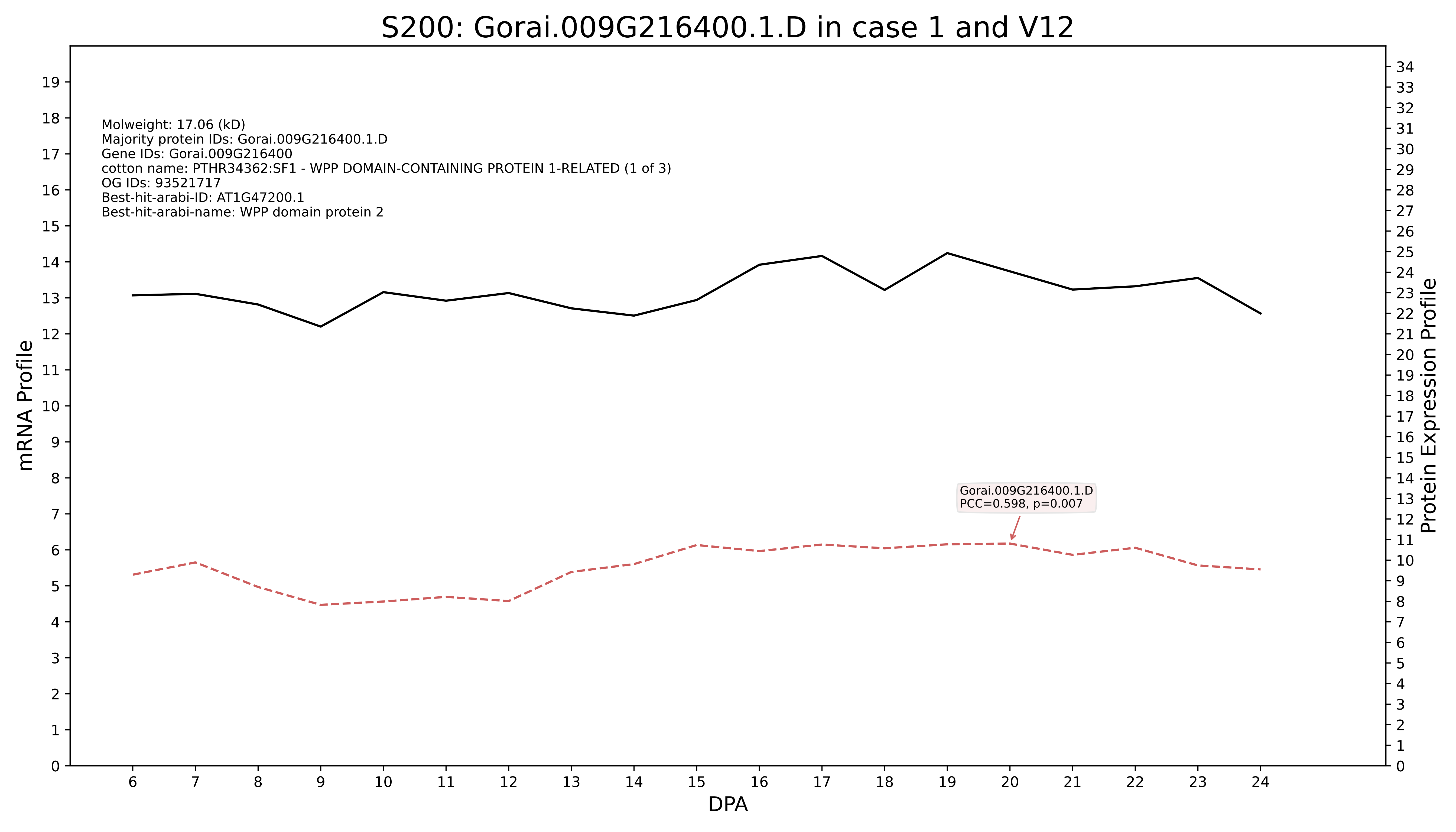

S200: Gorai.009G216400.1.D in case 1 and V12
Molweight: 17.06 (kD)
Majority protein IDs: Gorai.009G216400.1.D
Gene IDs: Gorai.009G216400
cotton name: PTHR34362:SF1 - WPP DOMAIN-CONTAINING PROTEIN 1-RELATED (1 of 3)
OG IDs: 93521717
Best-hit-arabi-ID: AT1G47200.1
Best-hit-arabi-name: WPP domain protein 2
Gorai.009G216400.1.D
PCC=0.598, p=0.007
mRNA Profile
Protein Expression Profile
DPA

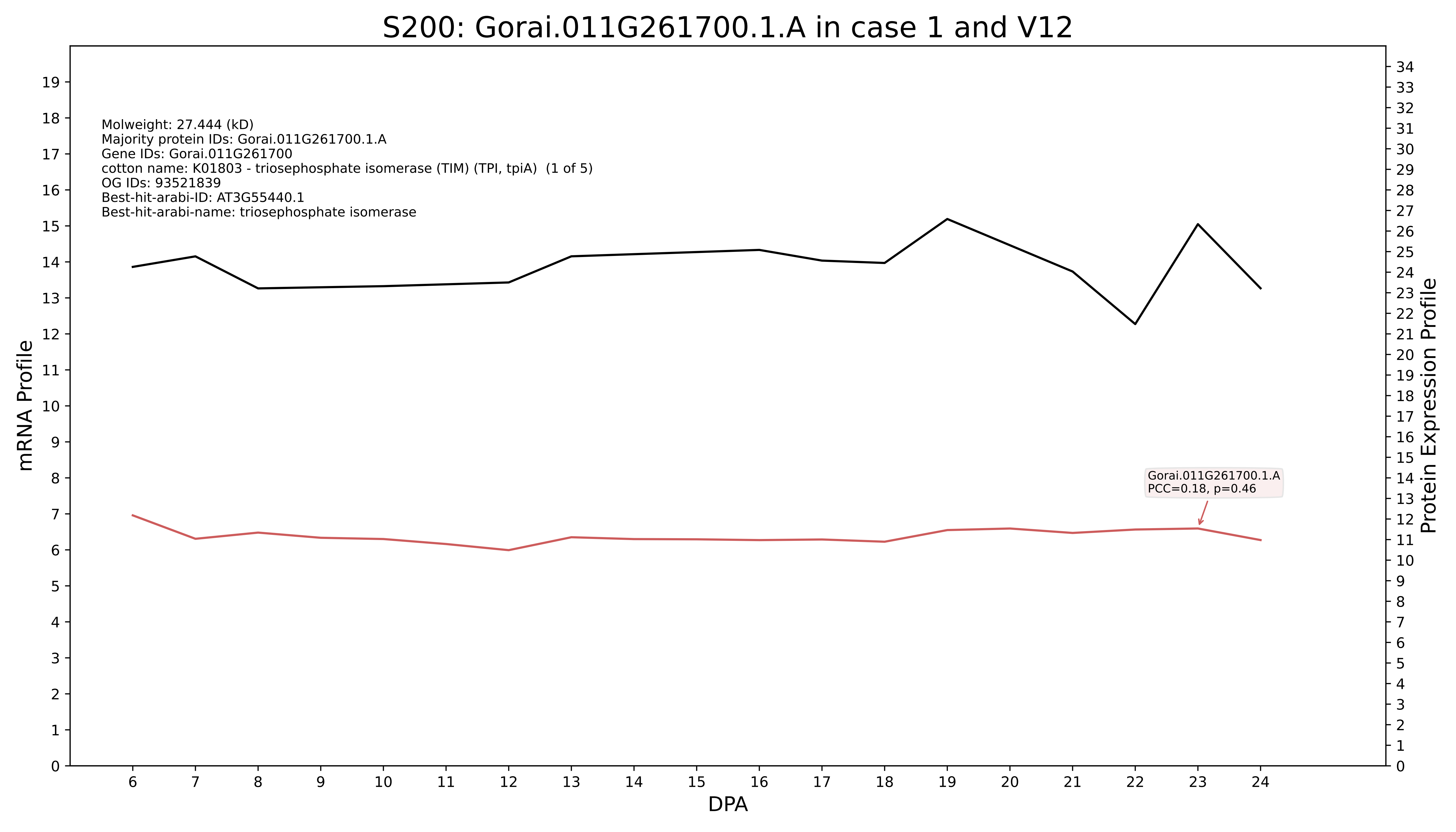

S200: Gorai.011G261700.1.A in case 1 and V12
Molweight: 27.444 (kD)
Majority protein IDs: Gorai.011G261700.1.A
Gene IDs: Gorai.011G261700
cotton name: K01803 - triosephosphate isomerase (TIM) (TPI, tpiA) (1 of 5)
OG IDs: 93521839
Best-hit-arabi-ID: AT3G55440.1
Best-hit-arabi-name: triosephosphate isomerase
Gorai.011G261700.1.A
PCC=0.18, p=0.46
mRNA Profile
Protein Expression Profile
DPA

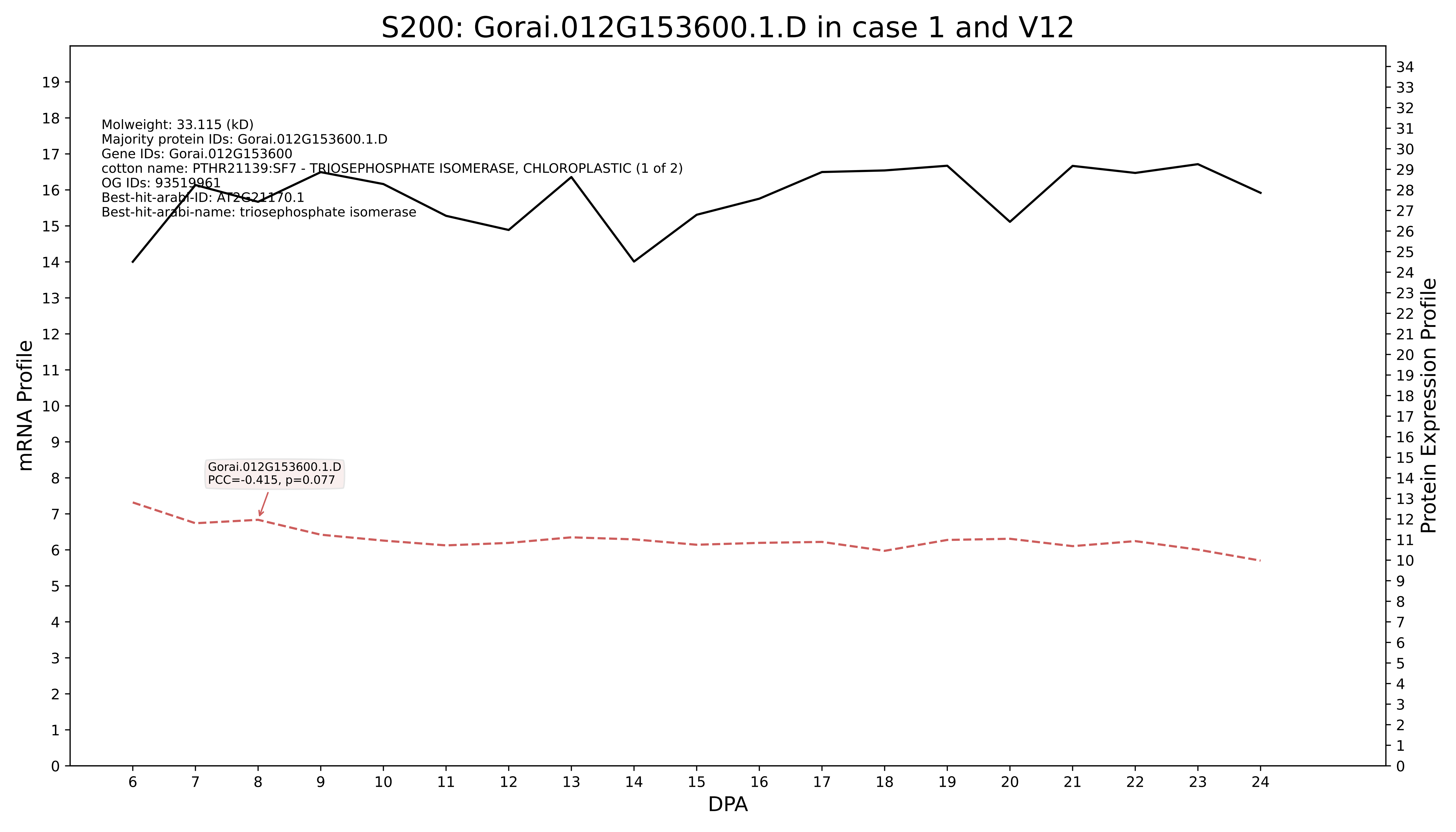
S200: Gorai.012G153600.1.D in case 1 and V12
Molweight: 33.115 (kD)
Majority protein IDs: Gorai.012G153600.1.D
Gene IDs: Gorai.012G153600
cotton name: PTHR21139:SF7 - TRIOSEPHOSPHATE ISOMERASE, CHLOROPLASTIC (1 of 2)
OG IDs: 93519961
Best-hit-arabi-ID: AT2G21170.1
Best-hit-arabi-name: triosephosphate isomerase
Gorai.012G153600.1.D
PCC=-0.415, p=0.077
mRNA Profile
Protein Expression Profile
DPA

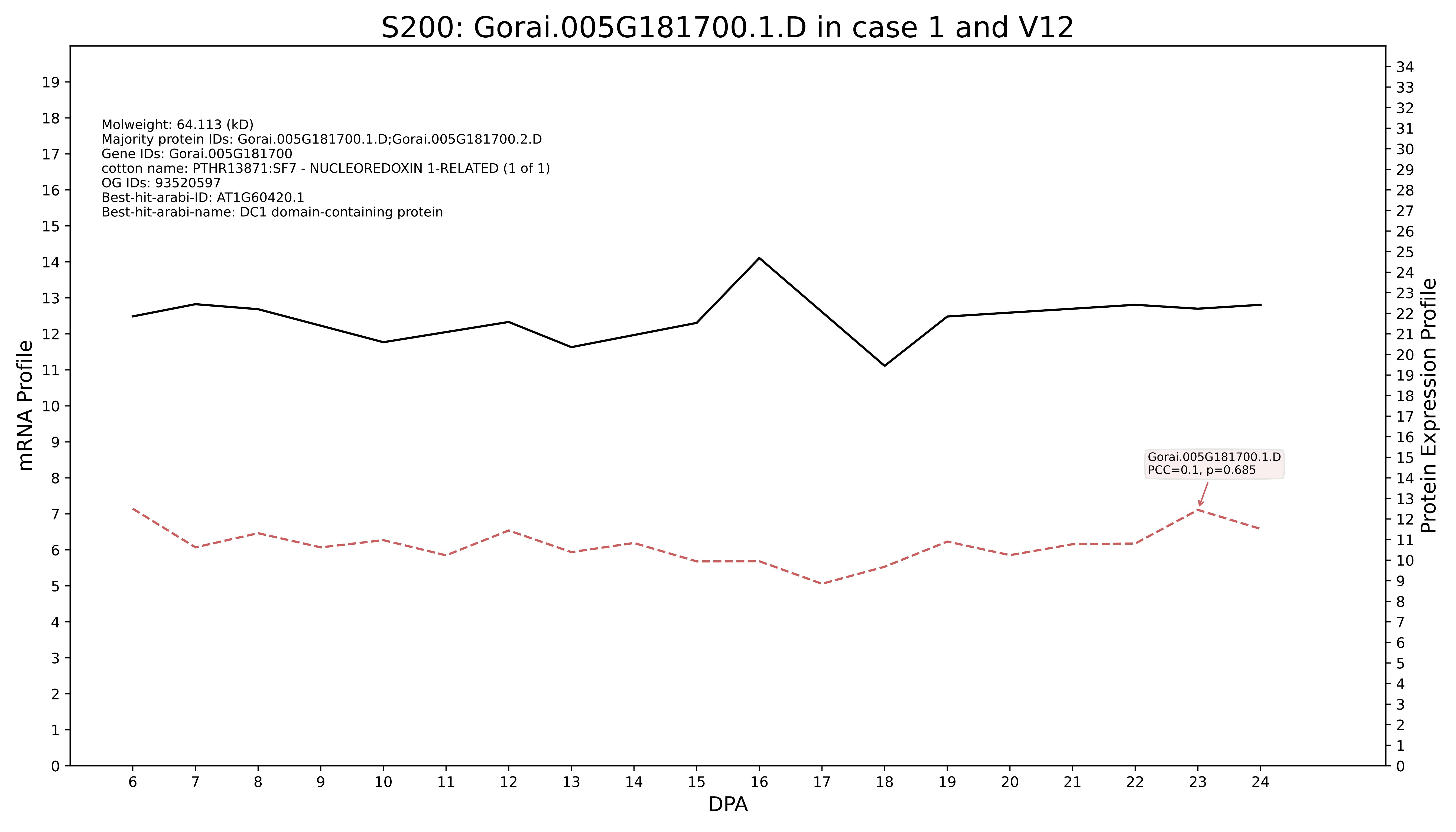

S200: Gorai.005G181700.1.D in case 1 and V12
Molweight: 64.113 (kD)
Majority protein IDs: Gorai.005G181700.1.D;Gorai.005G181700.2.D
Gene IDs: Gorai.005G181700
cotton name: PTHR13871:SF7 - NUCLEOREDOXIN 1-RELATED (1 of 1)
OG IDs: 93520597
Best-hit-arabi-ID: AT1G60420.1
Best-hit-arabi-name: DC1 domain-containing protein
Gorai.005G181700.1.D
PCC=0.1, p=0.685
mRNA Profile
Protein Expression Profile
DPA

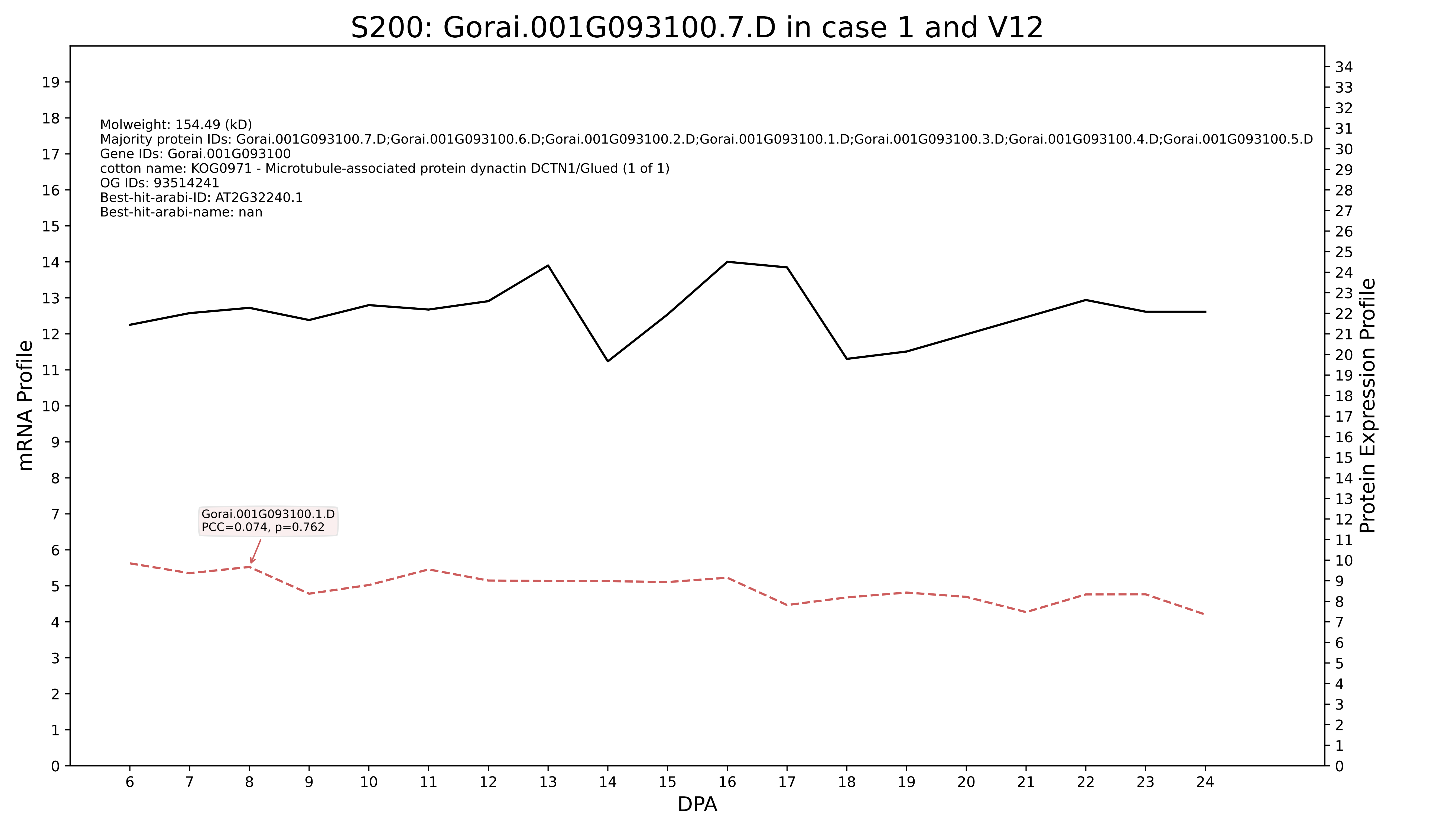
S200: Gorai.001G093100.7.D in case 1 and V12
Molweight: 154.49 (kD)
Majority protein IDs: Gorai.001G093100.7.D;Gorai.001G093100.6.D;Gorai.001G093100.2.D;Gorai.001G093100.1.D;Gorai.001G093100.3.D;Gorai.001G093100.4.D;Gorai.001G093100.5.D
Gene IDs: Gorai.001G093100
cotton name: KOG0971 - Microtubule-associated protein dynactin DCTN1/Glued (1 of 1)
OG IDs: 93514241
Best-hit-arabi-ID: AT2G32240.1
Best-hit-arabi-name: nan
Gorai.001G093100.1.D
PCC=0.074, p=0.762
mRNA Profile
Protein Expression Profile
DPA

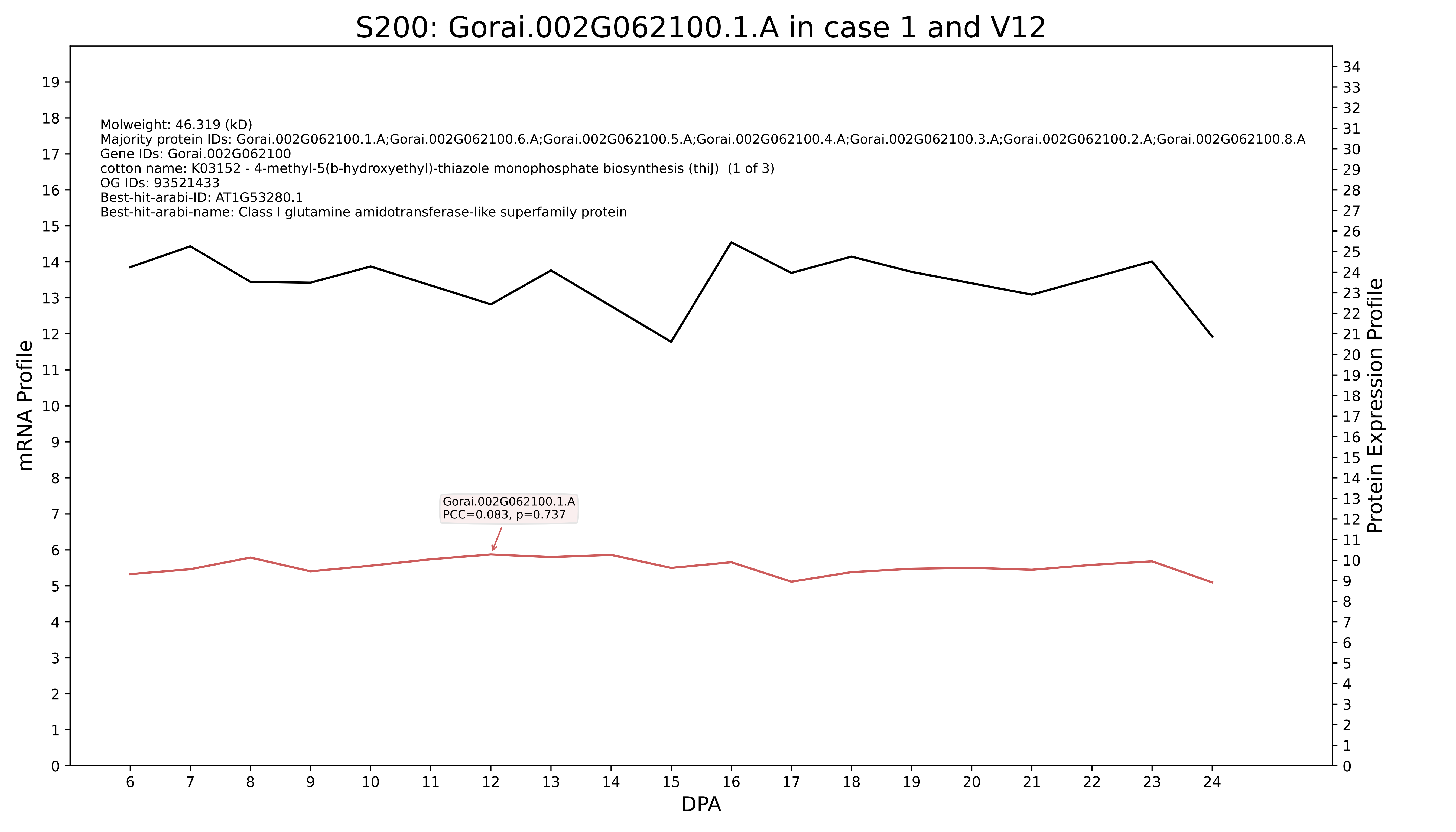
S200: Gorai.002G062100.1.A in case 1 and V12
Molweight: 46.319 (kD)
Majority protein IDs: Gorai.002G062100.1.A;Gorai.002G062100.6.A;Gorai.002G062100.5.A;Gorai.002G062100.4.A;Gorai.002G062100.3.A;Gorai.002G062100.2.A;Gorai.002G062100.8.A
Gene IDs: Gorai.002G062100
cotton name: K03152 - 4-methyl-5(b-hydroxyethyl)-thiazole monophosphate biosynthesis (thiJ) (1 of 3)
OG IDs: 93521433
Best-hit-arabi-ID: AT1G53280.1
Best-hit-arabi-name: Class I glutamine amidotransferase-like superfamily protein
Gorai.002G062100.1.A
PCC=0.083, p=0.737
mRNA Profile
Protein Expression Profile
DPA

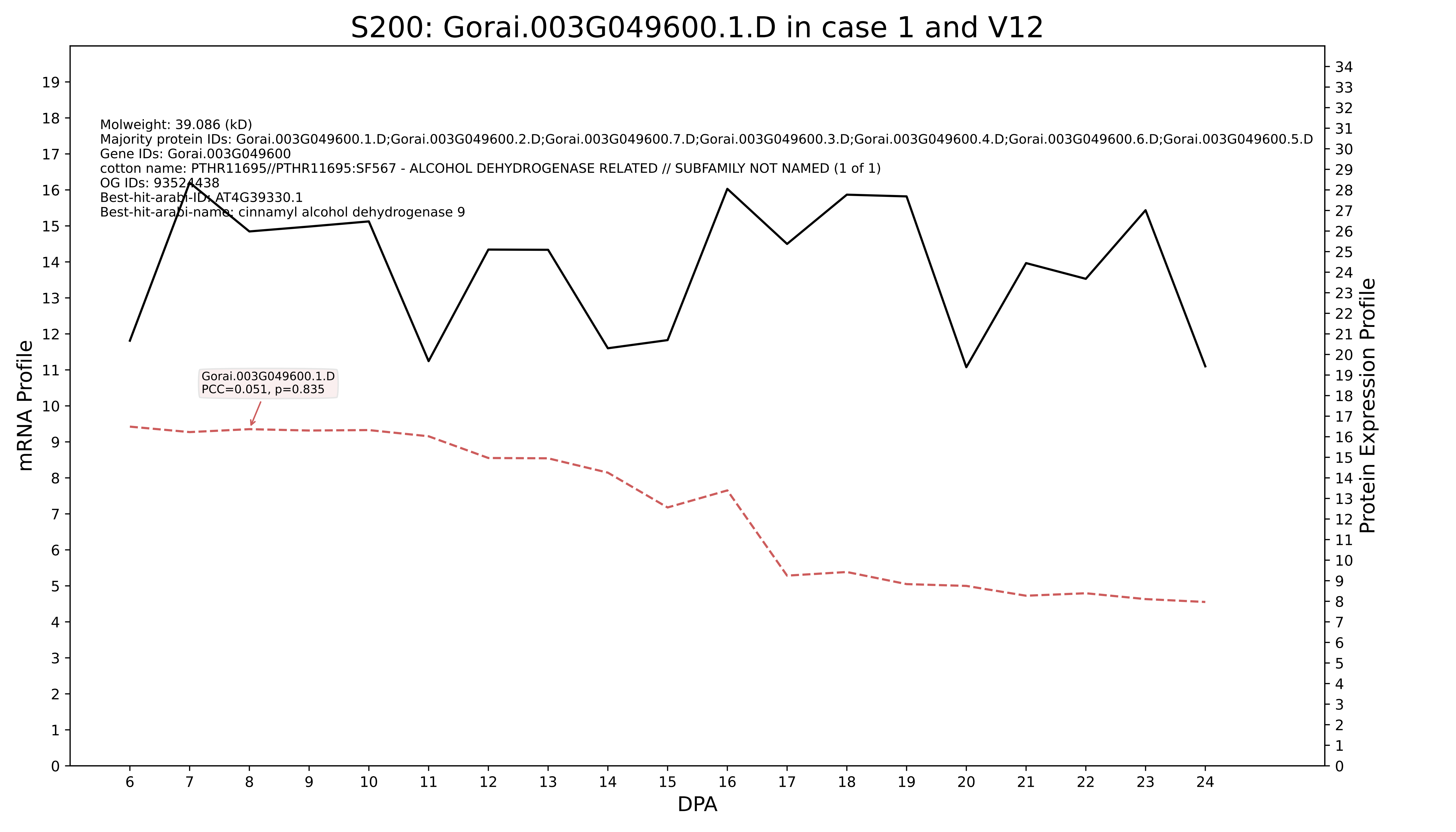
S200: Gorai.003G049600.1.D in case 1 and V12
Molweight: 39.086 (kD)
Majority protein IDs: Gorai.003G049600.1.D;Gorai.003G049600.2.D;Gorai.003G049600.7.D;Gorai.003G049600.3.D;Gorai.003G049600.4.D;Gorai.003G049600.6.D;Gorai.003G049600.5.D
Gene IDs: Gorai.003G049600
cotton name: PTHR11695//PTHR11695:SF567 - ALCOHOL DEHYDROGENASE RELATED // SUBFAMILY NOT NAMED (1 of 1)
OG IDs: 93524438
Best-hit-arabi-ID: AT4G39330.1
Best-hit-arabi-name: cinnamyl alcohol dehydrogenase 9
Gorai.003G049600.1.D
PCC=0.051, p=0.835
mRNA Profile
Protein Expression Profile
DPA

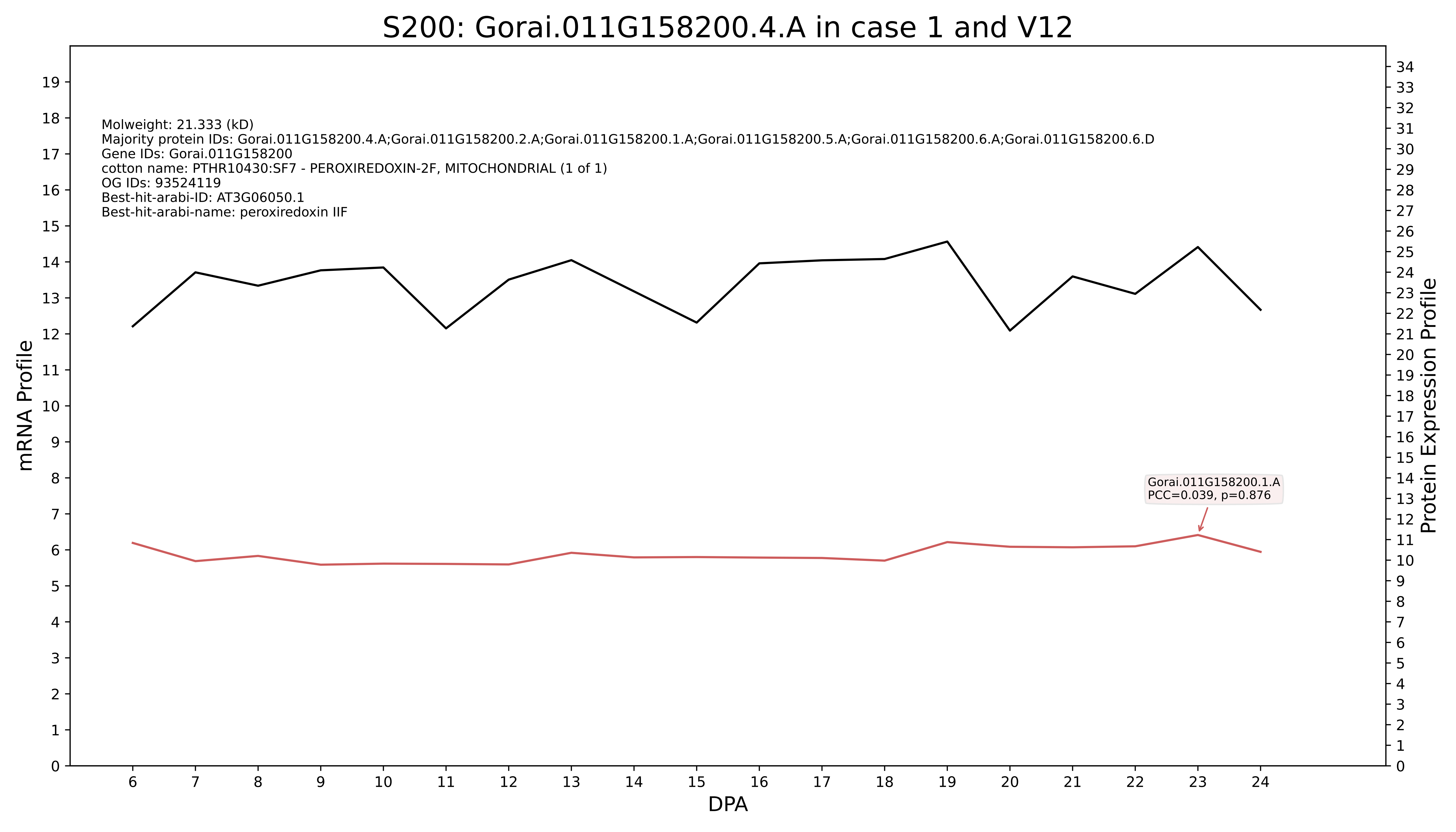
S200: Gorai.011G158200.4.A in case 1 and V12
Molweight: 21.333 (kD)
Majority protein IDs: Gorai.011G158200.4.A;Gorai.011G158200.2.A;Gorai.011G158200.1.A;Gorai.011G158200.5.A;Gorai.011G158200.6.A;Gorai.011G158200.6.D
Gene IDs: Gorai.011G158200
cotton name: PTHR10430:SF7 - PEROXIREDOXIN-2F, MITOCHONDRIAL (1 of 1)
OG IDs: 93524119
Best-hit-arabi-ID: AT3G06050.1
Best-hit-arabi-name: peroxiredoxin IIF
Gorai.011G158200.1.A
PCC=0.039, p=0.876
mRNA Profile
Protein Expression Profile
DPA

S200: Gorai.007G022600.5.D in case 2 and V12

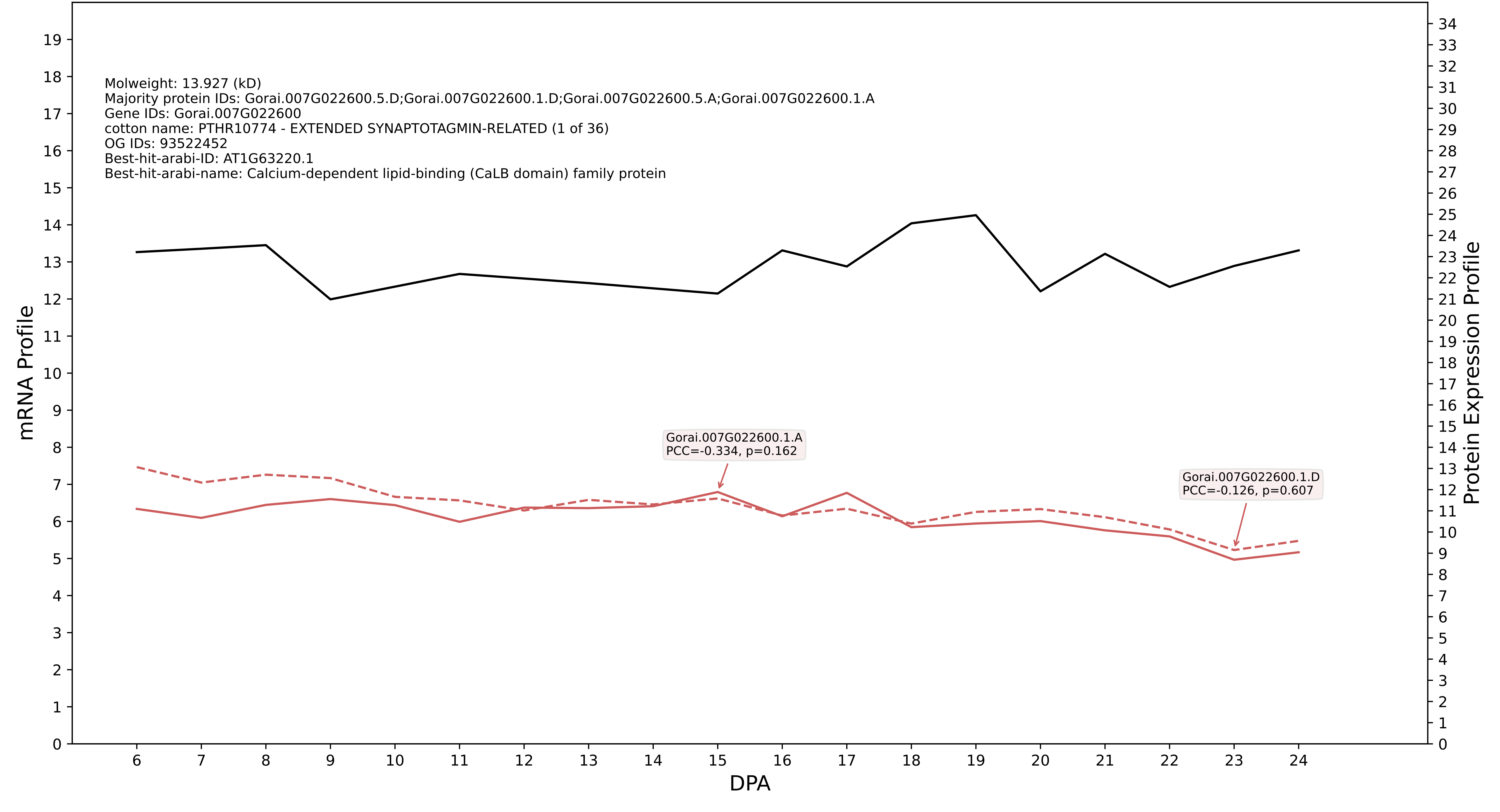

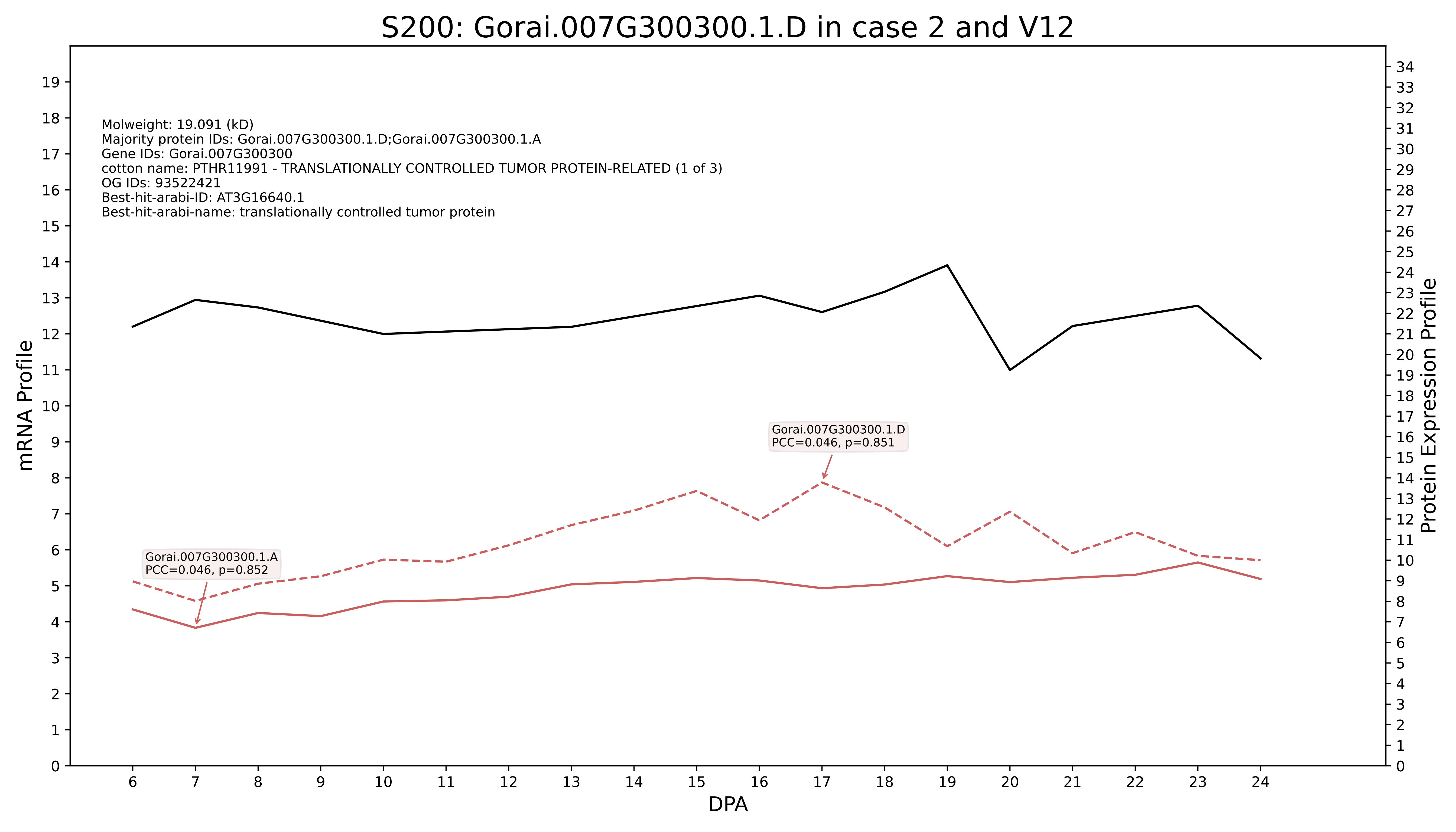

S200: Gorai.007G300300.1.D in case 2 and V12
Molweight: 19.091 (kD)
Majority protein IDs: Gorai.007G300300.1.D;Gorai.007G300300.1.A
Gene IDs: Gorai.007G300300
cotton name: PTHR11991 - TRANSLATIONALLY CONTROLLED TUMOR PROTEIN-RELATED (1 of 3)
OG IDs: 93522421
Best-hit-arabi-ID: AT3G16640.1
Best-hit-arabi-name: translationally controlled tumor protein
Gorai.007G300300.1.D
PCC=0.046, p=0.851
Gorai.007G300300.1.A
PCC=0.046, p=0.852
mRNA Profile
Protein Expression Profile
DPA

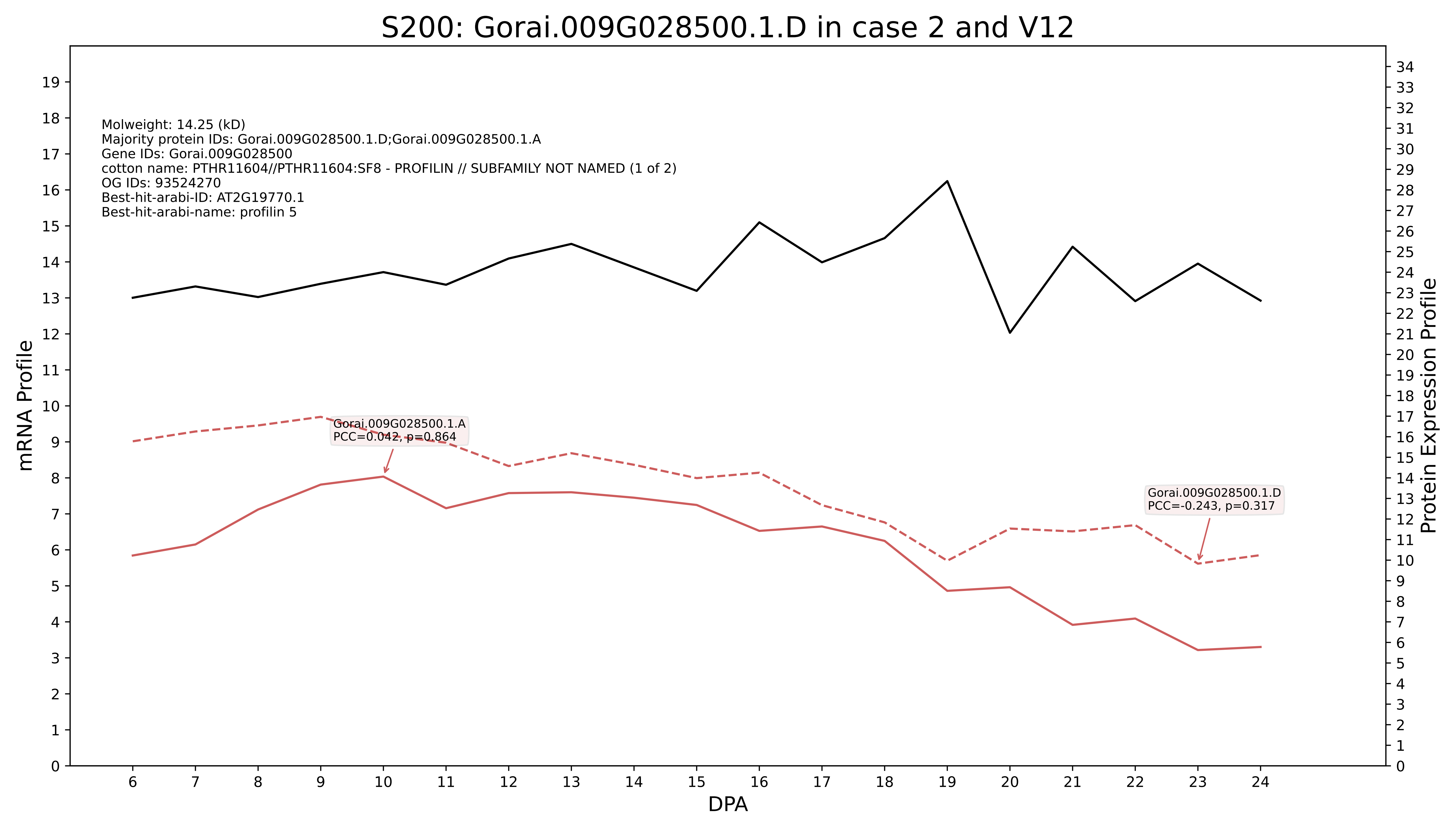
S200: Gorai.009G028500.1.D in case 2 and V12
Molweight: 14.25 (kD)
Majority protein IDs: Gorai.009G028500.1.D;Gorai.009G028500.1.A
Gene IDs: Gorai.009G028500
cotton name: PTHR11604//PTHR11604:SF8 - PROFILIN // SUBFAMILY NOT NAMED (1 of 2)
OG IDs: 93524270
Best-hit-arabi-ID: AT2G19770.1
Best-hit-arabi-name: profilin 5
Gorai.009G028500.1.A
PCC=0.042, p=0.864
Gorai.009G028500.1.D
PCC=-0.243, p=0.317
mRNA Profile
Protein Expression Profile
DPA
0
1
2
3
4
5
6
7
8
9
10
11
12
13
14
15
16
17
18
19
20
21
22
23
24
25
26
27
28
29
30
31
32
33
34

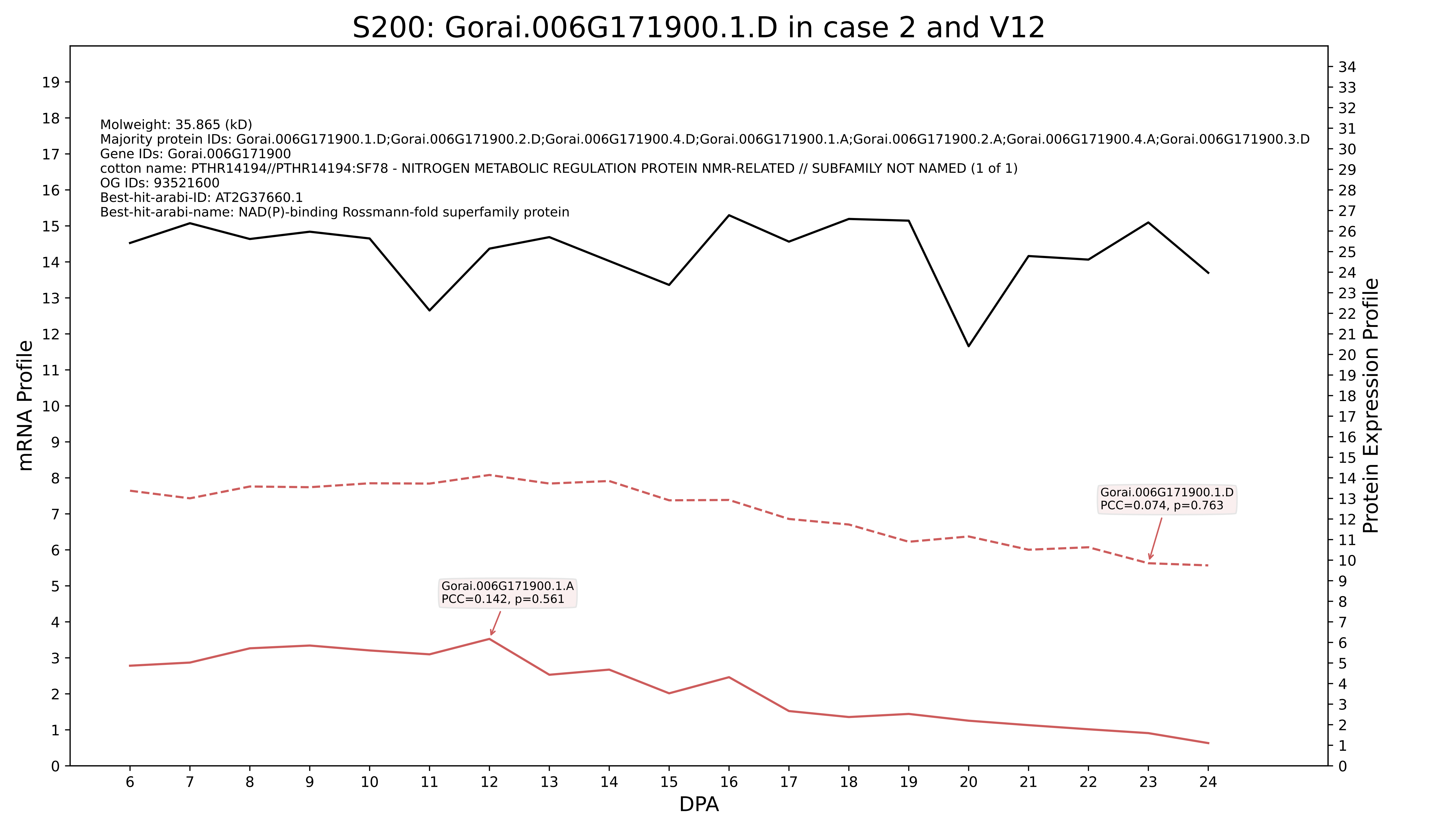
S200: Gorai.006G171900.1.D in case 2 and V12
Molweight: 35.865 (kD)
Majority protein IDs: Gorai.006G171900.1.D;Gorai.006G171900.2.D;Gorai.006G171900.4.D;Gorai.006G171900.1.A;Gorai.006G171900.2.A;Gorai.006G171900.4.A;Gorai.006G171900.3.D
Gene IDs: Gorai.006G171900
cotton name: PTHR14194//PTHR14194:SF78 - NITROGEN METABOLIC REGULATION PROTEIN NMR-RELATED // SUBFAMILY NOT NAMED (1 of 1)
OG IDs: 93521600
Best-hit-arabi-ID: AT2G37660.1
Best-hit-arabi-name: NAD(P)-binding Rossmann-fold superfamily protein
Gorai.006G171900.1.D
PCC=0.074, p=0.763
Gorai.006G171900.1.A
PCC=0.142, p=0.561
mRNA Profile
Protein Expression Profile
DPA

S200: Gorai.005G184600.1.D in case 2 and V12

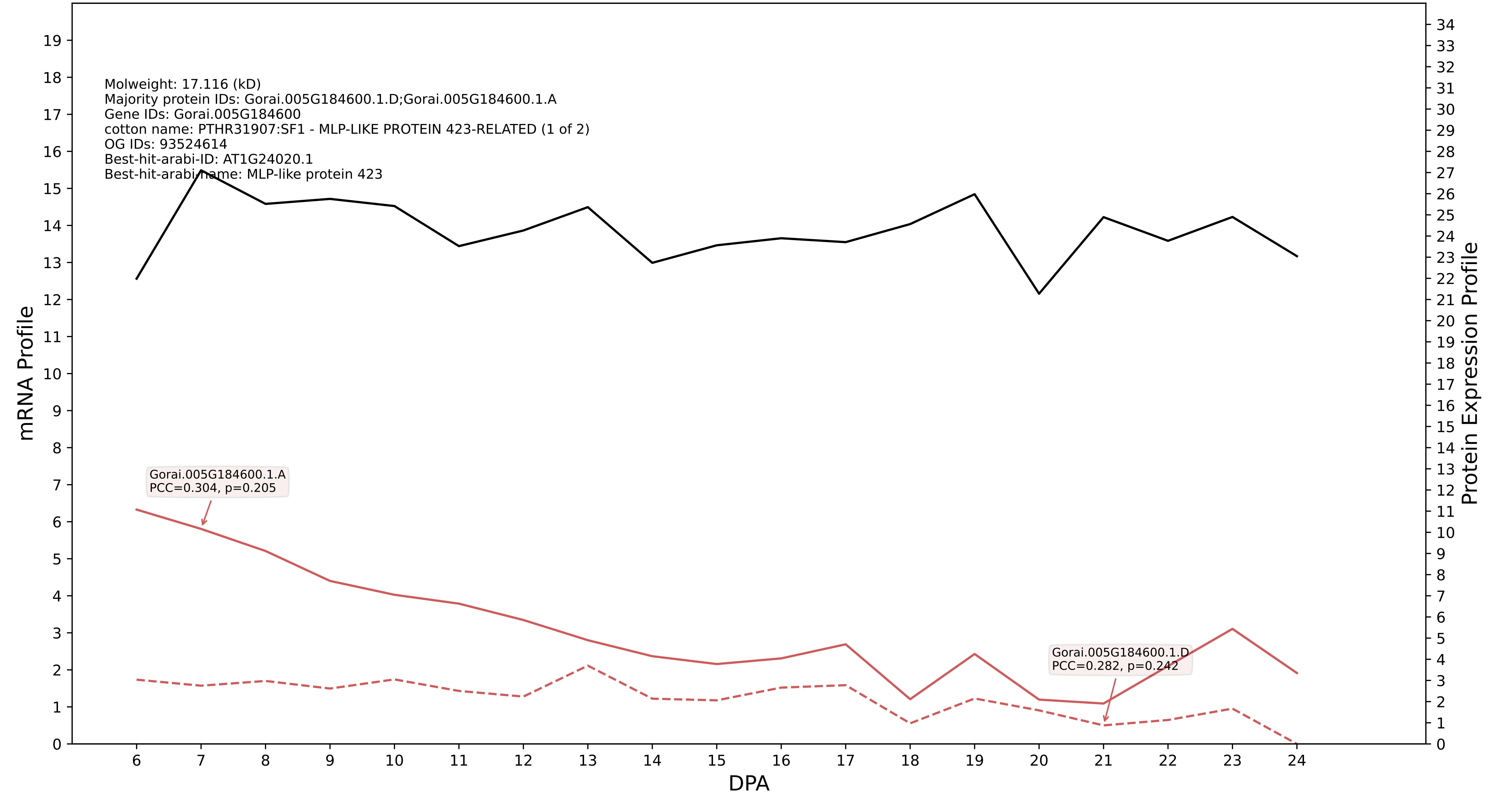

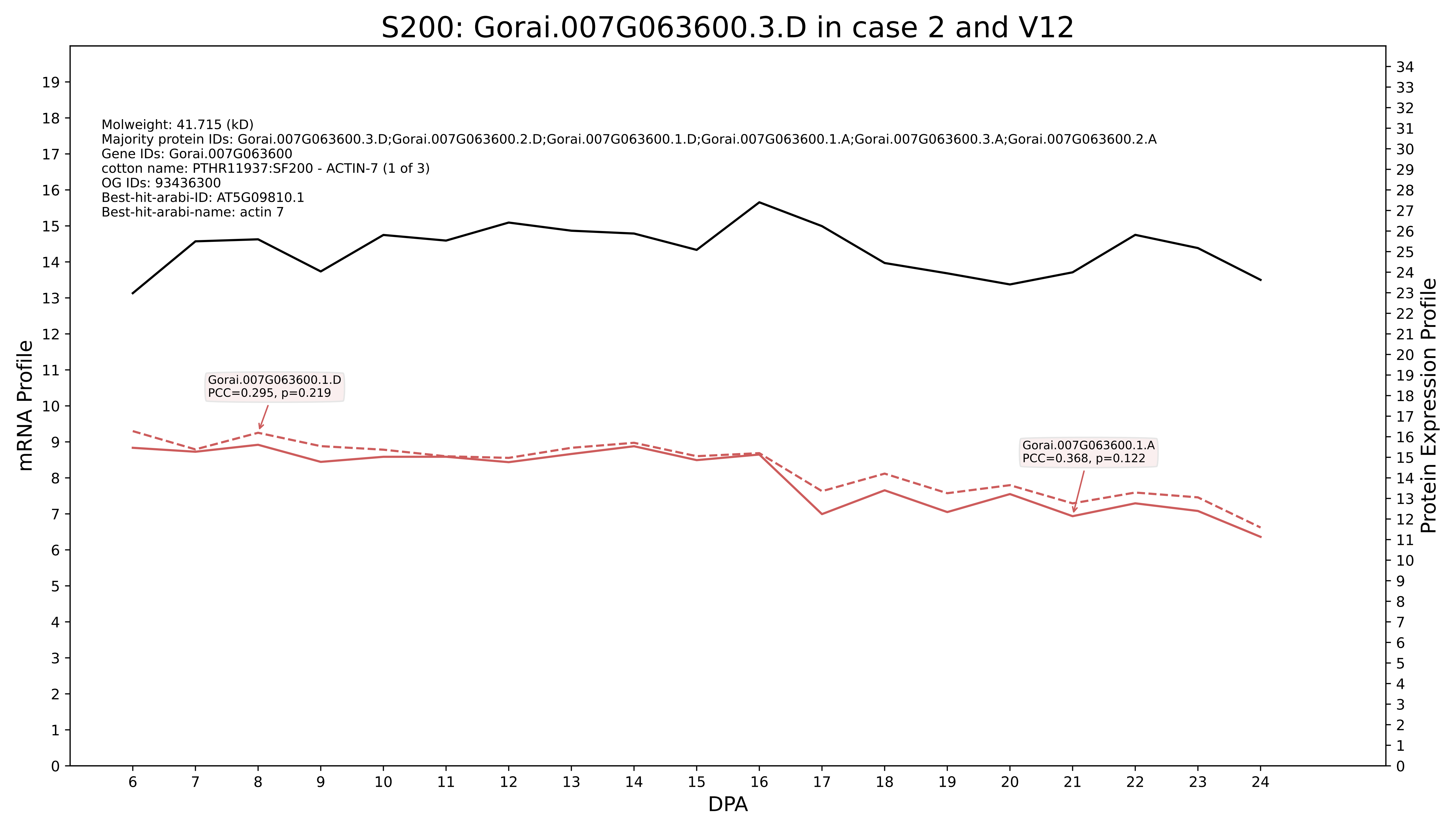
S200: Gorai.007G063600.3.D in case 2 and V12
Molweight: 41.715 (kD)
Majority protein IDs: Gorai.007G063600.3.D;Gorai.007G063600.2.D;Gorai.007G063600.1.D;Gorai.007G063600.1.A;Gorai.007G063600.3.A;Gorai.007G063600.2.A
Gene IDs: Gorai.007G063600
cotton name: PTHR11937:SF200 - ACTIN-7 (1 of 3)
OG IDs: 93436300
Best-hit-arabi-ID: AT5G09810.1
Best-hit-arabi-name: actin 7
Gorai.007G063600.1.D
PCC=0.295, p=0.219
Gorai.007G063600.1.A
PCC=0.368, p=0.122
mRNA Profile
Protein Expression Profile
DPA
0
1
2
3
4
5
6
7
8
9
10
11
12
13
14
15
16
17
18
19
20
21
22
23
24
25
26
27
28
29
30
31
32
33
34

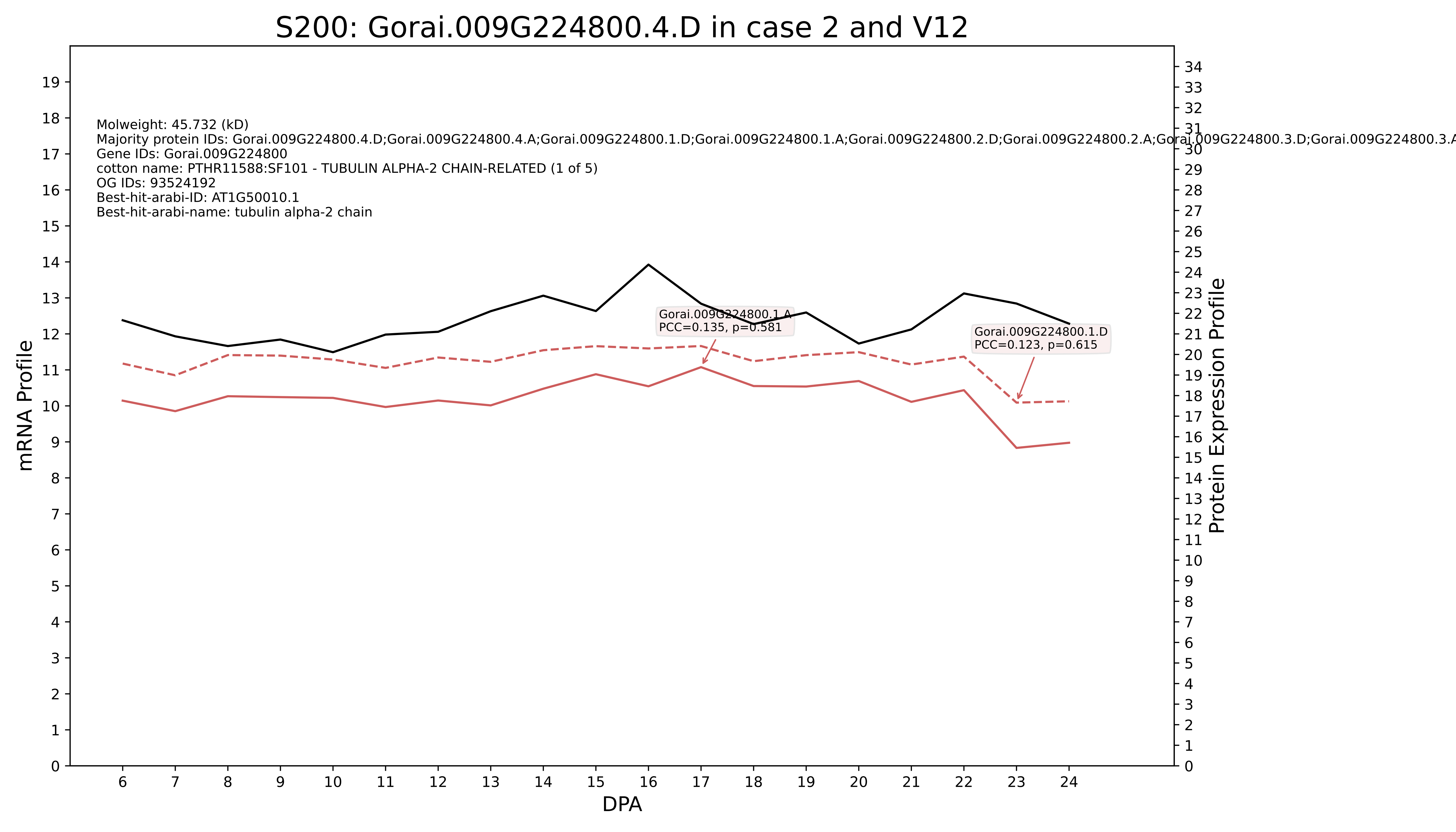

S200: Gorai.009G224800.4.D in case 2 and V12
Molweight: 45.732 (kD)
Majority protein IDs: Gorai.009G224800.4.D;Gorai.009G224800.4.A;Gorai.009G224800.1.D;Gorai.009G224800.1.A;Gorai.009G224800.2.D;Gorai.009G224800.2.A;Gorai.009G224800.3.D;Gorai.009G224800.3.A
Gene IDs: Gorai.009G224800
cotton name: PTHR11588:SF101 - TUBULIN ALPHA-2 CHAIN-RELATED (1 of 5)
OG IDs: 93524192
Best-hit-arabi-ID: AT1G50010.1
Best-hit-arabi-name: tubulin alpha-2 chain
Gorai.009G224800.1.A
PCC=0.135, p=0.581
Gorai.009G224800.1.D
PCC=0.123, p=0.615
mRNA Profile
Protein Expression Profile
DPA

S200: Gorai.006G165800.1.D in case 2 and V12

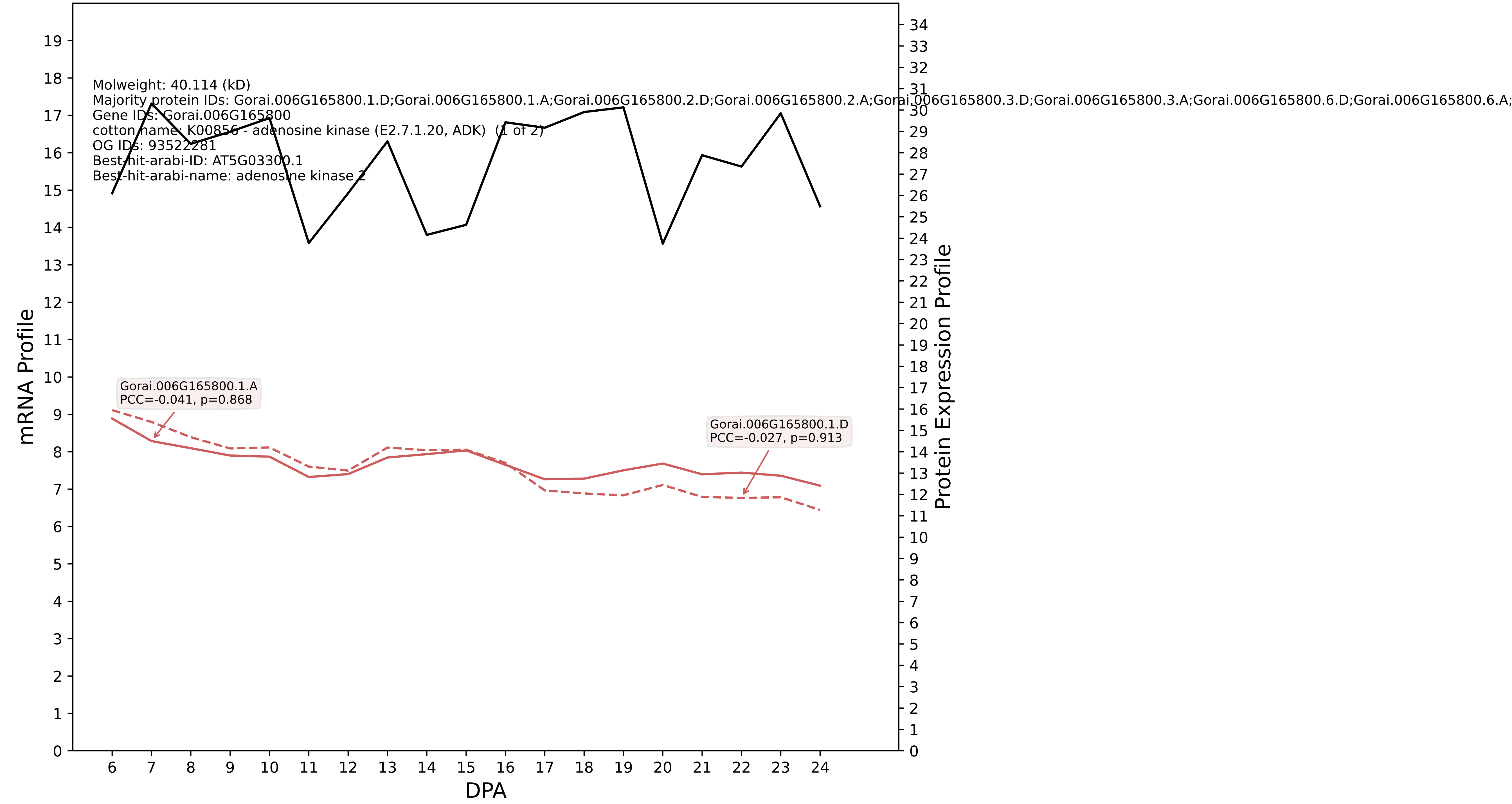

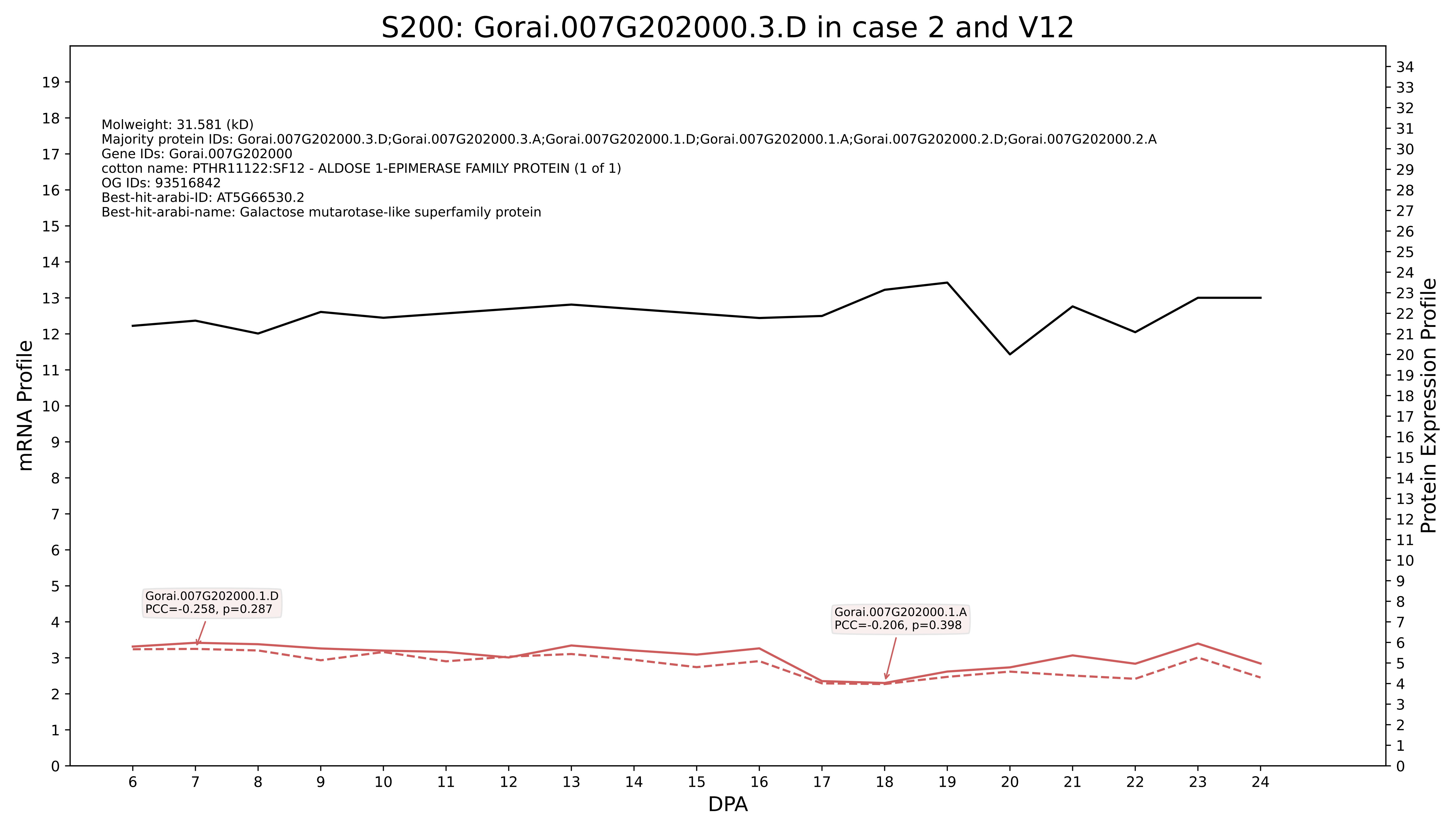
S200: Gorai.007G202000.3.D in case 2 and V12
Molweight: 31.581 (kD)
Majority protein IDs: Gorai.007G202000.3.D;Gorai.007G202000.3.A;Gorai.007G202000.1.D;Gorai.007G202000.1.A;Gorai.007G202000.2.D;Gorai.007G202000.2.A
Gene IDs: Gorai.007G202000
cotton name: PTHR11122:SF12 - ALDOSE 1-EPIMERASE FAMILY PROTEIN (1 of 1)
OG IDs: 93516842
Best-hit-arabi-ID: AT5G66530.2
Best-hit-arabi-name: Galactose mutarotase-like superfamily protein
Gorai.007G202000.1.D
PCC=-0.258, p=0.287
Gorai.007G202000.1.A
PCC=-0.206, p=0.398
mRNA Profile
Protein Expression Profile
DPA

S200: Gorai.002G183100.4.D in case 1 and V16

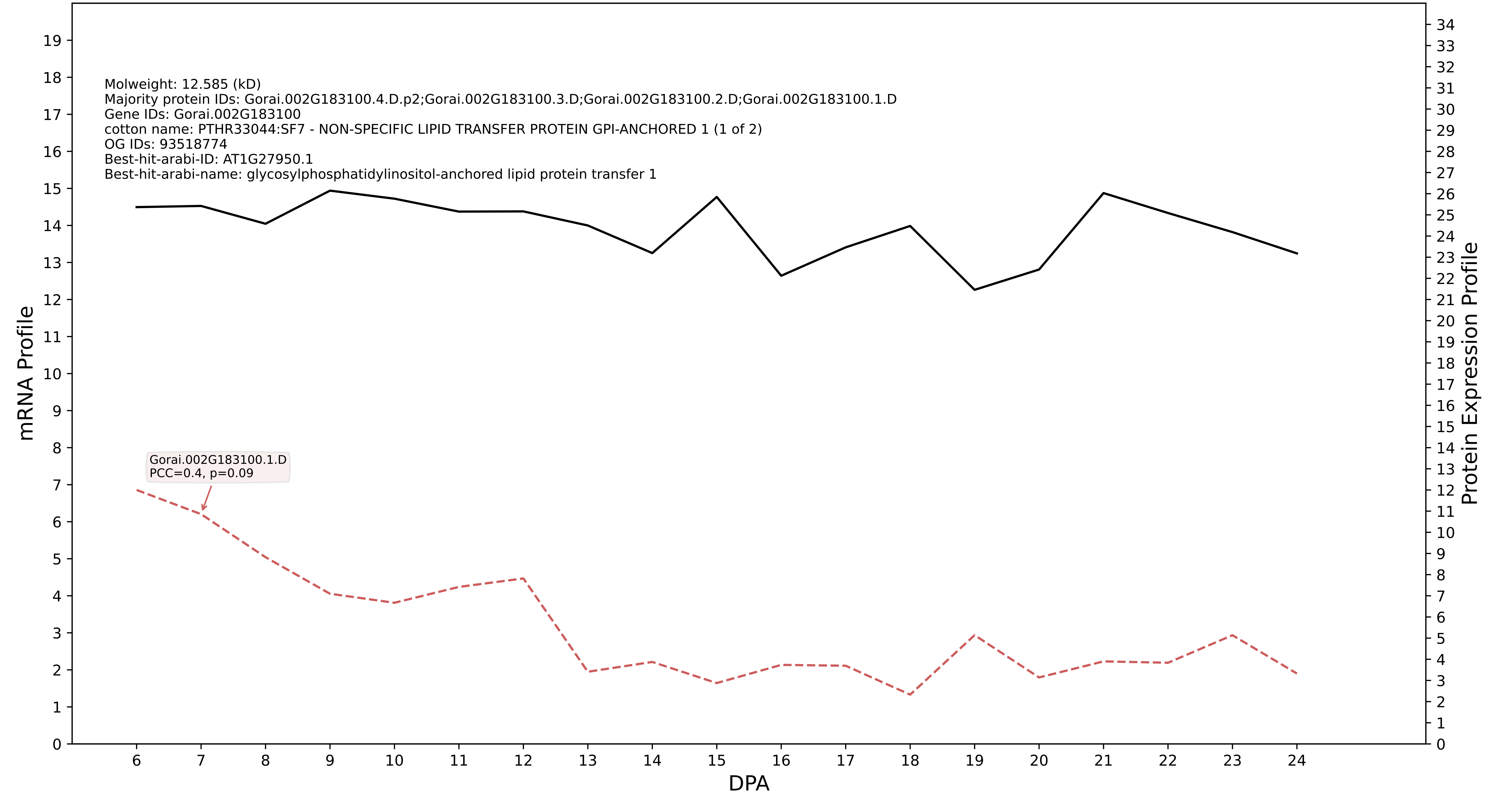

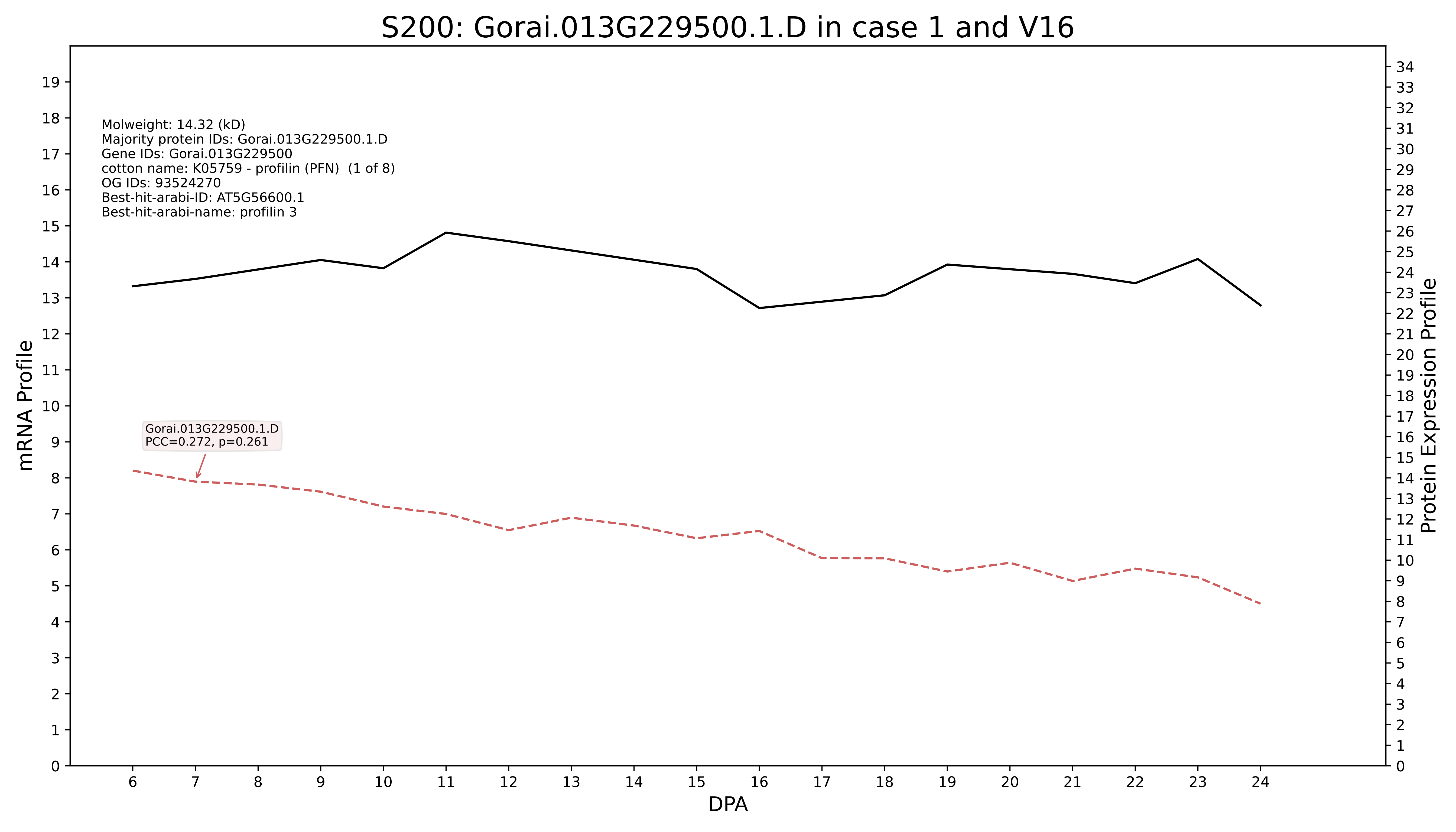
S200: Gorai.013G229500.1.D in case 1 and V16
Molweight: 14.32 (kD)
Majority protein IDs: Gorai.013G229500.1.D
Gene IDs: Gorai.013G229500
cotton name: K05759 - profilin (PFN) (1 of 8)
OG IDs: 93524270
Best-hit-arabi-ID: AT5G56600.1
Best-hit-arabi-name: profilin 3
Gorai.013G229500.1.D
PCC=0.272, p=0.261
mRNA Profile
Protein Expression Profile
DPA

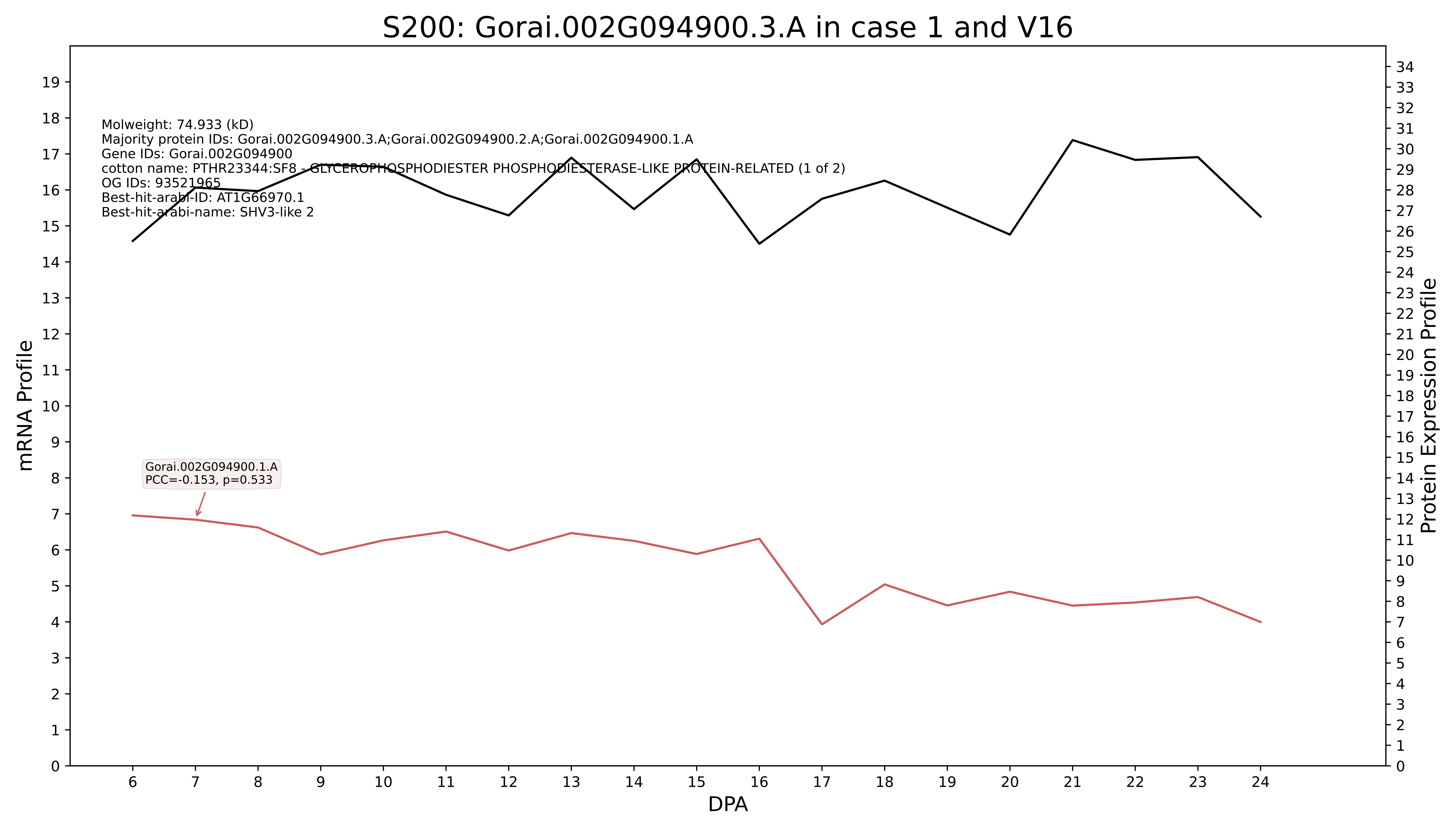

S200: Gorai.002G094900.3.A in case 1 and V16
Molweight: 74.933 (kD)
Majority protein IDs: Gorai.002G094900.3.A;Gorai.002G094900.2.A;Gorai.002G094900.1.A
Gene IDs: Gorai.002G094900
cotton name: PTHR23344:SF8 - GLYCEROPHOSPHODIESTER PHOSPHODIESTERASE-LIKE PROTEIN-RELATED (1 of 2)
OG IDs: 93521965
Best-hit-arabi-ID: AT1G66970.1
Best-hit-arabi-name: SHV3-like 2
Gorai.002G094900.1.A
PCC=-0.153, p=0.533
mRNA Profile
Protein Expression Profile
DPA

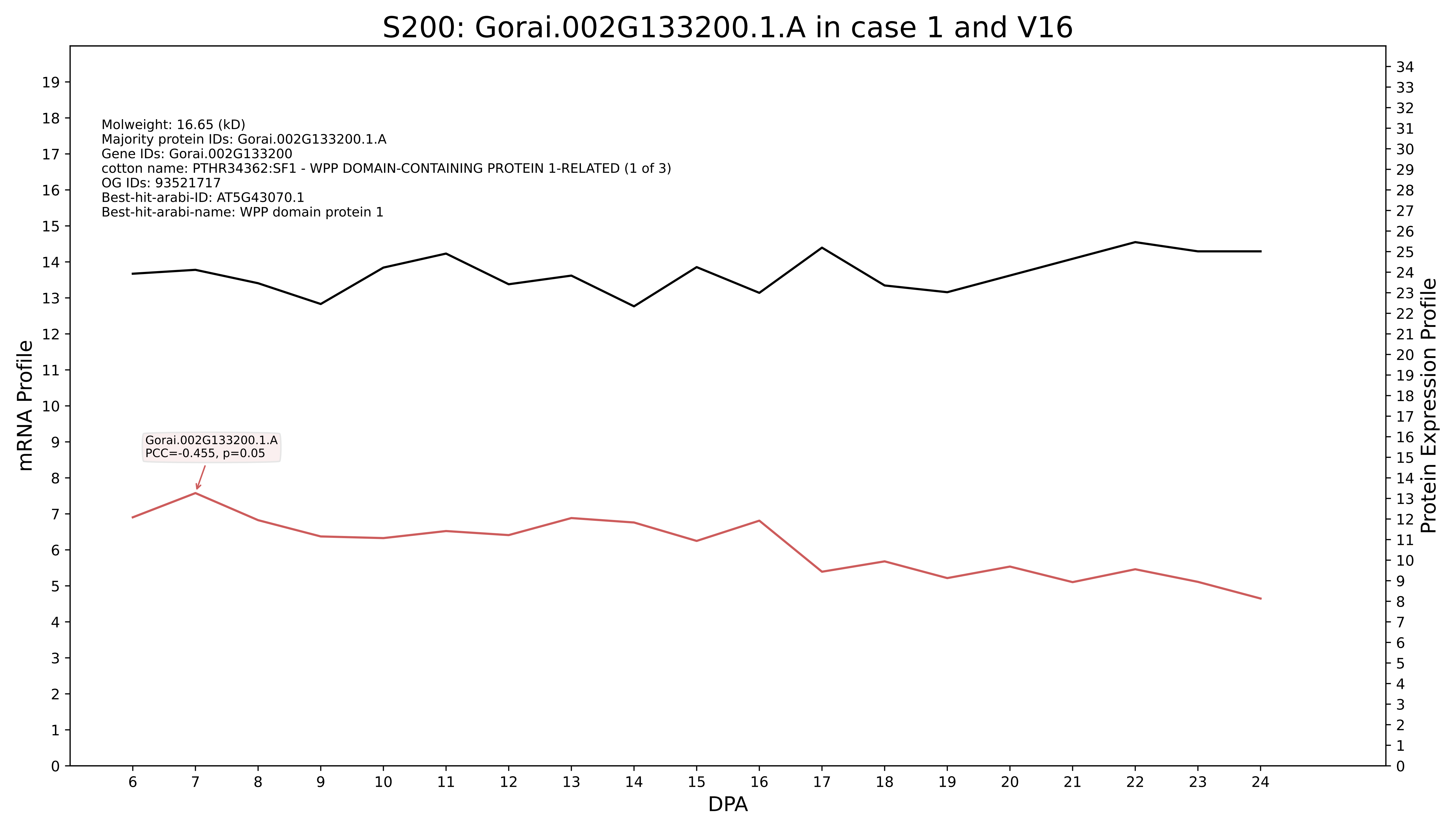

S200: Gorai.002G133200.1.A in case 1 and V16
Molweight: 16.65 (kD)
Majority protein IDs: Gorai.002G133200.1.A
Gene IDs: Gorai.002G133200
cotton name: PTHR34362:SF1 - WPP DOMAIN-CONTAINING PROTEIN 1-RELATED (1 of 3)
OG IDs: 93521717
Best-hit-arabi-ID: AT5G43070.1
Best-hit-arabi-name: WPP domain protein 1
Gorai.002G133200.1.A
PCC=-0.455, p=0.05
mRNA Profile
Protein Expression Profile
DPA

S200: Gorai.002G094900.3.D in case 1 and V16

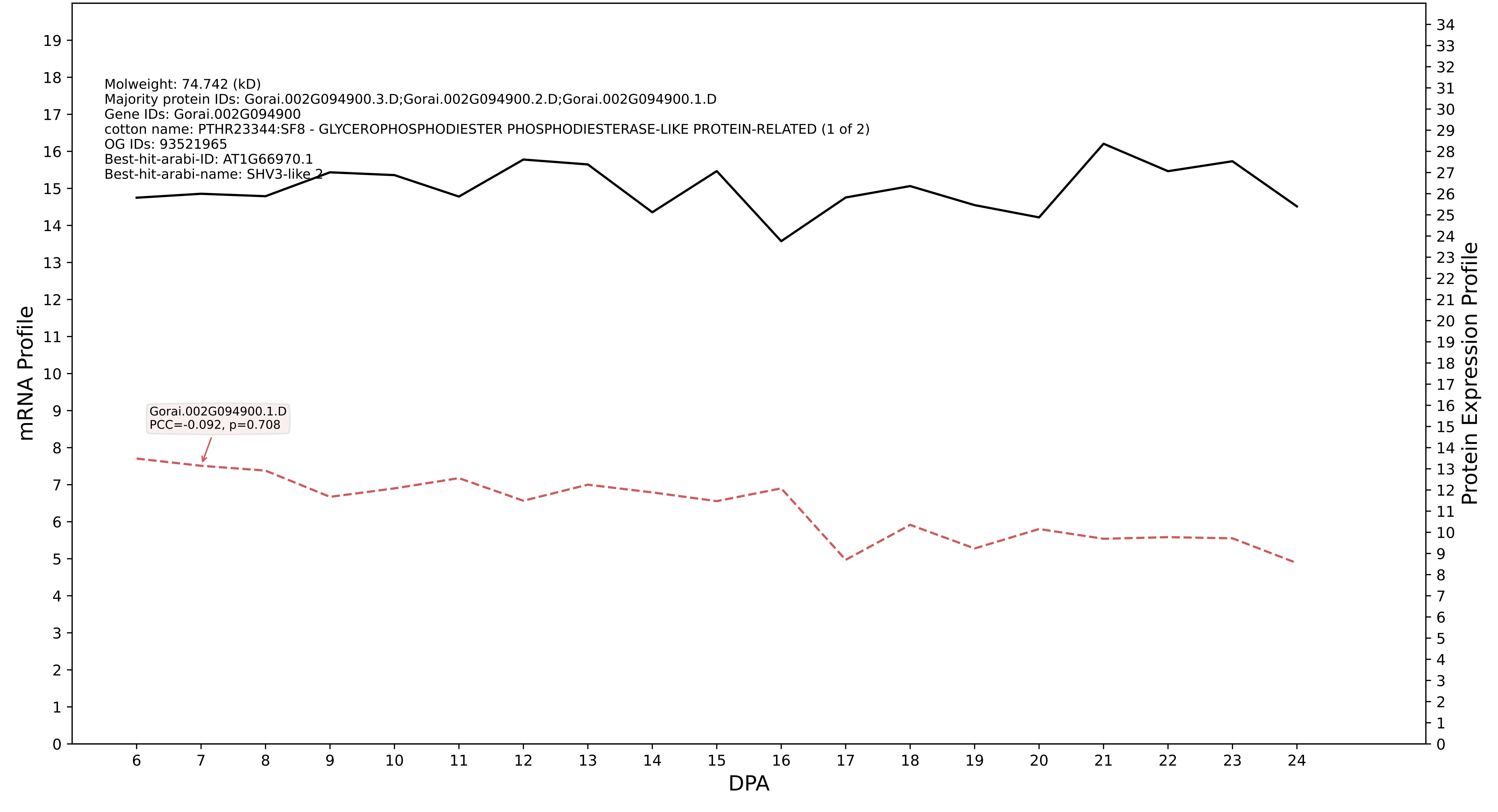

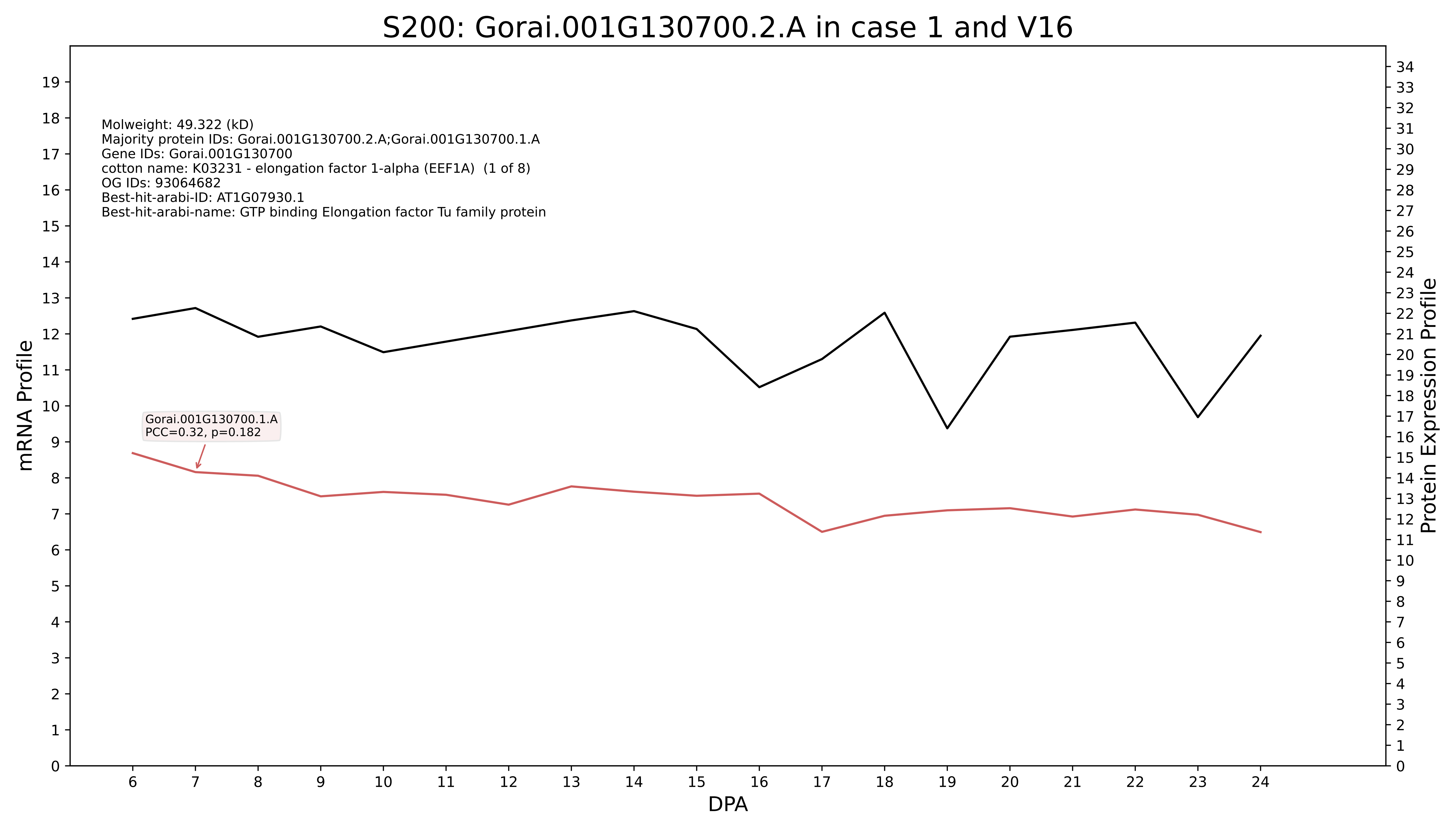
S200: Gorai.001G130700.2.A in case 1 and V16
Molweight: 49.322 (kD)
Majority protein IDs: Gorai.001G130700.2.A;Gorai.001G130700.1.A
Gene IDs: Gorai.001G130700
cotton name: K03231 - elongation factor 1-alpha (EEF1A) (1 of 8)
OG IDs: 93064682
Best-hit-arabi-ID: AT1G07930.1
Best-hit-arabi-name: GTP binding Elongation factor Tu family protein
Gorai.001G130700.1.A
PCC=0.32, p=0.182
mRNA Profile
Protein Expression Profile
DPA

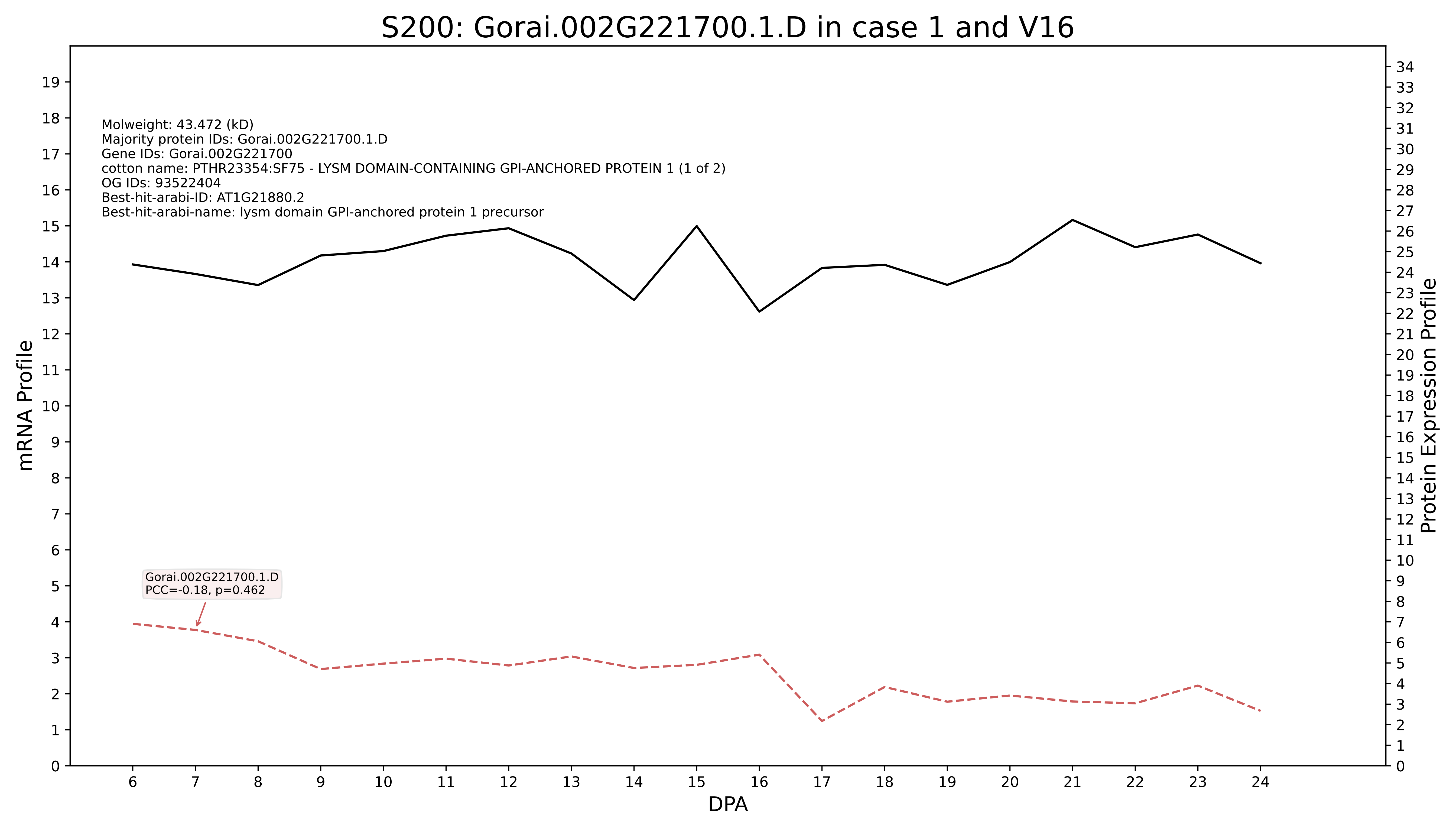
S200: Gorai.002G221700.1.D in case 1 and V16
Molweight: 43.472 (kD)
Majority protein IDs: Gorai.002G221700.1.D
Gene IDs: Gorai.002G221700
cotton name: PTHR23354:SF75 - LYSM DOMAIN-CONTAINING GPI-ANCHORED PROTEIN 1 (1 of 2)
OG IDs: 93522404
Best-hit-arabi-ID: AT1G21880.2
Best-hit-arabi-name: lysm domain GPI-anchored protein 1 precursor
Gorai.002G221700.1.D
PCC=-0.18, p=0.462
mRNA Profile
Protein Expression Profile
DPA

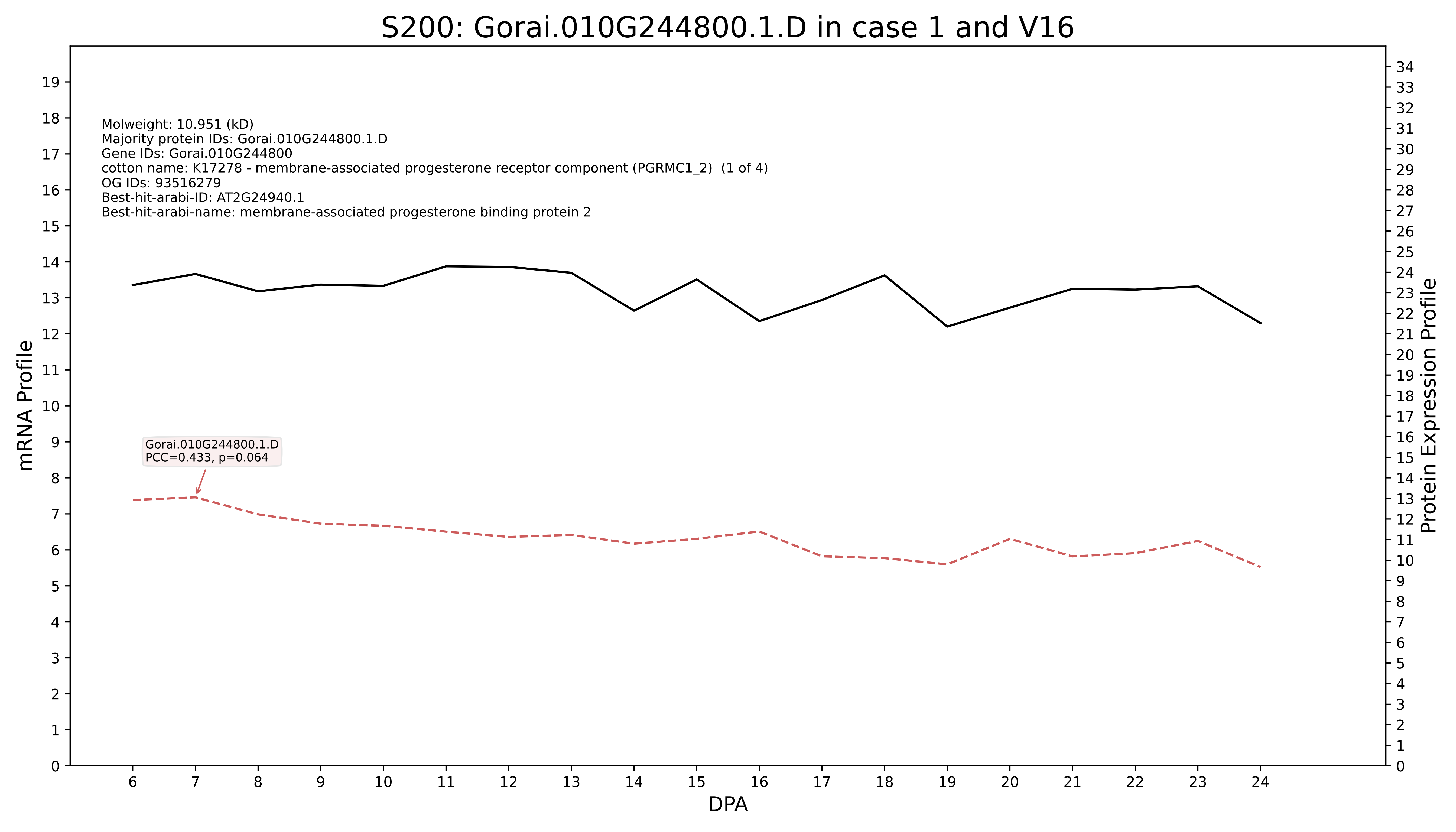
S200: Gorai.010G244800.1.D in case 1 and V16
Molweight: 10.951 (kD)
Majority protein IDs: Gorai.010G244800.1.D
Gene IDs: Gorai.010G244800
cotton name: K17278 - membrane-associated progesterone receptor component (PGRMC1_2) (1 of 4)
OG IDs: 93516279
Best-hit-arabi-ID: AT2G24940.1
Best-hit-arabi-name: membrane-associated progesterone binding protein 2
Gorai.010G244800.1.D
PCC=0.433, p=0.064
mRNA Profile
Protein Expression Profile
DPA

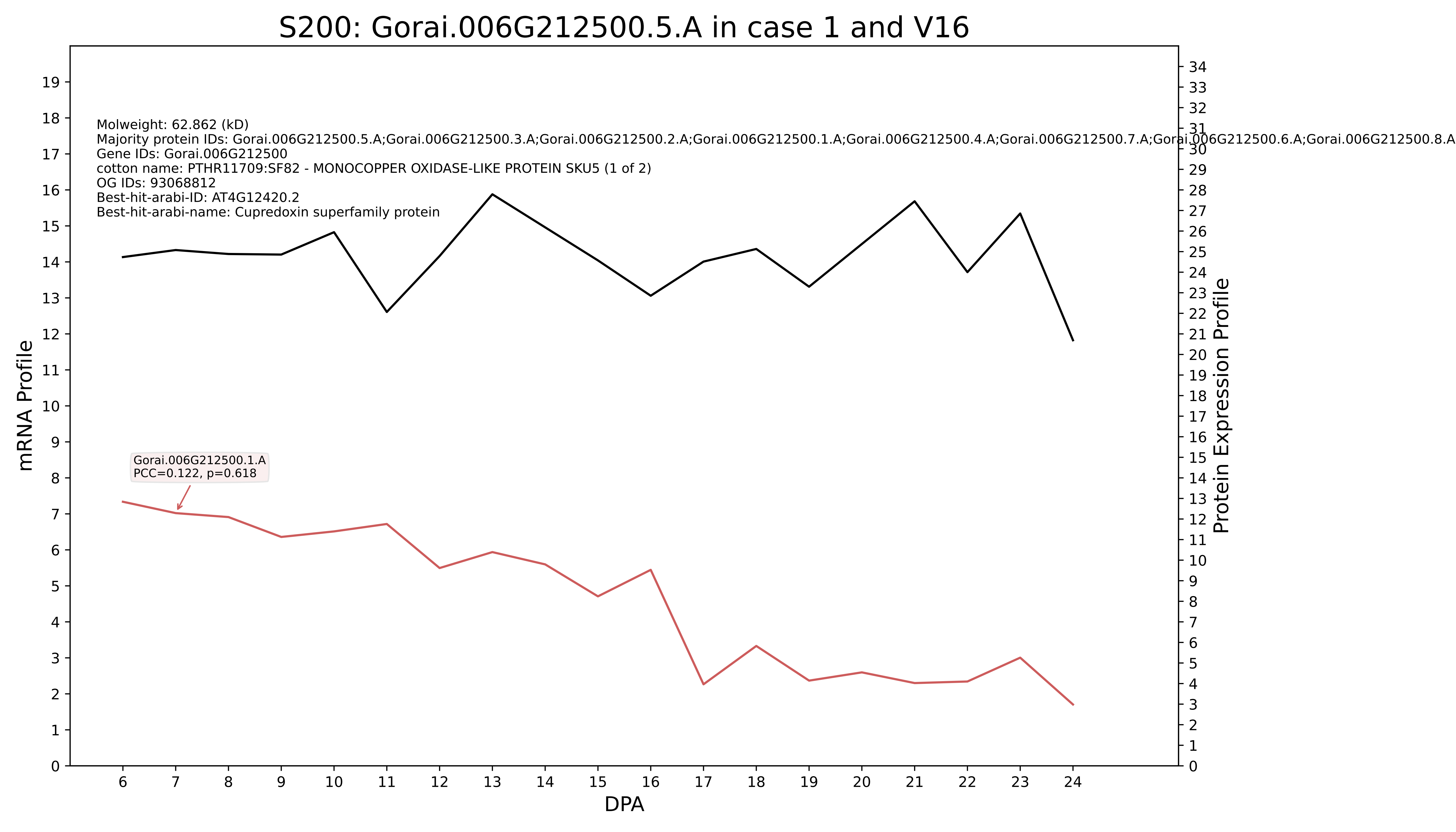
S200: Gorai.006G212500.5.A in case 1 and V16
Molweight: 62.862 (kD)
Majority protein IDs: Gorai.006G212500.5.A;Gorai.006G212500.3.A;Gorai.006G212500.2.A;Gorai.006G212500.1.A;Gorai.006G212500.4.A;Gorai.006G212500.7.A;Gorai.006G212500.6.A;Gorai.006G212500.8.A
Gene IDs: Gorai.006G212500
cotton name: PTHR11709:SF82 - MONOCOPPER OXIDASE-LIKE PROTEIN SKU5 (1 of 2)
OG IDs: 93068812
Best-hit-arabi-ID: AT4G12420.2
Best-hit-arabi-name: Cupredoxin superfamily protein
Gorai.006G212500.1.A
PCC=0.122, p=0.618
mRNA Profile
Protein Expression Profile
DPA

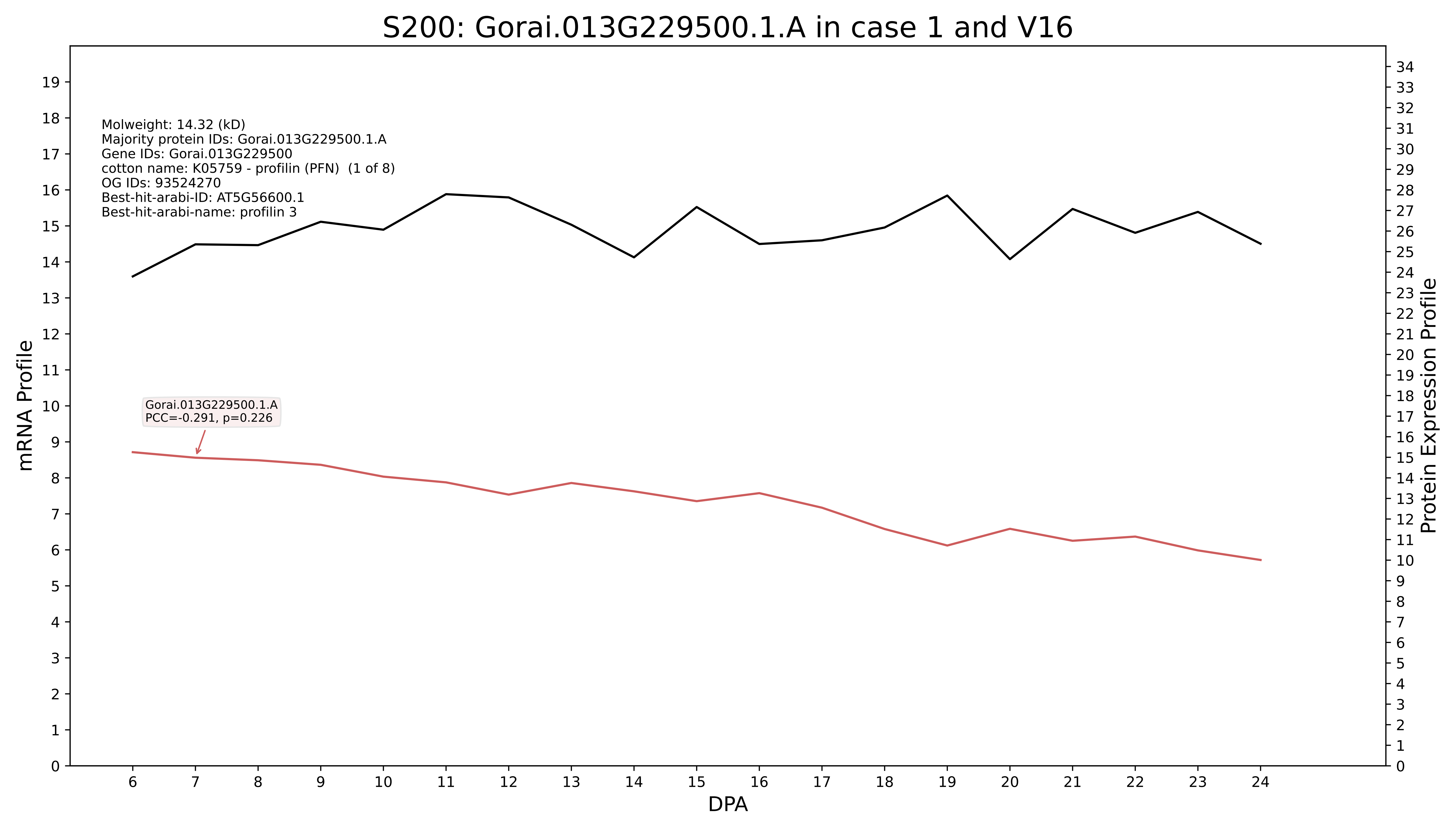
S200: Gorai.013G229500.1.A in case 1 and V16
Molweight: 14.32 (kD)
Majority protein IDs: Gorai.013G229500.1.A
Gene IDs: Gorai.013G229500
cotton name: K05759 - profilin (PFN) (1 of 8)
OG IDs: 93524270
Best-hit-arabi-ID: AT5G56600.1
Best-hit-arabi-name: profilin 3
Gorai.013G229500.1.A
PCC=-0.291, p=0.226
mRNA Profile
Protein Expression Profile
DPA

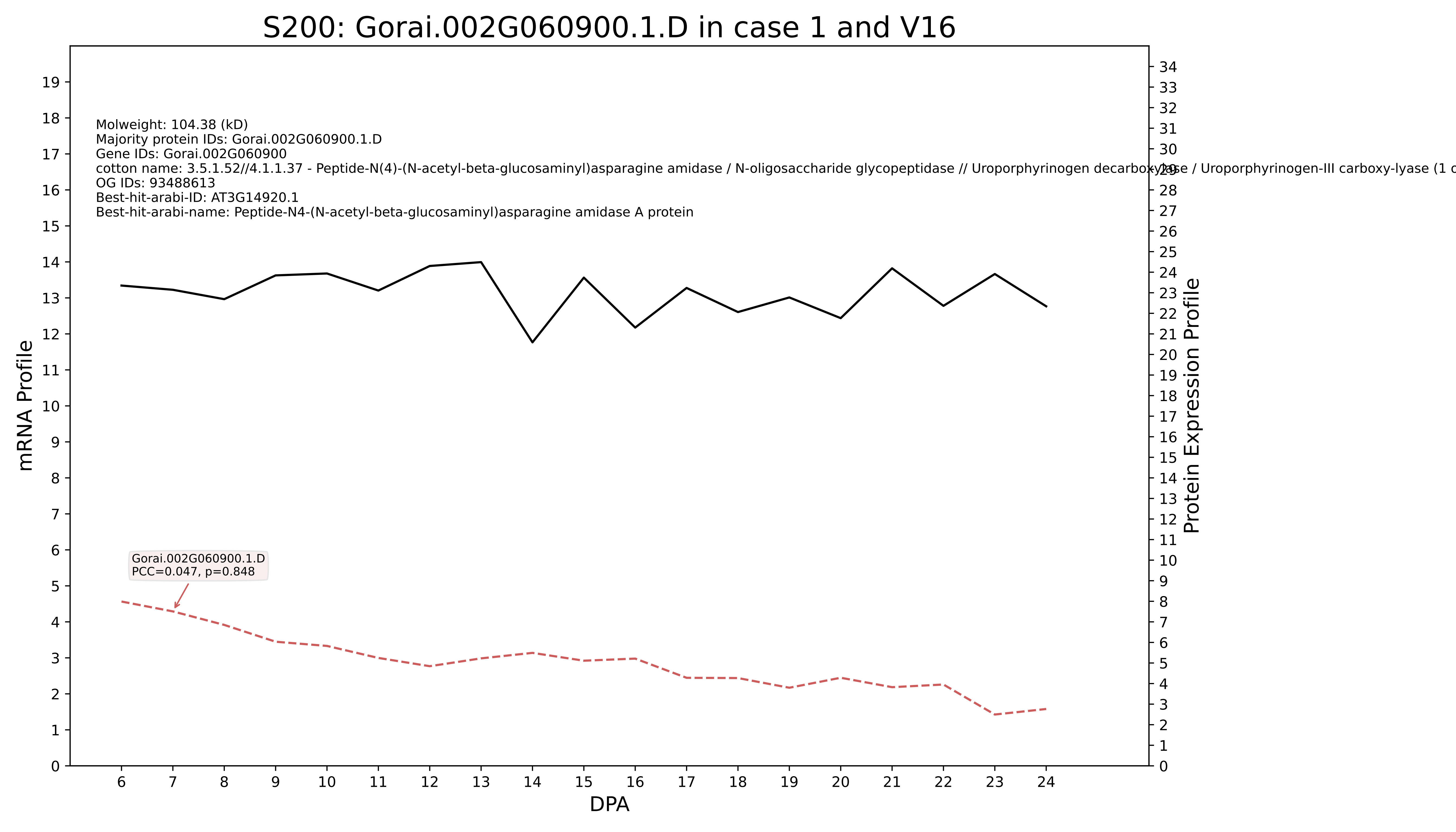

S200: Gorai.002G060900.1.D in case 1 and V16
Molweight: 104.38 (kD)
Majority protein IDs: Gorai.002G060900.1.D
Gene IDs: Gorai.002G060900
cotton name: 3.5.1.52//4.1.1.37 - Peptide-N(4)-(N-acetyl-beta-glucosaminyl)asparagine amidase / N-oligosaccharide glycopeptidase // Uroporphyrinogen decarboxylase / Uroporphyrinogen-III carboxy-lyase (1 c
OG IDs: 93488613
Best-hit-arabi-ID: AT3G14920.1
Best-hit-arabi-name: Peptide-N4-(N-acetyl-beta-glucosaminyl)asparagine amidase A protein
Gorai.002G060900.1.D
PCC=0.047, p=0.848
mRNA Profile
Protein Expression Profile
DPA

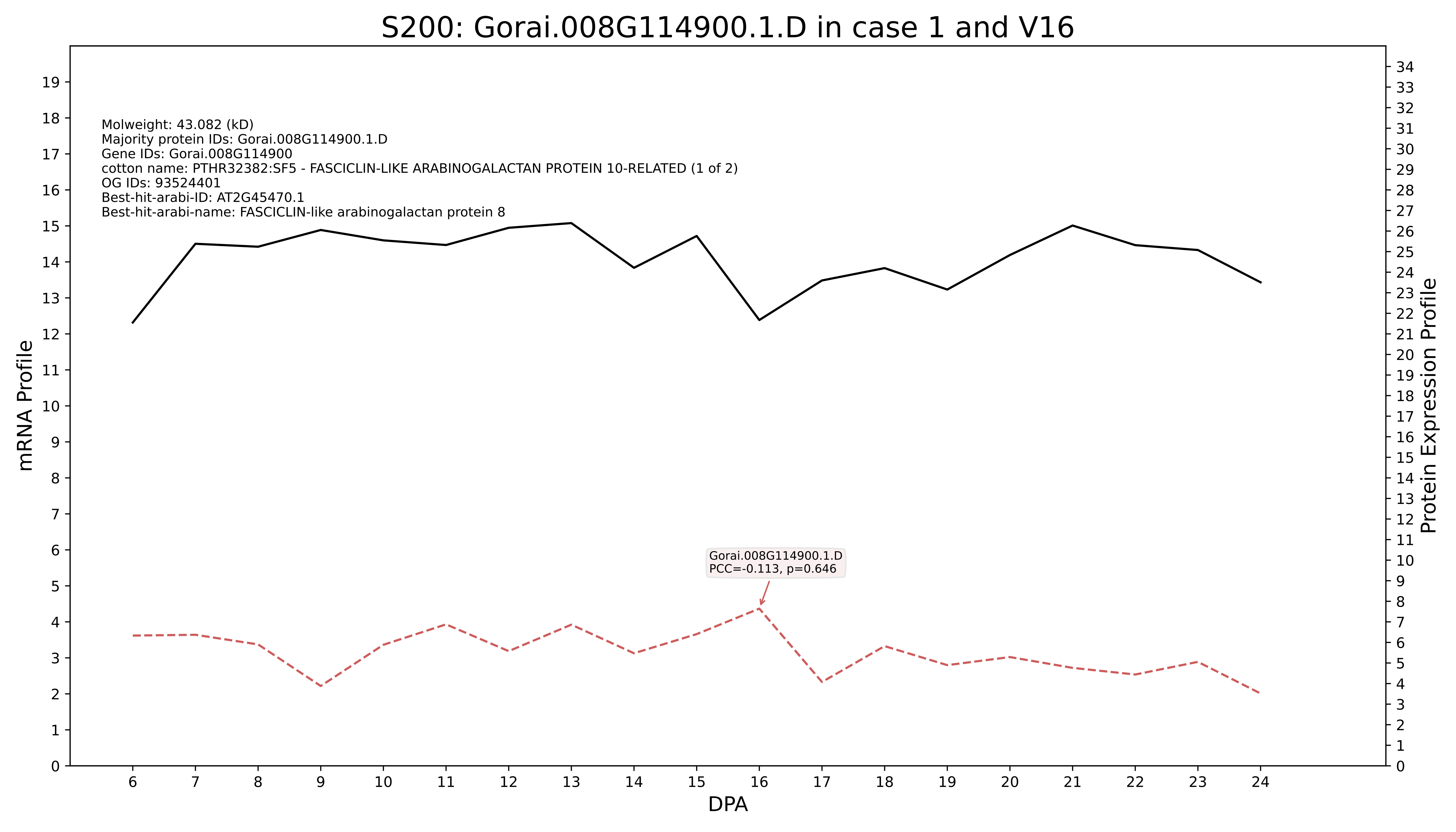

S200: Gorai.008G114900.1.D in case 1 and V16
Molweight: 43.082 (kD)
Majority protein IDs: Gorai.008G114900.1.D
Gene IDs: Gorai.008G114900
cotton name: PTHR32382:SF5 - FASCICLIN-LIKE ARABINOGALACTAN PROTEIN 10-RELATED (1 of 2)
OG IDs: 93524401
Best-hit-arabi-ID: AT2G45470.1
Best-hit-arabi-name: FASCICLIN-like arabinogalactan protein 8
Gorai.008G114900.1.D
PCC=-0.113, p=0.646
mRNA Profile
Protein Expression Profile
DPA

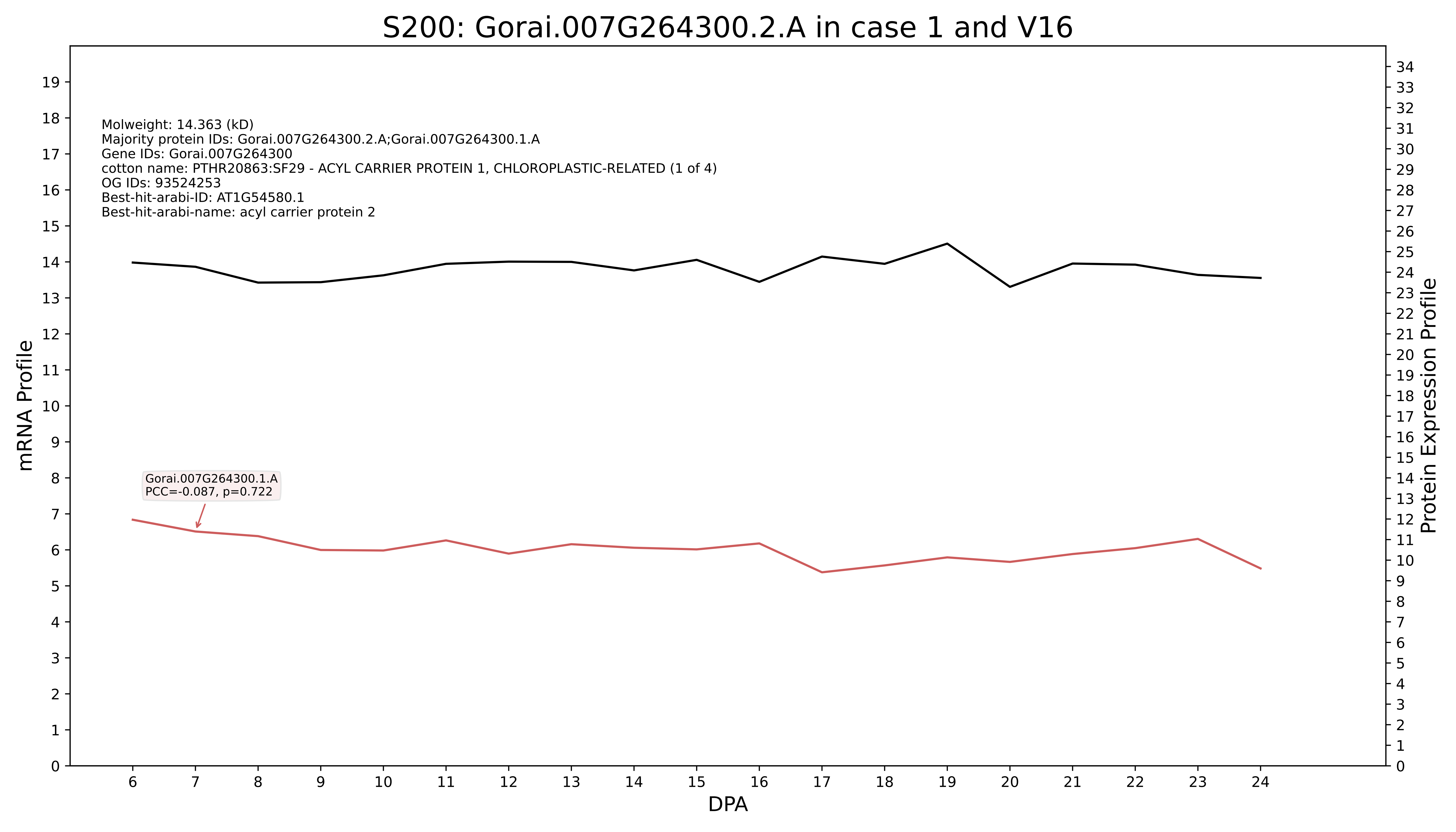
S200: Gorai.007G264300.2.A in case 1 and V16
Molweight: 14.363 (kD)
Majority protein IDs: Gorai.007G264300.2.A;Gorai.007G264300.1.A
Gene IDs: Gorai.007G264300
cotton name: PTHR20863:SF29 - ACYL CARRIER PROTEIN 1, CHLOROPLASTIC-RELATED (1 of 4)
OG IDs: 93524253
Best-hit-arabi-ID: AT1G54580.1
Best-hit-arabi-name: acyl carrier protein 2
Gorai.007G264300.1.A
PCC=-0.087, p=0.722
mRNA Profile
Protein Expression Profile
DPA

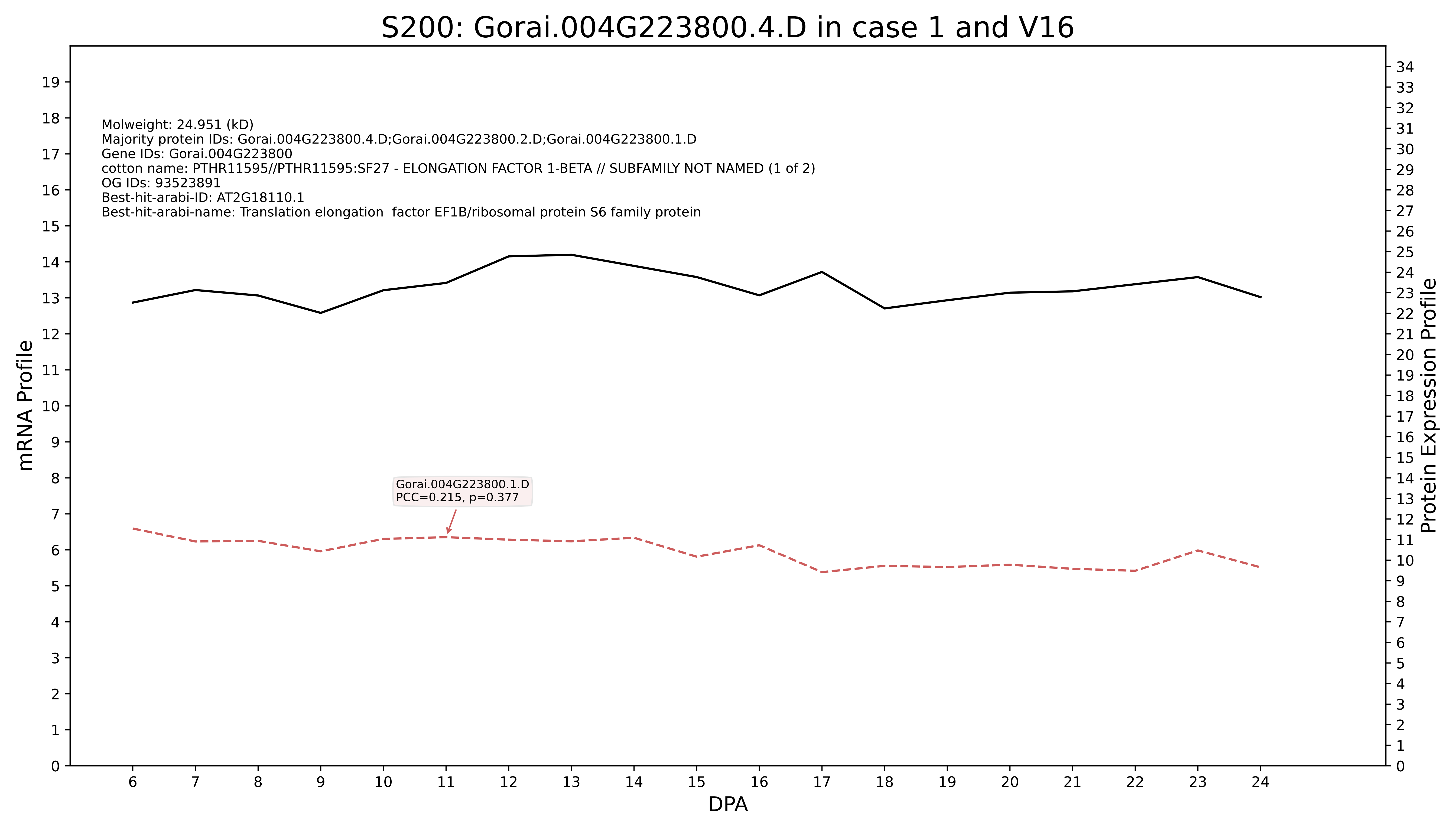

S200: Gorai.004G223800.4.D in case 1 and V16
Molweight: 24.951 (kD)
Majority protein IDs: Gorai.004G223800.4.D;Gorai.004G223800.2.D;Gorai.004G223800.1.D
Gene IDs: Gorai.004G223800
cotton name: PTHR11595//PTHR11595:SF27 - ELONGATION FACTOR 1-BETA // SUBFAMILY NOT NAMED (1 of 2)
OG IDs: 93523891
Best-hit-arabi-ID: AT2G18110.1
Best-hit-arabi-name: Translation elongation factor EF1B/ribosomal protein S6 family protein
Gorai.004G223800.1.D
PCC=0.215, p=0.377
mRNA Profile
Protein Expression Profile
DPA

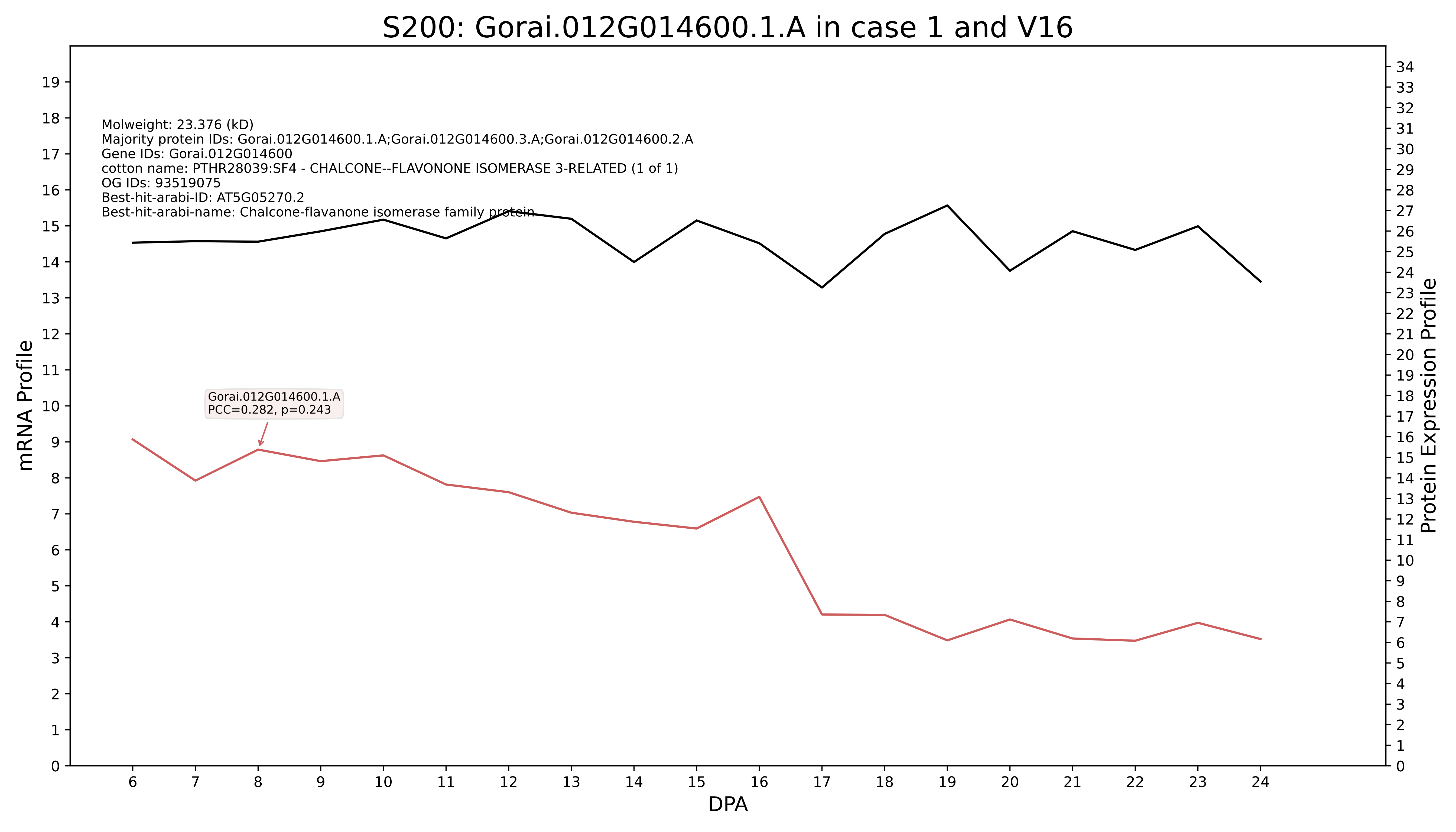
S200: Gorai.012G014600.1.A in case 1 and V16
Molweight: 23.376 (kD)
Majority protein IDs: Gorai.012G014600.1.A;Gorai.012G014600.3.A;Gorai.012G014600.2.A
Gene IDs: Gorai.012G014600
cotton name: PTHR28039:SF4 - CHALCONE--FLAVONONE ISOMERASE 3-RELATED (1 of 1)
OG IDs: 93519075
Best-hit-arabi-ID: AT5G05270.2
Best-hit-arabi-name: Chalcone-flavanone isomerase family protein
Gorai.012G014600.1.A
PCC=0.282, p=0.243
mRNA Profile
Protein Expression Profile
DPA

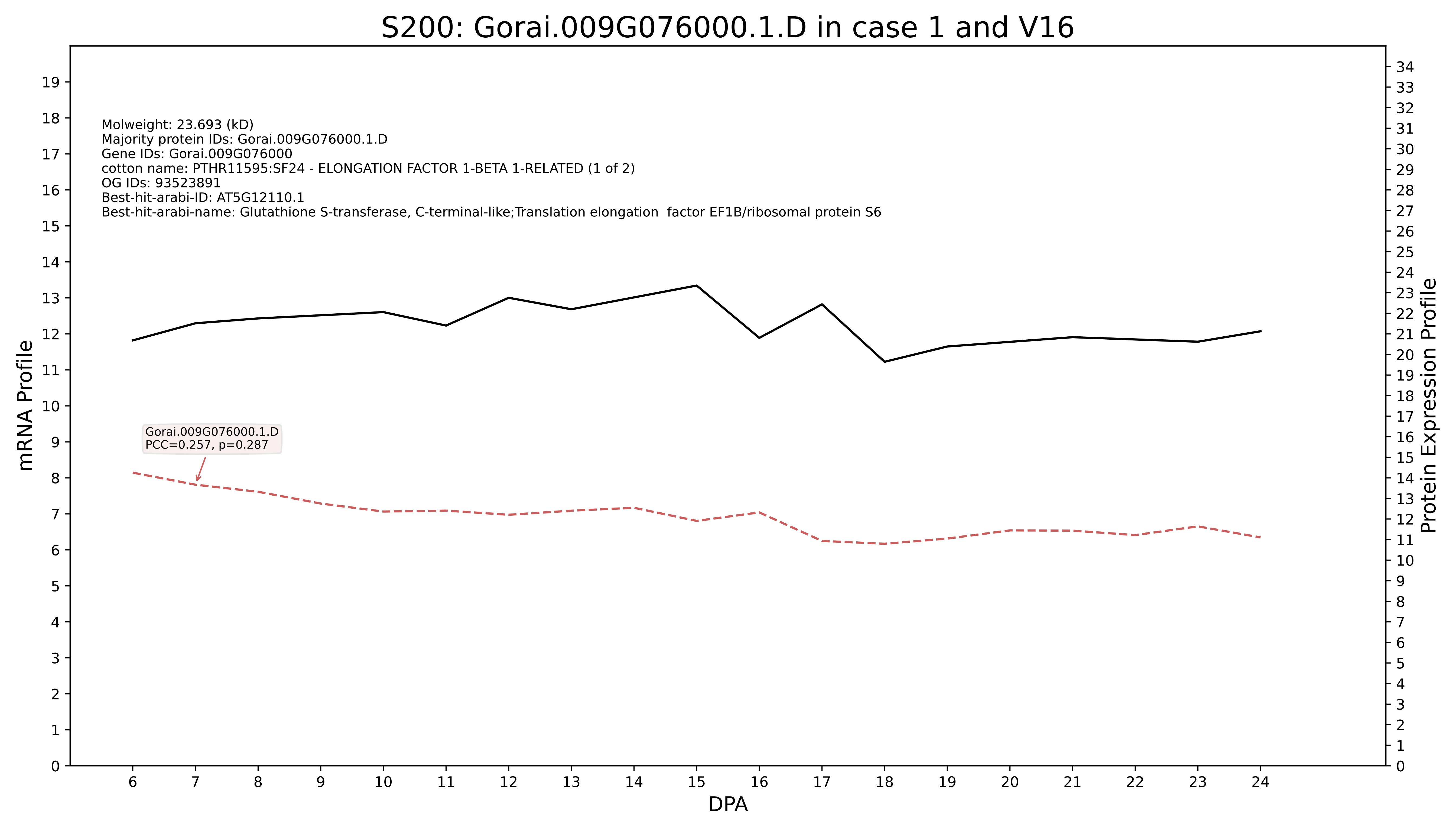
S200: Gorai.009G076000.1.D in case 1 and V16
Molweight: 23.693 (kD)
Majority protein IDs: Gorai.009G076000.1.D
Gene IDs: Gorai.009G076000
cotton name: PTHR11595:SF24 - ELONGATION FACTOR 1-BETA 1-RELATED (1 of 2)
OG IDs: 93523891
Best-hit-arabi-ID: AT5G12110.1
Best-hit-arabi-name: Glutathione S-transferase, C-terminal-like;Translation elongation factor EF1B/ribosomal protein S6
Gorai.009G076000.1.D
PCC=0.257, p=0.287
mRNA Profile
Protein Expression Profile
DPA

S200: Gorai.002G126200.1.D in case 1 and V16

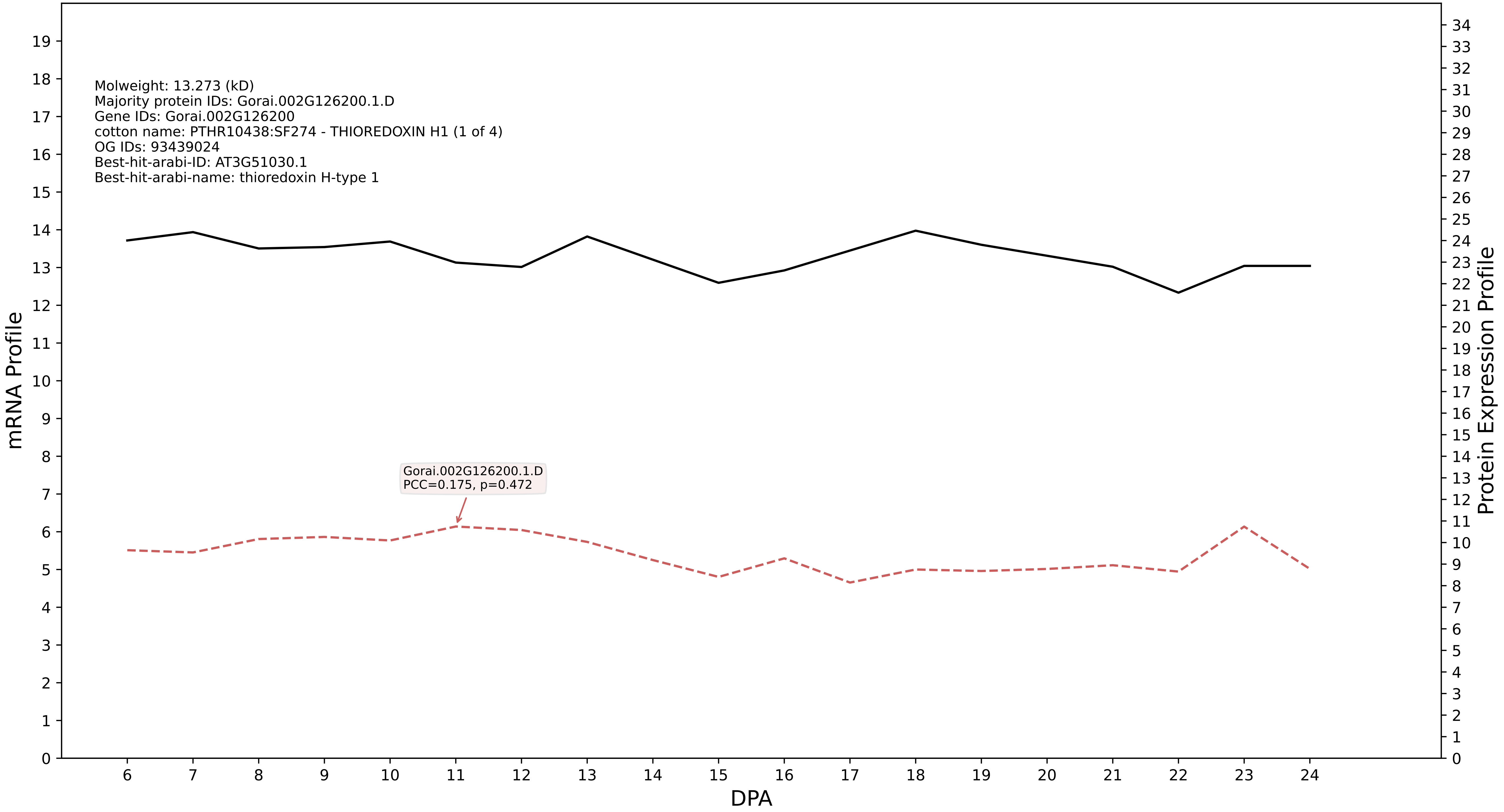

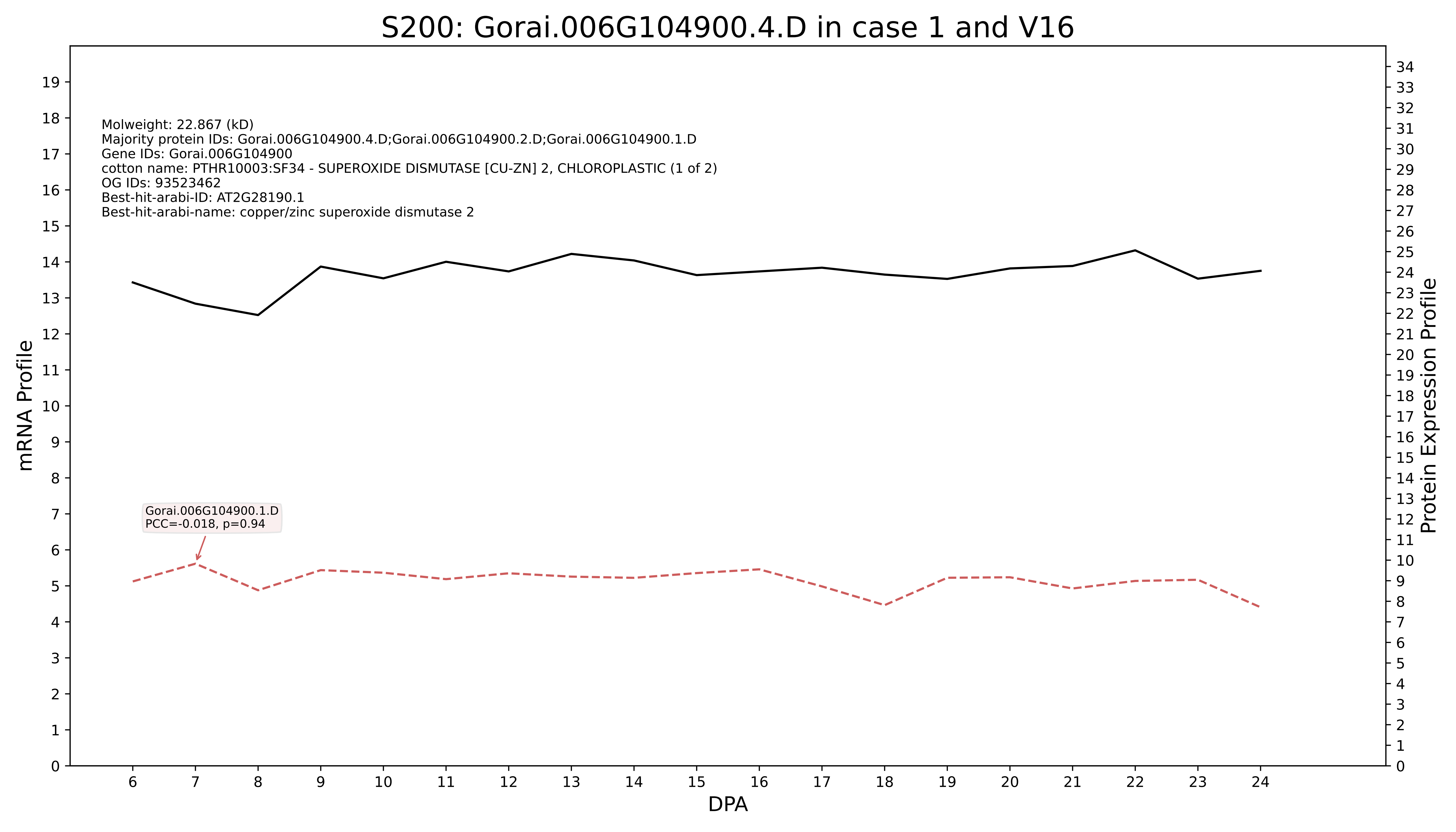
S200: Gorai.006G104900.4.D in case 1 and V16
Molweight: 22.867 (kD)
Majority protein IDs: Gorai.006G104900.4.D;Gorai.006G104900.2.D;Gorai.006G104900.1.D
Gene IDs: Gorai.006G104900
cotton name: PTHR10003:SF34 - SUPEROXIDE DISMUTASE [CU-ZN] 2, CHLOROPLASTIC (1 of 2)
OG IDs: 93523462
Best-hit-arabi-ID: AT2G28190.1
Best-hit-arabi-name: copper/zinc superoxide dismutase 2
Gorai.006G104900.1.D
PCC=-0.018, p=0.94
mRNA Profile
Protein Expression Profile
DPA

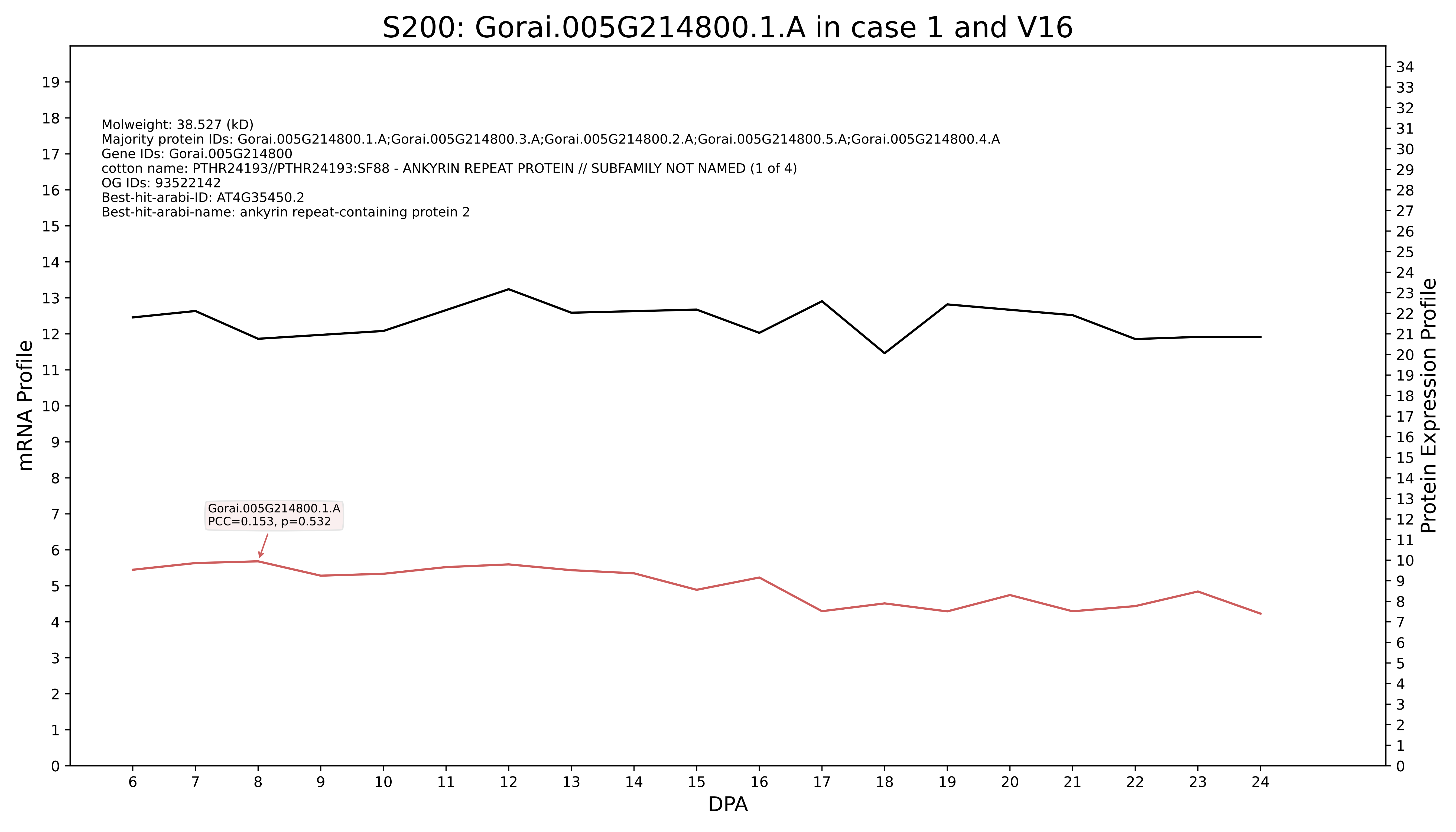

S200: Gorai.005G214800.1.A in case 1 and V16
Molweight: 38.527 (kD)
Majority protein IDs: Gorai.005G214800.1.A;Gorai.005G214800.3.A;Gorai.005G214800.2.A;Gorai.005G214800.5.A;Gorai.005G214800.4.A
Gene IDs: Gorai.005G214800
cotton name: PTHR24193//PTHR24193:SF88 - ANKYRIN REPEAT PROTEIN // SUBFAMILY NOT NAMED (1 of 4)
OG IDs: 93522142
Best-hit-arabi-ID: AT4G35450.2
Best-hit-arabi-name: ankyrin repeat-containing protein 2
Gorai.005G214800.1.A
PCC=0.153, p=0.532
mRNA Profile
Protein Expression Profile
DPA

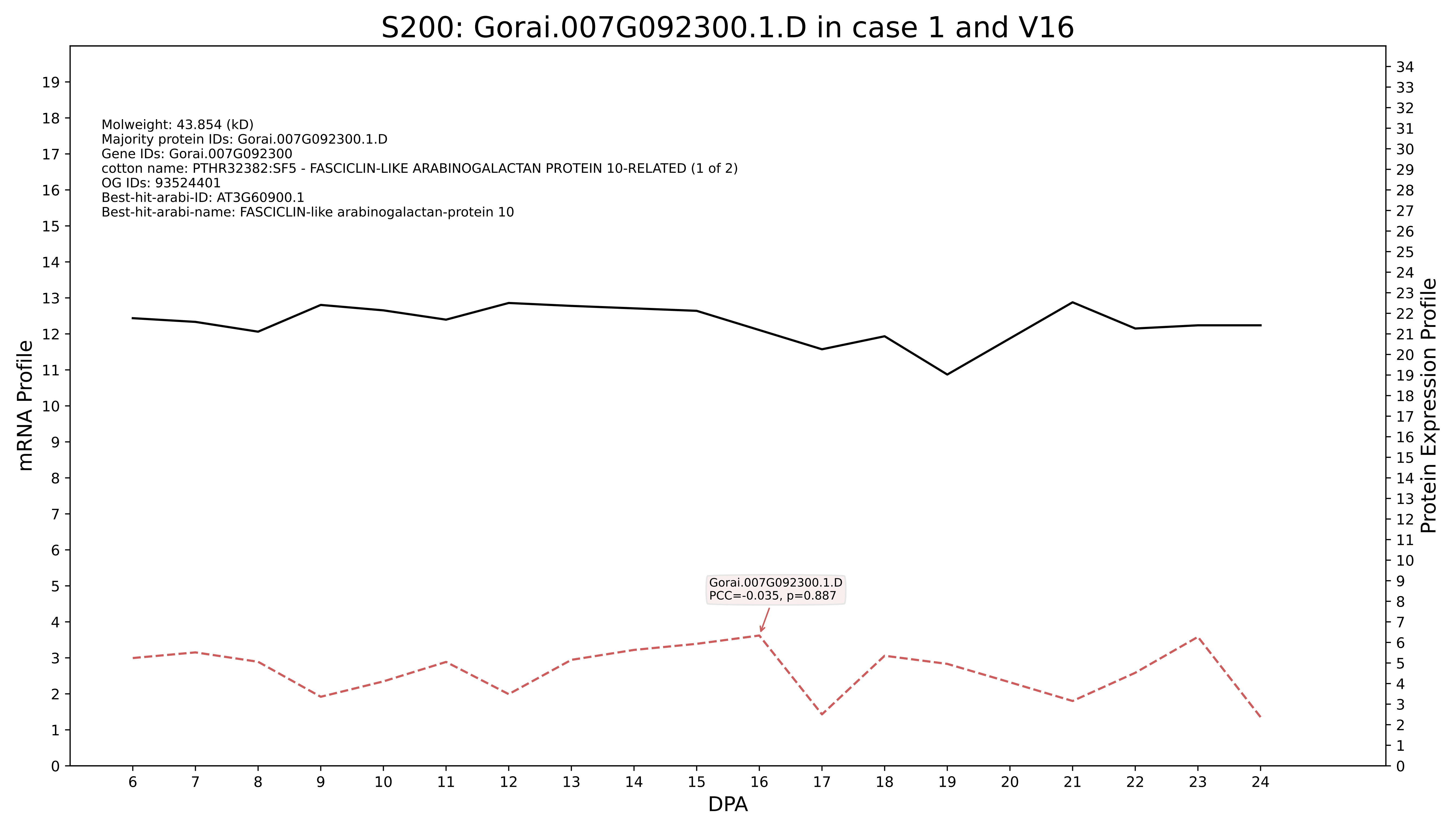
S200: Gorai.007G092300.1.D in case 1 and V16
Molweight: 43.854 (kD)
Majority protein IDs: Gorai.007G092300.1.D
Gene IDs: Gorai.007G092300
cotton name: PTHR32382:SF5 - FASCICLIN-LIKE ARABINOGALACTAN PROTEIN 10-RELATED (1 of 2)
OG IDs: 93524401
Best-hit-arabi-ID: AT3G60900.1
Best-hit-arabi-name: FASCICLIN-like arabinogalactan-protein 10
Gorai.007G092300.1.D
PCC=-0.035, p=0.887
mRNA Profile
Protein Expression Profile
DPA

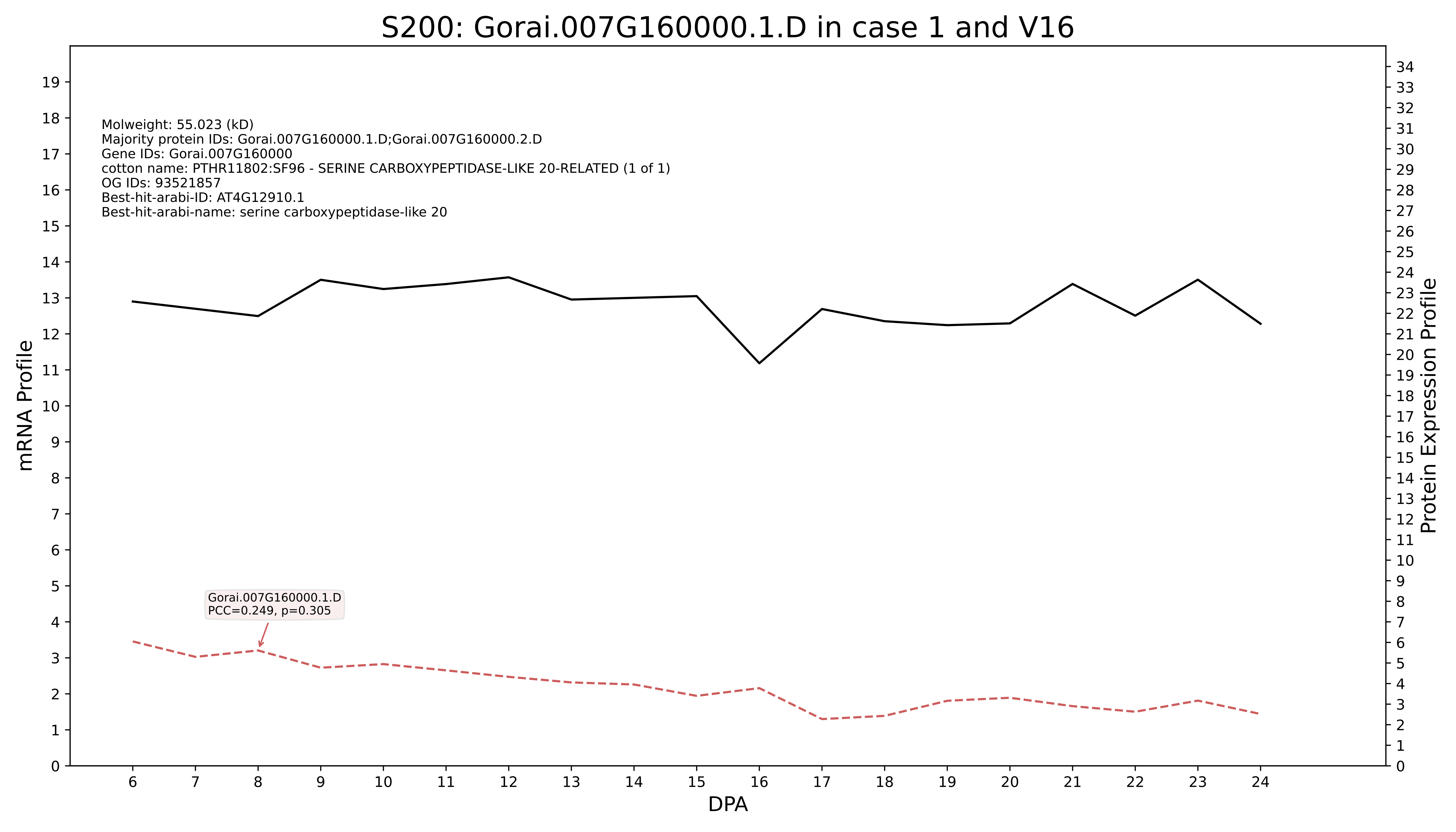

S200: Gorai.007G160000.1.D in case 1 and V16
Molweight: 55.023 (kD)
Majority protein IDs: Gorai.007G160000.1.D;Gorai.007G160000.2.D
Gene IDs: Gorai.007G160000
cotton name: PTHR11802:SF96 - SERINE CARBOXYPEPTIDASE-LIKE 20-RELATED (1 of 1)
OG IDs: 93521857
Best-hit-arabi-ID: AT4G12910.1
Best-hit-arabi-name: serine carboxypeptidase-like 20
Gorai.007G160000.1.D
PCC=0.249, p=0.305
mRNA Profile
Protein Expression Profile
DPA

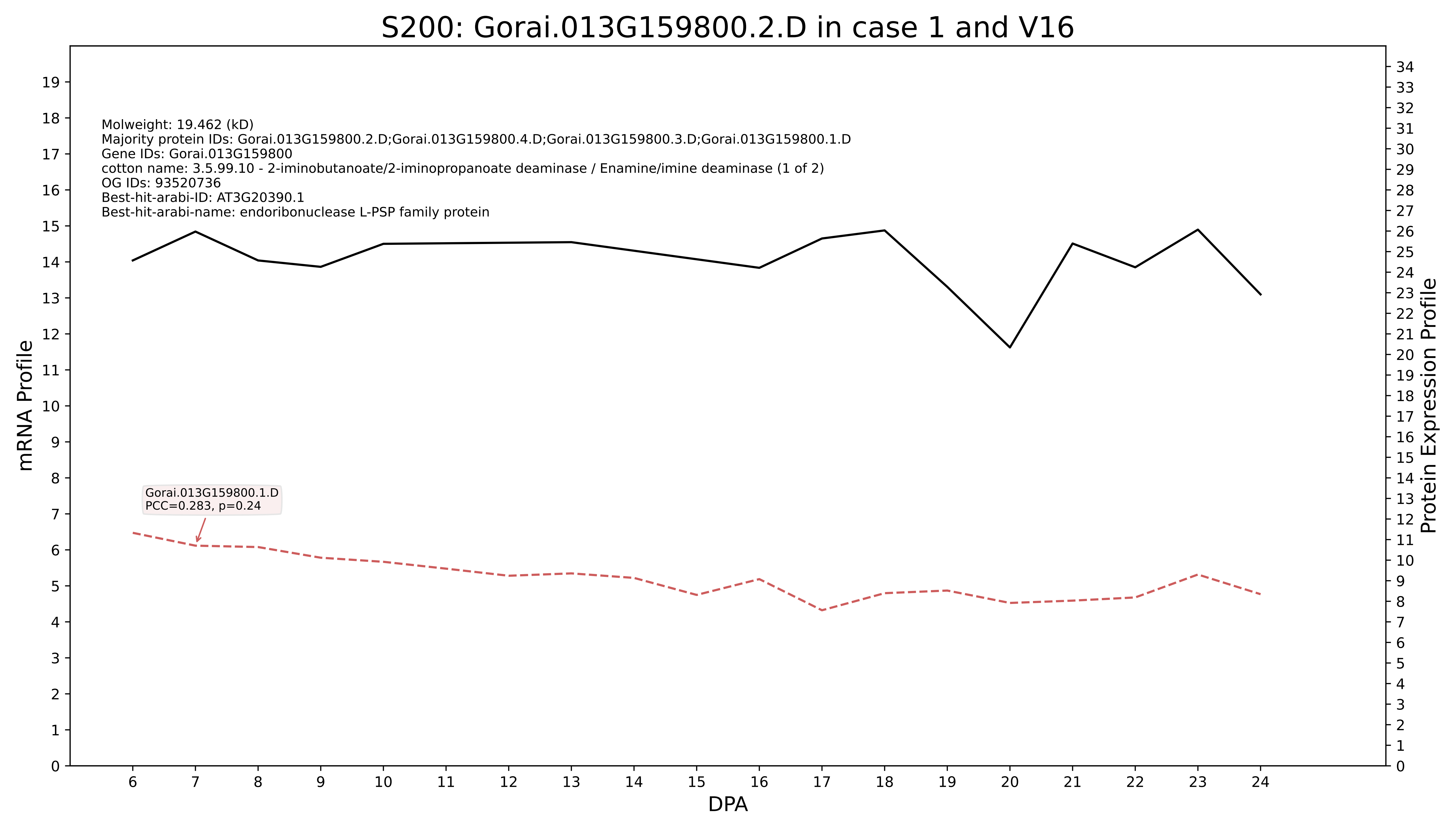
S200: Gorai.013G159800.2.D in case 1 and V16
Molweight: 19.462 (kD)
Majority protein IDs: Gorai.013G159800.2.D;Gorai.013G159800.4.D;Gorai.013G159800.3.D;Gorai.013G159800.1.D
Gene IDs: Gorai.013G159800
cotton name: 3.5.99.10 - 2-iminobutanoate/2-iminopropanoate deaminase / Enamine/imine deaminase (1 of 2)
OG IDs: 93520736
Best-hit-arabi-ID: AT3G20390.1
Best-hit-arabi-name: endoribonuclease L-PSP family protein
Gorai.013G159800.1.D
PCC=0.283, p=0.24
mRNA Profile
Protein Expression Profile
DPA
6
7
8
9
10
11
12
13
14
15
16
17
18
19
20
21
22
23
24

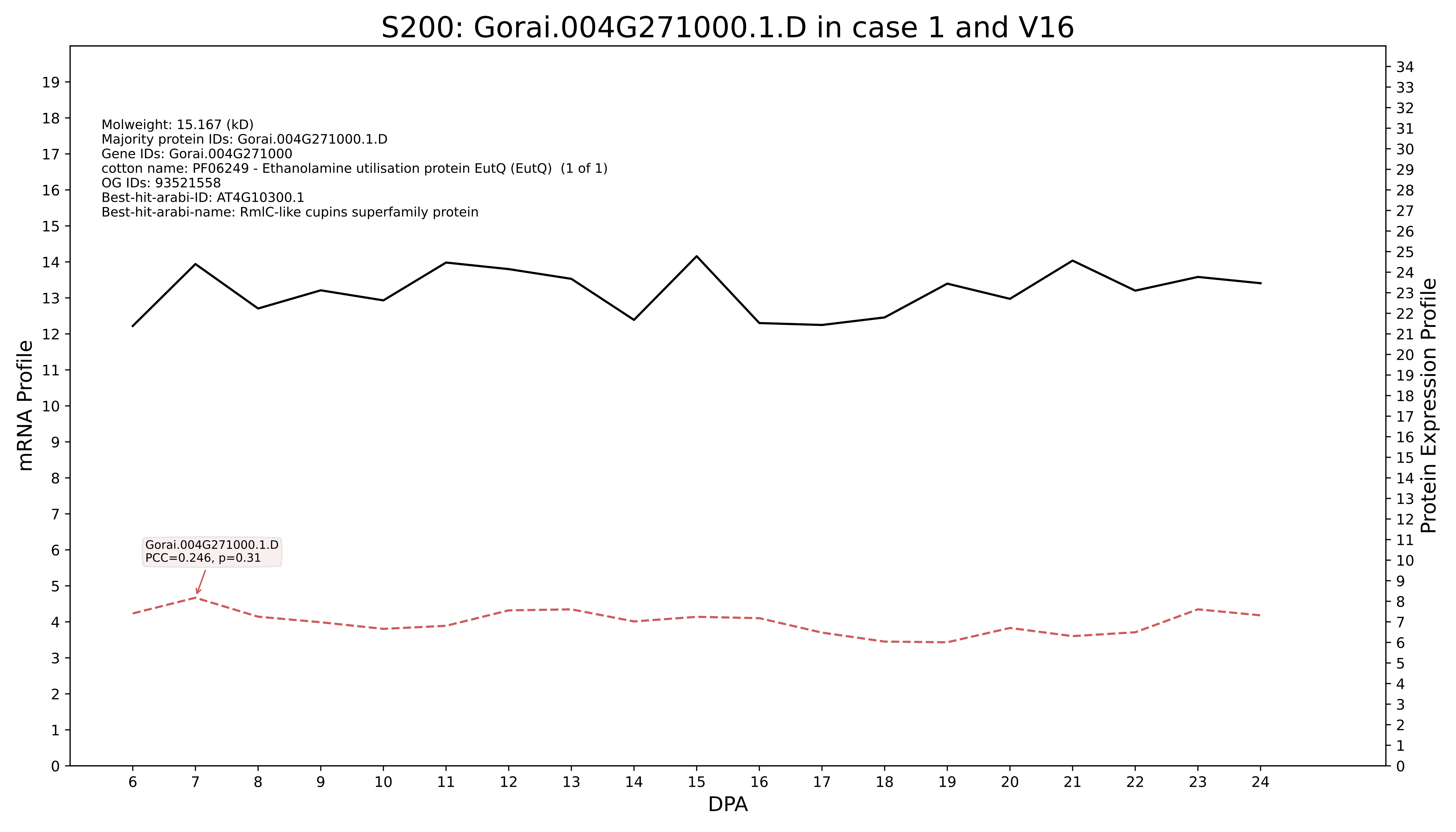
S200: Gorai.004G271000.1.D in case 1 and V16
Molweight: 15.167 (kD)
Majority protein IDs: Gorai.004G271000.1.D
Gene IDs: Gorai.004G271000
cotton name: PF06249 - Ethanolamine utilisation protein EutQ (EutQ) (1 of 1)
OG IDs: 93521558
Best-hit-arabi-ID: AT4G10300.1
Best-hit-arabi-name: RmlC-like cupins superfamily protein
Gorai.004G271000.1.D
PCC=0.246, p=0.31
mRNA Profile
Protein Expression Profile
DPA

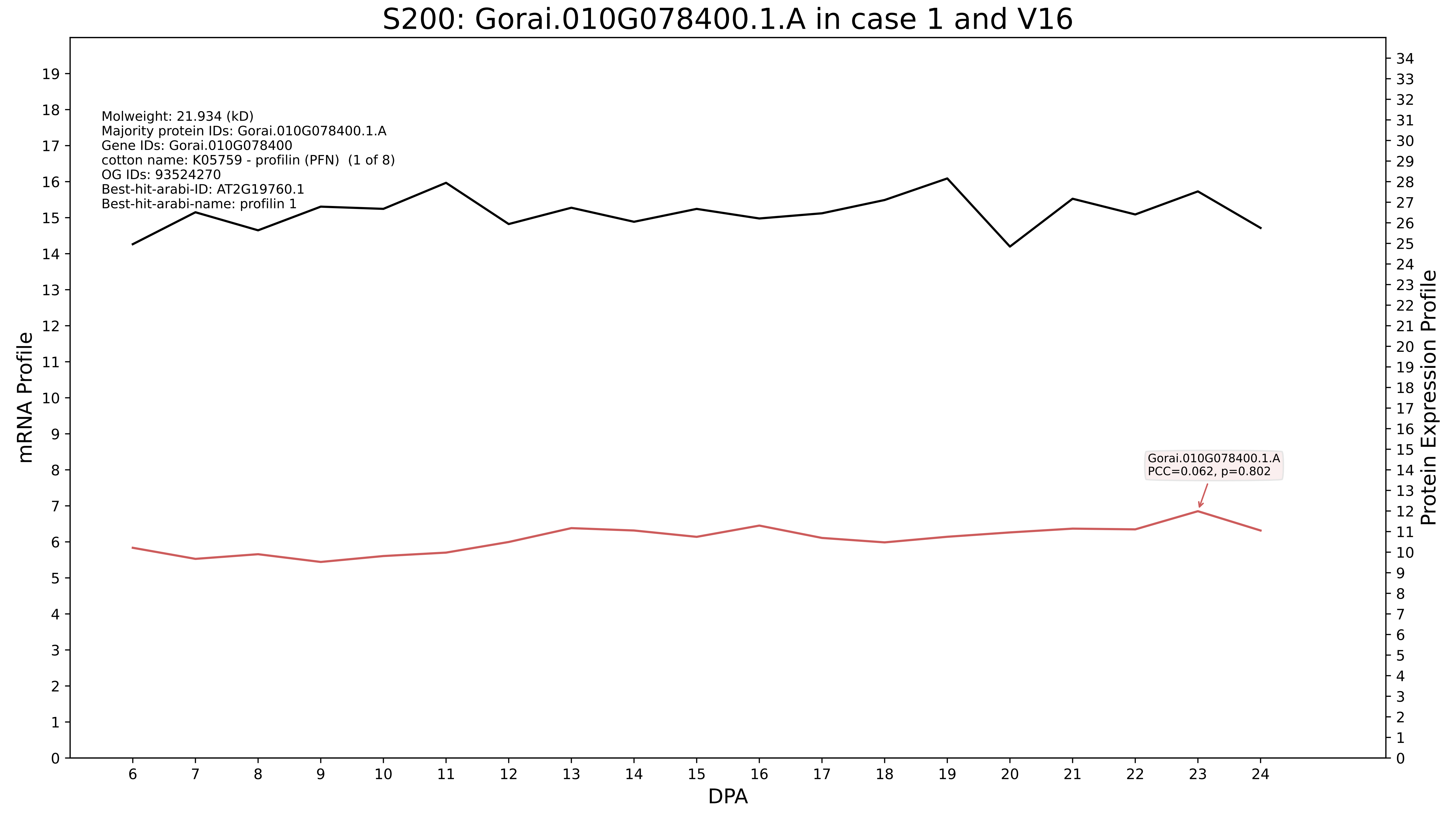

S200: Gorai.010G078400.1.A in case 1 and V16
Molweight: 21.934 (kD)
Majority protein IDs: Gorai.010G078400.1.A
Gene IDs: Gorai.010G078400
cotton name: K05759 - profilin (PFN) (1 of 8)
OG IDs: 93524270
Best-hit-arabi-ID: AT2G19760.1
Best-hit-arabi-name: profilin 1
Gorai.010G078400.1.A
PCC=0.062, p=0.802
mRNA Profile
Protein Expression Profile
DPA

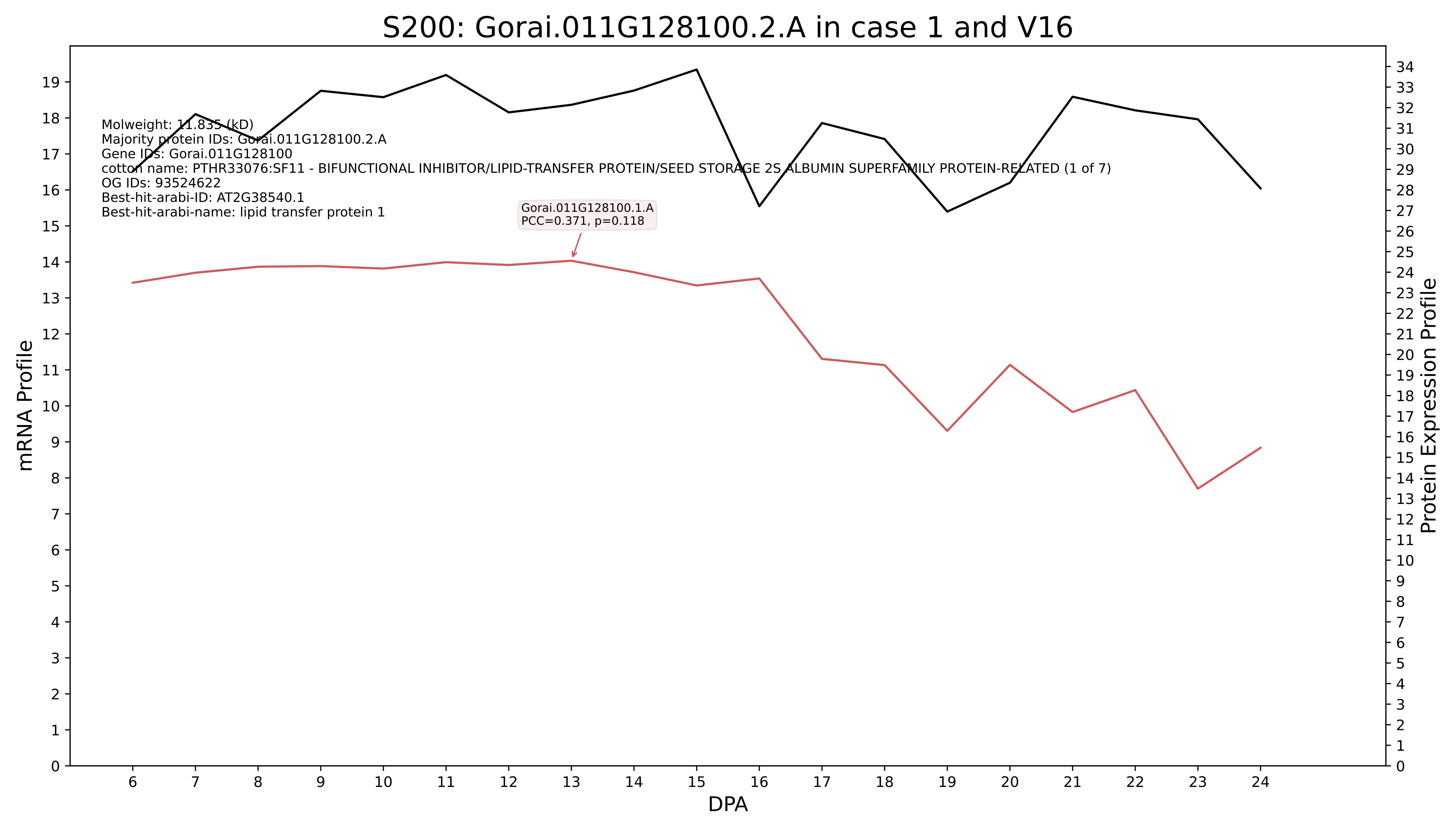
S200: Gorai.011G128100.2.A in case 1 and V16
Molweight: 11.835 (kD)
Majority protein IDs: Gorai.011G128100.2.A
Gene IDs: Gorai.011G128100
cotton name: PTHR33076:SF11 - BIFUNCTIONAL INHIBITOR/LIPID-TRANSFER PROTEIN/SEED STORAGE 2S ALBUMIN SUPERFAMILY PROTEIN-RELATED (1 of 7)
OG IDs: 93524622
Best-hit-arabi-ID: AT2G38540.1
Best-hit-arabi-name: lipid transfer protein 1
Gorai.011G128100.1.A
PCC=0.371, p=0.118
mRNA Profile
Protein Expression Profile
DPA

0: Gorai.012G064000.2.D in case 1 and V16

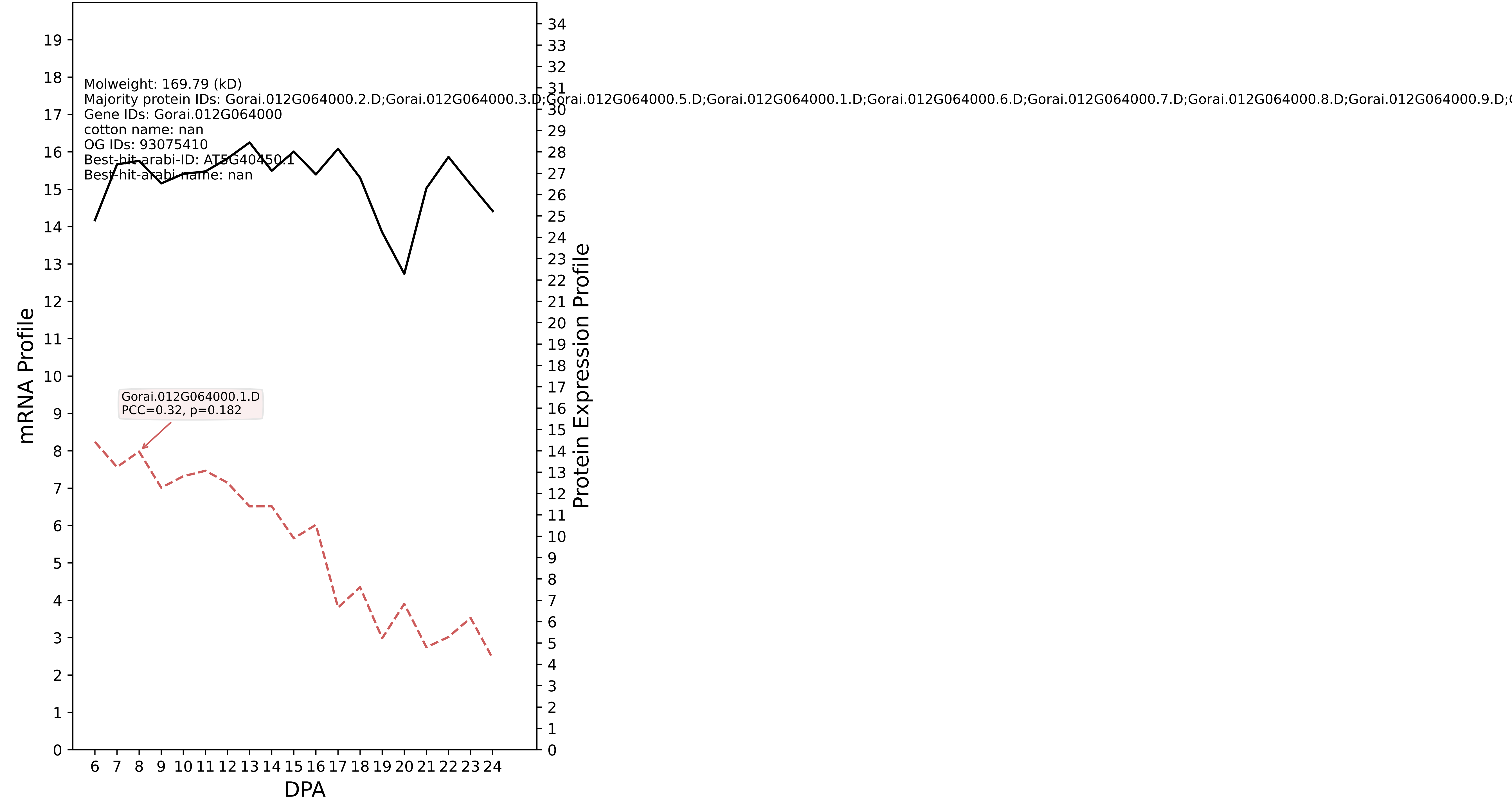

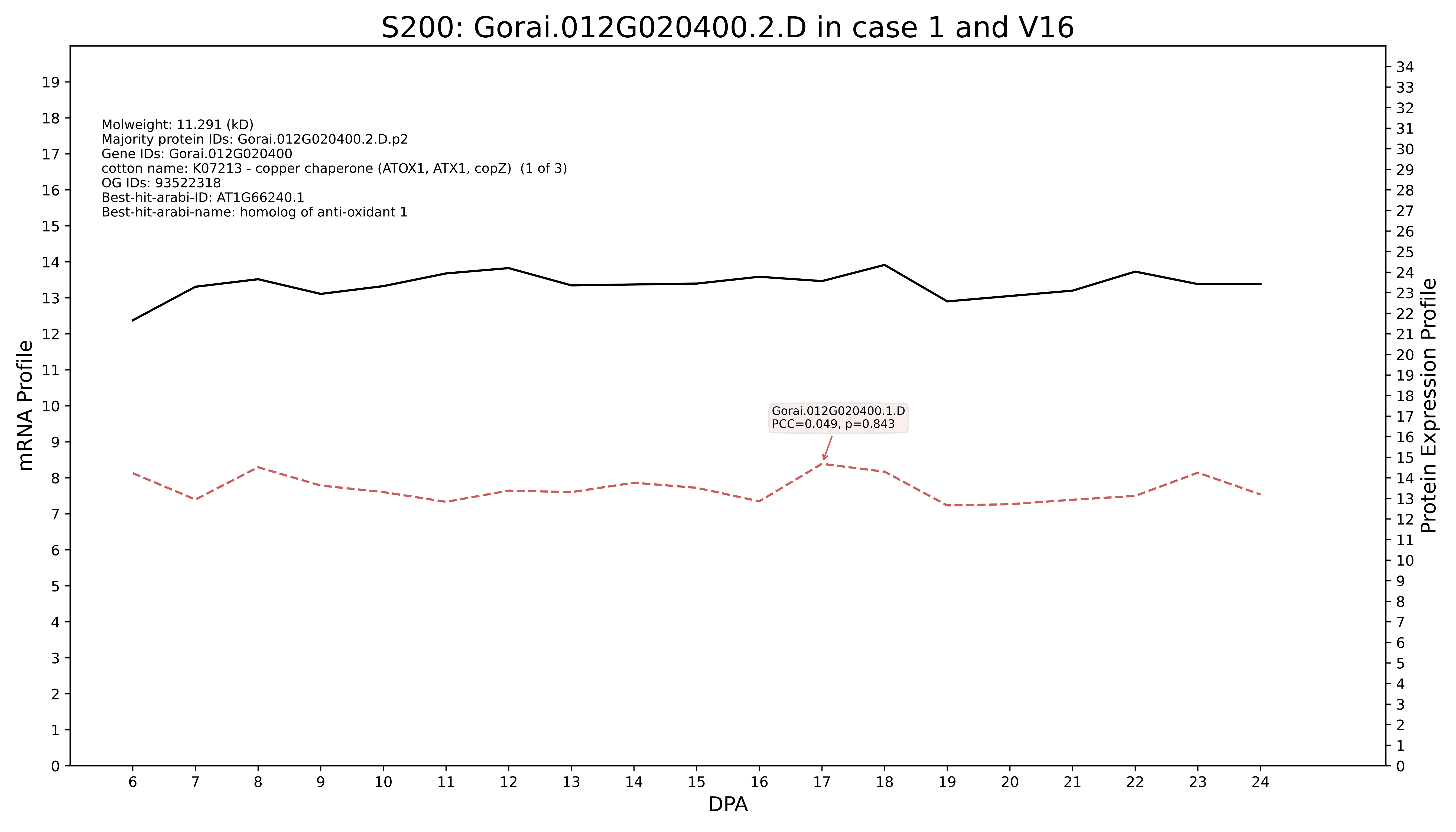
S200: Gorai.012G020400.2.D in case 1 and V16
Molweight: 11.291 (kD)
Majority protein IDs: Gorai.012G020400.2.D.p2
Gene IDs: Gorai.012G020400
cotton name: K07213 - copper chaperone (ATOX1, ATX1, copZ) (1 of 3)
OG IDs: 93522318
Best-hit-arabi-ID: AT1G66240.1
Best-hit-arabi-name: homolog of anti-oxidant 1
Gorai.012G020400.1.D
PCC=0.049, p=0.843
mRNA Profile
Protein Expression Profile
DPA
0
1
2
3
4
5
6
7
8
9
10
11
12
13
14
15
16
17
18
19
20
21
22
23
24
25
26
27
28
29
30
31
32
33
34

S200: Gorai.013G023400.1.A in case 1 and V16

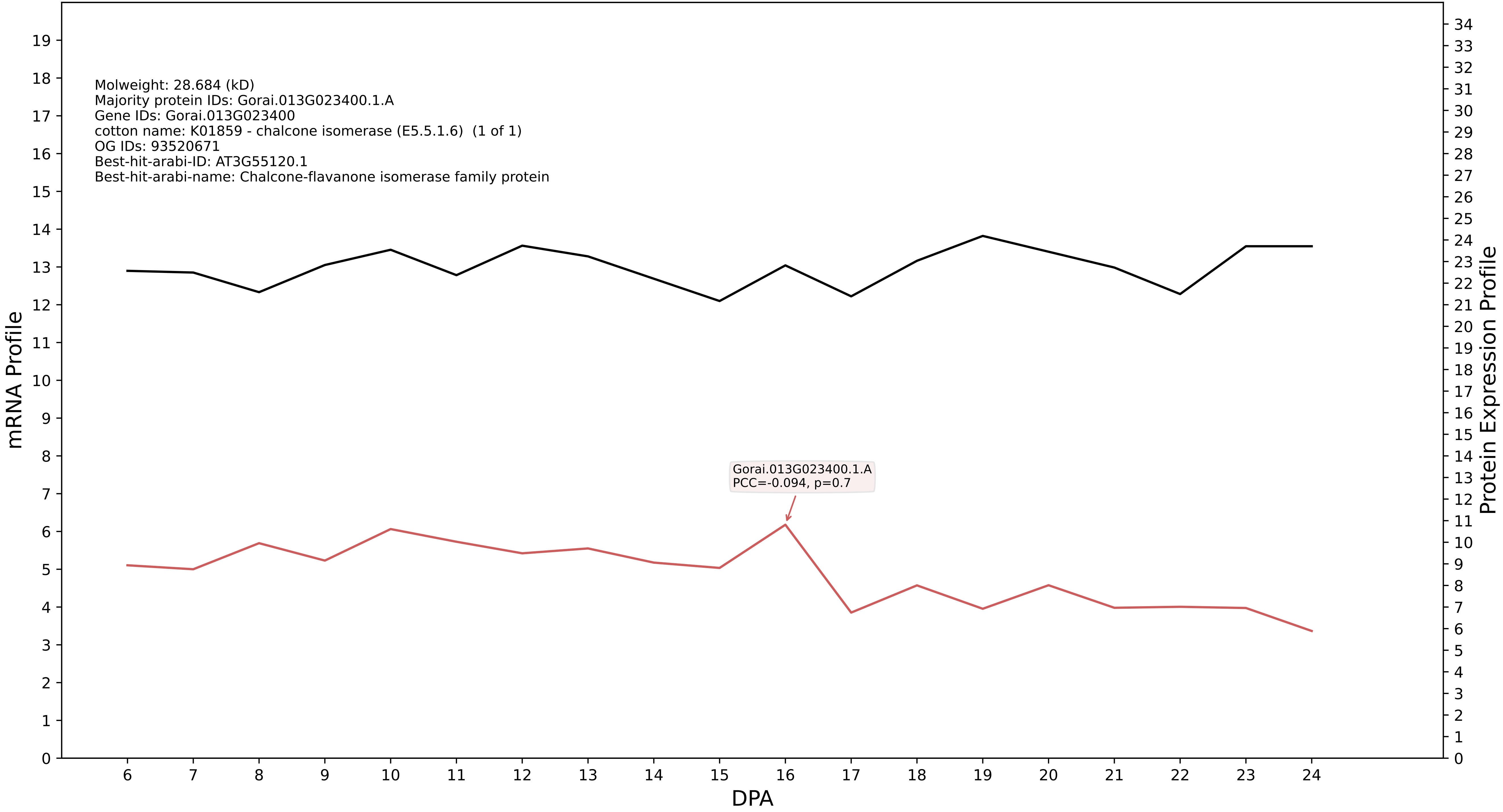

S200: Gorai.006G147500.2.A in case 1 and V16

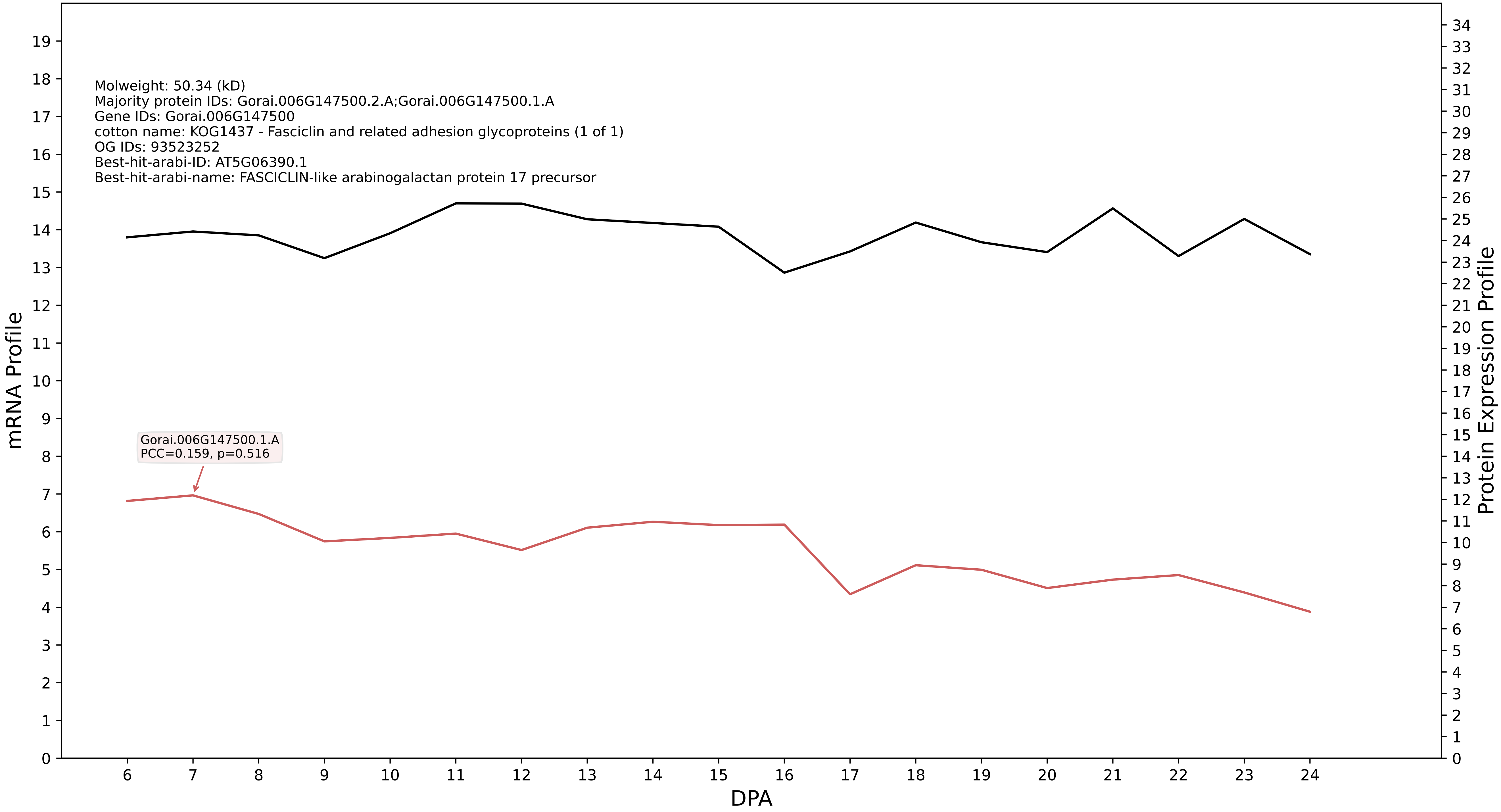

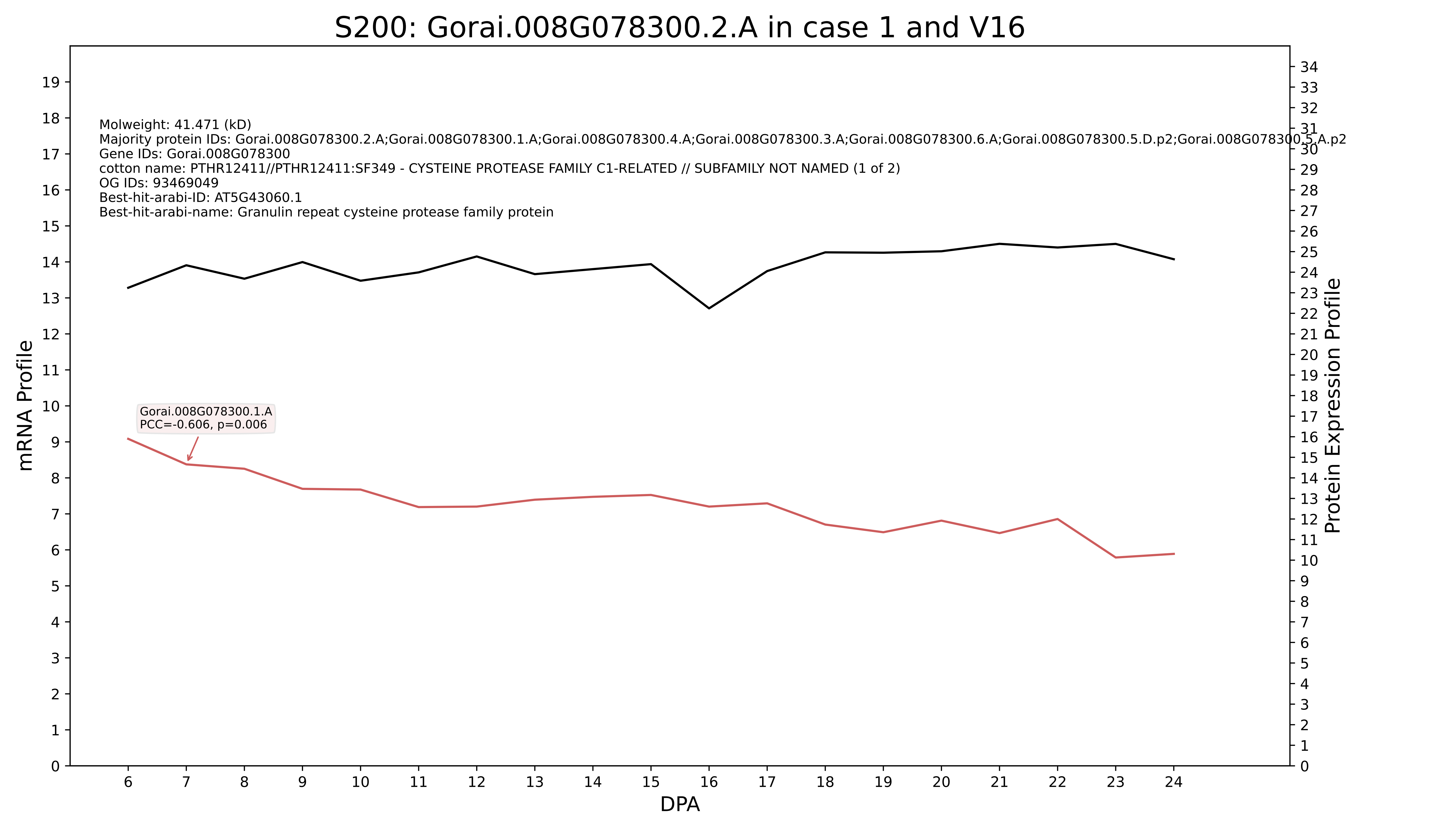
S200: Gorai.008G078300.2.A in case 1 and V16
Molweight: 41.471 (kD)
Majority protein IDs: Gorai.008G078300.2.A;Gorai.008G078300.1.A;Gorai.008G078300.4.A;Gorai.008G078300.3.A;Gorai.008G078300.6.A;Gorai.008G078300.5.D.p2;Gorai.008G078300.5.A.p2
Gene IDs: Gorai.008G078300
cotton name: PTHR12411//PTHR12411:SF349 - CYSTEINE PROTEASE FAMILY C1-RELATED // SUBFAMILY NOT NAMED (1 of 2)
OG IDs: 93469049
Best-hit-arabi-ID: AT5G43060.1
Best-hit-arabi-name: Granulin repeat cysteine protease family protein
Gorai.008G078300.1.A
PCC=-0.606, p=0.006
mRNA Profile
Protein Expression Profile
DPA

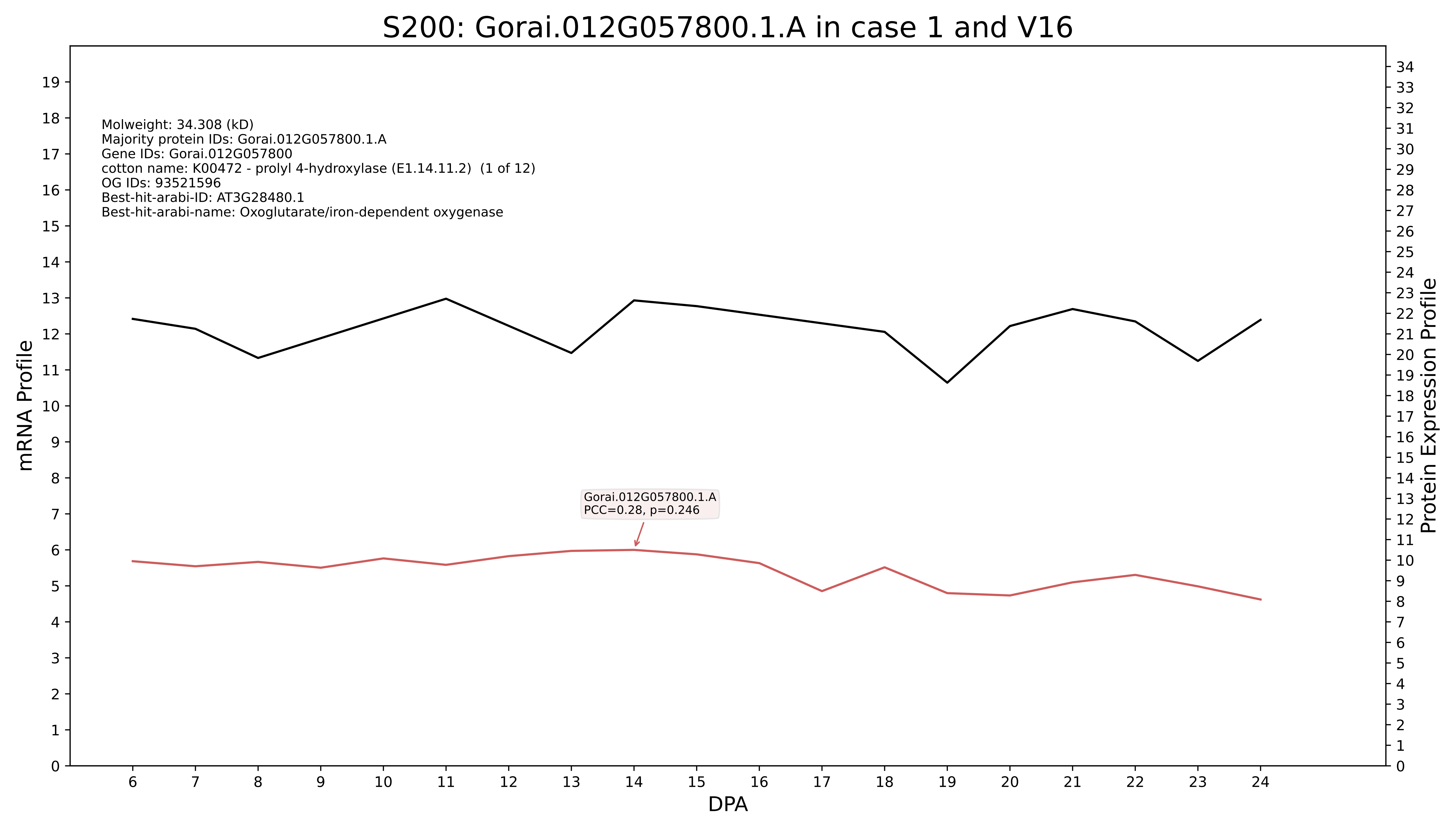
S200: Gorai.012G057800.1.A in case 1 and V16
Molweight: 34.308 (kD)
Majority protein IDs: Gorai.012G057800.1.A
Gene IDs: Gorai.012G057800
cotton name: K00472 - prolyl 4-hydroxylase (E1.14.11.2) (1 of 12)
OG IDs: 93521596
Best-hit-arabi-ID: AT3G28480.1
Best-hit-arabi-name: Oxoglutarate/iron-dependent oxygenase
Gorai.012G057800.1.A
PCC=0.28, p=0.246
mRNA Profile
Protein Expression Profile
DPA

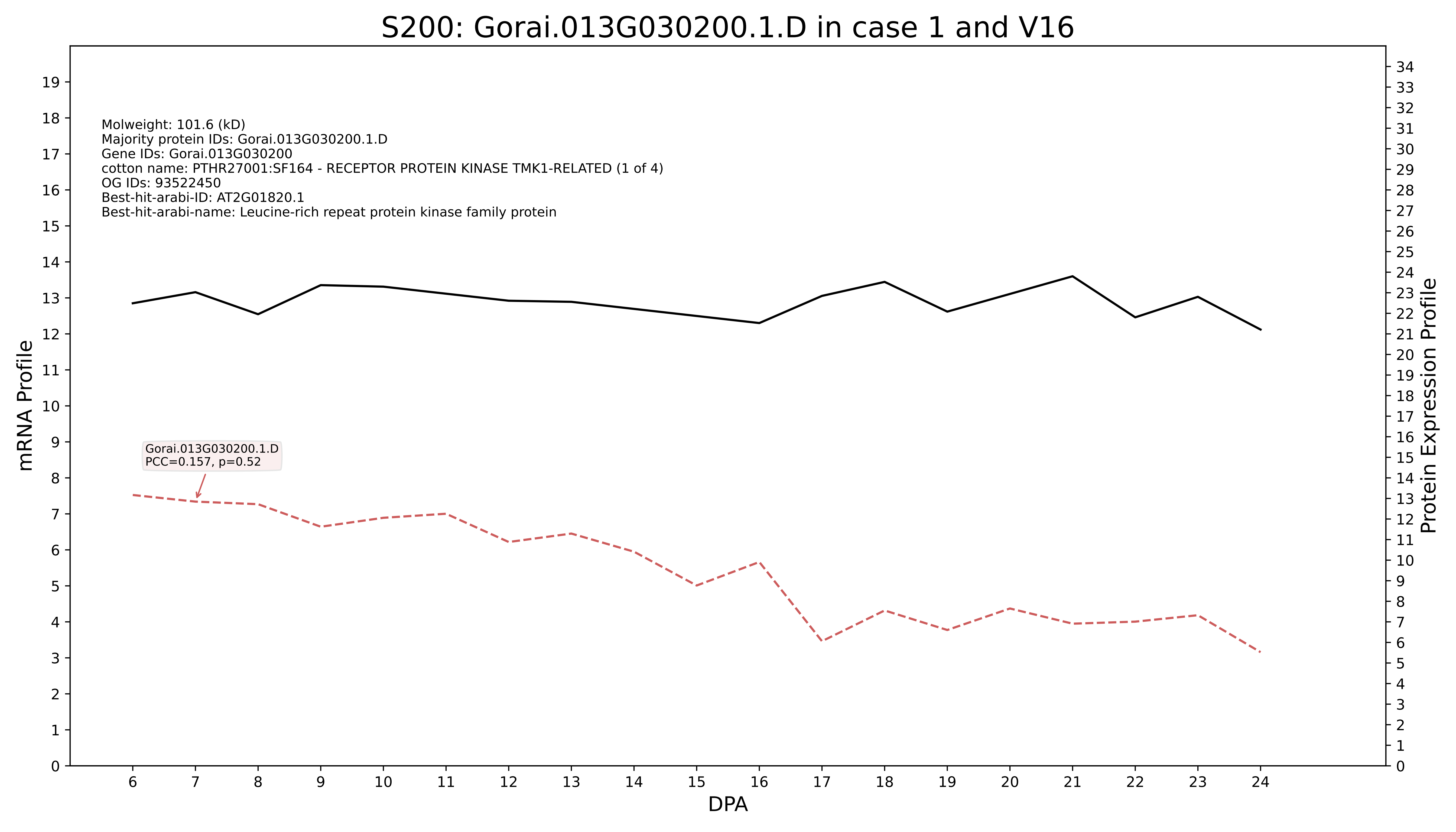
S200: Gorai.013G030200.1.D in case 1 and V16
Molweight: 101.6 (kD)
Majority protein IDs: Gorai.013G030200.1.D
Gene IDs: Gorai.013G030200
cotton name: PTHR27001:SF164 - RECEPTOR PROTEIN KINASE TMK1-RELATED (1 of 4)
OG IDs: 93522450
Best-hit-arabi-ID: AT2G01820.1
Best-hit-arabi-name: Leucine-rich repeat protein kinase family protein
Gorai.013G030200.1.D
PCC=0.157, p=0.52
mRNA Profile
Protein Expression Profile
DPA
0 1 2 3 4 5 6 7 8 9 10 11 12 13 14 15 16 17 18 19
0 1 2 3 4 5 6 7 8 9 10 11 12 13 14 15 16 17 18 19 20 21 22 23 24 25 26 27 28 29 30 31 32 33 34
6 7 8 9 10 11 12 13 14 15 16 17 18 19 20 21 22 23 24

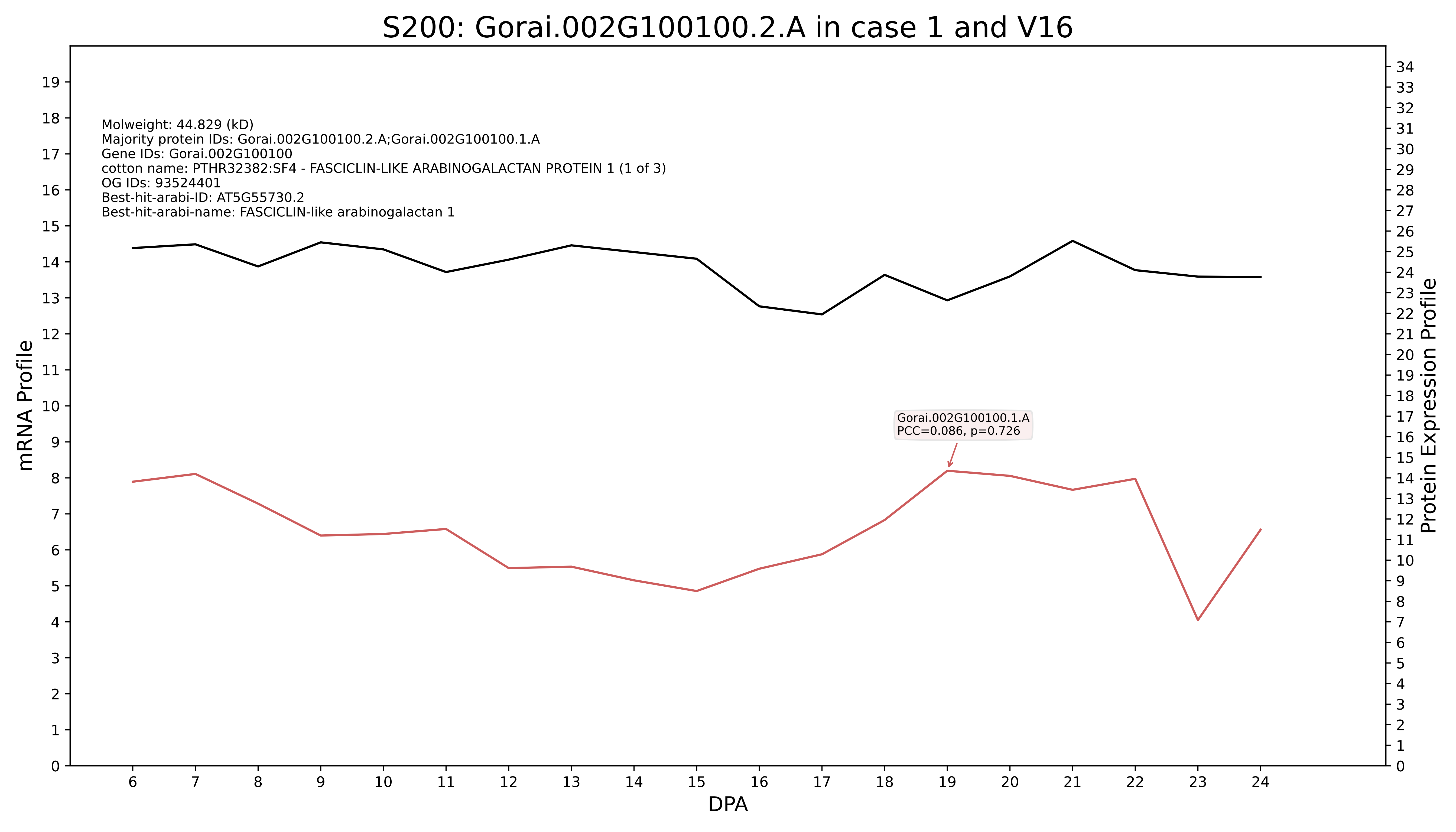
S200: Gorai.002G100100.2.A in case 1 and V16
Molweight: 44.829 (kD)
Majority protein IDs: Gorai.002G100100.2.A;Gorai.002G100100.1.A
Gene IDs: Gorai.002G100100
cotton name: PTHR32382:SF4 - FASCICLIN-LIKE ARABINOGALACTAN PROTEIN 1 (1 of 3)
OG IDs: 93524401
Best-hit-arabi-ID: AT5G55730.2
Best-hit-arabi-name: FASCICLIN-like arabinogalactan 1
Gorai.002G100100.1.A
PCC=0.086, p=0.726
mRNA Profile
Protein Expression Profile
DPA

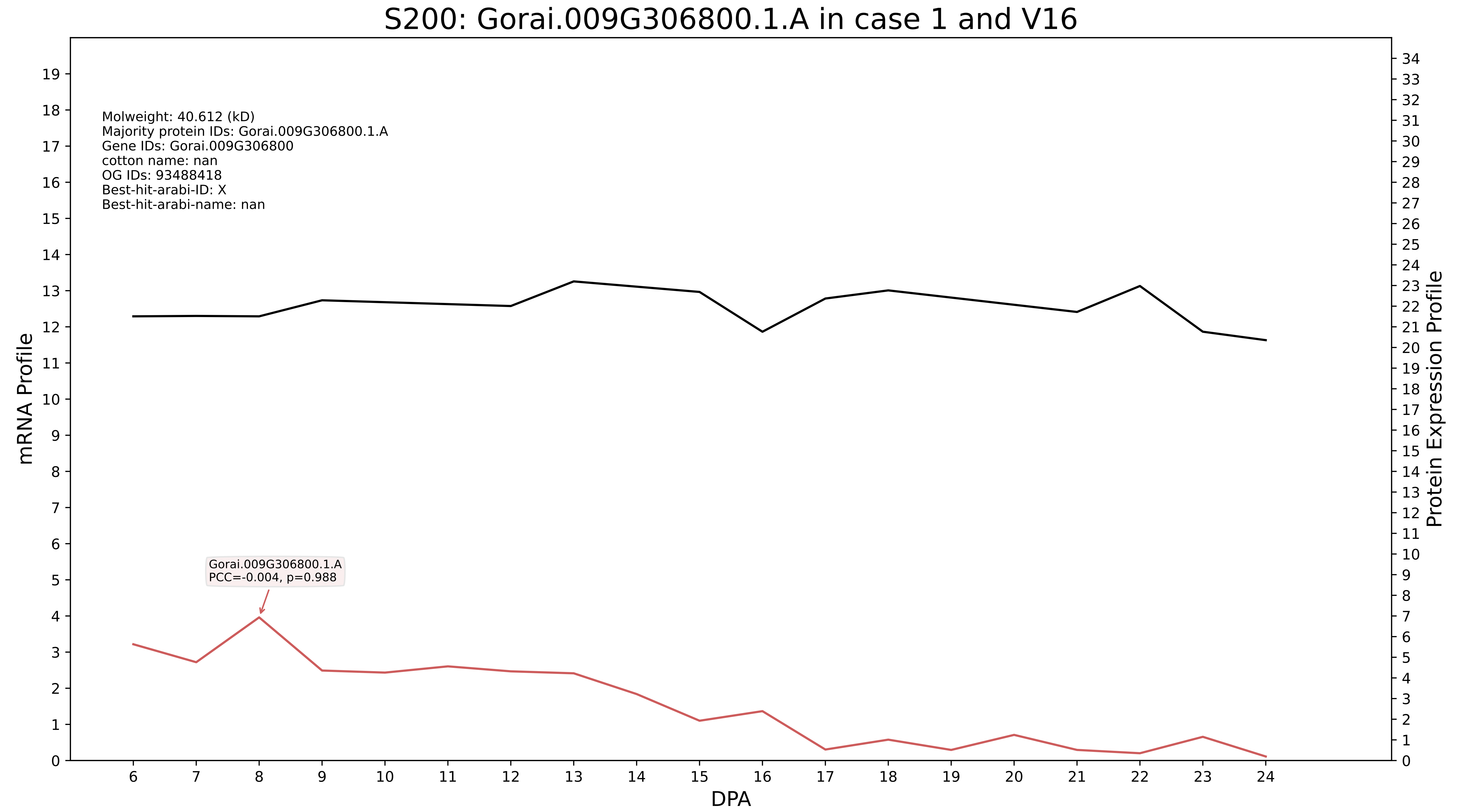

S200: Gorai.009G306800.1.A in case 1 and V16
Molweight: 40.612 (kD)
Majority protein IDs: Gorai.009G306800.1.A
Gene IDs: Gorai.009G306800
cotton name: nan
OG IDs: 93488418
Best-hit-arabi-ID: X
Best-hit-arabi-name: nan
Gorai.009G306800.1.A
PCC=-0.004, p=0.988
mRNA Profile
Protein Expression Profile
DPA

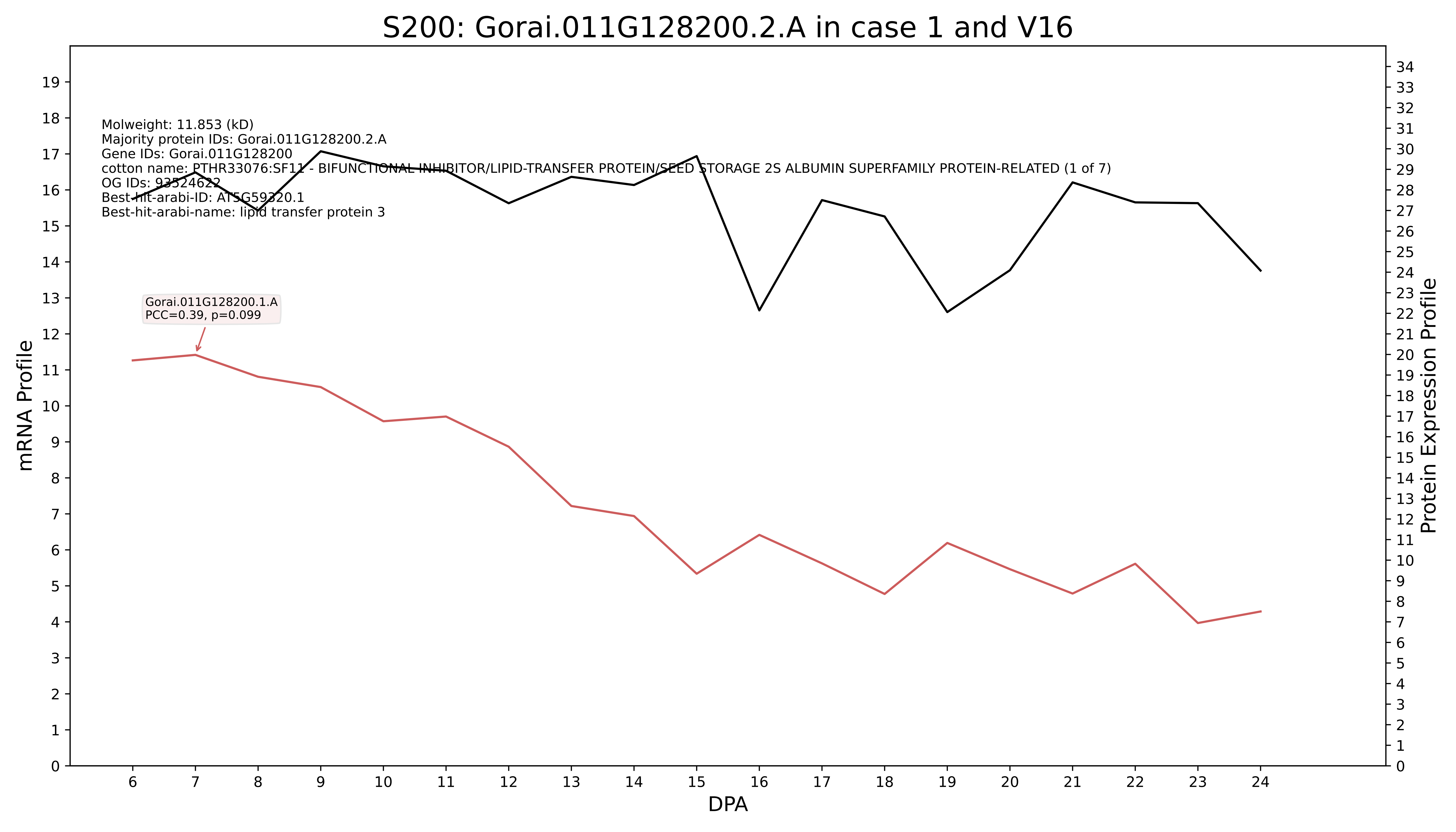

S200: Gorai.011G128200.2.A in case 1 and V16
Molweight: 11.853 (kD)
Majority protein IDs: Gorai.011G128200.2.A
Gene IDs: Gorai.011G128200
cotton name: PTHR33076:SF11 - BIFUNCTIONAL INHIBITOR/LIPID-TRANSFER PROTEIN/SEED STORAGE 2S ALBUMIN SUPERFAMILY PROTEIN-RELATED (1 of 7)
OG IDs: 93524622
Best-hit-arabi-ID: AT5G59320.1
Best-hit-arabi-name: lipid transfer protein 3
Gorai.011G128200.1.A
PCC=0.39, p=0.099
mRNA Profile
Protein Expression Profile
DPA

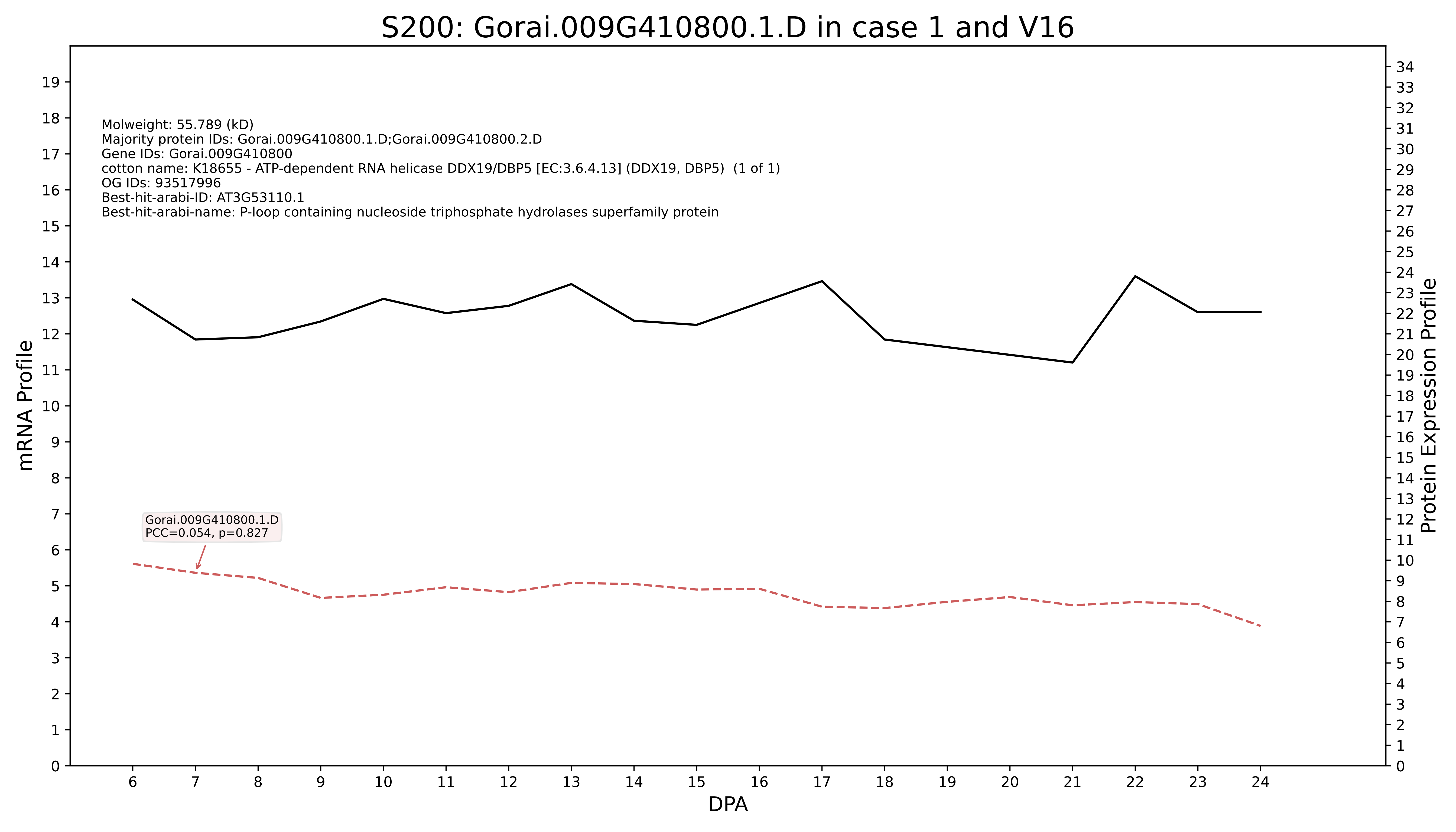
S200: Gorai.009G410800.1.D in case 1 and V16
Molweight: 55.789 (kD)
Majority protein IDs: Gorai.009G410800.1.D;Gorai.009G410800.2.D
Gene IDs: Gorai.009G410800
cotton name: K18655 - ATP-dependent RNA helicase DDX19/DBP5 [EC:3.6.4.13] (DDX19, DBP5) (1 of 1)
OG IDs: 93517996
Best-hit-arabi-ID: AT3G53110.1
Best-hit-arabi-name: P-loop containing nucleoside triphosphate hydrolases superfamily protein
Gorai.009G410800.1.D
PCC=0.054, p=0.827
mRNA Profile
Protein Expression Profile
DPA
6
7
8
9
10
11
12
13
14
15
16
17
18
19
20
21
22
23
24

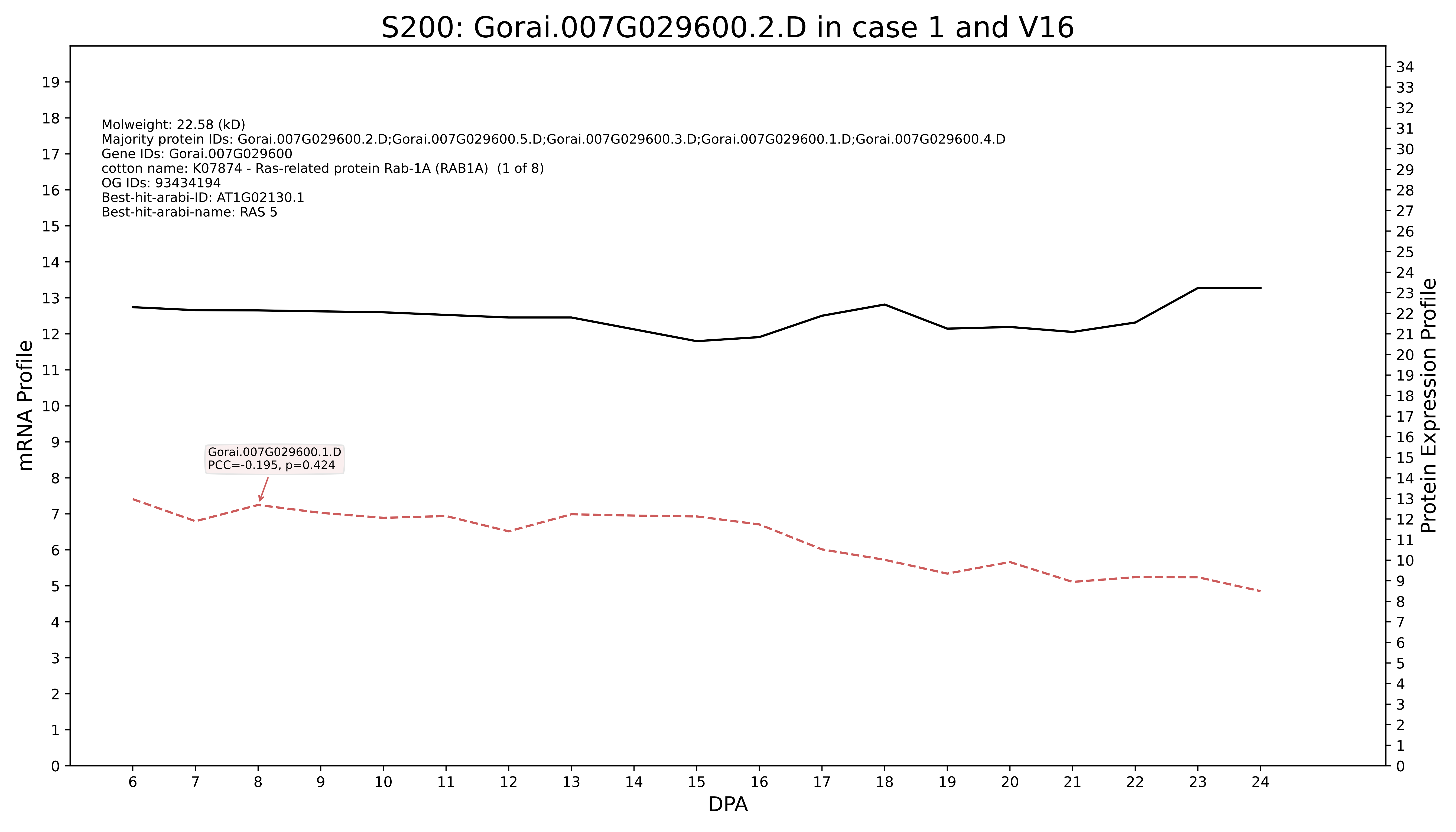
S200: Gorai.007G029600.2.D in case 1 and V16
Molweight: 22.58 (kD)
Majority protein IDs: Gorai.007G029600.2.D;Gorai.007G029600.5.D;Gorai.007G029600.3.D;Gorai.007G029600.1.D;Gorai.007G029600.4.D
Gene IDs: Gorai.007G029600
cotton name: K07874 - Ras-related protein Rab-1A (RAB1A) (1 of 8)
OG IDs: 93434194
Best-hit-arabi-ID: AT1G02130.1
Best-hit-arabi-name: RAS 5
Gorai.007G029600.1.D
PCC=-0.195, p=0.424
mRNA Profile
Protein Expression Profile
DPA

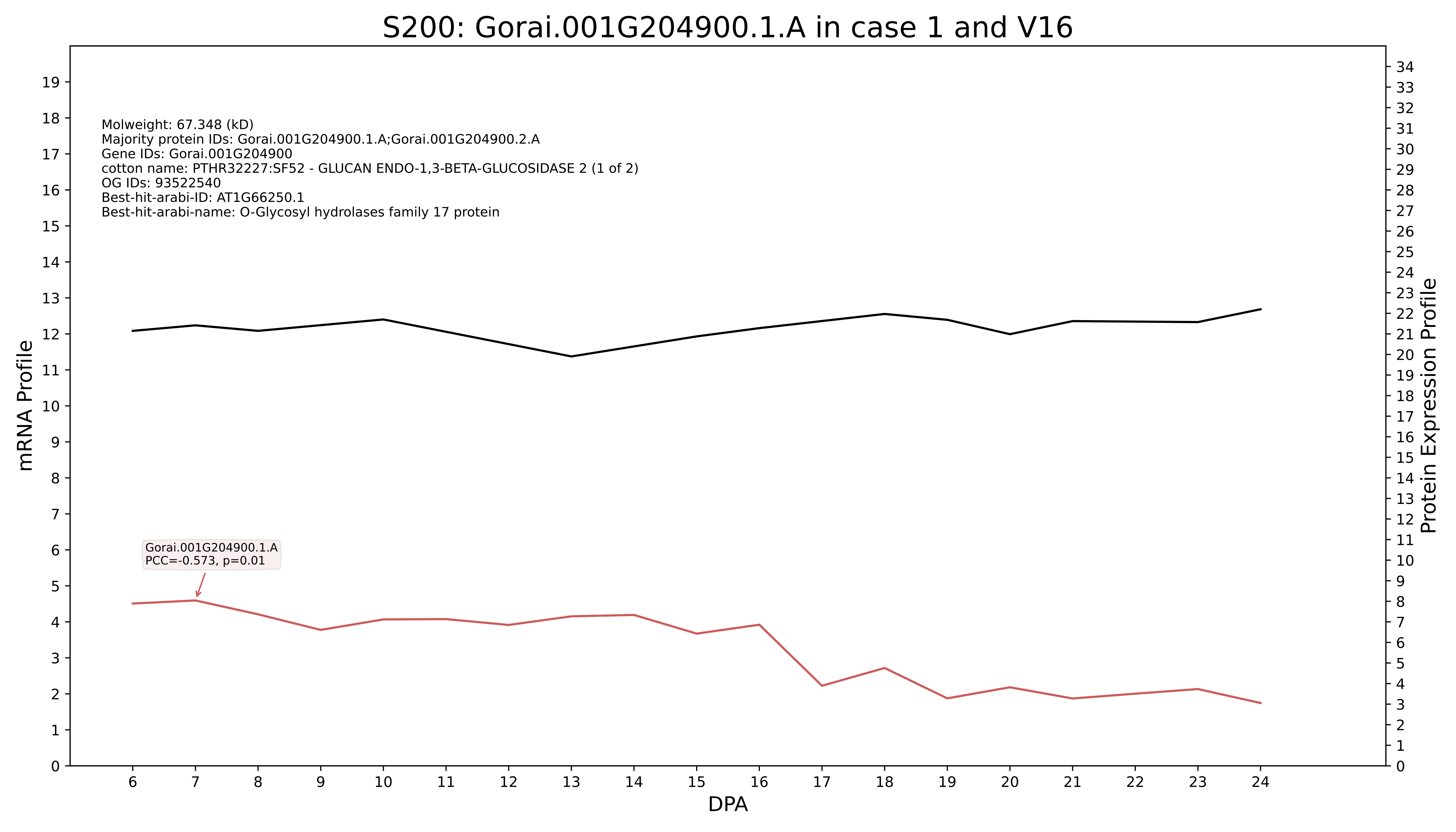
S200: Gorai.001G204900.1.A in case 1 and V16
Molweight: 67.348 (kD)
Majority protein IDs: Gorai.001G204900.1.A;Gorai.001G204900.2.A
Gene IDs: Gorai.001G204900
cotton name: PTHR32227:SF52 - GLUCAN ENDO-1,3-BETA-GLUCOSIDASE 2 (1 of 2)
OG IDs: 93522540
Best-hit-arabi-ID: AT1G66250.1
Best-hit-arabi-name: O-Glycosyl hydrolases family 17 protein
Gorai.001G204900.1.A
PCC=-0.573, p=0.01
mRNA Profile
Protein Expression Profile
DPA
0
1
2
3
4
5
6
7
8
9
10
11
12
13
14
15
16
17
18
19
20
21
22
23
24
25
26
27
28
29
30
31
32
33
34

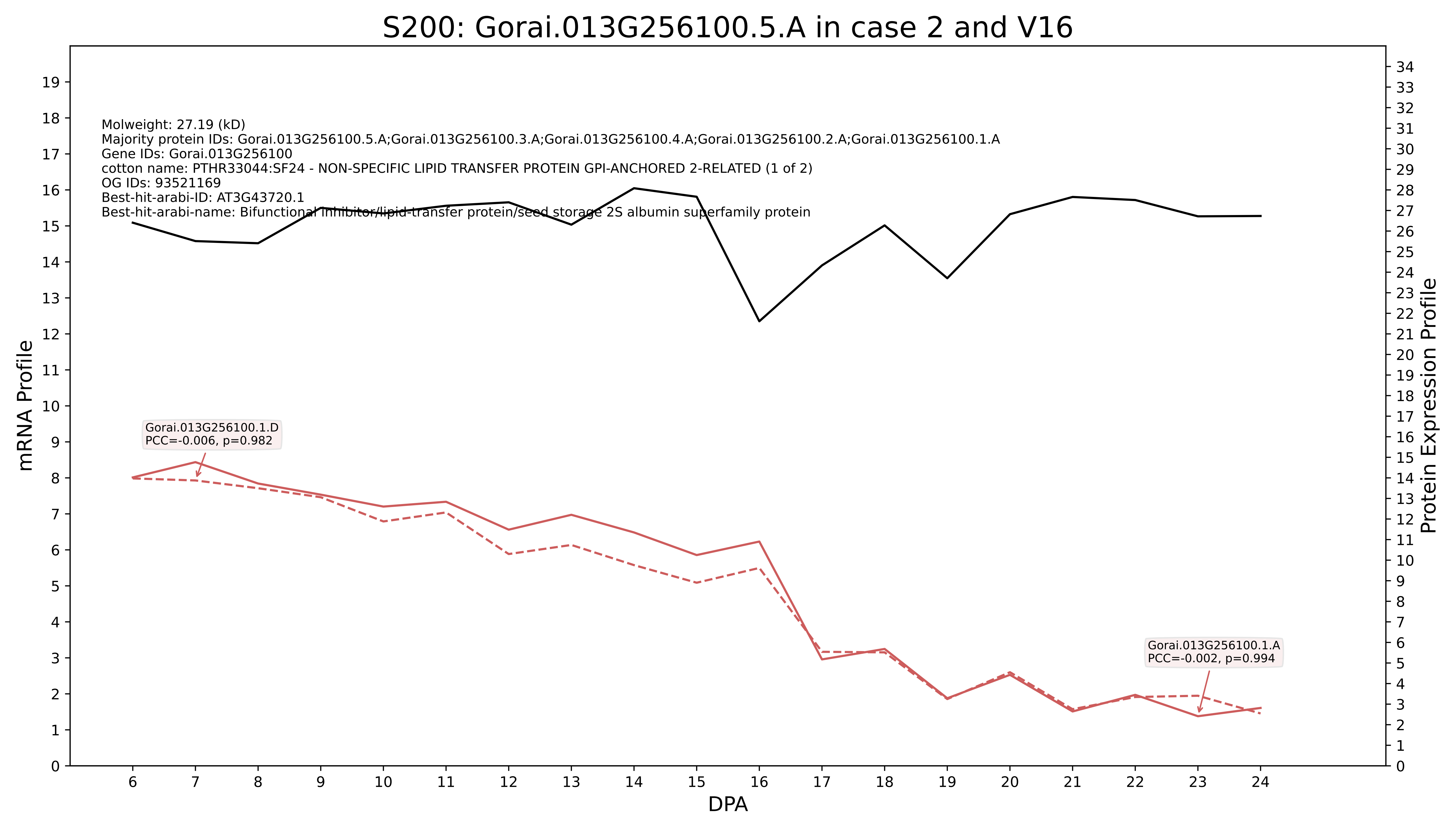
S200: Gorai.013G256100.5.A in case 2 and V16
Molweight: 27.19 (kD)
Majority protein IDs: Gorai.013G256100.5.A;Gorai.013G256100.3.A;Gorai.013G256100.4.A;Gorai.013G256100.2.A;Gorai.013G256100.1.A
Gene IDs: Gorai.013G256100
cotton name: PTHR33044:SF24 - NON-SPECIFIC LIPID TRANSFER PROTEIN GPI-ANCHORED 2-RELATED (1 of 2)
OG IDs: 93521169
Best-hit-arabi-ID: AT3G43720.1
Best-hit-arabi-name: Bifunctional inhibitor/lipid-transfer protein/seed storage 2S albumin superfamily protein
Gorai.013G256100.1.D
PCC=-0.006, p=0.982
Gorai.013G256100.1.A
PCC=-0.002, p=0.994
mRNA Profile
Protein Expression Profile
DPA

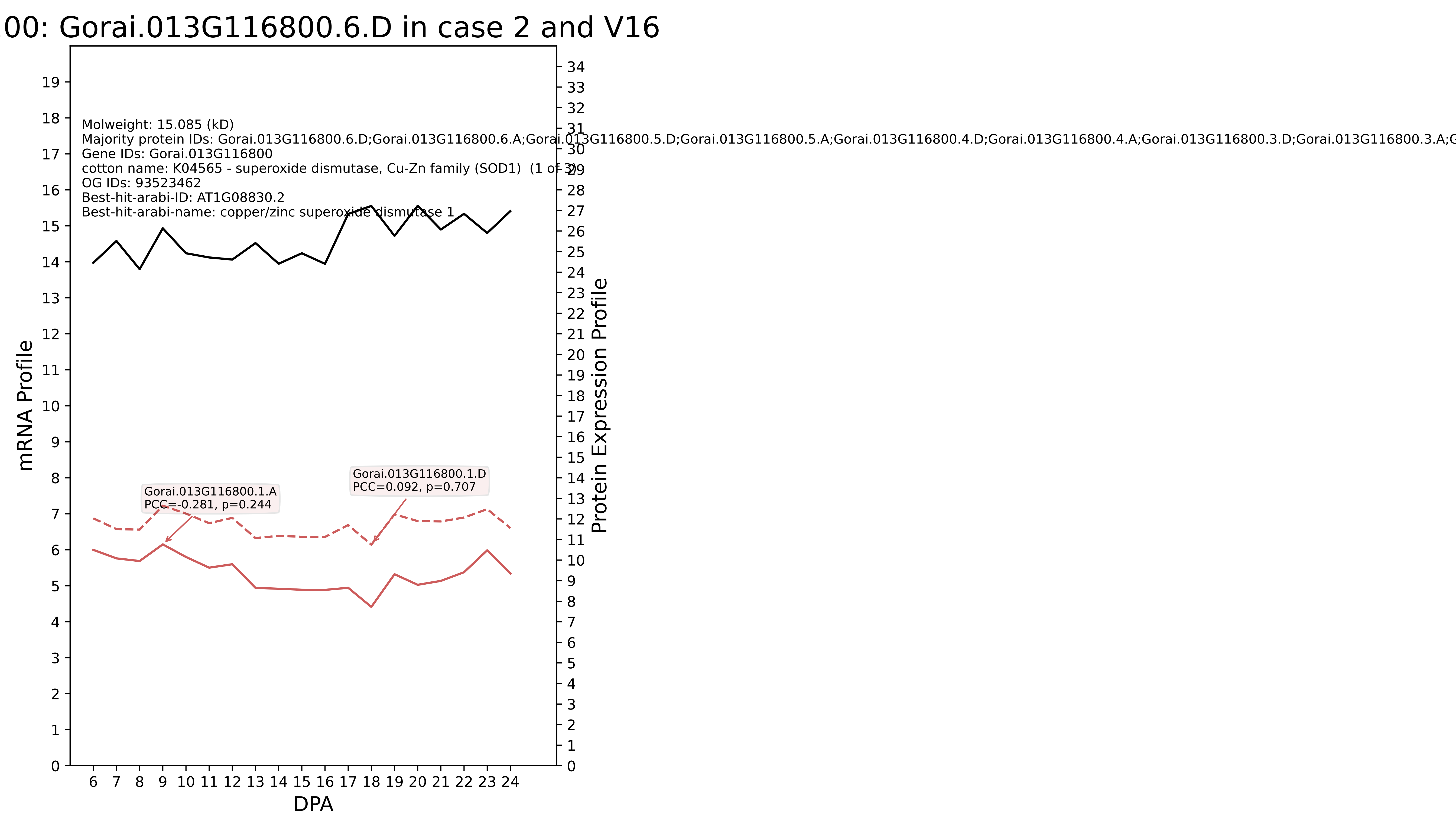

00: Gorai.013G116800.6.D in case 2 and V16
Molweight: 15.085 (kD)
Majority protein IDs: Gorai.013G116800.6.D;Gorai.013G116800.6.A;Gorai.013G116800.5.D;Gorai.013G116800.5.A;Gorai.013G116800.4.D;Gorai.013G116800.4.A;Gorai.013G116800.3.D;Gorai.013G116800.3.A;G
Gene IDs: Gorai.013G116800
cotton name: K04565 - superoxide dismutase, Cu-Zn family (SOD1) (1 of 3)
OG IDs: 93523462
Best-hit-arabi-ID: AT1G08830.2
Best-hit-arabi-name: copper/zinc superoxide dismutase 1
Gorai.013G116800.1.A
PCC=-0.281, p=0.244
Gorai.013G116800.1.D
PCC=0.092, p=0.707
mRNA Profile
Protein Expression Profile
DPA

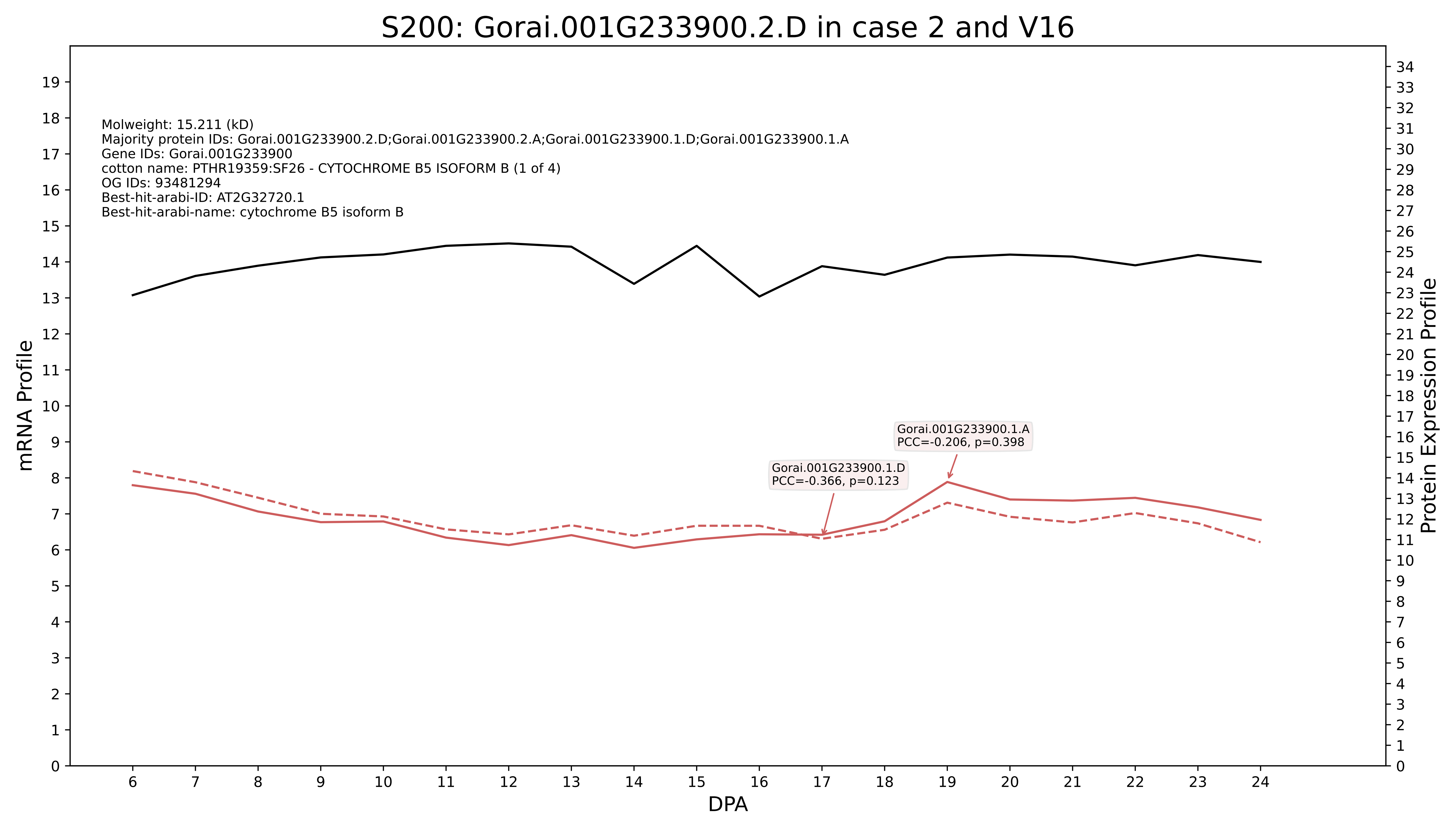
S200: Gorai.001G233900.2.D in case 2 and V16
Molweight: 15.211 (kD)
Majority protein IDs: Gorai.001G233900.2.D;Gorai.001G233900.2.A;Gorai.001G233900.1.D;Gorai.001G233900.1.A
Gene IDs: Gorai.001G233900
cotton name: PTHR19359:SF26 - CYTOCHROME B5 ISOFORM B (1 of 4)
OG IDs: 93481294
Best-hit-arabi-ID: AT2G32720.1
Best-hit-arabi-name: cytochrome B5 isoform B
Gorai.001G233900.1.A
PCC=-0.206, p=0.398
Gorai.001G233900.1.D
PCC=-0.366, p=0.123
mRNA Profile
Protein Expression Profile
DPA
0
1
2
3
4
5
6
7
8
9
10
11
12
13
14
15
16
17
18
19
20
21
22
23
24
25
26
27
28
29
30
31
32
33
34

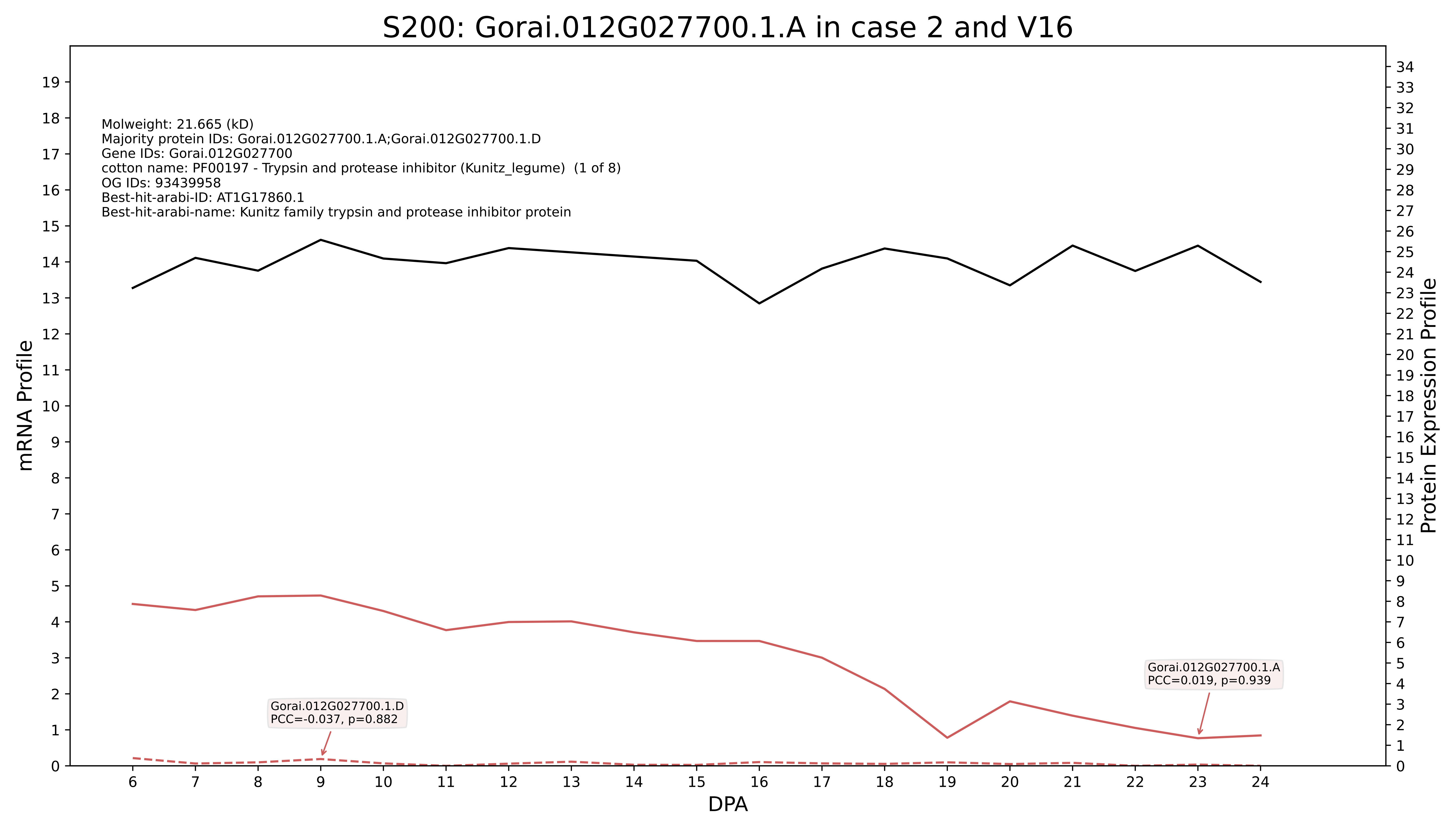
S200: Gorai.012G027700.1.A in case 2 and V16
Molweight: 21.665 (kD)
Majority protein IDs: Gorai.012G027700.1.A;Gorai.012G027700.1.D
Gene IDs: Gorai.012G027700
cotton name: PF00197 - Trypsin and protease inhibitor (Kunitz_legume) (1 of 8)
OG IDs: 93439958
Best-hit-arabi-ID: AT1G17860.1
Best-hit-arabi-name: Kunitz family trypsin and protease inhibitor protein
Gorai.012G027700.1.A
PCC=0.019, p=0.939
Gorai.012G027700.1.D
PCC=-0.037, p=0.882
mRNA Profile
Protein Expression Profile
DPA

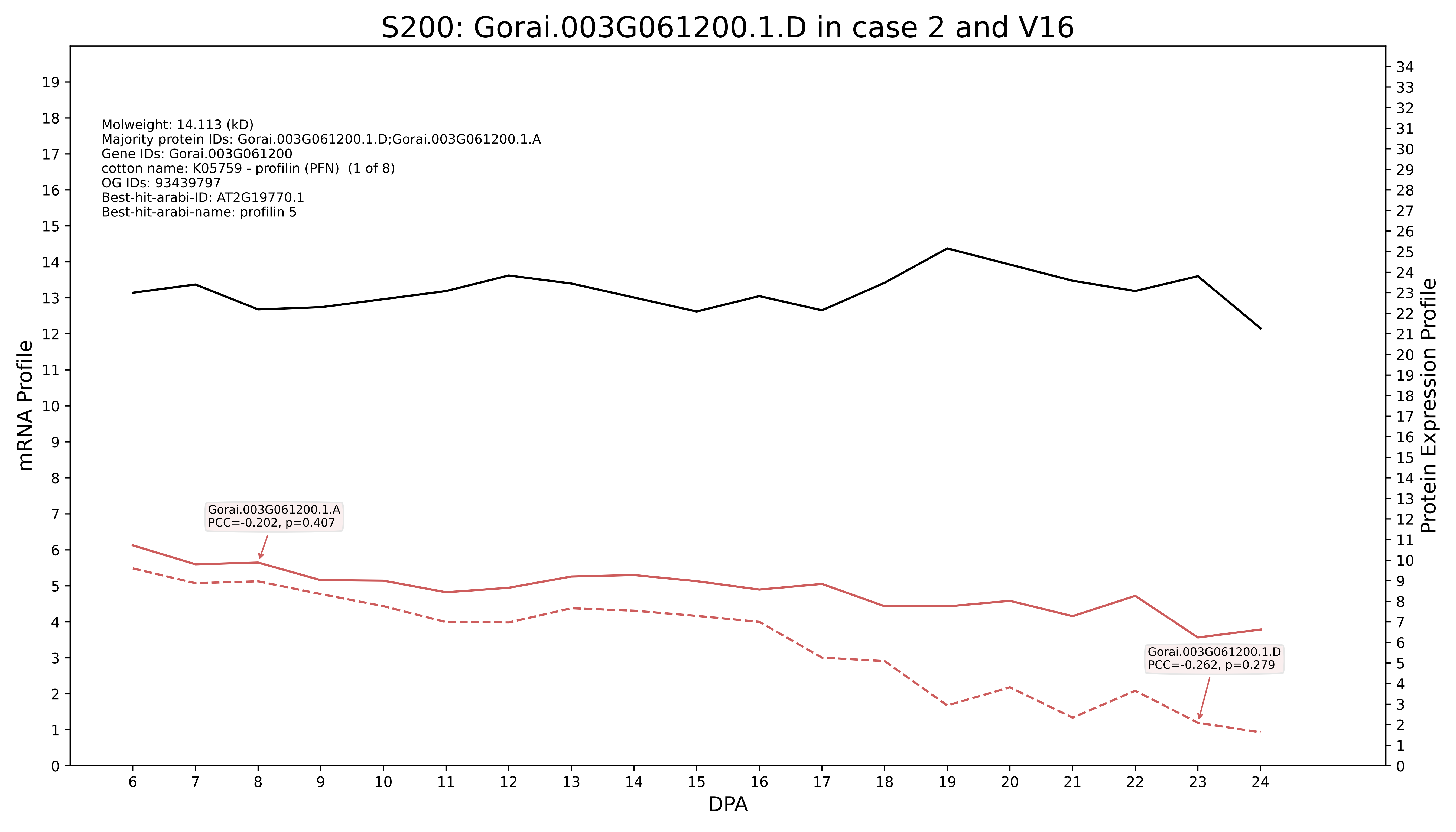
S200: Gorai.003G061200.1.D in case 2 and V16
Molweight: 14.113 (kD)
Majority protein IDs: Gorai.003G061200.1.D;Gorai.003G061200.1.A
Gene IDs: Gorai.003G061200
cotton name: K05759 - profilin (PFN) (1 of 8)
OG IDs: 93439797
Best-hit-arabi-ID: AT2G19770.1
Best-hit-arabi-name: profilin 5
Gorai.003G061200.1.A
PCC=-0.202, p=0.407
Gorai.003G061200.1.D
PCC=-0.262, p=0.279
mRNA Profile
Protein Expression Profile
DPA

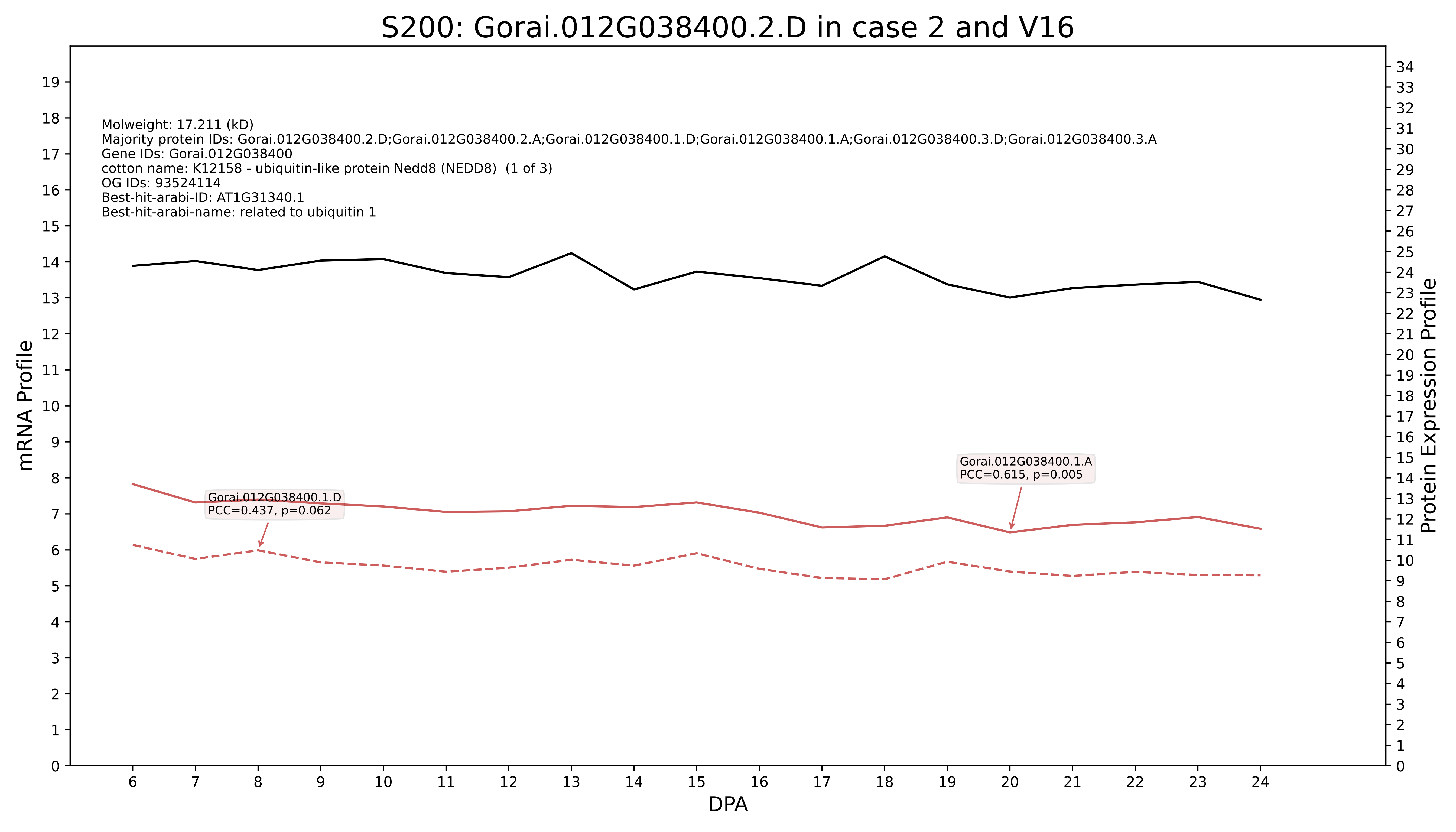

S200: Gorai.012G038400.2.D in case 2 and V16
Molweight: 17.211 (kD)
Majority protein IDs: Gorai.012G038400.2.D;Gorai.012G038400.2.A;Gorai.012G038400.1.D;Gorai.012G038400.1.A;Gorai.012G038400.3.D;Gorai.012G038400.3.A
Gene IDs: Gorai.012G038400
cotton name: K12158 - ubiquitin-like protein Nedd8 (NEDD8) (1 of 3)
OG IDs: 93524114
Best-hit-arabi-ID: AT1G31340.1
Best-hit-arabi-name: related to ubiquitin 1
Gorai.012G038400.1.A
PCC=0.615, p=0.005
Gorai.012G038400.1.D
PCC=0.437, p=0.062
mRNA Profile
Protein Expression Profile
DPA

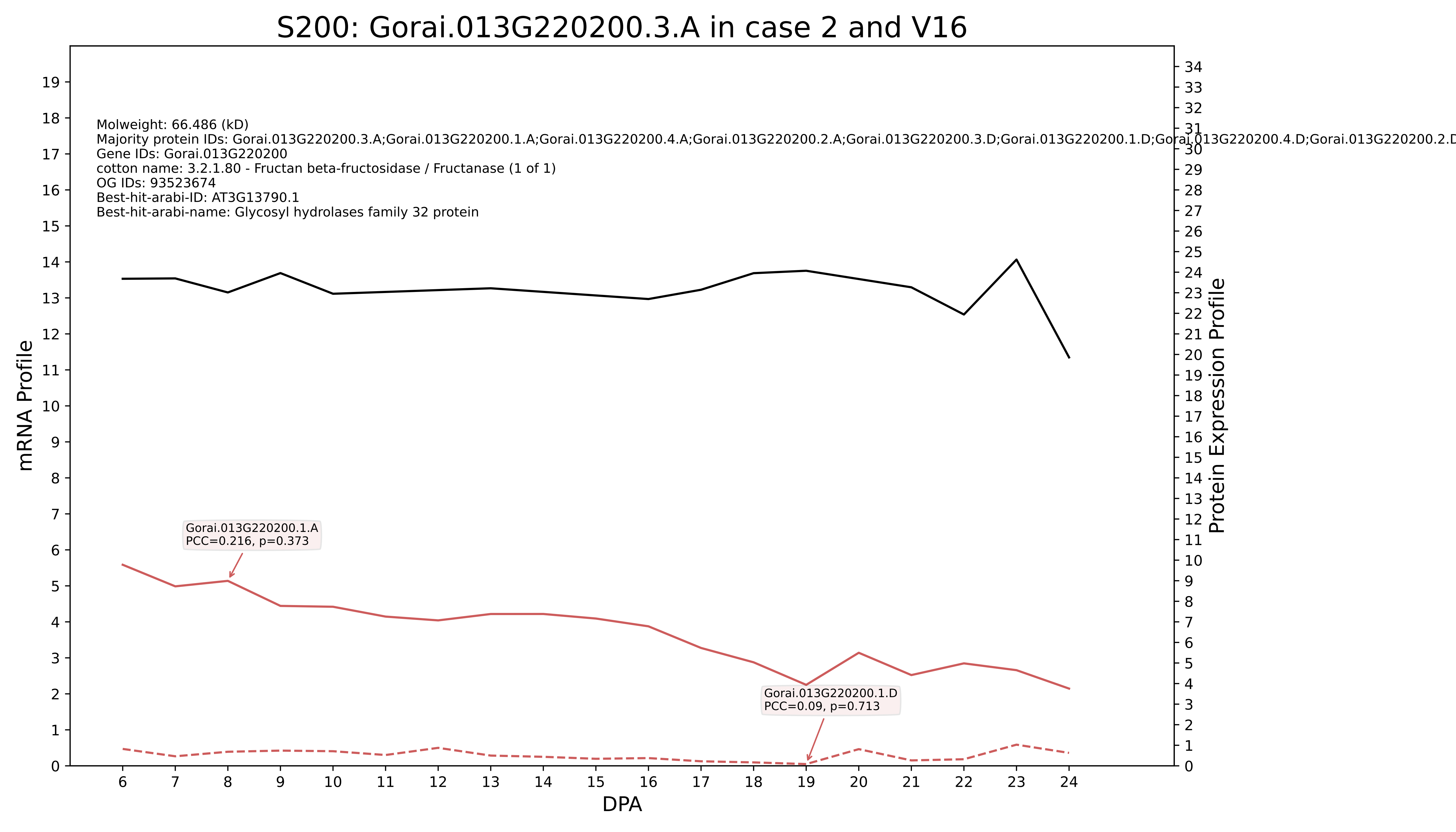
S200: Gorai.013G220200.3.A in case 2 and V16
Molweight: 66.486 (kD)
Majority protein IDs: Gorai.013G220200.3.A;Gorai.013G220200.1.A;Gorai.013G220200.4.A;Gorai.013G220200.2.A;Gorai.013G220200.3.D;Gorai.013G220200.1.D;Gorai.013G220200.4.D;Gorai.013G220200.2.D
Gene IDs: Gorai.013G220200
cotton name: 3.2.1.80 - Fructan beta-fructosidase / Fructanase (1 of 1)
OG IDs: 93523674
Best-hit-arabi-ID: AT3G13790.1
Best-hit-arabi-name: Glycosyl hydrolases family 32 protein
Gorai.013G220200.1.A
PCC=0.216, p=0.373
Gorai.013G220200.1.D
PCC=0.09, p=0.713
mRNA Profile
Protein Expression Profile
DPA

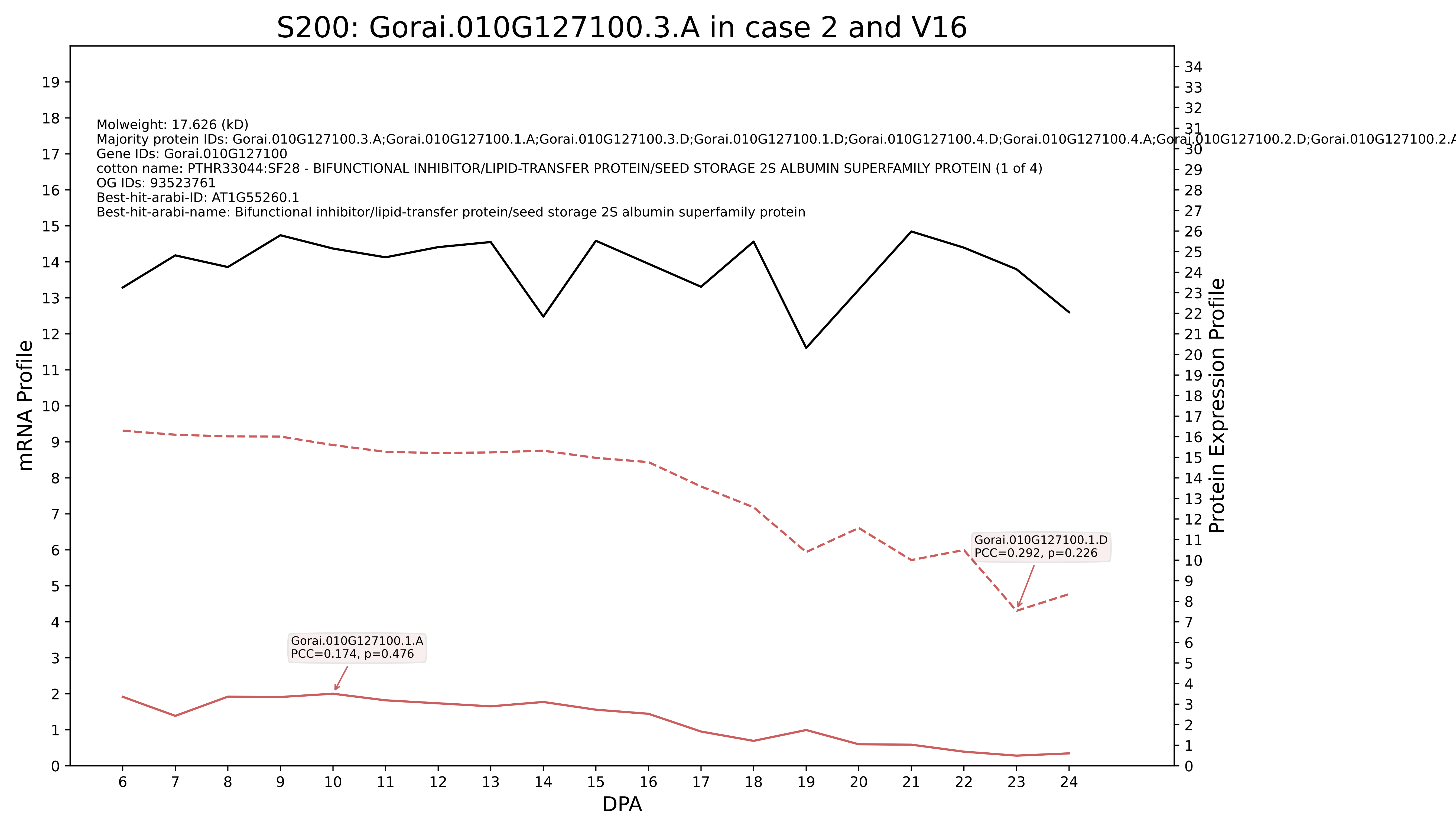
S200: Gorai.010G127100.3.A in case 2 and V16
Molweight: 17.626 (kD)
Majority protein IDs: Gorai.010G127100.3.A;Gorai.010G127100.1.A;Gorai.010G127100.3.D;Gorai.010G127100.1.D;Gorai.010G127100.4.D;Gorai.010G127100.4.A;Gorai.010G127100.2.D;Gorai.010G127100.2.A
Gene IDs: Gorai.010G127100
cotton name: PTHR33044:SF28 - BIFUNCTIONAL INHIBITOR/LIPID-TRANSFER PROTEIN/SEED STORAGE 2S ALBUMIN SUPERFAMILY PROTEIN (1 of 4)
OG IDs: 93523761
Best-hit-arabi-ID: AT1G55260.1
Best-hit-arabi-name: Bifunctional inhibitor/lipid-transfer protein/seed storage 2S albumin superfamily protein
Gorai.010G127100.1.D
PCC=0.292, p=0.226
Gorai.010G127100.1.A
PCC=0.174, p=0.476
mRNA Profile
Protein Expression Profile
DPA

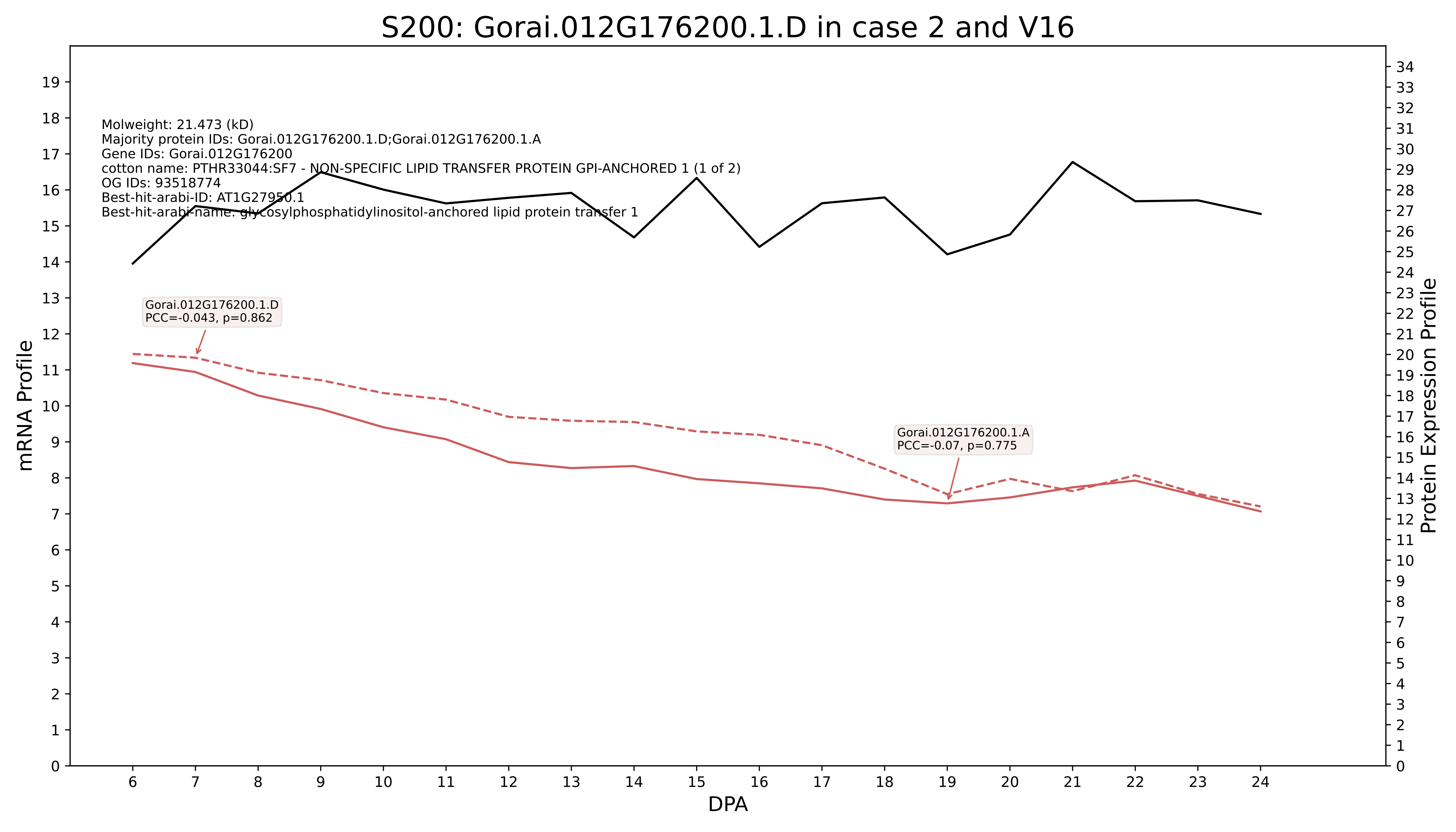
S200: Gorai.012G176200.1.D in case 2 and V16
Molweight: 21.473 (kD)
Majority protein IDs: Gorai.012G176200.1.D;Gorai.012G176200.1.A
Gene IDs: Gorai.012G176200
cotton name: PTHR33044:SF7 - NON-SPECIFIC LIPID TRANSFER PROTEIN GPI-ANCHORED 1 (1 of 2)
OG IDs: 93518774
Best-hit-arabi-ID: AT1G27950.1
Best-hit-arabi-name: glycosylphosphatidylinositol-anchored lipid protein transfer 1
Gorai.012G176200.1.D
PCC=-0.043, p=0.862
Gorai.012G176200.1.A
PCC=-0.07, p=0.775
mRNA Profile
Protein Expression Profile
DPA

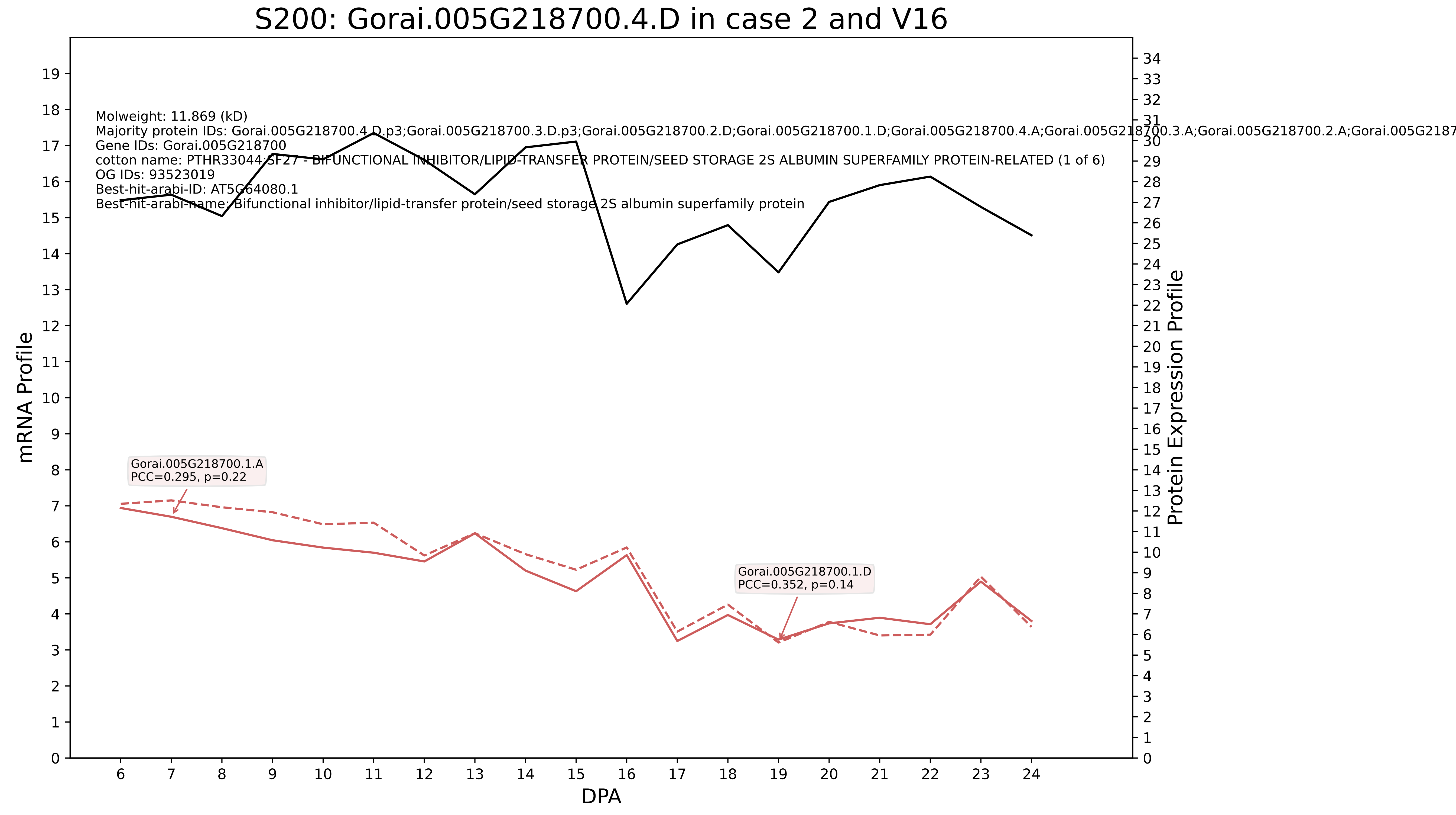
S200: Gorai.005G218700.4.D in case 2 and V16
Molweight: 11.869 (kD)
Majority protein IDs: Gorai.005G218700.4.D.p3;Gorai.005G218700.3.D.p3;Gorai.005G218700.2.D;Gorai.005G218700.1.D;Gorai.005G218700.4.A;Gorai.005G218700.3.A;Gorai.005G218700.2.A;Gorai.005G2187
Gene IDs: Gorai.005G218700
cotton name: PTHR33044:SF27 - BIFUNCTIONAL INHIBITOR/LIPID-TRANSFER PROTEIN/SEED STORAGE 2S ALBUMIN SUPERFAMILY PROTEIN-RELATED (1 of 6)
OG IDs: 93523019
Best-hit-arabi-ID: AT5G64080.1
Best-hit-arabi-name: Bifunctional inhibitor/lipid-transfer protein/seed storage 2S albumin superfamily protein
Gorai.005G218700.1.A
PCC=0.295, p=0.22
Gorai.005G218700.1.D
PCC=0.352, p=0.14
mRNA Profile
Protein Expression Profile
DPA
0
1
2
3
4
5
6
7
8
9
10
11
12
13
14
15
16
17
18
19
20
21
22
23
24
25
26
27
28
29
30
31
32
33
34

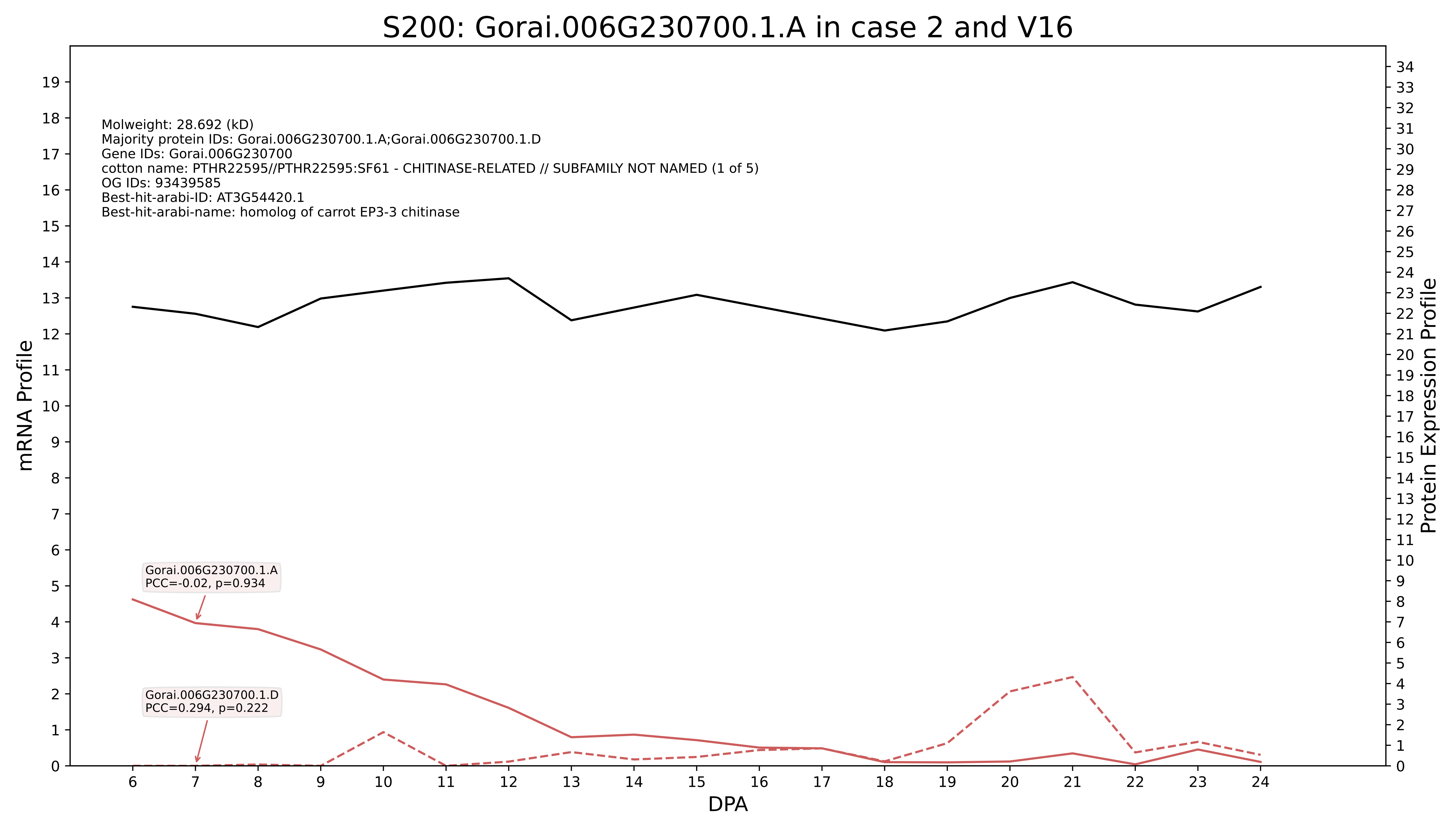
S200: Gorai.006G230700.1.A in case 2 and V16
Molweight: 28.692 (kD)
Majority protein IDs: Gorai.006G230700.1.A;Gorai.006G230700.1.D
Gene IDs: Gorai.006G230700
cotton name: PTHR22595//PTHR22595:SF61 - CHITINASE-RELATED // SUBFAMILY NOT NAMED (1 of 5)
OG IDs: 93439585
Best-hit-arabi-ID: AT3G54420.1
Best-hit-arabi-name: homolog of carrot EP3-3 chitinase
Gorai.006G230700.1.A
PCC=-0.02, p=0.934
Gorai.006G230700.1.D
PCC=0.294, p=0.222
mRNA Profile
Protein Expression Profile
DPA

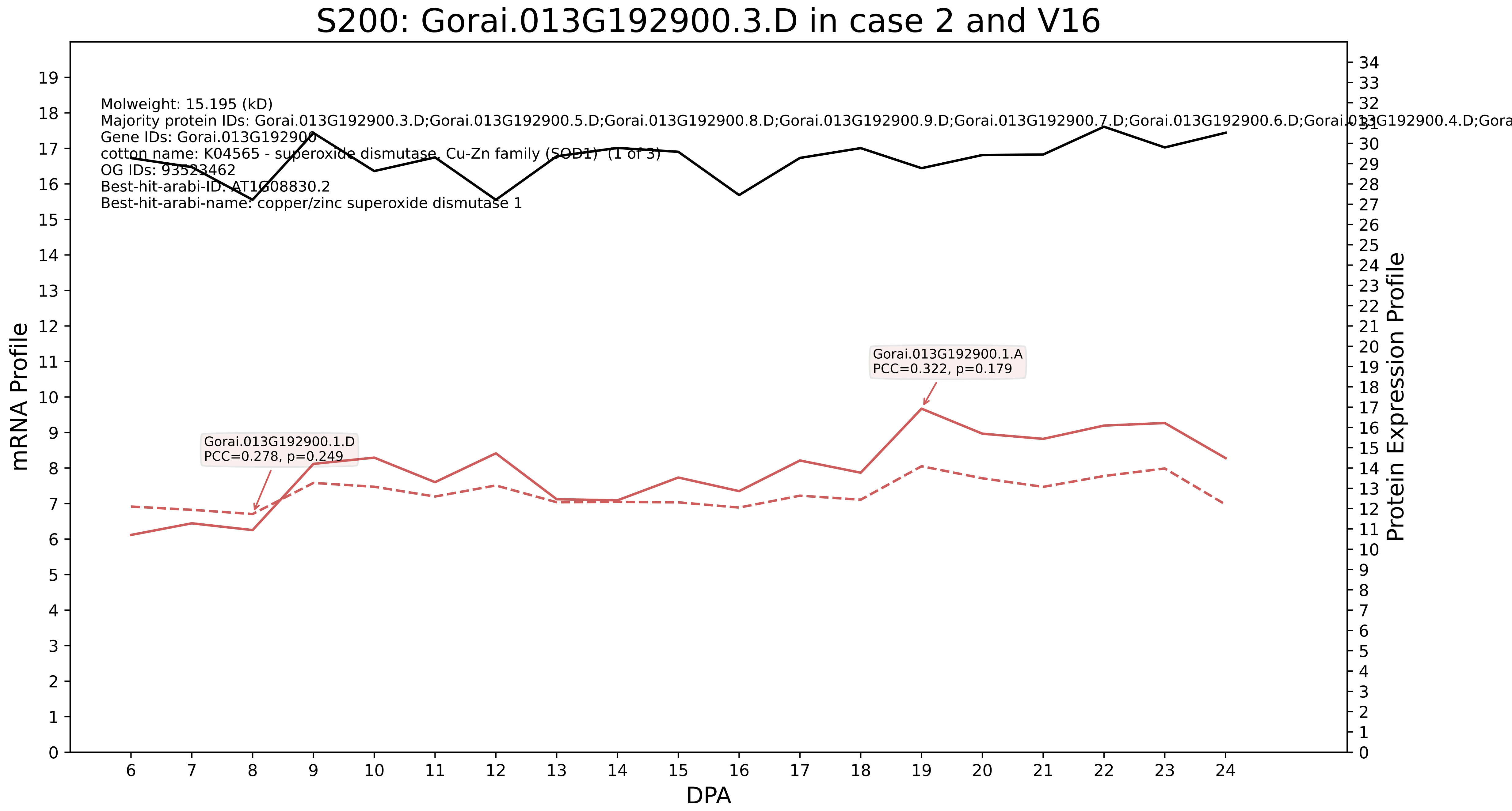
S200: Gorai.013G192900.3.D in case 2 and V16
Molweight: 15.195 (kD)
Majority protein IDs: Gorai.013G192900.3.D;Gorai.013G192900.5.D;Gorai.013G192900.8.D;Gorai.013G192900.9.D;Gorai.013G192900.7.D;Gorai.013G192900.6.D;Gorai.013G192900.4.D;Gora
Gene IDs: Gorai.013G192900
cotton name: K04565 - superoxide dismutase, Cu-Zn family (SOD1) (1 of 3)
OG IDs: 93523462
Best-hit-arabi-ID: AT1G08830.2
Best-hit-arabi-name: copper/zinc superoxide dismutase 1
Gorai.013G192900.1.A
PCC=0.322, p=0.179
Gorai.013G192900.1.D
PCC=0.278, p=0.249
mRNA Profile
Protein Expression Profile
DPA

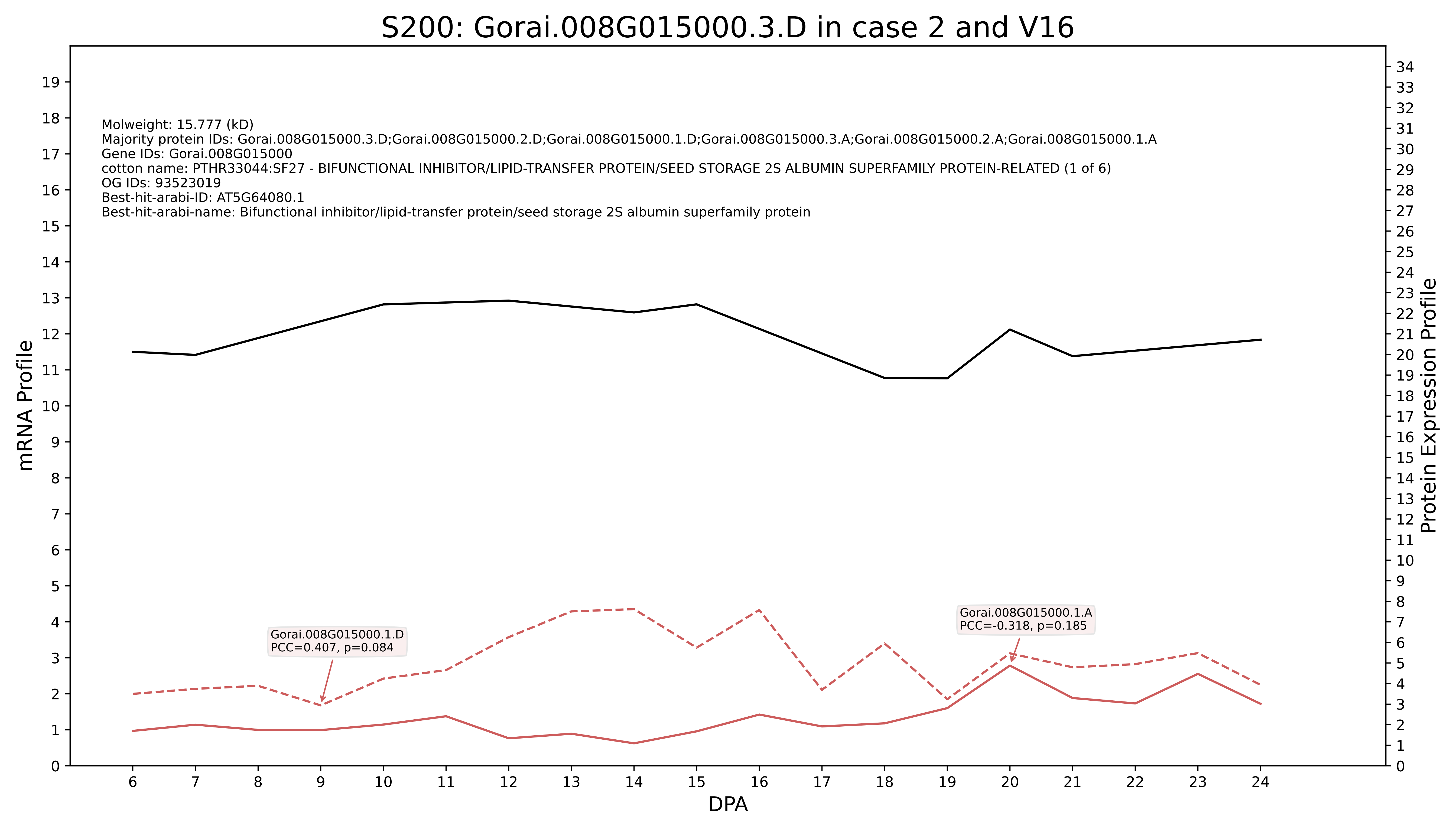
S200: Gorai.008G015000.3.D in case 2 and V16
Molweight: 15.777 (kD)
Majority protein IDs: Gorai.008G015000.3.D;Gorai.008G015000.2.D;Gorai.008G015000.1.D;Gorai.008G015000.3.A;Gorai.008G015000.2.A;Gorai.008G015000.1.A
Gene IDs: Gorai.008G015000
cotton name: PTHR33044:SF27 - BIFUNCTIONAL INHIBITOR/LIPID-TRANSFER PROTEIN/SEED STORAGE 2S ALBUMIN SUPERFAMILY PROTEIN-RELATED (1 of 6)
OG IDs: 93523019
Best-hit-arabi-ID: AT5G64080.1
Best-hit-arabi-name: Bifunctional inhibitor/lipid-transfer protein/seed storage 2S albumin superfamily protein
Gorai.008G015000.1.D
PCC=0.407, p=0.084
Gorai.008G015000.1.A
PCC=-0.318, p=0.185
mRNA Profile
Protein Expression Profile
DPA

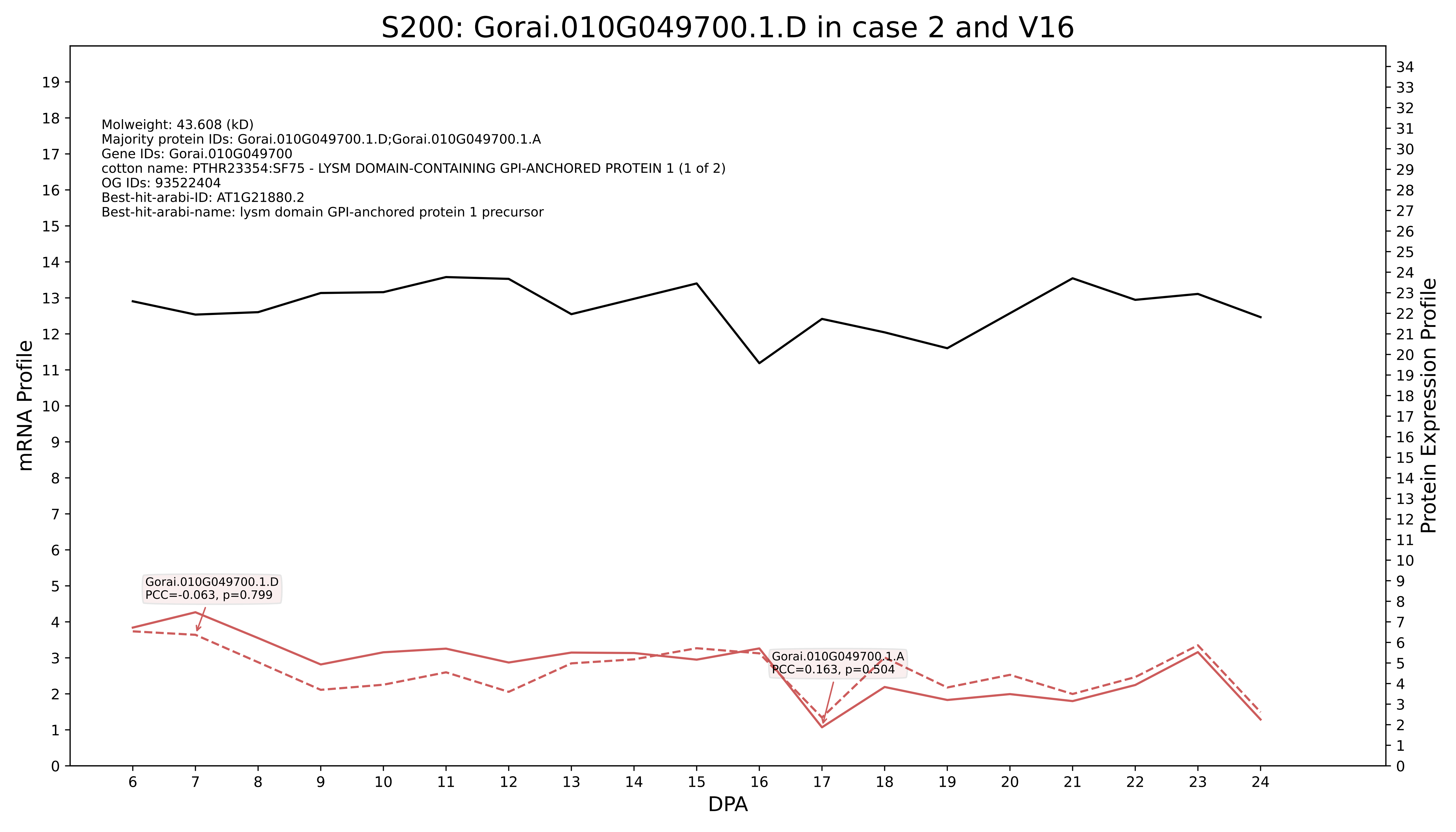
S200: Gorai.010G049700.1.D in case 2 and V16
Molweight: 43.608 (kD)
Majority protein IDs: Gorai.010G049700.1.D;Gorai.010G049700.1.A
Gene IDs: Gorai.010G049700
cotton name: PTHR23354:SF75 - LYSM DOMAIN-CONTAINING GPI-ANCHORED PROTEIN 1 (1 of 2)
OG IDs: 93522404
Best-hit-arabi-ID: AT1G21880.2
Best-hit-arabi-name: lysm domain GPI-anchored protein 1 precursor
Gorai.010G049700.1.D
PCC=-0.063, p=0.799
Gorai.010G049700.1.A
PCC=0.163, p=0.504
mRNA Profile
Protein Expression Profile
DPA

S200: Gorai.001G222400.1.D in case 2 and V16

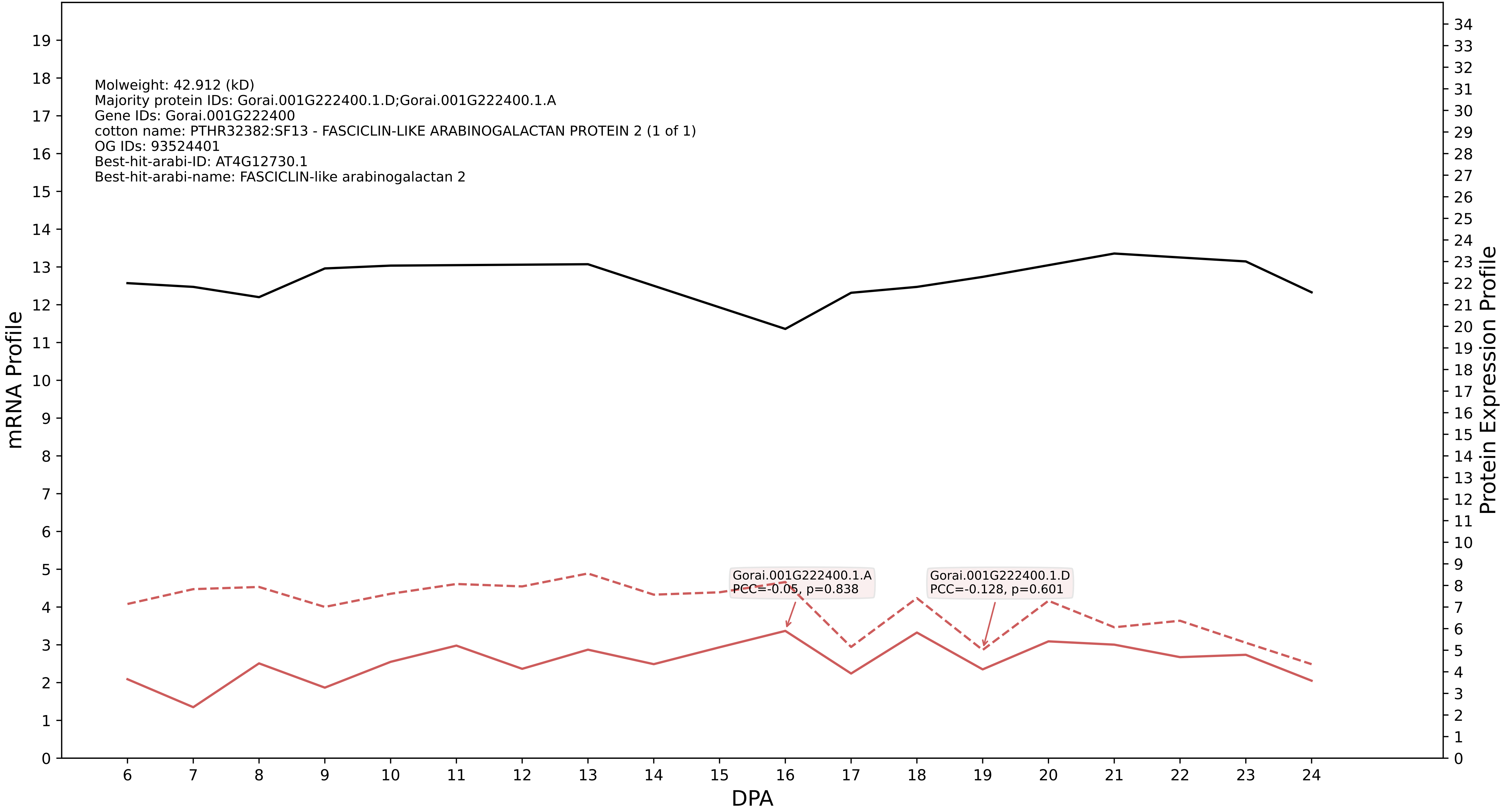

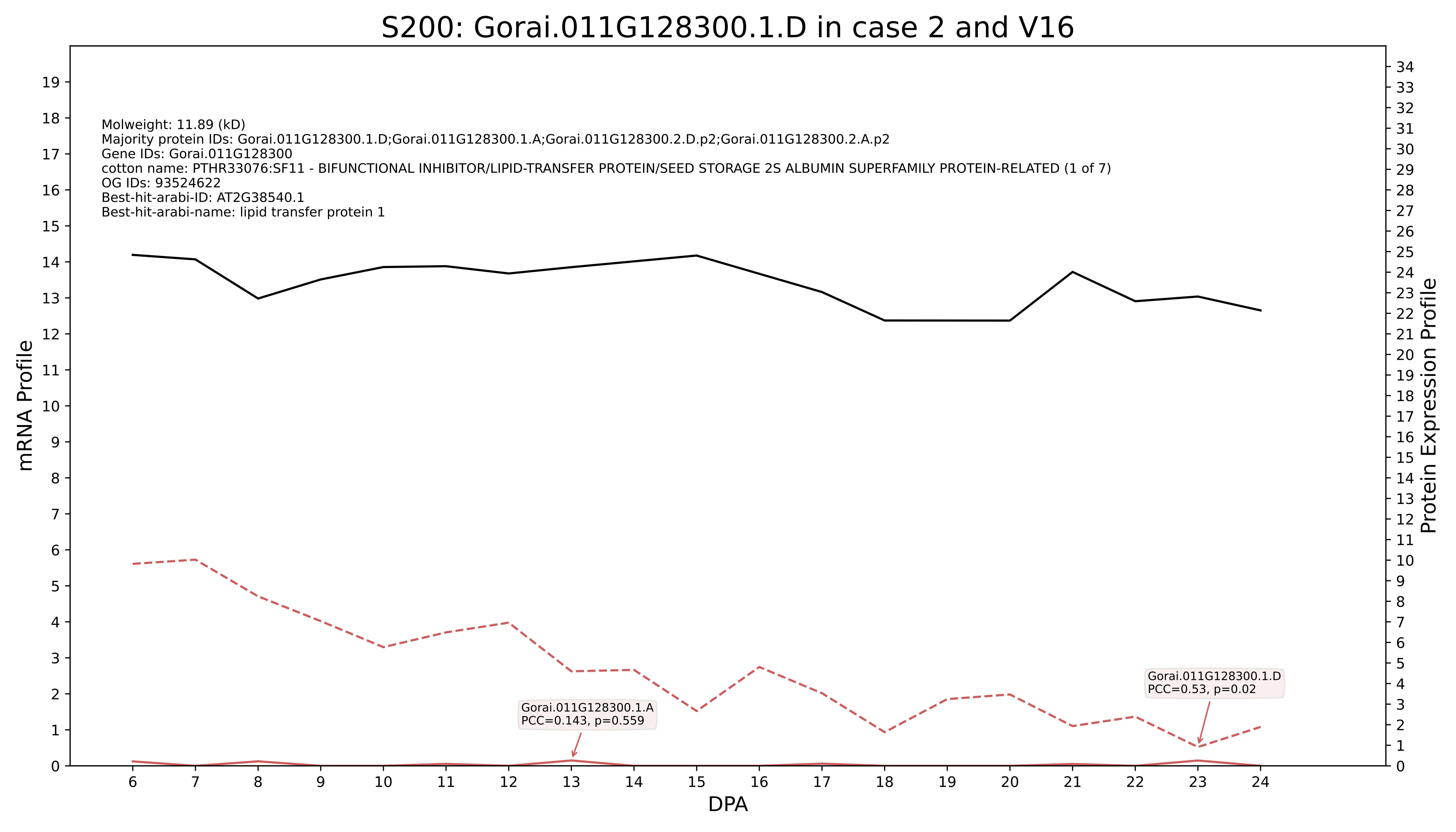

S200: Gorai.011G128300.1.D in case 2 and V16
Molweight: 11.89 (kD)
Majority protein IDs: Gorai.011G128300.1.D;Gorai.011G128300.1.A;Gorai.011G128300.2.D.p2;Gorai.011G128300.2.A.p2
Gene IDs: Gorai.011G128300
cotton name: PTHR33076:SF11 - BIFUNCTIONAL INHIBITOR/LIPID-TRANSFER PROTEIN/SEED STORAGE 2S ALBUMIN SUPERFAMILY PROTEIN-RELATED (1 of 7)
OG IDs: 93524622
Best-hit-arabi-ID: AT2G38540.1
Best-hit-arabi-name: lipid transfer protein 1
Gorai.011G128300.1.A
PCC=0.143, p=0.559
Gorai.011G128300.1.D
PCC=0.53, p=0.02
mRNA Profile
Protein Expression Profile
DPA

S200: Gorai.009G080400.1.D in case 3 and V16

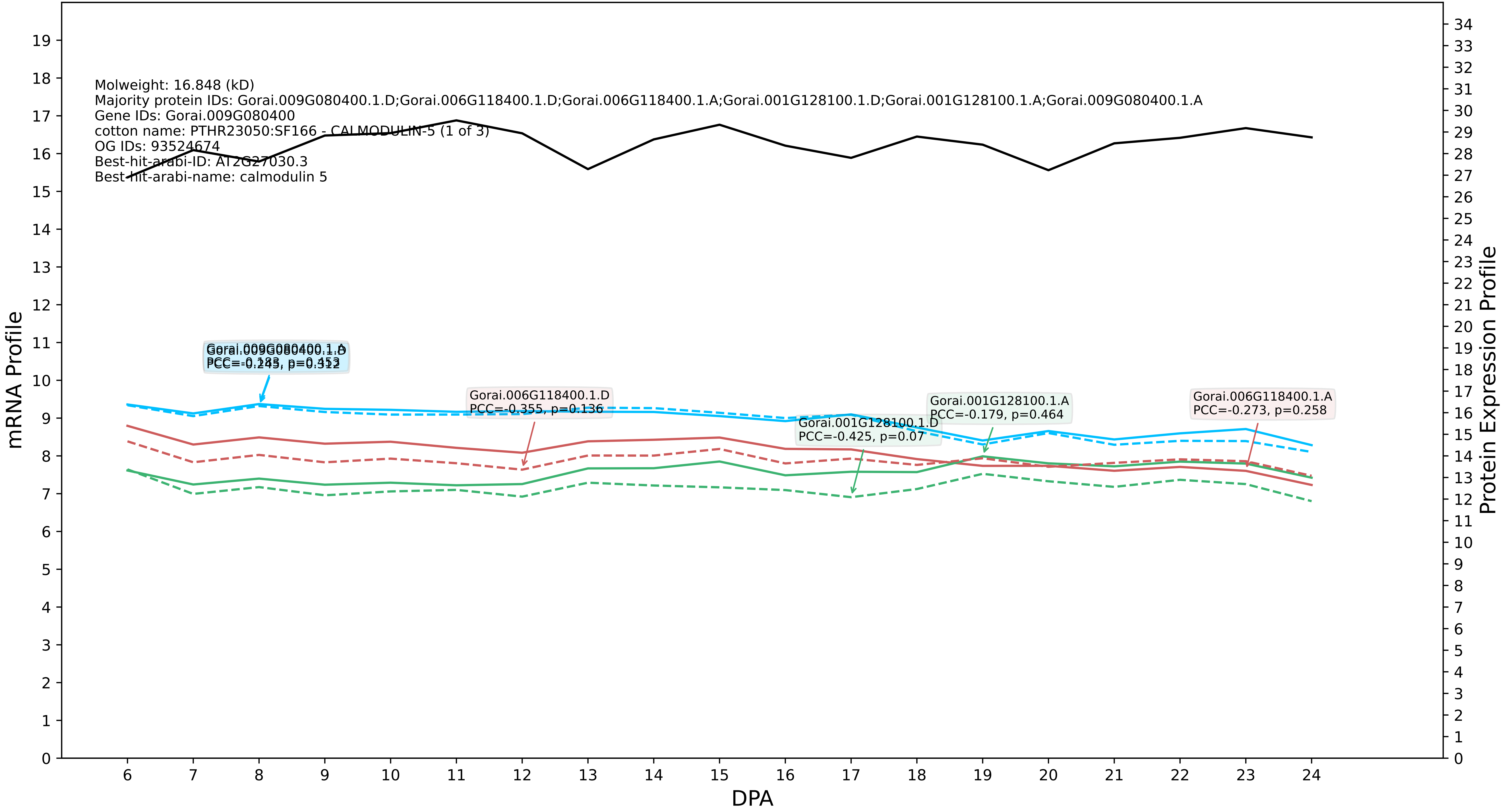

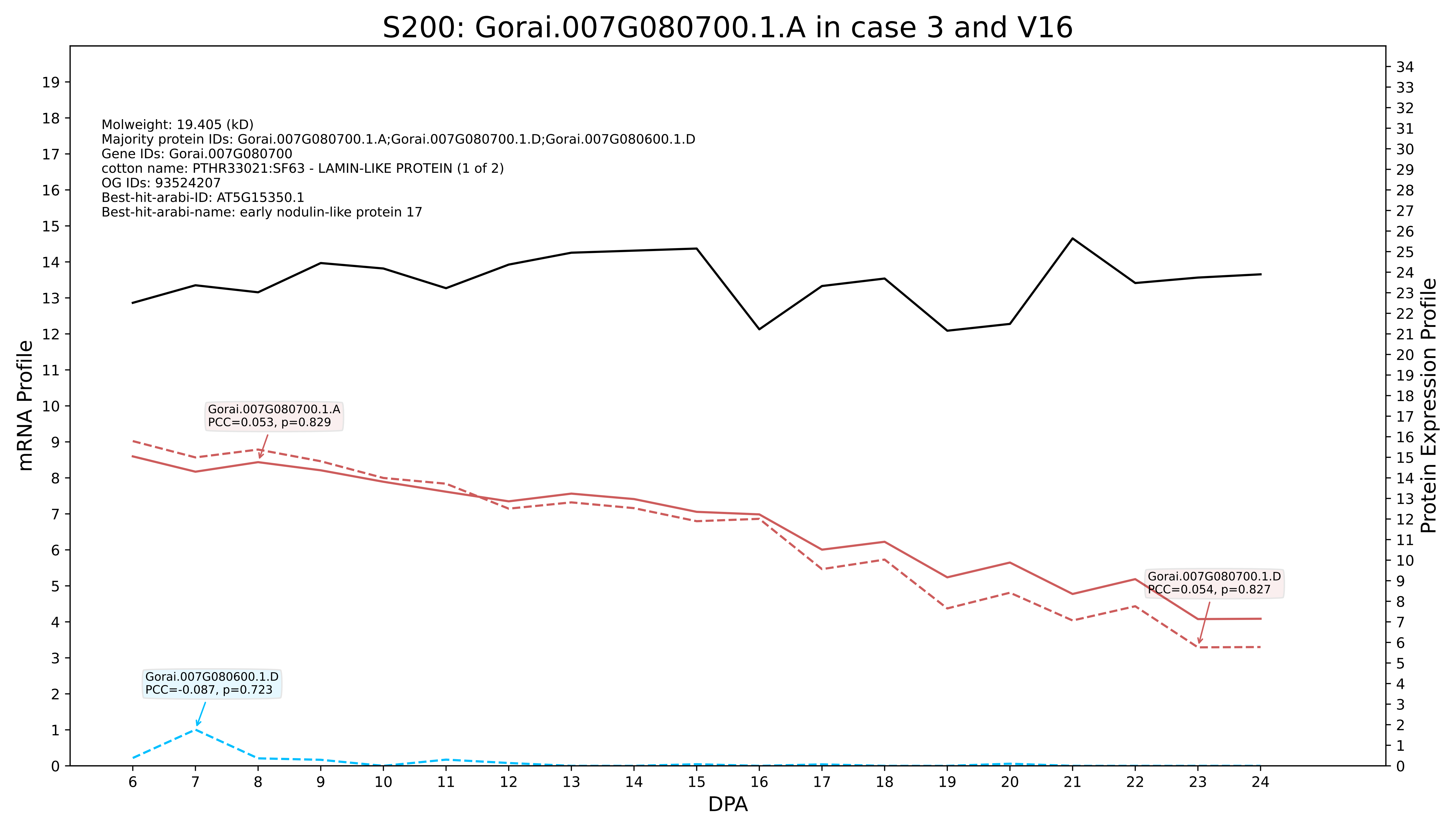
S200: Gorai.007G080700.1.A in case 3 and V16
Molweight: 19.405 (kD)
Majority protein IDs: Gorai.007G080700.1.A;Gorai.007G080700.1.D;Gorai.007G080600.1.D
Gene IDs: Gorai.007G080700
cotton name: PTHR33021:SF63 - LAMIN-LIKE PROTEIN (1 of 2)
OG IDs: 93524207
Best-hit-arabi-ID: AT5G15350.1
Best-hit-arabi-name: early nodulin-like protein 17
Gorai.007G080700.1.A
PCC=0.053, p=0.829
Gorai.007G080700.1.D
PCC=0.054, p=0.827
Gorai.007G080600.1.D
PCC=-0.087, p=0.723
mRNA Profile
Protein Expression Profile
DPA

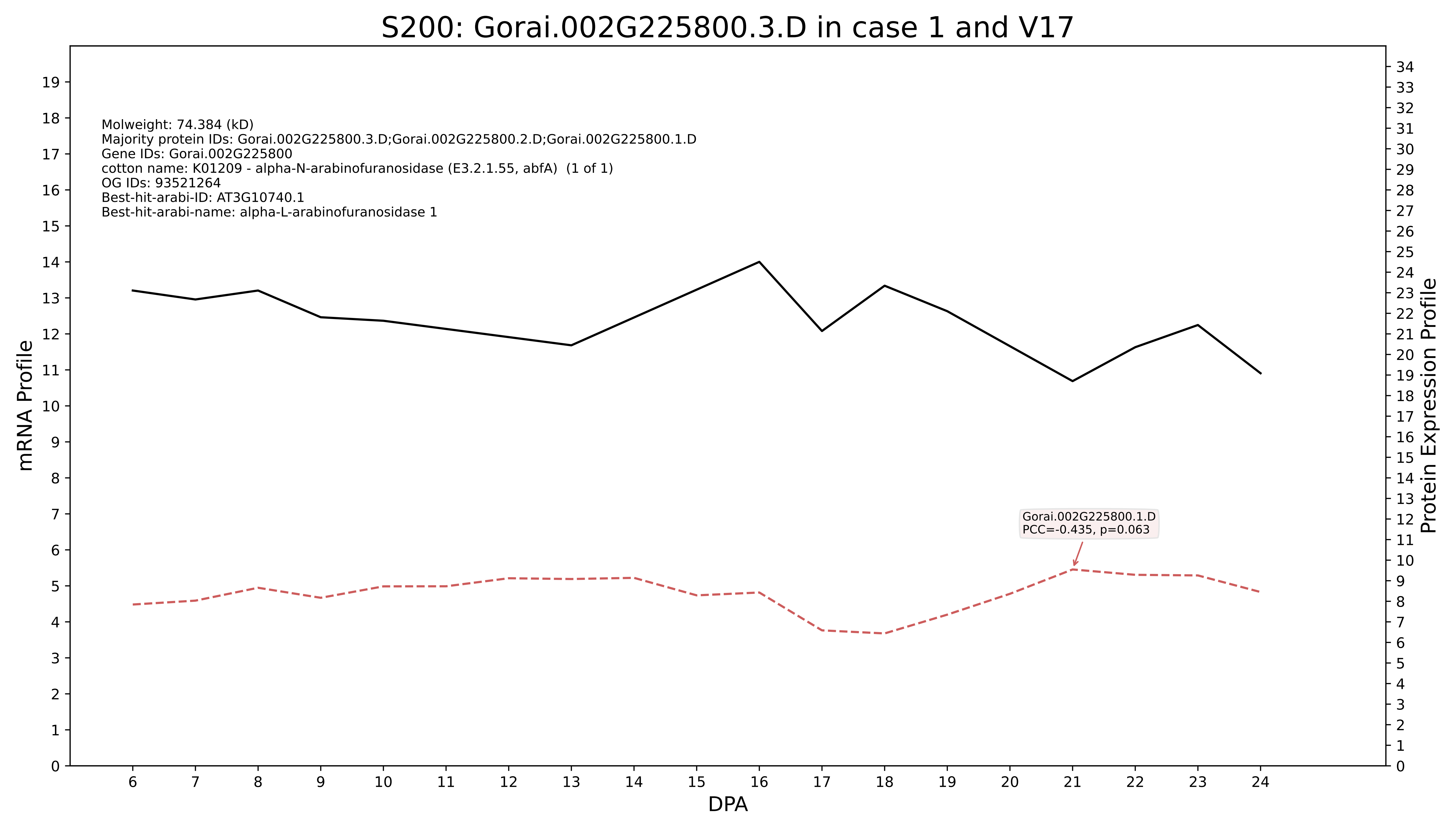

S200: Gorai.002G225800.3.D in case 1 and V17
Molweight: 74.384 (kD)
Majority protein IDs: Gorai.002G225800.3.D;Gorai.002G225800.2.D;Gorai.002G225800.1.D
Gene IDs: Gorai.002G225800
cotton name: K01209 - alpha-N-arabinofuranosidase (E3.2.1.55, abfA) (1 of 1)
OG IDs: 93521264
Best-hit-arabi-ID: AT3G10740.1
Best-hit-arabi-name: alpha-L-arabinofuranosidase 1
Gorai.002G225800.1.D
PCC=-0.435, p=0.063
mRNA Profile
Protein Expression Profile
DPA

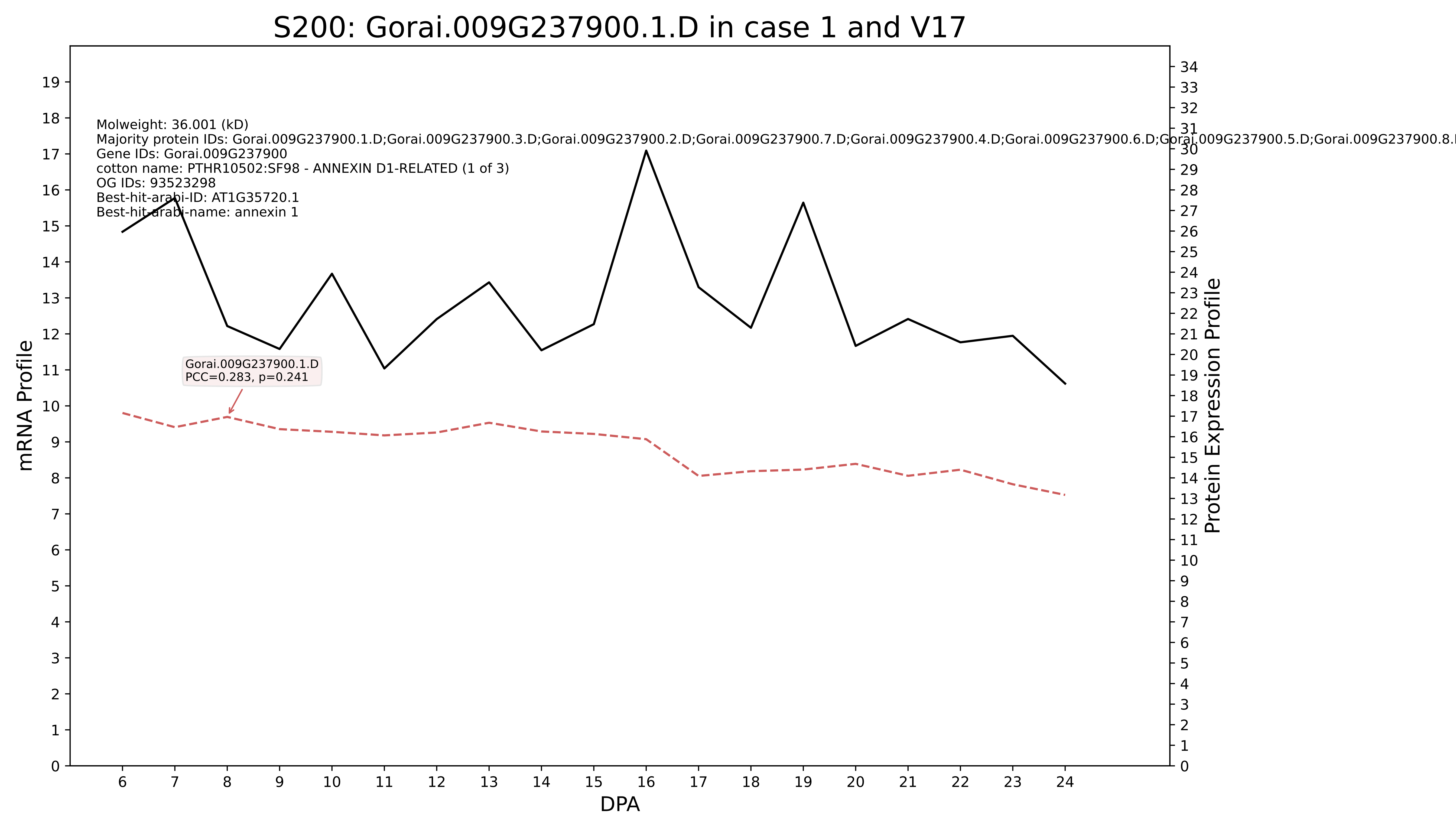
S200: Gorai.009G237900.1.D in case 1 and V17
Molweight: 36.001 (kD)
Majority protein IDs: Gorai.009G237900.1.D;Gorai.009G237900.3.D;Gorai.009G237900.2.D;Gorai.009G237900.7.D;Gorai.009G237900.4.D;Gorai.009G237900.6.D;Gorai.009G237900.5.D;Gorai.009G237900.8.
Gene IDs: Gorai.009G237900
cotton name: PTHR10502:SF98 - ANNEXIN D1-RELATED (1 of 3)
OG IDs: 93523298
Best-hit-arabi-ID: AT1G35720.1
Best-hit-arabi-name: annexin 1
Gorai.009G237900.1.D
PCC=0.283, p=0.241
mRNA Profile
Protein Expression Profile
DPA

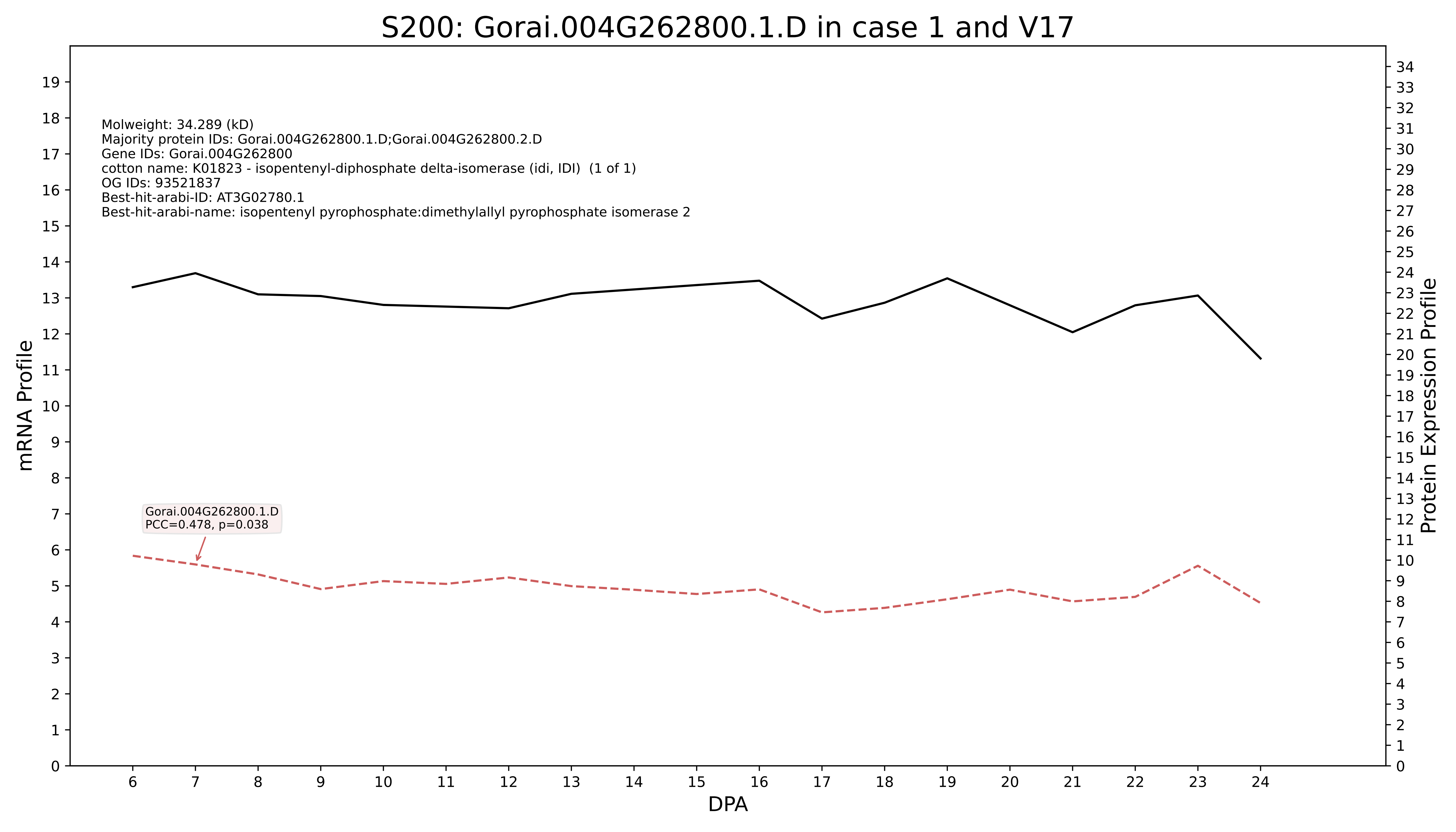
S200: Gorai.004G262800.1.D in case 1 and V17
Molweight: 34.289 (kD)
Majority protein IDs: Gorai.004G262800.1.D;Gorai.004G262800.2.D
Gene IDs: Gorai.004G262800
cotton name: K01823 - isopentenyl-diphosphate delta-isomerase (idi, IDI) (1 of 1)
OG IDs: 93521837
Best-hit-arabi-ID: AT3G02780.1
Best-hit-arabi-name: isopentenyl pyrophosphate:dimethylallyl pyrophosphate isomerase 2
Gorai.004G262800.1.D
PCC=0.478, p=0.038
mRNA Profile
Protein Expression Profile
DPA
0
1
2
3
4
5
6
7
8
9
10
11
12
13
14
15
16
17
18
19
20
21
22
23
24
25
26
27
28
29
30
31
32
33
34

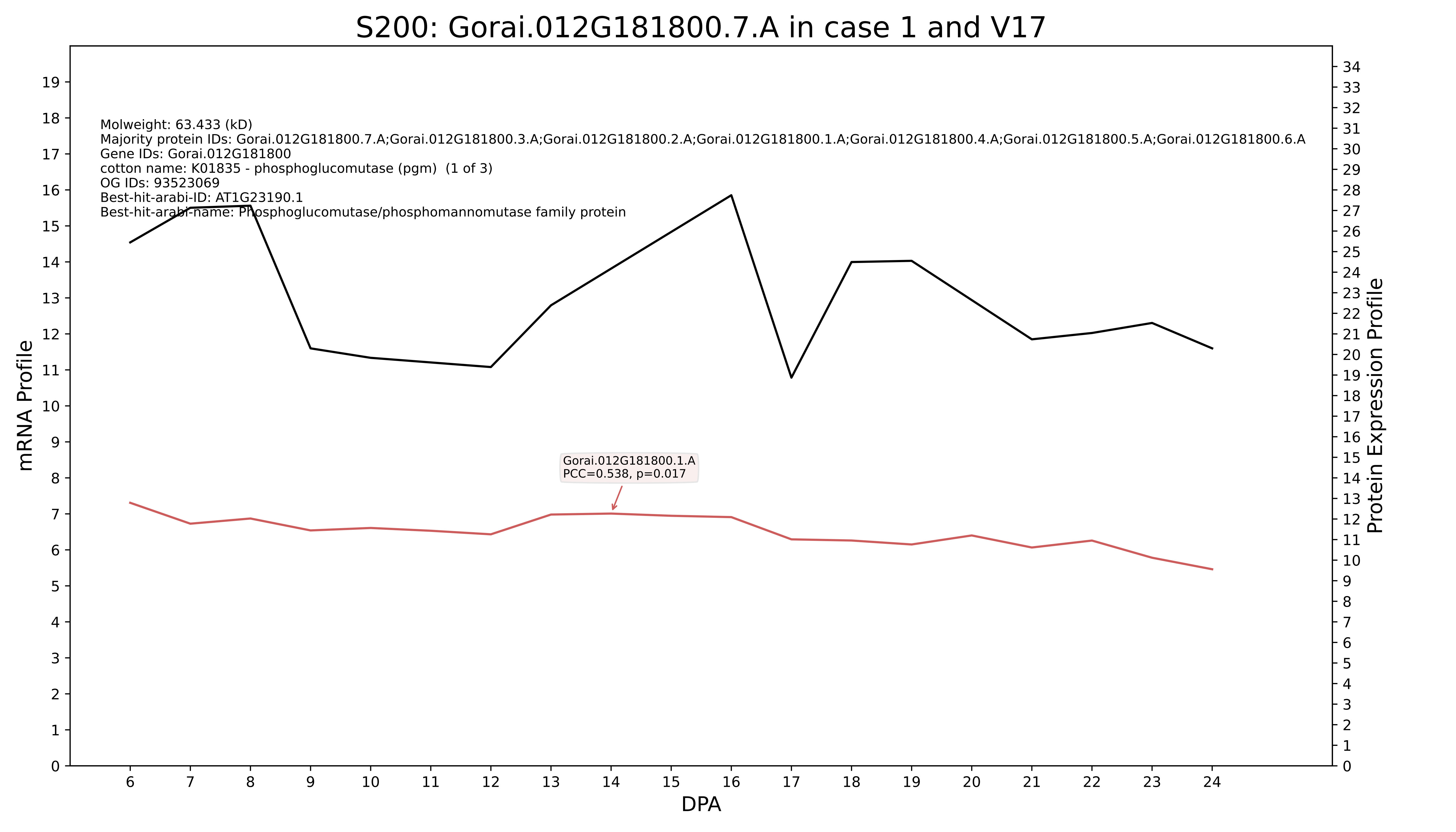

S200: Gorai.012G181800.7.A in case 1 and V17
Molweight: 63.433 (kD)
Majority protein IDs: Gorai.012G181800.7.A;Gorai.012G181800.3.A;Gorai.012G181800.2.A;Gorai.012G181800.1.A;Gorai.012G181800.4.A;Gorai.012G181800.5.A;Gorai.012G181800.6.A
Gene IDs: Gorai.012G181800
cotton name: K01835 - phosphoglucomutase (pgm) (1 of 3)
OG IDs: 93523069
Best-hit-arabi-ID: AT1G23190.1
Best-hit-arabi-name: Phosphoglucomutase/phosphomannomutase family protein
Gorai.012G181800.1.A
PCC=0.538, p=0.017
mRNA Profile
Protein Expression Profile
DPA

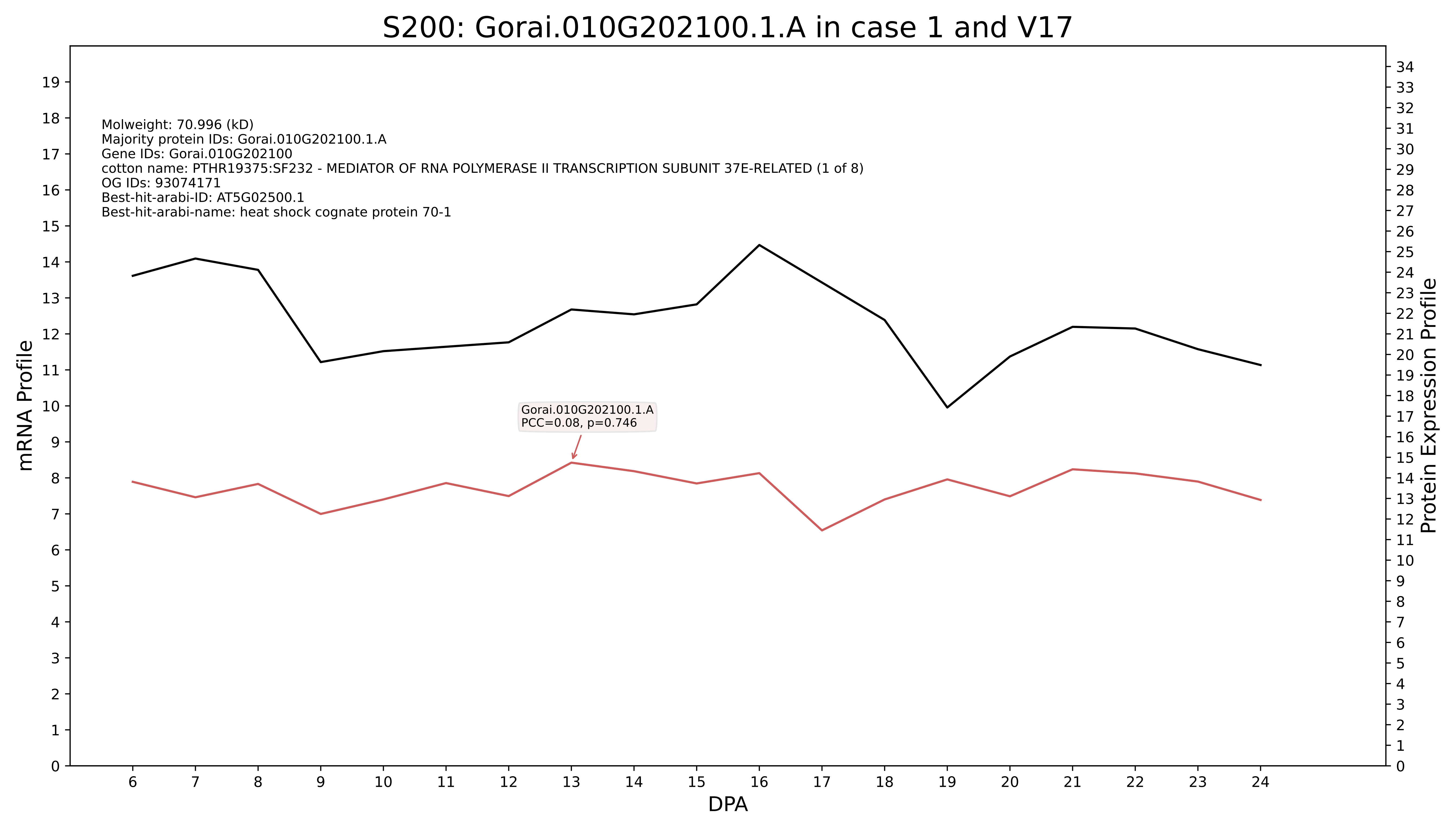
S200: Gorai.010G202100.1.A in case 1 and V17
Molweight: 70.996 (kD)
Majority protein IDs: Gorai.010G202100.1.A
Gene IDs: Gorai.010G202100
cotton name: PTHR19375:SF232 - MEDIATOR OF RNA POLYMERASE II TRANSCRIPTION SUBUNIT 37E-RELATED (1 of 8)
OG IDs: 93074171
Best-hit-arabi-ID: AT5G02500.1
Best-hit-arabi-name: heat shock cognate protein 70-1
Gorai.010G202100.1.A
PCC=0.08, p=0.746
mRNA Profile
Protein Expression Profile
DPA

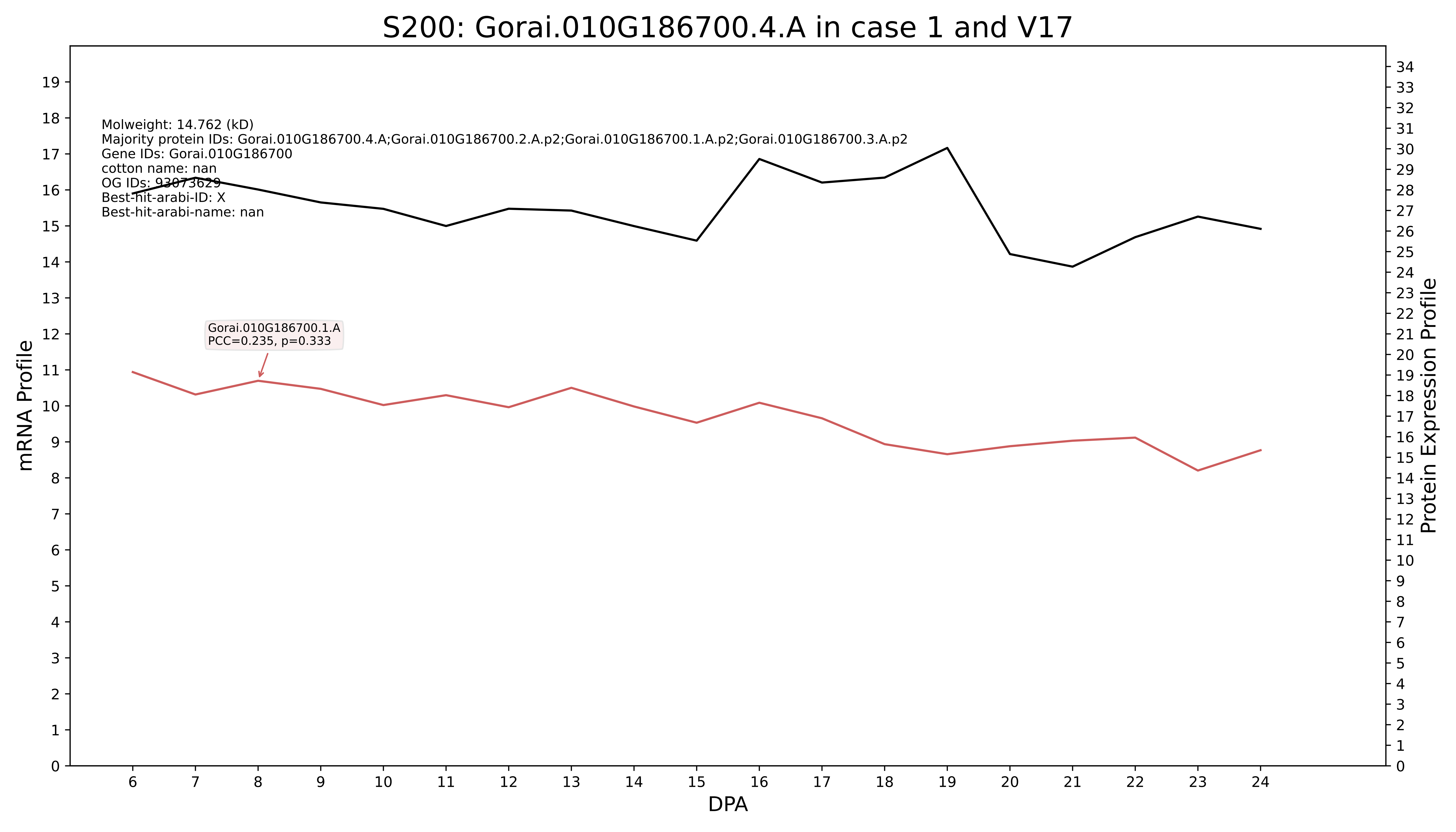
S200: Gorai.010G186700.4.A in case 1 and V17
Molweight: 14.762 (kD)
Majority protein IDs: Gorai.010G186700.4.A;Gorai.010G186700.2.A.p2;Gorai.010G186700.1.A.p2;Gorai.010G186700.3.A.p2
Gene IDs: Gorai.010G186700
cotton name: nan
OG IDs: 93073629
Best-hit-arabi-ID: X
Best-hit-arabi-name: nan
Gorai.010G186700.1.A
PCC=0.235, p=0.333
mRNA Profile
Protein Expression Profile
DPA

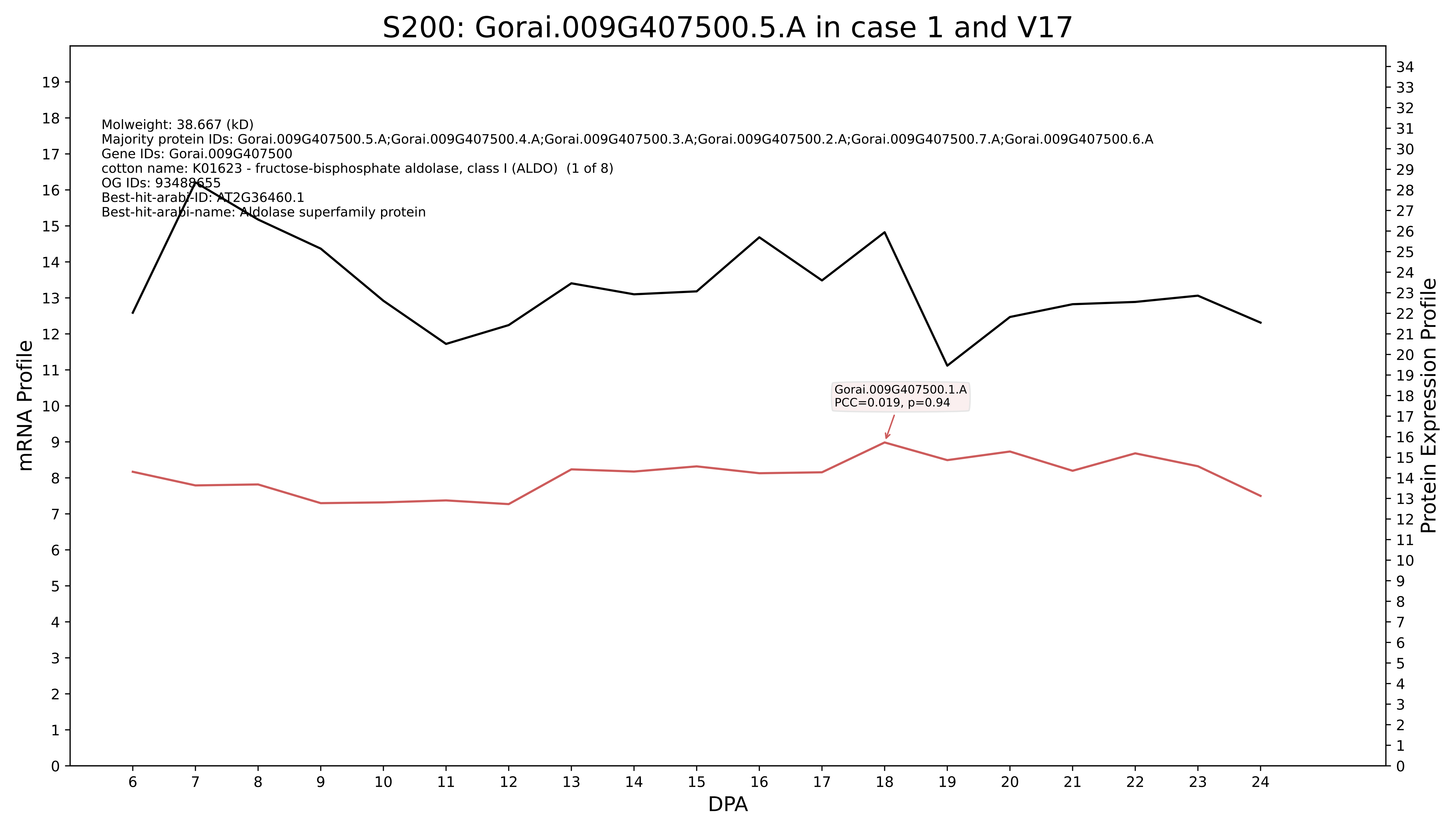
S200: Gorai.009G407500.5.A in case 1 and V17
Molweight: 38.667 (kD)
Majority protein IDs: Gorai.009G407500.5.A;Gorai.009G407500.4.A;Gorai.009G407500.3.A;Gorai.009G407500.2.A;Gorai.009G407500.7.A;Gorai.009G407500.6.A
Gene IDs: Gorai.009G407500
cotton name: K01623 - fructose-bisphosphate aldolase, class I (ALDO) (1 of 8)
OG IDs: 93488655
Best-hit-arabi-ID: AT2G36460.1
Best-hit-arabi-name: Aldolase superfamily protein
Gorai.009G407500.1.A
PCC=0.019, p=0.94
mRNA Profile
Protein Expression Profile
DPA

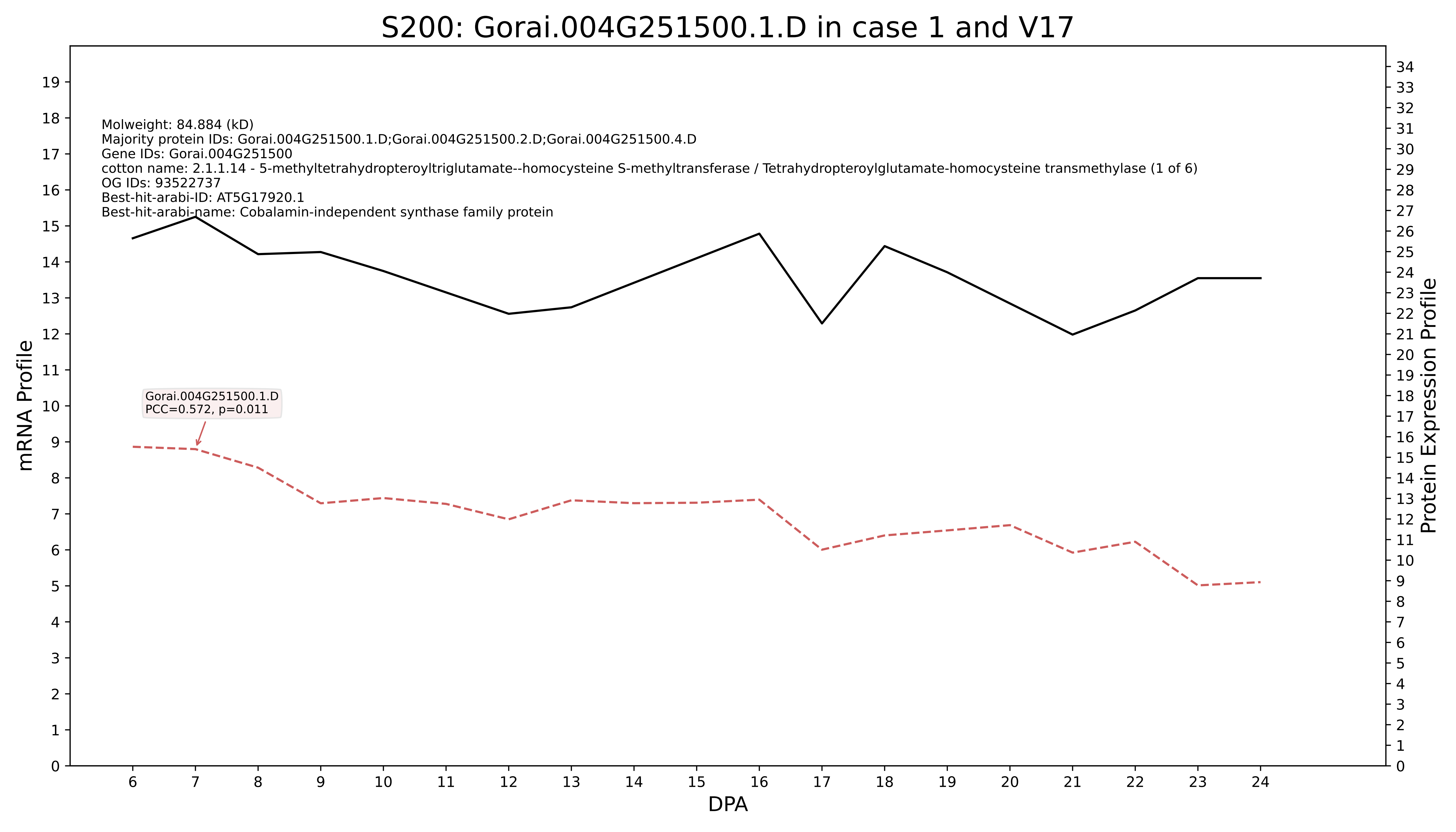
S200: Gorai.004G251500.1.D in case 1 and V17
Molweight: 84.884 (kD)
Majority protein IDs: Gorai.004G251500.1.D;Gorai.004G251500.2.D;Gorai.004G251500.4.D
Gene IDs: Gorai.004G251500
cotton name: 2.1.1.14 - 5-methyltetrahydropteroyltriglutamate--homocysteine S-methyltransferase / Tetrahydropteroylglutamate-homocysteine transmethylase (1 of 6)
OG IDs: 93522737
Best-hit-arabi-ID: AT5G17920.1
Best-hit-arabi-name: Cobalamin-independent synthase family protein
Gorai.004G251500.1.D
PCC=0.572, p=0.011
mRNA Profile
Protein Expression Profile
DPA
0
1
2
3
4
5
6
7
8
9
10
11
12
13
14
15
16
17
18
19
20
21
22
23
24
25
26
27
28
29
30
31
32
33
34

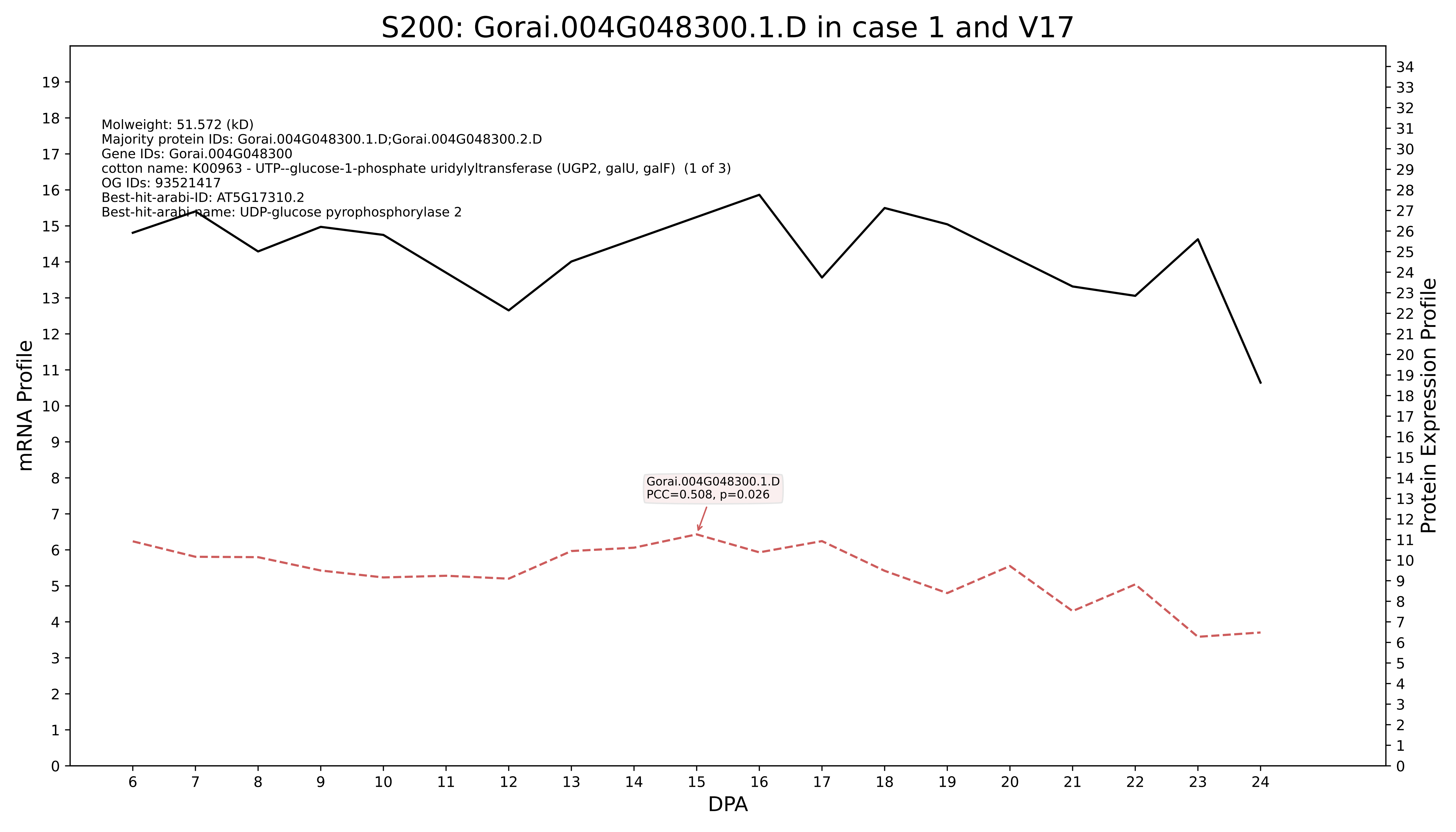
S200: Gorai.004G048300.1.D in case 1 and V17
Molweight: 51.572 (kD)
Majority protein IDs: Gorai.004G048300.1.D;Gorai.004G048300.2.D
Gene IDs: Gorai.004G048300
cotton name: K00963 - UTP--glucose-1-phosphate uridylyltransferase (UGP2, galU, galF) (1 of 3)
OG IDs: 93521417
Best-hit-arabi-ID: AT5G17310.2
Best-hit-arabi-name: UDP-glucose pyrophosphorylase 2
Gorai.004G048300.1.D
PCC=0.508, p=0.026
mRNA Profile
Protein Expression Profile
DPA
0
1
2
3
4
5
6
7
8
9
10
11
12
13
14
15
16
17
18
19
20
21
22
23
24
25
26
27
28
29
30
31
32
33
34

S200: Gorai.006G156800.1.A in case 1 and V17

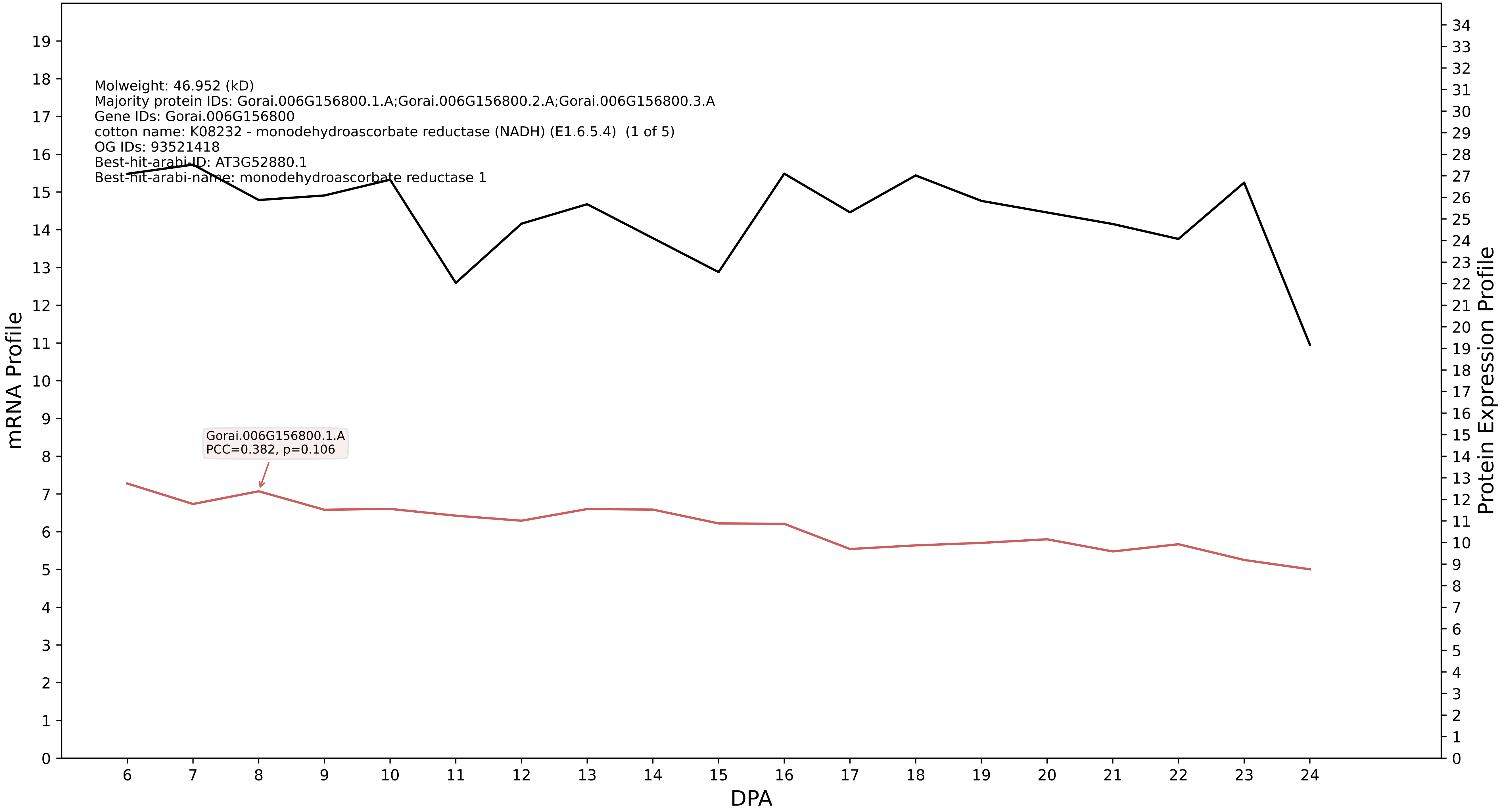

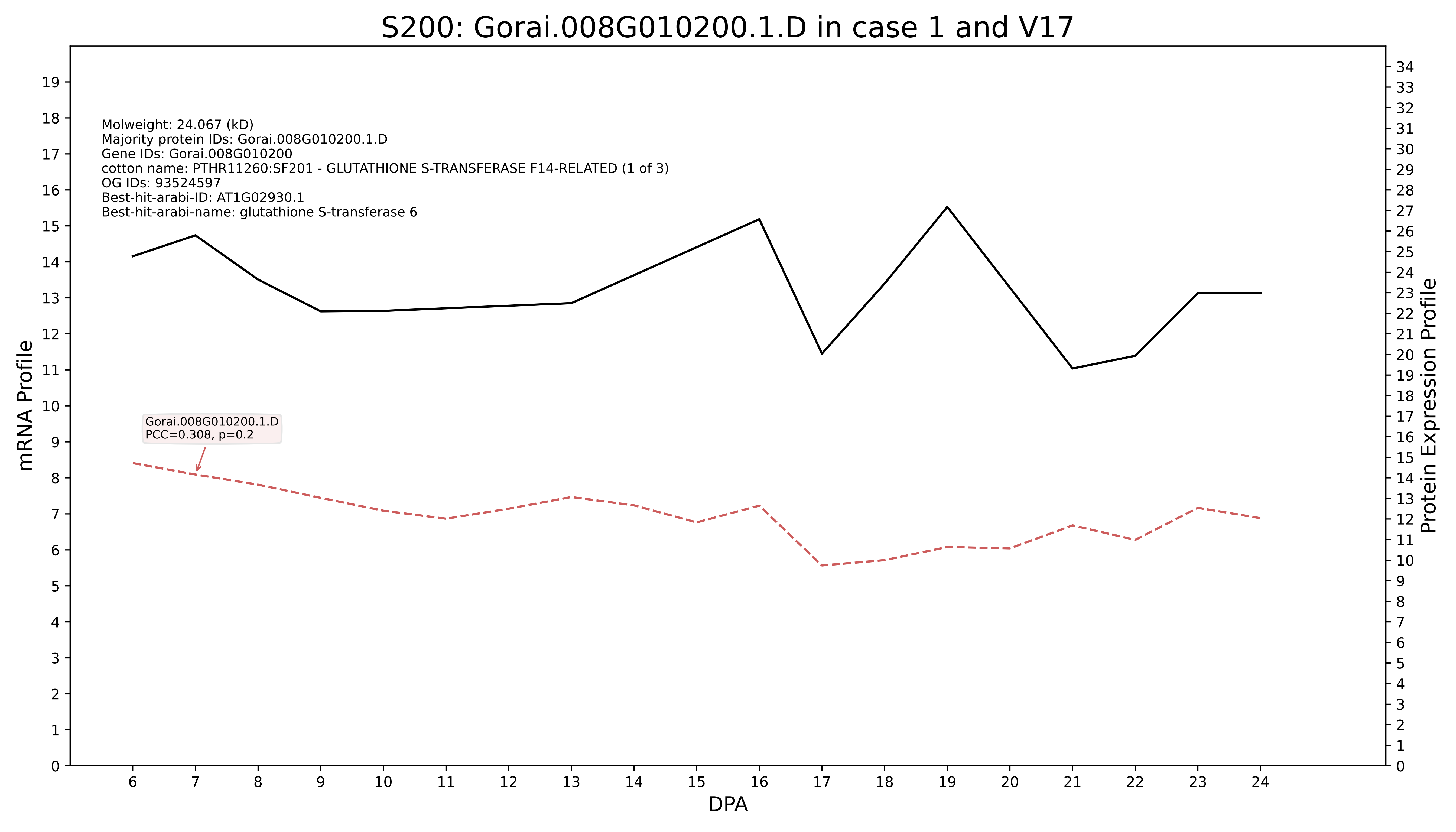
S200: Gorai.008G010200.1.D in case 1 and V17
Molweight: 24.067 (kD)
Majority protein IDs: Gorai.008G010200.1.D
Gene IDs: Gorai.008G010200
cotton name: PTHR11260:SF201 - GLUTATHIONE S-TRANSFERASE F14-RELATED (1 of 3)
OG IDs: 93524597
Best-hit-arabi-ID: AT1G02930.1
Best-hit-arabi-name: glutathione S-transferase 6
Gorai.008G010200.1.D
PCC=0.308, p=0.2
mRNA Profile
Protein Expression Profile
DPA

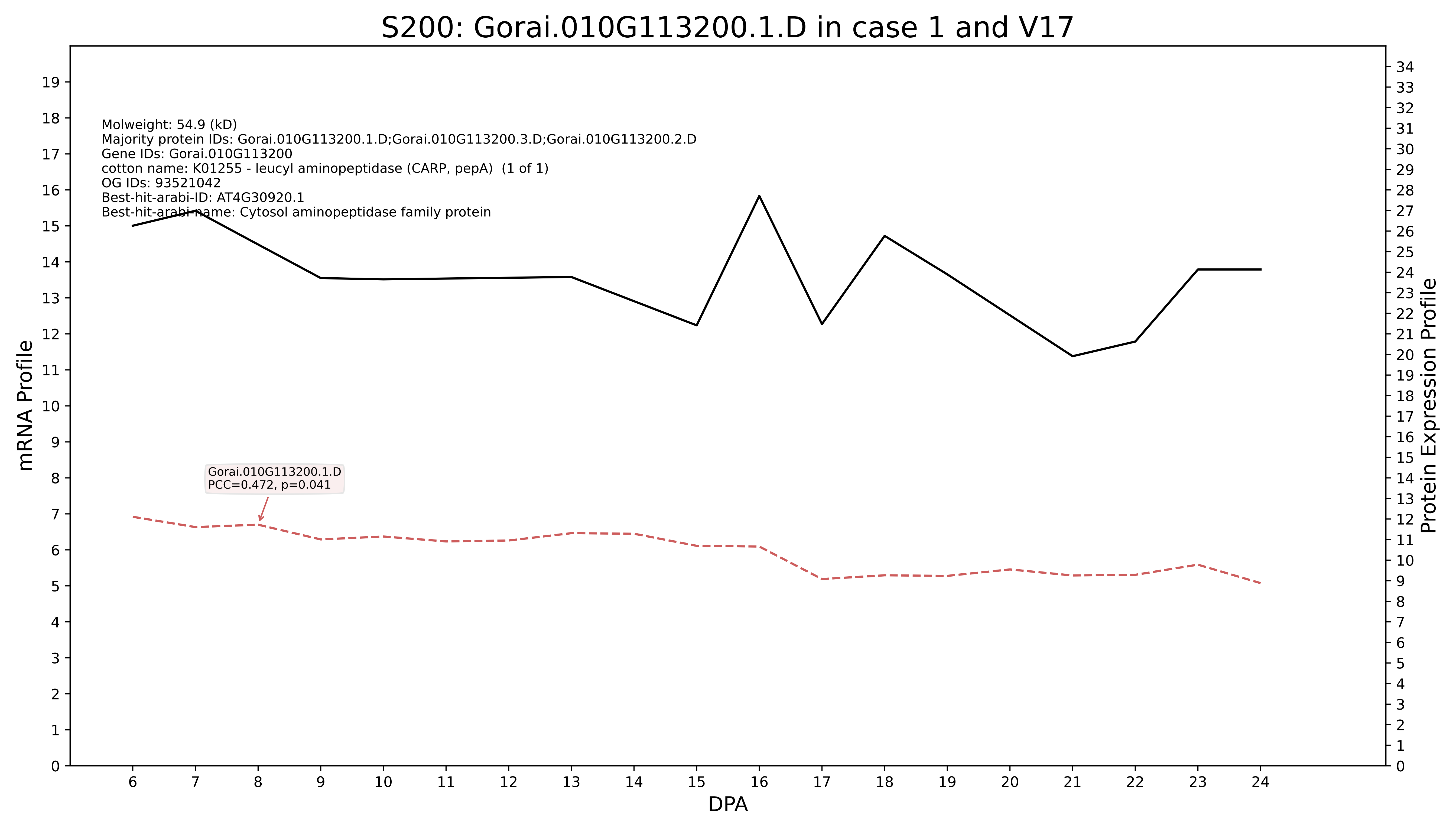
S200: Gorai.010G113200.1.D in case 1 and V17
Molweight: 54.9 (kD)
Majority protein IDs: Gorai.010G113200.1.D;Gorai.010G113200.3.D;Gorai.010G113200.2.D
Gene IDs: Gorai.010G113200
cotton name: K01255 - leucyl aminopeptidase (CARP, pepA) (1 of 1)
OG IDs: 93521042
Best-hit-arabi-ID: AT4G30920.1
Best-hit-arabi-name: Cytosol aminopeptidase family protein
Gorai.010G113200.1.D
PCC=0.472, p=0.041
mRNA Profile
Protein Expression Profile
DPA

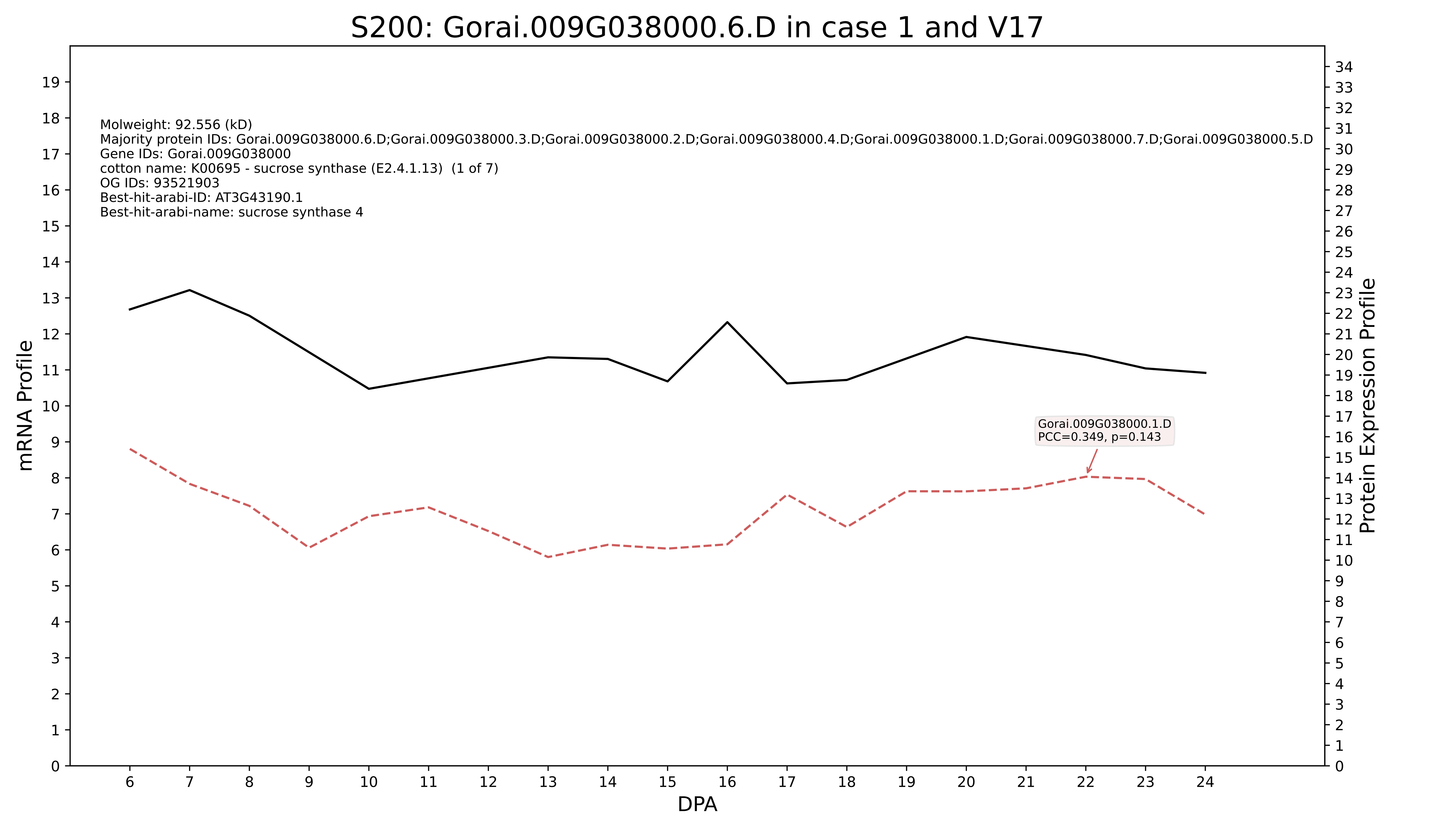
S200: Gorai.009G038000.6.D in case 1 and V17
Molweight: 92.556 (kD)
Majority protein IDs: Gorai.009G038000.6.D;Gorai.009G038000.3.D;Gorai.009G038000.2.D;Gorai.009G038000.4.D;Gorai.009G038000.1.D;Gorai.009G038000.7.D;Gorai.009G038000.5.D
Gene IDs: Gorai.009G038000
cotton name: K00695 - sucrose synthase (E2.4.1.13) (1 of 7)
OG IDs: 93521903
Best-hit-arabi-ID: AT3G43190.1
Best-hit-arabi-name: sucrose synthase 4
Gorai.009G038000.1.D
PCC=0.349, p=0.143
mRNA Profile
Protein Expression Profile
DPA
0 1 2 3 4 5 6 7 8 9 10 11 12 13 14 15 16 17 18 19
0 1 2 3 4 5 6 7 8 9 10 11 12 13 14 15 16 17 18 19 20 21 22 23 24 25 26 27 28 29 30 31 32 33 34
6 7 8 9 10 11 12 13 14 15 16 17 18 19 20 21 22 23 24

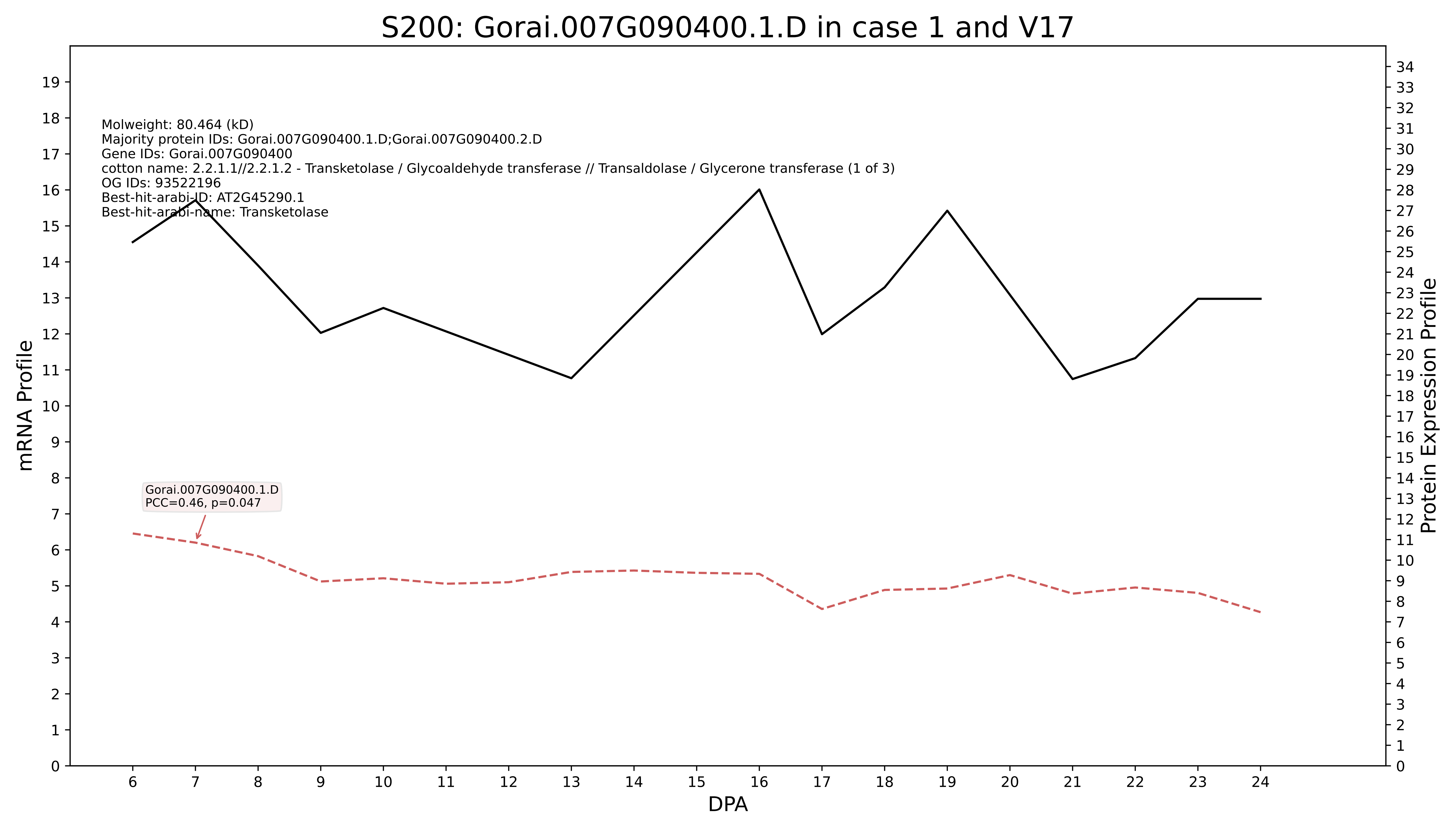
S200: Gorai.007G090400.1.D in case 1 and V17
Molweight: 80.464 (kD)
Majority protein IDs: Gorai.007G090400.1.D;Gorai.007G090400.2.D
Gene IDs: Gorai.007G090400
cotton name: 2.2.1.1//2.2.1.2 - Transketolase / Glycoaldehyde transferase // Transaldolase / Glycerone transferase (1 of 3)
OG IDs: 93522196
Best-hit-arabi-ID: AT2G45290.1
Best-hit-arabi-name: Transketolase
Gorai.007G090400.1.D
PCC=0.46, p=0.047
mRNA Profile
Protein Expression Profile
DPA
0
1
2
3
4
5
6
7
8
9
10
11
12
13
14
15
16
17
18
19
20
21
22
23
24
25
26
27
28
29
30
31
32
33
34

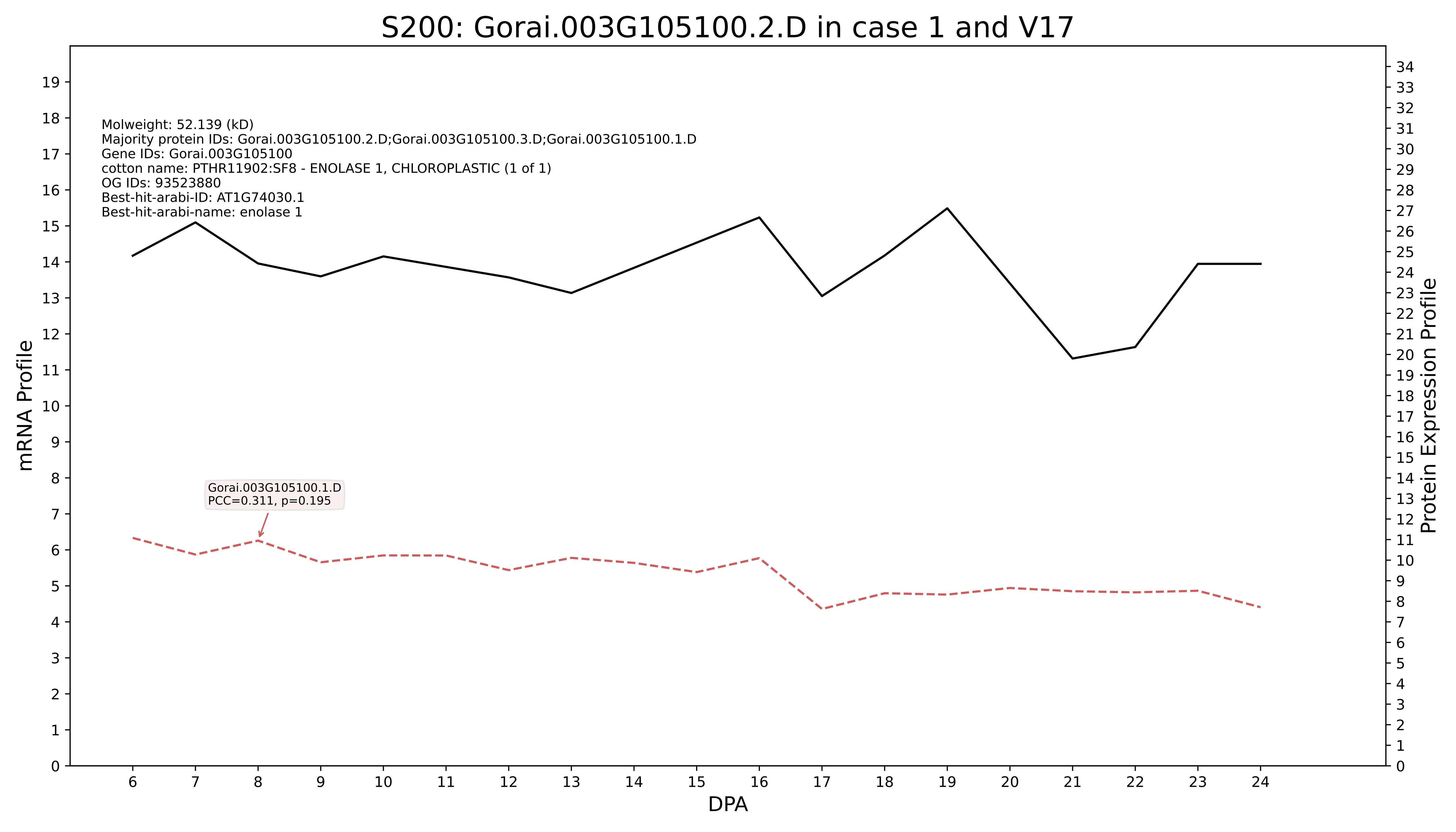
S200: Gorai.003G105100.2.D in case 1 and V17
Molweight: 52.139 (kD)
Majority protein IDs: Gorai.003G105100.2.D;Gorai.003G105100.3.D;Gorai.003G105100.1.D
Gene IDs: Gorai.003G105100
cotton name: PTHR11902:SF8 - ENOLASE 1, CHLOROPLASTIC (1 of 1)
OG IDs: 93523880
Best-hit-arabi-ID: AT1G74030.1
Best-hit-arabi-name: enolase 1
Gorai.003G105100.1.D
PCC=0.311, p=0.195
mRNA Profile
Protein Expression Profile
DPA

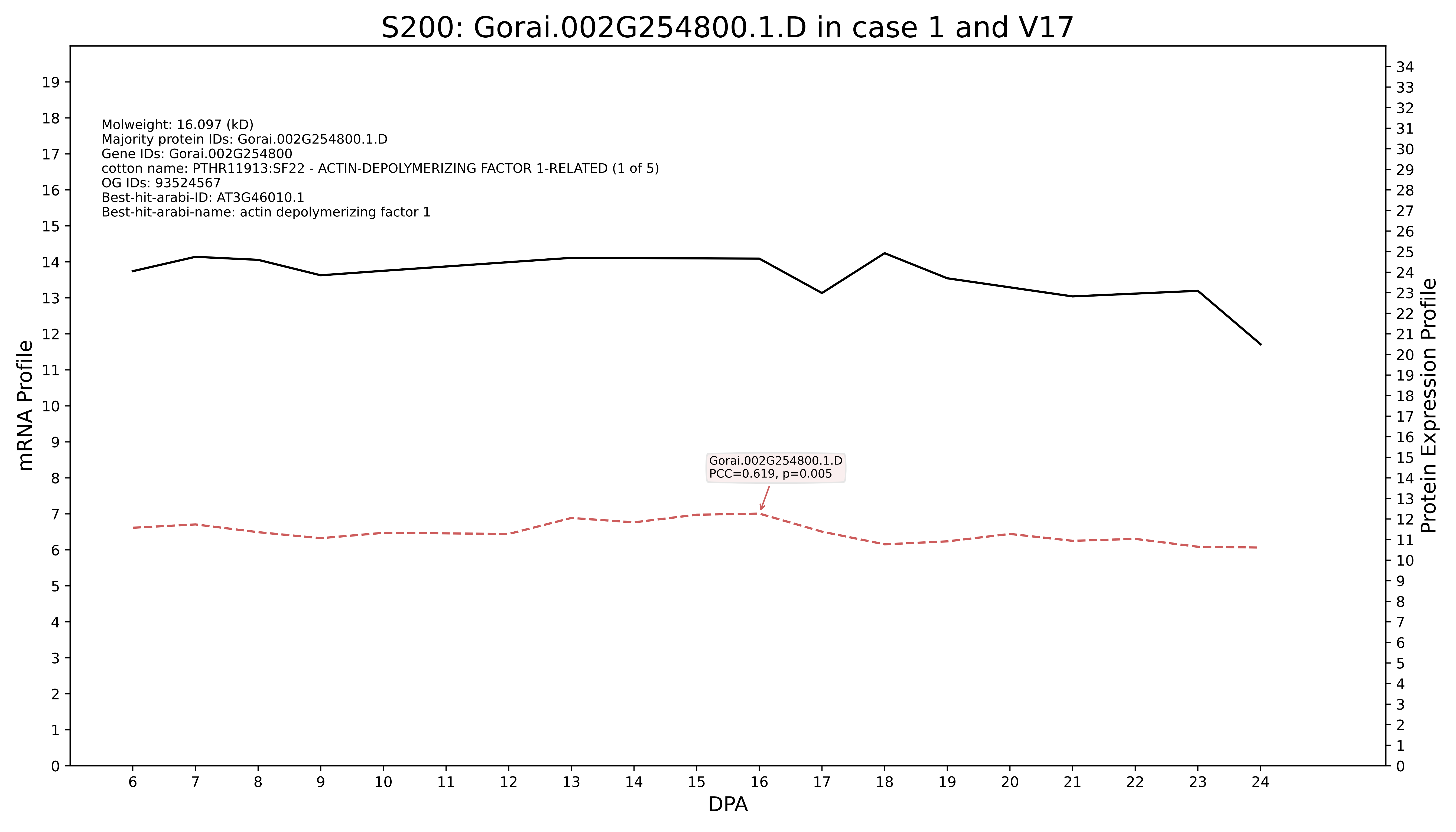
S200: Gorai.002G254800.1.D in case 1 and V17
Molweight: 16.097 (kD)
Majority protein IDs: Gorai.002G254800.1.D
Gene IDs: Gorai.002G254800
cotton name: PTHR11913:SF22 - ACTIN-DEPOLYMERIZING FACTOR 1-RELATED (1 of 5)
OG IDs: 93524567
Best-hit-arabi-ID: AT3G46010.1
Best-hit-arabi-name: actin depolymerizing factor 1
Gorai.002G254800.1.D
PCC=0.619, p=0.005
mRNA Profile
Protein Expression Profile
DPA

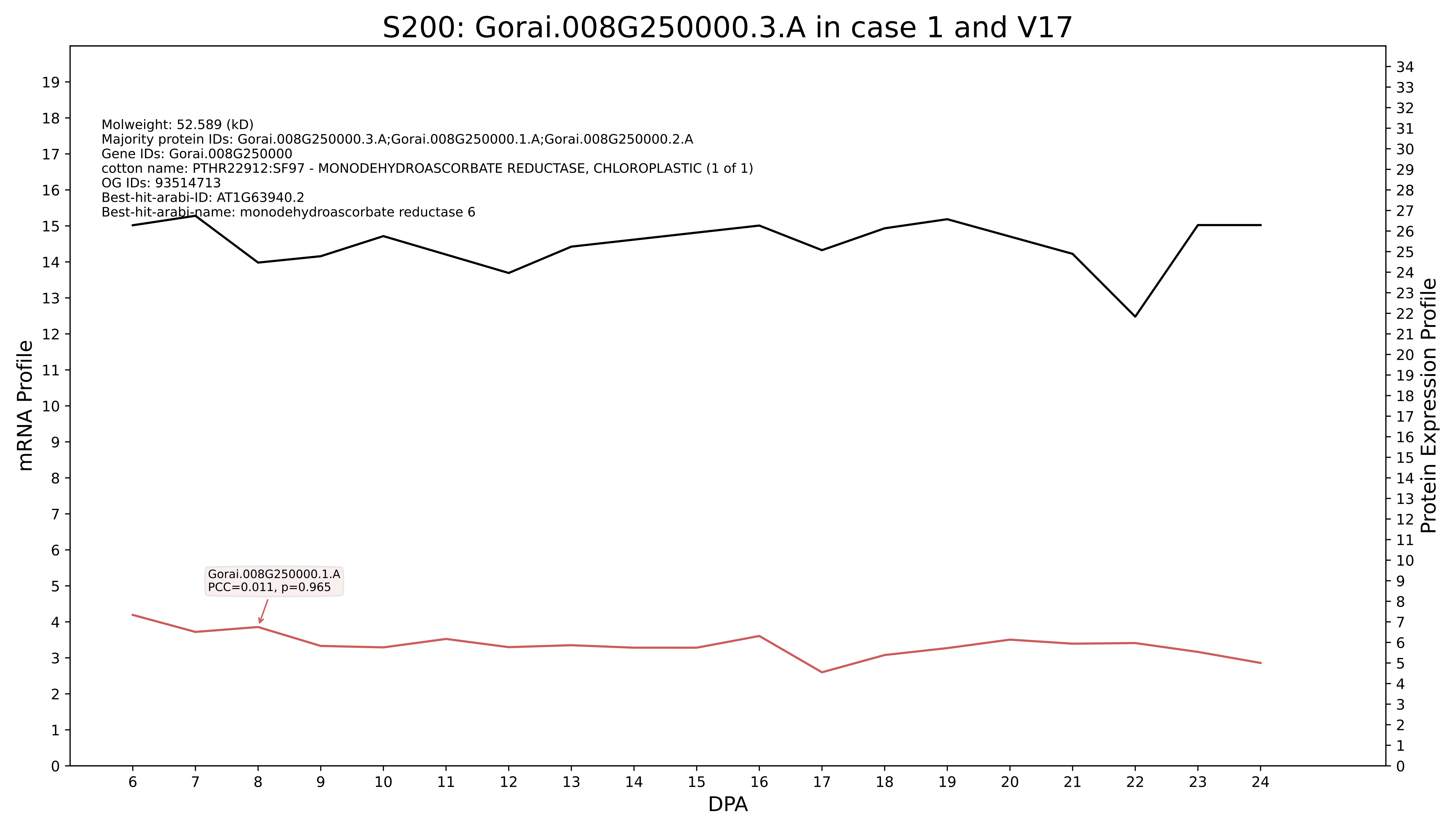

S200: Gorai.008G250000.3.A in case 1 and V17
Molweight: 52.589 (kD)
Majority protein IDs: Gorai.008G250000.3.A;Gorai.008G250000.1.A;Gorai.008G250000.2.A
Gene IDs: Gorai.008G250000
cotton name: PTHR22912:SF97 - MONODEHYDROASCORBATE REDUCTASE, CHLOROPLASTIC (1 of 1)
OG IDs: 93514713
Best-hit-arabi-ID: AT1G63940.2
Best-hit-arabi-name: monodehydroascorbate reductase 6
Gorai.008G250000.1.A
PCC=0.011, p=0.965
mRNA Profile
Protein Expression Profile
DPA

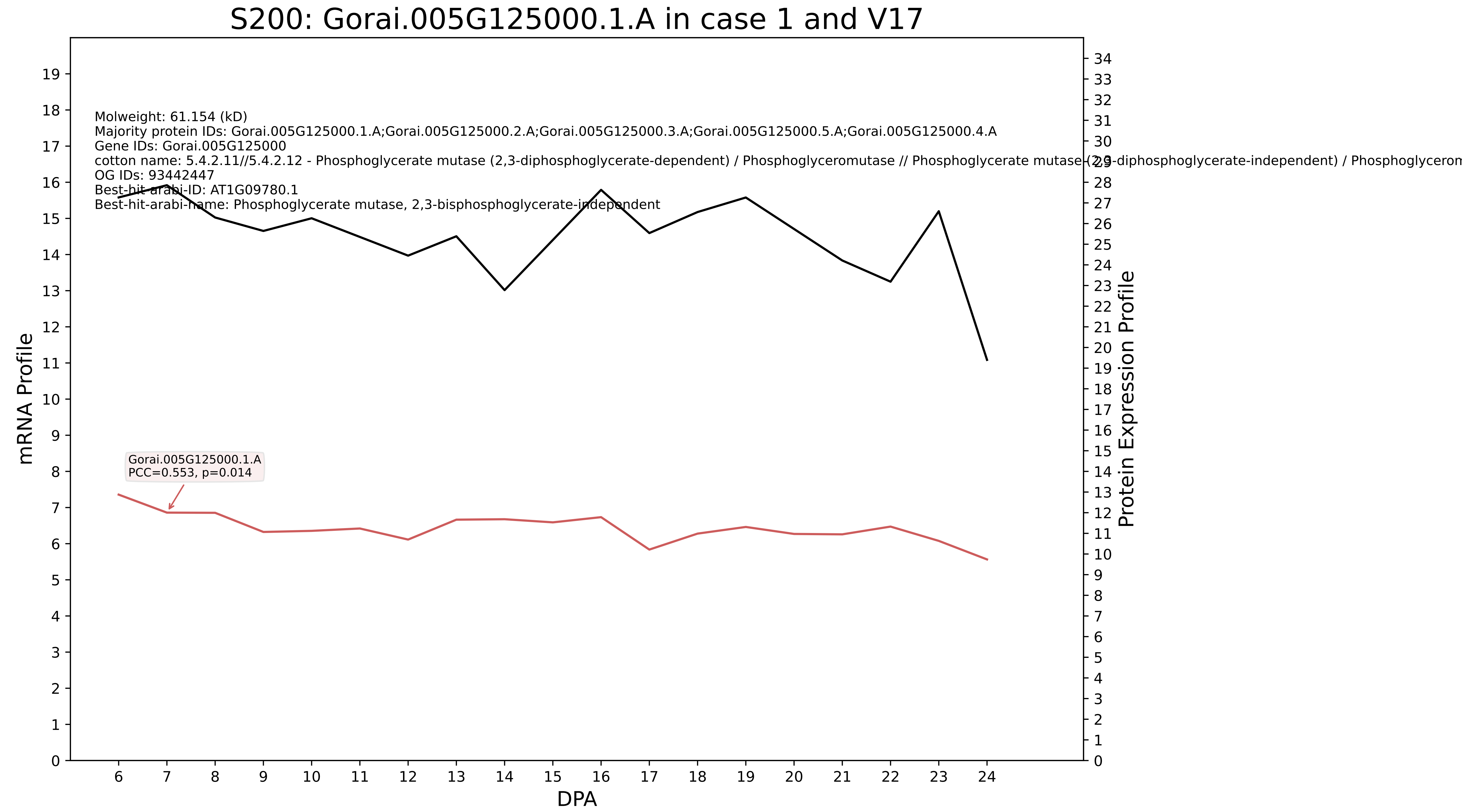
S200: Gorai.005G125000.1.A in case 1 and V17
Molweight: 61.154 (kD)
Majority protein IDs: Gorai.005G125000.1.A;Gorai.005G125000.2.A;Gorai.005G125000.3.A;Gorai.005G125000.5.A;Gorai.005G125000.4.A
Gene IDs: Gorai.005G125000
cotton name: 5.4.2.11//5.4.2.12 - Phosphoglycerate mutase (2,3-diphosphoglycerate-dependent) / Phosphoglyceromutase // Phosphoglycerate mutase (2,3-diphosphoglycerate-independent) / Phosphoglycerom
OG IDs: 93442447
Best-hit-arabi-ID: AT1G09780.1
Best-hit-arabi-name: Phosphoglycerate mutase, 2,3-bisphosphoglycerate-independent
Gorai.005G125000.1.A
PCC=0.553, p=0.014
mRNA Profile
Protein Expression Profile
DPA

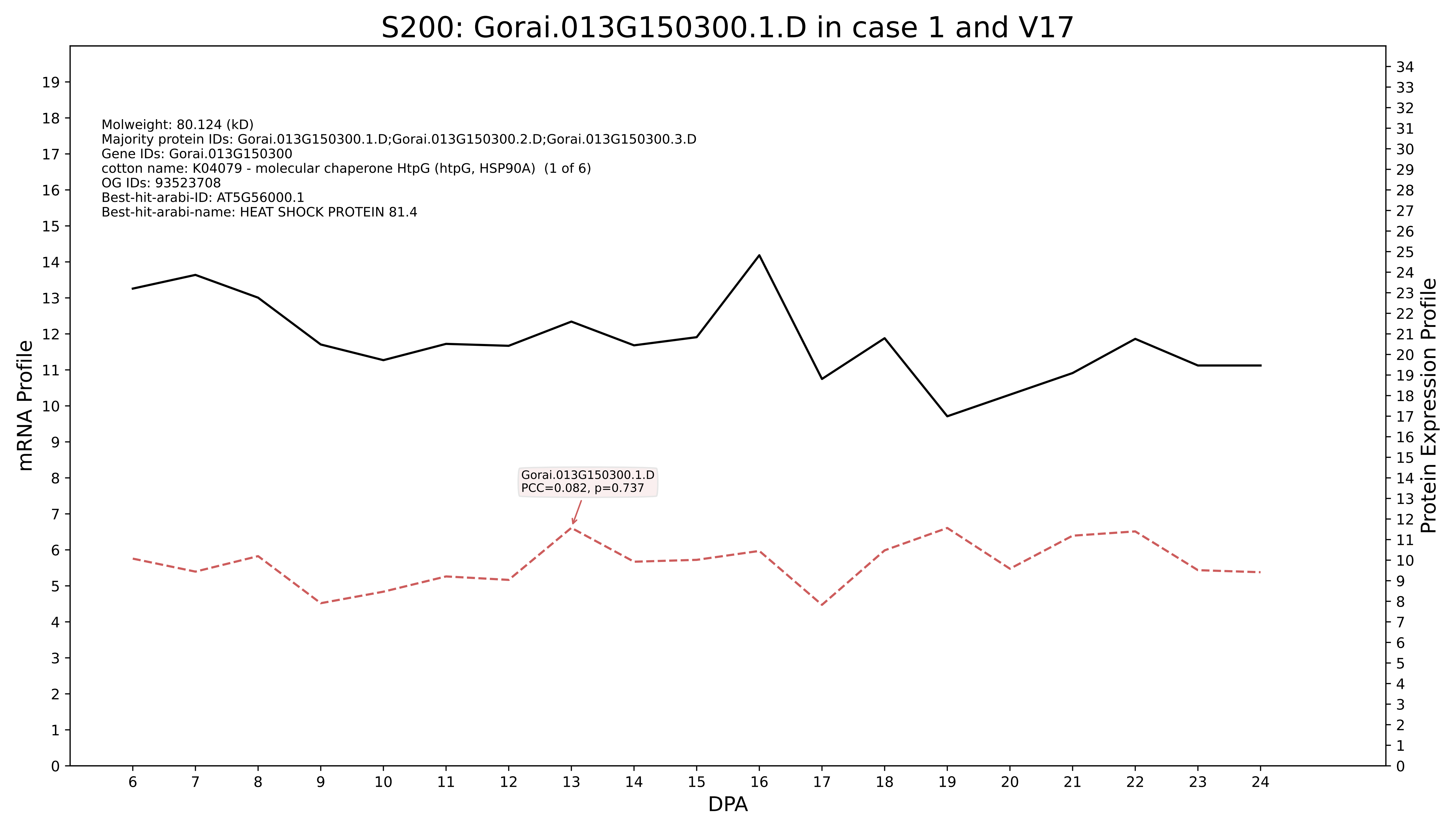
S200: Gorai.013G150300.1.D in case 1 and V17
Molweight: 80.124 (kD)
Majority protein IDs: Gorai.013G150300.1.D;Gorai.013G150300.2.D;Gorai.013G150300.3.D
Gene IDs: Gorai.013G150300
cotton name: K04079 - molecular chaperone HtpG (htpG, HSP90A) (1 of 6)
OG IDs: 93523708
Best-hit-arabi-ID: AT5G56000.1
Best-hit-arabi-name: HEAT SHOCK PROTEIN 81.4
Gorai.013G150300.1.D
PCC=0.082, p=0.737
mRNA Profile
Protein Expression Profile
DPA

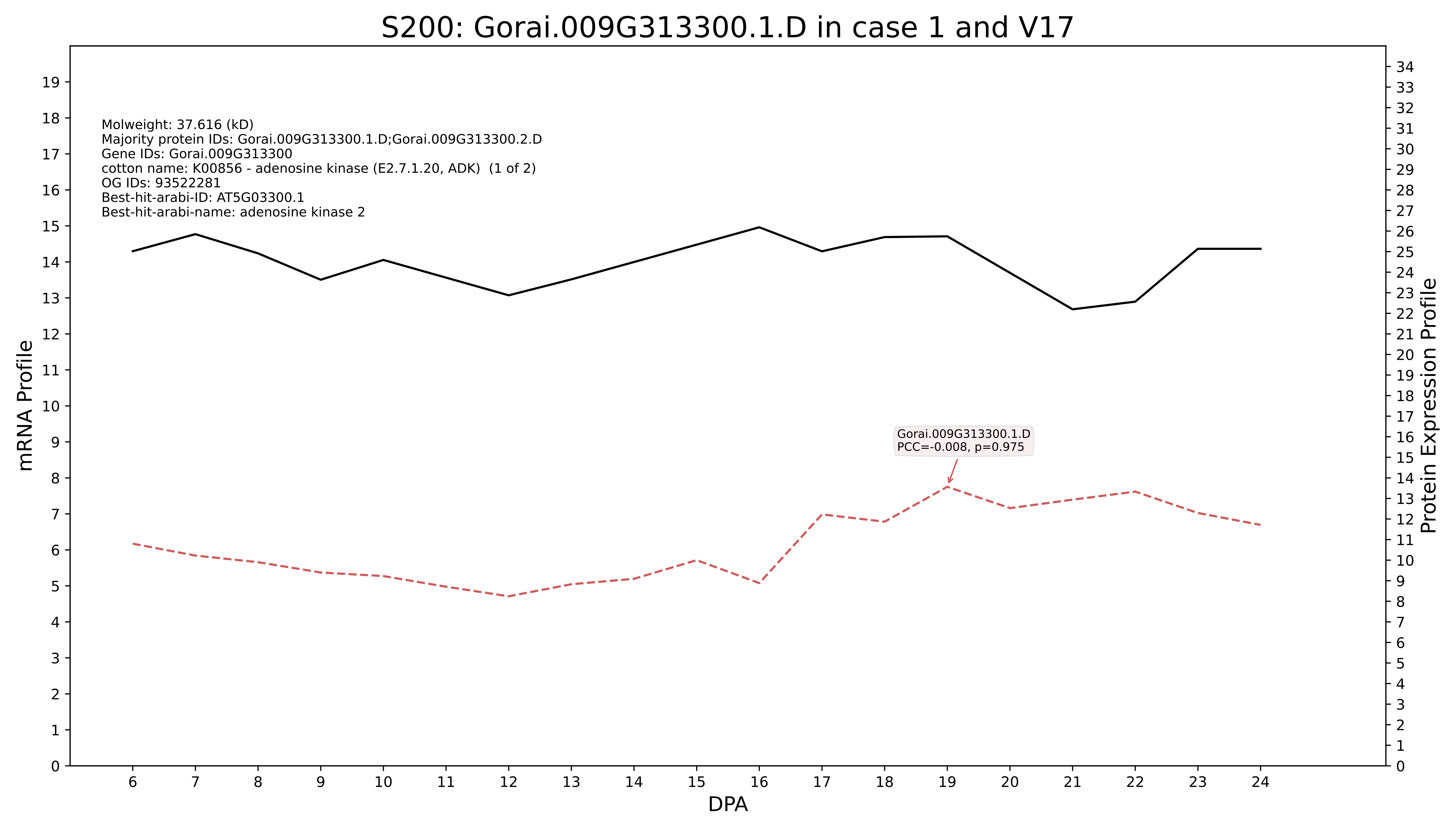
S200: Gorai.009G313300.1.D in case 1 and V17
Molweight: 37.616 (kD)
Majority protein IDs: Gorai.009G313300.1.D;Gorai.009G313300.2.D
Gene IDs: Gorai.009G313300
cotton name: K00856 - adenosine kinase (E2.7.1.20, ADK) (1 of 2)
OG IDs: 93522281
Best-hit-arabi-ID: AT5G03300.1
Best-hit-arabi-name: adenosine kinase 2
Gorai.009G313300.1.D
PCC=-0.008, p=0.975
mRNA Profile
Protein Expression Profile
DPA

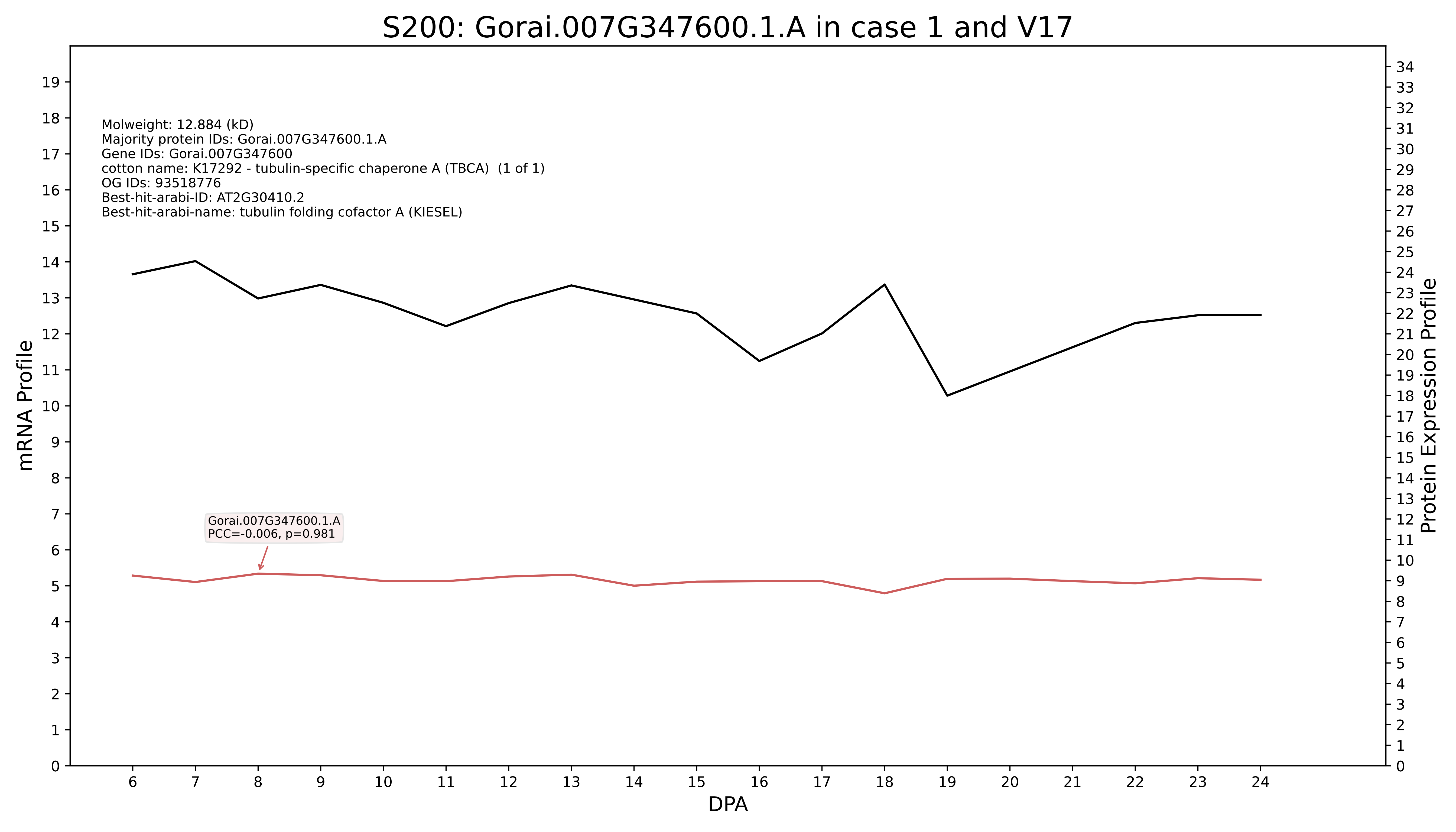
S200: Gorai.007G347600.1.A in case 1 and V17
Molweight: 12.884 (kD)
Majority protein IDs: Gorai.007G347600.1.A
Gene IDs: Gorai.007G347600
cotton name: K17292 - tubulin-specific chaperone A (TBCA) (1 of 1)
OG IDs: 93518776
Best-hit-arabi-ID: AT2G30410.2
Best-hit-arabi-name: tubulin folding cofactor A (KIESEL)
Gorai.007G347600.1.A
PCC=-0.006, p=0.981
mRNA Profile
Protein Expression Profile
DPA

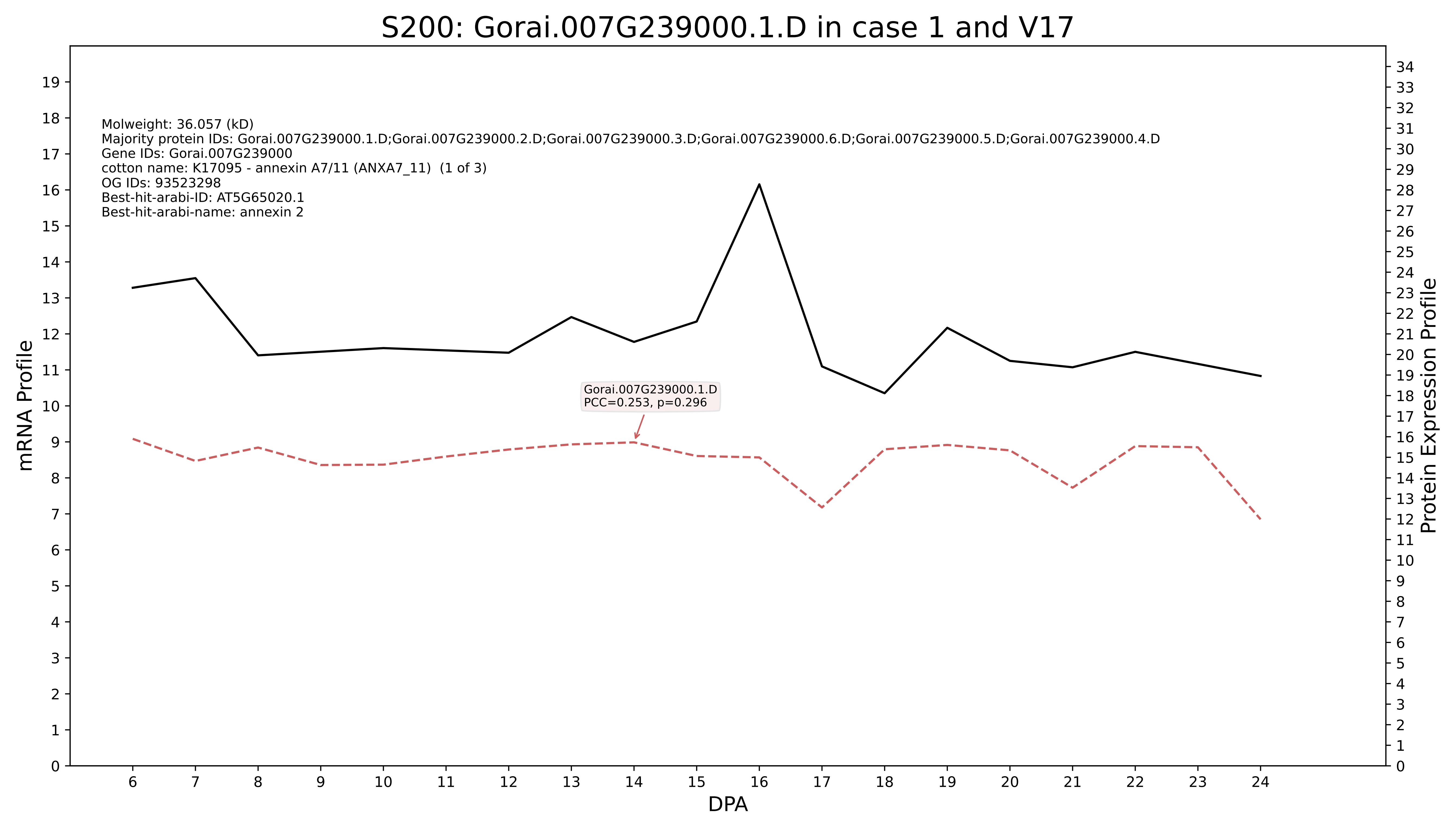
S200: Gorai.007G239000.1.D in case 1 and V17
Molweight: 36.057 (kD)
Majority protein IDs: Gorai.007G239000.1.D;Gorai.007G239000.2.D;Gorai.007G239000.3.D;Gorai.007G239000.6.D;Gorai.007G239000.5.D;Gorai.007G239000.4.D
Gene IDs: Gorai.007G239000
cotton name: K17095 - annexin A7/11 (ANXA7_11) (1 of 3)
OG IDs: 93523298
Best-hit-arabi-ID: AT5G65020.1
Best-hit-arabi-name: annexin 2
Gorai.007G239000.1.D
PCC=0.253, p=0.296
mRNA Profile
Protein Expression Profile
DPA
0
1
2
3
4
5
6
7
8
9
10
11
12
13
14
15
16
17
18
19
20
21
22
23
24
25
26
27
28
29
30
31
32
33
34

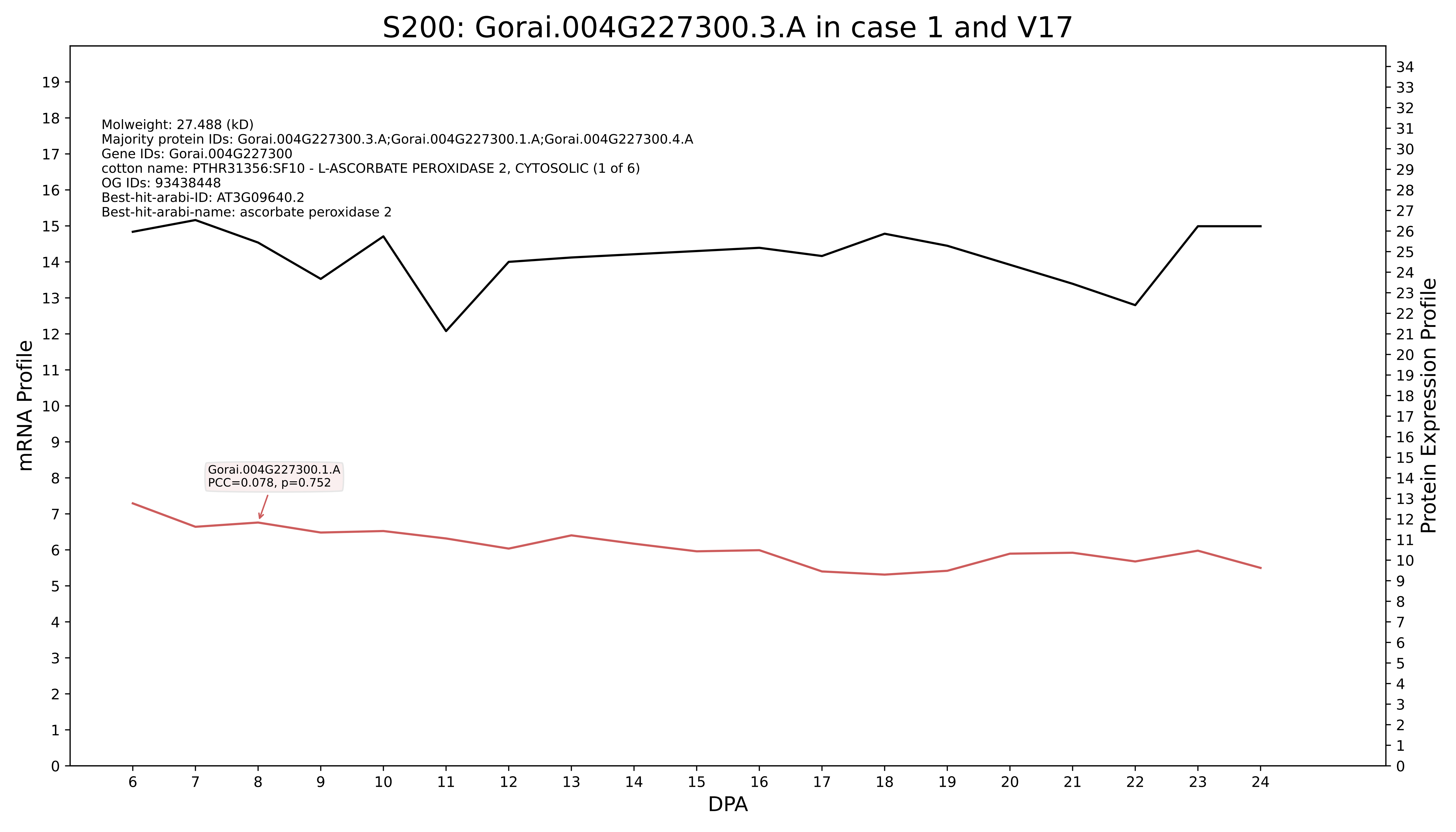
S200: Gorai.004G227300.3.A in case 1 and V17
Molweight: 27.488 (kD)
Majority protein IDs: Gorai.004G227300.3.A;Gorai.004G227300.1.A;Gorai.004G227300.4.A
Gene IDs: Gorai.004G227300
cotton name: PTHR31356:SF10 - L-ASCORBATE PEROXIDASE 2, CYTOSOLIC (1 of 6)
OG IDs: 93438448
Best-hit-arabi-ID: AT3G09640.2
Best-hit-arabi-name: ascorbate peroxidase 2
Gorai.004G227300.1.A
PCC=0.078, p=0.752
mRNA Profile
Protein Expression Profile
DPA

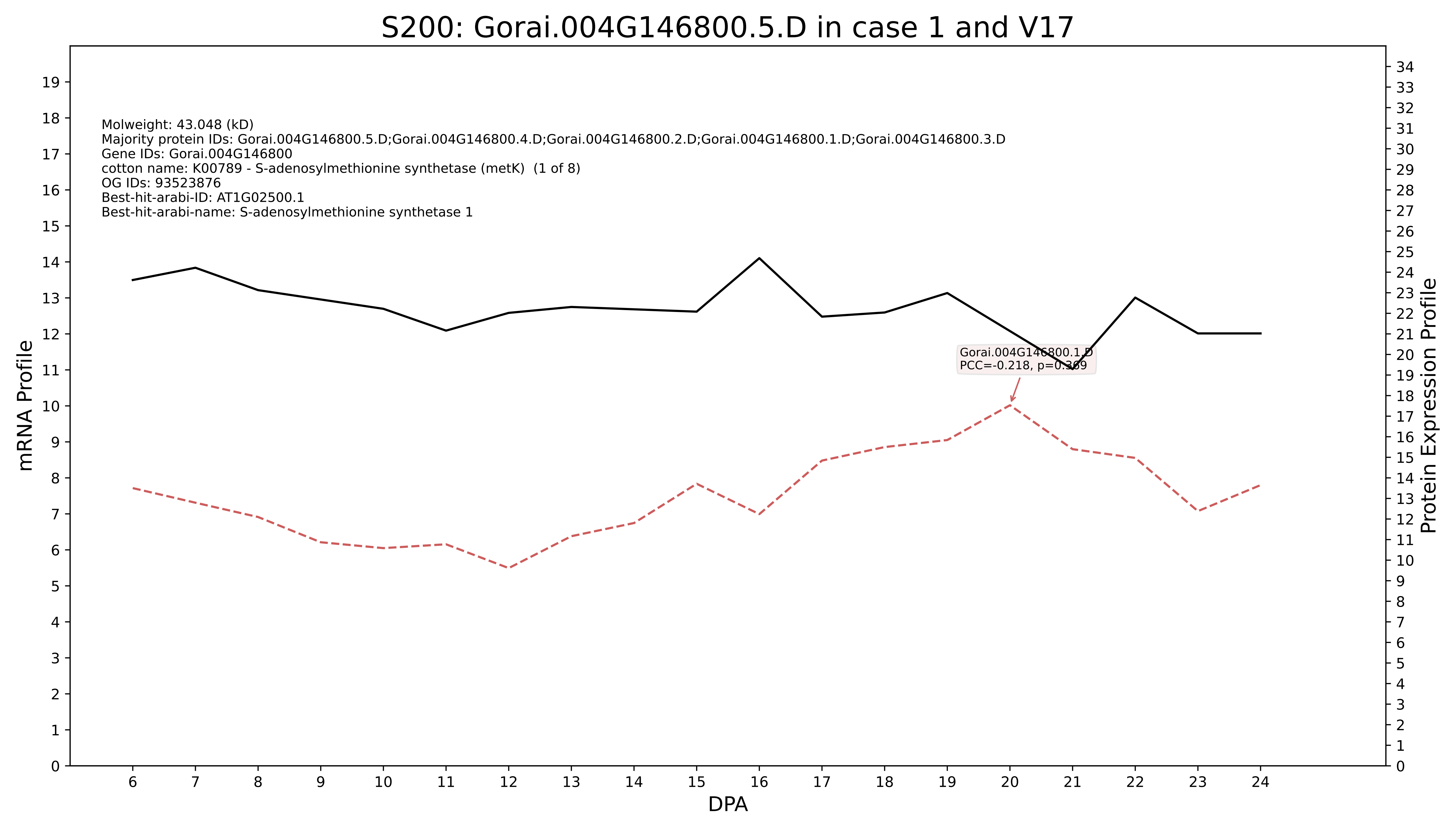
S200: Gorai.004G146800.5.D in case 1 and V17
Molweight: 43.048 (kD)
Majority protein IDs: Gorai.004G146800.5.D;Gorai.004G146800.4.D;Gorai.004G146800.2.D;Gorai.004G146800.1.D;Gorai.004G146800.3.D
Gene IDs: Gorai.004G146800
cotton name: K00789 - S-adenosylmethionine synthetase (metK) (1 of 8)
OG IDs: 93523876
Best-hit-arabi-ID: AT1G02500.1
Best-hit-arabi-name: S-adenosylmethionine synthetase 1
Gorai.004G146800.1.D
PCC=-0.218, p=0.369
mRNA Profile
Protein Expression Profile
DPA

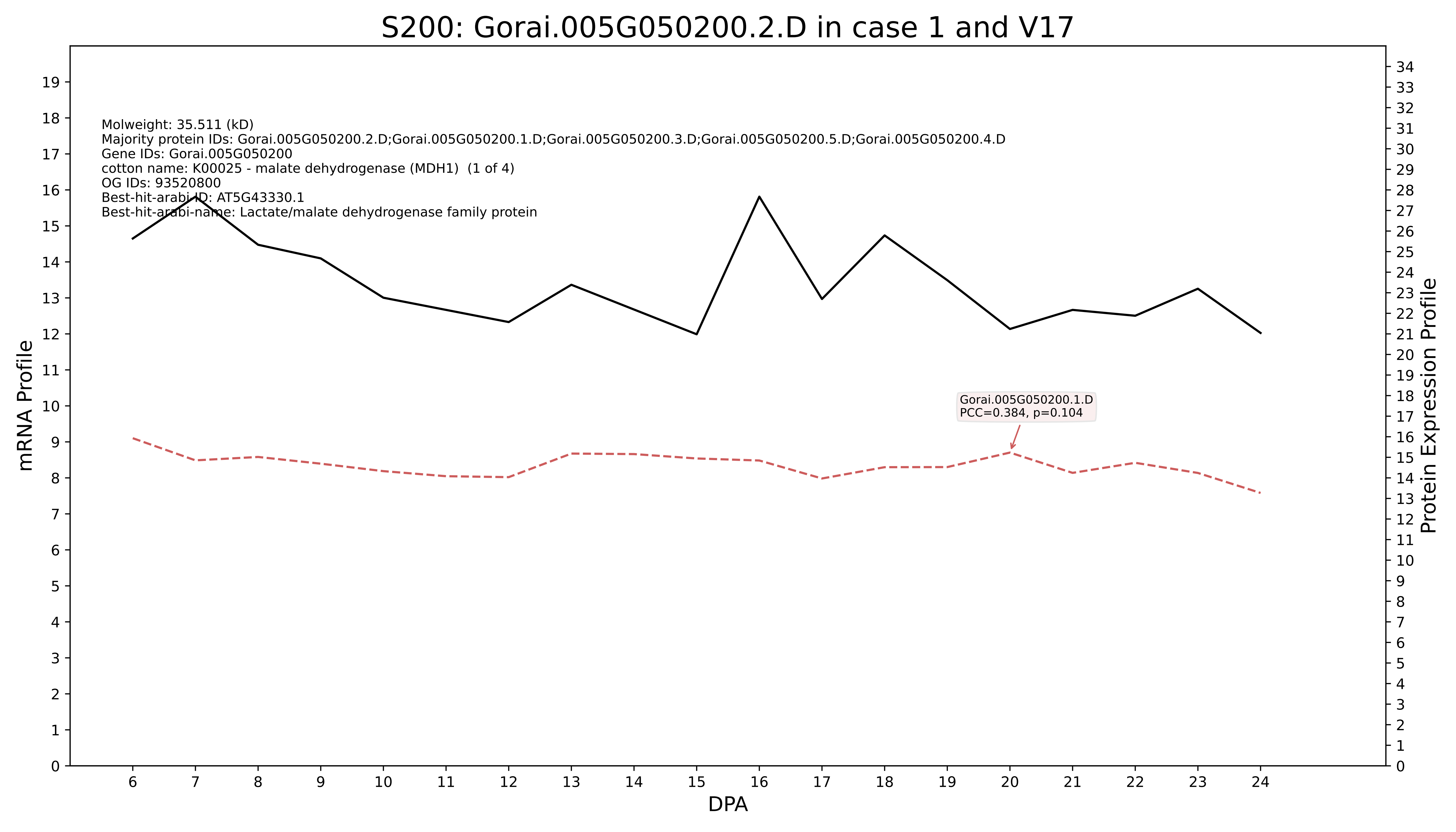
S200: Gorai.005G050200.2.D in case 1 and V17
Molweight: 35.511 (kD)
Majority protein IDs: Gorai.005G050200.2.D;Gorai.005G050200.1.D;Gorai.005G050200.3.D;Gorai.005G050200.5.D;Gorai.005G050200.4.D
Gene IDs: Gorai.005G050200
cotton name: K00025 - malate dehydrogenase (MDH1) (1 of 4)
OG IDs: 93520800
Best-hit-arabi-ID: AT5G43330.1
Best-hit-arabi-name: Lactate/malate dehydrogenase family protein
Gorai.005G050200.1.D
PCC=0.384, p=0.104
mRNA Profile
Protein Expression Profile
DPA

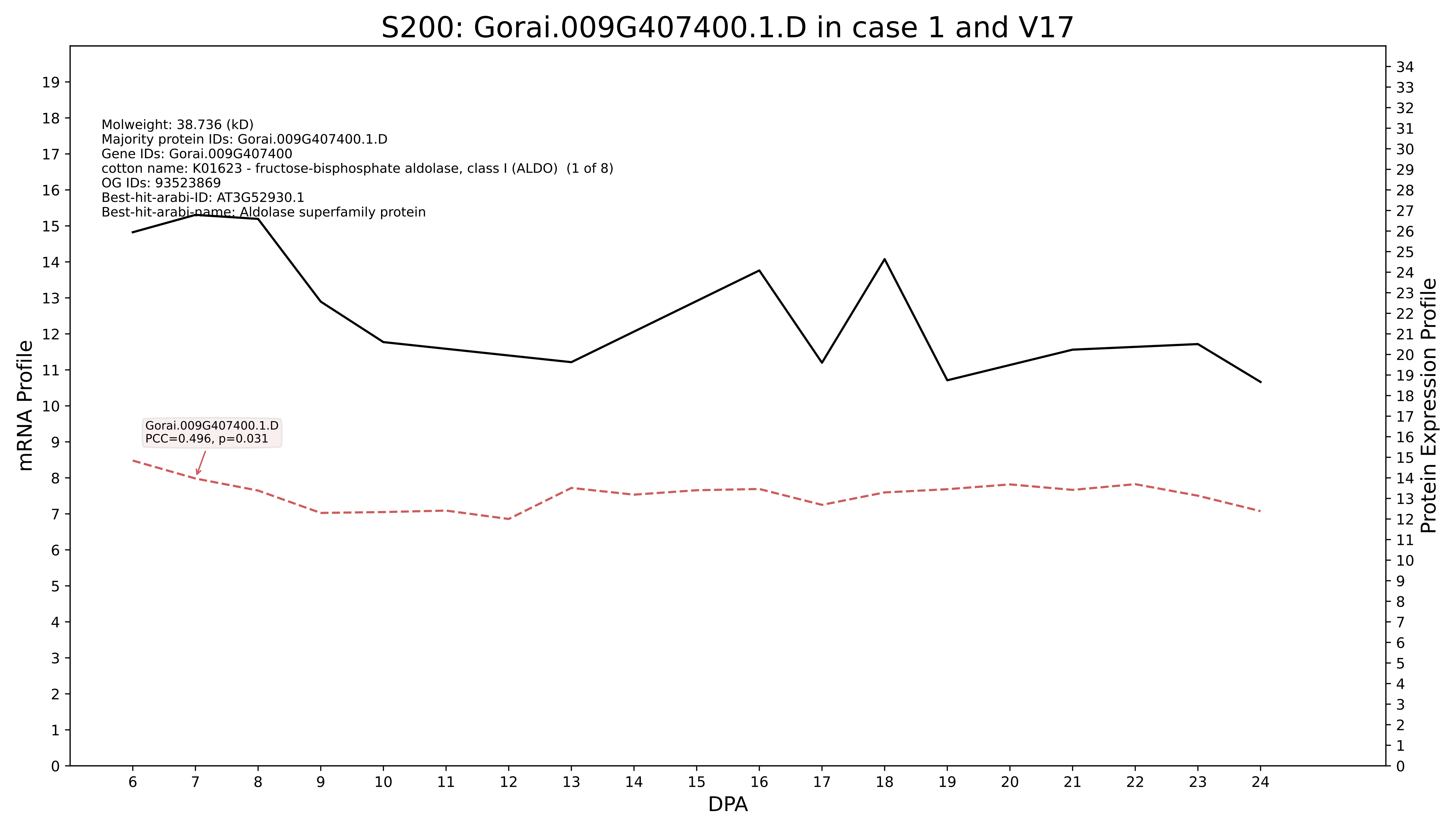
S200: Gorai.009G407400.1.D in case 1 and V17
Molweight: 38.736 (kD)
Majority protein IDs: Gorai.009G407400.1.D
Gene IDs: Gorai.009G407400
cotton name: K01623 - fructose-bisphosphate aldolase, class I (ALDO) (1 of 8)
OG IDs: 93523869
Best-hit-arabi-ID: AT3G52930.1
Best-hit-arabi-name: Aldolase superfamily protein
Gorai.009G407400.1.D
PCC=0.496, p=0.031
mRNA Profile
Protein Expression Profile
DPA

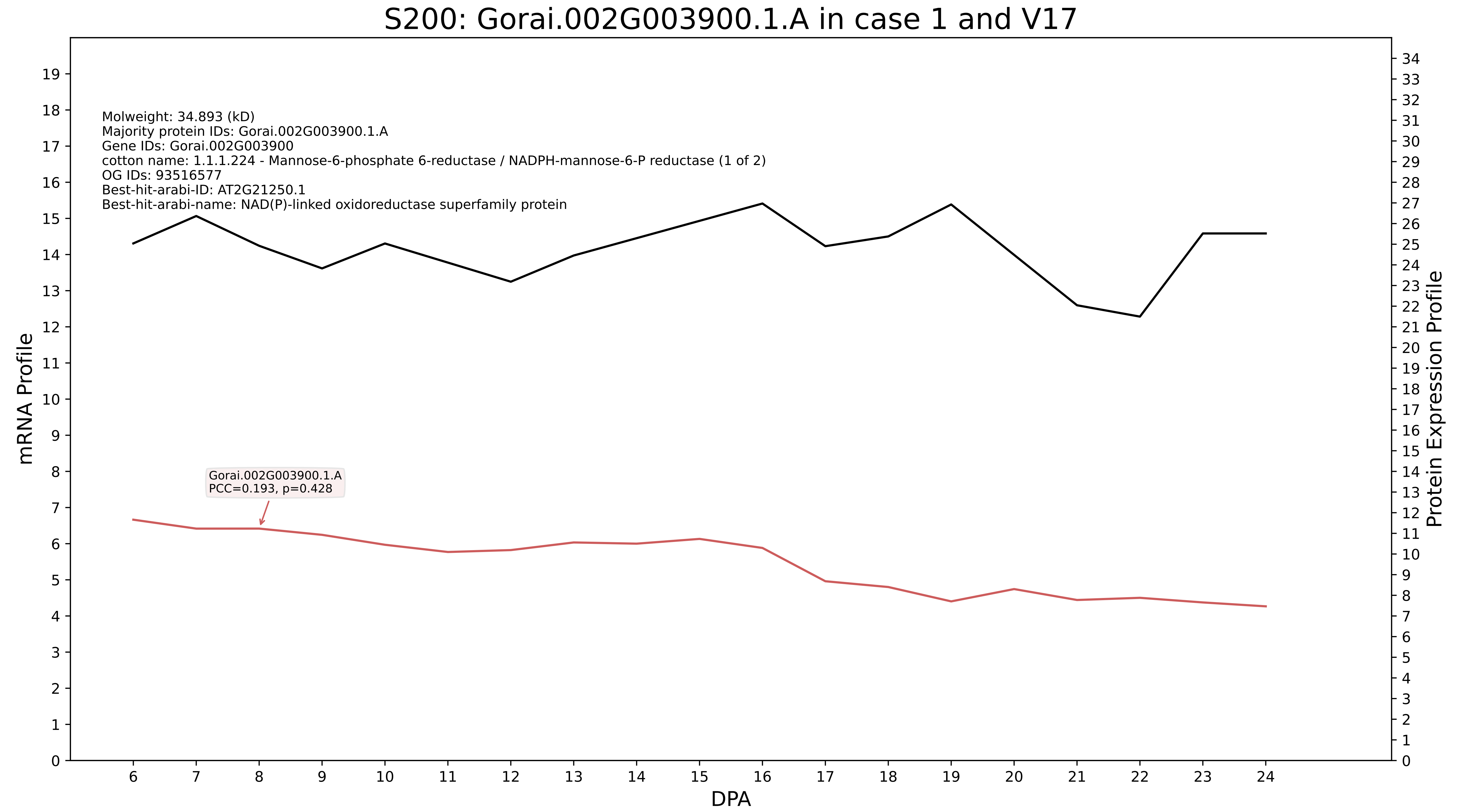
S200: Gorai.002G003900.1.A in case 1 and V17
Molweight: 34.893 (kD)
Majority protein IDs: Gorai.002G003900.1.A
Gene IDs: Gorai.002G003900
cotton name: 1.1.1.224 - Mannose-6-phosphate 6-reductase / NADPH-mannose-6-P reductase (1 of 2)
OG IDs: 93516577
Best-hit-arabi-ID: AT2G21250.1
Best-hit-arabi-name: NAD(P)-linked oxidoreductase superfamily protein
Gorai.002G003900.1.A
PCC=0.193, p=0.428
mRNA Profile
Protein Expression Profile
DPA
0
1
2
3
4
5
6
7
8
9
10
11
12
13
14
15
16
17
18
19
20
21
22
23
24
25
26
27
28
29
30
31
32
33
34

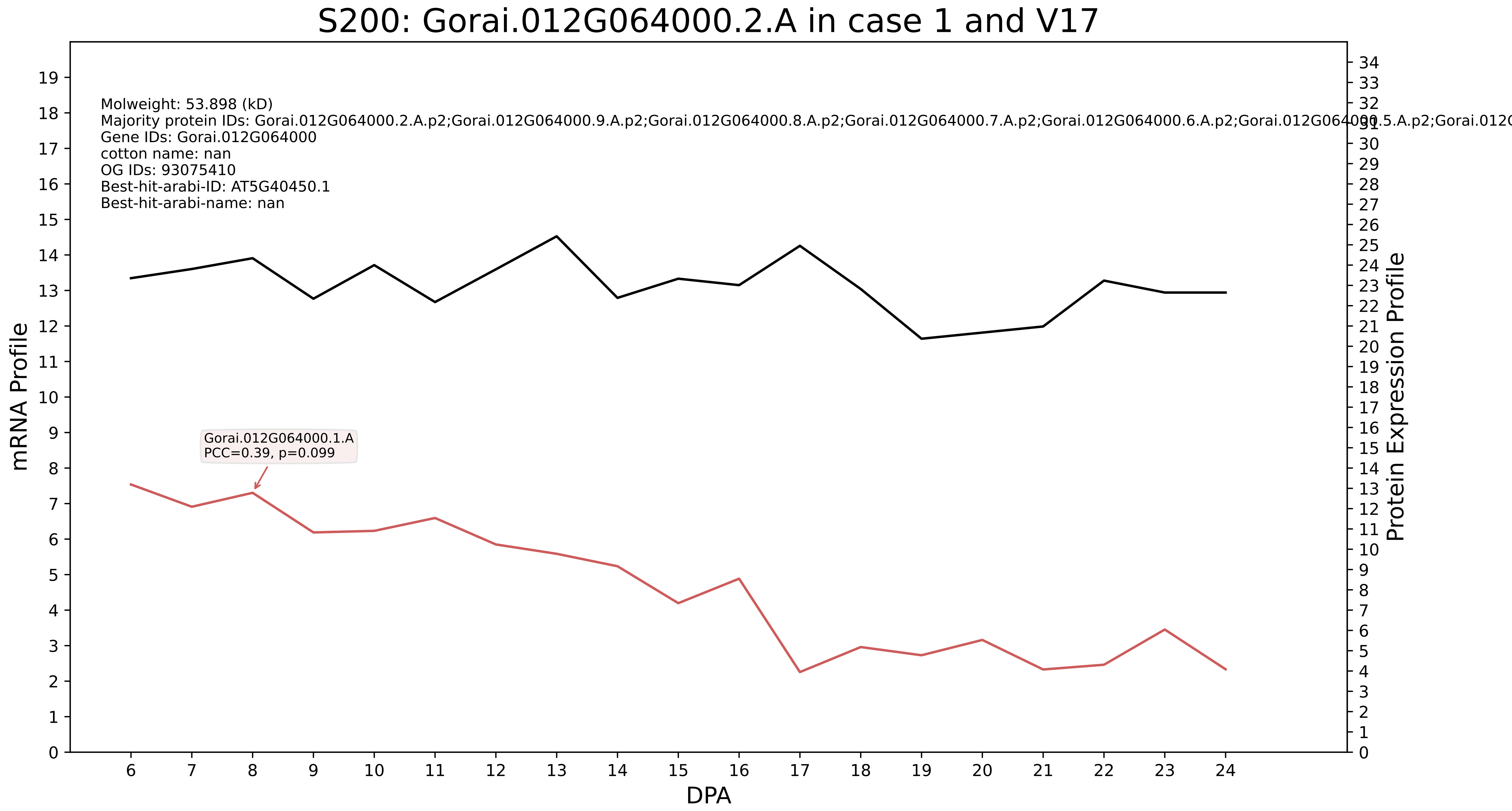

S200: Gorai.012G064000.2.A in case 1 and V17
Molweight: 53.898 (kD)
Majority protein IDs: Gorai.012G064000.2.A.p2;Gorai.012G064000.9.A.p2;Gorai.012G064000.8.A.p2;Gorai.012G064000.7.A.p2;Gorai.012G064000.6.A.p2;Gorai.012G064000.5.A.p2;Gorai.012G
Gene IDs: Gorai.012G064000
cotton name: nan
OG IDs: 93075410
Best-hit-arabi-ID: AT5G40450.1
Best-hit-arabi-name: nan
Gorai.012G064000.1.A
PCC=0.39, p=0.099
mRNA Profile
Protein Expression Profile
DPA

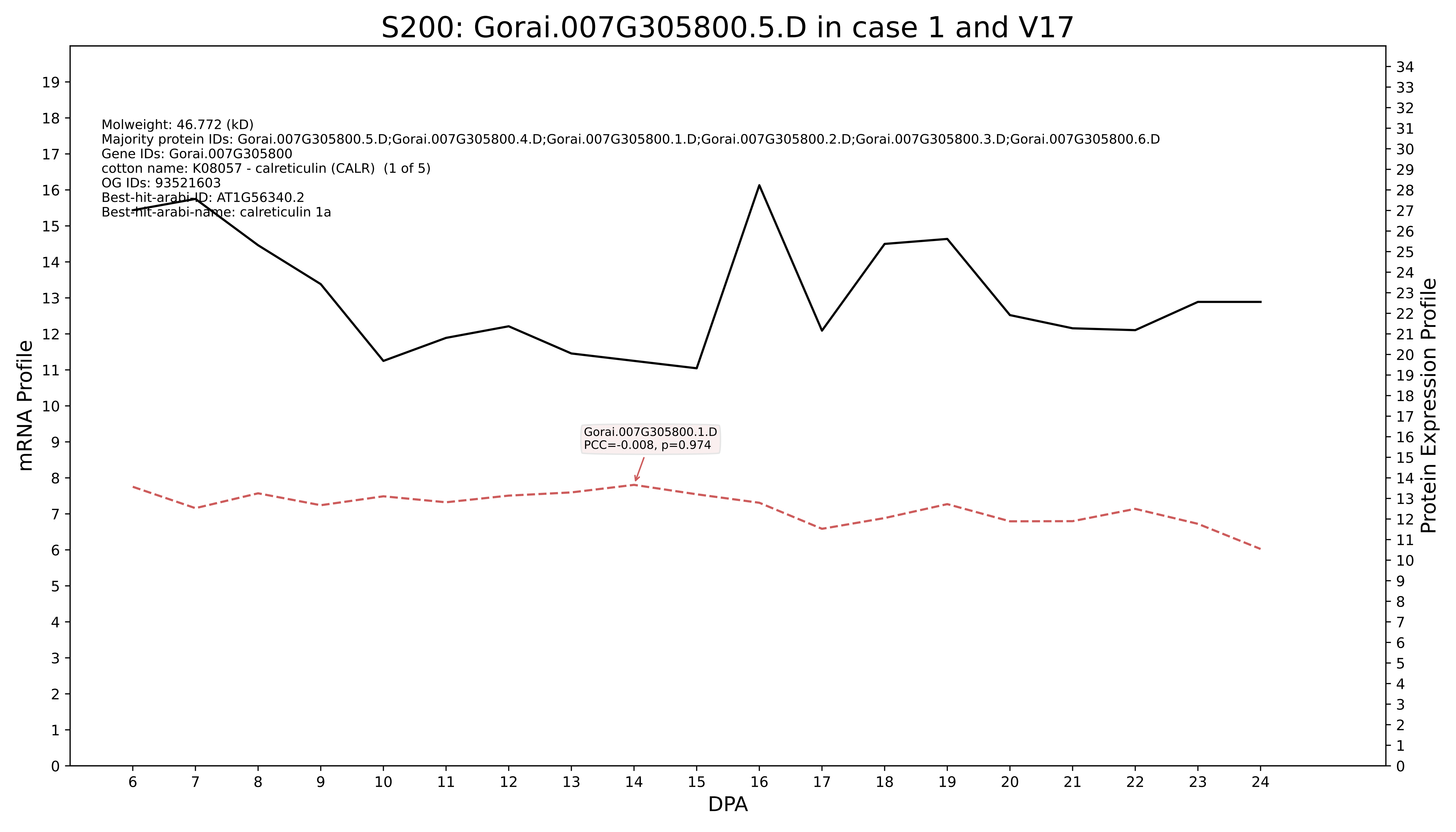
S200: Gorai.007G305800.5.D in case 1 and V17
Molweight: 46.772 (kD)
Majority protein IDs: Gorai.007G305800.5.D;Gorai.007G305800.4.D;Gorai.007G305800.1.D;Gorai.007G305800.2.D;Gorai.007G305800.3.D;Gorai.007G305800.6.D
Gene IDs: Gorai.007G305800
cotton name: K08057 - calreticulin (CALR) (1 of 5)
OG IDs: 93521603
Best-hit-arabi-ID: AT1G56340.2
Best-hit-arabi-name: calreticulin 1a
Gorai.007G305800.1.D
PCC=-0.008, p=0.974
mRNA Profile
Protein Expression Profile
DPA

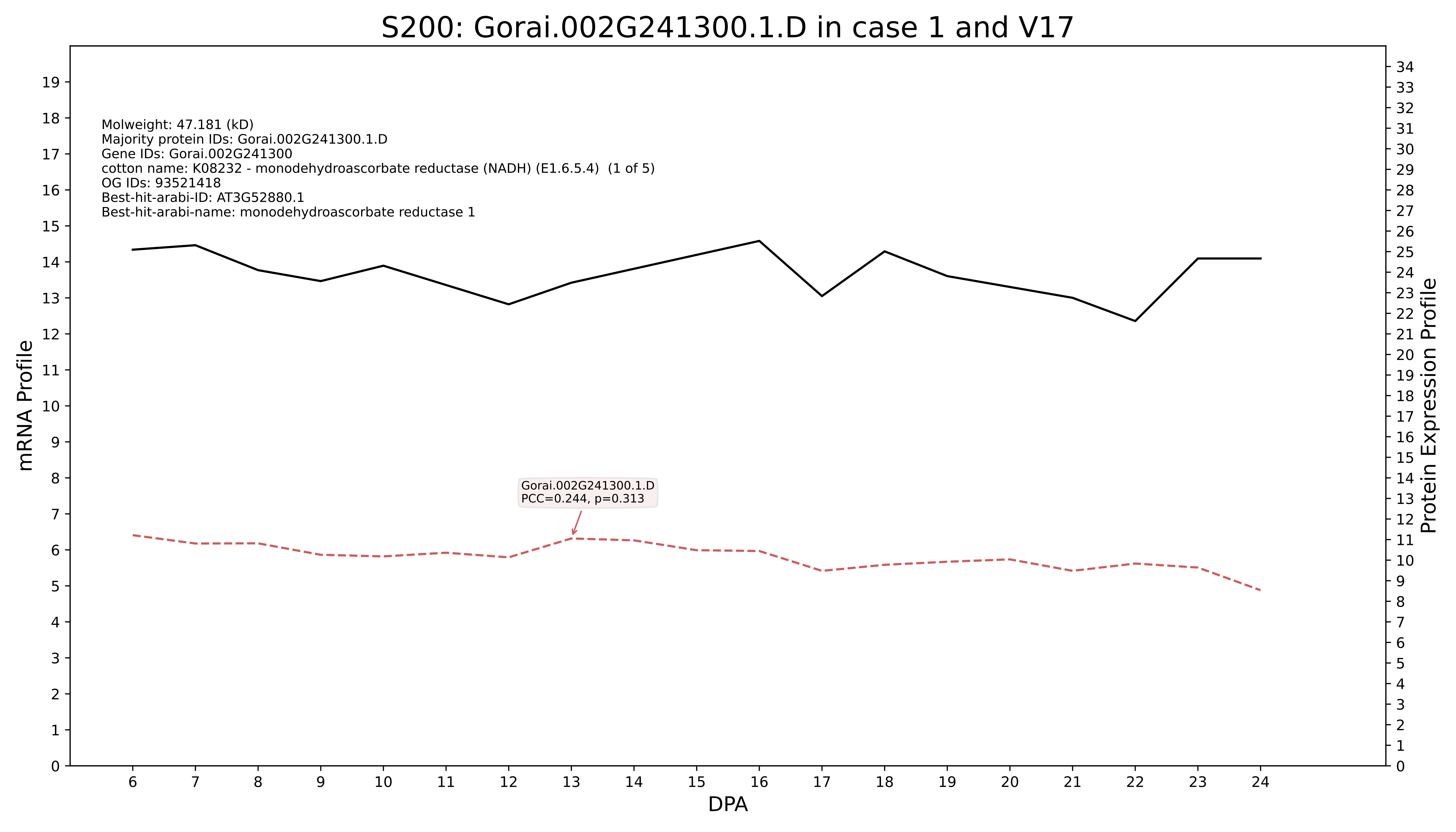
S200: Gorai.002G241300.1.D in case 1 and V17
Molweight: 47.181 (kD)
Majority protein IDs: Gorai.002G241300.1.D
Gene IDs: Gorai.002G241300
cotton name: K08232 - monodehydroascorbate reductase (NADH) (E1.6.5.4) (1 of 5)
OG IDs: 93521418
Best-hit-arabi-ID: AT3G52880.1
Best-hit-arabi-name: monodehydroascorbate reductase 1
Gorai.002G241300.1.D
PCC=0.244, p=0.313
mRNA Profile
Protein Expression Profile
DPA
0
1
2
3
4
5
6
7
8
9
10
11
12
13
14
15
16
17
18
19
20
21
22
23
24
25
26
27
28
29
30
31
32
33
34

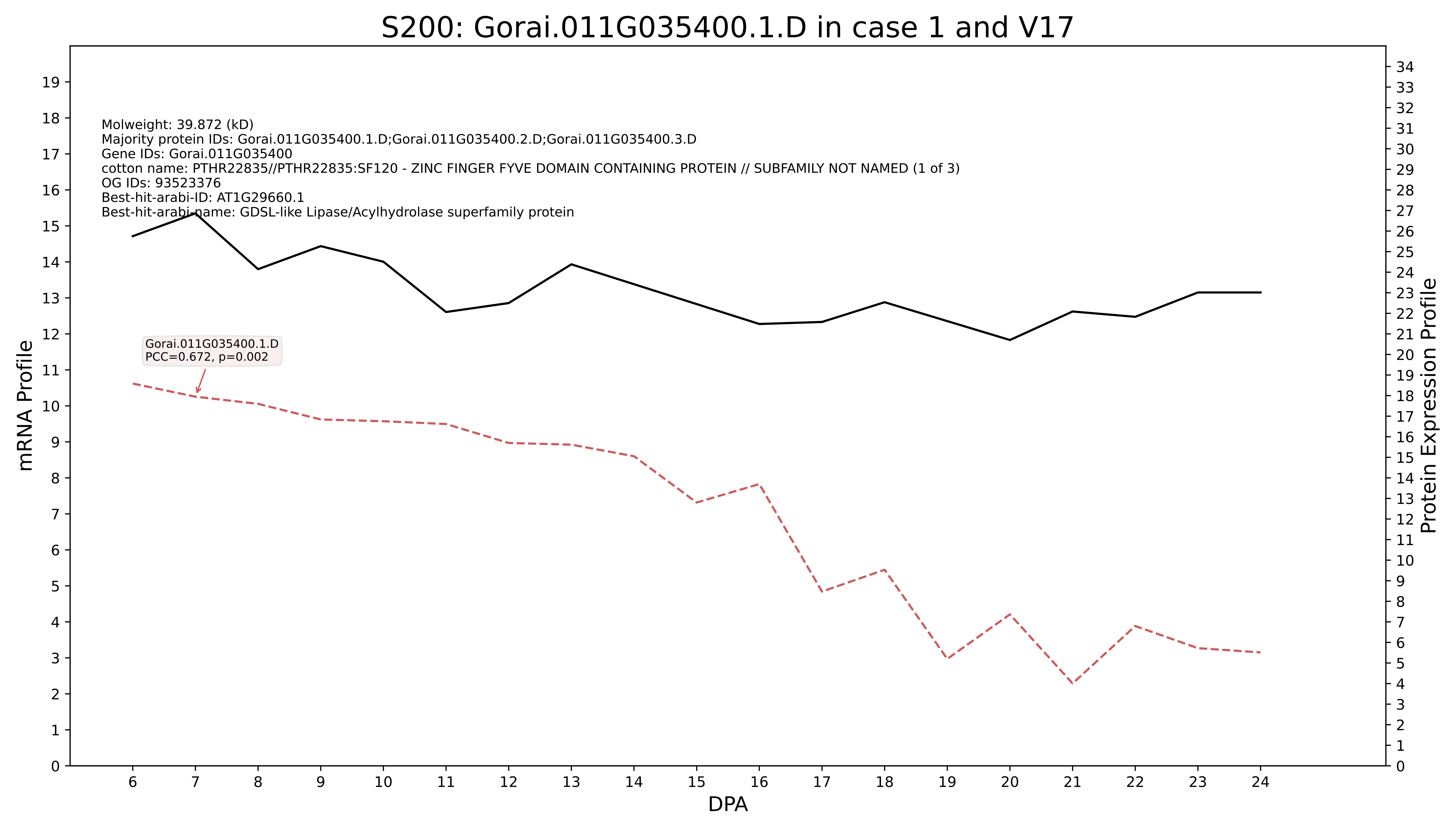

S200: Gorai.011G035400.1.D in case 1 and V17
Molweight: 39.872 (kD)
Majority protein IDs: Gorai.011G035400.1.D;Gorai.011G035400.2.D;Gorai.011G035400.3.D
Gene IDs: Gorai.011G035400
cotton name: PTHR22835//PTHR22835:SF120 - ZINC FINGER FYVE DOMAIN CONTAINING PROTEIN // SUBFAMILY NOT NAMED (1 of 3)
OG IDs: 93523376
Best-hit-arabi-ID: AT1G29660.1
Best-hit-arabi-name: GDSL-like Lipase/Acylhydrolase superfamily protein
Gorai.011G035400.1.D
PCC=0.672, p=0.002
mRNA Profile
Protein Expression Profile
DPA

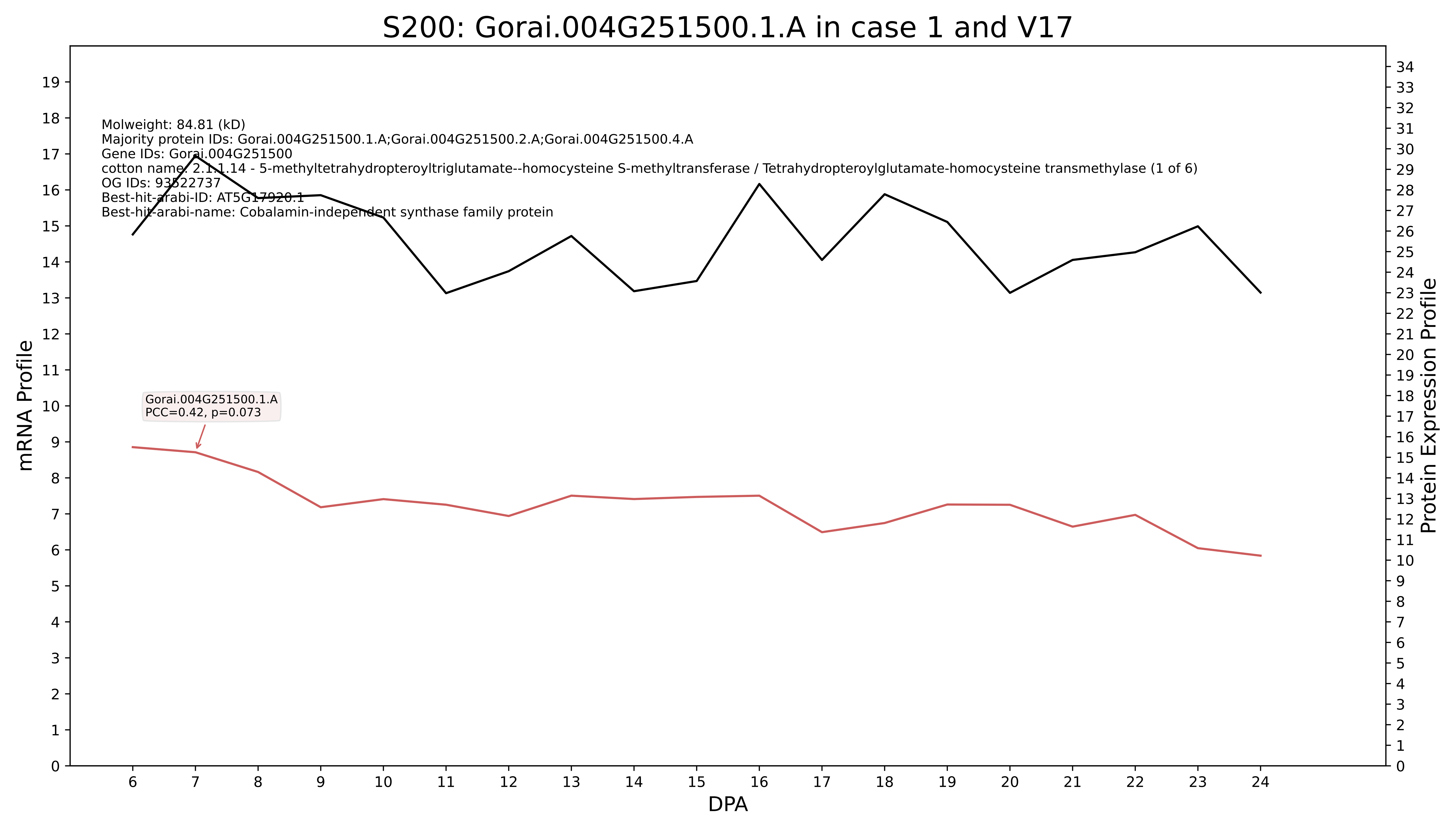

S200: Gorai.004G251500.1.A in case 1 and V17
Molweight: 84.81 (kD)
Majority protein IDs: Gorai.004G251500.1.A;Gorai.004G251500.2.A;Gorai.004G251500.4.A
Gene IDs: Gorai.004G251500
cotton name: 2.1.1.14 - 5-methyltetrahydropteroyltriglutamate--homocysteine S-methyltransferase / Tetrahydropteroylglutamate-homocysteine transmethylase (1 of 6)
OG IDs: 93522737
Best-hit-arabi-ID: AT5G17920.1
Best-hit-arabi-name: Cobalamin-independent synthase family protein
Gorai.004G251500.1.A
PCC=0.42, p=0.073
mRNA Profile
Protein Expression Profile
DPA

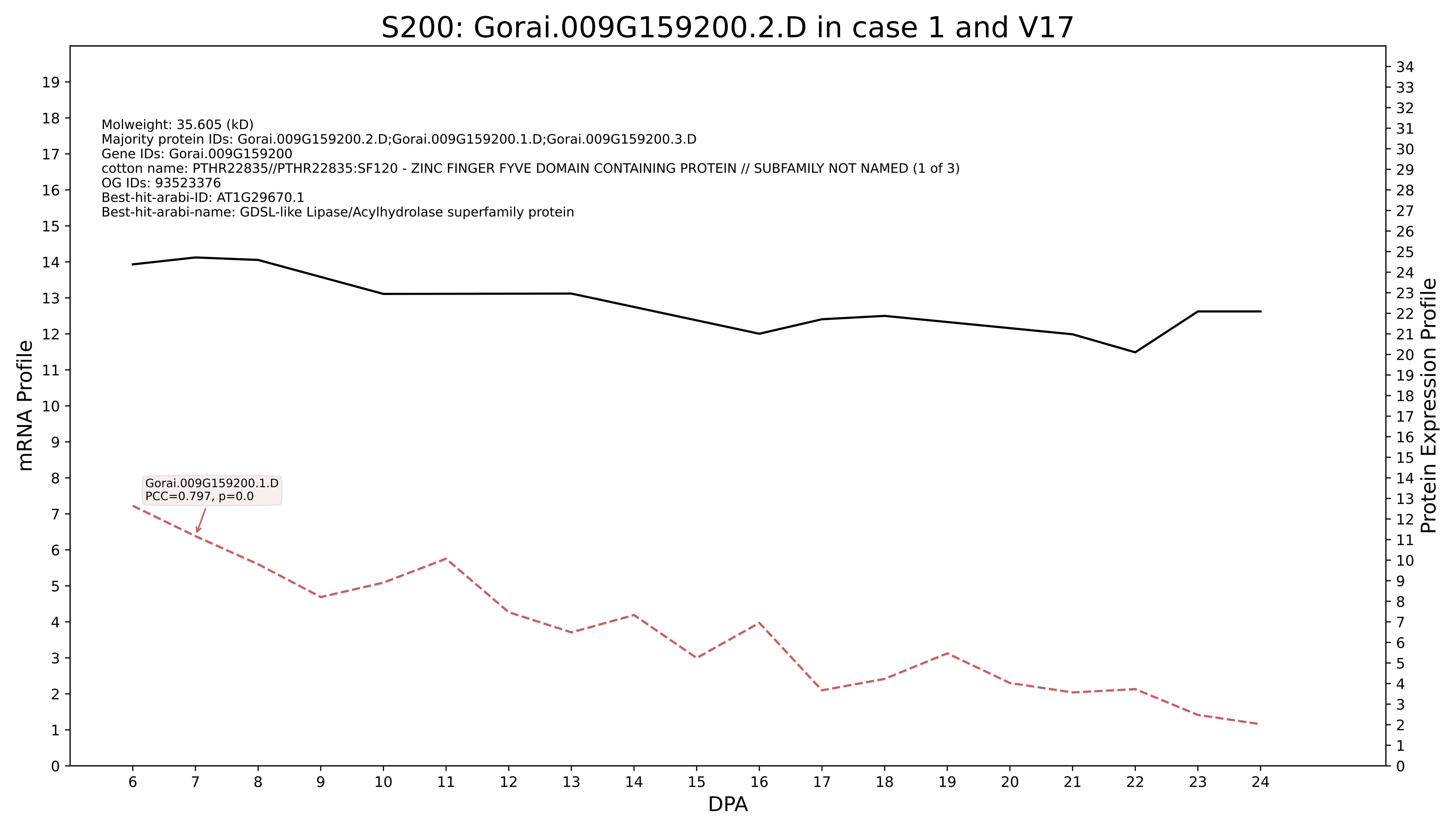
S200: Gorai.009G159200.2.D in case 1 and V17
Molweight: 35.605 (kD)
Majority protein IDs: Gorai.009G159200.2.D;Gorai.009G159200.1.D;Gorai.009G159200.3.D
Gene IDs: Gorai.009G159200
cotton name: PTHR22835//PTHR22835:SF120 - ZINC FINGER FYVE DOMAIN CONTAINING PROTEIN // SUBFAMILY NOT NAMED (1 of 3)
OG IDs: 93523376
Best-hit-arabi-ID: AT1G29670.1
Best-hit-arabi-name: GDSL-like Lipase/Acylhydrolase superfamily protein
Gorai.009G159200.1.D
PCC=0.797, p=0.0
mRNA Profile
Protein Expression Profile
DPA

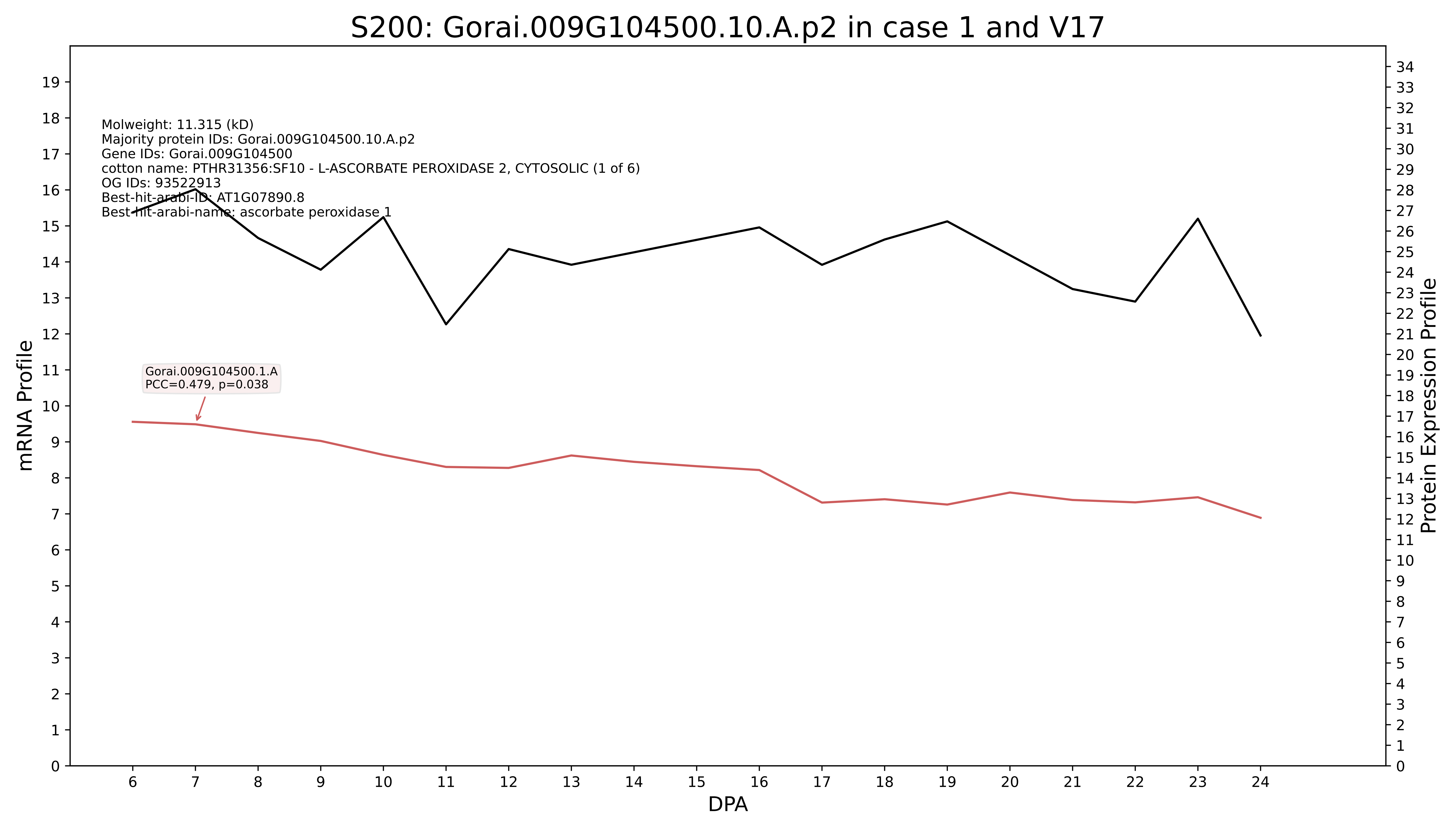
S200: Gorai.009G104500.10.A.p2 in case 1 and V17
Molweight: 11.315 (kD)
Majority protein IDs: Gorai.009G104500.10.A.p2
Gene IDs: Gorai.009G104500
cotton name: PTHR31356:SF10 - L-ASCORBATE PEROXIDASE 2, CYTOSOLIC (1 of 6)
OG IDs: 93522913
Best-hit-arabi-ID: AT1G07890.8
Best-hit-arabi-name: ascorbate peroxidase 1
Gorai.009G104500.1.A
PCC=0.479, p=0.038
mRNA Profile
Protein Expression Profile
DPA
0
1
2
3
4
5
6
7
8
9
10
11
12
13
14
15
16
17
18
19
20
21
22
23
24
25
26
27
28
29
30
31
32
33
34

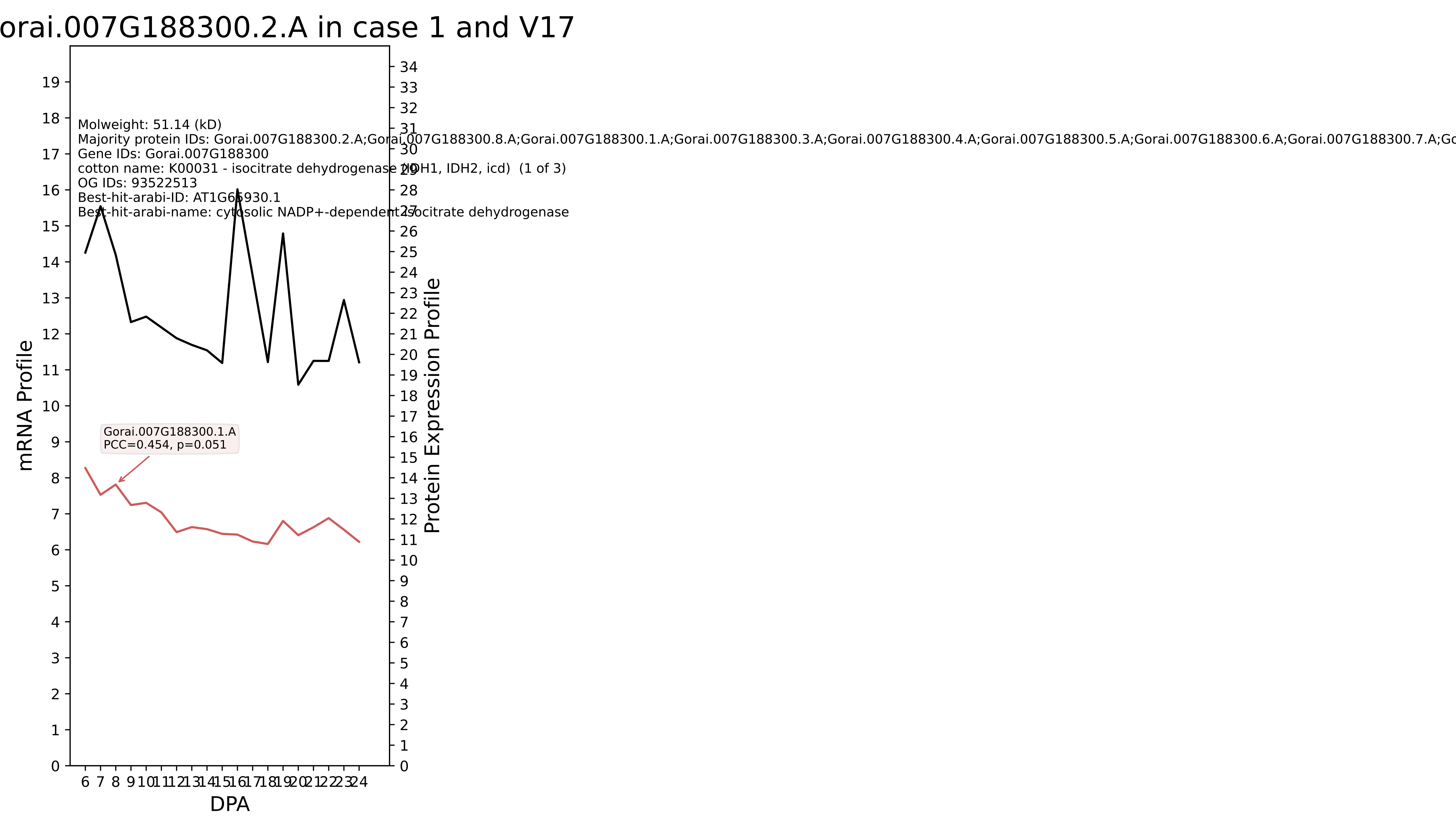

orai.007G188300.2.A in case 1 and V17
Molweight: 51.14 (kD)
Majority protein IDs: Gorai.007G188300.2.A;Gorai.007G188300.8.A;Gorai.007G188300.1.A;Gorai.007G188300.3.A;Gorai.007G188300.4.A;Gorai.007G188300.5.A;Gorai.007G188300.6.A;Gorai.007G188300.7.A;Go
Gene IDs: Gorai.007G188300
cotton name: K00031 - isocitrate dehydrogenase (IDH1, IDH2, icd) (1 of 3)
OG IDs: 93522513
Best-hit-arabi-ID: AT1G65930.1
Best-hit-arabi-name: cytosolic NADP+-dependent isocitrate dehydrogenase
Gorai.007G188300.1.A
PCC=0.454, p=0.051
mRNA Profile
Protein Expression Profile
DPA

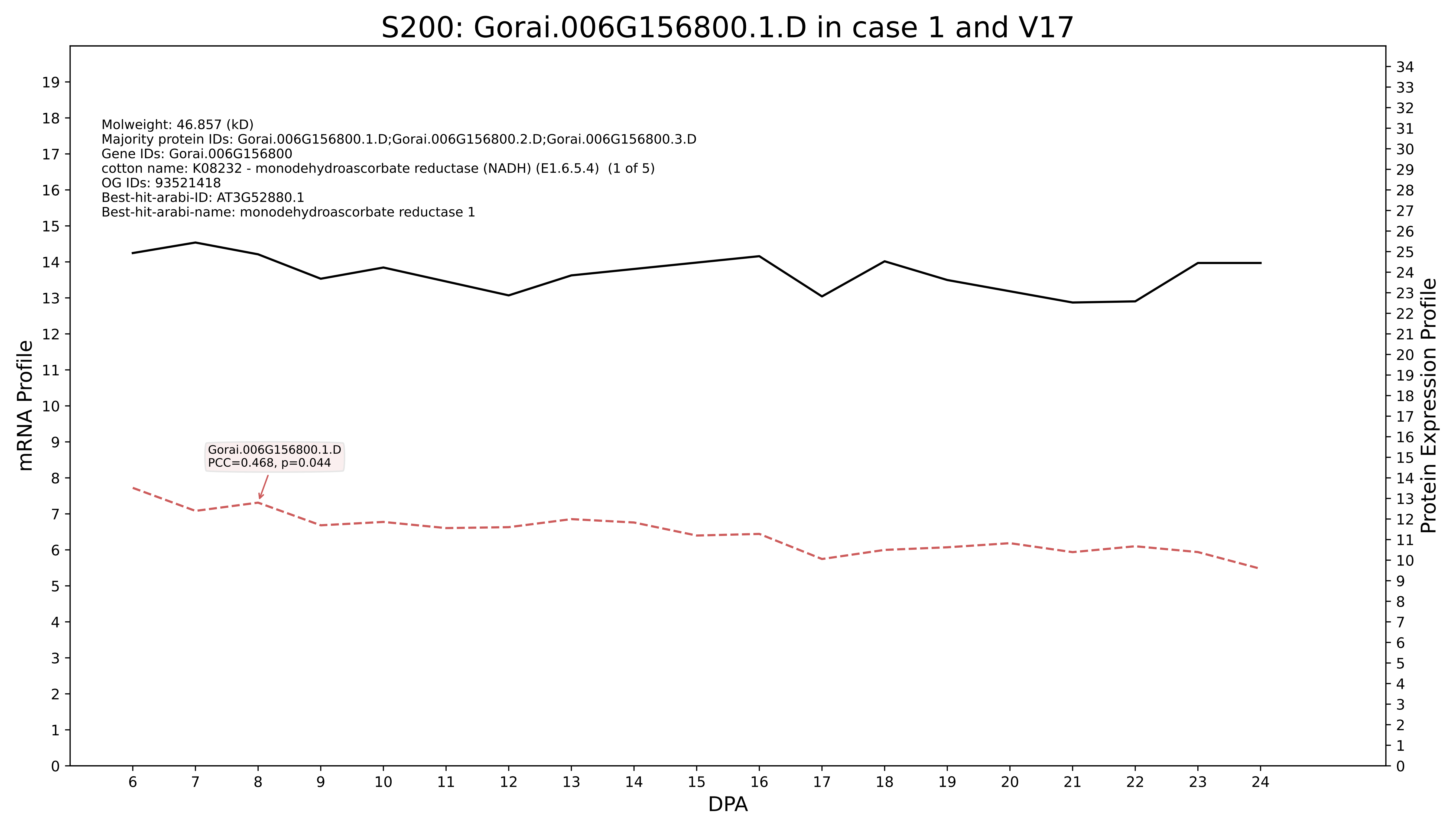

S200: Gorai.006G156800.1.D in case 1 and V17
Molweight: 46.857 (kD)
Majority protein IDs: Gorai.006G156800.1.D;Gorai.006G156800.2.D;Gorai.006G156800.3.D
Gene IDs: Gorai.006G156800
cotton name: K08232 - monodehydroascorbate reductase (NADH) (E1.6.5.4) (1 of 5)
OG IDs: 93521418
Best-hit-arabi-ID: AT3G52880.1
Best-hit-arabi-name: monodehydroascorbate reductase 1
Gorai.006G156800.1.D
PCC=0.468, p=0.044
mRNA Profile
Protein Expression Profile
DPA

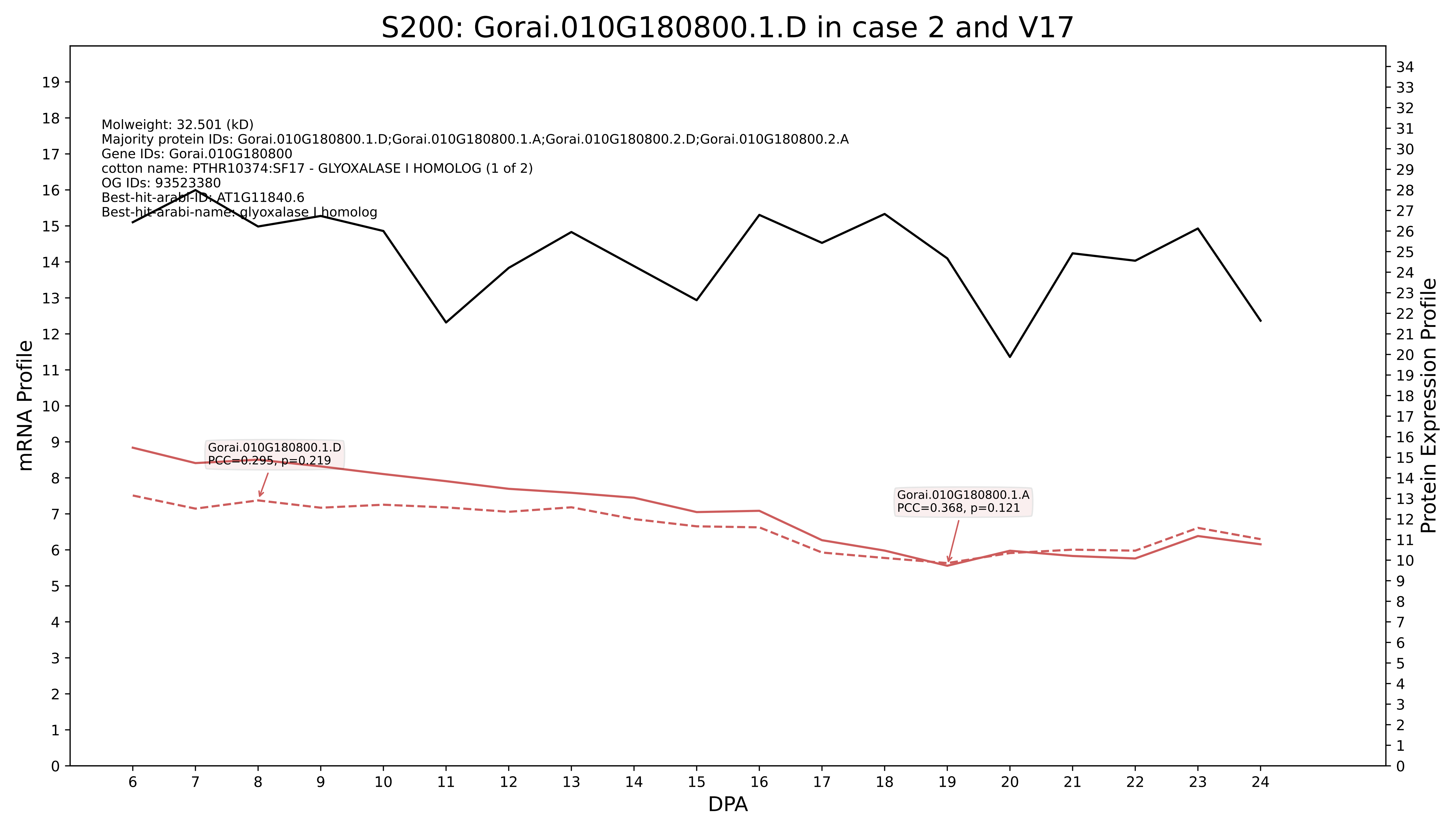
S200: Gorai.010G180800.1.D in case 2 and V17
Molweight: 32.501 (kD)
Majority protein IDs: Gorai.010G180800.1.D;Gorai.010G180800.1.A;Gorai.010G180800.2.D;Gorai.010G180800.2.A
Gene IDs: Gorai.010G180800
cotton name: PTHR10374:SF17 - GLYOXALASE I HOMOLOG (1 of 2)
OG IDs: 93523380
Best-hit-arabi-ID: AT1G11840.6
Best-hit-arabi-name: glyoxalase I homolog
Gorai.010G180800.1.D
PCC=0.295, p=0.219
Gorai.010G180800.1.A
PCC=0.368, p=0.121
mRNA Profile
Protein Expression Profile
DPA
0
1
2
3
4
5
6
7
8
9
10
11
12
13
14
15
16
17
18
19
20
21
22
23
24
25
26
27
28
29
30
31
32
33
34

S200: Gorai.012G027100.5.D in case 2 and V17

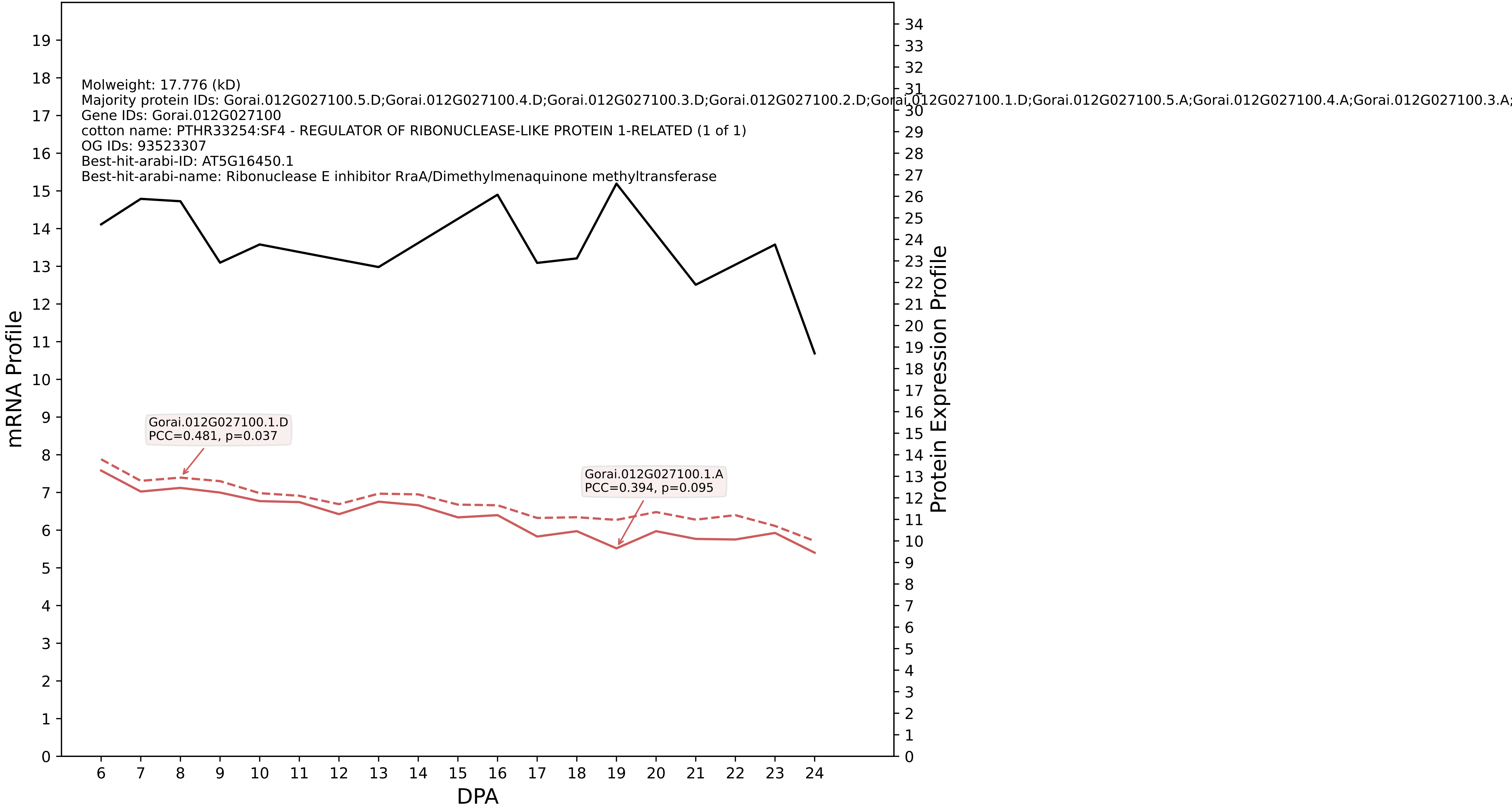

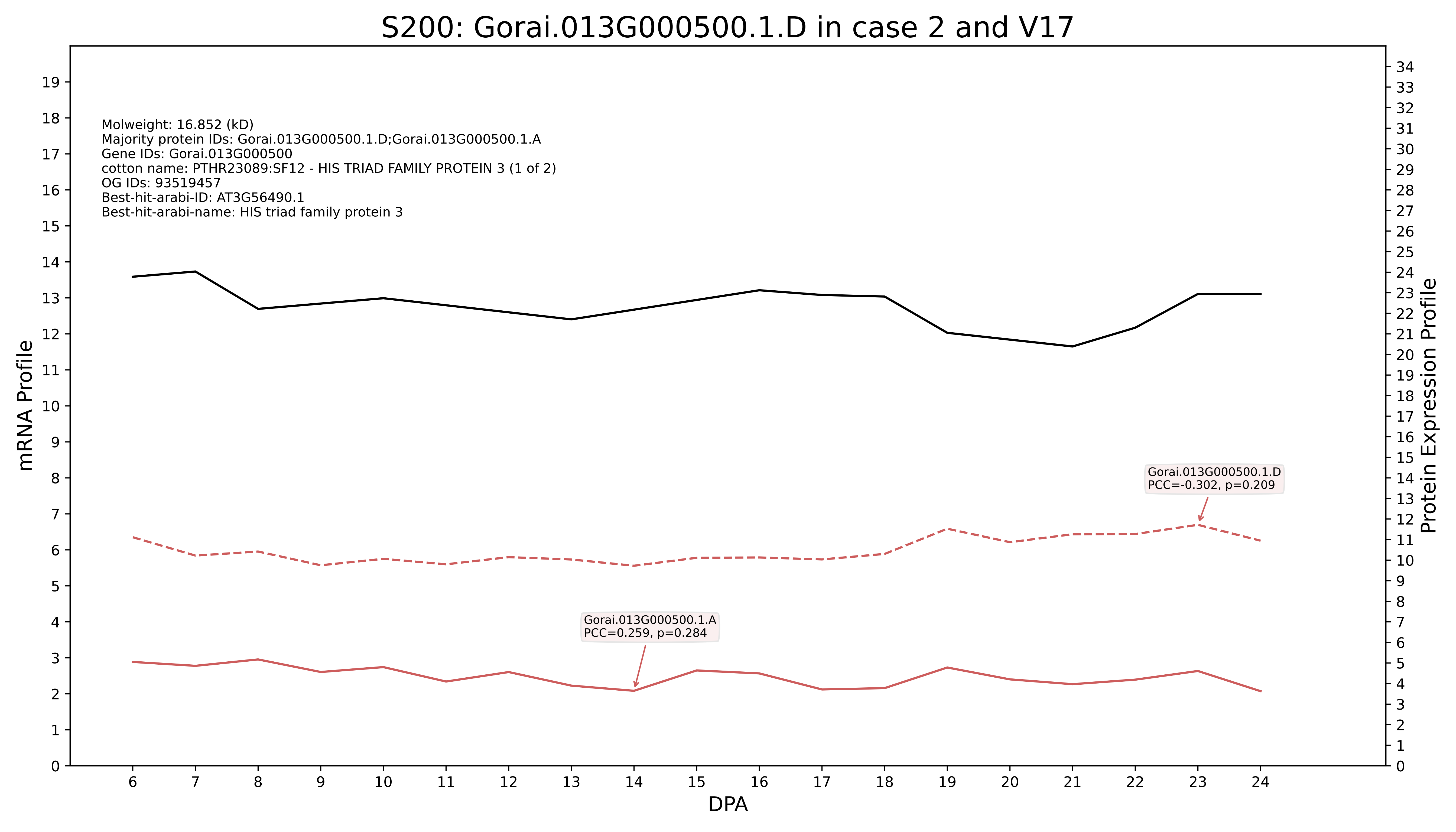
S200: Gorai.013G000500.1.D in case 2 and V17
Molweight: 16.852 (kD)
Majority protein IDs: Gorai.013G000500.1.D;Gorai.013G000500.1.A
Gene IDs: Gorai.013G000500
cotton name: PTHR23089:SF12 - HIS TRIAD FAMILY PROTEIN 3 (1 of 2)
OG IDs: 93519457
Best-hit-arabi-ID: AT3G56490.1
Best-hit-arabi-name: HIS triad family protein 3
Gorai.013G000500.1.D
PCC=-0.302, p=0.209
Gorai.013G000500.1.A
PCC=0.259, p=0.284
mRNA Profile
Protein Expression Profile
DPA

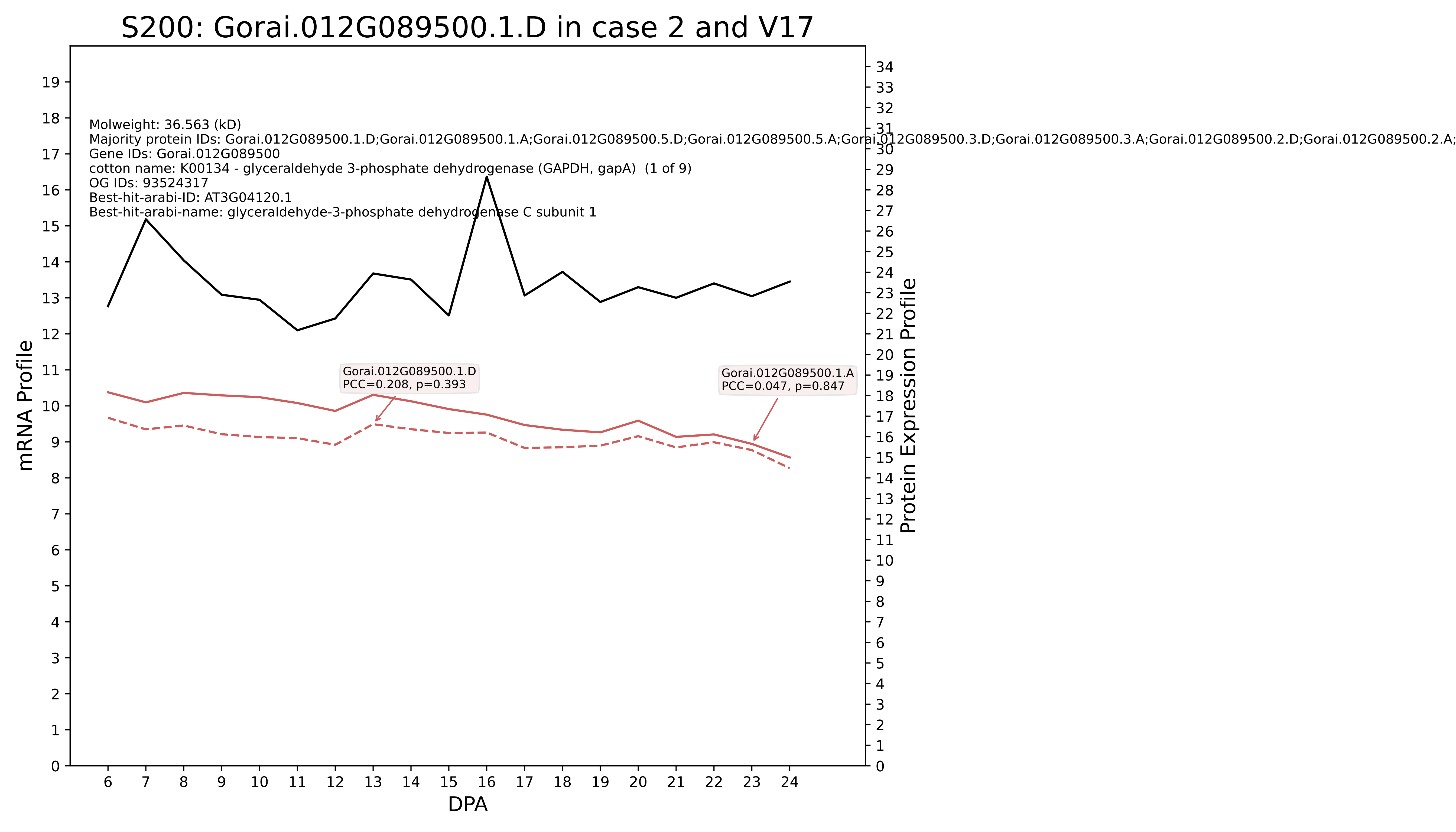
S200: Gorai.012G089500.1.D in case 2 and V17
Molweight: 36.563 (kD)
Majority protein IDs: Gorai.012G089500.1.D;Gorai.012G089500.1.A;Gorai.012G089500.5.D;Gorai.012G089500.5.A;Gorai.012G089500.3.D;Gorai.012G089500.3.A;Gorai.012G089500.2.D;Gorai.012G089500.2.A;
Gene IDs: Gorai.012G089500
cotton name: K00134 - glyceraldehyde 3-phosphate dehydrogenase (GAPDH, gapA) (1 of 9)
OG IDs: 93524317
Best-hit-arabi-ID: AT3G04120.1
Best-hit-arabi-name: glyceraldehyde-3-phosphate dehydrogenase C subunit 1
Gorai.012G089500.1.D
PCC=0.208, p=0.393
Gorai.012G089500.1.A
PCC=0.047, p=0.847
mRNA Profile
Protein Expression Profile
DPA

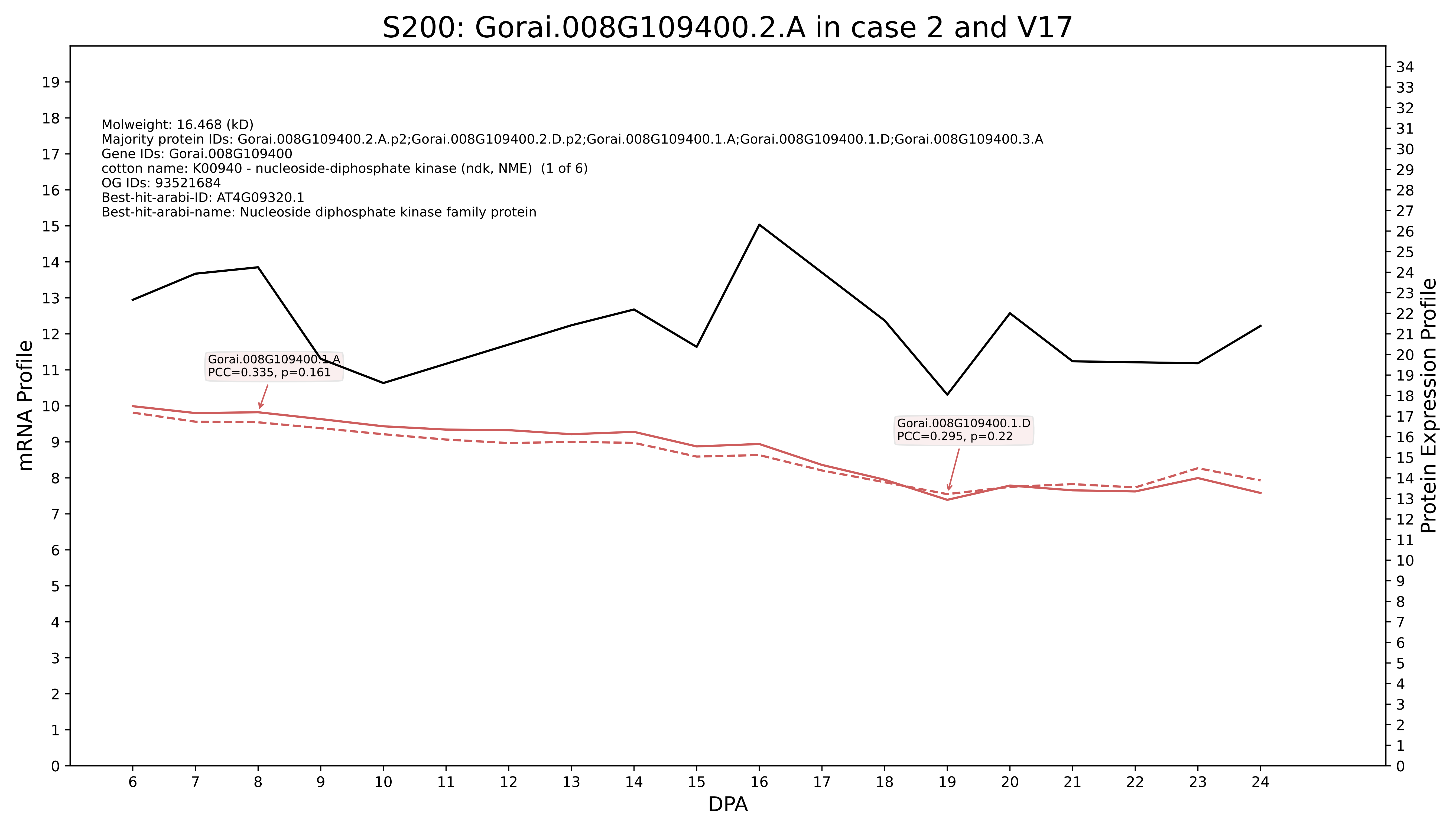
S200: Gorai.008G109400.2.A in case 2 and V17
Molweight: 16.468 (kD)
Majority protein IDs: Gorai.008G109400.2.A.p2;Gorai.008G109400.2.D.p2;Gorai.008G109400.1.A;Gorai.008G109400.1.D;Gorai.008G109400.3.A
Gene IDs: Gorai.008G109400
cotton name: K00940 - nucleoside-diphosphate kinase (ndk, NME) (1 of 6)
OG IDs: 93521684
Best-hit-arabi-ID: AT4G09320.1
Best-hit-arabi-name: Nucleoside diphosphate kinase family protein
Gorai.008G109400.1.A
PCC=0.335, p=0.161
Gorai.008G109400.1.D
PCC=0.295, p=0.22
mRNA Profile
Protein Expression Profile
DPA

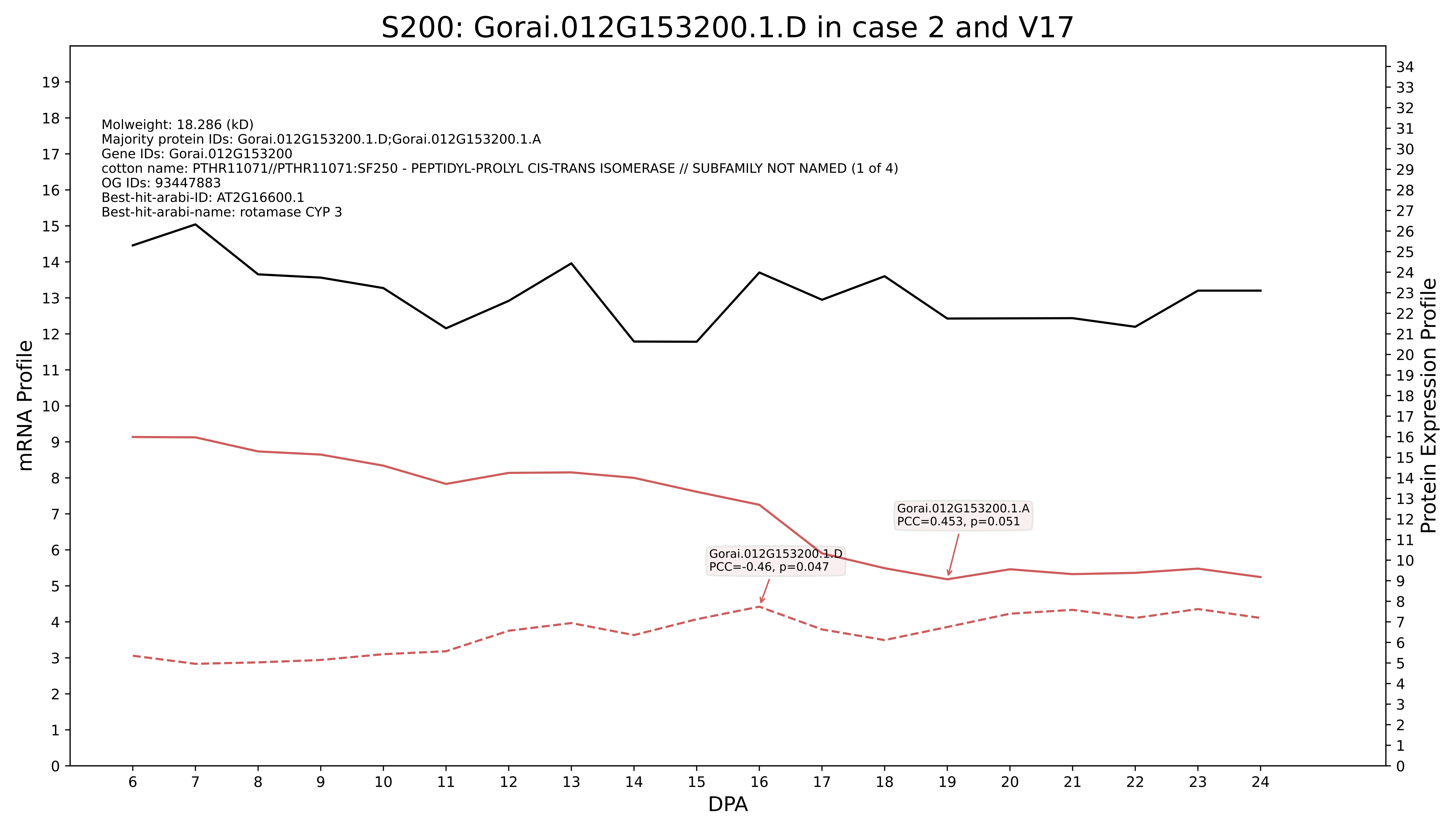
S200: Gorai.012G153200.1.D in case 2 and V17
Molweight: 18.286 (kD)
Majority protein IDs: Gorai.012G153200.1.D;Gorai.012G153200.1.A
Gene IDs: Gorai.012G153200
cotton name: PTHR11071//PTHR11071:SF250 - PEPTIDYL-PROLYL CIS-TRANS ISOMERASE // SUBFAMILY NOT NAMED (1 of 4)
OG IDs: 93447883
Best-hit-arabi-ID: AT2G16600.1
Best-hit-arabi-name: rotamase CYP 3
Gorai.012G153200.1.A
PCC=0.453, p=0.051
Gorai.012G153200.1.D
PCC=-0.46, p=0.047
mRNA Profile
Protein Expression Profile
DPA

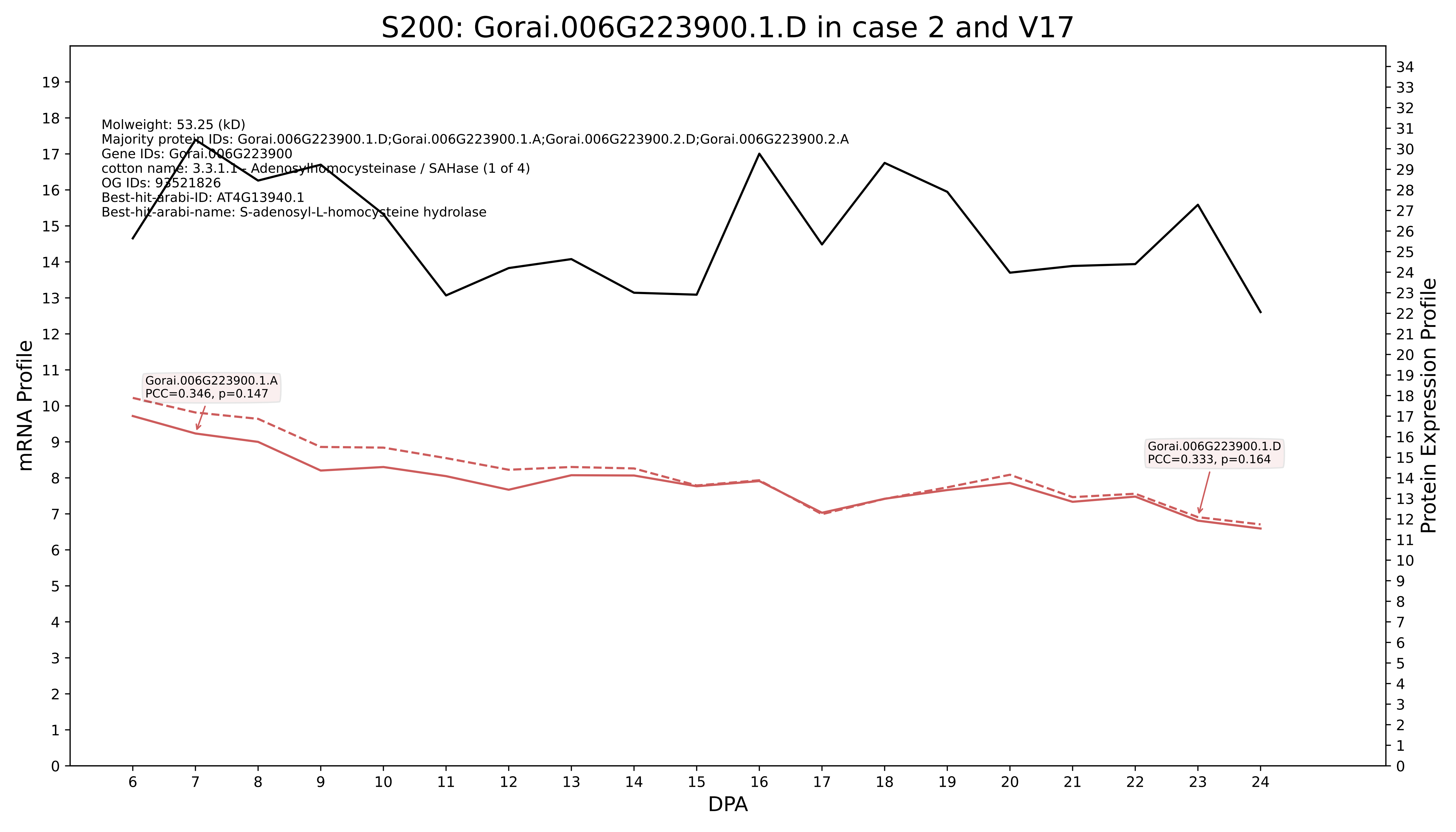
S200: Gorai.006G223900.1.D in case 2 and V17
Molweight: 53.25 (kD)
Majority protein IDs: Gorai.006G223900.1.D;Gorai.006G223900.1.A;Gorai.006G223900.2.D;Gorai.006G223900.2.A
Gene IDs: Gorai.006G223900
cotton name: 3.3.1.1 - Adenosylhomocysteinase / SAHase (1 of 4)
OG IDs: 93521826
Best-hit-arabi-ID: AT4G13940.1
Best-hit-arabi-name: S-adenosyl-L-homocysteine hydrolase
Gorai.006G223900.1.A
PCC=0.346, p=0.147
Gorai.006G223900.1.D
PCC=0.333, p=0.164
mRNA Profile
Protein Expression Profile
DPA

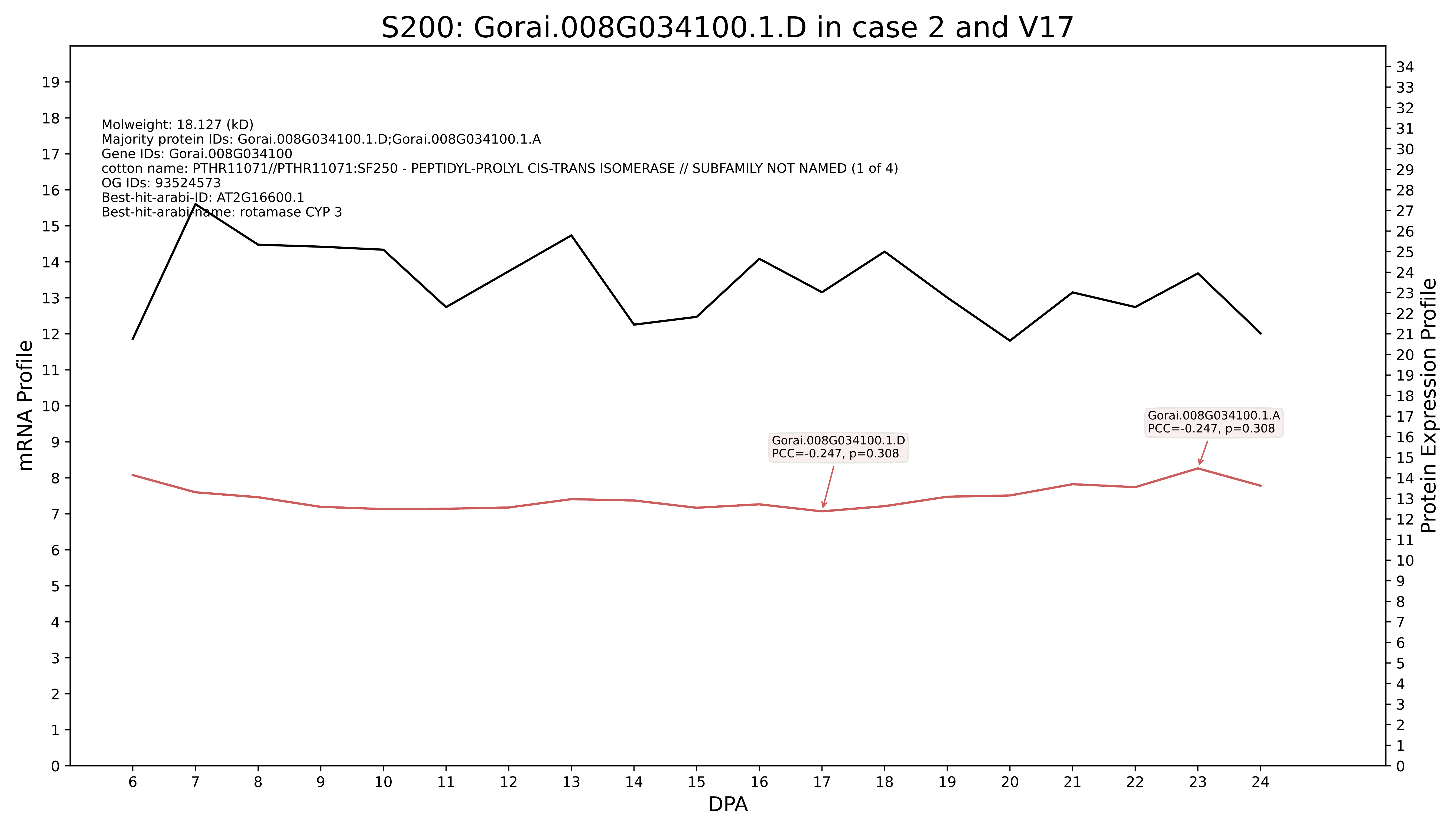
S200: Gorai.008G034100.1.D in case 2 and V17
Molweight: 18.127 (kD)
Majority protein IDs: Gorai.008G034100.1.D;Gorai.008G034100.1.A
Gene IDs: Gorai.008G034100
cotton name: PTHR11071//PTHR11071:SF250 - PEPTIDYL-PROLYL CIS-TRANS ISOMERASE // SUBFAMILY NOT NAMED (1 of 4)
OG IDs: 93524573
Best-hit-arabi-ID: AT2G16600.1
Best-hit-arabi-name: rotamase CYP 3
Gorai.008G034100.1.D
PCC=-0.247, p=0.308
Gorai.008G034100.1.A
PCC=-0.247, p=0.308
mRNA Profile
Protein Expression Profile
DPA
0
1
2
3
4
5
6
7
8
9
10
11
12
13
14
15
16
17
18
19
20
21
22
23
24
25
26
27
28
29
30
31
32
33
34

S200: Gorai.005G259200.1.D in case 3 and V17

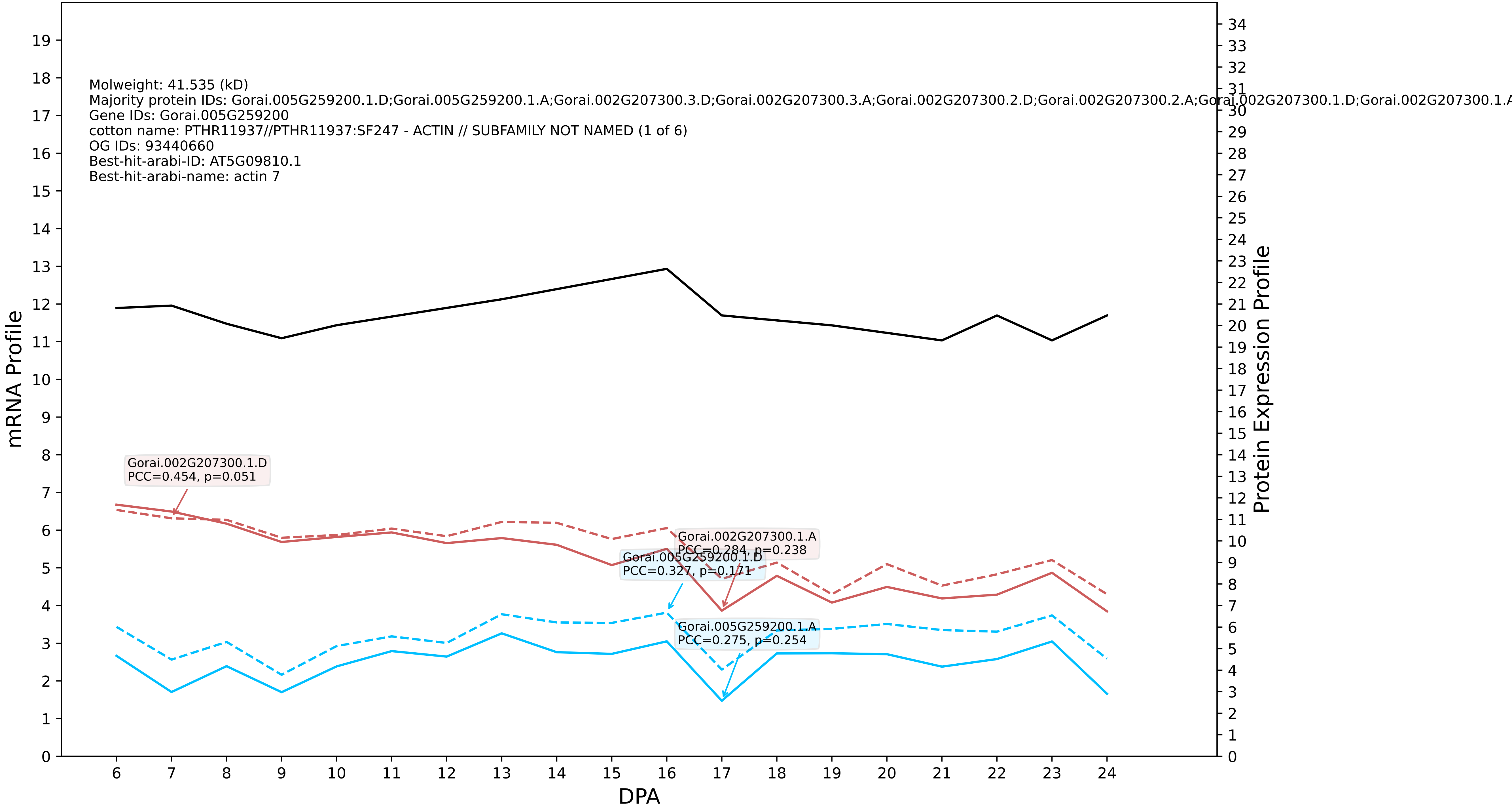

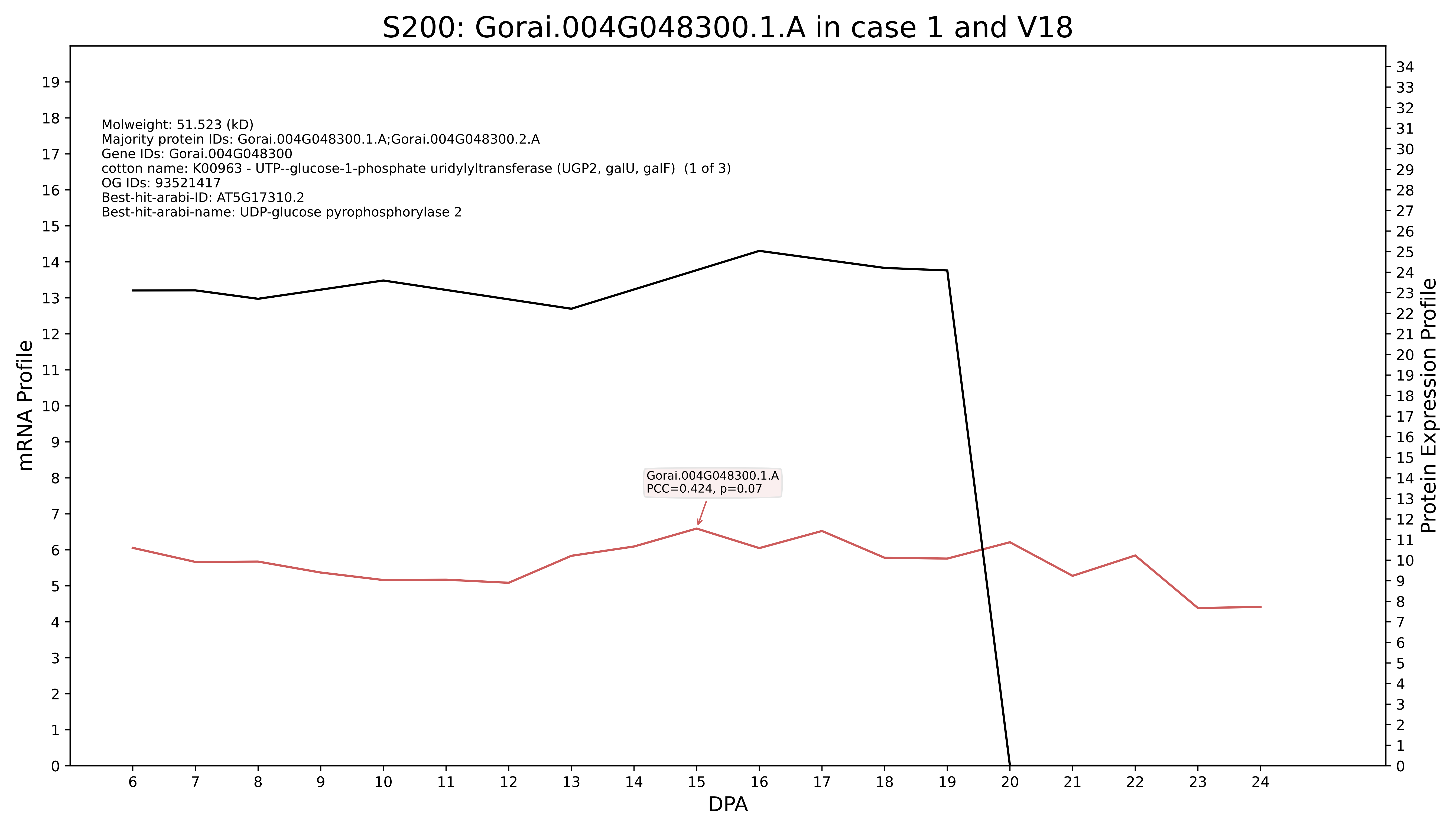
S200: Gorai.004G048300.1.A in case 1 and V18
Molweight: 51.523 (kD)
Majority protein IDs: Gorai.004G048300.1.A;Gorai.004G048300.2.A
Gene IDs: Gorai.004G048300
cotton name: K00963 - UTP--glucose-1-phosphate uridylyltransferase (UGP2, galU, galF) (1 of 3)
OG IDs: 93521417
Best-hit-arabi-ID: AT5G17310.2
Best-hit-arabi-name: UDP-glucose pyrophosphorylase 2
Gorai.004G048300.1.A
PCC=0.424, p=0.07
mRNA Profile
Protein Expression Profile
DPA

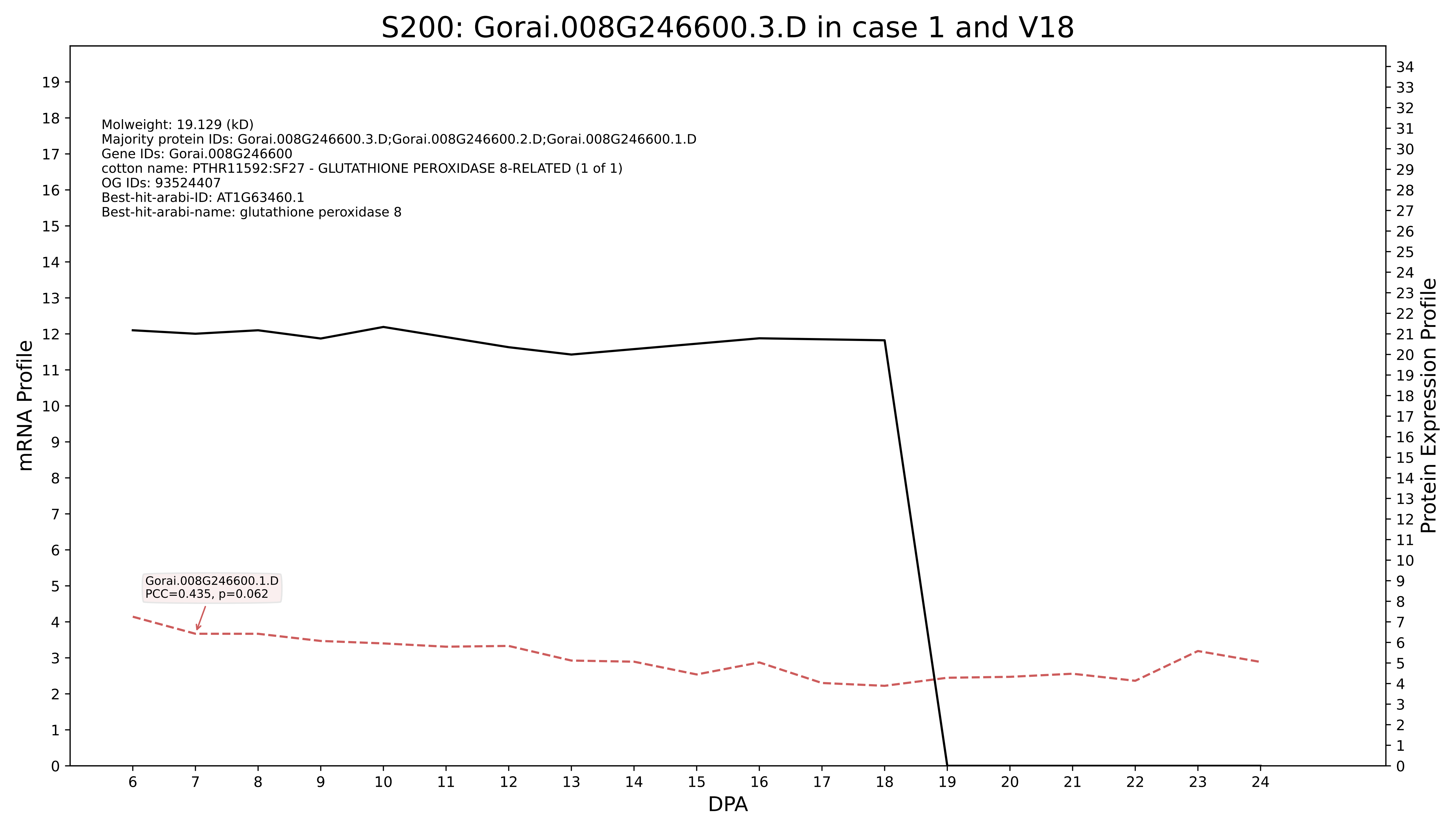

S200: Gorai.008G246600.3.D in case 1 and V18
Molweight: 19.129 (kD)
Majority protein IDs: Gorai.008G246600.3.D;Gorai.008G246600.2.D;Gorai.008G246600.1.D
Gene IDs: Gorai.008G246600
cotton name: PTHR11592:SF27 - GLUTATHIONE PEROXIDASE 8-RELATED (1 of 1)
OG IDs: 93524407
Best-hit-arabi-ID: AT1G63460.1
Best-hit-arabi-name: glutathione peroxidase 8
Gorai.008G246600.1.D
PCC=0.435, p=0.062
mRNA Profile
Protein Expression Profile
DPA

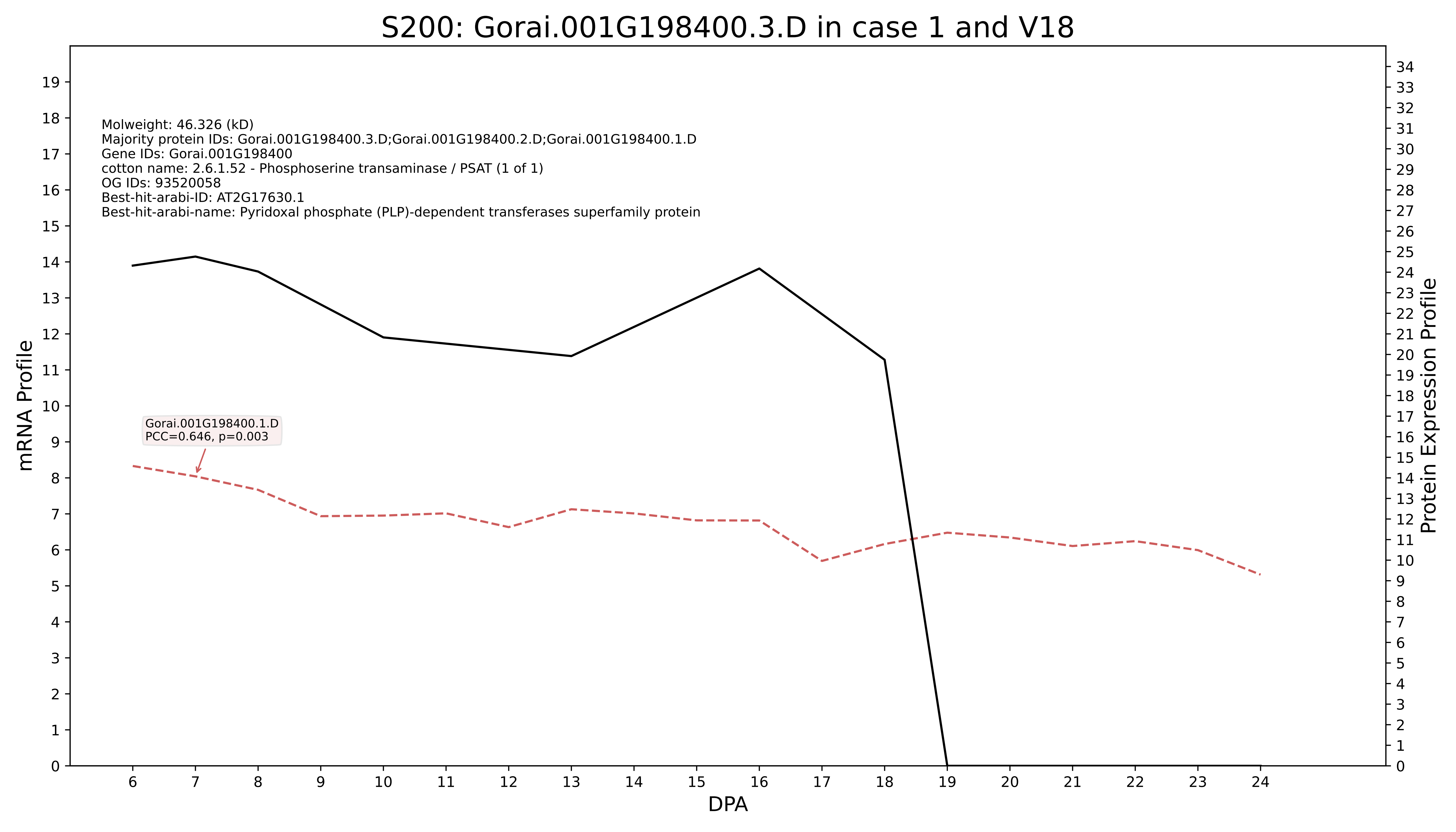
S200: Gorai.001G198400.3.D in case 1 and V18
Molweight: 46.326 (kD)
Majority protein IDs: Gorai.001G198400.3.D;Gorai.001G198400.2.D;Gorai.001G198400.1.D
Gene IDs: Gorai.001G198400
cotton name: 2.6.1.52 - Phosphoserine transaminase / PSAT (1 of 1)
OG IDs: 93520058
Best-hit-arabi-ID: AT2G17630.1
Best-hit-arabi-name: Pyridoxal phosphate (PLP)-dependent transferases superfamily protein
Gorai.001G198400.1.D
PCC=0.646, p=0.003
mRNA Profile
Protein Expression Profile
DPA

[illegible]00: Gorai.012G070300.5.D in case 2 and V18

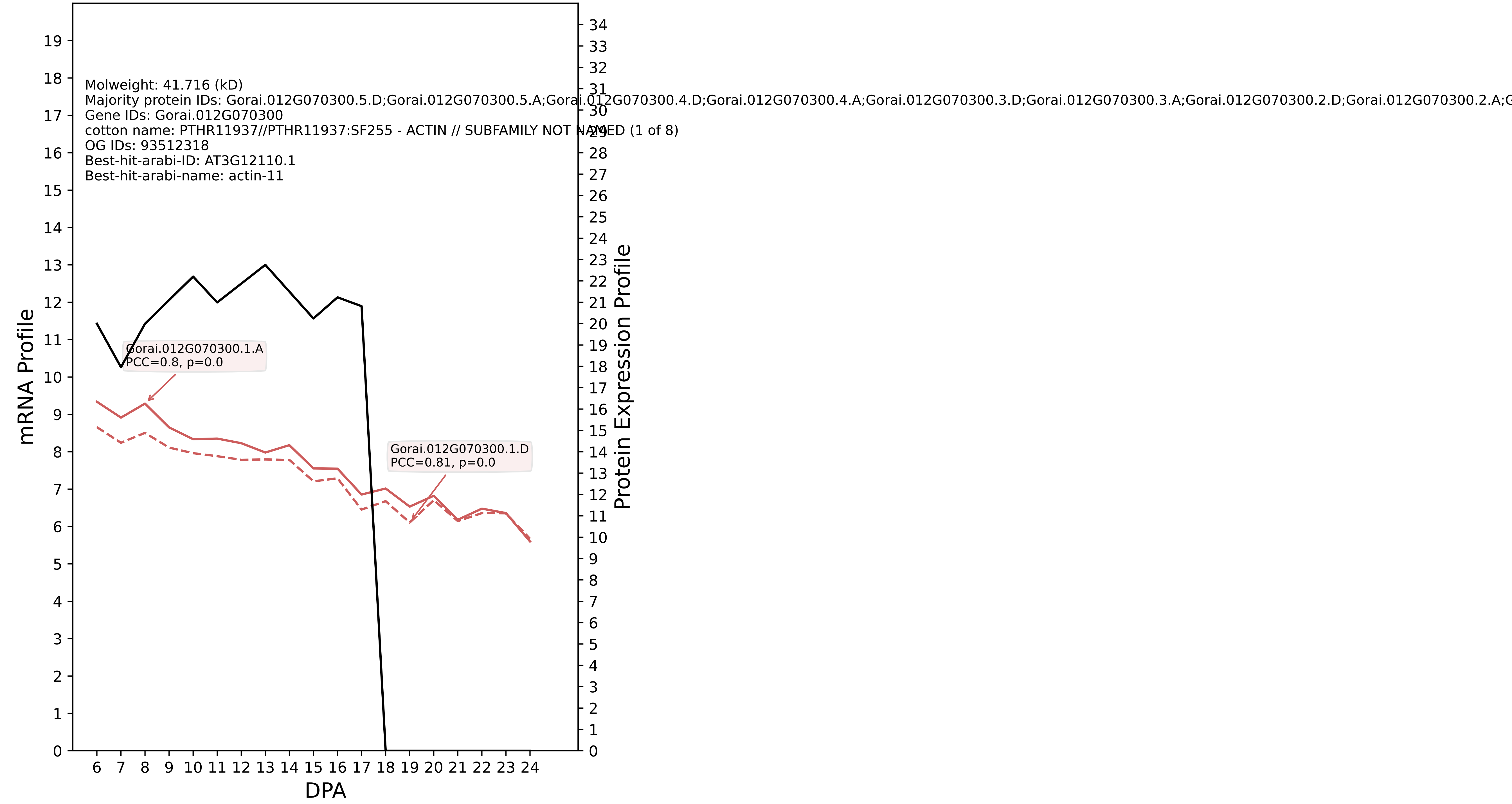

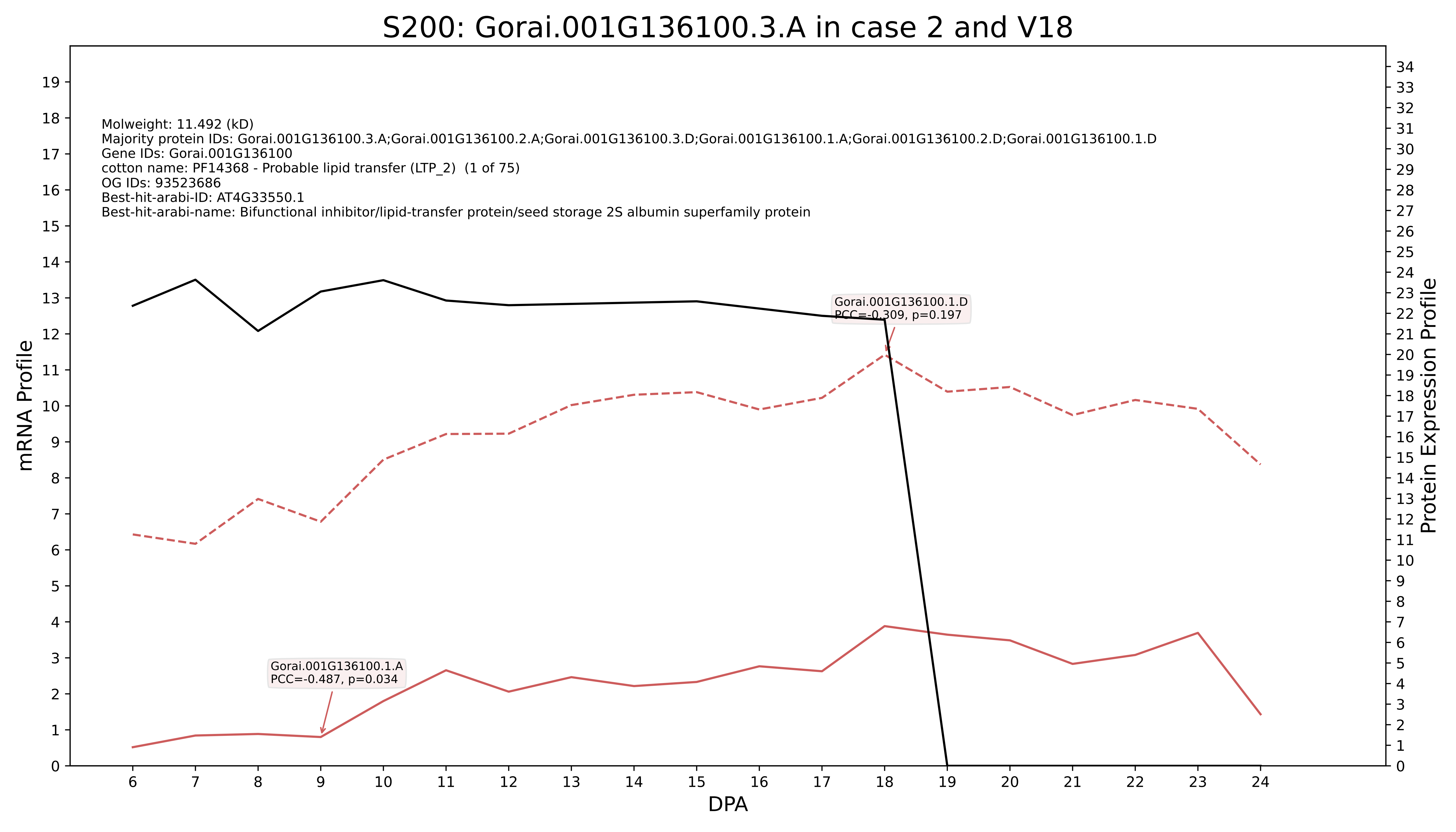

S200: Gorai.001G136100.3.A in case 2 and V18
Molweight: 11.492 (kD)
Majority protein IDs: Gorai.001G136100.3.A;Gorai.001G136100.2.A;Gorai.001G136100.3.D;Gorai.001G136100.1.A;Gorai.001G136100.2.D;Gorai.001G136100.1.D
Gene IDs: Gorai.001G136100
cotton name: PF14368 - Probable lipid transfer (LTP_2) (1 of 75)
OG IDs: 93523686
Best-hit-arabi-ID: AT4G33550.1
Best-hit-arabi-name: Bifunctional inhibitor/lipid-transfer protein/seed storage 2S albumin superfamily protein
Gorai.001G136100.1.D
PCC=-0.309, p=0.197
Gorai.001G136100.1.A
PCC=-0.487, p=0.034
mRNA Profile
Protein Expression Profile
DPA

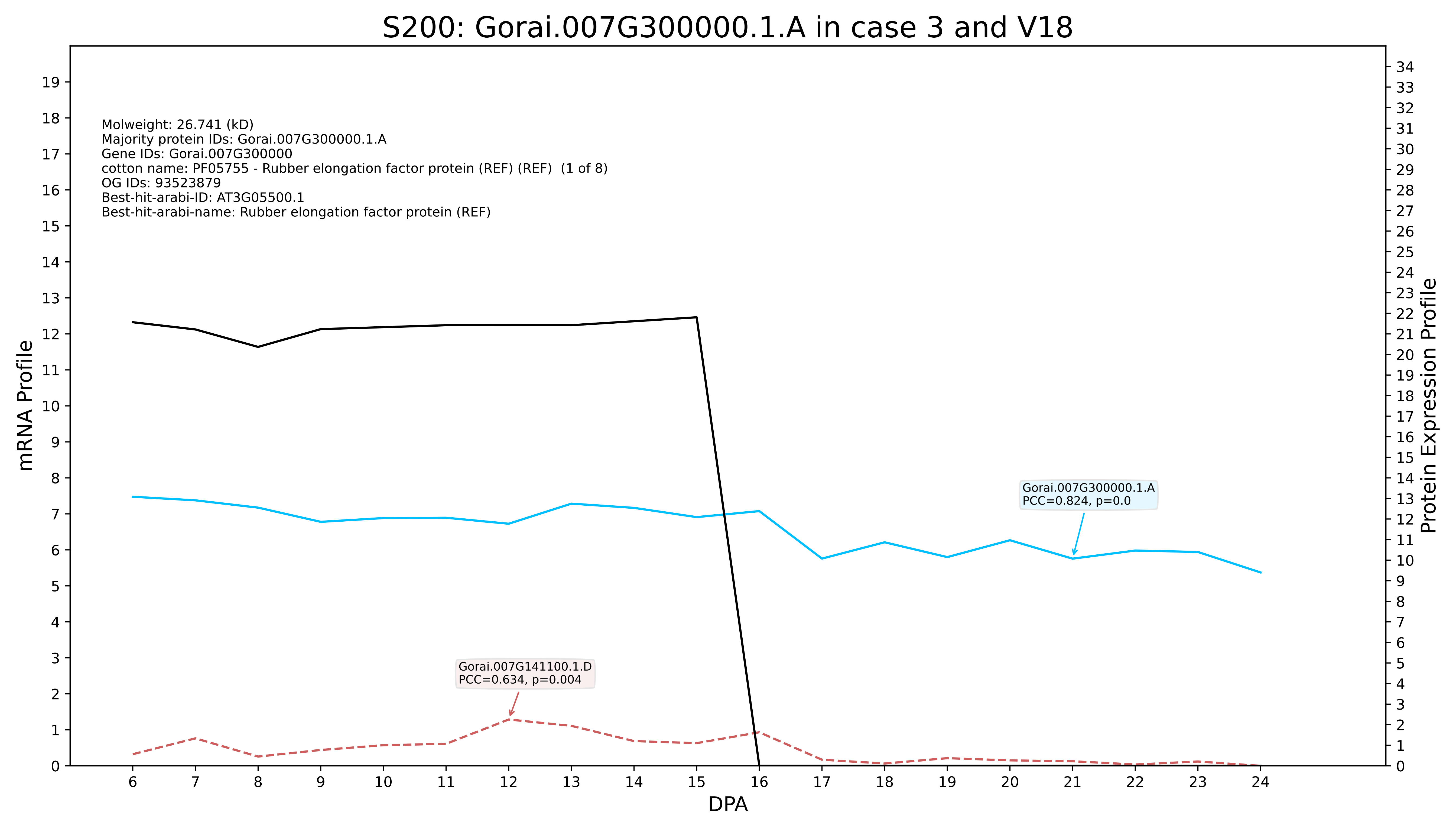
S200: Gorai.007G300000.1.A in case 3 and V18
Molweight: 26.741 (kD)
Majority protein IDs: Gorai.007G300000.1.A
Gene IDs: Gorai.007G300000
cotton name: PF05755 - Rubber elongation factor protein (REF) (REF) (1 of 8)
OG IDs: 93523879
Best-hit-arabi-ID: AT3G05500.1
Best-hit-arabi-name: Rubber elongation factor protein (REF)
Gorai.007G300000.1.A
PCC=0.824, p=0.0
Gorai.007G141100.1.D
PCC=0.634, p=0.004
mRNA Profile
Protein Expression Profile
DPA

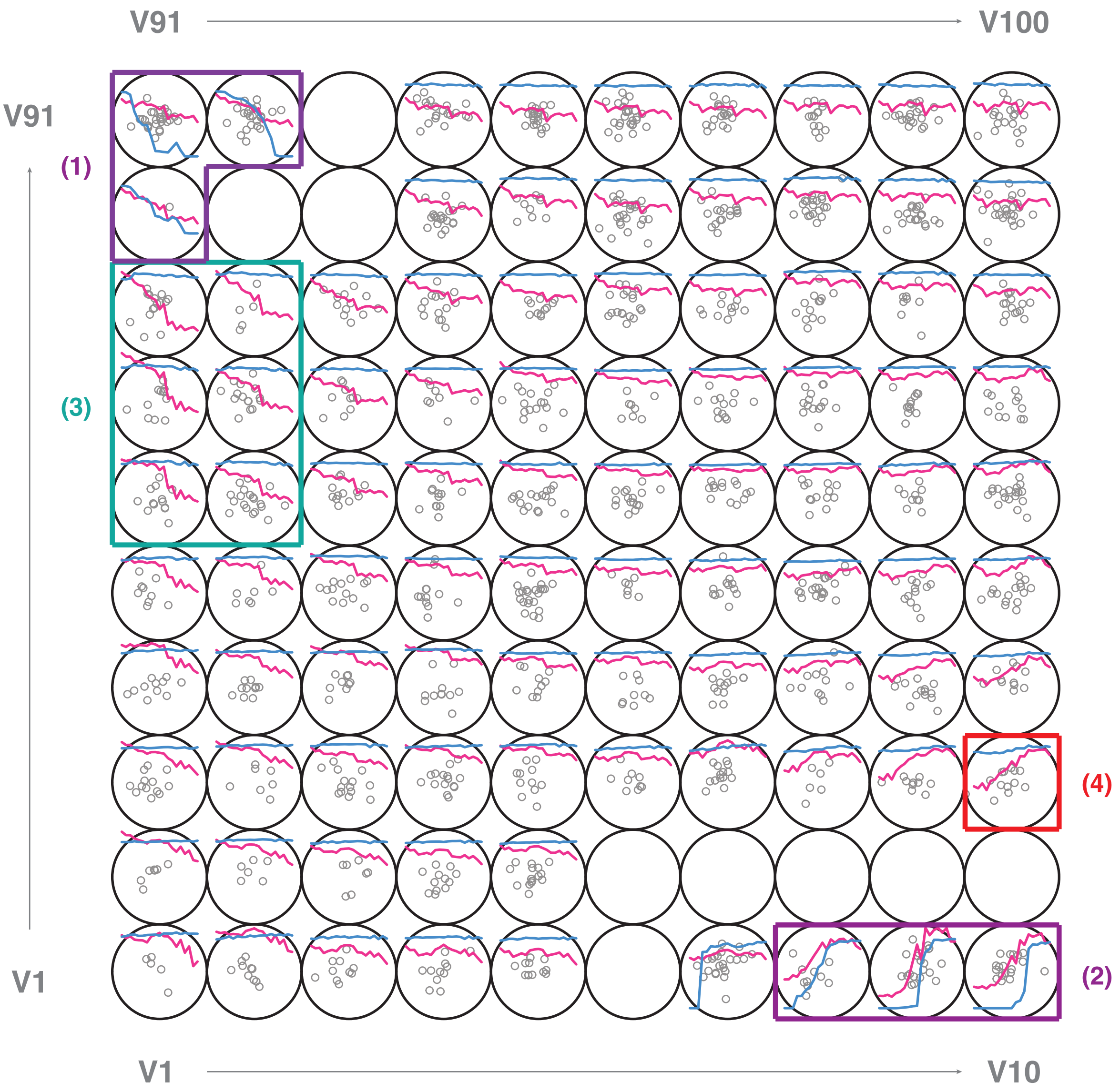

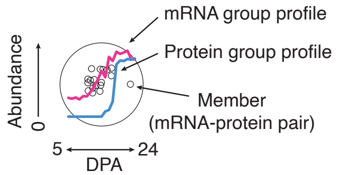

Supplementary Figure S13. Weighted cross-correlation SOM of paired mRNAs and proteins (P200).

SOM-based weighted cross correlation of paired mRNA and proteins that have flat profiles across development. Profiles of P200 proteins in the case 1 (1:1 mRNA and protein match) were paired with profiles of their corresponding mRNAs. (1) & (2) Groups of mRNA and protein pairs that show the similar trends (purple boxes). (3) Groups including 8-times decreasing mRNA profiles with flat protein profiles (teal box). (4) A group containing 8-times increasing mRNA profiles with flat protein profiles (red box).
